# Proteomic analysis of middle and late stages of bread wheat (*Triticum aestivum* L.) grain development

**DOI:** 10.3389/fpls.2015.00735

**Published:** 2015-09-15

**Authors:** Ning Zhang, Feng Chen, Wang Huo, Dangqun Cui

**Affiliations:** Agronomy College/Collaborative Innovation Center of Henan Grain Crops/National Key Laboratory of Wheat and Maize Crop Science, Henan Agricultural UniversityZhengzhou, China

**Keywords:** *Triticum aestivum* L., EMS, grain development, grain size, proteome

## Abstract

Proteomic approaches were applied in four grain developmental stages of the Chinese bread wheat Yunong 201 and its ethyl methanesulfonate (EMS) mutant line Yunong 3114. 2-DE and tandem MALDI-TOF/TOF-MS analyzed proteome characteristics during middle and late grain development of the Chinese bread wheat Yunong 201 and its EMS mutant line Yunong 3114 with larger grain sizes. We identified 130 differentially accumulated protein spots representing 88 unique proteins, and four main expression patterns displayed a dynamic description of middle and late grain formation. Those identified protein species participated in eight biochemical processes: stress/defense, carbohydrate metabolism, protein synthesis/assembly/degradation, storage proteins, energy production and transportation, photosynthesis, transcription/translation, signal transduction. Comparative proteomic characterization demonstrated 12 protein spots that co-accumulated in the two wheat cultivars with different expression patterns, and six cultivar-specific protein spots including serpin, small heat shock protein, β-amylase, α-amylase inhibitor, dimeric α-amylase inhibitor precursor, and cold regulated protein. These cultivar-specific protein spots possibly resulted in differential yield-related traits of the two wheat cultivars. Our results provide valuable information for dissection of molecular and genetics basis of yield-related traits in bread wheat and the proteomic characterization in this study could also provide insights in the biology of middle and late grain development.

## Introduction

Hexaploid wheat (*Triticum aestivum*, 2*n* = 6 × = 42, AABBDD) is one of the most important cereals that provides a large proportion of essential nutrients in the human diet. The major constituents of wheat grain are starch (70-80% dry weight) and proteins (10-15% dry weight; Tasleem-Tahir et al., 2012). Of the total wheat grain proteins, the major protein (80%) reserves are the prolamins, which are a mixture of monomeric gliadins and polymeric glutenins located in the starchy endosperm. In contrast to the gliadins and glutenins, the other major protein families of the wheat endosperm, are the non-prolamins, including albumins and globulins (Vensel et al., 2005a).

Wheat grain development is divided into two main stages: (1) grain enlargement, and (2) grain filling and desiccation/maturation. Grain enlargement involves early and rapid division of the zygote and triploid nucleus. Cell division is followed by the influx of water, which drives cell extension. This stage occurs at approximately 3–20 days post-anthesis (dpa). During the grain filling stage, cell division slows and then ceases and beginning at around 10 dpa until maturity, storage products are accumulated, at which point the endosperm serves its function as a carbohydrate store (Nadaud et al., 2010). In recent years, different approaches including transcriptomics, proteomics, and metabolomics have been used to understand the diversity and development of grain. However, the expression profiles of accumulated proteins are often poorly correlated with their corresponding mRNAs; e.g., in Arabidopsis (Ruuska et al., 2002), rice (Zhang et al., 2010), and wheat (Dong et al., 2012; Ma et al., 2014). Two-dimensional electrophoresis (2-DE) and mass spectrometry (MS) proteomic approaches have been broadly applied to investigate the dynamic expression profiles of proteins during grain development in different plant species, including Arabidopsis (Ruuska et al., 2002; Li et al., 2007), soybean (Li et al., 2012), maize (Méchin et al., 2007), and rice (Thelen, 2009; Zhang et al., 2012). Further, a significant study on the proteomics of the wheat grain developmental period has been carried out. Proteomic studies on the response to heat stress during grain filling in 10 wheat cultivars indicated that primarily changes in both the amount and activities of enzymes involved in photosynthesis and antioxidant activities contributed to relatively higher heat tolerance (Wang et al., 2015). Identification of proteins in the first 2 weeks of grain development stages showed that a total of 10 clusters of genes were examined in bread wheat (Nadaud et al., 2010). The proteomes of hard and soft near-isogenic wheat lines at four grain developmental stages revealed that kernel hardness is related to the amplification of a stress response during endosperm development (Lesage et al., 2012). Proteome characterization of four grain developmental phases in wheat cultivars Jimai 20 and Zhoumai 16 indicated that differences in seed storage proteins were related to different flour quality performance from these wheat cultivars (Guo et al., 2012).

Ethyl methanesulfonate (EMS) mutants have been widely used as an important method to develop new germplasms in wheat breeding programs due to its high mutant frequency (Henry et al., 2014). The key reasons for this include their highly beneficial mutations, excellent phenotypic characteristics, and novel gene traits. The EMS mutation technique has also reached a mature stage, in which damage in plants is reduced and abundant plant mutations are generated by controlling EMS use. Additionally, EMS mutants have been employed as basic materials in some studies (Botticella et al., 2011; Bonchev et al., 2012; Henry et al., 2014). For example, six EMS-mutagenized lines were validated to improve lodging resistance in Tef (*Eragrostis tef*; Zhu et al., 2012). Stay-green and fast-senescing EMS mutated wheat lines with similar anthesis were characterized to investigate the impact on yield and nitrogen partitioning (Derkx et al., 2012).

A combination of 2-DE and EMS mutants in wheat proteomic studies were rarely applied. The Chinese winter wheat cultivar Yunong 201, developed by Agronomy College of Henan Agricultural University, was released as a high-quality noodle wheat cultivar by Henan province in 2006. An elite M_2_ line was screened from a large EMS-mutagenized population because of its different plant architecture, larger kernel size, and higher grain weight. This line was self-crossed four times into Yunong 3114. Compared with Yunong 201, Yunong 3114 showed relatively larger kernel size, higher thousand grain weight and higher yield per plot. Therefore, comparison of proteomics of mid and late grain developmental stages of the bread wheat Yunong 201 and Yunong 3114 could provide valuable information for dissection of molecular and genetics basis of yield-related traits in bread wheat, and the proteomic characterization could also provide insights in the biology of middle and late grain development.

## Materials and methods

### Plant materials

**Figure 1:**
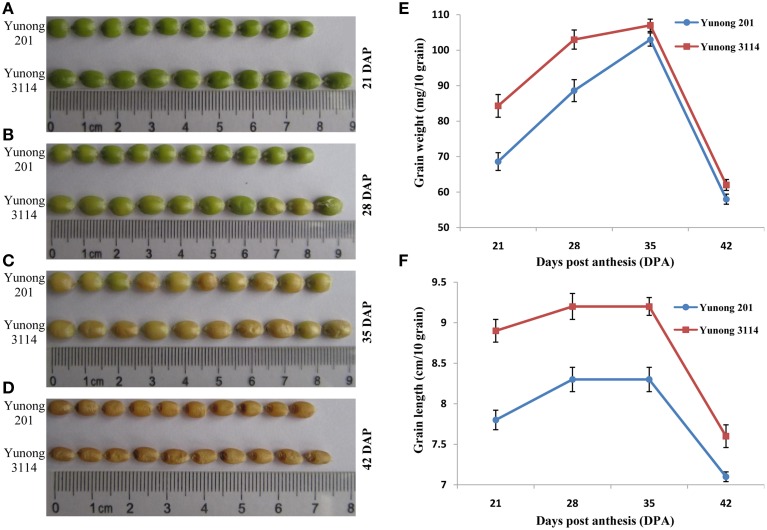
**Grain development during four grain developmental stages (21, 28, 35, 42) in Yunong 3114 and Yunong 201**. **(A–D)** Grain morphological development; **(E)** Grain weight accumulation; **(F)** Grain length accumulation.

A Chinese winter wheat cultivar Yunong 201 (released no. Yushenmai 2006006) was treated by 0.8% EMS (ethyl methanesulfonate) in 2007. An elite M_2_ line was screened from the EMS mutated population containing 2000 lines due to its differential plant architecture, larger kernel size and higher grain weight, which was self-crossed four times into an M_6_ line Yunong 3114. Yunong 201 and Yunong 3114 were planted at the Zhengzhou Scientific Research and Education Center of Henan Agricultural University (longitude 113.6°E; latitude 34.9°N) during the 2013–2014 cropping seasons under non-stressed natural soil conditions. Differently developmental seeds of Yunong 201 and Yunong 3114 were collected during the post-anthesis period based on thermal times that corresponded to the cumulative average daily temperatures as shown in Table 1, and grain size and weight of each sample were investigated (Figure 1). Sampled grains were stored at −80°C prior to analysis.

**Table 1:** **Details of grain samples harvested during the post-anthesis period based on thermal time corresponding to cumulative average daily temperatures**.

**Batch/No**.	**Date**	Dpa^*^	**°Cd**
I	2014.05.02-05.09	21	167°C
II	2014.05.09-05.16	28	175°C
III	2014.05.16-05.23	35	201°C
IV	2014.05.23-05.30	42	221°C

* *Days post-anthesis*.

### Protein preparation

For two-dimensional gel electrophoresis (2-DE), protein samples with three biological replicates were prepared according to the method of Gao et al. (2009). Grain samples of 500 mg were extracted in the mid-ear region of each spike, and were ground into a powder in liquid nitrogen with a mortar and pestle. Ten volumes of cold extraction buffer containing 100 mM Tris–HCl (pH 8.8), 10 mM fresh dithiothreitol (DTT), and 10% sodium dodecyl sulfate (SDS) were added, and further ground for 1 h on ice. After centrifuging at 10,000 g for 10 min at 4°C, the supernatants were collected to new tubes, and an equal volume of phenol was added, following which samples were shaken gently for 30 min, and centrifuged at 14,000 g for 10 min at 25°C. Below the top phenol phase, the samples were collected to new tubes, and then the above cold extraction buffer was added again to extract once more. The phase of phenol was acquired again, and samples were precipitated with five-fold volumes of cold ammonium acetate/methanol at −20°C for 2 h. After centrifugation at 14,000 g for 15 min at 4°C, the supernatants were discarded and the pellets were washed three times in ice-cold acetone containing 5 mM DTT. The pellets were vacuum-dried and resuspended in lysis buffer containing 8 M urea, 2 M thiourea, 4% 3-[(3-cholamidopropyl) dimethylammonio]-1-propanesulfonate (CHAPS), and 20 mM DTT at 25°C for 2 h according to method of Li et al. (2013). The suspension was centrifuged at 14,000 g for 40 min at 25°C to remove insoluble materials. Concentrations of total protein were determined by the Bradford assay (Bio-Rad) based on a bovine serum albumin standard (Li et al., 2013). Detailed standard curves with seven different concentrations of BSA (0–100 μg) resuspended in lysis buffer and water in triplicate were shown in Additional file 2 (Data sheets 11, 12).

### 2-DE and images analysis

For 2-DE, 800 μg of protein samples were loaded onto an ReadyStripTM IPG Strip (24 cm, pH 4-7, BIO-RAD, USA) and hydrated passively with 450 μL of protein solution containing 0.5% (v/v) immobilized pH gradient (IPG) buffer (pH 4-7) for 12-18 h at 20°C using a PROTEAN IEF Cell (BIO-RAD, USA). The first-dimension isoelectric focusing (IEF) was performed with six steps: 250 V for 130 min, 250 V for 90 min, 500 V for 90 min, 1000 V for 2 h, 9000 V for 5 h, and 9000 V for 10 h with a total of 99 kVh and a constant 500 V for the last 12 h. After IEF, the strips were incubated for 15 min in “equilibration buffer I” consisting of 6 M urea, 2% (w/v) SDS, 1.5 M Tris-HCl (pH 8.8), 20% (v/v) glycerol, 0.01% (w/v) bromophenol blue, and 2% (w/v) DTT and then in “buffer II” consisting 6 M urea, 2% (w/v) SDS, 1.5 M Tris-HCl (pH 8.8), 20% (v/v) glycerol, 0.01% (w/v) bromophenol blue, and 2.5% (w/v) iodoacetamide for 15 min.

For second-dimension electrophoresis, the strips were transferred to 12% vertical sodium dodecyl sulfate-polyacrylamide gel electrophoresis (SDS-PAGE) gels. All seed samples were run in triplicate to obtain statistically reliable results. After electrophoresis, gels were fixed in 40% (v/v) methanol and 10% (v/v) acetic acid for 40 min. To visualize the gels, they were stained with staining solution consisting of 0.12% (v/v) Coomassie brilliant blue (CBB) G-250, 20% (v/v) alcohol, 10% (v/v) phosphoric acid, and 10% (w/v) ammonium sulfate, and then destained in double-distilled water (Wang et al., 2012). The 2-DE images were scanned at 300 dpi with a UMAX Power Look 2, 100XL scanner (Maximum Tech, Taiwan, China), and quantitative intensity analysis was performed using PDQuest software (version 8.0.1, Bio-Rad, USA). First, the 2-DE gel of 21 dpa seed samples of Yunong 210 and Yunong 3114 was selected as the reference gel. All gels of other stages were matched to the reference gel. Automatic groups formed, and single spots that differed between replicates were manually checked and corrected when necessary. The spots that existed in three independent sample sets were selected. Image quantitative analysis revealed significant differences in protein spot abundance by Student's *t*-test (abundance variation at least two-fold, *P* < 0.05).

### Two-dimensional gel excision, tryptic digestion, and desalting

Protein extracts were separated on preparative gels and 130 proteins of interest were recovered from the gels for identification. Proteins (800 μg) from samples were resolved on separate preparative polyacrylamide gels and were visualized by staining with a modified silver staining method that was compatible with subsequent mass spectrometric analysis (Yan et al., 2000). Protein spots of interest were cut from the preparative gels, destained for 20 min in 30 mM potassium ferricyanide/100 mM sodium thiosulfate (1:1 v/v) and washed with Milli-Q water until the gels were destained. The spots were incubated in 0.2 M NH_4_HCO_3_ for 20 min and then lyophilized. Each spot was digested overnight in 12.5 ng/μl trypsin in 25 mM NH_4_HCO_3_. The peptides were extracted three times with 60% acetonitrile (ACN)/0.1% trifluoroacetic acid (TFA). The extracts were pooled and dried completely by a vacuum centrifuge.

### MALDI-TOF/TOF analysis

MS and MS/MS data for protein identification were obtained using a MALDI-TOF-TOF instrument (4800 proteomics analyzer; Applied Biosystems). Instrument parameters were set using the 4000 Series Explorer software (Applied Biosystems). The MS spectra were recorded in reflector mode and a mass range from 800 to 4000 and a focus mass of 2000. MS was used using a CalMix5 standard to calibrate the instrument (ABI 4700 Calibration Mixture).

For one main MS spectrum 25 sub-spectra with 125 shots per sub-spectrum were accumulated using a random search pattern. For MS calibration, autolysis peaks of trypsin ([M+H] + 842.5100 and 2, 211.1046) were used as internal calibrates, and up to 10 of the most intense ion signals were selected as precursors for MS/MS acquisition, excluding the trypsin autolysis peaks and the matrix ion signals. In MS/MS positive ion mode, for one main MS spectrum 50 sub-spectra with 50 shots per sub-spectrum were accumulated using a random search pattern. Collision energy was 2 kV, the collision gas was air, and the default calibration was set using the Glu1-Fibrino-peptide B ([M+H] + 1570.6696) spotted onto Cal 7 positions of the MALDI target. Combined peptide mass fingerprinting (PMF) and MS/MS queries were performed using the MASCOT search engine 2.2 (Matrix Science, Ltd.) that was embedded into the GPS-Explorer Software 3.6 (Applied Biosystems) on the NCBI database with the following parameter settings: 100 ppm mass accuracy, with trypsin cleavage and one missed cleavage allowed, carbamido methylation set as a fixed modification, and oxidation of methionine allowed as a variable modification. Additionally, the MS/MS fragment tolerance was set to 0.4 Da. A GPS explorer protein confidence index ≥95% was used for further manual validation.

## Results

### Comparison of grain size of Yunong 201 and Yunong 3114

Kernel sizes and weights of the grain in both Yunong 201 and Yunong 3114 increased gradually from 21 to 35 dpa, and then decreased from 35 to 42 dpa (Figures 1E,F). Compared with Yunong 201, Yunong 3114 possessed longer kernel length and higher grain weight at the four stages of development (detailed data in Figures 1A–D) but there was no obvious difference on grain width between the two cultivars.

### Identification, and classification of differentially accumulated proteins during grain development

**Figure 2:**
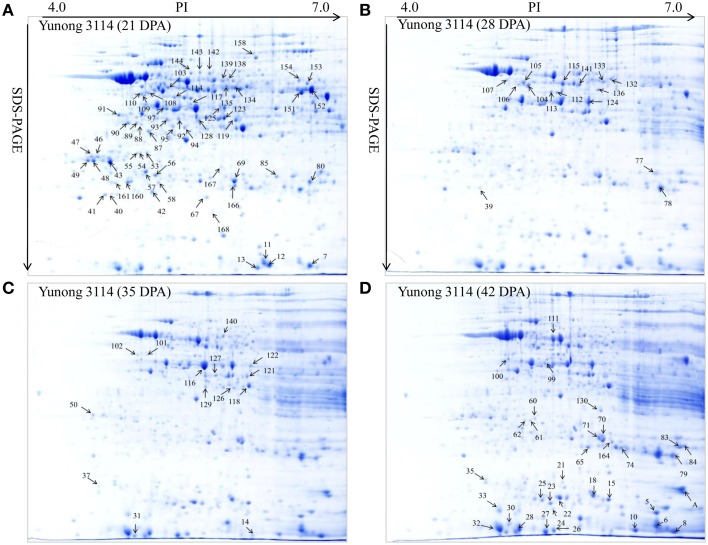
**Showing 2-DE maps of proteins extracted from the first sample of Yunong 3114**. **(A–D)** 2-DE maps during four grain development stages for 21, 28, 35, and 42 DPA in Yunong 3114.

**Figure 3:**
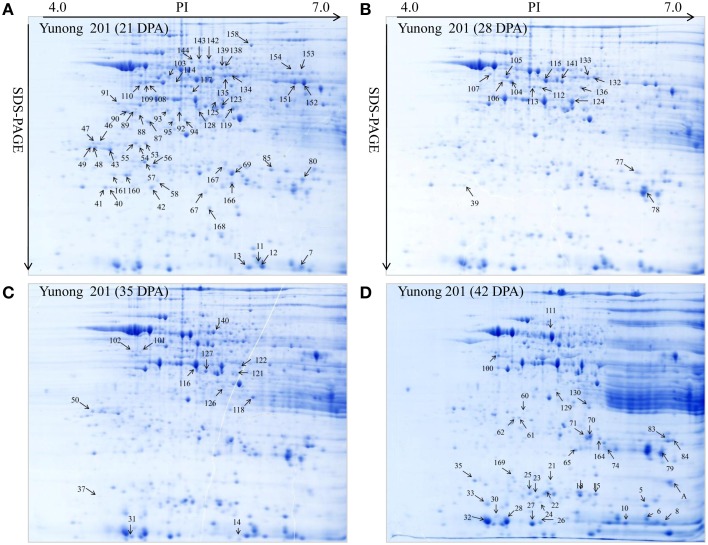
**Showing Yunong 201 at four stages of grain development**. **(A–D)** 2-DE maps during four grain development stages for 21, 28, 35, and 42 DPA in Yunong 201.

Yunong 201 and Yunong 3114 had similar proteomic profiles at the four stages according to the 2-DE protein maps that were extracted from both samples (Figures 2, 3). There were more than 1000 gel spots detected over the gel, and 173 spots were detected that displayed altered abundance, which were then analyzed by mass spectrometry. Finally, 130 spots out of the 173 spots, representing 88 unique proteins, were successfully identified based on BLASTp analyses of NCBI databases (Additional file 1: Data sheets 1–10; Table 2).

**Table 2:** **Differentially expressed proteins identified by MALDI-TOF/TOF-MS at four grain developmental stages in bread wheat cultivars Yunong 201 and Yunong 3114**.

**Spot no.^a^**	**Protein species**	**Accession no.^b^**	**Score^c^**	**Protein M_t_/pl^d^**	**Protein score C.1.%^c^**	**NP^e^**	**Plant species**
**OTHERS**							
18	Hypothetical protein TRIUR3_03549	gi|474071007	233	16.82/6.19	100	7	*Triticum urartu*
43	Unnamed protein product	gi|227473229	693	29.36/4.83	100	22	*Triticum aestivum*
63-1	Hypothetical protein TRIUR3_05104	gi|474427757	275	29.23/5.54	100	6	*Triticum urartu*
63-2	Hypothetical protein TRIUR3_05104	gi|474427757	320	29.23/5.54	100	8	*Triticum urartu*
65	Hypothetical protein TRIUR3_31593	gi|473979984	372	19.79/5.63	100	12	*Triticum urartu*
69	Unnamed protein product	gi|300586307	386	27.01/5.38	100	12	*Triticum aestivum*
79	Hypothetical protein TRIUR3_28410	gi|474060617	86	28.39/5.53	100	7	*Triticum urartu*
93	Unnamed protein product	gi|227478321	210	30.99/5.35	100	11	*Triticum aestivum*
97	Unnamed protein product	gi|296511811	573	46.52/5.23	100	11	*Triticum aestivum*
140	Hypothetical protein TRIUR3_12214	gi|473926526	63	87.85/6.54	100	19	*Triticum urartu*
157-1	Hypothetical protein TRIUR3_30168	gi|473888425	64	75.32/5.21	100	26	*Triticum urartu*
158	5-Methyltetra hydropteroyltriglutamate-homocysteine methyltransferase	gi|473993302	1120	84.84/5.74	100	20	*Triticum urartu*
167	Unnamed protein product	gi|296520469	78	28.25/5.58	99.93	6	*Triticum aestivum*
168	Unnamed protein product	gi|259439698	216	19.56/5.63	100	4	*Triticum aestivum*
**STORAGE PROTEIN**							
5	Globulin 3	gi|215398470	397	66.65/7.78	100	9	*Triticum aestivum*
15	Globulin 3	gi|215398470	286	66.65/7.78	100	8	*Triticum aestivum*
22	Globulin-3A	gi|390979705	505	66.63/8.48	100	12	*Triticum aestivum*
23	Globulin 3B	gi|215398472	142	57.07/7.36	100	10	*Triticum aestivum*
25	Globulin-3A	gi|390979705	396	66.63/8.48	100	11	*Triticum aestivum*
74	Gliadin/Avenin-like seed protein	gi|281335538	174	22.81/6.2	100	4	*Triticum aestivum*
78	Gamma gliadin	gi|133741924	82	16.54/8.86	99.91	3	*Triticum aestivum*
24	Globulin-3A	gi|390979705	137	66.63/8.48	100	8	*Triticum aestivum*
130	Globulin-3A	gi|390979705	325	66.63/8.48	100	14	*Triticum aestivum*
**STRESS/DEFENSE**							
6	α-Amylase inhibitor CM3	gi|39578552	503	18.89/7.44	100	8	*Triticum durum*
8	Dimeric α-amylase inhibitor precursor, partial	gi|108597921	375	14.03/6.69	100	5	*Triticum aestivum*
10	α-Amylase inhibitor 0.19	gi|66841026	192	13.34/6.86	100	6	*Triticum aestivum*
11	α-Amylase inhibitor CM3	gi|39578552	583	18.89/7.44	100	8	*Triticum durum*
12	Dimeric α-amylase inhibitor precursor, partial	gi|108597921	282	14.03/6.69	100	6	*Triticum aestivum*
19-1	α-amylase inhibitor 0.19	gi|66841026	351	13.34/6.86	100	6	*Triticum aestivum*
26	0.19 Dimeric α-amylase inhibitor	gi|65993852	253	15.56/5.73	100	6	*Triticum aestivum*
27	Dimeric α-amylase inhibitor	gi|114215794	353	13.75/5.23	100	5	*Triticum dicoccoides*
30	Dimeric α-amylase inhibitor	gi|114215794	308	13.75/5.23	100	6	*Triticum dicoccoides*
31	Dimeric α-amylase inhibitor	gi|114215794	430	13.75/5.23	100	7	*Triticum dicoccoides*
28	α-Amylase inhibitor CM16 subunit	gi|221855644	296	16.27/5.31	100	5	*Triticum macha*
29	α-Amylase inhibitor CM16 subunit	gi|221855644	178	16.27/5.31	100	5	*Triticum macha*
32	CM 17 Protein precursor	gi|21711	237	16.55/5.07	100	4	*Triticum aestivum*
33	CM 17 Protein precursor	gi|21711	284	16.55/5.07	100	4	*Triticum aestivum*
35	Cold regulated protein	gi|26017213	224	17.79/4.84	100	8	*Triticum aestivum*
37	Dehydrin	gi|61657604	226	16.31/4.39	100	6	*Triticum durum*
41	Thiol-specific antioxidant protein	gi|379060943	278	43.02/5.18	100	11	*Triticum aestivum*
39	Thiol-specific antioxidant protein	gi|1805351	208	23.43/5.71	100	6	*Triticum aestivum*
83	1-Cys peroxiredoxin	gi|12247762	195	24.11/6.3	100	12	*Triticum durum*
84	1-Cys peroxiredoxin	gi|12247762	320	24.11/6.3	100	10	*Triticum durum*
56	Ascorbate peroxidase	gi|226897533	479	26.78/5.54	100	9	*Triticum aestivum*
57	Ascorbate peroxidase	gi|226897533	510	26.78/5.54	100	11	*Triticum aestivum*
58	Aci-reductone-dioxygenase-like protein	gi|237512521	518	23.62/5.08	100	15	*Triticum aestivum*
60	Chitinase 2	gi|474441224	572	24.93/4.95	100	9	*Triticum urartu*
61	Chitinase 2	gi|474441224	144	24.93/4.95	100	5	*Triticum urartu*
62	Chitinase 2	gi|474441224	74	24.93/4.95	99.84	5	*Triticum urartu*
70	Disease resistance protein RPP13	gi|473786130	69	11.56/8.15	99.42	24	*Triticum urartu*
71	Vicilin-like antimicrobial peptides 2-2	gi|473890163	193	75.30/5.79	100	7	*Triticum urartu*
77	L-ascorbate peroxidase 1, cytosolic	gi|474311703	417	27.56/5.85	100	12	*Triticum urartu*
85	L-ascorbate peroxidase 1, cytosolic	gi|474311703	673	27.56/5.85	100	15	*Triticum urartu*
100	Vicilin-like antimicrobial peptides 2-2	gi|473890163	131	75.30/5.79	100	12	*Triticum urartu*
117	Bifunctional polymyxin resistance protein ArnA	gi|474224464	307	43.51/7.53	100	16	*Triticum urartu*
126	Putative NADP-dependent oxidoreductase P1	gi|473799043	348	38.36/5.53	100	12	*Triticum urartu*
129	Putative NADP-dependent oxidoreductase P1	gi|473799043	430	38.36/5.53	100	15	*Triticum urartu*
139	Betaine-aldehyde dehydrogenase	gi|21747870	344	55.23/5.44	100	12	*Triticum aestivum*
164	Dehydroascorbate reductase	gi|259017810	233	23.46/5.88	100	7	*Triticum aestivum*
169	Small heat shock protein Hsp23.5	gi|4138869	224	23.45/6.22	100	6	*Triticum aestivum*
A/1	α-Amylase inhibitor	gi|225042	464	19.85/6.77	100	14	
40	Serpin-N3.2	gi|379060943	631	43.02/5.18	100	12	*Triticum aestivum*
42	Serpin 1	gi|224589266	593	43.26/5.44	100	7	*Triticum aestivum*
116	Serpin 1	gi|224589266	649	43.26/5.44	100	13	*Triticum aestivum*
67	Serpin-N3.2	gi|379060943	631	43.03/5.18	100	12	*Triticum aestivum*
99	Serpin-N3.2	gi|379060943	577	43.03/5.18	100	15	*Triticum aestivum*
121	serpin-Z2B	gi|473793747	318	45.23/6.03	100	12	*Triticum urartu*
122	Serpin-Z1C	gi|474075261	141	42.96/5.62	100	9	*Triticum urartu*
124	Serpin-Z1C	gi|474075261	337	42.96/5.62	100	13	*Triticum urartu*
**Photosynthesis**							
13	Ribulose bisphosphate carboxylase small chain, chloroplastic	gi|474416311	338	15.09/5.85	100	14	*Triticum urartu*
14	Ribulose-1,5-bisphosphate carboxylase/oxygenase small subunit	gi|11990897	161	19.73/8.8	100	7	*Triticum aestivum*
53	Oxygen-evolving enhancer protein 1, chloroplastic	gi|474352688	749	36.64/5.75	100	18	*Triticum urartu*
55	Oxygen-evolving enhancer protein 1, chloroplastic	gi|474352688	667	36.64/5.75	100	16	*Triticum urartu*
152	Ribulose-1,5-bisphosphate carboxylase/oxygenase largesubunit (chloroplast)	gi|525778513	850	53.34/6.04	100	27	*Triticum monococcum*
159-2	Chlorophyll a-b binding protein 1, chloroplastic	gi|473965828	149	30.44/5.25	100	6	*Triticum urartu*
161	Chlorophyll a-b binding protein, chloroplastic	gi|473952980	102	28.46/5.51	100	7	*Triticum urartu*
166	23 kDa oxygen evolving protein of photosystem II	gi|21837	525	27.42/8.84	100	15	*Triticum aestivum*
**PROTEIN SYNTHESIS/ASSEMBLY/DEGRADATION**							
21	E3 ubiquitin-protein ligase BRE1-like 1-like isoform X1	gi|514816085	76	10.26/8	97.56	8	*Setaria italica*
134	Adenosylhomocysteinase	gi|474154141	354	46.13/6.48	100	16	*Triticum urartu*
108	Tubulin alpha chain	gi|474224323	470	50.37/4.89	100	16	*Triticum urartu*
110	Tubulin alpha chain	gi|474224323	350	50.37/4.89	100	10	*Triticum urartu*
**STARCH METABOLISM**							
54	ADP glucose pyrophosphorylase	gi|469952290	465	53.40/5.54	100	20	*Triticum aestivum*
92	ADP-glucose pyrophosphorylase large subunit	gi|110729318	70	58.40/6.12	99.57	13	*Triticum gi*
109	β-Amylase	gi|474451266	166	59.00/5.34	100	13	*Triticum urartu*
111	β-Amylase	gi|474451266	720	59.00/5.34	100	19	*Triticum urartu*
114	β-Amylase	gi|474451266	177	59.00/5.34	100	11	*Triticum urartu*
119	β-Amylase	gi|474451266	549	59.00/5.34	100	17	*Triticum urartu*
125	β-Amylase	gi|474451266	445	59.00/5.34	100	17	*Triticum urartu*
128	β-Amylase	gi|474451266	549	59.00/5.34	100	17	*Triticum urartu*
133	β-Amylase	gi|474451266	449	59.00/5.34	100	15	*Triticum urartu*
138	Granule bound starch synthase	gi|262385348	99	64.48/8.42	100	16	*Triticum turgidum subsp. dicoccon*
141	Small subunit ADP glucose pyrophosphorylase	gi|7340287	954	52.31/5.53	100	22	*Triticum aestivum*
153	ADP-glucose pyrophosphorylase large subunit	gi|110729318	1140	58.40/6.12	100	29	*Triticum aestivum*
**SIGNAL TRANSDUCTION**							
46	14-3-3 Protein	gi|390195996	684	30.07/4.73	100	20	*Triticum aestivum*
47	14-3-3 Protein	gi|390195996	529	30.07/4.73	100	24	*Triticum aestivum*
48	14-3-3 Protein	gi|390195996	134	30.07/4.73	100	15	*Triticum aestivum*
49	14-3-3-Like protein B	gi|474253094	416	29.79/4.67	100	22	*Triticum urartu*
50	14-3-3-Like protein B	gi|474253094	206	29.79/4.67	100	12	*Triticum urartu*
7	Nucleoside diphosphate kinase 1	gi|474369382	516	16.58/6.3	100	10	*Triticum urartu*
**GLYCOLYSIS**							
87	Fructokinase-2	gi|474190636	212	42.15/4.78	100	11	*Triticum urartu*
88	Transaldolase	gi|473926683	265	30.86/5.24	100	6	*Triticum urartu*
94	Fructose-bisphosphate aldolase, chloroplastic	gi|473848356	144	42.20/5.94	100	14	*Triticum urartu*
95	Chloroplast fructose-bisphosphate aldolase	gi|223018643	602	42.22/5.94	100	18	*Triticum aestivum*
104	UTP-glucose-1-phosphate uridylyltransferase	gi|473993048	516	51.08/5.76	100	18	*Triticum urartu*
106	UTP-glucose-1-phosphate uridylyltransferase	gi|473993048	511	51.08/5.76	100	23	*Triticum urartu*
115	Enolase	gi|461744058	627	48.46/5.49	100	17	*Triticum aestivum*
123	Phosphoglycerate kinase, cytosolic	gi|473781647	642	45.29/5.9	100	18	*Triticum urartu*
136	6-Phosphogluconate dehydrogenase, decarboxylating	gi|474156904	500	53.71/9.45	100	19	*Triticum urartu*
127	Cytosolic 3-phosphoglycerate kinase	gi|28172905	650	31.37/4.98	100	16	*Triticum urartu*
142	2,3-Bisphosphoglycerate-independent phosphoglycerate mutase	gi|473886714	657	57.76/5.28	100	15	*Triticum urartu*
143	2,3-Bisphosphoglycerate-independent phosphoglycerate mutase	gi|473886714	1020	57.76/5.28	100	21	*Triticum urartu*
144	2,3-Bisphosphoglycerate-independent phosphoglycerate mutase	gi|473886714	744	57.76/5.28	100	18	*Triticum urartu*
**TCA PATHWAY**							
118	Cytosolic malate dehydrogenase	gi|49343245	486	35.81/5.75	100	13	*Triticum aestivum*
**TRANSCRIPTION/TRANSLATION**							
76-2	27 K Protein	gi|30793446	148	2.327/6.06	100	4	*Triticum aestivum*
80	27 K Protein	gi|290350670	215	24.40/6.06	100	5	*Triticum aestivum*
91	40S Ribosomal protein SA	gi|474222337	458	33.93/4.97	100	14	*Triticum urartu*
112	Eukaryotic initiation factor 4A-1	gi|474441074	1120	47.16/5.38	100	24	*Triticum urartu*
113	Eukaryotic initiation factor 4A-1	gi|474441074	875	47.16/5.38	100	23	*Triticum urartu*
**ENERGY PRODUCTION AND TRANSPORTATION**							
103	ATP synthase subunit beta, mitochondrial	gi|473798701	933	57.83/5.25	100	25	*Triticum urartu*
105	ATP synthase beta subunit	gi|525291	1310	59.33/5.56	100	22	*Triticum aestivum*
107	ATP synthase CF1 beta subunit	gi|14017579	1160	53.88/5.06	100	25	*Triticum aestivum*
132	ATP synthase subunit alpha, mitochondrial	gi|474033641	564	44.89/5.54	100	16	*Triticum urartu*
135	ATP synthase subunit alpha, mitochondrial	gi|474033641	578	44.89/5.54	100	14	*Triticum urartu*
157-2	Cleavage stimulation factor subunit 1	gi|474365350	68	70.97/9.27	99.30	19	*Triticum urartu*
160	20 kDa Chaperonin, chloroplastic	gi|474407512	389	29.81/6.77	100	8	*Triticum urartu*
89	Adenosine kinase 2	gi|474049015	235	36.68/5.01	100	8	*Triticum urartu*
90	Adenosine kinase 2	gi|474049015	462	36.68/5.01	100	12	*Triticum urartu*
154	ATP synthase CF1 alpha subunit (chloroplast)	gi|521301484	932	55.32/6.11	100	27	*Triticum aestivum*
**NITROGEN METABOLISM**							
101	Glutaminyl-tRNA synthetase	gi|474021464	77	90.54/6.65	99.92	25	*Triticum aestivum*
151	Alanine aminotransferase 2	gi|473789790	437	57.75/6.77	100	20	*Triticum urartu*

a *Spot no. corresponds to protein spot on gels shown in Figures 2, 3*.

b *Accession no. predicted protein in NCBInr database*.

c *Scores were searched against the database NCBInr*.

d *M_r_/pI: M_r_ of molecular mass of predicted protein/pI of predicted protein*.

e *NP: Number of matched peptides*.

**Figure 4:**
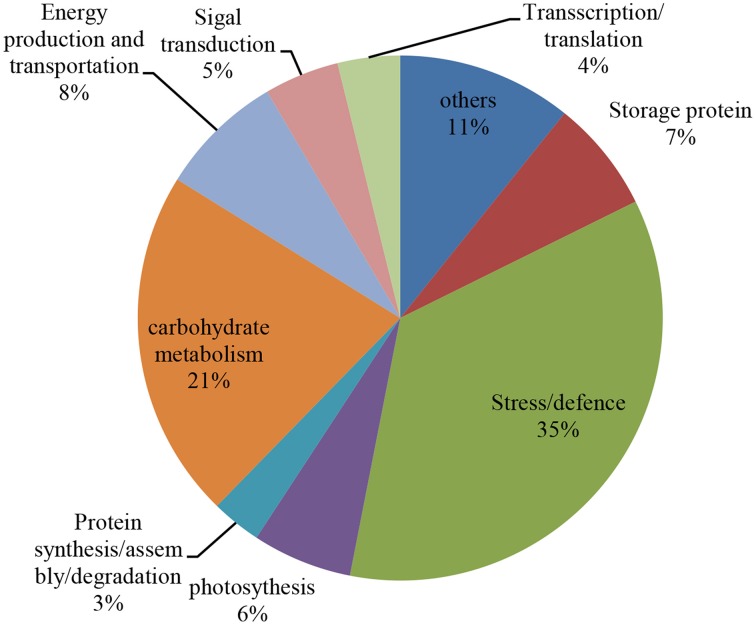
**Distribution of the proteins that were identified during four grain development stages in Yunong 201 and Yunong 3114**. Nine protein groups were categorized based on their putative functions.

According to the differential functions, the identified 130 protein spots were classified into nine main groups, including stress/defense (35.4%, 46), carbohydrate metabolism (21.5%, 28), protein synthesis/assembly/degradation (3.1%, 4), storage proteins (6.9%, 9), energy production and transportation (7.7%, 10), photosynthesis (6.2%, 8), transcription/translation (3.9%, 5), signal transduction (4.6%, 6), and unknown function groups (10.8%, 13) as shown as in Figure 4. Proteins associated with carbohydrate metabolism included four sub-categories: (1) starch metabolism (9.2%, 12), (2) glycolysis (10%, 13), (3) nitrogen metabolism (1.5%, 2), and (4) the TCA pathway (0.8%, 1). Some identified spots from different positions of the same gel and with the same isoelectric point (p*I*) and molecular mass (Mr) were expected to have the same name, and these spots referred to 27 different groups, including globulin-3A (i.e., protein spots 22, 25, and 130), α-amylase inhibitor CM3 (i.e., protein spots 8 and 12), dimeric α-amylase inhibitor (i.e., protein spots 27 and 31), serpin-N3.2 (i.e., spots 38-1, 67, and 99), ascorbate peroxidase (i.e., protein spots 56 and 57), and chitinase 2 (protein spots 60, 21, and 62; see Table 2). Those protein spots may be recognized as different products due to nucleotide gene polymorphisms, alternative splicing, proteolytic cleavage, or post-translational modifications of a single gene or protein, and might thus be associated with different cellular functions (Schlüter et al., 2009).

### Protein expression profiles during grain development

**Figure 5:**
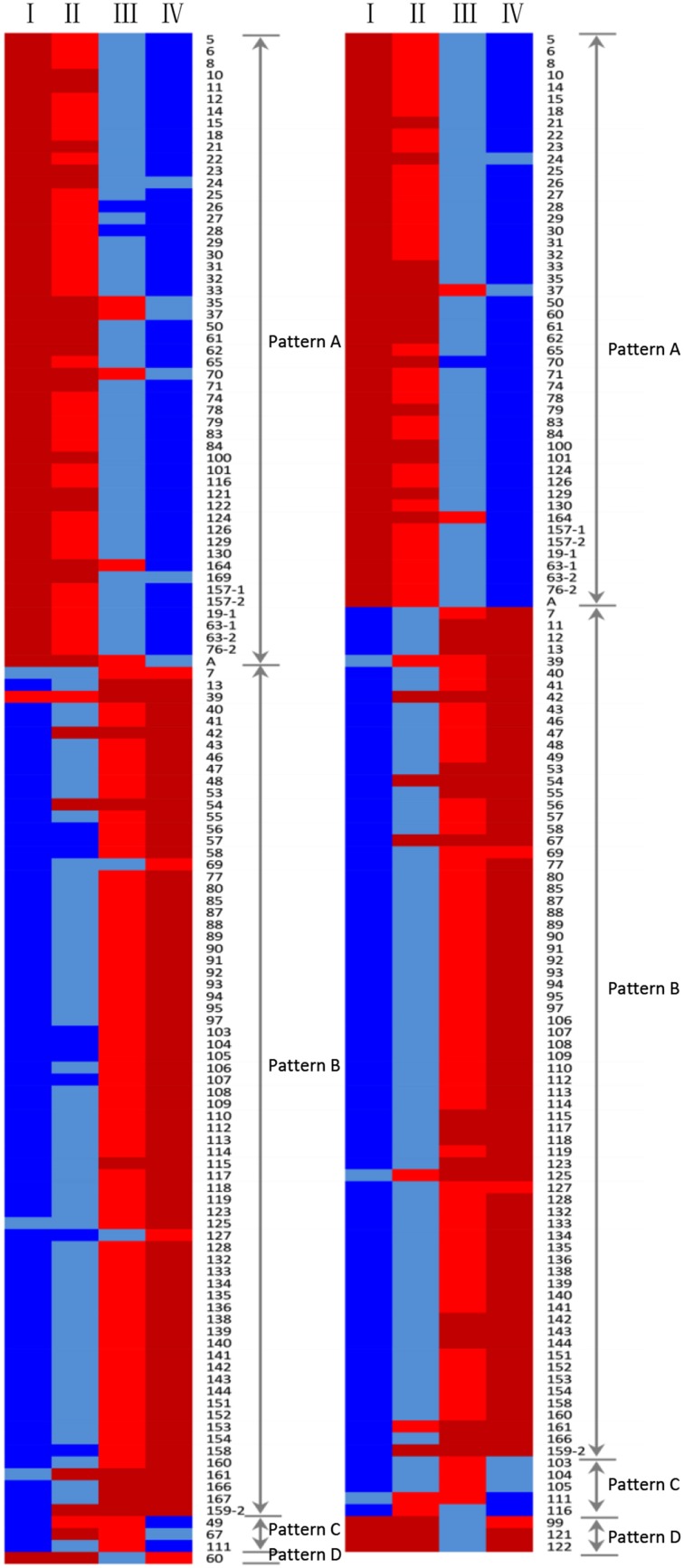
**Dynamic analysis of differentially accumulated protein spots during the grain developmental stages (I, II, III, and IV) in Yunong 201 (left) and Yunong 3114 (right)**. Red color, the lower abundance of protein spots; blue color, the higher abundance of protein spots.

The expression profiles of the 130 protein spots were investigated by hierarchical cluster analysis (Figure 5). Four main expression patterns (A–D) were presented and clearly reflected two distinct grain development phases: grain filling (21, 28), and desiccation/maturation (28–42), as shown in Figure 5.

Expression pattern A included 54 protein spots in Yunong 201, and 48 spots in Yunong 3114 that exhibited up-regulation during the four grain developmental stages, which contained many stress/defense-related proteins, such as α-amylase inhibitor 0.19 (protein spots 10 and 19-1), CM 17 protein precursor (protein spots 32 and 33), disease resistance protein RPP13 (protein spot 70), vicilin-like antimicrobial peptides 2-2 (protein spot 71), and 1-Cys peroxiredoxin (protein spot 83), which all accumulated significantly at the desiccation/maturation developmental stages in Yunong 201 and Yunong 3114.

Almost all of the storage proteins including globulin 3 (protein spots 5 and 15), globulin-3A (protein spots 22, 25, and 130), globulin 3B (protein spot 23), and gamma gliadin (protein spot 78) displayed this pattern in both Yunong 201 and Yunong 3114. Besides, the same responses were seen for glutaminyl-tRNA synthetase (i.e., protein spot 101), serpin-Z1C (i.e., protein spot 104), which is involved in protein synthesis/assembly/degradation (Fernando et al., 2015; Kodera et al., 2015) and 27 K protein (Kimoto et al., 2009; i.e., protein spot 76-2), which is involved in transcription/translation.

Expression pattern B included the largest proportion of identified proteins whose expression was down-regulated during the mid and late grain developmental stages. Moreover, 71 protein spots in Yunong 201 and Yunong 3114 belonged to this expression group, respectively. All of the proteins associated with glycolysis and most of the proteins involved in starch metabolism, photosynthesis, and energy production and transportation/signal transduction displayed this expression pattern in two samples; for example, ATP synthase CF1 beta subunit (protein spot 107), 2,3-bisphosphoglycerate-independent phosphoglycerate mutase (protein spots 142, 143, and 144), adenosine kinase 2 (protein spots 89 and 90), granule bound starch synthase (protein spot 138), and the 23 kDa oxygen evolving protein of photosystem II (protein spot 166). Expression pattern C showed both down- and up-regulated expression trends, including protein spots 49, 67, and 111, which were seen in the 14-3-3-like protein B, serpin-N3.2, and β-amylase in Yunong 201. There were five protein spots (i.e., 103, 104, 105, 106, and 111) that showed this pattern of expression in Yunong 3114. Unlike expression pattern C, expression pattern D displayed both an up- and down-regulated expression trend. Only protein spot 60 (chitinase 2) belonged to this pattern in Yunong 201, as did protein spots 121 (cerpin-Z2B), 122 (cerpin-Z1C), and protein spot 99 (serpin-N3.2) in Yunong 3114. Protein spots 168 (unnamed) and 167 (unnamed) displayed higher expression levels in Yunong 201 than did Yunong 3114, and protein spot 168 accumulated a single pattern E (not shown in Figure 5) that remained constant during the four grain developmental stage of both cultivars. In addition, protein spot 167 also belonged to this pattern in Yunong 3114, while it displayed expression pattern B in Yunong 201.

### Comparative proteomic characterization in Yunong 201 and Yunong 3114 during grain development

A total of 12 protein spots with different expression patterns co-accumulated in both samples (Figure 5), including stress/defense, protein synthesis/assembly/degradation, signal transduction, starch metabolism, photosynthesis, and the presence of two unnamed proteins. For example, protein spot 67 was identified as serpin-N3.2 that displayed expression pattern C in Yunong 201, and pattern B in Yunong 3114. Protein spot 7 (Nucleoside diphosphate kinase 1) accumulated steadily at the four developmental stages in Yunong 201, and showed pattern E, although it displayed a down-regulated trend in Yunong 3114. Serpin-Z2B, Serpin-Z1C (protein spots 121 and 122) showed expression pattern A in Yunong 201, but displayed expression pattern D in Yunong 3114. In addition, protein spots 103 (ATP synthase subunit beta, mitochondrial), 104 (UTP–glucose-1-phosphate uridylyltransferase) and 105 (ATP synthase beta subunit) showed expression pattern C in Yunong 201, and showed a down-regulated trend in expression in Jimai 20 during the four developmental stages.

Protein spots with two-fold changes or greater in abundance at particular times between the two cultivars were considered as cultivar-different proteins (Guo et al., 2012). Altogether six protein spots displayed cultivar-different proteins during the four developmental stages, which involved three groups: stress/defense, starch metabolism, protein synthesis/assembly/degradation. Among them, protein spot 99 (serpin-N3.2) was only identified in Yunong 3114; however, this protein spot was absent in Yunong 201. Meanwhile, protein spot 169 (i.e., small heat shock protein Hsp 23.5) was not detected in Yunong 3114, which was only identified in Yunong 201.

**Figure 6:**
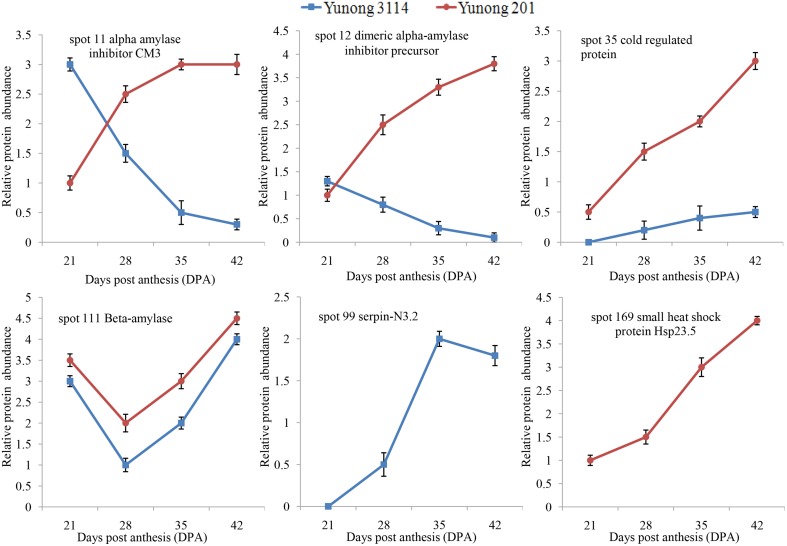
**Differential expression of six protein spots in Yunong 201 and Yunong 3114 during four grain developmental stages**.

A higher abundance of protein species 111 (β-amylase) occurred in Yunong 201 as compared that of Yunong 3114, which might be related to differential grain sizes of both wheat samples. Protein spot 35 (i.e., cold regulated protein) was also more abundantly accumulated in Yunong 201. Besides, compared with Yunong 201, α-amylase inhibitor CM3 (i.e., protein spot 11), and dimeric α-amylase inhibitor precursor (i.e., protein spot 12) were down-regulated in Yunong 3114, which displayed expression pattern B, but gradually accumulated in Yunong 201 (Figure 6).

## Discussion

In this study, a Chinese winter wheat cultivar Yunong 201 and its EMS mutant line Yunong 3114 were selected to study the proteomic expression differences during mid and late stages of grain development. Proteomic expression profiles during four grain development stages and cultivar-variable proteins of the Yunong 201 and Yunong 3114 were investigated by 2-DE and MALDI-TOF/TOF-MS. Proteomic characterization in this study could provide insights in the biology of middle and late grain development.

### Analysis of cultivar-different proteins in developmental seeds of Yunong 201 and Yunong 3114

Up to date, a considerable work has been carried out on wheat grain proteomics through different wheat cultivars (Majoul et al., 2004; Kim et al., 2010), for instance, grain storage proteins (Mamone et al., 2009; Dupont et al., 2011), endosperm and endosperm amyloplasts (Vensel et al., 2005b; Dupont, 2008), and kernel peripheral and aleurone layers (Tasleem-Tahir et al., 2011; Nadaud et al., 2015). Those studies provided the important information on biochemical processes of wheat grain development, however, few studies on proteomic of EMS-mutagenized cultivars were conducted in bread wheat. Due to the significant differences on kernel size, thousand grain weight and higher yield per plot of Yunong 201 and Yunong 3114, proteomics analysis of Yunong 201 and Yunong 3114 could provide valuable information for further understanding function of candidate cultivar-different proteins (e.g., serpin for spot 99, small heat shock protein for spot 169, β-amylase for spot 111, α-amylase inhibitor for spot 11, dimeric α-amylase inhibitor precursor for spot 12, and cold regulated protein for spot 35) which were possibly associated with yield-related traits in bread wheat.

Heightened stress interrupts normal protein functions. Small heat shock proteins (sHSPs) are produced in seeds during maturation and under various stress conditions, which can form large multimeric structures and display a wide range of cellular functions, as well as being able to act as molecular chaperones. These sHSPs do this by forming stable complexes with folding intermediates of their protein substrates (Omar et al., 2012; Wu et al., 2014). In our study, the abundance of a sHSPs (spot 169) was up-regulated in Yunong 201, but was absent in Yunong 3114. In addition, stress-related cold regulated protein (spot 35) had a higher abundance in Yunong 201 than in Yunong 3114 during the four grain developmental stages. α-Amylase inhibitors are high molecular weight macromolecules that are particularly abundant in certain cereals and leguminosae, which specifically involved in the degradation of α-1,4-linked sugar polymers, such as starch and glycogen, into oligosaccharides (Franco et al., 2000). α-Amylase inhibitors play important roles in protecting starch and protein reserves in the endosperm against degradation, particularly that caused by biotic stresses like insect attack (Franco et al., 2002). In our study, α-amylase inhibitors (spots 11 and 12) accumulated gradually from 21 to 42 dpa in Yunong 201, however, displayed down-regulated trends during the four developmental stages of Yunong 3114. Moreover, Spot 99 (serpin-N3.2) showed up-regulated expression in Yunong 3114, but absence in Yunong 201. The difference in accumulation indicated that Yunong 201 and Yunong 3114 possibly possess differential adaptability to abiotic stress.

β-Amylase is a starch-degrading enzyme that hydrolytically cleaves α-1,4-D-glucosidic bonds to liberate β-maltose from the non-reducing ends of a variety of polyglucans that are synthesized during grain development, and is one of the major proteins in the starchy endosperm (Yin et al., 2002; Vinje et al., 2011). They can only contribute to starch granule hydrolysis by degrading solubilized intermediates that are released from the granules by α-amylase (Sun and Henson, 1991). The identified protein spot 111 (β-amylase), which displayed pattern C, accumulated at a higher level of abundance in Yunong 201 than in Yunong 3114. This is possibly one of the important reasons to result in differences of grain size and weight between Yunong 201 and Yunong 3114.

### Analysis of protein spots during the developmental stage of Yunong 201 and Yunong 3114

A total of 173 identified protein spots showed more than a two-fold difference in abundance in Yunong 201 and Yunong 3114 at the four stages by means of the classic 2-DE method in this study. Of them, 130 were successfully identified by MALDI-TOF/TOF analysis. The identified protein spots had specific functions in stress/defense, carbohydrate metabolism, protein synthesis/assembly/degradation, storage proteins, energy production and transportation, photosynthesis, transcription/translation, signal transduction, and unknown functional groups. Protein spots with unknown functions were those not identified by interrogating three database including NCBI *Triticum*, NCBI Viridiplantae, and the Universal Protein Resource (UniProt), probably owing to the lack of a genomic sequence of bread wheat until now. A vast majority of the 130 protein spots had similar proteomic profiles at four stages of development in Yunong 201 and Yunong 3114. However, there were still 12 protein spots exhibiting differential expression patterns and six protein spots exhibiting cultivar-differential expression. It suggests that these six protein spots above-mentioned possibly contributed to difference of yield-related traits between Yunong 201 and Yunong 3114. Therefore, the identified protein spots showing differential expression could be used for further digging genes related to yield-related traits in bread wheat and characterization of these protein could also provide new insights into the biology of middle and late grain development in bread wheat.

### Stress/defense

Plants responsiveness to stress entails a complex mechanism and is involved in a large number of enzymes. Chitinase has been proven to play important physiological roles including defense from attack morphological changes, and digestion (Suzuki et al., 2014). Plants with over-expressed Chitinase genes showed stronger disease resistance in different crops (Cletus et al., 2013). In our study, the proteins for spots 60, 61, and 62 were identified as Chitinase 2, which gradually accumulated at four stages of development in Yunong 201 and Yunong 3114, suggesting a vital role of Chitinase in the response to different stress/defense challenges during the mid and late grain developmental periods. The *R*-proteins recognize pathogenic effectors and activate an efficient defense system that includes a “hypersensitive response (HR)” of programmed cell death or apoptosis at the infection site (Jones and Dangl, 2006). Disease resistance protein RPP13 (i.e., spot 70) is an R-protein, and our proteomic analysis showed that the abundance of RPP13 increased during the grain phase of development, which probably suggested a protective role from pathogens during the grain phase of development during mid and late developmental stages. Vicilin-like antimicrobial peptide 2–3 is a processing product of the 7S globulin precursor that is found in *Macadamia integrifolia* kernels that display anti-microbial activity (Marcus et al., 1999), the abundance of which (i.e., spots 71 and 100) also gradually increased, which was consistent with RPP13, and might indicate positive roles in anti-pathogen defense. 1-Cys peroxiredoxin (1-cysPrx) is a novel antioxidant enzyme that reduces phospholipid hydroperoxides, playing an important role in cellular defense mechanisms against oxidant stress (Manevich et al., 2002). The identified protein spots 83 and 84, (1-Cys peroxiredoxin) showed an enhanced trend in expression at the grain phases in Yunong 201 and Yunong 3114. Serpins are likely to participate in a range of biochemical pathways in distinct cell types, tissues and organs in plants to protect cells from oxidative stress, and are highly expressed during seed maturation and occur in tissues during all development stages (Roberts and Hejgaard, 2008). Four types of serpins including serpin-N3.2 (i.e., spots 40, 67, and 99), serpin 1 (i.e., spots 42 and 116), serpin-Z2B (spot 121), and serpin-Z1C (spots 122 and 124) were identified in this study, and spots 121 and 122 showed up-regulated trends in Yunong 201 but down-regulated trends in Yunong 3114. Moreover, spot 67 showed down-regulated trends in Yunong 3114, while it presented a C expression pattern in Yunong 201. In addition, spots 40 and 42 displayed down-regulated patterns of expression during the mid and late grain developmental stages in both cultivars. However, the abundance of spot 124 was increased, which was because of EMS mutagenesis that contributed to four types of serpins displaying differential trends in expression in the two wheat cultivars. Therefore, proteomic studies in this study could also be used in practical applications such as breeding for an enhanced stress tolerance as suggested by Kosova et al. (2014).

### Carbohydrate metabolism

Glycolysis provides energy and intermediates for the synthesis of metabolites, of which, we identified nine protein spots representing six types of proteins. These were referred to fructokinase-2 (spot 87), phosphoglycerate kinase, cytosolic (spot 123), enolase (spot 115), cytosolic 3-phosphoglycerate kinase (spot 127), UTP-glucose-1-phosphate uridylyl transferase (spot 104 and 106), and 2,3-bisphosphoglycerate-independent phosphoglycerate mutase (spot 142, 143, and 144), with the exception of the abundance of spot 104 (UTP-glucose-1-phosphate uridylyl transferase), which decreased in Yunong 201, and showed an expression pattern C in Yunong 3114. The remaining spots all showed down-regulated patterns of expression from the 21 to 42 dpa. Moreover, 21 dpa belonged to the late stage of wheat filling, which has enhanced glycolysis that is a significant source of energy for grain filling and accumulation of dry matter. Thus, this coincided with the synthesis stage of starch. During the filling stage, and due to the sharp accumulation of starch, ATPases are activated to provide more energy demands for an organism. Our research suggested that the reduced abundance of energy production from 21 dpa, as indicated by the down-regulated trend of the ATP synthase CF1 beta subunit (spot 107), ATP synthase subunit alpha, mitochondrial (spot 132 and 135), adenosine kinase 2 (spot 89 and 90), and the ATP synthase CF1 alpha subunit (spot 154). However, spot 103 (ATP synthase subunit beta, mitochondrial), and spot 105 (ATP synthase beta subunit) displayed an enhanced trend at the grain mature period in Yunong 3114.

### Starch synthesis and storage proteins

Starch is the major energy reserve for a large variety of higher green plants, such as cereals, legumes, and tubers (Miao et al., 2015). The biosynthesis of starch is the major determinant of overall yield in cereal grains (Emes et al., 2003). In all plant tissues capable of starch biosynthesis, adenosine diphosphate glucose (ADPGlc) pyrophosphorylase (AGPase, EC 2.7.7.27) is the enzyme that is responsible for the production of ADPGlc, the soluble precursor and substrate for starch synthases. The AGPase reaction is the first committed step in the biosynthesis of stored starch in amyloplasts (Tetlow et al., 2004). Our results demonstrated that ADP glucose pyrophosphorylase (spot 54), ADP-glucose pyrophosphorylase large subunit (spots 92 and 153), and small subunit ADP glucose pyrophosphorylase (spot 141) were all down-regulated from the filling stage to trace levels at the desiccation phase, which matched the increase in starch content and grain weight during the mid and late grain developmental stages. Glycogen synthase catalyzes the formation and elongation of the α-1,4-glucose backbone using ADP-glucose, the second and key step of glycogen biosynthesis. Elongation and branching of amylopectin is a complex process and it requires an array of enzymes including starch synthases (SS), starch branching enzymes (SBE), and debranching enzymes (DBE). However, synthesis of amylose is brought about solely by the enzyme granule-bound starch synthase I (GBSSI) or waxy protein (Ahuja et al., 2014).

In our study, the abundance of granule bound starch synthase (spot 138) was decreased from the 21 dpa, which belonged to the late stage of grain filling, which was consistent with the filling phase, and the most vital stage of starch accumulation. We identified one β-amylase that was involved in seven spots (i.e., 109, 111, 114, 119, 125, 128, and 133), except for spot 111. The remaining six spots showed a down-regulated trends. There were three types of globulin that were identified at the four grain stages, including the globulin 3 (spot 5 and 15), globulin-3A (spot 22, 24 and 25), and globulin 3B (spot 23). Further, with the exception of gamma gliadin (spot 78) and avenin-like seed protein (spot 74), they accumulated gradually at the mid and late developmental periods in both samples. In general, biosynthesis of seed storage protein is dependent of amino acid synthesis and the transport of nitrogen metabolism (Hernández-Sebastià et al., 2005). We identified one alanine aminotransferase 2 (i.e., spot 151) and one glutaminyl-tRNA synthetase (i.e., spot 101). However, they displayed the opposite expression trend for the two wheat cultivars.

### Other functional proteins

14-3-3 Proteins function as homodimers or heterodimers and bind a large number of differentially phosphorylated substrates to regulate a wide array of cellular signaling and physiological processes (Lozano-Durán and Robatzek, 2015). We identified one 14-3-3 protein (i.e., spots 46, 47, and 48), and one 14-3-3-like protein B (i.e., spots 49 and 50), among them, 14-3-3 proteins were down-regulated in two samples, while the abundance of protein spot 50 increased in Yunong 201 and Yunong 3114. Nevertheless, the other 14-3-3-like protein B (i.e., spot 49) displayed a steady expression trend in Yunong 201, but was gradually down-regulated at the four stages of development in Yunong 3114. The cytosolic NDPK1 is the main nucleoside diphosphate kinase (NDPK) isoform in plants, which operates in the context of homeostasis of cellular nucleoside triphosphate (NTP) pools that accounts for more than 70% of total NDPK activity in plants (Prabu et al., 2012). In our study, the abundance of nucleoside diphosphate kinase 1 (spot 7) was gradually reduced in Yunong 3114; however, it showed a steady expression trend during the four developmental grain stages in Yunong 201. Thus, nucleoside diphosphate kinase 1 might play a different role in signal regulation in the two wheat cultivars.

## Conclusions

In our study, 2-DE and tandem MALDI-TOF/TOF-MS were implemented to characterize protein accumulation at the middle and late stages of grain development in Yunong 201 and Yunong 3114, which differ by grain weight and size. Totals of 130 differentially accumulated protein spots representing 88 unique proteins were identified and they showed four main expression patterns in Yunong 201 and Yunong 3114. Moreover, six cultivar-different protein spots were examined. These included cultivar-different protein spot 111 (β-amylase), which accumulated at much higher abundance in Yunong 201 than in Yunong 3114. This difference was possibly related to the difference in grain size and weight between the two wheat cultivars. In addition, the absence or down-regulation of three protein spots identified as 11, 12, and 169 in Yunong 3114 were all related to stress/defense, the results possibly revealed that Yunong 201 and Yunong 3114 possessed differential adaptation to abiotic stress. Our results could provide valuable information for dissection of molecular and genetics basis of yield-related traits in bread wheat as well as new insights into the biology of late grain development.

## Author contributions

FC and DC designed the project. NZ and WH performed the experiments. NZ and FC wrote the paper. NZ performed the analyzed the data. All authors read and approved the manuscript.

### Conflict of interest statement

The authors declare that the research was conducted in the absence of any commercial or financial relationships that could be construed as a potential conflict of interest.

## Supplementary material

Additional file 1: Data sheets 1–10 **Result of MALDI-TOF/TOF-MS in Yunong 201 and Yunong 3114**.

Additional file 2: Data sheets 11,12 **Standard curves with SEVEN different concentrations of BSA (0–100 μg) resuspended in lysis buffer and water**.

## Abbreviations

EMS: ethyl methanesulfonate
dpa: day post-anthesis
2-DE: two-dimensional gel electrophoresis
MS: mass spectrometry
DTT: dithiothreitol
SDS: sodium dodecyl sulfate
CHAPS: 3-[(3-cholamidopropyl) dimethylammonio]-1-propanesulfonate
IPG: immobilized pH gradient
BSA: bovine serum albumin
IEF: isoelectric focusing
SDS-PAGE: sodium dodecyl sulfate-polyacrylamide gel electrophoresis
CBB: Coomassie brilliant blue
sHSPs: small heat shock proteins
UniProt: Universal Protein Resource
HR: hypersensitive response
ADPGlc: adenosine diphosphate glucose
AGPase: adenosine diphosphate glucose pyrophosphorylase
SS: starch synthases
SBE: starch branching enzymes
DBE: debranching enzymes
GBSSI: granule-bound starch synthase I
NDPK: nucleoside diphosphate kinase
NTP: nucleoside triphosphate.
