# Supplementary material for: Proteomic analysis of middle and late stages of bread wheat (Triticum aestivum L.) grain development
Source: Front Plant Sci. 2015 Sep 15;6:735. doi: 10.3389/fpls.2015.00735 (PMC4569854; doi:10.3389/fpls.2015.00735)
Supplement: Supplementary file 2 [file DataSheet1.PDF]

Analysis Information

Report Type	Protein-Peptide Summary by Spot	Analysis Type	Combined (MS+MS/MS)
Sample Set Name	Sample set_20140814	Database	triticum
Analysis Name	R14026-4-T	Creation Date	09/22/2014 13:48:03
Reported By	09/30/2014 15:01:40 - admin	Last Modified	09/22/2014 13:52:43

MS Acq. : Proc. Methods	(Unspecified) : (Unspecified)
Interpretation Method	(Unspecified)

**Gel Idx/Pos**	110/E9	**Instr./Gel Origin**	BA2151/Sample Project 20140814	**Process Status**	Analysis Succeeded
**Plate [#] Name**	[1] Sample Project 20140814	**Instrument Sample Name**		**Spectra**	11

Rank	Protein Name	Accession No.	Protein MW	Protein PI	Pep. Count	Protein Score	Protein Score C. I. %	Intensity Matched	Total Ion Score	Total Ion C. I. %	Confirmed
1	G protein, alpha subunit [Triticum durum]	gi|109804165	9929.2	6.88	3	21	0	.85			

Peptide Information

Calc. Mass	Obsrv. Mass	± da	± ppm	Start Seq.	End Seq.	Sequence	Ion Score	C. I. %	Modification	Rank	Result Type
832.4271	832.3669	-0.0602	-72	17	23	SADIDRR					Mascot
834.4216	834.3561	-0.0655	-78	30	36	ADQHVHK					Mascot
2724.3701	2724.4023	0.0322	12	60	83	TGFDEAELKGYMPVIHAN VFQTIK			Oxidation (M)[12]		Mascot

Rank	Protein Name	Accession No.	Protein MW	Protein PI	Pep. Count	Protein Score	Protein Score C. I. %	Intensity Matched	Total Ion Score	Total Ion C. I. %	Confirmed
2	G protein, alpha subunit [Triticum durum]	gi|109804167	10570.6	8.11	3	20	0	.85			

Peptide Information

Calc. Mass	Obsrv. Mass	± da	± ppm	Start Seq.	End Seq.	Sequence	Ion Score	C. I. %	Modification	Rank	Result Type
832.4271	832.3669	-0.0602	-72	17	23	SADIDRR					Mascot
834.4216	834.3561	-0.0655	-78	30	36	ADQHVHK					Mascot
2724.3701	2724.4023	0.0322	12	60	83	TGFDEAELKGYMPVIHAN VFQTIK			Oxidation (M)[12]		Mascot

Rank	Protein Name	Accession No.	Protein MW	Protein PI	Pep. Count	Protein Score	Protein Score C. I. %	Intensity Matched	Total Ion Score	Total Ion C. I. %	Confirmed
3	hypothetical protein TRIUR3_21604 [Triticum urartu]	gi|474425700	17009.2	7.08	4	20	0	1.224			

Peptide Information

Calc. Mass	Obsrv. Mass	± da	± ppm	Start Seq.	End Seq.	Sequence	Ion Score	C. I. %	Modification	Rank	Result Type

822.3563	822.375	0.0187	23	80	85	EWATCR			Carbamidomethyl (C)[5]		Mascot
834.3774	834.3561	-0.0213	-26	11	17	DCLPSSR			Carbamidomethyl (C)[2]		Mascot
1015.5167	1015.5669	0.0502	49	18	26	STSINTQHK					Mascot
1182.6776	1182.6244	-0.0532	-45	1	10	MAIAHLLNRK			Oxidation (M)[1]		Mascot

4	Homeobox-leucine zipper protein ROC8 [Triticum urartu]	gi|473842771	7367.4	4.85	3	20	0	.692

Peptide Information

Calc. Mass	Obsrv. Mass	± da	± ppm	Start Seq.	End Seq.	Sequence	Ion Score	C. I. %	Modification	Rank	Result Type
833.441	833.363	-0.078	-94	31	37	AQMTRAR					Mascot
849.4366	849.4496	0.013	15	24	29	LWFNNR					Mascot
850.4781	850.4095	-0.0686	-81	17	23	VEPQHIK					Mascot

5	conserved hypothetical protein, expressed [Triticum aestivum]	gi|299109311	24861.7	8.76	4	19	0	.855

Peptide Information

Calc. Mass	Obsrv. Mass	± da	± ppm	Start Seq.	End Seq.	Sequence	Ion Score	C. I. %	Modification	Rank	Result Type
834.4218	834.3561	-0.0657	-79	1	6	MVFYFK					Mascot
849.3883	849.4496	0.0613	72	168	173	REEMER					Mascot
850.4167	850.4095	-0.0072	-8	1	6	MVFYFK			Oxidation (M)[1]		Mascot
1799.8606	1799.9218	0.0612	34	99	114	KATSMDVGQVGFHNHR			Oxidation (M)[5]		Mascot
2724.3345	2724.4023	0.0678	25	100	123	ATSMDVGQVGFHNHRM VHVLTVEK			Oxidation (M)[4,16]		Mascot

6	Auxin-responsive protein IAA12 [Triticum urartu]	gi|474384898	22269	7.63	4	19	0	.687

Peptide Information

Calc. Mass	Obsrv. Mass	± da	± ppm	Start Seq.	End Seq.	Sequence	Ion Score	C. I. %	Modification	Rank	Result Type
833.3974	833.363	-0.0344	-41	129	134	MYKGYR			Oxidation (M)[1]		Mascot
849.4312	849.4496	0.0184	22	200	208	SEATGLGSK					Mascot
942.4536	942.4318	-0.0218	-23	125	131	VDMRMYK					Mascot
2724.3872	2724.4023	0.0151	6	2	26	EATDSLLMATELRLGLPG TDDKPHK			Oxidation (M)[8]		Mascot

7	hypothetical protein TRIUR3_28460 [Triticum urartu]	gi|473999752	14237	4.7	4	19	0	1.361

Peptide Information

Calc. Mass	Obsrv. Mass	± da	± ppm	Start Seq.	End Seq.	Sequence	Ion Score	C. I. %	Modification	Rank	Result Type

Calc. Mass	Obsrv. Mass	± da	± ppm	Start Seq.	End Seq.	Sequence	Ion Score	C. I. %	Modification	Rank	Result Type
814.5509	814.5818	0.0309	38	62	68	VKVVLTR					Mascot
933.416	933.3732	-0.0428	-46	27	33	EEEEELR					Mascot
1331.7028	1331.8112	0.1084	81	69	79	AELEWLMAQLK					Mascot
1376.6699	1376.8007	0.1308	95	2	13	GNCLNKASTQQR			Carbamidomethyl (C)[3]		Mascot

8	wuschel-related homeobox protein [Triticum monococcum subsp. aegilopoides]	gi|534291283	24780.3	7.67	4	18	0	.578

Protein Group

wuschel-related homeobox protein [Triticum aestivum]	gi|534291010	24838.4	7.6700000762 9395
wuschel-related homeobox protein [Triticum durum]	gi|534291076	24839.3	6.6700000762 9395
wuschel-related homeobox protein [Triticum monococcum subsp. aegilopoides]	gi|534291308	24780.3	7.6700000762 9395
wuschel-related homeobox protein [Triticum monococcum subsp. aegilopoides]	gi|534291223	24780.3	7.6700000762 9395
wuschel-related homeobox protein [Triticum turgidum subsp. dicoccon]	gi|534291116	24868.4	7.6700000762 9395
wuschel-related homeobox protein [Triticum turgidum subsp. dicoccon]	gi|534291104	24838.4	7.6700000762 9395
wuschel-related homeobox protein [Triticum turgidum subsp. dicoccon]	gi|534291096	24839.3	6.6700000762 9395
wuschel-related homeobox protein [Triticum urartu]	gi|534291177	24838.4	7.6700000762 9395

Peptide Information

Calc. Mass	Obsrv. Mass	± da	± ppm	Start Seq.	End Seq.	Sequence	Ion Score	C. I. %	Modification	Rank	Result Type
834.3522	834.3561	0.0039	5	179	185	GGEQQCR			Carbamidomethyl (C)[6]		Mascot
942.4536	942.4318	-0.0218	-23	127	133	IMTCYVR			Carbamidomethyl (C)[4]		Mascot
1015.5895	1015.5669	-0.0226	-22	2	11	EALSGRVGVK					Mascot
2724.3438	2724.4023	0.0585	21	150	172	EVETLELFPLKAACYDLE LEADR			Carbamidomethyl (C)[14]		Mascot

9	hypothetical protein TRIUR3_04990 [Triticum urartu]	gi|474111827	5537.6	6.13	2	18	0	.506

Peptide Information

Calc. Mass	Obsrv. Mass	± da	± ppm	Start Seq.	End Seq.	Sequence	Ion Score	C. I. %	Modification	Rank	Result Type
833.3934	833.363	-0.0304	-36	1	9	MTGSAPGGR					Mascot

849.3883	849.4496	0.0613	72	1	9	MTGSAPGGR			Oxidation (M)[1]		Mascot
2840.3672	2840.448	0.0808	28	14	40	STAAVELDGSQGVEWAP PPPPSFCGKR			Carbamidomethyl (C)[24]		Mascot

10 hga5 [Triticum aestivum] gi|300681531 46590.9 9.53 5 17 0 1.266

Peptide Information

Calc. Mass	Obsrv. Mass	± da	± ppm	Start Seq.	End Seq.	Sequence	Ion Score	C. I. %	Modification	Rank	Result Type
849.4689	849.4496	-0.0193	-23	206	212	HPTRNPK					Mascot
1269.6045	1269.6726	0.0681	54	371	381	DPMAIHAQGWK			Oxidation (M)[3]		Mascot
1331.6989	1331.8112	0.1123	84	382	393	ALAEVVMTQDVR					Mascot
1960.0168	1960.1753	0.1585	81	382	398	ALAEVVMTQDVRLDLDR			Oxidation (M)[7]		Mascot
2840.3918	2840.448	0.0562	20	47	73	MSGDVRIAPGSSSVILSM PLYQSAEGR			Oxidation (M)[1,18]		Mascot

Gel Idx/Pos	111/E10	Instr./Gel Origin	BA2151/Sample Project 20140814	Process Status	Analysis Succeeded
Plate [#] Name	[1] Sample Project 20140814	Instrument Sample Name		Spectra	11

Rank	Protein Name	Accession No.	Protein MW	Protein PI	Pep. Count	Protein Score	Protein Score C. I. %	Intensity Matched	Total Ion Score	Total Ion C. I. %	Confirmed
1	Ribulose bisphosphate carboxylase small chain PWS4.3, chloroplastic [Triticum urartu]	gi|473939671	23054.8	7.66	9	51	66.454	5.631			

Peptide Information

Calc. Mass	Obsrv. Mass	± da	± ppm	Start Seq.	End Seq.	Sequence	Ion Score	C. I. %	Modification	Rank	Result Type
802.4669	802.4954	0.0285	36	183	189	EVAEVKK					Mascot
1140.5684	1140.6317	0.0633	55	137	145	KEYPDAYVR					Mascot
1165.571	1165.6493	0.0783	67	84	92	WVPCLEFSK			Carbamidomethyl (C)[4]		Mascot
1320.6615	1320.6729	0.0114	9	30	43	RSSGSLGSVSNGGR					Mascot
1376.6702	1376.7865	0.1163	84	46	56	CMQVWPIEGIK			Carbamidomethyl (C)[1], Oxidation (M)[2]		Mascot
1742.8273	1742.9563	0.129	74	190	204	EYPDAYVLINGFDNL					Mascot
1846.9731	1846.8806	-0.0925	-50	2	20	APAVMASSATTVAPFQGLK					Mascot
1926.9708	1926.9812	0.0104	5	138	153	EYPDAYVRVIGFDNLR					Mascot
1994.0085	1994.1018	0.0933	47	1	20	MAPAVMASSATTVAPFQGLK			Oxidation (M)[1]		Mascot
1994.0085	1994.1018	0.0933	47	1	20	MAPAVMASSATTVAPFQGLK			Oxidation (M)[1]		Mascot

Rank	Protein Name	Accession No.	Protein MW	Protein PI	Pep. Count	Protein Score	Protein Score C. I. %	Intensity Matched	Total Ion Score	Total Ion C. I. %	Confirmed
2	Secologanin synthase [Triticum urartu]	gi|474401653	48633.9	8.73	12	48	31.509	8.129			

Peptide Information

Calc. Mass	Obsrv. Mass	± da	± ppm	Start Seq.	End Seq.	Sequence	Ion Score	C. I. %	Modification	Rank	Result Type
1057.5314	1057.5587	0.0273	26	109	117	SFSFDVISR					Mascot
1065.551	1065.5627	0.0117	11	340	349	FKNGVAAACK			Carbamidomethyl (C)[9]		Mascot
1107.5834	1107.6064	0.023	21	13	22	LFGTEGVAWK					Mascot
1127.6896	1127.5874	-0.1022	-91	173	182	SLILEIARGR					Mascot
1158.6378	1158.65	0.0122	11	2	10	DVSRYIHIR					Mascot
1232.6667	1232.6776	0.0109	9	275	285	TVGMVVQETLR					Mascot
1265.6373	1265.688	0.0507	40	417	428	IDATATQDGFVK					Mascot
1344.6583	1344.7557	0.0974	72	23	33	TGGDTYLYWLR					Mascot
1421.7384	1421.7313	-0.0071	-5	416	428	RIDATATQDGFVK					Mascot
1491.8022	1491.833	0.0308	21	273	285	MKTVGMVVQETLR					Mascot
1507.7971	1507.8231	0.026	17	273	285	MKTVGMVVQETLR			Oxidation (M)[1]		Mascot

1507.7971	1507.8231	0.026	17	273	285	MKTVGMVVQETLR			Oxidation (M)[1]		Mascot
1657.9272	1657.8982	-0.029	-17	296	310	ETFGDIRLGQLLAPK					Mascot
1657.9272	1657.8982	-0.029	-17	296	310	ETFGDIRLGQLLAPK					Mascot
3312.5645	3312.5403	-0.0242	-7	311	339	GTYLFVPVSTMHHDAAS WGPTVHRFDPDR			Oxidation (M)[11]		Mascot
3312.5645	3312.5403	-0.0242	-7	311	339	GTYLFVPVSTMHHDAAS WGPTVHRFDPDR			Oxidation (M)[11]		Mascot

3 hypothetical protein TRIUR3_30879 [Triticum urartu] gi|472909785 11663.1 9.09 8 48 31.509 4.845

Peptide Information

Calc. Mass	Obsrv. Mass	± da	± ppm	Start Seq.	End Seq.	Sequence	Ion Score	C. I. %	Modification	Rank	Result Type
807.4108	807.4536	0.0428	53	92	97	NRDQFK					Mascot
973.5425	973.5909	0.0484	50	37	44	SLEALERR					Mascot
973.5425	973.5909	0.0484	50	37	44	SLEALERR					Mascot
1065.5551	1065.5627	0.0076	7	1	10	MAAGGVWVFK					Mascot
1081.5499	1081.5435	-0.0064	-6	1	10	MAAGGVWVFK			Oxidation (M)[1]		Mascot
1182.6953	1182.6475	-0.0478	-40	65	75	RGGVDLISIPR					Mascot
1194.5824	1194.655	0.0726	61	82	91	STHMYDVVVK			Oxidation (M)[4]		Mascot
1379.6987	1379.7948	0.0961	70	25	36	ALVYVPANETMR			Oxidation (M)[11]		Mascot
1491.7988	1491.833	0.0342	23	24	36	KALVYVPANETMR					Mascot
1507.7937	1507.8231	0.0294	19	24	36	KALVYVPANETMR			Oxidation (M)[12]		Mascot
1507.7937	1507.8231	0.0294	19	24	36	KALVYVPANETMR			Oxidation (M)[12]		Mascot
2510.2168	2510.2524	0.0356	14	1	23	MAAGGVWVFKNGVMEL EQEATSR					Mascot

4 Dihydroflavonol-4-reductase [Triticum urartu] gi|474348521 38019.3 8.49 11 48 26.61 7.986

Peptide Information

Calc. Mass	Obsrv. Mass	± da	± ppm	Start Seq.	End Seq.	Sequence	Ion Score	C. I. %	Modification	Rank	Result Type
973.5425	973.5909	0.0484	50	318	327	ASGTKVPASR					Mascot
973.5425	973.5909	0.0484	50	318	327	ASGTKVPASR					Mascot
1036.5786	1036.5798	0.0012	1	313	322	GFIQKASGTK					Mascot
1141.5895	1141.6199	0.0304	27	247	255	TLHRGELCR			Carbamidomethyl (C)[8]		Mascot
1165.651	1165.6493	-0.0017	-1	21	30	MLLARGYAVR			Oxidation (M)[1]		Mascot
1405.7509	1405.7604	0.0095	7	332	344	LQNSAAPVFMSKL					Mascot
1421.7457	1421.7313	-0.0144	-10	332	344	LQNSAAPVFMSKL			Oxidation (M)[10]		Mascot
1507.754	1507.8231	0.0691	46	209	221	AYVNESQAYVHVK					Mascot
1507.754	1507.8231	0.0691	46	209	221	AYVNESQAYVHVK					Mascot

Calc. Mass	Obsrv. Mass	± da	± ppm	Start Seq.	End Seq.	Sequence	Ion Score	C. I. %	Modification	Rank	Result Type
1791.8881	1791.8446	-0.0435	-24	114	129	VVLSSSIGTMYMNPHR					Mascot
1791.8881	1791.8446	-0.0435	-24	114	129	VVLSSSIGTMYMNPHR					Mascot
1816.8759	1816.9534	0.0775	43	230	246	VLEAPNAGGPRYGCAER			Carbamidomethyl (C)[14]		Mascot
1947.9891	1948.0273	0.0382	20	113	129	RVVLSSSIGTMYMNPHR					Mascot
2023.0504	2023.017	-0.0334	-17	1	20	MAAVVCVTGAGGFIGSWIVK			Carbamidomethyl (C)[6]		Mascot
2341.2795	2341.3452	0.0657	28	176	197	GVVIPVVTLGELLQPTMNTSTR			Oxidation (M)[17]		Mascot

5 60S ribosomal protein L15 [Triticum urartu] gi|474021740 31723.2 11.5 10 47 13.775 4.544

Peptide Information

Calc. Mass	Obsrv. Mass	± da	± ppm	Start Seq.	End Seq.	Sequence	Ion Score	C. I. %	Modification	Rank	Result Type
897.5013	897.4689	-0.0324	-36	2	10	GARGQPGVR					Mascot
973.5537	973.5909	0.0372	38	259	266	NQTVSLRR					Mascot
973.5537	973.5909	0.0372	38	259	266	NQTVSLRR	14	0			Mascot
1066.5792	1066.5685	-0.0107	-10	11	19	ASTPWHVLR					Mascot
1068.5044	1068.5731	0.0687	64	72	78	VRCWEYR			Carbamidomethyl (C)[3]		Mascot
1092.595	1092.5721	-0.0229	-21	53	60	FVSELWRR					Mascot
1265.642	1265.688	0.046	36	62	71	QSDVMRFVQR					Mascot
1605.7955	1605.8174	0.0219	14	74	85	CWEYRQQPAIVR			Carbamidomethyl (C)[1]		Mascot
1699.9088	1699.9323	0.0235	14	102	115	AKQILFLCDSYTLK			Carbamidomethyl (C)[8]		Mascot
1742.9371	1742.9563	0.0192	11	20	35	HIGGPHVQCLVTSPLK			Carbamidomethyl (C)[9]		Mascot
1867.9436	1867.9961	0.0525	28	36	52	LQAPKEYTDVSTPGAYK					Mascot

6 hypothetical protein TAANSRALLhA_567H13.g00003 [Triticum aestivum] gi|332079226 21508.7 9.26 8 44 0 7.727

Peptide Information

Calc. Mass	Obsrv. Mass	± da	± ppm	Start Seq.	End Seq.	Sequence	Ion Score	C. I. %	Modification	Rank	Result Type
1127.6433	1127.5874	-0.0559	-50	64	72	QQLWARVAR					Mascot
1165.6324	1165.6493	0.0169	14	168	177	EAALLENHLR					Mascot
1323.6216	1323.7312	0.1096	83	24	34	AYGDLVWDEQK					Mascot
1323.6216	1323.7312	0.1096	83	24	34	AYGDLVWDEQK					Mascot
1434.7378	1434.8431	0.1053	73	1	12	MAMGERMVQLLR					Mascot
1699.9458	1699.9323	-0.0135	-8	106	119	QCRLKPMLTLGLNR			Carbamidomethyl (C)[2]		Mascot
1715.9408	1715.9122	-0.0286	-17	106	119	QCRLKPMLTLGLNR			Carbamidomethyl (C)[2], Oxidation (M)[7]		Mascot
1959.0891	1958.9752	-0.1139	-58	109	124	LKPMLTLGLNRCHIHR			Carbamidomethyl (C)[12]		Mascot
1993.9801	1994.1018	0.1217	61	24	40	AYGDLVWDEQKTGRPMK					Mascot

1993.9801	1994.1018	0.1217	61	24	40	AYGDLVWDEQKTGRPM K					Mascot
3312.7039	3312.5403	-0.1636	-49	35	63	TGRPMKDLSNIEVVRPCL ELVDSSAERPK			Carbamidomethyl (C)[17], Oxidation (M)[5]		Mascot
3312.7039	3312.5403	-0.1636	-49	35	63	TGRPMKDLSNIEVVRPCL ELVDSSAERPK			Carbamidomethyl (C)[17], Oxidation (M)[5]		Mascot

7 hypothetical protein TRIUR3_27212 [Triticum urartu] gi|474450960 58619.2 9.03 14 44 0 3.762

Peptide Information

Calc. Mass	Obsrv. Mass	± da	± ppm	Start Seq.	End Seq.	Sequence	Ion Score	C. I. %	Modification	Rank	Result Type
847.4883	847.4894	0.0011	1	331	338	SATALEKK					Mascot
897.4788	897.4689	-0.0099	-11	447	453	QHLEDKK					Mascot
951.4352	951.5168	0.0816	86	223	230	APEYVCGR			Carbamidomethyl (C)[6]		Mascot
1033.571	1033.5781	0.0071	7	365	373	AKEMVSQIK					Mascot
1165.575	1165.6493	0.0743	64	151	159	EFWDGIRSR					Mascot
1183.6868	1183.6428	-0.044	-37	250	261	LQGVLGMIPGAK					Mascot
1339.7878	1339.6949	-0.0929	-69	250	262	LQGVLGMIPGAKR					Mascot
1405.8386	1405.7604	-0.0782	-56	160	172	LAALRPDGPIARR					Mascot
1421.8264	1421.7313	-0.0951	-67	60	71	GAAELRLLYFLR					Mascot
1605.8153	1605.8174	0.0021	1	416	429	TGVIVEKLMDENNK			Oxidation (M)[9]		Mascot
1707.8701	1707.8805	0.0104	6	312	325	VITTDPTEAWQYRK					Mascot
1715.9175	1715.9122	-0.0053	-3	399	414	IVGSAEQQEQTTALLK					Mascot
1790.9008	1790.8622	-0.0386	-22	223	237	APEYVCGRDAVVWLR			Carbamidomethyl (C)[6]		Mascot
2501.217	2501.4143	0.1973	79	262	284	RMVMGHTIQSEGINAVC GAQAVR			Carbamidomethyl (C)[17], Oxidation (M)[2]		Mascot

8 Callose synthase 9 [Triticum urartu] gi|474139753 223034.8 8.83 27 42 0 14.384

Peptide Information

Calc. Mass	Obsrv. Mass	± da	± ppm	Start Seq.	End Seq.	Sequence	Ion Score	C. I. %	Modification	Rank	Result Type
802.4417	802.4954	0.0537	67	258	264	EQGLISR					Mascot
807.4067	807.4536	0.0469	58	719	726	SGRTSSGR					Mascot
948.4429	948.4907	0.0478	50	1137	1143	GMMYYRK					Mascot
1018.5527	1018.5725	0.0198	19	727	735	KTSNVDQVK					Mascot
1033.5902	1033.5781	-0.0121	-12	1094	1101	NFLTRINR					Mascot
1057.6616	1057.5587	-0.1029	-97	1758	1766	ILETILSLR					Mascot
1065.4994	1065.5627	0.0633	59	349	358	ASMDDKGVSR					Mascot
1081.4943	1081.5435	0.0492	45	349	358	ASMDDKGVSR			Oxidation (M)[3]		Mascot
1182.6477	1182.6475	-0.0002	0	110	120	LPNTISVPDAR					Mascot

Calc. Mass	Obsrv. Mass	± da	± ppm	Start Seq.	End Seq.	Sequence	Ion Score	C. I. %	Modification	Rank	Result Type
1201.6899	1201.6703	-0.0196	-16	616	625	LRELVDTTVR					Mascot
1227.7031	1227.6412	-0.0619	-50	1813	1822	AMVHFQLLLR					Mascot
1235.6882	1235.6145	-0.0737	-60	1222	1232	IAYIDVVESVK					Mascot
1300.611	1300.6179	0.0069	5	1721	1729	DWTNWLFYR					Mascot
1344.7522	1344.7557	0.0035	3	1255	1266	EIYSVKLPGNPK					Mascot
1365.7598	1365.7278	-0.032	-23	393	404	LGEIRSVEAVHR					Mascot
1421.7648	1421.7313	-0.0335	-24	1122	1133	LWASYRGQTLAR					Mascot
1434.7853	1434.8431	0.0578	40	246	257	LSNPFFSKPNRK					Mascot
1440.7014	1440.7537	0.0523	36	821	831	EMHMVFIDFKK			Oxidation (M)[2]		Mascot
1456.6964	1456.7714	0.075	51	821	831	EMHMVFIDFKK			Oxidation (M)[2,4]		Mascot
1458.8176	1458.812	-0.0056	-4	602	614	IRLGSWNVGSLTR					Mascot
1493.7231	1493.8334	0.1103	74	5	18	ESGVFSGNLGELER					Mascot
1507.7329	1507.8231	0.0902	60	314	325	GLQYQWHDFVSK					Mascot
1507.7329	1507.8231	0.0902	60	314	325	GLQYQWHDFVSK					Mascot
1698.8843	1698.9609	0.0766	45	1201	1216	GEGKPEAADIALLMQR					Mascot
1791.8317	1791.8446	0.0129	7	43	58	EISPEDAANLISEEMK			Oxidation (M)[15]		Mascot
1791.8317	1791.8446	0.0129	7	43	58	EISPEDAANLISEEMK			Oxidation (M)[15]		Mascot
1837.957	1838.0459	0.0889	48	836	849	IPWNVMWWALEKHK					Mascot
1958.8914	1958.9752	0.0838	43	497	512	DIAVDCNDPQDELWLR			Carbamidomethyl (C)[6]		Mascot
1993.9662	1994.1018	0.1356	68	1284	1300	GNAVQTIDMNQIHHPFR			Oxidation (M)[9]		Mascot
1993.9662	1994.1018	0.1356	68	1284	1300	GNAVQTIDMNQIHHPFR			Oxidation (M)[9]		Mascot
2022.994	2023.017	0.023	11	457	473	EEDYISNTELDLLLMPK					Mascot
2406.2222	2406.0745	-0.1477	-61	454	473	NLREEDYISNTELDLLLMPK					Mascot

9 hypothetical protein TRIUR3_12459 [Triticum urartu] gi|474380785 19953.1 6.33 7 42 0 2.132

Peptide Information

Calc. Mass	Obsrv. Mass	± da	± ppm	Start Seq.	End Seq.	Sequence	Ion Score	C. I. %	Modification	Rank	Result Type
1057.5314	1057.5587	0.0273	26	82	90	ETPEAHVFK					Mascot
1201.5597	1201.6703	0.1106	92	122	130	EEKTDTWHR					Mascot
1227.658	1227.6412	-0.0168	-14	144	154	LPEDAKADQIK					Mascot
1708.8654	1708.8435	-0.0219	-13	66	81	ISSESAAFAGARIDWK					Mascot
1816.9474	1816.9534	0.006	3	155	172	AAMDNGVLTVTVPKEGAK			Oxidation (M)[3]		Mascot
1987.9316	1988.129	0.1974	99	37	57	TTSSDTASSAHPSPSGLGATR					Mascot
3223.4434	3223.5027	0.0593	18	7	36	GNAFDPFSLDLWDPFDGFPFGSGGSSSFPR					Mascot

10 hypothetical protein TRIUR3_12126 [Triticum urartu] gi|474389265 85064.4 6.3 12 42 0 10.425

Peptide Information

Calc. Mass	Obsrv. Mass	± da	± ppm	Start Seq.	End Seq.	Sequence	Ion Score	C. I. %	Modification	Rank	Result Type
973.5425	973.5909	0.0484	50	111	118	EETLVARR					Mascot
973.5425	973.5909	0.0484	50	111	118	EETLVARR	10	0			Mascot
1057.5394	1057.5587	0.0193	18	129	136	ICMPPQRR			Carbamidomethyl (C)[2]		Mascot
1127.5579	1127.5874	0.0295	26	319	328	LSSDINSYTK					Mascot
1179.6224	1179.6729	0.0505	43	649	657	LEVIMMQKR			Oxidation (M)[5,6]		Mascot
1490.7487	1490.8395	0.0908	61	2	16	LGGVDGSLSGQEPFK					Mascot
1638.8997	1638.9562	0.0565	34	379	393	GLRDVKPELGPQAMK					Mascot
1698.8367	1698.9609	0.1242	73	551	565	SCLSELGEINASYKK			Carbamidomethyl (C)[2]		Mascot
1738.985	1738.8689	-0.1161	-67	566	581	ILYSGLEHLVASIAPR					Mascot
1816.9124	1816.9534	0.041	23	672	686	SLINHFSEMSQRPVR			Oxidation (M)[9]		Mascot
1987.9915	1988.129	0.1375	69	120	135	SPQSWILMKICMPPQR			Carbamidomethyl (C)[11], Oxidation (M)[8]		Mascot
2022.9338	2023.017	0.0832	41	530	546	HEVEELCAEIFHAPADR			Carbamidomethyl (C)[7]		Mascot
3312.7031	3312.5403	-0.1628	-49	497	527	LFLGGVGVQKTGEEITTA LNNMDVSSEYVLK					Mascot
3312.7031	3312.5403	-0.1628	-49	497	527	LFLGGVGVQKTGEEITTA LNNMDVSSEYVLK					Mascot

Gel Idx/Pos	112/E11	Instr./Gel Origin	BA2151/Sample Project 20140814	Process Status	Analysis Succeeded
Plate [#] Name	[1] Sample Project 20140814	Instrument Sample Name		Spectra	11

Rank	Protein Name	Accession No.	Protein MW	Protein PI	Pep. Count	Protein Score	Protein Score C. I. %	Intensity Matched	Total Ion Score	Total Ion C. I. %	Confirmed
1	unnamed protein product [Triticum aestivum]	gi|296511581	17468.9	6.34	8	374	100	10.911	334	100	

Protein Group

unnamed protein product [Triticum aestivum]	gi|296512797	17468.9	6.3400 001525 8789

Peptide Information

Calc. Mass	Obsrv. Mass	± da	± ppm	Start Seq.	End Seq.	Sequence	Ion Score	C. I. %	Modification	Rank	Result Type
815.3795	815.4232	0.0437	54	99	104	TDTWHR					Mascot
827.4985	827.499	0.0005	1	65	72	ADVPGLKK					Mascot
974.5417	974.5937	0.052	53	116	123	FRLPENAK					Mascot
974.5417	974.5937	0.052	53	116	123	FRLPENAK	18	2.203			Mascot
1027.6146	1027.5504	-0.0642	-62	143	151	VEAKKPEVK					Mascot
1057.5314	1057.5701	0.0387	37	56	64	ETPEAHVFK					Mascot
1154.5437	1154.6094	0.0657	57	40	51	TSSDTAAFAGAR					Mascot
1154.5437	1154.6094	0.0657	57	40	51	TSSDTAAFAGAR	72	100			Mascot
1657.8392	1657.9315	0.0923	56	77	91	VEVEDGNILQISGER					Mascot
1657.8392	1657.9315	0.0923	56	77	91	VEVEDGNILQISGER	139	100			Mascot
2143.0876	2143.2131	0.1255	59	73	91	EEVKVEVEDGNILQISGE R					Mascot
2143.0876	2143.2131	0.1255	59	73	91	EEVKVEVEDGNILQISGE R	104	100			Mascot

Rank	Protein Name	Accession No.	Protein MW	Protein PI	Pep. Count	Protein Score	Protein Score C. I. %	Intensity Matched	Total Ion Score	Total Ion C. I. %	Confirmed
2	unnamed protein product [Triticum aestivum]	gi|296511569	17548.9	5.8	7	368	100	10.755	334	100	

Protein Group

unnamed protein product [Triticum aestivum]	gi|296512785	17548.9	5.8000 001907 3486

Peptide Information

Calc. Mass	Obsrv. Mass	± da	± ppm	Start Seq.	End Seq.	Sequence	Ion Score	C. I. %	Modification	Rank	Result Type
815.3795	815.4232	0.0437	54	100	105	TDTWHR					Mascot
827.4985	827.499	0.0005	1	66	73	ADVPGLKK					Mascot
974.5417	974.5937	0.052	53	117	124	FRLPENAK					Mascot
974.5417	974.5937	0.052	53	117	124	FRLPENAK	18	2.203			Mascot

1057.5314	1057.5701	0.0387	37	57	65	ETPEAHVFK					Mascot
1154.5437	1154.6094	0.0657	57	41	52	TSSDTAAFAGAR					Mascot
1154.5437	1154.6094	0.0657	57	41	52	TSSDTAAFAGAR	72	100			Mascot
1657.8392	1657.9315	0.0923	56	78	92	VEVEDGNILQISGER					Mascot
1657.8392	1657.9315	0.0923	56	78	92	VEVEDGNILQISGER	139	100			Mascot
2143.0876	2143.2131	0.1255	59	74	92	EEVKVEVEDGNILQISGE R					Mascot
2143.0876	2143.2131	0.1255	59	74	92	EEVKVEVEDGNILQISGE R	104	100			Mascot

3 unnamed protein product [Triticum aestivum] gi|296511567 17519.9 5.81 7 367 100 10.755 334 100

Protein Group

unnamed protein product [Triticum aestivum] gi|296512783 17519.9 5.8099 999427 7954

Peptide Information

Calc. Mass	Obsrv. Mass	± da	± ppm	Start Seq.	End Seq.	Sequence	Ion Score	C. I. %	Modification	Rank	Result Type
815.3795	815.4232	0.0437	54	99	104	TDTWHR					Mascot
827.4985	827.499	0.0005	1	65	72	ADVPGLKK					Mascot
974.5417	974.5937	0.052	53	116	123	FRLPENAK					Mascot
974.5417	974.5937	0.052	53	116	123	FRLPENAK	18	2.203			Mascot
1057.5889	1057.5701	-0.0188	-18	143	151	EEAKKPEVK					Mascot
1154.5437	1154.6094	0.0657	57	40	51	TSSDTAAFAGAR					Mascot
1154.5437	1154.6094	0.0657	57	40	51	TSSDTAAFAGAR	72	100			Mascot
1657.8392	1657.9315	0.0923	56	77	91	VEVEDGNILQISGER					Mascot
1657.8392	1657.9315	0.0923	56	77	91	VEVEDGNILQISGER	139	100			Mascot
2143.0876	2143.2131	0.1255	59	73	91	EEVKVEVEDGNILQISGE R					Mascot
2143.0876	2143.2131	0.1255	59	73	91	EEVKVEVEDGNILQISGE R	104	100			Mascot

4 hypothetical protein TRIUR3_16950 [Triticum urartu] gi|473794549 27510.1 6.25 7 355 100 10.755 334 100

Peptide Information

Calc. Mass	Obsrv. Mass	± da	± ppm	Start Seq.	End Seq.	Sequence	Ion Score	C. I. %	Modification	Rank	Result Type
815.3795	815.4232	0.0437	54	99	104	TDTWHR					Mascot
827.4985	827.499	0.0005	1	65	72	ADVPGLKK					Mascot
974.5417	974.5937	0.052	53	116	123	FRLPENAK					Mascot
974.5417	974.5937	0.052	53	116	123	FRLPENAK	18	2.203			Mascot
1057.5314	1057.5701	0.0387	37	56	64	ETPEAHVFK					Mascot

1154.5437	1154.6094	0.0657	57	40	51	TSSDTAAFAGAR					Mascot
1154.5437	1154.6094	0.0657	57	40	51	TSSDTAAFAGAR	72	100			Mascot
1657.8392	1657.9315	0.0923	56	77	91	VEVEDGNILQISGER					Mascot
1657.8392	1657.9315	0.0923	56	77	91	VEVEDGNILQISGER	139	100			Mascot
2143.0876	2143.2131	0.1255	59	73	91	EEVKVEVEDGNILQISGE R					Mascot
2143.0876	2143.2131	0.1255	59	73	91	EEVKVEVEDGNILQISGE R	104	100			Mascot

5	unnamed protein product [Triticum aestivum]	gi|296511507	17513.9	5.81	6	342	100	9.694	315	100	

Protein Group

unnamed protein product [Triticum aestivum]	gi|296512731	17513.9	5.8099 999427 7954

Peptide Information

Calc. Mass	Obsrv. Mass	± da	± ppm	Start Seq.	End Seq.	Sequence	Ion Score	C. I. %	Modification	Rank	Result Type
815.3795	815.4232	0.0437	54	99	104	TDTWHR					Mascot
827.4985	827.499	0.0005	1	65	72	ADVPGLKK					Mascot
1057.5889	1057.5701	-0.0188	-18	143	151	EEAKKPEVK					Mascot
1154.5437	1154.6094	0.0657	57	40	51	TSSDTAAFAGAR					Mascot
1154.5437	1154.6094	0.0657	57	40	51	TSSDTAAFAGAR	72	100			Mascot
1657.8392	1657.9315	0.0923	56	77	91	VEVEDGNILQISGER					Mascot
1657.8392	1657.9315	0.0923	56	77	91	VEVEDGNILQISGER	139	100			Mascot
2143.0876	2143.2131	0.1255	59	73	91	EEVKVEVEDGNILQISGE R					Mascot
2143.0876	2143.2131	0.1255	59	73	91	EEVKVEVEDGNILQISGE R	104	100			Mascot

6	unnamed protein product [Triticum aestivum]	gi|296511579	17365.9	5.8	9	312	100	10.043	262	100	

Protein Group

unnamed protein product [Triticum aestivum]	gi|296512795	17365.9	5.8000 001907 3486

Peptide Information

Calc. Mass	Obsrv. Mass	± da	± ppm	Start Seq.	End Seq.	Sequence	Ion Score	C. I. %	Modification	Rank	Result Type
815.3795	815.4232	0.0437	54	97	102	TDTWHR					Mascot
827.4985	827.499	0.0005	1	63	70	ADVPGLKK					Mascot
974.5417	974.5937	0.052	53	114	121	FRLPENAK					Mascot
974.5417	974.5937	0.052	53	114	121	FRLPENAK	18	2.203			Mascot
1027.5571	1027.5504	-0.0067	-7	54	62	ETPVAHVFK					Mascot

1057.5889	1057.5701	-0.0188	-18	141	149	EEAKKPEVK					Mascot
1182.575	1182.6276	0.0526	44	38	49	TSSDTVAFAGAR					Mascot
1657.8392	1657.9315	0.0923	56	75	89	VEVEDGNILQISGER					Mascot
1657.8392	1657.9315	0.0923	56	75	89	VEVEDGNILQISGER	139	100			Mascot
1707.9429	1707.8722	-0.0707	-41	54	69	ETPVAHVFKADVPGLK					Mascot
2143.0876	2143.2131	0.1255	59	71	89	EEVKVEVEDGNILQISGER					Mascot
2143.0876	2143.2131	0.1255	59	71	89	EEVKVEVEDGNILQISGER	104	100			Mascot

7	hypothetical protein TRIUR3_24152 [Triticum urartu]	gi|474174891	12287.5	6.75	5	286	100	8.228	262	100

Peptide Information

Calc. Mass	Obsrv. Mass	± da	± ppm	Start Seq.	End Seq.	Sequence	Ion Score	C. I. %	Modification	Rank	Result Type
815.3795	815.4232	0.0437	54	53	58	TDTWHR					Mascot
827.4985	827.499	0.0005	1	19	26	ADVPGLKK					Mascot
974.5417	974.5937	0.052	53	70	77	FRLPENAK					Mascot
974.5417	974.5937	0.052	53	70	77	FRLPENAK	18	2.203			Mascot
1657.8392	1657.9315	0.0923	56	31	45	VEVEDGNILQISGER					Mascot
1657.8392	1657.9315	0.0923	56	31	45	VEVEDGNILQISGER	139	100			Mascot
2143.0876	2143.2131	0.1255	59	27	45	EEVKVEVEDGNILQISGER					Mascot
2143.0876	2143.2131	0.1255	59	27	45	EEVKVEVEDGNILQISGER	104	100			Mascot

8	putative rhamnose biosynthetic enzyme 1 [Triticum urartu]	gi|473775808	76545.8	6.38	15	53	77.837	7.609		

Peptide Information

Calc. Mass	Obsrv. Mass	± da	± ppm	Start Seq.	End Seq.	Sequence	Ion Score	C. I. %	Modification	Rank	Result Type
829.5002	829.4546	-0.0456	-55	120	126	VTGQIRR					Mascot
835.511	835.4603	-0.0507	-61	203	209	FILLAMK					Mascot
859.4785	859.5113	0.0328	38	299	305	KLGWAER					Mascot
922.488	922.5053	0.0173	19	268	275	LFGLDTEK					Mascot
948.507	948.499	-0.008	-8	524	531	AMVEELLK			Oxidation (M)[2]		Mascot
960.4996	960.5528	0.0532	55	356	365	SLASSPAEAK					Mascot
1033.517	1033.5695	0.0525	51	339	347	MLMTPGVER					Mascot
1065.5068	1065.5698	0.063	59	339	347	MLMTPGVER			Oxidation (M)[1,3]		Mascot
1106.6205	1106.5785	-0.042	-38	176	185	SYGLPVITTR					Mascot
1232.594	1232.671	0.077	62	543	553	MPISSDLSNPR			Oxidation (M)[1]		Mascot
1252.5837	1252.6559	0.0722	58	638	648	SNNEMDASKLK			Oxidation (M)[5]		Mascot

Calc. Mass	Obsrv. Mass	± da	± ppm	Start Seq.	End Seq.	Sequence	Ion Score	C. I. %	Modification	Rank	Result Type
1300.6024	1300.6263	0.0239	18	164	175	AGAEMLVMAYGR			Oxidation (M)[5,8]		Mascot
1302.8218	1302.7207	-0.1011	-78	199	209	LIPKFILLAMK			Oxidation (M)[10]		Mascot
1584.7727	1584.8104	0.0377	24	406	418	ICEKQGIPYEYGK			Carbamidomethyl (C)[2]		Mascot
1657.8657	1657.9315	0.0658	40	242	256	GEVGHVYNIGTVKER					Mascot
1657.8657	1657.9315	0.0658	40	242	256	GEVGHVYNIGTVKER	7	0			Mascot
2023.0416	2023.0266	-0.015	-7	258	275	VIDVASDICKLFGLDTEK			Carbamidomethyl (C)[9]		Mascot

9	Telomeric repeat-binding factor 2 [Triticum urartu]	gi|474445687	29996.6	9.61	12	52	73.96	3.956		

Peptide Information

Calc. Mass	Obsrv. Mass	± da	± ppm	Start Seq.	End Seq.	Sequence	Ion Score	C. I. %	Modification	Rank	Result Type
830.4618	830.4638	0.002	2	216	222	EPTKVEK					Mascot
948.4996	948.499	-0.0006	-1	175	183	LNELTSSGK					Mascot
960.5434	960.5528	0.0094	10	207	214	MLLLDDIK					Mascot
1090.5527	1090.5891	0.0364	33	34	42	DPEFSNILR					Mascot
1109.5474	1109.561	0.0136	12	141	150	TLNEPTGSYK					Mascot
1263.6111	1263.7345	0.1234	98	55	66	NMNVTVNASGTR					Mascot
1277.7061	1277.7808	0.0747	58	69	80	VRTTTTTTPTAK					Mascot
1320.7191	1320.6658	-0.0533	-40	175	186	LNELTSSGKLMK					Mascot
1320.7191	1320.6658	-0.0533	-40	175	186	LNELTSSGKLMK					Mascot
1482.77	1482.8297	0.0597	40	191	203	YRIAPSSSFLEGR					Mascot
1522.7279	1522.8512	0.1233	81	55	68	NMNVTVNASGTRDK			Oxidation (M)[2]		Mascot
1584.9319	1584.8104	-0.1215	-77	127	140	SLSRLENIILEAVK					Mascot
1605.7915	1605.8486	0.0571	36	53	66	WRNMNVTVNASGTR					Mascot

10	Mitochondrial division protein 1 [Triticum urartu]	gi|473964525	126904.3	7.29	10	50	57.769	7.662	33	96.593		

Peptide Information

Calc. Mass	Obsrv. Mass	± da	± ppm	Start Seq.	End Seq.	Sequence	Ion Score	C. I. %	Modification	Rank	Result Type
823.54	823.4761	-0.0639	-78	940	946	IIVLTHK					Mascot
827.5098	827.499	-0.0108	-13	820	826	KPLSVQR					Mascot
973.5214	973.5822	0.0608	62	866	873	AWDIKGQR					Mascot
1154.5446	1154.6094	0.0648	56	1	9	MDRCFQLGK			Carbamidomethyl (C)[4]		Mascot
1154.5446	1154.6094	0.0648	56	1	9	MDRCFQLGK			Carbamidomethyl (C)[4]		Mascot
1235.6412	1235.7067	0.0655	53	783	793	QKLMSLSEGSR					Mascot
1277.8304	1277.7808	-0.0496	-39	940	950	IIVLTHKNVLK					Mascot
1365.6394	1365.7185	0.0791	58	287	298	SGNNSQVDFLER					Mascot

1365.6394	1365.7185	0.0791	58	287	298	SGNNSQVDFLER	4	99.592		Mascot
1791.9025	1791.8383	-0.0642	-36	63	80	STALVSNFGGHGGFAEL K				Mascot
2143.1111	2143.2131	0.102	48	489	507	NNNLEEGMALAAILVRCV R			Carbamidomethyl (C)[17]	Mascot
2143.1111	2143.2131	0.102	48	489	507	NNNLEEGMALAAILVRCV R	33	100	Carbamidomethyl (C)[17]	Mascot
2369.2856	2369.1677	-0.1179	-50	483	504	LVDVAKNNNLEEGMALA AILVR			Oxidation (M)[14]	Mascot

Rank	Protein Name	Accession No.	Protein MW	Protein PI	Pep. Count	Protein Score	Protein Score C. I. %	Intensity Matched	Total Ion Score	Total Ion C. I. %	Confirmed
1	hypothetical protein TRIUR3_06778 [Triticum urartu]	gi|474115370	14049.2	10.75	2	9	0	.841			

Peptide Information

Calc. Mass	Obsrv. Mass	± da	± ppm	Start Seq.	End Seq.	Sequence	Ion Score	C. I. %	Modification	Rank	Result Type
832.4457	832.3728	-0.0729	-88	58	64	SVRPSMR					Mascot
889.4421	889.3856	-0.0565	-64	50	57	GMGGQRQR					Mascot

Rank	Protein Name	Accession No.	Protein MW	Protein PI	Pep. Count	Protein Score	Protein Score C. I. %	Intensity Matched	Total Ion Score	Total Ion C. I. %	Confirmed
2	50S ribosomal protein L28, chloroplastic [Triticum urartu]	gi|473889696	15184.3	10.72	2	8	0	.731			

Peptide Information

Calc. Mass	Obsrv. Mass	± da	± ppm	Start Seq.	End Seq.	Sequence	Ion Score	C. I. %	Modification	Rank	Result Type
818.4155	818.3382	-0.0773	-94	77	83	VSFSNHK					Mascot
889.4566	889.3856	-0.071	-80	97	103	LWWEAGK					Mascot

Rank	Protein Name	Accession No.	Protein MW	Protein PI	Pep. Count	Protein Score	Protein Score C. I. %	Intensity Matched	Total Ion Score	Total Ion C. I. %	Confirmed
3	U-box domain-containing protein 15 [Triticum urartu]	gi|473945241	60969.4	7.55	3	8	0	.685			

Peptide Information

Calc. Mass	Obsrv. Mass	± da	± ppm	Start Seq.	End Seq.	Sequence	Ion Score	C. I. %	Modification	Rank	Result Type
889.4598	889.3856	-0.0742	-83	528	535	NGTSRAQR					Mascot
1653.9608	1654.1134	0.1526	92	387	403	VAIGALGGMAPLVDLLK			Oxidation (M)[9]		Mascot
1961.0702	1961.2361	0.1659	85	151	168	EVAGIDEKNILGEVHIPK					Mascot

Rank	Protein Name	Accession No.	Protein MW	Protein PI	Pep. Count	Protein Score	Protein Score C. I. %	Intensity Matched	Total Ion Score	Total Ion C. I. %	Confirmed
4	unnamed protein product [Triticum aestivum]	gi|311813503	16423.1	10	2	8	0	1.173			

Protein Group

RecName: Full=Histone H2B.1	gi|122022	16423.1	10
histone H2B [Triticum aestivum]	gi|21801	16423.1	10
unnamed protein product [Triticum aestivum]	gi|207008838	16423.1	10

Peptide Information

Calc. Mass	Obsrv. Mass	± da	± ppm	Start Seq.	End Seq.	Sequence	Ion Score	C. I. %	Modification	Rank	Result Type
832.4523	832.3728	-0.0795	-96	121	127	EIQTSVR					Mascot
842.5094	842.5699	0.0605	72	39	47	LPAGKSAAK					Mascot

5	hypothetical protein TRIUR3_21824 [Triticum urartu]	gi|474169678	17009.9	9.51	2	8	0	1.173	

Peptide Information

Calc. Mass	Obsrv. Mass	± da	± ppm	Start Seq.	End Seq.	Sequence	Ion Score	C. I. %	Modification	Rank	Result Type
832.4047	832.3728	-0.0319	-38	125	132	VSADPDTK					Mascot
842.4982	842.5699	0.0717	85	139	146	IVGEPLSK					Mascot

6	Histone H2B.2 [Triticum urartu]	gi|474106628	16469.1	10	2	8	0	1.173	

Peptide Information

Calc. Mass	Obsrv. Mass	± da	± ppm	Start Seq.	End Seq.	Sequence	Ion Score	C. I. %	Modification	Rank	Result Type
832.4523	832.3728	-0.0795	-96	121	127	EIQTSVR					Mascot
842.5094	842.5699	0.0605	72	5	12	AEKKPAAK					Mascot

7	Histone H2B.2 [Triticum urartu]	gi|474404313	16763.2	10.11	2	8	0	1.173	

Peptide Information

Calc. Mass	Obsrv. Mass	± da	± ppm	Start Seq.	End Seq.	Sequence	Ion Score	C. I. %	Modification	Rank	Result Type
832.4523	832.3728	-0.0795	-96	125	131	EIQTSVR					Mascot
842.5094	842.5699	0.0605	72	5	12	AEKKPAAK					Mascot

8	hypothetical protein TRIUR3_23173 [Triticum urartu]	gi|474241562	17776.8	5.29	2	8	0	.947	

Peptide Information

Calc. Mass	Obsrv. Mass	± da	± ppm	Start Seq.	End Seq.	Sequence	Ion Score	C. I. %	Modification	Rank	Result Type
889.456	889.3856	-0.0704	-79	36	42	ACLQEIR			Carbamidomethyl (C)[2]		Mascot
931.4367	931.3613	-0.0754	-81	28	35	DPISEQDK					Mascot

9	putative glycine decarboxylase P subunit [Triticum durum]	gi|62176928	3168.6	10.39	1	8	0	.195	

Peptide Information

Calc. Mass	Obsrv. Mass	± da	± ppm	Start Seq.	End Seq.	Sequence	Ion Score	C. I. %	Modification	Rank	Result Type

856.4821	856.5657	0.0836	98	1	7	MLRSPPR					Mascot

10 hypothetical protein TRIUR3_09739 [Triticum urartu] gi|473907770 18488.1 4.95 2 7 0 .756

Peptide Information

Calc. Mass	Obsrv. Mass	± da	± ppm	Start Seq.	End Seq.	Sequence	Ion Score	C. I. %	Modification	Rank	Result Type
832.4531	832.3728	-0.0803	-96	123	129	MMKLPGR					Mascot
931.4479	931.3613	-0.0866	-93	59	66	EEENAALR					Mascot

Gel Idx/Pos	114/E13	Instr./Gel Origin	BA2151/Sample Project 20140814	Process Status	Analysis Succeeded
Plate [#] Name	[1] Sample Project 20140814	Instrument Sample Name		Spectra	11

Rank	Protein Name	Accession No.	Protein MW	Protein PI	Pep. Count	Protein Score	Protein Score C. I. %	Intensity Matched	Total Ion Score	Total Ion C. I. %	Confirmed
1	Globulin-1 S allele [Triticum urartu]	gi|474411419	57108.4	9.1	8	479	100	53.021	458	100	

Peptide Information

Calc. Mass	Obsrv. Mass	± da	± ppm	Start Seq.	End Seq.	Sequence	Ion Score	C. I. %	Modification	Rank	Result Type
906.468	906.5256	0.0576	64	457	463	EVQEVFR					Mascot
1018.5098	1018.5989	0.0891	87	40	47	SLQQCVQR			Carbamidomethyl (C)[5]		Mascot
1376.7169	1376.7614	0.0445	32	258	268	DTYNLLEQRPK					Mascot
1822.8752	1823.0076	0.1324	73	411	426	GSSNLQVVCFEINAER			Carbamidomethyl (C)[9]		Mascot
1822.8752	1823.0076	0.1324	73	411	426	GSSNLQVVCFEINAER	149	100	Carbamidomethyl (C)[9]		Mascot
1906.0182	1906.1533	0.1351	71	392	410	GSAFVVPPGHPVVEIASSR					Mascot
1906.0182	1906.1533	0.1351	71	392	410	GSAFVVPPGHPVVEIASSR	147	100			Mascot
1960.0035	1959.9902	-0.0133	-7	66	82	SDHGFVKALRPFDEVSR					Mascot
2089.9058	2090.0564	0.1506	72	466	483	DQQDEGFVAGPEQQEQER					Mascot
2089.9058	2090.0564	0.1506	72	466	483	DQQDEGFVAGPEQQEQER	162	100			Mascot
2289.0378	2289.2073	0.1695	74	464	483	AKDQQDEGFVAGPEQQEQER					Mascot

Rank	Protein Name	Accession No.	Protein MW	Protein PI	Pep. Count	Protein Score	Protein Score C. I. %	Intensity Matched	Total Ion Score	Total Ion C. I. %	Confirmed
2	globulin-3A [Triticum aestivum]	gi|390979705	66626.8	8.48	8	399	100	62.559	381	100	

Peptide Information

Calc. Mass	Obsrv. Mass	± da	± ppm	Start Seq.	End Seq.	Sequence	Ion Score	C. I. %	Modification	Rank	Result Type
832.4159	832.3812	-0.0347	-42	307	313	ASEEQLR					Mascot
906.468	906.5256	0.0576	64	535	541	EVQEVFR					Mascot
1360.7219	1360.7731	0.0512	38	339	349	DTFNLLEQRPK					Mascot
1360.7219	1360.7731	0.0512	38	339	349	DTFNLLEQRPK					Mascot
1565.631	1565.736	0.105	67	446	459	GSGSESEEEQDQQR					Mascot
1565.631	1565.736	0.105	67	446	459	GSGSESEEEQDQQR	85	100			Mascot
1721.7322	1721.8431	0.1109	64	445	459	RGSGSESEEEQDQQR					Mascot
1822.8752	1823.0076	0.1324	73	489	504	GSSNLQVVCFEINAER			Carbamidomethyl (C)[9]		Mascot
1822.8752	1823.0076	0.1324	73	489	504	GSSNLQVVCFEINAER	149	100	Carbamidomethyl (C)[9]		Mascot
1906.0182	1906.1533	0.1351	71	470	488	GSAFVVPPGHPVVEIASSR					Mascot

Calc. Mass	Obsrv. Mass	± da	± ppm	Start Seq.	End Seq.	Sequence	Ion Score	C. I. %	Modification	Rank	Result Type
1906.0182	1906.1533	0.1351	71	470	488	GSAFVVPPGHPVVEIASS R	147	100			Mascot
1960.0035	1959.9902	-0.0133	-7	147	163	SDHGFVKALRPFDEVSR					Mascot

3	globulin 3 [Triticum aestivum]	gi|215398470	66651.7	7.78	9	397	100	62.704	381	100	

Peptide Information

Calc. Mass	Obsrv. Mass	± da	± ppm	Start Seq.	End Seq.	Sequence	Ion Score	C. I. %	Modification	Rank	Result Type
832.4159	832.3812	-0.0347	-42	307	313	ASEEQLR					Mascot
906.468	906.5256	0.0576	64	535	541	EVQEVFR					Mascot
1018.5098	1018.5989	0.0891	87	40	47	SLQQCVQR			Carbamidomethyl (C)[5]		Mascot
1360.7219	1360.7731	0.0512	38	339	349	DTFNLLEQRPK					Mascot
1360.7219	1360.7731	0.0512	38	339	349	DTFNLLEQRPK					Mascot
1565.631	1565.736	0.105	67	446	459	GSGSESEEEQDQQR					Mascot
1565.631	1565.736	0.105	67	446	459	GSGSESEEEQDQQR	85	100			Mascot
1721.7322	1721.8431	0.1109	64	445	459	RGSGSESEEEQDQQR					Mascot
1822.8752	1823.0076	0.1324	73	489	504	GSSNLQVVCFEINAER			Carbamidomethyl (C)[9]		Mascot
1822.8752	1823.0076	0.1324	73	489	504	GSSNLQVVCFEINAER	149	100	Carbamidomethyl (C)[9]		Mascot
1906.0182	1906.1533	0.1351	71	470	488	GSAFVVPPGHPVVEIASS R					Mascot
1906.0182	1906.1533	0.1351	71	470	488	GSAFVVPPGHPVVEIASS R	147	100			Mascot
1960.0035	1959.9902	-0.0133	-7	147	163	SDHGFVKALRPFDEVSR					Mascot

4	globulin 3B [Triticum aestivum]	gi|215398472	57067.8	7.36	10	190	100	18.226	162	100	

Peptide Information

Calc. Mass	Obsrv. Mass	± da	± ppm	Start Seq.	End Seq.	Sequence	Ion Score	C. I. %	Modification	Rank	Result Type
832.4159	832.3812	-0.0347	-42	265	271	ASEEQLR					Mascot
878.5206	878.509	-0.0116	-13	133	139	SFRSIIR					Mascot
906.468	906.5256	0.0576	64	452	458	EVQEVFR					Mascot
944.5272	944.4857	-0.0415	-44	381	389	QGKQGSALR					Mascot
1376.7169	1376.7614	0.0445	32	297	307	DTYNLLEQRPK					Mascot
1612.7272	1612.8604	0.1332	83	408	421	GSSNLEMFEINAER			Oxidation (M)[7]		Mascot
1960.0035	1959.9902	-0.0133	-7	140	156	SDHGFVKALRPFDEVSR					Mascot
2089.9058	2090.0564	0.1506	72	461	478	DQQDEGFVAGPEQQEQ ER					Mascot
2089.9058	2090.0564	0.1506	72	461	478	DQQDEGFVAGPEQQEQ ER	162	100			Mascot
2289.0378	2289.2073	0.1695	74	459	478	AKDQQDEGFVAGPEQQ EQER					Mascot
2317.2188	2317.2341	0.0153	7	209	229	SYSVRQGDYFSAKPLLA					Mascot

						SLSK					
	2317.2188	2317.2341	0.0153	7	209	229 SYSVRQGDYFSAKPLLA SLSK					Mascot

5 Regulatory protein NPR1 [Triticum urartu] gi|473786885 171630 6.24 23 49 43.032 17.816

Peptide Information

Calc. Mass	Obsrv. Mass	± da	± ppm	Start Seq.	End Seq.	Sequence	Ion Score	C. I. %	Modification	Rank	Result Type
886.5972	886.5752	-0.022	-25	1280	1287	ILVSLLTK					Mascot
888.4646	888.5235	0.0589	66	1020	1027	RASVEGNR					Mascot
905.4761	905.5005	0.0244	27	628	635	IASDVCIK			Carbamidomethyl (C)[6]		Mascot
906.4536	906.5256	0.072	79	1155	1161	CLEMVVR			Carbamidomethyl (C)[1]		Mascot
1059.5867	1059.6454	0.0587	55	791	799	ELGNLCILK			Carbamidomethyl (C)[6]		Mascot
1136.6423	1136.6324	-0.0099	-9	736	746	NIVVSHGPSVK					Mascot
1179.6368	1179.6738	0.037	31	313	322	TTLANVLYER					Mascot
1359.6937	1359.7504	0.0567	42	1329	1339	LCIEVLEQAER			Carbamidomethyl (C)[2]		Mascot
1382.6886	1382.7468	0.0582	42	1442	1452	FFPRCSNVLDK			Carbamidomethyl (C)[5]		Mascot
1528.8119	1528.8854	0.0735	48	193	206	QIAFIAQVSEHGTK					Mascot
1704.92	1704.912	-0.008	-5	1209	1223	ALDSSDVELVRMLLK			Oxidation (M)[12]		Mascot
1721.8197	1721.8431	0.0234	14	642	656	GLQCLETLDVMDATR			Carbamidomethyl (C)[4]		Mascot
1749.9421	1749.9315	-0.0106	-6	1186	1201	LSLGLVSPEDKGFPYK					Mascot
1838.892	1839.0151	0.1231	67	1313	1328	DYFGITEEGKPSPKDR					Mascot
1844.9866	1844.9812	-0.0054	-3	193	209	QIAFIAQVSEHGTKTSK					Mascot
1860.924	1860.9644	0.0404	22	1311	1326	HRDYFGITEEGKPSPK					Mascot
1891.0323	1891.1592	0.1269	67	756	771	VIISWDDLEPPPLLQR					Mascot
1902.8981	1903.0487	0.1506	79	1474	1489	FHDVHDSLQKAFSEDK					Mascot
1918.0182	1918.1418	0.1236	64	898	915	EISVKFGGAAAHIEYAVR					Mascot
1958.0106	1957.9847	-0.0259	-13	1004	1019	YMLSVRSPFLHAFFAR			Oxidation (M)[2]		Mascot
2215.1646	2215.1089	-0.0557	-25	1038	1056	VEVGYEALQLVLEYLYSA R					Mascot
2271.1714	2271.2261	0.0547	24	46	67	NVLIDNPTTATVSDPVIES SAK					Mascot
2289.1003	2289.2073	0.107	47	313	330	TTLANVLYERIEMQFECR			Carbamidomethyl (C)[17], Oxidation (M)[13]		Mascot

6 Disease resistance protein RPM1 [Triticum urartu] gi|474122476 118615 8 21 46 1 5.203

Peptide Information

Calc. Mass	Obsrv. Mass	± da	± ppm	Start Seq.	End Seq.	Sequence	Ion Score	C. I. %	Modification	Rank	Result Type
886.5032	886.5752	0.072	81	1040	1046	WPKIITE					Mascot
888.4719	888.5235	0.0516	58	781	787	EPMRALR			Oxidation (M)[3]		Mascot

Calc. Mass	Obsrv. Mass	± da	± ppm	Start Seq.	End Seq.	Sequence	Ion Score	C. I. %	Modification	Rank	Result Type
904.4669	904.5082	0.0413	46	658	665	GMNLSQVR					Mascot
944.4869	944.4857	-0.0012	-1	976	983	EVMPALER					Mascot
1006.5428	1006.5041	-0.0387	-38	130	137	AQYIAERR					Mascot
1359.7366	1359.7504	0.0138	10	720	731	TEVSEIPSKIEK					Mascot
1398.8356	1398.7209	-0.1147	-82	944	956	EIIIPPGGFKSLK					Mascot
1475.7345	1475.833	0.0985	67	976	987	EVMPALERIDMR			Oxidation (M)[3]		Mascot
1528.7649	1528.8854	0.1205	79	652	665	HGNSTKGMNLSQVR					Mascot
1593.9211	1593.7689	-0.1522	-95	893	906	LTLSVTVLRTDTFK					Mascot
1698.9061	1699.027	0.1209	71	914	928	LFTLTFTLSAAKDDR					Mascot
1731.8945	1731.9838	0.0893	52	313	326	IETMNRDQLIEELK					Mascot
1731.8945	1731.9838	0.0893	52	313	326	IETMNRDQLIEELK					Mascot
1791.0051	1790.9825	-0.0226	-13	603	617	SFKVHDLILEYIVSK					Mascot
1838.859	1839.0151	0.1561	85	234	249	ASVMVSQNFDEDEVLR					Mascot
1876.0902	1876.1548	0.0646	34	886	901	ELNNLYKLTLSVTVLR					Mascot
1957.9722	1957.9847	0.0125	6	1	18	MEFVVGASEATMRSLLG K			Oxidation (M)[1,12]		Mascot
1965.9923	1966.1001	0.1078	55	658	675	GMNLSQVRSLTAFGSQN R					Mascot
1968.0033	1968.0902	0.0869	44	1002	1019	SLQEVHLSVGNQADEITK					Mascot
2059.0793	2059.2629	0.1836	89	877	892	MERPPQWIKELNNLYK					Mascot
2271.1802	2271.2261	0.0459	20	176	195	TQNPVGVQVDMEKLQG WLTK					Mascot
2327.2183	2327.1606	-0.0577	-25	677	696	LPFHSFNNGIIQVLDLEG WK					Mascot

7 Secologanin synthase [Triticum urartu] gi|474429248 102759.4 8.48 18 43 0 6.02

Peptide Information

Calc. Mass	Obsrv. Mass	± da	± ppm	Start Seq.	End Seq.	Sequence	Ion Score	C. I. %	Modification	Rank	Result Type
805.4679	805.4813	0.0134	17	254	259	YLPTRR					Mascot
876.4608	876.5251	0.0643	73	61	67	QMLIAER			Oxidation (M)[2]		Mascot
905.5414	905.5005	-0.0409	-45	269	276	SITSKITR					Mascot
914.5417	914.5992	0.0575	63	348	354	LREEVLR					Mascot
934.5145	934.5518	0.0373	40	217	224	VIFGAHYK					Mascot
1358.691	1358.76	0.069	51	849	859	DQVIQERDDLK					Mascot
1359.6764	1359.7504	0.074	54	772	783	HVAFESVDSGRR					Mascot
1376.6442	1376.7614	0.1172	85	143	154	GLLTTNGDDWER					Mascot
1565.8469	1565.736	-0.1109	-71	524	538	HSLILDAPMLSGGVR					Mascot
1565.8469	1565.736	-0.1109	-71	524	538	HSLILDAPMLSGGVR					Mascot
1684.9237	1684.9836	0.0599	36	413	427	GTMITIPVMTLHRSK					Mascot

Calc. Mass	Obsrv. Mass	± da	± ppm	Start Seq.	End Seq.	Sequence	Ion Score	C. I. %	Modification	Rank	Result Type
1684.9237	1684.9836	0.0599	36	413	427	GTMITIPVMTLHRSK					Mascot
1821.9131	1821.9874	0.0743	41	139	154	IFGKGLLTTNGDDWER					Mascot
1874.91	1875.0791	0.1691	90	548	564	SVVVPLIGCSDCGEQVR			Carbamidomethyl (C)[9,12]		Mascot
1875.9845	1876.1548	0.1703	91	817	834	EQAMSQLGSTQLALTGLK					Mascot
1902.9192	1903.0487	0.1295	68	648	666	GTAGGANYASSIENKYATK					Mascot
1922.0641	1922.13	0.0659	34	524	541	HSLILDAPMLSGGVRSIR					Mascot
1958.0376	1957.9847	-0.0529	-27	282	300	LASGVDGDDLIGLMLRANK					Mascot
2103.1479	2103.3076	0.1597	76	817	836	EQAMSQLGSTQLALTGLKVK					Mascot
2287.9905	2288.2026	0.2121	93	298	317	ANKSEEVESLSSDEMISECK			Carbamidomethyl (C)[19], Oxidation (M)[15]		Mascot

8	hypothetical protein TRIUR3_30079 [Triticum urartu]	gi|474234215	17358.8	6.74	7	43	0	1.875	9	0

Peptide Information

Calc. Mass	Obsrv. Mass	± da	± ppm	Start Seq.	End Seq.	Sequence	Ion Score	C. I. %	Modification	Rank	Result Type
830.4843	830.4728	-0.0115	-14	15	21	QSKSRPK					Mascot
1136.631	1136.6324	0.0014	1	112	121	ATFVLETLSR					Mascot
1641.8418	1641.9535	0.1117	68	34	49	AAPSARTMPYPPSAPK					Mascot
1731.8921	1731.9838	0.0917	53	87	101	EGHFMVMVVLEAVVR			Oxidation (M)[5]		Mascot
1731.8921	1731.9838	0.0917	53	87	101	EGHFMVMVVLEAVVR	9	0	Oxidation (M)[7]		Mascot
1804.915	1805.0304	0.1154	64	40	56	TMPYPPSAPKALETGTK			Oxidation (M)[2]		Mascot
1820.9866	1821.0348	0.0482	26	112	128	ATFVLETLSRTVVGGDR					Mascot
1866.9299	1866.9907	0.0608	33	1	17	MSSGLNMSLDDLIKQSK					Mascot

9	hypothetical protein TRIUR3_25639 [Triticum urartu]	gi|474172357	126958.9	6.71	13	42	0	18.018	23	65.819

Peptide Information

Calc. Mass	Obsrv. Mass	± da	± ppm	Start Seq.	End Seq.	Sequence	Ion Score	C. I. %	Modification	Rank	Result Type
805.4679	805.4813	0.0134	17	313	318	RFQNLK					Mascot
905.4509	905.5005	0.0496	55	640	647	ISLEGCAR			Carbamidomethyl (C)[6]		Mascot
1059.5793	1059.6454	0.0661	62	169	178	IGSEINASLR					Mascot
1398.77	1398.7209	-0.0491	-35	702	713	NPSLEVLRVDTR					Mascot
1513.7944	1513.8973	0.1029	68	115	126	IIHVDCSLWKSR			Carbamidomethyl (C)[6]		Mascot
1565.7853	1565.736	-0.0493	-31	375	386	TQLLTRCFDQQR			Carbamidomethyl (C)[7]		Mascot
1565.7853	1565.736	-0.0493	-31	375	386	TQLLTRCFDQQR	23	65.819	Carbamidomethyl (C)[7]		Mascot
1642.0336	1641.9535	-0.0801	-49	15	28	MLLLLLLVIQVTTR			Oxidation (M)[1]		Mascot
1667.9438	1668.0333	0.0895	54	164	178	LEIPRIGSEINASLR					Mascot

Calc. Mass	Obsrv. Mass	± da	± ppm	Start Seq.	End Seq.	Sequence	Ion Score	C. I. %	Modification	Rank	Result Type
1703.8235	1703.9395	0.116	68	60	74	LIDHLSESYDNQAAK					Mascot
1707.8768	1707.9159	0.0391	23	499	512	MIDLMTNLREINTK			Oxidation (M)[1]		Mascot
1822.9004	1823.0076	0.1072	59	296	311	GMPRSIYDIDAQAELK			Oxidation (M)[2]		Mascot
1822.9004	1823.0076	0.1072	59	296	311	GMPRSIYDIDAQAELK			Oxidation (M)[2]		Mascot
2103.1282	2103.3076	0.1794	85	990	1006	HISLYHLHRLEQICGVK			Carbamidomethyl (C)[14]		Mascot
2327.1853	2327.1606	-0.0247	-11	75	97	SFFLAGWCGEGVGASAV LKATAK			Carbamidomethyl (C)[8]		Mascot

10	powdery mildew resistance protein PM3 variant [Triticum aestivum]	gi|225580371	161522.2	6.55	19	39	0	42.197

Peptide Information

Calc. Mass	Obsrv. Mass	± da	± ppm	Start Seq.	End Seq.	Sequence	Ion Score	C. I. %	Modification	Rank	Result Type
832.4312	832.3812	-0.05	-60	1035	1040	EKWNQK					Mascot
904.4709	904.5082	0.0373	41	534	540	HLFLSCK			Carbamidomethyl (C)[6]		Mascot
944.5047	944.4857	-0.019	-20	249	257	SIVEASPNK					Mascot
1360.6777	1360.7731	0.0954	70	703	713	LELCQVENVEK			Carbamidomethyl (C)[4]		Mascot
1360.6777	1360.7731	0.0954	70	703	713	LELCQVENVEK			Carbamidomethyl (C)[4]		Mascot
1376.7057	1376.7614	0.0557	40	767	778	WTEVGDSKVLDK					Mascot
1678.8945	1678.9332	0.0387	23	1256	1272	SPIMPQPLAAATAPAAR			Oxidation (M)[4]		Mascot
1730.8345	1730.9679	0.1334	77	163	177	HTDYVSIDPQEIASR					Mascot
1731.8776	1731.9838	0.1062	61	815	829	LQVLFSCGTSFTFPK			Carbamidomethyl (C)[7]		Mascot
1731.8776	1731.9838	0.1062	61	815	829	LQVLFSCGTSFTFPK			Carbamidomethyl (C)[7]		Mascot
1805.8925	1806.0344	0.1419	79	1134	1149	NCPSLVEMFNVPASLK			Carbamidomethyl (C)[2]		Mascot
1821.0309	1821.0348	0.0039	2	445	459	LIQLWIANGFILEYK					Mascot
1821.8875	1821.9874	0.0999	55	1134	1149	NCPSLVEMFNVPASLK			Carbamidomethyl (C)[2], Oxidation (M)[8]		Mascot
1867.0786	1866.9907	-0.0879	-47	353	368	KEKPIELVEVVDEIVK					Mascot
1890.996	1891.1592	0.1632	86	278	292	YLLVLDDVWDNKELR					Mascot
1906.0327	1906.1533	0.1206	63	1254	1272	SRSPIMPQPLAAATAPAA R					Mascot
1906.0327	1906.1533	0.1206	63	1254	1272	SRSPIMPQPLAAATAPAA R					Mascot
1922.0277	1922.13	0.1023	53	1254	1272	SRSPIMPQPLAAATAPAA R			Oxidation (M)[6]		Mascot
1933.9874	1934.167	0.1796	93	1134	1150	NCPSLVEMFNVPASLKK			Carbamidomethyl (C)[2]		Mascot
1949.9824	1950.1167	0.1343	69	1134	1150	NCPSLVEMFNVPASLKK			Carbamidomethyl (C)[2], Oxidation (M)[8]		Mascot
1958.0317	1957.9847	-0.047	-24	588	603	GTESFLLKPMYLHHLR			Oxidation (M)[10]		Mascot
1960.0974	1959.9902	-0.1072	-55	871	889	LIALPEAPLLGEPSRGGN R					Mascot
1994.0483	1994.0939	0.0456	23	1	18	MAERVVTMAIGPLVSMLK			Oxidation (M)[1,8,16]		Mascot
2112.1006	2112.0408	-0.0598	-28	46	64	LPAILDVITDVEEQAMAQ					Mascot

2311.1604	2311.1897	0.0293	13	469	487	R<br>HIFDELVSRSFFLDLEESK	Mascot

Gel Idx/Pos	115/E14	Instr./Gel Origin	BA2151/Sample Project 20140814	Process Status	Analysis Succeeded
Plate [#] Name	[1] Sample Project 20140814	Instrument Sample Name		Spectra	11

Rank	Protein Name	Accession No.	Protein MW	Protein PI	Pep. Count	Protein Score	Protein Score C. I. %	Intensity Matched	Total Ion Score	Total Ion C. I. %	Confirmed
1	alpha amylase inhibitor CM3 [Triticum durum]	gi|39578552	18893.3	7.44	8	503	100	57.66	450	100	

Protein Group

CM3 protein [Triticum durum]	gi|21922	18893.3	7.4400 000572 2046
RecName: Full=Alpha-amylase/trypsin inhibitor CM3; AltName: Full=Chloroform/methanol-soluble protein CM3; Flags: Precursor	gi|123957	18893.3	7.4400 000572 2046
alpha amylase inhibitor protein [Triticum aestivum]	gi|38098487	18893.3	7.4400 000572 2046
alpha-amylase inhibitor, tetrameric, chain CM3 precursor - durum wheat	gi|100834	18893.3	7.4400 000572 2046
unnamed protein product [Triticum aestivum]	gi|21713	18893.3	7.4400 000572 2046
unnamed protein product [Triticum durum]	gi|57997836	18893.3	7.4400 000572 2046

Peptide Information

Calc. Mass	Obsrv. Mass	± da	± ppm	Start Seq.	End Seq.	Sequence	Ion Score	C. I. %	Modification	Rank	Result Type
1010.52	1010.6013	0.0813	80	37	44	TNLLPHCR			Carbamidomethyl (C)[7]		Mascot
1010.52	1010.6013	0.0813	80	37	44	TNLLPHCR	42	99.641	Carbamidomethyl (C)[7]		Mascot
1110.5038	1110.5927	0.0889	80	133	140	EMQWDFVR					Mascot
1126.4987	1126.579	0.0803	71	133	140	EMQWDFVR			Oxidation (M)[2]		Mascot
1126.4987	1126.579	0.0803	71	133	140	EMQWDFVR	18	2.769	Oxidation (M)[2]		Mascot
1698.9214	1699.0631	0.1417	83	101	115	YFIALPVPSQPVDPR					Mascot
1698.9214	1699.0631	0.1417	83	101	115	YFIALPVPSQPVDPR	90	100			Mascot
1727.8381	1727.9855	0.1474	85	116	132	SGNVGESGLIDLPGCPR			Carbamidomethyl (C)[15]		Mascot
1727.8381	1727.9855	0.1474	85	116	132	SGNVGESGLIDLPGCPR	97	100	Carbamidomethyl (C)[15]		Mascot
1801.8427	1801.9651	0.1224	68	45	60	DYVLQQTCGTFTPGSK			Carbamidomethyl (C)[8]		Mascot
1876.0222	1876.1882	0.166	88	141	157	LLVAPGQCNLATIHNVR			Carbamidomethyl (C)[8]		Mascot
1876.0222	1876.1882	0.166	88	141	157	LLVAPGQCNLATIHNVR	108	100	Carbamidomethyl (C)[8]		Mascot
1957.8564	1958.0225	0.1661	85	81	95	LYCCQELAEISQQCR			Carbamidomethyl (C)[3,4,14]		Mascot
1957.8564	1958.0225	0.1661	85	81	95	LYCCQELAEISQQCR	96	100	Carbamidomethyl (C)[3,4,14]		Mascot

2255.1416	2255.3093	0.1677	74	61	80	LPEWMTSASIYSPGKPYL AK			Oxidation (M)[5]		Mascot

2	Disease resistance protein RPM1 [Triticum urartu]	gi|474086298	129568	6.87	20	46	0	18.416		

Peptide Information

Calc. Mass	Obsrv. Mass	± da	± ppm	Start Seq.	End Seq.	Sequence	Ion Score	C. I. %	Modification	Rank	Result Type
1091.6824	1091.6376	-0.0448	-41	641	650	YLGLVKTAVK					Mascot
1098.579	1098.5846	0.0056	5	87	96	VEGNEPTKPK					Mascot
1142.6317	1142.5779	-0.0538	-47	895	904	KDAASLVPWR					Mascot
1146.6882	1146.6616	-0.0266	-23	193	204	IVSIVGFGGLGK					Mascot
1150.6102	1150.5441	-0.0661	-57	651	660	IPEEIGHDLK					Mascot
1158.5175	1158.5685	0.051	44	519	528	WIDPEDNGGR					Mascot
1158.5175	1158.5685	0.051	44	519	528	WIDPEDNGGR					Mascot
1198.6619	1198.5872	-0.0747	-62	278	286	IYHFLKYSK					Mascot
1670.8094	1670.9711	0.1617	97	732	746	VLNIFFGEMEESAGK					Mascot
1710.8619	1710.974	0.1121	66	703	716	LTCLEELQLYSVDK			Carbamidomethyl (C)[3]		Mascot
1722.9901	1723.0195	0.0294	17	802	816	VPSWINQLTVPLLSR					Mascot
1775.8997	1775.9758	0.0761	43	251	265	ATETMAEWQLINQLK					Mascot
1785.0116	1785.1614	0.1498	84	236	250	VLRDILLELDTELSR					Mascot
1785.0116	1785.1614	0.1498	84	236	250	VLRDILLELDTELSR					Mascot
1802.0463	1801.9651	-0.0812	-45	835	849	LPSLLFLLLWSKDEK					Mascot
1895.1212	1895.0681	-0.0531	-28	162	178	KLSDLVGIDKPINELIK					Mascot
1898.1144	1898.1403	0.0259	14	403	421	KCGGVPLAITTIASLLVGK			Carbamidomethyl (C)[2]		Mascot
1900.9084	1900.9973	0.0889	47	1104	1119	GFWLGPAENKMYLCAK			Carbamidomethyl (C)[14], Oxidation (M)[11]		Mascot
1915.9617	1915.9886	0.0269	14	669	686	GGMISELPPSVGELMNL R			Oxidation (M)[3]		Mascot
1939.9946	1940.0775	0.0829	43	730	746	LRVLNIFFGEMEESAGK					Mascot
1955.9895	1956.0698	0.0803	41	730	746	LRVLNIFFGEMEESAGK			Oxidation (M)[11]		Mascot
1958.093	1958.0225	-0.0705	-36	937	954	QASGSRPNAVYLNLRIAK					Mascot
1958.093	1958.0225	-0.0705	-36	937	954	QASGSRPNAVYLNLRIAK					Mascot
1986.059	1986.0487	-0.0103	-5	341	357	LAFMDSHPQSRLIITTR					Mascot

3	Jasmonate O-methyltransferase [Triticum urartu]	gi|474419077	28967.7	5.53	7	45	0	6.014	18	2.769

Peptide Information

Calc. Mass	Obsrv. Mass	± da	± ppm	Start Seq.	End Seq.	Sequence	Ion Score	C. I. %	Modification	Rank	Result Type
1045.5935	1045.6263	0.0328	31	110	118	MVVSLQGRR					Mascot
1061.5885	1061.62	0.0315	30	110	118	MVVSLQGRR			Oxidation (M)[1]		Mascot

Calc. Mass	Obsrv. Mass	± da	± ppm	Start Seq.	End Seq.	Sequence	Ion Score	C. I. %	Modification	Rank	Result Type
1076.6384	1076.5725	-0.0659	-61	250	259	VMLVVSLTKA			Oxidation (M)[2]		Mascot
1126.531	1126.579	0.048	43	183	192	VHDPISGMDR					Mascot
1126.531	1126.579	0.048	43	183	192	VHDPISGMDR	18	2.769			Mascot
1142.526	1142.5779	0.0519	45	183	192	VHDPISGMDR			Oxidation (M)[8]		Mascot
1152.6412	1152.5813	-0.0599	-52	92	100	DFTLFLQLR					Mascot
1704.9181	1705.0746	0.1565	92	76	90	ERLPVVAGAYAQQFR					Mascot
1891.9807	1892.1689	0.1882	99	183	199	VHDPISGMDRALLTPNR					Mascot
1939.9761	1940.0775	0.1014	52	151	167	LDSLYIPVHGPSDEELR					Mascot

4 Polyubiquitin [Triticum urartu] gi|474390564 56966.4 6.78 13 42 0 10.413

Peptide Information

Calc. Mass	Obsrv. Mass	± da	± ppm	Start Seq.	End Seq.	Sequence	Ion Score	C. I. %	Modification	Rank	Result Type
919.5281	919.5851	0.057	62	178	185	TQVLSMLK					Mascot
1067.6685	1067.5972	-0.0713	-67	6	14	IKLAVDRPR					Mascot
1076.5219	1076.5725	0.0506	47	291	300	SVTDLDDVGR					Mascot
1079.5632	1079.6042	0.041	38	237	244	VFYHTREK					Mascot
1091.6056	1091.6376	0.032	29	438	447	SDTIATIKSR					Mascot
1136.6021	1136.6466	0.0445	39	416	424	LEEMQIFVK					Mascot
1136.6021	1136.6466	0.0445	39	416	424	LEEMQIFVK					Mascot
1152.5969	1152.5813	-0.0156	-14	416	424	LEEMQIFVK			Oxidation (M)[4]		Mascot
1154.6681	1154.5759	-0.0922	-80	462	470	LLYASRYLR					Mascot
1176.583	1176.6691	0.0861	73	450	459	DKVSMPTGWR					Mascot
1176.583	1176.6691	0.0861	73	450	459	DKVSMPTGWR					Mascot
1198.615	1198.5872	-0.0278	-23	452	461	VSMPTGWRHK					Mascot
1714.8899	1715.0414	0.1515	88	186	201	AFLSSDTVLTDAFLSK					Mascot
1736.943	1736.9894	0.0464	27	400	415	ALLTSETALTDVFISR					Mascot
1745.0167	1744.963	-0.0537	-31	430	445	TITVQVVRSDTIATIK					Mascot
1751.9189	1751.9327	0.0138	8	416	429	LEEMQIFVKFPWGK					Mascot

5 hypothetical protein TRIUR3_23148 [Triticum urartu] gi|474256756 15074.7 12 7 41 0 3.962

Peptide Information

Calc. Mass	Obsrv. Mass	± da	± ppm	Start Seq.	End Seq.	Sequence	Ion Score	C. I. %	Modification	Rank	Result Type
1085.6075	1085.6619	0.0544	50	118	127	GGARPHVVHR					Mascot
1140.5579	1140.6074	0.0495	43	17	28	APPCAGGQIGGR			Carbamidomethyl (C)[4]		Mascot
1154.6892	1154.5759	-0.1133	-98	43	53	LLALEAKSGPR					Mascot

Calc. Mass	Obsrv. Mass	± da	± ppm	Start Seq.	End Seq.	Sequence	Ion Score	C. I. %	Modification	Rank	Result Type
1158.5862	1158.5685	-0.0177	-15	84	96	GGDGGGQIVATAR					Mascot
1158.5862	1158.5685	-0.0177	-15	84	96	GGDGGGQIVATAR					Mascot
1709.9069	1709.9904	0.0835	49	118	132	GGARPHVVHRGHHHR					Mascot
1714.8806	1715.0414	0.1608	94	54	68	HGIRQPTLGIYSCGR			Carbamidomethyl (C)[13]		Mascot
1956.0662	1956.0698	0.0036	2	31	49	HPSPDPAANVGRLLALEAK					Mascot

6	leaf rust resistance protein Lr10 [Triticum dicoccoides]	gi|305691198	56237.3	5.77	6	37	0	4.501	23	74.13	

Protein Group

leaf rust resistance protein Lr10 [Triticum dicoccoides]	gi|305691200	56237.3	5.7699999809 2651

Peptide Information

Calc. Mass	Obsrv. Mass	± da	± ppm	Start Seq.	End Seq.	Sequence	Ion Score	C. I. %	Modification	Rank	Result Type
1010.5377	1010.6013	0.0636	63	120	128	SQLLEAHGR					Mascot
1010.5377	1010.6013	0.0636	63	120	128	SQLLEAHGR	23	74.13			Mascot
1106.6357	1106.62	-0.0157	-14	167	174	YELVKWLR					Mascot
1744.8654	1744.963	0.0976	56	199	211	QVYDELRINFEYR					Mascot
1751.936	1751.9327	-0.0033	-2	212	227	AFVSISRSPDMATILK			Oxidation (M)[11]		Mascot
1898.0416	1898.1403	0.0987	52	352	370	CGGLPLAINAISSLLAAEK			Carbamidomethyl (C)[1]		Mascot
1915.9623	1915.9886	0.0263	14	398	414	FSFSQLTFEVGAMPNLK					Mascot

7	Armadillo repeat-containing kinesin-like protein 2 [Triticum urartu]	gi|474450999	94481.4	6.27	11	36	0	4.918	18	2.769	

Peptide Information

Calc. Mass	Obsrv. Mass	± da	± ppm	Start Seq.	End Seq.	Sequence	Ion Score	C. I. %	Modification	Rank	Result Type
1077.4882	1077.5859	0.0977	91	571	581	MSLDTGDGPGK					Mascot
1091.5765	1091.6376	0.0611	56	446	454	ISLENEKMK					Mascot
1092.5281	1092.61	0.0819	75	637	645	SSEDETIRR					Mascot
1093.6365	1093.6249	-0.0116	-11	27	35	TFKSQTILR					Mascot
1110.634	1110.5927	-0.0413	-37	7	15	VTLKTVYMR					Mascot
1126.6289	1126.579	-0.0499	-44	7	15	VTLKTVYMR			Oxidation (M)[8]		Mascot
1126.6289	1126.579	-0.0499	-44	7	15	VTLKTVYMR	18	2.769	Oxidation (M)[8]		Mascot
1146.6589	1146.6616	0.0027	2	30	39	SQTILRVSSR					Mascot
1148.6344	1148.6239	-0.0105	-9	1	10	MQGLDKVTLK			Oxidation (M)[1]		Mascot
1164.6372	1164.6622	0.025	21	345	355	LLKDSFGGTAR					Mascot
1182.5936	1182.5334	-0.0602	-51	816	824	EDIRMLAYR			Oxidation (M)[5]		Mascot

1670.896	1670.9711	0.0751	45	404	417	LDIELDKLIAENER					Mascot
1870.9869	1871.0168	0.0299	16	197	214	TGDVSLPGATVVEVRDQK					Mascot

8	TPR repeat-containing thioredoxin TTL1 [Triticum urartu]	gi|473951385	58817	8.74	12	36	0	16.527

Peptide Information

Calc. Mass	Obsrv. Mass	± da	± ppm	Start Seq.	End Seq.	Sequence	Ion Score	C. I. %	Modification	Rank	Result Type
1045.579	1045.6263	0.0473	45	158	166	IGDWKSALR					Mascot
1066.6367	1066.5836	-0.0531	-50	187	195	SEALLRLHK					Mascot
1108.6765	1108.5718	-0.1047	-94	491	499	IVPTFKIYK					Mascot
1158.6001	1158.5685	-0.0316	-27	475	485	VNVEDSPTVAK					Mascot
1158.6001	1158.5685	-0.0316	-27	475	485	VNVEDSPTVAK					Mascot
1176.6372	1176.6691	0.0319	27	295	305	AQGNELFKAAK					Mascot
1176.6372	1176.6691	0.0319	27	295	305	AQGNELFKAAK					Mascot
1190.5735	1190.6523	0.0788	66	371	379	LDRWADCVR			Carbamidomethyl (C)[7]		Mascot
1727.8494	1727.9855	0.1361	79	142	156	LQDVEMHLGRSTDAR					Mascot
1727.8923	1727.9855	0.0932	54	475	490	VNVEDSPTVAKAENVR					Mascot
1743.8442	1743.9559	0.1117	64	142	156	LQDVEMHLGRSTDAR			Oxidation (M)[6]		Mascot
1775.9637	1775.9758	0.0121	7	196	211	LEEADSTLTSLLKLDK					Mascot
1873.9775	1874.1575	0.18	96	506	520	EMICPTLHVLRYSVR			Carbamidomethyl (C)[4]		Mascot
1940.0535	1940.0775	0.024	12	244	259	LQQKQIIAEVGIHFCR			Carbamidomethyl (C)[15]		Mascot
1955.9491	1956.0698	0.1207	62	20	39	SPASSPVAPPSGGSLQEMTR					Mascot
1971.9441	1972.0355	0.0914	46	20	39	SPASSPVAPPSGGSLQEMTR			Oxidation (M)[18]		Mascot

9	hypothetical protein TRIUR3_24120 [Triticum urartu]	gi|473987456	25283	9.33	8	36	0	10.033

Peptide Information

Calc. Mass	Obsrv. Mass	± da	± ppm	Start Seq.	End Seq.	Sequence	Ion Score	C. I. %	Modification	Rank	Result Type
992.4942	992.5699	0.0757	76	152	160	GKVSNATCR			Carbamidomethyl (C)[8]		Mascot
1106.5663	1106.62	0.0537	49	143	151	MLNDVYPVR					Mascot
1130.6416	1130.5756	-0.066	-58	133	142	LQLAKDSLDK					Mascot
1136.543	1136.6466	0.1036	91	122	132	VSSESGEKEGK					Mascot
1136.543	1136.6466	0.1036	91	122	132	VSSESGEKEGK					Mascot
1150.5334	1150.5441	0.0107	9	84	95	DQASAANSGTTK					Mascot
1190.6562	1190.6523	-0.0039	-3	25	34	SKDSLLVCLR			Carbamidomethyl (C)[8]		Mascot

1736.8297	1736.9894	0.1597	92	79	95	EDTLKDQASAANSGTTK				Mascot
1785.0421	1785.1614	0.1193	67	65	78	LLTLLLQKFQPEWR				Mascot
1785.0421	1785.1614	0.1193	67	65	78	LLTLLLQKFQPEWR				Mascot

10	truncated leaf rust resistance protein Lr10 [Triticum dicoccoides]	gi|305691204	47241.9	5.86	5	35	0	4.425	23	74.13	

Protein Group

truncated leaf rust resistance protein Lr10 [Triticum dicoccoides]	gi|305691202	47241.9	5.8600 001335 144
truncated leaf rust resistance protein Lr10 [Triticum dicoccoides]	gi|305691206	47241.9	5.8600 001335 144

Peptide Information

Calc. Mass	Obsrv. Mass	± da	± ppm	Start Seq.	End Seq.	Sequence	Ion Score	C. I. %	Modification	Rank	Result Type
1010.5377	1010.6013	0.0636	63	120	128	SQLLEAHGR					Mascot
1010.5377	1010.6013	0.0636	63	120	128	SQLLEAHGR	23	74.13			Mascot
1106.6357	1106.62	-0.0157	-14	167	174	YELVKWLR					Mascot
1744.8654	1744.963	0.0976	56	199	211	QVYDELRINFEYR					Mascot
1751.936	1751.9327	-0.0033	-2	212	227	AFVSISRSPDMATILK			Oxidation (M)[11]		Mascot
1898.0416	1898.1403	0.0987	52	352	370	CGGLPLAINAISSLLAAEK			Carbamidomethyl (C)[1]		Mascot

Gel Idx/Pos	116/E15	Instr./Gel Origin	BA2151/Sample Project 20140814	Process Status	Analysis Succeeded
Plate [#] Name	[1] Sample Project 20140814	Instrument Sample Name		Spectra	11

Rank	Protein Name	Accession No.	Protein MW	Protein PI	Pep. Count	Protein Score	Protein Score C. I. %	Intensity Matched	Total Ion Score	Total Ion C. I. %	Confirmed
1	Nucleoside diphosphate kinase 1 [Triticum urartu]	gi|474369382	16578.6	6.3	10	516	100	50.406	440	100	

Peptide Information

Calc. Mass	Obsrv. Mass	± da	± ppm	Start Seq.	End Seq.	Sequence	Ion Score	C. I. %	Modification	Rank	Result Type
943.5571	943.6226	0.0655	69	17	25	GLIGEVISR					Mascot
943.5571	943.6226	0.0655	69	17	25	GLIGEVISR	64	99.998			Mascot
949.4738	949.5352	0.0614	65	104	112	GDFAVDIGR					Mascot
949.4738	949.5352	0.0614	65	104	112	GDFAVDIGR	47	99.901			Mascot
1234.5487	1234.5994	0.0507	41	141	150	SSQHNWIYEA					Mascot
1370.6659	1370.7637	0.0978	71	113	125	NVIHGSDSVESAR					Mascot
1370.6659	1370.7637	0.0978	71	113	125	NVIHGSDSVESAR	106	100			Mascot
1498.7609	1498.7765	0.0156	10	113	126	NVIHGSDSVESARK					Mascot
1609.8909	1610.0118	0.1209	75	88	103	IIGATNPLASEPGTIR					Mascot
1609.8909	1610.0118	0.1209	75	88	103	IIGATNPLASEPGTIR	102	100			Mascot
1716.8744	1717.0065	0.1321	77	127	140	EIALWFPEGIAEWR					Mascot
1716.8744	1717.0065	0.1321	77	127	140	EIALWFPEGIAEWR	91	100			Mascot
1732.9052	1733.0016	0.0964	56	2	16	AEQTFIMIKPDGVQR					Mascot
1737.9858	1738.1172	0.1314	76	87	103	KIIGATNPLASEPGTIR					Mascot
1737.9858	1738.1172	0.1314	76	87	103	KIIGATNPLASEPGTIR	30	94.755			Mascot
1748.9	1748.9937	0.0937	54	2	16	AEQTFIMIKPDGVQR			Oxidation (M)[7]		Mascot
1844.9694	1845.1071	0.1377	75	126	140	KEIALWFPEGIAEWR					Mascot

Rank	Protein Name	Accession No.	Protein MW	Protein PI	Pep. Count	Protein Score	Protein Score C. I. %	Intensity Matched	Total Ion Score	Total Ion C. I. %	Confirmed
2	WD repeat-containing protein 6 [Triticum urartu]	gi|473998603	166161.1	5.8	6	42	0	10.647	42	99.66	

Peptide Information

Calc. Mass	Obsrv. Mass	± da	± ppm	Start Seq.	End Seq.	Sequence	Ion Score	C. I. %	Modification	Rank	Result Type
943.5458	943.6226	0.0768	81	109	117	VVIDAALDK					Mascot
943.5458	943.6226	0.0768	81	94	102	VVIDAALDK	43	99.755			Mascot
1304.6189	1304.5782	-0.0407	-31	15	25	NEEASDQRSIR					Mascot
1579.8492	1579.9607	0.1115	71	914	926	LLPQVFHTQFHGR					Mascot
1631.8687	1631.9823	0.1136	70	1397	1412	ILGQHMVPSAHGSAVK					Mascot

1647.8635	1647.944	0.0805	49	1397	1412	ILGQHMVPSAHGSAVK			Oxidation (M)[6]		Mascot
1780.8171	1780.9832	0.1661	93	945	960	STDLWIATGCEDGTVR			Carbamidomethyl (C)[10]		Mascot
1842.928	1843.1001	0.1721	93	972	988	WCSSKLLGEHVGGSAVR			Carbamidomethyl (C)[2]		Mascot

3	L-type lectin-domain containing receptor kinase IX.1 [Triticum urartu]	gi|474340702	70189.9	6.56	4	41	0	12.518	34	97.668		

Peptide Information

Calc. Mass	Obsrv. Mass	± da	± ppm	Start Seq.	End Seq.	Sequence	Ion Score	C. I. %	Modification	Rank	Result Type
943.5683	943.6226	0.0543	58	413	420	AEVRIISR					Mascot
943.5683	943.6226	0.0543	58	413	420	AEVRIISR	34	97.668			Mascot
1748.968	1748.9937	0.0257	15	438	453	GLLLVYELVAESSLDK					Mascot
1861.9344	1862.0841	0.1497	80	25	39	RAFSLFFDLNFSNQR					Mascot
1877.063	1877.1149	0.0519	28	437	453	KGLLLVYELVAESSLDK					Mascot

4	NADH-dependent glutamate synthase [Triticum turgidum]	gi|530452549	237135.1	6.26	16	39	0	28.614	25	82.023		

Peptide Information

Calc. Mass	Obsrv. Mass	± da	± ppm	Start Seq.	End Seq.	Sequence	Ion Score	C. I. %	Modification	Rank	Result Type
943.6047	943.6226	0.0179	19	247	254	RLSIVSIR					Mascot
943.6047	943.6226	0.0179	19	247	254	RLSIVSIR	26	87.036			Mascot
948.54	948.5378	-0.0022	-2	690	697	VLDITYPK					Mascot
965.505	965.5719	0.0669	69	717	724	EAIREGYK					Mascot
1234.5699	1234.5994	0.0295	24	2001	2011	VDYGHQEASTK					Mascot
1256.6534	1256.6239	-0.0295	-23	517	526	AHPYGEWLKR					Mascot
1571.8652	1571.9036	0.0384	24	2117	2130	RGQSLVVWAITEGR					Mascot
1708.8687	1708.913	0.0443	26	869	883	IEGATFEMLARDALR			Oxidation (M)[8]		Mascot
1716.9042	1717.0065	0.1023	60	452	466	NGLRPGRFYVTHSGR					Mascot
1716.9042	1717.0065	0.1023	60	452	466	NGLRPGRFYVTHSGR					Mascot
1726.8945	1727.031	0.1365	79	621	635	QMFAQVTNPPIDPIR					Mascot
1726.8945	1727.031	0.1365	79	621	635	QMFAQVTNPPIDPIR					Mascot
1742.8894	1742.9521	0.0627	36	621	635	QMFAQVTNPPIDPIR			Oxidation (M)[2]		Mascot
1754.8638	1754.9991	0.1353	77	1838	1853	IGGLMMYGVPNMKTDK					Mascot
1764.928	1764.9728	0.0448	25	232	246	SPNSKADFEQLFILR					Mascot
1794.8943	1795.0537	0.1594	89	529	543	MYLKDIVESVPETDR					Mascot
1810.8892	1811.0403	0.1511	83	529	543	MYLKDIVESVPETDR			Oxidation (M)[1]		Mascot
1818.879	1819.0564	0.1774	98	1299	1314	SDMLEVDPEVVKSNEK					Mascot

1860.981	1861.1113	0.1303	70	663	679	LALKGPLVSMDEMESIK					Mascot
1862.0066	1862.0841	0.0775	42	1	19	MPTAQGIGLKHAAPTGVGR					Mascot
1876.9758	1877.1149	0.1391	74	663	679	LALKGPLVSMDEMESIK			Oxidation (M)[10]		Mascot
1878.0015	1878.0645	0.063	34	1	19	MPTAQGIGLKHAAPTGVGR			Oxidation (M)[1]		Mascot
1878.0015	1878.0645	0.063	34	1	19	MPTAQGIGLKHAAPTGVGR			Oxidation (M)[1]		Mascot
1894.0757	1894.0741	-0.0016	-1	1805	1824	KIAIVGSGPAGLAAADQLNK					Mascot

5	NADH-dependent glutamate synthase [Triticum turgidum]	gi|530452547	237084.1	6.21	18	39	0	26.931	25	82.023	

Peptide Information

Calc. Mass	Obsrv. Mass	± da	± ppm	Start Seq.	End Seq.	Sequence	Ion Score	C. I. %	Modification	Rank	Result Type
943.6047	943.6226	0.0179	19	247	254	RLSIVSIR					Mascot
943.6047	943.6226	0.0179	19	247	254	RLSIVSIR	26	87.036			Mascot
948.54	948.5378	-0.0022	-2	690	697	VLDITYPK					Mascot
1234.5699	1234.5994	0.0295	24	2001	2011	VDYGHQEASTK					Mascot
1256.6534	1256.6239	-0.0295	-23	517	526	AHPYGEWLKR					Mascot
1571.8652	1571.9036	0.0384	24	2117	2130	RGQSLVVWAITEGR					Mascot
1698.9398	1699.0251	0.0853	50	2	19	PTAQGIGLKHAAPPGAGR					Mascot
1708.8687	1708.913	0.0443	26	869	883	IEGATFEMLARDALR			Oxidation (M)[8]		Mascot
1716.9042	1717.0065	0.1023	60	452	466	NGLRPGRFYVTHSGR					Mascot
1716.9042	1717.0065	0.1023	60	452	466	NGLRPGRFYVTHSGR					Mascot
1726.8945	1727.031	0.1365	79	621	635	QMFAQVTNPPIDPIR					Mascot
1726.8945	1727.031	0.1365	79	621	635	QMFAQVTNPPIDPIR					Mascot
1742.8894	1742.9521	0.0627	36	621	635	QMFAQVTNPPIDPIR			Oxidation (M)[2]		Mascot
1754.8638	1754.9991	0.1353	77	1838	1853	IGGLMMYGVPNMKTDK					Mascot
1755.8715	1756.0209	0.1494	85	1987	2000	ASDNPWPQWPRVFR					Mascot
1788.8446	1788.9902	0.1456	81	36	53	QAHGAMSLDGGFLGGAQR			Oxidation (M)[6]		Mascot
1794.8943	1795.0537	0.1594	89	529	543	MYLKDIVESVPETDR					Mascot
1810.8892	1811.0403	0.1511	83	529	543	MYLKDIVESVPETDR			Oxidation (M)[1]		Mascot
1818.879	1819.0564	0.1774	98	1299	1314	SDMLEVDPEVVKSNEK					Mascot
1829.9803	1830.0583	0.078	43	1	19	MPTAQGIGLKHAAPPGAGR					Mascot
1860.981	1861.1113	0.1303	70	663	679	LALKGPLVSMDEMESIK					Mascot
1876.9758	1877.1149	0.1391	74	663	679	LALKGPLVSMDEMESIK			Oxidation (M)[10]		Mascot
1878.0015	1878.0645	0.063	34	323	339	AQPMRVLGHNGEINTLK					Mascot

1878.0015	1878.0645	0.063	34	323	339	AQPMRVLGHNGEINTLK					Mascot
1894.0757	1894.0741	-0.0016	-1	1805	1824	KIAIVGSGPAGLAAADQLNK					Mascot

6 Cytochrome P450 71D8 [Triticum urartu] gi|473860769 31803.3 6.2 9 36 0 4.255

Peptide Information

Calc. Mass	Obsrv. Mass	± da	± ppm	Start Seq.	End Seq.	Sequence	Ion Score	C. I. %	Modification	Rank	Result Type
971.6248	971.6266	0.0018	2	127	134	LVIKETLR					Mascot
1248.6406	1248.6604	0.0198	16	261	270	NDLYLCPVVR			Carbamidomethyl (C)[6]		Mascot
1288.6069	1288.6005	-0.0064	-5	174	183	DPKHWDHPEK					Mascot
1716.9941	1717.0065	0.0124	7	135	148	LHTVLPLLLPRECR			Carbamidomethyl (C)[13]		Mascot
1716.9941	1717.0065	0.0124	7	135	148	LHTVLPLLLPRECR			Carbamidomethyl (C)[13]		Mascot
1761.9283	1762.0172	0.0889	50	161	176	GTTVFVNAWAISRDPK					Mascot
1771.0953	1771.1464	0.0511	29	131	145	ETLRLHTVLPLLLPR					Mascot
1797.0117	1797.0659	0.0542	30	49	65	IQKEGGLEVPLTTGNIK					Mascot
1827.9698	1828.0493	0.0795	43	37	51	EEEEDLVDVLLRIQK					Mascot
1875.9658	1876.1487	0.1829	97	106	123	NSLQGKSTVTEDDLAGLK					Mascot

7 Formin-like protein 5 [Triticum urartu] gi|474139389 98831.3 6.36 13 34 0 11.177

Peptide Information

Calc. Mass	Obsrv. Mass	± da	± ppm	Start Seq.	End Seq.	Sequence	Ion Score	C. I. %	Modification	Rank	Result Type
912.5149	912.5869	0.072	79	704	711	LVIHSSEK					Mascot
948.5182	948.5378	0.0196	21	213	221	DKGSLIGMK					Mascot
949.5101	949.5352	0.0251	26	175	183	ATLDAVFGR					Mascot
949.5101	949.5352	0.0251	26	134	142	ATLDAVFGR	6	0			Mascot
955.4996	955.5574	0.0578	60	275	281	QQFKNYK					Mascot
1156.6797	1156.7271	0.0474	41	616	626	LKQGSAAQVVR					Mascot
1172.6998	1172.7006	0.0008	1	757	767	IRTTTSLPGVK					Mascot
1304.6594	1304.5782	-0.0812	-62	672	684	QASSSFAPSPLGR					Mascot
1394.7386	1394.7018	-0.0368	-26	720	731	QESPVVETPPRR					Mascot
1716.9028	1717.0065	0.1037	60	789	806	HSVTPPSSPGGSPLAGLR					Mascot
1716.9028	1717.0065	0.1037	60	789	806	HSVTPPSSPGGSPLAGLR					Mascot
1755.9025	1756.0209	0.1184	67	873	888	SSSPSSPRLTTQPWPK					Mascot
1771.8895	1771.9921	0.1026	58	738	756	SMSSPAISATPAVPSSPGK					Mascot
1796.0906	1796.0905	-0.0001	0	2	17	STHLPPHLSLLALLRK					Mascot

1908.9662	1909.0861	0.1199	63	256	272	ERSTDASLTEVLFSTPR					Mascot

8	Ent-copalyl diphosphate synthase 1, chloroplastic [Triticum urartu]	gi|474093673	92011.8	5.61	7	34	0	14.257	25	81.219

Peptide Information

Calc. Mass	Obsrv. Mass	± da	± ppm	Start Seq.	End Seq.	Sequence	Ion Score	C. I. %	Modification	Rank	Result Type
931.5029	931.5254	0.0225	24	691	698	IIEICAGR			Carbamidomethyl (C)[5]		Mascot
943.5683	943.6226	0.0543	58	321	328	LERLGISR					Mascot
943.5683	943.6226	0.0543	58	321	328	LERLGISR	25	81.219			Mascot
955.5247	955.5574	0.0327	34	329	335	YFKQEIK					Mascot
1579.869	1579.9607	0.0917	58	68	81	VIPGTELEQPLIDR					Mascot
1796.908	1797.0659	0.1579	88	206	220	SLDVVFPYDHHALQR					Mascot
1807.0323	1807.0348	0.0025	1	68	83	VIPGTELEQPLIDRVK					Mascot
1814.0569	1814.0938	0.0369	20	683	698	QTSLLLVKIIEICAGR			Carbamidomethyl (C)[13]		Mascot
1814.0569	1814.0938	0.0369	20	683	698	QTSLLLVKIIEICAGR			Carbamidomethyl (C)[13]		Mascot

9	CPS protein [Triticum urartu]	gi|440577573	94626.1	5.61	8	33	0	14.881	23	73.955

Peptide Information

Calc. Mass	Obsrv. Mass	± da	± ppm	Start Seq.	End Seq.	Sequence	Ion Score	C. I. %	Modification	Rank	Result Type
943.5683	943.6226	0.0543	58	344	351	LERLGISR	25	81.219			Mascot
943.6047	943.6226	0.0179	19	797	804	RTLLSVVR					Mascot
955.5247	955.5574	0.0327	34	352	358	YFKQEIK					Mascot
971.5455	971.6266	0.0811	83	24	32	ALAVKGPCR			Carbamidomethyl (C)[8]		Mascot
1248.5646	1248.6604	0.0958	77	254	262	RIPMEMMHR			Oxidation (M)[4,6,7]		Mascot
1790.8755	1791.0219	0.1464	82	538	551	CQVQHQLEWHGLQK			Carbamidomethyl (C)[1]		Mascot
1790.8755	1791.0219	0.1464	82	538	551	CQVQHQLEWHGLQK			Carbamidomethyl (C)[1]		Mascot
1860.0776	1860.1283	0.0507	27	706	721	QTFLLLVKIIEICGGR			Carbamidomethyl (C)[13]		Mascot
1893.9634	1894.0741	0.1107	58	396	411	LYGYTVSPSVFEKFEK					Mascot

10	Rop guanine nucleotide exchange factor 1 [Triticum urartu]	gi|474207683	65442.2	6.49	8	33	0	11.672

Peptide Information

Calc. Mass	Obsrv. Mass	± da	± ppm	Start Seq.	End Seq.	Sequence	Ion Score	C. I. %	Modification	Rank	Result Type
842.5206	842.5699	0.0493	59	421	427	ALLVDRR					Mascot
943.5822	943.6226	0.0404	43	297	305	AEGLLISLK					Mascot

943.5822	943.6226	0.0404	43	297	305	AEGLLISLK	17	0		Mascot
1102.6038	1102.656	0.0522	47	86	94	LEIMTSRPR				Mascot
1248.6848	1248.6604	-0.0244	-20	252	261	VEAAVYVWRR				Mascot
1353.7009	1353.7833	0.0824	61	326	337	DVGKSILESYSR				Mascot
1498.7999	1498.7765	-0.0234	-16	572	585	GLPLLTIDDVESNL				Mascot
1708.8688	1708.913	0.0442	26	306	320	QRFPGLTQTSLDMSK				Mascot
1724.8636	1724.9258	0.0622	36	306	320	QRFPGLTQTSLDMSK			Oxidation (M)[13]	Mascot
1731.9098	1731.9874	0.0776	45	106	119	KLDHMLLETLESFR				Mascot

Gel Idx/Pos	117/E16	Instr./Gel Origin	BA2151/Sample Project 20140814	Process Status	Analysis Succeeded
Plate [#] Name	[1] Sample Project 20140814	Instrument Sample Name		Spectra	11

Rank	Protein Name	Accession No.	Protein MW	Protein PI	Pep. Count	Protein Score	Protein Score C. I. %	Intensity Matched	Total Ion Score	Total Ion C. I. %	Confirmed
1	RecName: Full=Alpha-amylase inhibitor 0.19; AltName: Full=0.19 alpha-AI; Short=0.19 AI	gi|123963	13898.6	6.66	5	376	100	38.784	345	100	

Protein Group

0.19 alpha-amylase inhibitor [Triticum aestivum]	gi|2116581	13898.6	6.6599998474 1211
0.19 dimeric alpha-amylase inhibitor [Triticum aestivum]	gi|54778509	13898.6	6.6599998474 1211
0.19 dimeric alpha-amylase inhibitor [Triticum aestivum]	gi|54778501	13898.6	6.6599998474 1211
Chain A, 0.19 Alpha-Amylase Inhibitor From Wheat	gi|3318681	13898.6	6.6599998474 1211
Chain B, 0.19 Alpha-Amylase Inhibitor From Wheat	gi|3318682	13898.6	6.6599998474 1211
Chain C, 0.19 Alpha-Amylase Inhibitor From Wheat	gi|3318683	13898.6	6.6599998474 1211
Chain D, 0.19 Alpha-Amylase Inhibitor From Wheat	gi|3318684	13898.6	6.6599998474 1211
alpha-amylase inhibitor 0.19 [Triticum aestivum]	gi|66841026	13340.4	6.8600001335 144

Peptide Information

Calc. Mass	Obsrv. Mass	± da	± ppm	Start Seq.	End Seq.	Sequence	Ion Score	C. I. %	Modification	Rank	Result Type
1162.6249	1162.7083	0.0834	72	90	100	LTAASITAVCR			Carbamidomethyl (C)[10]		Mascot
1162.6249	1162.7083	0.0834	72	90	100	LTAASITAVCR	73	100	Carbamidomethyl (C)[10]		Mascot
1570.8007	1570.9241	0.1234	79	26	39	LQCNGSQVPEAVLR			Carbamidomethyl (C)[3]		Mascot
1612.7463	1612.8733	0.127	79	67	82	EHGAQEGQAGTGAFPR					Mascot
1612.7463	1612.8733	0.127	79	67	82	EHGAQEGQAGTGAFPR	138	100			Mascot
1663.8361	1663.9354	0.0993	60	101	116	LPIVVDASGDGAYVCK			Carbamidomethyl (C)[15]		Mascot
1862.7731	1862.9274	0.1543	83	40	53	DCCQQLAHISEWCR			Carbamidomethyl (C)[2,3,13]		Mascot
1862.7731	1862.9274	0.1543	83	40	53	DCCQQLAHISEWCR	135	100	Carbamidomethyl (C)[2,3,13]		Mascot

Rank	Protein Name	Accession No.	Protein MW	Protein PI	Pep. Count	Protein Score	Protein Score C. I. %	Intensity Matched	Total Ion Score	Total Ion C. I. %	Confirmed
2	dimeric alpha-amylase inhibitor precursor, partial [Triticum aestivum]	gi|108597921	14029.7	6.69	5	375	100	38.784	345	100	

Peptide Information

Calc. Mass	Obsrv. Mass	± da	± ppm	Start Seq.	End Seq.	Sequence	Ion Score	C. I. %	Modification	Rank	Result Type
1162.6249	1162.7083	0.0834	72	91	101	LTAASITAVCR			Carbamidomethyl (C)[10]		Mascot
1162.6249	1162.7083	0.0834	72	91	101	LTAASITAVCR	73	100	Carbamidomethyl (C)[10]		Mascot
1570.8007	1570.9241	0.1234	79	27	40	LQCNGSQVPEAVLR			Carbamidomethyl (C)[3]		Mascot
1612.7463	1612.8733	0.127	79	68	83	EHGAQEGQAGTGAFPR					Mascot
1612.7463	1612.8733	0.127	79	68	83	EHGAQEGQAGTGAFPR	138	100			Mascot
1663.8361	1663.9354	0.0993	60	102	117	LPIVVDASGDGAYVCK			Carbamidomethyl (C)[15]		Mascot
1862.7731	1862.9274	0.1543	83	41	54	DCCQQLAHISEWCR			Carbamidomethyl (C)[2,3,13]		Mascot
1862.7731	1862.9274	0.1543	83	41	54	DCCQQLAHISEWCR	135	100	Carbamidomethyl (C)[2,3,13]		Mascot

3	dimeric alpha-amylase inhibitor [Triticum dicoccoides]	gi|227809009	15730.5	5.58	5	373	100	38.784	345	100	

Protein Group

dimeric alpha-amylase inhibitor [Triticum aestivum]	gi|65993781	15688.5	5.5799 999237 0605
dimeric alpha-amylase inhibitor [Triticum aestivum]	gi|386877038	15702.5	5.5799 999237 0605
dimeric alpha-amylase inhibitor [Triticum dicoccoides]	gi|227809005	15730.5	5.5799 999237 0605

Peptide Information

Calc. Mass	Obsrv. Mass	± da	± ppm	Start Seq.	End Seq.	Sequence	Ion Score	C. I. %	Modification	Rank	Result Type
1162.6249	1162.7083	0.0834	72	107	117	LTAASITAVCR			Carbamidomethyl (C)[10]		Mascot
1162.6249	1162.7083	0.0834	72	107	117	LTAASITAVCR	73	100	Carbamidomethyl (C)[10]		Mascot
1570.8007	1570.9241	0.1234	79	43	56	LQCNGSQVPEAVLR			Carbamidomethyl (C)[3]		Mascot
1612.7463	1612.8733	0.127	79	84	99	EHGAQEGQAGTGAFPR					Mascot
1612.7463	1612.8733	0.127	79	84	99	EHGAQEGQAGTGAFPR	138	100			Mascot
1663.8361	1663.9354	0.0993	60	118	133	LPIVVDASGDGAYVCK			Carbamidomethyl (C)[15]		Mascot
1862.7731	1862.9274	0.1543	83	57	70	DCCQQLAHISEWCR			Carbamidomethyl (C)[2,3,13]		Mascot
1862.7731	1862.9274	0.1543	83	57	70	DCCQQLAHISEWCR	135	100	Carbamidomethyl (C)[2,3,13]		Mascot

4	0.19 dimeric alpha-amylase inhibitor [Triticum aestivum]	gi|54778503	13826.6	7.45	4	367	100	35.344	345	100	

Peptide Information

Calc. Mass	Obsrv. Mass	± da	± ppm	Start Seq.	End Seq.	Sequence	Ion Score	C. I. %	Modification	Rank	Result Type

Calc. Mass	Obsrv. Mass	± da	± ppm	Start Seq.	End Seq.	Sequence	Ion Score	C. I. %	Modification	Rank	Result Type
1162.6249	1162.7083	0.0834	72	90	100	LTAASITAVCR			Carbamidomethyl (C)[10]		Mascot
1162.6249	1162.7083	0.0834	72	90	100	LTAASITAVCR	73	100	Carbamidomethyl (C)[10]		Mascot
1612.7463	1612.8733	0.127	79	67	82	EHGAQEGQAGTGAFPR					Mascot
1612.7463	1612.8733	0.127	79	67	82	EHGAQEGQAGTGAFPR	138	100			Mascot
1663.8361	1663.9354	0.0993	60	101	116	LPIVVDASGDGAYVCK			Carbamidomethyl (C)[15]		Mascot
1862.7731	1862.9274	0.1543	83	40	53	DCCQQLAHISEWCR			Carbamidomethyl (C)[2,3,13]		Mascot
1862.7731	1862.9274	0.1543	83	40	53	DCCQQLAHISEWCR	135	100	Carbamidomethyl (C)[2,3,13]		Mascot

5	dimeric alpha-amylase inhibitor, partial [Triticum aestivum]	gi|386877068	14415.8	6.88	4	366	100	37.992	345	100

Peptide Information

Calc. Mass	Obsrv. Mass	± da	± ppm	Start Seq.	End Seq.	Sequence	Ion Score	C. I. %	Modification	Rank	Result Type
1162.6249	1162.7083	0.0834	72	95	105	LTAASITAVCR			Carbamidomethyl (C)[10]		Mascot
1162.6249	1162.7083	0.0834	72	95	105	LTAASITAVCR	73	100	Carbamidomethyl (C)[10]		Mascot
1570.8007	1570.9241	0.1234	79	31	44	LQCNGSQVPEAVLR			Carbamidomethyl (C)[3]		Mascot
1612.7463	1612.8733	0.127	79	72	87	EHGAQEGQAGTGAFPR					Mascot
1612.7463	1612.8733	0.127	79	72	87	EHGAQEGQAGTGAFPR	138	100			Mascot
1862.7731	1862.9274	0.1543	83	45	58	DCCQQLAHISEWCR			Carbamidomethyl (C)[2,3,13]		Mascot
1862.7731	1862.9274	0.1543	83	45	58	DCCQQLAHISEWCR	135	100	Carbamidomethyl (C)[2,3,13]		Mascot

6	dimeric alpha-amylase inhibitor [Triticum dicoccoides]	gi|227809180	15716.5	5.58	4	366	100	35.344	345	100

Protein Group

dimeric alpha-amylase inhibitor [Triticum aestivum]	gi|65993925	15702.5	5.5799 999237 0605
dimeric alpha-amylase inhibitor [Triticum dicoccoides]	gi|227809156	15716.5	5.5799 999237 0605
dimeric alpha-amylase inhibitor [Triticum timopheevii subsp. armeniacum]	gi|227809268	15702.5	5.5799 999237 0605

Peptide Information

Calc. Mass	Obsrv. Mass	± da	± ppm	Start Seq.	End Seq.	Sequence	Ion Score	C. I. %	Modification	Rank	Result Type
1162.6249	1162.7083	0.0834	72	107	117	LTAASITAVCR			Carbamidomethyl (C)[10]		Mascot
1162.6249	1162.7083	0.0834	72	107	117	LTAASITAVCR	73	100	Carbamidomethyl (C)[10]		Mascot
1612.7463	1612.8733	0.127	79	84	99	EHGAQEGQAGTGAFPR					Mascot
1612.7463	1612.8733	0.127	79	84	99	EHGAQEGQAGTGAFPR	138	100			Mascot
1663.8361	1663.9354	0.0993	60	118	133	LPIVVDASGDGAYVCK			Carbamidomethyl (C)[15]		Mascot
1862.7731	1862.9274	0.1543	83	57	70	DCCQQLAHISEWCR			Carbamidomethyl (C)[2,3,13]		Mascot

1862.7731	1862.9274	0.1543	83	57	70	DCCQQLAHISEWCR	135	100	Carbamidomethyl (C)[2,3,13]		Mascot

7	dimeric alpha-amylase inhibitor [Triticum aestivum]	gi|255988225	15665.5	6.69	3	286	100	35.395	272	100	

Peptide Information

Calc. Mass	Obsrv. Mass	± da	± ppm	Start Seq.	End Seq.	Sequence	Ion Score	C. I. %	Modification	Rank	Result Type
1570.8007	1570.9241	0.1234	79	43	56	LQCNGSQVPEAVLR			Carbamidomethyl (C)[3]		Mascot
1612.7463	1612.8733	0.127	79	84	99	EHGAQEGQAGTGAFPR					Mascot
1612.7463	1612.8733	0.127	79	84	99	EHGAQEGQAGTGAFPR	138	100			Mascot
1862.7731	1862.9274	0.1543	83	57	70	DCCQQLAHISEWCR			Carbamidomethyl (C)[2,3,13]		Mascot
1862.7731	1862.9274	0.1543	83	57	70	DCCQQLAHISEWCR	135	100	Carbamidomethyl (C)[2,3,13]		Mascot

8	dimeric alpha-amylase inhibitor [Triticum dicoccoides]	gi|114215806	13862.5	5.25	4	233	100	26.236	211	100	

Protein Group

dimeric alpha-amylase inhibitor [Triticum dicoccoides]	gi|114215804	13862.5	5.25
dimeric alpha-amylase inhibitor [Triticum dicoccoides]	gi|114215808	13921.6	5.25

Peptide Information

Calc. Mass	Obsrv. Mass	± da	± ppm	Start Seq.	End Seq.	Sequence	Ion Score	C. I. %	Modification	Rank	Result Type
1162.6249	1162.7083	0.0834	72	90	100	LTAASITAVCR			Carbamidomethyl (C)[10]		Mascot
1162.6249	1162.7083	0.0834	72	90	100	LTAASITAVCR	73	100	Carbamidomethyl (C)[10]		Mascot
1570.8007	1570.9241	0.1234	79	26	39	LQCNGSQVPEAVLR			Carbamidomethyl (C)[3]		Mascot
1612.7463	1612.8733	0.127	79	67	82	EHGAQEGQAGTGAFPR					Mascot
1612.7463	1612.8733	0.127	79	67	82	EHGAQEGQAGTGAFPR	138	100			Mascot
1663.8361	1663.9354	0.0993	60	101	116	LPIVVDASGDGAYVCK			Carbamidomethyl (C)[15]		Mascot

9	dimeric alpha-amylase inhibitor [Triticum dicoccoides]	gi|227809252	15716.4	5.01	4	231	100	26.236	211	100	

Protein Group

dimeric alpha-amylase inhibitor [Triticum aestivum]	gi|65993829	15722.4	5.5799 999237 0605
dimeric alpha-amylase inhibitor [Triticum dicoccoides]	gi|227809250	15694.4	4.8299 999237 0605
dimeric alpha-amylase inhibitor [Triticum dicoccoides]	gi|227809102	15678.4	5.25
dimeric alpha-amylase inhibitor [Triticum dicoccoides]	gi|227809254	15753.5	4.8299

dimeric alpha-amylase inhibitor [Triticum dicoccoides]	gi|227809078	15722.4	999237 0605 4.8299 999237 0605

Peptide Information

Calc. Mass	Obsrv. Mass	± da	± ppm	Start Seq.	End Seq.	Sequence	Ion Score	C. I. %	Modification	Rank	Result Type
1162.6249	1162.7083	0.0834	72	107	117	LTAASITAVCR			Carbamidomethyl (C)[10]		Mascot
1162.6249	1162.7083	0.0834	72	107	117	LTAASITAVCR	73	100	Carbamidomethyl (C)[10]		Mascot
1570.8007	1570.9241	0.1234	79	43	56	LQCNGSQVPEAVLR			Carbamidomethyl (C)[3]		Mascot
1612.7463	1612.8733	0.127	79	84	99	EHGAQEGQAGTGAFPR					Mascot
1612.7463	1612.8733	0.127	79	84	99	EHGAQEGQAGTGAFPR	138	100			Mascot
1663.8361	1663.9354	0.0993	60	118	133	LPIVVDASGDGAYVCK			Carbamidomethyl (C)[15]		Mascot

10	dimeric alpha-amylase inhibitor [Triticum dicoccoides]	gi|114215786	13832.5	5.71	3	225	100	25.443	211	100	

Peptide Information

Calc. Mass	Obsrv. Mass	± da	± ppm	Start Seq.	End Seq.	Sequence	Ion Score	C. I. %	Modification	Rank	Result Type
1162.6249	1162.7083	0.0834	72	90	100	LTAASITAVCR			Carbamidomethyl (C)[10]		Mascot
1162.6249	1162.7083	0.0834	72	90	100	LTAASITAVCR	73	100	Carbamidomethyl (C)[10]		Mascot
1570.8007	1570.9241	0.1234	79	26	39	LQCNGSQVPEAVLR			Carbamidomethyl (C)[3]		Mascot
1612.7463	1612.8733	0.127	79	67	82	EHGAQEGQAGTGAFPR					Mascot
1612.7463	1612.8733	0.127	79	67	82	EHGAQEGQAGTGAFPR	138	100			Mascot

Gel Idx/Pos	118/E17	Instr./Gel Origin	BA2151/Sample Project 20140814	Process Status	Analysis Succeeded
Plate [#] Name	[1] Sample Project 20140814	Instrument Sample Name		Spectra	11

Rank	Protein Name	Accession No.	Protein MW	Protein PI	Pep. Count	Protein Score	Protein Score C. I. %	Intensity Matched	Total Ion Score	Total Ion C. I. %	Confirmed
1	hypothetical protein TRIUR3_03549 [Triticum urartu]	gi|474071007	16824.8	6.19	6	324	100	27.362	290	100	

Protein Group

Protein Name	Accession No.	Protein MW	Protein PI
RecName: Full=16.9 kDa class I heat shock protein 1; AltName: Full=HSP 16.9; AltName: Full=Heat shock protein 16.9A; AltName: Full=Heat shock protein 17; AltName: Full=Low molecular weight heat shock protein	gi|123545	16867.8	5.8299 999237 0605
heat shock protein 16.8	gi|445135	16867.8	5.8299 999237 0605
unnamed protein product [Triticum aestivum]	gi|296512688	16867.8	5.8299 999237 0605
unnamed protein product [Triticum aestivum]	gi|296511073	16867.8	5.8299 999237 0605
unnamed protein product [Triticum aestivum]	gi|21813	16867.8	5.8299 999237 0605

Peptide Information

Calc. Mass	Obsrv. Mass	± da	± ppm	Start Seq.	End Seq.	Sequence	Ion Score	C. I. %	Modification	Rank	Result Type
975.5258	975.5861	0.0603	62	110	117	FRLPEDAK					Mascot
975.5258	975.5861	0.0603	62	110	117	FRLPEDAK	30	94.077			Mascot
1027.6146	1027.5669	-0.0477	-46	137	145	AEVKKPEVK					Mascot
1057.5314	1057.5671	0.0357	34	50	58	ETPEAHVFK					Mascot
1600.8177	1600.9133	0.0956	60	71	85	VEVEDGNVLVVSGER					Mascot
1600.8177	1600.9133	0.0956	60	71	85	VEVEDGNVLVVSGER	97	100			Mascot
1905.9666	1906.083	0.1164	61	26	45	SIVPAISGGSSETAAFAN AR					Mascot
1905.9666	1906.083	0.1164	61	26	45	SIVPAISGGSSETAAFAN AR	164	100			Mascot
2086.0664	2086.1868	0.1204	58	67	85	EEVKVEVEDGNVLVVSG ER					Mascot
2086.0664	2086.1868	0.1204	58	67	85	EEVKVEVEDGNVLVVSG ER					Mascot

Rank	Protein Name	Accession No.	Protein MW	Protein PI	Pep. Count	Protein Score	Protein Score C. I. %	Intensity Matched	Total Ion Score	Total Ion C. I. %	Confirmed
2	unnamed protein product [Triticum aestivum]	gi|296512787	16857.8	5.83	6	323	100	27.362	290	100	

Protein Group

Protein Name	Accession No.	Protein MW	Protein PI
unnamed protein product [Triticum aestivum]	gi|296511575	16856.8	6.1900 000572 2046

unnamed protein product [Triticum aestivum] gi|296511571 16857.8 5.8299999237 0605

unnamed protein product [Triticum aestivum] gi|296512791 16856.8 6.1900000572 2046

Peptide Information

Calc. Mass	Obsrv. Mass	± da	± ppm	Start Seq.	End Seq.	Sequence	Ion Score	C. I. %	Modification	Rank	Result Type
975.5258	975.5861	0.0603	62	110	117	FRLPEDAK					Mascot
975.5258	975.5861	0.0603	62	110	117	FRLPEDAK	30	94.077			Mascot
1027.6146	1027.5669	-0.0477	-46	137	145	AEVKKPEVK					Mascot
1057.5314	1057.5671	0.0357	34	50	58	ETPEAHVFK					Mascot
1600.8177	1600.9133	0.0956	60	71	85	VEVEDGNVLVVSGER					Mascot
1600.8177	1600.9133	0.0956	60	71	85	VEVEDGNVLVVSGER	97	100			Mascot
1905.9666	1906.083	0.1164	61	26	45	SIVPAISGGSSETAAFANAR					Mascot
1905.9666	1906.083	0.1164	61	26	45	SIVPAISGGSSETAAFANAR	164	100			Mascot
2086.0664	2086.1868	0.1204	58	67	85	EEVKVEVEDGNVLVVSGER					Mascot
2086.0664	2086.1868	0.1204	58	67	85	EEVKVEVEDGNVLVVSGER					Mascot

3 unnamed protein product [Triticum dicoccoides] gi|296510911 16838.8 5.82 5 318 100 27.17 290 100

Protein Group

16.8 kDa heat-shock protein [Triticum dicoccoides] gi|186886530 16838.8 5.8200001716 6138

unnamed protein product [Triticum dicoccoides] gi|296512518 16838.8 5.8200001716 6138

Peptide Information

Calc. Mass	Obsrv. Mass	± da	± ppm	Start Seq.	End Seq.	Sequence	Ion Score	C. I. %	Modification	Rank	Result Type
975.5258	975.5861	0.0603	62	110	117	FRLPEDAK					Mascot
975.5258	975.5861	0.0603	62	110	117	FRLPEDAK	30	94.077			Mascot
1057.5314	1057.5671	0.0357	34	50	58	ETPEAHVFK					Mascot
1600.8177	1600.9133	0.0956	60	71	85	VEVEDGNVLVVSGER					Mascot
1600.8177	1600.9133	0.0956	60	71	85	VEVEDGNVLVVSGER	97	100			Mascot
1905.9666	1906.083	0.1164	61	26	45	SIVPAISGGSSETAAFANAR					Mascot
1905.9666	1906.083	0.1164	61	26	45	SIVPAISGGSSETAAFANAR	164	100			Mascot
2086.0664	2086.1868	0.1204	58	67	85	EEVKVEVEDGNVLVVSGER					Mascot

Calc. Mass	Obsrv. Mass	± da	± ppm	Start Seq.	End Seq.	Sequence	Ion Score	C. I. %	Modification	Rank	Result Type
2086.0664	2086.1868	0.1204	58	67	85	EEVKVEVEDGNVLVVSGER					Mascot

4	unnamed protein product [Triticum durum]	gi|296510917	16960.8	5.83	4	282	100	25.302	261	100	

Protein Group

16.9 kDa heat-shock protein [Triticum durum]	gi|186886536	16960.8	5.8299999237 0605
unnamed protein product [Triticum durum]	gi|296512524	16960.8	5.8299999237 0605

Peptide Information

Calc. Mass	Obsrv. Mass	± da	± ppm	Start Seq.	End Seq.	Sequence	Ion Score	C. I. %	Modification	Rank	Result Type
1027.6146	1027.5669	-0.0477	-46	137	145	AEVKKPEVK					Mascot
1600.8177	1600.9133	0.0956	60	71	85	VEVEDGNVLVVSGER					Mascot
1600.8177	1600.9133	0.0956	60	71	85	VEVEDGNVLVVSGER	97	100			Mascot
1905.9666	1906.083	0.1164	61	26	45	SIVPAISGGSSETAAFANAR					Mascot
1905.9666	1906.083	0.1164	61	26	45	SIVPAISGGSSETAAFANAR	164	100			Mascot
2086.0664	2086.1868	0.1204	58	67	85	EEVKVEVEDGNVLVVSGER					Mascot
2086.0664	2086.1868	0.1204	58	67	85	EEVKVEVEDGNVLVVSGER					Mascot

5	unnamed protein product [Triticum turgidum subsp. dicoccon]	gi|296510913	16852.9	6.77	5	213	100	23.892	194	100	

Protein Group

16.9a kDa heat-shock protein [Triticum turgidum subsp. dicoccon]	gi|186886532	16852.9	6.7699999809 2651
unnamed protein product [Triticum turgidum subsp. dicoccon]	gi|296512520	16852.9	6.7699999809 2651

Peptide Information

Calc. Mass	Obsrv. Mass	± da	± ppm	Start Seq.	End Seq.	Sequence	Ion Score	C. I. %	Modification	Rank	Result Type
858.5043	858.5243	0.02	23	78	85	VLVVSGER					Mascot
975.5258	975.5861	0.0603	62	110	117	FRLPEDAK					Mascot
975.5258	975.5861	0.0603	62	110	117	FRLPEDAK	30	94.077			Mascot
1027.6146	1027.5669	-0.0477	-46	137	145	AEVKKPEVK					Mascot
1057.5314	1057.5671	0.0357	34	50	58	ETPEAHVFK					Mascot
1905.9666	1906.083	0.1164	61	26	45	SIVPAISGGSSETAAFANAR					Mascot
1905.9666	1906.083	0.1164	61	26	45	SIVPAISGGSSETAAFANAR	164	100			Mascot

6	heat shock protein 16.9C, partial [Triticum aestivum]	gi|295501	14375.6	6.23	6	165	100	6.145	127	100

Peptide Information

Calc. Mass	Obsrv. Mass	± da	± ppm	Start Seq.	End Seq.	Sequence	Ion Score	C. I. %	Modification	Rank	Result Type
975.5258	975.5861	0.0603	62	89	96	FRLPEDAK					Mascot
975.5258	975.5861	0.0603	62	89	96	FRLPEDAK	30	94.077			Mascot
1027.6146	1027.5669	-0.0477	-46	116	124	AEVKKPEVK					Mascot
1057.5314	1057.5671	0.0357	34	29	37	ETPEAHVFK					Mascot
1600.8177	1600.9133	0.0956	60	50	64	VEVEDGNVLVVSGER					Mascot
1600.8177	1600.9133	0.0956	60	50	64	VEVEDGNVLVVSGER	97	100			Mascot
1919.9822	1920.0708	0.0886	46	5	24	SIVPAISGGTSETAAFANA R					Mascot
2086.0664	2086.1868	0.1204	58	46	64	EEVKVEVEDGNVLVVSG ER					Mascot
2086.0664	2086.1868	0.1204	58	46	64	EEVKVEVEDGNVLVVSG ER					Mascot

7	unnamed protein product [Triticum monococcum]	gi|296510919	16794.8	6.19	6	162	100	6.021	127	100

Protein Group

16.8 kDa heat-shock protein [Triticum monococcum]	gi|186886538	16794.8	6.1900 000572 2046
unnamed protein product [Triticum monococcum]	gi|296512526	16794.8	6.1900 000572 2046

Peptide Information

Calc. Mass	Obsrv. Mass	± da	± ppm	Start Seq.	End Seq.	Sequence	Ion Score	C. I. %	Modification	Rank	Result Type
975.5258	975.5861	0.0603	62	110	117	FRLPEDAK					Mascot
975.5258	975.5861	0.0603	62	110	117	FRLPEDAK	30	94.077			Mascot
1027.5208	1027.5669	0.0461	45	50	58	EAPEAHVFK					Mascot
1600.8177	1600.9133	0.0956	60	71	85	VEVEDGNVLVVSGER					Mascot
1600.8177	1600.9133	0.0956	60	71	85	VEVEDGNVLVVSGER	97	100			Mascot
1707.9065	1707.886	-0.0205	-12	50	65	EAPEAHVFKADLPGVK					Mascot
1875.9559	1876.0944	0.1385	74	26	45	SIVPAISGGGSETAAFAN AR					Mascot
2086.0664	2086.1868	0.1204	58	67	85	EEVKVEVEDGNVLVVSG ER					Mascot
2086.0664	2086.1868	0.1204	58	67	85	EEVKVEVEDGNVLVVSG ER					Mascot

8	heat shock protein 16.9	gi|445136	16824.8	5.56	6	160	100	6.145	127	100

Peptide Information

Calc. Mass	Obsrv. Mass	± da	± ppm	Start Seq.	End Seq.	Sequence	Ion Score	C. I. %	Modification	Rank	Result Type
975.5258	975.5861	0.0603	62	110	117	FRLPEDAK					Mascot
975.5258	975.5861	0.0603	62	110	117	FRLPEDAK	30	94.077			Mascot
1027.6146	1027.5669	-0.0477	-46	137	145	AEVKKPEVK					Mascot
1057.5314	1057.5671	0.0357	34	50	58	ETPEAHVFK					Mascot
1600.8177	1600.9133	0.0956	60	71	85	VEVEDGNVLVVSGER					Mascot
1600.8177	1600.9133	0.0956	60	71	85	VEVEDGNVLVVSGER	97	100			Mascot
1919.9822	1920.0708	0.0886	46	26	45	SIVPAISGGTSETAAFANA R					Mascot
2086.0664	2086.1868	0.1204	58	67	85	EEVKVEVEDGNVLVVSG ER					Mascot
2086.0664	2086.1868	0.1204	58	67	85	EEVKVEVEDGNVLVVSG ER					Mascot

9 unnamed protein product [Triticum aestivum] gi|296512536 16838.8 5.56 6 153 100 6.248 127 100

Protein Group

small heat shock protein 16.9 kDa [Triticum aestivum]	gi|187384869	16838.8	5.5599 999427 7954
unnamed protein product [Triticum aestivum]	gi|296510929	16838.8	5.5599 999427 7954

Peptide Information

Calc. Mass	Obsrv. Mass	± da	± ppm	Start Seq.	End Seq.	Sequence	Ion Score	C. I. %	Modification	Rank	Result Type
837.4577	837.4843	0.0266	32	102	109	SSGKFVGR					Mascot
975.5258	975.5861	0.0603	62	110	117	FRLPEDAK					Mascot
975.5258	975.5861	0.0603	62	110	117	FRLPEDAK	30	94.077			Mascot
1027.6146	1027.5669	-0.0477	-46	137	145	AEVKKPEVK					Mascot
1057.5314	1057.5671	0.0357	34	50	58	ETPEAHVFK					Mascot
1600.8177	1600.9133	0.0956	60	71	85	VEVEDGNVLVVSGER					Mascot
1600.8177	1600.9133	0.0956	60	71	85	VEVEDGNVLVVSGER	97	100			Mascot
2086.0664	2086.1868	0.1204	58	67	85	EEVKVEVEDGNVLVVSG ER					Mascot
2086.0664	2086.1868	0.1204	58	67	85	EEVKVEVEDGNVLVVSG ER					Mascot

10 unnamed protein product [Triticum monococcum] gi|296510925 16866.8 6.19 5 153 100 5.746 127 100

Protein Group

16.9b kDa heat-shock protein [Triticum monococcum]	gi|186886544	16866.8	6.1900 000572 2046
unnamed protein product [Triticum monococcum]	gi|296512532	16866.8	6.1900

Peptide Information

Calc. Mass	Obsrv. Mass	± da	± ppm	Start Seq.	End Seq.	Sequence	Ion Score	C. I. %	Modification	Rank	Result Type
975.5258	975.5861	0.0603	62	110	117	FRLPEDAK					Mascot
975.5258	975.5861	0.0603	62	110	117	FRLPEDAK	30	93.375			Mascot
1027.6146	1027.5669	-0.0477	-46	137	145	AEVKKPEVK					Mascot
1600.8177	1600.9133	0.0956	60	71	85	VEVEDGNVLVVSGER					Mascot
1600.8177	1600.9133	0.0956	60	71	85	VEVEDGNVLVVSGER	97	100			Mascot
1875.9559	1876.0944	0.1385	74	26	45	SIVPAISGGGSETAAFANAR					Mascot
2086.0664	2086.1868	0.1204	58	67	85	EEVKVEVEDGNVLVVSGER					Mascot
2086.0664	2086.1868	0.1204	58	67	85	EEVKVEVEDGNVLVVSGER					Mascot

Gel Idx/Pos	119/E18	Instr./Gel Origin	BA2151/Sample Project 20140814	Process Status	Analysis Succeeded
Plate [#] Name	[1] Sample Project 20140814	Instrument Sample Name		Spectra	11

Rank	Protein Name	Accession No.	Protein MW	Protein PI	Pep. Count	Protein Score	Protein Score C. I. %	Intensity Matched	Total Ion Score	Total Ion C. I. %	Confirmed
1	alpha-amylase inhibitor 0.19 [Triticum aestivum]	gi|66841026	13340.4	6.86	6	192	100	7.628	148	100	

Peptide Information

Calc. Mass	Obsrv. Mass	± da	± ppm	Start Seq.	End Seq.	Sequence	Ion Score	C. I. %	Modification	Rank	Result Type
1162.6249	1162.7152	0.0903	78	85	95	LTAASITAVCR			Carbamidomethyl (C)[10]		Mascot
1162.6249	1162.7152	0.0903	78	85	95	LTAASITAVCR			Carbamidomethyl (C)[10]		Mascot
1570.8007	1570.8739	0.0732	47	21	34	LQCNGSQVPEAVLR			Carbamidomethyl (C)[3]		Mascot
1570.8007	1570.8739	0.0732	47	21	34	LQCNGSQVPEAVLR	44	99.764	Carbamidomethyl (C)[3]		Mascot
1612.7463	1612.8792	0.1329	82	62	77	EHGAQEGQAGTGAFPR					Mascot
1612.7463	1612.8792	0.1329	82	62	77	EHGAQEGQAGTGAFPR	104	100			Mascot
1663.8361	1663.931	0.0949	57	96	111	LPIVVDASGDGAYVCK			Carbamidomethyl (C)[15]		Mascot
1862.7731	1862.9211	0.148	79	35	48	DCCQQLAHISEWCR			Carbamidomethyl (C)[2,3,13]		Mascot
1862.7731	1862.9211	0.148	79	35	48	DCCQQLAHISEWCR			Carbamidomethyl (C)[2,3,13]		Mascot
2807.4431	2807.5427	0.0996	35	85	111	LTAASITAVCRLPIVVDAS GDGAYVCK			Carbamidomethyl (C)[10,26]		Mascot

Rank	Protein Name	Accession No.	Protein MW	Protein PI	Pep. Count	Protein Score	Protein Score C. I. %	Intensity Matched	Total Ion Score	Total Ion C. I. %	Confirmed
2	Chain D, 0.19 Alpha-Amylase Inhibitor From Wheat	gi|3318684	13898.6	6.66	6	191	100	7.628	148	100	

Protein Group

0.19 alpha-amylase inhibitor [Triticum aestivum]	gi|2116581	13898.6	6.6599 998474 1211
0.19 dimeric alpha-amylase inhibitor [Triticum aestivum]	gi|54778509	13898.6	6.6599 998474 1211
0.19 dimeric alpha-amylase inhibitor [Triticum aestivum]	gi|54778501	13898.6	6.6599 998474 1211
Chain A, 0.19 Alpha-Amylase Inhibitor From Wheat	gi|3318681	13898.6	6.6599 998474 1211
Chain B, 0.19 Alpha-Amylase Inhibitor From Wheat	gi|3318682	13898.6	6.6599 998474 1211
Chain C, 0.19 Alpha-Amylase Inhibitor From Wheat	gi|3318683	13898.6	6.6599 998474 1211
RecName: Full=Alpha-amylase inhibitor 0.19; AltName: Full=0.19 alpha-AI; Short=0.19 AI	gi|123963	13898.6	6.6599 998474 1211

Peptide Information

Calc. Mass	Obsrv. Mass	± da	± ppm	Start Seq.	End Seq.	Sequence	Ion Score	C. I. %	Modification	Rank	Result Type
1162.6249	1162.7152	0.0903	78	90	100	LTAASITAVCR			Carbamidomethyl (C)[10]		Mascot
1162.6249	1162.7152	0.0903	78	90	100	LTAASITAVCR			Carbamidomethyl (C)[10]		Mascot
1570.8007	1570.8739	0.0732	47	26	39	LQCNGSQVPEAVLR			Carbamidomethyl (C)[3]		Mascot
1570.8007	1570.8739	0.0732	47	26	39	LQCNGSQVPEAVLR	44	99.764	Carbamidomethyl (C)[3]		Mascot
1612.7463	1612.8792	0.1329	82	67	82	EHGAQEGQAGTGAFPR					Mascot
1612.7463	1612.8792	0.1329	82	67	82	EHGAQEGQAGTGAFPR	104	100			Mascot
1663.8361	1663.931	0.0949	57	101	116	LPIVVDASGDGAYVCK			Carbamidomethyl (C)[15]		Mascot
1862.7731	1862.9211	0.148	79	40	53	DCCQQLAHISEWCR			Carbamidomethyl (C)[2,3,13]		Mascot
1862.7731	1862.9211	0.148	79	40	53	DCCQQLAHISEWCR			Carbamidomethyl (C)[2,3,13]		Mascot
2807.4431	2807.5427	0.0996	35	90	116	LTAASITAVCRLPIVVDAS GDGAYVCK			Carbamidomethyl (C)[10,26]		Mascot

3	dimeric alpha-amylase inhibitor precursor, partial [Triticum aestivum]	gi|108597921	14029.7	6.69	6	190	100	7.628	148	100	

Peptide Information

Calc. Mass	Obsrv. Mass	± da	± ppm	Start Seq.	End Seq.	Sequence	Ion Score	C. I. %	Modification	Rank	Result Type
1162.6249	1162.7152	0.0903	78	91	101	LTAASITAVCR			Carbamidomethyl (C)[10]		Mascot
1162.6249	1162.7152	0.0903	78	91	101	LTAASITAVCR			Carbamidomethyl (C)[10]		Mascot
1570.8007	1570.8739	0.0732	47	27	40	LQCNGSQVPEAVLR			Carbamidomethyl (C)[3]		Mascot
1570.8007	1570.8739	0.0732	47	27	40	LQCNGSQVPEAVLR	44	99.764	Carbamidomethyl (C)[3]		Mascot
1612.7463	1612.8792	0.1329	82	68	83	EHGAQEGQAGTGAFPR					Mascot
1612.7463	1612.8792	0.1329	82	68	83	EHGAQEGQAGTGAFPR	104	100			Mascot
1663.8361	1663.931	0.0949	57	102	117	LPIVVDASGDGAYVCK			Carbamidomethyl (C)[15]		Mascot
1862.7731	1862.9211	0.148	79	41	54	DCCQQLAHISEWCR			Carbamidomethyl (C)[2,3,13]		Mascot
1862.7731	1862.9211	0.148	79	41	54	DCCQQLAHISEWCR			Carbamidomethyl (C)[2,3,13]		Mascot
2807.4431	2807.5427	0.0996	35	91	117	LTAASITAVCRLPIVVDAS GDGAYVCK			Carbamidomethyl (C)[10,26]		Mascot

4	dimeric alpha-amylase inhibitor [Triticum dicoccoides]	gi|227809009	15730.5	5.58	6	188	100	7.628	148	100	

Protein Group

dimeric alpha-amylase inhibitor [Triticum aestivum]	gi|65993781	15688.5	5.5799 999237 0605
dimeric alpha-amylase inhibitor [Triticum aestivum]	gi|386877038	15702.5	5.5799 999237 0605
dimeric alpha-amylase inhibitor [Triticum dicoccoides]	gi|227809005	15730.5	5.5799

999237
0605

Peptide Information

Calc. Mass	Obsrv. Mass	± da	± ppm	Start Seq.	End Seq.	Sequence	Ion Score	C. I. %	Modification	Rank	Result Type
1162.6249	1162.7152	0.0903	78	107	117	LTAASITAVCR			Carbamidomethyl (C)[10]		Mascot
1162.6249	1162.7152	0.0903	78	107	117	LTAASITAVCR			Carbamidomethyl (C)[10]		Mascot
1570.8007	1570.8739	0.0732	47	43	56	LQCNGSQVPEAVLR			Carbamidomethyl (C)[3]		Mascot
1570.8007	1570.8739	0.0732	47	43	56	LQCNGSQVPEAVLR	44	99.764	Carbamidomethyl (C)[3]		Mascot
1612.7463	1612.8792	0.1329	82	84	99	EHGAQEGQAGTGAFPR					Mascot
1612.7463	1612.8792	0.1329	82	84	99	EHGAQEGQAGTGAFPR	104	100			Mascot
1663.8361	1663.931	0.0949	57	118	133	LPIVVDASGDGAYVCK			Carbamidomethyl (C)[15]		Mascot
1862.7731	1862.9211	0.148	79	57	70	DCCQQLAHISEWCR			Carbamidomethyl (C)[2,3,13]		Mascot
1862.7731	1862.9211	0.148	79	57	70	DCCQQLAHISEWCR			Carbamidomethyl (C)[2,3,13]		Mascot
2807.4431	2807.5427	0.0996	35	107	133	LTAASITAVCRLPIVVDAS GDGAYVCK			Carbamidomethyl (C)[10,26]		Mascot

5 dimeric alpha-amylase inhibitor [Triticum dicoccoides] gi|114215806 13862.5 5.25 5 181 100 6.641 148 100

Protein Group

dimeric alpha-amylase inhibitor [Triticum dicoccoides] gi|114215804 13862.5 5.25

dimeric alpha-amylase inhibitor [Triticum dicoccoides] gi|114215808 13921.6 5.25

Peptide Information

Calc. Mass	Obsrv. Mass	± da	± ppm	Start Seq.	End Seq.	Sequence	Ion Score	C. I. %	Modification	Rank	Result Type
1162.6249	1162.7152	0.0903	78	90	100	LTAASITAVCR			Carbamidomethyl (C)[10]		Mascot
1162.6249	1162.7152	0.0903	78	90	100	LTAASITAVCR			Carbamidomethyl (C)[10]		Mascot
1570.8007	1570.8739	0.0732	47	26	39	LQCNGSQVPEAVLR			Carbamidomethyl (C)[3]		Mascot
1570.8007	1570.8739	0.0732	47	26	39	LQCNGSQVPEAVLR	44	99.764	Carbamidomethyl (C)[3]		Mascot
1612.7463	1612.8792	0.1329	82	67	82	EHGAQEGQAGTGAFPR					Mascot
1612.7463	1612.8792	0.1329	82	67	82	EHGAQEGQAGTGAFPR	104	100			Mascot
1663.8361	1663.931	0.0949	57	101	116	LPIVVDASGDGAYVCK			Carbamidomethyl (C)[15]		Mascot
2807.4431	2807.5427	0.0996	35	90	116	LTAASITAVCRLPIVVDAS GDGAYVCK			Carbamidomethyl (C)[10,26]		Mascot

6 dimeric alpha-amylase inhibitor [Triticum dicoccoides] gi|227809252 15716.4 5.01 5 179 100 6.641 148 100

Protein Group

dimeric alpha-amylase inhibitor [Triticum aestivum] gi|65993829 15722.4 5.5799
999237

				0605
	dimeric alpha-amylase inhibitor [Triticum dicoccoides]	gi|227809250	15694.4	4.8299999237 0605
	dimeric alpha-amylase inhibitor [Triticum dicoccoides]	gi|227809102	15678.4	5.25
	dimeric alpha-amylase inhibitor [Triticum dicoccoides]	gi|227809254	15753.5	4.8299999237 0605
	dimeric alpha-amylase inhibitor [Triticum dicoccoides]	gi|227809078	15722.4	4.8299999237 0605

Peptide Information

Calc. Mass	Obsrv. Mass	± da	± ppm	Start Seq.	End Seq.	Sequence	Ion Score	C. I. %	Modification	Rank	Result Type
1162.6249	1162.7152	0.0903	78	107	117	LTAASITAVCR			Carbamidomethyl (C)[10]		Mascot
1162.6249	1162.7152	0.0903	78	107	117	LTAASITAVCR			Carbamidomethyl (C)[10]		Mascot
1570.8007	1570.8739	0.0732	47	43	56	LQCNGSQVPEAVLR			Carbamidomethyl (C)[3]		Mascot
1570.8007	1570.8739	0.0732	47	43	56	LQCNGSQVPEAVLR	44	99.764	Carbamidomethyl (C)[3]		Mascot
1612.7463	1612.8792	0.1329	82	84	99	EHGAQEGQAGTGAFPR					Mascot
1612.7463	1612.8792	0.1329	82	84	99	EHGAQEGQAGTGAFPR	104	100			Mascot
1663.8361	1663.931	0.0949	57	118	133	LPIVVDASGDGAYVCK			Carbamidomethyl (C)[15]		Mascot
2807.4431	2807.5427	0.0996	35	107	133	LTAASITAVCRLPIVVDAS GDGAYVCK			Carbamidomethyl (C)[10,26]		Mascot

7	Globulin-1 S allele [Triticum urartu]	gi|474323981	55586	7.77	4	176	100	59.944	169	100

Peptide Information

Calc. Mass	Obsrv. Mass	± da	± ppm	Start Seq.	End Seq.	Sequence	Ion Score	C. I. %	Modification	Rank	Result Type
842.5206	842.5801	0.0595	71	395	401	QVRAQIK					Mascot
1001.5527	1001.6293	0.0766	76	457	465	ALAFPQQAR					Mascot
1001.5527	1001.6293	0.0766	76	457	465	ALAFPQQAR	53	99.966			Mascot
1641.8344	1641.9668	0.1324	81	474	488	AQPESVFVAGPQQQR					Mascot
1641.8344	1641.9668	0.1324	81	474	488	AQPESVFVAGPQQQR	116	100			Mascot
1723.8762	1724.0227	0.1465	85	86	101	GSIGDYRVAYLDAAPR					Mascot

8	dimeric alpha-amylase inhibitor, partial [Triticum aestivum]	gi|386877068	14415.8	6.88	4	170	100	7.033	148	100

Peptide Information

Calc. Mass	Obsrv. Mass	± da	± ppm	Start Seq.	End Seq.	Sequence	Ion Score	C. I. %	Modification	Rank	Result Type

Calc. Mass	Obsrv. Mass	± da	± ppm	Start Seq.	End Seq.	Sequence	Ion Score	C. I. %	Modification	Rank	Result Type
1162.6249	1162.7152	0.0903	78	95	105	LTAASITAVCR			Carbamidomethyl (C)[10]		Mascot
1162.6249	1162.7152	0.0903	78	95	105	LTAASITAVCR			Carbamidomethyl (C)[10]		Mascot
1570.8007	1570.8739	0.0732	47	31	44	LQCNGSQVPEAVLR			Carbamidomethyl (C)[3]		Mascot
1570.8007	1570.8739	0.0732	47	31	44	LQCNGSQVPEAVLR	44	99.764	Carbamidomethyl (C)[3]		Mascot
1612.7463	1612.8792	0.1329	82	72	87	EHGAQEGQAGTGAFPR					Mascot
1612.7463	1612.8792	0.1329	82	72	87	EHGAQEGQAGTGAFPR	104	100			Mascot
1862.7731	1862.9211	0.148	79	45	58	DCCQQLAHISEWCR			Carbamidomethyl (C)[2,3,13]		Mascot
1862.7731	1862.9211	0.148	79	45	58	DCCQQLAHISEWCR			Carbamidomethyl (C)[2,3,13]		Mascot

9	dimeric alpha-amylase inhibitor [Triticum dicoccoides]	gi|114215786	13832.5	5.71	3	163	100	6.046	148	100	

Peptide Information

Calc. Mass	Obsrv. Mass	± da	± ppm	Start Seq.	End Seq.	Sequence	Ion Score	C. I. %	Modification	Rank	Result Type
1162.6249	1162.7152	0.0903	78	90	100	LTAASITAVCR			Carbamidomethyl (C)[10]		Mascot
1162.6249	1162.7152	0.0903	78	90	100	LTAASITAVCR			Carbamidomethyl (C)[10]		Mascot
1570.8007	1570.8739	0.0732	47	26	39	LQCNGSQVPEAVLR			Carbamidomethyl (C)[3]		Mascot
1570.8007	1570.8739	0.0732	47	26	39	LQCNGSQVPEAVLR	44	99.764	Carbamidomethyl (C)[3]		Mascot
1612.7463	1612.8792	0.1329	82	67	82	EHGAQEGQAGTGAFPR					Mascot
1612.7463	1612.8792	0.1329	82	67	82	EHGAQEGQAGTGAFPR	104	100			Mascot

10	dimeric alpha-amylase inhibitor [Triticum dicoccoides]	gi|227809096	15664.4	5.01	3	162	100	6.046	148	100	

Protein Group

dimeric alpha-amylase inhibitor, partial [Triticum aestivum]	gi|386877040	15014.1	5.2800 002098 0835

Peptide Information

Calc. Mass	Obsrv. Mass	± da	± ppm	Start Seq.	End Seq.	Sequence	Ion Score	C. I. %	Modification	Rank	Result Type
1162.6249	1162.7152	0.0903	78	107	117	LTAASITAVCR			Carbamidomethyl (C)[10]		Mascot
1162.6249	1162.7152	0.0903	78	107	117	LTAASITAVCR			Carbamidomethyl (C)[10]		Mascot
1570.8007	1570.8739	0.0732	47	43	56	LQCNGSQVPEAVLR			Carbamidomethyl (C)[3]		Mascot
1570.8007	1570.8739	0.0732	47	43	56	LQCNGSQVPEAVLR	44	99.764	Carbamidomethyl (C)[3]		Mascot
1612.7463	1612.8792	0.1329	82	84	99	EHGAQEGQAGTGAFPR					Mascot
1612.7463	1612.8792	0.1329	82	84	99	EHGAQEGQAGTGAFPR	104	100			Mascot

Gel Idx/Pos	120/E19	Instr./Gel Origin	BA2151/Sample Project 20140814	Process Status	Analysis Succeeded
Plate [#] Name	[1] Sample Project 20140814	Instrument Sample Name		Spectra	11

Rank	Protein Name	Accession No.	Protein MW	Protein PI	Pep. Count	Protein Score	Protein Score C. I. %	Intensity Matched	Total Ion Score	Total Ion C. I. %	Confirmed
1	alpha amylase inhibitor CM3 [Triticum durum]	gi|39578552	18893.3	7.44	8	583	100	57.249	531	100	

Protein Group

CM3 protein [Triticum durum]	gi|21922	18893.3	7.4400 000572 2046
RecName: Full=Alpha-amylase/trypsin inhibitor CM3; AltName: Full=Chloroform/methanol-soluble protein CM3; Flags: Precursor	gi|123957	18893.3	7.4400 000572 2046
alpha amylase inhibitor protein [Triticum aestivum]	gi|38098487	18893.3	7.4400 000572 2046
alpha-amylase inhibitor, tetrameric, chain CM3 precursor - durum wheat	gi|100834	18893.3	7.4400 000572 2046
unnamed protein product [Triticum aestivum]	gi|21713	18893.3	7.4400 000572 2046
unnamed protein product [Triticum durum]	gi|57997836	18893.3	7.4400 000572 2046

Peptide Information

Calc. Mass	Obsrv. Mass	± da	± ppm	Start Seq.	End Seq.	Sequence	Ion Score	C. I. %	Modification	Rank	Result Type
1010.52	1010.5914	0.0714	71	37	44	TNLLPHCR			Carbamidomethyl (C)[7]		Mascot
1010.52	1010.5914	0.0714	71	37	44	TNLLPHCR	42	99.569	Carbamidomethyl (C)[7]		Mascot
1110.5038	1110.5851	0.0813	73	133	140	EMQWDFVR					Mascot
1126.4987	1126.5664	0.0677	60	133	140	EMQWDFVR			Oxidation (M)[2]		Mascot
1126.4987	1126.5664	0.0677	60	133	140	EMQWDFVR	57	99.987	Oxidation (M)[2]		Mascot
1698.9214	1699.0544	0.133	78	101	115	YFIALPVPSQPVDPR					Mascot
1698.9214	1699.0544	0.133	78	101	115	YFIALPVPSQPVDPR	88	100			Mascot
1727.8381	1727.9695	0.1314	76	116	132	SGNVGESGLIDLPGCPR			Carbamidomethyl (C)[15]		Mascot
1727.8381	1727.9695	0.1314	76	116	132	SGNVGESGLIDLPGCPR	122	100	Carbamidomethyl (C)[15]		Mascot
1801.8427	1801.9408	0.0981	54	45	60	DYVLQQTCGTFTPGSK			Carbamidomethyl (C)[8]		Mascot
1876.0222	1876.1837	0.1615	86	141	157	LLVAPGQCNLATIHNVR			Carbamidomethyl (C)[8]		Mascot
1876.0222	1876.1837	0.1615	86	141	157	LLVAPGQCNLATIHNVR	98	100	Carbamidomethyl (C)[8]		Mascot
1957.8564	1958.0208	0.1644	84	81	95	LYCCQELAEISQQCR			Carbamidomethyl (C)[3,4,14]		Mascot
1957.8564	1958.0208	0.1644	84	81	95	LYCCQELAEISQQCR	124	100	Carbamidomethyl (C)[3,4,14]		Mascot

Calc. Mass	Obsrv. Mass	± da	± ppm	Start Seq.	End Seq.	Sequence	Ion Score	C. I. %	Modification	Rank	Result Type
2255.1416	2255.2786	0.137	61	61	80	LPEWMTSASIYSPGKPYLAK			Oxidation (M)[5]		Mascot

2 Disease resistance protein RPM1 [Triticum urartu] gi|474086298 129568 6.87 19 45 0 23.468

Peptide Information

Calc. Mass	Obsrv. Mass	± da	± ppm	Start Seq.	End Seq.	Sequence	Ion Score	C. I. %	Modification	Rank	Result Type
1142.6317	1142.5651	-0.0666	-58	895	904	KDAASLVPWR					Mascot
1146.6882	1146.6166	-0.0716	-62	193	204	IVSIVGFGGLGK					Mascot
1158.5175	1158.5547	0.0372	32	519	528	WIDPEDNGGR					Mascot
1158.5175	1158.5547	0.0372	32	519	528	WIDPEDNGGR					Mascot
1173.611	1173.5831	-0.0279	-24	927	936	EVKEAEEALR					Mascot
1198.6619	1198.6265	-0.0354	-30	278	286	IYHFLKYSK					Mascot
1649.8422	1649.9917	0.1495	91	449	462	ILSFSYYDLSSNLK					Mascot
1670.8094	1670.9606	0.1512	90	732	746	VLNIFFGEMEESAGK					Mascot
1710.8619	1710.9543	0.0924	54	703	716	LTCLEELQLYSVDK			Carbamidomethyl (C)[3]		Mascot
1775.8997	1775.9755	0.0758	43	251	265	ATETMAEWQLINQLK					Mascot
1785.0116	1785.1517	0.1401	78	236	250	VLRDILLELDTELSR					Mascot
1785.0116	1785.1517	0.1401	78	236	250	VLRDILLELDTELSR					Mascot
1802.0463	1801.9408	-0.1055	-59	835	849	LPSLLFLLLWSKDEK					Mascot
1802.0463	1802.1733	0.127	70	835	849	LPSLLFLLLWSKDEK					Mascot
1810.0143	1809.9728	-0.0415	-23	871	887	IEIAVGEGALPMLEVLR					Mascot
1895.1212	1895.028	-0.0932	-49	162	178	KLSDLVGIDKPINELIK					Mascot
1898.1144	1898.1085	-0.0059	-3	403	421	KCGGVPLAITTIASLLVGK			Carbamidomethyl (C)[2]		Mascot
1900.9084	1900.9803	0.0719	38	1104	1119	GFWLGPAENKMYLCAK			Carbamidomethyl (C)[14], Oxidation (M)[11]		Mascot
1915.9617	1916.0728	0.1111	58	669	686	GGMISELPPSVGELMNLR			Oxidation (M)[3]		Mascot
1939.9946	1940.0787	0.0841	43	730	746	LRVLNIFFGEMEESAGK					Mascot
1955.9895	1956.0895	0.1	51	730	746	LRVLNIFFGEMEESAGK			Oxidation (M)[11]		Mascot
1958.093	1958.0208	-0.0722	-37	937	954	QASGSRPNAVYLNLRIAK					Mascot
1958.093	1958.0208	-0.0722	-37	937	954	QASGSRPNAVYLNLRIAK					Mascot
1986.059	1986.0424	-0.0166	-8	341	357	LAFMDSHPQSRLIITTR					Mascot

3 TPR repeat-containing thioredoxin TTL1 [Triticum urartu] gi|473951385 58817 8.74 13 42 0 18.254

Peptide Information

Calc. Mass	Obsrv. Mass	± da	± ppm	Start Seq.	End Seq.	Sequence	Ion Score	C. I. %	Modification	Rank	Result Type
1045.579	1045.6168	0.0378	36	158	166	IGDWKSALR					Mascot

Calc. Mass	Obsrv. Mass	± da	± ppm	Start Seq.	End Seq.	Sequence	Ion Score	C. I. %	Modification	Rank	Result Type
1066.6367	1066.5786	-0.0581	-54	187	195	SEALLRLHK					Mascot
1108.6765	1108.5729	-0.1036	-93	491	499	IVPTFKIYK					Mascot
1158.6001	1158.5547	-0.0454	-39	475	485	VNVEDSPTVAK					Mascot
1158.6001	1158.5547	-0.0454	-39	475	485	VNVEDSPTVAK					Mascot
1162.5157	1162.6079	0.0922	79	343	352	AVDDCNEALR			Carbamidomethyl (C)[5]		Mascot
1176.6372	1176.6429	0.0057	5	295	305	AQGNELFKAAK					Mascot
1176.6372	1176.6429	0.0057	5	295	305	AQGNELFKAAK					Mascot
1727.8494	1727.9695	0.1201	70	142	156	LQDVEMHLGRSTDAR					Mascot
1727.8923	1727.9695	0.0772	45	475	490	VNVEDSPTVAKAENVR					Mascot
1743.8442	1743.9325	0.0883	51	142	156	LQDVEMHLGRSTDAR			Oxidation (M)[6]		Mascot
1775.9637	1775.9755	0.0118	7	196	211	LEEADSTLTSLLKLDK					Mascot
1873.9775	1874.1099	0.1324	71	506	520	EMICPTLHVLRYSVR			Carbamidomethyl (C)[4]		Mascot
1889.9725	1890.1569	0.1844	98	506	520	EMICPTLHVLRYSVR			Carbamidomethyl (C)[4], Oxidation (M)[2]		Mascot
1940.0535	1940.0787	0.0252	13	244	259	LQQKQIIAEVGIHFCR			Carbamidomethyl (C)[15]		Mascot
1955.9491	1956.0895	0.1404	72	20	39	SPASSPVAPPSGGSLQEMTR					Mascot
1971.9441	1972.0272	0.0831	42	20	39	SPASSPVAPPSGGSLQEMTR			Oxidation (M)[18]		Mascot
1993.9648	1994.1057	0.1409	71	343	359	AVDDCNEALRIQPTYTK			Carbamidomethyl (C)[5]		Mascot

4 hypothetical protein TRIUR3_23148 [Triticum urartu] gi|474256756 15074.7 12 7 40 0 5.115

Peptide Information

Calc. Mass	Obsrv. Mass	± da	± ppm	Start Seq.	End Seq.	Sequence	Ion Score	C. I. %	Modification	Rank	Result Type
1085.6075	1085.6533	0.0458	42	118	127	GGARPHVVHR					Mascot
1140.5579	1140.5767	0.0188	16	17	28	APPCAGGQIGGR			Carbamidomethyl (C)[4]		Mascot
1158.5862	1158.5547	-0.0315	-27	84	96	GGDGGGQIVATAR					Mascot
1158.5862	1158.5547	-0.0315	-27	84	96	GGDGGGQIVATAR					Mascot
1173.5443	1173.5831	0.0388	33	128	137	GHHHRGGMER					Mascot
1709.9069	1709.9583	0.0514	30	118	132	GGARPHVVHRGHHHR					Mascot
1714.8806	1715.0264	0.1458	85	54	68	HGIRQPTLGIYSCGR			Carbamidomethyl (C)[13]		Mascot
1956.0662	1956.0895	0.0233	12	31	49	HPSPDPAANVGRLLALEAK					Mascot

5 Serpin-Z2A [Triticum urartu] gi|474291222 32695.3 5.12 5 38 0 4.014 24 76.644

Peptide Information

Calc. Mass	Obsrv. Mass	± da	± ppm	Start Seq.	End Seq.	Sequence	Ion Score	C. I. %	Modification	Rank	Result Type
1076.5259	1076.5583	0.0324	30	149	157	DGLWELTDK					Mascot

Calc. Mass	Obsrv. Mass	± da	± ppm	Start Seq.	End Seq.	Sequence	Ion Score	C. I. %	Modification	Rank	Result Type
1092.6888	1092.592	-0.0968	-89	223	231	LALEKIIHR					Mascot
1093.5314	1093.6212	0.0898	82	72	80	GSWEKPFDK					Mascot
1126.4657	1126.5664	0.1007	89	158	166	MACDPDFVR			Carbamidomethyl (C)[3], Oxidation (M)[1]		Mascot
1126.4657	1126.5664	0.1007	89	158	166	MACDPDFVR	24	76.644	Carbamidomethyl (C)[3], Oxidation (M)[1]		Mascot
1870.9303	1871.0052	0.0749	40	168	183	HLPCGDVMVSDFRLPK			Carbamidomethyl (C)[4]		Mascot

6	hypothetical protein TRIUR3_13124 [Triticum urartu]	gi|473996456	42874.5	4.93	6	37	0	13.639	21	46.495	

Peptide Information

Calc. Mass	Obsrv. Mass	± da	± ppm	Start Seq.	End Seq.	Sequence	Ion Score	C. I. %	Modification	Rank	Result Type
1076.5558	1076.5583	0.0025	2	24	33	DAVMGIAPFR					Mascot
1085.7115	1085.6533	-0.0582	-54	273	281	VLLLKICVK			Carbamidomethyl (C)[7]		Mascot
1092.5507	1092.592	0.0413	38	24	33	DAVMGIAPFR			Oxidation (M)[4]		Mascot
1154.5333	1154.5677	0.0344	30	367	375	LECVYMPSR			Carbamidomethyl (C)[3]		Mascot
1699.049	1699.0544	0.0054	3	41	56	AAVRPTPRLLLPAAPR					Mascot
1699.049	1699.0544	0.0054	3	41	56	AAVRPTPRLLLPAAPR	21	46.495			Mascot
1709.8606	1709.9583	0.0977	57	57	73	GGSEHALVFPLSPGDAR					Mascot
1979.9458	1979.9819	0.0361	18	282	299	SSRPEGFHVFELSSGSEK					Mascot

7	hypothetical protein TRIUR3_01336 [Triticum urartu]	gi|473961271	34392.3	5.31	10	37	0	6.544			

Peptide Information

Calc. Mass	Obsrv. Mass	± da	± ppm	Start Seq.	End Seq.	Sequence	Ion Score	C. I. %	Modification	Rank	Result Type
832.4159	832.3792	-0.0367	-44	209	215	DDSLNLR					Mascot
1066.5891	1066.5786	-0.0105	-10	49	58	DQKSAPPPVK					Mascot
1077.5171	1077.5797	0.0626	58	81	91	DSSEAALSAAR					Mascot
1114.5891	1114.5723	-0.0168	-15	216	224	ITYARAEYK					Mascot
1125.615	1125.5731	-0.0419	-37	9	18	DDPLALPEKK					Mascot
1154.5854	1154.5677	-0.0177	-15	231	238	RYHNVYFR					Mascot
1158.6226	1158.5547	-0.0679	-59	206	215	LGRDDSLNLR					Mascot
1158.6226	1158.5547	-0.0679	-59	206	215	LGRDDSLNLR					Mascot
1176.6161	1176.6429	0.0268	23	71	80	FHTAEVFAVR					Mascot
1176.6161	1176.6429	0.0268	23	71	80	FHTAEVFAVR					Mascot
1612.7312	1612.8743	0.1431	89	108	120	CSGFLVDWDEEKK			Carbamidomethyl (C)[1]		Mascot
1725.8654	1726.0194	0.154	89	2	17	TADPTSRDDPLALPEK					Mascot

8	hypothetical protein TRIUR3_28570 [Triticum urartu]	gi|474443423	62907.9	6.81	13	37	0	3.187			

Peptide Information

Calc. Mass	Obsrv. Mass	± da	± ppm	Start Seq.	End Seq.	Sequence	Ion Score	C. I. %	Modification	Rank	Result Type
981.4822	981.5356	0.0534	54	327	334	QLCTAFNK			Carbamidomethyl (C)[3]		Mascot
1077.5687	1077.5797	0.011	10	99	108	ATSSFLAQPR					Mascot
1078.4834	1078.5765	0.0931	86	158	166	DSDNEVMLR					Mascot
1085.6136	1085.6533	0.0397	37	247	255	RAEVLCLPK			Carbamidomethyl (C)[6]		Mascot
1094.4783	1094.5773	0.099	90	158	166	DSDNEVMLR			Oxidation (M)[7]		Mascot
1108.5303	1108.5729	0.0426	38	13	22	ILSMESDSAR					Mascot
1109.5773	1109.5797	0.0024	2	326	334	KQLCTAFNK			Carbamidomethyl (C)[4]		Mascot
1124.5253	1124.5582	0.0329	29	13	22	ILSMESDSAR			Oxidation (M)[4]		Mascot
1162.5699	1162.6079	0.038	33	468	477	DRDTQEATVK					Mascot
1550.8842	1550.9021	0.0179	12	61	72	WRNLPWLLPDLK					Mascot
1590.8633	1590.9554	0.0921	58	46	60	VDLAMAARTSVLSTR					Mascot
1892.0323	1892.1588	0.1265	67	421	437	HHLLFASTVMGRAPNLK					Mascot
1971.8511	1972.0272	0.1761	89	195	209	FAERDMNHLLFDCCK			Carbamidomethyl (C)[13,14], Oxidation (M)[6]		Mascot
1985.9716	1986.0424	0.0708	36	167	183	QARDVDGFFSEYPSVLR					Mascot
1994.1011	1994.1057	0.0046	2	405	420	WQLKEFQFGGFRPLLK					Mascot

9	Dynamin-related protein 5A [Triticum urartu]	gi|473813192	65475	8.5	10	36	0	6.752	20	29.304

Peptide Information

Calc. Mass	Obsrv. Mass	± da	± ppm	Start Seq.	End Seq.	Sequence	Ion Score	C. I. %	Modification	Rank	Result Type
832.4159	832.3792	-0.0367	-44	208	214	SQQDINK					Mascot
873.5152	873.5439	0.0287	33	532	538	EIRQLSK					Mascot
1076.5219	1076.5583	0.0364	34	69	77	KEIADETDR					Mascot
1107.6157	1107.644	0.0283	26	167	176	GERTFGVLTK					Mascot
1114.5714	1114.5723	0.0009	1	61	69	FTDFAMVRK					Mascot
1130.5664	1130.563	-0.0034	-3	61	69	FTDFAMVRK			Oxidation (M)[6]		Mascot
1140.6372	1140.5767	-0.0605	-53	284	295	LGKPIANDAGGK					Mascot
1142.5775	1142.5651	-0.0124	-11	60	68	RFTDFAMVR					Mascot
1158.5725	1158.5547	-0.0178	-15	60	68	RFTDFAMVR			Oxidation (M)[7]		Mascot
1158.5725	1158.5547	-0.0178	-15	60	68	RFTDFAMVR	20	29.304	Oxidation (M)[7]		Mascot
1198.5959	1198.6265	0.0306	26	296	304	LYTIMEICR			Carbamidomethyl (C)[8]		Mascot
1743.8945	1743.9325	0.038	22	535	549	QLSKLLDEDPAVMER					Mascot
1979.9784	1979.9819	0.0035	2	443	458	LVDMETSYLTVDFFRK			Oxidation (M)[4]		Mascot

10	hypothetical protein TRIUR3_16373 [Triticum urartu]	gi|474290052	25566.2	8.74	7	35	0	12.855	

Peptide Information

Calc. Mass	Obsrv. Mass	± da	± ppm	Start Seq.	End Seq.	Sequence	Ion Score	C. I. %	Modification	Rank	Result Type
1076.6211	1076.5583	-0.0628	-58	122	130	RYIITASPR					Mascot
1550.781	1550.9021	0.1211	78	18	32	SGQTVAADFDGTLLR					Mascot
1685.9044	1685.9717	0.0673	40	159	174	ATGFMVKPGVLVGEHK			Oxidation (M)[5]		Mascot
1736.8789	1736.9744	0.0955	55	131	145	VMVEPFAREFLGADR					Mascot
1752.8738	1752.9395	0.0657	37	131	145	VMVEPFAREFLGADR			Oxidation (M)[2]		Mascot
1865.9943	1865.9882	-0.0061	-3	123	138	YIITASPRVMVEPFAR			Oxidation (M)[10]		Mascot
1955.9856	1956.0895	0.1039	53	176	194	QAVVKELGDAVPDVGMG DR					Mascot
1958.0529	1958.0208	-0.0321	-16	156	174	SGKATGFMVKPGVLVGE HK			Oxidation (M)[8]		Mascot
1958.0529	1958.0208	-0.0321	-16	156	174	SGKATGFMVKPGVLVGE HK			Oxidation (M)[8]		Mascot
1971.9805	1972.0272	0.0467	24	176	194	QAVVKELGDAVPDVGMG DR			Oxidation (M)[16]		Mascot

Gel Idx/Pos	121/E20	Instr./Gel Origin	BA2151/Sample Project 20140814	Process Status	Analysis Succeeded
Plate [#] Name	[1] Sample Project 20140814	Instrument Sample Name		Spectra	11

Rank	Protein Name	Accession No.	Protein MW	Protein PI	Pep. Count	Protein Score	Protein Score C. I. %	Intensity Matched	Total Ion Score	Total Ion C. I. %	Confirmed
1	RecName: Full=Alpha-amylase inhibitor 0.19; AltName: Full=0.19 alpha-AI; Short=0.19 AI	gi|123963	13898.6	6.66	6	283	100	27.661	243	100	

Protein Group

Protein Name	Accession No.	Protein MW	Protein PI
0.19 alpha-amylase inhibitor [Triticum aestivum]	gi|2116581	13898.6	6.6599998474 1211
0.19 dimeric alpha-amylase inhibitor [Triticum aestivum]	gi|54778509	13898.6	6.6599998474 1211
0.19 dimeric alpha-amylase inhibitor [Triticum aestivum]	gi|54778501	13898.6	6.6599998474 1211
Chain A, 0.19 Alpha-Amylase Inhibitor From Wheat	gi|3318681	13898.6	6.6599998474 1211
Chain B, 0.19 Alpha-Amylase Inhibitor From Wheat	gi|3318682	13898.6	6.6599998474 1211
Chain C, 0.19 Alpha-Amylase Inhibitor From Wheat	gi|3318683	13898.6	6.6599998474 1211
Chain D, 0.19 Alpha-Amylase Inhibitor From Wheat	gi|3318684	13898.6	6.6599998474 1211
alpha-amylase inhibitor 0.19 [Triticum aestivum]	gi|66841026	13340.4	6.8600001335 144

Peptide Information

Calc. Mass	Obsrv. Mass	± da	± ppm	Start Seq.	End Seq.	Sequence	Ion Score	C. I. %	Modification	Rank	Result Type
1162.6249	1162.7305	0.1056	91	90	100	LTAASITAVCR			Carbamidomethyl (C)[10]		Mascot
1570.8007	1570.9443	0.1436	91	26	39	LQCNGSQVPEAVLR			Carbamidomethyl (C)[3]		Mascot
1612.7463	1612.8998	0.1535	95	67	82	EHGAQEGQAGTGAFPR					Mascot
1612.7463	1612.8998	0.1535	95	67	82	EHGAQEGQAGTGAFPR	141	100			Mascot
1617.8993	1617.8964	-0.0029	-2	86	100	EVVKLTAASITAVCR			Carbamidomethyl (C)[14]		Mascot
1663.8361	1663.9633	0.1272	76	101	116	LPIVVDASGDGAYVCK			Carbamidomethyl (C)[15]		Mascot
1862.7731	1862.9509	0.1778	95	40	53	DCCQQLAHISEWCR			Carbamidomethyl (C)[2,3,13]		Mascot
1862.7731	1862.9509	0.1778	95	40	53	DCCQQLAHISEWCR	102	100	Carbamidomethyl (C)[2,3,13]		Mascot

Rank	Protein Name	Accession No.	Protein MW	Protein PI	Pep. Count	Protein Score	Protein Score C. I. %	Intensity Matched	Total Ion Score	Total Ion C. I. %	Confirmed
2	dimeric alpha-amylase inhibitor precursor, partial [Triticum aestivum]	gi|108597921	14029.7	6.69	6	282	100	27.661	243	100	

Peptide Information

Calc. Mass	Obsrv. Mass	± da	± ppm	Start Seq.	End Seq.	Sequence	Ion Score	C. I. %	Modification	Rank	Result Type
1162.6249	1162.7305	0.1056	91	91	101	LTAASITAVCR			Carbamidomethyl (C)[10]		Mascot
1570.8007	1570.9443	0.1436	91	27	40	LQCNGSQVPEAVLR			Carbamidomethyl (C)[3]		Mascot
1612.7463	1612.8998	0.1535	95	68	83	EHGAQEGQAGTGAFPR					Mascot
1612.7463	1612.8998	0.1535	95	68	83	EHGAQEGQAGTGAFPR	141	100			Mascot
1617.8993	1617.8964	-0.0029	-2	87	101	EVVKLTAASITAVCR			Carbamidomethyl (C)[14]		Mascot
1663.8361	1663.9633	0.1272	76	102	117	LPIVVDASGDGAYVCK			Carbamidomethyl (C)[15]		Mascot
1862.7731	1862.9509	0.1778	95	41	54	DCCQQLAHISEWCR			Carbamidomethyl (C)[2,3,13]		Mascot
1862.7731	1862.9509	0.1778	95	41	54	DCCQQLAHISEWCR	102	100	Carbamidomethyl (C)[2,3,13]		Mascot

3 dimeric alpha-amylase inhibitor [Triticum dicoccoides] gi|227809009 15730.5 5.58 6 280 100 27.661 243 100

Protein Group

dimeric alpha-amylase inhibitor [Triticum aestivum]	gi|65993781	15688.5	5.5799 999237 0605
dimeric alpha-amylase inhibitor [Triticum aestivum]	gi|386877038	15702.5	5.5799 999237 0605
dimeric alpha-amylase inhibitor [Triticum dicoccoides]	gi|227809005	15730.5	5.5799 999237 0605

Peptide Information

Calc. Mass	Obsrv. Mass	± da	± ppm	Start Seq.	End Seq.	Sequence	Ion Score	C. I. %	Modification	Rank	Result Type
1162.6249	1162.7305	0.1056	91	107	117	LTAASITAVCR			Carbamidomethyl (C)[10]		Mascot
1570.8007	1570.9443	0.1436	91	43	56	LQCNGSQVPEAVLR			Carbamidomethyl (C)[3]		Mascot
1612.7463	1612.8998	0.1535	95	84	99	EHGAQEGQAGTGAFPR					Mascot
1612.7463	1612.8998	0.1535	95	84	99	EHGAQEGQAGTGAFPR	141	100			Mascot
1617.8993	1617.8964	-0.0029	-2	103	117	EVVKLTAASITAVCR			Carbamidomethyl (C)[14]		Mascot
1663.8361	1663.9633	0.1272	76	118	133	LPIVVDASGDGAYVCK			Carbamidomethyl (C)[15]		Mascot
1862.7731	1862.9509	0.1778	95	57	70	DCCQQLAHISEWCR			Carbamidomethyl (C)[2,3,13]		Mascot
1862.7731	1862.9509	0.1778	95	57	70	DCCQQLAHISEWCR	102	100	Carbamidomethyl (C)[2,3,13]		Mascot

4 0.19 dimeric alpha-amylase inhibitor [Triticum aestivum] gi|54778503 13826.6 7.45 5 273 100 25.903 243 100

Peptide Information

Calc. Mass	Obsrv. Mass	± da	± ppm	Start Seq.	End Seq.	Sequence	Ion Score	C. I. %	Modification	Rank	Result Type

1162.6249	1162.7305	0.1056	91	90	100	LTAASITAVCR			Carbamidomethyl (C)[10]		Mascot
1612.7463	1612.8998	0.1535	95	67	82	EHGAQEGQAGTGAFPR					Mascot
1612.7463	1612.8998	0.1535	95	67	82	EHGAQEGQAGTGAFPR	141	100			Mascot
1617.8993	1617.8964	-0.0029	-2	86	100	EVVKLTAASITAVCR			Carbamidomethyl (C)[14]		Mascot
1663.8361	1663.9633	0.1272	76	101	116	LPIVVDASGDGAYVCK			Carbamidomethyl (C)[15]		Mascot
1862.7731	1862.9509	0.1778	95	40	53	DCCQQLAHISEWCR			Carbamidomethyl (C)[2,3,13]		Mascot
1862.7731	1862.9509	0.1778	95	40	53	DCCQQLAHISEWCR	102	100	Carbamidomethyl (C)[2,3,13]		Mascot

5	dimeric alpha-amylase inhibitor, partial [Triticum aestivum]	gi|386877068	14415.8	6.88	5	272	100	27.2	243	100	

Peptide Information

Calc. Mass	Obsrv. Mass	± da	± ppm	Start Seq.	End Seq.	Sequence	Ion Score	C. I. %	Modification	Rank	Result Type
1162.6249	1162.7305	0.1056	91	95	105	LTAASITAVCR			Carbamidomethyl (C)[10]		Mascot
1570.8007	1570.9443	0.1436	91	31	44	LQCNGSQVPEAVLR			Carbamidomethyl (C)[3]		Mascot
1612.7463	1612.8998	0.1535	95	72	87	EHGAQEGQAGTGAFPR					Mascot
1612.7463	1612.8998	0.1535	95	72	87	EHGAQEGQAGTGAFPR	141	100			Mascot
1617.8993	1617.8964	-0.0029	-2	91	105	EVVKLTAASITAVCR			Carbamidomethyl (C)[14]		Mascot
1862.7731	1862.9509	0.1778	95	45	58	DCCQQLAHISEWCR			Carbamidomethyl (C)[2,3,13]		Mascot
1862.7731	1862.9509	0.1778	95	45	58	DCCQQLAHISEWCR	102	100	Carbamidomethyl (C)[2,3,13]		Mascot

6	dimeric alpha-amylase inhibitor [Triticum dicoccoides]	gi|227809180	15716.5	5.58	5	271	100	25.903	243	100	

Protein Group

dimeric alpha-amylase inhibitor [Triticum aestivum]	gi|65993925	15702.5	5.5799 999237 0605
dimeric alpha-amylase inhibitor [Triticum dicoccoides]	gi|227809156	15716.5	5.5799 999237 0605
dimeric alpha-amylase inhibitor [Triticum timopheevii subsp. armeniacum]	gi|227809268	15702.5	5.5799 999237 0605

Peptide Information

Calc. Mass	Obsrv. Mass	± da	± ppm	Start Seq.	End Seq.	Sequence	Ion Score	C. I. %	Modification	Rank	Result Type
1162.6249	1162.7305	0.1056	91	107	117	LTAASITAVCR			Carbamidomethyl (C)[10]		Mascot
1612.7463	1612.8998	0.1535	95	84	99	EHGAQEGQAGTGAFPR					Mascot
1612.7463	1612.8998	0.1535	95	84	99	EHGAQEGQAGTGAFPR	141	100			Mascot
1617.8993	1617.8964	-0.0029	-2	103	117	EVVKLTAASITAVCR			Carbamidomethyl (C)[14]		Mascot
1663.8361	1663.9633	0.1272	76	118	133	LPIVVDASGDGAYVCK			Carbamidomethyl (C)[15]		Mascot
1862.7731	1862.9509	0.1778	95	57	70	DCCQQLAHISEWCR			Carbamidomethyl (C)[2,3,13]		Mascot

1862.7731	1862.9509	0.1778	95	57	70	DCCQQLAHISEWCR	102	100	Carbamidomethyl (C)[2,3,13]		Mascot

7	dimeric alpha-amylase inhibitor [Triticum aestivum]	gi|255988225	15665.5	6.69	3	257	100	25.723	243	100	

Peptide Information

Calc. Mass	Obsrv. Mass	± da	± ppm	Start Seq.	End Seq.	Sequence	Ion Score	C. I. %	Modification	Rank	Result Type
1570.8007	1570.9443	0.1436	91	43	56	LQCNGSQVPEAVLR			Carbamidomethyl (C)[3]		Mascot
1612.7463	1612.8998	0.1535	95	84	99	EHGAQEGQAGTGAFPR					Mascot
1612.7463	1612.8998	0.1535	95	84	99	EHGAQEGQAGTGAFPR	141	100			Mascot
1862.7731	1862.9509	0.1778	95	57	70	DCCQQLAHISEWCR			Carbamidomethyl (C)[2,3,13]		Mascot
1862.7731	1862.9509	0.1778	95	57	70	DCCQQLAHISEWCR	102	100	Carbamidomethyl (C)[2,3,13]		Mascot

8	dimeric alpha-amylase inhibitor [Triticum dicoccoides]	gi|114215806	13862.5	5.25	5	171	100	21.243	141	100	

Protein Group

dimeric alpha-amylase inhibitor [Triticum dicoccoides]	gi|114215808	13921.6	5.25

Peptide Information

Calc. Mass	Obsrv. Mass	± da	± ppm	Start Seq.	End Seq.	Sequence	Ion Score	C. I. %	Modification	Rank	Result Type
1162.6249	1162.7305	0.1056	91	90	100	LTAASITAVCR			Carbamidomethyl (C)[10]		Mascot
1570.8007	1570.9443	0.1436	91	26	39	LQCNGSQVPEAVLR			Carbamidomethyl (C)[3]		Mascot
1612.7463	1612.8998	0.1535	95	67	82	EHGAQEGQAGTGAFPR					Mascot
1612.7463	1612.8998	0.1535	95	67	82	EHGAQEGQAGTGAFPR	141	100			Mascot
1617.8993	1617.8964	-0.0029	-2	86	100	EVVKLTAASITAVCR			Carbamidomethyl (C)[14]		Mascot
1663.8361	1663.9633	0.1272	76	101	116	LPIVVDASGDGAYVCK			Carbamidomethyl (C)[15]		Mascot

9	dimeric alpha-amylase inhibitor [Triticum dicoccoides]	gi|227809252	15716.4	5.01	5	169	100	21.243	141	100	

Protein Group

dimeric alpha-amylase inhibitor [Triticum aestivum]	gi|65993829	15722.4	5.5799 999237 0605
dimeric alpha-amylase inhibitor [Triticum dicoccoides]	gi|227809102	15678.4	5.25
dimeric alpha-amylase inhibitor [Triticum dicoccoides]	gi|227809254	15753.5	4.8299 999237 0605
dimeric alpha-amylase inhibitor [Triticum dicoccoides]	gi|227809078	15722.4	4.8299 999237 0605

Peptide Information

Calc. Mass	Obsrv. Mass	± da	± ppm	Start Seq.	End Seq.	Sequence	Ion Score	C. I. %	Modification	Rank	Result Type
1162.6249	1162.7305	0.1056	91	107	117	LTAASITAVCR			Carbamidomethyl (C)[10]		Mascot
1570.8007	1570.9443	0.1436	91	43	56	LQCNGSQVPEAVLR			Carbamidomethyl (C)[3]		Mascot
1612.7463	1612.8998	0.1535	95	84	99	EHGAQEGQAGTGAFPR					Mascot
1612.7463	1612.8998	0.1535	95	84	99	EHGAQEGQAGTGAFPR	141	100			Mascot
1617.8993	1617.8964	-0.0029	-2	103	117	EVVKLTAASITAVCR			Carbamidomethyl (C)[14]		Mascot
1663.8361	1663.9633	0.1272	76	118	133	LPIVVDASGDGAYVCK			Carbamidomethyl (C)[15]		Mascot

10 dimeric alpha-amylase inhibitor [Triticum dicoccoides] gi|114215804 13862.5 5.25 4 163 100 21.052 141 100

Peptide Information

Calc. Mass	Obsrv. Mass	± da	± ppm	Start Seq.	End Seq.	Sequence	Ion Score	C. I. %	Modification	Rank	Result Type
1162.6249	1162.7305	0.1056	91	90	100	LTAASITAVCR			Carbamidomethyl (C)[10]		Mascot
1570.8007	1570.9443	0.1436	91	26	39	LQCNGSQVPEAVLR			Carbamidomethyl (C)[3]		Mascot
1612.7463	1612.8998	0.1535	95	67	82	EHGAQEGQAGTGAFPR					Mascot
1612.7463	1612.8998	0.1535	95	67	82	EHGAQEGQAGTGAFPR	141	100			Mascot
1663.8361	1663.9633	0.1272	76	101	116	LPIVVDASGDGAYVCK			Carbamidomethyl (C)[15]		Mascot

Gel Idx/Pos	122/E21	Instr./Gel Origin	BA2151/Sample Project 20140814	Process Status	Analysis Succeeded
Plate [#] Name	[1] Sample Project 20140814	Instrument Sample Name		Spectra	11

Rank	Protein Name	Accession No.	Protein MW	Protein PI	Pep. Count	Protein Score	Protein Score C. I. %	Intensity Matched	Total Ion Score	Total Ion C. I. %	Confirmed
1	Ribulose bisphosphate carboxylase small chain, chloroplastic [Triticum urartu]	gi|474416311	15090.5	5.85	14	338	100	37.781	212	100	

Peptide Information

Calc. Mass	Obsrv. Mass	± da	± ppm	Start Seq.	End Seq.	Sequence	Ion Score	C. I. %	Modification	Rank	Result Type
906.5043	906.5795	0.0752	83	29	35	QVDYLIR					Mascot
914.4229	914.481	0.0581	64	65	70	YWTMWK					Mascot
930.4178	930.4788	0.061	66	65	70	YWTMWK			Oxidation (M)[4]		Mascot
965.4873	965.5659	0.0786	81	100	107	IIGFDNMR					Mascot
965.4873	965.5659	0.0786	81	100	107	IIGFDNMR	25	77.149			Mascot
981.4822	981.5511	0.0689	70	100	107	IIGFDNMR			Oxidation (M)[7]		Mascot
981.4822	981.5511	0.0689	70	100	107	IIGFDNMR	15	0	Oxidation (M)[7]		Mascot
1012.4734	1012.5549	0.0815	80	92	99	EYPDAYVR					Mascot
1012.4734	1012.5549	0.0815	80	92	99	EYPDAYVR	59	99.99			Mascot
1140.5684	1140.6562	0.0878	77	91	99	KEYPDAYVR					Mascot
1165.571	1165.6488	0.0778	67	38	46	WVPCLEFSK			Carbamidomethyl (C)[4]		Mascot
1165.571	1165.6488	0.0778	67	38	46	WVPCLEFSK	29	90.669	Carbamidomethyl (C)[4]		Mascot
1197.6991	1197.6556	-0.0435	-36	2	11	QVWPIEGIKK					Mascot
1365.5819	1365.696	0.1141	84	53	64	EHNASPGYYDGR	100	100			Mascot
1922.0521	1922.1836	0.1315	68	12	28	FETLSYLPPLSTEALLK					Mascot
2050.147	2050.2974	0.1504	73	11	28	KFETLSYLPPLSTEALLK					Mascot
2268.0789	2268.2593	0.1804	80	108	127	QVQCVSFIAFKPPGCEESGK			Carbamidomethyl (C)[4,15]		Mascot
2296.0837	2296.2825	0.1988	87	71	90	LPMFGCTDATQVINEVEEVK			Carbamidomethyl (C)[6], Oxidation (M)[3]		Mascot
2339.116	2339.302	0.186	80	108	128	QVQCVSFIAFKPPGCEESGKA			Carbamidomethyl (C)[4,15]		Mascot
2408.1836	2408.375	0.1914	79	71	91	LPMFGCTDATQVINEVEEVKK			Carbamidomethyl (C)[6]		Mascot
2424.1785	2424.363	0.1845	76	71	91	LPMFGCTDATQVINEVEEVKK			Carbamidomethyl (C)[6], Oxidation (M)[3]		Mascot

Rank	Protein Name	Accession No.	Protein MW	Protein PI	Pep. Count	Protein Score	Protein Score C. I. %	Intensity Matched	Total Ion Score	Total Ion C. I. %	Confirmed
2	Ribulose bisphosphate carboxylase small chain, chloroplastic [Triticum urartu]	gi|473882355	18742.4	8.65	15	332	100	37.854	212	100	

Peptide Information

Calc. Mass	Obsrv. Mass	± da	± ppm	Start Seq.	End Seq.	Sequence	Ion Score	C. I. %	Modification	Rank	Result Type

Calc. Mass	Obsrv. Mass	± da	± ppm	Start Seq.	End Seq.	Sequence	Ion Score	C. I. %	Modification	Rank	Result Type
906.5043	906.5795	0.0752	83	65	71	QVDYLIR					Mascot
914.4229	914.481	0.0581	64	101	106	YWTMWK					Mascot
928.5323	928.5342	0.0019	2	21	29	STAGLPARR					Mascot
930.4178	930.4788	0.061	66	101	106	YWTMWK			Oxidation (M)[4]		Mascot
965.4873	965.5659	0.0786	81	136	143	IIGFDNMR					Mascot
965.4873	965.5659	0.0786	81	136	143	IIGFDNMR	25	77.149			Mascot
981.4822	981.5511	0.0689	70	136	143	IIGFDNMR			Oxidation (M)[7]		Mascot
981.4822	981.5511	0.0689	70	136	143	IIGFDNMR	15	0	Oxidation (M)[7]		Mascot
1012.4734	1012.5549	0.0815	80	128	135	EYPDAYVR					Mascot
1012.4734	1012.5549	0.0815	80	128	135	EYPDAYVR	59	99.99			Mascot
1140.5684	1140.6562	0.0878	77	127	135	KEYPDAYVR					Mascot
1165.571	1165.6488	0.0778	67	74	82	WVPCLEFSK			Carbamidomethyl (C)[4]		Mascot
1165.571	1165.6488	0.0778	67	74	82	WVPCLEFSK	29	90.669	Carbamidomethyl (C)[4]		Mascot
1365.5819	1365.696	0.1141	84	89	100	EHNASPGYYDGR	100	100			Mascot
1862.9681	1862.9376	-0.0305	-16	2	20	APTVMASSATSVAPFQG LK					Mascot
1922.0521	1922.1836	0.1315	68	48	64	FETLSYLPPLSTEALLK					Mascot
2050.147	2050.2974	0.1504	73	47	64	KFETLSYLPPLSTEALLK					Mascot
2268.0789	2268.2593	0.1804	80	144	163	QVQCVSFIAFKPPGCEES GK			Carbamidomethyl (C)[4,15]		Mascot
2296.0837	2296.2825	0.1988	87	107	126	LPMFGCTDATQVINEVEE VK			Carbamidomethyl (C)[6], Oxidation (M)[3]		Mascot
2339.116	2339.302	0.186	80	144	164	QVQCVSFIAFKPPGCEES GKA			Carbamidomethyl (C)[4,15]		Mascot
2408.1836	2408.375	0.1914	79	107	127	LPMFGCTDATQVINEVEE VKK			Carbamidomethyl (C)[6]		Mascot
2424.1785	2424.363	0.1845	76	107	127	LPMFGCTDATQVINEVEE VKK			Carbamidomethyl (C)[6], Oxidation (M)[3]		Mascot

3	ribulose-1,5-bisphosphate carboxylase/oxygenase small subunit [Triticum aestivum]	gi|11990897	19732.9	8.8	14	321	100	38.883	212	100

Peptide Information

Calc. Mass	Obsrv. Mass	± da	± ppm	Start Seq.	End Seq.	Sequence	Ion Score	C. I. %	Modification	Rank	Result Type
906.5043	906.5795	0.0752	83	76	82	QVDYLIR					Mascot
914.4229	914.481	0.0581	64	112	117	YWTMWK					Mascot
930.4178	930.4788	0.061	66	112	117	YWTMWK			Oxidation (M)[4]		Mascot
965.4873	965.5659	0.0786	81	147	154	IIGFDNMR					Mascot
965.4873	965.5659	0.0786	81	147	154	IIGFDNMR	25	77.149			Mascot
981.4822	981.5511	0.0689	70	147	154	IIGFDNMR			Oxidation (M)[7]		Mascot
981.4822	981.5511	0.0689	70	147	154	IIGFDNMR	15	0	Oxidation (M)[7]		Mascot

1012.4734	1012.5549	0.0815	80	139	146	EYPDAYVR					Mascot
1012.4734	1012.5549	0.0815	80	139	146	EYPDAYVR	59	99.99			Mascot
1140.5684	1140.6562	0.0878	77	138	146	KEYPDAYVR					Mascot
1165.571	1165.6488	0.0778	67	85	93	WVPCLEFSK			Carbamidomethyl (C)[4]		Mascot
1165.571	1165.6488	0.0778	67	85	93	WVPCLEFSK	29	90.669	Carbamidomethyl (C)[4]		Mascot
1262.6084	1262.7251	0.1167	92	31	44	SNGASLGSVSNGGR					Mascot
1262.6084	1262.7251	0.1167	92	31	44	SNGASLGSVSNGGR					Mascot
1365.5819	1365.696	0.1141	84	100	111	EHNASPGYYDGR	100	100			Mascot
1922.0521	1922.1836	0.1315	68	59	75	FETLSYLPPLSTEALLK					Mascot
2050.147	2050.2974	0.1504	73	58	75	KFETLSYLPPLSTEALLK					Mascot
2268.0789	2268.2593	0.1804	80	155	174	QVQCVSFIAFKPPGCEESGK			Carbamidomethyl (C)[4,15]		Mascot
2296.0837	2296.2825	0.1988	87	118	137	LPMFGCTDATQVINEVEEVK			Carbamidomethyl (C)[6], Oxidation (M)[3]		Mascot
2339.116	2339.302	0.186	80	155	175	QVQCVSFIAFKPPGCEESGKA			Carbamidomethyl (C)[4,15]		Mascot
2408.1836	2408.375	0.1914	79	118	138	LPMFGCTDATQVINEVEEVKK			Carbamidomethyl (C)[6]		Mascot
2424.1785	2424.363	0.1845	76	118	138	LPMFGCTDATQVINEVEEVKK			Carbamidomethyl (C)[6], Oxidation (M)[3]		Mascot

4	ribulose-1,5-bisphosphate carboxylase/oxygenase small subunit [Triticum aestivum]	gi|11990893	19728.9	9.06	14	317	100	38.883	212	100

Peptide Information

Calc. Mass	Obsrv. Mass	± da	± ppm	Start Seq.	End Seq.	Sequence	Ion Score	C. I. %	Modification	Rank	Result Type
906.5043	906.5795	0.0752	83	76	82	QVDYLIR					Mascot
914.4229	914.481	0.0581	64	112	117	YWTMWK					Mascot
930.4178	930.4788	0.061	66	112	117	YWTMWK			Oxidation (M)[4]		Mascot
965.4873	965.5659	0.0786	81	147	154	IIGFDNMR					Mascot
965.4873	965.5659	0.0786	81	147	154	IIGFDNMR	25	77.149			Mascot
981.4822	981.5511	0.0689	70	147	154	IIGFDNMR			Oxidation (M)[7]		Mascot
981.4822	981.5511	0.0689	70	147	154	IIGFDNMR	15	0	Oxidation (M)[7]		Mascot
1012.4734	1012.5549	0.0815	80	139	146	EYPDAYVR					Mascot
1012.4734	1012.5549	0.0815	80	139	146	EYPDAYVR	59	99.99			Mascot
1140.5684	1140.6562	0.0878	77	138	146	KEYPDAYVR					Mascot
1165.571	1165.6488	0.0778	67	85	93	WVPCLEFSK			Carbamidomethyl (C)[4]		Mascot
1165.571	1165.6488	0.0778	67	85	93	WVPCLEFSK	29	90.669	Carbamidomethyl (C)[4]		Mascot
1262.6084	1262.7251	0.1167	92	31	44	SNGASLGSVSNGGR					Mascot
1262.6084	1262.7251	0.1167	92	31	44	SNGASLGSVSNGGR					Mascot
1365.5819	1365.696	0.1141	84	100	111	EHNASPGYYDGR	100	100			Mascot

Calc. Mass	Obsrv. Mass	± da	± ppm	Start Seq.	End Seq.	Sequence	Ion Score	C. I. %	Modification	Rank	Result Type
1922.0521	1922.1836	0.1315	68	59	75	FETLSYLPPLSTEALLK					Mascot
2050.147	2050.2974	0.1504	73	58	75	KFETLSYLPPLSTEALLK					Mascot
2268.0789	2268.2593	0.1804	80	155	174	QVQCVSFIAFKPPGCEES GK			Carbamidomethyl (C)[4,15]		Mascot
2296.0837	2296.2825	0.1988	87	118	137	LPMFGCTDATQVINEVEE VK			Carbamidomethyl (C)[6], Oxidation (M)[3]		Mascot
2339.116	2339.302	0.186	80	155	175	QVQCVSFIAFKPPGCEES GKA			Carbamidomethyl (C)[4,15]		Mascot
2408.1836	2408.375	0.1914	79	118	138	LPMFGCTDATQVINEVEE VKK			Carbamidomethyl (C)[6]		Mascot
2424.1785	2424.363	0.1845	76	118	138	LPMFGCTDATQVINEVEE VKK			Carbamidomethyl (C)[6], Oxidation (M)[3]		Mascot

5	unnamed protein product [Triticum aestivum]	gi|21866	13274.5	5.84	11	309	100	36.132	212	100	

Protein Group

RecName: Full=Ribulose bisphosphate carboxylase small chain clone 512; Short=RuBisCO small subunit	gi|132107	13274.5	5.8400 001525 8789

Peptide Information

Calc. Mass	Obsrv. Mass	± da	± ppm	Start Seq.	End Seq.	Sequence	Ion Score	C. I. %	Modification	Rank	Result Type
906.5043	906.5795	0.0752	83	14	20	QVDYLIR					Mascot
914.4229	914.481	0.0581	64	50	55	YWTMWK					Mascot
930.4178	930.4788	0.061	66	50	55	YWTMWK			Oxidation (M)[4]		Mascot
965.4873	965.5659	0.0786	81	85	92	IIGFDNMR					Mascot
965.4873	965.5659	0.0786	81	85	92	IIGFDNMR	25	77.149			Mascot
981.4822	981.5511	0.0689	70	85	92	IIGFDNMR			Oxidation (M)[7]		Mascot
981.4822	981.5511	0.0689	70	85	92	IIGFDNMR	15	0	Oxidation (M)[7]		Mascot
1012.4734	1012.5549	0.0815	80	77	84	EYPDAYVR					Mascot
1012.4734	1012.5549	0.0815	80	77	84	EYPDAYVR	59	99.99			Mascot
1140.5684	1140.6562	0.0878	77	76	84	KEYPDAYVR					Mascot
1165.571	1165.6488	0.0778	67	23	31	WVPCLEFSK			Carbamidomethyl (C)[4]		Mascot
1165.571	1165.6488	0.0778	67	23	31	WVPCLEFSK	29	90.669	Carbamidomethyl (C)[4]		Mascot
1365.5819	1365.696	0.1141	84	38	49	EHNASPGYYDGR	100	100			Mascot
2268.0789	2268.2593	0.1804	80	93	112	QVQCVSFIAFKPPGCEES GK			Carbamidomethyl (C)[4,15]		Mascot
2296.0837	2296.2825	0.1988	87	56	75	LPMFGCTDATQVINEVEE VK			Carbamidomethyl (C)[6], Oxidation (M)[3]		Mascot
2339.116	2339.302	0.186	80	93	113	QVQCVSFIAFKPPGCEES GKA			Carbamidomethyl (C)[4,15]		Mascot
2408.1836	2408.375	0.1914	79	56	76	LPMFGCTDATQVINEVEE VKK			Carbamidomethyl (C)[6]		Mascot
2424.1785	2424.363	0.1845	76	56	76	LPMFGCTDATQVINEVEE VKK			Carbamidomethyl (C)[6], Oxidation (M)[3]		Mascot

6	ribulose-bisphosphate carboxylase (EC 4.1.1.39) small chain precursor (clone 234) - wheat (fragment)	gi|82619	15573	8.95	11	201	100	37.491	112	100	

Peptide Information

Calc. Mass	Obsrv. Mass	± da	± ppm	Start Seq.	End Seq.	Sequence	Ion Score	C. I. %	Modification	Rank	Result Type
906.5043	906.5795	0.0752	83	64	70	QVDYLIR					Mascot
965.4873	965.5659	0.0786	81	110	117	IIGFDNMR					Mascot
965.4873	965.5659	0.0786	81	110	117	IIGFDNMR	25	77.149			Mascot
981.4822	981.5511	0.0689	70	110	117	IIGFDNMR			Oxidation (M)[7]		Mascot
981.4822	981.5511	0.0689	70	110	117	IIGFDNMR	15	0	Oxidation (M)[7]		Mascot
1012.4734	1012.5549	0.0815	80	102	109	EYPDAYVR					Mascot
1012.4734	1012.5549	0.0815	80	102	109	EYPDAYVR	59	99.99			Mascot
1140.5684	1140.6562	0.0878	77	101	109	KEYPDAYVR					Mascot
1165.571	1165.6488	0.0778	67	73	81	WVPCLEFSK			Carbamidomethyl (C)[4]		Mascot
1165.571	1165.6488	0.0778	67	73	81	WVPCLEFSK	29	90.669	Carbamidomethyl (C)[4]		Mascot
1262.6084	1262.7251	0.1167	92	19	32	SNGASLGSVSNGGR					Mascot
1262.6084	1262.7251	0.1167	92	19	32	SNGASLGSVSNGGR					Mascot
1727.9803	1727.9868	0.0065	4	1	17	VAPFQGLKSTAGLPVSR					Mascot
1922.0521	1922.1836	0.1315	68	47	63	FETLSYLPPLSTEALLK					Mascot
2050.147	2050.2974	0.1504	73	46	63	KFETLSYLPPLSTEALLK					Mascot
2268.0789	2268.2593	0.1804	80	118	137	QVQCVSFIAFKPPGCEES GK			Carbamidomethyl (C)[4,15]		Mascot
2339.116	2339.302	0.186	80	118	138	QVQCVSFIAFKPPGCEES GKA			Carbamidomethyl (C)[4,15]		Mascot

7 putative rubisco small subunit [Triticum durum] gi|62176930 19322.7 8.59 14 200 100 26.118 87 100

Peptide Information

Calc. Mass	Obsrv. Mass	± da	± ppm	Start Seq.	End Seq.	Sequence	Ion Score	C. I. %	Modification	Rank	Result Type
906.5043	906.5795	0.0752	83	71	77	QVDYLIR					Mascot
914.4229	914.481	0.0581	64	107	112	YWTMWK					Mascot
930.4178	930.4788	0.061	66	107	112	YWTMWK			Oxidation (M)[4]		Mascot
933.5152	933.5845	0.0693	74	142	149	VIGFDNLR					Mascot
945.4999	945.5121	0.0122	13	16	24	STDGLPISR					Mascot
1012.4734	1012.5549	0.0815	80	134	141	EYPDAYVR					Mascot
1012.4734	1012.5549	0.0815	80	134	141	EYPDAYVR	59	99.99			Mascot
1140.5684	1140.6562	0.0878	77	133	141	KEYPDAYVR					Mascot
1165.571	1165.6488	0.0778	67	80	88	WVPCLEFSK			Carbamidomethyl (C)[4]		Mascot
1165.571	1165.6488	0.0778	67	80	88	WVPCLEFSK	29	90.669	Carbamidomethyl (C)[4]		Mascot

Calc. Mass	Obsrv. Mass	± da	± ppm	Start Seq.	End Seq.	Sequence	Ion Score	C. I. %	Modification	Rank	Result Type
1363.7217	1363.6809	-0.0408	-30	2	15	ASSATSVAPFQGLK					Mascot
1381.5768	1381.6897	0.1129	82	95	106	EHNSSPGYYDGR					Mascot
1922.0521	1922.1836	0.1315	68	54	70	FETLSYLPPLSTEALLK					Mascot
2050.147	2050.2974	0.1504	73	53	70	KFETLSYLPPLSTEALLK					Mascot
2290.2039	2290.2556	0.0517	23	2	24	ASSATSVAPFQGLKSTD GLPISR					Mascot
2296.085	2296.2825	0.1975	86	150	169	QVQCVSFIAFRPPGCEES GK			Carbamidomethyl (C)[4,15]		Mascot
2408.1836	2408.375	0.1914	79	113	133	LPMFGCTDATQVLNEVE EVKK			Carbamidomethyl (C)[6]		Mascot
2424.1785	2424.363	0.1845	76	113	133	LPMFGCTDATQVLNEVE EVKK			Carbamidomethyl (C)[6], Oxidation (M)[3]		Mascot

8	ribulosebisphosphate carboxylase [Triticum aestivum]	gi|755804	18348	8.95	11	196	100	37.491	112	100	

Peptide Information

Calc. Mass	Obsrv. Mass	± da	± ppm	Start Seq.	End Seq.	Sequence	Ion Score	C. I. %	Modification	Rank	Result Type
906.5043	906.5795	0.0752	83	64	70	QVDYLIR					Mascot
965.4873	965.5659	0.0786	81	135	142	IIGFDNMR					Mascot
965.4873	965.5659	0.0786	81	135	142	IIGFDNMR	25	77.149			Mascot
981.4822	981.5511	0.0689	70	135	142	IIGFDNMR			Oxidation (M)[7]		Mascot
981.4822	981.5511	0.0689	70	135	142	IIGFDNMR	15	0	Oxidation (M)[7]		Mascot
1012.4734	1012.5549	0.0815	80	127	134	EYPDAYVR					Mascot
1012.4734	1012.5549	0.0815	80	127	134	EYPDAYVR	59	99.99			Mascot
1140.5684	1140.6562	0.0878	77	126	134	KEYPDAYVR					Mascot
1165.571	1165.6488	0.0778	67	73	81	WVPCLEFSK			Carbamidomethyl (C)[4]		Mascot
1165.571	1165.6488	0.0778	67	73	81	WVPCLEFSK	29	90.669	Carbamidomethyl (C)[4]		Mascot
1262.6084	1262.7251	0.1167	92	19	32	SNGASLGSVSNGGR					Mascot
1262.6084	1262.7251	0.1167	92	19	32	SNGASLGSVSNGGR					Mascot
1727.9803	1727.9868	0.0065	4	1	17	VAPFQGLKSTAGLPVSR					Mascot
1922.0521	1922.1836	0.1315	68	47	63	FETLSYLPPLSTEALLK					Mascot
2050.147	2050.2974	0.1504	73	46	63	KFETLSYLPPLSTEALLK					Mascot
2268.0789	2268.2593	0.1804	80	143	162	QVQCVSFIAFKPPGCEES GK			Carbamidomethyl (C)[4,15]		Mascot
2339.116	2339.302	0.186	80	143	163	QVQCVSFIAFKPPGCEES GKA			Carbamidomethyl (C)[4,15]		Mascot

9	Ribulose bisphosphate carboxylase small chain PW9, chloroplastic [Triticum urartu]	gi|473721334	19806.9	8.81	13	182	100	26.354	87	100	

Peptide Information

Calc. Mass	Obsrv. Mass	± da	± ppm	Start Seq.	End Seq.	Sequence	Ion Score	C. I. %	Modification	Rank	Result Type

906.5043	906.5795	0.0752	83	76	82	QVDYLIR					Mascot
914.4229	914.481	0.0581	64	112	117	YWTMWK					Mascot
930.4178	930.4788	0.061	66	112	117	YWTMWK			Oxidation (M)[4]		Mascot
951.4717	951.5367	0.065	68	147	154	VIGFDNMR					Mascot
1012.4734	1012.5549	0.0815	80	139	146	EYPDAYVR					Mascot
1012.4734	1012.5549	0.0815	80	139	146	EYPDAYVR	59	99.99			Mascot
1140.5684	1140.6562	0.0878	77	138	146	KEYPDAYVR					Mascot
1165.571	1165.6488	0.0778	67	85	93	WVPCLEFSK			Carbamidomethyl (C)[4]		Mascot
1165.571	1165.6488	0.0778	67	85	93	WVPCLEFSK	29	90.669	Carbamidomethyl (C)[4]		Mascot
1262.6084	1262.7251	0.1167	92	31	44	SGSAGLSNVSNGGR					Mascot
1262.6084	1262.7251	0.1167	92	31	44	SGSAGLSNVSNGGR					Mascot
1381.5768	1381.6897	0.1129	82	100	111	EHNSSPGYYDGR					Mascot
1862.9681	1862.9376	-0.0305	-16	2	20	APAVMASSATTVAPFQG LK			Oxidation (M)[5]		Mascot
1922.0521	1922.1836	0.1315	68	59	75	FETLSYLPPLSTEALLK					Mascot
2050.147	2050.2974	0.1504	73	58	75	KFETLSYLPPLSTEALLK					Mascot
2296.085	2296.2825	0.1975	86	155	174	QVQCVSFIAFRPPGCEES GK			Carbamidomethyl (C)[4,15]		Mascot
2408.1836	2408.375	0.1914	79	118	138	LPMFGCTDATQVLNEVE EVKK			Carbamidomethyl (C)[6]		Mascot
2424.1785	2424.363	0.1845	76	118	138	LPMFGCTDATQVLNEVE EVKK			Carbamidomethyl (C)[6], Oxidation (M)[3]		Mascot

10 Ribulose bisphosphate carboxylase small chain PWS4.3, chloroplastic [Triticum urartu] gi|473868752 20787.4 8.98 13 180 100 25.244 87 100

Peptide Information

Calc. Mass	Obsrv. Mass	± da	± ppm	Start Seq.	End Seq.	Sequence	Ion Score	C. I. %	Modification	Rank	Result Type
906.5043	906.5795	0.0752	83	87	93	QVDYLIR					Mascot
914.4229	914.481	0.0581	64	123	128	YWTMWK					Mascot
930.4178	930.4788	0.061	66	123	128	YWTMWK			Oxidation (M)[4]		Mascot
951.4717	951.5367	0.065	68	158	165	VIGFDNMR					Mascot
1012.4734	1012.5549	0.0815	80	150	157	EYPDAYVR					Mascot
1012.4734	1012.5549	0.0815	80	150	157	EYPDAYVR	59	99.99			Mascot
1140.5684	1140.6562	0.0878	77	149	157	KEYPDAYVR					Mascot
1165.571	1165.6488	0.0778	67	96	104	WVPCLEFSK			Carbamidomethyl (C)[4]		Mascot
1165.571	1165.6488	0.0778	67	96	104	WVPCLEFSK	29	90.669	Carbamidomethyl (C)[4]		Mascot
1187.6168	1187.6545	0.0377	32	21	32	STPGPPANPPPR					Mascot
1381.5768	1381.6897	0.1129	82	111	122	EHNSSPGYYDGR					Mascot
1862.9681	1862.9376	-0.0305	-16	2	20	APAVMASSATTVAPFQG LK			Oxidation (M)[5]		Mascot

1922.0521	1922.1836	0.1315	68	70	86	FETLSYLPPLSTEALLK		Mascot
2050.147	2050.2974	0.1504	73	69	86	KFETLSYLPPLSTEALLK		Mascot
2296.085	2296.2825	0.1975	86	166	185	QVQCVSFIAFRPPGCEES GK	Carbamidomethyl (C)[4,15]	Mascot
2408.1836	2408.375	0.1914	79	129	149	LPMFGCTDATQVLNEVE EVKK	Carbamidomethyl (C)[6]	Mascot
2424.1785	2424.363	0.1845	76	129	149	LPMFGCTDATQVLNEVE EVKK	Carbamidomethyl (C)[6], Oxidation (M)[3]	Mascot

Gel Idx/Pos	123/E22	Instr./Gel Origin	BA2151/Sample Project 20140814	Process Status	Analysis Succeeded
Plate [#] Name	[1] Sample Project 20140814	Instrument Sample Name		Spectra	11

Rank	Protein Name	Accession No.	Protein MW	Protein PI	Pep. Count	Protein Score	Protein Score C. I. %	Intensity Matched	Total Ion Score	Total Ion C. I. %	Confirmed
1	unnamed protein product [Triticum aestivum]	gi|21866	13274.5	5.84	6	165	100	3.098	128	100	

Protein Group

Protein Name	Accession No.	Protein MW	Protein PI
RecName: Full=Ribulose bisphosphate carboxylase small chain clone 512; Short=RuBisCO small subunit	gi|132107	13274.5	5.8400 001525 8789

Peptide Information

Calc. Mass	Obsrv. Mass	± da	± ppm	Start Seq.	End Seq.	Sequence	Ion Score	C. I. %	Modification	Rank	Result Type
906.5043	906.5706	0.0663	73	14	20	QVDYLIR					Mascot
914.4229	914.4921	0.0692	76	50	55	YWTMWK					Mascot
965.4873	965.5562	0.0689	71	85	92	IIGFDNMR					Mascot
981.4822	981.5389	0.0567	58	85	92	IIGFDNMR			Oxidation (M)[7]		Mascot
1012.4734	1012.5547	0.0813	80	77	84	EYPDAYVR					Mascot
1012.4734	1012.5547	0.0813	80	77	84	EYPDAYVR	26	81.546			Mascot
1165.571	1165.6528	0.0818	70	23	31	WVPCLEFSK			Carbamidomethyl (C)[4]		Mascot
1365.5819	1365.6908	0.1089	80	38	49	EHNASPGYYDGR	103	100			Mascot

Rank	Protein Name	Accession No.	Protein MW	Protein PI	Pep. Count	Protein Score	Protein Score C. I. %	Intensity Matched	Total Ion Score	Total Ion C. I. %	Confirmed
2	ribulose-1,5-bisphosphate carboxylase/oxygenase small subunit [Triticum aestivum]	gi|11990897	19732.9	8.8	7	161	100	3.218	128	100	

Peptide Information

Calc. Mass	Obsrv. Mass	± da	± ppm	Start Seq.	End Seq.	Sequence	Ion Score	C. I. %	Modification	Rank	Result Type
906.5043	906.5706	0.0663	73	76	82	QVDYLIR					Mascot
914.4229	914.4921	0.0692	76	112	117	YWTMWK					Mascot
965.4873	965.5562	0.0689	71	147	154	IIGFDNMR					Mascot
981.4822	981.5389	0.0567	58	147	154	IIGFDNMR			Oxidation (M)[7]		Mascot
1012.4734	1012.5547	0.0813	80	139	146	EYPDAYVR					Mascot
1012.4734	1012.5547	0.0813	80	139	146	EYPDAYVR	26	81.546			Mascot
1165.571	1165.6528	0.0818	70	85	93	WVPCLEFSK			Carbamidomethyl (C)[4]		Mascot
1262.6084	1262.6996	0.0912	72	31	44	SNGASLGSVSNGGR					Mascot
1365.5819	1365.6908	0.1089	80	100	111	EHNASPGYYDGR	103	100			Mascot

Rank	Protein Name	Accession No.	Protein MW	Protein PI	Pep. Count	Protein Score	Protein Score C. I. %	Intensity Matched	Total Ion Score	Total Ion C. I. %	Confirmed
3	Ribulose bisphosphate carboxylase small chain, chloroplastic [Triticum urartu]	gi|473882355	18742.4	8.65	7	160	100	3.382	128	100	

Peptide Information

Calc. Mass	Obsrv. Mass	± da	± ppm	Start Seq.	End Seq.	Sequence	Ion Score	C. I. %	Modification	Rank	Result Type
906.5043	906.5706	0.0663	73	65	71	QVDYLIR					Mascot
914.4229	914.4921	0.0692	76	101	106	YWTMWK					Mascot
965.4873	965.5562	0.0689	71	136	143	IIGFDNMR					Mascot
981.4822	981.5389	0.0567	58	136	143	IIGFDNMR			Oxidation (M)[7]		Mascot
1012.4734	1012.5547	0.0813	80	128	135	EYPDAYVR					Mascot
1012.4734	1012.5547	0.0813	80	128	135	EYPDAYVR	26	81.546			Mascot
1165.571	1165.6528	0.0818	70	74	82	WVPCLEFSK			Carbamidomethyl (C)[4]		Mascot
1365.5819	1365.6908	0.1089	80	89	100	EHNASPGYYDGR	103	100			Mascot
1862.9681	1862.929	-0.0391	-21	2	20	APTVMASSATSVAPFQG LK					Mascot

4 Ribulose bisphosphate carboxylase small chain, chloroplastic [Triticum urartu] gi|474416311 15090.5 5.85 6 160 100 3.098 128 100

Peptide Information

Calc. Mass	Obsrv. Mass	± da	± ppm	Start Seq.	End Seq.	Sequence	Ion Score	C. I. %	Modification	Rank	Result Type
906.5043	906.5706	0.0663	73	29	35	QVDYLIR					Mascot
914.4229	914.4921	0.0692	76	65	70	YWTMWK					Mascot
965.4873	965.5562	0.0689	71	100	107	IIGFDNMR					Mascot
981.4822	981.5389	0.0567	58	100	107	IIGFDNMR			Oxidation (M)[7]		Mascot
1012.4734	1012.5547	0.0813	80	92	99	EYPDAYVR					Mascot
1012.4734	1012.5547	0.0813	80	92	99	EYPDAYVR	26	81.546			Mascot
1165.571	1165.6528	0.0818	70	38	46	WVPCLEFSK			Carbamidomethyl (C)[4]		Mascot
1365.5819	1365.6908	0.1089	80	53	64	EHNASPGYYDGR	103	100			Mascot

5 ribulose-1,5-bisphosphate carboxylase/oxygenase small subunit [Triticum aestivum] gi|11990893 19728.9 9.06 7 159 100 3.218 128 100

Peptide Information

Calc. Mass	Obsrv. Mass	± da	± ppm	Start Seq.	End Seq.	Sequence	Ion Score	C. I. %	Modification	Rank	Result Type
906.5043	906.5706	0.0663	73	76	82	QVDYLIR					Mascot
914.4229	914.4921	0.0692	76	112	117	YWTMWK					Mascot
965.4873	965.5562	0.0689	71	147	154	IIGFDNMR					Mascot
981.4822	981.5389	0.0567	58	147	154	IIGFDNMR			Oxidation (M)[7]		Mascot
1012.4734	1012.5547	0.0813	80	139	146	EYPDAYVR					Mascot

1012.4734	1012.5547	0.0813	80	139	146	EYPDAYVR	26	81.546			Mascot
1165.571	1165.6528	0.0818	70	85	93	WVPCLEFSK			Carbamidomethyl (C)[4]		Mascot
1262.6084	1262.6996	0.0912	72	31	44	SNGASLGSVSNGGR					Mascot
1365.5819	1365.6908	0.1089	80	100	111	EHNASPGYYDGR	103	100			Mascot

6	alpha-amylase inhibitor 0.19 [Triticum aestivum]	gi|66841026	13340.4	6.86	5	134	100	4.086	103	100	

Peptide Information

Calc. Mass	Obsrv. Mass	± da	± ppm	Start Seq.	End Seq.	Sequence	Ion Score	C. I. %	Modification	Rank	Result Type
1162.6249	1162.7157	0.0908	78	85	95	LTAASITAVCR			Carbamidomethyl (C)[10]		Mascot
1570.8007	1570.91	0.1093	70	21	34	LQCNGSQVPEAVLR			Carbamidomethyl (C)[3]		Mascot
1612.7463	1612.8781	0.1318	82	62	77	EHGAQEGQAGTGAFPR					Mascot
1612.7463	1612.8781	0.1318	82	62	77	EHGAQEGQAGTGAFPR	103	100			Mascot
1663.8361	1663.9304	0.0943	57	96	111	LPIVVDASGDGAYVCK			Carbamidomethyl (C)[15]		Mascot
1862.7731	1862.929	0.1559	84	35	48	DCCQQLAHISEWCR			Carbamidomethyl (C)[2,3,13]		Mascot

7	Chain D, 0.19 Alpha-Amylase Inhibitor From Wheat	gi|3318684	13898.6	6.66	5	133	100	4.086	103	100	

Protein Group

0.19 alpha-amylase inhibitor [Triticum aestivum]	gi|2116581	13898.6	6.6599998474 1211
0.19 dimeric alpha-amylase inhibitor [Triticum aestivum]	gi|54778509	13898.6	6.6599998474 1211
0.19 dimeric alpha-amylase inhibitor [Triticum aestivum]	gi|54778501	13898.6	6.6599998474 1211
Chain A, 0.19 Alpha-Amylase Inhibitor From Wheat	gi|3318681	13898.6	6.6599998474 1211
Chain B, 0.19 Alpha-Amylase Inhibitor From Wheat	gi|3318682	13898.6	6.6599998474 1211
Chain C, 0.19 Alpha-Amylase Inhibitor From Wheat	gi|3318683	13898.6	6.6599998474 1211
RecName: Full=Alpha-amylase inhibitor 0.19; AltName: Full=0.19 alpha-AI; Short=0.19 AI	gi|123963	13898.6	6.6599998474 1211
dimeric alpha-amylase inhibitor precursor, partial [Triticum aestivum]	gi|108597921	14029.7	6.6900000572 2046

Peptide Information

Calc. Mass	Obsrv. Mass	± da	± ppm	Start Seq.	End Seq.	Sequence	Ion Score	C. I. %	Modification	Rank	Result Type

Calc. Mass	Obsrv. Mass	± da	± ppm	Start Seq.	End Seq.	Sequence	Ion Score	C. I. %	Modification	Rank	Result Type
1162.6249	1162.7157	0.0908	78	90	100	LTAASITAVCR			Carbamidomethyl (C)[10]		Mascot
1570.8007	1570.91	0.1093	70	26	39	LQCNGSQVPEAVLR			Carbamidomethyl (C)[3]		Mascot
1612.7463	1612.8781	0.1318	82	67	82	EHGAQEGQAGTGAFPR					Mascot
1612.7463	1612.8781	0.1318	82	67	82	EHGAQEGQAGTGAFPR	103	100			Mascot
1663.8361	1663.9304	0.0943	57	101	116	LPIVVDASGDGAYVCK			Carbamidomethyl (C)[15]		Mascot
1862.7731	1862.929	0.1559	84	40	53	DCCQQLAHISEWCR			Carbamidomethyl (C)[2,3,13]		Mascot

8	dimeric alpha-amylase inhibitor [Triticum dicoccoides]	gi|227809009	15730.5	5.58	5	131	100	4.086	103	100	

Protein Group

dimeric alpha-amylase inhibitor [Triticum aestivum]	gi|65993781	15688.5	5.5799 999237 0605
dimeric alpha-amylase inhibitor [Triticum aestivum]	gi|386877038	15702.5	5.5799 999237 0605
dimeric alpha-amylase inhibitor [Triticum dicoccoides]	gi|227809005	15730.5	5.5799 999237 0605

Peptide Information

Calc. Mass	Obsrv. Mass	± da	± ppm	Start Seq.	End Seq.	Sequence	Ion Score	C. I. %	Modification	Rank	Result Type
1162.6249	1162.7157	0.0908	78	107	117	LTAASITAVCR			Carbamidomethyl (C)[10]		Mascot
1570.8007	1570.91	0.1093	70	43	56	LQCNGSQVPEAVLR			Carbamidomethyl (C)[3]		Mascot
1612.7463	1612.8781	0.1318	82	84	99	EHGAQEGQAGTGAFPR					Mascot
1612.7463	1612.8781	0.1318	82	84	99	EHGAQEGQAGTGAFPR	103	100			Mascot
1663.8361	1663.9304	0.0943	57	118	133	LPIVVDASGDGAYVCK			Carbamidomethyl (C)[15]		Mascot
1862.7731	1862.929	0.1559	84	57	70	DCCQQLAHISEWCR			Carbamidomethyl (C)[2,3,13]		Mascot

9	0.19 dimeric alpha-amylase inhibitor [Triticum aestivum]	gi|54778503	13826.6	7.45	4	125	100	3.855	103	100	

Peptide Information

Calc. Mass	Obsrv. Mass	± da	± ppm	Start Seq.	End Seq.	Sequence	Ion Score	C. I. %	Modification	Rank	Result Type
1162.6249	1162.7157	0.0908	78	90	100	LTAASITAVCR			Carbamidomethyl (C)[10]		Mascot
1612.7463	1612.8781	0.1318	82	67	82	EHGAQEGQAGTGAFPR					Mascot
1612.7463	1612.8781	0.1318	82	67	82	EHGAQEGQAGTGAFPR	103	100			Mascot
1663.8361	1663.9304	0.0943	57	101	116	LPIVVDASGDGAYVCK			Carbamidomethyl (C)[15]		Mascot
1862.7731	1862.929	0.1559	84	40	53	DCCQQLAHISEWCR			Carbamidomethyl (C)[2,3,13]		Mascot

10	dimeric alpha-amylase inhibitor [Triticum dicoccoides]	gi|114215806	13862.5	5.25	4	125	100	3.802	103	100	

Protein Group

dimeric alpha-amylase inhibitor [Triticum dicoccoides]	gi|114215804	13862.5	5.25

dimeric alpha-amylase inhibitor [Triticum dicoccoides] gi|114215808 13921.6 5.25

Peptide Information

Calc. Mass	Obsrv. Mass	± da	± ppm	Start Seq.	End Seq.	Sequence	Ion Score	C. I. %	Modification	Rank	Result Type
1162.6249	1162.7157	0.0908	78	90	100	LTAASITAVCR			Carbamidomethyl (C)[10]		Mascot
1570.8007	1570.91	0.1093	70	26	39	LQCNGSQVPEAVLR			Carbamidomethyl (C)[3]		Mascot
1612.7463	1612.8781	0.1318	82	67	82	EHGAQEGQAGTGAFPR					Mascot
1612.7463	1612.8781	0.1318	82	67	82	EHGAQEGQAGTGAFPR	103	100			Mascot
1663.8361	1663.9304	0.0943	57	101	116	LPIVVDASGDGAYVCK			Carbamidomethyl (C)[15]		Mascot

Gel Idx/Pos	124/E23	Instr./Gel Origin	BA2151/Sample Project 20140814	Process Status	Analysis Succeeded
Plate [#] Name	[1] Sample Project 20140814	Instrument Sample Name		Spectra	11

Rank	Protein Name	Accession No.	Protein MW	Protein PI	Pep. Count	Protein Score	Protein Score C. I. %	Intensity Matched	Total Ion Score	Total Ion C. I. %	Confirmed
1	globulin 3 [Triticum aestivum]	gi|215398470	66651.7	7.78	8	286	100	42.27	269	100	

Protein Group

Protein Name	Accession No.	Protein MW	Protein PI
globulin-3A [Triticum aestivum]	gi|390979705	66626.8	8.4799 995422 3633

Peptide Information

Calc. Mass	Obsrv. Mass	± da	± ppm	Start Seq.	End Seq.	Sequence	Ion Score	C. I. %	Modification	Rank	Result Type
832.4159	832.3878	-0.0281	-34	307	313	ASEEQLR					Mascot
906.468	906.5512	0.0832	92	535	541	EVQEVFR					Mascot
959.4476	959.4211	-0.0265	-28	48	54	CQQDRPR			Carbamidomethyl (C)[1]		Mascot
1105.6001	1105.6703	0.0702	63	535	543	EVQEVFRAK					Mascot
1164.4763	1164.4379	-0.0384	-33	432	440	SEEEEDDRR					Mascot
1814.9872	1814.9138	-0.0734	-40	339	353	DTFNLLEQRPKIANR					Mascot
1822.8752	1823.0492	0.174	95	489	504	GSSNLQVVCFEINAER			Carbamidomethyl (C)[9]		Mascot
1822.8752	1823.0492	0.174	95	489	504	GSSNLQVVCFEINAER	119	100	Carbamidomethyl (C)[9]		Mascot
1906.0182	1906.2008	0.1826	96	470	488	GSAFVVPPGHPVVEIASS R					Mascot
1906.0182	1906.2008	0.1826	96	470	488	GSAFVVPPGHPVVEIASS R	150	100			Mascot

Rank	Protein Name	Accession No.	Protein MW	Protein PI	Pep. Count	Protein Score	Protein Score C. I. %	Intensity Matched	Total Ion Score	Total Ion C. I. %	Confirmed
2	Globulin-1 S allele [Triticum urartu]	gi|474411419	57108.4	9.1	6	281	100	41.908	269	100	

Peptide Information

Calc. Mass	Obsrv. Mass	± da	± ppm	Start Seq.	End Seq.	Sequence	Ion Score	C. I. %	Modification	Rank	Result Type
906.468	906.5512	0.0832	92	457	463	EVQEVFR					Mascot
959.4476	959.4211	-0.0265	-28	48	54	CQQDRPR			Carbamidomethyl (C)[1]		Mascot
1105.6001	1105.6703	0.0702	63	457	465	EVQEVFRAK					Mascot
1164.444	1164.4379	-0.0061	-5	349	357	WGEEEEDDR					Mascot
1822.8752	1823.0492	0.174	95	411	426	GSSNLQVVCFEINAER			Carbamidomethyl (C)[9]		Mascot
1822.8752	1823.0492	0.174	95	411	426	GSSNLQVVCFEINAER	119	100	Carbamidomethyl (C)[9]		Mascot
1906.0182	1906.2008	0.1826	96	392	410	GSAFVVPPGHPVVEIASS R					Mascot
1906.0182	1906.2008	0.1826	96	392	410	GSAFVVPPGHPVVEIASS R	150	100			Mascot

3 hypothetical protein TRIUR3_14290 [Triticum urartu] gi|474100670 39386.1 9.2 8 31 0 1.487

Peptide Information

Calc. Mass	Obsrv. Mass	± da	± ppm	Start Seq.	End Seq.	Sequence	Ion Score	C. I. %	Modification	Rank	Result Type
979.5142	979.4661	-0.0481	-49	77	84	HHLLGMQK			Oxidation (M)[6]		Mascot
1361.804	1361.8301	0.0261	19	252	263	KSAVDIVEILFK					Mascot
1847.0314	1846.9899	-0.0415	-22	253	268	SAVDIVEILFKDIAWK					Mascot
1848.9537	1849.1207	0.167	90	38	53	GFSTMKTWLPQGRPAR			Oxidation (M)[5]		Mascot
1922.0719	1922.1959	0.124	65	44	60	TWLPQGRPARNVLQTGK					Mascot
1928.0487	1928.1366	0.0879	46	185	200	IEQLFEELPNILINSR					Mascot
1948.0094	1948.199	0.1896	97	269	287	TLSDQIIVSSSPRGASSS R					Mascot
2025.991	2026.0072	0.0162	8	20	37	NKPELLESADMHEAGKE K					Mascot

4 hypothetical protein TRIUR3_32146 [Triticum urartu] gi|474402981 19820.3 10.63 3 29 0 10.438 19 54.058

Peptide Information

Calc. Mass	Obsrv. Mass	± da	± ppm	Start Seq.	End Seq.	Sequence	Ion Score	C. I. %	Modification	Rank	Result Type
906.5447	906.5512	0.0065	7	44	50	LWVFTLK					Mascot
944.5022	944.5067	0.0045	5	35	41	CWIQPIK			Carbamidomethyl (C)[1]		Mascot
955.4228	955.5062	0.0834	87	69	76	HEVDNEGR					Mascot
955.4228	955.5062	0.0834	87	69	76	HEVDNEGR	19	54.058			Mascot

5 hypothetical protein TRIUR3_28377 [Triticum urartu] gi|474271349 10698.7 11.1 4 26 0 .411

Peptide Information

Calc. Mass	Obsrv. Mass	± da	± ppm	Start Seq.	End Seq.	Sequence	Ion Score	C. I. %	Modification	Rank	Result Type
876.4495	876.5278	0.0783	89	83	90	ELTSMPAK					Mascot
1182.6702	1182.6604	-0.0098	-8	38	49	SAARSAAPRPAK					Mascot
1349.6995	1349.7954	0.0959	71	1	12	MEVVASPSRFAR					Mascot
1365.6943	1365.7241	0.0298	22	1	12	MEVVASPSRFAR			Oxidation (M)[1]		Mascot
2807.374	2807.5627	0.1887	67	14	41	GIHDGLLQGPAMEVAAG STAEEPRSAAR			Oxidation (M)[12]		Mascot

6 GPN-loop GTPase 3-like protein [Triticum urartu] gi|474059663 45475.6 5.56 3 26 0 1.669 19 54.058

Peptide Information

Calc. Mass	Obsrv. Mass	± da	± ppm	Start Seq.	End Seq.	Sequence	Ion Score	C. I. %	Modification	Rank	Result Type

Calc. Mass	Obsrv. Mass	± da	± ppm	Start Seq.	End Seq.	Sequence	Ion Score	C. I. %	Modification	Rank	Result Type
934.5026	934.582	0.0794	85	156	163	MDLVSNKK					Mascot
955.489	955.5062	0.0172	18	353	359	EHRMVQR					Mascot
955.489	955.5062	0.0172	18	353	359	EHRMVQR	19	54.058			Mascot
2017.955	2018.1365	0.1815	90	373	391	YPMSNVNFDPGGGAGLHIR			Oxidation (M)[3]		Mascot

7	hypothetical protein TRIUR3_03744 [Triticum urartu]	gi|474365672	51364	5.28	10	25	0	22.989		

Peptide Information

Calc. Mass	Obsrv. Mass	± da	± ppm	Start Seq.	End Seq.	Sequence	Ion Score	C. I. %	Modification	Rank	Result Type
832.4159	832.3878	-0.0281	-34	113	119	KDDEIGR					Mascot
888.4785	888.5419	0.0634	71	147	154	ANDIQSLK					Mascot
906.4965	906.5512	0.0547	60	414	421	MVLTLEGK			Oxidation (M)[1]		Mascot
944.5159	944.5067	-0.0092	-10	357	364	NKDIEIGR					Mascot
959.4792	959.4211	-0.0581	-61	292	300	EVEAAQANK					Mascot
1333.7396	1333.7976	0.058	43	422	432	LLEMQSLVKEK			Oxidation (M)[4]		Mascot
1333.7396	1333.7976	0.058	43	422	432	LLEMQSLVKEK			Oxidation (M)[4]		Mascot
1814.8879	1814.9138	0.0259	14	114	130	DDEIGRLQAEAPASTNK					Mascot
1928.0334	1928.1366	0.1032	54	120	137	LQAEAPASTNKTLVLEDK					Mascot
1948.0857	1948.199	0.1133	58	414	430	MVLTLEGKLLEMQSLVK			Oxidation (M)[1]		Mascot
1971.9805	1972.1152	0.1347	68	138	154	LNEMHSLDKANDIQSLK			Oxidation (M)[4]		Mascot

8	40S ribosomal protein S27 [Triticum urartu]	gi|473827062	18777.4	8.73	2	24	0	11.276	15	0

Peptide Information

Calc. Mass	Obsrv. Mass	± da	± ppm	Start Seq.	End Seq.	Sequence	Ion Score	C. I. %	Modification	Rank	Result Type
1822.9303	1823.0492	0.1189	65	1	15	MATSLSWTCVLELRR			Carbamidomethyl (C)[9]		Mascot
1822.9303	1823.0492	0.1189	65	1	15	MATSLSWTCVLELRR	15	0	Carbamidomethyl (C)[9]		Mascot
1838.9252	1839.0602	0.135	73	1	15	MATSLSWTCVLELRR			Carbamidomethyl (C)[9], Oxidation (M)[1]		Mascot
1921.0277	1921.1929	0.1652	86	78	94	VLQNDIDLLNPPAELEK					Mascot

9	Ribulose bisphosphate carboxylase small chain PWS4.3, chloroplastic [Triticum urartu]	gi|473721335	19632.8	8.81	5	24	0	9.874		

Peptide Information

Calc. Mass	Obsrv. Mass	± da	± ppm	Start Seq.	End Seq.	Sequence	Ion Score	C. I. %	Modification	Rank	Result Type
906.5043	906.5512	0.0469	52	75	81	QVDYLIR					Mascot
930.4178	930.5039	0.0861	93	111	116	YWTMWK			Oxidation (M)[4]		Mascot

1848.9524	1849.1207	0.1683	91	2	20	APAVMASSASTVAPFQGLK			Oxidation (M)[5]		Mascot
1922.0521	1922.1959	0.1438	75	58	74	FETLSYLPPLSTEALLK					Mascot
1995.9879	1996.1115	0.1236	62	1	20	MAPAVMASSASTVAPFQGLK			Oxidation (M)[1,6]		Mascot

10	ribulose-1,5-bisphosphate carboxylase/oxygenase small subunit [Triticum aestivum]	gi|11990901	19733.9	8.81	5	24	0	9.874

Peptide Information

Calc. Mass	Obsrv. Mass	± da	± ppm	Start Seq.	End Seq.	Sequence	Ion Score	C. I. %	Modification	Rank	Result Type
906.5043	906.5512	0.0469	52	76	82	QVDYLIR					Mascot
930.4178	930.5039	0.0861	93	112	117	YWTMWK			Oxidation (M)[4]		Mascot
1848.9524	1849.1207	0.1683	91	2	20	APAVMASSATSVAPFQGLK			Oxidation (M)[5]		Mascot
1922.0521	1922.1959	0.1438	75	59	75	FETLSYLPPLSTEALLK					Mascot
1995.9879	1996.1115	0.1236	62	1	20	MAPAVMASSATSVAPFQGLK			Oxidation (M)[1,6]		Mascot

Gel Idx/Pos	125/E24	Instr./Gel Origin	BA2151/Sample Project 20140814	Process Status	Analysis Succeeded
Plate [#] Name	[1] Sample Project 20140814	Instrument Sample Name		Spectra	11

Rank	Protein Name	Accession No.	Protein MW	Protein PI	Pep. Count	Protein Score	Protein Score C. I. %	Intensity Matched	Total Ion Score	Total Ion C. I. %	Confirmed
1	globulin 3 [Triticum aestivum]	gi|215398470	66651.7	7.78	10	59	94.17	4.81	35	98.327	

Protein Group

Protein Name	Accession No.	Protein MW	Protein PI
globulin-3A [Triticum aestivum]	gi|390979705	66626.8	8.4799 995422 3633

Peptide Information

Calc. Mass	Obsrv. Mass	± da	± ppm	Start Seq.	End Seq.	Sequence	Ion Score	C. I. %	Modification	Rank	Result Type
823.4645	823.5065	0.042	51	350	356	IANRHGR					Mascot
832.4159	832.3934	-0.0225	-27	307	313	ASEEQLR					Mascot
837.4101	837.4911	0.081	97	357	363	LYEADAR					Mascot
906.468	906.5355	0.0675	74	535	541	EVQEVFR					Mascot
1303.7217	1303.7542	0.0325	25	273	284	VLTAALKTSDER					Mascot
1390.7285	1390.797	0.0685	49	280	292	TSDERLGSLLGSR					Mascot
1487.8176	1487.8589	0.0413	28	301	313	SISIVRASEEQLR					Mascot
1699.9418	1699.9738	0.032	19	257	271	FQYFSAKPLLASLSK					Mascot
1822.8752	1823.0372	0.162	89	489	504	GSSNLQVVCFEINAER			Carbamidomethyl (C)[9]		Mascot
1906.0182	1906.1669	0.1487	78	470	488	GSAFVVPPGHPVVEIASS R					Mascot
1906.0182	1906.1669	0.1487	78	470	488	GSAFVVPPGHPVVEIASS R	35	98.327			Mascot

Rank	Protein Name	Accession No.	Protein MW	Protein PI	Pep. Count	Protein Score	Protein Score C. I. %	Intensity Matched	Total Ion Score	Total Ion C. I. %	Confirmed
2	Globulin-1 S allele [Triticum urartu]	gi|474411419	57108.4	9.1	9	58	92.315	4.188	35	98.327	

Peptide Information

Calc. Mass	Obsrv. Mass	± da	± ppm	Start Seq.	End Seq.	Sequence	Ion Score	C. I. %	Modification	Rank	Result Type
823.4645	823.5065	0.042	51	269	275	IANRHGR					Mascot
837.4101	837.4911	0.081	97	276	282	LYEADAR					Mascot
906.468	906.5355	0.0675	74	457	463	EVQEVFR					Mascot
990.5214	990.5675	0.0461	47	195	203	AALKTSDER					Mascot
1390.7285	1390.797	0.0685	49	199	211	TSDERLGSLLGSR					Mascot
1699.9418	1699.9738	0.032	19	176	190	FQYFSAKPLLASLSK					Mascot
1791.8984	1791.8975	-0.0009	-1	254	268	GDSRDTYNLLEQRPK					Mascot
1822.8752	1823.0372	0.162	89	411	426	GSSNLQVVCFEINAER			Carbamidomethyl (C)[9]		Mascot

Calc. Mass	Obsrv. Mass	± da	± ppm	Start Seq.	End Seq.	Sequence	Ion Score	C. I. %	Modification	Rank	Result Type
1906.0182	1906.1669	0.1487	78	392	410	GSAFVVPPGHPVVEIASSR					Mascot
1906.0182	1906.1669	0.1487	78	392	410	GSAFVVPPGHPVVEIASSR	35	98.327			Mascot

3	hypothetical protein TRIUR3_21665 [Triticum urartu]	gi|474086763	120630.8	9.3	22	44	0	7.216	17	0	

Peptide Information

Calc. Mass	Obsrv. Mass	± da	± ppm	Start Seq.	End Seq.	Sequence	Ion Score	C. I. %	Modification	Rank	Result Type
800.4736	800.4716	-0.002	-2	50	56	AQNRLAK					Mascot
823.4421	823.5065	0.0644	78	126	131	YTERVR					Mascot
857.5567	857.5762	0.0195	23	130	137	VRSAILAK					Mascot
858.5043	858.5594	0.0551	64	1030	1039	GVLLGGSGAK					Mascot
868.5475	868.5245	-0.023	-26	326	333	VAAKRPAR					Mascot
888.4785	888.5284	0.0499	56	451	458	SLEGDLVR					Mascot
905.5607	905.5175	-0.0432	-48	441	447	FVVWKVK					Mascot
943.5319	943.55	0.0181	19	36	43	LQAAEQKR					Mascot
955.5029	955.5417	0.0388	41	97	104	LLHADMQK					Mascot
973.5214	973.6044	0.083	85	217	224	HADFLSRK					Mascot
1033.516	1033.5986	0.0826	80	75	82	EKEELETR					Mascot
1036.5382	1036.6017	0.0635	61	334	342	TSETSRLSR					Mascot
1201.6536	1201.7164	0.0628	52	492	501	QVSDDQKLLR					Mascot
1259.6591	1259.7604	0.1013	80	488	498	AIQKQVSDDQK					Mascot
1307.7029	1307.785	0.0821	63	776	786	AYVQLMQPTIK			Oxidation (M)[6]		Mascot
1379.7278	1379.8333	0.1055	76	663	674	EVRDSLHDLAPK					Mascot
1600.8483	1600.9283	0.08	50	1070	1083	VATVSEKVHGPWYK					Mascot
1699.9061	1699.9738	0.0677	40	1023	1039	AVHCAFRGVLLGGSGAK			Carbamidomethyl (C)[4]		Mascot
1716.8521	1716.9916	0.1395	81	160	174	LMHIQQAAMTVSSQR			Oxidation (M)[2]		Mascot
1716.8521	1716.9916	0.1395	81	160	174	LMHIQQAAMTVSSQR	17	0	Oxidation (M)[2]		Mascot
1790.9899	1790.9393	-0.0506	-28	761	775	FTLEEIKQLQVEVSK					Mascot
1791.9039	1791.8975	-0.0064	-4	433	445	AWCTYLYRFVVWK			Carbamidomethyl (C)[3]		Mascot
1993.9761	1994.1547	0.1786	90	855	876	AGHGAPAAVVASAGSSGLPECK			Carbamidomethyl (C)[21]		Mascot

4	hypothetical protein TRIUR3_25195 [Triticum urartu]	gi|474095946	37119.2	8.5	11	43	0	7.283	

Peptide Information

Calc. Mass	Obsrv. Mass	± da	± ppm	Start Seq.	End Seq.	Sequence	Ion Score	C. I. %	Modification	Rank	Result Type
844.5039	844.5616	0.0577	68	69	75	ALLPFQR					Mascot

990.5149	990.5675	0.0526	53	136	143	MTSRDRPK					Mascot
1033.579	1033.5986	0.0196	19	325	333	LSNNFGLIR					Mascot
1036.5939	1036.6017	0.0078	8	287	296	GAPGWPPVKK					Mascot
1073.5885	1073.6378	0.0493	46	197	205	CLVAAVERR			Carbamidomethyl (C)[1]		Mascot
1118.5874	1118.6057	0.0183	16	46	55	DGVLLEMIGR			Oxidation (M)[7]		Mascot
1232.6555	1232.6953	0.0398	32	275	286	VAEMGISLDVAK					Mascot
1259.689	1259.7604	0.0714	57	194	204	VSRCLVAAVER			Carbamidomethyl (C)[4]		Mascot
1300.7372	1300.682	-0.0552	-42	69	80	ALLPFQRSGSPK					Mascot
1434.7336	1434.8754	0.1418	99	216	228	APDRSGLLSDFTR					Mascot
2399.3193	2399.2385	-0.0808	-34	312	333	SSLGSLLWSHLGKLSNN FGLIR					Mascot

5 Nuclease S1 [Triticum urartu] gi|473797598 34678.6 9.32 10 42 0 4.651

Peptide Information

Calc. Mass	Obsrv. Mass	± da	± ppm	Start Seq.	End Seq.	Sequence	Ion Score	C. I. %	Modification	Rank	Result Type
800.4301	800.4716	0.0415	52	113	118	TNFKYK					Mascot
812.5352	812.4952	-0.04	-49	283	289	LALILNR					Mascot
868.4523	868.5245	0.0722	83	210	217	ATTVSAYR					Mascot
870.5268	870.6011	0.0743	85	275	282	RLAQAGVR					Mascot
890.5094	890.5129	0.0035	4	269	275	YPVVEKR					Mascot
1165.5922	1165.6805	0.0883	76	295	304	KADTMPLYVQ					Mascot
1165.5922	1165.6805	0.0883	76	295	304	KADTMPLYVQ					Mascot
1507.9431	1507.8632	-0.0799	-53	276	289	LAQAGVRLALILNR					Mascot
1716.8949	1716.9916	0.0967	56	89	104	SLDTMVGALQTNLTPR					Mascot
1716.8949	1716.9916	0.0967	56	89	104	SLDTMVGALQTNLTPR					Mascot
1994.0165	1994.1547	0.1382	69	67	83	ANLHHVWDVSIIDTVMK			Oxidation (M)[16]		Mascot
2384.1914	2384.1689	-0.0225	-9	84	104	DFYNKSLDTMVGALQTN LTPR					Mascot

6 hypothetical protein TRIUR3_29372 [Triticum urartu] gi|474151499 39765.1 5.16 10 41 0 2.783

Peptide Information

Calc. Mass	Obsrv. Mass	± da	± ppm	Start Seq.	End Seq.	Sequence	Ion Score	C. I. %	Modification	Rank	Result Type
806.4519	806.488	0.0361	45	343	349	SIPQPHK					Mascot
815.4482	815.5014	0.0532	65	284	291	ERLAGGGR					Mascot
826.5032	826.5173	0.0141	17	244	251	AVAPLVEK					Mascot
853.5618	853.5295	-0.0323	-38	209	215	KIPVQLR					Mascot
1037.5044	1037.6063	0.1019	98	327	335	SNTGIDRMK			Oxidation (M)[8]		Mascot

1066.4946	1066.5927	0.0981	92	1	11	MASAGGDTKGR			Oxidation (M)[1]		Mascot
1800.8862	1800.9937	0.1075	60	187	202	ASNDKPIDDLIDEDLK					Mascot
1822.9229	1823.0372	0.1143	63	263	280	LCRFSITGSLGEAAAGGR			Carbamidomethyl (C)[2]		Mascot
1990.0433	1990.0985	0.0552	28	14	30	KFLPHGKPFYEDLVDGK					Mascot
2705.3975	2705.4072	0.0097	4	15	37	FLPHGKPFYEDLVDGKVFDEKPK					Mascot

7	hypothetical protein TRIUR3_11888 [Triticum urartu]	gi|474306987	34313.9	9.3	7	41	0	3.112	18	.803	

Peptide Information

Calc. Mass	Obsrv. Mass	± da	± ppm	Start Seq.	End Seq.	Sequence	Ion Score	C. I. %	Modification	Rank	Result Type
807.4103	807.4669	0.0566	70	98	104	DMAIVMK					Mascot
857.5203	857.5762	0.0559	65	67	73	RVQLVDK					Mascot
900.5261	900.6081	0.082	91	235	242	QGRVLEAK					Mascot
973.52	973.6044	0.0844	87	109	117	AEEAIEAIK					Mascot
1329.7372	1329.7509	0.0137	10	109	120	AEEAIEAIKSLR					Mascot
1333.6715	1333.7825	0.111	83	98	108	DMAIVMKQQNR					Mascot
1365.6614	1365.7504	0.089	65	98	108	DMAIVMKQQNR			Oxidation (M)[2,6]		Mascot
1365.6614	1365.7504	0.089	65	98	108	DMAIVMKQQNR	18	0.803	Oxidation (M)[2,6]		Mascot
2088.1238	2088.2114	0.0876	42	78	97	AIALFWGAINAGDRVDSALK					Mascot

8	Dihydroflavonol-4-reductase [Triticum urartu]	gi|474348521	38019.3	8.49	8	39	0	3.028	18	12.199	

Peptide Information

Calc. Mass	Obsrv. Mass	± da	± ppm	Start Seq.	End Seq.	Sequence	Ion Score	C. I. %	Modification	Rank	Result Type
863.4621	863.5358	0.0737	85	283	289	YTNQPLK					Mascot
973.5425	973.6044	0.0619	64	318	327	ASGTKVPASR					Mascot
1036.5786	1036.6017	0.0231	22	313	322	GFIQKASGTK					Mascot
1165.651	1165.6805	0.0295	25	21	30	MLLARGYAVR			Oxidation (M)[1]		Mascot
1165.651	1165.6805	0.0295	25	21	30	MLLARGYAVR	18	12.199	Oxidation (M)[1]		Mascot
1308.6617	1308.761	0.0993	76	332	343	LQNSAAPVFMSK			Oxidation (M)[10]		Mascot
1407.7301	1407.8213	0.0912	65	283	294	YTNQPLKDLGMK					Mascot
1507.754	1507.8632	0.1092	72	209	221	AYVNESQAYVHVK					Mascot
1791.8881	1791.8975	0.0094	5	114	129	VVLSSSIGTMYMNPHR					Mascot

9	hypothetical protein TRIUR3_32941 [Triticum urartu]	gi|474417939	122949.1	9.12	17	38	0	5.514	17	0	

Peptide Information

Calc. Mass	Obsrv. Mass	± da	± ppm	Start	End	Sequence	Ion	C. I. %	Modification	Rank	Result Type

				Seq.	Seq.		Score				
800.4736	800.4716	-0.002	-2	106	112	AQNRLAK					Mascot
823.4421	823.5065	0.0644	78	182	187	YTERVR					Mascot
868.5475	868.5245	-0.023	-26	382	389	VAAKRPAR					Mascot
888.4785	888.5284	0.0499	56	507	514	SLEGDLVR					Mascot
905.5607	905.5175	-0.0432	-48	497	503	FVVWKVK					Mascot
943.5319	943.55	0.0181	19	92	99	LQAAEQKR					Mascot
973.5214	973.6044	0.083	85	273	280	HADFLSRK					Mascot
1033.516	1033.5986	0.0826	80	131	138	EKEELETR					Mascot
1036.5382	1036.6017	0.0635	61	390	398	TSETSRLSR					Mascot
1307.7029	1307.785	0.0821	63	815	825	AYVQLMQPTIK			Oxidation (M)[6]		Mascot
1379.7278	1379.8333	0.1055	76	702	713	EVRDSLHDLAPK					Mascot
1407.7096	1407.8213	0.1117	79	151	161	MHLLHADMQRR					Mascot
1600.8483	1600.9283	0.08	50	1089	1102	VATVSEKVHGPWYK					Mascot
1716.8521	1716.9916	0.1395	81	216	230	IMHIQQAAMTVSSQR			Oxidation (M)[2]		Mascot
1716.8521	1716.9916	0.1395	81	216	230	IMHIQQAAMTVSSQR	17	0	Oxidation (M)[2]		Mascot
1790.9899	1790.9393	-0.0506	-28	800	814	FTLEEIKQLQVEVSK					Mascot
1791.9039	1791.8975	-0.0064	-4	489	501	AWCTYLYRFVVWK			Carbamidomethyl (C)[3]		Mascot
2152.0862	2152.2019	0.1157	54	212	230	AHARIMHIQQAAMTVSSQR			Oxidation (M)[6]		Mascot

10	Disease resistance protein RGA2 [Triticum urartu]	gi|473850875	146087.3	6.81	16	37	0	6.96	17	0	

Peptide Information

Calc. Mass	Obsrv. Mass	± da	± ppm	Start Seq.	End Seq.	Sequence	Ion Score	C. I. %	Modification	Rank	Result Type
830.5094	830.4927	-0.0167	-20	987	993	SDIRVLK					Mascot
832.4597	832.3934	-0.0663	-80	1207	1213	LALVECK			Carbamidomethyl (C)[6]		Mascot
906.4792	906.5355	0.0563	62	327	334	TGFLANQR					Mascot
1060.6038	1060.6432	0.0394	37	627	636	YIDAAGLPIK					Mascot
1068.6412	1068.6014	-0.0398	-37	577	586	DLGGLQLPKK					Mascot
1165.7052	1165.6805	-0.0247	-21	1009	1018	SVLERLVPPR					Mascot
1165.7052	1165.6805	-0.0247	-21	1009	1018	SVLERLVPPR					Mascot
1303.7217	1303.7542	0.0325	25	216	227	TTLAESVLADKR					Mascot
1329.6434	1329.7509	0.1075	81	972	982	VWDPEDADRVK					Mascot
1333.7145	1333.7825	0.068	51	2	16	AMSGIGSVIAGAVGK			Oxidation (M)[2]		Mascot
1390.6962	1390.797	0.1008	72	403	414	TVGAWEDIRDTK					Mascot
1493.8046	1493.8571	0.0525	35	862	872	LRYLHLSYCLR			Carbamidomethyl (C)[9]		Mascot

1707.8557	1707.9216	0.0659	39	801	815	VSMLPGSFCQLNNLK			Carbamidomethyl (C)[9]	Mascot
1716.9504	1716.9916	0.0412	24	320	334	SVVQQLRTGFLANQR				Mascot
1716.9504	1716.9916	0.0412	24	320	334	SVVQQLRTGFLANQR	17	0		Mascot
1791.9963	1791.8975	-0.0988	-55	606	623	GQPAPSGVVLPSSINQLK				Mascot
1990.0824	1990.0985	0.0161	8	1	21	MAMSGIGSVIAGAVGKQI VGK			Oxidation (M)[1]	Mascot
2399.2654	2399.2385	-0.0269	-11	109	128	VSLWFSSNNQLLQRITMP HK				Mascot

Gel Idx/Pos	126/F1	Instr./Gel Origin	BA2151/Sample Project 20140814	Process Status	Analysis Succeeded
Plate [#] Name	[1] Sample Project 20140814	Instrument Sample Name		Spectra	11

Rank	Protein Name	Accession No.	Protein MW	Protein PI	Pep. Count	Protein Score	Protein Score C. I. %	Intensity Matched	Total Ion Score	Total Ion C. I. %	Confirmed
1	putative prefoldin subunit 2 [Triticum urartu]	gi|473960164	16389.4	5.83	8	68	99.283	2.893	40	99.215	

Peptide Information

Calc. Mass	Obsrv. Mass	± da	± ppm	Start Seq.	End Seq.	Sequence	Ion Score	C. I. %	Modification	Rank	Result Type
892.4556	892.5085	0.0529	59	96	102	MKEALER			Oxidation (M)[1]		Mascot
920.5312	920.5696	0.0384	42	77	84	EVLPAVHR					Mascot
920.5312	920.5696	0.0384	42	77	84	EVLPAVHR	28	86.606			Mascot
973.5499	973.568	0.0181	19	65	73	MIGGVLVER					Mascot
989.5448	989.5548	0.01	10	65	73	MIGGVLVER			Oxidation (M)[1]		Mascot
1001.5262	1001.5497	0.0235	23	87	95	EGLEEVVAR					Mascot
1136.5834	1136.5999	0.0165	15	105	113	QEITEFELK					Mascot
1143.535	1143.5796	0.0446	39	27	35	TEMNQLYTK			Oxidation (M)[3]		Mascot
1162.6692	1162.6561	-0.0131	-11	77	86	EVLPAVHRNK					Mascot
1243.6641	1243.714	0.0499	40	85	95	NKEGLEEVVAR					Mascot
1243.6641	1243.714	0.0499	40	85	95	NKEGLEEVVAR	12	0			Mascot

Rank	Protein Name	Accession No.	Protein MW	Protein PI	Pep. Count	Protein Score	Protein Score C. I. %	Intensity Matched	Total Ion Score	Total Ion C. I. %	Confirmed
2	hypothetical protein TRIUR3_33395 [Triticum urartu]	gi|473794188	67193	6.41	20	58	92.828	12.316	12	0	

Peptide Information

Calc. Mass	Obsrv. Mass	± da	± ppm	Start Seq.	End Seq.	Sequence	Ion Score	C. I. %	Modification	Rank	Result Type
829.4162	829.4451	0.0289	35	80	86	EEPNRGK					Mascot
846.4427	846.5065	0.0638	75	364	371	NSPSSRAK					Mascot
886.4741	886.4849	0.0108	12	332	340	ASGSPPSKR					Mascot
890.4214	890.5004	0.079	89	302	309	IDQNEGSK					Mascot
892.4886	892.5085	0.0199	22	511	517	YTEPVRK					Mascot
906.46	906.5106	0.0506	56	190	196	LEEEMKK					Mascot
925.4598	925.5241	0.0643	69	248	254	EREAHQR					Mascot
963.4676	963.5128	0.0452	47	149	156	DAEMSRVR					Mascot
973.5312	973.568	0.0368	38	141	148	LEREIGEK					Mascot
1001.5374	1001.5497	0.0123	12	70	79	QGAAGLLSER					Mascot
1003.5418	1003.5793	0.0375	37	350	358	NESIQVVSK					Mascot

Calc. Mass	Obsrv. Mass	± da	± ppm	Start Seq.	End Seq.	Sequence	Ion Score	C. I. %	Modification	Rank	Result Type
1060.5422	1060.5878	0.0456	43	285	293	SEHNYLLGK					Mascot
1136.5253	1136.5999	0.0746	66	219	227	GRLEEEMEK			Oxidation (M)[7]		Mascot
1152.5685	1152.6266	0.0581	50	535	544	FYDAVLPDGR					Mascot
1154.5841	1154.6056	0.0215	19	508	516	DFKYTEPVR					Mascot
1154.5841	1154.6056	0.0215	19	508	516	DFKYTEPVR	12	0			Mascot
1248.5815	1248.6755	0.094	75	359	369	TEDQKNSPSSR					Mascot
1262.6223	1262.6655	0.0432	34	175	185	EIGEKDSEISR					Mascot
1883.0195	1882.9701	-0.0494	-26	24	40	YITGMSTILVATIQEVK			Oxidation (M)[5]		Mascot
1905.9375	1906.0681	0.1306	69	285	301	SEHNYLLGKIAEMEGSK					Mascot
1918.9539	1918.8678	-0.0861	-45	89	104	AEDRLLQLESSLEEMR					Mascot
1918.9539	1918.8678	-0.0861	-45	89	104	AEDRLLQLESSLEEMR					Mascot

3 hypothetical protein TRIUR3_08550 [Triticum urartu] gi|474006911 68701.4 4.86 18 56 90.325 5.286

Peptide Information

Calc. Mass	Obsrv. Mass	± da	± ppm	Start Seq.	End Seq.	Sequence	Ion Score	C. I. %	Modification	Rank	Result Type
802.4781	802.4725	-0.0056	-7	538	544	KSVAELR					Mascot
806.4076	806.4321	0.0245	30	243	250	GEMALAAK			Oxidation (M)[3]		Mascot
821.4008	821.4584	0.0576	70	598	604	RPMVMAT			Oxidation (M)[3]		Mascot
888.4785	888.5007	0.0222	25	206	212	DLEEKVR					Mascot
920.4869	920.5696	0.0827	90	84	91	KNMPSSLK			Oxidation (M)[3]		Mascot
920.4869	920.5696	0.0827	90	84	91	KNMPSSLK			Oxidation (M)[3]		Mascot
942.5731	942.4948	-0.0783	-83	339	346	VIKDLQAR					Mascot
993.5615	993.5475	-0.014	-14	417	424	LEKFSLEK					Mascot
1066.5164	1066.5559	0.0395	37	75	83	YDNVTGELR					Mascot
1073.5474	1073.5959	0.0485	45	390	399	ADLEGALQEK					Mascot
1223.5791	1223.6602	0.0811	66	35	45	LIEDEGDSFAK					Mascot
1232.6304	1232.6476	0.0172	14	174	183	LEALEEKNMR					Mascot
1240.5991	1240.6931	0.094	76	559	569	LMAFDSAEGKR			Oxidation (M)[2]		Mascot
1248.6252	1248.6755	0.0503	40	174	183	LEALEEKNMR			Oxidation (M)[9]		Mascot
1314.7263	1314.7166	-0.0097	-7	388	399	LKADLEGALQEK					Mascot
1349.7158	1349.7333	0.0175	13	213	224	SLESIKEISSEK					Mascot
1435.6985	1435.7716	0.0731	51	169	180	EAMEKLEALEEK			Oxidation (M)[3]		Mascot
1436.7454	1436.7327	-0.0127	-9	238	250	FEELKGEMALAAK					Mascot
1436.7454	1436.7327	-0.0127	-9	238	250	FEELKGEMALAAK					Mascot
1507.731	1507.7996	0.0686	45	498	511	VSGLLSDLATCDEK			Carbamidomethyl (C)[11]		Mascot
1691.8408	1691.913	0.0722	43	160	173	VIELEDELKEAMEK			Oxidation (M)[12]		Mascot

4 hypothetical protein TRIUR3_29900 [Triticum urartu] gi|473754167 71411.5 5.69 16 56 88.892 8.959

Peptide Information

Calc. Mass	Obsrv. Mass	± da	± ppm	Start Seq.	End Seq.	Sequence	Ion Score	C. I. %	Modification	Rank	Result Type
892.4774	892.5085	0.0311	35	116	123	LFPSTAEK					Mascot
928.5462	928.4855	-0.0607	-65	32	39	DVLAEILR					Mascot
1060.5382	1060.5878	0.0496	47	569	580	TSAAGVGGEGVR					Mascot
1084.6473	1084.6068	-0.0405	-37	32	40	DVLAEILRR					Mascot
1115.6532	1115.5979	-0.0553	-50	544	553	GSITALEIRR					Mascot
1126.6481	1126.571	-0.0771	-68	295	303	KPWDGIVRR					Mascot
1136.6245	1136.5999	-0.0246	-22	444	453	FTGRCGVVLK			Carbamidomethyl (C)[5]		Mascot
1205.5481	1205.6478	0.0997	83	140	149	MHLHPDQGDR					Mascot
1232.613	1232.6476	0.0346	28	40	49	RNSHTEYLGR					Mascot
1308.7059	1308.7041	-0.0018	-1	259	269	LSDWITHAPLR					Mascot
1333.6431	1333.7375	0.0944	71	139	149	KMHLHPDQGDR					Mascot
1333.6431	1333.7375	0.0944	71	139	149	KMHLHPDQGDR					Mascot
1349.6379	1349.7333	0.0954	71	139	149	KMHLHPDQGDR			Oxidation (M)[2]		Mascot
1361.6492	1361.7535	0.1043	77	140	150	MHLHPDQGDRR					Mascot
1527.7803	1527.791	0.0107	7	2	16	ALLPEFDPADVGAGR					Mascot
1636.8218	1636.8833	0.0615	38	71	84	VPVSAYEDIEPYVR					Mascot
1707.9351	1707.8776	-0.0575	-34	604	618	FLTDCKTLAIAVEVK			Carbamidomethyl (C)[5]		Mascot
1806.0021	1805.9062	-0.0959	-53	124	138	LDQRLFYNGVQALLR					Mascot

5 hypothetical protein TRIUR3_34329 [Triticum urartu] gi|473847480 94344.8 5.57 23 50 61.485 10.688

Peptide Information

Calc. Mass	Obsrv. Mass	± da	± ppm	Start Seq.	End Seq.	Sequence	Ion Score	C. I. %	Modification	Rank	Result Type
806.3712	806.4321	0.0609	76	680	687	AAMAEEGK					Mascot
821.4879	821.4584	-0.0295	-36	805	810	IYLEKR					Mascot
827.4622	827.478	0.0158	19	190	196	QDIPQVK					Mascot
847.4155	847.448	0.0325	38	309	317	AEEAASAAK					Mascot
886.5356	886.4849	-0.0507	-57	218	224	RLVEELK					Mascot
888.5149	888.5007	-0.0142	-16	811	818	TQANLTLK					Mascot
897.4901	897.4681	-0.022	-25	699	705	SEHKQLR					Mascot
995.5884	995.5937	0.0053	5	219	226	LVEELKHK					Mascot
1001.5738	1001.5497	-0.0241	-24	671	679	DALKIASQR					Mascot

Calc. Mass	Obsrv. Mass	± da	± ppm	Start Seq.	End Seq.	Sequence	Ion Score	C. I. %	Modification	Rank	Result Type
1003.5167	1003.5793	0.0626	62	308	317	RAEEAASAAK					Mascot
1033.4558	1033.5587	0.1029	100	115	123	SNGHNPDHR					Mascot
1060.6071	1060.5878	-0.0193	-18	277	285	AIEELKMVK					Mascot
1073.5837	1073.5959	0.0122	11	378	386	QLDEQILSK					Mascot
1078.5123	1078.5739	0.0616	57	650	658	ESESRSLDR					Mascot
1243.6641	1243.714	0.0499	40	368	377	ELQQAKEELR					Mascot
1243.6641	1243.714	0.0499	40	368	377	ELQQAKEELR					Mascot
1262.6475	1262.6655	0.018	14	207	217	SQVVEELETTK					Mascot
1331.7166	1331.7196	0.003	2	467	478	AVAESLRTELDK					Mascot
1333.6998	1333.7375	0.0377	28	143	155	GLVDTAAPFESVK					Mascot
1333.6998	1333.7375	0.0377	28	143	155	GLVDTAAPFESVK					Mascot
1361.6842	1361.7535	0.0693	51	675	687	IASQRAAMAEEGK					Mascot
1435.7581	1435.7716	0.0135	9	156	168	DAVTKFGGIVDWK					Mascot
1562.8538	1562.8428	-0.011	-7	322	334	RVEELTLELFASR					Mascot
1600.8541	1600.8834	0.0293	18	374	386	EELRQLDEQILSK					Mascot
1819.8604	1819.9797	0.1193	66	239	253	QDSELAQLRAQEMER			Oxidation (M)[13]		Mascot

6 unnamed protein product [Triticum aestivum] gi|296511569 17548.9 5.8 6 49 48.044 2.459 21 40.309

Protein Group

unnamed protein product [Triticum aestivum] gi|296512785 17548.9 5.8000 001907 3486

Peptide Information

Calc. Mass	Obsrv. Mass	± da	± ppm	Start Seq.	End Seq.	Sequence	Ion Score	C. I. %	Modification	Rank	Result Type
815.3795	815.4153	0.0358	44	100	105	TDTWHR					Mascot
827.4985	827.478	-0.0205	-25	66	73	ADVPGLKK					Mascot
1057.5314	1057.5532	0.0218	21	57	65	ETPEAHVFK					Mascot
1154.5437	1154.6056	0.0619	54	41	52	TSSDTAAFAGAR					Mascot
1154.5437	1154.6056	0.0619	54	41	52	TSSDTAAFAGAR	12	0			Mascot
1657.8392	1657.9304	0.0912	55	78	92	VEVEDGNILQISGER					Mascot
1657.8392	1657.9304	0.0912	55	78	92	VEVEDGNILQISGER	10	0			Mascot
1918.979	1918.8678	-0.1112	-58	130	147	ASMENGVLTVTVPKEEAK			Oxidation (M)[3]		Mascot
1918.979	1918.8678	-0.1112	-58	130	147	ASMENGVLTVTVPKEEAK			Oxidation (M)[3]		Mascot

7 unnamed protein product [Triticum aestivum] gi|296511507 17513.9 5.81 6 49 40.347 2.459 21 40.309

Protein Group

unnamed protein product [Triticum aestivum] gi|296512731 17513.9 5.8099

999427
7954

Peptide Information

Calc. Mass	Obsrv. Mass	± da	± ppm	Start Seq.	End Seq.	Sequence	Ion Score	C. I. %	Modification	Rank	Result Type
815.3795	815.4153	0.0358	44	99	104	TDTWHR					Mascot
827.4985	827.478	-0.0205	-25	65	72	ADVPGLKK					Mascot
1057.5889	1057.5532	-0.0357	-34	143	151	EEAKKPEVK					Mascot
1154.5437	1154.6056	0.0619	54	40	51	TSSDTAAFAGAR					Mascot
1154.5437	1154.6056	0.0619	54	40	51	TSSDTAAFAGAR	12	0			Mascot
1657.8392	1657.9304	0.0912	55	77	91	VEVEDGNILQISGER					Mascot
1657.8392	1657.9304	0.0912	55	77	91	VEVEDGNILQISGER	10	0			Mascot
1918.979	1918.8678	-0.1112	-58	129	146	ASMENGVLTVTVPKEEAK			Oxidation (M)[3]		Mascot
1918.979	1918.8678	-0.1112	-58	129	146	ASMENGVLTVTVPKEEAK			Oxidation (M)[3]		Mascot

8 unnamed protein product [Triticum aestivum] gi|296511567 17519.9 5.81 6 48 36.08 2.459 21 40.309

Protein Group

unnamed protein product [Triticum aestivum] gi|296512783 17519.9 5.8099999427 7954

Peptide Information

Calc. Mass	Obsrv. Mass	± da	± ppm	Start Seq.	End Seq.	Sequence	Ion Score	C. I. %	Modification	Rank	Result Type
815.3795	815.4153	0.0358	44	99	104	TDTWHR					Mascot
827.4985	827.478	-0.0205	-25	65	72	ADVPGLKK					Mascot
1057.5314	1057.5532	0.0218	21	56	64	ETPEAHVFK					Mascot
1154.5437	1154.6056	0.0619	54	40	51	TSSDTAAFAGAR					Mascot
1154.5437	1154.6056	0.0619	54	40	51	TSSDTAAFAGAR	12	0			Mascot
1657.8392	1657.9304	0.0912	55	77	91	VEVEDGNILQISGER					Mascot
1657.8392	1657.9304	0.0912	55	77	91	VEVEDGNILQISGER	10	0			Mascot
1918.979	1918.8678	-0.1112	-58	129	146	ASMENGVLTVTVPKEEAK			Oxidation (M)[3]		Mascot
1918.979	1918.8678	-0.1112	-58	129	146	ASMENGVLTVTVPKEEAK			Oxidation (M)[3]		Mascot

9 Glutaminyl-tRNA synthetase [Triticum urartu] gi|474021464 90535.6 6.65 17 48 29.914 15.519

Peptide Information

Calc. Mass	Obsrv. Mass	± da	± ppm	Start Seq.	End Seq.	Sequence	Ion Score	C. I. %	Modification	Rank	Result Type
811.4785	811.45	-0.0285	-35	257	264	HLKATGGK					Mascot

Calc. Mass	Obsrv. Mass	± da	± ppm	Start Seq.	End Seq.	Sequence	Ion Score	C. I. %	Modification	Rank	Result Type
829.5029	829.4451	-0.0578	-70	2	8	EEVIVLK					Mascot
836.4698	836.4729	0.0031	4	18	24	CFLLVGK			Carbamidomethyl (C)[1]		Mascot
856.5615	856.5681	0.0066	8	522	529	LLTLAGLR					Mascot
920.4982	920.5696	0.0714	78	260	268	ATGGKVMTR					Mascot
920.4982	920.5696	0.0714	78	260	268	ATGGKVMTR	3	0			Mascot
1001.5812	1001.5497	-0.0315	-31	25	32	QMLLLDLR					Mascot
1033.6041	1033.5587	-0.0454	-44	702	709	VEIRLFEK					Mascot
1037.6177	1037.5587	-0.059	-57	575	583	TMVVLHPLK					Mascot
1057.5889	1057.5532	-0.0357	-34	195	203	EKPAKVEEK					Mascot
1082.5299	1082.551	0.0211	19	417	425	NMSDLIAYR					Mascot
1105.6517	1105.6265	-0.0252	-23	9	17	AIVFFPQRK					Mascot
1105.6517	1105.6265	-0.0252	-23	9	17	AIVFFPQRK					Mascot
1182.6477	1182.6128	-0.0349	-30	247	256	AHNTKEILEK					Mascot
1182.6477	1182.6128	-0.0349	-30	247	256	AHNTKEILEK					Mascot
1193.5653	1193.6578	0.0925	77	493	502	LNISHTMMSK			Oxidation (M)[7,8]		Mascot
1227.7168	1227.676	-0.0408	-33	395	406	RGLIAEGAATLR					Mascot
1232.594	1232.6476	0.0536	43	133	142	ENMEAILEQR					Mascot
1248.5889	1248.6755	0.0866	69	133	142	ENMEAILEQR			Oxidation (M)[3]		Mascot
1333.6715	1333.7375	0.066	49	493	503	LNISHTMMSKR			Oxidation (M)[7]		Mascot
1333.6715	1333.7375	0.066	49	493	503	LNISHTMMSKR			Oxidation (M)[7]		Mascot
1349.6665	1349.7333	0.0668	49	493	503	LNISHTMMSKR			Oxidation (M)[7,8]		Mascot
1600.8376	1600.8834	0.0458	29	143	156	YHINVGGLCGQVRK			Carbamidomethyl (C)[9]		Mascot

10	unnamed protein product [Triticum aestivum]	gi|296511579	17365.9	5.8	7	46	0	3.167	10	0	

Protein Group

unnamed protein product [Triticum aestivum]	gi|296512795	17365.9	5.8000 001907 3486

Peptide Information

Calc. Mass	Obsrv. Mass	± da	± ppm	Start Seq.	End Seq.	Sequence	Ion Score	C. I. %	Modification	Rank	Result Type
815.3795	815.4153	0.0358	44	97	102	TDTWHR					Mascot
827.4985	827.478	-0.0205	-25	63	70	ADVPGLKK					Mascot
1057.5889	1057.5532	-0.0357	-34	141	149	EEAKKPEVK					Mascot
1182.575	1182.6128	0.0378	32	38	49	TSSDTVAFAGAR					Mascot
1182.575	1182.6128	0.0378	32	38	49	TSSDTVAFAGAR					Mascot
1657.8392	1657.9304	0.0912	55	75	89	VEVEDGNILQISGER					Mascot
1657.8392	1657.9304	0.0912	55	75	89	VEVEDGNILQISGER	10	0			Mascot

1707.9429	1707.8776	-0.0653	-38	54	69	ETPVAHVFKADVPGLK		Mascot
1918.979	1918.8678	-0.1112	-58	127	144	ASMENGVLTVTVPKEEA K	Oxidation (M)[3]	Mascot
1918.979	1918.8678	-0.1112	-58	127	144	ASMENGVLTVTVPKEEA K	Oxidation (M)[3]	Mascot

Rank	Protein Name	Accession No.	Protein MW	Protein PI	Pep. Count	Protein Score	Protein Score C. I. %	Intensity Matched	Total Ion Score	Total Ion C. I. %	Confirmed
1	hypothetical protein TRIUR3_03549 [Triticum urartu]	gi|474071007	16824.8	6.19	7	233	100	26.074	189	100	

Peptide Information

Calc. Mass	Obsrv. Mass	± da	± ppm	Start Seq.	End Seq.	Sequence	Ion Score	C. I. %	Modification	Rank	Result Type
827.4985	827.4829	-0.0156	-19	59	66	ADLPGVKK					Mascot
975.5258	975.5746	0.0488	50	110	117	FRLPEDAK					Mascot
975.5258	975.5746	0.0488	50	110	117	FRLPEDAK	24	68.086			Mascot
1057.5314	1057.5516	0.0202	19	50	58	ETPEAHVFK					Mascot
1057.5314	1057.5516	0.0202	19	50	58	ETPEAHVFK	14	0			Mascot
1600.8177	1600.8934	0.0757	47	71	85	VEVEDGNVLVVSGER					Mascot
1600.8177	1600.8934	0.0757	47	71	85	VEVEDGNVLVVSGER	9	0			Mascot
1905.9666	1906.0603	0.0937	49	26	45	SIVPAISGGSSETAAFAN AR					Mascot
1905.9666	1906.0603	0.0937	49	26	45	SIVPAISGGSSETAAFAN AR	142	100			Mascot
2086.0664	2086.155	0.0886	42	67	85	EEVKVEVEDGNVLVVSG ER					Mascot
2260.0347	2260.1248	0.0901	40	7	25	SNVFDPFADLWADPFDT FR					Mascot

Rank	Protein Name	Accession No.	Protein MW	Protein PI	Pep. Count	Protein Score	Protein Score C. I. %	Intensity Matched	Total Ion Score	Total Ion C. I. %	Confirmed
2	unnamed protein product [Triticum aestivum]	gi|296512787	16857.8	5.83	7	231	100	26.074	189	100	

Protein Group

unnamed protein product [Triticum aestivum]	gi|296511575	16856.8	6.1900 000572 2046
unnamed protein product [Triticum aestivum]	gi|296511571	16857.8	5.8299 999237 0605
unnamed protein product [Triticum aestivum]	gi|296512791	16856.8	6.1900 000572 2046

Peptide Information

Calc. Mass	Obsrv. Mass	± da	± ppm	Start Seq.	End Seq.	Sequence	Ion Score	C. I. %	Modification	Rank	Result Type
827.4985	827.4829	-0.0156	-19	59	66	ADLPGVKK					Mascot
975.5258	975.5746	0.0488	50	110	117	FRLPEDAK					Mascot
975.5258	975.5746	0.0488	50	110	117	FRLPEDAK	24	68.086			Mascot

1057.5314	1057.5516	0.0202	19	50	58	ETPEAHVFK						Mascot
1057.5314	1057.5516	0.0202	19	50	58	ETPEAHVFK	14	0				Mascot
1600.8177	1600.8934	0.0757	47	71	85	VEVEDGNVLVVSGER						Mascot
1600.8177	1600.8934	0.0757	47	71	85	VEVEDGNVLVVSGER	9	0				Mascot
1905.9666	1906.0603	0.0937	49	26	45	SIVPAISGGSSETAAFAN AR						Mascot
1905.9666	1906.0603	0.0937	49	26	45	SIVPAISGGSSETAAFAN AR	142	100				Mascot
2086.0664	2086.155	0.0886	42	67	85	EEVKVEVEDGNVLVVSG ER						Mascot
2260.0347	2260.1248	0.0901	40	7	25	SNVFDPFADLWADPFDT FR						Mascot

3 unnamed protein product [Triticum dicoccoides] gi|296510911 16838.8 5.82 7 228 100 25.951 189 100

Protein Group

16.8 kDa heat-shock protein [Triticum dicoccoides]	gi|186886530	16838.8	5.8200 001716 6138
unnamed protein product [Triticum dicoccoides]	gi|296512518	16838.8	5.8200 001716 6138

Peptide Information

Calc. Mass	Obsrv. Mass	± da	± ppm	Start Seq.	End Seq.	Sequence	Ion Score	C. I. %	Modification	Rank	Result Type
827.4985	827.4829	-0.0156	-19	59	66	ADLPGVKK					Mascot
975.5258	975.5746	0.0488	50	110	117	FRLPEDAK					Mascot
975.5258	975.5746	0.0488	50	110	117	FRLPEDAK	24	68.086			Mascot
1006.4323	1006.4822	0.0499	50	88	95	EEEDKNDK					Mascot
1057.5314	1057.5516	0.0202	19	50	58	ETPEAHVFK					Mascot
1057.5314	1057.5516	0.0202	19	50	58	ETPEAHVFK	14	0			Mascot
1600.8177	1600.8934	0.0757	47	71	85	VEVEDGNVLVVSGER					Mascot
1600.8177	1600.8934	0.0757	47	71	85	VEVEDGNVLVVSGER	9	0			Mascot
1905.9666	1906.0603	0.0937	49	26	45	SIVPAISGGSSETAAFAN AR					Mascot
1905.9666	1906.0603	0.0937	49	26	45	SIVPAISGGSSETAAFAN AR	142	100			Mascot
2086.0664	2086.155	0.0886	42	67	85	EEVKVEVEDGNVLVVSG ER					Mascot

4 unnamed protein product [Triticum aestivum] gi|296512688 16867.8 5.83 6 226 100 25.752 189 100

Protein Group

RecName: Full=16.9 kDa class I heat shock protein 1; AltName: Full=HSP 16.9; AltName: Full=Heat shock protein 16.9A; AltName: Full=Heat shock protein 17; AltName: Full=Low molecular weight heat shock protein	gi|123545	16867.8	5.8299 999237 0605
heat shock protein 16.8	gi|445135	16867.8	5.8299 999237

			0605
unnamed protein product [Triticum aestivum]	gi|296511073	16867.8	5.8299 999237 0605
unnamed protein product [Triticum aestivum]	gi|21813	16867.8	5.8299 999237 0605

Peptide Information

Calc. Mass	Obsrv. Mass	± da	± ppm	Start Seq.	End Seq.	Sequence	Ion Score	C. I. %	Modification	Rank	Result Type
975.5258	975.5746	0.0488	50	110	117	FRLPEDAK					Mascot
975.5258	975.5746	0.0488	50	110	117	FRLPEDAK	24	68.086			Mascot
1057.5314	1057.5516	0.0202	19	50	58	ETPEAHVFK					Mascot
1057.5314	1057.5516	0.0202	19	50	58	ETPEAHVFK	14	0			Mascot
1600.8177	1600.8934	0.0757	47	71	85	VEVEDGNVLVVSGER					Mascot
1600.8177	1600.8934	0.0757	47	71	85	VEVEDGNVLVVSGER	9	0			Mascot
1905.9666	1906.0603	0.0937	49	26	45	SIVPAISGGSSETAAFAN AR					Mascot
1905.9666	1906.0603	0.0937	49	26	45	SIVPAISGGSSETAAFAN AR	142	100			Mascot
2086.0664	2086.155	0.0886	42	67	85	EEVKVEVEDGNVLVVSG ER					Mascot
2260.0347	2260.1248	0.0901	40	7	25	SNVFDPFADLWADPFDT FR					Mascot

5	unnamed protein product [Triticum aestivum]	gi|296511573	16884.8	5.83	7	213	100	12.481	171	100

Protein Group

unnamed protein product [Triticum aestivum]	gi|296512789	16884.8	5.8299 999237 0605

Peptide Information

Calc. Mass	Obsrv. Mass	± da	± ppm	Start Seq.	End Seq.	Sequence	Ion Score	C. I. %	Modification	Rank	Result Type
827.4985	827.4829	-0.0156	-19	59	66	ADLPGVKK					Mascot
975.5258	975.5746	0.0488	50	110	117	FRLPEDAK					Mascot
975.5258	975.5746	0.0488	50	110	117	FRLPEDAK	24	68.086			Mascot
1057.5314	1057.5516	0.0202	19	50	58	ETPEAHVFK					Mascot
1057.5314	1057.5516	0.0202	19	50	58	ETPEAHVFK	14	0			Mascot
1600.8177	1600.8934	0.0757	47	71	85	VEVEDGNVLVVSGER					Mascot
1600.8177	1600.8934	0.0757	47	71	85	VEVEDGNVLVVSGER	9	0			Mascot
1932.9774	1933.0692	0.0918	47	26	45	SIVPAISGGNSETAAFAN AR					Mascot
1932.9774	1933.0692	0.0918	47	26	45	SIVPAISGGNSETAAFAN AR	124	100			Mascot
2086.0664	2086.155	0.0886	42	67	85	EEVKVEVEDGNVLVVSG					Mascot

2260.0347	2260.1248	0.0901	40	7	25	ER SNVFDPFADLWADPFDT FR					Mascot

6	unnamed protein product [Triticum turgidum subsp. dicoccon]	gi|296510913	16852.9	6.77	4	199	100	24.415	180	100

Protein Group

16.9a kDa heat-shock protein [Triticum turgidum subsp. dicoccon]	gi|186886532	16852.9	6.7699 999809 2651
unnamed protein product [Triticum turgidum subsp. dicoccon]	gi|296512520	16852.9	6.7699 999809 2651

Peptide Information

Calc. Mass	Obsrv. Mass	± da	± ppm	Start Seq.	End Seq.	Sequence	Ion Score	C. I. %	Modification	Rank	Result Type
827.4985	827.4829	-0.0156	-19	59	66	ADLPGVKK					Mascot
975.5258	975.5746	0.0488	50	110	117	FRLPEDAK					Mascot
975.5258	975.5746	0.0488	50	110	117	FRLPEDAK	24	68.086			Mascot
1057.5314	1057.5516	0.0202	19	50	58	ETPEAHVFK					Mascot
1057.5314	1057.5516	0.0202	19	50	58	ETPEAHVFK	14	0			Mascot
1905.9666	1906.0603	0.0937	49	26	45	SIVPAISGGSSETAAFAN AR					Mascot
1905.9666	1906.0603	0.0937	49	26	45	SIVPAISGGSSETAAFAN AR	142	100			Mascot

7	unnamed protein product [Triticum durum]	gi|296510917	16960.8	5.83	3	166	100	22.716	151	100

Protein Group

16.9 kDa heat-shock protein [Triticum durum]	gi|186886536	16960.8	5.8299 999237 0605
unnamed protein product [Triticum durum]	gi|296512524	16960.8	5.8299 999237 0605

Peptide Information

Calc. Mass	Obsrv. Mass	± da	± ppm	Start Seq.	End Seq.	Sequence	Ion Score	C. I. %	Modification	Rank	Result Type
1600.8177	1600.8934	0.0757	47	71	85	VEVEDGNVLVVSGER					Mascot
1600.8177	1600.8934	0.0757	47	71	85	VEVEDGNVLVVSGER	9	0			Mascot
1905.9666	1906.0603	0.0937	49	26	45	SIVPAISGGSSETAAFAN AR					Mascot
1905.9666	1906.0603	0.0937	49	26	45	SIVPAISGGSSETAAFAN AR	142	100			Mascot
2086.0664	2086.155	0.0886	42	67	85	EEVKVEVEDGNVLVVSG ER					Mascot

8	unnamed protein product [Triticum aestivum]	gi|296511581	17468.9	6.34	10	110	100	7.56	51	99.946

Protein Group

unnamed protein product [Triticum aestivum]	gi|296512797	17468.9	6.3400 001525 8789

Peptide Information

Calc. Mass	Obsrv. Mass	± da	± ppm	Start Seq.	End Seq.	Sequence	Ion Score	C. I. %	Modification	Rank	Result Type
815.3795	815.4125	0.033	40	99	104	TDTWHR					Mascot
827.4985	827.4829	-0.0156	-19	65	72	ADVPGLKK					Mascot
904.437	904.4432	0.0062	7	92	98	NKEQEEK					Mascot
974.5417	974.5853	0.0436	45	116	123	FRLPENAK					Mascot
1057.5314	1057.5516	0.0202	19	56	64	ETPEAHVFK					Mascot
1057.5314	1057.5516	0.0202	19	56	64	ETPEAHVFK	14	0			Mascot
1154.5437	1154.5985	0.0548	47	40	51	TSSDTAAFAGAR					Mascot
1154.5437	1154.5985	0.0548	47	40	51	TSSDTAAFAGAR	20	20.204			Mascot
1194.6841	1194.6484	-0.0357	-30	147	157	KPEVKSIHISG					Mascot
1657.8392	1657.9052	0.066	40	77	91	VEVEDGNILQISGER					Mascot
1657.8392	1657.9052	0.066	40	77	91	VEVEDGNILQISGER	17	0			Mascot
1889.0049	1889.0582	0.0533	28	129	146	ASMENGVLTVTVPKVEAK			Oxidation (M)[3]		Mascot
2143.0876	2143.1763	0.0887	41	73	91	EEVKVEVEDGNILQISGER					Mascot

9	unnamed protein product [Triticum aestivum]	gi|296511569	17548.9	5.8	8	92	99.997	7.284	51	99.946	

Protein Group

unnamed protein product [Triticum aestivum]	gi|296512785	17548.9	5.8000 001907 3486

Peptide Information

Calc. Mass	Obsrv. Mass	± da	± ppm	Start Seq.	End Seq.	Sequence	Ion Score	C. I. %	Modification	Rank	Result Type
815.3795	815.4125	0.033	40	100	105	TDTWHR					Mascot
827.4985	827.4829	-0.0156	-19	66	73	ADVPGLKK					Mascot
904.437	904.4432	0.0062	7	93	99	NKEQEEK					Mascot
974.5417	974.5853	0.0436	45	117	124	FRLPENAK					Mascot
1057.5314	1057.5516	0.0202	19	57	65	ETPEAHVFK					Mascot
1057.5314	1057.5516	0.0202	19	57	65	ETPEAHVFK	14	0			Mascot
1154.5437	1154.5985	0.0548	47	41	52	TSSDTAAFAGAR					Mascot
1154.5437	1154.5985	0.0548	47	41	52	TSSDTAAFAGAR	20	20.204			Mascot
1657.8392	1657.9052	0.066	40	78	92	VEVEDGNILQISGER					Mascot

1657.8392	1657.9052	0.066	40	78	92	VEVEDGNILQISGER	17	0			Mascot
2143.0876	2143.1763	0.0887	41	74	92	EEVKVEVEDGNILQISGE R					Mascot

10	unnamed protein product [Triticum aestivum]	gi|296511567	17519.9	5.81	8	89	99.994	7.284	50	99.925	

Protein Group

unnamed protein product [Triticum aestivum]	gi|296512783	17519.9	5.8099 999427 7954

Peptide Information

Calc. Mass	Obsrv. Mass	± da	± ppm	Start Seq.	End Seq.	Sequence	Ion Score	C. I. %	Modification	Rank	Result Type
815.3795	815.4125	0.033	40	99	104	TDTWHR					Mascot
827.4985	827.4829	-0.0156	-19	65	72	ADVPGLKK					Mascot
904.437	904.4432	0.0062	7	92	98	NKEQEEK					Mascot
974.5417	974.5853	0.0436	45	116	123	FRLPENAK					Mascot
1057.5314	1057.5516	0.0202	19	56	64	ETPEAHVFK					Mascot
1057.5314	1057.5516	0.0202	19	56	64	ETPEAHVFK	14	0			Mascot
1154.5437	1154.5985	0.0548	47	40	51	TSSDTAAFAGAR					Mascot
1154.5437	1154.5985	0.0548	47	40	51	TSSDTAAFAGAR	20	20.204			Mascot
1657.8392	1657.9052	0.066	40	77	91	VEVEDGNILQISGER					Mascot
1657.8392	1657.9052	0.066	40	77	91	VEVEDGNILQISGER	17	0			Mascot
2143.0876	2143.1763	0.0887	41	73	91	EEVKVEVEDGNILQISGE R					Mascot

Gel Idx/Pos	128/F3	Instr./Gel Origin	BA2151/Sample Project 20140814	Process Status	Analysis Succeeded
Plate [#] Name	[1] Sample Project 20140814	Instrument Sample Name		Spectra	11

Rank	Protein Name	Accession No.	Protein MW	Protein PI	Pep. Count	Protein Score	Protein Score C. I. %	Intensity Matched	Total Ion Score	Total Ion C. I. %	Confirmed
1	alpha-amylase inhibitor 0.19 [Triticum aestivum]	gi|66841026	13340.4	6.86	6	351	100	35.685	310	100	

Peptide Information

Calc. Mass	Obsrv. Mass	± da	± ppm	Start Seq.	End Seq.	Sequence	Ion Score	C. I. %	Modification	Rank	Result Type
1162.6249	1162.6868	0.0619	53	85	95	LTAASITAVCR			Carbamidomethyl (C)[10]		Mascot
1162.6249	1162.6868	0.0619	53	85	95	LTAASITAVCR	36	98.061	Carbamidomethyl (C)[10]		Mascot
1570.8007	1570.892	0.0913	58	21	34	LQCNGSQVPEAVLR			Carbamidomethyl (C)[3]		Mascot
1612.7463	1612.8384	0.0921	57	62	77	EHGAQEGQAGTGAFPR					Mascot
1612.7463	1612.8384	0.0921	57	62	77	EHGAQEGQAGTGAFPR	144	100			Mascot
1617.8993	1617.8442	-0.0551	-34	81	95	EVVKLTAASITAVCR			Carbamidomethyl (C)[14]		Mascot
1663.8361	1663.8826	0.0465	28	96	111	LPIVVDASGDGAYVCK			Carbamidomethyl (C)[15]		Mascot
1862.7731	1862.8889	0.1158	62	35	48	DCCQQLAHISEWCR			Carbamidomethyl (C)[2,3,13]		Mascot
1862.7731	1862.8889	0.1158	62	35	48	DCCQQLAHISEWCR	130	100	Carbamidomethyl (C)[2,3,13]		Mascot

Rank	Protein Name	Accession No.	Protein MW	Protein PI	Pep. Count	Protein Score	Protein Score C. I. %	Intensity Matched	Total Ion Score	Total Ion C. I. %	Confirmed
2	Chain D, 0.19 Alpha-Amylase Inhibitor From Wheat	gi|3318684	13898.6	6.66	6	350	100	35.685	310	100	

Protein Group

0.19 alpha-amylase inhibitor [Triticum aestivum]	gi|2116581	13898.6	6.6599998474 1211
0.19 dimeric alpha-amylase inhibitor [Triticum aestivum]	gi|54778509	13898.6	6.6599998474 1211
0.19 dimeric alpha-amylase inhibitor [Triticum aestivum]	gi|54778501	13898.6	6.6599998474 1211
Chain A, 0.19 Alpha-Amylase Inhibitor From Wheat	gi|3318681	13898.6	6.6599998474 1211
Chain B, 0.19 Alpha-Amylase Inhibitor From Wheat	gi|3318682	13898.6	6.6599998474 1211
Chain C, 0.19 Alpha-Amylase Inhibitor From Wheat	gi|3318683	13898.6	6.6599998474 1211
RecName: Full=Alpha-amylase inhibitor 0.19; AltName: Full=0.19 alpha-AI; Short=0.19 AI	gi|123963	13898.6	6.6599998474 1211
dimeric alpha-amylase inhibitor precursor, partial [Triticum aestivum]	gi|108597921	14029.7	6.6900000572

Peptide Information

Calc. Mass	Obsrv. Mass	± da	± ppm	Start Seq.	End Seq.	Sequence	Ion Score	C. I. %	Modification	Rank	Result Type
1162.6249	1162.6868	0.0619	53	90	100	LTAASITAVCR			Carbamidomethyl (C)[10]		Mascot
1162.6249	1162.6868	0.0619	53	90	100	LTAASITAVCR	36	98.061	Carbamidomethyl (C)[10]		Mascot
1570.8007	1570.892	0.0913	58	26	39	LQCNGSQVPEAVLR			Carbamidomethyl (C)[3]		Mascot
1612.7463	1612.8384	0.0921	57	67	82	EHGAQEGQAGTGAFPR					Mascot
1612.7463	1612.8384	0.0921	57	67	82	EHGAQEGQAGTGAFPR	144	100			Mascot
1617.8993	1617.8442	-0.0551	-34	86	100	EVVKLTAASITAVCR			Carbamidomethyl (C)[14]		Mascot
1663.8361	1663.8826	0.0465	28	101	116	LPIVVDASGDGAYVCK			Carbamidomethyl (C)[15]		Mascot
1862.7731	1862.8889	0.1158	62	40	53	DCCQQLAHISEWCR			Carbamidomethyl (C)[2,3,13]		Mascot
1862.7731	1862.8889	0.1158	62	40	53	DCCQQLAHISEWCR	130	100	Carbamidomethyl (C)[2,3,13]		Mascot

3	dimeric alpha-amylase inhibitor [Triticum dicoccoides]	gi|227809009	15730.5	5.58	6	347	100	35.685	310	100	

Protein Group

dimeric alpha-amylase inhibitor [Triticum aestivum]	gi|65993781	15688.5	5.5799 999237 0605
dimeric alpha-amylase inhibitor [Triticum aestivum]	gi|386877038	15702.5	5.5799 999237 0605
dimeric alpha-amylase inhibitor [Triticum dicoccoides]	gi|227809005	15730.5	5.5799 999237 0605

Peptide Information

Calc. Mass	Obsrv. Mass	± da	± ppm	Start Seq.	End Seq.	Sequence	Ion Score	C. I. %	Modification	Rank	Result Type
1162.6249	1162.6868	0.0619	53	107	117	LTAASITAVCR			Carbamidomethyl (C)[10]		Mascot
1162.6249	1162.6868	0.0619	53	107	117	LTAASITAVCR	36	98.061	Carbamidomethyl (C)[10]		Mascot
1570.8007	1570.892	0.0913	58	43	56	LQCNGSQVPEAVLR			Carbamidomethyl (C)[3]		Mascot
1612.7463	1612.8384	0.0921	57	84	99	EHGAQEGQAGTGAFPR					Mascot
1612.7463	1612.8384	0.0921	57	84	99	EHGAQEGQAGTGAFPR	144	100			Mascot
1617.8993	1617.8442	-0.0551	-34	103	117	EVVKLTAASITAVCR			Carbamidomethyl (C)[14]		Mascot
1663.8361	1663.8826	0.0465	28	118	133	LPIVVDASGDGAYVCK			Carbamidomethyl (C)[15]		Mascot
1862.7731	1862.8889	0.1158	62	57	70	DCCQQLAHISEWCR			Carbamidomethyl (C)[2,3,13]		Mascot
1862.7731	1862.8889	0.1158	62	57	70	DCCQQLAHISEWCR	130	100	Carbamidomethyl (C)[2,3,13]		Mascot

4	0.19 dimeric alpha-amylase inhibitor [Triticum aestivum]	gi|54778503	13826.6	7.45	5	341	100	33.655	310	100	

Peptide Information

Calc. Mass	Obsrv. Mass	± da	± ppm	Start Seq.	End Seq.	Sequence	Ion Score	C. I. %	Modification	Rank	Result Type
1162.6249	1162.6868	0.0619	53	90	100	LTAASITAVCR			Carbamidomethyl (C)[10]		Mascot
1162.6249	1162.6868	0.0619	53	90	100	LTAASITAVCR	36	98.061	Carbamidomethyl (C)[10]		Mascot
1612.7463	1612.8384	0.0921	57	67	82	EHGAQEGQAGTGAFPR					Mascot
1612.7463	1612.8384	0.0921	57	67	82	EHGAQEGQAGTGAFPR	144	100			Mascot
1617.8993	1617.8442	-0.0551	-34	86	100	EVVKLTAASITAVCR			Carbamidomethyl (C)[14]		Mascot
1663.8361	1663.8826	0.0465	28	101	116	LPIVVDASGDGAYVCK			Carbamidomethyl (C)[15]		Mascot
1862.7731	1862.8889	0.1158	62	40	53	DCCQQLAHISEWCR			Carbamidomethyl (C)[2,3,13]		Mascot
1862.7731	1862.8889	0.1158	62	40	53	DCCQQLAHISEWCR	130	100	Carbamidomethyl (C)[2,3,13]		Mascot

5	dimeric alpha-amylase inhibitor, partial [Triticum aestivum]	gi|386877068	14415.8	6.88	5	340	100	34.955	310	100

Peptide Information

Calc. Mass	Obsrv. Mass	± da	± ppm	Start Seq.	End Seq.	Sequence	Ion Score	C. I. %	Modification	Rank	Result Type
1162.6249	1162.6868	0.0619	53	95	105	LTAASITAVCR			Carbamidomethyl (C)[10]		Mascot
1162.6249	1162.6868	0.0619	53	95	105	LTAASITAVCR	36	98.061	Carbamidomethyl (C)[10]		Mascot
1570.8007	1570.892	0.0913	58	31	44	LQCNGSQVPEAVLR			Carbamidomethyl (C)[3]		Mascot
1612.7463	1612.8384	0.0921	57	72	87	EHGAQEGQAGTGAFPR					Mascot
1612.7463	1612.8384	0.0921	57	72	87	EHGAQEGQAGTGAFPR	144	100			Mascot
1617.8993	1617.8442	-0.0551	-34	91	105	EVVKLTAASITAVCR			Carbamidomethyl (C)[14]		Mascot
1862.7731	1862.8889	0.1158	62	45	58	DCCQQLAHISEWCR			Carbamidomethyl (C)[2,3,13]		Mascot
1862.7731	1862.8889	0.1158	62	45	58	DCCQQLAHISEWCR	130	100	Carbamidomethyl (C)[2,3,13]		Mascot

6	dimeric alpha-amylase inhibitor [Triticum dicoccoides]	gi|227809180	15716.5	5.58	5	338	100	33.655	310	100

Protein Group

dimeric alpha-amylase inhibitor [Triticum aestivum]	gi|65993925	15702.5	5.5799 999237 0605
dimeric alpha-amylase inhibitor [Triticum dicoccoides]	gi|227809156	15716.5	5.5799 999237 0605
dimeric alpha-amylase inhibitor [Triticum timopheevii subsp. armeniacum]	gi|227809268	15702.5	5.5799 999237 0605

Peptide Information

Calc. Mass	Obsrv. Mass	± da	± ppm	Start Seq.	End Seq.	Sequence	Ion Score	C. I. %	Modification	Rank	Result Type
1162.6249	1162.6868	0.0619	53	107	117	LTAASITAVCR			Carbamidomethyl (C)[10]		Mascot

Calc. Mass	Obsrv. Mass	± da	± ppm	Start Seq.	End Seq.	Sequence	Ion Score	C. I. %	Modification	Rank	Result Type
1162.6249	1162.6868	0.0619	53	107	117	LTAASITAVCR	36	98.061	Carbamidomethyl (C)[10]		Mascot
1612.7463	1612.8384	0.0921	57	84	99	EHGAQEGQAGTGAFPR					Mascot
1612.7463	1612.8384	0.0921	57	84	99	EHGAQEGQAGTGAFPR	144	100			Mascot
1617.8993	1617.8442	-0.0551	-34	103	117	EVVKLTAASITAVCR			Carbamidomethyl (C)[14]		Mascot
1663.8361	1663.8826	0.0465	28	118	133	LPIVVDASGDGAYVCK			Carbamidomethyl (C)[15]		Mascot
1862.7731	1862.8889	0.1158	62	57	70	DCCQQLAHISEWCR			Carbamidomethyl (C)[2,3,13]		Mascot
1862.7731	1862.8889	0.1158	62	57	70	DCCQQLAHISEWCR	130	100	Carbamidomethyl (C)[2,3,13]		Mascot

7	dimeric alpha-amylase inhibitor [Triticum aestivum]	gi|255988225	15665.5	6.69	3	288	100	32.931	274	100	

Peptide Information

Calc. Mass	Obsrv. Mass	± da	± ppm	Start Seq.	End Seq.	Sequence	Ion Score	C. I. %	Modification	Rank	Result Type
1570.8007	1570.892	0.0913	58	43	56	LQCNGSQVPEAVLR			Carbamidomethyl (C)[3]		Mascot
1612.7463	1612.8384	0.0921	57	84	99	EHGAQEGQAGTGAFPR					Mascot
1612.7463	1612.8384	0.0921	57	84	99	EHGAQEGQAGTGAFPR	144	100			Mascot
1862.7731	1862.8889	0.1158	62	57	70	DCCQQLAHISEWCR			Carbamidomethyl (C)[2,3,13]		Mascot
1862.7731	1862.8889	0.1158	62	57	70	DCCQQLAHISEWCR	130	100	Carbamidomethyl (C)[2,3,13]		Mascot

8	dimeric alpha-amylase inhibitor [Triticum dicoccoides]	gi|114215806	13862.5	5.25	5	210	100	28.475	180	100	

Protein Group

dimeric alpha-amylase inhibitor [Triticum dicoccoides]	gi|114215808	13921.6	5.25

Peptide Information

Calc. Mass	Obsrv. Mass	± da	± ppm	Start Seq.	End Seq.	Sequence	Ion Score	C. I. %	Modification	Rank	Result Type
1162.6249	1162.6868	0.0619	53	90	100	LTAASITAVCR			Carbamidomethyl (C)[10]		Mascot
1162.6249	1162.6868	0.0619	53	90	100	LTAASITAVCR	36	98.061	Carbamidomethyl (C)[10]		Mascot
1570.8007	1570.892	0.0913	58	26	39	LQCNGSQVPEAVLR			Carbamidomethyl (C)[3]		Mascot
1612.7463	1612.8384	0.0921	57	67	82	EHGAQEGQAGTGAFPR					Mascot
1612.7463	1612.8384	0.0921	57	67	82	EHGAQEGQAGTGAFPR	144	100			Mascot
1617.8993	1617.8442	-0.0551	-34	86	100	EVVKLTAASITAVCR			Carbamidomethyl (C)[14]		Mascot
1663.8361	1663.8826	0.0465	28	101	116	LPIVVDASGDGAYVCK			Carbamidomethyl (C)[15]		Mascot

9	dimeric alpha-amylase inhibitor [Triticum dicoccoides]	gi|227809252	15716.4	5.01	5	208	100	28.475	180	100	

Protein Group

dimeric alpha-amylase inhibitor [Triticum aestivum]	gi|65993829	15722.4	5.5799 999237 0605
dimeric alpha-amylase inhibitor [Triticum dicoccoides]	gi|227809102	15678.4	5.25

dimeric alpha-amylase inhibitor [Triticum dicoccoides]	gi|227809254	15753.5	4.8299 999237 0605
dimeric alpha-amylase inhibitor [Triticum dicoccoides]	gi|227809078	15722.4	4.8299 999237 0605

Peptide Information

Calc. Mass	Obsrv. Mass	± da	± ppm	Start Seq.	End Seq.	Sequence	Ion Score	C. I. %	Modification	Rank	Result Type
1162.6249	1162.6868	0.0619	53	107	117	LTAASITAVCR			Carbamidomethyl (C)[10]		Mascot
1162.6249	1162.6868	0.0619	53	107	117	LTAASITAVCR	36	98.061	Carbamidomethyl (C)[10]		Mascot
1570.8007	1570.892	0.0913	58	43	56	LQCNGSQVPEAVLR			Carbamidomethyl (C)[3]		Mascot
1612.7463	1612.8384	0.0921	57	84	99	EHGAQEGQAGTGAFPR					Mascot
1612.7463	1612.8384	0.0921	57	84	99	EHGAQEGQAGTGAFPR	144	100			Mascot
1617.8993	1617.8442	-0.0551	-34	103	117	EVVKLTAASITAVCR			Carbamidomethyl (C)[14]		Mascot
1663.8361	1663.8826	0.0465	28	118	133	LPIVVDASGDGAYVCK			Carbamidomethyl (C)[15]		Mascot

10	dimeric alpha-amylase inhibitor [Triticum dicoccoides]	gi|114215804	13862.5	5.25	4	202	100	28.197	180	100	

Peptide Information

Calc. Mass	Obsrv. Mass	± da	± ppm	Start Seq.	End Seq.	Sequence	Ion Score	C. I. %	Modification	Rank	Result Type
1162.6249	1162.6868	0.0619	53	90	100	LTAASITAVCR			Carbamidomethyl (C)[10]		Mascot
1162.6249	1162.6868	0.0619	53	90	100	LTAASITAVCR	36	98.061	Carbamidomethyl (C)[10]		Mascot
1570.8007	1570.892	0.0913	58	26	39	LQCNGSQVPEAVLR			Carbamidomethyl (C)[3]		Mascot
1612.7463	1612.8384	0.0921	57	67	82	EHGAQEGQAGTGAFPR					Mascot
1612.7463	1612.8384	0.0921	57	67	82	EHGAQEGQAGTGAFPR	144	100			Mascot
1663.8361	1663.8826	0.0465	28	101	116	LPIVVDASGDGAYVCK			Carbamidomethyl (C)[15]		Mascot

Gel Idx/Pos	129/F4	Instr./Gel Origin	BA2151/Sample Project 20140814	Process Status	Analysis Succeeded
Plate [#] Name	[1] Sample Project 20140814	Instrument Sample Name		Spectra	11

Rank	Protein Name	Accession No.	Protein MW	Protein PI	Pep. Count	Protein Score	Protein Score C. I. %	Intensity Matched	Total Ion Score	Total Ion C. I. %	Confirmed
1	alpha-amylase inhibitor 0.19 [Triticum aestivum]	gi|66841026	13340.4	6.86	6	318	100	36.012	278	100	

Peptide Information

Calc. Mass	Obsrv. Mass	± da	± ppm	Start Seq.	End Seq.	Sequence	Ion Score	C. I. %	Modification	Rank	Result Type
1162.6249	1162.6742	0.0493	42	85	95	LTAASITAVCR			Carbamidomethyl (C)[10]		Mascot
1570.8007	1570.8644	0.0637	41	21	34	LQCNGSQVPEAVLR			Carbamidomethyl (C)[3]		Mascot
1612.7463	1612.8197	0.0734	46	62	77	EHGAQEGQAGTGAFPR					Mascot
1612.7463	1612.8197	0.0734	46	62	77	EHGAQEGQAGTGAFPR	147	100			Mascot
1617.8993	1617.8162	-0.0831	-51	81	95	EVVKLTAASITAVCR			Carbamidomethyl (C)[14]		Mascot
1663.8361	1663.8524	0.0163	10	96	111	LPIVVDASGDGAYVCK			Carbamidomethyl (C)[15]		Mascot
1862.7731	1862.8665	0.0934	50	35	48	DCCQQLAHISEWCR			Carbamidomethyl (C)[2,3,13]		Mascot
1862.7731	1862.8665	0.0934	50	35	48	DCCQQLAHISEWCR	131	100	Carbamidomethyl (C)[2,3,13]		Mascot

Rank	Protein Name	Accession No.	Protein MW	Protein PI	Pep. Count	Protein Score	Protein Score C. I. %	Intensity Matched	Total Ion Score	Total Ion C. I. %	Confirmed
2	Chain D, 0.19 Alpha-Amylase Inhibitor From Wheat	gi|3318684	13898.6	6.66	6	317	100	36.012	278	100	

Protein Group

0.19 alpha-amylase inhibitor [Triticum aestivum]	gi|2116581	13898.6	6.6599998474 1211
0.19 dimeric alpha-amylase inhibitor [Triticum aestivum]	gi|54778509	13898.6	6.6599998474 1211
0.19 dimeric alpha-amylase inhibitor [Triticum aestivum]	gi|54778501	13898.6	6.6599998474 1211
Chain A, 0.19 Alpha-Amylase Inhibitor From Wheat	gi|3318681	13898.6	6.6599998474 1211
Chain B, 0.19 Alpha-Amylase Inhibitor From Wheat	gi|3318682	13898.6	6.6599998474 1211
Chain C, 0.19 Alpha-Amylase Inhibitor From Wheat	gi|3318683	13898.6	6.6599998474 1211
RecName: Full=Alpha-amylase inhibitor 0.19; AltName: Full=0.19 alpha-AI; Short=0.19 AI	gi|123963	13898.6	6.6599998474 1211
dimeric alpha-amylase inhibitor precursor, partial [Triticum aestivum]	gi|108597921	14029.7	6.6900000572 2046

Peptide Information

Calc. Mass	Obsrv. Mass	± da	± ppm	Start Seq.	End Seq.	Sequence	Ion Score	C. I. %	Modification	Rank	Result Type
1162.6249	1162.6742	0.0493	42	90	100	LTAASITAVCR			Carbamidomethyl (C)[10]		Mascot
1570.8007	1570.8644	0.0637	41	26	39	LQCNGSQVPEAVLR			Carbamidomethyl (C)[3]		Mascot
1612.7463	1612.8197	0.0734	46	67	82	EHGAQEGQAGTGAFPR					Mascot
1612.7463	1612.8197	0.0734	46	67	82	EHGAQEGQAGTGAFPR	147	100			Mascot
1617.8993	1617.8162	-0.0831	-51	86	100	EVVKLTAASITAVCR			Carbamidomethyl (C)[14]		Mascot
1663.8361	1663.8524	0.0163	10	101	116	LPIVVDASGDGAYVCK			Carbamidomethyl (C)[15]		Mascot
1862.7731	1862.8665	0.0934	50	40	53	DCCQQLAHISEWCR			Carbamidomethyl (C)[2,3,13]		Mascot
1862.7731	1862.8665	0.0934	50	40	53	DCCQQLAHISEWCR	131	100	Carbamidomethyl (C)[2,3,13]		Mascot

3	dimeric alpha-amylase inhibitor [Triticum dicoccoides]	gi|227809009	15730.5	5.58	6	314	100	36.012	278	100	

Protein Group

dimeric alpha-amylase inhibitor [Triticum aestivum]	gi|65993781	15688.5	5.5799 999237 0605
dimeric alpha-amylase inhibitor [Triticum aestivum]	gi|386877038	15702.5	5.5799 999237 0605
dimeric alpha-amylase inhibitor [Triticum dicoccoides]	gi|227809005	15730.5	5.5799 999237 0605

Peptide Information

Calc. Mass	Obsrv. Mass	± da	± ppm	Start Seq.	End Seq.	Sequence	Ion Score	C. I. %	Modification	Rank	Result Type
1162.6249	1162.6742	0.0493	42	107	117	LTAASITAVCR			Carbamidomethyl (C)[10]		Mascot
1570.8007	1570.8644	0.0637	41	43	56	LQCNGSQVPEAVLR			Carbamidomethyl (C)[3]		Mascot
1612.7463	1612.8197	0.0734	46	84	99	EHGAQEGQAGTGAFPR					Mascot
1612.7463	1612.8197	0.0734	46	84	99	EHGAQEGQAGTGAFPR	147	100			Mascot
1617.8993	1617.8162	-0.0831	-51	103	117	EVVKLTAASITAVCR			Carbamidomethyl (C)[14]		Mascot
1663.8361	1663.8524	0.0163	10	118	133	LPIVVDASGDGAYVCK			Carbamidomethyl (C)[15]		Mascot
1862.7731	1862.8665	0.0934	50	57	70	DCCQQLAHISEWCR			Carbamidomethyl (C)[2,3,13]		Mascot
1862.7731	1862.8665	0.0934	50	57	70	DCCQQLAHISEWCR	131	100	Carbamidomethyl (C)[2,3,13]		Mascot

4	0.19 dimeric alpha-amylase inhibitor [Triticum aestivum]	gi|54778503	13826.6	7.45	5	308	100	34.652	278	100	

Peptide Information

Calc. Mass	Obsrv. Mass	± da	± ppm	Start Seq.	End Seq.	Sequence	Ion Score	C. I. %	Modification	Rank	Result Type
1162.6249	1162.6742	0.0493	42	90	100	LTAASITAVCR			Carbamidomethyl (C)[10]		Mascot

Calc. Mass	Obsrv. Mass	± da	± ppm	Start Seq.	End Seq.	Sequence	Ion Score	C. I. %	Modification	Rank	Result Type
1612.7463	1612.8197	0.0734	46	67	82	EHGAQEGQAGTGAFPR					Mascot
1612.7463	1612.8197	0.0734	46	67	82	EHGAQEGQAGTGAFPR	147	100			Mascot
1617.8993	1617.8162	-0.0831	-51	86	100	EVVKLTAASITAVCR			Carbamidomethyl (C)[14]		Mascot
1663.8361	1663.8524	0.0163	10	101	116	LPIVVDASGDGAYVCK			Carbamidomethyl (C)[15]		Mascot
1862.7731	1862.8665	0.0934	50	40	53	DCCQQLAHISEWCR			Carbamidomethyl (C)[2,3,13]		Mascot
1862.7731	1862.8665	0.0934	50	40	53	DCCQQLAHISEWCR	131	100	Carbamidomethyl (C)[2,3,13]		Mascot

5	dimeric alpha-amylase inhibitor, partial [Triticum aestivum]	gi|386877068	14415.8	6.88	5	307	100	35.74	278	100	

Peptide Information

Calc. Mass	Obsrv. Mass	± da	± ppm	Start Seq.	End Seq.	Sequence	Ion Score	C. I. %	Modification	Rank	Result Type
1162.6249	1162.6742	0.0493	42	95	105	LTAASITAVCR			Carbamidomethyl (C)[10]		Mascot
1570.8007	1570.8644	0.0637	41	31	44	LQCNGSQVPEAVLR			Carbamidomethyl (C)[3]		Mascot
1612.7463	1612.8197	0.0734	46	72	87	EHGAQEGQAGTGAFPR					Mascot
1612.7463	1612.8197	0.0734	46	72	87	EHGAQEGQAGTGAFPR	147	100			Mascot
1617.8993	1617.8162	-0.0831	-51	91	105	EVVKLTAASITAVCR			Carbamidomethyl (C)[14]		Mascot
1862.7731	1862.8665	0.0934	50	45	58	DCCQQLAHISEWCR			Carbamidomethyl (C)[2,3,13]		Mascot
1862.7731	1862.8665	0.0934	50	45	58	DCCQQLAHISEWCR	131	100	Carbamidomethyl (C)[2,3,13]		Mascot

6	dimeric alpha-amylase inhibitor [Triticum dicoccoides]	gi|227809180	15716.5	5.58	5	306	100	34.652	278	100	

Protein Group

dimeric alpha-amylase inhibitor [Triticum aestivum]	gi|65993925	15702.5	5.5799 999237 0605
dimeric alpha-amylase inhibitor [Triticum dicoccoides]	gi|227809156	15716.5	5.5799 999237 0605
dimeric alpha-amylase inhibitor [Triticum timopheevii subsp. armeniacum]	gi|227809268	15702.5	5.5799 999237 0605

Peptide Information

Calc. Mass	Obsrv. Mass	± da	± ppm	Start Seq.	End Seq.	Sequence	Ion Score	C. I. %	Modification	Rank	Result Type
1162.6249	1162.6742	0.0493	42	107	117	LTAASITAVCR			Carbamidomethyl (C)[10]		Mascot
1612.7463	1612.8197	0.0734	46	84	99	EHGAQEGQAGTGAFPR					Mascot
1612.7463	1612.8197	0.0734	46	84	99	EHGAQEGQAGTGAFPR	147	100			Mascot
1617.8993	1617.8162	-0.0831	-51	103	117	EVVKLTAASITAVCR			Carbamidomethyl (C)[14]		Mascot
1663.8361	1663.8524	0.0163	10	118	133	LPIVVDASGDGAYVCK			Carbamidomethyl (C)[15]		Mascot
1862.7731	1862.8665	0.0934	50	57	70	DCCQQLAHISEWCR			Carbamidomethyl (C)[2,3,13]		Mascot
1862.7731	1862.8665	0.0934	50	57	70	DCCQQLAHISEWCR	131	100	Carbamidomethyl (C)[2,3,13]		Mascot

7	dimeric alpha-amylase inhibitor [Triticum aestivum]	gi|255988225	15665.5	6.69	3	291	100	34.677	278	100	

Peptide Information

Calc. Mass	Obsrv. Mass	± da	± ppm	Start Seq.	End Seq.	Sequence	Ion Score	C. I. %	Modification	Rank	Result Type
1570.8007	1570.8644	0.0637	41	43	56	LQCNGSQVPEAVLR			Carbamidomethyl (C)[3]		Mascot
1612.7463	1612.8197	0.0734	46	84	99	EHGAQEGQAGTGAFPR					Mascot
1612.7463	1612.8197	0.0734	46	84	99	EHGAQEGQAGTGAFPR	147	100			Mascot
1862.7731	1862.8665	0.0934	50	57	70	DCCQQLAHISEWCR			Carbamidomethyl (C)[2,3,13]		Mascot
1862.7731	1862.8665	0.0934	50	57	70	DCCQQLAHISEWCR	131	100	Carbamidomethyl (C)[2,3,13]		Mascot

8	alpha amylase inhibitor CM3 [Triticum durum]	gi|39578552	18893.3	7.44	7	236	100	13.203	195	100	

Protein Group

CM3 protein [Triticum durum]	gi|21922	18893.3	7.4400 000572 2046
RecName: Full=Alpha-amylase/trypsin inhibitor CM3; AltName: Full=Chloroform/methanol-soluble protein CM3; Flags: Precursor	gi|123957	18893.3	7.4400 000572 2046
alpha amylase inhibitor protein [Triticum aestivum]	gi|38098487	18893.3	7.4400 000572 2046
alpha-amylase inhibitor, tetrameric, chain CM3 precursor - durum wheat	gi|100834	18893.3	7.4400 000572 2046
unnamed protein product [Triticum aestivum]	gi|21713	18893.3	7.4400 000572 2046
unnamed protein product [Triticum durum]	gi|57997836	18893.3	7.4400 000572 2046

Peptide Information

Calc. Mass	Obsrv. Mass	± da	± ppm	Start Seq.	End Seq.	Sequence	Ion Score	C. I. %	Modification	Rank	Result Type
1010.52	1010.5569	0.0369	37	37	44	TNLLPHCR			Carbamidomethyl (C)[7]		Mascot
1110.5038	1110.5485	0.0447	40	133	140	EMQWDFVR					Mascot
1126.4987	1126.5249	0.0262	23	133	140	EMQWDFVR			Oxidation (M)[2]		Mascot
1698.9214	1698.9817	0.0603	35	101	115	YFIALPVPSQPVDPR					Mascot
1698.9214	1698.9817	0.0603	35	101	115	YFIALPVPSQPVDPR	75	100			Mascot
1727.8381	1727.9132	0.0751	43	116	132	SGNVGESGLIDLPGCPR			Carbamidomethyl (C)[15]		Mascot
1801.8427	1801.869	0.0263	15	45	60	DYVLQQTCGTFTPGSK			Carbamidomethyl (C)[8]		Mascot
1876.0222	1876.1145	0.0923	49	141	157	LLVAPGQCNLATIHNVR			Carbamidomethyl (C)[8]		Mascot
1876.0222	1876.1145	0.0923	49	141	157	LLVAPGQCNLATIHNVR	35	97.541	Carbamidomethyl (C)[8]		Mascot

Calc. Mass	Obsrv. Mass	± da	± ppm	Start Seq.	End Seq.	Sequence	Ion Score	C. I. %	Modification	Rank	Result Type
1957.8564	1957.9537	0.0973	50	81	95	LYCCQELAEISQQCR			Carbamidomethyl (C)[3,4,14]		Mascot
1957.8564	1957.9537	0.0973	50	81	95	LYCCQELAEISQQCR	85	100	Carbamidomethyl (C)[3,4,14]		Mascot

9	dimeric alpha-amylase inhibitor [Triticum dicoccoides]	gi|114215806	13862.5	5.25	5	177	100	22.941	147	100	

Protein Group

dimeric alpha-amylase inhibitor [Triticum dicoccoides]	gi|114215808	13921.6	5.25

Peptide Information

Calc. Mass	Obsrv. Mass	± da	± ppm	Start Seq.	End Seq.	Sequence	Ion Score	C. I. %	Modification	Rank	Result Type
1162.6249	1162.6742	0.0493	42	90	100	LTAASITAVCR			Carbamidomethyl (C)[10]		Mascot
1570.8007	1570.8644	0.0637	41	26	39	LQCNGSQVPEAVLR			Carbamidomethyl (C)[3]		Mascot
1612.7463	1612.8197	0.0734	46	67	82	EHGAQEGQAGTGAFPR					Mascot
1612.7463	1612.8197	0.0734	46	67	82	EHGAQEGQAGTGAFPR	147	100			Mascot
1617.8993	1617.8162	-0.0831	-51	86	100	EVVKLTAASITAVCR			Carbamidomethyl (C)[14]		Mascot
1663.8361	1663.8524	0.0163	10	101	116	LPIVVDASGDGAYVCK			Carbamidomethyl (C)[15]		Mascot

10	dimeric alpha-amylase inhibitor [Triticum dicoccoides]	gi|227809252	15716.4	5.01	5	175	100	22.941	147	100	

Protein Group

dimeric alpha-amylase inhibitor [Triticum aestivum]	gi|65993829	15722.4	5.5799999237 0605
dimeric alpha-amylase inhibitor [Triticum dicoccoides]	gi|227809102	15678.4	5.25
dimeric alpha-amylase inhibitor [Triticum dicoccoides]	gi|227809254	15753.5	4.8299999237 0605
dimeric alpha-amylase inhibitor [Triticum dicoccoides]	gi|227809078	15722.4	4.8299999237 0605

Peptide Information

Calc. Mass	Obsrv. Mass	± da	± ppm	Start Seq.	End Seq.	Sequence	Ion Score	C. I. %	Modification	Rank	Result Type
1162.6249	1162.6742	0.0493	42	107	117	LTAASITAVCR			Carbamidomethyl (C)[10]		Mascot
1570.8007	1570.8644	0.0637	41	43	56	LQCNGSQVPEAVLR			Carbamidomethyl (C)[3]		Mascot
1612.7463	1612.8197	0.0734	46	84	99	EHGAQEGQAGTGAFPR					Mascot
1612.7463	1612.8197	0.0734	46	84	99	EHGAQEGQAGTGAFPR	147	100			Mascot
1617.8993	1617.8162	-0.0831	-51	103	117	EVVKLTAASITAVCR			Carbamidomethyl (C)[14]		Mascot
1663.8361	1663.8524	0.0163	10	118	133	LPIVVDASGDGAYVCK			Carbamidomethyl (C)[15]		Mascot

Rank	Protein Name	Accession No.	Protein MW	Protein PI	Pep. Count	Protein Score	Protein Score C. I. %	Intensity Matched	Total Ion Score	Total Ion C. I. %	Confirmed
1	hypothetical protein TRIUR3_03549 [Triticum urartu]	gi|474071007	16824.8	6.19	4	43	0	3.766	26	90.891	

Peptide Information

Calc. Mass	Obsrv. Mass	± da	± ppm	Start Seq.	End Seq.	Sequence	Ion Score	C. I. %	Modification	Rank	Result Type
827.4985	827.458	-0.0405	-49	59	66	ADLPGVKK					Mascot
975.5258	975.5727	0.0469	48	110	117	FRLPEDAK					Mascot
1027.6146	1027.5535	-0.0611	-59	137	145	AEVKKPEVK					Mascot
1905.9666	1906.0742	0.1076	56	26	45	SIVPAISGGSSETAAFANAR					Mascot
1905.9666	1906.0742	0.1076	56	26	45	SIVPAISGGSSETAAFANAR	26	90.891			Mascot

Rank	Protein Name	Accession No.	Protein MW	Protein PI	Pep. Count	Protein Score	Protein Score C. I. %	Intensity Matched	Total Ion Score	Total Ion C. I. %	Confirmed
2	unnamed protein product [Triticum turgidum subsp. dicoccon]	gi|296510913	16852.9	6.77	4	42	0	3.766	26	90.891	

Protein Group

Protein Name	Accession No.	Protein MW	Protein PI
16.9a kDa heat-shock protein [Triticum turgidum subsp. dicoccon]	gi|186886532	16852.9	6.76999998092651
unnamed protein product [Triticum turgidum subsp. dicoccon]	gi|296512520	16852.9	6.76999998092651

Peptide Information

Calc. Mass	Obsrv. Mass	± da	± ppm	Start Seq.	End Seq.	Sequence	Ion Score	C. I. %	Modification	Rank	Result Type
827.4985	827.458	-0.0405	-49	59	66	ADLPGVKK					Mascot
975.5258	975.5727	0.0469	48	110	117	FRLPEDAK					Mascot
1027.6146	1027.5535	-0.0611	-59	137	145	AEVKKPEVK					Mascot
1905.9666	1906.0742	0.1076	56	26	45	SIVPAISGGSSETAAFANAR					Mascot
1905.9666	1906.0742	0.1076	56	26	45	SIVPAISGGSSETAAFANAR	26	90.891			Mascot

Rank	Protein Name	Accession No.	Protein MW	Protein PI	Pep. Count	Protein Score	Protein Score C. I. %	Intensity Matched	Total Ion Score	Total Ion C. I. %	Confirmed
3	unnamed protein product [Triticum aestivum]	gi|296512787	16857.8	5.83	4	42	0	3.766	26	90.891	

Protein Group

Protein Name	Accession No.	Protein MW	Protein PI
unnamed protein product [Triticum aestivum]	gi|296511575	16856.8	6.19000005722046
unnamed protein product [Triticum aestivum]	gi|296511571	16857.8	5.8299999237

			0605
unnamed protein product [Triticum aestivum]	gi|296512791	16856.8	6.19000005722046

Peptide Information

Calc. Mass	Obsrv. Mass	± da	± ppm	Start Seq.	End Seq.	Sequence	Ion Score	C. I. %	Modification	Rank	Result Type
827.4985	827.458	-0.0405	-49	59	66	ADLPGVKK					Mascot
975.5258	975.5727	0.0469	48	110	117	FRLPEDAK					Mascot
1027.6146	1027.5535	-0.0611	-59	137	145	AEVKKPEVK					Mascot
1905.9666	1906.0742	0.1076	56	26	45	SIVPAISGGSSETAAFANAR					Mascot
1905.9666	1906.0742	0.1076	56	26	45	SIVPAISGGSSETAAFANAR	26	90.891			Mascot

4	unnamed protein product [Triticum aestivum]	gi|296512688	16867.8	5.83	3	40	0	3.592	26	90.891

Protein Group

RecName: Full=16.9 kDa class I heat shock protein 1; AltName: Full=HSP 16.9; AltName: Full=Heat shock protein 16.9A; AltName: Full=Heat shock protein 17; AltName: Full=Low molecular weight heat shock protein	gi|123545	16867.8	5.82999992370605
heat shock protein 16.8	gi|445135	16867.8	5.82999992370605
unnamed protein product [Triticum aestivum]	gi|296511073	16867.8	5.82999992370605
unnamed protein product [Triticum aestivum]	gi|21813	16867.8	5.82999992370605

Peptide Information

Calc. Mass	Obsrv. Mass	± da	± ppm	Start Seq.	End Seq.	Sequence	Ion Score	C. I. %	Modification	Rank	Result Type
975.5258	975.5727	0.0469	48	110	117	FRLPEDAK					Mascot
1027.6146	1027.5535	-0.0611	-59	137	145	AEVKKPEVK					Mascot
1905.9666	1906.0742	0.1076	56	26	45	SIVPAISGGSSETAAFANAR					Mascot
1905.9666	1906.0742	0.1076	56	26	45	SIVPAISGGSSETAAFANAR	26	90.891			Mascot

5	unnamed protein product [Triticum dicoccoides]	gi|296510911	16838.8	5.82	3	40	0	3.355	26	90.891

Protein Group

16.8 kDa heat-shock protein [Triticum dicoccoides]	gi|186886530	16838.8	5.82000017166138
unnamed protein product [Triticum dicoccoides]	gi|296512518	16838.8	5.82000017166138

Peptide Information

Calc. Mass	Obsrv. Mass	± da	± ppm	Start Seq.	End Seq.	Sequence	Ion Score	C. I. %	Modification	Rank	Result Type
827.4985	827.458	-0.0405	-49	59	66	ADLPGVKK					Mascot
975.5258	975.5727	0.0469	48	110	117	FRLPEDAK					Mascot
1905.9666	1906.0742	0.1076	56	26	45	SIVPAISGGSSETAAFANAR					Mascot
1905.9666	1906.0742	0.1076	56	26	45	SIVPAISGGSSETAAFANAR	26	90.891			Mascot

6	unnamed protein product [Triticum durum]	gi|296510917	16960.8	5.83	2	35	0	3.273	26	90.891

Protein Group

16.9 kDa heat-shock protein [Triticum durum]	gi|186886536	16960.8	5.82999992370605
unnamed protein product [Triticum durum]	gi|296512524	16960.8	5.82999992370605

Peptide Information

Calc. Mass	Obsrv. Mass	± da	± ppm	Start Seq.	End Seq.	Sequence	Ion Score	C. I. %	Modification	Rank	Result Type
1027.6146	1027.5535	-0.0611	-59	137	145	AEVKKPEVK					Mascot
1905.9666	1906.0742	0.1076	56	26	45	SIVPAISGGSSETAAFANAR					Mascot
1905.9666	1906.0742	0.1076	56	26	45	SIVPAISGGSSETAAFANAR	26	90.891			Mascot

7	putative 38.1 kDa protein [Triticum urartu]	gi|474392608	63599	5.73	10	31	0	4.269

Peptide Information

Calc. Mass	Obsrv. Mass	± da	± ppm	Start Seq.	End Seq.	Sequence	Ion Score	C. I. %	Modification	Rank	Result Type
1027.6411	1027.5535	-0.0876	-85	543	550	KPTVWRIK					Mascot
1033.516	1033.5718	0.0558	54	423	431	LEGSEEKNK					Mascot
1090.6144	1090.5977	-0.0167	-15	132	141	VSILVYGPDK					Mascot
1109.595	1109.5765	-0.0185	-17	286	294	LHDQVQELK					Mascot
1182.6412	1182.6407	-0.0005	0	1	11	MGNIHQAGVKK					Mascot
1254.5558	1254.6699	0.1141	91	59	71	TNSTATDGGTSSR					Mascot
1320.673	1320.6605	-0.0125	-9	538	548	DSGMKKPTVWR			Oxidation (M)[4]		Mascot
1707.7854	1707.8678	0.0824	48	376	390	EAESEMESVVAENRK					Mascot
1993.9384	1994.1021	0.1637	82	524	542	SEQIAETPSATPTKDSGMK			Oxidation (M)[18]		Mascot
2367.2588	2367.3945	0.1357	57	243	263	QVEPQNQVNESSIALPLLIMK			Oxidation (M)[20]		Mascot

8	hypothetical protein TRIUR3_08231 [Triticum urartu]	gi|474045401	15699.5	10.55	7	29	0	4.35

Peptide Information

Calc. Mass	Obsrv. Mass	± da	± ppm	Start Seq.	End Seq.	Sequence	Ion Score	C. I. %	Modification	Rank	Result Type
975.5291	975.5727	0.0436	45	112	119	AIKDLMER					Mascot
999.6057	999.5295	-0.0762	-76	102	110	LRINGSLAR					Mascot
1090.6508	1090.5977	-0.0531	-49	83	91	LLTEVPKYK					Mascot
1118.5986	1118.5695	-0.0291	-26	115	123	DLMERGLIR			Oxidation (M)[3]		Mascot
1184.6633	1184.6407	-0.0226	-19	38	49	EKAPAASSKPAK					Mascot
1234.6791	1234.7545	0.0754	61	2	12	TIAAIQDFLSR					Mascot
1365.7195	1365.7216	0.0021	2	1	12	MTIAAIQDFLSR					Mascot

9	hypothetical protein TRIUR3_12727 [Triticum urartu]	gi|474409740	52597.7	5.51	8	28	0	5.41

Peptide Information

Calc. Mass	Obsrv. Mass	± da	± ppm	Start Seq.	End Seq.	Sequence	Ion Score	C. I. %	Modification	Rank	Result Type
827.4622	827.458	-0.0042	-5	336	343	GDPQLGIK					Mascot
973.5254	973.5884	0.063	65	236	242	WIKDHFK					Mascot
1090.6409	1090.5977	-0.0432	-40	347	354	RLLWVEFK					Mascot
1184.5472	1184.6407	0.0935	79	209	220	MASIVGMAGMGK			Oxidation (M)[1,7]		Mascot
1201.6357	1201.699	0.0633	53	295	304	ELVVDSRMPR					Mascot
1707.9752	1707.8678	-0.1074	-63	2	19	ERAPVTAATGALGPVVA K					Mascot
1839.0157	1839.0247	0.009	5	1	19	MERAPVTAATGALGPVV AK					Mascot
1839.0157	1839.0247	0.009	5	1	19	MERAPVTAATGALGPVV AK					Mascot
2831.3403	2831.3674	0.0271	10	70	94	KEALDVADDVHDAIDDFIL TMEPSR			Oxidation (M)[21]		Mascot

10	hypothetical protein TRIUR3_07263 [Triticum urartu]	gi|473865069	8268.1	8.89	5	28	0	3.486

Peptide Information

Calc. Mass	Obsrv. Mass	± da	± ppm	Start Seq.	End Seq.	Sequence	Ion Score	C. I. %	Modification	Rank	Result Type
993.4669	993.5597	0.0928	93	42	49	EMAKEETR					Mascot
1090.6104	1090.5977	-0.0127	-12	6	16	VSVKDAVSSAK					Mascot
1201.6172	1201.699	0.0818	68	30	41	TGKATATTHGEK					Mascot
1365.6328	1365.7216	0.0888	65	57	67	AEMHQEKAEHR					Mascot
1390.6631	1390.7526	0.0895	64	33	45	ATATTHGEKEMAK			Oxidation (M)[11]		Mascot

Gel Idx/Pos	131/F6	Instr./Gel Origin	BA2151/Sample Project 20140814	Process Status	Analysis Succeeded
Plate [#] Name	[1] Sample Project 20140814	Instrument Sample Name		Spectra	11

Rank	Protein Name	Accession No.	Protein MW	Protein PI	Pep. Count	Protein Score	Protein Score C. I. %	Intensity Matched	Total Ion Score	Total Ion C. I. %	Confirmed
1	hypothetical protein TRIUR3_30879 [Triticum urartu]	gi|472909785	11663.1	9.09	9	56	88.892	4.997			

Peptide Information

Calc. Mass	Obsrv. Mass	± da	± ppm	Start Seq.	End Seq.	Sequence	Ion Score	C. I. %	Modification	Rank	Result Type
807.4108	807.4395	0.0287	36	92	97	NRDQFK					Mascot
808.5039	808.426	-0.0779	-96	58	64	AIVQLHK					Mascot
817.4413	817.4223	-0.019	-23	37	43	SLEALER					Mascot
1065.5551	1065.5483	-0.0068	-6	1	10	MAAGGVWVFK					Mascot
1081.5499	1081.5455	-0.0044	-4	1	10	MAAGGVWVFK			Oxidation (M)[1]		Mascot
1182.6953	1182.6262	-0.0691	-58	65	75	RGGVDLISIPR					Mascot
1194.5824	1194.6404	0.058	49	82	91	STHMYDVVVK			Oxidation (M)[4]		Mascot
1379.6987	1379.7723	0.0736	53	25	36	ALVYVPANETMR			Oxidation (M)[11]		Mascot
1491.7988	1491.8051	0.0063	4	24	36	KALVYVPANETMR					Mascot
1507.7937	1507.8065	0.0128	8	24	36	KALVYVPANETMR			Oxidation (M)[12]		Mascot
2510.2168	2510.239	0.0222	9	1	23	MAAGGVWVFKNGVMEL EQEATSR					Mascot

Rank	Protein Name	Accession No.	Protein MW	Protein PI	Pep. Count	Protein Score	Protein Score C. I. %	Intensity Matched	Total Ion Score	Total Ion C. I. %	Confirmed
2	hypothetical protein TRIUR3_08638 [Triticum urartu]	gi|474241595	40046.2	6.16	13	55	85.016	6.544			

Peptide Information

Calc. Mass	Obsrv. Mass	± da	± ppm	Start Seq.	End Seq.	Sequence	Ion Score	C. I. %	Modification	Rank	Result Type
806.3937	806.4064	0.0127	16	1	6	MNRTER					Mascot
832.4159	832.3676	-0.0483	-58	255	261	ISNLDDR					Mascot
847.3792	847.4191	0.0399	47	180	187	GESPSQDK					Mascot
982.4662	982.4804	0.0142	14	262	269	DMVLFDSR					Mascot
1032.511	1032.5415	0.0305	30	238	246	VTEGSWPTR					Mascot
1033.5902	1033.5616	-0.0286	-28	135	142	NLLFNRTR					Mascot
1165.5883	1165.6404	0.0521	45	228	237	QTVEATMTIR			Oxidation (M)[7]		Mascot
1201.6536	1201.6404	-0.0132	-11	287	297	AVSVEKNGELR					Mascot
1277.6597	1277.765	0.1053	82	251	261	FAARISNLDDR					Mascot
1306.7002	1306.6534	-0.0468	-36	167	177	VRAVDYDVELK					Mascot
1485.8424	1485.787	-0.0554	-37	340	352	IEVTVAWSLVVNR					Mascot

Calc. Mass	Obsrv. Mass	± da	± ppm	Start Seq.	End Seq.	Sequence	Ion Score	C. I. %	Modification	Rank	Result Type
1708.7959	1708.8358	0.0399	23	68	82	GRESYEDATSIPPMR					Mascot
2717.3562	2717.2246	-0.1316	-48	36	58	RVARPEEEEEPVDLMAL VATQYR			Oxidation (M)[15]		Mascot

3	hypothetical protein TRIUR3_21665 [Triticum urartu]	gi|474086763	120630.8	9.3	18	48	31.509	11.986	17	0	

Peptide Information

Calc. Mass	Obsrv. Mass	± da	± ppm	Start Seq.	End Seq.	Sequence	Ion Score	C. I. %	Modification	Rank	Result Type
819.3843	819.3833	-0.001	-1	492	498	QVSDDQK					Mascot
982.4927	982.4804	-0.0123	-13	228	234	CWRSFVK			Carbamidomethyl (C)[1]		Mascot
1033.516	1033.5616	0.0456	44	75	82	EKEELETR					Mascot
1036.5382	1036.5635	0.0253	24	334	342	TSETSRLSR					Mascot
1127.599	1127.572	-0.027	-24	97	105	LLHADMQKR			Oxidation (M)[6]		Mascot
1141.6154	1141.5803	-0.0351	-31	1077	1086	VHGPWYKAIA					Mascot
1194.5671	1194.6404	0.0733	61	717	727	LSAAAKDDEMK			Oxidation (M)[10]		Mascot
1201.6536	1201.6404	-0.0132	-11	492	501	QVSDDQKLLR					Mascot
1259.6591	1259.6901	0.031	25	488	498	AIQKQVSDDQK					Mascot
1379.7278	1379.7723	0.0445	32	663	674	EVRDSLHDLAPK					Mascot
1485.8635	1485.787	-0.0765	-51	1063	1076	AAEVLIRVATVSEK					Mascot
1507.6693	1507.8065	0.1372	91	400	413	QPSLDAAESSSCQK			Carbamidomethyl (C)[12]		Mascot
1699.9061	1699.9058	-0.0003	0	1023	1039	AVHCAFRGVLLGGSGAK			Carbamidomethyl (C)[4]		Mascot
1716.8521	1716.9358	0.0837	49	160	174	LMHIQQAAMTVSSQR	17	0	Oxidation (M)[2]		Mascot
1791.9039	1791.8201	-0.0838	-47	433	445	AWCTYLYRFVVWK			Carbamidomethyl (C)[3]		Mascot
1791.9487	1791.8201	-0.1286	-72	238	254	TTLALAQAYDALGINEK					Mascot
1993.9761	1994.0825	0.1064	53	855	876	AGHGAPAAVVASAGSSG LPECK			Carbamidomethyl (C)[21]		Mascot
1993.9761	1994.0825	0.1064	53	855	876	AGHGAPAAVVASAGSSG LPECK			Carbamidomethyl (C)[21]		Mascot
2286.0703	2286.0864	0.0161	7	258	278	SMPFEELAMLMGSPTAL EATK			Oxidation (M)[2,9]		Mascot

4	Chain P, Localization Of The Small Subunit Ribosomal Proteins Into A 5.5 A Cryo-Em Map Of Triticum Aestivum Translating 80s Ribosome	gi|313103641	18052.7	10.59	11	48	26.61	4.953			

Peptide Information

Calc. Mass	Obsrv. Mass	± da	± ppm	Start Seq.	End Seq.	Sequence	Ion Score	C. I. %	Modification	Rank	Result Type
808.4927	808.426	-0.0667	-82	15	21	VFLSSKK					Mascot
831.5087	831.4349	-0.0738	-89	8	14	AFLKQPK					Mascot
836.3818	836.4019	0.0201	24	1	7	MAEQTEK					Mascot
864.4474	864.4551	0.0077	9	31	37	GGNRFWK					Mascot

1002.5876	1002.6257	0.0381	38	81	88	MNRTIIVR					Mascot
1002.5876	1002.6257	0.0381	38	81	88	MNRTIIVR					Mascot
1033.6041	1033.5616	-0.0425	-41	12	20	QPKVFLSSK					Mascot
1057.5459	1057.5515	0.0056	5	71	80	IIAGTCHSAK			Carbamidomethyl (C)[6]		Mascot
1182.667	1182.6262	-0.0408	-34	35	44	FWKSIGLGFK					Mascot
1265.6671	1265.6681	0.001	1	58	68	KCPFTGTVSIR			Carbamidomethyl (C)[2]		Mascot
1458.7305	1458.8102	0.0797	55	71	83	IIAGTCHSAKMNR			Carbamidomethyl (C)[6]		Mascot
1708.88	1708.8358	-0.0442	-26	120	134	EGDHVIIGQCRPLSK			Carbamidomethyl (C)[10]		Mascot

5 hypothetical protein TRIUR3_31008 [Triticum urartu] gi|474239039 53482.1 8.57 13 48 26.61 9.528

Peptide Information

Calc. Mass	Obsrv. Mass	± da	± ppm	Start Seq.	End Seq.	Sequence	Ion Score	C. I. %	Modification	Rank	Result Type
993.4669	993.5444	0.0775	78	52	60	EICSSGNVK			Carbamidomethyl (C)[3]		Mascot
1036.6011	1036.5635	-0.0376	-36	292	299	ENLHIRVR					Mascot
1107.5615	1107.5959	0.0344	31	61	69	AQEAFICLR			Carbamidomethyl (C)[7]		Mascot
1165.6226	1165.6404	0.0178	15	87	96	SPHGFSHIKR					Mascot
1235.6565	1235.6068	-0.0497	-40	61	70	AQEAFICLRK			Carbamidomethyl (C)[7]		Mascot
1390.7689	1390.746	-0.0229	-16	430	443	ILLGLAFGSDSAAR					Mascot
1458.7634	1458.8102	0.0468	32	387	399	REVAWNVAGLMGR					Mascot
1674.9286	1674.816	-0.1126	-67	430	445	ILLGLAFGSDSAARQR					Mascot
1873.9114	1874.0637	0.1523	81	306	322	GLSIGAINPDTDYHCLK			Carbamidomethyl (C)[15]		Mascot
1891.0397	1891.046	0.0063	3	96	113	RVTSGVQNLVGSQFASLK					Mascot
1993.9042	1994.0825	0.1783	89	388	405	EVAWNVAGLMGRCGGCEK			Carbamidomethyl (C)[13,16]		Mascot
1993.9042	1994.0825	0.1783	89	388	405	EVAWNVAGLMGRCGGCEK			Carbamidomethyl (C)[13,16]		Mascot
2243.1606	2243.2505	0.0899	40	444	461	QRNSSELPFSALWLYLYR					Mascot
2286.1917	2286.0864	-0.1053	-46	446	464	NSSELPFSALWLYLYRAQK					Mascot

6 Acetyl-CoA carboxylase [Triticum urartu] gi|474238076 235847 6.13 29 47 17.656 16.372

Peptide Information

Calc. Mass	Obsrv. Mass	± da	± ppm	Start Seq.	End Seq.	Sequence	Ion Score	C. I. %	Modification	Rank	Result Type
817.4275	817.4223	-0.0052	-6	778	784	DRVTNGR					Mascot
832.441	832.3676	-0.0734	-88	2029	2036	VAEDSLAK					Mascot
836.4182	836.4019	-0.0163	-19	365	372	EMSAVATK					Mascot
864.4574	864.4551	-0.0023	-3	593	600	FLVADGSR					Mascot

1016.5775	1016.5536	-0.0239	-24	1584	1592	WVVDTIVGK					Mascot
1033.495	1033.5616	0.0666	64	2011	2018	VVEWEESR					Mascot
1060.5422	1060.6061	0.0639	60	1471	1480	LSTPEFPGGR					Mascot
1081.6001	1081.5455	-0.0546	-50	836	844	LQHAKDLEK					Mascot
1140.5201	1140.6119	0.0918	80	1960	1969	MGTSETETIR			Oxidation (M)[1]		Mascot
1165.5895	1165.6404	0.0509	44	1213	1221	SNNQRMFLR					Mascot
1299.7454	1299.6729	-0.0725	-56	582	592	LLADTPCKLLR			Carbamidomethyl (C)[7]		Mascot
1299.7454	1299.6729	-0.0725	-56	582	592	LLADTPCKLLR			Carbamidomethyl (C)[7]		Mascot
1306.6936	1306.6534	-0.0402	-31	538	548	YMIEIVRGGPR			Oxidation (M)[2]		Mascot
1323.658	1323.703	0.045	34	1761	1772	ESFVETLGGWAK					Mascot
1345.7838	1345.7233	-0.0605	-45	1516	1528	IPLIYLAATSGAR					Mascot
1379.724	1379.7723	0.0483	35	1481	1492	EIMVVANDITFK					Mascot
1405.7322	1405.7343	0.0021	1	1665	1677	EVYSSQLQLGGPK					Mascot
1458.7853	1458.8102	0.0249	17	1196	1206	DRQWHIYTLVK					Mascot
1490.8228	1490.7974	-0.0254	-17	982	993	LYQHYLVRGSVR					Mascot
1513.7281	1513.8087	0.0806	53	1183	1195	LEGYNDAKYTPSR					Mascot
1605.8054	1605.7875	-0.0179	-11	1992	2005	FAELHDTTSRMVAK					Mascot
1629.9033	1629.8849	-0.0184	-11	207	221	KVQGEVPGSPIFIMK					Mascot
1629.9033	1629.8849	-0.0184	-11	207	221	KVQGEVPGSPIFIMK					Mascot
1657.9307	1657.8805	-0.0502	-30	780	794	VTNGRLVDPLMSLVK			Oxidation (M)[11]		Mascot
1707.9033	1707.861	-0.0423	-25	29	44	SVLVANNGMAAVKFMR					Mascot
1707.9033	1707.861	-0.0423	-25	29	44	SVLVANNGMAAVKFMR					Mascot
1791.7391	1791.8201	0.081	45	1537	1551	ACFHVGWSDDESPER			Carbamidomethyl (C)[2]		Mascot
1791.8337	1791.8201	-0.0136	-8	1552	1565	GFHYIYLTEQDYSR					Mascot
2022.9285	2022.9906	0.0621	31	1278	1294	RTDEVDQDEETACTLLK			Carbamidomethyl (C)[13]		Mascot
2239.103	2239.1973	0.0943	42	480	500	AESPPWYLSVVGGALYE ASSR					Mascot
2243.2183	2243.2505	0.0322	14	1617	1637	ETFTLTFVTGRAVGIGAY LAR					Mascot
2286.1013	2286.0864	-0.0149	-7	1753	1772	WLGGMFDRESFVETLGG WAK					Mascot

7	unnamed protein product [Triticum aestivum]	gi|296511505	17280.8	5.78	5	47	9.711	4.474	27	85.945

Protein Group

unnamed protein product [Triticum aestivum]	gi|296512729	17280.8	5.7800 002098 0835

Peptide Information

Calc. Mass	Obsrv. Mass	± da	± ppm	Start Seq.	End Seq.	Sequence	Ion Score	C. I. %	Modification	Rank	Result Type

Calc. Mass	Obsrv. Mass	± da	± ppm	Start Seq.	End Seq.	Sequence	Ion Score	C. I. %	Modification	Rank	Result Type
815.3795	815.4041	0.0246	30	97	102	TDTWHR					Mascot
827.4985	827.4303	-0.0682	-82	63	70	ADVPGLKK					Mascot
1057.5314	1057.5515	0.0201	19	54	62	ETPEAHVFK					Mascot
1629.8079	1629.8849	0.077	47	75	89	VEVDDGNVLQISGER					Mascot
1629.8079	1629.8849	0.077	47	75	89	VEVDDGNVLQISGER	27	85.945			Mascot
2115.0564	2115.1475	0.0911	43	71	89	EEVKVEVDDGNVLQISGER					Mascot

8	Putative disease resistance RPP13-like protein 2 [Triticum urartu]	gi|474060631	82914.2	8.56	16	47	7.608	8.099		

Peptide Information

Calc. Mass	Obsrv. Mass	± da	± ppm	Start Seq.	End Seq.	Sequence	Ion Score	C. I. %	Modification	Rank	Result Type
808.3869	808.426	0.0391	48	638	644	IEQGSMK			Oxidation (M)[6]		Mascot
952.4404	952.4902	0.0498	52	176	184	STACTLGDK			Carbamidomethyl (C)[4]		Mascot
1033.5637	1033.5616	-0.0021	-2	321	329	RLSVQDSTK					Mascot
1118.6066	1118.5629	-0.0437	-39	719	728	RQPQPTIHAA					Mascot
1306.7035	1306.6534	-0.0501	-38	176	187	STACTLGDKILK			Carbamidomethyl (C)[4]		Mascot
1320.6213	1320.6505	0.0292	22	1	13	MSATNDDAGILGR					Mascot
1345.7587	1345.7233	-0.0354	-26	411	421	HLVHLETLDIR					Mascot
1373.7457	1373.729	-0.0167	-12	633	644	LPQVKIEQGSMK			Oxidation (M)[11]		Mascot
1390.7438	1390.746	0.0022	2	399	410	GTDVDHLHQKIK					Mascot
1407.8066	1407.7469	-0.0597	-42	555	567	GQLDRLPATPALR					Mascot
1427.7676	1427.8231	0.0555	39	264	275	CIVEPVEIKNAR			Carbamidomethyl (C)[1]		Mascot
1716.9465	1716.9358	-0.0107	-6	141	156	CGGLPLALISVANYLR			Carbamidomethyl (C)[1]		Mascot
1738.9269	1738.9048	-0.0221	-13	560	574	LPATPALRELCDLNR			Carbamidomethyl (C)[11]		Mascot
1838.8451	1839.0095	0.1644	89	159	175	GQTENHVAGGLTTEHCK			Carbamidomethyl (C)[16]		Mascot
1873.9689	1874.0637	0.0948	51	368	384	VLDLEGCKGVNNDTVLK			Carbamidomethyl (C)[7]		Mascot
1994.0804	1994.0825	0.0021	1	518	536	FTGSKALQILSIDSSDLAK					Mascot
1994.0804	1994.0825	0.0021	1	518	536	FTGSKALQILSIDSSDLAK					Mascot

9	hypothetical protein TRIUR3_21260 [Triticum urartu]	gi|474429490	17279.9	6.75	5	46	0	4.474	27	85.945

Peptide Information

Calc. Mass	Obsrv. Mass	± da	± ppm	Start Seq.	End Seq.	Sequence	Ion Score	C. I. %	Modification	Rank	Result Type
815.3795	815.4041	0.0246	30	97	102	TDTWHR					Mascot
827.4985	827.4303	-0.0682	-82	63	70	ADVPGLKK					Mascot
1057.5314	1057.5515	0.0201	19	54	62	ETPEAHVFK					Mascot

Calc. Mass	Obsrv. Mass	± da	± ppm	Start Seq.	End Seq.	Sequence	Ion Score	C. I. %	Modification	Rank	Result Type
1629.8079	1629.8849	0.077	47	75	89	VEVDDGNVLQISGER					Mascot
1629.8079	1629.8849	0.077	47	75	89	VEVDDGNVLQISGER	27	85.945			Mascot
2115.0564	2115.1475	0.0911	43	71	89	EEVKVEVDDGNVLQISGE R					Mascot

10 Programmed cell death protein 4 [Triticum urartu] gi|473951553 71006.1 4.88 12 46 0 10.008 17 0

Peptide Information

Calc. Mass	Obsrv. Mass	± da	± ppm	Start Seq.	End Seq.	Sequence	Ion Score	C. I. %	Modification	Rank	Result Type
808.4047	808.426	0.0213	26	179	186	GSLTESSK					Mascot
951.5482	951.5015	-0.0467	-49	500	507	HAGERLLR					Mascot
1036.553	1036.5635	0.0105	10	560	568	ALVMAMEKK			Oxidation (M)[4]		Mascot
1037.5626	1037.5621	-0.0005	0	318	326	SQFQTLVSK					Mascot
1060.6184	1060.6061	-0.0123	-12	397	405	LITIAMDRK					Mascot
1066.5385	1066.559	0.0205	19	98	106	KLVSMAMDR			Oxidation (M)[5]		Mascot
1082.5333	1082.5272	-0.0061	-6	98	106	KLVSMAMDR			Oxidation (M)[5,7]		Mascot
1235.7246	1235.6068	-0.1178	-95	227	236	ITDLLKEYIK					Mascot
1405.7111	1405.7343	0.0232	17	625	638	NGWLVPSFGVAAST					Mascot
1707.933	1707.861	-0.072	-42	249	262	ELAVPFFHHEVVKR					Mascot
1707.933	1707.861	-0.072	-42	249	262	ELAVPFFHHEVVKR	17	0			Mascot
1708.8186	1708.8358	0.0172	10	546	559	EMGMPFFNHEVVKK			Oxidation (M)[2]		Mascot
1993.9446	1994.0825	0.1379	69	543	558	CIREMGMPFFNHEVVK			Carbamidomethyl (C)[1]		Mascot
1993.9446	1994.0825	0.1379	69	543	558	CIREMGMPFFNHEVVK			Carbamidomethyl (C)[1]		Mascot
2243.1477	2243.2505	0.1028	46	112	132	EMVSVLLSSLYGDGLSST QIK			Oxidation (M)[2]		Mascot

Gel Idx/Pos	132/F7	Instr./Gel Origin	BA2151/Sample Project 20140814	Process Status	Analysis Succeeded
Plate [#] Name	[1] Sample Project 20140814	Instrument Sample Name		Spectra	11

Rank	Protein Name	Accession No.	Protein MW	Protein PI	Pep. Count	Protein Score	Protein Score C. I. %	Intensity Matched	Total Ion Score	Total Ion C. I. %	Confirmed
1	globulin-3A [Triticum aestivum]	gi|390979705	66626.8	8.48	12	505	100	39.548	467	100	

Peptide Information

Calc. Mass	Obsrv. Mass	± da	± ppm	Start Seq.	End Seq.	Sequence	Ion Score	C. I. %	Modification	Rank	Result Type
832.4159	832.3693	-0.0466	-56	307	313	ASEEQLR					Mascot
906.468	906.5155	0.0475	52	535	541	EVQEVFR					Mascot
1390.7285	1390.7463	0.0178	13	280	292	TSDERLGSLLGSR					Mascot
1412.615	1412.6616	0.0466	33	86	97	HGEGEREEEQGR					Mascot
1487.8176	1487.8179	0.0003	0	301	313	SISIVRASEEQLR					Mascot
1565.631	1565.7141	0.0831	53	446	459	GSGSESEEEQDQQR					Mascot
1655.85	1655.9286	0.0786	47	520	534	LDDPAQELAFGRPAR					Mascot
1655.85	1655.9286	0.0786	47	520	534	LDDPAQELAFGRPAR	49	99.925			Mascot
1699.9418	1699.9164	-0.0254	-15	257	271	FQYFSAKPLLASLSK					Mascot
1822.8752	1822.9807	0.1055	58	489	504	GSSNLQVVCFEINAER			Carbamidomethyl (C)[9]		Mascot
1822.8752	1822.9807	0.1055	58	489	504	GSSNLQVVCFEINAER	145	100	Carbamidomethyl (C)[9]		Mascot
1906.0182	1906.1257	0.1075	56	470	488	GSAFVVPPGHPVVEIASS R					Mascot
1906.0182	1906.1257	0.1075	56	470	488	GSAFVVPPGHPVVEIASS R	145	100			Mascot
2226.9646	2227.0925	0.1279	57	544	562	DQQDEGFVAGPEQQQE HER					Mascot
2426.0967	2426.2397	0.143	59	542	562	AKDQQDEGFVAGPEQQ QEHER					Mascot
2426.0967	2426.2397	0.143	59	542	562	AKDQQDEGFVAGPEQQ QEHER	128	100			Mascot

Rank	Protein Name	Accession No.	Protein MW	Protein PI	Pep. Count	Protein Score	Protein Score C. I. %	Intensity Matched	Total Ion Score	Total Ion C. I. %	Confirmed
2	globulin 3 [Triticum aestivum]	gi|215398470	66651.7	7.78	12	478	100	38.501	440	100	

Peptide Information

Calc. Mass	Obsrv. Mass	± da	± ppm	Start Seq.	End Seq.	Sequence	Ion Score	C. I. %	Modification	Rank	Result Type
832.4159	832.3693	-0.0466	-56	307	313	ASEEQLR					Mascot
906.468	906.5155	0.0475	52	535	541	EVQEVFR					Mascot
1390.7285	1390.7463	0.0178	13	280	292	TSDERLGSLLGSR					Mascot
1412.615	1412.6616	0.0466	33	86	97	HGEGEREEEQGR					Mascot
1487.8176	1487.8179	0.0003	0	301	313	SISIVRASEEQLR					Mascot

Calc. Mass	Obsrv. Mass	± da	± ppm	Start Seq.	End Seq.	Sequence	Ion Score	C. I. %	Modification	Rank	Result Type
1565.631	1565.7141	0.0831	53	446	459	GSGSESEEEQDQQR					Mascot
1685.8606	1685.9408	0.0802	48	520	534	LDDPAQELTFGRPAR					Mascot
1685.8606	1685.9408	0.0802	48	520	534	LDDPAQELTFGRPAR	22	64.096			Mascot
1699.9418	1699.9164	-0.0254	-15	257	271	FQYFSAKPLLASLSK					Mascot
1822.8752	1822.9807	0.1055	58	489	504	GSSNLQVVCFEINAER			Carbamidomethyl (C)[9]		Mascot
1822.8752	1822.9807	0.1055	58	489	504	GSSNLQVVCFEINAER	145	100	Carbamidomethyl (C)[9]		Mascot
1906.0182	1906.1257	0.1075	56	470	488	GSAFVVPPGHPVVEIASSR					Mascot
1906.0182	1906.1257	0.1075	56	470	488	GSAFVVPPGHPVVEIASSR	145	100			Mascot
2226.9646	2227.0925	0.1279	57	544	562	DQQDEGFVAGPEQQQEHER					Mascot
2426.0967	2426.2397	0.143	59	542	562	AKDQQDEGFVAGPEQQQEHER					Mascot
2426.0967	2426.2397	0.143	59	542	562	AKDQQDEGFVAGPEQQQEHER	128	100			Mascot

3	Globulin-1 S allele [Triticum urartu]	gi|474411419	57108.4	9.1	9	336	100	36.867	312	100

Peptide Information

Calc. Mass	Obsrv. Mass	± da	± ppm	Start Seq.	End Seq.	Sequence	Ion Score	C. I. %	Modification	Rank	Result Type
906.468	906.5155	0.0475	52	457	463	EVQEVFR					Mascot
1320.5452	1320.6606	0.1154	87	349	358	WGEEEEDDRR					Mascot
1390.7285	1390.7463	0.0178	13	199	211	TSDERLGSLLGSR					Mascot
1473.802	1473.7731	-0.0289	-20	220	232	SISIVRASEEQVR					Mascot
1685.8606	1685.9408	0.0802	48	442	456	LDDPAQELTFGRPAR					Mascot
1685.8606	1685.9408	0.0802	48	442	456	LDDPAQELTFGRPAR	22	64.096			Mascot
1699.9418	1699.9164	-0.0254	-15	176	190	FQYFSAKPLLASLSK					Mascot
1791.8984	1791.8416	-0.0568	-32	254	268	GDSRDTYNLLEQRPK					Mascot
1822.8752	1822.9807	0.1055	58	411	426	GSSNLQVVCFEINAER			Carbamidomethyl (C)[9]		Mascot
1822.8752	1822.9807	0.1055	58	411	426	GSSNLQVVCFEINAER	145	100	Carbamidomethyl (C)[9]		Mascot
1906.0182	1906.1257	0.1075	56	392	410	GSAFVVPPGHPVVEIASSR					Mascot
1906.0182	1906.1257	0.1075	56	392	410	GSAFVVPPGHPVVEIASSR	145	100			Mascot

4	Transcription initiation factor TFIID subunit 5 [Triticum urartu]	gi|473904754	67275.7	8.31	15	49	48.044	5.884	

Peptide Information

Calc. Mass	Obsrv. Mass	± da	± ppm	Start Seq.	End Seq.	Sequence	Ion Score	C. I. %	Modification	Rank	Result Type
888.5149	888.5143	-0.0006	-1	262	270	DKLVGATGK					Mascot
988.5786	988.5723	-0.0063	-6	264	273	LVGATGKNTK					Mascot

Calc. Mass	Obsrv. Mass	± da	± ppm	Start Seq.	End Seq.	Sequence	Ion Score	C. I. %	Modification	Rank	Result Type
1006.5204	1006.4913	-0.0291	-29	538	546	LWDVASSTK					Mascot
1060.5131	1060.6169	0.1038	98	27	35	NDRVESGQR					Mascot
1107.5615	1107.5984	0.0369	33	409	417	TARIWSMDK					Mascot
1165.5518	1165.627	0.0752	65	274	284	SETSMISAAPR			Oxidation (M)[5]		Mascot
1165.5518	1165.627	0.0752	65	274	284	SETSMISAAPR			Oxidation (M)[5]		Mascot
1359.709	1359.7566	0.0476	35	355	366	VWDMAKIGQPAK			Oxidation (M)[4]		Mascot
1379.6658	1379.7778	0.112	81	471	483	SMVLSLAMSPDGR			Oxidation (M)[2]		Mascot
1457.8145	1457.7926	-0.0219	-15	155	167	TQALVMLGIINER					Mascot
1473.8094	1473.7731	-0.0363	-25	155	167	TQALVMLGIINER			Oxidation (M)[6]		Mascot
1487.7377	1487.8179	0.0802	54	199	211	FGPPLLEDSVEER					Mascot
1637.77	1637.9283	0.1583	97	100	112	EDHELMHSRDLQK					Mascot
1875.9156	1876.0709	0.1553	83	199	214	FGPPLLEDSVEERMEK					Mascot
2023.0032	2023.0099	0.0067	3	54	69	SWAYNSLDQYKHELLR					Mascot
2251.9304	2252.1042	0.1738	77	484	503	YMASGDEDGTIMMWDLS TGR			Oxidation (M)[2]		Mascot
2705.3215	2705.3333	0.0118	4	70	92	VLYPAFIHCFMDLVSEGH TLEAR			Carbamidomethyl (C)[9]		Mascot
2705.3215	2705.3333	0.0118	4	70	92	VLYPAFIHCFMDLVSEGH TLEAR			Carbamidomethyl (C)[9]		Mascot

5 Ethylene receptor 1 [Triticum urartu] gi|474407003 82533.8 8.13 15 47 11.766 10.138

Peptide Information

Calc. Mass	Obsrv. Mass	± da	± ppm	Start Seq.	End Seq.	Sequence	Ion Score	C. I. %	Modification	Rank	Result Type
888.5149	888.5143	-0.0006	-1	280	287	TTLVELGR					Mascot
906.5407	906.5155	-0.0252	-28	232	238	TRELFLK					Mascot
975.504	975.5868	0.0828	85	450	457	REAEMAIR					Mascot
982.5026	982.4886	-0.014	-14	691	699	GCTATFVVK			Carbamidomethyl (C)[2]		Mascot
1307.7001	1307.7468	0.0467	36	260	269	HVRMLTHEIR			Oxidation (M)[4]		Mascot
1417.7104	1417.7914	0.081	57	241	252	ADELDREMGLIR					Mascot
1487.7635	1487.8179	0.0544	37	263	274	MLTHEIRSTLDR			Oxidation (M)[1]		Mascot
1628.8326	1628.9452	0.1126	69	723	737	VGSPLQHAAKHQQPM					Mascot
1685.8237	1685.9408	0.1171	69	387	401	SYAIMVLMLPSDSAR			Oxidation (M)[5,8]		Mascot
1685.8237	1685.9408	0.1171	69	387	401	SYAIMVLMLPSDSAR			Oxidation (M)[5,8]		Mascot
1716.9248	1716.9432	0.0184	11	574	588	RLMQTILNVCGNAVK			Carbamidomethyl (C)[10]		Mascot
1716.9248	1716.9432	0.0184	11	574	588	RLMQTILNVCGNAVK			Carbamidomethyl (C)[10]		Mascot
1805.028	1805.0232	-0.0048	-3	592	608	EGHISLLASVVKPDALR					Mascot
1903.9371	1904.0781	0.141	74	673	690	FVSLMGGHIWLESDGAG K					Mascot
2227.2002	2227.0925	-0.1077	-48	554	574	LSVSVMLSPDLPLSAIGD					Mascot

Calc. Mass	Obsrv. Mass	± da	± ppm	Start Seq.	End Seq.	Sequence	Ion Score	C. I. %	Modification	Rank	Result Type
						EKR					
2252.1235	2252.1042	-0.0193	-9	520	539	LEDGSFELDISAFNLHAVFK					Mascot
2369.0498	2369.1567	0.1069	45	116	134	MEGCDCIEPFWPTDELLIK			Carbamidomethyl (C)[4,6], Oxidation (M)[1]		Mascot

6	hypothetical protein TRIUR3_03726 [Triticum urartu]	gi|474377086	73738.8	9.44	16	45	0	5.96	

Peptide Information

Calc. Mass	Obsrv. Mass	± da	± ppm	Start Seq.	End Seq.	Sequence	Ion Score	C. I. %	Modification	Rank	Result Type
888.4785	888.5143	0.0358	40	112	119	VLAENNTK					Mascot
1033.5823	1033.564	-0.0183	-18	576	584	GCTSRVILK			Carbamidomethyl (C)[2]		Mascot
1060.5634	1060.6169	0.0535	50	390	399	IIINSDNGSK					Mascot
1092.5255	1092.5701	0.0446	41	342	351	AAHMVGTSYR					Mascot
1262.6774	1262.6707	-0.0067	-5	163	173	VLTKDMEGVVR			Oxidation (M)[6]		Mascot
1427.8005	1427.8638	0.0633	44	155	166	ELHGDIFRVLTK					Mascot
1475.7596	1475.824	0.0644	44	135	147	LNEMSLVVSVCPK			Carbamidomethyl (C)[11]		Mascot
1475.7596	1475.824	0.0644	44	135	147	LNEMSLVVSVCPK			Carbamidomethyl (C)[11]		Mascot
1493.7708	1493.8148	0.044	29	642	655	DEAVHTVEAAPAKR					Mascot
1543.8075	1543.9371	0.1296	84	106	119	GQNDLKVLAENNTK					Mascot
1613.8494	1613.9102	0.0608	38	547	560	EVLNQKVNSQEPTK					Mascot
1628.849	1628.9452	0.0962	59	561	575	KEPVNIIIGSDNDSK					Mascot
1638.8558	1638.9734	0.1176	72	286	299	DLTPEINGRPQKDR					Mascot
1699.8796	1699.9164	0.0368	22	271	285	SQLKQTPVTDAMPQR					Mascot
1740.9491	1740.823	-0.1261	-72	384	399	VQEPVKIIINSDNGSK					Mascot
1759.8909	1760.0138	0.1229	70	531	546	ANHMVGTSYRVAPTQK					Mascot
1791.9343	1791.8416	-0.0927	-52	132	147	TSKLNEMSLVVSVCPK			Carbamidomethyl (C)[14]		Mascot

7	hypothetical protein TRIUR3_26364 [Triticum urartu]	gi|474451320	14627.5	10.14	7	44	0	1.921	

Peptide Information

Calc. Mass	Obsrv. Mass	± da	± ppm	Start Seq.	End Seq.	Sequence	Ion Score	C. I. %	Modification	Rank	Result Type
897.5152	897.4661	-0.0491	-55	81	88	INTHTAIK					Mascot
1359.6528	1359.7566	0.1038	76	64	74	VMPGHGWWHPR					Mascot
1565.804	1565.7141	-0.0899	-57	5	18	HCVMIGSLAGIVHR			Carbamidomethyl (C)[2], Oxidation (M)[4]		Mascot
1857.0706	1857.0701	-0.0005	0	47	63	LIREAITLVDIPGPHGR					Mascot
1890.9902	1891.0885	0.0983	52	2	18	GQRHCVMIGSLAGIVHR			Carbamidomethyl (C)[5]		Mascot
1995.0369	1995.0848	0.0479	24	113	130	LYPGVTDPLIAEPMGHLR			Oxidation (M)[14]		Mascot
2831.4312	2831.376	-0.0552	-19	50	74	EAITLVDIPGPHGRVMPG			Oxidation (M)[16]		Mascot

		HGWWHPR						
8	Putative disease resistance RPP13-like protein 2 [Triticum urartu]	gi|474060631	82914.2	8.56	15	43	0	4.916

Peptide Information

Calc. Mass	Obsrv. Mass	± da	± ppm	Start Seq.	End Seq.	Sequence	Ion Score	C. I. %	Modification	Rank	Result Type
1033.5637	1033.564	0.0003	0	321	329	RLSVQDSTK					Mascot
1320.6213	1320.6606	0.0393	30	1	13	MSATNDDAGILGR					Mascot
1329.6732	1329.7091	0.0359	27	505	515	WMNPRSNSPLK					Mascot
1373.7457	1373.7445	-0.0012	-1	633	644	LPQVKIEQGSMK			Oxidation (M)[11]		Mascot
1390.7438	1390.7463	0.0025	2	399	410	GTDVDHLHQKIK					Mascot
1427.7676	1427.8638	0.0962	67	264	275	CIVEPVEIKNAR			Carbamidomethyl (C)[1]		Mascot
1457.7933	1457.7926	-0.0007	0	626	637	LCFEAPRLPQVK			Carbamidomethyl (C)[2]		Mascot
1473.8159	1473.7731	-0.0428	-29	523	536	ALQILSIDSSDLAK					Mascot
1600.7748	1600.8983	0.1235	77	489	502	VKICQATSYSSNSR			Carbamidomethyl (C)[4]		Mascot
1716.9465	1716.9432	-0.0033	-2	141	156	CGGLPLALISVANYLR			Carbamidomethyl (C)[1]		Mascot
1716.9465	1716.9432	-0.0033	-2	141	156	CGGLPLALISVANYLR			Carbamidomethyl (C)[1]		Mascot
1838.8451	1839.0139	0.1688	92	159	175	GQTENHVAGGLTTEHCK			Carbamidomethyl (C)[16]		Mascot
1845.0415	1844.9774	-0.0641	-35	140	156	KCGGLPLALISVANYLR			Carbamidomethyl (C)[2]		Mascot
1873.9689	1874.0587	0.0898	48	368	384	VLDLEGCKGVNNDTVLK			Carbamidomethyl (C)[7]		Mascot
1994.0804	1994.092	0.0116	6	518	536	FTGSKALQILSIDSSDLAK					Mascot
1994.9462	1995.0848	0.1386	69	158	175	RGQTENHVAGGLTTEHC K			Carbamidomethyl (C)[17]		Mascot

9	hypothetical protein TRIUR3_13181 [Triticum urartu]	gi|474069655	32404.3	4.89	9	42	0	10.996

Peptide Information

Calc. Mass	Obsrv. Mass	± da	± ppm	Start Seq.	End Seq.	Sequence	Ion Score	C. I. %	Modification	Rank	Result Type
1037.5779	1037.5734	-0.0045	-4	25	33	LAQVFQGFK					Mascot
1107.6595	1107.5984	-0.0611	-55	139	148	KLGIIGTFMK					Mascot
1193.679	1193.6736	-0.0054	-5	25	34	LAQVFQGFKR					Mascot
1417.7734	1417.7914	0.018	13	1	11	MTLLVPWMIWK					Mascot
1507.8124	1507.8145	0.0021	1	149	161	GCILTKLPAMFTR			Carbamidomethyl (C)[2]		Mascot
1523.8074	1523.8939	0.0865	57	149	161	GCILTKLPAMFTR			Carbamidomethyl (C)[2], Oxidation (M)[10]		Mascot
1605.8975	1605.7992	-0.0983	-61	2	13	TLLVPWMIWKYR					Mascot
1804.9263	1805.0232	0.0969	54	65	80	LTSFEGINCLNIQAPK			Carbamidomethyl (C)[9]		Mascot
1822.8826	1822.9807	0.0981	54	255	270	IEWEAEMARSMVAGVK			Oxidation (M)[7]		Mascot
1822.8826	1822.9807	0.0981	54	255	270	IEWEAEMARSMVAGVK			Oxidation (M)[7]		Mascot

1838.8776	1839.0139	0.1363	74	255	270	IEWEAEMARSMVAGVK			Oxidation (M)[7,11]		Mascot
1933.9689	1934.1437	0.1748	90	49	64	DIQNLISFCPELTDLR			Carbamidomethyl (C)[9]		Mascot

10	WW domain-binding protein 4 [Triticum urartu]	gi|473797577	30998.9	9.75	9	42	0	4.046	

Peptide Information

Calc. Mass	Obsrv. Mass	± da	± ppm	Start Seq.	End Seq.	Sequence	Ion Score	C. I. %	Modification	Rank	Result Type
1055.5956	1055.6652	0.0696	66	225	235	GAPSALAANKR					Mascot
1165.5881	1165.627	0.0389	33	78	88	LSTMQKDGAAK			Oxidation (M)[4]		Mascot
1165.5881	1165.627	0.0389	33	78	88	LSTMQKDGAAK			Oxidation (M)[4]		Mascot
1197.6949	1197.5919	-0.103	-86	222	234	AVKGAPSALAANK					Mascot
1307.7107	1307.7468	0.0361	28	52	62	IFISNNPFSIR					Mascot
1593.8306	1593.7484	-0.0822	-52	264	276	VEDREKPLMGLYK			Oxidation (M)[9]		Mascot
1668.9279	1668.9749	0.047	28	91	105	EQQQAAKALQLIEAK					Mascot
1716.9941	1716.9432	-0.0509	-30	205	221	GGPAPGVVVKKPLNPMR					Mascot
1716.9941	1716.9432	-0.0509	-30	205	221	GGPAPGVVVKKPLNPMR					Mascot
1791.9463	1791.8416	-0.1047	-58	1	15	MTEVLPHLPPPFEIR			Oxidation (M)[1]		Mascot
3312.5007	3312.5312	0.0305	9	146	174	ATGLYYDSNSGFYYSDG LGKWVTQEEAYK					Mascot

Gel Idx/Pos	133/F8	Instr./Gel Origin	BA2151/Sample Project 20140814	Process Status	Analysis Succeeded
Plate [#] Name	[1] Sample Project 20140814	Instrument Sample Name		Spectra	11

Rank	Protein Name	Accession No.	Protein MW	Protein PI	Pep. Count	Protein Score	Protein Score C. I. %	Intensity Matched	Total Ion Score	Total Ion C. I. %	Confirmed
1	Globulin-1 S allele [Triticum urartu]	gi|474411419	57108.4	9.1	10	144	100	20.578	106	100	

Peptide Information

Calc. Mass	Obsrv. Mass	± da	± ppm	Start Seq.	End Seq.	Sequence	Ion Score	C. I. %	Modification	Rank	Result Type
837.4101	837.3646	-0.0455	-54	276	282	LYEADAR					Mascot
906.468	906.5266	0.0586	65	457	463	EVQEVFR					Mascot
1477.7581	1477.835	0.0769	52	89	100	NYRVAIMEVNPR			Oxidation (M)[7]		Mascot
1822.8752	1822.9938	0.1186	65	411	426	GSSNLQVVCFEINAER			Carbamidomethyl (C)[9]		Mascot
1906.0182	1906.1251	0.1069	56	392	410	GSAFVVPPGHPVVEIASS R					Mascot
1958.9396	1958.993	0.0534	27	40	54	SLQQCVQRCQQDRPR			Carbamidomethyl (C)[5,9]		Mascot
2089.9058	2090.0396	0.1338	64	466	483	DQQDEGFVAGPEQQEQ ER					Mascot
2089.9058	2090.0396	0.1338	64	466	483	DQQDEGFVAGPEQQEQ ER	106	100			Mascot
2289.0378	2289.1877	0.1499	65	464	483	AKDQQDEGFVAGPEQQ EQER					Mascot
2663.3457	2663.5601	0.2144	81	316	339	LAVVLEGEGEVEIVCPHL GRDSER			Carbamidomethyl (C)[15]		Mascot
3709.8755	3710.1519	0.2764	75	392	426	GSAFVVPPGHPVVEIASS RGSSNLQVVCFEINAER			Carbamidomethyl (C)[28]		Mascot

Rank	Protein Name	Accession No.	Protein MW	Protein PI	Pep. Count	Protein Score	Protein Score C. I. %	Intensity Matched	Total Ion Score	Total Ion C. I. %	Confirmed
2	globulin 3B [Triticum aestivum]	gi|215398472	57067.8	7.36	10	142	100	41.584	106	100	

Peptide Information

Calc. Mass	Obsrv. Mass	± da	± ppm	Start Seq.	End Seq.	Sequence	Ion Score	C. I. %	Modification	Rank	Result Type
832.4159	832.3776	-0.0383	-46	265	271	ASEEQLR					Mascot
837.4101	837.3646	-0.0455	-54	315	321	LYEADAR					Mascot
849.3995	849.3922	-0.0073	-9	34	40	RTGAECR			Carbamidomethyl (C)[6]		Mascot
906.468	906.5266	0.0586	65	452	458	EVQEVFR					Mascot
2089.9058	2090.0396	0.1338	64	461	478	DQQDEGFVAGPEQQEQ ER					Mascot
2089.9058	2090.0396	0.1338	64	461	478	DQQDEGFVAGPEQQEQ ER	106	100			Mascot
2289.0378	2289.1877	0.1499	65	459	478	AKDQQDEGFVAGPEQQ EQER					Mascot
2317.1267	2317.2192	0.0925	40	252	271	QGQEEEESISIVRASEEQ LR	3	0			Mascot
2317.2188	2317.2192	0.0004	0	209	229	SYSVRQGDYFSAKPLLA SLSK					Mascot

2442.281	2442.301	0.02	8	166	188	VAIMEVNPRSFVVPGLTD ADGVR					Mascot
2663.4014	2663.5601	0.1587	60	1	24	MAIRAIPLSFLSSSSWEP AFSLPR					Mascot

3 putative leucine-rich repeat protein [Triticum aestivum] gi|66840996 15413.8 8.83 7 46 1 4.118

Peptide Information

Calc. Mass	Obsrv. Mass	± da	± ppm	Start Seq.	End Seq.	Sequence	Ion Score	C. I. %	Modification	Rank	Result Type
874.4489	874.4152	-0.0337	-39	47	53	DNNRVTR					Mascot
958.568	958.5798	0.0118	12	51	58	VTRIDLNK					Mascot
1342.7035	1342.7738	0.0703	52	59	71	MNLSGPLAPELGK			Oxidation (M)[1]		Mascot
1667.9076	1668.0143	0.1067	64	1	16	KPVAANQDVDALSALR					Mascot
1678.985	1678.943	-0.042	-25	85	100	LTGPIPRELAGLSNLK					Mascot
1684.8766	1684.9712	0.0946	56	72	84	LDRLQYLEIDHNR					Mascot
1684.8766	1684.9712	0.0946	56	72	84	LDRLQYLEIDHNR					Mascot
2317.0779	2317.2192	0.1413	61	101	121	HADFSNNNLCGPIPTTGA FQR			Carbamidomethyl (C)[10]		Mascot
2317.0779	2317.2192	0.1413	61	101	121	HADFSNNNLCGPIPTTGA FQR			Carbamidomethyl (C)[10]		Mascot

4 CBL-interacting protein kinase 17 [Triticum urartu] gi|473891275 51985.7 7.22 10 38 0 12.858

Peptide Information

Calc. Mass	Obsrv. Mass	± da	± ppm	Start Seq.	End Seq.	Sequence	Ion Score	C. I. %	Modification	Rank	Result Type
834.4389	834.3815	-0.0574	-69	269	275	INMAEIK			Oxidation (M)[3]		Mascot
846.4427	846.3602	-0.0825	-97	120	126	LSEREGR					Mascot
1360.7695	1360.7733	0.0038	3	16	28	ARAALLGAYELGR					Mascot
1360.7695	1360.7733	0.0038	3	16	28	ARAALLGAYELGR					Mascot
1382.7865	1382.7384	-0.0481	-35	230	240	NMVVLYQKIFK					Mascot
1439.8217	1439.8177	-0.004	-3	148	159	DLKPENVLIDRK					Mascot
1967.0597	1967.1133	0.0536	27	241	258	GDTKIPEWLSPGAQNLLK					Mascot
2345.2034	2345.2429	0.0395	17	98	117	IYMVLEFVNGGELFDRIA MK					Mascot
2839.2483	2839.4954	0.2471	87	276	298	LHEWFQKDYTPVGPYDD DDEDVR					Mascot
3319.6013	3319.9202	0.3189	96	201	229	GYDGSLSDIWSCGVILYI MLIGQLPFDDR			Carbamidomethyl (C)[12], Oxidation (M)[19]		Mascot
3709.8418	3710.1519	0.3101	84	432	466	ISSDLGIDNIFGMGSLFDE NLPNFDSRAATPLVAL					Mascot

5 Fructokinase-1 [Triticum urartu] gi|474378351 18707.7 5 6 34 0 1.463

Peptide Information

Calc. Mass	Obsrv. Mass	± da	± ppm	Start	End	Sequence	Ion	C. I. %	Modification	Rank	Result Type

				Seq.	Seq.		Score				
848.4261	848.3487	-0.0774	-91	19	25	EALWSSR					Mascot
1333.6495	1333.7251	0.0756	57	19	29	EALWSSREEAR					Mascot
1382.7097	1382.7384	0.0287	21	138	150	FANACGAITTTKK			Carbamidomethyl (C)[5]		Mascot
2025.0573	2024.9546	-0.1027	-51	151	169	GAIPSLPTEIEVLQLMENA					Mascot
2153.1523	2153.009	-0.1433	-67	150	169	KGAIPSLPTEIEVLQLMEN A					Mascot
2442.1592	2442.301	0.1418	58	44	65	VSESEVEFLTGINSVEDD VVMK			Oxidation (M)[21]		Mascot

6 putative signal peptidase complex subunit 3 [Triticum urartu] gi|474419829 15506 9.4 6 34 0 2.159

Peptide Information

Calc. Mass	Obsrv. Mass	± da	± ppm	Start Seq.	End Seq.	Sequence	Ion Score	C. I. %	Modification	Rank	Result Type
888.4509	888.52	0.0691	78	1	7	MQLGHFR					Mascot
1359.7114	1359.7456	0.0342	25	77	88	EEANVQVEVKSK					Mascot
2118.1099	2118.073	-0.0369	-17	103	120	VQLVLHWHIMPNAGAMI R			Oxidation (M)[10,16]		Mascot
2118.1099	2118.073	-0.0369	-17	103	120	VQLVLHWHIMPNAGAMI R			Oxidation (M)[10,16]		Mascot
2230.21	2230.3457	0.1357	61	102	120	KVQLVLHWHIMPNAGAMI R			Oxidation (M)[11]		Mascot
2271.2366	2271.1941	-0.0425	-19	103	122	VQLVLHWHIMPNAGAMI RGK					Mascot
2272.186	2272.2039	0.0179	8	27	46	VTLTFSLSANLESLFTWN TK					Mascot

7 PTI1-like tyrosine-protein kinase 3 [Triticum urartu] gi|473892834 40243.8 6.09 8 32 0 2.058

Peptide Information

Calc. Mass	Obsrv. Mass	± da	± ppm	Start Seq.	End Seq.	Sequence	Ion Score	C. I. %	Modification	Rank	Result Type
1333.7699	1333.7251	-0.0448	-34	187	197	VQPSIIHRDIR					Mascot
1684.9269	1684.9712	0.0443	26	172	186	IAIEAAKGIEYLHEK					Mascot
1684.9269	1684.9712	0.0443	26	172	186	IAIEAAKGIEYLHEK					Mascot
1738.8694	1738.9376	0.0682	39	154	169	GVQGAQPGPVLDWMQR					Mascot
1959.0116	1958.993	-0.0186	-9	209	226	AKIADFNLLNQAPDMAAR					Mascot
2271.2456	2271.1941	-0.0515	-23	340	363	ALSPLLQQRAAAPAASEL APAPGA					Mascot
2274.1699	2274.2271	0.0572	25	134	153	ILAYEFATMGSLHDVLHG RK			Oxidation (M)[9]		Mascot
2312.1379	2312.1604	0.0225	10	232	252	VLGTFGYHAPEYAMTGQ LTQK					Mascot
2327.1489	2327.1411	-0.0078	-3	115	133	LKHENLVEMLGYYVEGN YR					Mascot

8	globulin-3A [Triticum aestivum]	gi|390979705	66626.8	8.48	10	31	0	35.094

Peptide Information

Calc. Mass	Obsrv. Mass	± da	± ppm	Start Seq.	End Seq.	Sequence	Ion Score	C. I. %	Modification	Rank	Result Type
832.4159	832.3776	-0.0383	-46	307	313	ASEEQLR					Mascot
837.4101	837.3646	-0.0455	-54	357	363	LYEADAR					Mascot
906.468	906.5266	0.0586	65	535	541	EVQEVFR					Mascot
1360.7219	1360.7733	0.0514	38	339	349	DTFNLLEQRPK					Mascot
1360.7219	1360.7733	0.0514	38	339	349	DTFNLLEQRPK					Mascot
1477.7581	1477.835	0.0769	52	170	181	NYRVAIMEVNPR			Oxidation (M)[7]		Mascot
1822.8752	1822.9938	0.1186	65	489	504	GSSNLQVVCFEINAER			Carbamidomethyl (C)[9]		Mascot
1906.0182	1906.1251	0.1069	56	470	488	GSAFVVPPGHPVVEIASS R					Mascot
1958.9257	1958.993	0.0673	34	318	334	QASEGDQGHHWPLPPF R					Mascot
2663.3457	2663.5601	0.2144	81	397	420	LAVVLEGEGEVEIVCPHL GRDSER			Carbamidomethyl (C)[15]		Mascot
3709.8755	3710.1519	0.2764	75	470	504	GSAFVVPPGHPVVEIASS RGSSNLQVVCFEINAER			Carbamidomethyl (C)[28]		Mascot

9	globulin 3 [Triticum aestivum]	gi|215398470	66651.7	7.78	10	31	0	35.094

Peptide Information

Calc. Mass	Obsrv. Mass	± da	± ppm	Start Seq.	End Seq.	Sequence	Ion Score	C. I. %	Modification	Rank	Result Type
832.4159	832.3776	-0.0383	-46	307	313	ASEEQLR					Mascot
837.4101	837.3646	-0.0455	-54	357	363	LYEADAR					Mascot
906.468	906.5266	0.0586	65	535	541	EVQEVFR					Mascot
1360.7219	1360.7733	0.0514	38	339	349	DTFNLLEQRPK					Mascot
1360.7219	1360.7733	0.0514	38	339	349	DTFNLLEQRPK					Mascot
1477.7581	1477.835	0.0769	52	170	181	NYRVAIMEVNPR			Oxidation (M)[7]		Mascot
1822.8752	1822.9938	0.1186	65	489	504	GSSNLQVVCFEINAER			Carbamidomethyl (C)[9]		Mascot
1906.0182	1906.1251	0.1069	56	470	488	GSAFVVPPGHPVVEIASS R					Mascot
1958.9396	1958.993	0.0534	27	40	54	SLQQCVQRCQQDRPR			Carbamidomethyl (C)[5,9]		Mascot
2663.3457	2663.5601	0.2144	81	397	420	LAVVLEGEGEVEIVCPHL GRDSER			Carbamidomethyl (C)[15]		Mascot
3709.8755	3710.1519	0.2764	75	470	504	GSAFVVPPGHPVVEIASS RGSSNLQVVCFEINAER			Carbamidomethyl (C)[28]		Mascot

10	hypothetical protein TRIUR3_04720 [Triticum urartu]	gi|473895821	8032.2	7.93	4	30	0	1.912

Peptide Information

Calc. Mass	Obsrv. Mass	± da	± ppm	Start Seq.	End Seq.	Sequence	Ion Score	C. I. %	Modification	Rank	Result Type

804.3734	804.3424	-0.031	-39	42	48	DQAIDDK		Mascot
1641.8741	1641.9208	0.0467	28	27	41	EASVMRPLRPAGDVK	Oxidation (M)[5]	Mascot
2073.105	2073.0957	-0.0093	-4	42	59	DQAIDDKYAPLLLTMLPR		Mascot
2839.3752	2839.4954	0.1202	42	49	76	YAPLLLTMLPRGPAPPSA PSGGMNEDGN	Oxidation (M)[8]	Mascot

Gel Idx/Pos	134/F9	Instr./Gel Origin	BA2151/Sample Project 20140814	Process Status	Analysis Succeeded
Plate [#] Name	[1] Sample Project 20140814	Instrument Sample Name		Spectra	11

Rank	Protein Name	Accession No.	Protein MW	Protein PI	Pep. Count	Protein Score	Protein Score C. I. %	Intensity Matched	Total Ion Score	Total Ion C. I. %	Confirmed
1	Globulin-1 S allele [Triticum urartu]	gi|474411419	57108.4	9.1	8	141	100	27.186	116	100	

Peptide Information

Calc. Mass	Obsrv. Mass	± da	± ppm	Start Seq.	End Seq.	Sequence	Ion Score	C. I. %	Modification	Rank	Result Type
818.4003	818.3643	-0.036	-44	226	232	ASEEQVR					Mascot
906.468	906.5375	0.0695	77	457	463	EVQEVFR					Mascot
1164.444	1164.4258	-0.0182	-16	349	357	WGEEEEDDR					Mascot
1791.8984	1791.8669	-0.0315	-18	254	268	GDSRDTYNLLEQRPK					Mascot
1822.8752	1823.0137	0.1385	76	411	426	GSSNLQVVCFEINAER			Carbamidomethyl (C)[9]		Mascot
1822.8752	1823.0137	0.1385	76	411	426	GSSNLQVVCFEINAER	21	75.501	Carbamidomethyl (C)[9]		Mascot
1906.0182	1906.1636	0.1454	76	392	410	GSAFVVPPGHPVVEIASSR					Mascot
1906.0182	1906.1636	0.1454	76	392	410	GSAFVVPPGHPVVEIASSR	95	100			Mascot
1958.9396	1959.0035	0.0639	33	40	54	SLQQCVQRCQQDRPR			Carbamidomethyl (C)[5,9]		Mascot
2289.0378	2289.1997	0.1619	71	464	483	AKDQQDEGFVAGPEQQEQER					Mascot

Rank	Protein Name	Accession No.	Protein MW	Protein PI	Pep. Count	Protein Score	Protein Score C. I. %	Intensity Matched	Total Ion Score	Total Ion C. I. %	Confirmed
2	globulin-3A [Triticum aestivum]	gi|390979705	66626.8	8.48	8	137	100	33.167	116	100	

Peptide Information

Calc. Mass	Obsrv. Mass	± da	± ppm	Start Seq.	End Seq.	Sequence	Ion Score	C. I. %	Modification	Rank	Result Type
832.4159	832.3834	-0.0325	-39	307	313	ASEEQLR					Mascot
906.468	906.5375	0.0695	77	535	541	EVQEVFR					Mascot
1164.4763	1164.4258	-0.0505	-43	432	440	SEEEEDDRR					Mascot
1360.7219	1360.7939	0.072	53	339	349	DTFNLLEQRPK					Mascot
1360.7219	1360.7939	0.072	53	339	349	DTFNLLEQRPK					Mascot
1565.631	1565.7477	0.1167	75	446	459	GSGSESEEEQDQQR					Mascot
1822.8752	1823.0137	0.1385	76	489	504	GSSNLQVVCFEINAER			Carbamidomethyl (C)[9]		Mascot
1822.8752	1823.0137	0.1385	76	489	504	GSSNLQVVCFEINAER	21	75.501	Carbamidomethyl (C)[9]		Mascot
1906.0182	1906.1636	0.1454	76	470	488	GSAFVVPPGHPVVEIASSR					Mascot
1906.0182	1906.1636	0.1454	76	470	488	GSAFVVPPGHPVVEIASSR	95	100			Mascot
1958.9257	1959.0035	0.0778	40	318	334	QASEGDQGHHWPLPPFR					Mascot

3	globulin 3 [Triticum aestivum]	gi|215398470	66651.7	7.78	8	136	100	33.167	116	100

Peptide Information

Calc. Mass	Obsrv. Mass	± da	± ppm	Start Seq.	End Seq.	Sequence	Ion Score	C. I. %	Modification	Rank	Result Type
832.4159	832.3834	-0.0325	-39	307	313	ASEEQLR					Mascot
906.468	906.5375	0.0695	77	535	541	EVQEVFR					Mascot
1164.4763	1164.4258	-0.0505	-43	432	440	SEEEEDDRR					Mascot
1360.7219	1360.7939	0.072	53	339	349	DTFNLLEQRPK					Mascot
1360.7219	1360.7939	0.072	53	339	349	DTFNLLEQRPK					Mascot
1565.631	1565.7477	0.1167	75	446	459	GSGSESEEEQDQQR					Mascot
1822.8752	1823.0137	0.1385	76	489	504	GSSNLQVVCFEINAER			Carbamidomethyl (C)[9]		Mascot
1822.8752	1823.0137	0.1385	76	489	504	GSSNLQVVCFEINAER	21	75.501	Carbamidomethyl (C)[9]		Mascot
1906.0182	1906.1636	0.1454	76	470	488	GSAFVVPPGHPVVEIASS R					Mascot
1906.0182	1906.1636	0.1454	76	470	488	GSAFVVPPGHPVVEIASS R	95	100			Mascot
1958.9396	1959.0035	0.0639	33	40	54	SLQQCVQRCQQDRPR			Carbamidomethyl (C)[5,9]		Mascot

4	NBS-LRR type RGA [Triticum aestivum]	gi|74121593	20412.7	8.97	7	41	0	23.23		

Peptide Information

Calc. Mass	Obsrv. Mass	± da	± ppm	Start Seq.	End Seq.	Sequence	Ion Score	C. I. %	Modification	Rank	Result Type
818.4301	818.3643	-0.0658	-80	167	172	VVERCR			Carbamidomethyl (C)[5]		Mascot
842.5134	842.5831	0.0697	83	173	180	GLPFALKP					Mascot
888.4495	888.5308	0.0813	92	155	161	ECPLELK			Carbamidomethyl (C)[2]		Mascot
1388.7454	1388.8204	0.075	54	155	166	ECPLELKTVATK			Carbamidomethyl (C)[2]		Mascot
1475.7927	1475.8553	0.0626	42	81	92	KCLIVLDDVWSK			Carbamidomethyl (C)[2]		Mascot
1905.9773	1906.1636	0.1863	98	110	126	VMITTRMEDVAALAQPK			Oxidation (M)[2,7]		Mascot
1905.9773	1906.1636	0.1863	98	110	126	VMITTRMEDVAALAQPK			Oxidation (M)[2,7]		Mascot
3313.5188	3313.5818	0.063	19	82	109	CLIVLDDVWSKDAYNQM CNAFQGIHGSR			Carbamidomethyl (C)[1,18], Oxidation (M)[17]		Mascot

5	hypothetical protein TRIUR3_30438 [Triticum urartu]	gi|473944792	22130	10.47	4	40	0	9.704	21	75.501

Peptide Information

Calc. Mass	Obsrv. Mass	± da	± ppm	Start Seq.	End Seq.	Sequence	Ion Score	C. I. %	Modification	Rank	Result Type
842.5206	842.5831	0.0625	74	55	62	AIRGLQGK					Mascot
1320.7634	1320.6969	-0.0665	-50	58	69	GLQGKIVYSLSR					Mascot
1822.9303	1823.0137	0.0834	46	10	25	VVNSAACKMFPLNSLR			Carbamidomethyl (C)[7], Oxidation (M)[9]		Mascot

Calc. Mass	Obsrv. Mass	± da	± ppm	Start Seq.	End Seq.	Sequence	Ion Score	C. I. %	Modification	Rank	Result Type
1822.9303	1823.0137	0.0834	46	10	25	VVNSAACKMFPLNSLR	21	75.501	Carbamidomethyl (C)[7], Oxidation (M)[9]		Mascot
1958.9066	1959.0035	0.0969	49	126	142	MPPGYYTGSPQLEEHPR					Mascot

6 Delta(24)-sterol reductase [Triticum urartu] gi|474169153 56396.9 8.64 8 38 0 6.639 16 26.861

Peptide Information

Calc. Mass	Obsrv. Mass	± da	± ppm	Start Seq.	End Seq.	Sequence	Ion Score	C. I. %	Modification	Rank	Result Type
891.4352	891.3884	-0.0468	-52	82	89	DGLVCTAR			Carbamidomethyl (C)[5]		Mascot
1342.7147	1342.7579	0.0432	32	130	141	VEPLVNMGQISR					Mascot
1360.6855	1360.7939	0.1084	80	249	261	EVAQAYADAVAPR					Mascot
1360.6855	1360.7939	0.1084	80	249	261	EVAQAYADAVAPR	16	26.861			Mascot
1475.7754	1475.8553	0.0799	54	105	116	VRHFEVDLSAFR					Mascot
1792.0231	1791.8669	-0.1562	-87	361	375	FLFGWLMPPKVSLLK			Oxidation (M)[7]		Mascot
1930.015	1930.1239	0.1089	56	82	98	DGLVCTARKPWIAVGMR			Carbamidomethyl (C)[5]		Mascot
1958.9396	1959.0035	0.0639	33	324	338	GEFVEYIPTREYYHR					Mascot
2262.1013	2262.1145	0.0132	6	384	401	NYYHDNHVIQDMLVPLYK					Mascot

7 NBS-LRR-like protein [Triticum aestivum] gi|17940782 20160.4 8.8 6 33 0 19.071

Peptide Information

Calc. Mass	Obsrv. Mass	± da	± ppm	Start Seq.	End Seq.	Sequence	Ion Score	C. I. %	Modification	Rank	Result Type
818.4301	818.3643	-0.0658	-80	167	172	VVERCR			Carbamidomethyl (C)[5]		Mascot
888.4495	888.5308	0.0813	92	155	161	ECPLELK			Carbamidomethyl (C)[2]		Mascot
1388.7454	1388.8204	0.075	54	155	166	ECPLELKTVATK			Carbamidomethyl (C)[2]		Mascot
1475.7927	1475.8553	0.0626	42	81	92	KCLIVLDDVWSK			Carbamidomethyl (C)[2]		Mascot
1905.9773	1906.1636	0.1863	98	110	126	VMITTRMEDVAALAQPK			Oxidation (M)[2,7]		Mascot
1905.9773	1906.1636	0.1863	98	110	126	VMITTRMEDVAALAQPK			Oxidation (M)[2,7]		Mascot
3313.5188	3313.5818	0.063	19	82	109	CLIVLDDVWSKDAYNQM CNAFQGIHGSR			Carbamidomethyl (C)[1,18], Oxidation (M)[17]		Mascot

8 hypothetical protein TRIUR3_31101 [Triticum urartu] gi|473999512 76855.9 9.13 11 29 0 25.439

Peptide Information

Calc. Mass	Obsrv. Mass	± da	± ppm	Start Seq.	End Seq.	Sequence	Ion Score	C. I. %	Modification	Rank	Result Type
832.4346	832.3834	-0.0512	-62	369	375	LQGQCVK			Carbamidomethyl (C)[5]		Mascot
1164.5062	1164.4258	-0.0804	-69	202	211	QSMVNGNQDR			Oxidation (M)[3]		Mascot
1169.6249	1169.7087	0.0838	72	118	126	VQFMGLYRR					Mascot
1320.677	1320.6969	0.0199	15	81	91	TFFFSAVRAMK			Oxidation (M)[10]		Mascot

Calc. Mass	Obsrv. Mass	± da	± ppm	Start Seq.	End Seq.	Sequence	Ion Score	C. I. %	Modification	Rank	Result Type
1822.866	1823.0137	0.1477	81	327	340	YGHNLHIYYDVWSR					Mascot
1822.866	1823.0137	0.1477	81	327	340	YGHNLHIYYDVWSR					Mascot
1838.9283	1839.0477	0.1194	65	27	43	IFSDARSIGGDVPVDYK					Mascot
1889.0392	1889.1967	0.1575	83	309	324	AQKLALQHWLEAIDPR					Mascot
1905.974	1906.1636	0.1896	99	394	410	LMFKQTGVLVHTSDDSK					Mascot
1905.974	1906.1636	0.1896	99	394	410	LMFKQTGVLVHTSDDSK					Mascot
1927.9873	1928.1187	0.1314	68	127	143	TWSEVLGTEISSPRTHK					Mascot
1931.0645	1931.1014	0.0369	19	636	653	RAPVPSPRPSPGMILSPR			Oxidation (M)[13]		Mascot
2262.1335	2262.1145	-0.019	-8	165	184	SQGQEHLFSKATLMHSV SFK					Mascot

9	hypothetical protein TRIUR3_29123 [Triticum urartu]	gi|474150393	5868.2	11.63	2	25	0	5.397	9	0	

Peptide Information

Calc. Mass	Obsrv. Mass	± da	± ppm	Start Seq.	End Seq.	Sequence	Ion Score	C. I. %	Modification	Rank	Result Type
1060.5786	1060.6289	0.0503	47	2	13	AGLAPGSFAAAK					Mascot
1822.948	1823.0137	0.0657	36	1	19	MAGLAPGSFAAAKTTGS LR			Oxidation (M)[1]		Mascot
1822.948	1823.0137	0.0657	36	1	19	MAGLAPGSFAAAKTTGS LR	9	0	Oxidation (M)[1]		Mascot

10	hypothetical protein TRIUR3_24873 [Triticum urartu]	gi|474300770	11382	7.85	4	23	0	4.243	

Peptide Information

Calc. Mass	Obsrv. Mass	± da	± ppm	Start Seq.	End Seq.	Sequence	Ion Score	C. I. %	Modification	Rank	Result Type
805.4488	805.4794	0.0306	38	39	45	TDIVVMK					Mascot
906.5043	906.5375	0.0332	37	72	78	VLDFERK					Mascot
1839.1479	1839.0477	-0.1002	-54	79	93	RQHHAVLIILWLIVK					Mascot
2384.3271	2384.1333	-0.1938	-81	80	100	QHHAVLIILWLIVKGCVAE AN			Carbamidomethyl (C)[16]		Mascot

Gel Idx/Pos	135/F10	Instr./Gel Origin	BA2151/Sample Project 20140814	Process Status	Analysis Succeeded
Plate [#] Name	[1] Sample Project 20140814	Instrument Sample Name		Spectra	11

Rank	Protein Name	Accession No.	Protein MW	Protein PI	Pep. Count	Protein Score	Protein Score C. I. %	Intensity Matched	Total Ion Score	Total Ion C. I. %	Confirmed
1	globulin-3A [Triticum aestivum]	gi|390979705	66626.8	8.48	11	396	100	55.631	361	100	

Peptide Information

Calc. Mass	Obsrv. Mass	± da	± ppm	Start Seq.	End Seq.	Sequence	Ion Score	C. I. %	Modification	Rank	Result Type
832.4159	832.378	-0.0379	-46	307	313	ASEEQLR					Mascot
906.468	906.5305	0.0625	69	535	541	EVQEVFR					Mascot
1105.6001	1105.587	-0.0131	-12	535	543	EVQEVFRAK					Mascot
1565.631	1565.7424	0.1114	71	446	459	GSGSESEEEQDQQR					Mascot
1655.85	1655.9434	0.0934	56	520	534	LDDPAQELAFGRPAR					Mascot
1655.85	1655.9434	0.0934	56	520	534	LDDPAQELAFGRPAR	7	0			Mascot
1721.7322	1721.8359	0.1037	60	445	459	RGSGSESEEEQDQQR					Mascot
1822.8752	1823.0027	0.1275	70	489	504	GSSNLQVVCFEINAER			Carbamidomethyl (C)[9]		Mascot
1822.8752	1823.0027	0.1275	70	489	504	GSSNLQVVCFEINAER	89	100	Carbamidomethyl (C)[9]		Mascot
1906.0182	1906.1558	0.1376	72	470	488	GSAFVVPPGHPVVEIASS R					Mascot
1906.0182	1906.1558	0.1376	72	470	488	GSAFVVPPGHPVVEIASS R	150	100			Mascot
1958.9257	1959.004	0.0783	40	318	334	QASEGDQGHHWPLPPF R					Mascot
2226.9646	2227.1238	0.1592	71	544	562	DQQDEGFVAGPEQQQE HER					Mascot
2226.9646	2227.1238	0.1592	71	544	562	DQQDEGFVAGPEQQQE HER	13	0			Mascot
2426.0967	2426.2661	0.1694	70	542	562	AKDQQDEGFVAGPEQQ QEHER					Mascot
2426.0967	2426.2661	0.1694	70	542	562	AKDQQDEGFVAGPEQQ QEHER	102	100			Mascot

Rank	Protein Name	Accession No.	Protein MW	Protein PI	Pep. Count	Protein Score	Protein Score C. I. %	Intensity Matched	Total Ion Score	Total Ion C. I. %	Confirmed
2	globulin 3 [Triticum aestivum]	gi|215398470	66651.7	7.78	11	387	100	55.022	353	100	

Peptide Information

Calc. Mass	Obsrv. Mass	± da	± ppm	Start Seq.	End Seq.	Sequence	Ion Score	C. I. %	Modification	Rank	Result Type
832.4159	832.378	-0.0379	-46	307	313	ASEEQLR					Mascot
906.468	906.5305	0.0625	69	535	541	EVQEVFR					Mascot
1105.6001	1105.587	-0.0131	-12	535	543	EVQEVFRAK					Mascot
1565.631	1565.7424	0.1114	71	446	459	GSGSESEEEQDQQR					Mascot
1685.8606	1685.9565	0.0959	57	520	534	LDDPAQELTFGRPAR					Mascot

Calc. Mass	Obsrv. Mass	± da	± ppm	Start Seq.	End Seq.	Sequence	Ion Score	C. I. %	Modification	Rank	Result Type
1721.7322	1721.8359	0.1037	60	445	459	RGSGSESEEEQDQQR					Mascot
1822.8752	1823.0027	0.1275	70	489	504	GSSNLQVVCFEINAER			Carbamidomethyl (C)[9]		Mascot
1822.8752	1823.0027	0.1275	70	489	504	GSSNLQVVCFEINAER	89	100	Carbamidomethyl (C)[9]		Mascot
1906.0182	1906.1558	0.1376	72	470	488	GSAFVVPPGHPVVEIASSR					Mascot
1906.0182	1906.1558	0.1376	72	470	488	GSAFVVPPGHPVVEIASSR	150	100			Mascot
1958.9396	1959.004	0.0644	33	40	54	SLQQCVQRCQQDRPR			Carbamidomethyl (C)[5,9]		Mascot
2226.9646	2227.1238	0.1592	71	544	562	DQQDEGFVAGPEQQQEHER					Mascot
2226.9646	2227.1238	0.1592	71	544	562	DQQDEGFVAGPEQQQEHER	13	0			Mascot
2426.0967	2426.2661	0.1694	70	542	562	AKDQQDEGFVAGPEQQQEHER					Mascot
2426.0967	2426.2661	0.1694	70	542	562	AKDQQDEGFVAGPEQQQEHER	102	100			Mascot

3 Globulin-1 S allele [Triticum urartu] gi|474411419 57108.4 9.1 8 257 100 50.95 238 100

Peptide Information

Calc. Mass	Obsrv. Mass	± da	± ppm	Start Seq.	End Seq.	Sequence	Ion Score	C. I. %	Modification	Rank	Result Type
906.468	906.5305	0.0625	69	457	463	EVQEVFR					Mascot
1105.6001	1105.587	-0.0131	-12	457	465	EVQEVFRAK					Mascot
1320.5452	1320.672	0.1268	96	349	358	WGEEEEDDRR					Mascot
1685.8606	1685.9565	0.0959	57	442	456	LDDPAQELTFGRPAR					Mascot
1791.8984	1791.864	-0.0344	-19	254	268	GDSRDTYNLLEQRPK					Mascot
1822.8752	1823.0027	0.1275	70	411	426	GSSNLQVVCFEINAER			Carbamidomethyl (C)[9]		Mascot
1822.8752	1823.0027	0.1275	70	411	426	GSSNLQVVCFEINAER	89	100	Carbamidomethyl (C)[9]		Mascot
1906.0182	1906.1558	0.1376	72	392	410	GSAFVVPPGHPVVEIASSR					Mascot
1906.0182	1906.1558	0.1376	72	392	410	GSAFVVPPGHPVVEIASSR	150	100			Mascot
1958.9396	1959.004	0.0644	33	40	54	SLQQCVQRCQQDRPR			Carbamidomethyl (C)[5,9]		Mascot

4 Ribulose bisphosphate carboxylase small chain PWS4.3, chloroplastic [Triticum urartu] gi|473721335 19632.8 8.81 8 47 9.711 6.767

Peptide Information

Calc. Mass	Obsrv. Mass	± da	± ppm	Start Seq.	End Seq.	Sequence	Ion Score	C. I. %	Modification	Rank	Result Type
887.4945	887.5483	0.0538	61	21	29	STAGLPVSR					Mascot
906.5043	906.5305	0.0262	29	75	81	QVDYLIR					Mascot
930.4178	930.5034	0.0856	92	111	116	YWTMWK			Oxidation (M)[4]		Mascot
1320.6615	1320.672	0.0105	8	30	43	RSSGSLGSVSNGGR					Mascot

1848.9524	1849.1241	0.1717	93	2	20	APAVMASSASTVAPFQG LK			Oxidation (M)[5]		Mascot
1922.0521	1922.1403	0.0882	46	58	74	FETLSYLPPLSTEALLK					Mascot
1926.9708	1927.012	0.0412	21	138	153	EYPDAYVRVIGFDNLR					Mascot
2408.1836	2408.2317	0.0481	20	117	137	LPMFGCTDATQVLNEVE EVKK			Carbamidomethyl (C)[6]		Mascot

5 ribulose-1,5-bisphosphate carboxylase/oxygenase small subunit [Triticum aestivum] gi|11990899 19832.9 8.99 8 44 0 7.121

Peptide Information

Calc. Mass	Obsrv. Mass	± da	± ppm	Start Seq.	End Seq.	Sequence	Ion Score	C. I. %	Modification	Rank	Result Type
887.4945	887.5483	0.0538	61	21	29	STAGLPVSR					Mascot
906.5043	906.5305	0.0262	29	74	80	QVDYLIR					Mascot
930.4178	930.5034	0.0856	92	110	115	YWTMWK			Oxidation (M)[4]		Mascot
934.4741	934.5605	0.0864	92	145	152	VNGFDNLR					Mascot
1922.0521	1922.1403	0.0882	46	57	73	FETLSYLPPLSTEALLK					Mascot
1927.9297	1928.0995	0.1698	88	137	152	EYPDAYVRVNGFDNLR					Mascot
1994.0085	1994.1165	0.108	54	1	20	MAPAVMASSATTVAPFQ GLK			Oxidation (M)[1]		Mascot
2408.1836	2408.2317	0.0481	20	116	136	LPMFGCTDATQVLNEVE EVKK			Carbamidomethyl (C)[6]		Mascot

6 ribulose-1,5-bisphosphate carboxylase/oxygenase small subunit [Triticum aestivum] gi|11990895 19705.9 8.99 8 44 0 6.583

Protein Group

ribulose-1,5-bisphosphate carboxylase/oxygenase small subunit [Triticum aestivum] gi|11990903 19705.9 8.9899997711816

Peptide Information

Calc. Mass	Obsrv. Mass	± da	± ppm	Start Seq.	End Seq.	Sequence	Ion Score	C. I. %	Modification	Rank	Result Type
819.4431	819.4332	-0.0099	-12	30	37	RSSGSLGR					Mascot
887.4945	887.5483	0.0538	61	21	29	STAGLPVSR					Mascot
906.5043	906.5305	0.0262	29	75	81	QVDYLIR					Mascot
930.4178	930.5034	0.0856	92	111	116	YWTMWK			Oxidation (M)[4]		Mascot
1922.0521	1922.1403	0.0882	46	58	74	FETLSYLPPLSTEALLK					Mascot
1926.9708	1927.012	0.0412	21	138	153	EYPDAYVRVIGFDNLR					Mascot
1994.0085	1994.1165	0.108	54	1	20	MAPAVMASSATTVAPFQ GLK			Oxidation (M)[1]		Mascot
2408.1836	2408.2317	0.0481	20	117	137	LPMFGCTDATQVLNEVE EVKK			Carbamidomethyl (C)[6]		Mascot

7 Ribulose bisphosphate carboxylase small chain PWS4.3, chloroplastic [Triticum urartu] gi|473939671 23054.8 7.66 8 44 0 6.593

Peptide Information

Calc. Mass	Obsrv. Mass	± da	± ppm	Start Seq.	End Seq.	Sequence	Ion Score	C. I. %	Modification	Rank	Result Type
887.4945	887.5483	0.0538	61	21	29	STAGLPVSR					Mascot
906.5043	906.5305	0.0262	29	75	81	QVDYLIR					Mascot
930.4178	930.5034	0.0856	92	111	116	YWTMWK			Oxidation (M)[4]		Mascot
1320.6615	1320.672	0.0105	8	30	43	RSSGSLGSVSNGGR					Mascot
1922.0521	1922.1403	0.0882	46	58	74	FETLSYLPPLSTEALLK					Mascot
1926.9708	1927.012	0.0412	21	138	153	EYPDAYVRVIGFDNLR					Mascot
1994.0085	1994.1165	0.108	54	1	20	MAPAVMASSATTVAPFQ GLK			Oxidation (M)[1]		Mascot
2408.1836	2408.2317	0.0481	20	117	137	LPMFGCTDATQVLNEVE EVKK			Carbamidomethyl (C)[6]		Mascot

8 ribulose-1,5-bisphosphate carboxylase/oxygenase small subunit [Triticum urartu] gi|4038713 18853.5 8.82 7 40 0 6.415

Protein Group

ribulose-1,5-bisphosphate carboxylase/oxygenase small subunit [Triticum timopheevii subsp. armeniacum] gi|4038715 18804.4 8.8299999237 0605

Peptide Information

Calc. Mass	Obsrv. Mass	± da	± ppm	Start Seq.	End Seq.	Sequence	Ion Score	C. I. %	Modification	Rank	Result Type
887.4945	887.5483	0.0538	61	17	25	STAGLPVSR					Mascot
906.5043	906.5305	0.0262	29	71	77	QVDYLIR					Mascot
930.4178	930.5034	0.0856	92	107	112	YWTMWK			Oxidation (M)[4]		Mascot
1320.6615	1320.672	0.0105	8	26	39	RSSGSLGSVSNGGR					Mascot
1922.0521	1922.1403	0.0882	46	54	70	FETLSYLPPLSTEALLK					Mascot
1926.9708	1927.012	0.0412	21	134	149	EYPDAYVRVIGFDNLR					Mascot
2408.1836	2408.2317	0.0481	20	113	133	LPMFGCTDATQVLNEVE EVKK			Carbamidomethyl (C)[6]		Mascot

9 hypothetical protein TRIUR3_10048 [Triticum urartu] gi|474369396 20865.7 5.25 7 40 0 12.068

Peptide Information

Calc. Mass	Obsrv. Mass	± da	± ppm	Start Seq.	End Seq.	Sequence	Ion Score	C. I. %	Modification	Rank	Result Type
893.4332	893.4988	0.0656	73	29	35	KMVEACR			Carbamidomethyl (C)[6]		Mascot
1060.5521	1060.5781	0.026	25	36	44	NEEIESLVK					Mascot
1359.7056	1359.7766	0.071	52	46	56	KYGQEAFTIFR					Mascot
1707.8986	1707.8939	-0.0047	-3	136	150	QMVNYIAELLLEGSK					Mascot

1822.8673	1823.0027	0.1354	74	30	44	MVEACRNEEIESLVK			Carbamidomethyl (C)[5], Oxidation (M)[1]		Mascot
1822.8673	1823.0027	0.1354	74	30	44	MVEACRNEEIESLVK			Carbamidomethyl (C)[5], Oxidation (M)[1]		Mascot
1944.0127	1944.0599	0.0472	24	99	116	IASAAGTGYAQFFVWRA K					Mascot
2384.2432	2384.1318	-0.1114	-47	160	179	NILILALTRHDDSLMLFHD F					Mascot

10	ribulose-1,5-bisphosphate carboxylase/oxygenase small subunit [Triticum aestivum]	gi|11990901	19733.9	8.81	7	38	0	6.659

Peptide Information

Calc. Mass	Obsrv. Mass	± da	± ppm	Start Seq.	End Seq.	Sequence	Ion Score	C. I. %	Modification	Rank	Result Type
887.4945	887.5483	0.0538	61	21	29	STAGLPVSR					Mascot
906.5043	906.5305	0.0262	29	76	82	QVDYLIR					Mascot
930.4178	930.5034	0.0856	92	112	117	YWTMWK			Oxidation (M)[4]		Mascot
1848.9524	1849.1241	0.1717	93	2	20	APAVMASSATSVAPFQG LK			Oxidation (M)[5]		Mascot
1922.0521	1922.1403	0.0882	46	59	75	FETLSYLPPLSTEALLK					Mascot
1926.9708	1927.012	0.0412	21	139	154	EYPDAYVRVIGFDNLR					Mascot
2408.1836	2408.2317	0.0481	20	118	138	LPMFGCTDATQVLNEVE EVKK			Carbamidomethyl (C)[6]		Mascot

Rank	Protein Name	Accession No.	Protein MW	Protein PI	Pep. Count	Protein Score	Protein Score C. I. %	Intensity Matched	Total Ion Score	Total Ion C. I. %	Confirmed
1	0.19 dimeric alpha-amylase inhibitor [Triticum aestivum]	gi|54778507	13814.6	5.73	6	253	100	27.876	210	100	

Protein Group

0.19 dimeric alpha-amylase inhibitor [Triticum aestivum]	gi|54778511	13756.6	6.4899 997711 1816

Peptide Information

Calc. Mass	Obsrv. Mass	± da	± ppm	Start Seq.	End Seq.	Sequence	Ion Score	C. I. %	Modification	Rank	Result Type
1134.6188	1134.6531	0.0343	30	90	100	LTAASITAVCK			Carbamidomethyl (C)[10]		Mascot
1306.7665	1306.8643	0.0978	75	14	25	VPALPGCRPVLK			Carbamidomethyl (C)[7]		Mascot
1306.7665	1306.8643	0.0978	75	14	25	VPALPGCRPVLK	23	79.673	Carbamidomethyl (C)[7]		Mascot
1570.8007	1570.9113	0.1106	70	26	39	LQCNGSQVPEAVLR			Carbamidomethyl (C)[3]		Mascot
1677.8517	1677.9301	0.0784	47	101	116	LPIVIDASGDGAYVCK			Carbamidomethyl (C)[15]		Mascot
1854.7568	1854.8948	0.138	74	40	53	ECCQQLADISEWCR			Carbamidomethyl (C)[2,3,13]		Mascot
1854.7568	1854.8948	0.138	74	40	53	ECCQQLADISEWCR	26	89.623	Carbamidomethyl (C)[2,3,13]		Mascot
1887.8403	1887.9731	0.1328	70	67	84	EHGVQEGGQAGTGAFPSC R			Carbamidomethyl (C)[17]		Mascot
1887.8403	1887.9731	0.1328	70	67	84	EHGVQEGGQAGTGAFPSC R	160	100	Carbamidomethyl (C)[17]		Mascot

Rank	Protein Name	Accession No.	Protein MW	Protein PI	Pep. Count	Protein Score	Protein Score C. I. %	Intensity Matched	Total Ion Score	Total Ion C. I. %	Confirmed
2	dimeric alpha-amylase inhibitor [Triticum aestivum]	gi|65993852	15558.5	5.3	6	250	100	27.876	210	100	

Protein Group

dimeric alpha-amylase inhibitor [Triticum aestivum]	gi|65993807	15588.5	5.3000 001907 3486
dimeric alpha-amylase inhibitor [Triticum aestivum]	gi|65993941	15631.5	5.7699 999809 2651

Peptide Information

Calc. Mass	Obsrv. Mass	± da	± ppm	Start Seq.	End Seq.	Sequence	Ion Score	C. I. %	Modification	Rank	Result Type
1134.6188	1134.6531	0.0343	30	107	117	LTAASITAVCK			Carbamidomethyl (C)[10]		Mascot
1306.7665	1306.8643	0.0978	75	31	42	VPALPGCRPVLK			Carbamidomethyl (C)[7]		Mascot
1306.7665	1306.8643	0.0978	75	31	42	VPALPGCRPVLK	23	79.673	Carbamidomethyl (C)[7]		Mascot
1570.8007	1570.9113	0.1106	70	43	56	LQCNGSQVPEAVLR			Carbamidomethyl (C)[3]		Mascot
1677.8517	1677.9301	0.0784	47	118	133	LPIVIDASGDGAYVCK			Carbamidomethyl (C)[15]		Mascot

1854.7568	1854.8948	0.138	74	57	70	ECCQQLADISEWCR			Carbamidomethyl (C)[2,3,13]		Mascot
1854.7568	1854.8948	0.138	74	57	70	ECCQQLADISEWCR	26	89.623	Carbamidomethyl (C)[2,3,13]		Mascot
1887.8403	1887.9731	0.1328	70	84	101	EHGVQEGQAGTGAFPSCR			Carbamidomethyl (C)[17]		Mascot
1887.8403	1887.9731	0.1328	70	84	101	EHGVQEGQAGTGAFPSCR	160	100	Carbamidomethyl (C)[17]		Mascot

3	dimeric alpha-amylase inhibitor [Triticum aestivum]	gi|65993756	15820.6	6.54	5	240	100	27.114	210	100

Peptide Information

Calc. Mass	Obsrv. Mass	± da	± ppm	Start Seq.	End Seq.	Sequence	Ion Score	C. I. %	Modification	Rank	Result Type
1134.6188	1134.6531	0.0343	30	107	117	LTAASITAVCK			Carbamidomethyl (C)[10]		Mascot
1306.7665	1306.8643	0.0978	75	31	42	VPALPGCRPVLK			Carbamidomethyl (C)[7]		Mascot
1306.7665	1306.8643	0.0978	75	31	42	VPALPGCRPVLK	23	79.673	Carbamidomethyl (C)[7]		Mascot
1570.8007	1570.9113	0.1106	70	43	56	LQCNGSQVPEAVLR			Carbamidomethyl (C)[3]		Mascot
1854.7568	1854.8948	0.138	74	57	70	ECCQQLADISEWCR			Carbamidomethyl (C)[2,3,13]		Mascot
1854.7568	1854.8948	0.138	74	57	70	ECCQQLADISEWCR	26	89.623	Carbamidomethyl (C)[2,3,13]		Mascot
1887.8403	1887.9731	0.1328	70	84	101	EHGVQEGQAGTGAFPSCR			Carbamidomethyl (C)[17]		Mascot
1887.8403	1887.9731	0.1328	70	84	101	EHGVQEGQAGTGAFPSCR	160	100	Carbamidomethyl (C)[17]		Mascot

4	dimeric alpha-amylase inhibitor [Triticum dicoccoides]	gi|114215776	13742.5	6.49	5	216	100	26.15	183	100

Protein Group

dimeric alpha-amylase inhibitor [Triticum dicoccoides]	gi|114215772	13742.5	6.48999977111816
dimeric alpha-amylase inhibitor [Triticum dicoccoides]	gi|114215768	13742.5	6.48999977111816
dimeric alpha-amylase inhibitor [Triticum dicoccoides]	gi|114215762	13742.5	6.48999977111816
dimeric alpha-amylase inhibitor [Triticum dicoccoides]	gi|114215790	13742.5	6.48999977111816

Peptide Information

Calc. Mass	Obsrv. Mass	± da	± ppm	Start Seq.	End Seq.	Sequence	Ion Score	C. I. %	Modification	Rank	Result Type
1134.6188	1134.6531	0.0343	30	90	100	LTAASITAVCK			Carbamidomethyl (C)[10]		Mascot
1306.7665	1306.8643	0.0978	75	14	25	VPALPGCRPVLK			Carbamidomethyl (C)[7]		Mascot
1306.7665	1306.8643	0.0978	75	14	25	VPALPGCRPVLK	23	79.673	Carbamidomethyl (C)[7]		Mascot
1570.8007	1570.9113	0.1106	70	26	39	LQCNGSQVPEAVLR			Carbamidomethyl (C)[3]		Mascot
1677.8517	1677.9301	0.0784	47	101	116	LPIVIDASGDGAYVCK			Carbamidomethyl (C)[15]		Mascot

	1887.8403	1887.9731	0.1328	70	67	84	EHGVQEGQAGTGAFPSC R				Carbamidomethyl (C)[17]		Mascot
	1887.8403	1887.9731	0.1328	70	67	84	EHGVQEGQAGTGAFPSC R		160	100	Carbamidomethyl (C)[17]		Mascot

5	dimeric alpha-amylase inhibitor [Triticum timopheevii subsp. armeniacum]	gi|227809294	15588.4	5.28	5	214	100	26.15	183	100

Protein Group

dimeric alpha-amylase inhibitor [Triticum dicoccoides]	gi|227809226	15574.4	5.2800 002098 0835
dimeric alpha-amylase inhibitor [Triticum dicoccoides]	gi|227809216	15574.4	5.2800 002098 0835
dimeric alpha-amylase inhibitor [Triticum dicoccoides]	gi|227809212	15574.4	5.2800 002098 0835
dimeric alpha-amylase inhibitor [Triticum dicoccoides]	gi|227809196	15574.4	5.2800 002098 0835
dimeric alpha-amylase inhibitor [Triticum dicoccoides]	gi|227809192	15604.4	5.2800 002098 0835
dimeric alpha-amylase inhibitor [Triticum dicoccoides]	gi|227809174	15574.4	5.2800 002098 0835
dimeric alpha-amylase inhibitor [Triticum dicoccoides]	gi|227809172	15548.4	5.5799 999237 0605
dimeric alpha-amylase inhibitor [Triticum dicoccoides]	gi|227809154	15574.4	5.2800 002098 0835
dimeric alpha-amylase inhibitor [Triticum dicoccoides]	gi|227809140	15574.4	5.2800 002098 0835
dimeric alpha-amylase inhibitor [Triticum dicoccoides]	gi|227809092	15574.4	5.2800 002098 0835
dimeric alpha-amylase inhibitor [Triticum dicoccoides]	gi|227809084	15574.4	5.2800 002098 0835
dimeric alpha-amylase inhibitor [Triticum dicoccoides]	gi|227809082	15574.4	5.2800 002098 0835
dimeric alpha-amylase inhibitor [Triticum dicoccoides]	gi|227809055	15558.5	5.7399 997711 1816
dimeric alpha-amylase inhibitor [Triticum dicoccoides]	gi|227809047	15574.4	5.2800 002098 0835
dimeric alpha-amylase inhibitor [Triticum dicoccoides]	gi|227809039	15574.4	5.2800 002098 0835
dimeric alpha-amylase inhibitor [Triticum dicoccoides]	gi|227809037	15574.4	5.2800

			002098 0835
dimeric alpha-amylase inhibitor [Triticum dicoccoides]	gi|227809033	15574.4	5.2800 002098 0835
dimeric alpha-amylase inhibitor [Triticum dicoccoides]	gi|227809025	15574.4	5.2800 002098 0835
dimeric alpha-amylase inhibitor [Triticum dicoccoides]	gi|227809003	15574.4	5.2800 002098 0835
dimeric alpha-amylase inhibitor [Triticum dicoccoides]	gi|227808981	15574.4	5.2800 002098 0835
dimeric alpha-amylase inhibitor [Triticum dicoccoides]	gi|227808979	15574.4	5.2800 002098 0835
dimeric alpha-amylase inhibitor [Triticum dicoccoides]	gi|227808972	15574.4	5.2800 002098 0835
dimeric alpha-amylase inhibitor [Triticum dicoccoides]	gi|227808962	15574.4	5.2800 002098 0835
dimeric alpha-amylase inhibitor [Triticum dicoccoides]	gi|227808960	15574.4	5.2800 002098 0835
dimeric alpha-amylase inhibitor [Triticum dicoccoides]	gi|227808948	15574.4	5.2800 002098 0835
dimeric alpha-amylase inhibitor [Triticum dicoccoides]	gi|227808942	15574.4	5.2800 002098 0835
dimeric alpha-amylase inhibitor [Triticum dicoccoides]	gi|227808940	15574.4	5.2800 002098 0835
dimeric alpha-amylase inhibitor [Triticum dicoccoides]	gi|227808932	15574.4	5.2800 002098 0835
dimeric alpha-amylase inhibitor [Triticum dicoccoides]	gi|227808926	15574.4	5.2800 002098 0835
dimeric alpha-amylase inhibitor [Triticum dicoccoides]	gi|227808924	15574.4	5.2800 002098 0835
dimeric alpha-amylase inhibitor [Triticum dicoccoides]	gi|227808908	15574.4	5.2800 002098 0835
dimeric alpha-amylase inhibitor [Triticum dicoccoides]	gi|227808906	15574.4	5.2800 002098 0835
dimeric alpha-amylase inhibitor [Triticum dicoccoides]	gi|227808896	15588.5	5.2800 002098 0835
dimeric alpha-amylase inhibitor [Triticum dicoccoides]	gi|227808889	15588.5	5.2800 002098

			0835
dimeric alpha-amylase inhibitor [Triticum dicoccoides]	gi|227808887	15588.5	5.2800<br>002098<br>0835
dimeric alpha-amylase inhibitor [Triticum dicoccoides]	gi|227808884	15588.5	5.2800<br>002098<br>0835
dimeric alpha-amylase inhibitor [Triticum timopheevii subsp. armeniacum]	gi|227809296	15574.4	5.2800<br>002098<br>0835

Peptide Information

Calc. Mass	Obsrv. Mass	± da	± ppm	Start Seq.	End Seq.	Sequence	Ion Score	C. I. %	Modification	Rank	Result Type
1134.6188	1134.6531	0.0343	30	107	117	LTAASITAVCK			Carbamidomethyl (C)[10]		Mascot
1306.7665	1306.8643	0.0978	75	31	42	VPALPGCRPVLK			Carbamidomethyl (C)[7]		Mascot
1306.7665	1306.8643	0.0978	75	31	42	VPALPGCRPVLK	23	79.673	Carbamidomethyl (C)[7]		Mascot
1570.8007	1570.9113	0.1106	70	43	56	LQCNGSQVPEAVLR			Carbamidomethyl (C)[3]		Mascot
1677.8517	1677.9301	0.0784	47	118	133	LPIVIDASGDGAYVCK			Carbamidomethyl (C)[15]		Mascot
1887.8403	1887.9731	0.1328	70	84	101	EHGVQEGQAGTGAFPSC<br>R			Carbamidomethyl (C)[17]		Mascot
1887.8403	1887.9731	0.1328	70	84	101	EHGVQEGQAGTGAFPSC<br>R	160	100	Carbamidomethyl (C)[17]		Mascot

6	dimeric alpha-amylase inhibitor [Triticum dicoccoides]	gi|227809222	15602.5	5.75	5	214	100	23.233	183	100

Peptide Information

Calc. Mass	Obsrv. Mass	± da	± ppm	Start Seq.	End Seq.	Sequence	Ion Score	C. I. %	Modification	Rank	Result Type
1134.6188	1134.6531	0.0343	30	107	117	LTAASITAVCK			Carbamidomethyl (C)[10]		Mascot
1306.7665	1306.8643	0.0978	75	31	42	VPALPGCRPVLK			Carbamidomethyl (C)[7]		Mascot
1306.7665	1306.8643	0.0978	75	31	42	VPALPGCRPVLK	23	79.673	Carbamidomethyl (C)[7]		Mascot
1598.8431	1598.8875	0.0444	28	43	56	LQCNGSRVPEAVLR			Carbamidomethyl (C)[3]		Mascot
1677.8517	1677.9301	0.0784	47	118	133	LPIVIDASGDGAYVCK			Carbamidomethyl (C)[15]		Mascot
1887.8403	1887.9731	0.1328	70	84	101	EHGVQEGQAGTGAFPSC<br>R			Carbamidomethyl (C)[17]		Mascot
1887.8403	1887.9731	0.1328	70	84	101	EHGVQEGQAGTGAFPSC<br>R	160	100	Carbamidomethyl (C)[17]		Mascot

7	hypothetical protein TRIUR3_00718 [Triticum urartu]	gi|474190769	27498.3	9.02	1			0	19	37.472

Peptide Information

Calc. Mass	Obsrv. Mass	± da	± ppm	Start Seq.	End Seq.	Sequence	Ion Score	C. I. %	Modification	Rank	Result Type
1571.7708	1571.8998	0.129	82	29	42	MGKSPGRPVNQNDR	19	37.472	Oxidation (M)[1]	1	Mascot

8	hypothetical protein TRIUR3_07139 [Triticum urartu]	gi|473889425	88871	7.19	1			0	17	10.448

Peptide Information

Calc. Mass	Obsrv. Mass	± da	± ppm	Start Seq.	End Seq.	Sequence	Ion Score	C. I. %	Modification	Rank	Result Type
1869.8403	1869.9636	0.1233	66	17	33	GAESSRFDSGQYSFFGK	17	10.448		1	Mascot

9	hypothetical protein TRIUR3_21450 [Triticum urartu]	gi|473753967	48785.8	5.54	1	0	17	9.202	

Peptide Information

Calc. Mass	Obsrv. Mass	± da	± ppm	Start Seq.	End Seq.	Sequence	Ion Score	C. I. %	Modification	Rank	Result Type
1887.8575	1887.9731	0.1156	61	106	122	AGLMLMNTADTYQEAAR	17	9.202	Oxidation (M)[4,6]	2	Mascot

10	hypothetical protein TRIUR3_09519 [Triticum urartu]	gi|473894932	54375.6	9.57	1	0	14	0	

Peptide Information

Calc. Mass	Obsrv. Mass	± da	± ppm	Start Seq.	End Seq.	Sequence	Ion Score	C. I. %	Modification	Rank	Result Type
1571.7772	1571.8998	0.1226	78	9	23	RLPSDDNVASDGGLR	15	0		2	Mascot

Gel Idx/Pos	137/F12	Instr./Gel Origin	BA2151/Sample Project 20140814	Process Status	Analysis Succeeded
Plate [#] Name	[1] Sample Project 20140814	Instrument Sample Name		Spectra	11

Rank	Protein Name	Accession No.	Protein MW	Protein PI	Pep. Count	Protein Score	Protein Score C. I. %	Intensity Matched	Total Ion Score	Total Ion C. I. %	Confirmed
1	dimeric alpha-amylase inhibitor [Triticum dicoccoides]	gi|114215794	13752.5	5.23	5	353	100	45.92	322	100	

Protein Group

0.19 dimeric alpha-amylase inhibitor [Triticum aestivum]	gi|56480630	13752.5	5.23000001907349
dimeric alpha-amylase inhibitor [Triticum dicoccoides]	gi|114215792	13752.5	5.23000001907349
dimeric alpha-amylase inhibitor [Triticum dicoccoides]	gi|114215788	13752.5	5.23000001907349
dimeric alpha-amylase inhibitor [Triticum dicoccoides]	gi|114215796	13752.5	5.23000001907349
dimeric alpha-amylase inhibitor [Triticum dicoccoides]	gi|114215770	13752.5	5.23000001907349

Peptide Information

Calc. Mass	Obsrv. Mass	± da	± ppm	Start Seq.	End Seq.	Sequence	Ion Score	C. I. %	Modification	Rank	Result Type
1162.6249	1162.7124	0.0875	75	90	100	LTAASITAVCR			Carbamidomethyl (C)[10]		Mascot
1162.6249	1162.7124	0.0875	75	90	100	LTAASITAVCR	58	99.99	Carbamidomethyl (C)[10]		Mascot
1570.8007	1570.9186	0.1179	75	26	39	LQCNGSQVPEAVLR			Carbamidomethyl (C)[3]		Mascot
1663.8361	1663.9296	0.0935	56	101	116	LPIVVDASGDGAYVCK			Carbamidomethyl (C)[15]		Mascot
1663.8361	1663.9296	0.0935	56	101	116	LPIVVDASGDGAYVCK	104	100	Carbamidomethyl (C)[15]		Mascot
1840.7412	1840.8787	0.1375	75	40	53	DCCQQLADISEWCR			Carbamidomethyl (C)[2,3,13]		Mascot
1846.8137	1846.9543	0.1406	76	67	84	EHGVSEGGQAGTGAFPSCR			Carbamidomethyl (C)[17]		Mascot
1846.8137	1846.9543	0.1406	76	67	84	EHGVSEGGQAGTGAFPSCR	159	100	Carbamidomethyl (C)[17]		Mascot

Rank	Protein Name	Accession No.	Protein MW	Protein PI	Pep. Count	Protein Score	Protein Score C. I. %	Intensity Matched	Total Ion Score	Total Ion C. I. %	Confirmed
2	dimeric alpha-amylase inhibitor precursor [Triticum aestivum]	gi|108597903	13823.5	5.23	5	352	100	45.92	322	100	

Peptide Information

Calc. Mass	Obsrv. Mass	± da	± ppm	Start Seq.	End Seq.	Sequence	Ion Score	C. I. %	Modification	Rank	Result Type
1162.6249	1162.7124	0.0875	75	91	101	LTAASITAVCR			Carbamidomethyl (C)[10]		Mascot
1162.6249	1162.7124	0.0875	75	91	101	LTAASITAVCR	58	99.99	Carbamidomethyl (C)[10]		Mascot

Calc. Mass	Obsrv. Mass	± da	± ppm	Start Seq.	End Seq.	Sequence	Ion Score	C. I. %	Modification	Rank	Result Type
1570.8007	1570.9186	0.1179	75	27	40	LQCNGSQVPEAVLR			Carbamidomethyl (C)[3]		Mascot
1663.8361	1663.9296	0.0935	56	102	117	LPIVVDASGDGAYVCK			Carbamidomethyl (C)[15]		Mascot
1663.8361	1663.9296	0.0935	56	102	117	LPIVVDASGDGAYVCK	104	100	Carbamidomethyl (C)[15]		Mascot
1840.7412	1840.8787	0.1375	75	41	54	DCCQQLADISEWCR			Carbamidomethyl (C)[2,3,13]		Mascot
1846.8137	1846.9543	0.1406	76	68	85	EHGVSEGGQAGTGAFPSCR			Carbamidomethyl (C)[17]		Mascot
1846.8137	1846.9543	0.1406	76	68	85	EHGVSEGGQAGTGAFPSCR	159	100	Carbamidomethyl (C)[17]		Mascot

3 dimeric alpha-amylase inhibitor [Triticum dicoccoides] gi|227809146 15605.4 5.3 5 351 100 41.938 322 100

Peptide Information

Calc. Mass	Obsrv. Mass	± da	± ppm	Start Seq.	End Seq.	Sequence	Ion Score	C. I. %	Modification	Rank	Result Type
1162.6249	1162.7124	0.0875	75	107	117	LTAASITAVCR			Carbamidomethyl (C)[10]		Mascot
1162.6249	1162.7124	0.0875	75	107	117	LTAASITAVCR	58	99.99	Carbamidomethyl (C)[10]		Mascot
1570.8007	1570.9186	0.1179	75	43	56	LQCNGSQVPEAVLR			Carbamidomethyl (C)[3]		Mascot
1663.8361	1663.9296	0.0935	56	118	133	LPIVVDASGDGAYVCK			Carbamidomethyl (C)[15]		Mascot
1663.8361	1663.9296	0.0935	56	118	133	LPIVVDASGDGAYVCK	104	100	Carbamidomethyl (C)[15]		Mascot
1846.8137	1846.9543	0.1406	76	84	101	EHGVSEGGQAGTGAFPSCR			Carbamidomethyl (C)[17]		Mascot
1846.8137	1846.9543	0.1406	76	84	101	EHGVSEGGQAGTGAFPSCR	159	100	Carbamidomethyl (C)[17]		Mascot
1884.8112	1884.8907	0.0795	42	69	83	CRCSALYSMLDSMYK			Carbamidomethyl (C)[1,3]		Mascot
1900.806	1900.8883	0.0823	43	69	83	CRCSALYSMLDSMYK			Carbamidomethyl (C)[1,3], Oxidation (M)[9]		Mascot

4 dimeric alpha-amylase inhibitor [Triticum dicoccoides] gi|227809234 15606.4 4.99 5 350 100 45.92 322 100

Protein Group

dimeric alpha-amylase inhibitor [Triticum dicoccoides]	gi|227809206	15606.4	4.9899997711816
dimeric alpha-amylase inhibitor [Triticum dicoccoides]	gi|227809202	15606.4	4.9899997711816
dimeric alpha-amylase inhibitor [Triticum dicoccoides]	gi|227809200	15606.4	4.9899997711816
dimeric alpha-amylase inhibitor [Triticum dicoccoides]	gi|227809194	15606.4	4.9899997711816
dimeric alpha-amylase inhibitor [Triticum dicoccoides]	gi|227809190	15606.4	4.9899997711816
dimeric alpha-amylase inhibitor [Triticum dicoccoides]	gi|227809184	15606.4	4.9899997711816
dimeric alpha-amylase inhibitor [Triticum dicoccoides]	gi|227809182	15606.4	4.9899997711

			1816
dimeric alpha-amylase inhibitor [Triticum dicoccoides]	gi|227809176	15606.4	4.9899<br>997711<br>1816
dimeric alpha-amylase inhibitor [Triticum dicoccoides]	gi|227809170	15606.4	4.9899<br>997711<br>1816
dimeric alpha-amylase inhibitor [Triticum dicoccoides]	gi|227809168	15606.4	4.9899<br>997711<br>1816
dimeric alpha-amylase inhibitor [Triticum dicoccoides]	gi|227809166	15606.4	4.9899<br>997711<br>1816
dimeric alpha-amylase inhibitor [Triticum dicoccoides]	gi|227809164	15606.4	4.9899<br>997711<br>1816
dimeric alpha-amylase inhibitor [Triticum dicoccoides]	gi|227809158	15606.4	4.9899<br>997711<br>1816
dimeric alpha-amylase inhibitor [Triticum dicoccoides]	gi|227809152	15606.4	4.9899<br>997711<br>1816
dimeric alpha-amylase inhibitor [Triticum dicoccoides]	gi|227809150	15578.3	4.9899<br>997711<br>1816
dimeric alpha-amylase inhibitor [Triticum dicoccoides]	gi|227809138	15606.4	4.9899<br>997711<br>1816
dimeric alpha-amylase inhibitor [Triticum dicoccoides]	gi|227809134	15606.4	4.9899<br>997711<br>1816
dimeric alpha-amylase inhibitor [Triticum dicoccoides]	gi|227809127	15606.4	4.9899<br>997711<br>1816
dimeric alpha-amylase inhibitor [Triticum dicoccoides]	gi|227809125	15606.4	4.9899<br>997711<br>1816
dimeric alpha-amylase inhibitor [Triticum dicoccoides]	gi|227809123	15606.4	4.9899<br>997711<br>1816
dimeric alpha-amylase inhibitor [Triticum dicoccoides]	gi|227809098	15606.4	4.9899<br>997711<br>1816
dimeric alpha-amylase inhibitor [Triticum dicoccoides]	gi|227809094	15606.4	4.9899<br>997711<br>1816
dimeric alpha-amylase inhibitor [Triticum dicoccoides]	gi|227809088	15606.4	4.9899<br>997711<br>1816
dimeric alpha-amylase inhibitor [Triticum dicoccoides]	gi|227809071	15606.4	4.9899<br>997711<br>1816
dimeric alpha-amylase inhibitor [Triticum dicoccoides]	gi|227809069	15606.4	4.9899<br>997711<br>1816

dimeric alpha-amylase inhibitor [Triticum dicoccoides]	gi|227809063	15606.4	4.9899 997711 1816
dimeric alpha-amylase inhibitor [Triticum dicoccoides]	gi|227809061	15606.4	4.9899 997711 1816
dimeric alpha-amylase inhibitor [Triticum dicoccoides]	gi|227809059	15606.4	4.9899 997711 1816
dimeric alpha-amylase inhibitor [Triticum dicoccoides]	gi|227809057	15606.4	4.9899 997711 1816
dimeric alpha-amylase inhibitor [Triticum dicoccoides]	gi|227809053	15606.4	4.9899 997711 1816
dimeric alpha-amylase inhibitor [Triticum dicoccoides]	gi|227809051	15606.4	4.9899 997711 1816
dimeric alpha-amylase inhibitor [Triticum dicoccoides]	gi|227808999	15584.4	4.8099 999427 7954
dimeric alpha-amylase inhibitor [Triticum dicoccoides]	gi|227808995	15605.4	5.7100 000381 4697
dimeric alpha-amylase inhibitor [Triticum dicoccoides]	gi|227808987	15606.4	4.9899 997711 1816
dimeric alpha-amylase inhibitor [Triticum dicoccoides]	gi|227808985	15606.4	4.9899 997711 1816
dimeric alpha-amylase inhibitor [Triticum dicoccoides]	gi|227808983	15606.4	4.9899 997711 1816
dimeric alpha-amylase inhibitor [Triticum dicoccoides]	gi|227808974	15592.3	4.9899 997711 1816
dimeric alpha-amylase inhibitor [Triticum dicoccoides]	gi|227808958	15606.4	4.9899 997711 1816
dimeric alpha-amylase inhibitor [Triticum dicoccoides]	gi|227808952	15606.4	4.9899 997711 1816
dimeric alpha-amylase inhibitor [Triticum dicoccoides]	gi|227808944	15606.4	4.9899 997711 1816
dimeric alpha-amylase inhibitor [Triticum dicoccoides]	gi|227809244	15606.4	4.9899 997711 1816

Peptide Information

Calc. Mass	Obsrv. Mass	± da	± ppm	Start Seq.	End Seq.	Sequence	Ion Score	C. I. %	Modification	Rank	Result Type
1162.6249	1162.7124	0.0875	75	107	117	LTAASITAVCR			Carbamidomethyl (C)[10]		Mascot

	1162.6249	1162.7124	0.0875	75	107	117	LTAASITAVCR	58	99.99	Carbamidomethyl (C)[10]	Mascot
	1570.8007	1570.9186	0.1179	75	43	56	LQCNGSQVPEAVLR			Carbamidomethyl (C)[3]	Mascot
	1663.8361	1663.9296	0.0935	56	118	133	LPIVVDASGDGAYVCK			Carbamidomethyl (C)[15]	Mascot
	1663.8361	1663.9296	0.0935	56	118	133	LPIVVDASGDGAYVCK	104	100	Carbamidomethyl (C)[15]	Mascot
	1840.7412	1840.8787	0.1375	75	57	70	DCCQQLADISEWCR			Carbamidomethyl (C)[2,3,13]	Mascot
	1846.8137	1846.9543	0.1406	76	84	101	EHGVSEGGQAGTGAFPSC R			Carbamidomethyl (C)[17]	Mascot
	1846.8137	1846.9543	0.1406	76	84	101	EHGVSEGGQAGTGAFPSC R	159	100	Carbamidomethyl (C)[17]	Mascot

5	dimeric alpha-amylase inhibitor [Triticum timopheevii subsp. armeniacum]	gi|227809288	15533.4	5.7	3	0	322	100

Protein Group

RecName: Full=Alpha-amylase inhibitor 0.53	gi|123968	13689.5	5.2300 000190 7349
dimeric alpha-amylase inhibitor [Triticum aestivum]	gi|65993898	15533.4	5.6999 998092 6514
dimeric alpha-amylase inhibitor [Triticum aestivum]	gi|65993731	15606.4	4.9899 997711 1816
dimeric alpha-amylase inhibitor [Triticum aestivum]	gi|65993709	15578.3	4.9899 997711 1816
dimeric alpha-amylase inhibitor [Triticum dicoccoides]	gi|227808966	15664.4	4.8099 999427 7954
dimeric alpha-amylase inhibitor [Triticum dicoccoides]	gi|227808934	15606.4	4.9899 997711 1816
dimeric alpha-amylase inhibitor [Triticum dicoccoides]	gi|227808928	15605.4	5.0100 002288 8184
dimeric alpha-amylase inhibitor [Triticum dicoccoides]	gi|227808922	15606.4	4.9899 997711 1816
dimeric alpha-amylase inhibitor [Triticum dicoccoides]	gi|227808920	15606.4	4.9899 997711 1816
dimeric alpha-amylase inhibitor [Triticum dicoccoides]	gi|227808914	15606.4	4.9899 997711 1816
dimeric alpha-amylase inhibitor [Triticum dicoccoides]	gi|227808912	15638.3	4.9899 997711 1816
dimeric alpha-amylase inhibitor [Triticum dicoccoides]	gi|227808900	15578.3	4.9899 997711 1816
dimeric alpha-amylase inhibitor [Triticum dicoccoides]	gi|227808898	15606.4	4.9899 997711 1816

dimeric alpha-amylase inhibitor [Triticum dicoccoides] gi|227808869 15606.4 4.98999977111816

dimeric alpha-amylase inhibitor [Triticum dicoccoides] gi|227808867 15578.3 4.98999977111816

dimeric alpha-amylase inhibitor [Triticum dicoccoides] gi|227808861 15658.4 5.57999992370605

dimeric alpha-amylase inhibitor [Triticum dicoccoides] gi|227808853 15618.3 4.98999977111816

dimeric alpha-amylase inhibitor [Triticum dicoccoides] gi|227808851 15658.4 5.57999992370605

Peptide Information

Calc. Mass	Obsrv. Mass	± da	± ppm	Start Seq.	End Seq.	Sequence	Ion Score	C. I. %	Modification	Rank	Result Type
1162.6249	1162.7124	0.0875	75	107	117	LTAASITAVCR	58	99.99	Carbamidomethyl (C)[10]	1	Mascot
1663.8361	1663.9296	0.0935	56	118	133	LPIVVDASGDGAYVCK	104	100	Carbamidomethyl (C)[15]	1	Mascot
1846.8137	1846.9543	0.1406	76	84	101	EHGVSEGGQAGTGAFPSCR	159	100	Carbamidomethyl (C)[17]	1	Mascot

6 dimeric alpha-amylase inhibitor [Triticum dicoccoides] gi|227809023 15604.3 4.99 3 0 170 100

Peptide Information

Calc. Mass	Obsrv. Mass	± da	± ppm	Start Seq.	End Seq.	Sequence	Ion Score	C. I. %	Modification	Rank	Result Type
1162.6249	1162.7124	0.0875	75	107	117	LTAASITAVCR	58	99.99	Carbamidomethyl (C)[10]	1	Mascot
1663.8361	1663.9296	0.0935	56	118	133	LPIVVDASGDGAYVCK	104	100	Carbamidomethyl (C)[15]	1	Mascot
1872.8295	1872.869	0.0395	21	84	101	EHGVSEGGQAGTGPFPSCR	7	0	Carbamidomethyl (C)[17]	1	Mascot

7 dimeric alpha-amylase inhibitor [Triticum dicoccoides] gi|227809121 15555.3 4.99 2 0 163 100

Peptide Information

Calc. Mass	Obsrv. Mass	± da	± ppm	Start Seq.	End Seq.	Sequence	Ion Score	C. I. %	Modification	Rank	Result Type
1162.6249	1162.7124	0.0875	75	107	117	LTAASITAVXR	58	99.99		1	Mascot
1663.8361	1663.9296	0.0935	56	118	133	LPIVVDASGDGAYVCK	104	100	Carbamidomethyl (C)[15]	1	Mascot

8 hypothetical protein TRIUR3_32567 [Triticum urartu] gi|473837434 60255.1 5.25 1 0 25 75.993

Peptide Information

Calc. Mass	Obsrv. Mass	± da	± ppm	Start Seq.	End Seq.	Sequence	Ion Score	C. I. %	Modification	Rank	Result Type
997.5676	997.6003	0.0327	33	181	189	LNNAIPLDK	25	75.993		1	Mascot

9	unnamed protein product [Triticum aestivum]	gi|219918350	51710.3	8.64	1		0	22	51.434

Protein Group

unnamed protein product [Triticum aestivum]	gi|257661794	51710.3	8.6400 003433 2275

Peptide Information

Calc. Mass	Obsrv. Mass	± da	± ppm	Start Seq.	End Seq.	Sequence	Ion Score	C. I. %	Modification	Rank	Result Type
997.5499	997.6003	0.0504	51	1	9	MAEAPLLPR	22	51.434		2	Mascot

10	hypothetical protein TRIUR3_09992 [Triticum urartu]	gi|474103565	46125.6	8.51	1		0	19	11.216

Peptide Information

Calc. Mass	Obsrv. Mass	± da	± ppm	Start Seq.	End Seq.	Sequence	Ion Score	C. I. %	Modification	Rank	Result Type
997.6152	997.6003	-0.0149	-15	394	402	VKPLAKEGR	19	11.216		3	Mascot

Gel Idx/Pos	138/F13	Instr./Gel Origin	BA2151/Sample Project 20140814	Process Status	Analysis Succeeded
Plate [#] Name	[1] Sample Project 20140814	Instrument Sample Name		Spectra	11

Rank	Protein Name	Accession No.	Protein MW	Protein PI	Pep. Count	Protein Score	Protein Score C. I. %	Intensity Matched	Total Ion Score	Total Ion C. I. %	Confirmed
1	alpha-amylase inhibitor CM16 subunit [Triticum macha]	gi|221855644	16267.8	5.31	5	296	100	37.2	268	100	

Protein Group

CM16 protein [Triticum aestivum]	gi|21709	16398.8	5.3099999427 7954
RecName: Full=Alpha-amylase/trypsin inhibitor CM16; AltName: Full=Chloroform/methanol-soluble protein CM16; Flags: Precursor	gi|123958	16398.8	5.3099999427 7954
alpha-amylase inhibitor CM16 subunit [Triticum macha]	gi|221855632	16267.8	5.3099999427 7954
alpha-amylase inhibitor CM16 subunit [Triticum macha]	gi|221855656	16267.8	5.3099999427 7954
alpha-amylase inhibitor, tetrameric, chain CM16 precursor - durum wheat	gi|100832	16398.8	5.3099999427 7954
major allergen CM16 [Triticum aestivum]	gi|195957140	16399.8	4.8600001335 144
precursor (AA -24 to 119) [Triticum durum]	gi|21916	16398.8	5.3099999427 7954
unnamed protein product [Triticum aestivum]	gi|21705	16398.8	5.3099999427 7954

Peptide Information

Calc. Mass	Obsrv. Mass	± da	± ppm	Start Seq.	End Seq.	Sequence	Ion Score	C. I. %	Modification	Rank	Result Type
1023.4928	1023.5602	0.0674	66	107	114	EVQMDFVR					Mascot
1023.4928	1023.5602	0.0674	66	107	114	EVQMDFVR	20	14.131			Mascot
1039.4878	1039.5409	0.0531	51	107	114	EVQMDFVR			Oxidation (M)[4]		Mascot
1039.4878	1039.5409	0.0531	51	107	114	EVQMDFVR	36	97.887	Oxidation (M)[4]		Mascot
1168.5052	1168.5846	0.0794	68	45	53	DYVEQQACR			Carbamidomethyl (C)[8]		Mascot
1168.5052	1168.5846	0.0794	68	45	53	DYVEQQACR	74	100	Carbamidomethyl (C)[8]		Mascot
1175.6307	1175.6776	0.0469	40	54	64	IETPGSPYLAK					Mascot
1799.8528	1799.9772	0.1244	69	91	106	SRPDQSGLMELPGCPR			Carbamidomethyl (C)[14]		Mascot
1815.8477	1815.9524	0.1047	58	91	106	SRPDQSGLMELPGCPR			Carbamidomethyl (C)[14], Oxidation (M)[9]		Mascot
1815.8477	1815.9524	0.1047	58	91	106	SRPDQSGLMELPGCPR	46	99.82	Carbamidomethyl (C)[14], Oxidation (M)[9]		Mascot

1861.8102	1861.9398	0.1296	70	65	79	QQCCGELANIPQQCR			Carbamidomethyl (C)[3,4,14]		Mascot
1861.8102	1861.9398	0.1296	70	65	79	QQCCGELANIPQQCR	112	100	Carbamidomethyl (C)[3,4,14]		Mascot

2	CM 17 protein precursor [Triticum aestivum]	gi|21711	16548.8	5.07	2	68	99.266	19.614	60	99.992	

Peptide Information

Calc. Mass	Obsrv. Mass	± da	± ppm	Start Seq.	End Seq.	Sequence	Ion Score	C. I. %	Modification	Rank	Result Type
1168.5051	1168.5846	0.0795	68	46	54	NYVEEQACR			Carbamidomethyl (C)[8]		Mascot
1168.5051	1168.5846	0.0795	68	46	54	NYVEEQACR	14	0	Carbamidomethyl (C)[8]		Mascot
1799.8528	1799.9772	0.1244	69	92	107	SRPDQSGLMELPGCPR			Carbamidomethyl (C)[14]		Mascot
1815.8477	1815.9524	0.1047	58	92	107	SRPDQSGLMELPGCPR			Carbamidomethyl (C)[14], Oxidation (M)[9]		Mascot
1815.8477	1815.9524	0.1047	58	92	107	SRPDQSGLMELPGCPR	46	99.82	Carbamidomethyl (C)[14], Oxidation (M)[9]		Mascot

3	Myosin-J heavy chain [Triticum urartu]	gi|474114531	236907.2	6.35	32	52	73.354	17.271			

Peptide Information

Calc. Mass	Obsrv. Mass	± da	± ppm	Start Seq.	End Seq.	Sequence	Ion Score	C. I. %	Modification	Rank	Result Type
800.4875	800.5052	0.0177	22	390	396	LENALIK					Mascot
804.3846	804.3448	-0.0398	-49	1340	1346	QENNATK					Mascot
874.4265	874.3775	-0.049	-56	1669	1675	NEDLLDR					Mascot
908.4472	908.4642	0.017	19	1474	1480	RFEDVDK					Mascot
956.5887	956.5419	-0.0468	-49	390	397	LENALIKR					Mascot
975.5655	975.5636	-0.0019	-2	926	933	LGLEMKLR			Oxidation (M)[5]		Mascot
991.5128	991.5533	0.0405	41	1499	1506	DTLLLCEK			Carbamidomethyl (C)[6]		Mascot
1023.4966	1023.5602	0.0636	62	231	239	NNNSSRFGK					Mascot
1023.6057	1023.5602	-0.0455	-44	854	862	KASIHIQAR					Mascot
1061.5044	1061.5189	0.0145	14	742	751	AGQMAELDAR					Mascot
1077.4994	1077.5746	0.0752	70	742	751	AGQMAELDAR			Oxidation (M)[4]		Mascot
1077.4994	1077.5746	0.0752	70	742	751	AGQMAELDAR			Oxidation (M)[4]		Mascot
1111.6259	1111.558	-0.0679	-61	194	203	YLAFLGGRSK					Mascot
1175.6453	1175.6776	0.0323	27	1257	1266	SINKLMENVK					Mascot
1237.6536	1237.6249	-0.0287	-23	781	791	NTSVSFQSIVR					Mascot
1321.7223	1321.713	-0.0093	-7	726	737	TGLQGYQIGRTK					Mascot
1355.6914	1355.7325	0.0411	30	1228	1239	QEHGQTKEALSK					Mascot
1566.6952	1566.777	0.0818	52	79	92	DTEVLSDGIDDMTR					Mascot
1571.8387	1571.889	0.0503	32	2051	2064	ENQSLALILQRGTE					Mascot
1572.7864	1572.8451	0.0587	37	1669	1681	NEDLLDRNDDLIK					Mascot

1679.8851	1679.9097	0.0246	15	1638	1652	LSSFVLEKQESDAVK					Mascot
1753.908	1753.9288	0.0208	12	1212	1227	LEETAATRDALHVAEK					Mascot
1769.9944	1769.9449	-0.0495	-28	46	60	TFGIMIYIDMLLVLK					Mascot
1773.8654	1773.9258	0.0604	34	1130	1144	GDNEELVHKTEVFEK					Mascot
1781.9167	1781.9882	0.0715	40	1515	1529	ALTETEYKNEELTIK					Mascot
1785.9894	1785.963	-0.0264	-15	46	60	TFGIMIYIDMLLVLK			Oxidation (M)[5]		Mascot
1789.8716	1789.9257	0.0541	30	1588	1602	DSEYKAHQLQDTVQK					Mascot
1795.9701	1795.907	-0.0631	-35	93	108	LSYLHEPGVLDNLAVR					Mascot
1805.0167	1804.9165	-0.1002	-56	1630	1645	LQVDAISRLSSFVLEK					Mascot
1837.984	1837.9198	-0.0642	-35	1894	1908	KELNPLLELCIQDPR			Carbamidomethyl (C)[10]		Mascot
1844.8484	1844.9202	0.0718	39	465	479	TNSFEQLCINFTNEK			Carbamidomethyl (C)[8]		Mascot
1844.8484	1844.9202	0.0718	39	465	479	TNSFEQLCINFTNEK			Carbamidomethyl (C)[8]		Mascot
1846.8851	1846.9163	0.0312	17	1020	1035	CEDLNGKIEVADENIK			Carbamidomethyl (C)[1]		Mascot
1859.9255	1859.9359	0.0104	6	372	387	AKFHLNAAAELLMCDR			Carbamidomethyl (C)[14]		Mascot
1872.9774	1872.9381	-0.0393	-21	1082	1099	QVADADGKSAVLQSTVQ R					Mascot
1875.9204	1875.9397	0.0193	10	372	387	AKFHLNAAAELLMCDR			Carbamidomethyl (C)[14], Oxidation (M)[13]		Mascot
2400.1677	2400.3511	0.1834	76	1777	1798	TGVFDSILQAINSATEAQ YDTR					Mascot

4 Disease resistance protein RPM1 [Triticum urartu] gi|474431373 108524.2 8.73 18 45 0 14.198

Peptide Information

Calc. Mass	Obsrv. Mass	± da	± ppm	Start Seq.	End Seq.	Sequence	Ion Score	C. I. %	Modification	Rank	Result Type
801.4213	801.4696	0.0483	60	133	139	AQQIGER					Mascot
804.425	804.3448	-0.0802	-100	438	444	SAAEWLK					Mascot
870.5407	870.5955	0.0548	63	654	661	QLPKSIGK					Mascot
975.4677	975.5636	0.0959	98	257	264	NQVMPQSR			Oxidation (M)[4]		Mascot
1021.4486	1021.5346	0.086	84	230	237	FGDQFDHR					Mascot
1126.5198	1126.5582	0.0384	34	184	193	DPVGVEDHMK					Mascot
1424.7493	1424.7662	0.0169	12	946	957	EVQHEVETAVKR					Mascot
1515.806	1515.7802	-0.0258	-17	265	279	GQMQQAGGGVLGRLK			Oxidation (M)[3]		Mascot
1663.8658	1663.8829	0.0171	10	535	548	CIVHDMVLEHIVAK			Carbamidomethyl (C)[1]		Mascot
1679.8608	1679.9097	0.0489	29	535	548	CIVHDMVLEHIVAK			Carbamidomethyl (C)[1], Oxidation (M)[6]		Mascot
1789.9178	1789.9257	0.0079	4	851	866	AEKQDSETLTILAENK					Mascot
1791.9608	1791.8873	-0.0735	-41	534	548	KCIVHDMVLEHIVAK			Carbamidomethyl (C)[2]		Mascot
1798.0474	1798.0042	-0.0432	-24	805	820	IVQLPSWITQLSALTK					Mascot
1815.9198	1815.9524	0.0326	18	741	756	LAIYKLSTMSDDPSFK					Mascot

1815.9198	1815.9524	0.0326	18	741	756	LAIYKLSTMSDDPSFK						Mascot
1831.9147	1831.9338	0.0191	10	741	756	LAIYKLSTMSDDPSFK				Oxidation (M)[9]		Mascot
1831.9147	1831.9338	0.0191	10	741	756	LAIYKLSTMSDDPSFK				Oxidation (M)[9]		Mascot
1846.991	1846.9163	-0.0747	-40	867	884	LSSDGEITIPNAGFKGLK						Mascot
1883.9275	1883.8893	-0.0382	-20	1	18	MELVVGASEATMKSVMGK				Oxidation (M)[1]		Mascot
1887.9845	1887.9604	-0.0241	-13	672	687	ETSVVELPKTVCQLER				Carbamidomethyl (C)[12]		Mascot
2384.1074	2384.1272	0.0198	8	184	203	DPVGVEDHMKELEEWLTNDK						Mascot
2400.281	2400.3511	0.0701	29	830	850	TDNLLLLSNLDALFSLTFSFR						Mascot

5 hypothetical protein TRIUR3_02653 [Triticum urartu] gi|473895929 88455.4 5.43 20 44 0 19.52

Peptide Information

Calc. Mass	Obsrv. Mass	± da	± ppm	Start Seq.	End Seq.	Sequence	Ion Score	C. I. %	Modification	Rank	Result Type
804.3846	804.3448	-0.0398	-49	639	645	EADDRAK					Mascot
828.5302	828.468	-0.0622	-75	232	238	RIIEGLK					Mascot
832.441	832.3814	-0.0596	-72	483	489	ADLEKEK					Mascot
858.4753	858.4742	-0.0011	-1	532	538	MPIELQK					Mascot
974.5516	974.5739	0.0223	23	490	498	ADLTALKDK					Mascot
1005.5033	1005.5474	0.0441	44	698	705	LTMENELR					Mascot
1021.4982	1021.5346	0.0364	36	698	705	LTMENELR			Oxidation (M)[3]		Mascot
1077.58	1077.5746	-0.0054	-5	308	316	EHTALITHR					Mascot
1077.58	1077.5746	-0.0054	-5	308	316	EHTALITHR					Mascot
1179.6256	1179.6786	0.053	45	213	222	FIAEEVEKSK					Mascot
1182.6841	1182.6317	-0.0524	-44	665	674	SLDKLHQLTK					Mascot
1190.5946	1190.559	-0.0356	-30	550	559	ARLACDEVTR			Carbamidomethyl (C)[5]		Mascot
1196.6127	1196.6194	0.0067	6	440	449	VRSMVSVQMK			Oxidation (M)[4,9]		Mascot
1237.6643	1237.6249	-0.0394	-32	442	452	SMVSVQMKLAK			Oxidation (M)[2]		Mascot
1355.7781	1355.7325	-0.0456	-34	164	176	VLVETAAPIESVK					Mascot
1734.8278	1734.9948	0.167	96	771	784	SFFPRSIAMMFMNR					Mascot
1754.8014	1754.9159	0.1145	65	102	116	NFSEDIGSLTINECR			Carbamidomethyl (C)[14]		Mascot
1770.8076	1770.9294	0.1218	69	117	131	ANKVEENCHDQLEGK			Carbamidomethyl (C)[8]		Mascot
1770.8076	1770.9294	0.1218	69	117	131	ANKVEENCHDQLEGK			Carbamidomethyl (C)[8]		Mascot
1781.8561	1781.9882	0.1321	74	622	636	SMMVPLEDYDALNKR					Mascot
1789.9304	1789.9257	-0.0047	-3	308	322	EHTALITHRENAEIR					Mascot
1791.9348	1791.8873	-0.0475	-27	499	514	VHHATVSVSSLQEELR					Mascot
1797.851	1798.0042	0.1532	85	622	636	SMMVPLEDYDALNKR			Oxidation (M)[2]		Mascot

Calc. Mass	Obsrv. Mass	± da	± ppm	Start Seq.	End Seq.	Sequence	Ion Score	C. I. %	Modification	Rank	Result Type
1813.8459	1813.9893	0.1434	79	622	636	SMMVPLEDYDALNKR			Oxidation (M)[2,3]		Mascot
1844.9786	1844.9202	-0.0584	-32	532	547	MPIELQKATQETQLAK			Oxidation (M)[1]		Mascot
1844.9786	1844.9202	-0.0584	-32	532	547	MPIELQKATQETQLAK			Oxidation (M)[1]		Mascot

6	hypothetical protein TRIUR3_28256 [Triticum urartu]	gi|473732516	18523.4	10.77	9	44	0	8.582	

Peptide Information

Calc. Mass	Obsrv. Mass	± da	± ppm	Start Seq.	End Seq.	Sequence	Ion Score	C. I. %	Modification	Rank	Result Type
911.5349	911.5076	-0.0273	-30	20	26	FQKVVYK					Mascot
1039.5208	1039.5409	0.0201	19	59	67	SIFSAYHSK					Mascot
1039.5208	1039.5409	0.0201	19	59	67	SIFSAYHSK					Mascot
1111.5565	1111.558	0.0015	1	144	152	YIGMKSEQR					Mascot
1196.6497	1196.6194	-0.0303	-25	98	106	YVILMWSLR			Oxidation (M)[5]		Mascot
1570.7748	1570.8727	0.0979	62	27	39	DVPTRYSDVDYLK					Mascot
1572.8356	1572.8451	0.0095	6	95	106	GYRYVILMWSLR			Oxidation (M)[8]		Mascot
1724.9653	1724.9501	-0.0152	-9	129	143	VVANPSIEELTQRLR					Mascot
1846.9657	1846.9163	-0.0494	-27	40	55	ERAILTPTNQVAENYK					Mascot
2063.0881	2063.1348	0.0467	23	1	17	MEFVYFSSILVIKDVTR			Oxidation (M)[1]		Mascot

7	hypothetical protein TRIUR3_27355 [Triticum urartu]	gi|474416442	10213.2	5.25	6	41	0	14.152	

Peptide Information

Calc. Mass	Obsrv. Mass	± da	± ppm	Start Seq.	End Seq.	Sequence	Ion Score	C. I. %	Modification	Rank	Result Type
832.441	832.3814	-0.0596	-72	68	74	LTVENEK					Mascot
974.4975	974.5739	0.0764	78	1	10	MAAGPAEALK			Oxidation (M)[1]		Mascot
1168.4899	1168.5846	0.0947	81	34	45	DGASEASSSAMR					Mascot
1168.4899	1168.5846	0.0947	81	34	45	DGASEASSSAMR	3	0			Mascot
1184.4849	1184.5673	0.0824	70	34	45	DGASEASSSAMR			Oxidation (M)[11]		Mascot
1190.6263	1190.559	-0.0673	-57	46	56	DLESKLDAATK					Mascot
1475.7489	1475.8292	0.0803	54	2	15	AAGPAEALKSFEER					Mascot
2063.0437	2063.1348	0.0911	44	26	45	LEALLLNKDGASEASSSAMR					Mascot

8	SNW domain-containing protein 1 [Triticum urartu]	gi|473954123	62991.9	8.97	10	41	0	11.171	10	0	

Peptide Information

Calc. Mass	Obsrv. Mass	± da	± ppm	Start Seq.	End Seq.	Sequence	Ion Score	C. I. %	Modification	Rank	Result Type
1005.4999	1005.5474	0.0475	47	457	464	DRPVEFDK					Mascot

1055.5667	1055.5548	-0.0119	-11	357	367	VALGMAHTGAK					Mascot
1424.6952	1424.7662	0.071	50	262	277	SGAPPPSTGMPVGGGR					Mascot
1475.7489	1475.8292	0.0803	54	200	212	GLQEVQINDNFAK					Mascot
1724.787	1724.9501	0.1631	95	126	140	MSEMASDPLDPPKFK			Oxidation (M)[1,4]		Mascot
1799.9031	1799.9772	0.0741	41	123	138	IIRMSEMASDPLDPPK					Mascot
1813.824	1813.9893	0.1653	91	505	522	DDYEGGGSGSAFSNIIPK					Mascot
1815.8979	1815.9524	0.0545	30	123	138	IIRMSEMASDPLDPPK			Oxidation (M)[4]		Mascot
1815.8979	1815.9524	0.0545	30	123	138	IIRMSEMASDPLDPPK	10	0	Oxidation (M)[4]		Mascot
1831.8928	1831.9338	0.041	22	123	138	IIRMSEMASDPLDPPK			Oxidation (M)[4,7]		Mascot
1831.8928	1831.9338	0.041	22	123	138	IIRMSEMASDPLDPPK			Oxidation (M)[4,7]		Mascot
1843.8644	1843.972	0.1076	58	368	382	TGEVMYDQRLFNQDK					Mascot
1859.8593	1859.9359	0.0766	41	368	382	TGEVMYDQRLFNQDK			Oxidation (M)[5]		Mascot
1872.8691	1872.9381	0.069	37	259	277	MERSGAPPPSTGMPVG GGR			Oxidation (M)[1,13]		Mascot
1883.7753	1883.8893	0.114	61	383	399	GMDSGFGADDQYNLYSK			Oxidation (M)[2]		Mascot

9 Phospholipase A1-II 7 [Triticum urartu] gi|474435629 42489.7 6.76 5 40 0 9.135 28 86.792

Peptide Information

Calc. Mass	Obsrv. Mass	± da	± ppm	Start Seq.	End Seq.	Sequence	Ion Score	C. I. %	Modification	Rank	Result Type
858.4832	858.4742	-0.009	-10	124	130	DIVVAWR					Mascot
1023.5404	1023.5602	0.0198	19	301	308	TIRSPYMR					Mascot
1023.5404	1023.5602	0.0198	19	301	308	TIRSPYMR					Mascot
1039.5354	1039.5409	0.0055	5	301	308	TIRSPYMR			Oxidation (M)[7]		Mascot
1039.5354	1039.5409	0.0055	5	301	308	TIRSPYMR	28	86.792	Oxidation (M)[7]		Mascot
1798.0183	1798.0042	-0.0141	-8	1	17	MSFLPIPLPIVGDIASK					Mascot
1799.944	1799.9772	0.0332	18	242	258	SFPVTAIVFASPHVGDR					Mascot
1814.0133	1813.9893	-0.024	-13	1	17	MSFLPIPLPIVGDIASK			Oxidation (M)[1]		Mascot
1912.0076	1911.9377	-0.0699	-37	162	178	FAVVHRGFLSVYTSSNK					Mascot

10 Phospholipase A1-II 7 [Triticum urartu] gi|474369590 56433.6 8.59 6 40 0 13.001 28 86.792

Peptide Information

Calc. Mass	Obsrv. Mass	± da	± ppm	Start Seq.	End Seq.	Sequence	Ion Score	C. I. %	Modification	Rank	Result Type
858.4832	858.4742	-0.009	-10	170	176	DIVVAWR					Mascot
1023.5404	1023.5602	0.0198	19	347	354	TIRSPYMR					Mascot
1023.5404	1023.5602	0.0198	19	347	354	TIRSPYMR					Mascot
1039.5354	1039.5409	0.0055	5	347	354	TIRSPYMR			Oxidation (M)[7]		Mascot

1039.5354	1039.5409	0.0055	5	347	354	TIRSPYMR	28	88.44	Oxidation (M)[7]	Mascot
1196.6271	1196.6194	-0.0077	-6	470	480	QDLAPPPTTTR				Mascot
1515.7889	1515.7802	-0.0087	-6	481	492	RPPIYAWIPSCR			Carbamidomethyl (C)[11]	Mascot
1844.9476	1844.9202	-0.0274	-15	288	304	SFPVTAIVFASPHVGCR			Carbamidomethyl (C)[16]	Mascot
1844.9476	1844.9202	-0.0274	-15	288	304	SFPVTAIVFASPHVGCR			Carbamidomethyl (C)[16]	Mascot
1846.9154	1846.9163	0.0009	0	497	514	TEEHRAEPPASGGAKPG R				Mascot

Gel Idx/Pos	139/F14	Instr./Gel Origin	BA2151/Sample Project 20140814	Process Status	Analysis Succeeded
Plate [#] Name	[1] Sample Project 20140814	Instrument Sample Name		Spectra	11

Rank	Protein Name	Accession No.	Protein MW	Protein PI	Pep. Count	Protein Score	Protein Score C. I. %	Intensity Matched	Total Ion Score	Total Ion C. I. %	Confirmed
1	alpha-amylase inhibitor CM16 subunit [Triticum macha]	gi|221855644	16267.8	5.31	5	178	100	16.726	151	100	

Protein Group

CM16 protein [Triticum aestivum]	gi|21709	16398.8	5.3099999427 7954
RecName: Full=Alpha-amylase/trypsin inhibitor CM16; AltName: Full=Chloroform/methanol-soluble protein CM16; Flags: Precursor	gi|123958	16398.8	5.3099999427 7954
alpha-amylase inhibitor CM16 subunit [Triticum macha]	gi|221855632	16267.8	5.3099999427 7954
alpha-amylase inhibitor CM16 subunit [Triticum macha]	gi|221855656	16267.8	5.3099999427 7954
alpha-amylase inhibitor, tetrameric, chain CM16 precursor - durum wheat	gi|100832	16398.8	5.3099999427 7954
major allergen CM16 [Triticum aestivum]	gi|195957140	16399.8	4.8600001335 144
precursor (AA -24 to 119) [Triticum durum]	gi|21916	16398.8	5.3099999427 7954
unnamed protein product [Triticum aestivum]	gi|21705	16398.8	5.3099999427 7954

Peptide Information

Calc. Mass	Obsrv. Mass	± da	± ppm	Start Seq.	End Seq.	Sequence	Ion Score	C. I. %	Modification	Rank	Result Type
1023.4928	1023.5446	0.0518	51	107	114	EVQMDFVR					Mascot
1039.4878	1039.5559	0.0681	66	107	114	EVQMDFVR			Oxidation (M)[4]		Mascot
1039.4878	1039.5559	0.0681	66	107	114	EVQMDFVR	8	0	Oxidation (M)[4]		Mascot
1168.5052	1168.6005	0.0953	82	45	53	DYVEQQACR			Carbamidomethyl (C)[8]		Mascot
1168.5052	1168.6005	0.0953	82	45	53	DYVEQQACR	57	99.986	Carbamidomethyl (C)[8]		Mascot
1175.6307	1175.6857	0.055	47	54	64	IETPGSPYLAK					Mascot
1799.8528	1800.002	0.1492	83	91	106	SRPDQSGLMELPGCPR			Carbamidomethyl (C)[14]		Mascot
1815.8477	1815.9718	0.1241	68	91	106	SRPDQSGLMELPGCPR			Carbamidomethyl (C)[14], Oxidation (M)[9]		Mascot
1815.8477	1815.9718	0.1241	68	91	106	SRPDQSGLMELPGCPR	10	0	Carbamidomethyl (C)[14], Oxidation (M)[9]		Mascot
1861.8102	1861.9623	0.1521	82	65	79	QQCCGELANIPQQCR			Carbamidomethyl (C)[3,4,14]		Mascot

1861.8102	1861.9623	0.1521	82	65	79	QQCCGELANIPQQCR	76	100	Carbamidomethyl (C)[3,4,14]		Mascot

2 Disease resistance protein RPM1 [Triticum urartu] gi|474431373 108524.2 8.73 25 73 99.773 17.444

Peptide Information

Calc. Mass	Obsrv. Mass	± da	± ppm	Start Seq.	End Seq.	Sequence	Ion Score	C. I. %	Modification	Rank	Result Type
801.4213	801.452	0.0307	38	133	139	AQQIGER					Mascot
804.425	804.4014	-0.0236	-29	438	444	SAAEWLK					Mascot
806.4016	806.3947	-0.0069	-9	62	68	AQTHGHR					Mascot
807.4835	807.406	-0.0775	-96	644	649	YLSLRR					Mascot
817.4163	817.4033	-0.013	-16	53	61	DLTAGGAGR					Mascot
818.4366	818.3757	-0.0609	-74	300	306	GETKLDR					Mascot
833.4111	833.3818	-0.0293	-35	583	589	RANDTEK					Mascot
864.4461	864.4405	-0.0056	-6	448	455	SLFPESGK					Mascot
870.5407	870.5858	0.0451	52	654	661	QLPKSIGK					Mascot
872.4407	872.4678	0.0271	31	590	596	MNLSHVR			Oxidation (M)[1]		Mascot
1001.5513	1001.5838	0.0325	32	672	680	ETSVVELPK					Mascot
1021.4486	1021.5485	0.0999	98	230	237	FGDQFDHR					Mascot
1126.5198	1126.5883	0.0685	61	184	193	DPVGVEDHMK					Mascot
1162.6063	1162.6938	0.0875	75	574	583	LSLQESDSKR					Mascot
1365.6753	1365.7491	0.0738	54	1	13	MELVVGASEATMK					Mascot
1679.8608	1679.9415	0.0807	48	535	548	CIVHDMVLEHIVAK			Carbamidomethyl (C)[1], Oxidation (M)[6]		Mascot
1791.9608	1791.9139	-0.0469	-26	534	548	KCIVHDMVLEHIVAK			Carbamidomethyl (C)[2]		Mascot
1812.9023	1813.017	0.1147	63	614	629	FGIVQVLDLEGCMGFK			Carbamidomethyl (C)[12]		Mascot
1815.9198	1815.9718	0.052	29	741	756	LAIYKLSTMSDDPSFK					Mascot
1815.9198	1815.9718	0.052	29	741	756	LAIYKLSTMSDDPSFK					Mascot
1831.9147	1831.9639	0.0492	27	741	756	LAIYKLSTMSDDPSFK			Oxidation (M)[9]		Mascot
1846.991	1846.9449	-0.0461	-25	867	884	LSSDGEITIPNAGFKGLK					Mascot
1862.1302	1861.9623	-0.1679	-90	885	900	LLRFFAPLLPVLTFSK					Mascot
1862.1302	1861.9623	-0.1679	-90	885	900	LLRFFAPLLPVLTFSK					Mascot
1883.9275	1883.9255	-0.002	-1	1	18	MELVVGASEATMKSVMGK			Oxidation (M)[1]		Mascot
1887.9845	1887.9917	0.0072	4	672	687	ETSVVELPKTVCQLER			Carbamidomethyl (C)[12]		Mascot
1887.9845	1887.9917	0.0072	4	672	687	ETSVVELPKTVCQLER			Carbamidomethyl (C)[12]		Mascot
1926.8644	1926.9982	0.1338	69	281	299	GTSALAAKCCSAGASEETR			Carbamidomethyl (C)[9,10]		Mascot
2384.1074	2384.1584	0.051	21	184	203	DPVGVEDHMKELEEWLTNDK					Mascot

3 T-complex protein 1 subunit theta [Triticum urartu] gi|474142427 73650 6.85 16 49 43.032 5.724

Peptide Information

Calc. Mass	Obsrv. Mass	± da	± ppm	Start Seq.	End Seq.	Sequence	Ion Score	C. I. %	Modification	Rank	Result Type
803.4257	803.4114	-0.0143	-18	223	230	NDAVGSIK					Mascot
808.4424	808.39	-0.0524	-65	544	550	RHPDVGK					Mascot
837.4498	837.4123	-0.0375	-45	161	168	MKSAVASK			Oxidation (M)[1]		Mascot
864.5189	864.4405	-0.0784	-91	421	427	LKEFSLK					Mascot
1037.5667	1037.5526	-0.0141	-14	493	500	IWDLYVTK					Mascot
1184.6859	1184.6152	-0.0707	-60	620	631	TAGAARVVVANR					Mascot
1320.6278	1320.694	0.0662	50	377	388	GSTDSILDDLER					Mascot
1445.7847	1445.7733	-0.0114	-8	274	287	VEELIKSVADSGAK					Mascot
1571.8104	1571.9174	0.107	68	571	583	IIDFSPFFSITER					Mascot
1571.8104	1571.9174	0.107	68	571	583	IIDFSPFFSITER					Mascot
1679.8245	1679.9415	0.117	70	1	15	MVGYGIQSMLKDGHK			Oxidation (M)[1]		Mascot
1754.9647	1754.9247	-0.04	-23	403	419	DSRIIPGAAATEIELAK					Mascot
1772.9177	1772.9491	0.0314	18	253	268	GTVLIHSAEQLENYAK					Mascot
1779.9487	1780.0184	0.0697	39	235	252	VKVAVFAGGVDTSATET K					Mascot
1804.9222	1804.9391	0.0169	9	33	49	ELSAITRTSLGPNGMNK			Oxidation (M)[15]		Mascot
1812.9379	1813.017	0.0791	44	423	438	EFSLKETGLDQYAIAK					Mascot
1839.027	1839.0522	0.0252	14	205	222	LVGGGLHNSSVVRGMVL K			Oxidation (M)[15]		Mascot

4 unnamed protein product [Triticum aestivum] gi|295422633 26435.8 6.96 10 47 7.608 8.769

Peptide Information

Calc. Mass	Obsrv. Mass	± da	± ppm	Start Seq.	End Seq.	Sequence	Ion Score	C. I. %	Modification	Rank	Result Type
808.3869	808.39	0.0031	4	77	83	AKEAMDK			Oxidation (M)[5]		Mascot
819.428	819.3775	-0.0505	-62	124	130	LDEMLAK					Mascot
860.4724	860.4125	-0.0599	-70	190	197	DDTAVVLK					Mascot
1021.5465	1021.5485	0.002	2	108	116	GFILDGFPR					Mascot
1037.6354	1037.5526	-0.0828	-80	31	41	IILVGPPGSGK					Mascot
1175.6208	1175.6857	0.0649	55	164	173	SYHTKFAPPK					Mascot
1555.7765	1555.9265	0.15	96	156	168	WIHPSSGRSYHTK					Mascot
1862.111	1861.9623	-0.1487	-80	31	49	IILVGPPGSGKGTQSPLIK					Mascot
1862.111	1861.9623	-0.1487	-80	31	49	IILVGPPGSGKGTQSPLIK					Mascot
1869.7928	1869.9614	0.1686	90	50	64	DEYCLCHLATGDMLR			Carbamidomethyl (C)[4,6], Oxidation (M)[13]		Mascot
2045.1027	2045.1516	0.0489	24	138	155	VLNFAIDDAILEERITGR					Mascot

5 ABC transporter C family member 2 [Triticum urartu] gi|474339813 183903.2 6.43 24 43 0 14.311

Peptide Information

Calc. Mass	Obsrv. Mass	± da	± ppm	Start Seq.	End Seq.	Sequence	Ion Score	C. I. %	Modification	Rank	Result Type
803.3893	803.4114	0.0221	28	1028	1034	DLGDIDR					Mascot
804.4727	804.4014	-0.0713	-89	54	59	IWRTTK					Mascot
808.4312	808.39	-0.0412	-51	60	65	DYKVQR					Mascot
811.442	811.4166	-0.0254	-31	791	796	EELRHK					Mascot
820.441	820.3961	-0.0449	-55	904	911	ETGVVSTK					Mascot
832.4424	832.3828	-0.0596	-72	1516	1522	KWAASNR					Mascot
833.4475	833.3818	-0.0657	-79	390	396	LTNDSRK					Mascot
844.4424	844.3815	-0.0609	-72	234	240	HANIFSR					Mascot
847.4567	847.4274	-0.0293	-35	752	758	QRVSMAR					Mascot
849.3883	849.4266	0.0383	45	1130	1136	SMDNNIR					Mascot
906.4713	906.4583	-0.013	-14	840	847	KLMENAGK			Oxidation (M)[3]		Mascot
1001.5244	1001.5838	0.0594	59	306	313	FWLGGFFK					Mascot
1037.5449	1037.5526	0.0077	7	783	790	QVFDKCIK			Carbamidomethyl (C)[6]		Mascot
1054.535	1054.5491	0.0141	13	1288	1296	SSMLNALFR			Oxidation (M)[3]		Mascot
1106.6932	1106.589	-0.1042	-94	814	823	ILLIHDGVVK					Mascot
1165.5848	1165.6744	0.0896	77	1025	1034	FSKDLGDIDR					Mascot
1168.578	1168.6005	0.0225	19	1137	1146	FTLVNMSSNR					Mascot
1168.578	1168.6005	0.0225	19	1137	1146	FTLVNMSSNR					Mascot
1184.5729	1184.6152	0.0423	36	1137	1146	FTLVNMSSNR			Oxidation (M)[6]		Mascot
1232.682	1232.6945	0.0125	10	156	166	AAMFNVVLPVR			Oxidation (M)[3]		Mascot
1571.9004	1571.9174	0.017	11	1557	1571	TKDAVITLQGVLEGK					Mascot
1571.9004	1571.9174	0.017	11	1557	1571	TKDAVITLQGVLEGK					Mascot
1755.0356	1754.9247	-0.1109	-63	142	155	WYIRFVVIYVLVGK					Mascot
1804.9659	1804.9391	-0.0268	-15	481	496	RISLMNEILAAMDTVK					Mascot
1992.8723	1993.0671	0.1948	98	1614	1630	QPGYSFENHGSIDWDQI					Mascot
2044.9546	2045.1516	0.197	96	497	512	CYAWEQSFQSKVQDIR			Carbamidomethyl (C)[1]		Mascot
2067.0215	2067.1704	0.1489	72	1305	1321	ILIDDCDTSKFGIWDLR			Carbamidomethyl (C)[6]		Mascot

6 Myosin-J heavy chain [Triticum urartu] gi|474114531 236907.2 6.35 29 42 0 13.448

Peptide Information

Calc. Mass	Obsrv. Mass	± da	± ppm	Start Seq.	End Seq.	Sequence	Ion Score	C. I. %	Modification	Rank	Result Type

800.4875	800.411	-0.0765	-96	390	396	LENALIK					Mascot
803.3893	803.4114	0.0221	28	1082	1089	QVADADGK					Mascot
804.4097	804.4014	-0.0083	-10	1116	1122	QESEAIK					Mascot
814.4053	814.4435	0.0382	47	1396	1402	QTHEATK					Mascot
833.4363	833.3818	-0.0545	-65	1418	1424	KIQDSDK					Mascot
834.4203	834.3803	-0.04	-48	361	368	DADSSVLK					Mascot
845.4727	845.4466	-0.0261	-31	1676	1682	NDDLIKK					Mascot
860.4737	860.4125	-0.0612	-71	1957	1962	TWREIR					Mascot
864.4574	864.4405	-0.0169	-20	1823	1830	TAASVPYR					Mascot
874.4265	874.3839	-0.0426	-49	1669	1675	NEDLLDR					Mascot
975.5655	975.5757	0.0102	10	926	933	LGLEMKLR			Oxidation (M)[5]		Mascot
1001.5625	1001.5838	0.0213	21	1371	1378	KQLEENIK					Mascot
1023.4966	1023.5446	0.048	47	231	239	NNNSSRFGK					Mascot
1024.488	1024.5574	0.0694	68	683	691	ISCAGYPTR			Carbamidomethyl (C)[3]		Mascot
1037.5739	1037.5526	-0.0213	-21	258	265	TYLLERSR					Mascot
1077.4994	1077.5876	0.0882	82	742	751	AGQMAELDAR			Oxidation (M)[4]		Mascot
1077.4994	1077.5876	0.0882	82	742	751	AGQMAELDAR			Oxidation (M)[4]		Mascot
1165.6113	1165.6744	0.0631	54	767	775	YHTYVARQK					Mascot
1175.6453	1175.6857	0.0404	34	1257	1266	SINKLMENVK					Mascot
1320.6583	1320.694	0.0357	27	21	31	DLAWIDGEVFR					Mascot
1571.8387	1571.9174	0.0787	50	2051	2064	ENQSLALILQRGTE					Mascot
1571.8387	1571.9174	0.0787	50	2051	2064	ENQSLALILQRGTE					Mascot
1679.8851	1679.9415	0.0564	34	1638	1652	LSSFVLEKQESDAVK					Mascot
1753.908	1753.9495	0.0415	24	1212	1227	LEETAATRDALHVAEK					Mascot
1769.9944	1770.0162	0.0218	12	46	60	TFGIMIYIDMLLVLK					Mascot
1772.896	1772.9491	0.0531	30	1358	1371	ELVNELQNCQEIRK			Carbamidomethyl (C)[9]		Mascot
1805.0167	1804.9391	-0.0776	-43	1630	1645	LQVDAISRLSSFVLEK					Mascot
1838.8879	1839.0522	0.1643	89	1195	1210	FTDANRTNDTLQDSLK					Mascot
1844.8484	1844.9467	0.0983	53	465	479	TNSFEQLCINFTNEK			Carbamidomethyl (C)[8]		Mascot
1844.8484	1844.9467	0.0983	53	465	479	TNSFEQLCINFTNEK			Carbamidomethyl (C)[8]		Mascot
1846.8851	1846.9449	0.0598	32	1020	1035	CEDLNGKIEVADENIK			Carbamidomethyl (C)[1]		Mascot
1859.9758	1859.9629	-0.0129	-7	521	537	KPGGIIALLDEACMFPK			Carbamidomethyl (C)[13]		Mascot

7 hypothetical protein TRIUR3_02653 [Triticum urartu] gi|473895929 88455.4 5.43 18 42 0 11.647

Peptide Information

Calc. Mass	Obsrv. Mass	± da	± ppm	Start Seq.	End Seq.	Sequence	Ion Score	C. I. %	Modification	Rank	Result Type

804.3846	804.4014	0.0168	21	639	645	EADDRAK					Mascot
830.4618	830.4372	-0.0246	-30	12	18	VEELNVK					Mascot
832.441	832.3828	-0.0582	-70	483	489	ADLEKEK					Mascot
874.4702	874.3839	-0.0863	-99	532	538	MPIELQK			Oxidation (M)[1]		Mascot
906.4349	906.4583	0.0234	26	455	461	KELDDMR					Mascot
914.5054	914.4559	-0.0495	-54	264	271	EIQQGIAR					Mascot
974.5516	974.564	0.0124	13	490	498	ADLTALKDK					Mascot
1021.4982	1021.5485	0.0503	49	698	705	LTMENELR			Oxidation (M)[3]		Mascot
1077.58	1077.5876	0.0076	7	308	316	EHTALITHR					Mascot
1077.58	1077.5876	0.0076	7	308	316	EHTALITHR					Mascot
1179.6256	1179.6975	0.0719	61	213	222	FIAEEVEKSK					Mascot
1182.6841	1182.6459	-0.0382	-32	665	674	SLDKLHQLTK					Mascot
1196.6127	1196.637	0.0243	20	440	449	VRSMVSVQMK			Oxidation (M)[4,9]		Mascot
1320.6941	1320.694	-0.0001	0	221	231	SKVLQELCSTR			Carbamidomethyl (C)[8]		Mascot
1716.9027	1716.985	0.0823	48	149	163	AAEIAERFIQALDNR					Mascot
1754.8014	1754.9247	0.1233	70	102	116	NFSEDIGSLTINECR			Carbamidomethyl (C)[14]		Mascot
1770.8076	1770.9562	0.1486	84	117	131	ANKVEENCHDQLEGK			Carbamidomethyl (C)[8]		Mascot
1770.8076	1770.9562	0.1486	84	117	131	ANKVEENCHDQLEGK			Carbamidomethyl (C)[8]		Mascot
1791.9348	1791.9139	-0.0209	-12	499	514	VHHATVSVSSLQEELR					Mascot
1844.9786	1844.9467	-0.0319	-17	532	547	MPIELQKATQETQLAK			Oxidation (M)[1]		Mascot
1844.9786	1844.9467	-0.0319	-17	532	547	MPIELQKATQETQLAK			Oxidation (M)[1]		Mascot

8 unnamed protein product [Triticum aestivum] gi|295422149 26509.8 6.54 9 40 0 8.448

Peptide Information

Calc. Mass	Obsrv. Mass	± da	± ppm	Start Seq.	End Seq.	Sequence	Ion Score	C. I. %	Modification	Rank	Result Type
808.3869	808.39	0.0031	4	77	83	AKEAMDK			Oxidation (M)[5]		Mascot
819.428	819.3775	-0.0505	-62	124	130	LDEMLAK					Mascot
1021.5465	1021.5485	0.002	2	108	116	GFILDGFPR					Mascot
1037.6354	1037.5526	-0.0828	-80	31	41	IILVGPPGSGK					Mascot
1175.6208	1175.6857	0.0649	55	164	173	SYHTKFAPPK					Mascot
1555.7765	1555.9265	0.15	96	156	168	WIHPSSGRSYHTK					Mascot
1862.111	1861.9623	-0.1487	-80	31	49	IILVGPPGSGKGTQSPLIK					Mascot
1862.111	1861.9623	-0.1487	-80	31	49	IILVGPPGSGKGTQSPLIK					Mascot
1869.7928	1869.9614	0.1686	90	50	64	DEYCLCHLATGDMLR			Carbamidomethyl (C)[4,6], Oxidation (M)[13]		Mascot
2045.1027	2045.1516	0.0489	24	138	155	VLNFAIDDAILEERITGR					Mascot

9 Aspartyl/glutamyl-tRNA(Asn/Gln) amidotransferase subunit B [Triticum urartu] gi|473895162 60940.2 5.84 12 40 0 9.171

Peptide Information

Calc. Mass	Obsrv. Mass	± da	± ppm	Start Seq.	End Seq.	Sequence	Ion Score	C. I. %	Modification	Rank	Result Type
804.4284	804.4014	-0.027	-34	119	125	VVECAVK			Carbamidomethyl (C)[4]		Mascot
817.4665	817.4033	-0.0632	-77	428	434	LSIDEIK					Mascot
836.4261	836.3975	-0.0286	-34	503	508	QLEQYR					Mascot
974.5629	974.564	0.0011	1	449	458	NGTISGKIGK					Mascot
1165.6589	1165.6744	0.0155	13	27	36	RPPLAHFTAR					Mascot
1184.5365	1184.6152	0.0787	66	283	292	NMNSFSEISR					Mascot
1445.7595	1445.7733	0.0138	10	37	49	VESVQTSEPKSVR					Mascot
1641.8517	1641.9644	0.1127	69	214	228	AGVPLLEIVSEPDMR			Oxidation (M)[14]		Mascot
1679.8826	1679.9415	0.0589	35	410	424	LAANWIMGDITAYLK					Mascot
1861.9767	1861.9623	-0.0144	-8	229	245	TGIEAAEYGAEIQRVVR					Mascot
1861.9767	1861.9623	-0.0144	-8	229	245	TGIEAAEYGAEIQRVVR					Mascot
1992.9808	1993.0671	0.0863	43	261	278	CDVNVSVRPIGQSEFGTK			Carbamidomethyl (C)[1]		Mascot
2067.0579	2067.1704	0.1125	54	410	427	LAANWIMGDITAYLKNEK			Oxidation (M)[7]		Mascot

10 hypothetical protein TRIUR3_09010 [Triticum urartu] gi|474096028 32996.2 9.94 10 38 0 5.827

Peptide Information

Calc. Mass	Obsrv. Mass	± da	± ppm	Start Seq.	End Seq.	Sequence	Ion Score	C. I. %	Modification	Rank	Result Type
811.3832	811.4166	0.0334	41	273	278	YEDEKK					Mascot
830.473	830.4372	-0.0358	-43	236	242	EVALDRK					Mascot
974.4902	974.564	0.0738	76	110	119	KPGGTGSDQK					Mascot
1023.6057	1023.5446	-0.0611	-60	102	109	RPLKPEQR					Mascot
1175.595	1175.6857	0.0907	77	260	271	IAGGARSMAGER					Mascot
1182.6841	1182.6459	-0.0382	-32	54	64	QKASPVQLPSK					Mascot
1753.8538	1753.9495	0.0957	55	246	259	QLAEYNQEMTRINK			Oxidation (M)[9]		Mascot
1772.9728	1772.9491	-0.0237	-13	120	136	VPLPLPPKMHDSSVGAK					Mascot
1816.0327	1815.9718	-0.0609	-34	110	127	KPGGTGSDQKVPLPLPPK					Mascot
1816.0327	1815.9718	-0.0609	-34	110	127	KPGGTGSDQKVPLPLPPK					Mascot
1926.9515	1926.9982	0.0467	24	19	37	DKGGEASTILPQENSAPGR					Mascot
