# Supplementary material for: Proteomic analysis of middle and late stages of bread wheat (Triticum aestivum L.) grain development
Source: Front Plant Sci. 2015 Sep 15;6:735. doi: 10.3389/fpls.2015.00735 (PMC4569854; doi:10.3389/fpls.2015.00735)
Supplement: Supplementary file 3 [file DataSheet2.PDF]

Analysis Information

Report Type	Protein-Peptide Summary by Spot	Analysis Type	Combined (MS+MS/MS)
Sample Set Name	Sample set_20140814	Database	triticum
Analysis Name	R14026-4-T1	Creation Date	09/29/2014 15:04:02
Reported By	09/30/2014 14:56:43 - admin	Last Modified	09/29/2014 15:17:02

MS Acq. : Proc. Methods	(Unspecified) : (Unspecified)
Interpretation Method	(Unspecified)

**Gel Idx/Pos**	151/G2	**Instr./Gel Origin**	BA2151/Sample Project 20140814	**Process Status**	Analysis Succeeded
**Plate [#] Name**	[1] Sample Project 20140814	**Instrument Sample Name**		**Spectra**	11

Rank	Protein Name	Accession No.	Protein MW	Protein PI	Pep. Count	Protein Score	Protein Score C. I. %	Intensity Matched	Total Ion Score	Total Ion C. I. %	Confirmed
1	dimeric alpha-amylase inhibitor [Triticum dicoccoides]	gi|114215794	13752.5	5.23	6	308	100	28.322	268	100	

Protein Group

0.19 dimeric alpha-amylase inhibitor [Triticum aestivum]	gi|56480630	13752.5	5.2300000190 7349
dimeric alpha-amylase inhibitor [Triticum dicoccoides]	gi|114215792	13752.5	5.2300000190 7349
dimeric alpha-amylase inhibitor [Triticum dicoccoides]	gi|114215788	13752.5	5.2300000190 7349
dimeric alpha-amylase inhibitor [Triticum dicoccoides]	gi|114215796	13752.5	5.2300000190 7349
dimeric alpha-amylase inhibitor [Triticum dicoccoides]	gi|114215770	13752.5	5.2300000190 7349
dimeric alpha-amylase inhibitor precursor [Triticum aestivum]	gi|108597903	13823.5	5.2300000190 7349

Peptide Information

Calc. Mass	Obsrv. Mass	± da	± ppm	Start Seq.	End Seq.	Sequence	Ion Score	C. I. %	Modification	Rank	Result Type
1162.6249	1162.6165	-0.0084	-7	90	100	LTAASITAVCR			Carbamidomethyl (C)[10]		Mascot
1162.6249	1162.6165	-0.0084	-7	90	100	LTAASITAVCR	49	99.91	Carbamidomethyl (C)[10]		Mascot
1554.6637	1554.7523	0.0886	57	54	66	CGALYSMLDSMYK			Carbamidomethyl (C)[1], Oxidation (M)[7]		Mascot

Calc. Mass	Obsrv. Mass	± da	± ppm	Start Seq.	End Seq.	Sequence	Ion Score	C. I. %	Modification	Rank	Result Type
1570.8007	1570.7888	-0.0119	-8	26	39	LQCNGSQVPEAVLR			Carbamidomethyl (C)[3]		Mascot
1663.8361	1663.7917	-0.0444	-27	101	116	LPIVVDASGDGAYVCK			Carbamidomethyl (C)[15]		Mascot
1663.8361	1663.7917	-0.0444	-27	101	116	LPIVVDASGDGAYVCK	86	100	Carbamidomethyl (C)[15]		Mascot
1840.7412	1840.7278	-0.0134	-7	40	53	DCCQQLADISEWCR			Carbamidomethyl (C)[2,3,13]		Mascot
1846.8137	1846.8	-0.0137	-7	67	84	EHGVSEGGQAGTGAFPSCR			Carbamidomethyl (C)[17]		Mascot
1846.8137	1846.8	-0.0137	-7	67	84	EHGVSEGGQAGTGAFPSCR	134	100	Carbamidomethyl (C)[17]		Mascot

2	dimeric alpha-amylase inhibitor [Triticum dicoccoides]	gi|227809146	15605.4	5.3	6	306	100	22.024	268	100	

Peptide Information

Calc. Mass	Obsrv. Mass	± da	± ppm	Start Seq.	End Seq.	Sequence	Ion Score	C. I. %	Modification	Rank	Result Type
1162.6249	1162.6165	-0.0084	-7	107	117	LTAASITAVCR			Carbamidomethyl (C)[10]		Mascot
1162.6249	1162.6165	-0.0084	-7	107	117	LTAASITAVCR	49	99.91	Carbamidomethyl (C)[10]		Mascot
1570.8007	1570.7888	-0.0119	-8	43	56	LQCNGSQVPEAVLR			Carbamidomethyl (C)[3]		Mascot
1663.8361	1663.7917	-0.0444	-27	118	133	LPIVVDASGDGAYVCK			Carbamidomethyl (C)[15]		Mascot
1663.8361	1663.7917	-0.0444	-27	118	133	LPIVVDASGDGAYVCK	86	100	Carbamidomethyl (C)[15]		Mascot
1810.7629	1810.7822	0.0193	11	57	70	DCCQQLADISERCR			Carbamidomethyl (C)[2,3,13]		Mascot
1846.8137	1846.8	-0.0137	-7	84	101	EHGVSEGGQAGTGAFPSCR			Carbamidomethyl (C)[17]		Mascot
1846.8137	1846.8	-0.0137	-7	84	101	EHGVSEGGQAGTGAFPSCR	134	100	Carbamidomethyl (C)[17]		Mascot
1884.8112	1884.7338	-0.0774	-41	69	83	CRCSALYSMLDSMYK			Carbamidomethyl (C)[1,3]		Mascot
1900.806	1900.759	-0.047	-25	69	83	CRCSALYSMLDSMYK			Carbamidomethyl (C)[1,3], Oxidation (M)[9]		Mascot

3	dimeric alpha-amylase inhibitor [Triticum dicoccoides]	gi|227809234	15606.4	4.99	6	305	100	28.322	268	100	

Protein Group

dimeric alpha-amylase inhibitor [Triticum dicoccoides]	gi|227809206	15606.4	4.98999977111816
dimeric alpha-amylase inhibitor [Triticum dicoccoides]	gi|227809202	15606.4	4.98999977111816
dimeric alpha-amylase inhibitor [Triticum dicoccoides]	gi|227809200	15606.4	4.98999977111816
dimeric alpha-amylase inhibitor [Triticum dicoccoides]	gi|227809194	15606.4	4.98999977111816
dimeric alpha-amylase inhibitor [Triticum dicoccoides]	gi|227809190	15606.4	4.98999977111816
dimeric alpha-amylase inhibitor [Triticum dicoccoides]	gi|227809184	15606.4	4.98999977111816

dimeric alpha-amylase inhibitor [Triticum dicoccoides]	gi|227809182	15606.4	4.9899 997711 1816
dimeric alpha-amylase inhibitor [Triticum dicoccoides]	gi|227809176	15606.4	4.9899 997711 1816
dimeric alpha-amylase inhibitor [Triticum dicoccoides]	gi|227809170	15606.4	4.9899 997711 1816
dimeric alpha-amylase inhibitor [Triticum dicoccoides]	gi|227809168	15606.4	4.9899 997711 1816
dimeric alpha-amylase inhibitor [Triticum dicoccoides]	gi|227809166	15606.4	4.9899 997711 1816
dimeric alpha-amylase inhibitor [Triticum dicoccoides]	gi|227809164	15606.4	4.9899 997711 1816
dimeric alpha-amylase inhibitor [Triticum dicoccoides]	gi|227809158	15606.4	4.9899 997711 1816
dimeric alpha-amylase inhibitor [Triticum dicoccoides]	gi|227809152	15606.4	4.9899 997711 1816
dimeric alpha-amylase inhibitor [Triticum dicoccoides]	gi|227809150	15578.3	4.9899 997711 1816
dimeric alpha-amylase inhibitor [Triticum dicoccoides]	gi|227809138	15606.4	4.9899 997711 1816
dimeric alpha-amylase inhibitor [Triticum dicoccoides]	gi|227809134	15606.4	4.9899 997711 1816
dimeric alpha-amylase inhibitor [Triticum dicoccoides]	gi|227809127	15606.4	4.9899 997711 1816
dimeric alpha-amylase inhibitor [Triticum dicoccoides]	gi|227809125	15606.4	4.9899 997711 1816
dimeric alpha-amylase inhibitor [Triticum dicoccoides]	gi|227809123	15606.4	4.9899 997711 1816
dimeric alpha-amylase inhibitor [Triticum dicoccoides]	gi|227809098	15606.4	4.9899 997711 1816
dimeric alpha-amylase inhibitor [Triticum dicoccoides]	gi|227809094	15606.4	4.9899 997711 1816
dimeric alpha-amylase inhibitor [Triticum dicoccoides]	gi|227809088	15606.4	4.9899 997711 1816
dimeric alpha-amylase inhibitor [Triticum dicoccoides]	gi|227809071	15606.4	4.9899 997711 1816
dimeric alpha-amylase inhibitor [Triticum dicoccoides]	gi|227809069	15606.4	4.9899

			997711 1816
dimeric alpha-amylase inhibitor [Triticum dicoccoides]	gi|227809063	15606.4	4.9899 997711 1816
dimeric alpha-amylase inhibitor [Triticum dicoccoides]	gi|227809061	15606.4	4.9899 997711 1816
dimeric alpha-amylase inhibitor [Triticum dicoccoides]	gi|227809059	15606.4	4.9899 997711 1816
dimeric alpha-amylase inhibitor [Triticum dicoccoides]	gi|227809057	15606.4	4.9899 997711 1816
dimeric alpha-amylase inhibitor [Triticum dicoccoides]	gi|227809053	15606.4	4.9899 997711 1816
dimeric alpha-amylase inhibitor [Triticum dicoccoides]	gi|227809051	15606.4	4.9899 997711 1816
dimeric alpha-amylase inhibitor [Triticum dicoccoides]	gi|227808999	15584.4	4.8099 999427 7954
dimeric alpha-amylase inhibitor [Triticum dicoccoides]	gi|227808995	15605.4	5.7100 000381 4697
dimeric alpha-amylase inhibitor [Triticum dicoccoides]	gi|227808987	15606.4	4.9899 997711 1816
dimeric alpha-amylase inhibitor [Triticum dicoccoides]	gi|227808985	15606.4	4.9899 997711 1816
dimeric alpha-amylase inhibitor [Triticum dicoccoides]	gi|227808983	15606.4	4.9899 997711 1816
dimeric alpha-amylase inhibitor [Triticum dicoccoides]	gi|227808974	15592.3	4.9899 997711 1816
dimeric alpha-amylase inhibitor [Triticum dicoccoides]	gi|227808958	15606.4	4.9899 997711 1816
dimeric alpha-amylase inhibitor [Triticum dicoccoides]	gi|227808952	15606.4	4.9899 997711 1816
dimeric alpha-amylase inhibitor [Triticum dicoccoides]	gi|227808944	15606.4	4.9899 997711 1816
dimeric alpha-amylase inhibitor [Triticum dicoccoides]	gi|227809244	15606.4	4.9899 997711 1816

Peptide Information

Calc. Mass	Obsrv. Mass	± da	± ppm	Start Seq.	End Seq.	Sequence	Ion Score	C. I. %	Modification	Rank	Result Type

1162.6249	1162.6165	-0.0084	-7	107	117	LTAASITAVCR			Carbamidomethyl (C)[10]	Mascot
1162.6249	1162.6165	-0.0084	-7	107	117	LTAASITAVCR	49	99.91	Carbamidomethyl (C)[10]	Mascot
1554.6637	1554.7523	0.0886	57	71	83	CGALYSMLDSMYK			Carbamidomethyl (C)[1], Oxidation (M)[7]	Mascot
1570.8007	1570.7888	-0.0119	-8	43	56	LQCNGSQVPEAVLR			Carbamidomethyl (C)[3]	Mascot
1663.8361	1663.7917	-0.0444	-27	118	133	LPIVVDASGDGAYVCK			Carbamidomethyl (C)[15]	Mascot
1663.8361	1663.7917	-0.0444	-27	118	133	LPIVVDASGDGAYVCK	86	100	Carbamidomethyl (C)[15]	Mascot
1840.7412	1840.7278	-0.0134	-7	57	70	DCCQQLADISEWCR			Carbamidomethyl (C)[2,3,13]	Mascot
1846.8137	1846.8	-0.0137	-7	84	101	EHGVSEGGQAGTGAFPSC R			Carbamidomethyl (C)[17]	Mascot
1846.8137	1846.8	-0.0137	-7	84	101	EHGVSEGGQAGTGAFPSC R	134	100	Carbamidomethyl (C)[17]	Mascot

4	dimeric alpha-amylase inhibitor [Triticum timopheevii subsp. armeniacum]	gi|227809288	15533.4	5.7	3	0	268	100

Protein Group

RecName: Full=Alpha-amylase inhibitor 0.53	gi|123968	13689.5	5.2300 000190 7349
dimeric alpha-amylase inhibitor [Triticum aestivum]	gi|65993898	15533.4	5.6999 998092 6514
dimeric alpha-amylase inhibitor [Triticum aestivum]	gi|65993731	15606.4	4.9899 997711 1816
dimeric alpha-amylase inhibitor [Triticum aestivum]	gi|65993709	15578.3	4.9899 997711 1816
dimeric alpha-amylase inhibitor [Triticum dicoccoides]	gi|227808966	15664.4	4.8099 999427 7954
dimeric alpha-amylase inhibitor [Triticum dicoccoides]	gi|227808934	15606.4	4.9899 997711 1816
dimeric alpha-amylase inhibitor [Triticum dicoccoides]	gi|227808928	15605.4	5.0100 002288 8184
dimeric alpha-amylase inhibitor [Triticum dicoccoides]	gi|227808922	15606.4	4.9899 997711 1816
dimeric alpha-amylase inhibitor [Triticum dicoccoides]	gi|227808920	15606.4	4.9899 997711 1816
dimeric alpha-amylase inhibitor [Triticum dicoccoides]	gi|227808914	15606.4	4.9899 997711 1816
dimeric alpha-amylase inhibitor [Triticum dicoccoides]	gi|227808912	15638.3	4.9899 997711 1816
dimeric alpha-amylase inhibitor [Triticum dicoccoides]	gi|227808900	15578.3	4.9899 997711 1816

dimeric alpha-amylase inhibitor [Triticum dicoccoides]	gi|227808898	15606.4	4.9899 997711 1816
dimeric alpha-amylase inhibitor [Triticum dicoccoides]	gi|227808869	15606.4	4.9899 997711 1816
dimeric alpha-amylase inhibitor [Triticum dicoccoides]	gi|227808867	15578.3	4.9899 997711 1816
dimeric alpha-amylase inhibitor [Triticum dicoccoides]	gi|227808861	15658.4	5.5799 999237 0605
dimeric alpha-amylase inhibitor [Triticum dicoccoides]	gi|227808853	15618.3	4.9899 997711 1816
dimeric alpha-amylase inhibitor [Triticum dicoccoides]	gi|227808851	15658.4	5.5799 999237 0605

Peptide Information

Calc. Mass	Obsrv. Mass	± da	± ppm	Start Seq.	End Seq.	Sequence	Ion Score	C. I. %	Modification	Rank	Result Type
1162.6249	1162.6165	-0.0084	-7	107	117	LTAASITAVCR	49	99.91	Carbamidomethyl (C)[10]	1	Mascot
1663.8361	1663.7917	-0.0444	-27	118	133	LPIVVDASGDGAYVCK	86	100	Carbamidomethyl (C)[15]	1	Mascot
1846.8137	1846.8	-0.0137	-7	84	101	EHGVSEGQAGTGAFPSC R	134	100	Carbamidomethyl (C)[17]	1	Mascot

5	dimeric alpha-amylase inhibitor [Triticum dicoccoides]	gi|227809121	15555.3	4.99	2	0	135	100

Peptide Information

Calc. Mass	Obsrv. Mass	± da	± ppm	Start Seq.	End Seq.	Sequence	Ion Score	C. I. %	Modification	Rank	Result Type
1162.6249	1162.6165	-0.0084	-7	107	117	LTAASITAVXR	49	99.91		1	Mascot
1663.8361	1663.7917	-0.0444	-27	118	133	LPIVVDASGDGAYVCK	86	100	Carbamidomethyl (C)[15]	1	Mascot

6	alpha amylase inhibitor CM3 [Triticum durum]	gi|39578552	18893.3	7.44	1	0	18	0

Protein Group

CM3 protein [Triticum durum]	gi|21922	18893.3	7.4400 000572 2046
RecName: Full=Alpha-amylase/trypsin inhibitor CM3; AltName: Full=Chloroform/methanol-soluble protein CM3; Flags: Precursor	gi|123957	18893.3	7.4400 000572 2046
alpha amylase inhibitor protein [Triticum aestivum]	gi|38098487	18893.3	7.4400 000572 2046
alpha-amylase inhibitor, tetrameric, chain CM3 precursor - durum wheat	gi|100834	18893.3	7.4400 000572 2046
unnamed protein product [Triticum aestivum]	gi|21713	18893.3	7.4400

	unnamed protein product [Triticum durum]	gi|57997836	18893.3	000572 2046 7.4400 000572 2046

Peptide Information

Calc. Mass	Obsrv. Mass	± da	± ppm	Start Seq.	End Seq.	Sequence	Ion Score	C. I. %	Modification	Rank	Result Type
1698.9214	1698.8981	-0.0233	-14	101	115	YFIALPVPSQPVDPR	18	0		1	Mascot

7	rpl16 [Triticum aestivum]	gi|291498607	21419.2	10.88	1	0	18	0	

Protein Group

rpl16 [Triticum aestivum]	gi|169649057	21419.2	10.880 000114 4409
rpl16 [Triticum aestivum]	gi|78675244	21419.2	10.880 000114 4409
rpl16 [Triticum aestivum]	gi|81176520	21419.2	10.880 000114 4409

Peptide Information

Calc. Mass	Obsrv. Mass	± da	± ppm	Start Seq.	End Seq.	Sequence	Ion Score	C. I. %	Modification	Rank	Result Type
1846.9269	1846.8	-0.1269	-69	170	185	LAAHKPCSSTKFVQWS	18	0	Carbamidomethyl (C)[7]	2	Mascot

8	hypothetical protein TRIUR3_13124 [Triticum urartu]	gi|473996456	42874.5	4.93	1	0	16	0

Peptide Information

Calc. Mass	Obsrv. Mass	± da	± ppm	Start Seq.	End Seq.	Sequence	Ion Score	C. I. %	Modification	Rank	Result Type
1699.049	1698.8981	-0.1509	-89	41	56	AAVRPTPRLLLPAAPR	16	0		2	Mascot

9	Disease resistance protein RPP13 [Triticum urartu]	gi|473807091	93807.1	6	1	0	14	0

Protein Group

CNL2 [Triticum monococcum subsp. monococcum]	gi|521311581	106129.4	5.8499 999046 3257

Peptide Information

Calc. Mass	Obsrv. Mass	± da	± ppm	Start Seq.	End Seq.	Sequence	Ion Score	C. I. %	Modification	Rank	Result Type
1781.8739	1781.7147	-0.1592	-89	320	334	DDIYKMKPLSDDVSR	14	0		1	Mascot

10	Disease resistance protein RGA2 [Triticum urartu]	gi|474359601	100106.7	9.33	1	0	13	0

Peptide Information

Calc. Mass	Obsrv. Mass	± da	± ppm	Start Seq.	End Seq.	Sequence	Ion Score	C. I. %	Modification	Rank	Result Type
1663.9014	1663.7917	-0.1097	-66	31	46	TIPIPDDASAPALLNR	13	0		2	Mascot

Gel Idx/Pos	152/G3	Instr./Gel Origin	BA2151/Sample Project 20140814	Process Status	Analysis Succeeded
Plate [#] Name	[1] Sample Project 20140814	Instrument Sample Name		Spectra	11

Rank	Protein Name	Accession No.	Protein MW	Protein PI	Pep. Count	Protein Score	Protein Score C. I. %	Intensity Matched	Total Ion Score	Total Ion C. I. %	Confirmed
1	dimeric alpha-amylase inhibitor [Triticum dicoccoides]	gi|114215794	13752.5	5.23	7	430	100	38.859	378	100	

Protein Group

0.19 dimeric alpha-amylase inhibitor [Triticum aestivum]	gi|56480630	13752.5	5.2300 000190 7349
dimeric alpha-amylase inhibitor [Triticum dicoccoides]	gi|114215792	13752.5	5.2300 000190 7349
dimeric alpha-amylase inhibitor [Triticum dicoccoides]	gi|114215788	13752.5	5.2300 000190 7349
dimeric alpha-amylase inhibitor [Triticum dicoccoides]	gi|114215796	13752.5	5.2300 000190 7349
dimeric alpha-amylase inhibitor [Triticum dicoccoides]	gi|114215770	13752.5	5.2300 000190 7349
dimeric alpha-amylase inhibitor precursor [Triticum aestivum]	gi|108597903	13823.5	5.2300 000190 7349

Peptide Information

Calc. Mass	Obsrv. Mass	± da	± ppm	Start Seq.	End Seq.	Sequence	Ion Score	C. I. %	Modification	Rank	Result Type
1162.6249	1162.6138	-0.0111	-10	90	100	LTAASITAVCR			Carbamidomethyl (C)[10]		Mascot
1162.6249	1162.6138	-0.0111	-10	90	100	LTAASITAVCR	70	100	Carbamidomethyl (C)[10]		Mascot
1554.6637	1554.6335	-0.0302	-19	54	66	CGALYSMLDSMYK			Carbamidomethyl (C)[1], Oxidation (M)[7]		Mascot
1570.8007	1570.7839	-0.0168	-11	26	39	LQCNGSQVPEAVLR			Carbamidomethyl (C)[3]		Mascot
1663.8361	1663.7701	-0.066	-40	101	116	LPIVVDASGDGAYVCK			Carbamidomethyl (C)[15]		Mascot
1663.8361	1663.7701	-0.066	-40	101	116	LPIVVDASGDGAYVCK	141	100	Carbamidomethyl (C)[15]		Mascot
1840.7412	1840.724	-0.0172	-9	40	53	DCCQQLADISEWCR			Carbamidomethyl (C)[2,3,13]		Mascot
1846.8137	1846.7911	-0.0226	-12	67	84	EHGVSEGGQAGTGAFPSC R			Carbamidomethyl (C)[17]		Mascot
1846.8137	1846.7911	-0.0226	-12	67	84	EHGVSEGGQAGTGAFPSC R	167	100	Carbamidomethyl (C)[17]		Mascot
2807.4431	2807.3105	-0.1326	-47	90	116	LTAASITAVCRLPIVVDAS GDGAYVCK			Carbamidomethyl (C)[10,26]		Mascot

Rank	Protein Name	Accession No.	Protein MW	Protein PI	Pep. Count	Protein Score	Protein Score C. I. %	Intensity Matched	Total Ion Score	Total Ion C. I. %	Confirmed
2	dimeric alpha-amylase inhibitor [Triticum dicoccoides]	gi|227809234	15606.4	4.99	7	426	100	38.859	378	100	

Protein Group

dimeric alpha-amylase inhibitor [Triticum dicoccoides]	gi|227809206	15606.4	4.9899

			997711 1816
dimeric alpha-amylase inhibitor [Triticum dicoccoides]	gi|227809202	15606.4	4.9899 997711 1816
dimeric alpha-amylase inhibitor [Triticum dicoccoides]	gi|227809200	15606.4	4.9899 997711 1816
dimeric alpha-amylase inhibitor [Triticum dicoccoides]	gi|227809194	15606.4	4.9899 997711 1816
dimeric alpha-amylase inhibitor [Triticum dicoccoides]	gi|227809190	15606.4	4.9899 997711 1816
dimeric alpha-amylase inhibitor [Triticum dicoccoides]	gi|227809184	15606.4	4.9899 997711 1816
dimeric alpha-amylase inhibitor [Triticum dicoccoides]	gi|227809182	15606.4	4.9899 997711 1816
dimeric alpha-amylase inhibitor [Triticum dicoccoides]	gi|227809176	15606.4	4.9899 997711 1816
dimeric alpha-amylase inhibitor [Triticum dicoccoides]	gi|227809170	15606.4	4.9899 997711 1816
dimeric alpha-amylase inhibitor [Triticum dicoccoides]	gi|227809168	15606.4	4.9899 997711 1816
dimeric alpha-amylase inhibitor [Triticum dicoccoides]	gi|227809166	15606.4	4.9899 997711 1816
dimeric alpha-amylase inhibitor [Triticum dicoccoides]	gi|227809164	15606.4	4.9899 997711 1816
dimeric alpha-amylase inhibitor [Triticum dicoccoides]	gi|227809158	15606.4	4.9899 997711 1816
dimeric alpha-amylase inhibitor [Triticum dicoccoides]	gi|227809152	15606.4	4.9899 997711 1816
dimeric alpha-amylase inhibitor [Triticum dicoccoides]	gi|227809150	15578.3	4.9899 997711 1816
dimeric alpha-amylase inhibitor [Triticum dicoccoides]	gi|227809138	15606.4	4.9899 997711 1816
dimeric alpha-amylase inhibitor [Triticum dicoccoides]	gi|227809134	15606.4	4.9899 997711 1816
dimeric alpha-amylase inhibitor [Triticum dicoccoides]	gi|227809127	15606.4	4.9899 997711 1816
dimeric alpha-amylase inhibitor [Triticum dicoccoides]	gi|227809125	15606.4	4.9899 997711

			1816
dimeric alpha-amylase inhibitor [Triticum dicoccoides]	gi|227809123	15606.4	4.9899<br>997711<br>1816
dimeric alpha-amylase inhibitor [Triticum dicoccoides]	gi|227809098	15606.4	4.9899<br>997711<br>1816
dimeric alpha-amylase inhibitor [Triticum dicoccoides]	gi|227809094	15606.4	4.9899<br>997711<br>1816
dimeric alpha-amylase inhibitor [Triticum dicoccoides]	gi|227809088	15606.4	4.9899<br>997711<br>1816
dimeric alpha-amylase inhibitor [Triticum dicoccoides]	gi|227809071	15606.4	4.9899<br>997711<br>1816
dimeric alpha-amylase inhibitor [Triticum dicoccoides]	gi|227809069	15606.4	4.9899<br>997711<br>1816
dimeric alpha-amylase inhibitor [Triticum dicoccoides]	gi|227809063	15606.4	4.9899<br>997711<br>1816
dimeric alpha-amylase inhibitor [Triticum dicoccoides]	gi|227809061	15606.4	4.9899<br>997711<br>1816
dimeric alpha-amylase inhibitor [Triticum dicoccoides]	gi|227809059	15606.4	4.9899<br>997711<br>1816
dimeric alpha-amylase inhibitor [Triticum dicoccoides]	gi|227809057	15606.4	4.9899<br>997711<br>1816
dimeric alpha-amylase inhibitor [Triticum dicoccoides]	gi|227809053	15606.4	4.9899<br>997711<br>1816
dimeric alpha-amylase inhibitor [Triticum dicoccoides]	gi|227809051	15606.4	4.9899<br>997711<br>1816
dimeric alpha-amylase inhibitor [Triticum dicoccoides]	gi|227808999	15584.4	4.8099<br>999427<br>7954
dimeric alpha-amylase inhibitor [Triticum dicoccoides]	gi|227808995	15605.4	5.7100<br>000381<br>4697
dimeric alpha-amylase inhibitor [Triticum dicoccoides]	gi|227808987	15606.4	4.9899<br>997711<br>1816
dimeric alpha-amylase inhibitor [Triticum dicoccoides]	gi|227808985	15606.4	4.9899<br>997711<br>1816
dimeric alpha-amylase inhibitor [Triticum dicoccoides]	gi|227808983	15606.4	4.9899<br>997711<br>1816
dimeric alpha-amylase inhibitor [Triticum dicoccoides]	gi|227808974	15592.3	4.9899<br>997711<br>1816

dimeric alpha-amylase inhibitor [Triticum dicoccoides]	gi|227808958	15606.4	4.9899 997711 1816
dimeric alpha-amylase inhibitor [Triticum dicoccoides]	gi|227808952	15606.4	4.9899 997711 1816
dimeric alpha-amylase inhibitor [Triticum dicoccoides]	gi|227808944	15606.4	4.9899 997711 1816
dimeric alpha-amylase inhibitor [Triticum dicoccoides]	gi|227808934	15606.4	4.9899 997711 1816
dimeric alpha-amylase inhibitor [Triticum dicoccoides]	gi|227809244	15606.4	4.9899 997711 1816

Peptide Information

Calc. Mass	Obsrv. Mass	± da	± ppm	Start Seq.	End Seq.	Sequence	Ion Score	C. I. %	Modification	Rank	Result Type
1162.6249	1162.6138	-0.0111	-10	107	117	LTAASITAVCR			Carbamidomethyl (C)[10]		Mascot
1162.6249	1162.6138	-0.0111	-10	107	117	LTAASITAVCR	70	100	Carbamidomethyl (C)[10]		Mascot
1554.6637	1554.6335	-0.0302	-19	71	83	CGALYSMLDSMYK			Carbamidomethyl (C)[1], Oxidation (M)[7]		Mascot
1570.8007	1570.7839	-0.0168	-11	43	56	LQCNGSQVPEAVLR			Carbamidomethyl (C)[3]		Mascot
1663.8361	1663.7701	-0.066	-40	118	133	LPIVVDASGDGAYVCK			Carbamidomethyl (C)[15]		Mascot
1663.8361	1663.7701	-0.066	-40	118	133	LPIVVDASGDGAYVCK	141	100	Carbamidomethyl (C)[15]		Mascot
1840.7412	1840.724	-0.0172	-9	57	70	DCCQQLADISEWCR			Carbamidomethyl (C)[2,3,13]		Mascot
1846.8137	1846.7911	-0.0226	-12	84	101	EHGVSEGGQAGTGAFPSC R			Carbamidomethyl (C)[17]		Mascot
1846.8137	1846.7911	-0.0226	-12	84	101	EHGVSEGGQAGTGAFPSC R	167	100	Carbamidomethyl (C)[17]		Mascot
2807.4431	2807.3105	-0.1326	-47	107	133	LTAASITAVCRLPIVVDAS GDGAYVCK			Carbamidomethyl (C)[10,26]		Mascot

3	dimeric alpha-amylase inhibitor [Triticum timopheevii subsp. armeniacum]	gi|227809288	15533.4	5.7	3	0	378	100	

Protein Group

RecName: Full=Alpha-amylase inhibitor 0.53	gi|123968	13689.5	5.2300 000190 7349
dimeric alpha-amylase inhibitor [Triticum aestivum]	gi|65993898	15533.4	5.6999 998092 6514
dimeric alpha-amylase inhibitor [Triticum aestivum]	gi|65993731	15606.4	4.9899 997711 1816
dimeric alpha-amylase inhibitor [Triticum aestivum]	gi|65993709	15578.3	4.9899 997711 1816
dimeric alpha-amylase inhibitor [Triticum dicoccoides]	gi|227809146	15605.4	5.3000 001907

			3486
dimeric alpha-amylase inhibitor [Triticum dicoccoides]	gi|227808966	15664.4	4.8099 999427 7954
dimeric alpha-amylase inhibitor [Triticum dicoccoides]	gi|227808928	15605.4	5.0100 002288 8184
dimeric alpha-amylase inhibitor [Triticum dicoccoides]	gi|227808922	15606.4	4.9899 997711 1816
dimeric alpha-amylase inhibitor [Triticum dicoccoides]	gi|227808920	15606.4	4.9899 997711 1816
dimeric alpha-amylase inhibitor [Triticum dicoccoides]	gi|227808914	15606.4	4.9899 997711 1816
dimeric alpha-amylase inhibitor [Triticum dicoccoides]	gi|227808912	15638.3	4.9899 997711 1816
dimeric alpha-amylase inhibitor [Triticum dicoccoides]	gi|227808900	15578.3	4.9899 997711 1816
dimeric alpha-amylase inhibitor [Triticum dicoccoides]	gi|227808898	15606.4	4.9899 997711 1816
dimeric alpha-amylase inhibitor [Triticum dicoccoides]	gi|227808869	15606.4	4.9899 997711 1816
dimeric alpha-amylase inhibitor [Triticum dicoccoides]	gi|227808867	15578.3	4.9899 997711 1816
dimeric alpha-amylase inhibitor [Triticum dicoccoides]	gi|227808861	15658.4	5.5799 999237 0605
dimeric alpha-amylase inhibitor [Triticum dicoccoides]	gi|227808853	15618.3	4.9899 997711 1816
dimeric alpha-amylase inhibitor [Triticum dicoccoides]	gi|227808851	15658.4	5.5799 999237 0605

Peptide Information

Calc. Mass	Obsrv. Mass	± da	± ppm	Start Seq.	End Seq.	Sequence	Ion Score	C. I. %	Modification	Rank	Result Type
1162.6249	1162.6138	-0.0111	-10	107	117	LTAASITAVCR	70	100	Carbamidomethyl (C)[10]	1	Mascot
1663.8361	1663.7701	-0.066	-40	118	133	LPIVVDASGDGAYVCK	141	100	Carbamidomethyl (C)[15]	1	Mascot
1846.8137	1846.7911	-0.0226	-12	84	101	EHGVSEGQAGTGAFPSC R	167	100	Carbamidomethyl (C)[17]	1	Mascot

4	dimeric alpha-amylase inhibitor [Triticum urartu]	gi|146214722	13876.6	6.49	3	0	223	100

Peptide Information

Calc. Mass	Obsrv. Mass	± da	± ppm	Start	End	Sequence	Ion	C. I. %	Modification	Rank	Result Type

				Seq.	Seq.		Score				
1162.6249	1162.6138	-0.0111	-10	90	100	LTAASITAVCR	70	100	Carbamidomethyl (C)[10]	1	Mascot
1598.8431	1598.8308	-0.0123	-8	26	39	LQCNGSRVPEAVLR	12	0	Carbamidomethyl (C)[3]	1	Mascot
1663.8361	1663.7701	-0.066	-40	101	116	LPIVVDASGDGAYVCK	141	100	Carbamidomethyl (C)[15]	1	Mascot

5	dimeric alpha-amylase inhibitor [Triticum dicoccoides]	gi|227809121	15555.3	4.99	2	0	211	100

Peptide Information

Calc. Mass	Obsrv. Mass	± da	± ppm	Start Seq.	End Seq.	Sequence	Ion Score	C. I. %	Modification	Rank	Result Type
1162.6249	1162.6138	-0.0111	-10	107	117	LTAASITAVXR	70	100		1	Mascot
1663.8361	1663.7701	-0.066	-40	118	133	LPIVVDASGDGAYVCK	141	100	Carbamidomethyl (C)[15]	1	Mascot

6	hypothetical protein TRIUR3_00718 [Triticum urartu]	gi|474190769	27498.3	9.02	1	0	16	0

Peptide Information

Calc. Mass	Obsrv. Mass	± da	± ppm	Start Seq.	End Seq.	Sequence	Ion Score	C. I. %	Modification	Rank	Result Type
1571.7708	1571.7659	-0.0049	-3	29	42	MGKSPGRPVNQNDR	16	0	Oxidation (M)[1]	1	Mascot

7	rpl16 [Triticum aestivum]	gi|291498607	21419.2	10.88	1	0	16	0

Protein Group

rpl16 [Triticum aestivum]	gi|169649057	21419.2	10.8800001144409
rpl16 [Triticum aestivum]	gi|78675244	21419.2	10.8800001144409
rpl16 [Triticum aestivum]	gi|81176520	21419.2	10.8800001144409

Peptide Information

Calc. Mass	Obsrv. Mass	± da	± ppm	Start Seq.	End Seq.	Sequence	Ion Score	C. I. %	Modification	Rank	Result Type
1846.9269	1846.7911	-0.1358	-74	170	185	LAAHKPCSSTKFVQWS	16	0	Carbamidomethyl (C)[7]	2	Mascot

8	Serine/threonine-protein kinase AFC2 [Triticum urartu]	gi|473983274	41776.4	9.08	1	0	15	0

Peptide Information

Calc. Mass	Obsrv. Mass	± da	± ppm	Start Seq.	End Seq.	Sequence	Ion Score	C. I. %	Modification	Rank	Result Type
1663.7856	1663.7701	-0.0155	-9	307	320	LNWPEGATTRESMR	15	0	Oxidation (M)[13]	2	Mascot

9	hypothetical protein TRIUR3_09519 [Triticum urartu]	gi|473894932	54375.6	9.57	1	0	13	0

Peptide Information

Calc. Mass	Obsrv. Mass	± da	± ppm	Start Seq.	End Seq.	Sequence	Ion Score	C. I. %	Modification	Rank	Result Type
1571.7772	1571.7659	-0.0113	-7	9	23	RLPSDDNVASDGGLR	14	0		2	Mascot

10	hypothetical protein TRIUR3_35068 [Triticum urartu]	gi|474433790	43906.8	6.82	1	0	12	0

Peptide Information

Calc. Mass	Obsrv. Mass	± da	± ppm	Start Seq.	End Seq.	Sequence	Ion Score	C. I. %	Modification	Rank	Result Type
1846.7629	1846.7911	0.0282	15	1	17	MAPPPEEERGGGGCCA R	12	0	Carbamidomethyl (C)[14,15], Oxidation (M)[1]	3	Mascot

Gel Idx/Pos	153/G4	Instr./Gel Origin	BA2151/Sample Project 20140814	Process Status	Analysis Succeeded
Plate [#] Name	[1] Sample Project 20140814	Instrument Sample Name		Spectra	11

Rank	Protein Name	Accession No.	Protein MW	Protein PI	Pep. Count	Protein Score	Protein Score C. I. %	Intensity Matched	Total Ion Score	Total Ion C. I. %	Confirmed
1	CM 17 protein precursor [Triticum aestivum]	gi|21711	16548.8	5.07	4	237	100	44.455	218	100	

Peptide Information

Calc. Mass	Obsrv. Mass	± da	± ppm	Start Seq.	End Seq.	Sequence	Ion Score	C. I. %	Modification	Rank	Result Type
1168.5051	1168.486	-0.0191	-16	46	54	NYVEEQACR			Carbamidomethyl (C)[8]		Mascot
1168.5051	1168.486	-0.0191	-16	46	54	NYVEEQACR	48	99.924	Carbamidomethyl (C)[8]		Mascot
1215.6443	1215.6046	-0.0397	-33	55	65	IEMPGPPYLAK					Mascot
1231.6392	1231.6036	-0.0356	-29	55	65	IEMPGPPYLAK			Oxidation (M)[3]		Mascot
1799.8528	1799.8123	-0.0405	-23	92	107	SRPDQSGLMELPGCPR			Carbamidomethyl (C)[14]		Mascot
1799.8528	1799.8123	-0.0405	-23	92	107	SRPDQSGLMELPGCPR	41	99.625	Carbamidomethyl (C)[14]		Mascot
1815.8477	1815.7909	-0.0568	-31	92	107	SRPDQSGLMELPGCPR			Carbamidomethyl (C)[14], Oxidation (M)[9]		Mascot
1815.8477	1815.7909	-0.0568	-31	92	107	SRPDQSGLMELPGCPR	28	91.492	Carbamidomethyl (C)[14], Oxidation (M)[9]		Mascot
1933.8314	1933.7983	-0.0331	-17	66	80	QECCEQLANIPQQCR			Carbamidomethyl (C)[3,4,14]		Mascot
1933.8314	1933.7983	-0.0331	-17	66	80	QECCEQLANIPQQCR	128	100	Carbamidomethyl (C)[3,4,14]		Mascot

Rank	Protein Name	Accession No.	Protein MW	Protein PI	Pep. Count	Protein Score	Protein Score C. I. %	Intensity Matched	Total Ion Score	Total Ion C. I. %	Confirmed
2	alpha-amylase inhibitor CM16 subunit [Triticum macha]	gi|221855644	16267.8	5.31	4	80	99.953	13.591	60	99.995	

Protein Group

alpha-amylase inhibitor CM16 subunit [Triticum macha]	gi|221855632	16267.8	5.3099 999427 7954
alpha-amylase inhibitor CM16 subunit [Triticum macha]	gi|221855656	16267.8	5.3099 999427 7954

Peptide Information

Calc. Mass	Obsrv. Mass	± da	± ppm	Start Seq.	End Seq.	Sequence	Ion Score	C. I. %	Modification	Rank	Result Type
1023.4928	1023.462	-0.0308	-30	107	114	EVQMDFVR					Mascot
1039.4878	1039.4647	-0.0231	-22	107	114	EVQMDFVR			Oxidation (M)[4]		Mascot
1168.5052	1168.486	-0.0192	-16	45	53	DYVEQQACR			Carbamidomethyl (C)[8]		Mascot
1168.5052	1168.486	-0.0192	-16	45	53	DYVEQQACR	19	35.163	Carbamidomethyl (C)[8]		Mascot
1799.8528	1799.8123	-0.0405	-23	91	106	SRPDQSGLMELPGCPR			Carbamidomethyl (C)[14]		Mascot
1799.8528	1799.8123	-0.0405	-23	91	106	SRPDQSGLMELPGCPR	41	99.625	Carbamidomethyl (C)[14]		Mascot
1815.8477	1815.7909	-0.0568	-31	91	106	SRPDQSGLMELPGCPR			Carbamidomethyl (C)[14], Oxidation (M)[9]		Mascot

1815.8477	1815.7909	-0.0568	-31	91	106	SRPDQSGLMELPGCPR	28	91.492	Carbamidomethyl (C)[14], Oxidation (M)[9]		Mascot
1861.8102	1861.7733	-0.0369	-20	65	79	QQCCGELANIPQQCR			Carbamidomethyl (C)[3,4,14]		Mascot

<table>
<tr><td>3</td><td>major allergen CM16 [Triticum aestivum]</td><td>gi|195957140</td><td>16399.8</td><td>4.86</td><td>4</td><td>80</td><td>99.952</td><td>13.591</td><td>60</td><td>99.995</td></tr>
</table>

Protein Group

<table>
<tr><td>CM16 protein [Triticum aestivum]</td><td>gi|21709</td><td>16398.8</td><td>5.3099999427 7954</td></tr>
<tr><td>RecName: Full=Alpha-amylase/trypsin inhibitor CM16; AltName: Full=Chloroform/methanol-soluble protein CM16; Flags: Precursor</td><td>gi|123958</td><td>16398.8</td><td>5.3099999427 7954</td></tr>
<tr><td>alpha-amylase inhibitor, tetrameric, chain CM16 precursor - durum wheat</td><td>gi|100832</td><td>16398.8</td><td>5.3099999427 7954</td></tr>
<tr><td>precursor (AA -24 to 119) [Triticum durum]</td><td>gi|21916</td><td>16398.8</td><td>5.3099999427 7954</td></tr>
<tr><td>unnamed protein product [Triticum aestivum]</td><td>gi|21705</td><td>16398.8</td><td>5.3099999427 7954</td></tr>
</table>

Peptide Information

Calc. Mass	Obsrv. Mass	± da	± ppm	Start Seq.	End Seq.	Sequence	Ion Score	C. I. %	Modification	Rank	Result Type
1023.4928	1023.462	-0.0308	-30	108	115	EVQMDFVR					Mascot
1039.4878	1039.4647	-0.0231	-22	108	115	EVQMDFVR			Oxidation (M)[4]		Mascot
1168.5052	1168.486	-0.0192	-16	46	54	DYVEQQACR			Carbamidomethyl (C)[8]		Mascot
1168.5052	1168.486	-0.0192	-16	46	54	DYVEQQACR	19	35.163	Carbamidomethyl (C)[8]		Mascot
1799.8528	1799.8123	-0.0405	-23	92	107	SRPDQSGLMELPGCPR			Carbamidomethyl (C)[14]		Mascot
1799.8528	1799.8123	-0.0405	-23	92	107	SRPDQSGLMELPGCPR	41	99.625	Carbamidomethyl (C)[14]		Mascot
1815.8477	1815.7909	-0.0568	-31	92	107	SRPDQSGLMELPGCPR			Carbamidomethyl (C)[14], Oxidation (M)[9]		Mascot
1815.8477	1815.7909	-0.0568	-31	92	107	SRPDQSGLMELPGCPR	28	91.492	Carbamidomethyl (C)[14], Oxidation (M)[9]		Mascot
1861.8102	1861.7733	-0.0369	-20	66	80	QQCCGELANIPQQCR			Carbamidomethyl (C)[3,4,14]		Mascot

<table>
<tr><td>4</td><td>hypothetical protein TRIUR3_13738 [Triticum urartu]</td><td>gi|474140435</td><td>67934.1</td><td>8.83</td><td>13</td><td>46</td><td>0</td><td>13.001</td></tr>
</table>

Peptide Information

Calc. Mass	Obsrv. Mass	± da	± ppm	Start Seq.	End Seq.	Sequence	Ion Score	C. I. %	Modification	Rank	Result Type
809.4152	809.3411	-0.0741	-92	366	371	YETQLR					Mascot
820.3696	820.3605	-0.0091	-11	608	614	SQQHPHS					Mascot
1023.5543	1023.462	-0.0923	-90	439	447	ITIEAMAFK					Mascot
1039.5493	1039.4647	-0.0846	-81	439	447	ITIEAMAFK			Oxidation (M)[6]		Mascot
1345.6594	1345.6583	-0.0011	-1	284	295	ELTEVAEDNVAR					Mascot

1544.7738	1544.6913	-0.0825	-53	351	365	VHAVVVGSDMSAQTK			Oxidation (M)[10]		Mascot
1782.9055	1782.859	-0.0465	-26	310	325	SEDLLFAMINSVSRGK			Oxidation (M)[8]		Mascot
1815.9244	1815.7909	-0.1335	-74	418	432	VHACKEMLLDIPYAR			Carbamidomethyl (C)[4]		Mascot
1815.9244	1815.7909	-0.1335	-74	418	432	VHACKEMLLDIPYAR			Carbamidomethyl (C)[4]		Mascot
1831.9193	1831.7776	-0.1417	-77	418	432	VHACKEMLLDIPYAR			Carbamidomethyl (C)[4], Oxidation (M)[7]		Mascot
1842.9353	1842.7883	-0.147	-80	588	602	FMEHHMAERIAVVLK			Oxidation (M)[2,6]		Mascot
1842.9353	1842.7883	-0.147	-80	588	602	FMEHHMAERIAVVLK			Oxidation (M)[2,6]		Mascot
1915.9789	1915.8358	-0.1431	-75	222	235	ILWWIHEMRGHYFK					Mascot
1931.9738	1931.7888	-0.185	-96	222	235	ILWWIHEMRGHYFK			Oxidation (M)[8]		Mascot
1994.9713	1994.9395	-0.0318	-16	83	102	GGPVGPCDTRGDPVDVV AAR			Carbamidomethyl (C)[7]		Mascot
2173.1072	2172.9834	-0.1238	-57	93	115	GDPVDVVAARAGGAASS PLGFMK					Mascot
2189.1021	2188.9839	-0.1182	-54	93	115	GDPVDVVAARAGGAASS PLGFMK			Oxidation (M)[22]		Mascot
2280.1846	2279.9844	-0.2002	-88	241	260	HLPLVAGAMIDSHITVEY WK					Mascot
2280.1846	2279.9844	-0.2002	-88	241	260	HLPLVAGAMIDSHITVEY WK					Mascot
2290.2166	2290.0178	-0.1988	-87	213	230	DNVPQVLPKILWWIHEM R			Oxidation (M)[17]		Mascot

5	heat shock protein 90 [Triticum dicoccoides]	gi|294717865	80585	4.99	14	42	0	36.793	

Protein Group

Heat shock protein 81-3 [Triticum urartu]	gi|474225055	80657	4.9800 000190 7349
heat shock protein 90 [Triticum aestivum]	gi|294717828	80585	4.9899 997711 1816
heat shock protein 90 [Triticum aestivum]	gi|294717810	80585	4.9899 997711 1816
heat shock protein 90 [Triticum urartu]	gi|294717842	80626	4.9800 000190 7349

Peptide Information

Calc. Mass	Obsrv. Mass	± da	± ppm	Start Seq.	End Seq.	Sequence	Ion Score	C. I. %	Modification	Rank	Result Type
876.4057	876.3215	-0.0842	-96	623	630	ADADKNDK					Mascot
1054.5415	1054.4462	-0.0953	-90	49	57	FESLTDKSK					Mascot
1231.6278	1231.6036	-0.0242	-20	163	174	DTTGEPLGRGTK					Mascot
1237.6324	1237.6034	-0.029	-23	319	328	RAPFDLFDTR					Mascot
1544.8279	1544.6913	-0.1366	-88	35	48	ELISNASDALDKIR					Mascot
1734.7786	1734.7881	0.0095	5	589	604	AQALRDTSMGGYMSSK			Oxidation (M)[9,13]		Mascot
1782.74	1782.859	0.119	67	214	228	TTEKEISDDEDEDEK					Mascot

Calc. Mass	Obsrv. Mass	± da	± ppm	Start Seq.	End Seq.	Sequence	Ion Score	C. I. %	Modification	Rank	Result Type
1799.9836	1799.8123	-0.1713	-95	266	279	QKPIWMRKPEEITK			Oxidation (M)[6]		Mascot
1799.9836	1799.8123	-0.1713	-95	266	279	QKPIWMRKPEEITK			Oxidation (M)[6]		Mascot
1831.9113	1831.7776	-0.1337	-73	273	287	KPEEITKDEYAAFYK					Mascot
1875.8793	1875.7806	-0.0987	-53	457	472	MKEGQNDIYYITGESK					Mascot
1933.9358	1933.7983	-0.1375	-71	606	621	TMEINPENAIMEELRK			Oxidation (M)[2]		Mascot
1933.9358	1933.7983	-0.1375	-71	605	620	KTMEINPENAIMEELR			Oxidation (M)[3]		Mascot
2306.126	2306.0156	-0.1104	-48	488	507	GYEVLYMVDAIDEYSIGQLK					Mascot
3039.4443	3039.4082	-0.0361	-12	136	162	VVVTSKHNDDEQYVWESQAGGSFTVTR					Mascot

6 hypothetical protein TRIUR3_31995 [Triticum urartu] gi|474036856 8713.2 6.34 5 40 0 .743

Peptide Information

Calc. Mass	Obsrv. Mass	± da	± ppm	Start Seq.	End Seq.	Sequence	Ion Score	C. I. %	Modification	Rank	Result Type
1023.4377	1023.462	0.0243	24	70	78	DEDVGGFER					Mascot
1215.6189	1215.6046	-0.0143	-12	34	46	ARGGGESLQGAGR					Mascot
1231.5009	1231.6036	0.1027	83	57	69	MEDGGGSGGPPDR					Mascot
1960.8414	1960.7787	-0.0627	-32	49	69	DSAAAASRMEDGGGSGGPPDR					Mascot
2235.9209	2235.9932	0.0723	32	57	78	MEDGGGSGGPPDRDEDVGGFER					Mascot

7 hypothetical protein TRIUR3_08536 [Triticum urartu] gi|474407060 49568.3 5.63 11 36 0 8.404

Peptide Information

Calc. Mass	Obsrv. Mass	± da	± ppm	Start Seq.	End Seq.	Sequence	Ion Score	C. I. %	Modification	Rank	Result Type
1039.4547	1039.4647	0.01	10	268	276	DMTAQTMNK					Mascot
1054.447	1054.4462	-0.0008	-1	330	338	DTTEQTMGR			Oxidation (M)[7]		Mascot
1237.6206	1237.6034	-0.0172	-14	414	427	SATGGGMTGTAKAK					Mascot
1253.5791	1253.5651	-0.014	-11	330	340	DTTEQTMGRAK			Oxidation (M)[7]		Mascot
1781.8521	1781.8518	-0.0003	0	350	367	TGSMAAQVKDATGAMAQK			Oxidation (M)[4]		Mascot
1782.8691	1782.859	-0.0101	-6	359	376	DATGAMAQKAGDTAAYIK					Mascot
1797.847	1797.8259	-0.0211	-12	350	367	TGSMAAQVKDATGAMAQK			Oxidation (M)[4,15]		Mascot
1798.864	1798.8446	-0.0194	-11	359	376	DATGAMAQKAGDTAAYIK			Oxidation (M)[6]		Mascot
1827.8575	1827.7761	-0.0814	-45	288	305	TGSMAAQVKDTTGAMAQK			Oxidation (M)[4,15]		Mascot
1842.8903	1842.7883	-0.102	-55	297	314	DTTGAMAQKATDTAAYVK					Mascot
1842.9154	1842.7883	-0.1271	-69	119	135	AKEVTLTTGEMTAEYAK					Mascot
1944.9709	1944.8005	-0.1704	-88	1	18	MAAWHVDTVSRATVASS					Mascot

Calc. Mass	Obsrv. Mass	± da	± ppm	Start Seq.	End Seq.	Sequence	Ion Score	C. I. %	Modification	Rank	Result Type
						R					
1960.9658	1960.7787	-0.1871	-95	1	18	MAAWHVDTVSRATVASSR			Oxidation (M)[1]		Mascot
2157.0745	2156.9851	-0.0894	-41	121	140	EVTLTTGEMTAEYAKQAAVK			Oxidation (M)[9]		Mascot

8	Sex determination protein tasselseed-2 [Triticum urartu]	gi|474113021	10552.3	4.85	5	35	0	.48

Peptide Information

Calc. Mass	Obsrv. Mass	± da	± ppm	Start Seq.	End Seq.	Sequence	Ion Score	C. I. %	Modification	Rank	Result Type
1077.5615	1077.4797	-0.0818	-76	24	31	ILEEWYPK					Mascot
1151.5942	1151.4944	-0.0998	-87	61	71	AALYLASDEAK					Mascot
1770.9572	1770.7838	-0.1734	-98	8	23	VNAVSPNYIPTPLVMR					Mascot
1857.913	1857.774	-0.139	-75	24	38	ILEEWYPKASAEEHR					Mascot
2186.175	2186.0232	-0.1518	-69	4	23	SGVRVNAVSPNYIPTPLVMR			Oxidation (M)[19]		Mascot

9	heat shock protein 90 [Triticum aestivum]	gi|294717814	80672	4.98	12	34	0	36.746

Protein Group

heat shock protein 90 [Triticum aestivum]	gi|294717832	80672	4.98000001907349

Peptide Information

Calc. Mass	Obsrv. Mass	± da	± ppm	Start Seq.	End Seq.	Sequence	Ion Score	C. I. %	Modification	Rank	Result Type
1054.5415	1054.4462	-0.0953	-90	49	57	FESLTDKSK					Mascot
1231.6278	1231.6036	-0.0242	-20	163	174	DTTGEPLGRGTK					Mascot
1237.6324	1237.6034	-0.029	-23	319	328	RAPFDLFDTR					Mascot
1544.8279	1544.6913	-0.1366	-88	35	48	ELISNASDALDKIR					Mascot
1734.7786	1734.7881	0.0095	5	589	604	AQALRDTSMGGYMSSK			Oxidation (M)[9,13]		Mascot
1782.74	1782.859	0.119	67	214	228	TTEKEISDDEDEDEK					Mascot
1799.9836	1799.8123	-0.1713	-95	266	279	QKPIWMRKPEEITK			Oxidation (M)[6]		Mascot
1799.9836	1799.8123	-0.1713	-95	266	279	QKPIWMRKPEEITK			Oxidation (M)[6]		Mascot
1845.9269	1845.7786	-0.1483	-80	273	287	KPEEITKEEYAAFYK					Mascot
1933.9358	1933.7983	-0.1375	-71	606	621	TMEINPENAIMEELRK			Oxidation (M)[2]		Mascot
1933.9358	1933.7983	-0.1375	-71	605	620	KTMEINPENAIMEELR			Oxidation (M)[3]		Mascot
2306.126	2306.0156	-0.1104	-48	488	507	GYEVLYMVDAIDEYSIGQLK					Mascot
3039.4443	3039.4082	-0.0361	-12	136	162	VVVTSKHNDDEQYVWESQAGGSFTVTR					Mascot

10	hypothetical protein TRIUR3_01294 [Triticum urartu]	gi|474317374	28037.5	9.83	7	33	0	1.894

Peptide Information

Calc. Mass	Obsrv. Mass	± da	± ppm	Start Seq.	End Seq.	Sequence	Ion Score	C. I. %	Modification	Rank	Result Type
805.3686	805.3469	-0.0217	-27	162	169	EGNTDAAK					Mascot
1544.7704	1544.6913	-0.0791	-51	96	111	EAAGTLAAAHYAAAEK					Mascot
1825.8749	1825.7653	-0.1096	-60	260	277	SEADAMKYGAISQQAAGK					Mascot
1845.8824	1845.7786	-0.1038	-56	50	68	AESATTPEEAAAAKQDGAK					Mascot
1872.9813	1872.8197	-0.1616	-86	96	114	EAAGTLAAAHYAAAEKSIK					Mascot
2279.0649	2279.0071	-0.0578	-25	1	22	MGGWAPTGPAETYGVAEENTLK					Mascot
2807.3306	2807.2622	-0.0684	-24	2	28	GGWAPTGPAETYGVAEENTLKMATPSR			Oxidation (M)[22]		Mascot

Gel Idx/Pos	154/G5	Instr./Gel Origin	BA2151/Sample Project 20140814	Process Status	Analysis Succeeded
Plate [#] Name	[1] Sample Project 20140814	Instrument Sample Name		Spectra	11

Rank	Protein Name	Accession No.	Protein MW	Protein PI	Pep. Count	Protein Score	Protein Score C. I. %	Intensity Matched	Total Ion Score	Total Ion C. I. %	Confirmed
1	CM 17 protein precursor [Triticum aestivum]	gi|21711	16548.8	5.07	4	284	100	27.881	264	100	

Peptide Information

Calc. Mass	Obsrv. Mass	± da	± ppm	Start Seq.	End Seq.	Sequence	Ion Score	C. I. %	Modification	Rank	Result Type
1168.5051	1168.489	-0.0161	-14	46	54	NYVEEQACR			Carbamidomethyl (C)[8]		Mascot
1168.5051	1168.489	-0.0161	-14	46	54	NYVEEQACR	62	99.997	Carbamidomethyl (C)[8]		Mascot
1231.6392	1231.59	-0.0492	-40	55	65	IEMPGPPYLAK			Oxidation (M)[3]		Mascot
1231.6392	1231.59	-0.0492	-40	55	65	IEMPGPPYLAK	34	97.643	Oxidation (M)[3]		Mascot
1799.8528	1799.8337	-0.0191	-11	92	107	SRPDQSGLMELPGCPR			Carbamidomethyl (C)[14]		Mascot
1815.8477	1815.7843	-0.0634	-35	92	107	SRPDQSGLMELPGCPR			Carbamidomethyl (C)[14], Oxidation (M)[9]		Mascot
1815.8477	1815.7843	-0.0634	-35	92	107	SRPDQSGLMELPGCPR	61	99.995	Carbamidomethyl (C)[14], Oxidation (M)[9]		Mascot
1933.8314	1933.8021	-0.0293	-15	66	80	QECCEQLANIPQQCR			Carbamidomethyl (C)[3,4,14]		Mascot
1933.8314	1933.8021	-0.0293	-15	66	80	QECCEQLANIPQQCR	107	100	Carbamidomethyl (C)[3,4,14]		Mascot

Rank	Protein Name	Accession No.	Protein MW	Protein PI	Pep. Count	Protein Score	Protein Score C. I. %	Intensity Matched	Total Ion Score	Total Ion C. I. %	Confirmed
2	alpha-amylase inhibitor CM16 subunit [Triticum macha]	gi|221855644	16267.8	5.31	4	105	100	20.146	86	100	

Protein Group

CM16 protein [Triticum aestivum]	gi|21709	16398.8	5.3099999427 7954
RecName: Full=Alpha-amylase/trypsin inhibitor CM16; AltName: Full=Chloroform/methanol-soluble protein CM16; Flags: Precursor	gi|123958	16398.8	5.3099999427 7954
alpha-amylase inhibitor CM16 subunit [Triticum macha]	gi|221855632	16267.8	5.3099999427 7954
alpha-amylase inhibitor CM16 subunit [Triticum macha]	gi|221855656	16267.8	5.3099999427 7954
alpha-amylase inhibitor, tetrameric, chain CM16 precursor - durum wheat	gi|100832	16398.8	5.3099999427 7954
major allergen CM16 [Triticum aestivum]	gi|195957140	16399.8	4.8600001335 144
precursor (AA -24 to 119) [Triticum durum]	gi|21916	16398.8	5.3099999427 7954
unnamed protein product [Triticum aestivum]	gi|21705	16398.8	5.3099999427

Peptide Information

Calc. Mass	Obsrv. Mass	± da	± ppm	Start Seq.	End Seq.	Sequence	Ion Score	C. I. %	Modification	Rank	Result Type
1039.4878	1039.4535	-0.0343	-33	107	114	EVQMDFVR			Oxidation (M)[4]		Mascot
1168.5052	1168.489	-0.0162	-14	45	53	DYVEQQACR			Carbamidomethyl (C)[8]		Mascot
1168.5052	1168.489	-0.0162	-14	45	53	DYVEQQACR	25	83.196	Carbamidomethyl (C)[8]		Mascot
1799.8528	1799.8337	-0.0191	-11	91	106	SRPDQSGLMELPGCPR			Carbamidomethyl (C)[14]		Mascot
1815.8477	1815.7843	-0.0634	-35	91	106	SRPDQSGLMELPGCPR			Carbamidomethyl (C)[14], Oxidation (M)[9]		Mascot
1815.8477	1815.7843	-0.0634	-35	91	106	SRPDQSGLMELPGCPR	61	99.995	Carbamidomethyl (C)[14], Oxidation (M)[9]		Mascot
1861.8102	1861.7701	-0.0401	-22	65	79	QQCCGELANIPQQCR			Carbamidomethyl (C)[3,4,14]		Mascot

3 hypothetical protein TRIUR3_08536 [Triticum urartu] gi|474407060 49568.3 5.63 14 48 31.509 6.649

Peptide Information

Calc. Mass	Obsrv. Mass	± da	± ppm	Start Seq.	End Seq.	Sequence	Ion Score	C. I. %	Modification	Rank	Result Type
808.3505	808.3455	-0.005	-6	217	223	EMAEQGK			Oxidation (M)[2]		Mascot
836.3632	836.3044	-0.0588	-70	428	435	GEGTEDTK					Mascot
890.3962	890.371	-0.0252	-28	341	349	EATGDAGNR					Mascot
908.4142	908.384	-0.0302	-33	359	367	DATGAMAQK			Oxidation (M)[6]		Mascot
909.4676	909.3979	-0.0697	-77	368	376	AGDTAAYIK					Mascot
1039.4547	1039.4535	-0.0012	-1	268	276	DMTAQTMNK					Mascot
1054.447	1054.4471	0.0001	0	330	338	DTTEQTMGR			Oxidation (M)[7]		Mascot
1193.5468	1193.5889	0.0421	35	377	389	DSVMGAAGGAVDK			Oxidation (M)[4]		Mascot
1253.5791	1253.5657	-0.0134	-11	330	340	DTTEQTMGRAK			Oxidation (M)[7]		Mascot
1797.847	1797.8691	0.0221	12	350	367	TGSMAAQVKDATGAMA QK			Oxidation (M)[4,15]		Mascot
1827.8575	1827.7661	-0.0914	-50	288	305	TGSMAAQVKDTTGAMAQ K			Oxidation (M)[4,15]		Mascot
1842.8903	1842.7971	-0.0932	-51	297	314	DTTGAMAQKATDTAAYV K					Mascot
1842.9154	1842.7971	-0.1183	-64	119	135	AKEVTLTTGEMTAEYAK					Mascot
2157.0745	2156.989	-0.0855	-40	121	140	EVTLTTGEMTAEYAKQA AVK			Oxidation (M)[9]		Mascot

4 ribulose-1,5-bisphosphate carboxylase/oxygenase small subunit [Triticum urartu] gi|4038713 18853.5 8.82 7 39 0 2.616

Protein Group

ribulose-1,5-bisphosphate carboxylase/oxygenase small subunit [Triticum timopheevii subsp. armeniacum] gi|4038715 18804.4 8.8299 999237 0605

Peptide Information

Calc. Mass	Obsrv. Mass	± da	± ppm	Start Seq.	End Seq.	Sequence	Ion Score	C. I. %	Modification	Rank	Result Type
906.5043	906.4412	-0.0631	-70	71	77	QVDYLIR					Mascot
914.4229	914.4219	-0.001	-1	107	112	YWTMWK					Mascot
1140.5684	1140.5039	-0.0645	-57	133	141	KEYPDAYVR					Mascot
1320.6615	1320.5688	-0.0927	-70	26	39	RSSGSLGSVSNGGR					Mascot
1870.9674	1870.8123	-0.1551	-83	80	94	WVPCLEFSKVGFVFR			Carbamidomethyl (C)[4]		Mascot
1926.9708	1926.8394	-0.1314	-68	134	149	EYPDAYVRVIGFDNLR					Mascot
2280.0886	2279.9905	-0.0981	-43	113	132	LPMFGCTDATQVLNEVE EVK			Carbamidomethyl (C)[6]		Mascot
2280.0886	2279.9905	-0.0981	-43	113	132	LPMFGCTDATQVLNEVE EVK			Carbamidomethyl (C)[6]		Mascot

5 putative rubisco small subunit [Triticum durum] gi|62176930 19322.7 8.59 7 38 0 2.496

Peptide Information

Calc. Mass	Obsrv. Mass	± da	± ppm	Start Seq.	End Seq.	Sequence	Ion Score	C. I. %	Modification	Rank	Result Type
906.5043	906.4412	-0.0631	-70	71	77	QVDYLIR					Mascot
914.4229	914.4219	-0.001	-1	107	112	YWTMWK					Mascot
1140.5684	1140.5039	-0.0645	-57	133	141	KEYPDAYVR					Mascot
1265.608	1265.582	-0.026	-21	26	39	SSSAGLSSVSNGGR					Mascot
1870.9674	1870.8123	-0.1551	-83	80	94	WVPCLEFSKVGFVFR			Carbamidomethyl (C)[4]		Mascot
1926.9708	1926.8394	-0.1314	-68	134	149	EYPDAYVRVIGFDNLR					Mascot
2280.0886	2279.9905	-0.0981	-43	113	132	LPMFGCTDATQVLNEVE EVK			Carbamidomethyl (C)[6]		Mascot
2280.0886	2279.9905	-0.0981	-43	113	132	LPMFGCTDATQVLNEVE EVK			Carbamidomethyl (C)[6]		Mascot

6 Ribulose bisphosphate carboxylase small chain PW9, chloroplastic [Triticum urartu] gi|473939672 21992 7.63 7 38 0 2.519

Peptide Information

Calc. Mass	Obsrv. Mass	± da	± ppm	Start Seq.	End Seq.	Sequence	Ion Score	C. I. %	Modification	Rank	Result Type
906.5043	906.4412	-0.0631	-70	91	97	QVDYLIR					Mascot
914.4229	914.4219	-0.001	-1	127	132	YWTMWK					Mascot
1140.5684	1140.5039	-0.0645	-57	153	161	KEYPDAYVR					Mascot
1870.9674	1870.8123	-0.1551	-83	100	114	WVPCLEFSKVGFVFR			Carbamidomethyl (C)[4]		Mascot
1926.9708	1926.8394	-0.1314	-68	154	169	EYPDAYVRVIGFDNLR					Mascot
2263.1096	2263.019	-0.0906	-40	36	59	STAGMPISRPSSSAGLSS VSNGGR					Mascot
2280.0886	2279.9905	-0.0981	-43	133	152	LPMFGCTDATQVLNEVE EVK			Carbamidomethyl (C)[6]		Mascot

Calc. Mass	Obsrv. Mass	± da	± ppm	Start Seq.	End Seq.	Sequence	Ion Score	C. I. %	Modification	Rank	Result Type
2280.0886	2279.9905	-0.0981	-43	133	152	LPMFGCTDATQVLNEVEEVK			Carbamidomethyl (C)[6]		Mascot

7	unnamed protein product [Triticum aestivum]	gi|295415754	50966.1	9.26	12	38	0	17.712

Protein Group

geranylgeranyl hydrogenase [Triticum aestivum]	gi|288904218	50992.1	9.2600002288 8184
unnamed protein product [Triticum aestivum]	gi|295421995	50966.1	9.2600002288 8184

Peptide Information

Calc. Mass	Obsrv. Mass	± da	± ppm	Start Seq.	End Seq.	Sequence	Ion Score	C. I. %	Modification	Rank	Result Type
890.44	890.371	-0.069	-77	234	240	IPDDKMR			Oxidation (M)[6]		Mascot
913.4414	913.3907	-0.0507	-56	374	380	YLAEFDR					Mascot
1167.5391	1167.6013	0.0622	53	418	426	MTFDSYLYK					Mascot
1168.5627	1168.489	-0.0737	-63	1	12	MTSLSSSAAAAR			Oxidation (M)[1]		Mascot
1168.5627	1168.489	-0.0737	-63	1	12	MTSLSSSAAAAR			Oxidation (M)[1]		Mascot
1323.6403	1323.618	-0.0223	-17	418	427	MTFDSYLYKR					Mascot
1507.6992	1507.7124	0.0132	9	350	364	MCAEAIVAGSANGTR			Carbamidomethyl (C)[2]		Mascot
1649.835	1649.8076	-0.0274	-17	114	128	MKMISPSNVAVDIGR			Oxidation (M)[1,3]		Mascot
1823.8488	1823.8304	-0.0184	-10	347	364	SGRMCAEAIVAGSANGTR			Carbamidomethyl (C)[5], Oxidation (M)[4]		Mascot
1905.9666	1905.9261	-0.0405	-21	197	215	SFEVDAIVGADGANSRVAK					Mascot
1908.9597	1908.7847	-0.175	-92	266	283	CDHVAVGTGTVTHKADIK			Carbamidomethyl (C)[1]		Mascot
2188.0486	2187.9866	-0.062	-28	1	21	MTSLSSSAAAARATFLPSSCR			Carbamidomethyl (C)[20], Oxidation (M)[1]		Mascot
2247.9758	2248.0044	0.0286	13	399	417	SNAAREAFVEMCADDYVQR			Carbamidomethyl (C)[12], Oxidation (M)[11]		Mascot

8	Ribulose bisphosphate carboxylase small chain PWS4.3, chloroplastic [Triticum urartu]	gi|473721335	19632.8	8.81	7	37	0	2.616

Peptide Information

Calc. Mass	Obsrv. Mass	± da	± ppm	Start Seq.	End Seq.	Sequence	Ion Score	C. I. %	Modification	Rank	Result Type
906.5043	906.4412	-0.0631	-70	75	81	QVDYLIR					Mascot
914.4229	914.4219	-0.001	-1	111	116	YWTMWK					Mascot
1140.5684	1140.5039	-0.0645	-57	137	145	KEYPDAYVR					Mascot
1320.6615	1320.5688	-0.0927	-70	30	43	RSSGSLGSVSNGGR					Mascot
1870.9674	1870.8123	-0.1551	-83	84	98	WVPCLEFSKVGFVFR			Carbamidomethyl (C)[4]		Mascot
1926.9708	1926.8394	-0.1314	-68	138	153	EYPDAYVRVIGFDNLR					Mascot

2280.0886	2279.9905	-0.0981	-43	117	136	LPMFGCTDATQVLNEVE EVK			Carbamidomethyl (C)[6]		Mascot
2280.0886	2279.9905	-0.0981	-43	117	136	LPMFGCTDATQVLNEVE EVK			Carbamidomethyl (C)[6]		Mascot

9	hypothetical protein TRIUR3_33407 [Triticum urartu]	gi|474267229	15066.7	9.66	6	37	0	1.784

Peptide Information

Calc. Mass	Obsrv. Mass	± da	± ppm	Start Seq.	End Seq.	Sequence	Ion Score	C. I. %	Modification	Rank	Result Type
823.2886	823.3573	0.0687	83	69	74	EDECDR			Carbamidomethyl (C)[4]		Mascot
1118.559	1118.4944	-0.0646	-58	114	123	LPSTSTHGYR					Mascot
1308.5936	1308.6238	0.0302	23	59	68	SSWCPRLCSR			Carbamidomethyl (C)[4,8]		Mascot
1861.865	1861.7701	-0.0949	-51	94	108	YHRCHVDSSAPEYLK			Carbamidomethyl (C)[4]		Mascot
1915.9807	1915.83	-0.1507	-79	2	18	CIEGLGAVNEFPRGQLR			Carbamidomethyl (C)[1]		Mascot
2263.2056	2263.019	-0.1866	-82	22	41	SLAFLLYATCPPAGPRPY LR			Carbamidomethyl (C)[10]		Mascot

10	ribulose-1,5-bisphosphate carboxylase/oxygenase small subunit [Triticum aestivum]	gi|11990901	19733.9	8.81	7	37	0	2.496

Peptide Information

Calc. Mass	Obsrv. Mass	± da	± ppm	Start Seq.	End Seq.	Sequence	Ion Score	C. I. %	Modification	Rank	Result Type
906.5043	906.4412	-0.0631	-70	76	82	QVDYLIR					Mascot
914.4229	914.4219	-0.001	-1	112	117	YWTMWK					Mascot
1140.5684	1140.5039	-0.0645	-57	138	146	KEYPDAYVR					Mascot
1265.608	1265.582	-0.026	-21	31	44	SSSAGLSSVSNGGR					Mascot
1870.9674	1870.8123	-0.1551	-83	85	99	WVPCLEFSKVGFVFR			Carbamidomethyl (C)[4]		Mascot
1926.9708	1926.8394	-0.1314	-68	139	154	EYPDAYVRVIGFDNLR					Mascot
2280.0886	2279.9905	-0.0981	-43	118	137	LPMFGCTDATQVLNEVE EVK			Carbamidomethyl (C)[6]		Mascot
2280.0886	2279.9905	-0.0981	-43	118	137	LPMFGCTDATQVLNEVE EVK			Carbamidomethyl (C)[6]		Mascot

Gel Idx/Pos	155/G6	Instr./Gel Origin	BA2151/Sample Project 20140814	Process Status	Analysis Succeeded
Plate [#] Name	[1] Sample Project 20140814	Instrument Sample Name		Spectra	11

Rank	Protein Name	Accession No.	Protein MW	Protein PI	Pep. Count	Protein Score	Protein Score C. I. %	Intensity Matched	Total Ion Score	Total Ion C. I. %	Confirmed
1	hypothetical protein TRIUR3_28083 [Triticum urartu]	gi|474244636	22586.2	8.41	4	18	0	1.147			

Peptide Information

Calc. Mass	Obsrv. Mass	± da	± ppm	Start Seq.	End Seq.	Sequence	Ion Score	C. I. %	Modification	Rank	Result Type
856.4635	856.5172	0.0537	63	148	155	APAQGKER					Mascot
1168.58	1168.498	-0.082	-70	139	147	HGGAWWWLR					Mascot
1308.6808	1308.64	-0.0408	-31	99	110	WLGLAAEANHAR					Mascot
1855.1277	1855.2732	0.1455	78	9	24	VNLTNTKPFRVVVVLR					Mascot

Rank	Protein Name	Accession No.	Protein MW	Protein PI	Pep. Count	Protein Score	Protein Score C. I. %	Intensity Matched	Total Ion Score	Total Ion C. I. %	Confirmed
2	putative exosome complex exonuclease 1 [Triticum urartu]	gi|474445467	16819.8	10.66	3	16	0	.679			

Peptide Information

Calc. Mass	Obsrv. Mass	± da	± ppm	Start Seq.	End Seq.	Sequence	Ion Score	C. I. %	Modification	Rank	Result Type
1168.5454	1168.498	-0.0474	-41	14	23	HDPRTSDGQR					Mascot
1182.6055	1182.548	-0.0575	-49	71	80	SFPSFAWRGK					Mascot
2399.1528	2399.0247	-0.1281	-53	57	78	AWASTSTGGGQIERSFP SFAWR					Mascot

Rank	Protein Name	Accession No.	Protein MW	Protein PI	Pep. Count	Protein Score	Protein Score C. I. %	Intensity Matched	Total Ion Score	Total Ion C. I. %	Confirmed
3	hypothetical protein TRIUR3_00761 [Triticum urartu]	gi|474057027	14690.2	7.6	3	16	0	.725			

Peptide Information

Calc. Mass	Obsrv. Mass	± da	± ppm	Start Seq.	End Seq.	Sequence	Ion Score	C. I. %	Modification	Rank	Result Type
1107.5649	1107.5387	-0.0262	-24	1	9	MACEVLRTK			Carbamidomethyl (C)[3]		Mascot
1182.6014	1182.548	-0.0534	-45	117	126	YGLPRGNSYR					Mascot
1308.6001	1308.64	0.0399	30	27	37	ALDQCWSTTAR			Carbamidomethyl (C)[5]		Mascot

Rank	Protein Name	Accession No.	Protein MW	Protein PI	Pep. Count	Protein Score	Protein Score C. I. %	Intensity Matched	Total Ion Score	Total Ion C. I. %	Confirmed
4	hypothetical protein TRIUR3_29662 [Triticum urartu]	gi|474270445	17269	10.88	3	16	0	.608			

Peptide Information

Calc. Mass	Obsrv. Mass	± da	± ppm	Start Seq.	End Seq.	Sequence	Ion Score	C. I. %	Modification	Rank	Result Type
1194.7318	1194.6129	-0.1189	-100	114	124	GLVPVDLIRGR					Mascot
1320.6729	1320.5625	-0.1104	-84	1	12	MGTSIRAWSPSK					Mascot

1729.0483	1729.1053	0.057	33	106	122	AAHAAVVKGLVPVDLIR					Mascot

5	ribosomal Pr 117, partial [Triticum aestivum]	gi|32400871	15821.4	10.48	3	16	0	1.195

Peptide Information

Calc. Mass	Obsrv. Mass	± da	± ppm	Start Seq.	End Seq.	Sequence	Ion Score	C. I. %	Modification	Rank	Result Type
870.5057	870.5399	0.0342	39	91	96	QRKPWR					Mascot
1182.63	1182.548	-0.082	-69	1	11	GPPPLPSMSKR			Oxidation (M)[8]		Mascot
1927.9695	1927.8556	-0.1139	-59	121	138	GSAITGPIGKECADLWPR			Carbamidomethyl (C)[12]		Mascot

6	hypothetical protein TRIUR3_10047 [Triticum urartu]	gi|473999812	17799.1	9.33	3	15	0	.305

Peptide Information

Calc. Mass	Obsrv. Mass	± da	± ppm	Start Seq.	End Seq.	Sequence	Ion Score	C. I. %	Modification	Rank	Result Type
1194.499	1194.6129	0.1139	95	37	45	IDCEGCERR			Carbamidomethyl (C)[3,6]		Mascot
1308.6907	1308.64	-0.0507	-39	123	135	NVVADPDAAPLAR					Mascot
1927.8381	1927.8556	0.0175	9	1	17	MGILDAVSDMCACPTVR			Carbamidomethyl (C)[11,13], Oxidation (M)[1,10]		Mascot

7	hypothetical protein TRIUR3_06999 [Triticum urartu]	gi|474239014	32634.9	9.1	4	15	0	.447

Peptide Information

Calc. Mass	Obsrv. Mass	± da	± ppm	Start Seq.	End Seq.	Sequence	Ion Score	C. I. %	Modification	Rank	Result Type
1107.5793	1107.5387	-0.0406	-37	44	52	SQLEEFARK					Mascot
1194.5031	1194.6129	0.1098	92	104	112	QQFHEMCAK			Carbamidomethyl (C)[7], Oxidation (M)[6]		Mascot
1308.6907	1308.64	-0.0507	-39	217	228	DHNGILELAQAK					Mascot
2399.166	2399.0247	-0.1413	-59	32	51	TDVMQEQLATFRSQLEE FAR					Mascot

8	hypothetical protein TRIUR3_34528 [Triticum urartu]	gi|474244850	6182.1	11.36	2	15	0	.262

Peptide Information

Calc. Mass	Obsrv. Mass	± da	± ppm	Start Seq.	End Seq.	Sequence	Ion Score	C. I. %	Modification	Rank	Result Type
1107.5762	1107.5387	-0.0375	-34	33	41	IMMQRLGAR			Oxidation (M)[2,3]		Mascot
1308.6465	1308.64	-0.0065	-5	1	12	MEISTSSVPSVR			Oxidation (M)[1]		Mascot

9	hypothetical protein TRIUR3_31825 [Triticum urartu]	gi|474259051	27233.9	11.35	4	15	0	1.333

Peptide Information

Calc. Mass	Obsrv. Mass	± da	± ppm	Start	End	Sequence	Ion	C. I. %	Modification	Rank	Result Type

				Seq.	Seq.		Score				
832.3907	832.3159	-0.0748	-90	209	215	EEGDARR					Mascot
870.5268	870.5399	0.0131	15	25	31	RNLSRPK					Mascot
917.4145	917.3455	-0.069	-75	1	7	MDLTHER			Oxidation (M)[1]		Mascot
1194.6226	1194.6129	-0.0097	-8	65	74	NLAFTETRSR					Mascot

10 Chain O, Cryo-Em Structure Of The Mammalian Sec61 Complex Bound To The Actively Translating Wheat Germ 80s Ribosome gi|270346382 6337.5 12.55 2 15 0 .214

Peptide Information

Calc. Mass	Obsrv. Mass	± da	± ppm	Start Seq.	End Seq.	Sequence	Ion Score	C. I. %	Modification	Rank	Result Type
807.4471	807.3845	-0.0626	-78	2	8	AAQKSFR					Mascot
1194.6226	1194.6129	-0.0097	-8	31	40	TNNTIRYNAK					Mascot

Gel Idx/Pos	156/G7	Instr./Gel Origin	BA2151/Sample Project 20140814	Process Status	Analysis Succeeded
Plate [#] Name	[1] Sample Project 20140814	Instrument Sample Name		Spectra	11

Rank	Protein Name	Accession No.	Protein MW	Protein PI	Pep. Count	Protein Score	Protein Score C. I. %	Intensity Matched	Total Ion Score	Total Ion C. I. %	Confirmed
1	cold regulated protein [Triticum aestivum]	gi|26017213	17789.9	4.84	8	224	100	6.939	173	100	

Peptide Information

Calc. Mass	Obsrv. Mass	± da	± ppm	Start Seq.	End Seq.	Sequence	Ion Score	C. I. %	Modification	Rank	Result Type
834.4138	834.3375	-0.0763	-91	1	7	MAQESLR					Mascot
1058.5127	1058.5005	-0.0122	-12	115	124	GPVPDSHGHR					Mascot
1321.6682	1321.6213	-0.0469	-35	8	19	LVCPPVSAHEGR			Carbamidomethyl (C)[3]		Mascot
1321.6682	1321.6213	-0.0469	-35	8	19	LVCPPVSAHEGR	44	99.696	Carbamidomethyl (C)[3]		Mascot
1460.7856	1460.7269	-0.0587	-40	20	32	LPRQYTLEGQGAK					Mascot
1515.765	1515.7372	-0.0278	-18	50	63	SLAVVVQDVDADER					Mascot
1515.765	1515.7372	-0.0278	-18	50	63	SLAVVVQDVDADER	63	99.996			Mascot
1801.8756	1801.845	-0.0306	-17	34	49	DISPPLEWYGVPDGTR					Mascot
1801.8756	1801.845	-0.0306	-17	34	49	DISPPLEWYGVPDGTR	66	99.998			Mascot
1929.9706	1929.907	-0.0636	-33	33	49	KDISPPLEWYGVPDGTR					Mascot
2031.0104	2030.9117	-0.0987	-49	147	165	VMEAIEGHVLGEAEITAVF			Oxidation (M)[2]		Mascot

Rank	Protein Name	Accession No.	Protein MW	Protein PI	Pep. Count	Protein Score	Protein Score C. I. %	Intensity Matched	Total Ion Score	Total Ion C. I. %	Confirmed
2	hypothetical protein TRIUR3_03726 [Triticum urartu]	gi|474377086	73738.8	9.44	17	55	85.016	7.106			

Peptide Information

Calc. Mass	Obsrv. Mass	± da	± ppm	Start Seq.	End Seq.	Sequence	Ion Score	C. I. %	Modification	Rank	Result Type
869.474	869.426	-0.048	-55	656	662	AHRFNPK					Mascot
886.4628	886.4082	-0.0546	-62	352	359	LAPSQNEK					Mascot
888.4785	888.4403	-0.0382	-43	112	119	VLAENNTK					Mascot
992.4102	992.464	0.0538	54	43	50	VNEDAECR			Carbamidomethyl (C)[7]		Mascot
1033.5823	1033.4937	-0.0886	-86	576	584	GCTSRVILK			Carbamidomethyl (C)[2]		Mascot
1262.6774	1262.5918	-0.0856	-68	163	173	VLTKDMEGVVR			Oxidation (M)[6]		Mascot
1308.542	1308.619	0.077	59	41	50	CRVNEDAECR			Carbamidomethyl (C)[1,9]		Mascot
1427.8005	1427.7582	-0.0423	-30	155	166	ELHGDIFRVLTK					Mascot
1434.7153	1434.7395	0.0242	17	405	416	MILNMEPVSCLK			Carbamidomethyl (C)[10]		Mascot
1475.7596	1475.7263	-0.0333	-23	135	147	LNEMSLVVSVCPK			Carbamidomethyl (C)[11]		Mascot
1479.8427	1479.7128	-0.1299	-88	372	383	KCVIVALYLCLK			Carbamidomethyl (C)[2,10]		Mascot

Calc. Mass	Obsrv. Mass	± da	± ppm	Start Seq.	End Seq.	Sequence	Ion Score	C. I. %	Modification	Rank	Result Type
1493.7708	1493.7051	-0.0657	-44	642	655	DEAVHTVEAAPAKR					Mascot
1543.8075	1543.7606	-0.0469	-30	106	119	GQNDLKVLAENNTK					Mascot
1628.849	1628.8151	-0.0339	-21	561	575	KEPVNIIIGSDNDSK					Mascot
1699.8796	1699.7977	-0.0819	-48	271	285	SQLKQTPVTDAMPQR					Mascot
1801.9048	1801.845	-0.0598	-33	203	219	MSVANGRPTMNQTPVAK					Mascot
1801.9048	1801.845	-0.0598	-33	203	219	MSVANGRPTMNQTPVAK					Mascot
1817.8997	1817.8295	-0.0702	-39	203	219	MSVANGRPTMNQTPVAK			Oxidation (M)[1]		Mascot
1833.8947	1833.8386	-0.0561	-31	203	219	MSVANGRPTMNQTPVAK			Oxidation (M)[1,10]		Mascot
1833.8947	1833.8386	-0.0561	-31	203	219	MSVANGRPTMNQTPVAK			Oxidation (M)[1,10]		Mascot
3052.5232	3052.573	0.0498	16	300	327	IPLADGSPHMEQITPTKP LPACSTYATR			Carbamidomethyl (C)[22]		Mascot

3 DEAD-box ATP-dependent RNA helicase 52A [Triticum urartu] gi|474239079 47632.3 8.92 13 50 57.769 6.937

Peptide Information

Calc. Mass	Obsrv. Mass	± da	± ppm	Start Seq.	End Seq.	Sequence	Ion Score	C. I. %	Modification	Rank	Result Type
834.4025	834.3375	-0.065	-78	1	7	MINEEAK					Mascot
886.4457	886.4082	-0.0375	-42	215	220	YWLYNK					Mascot
891.4604	891.4424	-0.018	-20	75	81	LVDMIER			Oxidation (M)[4]		Mascot
1037.4243	1037.499	0.0747	72	348	359	SNGDGGSSSGGR					Mascot
1104.5969	1104.5208	-0.0761	-69	311	320	GLLELMTEAK					Mascot
1141.5194	1141.4553	-0.0641	-56	91	99	YLIMDEADR			Oxidation (M)[4]		Mascot
1193.5254	1193.5961	0.0707	59	347	359	RSNGDGGSSSGGR					Mascot
1336.6388	1336.6111	-0.0277	-21	100	110	MLDMGFEPQIR					Mascot
1390.5619	1390.6545	0.0926	67	363	378	GGSDYSGYSGGGGGGR					Mascot
1471.6157	1471.6912	0.0755	51	348	362	SNGDGGSSSGGRDYR					Mascot
1515.8199	1515.7372	-0.0827	-55	245	259	SGLTPVMVATDVVAR					Mascot
1515.8199	1515.7372	-0.0827	-55	245	259	SGLTPVMVATDVVAR					Mascot
1830.016	1829.8708	-0.1452	-79	140	155	LASDFLSKYIFITVGR					Mascot
1942.0426	1941.8958	-0.1468	-76	64	81	GADILVATPGRLVDMIER			Oxidation (M)[15]		Mascot

4 hypothetical protein TRIUR3_29577 [Triticum urartu] gi|473951269 69405.6 5.41 17 46 0 3.606

Peptide Information

Calc. Mass	Obsrv. Mass	± da	± ppm	Start Seq.	End Seq.	Sequence	Ion Score	C. I. %	Modification	Rank	Result Type
822.4468	822.3892	-0.0576	-70	59	64	ELNKYR					Mascot
888.5149	888.4403	-0.0746	-84	95	102	LDAINKSK					Mascot

Calc. Mass	Obsrv. Mass	± da	± ppm	Start Seq.	End Seq.	Sequence	Ion Score	C. I. %	Modification	Rank	Result Type
992.4829	992.464	-0.0189	-19	532	540	RAEMAEAAK			Oxidation (M)[4]		Mascot
1068.5684	1068.4973	-0.0711	-67	50	58	ETELHLAQK					Mascot
1118.5437	1118.4897	-0.054	-48	65	74	EQLSNAETAR					Mascot
1286.7063	1286.6093	-0.097	-75	441	451	ALDQIKQLSDR					Mascot
1316.7056	1316.5868	-0.1188	-90	424	435	LQSALKEAEEAK					Mascot
1460.772	1460.7269	-0.0451	-31	603	615	GFVLPNITGMFHK					Mascot
1479.8529	1479.7128	-0.1401	-95	46	58	VLAKETELHLAQK					Mascot
1493.7264	1493.7051	-0.0213	-14	103	115	EMAIQATEDTKTR					Mascot
1542.7646	1542.7236	-0.041	-27	340	353	DTDTESIVADLHVK					Mascot
1605.8092	1605.7351	-0.0741	-46	580	594	APAGKPTEKNDGHQR					Mascot
1657.9119	1657.7573	-0.1546	-93	415	429	VALTEAEERLQSALK					Mascot
1738.8164	1738.8005	-0.0159	-9	264	278	EKLDETNSEISSMQK					Mascot
1799.9022	1799.8578	-0.0444	-25	338	353	EKDTDTESIVADLHVK					Mascot
1817.9603	1817.8295	-0.1308	-72	452	470	ASAVQASTSEPGGKVTIS K					Mascot
1983.9982	1983.9072	-0.091	-46	174	191	LSAAQKEEESLHSIEANK					Mascot

5	Cell division control protein 48-like protein B [Triticum urartu]	gi|473886645	61577.5	8.92	13	44	0	3.493	

Peptide Information

Calc. Mass	Obsrv. Mass	± da	± ppm	Start Seq.	End Seq.	Sequence	Ion Score	C. I. %	Modification	Rank	Result Type
802.4603	802.4217	-0.0386	-48	35	41	MALQALR					Mascot
896.3964	896.4196	0.0232	26	1	8	MGEVMAAR			Oxidation (M)[1,5]		Mascot
992.4941	992.464	-0.0301	-30	2	10	GEVMAARSR			Oxidation (M)[4]		Mascot
1033.5823	1033.4937	-0.0886	-86	305	313	SQIKASMIR					Mascot
1106.5841	1106.5032	-0.0809	-73	399	408	YVGEGEALLR					Mascot
1107.5398	1107.5212	-0.0186	-17	1	10	MGEVMAARSR					Mascot
1262.6852	1262.5918	-0.0934	-74	399	409	YVGEGEALLRR					Mascot
1471.6885	1471.6912	0.0027	2	9	23	SRGGANGEDGEPVAR					Mascot
1487.7377	1487.7126	-0.0251	-17	318	331	EAPTVSWDDIGGLK					Mascot
1674.8368	1674.7028	-0.134	-80	514	528	MKLGEDVDLGEIAER					Mascot
1699.8909	1699.7977	-0.0932	-55	26	41	AEEAVAGNRMALQALR					Mascot
2031.054	2030.9117	-0.1423	-70	172	189	EQESRIVGQLLTLMDGNK					Mascot
2369.207	2368.9724	-0.2346	-99	489	508	FDMVLYVPPPDAQGRHE ILR			Oxidation (M)[3]		Mascot

6	hypothetical protein TRIUR3_09872 [Triticum urartu]	gi|473860383	8133.9	5.01	7	44	0	3.325	

Peptide Information

Calc. Mass	Obsrv. Mass	± da	± ppm	Start Seq.	End Seq.	Sequence	Ion Score	C. I. %	Modification	Rank	Result Type
869.4879	869.426	-0.0619	-71	55	61	KNLYGFK					Mascot
922.3723	922.4426	0.0703	76	11	17	MWEGDER					Mascot
1106.5325	1106.5032	-0.0293	-26	62	72	GLDSSGEQVSK					Mascot
1193.5831	1193.5961	0.013	11	45	54	DLQMQNISTK			Oxidation (M)[4]		Mascot
1286.6199	1286.6093	-0.0106	-8	1	10	MEEHWSPVKK			Oxidation (M)[1]		Mascot
1321.6781	1321.6213	-0.0568	-43	45	55	DLQMQNISTKK			Oxidation (M)[4]		Mascot
1321.6781	1321.6213	-0.0568	-43	45	55	DLQMQNISTKK			Oxidation (M)[4]		Mascot
1542.6224	1542.7236	0.1012	66	18	32	STDQGESSGMVDDAK			Oxidation (M)[10]		Mascot

7 Serine/threonine-protein kinase ATR [Triticum urartu] gi|473878625 222281.3 7.03 25 42 0 12.622

Peptide Information

Calc. Mass	Obsrv. Mass	± da	± ppm	Start Seq.	End Seq.	Sequence	Ion Score	C. I. %	Modification	Rank	Result Type
802.4669	802.4217	-0.0452	-56	370	376	LLNSIDK					Mascot
826.4893	826.4093	-0.08	-97	45	51	VLRHSSK					Mascot
829.4778	829.3951	-0.0827	-100	1514	1521	EAAGQILK					Mascot
848.3818	848.4283	0.0465	55	1431	1438	EGPSAEMK					Mascot
870.5117	870.5199	0.0082	9	236	243	MVPSVIPK					Mascot
926.4651	926.4061	-0.059	-64	109	115	ELKFMDK			Oxidation (M)[5]		Mascot
992.4427	992.464	0.0213	21	1743	1750	IPEDEMMK					Mascot
999.4597	999.4437	-0.016	-16	1725	1732	MKTNSQMK			Oxidation (M)[1,7]		Mascot
1037.6003	1037.499	-0.1013	-98	1241	1248	AKHLWNIR					Mascot
1263.7382	1263.624	-0.1142	-90	412	422	IMVLLIFATDK					Mascot
1316.6879	1316.5868	-0.1011	-77	48	58	HSSKEVLLEMK			Oxidation (M)[10]		Mascot
1357.6455	1357.6669	0.0214	16	1896	1908	SSGGEVQNPQAQR					Mascot
1460.7567	1460.7269	-0.0298	-20	1710	1721	HILQDIYITCGK			Carbamidomethyl (C)[10]		Mascot
1493.6777	1493.7051	0.0274	18	1038	1049	TWCMQGVQAAWR			Carbamidomethyl (C)[3]		Mascot
1515.7183	1515.7372	0.0189	12	1088	1100	AMTTKDQFMVAEK			Oxidation (M)[2]		Mascot
1515.7183	1515.7372	0.0189	12	1088	1100	AMTTKDQFMVAEK			Oxidation (M)[2]		Mascot
1605.7649	1605.7351	-0.0298	-19	849	863	QEIALSQSNNAMAGR			Oxidation (M)[12]		Mascot
1738.8656	1738.8005	-0.0651	-37	61	74	WVNCIDFLLLNGMK			Carbamidomethyl (C)[4], Oxidation (M)[13]		Mascot
1801.934	1801.845	-0.089	-49	396	411	KLVEMMGPYLSTHAPK					Mascot
1801.934	1801.845	-0.089	-49	396	411	KLVEMMGPYLSTHAPK					Mascot
1805.903	1805.8442	-0.0588	-33	1364	1384	FTGGVGSVTPGSAGSAS APAK					Mascot

Calc. Mass	Obsrv. Mass	± da	± ppm	Start Seq.	End Seq.	Sequence	Ion Score	C. I. %	Modification	Rank	Result Type
1817.9288	1817.8295	-0.0993	-55	396	411	KLVEMMGPYLSTHAPK			Oxidation (M)[5]		Mascot
1829.9976	1829.8708	-0.1268	-69	33	47	ECIISMVEVLPRVLR			Carbamidomethyl (C)[2], Oxidation (M)[6]		Mascot
1833.9238	1833.8386	-0.0852	-46	396	411	KLVEMMGPYLSTHAPK			Oxidation (M)[5,6]		Mascot
1833.9238	1833.8386	-0.0852	-46	396	411	KLVEMMGPYLSTHAPK			Oxidation (M)[5,6]		Mascot
1838.9139	1838.8856	-0.0283	-15	1106	1122	QALLVPLAAAGMDSYMR			Oxidation (M)[12,16]		Mascot
1933.9978	1933.8875	-0.1103	-57	1363	1384	KFTGGVGSVTPGSAGSA SAPAK					Mascot
1966.9215	1966.9036	-0.0179	-9	1050	1065	LGRWDLMDEYLPEADK			Oxidation (M)[7]		Mascot
1989.9771	1989.9128	-0.0643	-32	849	867	QEIALSQSNNAMAGRQA GK			Oxidation (M)[12]		Mascot
2904.4058	2904.3218	-0.084	-29	1214	1240	LAGHYETAHRAILEADAS GAPNVHMEK			Oxidation (M)[25]		Mascot

8 THO complex subunit 4 [Triticum urartu] gi|474314133 23909.1 10.39 9 41 0 1.977

Peptide Information

Calc. Mass	Obsrv. Mass	± da	± ppm	Start Seq.	End Seq.	Sequence	Ion Score	C. I. %	Modification	Rank	Result Type
1033.5287	1033.4937	-0.035	-34	157	167	GAFQAGRGGGR					Mascot
1106.5511	1106.5032	-0.0479	-43	121	130	TVVMTPETGR			Oxidation (M)[4]		Mascot
1168.5535	1168.5148	-0.0387	-33	50	59	FAVHFDGYGR					Mascot
1262.6522	1262.5918	-0.0604	-48	121	131	TVVMTPETGRR			Oxidation (M)[4]		Mascot
1381.7145	1381.6163	-0.0982	-71	81	92	YNNVLLDGKSMK					Mascot
1434.7369	1434.7395	0.0026	2	118	130	ATRTVVMTPETGR			Oxidation (M)[7]		Mascot
1487.7561	1487.7126	-0.0435	-29	132	146	GGGSSTRPLSNPTTR					Mascot
1934.0085	1933.8875	-0.121	-63	90	107	SMKIEVIGSDLGLSMTPR					Mascot
1941.9917	1941.8958	-0.0959	-49	23	38	LYISNLDYRVSNEDIK					Mascot

9 Phospholipase A1-II 1 [Triticum urartu] gi|473780234 19614.8 9.06 9 41 0 2.548

Peptide Information

Calc. Mass	Obsrv. Mass	± da	± ppm	Start Seq.	End Seq.	Sequence	Ion Score	C. I. %	Modification	Rank	Result Type
800.4009	800.374	-0.0269	-34	115	121	EDGAPRR					Mascot
810.3563	810.3807	0.0244	30	107	113	CAAGEFR			Carbamidomethyl (C)[1]		Mascot
841.4454	841.4343	-0.0111	-13	35	41	LVDYFGK					Mascot
848.4625	848.4283	-0.0342	-40	78	85	AVTFSAPR					Mascot
859.4492	859.4316	-0.0176	-20	99	106	NGSAQRAR					Mascot
1037.4945	1037.499	0.0045	4	105	113	ARCAAGEFR			Carbamidomethyl (C)[3]		Mascot
1373.6768	1373.6379	-0.0389	-28	23	34	SARDQVADELNR					Mascot
1929.9011	1929.907	0.0059	3	1	18	MVAEGFHTLYTSSNAGT K			Oxidation (M)[1]		Mascot

1990.0618	1989.9128	-0.149	-75	66	85	DAAAAHPGVPVRAVTFS APR					Mascot

10	LL-diaminopimelate aminotransferase, chloroplastic [Triticum urartu]	gi|474069757	20284	6.19	8	40	0	1.787

Peptide Information

Calc. Mass	Obsrv. Mass	± da	± ppm	Start Seq.	End Seq.	Sequence	Ion Score	C. I. %	Modification	Rank	Result Type
822.3774	822.3892	0.0118	14	13	19	GDQISMR			Oxidation (M)[6]		Mascot
886.4053	886.4082	0.0029	3	151	157	YDNAFTR					Mascot
914.4222	914.3859	-0.0363	-40	109	115	LQMMFGR			Oxidation (M)[3,4]		Mascot
992.4771	992.464	-0.0131	-13	2	8	SFHWMLR			Oxidation (M)[5]		Mascot
1107.5227	1107.5212	-0.0015	-1	1	8	MSFHWMLR					Mascot
1316.7103	1316.5868	-0.1235	-94	9	19	LLQRGDQISMR					Mascot
1434.741	1434.7395	-0.0015	-1	80	92	AIADVVYPNMGIR			Oxidation (M)[10]		Mascot
1796.812	1796.92	0.108	60	93	108	DTEVFISDGAQCDIAR			Carbamidomethyl (C)[12]		Mascot

Gel Idx/Pos	157/G8	Instr./Gel Origin	BA2151/Sample Project 20140814	Process Status	Analysis Succeeded
Plate [#] Name	[1] Sample Project 20140814	Instrument Sample Name		Spectra	11

Rank	Protein Name	Accession No.	Protein MW	Protein PI	Pep. Count	Protein Score	Protein Score C. I. %	Intensity Matched	Total Ion Score	Total Ion C. I. %	Confirmed
1	cold regulated protein [Triticum aestivum]	gi|26017213	17789.9	4.84	8	185	100	10.828	129	100	

Peptide Information

Calc. Mass	Obsrv. Mass	± da	± ppm	Start Seq.	End Seq.	Sequence	Ion Score	C. I. %	Modification	Rank	Result Type
834.4138	834.4421	0.0283	34	1	7	MAQESLR					Mascot
850.4087	850.3942	-0.0145	-17	1	7	MAQESLR			Oxidation (M)[1]		Mascot
1058.5127	1058.4834	-0.0293	-28	115	124	GPVPDSHGHR					Mascot
1321.6682	1321.6199	-0.0483	-37	8	19	LVCPPVSAHEGR			Carbamidomethyl (C)[3]		Mascot
1321.6682	1321.6199	-0.0483	-37	8	19	LVCPPVSAHEGR	58	99.988	Carbamidomethyl (C)[3]		Mascot
1420.7682	1420.6823	-0.0859	-60	129	141	LYALDDVLSLGNK					Mascot
1515.765	1515.7192	-0.0458	-30	50	63	SLAVVVQDVDADER					Mascot
1515.765	1515.7192	-0.0458	-30	50	63	SLAVVVQDVDADER	71	100			Mascot
1801.8756	1801.8271	-0.0485	-27	34	49	DISPPLEWYGVPDGTR					Mascot
1929.9706	1929.9089	-0.0617	-32	33	49	KDISPPLEWYGVPDGTR					Mascot
2031.0104	2030.8981	-0.1123	-55	147	165	VMEAIEGHVLGEAEITAV F			Oxidation (M)[2]		Mascot

Rank	Protein Name	Accession No.	Protein MW	Protein PI	Pep. Count	Protein Score	Protein Score C. I. %	Intensity Matched	Total Ion Score	Total Ion C. I. %	Confirmed
2	hypothetical protein TRIUR3_14206 [Triticum urartu]	gi|474314641	18385.3	4.93	7	46	0	6.464			

Peptide Information

Calc. Mass	Obsrv. Mass	± da	± ppm	Start Seq.	End Seq.	Sequence	Ion Score	C. I. %	Modification	Rank	Result Type
801.4213	801.4565	0.0352	44	75	82	GAPRSDAK					Mascot
831.4683	831.4418	-0.0265	-32	125	132	ATGVRAEK					Mascot
1193.5281	1193.5756	0.0475	40	64	74	SGDESEAETIR					Mascot
1349.6292	1349.6318	0.0026	2	63	74	RSGDESEAETIR					Mascot
1515.7802	1515.7192	-0.061	-40	113	124	LEKAWQEVEQQK					Mascot
1515.7802	1515.7192	-0.061	-40	113	124	LEKAWQEVEQQK					Mascot
2222.116	2222.094	-0.022	-10	144	162	DQARVLEVEETLNGVHQ ER					Mascot
2239.0774	2239.0542	-0.0232	-10	86	105	VGLSFNANAELFYMTAV MTK			Oxidation (M)[14,18]		Mascot

Rank	Protein Name	Accession No.	Protein MW	Protein PI	Pep. Count	Protein Score	Protein Score C. I. %	Intensity Matched	Total Ion Score	Total Ion C. I. %	Confirmed
3	Phospholipase A1-II 1 [Triticum urartu]	gi|473780234	19614.8	9.06	8	41	0	4.699			

Peptide Information

Calc. Mass	Obsrv. Mass	± da	± ppm	Start Seq.	End Seq.	Sequence	Ion Score	C. I. %	Modification	Rank	Result Type
800.4009	800.3549	-0.046	-57	115	121	EDGAPRR					Mascot
810.3563	810.3676	0.0113	14	107	113	CAAGEFR			Carbamidomethyl (C)[1]		Mascot
848.4625	848.3998	-0.0627	-74	78	85	AVTFSAPR					Mascot
859.4492	859.4466	-0.0026	-3	99	106	NGSAQRAR					Mascot
864.4032	864.4283	0.0251	29	143	149	WSQMANK					Mascot
1929.9011	1929.9089	0.0078	4	1	18	MVAEGFHTLYTSSNAGT K			Oxidation (M)[1]		Mascot
1944.8855	1944.8307	-0.0548	-28	165	181	ERDDMPANDVLPLSELD			Oxidation (M)[5]		Mascot
2239.0369	2239.0542	0.0173	8	130	149	SAMLSDTEGIPEKWSQM ANK			Oxidation (M)[3]		Mascot

4 G-type lectin S-receptor-like serine/threonine-protein kinase SD2-5 [Triticum urartu] gi|473905253 44913.3 6.53 9 38 0 5.961

Peptide Information

Calc. Mass	Obsrv. Mass	± da	± ppm	Start Seq.	End Seq.	Sequence	Ion Score	C. I. %	Modification	Rank	Result Type
848.4625	848.3998	-0.0627	-74	195	202	VADFGLAR					Mascot
1193.6888	1193.5756	-0.1132	-95	95	106	GELPNGLPVAVK					Mascot
1420.7048	1420.6823	-0.0225	-16	268	277	EWFPKWVWDK					Mascot
1507.7573	1507.7032	-0.0541	-36	116	129	VQEGFMAEIGTIGR					Mascot
1801.861	1801.8271	-0.0339	-19	203	219	LGERENTHMSSLTGGGR					Mascot
1817.856	1817.822	-0.034	-19	203	219	LGERENTHMSSLTGGGR			Oxidation (M)[9]		Mascot
1817.856	1817.822	-0.034	-19	203	219	LGERENTHMSSLTGGGR			Oxidation (M)[9]		Mascot
1941.995	1941.8744	-0.1206	-62	373	392	DSEVSAASAPAPKPSVA MVK					Mascot
2222.1299	2222.094	-0.0359	-16	34	55	RAAPDDGAGVDDVVIEIG PVEK					Mascot
2257.97	2258.1106	0.1406	62	278	298	YEQGDMECIVSAAAGIGE ADR			Carbamidomethyl (C)[8], Oxidation (M)[6]		Mascot
2368.1543	2368.2007	0.0464	20	130	148	TYHVHLVRLYGFCFDPDT K			Carbamidomethyl (C)[13]		Mascot

5 hypothetical protein TRIUR3_20602 [Triticum urartu] gi|474043553 7085.5 8.2 2 37 0 1.843 23 65.273

Peptide Information

Calc. Mass	Obsrv. Mass	± da	± ppm	Start Seq.	End Seq.	Sequence	Ion Score	C. I. %	Modification	Rank	Result Type
859.442	859.4466	0.0046	5	60	66	RYELHAA					Mascot
1817.8373	1817.822	-0.0153	-8	27	44	EADGPERAQSSTAQGSA R					Mascot
1817.8373	1817.822	-0.0153	-8	27	44	EADGPERAQSSTAQGSA R	23	65.273			Mascot

6	Fructokinase-2 [Triticum urartu]	gi|474190636	42253.2	4.78	9	36	0	5.528

Peptide Information

Calc. Mass	Obsrv. Mass	± da	± ppm	Start Seq.	End Seq.	Sequence	Ion Score	C. I. %	Modification	Rank	Result Type
817.3839	817.3376	-0.0463	-57	2	8	AHYPSDK					Mascot
858.425	858.4929	0.0679	79	251	258	SAHVAAMR			Oxidation (M)[7]		Mascot
908.3971	908.3735	-0.0236	-26	208	213	EFMFYR			Oxidation (M)[3]		Mascot
948.4244	948.4483	0.0239	25	1	8	MAHYPSDK					Mascot
1253.6637	1253.6033	-0.0604	-48	275	285	LPLWPSDQAAR					Mascot
1320.6332	1320.5499	-0.0833	-63	2	12	AHYPSDKEAFR					Mascot
1383.705	1383.6206	-0.0844	-61	138	152	APGGAPANVACAISK			Carbamidomethyl (C)[11]		Mascot
1792.9052	1792.9717	0.0665	37	236	250	IFHYGSISLITEPCR			Carbamidomethyl (C)[14]		Mascot
1792.9052	1792.9717	0.0665	37	236	250	IFHYGSISLITEPCR			Carbamidomethyl (C)[14]		Mascot
1962.0178	1961.9135	-0.1043	-53	25	40	LEEQNLEIPEIHQELK					Mascot

7	Disease resistance protein RPM1 [Triticum urartu]	gi|474147744	139152.2	8	16	34	0	10.886

Peptide Information

Calc. Mass	Obsrv. Mass	± da	± ppm	Start Seq.	End Seq.	Sequence	Ion Score	C. I. %	Modification	Rank	Result Type
826.4781	826.397	-0.0811	-98	60	66	LENPVVR					Mascot
835.423	835.3668	-0.0562	-67	1072	1078	MDLEIAK			Oxidation (M)[1]		Mascot
858.4904	858.4929	0.0025	3	30	36	LRQSAQR					Mascot
859.4091	859.4466	0.0375	44	536	542	NPIAECR			Carbamidomethyl (C)[6]		Mascot
903.4968	903.4227	-0.0741	-82	2	10	ADLVVGMAK					Mascot
948.4686	948.4483	-0.0203	-21	92	98	NSSWWLR					Mascot
955.4818	955.4586	-0.0232	-24	349	355	RFFPNMK			Oxidation (M)[6]		Mascot
993.4782	993.4468	-0.0314	-32	794	802	GSCALTTQR			Carbamidomethyl (C)[3]		Mascot
1182.561	1182.5209	-0.0401	-34	1087	1097	HSDGATPEGRR					Mascot
1182.561	1182.5209	-0.0401	-34	1087	1097	HSDGATPEGRR					Mascot
1420.6501	1420.6823	0.0322	23	766	776	MVFCQVNGFFR			Carbamidomethyl (C)[4], Oxidation (M)[1]		Mascot
1442.8214	1442.6907	-0.1307	-91	183	195	TTQKGDLTQLLPK					Mascot
1792.8582	1792.9717	0.1135	63	882	897	GCHEICHLPSSIGGLR			Carbamidomethyl (C)[2,6]		Mascot
1793.0531	1792.9717	-0.0814	-45	906	921	HTSIVTLPVNITKLEK					Mascot
1801.8313	1801.8271	-0.0042	-2	666	680	FMLELENNGEFDSLK			Oxidation (M)[2]		Mascot
1946.0077	1945.8882	-0.1195	-61	11	29	SVVDGALTKAQAAIEEES K					Mascot
2222.1235	2222.094	-0.0295	-13	1029	1048	LEDNNQGCLNGIPLPLEG			Carbamidomethyl (C)[8]		Mascot

LR

8	Cellulose synthase-like protein E6 [Triticum urartu]	gi|474311858	76464.4	6.45	11	33	0	5.77	

Peptide Information

Calc. Mass	Obsrv. Mass	± da	± ppm	Start Seq.	End Seq.	Sequence	Ion Score	C. I. %	Modification	Rank	Result Type
848.4811	848.3998	-0.0813	-96	528	533	MWLVKR			Oxidation (M)[1]		Mascot
903.422	903.4227	0.0007	1	521	527	GWWNGQR					Mascot
908.3843	908.3735	-0.0108	-12	562	569	VSDEDESK					Mascot
1193.5586	1193.5756	0.017	14	394	403	GWESVYNNPK					Mascot
1320.6166	1320.5499	-0.0667	-51	344	354	LQESIDETEEK					Mascot
1338.5961	1338.6079	0.0118	9	331	341	DYQEDWNAGIK					Mascot
1475.6914	1475.703	0.0116	8	103	114	GFHEWNQEITSK					Mascot
1929.8721	1929.9089	0.0368	19	69	83	EWSLIKDMFDEMTER					Mascot
1933.9259	1933.8823	-0.0436	-23	377	393	YGCAVEDVITGLAIHCR			Carbamidomethyl (C)[3,16]		Mascot
1944.9994	1944.8307	-0.1687	-87	195	211	LIDAQSVTCGMSIKLHR			Carbamidomethyl (C)[9], Oxidation (M)[11]		Mascot
1945.8671	1945.8882	0.0211	11	69	83	EWSLIKDMFDEMTER			Oxidation (M)[8]		Mascot
1961.8619	1961.9135	0.0516	26	69	83	EWSLIKDMFDEMTER			Oxidation (M)[8,12]		Mascot
2222.0183	2222.094	0.0757	34	357	376	SLAACTYEHGTQWGDEI GVK			Carbamidomethyl (C)[5]		Mascot

9	Transcription initiation factor TFIID subunit 1 [Triticum urartu]	gi|474156043	226172.6	5.62	21	32	0	14.726	

Peptide Information

Calc. Mass	Obsrv. Mass	± da	± ppm	Start Seq.	End Seq.	Sequence	Ion Score	C. I. %	Modification	Rank	Result Type
801.4213	801.4565	0.0352	44	1745	1751	SGQEPRK					Mascot
827.437	827.387	-0.05	-60	256	262	AKTDQHK					Mascot
834.4614	834.4421	-0.0193	-23	772	778	GMLSVRR			Oxidation (M)[2]		Mascot
847.4454	847.3937	-0.0517	-61	1453	1460	ADGMRGLK					Mascot
850.3835	850.3942	0.0107	13	1	7	MSDGERR					Mascot
856.4635	856.4998	0.0363	42	410	417	NNGSPVIR					Mascot
890.473	890.3884	-0.0846	-95	730	737	SPFLGDVR					Mascot
895.3938	895.4143	0.0205	23	1450	1457	SDKADGMR			Oxidation (M)[7]		Mascot
903.4465	903.4227	-0.0238	-26	1142	1149	TPMGDARR					Mascot
916.4159	916.3589	-0.057	-62	396	403	DYGYVSGR					Mascot
932.4333	932.4264	-0.0069	-7	1160	1166	GNERDWR					Mascot
1323.7419	1323.6218	-0.1201	-91	1280	1291	GLGFSYVRVTPK					Mascot
1383.6097	1383.6206	0.0109	8	1873	1885	TPADMTDYAPSAK			Oxidation (M)[5]		Mascot

1442.6646	1442.6907	0.0261	18	1530	1543	EVDSSFTEGGLSSK					Mascot
1507.7904	1507.7032	-0.0872	-58	243	255	FSEIFGAQEPVRK					Mascot
1515.7883	1515.7192	-0.0691	-46	528	540	SHARAMIVSRPMK			Oxidation (M)[6,12]		Mascot
1515.7883	1515.7192	-0.0691	-46	528	540	SHARAMIVSRPMK			Oxidation (M)[6,12]		Mascot
1837.8572	1837.8566	-0.0006	0	1068	1085	SSVAGQTGWGCGMPTV VK			Carbamidomethyl (C)[11], Oxidation (M)[13]		Mascot
1945.0125	1944.8307	-0.1818	-93	1355	1373	LSSEQAASGITIDEIPVSK					Mascot
1961.8771	1961.9135	0.0364	19	1957	1973	NDVAQIADNAHMYNETR					Mascot
2222.1274	2222.094	-0.0334	-15	1923	1941	VAPDYYDVIQRPMDLGTI R					Mascot
2246.1123	2246.1206	0.0083	4	694	712	LCTYYQKTSPTDQTAISL R			Carbamidomethyl (C)[2]		Mascot

10	hypothetical protein TRIUR3_32091 [Triticum urartu]	gi|474431491	34798.9	6.28	5	32	0	3.682	15	0	

Peptide Information

Calc. Mass	Obsrv. Mass	± da	± ppm	Start Seq.	End Seq.	Sequence	Ion Score	C. I. %	Modification	Rank	Result Type
848.4658	848.3998	-0.066	-78	4	10	TRVVMDK					Mascot
864.4608	864.4283	-0.0325	-38	4	10	TRVVMDK			Oxidation (M)[5]		Mascot
1107.5178	1107.5027	-0.0151	-14	89	97	RHDNSTSYK					Mascot
1107.5178	1107.5027	-0.0151	-14	89	97	RHDNSTSYK					Mascot
1349.6189	1349.6318	0.0129	10	160	171	TCPLCADATVNK			Carbamidomethyl (C)[2,5]		Mascot
1475.774	1475.703	-0.071	-48	277	289	TIDNIVTGWEISK					Mascot
1817.91	1817.822	-0.088	-48	172	188	NKQIGATQLSTNGDNTR					Mascot
1817.91	1817.822	-0.088	-48	172	188	NKQIGATQLSTNGDNTR	15	0			Mascot

Gel Idx/Pos	158/G9	Instr./Gel Origin	BA2151/Sample Project 20140814	Process Status	Analysis Succeeded
Plate [#] Name	[1] Sample Project 20140814	Instrument Sample Name		Spectra	11

Rank	Protein Name	Accession No.	Protein MW	Protein PI	Pep. Count	Protein Score	Protein Score C. I. %	Intensity Matched	Total Ion Score	Total Ion C. I. %	Confirmed
1	dehydrin [Triticum durum]	gi|61657604	16305.3	4.79	6	226	100	21.429	192	100	

Peptide Information

Calc. Mass	Obsrv. Mass	± da	± ppm	Start Seq.	End Seq.	Sequence	Ion Score	C. I. %	Modification	Rank	Result Type
905.4979	905.4672	-0.0307	-34	144	151	LPTLSDLF					Mascot
905.4979	905.4672	-0.0307	-34	144	151	LPTLSDLF	45	99.747			Mascot
1259.6703	1259.6483	-0.022	-17	37	48	DGATLSGRLDVR					Mascot
1327.6967	1327.6471	-0.0496	-37	96	106	VPYDFLMSLVK			Oxidation (M)[7]		Mascot
1403.7781	1403.6831	-0.095	-68	139	151	AGELKLPTLSDLF					Mascot
1629.6849	1629.6421	-0.0428	-26	107	119	DAGKDWDLDYEMR			Oxidation (M)[12]		Mascot
2235.1729	2235.1289	-0.044	-20	16	36	ISGVQKPEADLSDLSVQH VGR					Mascot
2235.1729	2235.1289	-0.044	-20	16	36	ISGVQKPEADLSDLSVQH VGR	147	100			Mascot

Rank	Protein Name	Accession No.	Protein MW	Protein PI	Pep. Count	Protein Score	Protein Score C. I. %	Intensity Matched	Total Ion Score	Total Ion C. I. %	Confirmed
2	Late embryogenesis abundant protein Lea14-A [Triticum urartu]	gi|474110039	16319.3	4.79	6	105	100	3.827	71	100	

Peptide Information

Calc. Mass	Obsrv. Mass	± da	± ppm	Start Seq.	End Seq.	Sequence	Ion Score	C. I. %	Modification	Rank	Result Type
905.4979	905.4672	-0.0307	-34	144	151	LPTLSDLF					Mascot
905.4979	905.4672	-0.0307	-34	144	151	LPTLSDLF	45	99.747			Mascot
1259.6703	1259.6483	-0.022	-17	37	48	DGATLSGRLDVR					Mascot
1327.6967	1327.6471	-0.0496	-37	96	106	VPYDFLMSLVK			Oxidation (M)[7]		Mascot
1403.7781	1403.6831	-0.095	-68	139	151	AGELKLPTLSDLF					Mascot
1629.6849	1629.6421	-0.0428	-26	107	119	DAGKDWDLDYEMR			Oxidation (M)[12]		Mascot
2022.0001	2021.9229	-0.0772	-38	49	65	NPYSHTIPICEISYSLK			Carbamidomethyl (C)[10]		Mascot
2022.0001	2021.9229	-0.0772	-38	49	65	NPYSHTIPICEISYSLK	26	78.092	Carbamidomethyl (C)[10]		Mascot

Rank	Protein Name	Accession No.	Protein MW	Protein PI	Pep. Count	Protein Score	Protein Score C. I. %	Intensity Matched	Total Ion Score	Total Ion C. I. %	Confirmed
3	Mitochondrial import inner membrane translocase subunit Tim8 [Triticum urartu]	gi|474250473	12948.1	6.56	8	48	24.901	3.797			

Peptide Information

Calc. Mass	Obsrv. Mass	± da	± ppm	Start Seq.	End Seq.	Sequence	Ion Score	C. I. %	Modification	Rank	Result Type

948.4316	948.3887	-0.0429	-45	100	106	EDNRQMR					Mascot
964.4265	964.4389	0.0124	13	100	106	EDNRQMR			Oxidation (M)[6]		Mascot
1103.4604	1103.5062	0.0458	42	18	26	MMMSEMVTK			Oxidation (M)[1]		Mascot
1193.5586	1193.5924	0.0338	28	90	99	GSWEDFSLPR					Mascot
1308.7345	1308.6294	-0.1051	-80	59	69	YLDMSVIIAKR					Mascot
1491.7413	1491.7136	-0.0277	-19	104	116	QMRVGVDFAPQPF					Mascot
1507.7363	1507.7065	-0.0298	-20	104	116	QMRVGVDFAPQPF			Oxidation (M)[2]		Mascot
1661.8018	1661.6364	-0.1654	-100	2	15	DASPELQQFLEQEK					Mascot
1707.7722	1707.7434	-0.0288	-17	90	103	GSWEDFSLPREDNR					Mascot
1926.9556	1926.8328	-0.1228	-64	2	17	DASPELQQFLEQEKHK					Mascot

4	hypothetical protein TRIUR3_21085 [Triticum urartu]	gi|474128168	41644.8	6.37	11	48	24.901	2.652	

Peptide Information

Calc. Mass	Obsrv. Mass	± da	± ppm	Start Seq.	End Seq.	Sequence	Ion Score	C. I. %	Modification	Rank	Result Type
820.4199	820.3835	-0.0364	-44	192	198	TNFPDVK					Mascot
1126.578	1126.4785	-0.0995	-88	353	363	VPLGGGYTSYL					Mascot
1165.5154	1165.5651	0.0497	43	270	279	SMEENQNVSK					Mascot
1259.6089	1259.6483	0.0394	31	50	59	SAQLFMDYIR			Oxidation (M)[6]		Mascot
1349.6995	1349.6364	-0.0631	-47	293	303	HSRYLQLSAMK			Oxidation (M)[10]		Mascot
1486.8112	1486.7281	-0.0831	-56	280	292	VSNNKLEDDVLLK					Mascot
1627.7971	1627.6421	-0.155	-95	65	79	GALTVAGCYLCAFPK			Carbamidomethyl (C)[8,11]		Mascot
1707.7966	1707.7434	-0.0532	-31	270	284	SMEENQNVSKVSNNK					Mascot
1838.8783	1838.8928	0.0145	8	253	267	HMFDTDWLFLAEVSK					Mascot
1867.8871	1867.8401	-0.047	-25	199	213	VLFLWPGETCHFMSK			Carbamidomethyl (C)[10], Oxidation (M)[13]		Mascot
1943.0009	1942.8329	-0.168	-86	5	21	QSITITEYGYAVIDLEK					Mascot

5	hypothetical protein TRIUR3_08211 [Triticum urartu]	gi|473933856	18391.7	7.66	6	47	5.456	2.679	17	0

Peptide Information

Calc. Mass	Obsrv. Mass	± da	± ppm	Start Seq.	End Seq.	Sequence	Ion Score	C. I. %	Modification	Rank	Result Type
870.4614	870.5299	0.0685	79	146	153	CKGHGLAK			Carbamidomethyl (C)[1]		Mascot
905.4662	905.4672	0.001	1	139	145	RMWTPSK					Mascot
905.4662	905.4672	0.001	1	139	145	RMWTPSK					Mascot
1037.4907	1037.5099	0.0192	19	140	147	MWTPSKCK			Carbamidomethyl (C)[7]		Mascot
1379.6526	1379.6575	0.0049	4	94	104	WTSWMNDGVKR					Mascot
1423.6754	1423.6981	0.0227	16	125	136	EFAYKSNGWHGK					Mascot

1707.8231	1707.7434	-0.0797	-47	70	86	AHVMLPTASHGSGEAAR	17	0	Oxidation (M)[4]		Mascot

6	hypothetical protein TRIUR3_10866 [Triticum urartu]	gi|473825696	13580.7	5.58	8	44	0	2.895

Peptide Information

Calc. Mass	Obsrv. Mass	± da	± ppm	Start Seq.	End Seq.	Sequence	Ion Score	C. I. %	Modification	Rank	Result Type
878.4214	878.4086	-0.0128	-15	2	9	QENATTSK					Mascot
894.3622	894.4447	0.0825	92	69	76	MDGDDIGR			Oxidation (M)[1]		Mascot
898.4669	898.3996	-0.0673	-75	40	46	FNEIFTK					Mascot
1235.5685	1235.5178	-0.0507	-41	69	80	MDGDDIGRAASK					Mascot
1263.6732	1263.642	-0.0312	-25	10	19	FSIYIENIHK					Mascot
1308.6835	1308.6294	-0.0541	-41	81	91	AEWDLLYSLAK					Mascot
1320.5961	1320.5637	-0.0324	-25	65	76	TANRMDGDDIGR					Mascot
1349.692	1349.6364	-0.0556	-41	47	58	HGKVEPNAVNER					Mascot

7	hypothetical protein TRIUR3_30944 [Triticum urartu]	gi|474302406	40651.1	9.12	13	43	0	5.609

Peptide Information

Calc. Mass	Obsrv. Mass	± da	± ppm	Start Seq.	End Seq.	Sequence	Ion Score	C. I. %	Modification	Rank	Result Type
864.4283	864.4537	0.0254	29	161	167	CPAEIFK			Carbamidomethyl (C)[1]		Mascot
878.495	878.4086	-0.0864	-98	308	314	MTMRLVK					Mascot
894.4899	894.4447	-0.0452	-51	308	314	MTMRLVK			Oxidation (M)[1]		Mascot
927.5046	927.4594	-0.0452	-49	62	69	SFVFSRGK					Mascot
1037.5851	1037.5099	-0.0752	-72	76	83	HLQQDLRK					Mascot
1103.603	1103.5062	-0.0968	-88	34	42	WKMPLSVAR			Oxidation (M)[3]		Mascot
1193.6963	1193.5924	-0.1039	-87	83	92	KLMLPYTALK			Oxidation (M)[3]		Mascot
1308.7158	1308.6294	-0.0864	-66	193	204	HLVPAIDTDTVK					Mascot
1327.6787	1327.6471	-0.0316	-24	244	254	LMQNNQVPDLR					Mascot
1349.6665	1349.6364	-0.0301	-22	301	311	LQEVGPRMTMR			Oxidation (M)[8,10]		Mascot
1497.8536	1497.7119	-0.1417	-95	227	239	HYSIKLQPVGISR					Mascot
1699.8796	1699.7897	-0.0899	-53	244	257	LMQNNQVPDLRDLK			Oxidation (M)[2]		Mascot
1908.9373	1908.7704	-0.1669	-87	315	332	VESGLCSGDILYPQSVGK			Carbamidomethyl (C)[6]		Mascot
2054.0701	2054.0684	-0.0017	-1	193	210	HLVPAIDTDTVKLSTCQR			Carbamidomethyl (C)[16]		Mascot

8	hypothetical protein TRIUR3_21202 [Triticum urartu]	gi|474375160	9230.5	5.62	6	43	0	2.379

Peptide Information

Calc. Mass	Obsrv. Mass	± da	± ppm	Start	End	Sequence	Ion	C. I. %	Modification	Rank	Result Type

Calc. Mass	Obsrv. Mass	± da	± ppm	Start Seq.	End Seq.	Sequence	Ion Score	C. I. %	Modification	Rank	Result Type
820.4523	820.3835	-0.0688	-84	39	45	TELRSSK					Mascot
858.4064	858.4236	0.0172	20	43	49	SSKDEHR					Mascot
993.5476	993.4618	-0.0858	-86	46	53	DEHRLPVK					Mascot
1393.6628	1393.6746	0.0118	8	54	65	MLEERVEDATGK			Oxidation (M)[1]		Mascot
1507.7725	1507.7065	-0.066	-44	31	42	HQQQQSPRTELR					Mascot
1942.944	1942.8329	-0.1111	-57	1	19	MSGAQGAQPKGAFTATT YR					Mascot
1958.939	1958.8219	-0.1171	-60	1	19	MSGAQGAQPKGAFTATT YR			Oxidation (M)[1]		Mascot

9 Wall-associated receptor kinase 5 [Triticum urartu] gi|474402869 105746.9 6.1 17 42 0 6.935

Peptide Information

Calc. Mass	Obsrv. Mass	± da	± ppm	Start Seq.	End Seq.	Sequence	Ion Score	C. I. %	Modification	Rank	Result Type
813.3624	813.3796	0.0172	21	500	506	TEYDSAK					Mascot
822.429	822.3696	-0.0594	-72	156	162	MPGRSFK					Mascot
829.4138	829.3697	-0.0441	-53	1	6	MGFRYR					Mascot
838.4053	838.3748	-0.0305	-36	737	744	VSDFGASR					Mascot
864.4033	864.4537	0.0504	58	604	611	GGHGMVYK			Oxidation (M)[5]		Mascot
1037.5085	1037.5099	0.0014	1	240	247	MTSLLWDR			Oxidation (M)[1]		Mascot
1107.5933	1107.521	-0.0723	-65	582	590	IFSLEELEK					Mascot
1140.5354	1140.5441	0.0087	8	927	936	EFMSSASLPR			Oxidation (M)[3]		Mascot
1182.5056	1182.5366	0.031	26	873	882	QMDSSQVDTR			Oxidation (M)[2]		Mascot
1182.5056	1182.5366	0.031	26	873	882	QMDSSQVDTR			Oxidation (M)[2]		Mascot
1287.6991	1287.6256	-0.0735	-57	600	611	IIGRGGHGMVYK					Mascot
1308.6075	1308.6294	0.0219	17	188	198	TTWVCGTVCPK			Carbamidomethyl (C)[5,9]		Mascot
1403.677	1403.6831	0.0061	4	262	273	MVDQPNCVVAVR			Carbamidomethyl (C)[7], Oxidation (M)[1]		Mascot
1507.7573	1507.7065	-0.0508	-34	248	261	ISISDGSGSMIVWR					Mascot
1633.8115	1633.6504	-0.1611	-99	604	618	GGHGMVYKGILSDQR			Oxidation (M)[5]		Mascot
1657.8262	1657.7297	-0.0965	-58	262	275	MVDQPNCVVAVRNR			Carbamidomethyl (C)[7]		Mascot
1838.7933	1838.8928	0.0995	54	873	887	QMDSSQVDTRNEEQR			Oxidation (M)[2]		Mascot
2257.146	2257.0898	-0.0562	-25	724	744	SSNILLDVNYTAKVSDFG ASR					Mascot

10 hypothetical protein TRIUR3_15512 [Triticum urartu] gi|473952824 98289.7 9.61 16 40 0 6.011

Peptide Information

Calc. Mass	Obsrv. Mass	± da	± ppm	Start Seq.	End Seq.	Sequence	Ion Score	C. I. %	Modification	Rank	Result Type

820.4271	820.3835	-0.0436	-53	860	867	RGSSTANK		Mascot
827.3893	827.4014	0.0121	15	673	680	TNSFSGSK		Mascot
870.4866	870.5299	0.0433	50	1	8	MAQPPAKK		Mascot
894.4639	894.4447	-0.0192	-21	868	876	ASSSSTAKR		Mascot
1081.5558	1081.4514	-0.1044	-97	534	543	SVLMTQSTSK		Mascot
1107.5793	1107.521	-0.0583	-53	782	791	TSVPTDFGKR		Mascot
1118.5624	1118.495	-0.0674	-60	725	733	DQMVQNLVR	Oxidation (M)[3]	Mascot
1277.6307	1277.683	0.0523	41	610	619	QESVHKYMQK		Mascot
1327.6602	1327.6471	-0.0131	-10	661	672	SVQHSTPSQQTK		Mascot
1403.7061	1403.6831	-0.023	-16	725	735	DQMVQNLVRER	Oxidation (M)[3]	Mascot
1427.7577	1427.7651	0.0074	5	458	471	GPAPRSMAPPPPPR		Mascot
1629.736	1629.6421	-0.0939	-58	115	128	IMNINTNYANCASK	Carbamidomethyl (C)[11], Oxidation (M)[2]	Mascot
1657.8545	1657.7297	-0.1248	-75	472	487	AAPPLATEASPDFSRK		Mascot
1661.6926	1661.6364	-0.0562	-34	581	594	IYFVDSDTDSDDNR		Mascot
1699.832	1699.7897	-0.0423	-25	323	339	SPSCGLSQSPPSSPALK	Carbamidomethyl (C)[4]	Mascot
1931.0193	1930.8529	-0.1664	-86	620	637	KPVSQQNSKSEGSTVVQ K		Mascot

Gel Idx/Pos	159/G10	Instr./Gel Origin	BA2151/Sample Project 20140814	Process Status	Analysis Succeeded
Plate [#] Name	[1] Sample Project 20140814	Instrument Sample Name		Spectra	11

Rank	Protein Name	Accession No.	Protein MW	Protein PI	Pep. Count	Protein Score	Protein Score C. I. %	Intensity Matched	Total Ion Score	Total Ion C. I. %	Confirmed
1	hypothetical protein TRIUR3_30879 [Triticum urartu]	gi|472909785	11663.1	9.09	9	57	90.971	5.625			

Peptide Information

Calc. Mass	Obsrv. Mass	± da	± ppm	Start Seq.	End Seq.	Sequence	Ion Score	C. I. %	Modification	Rank	Result Type
807.4108	807.3778	-0.033	-41	92	97	NRDQFK					Mascot
973.5425	973.508	-0.0345	-35	37	44	SLEALERR					Mascot
1065.5551	1065.4773	-0.0778	-73	1	10	MAAGGVWVFK					Mascot
1073.5851	1073.5188	-0.0663	-62	44	52	RLGSLGWER					Mascot
1081.5499	1081.4749	-0.075	-69	1	10	MAAGGVWVFK			Oxidation (M)[1]		Mascot
1194.5824	1194.5734	-0.009	-8	82	91	STHMYDVVVK			Oxidation (M)[4]		Mascot
1379.6987	1379.6708	-0.0279	-20	25	36	ALVYVPANETMR			Oxidation (M)[11]		Mascot
1479.6744	1479.7141	0.0397	27	11	23	NGVMELEQEATSR			Oxidation (M)[4]		Mascot
1491.7988	1491.7074	-0.0914	-61	24	36	KALVYVPANETMR					Mascot
1507.7937	1507.7042	-0.0895	-59	24	36	KALVYVPANETMR			Oxidation (M)[12]		Mascot
1507.7937	1507.7042	-0.0895	-59	24	36	KALVYVPANETMR			Oxidation (M)[12]		Mascot
2510.2168	2510.0449	-0.1719	-68	1	23	MAAGGVWVFKNGVMEL EQEATSR					Mascot

Rank	Protein Name	Accession No.	Protein MW	Protein PI	Pep. Count	Protein Score	Protein Score C. I. %	Intensity Matched	Total Ion Score	Total Ion C. I. %	Confirmed
2	hypothetical protein TRIUR3_12298 [Triticum urartu]	gi|473734609	9794.7	4.47	7	56	90.325	2.733			

Peptide Information

Calc. Mass	Obsrv. Mass	± da	± ppm	Start Seq.	End Seq.	Sequence	Ion Score	C. I. %	Modification	Rank	Result Type
993.4999	993.476	-0.0239	-24	59	67	AKDFGEQAK					Mascot
1033.516	1033.4932	-0.0228	-22	51	60	GASEVDEKAK					Mascot
1390.6445	1390.644	-0.0005	0	29	43	GVAEAAEAGSGNTEK					Mascot
1473.7544	1473.6688	-0.0856	-58	79	93	DAAQGITDKVADAAK					Mascot
1490.6969	1490.6838	-0.0131	-9	44	58	AAEEAGKGASEVDEK					Mascot
1976.9196	1976.9419	0.0223	11	69	87	ATEEAWDGAKDAAQGIT DK					Mascot
2053.9495	2054.0728	0.1233	60	1	20	MGQAYNDAADATDKAID GVK					Mascot

Rank	Protein Name	Accession No.	Protein MW	Protein PI	Pep. Count	Protein Score	Protein Score C. I. %	Intensity Matched	Total Ion Score	Total Ion C. I. %	Confirmed
3	unnamed protein product [Triticum aestivum]	gi|296522899	67458.7	7.69	13	47	15.737	4.187			

Peptide Information

Calc. Mass	Obsrv. Mass	± da	± ppm	Start Seq.	End Seq.	Sequence	Ion Score	C. I. %	Modification	Rank	Result Type
905.4761	905.4398	-0.0363	-40	315	321	LMDLLER			Oxidation (M)[2]		Mascot
913.4778	913.3878	-0.09	-99	277	284	FAYQTGVK					Mascot
993.5701	993.476	-0.0941	-95	242	249	SRPPQRPR					Mascot
1343.681	1343.6594	-0.0216	-16	351	361	KIVEQMDMPPR					Mascot
1490.7784	1490.6838	-0.0946	-63	416	427	RSYLMDLIHAQK			Oxidation (M)[5]		Mascot
1491.8068	1491.7074	-0.0994	-67	86	101	WAAPPGAPSAGVGVVR					Mascot
1527.7771	1527.679	-0.0981	-64	352	364	IVEQMDMPPRGVR					Mascot
1796.9364	1796.9375	0.0011	1	365	379	QTMLFSATFPKEIQR					Mascot
1856.8536	1856.9265	0.0729	39	108	127	GAVGGGGGGWNSRPGG WGDR					Mascot
2004.0522	2003.9404	-0.1118	-56	86	107	WAAPPGAPSAGVGVVR QGGGGR					Mascot
2252.1638	2251.9436	-0.2202	-98	206	227	HAIPIVIGGRDLMACAQT GSGK			Carbamidomethyl (C)[15]		Mascot
2369.0991	2368.9504	-0.1487	-63	102	127	QGGGGRGAVGGGGGG WNSRPGGWGDR					Mascot
2509.9626	2510.0449	0.0823	33	604	636	GGGGGGGGGYGGGGG GGYGGGGGYGGGASSS WD					Mascot

4	putative GTP-binding protein EngB [Triticum urartu]	gi|473970529	68948.1	9.72	9	46	0	4.217	26	78.826

Peptide Information

Calc. Mass	Obsrv. Mass	± da	± ppm	Start Seq.	End Seq.	Sequence	Ion Score	C. I. %	Modification	Rank	Result Type
832.4271	832.4731	0.046	55	55	62	GGGTEQKR					Mascot
1027.5055	1027.4796	-0.0259	-25	174	183	DSSKVYGGSK					Mascot
1141.4613	1141.5021	0.0408	36	23	35	DMSGGGTGMAGGK			Oxidation (M)[2]		Mascot
1327.6199	1327.6224	0.0025	2	280	290	MKLDPYDTSNK			Oxidation (M)[1]		Mascot
1373.6431	1373.6257	-0.0174	-13	263	275	DSVSDSEEIAPPK					Mascot
1483.619	1483.7032	0.0842	57	143	157	SSSNGSGMGRGEGER			Oxidation (M)[8]		Mascot
1487.64	1487.7064	0.0664	45	187	199	GRSMVCMNSQASK			Carbamidomethyl (C)[6], Oxidation (M)[4,7]		Mascot
1513.6853	1513.7052	0.0199	13	158	169	FKPRCSEDDGFR			Carbamidomethyl (C)[5]		Mascot
1838.9027	1838.8805	-0.0222	-12	500	514	GMKPLDYELIDLMER			Oxidation (M)[2]		Mascot
1838.9027	1838.8805	-0.0222	-12	500	514	GMKPLDYELIDLMER	26	78.826	Oxidation (M)[2]		Mascot

5	Putative disease resistance RPP13-like protein 2 [Triticum urartu]	gi|474060631	82914.2	8.56	16	46	0	5.778		

Peptide Information

Calc. Mass	Obsrv. Mass	± da	± ppm	Start Seq.	End Seq.	Sequence	Ion Score	C. I. %	Modification	Rank	Result Type

Calc. Mass	Obsrv. Mass	± da	± ppm	Start Seq.	End Seq.	Sequence	Ion Score	C. I. %	Modification	Rank	Result Type
948.4996	948.411	-0.0886	-93	453	460	EEISKQSK					Mascot
973.4785	973.508	0.0295	30	503	509	NRWMNPR	2	0			Mascot
1033.5637	1033.4932	-0.0705	-68	321	329	RLSVQDSTK					Mascot
1306.7035	1306.6328	-0.0707	-54	176	187	STACTLGDKILK			Carbamidomethyl (C)[4]		Mascot
1320.6213	1320.5592	-0.0621	-47	1	13	MSATNDDAGILGR					Mascot
1327.7482	1327.6224	-0.1258	-95	475	485	SFENIILHARK					Mascot
1357.7509	1357.6211	-0.1298	-96	633	644	LPQVKIEQGSMK					Mascot
1373.6115	1373.6257	0.0142	10	491	502	ICQATSYSSNSR			Carbamidomethyl (C)[2]		Mascot
1390.7438	1390.644	-0.0998	-72	399	410	GTDVDHLHQKIK					Mascot
1427.7676	1427.7507	-0.0169	-12	264	275	CIVEPVEIKNAR			Carbamidomethyl (C)[1]		Mascot
1473.8159	1473.6688	-0.1471	-100	523	536	ALQILSIDSSDLAK					Mascot
1716.9465	1716.8074	-0.1391	-81	141	156	CGGLPLALISVANYLR			Carbamidomethyl (C)[1]		Mascot
1838.8451	1838.8805	0.0354	19	159	175	GQTENHVAGGLTTEHCK			Carbamidomethyl (C)[16]		Mascot
1838.8451	1838.8805	0.0354	19	159	175	GQTENHVAGGLTTEHCK			Carbamidomethyl (C)[16]		Mascot
1852.0514	1851.8669	-0.1845	-100	281	296	VHSIMLEFIIHKAVSK					Mascot
1873.9689	1873.9034	-0.0655	-35	368	384	VLDLEGCKGVNNDTVLK			Carbamidomethyl (C)[7]		Mascot
3052.4392	3052.5518	0.1126	37	46	71	HGSPMEDTYFVVIDDVD RLEVCQAIK			Carbamidomethyl (C)[22], Oxidation (M)[5]		Mascot

6 Cytochrome P450 71D8 [Triticum urartu] gi|473860769 31803.3 6.2 10 45 0 2.87

Peptide Information

Calc. Mass	Obsrv. Mass	± da	± ppm	Start Seq.	End Seq.	Sequence	Ion Score	C. I. %	Modification	Rank	Result Type
948.4322	948.411	-0.0212	-22	177	183	HWDHPEK					Mascot
1073.515	1073.5188	0.0038	4	189	197	FESTTVDFK					Mascot
1412.6185	1412.5449	-0.0736	-52	149	160	ETCNVMGYDVPK			Carbamidomethyl (C)[3]		Mascot
1427.774	1427.7507	-0.0233	-16	52	65	EGGLEVPLTTGNIK					Mascot
1458.7323	1458.6808	-0.0515	-35	37	48	EEEEDLVDVLLR					Mascot
1605.7921	1605.675	-0.1171	-73	177	188	HWDHPEKFKPER					Mascot
1797.0117	1796.9375	-0.0742	-41	49	65	IQKEGGLEVPLTTGNIK					Mascot
1851.8285	1851.8669	0.0384	21	242	258	AMPGELDMAEEMGISVR			Oxidation (M)[2]		Mascot
1873.7878	1873.9034	0.1156	62	146	160	ECRETCNVMGYDVPK			Carbamidomethyl (C)[2,6], Oxidation (M)[9]		Mascot
2831.3491	2831.1287	-0.2204	-78	149	173	ETCNVMGYDVPKGTTVF VNAWAISR			Carbamidomethyl (C)[3], Oxidation (M)[6]		Mascot

7 Tetratricopeptide repeat protein 35-A [Triticum urartu] gi|474100421 23154 8.18 8 43 0 3.847

Peptide Information

Calc. Mass	Obsrv. Mass	± da	± ppm	Start Seq.	End Seq.	Sequence	Ion Score	C. I. %	Modification	Rank	Result Type

Calc. Mass	Obsrv. Mass	± da	± ppm	Start Seq.	End Seq.	Sequence	Ion Score	C. I. %	Modification	Rank	Result Type
905.4761	905.4398	-0.0363	-40	11	18	DCIGILSK			Carbamidomethyl (C)[2]		Mascot
1033.5928	1033.4932	-0.0996	-96	29	37	LEALLFEAK					Mascot
1074.5803	1074.4915	-0.0888	-83	19	28	QFPGSARVGR					Mascot
1235.5872	1235.5109	-0.0763	-62	1	10	MDCQRLDVAK			Carbamidomethyl (C)[3]		Mascot
1473.7916	1473.6688	-0.1228	-83	188	201	RAPSMEALVAGMLK					Mascot
1838.9503	1838.8805	-0.0698	-38	146	162	ALFGVCLCSAAISQLTK			Carbamidomethyl (C)[6,8]		Mascot
1838.9503	1838.8805	-0.0698	-38	146	162	ALFGVCLCSAAISQLTK			Carbamidomethyl (C)[6,8]		Mascot
1947.9811	1947.9103	-0.0708	-36	29	45	LEALLFEAKGDWAEAER					Mascot
2021.0736	2020.9701	-0.1035	-51	65	83	IAIAKAQGDMSLAVDYLNK					Mascot

8	Phosphatidylinositol-4-phosphate 5-kinase 9 [Triticum urartu]	gi|474386334	106530.9	9.26	18	42	0	5.314	

Peptide Information

Calc. Mass	Obsrv. Mass	± da	± ppm	Start Seq.	End Seq.	Sequence	Ion Score	C. I. %	Modification	Rank	Result Type
809.4668	809.4012	-0.0656	-81	925	930	FLNFIR					Mascot
847.4394	847.439	-0.0004	0	213	220	GARHGHGR					Mascot
1074.5942	1074.4915	-0.1027	-96	899	907	VEHAYKSIK					Mascot
1232.6383	1232.5718	-0.0665	-54	568	578	RQVQASDFGPK					Mascot
1287.6692	1287.578	-0.0912	-71	9	18	SYLYLTSERR					Mascot
1309.7158	1309.6316	-0.0842	-64	533	543	RPGEMIIKGHR			Oxidation (M)[5]		Mascot
1343.7828	1343.6594	-0.1234	-92	667	677	SEVQVLLRMLR					Mascot
1357.6958	1357.6211	-0.0747	-55	773	785	GGSLPESILQDNK					Mascot
1405.6053	1405.6198	0.0145	10	647	658	SGSMFFLSQDDR			Oxidation (M)[4]		Mascot
1427.7278	1427.7507	0.0229	16	682	693	HVHTYDNTLVTK					Mascot
1491.7743	1491.7074	-0.0669	-45	920	930	FYSERFLNFIR					Mascot
1716.928	1716.8074	-0.1206	-70	905	919	SIKYNPQSISVVEPR	4	0			Mascot
1908.8983	1908.7643	-0.134	-70	647	662	SGSMFFLSQDDRFMIK					Mascot
1926.9531	1926.8184	-0.1347	-70	750	765	NQGIMDYSLLLGFHYR					Mascot
1947.9891	1947.9103	-0.0788	-40	541	556	GHRSYDLMLCLQLGIR			Carbamidomethyl (C)[10], Oxidation (M)[8]		Mascot
2286.1013	2285.938	-0.1633	-71	569	588	QVQASDFGPKASFWMNFPTK					Mascot
2369.0803	2368.9504	-0.1299	-55	302	322	YTWANGNTYVGTMRNGTMFGK					Mascot
2902.5383	2902.3367	-0.2016	-69	873	896	SLRQVYDVVLYIGIIDILQEYSMR			Oxidation (M)[23]		Mascot

9	hypothetical protein TRIUR3_08211 [Triticum urartu]	gi|473933856	18391.7	7.66	5	41	0	.993	16	0	

Peptide Information

Calc. Mass	Obsrv. Mass	± da	± ppm	Start Seq.	End Seq.	Sequence	Ion Score	C. I. %	Modification	Rank	Result Type
905.4662	905.4398	-0.0264	-29	139	145	RMWTPSK					Mascot
1037.4907	1037.4971	0.0064	6	140	147	MWTPSKCK			Carbamidomethyl (C)[7]		Mascot
1379.6526	1379.6708	0.0182	13	94	104	WTSWMNDGVKR					Mascot
1707.8231	1707.7317	-0.0914	-54	70	86	AHVMLPTASHGSGEAAR	16	0	Oxidation (M)[4]		Mascot
1947.8767	1947.9103	0.0336	17	87	103	DHGVGSRWTSWMNDGV K			Oxidation (M)[12]		Mascot

10 Secologanin synthase [Triticum urartu] gi|474401653 48633.9 8.73 10 40 0 6.48

Peptide Information

Calc. Mass	Obsrv. Mass	± da	± ppm	Start Seq.	End Seq.	Sequence	Ion Score	C. I. %	Modification	Rank	Result Type
1057.5314	1057.4642	-0.0672	-64	109	117	SFSFDVISR					Mascot
1065.551	1065.4773	-0.0737	-69	340	349	FKNGVAAACK			Carbamidomethyl (C)[9]		Mascot
1107.5834	1107.5167	-0.0667	-60	13	22	LFGTEGVAWK					Mascot
1158.6378	1158.5542	-0.0836	-72	2	10	DVSRYIHIR					Mascot
1232.6667	1232.5718	-0.0949	-77	275	285	TVGMVVQETLR					Mascot
1265.6373	1265.6135	-0.0238	-19	417	428	IDATATQDGFVK					Mascot
1491.8022	1491.7074	-0.0948	-64	273	285	MKTVGMVVQETLR					Mascot
1507.7971	1507.7042	-0.0929	-62	273	285	MKTVGMVVQETLR			Oxidation (M)[1]		Mascot
1507.7971	1507.7042	-0.0929	-62	273	285	MKTVGMVVQETLR			Oxidation (M)[1]		Mascot
2367.0593	2367.2104	0.1511	64	250	272	AEVLDVCGGATGAADPD FDMISR			Carbamidomethyl (C)[7]		Mascot
2902.5066	2902.3367	-0.1699	-59	350	376	NPQAAFMPFGLGARTCL GQNLAIVEVK			Carbamidomethyl (C)[16]		Mascot
3312.5645	3312.2476	-0.3169	-96	311	339	GTYLFVPVSTMHHDAAS WGPTVHRFDPDR			Oxidation (M)[11]		Mascot

Gel Idx/Pos	160/G11	Instr./Gel Origin	BA2151/Sample Project 20140814	Process Status	Analysis Succeeded
Plate [#] Name	[1] Sample Project 20140814	Instrument Sample Name		Spectra	11

Rank	Protein Name	Accession No.	Protein MW	Protein PI	Pep. Count	Protein Score	Protein Score C. I. %	Intensity Matched	Total Ion Score	Total Ion C. I. %	Confirmed
1	translationally-controlled tumor protein [Triticum aestivum]	gi|146285306	18823.3	4.55	11	459	100	78.457	383	100	

Protein Group

hypothetical protein TRIUR3_27725 [Triticum urartu]	gi|473741089	18823.3	4.5500 001907 3486

Peptide Information

Calc. Mass	Obsrv. Mass	± da	± ppm	Start Seq.	End Seq.	Sequence	Ion Score	C. I. %	Modification	Rank	Result Type
890.4553	890.4107	-0.0446	-50	86	92	QFISHMK					Mascot
896.4546	896.4383	-0.0163	-18	1	7	MLVYQDK					Mascot
906.4502	906.4373	-0.0129	-14	86	92	QFISHMK			Oxidation (M)[6]		Mascot
912.4495	912.4365	-0.013	-14	1	7	MLVYQDK			Oxidation (M)[1]		Mascot
912.4495	912.4365	-0.013	-14	1	7	MLVYQDK	25	74.635	Oxidation (M)[1]		Mascot
1046.5564	1046.5015	-0.0549	-52	86	93	QFISHMKR					Mascot
1062.5514	1062.5211	-0.0303	-29	86	93	QFISHMKR			Oxidation (M)[6]		Mascot
1063.5784	1063.5646	-0.0138	-13	67	75	VVDIVDTFR					Mascot
1063.5784	1063.5646	-0.0138	-13	67	75	VVDIVDTFR	72	100			Mascot
1075.5419	1075.5221	-0.0198	-18	76	84	LQEQPAFDK					Mascot
1122.5314	1122.5162	-0.0152	-14	102	111	LEGDDLDAFK					Mascot
1203.6368	1203.6132	-0.0236	-20	76	85	LQEQPAFDKK					Mascot
1203.6368	1203.6132	-0.0236	-20	76	85	LQEQPAFDKK	48	99.863			Mascot
1250.6263	1250.6012	-0.0251	-20	102	112	LEGDDLDAFKK					Mascot
1598.7697	1598.7454	-0.0243	-15	8	21	LSGDELLSDSFPYR					Mascot
1598.7697	1598.7454	-0.0243	-15	8	21	LSGDELLSDSFPYR	139	100			Mascot
1736.8643	1736.8264	-0.0379	-22	149	164	EGAADPTFLYFAHGLK					Mascot
1736.8643	1736.8264	-0.0379	-22	149	164	EGAADPTFLYFAHGLK	99	100			Mascot
2781.323	2781.2561	-0.0669	-24	125	148	LKDLQFFVGESMHDDGG VVFAYYK			Oxidation (M)[12]		Mascot

Rank	Protein Name	Accession No.	Protein MW	Protein PI	Pep. Count	Protein Score	Protein Score C. I. %	Intensity Matched	Total Ion Score	Total Ion C. I. %	Confirmed
2	translationally controlled tumor protein [Triticum aestivum]	gi|21070379	18851.3	4.55	11	459	100	78.453	383	100	

Protein Group

RecName: Full=Translationally-controlled tumor protein	gi|75246527	18851.3	4.5500

homolog; Short=TCTP 001907 3486

Peptide Information

Calc. Mass	Obsrv. Mass	± da	± ppm	Start Seq.	End Seq.	Sequence	Ion Score	C. I. %	Modification	Rank	Result Type
890.4553	890.4107	-0.0446	-50	86	92	QFISHMK					Mascot
896.4546	896.4383	-0.0163	-18	1	7	MLVYQDK					Mascot
906.4502	906.4373	-0.0129	-14	86	92	QFISHMK			Oxidation (M)[6]		Mascot
912.4495	912.4365	-0.013	-14	1	7	MLVYQDK			Oxidation (M)[1]		Mascot
912.4495	912.4365	-0.013	-14	1	7	MLVYQDK	25	74.635	Oxidation (M)[1]		Mascot
1046.5564	1046.5015	-0.0549	-52	86	93	QFISHMKR					Mascot
1062.5514	1062.5211	-0.0303	-29	86	93	QFISHMKR			Oxidation (M)[6]		Mascot
1063.5784	1063.5646	-0.0138	-13	67	75	VVDIVDTFR					Mascot
1063.5784	1063.5646	-0.0138	-13	67	75	VVDIVDTFR	72	100			Mascot
1075.5419	1075.5221	-0.0198	-18	76	84	LQEQPAFDK					Mascot
1150.5627	1150.5693	0.0066	6	102	111	LEGDDLDVFK					Mascot
1203.6368	1203.6132	-0.0236	-20	76	85	LQEQPAFDKK					Mascot
1203.6368	1203.6132	-0.0236	-20	76	85	LQEQPAFDKK	48	99.863			Mascot
1278.6577	1278.6486	-0.0091	-7	102	112	LEGDDLDVFKK					Mascot
1598.7697	1598.7454	-0.0243	-15	8	21	LSGDELLSDSFPYR					Mascot
1598.7697	1598.7454	-0.0243	-15	8	21	LSGDELLSDSFPYR	139	100			Mascot
1736.8643	1736.8264	-0.0379	-22	149	164	EGAADPTFLYFAHGLK					Mascot
1736.8643	1736.8264	-0.0379	-22	149	164	EGAADPTFLYFAHGLK	99	100			Mascot
2781.323	2781.2561	-0.0669	-24	125	148	LKDLQFFVGESMHDDGG VVFAYYK			Oxidation (M)[12]		Mascot

3 hypothetical protein TRIUR3_11094 [Triticum urartu] gi|473814309 16304 6.62 10 61 96.796 40.811

Peptide Information

Calc. Mass	Obsrv. Mass	± da	± ppm	Start Seq.	End Seq.	Sequence	Ion Score	C. I. %	Modification	Rank	Result Type
889.4738	889.4106	-0.0632	-71	52	60	TAQTAQAAK					Mascot
890.4578	890.4107	-0.0471	-53	105	113	DSAVAGKDK					Mascot
934.5026	934.4243	-0.0783	-84	23	31	TGQMVGITK					Mascot
1193.6195	1193.6007	-0.0188	-16	23	33	TGQMVGITKDK			Oxidation (M)[4]		Mascot
1206.5961	1206.5785	-0.0176	-15	34	44	AGQDTETTKQK					Mascot
1539.7034	1539.6886	-0.0148	-10	2	16	ASNQNQASYAAGETK					Mascot
1580.7222	1580.748	0.0258	16	134	149	DAVANTLGMGGDNTTK			Oxidation (M)[9]		Mascot
1580.7222	1580.748	0.0258	16	134	149	DAVANTLGMGGDNTTK			Oxidation (M)[9]		Mascot

1583.7548	1583.7294	-0.0254	-16	63	77	AAESKDQTGSYLGEK					Mascot
1597.7704	1597.7289	-0.0415	-26	68	82	DQTGSYLGEKTEAAK					Mascot
1598.7657	1598.7454	-0.0203	-13	96	111	SSDAAQYTKDSAVAGK					Mascot
1598.7657	1598.7454	-0.0203	-13	96	111	SSDAAQYTKDSAVAGK					Mascot

4 Metacaspase-4 [Triticum urartu] gi|473895598 45153.9 5.22 12 50 51.512 2.994

Peptide Information

Calc. Mass	Obsrv. Mass	± da	± ppm	Start Seq.	End Seq.	Sequence	Ion Score	C. I. %	Modification	Rank	Result Type
948.4169	948.4884	0.0715	75	195	202	DAFESHSR					Mascot
1027.5315	1027.4692	-0.0623	-61	265	272	FMKVMLDK			Oxidation (M)[2]		Mascot
1043.5265	1043.4917	-0.0348	-33	265	272	FMKVMLDK			Oxidation (M)[2,5]		Mascot
1075.5742	1075.5221	-0.0521	-48	156	165	AKEQIGNSTK					Mascot
1077.5973	1077.532	-0.0653	-61	375	383	ITNKDLVMK			Oxidation (M)[8]		Mascot
1150.6692	1150.5693	-0.0999	-87	72	82	HALAKLVGDAR					Mascot
1576.745	1576.7706	0.0256	16	234	247	EQTGKDDIEEGSIR					Mascot
1610.7704	1610.7506	-0.0198	-12	1	14	MIQSRPSSPHPSDR			Oxidation (M)[1]		Mascot
1620.7208	1620.7158	-0.005	-3	166	178	QNQTQSRESEEER					Mascot
1640.7605	1640.74	-0.0205	-12	84	97	GDFLFFHYSGHGTR					Mascot
1718.8417	1718.8237	-0.018	-10	158	172	EQIGNSTKQNQTQSR					Mascot
1718.8417	1718.8237	-0.018	-10	158	172	EQIGNSTKQNQTQSR					Mascot
1764.9531	1764.8464	-0.1067	-60	248	263	LTLFNVFGDDASPKIK					Mascot
1796.8616	1796.7733	-0.0883	-49	83	97	RGDFLFFHYSGHGTR					Mascot

5 Disease resistance protein RPP13 [Triticum urartu] gi|473786130 115619.3 8.15 20 45 0 6.111

Peptide Information

Calc. Mass	Obsrv. Mass	± da	± ppm	Start Seq.	End Seq.	Sequence	Ion Score	C. I. %	Modification	Rank	Result Type
870.468	870.535	0.067	77	59	67	GQPSPSAVK					Mascot
890.4764	890.4107	-0.0657	-74	457	463	LDKLMDR					Mascot
906.4713	906.4373	-0.034	-38	457	463	LDKLMDR			Oxidation (M)[5]		Mascot
912.4897	912.4365	-0.0532	-58	217	224	QKHDISGK					Mascot
912.4897	912.4365	-0.0532	-58	217	224	QKHDISGK					Mascot
1016.5748	1016.5001	-0.0747	-73	514	521	HLVIQNHR					Mascot
1019.4752	1019.5228	0.0476	47	964	972	SQSDEAQVR					Mascot
1027.5393	1027.4692	-0.0701	-68	273	280	MELWHAIK					Mascot
1043.5343	1043.4917	-0.0426	-41	273	280	MELWHAIK			Oxidation (M)[1]		Mascot

Calc. Mass	Obsrv. Mass	± da	± ppm	Start Seq.	End Seq.	Sequence	Ion Score	C. I. %	Modification	Rank	Result Type
1073.6024	1073.4961	-0.1063	-99	264	272	CLVVIDNIK			Carbamidomethyl (C)[1]		Mascot
1106.6205	1106.5135	-0.107	-97	177	188	VVSIVGFGGSGK					Mascot
1141.5969	1141.4832	-0.1137	-100	675	683	LMVHMNQLR					Mascot
1182.6552	1182.5425	-0.1127	-95	915	926	VVAPAEAMAPVK					Mascot
1182.6552	1182.5425	-0.1127	-95	915	926	VVAPAEAMAPVK					Mascot
1193.6022	1193.6007	-0.0015	-1	447	456	RSAQQVAYDR					Mascot
1194.5758	1194.5967	0.0209	17	209	218	AWATMACKQK			Carbamidomethyl (C)[7]		Mascot
1232.6304	1232.5742	-0.0562	-46	59	70	GQPSPSAVKSMK			Oxidation (M)[11]		Mascot
1491.8026	1491.7008	-0.1018	-68	464	476	NIIRPIDAHNNSK					Mascot
1642.8184	1642.7085	-0.1099	-67	496	509	SLNFISTSFNDKNR					Mascot
1660.7782	1660.6646	-0.1136	-68	973	987	NGLALLSCCEAPEAR			Carbamidomethyl (C)[8,9]		Mascot
1707.8669	1707.8942	0.0273	16	482	495	THGIMNQLMLYKSR			Oxidation (M)[5]		Mascot
1718.9661	1718.8237	-0.1424	-83	464	478	NIIRPIDAHNNSKVK					Mascot
1718.9661	1718.8237	-0.1424	-83	464	478	NIIRPIDAHNNSKVK					Mascot
1758.9572	1758.8143	-0.1429	-81	915	931	VVAPAEAMAPVKYVASR					Mascot
1774.952	1774.778	-0.174	-98	915	931	VVAPAEAMAPVKYVASR			Oxidation (M)[8]		Mascot
1796.9138	1796.7733	-0.1405	-78	719	735	AGIDTPVRDLDTPVGDR					Mascot

6	putative WRKY transcription factor 7 [Triticum urartu]	gi|473891047	28294.9	7.08	9	44	0	1.297

Peptide Information

Calc. Mass	Obsrv. Mass	± da	± ppm	Start Seq.	End Seq.	Sequence	Ion Score	C. I. %	Modification	Rank	Result Type
890.4876	890.4107	-0.0769	-86	10	17	AAVVSCKR			Carbamidomethyl (C)[6]		Mascot
1027.4803	1027.4692	-0.0111	-11	134	143	AAHKADDDGK					Mascot
1062.5579	1062.5211	-0.0368	-35	22	31	LSLSAGDPFR					Mascot
1184.606	1184.5504	-0.0556	-47	196	206	VVINSGGGYYR					Mascot
1193.5402	1193.6007	0.0605	51	207	216	CSSIKDCPAR			Carbamidomethyl (C)[1,7]		Mascot
1552.8483	1552.7432	-0.1051	-68	173	184	YTFRQLLVEEVR					Mascot
1707.9137	1707.8942	-0.0195	-11	177	190	QLLVEEVRAETHQR					Mascot
1765.8208	1765.8497	0.0289	16	1	16	MEGVEEANRAAVVSCK			Carbamidomethyl (C)[15], Oxidation (M)[1]		Mascot
1774.7362	1774.778	0.0418	24	151	168	SVPEAEGEEGGEDAGGE R					Mascot

7	hypothetical protein TRIUR3_29919 [Triticum urartu]	gi|474267353	10100.9	9.39	7	42	0	.766

Peptide Information

Calc. Mass	Obsrv. Mass	± da	± ppm	Start Seq.	End Seq.	Sequence	Ion Score	C. I. %	Modification	Rank	Result Type
1016.4175	1016.5001	0.0826	81	2	9	ASYEQCMK			Carbamidomethyl (C)[6]		Mascot

1048.5018	1048.5326	0.0308	29	20	29	QSEVQSGGTR					Mascot
1222.6791	1222.632	-0.0471	-39	74	84	EKSGFISLVSR					Mascot
1642.8041	1642.7085	-0.0956	-58	52	65	CVDPSKINCTPVPR			Carbamidomethyl (C)[1,9]		Mascot
1780.7517	1780.7764	0.0247	14	2	15	ASYEQCMKSYEQEK			Carbamidomethyl (C)[6]		Mascot
1796.7466	1796.7733	0.0267	15	2	15	ASYEQCMKSYEQEK			Carbamidomethyl (C)[6], Oxidation (M)[7]		Mascot
1908.8513	1908.7853	-0.066	-35	35	51	AMQCSAIHPSAMQFTGR			Carbamidomethyl (C)[4], Oxidation (M)[2]		Mascot
1927.7871	1927.8405	0.0534	28	1	15	MASYEQCMKSYEQEK			Carbamidomethyl (C)[7], Oxidation (M)[1]		Mascot

8 Ras-related protein RABD1 [Triticum urartu] gi|473937131 24869.3 5.69 9 41 0 1.84

Peptide Information

Calc. Mass	Obsrv. Mass	± da	± ppm	Start Seq.	End Seq.	Sequence	Ion Score	C. I. %	Modification	Rank	Result Type
890.4465	890.4107	-0.0358	-40	131	138	VVDTEEAK					Mascot
1046.5266	1046.5015	-0.0251	-24	102	109	QWLSEIDR					Mascot
1075.5994	1075.5221	-0.0773	-72	50	59	TVELDGKSVK					Mascot
1085.5925	1085.5331	-0.0594	-55	139	146	FRSMLFIR			Oxidation (M)[4]		Mascot
1193.6161	1193.6007	-0.0154	-13	131	140	VVDTEEAKFR					Mascot
1202.5946	1202.5786	-0.016	-13	186	196	NKMASQPAAER					Mascot
1316.6594	1316.6167	-0.0427	-32	60	70	LQIWDTAGQER					Mascot
1580.6428	1580.748	0.1052	67	206	219	GQPIQQQNSSCCSS			Carbamidomethyl (C)[11,12]		Mascot
1580.6428	1580.748	0.1052	67	206	219	GQPIQQQNSSCCSS			Carbamidomethyl (C)[11,12]		Mascot
1707.8107	1707.8942	0.0835	49	124	138	CDLVDSKVVDTEEAK			Carbamidomethyl (C)[1]		Mascot

9 hypothetical protein TRIUR3_20753 [Triticum urartu] gi|473968637 66592.2 4.54 15 40 0 3.56

Peptide Information

Calc. Mass	Obsrv. Mass	± da	± ppm	Start Seq.	End Seq.	Sequence	Ion Score	C. I. %	Modification	Rank	Result Type
819.3843	819.3888	0.0045	5	477	484	KEEGGDGK					Mascot
869.4475	869.4117	-0.0358	-41	261	269	TGGVAAEHK					Mascot
896.3665	896.4383	0.0718	80	35	42	MEEAGESK			Oxidation (M)[1]		Mascot
1016.5622	1016.5001	-0.0621	-61	118	127	TAAIEDVVAK					Mascot
1078.4722	1078.5203	0.0481	45	321	330	MVDVDDADAK					Mascot
1085.5222	1085.5331	0.0109	10	205	214	AKAAEHEDSK					Mascot
1106.5398	1106.5135	-0.0263	-24	237	246	MVIVEDADAK			Oxidation (M)[1]		Mascot
1193.5355	1193.6007	0.0652	55	412	421	EEMDVNTEVK					Mascot
1203.5852	1203.6132	0.028	23	485	494	ANENEEKLEK					Mascot
1203.5852	1203.6132	0.028	23	485	494	ANENEEKLEK					Mascot

1316.6328	1316.6167	-0.0161	-12	517	527	NVEENKEETPK					Mascot
1507.6759	1507.7018	0.0259	17	503	516	DGKVTEEEASEADK					Mascot
1718.8444	1718.8237	-0.0207	-12	118	133	TAAIEDVVAKEDDNTK					Mascot
1718.8444	1718.8237	-0.0207	-12	118	133	TAAIEDVVAKEDDNTK					Mascot
1735.7692	1735.7872	0.018	10	72	87	MEEAEDAKEDDVGAVK					Mascot
1800.8373	1800.7603	-0.077	-43	253	269	AAEHDDHRTGGVAAEHK					Mascot
1908.8491	1908.7853	-0.0638	-33	188	204	DSKMVIVEDANANEDDK			Oxidation (M)[4]		Mascot

10	glycogen synthase kinase [Triticum monococcum subsp. aegilopoides]	gi|90797273	40886	8.61	9	40	0	27.902	9	0

Peptide Information

Calc. Mass	Obsrv. Mass	± da	± ppm	Start Seq.	End Seq.	Sequence	Ion Score	C. I. %	Modification	Rank	Result Type
1063.5314	1063.5646	0.0332	31	84	91	NRELQTMR			Oxidation (M)[7]		Mascot
1063.5314	1063.5646	0.0332	31	84	91	NRELQTMR	11	0	Oxidation (M)[7]		Mascot
1106.516	1106.5135	-0.0025	-2	133	140	HYNKMNQR			Oxidation (M)[5]		Mascot
1194.5354	1194.5967	0.0613	51	1	10	MGNMSIRDDR					Mascot
1203.6039	1203.6132	0.0093	8	298	308	MPAEAVDLVSR			Oxidation (M)[1]		Mascot
1203.6039	1203.6132	0.0093	8	298	308	MPAEAVDLVSR			Oxidation (M)[1]		Mascot
1348.7141	1348.644	-0.0701	-52	64	75	CLETGETVAIKK			Carbamidomethyl (C)[1]		Mascot
1596.7799	1596.7365	-0.0434	-27	36	49	NGQAKQTISYMAER					Mascot
1610.8499	1610.7506	-0.0993	-62	341	355	TAVFFLPSLTLSPMS					Mascot
1612.7748	1612.7168	-0.058	-36	36	49	NGQAKQTISYMAER			Oxidation (M)[11]		Mascot
1626.8169	1626.7794	-0.0375	-23	156	169	SLAYIHNSIGVCHR			Carbamidomethyl (C)[12]		Mascot
1626.8169	1626.7794	-0.0375	-23	156	169	SLAYIHNSIGVCHR			Carbamidomethyl (C)[12]		Mascot
1802.8088	1802.7573	-0.0515	-29	268	281	EEIKCMNPNYTEFK			Carbamidomethyl (C)[5]		Mascot

Gel Idx/Pos	161/G12	Instr./Gel Origin	BA2151/Sample Project 20140814	Process Status	Analysis Succeeded
Plate [#] Name	[1] Sample Project 20140814	Instrument Sample Name		Spectra	11

Rank	Protein Name	Accession No.	Protein MW	Protein PI	Pep. Count	Protein Score	Protein Score C. I. %	Intensity Matched	Total Ion Score	Total Ion C. I. %	Confirmed
1	Thiol-specific antioxidant protein [Triticum aestivum]	gi|1805351	23426.2	5.71	6	208	100	50.838	146	100	

Protein Group

RecName: Full=2-Cys peroxiredoxin BAS1, chloroplastic; AltName: Full=Thiol-specific antioxidant protein; Flags: Precursor	gi|2829687	23426.2	5.7100 000381 4697

Peptide Information

Calc. Mass	Obsrv. Mass	± da	± ppm	Start Seq.	End Seq.	Sequence	Ion Score	C. I. %	Modification	Rank	Result Type
805.4818	805.4572	-0.0246	-31	141	147	GLFIIDK					Mascot
819.4207	819.4034	-0.0173	-21	164	170	SVDETLR					Mascot
1021.5565	1021.5298	-0.0267	-26	114	122	YPLVSDVTK					Mascot
1360.7107	1360.6843	-0.0264	-19	11	23	AAAEYDLPLVGNK					Mascot
1485.8424	1485.8036	-0.0388	-26	127	140	SFGVLIPDQGIALR					Mascot
1485.8424	1485.8036	-0.0388	-26	127	140	SFGVLIPDQGIALR	109	100			Mascot
1707.9137	1707.8635	-0.0502	-29	148	163	EGVIQHSTINNLGIGR					Mascot
1707.9137	1707.8635	-0.0502	-29	148	163	EGVIQHSTINNLGIGR	37	98.79			Mascot

Rank	Protein Name	Accession No.	Protein MW	Protein PI	Pep. Count	Protein Score	Protein Score C. I. %	Intensity Matched	Total Ion Score	Total Ion C. I. %	Confirmed
2	unnamed protein product [Triticum aestivum]	gi|296514492	28166.5	6.33	6	203	100	50.838	146	100	

Peptide Information

Calc. Mass	Obsrv. Mass	± da	± ppm	Start Seq.	End Seq.	Sequence	Ion Score	C. I. %	Modification	Rank	Result Type
805.4818	805.4572	-0.0246	-31	188	194	GLFIIDK					Mascot
819.4207	819.4034	-0.0173	-21	211	217	SVDETLR					Mascot
1021.5565	1021.5298	-0.0267	-26	161	169	YPLVSDVTK					Mascot
1360.7107	1360.6843	-0.0264	-19	58	70	AAAEYDLPLVGNK					Mascot
1485.8424	1485.8036	-0.0388	-26	174	187	SFGVLIPDQGIALR					Mascot
1485.8424	1485.8036	-0.0388	-26	174	187	SFGVLIPDQGIALR	109	100			Mascot
1707.9137	1707.8635	-0.0502	-29	195	210	EGVIQHSTINNLGIGR					Mascot
1707.9137	1707.8635	-0.0502	-29	195	210	EGVIQHSTINNLGIGR	37	98.79			Mascot

Rank	Protein Name	Accession No.	Protein MW	Protein PI	Pep. Count	Protein Score	Protein Score C. I. %	Intensity Matched	Total Ion Score	Total Ion C. I. %	Confirmed
3	Secologanin synthase [Triticum urartu]	gi|474429248	102759.4	8.48	3	30	0	33.373	21	49.321	

Peptide Information

Calc. Mass	Obsrv. Mass	± da	± ppm	Start	End	Sequence	Ion	C. I. %	Modification	Rank	Result Type

Calc. Mass	Obsrv. Mass	± da	± ppm	Start Seq.	End Seq.	Sequence	Ion Score	C. I. %	Modification	Rank	Result Type
805.4679	805.4572	-0.0107	-13	254	259	YLPTRR					Mascot
1251.6654	1251.6959	0.0305	24	886	896	LKGMLDEFVSL					Mascot
1485.7916	1485.8036	0.012	8	413	425	GTMITIPVMTLHR			Oxidation (M)[3]		Mascot
1485.7916	1485.8036	0.012	8	413	425	GTMITIPVMTLHR	21	49.321	Oxidation (M)[9]		Mascot

4	hypothetical protein TRIUR3_19789 [Triticum urartu]	gi|473911338	18591.3	5.27	1	24	0	12.252	17	0

Peptide Information

Calc. Mass	Obsrv. Mass	± da	± ppm	Start Seq.	End Seq.	Sequence	Ion Score	C. I. %	Modification	Rank	Result Type
1707.8846	1707.8635	-0.0211	-12	45	60	YCLRTLSANPAAAATK			Carbamidomethyl (C)[2]		Mascot
1707.8846	1707.8635	-0.0211	-12	45	60	YCLRTLSANPAAAATK	17	0	Carbamidomethyl (C)[2]		Mascot

5	HMG-Y-related protein A [Triticum urartu]	gi|474391007	13030.9	10.47	3	23	0	5.539

Peptide Information

Calc. Mass	Obsrv. Mass	± da	± ppm	Start Seq.	End Seq.	Sequence	Ion Score	C. I. %	Modification	Rank	Result Type
819.3665	819.4034	0.0369	45	1	8	MANDGSPK					Mascot
1251.6362	1251.6959	0.0597	48	73	84	EAVAKATTGMTR			Oxidation (M)[10]		Mascot
1360.6777	1360.6843	0.0066	5	65	77	DPMADAVKEAVAK			Oxidation (M)[3]		Mascot

6	hypothetical protein TRIUR3_12433 [Triticum urartu]	gi|474443430	67967.8	7.28	1	23	0	12.252	18	0

Peptide Information

Calc. Mass	Obsrv. Mass	± da	± ppm	Start Seq.	End Seq.	Sequence	Ion Score	C. I. %	Modification	Rank	Result Type
1707.8735	1707.8635	-0.01	-6	83	98	IMQGLLDQYTVAGNGK					Mascot
1707.8735	1707.8635	-0.01	-6	83	98	IMQGLLDQYTVAGNGK	19	27.751			Mascot

7	hypothetical protein TRIUR3_08291 [Triticum urartu]	gi|473945264	9841.1	11.7	2	22	0	14.942

Peptide Information

Calc. Mass	Obsrv. Mass	± da	± ppm	Start Seq.	End Seq.	Sequence	Ion Score	C. I. %	Modification	Rank	Result Type
1707.8219	1707.8635	0.0416	24	1	17	MAVTAATATTTTSPPDR			Oxidation (M)[1]		Mascot
1707.8219	1707.8635	0.0416	24	1	17	MAVTAATATTTTSPPDR			Oxidation (M)[1]		Mascot
2034.0284	2033.932	-0.0964	-47	1	20	MAVTAATATTTTSPPDRIGR			Oxidation (M)[1]		Mascot

8	hypothetical protein TRIUR3_22295 [Triticum urartu]	gi|474187389	7446.8	8.65	2	22	0	1.606

Peptide Information

Calc. Mass	Obsrv. Mass	± da	± ppm	Start Seq.	End Seq.	Sequence	Ion Score	C. I. %	Modification	Rank	Result Type
1021.5499	1021.5298	-0.0201	-20	54	62	YAMIAPLAR			Oxidation (M)[3]		Mascot
1251.7096	1251.6959	-0.0137	-11	32	41	ILENIVTFFR					Mascot

9 hypothetical protein TRIUR3_26808 [Triticum urartu] gi|474016240 8234.2 5.66 2 21 0 12.688

Peptide Information

Calc. Mass	Obsrv. Mass	± da	± ppm	Start Seq.	End Seq.	Sequence	Ion Score	C. I. %	Modification	Rank	Result Type
1410.8719	1410.9866	0.1147	81	3	15	VPVVKPAAIFLEK					Mascot
1707.8225	1707.8635	0.041	24	16	29	DDIDLDEWIGFTLR					Mascot
1707.8225	1707.8635	0.041	24	16	29	DDIDLDEWIGFTLR					Mascot

10 hypothetical protein TRIUR3_09741 [Triticum urartu] gi|473781913 7289 10.02 2 21 0 1.164

Peptide Information

Calc. Mass	Obsrv. Mass	± da	± ppm	Start Seq.	End Seq.	Sequence	Ion Score	C. I. %	Modification	Rank	Result Type
1251.6449	1251.6959	0.051	41	1	9	MCRPLRQFK			Carbamidomethyl (C)[2], Oxidation (M)[1]		Mascot
1360.7219	1360.6843	-0.0376	-28	7	17	QFKLDPQSELR					Mascot

Gel Idx/Pos	162/G13	Instr./Gel Origin	BA2151/Sample Project 20140814	Process Status	Analysis Succeeded
Plate [#] Name	[1] Sample Project 20140814	Instrument Sample Name		Spectra	11

Rank	Protein Name	Accession No.	Protein MW	Protein PI	Pep. Count	Protein Score	Protein Score C. I. %	Intensity Matched	Total Ion Score	Total Ion C. I. %	Confirmed
1	serpin-N3.2 [Triticum aestivum]	gi|379060943	43026.4	5.18	12	631	100	44.5	571	100	

Protein Group

Protein Name	Accession No.	Protein MW	Protein PI
RecName: Full=Serpin-Z2B; AltName: Full=TriaeZ2b; AltName: Full=WSZ2b; AltName: Full=WZS3	gi|75279909	43011.4	5.1799998283862
serpin [Triticum aestivum]	gi|1885346	43011.4	5.1799998283862

Peptide Information

Calc. Mass	Obsrv. Mass	± da	± ppm	Start Seq.	End Seq.	Sequence	Ion Score	C. I. %	Modification	Rank	Result Type
925.5214	925.5009	-0.0205	-22	11	18	LSIAHQTR					Mascot
925.5214	925.5009	-0.0205	-22	11	18	LSIAHQTR	47	99.854			Mascot
947.5156	947.4725	-0.0431	-45	2	10	ATTLATDVR					Mascot
1137.6667	1137.6322	-0.0345	-30	172	181	LVLGNALYFK					Mascot
1137.6667	1137.6322	-0.0345	-30	172	181	LVLGNALYFK	56	99.981			Mascot
1192.5382	1192.5111	-0.0271	-23	182	191	GAWTDQFDPR					Mascot
1192.5382	1192.5111	-0.0271	-23	182	191	GAWTDQFDPR	71	100			Mascot
1223.5903	1223.5408	-0.0495	-40	127	137	AEAQSVDFQTK					Mascot
1372.7068	1372.6772	-0.0296	-22	159	171	DILPAGSIDNTTR					Mascot
1372.7068	1372.6772	-0.0296	-22	159	171	DILPAGSIDNTTR	91	100			Mascot
1446.7965	1446.7487	-0.0478	-33	11	22	LSIAHQTRFAFR					Mascot
1514.7485	1514.7039	-0.0446	-29	125	137	YKAEAQSVDFQTK					Mascot
1514.7485	1514.7039	-0.0446	-29	125	137	YKAEAQSVDFQTK	106	100			Mascot
1531.7751	1531.7251	-0.05	-33	138	151	AAEVTAQVNSWVEK					Mascot
2085.155	2085.105	-0.05	-24	152	171	VTTGLIKDILPAGSIDNTTR					Mascot
2085.155	2085.105	-0.05	-24	152	171	VTTGLIKDILPAGSIDNTTR	200	100			Mascot
2838.4858	2838.4041	-0.0817	-29	99	124	VAFANGVFVDASLQLKPSFQELAVCK			Carbamidomethyl (C)[25]		Mascot
3751.9614	3751.9146	-0.0468	-12	23	61	LASAISSNPESTVNNAAFSPVSLHVALSLITAGAGGATR					Mascot

Rank	Protein Name	Accession No.	Protein MW	Protein PI	Pep. Count	Protein Score	Protein Score C. I. %	Intensity Matched	Total Ion Score	Total Ion C. I. %	Confirmed
2	Serpin-Z2B [Triticum urartu]	gi|473793747	45225.7	6.03	9	312	100	30.998	280	100	

Peptide Information

Calc. Mass	Obsrv. Mass	± da	± ppm	Start Seq.	End Seq.	Sequence	Ion Score	C. I. %	Modification	Rank	Result Type
925.5214	925.5009	-0.0205	-22	11	18	LSIAHQTR					Mascot
925.5214	925.5009	-0.0205	-22	11	18	LSIAHQTR	47	99.854			Mascot
947.5156	947.4725	-0.0431	-45	2	10	ATTLATDVR					Mascot
1137.6667	1137.6322	-0.0345	-30	189	198	LVLGNALYFK					Mascot
1137.6667	1137.6322	-0.0345	-30	189	198	LVLGNALYFK	56	99.981			Mascot
1192.5382	1192.5111	-0.0271	-23	199	208	GAWTDQFDPR					Mascot
1192.5382	1192.5111	-0.0271	-23	199	208	GAWTDQFDPR	71	100			Mascot
1223.5903	1223.5408	-0.0495	-40	127	137	AEAQSVDFQTK					Mascot
1446.7965	1446.7487	-0.0478	-33	11	22	LSIAHQTRFAFR					Mascot
1514.7485	1514.7039	-0.0446	-29	125	137	YKAEAQSVDFQTK					Mascot
1514.7485	1514.7039	-0.0446	-29	125	137	YKAEAQSVDFQTK	106	100			Mascot
1531.7751	1531.7251	-0.05	-33	155	168	AAEVTAQVNSWVEK					Mascot
2838.4858	2838.4041	-0.0817	-29	99	124	VAFANGVFVDASLQLKPS FQELAVCK			Carbamidomethyl (C)[25]		Mascot

3	serpin [Triticum aestivum]	gi|5734506	43341.5	5.46	7	228	100	18.112	209	100	

Protein Group

RecName: Full=Serpin-Z2A; AltName: Full=TriaeZ2a; AltName: Full=WSZ2a	gi|75313847	43341.5	5.4600 000381 4697

Peptide Information

Calc. Mass	Obsrv. Mass	± da	± ppm	Start Seq.	End Seq.	Sequence	Ion Score	C. I. %	Modification	Rank	Result Type
925.5214	925.5009	-0.0205	-22	11	18	LSIAHQTR					Mascot
925.5214	925.5009	-0.0205	-22	11	18	LSIAHQTR	47	99.854			Mascot
947.5156	947.4725	-0.0431	-45	2	10	ATTLATDVR					Mascot
1137.6667	1137.6322	-0.0345	-30	172	181	LVLGNALYFK					Mascot
1137.6667	1137.6322	-0.0345	-30	172	181	LVLGNALYFK	56	99.981			Mascot
1182.5175	1182.5314	0.0139	12	182	191	GAWTDQFDSR					Mascot
1223.5903	1223.5408	-0.0495	-40	127	137	AEAQSVDFQTK					Mascot
1514.7485	1514.7039	-0.0446	-29	125	137	YKAEAQSVDFQTK					Mascot
1514.7485	1514.7039	-0.0446	-29	125	137	YKAEAQSVDFQTK	106	100			Mascot
1531.7751	1531.7251	-0.05	-33	138	151	AAEVTAQVNSWVEK					Mascot

4	Serpin-ZX [Triticum urartu]	gi|474139641	42666.2	5.89	3	80	99.954	6.164	74	100	

Peptide Information

Calc. Mass	Obsrv. Mass	± da	± ppm	Start	End	Sequence	Ion	C. I. %	Modification	Rank	Result Type

Calc. Mass	Obsrv. Mass	± da	± ppm	Start Seq.	End Seq.	Sequence	Ion Score	C. I. %	Modification	Rank	Result Type
868.5138	868.5022	-0.0116	-13	358	365	SLPVEPVK					Mascot
1137.6667	1137.6322	-0.0345	-30	174	183	LVLGNALYFK					Mascot
1137.6667	1137.6322	-0.0345	-30	174	183	LVLGNALYFK	56	99.981			Mascot
1372.7068	1372.6772	-0.0296	-22	161	173	EILPAGSVDNTTR					Mascot
1372.7068	1372.6772	-0.0296	-22	161	173	EILPAGSVDNTTR	18	0			Mascot

5	serpin 2 [Triticum aestivum]	gi|224589268	43518.4	5.11	6	72	99.751	4.31	56	99.981	

Protein Group

serpin 4 [Triticum aestivum]	gi|224589272	43587.4	5.1900<br>000572<br>2046

Peptide Information

Calc. Mass	Obsrv. Mass	± da	± ppm	Start Seq.	End Seq.	Sequence	Ion Score	C. I. %	Modification	Rank	Result Type
1137.6667	1137.6322	-0.0345	-30	175	184	LVLGNALYFK					Mascot
1137.6667	1137.6322	-0.0345	-30	175	184	LVLGNALYFK	56	99.981			Mascot
1175.6494	1175.5488	-0.1006	-86	280	289	VVVDQFMLPK					Mascot
1182.5889	1182.5314	-0.0575	-49	264	273	LSTESEFIEK					Mascot
1552.8304	1552.71	-0.1204	-78	243	254	QFSMYILLPERR					Mascot
1895.9432	1895.981	0.0378	20	238	253	GGDNRQFSMYILLPER					Mascot
2494.2283	2494.3174	0.0891	36	314	335	EANLSEMVNSQVDLFLS<br>SVFHK					Mascot

6	Serpin-ZX [Triticum urartu]	gi|474274187	43393.1	5.52	6	71	99.68	17.848	56	99.981	

Peptide Information

Calc. Mass	Obsrv. Mass	± da	± ppm	Start Seq.	End Seq.	Sequence	Ion Score	C. I. %	Modification	Rank	Result Type
1137.6667	1137.6322	-0.0345	-30	204	213	LVLGNALYFK					Mascot
1137.6667	1137.6322	-0.0345	-30	204	213	LVLGNALYFK	56	99.981			Mascot
1192.5892	1192.5111	-0.0781	-65	262	271	LPYRQGGDMR					Mascot
1192.5892	1192.5111	-0.0781	-65	262	271	LPYRQGGDMR					Mascot
1208.5841	1208.504	-0.0801	-66	262	271	LPYRQGGDMR			Oxidation (M)[9]		Mascot
1208.5841	1208.504	-0.0801	-66	262	271	LPYRQGGDMR			Oxidation (M)[9]		Mascot
1262.6012	1262.5374	-0.0638	-51	159	169	AETHSVDFQTK					Mascot
1389.6857	1389.6606	-0.0251	-18	191	203	EILPDGSVDSTTR					Mascot
1468.7068	1468.6671	-0.0397	-27	214	226	GAWTQEFDASKTK					Mascot
1553.7595	1553.7238	-0.0357	-23	157	169	YKAETHSVDFQTK					Mascot

7 Serpin-ZX [Triticum urartu] gi|474111625 47368.2 8.71 6 70 99.547 17.259 56 99.981

Peptide Information

Calc. Mass	Obsrv. Mass	± da	± ppm	Start Seq.	End Seq.	Sequence	Ion Score	C. I. %	Modification	Rank	Result Type
1137.6667	1137.6322	-0.0345	-30	188	197	LVLGNALYFK					Mascot
1137.6667	1137.6322	-0.0345	-30	188	197	LVLGNALYFK	56	99.981			Mascot
1192.6096	1192.5111	-0.0985	-83	284	293	LTSEPEFLEK					Mascot
1192.6096	1192.5111	-0.0985	-83	284	293	LTSEPEFLEK					Mascot
1262.6376	1262.5374	-0.1002	-79	131	141	AETHSVDFKTK					Mascot
1320.7046	1320.5927	-0.1119	-85	283	293	KLTSEPEFLEK					Mascot
1485.7843	1485.8077	0.0234	16	44	55	ENQKPVKQCVQK			Carbamidomethyl (C)[9]		Mascot
1485.7843	1485.8077	0.0234	16	44	55	ENQKPVKQCVQK			Carbamidomethyl (C)[9]		Mascot
1492.7465	1492.6941	-0.0524	-35	226	238	HKTIEAPFMSSTK			Oxidation (M)[9]		Mascot

8 Thiol-specific antioxidant protein [Triticum aestivum] gi|1805351 23426.2 5.71 6 69 99.456 2.473 41 99.447

Protein Group

RecName: Full=2-Cys peroxiredoxin BAS1, chloroplastic; AltName: Full=Thiol-specific antioxidant protein; Flags: Precursor gi|2829687 23426.2 5.7100000381 4697

Peptide Information

Calc. Mass	Obsrv. Mass	± da	± ppm	Start Seq.	End Seq.	Sequence	Ion Score	C. I. %	Modification	Rank	Result Type
818.4077	818.3915	-0.0162	-20	195	201	SMKPDPK			Oxidation (M)[2]		Mascot
819.4207	819.399	-0.0217	-26	164	170	SVDETLR					Mascot
923.5197	923.4603	-0.0594	-64	42	49	LSDYIGKK					Mascot
1485.8424	1485.8077	-0.0347	-23	127	140	SFGVLIPDQGIALR					Mascot
1485.8424	1485.8077	-0.0347	-23	127	140	SFGVLIPDQGIALR	41	99.447			Mascot
1707.9137	1707.8701	-0.0436	-26	148	163	EGVIQHSTINNLGIGR					Mascot
2494.3777	2494.3174	-0.0603	-24	141	163	GLFIIDKEGVIQHSTINNLGIGR					Mascot

9 unnamed protein product [Triticum aestivum] gi|296514492 28166.5 6.33 6 65 98.602 2.473 41 99.447

Peptide Information

Calc. Mass	Obsrv. Mass	± da	± ppm	Start Seq.	End Seq.	Sequence	Ion Score	C. I. %	Modification	Rank	Result Type
818.4077	818.3915	-0.0162	-20	243	249	SMKPDPK			Oxidation (M)[2]		Mascot
819.4207	819.399	-0.0217	-26	211	217	SVDETLR					Mascot
923.5197	923.4603	-0.0594	-64	89	96	LSDYIGKK					Mascot

1485.8424	1485.8077	-0.0347	-23	174	187	SFGVLIPDQGIALR					Mascot
1485.8424	1485.8077	-0.0347	-23	174	187	SFGVLIPDQGIALR	41	99.447			Mascot
1707.9137	1707.8701	-0.0436	-26	195	210	EGVIQHSTINNLGIGR					Mascot
2494.3777	2494.3174	-0.0603	-24	188	210	GLFIIDKEGVIQHSTINNL GIGR					Mascot

10	serpin [Triticum aestivum]	gi|1885350	43119.9	5.44	5	58	92.828	10.261	47	99.854	

Protein Group

RecName: Full=Serpin-Z1B; AltName: Full=TriaeZ1b; AltName: Full=WSZ1b; AltName: Full=WZS2	gi|75279910	43119.9	5.4400 000572 2046

Peptide Information

Calc. Mass	Obsrv. Mass	± da	± ppm	Start Seq.	End Seq.	Sequence	Ion Score	C. I. %	Modification	Rank	Result Type
925.5214	925.5009	-0.0205	-22	11	18	LSIAHQTR					Mascot
925.5214	925.5009	-0.0205	-22	11	18	LSIAHQTR	47	99.854			Mascot
947.5156	947.4725	-0.0431	-45	2	10	ATTLATDVR					Mascot
1176.5896	1176.5562	-0.0334	-28	262	271	LSAEPDFLER					Mascot
1345.6958	1345.619	-0.0768	-57	159	171	NILPSGSVDNTTK					Mascot
1585.8295	1585.7068	-0.1227	-77	288	301	FKISFGMEASDLLK					Mascot

Gel Idx/Pos	163/G14	Instr./Gel Origin	BA2151/Sample Project 20140814	Process Status	Analysis Succeeded
Plate [#] Name	[1] Sample Project 20140814	Instrument Sample Name		Spectra	11

Rank	Protein Name	Accession No.	Protein MW	Protein PI	Pep. Count	Protein Score	Protein Score C. I. %	Intensity Matched	Total Ion Score	Total Ion C. I. %	Confirmed
1	unnamed protein product [Triticum aestivum]	gi|296514492	28166.5	6.33	12	282	100	69.035	186	100	

Peptide Information

Calc. Mass	Obsrv. Mass	± da	± ppm	Start Seq.	End Seq.	Sequence	Ion Score	C. I. %	Modification	Rank	Result Type
805.4818	805.4611	-0.0207	-26	188	194	GLFIIDK					Mascot
818.4077	818.3916	-0.0161	-20	243	249	SMKPDPK			Oxidation (M)[2]		Mascot
819.4207	819.4026	-0.0181	-22	211	217	SVDETLR					Mascot
1021.5565	1021.5464	-0.0101	-10	161	169	YPLVSDVTK					Mascot
1360.7107	1360.6747	-0.036	-26	58	70	AAAEYDLPLVGNK					Mascot
1485.8424	1485.8185	-0.0239	-16	174	187	SFGVLIPDQGIALR					Mascot
1485.8424	1485.8185	-0.0239	-16	174	187	SFGVLIPDQGIALR	134	100			Mascot
1707.9137	1707.8865	-0.0272	-16	195	210	EGVIQHSTINNLGIGR					Mascot
1707.9137	1707.8865	-0.0272	-16	195	210	EGVIQHSTINNLGIGR	17	0			Mascot
1748.943	1748.8667	-0.0763	-44	153	169	SGGLGDLKYPLVSDVTK					Mascot
2010.9808	2010.9224	-0.0584	-29	71	88	APDFAAEAVFDQEFINVK					Mascot
2010.9808	2010.9224	-0.0584	-29	71	88	APDFAAEAVFDQEFINVK					Mascot
2494.3777	2494.3333	-0.0444	-18	188	210	GLFIIDKEGVIQHSTINNL GIGR					Mascot
2494.3777	2494.3333	-0.0444	-18	188	210	GLFIIDKEGVIQHSTINNL GIGR	34	97.093			Mascot
2700.3992	2700.3516	-0.0476	-18	128	151	INTEILGVSVDSVFSHLA WVQTER					Mascot
2700.3992	2700.3516	-0.0476	-18	128	151	INTEILGVSVDSVFSHLA WVQTER					Mascot
2857.3826	2857.3193	-0.0633	-22	218	242	TLQALQYVQENPDEVCP AGWKPGEK			Carbamidomethyl (C)[16]		Mascot

Rank	Protein Name	Accession No.	Protein MW	Protein PI	Pep. Count	Protein Score	Protein Score C. I. %	Intensity Matched	Total Ion Score	Total Ion C. I. %	Confirmed
2	Thiol-specific antioxidant protein [Triticum aestivum]	gi|1805351	23426.2	5.71	11	278	100	68.743	186	100	

Protein Group

Protein Name	Accession No.	Protein MW	Protein PI
RecName: Full=2-Cys peroxiredoxin BAS1, chloroplastic; AltName: Full=Thiol-specific antioxidant protein; Flags: Precursor	gi|2829687	23426.2	5.7100 000381 4697

Peptide Information

Calc. Mass	Obsrv. Mass	± da	± ppm	Start Seq.	End Seq.	Sequence	Ion Score	C. I. %	Modification	Rank	Result Type
805.4818	805.4611	-0.0207	-26	141	147	GLFIIDK					Mascot

818.4077	818.3916	-0.0161	-20	195	201	SMKPDPK			Oxidation (M)[2]		Mascot
819.4207	819.4026	-0.0181	-22	164	170	SVDETLR					Mascot
1021.5565	1021.5464	-0.0101	-10	114	122	YPLVSDVTK					Mascot
1360.7107	1360.6747	-0.036	-26	11	23	AAAEYDLPLVGNK					Mascot
1485.8424	1485.8185	-0.0239	-16	127	140	SFGVLIPDQGIALR					Mascot
1485.8424	1485.8185	-0.0239	-16	127	140	SFGVLIPDQGIALR	134	100			Mascot
1707.9137	1707.8865	-0.0272	-16	148	163	EGVIQHSTINNLGIGR					Mascot
1707.9137	1707.8865	-0.0272	-16	148	163	EGVIQHSTINNLGIGR	17	0			Mascot
1748.943	1748.8667	-0.0763	-44	106	122	SGGLGDLKYPLVSDVTK					Mascot
2010.9808	2010.9224	-0.0584	-29	24	41	APDFAAEAVFDQEFINVK					Mascot
2010.9808	2010.9224	-0.0584	-29	24	41	APDFAAEAVFDQEFINVK					Mascot
2494.3777	2494.3333	-0.0444	-18	141	163	GLFIIDKEGVIQHSTINNL GIGR					Mascot
2494.3777	2494.3333	-0.0444	-18	141	163	GLFIIDKEGVIQHSTINNL GIGR	34	97.093			Mascot
2700.3992	2700.3516	-0.0476	-18	81	104	INTEILGVSVDSVFSHLA WVQTER					Mascot
2700.3992	2700.3516	-0.0476	-18	81	104	INTEILGVSVDSVFSHLA WVQTER					Mascot

3 putative glutathione S-transferase GSTF1 [Triticum urartu] gi|474117067 6007 6.03 5 54 84.31 6.651

Peptide Information

Calc. Mass	Obsrv. Mass	± da	± ppm	Start Seq.	End Seq.	Sequence	Ion Score	C. I. %	Modification	Rank	Result Type
1251.6151	1251.7169	0.1018	81	5	16	VFGPATSTNMAR					Mascot
1251.6151	1251.7169	0.1018	81	5	16	VFGPATSTNMAR					Mascot
1547.7999	1547.7235	-0.0764	-49	2	16	APKVFGPATSTNMAR					Mascot
1550.8187	1550.8054	-0.0133	-9	42	54	RPEHLTRNPFGQV					Mascot
1694.8353	1694.8005	-0.0348	-21	1	16	MAPKVFGPATSTNMAR			Oxidation (M)[1]		Mascot
2494.2422	2494.3333	0.0911	37	17	38	VLVCLEEVGAEYELVDID FPGK			Carbamidomethyl (C)[4]		Mascot
2494.2422	2494.3333	0.0911	37	17	38	VLVCLEEVGAEYELVDID FPGK			Carbamidomethyl (C)[4]		Mascot

4 eukaryotic translation initiation factor,putative, expressed [Triticum aestivum] gi|300681560 117424.6 9.36 17 45 0 10.674

Peptide Information

Calc. Mass	Obsrv. Mass	± da	± ppm	Start Seq.	End Seq.	Sequence	Ion Score	C. I. %	Modification	Rank	Result Type
800.376	800.301	-0.075	-94	182	187	AFQFCK			Carbamidomethyl (C)[5]		Mascot
828.4032	828.4495	0.0463	56	936	942	QEAPPMR					Mascot
1043.519	1043.5166	-0.0024	-2	649	657	IHIEGDMTK					Mascot

1411.7006	1411.6674	-0.0332	-24	142	152	SDREFVTPWFK					Mascot
1468.7651	1468.7974	0.0323	22	461	472	IFQSMKIDMLSR					Mascot
1499.7604	1499.8033	0.0429	29	473	484	MIPFFEFNVVEK					Mascot
1539.7948	1539.7283	-0.0665	-43	658	670	QHAMEVVLNQQVK			Oxidation (M)[4]		Mascot
1550.807	1550.8054	-0.0016	-1	41	52	SWQKPLEKIMMK			Oxidation (M)[10,11]		Mascot
1551.8312	1551.7709	-0.0603	-39	454	466	VLQQASRIFQSMK			Oxidation (M)[12]		Mascot
1639.7996	1639.8872	0.0876	53	124	137	RPEDLMLSYVSGEK			Oxidation (M)[6]		Mascot
1689.8013	1689.8751	0.0738	44	91	104	HFMQLSNEKAEEAR					Mascot
1689.8013	1689.8751	0.0738	44	91	104	HFMQLSNEKAEEAR					Mascot
1694.8894	1694.8005	-0.0889	-52	351	365	SLRMANLVNFSLDSK					Mascot
1707.9323	1707.8865	-0.0458	-27	198	211	LCEIIRNHLANLNK			Carbamidomethyl (C)[2]		Mascot
1707.9323	1707.8865	-0.0458	-27	198	211	LCEIIRNHLANLNK			Carbamidomethyl (C)[2]		Mascot
1824.916	1824.8235	-0.0925	-51	124	139	RPEDLMLSYVSGEKGK			Oxidation (M)[6]		Mascot
1839.0164	1838.8975	-0.1189	-65	153	166	FLWETYRTVLEILR					Mascot
1927.9216	1927.8445	-0.0771	-40	906	923	REGDSSSQRPAVAAEPD R					Mascot
2049.0269	2048.9133	-0.1136	-55	145	159	EFVTPWFKFLWETYR					Mascot

5 Eukaryotic translation initiation factor 3 subunit A [Triticum urartu] gi|474111197 117354.8 9.4 15 37 0 10.44

Peptide Information

Calc. Mass	Obsrv. Mass	± da	± ppm	Start Seq.	End Seq.	Sequence	Ion Score	C. I. %	Modification	Rank	Result Type
800.376	800.301	-0.075	-94	182	187	AFQFCK			Carbamidomethyl (C)[5]		Mascot
1043.519	1043.5166	-0.0024	-2	649	657	IHIEGDMTK					Mascot
1411.7006	1411.6674	-0.0332	-24	142	152	SDREFVTPWFK					Mascot
1468.7651	1468.7974	0.0323	22	461	472	IFQSMKIDMLSR					Mascot
1499.7604	1499.8033	0.0429	29	473	484	MIPFFEFNVVEK					Mascot
1539.7948	1539.7283	-0.0665	-43	658	670	QHAMEVVLNQQVK			Oxidation (M)[4]		Mascot
1550.807	1550.8054	-0.0016	-1	41	52	SWQKPLEKIMMK			Oxidation (M)[10,11]		Mascot
1551.8312	1551.7709	-0.0603	-39	454	466	VLQQASRIFQSMK			Oxidation (M)[12]		Mascot
1639.7996	1639.8872	0.0876	53	124	137	RPEDLMLSYVSGEK			Oxidation (M)[6]		Mascot
1689.8013	1689.8751	0.0738	44	91	104	HFMQLSNEKAEEAR					Mascot
1689.8013	1689.8751	0.0738	44	91	104	HFMQLSNEKAEEAR					Mascot
1694.8894	1694.8005	-0.0889	-52	351	365	SLRMANLVNFSLDSK					Mascot
1707.9323	1707.8865	-0.0458	-27	198	211	LCEIIRNHLANLNK			Carbamidomethyl (C)[2]		Mascot
1707.9323	1707.8865	-0.0458	-27	198	211	LCEIIRNHLANLNK			Carbamidomethyl (C)[2]		Mascot
1824.916	1824.8235	-0.0925	-51	124	139	RPEDLMLSYVSGEKGK			Oxidation (M)[6]		Mascot

1839.0164	1838.8975	-0.1189	-65	153	166	FLWETYRTVLEILR						Mascot
2049.0269	2048.9133	-0.1136	-55	145	159	EFVTPWFKFLWETYR						Mascot

6	eukaryotic translation initiation factor 3 subunit A, putative, expressed [Triticum aestivum]	gi|383100786	117173.6	9.38	15	37	0	10.44

Peptide Information

Calc. Mass	Obsrv. Mass	± da	± ppm	Start Seq.	End Seq.	Sequence	Ion Score	C. I. %	Modification	Rank	Result Type
800.376	800.301	-0.075	-94	182	187	AFQFCK			Carbamidomethyl (C)[5]		Mascot
1043.519	1043.5166	-0.0024	-2	649	657	IHIEGDMTK					Mascot
1411.7006	1411.6674	-0.0332	-24	142	152	SDREFVTPWFK					Mascot
1468.7651	1468.7974	0.0323	22	461	472	IFQSMKIDMLSR					Mascot
1499.7604	1499.8033	0.0429	29	473	484	MIPFFEFNVVEK					Mascot
1539.7948	1539.7283	-0.0665	-43	658	670	QHAMEVVLNQQVK			Oxidation (M)[4]		Mascot
1550.807	1550.8054	-0.0016	-1	41	52	SWQKPLEKIMMK			Oxidation (M)[10,11]		Mascot
1551.8312	1551.7709	-0.0603	-39	454	466	VLQQASRIFQSMK			Oxidation (M)[12]		Mascot
1639.7996	1639.8872	0.0876	53	124	137	RPEDLMLSYVSGEK			Oxidation (M)[6]		Mascot
1689.8013	1689.8751	0.0738	44	91	104	HFMQLSNEKAEEAR					Mascot
1689.8013	1689.8751	0.0738	44	91	104	HFMQLSNEKAEEAR					Mascot
1694.8894	1694.8005	-0.0889	-52	351	365	SLRMANLVNFSLDSK					Mascot
1707.9323	1707.8865	-0.0458	-27	198	211	LCEIIRNHLANLNK			Carbamidomethyl (C)[2]		Mascot
1707.9323	1707.8865	-0.0458	-27	198	211	LCEIIRNHLANLNK			Carbamidomethyl (C)[2]		Mascot
1824.916	1824.8235	-0.0925	-51	124	139	RPEDLMLSYVSGEKGK			Oxidation (M)[6]		Mascot
1839.0164	1838.8975	-0.1189	-65	153	166	FLWETYRTVLEILR					Mascot
2049.0269	2048.9133	-0.1136	-55	145	159	EFVTPWFKFLWETYR					Mascot

7	Replication protein A 70 kDa DNA-binding subunit [Triticum urartu]	gi|474051756	65708.6	5.6	9	37	0	6.684

Peptide Information

Calc. Mass	Obsrv. Mass	± da	± ppm	Start Seq.	End Seq.	Sequence	Ion Score	C. I. %	Modification	Rank	Result Type
858.5155	858.4847	-0.0308	-36	225	232	GSLRIANK					Mascot
1043.5731	1043.5166	-0.0565	-54	301	309	KIDNESIPK					Mascot
1382.6621	1382.6809	0.0188	14	550	561	VSVVQNEYMGEK					Mascot
1398.657	1398.7511	0.0941	67	550	561	VSVVQNEYMGEK			Oxidation (M)[9]		Mascot
1468.6989	1468.7974	0.0985	67	528	539	EEGDDMFQLKLK			Oxidation (M)[6]		Mascot
1539.8265	1539.7283	-0.0982	-64	369	382	STLVINPELPEAEK					Mascot

Calc. Mass	Obsrv. Mass	± da	± ppm	Start Seq.	End Seq.	Sequence	Ion Score	C. I. %	Modification	Rank	Result Type
1936.0439	1935.8794	-0.1645	-85	178	194	GHPLISLNPYQGNWVIK					Mascot
2477.3623	2477.3237	-0.0386	-16	56	78	AILSTHFASEVHSGKLQN LGLVR					Mascot
2700.4866	2700.3516	-0.135	-50	128	153	EEAAGLSKPIGAAPATIML KPRHDVK					Mascot
2700.4866	2700.3516	-0.135	-50	128	153	EEAAGLSKPIGAAPATIML KPRHDVK					Mascot
2716.4814	2716.344	-0.1374	-51	128	153	EEAAGLSKPIGAAPATIML KPRHDVK			Oxidation (M)[18]		Mascot
2879.2546	2879.3286	0.074	26	465	488	VTEALGSGFWCEGCQKN YEQCSLR			Carbamidomethyl (C)[11,14,21]		Mascot
2879.2546	2879.3286	0.074	26	465	488	VTEALGSGFWCEGCQKN YEQCSLR			Carbamidomethyl (C)[11,14,21]		Mascot

8 Cysteine synthase [Triticum urartu] gi|474315986 35674.8 5.83 7 36 0 12.286

Peptide Information

Calc. Mass	Obsrv. Mass	± da	± ppm	Start Seq.	End Seq.	Sequence	Ion Score	C. I. %	Modification	Rank	Result Type
1021.487	1021.5464	0.0594	58	330	338	KEAESMVVE					Mascot
1282.5654	1282.6028	0.0374	29	40	50	LESMEPCSSVK			Carbamidomethyl (C)[7], Oxidation (M)[4]		Mascot
1553.6935	1553.7893	0.0958	62	40	52	LESMEPCSSVKDR			Carbamidomethyl (C)[7], Oxidation (M)[4]		Mascot
1689.9019	1689.8751	-0.0268	-16	177	195	IDGLVSGIGTGGTITGSG K					Mascot
1689.9019	1689.8751	-0.0268	-16	177	195	IDGLVSGIGTGGTITGSG K					Mascot
1707.8833	1707.8865	0.0032	2	263	278	VSSDESIEMAKSLALK					Mascot
1707.8833	1707.8865	0.0032	2	263	278	VSSDESIEMAKSLALK					Mascot
2055.0466	2054.9089	-0.1377	-67	53	71	IGYSMITDAEEKGFIVPGK					Mascot
2700.4456	2700.3516	-0.094	-35	2	27	GEASSPAIAKDVTELIGNT PLVYLNK					Mascot
2700.4456	2700.3516	-0.094	-35	2	27	GEASSPAIAKDVTELIGNT PLVYLNK					Mascot

9 Constitutive photomorphogenesis protein 10 [Triticum urartu] gi|474051855 63180.8 4.75 9 34 0 1.792

Peptide Information

Calc. Mass	Obsrv. Mass	± da	± ppm	Start Seq.	End Seq.	Sequence	Ion Score	C. I. %	Modification	Rank	Result Type
805.4454	805.4611	0.0157	19	535	541	SVPFKEV					Mascot
815.4774	815.4952	0.0178	22	162	168	YVPPLAR					Mascot
1136.6423	1136.5439	-0.0984	-87	442	452	KPTAPVGDVPR					Mascot
1539.7914	1539.7283	-0.0631	-41	88	100	AKHDEIAAEWTLR					Mascot
1550.6969	1550.8054	0.1085	70	228	239	KPQQYEDEEQEK					Mascot
1748.8099	1748.8667	0.0568	32	471	486	AGRDFGYGENAIADHR					Mascot
2055.1128	2054.9089	-0.2039	-99	487	508	AAAAAAGGTTLAMVIRRP DGGK					Mascot

2730.446	2730.3684	-0.0776	-28	64	87	AIITNPDPYNPLVDSIARL YLTDR					Mascot
2857.2944	2857.3193	0.0249	9	358	381	IAQQYQDEKAPEECHVE EQTAPEK			Carbamidomethyl (C)[14]		Mascot

10	Peroxidase 1 [Triticum urartu]	gi|474368431	32677.3	7.07	7	34	0	4.128

Peptide Information

Calc. Mass	Obsrv. Mass	± da	± ppm	Start Seq.	End Seq.	Sequence	Ion Score	C. I. %	Modification	Rank	Result Type
818.4407	818.3916	-0.0491	-60	94	100	DAVWLSK					Mascot
841.4091	841.392	-0.0171	-20	63	69	GFDFVEK					Mascot
870.4468	870.5106	0.0638	73	169	175	LYNFTGR					Mascot
1471.7864	1471.8124	0.026	18	50	62	TAEKDAKPNQTLR					Mascot
1507.6774	1507.7943	0.1169	78	258	270	DEFFADFAASMIK			Oxidation (M)[11]		Mascot
1547.7483	1547.7235	-0.0248	-16	271	285	MGNADVLTGSQGEIR					Mascot
2857.3713	2857.3193	-0.052	-18	169	192	LYNFTGRVNPTDIDPTLE PQYMEK			Oxidation (M)[22]		Mascot

Gel Idx/Pos	164/G15	Instr./Gel Origin	BA2151/Sample Project 20140814	Process Status	Analysis Succeeded
Plate [#] Name	[1] Sample Project 20140814	Instrument Sample Name		Spectra	11

Rank	Protein Name	Accession No.	Protein MW	Protein PI	Pep. Count	Protein Score	Protein Score C. I. %	Intensity Matched	Total Ion Score	Total Ion C. I. %	Confirmed
1	serpin 1 [Triticum aestivum]	gi|224589266	43261.1	5.44	7	593	100	52.559	568	100	

Peptide Information

Calc. Mass	Obsrv. Mass	± da	± ppm	Start Seq.	End Seq.	Sequence	Ion Score	C. I. %	Modification	Rank	Result Type
1176.5896	1176.5861	-0.0035	-3	262	271	LSAEPDFLER					Mascot
1176.5896	1176.5861	-0.0035	-3	262	271	LSAEPDFLER	94	100			Mascot
1292.7097	1292.6851	-0.0246	-19	290	301	ISFGIEASDLLK					Mascot
1292.7097	1292.6851	-0.0246	-19	290	301	ISFGIEASDLLK	83	100			Mascot
1567.873	1567.8324	-0.0406	-26	288	301	FKISFGIEASDLLK					Mascot
1679.8864	1679.8699	-0.0165	-10	262	275	LSAEPDFLERHIPR					Mascot
2113.0999	2113.052	-0.0479	-23	380	399	EDISGVVLFMGHVVNPLLSS					Mascot
2113.0999	2113.052	-0.0479	-23	380	399	EDISGVVLFMGHVVNPLLSS	63	99.996			Mascot
2129.0947	2129.0298	-0.0649	-30	380	399	EDISGVVLFMGHVVNPLLSS			Oxidation (M)[10]		Mascot
2129.0947	2129.0298	-0.0649	-30	380	399	EDISGVVLFMGHVVNPLLSS	73	100	Oxidation (M)[10]		Mascot
2720.3525	2720.3323	-0.0202	-7	329	354	VSSVFHQAFVEVNEQGTEAAASTAIK					Mascot
2720.3525	2720.3323	-0.0202	-7	329	354	VSSVFHQAFVEVNEQGTEAAASTAIK	259	100			Mascot
3039.3896	3039.376	-0.0136	-4	302	328	CLGLQLPFSDEADFSEMVDSPMPQGLR			Carbamidomethyl (C)[1]		Mascot
3055.3845	3055.3569	-0.0276	-9	302	328	CLGLQLPFSDEADFSEMVDSPMPQGLR			Carbamidomethyl (C)[1], Oxidation (M)[17]		Mascot
3071.3796	3071.3579	-0.0217	-7	302	328	CLGLQLPFSDEADFSEMVDSPMPQGLR			Carbamidomethyl (C)[1], Oxidation (M)[17,22]		Mascot
3071.3796	3071.3579	-0.0217	-7	302	328	CLGLQLPFSDEADFSEMVDSPMPQGLR	60	99.993	Carbamidomethyl (C)[1], Oxidation (M)[17,22]		Mascot

Rank	Protein Name	Accession No.	Protein MW	Protein PI	Pep. Count	Protein Score	Protein Score C. I. %	Intensity Matched	Total Ion Score	Total Ion C. I. %	Confirmed
2	serpin [Triticum aestivum]	gi|871551	43262.2	5.6	7	534	100	51.094	508	100	

Protein Group

RecName: Full=Serpin-Z1A; AltName: Full=TriaeZ1a; AltName: Full=WSZ1a; Short=WSZ1; AltName: Full=WSZCI	gi|75282265	43262.2	5.59999990463257

Peptide Information

Calc. Mass	Obsrv. Mass	± da	± ppm	Start Seq.	End Seq.	Sequence	Ion Score	C. I. %	Modification	Rank	Result Type
1176.5896	1176.5861	-0.0035	-3	261	270	LSAEPDFLER					Mascot

Calc. Mass	Obsrv. Mass	± da	± ppm	Start Seq.	End Seq.	Sequence	Ion Score	C. I. %	Modification	Rank	Result Type
1176.5896	1176.5861	-0.0035	-3	261	270	LSAEPDFLER	94	100			Mascot
1292.7097	1292.6851	-0.0246	-19	289	300	ISFGIEASDLLK					Mascot
1292.7097	1292.6851	-0.0246	-19	289	300	ISFGIEASDLLK	83	100			Mascot
1567.873	1567.8324	-0.0406	-26	287	300	FKISFGIEASDLLK					Mascot
1679.8864	1679.8699	-0.0165	-10	261	274	LSAEPDFLERHIPR					Mascot
2113.0999	2113.052	-0.0479	-23	379	398	EDISGVVLFMGHVVNPLL SS					Mascot
2113.0999	2113.052	-0.0479	-23	379	398	EDISGVVLFMGHVVNPLL SS	63	99.996			Mascot
2129.0947	2129.0298	-0.0649	-30	379	398	EDISGVVLFMGHVVNPLL SS			Oxidation (M)[10]		Mascot
2129.0947	2129.0298	-0.0649	-30	379	398	EDISGVVLFMGHVVNPLL SS	73	100	Oxidation (M)[10]		Mascot
2720.3525	2720.3323	-0.0202	-7	328	353	VSSVFHQAFVEVNEQGT EAAASTAIK					Mascot
2720.3525	2720.3323	-0.0202	-7	328	353	VSSVFHQAFVEVNEQGT EAAASTAIK	259	100			Mascot
2944.5642	2944.7695	0.2053	70	101	126	FANGVFVDASLLLKPSFQ EIAVCKYK			Carbamidomethyl (C)[23]		Mascot

3 serpin 3 [Triticum aestivum] gi|224589270 43227.1 5.56 7 521 100 51.078 495 100

Protein Group

serpin-N3.7 [Triticum aestivum] gi|379060945 42639.8 5.5199 999809 2651

Peptide Information

Calc. Mass	Obsrv. Mass	± da	± ppm	Start Seq.	End Seq.	Sequence	Ion Score	C. I. %	Modification	Rank	Result Type
1176.5896	1176.5861	-0.0035	-3	262	271	LSAEPDFLER					Mascot
1176.5896	1176.5861	-0.0035	-3	262	271	LSAEPDFLER	94	100			Mascot
1292.7097	1292.6851	-0.0246	-19	290	301	ISFGIEASDLLK					Mascot
1292.7097	1292.6851	-0.0246	-19	290	301	ISFGIEASDLLK	83	100			Mascot
1567.873	1567.8324	-0.0406	-26	288	301	FKISFGIEASDLLK					Mascot
1679.8864	1679.8699	-0.0165	-10	262	275	LSAEPDFLERHIPR					Mascot
2267.1628	2267.1016	-0.0612	-27	241	261	QFSMYILLPEAPGGLSSL AEK			Oxidation (M)[4]		Mascot
2720.3525	2720.3323	-0.0202	-7	329	354	VSSVFHQAFVEVNEQGT EAAASTAIK					Mascot
2720.3525	2720.3323	-0.0202	-7	329	354	VSSVFHQAFVEVNEQGT EAAASTAIK	259	100			Mascot
3039.3896	3039.376	-0.0136	-4	302	328	CLGLQLPFSDEADFSEM VDSPMPQGLR			Carbamidomethyl (C)[1]		Mascot
3055.3845	3055.3569	-0.0276	-9	302	328	CLGLQLPFSDEADFSEM VDSPMPQGLR			Carbamidomethyl (C)[1], Oxidation (M)[17]		Mascot
3071.3796	3071.3579	-0.0217	-7	302	328	CLGLQLPFSDEADFSEM VDSPMPQGLR			Carbamidomethyl (C)[1], Oxidation (M)[17,22]		Mascot
3071.3796	3071.3579	-0.0217	-7	302	328	CLGLQLPFSDEADFSEM VDSPMPQGLR	60	99.993	Carbamidomethyl (C)[1], Oxidation (M)[17,22]		Mascot

4 serpin [Triticum aestivum] gi|1885350 43119.9 5.44 6 506 100 46.058 486 100

Protein Group

RecName: Full=Serpin-Z1B; AltName: Full=TriaeZ1b; AltName: Full=WSZ1b; AltName: Full=WZS2	gi|75279910	43119.9	5.4400 000572 2046

Peptide Information

Calc. Mass	Obsrv. Mass	± da	± ppm	Start Seq.	End Seq.	Sequence	Ion Score	C. I. %	Modification	Rank	Result Type
1176.5896	1176.5861	-0.0035	-3	262	271	LSAEPDFLER					Mascot
1176.5896	1176.5861	-0.0035	-3	262	271	LSAEPDFLER	94	100			Mascot
1679.8864	1679.8699	-0.0165	-10	262	275	LSAEPDFLERHIPR					Mascot
2113.0999	2113.052	-0.0479	-23	380	399	EDISGVVLFMGHVVNPLL SS					Mascot
2113.0999	2113.052	-0.0479	-23	380	399	EDISGVVLFMGHVVNPLL SS	63	99.996			Mascot
2129.0947	2129.0298	-0.0649	-30	380	399	EDISGVVLFMGHVVNPLL SS			Oxidation (M)[10]		Mascot
2129.0947	2129.0298	-0.0649	-30	380	399	EDISGVVLFMGHVVNPLL SS	73	100	Oxidation (M)[10]		Mascot
2267.1628	2267.1016	-0.0612	-27	241	261	QFSMYILLPEAPGGLSSL AEK			Oxidation (M)[4]		Mascot
2720.3525	2720.3323	-0.0202	-7	329	354	VSSVFHQAFVEVNEQGT EAAASTAIK					Mascot
2720.3525	2720.3323	-0.0202	-7	329	354	VSSVFHQAFVEVNEQGT EAAASTAIK	259	100			Mascot
3039.3896	3039.376	-0.0136	-4	302	328	CLGLQLPFSDEADFSEM VDSPMPQGLR			Carbamidomethyl (C)[1]		Mascot
3055.3845	3055.3569	-0.0276	-9	302	328	CLGLQLPFSDEADFSEM VDSPMPQGLR			Carbamidomethyl (C)[1], Oxidation (M)[17]		Mascot
3071.3796	3071.3579	-0.0217	-7	302	328	CLGLQLPFSDEADFSEM VDSPMPQGLR			Carbamidomethyl (C)[1], Oxidation (M)[17,22]		Mascot
3071.3796	3071.3579	-0.0217	-7	302	328	CLGLQLPFSDEADFSEM VDSPMPQGLR	60	99.993	Carbamidomethyl (C)[1], Oxidation (M)[17,22]		Mascot

5	Serpin-Z1C [Triticum urartu]	gi|474075261	42956	5.62	6	447	100	44.544	426	100

Peptide Information

Calc. Mass	Obsrv. Mass	± da	± ppm	Start Seq.	End Seq.	Sequence	Ion Score	C. I. %	Modification	Rank	Result Type
1176.5896	1176.5861	-0.0035	-3	261	270	LSAEPDFLER					Mascot
1176.5896	1176.5861	-0.0035	-3	261	270	LSAEPDFLER	94	100			Mascot
1679.8864	1679.8699	-0.0165	-10	261	274	LSAEPDFLERHIPR					Mascot
2113.0999	2113.052	-0.0479	-23	379	398	EDISGVVLFMGHVVNPLL SS					Mascot
2113.0999	2113.052	-0.0479	-23	379	398	EDISGVVLFMGHVVNPLL SS	63	99.996			Mascot
2129.0947	2129.0298	-0.0649	-30	379	398	EDISGVVLFMGHVVNPLL SS			Oxidation (M)[10]		Mascot
2129.0947	2129.0298	-0.0649	-30	379	398	EDISGVVLFMGHVVNPLL SS	73	100	Oxidation (M)[10]		Mascot
2267.1628	2267.1016	-0.0612	-27	240	260	QFSMYILLPEAPGGLSSL			Oxidation (M)[4]		Mascot

						AEK					
2720.3525	2720.3323	-0.0202	-7	328	353	VSSVFHQAFVEVNEQGT EAAASTAIK					Mascot
2720.3525	2720.3323	-0.0202	-7	328	353	VSSVFHQAFVEVNEQGT EAAASTAIK	259	100			Mascot
3037.3853	3037.4165	0.0312	10	301	327	CLGLQLPFSNEADFSEM VDSPMAHGLR			Carbamidomethyl (C)[1], Oxidation (M)[17]		Mascot

6	serpin [Triticum aestivum]	gi|5734504	42969	5.62	5	442	100	44.052	426	100	

Protein Group

RecName: Full=Serpin-Z1C; AltName: Full=TriaeZ1c; AltName: Full=WSZ1c	gi|75313848	42969	5.6199 998855 5908

Peptide Information

Calc. Mass	Obsrv. Mass	± da	± ppm	Start Seq.	End Seq.	Sequence	Ion Score	C. I. %	Modification	Rank	Result Type
1176.5896	1176.5861	-0.0035	-3	261	270	LSAEPDFLER					Mascot
1176.5896	1176.5861	-0.0035	-3	261	270	LSAEPDFLER	94	100			Mascot
1679.8864	1679.8699	-0.0165	-10	261	274	LSAEPDFLERHIPR					Mascot
2113.0999	2113.052	-0.0479	-23	379	398	EDISGVVLFMGHVVNPLL SS					Mascot
2113.0999	2113.052	-0.0479	-23	379	398	EDISGVVLFMGHVVNPLL SS	63	99.996			Mascot
2129.0947	2129.0298	-0.0649	-30	379	398	EDISGVVLFMGHVVNPLL SS			Oxidation (M)[10]		Mascot
2129.0947	2129.0298	-0.0649	-30	379	398	EDISGVVLFMGHVVNPLL SS	73	100	Oxidation (M)[10]		Mascot
2720.3525	2720.3323	-0.0202	-7	328	353	VSSVFHQAFVEVNEQGT EAAASTAIK					Mascot
2720.3525	2720.3323	-0.0202	-7	328	353	VSSVFHQAFVEVNEQGT EAAASTAIK	259	100			Mascot
3037.3853	3037.4165	0.0312	10	301	327	CLGLQLPFSNEADFSEM VDSPMAHGLR			Carbamidomethyl (C)[1], Oxidation (M)[17]		Mascot

7	Serpin-Z2B [Triticum urartu]	gi|473793747	45225.7	6.03	6	96	100	7.418	83	100	

Peptide Information

Calc. Mass	Obsrv. Mass	± da	± ppm	Start Seq.	End Seq.	Sequence	Ion Score	C. I. %	Modification	Rank	Result Type
1154.6278	1154.5731	-0.0547	-47	146	154	YVMQLFLPK			Oxidation (M)[3]		Mascot
1192.5382	1192.5776	0.0394	33	199	208	GAWTDQFDPR					Mascot
1292.7097	1292.6851	-0.0246	-19	306	317	ISFGIEASDLLK					Mascot
1292.7097	1292.6851	-0.0246	-19	306	317	ISFGIEASDLLK	83	100			Mascot
1385.7019	1385.7474	0.0455	33	176	188	DILPAGSIDNNTR					Mascot
1567.873	1567.8324	-0.0406	-26	304	317	FKISFGIEASDLLK					Mascot
1665.8595	1665.8348	-0.0247	-15	278	291	LSAEPEFLEQHIPR					Mascot

8	serpin [Triticum aestivum]	gi|5734506	43341.5	5.46	3	88	99.993	7.327	83	100	

Protein Group

RecName: Full=Serpin-Z2A; AltName: Full=TriaeZ2a; AltName: Full=WSZ2a	gi|75313847	43341.5	5.4600 000381 4697

Peptide Information

Calc. Mass	Obsrv. Mass	± da	± ppm	Start Seq.	End Seq.	Sequence	Ion Score	C. I. %	Modification	Rank	Result Type
1182.5175	1182.5504	0.0329	28	182	191	GAWTDQFDSR					Mascot
1292.7097	1292.6851	-0.0246	-19	289	300	ISFGIEASDLLK					Mascot
1292.7097	1292.6851	-0.0246	-19	289	300	ISFGIEASDLLK	83	100			Mascot
1567.873	1567.8324	-0.0406	-26	287	300	FKISFGIEASDLLK					Mascot

9	unnamed protein product [Triticum aestivum]	gi|227473229	29360.7	4.83	11	57	90.546	1.674	

Protein Group

14-3-3 protein [Triticum aestivum]	gi|431822520	29360.7	4.8299 999237 0605

Peptide Information

Calc. Mass	Obsrv. Mass	± da	± ppm	Start Seq.	End Seq.	Sequence	Ion Score	C. I. %	Modification	Rank	Result Type
819.4458	819.4134	-0.0324	-40	95	101	IETELSK					Mascot
922.4199	922.4544	0.0345	37	130	136	MKGDYHR			Oxidation (M)[1]		Mascot
1051.5419	1051.516	-0.0259	-25	80	89	GNEAYVASIK					Mascot
1336.7107	1336.6428	-0.0679	-51	148	159	KEAAENTLVAYK					Mascot
1616.849	1616.8638	0.0148	9	34	48	VAKTADVGELTVEER					Mascot
1632.7648	1632.84	0.0752	46	24	36	YEEMVEFMEKVAK					Mascot
1699.7955	1699.7889	-0.0066	-4	2	16	STAEATREENVYMAK					Mascot
1846.8309	1846.7903	-0.0406	-22	1	16	MSTAEATREENVYMAK			Oxidation (M)[1]		Mascot
2131.9675	2132.0564	0.0889	42	17	33	LAEQAERYEEMVEFMEK					Mascot
2289.249	2289.0933	-0.1557	-68	109	129	LLDSHLVPSATAAESKVF YLK					Mascot
3008.5688	3008.7617	0.1929	64	149	176	EAAENTLVAYKSAQDIAL ADLPTTHPIR					Mascot

10	hypothetical protein TRIUR3_25252 [Triticum urartu]	gi|473936455	12730.6	6.41	8	52	75.698	1.961	

Peptide Information

Calc. Mass	Obsrv. Mass	± da	± ppm	Start Seq.	End Seq.	Sequence	Ion Score	C. I. %	Modification	Rank	Result Type
901.4746	901.4532	-0.0214	-24	42	48	CPMKPIR			Carbamidomethyl (C)[1]		Mascot
1029.5696	1029.5459	-0.0237	-23	42	49	CPMKPIRK			Carbamidomethyl (C)[1]		Mascot

1130.5922	1130.5557	-0.0365	-32	40	48	GRCPMKPIR	Carbamidomethyl (C)[3], Oxidation (M)[5]	Mascot
1290.6219	1290.6093	-0.0126	-10	1	13	MAGRDELASGGAR		Mascot
1306.6168	1306.5713	-0.0455	-35	1	13	MAGRDELASGGAR	Oxidation (M)[1]	Mascot
1320.6254	1320.6599	0.0345	26	19	29	GDPMDPFLKER	Oxidation (M)[4]	Mascot
1336.7471	1336.6428	-0.1043	-78	104	115	VAHQLDVELLLS		Mascot
2151.1233	2151.033	-0.0903	-42	84	103	DWANVVVAHGPVYSPLA LDK		Mascot
2991.5352	2991.7852	0.25	84	56	81	LTITCGIKCDVLPEVQAE MIVLFADR	Carbamidomethyl (C)[5,9]	Mascot
3007.5303	3007.3887	-0.1416	-47	56	81	LTITCGIKCDVLPEVQAE MIVLFADR	Carbamidomethyl (C)[5,9], Oxidation (M)[19]	Mascot

Gel Idx/Pos	165/G16	Instr./Gel Origin	BA2151/Sample Project 20140814	Process Status	Analysis Succeeded
Plate [#] Name	[1] Sample Project 20140814	Instrument Sample Name		Spectra	11

Rank	Protein Name	Accession No.	Protein MW	Protein PI	Pep. Count	Protein Score	Protein Score C. I. %	Intensity Matched	Total Ion Score	Total Ion C. I. %	Confirmed
1	unnamed protein product [Triticum aestivum]	gi|227473229	29360.7	4.83	22	693	100	68.468	515	100	

Protein Group

Protein Name	Accession No.	Protein MW	Protein PI
14-3-3 protein [Triticum aestivum]	gi|431822520	29360.7	4.8299 999237 0605

Peptide Information

Calc. Mass	Obsrv. Mass	± da	± ppm	Start Seq.	End Seq.	Sequence	Ion Score	C. I. %	Modification	Rank	Result Type
816.421	816.4076	-0.0134	-16	17	23	LAEQAER					Mascot
907.5247	907.4792	-0.0455	-50	49	56	NLLSVAYK					Mascot
917.5302	917.5087	-0.0215	-23	68	75	IISSIEQK					Mascot
922.4199	922.4067	-0.0132	-14	130	136	MKGDYHR			Oxidation (M)[1]		Mascot
1051.5419	1051.522	-0.0199	-19	80	89	GNEAYVASIK					Mascot
1076.5946	1076.5731	-0.0215	-20	93	101	TRIETELSK					Mascot
1189.6609	1189.6497	-0.0112	-9	222	231	DSTLIMQLLR					Mascot
1189.6609	1189.6497	-0.0112	-9	222	231	DSTLIMQLLR	87	100			Mascot
1205.6559	1205.6342	-0.0217	-18	222	231	DSTLIMQLLR			Oxidation (M)[6]		Mascot
1205.6559	1205.6342	-0.0217	-18	222	231	DSTLIMQLLR	35	97.738	Oxidation (M)[6]		Mascot
1208.6157	1208.611	-0.0047	-4	149	159	EAAENTLVAYK					Mascot
1318.6486	1318.6364	-0.0122	-9	37	48	TADVGELTVEER					Mascot
1318.6486	1318.6364	-0.0122	-9	37	48	TADVGELTVEER	118	100			Mascot
1334.5643	1334.542	-0.0223	-17	24	33	YEEMVEFMEK					Mascot
1336.7107	1336.6345	-0.0762	-57	148	159	KEAAENTLVAYK					Mascot
1350.5592	1350.5243	-0.0349	-26	24	33	YEEMVEFMEK			Oxidation (M)[4]		Mascot
1366.5542	1366.511	-0.0432	-32	24	33	YEEMVEFMEK			Oxidation (M)[4,8]		Mascot
1418.7485	1418.7345	-0.014	-10	68	79	IISSIEQKEESR					Mascot
1517.8799	1517.8373	-0.0426	-28	49	62	NLLSVAYKNVIGAR					Mascot
1552.7601	1552.745	-0.0151	-10	76	89	EESRGNEAYVASIK					Mascot
1818.9708	1818.9503	-0.0205	-11	160	176	SAQDIALADLPTTHPIR					Mascot
1818.9708	1818.9503	-0.0205	-11	160	176	SAQDIALADLPTTHPIR	140	100			Mascot
1846.8309	1846.968	0.1371	74	1	16	MSTAEATREENVYMAK			Oxidation (M)[1]		Mascot
2106.9463	2106.8904	-0.0559	-27	232	250	DNLTLWTSDNAEEGGDE IK					Mascot

2114.9763	2114.9133	-0.063	-30	203	221	QAFDEAIAELDSLGEESY K					Mascot
2131.9675	2131.9492	-0.0183	-9	17	33	LAEQAERYEEMVEFMEK					Mascot
2131.9675	2131.9492	-0.0183	-9	17	33	LAEQAERYEEMVEFMEK	34	96.962			Mascot
2147.9624	2147.9199	-0.0425	-20	17	33	LAEQAERYEEMVEFMEK			Oxidation (M)[11]		Mascot
2147.9624	2147.9199	-0.0425	-20	17	33	LAEQAERYEEMVEFMEK	19	5.911	Oxidation (M)[15]		Mascot
2163.9573	2163.8999	-0.0574	-27	17	33	LAEQAERYEEMVEFMEK			Oxidation (M)[11,15]		Mascot
2163.9573	2163.8999	-0.0574	-27	17	33	LAEQAERYEEMVEFMEK	44	99.704	Oxidation (M)[11,15]		Mascot
2331.2019	2331.1804	-0.0215	-9	177	196	LGLALNFSVFYYEILNSPD R					Mascot
2331.2019	2331.1804	-0.0215	-9	177	196	LGLALNFSVFYYEILNSPD R	125	100			Mascot
3199.4297	3199.406	-0.0237	-7	232	261	DNLTLWTSDNAEEGGDE IKEAASKPEGEGH					Mascot
3285.6196	3285.6016	-0.018	-5	203	231	QAFDEAIAELDSLGEESY KDSTLIMQLLR					Mascot
3301.6145	3301.5984	-0.0161	-5	203	231	QAFDEAIAELDSLGEESY KDSTLIMQLLR			Oxidation (M)[25]		Mascot

2	unnamed protein product [Triticum aestivum]	gi|227471938	29387.7	4.83	21	683	100	68.22	515	100	

Protein Group

14-3-3 protein [Triticum aestivum]	gi|40781605	29387.7	4.8299 999237 0605

Peptide Information

Calc. Mass	Obsrv. Mass	± da	± ppm	Start Seq.	End Seq.	Sequence	Ion Score	C. I. %	Modification	Rank	Result Type
816.421	816.4076	-0.0134	-16	17	23	LAEQAER					Mascot
907.5247	907.4792	-0.0455	-50	49	56	NLLSVAYK					Mascot
917.5302	917.5087	-0.0215	-23	68	75	IISSIEQK					Mascot
922.4199	922.4067	-0.0132	-14	130	136	MKGDYHR			Oxidation (M)[1]		Mascot
1051.5419	1051.522	-0.0199	-19	80	89	GNEAYVASIK					Mascot
1189.6609	1189.6497	-0.0112	-9	222	231	DSTLIMQLLR					Mascot
1189.6609	1189.6497	-0.0112	-9	222	231	DSTLIMQLLR	87	100			Mascot
1205.6559	1205.6342	-0.0217	-18	222	231	DSTLIMQLLR			Oxidation (M)[6]		Mascot
1205.6559	1205.6342	-0.0217	-18	222	231	DSTLIMQLLR	35	97.738	Oxidation (M)[6]		Mascot
1208.6157	1208.611	-0.0047	-4	149	159	EAAENTLVAYK					Mascot
1318.6486	1318.6364	-0.0122	-9	37	48	TADVGELTVEER					Mascot
1318.6486	1318.6364	-0.0122	-9	37	48	TADVGELTVEER	118	100			Mascot
1334.5643	1334.542	-0.0223	-17	24	33	YEEMVEFMEK					Mascot
1336.7107	1336.6345	-0.0762	-57	148	159	KEAAENTLVAYK					Mascot
1350.5592	1350.5243	-0.0349	-26	24	33	YEEMVEFMEK			Oxidation (M)[4]		Mascot
1366.5542	1366.511	-0.0432	-32	24	33	YEEMVEFMEK			Oxidation (M)[4,8]		Mascot

1418.7485	1418.7345	-0.014	-10	68	79	IISSIEQKEESR					Mascot
1517.8799	1517.8373	-0.0426	-28	49	62	NLLSVAYKNVIGAR					Mascot
1552.7601	1552.745	-0.0151	-10	76	89	EESRGNEAYVASIK					Mascot
1818.9708	1818.9503	-0.0205	-11	160	176	SAQDIALADLPTTHPIR					Mascot
1818.9708	1818.9503	-0.0205	-11	160	176	SAQDIALADLPTTHPIR	140	100			Mascot
1846.8309	1846.968	0.1371	74	1	16	MSTAEATREENVYMAK			Oxidation (M)[1]		Mascot
2106.9463	2106.8904	-0.0559	-27	232	250	DNLTLWTSDNAEEGGDEIK					Mascot
2114.9763	2114.9133	-0.063	-30	203	221	QAFDEAIAELDSLGEESYK					Mascot
2131.9675	2131.9492	-0.0183	-9	17	33	LAEQAERYEEMVEFMEK					Mascot
2131.9675	2131.9492	-0.0183	-9	17	33	LAEQAERYEEMVEFMEK	34	96.962			Mascot
2147.9624	2147.9199	-0.0425	-20	17	33	LAEQAERYEEMVEFMEK			Oxidation (M)[11]		Mascot
2147.9624	2147.9199	-0.0425	-20	17	33	LAEQAERYEEMVEFMEK	19	5.911	Oxidation (M)[15]		Mascot
2163.9573	2163.8999	-0.0574	-27	17	33	LAEQAERYEEMVEFMEK			Oxidation (M)[11,15]		Mascot
2163.9573	2163.8999	-0.0574	-27	17	33	LAEQAERYEEMVEFMEK	44	99.704	Oxidation (M)[11,15]		Mascot
2331.2019	2331.1804	-0.0215	-9	177	196	LGLALNFSVFYYEILNSPDR					Mascot
2331.2019	2331.1804	-0.0215	-9	177	196	LGLALNFSVFYYEILNSPDR	125	100			Mascot
3199.4297	3199.406	-0.0237	-7	232	261	DNLTLWTSDNAEEGGDEIKEAASKPEGEGH					Mascot
3285.6196	3285.6016	-0.018	-5	203	231	QAFDEAIAELDSLGEESYKDSTLIMQLLR					Mascot
3301.6145	3301.5984	-0.0161	-5	203	231	QAFDEAIAELDSLGEESYKDSTLIMQLLR			Oxidation (M)[25]		Mascot

3 14-3-3-like protein A [Triticum urartu] gi|474293618 28778.5 5.2 11 314 100 30.455 256 100

Peptide Information

Calc. Mass	Obsrv. Mass	± da	± ppm	Start Seq.	End Seq.	Sequence	Ion Score	C. I. %	Modification	Rank	Result Type
816.421	816.4076	-0.0134	-16	17	23	LAEQAER					Mascot
907.5247	907.4792	-0.0455	-50	49	56	NLLSVAYK					Mascot
922.4199	922.4067	-0.0132	-14	130	136	MKGDYHR			Oxidation (M)[1]		Mascot
1189.6609	1189.6497	-0.0112	-9	222	231	DSTLIMQLLR					Mascot
1189.6609	1189.6497	-0.0112	-9	222	231	DSTLIMQLLR	87	100			Mascot
1205.6559	1205.6342	-0.0217	-18	222	231	DSTLIMQLLR			Oxidation (M)[6]		Mascot
1205.6559	1205.6342	-0.0217	-18	222	231	DSTLIMQLLR	35	97.738	Oxidation (M)[6]		Mascot
1334.5643	1334.542	-0.0223	-17	24	33	YEEMVEFMEK					Mascot
1350.5592	1350.5243	-0.0349	-26	24	33	YEEMVEFMEK			Oxidation (M)[4]		Mascot
1366.5542	1366.511	-0.0432	-32	24	33	YEEMVEFMEK			Oxidation (M)[4,8]		Mascot
1374.7224	1374.6567	-0.0657	-48	68	79	IVSSIEQKEEGR					Mascot

Calc. Mass	Obsrv. Mass	± da	± ppm	Start Seq.	End Seq.	Sequence	Ion Score	C. I. %	Modification	Rank	Result Type
1517.8799	1517.8373	-0.0426	-28	49	62	NLLSVAYKNVIGAR					Mascot
2083.9966	2084.0505	0.0539	26	148	167	KDAADSTLGAYQAAQDIAMK			Oxidation (M)[19]		Mascot
2100.9607	2100.9849	0.0242	12	203	221	QAFDEAIAELDSLGEDSYK					Mascot
2131.9675	2131.9492	-0.0183	-9	17	33	LAEQAERYEEMVEFMEK					Mascot
2131.9675	2131.9492	-0.0183	-9	17	33	LAEQAERYEEMVEFMEK	34	96.962			Mascot
2147.9624	2147.9199	-0.0425	-20	17	33	LAEQAERYEEMVEFMEK			Oxidation (M)[11]		Mascot
2147.9624	2147.9199	-0.0425	-20	17	33	LAEQAERYEEMVEFMEK	19	5.911	Oxidation (M)[15]		Mascot
2163.9573	2163.8999	-0.0574	-27	17	33	LAEQAERYEEMVEFMEK			Oxidation (M)[11,15]		Mascot
2163.9573	2163.8999	-0.0574	-27	17	33	LAEQAERYEEMVEFMEK	44	99.704	Oxidation (M)[11,15]		Mascot
2331.2019	2331.1804	-0.0215	-9	177	196	LGLALNFSVFYYEILNSPDR					Mascot
2331.2019	2331.1804	-0.0215	-9	177	196	LGLALNFSVFYYEILNSPDR	125	100			Mascot

4 14-3-3 protein [Triticum aestivum] gi|390195996 30066.1 4.73 12 313 100 30.125 256 100

Protein Group

14-3-3 protein [Triticum aestivum] gi|351602255 30066.1 4.7300000190 7349

14-3-3-like protein GF14-B [Triticum urartu] gi|474147722 30043.1 4.6900000572 2046

Peptide Information

Calc. Mass	Obsrv. Mass	± da	± ppm	Start Seq.	End Seq.	Sequence	Ion Score	C. I. %	Modification	Rank	Result Type
816.421	816.4076	-0.0134	-16	18	24	LAEQAER					Mascot
844.4523	844.448	-0.0043	-5	2	9	TAPAELSR					Mascot
907.5247	907.4792	-0.0455	-50	50	57	NLLSVAYK					Mascot
917.5302	917.5087	-0.0215	-23	69	76	IISSIEQK					Mascot
1189.6609	1189.6497	-0.0112	-9	223	232	DSTLIMQLLR					Mascot
1189.6609	1189.6497	-0.0112	-9	223	232	DSTLIMQLLR	87	100			Mascot
1205.6559	1205.6342	-0.0217	-18	223	232	DSTLIMQLLR			Oxidation (M)[6]		Mascot
1205.6559	1205.6342	-0.0217	-18	223	232	DSTLIMQLLR	35	97.738	Oxidation (M)[6]		Mascot
1334.5643	1334.542	-0.0223	-17	25	34	YEEMVEFMEK					Mascot
1340.6515	1340.6027	-0.0488	-36	149	160	KDAAENTMVAYK					Mascot
1350.5592	1350.5243	-0.0349	-26	25	34	YEEMVEFMEK			Oxidation (M)[4]		Mascot
1356.6464	1356.5636	-0.0828	-61	149	160	KDAAENTMVAYK			Oxidation (M)[8]		Mascot
1366.5542	1366.511	-0.0432	-32	25	34	YEEMVEFMEK			Oxidation (M)[4,8]		Mascot
1418.7485	1418.7345	-0.014	-10	69	80	IISSIEQKEESR					Mascot
1517.8799	1517.8373	-0.0426	-28	50	63	NLLSVAYKNVIGAR					Mascot

1786.9811	1786.9465	-0.0346	-19	161	177	AAQDIALAELAPTHPIR					Mascot
2131.9675	2131.9492	-0.0183	-9	18	34	LAEQAERYEEMVEFMEK					Mascot
2131.9675	2131.9492	-0.0183	-9	18	34	LAEQAERYEEMVEFMEK	34	96.962			Mascot
2147.9624	2147.9199	-0.0425	-20	18	34	LAEQAERYEEMVEFMEK			Oxidation (M)[11]		Mascot
2147.9624	2147.9199	-0.0425	-20	18	34	LAEQAERYEEMVEFMEK	19	5.911	Oxidation (M)[15]		Mascot
2163.9573	2163.8999	-0.0574	-27	18	34	LAEQAERYEEMVEFMEK			Oxidation (M)[11,15]		Mascot
2163.9573	2163.8999	-0.0574	-27	18	34	LAEQAERYEEMVEFMEK	44	99.704	Oxidation (M)[11,15]		Mascot
2331.2019	2331.1804	-0.0215	-9	178	197	LGLALNFSVFYYEILNSPD R					Mascot
2331.2019	2331.1804	-0.0215	-9	178	197	LGLALNFSVFYYEILNSPD R	125	100			Mascot

5	14-3-3-like protein B [Triticum urartu]	gi|474253094	29786.9	4.67	12	312	100	30.027	255	100

Peptide Information

Calc. Mass	Obsrv. Mass	± da	± ppm	Start Seq.	End Seq.	Sequence	Ion Score	C. I. %	Modification	Rank	Result Type
816.421	816.4076	-0.0134	-16	18	24	LAEQAER					Mascot
907.5247	907.4792	-0.0455	-50	50	57	NLLSVAYK					Mascot
917.5302	917.5087	-0.0215	-23	69	76	IISSIEQK					Mascot
1189.6609	1189.6497	-0.0112	-9	223	232	DSTLIMQLLR					Mascot
1189.6609	1189.6497	-0.0112	-9	223	232	DSTLIMQLLR	87	100			Mascot
1205.6559	1205.6342	-0.0217	-18	223	232	DSTLIMQLLR			Oxidation (M)[6]		Mascot
1205.6559	1205.6342	-0.0217	-18	223	232	DSTLIMQLLR	35	97.738	Oxidation (M)[6]		Mascot
1300.6532	1300.6218	-0.0314	-24	138	148	YLAEFKSGTER					Mascot
1334.5643	1334.542	-0.0223	-17	25	34	YEEMVEFMEK					Mascot
1340.6515	1340.6027	-0.0488	-36	149	160	KDAAENTMVAYK					Mascot
1350.5592	1350.5243	-0.0349	-26	25	34	YEEMVEFMEK			Oxidation (M)[4]		Mascot
1356.6464	1356.5636	-0.0828	-61	149	160	KDAAENTMVAYK			Oxidation (M)[8]		Mascot
1366.5542	1366.511	-0.0432	-32	25	34	YEEMVEFMEK			Oxidation (M)[4,8]		Mascot
1418.7485	1418.7345	-0.014	-10	69	80	IISSIEQKEESR					Mascot
1517.8799	1517.8373	-0.0426	-28	50	63	NLLSVAYKNVIGAR					Mascot
2131.9675	2131.9492	-0.0183	-9	18	34	LAEQAERYEEMVEFMEK					Mascot
2131.9675	2131.9492	-0.0183	-9	18	34	LAEQAERYEEMVEFMEK	34	96.962			Mascot
2147.9624	2147.9199	-0.0425	-20	18	34	LAEQAERYEEMVEFMEK			Oxidation (M)[11]		Mascot
2147.9624	2147.9199	-0.0425	-20	18	34	LAEQAERYEEMVEFMEK	19	5.911	Oxidation (M)[15]		Mascot
2160.9819	2160.9421	-0.0398	-18	204	222	QAFDEAISELDSLSEESY K					Mascot
2163.9573	2163.8999	-0.0574	-27	18	34	LAEQAERYEEMVEFMEK			Oxidation (M)[11,15]		Mascot
2163.9573	2163.8999	-0.0574	-27	18	34	LAEQAERYEEMVEFMEK	44	99.704	Oxidation (M)[11,15]		Mascot

2331.2019	2331.1804	-0.0215	-9	178	197	LGLALNFSVFYYEILNSPDR					Mascot
2331.2019	2331.1804	-0.0215	-9	178	197	LGLALNFSVFYYEILNSPDR	125	100			Mascot

6 14-3-3 protein [Triticum aestivum] gi|431822518 29843.9 4.71 12 311 100 30.027 255 100

Peptide Information

Calc. Mass	Obsrv. Mass	± da	± ppm	Start Seq.	End Seq.	Sequence	Ion Score	C. I. %	Modification	Rank	Result Type
816.421	816.4076	-0.0134	-16	18	24	LAEQAER					Mascot
907.5247	907.4792	-0.0455	-50	50	57	NLLSVAYK					Mascot
917.5302	917.5087	-0.0215	-23	69	76	IISSIEQK					Mascot
1189.6609	1189.6497	-0.0112	-9	223	232	DSTLIMQLLR					Mascot
1189.6609	1189.6497	-0.0112	-9	223	232	DSTLIMQLLR	87	100			Mascot
1205.6559	1205.6342	-0.0217	-18	223	232	DSTLIMQLLR			Oxidation (M)[6]		Mascot
1205.6559	1205.6342	-0.0217	-18	223	232	DSTLIMQLLR	35	97.738	Oxidation (M)[6]		Mascot
1300.6532	1300.6218	-0.0314	-24	138	148	YLAEFKSGTER					Mascot
1334.5643	1334.542	-0.0223	-17	25	34	YEEMVEFMEK					Mascot
1340.6515	1340.6027	-0.0488	-36	149	160	KDAAENTMVAYK					Mascot
1350.5592	1350.5243	-0.0349	-26	25	34	YEEMVEFMEK			Oxidation (M)[4]		Mascot
1356.6464	1356.5636	-0.0828	-61	149	160	KDAAENTMVAYK			Oxidation (M)[8]		Mascot
1366.5542	1366.511	-0.0432	-32	25	34	YEEMVEFMEK			Oxidation (M)[4,8]		Mascot
1418.7485	1418.7345	-0.014	-10	69	80	IISSIEQKEESR					Mascot
1517.8799	1517.8373	-0.0426	-28	50	63	NLLSVAYKNVIGAR					Mascot
2131.9675	2131.9492	-0.0183	-9	18	34	LAEQAERYEEMVEFMEK					Mascot
2131.9675	2131.9492	-0.0183	-9	18	34	LAEQAERYEEMVEFMEK	34	96.962			Mascot
2147.9624	2147.9199	-0.0425	-20	18	34	LAEQAERYEEMVEFMEK			Oxidation (M)[11]		Mascot
2147.9624	2147.9199	-0.0425	-20	18	34	LAEQAERYEEMVEFMEK	19	5.911	Oxidation (M)[15]		Mascot
2160.9819	2160.9421	-0.0398	-18	204	222	QAFDEAISELDSLSEESYK					Mascot
2163.9573	2163.8999	-0.0574	-27	18	34	LAEQAERYEEMVEFMEK			Oxidation (M)[11,15]		Mascot
2163.9573	2163.8999	-0.0574	-27	18	34	LAEQAERYEEMVEFMEK	44	99.704	Oxidation (M)[11,15]		Mascot
2331.2019	2331.1804	-0.0215	-9	178	197	LGLALNFSVFYYEILNSPDR					Mascot
2331.2019	2331.1804	-0.0215	-9	178	197	LGLALNFSVFYYEILNSPDR	125	100			Mascot

7 unnamed protein product [Triticum aestivum] gi|227472076 28794.4 4.8 11 311 100 30.455 256 100

Protein Group

TaWIN2 [Triticum aestivum] gi|9798605 28794.4 4.80000019073486

unnamed protein product [Triticum aestivum]	gi|219911924	28794.4	4.8000 001907 3486
unnamed protein product [Triticum aestivum]	gi|257664806	28794.4	4.8000 001907 3486

Peptide Information

Calc. Mass	Obsrv. Mass	± da	± ppm	Start Seq.	End Seq.	Sequence	Ion Score	C. I. %	Modification	Rank	Result Type
816.421	816.4076	-0.0134	-16	17	23	LAEQAER					Mascot
907.5247	907.4792	-0.0455	-50	49	56	NLLSVAYK					Mascot
922.4199	922.4067	-0.0132	-14	130	136	MKGDYHR			Oxidation (M)[1]		Mascot
1189.6609	1189.6497	-0.0112	-9	222	231	DSTLIMQLLR					Mascot
1189.6609	1189.6497	-0.0112	-9	222	231	DSTLIMQLLR	87	100			Mascot
1205.6559	1205.6342	-0.0217	-18	222	231	DSTLIMQLLR			Oxidation (M)[6]		Mascot
1205.6559	1205.6342	-0.0217	-18	222	231	DSTLIMQLLR	35	97.738	Oxidation (M)[6]		Mascot
1334.5643	1334.542	-0.0223	-17	24	33	YEEMVEFMEK					Mascot
1350.5592	1350.5243	-0.0349	-26	24	33	YEEMVEFMEK			Oxidation (M)[4]		Mascot
1366.5542	1366.511	-0.0432	-32	24	33	YEEMVEFMEK			Oxidation (M)[4,8]		Mascot
1374.7224	1374.6567	-0.0657	-48	68	79	IVSSIEQKEEGR					Mascot
1517.8799	1517.8373	-0.0426	-28	49	62	NLLSVAYKNVIGAR					Mascot
2083.9966	2084.0505	0.0539	26	148	167	KDAADSTLGAYQAAQDIA MK			Oxidation (M)[19]		Mascot
2100.9607	2100.9849	0.0242	12	203	221	QAFDEAIAELDSLGEDSY K					Mascot
2131.9675	2131.9492	-0.0183	-9	17	33	LAEQAERYEEMVEFMEK					Mascot
2131.9675	2131.9492	-0.0183	-9	17	33	LAEQAERYEEMVEFMEK	34	96.962			Mascot
2147.9624	2147.9199	-0.0425	-20	17	33	LAEQAERYEEMVEFMEK			Oxidation (M)[11]		Mascot
2147.9624	2147.9199	-0.0425	-20	17	33	LAEQAERYEEMVEFMEK	19	5.911	Oxidation (M)[15]		Mascot
2163.9573	2163.8999	-0.0574	-27	17	33	LAEQAERYEEMVEFMEK			Oxidation (M)[11,15]		Mascot
2163.9573	2163.8999	-0.0574	-27	17	33	LAEQAERYEEMVEFMEK	44	99.704	Oxidation (M)[11,15]		Mascot
2331.2019	2331.1804	-0.0215	-9	177	196	LGLALNFSVFYYEILNSPD R					Mascot
2331.2019	2331.1804	-0.0215	-9	177	196	LGLALNFSVFYYEILNSPD R	125	100			Mascot

8	unnamed protein product [Triticum aestivum]	gi|227473231	28898.4	4.8	11	309	100	30.455	256	100	

Protein Group

unnamed protein product [Triticum aestivum]	gi|257710883	28898.4	4.8000 001907 3486

Peptide Information

Calc. Mass	Obsrv. Mass	± da	± ppm	Start	End	Sequence	Ion	C. I. %	Modification	Rank	Result Type

				Seq.	Seq.		Score				
816.421	816.4076	-0.0134	-16	19	25	LAEQAER					Mascot
907.5247	907.4792	-0.0455	-50	51	58	NLLSVAYK					Mascot
922.4199	922.4067	-0.0132	-14	132	138	MKGDYHR			Oxidation (M)[1]		Mascot
1189.6609	1189.6497	-0.0112	-9	224	233	DSTLIMQLLR					Mascot
1189.6609	1189.6497	-0.0112	-9	224	233	DSTLIMQLLR	87	100			Mascot
1205.6559	1205.6342	-0.0217	-18	224	233	DSTLIMQLLR			Oxidation (M)[6]		Mascot
1205.6559	1205.6342	-0.0217	-18	224	233	DSTLIMQLLR	35	97.738	Oxidation (M)[6]		Mascot
1334.5643	1334.542	-0.0223	-17	26	35	YEEMVEFMEK					Mascot
1350.5592	1350.5243	-0.0349	-26	26	35	YEEMVEFMEK			Oxidation (M)[4]		Mascot
1366.5542	1366.511	-0.0432	-32	26	35	YEEMVEFMEK			Oxidation (M)[4,8]		Mascot
1374.7224	1374.6567	-0.0657	-48	70	81	IVSSIEQKEEGR					Mascot
1517.8799	1517.8373	-0.0426	-28	51	64	NLLSVAYKNVIGAR					Mascot
2083.9966	2084.0505	0.0539	26	150	169	KDAADSTLGAYQAAQDIA MK			Oxidation (M)[19]		Mascot
2100.9607	2100.9849	0.0242	12	205	223	QAFDEAIAELDSLGEDSY K					Mascot
2131.9675	2131.9492	-0.0183	-9	19	35	LAEQAERYEEMVEFMEK					Mascot
2131.9675	2131.9492	-0.0183	-9	19	35	LAEQAERYEEMVEFMEK	34	96.962			Mascot
2147.9624	2147.9199	-0.0425	-20	19	35	LAEQAERYEEMVEFMEK			Oxidation (M)[11]		Mascot
2147.9624	2147.9199	-0.0425	-20	19	35	LAEQAERYEEMVEFMEK	19	5.911	Oxidation (M)[15]		Mascot
2163.9573	2163.8999	-0.0574	-27	19	35	LAEQAERYEEMVEFMEK			Oxidation (M)[11,15]		Mascot
2163.9573	2163.8999	-0.0574	-27	19	35	LAEQAERYEEMVEFMEK	44	99.704	Oxidation (M)[11,15]		Mascot
2331.2019	2331.1804	-0.0215	-9	179	198	LGLALNFSVFYYEILNSPD R					Mascot
2331.2019	2331.1804	-0.0215	-9	179	198	LGLALNFSVFYYEILNSPD R	125	100			Mascot

9	unnamed protein product [Triticum aestivum]	gi|257664788	28475.3	4.78	11	305	100	29.975	256	100	

Protein Group

14-3-3 protein [Triticum aestivum]	gi|32401388	28475.3	4.7800 002098 0835
unnamed protein product [Triticum aestivum]	gi|227472036	28475.3	4.7800 002098 0835
unnamed protein product [Triticum aestivum]	gi|219911730	28475.3	4.7800 002098 0835

Peptide Information

Calc. Mass	Obsrv. Mass	± da	± ppm	Start Seq.	End Seq.	Sequence	Ion Score	C. I. %	Modification	Rank	Result Type

Calc. Mass	Obsrv. Mass	± da	± ppm	Start Seq.	End Seq.	Sequence	Ion Score	C. I. %	Modification	Rank	Result Type
816.421	816.4076	-0.0134	-16	4	10	LAEQAER					Mascot
907.5247	907.4792	-0.0455	-50	36	43	NLLSVAYK					Mascot
917.5302	917.5087	-0.0215	-23	55	62	IISSIEQK					Mascot
1189.6609	1189.6497	-0.0112	-9	209	218	DSTLIMQLLR					Mascot
1189.6609	1189.6497	-0.0112	-9	209	218	DSTLIMQLLR	87	100			Mascot
1205.6559	1205.6342	-0.0217	-18	209	218	DSTLIMQLLR			Oxidation (M)[6]		Mascot
1205.6559	1205.6342	-0.0217	-18	209	218	DSTLIMQLLR	35	97.738	Oxidation (M)[6]		Mascot
1334.5643	1334.542	-0.0223	-17	11	20	YEEMVEFMEK					Mascot
1340.6515	1340.6027	-0.0488	-36	135	146	KDAAENTMVAYK					Mascot
1350.5592	1350.5243	-0.0349	-26	11	20	YEEMVEFMEK			Oxidation (M)[4]		Mascot
1356.6464	1356.5636	-0.0828	-61	135	146	KDAAENTMVAYK			Oxidation (M)[8]		Mascot
1366.5542	1366.511	-0.0432	-32	11	20	YEEMVEFMEK			Oxidation (M)[4,8]		Mascot
1418.7485	1418.7345	-0.014	-10	55	66	IISSIEQKEESR					Mascot
1517.8799	1517.8373	-0.0426	-28	36	49	NLLSVAYKNVIGAR					Mascot
1786.9811	1786.9465	-0.0346	-19	147	163	AAQDIALAELAPTHPIR					Mascot
2131.9675	2131.9492	-0.0183	-9	4	20	LAEQAERYEEMVEFMEK					Mascot
2131.9675	2131.9492	-0.0183	-9	4	20	LAEQAERYEEMVEFMEK	34	96.962			Mascot
2147.9624	2147.9199	-0.0425	-20	4	20	LAEQAERYEEMVEFMEK			Oxidation (M)[11]		Mascot
2147.9624	2147.9199	-0.0425	-20	4	20	LAEQAERYEEMVEFMEK	19	5.911	Oxidation (M)[15]		Mascot
2163.9573	2163.8999	-0.0574	-27	4	20	LAEQAERYEEMVEFMEK			Oxidation (M)[11,15]		Mascot
2163.9573	2163.8999	-0.0574	-27	4	20	LAEQAERYEEMVEFMEK	44	99.704	Oxidation (M)[11,15]		Mascot
2331.2019	2331.1804	-0.0215	-9	164	183	LGLALNFSVFYYEILNSPD R					Mascot
2331.2019	2331.1804	-0.0215	-9	164	183	LGLALNFSVFYYEILNSPD R	125	100			Mascot

10	unnamed protein product [Triticum aestivum]	gi|227473233	29549.7	4.75	12	277	100	22.15	212	100	

Protein Group

unnamed protein product [Triticum aestivum]	gi|257710885	29549.7	4.75

Peptide Information

Calc. Mass	Obsrv. Mass	± da	± ppm	Start Seq.	End Seq.	Sequence	Ion Score	C. I. %	Modification	Rank	Result Type
816.421	816.4076	-0.0134	-16	17	23	LAEQAER					Mascot
907.5247	907.4792	-0.0455	-50	53	60	NLLSVAYK					Mascot
917.5302	917.5087	-0.0215	-23	72	79	IISSIEQK					Mascot
922.4199	922.4067	-0.0132	-14	134	140	MKGDYHR			Oxidation (M)[1]		Mascot
1189.6609	1189.6497	-0.0112	-9	226	235	DSTLIMQLLR					Mascot
1189.6609	1189.6497	-0.0112	-9	226	235	DSTLIMQLLR	87	100			Mascot

1205.6559	1205.6342	-0.0217	-18	226	235	DSTLIMQLLR			Oxidation (M)[6]	Mascot
1205.6559	1205.6342	-0.0217	-18	226	235	DSTLIMQLLR	35	97.644	Oxidation (M)[6]	Mascot
1394.5603	1394.5444	-0.0159	-11	24	33	YEEMVEFMER			Oxidation (M)[4,8]	Mascot
1517.8799	1517.8373	-0.0426	-28	53	66	NLLSVAYKNVIGAR				Mascot
1800.9967	1800.9512	-0.0455	-25	164	180	AAQDIALVDLAPTHPIR				Mascot
1856.9348	1856.8937	-0.0411	-22	34	52	VAKATGGAGPGEELSVE ER				Mascot
2159.9736	2159.9712	-0.0024	-1	17	33	LAEQAERYEEMVEFMER				Mascot
2175.9685	2175.9294	-0.0391	-18	17	33	LAEQAERYEEMVEFMER			Oxidation (M)[11]	Mascot
2191.9634	2191.9258	-0.0376	-17	17	33	LAEQAERYEEMVEFMER			Oxidation (M)[11,15]	Mascot
2331.2019	2331.1804	-0.0215	-9	181	200	LGLALNFSVFYYEILNSPD R				Mascot
2331.2019	2331.1804	-0.0215	-9	181	200	LGLALNFSVFYYEILNSPD R	125	100		Mascot
3301.6145	3301.5984	-0.0161	-5	207	235	QAFDEAISELDSLGEESY KDSTLIMQLLR				Mascot

Gel Idx/Pos	166/G17	Instr./Gel Origin	BA2151/Sample Project 20140814	Process Status	Analysis Succeeded
Plate [#] Name	[1] Sample Project 20140814	Instrument Sample Name		Spectra	11

Rank	Protein Name	Accession No.	Protein MW	Protein PI	Pep. Count	Protein Score	Protein Score C. I. %	Intensity Matched	Total Ion Score	Total Ion C. I. %	Confirmed
1	14-3-3 protein [Triticum aestivum]	gi|390195996	30066.1	4.73	21	513	100	44.306	365	100	

Protein Group

14-3-3 protein [Triticum aestivum]	gi|351602255	30066.1	4.7300 000190 7349
14-3-3-like protein GF14-B [Triticum urartu]	gi|474147722	30043.1	4.6900 000572 2046

Peptide Information

Calc. Mass	Obsrv. Mass	± da	± ppm	Start Seq.	End Seq.	Sequence	Ion Score	C. I. %	Modification	Rank	Result Type
816.421	816.4134	-0.0076	-9	18	24	LAEQAER					Mascot
818.444	818.4239	-0.0201	-25	103	109	ICDGILK			Carbamidomethyl (C)[2]		Mascot
844.4523	844.4604	0.0081	10	2	9	TAPAELSR					Mascot
907.5247	907.4822	-0.0425	-47	50	57	NLLSVAYK					Mascot
917.5302	917.5153	-0.0149	-16	69	76	IISSIEQK					Mascot
932.4294	932.4268	-0.0026	-3	131	137	MKGDYYR					Mascot
948.4244	948.4129	-0.0115	-12	131	137	MKGDYYR			Oxidation (M)[1]		Mascot
999.4451	999.4496	0.0045	5	10	17	EENVYMAK			Oxidation (M)[6]		Mascot
1144.6321	1144.6272	-0.0049	-4	81	90	GNEDRVTLIK					Mascot
1189.6609	1189.6603	-0.0006	-1	223	232	DSTLIMQLLR					Mascot
1189.6609	1189.6603	-0.0006	-1	223	232	DSTLIMQLLR	22	55.816			Mascot
1205.6559	1205.6425	-0.0134	-11	223	232	DSTLIMQLLR			Oxidation (M)[6]		Mascot
1205.6559	1205.6425	-0.0134	-11	223	232	DSTLIMQLLR	23	57.998	Oxidation (M)[6]		Mascot
1212.5565	1212.6031	0.0466	38	150	160	DAAENTMVAYK					Mascot
1228.5514	1228.5819	0.0305	25	150	160	DAAENTMVAYK			Oxidation (M)[7]		Mascot
1366.5542	1366.5227	-0.0315	-23	25	34	YEEMVEFMEK			Oxidation (M)[4,8]		Mascot
1406.6646	1406.6599	-0.0047	-3	38	49	TVDSEELTVEER					Mascot
1406.6646	1406.6599	-0.0047	-3	38	49	TVDSEELTVEER	109	100			Mascot
1418.7485	1418.7394	-0.0091	-6	69	80	IISSIEQKEESR					Mascot
1418.7485	1418.7394	-0.0091	-6	69	80	IISSIEQKEESR	69	100			Mascot
1708.9116	1708.85	-0.0616	-36	110	125	LLETHLVPSSTAPESK					Mascot
1786.9811	1786.9723	-0.0088	-5	161	177	AAQDIALAELAPTHPIR					Mascot

1786.9811	1786.9723	-0.0088	-5	161	177	AAQDIALAELAPTHPIR	156	100			Mascot
1808.8848	1808.9418	0.057	32	2	17	TAPAELSREENVYMAK					Mascot
1824.8796	1824.8773	-0.0023	-1	2	17	TAPAELSREENVYMAK			Oxidation (M)[14]		Mascot
2147.9624	2147.9368	-0.0256	-12	18	34	LAEQAERYEEMVEFMEK			Oxidation (M)[11]		Mascot
2163.9573	2163.9104	-0.0469	-22	18	34	LAEQAERYEEMVEFMEK			Oxidation (M)[11,15]		Mascot
2163.9573	2163.9104	-0.0469	-22	18	34	LAEQAERYEEMVEFMEK	8	0	Oxidation (M)[11,15]		Mascot
2174.9976	2174.946	-0.0516	-24	204	222	QAFDEAISELDTLSEESY K					Mascot
2331.2019	2331.2019	0	0	178	197	LGLALNFSVFYYEILNSPD R					Mascot
2351.0886	2351.0681	-0.0205	-9	233	252	DNLTLWTSDITEDTAEEEI R					Mascot
2776.3159	2776.304	-0.0119	-4	233	256	DNLTLWTSDITEDTAEEEI REAPK					Mascot

2	unnamed protein product [Triticum aestivum]	gi|257664788	28475.3	4.78	18	480	100	42.954	365	100	

Protein Group

14-3-3 protein [Triticum aestivum]	gi|32401388	28475.3	4.7800 002098 0835
unnamed protein product [Triticum aestivum]	gi|227472036	28475.3	4.7800 002098 0835
unnamed protein product [Triticum aestivum]	gi|219911730	28475.3	4.7800 002098 0835

Peptide Information

Calc. Mass	Obsrv. Mass	± da	± ppm	Start Seq.	End Seq.	Sequence	Ion Score	C. I. %	Modification	Rank	Result Type
816.421	816.4134	-0.0076	-9	4	10	LAEQAER					Mascot
818.444	818.4239	-0.0201	-25	89	95	ICDGILK			Carbamidomethyl (C)[2]		Mascot
907.5247	907.4822	-0.0425	-47	36	43	NLLSVAYK					Mascot
917.5302	917.5153	-0.0149	-16	55	62	IISSIEQK					Mascot
932.4294	932.4268	-0.0026	-3	117	123	MKGDYYR					Mascot
948.4244	948.4129	-0.0115	-12	117	123	MKGDYYR			Oxidation (M)[1]		Mascot
1144.6321	1144.6272	-0.0049	-4	67	76	GNEDRVTLIK					Mascot
1189.6609	1189.6603	-0.0006	-1	209	218	DSTLIMQLLR					Mascot
1189.6609	1189.6603	-0.0006	-1	209	218	DSTLIMQLLR	22	55.816			Mascot
1205.6559	1205.6425	-0.0134	-11	209	218	DSTLIMQLLR			Oxidation (M)[6]		Mascot
1205.6559	1205.6425	-0.0134	-11	209	218	DSTLIMQLLR	23	57.998	Oxidation (M)[6]		Mascot
1212.5565	1212.6031	0.0466	38	136	146	DAAENTMVAYK					Mascot
1228.5514	1228.5819	0.0305	25	136	146	DAAENTMVAYK			Oxidation (M)[7]		Mascot
1366.5542	1366.5227	-0.0315	-23	11	20	YEEMVEFMEK			Oxidation (M)[4,8]		Mascot

1406.6646	1406.6599	-0.0047	-3	24	35	TVDSEELTVEER					Mascot
1406.6646	1406.6599	-0.0047	-3	24	35	TVDSEELTVEER	109	100			Mascot
1418.7485	1418.7394	-0.0091	-6	55	66	IISSIEQKEESR					Mascot
1418.7485	1418.7394	-0.0091	-6	55	66	IISSIEQKEESR	69	100			Mascot
1708.9116	1708.85	-0.0616	-36	96	111	LLETHLVPSSTAPESK					Mascot
1786.9811	1786.9723	-0.0088	-5	147	163	AAQDIALAELAPTHPIR					Mascot
1786.9811	1786.9723	-0.0088	-5	147	163	AAQDIALAELAPTHPIR	156	100			Mascot
2147.9624	2147.9368	-0.0256	-12	4	20	LAEQAERYEEMVEFMEK			Oxidation (M)[11]		Mascot
2163.9573	2163.9104	-0.0469	-22	4	20	LAEQAERYEEMVEFMEK			Oxidation (M)[11,15]		Mascot
2163.9573	2163.9104	-0.0469	-22	4	20	LAEQAERYEEMVEFMEK	8	0	Oxidation (M)[11,15]		Mascot
2174.9976	2174.946	-0.0516	-24	190	208	QAFDEAISELDTLSEESY K					Mascot
2331.2019	2331.2019	0	0	164	183	LGLALNFSVFYYEILNSPD R					Mascot
2351.0886	2351.0681	-0.0205	-9	219	238	DNLTLWTSDITEDTAEEEI R					Mascot
2776.3159	2776.304	-0.0119	-4	219	242	DNLTLWTSDITEDTAEEEI REAPK					Mascot

3 unnamed protein product [Triticum aestivum] gi|257664756 29983 4.62 16 453 100 42.037 365 100

Protein Group

14-3-3 protein [Triticum aestivum] gi|52548256 29983 4.6199998855 5908

unnamed protein product [Triticum aestivum] gi|227471994 29983 4.6199998855 5908

unnamed protein product [Triticum aestivum] gi|219911698 29983 4.6199998855 5908

Peptide Information

Calc. Mass	Obsrv. Mass	± da	± ppm	Start Seq.	End Seq.	Sequence	Ion Score	C. I. %	Modification	Rank	Result Type
816.421	816.4134	-0.0076	-9	18	24	LAEQAER					Mascot
818.444	818.4239	-0.0201	-25	103	109	ICDGILK			Carbamidomethyl (C)[2]		Mascot
844.4523	844.4604	0.0081	10	2	9	TAPAELSR					Mascot
907.5247	907.4822	-0.0425	-47	50	57	NLLSVAYK					Mascot
917.5302	917.5153	-0.0149	-16	69	76	IISSIEQK					Mascot
932.4294	932.4268	-0.0026	-3	131	137	MKGDYYR					Mascot
948.4244	948.4129	-0.0115	-12	131	137	MKGDYYR			Oxidation (M)[1]		Mascot
999.4451	999.4496	0.0045	5	10	17	EENVYMAK			Oxidation (M)[6]		Mascot
1144.6321	1144.6272	-0.0049	-4	81	90	GNEDRVTLIK					Mascot
1189.6609	1189.6603	-0.0006	-1	223	232	DSTLIMQLLR					Mascot

Calc. Mass	Obsrv. Mass	± da	± ppm	Start Seq.	End Seq.	Sequence	Ion Score	C. I. %	Modification	Rank	Result Type
1189.6609	1189.6603	-0.0006	-1	223	232	DSTLIMQLLR	22	55.816			Mascot
1205.6559	1205.6425	-0.0134	-11	223	232	DSTLIMQLLR			Oxidation (M)[6]		Mascot
1205.6559	1205.6425	-0.0134	-11	223	232	DSTLIMQLLR	23	57.998	Oxidation (M)[6]		Mascot
1366.5542	1366.5227	-0.0315	-23	25	34	YEEMVEFMEK			Oxidation (M)[4,8]		Mascot
1406.6646	1406.6599	-0.0047	-3	38	49	TVDSEELTVEER					Mascot
1406.6646	1406.6599	-0.0047	-3	38	49	TVDSEELTVEER	109	100			Mascot
1418.7485	1418.7394	-0.0091	-6	69	80	IISSIEQKEESR					Mascot
1418.7485	1418.7394	-0.0091	-6	69	80	IISSIEQKEESR	69	100			Mascot
1708.9116	1708.85	-0.0616	-36	110	125	LLETHLVPSSTAPESK					Mascot
1786.9811	1786.9723	-0.0088	-5	161	177	AAQDIALAELAPTHPIR					Mascot
1786.9811	1786.9723	-0.0088	-5	161	177	AAQDIALAELAPTHPIR	156	100			Mascot
1808.8848	1808.9418	0.057	32	2	17	TAPAELSREENVYMAK					Mascot
1824.8796	1824.8773	-0.0023	-1	2	17	TAPAELSREENVYMAK			Oxidation (M)[14]		Mascot
2147.9624	2147.9368	-0.0256	-12	18	34	LAEQAERYEEMVEFMEK			Oxidation (M)[11]		Mascot
2163.9573	2163.9104	-0.0469	-22	18	34	LAEQAERYEEMVEFMEK			Oxidation (M)[11,15]		Mascot
2163.9573	2163.9104	-0.0469	-22	18	34	LAEQAERYEEMVEFMEK	8	0	Oxidation (M)[11,15]		Mascot

4	unnamed protein product [Triticum aestivum]	gi|227472078	29490.6	4.75	14	347	100	13.485	269	100	

Protein Group

TaWIN1 [Triticum aestivum]	gi|9798603	29490.6	4.75
unnamed protein product [Triticum aestivum]	gi|219911926	29490.6	4.75
unnamed protein product [Triticum aestivum]	gi|257664808	29490.6	4.75

Peptide Information

Calc. Mass	Obsrv. Mass	± da	± ppm	Start Seq.	End Seq.	Sequence	Ion Score	C. I. %	Modification	Rank	Result Type
816.421	816.4134	-0.0076	-9	17	23	LAEQAER					Mascot
907.5247	907.4822	-0.0425	-47	53	60	NLLSVAYK					Mascot
917.5302	917.5153	-0.0149	-16	72	79	IISSIEQK					Mascot
1025.5123	1025.5107	-0.0016	-2	84	93	GNDAHAATIR					Mascot
1189.6609	1189.6603	-0.0006	-1	226	235	DSTLIMQLLR					Mascot
1189.6609	1189.6603	-0.0006	-1	226	235	DSTLIMQLLR	22	55.816			Mascot
1205.6559	1205.6425	-0.0134	-11	226	235	DSTLIMQLLR			Oxidation (M)[6]		Mascot
1205.6559	1205.6425	-0.0134	-11	226	235	DSTLIMQLLR	23	57.998	Oxidation (M)[6]		Mascot
1358.6256	1358.7042	0.0786	58	152	163	KEAAESTMNAYK			Oxidation (M)[8]		Mascot

1388.738	1388.7262	-0.0118	-8	72	83	IISSIEQKEEGR					Mascot
1388.738	1388.7262	-0.0118	-8	72	83	IISSIEQKEEGR	54	99.971			Mascot
1394.5603	1394.526	-0.0343	-25	24	33	YEEMVEFMER			Oxidation (M)[4,8]		Mascot
1558.7344	1558.7302	-0.0042	-3	37	52	ATGGAGPGEELSVEER					Mascot
1558.7344	1558.7302	-0.0042	-3	37	52	ATGGAGPGEELSVEER	86	100			Mascot
1660.771	1660.8453	0.0743	45	24	36	YEEMVEFMERVAK					Mascot
1696.7847	1696.7582	-0.0265	-16	2	16	SPAEPTRDESVYMAK			Oxidation (M)[13]		Mascot
1772.9653	1772.9572	-0.0081	-5	164	180	AAQDIALADLAPTHPIR					Mascot
1772.9653	1772.9572	-0.0081	-5	164	180	AAQDIALADLAPTHPIR	106	100			Mascot
2191.9634	2191.9226	-0.0408	-19	17	33	LAEQAERYEEMVEFMER			Oxidation (M)[11,15]		Mascot
2331.2019	2331.2019	0	0	181	200	LGLALNFSVFYYEILNSPD R					Mascot

5	14-3-3-like protein B [Triticum urartu]	gi|474253094	29786.9	4.67	14	276	100	17.653	209	100	

Peptide Information

Calc. Mass	Obsrv. Mass	± da	± ppm	Start Seq.	End Seq.	Sequence	Ion Score	C. I. %	Modification	Rank	Result Type
816.421	816.4134	-0.0076	-9	18	24	LAEQAER					Mascot
818.444	818.4239	-0.0201	-25	103	109	ICDGILK			Carbamidomethyl (C)[2]		Mascot
907.5247	907.4822	-0.0425	-47	50	57	NLLSVAYK					Mascot
917.5302	917.5153	-0.0149	-16	69	76	IISSIEQK					Mascot
932.4294	932.4268	-0.0026	-3	131	137	MKGDYYR					Mascot
948.4244	948.4129	-0.0115	-12	131	137	MKGDYYR			Oxidation (M)[1]		Mascot
999.4451	999.4496	0.0045	5	10	17	EENVYMAK			Oxidation (M)[6]		Mascot
1144.6321	1144.6272	-0.0049	-4	81	90	GNEDRVTLIK					Mascot
1189.6609	1189.6603	-0.0006	-1	223	232	DSTLIMQLLR					Mascot
1189.6609	1189.6603	-0.0006	-1	223	232	DSTLIMQLLR	22	55.816			Mascot
1205.6559	1205.6425	-0.0134	-11	223	232	DSTLIMQLLR			Oxidation (M)[6]		Mascot
1205.6559	1205.6425	-0.0134	-11	223	232	DSTLIMQLLR	23	57.998	Oxidation (M)[6]		Mascot
1212.5565	1212.6031	0.0466	38	150	160	DAAENTMVAYK					Mascot
1228.5514	1228.5819	0.0305	25	150	160	DAAENTMVAYK			Oxidation (M)[7]		Mascot
1366.5542	1366.5227	-0.0315	-23	25	34	YEEMVEFMEK			Oxidation (M)[4,8]		Mascot
1406.6646	1406.6599	-0.0047	-3	38	49	TVDSEELTVEER					Mascot
1406.6646	1406.6599	-0.0047	-3	38	49	TVDSEELTVEER	109	100			Mascot
1418.7485	1418.7394	-0.0091	-6	69	80	IISSIEQKEESR					Mascot
1418.7485	1418.7394	-0.0091	-6	69	80	IISSIEQKEESR	69	100			Mascot
2147.9624	2147.9368	-0.0256	-12	18	34	LAEQAERYEEMVEFMEK			Oxidation (M)[11]		Mascot

Calc. Mass	Obsrv. Mass	± da	± ppm	Start Seq.	End Seq.	Sequence	Ion Score	C. I. %	Modification	Rank	Result Type
2163.9573	2163.9104	-0.0469	-22	18	34	LAEQAERYEEMVEFMEK			Oxidation (M)[11,15]		Mascot
2163.9573	2163.9104	-0.0469	-22	18	34	LAEQAERYEEMVEFMEK	8	0	Oxidation (M)[11,15]		Mascot
2331.2019	2331.2019	0	0	178	197	LGLALNFSVFYYEILNSPD R					Mascot

6	14-3-3 protein [Triticum aestivum]	gi|431822518	29843.9	4.71	14	275	100	17.653	209	100	

Peptide Information

Calc. Mass	Obsrv. Mass	± da	± ppm	Start Seq.	End Seq.	Sequence	Ion Score	C. I. %	Modification	Rank	Result Type
816.421	816.4134	-0.0076	-9	18	24	LAEQAER					Mascot
818.444	818.4239	-0.0201	-25	103	109	ICDGILK			Carbamidomethyl (C)[2]		Mascot
907.5247	907.4822	-0.0425	-47	50	57	NLLSVAYK					Mascot
917.5302	917.5153	-0.0149	-16	69	76	IISSIEQK					Mascot
932.4294	932.4268	-0.0026	-3	131	137	MKGDYYR					Mascot
948.4244	948.4129	-0.0115	-12	131	137	MKGDYYR			Oxidation (M)[1]		Mascot
999.4451	999.4496	0.0045	5	10	17	EENVYMAK			Oxidation (M)[6]		Mascot
1144.6321	1144.6272	-0.0049	-4	81	90	GNEDRVTLIK					Mascot
1189.6609	1189.6603	-0.0006	-1	223	232	DSTLIMQLLR					Mascot
1189.6609	1189.6603	-0.0006	-1	223	232	DSTLIMQLLR	22	55.816			Mascot
1205.6559	1205.6425	-0.0134	-11	223	232	DSTLIMQLLR			Oxidation (M)[6]		Mascot
1205.6559	1205.6425	-0.0134	-11	223	232	DSTLIMQLLR	23	57.998	Oxidation (M)[6]		Mascot
1212.5565	1212.6031	0.0466	38	150	160	DAAENTMVAYK					Mascot
1228.5514	1228.5819	0.0305	25	150	160	DAAENTMVAYK			Oxidation (M)[7]		Mascot
1366.5542	1366.5227	-0.0315	-23	25	34	YEEMVEFMEK			Oxidation (M)[4,8]		Mascot
1406.6646	1406.6599	-0.0047	-3	38	49	TVDSEELTVEER					Mascot
1406.6646	1406.6599	-0.0047	-3	38	49	TVDSEELTVEER	109	100			Mascot
1418.7485	1418.7394	-0.0091	-6	69	80	IISSIEQKEESR					Mascot
1418.7485	1418.7394	-0.0091	-6	69	80	IISSIEQKEESR	69	100			Mascot
2147.9624	2147.9368	-0.0256	-12	18	34	LAEQAERYEEMVEFMEK			Oxidation (M)[11]		Mascot
2163.9573	2163.9104	-0.0469	-22	18	34	LAEQAERYEEMVEFMEK			Oxidation (M)[11,15]		Mascot
2163.9573	2163.9104	-0.0469	-22	18	34	LAEQAERYEEMVEFMEK	8	0	Oxidation (M)[11,15]		Mascot
2331.2019	2331.2019	0	0	178	197	LGLALNFSVFYYEILNSPD R					Mascot

7	unnamed protein product [Triticum aestivum]	gi|227473233	29549.7	4.75	13	231	100	11.777	163	100	

Protein Group

unnamed protein product [Triticum aestivum]	gi|257710885	29549.7	4.75

Peptide Information

Calc. Mass	Obsrv. Mass	± da	± ppm	Start Seq.	End Seq.	Sequence	Ion Score	C. I. %	Modification	Rank	Result Type
816.421	816.4134	-0.0076	-9	17	23	LAEQAER					Mascot
907.5247	907.4822	-0.0425	-47	53	60	NLLSVAYK					Mascot
917.5302	917.5153	-0.0149	-16	72	79	IISSIEQK					Mascot
1025.5123	1025.5107	-0.0016	-2	84	93	GNDAHAATIR					Mascot
1189.6609	1189.6603	-0.0006	-1	226	235	DSTLIMQLLR					Mascot
1189.6609	1189.6603	-0.0006	-1	226	235	DSTLIMQLLR	22	55.816			Mascot
1205.6559	1205.6425	-0.0134	-11	226	235	DSTLIMQLLR			Oxidation (M)[6]		Mascot
1205.6559	1205.6425	-0.0134	-11	226	235	DSTLIMQLLR	23	57.998	Oxidation (M)[6]		Mascot
1358.6256	1358.7042	0.0786	58	152	163	KEAAESTMNAYK			Oxidation (M)[8]		Mascot
1388.738	1388.7262	-0.0118	-8	72	83	IISSIEQKEEGR					Mascot
1388.738	1388.7262	-0.0118	-8	72	83	IISSIEQKEEGR	54	99.971			Mascot
1394.5603	1394.526	-0.0343	-25	24	33	YEEMVEFMER			Oxidation (M)[4,8]		Mascot
1558.7344	1558.7302	-0.0042	-3	37	52	ATGGAGPGEELSVEER					Mascot
1558.7344	1558.7302	-0.0042	-3	37	52	ATGGAGPGEELSVEER	86	100			Mascot
1660.771	1660.8453	0.0743	45	24	36	YEEMVEFMERVAK					Mascot
1800.9967	1800.9705	-0.0262	-15	164	180	AAQDIALVDLAPTHPIR					Mascot
2191.9634	2191.9226	-0.0408	-19	17	33	LAEQAERYEEMVEFMER			Oxidation (M)[11,15]		Mascot
2331.2019	2331.2019	0	0	181	200	LGLALNFSVFYYEILNSPDR					Mascot

8	14-3-3-like protein GF14-D [Triticum urartu]	gi|474137097	21391.7	4.86	7	188	100	8.832	163	100	

Peptide Information

Calc. Mass	Obsrv. Mass	± da	± ppm	Start Seq.	End Seq.	Sequence	Ion Score	C. I. %	Modification	Rank	Result Type
907.5247	907.4822	-0.0425	-47	23	30	NLLSVAYK					Mascot
917.5302	917.5153	-0.0149	-16	42	49	IISSIEQK					Mascot
1025.5123	1025.5107	-0.0016	-2	54	63	GNDAHAATIR					Mascot
1189.6609	1189.6603	-0.0006	-1	155	164	DSTLIMQLLR					Mascot
1189.6609	1189.6603	-0.0006	-1	155	164	DSTLIMQLLR	22	55.816			Mascot
1205.6559	1205.6425	-0.0134	-11	155	164	DSTLIMQLLR			Oxidation (M)[6]		Mascot
1205.6559	1205.6425	-0.0134	-11	155	164	DSTLIMQLLR	23	57.998	Oxidation (M)[6]		Mascot
1358.6256	1358.7042	0.0786	58	122	133	KEAAESTMNAYK			Oxidation (M)[8]		Mascot
1388.738	1388.7262	-0.0118	-8	42	53	IISSIEQKEEGR					Mascot
1388.738	1388.7262	-0.0118	-8	42	53	IISSIEQKEEGR	54	99.971			Mascot
1558.7344	1558.7302	-0.0042	-3	7	22	ATGGAGPGEELSVEER					Mascot

1558.7344	1558.7302	-0.0042	-3	7	22	ATGGAGPGEELSVEER	86	100			Mascot

9	14-3-3 protein [Triticum aestivum]	gi|40781605	29387.7	4.83	14	172	100	12.698	100	100	

Protein Group

14-3-3 protein [Triticum aestivum]	gi|431822520	29360.7	4.8299 999237 0605
unnamed protein product [Triticum aestivum]	gi|227473229	29360.7	4.8299 999237 0605
unnamed protein product [Triticum aestivum]	gi|227471938	29387.7	4.8299 999237 0605

Peptide Information

Calc. Mass	Obsrv. Mass	± da	± ppm	Start Seq.	End Seq.	Sequence	Ion Score	C. I. %	Modification	Rank	Result Type
816.421	816.4134	-0.0076	-9	17	23	LAEQAER					Mascot
818.444	818.4239	-0.0201	-25	102	108	ICDGILK			Carbamidomethyl (C)[2]		Mascot
882.3986	882.3768	-0.0218	-25	1	8	MSTAEATR			Oxidation (M)[1]		Mascot
907.5247	907.4822	-0.0425	-47	49	56	NLLSVAYK					Mascot
917.5302	917.5153	-0.0149	-16	68	75	IISSIEQK					Mascot
999.4451	999.4496	0.0045	5	9	16	EENVYMAK			Oxidation (M)[6]		Mascot
1051.5419	1051.5254	-0.0165	-16	80	89	GNEAYVASIK					Mascot
1189.6609	1189.6603	-0.0006	-1	222	231	DSTLIMQLLR					Mascot
1189.6609	1189.6603	-0.0006	-1	222	231	DSTLIMQLLR	22	55.816			Mascot
1205.6559	1205.6425	-0.0134	-11	222	231	DSTLIMQLLR			Oxidation (M)[6]		Mascot
1205.6559	1205.6425	-0.0134	-11	222	231	DSTLIMQLLR	23	57.998	Oxidation (M)[6]		Mascot
1318.6486	1318.6445	-0.0041	-3	37	48	TADVGELTVEER					Mascot
1366.5542	1366.5227	-0.0315	-23	24	33	YEEMVEFMEK			Oxidation (M)[4,8]		Mascot
1418.7485	1418.7394	-0.0091	-6	68	79	IISSIEQKEESR					Mascot
1418.7485	1418.7394	-0.0091	-6	68	79	IISSIEQKEESR	69	100			Mascot
1818.9708	1818.9535	-0.0173	-10	160	176	SAQDIALADLPTTHPIR					Mascot
2147.9624	2147.9368	-0.0256	-12	17	33	LAEQAERYEEMVEFMEK			Oxidation (M)[11]		Mascot
2163.9573	2163.9104	-0.0469	-22	17	33	LAEQAERYEEMVEFMEK			Oxidation (M)[11,15]		Mascot
2163.9573	2163.9104	-0.0469	-22	17	33	LAEQAERYEEMVEFMEK	8	0	Oxidation (M)[11,15]		Mascot
2331.2019	2331.2019	0	0	177	196	LGLALNFSVFYYEILNSPD R					Mascot

10	14-3-3-like protein A [Triticum urartu]	gi|474293618	28778.5	5.2	10	73	99.778	9.39	31	93.374	

Peptide Information

Calc. Mass	Obsrv. Mass	± da	± ppm	Start Seq.	End Seq.	Sequence	Ion Score	C. I. %	Modification	Rank	Result Type

816.421	816.4134	-0.0076	-9	17	23	LAEQAER				Mascot
835.4454	835.3749	-0.0705	-84	1	8	MAKAAATR			Oxidation (M)[1]	Mascot
907.5247	907.4822	-0.0425	-47	49	56	NLLSVAYK				Mascot
1025.599	1025.5107	-0.0883	-86	114	124	LVPAAAAVDAK				Mascot
1189.6609	1189.6603	-0.0006	-1	222	231	DSTLIMQLLR				Mascot
1189.6609	1189.6603	-0.0006	-1	222	231	DSTLIMQLLR	22	52.408		Mascot
1205.6559	1205.6425	-0.0134	-11	222	231	DSTLIMQLLR			Oxidation (M)[6]	Mascot
1205.6559	1205.6425	-0.0134	-11	222	231	DSTLIMQLLR	23	54.759	Oxidation (M)[6]	Mascot
1228.6294	1228.5819	-0.0475	-39	80	92	GAAGHAAAARGYR				Mascot
1323.6512	1323.6381	-0.0131	-10	76	89	EEGRGAAGHAAAAR				Mascot
1366.5542	1366.5227	-0.0315	-23	24	33	YEEMVEFMEK			Oxidation (M)[4,8]	Mascot
2147.9624	2147.9368	-0.0256	-12	17	33	LAEQAERYEEMVEFMEK			Oxidation (M)[11]	Mascot
2163.9573	2163.9104	-0.0469	-22	17	33	LAEQAERYEEMVEFMEK			Oxidation (M)[11,15]	Mascot
2163.9573	2163.9104	-0.0469	-22	17	33	LAEQAERYEEMVEFMEK	8	0	Oxidation (M)[11,15]	Mascot
2331.2019	2331.2019	0	0	177	196	LGLALNFSVFYYEILNSPD R				Mascot

Gel Idx/Pos	167/G18	Instr./Gel Origin	BA2151/Sample Project 20140814	Process Status	Analysis Succeeded
Plate [#] Name	[1] Sample Project 20140814	Instrument Sample Name		Spectra	11

Rank	Protein Name	Accession No.	Protein MW	Protein PI	Pep. Count	Protein Score	Protein Score C. I. %	Intensity Matched	Total Ion Score	Total Ion C. I. %	Confirmed
1	unnamed protein product [Triticum aestivum]	gi|227473229	29360.7	4.83	19	346	100	41.698	219	100	

Protein Group

14-3-3 protein [Triticum aestivum]	gi|431822520	29360.7	4.8299 999237 0605

Peptide Information

Calc. Mass	Obsrv. Mass	± da	± ppm	Start Seq.	End Seq.	Sequence	Ion Score	C. I. %	Modification	Rank	Result Type
816.421	816.4305	0.0095	12	17	23	LAEQAER					Mascot
819.4458	819.4396	-0.0062	-8	95	101	IETELSK					Mascot
906.425	906.4412	0.0162	18	130	136	MKGDYHR					Mascot
907.5247	907.4802	-0.0445	-49	49	56	NLLSVAYK					Mascot
917.5302	917.5102	-0.02	-22	68	75	IISSIEQK					Mascot
922.4199	922.43	0.0101	11	130	136	MKGDYHR			Oxidation (M)[1]		Mascot
922.4199	922.43	0.0101	11	130	136	MKGDYHR			Oxidation (M)[1]		Mascot
928.5324	928.4614	-0.071	-76	125	131	VFYLKMK					Mascot
1051.5419	1051.551	0.0091	9	80	89	GNEAYVASIK					Mascot
1051.5419	1051.551	0.0091	9	80	89	GNEAYVASIK					Mascot
1076.5946	1076.6132	0.0186	17	93	101	TRIETELSK					Mascot
1076.5946	1076.6132	0.0186	17	93	101	TRIETELSK					Mascot
1111.5015	1111.4783	-0.0232	-21	251	261	EAASKPEGEGH					Mascot
1189.6609	1189.6735	0.0126	11	222	231	DSTLIMQLLR					Mascot
1205.6559	1205.6677	0.0118	10	222	231	DSTLIMQLLR			Oxidation (M)[6]		Mascot
1205.6559	1205.6677	0.0118	10	222	231	DSTLIMQLLR	13	0	Oxidation (M)[6]		Mascot
1208.6157	1208.6475	0.0318	26	149	159	EAAENTLVAYK					Mascot
1318.6486	1318.6693	0.0207	16	37	48	TADVGELTVEER					Mascot
1318.6486	1318.6693	0.0207	16	37	48	TADVGELTVEER	89	100			Mascot
1336.7107	1336.722	0.0113	8	148	159	KEAAENTLVAYK					Mascot
1366.5542	1366.5656	0.0114	8	24	33	YEEMVEFMEK			Oxidation (M)[4,8]		Mascot
1418.7485	1418.7694	0.0209	15	68	79	IISSIEQKEESR					Mascot
1418.7485	1418.7694	0.0209	15	68	79	IISSIEQKEESR	28	85.987			Mascot
1552.7601	1552.7839	0.0238	15	76	89	EESRGNEAYVASIK					Mascot

Calc. Mass	Obsrv. Mass	± da	± ppm	Start Seq.	End Seq.	Sequence	Ion Score	C. I. %	Modification	Rank	Result Type
1664.7546	1664.8337	0.0791	48	24	36	YEEMVEFMEKVAK			Oxidation (M)[4,8]		Mascot
1818.9708	1818.9923	0.0215	12	160	176	SAQDIALADLPTTHPIR					Mascot
1818.9708	1818.9923	0.0215	12	160	176	SAQDIALADLPTTHPIR	90	100			Mascot
2163.9573	2163.9624	0.0051	2	17	33	LAEQAERYEEMVEFMEK			Oxidation (M)[11,15]		Mascot

2	unnamed protein product [Triticum aestivum]	gi|227471938	29387.7	4.83	18	336	100	39.317	219	100		

Protein Group

14-3-3 protein [Triticum aestivum]	gi|40781605	29387.7	4.8299999237 0605

Peptide Information

Calc. Mass	Obsrv. Mass	± da	± ppm	Start Seq.	End Seq.	Sequence	Ion Score	C. I. %	Modification	Rank	Result Type
816.421	816.4305	0.0095	12	17	23	LAEQAER					Mascot
846.4567	846.446	-0.0107	-13	95	101	IETELNK					Mascot
906.425	906.4412	0.0162	18	130	136	MKGDYHR					Mascot
907.5247	907.4802	-0.0445	-49	49	56	NLLSVAYK					Mascot
917.5302	917.5102	-0.02	-22	68	75	IISSIEQK					Mascot
922.4199	922.43	0.0101	11	130	136	MKGDYHR			Oxidation (M)[1]		Mascot
922.4199	922.43	0.0101	11	130	136	MKGDYHR			Oxidation (M)[1]		Mascot
928.5324	928.4614	-0.071	-76	125	131	VFYLKMK					Mascot
1051.5419	1051.551	0.0091	9	80	89	GNEAYVASIK					Mascot
1051.5419	1051.551	0.0091	9	80	89	GNEAYVASIK					Mascot
1111.5015	1111.4783	-0.0232	-21	251	261	EAASKPEGEGH					Mascot
1189.6609	1189.6735	0.0126	11	222	231	DSTLIMQLLR					Mascot
1205.6559	1205.6677	0.0118	10	222	231	DSTLIMQLLR			Oxidation (M)[6]		Mascot
1205.6559	1205.6677	0.0118	10	222	231	DSTLIMQLLR	13	0	Oxidation (M)[6]		Mascot
1208.6157	1208.6475	0.0318	26	149	159	EAAENTLVAYK					Mascot
1318.6486	1318.6693	0.0207	16	37	48	TADVGELTVEER					Mascot
1318.6486	1318.6693	0.0207	16	37	48	TADVGELTVEER	89	100			Mascot
1336.7107	1336.722	0.0113	8	148	159	KEAAENTLVAYK					Mascot
1366.5542	1366.5656	0.0114	8	24	33	YEEMVEFMEK			Oxidation (M)[4,8]		Mascot
1418.7485	1418.7694	0.0209	15	68	79	IISSIEQKEESR					Mascot
1418.7485	1418.7694	0.0209	15	68	79	IISSIEQKEESR	28	85.987			Mascot
1552.7601	1552.7839	0.0238	15	76	89	EESRGNEAYVASIK					Mascot
1664.7546	1664.8337	0.0791	48	24	36	YEEMVEFMEKVAK			Oxidation (M)[4,8]		Mascot
1818.9708	1818.9923	0.0215	12	160	176	SAQDIALADLPTTHPIR					Mascot
1818.9708	1818.9923	0.0215	12	160	176	SAQDIALADLPTTHPIR	90	100			Mascot

2163.9573	2163.9624	0.0051	2	17	33	LAEQAERYEEMVEFMEK			Oxidation (M)[11,15]		Mascot

3	14-3-3 protein [Triticum aestivum]	gi|390195996	30066.1	4.73	17	135	100	13.93	41	99.286

Protein Group

14-3-3 protein [Triticum aestivum]	gi|351602255	30066.1	4.7300 000190 7349
14-3-3-like protein GF14-B [Triticum urartu]	gi|474147722	30043.1	4.6900 000572 2046

Peptide Information

Calc. Mass	Obsrv. Mass	± da	± ppm	Start Seq.	End Seq.	Sequence	Ion Score	C. I. %	Modification	Rank	Result Type
816.421	816.4305	0.0095	12	18	24	LAEQAER					Mascot
819.4458	819.4396	-0.0062	-8	96	102	IETELSK					Mascot
844.4523	844.4958	0.0435	52	2	9	TAPAELSR					Mascot
907.5247	907.4802	-0.0445	-49	50	57	NLLSVAYK					Mascot
917.5302	917.5102	-0.02	-22	69	76	IISSIEQK					Mascot
928.5324	928.4614	-0.071	-76	126	132	VFYLKMK					Mascot
948.4244	948.4443	0.0199	21	131	137	MKGDYYR			Oxidation (M)[1]		Mascot
1091.4712	1091.5502	0.079	72	77	85	EESRGNEDR					Mascot
1189.6609	1189.6735	0.0126	11	223	232	DSTLIMQLLR					Mascot
1205.6559	1205.6677	0.0118	10	223	232	DSTLIMQLLR			Oxidation (M)[6]		Mascot
1205.6559	1205.6677	0.0118	10	223	232	DSTLIMQLLR	13	0	Oxidation (M)[6]		Mascot
1212.5565	1212.6271	0.0706	58	150	160	DAAENTMVAYK					Mascot
1228.5514	1228.6252	0.0738	60	150	160	DAAENTMVAYK			Oxidation (M)[7]		Mascot
1340.6515	1340.6578	0.0063	5	149	160	KDAAENTMVAYK					Mascot
1356.6464	1356.6136	-0.0328	-24	149	160	KDAAENTMVAYK			Oxidation (M)[8]		Mascot
1366.5542	1366.5656	0.0114	8	25	34	YEEMVEFMEK			Oxidation (M)[4,8]		Mascot
1406.6646	1406.6847	0.0201	14	38	49	TVDSEELTVEER					Mascot
1418.7485	1418.7694	0.0209	15	69	80	IISSIEQKEESR					Mascot
1418.7485	1418.7694	0.0209	15	69	80	IISSIEQKEESR	28	85.987			Mascot
1664.7546	1664.8337	0.0791	48	25	37	YEEMVEFMEKVAK			Oxidation (M)[4,8]		Mascot
1786.9811	1787.0072	0.0261	15	161	177	AAQDIALAELAPTHPIR					Mascot
2163.9573	2163.9624	0.0051	2	18	34	LAEQAERYEEMVEFMEK			Oxidation (M)[11,15]		Mascot

4	unnamed protein product [Triticum aestivum]	gi|257664788	28475.3	4.78	16	121	100	13.619	41	99.286

Protein Group

14-3-3 protein [Triticum aestivum]	gi|32401388	28475.3	4.7800 002098

			0835
unnamed protein product [Triticum aestivum]	gi|227472036	28475.3	4.7800<br>002098<br>0835
unnamed protein product [Triticum aestivum]	gi|219911730	28475.3	4.7800<br>002098<br>0835

Peptide Information

Calc. Mass	Obsrv. Mass	± da	± ppm	Start Seq.	End Seq.	Sequence	Ion Score	C. I. %	Modification	Rank	Result Type
816.421	816.4305	0.0095	12	4	10	LAEQAER					Mascot
819.4458	819.4396	-0.0062	-8	82	88	IETELSK					Mascot
907.5247	907.4802	-0.0445	-49	36	43	NLLSVAYK					Mascot
917.5302	917.5102	-0.02	-22	55	62	IISSIEQK					Mascot
928.5324	928.4614	-0.071	-76	112	118	VFYLKMK					Mascot
948.4244	948.4443	0.0199	21	117	123	MKGDYYR			Oxidation (M)[1]		Mascot
1091.4712	1091.5502	0.079	72	63	71	EESRGNEDR					Mascot
1189.6609	1189.6735	0.0126	11	209	218	DSTLIMQLLR					Mascot
1205.6559	1205.6677	0.0118	10	209	218	DSTLIMQLLR			Oxidation (M)[6]		Mascot
1205.6559	1205.6677	0.0118	10	209	218	DSTLIMQLLR	13	0	Oxidation (M)[6]		Mascot
1212.5565	1212.6271	0.0706	58	136	146	DAAENTMVAYK					Mascot
1228.5514	1228.6252	0.0738	60	136	146	DAAENTMVAYK			Oxidation (M)[7]		Mascot
1340.6515	1340.6578	0.0063	5	135	146	KDAAENTMVAYK					Mascot
1356.6464	1356.6136	-0.0328	-24	135	146	KDAAENTMVAYK			Oxidation (M)[8]		Mascot
1366.5542	1366.5656	0.0114	8	11	20	YEEMVEFMEK			Oxidation (M)[4,8]		Mascot
1406.6646	1406.6847	0.0201	14	24	35	TVDSEELTVEER					Mascot
1418.7485	1418.7694	0.0209	15	55	66	IISSIEQKEESR					Mascot
1418.7485	1418.7694	0.0209	15	55	66	IISSIEQKEESR	28	85.987			Mascot
1664.7546	1664.8337	0.0791	48	11	23	YEEMVEFMEKVAK			Oxidation (M)[4,8]		Mascot
1786.9811	1787.0072	0.0261	15	147	163	AAQDIALAELAPTHPIR					Mascot
2163.9573	2163.9624	0.0051	2	4	20	LAEQAERYEEMVEFMEK			Oxidation (M)[11,15]		Mascot

5	14-3-3-like protein B [Triticum urartu]	gi|474253094	29786.9	4.67	15	118	100	13.429	41	99.286	

Peptide Information

Calc. Mass	Obsrv. Mass	± da	± ppm	Start Seq.	End Seq.	Sequence	Ion Score	C. I. %	Modification	Rank	Result Type
816.421	816.4305	0.0095	12	18	24	LAEQAER					Mascot
907.5247	907.4802	-0.0445	-49	50	57	NLLSVAYK					Mascot
917.5302	917.5102	-0.02	-22	69	76	IISSIEQK					Mascot

928.5324	928.4614	-0.071	-76	126	132	VFYLKMK					Mascot
948.4244	948.4443	0.0199	21	131	137	MKGDYYR			Oxidation (M)[1]		Mascot
1091.4712	1091.5502	0.079	72	77	85	EESRGNEDR					Mascot
1189.6609	1189.6735	0.0126	11	223	232	DSTLIMQLLR					Mascot
1205.6559	1205.6677	0.0118	10	223	232	DSTLIMQLLR			Oxidation (M)[6]		Mascot
1205.6559	1205.6677	0.0118	10	223	232	DSTLIMQLLR	13	0	Oxidation (M)[6]		Mascot
1212.5565	1212.6271	0.0706	58	150	160	DAAENTMVAYK					Mascot
1228.5514	1228.6252	0.0738	60	150	160	DAAENTMVAYK			Oxidation (M)[7]		Mascot
1340.6515	1340.6578	0.0063	5	149	160	KDAAENTMVAYK					Mascot
1356.6464	1356.6136	-0.0328	-24	149	160	KDAAENTMVAYK			Oxidation (M)[8]		Mascot
1366.5542	1366.5656	0.0114	8	25	34	YEEMVEFMEK			Oxidation (M)[4,8]		Mascot
1406.6646	1406.6847	0.0201	14	38	49	TVDSEELTVEER					Mascot
1418.7485	1418.7694	0.0209	15	69	80	IISSIEQKEESR					Mascot
1418.7485	1418.7694	0.0209	15	69	80	IISSIEQKEESR	28	85.987			Mascot
1664.7546	1664.8337	0.0791	48	25	37	YEEMVEFMEKVAK			Oxidation (M)[4,8]		Mascot
1827.0123	1827.0353	0.023	13	161	177	AAQEIALAELPPTHPIR					Mascot
2163.9573	2163.9624	0.0051	2	18	34	LAEQAERYEEMVEFMEK			Oxidation (M)[11,15]		Mascot

6 14-3-3 protein [Triticum aestivum] gi|431822518 29843.9 4.71 15 115 100 13.84 41 99.286

Peptide Information

Calc. Mass	Obsrv. Mass	± da	± ppm	Start Seq.	End Seq.	Sequence	Ion Score	C. I. %	Modification	Rank	Result Type
816.421	816.4305	0.0095	12	18	24	LAEQAER					Mascot
907.5247	907.4802	-0.0445	-49	50	57	NLLSVAYK					Mascot
917.5302	917.5102	-0.02	-22	69	76	IISSIEQK					Mascot
928.5324	928.4614	-0.071	-76	126	132	VFYLKMK					Mascot
948.4244	948.4443	0.0199	21	131	137	MKGDYYR			Oxidation (M)[1]		Mascot
1059.5946	1059.6057	0.0111	10	169	177	ELPPTHPIR					Mascot
1091.4712	1091.5502	0.079	72	77	85	EESRGNEDR					Mascot
1189.6609	1189.6735	0.0126	11	223	232	DSTLIMQLLR					Mascot
1205.6559	1205.6677	0.0118	10	223	232	DSTLIMQLLR			Oxidation (M)[6]		Mascot
1205.6559	1205.6677	0.0118	10	223	232	DSTLIMQLLR	13	0	Oxidation (M)[6]		Mascot
1212.5565	1212.6271	0.0706	58	150	160	DAAENTMVAYK					Mascot
1228.5514	1228.6252	0.0738	60	150	160	DAAENTMVAYK			Oxidation (M)[7]		Mascot
1340.6515	1340.6578	0.0063	5	149	160	KDAAENTMVAYK					Mascot
1356.6464	1356.6136	-0.0328	-24	149	160	KDAAENTMVAYK			Oxidation (M)[8]		Mascot

Calc. Mass	Obsrv. Mass	± da	± ppm	Start Seq.	End Seq.	Sequence	Ion Score	C. I. %	Modification	Rank	Result Type
1366.5542	1366.5656	0.0114	8	25	34	YEEMVEFMEK			Oxidation (M)[4,8]		Mascot
1406.6646	1406.6847	0.0201	14	38	49	TVDSEELTVEER					Mascot
1418.7485	1418.7694	0.0209	15	69	80	IISSIEQKEESR					Mascot
1418.7485	1418.7694	0.0209	15	69	80	IISSIEQKEESR	28	85.987			Mascot
1664.7546	1664.8337	0.0791	48	25	37	YEEMVEFMEKVAK			Oxidation (M)[4,8]		Mascot
2163.9573	2163.9624	0.0051	2	18	34	LAEQAERYEEMVEFMEK			Oxidation (M)[11,15]		Mascot

7	unnamed protein product [Triticum aestivum]	gi|257664756	29983	4.62	15	115	100	13.071	41	99.286

Protein Group

14-3-3 protein [Triticum aestivum]	gi|52548256	29983	4.61999988555908
unnamed protein product [Triticum aestivum]	gi|227471994	29983	4.61999988555908
unnamed protein product [Triticum aestivum]	gi|219911698	29983	4.61999988555908

Peptide Information

Calc. Mass	Obsrv. Mass	± da	± ppm	Start Seq.	End Seq.	Sequence	Ion Score	C. I. %	Modification	Rank	Result Type
816.421	816.4305	0.0095	12	18	24	LAEQAER					Mascot
819.4458	819.4396	-0.0062	-8	96	102	IETELSK					Mascot
844.4523	844.4958	0.0435	52	2	9	TAPAELSR					Mascot
907.5247	907.4802	-0.0445	-49	50	57	NLLSVAYK					Mascot
917.5302	917.5102	-0.02	-22	69	76	IISSIEQK					Mascot
928.5324	928.4614	-0.071	-76	126	132	VFYLKMK					Mascot
948.4244	948.4443	0.0199	21	131	137	MKGDYYR			Oxidation (M)[1]		Mascot
1091.4712	1091.5502	0.079	72	77	85	EESRGNEDR					Mascot
1189.6609	1189.6735	0.0126	11	223	232	DSTLIMQLLR					Mascot
1205.6559	1205.6677	0.0118	10	223	232	DSTLIMQLLR			Oxidation (M)[6]		Mascot
1205.6559	1205.6677	0.0118	10	223	232	DSTLIMQLLR	13	0	Oxidation (M)[6]		Mascot
1366.5542	1366.5656	0.0114	8	25	34	YEEMVEFMEK			Oxidation (M)[4,8]		Mascot
1406.6646	1406.6847	0.0201	14	38	49	TVDSEELTVEER					Mascot
1418.7485	1418.7694	0.0209	15	69	80	IISSIEQKEESR					Mascot
1418.7485	1418.7694	0.0209	15	69	80	IISSIEQKEESR	28	85.987			Mascot
1664.7546	1664.8337	0.0791	48	25	37	YEEMVEFMEKVAK			Oxidation (M)[4,8]		Mascot
1786.9811	1787.0072	0.0261	15	161	177	AAQDIALAELAPTHPIR					Mascot
2163.9573	2163.9624	0.0051	2	18	34	LAEQAERYEEMVEFMEK			Oxidation (M)[11,15]		Mascot

8	14-3-3-like protein A [Triticum urartu]	gi|474293618	28778.5	5.2	13	78	99.935	15.075	13	0

Peptide Information

Calc. Mass	Obsrv. Mass	± da	± ppm	Start Seq.	End Seq.	Sequence	Ion Score	C. I. %	Modification	Rank	Result Type
816.421	816.4305	0.0095	12	17	23	LAEQAER					Mascot
819.4505	819.4396	-0.0109	-13	1	8	MAKAAATR					Mascot
903.5145	903.4609	-0.0536	-59	68	75	IVSSIEQK					Mascot
906.425	906.4412	0.0162	18	130	136	MKGDYHR					Mascot
907.5247	907.4802	-0.0445	-49	49	56	NLLSVAYK					Mascot
922.4199	922.43	0.0101	11	130	136	MKGDYHR			Oxidation (M)[1]		Mascot
922.4199	922.43	0.0101	11	130	136	MKGDYHR			Oxidation (M)[1]		Mascot
928.5324	928.4614	-0.071	-76	125	131	VFYLKMK					Mascot
982.4913	982.4517	-0.0396	-40	9	16	EEMVYLAK					Mascot
1059.5946	1059.6057	0.0111	10	168	176	ELPPTHPIR					Mascot
1189.6609	1189.6735	0.0126	11	222	231	DSTLIMQLLR					Mascot
1205.6559	1205.6677	0.0118	10	222	231	DSTLIMQLLR			Oxidation (M)[6]		Mascot
1205.6559	1205.6677	0.0118	10	222	231	DSTLIMQLLR	13	0	Oxidation (M)[6]		Mascot
1228.6294	1228.6252	-0.0042	-3	80	92	GAAGHAAAARGYR					Mascot
1323.6512	1323.6761	0.0249	19	76	89	EEGRGAAGHAAAAR					Mascot
1366.5542	1366.5656	0.0114	8	24	33	YEEMVEFMEK			Oxidation (M)[4,8]		Mascot
2163.9573	2163.9624	0.0051	2	17	33	LAEQAERYEEMVEFMEK			Oxidation (M)[11,15]		Mascot

9	unnamed protein product [Triticum aestivum]	gi|227472076	28794.4	4.8	13	75	99.872	15.075	13	0	

Protein Group

TaWIN2 [Triticum aestivum]	gi|9798605	28794.4	4.8000 001907 3486
unnamed protein product [Triticum aestivum]	gi|219911924	28794.4	4.8000 001907 3486
unnamed protein product [Triticum aestivum]	gi|257664806	28794.4	4.8000 001907 3486

Peptide Information

Calc. Mass	Obsrv. Mass	± da	± ppm	Start Seq.	End Seq.	Sequence	Ion Score	C. I. %	Modification	Rank	Result Type
816.421	816.4305	0.0095	12	17	23	LAEQAER					Mascot
819.4505	819.4396	-0.0109	-13	1	8	MAKAAATR					Mascot
903.5145	903.4609	-0.0536	-59	68	75	IVSSIEQK					Mascot
906.425	906.4412	0.0162	18	130	136	MKGDYHR					Mascot

Calc. Mass	Obsrv. Mass	± da	± ppm	Start Seq.	End Seq.	Sequence	Ion Score	C. I. %	Modification	Rank	Result Type
907.5247	907.4802	-0.0445	-49	49	56	NLLSVAYK					Mascot
922.4199	922.43	0.0101	11	130	136	MKGDYHR			Oxidation (M)[1]		Mascot
922.4199	922.43	0.0101	11	130	136	MKGDYHR			Oxidation (M)[1]		Mascot
928.5324	928.4614	-0.071	-76	125	131	VFYLKMK					Mascot
982.4913	982.4517	-0.0396	-40	9	16	EEMVYLAK					Mascot
1059.5946	1059.6057	0.0111	10	168	176	ELPPTHPIR					Mascot
1189.6609	1189.6735	0.0126	11	222	231	DSTLIMQLLR					Mascot
1205.6559	1205.6677	0.0118	10	222	231	DSTLIMQLLR			Oxidation (M)[6]		Mascot
1205.6559	1205.6677	0.0118	10	222	231	DSTLIMQLLR	13	0	Oxidation (M)[6]		Mascot
1228.6294	1228.6252	-0.0042	-3	80	92	GAAGHAAAARGYR					Mascot
1323.6512	1323.6761	0.0249	19	76	89	EEGRGAAGHAAAAR					Mascot
1366.5542	1366.5656	0.0114	8	24	33	YEEMVEFMEK			Oxidation (M)[4,8]		Mascot
2163.9573	2163.9624	0.0051	2	17	33	LAEQAERYEEMVEFMEK			Oxidation (M)[11,15]		Mascot

10	unnamed protein product [Triticum aestivum]	gi|227473231	28898.4	4.8	11	60	95.578	14.654	13	0	

Protein Group

unnamed protein product [Triticum aestivum]	gi|257710883	28898.4	4.8000 001907 3486

Peptide Information

Calc. Mass	Obsrv. Mass	± da	± ppm	Start Seq.	End Seq.	Sequence	Ion Score	C. I. %	Modification	Rank	Result Type
816.421	816.4305	0.0095	12	19	25	LAEQAER					Mascot
903.5145	903.4609	-0.0536	-59	70	77	IVSSIEQK					Mascot
906.425	906.4412	0.0162	18	132	138	MKGDYHR					Mascot
907.5247	907.4802	-0.0445	-49	51	58	NLLSVAYK					Mascot
922.4199	922.43	0.0101	11	132	138	MKGDYHR			Oxidation (M)[1]		Mascot
922.4199	922.43	0.0101	11	132	138	MKGDYHR			Oxidation (M)[1]		Mascot
928.5324	928.4614	-0.071	-76	127	133	VFYLKMK					Mascot
1059.5946	1059.6057	0.0111	10	170	178	ELPPTHPIR					Mascot
1189.6609	1189.6735	0.0126	11	224	233	DSTLIMQLLR					Mascot
1205.6559	1205.6677	0.0118	10	224	233	DSTLIMQLLR			Oxidation (M)[6]		Mascot
1205.6559	1205.6677	0.0118	10	224	233	DSTLIMQLLR	13	0	Oxidation (M)[6]		Mascot
1228.6294	1228.6252	-0.0042	-3	82	94	GAAGHAAAARGYR					Mascot
1323.6512	1323.6761	0.0249	19	78	91	EEGRGAAGHAAAAR					Mascot
1366.5542	1366.5656	0.0114	8	26	35	YEEMVEFMEK			Oxidation (M)[4,8]		Mascot
2163.9573	2163.9624	0.0051	2	19	35	LAEQAERYEEMVEFMEK			Oxidation (M)[11,15]		Mascot

Gel Idx/Pos	168/G19	Instr./Gel Origin	BA2151/Sample Project 20140814	Process Status	Analysis Succeeded
Plate [#] Name	[1] Sample Project 20140814	Instrument Sample Name		Spectra	11

Rank	Protein Name	Accession No.	Protein MW	Protein PI	Pep. Count	Protein Score	Protein Score C. I. %	Intensity Matched	Total Ion Score	Total Ion C. I. %	Confirmed
1	14-3-3 protein [Triticum aestivum]	gi|390195996	30066.1	4.73	20	684	100	62.719	545	100	

Protein Group

Protein Name	Accession No.	Protein MW	Protein PI
14-3-3 protein [Triticum aestivum]	gi|351602255	30066.1	4.7300 000190 7349
14-3-3-like protein GF14-B [Triticum urartu]	gi|474147722	30043.1	4.6900 000572 2046

Peptide Information

Calc. Mass	Obsrv. Mass	± da	± ppm	Start Seq.	End Seq.	Sequence	Ion Score	C. I. %	Modification	Rank	Result Type
816.421	816.4236	0.0026	3	18	24	LAEQAER					Mascot
844.4523	844.465	0.0127	15	2	9	TAPAELSR					Mascot
907.5247	907.5185	-0.0062	-7	50	57	NLLSVAYK					Mascot
917.5302	917.5294	-0.0008	-1	69	76	IISSIEQK					Mascot
917.5302	917.5294	-0.0008	-1	69	76	IISSIEQK	43	99.58			Mascot
932.4294	932.4377	0.0083	9	131	137	MKGDYYR					Mascot
948.4244	948.4241	-0.0003	0	131	137	MKGDYYR			Oxidation (M)[1]		Mascot
948.4244	948.4241	-0.0003	0	131	137	MKGDYYR	15	0	Oxidation (M)[1]		Mascot
999.4451	999.4538	0.0087	9	10	17	EENVYMAK			Oxidation (M)[6]		Mascot
1091.4712	1091.5469	0.0757	69	77	85	EESRGNEDR					Mascot
1189.6609	1189.6698	0.0089	7	223	232	DSTLIMQLLR					Mascot
1189.6609	1189.6698	0.0089	7	223	232	DSTLIMQLLR	80	100			Mascot
1205.6559	1205.6586	0.0027	2	223	232	DSTLIMQLLR			Oxidation (M)[6]		Mascot
1205.6559	1205.6586	0.0027	2	223	232	DSTLIMQLLR	37	98.291	Oxidation (M)[6]		Mascot
1212.5565	1212.5978	0.0413	34	150	160	DAAENTMVAYK					Mascot
1228.5514	1228.6074	0.056	46	150	160	DAAENTMVAYK			Oxidation (M)[7]		Mascot
1366.5542	1366.5485	-0.0057	-4	25	34	YEEMVEFMEK			Oxidation (M)[4,8]		Mascot
1366.5542	1366.5485	-0.0057	-4	25	34	YEEMVEFMEK	1	0	Oxidation (M)[4,8]		Mascot
1406.6646	1406.6774	0.0128	9	38	49	TVDSEELTVEER					Mascot
1406.6646	1406.6774	0.0128	9	38	49	TVDSEELTVEER	94	100			Mascot
1418.7485	1418.7595	0.011	8	69	80	IISSIEQKEESR					Mascot
1708.9116	1708.8749	-0.0367	-21	110	125	LLETHLVPSSTAPESK					Mascot

1786.9811	1786.9965	0.0154	9	161	177	AAQDIALAELAPTHPIR					Mascot
1786.9811	1786.9965	0.0154	9	161	177	AAQDIALAELAPTHPIR	156	100			Mascot
1808.8848	1808.9656	0.0808	45	2	17	TAPAELSREENVYMAK					Mascot
1824.8796	1824.9293	0.0497	27	2	17	TAPAELSREENVYMAK			Oxidation (M)[14]		Mascot
2163.9573	2163.936	-0.0213	-10	18	34	LAEQAERYEEMVEFMEK			Oxidation (M)[11,15]		Mascot
2174.9976	2174.9839	-0.0137	-6	204	222	QAFDEAISELDTLSEESYK					Mascot
2331.2019	2331.2153	0.0134	6	178	197	LGLALNFSVFYYEILNSPDR					Mascot
2351.0886	2351.105	0.0164	7	233	252	DNLTLWTSDITEDTAEEEIR					Mascot
2351.0886	2351.105	0.0164	7	233	252	DNLTLWTSDITEDTAEEEIR	155	100			Mascot
2776.3159	2776.3403	0.0244	9	233	256	DNLTLWTSDITEDTAEEEIREAPK					Mascot

2 unnamed protein product [Triticum aestivum] gi|257664788 28475.3 4.78 17 651 100 60.073 545 100

Protein Group

14-3-3 protein [Triticum aestivum]	gi|32401388	28475.3	4.7800002098 0835
unnamed protein product [Triticum aestivum]	gi|227472036	28475.3	4.7800002098 0835
unnamed protein product [Triticum aestivum]	gi|219911730	28475.3	4.7800002098 0835

Peptide Information

Calc. Mass	Obsrv. Mass	± da	± ppm	Start Seq.	End Seq.	Sequence	Ion Score	C. I. %	Modification	Rank	Result Type
816.421	816.4236	0.0026	3	4	10	LAEQAER					Mascot
907.5247	907.5185	-0.0062	-7	36	43	NLLSVAYK					Mascot
917.5302	917.5294	-0.0008	-1	55	62	IISSIEQK					Mascot
917.5302	917.5294	-0.0008	-1	55	62	IISSIEQK	43	99.58			Mascot
932.4294	932.4377	0.0083	9	117	123	MKGDYYR					Mascot
948.4244	948.4241	-0.0003	0	117	123	MKGDYYR			Oxidation (M)[1]		Mascot
948.4244	948.4241	-0.0003	0	117	123	MKGDYYR	15	0	Oxidation (M)[1]		Mascot
1091.4712	1091.5469	0.0757	69	63	71	EESRGNEDR					Mascot
1189.6609	1189.6698	0.0089	7	209	218	DSTLIMQLLR					Mascot
1189.6609	1189.6698	0.0089	7	209	218	DSTLIMQLLR	80	100			Mascot
1205.6559	1205.6586	0.0027	2	209	218	DSTLIMQLLR			Oxidation (M)[6]		Mascot
1205.6559	1205.6586	0.0027	2	209	218	DSTLIMQLLR	37	98.291	Oxidation (M)[6]		Mascot
1212.5565	1212.5978	0.0413	34	136	146	DAAENTMVAYK					Mascot
1228.5514	1228.6074	0.056	46	136	146	DAAENTMVAYK			Oxidation (M)[7]		Mascot

1366.5542	1366.5485	-0.0057	-4	11	20	YEEMVEFMEK			Oxidation (M)[4,8]		Mascot
1366.5542	1366.5485	-0.0057	-4	11	20	YEEMVEFMEK	1	0	Oxidation (M)[4,8]		Mascot
1406.6646	1406.6774	0.0128	9	24	35	TVDSEELTVEER					Mascot
1406.6646	1406.6774	0.0128	9	24	35	TVDSEELTVEER	94	100			Mascot
1418.7485	1418.7595	0.011	8	55	66	IISSIEQKEESR					Mascot
1708.9116	1708.8749	-0.0367	-21	96	111	LLETHLVPSSTAPESK					Mascot
1786.9811	1786.9965	0.0154	9	147	163	AAQDIALAELAPTHPIR					Mascot
1786.9811	1786.9965	0.0154	9	147	163	AAQDIALAELAPTHPIR	156	100			Mascot
2163.9573	2163.936	-0.0213	-10	4	20	LAEQAERYEEMVEFMEK			Oxidation (M)[11,15]		Mascot
2174.9976	2174.9839	-0.0137	-6	190	208	QAFDEAISELDTLSEESY K					Mascot
2331.2019	2331.2153	0.0134	6	164	183	LGLALNFSVFYYEILNSPD R					Mascot
2351.0886	2351.105	0.0164	7	219	238	DNLTLWTSDITEDTAEEEI R					Mascot
2351.0886	2351.105	0.0164	7	219	238	DNLTLWTSDITEDTAEEEI R	155	100			Mascot
2776.3159	2776.3403	0.0244	9	219	242	DNLTLWTSDITEDTAEEEI REAPK					Mascot

3 unnamed protein product [Triticum aestivum] gi|257664756 29983 4.62 15 468 100 61.105 389 100

Protein Group

14-3-3 protein [Triticum aestivum]	gi|52548256	29983	4.6199 998855 5908
unnamed protein product [Triticum aestivum]	gi|227471994	29983	4.6199 998855 5908
unnamed protein product [Triticum aestivum]	gi|219911698	29983	4.6199 998855 5908

Peptide Information

Calc. Mass	Obsrv. Mass	± da	± ppm	Start Seq.	End Seq.	Sequence	Ion Score	C. I. %	Modification	Rank	Result Type
816.421	816.4236	0.0026	3	18	24	LAEQAER					Mascot
844.4523	844.465	0.0127	15	2	9	TAPAELSR					Mascot
907.5247	907.5185	-0.0062	-7	50	57	NLLSVAYK					Mascot
917.5302	917.5294	-0.0008	-1	69	76	IISSIEQK					Mascot
917.5302	917.5294	-0.0008	-1	69	76	IISSIEQK	43	99.58			Mascot
932.4294	932.4377	0.0083	9	131	137	MKGDYYR					Mascot
948.4244	948.4241	-0.0003	0	131	137	MKGDYYR			Oxidation (M)[1]		Mascot
948.4244	948.4241	-0.0003	0	131	137	MKGDYYR	15	0	Oxidation (M)[1]		Mascot
999.4451	999.4538	0.0087	9	10	17	EENVYMAK			Oxidation (M)[6]		Mascot

1091.4712	1091.5469	0.0757	69	77	85	EESRGNEDR					Mascot
1189.6609	1189.6698	0.0089	7	223	232	DSTLIMQLLR					Mascot
1189.6609	1189.6698	0.0089	7	223	232	DSTLIMQLLR	80	100			Mascot
1205.6559	1205.6586	0.0027	2	223	232	DSTLIMQLLR			Oxidation (M)[6]		Mascot
1205.6559	1205.6586	0.0027	2	223	232	DSTLIMQLLR	37	98.291	Oxidation (M)[6]		Mascot
1366.5542	1366.5485	-0.0057	-4	25	34	YEEMVEFMEK			Oxidation (M)[4,8]		Mascot
1366.5542	1366.5485	-0.0057	-4	25	34	YEEMVEFMEK	1	0	Oxidation (M)[4,8]		Mascot
1406.6646	1406.6774	0.0128	9	38	49	TVDSEELTVEER					Mascot
1406.6646	1406.6774	0.0128	9	38	49	TVDSEELTVEER	94	100			Mascot
1418.7485	1418.7595	0.011	8	69	80	IISSIEQKEESR					Mascot
1708.9116	1708.8749	-0.0367	-21	110	125	LLETHLVPSSTAPESK					Mascot
1786.9811	1786.9965	0.0154	9	161	177	AAQDIALAELAPTHPIR					Mascot
1786.9811	1786.9965	0.0154	9	161	177	AAQDIALAELAPTHPIR	156	100			Mascot
1808.8848	1808.9656	0.0808	45	2	17	TAPAELSREENVYMAK					Mascot
1824.8796	1824.9293	0.0497	27	2	17	TAPAELSREENVYMAK			Oxidation (M)[14]		Mascot
2163.9573	2163.936	-0.0213	-10	18	34	LAEQAERYEEMVEFMEK			Oxidation (M)[11,15]		Mascot

4 14-3-3 protein [Triticum aestivum] gi|431822518 29843.9 4.71 14 300 100 18.79 233 100

Peptide Information

Calc. Mass	Obsrv. Mass	± da	± ppm	Start Seq.	End Seq.	Sequence	Ion Score	C. I. %	Modification	Rank	Result Type
816.421	816.4236	0.0026	3	18	24	LAEQAER					Mascot
907.5247	907.5185	-0.0062	-7	50	57	NLLSVAYK					Mascot
917.5302	917.5294	-0.0008	-1	69	76	IISSIEQK					Mascot
917.5302	917.5294	-0.0008	-1	69	76	IISSIEQK	43	99.58			Mascot
932.4294	932.4377	0.0083	9	131	137	MKGDYYR					Mascot
948.4244	948.4241	-0.0003	0	131	137	MKGDYYR			Oxidation (M)[1]		Mascot
948.4244	948.4241	-0.0003	0	131	137	MKGDYYR	15	0	Oxidation (M)[1]		Mascot
999.4451	999.4538	0.0087	9	10	17	EENVYMAK			Oxidation (M)[6]		Mascot
1059.5946	1059.579	-0.0156	-15	169	177	ELPPTHPIR					Mascot
1091.4712	1091.5469	0.0757	69	77	85	EESRGNEDR					Mascot
1189.6609	1189.6698	0.0089	7	223	232	DSTLIMQLLR					Mascot
1189.6609	1189.6698	0.0089	7	223	232	DSTLIMQLLR	80	100			Mascot
1205.6559	1205.6586	0.0027	2	223	232	DSTLIMQLLR			Oxidation (M)[6]		Mascot
1205.6559	1205.6586	0.0027	2	223	232	DSTLIMQLLR	37	98.291	Oxidation (M)[6]		Mascot
1212.5565	1212.5978	0.0413	34	150	160	DAAENTMVAYK					Mascot
1228.5514	1228.6074	0.056	46	150	160	DAAENTMVAYK			Oxidation (M)[7]		Mascot

Calc. Mass	Obsrv. Mass	± da	± ppm	Start Seq.	End Seq.	Sequence	Ion Score	C. I. %	Modification	Rank	Result Type
1366.5542	1366.5485	-0.0057	-4	25	34	YEEMVEFMEK			Oxidation (M)[4,8]		Mascot
1366.5542	1366.5485	-0.0057	-4	25	34	YEEMVEFMEK	1	0	Oxidation (M)[4,8]		Mascot
1406.6646	1406.6774	0.0128	9	38	49	TVDSEELTVEER					Mascot
1406.6646	1406.6774	0.0128	9	38	49	TVDSEELTVEER	94	100			Mascot
1418.7485	1418.7595	0.011	8	69	80	IISSIEQKEESR					Mascot
2163.9573	2163.936	-0.0213	-10	18	34	LAEQAERYEEMVEFMEK			Oxidation (M)[11,15]		Mascot
2331.2019	2331.2153	0.0134	6	178	197	LGLALNFSVFYYEILNSPD R					Mascot

5 14-3-3-like protein B [Triticum urartu] gi|474253094 29786.9 4.67 13 293 100 18.674 233 100

Peptide Information

Calc. Mass	Obsrv. Mass	± da	± ppm	Start Seq.	End Seq.	Sequence	Ion Score	C. I. %	Modification	Rank	Result Type
816.421	816.4236	0.0026	3	18	24	LAEQAER					Mascot
907.5247	907.5185	-0.0062	-7	50	57	NLLSVAYK					Mascot
917.5302	917.5294	-0.0008	-1	69	76	IISSIEQK					Mascot
917.5302	917.5294	-0.0008	-1	69	76	IISSIEQK	43	99.58			Mascot
932.4294	932.4377	0.0083	9	131	137	MKGDYYR					Mascot
948.4244	948.4241	-0.0003	0	131	137	MKGDYYR			Oxidation (M)[1]		Mascot
948.4244	948.4241	-0.0003	0	131	137	MKGDYYR	15	0	Oxidation (M)[1]		Mascot
999.4451	999.4538	0.0087	9	10	17	EENVYMAK			Oxidation (M)[6]		Mascot
1091.4712	1091.5469	0.0757	69	77	85	EESRGNEDR					Mascot
1189.6609	1189.6698	0.0089	7	223	232	DSTLIMQLLR					Mascot
1189.6609	1189.6698	0.0089	7	223	232	DSTLIMQLLR	80	100			Mascot
1205.6559	1205.6586	0.0027	2	223	232	DSTLIMQLLR			Oxidation (M)[6]		Mascot
1205.6559	1205.6586	0.0027	2	223	232	DSTLIMQLLR	37	98.291	Oxidation (M)[6]		Mascot
1212.5565	1212.5978	0.0413	34	150	160	DAAENTMVAYK					Mascot
1228.5514	1228.6074	0.056	46	150	160	DAAENTMVAYK			Oxidation (M)[7]		Mascot
1366.5542	1366.5485	-0.0057	-4	25	34	YEEMVEFMEK			Oxidation (M)[4,8]		Mascot
1366.5542	1366.5485	-0.0057	-4	25	34	YEEMVEFMEK	1	0	Oxidation (M)[4,8]		Mascot
1406.6646	1406.6774	0.0128	9	38	49	TVDSEELTVEER					Mascot
1406.6646	1406.6774	0.0128	9	38	49	TVDSEELTVEER	94	100			Mascot
1418.7485	1418.7595	0.011	8	69	80	IISSIEQKEESR					Mascot
2163.9573	2163.936	-0.0213	-10	18	34	LAEQAERYEEMVEFMEK			Oxidation (M)[11,15]		Mascot
2331.2019	2331.2153	0.0134	6	178	197	LGLALNFSVFYYEILNSPD R					Mascot

6 unnamed protein product [Triticum aestivum] gi|227472078 29490.6 4.75 11 231 100 15.452 179 100

Protein Group

TaWIN1 [Triticum aestivum]	gi|9798603	29490.6	4.75
unnamed protein product [Triticum aestivum]	gi|219911926	29490.6	4.75
unnamed protein product [Triticum aestivum]	gi|257664808	29490.6	4.75

Peptide Information

Calc. Mass	Obsrv. Mass	± da	± ppm	Start Seq.	End Seq.	Sequence	Ion Score	C. I. %	Modification	Rank	Result Type
816.421	816.4236	0.0026	3	17	23	LAEQAER					Mascot
907.5247	907.5185	-0.0062	-7	53	60	NLLSVAYK					Mascot
917.5302	917.5294	-0.0008	-1	72	79	IISSIEQK					Mascot
917.5302	917.5294	-0.0008	-1	72	79	IISSIEQK	43	99.58			Mascot
1025.5123	1025.5297	0.0174	17	84	93	GNDAHAATIR					Mascot
1189.6609	1189.6698	0.0089	7	226	235	DSTLIMQLLR					Mascot
1189.6609	1189.6698	0.0089	7	226	235	DSTLIMQLLR	80	100			Mascot
1205.6559	1205.6586	0.0027	2	226	235	DSTLIMQLLR			Oxidation (M)[6]		Mascot
1205.6559	1205.6586	0.0027	2	226	235	DSTLIMQLLR	37	98.291	Oxidation (M)[6]		Mascot
1378.5654	1378.6094	0.044	32	24	33	YEEMVEFMER			Oxidation (M)[4]		Mascot
1388.738	1388.7393	0.0013	1	72	83	IISSIEQKEEGR					Mascot
1394.5603	1394.5698	0.0095	7	24	33	YEEMVEFMER			Oxidation (M)[4,8]		Mascot
1558.7344	1558.7461	0.0117	8	37	52	ATGGAGPGEELSVEER					Mascot
1772.9653	1772.9747	0.0094	5	164	180	AAQDIALADLAPTHPIR					Mascot
1772.9653	1772.9747	0.0094	5	164	180	AAQDIALADLAPTHPIR	56	99.98			Mascot
1827.8252	1827.9589	0.1337	73	1	16	MSPAEPTRDESVYMAK			Oxidation (M)[1]		Mascot
1843.8201	1844.004	0.1839	100	1	16	MSPAEPTRDESVYMAK			Oxidation (M)[1,14]		Mascot
2331.2019	2331.2153	0.0134	6	181	200	LGLALNFSVFYYEILNSPDR					Mascot

7	14-3-3 protein [Triticum aestivum]	gi|40781605	29387.7	4.83	13	192	100	15.545	124	100

Protein Group

14-3-3 protein [Triticum aestivum]	gi|431822520	29360.7	4.8299999237 0605
unnamed protein product [Triticum aestivum]	gi|227473229	29360.7	4.8299999237 0605
unnamed protein product [Triticum aestivum]	gi|227471938	29387.7	4.8299999237

Peptide Information

Calc. Mass	Obsrv. Mass	± da	± ppm	Start Seq.	End Seq.	Sequence	Ion Score	C. I. %	Modification	Rank	Result Type
816.421	816.4236	0.0026	3	17	23	LAEQAER					Mascot
907.5247	907.5185	-0.0062	-7	49	56	NLLSVAYK					Mascot
917.5302	917.5294	-0.0008	-1	68	75	IISSIEQK					Mascot
917.5302	917.5294	-0.0008	-1	68	75	IISSIEQK	43	99.58			Mascot
999.4451	999.4538	0.0087	9	9	16	EENVYMAK			Oxidation (M)[6]		Mascot
1189.6609	1189.6698	0.0089	7	222	231	DSTLIMQLLR					Mascot
1189.6609	1189.6698	0.0089	7	222	231	DSTLIMQLLR	80	100			Mascot
1205.6559	1205.6586	0.0027	2	222	231	DSTLIMQLLR			Oxidation (M)[6]		Mascot
1205.6559	1205.6586	0.0027	2	222	231	DSTLIMQLLR	37	98.291	Oxidation (M)[6]		Mascot
1318.6486	1318.6327	-0.0159	-12	37	48	TADVGELTVEER					Mascot
1366.5542	1366.5485	-0.0057	-4	24	33	YEEMVEFMEK			Oxidation (M)[4,8]		Mascot
1366.5542	1366.5485	-0.0057	-4	24	33	YEEMVEFMEK	1	0	Oxidation (M)[4,8]		Mascot
1418.7485	1418.7595	0.011	8	68	79	IISSIEQKEESR					Mascot
1715.7905	1715.9297	0.1392	81	2	16	STAEATREENVYMAK			Oxidation (M)[13]		Mascot
1818.9708	1818.9784	0.0076	4	160	176	SAQDIALADLPTTHPIR					Mascot
1830.8361	1830.964	0.1279	70	1	16	MSTAEATREENVYMAK					Mascot
1846.8309	1846.9508	0.1199	65	1	16	MSTAEATREENVYMAK			Oxidation (M)[1]		Mascot
1862.8259	1862.8651	0.0392	21	1	16	MSTAEATREENVYMAK			Oxidation (M)[1,14]		Mascot
2163.9573	2163.936	-0.0213	-10	17	33	LAEQAERYEEMVEFMEK			Oxidation (M)[11,15]		Mascot
2331.2019	2331.2153	0.0134	6	177	196	LGLALNFSVFYYEILNSPD R					Mascot

8	unnamed protein product [Triticum aestivum]	gi|227473233	29549.7	4.75	10	166	100	14.745	123	100		

Protein Group

unnamed protein product [Triticum aestivum] gi|257710885 29549.7 4.75

Peptide Information

Calc. Mass	Obsrv. Mass	± da	± ppm	Start Seq.	End Seq.	Sequence	Ion Score	C. I. %	Modification	Rank	Result Type
816.421	816.4236	0.0026	3	17	23	LAEQAER					Mascot
907.5247	907.5185	-0.0062	-7	53	60	NLLSVAYK					Mascot
917.5302	917.5294	-0.0008	-1	72	79	IISSIEQK					Mascot
917.5302	917.5294	-0.0008	-1	72	79	IISSIEQK	43	99.58			Mascot
1025.5123	1025.5297	0.0174	17	84	93	GNDAHAATIR					Mascot

1189.6609	1189.6698	0.0089	7	226	235	DSTLIMQLLR					Mascot
1189.6609	1189.6698	0.0089	7	226	235	DSTLIMQLLR	80	100			Mascot
1205.6559	1205.6586	0.0027	2	226	235	DSTLIMQLLR			Oxidation (M)[6]		Mascot
1205.6559	1205.6586	0.0027	2	226	235	DSTLIMQLLR	37	98.291	Oxidation (M)[6]		Mascot
1378.5654	1378.6094	0.044	32	24	33	YEEMVEFMER			Oxidation (M)[4]		Mascot
1388.738	1388.7393	0.0013	1	72	83	IISSIEQKEEGR					Mascot
1394.5603	1394.5698	0.0095	7	24	33	YEEMVEFMER			Oxidation (M)[4,8]		Mascot
1558.7344	1558.7461	0.0117	8	37	52	ATGGAGPGEELSVEER					Mascot
1800.9967	1800.9907	-0.006	-3	164	180	AAQDIALVDLAPTHPIR					Mascot
2331.2019	2331.2153	0.0134	6	181	200	LGLALNFSVFYYEILNSPD R					Mascot

9	unnamed protein product [Triticum aestivum]	gi|227473235	30434.1	4.9	6	143	100	11.689	123	100	

Protein Group

unnamed protein product [Triticum aestivum]	gi|219912012	30434.1	4.9000 000953 6743
unnamed protein product [Triticum aestivum]	gi|257673408	30434.1	4.9000 000953 6743

Peptide Information

Calc. Mass	Obsrv. Mass	± da	± ppm	Start Seq.	End Seq.	Sequence	Ion Score	C. I. %	Modification	Rank	Result Type
907.5247	907.5185	-0.0062	-7	44	51	NLLSVAYK					Mascot
917.5302	917.5294	-0.0008	-1	63	70	IISSIEQK					Mascot
917.5302	917.5294	-0.0008	-1	63	70	IISSIEQK	43	99.58			Mascot
1189.6609	1189.6698	0.0089	7	217	226	DSTLIMQLLR					Mascot
1189.6609	1189.6698	0.0089	7	217	226	DSTLIMQLLR	80	100			Mascot
1205.6559	1205.6586	0.0027	2	217	226	DSTLIMQLLR			Oxidation (M)[6]		Mascot
1205.6559	1205.6586	0.0027	2	217	226	DSTLIMQLLR	37	98.291	Oxidation (M)[6]		Mascot
1742.8132	1742.9738	0.1606	92	127	141	GDYHRYLAEFASGEK					Mascot
1836.9451	1836.9177	-0.0274	-15	155	171	SATDVAQTELTPTHPIR					Mascot
2331.2019	2331.2153	0.0134	6	172	191	LGLALNFSVFYYEILNSPD R					Mascot

10	14-3-3-like protein GF14-D [Triticum urartu]	gi|474137097	21391.7	4.86	6	142	100	11.851	123	100	

Peptide Information

Calc. Mass	Obsrv. Mass	± da	± ppm	Start Seq.	End Seq.	Sequence	Ion Score	C. I. %	Modification	Rank	Result Type
907.5247	907.5185	-0.0062	-7	23	30	NLLSVAYK					Mascot

917.5302	917.5294	-0.0008	-1	42	49	IISSIEQK				Mascot
917.5302	917.5294	-0.0008	-1	42	49	IISSIEQK	43	99.556		Mascot
1025.5123	1025.5297	0.0174	17	54	63	GNDAHAATIR				Mascot
1189.6609	1189.6698	0.0089	7	155	164	DSTLIMQLLR				Mascot
1189.6609	1189.6698	0.0089	7	155	164	DSTLIMQLLR	80	100		Mascot
1205.6559	1205.6586	0.0027	2	155	164	DSTLIMQLLR			Oxidation (M)[6]	Mascot
1205.6559	1205.6586	0.0027	2	155	164	DSTLIMQLLR	37	98.195	Oxidation (M)[6]	Mascot
1388.738	1388.7393	0.0013	1	42	53	IISSIEQKEEGR				Mascot
1558.7344	1558.7461	0.0117	8	7	22	ATGGAGPGEELSVEER				Mascot

Gel Idx/Pos	169/G20	Instr./Gel Origin	BA2151/Sample Project 20140814	Process Status	Analysis Succeeded
Plate [#] Name	[1] Sample Project 20140814	Instrument Sample Name		Spectra	11

Rank	Protein Name	Accession No.	Protein MW	Protein PI	Pep. Count	Protein Score	Protein Score C. I. %	Intensity Matched	Total Ion Score	Total Ion C. I. %	Confirmed
1	14-3-3 protein [Triticum aestivum]	gi|390195996	30066.1	4.73	24	529	100	67.813	352	100	

Protein Group

Protein Name	Accession No.	Protein MW	Protein PI
14-3-3 protein [Triticum aestivum]	gi|351602255	30066.1	4.7300 000190 7349
14-3-3-like protein GF14-B [Triticum urartu]	gi|474147722	30043.1	4.6900 000572 2046

Peptide Information

Calc. Mass	Obsrv. Mass	± da	± ppm	Start Seq.	End Seq.	Sequence	Ion Score	C. I. %	Modification	Rank	Result Type
816.421	816.4315	0.0105	13	18	24	LAEQAER					Mascot
818.444	818.4407	-0.0033	-4	103	109	ICDGILK			Carbamidomethyl (C)[2]		Mascot
819.4458	819.4365	-0.0093	-11	96	102	IETELSK					Mascot
844.4523	844.4795	0.0272	32	2	9	TAPAELSR					Mascot
907.5247	907.5327	0.008	9	50	57	NLLSVAYK					Mascot
917.5302	917.538	0.0078	9	69	76	IISSIEQK					Mascot
917.5302	917.538	0.0078	9	69	76	IISSIEQK	25	74.792			Mascot
932.4294	932.4701	0.0407	44	131	137	MKGDYYR					Mascot
948.4244	948.4344	0.01	11	131	137	MKGDYYR			Oxidation (M)[1]		Mascot
948.4244	948.4344	0.01	11	131	137	MKGDYYR	15	0	Oxidation (M)[1]		Mascot
999.4451	999.4573	0.0122	12	10	17	EENVYMAK			Oxidation (M)[6]		Mascot
1004.5622	1004.5597	-0.0025	-2	94	102	GKIETELSK					Mascot
1091.4712	1091.5339	0.0627	57	77	85	EESRGNEDR					Mascot
1144.6321	1144.6542	0.0221	19	81	90	GNEDRVTLIK					Mascot
1189.6609	1189.678	0.0171	14	223	232	DSTLIMQLLR					Mascot
1205.6559	1205.6694	0.0135	11	223	232	DSTLIMQLLR			Oxidation (M)[6]		Mascot
1205.6559	1205.6694	0.0135	11	223	232	DSTLIMQLLR	18	0	Oxidation (M)[6]		Mascot
1212.5565	1212.6193	0.0628	52	150	160	DAAENTMVAYK					Mascot
1228.5514	1228.6051	0.0537	44	150	160	DAAENTMVAYK			Oxidation (M)[7]		Mascot
1366.5542	1366.5502	-0.004	-3	25	34	YEEMVEFMEK			Oxidation (M)[4,8]		Mascot
1406.6646	1406.6904	0.0258	18	38	49	TVDSEELTVEER					Mascot
1406.6646	1406.6904	0.0258	18	38	49	TVDSEELTVEER	101	100			Mascot

1418.7485	1418.777	0.0285	20	69	80	IISSIEQKEESR					Mascot
1418.7485	1418.777	0.0285	20	69	80	IISSIEQKEESR	33	95.996			Mascot
1708.9116	1708.9102	-0.0014	-1	110	125	LLETHLVPSSTAPESK					Mascot
1786.9811	1787.0148	0.0337	19	161	177	AAQDIALAELAPTHPIR					Mascot
1786.9811	1787.0148	0.0337	19	161	177	AAQDIALAELAPTHPIR	146	100			Mascot
1808.8848	1808.9774	0.0926	51	2	17	TAPAELSREENVYMAK					Mascot
1824.8796	1824.9255	0.0459	25	2	17	TAPAELSREENVYMAK			Oxidation (M)[14]		Mascot
2163.9573	2163.9636	0.0063	3	18	34	LAEQAERYEEMVEFMEK			Oxidation (M)[11,15]		Mascot
2163.9573	2163.9636	0.0063	3	18	34	LAEQAERYEEMVEFMEK	13	0	Oxidation (M)[11,15]		Mascot
2174.9976	2175.0115	0.0139	6	204	222	QAFDEAISELDTLSEESY K					Mascot
2331.2019	2331.2488	0.0469	20	178	197	LGLALNFSVFYYEILNSPD R					Mascot
2351.0886	2351.1287	0.0401	17	233	252	DNLTLWTSDITEDTAEEEI R					Mascot
2776.3159	2776.377	0.0611	22	233	256	DNLTLWTSDITEDTAEEEI REAPK					Mascot

2	unnamed protein product [Triticum aestivum]	gi|257664788	28475.3	4.78	21	493	100	66.408	352	100	

Protein Group

14-3-3 protein [Triticum aestivum]	gi|32401388	28475.3	4.7800 002098 0835
unnamed protein product [Triticum aestivum]	gi|227472036	28475.3	4.7800 002098 0835
unnamed protein product [Triticum aestivum]	gi|219911730	28475.3	4.7800 002098 0835

Peptide Information

Calc. Mass	Obsrv. Mass	± da	± ppm	Start Seq.	End Seq.	Sequence	Ion Score	C. I. %	Modification	Rank	Result Type
816.421	816.4315	0.0105	13	4	10	LAEQAER					Mascot
818.444	818.4407	-0.0033	-4	89	95	ICDGILK			Carbamidomethyl (C)[2]		Mascot
819.4458	819.4365	-0.0093	-11	82	88	IETELSK					Mascot
907.5247	907.5327	0.008	9	36	43	NLLSVAYK					Mascot
917.5302	917.538	0.0078	9	55	62	IISSIEQK					Mascot
917.5302	917.538	0.0078	9	55	62	IISSIEQK	25	74.792			Mascot
932.4294	932.4701	0.0407	44	117	123	MKGDYYR					Mascot
948.4244	948.4344	0.01	11	117	123	MKGDYYR			Oxidation (M)[1]		Mascot
948.4244	948.4344	0.01	11	117	123	MKGDYYR	15	0	Oxidation (M)[1]		Mascot
1004.5622	1004.5597	-0.0025	-2	80	88	GKIETELSK					Mascot
1091.4712	1091.5339	0.0627	57	63	71	EESRGNEDR					Mascot

1144.6321	1144.6542	0.0221	19	67	76	GNEDRVTLIK					Mascot
1189.6609	1189.678	0.0171	14	209	218	DSTLIMQLLR					Mascot
1205.6559	1205.6694	0.0135	11	209	218	DSTLIMQLLR			Oxidation (M)[6]		Mascot
1205.6559	1205.6694	0.0135	11	209	218	DSTLIMQLLR	18	0	Oxidation (M)[6]		Mascot
1212.5565	1212.6193	0.0628	52	136	146	DAAENTMVAYK					Mascot
1228.5514	1228.6051	0.0537	44	136	146	DAAENTMVAYK			Oxidation (M)[7]		Mascot
1366.5542	1366.5502	-0.004	-3	11	20	YEEMVEFMEK			Oxidation (M)[4,8]		Mascot
1406.6646	1406.6904	0.0258	18	24	35	TVDSEELTVEER					Mascot
1406.6646	1406.6904	0.0258	18	24	35	TVDSEELTVEER	101	100			Mascot
1418.7485	1418.777	0.0285	20	55	66	IISSIEQKEESR					Mascot
1418.7485	1418.777	0.0285	20	55	66	IISSIEQKEESR	33	95.996			Mascot
1708.9116	1708.9102	-0.0014	-1	96	111	LLETHLVPSSTAPESK					Mascot
1786.9811	1787.0148	0.0337	19	147	163	AAQDIALAELAPTHPIR					Mascot
1786.9811	1787.0148	0.0337	19	147	163	AAQDIALAELAPTHPIR	146	100			Mascot
2163.9573	2163.9636	0.0063	3	4	20	LAEQAERYEEMVEFMEK			Oxidation (M)[11,15]		Mascot
2163.9573	2163.9636	0.0063	3	4	20	LAEQAERYEEMVEFMEK	13	0	Oxidation (M)[11,15]		Mascot
2174.9976	2175.0115	0.0139	6	190	208	QAFDEAISELDTLSEESYK					Mascot
2331.2019	2331.2488	0.0469	20	164	183	LGLALNFSVFYYEILNSPDR					Mascot
2351.0886	2351.1287	0.0401	17	219	238	DNLTLWTSDITEDTAEEEIR					Mascot
2776.3159	2776.377	0.0611	22	219	242	DNLTLWTSDITEDTAEEEIREAPK					Mascot

3 unnamed protein product [Triticum aestivum] gi|257664756 29983 4.62 19 465 100 66.121 352 100

Protein Group

14-3-3 protein [Triticum aestivum]	gi|52548256	29983	4.61999988555908
unnamed protein product [Triticum aestivum]	gi|227471994	29983	4.61999988555908
unnamed protein product [Triticum aestivum]	gi|219911698	29983	4.61999988555908

Peptide Information

Calc. Mass	Obsrv. Mass	± da	± ppm	Start Seq.	End Seq.	Sequence	Ion Score	C. I. %	Modification	Rank	Result Type
816.421	816.4315	0.0105	13	18	24	LAEQAER					Mascot
818.444	818.4407	-0.0033	-4	103	109	ICDGILK			Carbamidomethyl (C)[2]		Mascot
819.4458	819.4365	-0.0093	-11	96	102	IETELSK					Mascot
844.4523	844.4795	0.0272	32	2	9	TAPAELSR					Mascot

Calc. Mass	Obsrv. Mass	± da	± ppm	Start Seq.	End Seq.	Sequence	Ion Score	C. I. %	Modification	Rank	Result Type
907.5247	907.5327	0.008	9	50	57	NLLSVAYK					Mascot
917.5302	917.538	0.0078	9	69	76	IISSIEQK					Mascot
917.5302	917.538	0.0078	9	69	76	IISSIEQK	25	74.792			Mascot
932.4294	932.4701	0.0407	44	131	137	MKGDYYR					Mascot
948.4244	948.4344	0.01	11	131	137	MKGDYYR			Oxidation (M)[1]		Mascot
948.4244	948.4344	0.01	11	131	137	MKGDYYR	15	0	Oxidation (M)[1]		Mascot
999.4451	999.4573	0.0122	12	10	17	EENVYMAK			Oxidation (M)[6]		Mascot
1004.5622	1004.5597	-0.0025	-2	94	102	GKIETELSK					Mascot
1091.4712	1091.5339	0.0627	57	77	85	EESRGNEDR					Mascot
1144.6321	1144.6542	0.0221	19	81	90	GNEDRVTLIK					Mascot
1189.6609	1189.678	0.0171	14	223	232	DSTLIMQLLR					Mascot
1205.6559	1205.6694	0.0135	11	223	232	DSTLIMQLLR			Oxidation (M)[6]		Mascot
1205.6559	1205.6694	0.0135	11	223	232	DSTLIMQLLR	18	0	Oxidation (M)[6]		Mascot
1366.5542	1366.5502	-0.004	-3	25	34	YEEMVEFMEK			Oxidation (M)[4,8]		Mascot
1406.6646	1406.6904	0.0258	18	38	49	TVDSEELTVEER					Mascot
1406.6646	1406.6904	0.0258	18	38	49	TVDSEELTVEER	101	100			Mascot
1418.7485	1418.777	0.0285	20	69	80	IISSIEQKEESR					Mascot
1418.7485	1418.777	0.0285	20	69	80	IISSIEQKEESR	33	95.996			Mascot
1708.9116	1708.9102	-0.0014	-1	110	125	LLETHLVPSSTAPESK					Mascot
1786.9811	1787.0148	0.0337	19	161	177	AAQDIALAELAPTHPIR					Mascot
1786.9811	1787.0148	0.0337	19	161	177	AAQDIALAELAPTHPIR	146	100			Mascot
1808.8848	1808.9774	0.0926	51	2	17	TAPAELSREENVYMAK					Mascot
1824.8796	1824.9255	0.0459	25	2	17	TAPAELSREENVYMAK			Oxidation (M)[14]		Mascot
2163.9573	2163.9636	0.0063	3	18	34	LAEQAERYEEMVEFMEK			Oxidation (M)[11,15]		Mascot
2163.9573	2163.9636	0.0063	3	18	34	LAEQAERYEEMVEFMEK	13	0	Oxidation (M)[11,15]		Mascot

4 14-3-3-like protein B [Triticum urartu] gi|474253094 29786.9 4.67 16 291 100 23.077 206 100

Peptide Information

Calc. Mass	Obsrv. Mass	± da	± ppm	Start Seq.	End Seq.	Sequence	Ion Score	C. I. %	Modification	Rank	Result Type
816.421	816.4315	0.0105	13	18	24	LAEQAER					Mascot
818.444	818.4407	-0.0033	-4	103	109	ICDGILK			Carbamidomethyl (C)[2]		Mascot
907.5247	907.5327	0.008	9	50	57	NLLSVAYK					Mascot
917.5302	917.538	0.0078	9	69	76	IISSIEQK					Mascot
917.5302	917.538	0.0078	9	69	76	IISSIEQK	25	74.792			Mascot
932.4294	932.4701	0.0407	44	131	137	MKGDYYR					Mascot
948.4244	948.4344	0.01	11	131	137	MKGDYYR			Oxidation (M)[1]		Mascot

948.4244	948.4344	0.01	11	131	137	MKGDYYR	15	0	Oxidation (M)[1]		Mascot
999.4451	999.4573	0.0122	12	10	17	EENVYMAK			Oxidation (M)[6]		Mascot
1091.4712	1091.5339	0.0627	57	77	85	EESRGNEDR					Mascot
1144.6321	1144.6542	0.0221	19	81	90	GNEDRVTLIK					Mascot
1189.6609	1189.678	0.0171	14	223	232	DSTLIMQLLR					Mascot
1205.6559	1205.6694	0.0135	11	223	232	DSTLIMQLLR			Oxidation (M)[6]		Mascot
1205.6559	1205.6694	0.0135	11	223	232	DSTLIMQLLR	18	0	Oxidation (M)[6]		Mascot
1212.5565	1212.6193	0.0628	52	150	160	DAAENTMVAYK					Mascot
1228.5514	1228.6051	0.0537	44	150	160	DAAENTMVAYK			Oxidation (M)[7]		Mascot
1366.5542	1366.5502	-0.004	-3	25	34	YEEMVEFMEK			Oxidation (M)[4,8]		Mascot
1406.6646	1406.6904	0.0258	18	38	49	TVDSEELTVEER					Mascot
1406.6646	1406.6904	0.0258	18	38	49	TVDSEELTVEER	101	100			Mascot
1418.7485	1418.777	0.0285	20	69	80	IISSIEQKEESR					Mascot
1418.7485	1418.777	0.0285	20	69	80	IISSIEQKEESR	33	95.996			Mascot
1827.0123	1827.0242	0.0119	7	161	177	AAQEIALAELPPTHPIR					Mascot
2163.9573	2163.9636	0.0063	3	18	34	LAEQAERYEEMVEFMEK			Oxidation (M)[11,15]		Mascot
2163.9573	2163.9636	0.0063	3	18	34	LAEQAERYEEMVEFMEK	13	0	Oxidation (M)[11,15]		Mascot
2331.2595	2331.2488	-0.0107	-5	110	130	LLDSHLVPSSTAPESKVF YLK					Mascot

5 14-3-3 protein [Triticum aestivum] gi|431822518 29843.9 4.71 16 289 100 23.348 206 100

Peptide Information

Calc. Mass	Obsrv. Mass	± da	± ppm	Start Seq.	End Seq.	Sequence	Ion Score	C. I. %	Modification	Rank	Result Type
816.421	816.4315	0.0105	13	18	24	LAEQAER					Mascot
818.444	818.4407	-0.0033	-4	103	109	ICDGILK			Carbamidomethyl (C)[2]		Mascot
907.5247	907.5327	0.008	9	50	57	NLLSVAYK					Mascot
917.5302	917.538	0.0078	9	69	76	IISSIEQK					Mascot
917.5302	917.538	0.0078	9	69	76	IISSIEQK	25	74.792			Mascot
932.4294	932.4701	0.0407	44	131	137	MKGDYYR					Mascot
948.4244	948.4344	0.01	11	131	137	MKGDYYR			Oxidation (M)[1]		Mascot
948.4244	948.4344	0.01	11	131	137	MKGDYYR	15	0	Oxidation (M)[1]		Mascot
999.4451	999.4573	0.0122	12	10	17	EENVYMAK			Oxidation (M)[6]		Mascot
1059.5946	1059.616	0.0214	20	169	177	ELPPTHPIR					Mascot
1091.4712	1091.5339	0.0627	57	77	85	EESRGNEDR					Mascot
1144.6321	1144.6542	0.0221	19	81	90	GNEDRVTLIK					Mascot
1189.6609	1189.678	0.0171	14	223	232	DSTLIMQLLR					Mascot

1205.6559	1205.6694	0.0135	11	223	232	DSTLIMQLLR			Oxidation (M)[6]		Mascot
1205.6559	1205.6694	0.0135	11	223	232	DSTLIMQLLR	18	0	Oxidation (M)[6]		Mascot
1212.5565	1212.6193	0.0628	52	150	160	DAAENTMVAYK					Mascot
1228.5514	1228.6051	0.0537	44	150	160	DAAENTMVAYK			Oxidation (M)[7]		Mascot
1366.5542	1366.5502	-0.004	-3	25	34	YEEMVEFMEK			Oxidation (M)[4,8]		Mascot
1406.6646	1406.6904	0.0258	18	38	49	TVDSEELTVEER					Mascot
1406.6646	1406.6904	0.0258	18	38	49	TVDSEELTVEER	101	100			Mascot
1418.7485	1418.777	0.0285	20	69	80	IISSIEQKEESR					Mascot
1418.7485	1418.777	0.0285	20	69	80	IISSIEQKEESR	33	95.996			Mascot
2163.9573	2163.9636	0.0063	3	18	34	LAEQAERYEEMVEFMEK			Oxidation (M)[11,15]		Mascot
2163.9573	2163.9636	0.0063	3	18	34	LAEQAERYEEMVEFMEK	13	0	Oxidation (M)[11,15]		Mascot
2331.2595	2331.2488	-0.0107	-5	110	130	LLDSHLVPSSTAPESKVFYLK					Mascot

6 unnamed protein product [Triticum aestivum] gi|227473229 29360.7 4.83 13 154 100 13.784 90 100

Protein Group

14-3-3 protein [Triticum aestivum]	gi|431822520	29360.7	4.82999992370605

Peptide Information

Calc. Mass	Obsrv. Mass	± da	± ppm	Start Seq.	End Seq.	Sequence	Ion Score	C. I. %	Modification	Rank	Result Type
816.421	816.4315	0.0105	13	17	23	LAEQAER					Mascot
818.444	818.4407	-0.0033	-4	102	108	ICDGILK			Carbamidomethyl (C)[2]		Mascot
819.4458	819.4365	-0.0093	-11	95	101	IETELSK					Mascot
907.5247	907.5327	0.008	9	49	56	NLLSVAYK					Mascot
917.5302	917.538	0.0078	9	68	75	IISSIEQK					Mascot
917.5302	917.538	0.0078	9	68	75	IISSIEQK	25	74.792			Mascot
999.4451	999.4573	0.0122	12	9	16	EENVYMAK			Oxidation (M)[6]		Mascot
1189.6609	1189.678	0.0171	14	222	231	DSTLIMQLLR					Mascot
1205.6559	1205.6694	0.0135	11	222	231	DSTLIMQLLR			Oxidation (M)[6]		Mascot
1205.6559	1205.6694	0.0135	11	222	231	DSTLIMQLLR	18	0	Oxidation (M)[6]		Mascot
1366.5542	1366.5502	-0.004	-3	24	33	YEEMVEFMEK			Oxidation (M)[4,8]		Mascot
1418.7485	1418.777	0.0285	20	68	79	IISSIEQKEESR					Mascot
1418.7485	1418.777	0.0285	20	68	79	IISSIEQKEESR	33	95.996			Mascot
1818.9708	1818.988	0.0172	9	160	176	SAQDIALADLPTTHPIR					Mascot
1846.8309	1846.9272	0.0963	52	1	16	MSTAEATREENVYMAK			Oxidation (M)[1]		Mascot
2163.9573	2163.9636	0.0063	3	17	33	LAEQAERYEEMVEFMEK			Oxidation (M)[11,15]		Mascot
2163.9573	2163.9636	0.0063	3	17	33	LAEQAERYEEMVEFMEK	13	0	Oxidation (M)[11,15]		Mascot

2331.2019	2331.2488	0.0469	20	177	196	LGLALNFSVFYYEILNSPD R						Mascot

7	unnamed protein product [Triticum aestivum]	gi|227471938	29387.7	4.83	12	147	100	13.69	90	100

Protein Group

14-3-3 protein [Triticum aestivum]	gi|40781605	29387.7	4.8299 999237 0605

Peptide Information

Calc. Mass	Obsrv. Mass	± da	± ppm	Start Seq.	End Seq.	Sequence	Ion Score	C. I. %	Modification	Rank	Result Type
816.421	816.4315	0.0105	13	17	23	LAEQAER					Mascot
818.444	818.4407	-0.0033	-4	102	108	ICDGILK			Carbamidomethyl (C)[2]		Mascot
907.5247	907.5327	0.008	9	49	56	NLLSVAYK					Mascot
917.5302	917.538	0.0078	9	68	75	IISSIEQK					Mascot
917.5302	917.538	0.0078	9	68	75	IISSIEQK	25	74.792			Mascot
999.4451	999.4573	0.0122	12	9	16	EENVYMAK			Oxidation (M)[6]		Mascot
1189.6609	1189.678	0.0171	14	222	231	DSTLIMQLLR					Mascot
1205.6559	1205.6694	0.0135	11	222	231	DSTLIMQLLR			Oxidation (M)[6]		Mascot
1205.6559	1205.6694	0.0135	11	222	231	DSTLIMQLLR	18	0	Oxidation (M)[6]		Mascot
1366.5542	1366.5502	-0.004	-3	24	33	YEEMVEFMEK			Oxidation (M)[4,8]		Mascot
1418.7485	1418.777	0.0285	20	68	79	IISSIEQKEESR					Mascot
1418.7485	1418.777	0.0285	20	68	79	IISSIEQKEESR	33	95.996			Mascot
1818.9708	1818.988	0.0172	9	160	176	SAQDIALADLPTTHPIR					Mascot
1846.8309	1846.9272	0.0963	52	1	16	MSTAEATREENVYMAK			Oxidation (M)[1]		Mascot
2163.9573	2163.9636	0.0063	3	17	33	LAEQAERYEEMVEFMEK			Oxidation (M)[11,15]		Mascot
2163.9573	2163.9636	0.0063	3	17	33	LAEQAERYEEMVEFMEK	13	0	Oxidation (M)[11,15]		Mascot
2331.2019	2331.2488	0.0469	20	177	196	LGLALNFSVFYYEILNSPD R					Mascot

8	unnamed protein product [Triticum aestivum]	gi|227472078	29490.6	4.75	10	87	99.991	9.482	43	99.573

Protein Group

TaWIN1 [Triticum aestivum]	gi|9798603	29490.6	4.75
unnamed protein product [Triticum aestivum]	gi|219911926	29490.6	4.75
unnamed protein product [Triticum aestivum]	gi|257664808	29490.6	4.75

Peptide Information

Calc. Mass	Obsrv. Mass	± da	± ppm	Start	End	Sequence	Ion	C. I. %	Modification	Rank	Result Type

				Seq.	Seq.		Score				
816.421	816.4315	0.0105	13	17	23	LAEQAER					Mascot
907.5247	907.5327	0.008	9	53	60	NLLSVAYK					Mascot
917.5302	917.538	0.0078	9	72	79	IISSIEQK					Mascot
917.5302	917.538	0.0078	9	72	79	IISSIEQK	25	74.792			Mascot
1189.6609	1189.678	0.0171	14	226	235	DSTLIMQLLR					Mascot
1205.6559	1205.6694	0.0135	11	226	235	DSTLIMQLLR			Oxidation (M)[6]		Mascot
1205.6559	1205.6694	0.0135	11	226	235	DSTLIMQLLR	18	0	Oxidation (M)[6]		Mascot
1388.738	1388.7384	0.0004	0	72	83	IISSIEQKEEGR					Mascot
1394.5603	1394.5781	0.0178	13	24	33	YEEMVEFMER			Oxidation (M)[4,8]		Mascot
1558.7344	1558.7654	0.031	20	37	52	ATGGAGPGEELSVEER					Mascot
1772.9653	1772.9893	0.024	14	164	180	AAQDIALADLAPTHPIR					Mascot
2191.9634	2191.9998	0.0364	17	17	33	LAEQAERYEEMVEFMER			Oxidation (M)[11,15]		Mascot
2331.2019	2331.2488	0.0469	20	181	200	LGLALNFSVFYYEILNSPDR					Mascot

9	unnamed protein product [Triticum aestivum]	gi|227473233	29549.7	4.75	10	87	99.991	9.622	43	99.573	

Protein Group

unnamed protein product [Triticum aestivum]	gi|257710885	29549.7	4.75

Peptide Information

Calc. Mass	Obsrv. Mass	± da	± ppm	Start Seq.	End Seq.	Sequence	Ion Score	C. I. %	Modification	Rank	Result Type
816.421	816.4315	0.0105	13	17	23	LAEQAER					Mascot
907.5247	907.5327	0.008	9	53	60	NLLSVAYK					Mascot
917.5302	917.538	0.0078	9	72	79	IISSIEQK					Mascot
917.5302	917.538	0.0078	9	72	79	IISSIEQK	25	74.792			Mascot
1189.6609	1189.678	0.0171	14	226	235	DSTLIMQLLR					Mascot
1205.6559	1205.6694	0.0135	11	226	235	DSTLIMQLLR			Oxidation (M)[6]		Mascot
1205.6559	1205.6694	0.0135	11	226	235	DSTLIMQLLR	18	0	Oxidation (M)[6]		Mascot
1388.738	1388.7384	0.0004	0	72	83	IISSIEQKEEGR					Mascot
1394.5603	1394.5781	0.0178	13	24	33	YEEMVEFMER			Oxidation (M)[4,8]		Mascot
1558.7344	1558.7654	0.031	20	37	52	ATGGAGPGEELSVEER					Mascot
1800.9967	1801.0056	0.0089	5	164	180	AAQDIALVDLAPTHPIR					Mascot
2191.9634	2191.9998	0.0364	17	17	33	LAEQAERYEEMVEFMER			Oxidation (M)[11,15]		Mascot
2331.2019	2331.2488	0.0469	20	181	200	LGLALNFSVFYYEILNSPDR					Mascot

10	14-3-3-like protein A [Triticum urartu]	gi|474293618	28778.5	5.2	11	84	99.982	11.641	31	93.415	

Peptide Information

Calc. Mass	Obsrv. Mass	± da	± ppm	Start Seq.	End Seq.	Sequence	Ion Score	C. I. %	Modification	Rank	Result Type
816.421	816.4315	0.0105	13	17	23	LAEQAER					Mascot
819.4505	819.4365	-0.014	-17	1	8	MAKAAATR					Mascot
907.5247	907.5327	0.008	9	49	56	NLLSVAYK					Mascot
1059.5946	1059.616	0.0214	20	168	176	ELPPTHPIR					Mascot
1189.6609	1189.678	0.0171	14	222	231	DSTLIMQLLR					Mascot
1205.6559	1205.6694	0.0135	11	222	231	DSTLIMQLLR			Oxidation (M)[6]		Mascot
1205.6559	1205.6694	0.0135	11	222	231	DSTLIMQLLR	18	0	Oxidation (M)[6]		Mascot
1228.6294	1228.6051	-0.0243	-20	80	92	GAAGHAAAARGYR					Mascot
1323.6512	1323.6851	0.0339	26	76	89	EEGRGAAGHAAAAR					Mascot
1366.5542	1366.5502	-0.004	-3	24	33	YEEMVEFMEK			Oxidation (M)[4,8]		Mascot
2163.9573	2163.9636	0.0063	3	17	33	LAEQAERYEEMVEFMEK			Oxidation (M)[11,15]		Mascot
2163.9573	2163.9636	0.0063	3	17	33	LAEQAERYEEMVEFMEK	13	0	Oxidation (M)[11,15]		Mascot
2331.2019	2331.2488	0.0469	20	177	196	LGLALNFSVFYYEILNSPD R					Mascot
2355.27	2355.1184	-0.1516	-64	93	113	ALVEAELSNICAGILRLLD ER			Carbamidomethyl (C)[11]		Mascot

Gel Idx/Pos	170/G21	Instr./Gel Origin	BA2151/Sample Project 20140814	Process Status	Analysis Succeeded
Plate [#] Name	[1] Sample Project 20140814	Instrument Sample Name		Spectra	11

Rank	Protein Name	Accession No.	Protein MW	Protein PI	Pep. Count	Protein Score	Protein Score C. I. %	Intensity Matched	Total Ion Score	Total Ion C. I. %	Confirmed
1	14-3-3 protein [Triticum aestivum]	gi|431822518	29843.9	4.71	15	134	100	16.02	38	99.06	

Peptide Information

Calc. Mass	Obsrv. Mass	± da	± ppm	Start Seq.	End Seq.	Sequence	Ion Score	C. I. %	Modification	Rank	Result Type
816.421	816.4322	0.0112	14	18	24	LAEQAER					Mascot
818.444	818.4481	0.0041	5	103	109	ICDGILK			Carbamidomethyl (C)[2]		Mascot
843.4934	843.517	0.0236	28	161	168	AAQEIALK					Mascot
907.5247	907.5406	0.0159	18	50	57	NLLSVAYK					Mascot
917.5302	917.5402	0.01	11	69	76	IISSIEQK					Mascot
932.4294	932.4441	0.0147	16	131	137	MKGDYYR					Mascot
948.4244	948.4377	0.0133	14	131	137	MKGDYYR			Oxidation (M)[1]		Mascot
999.4451	999.462	0.0169	17	10	17	EENVYMAK			Oxidation (M)[6]		Mascot
1016.5986	1016.6001	0.0015	1	94	102	GKIEVELTK					Mascot
1059.5946	1059.6146	0.02	19	169	177	ELPPTHPIR					Mascot
1059.5946	1059.6146	0.02	19	169	177	ELPPTHPIR	38	99.06			Mascot
1189.6609	1189.6863	0.0254	21	223	232	DSTLIMQLLR					Mascot
1189.6609	1189.6863	0.0254	21	223	232	DSTLIMQLLR					Mascot
1205.6559	1205.6742	0.0183	15	223	232	DSTLIMQLLR			Oxidation (M)[6]		Mascot
1366.5542	1366.5648	0.0106	8	25	34	YEEMVEFMEK			Oxidation (M)[4,8]		Mascot
1406.6646	1406.691	0.0264	19	38	49	TVDSEELTVEER					Mascot
1406.6646	1406.691	0.0264	19	38	49	TVDSEELTVEER					Mascot
1418.7485	1418.7678	0.0193	14	69	80	IISSIEQKEESR					Mascot
2131.9675	2132.0005	0.033	15	18	34	LAEQAERYEEMVEFMEK					Mascot
2163.9573	2163.9697	0.0124	6	18	34	LAEQAERYEEMVEFMEK			Oxidation (M)[11,15]		Mascot
2331.2019	2331.2139	0.012	5	178	197	LGLALNFSVFYYEILNSPDR					Mascot

Rank	Protein Name	Accession No.	Protein MW	Protein PI	Pep. Count	Protein Score	Protein Score C. I. %	Intensity Matched	Total Ion Score	Total Ion C. I. %	Confirmed
2	14-3-3-like protein B [Triticum urartu]	gi|474253094	29786.9	4.67	14	87	99.991	11.057			

Peptide Information

Calc. Mass	Obsrv. Mass	± da	± ppm	Start Seq.	End Seq.	Sequence	Ion Score	C. I. %	Modification	Rank	Result Type
816.421	816.4322	0.0112	14	18	24	LAEQAER					Mascot

818.444	818.4481	0.0041	5	103	109	ICDGILK			Carbamidomethyl (C)[2]		Mascot
907.5247	907.5406	0.0159	18	50	57	NLLSVAYK					Mascot
917.5302	917.5402	0.01	11	69	76	IISSIEQK					Mascot
932.4294	932.4441	0.0147	16	131	137	MKGDYYR					Mascot
948.4244	948.4377	0.0133	14	131	137	MKGDYYR			Oxidation (M)[1]		Mascot
999.4451	999.462	0.0169	17	10	17	EENVYMAK			Oxidation (M)[6]		Mascot
1016.5986	1016.6001	0.0015	1	94	102	GKIEVELTK					Mascot
1189.6609	1189.6863	0.0254	21	223	232	DSTLIMQLLR					Mascot
1189.6609	1189.6863	0.0254	21	223	232	DSTLIMQLLR					Mascot
1205.6559	1205.6742	0.0183	15	223	232	DSTLIMQLLR			Oxidation (M)[6]		Mascot
1366.5542	1366.5648	0.0106	8	25	34	YEEMVEFMEK			Oxidation (M)[4,8]		Mascot
1406.6646	1406.691	0.0264	19	38	49	TVDSEELTVEER					Mascot
1406.6646	1406.691	0.0264	19	38	49	TVDSEELTVEER					Mascot
1418.7485	1418.7678	0.0193	14	69	80	IISSIEQKEESR					Mascot
1827.0123	1827.0411	0.0288	16	161	177	AAQEIALAELPPTHPIR					Mascot
2131.9675	2132.0005	0.033	15	18	34	LAEQAERYEEMVEFMEK					Mascot
2163.9573	2163.9697	0.0124	6	18	34	LAEQAERYEEMVEFMEK			Oxidation (M)[11,15]		Mascot
2331.2019	2331.2139	0.012	5	178	197	LGLALNFSVFYYEILNSPDR					Mascot

3 14-3-3 protein [Triticum aestivum] gi|390195996 30066.1 4.73 13 76 99.894 10.837

Protein Group

14-3-3 protein [Triticum aestivum]	gi|351602255	30066.1	4.7300000190 7349
14-3-3-like protein GF14-B [Triticum urartu]	gi|474147722	30043.1	4.6900000572 2046

Peptide Information

Calc. Mass	Obsrv. Mass	± da	± ppm	Start Seq.	End Seq.	Sequence	Ion Score	C. I. %	Modification	Rank	Result Type
816.421	816.4322	0.0112	14	18	24	LAEQAER					Mascot
818.444	818.4481	0.0041	5	103	109	ICDGILK			Carbamidomethyl (C)[2]		Mascot
844.4523	844.4928	0.0405	48	2	9	TAPAELSR					Mascot
907.5247	907.5406	0.0159	18	50	57	NLLSVAYK					Mascot
917.5302	917.5402	0.01	11	69	76	IISSIEQK					Mascot
932.4294	932.4441	0.0147	16	131	137	MKGDYYR					Mascot
948.4244	948.4377	0.0133	14	131	137	MKGDYYR			Oxidation (M)[1]		Mascot
999.4451	999.462	0.0169	17	10	17	EENVYMAK			Oxidation (M)[6]		Mascot
1189.6609	1189.6863	0.0254	21	223	232	DSTLIMQLLR					Mascot

Calc. Mass	Obsrv. Mass	± da	± ppm	Start Seq.	End Seq.	Sequence	Ion Score	C. I. %	Modification	Rank	Result Type
1189.6609	1189.6863	0.0254	21	223	232	DSTLIMQLLR					Mascot
1205.6559	1205.6742	0.0183	15	223	232	DSTLIMQLLR			Oxidation (M)[6]		Mascot
1366.5542	1366.5648	0.0106	8	25	34	YEEMVEFMEK			Oxidation (M)[4,8]		Mascot
1406.6646	1406.691	0.0264	19	38	49	TVDSEELTVEER					Mascot
1406.6646	1406.691	0.0264	19	38	49	TVDSEELTVEER					Mascot
1418.7485	1418.7678	0.0193	14	69	80	IISSIEQKEESR					Mascot
2131.9675	2132.0005	0.033	15	18	34	LAEQAERYEEMVEFMEK					Mascot
2163.9573	2163.9697	0.0124	6	18	34	LAEQAERYEEMVEFMEK			Oxidation (M)[11,15]		Mascot
2331.2019	2331.2139	0.012	5	178	197	LGLALNFSVFYYEILNSPDR					Mascot

4	14-3-3-like protein A [Triticum urartu]	gi|474293618	28778.5	5.2	7	72	99.721	10.674	38	99.06	

Peptide Information

Calc. Mass	Obsrv. Mass	± da	± ppm	Start Seq.	End Seq.	Sequence	Ion Score	C. I. %	Modification	Rank	Result Type
816.421	816.4322	0.0112	14	17	23	LAEQAER					Mascot
907.5247	907.5406	0.0159	18	49	56	NLLSVAYK					Mascot
1059.5946	1059.6146	0.02	19	168	176	ELPPTHPIR					Mascot
1059.5946	1059.6146	0.02	19	168	176	ELPPTHPIR	38	99.06			Mascot
1189.6609	1189.6863	0.0254	21	222	231	DSTLIMQLLR					Mascot
1189.6609	1189.6863	0.0254	21	222	231	DSTLIMQLLR					Mascot
1205.6559	1205.6742	0.0183	15	222	231	DSTLIMQLLR			Oxidation (M)[6]		Mascot
1366.5542	1366.5648	0.0106	8	24	33	YEEMVEFMEK			Oxidation (M)[4,8]		Mascot
2131.9675	2132.0005	0.033	15	17	33	LAEQAERYEEMVEFMEK					Mascot
2163.9573	2163.9697	0.0124	6	17	33	LAEQAERYEEMVEFMEK			Oxidation (M)[11,15]		Mascot
2331.2019	2331.2139	0.012	5	177	196	LGLALNFSVFYYEILNSPDR					Mascot

5	unnamed protein product [Triticum aestivum]	gi|227472076	28794.4	4.8	7	70	99.578	10.674	38	99.06	

Protein Group

TaWIN2 [Triticum aestivum]	gi|9798605	28794.4	4.8000 001907 3486
unnamed protein product [Triticum aestivum]	gi|219911924	28794.4	4.8000 001907 3486
unnamed protein product [Triticum aestivum]	gi|257664806	28794.4	4.8000 001907 3486

Peptide Information

Calc. Mass	Obsrv. Mass	± da	± ppm	Start Seq.	End Seq.	Sequence	Ion Score	C. I. %	Modification	Rank	Result Type

816.421	816.4322	0.0112	14	17	23	LAEQAER					Mascot
907.5247	907.5406	0.0159	18	49	56	NLLSVAYK					Mascot
1059.5946	1059.6146	0.02	19	168	176	ELPPTHPIR					Mascot
1059.5946	1059.6146	0.02	19	168	176	ELPPTHPIR	38	99.06			Mascot
1189.6609	1189.6863	0.0254	21	222	231	DSTLIMQLLR					Mascot
1189.6609	1189.6863	0.0254	21	222	231	DSTLIMQLLR					Mascot
1205.6559	1205.6742	0.0183	15	222	231	DSTLIMQLLR			Oxidation (M)[6]		Mascot
1366.5542	1366.5648	0.0106	8	24	33	YEEMVEFMEK			Oxidation (M)[4,8]		Mascot
2131.9675	2132.0005	0.033	15	17	33	LAEQAERYEEMVEFMEK					Mascot
2163.9573	2163.9697	0.0124	6	17	33	LAEQAERYEEMVEFMEK			Oxidation (M)[11,15]		Mascot
2331.2019	2331.2139	0.012	5	177	196	LGLALNFSVFYYEILNSPD R					Mascot

6	unnamed protein product [Triticum aestivum]	gi|227473231	28898.4	4.8	7	69	99.468	10.674	38	99.06

Protein Group

unnamed protein product [Triticum aestivum]	gi|257710883	28898.4	4.8000 001907 3486

Peptide Information

Calc. Mass	Obsrv. Mass	± da	± ppm	Start Seq.	End Seq.	Sequence	Ion Score	C. I. %	Modification	Rank	Result Type
816.421	816.4322	0.0112	14	19	25	LAEQAER					Mascot
907.5247	907.5406	0.0159	18	51	58	NLLSVAYK					Mascot
1059.5946	1059.6146	0.02	19	170	178	ELPPTHPIR					Mascot
1059.5946	1059.6146	0.02	19	170	178	ELPPTHPIR	38	99.06			Mascot
1189.6609	1189.6863	0.0254	21	224	233	DSTLIMQLLR					Mascot
1189.6609	1189.6863	0.0254	21	224	233	DSTLIMQLLR					Mascot
1205.6559	1205.6742	0.0183	15	224	233	DSTLIMQLLR			Oxidation (M)[6]		Mascot
1366.5542	1366.5648	0.0106	8	26	35	YEEMVEFMEK			Oxidation (M)[4,8]		Mascot
2131.9675	2132.0005	0.033	15	19	35	LAEQAERYEEMVEFMEK					Mascot
2163.9573	2163.9697	0.0124	6	19	35	LAEQAERYEEMVEFMEK			Oxidation (M)[11,15]		Mascot
2331.2019	2331.2139	0.012	5	179	198	LGLALNFSVFYYEILNSPD R					Mascot

7	unnamed protein product [Triticum aestivum]	gi|257664756	29983	4.62	12	65	98.602	10.566

Protein Group

14-3-3 protein [Triticum aestivum]	gi|52548256	29983	4.6199 998855 5908
unnamed protein product [Triticum aestivum]	gi|227471994	29983	4.6199

unnamed protein product [Triticum aestivum]	gi|219911698	29983	998855 5908 4.6199 998855 5908

Peptide Information

Calc. Mass	Obsrv. Mass	± da	± ppm	Start Seq.	End Seq.	Sequence	Ion Score	C. I. %	Modification	Rank	Result Type
816.421	816.4322	0.0112	14	18	24	LAEQAER					Mascot
818.444	818.4481	0.0041	5	103	109	ICDGILK			Carbamidomethyl (C)[2]		Mascot
844.4523	844.4928	0.0405	48	2	9	TAPAELSR					Mascot
907.5247	907.5406	0.0159	18	50	57	NLLSVAYK					Mascot
917.5302	917.5402	0.01	11	69	76	IISSIEQK					Mascot
932.4294	932.4441	0.0147	16	131	137	MKGDYYR					Mascot
948.4244	948.4377	0.0133	14	131	137	MKGDYYR			Oxidation (M)[1]		Mascot
999.4451	999.462	0.0169	17	10	17	EENVYMAK			Oxidation (M)[6]		Mascot
1189.6609	1189.6863	0.0254	21	223	232	DSTLIMQLLR					Mascot
1189.6609	1189.6863	0.0254	21	223	232	DSTLIMQLLR					Mascot
1205.6559	1205.6742	0.0183	15	223	232	DSTLIMQLLR			Oxidation (M)[6]		Mascot
1366.5542	1366.5648	0.0106	8	25	34	YEEMVEFMEK			Oxidation (M)[4,8]		Mascot
1406.6646	1406.691	0.0264	19	38	49	TVDSEELTVEER					Mascot
1406.6646	1406.691	0.0264	19	38	49	TVDSEELTVEER					Mascot
1418.7485	1418.7678	0.0193	14	69	80	IISSIEQKKEESR					Mascot
2131.9675	2132.0005	0.033	15	18	34	LAEQAERYEEMVEFMEK					Mascot
2163.9573	2163.9697	0.0124	6	18	34	LAEQAERYEEMVEFMEK			Oxidation (M)[11,15]		Mascot

8	unnamed protein product [Triticum aestivum]	gi|257664788	28475.3	4.78	11	56	88.892	10.454

Protein Group

14-3-3 protein [Triticum aestivum]	gi|32401388	28475.3	4.7800 002098 0835
unnamed protein product [Triticum aestivum]	gi|227472036	28475.3	4.7800 002098 0835
unnamed protein product [Triticum aestivum]	gi|219911730	28475.3	4.7800 002098 0835

Peptide Information

Calc. Mass	Obsrv. Mass	± da	± ppm	Start Seq.	End Seq.	Sequence	Ion Score	C. I. %	Modification	Rank	Result Type
816.421	816.4322	0.0112	14	4	10	LAEQAER					Mascot

818.444	818.4481	0.0041	5	89	95	ICDGILK			Carbamidomethyl (C)[2]		Mascot
907.5247	907.5406	0.0159	18	36	43	NLLSVAYK					Mascot
917.5302	917.5402	0.01	11	55	62	IISSIEQK					Mascot
932.4294	932.4441	0.0147	16	117	123	MKGDYYR					Mascot
948.4244	948.4377	0.0133	14	117	123	MKGDYYR			Oxidation (M)[1]		Mascot
1189.6609	1189.6863	0.0254	21	209	218	DSTLIMQLLR					Mascot
1189.6609	1189.6863	0.0254	21	209	218	DSTLIMQLLR					Mascot
1205.6559	1205.6742	0.0183	15	209	218	DSTLIMQLLR			Oxidation (M)[6]		Mascot
1366.5542	1366.5648	0.0106	8	11	20	YEEMVEFMEK			Oxidation (M)[4,8]		Mascot
1406.6646	1406.691	0.0264	19	24	35	TVDSEELTVEER					Mascot
1406.6646	1406.691	0.0264	19	24	35	TVDSEELTVEER					Mascot
1418.7485	1418.7678	0.0193	14	55	66	IISSIEQKEESR					Mascot
2131.9675	2132.0005	0.033	15	4	20	LAEQAERYEEMVEFMEK					Mascot
2163.9573	2163.9697	0.0124	6	4	20	LAEQAERYEEMVEFMEK			Oxidation (M)[11,15]		Mascot
2331.2019	2331.2139	0.012	5	164	183	LGLALNFSVFYYEILNSPDR					Mascot

9	14-3-3 protein [Triticum aestivum]	gi|40781605	29387.7	4.83	10	52	74.553	8.216	

Protein Group

14-3-3 protein [Triticum aestivum]	gi|431822520	29360.7	4.82999992370605
unnamed protein product [Triticum aestivum]	gi|227473229	29360.7	4.82999992370605
unnamed protein product [Triticum aestivum]	gi|227471938	29387.7	4.82999992370605

Peptide Information

Calc. Mass	Obsrv. Mass	± da	± ppm	Start Seq.	End Seq.	Sequence	Ion Score	C. I. %	Modification	Rank	Result Type
816.421	816.4322	0.0112	14	17	23	LAEQAER					Mascot
818.444	818.4481	0.0041	5	102	108	ICDGILK			Carbamidomethyl (C)[2]		Mascot
907.5247	907.5406	0.0159	18	49	56	NLLSVAYK					Mascot
917.5302	917.5402	0.01	11	68	75	IISSIEQK					Mascot
999.4451	999.462	0.0169	17	9	16	EENVYMAK			Oxidation (M)[6]		Mascot
1189.6609	1189.6863	0.0254	21	222	231	DSTLIMQLLR					Mascot
1189.6609	1189.6863	0.0254	21	222	231	DSTLIMQLLR					Mascot
1205.6559	1205.6742	0.0183	15	222	231	DSTLIMQLLR			Oxidation (M)[6]		Mascot
1366.5542	1366.5648	0.0106	8	24	33	YEEMVEFMEK			Oxidation (M)[4,8]		Mascot
1418.7485	1418.7678	0.0193	14	68	79	IISSIEQKEESR					Mascot

2131.9675	2132.0005	0.033	15	17	33	LAEQAERYEEMVEFMEK						Mascot
2163.9573	2163.9697	0.0124	6	17	33	LAEQAERYEEMVEFMEK				Oxidation (M)[11,15]		Mascot
2331.2019	2331.2139	0.012	5	177	196	LGLALNFSVFYYEILNSPD R						Mascot

10	hypothetical protein TRIUR3_13999 [Triticum urartu]	gi|473799582	36319.3	5.65	2	33	0	4.195	27	88.746

Peptide Information

Calc. Mass	Obsrv. Mass	± da	± ppm	Start Seq.	End Seq.	Sequence	Ion Score	C. I. %	Modification	Rank	Result Type
913.5101	913.4936	-0.0165	-18	198	205	KLPAEEAR					Mascot
913.5101	913.4936	-0.0165	-18	198	205	KLPAEEAR	27	88.746			Mascot
937.4044	937.4534	0.049	52	1	9	MAETGDASR					Mascot

Gel Idx/Pos	171/G22	Instr./Gel Origin	BA2151/Sample Project 20140814	Process Status	Analysis Succeeded
Plate [#] Name	[1] Sample Project 20140814	Instrument Sample Name		Spectra	11

Rank	Protein Name	Accession No.	Protein MW	Protein PI	Pep. Count	Protein Score	Protein Score C. I. %	Intensity Matched	Total Ion Score	Total Ion C. I. %	Confirmed
1	14-3-3-like protein B [Triticum urartu]	gi|474253094	29786.9	4.67	22	416	100	72.463	263	100	

Peptide Information

Calc. Mass	Obsrv. Mass	± da	± ppm	Start Seq.	End Seq.	Sequence	Ion Score	C. I. %	Modification	Rank	Result Type
816.421	816.4395	0.0185	23	18	24	LAEQAER					Mascot
831.4822	831.5016	0.0194	23	96	102	IEVELTK					Mascot
907.5247	907.5403	0.0156	17	50	57	NLLSVAYK					Mascot
917.5302	917.5472	0.017	19	69	76	IISSIEQK					Mascot
932.4294	932.4494	0.02	21	131	137	MKGDYYR					Mascot
948.4244	948.4431	0.0187	20	131	137	MKGDYYR			Oxidation (M)[1]		Mascot
948.4244	948.4431	0.0187	20	131	137	MKGDYYR	15	0	Oxidation (M)[1]		Mascot
999.4451	999.4665	0.0214	21	10	17	EENVYMAK			Oxidation (M)[6]		Mascot
1016.5986	1016.6107	0.0121	12	94	102	GKIEVELTK					Mascot
1091.4712	1091.5564	0.0852	78	77	85	EESRGNEDR					Mascot
1189.6609	1189.6909	0.03	25	223	232	DSTLIMQLLR					Mascot
1205.6559	1205.6827	0.0268	22	223	232	DSTLIMQLLR			Oxidation (M)[6]		Mascot
1205.6559	1205.6827	0.0268	22	223	232	DSTLIMQLLR	32	95.48	Oxidation (M)[6]		Mascot
1212.5565	1212.6049	0.0484	40	150	160	DAAENTMVAYK					Mascot
1228.5514	1228.5693	0.0179	15	150	160	DAAENTMVAYK			Oxidation (M)[7]		Mascot
1300.6532	1300.6512	-0.002	-2	138	148	YLAEFKSGTER					Mascot
1356.6464	1356.6509	0.0045	3	149	160	KDAAENTMVAYK			Oxidation (M)[8]		Mascot
1366.5542	1366.5667	0.0125	9	25	34	YEEMVEFMEK			Oxidation (M)[4,8]		Mascot
1366.5542	1366.5667	0.0125	9	25	34	YEEMVEFMEK			Oxidation (M)[4,8]		Mascot
1406.6646	1406.7013	0.0367	26	38	49	TVDSEELTVEER					Mascot
1406.6646	1406.7013	0.0367	26	38	49	TVDSEELTVEER	102	100			Mascot
1418.7485	1418.7878	0.0393	28	69	80	IISSIEQKEESR					Mascot
1517.8799	1517.9158	0.0359	24	50	63	NLLSVAYKNVIGAR					Mascot
1680.8804	1680.9084	0.028	17	110	125	LLDSHLVPSSTAPESK					Mascot
1827.0123	1827.0615	0.0492	27	161	177	AAQEIALAELPPTHPIR					Mascot
1827.0123	1827.0615	0.0492	27	161	177	AAQEIALAELPPTHPIR	114	100			Mascot
2160.9819	2160.9971	0.0152	7	204	222	QAFDEAISELDSLSEESYK					Mascot

2163.9573	2163.9719	0.0146	7	18	34	LAEQAERYEEMVEFMEK			Oxidation (M)[11,15]		Mascot
2331.2595	2331.269	0.0095	4	110	130	LLDSHLVPSSTAPESKVF YLK					Mascot
2595.1768	2595.2207	0.0439	17	233	255	DNLTLWTSDISEDAAEEM KDAPK			Oxidation (M)[18]		Mascot

2 14-3-3 protein [Triticum aestivum] gi|431822518 29843.9 4.71 23 352 100 31.194 190 100

Peptide Information

Calc. Mass	Obsrv. Mass	± da	± ppm	Start Seq.	End Seq.	Sequence	Ion Score	C. I. %	Modification	Rank	Result Type
816.421	816.4395	0.0185	23	18	24	LAEQAER					Mascot
831.4822	831.5016	0.0194	23	96	102	IEVELTK					Mascot
907.5247	907.5403	0.0156	17	50	57	NLLSVAYK					Mascot
917.5302	917.5472	0.017	19	69	76	IISSIEQK					Mascot
932.4294	932.4494	0.02	21	131	137	MKGDYYR					Mascot
948.4244	948.4431	0.0187	20	131	137	MKGDYYR			Oxidation (M)[1]		Mascot
948.4244	948.4431	0.0187	20	131	137	MKGDYYR	15	0	Oxidation (M)[1]		Mascot
999.4451	999.4665	0.0214	21	10	17	EENVYMAK			Oxidation (M)[6]		Mascot
1016.5986	1016.6107	0.0121	12	94	102	GKIEVELTK					Mascot
1059.5946	1059.6219	0.0273	26	169	177	ELPPTHPIR					Mascot
1059.5946	1059.6219	0.0273	26	169	177	ELPPTHPIR	41	99.458			Mascot
1091.4712	1091.5564	0.0852	78	77	85	EESRGNEDR					Mascot
1189.6609	1189.6909	0.03	25	223	232	DSTLIMQLLR					Mascot
1205.6559	1205.6827	0.0268	22	223	232	DSTLIMQLLR			Oxidation (M)[6]		Mascot
1205.6559	1205.6827	0.0268	22	223	232	DSTLIMQLLR	32	95.48	Oxidation (M)[6]		Mascot
1212.5565	1212.6049	0.0484	40	150	160	DAAENTMVAYK					Mascot
1228.5514	1228.5693	0.0179	15	150	160	DAAENTMVAYK			Oxidation (M)[7]		Mascot
1300.6532	1300.6512	-0.002	-2	138	148	YLAEFKSGTER					Mascot
1356.6464	1356.6509	0.0045	3	149	160	KDAAENTMVAYK			Oxidation (M)[8]		Mascot
1366.5542	1366.5667	0.0125	9	25	34	YEEMVEFMEK			Oxidation (M)[4,8]		Mascot
1366.5542	1366.5667	0.0125	9	25	34	YEEMVEFMEK			Oxidation (M)[4,8]		Mascot
1406.6646	1406.7013	0.0367	26	38	49	TVDSEELTVEER					Mascot
1406.6646	1406.7013	0.0367	26	38	49	TVDSEELTVEER	102	100			Mascot
1418.7485	1418.7878	0.0393	28	69	80	IISSIEQKEESR					Mascot
1517.8799	1517.9158	0.0359	24	50	63	NLLSVAYKNVIGAR					Mascot
1680.8804	1680.9084	0.028	17	110	125	LLDSHLVPSSTAPESK					Mascot
1884.0702	1884.0795	0.0093	5	161	177	AAQEIALKELPPTHPIR					Mascot
2160.9819	2160.9971	0.0152	7	204	222	QAFDEAISELDSLSEESY K					Mascot

2163.9573	2163.9719	0.0146	7	18	34	LAEQAERYEEMVEFMEK			Oxidation (M)[11,15]		Mascot
2331.2595	2331.269	0.0095	4	110	130	LLDSHLVPSSTAPESKVF YLK					Mascot
2595.1768	2595.2207	0.0439	17	233	255	DNLTLWTSDISEDAAEEM KDAPK			Oxidation (M)[18]		Mascot

3	14-3-3 protein [Triticum aestivum]	gi|390195996	30066.1	4.73	17	246	100	28.463	148	100	

Protein Group

14-3-3 protein [Triticum aestivum]	gi|351602255	30066.1	4.7300 000190 7349
14-3-3-like protein GF14-B [Triticum urartu]	gi|474147722	30043.1	4.6900 000572 2046

Peptide Information

Calc. Mass	Obsrv. Mass	± da	± ppm	Start Seq.	End Seq.	Sequence	Ion Score	C. I. %	Modification	Rank	Result Type
816.421	816.4395	0.0185	23	18	24	LAEQAER					Mascot
844.4523	844.4822	0.0299	35	2	9	TAPAELSR					Mascot
907.5247	907.5403	0.0156	17	50	57	NLLSVAYK					Mascot
917.5302	917.5472	0.017	19	69	76	IISSIEQK					Mascot
932.4294	932.4494	0.02	21	131	137	MKGDYYR					Mascot
948.4244	948.4431	0.0187	20	131	137	MKGDYYR			Oxidation (M)[1]		Mascot
948.4244	948.4431	0.0187	20	131	137	MKGDYYR	15	0	Oxidation (M)[1]		Mascot
999.4451	999.4665	0.0214	21	10	17	EENVYMAK			Oxidation (M)[6]		Mascot
1091.4712	1091.5564	0.0852	78	77	85	EESRGNEDR					Mascot
1189.6609	1189.6909	0.03	25	223	232	DSTLIMQLLR					Mascot
1205.6559	1205.6827	0.0268	22	223	232	DSTLIMQLLR			Oxidation (M)[6]		Mascot
1205.6559	1205.6827	0.0268	22	223	232	DSTLIMQLLR	32	95.48	Oxidation (M)[6]		Mascot
1212.5565	1212.6049	0.0484	40	150	160	DAAENTMVAYK					Mascot
1228.5514	1228.5693	0.0179	15	150	160	DAAENTMVAYK			Oxidation (M)[7]		Mascot
1356.6464	1356.6509	0.0045	3	149	160	KDAAENTMVAYK			Oxidation (M)[8]		Mascot
1366.5542	1366.5667	0.0125	9	25	34	YEEMVEFMEK			Oxidation (M)[4,8]		Mascot
1366.5542	1366.5667	0.0125	9	25	34	YEEMVEFMEK			Oxidation (M)[4,8]		Mascot
1406.6646	1406.7013	0.0367	26	38	49	TVDSEELTVEER					Mascot
1406.6646	1406.7013	0.0367	26	38	49	TVDSEELTVEER	102	100			Mascot
1418.7485	1418.7878	0.0393	28	69	80	IISSIEQKEESR					Mascot
1517.8799	1517.9158	0.0359	24	50	63	NLLSVAYKNVIGAR					Mascot
1824.8796	1825.0389	0.1593	87	2	17	TAPAELSREENVYMAK			Oxidation (M)[14]		Mascot
2163.9573	2163.9719	0.0146	7	18	34	LAEQAERYEEMVEFMEK			Oxidation (M)[11,15]		Mascot
2331.2019	2331.269	0.0671	29	178	197	LGLALNFSVFYYEILNSPD					Mascot

R

4	unnamed protein product [Triticum aestivum]	gi|257664756	29983	4.62	16	235	100	28.041	148	100	

Protein Group

14-3-3 protein [Triticum aestivum]	gi|52548256	29983	4.6199 998855 5908
unnamed protein product [Triticum aestivum]	gi|227471994	29983	4.6199 998855 5908
unnamed protein product [Triticum aestivum]	gi|219911698	29983	4.6199 998855 5908

Peptide Information

Calc. Mass	Obsrv. Mass	± da	± ppm	Start Seq.	End Seq.	Sequence	Ion Score	C. I. %	Modification	Rank	Result Type
816.421	816.4395	0.0185	23	18	24	LAEQAER					Mascot
844.4523	844.4822	0.0299	35	2	9	TAPAELSR					Mascot
907.5247	907.5403	0.0156	17	50	57	NLLSVAYK					Mascot
917.5302	917.5472	0.017	19	69	76	IISSIEQK					Mascot
932.4294	932.4494	0.02	21	131	137	MKGDYYR					Mascot
948.4244	948.4431	0.0187	20	131	137	MKGDYYR			Oxidation (M)[1]		Mascot
948.4244	948.4431	0.0187	20	131	137	MKGDYYR	15	0	Oxidation (M)[1]		Mascot
999.4451	999.4665	0.0214	21	10	17	EENVYMAK			Oxidation (M)[6]		Mascot
1091.4712	1091.5564	0.0852	78	77	85	EESRGNEDR					Mascot
1189.6609	1189.6909	0.03	25	223	232	DSTLIMQLLR					Mascot
1205.6559	1205.6827	0.0268	22	223	232	DSTLIMQLLR			Oxidation (M)[6]		Mascot
1205.6559	1205.6827	0.0268	22	223	232	DSTLIMQLLR	32	95.48	Oxidation (M)[6]		Mascot
1229.5355	1229.6079	0.0724	59	150	160	DAAEDTMVAYK			Oxidation (M)[7]		Mascot
1357.6305	1357.703	0.0725	53	149	160	KDAAEDTMVAYK			Oxidation (M)[8]		Mascot
1366.5542	1366.5667	0.0125	9	25	34	YEEMVEFMEK			Oxidation (M)[4,8]		Mascot
1366.5542	1366.5667	0.0125	9	25	34	YEEMVEFMEK			Oxidation (M)[4,8]		Mascot
1406.6646	1406.7013	0.0367	26	38	49	TVDSEELTVEER					Mascot
1406.6646	1406.7013	0.0367	26	38	49	TVDSEELTVEER	102	100			Mascot
1418.7485	1418.7878	0.0393	28	69	80	IISSIEQKEESR					Mascot
1517.8799	1517.9158	0.0359	24	50	63	NLLSVAYKNVIGAR					Mascot
1824.8796	1825.0389	0.1593	87	2	17	TAPAELSREENVYMAK			Oxidation (M)[14]		Mascot
2163.9573	2163.9719	0.0146	7	18	34	LAEQAERYEEMVEFMEK			Oxidation (M)[11,15]		Mascot

5	unnamed protein product [Triticum aestivum]	gi|257664788	28475.3	4.78	14	216	100	27.485	148	100

Protein Group

14-3-3 protein [Triticum aestivum]	gi|32401388	28475.3	4.7800 002098 0835
unnamed protein product [Triticum aestivum]	gi|227472036	28475.3	4.7800 002098 0835
unnamed protein product [Triticum aestivum]	gi|219911730	28475.3	4.7800 002098 0835

Peptide Information

Calc. Mass	Obsrv. Mass	± da	± ppm	Start Seq.	End Seq.	Sequence	Ion Score	C. I. %	Modification	Rank	Result Type
816.421	816.4395	0.0185	23	4	10	LAEQAER					Mascot
907.5247	907.5403	0.0156	17	36	43	NLLSVAYK					Mascot
917.5302	917.5472	0.017	19	55	62	IISSIEQK					Mascot
932.4294	932.4494	0.02	21	117	123	MKGDYYR					Mascot
948.4244	948.4431	0.0187	20	117	123	MKGDYYR			Oxidation (M)[1]		Mascot
948.4244	948.4431	0.0187	20	117	123	MKGDYYR	15	0	Oxidation (M)[1]		Mascot
1091.4712	1091.5564	0.0852	78	63	71	EESRGNEDR					Mascot
1189.6609	1189.6909	0.03	25	209	218	DSTLIMQLLR					Mascot
1205.6559	1205.6827	0.0268	22	209	218	DSTLIMQLLR			Oxidation (M)[6]		Mascot
1205.6559	1205.6827	0.0268	22	209	218	DSTLIMQLLR	32	95.48	Oxidation (M)[6]		Mascot
1212.5565	1212.6049	0.0484	40	136	146	DAAENTMVAYK					Mascot
1228.5514	1228.5693	0.0179	15	136	146	DAAENTMVAYK			Oxidation (M)[7]		Mascot
1356.6464	1356.6509	0.0045	3	135	146	KDAAENTMVAYK			Oxidation (M)[8]		Mascot
1366.5542	1366.5667	0.0125	9	11	20	YEEMVEFMEK			Oxidation (M)[4,8]		Mascot
1366.5542	1366.5667	0.0125	9	11	20	YEEMVEFMEK			Oxidation (M)[4,8]		Mascot
1406.6646	1406.7013	0.0367	26	24	35	TVDSEELTVEER					Mascot
1406.6646	1406.7013	0.0367	26	24	35	TVDSEELTVEER	102	100			Mascot
1418.7485	1418.7878	0.0393	28	55	66	IISSIEQKEESR					Mascot
1517.8799	1517.9158	0.0359	24	36	49	NLLSVAYKNVIGAR					Mascot
2163.9573	2163.9719	0.0146	7	4	20	LAEQAERYEEMVEFMEK			Oxidation (M)[11,15]		Mascot
2331.2019	2331.269	0.0671	29	164	183	LGLALNFSVFYYEILNSPD R					Mascot

6	14-3-3-like protein A [Triticum urartu]	gi|474293618	28778.5	5.2	9	111	100	21.388	73	100	

Peptide Information

Calc. Mass	Obsrv. Mass	± da	± ppm	Start Seq.	End Seq.	Sequence	Ion Score	C. I. %	Modification	Rank	Result Type
816.421	816.4395	0.0185	23	17	23	LAEQAER					Mascot

Calc. Mass	Obsrv. Mass	± da	± ppm	Start Seq.	End Seq.	Sequence	Ion Score	C. I. %	Modification	Rank	Result Type
907.5247	907.5403	0.0156	17	49	56	NLLSVAYK					Mascot
1059.5946	1059.6219	0.0273	26	168	176	ELPPTHPIR					Mascot
1059.5946	1059.6219	0.0273	26	168	176	ELPPTHPIR	41	99.458			Mascot
1189.6609	1189.6909	0.03	25	222	231	DSTLIMQLLR					Mascot
1205.6559	1205.6827	0.0268	22	222	231	DSTLIMQLLR			Oxidation (M)[6]		Mascot
1205.6559	1205.6827	0.0268	22	222	231	DSTLIMQLLR	32	95.48	Oxidation (M)[6]		Mascot
1228.6294	1228.5693	-0.0601	-49	80	92	GAAGHAAAARGYR					Mascot
1366.5542	1366.5667	0.0125	9	24	33	YEEMVEFMEK			Oxidation (M)[4,8]		Mascot
1366.5542	1366.5667	0.0125	9	24	33	YEEMVEFMEK			Oxidation (M)[4,8]		Mascot
1517.8799	1517.9158	0.0359	24	49	62	NLLSVAYKNVIGAR					Mascot
2163.9573	2163.9719	0.0146	7	17	33	LAEQAERYEEMVEFMEK			Oxidation (M)[11,15]		Mascot
2331.2019	2331.269	0.0671	29	177	196	LGLALNFSVFYYEILNSPDR					Mascot

7	unnamed protein product [Triticum aestivum]	gi|227472076	28794.4	4.8	9	109	100	21.388	73	100

Protein Group

TaWIN2 [Triticum aestivum]	gi|9798605	28794.4	4.8000001907 3486
unnamed protein product [Triticum aestivum]	gi|219911924	28794.4	4.8000001907 3486
unnamed protein product [Triticum aestivum]	gi|257664806	28794.4	4.8000001907 3486

Peptide Information

Calc. Mass	Obsrv. Mass	± da	± ppm	Start Seq.	End Seq.	Sequence	Ion Score	C. I. %	Modification	Rank	Result Type
816.421	816.4395	0.0185	23	17	23	LAEQAER					Mascot
907.5247	907.5403	0.0156	17	49	56	NLLSVAYK					Mascot
1059.5946	1059.6219	0.0273	26	168	176	ELPPTHPIR					Mascot
1059.5946	1059.6219	0.0273	26	168	176	ELPPTHPIR	41	99.458			Mascot
1189.6609	1189.6909	0.03	25	222	231	DSTLIMQLLR					Mascot
1205.6559	1205.6827	0.0268	22	222	231	DSTLIMQLLR			Oxidation (M)[6]		Mascot
1205.6559	1205.6827	0.0268	22	222	231	DSTLIMQLLR	32	95.48	Oxidation (M)[6]		Mascot
1228.6294	1228.5693	-0.0601	-49	80	92	GAAGHAAAARGYR					Mascot
1366.5542	1366.5667	0.0125	9	24	33	YEEMVEFMEK			Oxidation (M)[4,8]		Mascot
1366.5542	1366.5667	0.0125	9	24	33	YEEMVEFMEK			Oxidation (M)[4,8]		Mascot
1517.8799	1517.9158	0.0359	24	49	62	NLLSVAYKNVIGAR					Mascot
2163.9573	2163.9719	0.0146	7	17	33	LAEQAERYEEMVEFMEK			Oxidation (M)[11,15]		Mascot

2331.2019	2331.269	0.0671	29	177	196	LGLALNFSVFYYEILNSPD R					Mascot

8	unnamed protein product [Triticum aestivum]	gi|227473231	28898.4	4.8	9	108	100	21.388	73	100		

Protein Group

unnamed protein product [Triticum aestivum]	gi|257710883	28898.4	4.8000 001907 3486

Peptide Information

Calc. Mass	Obsrv. Mass	± da	± ppm	Start Seq.	End Seq.	Sequence	Ion Score	C. I. %	Modification	Rank	Result Type
816.421	816.4395	0.0185	23	19	25	LAEQAER					Mascot
907.5247	907.5403	0.0156	17	51	58	NLLSVAYK					Mascot
1059.5946	1059.6219	0.0273	26	170	178	ELPPTHPIR					Mascot
1059.5946	1059.6219	0.0273	26	170	178	ELPPTHPIR	41	99.458			Mascot
1189.6609	1189.6909	0.03	25	224	233	DSTLIMQLLR					Mascot
1205.6559	1205.6827	0.0268	22	224	233	DSTLIMQLLR			Oxidation (M)[6]		Mascot
1205.6559	1205.6827	0.0268	22	224	233	DSTLIMQLLR	32	95.48	Oxidation (M)[6]		Mascot
1228.6294	1228.5693	-0.0601	-49	82	94	GAAGHAAAARGYR					Mascot
1366.5542	1366.5667	0.0125	9	26	35	YEEMVEFMEK			Oxidation (M)[4,8]		Mascot
1366.5542	1366.5667	0.0125	9	26	35	YEEMVEFMEK			Oxidation (M)[4,8]		Mascot
1517.8799	1517.9158	0.0359	24	51	64	NLLSVAYKNVIGAR					Mascot
2163.9573	2163.9719	0.0146	7	19	35	LAEQAERYEEMVEFMEK			Oxidation (M)[11,15]		Mascot
2331.2019	2331.269	0.0671	29	179	198	LGLALNFSVFYYEILNSPD R					Mascot

9	14-3-3 protein [Triticum aestivum]	gi|40781605	29387.7	4.83	14	106	100	22.217	32	95.48		

Protein Group

14-3-3 protein [Triticum aestivum]	gi|431822520	29360.7	4.8299 999237 0605
unnamed protein product [Triticum aestivum]	gi|227473229	29360.7	4.8299 999237 0605
unnamed protein product [Triticum aestivum]	gi|227471938	29387.7	4.8299 999237 0605

Peptide Information

Calc. Mass	Obsrv. Mass	± da	± ppm	Start Seq.	End Seq.	Sequence	Ion Score	C. I. %	Modification	Rank	Result Type
816.421	816.4395	0.0185	23	17	23	LAEQAER					Mascot
907.5247	907.5403	0.0156	17	49	56	NLLSVAYK					Mascot
917.5302	917.5472	0.017	19	68	75	IISSIEQK					Mascot

Calc. Mass	Obsrv. Mass	± da	± ppm	Start Seq.	End Seq.	Sequence	Ion Score	C. I. %	Modification	Rank	Result Type
999.4451	999.4665	0.0214	21	9	16	EENVYMAK			Oxidation (M)[6]		Mascot
1051.5419	1051.5702	0.0283	27	80	89	GNEAYVASIK					Mascot
1189.6609	1189.6909	0.03	25	222	231	DSTLIMQLLR					Mascot
1205.6559	1205.6827	0.0268	22	222	231	DSTLIMQLLR			Oxidation (M)[6]		Mascot
1205.6559	1205.6827	0.0268	22	222	231	DSTLIMQLLR	32	95.48	Oxidation (M)[6]		Mascot
1318.6486	1318.6783	0.0297	23	37	48	TADVGELTVEER					Mascot
1366.5542	1366.5667	0.0125	9	24	33	YEEMVEFMEK			Oxidation (M)[4,8]		Mascot
1366.5542	1366.5667	0.0125	9	24	33	YEEMVEFMEK			Oxidation (M)[4,8]		Mascot
1418.7485	1418.7878	0.0393	28	68	79	IISSIEQKEESR					Mascot
1517.8799	1517.9158	0.0359	24	49	62	NLLSVAYKNVIGAR					Mascot
1818.9708	1819.0199	0.0491	27	160	176	SAQDIALADLPTTHPIR					Mascot
1846.8309	1847.0034	0.1725	93	1	16	MSTAEATREENVYMAK			Oxidation (M)[1]		Mascot
2163.9573	2163.9719	0.0146	7	17	33	LAEQAERYEEMVEFMEK			Oxidation (M)[11,15]		Mascot
2331.2019	2331.269	0.0671	29	177	196	LGLALNFSVFYYEILNSPDR					Mascot

10	hypothetical protein TRIUR3_13999 [Triticum urartu]	gi|473799582	36319.3	5.65	7	61	96.796	7.529	41	99.482

Peptide Information

Calc. Mass	Obsrv. Mass	± da	± ppm	Start Seq.	End Seq.	Sequence	Ion Score	C. I. %	Modification	Rank	Result Type
806.3639	806.3615	-0.0024	-3	2	9	AETGDASR					Mascot
913.5101	913.499	-0.0111	-12	198	205	KLPAEEAR					Mascot
913.5101	913.499	-0.0111	-12	198	205	KLPAEEAR	41	99.482			Mascot
937.4044	937.4568	0.0524	56	1	9	MAETGDASR					Mascot
943.5459	943.5588	0.0129	14	229	237	TVTVEGIPK					Mascot
1203.5537	1203.637	0.0833	69	30	39	YAFMDCVGIK			Carbamidomethyl (C)[6]		Mascot
1219.5487	1219.6517	0.103	84	30	39	YAFMDCVGIK			Carbamidomethyl (C)[6], Oxidation (M)[4]		Mascot
1228.6532	1228.5693	-0.0839	-68	56	66	QNAIDQLVAEK					Mascot
1864.9552	1864.9927	0.0375	20	293	308	ATHSESIEYLFGQVKR					Mascot

Gel Idx/Pos	172/G23	Instr./Gel Origin	BA2151/Sample Project 20140814	Process Status	Analysis Succeeded
Plate [#] Name	[1] Sample Project 20140814	Instrument Sample Name		Spectra	11

Rank	Protein Name	Accession No.	Protein MW	Protein PI	Pep. Count	Protein Score	Protein Score C. I. %	Intensity Matched	Total Ion Score	Total Ion C. I. %	Confirmed
1	14-3-3-like protein B [Triticum urartu]	gi|474253094	29786.9	4.67	12	206	100	7.351	153	100	

Peptide Information

Calc. Mass	Obsrv. Mass	± da	± ppm	Start Seq.	End Seq.	Sequence	Ion Score	C. I. %	Modification	Rank	Result Type
816.421	816.4482	0.0272	33	18	24	LAEQAER					Mascot
818.444	818.468	0.024	29	103	109	ICDGILK			Carbamidomethyl (C)[2]		Mascot
917.5302	917.5295	-0.0007	-1	69	76	IISSIEQK					Mascot
948.4244	948.4635	0.0391	41	131	137	MKGDYYR			Oxidation (M)[1]		Mascot
1018.4985	1018.5401	0.0416	41	1	9	MAQPAELSR			Oxidation (M)[1]		Mascot
1189.6609	1189.7001	0.0392	33	223	232	DSTLIMQLLR					Mascot
1205.6559	1205.6904	0.0345	29	223	232	DSTLIMQLLR			Oxidation (M)[6]		Mascot
1212.5565	1212.6239	0.0674	56	150	160	DAAENTMVAYK					Mascot
1212.5565	1212.6239	0.0674	56	150	160	DAAENTMVAYK					Mascot
1228.5514	1228.6569	0.1055	86	150	160	DAAENTMVAYK			Oxidation (M)[7]		Mascot
1366.5542	1366.6586	0.1044	76	25	34	YEEMVEFMEK			Oxidation (M)[4,8]		Mascot
1406.6646	1406.7145	0.0499	35	38	49	TVDSEELTVEER					Mascot
1406.6646	1406.7145	0.0499	35	38	49	TVDSEELTVEER	49	99.908			Mascot
1418.7485	1418.7648	0.0163	11	69	80	IISSIEQKEESR					Mascot
1827.0123	1827.0712	0.0589	32	161	177	AAQEIALAELPPTHPIR					Mascot
1827.0123	1827.0712	0.0589	32	161	177	AAQEIALAELPPTHPIR	104	100			Mascot
2183.9648	2184.0884	0.1236	57	233	251	DNLTLWTSDISEDAAEEMK			Oxidation (M)[18]		Mascot

Rank	Protein Name	Accession No.	Protein MW	Protein PI	Pep. Count	Protein Score	Protein Score C. I. %	Intensity Matched	Total Ion Score	Total Ion C. I. %	Confirmed
2	14-3-3 protein [Triticum aestivum]	gi|431822518	29843.9	4.71	12	98	100	4.922	49	99.908	

Peptide Information

Calc. Mass	Obsrv. Mass	± da	± ppm	Start Seq.	End Seq.	Sequence	Ion Score	C. I. %	Modification	Rank	Result Type
816.421	816.4482	0.0272	33	18	24	LAEQAER					Mascot
818.444	818.468	0.024	29	103	109	ICDGILK			Carbamidomethyl (C)[2]		Mascot
917.5302	917.5295	-0.0007	-1	69	76	IISSIEQK					Mascot
948.4244	948.4635	0.0391	41	131	137	MKGDYYR			Oxidation (M)[1]		Mascot
1018.4985	1018.5401	0.0416	41	1	9	MAQPAELSR			Oxidation (M)[1]		Mascot

Calc. Mass	Obsrv. Mass	± da	± ppm	Start Seq.	End Seq.	Sequence	Ion Score	C. I. %	Modification	Rank	Result Type
1059.5946	1059.6129	0.0183	17	169	177	ELPPTHPIR					Mascot
1189.6609	1189.7001	0.0392	33	223	232	DSTLIMQLLR					Mascot
1205.6559	1205.6904	0.0345	29	223	232	DSTLIMQLLR			Oxidation (M)[6]		Mascot
1212.5565	1212.6239	0.0674	56	150	160	DAAENTMVAYK					Mascot
1212.5565	1212.6239	0.0674	56	150	160	DAAENTMVAYK					Mascot
1228.5514	1228.6569	0.1055	86	150	160	DAAENTMVAYK			Oxidation (M)[7]		Mascot
1366.5542	1366.6586	0.1044	76	25	34	YEEMVEFMEK			Oxidation (M)[4,8]		Mascot
1406.6646	1406.7145	0.0499	35	38	49	TVDSEELTVEER					Mascot
1406.6646	1406.7145	0.0499	35	38	49	TVDSEELTVEER	49	99.908			Mascot
1418.7485	1418.7648	0.0163	11	69	80	IISSIEQKEESR					Mascot
2183.9648	2184.0884	0.1236	57	233	251	DNLTLWTSDISEDAAEEMK			Oxidation (M)[18]		Mascot

3	unnamed protein product [Triticum aestivum]	gi|257664788	28475.3	4.78	12	93	99.998	4.739	49	99.908	

Protein Group

14-3-3 protein [Triticum aestivum]	gi|32401388	28475.3	4.78000020980835
unnamed protein product [Triticum aestivum]	gi|227472036	28475.3	4.78000020980835
unnamed protein product [Triticum aestivum]	gi|219911730	28475.3	4.78000020980835

Peptide Information

Calc. Mass	Obsrv. Mass	± da	± ppm	Start Seq.	End Seq.	Sequence	Ion Score	C. I. %	Modification	Rank	Result Type
816.421	816.4482	0.0272	33	4	10	LAEQAER					Mascot
818.444	818.468	0.024	29	89	95	ICDGILK			Carbamidomethyl (C)[2]		Mascot
819.4458	819.4619	0.0161	20	82	88	IETELSK					Mascot
917.5302	917.5295	-0.0007	-1	55	62	IISSIEQK					Mascot
948.4244	948.4635	0.0391	41	117	123	MKGDYYR			Oxidation (M)[1]		Mascot
1015.553	1015.5515	-0.0015	-1	2	10	AKLAEQAER					Mascot
1189.6609	1189.7001	0.0392	33	209	218	DSTLIMQLLR					Mascot
1205.6559	1205.6904	0.0345	29	209	218	DSTLIMQLLR			Oxidation (M)[6]		Mascot
1212.5565	1212.6239	0.0674	56	136	146	DAAENTMVAYK					Mascot
1212.5565	1212.6239	0.0674	56	136	146	DAAENTMVAYK					Mascot
1228.5514	1228.6569	0.1055	86	136	146	DAAENTMVAYK			Oxidation (M)[7]		Mascot
1366.5542	1366.6586	0.1044	76	11	20	YEEMVEFMEK			Oxidation (M)[4,8]		Mascot
1406.6646	1406.7145	0.0499	35	24	35	TVDSEELTVEER					Mascot
1406.6646	1406.7145	0.0499	35	24	35	TVDSEELTVEER	49	99.908			Mascot

1418.7485	1418.7648	0.0163	11	55	66	IISSIEQKEESR					Mascot
1786.9811	1787.038	0.0569	32	147	163	AAQDIALAELAPTHPIR					Mascot

4	14-3-3 protein [Triticum aestivum]	gi|390195996	30066.1	4.73	11	92	99.997	4.588	49	99.908

Protein Group

14-3-3 protein [Triticum aestivum]	gi|351602255	30066.1	4.7300 000190 7349
14-3-3-like protein GF14-B [Triticum urartu]	gi|474147722	30043.1	4.6900 000572 2046

Peptide Information

Calc. Mass	Obsrv. Mass	± da	± ppm	Start Seq.	End Seq.	Sequence	Ion Score	C. I. %	Modification	Rank	Result Type
816.421	816.4482	0.0272	33	18	24	LAEQAER					Mascot
818.444	818.468	0.024	29	103	109	ICDGILK			Carbamidomethyl (C)[2]		Mascot
819.4458	819.4619	0.0161	20	96	102	IETELSK					Mascot
917.5302	917.5295	-0.0007	-1	69	76	IISSIEQK					Mascot
948.4244	948.4635	0.0391	41	131	137	MKGDYYR			Oxidation (M)[1]		Mascot
1189.6609	1189.7001	0.0392	33	223	232	DSTLIMQLLR					Mascot
1205.6559	1205.6904	0.0345	29	223	232	DSTLIMQLLR			Oxidation (M)[6]		Mascot
1212.5565	1212.6239	0.0674	56	150	160	DAAENTMVAYK					Mascot
1212.5565	1212.6239	0.0674	56	150	160	DAAENTMVAYK					Mascot
1228.5514	1228.6569	0.1055	86	150	160	DAAENTMVAYK			Oxidation (M)[7]		Mascot
1366.5542	1366.6586	0.1044	76	25	34	YEEMVEFMEK			Oxidation (M)[4,8]		Mascot
1406.6646	1406.7145	0.0499	35	38	49	TVDSEELTVEER					Mascot
1406.6646	1406.7145	0.0499	35	38	49	TVDSEELTVEER	49	99.908			Mascot
1418.7485	1418.7648	0.0163	11	69	80	IISSIEQKEESR					Mascot
1786.9811	1787.038	0.0569	32	161	177	AAQDIALAELAPTHPIR					Mascot

5	unnamed protein product [Triticum aestivum]	gi|257664756	29983	4.62	10	85	99.985	3.898	49	99.908

Protein Group

14-3-3 protein [Triticum aestivum]	gi|52548256	29983	4.6199 998855 5908
unnamed protein product [Triticum aestivum]	gi|227471994	29983	4.6199 998855 5908
unnamed protein product [Triticum aestivum]	gi|219911698	29983	4.6199 998855 5908

Peptide Information

Calc. Mass	Obsrv. Mass	± da	± ppm	Start Seq.	End Seq.	Sequence	Ion Score	C. I. %	Modification	Rank	Result Type
816.421	816.4482	0.0272	33	18	24	LAEQAER					Mascot
818.444	818.468	0.024	29	103	109	ICDGILK			Carbamidomethyl (C)[2]		Mascot
819.4458	819.4619	0.0161	20	96	102	IETELSK					Mascot
917.5302	917.5295	-0.0007	-1	69	76	IISSIEQK					Mascot
948.4244	948.4635	0.0391	41	131	137	MKGDYYR			Oxidation (M)[1]		Mascot
1189.6609	1189.7001	0.0392	33	223	232	DSTLIMQLLR					Mascot
1205.6559	1205.6904	0.0345	29	223	232	DSTLIMQLLR			Oxidation (M)[6]		Mascot
1366.5542	1366.6586	0.1044	76	25	34	YEEMVEFMEK			Oxidation (M)[4,8]		Mascot
1406.6646	1406.7145	0.0499	35	38	49	TVDSEELTVEER					Mascot
1406.6646	1406.7145	0.0499	35	38	49	TVDSEELTVEER	49	99.908			Mascot
1418.7485	1418.7648	0.0163	11	69	80	IISSIEQKEESR					Mascot
1786.9811	1787.038	0.0569	32	161	177	AAQDIALAELAPTHPIR					Mascot

6 hypothetical protein TRIUR3_29935 [Triticum urartu] gi|473990946 76571.9 4.97 21 61 96.645 6.273

Peptide Information

Calc. Mass	Obsrv. Mass	± da	± ppm	Start Seq.	End Seq.	Sequence	Ion Score	C. I. %	Modification	Rank	Result Type
806.389	806.448	0.059	73	288	294	SDAEEKK					Mascot
815.5097	815.4548	-0.0549	-67	497	503	INATLKR					Mascot
838.4053	838.4747	0.0694	83	109	114	ENYEKR					Mascot
858.493	858.4931	0.0001	0	159	165	ELVEQLK					Mascot
881.4614	881.4678	0.0064	7	476	482	ELLDTYK					Mascot
917.5302	917.5295	-0.0007	-1	554	561	ISAIKEEK					Mascot
977.4786	977.5485	0.0699	72	428	436	VEEAESVSK					Mascot
1015.4955	1015.5515	0.056	55	183	190	HAFEENLR					Mascot
1022.5265	1022.5552	0.0287	28	384	391	ESRNELFK					Mascot
1032.5573	1032.5603	0.003	3	437	446	ALSDELASVK					Mascot
1037.556	1037.5547	-0.0013	-1	615	622	GEMLYLRR					Mascot
1189.592	1189.7001	0.1081	91	511	520	QSQVESEARR					Mascot
1201.5808	1201.6555	0.0747	62	323	332	DGQLNQLEER					Mascot
1201.5808	1201.6555	0.0747	62	323	332	DGQLNQLEER					Mascot
1205.6195	1205.6904	0.0709	59	359	368	TMLLNEVETR					Mascot
1320.6827	1320.6375	-0.0452	-34	146	156	TVKNLNEEVMK			Oxidation (M)[10]		Mascot
1324.7155	1324.6782	-0.0373	-28	581	591	NMVDAQHLIKR					Mascot
1324.7155	1324.6782	-0.0373	-28	581	591	NMVDAQHLIKR					Mascot

1333.6528	1333.6329	-0.0199	-15	592	602	LGMERENSEIR					Mascot
1349.7094	1349.6935	-0.0159	-12	359	369	TMLLNEVETRK			Oxidation (M)[2]		Mascot
1472.7454	1472.7424	-0.003	-2	252	263	MEQLEKYVYAAK					Mascot
1558.8071	1558.7932	-0.0139	-9	345	358	SIIAELNSELEANR					Mascot
1981.9535	1981.9778	0.0243	12	97	113	LAEDEAAMSLLRENYEK					Mascot

7 NBS-LRR protein [Triticum durum] gi|530452622 151907.9 8.71 24 53 78.834 5.287

Peptide Information

Calc. Mass	Obsrv. Mass	± da	± ppm	Start Seq.	End Seq.	Sequence	Ion Score	C. I. %	Modification	Rank	Result Type
806.3751	806.448	0.0729	90	852	858	SSRDDAR					Mascot
807.4471	807.4329	-0.0142	-18	90	95	YEALRR					Mascot
812.4638	812.4586	-0.0052	-6	704	709	HLHHLR					Mascot
859.4883	859.4933	0.005	6	1301	1307	LDNIEKK					Mascot
864.4244	864.499	0.0746	86	666	672	TLLCDSR			Carbamidomethyl (C)[4]		Mascot
866.5094	866.4695	-0.0399	-46	1292	1299	TLPASLHK					Mascot
884.4737	884.508	0.0343	39	113	119	LFPTHNR					Mascot
887.4944	887.4862	-0.0082	-9	1300	1306	RLDNIEK					Mascot
906.4237	906.4913	0.0676	75	1042	1049	ELVDGMDK					Mascot
917.5163	917.5295	0.0132	14	1170	1177	LESISGRR					Mascot
937.5465	937.5152	-0.0313	-33	684	691	YSSLRALK					Mascot
977.4897	977.5485	0.0588	60	1249	1257	SGELSSLER					Mascot
1022.6105	1022.5552	-0.0553	-54	1292	1300	TLPASLHKR					Mascot
1032.587	1032.5603	-0.0267	-26	994	1002	LSVLKMEGR					Mascot
1072.5092	1072.5725	0.0633	59	165	173	DHVMSDPKK			Oxidation (M)[4]		Mascot
1229.5831	1229.678	0.0949	77	35	44	GMEEQHEILK			Oxidation (M)[2]		Mascot
1349.6519	1349.6935	0.0416	31	637	647	DTTQHLFLSCK			Carbamidomethyl (C)[10]		Mascot
1366.6387	1366.6586	0.0199	15	840	851	WSFGSDNIVGER					Mascot
1369.6893	1369.7151	0.0258	19	35	45	GMEEQHEILKR					Mascot
1406.6055	1406.7145	0.109	77	493	503	QCFAFCAMFPK			Carbamidomethyl (C)[2,6]		Mascot
1406.6055	1406.7145	0.109	77	493	503	QCFAFCAMFPK			Carbamidomethyl (C)[2,6]		Mascot
1420.7471	1420.7468	-0.0003	0	344	354	YLLVLDDVWER					Mascot
1472.8431	1472.7424	-0.1007	-68	1249	1261	SGELSSLERLILR					Mascot
1926.9767	1926.931	-0.0457	-24	267	285	NDGNEVDALADSIVKAAPK					Mascot
2209.0706	2209.1475	0.0769	35	1262	1281	GCNSLASLPDGPQAYLYVQR			Carbamidomethyl (C)[2]		Mascot

8 14-3-3-like protein A [Triticum urartu] gi|474293618 28778.5 5.2 10 48 24.901 3.924

Peptide Information

Calc. Mass	Obsrv. Mass	± da	± ppm	Start Seq.	End Seq.	Sequence	Ion Score	C. I. %	Modification	Rank	Result Type
816.421	816.4482	0.0272	33	17	23	LAEQAER					Mascot
819.4505	819.4619	0.0114	14	1	8	MAKAAATR					Mascot
835.4454	835.4996	0.0542	65	1	8	MAKAAATR			Oxidation (M)[1]		Mascot
906.425	906.4913	0.0663	73	130	136	MKGDYHR					Mascot
1059.5946	1059.6129	0.0183	17	168	176	ELPPTHPIR					Mascot
1189.6609	1189.7001	0.0392	33	222	231	DSTLIMQLLR					Mascot
1205.6559	1205.6904	0.0345	29	222	231	DSTLIMQLLR			Oxidation (M)[6]		Mascot
1228.6294	1228.6569	0.0275	22	80	92	GAAGHAAAARGYR					Mascot
1323.6512	1323.6783	0.0271	20	76	89	EEGRGAAGHAAAAR					Mascot
1366.5542	1366.6586	0.1044	76	24	33	YEEMVEFMEK			Oxidation (M)[4,8]		Mascot
2083.9966	2084.1589	0.1623	78	148	167	KDAADSTLGAYQAAQDIA MK			Oxidation (M)[19]		Mascot
2100.9607	2101.1707	0.21	100	203	221	QAFDEAIAELDSLGEDSY K					Mascot
2100.9607	2101.1707	0.21	100	203	221	QAFDEAIAELDSLGEDSY K					Mascot

9 hypothetical protein TRIUR3_07942 [Triticum urartu] gi|473969902 38595.6 6.68 11 46 0 4.805

Peptide Information

Calc. Mass	Obsrv. Mass	± da	± ppm	Start Seq.	End Seq.	Sequence	Ion Score	C. I. %	Modification	Rank	Result Type
803.4774	803.4896	0.0122	15	47	53	AVTFLPR					Mascot
807.3995	807.4329	0.0334	41	182	188	ENAAFQK					Mascot
812.4988	812.4586	-0.0402	-49	2	8	RLADLPK					Mascot
937.5353	937.5152	-0.0201	-21	94	102	LSSAGLVYK					Mascot
1140.5354	1140.6013	0.0659	58	323	332	TYEGALEMAR					Mascot
1301.6372	1301.6438	0.0066	5	259	268	YVLYEDERSK					Mascot
1301.6372	1301.6438	0.0066	5	259	268	YVLYEDERSK					Mascot
1406.7791	1406.7145	-0.0646	-46	94	106	LSSAGLVYKHFGK					Mascot
1406.7791	1406.7145	-0.0646	-46	94	106	LSSAGLVYKHFGK					Mascot
1420.8523	1420.7468	-0.1055	-74	3	15	LADLPKAQNPLLK					Mascot
1501.7316	1501.8018	0.0702	47	223	236	GNVDPSGEIMVLDR					Mascot
2117.0889	2117.1597	0.0708	33	237	252	FCPWKLHLFELEQELK			Carbamidomethyl (C)[2]		Mascot
2117.0889	2117.1597	0.0708	33	237	252	FCPWKLHLFELEQELK			Carbamidomethyl (C)[2]		Mascot
2121.0256	2121.1135	0.0879	41	303	322	ETGIPGCVFIHMSGFIGG NK			Carbamidomethyl (C)[7]		Mascot

10	Dynamin-related protein 5A [Triticum urartu]	gi|473813192	65475	8.5	16	45	0	5.299

Peptide Information

Calc. Mass	Obsrv. Mass	± da	± ppm	Start Seq.	End Seq.	Sequence	Ion Score	C. I. %	Modification	Rank	Result Type
806.4519	806.448	-0.0039	-5	338	343	RLQFDK					Mascot
825.4828	825.4637	-0.0191	-23	254	260	HLESVIK					Mascot
828.4461	828.467	0.0209	25	459	465	LPQDVEK					Mascot
832.4159	832.347	-0.0689	-83	208	214	SQQDINK					Mascot
849.4941	849.4724	-0.0217	-26	556	561	RLELYR					Mascot
872.4294	872.4678	0.0384	44	242	249	MGSEHLAK					Mascot
948.4268	948.4635	0.0367	39	70	77	EIADETDR					Mascot
1018.5462	1018.5401	-0.0061	-6	215	223	SVDMIAARR					Mascot
1107.6157	1107.5754	-0.0403	-36	167	176	GERTFGVLTK					Mascot
1111.5531	1111.6084	0.0553	50	4	13	FGQIFSAGER					Mascot
1140.6372	1140.6013	-0.0359	-31	284	295	LGKPIANDAGGK					Mascot
1189.6609	1189.7001	0.0392	33	374	383	LIESCLVSIR			Carbamidomethyl (C)[5]		Mascot
1406.6886	1406.7145	0.0259	18	49	59	EYAEFMHVPRK					Mascot
1406.6886	1406.7145	0.0259	18	49	59	EYAEFMHVPRK					Mascot
1420.7067	1420.7468	0.0401	28	419	431	VEVGNAAFESLER					Mascot
2084.1499	2084.1589	0.009	4	384	403	GPAEAAVDTVHGILKELVHK					Mascot
2399.2354	2399.1946	-0.0408	-17	352	372	KLITEADGYQPHLIAPEQGYR					Mascot

Gel Idx/Pos	173/G24	Instr./Gel Origin	BA2151/Sample Project 20140814	Process Status	Analysis Succeeded
Plate [#] Name	[1] Sample Project 20140814	Instrument Sample Name		Spectra	11

Rank	Protein Name	Accession No.	Protein MW	Protein PI	Pep. Count	Protein Score	Protein Score C. I. %	Intensity Matched	Total Ion Score	Total Ion C. I. %	Confirmed
1	Alpha-soluble NSF attachment protein [Triticum urartu]	gi|474212649	42706.3	6.36	18	738	100	49.687	636	100	

Peptide Information

Calc. Mass	Obsrv. Mass	± da	± ppm	Start Seq.	End Seq.	Sequence	Ion Score	C. I. %	Modification	Rank	Result Type
853.405	853.4409	0.0359	42	220	226	ASDYLER					Mascot
855.4505	855.4409	-0.0096	-11	148	154	IANCHLK			Carbamidomethyl (C)[4]		Mascot
871.3615	871.397	0.0355	41	345	351	EFDGMTR			Oxidation (M)[5]		Mascot
1051.5571	1051.5884	0.0313	30	19	28	LSGWGLFGSK					Mascot
1178.6052	1178.6536	0.0484	41	260	269	ATEIFEEIAR					Mascot
1178.6052	1178.6536	0.0484	41	260	269	ATEIFEEIAR	86	100			Mascot
1202.5212	1202.5748	0.0536	45	29	38	YEDAADLYDK					Mascot
1286.6699	1286.7242	0.0543	42	296	307	ADAVAIQNSLER					Mascot
1286.6699	1286.7242	0.0543	42	296	307	ADAVAIQNSLER	97	100			Mascot
1413.6646	1413.7217	0.0571	40	308	319	YQEIDPTFSGTR					Mascot
1413.6646	1413.7217	0.0571	40	308	319	YQEIDPTFSGTR	112	100			Mascot
1459.7792	1459.8174	0.0382	26	247	259	VAEIAAQLEQYPK					Mascot
1459.7792	1459.8174	0.0382	26	247	259	VAEIAAQLEQYPK	73	100			Mascot
1487.7821	1487.844	0.0619	42	283	295	GILLNAGICQLCR			Carbamidomethyl (C)[9,12]		Mascot
1487.7821	1487.844	0.0619	42	283	295	GILLNAGICQLCR	96	100	Carbamidomethyl (C)[9,12]		Mascot
1530.7257	1530.813	0.0873	57	339	351	FTDAIKEFDGMTR					Mascot
1546.7207	1546.7697	0.049	32	339	351	FTDAIKEFDGMTR			Oxidation (M)[11]		Mascot
1620.7786	1620.8264	0.0478	29	323	338	LLADLAASMDDGDVAK			Oxidation (M)[9]		Mascot
1683.7432	1683.7976	0.0544	32	159	173	HEAASAYVEAANCYK			Carbamidomethyl (C)[13]		Mascot
1707.8073	1707.8562	0.0489	29	206	219	DIGEIYQQEQDLEK	52	99.954			Mascot
1811.8381	1811.9164	0.0783	43	159	174	HEAASAYVEAANCYKK			Carbamidomethyl (C)[13]		Mascot
1911.8931	1911.9473	0.0542	28	227	244	AADLFDSEGQTSQSNTIK					Mascot
1911.8931	1911.9473	0.0542	28	227	244	AADLFDSEGQTSQSNTIK	120	100			Mascot
2070.9185	2070.9846	0.0661	32	155	173	GDSKHEAASAYVEAANCYK			Carbamidomethyl (C)[17]		Mascot
2417.2458	2417.3477	0.1019	42	175	196	FSPQEAAQALDQAVNLFLEIGR					Mascot

Rank	Protein Name	Accession No.	Protein MW	Protein PI	Pep. Count	Protein Score	Protein Score C. I. %	Intensity Matched	Total Ion Score	Total Ion C. I. %	Confirmed
2	Actin-3 [Triticum urartu]	gi|474259583	44624.2	5.26	12	140	100	5.76	88	100	

Peptide Information

Calc. Mass	Obsrv. Mass	± da	± ppm	Start Seq.	End Seq.	Sequence	Ion Score	C. I. %	Modification	Rank	Result Type
976.4483	976.4879	0.0396	41	44	53	AGFAGDDAPR					Mascot
1132.527	1132.574	0.047	42	222	231	GYSFTTTAER					Mascot
1182.5273	1182.6241	0.0968	82	76	86	DAYVGDEAQSK					Mascot
1198.7056	1198.7493	0.0437	36	54	64	AVFPSIVGRPR					Mascot
1493.7703	1493.8156	0.0453	30	338	351	MSKEITALAPSSMK					Mascot
1509.7651	1509.8357	0.0706	47	338	351	MSKEITALAPSSMK			Oxidation (M)[1]		Mascot
1515.7491	1515.8109	0.0618	41	110	120	IWHHTFYNELR					Mascot
1525.76	1525.7906	0.0306	20	338	351	MSKEITALAPSSMK			Oxidation (M)[1,13]		Mascot
1620.8085	1620.8264	0.0179	11	203	216	LDLAGRDLTDCLMK			Carbamidomethyl (C)[11]		Mascot
1623.8081	1623.933	0.1249	77	209	221	DLTDCLMKILTER			Carbamidomethyl (C)[5], Oxidation (M)[7]		Mascot
1747.8861	1747.9551	0.069	39	264	279	SYELPDGQVITIGAER					Mascot
1747.8861	1747.9551	0.069	39	264	279	SYELPDGQVITIGAER	88	100			Mascot
1948.8746	1948.9633	0.0887	46	94	109	YPIEHGIVSNWDDMEK			Oxidation (M)[14]		Mascot
1954.0645	1954.1154	0.0509	26	121	138	VAPEEHPVLLTEAPLNPK					Mascot
3151.6423	3151.7866	0.1443	46	173	202	TTGIVLDSGDGVSHTVPI YEGYALPHAILR					Mascot

3	Actin-2 [Triticum urartu]	gi|474372381	41863.1	5.31	11	137	100	5.925	88	100	

Peptide Information

Calc. Mass	Obsrv. Mass	± da	± ppm	Start Seq.	End Seq.	Sequence	Ion Score	C. I. %	Modification	Rank	Result Type
976.4483	976.4879	0.0396	41	21	30	AGFAGDDAPR					Mascot
1132.527	1132.574	0.047	42	199	208	GYSFTTTAER					Mascot
1182.5273	1182.6241	0.0968	82	53	63	DAYVGDEAQSK					Mascot
1198.7056	1198.7493	0.0437	36	31	41	AVFPSIVGRPR					Mascot
1493.7703	1493.8156	0.0453	30	315	328	MSKEITALAPSSMK					Mascot
1509.7651	1509.8357	0.0706	47	315	328	MSKEITALAPSSMK			Oxidation (M)[1]		Mascot
1515.7491	1515.8109	0.0618	41	87	97	IWHHTFYNELR					Mascot
1525.76	1525.7906	0.0306	20	315	328	MSKEITALAPSSMK			Oxidation (M)[1,13]		Mascot
1547.8098	1547.7852	-0.0246	-16	180	193	LDLAGRDLTDSLMK					Mascot
1747.8861	1747.9551	0.069	39	241	256	SYELPDGQVITIGAER					Mascot
1747.8861	1747.9551	0.069	39	241	256	SYELPDGQVITIGAER	88	100			Mascot
1948.8746	1948.9633	0.0887	46	71	86	YPIEHGIVSNWDDMEK			Oxidation (M)[14]		Mascot
1954.0645	1954.1154	0.0509	26	98	115	VAPEEHPVLLTEAPLNPK					Mascot
3151.6423	3151.7866	0.1443	46	150	179	TTGIVLDSGDGVSHTVPI					Mascot

YEGYALPHAILR

4	actin [Triticum aestivum]	gi|58533119	41929	5.23	11	136	100	5.57	88	100

Protein Group

Actin-97 [Triticum urartu]	gi|474287474	41929	5.2300 000190 7349
actin [Triticum turgidum]	gi|58533114	41929	5.2300 000190 7349

Peptide Information

Calc. Mass	Obsrv. Mass	± da	± ppm	Start Seq.	End Seq.	Sequence	Ion Score	C. I. %	Modification	Rank	Result Type
976.4483	976.4879	0.0396	41	21	30	AGFAGDDAPR					Mascot
1118.5114	1118.5582	0.0468	42	199	208	GYSFTTSAER					Mascot
1182.5273	1182.6241	0.0968	82	53	63	DAYVGDEAQSK					Mascot
1198.7056	1198.7493	0.0437	36	31	41	AVFPSIVGRPR					Mascot
1493.7703	1493.8156	0.0453	30	315	328	MSKEITALAPSSMK					Mascot
1509.7651	1509.8357	0.0706	47	315	328	MSKEITALAPSSMK			Oxidation (M)[1]		Mascot
1515.7491	1515.8109	0.0618	41	87	97	IWHHTFYNELR					Mascot
1525.76	1525.7906	0.0306	20	315	328	MSKEITALAPSSMK			Oxidation (M)[1,13]		Mascot
1547.8098	1547.7852	-0.0246	-16	180	193	LDLAGRDLTDSLMK					Mascot
1747.8861	1747.9551	0.069	39	241	256	SYELPDGQVITIGAER					Mascot
1747.8861	1747.9551	0.069	39	241	256	SYELPDGQVITIGAER	88	100			Mascot
1948.8746	1948.9633	0.0887	46	71	86	YPIEHGIVSNWDDMEK			Oxidation (M)[14]		Mascot
1954.0645	1954.1154	0.0509	26	98	115	VAPEEHPVLLTEAPLNPK					Mascot
3151.6423	3151.7866	0.1443	46	150	179	TTGIVLDSGDGVSHTVPI YEGYALPHAILR					Mascot

5	actin [Triticum aestivum]	gi|255684860	28232.3	5.34	8	130	100	4.241	88	100

Peptide Information

Calc. Mass	Obsrv. Mass	± da	± ppm	Start Seq.	End Seq.	Sequence	Ion Score	C. I. %	Modification	Rank	Result Type
1132.527	1132.574	0.047	42	113	122	GYSFTTTAER					Mascot
1493.7703	1493.8156	0.0453	30	229	242	MSKEITALAPSSMK					Mascot
1509.7651	1509.8357	0.0706	47	229	242	MSKEITALAPSSMK			Oxidation (M)[1]		Mascot
1515.7491	1515.8109	0.0618	41	1	11	IWHHTFYNELR					Mascot
1525.76	1525.7906	0.0306	20	229	242	MSKEITALAPSSMK			Oxidation (M)[1,13]		Mascot
1620.8085	1620.8264	0.0179	11	94	107	LDLAGRDLTDCLMK			Carbamidomethyl (C)[11]		Mascot
1623.8081	1623.933	0.1249	77	100	112	DLTDCLMKILTER			Carbamidomethyl (C)[5], Oxidation (M)[7]		Mascot

Calc. Mass	Obsrv. Mass	± da	± ppm	Start Seq.	End Seq.	Sequence	Ion Score	C. I. %	Modification	Rank	Result Type
1747.8861	1747.9551	0.069	39	155	170	SYELPDGQVITIGAER					Mascot
1747.8861	1747.9551	0.069	39	155	170	SYELPDGQVITIGAER	88	100			Mascot
1954.0645	1954.1154	0.0509	26	12	29	VAPEEHPVLLTEAPLNPK					Mascot
3151.6423	3151.7866	0.1443	46	64	93	TTGIVLDSGDGVSHTVPI YEGYALPHAILR					Mascot

6	unnamed protein product [Triticum aestivum]	gi|298541521	42152.7	5.64	6	128	100	3.833	110	100	

Protein Group

RecName: Full=Phosphoglycerate kinase, cytosolic	gi|129916	42152.7	5.6399 998664 856
unnamed protein product [Triticum aestivum]	gi|21835	42152.7	5.6399 998664 856

Peptide Information

Calc. Mass	Obsrv. Mass	± da	± ppm	Start Seq.	End Seq.	Sequence	Ion Score	C. I. %	Modification	Rank	Result Type
1493.8297	1493.8156	-0.0141	-9	166	178	FLRPSVAGFLMQK					Mascot
1503.8629	1503.8003	-0.0626	-42	248	261	LELATSLIETAKSK					Mascot
1509.8247	1509.8357	0.011	7	166	178	FLRPSVAGFLMQK			Oxidation (M)[11]		Mascot
1543.8326	1543.7772	-0.0554	-36	233	247	AQGLAVGKSLVEEDK					Mascot
1720.9956	1721.0636	0.068	40	106	122	LAAALPDGGVLLLENVR					Mascot
1720.9956	1721.0636	0.068	40	106	122	LAAALPDGGVLLLENVR	110	100			Mascot
1919.9611	1920.0375	0.0764	40	138	155	LASVADLYVNDAFGTAH R					Mascot
2048.0559	2048.1406	0.0847	41	137	155	KLASVADLYVNDAFGTAH R					Mascot

7	cytosolic 3-phosphoglycerate kinase [Triticum aestivum]	gi|28172909	31320.7	4.91	5	127	100	3.338	110	100	

Peptide Information

Calc. Mass	Obsrv. Mass	± da	± ppm	Start Seq.	End Seq.	Sequence	Ion Score	C. I. %	Modification	Rank	Result Type
1503.8629	1503.8003	-0.0626	-42	174	187	LELATSLIETAKSK					Mascot
1543.8326	1543.7772	-0.0554	-36	159	173	AQGLAVGKSLVEEDK					Mascot
1720.9956	1721.0636	0.068	40	32	48	LAAALPDGGVLLLENVR					Mascot
1720.9956	1721.0636	0.068	40	32	48	LAAALPDGGVLLLENVR	110	100			Mascot
1919.9611	1920.0375	0.0764	40	64	81	LASVADLYVNDAFGTAH R					Mascot
2048.0559	2048.1406	0.0847	41	63	81	KLASVADLYVNDAFGTAH R					Mascot

8	cytosolic 3-phosphoglycerate kinase [Triticum urartu]	gi|28172905	31371.7	4.98	5	127	100	3.72	110	100	

Protein Group

cytosolic 3-phosphoglycerate kinase [Triticum aestivum]	gi|28172911	31371.7	4.9800

Peptide Information

Calc. Mass	Obsrv. Mass	± da	± ppm	Start Seq.	End Seq.	Sequence	Ion Score	C. I. %	Modification	Rank	Result Type
1493.8297	1493.8156	-0.0141	-9	92	104	FLRPSVAGFLMQK					Mascot
1503.8629	1503.8003	-0.0626	-42	174	187	LELATSLIETAKSK					Mascot
1509.8247	1509.8357	0.011	7	92	104	FLRPSVAGFLMQK			Oxidation (M)[11]		Mascot
1720.9956	1721.0636	0.068	40	32	48	LAAALPDGGVLLLENVR					Mascot
1720.9956	1721.0636	0.068	40	32	48	LAAALPDGGVLLLENVR	110	100			Mascot
1919.9611	1920.0375	0.0764	40	64	81	LASVADLYVNDAFGTAH R					Mascot
2048.0559	2048.1406	0.0847	41	63	81	KLASVADLYVNDAFGTAH R					Mascot

9	Phosphoglycerate kinase, cytosolic [Triticum urartu]	gi|473995124	55052.6	5.45	6	124	100	4.024	110	100	

Peptide Information

Calc. Mass	Obsrv. Mass	± da	± ppm	Start Seq.	End Seq.	Sequence	Ion Score	C. I. %	Modification	Rank	Result Type
1083.6772	1083.5822	-0.095	-88	291	300	LGLANLLIEK					Mascot
1493.8297	1493.8156	-0.0141	-9	209	221	YLRPAVAGFLMQK					Mascot
1509.8247	1509.8357	0.011	7	209	221	YLRPAVAGFLMQK			Oxidation (M)[11]		Mascot
1720.9956	1721.0636	0.068	40	149	165	LAAALPDGGVLLLENVR					Mascot
1720.9956	1721.0636	0.068	40	149	165	LAAALPDGGVLLLENVR	110	100			Mascot
1884.0688	1884.0081	-0.0607	-32	284	300	SLVEEDKLGLANLLIEK					Mascot
1919.9611	1920.0375	0.0764	40	181	198	LASVADLYVNDAFGTAH R					Mascot
2048.0559	2048.1406	0.0847	41	180	198	KLASVADLYVNDAFGTAH R					Mascot

10	Phosphoglycerate kinase, cytosolic [Triticum urartu]	gi|473781647	45286.1	5.9	5	123	100	3.72	110	100	

Peptide Information

Calc. Mass	Obsrv. Mass	± da	± ppm	Start Seq.	End Seq.	Sequence	Ion Score	C. I. %	Modification	Rank	Result Type
1493.8297	1493.8156	-0.0141	-9	166	178	FLRPSVAGFLMQK					Mascot
1503.8629	1503.8003	-0.0626	-42	248	261	LELATSLIETAKSK					Mascot
1509.8247	1509.8357	0.011	7	166	178	FLRPSVAGFLMQK			Oxidation (M)[11]		Mascot
1720.9956	1721.0636	0.068	40	106	122	LAAALPDGGVLLLENVR					Mascot
1720.9956	1721.0636	0.068	40	106	122	LAAALPDGGVLLLENVR	110	100			Mascot
1919.9611	1920.0375	0.0764	40	138	155	LASVADLYVNDAFGTAH R					Mascot

2048.0559	2048.1406	0.0847	41	137	155	KLASVADLYVNDAFGTAH<br>R	Mascot

Gel Idx/Pos	174/H1	Instr./Gel Origin	BA2151/Sample Project 20140814	Process Status	Analysis Succeeded
Plate [#] Name	[1] Sample Project 20140814	Instrument Sample Name		Spectra	11

Rank	Protein Name	Accession No.	Protein MW	Protein PI	Pep. Count	Protein Score	Protein Score C. I. %	Intensity Matched	Total Ion Score	Total Ion C. I. %	Confirmed
1	Spermidine synthase 1 [Triticum urartu]	gi|473890074	35788.8	4.99	11	787	100	49.629	727	100	

Peptide Information

Calc. Mass	Obsrv. Mass	± da	± ppm	Start Seq.	End Seq.	Sequence	Ion Score	C. I. %	Modification	Rank	Result Type
850.3975	850.4307	0.0332	39	1	8	MEAETAAK					Mascot
1211.7107	1211.7185	0.0078	6	112	124	VLVIGGGDGGVLR					Mascot
1211.7107	1211.7185	0.0078	6	112	124	VLVIGGGDGGVLR	126	100			Mascot
1339.8057	1339.8129	0.0072	5	111	124	KVLVIGGGDGGVLR					Mascot
1339.8057	1339.8129	0.0072	5	111	124	KVLVIGGGDGGVLR	94	100			Mascot
1355.7682	1355.7615	-0.0067	-5	164	176	VSLHIGDGVAFLK					Mascot
1355.7682	1355.7615	-0.0067	-5	164	176	VSLHIGDGVAFLK	90	100			Mascot
1440.8422	1440.8477	0.0055	4	77	89	VLVLDGVIQVTER					Mascot
1440.8422	1440.8477	0.0055	4	77	89	VLVLDGVIQVTER	92	100			Mascot
1672.7847	1672.7766	-0.0081	-5	129	142	HSSVEQIDICEIDK			Carbamidomethyl (C)[10]		Mascot
1673.8435	1673.8464	0.0029	2	150	163	QFFPHLALGFEDPR					Mascot
1673.8435	1673.8464	0.0029	2	150	163	QFFPHLALGFEDPR	79	100			Mascot
1835.8811	1835.8411	-0.04	-22	61	76	SDYQNVLVFQSSTYGK					Mascot
2180.9858	2181.0007	0.0149	7	299	316	FYNSEFHTASFCLPSFAR			Carbamidomethyl (C)[12]		Mascot
2180.9858	2181.0007	0.0149	7	299	316	FYNSEFHTASFCLPSFAR	121	100	Carbamidomethyl (C)[12]		Mascot
2447.1792	2447.1609	-0.0183	-7	129	149	HSSVEQIDICEIDKMVVD VSK			Carbamidomethyl (C)[10], Oxidation (M)[15]		Mascot
3797.8181	3797.9089	0.0908	24	177	211	NAPEGTYDAVIVDSSDPV GPAQELFEKPFFESVSR					Mascot
3797.8181	3797.9089	0.0908	24	177	211	NAPEGTYDAVIVDSSDPV GPAQELFEKPFFESVSR	125	100			Mascot

Rank	Protein Name	Accession No.	Protein MW	Protein PI	Pep. Count	Protein Score	Protein Score C. I. %	Intensity Matched	Total Ion Score	Total Ion C. I. %	Confirmed
2	Spermine synthase [Triticum urartu]	gi|474201758	42586.4	5.56	3	226	100	11.834	219	100	

Peptide Information

Calc. Mass	Obsrv. Mass	± da	± ppm	Start Seq.	End Seq.	Sequence	Ion Score	C. I. %	Modification	Rank	Result Type
1211.7107	1211.7185	0.0078	6	145	157	VLVIGGGDGGVLR					Mascot
1211.7107	1211.7185	0.0078	6	145	157	VLVIGGGDGGVLR	126	100			Mascot
1339.8057	1339.8129	0.0072	5	144	157	KVLVIGGGDGGVLR					Mascot
1339.8057	1339.8129	0.0072	5	144	157	KVLVIGGGDGGVLR	94	100			Mascot

1372.6703	1372.6873	0.017	12	361	373	GAQQEEKSAEPAK					Mascot

3	hypothetical protein TRIUR3_01278 [Triticum urartu]	gi|473794285	28029.9	5.78	9	48	26.61	3.007	

Peptide Information

Calc. Mass	Obsrv. Mass	± da	± ppm	Start Seq.	End Seq.	Sequence	Ion Score	C. I. %	Modification	Rank	Result Type
1182.5977	1182.5637	-0.034	-29	148	156	YYHINLTMK					Mascot
1198.5925	1198.6304	0.0379	32	148	156	YYHINLTMK			Oxidation (M)[8]		Mascot
1263.6692	1263.6536	-0.0156	-12	81	91	LSLNYPDGTRK					Mascot
1376.6838	1376.6951	0.0113	8	92	102	KLLESQQMDER					Mascot
1806.8868	1806.8237	-0.0631	-35	179	193	GGEYVELVLEEEERR					Mascot
1858.7913	1858.8549	0.0636	34	159	176	GADDSNCGGGDLFFAEV K			Carbamidomethyl (C)[7]		Mascot
1958.9131	1958.8721	-0.041	-21	196	211	RFYETQGLDDPDFVEK					Mascot
2033.9974	2033.9299	-0.0675	-33	139	156	SVAGGNGHRYYHINLTM K			Oxidation (M)[17]		Mascot
2087.9556	2088.0664	0.1108	53	197	213	FYETQGLDDPDFVEKER					Mascot
2090.074	2089.9922	-0.0818	-39	216	233	AQIPVKPFSPADCVLEYR			Carbamidomethyl (C)[13]		Mascot

4	acyl-desaturase, chloroplast precursor,putative,expressed [Triticum aestivum]	gi|300681441	46184	6.41	12	46	0	1.909	

Peptide Information

Calc. Mass	Obsrv. Mass	± da	± ppm	Start Seq.	End Seq.	Sequence	Ion Score	C. I. %	Modification	Rank	Result Type
833.39	833.4101	0.0201	24	280	286	HEAGYTR					Mascot
982.5615	982.4788	-0.0827	-84	302	310	ALAHVMRGK					Mascot
1288.6427	1288.7689	0.1262	98	390	400	MEELANQRAAR					Mascot
1308.6287	1308.6661	0.0374	29	311	322	VTMPGLLMSDGR			Oxidation (M)[3,8]		Mascot
1323.7321	1323.6692	-0.0629	-48	31	41	LGWILFPANHR					Mascot
1349.6484	1349.6171	-0.0313	-23	349	359	DYGDLVEHFVR					Mascot
1372.7042	1372.6873	-0.0169	-12	201	211	YLYLSGRVDMR					Mascot
1439.7278	1439.8011	0.0733	51	48	61	WTAAAAEAVEAPPR					Mascot
1545.7115	1545.7742	0.0627	41	14	27	LCFFSNKSDAGSGR			Carbamidomethyl (C)[2]		Mascot
1735.8507	1735.7729	-0.0778	-45	287	301	VCAKLFEVDPDGMVR			Carbamidomethyl (C)[2]		Mascot
2088.0366	2088.0664	0.0298	14	291	308	LFEVDPDGMVRALAHVM R			Oxidation (M)[9,17]		Mascot
2162.0149	2162.0503	0.0354	16	166	185	TEGTADDTGASSLPWAQ WIR					Mascot

5	1-Cys peroxiredoxin PER1 [Triticum urartu]	gi|474094006	24248.3	6.08	8	45	0	1.572	

Calc. Mass	Obsrv. Mass	± da	± ppm	Start Seq.	End Seq.	Sequence	Ion Score	C. I. %	Modification	Rank	Result Type
1107.5681	1107.5417	-0.0264	-24	84	93	DIEAYKPGSK					Mascot
1567.7462	1567.8618	0.1156	74	197	210	MFPQGFETADLPSK					Mascot
1695.8411	1695.8082	-0.0329	-19	197	211	MFPQGFETADLPSKK					Mascot
1931.0266	1930.9227	-0.1039	-54	151	168	NMDEVVVRAVDSLLTAAK					Mascot
2064.0608	2064.1116	0.0508	25	2	21	PGLTIGDTVPNLELDSTHGK					Mascot
2195.1013	2195.05	-0.0513	-23	1	21	MPGLTIGDTVPNLELDSTHGK					Mascot
2243.0947	2242.9478	-0.1469	-65	140	158	LSFLYPSCTGRNMDEVVVR			Carbamidomethyl (C)[8]		Mascot
2382.1646	2382.1924	0.0278	12	84	104	DIEAYKPGSKVTYPIMADPDR			Oxidation (M)[16]		Mascot

6 protein disulfide isomerase [Triticum aestivum] gi|222446344 56921 4.93 12 45 0 5.161

Calc. Mass	Obsrv. Mass	± da	± ppm	Start Seq.	End Seq.	Sequence	Ion Score	C. I. %	Modification	Rank	Result Type
1182.6589	1182.5637	-0.0952	-80	212	222	GDAAVERPLVR					Mascot
1308.5955	1308.6661	0.0706	54	158	169	APEDATYLEDGK					Mascot
1320.6107	1320.6083	-0.0024	-2	295	306	AYYGAVEEFSGK					Mascot
1440.7847	1440.8477	0.063	44	386	398	VVVADNVHDVVFK					Mascot
1440.7847	1440.8477	0.063	44	386	398	VVVADNVHDVVFK					Mascot
1647.8951	1647.826	-0.0691	-42	73	87	SLAPEYEKAAQLLSK					Mascot
1654.8688	1654.7916	-0.0772	-47	245	259	FIDASSTPKVVTFDK					Mascot
1678.817	1678.8322	0.0152	9	155	169	EIKAPEDATYLEDGK					Mascot
1735.8497	1735.7729	-0.0768	-44	88	103	HDPAIVLAEVDANDEK					Mascot
1799.9386	1799.8593	-0.0793	-44	329	344	EDQAPLILIQDSDSKK					Mascot
1835.9037	1835.8411	-0.0626	-34	278	293	AMLFLNFSTGPFESFK					Mascot
1851.8987	1851.9749	0.0762	41	278	293	AMLFLNFSTGPFESFK			Oxidation (M)[2]		Mascot
1899.9963	1899.9423	-0.054	-28	254	269	VVTFDKNPDNHPYLLK					Mascot
2447.2776	2447.1609	-0.1167	-48	81	103	AAQLLSKHDPAIVLAEVDANDEK					Mascot

7 Aldo-keto reductase family 4 member C9 [Triticum urartu] gi|473826004 65804.4 8.49 14 43 0 3.279

Calc. Mass	Obsrv. Mass	± da	± ppm	Start Seq.	End Seq.	Sequence	Ion Score	C. I. %	Modification	Rank	Result Type

Calc. Mass	Obsrv. Mass	± da	± ppm	Start Seq.	End Seq.	Sequence	Ion Score	C. I. %	Modification	Rank	Result Type
833.4111	833.4101	-0.001	-1	97	103	NETREGK					Mascot
872.4836	872.424	-0.0596	-68	202	209	AVQEAVQK					Mascot
976.4933	976.5054	0.0121	12	1	7	MAWRWAR					Mascot
1376.674	1376.6951	0.0211	15	291	303	MAAAHFTLNTGAR			Oxidation (M)[1]		Mascot
1516.7802	1516.7319	-0.0483	-32	290	303	RMAAAHFTLNTGAR					Mascot
1612.8727	1612.7802	-0.0925	-57	105	118	IEEPEQIAKAVVMR					Mascot
1647.837	1647.826	-0.011	-7	232	245	RNLSAMDQIVEEVK			Oxidation (M)[6]		Mascot
1672.8752	1672.7766	-0.0986	-59	501	517	DVASNPVVTSVAESLGK					Mascot
1799.8381	1799.8593	0.0212	12	426	440	TWQAMEQLYDSGKAR			Oxidation (M)[5]		Mascot
1859.0386	1858.8549	-0.1837	-99	314	332	AAPGVITDVLSAAVKAGYR					Mascot
1930.8456	1930.9227	0.0771	40	246	261	SMCSSLAECKITWDSR			Carbamidomethyl (C)[3,9]		Mascot
1958.931	1958.8721	-0.0589	-30	214	230	LHLETDCQTVAGMLNEK			Carbamidomethyl (C)[7]		Mascot
2203.0154	2202.9717	-0.0437	-20	28	43	LYHRFWACYHSAMFWK			Carbamidomethyl (C)[8]		Mascot
2243.1265	2242.9478	-0.1787	-80	370	389	IWCSDLAPEDVPLAIDSTLK			Carbamidomethyl (C)[3]		Mascot

8 Disease resistance protein RPM1 [Triticum urartu] gi|473965111 134539.5 7.15 20 42 0 8.899 7 0

Peptide Information

Calc. Mass	Obsrv. Mass	± da	± ppm	Start Seq.	End Seq.	Sequence	Ion Score	C. I. %	Modification	Rank	Result Type
966.4276	966.4673	0.0397	41	1148	1155	GTNDEQFR					Mascot
976.5284	976.5054	-0.023	-24	404	411	GVIKDWMK					Mascot
1017.5436	1017.5395	-0.0041	-4	122	130	ARVNDVSTR					Mascot
1083.5317	1083.6355	0.1038	96	134	143	YNLISDSGSK					Mascot
1107.5238	1107.5417	0.0179	16	834	842	DEDLIEMVK			Oxidation (M)[7]		Mascot
1194.5559	1194.6351	0.0792	66	1041	1050	LDVEMDSLEK			Oxidation (M)[5]		Mascot
1256.6593	1256.6315	-0.0278	-22	20	31	AQAAIGEEAQLR					Mascot
1263.625	1263.6536	0.0286	23	834	843	DEDLIEMVKR			Oxidation (M)[7]		Mascot
1323.6362	1323.6692	0.033	25	565	576	GLEANAAMDLFR			Oxidation (M)[8]		Mascot
1355.6511	1355.7615	0.1104	81	274	284	KMEATQEDLFK			Oxidation (M)[2]		Mascot
1355.6511	1355.7615	0.1104	81	274	284	KMEATQEDLFK	7	0	Oxidation (M)[2]		Mascot
1376.7645	1376.6951	-0.0694	-50	923	935	DLHGVEVPRGIGK					Mascot
1440.7078	1440.8477	0.1399	97	131	143	NSRYNLISDSGSK					Mascot
1440.7078	1440.8477	0.1399	97	131	143	NSRYNLISDSGSK					Mascot
1545.8483	1545.7742	-0.0741	-48	389	403	VSQISPLQTLSSASK					Mascot
1678.8031	1678.8322	0.0291	17	366	380	KEIPQSQGSQNYGDK					Mascot
1695.9065	1695.8082	-0.0983	-58	788	801	DIIVFDSIDFSRLR					Mascot

1701.8014	1701.8794	0.078	46	483	496	DFSRSLLMDHHSEK					Mascot
1717.7963	1717.8119	0.0156	9	483	496	DFSRSLLMDHHSEK			Oxidation (M)[8]		Mascot
1799.9539	1799.8593	-0.0946	-53	1005	1020	SLDGISLPWENLQSLK					Mascot
1858.9766	1858.8549	-0.1217	-65	1051	1066	TDIESLAMLPNLCILR			Carbamidomethyl (C)[13]		Mascot
1874.9714	1874.8928	-0.0786	-42	1051	1066	TDIESLAMLPNLCILR			Carbamidomethyl (C)[13], Oxidation (M)[8]		Mascot
2087.9678	2088.0664	0.0986	47	1077	1092	LHFYAEMCREQLDTFK			Carbamidomethyl (C)[8]		Mascot
2192.0559	2192.134	0.0781	36	470	486	YNWVDISYPFNLRDFSR					Mascot

9 hypothetical protein TRIUR3_27961 [Triticum urartu] gi|474021068 95074.5 6.23 16 42 0 11.214

Peptide Information

Calc. Mass	Obsrv. Mass	± da	± ppm	Start Seq.	End Seq.	Sequence	Ion Score	C. I. %	Modification	Rank	Result Type
856.525	856.5255	0.0005	1	68	75	SAKPPKTK					Mascot
870.4904	870.5482	0.0578	66	851	858	GGKPNNRK					Mascot
966.4097	966.4673	0.0576	60	520	527	WGMNNSNK			Oxidation (M)[3]		Mascot
976.4945	976.5054	0.0109	11	578	586	EKSVAEEGK					Mascot
1207.6001	1207.61	0.0099	8	1	11	MGFAENRAGVR					Mascot
1211.6631	1211.7185	0.0554	46	567	577	VNEINPDVLAK					Mascot
1211.6631	1211.7185	0.0554	46	567	577	VNEINPDVLAK					Mascot
1263.59	1263.6536	0.0636	50	517	527	GQKWGMNNSNK					Mascot
1377.7261	1377.7482	0.0221	16	266	277	ESKPFTIGIEEK					Mascot
1567.9279	1567.8618	-0.0661	-42	819	833	AQRSTALGPTLALLR					Mascot
1705.8505	1705.8705	0.02	12	731	746	VHSQNGTPVPALDENK					Mascot
1710.9609	1710.8057	-0.1552	-91	822	838	STALGPTLALLRGNNGR					Mascot
1782.9497	1782.8182	-0.1315	-74	22	38	AVSNHPSSIGIVGFELR					Mascot
1851.8971	1851.9749	0.0778	42	676	691	EDGSLEIEYSNLLDVR					Mascot
1930.7985	1930.9227	0.1242	64	193	207	HQEEEVHCDHQPEEK			Carbamidomethyl (C)[8]		Mascot
2192.1445	2192.134	-0.0105	-5	676	694	EDGSLEIEYSNLLDVRLVK					Mascot
2203.0659	2202.9717	-0.0942	-43	279	297	ADIQTECTVELDGKQKPDR			Carbamidomethyl (C)[7]		Mascot

10 hypothetical protein TRIUR3_32377 [Triticum urartu] gi|474190958 25087 6.35 8 42 0 13.07

Peptide Information

Calc. Mass	Obsrv. Mass	± da	± ppm	Start Seq.	End Seq.	Sequence	Ion Score	C. I. %	Modification	Rank	Result Type
872.5022	872.424	-0.0782	-90	2	9	VMGLLSPR					Mascot
1139.5765	1139.6283	0.0518	45	72	80	MENVKDYIK					Mascot
1155.5714	1155.592	0.0206	18	72	80	MENVKDYIK			Oxidation (M)[1]		Mascot

1372.7328	1372.6873	-0.0455	-33	46	58	IVIGVCDGPMLAK	Carbamidomethyl (C)[6]	Mascot
1516.8226	1516.7319	-0.0907	-60	46	59	IVIGVCDGPMLAKK	Carbamidomethyl (C)[6], Oxidation (M)[10]	Mascot
1673.8713	1673.8464	-0.0249	-15	44	58	ERIVIGVCDGPMLAK	Carbamidomethyl (C)[8], Oxidation (M)[12]	Mascot
1673.8713	1673.8464	-0.0249	-15	44	58	ERIVIGVCDGPMLAK	Carbamidomethyl (C)[8], Oxidation (M)[12]	Mascot
1683.7677	1683.8386	0.0709	42	10	25	GSQITTTTVAACGECK	Carbamidomethyl (C)[12,15]	Mascot
1756.8146	1756.8135	-0.0011	-1	133	146	CEIFNLSLFPCNSR	Carbamidomethyl (C)[1,11]	Mascot
2124.1548	2123.9849	-0.1699	-80	163	181	AERGLTQLEIEVAELVPE K		Mascot

Gel Idx/Pos	175/H2	Instr./Gel Origin	BA2151/Sample Project 20140814	Process Status	Analysis Succeeded
Plate [#] Name	[1] Sample Project 20140814	Instrument Sample Name		Spectra	11

Rank	Protein Name	Accession No.	Protein MW	Protein PI	Pep. Count	Protein Score	Protein Score C. I. %	Intensity Matched	Total Ion Score	Total Ion C. I. %	Confirmed
1	Oxygen-evolving enhancer protein 1, chloroplastic [Triticum urartu]	gi|474352688	34635.5	5.75	18	749	100	50.833	624	100	

Peptide Information

Calc. Mass	Obsrv. Mass	± da	± ppm	Start Seq.	End Seq.	Sequence	Ion Score	C. I. %	Modification	Rank	Result Type
850.4305	850.4356	0.0051	6	232	239	GSSFLDPK					Mascot
930.468	930.4703	0.0023	2	147	154	NEPPAFQK					Mascot
950.571	950.5679	-0.0031	-3	203	210	VPFLFTVK					Mascot
950.571	950.5679	-0.0031	-3	203	210	VPFLFTVK	59	99.991			Mascot
1080.5573	1080.563	0.0057	5	86	94	LTFDEIQSK					Mascot
1159.6106	1159.5874	-0.0232	-20	147	156	NEPPAFQKTK					Mascot
1236.6583	1236.6716	0.0133	11	85	94	RLTFDEIQSK					Mascot
1236.6583	1236.6716	0.0133	11	85	94	RLTFDEIQSK	34	97.506			Mascot
1328.6555	1328.6542	-0.0013	-1	130	140	FCLEPTSFTVK			Carbamidomethyl (C)[2]		Mascot
1456.7505	1456.749	-0.0015	-1	129	140	KFCLEPTSFTVK			Carbamidomethyl (C)[3]		Mascot
1506.7655	1506.7395	-0.026	-17	1	15	MAASLQAAATLMPAK			Oxidation (M)[1,12]		Mascot
1562.7559	1562.7727	0.0168	11	242	258	GGSTGYDNAVALPAGGR					Mascot
1562.7559	1562.7727	0.0168	11	242	258	GGSTGYDNAVALPAGGR	145	100			Mascot
1742.9581	1742.8796	-0.0785	-45	2	19	AASLQAAATLMPAKIGGR			Oxidation (M)[11]		Mascot
1760.8813	1760.9	0.0187	11	186	202	DGIDYAAVTVQLPGGER					Mascot
1760.8813	1760.9	0.0187	11	186	202	DGIDYAAVTVQLPGGER	136	100			Mascot
1775.8784	1775.8879	0.0095	5	240	258	GRGGSTGYDNAVALPAG GR					Mascot
2168.9917	2168.9822	-0.0095	-4	101	121	GTGTANQCPTIDGGVDS FPFK			Carbamidomethyl (C)[8]		Mascot
2168.9917	2168.9822	-0.0095	-4	101	121	GTGTANQCPTIDGGVDS FPFK	103	100	Carbamidomethyl (C)[8]		Mascot
2280.2024	2280.2263	0.0239	10	211	231	QLVATGKPESFSGPFLVP SYR					Mascot
2280.2024	2280.2263	0.0239	10	211	231	QLVATGKPESFSGPFLVP SYR	147	100			Mascot
2294.1299	2294.1667	0.0368	16	182	202	FEEKDGIDYAAVTVQLPG GER					Mascot
2434.1482	2434.1724	0.0242	10	242	266	GGSTGYDNAVALPAGGR GDEEELAK					Mascot
2590.2883	2590.2869	-0.0014	-1	286	310	SKPETGEVIGVFESVQPS DTDLGAK					Mascot

Rank	Protein Name	Accession No.	Protein MW	Protein PI	Pep. Count	Protein Score	Protein Score C. I. %	Intensity Matched	Total Ion Score	Total Ion C. I. %	Confirmed
2	photosystem II oxygen-evolving complex protein 1 -	gi|100831	34946.8	8.73	8	301	100	20.132	273	100	

common wheat x Sanduri wheat

Protein Group

33kDa oxygen evolving protein of photosystem II [Triticum aestivum]	gi|21844	34946.8	8.7299 995422 3633
RecName: Full=Oxygen-evolving enhancer protein 1, chloroplastic; Short=OEE1; AltName: Full=33 kDa subunit of oxygen evolving system of photosystem II; AltName: Full=33 kDa thylakoid membrane protein; AltName: Full=OEC 33 kDa subunit; Flags: Precurso	gi|131388	34946.8	8.7299 995422 3633

Peptide Information

Calc. Mass	Obsrv. Mass	± da	± ppm	Start Seq.	End Seq.	Sequence	Ion Score	C. I. %	Modification	Rank	Result Type
850.4305	850.4356	0.0051	6	231	238	GSSFLDPK					Mascot
1080.5573	1080.563	0.0057	5	86	94	LTFDEIQSK					Mascot
1236.6583	1236.6716	0.0133	11	85	94	RLTFDEIQSK					Mascot
1236.6583	1236.6716	0.0133	11	85	94	RLTFDEIQSK	34	97.506			Mascot
1328.6555	1328.6542	-0.0013	-1	130	140	FCLEPTSFTVK			Carbamidomethyl (C)[2]		Mascot
1456.7505	1456.749	-0.0015	-1	129	140	KFCLEPTSFTVK			Carbamidomethyl (C)[3]		Mascot
1760.8813	1760.9	0.0187	11	186	202	DGIDYAAVTVQLPGGER					Mascot
1760.8813	1760.9	0.0187	11	186	202	DGIDYAAVTVQLPGGER	136	100			Mascot
2168.9917	2168.9822	-0.0095	-4	101	121	GTGTANQCPTIDGGVDS FPFK			Carbamidomethyl (C)[8]		Mascot
2168.9917	2168.9822	-0.0095	-4	101	121	GTGTANQCPTIDGGVDS FPFK	103	100	Carbamidomethyl (C)[8]		Mascot
2294.1299	2294.1667	0.0368	16	182	202	FEEKDGIDYAAVTVQLPG GER					Mascot

3 ferritin 2B, partial [Triticum aestivum] gi|210061151 22706.2 4.99 9 69 99.456 11.012 18 0

Peptide Information

Calc. Mass	Obsrv. Mass	± da	± ppm	Start Seq.	End Seq.	Sequence	Ion Score	C. I. %	Modification	Rank	Result Type
850.3862	850.4356	0.0494	58	190	196	MLLEEEA			Oxidation (M)[1]		Mascot
1108.5997	1108.5779	-0.0218	-20	168	176	ISEYVTQLR					Mascot
1235.6671	1235.6375	-0.0296	-24	1	10	VVFQPFEELK					Mascot
1236.6947	1236.6716	-0.0231	-19	167	176	KISEYVTQLR					Mascot
1236.6947	1236.6716	-0.0231	-19	167	176	KISEYVTQLR	18	0			Mascot
1264.7008	1264.6732	-0.0276	-22	168	177	ISEYVTQLRR					Mascot
1582.6625	1582.8055	0.143	90	80	91	EHAEMLMEYQNR			Oxidation (M)[5,7]		Mascot
1582.6625	1582.8055	0.143	90	80	91	EHAEMLMEYQNR			Oxidation (M)[5,7]		Mascot
1607.835	1607.7776	-0.0574	-36	114	128	GDALYAMELALALEK					Mascot

Calc. Mass	Obsrv. Mass	± da	± ppm	Start Seq.	End Seq.	Sequence	Ion Score	C. I. %	Modification	Rank	Result Type
1738.7635	1738.8522	0.0887	51	80	92	EHAEMLMEYQNRR			Oxidation (M)[5,7]		Mascot
2191.168	2190.9871	-0.1809	-83	114	133	GDALYAMELALALEKLVN EK					Mascot
2207.1628	2206.9785	-0.1843	-84	114	133	GDALYAMELALALEKLVN EK			Oxidation (M)[7]		Mascot

4 ferritin 2B [Triticum aestivum] gi|210061149 28376.2 5.45 9 62 97.21 11.227 18 0

Peptide Information

Calc. Mass	Obsrv. Mass	± da	± ppm	Start Seq.	End Seq.	Sequence	Ion Score	C. I. %	Modification	Rank	Result Type
850.3862	850.4356	0.0494	58	248	254	MLLEEEA			Oxidation (M)[1]		Mascot
1108.5997	1108.5779	-0.0218	-20	226	234	ISEYVTQLR					Mascot
1236.6947	1236.6716	-0.0231	-19	225	234	KISEYVTQLR					Mascot
1236.6947	1236.6716	-0.0231	-19	225	234	KISEYVTQLR	18	0			Mascot
1264.7008	1264.6732	-0.0276	-22	226	235	ISEYVTQLRR					Mascot
1582.6625	1582.8055	0.143	90	138	149	EHAEMLMEYQNR			Oxidation (M)[5,7]		Mascot
1582.6625	1582.8055	0.143	90	138	149	EHAEMLMEYQNR			Oxidation (M)[5,7]		Mascot
1607.835	1607.7776	-0.0574	-36	172	186	GDALYAMELALALEK					Mascot
1738.7635	1738.8522	0.0887	51	138	150	EHAEMLMEYQNRR			Oxidation (M)[5,7]		Mascot
2191.168	2190.9871	-0.1809	-83	172	191	GDALYAMELALALEKLVN EK					Mascot
2207.1628	2206.9785	-0.1843	-84	172	191	GDALYAMELALALEKLVN EK			Oxidation (M)[7]		Mascot
2294.1738	2294.1667	-0.0071	-3	49	68	GNKEEVLLSGVMFQPFE ELK					Mascot

5 ferritin 2C, partial [Triticum aestivum] gi|210061155 22734.3 4.99 8 60 95.777 10.751 18 0

Peptide Information

Calc. Mass	Obsrv. Mass	± da	± ppm	Start Seq.	End Seq.	Sequence	Ion Score	C. I. %	Modification	Rank	Result Type
850.3862	850.4356	0.0494	58	190	196	MLLEEEA			Oxidation (M)[1]		Mascot
1108.5997	1108.5779	-0.0218	-20	168	176	ISEYVTQLR					Mascot
1235.6671	1235.6375	-0.0296	-24	1	10	VVFQPFEELK					Mascot
1236.6947	1236.6716	-0.0231	-19	167	176	KISEYVTQLR					Mascot
1236.6947	1236.6716	-0.0231	-19	167	176	KISEYVTQLR	18	0			Mascot
1264.7008	1264.6732	-0.0276	-22	168	177	ISEYVTQLRR					Mascot
1607.835	1607.7776	-0.0574	-36	114	128	GDALYAMELALALEK					Mascot
1610.6937	1610.8398	0.1461	91	80	91	EHVEMLMEYQNR			Oxidation (M)[5,7]		Mascot
2191.168	2190.9871	-0.1809	-83	114	133	GDALYAMELALALEKLVN EK					Mascot
2207.1628	2206.9785	-0.1843	-84	114	133	GDALYAMELALALEKLVN EK			Oxidation (M)[7]		Mascot

6 wheat 33K [Triticum aestivum] gi|579237 10683.4 9.44 4 56 90.325 9.461 34 97.506

Peptide Information

Calc. Mass	Obsrv. Mass	± da	± ppm	Start Seq.	End Seq.	Sequence	Ion Score	C. I. %	Modification	Rank	Result Type
1080.5573	1080.563	0.0057	5	86	94	LTFDEIQSK					Mascot
1236.6583	1236.6716	0.0133	11	85	94	RLTFDEIQSK					Mascot
1236.6583	1236.6716	0.0133	11	85	94	RLTFDEIQSK	34	97.506			Mascot
1506.7655	1506.7395	-0.026	-17	1	15	MAASLQAAATLMPAK			Oxidation (M)[1,12]		Mascot
1742.9581	1742.8796	-0.0785	-45	2	19	AASLQAAATLMPAKIGGR			Oxidation (M)[11]		Mascot

7 Disease resistance protein RPM1 [Triticum urartu] gi|473926895 104315.5 7.81 18 53 76.792 18.667 4 0

Peptide Information

Calc. Mass	Obsrv. Mass	± da	± ppm	Start Seq.	End Seq.	Sequence	Ion Score	C. I. %	Modification	Rank	Result Type
1011.5721	1011.5108	-0.0613	-61	199	207	TTLVTSVYK					Mascot
1035.6422	1035.567	-0.0752	-73	526	533	IRHLILDR					Mascot
1106.551	1106.5498	-0.0012	-1	516	525	CSPALESSKK			Carbamidomethyl (C)[1]		Mascot
1205.6121	1205.6331	0.021	17	307	318	SQDVASLAASTR					Mascot
1214.674	1214.5718	-0.1022	-84	781	792	GDSIGLLSSLPR					Mascot
1245.7314	1245.6614	-0.07	-56	41	50	DLELLRAFLR					Mascot
1264.6639	1264.6732	0.0093	7	1	12	MAEMIAISLSAK					Mascot
1346.6661	1346.6752	0.0091	7	913	923	TIYTNLFTVQM			Oxidation (M)[11]		Mascot
1428.577	1428.7076	0.1306	91	246	257	AGMPDIDEMDYR			Oxidation (M)[3]		Mascot
1456.7893	1456.749	-0.0403	-28	598	610	STLIEELPQELGK					Mascot
1544.8656	1544.7468	-0.1188	-77	258	271	SLVEALHGHLANKR					Mascot
1562.8009	1562.7727	-0.0282	-18	94	107	ACANFGAWVALARR			Carbamidomethyl (C)[2]		Mascot
1562.8009	1562.7727	-0.0282	-18	94	107	ACANFGAWVALARR	4	0	Carbamidomethyl (C)[2]		Mascot
1738.9963	1738.8522	-0.1441	-83	759	773	LPSWVGRLDSLVQLR					Mascot
1760.9575	1760.9	-0.0575	-33	766	780	LDSLVQLRLCSSELK			Carbamidomethyl (C)[10]		Mascot
1760.9575	1760.9	-0.0575	-33	766	780	LDSLVQLRLCSSELK			Carbamidomethyl (C)[10]		Mascot
1782.8943	1782.8804	-0.0139	-8	676	691	YVEADENMVKSLGSLK					Mascot
1788.801	1788.9297	0.1287	72	327	340	EQEAWSLFCNTTFR			Carbamidomethyl (C)[9]		Mascot
2169.1882	2168.9822	-0.206	-95	362	381	CCGLPLAIVSVGNLLALK DR			Carbamidomethyl (C)[1,2]		Mascot
2169.1882	2168.9822	-0.206	-95	362	381	CCGLPLAIVSVGNLLALK DR			Carbamidomethyl (C)[1,2]		Mascot
2269.1948	2269.1021	-0.0927	-41	151	171	LAEAAHFVEHGEIVGVAA HRR					Mascot

8	ferritin 2A [Triticum aestivum]	gi|210061143	28377.2	5.56	8	52	70.103	10.875	18	0

Protein Group

ferritin 2A [Triticum aestivum]	gi|210061147	28353.3	5.5599 999427 7954

Peptide Information

Calc. Mass	Obsrv. Mass	± da	± ppm	Start Seq.	End Seq.	Sequence	Ion Score	C. I. %	Modification	Rank	Result Type
850.3862	850.4356	0.0494	58	247	253	MLLEEEA			Oxidation (M)[1]		Mascot
1108.5997	1108.5779	-0.0218	-20	225	233	ISEYVTQLR					Mascot
1236.6947	1236.6716	-0.0231	-19	224	233	KISEYVTQLR					Mascot
1236.6947	1236.6716	-0.0231	-19	224	233	KISEYVTQLR	18	0			Mascot
1264.7008	1264.6732	-0.0276	-22	225	234	ISEYVTQLRR					Mascot
1582.6625	1582.8055	0.143	90	137	148	EHAEMLMEYQNR			Oxidation (M)[5,7]		Mascot
1582.6625	1582.8055	0.143	90	137	148	EHAEMLMEYQNR			Oxidation (M)[5,7]		Mascot
1607.835	1607.7776	-0.0574	-36	171	185	GDALYAMELALALEK					Mascot
1738.7635	1738.8522	0.0887	51	137	149	EHAEMLMEYQNRR			Oxidation (M)[5,7]		Mascot
2191.168	2190.9871	-0.1809	-83	171	190	GDALYAMELALALEKLVN EK					Mascot
2207.1628	2206.9785	-0.1843	-84	171	190	GDALYAMELALALEKLVN EK			Oxidation (M)[7]		Mascot

9	ferritin 2A, partial [Triticum aestivum]	gi|210061145	9083.7	5.47	5	51	64.874	9.368	18	0

Peptide Information

Calc. Mass	Obsrv. Mass	± da	± ppm	Start Seq.	End Seq.	Sequence	Ion Score	C. I. %	Modification	Rank	Result Type
850.3862	850.4356	0.0494	58	72	78	MLLEEEA			Oxidation (M)[1]		Mascot
1108.5997	1108.5779	-0.0218	-20	50	58	ISEYVTQLR					Mascot
1236.6947	1236.6716	-0.0231	-19	49	58	KISEYVTQLR					Mascot
1236.6947	1236.6716	-0.0231	-19	49	58	KISEYVTQLR	18	0			Mascot
1264.7008	1264.6732	-0.0276	-22	50	59	ISEYVTQLRR					Mascot
1715.9612	1715.8752	-0.086	-50	1	15	AMELVLALEKLVNEK			Oxidation (M)[2]		Mascot

10	hypothetical protein TRIUR3_09884 [Triticum urartu]	gi|473810121	34849.4	6.17	10	46	0	2.089		

Peptide Information

Calc. Mass	Obsrv. Mass	± da	± ppm	Start Seq.	End Seq.	Sequence	Ion Score	C. I. %	Modification	Rank	Result Type
930.4938	930.4703	-0.0235	-25	245	252	GRSNLMPR					Mascot

1128.5242	1128.5408	0.0166	15	117	126	LDGFLMDSSK	Oxidation (M)[6]	Mascot
1205.5619	1205.6331	0.0712	59	187	195	HCELYLENK	Carbamidomethyl (C)[2]	Mascot
1245.7328	1245.6614	-0.0714	-57	78	87	LLHVARPFHR		Mascot
1338.6479	1338.6709	0.023	17	1	10	MWIGMRMELR	Oxidation (M)[1]	Mascot
1346.7023	1346.6752	-0.0271	-20	281	292	DTTRDGQIVSVR		Mascot
1428.7706	1428.7076	-0.063	-44	235	246	NILDAQSWIRGR		Mascot
1456.827	1456.749	-0.078	-54	233	244	LKNILDAQSWIR		Mascot
1549.6985	1549.7435	0.045	29	65	77	DLSLCTDDPNMIR	Carbamidomethyl (C)[5]	Mascot
2279.1753	2279.2119	0.0366	16	36	54	HLQAPELLSLSATCLWFH R	Carbamidomethyl (C)[14]	Mascot

Gel Idx/Pos	176/H3	Instr./Gel Origin	BA2151/Sample Project 20140814	Process Status	Analysis Succeeded
Plate [#] Name	[1] Sample Project 20140814	Instrument Sample Name		Spectra	11

Rank	Protein Name	Accession No.	Protein MW	Protein PI	Pep. Count	Protein Score	Protein Score C. I. %	Intensity Matched	Total Ion Score	Total Ion C. I. %	Confirmed
1	ADP glucose pyrophosphorylase [Triticum aestivum]	gi|469952290	52399.6	5.54	20	465	100	40.291	348	100	

Protein Group

Protein Name	Accession No.	Protein MW	Protein PI
ADP-glucose pyrophosphorylase [Triticum aestivum]	gi|21687	52399.6	5.5399999618 5303
RecName: Full=Glucose-1-phosphate adenylyltransferase small subunit, chloroplastic/amyloplastic; AltName: Full=ADP-glucose pyrophosphorylase; AltName: Full=ADP-glucose synthase; AltName: Full=AGPase B; AltName: Full=Alpha-D-glucose-1-phosphate adeny	gi|232172	52399.6	5.5399999618 5303
unnamed protein product [Triticum aestivum]	gi|300634091	52399.6	5.5399999618 5303
unnamed protein product [Triticum aestivum]	gi|300544577	52399.6	5.5399999618 5303
unnamed protein product [Triticum aestivum]	gi|259471779	52399.6	5.5399999618 5303
unnamed protein product [Triticum aestivum]	gi|259439192	52399.6	5.5399999618 5303
unnamed protein product [Triticum aestivum]	gi|257671726	52399.6	5.5399999618 5303
unnamed protein product [Triticum aestivum]	gi|257306995	52399.6	5.5399999618 5303
unnamed protein product [Triticum aestivum]	gi|257308999	52399.6	5.5399999618 5303

unnamed protein product [Triticum aestivum]	gi|219764760	52399.6	5.5399<br>999618<br>5303
unnamed protein product [Triticum aestivum]	gi|219752173	52399.6	5.5399<br>999618<br>5303

Peptide Information

Calc. Mass	Obsrv. Mass	± da	± ppm	Start Seq.	End Seq.	Sequence	Ion Score	C. I. %	Modification	Rank	Result Type
854.444	854.4503	0.0063	7	200	207	ATAFGLMK			Oxidation (M)[7]		Mascot
972.4673	972.473	0.0057	6	448	455	ETDGYFIK					Mascot
1009.5975	1009.6042	0.0067	7	260	267	HVMLQLLR					Mascot
1017.5952	1017.603	0.0078	8	366	374	IHHSVVGLR					Mascot
1017.5952	1017.603	0.0078	8	366	374	IHHSVVGLR	69	99.999			Mascot
1025.5925	1025.5934	0.0009	1	260	267	HVMLQLLR			Oxidation (M)[3]		Mascot
1025.5925	1025.5934	0.0009	1	260	267	HVMLQLLR			Oxidation (M)[3]		Mascot
1032.5472	1032.5532	0.006	6	330	338	SAPIYTQPR					Mascot
1032.5472	1032.5532	0.006	6	330	338	SAPIYTQPR	43	99.569			Mascot
1074.6194	1074.6204	0.001	1	214	222	IIEFAEKPK					Mascot
1256.5801	1256.6061	0.026	21	18	27	REQCNIDGHK			Carbamidomethyl (C)[4]		Mascot
1384.6896	1384.7012	0.0116	8	319	329	KPIPDFSFYDR					Mascot
1384.6896	1384.7012	0.0116	8	319	329	KPIPDFSFYDR	103	100			Mascot
1390.8165	1390.6974	-0.1191	-86	407	420	GGIPIGIGKNSHIK					Mascot
1489.67	1489.7454	0.0754	51	19	31	EQCNIDGHKSSSK			Carbamidomethyl (C)[3]		Mascot
1522.7761	1522.7578	-0.0183	-12	106	119	HLSRAYGSNIGGYK					Mascot
1553.7628	1553.8525	0.0897	58	200	213	ATAFGLMKIDEEGR			Oxidation (M)[7]		Mascot
1621.7925	1621.7865	-0.006	-4	228	242	AMMVDTTILGLDDAR					Mascot
1637.7874	1637.7786	-0.0088	-5	228	242	AMMVDTTILGLDDAR			Oxidation (M)[2]		Mascot
1653.7822	1653.774	-0.0082	-5	228	242	AMMVDTTILGLDDAR			Oxidation (M)[2,3]		Mascot
1657.8796	1657.8049	-0.0747	-45	2	17	DVPLASKTFPSPSPSK					Mascot
1786.9368	1786.9772	0.0404	23	76	91	LIDIPVSNCLNSNISK			Carbamidomethyl (C)[9]		Mascot
1889.9525	1889.9342	-0.0183	-10	345	362	VLDADVTDSVIGEGCVIK			Carbamidomethyl (C)[15]		Mascot
1970.9965	1971.0115	0.015	8	430	447	IGDNVMIINVDNVQEAAR					Mascot
1970.9965	1971.0115	0.015	8	430	447	IGDNVMIINVDNVQEAAR	133	100			Mascot
1986.9913	1986.9746	-0.0167	-8	430	447	IGDNVMIINVDNVQEAAR			Oxidation (M)[6]		Mascot

Calc. Mass	Obsrv. Mass	± da	± ppm	Start Seq.	End Seq.	Sequence	Ion Score	C. I. %	Modification	Rank	Result Type
1986.9913	1986.9746	-0.0167	-8	430	447	IGDNVMIINVDNVQEAAR	96	100	Oxidation (M)[6]		Mascot
2318.2537	2318.1223	-0.1314	-57	76	95	LIDIPVSNCLNSNISKIYVR			Carbamidomethyl (C)[9]		Mascot
2368.0874	2368.1069	0.0195	8	268	290	EQFPGANDFGSEVIPGAT STGMR					Mascot
2398.219	2398.1899	-0.0291	-12	319	338	KPIPDFSFYDRSAPIYTQP R					Mascot

2	plastid ADP-glucose pyrophosphorylase small subunit [Triticum aestivum]	gi|224021585	56652.9	6.4	20	460	100	40.405	348	100	

Peptide Information

Calc. Mass	Obsrv. Mass	± da	± ppm	Start Seq.	End Seq.	Sequence	Ion Score	C. I. %	Modification	Rank	Result Type
854.444	854.4503	0.0063	7	241	248	ATAFGLMK			Oxidation (M)[7]		Mascot
972.4673	972.473	0.0057	6	489	496	ETDGYFIK					Mascot
1009.5975	1009.6042	0.0067	7	301	308	HVMLQLLR					Mascot
1017.5952	1017.603	0.0078	8	407	415	IHHSVVGLR					Mascot
1017.5952	1017.603	0.0078	8	407	415	IHHSVVGLR	69	99.999			Mascot
1025.5925	1025.5934	0.0009	1	301	308	HVMLQLLR			Oxidation (M)[3]		Mascot
1025.5925	1025.5934	0.0009	1	301	308	HVMLQLLR			Oxidation (M)[3]		Mascot
1032.5472	1032.5532	0.006	6	371	379	SAPIYTQPR					Mascot
1032.5472	1032.5532	0.006	6	371	379	SAPIYTQPR	43	99.569			Mascot
1074.6194	1074.6204	0.001	1	255	263	IIEFAEKPK					Mascot
1256.7109	1256.6061	-0.1048	-83	105	116	AKPAVPLGANYR					Mascot
1384.6896	1384.7012	0.0116	8	360	370	KPIPDFSFYDR					Mascot
1384.6896	1384.7012	0.0116	8	360	370	KPIPDFSFYDR	103	100			Mascot
1390.8165	1390.6974	-0.1191	-86	448	461	GGIPIGIGKNSHIK					Mascot
1522.7761	1522.7578	-0.0183	-12	147	160	HLSRAYGSNIGGYK					Mascot
1553.7628	1553.8525	0.0897	58	241	254	ATAFGLMKIDEEGR			Oxidation (M)[7]		Mascot
1573.6912	1573.7963	0.1051	67	19	34	ASAATAAASTSCDSFR			Carbamidomethyl (C)[12]		Mascot
1621.7925	1621.7865	-0.006	-4	269	283	AMMVDTTILGLDDAR					Mascot
1637.7874	1637.7786	-0.0088	-5	269	283	AMMVDTTILGLDDAR			Oxidation (M)[2]		Mascot
1653.7822	1653.774	-0.0082	-5	269	283	AMMVDTTILGLDDAR			Oxidation (M)[2,3]		Mascot
1786.9368	1786.9772	0.0404	23	117	132	LIDIPVSNCLNSNISK			Carbamidomethyl (C)[9]		Mascot
1889.9525	1889.9342	-0.0183	-10	386	403	VLDADVTDSVIGEGCVIK			Carbamidomethyl (C)[15]		Mascot
1894.0038	1894.0343	0.0305	16	1	18	MAMAAAASPSKILIPPHR			Oxidation (M)[1,3]		Mascot
1970.9965	1971.0115	0.015	8	471	488	IGDNVMIINVDNVQEAAR					Mascot
1970.9965	1971.0115	0.015	8	471	488	IGDNVMIINVDNVQEAAR	133	100			Mascot
1986.9913	1986.9746	-0.0167	-8	471	488	IGDNVMIINVDNVQEAAR			Oxidation (M)[6]		Mascot

1986.9913	1986.9746	-0.0167	-8	471	488	IGDNVMIINVDNVQEAAR	96	100	Oxidation (M)[6]		Mascot
2368.0874	2368.1069	0.0195	8	309	331	EQFPGANDFGSEVIPGAT STGMR					Mascot
2398.219	2398.1899	-0.0291	-12	360	379	KPIPDFSFYDRSAPIYTQP R					Mascot
2400.209	2400.1528	-0.0562	-23	12	34	ILIPPHRASAATAAASTSC DSFR			Carbamidomethyl (C)[19]		Mascot

3	cytosolic small subunit ADP glucose pyrophosphorylase [Triticum aestivum]	gi|125976023	52277.6	5.53	19	455	100	40.809	348	100	

Peptide Information

Calc. Mass	Obsrv. Mass	± da	± ppm	Start Seq.	End Seq.	Sequence	Ion Score	C. I. %	Modification	Rank	Result Type
854.444	854.4503	0.0063	7	200	207	ATAFGLMK			Oxidation (M)[7]		Mascot
972.4673	972.473	0.0057	6	448	455	ETDGYFIK					Mascot
1009.5975	1009.6042	0.0067	7	260	267	HVMLQLLR					Mascot
1017.5952	1017.603	0.0078	8	366	374	IHHSVVGLR					Mascot
1017.5952	1017.603	0.0078	8	366	374	IHHSVVGLR	69	99.999			Mascot
1025.5925	1025.5934	0.0009	1	260	267	HVMLQLLR			Oxidation (M)[3]		Mascot
1025.5925	1025.5934	0.0009	1	260	267	HVMLQLLR			Oxidation (M)[3]		Mascot
1032.5472	1032.5532	0.006	6	330	338	SAPIYTQPR					Mascot
1032.5472	1032.5532	0.006	6	330	338	SAPIYTQPR	43	99.569			Mascot
1074.6194	1074.6204	0.001	1	214	222	IIEFAEKPK					Mascot
1256.7109	1256.6061	-0.1048	-83	64	75	AKPAVPLGANYR					Mascot
1384.6896	1384.7012	0.0116	8	319	329	KPIPDFSFYDR					Mascot
1384.6896	1384.7012	0.0116	8	319	329	KPIPDFSFYDR	103	100			Mascot
1390.8165	1390.6974	-0.1191	-86	407	420	GGIPIGIGKNSHIK					Mascot
1475.6544	1475.7617	0.1073	73	19	31	EQCNVDGHKSSSK			Carbamidomethyl (C)[3]		Mascot
1522.7761	1522.7578	-0.0183	-12	106	119	HLSRAYGSNIGGYK					Mascot
1553.7628	1553.8525	0.0897	58	200	213	ATAFGLMKIDEEGR			Oxidation (M)[7]		Mascot
1621.7925	1621.7865	-0.006	-4	228	242	AMMVDTTILGLDDAR					Mascot
1637.7874	1637.7786	-0.0088	-5	228	242	AMMVDTTILGLDDAR			Oxidation (M)[2]		Mascot
1653.7822	1653.774	-0.0082	-5	228	242	AMMVDTTILGLDDAR			Oxidation (M)[2,3]		Mascot
1657.8796	1657.8049	-0.0747	-45	2	17	DVPLASKTFPSPSPSK					Mascot
1829.8763	1829.9368	0.0605	33	183	199	ESDADITVAALPVDEER					Mascot
1889.9525	1889.9342	-0.0183	-10	345	362	VLDADVTDSVIGEGCVIK			Carbamidomethyl (C)[15]		Mascot
1970.9965	1971.0115	0.015	8	430	447	IGDNVMIINVDNVQEAAR					Mascot
1970.9965	1971.0115	0.015	8	430	447	IGDNVMIINVDNVQEAAR	133	100			Mascot
1986.9913	1986.9746	-0.0167	-8	430	447	IGDNVMIINVDNVQEAAR			Oxidation (M)[6]		Mascot
1986.9913	1986.9746	-0.0167	-8	430	447	IGDNVMIINVDNVQEAAR	96	100	Oxidation (M)[6]		Mascot

2368.0874	2368.1069	0.0195	8	268	290	EQFPGANDFGSEVIPGAT STGMR						Mascot
2398.219	2398.1899	-0.0291	-12	319	338	KPIPDFSFYDRSAPIYTQP R						Mascot

4	small subunit ADP glucose pyrophosphorylase [Triticum aestivum]	gi|7340287	52313.6	5.53	19	455	100	40.04	348	100	

Protein Group

small subunit ADP glucose pyrophosphorylase [Triticum aestivum]	gi|20127139	52313.6	5.5300 002098 0835
unnamed protein product [Triticum aestivum]	gi|257671690	52313.6	5.5300 002098 0835
unnamed protein product [Triticum aestivum]	gi|257304117	52313.6	5.5300 002098 0835
unnamed protein product [Triticum aestivum]	gi|257308963	52313.6	5.5300 002098 0835
unnamed protein product [Triticum aestivum]	gi|219764724	52313.6	5.5300 002098 0835
unnamed protein product [Triticum aestivum]	gi|219752137	52313.6	5.5300 002098 0835

Peptide Information

Calc. Mass	Obsrv. Mass	± da	± ppm	Start Seq.	End Seq.	Sequence	Ion Score	C. I. %	Modification	Rank	Result Type
854.444	854.4503	0.0063	7	200	207	ATAFGLMK			Oxidation (M)[7]		Mascot
972.4673	972.473	0.0057	6	448	455	ETDGYFIK					Mascot
1009.5975	1009.6042	0.0067	7	260	267	HVMLQLLR					Mascot
1017.5952	1017.603	0.0078	8	366	374	IHHSVVGLR					Mascot
1017.5952	1017.603	0.0078	8	366	374	IHHSVVGLR	69	99.999			Mascot
1025.5925	1025.5934	0.0009	1	260	267	HVMLQLLR			Oxidation (M)[3]		Mascot
1025.5925	1025.5934	0.0009	1	260	267	HVMLQLLR			Oxidation (M)[3]		Mascot
1032.5472	1032.5532	0.006	6	330	338	SAPIYTQPR					Mascot

1032.5472	1032.5532	0.006	6	330	338	SAPIYTQPR	43	99.569			Mascot
1074.6194	1074.6204	0.001	1	214	222	IIEFAEKPK					Mascot
1256.7109	1256.6061	-0.1048	-83	64	75	AKPAVPLGANYR					Mascot
1384.6896	1384.7012	0.0116	8	319	329	KPIPDFSFYDR					Mascot
1384.6896	1384.7012	0.0116	8	319	329	KPIPDFSFYDR	103	100			Mascot
1390.8165	1390.6974	-0.1191	-86	407	420	GGIPIGIGKNSHIK					Mascot
1475.6544	1475.7617	0.1073	73	19	31	EQCNVDGHKSSSK			Carbamidomethyl (C)[3]		Mascot
1522.7761	1522.7578	-0.0183	-12	106	119	HLSRAYGSNIGGYK					Mascot
1553.7628	1553.8525	0.0897	58	200	213	ATAFGLMKIDEEGR			Oxidation (M)[7]		Mascot
1621.7925	1621.7865	-0.006	-4	228	242	AMMVDTTILGLDDAR					Mascot
1637.7874	1637.7786	-0.0088	-5	228	242	AMMVDTTILGLDDAR			Oxidation (M)[2]		Mascot
1653.7822	1653.774	-0.0082	-5	228	242	AMMVDTTILGLDDAR			Oxidation (M)[2,3]		Mascot
1657.8796	1657.8049	-0.0747	-45	2	17	DVPLASKTFPSPSPSK					Mascot
1786.9368	1786.9772	0.0404	23	76	91	LIDIPVSNCLNSNISK			Carbamidomethyl (C)[9]		Mascot
1889.9525	1889.9342	-0.0183	-10	345	362	VLDADVTDSVIGEGCVIK			Carbamidomethyl (C)[15]		Mascot
1970.9965	1971.0115	0.015	8	430	447	IGDNVMIINVDNVQEAAR					Mascot
1970.9965	1971.0115	0.015	8	430	447	IGDNVMIINVDNVQEAAR	133	100			Mascot
1986.9913	1986.9746	-0.0167	-8	430	447	IGDNVMIINVDNVQEAAR			Oxidation (M)[6]		Mascot
1986.9913	1986.9746	-0.0167	-8	430	447	IGDNVMIINVDNVQEAAR	96	100	Oxidation (M)[6]		Mascot
2368.0874	2368.1069	0.0195	8	268	290	EQFPGANDFGSEVIPGAT STGMR					Mascot
2398.219	2398.1899	-0.0291	-12	319	338	KPIPDFSFYDRSAPIYTQP R					Mascot

5	plastid ADP-glucose pyrophosphorylase small subunit [Triticum aestivum]	gi|182894563	52263.6	5.48	14	413	100	38.153	348	100	

Peptide Information

Calc. Mass	Obsrv. Mass	± da	± ppm	Start Seq.	End Seq.	Sequence	Ion Score	C. I. %	Modification	Rank	Result Type
972.4673	972.473	0.0057	6	450	457	ETDGYFIK					Mascot
1009.5975	1009.6042	0.0067	7	262	269	HVMLQLLR					Mascot
1017.5952	1017.603	0.0078	8	368	376	IHHSVVGLR					Mascot
1017.5952	1017.603	0.0078	8	368	376	IHHSVVGLR	69	99.999			Mascot
1025.5925	1025.5934	0.0009	1	262	269	HVMLQLLR			Oxidation (M)[3]		Mascot
1025.5925	1025.5934	0.0009	1	262	269	HVMLQLLR			Oxidation (M)[3]		Mascot
1032.5472	1032.5532	0.006	6	332	340	SAPIYTQPR					Mascot
1032.5472	1032.5532	0.006	6	332	340	SAPIYTQPR	43	99.569			Mascot
1256.7109	1256.6061	-0.1048	-83	66	77	AKPAVPLGANYR					Mascot

1384.6896	1384.7012	0.0116	8	321	331	KPIPDFSFYDR					Mascot
1384.6896	1384.7012	0.0116	8	321	331	KPIPDFSFYDR	103	100			Mascot
1390.8165	1390.6974	-0.1191	-86	409	422	GGIPIGIGKNSHIK					Mascot
1522.7761	1522.7578	-0.0183	-12	108	121	HLSRAYGSNIGGYK					Mascot
1621.7925	1621.7865	-0.006	-4	230	244	AMMVDTTILGLDDAR					Mascot
1637.7874	1637.7786	-0.0088	-5	230	244	AMMVDTTILGLDDAR			Oxidation (M)[2]		Mascot
1653.7822	1653.774	-0.0082	-5	230	244	AMMVDTTILGLDDAR			Oxidation (M)[2,3]		Mascot
1786.9368	1786.9772	0.0404	23	78	93	LIDIPVSNCLNSNISK			Carbamidomethyl (C)[9]		Mascot
1889.9525	1889.9342	-0.0183	-10	347	364	VLDADVTDSVIGEGCVIK			Carbamidomethyl (C)[15]		Mascot
1970.9965	1971.0115	0.015	8	432	449	IGDNVMIINVDNVQEAAR					Mascot
1970.9965	1971.0115	0.015	8	432	449	IGDNVMIINVDNVQEAAR	133	100			Mascot
1986.9913	1986.9746	-0.0167	-8	432	449	IGDNVMIINVDNVQEAAR			Oxidation (M)[6]		Mascot
1986.9913	1986.9746	-0.0167	-8	432	449	IGDNVMIINVDNVQEAAR	96	100	Oxidation (M)[6]		Mascot
2368.0874	2368.1069	0.0195	8	270	292	EQFPGANDFGSEVIPGAT STGMR					Mascot
2398.219	2398.1899	-0.0291	-12	321	340	KPIPDFSFYDRSAPIYTQP R					Mascot

6	Glucose-1-phosphate adenylyltransferase small subunit, chloroplastic/amyloplastic [Triticum urartu]	gi|474108293	65309.1	7.9	17	291	100	35.534	214	100	

Peptide Information

Calc. Mass	Obsrv. Mass	± da	± ppm	Start Seq.	End Seq.	Sequence	Ion Score	C. I. %	Modification	Rank	Result Type
854.444	854.4503	0.0063	7	241	248	ATAFGLMK			Oxidation (M)[7]		Mascot
1009.5975	1009.6042	0.0067	7	301	308	HVMLQLLR					Mascot
1017.5952	1017.603	0.0078	8	407	415	IHHSVVGLR					Mascot
1017.5952	1017.603	0.0078	8	407	415	IHHSVVGLR	69	99.999			Mascot
1025.5925	1025.5934	0.0009	1	301	308	HVMLQLLR			Oxidation (M)[3]		Mascot
1025.5925	1025.5934	0.0009	1	301	308	HVMLQLLR			Oxidation (M)[3]		Mascot
1032.5472	1032.5532	0.006	6	371	379	SAPIYTQPR					Mascot
1032.5472	1032.5532	0.006	6	371	379	SAPIYTQPR	43	99.569			Mascot
1074.6194	1074.6204	0.001	1	255	263	IIEFAEKPK					Mascot
1256.7109	1256.6061	-0.1048	-83	105	116	AKPAVPLGANYR					Mascot
1384.6896	1384.7012	0.0116	8	360	370	KPIPDFSFYDR					Mascot
1384.6896	1384.7012	0.0116	8	360	370	KPIPDFSFYDR	103	100			Mascot
1390.8165	1390.6974	-0.1191	-86	448	461	GGIPIGIGKNSHIK					Mascot
1522.7761	1522.7578	-0.0183	-12	147	160	HLSRAYGSNIGGYK					Mascot
1553.7628	1553.8525	0.0897	58	241	254	ATAFGLMKIDEEGR			Oxidation (M)[7]		Mascot
1621.7925	1621.7865	-0.006	-4	269	283	AMMVDTTILGLDDAR					Mascot

Calc. Mass	Obsrv. Mass	± da	± ppm	Start Seq.	End Seq.	Sequence	Ion Score	C. I. %	Modification	Rank	Result Type
1637.7874	1637.7786	-0.0088	-5	269	283	AMMVDTTILGLDDAR			Oxidation (M)[2]		Mascot
1653.7822	1653.774	-0.0082	-5	269	283	AMMVDTTILGLDDAR			Oxidation (M)[2,3]		Mascot
1786.9368	1786.9772	0.0404	23	117	132	LIDIPVSNCLNSNISK			Carbamidomethyl (C)[9]		Mascot
1889.9525	1889.9342	-0.0183	-10	386	403	VLDADVTDSVIGEGCVIK			Carbamidomethyl (C)[15]		Mascot
1894.0038	1894.0343	0.0305	16	1	18	MAMAAAASPSKILIPPHR			Oxidation (M)[1,3]		Mascot
2366.2246	2366.0886	-0.136	-57	12	34	ILIPPHRASAATAAASTSC DSLR			Carbamidomethyl (C)[19]		Mascot
2368.0874	2368.1069	0.0195	8	309	331	EQFPGANDFGSEVIPGAT STGMR					Mascot
2398.219	2398.1899	-0.0291	-12	360	379	KPIPDFSFYDRSAPIYTQP R					Mascot

7	chloroplast sigma factor [Triticum aestivum]	gi|4218586	52125.6	9.78	11	56	88.633	26.905	33	96.013

Peptide Information

Calc. Mass	Obsrv. Mass	± da	± ppm	Start Seq.	End Seq.	Sequence	Ion Score	C. I. %	Modification	Rank	Result Type
816.4825	816.4282	-0.0543	-67	328	334	LLNISEK					Mascot
950.4829	950.5363	0.0534	56	454	461	LDALLQDY					Mascot
960.5407	960.4539	-0.0868	-90	24	31	AQVMRALR			Oxidation (M)[4]		Mascot
992.5272	992.4458	-0.0814	-82	426	433	QFGLSRER					Mascot
1009.5247	1009.6042	0.0795	79	212	219	MRESFLAR					Mascot
1017.58	1017.603	0.023	23	99	108	GRSSSVVVAR					Mascot
1017.58	1017.603	0.023	23	99	108	GRSSSVVVAR	33	96.013			Mascot
1025.5197	1025.5934	0.0737	72	212	219	MRESFLAR			Oxidation (M)[1]		Mascot
1025.5197	1025.5934	0.0737	72	212	219	MRESFLAR			Oxidation (M)[1]		Mascot
1122.5902	1122.592	0.0018	2	285	294	ALADNSKTFR					Mascot
1245.6547	1245.6661	0.0114	9	280	291	QGVSRALADNSK					Mascot
1256.6998	1256.6061	-0.0937	-75	405	415	DIIQLYHGIGK					Mascot
1384.7695	1384.7012	-0.0683	-49	171	181	IKDGIWLQQQR					Mascot
1384.7695	1384.7012	-0.0683	-49	171	181	IKDGIWLQQQR					Mascot
1522.8951	1522.7578	-0.1373	-90	157	169	ELLTQKQVVHLSK					Mascot

8	Serine/threonine-protein kinase HT1 [Triticum urartu]	gi|473968294	76793.6	7.08	16	56	87.82	5.176

Peptide Information

Calc. Mass	Obsrv. Mass	± da	± ppm	Start Seq.	End Seq.	Sequence	Ion Score	C. I. %	Modification	Rank	Result Type
857.5567	857.4976	-0.0591	-69	590	596	ISKLIQR					Mascot
1060.5092	1060.5721	0.0629	59	65	74	LSGDDAPVMR					Mascot
1074.6418	1074.6204	-0.0214	-20	580	589	LVIPSGVHPR					Mascot

1108.6222	1108.5677	-0.0545	-49	170	178	AEDVLLHRR					Mascot
1120.6144	1120.568	-0.0464	-41	1	11	MTGTAAATKLR					Mascot
1193.6412	1193.6204	-0.0208	-17	384	394	GTYLDVDVAIK					Mascot
1205.6307	1205.6479	0.0172	14	238	247	LMEDLSLGRR			Oxidation (M)[2]		Mascot
1390.7764	1390.6974	-0.079	-57	407	417	VEFLQEIMILR					Mascot
1637.7952	1637.7786	-0.0166	-10	233	246	GFDERLMEDLSLGR					Mascot
1653.7902	1653.774	-0.0162	-10	233	246	GFDERLMEDLSLGR			Oxidation (M)[7]		Mascot
1669.9484	1669.7965	-0.1519	-91	488	503	DLKSANLLIGDGQVVK					Mascot
1731.9137	1731.8688	-0.0449	-26	127	143	VGTGARATWLALGTSDR					Mascot
1829.8083	1829.9368	0.1285	70	512	527	QRSQEGDMTAETGTYR					Mascot
1920.955	1920.9838	0.0288	15	357	372	IGESEFDRDLLQIEEK					Mascot
2003.1835	2002.9976	-0.1859	-93	638	656	MILNVVALGPVIITHLGSR					Mascot
2263.1104	2263.1924	0.082	36	437	455	YLIVTEYMAGGNLYDFLH K			Oxidation (M)[8]		Mascot
2397.2244	2397.1084	-0.116	-48	179	198	ILAECADPDNRPVFHARF LR			Carbamidomethyl (C)[5]		Mascot

9 hypothetical protein TRIUR3_26886 [Triticum urartu] gi|474169710 18643.6 9.74 8 48 37.535 22.686

Peptide Information

Calc. Mass	Obsrv. Mass	± da	± ppm	Start Seq.	End Seq.	Sequence	Ion Score	C. I. %	Modification	Rank	Result Type
945.5251	945.5195	-0.0056	-6	145	153	LALSTDTPK					Mascot
976.5132	976.4446	-0.0686	-70	161	169	SCVGIVVDK			Carbamidomethyl (C)[2]		Mascot
976.5132	976.4446	-0.0686	-70	161	169	SCVGIVVDK			Carbamidomethyl (C)[2]		Mascot
1065.4241	1065.5074	0.0833	78	76	84	MGCWPGGER			Carbamidomethyl (C)[3], Oxidation (M)[1]		Mascot
1221.6322	1221.63	-0.0022	-2	123	133	ETTTDNLTAKK					Mascot
1256.6416	1256.6061	-0.0355	-28	134	144	LIPTHSMSTNR					Mascot
1384.7366	1384.7012	-0.0354	-26	133	144	KLIPTHSMSTNR					Mascot
1384.7366	1384.7012	-0.0354	-26	133	144	KLIPTHSMSTNR					Mascot
1400.7314	1400.6965	-0.0349	-25	133	144	KLIPTHSMSTNR			Oxidation (M)[8]		Mascot
2369.1892	2369.1045	-0.0847	-36	47	70	AAARANRPASQGAAAEG ACVVWGK			Carbamidomethyl (C)[19]		Mascot
2398.2043	2398.1899	-0.0144	-6	51	73	ANRPASQGAAAEGACVV WGKEIR			Carbamidomethyl (C)[15]		Mascot

10 RNA polymerase sigma factor rpoD [Triticum urartu] gi|473782196 56720.9 9.81 10 47 5.456 26.762 33 96.013

Peptide Information

Calc. Mass	Obsrv. Mass	± da	± ppm	Start Seq.	End Seq.	Sequence	Ion Score	C. I. %	Modification	Rank	Result Type
816.4825	816.4282	-0.0543	-67	375	381	LLNISEK					Mascot

950.4829	950.5363	0.0534	56	501	508	LDALLQDY				Mascot
960.5407	960.4539	-0.0868	-90	74	81	AQVMRALR			Oxidation (M)[4]	Mascot
992.5272	992.4458	-0.0814	-82	473	480	QFGLSRER				Mascot
1009.5247	1009.6042	0.0795	79	259	266	MRESFLAR				Mascot
1017.58	1017.603	0.023	23	146	155	GRSSSVVVAR				Mascot
1017.58	1017.603	0.023	23	146	155	GRSSSVVVAR	33	96.692		Mascot
1025.5197	1025.5934	0.0737	72	259	266	MRESFLAR			Oxidation (M)[1]	Mascot
1025.5197	1025.5934	0.0737	72	259	266	MRESFLAR			Oxidation (M)[1]	Mascot
1122.5902	1122.592	0.0018	2	332	341	ALADNSKTFR				Mascot
1245.6547	1245.6661	0.0114	9	327	338	QGVSRALADNSK				Mascot
1256.6998	1256.6061	-0.0937	-75	452	462	DIIQLYHGIGK				Mascot
1384.7695	1384.7012	-0.0683	-49	218	228	IKDGIWLQQQR				Mascot
1384.7695	1384.7012	-0.0683	-49	218	228	IKDGIWLQQQR				Mascot

Gel Idx/Pos	177/H4	Instr./Gel Origin	BA2151/Sample Project 20140814	Process Status	Analysis Succeeded
Plate [#] Name	[1] Sample Project 20140814	Instrument Sample Name		Spectra	11

Rank	Protein Name	Accession No.	Protein MW	Protein PI	Pep. Count	Protein Score	Protein Score C. I. %	Intensity Matched	Total Ion Score	Total Ion C. I. %	Confirmed
1	Oxygen-evolving enhancer protein 1, chloroplastic [Triticum urartu]	gi|474352688	34635.5	5.75	16	667	100	48.012	560	100	

Peptide Information

Calc. Mass	Obsrv. Mass	± da	± ppm	Start Seq.	End Seq.	Sequence	Ion Score	C. I. %	Modification	Rank	Result Type
930.468	930.4618	-0.0062	-7	147	154	NEPPAFQK					Mascot
950.571	950.5623	-0.0087	-9	203	210	VPFLFTVK					Mascot
950.571	950.5623	-0.0087	-9	203	210	VPFLFTVK	58	99.99			Mascot
1080.5573	1080.5521	-0.0052	-5	86	94	LTFDEIQSK					Mascot
1236.6583	1236.6652	0.0069	6	85	94	RLTFDEIQSK					Mascot
1236.6583	1236.6652	0.0069	6	85	94	RLTFDEIQSK	21	50.391			Mascot
1328.6555	1328.6475	-0.008	-6	130	140	FCLEPTSFTVK			Carbamidomethyl (C)[2]		Mascot
1360.6591	1360.7015	0.0424	31	259	270	GDEEELAKENVK					Mascot
1456.7505	1456.7262	-0.0243	-17	129	140	KFCLEPTSFTVK			Carbamidomethyl (C)[3]		Mascot
1562.7559	1562.765	0.0091	6	242	258	GGSTGYDNAVALPAGGR					Mascot
1562.7559	1562.765	0.0091	6	242	258	GGSTGYDNAVALPAGGR	107	100			Mascot
1742.9581	1742.8563	-0.1018	-58	2	19	AASLQAAATLMPAKIGGR			Oxidation (M)[11]		Mascot
1760.8813	1760.8878	0.0065	4	186	202	DGIDYAAVTVQLPGGER					Mascot
1760.8813	1760.8878	0.0065	4	186	202	DGIDYAAVTVQLPGGER	140	100			Mascot
2168.9917	2168.9763	-0.0154	-7	101	121	GTGTANQCPTIDGGVDS FPFK			Carbamidomethyl (C)[8]		Mascot
2268.0952	2268.1057	0.0105	5	161	181	LTYTLDEMEGPLEVGAD GTLK			Oxidation (M)[8]		Mascot
2280.2024	2280.2117	0.0093	4	211	231	QLVATGKPESFSGPFLVP SYR					Mascot
2280.2024	2280.2117	0.0093	4	211	231	QLVATGKPESFSGPFLVP SYR	157	100			Mascot
2294.1299	2294.1372	0.0073	3	182	202	FEEKDGIDYAAVTVQLPG GER					Mascot
2434.1482	2434.1592	0.011	5	242	266	GGSTGYDNAVALPAGGR GDEEELAK					Mascot
2434.1482	2434.1592	0.011	5	242	266	GGSTGYDNAVALPAGGR GDEEELAK	18	0			Mascot
2590.2883	2590.271	-0.0173	-7	286	310	SKPETGEVIGVFESVQPS DTDLGAK					Mascot
2590.2883	2590.271	-0.0173	-7	286	310	SKPETGEVIGVFESVQPS DTDLGAK	58	99.99			Mascot

Rank	Protein Name	Accession No.	Protein MW	Protein PI	Pep. Count	Protein Score	Protein Score C. I. %	Intensity Matched	Total Ion Score	Total Ion C. I. %	Confirmed
2	photosystem II oxygen-evolving complex protein 1 - common wheat x Sanduri wheat	gi|100831	34946.8	8.73	9	199	100	24.731	162	100	

Protein Group

33kDa oxygen evolving protein of photosystem II [Triticum aestivum]	gi|21844	34946.8	8.7299995422 3633
RecName: Full=Oxygen-evolving enhancer protein 1, chloroplastic; Short=OEE1; AltName: Full=33 kDa subunit of oxygen evolving system of photosystem II; AltName: Full=33 kDa thylakoid membrane protein; AltName: Full=OEC 33 kDa subunit; Flags: Precurso	gi|131388	34946.8	8.7299995422 3633

Peptide Information

Calc. Mass	Obsrv. Mass	± da	± ppm	Start Seq.	End Seq.	Sequence	Ion Score	C. I. %	Modification	Rank	Result Type
1080.5573	1080.5521	-0.0052	-5	86	94	LTFDEIQSK					Mascot
1236.6583	1236.6652	0.0069	6	85	94	RLTFDEIQSK					Mascot
1236.6583	1236.6652	0.0069	6	85	94	RLTFDEIQSK	21	50.391			Mascot
1328.6555	1328.6475	-0.008	-6	130	140	FCLEPTSFTVK			Carbamidomethyl (C)[2]		Mascot
1360.6591	1360.7015	0.0424	31	258	269	GDEEELAKENVK					Mascot
1456.7505	1456.7262	-0.0243	-17	129	140	KFCLEPTSFTVK			Carbamidomethyl (C)[3]		Mascot
1760.8813	1760.8878	0.0065	4	186	202	DGIDYAAVTVQLPGGER					Mascot
1760.8813	1760.8878	0.0065	4	186	202	DGIDYAAVTVQLPGGER	140	100			Mascot
2168.9917	2168.9763	-0.0154	-7	101	121	GTGTANQCPTIDGGVDSFPFK			Carbamidomethyl (C)[8]		Mascot
2290.2224	2290.0703	-0.1521	-66	62	85	MAGFALATSALLVSGATAEGAPKR					Mascot
2294.1299	2294.1372	0.0073	3	182	202	FEEKDGIDYAAVTVQLPGGER					Mascot

3	hypothetical protein TRIUR3_12066 [Triticum urartu]	gi|474408932	34997.4	9.18	10	49	46.834	2.816

Peptide Information

Calc. Mass	Obsrv. Mass	± da	± ppm	Start Seq.	End Seq.	Sequence	Ion Score	C. I. %	Modification	Rank	Result Type
988.5574	988.5256	-0.0318	-32	271	279	LGWSSIAVR					Mascot
1144.6586	1144.5677	-0.0909	-79	270	279	RLGWSSIAVR					Mascot
1328.707	1328.6475	-0.0595	-45	18	28	ITEHADLARFR					Mascot
1443.6469	1443.6869	0.04	28	139	151	SPDAVMVSQGCHR			Carbamidomethyl (C)[11]		Mascot
1560.7588	1560.731	-0.0278	-18	291	303	RPSPSCQKWSEAK			Carbamidomethyl (C)[6]		Mascot
1645.793	1645.8356	0.0426	26	164	181	GSLAAGWSEVGGGGLGDR					Mascot
1699.865	1699.8367	-0.0283	-17	2	17	SWSDLPAELVAGIADR					Mascot
2245.99	2246.1628	0.1728	77	133	151	DMVWDRSPDAVMVSQGCHR			Carbamidomethyl (C)[17]		Mascot
2261.9849	2262.2004	0.2155	95	133	151	DMVWDRSPDAVMVSQG			Carbamidomethyl (C)[17], Oxidation (M)[2]		Mascot

						CHR						
	2277.9797	2278.1426	0.1629	72	133	151 DMVWDRSPDAVMVSQG CHR					Carbamidomethyl (C)[17], Oxidation (M)[2,12]	Mascot
	2302.1511	2302.1621	0.011	5	182	202 AVFVDHVRGFCVEANGV NGVR					Carbamidomethyl (C)[11]	Mascot
	2359.1533	2359.2227	0.0694	29	63	83 RLWSLADDSITEIPMPAA CGR					Carbamidomethyl (C)[19]	Mascot
4	ADP glucose pyrophosphorylase [Triticum aestivum]	gi|469952290	52399.6	5.54	12	49	40.347	3.663				

Protein Group

ADP-glucose pyrophosphorylase [Triticum aestivum]	gi|21687	52399.6	5.5399 999618 5303
RecName: Full=Glucose-1-phosphate adenylyltransferase small subunit, chloroplastic/amyloplastic; AltName: Full=ADP-glucose pyrophosphorylase; AltName: Full=ADP-glucose synthase; AltName: Full=AGPase B; AltName: Full=Alpha-D-glucose-1-phosphate adeny	gi|232172	52399.6	5.5399 999618 5303
unnamed protein product [Triticum aestivum]	gi|300634091	52399.6	5.5399 999618 5303
unnamed protein product [Triticum aestivum]	gi|300544577	52399.6	5.5399 999618 5303
unnamed protein product [Triticum aestivum]	gi|259471779	52399.6	5.5399 999618 5303
unnamed protein product [Triticum aestivum]	gi|259439192	52399.6	5.5399 999618 5303
unnamed protein product [Triticum aestivum]	gi|257671726	52399.6	5.5399 999618 5303
unnamed protein product [Triticum aestivum]	gi|257306995	52399.6	5.5399 999618 5303
unnamed protein product [Triticum aestivum]	gi|257308999	52399.6	5.5399 999618 5303

unnamed protein product [Triticum aestivum]	gi|219764760	52399.6	5.5399 999618 5303
unnamed protein product [Triticum aestivum]	gi|219752173	52399.6	5.5399 999618 5303

Peptide Information

Calc. Mass	Obsrv. Mass	± da	± ppm	Start Seq.	End Seq.	Sequence	Ion Score	C. I. %	Modification	Rank	Result Type
972.4673	972.5568	0.0895	92	448	455	ETDGYFIK					Mascot
1017.5952	1017.5939	-0.0013	-1	366	374	IHHSVVGLR					Mascot
1032.5472	1032.5483	0.0011	1	330	338	SAPIYTQPR					Mascot
1384.6896	1384.6921	0.0025	2	319	329	KPIPDFSFYDR					Mascot
1657.8796	1657.7946	-0.085	-51	2	17	DVPLASKTFPSPSPSK					Mascot
1786.9368	1786.9529	0.0161	9	76	91	LIDIPVSNCLNSNISK			Carbamidomethyl (C)[9]		Mascot
1788.92	1788.92	0	0	1	17	MDVPLASKTFPSPSPSK					Mascot
1852.9143	1852.9818	0.0675	36	228	244	AMMVDTTILGLDDARAK			Oxidation (M)[2,3]		Mascot
1986.9913	1986.9827	-0.0086	-4	430	447	IGDNVMIINVDNVQEAAR			Oxidation (M)[6]		Mascot
2318.2537	2318.1404	-0.1133	-49	76	95	LIDIPVSNCLNSNISKIYVR			Carbamidomethyl (C)[9]		Mascot
2398.219	2398.2019	-0.0171	-7	319	338	KPIPDFSFYDRSAPIYTQPR					Mascot
2628.2722	2628.2461	-0.0261	-10	177	199	FIQAHRETDADITVAALPMDEER					Mascot

5	hypothetical protein TRIUR3_33061 [Triticum urartu]	gi|472901098	13740.9	6.85	7	48	23.152	2.224	

Peptide Information

Calc. Mass	Obsrv. Mass	± da	± ppm	Start Seq.	End Seq.	Sequence	Ion Score	C. I. %	Modification	Rank	Result Type
988.5058	988.5256	0.0198	20	59	68	EAALDGAVSR					Mascot
1032.4858	1032.5483	0.0625	61	8	17	HGSSGTFSPR					Mascot
1445.7166	1445.6849	-0.0317	-22	20	32	DRVAVLDASPCSR			Carbamidomethyl (C)[11]		Mascot
1774.8832	1774.8921	0.0089	5	59	75	EAALDGAVSRGQTGTWR					Mascot
1838.0018	1837.8517	-0.1501	-82	76	91	LVQPAIDEEAKIEQVR					Mascot
2278.1575	2278.1426	-0.0149	-7	107	127	RPVLVEEVEAEVGHGASGTWR					Mascot
2434.2585	2434.1592	-0.0993	-41	106	127	RRPVLVEEVEAEVGHGASGTWR					Mascot
2434.2585	2434.1592	-0.0993	-41	106	127	RRPVLVEEVEAEVGHGASGTWR					Mascot

6	Nuclear-pore anchor [Triticum urartu]	gi|474360395	233406.6	5.24	34	47	11.766	36.056

Peptide Information

Calc. Mass	Obsrv. Mass	± da	± ppm	Start Seq.	End Seq.	Sequence	Ion Score	C. I. %	Modification	Rank	Result Type
849.4498	849.4397	-0.0101	-12	611	617	TELMSLR					Mascot
889.4778	889.4512	-0.0266	-30	756	762	DWAELKK					Mascot
930.5255	930.4618	-0.0637	-68	1473	1480	ADLIKENK					Mascot
945.4999	945.5346	0.0347	37	7	14	DGEVERLK					Mascot
972.5472	972.5568	0.0096	10	1244	1251	IEAERLNK					Mascot
988.5309	988.5256	-0.0053	-5	1440	1448	LNDVEASLK					Mascot
1035.5754	1035.5609	-0.0145	-14	686	694	LSMEVSILK			Oxidation (M)[3]		Mascot
1080.5908	1080.5521	-0.0387	-36	344	352	RHAEAVLER					Mascot
1105.5605	1105.5571	-0.0034	-3	1730	1738	RLMPSSQMR					Mascot
1236.5776	1236.6652	0.0876	71	926	936	CEEAASTIEVK			Carbamidomethyl (C)[1]		Mascot
1236.5776	1236.6652	0.0876	71	926	936	CEEAASTIEVK			Carbamidomethyl (C)[1]		Mascot
1245.6936	1245.6484	-0.0452	-36	1150	1160	EIAETEISLLK					Mascot
1247.5613	1247.6552	0.0939	75	1193	1202	SSMFKDEEFK					Mascot
1258.689	1258.6438	-0.0452	-36	83	92	IEQEKELLEK					Mascot
1284.6504	1284.5748	-0.0756	-59	1378	1388	GVDLKTYETMK					Mascot
1360.7318	1360.7015	-0.0303	-22	1074	1085	TSGEQEKIELVK					Mascot
1384.5831	1384.6921	0.109	79	114	125	ANMDEESRMSAK			Oxidation (M)[3]		Mascot
1415.7601	1415.6465	-0.1136	-80	2025	2037	LVRTSTPQPSSSR					Mascot
1428.7917	1428.6959	-0.0958	-67	2007	2018	TINLTERAIQNR					Mascot
1429.772	1429.6953	-0.0767	-54	686	697	LSMEVSILKHEK			Oxidation (M)[3]		Mascot
1445.821	1445.6849	-0.1361	-94	1166	1178	LQIELESALKSSK					Mascot
1491.7802	1491.7339	-0.0463	-31	1198	1209	DEEFKSLQLQVR					Mascot
1544.7737	1544.7515	-0.0222	-14	489	501	NKVHMLSTDLENK			Oxidation (M)[5]		Mascot
1547.8428	1547.7371	-0.1057	-68	502	514	GVELRESFQIELK					Mascot
1560.7727	1560.731	-0.0417	-27	655	667	NAELMHLVVDYEK					Mascot
1664.7246	1664.8744	0.1498	90	1877	1891	EEGQITTATDAEDER					Mascot
1699.803	1699.8367	0.0337	20	1257	1270	QVYAEVCTKDLDMK			Carbamidomethyl (C)[7]		Mascot
1711.8723	1711.8986	0.0263	15	1056	1069	AENILFEKYSLCPK			Carbamidomethyl (C)[12]		Mascot
1715.798	1715.8748	0.0768	45	1257	1270	QVYAEVCTKDLDMK			Carbamidomethyl (C)[7], Oxidation (M)[13]		Mascot
1760.8596	1760.8878	0.0282	16	1421	1436	NLDEKLAGCQSELGAR			Carbamidomethyl (C)[9]		Mascot
1760.8596	1760.8878	0.0282	16	1421	1436	NLDEKLAGCQSELGAR			Carbamidomethyl (C)[9]		Mascot
1798.9844	1798.8447	-0.1397	-78	426	440	QVAVLLKECQDVQLR			Carbamidomethyl (C)[9]		Mascot

2038.9452	2039.0614	0.1162	57	1859	1876	SEAVVESFDEDQKLEDAK					Mascot
2204.1592	2204.0771	-0.0821	-37	939	957	DITTLMNDITILRNEVSQK					Mascot
2263.1123	2263.187	0.0747	33	961	980	IENLEVELASSKSALDEQCK			Carbamidomethyl (C)[19]		Mascot
2263.1123	2263.187	0.0747	33	961	980	IENLEVELASSKSALDEQCK			Carbamidomethyl (C)[19]		Mascot
2290.1846	2290.0703	-0.1143	-50	1016	1035	IEPMVILQSETIQELTNTSK			Oxidation (M)[4]		Mascot
2302.1279	2302.1621	0.0342	15	1036	1055	QLSSLQQEMAMLRQTADAHK			Oxidation (M)[9]		Mascot
2318.1228	2318.1404	0.0176	8	1036	1055	QLSSLQQEMAMLRQTADAHK			Oxidation (M)[9,11]		Mascot

7 Disease resistance protein RPM1 [Triticum urartu] gi|473926895 104315.5 7.81 17 47 5.456 24.717 4 0

Peptide Information

Calc. Mass	Obsrv. Mass	± da	± ppm	Start Seq.	End Seq.	Sequence	Ion Score	C. I. %	Modification	Rank	Result Type
849.4577	849.4397	-0.018	-21	483	489	NEFGRVK					Mascot
1035.6422	1035.5609	-0.0813	-79	526	533	IRHLILDR					Mascot
1205.6121	1205.6165	0.0044	4	307	318	SQDVASLAASTR					Mascot
1245.7314	1245.6484	-0.083	-67	41	50	DLELLRAFLR					Mascot
1428.577	1428.6959	0.1189	83	246	257	AGMPDIDEMDYR			Oxidation (M)[3]		Mascot
1456.7893	1456.7262	-0.0631	-43	598	610	STLIEELPQELGK					Mascot
1500.7805	1500.7538	-0.0267	-18	57	70	GADALVSAWVDQIR					Mascot
1544.8656	1544.7515	-0.1141	-74	258	271	SLVEALHGHLANKR					Mascot
1562.8009	1562.765	-0.0359	-23	94	107	ACANFGAWVALARR			Carbamidomethyl (C)[2]		Mascot
1562.8009	1562.765	-0.0359	-23	94	107	ACANFGAWVALARR	4	0	Carbamidomethyl (C)[2]		Mascot
1738.9963	1738.881	-0.1153	-66	759	773	LPSWVGRLDSLVQLR					Mascot
1760.9575	1760.8878	-0.0697	-40	766	780	LDSLVQLRLCSSELK			Carbamidomethyl (C)[10]		Mascot
1760.9575	1760.8878	-0.0697	-40	766	780	LDSLVQLRLCSSELK			Carbamidomethyl (C)[10]		Mascot
1782.8943	1782.8711	-0.0232	-13	676	691	YVEADENMVKSLGSLK					Mascot
1788.801	1788.92	0.119	67	327	340	EQEAWSLFCNTTFR			Carbamidomethyl (C)[9]		Mascot
1798.8892	1798.8447	-0.0445	-25	676	691	YVEADENMVKSLGSLK			Oxidation (M)[8]		Mascot
1852.8308	1852.9818	0.151	81	880	895	IQDGETLQGNYEDSQR					Mascot
2169.1882	2168.9763	-0.2119	-98	362	381	CCGLPLAIVSVGNLLALKDR			Carbamidomethyl (C)[1,2]		Mascot
2268.1335	2268.1057	-0.0278	-12	272	289	YLLLLDDVWDANAWYEIR					Mascot
2269.1948	2269.0588	-0.136	-60	151	171	LAEAAHFVEHGEIVGVAAHRR					Mascot

8 Leucyl-tRNA synthetase [Triticum urartu] gi|473784820 104740.7 5.39 17 46 0 10.906

Peptide Information

Calc. Mass	Obsrv. Mass	± da	± ppm	Start	End	Sequence	Ion	C. I. %	Modification	Rank	Result Type

				Seq.	Seq.		Score				
820.4424	820.4235	-0.0189	-23	125	130	NIDRFR					Mascot
930.5043	930.4618	-0.0425	-46	659	666	VGDHYVLK					Mascot
1091.5746	1091.5718	-0.0028	-3	450	457	DWLFARQR					Mascot
1193.6776	1193.6132	-0.0644	-54	596	606	VLYDIGVVSTK					Mascot
1205.5845	1205.6165	0.032	27	238	246	EMQRNWIGR			Oxidation (M)[2]		Mascot
1245.6708	1245.6484	-0.0224	-18	207	215	KPMRQWMLR					Mascot
1328.7645	1328.6475	-0.117	-88	511	522	TTDLLTGKPARR					Mascot
1590.7904	1590.7104	-0.08	-50	769	781	CIARVSEEIQETR			Carbamidomethyl (C)[1]		Mascot
1715.8389	1715.8748	0.0359	21	131	144	TQLKSLGFSYDWDR					Mascot
1758.9537	1758.8762	-0.0775	-44	733	748	TWRLVVGAPLPDGSYK					Mascot
1837.8636	1837.8517	-0.0119	-6	749	764	DGTMAIDVEPTFEQLR			Oxidation (M)[4]		Mascot
1869.0957	1868.9587	-0.137	-73	901	916	IYVPGRILNVILDQQK					Mascot
1926.8386	1926.874	0.0354	18	634	650	WVSADSDSSLSDCIQEK			Carbamidomethyl (C)[13]		Mascot
1939.8928	1939.9985	0.1057	54	782	798	FNTAISAMMEFVNAAYK			Oxidation (M)[8,9]		Mascot
2151.9263	2152.1318	0.2055	95	523	539	ETSTMPQWAGSCWYYL R			Carbamidomethyl (C)[12], Oxidation (M)[5]		Mascot
2261.998	2262.2004	0.2024	89	247	267	SEGAELEFCAVDQEGHD LGAK			Carbamidomethyl (C)[9]		Mascot
2263.095	2263.187	0.092	41	683	703	SRGNVINPDDVVSEYGA DSLR					Mascot
2263.095	2263.187	0.092	41	683	703	SRGNVINPDDVVSEYGA DSLR					Mascot

9 hypothetical protein TRIUR3_13085 [Triticum urartu] gi|473909947 55680.7 7.44 12 45 0 3

Peptide Information

Calc. Mass	Obsrv. Mass	± da	± ppm	Start Seq.	End Seq.	Sequence	Ion Score	C. I. %	Modification	Rank	Result Type
820.4233	820.4235	0.0002	0	477	483	VTACEIK			Carbamidomethyl (C)[4]		Mascot
907.4163	907.4682	0.0519	57	2	9	GSACSRNR			Carbamidomethyl (C)[4]		Mascot
948.5182	948.4758	-0.0424	-45	477	484	VTACEIKK			Carbamidomethyl (C)[4]		Mascot
1105.567	1105.5571	-0.0099	-9	192	202	KCAAVTAEGAK			Carbamidomethyl (C)[2]		Mascot
1461.7697	1461.6758	-0.0939	-64	82	93	DLSQQIFNELVR					Mascot
1715.8058	1715.8748	0.069	40	68	81	EDISRYSDFSMLPR					Mascot
1748.0428	1747.8792	-0.1636	-94	484	499	KLQLAALPNLVSVRPE					Mascot
1758.9232	1758.8762	-0.047	-27	417	432	DLTLLNLSQNGNLTDK					Mascot
1775.0538	1774.8921	-0.1617	-91	284	299	IGDEGLLHLKGLLQLR					Mascot
1895.8514	1896.0393	0.1879	99	107	122	DCALQDICLDEYPGVK			Carbamidomethyl (C)[2,8]		Mascot
2434.1887	2434.1592	-0.0295	-12	223	243	LASLVLLNLNRCGICDEG CEK			Carbamidomethyl (C)[12,15,19]		Mascot

2434.1887	2434.1592	-0.0295	-12	223	243	LASLVLLNLNRCGICDEG CEK			Carbamidomethyl (C)[12,15,19]		Mascot
2612.4043	2612.2844	-0.1199	-46	355	379	QITDNGLAALTTLTGLTHL DLFGAR					Mascot

10	wheat 33K [Triticum aestivum]	gi|579237	10683.4	9.44	4	45	0	6.519	21	50.391

Peptide Information

Calc. Mass	Obsrv. Mass	± da	± ppm	Start Seq.	End Seq.	Sequence	Ion Score	C. I. %	Modification	Rank	Result Type
1080.5573	1080.5521	-0.0052	-5	86	94	LTFDEIQSK					Mascot
1236.6583	1236.6652	0.0069	6	85	94	RLTFDEIQSK					Mascot
1236.6583	1236.6652	0.0069	6	85	94	RLTFDEIQSK	21	50.391			Mascot
1742.9581	1742.8563	-0.1018	-58	2	19	AASLQAAATLMPAKIGGR			Oxidation (M)[11]		Mascot
2290.2224	2290.0703	-0.1521	-66	62	85	MAGFALATSALLVSGATA EGAPKR					Mascot

Gel Idx/Pos	178/H5	Instr./Gel Origin	BA2151/Sample Project 20140814	Process Status	Analysis Succeeded
Plate [#] Name	[1] Sample Project 20140814	Instrument Sample Name		Spectra	11

Rank	Protein Name	Accession No.	Protein MW	Protein PI	Pep. Count	Protein Score	Protein Score C. I. %	Intensity Matched	Total Ion Score	Total Ion C. I. %	Confirmed
1	ascorbate peroxidase [Triticum aestivum]	gi|226897533	26776.6	5.54	9	479	100	33.265	426	100	

Peptide Information

Calc. Mass	Obsrv. Mass	± da	± ppm	Start Seq.	End Seq.	Sequence	Ion Score	C. I. %	Modification	Rank	Result Type
911.4291	911.4346	0.0055	6	46	54	TGGPFGTMK			Oxidation (M)[8]		Mascot
974.491	974.4979	0.0069	7	24	31	NCAPLMLR			Carbamidomethyl (C)[2]		Mascot
990.4859	990.4873	0.0014	1	24	31	NCAPLMLR			Carbamidomethyl (C)[2], Oxidation (M)[6]		Mascot
1249.6172	1249.6227	0.0055	4	113	123	QDKPEPPPEGR					Mascot
1249.6172	1249.6227	0.0055	4	113	123	QDKPEPPPEGR	70	100			Mascot
1309.6495	1309.6583	0.0088	7	124	135	LPDATQGSDHLR					Mascot
1309.6495	1309.6583	0.0088	7	124	135	LPDATQGSDHLR	112	100			Mascot
1503.7592	1503.7513	-0.0079	-5	32	45	LAWHSAGTFDVATK					Mascot
1585.8552	1585.8997	0.0445	28	18	31	GLIAEKNCAPLMLR			Carbamidomethyl (C)[8]		Mascot
1585.8552	1585.8997	0.0445	28	18	31	GLIAEKNCAPLMLR			Carbamidomethyl (C)[8]		Mascot
1601.8502	1601.8862	0.036	22	18	31	GLIAEKNCAPLMLR			Carbamidomethyl (C)[8], Oxidation (M)[12]		Mascot
1834.9229	1834.9248	0.0019	1	55	72	CPAELAHGANAGLDIAVR	143	100	Carbamidomethyl (C)[1]		Mascot
2633.2988	2633.3027	0.0039	1	136	160	QVFSTQMGLSDQDIVALS GGHTLGR			Oxidation (M)[7]		Mascot
2633.2988	2633.3027	0.0039	1	136	160	QVFSTQMGLSDQDIVALS GGHTLGR	101	100	Oxidation (M)[7]		Mascot
3689.864	3689.927	0.063	17	79	112	EQFPILSYADFYQLAGVV AVEVTGGPEVPFHPGR					Mascot

Rank	Protein Name	Accession No.	Protein MW	Protein PI	Pep. Count	Protein Score	Protein Score C. I. %	Intensity Matched	Total Ion Score	Total Ion C. I. %	Confirmed
2	L-ascorbate peroxidase 1, cytosolic [Triticum urartu]	gi|474311703	27561.1	5.85	5	165	100	14.597	147	100	

Peptide Information

Calc. Mass	Obsrv. Mass	± da	± ppm	Start Seq.	End Seq.	Sequence	Ion Score	C. I. %	Modification	Rank	Result Type
911.4291	911.4346	0.0055	6	53	61	TGGPFGTMK			Oxidation (M)[8]		Mascot
990.4859	990.4873	0.0014	1	31	38	NCSPLMLR			Carbamidomethyl (C)[2]		Mascot
1006.4808	1006.4913	0.0105	10	31	38	NCSPLMLR			Carbamidomethyl (C)[2], Oxidation (M)[6]		Mascot
1249.6172	1249.6227	0.0055	4	120	130	EDKPQPPPEGR					Mascot
1249.6172	1249.6227	0.0055	4	120	130	EDKPQPPPEGR	36	98.141			Mascot
1309.6859	1309.6583	-0.0276	-21	131	142	LPDATKGSDHLR					Mascot
1309.6859	1309.6583	-0.0276	-21	131	142	LPDATKGSDHLR	112	100			Mascot

1831.9773	1831.8876	-0.0897	-49	62	79	KPAEQAHAANAGLDIAVR					Mascot

3 Xyloglucan endotransglucosylase/hydrolase protein 24 [Triticum urartu] gi|474308768 26347 9.04 8 48 34.591 6.607 10 0

Peptide Information

Calc. Mass	Obsrv. Mass	± da	± ppm	Start Seq.	End Seq.	Sequence	Ion Score	C. I. %	Modification	Rank	Result Type
859.4818	859.4189	-0.0629	-73	2	9	ACLVAVAR			Carbamidomethyl (C)[2]		Mascot
990.5223	990.4873	-0.035	-35	1	9	MACLVAVAR			Carbamidomethyl (C)[3]		Mascot
1006.5172	1006.4913	-0.0259	-26	1	9	MACLVAVAR			Carbamidomethyl (C)[3], Oxidation (M)[1]		Mascot
1241.6385	1241.6694	0.0309	25	176	186	NHEARGVSFPK					Mascot
1249.6359	1249.6227	-0.0132	-11	181	191	GVSFPKSQPMR			Oxidation (M)[10]		Mascot
1249.6359	1249.6227	-0.0132	-11	181	191	GVSFPKSQPMR	10	0	Oxidation (M)[10]		Mascot
1451.6334	1451.7046	0.0712	49	210	220	FMIYNYCTDPK			Carbamidomethyl (C)[7]		Mascot
1607.7345	1607.8595	0.125	78	210	221	FMIYNYCTDPKR			Carbamidomethyl (C)[7]		Mascot
1761.8411	1761.8989	0.0578	33	187	201	SQPMRLYASLGMGYR			Oxidation (M)[4,12]		Mascot
1970.8662	1970.9799	0.1137	58	10	27	GGNFIQDSEMTWGDGR GK			Oxidation (M)[10]		Mascot

4 hypothetical protein TRIUR3_22532 [Triticum urartu] gi|473797438 36043.2 5.13 11 46 0 2.254

Peptide Information

Calc. Mass	Obsrv. Mass	± da	± ppm	Start Seq.	End Seq.	Sequence	Ion Score	C. I. %	Modification	Rank	Result Type
856.4457	856.5272	0.0815	95	156	163	SAHIAAMR					Mascot
908.3971	908.3921	-0.005	-6	113	118	EFMFYR			Oxidation (M)[3]		Mascot
948.4825	948.3906	-0.0919	-97	246	252	YFTKDFK					Mascot
1143.5317	1143.6047	0.073	64	283	292	DGSIFYNEAK					Mascot
1209.674	1209.6808	0.0068	6	180	190	LPLWPSAQAAR					Mascot
1291.7046	1291.6434	-0.0612	-47	223	233	NVLSLWFDGLK					Mascot
1451.6948	1451.7046	0.0098	7	167	179	SAGILCSYDPNVR			Carbamidomethyl (C)[6]		Mascot
1792.9052	1792.913	0.0078	4	141	155	IFHYGSISLITEPCR			Carbamidomethyl (C)[14]		Mascot
1798.8463	1798.8157	-0.0306	-17	295	310	EVLQFSNACGAICTTK			Carbamidomethyl (C)[9,13]		Mascot
2144.0903	2144.0728	-0.0175	-8	119	137	NPSADMLLTEAELNLDLI R			Oxidation (M)[6]		Mascot
2646.3279	2646.3123	-0.0156	-6	141	163	IFHYGSISLITEPCRSAHIA AMR			Carbamidomethyl (C)[14], Oxidation (M)[22]		Mascot

5 hypothetical protein TRIUR3_00756 [Triticum urartu] gi|474043606 40046.4 9.41 7 44 0 16.926 23 66.015

Peptide Information

Calc. Mass	Obsrv. Mass	± da	± ppm	Start	End	Sequence	Ion	C. I. %	Modification	Rank	Result Type

Calc. Mass	Obsrv. Mass	± da	± ppm	Start Seq.	End Seq.	Sequence	Ion Score	C. I. %	Modification	Rank	Result Type
1241.7113	1241.6694	-0.0419	-34	47	56	LHYDLRLGVR					Mascot
1249.5963	1249.6227	0.0264	21	93	102	RPCMCSLLGR			Carbamidomethyl (C)[3,5]		Mascot
1249.5963	1249.6227	0.0264	21	93	102	RPCMCSLLGR	23	66.015	Carbamidomethyl (C)[3,5]		Mascot
1484.7413	1484.8512	0.1099	74	168	180	DNSINKASTMFLK			Oxidation (M)[10]		Mascot
1585.7872	1585.8997	0.1125	71	93	105	RPCMCSLLGRAHK			Carbamidomethyl (C)[3,5]		Mascot
1585.7872	1585.8997	0.1125	71	93	105	RPCMCSLLGRAHK			Carbamidomethyl (C)[3,5]		Mascot
1601.8898	1601.8862	-0.0036	-2	253	267	LPAGQEKFASVVDLK					Mascot
1738.9011	1738.9407	0.0396	23	201	215	GELVQDKLYVQGFDK					Mascot
2646.2876	2646.3123	0.0247	9	2	24	ALRQALGWSDGEVMRP ESKPCSR			Carbamidomethyl (C)[21], Oxidation (M)[14]		Mascot

6 hypothetical protein TRIUR3_03382 [Triticum urartu] gi|474437298 68499.1 6.12 17 43 0 8.081

Peptide Information

Calc. Mass	Obsrv. Mass	± da	± ppm	Start Seq.	End Seq.	Sequence	Ion Score	C. I. %	Modification	Rank	Result Type
814.5396	814.4724	-0.0672	-83	466	472	VLVKEVK					Mascot
876.4971	876.4435	-0.0536	-61	325	331	LLVTMQR			Oxidation (M)[5]		Mascot
900.5149	900.4582	-0.0567	-63	347	354	LNQEVAVK					Mascot
908.4254	908.3921	-0.0333	-37	476	482	NSQREMK			Oxidation (M)[6]		Mascot
1143.6481	1143.6047	-0.0434	-38	493	501	TELERIINR					Mascot
1232.7144	1232.5992	-0.1152	-93	322	331	LSRLLVTMQR			Oxidation (M)[8]		Mascot
1248.6179	1248.613	-0.0049	-4	421	432	VESEKATASGNR					Mascot
1249.6787	1249.6227	-0.056	-45	483	492	KVLNQYVEEK					Mascot
1249.6787	1249.6227	-0.056	-45	483	492	KVLNQYVEEK					Mascot
1331.7053	1331.647	-0.0583	-44	361	372	VKDLEGELEATK					Mascot
1533.7942	1533.7635	-0.0307	-20	333	346	IGTAKTDMEDLIAR					Mascot
1580.7762	1580.7927	0.0165	10	203	217	KDSSESLGSDLSSLR					Mascot
1607.8639	1607.8595	-0.0044	-3	347	360	LNQEVAVKDYLSTK					Mascot
1816.7905	1816.9022	0.1117	61	404	418	YSEMESNLKAEQDEK			Oxidation (M)[4]		Mascot
1847.8981	1847.951	0.0529	29	426	442	ATASGNREELAEELETK					Mascot
1850.8524	1850.8995	0.0471	25	388	401	ERITQMQWDMDELR					Mascot
1856.8192	1856.8738	0.0546	29	187	203	GVVSEEDCDSNPGHARK			Carbamidomethyl (C)[8]		Mascot
2568.3779	2568.3245	-0.0534	-21	83	106	LLSDIDLSRSAHVAAFLEL EAAAR					Mascot

7 U-box domain-containing protein 17 [Triticum urartu] gi|474011946 77580.9 8.93 15 42 0 20.226

Peptide Information

Calc. Mass	Obsrv. Mass	± da	± ppm	Start Seq.	End Seq.	Sequence	Ion Score	C. I. %	Modification	Rank	Result Type
900.5261	900.4582	-0.0679	-75	62	69	SKPDARVK					Mascot
990.4487	990.4873	0.0386	39	254	261	DGKEEEQR					Mascot
1052.5446	1052.5769	0.0323	31	320	327	EMVELFIR			Oxidation (M)[2]		Mascot
1054.5027	1054.5565	0.0538	51	689	697	MKALFGSWD					Mascot
1231.6754	1231.6073	-0.0681	-55	297	308	SEVVSARSAAVR					Mascot
1249.6423	1249.6227	-0.0196	-16	75	85	LFVEALDEGTR					Mascot
1249.6423	1249.6227	-0.0196	-16	75	85	LFVEALDEGTR					Mascot
1260.546	1260.6693	0.1233	98	580	590	CTAGCFSSKSR			Carbamidomethyl (C)[1,5]		Mascot
1503.765	1503.7513	-0.0137	-9	222	235	VLDAGLSVSEQTER					Mascot
1557.8054	1557.7363	-0.0691	-44	395	407	CLIDENVVPNTRK			Carbamidomethyl (C)[1]		Mascot
1580.9172	1580.7927	-0.1245	-79	41	53	LFRPGLSHLKAWR					Mascot
1585.8115	1585.8997	0.0882	56	394	406	RCLIDENVVPNTR			Carbamidomethyl (C)[2]		Mascot
1585.8115	1585.8997	0.0882	56	394	406	RCLIDENVVPNTR			Carbamidomethyl (C)[2]		Mascot
1697.9181	1697.8232	-0.0949	-56	614	629	SIQIQPIGTNISPSSR					Mascot
1845.9653	1845.9285	-0.0368	-20	236	251	ARHLMAIGGVDFHLHR			Oxidation (M)[5]		Mascot
1856.9396	1856.8738	-0.0658	-35	630	648	AGAHVMSASRTAAVAPT SR			Oxidation (M)[6]		Mascot
1872.9274	1872.8409	-0.0865	-46	514	530	DTHTDMQLAAFSALVPR					Mascot
1888.9222	1888.8297	-0.0925	-49	514	530	DTHTDMQLAAFSALVPR			Oxidation (M)[6]		Mascot

8 hypothetical protein TRIUR3_27053 [Triticum urartu] gi|474311378 79170.6 6.24 15 41 0 11.083

Peptide Information

Calc. Mass	Obsrv. Mass	± da	± ppm	Start Seq.	End Seq.	Sequence	Ion Score	C. I. %	Modification	Rank	Result Type
859.4996	859.4189	-0.0807	-94	515	521	RLSQLDK					Mascot
990.5037	990.4873	-0.0164	-17	337	345	GLANISEMR					Mascot
1006.4985	1006.4913	-0.0072	-7	337	345	GLANISEMR			Oxidation (M)[8]		Mascot
1056.4854	1056.5825	0.0971	92	188	195	KIDMDCFK			Carbamidomethyl (C)[6]		Mascot
1182.6365	1182.5625	-0.074	-63	516	525	LSQLDKTYSK					Mascot
1232.6886	1232.5992	-0.0894	-73	80	90	EIGFDGLLQIK					Mascot
1291.7593	1291.6434	-0.1159	-90	326	336	LGILLREHNAR					Mascot
1613.7812	1613.8496	0.0684	42	332	345	EHNARGLANISEMR			Oxidation (M)[13]		Mascot
1697.8275	1697.8232	-0.0043	-3	173	187	AAEAYLMRDITANSR			Oxidation (M)[7]		Mascot
1729.0007	1728.8999	-0.1008	-58	76	90	QLVREIGFDGLLQIK					Mascot
1743.8694	1743.9214	0.052	30	267	282	GIVCASTELANENIPR			Carbamidomethyl (C)[4]		Mascot

1761.9535	1761.8989	-0.0546	-31	80	94	EIGFDGLLQIKSWQK					Mascot
1790.9258	1790.9156	-0.0102	-6	222	236	GIYPRIALFEHESMK					Mascot
1835.0062	1834.9248	-0.0814	-44	499	514	AIKDWLAVSSTFVLER					Mascot
1894.856	1894.9009	0.0449	24	416	434	MAGQLENDGSSSANSSIAR					Mascot
2047.043	2046.8977	-0.1453	-71	581	597	INMHTYVSDLLHTALFR			Oxidation (M)[3]		Mascot
2047.043	2046.8977	-0.1453	-71	581	597	INMHTYVSDLLHTALFR			Oxidation (M)[3]		Mascot

9 Ubiquitin carboxyl-terminal hydrolase 17 [Triticum urartu] gi|473842831 107780.1 6.38 16 40 0 2.745

Peptide Information

Calc. Mass	Obsrv. Mass	± da	± ppm	Start Seq.	End Seq.	Sequence	Ion Score	C. I. %	Modification	Rank	Result Type
812.4373	812.429	-0.0083	-10	389	396	VSSIHGGR					Mascot
1054.6508	1054.5565	-0.0943	-89	379	388	LVTPVLAVDK					Mascot
1056.6161	1056.5825	-0.0336	-32	430	438	QQTTPKVVR					Mascot
1143.5687	1143.6047	0.036	31	769	778	CSPRAPNSVR			Carbamidomethyl (C)[1]		Mascot
1331.7278	1331.647	-0.0808	-61	413	426	GGSAANNLATSLKK					Mascot
1507.7032	1507.7562	0.053	35	779	791	HAMMVNDPSYAKK			Oxidation (M)[3]		Mascot
1557.8231	1557.7363	-0.0868	-56	48	61	LARLAAEESELAER					Mascot
1568.9258	1568.8622	-0.0636	-41	374	388	SEGLKLVTPVLAVDK					Mascot
1739.0426	1738.9407	-0.1019	-59	659	674	LTISEAPNVLTIALKR					Mascot
1744.8469	1744.9126	0.0657	38	556	570	HSIDAMQSVCIKEAR			Carbamidomethyl (C)[10]		Mascot
1792.8488	1792.913	0.0642	36	222	237	DTPFEEVSATTEIPEK					Mascot
1848.0702	1847.951	-0.1192	-64	379	396	LVTPVLAVDKVSSIHGGR					Mascot
1888.9685	1888.8297	-0.1388	-73	743	759	IDDSQVKPVSLENVMSK					Mascot
1894.8738	1894.9009	0.0271	14	911	928	SRSALGTSSSGQEVDEER					Mascot
2074.9548	2074.9314	-0.0234	-11	505	520	AWCFMCEFERLIVEGK			Carbamidomethyl (C)[3,6]		Mascot
2646.2651	2646.3123	0.0472	18	288	311	SQASAPKMSGLTSSIHEDIYVHCK			Carbamidomethyl (C)[23]		Mascot

10 heat shock protein 90, partial [Triticum aestivum] gi|4204861 19803.1 5.17 8 38 0 10.378

Peptide Information

Calc. Mass	Obsrv. Mass	± da	± ppm	Start Seq.	End Seq.	Sequence	Ion Score	C. I. %	Modification	Rank	Result Type
974.524	974.4979	-0.0261	-27	113	119	QKPIWMR			Oxidation (M)[6]		Mascot
1006.4516	1006.4913	0.0397	39	127	134	DEYAAFYK					Mascot
1231.6278	1231.6073	-0.0205	-17	10	21	DTTGEPLGRGTK					Mascot
1309.5907	1309.6583	0.0676	52	28	37	DDQLEYLEER					Mascot

1309.5907	1309.6583	0.0676	52	28	37	DDQLEYLEER	Mascot
1451.6021	1451.7046	0.1025	71	65	76	EISDDEDEDEKK	Mascot
1697.7389	1697.8232	0.0843	50	77	90	DTEEGKFEEIDEEK	Mascot
1831.9113	1831.8876	-0.0237	-13	120	134	KPEEITKDEYAAFYK	Mascot
2075.1326	2074.9314	-0.2012	-97	148	165	HFSVEGQLEFKAVLFVPK	Mascot

Gel Idx/Pos	179/H6	Instr./Gel Origin	BA2151/Sample Project 20140814	Process Status	Analysis Succeeded
Plate [#] Name	[1] Sample Project 20140814	Instrument Sample Name		Spectra	11

Rank	Protein Name	Accession No.	Protein MW	Protein PI	Pep. Count	Protein Score	Protein Score C. I. %	Intensity Matched	Total Ion Score	Total Ion C. I. %	Confirmed
1	ascorbate peroxidase [Triticum aestivum]	gi|226897533	26776.6	5.54	11	510	100	46.446	444	100	

Peptide Information

Calc. Mass	Obsrv. Mass	± da	± ppm	Start Seq.	End Seq.	Sequence	Ion Score	C. I. %	Modification	Rank	Result Type
899.5672	899.4792	-0.088	-98	16	23	LRGLIAEK					Mascot
911.4291	911.4317	0.0026	3	46	54	TGGPFGTMK			Oxidation (M)[8]		Mascot
974.491	974.4974	0.0064	7	24	31	NCAPLMLR			Carbamidomethyl (C)[2]		Mascot
990.4859	990.4855	-0.0004	0	24	31	NCAPLMLR			Carbamidomethyl (C)[2], Oxidation (M)[6]		Mascot
1249.6172	1249.6228	0.0056	4	113	123	QDKPEPPPEGR					Mascot
1249.6172	1249.6228	0.0056	4	113	123	QDKPEPPPEGR	67	99.999			Mascot
1309.6495	1309.656	0.0065	5	124	135	LPDATQGSDHLR					Mascot
1309.6495	1309.656	0.0065	5	124	135	LPDATQGSDHLR	110	100			Mascot
1503.7592	1503.7535	-0.0057	-4	32	45	LAWHSAGTFDVATK					Mascot
1525.7434	1525.757	0.0136	9	223	234	AFFEDYKEAHLR					Mascot
1585.8552	1585.8948	0.0396	25	18	31	GLIAEKNCAPLMLR			Carbamidomethyl (C)[8]		Mascot
1585.8552	1585.8948	0.0396	25	18	31	GLIAEKNCAPLMLR			Carbamidomethyl (C)[8]		Mascot
1596.7217	1596.7545	0.0328	21	217	229	YAADEKAFFEDYK					Mascot
1601.8502	1601.8773	0.0271	17	18	31	GLIAEKNCAPLMLR			Carbamidomethyl (C)[8], Oxidation (M)[12]		Mascot
1834.9229	1834.9218	-0.0011	-1	55	72	CPAELAHGANAGLDIAVR	153	100	Carbamidomethyl (C)[1]		Mascot
2617.304	2617.2988	-0.0052	-2	136	160	QVFSTQMGLSDQDIVALS GGHTLGR					Mascot
2633.2988	2633.3018	0.003	1	136	160	QVFSTQMGLSDQDIVALS GGHTLGR			Oxidation (M)[7]		Mascot
2633.2988	2633.3018	0.003	1	136	160	QVFSTQMGLSDQDIVALS GGHTLGR	114	100	Oxidation (M)[7]		Mascot

Rank	Protein Name	Accession No.	Protein MW	Protein PI	Pep. Count	Protein Score	Protein Score C. I. %	Intensity Matched	Total Ion Score	Total Ion C. I. %	Confirmed
2	L-ascorbate peroxidase 1, cytosolic [Triticum urartu]	gi|474311703	27561.1	5.85	7	175	100	21.892	146	100	

Peptide Information

Calc. Mass	Obsrv. Mass	± da	± ppm	Start Seq.	End Seq.	Sequence	Ion Score	C. I. %	Modification	Rank	Result Type
911.4291	911.4317	0.0026	3	53	61	TGGPFGTMK			Oxidation (M)[8]		Mascot
990.4859	990.4855	-0.0004	0	31	38	NCSPLMLR			Carbamidomethyl (C)[2]		Mascot
1006.4808	1006.4825	0.0017	2	31	38	NCSPLMLR			Carbamidomethyl (C)[2], Oxidation (M)[6]		Mascot
1249.6172	1249.6228	0.0056	4	120	130	EDKPQPPPEGR					Mascot

1249.6172	1249.6228	0.0056	4	120	130	EDKPQPPPEGR	36	98.024			Mascot
1309.6859	1309.656	-0.0299	-23	131	142	LPDATKGSDHLR					Mascot
1309.6859	1309.656	-0.0299	-23	131	142	LPDATKGSDHLR	110	100			Mascot
1525.7434	1525.757	0.0136	9	230	241	AFFEDYKEAHLR					Mascot
1596.7217	1596.7545	0.0328	21	224	236	YAADEKAFFEDYK					Mascot
1831.9773	1831.8951	-0.0822	-45	62	79	KPAEQAHAANAGLDIAVR					Mascot

3 Endoribonuclease Dicer-like protein 1 [Triticum urartu] gi|474213220 178911.7 6.23 25 49 45.596 47.895

Peptide Information

Calc. Mass	Obsrv. Mass	± da	± ppm	Start Seq.	End Seq.	Sequence	Ion Score	C. I. %	Modification	Rank	Result Type
859.4995	859.42	-0.0795	-92	101	107	IRNLESK					Mascot
911.4193	911.4317	0.0124	14	168	174	WQFMGAR			Oxidation (M)[4]		Mascot
990.4825	990.4855	0.003	3	631	638	GEWILCGR			Carbamidomethyl (C)[6]		Mascot
1056.5619	1056.5752	0.0133	13	62	71	MGIHISGVSR					Mascot
1121.5197	1121.5509	0.0312	28	1505	1512	QWPMPQYR			Oxidation (M)[4]		Mascot
1231.6569	1231.6105	-0.0464	-38	1022	1032	ETIDYPVPAVK					Mascot
1249.7012	1249.6228	-0.0784	-63	420	430	TVLAYVQSRGR					Mascot
1249.7012	1249.6228	-0.0784	-63	420	430	TVLAYVQSRGR					Mascot
1260.5492	1260.6383	0.0891	71	858	868	GYSSEYQNQGK					Mascot
1309.6429	1309.656	0.0131	10	1513	1524	CINEGGPAHAKR			Carbamidomethyl (C)[1]		Mascot
1309.6429	1309.656	0.0131	10	1513	1524	CINEGGPAHAKR			Carbamidomethyl (C)[1]		Mascot
1484.6976	1484.8405	0.1429	96	603	616	TEQNEGDPLPGTAR					Mascot
1525.641	1525.757	0.116	76	534	546	HEKPGGSMEYSCK			Carbamidomethyl (C)[12], Oxidation (M)[8]		Mascot
1585.8558	1585.8948	0.039	25	1322	1335	LHTHLRHGSSALEK					Mascot
1585.8558	1585.8948	0.039	25	1322	1335	LHTHLRHGSSALEK					Mascot
1601.8507	1601.8773	0.0266	17	1305	1319	AAAVNNENFARVAVR					Mascot
1669.916	1669.762	-0.154	-92	1272	1286	LEFVGDAVLDHLITK					Mascot
1686.8486	1686.7644	-0.0842	-50	1115	1129	WAAPGVLPVFDEETR					Mascot
1777.8577	1777.9078	0.0501	28	603	618	TEQNEGDPLPGTARHR					Mascot
1785.9957	1785.9296	-0.0661	-37	391	407	VTLLVATSVAEEGLDIR					Mascot
1815.9963	1815.9576	-0.0387	-21	838	855	GAIADFDIVKASGLVPSR					Mascot
1833.8511	1833.895	0.0439	24	1537	1552	GWTDECIGEPMPSVKK			Carbamidomethyl (C)[6]		Mascot
1834.8463	1834.9218	0.0755	41	581	595	LHEMNAFTDMLLPDR			Oxidation (M)[4,10]		Mascot
1849.8459	1849.9266	0.0807	44	1537	1552	GWTDECIGEPMPSVKK			Carbamidomethyl (C)[6], Oxidation (M)[11]		Mascot
1856.9	1856.8896	-0.0104	-6	206	221	AINYALGELGQWCAYK			Carbamidomethyl (C)[13]		Mascot

Calc. Mass	Obsrv. Mass	± da	± ppm	Start Seq.	End Seq.	Sequence	Ion Score	C. I. %	Modification	Rank	Result Type
2568.3782	2568.3601	-0.0181	-7	1432	1456	AGNVATVEVFVDGVQIG VAQNPQKK					Mascot
2585.1072	2585.3171	0.2099	81	858	879	GYSSEYQNQGKLFMADS CWDAK			Carbamidomethyl (C)[18]		Mascot
2600.3945	2600.3152	-0.0793	-30	978	999	TYYVYLPPELCLVHPLPG SLIR			Carbamidomethyl (C)[11]		Mascot
2655.45	2655.2732	-0.1768	-67	391	414	VTLLVATSVAEEGLDIRQ CNVVIR			Carbamidomethyl (C)[19]		Mascot

4 U-box domain-containing protein 17 [Triticum urartu] gi|474011946 77580.9 8.93 16 46 0 26.629

Peptide Information

Calc. Mass	Obsrv. Mass	± da	± ppm	Start Seq.	End Seq.	Sequence	Ion Score	C. I. %	Modification	Rank	Result Type
900.5261	900.4675	-0.0586	-65	62	69	SKPDARVK					Mascot
990.4487	990.4855	0.0368	37	254	261	DGKEEEQR					Mascot
1052.5446	1052.4873	-0.0573	-54	320	327	EMVELFIR			Oxidation (M)[2]		Mascot
1192.6508	1192.5811	-0.0697	-58	319	327	REMVELFIR					Mascot
1231.6754	1231.6105	-0.0649	-53	297	308	SEVVSARSAAVR					Mascot
1249.6423	1249.6228	-0.0195	-16	75	85	LFVEALDEGTR					Mascot
1249.6423	1249.6228	-0.0195	-16	75	85	LFVEALDEGTR					Mascot
1258.6498	1258.6843	0.0345	27	56	67	ESAAARSKPDAR					Mascot
1260.546	1260.6383	0.0923	73	580	590	CTAGCFSSKSR			Carbamidomethyl (C)[1,5]		Mascot
1503.765	1503.7535	-0.0115	-8	222	235	VLDAGLSVSEQTER					Mascot
1557.8054	1557.7429	-0.0625	-40	395	407	CLIDENVVPNTRK			Carbamidomethyl (C)[1]		Mascot
1580.9172	1580.7908	-0.1264	-80	41	53	LFRPGLSHLKAWR					Mascot
1585.8115	1585.8948	0.0833	53	394	406	RCLIDENVVPNTR			Carbamidomethyl (C)[2]		Mascot
1585.8115	1585.8948	0.0833	53	394	406	RCLIDENVVPNTR			Carbamidomethyl (C)[2]		Mascot
1697.9181	1697.8237	-0.0944	-56	614	629	SIQIQPIGTNISPSSR					Mascot
1845.9653	1845.9078	-0.0575	-31	236	251	ARHLMAIGGVDFHLHR			Oxidation (M)[5]		Mascot
1856.9396	1856.8896	-0.05	-27	630	648	AGAHVMSASRTAAVAPT SR			Oxidation (M)[6]		Mascot
1872.9274	1872.866	-0.0614	-33	514	530	DTHTDMQLAAFSALVPR					Mascot

5 hypothetical protein TRIUR3_16800 [Triticum urartu] gi|474353901 18480.5 7.9 7 45 0 2.142

Peptide Information

Calc. Mass	Obsrv. Mass	± da	± ppm	Start Seq.	End Seq.	Sequence	Ion Score	C. I. %	Modification	Rank	Result Type
1258.6063	1258.6843	0.078	62	81	91	FAGLEPDQEPR					Mascot
1456.7264	1456.7677	0.0413	28	38	49	DPNHRSHQRPGR					Mascot
1517.7344	1517.77	0.0356	23	55	69	VGASEVAGQWESAAR					Mascot
1815.9097	1815.9576	0.0479	26	52	69	AARVGASEVAGQWESAA					Mascot

					R						
1850.976	1850.8958	-0.0802	-43	157	171	EEEIFRLEIAVHHTK					Mascot
2590.2896	2590.3416	0.052	20	55	80	VGASEVAGQWESAARFS GALDPVTGK					Mascot
2632.3438	2632.2778	-0.066	-25	2	27	AVDTAPPSPRSATASATD QHQVLGVR					Mascot

6	putative disease resistance RPP8-like protein 4 [Triticum urartu]	gi|474298513	120357.3	6.07	16	44	0	17.849	9	0	

Peptide Information

Calc. Mass	Obsrv. Mass	± da	± ppm	Start Seq.	End Seq.	Sequence	Ion Score	C. I. %	Modification	Rank	Result Type
876.3958	876.4454	0.0496	57	615	620	DDWERR					Mascot
911.5057	911.4317	-0.074	-81	216	222	EHLQTKR					Mascot
948.4316	948.3896	-0.042	-44	330	336	NCNQKER			Carbamidomethyl (C)[2]		Mascot
960.4819	960.5406	0.0587	61	568	576	LPDGIGNMK			Oxidation (M)[8]		Mascot
1258.6863	1258.6843	-0.002	-2	448	459	LSIRLDGASNGR					Mascot
1260.6881	1260.6383	-0.0498	-40	660	668	LERLIMQWR			Oxidation (M)[6]		Mascot
1309.6747	1309.656	-0.0187	-14	141	152	IPPSSNVDIDPR					Mascot
1309.6747	1309.656	-0.0187	-14	141	152	IPPSSNVDIDPR	9	0			Mascot
1507.7798	1507.7518	-0.028	-19	940	952	YGLSLAQRIGCNR			Carbamidomethyl (C)[11]		Mascot
1743.9218	1743.9199	-0.0019	-1	577	590	SLRHLFGFDFTLYK					Mascot
1846.9766	1846.9564	-0.0202	-11	312	329	CGGLPLAVISISSMLANK			Carbamidomethyl (C)[1], Oxidation (M)[14]		Mascot
2590.272	2590.3416	0.0696	27	423	444	SAQENFLTIVDDPHAFTR LQCK			Carbamidomethyl (C)[21]		Mascot
2600.3408	2600.3152	-0.0256	-10	833	854	GYIWRVGNGQNIDIWVD AWIPK					Mascot
2631.3599	2631.2839	-0.076	-29	916	939	FIAGANEKIDWCADILAAE ALALR			Carbamidomethyl (C)[12]		Mascot
2649.1331	2649.3081	0.175	66	74	95	EMSYEIEDIIDDFMQSIGE NDR					Mascot
2655.4282	2655.2732	-0.155	-58	1	26	MAGIMVSASTGVMNYLL GKLTTLVGK					Mascot
2673.1128	2673.25	0.1372	51	335	356	ERWEYVQDSMGSESNH MLDGMR			Oxidation (M)[10]		Mascot

7	unnamed protein product [Triticum aestivum]	gi|219802696	35771.3	8.88	10	44	0	.812	

Protein Group

unnamed protein product [Triticum aestivum]	gi|257342052	35771.3	8.8800 001144 4092

Peptide Information

Calc. Mass	Obsrv. Mass	± da	± ppm	Start Seq.	End Seq.	Sequence	Ion Score	C. I. %	Modification	Rank	Result Type

Calc. Mass	Obsrv. Mass	± da	± ppm	Start Seq.	End Seq.	Sequence	Ion Score	C. I. %	Modification	Rank	Result Type
1006.4986	1006.4825	-0.0161	-16	105	113	AATEVICSR			Carbamidomethyl (C)[7]		Mascot
1052.516	1052.4873	-0.0287	-27	282	289	AEYHNKYK					Mascot
1271.6089	1271.5897	-0.0192	-15	239	249	CADTPAKYFAK			Carbamidomethyl (C)[1]		Mascot
1306.7155	1306.6155	-0.1	-77	306	316	TFLLSLVGWDR					Mascot
1691.7983	1691.7773	-0.021	-12	290	305	GSLAEAIHSETSGNYR					Mascot
1748.8385	1748.9331	0.0946	54	26	41	GFGCDSTTVTNILAHR			Carbamidomethyl (C)[4]		Mascot
1847.0175	1846.9564	-0.0611	-33	141	156	TYGDHQKLLLAYLGVR					Mascot
1872.9662	1872.866	-0.1002	-53	88	104	DATILNQALNSDITDLR					Mascot
2550.3496	2550.3416	-0.008	-3	65	87	LATELSGNHKNAMLLWV LDPVGR			Oxidation (M)[13]		Mascot
2590.1904	2590.3416	0.1512	58	158	181	NEGPEVDPSAVTDDARE LYQAGEK					Mascot

8	hypothetical protein TRIUR3_07243 [Triticum urartu]	gi|474114469	65380	6.34	13	44	0	7.008	

Peptide Information

Calc. Mass	Obsrv. Mass	± da	± ppm	Start Seq.	End Seq.	Sequence	Ion Score	C. I. %	Modification	Rank	Result Type
909.4523	909.4584	0.0061	7	281	289	GTSELSTSK					Mascot
1052.5194	1052.4873	-0.0321	-30	397	405	SVCPTSFVR			Carbamidomethyl (C)[3]		Mascot
1056.4746	1056.5752	0.1006	95	309	317	FDDFAGTQR					Mascot
1308.6478	1308.6586	0.0108	8	246	256	GKPDCTVGYRR			Carbamidomethyl (C)[5]		Mascot
1351.6498	1351.6484	-0.0014	-1	383	393	MDDPLKFCGLR			Carbamidomethyl (C)[8]		Mascot
1596.7653	1596.7545	-0.0108	-7	364	377	SGFPTSYNRPEVDK					Mascot
1648.8514	1648.8329	-0.0185	-11	453	467	LDTAHAKNSAITHNR					Mascot
1669.912	1669.762	-0.15	-90	71	85	QIVLLDEEGQVLGTR					Mascot
1686.8082	1686.7644	-0.0438	-26	318	333	GTSGFSTSYNRPEVGK					Mascot
1834.0011	1833.895	-0.1061	-58	231	245	FVSPFKLHVFTDLQR					Mascot
1872.8835	1872.866	-0.0175	-9	197	214	DVEHTHAAGEASSHTVP K					Mascot
2046.921	2046.9011	-0.0199	-10	542	561	EDKSGSADQLGANNTAE DPK					Mascot
2590.1699	2590.3416	0.1717	66	197	220	DVEHTHAAGEASSHTVP KNGENMR			Oxidation (M)[23]		Mascot

9	hypothetical protein TRIUR3_29952 [Triticum urartu]	gi|473727630	47274	6.19	11	44	0	7.735	

Peptide Information

Calc. Mass	Obsrv. Mass	± da	± ppm	Start Seq.	End Seq.	Sequence	Ion Score	C. I. %	Modification	Rank	Result Type
1033.4924	1033.5713	0.0789	76	356	363	FALCYGFR			Carbamidomethyl (C)[4]		Mascot
1056.6161	1056.5752	-0.0409	-39	32	41	NSAASRLPIK					Mascot
1081.5902	1081.5422	-0.048	-44	317	325	YAARALFNR					Mascot

1503.7108	1503.7535	0.0427	28	129	142	SMGVKAVYDTSSSR			Oxidation (M)[2]		Mascot
1648.8694	1648.8329	-0.0365	-22	321	334	ALFNREIEGPLDFK					Mascot
1669.7738	1669.762	-0.0118	-7	276	289	SVDFKTMEESPLDR			Oxidation (M)[7]		Mascot
1728.9069	1728.9133	0.0064	4	106	121	ASLAAFFGLSQSQVFR					Mascot
1857.0018	1856.8896	-0.1122	-60	106	122	ASLAAFFGLSQSQVFRK					Mascot
1873.0112	1872.866	-0.1452	-78	203	219	SPQQVIGAAIKHHMVEK					Mascot
2617.2563	2617.2988	0.0425	16	134	156	AVYDTSSSRDLALIEACN EFISR			Carbamidomethyl (C)[17]		Mascot
2633.4912	2633.3018	-0.1894	-72	189	213	TLGSYILPYISSVKSPQQV IGAAIK					Mascot
2633.4912	2633.3018	-0.1894	-72	189	213	TLGSYILPYISSVKSPQQV IGAAIK					Mascot

10 hypothetical protein TRIUR3_19949 [Triticum urartu] gi|474159822 59530.9 5.84 6 42 0 2.711 30 92.972

Peptide Information

Calc. Mass	Obsrv. Mass	± da	± ppm	Start Seq.	End Seq.	Sequence	Ion Score	C. I. %	Modification	Rank	Result Type
876.4906	876.4454	-0.0452	-52	495	501	MVLRAMR					Mascot
1232.6495	1232.5988	-0.0507	-41	220	230	RPPETPVHSGR					Mascot
1232.6495	1232.5988	-0.0507	-41	220	230	RPPETPVHSGR	30	92.972			Mascot
1323.6045	1323.6517	0.0472	36	1	11	MGAGQVCRSWR			Carbamidomethyl (C)[7], Oxidation (M)[1]		Mascot
1848.016	1847.9479	-0.0681	-37	197	212	VGVAPIEEKLVQHCLR			Carbamidomethyl (C)[14]		Mascot
1849.9756	1849.9266	-0.049	-26	206	219	LVQHCLRWFGHIQR			Carbamidomethyl (C)[5]		Mascot
2615.1145	2615.3591	0.2446	94	270	289	VELICNDCRMYYDVATC NYR			Carbamidomethyl (C)[5,8,17]		Mascot
2631.1096	2631.2839	0.1743	66	270	289	VELICNDCRMYYDVATC NYR			Carbamidomethyl (C)[5,8,17], Oxidation (M)[10]		Mascot

Rank	Protein Name	Accession No.	Protein MW	Protein PI	Pep. Count	Protein Score	Protein Score C. I. %	Intensity Matched	Total Ion Score	Total Ion C. I. %	Confirmed
1	aci-reductone-dioxygenase-like protein [Triticum aestivum]	gi|237512521	23621.6	5.08	15	518	100	32.857	412	100	

Peptide Information

Calc. Mass	Obsrv. Mass	± da	± ppm	Start Seq.	End Seq.	Sequence	Ion Score	C. I. %	Modification	Rank	Result Type
817.4818	817.4586	-0.0232	-28	35	41	EFIPLAK					Mascot
834.4355	834.4135	-0.022	-26	85	91	LPNYEAK					Mascot
931.4592	931.4581	-0.0011	-1	190	198	GGNQTVEAR					Mascot
931.4592	931.4581	-0.0011	-1	190	198	GGNQTVEAR	18	0			Mascot
1013.5639	1013.5618	-0.0021	-2	27	34	LPHHREPK					Mascot
1088.512	1088.5109	-0.0011	-1	120	127	DQNEQWIR					Mascot
1088.512	1088.5109	-0.0011	-1	120	127	DQNEQWIR	54	99.968			Mascot
1215.5892	1215.5856	-0.0036	-3	146	155	FTLDSDNYIK					Mascot
1417.7079	1417.6674	-0.0405	-29	133	145	GGMIVLPAGMYHR			Oxidation (M)[3]		Mascot
1433.7029	1433.6815	-0.0214	-15	133	145	GGMIVLPAGMYHR			Oxidation (M)[3,10]		Mascot
1433.7029	1433.6815	-0.0214	-15	133	145	GGMIVLPAGMYHR			Oxidation (M)[3,10]		Mascot
1465.6417	1465.6401	-0.0016	-1	108	119	YCLEGSGYFDVR			Carbamidomethyl (C)[2]		Mascot
1465.6417	1465.6401	-0.0016	-1	108	119	YCLEGSGYFDVR	103	100	Carbamidomethyl (C)[2]		Mascot
1561.7979	1561.755	-0.0429	-27	132	145	KGGMIVLPAGMYHR			Oxidation (M)[4,11]		Mascot
1573.7679	1573.7462	-0.0217	-14	146	158	FTLDSDNYIKAMR					Mascot
1589.7628	1589.777	0.0142	9	146	158	FTLDSDNYIKAMR			Oxidation (M)[12]		Mascot
1704.7245	1704.6937	-0.0308	-18	71	84	GYSYVDICDVCPEK			Carbamidomethyl (C)[8,11]		Mascot
1704.7245	1704.6937	-0.0308	-18	71	84	GYSYVDICDVCPEK	98	100	Carbamidomethyl (C)[8,11]		Mascot
1815.8297	1815.8312	0.0015	1	94	107	NFFEEHLHTDEEIR					Mascot
1815.8297	1815.8312	0.0015	1	94	107	NFFEEHLHTDEEIR	140	100			Mascot
2057.0088	2057.0054	-0.0034	-2	92	107	LKNFFEEHLHTDEEIR					Mascot
2515.2993	2515.2703	-0.029	-12	159	179	LFVGEPIWTPYNRPHDHLPAR					Mascot
2520.1421	2520.1194	-0.0227	-9	71	91	GYSYVDICDVCPEKLPNYEAK			Carbamidomethyl (C)[8,11]		Mascot

Rank	Protein Name	Accession No.	Protein MW	Protein PI	Pep. Count	Protein Score	Protein Score C. I. %	Intensity Matched	Total Ion Score	Total Ion C. I. %	Confirmed
2	Heat stress transcription factor A-2d [Triticum urartu]	gi|474273198	47791.6	4.68	8	164	100	13.081	140	100	

Peptide Information

Calc. Mass	Obsrv. Mass	± da	± ppm	Start Seq.	End Seq.	Sequence	Ion Score	C. I. %	Modification	Rank	Result Type
834.4355	834.4135	-0.022	-26	16	22	LPNYEAK					Mascot
1140.4773	1140.569	0.0917	80	285	293	ADMRAMEER			Oxidation (M)[3,6]		Mascot
1194.6122	1194.6138	0.0016	1	301	310	QLQMMGFLAR					Mascot
1212.6882	1212.6326	-0.0556	-46	134	144	GGMDRVLLPVR					Mascot
1212.6882	1212.6326	-0.0556	-46	134	144	GGMDRVLLPVR	1	0			Mascot
1234.562	1234.6134	0.0514	42	396	405	DEAEMLELER					Mascot
1351.6019	1351.6625	0.0606	45	278	288	QEQQSSRADMR			Oxidation (M)[10]		Mascot
1815.8297	1815.8312	0.0015	1	25	38	NFFEEHLHTDEEIR					Mascot
1815.8297	1815.8312	0.0015	1	25	38	NFFEEHLHTDEEIR	140	100			Mascot
2057.0088	2057.0054	-0.0034	-2	23	38	LKNFFEEHLHTDEEIR					Mascot

3 hypothetical protein TRIUR3_17720 [Triticum urartu] gi|474088140 71957.9 5.72 15 56 90.325 7.517

Peptide Information

Calc. Mass	Obsrv. Mass	± da	± ppm	Start Seq.	End Seq.	Sequence	Ion Score	C. I. %	Modification	Rank	Result Type
942.439	942.4789	0.0399	42	634	640	EFLCNFI			Carbamidomethyl (C)[4]		Mascot
1116.4813	1116.5323	0.051	46	236	244	CEMASAYIR			Carbamidomethyl (C)[1], Oxidation (M)[3]		Mascot
1146.6113	1146.5089	-0.1024	-89	344	355	ISTSKAPGSNGK					Mascot
1155.5464	1155.4463	-0.1001	-87	114	123	ECGFTSLSVR			Carbamidomethyl (C)[2]		Mascot
1177.6787	1177.5857	-0.093	-79	338	348	LSISNKISTSK					Mascot
1212.6332	1212.6326	-0.0006	0	64	76	VGPDGVNATLGGR					Mascot
1212.6332	1212.6326	-0.0006	0	64	76	VGPDGVNATLGGR	3	0			Mascot
1306.6307	1306.6331	0.0024	2	394	405	SIGQMEGSEQLK					Mascot
1320.5746	1320.635	0.0604	46	225	235	SIMMYCTGGIR			Carbamidomethyl (C)[6], Oxidation (M)[3,4]		Mascot
1413.6315	1413.6812	0.0497	35	598	610	ASTELAGMFEEGR			Oxidation (M)[8]		Mascot
1473.7012	1473.701	-0.0002	0	223	235	GKSIMMYCTGGIR			Carbamidomethyl (C)[8]		Mascot
1481.7529	1481.6083	-0.1446	-98	111	123	VARECGFTSLSVR			Carbamidomethyl (C)[5]		Mascot
1843.9219	1843.8586	-0.0633	-34	1	17	MDAKAALPPTSTQEQQK					Mascot
1968.9443	1968.9481	0.0038	2	593	610	QIASRASTELAGMFEEGR			Oxidation (M)[13]		Mascot
2520.2485	2520.1194	-0.1291	-51	611	631	CTIVEHDMGHIIPTRPPYIDR			Carbamidomethyl (C)[1]		Mascot
2849.301	2849.3877	0.0867	30	287	309	MLVLVCSTCQDSNKEYVCELCLK			Carbamidomethyl (C)[6,9,18,21]		Mascot
2865.2959	2865.3782	0.0823	29	287	309	MLVLVCSTCQDSNKEYVCELCLK			Carbamidomethyl (C)[6,9,18,21], Oxidation (M)[1]		Mascot

4 putative cadmium/zinc-transporting ATPase HMA1, chloroplastic [Triticum urartu] gi|474111210 77527.1 8.16 16 54 80.697 4.663

Peptide Information

Calc. Mass	Obsrv. Mass	± da	± ppm	Start Seq.	End Seq.	Sequence	Ion Score	C. I. %	Modification	Rank	Result Type
800.4625	800.4377	-0.0248	-31	689	695	QLVDGLR					Mascot
817.4778	817.4586	-0.0192	-23	510	516	EVISTLR					Mascot
834.4502	834.4135	-0.0367	-44	133	139	KSMVDVR					Mascot
1013.5374	1013.5618	0.0244	24	217	227	TVGDAIPGGAR					Mascot
1074.6154	1074.5231	-0.0923	-86	510	518	EVISTLREK					Mascot
1107.5066	1107.5133	0.0067	6	240	248	SWEDSTLNR					Mascot
1148.5769	1148.5343	-0.0426	-37	482	490	LKTYSAMYR			Oxidation (M)[7]		Mascot
1195.6351	1195.6021	-0.033	-28	42	52	SQSLMGKTSLK			Oxidation (M)[5]		Mascot
1269.5834	1269.5933	0.0099	8	295	304	WPFFGNSVCR			Carbamidomethyl (C)[9]		Mascot
1359.6726	1359.6223	-0.0503	-37	471	481	FEYIVSACSRK			Carbamidomethyl (C)[8]		Mascot
1374.6624	1374.6404	-0.022	-16	484	494	TYSAMYRHAFK					Mascot
1374.6624	1374.6404	-0.022	-16	484	494	TYSAMYRHAFK					Mascot
1491.6744	1491.6659	-0.0085	-6	421	433	SNGECEQVQEAIK			Carbamidomethyl (C)[5]		Mascot
1826.8993	1826.922	0.0227	12	434	450	SSAYGPEFVQAALLMDK					Mascot
2515.2312	2515.2703	0.0391	16	140	161	ELKENHPEFALLLETSGD ESTR					Mascot
2547.4043	2547.2698	-0.1345	-53	162	184	FSNLSHTKVPVQDLTVGS HILVR					Mascot
2833.4951	2833.3818	-0.1133	-40	310	338	GLGLMVAASPCALAAAPL AYATAISSLTR			Carbamidomethyl (C)[11], Oxidation (M)[5]		Mascot

5 hypothetical protein TRIUR3_09895 [Triticum urartu] gi|474201872 85144.3 4.94 13 52 69.406 18.968 19 0

Peptide Information

Calc. Mass	Obsrv. Mass	± da	± ppm	Start Seq.	End Seq.	Sequence	Ion Score	C. I. %	Modification	Rank	Result Type
953.4509	953.4387	-0.0122	-13	416	423	WKMNSSGK			Oxidation (M)[3]		Mascot
954.4163	954.4536	0.0373	39	586	594	DGAESNSFK					Mascot
1004.4894	1004.5128	0.0234	23	180	189	AVEGVATDDK					Mascot
1074.5677	1074.5231	-0.0446	-42	210	219	AIEVIATDDK					Mascot
1075.5378	1075.5006	-0.0372	-35	140	149	AIEGRATDDK					Mascot
1104.6082	1104.5066	-0.1016	-92	608	616	DMLLEKSIR					Mascot
1104.6082	1104.5066	-0.1016	-92	608	616	DMLLEKSIR	19	0			Mascot
1120.603	1120.5024	-0.1006	-90	608	616	DMLLEKSIR			Oxidation (M)[2]		Mascot
1120.603	1120.5024	-0.1006	-90	608	616	DMLLEKSIR	16	0	Oxidation (M)[2]		Mascot
1148.5114	1148.5343	0.0229	20	1	10	MGARENGEER					Mascot
1374.7012	1374.6404	-0.0608	-44	89	100	LQYISPNNSPNK					Mascot

1374.7012	1374.6404	-0.0608	-44	89	100	LQYISPNNSPNK					Mascot
1507.6508	1507.6957	0.0449	30	165	179	ADDNKASEGTAADDK					Mascot
1591.8268	1591.8345	0.0077	5	327	339	LYIHASKFWSPDK					Mascot
1604.6567	1604.8096	0.1529	95	558	571	LLTDMFGMDGDDSR			Oxidation (M)[5,8]		Mascot
1958.8574	1958.8679	0.0105	5	38	55	DSFSSQGESNSNEDTKV K					Mascot
2520.1196	2520.1194	-0.0002	0	711	732	RGYTSDDDLDDLGSPLM SLYDR			Oxidation (M)[17]		Mascot

6 stress responsive protein 3 [Triticum aestivum] gi|393689044 19795.6 5.82 9 47 15.737 1.889

Protein Group

group 3 late embryogenesis abundant protein [Triticum aestivum]	gi|320526040	19795.6	5.8200 001716 6138

Peptide Information

Calc. Mass	Obsrv. Mass	± da	± ppm	Start Seq.	End Seq.	Sequence	Ion Score	C. I. %	Modification	Rank	Result Type
908.4328	908.4153	-0.0175	-19	58	66	KMGGEAMGK					Mascot
986.5166	986.4793	-0.0373	-38	172	179	VWPSRGER					Mascot
1075.5491	1075.5006	-0.0485	-45	177	186	GERLSPSSSR					Mascot
1136.5793	1136.5194	-0.0599	-53	98	108	KTATETADATK					Mascot
1265.6219	1265.6313	0.0094	7	99	110	TATETADATKEK					Mascot
1306.6234	1306.6331	0.0097	7	15	26	TASETGQTIQDR					Mascot
1593.7966	1593.7756	-0.021	-13	42	57	SEAVTKAASETTEAAK					Mascot
1680.844	1680.8273	-0.0167	-10	32	47	DQTGAFLGEKSEAVTK					Mascot
1837.8352	1837.8636	0.0284	15	76	92	DHAVEGKDQTGSFFGDK					Mascot

7 hypothetical protein TRIUR3_29772 [Triticum urartu] gi|474397896 13456.3 8.57 7 47 9.711 1.946

Peptide Information

Calc. Mass	Obsrv. Mass	± da	± ppm	Start Seq.	End Seq.	Sequence	Ion Score	C. I. %	Modification	Rank	Result Type
1140.5718	1140.569	-0.0028	-2	106	114	CYLSLRESI			Carbamidomethyl (C)[1]		Mascot
1155.481	1155.4463	-0.0347	-30	5	13	CPPEYTCTK			Carbamidomethyl (C)[1,7]		Mascot
1320.5348	1320.635	0.1002	76	77	86	TQSMWFSMDR			Oxidation (M)[4,8]		Mascot
1448.6298	1448.6487	0.0189	13	76	86	KTQSMWFSMDR			Oxidation (M)[5,9]		Mascot
1455.6606	1455.6479	-0.0127	-9	2	13	ATKCPPEYTCTK			Carbamidomethyl (C)[4,10]		Mascot
1843.8466	1843.8586	0.012	7	77	92	TQSMWFSMDRVAGAEK					Mascot
1968.9167	1968.9481	0.0314	16	33	49	GAGEGQCFPQGCRLYLR			Carbamidomethyl (C)[7,12]		Mascot

8 Disease resistance protein RPM1 [Triticum urartu] gi|474431373 108524.2 8.73 15 42 0 16.52 19 0

Peptide Information

Calc. Mass	Obsrv. Mass	± da	± ppm	Start Seq.	End Seq.	Sequence	Ion Score	C. I. %	Modification	Rank	Result Type
806.4016	806.4012	-0.0004	0	62	68	AQTHGHR					Mascot
817.4163	817.4586	0.0423	52	53	61	DLTAGGAGR					Mascot
856.4457	856.5243	0.0786	92	590	596	MNLSHVR					Mascot
870.5407	870.5389	-0.0018	-2	654	661	QLPKSIGK					Mascot
959.4727	959.4835	0.0108	11	257	264	NQVMPQSR					Mascot
1088.563	1088.5109	-0.0521	-48	131	139	MRAQQIGER					Mascot
1088.563	1088.5109	-0.0521	-48	131	139	MRAQQIGER	19	0			Mascot
1104.5579	1104.5066	-0.0513	-46	131	139	MRAQQIGER			Oxidation (M)[1]		Mascot
1104.5579	1104.5066	-0.0513	-46	131	139	MRAQQIGER			Oxidation (M)[1]		Mascot
1142.5146	1142.4917	-0.0229	-20	184	193	DPVGVEDHMK			Oxidation (M)[9]		Mascot
1215.658	1215.5856	-0.0724	-60	662	671	LENLETLDIR					Mascot
1234.6348	1234.6134	-0.0214	-17	2	13	ELVVGASEATMK					Mascot
1301.6484	1301.572	-0.0764	-59	448	459	SLFPESGKDHGK					Mascot
1417.6991	1417.6674	-0.0317	-22	901	912	NAMPELESLELR			Oxidation (M)[3]		Mascot
1589.8534	1589.777	-0.0764	-48	373	386	VSPLGVEESERLFK					Mascot
1604.8	1604.8096	0.0096	6	53	68	DLTAGGAGRAQTHGHR					Mascot
1815.9198	1815.8312	-0.0886	-49	741	756	LAIYKLSTMSDDPSFK					Mascot
1815.9198	1815.8312	-0.0886	-49	741	756	LAIYKLSTMSDDPSFK					Mascot
1926.8644	1926.8634	-0.001	-1	281	299	GTSALAAKCCSAGASEE TR			Carbamidomethyl (C)[9,10]		Mascot

9 hypothetical protein [Triticum durum] gi|188038091 121493.3 5.4 18 42 0 37.293

Peptide Information

Calc. Mass	Obsrv. Mass	± da	± ppm	Start Seq.	End Seq.	Sequence	Ion Score	C. I. %	Modification	Rank	Result Type
834.4138	834.4135	-0.0003	0	1	8	MADNGKAK					Mascot
942.5003	942.4789	-0.0214	-23	1066	1074	SRPPPSSSK					Mascot
954.3832	954.4536	0.0704	74	478	485	SDNQEMSK			Oxidation (M)[6]		Mascot
989.5302	989.5115	-0.0187	-19	159	166	LLETWAEK					Mascot
1074.5367	1074.5231	-0.0136	-13	714	722	HAFFQPAEK					Mascot
1107.5793	1107.5133	-0.066	-60	364	374	SAGTGIGLEFR					Mascot
1120.5521	1120.5024	-0.0497	-44	899	907	EWLDTTDLK					Mascot
1120.5521	1120.5024	-0.0497	-44	899	907	EWLDTTDLK					Mascot

Calc. Mass	Obsrv. Mass	± da	± ppm	Start Seq.	End Seq.	Sequence	Ion Score	C. I. %	Modification	Rank	Result Type
1146.5942	1146.5089	-0.0853	-74	818	827	ELGFHGVPYK					Mascot
1359.7366	1359.6223	-0.1143	-84	886	898	IDSIPSTSLDAIK					Mascot
1413.6831	1413.6812	-0.0019	-1	785	795	INMEFQNYINK					Mascot
1465.6587	1465.6401	-0.0186	-13	478	489	SDNQEMSKEELR					Mascot
1465.6587	1465.6401	-0.0186	-13	478	489	SDNQEMSKEELR					Mascot
1481.6537	1481.6083	-0.0454	-31	478	489	SDNQEMSKEELR			Oxidation (M)[6]		Mascot
1600.8217	1600.7271	-0.0946	-59	153	166	EAPEGKLLETWAEK					Mascot
1680.928	1680.8273	-0.1007	-60	462	477	VEPNGVEALPSKATLR					Mascot
1699.822	1699.8047	-0.0173	-10	783	795	NRINMEFQNYINK			Oxidation (M)[5]		Mascot
1815.8356	1815.8312	-0.0044	-2	765	779	SALDPDEIEEEQRER					Mascot
1815.8356	1815.8312	-0.0044	-2	765	779	SALDPDEIEEEQRER					Mascot
1942.0228	1941.9205	-0.1023	-53	664	679	QMRLNDVWIRPPFGGR					Mascot
1968.9889	1968.9481	-0.0408	-21	586	602	IFFNVPGMPFSNDKDLK					Mascot
2025.1379	2024.9823	-0.1556	-77	345	363	AAVAVFEKNAPELLPNLTK					Mascot

10 Protein kinase 2B, chloroplastic [Triticum urartu] gi|474374929 51195.2 9.39 11 42 0 4.367

Peptide Information

Calc. Mass	Obsrv. Mass	± da	± ppm	Start Seq.	End Seq.	Sequence	Ion Score	C. I. %	Modification	Rank	Result Type
870.5771	870.5389	-0.0382	-44	237	245	LKVAIGAAK					Mascot
1140.6161	1140.569	-0.0471	-41	168	177	LKPEGFQGHK					Mascot
1155.5068	1155.4463	-0.0605	-52	1	10	MGNCMKSTAR			Carbamidomethyl (C)[4]		Mascot
1269.5529	1269.5933	0.0404	32	197	207	LIGYCSDGDNR			Carbamidomethyl (C)[5]		Mascot
1301.6671	1301.572	-0.0951	-73	446	458	SLGAMAPPSPAFR					Mascot
1433.6512	1433.6815	0.0303	21	11	23	VDHSMNTSAACLK			Carbamidomethyl (C)[11]		Mascot
1433.6512	1433.6815	0.0303	21	11	23	VDHSMNTSAACLK			Carbamidomethyl (C)[11]		Mascot
1473.7631	1473.701	-0.0621	-42	445	458	RSLGAMAPPSPAFR			Oxidation (M)[6]		Mascot
1837.9379	1837.8636	-0.0743	-40	446	463	SLGAMAPPSPAFRTAQVH					Mascot
1843.9396	1843.8586	-0.081	-44	98	114	SEPPRTEGEILSSSNLK					Mascot
2251.2698	2251.2656	-0.0042	-2	315	335	LSAKADVYSFGVVLLELLTGR					Mascot
2515.3779	2515.2703	-0.1076	-43	337	358	ALDKSKPGIEQNLVDWAKPHLR					Mascot

Gel Idx/Pos	181/H8	Instr./Gel Origin	BA2151/Sample Project 20140814	Process Status	Analysis Succeeded
Plate [#] Name	[1] Sample Project 20140814	Instrument Sample Name		Spectra	11

Rank	Protein Name	Accession No.	Protein MW	Protein PI	Pep. Count	Protein Score	Protein Score C. I. %	Intensity Matched	Total Ion Score	Total Ion C. I. %	Confirmed
1	VIL2 protein [Triticum monococcum subsp. aegilopoides]	gi|116562951	84613.7	8.15	18	66	98.81	13.573			

Protein Group

Protein Name	Accession No.	Protein MW	Protein PI
VIL2 protein [Triticum monococcum subsp. monococcum]	gi|116563031	84613.7	8.1499 996185 3027

Peptide Information

Calc. Mass	Obsrv. Mass	± da	± ppm	Start Seq.	End Seq.	Sequence	Ion Score	C. I. %	Modification	Rank	Result Type
803.3682	803.3351	-0.0331	-41	652	658	FEDAGHK					Mascot
821.4073	821.3494	-0.0579	-70	17	23	LMSVDEK					Mascot
824.4373	824.3596	-0.0777	-94	698	704	ATPHDRK					Mascot
832.3795	832.3132	-0.0663	-80	640	647	EPGNSSNK					Mascot
837.4022	837.4219	0.0197	24	17	23	LMSVDEK			Oxidation (M)[2]		Mascot
906.46	906.3746	-0.0854	-94	149	156	AILSMEDK					Mascot
921.402	921.4919	0.0899	98	464	472	TNSGGQSDR					Mascot
921.402	921.4919	0.0899	98	464	472	TNSGGQSDR					Mascot
1060.5457	1060.5209	-0.0248	-23	207	216	TGILPSGQCK			Carbamidomethyl (C)[9]		Mascot
1182.6378	1182.5597	-0.0781	-66	230	238	QHDLLRSWK					Mascot
1182.6378	1182.5597	-0.0781	-66	230	238	QHDLLRSWK					Mascot
1309.5437	1309.6454	0.1017	78	564	574	ESSNSMEQNQR					Mascot
1419.7994	1419.7095	-0.0899	-63	270	281	YSVLHKFVDIAK					Mascot
1518.8679	1518.7317	-0.1362	-90	257	269	IFLGHKVLFSTEK					Mascot
1556.7487	1556.7242	-0.0245	-16	284	299	LEAEVGSVAGHGSMGR					Mascot
1650.9174	1650.7777	-0.1397	-85	78	93	VVTGKSSGPVVHVQEK					Mascot
1812.9021	1812.8462	-0.0559	-31	282	299	QKLEAEVGSVAGHGSMG R					Mascot
1842.9857	1842.8755	-0.1102	-60	402	417	TFVVTALKPATCYMIK			Carbamidomethyl (C)[12]		Mascot
1901.8521	1901.9127	0.0606	32	174	189	DPTIWLSCSSDHPMQK			Carbamidomethyl (C)[8]		Mascot
2011.0204	2011.0222	0.0018	1	441	460	ESDLKGLAPGGAGLVDQ NNR					Mascot
2239.0737	2239.1106	0.0369	16	620	639	SFNTKPSDNIFQNGSSKP DR					Mascot

2	Protein VERNALIZATION INSENSITIVE 3 [Triticum urartu]	gi|474019031	84609.7	8.03	17	59	94.035	12.624					

Peptide Information

Calc. Mass	Obsrv. Mass	± da	± ppm	Start Seq.	End Seq.	Sequence	Ion Score	C. I. %	Modification	Rank	Result Type
803.3682	803.3351	-0.0331	-41	652	658	FEDAGHK					Mascot
821.4073	821.3494	-0.0579	-70	17	23	LMSVDEK					Mascot
832.3795	832.3132	-0.0663	-80	640	647	EPGNSSNK					Mascot
837.4022	837.4219	0.0197	24	17	23	LMSVDEK			Oxidation (M)[2]		Mascot
852.4435	852.399	-0.0445	-52	698	704	ATPHDRR					Mascot
906.46	906.3746	-0.0854	-94	149	156	AILSMEDK					Mascot
921.402	921.4919	0.0899	98	464	472	TNSGGQSDR					Mascot
921.402	921.4919	0.0899	98	464	472	TNSGGQSDR					Mascot
1060.5457	1060.5209	-0.0248	-23	207	216	TGILPSGQCK			Carbamidomethyl (C)[9]		Mascot
1182.6378	1182.5597	-0.0781	-66	230	238	QHDLLRSWK					Mascot
1182.6378	1182.5597	-0.0781	-66	230	238	QHDLLRSWK					Mascot
1309.5437	1309.6454	0.1017	78	564	574	ESSNSMEQNQR					Mascot
1419.7994	1419.7095	-0.0899	-63	270	281	YSVLHKFVDIAK					Mascot
1518.8679	1518.7317	-0.1362	-90	257	269	IFLGHKVLFSTEK					Mascot
1556.7487	1556.7242	-0.0245	-16	284	299	LEAEVGSVAGHGSMGR					Mascot
1650.9174	1650.7777	-0.1397	-85	78	93	VVTGKSSGPVVHVQEK					Mascot
1812.9021	1812.8462	-0.0559	-31	282	299	QKLEAEVGSVAGHGSMGR					Mascot
1901.8521	1901.9127	0.0606	32	174	189	DPTIWLSCSSDHPMQK			Carbamidomethyl (C)[8]		Mascot
2011.0204	2011.0222	0.0018	1	441	460	ESDLKGLAPGGAGLVDQNNR					Mascot
2239.0737	2239.1106	0.0369	16	620	639	SFNTKPSDNIFQNGSSKPDR					Mascot

3	hypothetical protein TRIUR3_26563 [Triticum urartu]	gi|474400087	13679.8	6.29	5	49	43.032	4.051	23	72.548			

Peptide Information

Calc. Mass	Obsrv. Mass	± da	± ppm	Start Seq.	End Seq.	Sequence	Ion Score	C. I. %	Modification	Rank	Result Type
1201.6536	1201.6393	-0.0143	-12	86	97	EDAVAVATAAKR					Mascot
1211.5652	1211.5447	-0.0205	-17	45	55	VTETDEPHAGR					Mascot
1527.7188	1527.7109	-0.0079	-5	10	24	TGDVYPPSAAAHDAR					Mascot
1527.7188	1527.7109	-0.0079	-5	10	24	TGDVYPPSAAAHDAR	23	72.548			Mascot
1556.7188	1556.7242	0.0054	3	28	40	DEVLTTHDDQQQK					Mascot
1840.8784	1840.8756	-0.0028	-2	26	40	QRDEVLTTHDDQQQK					Mascot

4	Ferredoxin-thioredoxin reductase, variable chain [Triticum urartu]	gi|474416354	10130.2	9.51	6	40	0	3.055

Peptide Information

Calc. Mass	Obsrv. Mass	± da	± ppm	Start Seq.	End Seq.	Sequence	Ion Score	C. I. %	Modification	Rank	Result Type
1027.5531	1027.4686	-0.0845	-82	31	39	LDGQDKPVR					Mascot
1391.7682	1391.6547	-0.1135	-82	2	13	EGVVKQYVGVWK					Mascot
1491.7036	1491.7024	-0.0012	-1	50	62	GDEMSILSVYSFK			Oxidation (M)[4]		Mascot
1582.7266	1582.7416	0.015	9	69	83	GVSGNELTMDTDLSK			Oxidation (M)[9]		Mascot
1582.7266	1582.7416	0.015	9	69	83	GVSGNELTMDTDLSK			Oxidation (M)[9]		Mascot
1799.9432	1799.8931	-0.0501	-28	31	45	LDGQDKPVRICTLER			Carbamidomethyl (C)[11]		Mascot
1959.8093	1959.9055	0.0962	49	69	86	GVSGNELTMDTDLSKGCC			Carbamidomethyl (C)[17,18], Oxidation (M)[9]		Mascot

5	hypothetical protein TRIUR3_16781 [Triticum urartu]	gi|473888629	23620	9.01	8	39	0	3.406

Peptide Information

Calc. Mass	Obsrv. Mass	± da	± ppm	Start Seq.	End Seq.	Sequence	Ion Score	C. I. %	Modification	Rank	Result Type
863.3928	863.3525	-0.0403	-47	177	183	MVDDDIR					Mascot
1062.5579	1062.5435	-0.0144	-14	23	32	EAVEVFGGVR					Mascot
1278.7053	1278.6652	-0.0401	-31	202	214	NAVSLVGGIFSSK					Mascot
1484.6322	1484.6619	0.0297	20	131	144	TAMVGAEEEYGDGR					Mascot
1491.7551	1491.7024	-0.0527	-35	10	22	GEIDSTRPFQSVR					Mascot
1594.837	1594.9169	0.0799	50	202	216	NAVSLVGGIFSSKCR			Carbamidomethyl (C)[14]		Mascot
1960.0321	1959.9055	-0.1266	-65	52	70	FSAPRAASPPSSTLLGCLK			Carbamidomethyl (C)[17]		Mascot
3166.4741	3166.5615	0.0874	28	90	118	QSQMEMAVSSIGMQFANSLGIFSDSVELK			Oxidation (M)[4,6]		Mascot

6	E3 ubiquitin-protein ligase SINA-like 2 [Triticum urartu]	gi|474375029	31020.8	6.59	9	39	0	3.694

Peptide Information

Calc. Mass	Obsrv. Mass	± da	± ppm	Start Seq.	End Seq.	Sequence	Ion Score	C. I. %	Modification	Rank	Result Type
838.4352	838.3524	-0.0828	-99	92	97	IRFSCR			Carbamidomethyl (C)[5]		Mascot
897.4788	897.4244	-0.0544	-61	225	232	HGLGEKEK					Mascot
1181.7001	1181.5842	-0.1159	-98	137	147	AVALTSHLTLR					Mascot
1184.5834	1184.5591	-0.0243	-21	205	213	EQEFVYELK					Mascot
1211.6492	1211.5447	-0.1045	-86	220	230	NTSLRHGLGEK					Mascot
1542.6498	1542.7349	0.0851	55	1	13	MADIMHDMPHANK			Oxidation (M)[1,5]		Mascot

1700.8934	1700.8585	-0.0349	-21	190	204	GIALSMICIRPENAR			Carbamidomethyl (C)[8]		Mascot
1901.8787	1901.9127	0.034	18	121	136	HEPIFCPVPQCGFASR			Carbamidomethyl (C)[6,11]		Mascot
2165.9929	2166.1641	0.1712	79	43	60	MLSPPVYQCPFAHVTCSR			Carbamidomethyl (C)[9,16], Oxidation (M)[1]		Mascot

7	Putative cysteine-rich receptor-like protein kinase 12 [Triticum urartu]	gi|474201971	66220.6	6.4	13	38	0	4.839	

Peptide Information

Calc. Mass	Obsrv. Mass	± da	± ppm	Start Seq.	End Seq.	Sequence	Ion Score	C. I. %	Modification	Rank	Result Type
865.4275	865.3643	-0.0632	-73	231	237	HTSHEVR					Mascot
903.4683	903.3887	-0.0796	-88	291	298	HITNGFSK					Mascot
921.4094	921.4919	0.0825	90	541	547	CVEDDRK			Carbamidomethyl (C)[1]		Mascot
921.4094	921.4919	0.0825	90	541	547	CVEDDRK			Carbamidomethyl (C)[1]		Mascot
1484.709	1484.6619	-0.0471	-32	461	472	GYMPPEYIDKQK			Oxidation (M)[3]		Mascot
1497.759	1497.7069	-0.0521	-35	339	350	NEVNNLMRVQHK			Oxidation (M)[7]		Mascot
1518.8428	1518.7317	-0.1111	-73	299	312	KNIIGHGGYGIVYK					Mascot
1542.7257	1542.7349	0.0092	6	458	470	GTRGYMPPEYIDK			Oxidation (M)[6]		Mascot
1569.7618	1569.7494	-0.0124	-8	66	78	LHPMPVLDDEEFK					Mascot
1584.8091	1584.7383	-0.0708	-45	164	176	TSLYMPPEYISKR					Mascot
1594.9387	1594.9169	-0.0218	-14	254	266	RPTINEIIQRLNK					Mascot
1834.9739	1834.8755	-0.0984	-54	116	130	GFEYPIYHLELKPTK					Mascot
1842.9491	1842.8755	-0.0736	-40	517	532	VRLQATMSSHVTEEVR					Mascot
1844.9364	1844.9204	-0.016	-9	325	338	KLHQMLWIEDEQFK					Mascot

8	putative beta-1,3-galactosyltransferase 20 [Triticum urartu]	gi|474210015	52190.2	8.57	12	36	0	5.53	

Peptide Information

Calc. Mass	Obsrv. Mass	± da	± ppm	Start Seq.	End Seq.	Sequence	Ion Score	C. I. %	Modification	Rank	Result Type
838.3723	838.3524	-0.0199	-24	79	85	RDMADSK			Oxidation (M)[3]		Mascot
897.4213	897.4244	0.0031	3	89	95	TSSWFNR					Mascot
907.4091	907.3831	-0.026	-29	73	78	CEKWER			Carbamidomethyl (C)[1]		Mascot
957.4272	957.3837	-0.0435	-45	14	22	ASEGEDPPR					Mascot
1027.415	1027.4686	0.0536	52	296	303	CDDDTFVR			Carbamidomethyl (C)[1]		Mascot
1119.5212	1119.516	-0.0052	-5	53	62	AQRCDGTPSK			Carbamidomethyl (C)[4]		Mascot
1255.6066	1255.5903	-0.0163	-13	86	95	ETKTSSWFNR					Mascot
1497.705	1497.7069	0.0019	1	438	449	LSLGRAQCCNYR			Carbamidomethyl (C)[8,9]		Mascot
1518.8275	1518.7317	-0.0958	-63	304	316	LDVVLQQVSTFNR					Mascot

1812.9868	1812.8462	-0.1406	-78	367	381	NIVSRHANQSLWLFK					Mascot
2003.1913	2003.0315	-0.1598	-80	317	333	TLPLYLGNLNLLHRPLR					Mascot
2011.0536	2011.0222	-0.0314	-16	253	269	KEAEYFGDVVILPFIDR					Mascot

9	unnamed protein product [Triticum aestivum]	gi|227248194	23269.6	5.78	8	36	0	4.385		

Peptide Information

Calc. Mass	Obsrv. Mass	± da	± ppm	Start Seq.	End Seq.	Sequence	Ion Score	C. I. %	Modification	Rank	Result Type
824.3897	824.3596	-0.0301	-37	109	115	YANDSVR					Mascot
957.3764	957.3837	0.0073	8	199	206	QKSSCCST			Carbamidomethyl (C)[5,6]		Mascot
1034.5663	1034.4829	-0.0834	-81	180	188	KASNLVQMK			Oxidation (M)[8]		Mascot
1062.5613	1062.5435	-0.0178	-17	47	55	IRTLEMDGK					Mascot
1316.6594	1316.5839	-0.0755	-57	59	69	LQIWDTAGQER					Mascot
1447.7765	1447.6946	-0.0819	-57	189	200	GQPIQQQQHKQK					Mascot
1823.8923	1823.8336	-0.0587	-32	101	115	QWLSEIDKYANDSVR					Mascot
1838.9204	1838.8862	-0.0342	-19	154	170	ESINVEEAFLAMSAAIK			Oxidation (M)[12]		Mascot

10	Wall-associated receptor kinase 3 [Triticum urartu]	gi|473901443	82753.3	6.64	8	36	0	7.042	23	71.188

Peptide Information

Calc. Mass	Obsrv. Mass	± da	± ppm	Start Seq.	End Seq.	Sequence	Ion Score	C. I. %	Modification	Rank	Result Type
826.4053	826.3533	-0.052	-63	250	256	SYVNSTR					Mascot
852.421	852.399	-0.022	-26	560	567	VTDFGASR					Mascot
921.4424	921.4919	0.0495	54	327	334	GFRSDDPK					Mascot
921.4424	921.4919	0.0495	54	327	334	GFRSDDPK					Mascot
1013.4839	1013.4067	-0.0772	-76	243	249	DWFKFDR					Mascot
1182.5386	1182.5597	0.0211	18	414	423	KATNDFDESR					Mascot
1182.5386	1182.5597	0.0211	18	414	423	KATNDFDESR	23	71.188			Mascot
1321.6934	1321.6331	-0.0603	-46	560	571	VTDFGASRMLPK					Mascot
1497.7982	1497.7069	-0.0913	-61	627	640	EGKSLASSFLLAMK			Oxidation (M)[13]		Mascot
1527.7181	1527.7109	-0.0072	-5	547	559	SMNILLDDNYMAK					Mascot
1527.7181	1527.7109	-0.0072	-5	547	559	SMNILLDDNYMAK					Mascot

Rank	Protein Name	Accession No.	Protein MW	Protein PI	Pep. Count	Protein Score	Protein Score C. I. %	Intensity Matched	Total Ion Score	Total Ion C. I. %	Confirmed
1	Chitinase 2 [Triticum urartu]	gi|474441224	24930.5	4.95	9	572	100	33.181	518	100	

Peptide Information

Calc. Mass	Obsrv. Mass	± da	± ppm	Start Seq.	End Seq.	Sequence	Ion Score	C. I. %	Modification	Rank	Result Type
816.3886	816.3838	-0.0048	-6	212	217	FTYETR					Mascot
916.5462	916.502	-0.0442	-48	160	168	VLASLQTGK					Mascot
996.4897	996.4883	-0.0014	-1	151	159	QTGFYPGAR					Mascot
996.4897	996.4883	-0.0014	-1	151	159	QTGFYPGAR	67	99.999			Mascot
1454.7314	1454.7075	-0.0239	-16	194	206	LPGFFIWSADSSK					Mascot
1813.9542	1813.8994	-0.0548	-30	169	186	TTEELGLLSPDQGIAAAK					Mascot
1869.8865	1869.8793	-0.0072	-4	55	70	LINEYGLDGVDVDYER					Mascot
1869.8865	1869.8793	-0.0072	-4	55	70	LINEYGLDGVDVDYER	133	100			Mascot
2134.0928	2134.092	-0.0008	0	34	54	VSFAPASVDSWVANAVA SLSR					Mascot
2134.0928	2134.092	-0.0008	0	34	54	VSFAPASVDSWVANAVA SLSR	146	100			Mascot
2193.0823	2193.0747	-0.0076	-3	93	112	AAFPNITTSIAPFEDDTVQ R					Mascot
2193.0823	2193.0747	-0.0076	-3	93	112	AAFPNITTSIAPFEDDTVQ R	171	100			Mascot
3580.6042	3580.6394	0.0352	10	121	150	YSGVIDYVNFQFYGYGA NTDVPTYVMFYDR			Oxidation (M)[26]		Mascot

Rank	Protein Name	Accession No.	Protein MW	Protein PI	Pep. Count	Protein Score	Protein Score C. I. %	Intensity Matched	Total Ion Score	Total Ion C. I. %	Confirmed
2	Protein IN2-1-like protein B [Triticum urartu]	gi|473887484	41166.5	8.84	11	240	100	5.834	191	100	

Peptide Information

Calc. Mass	Obsrv. Mass	± da	± ppm	Start Seq.	End Seq.	Sequence	Ion Score	C. I. %	Modification	Rank	Result Type
870.4791	870.4536	-0.0255	-29	208	214	DRPNIQK					Mascot
965.505	965.4653	-0.0397	-41	55	62	NYKGLQDK					Mascot
1086.5983	1086.59	-0.0083	-8	193	201	FQIFFSGIK					Mascot
1340.7209	1340.6887	-0.0322	-24	230	240	LDPQFLLEHTK					Mascot
1468.8159	1468.7197	-0.0962	-65	230	241	LDPQFLLEHTKK					Mascot
1492.7908	1492.7394	-0.0514	-34	325	337	IFLDIRDPGSFGR					Mascot
1540.7366	1540.7316	-0.005	-3	38	49	LYVAYHCPYAQR			Carbamidomethyl (C)[7]		Mascot
1540.7366	1540.7316	-0.005	-3	38	49	LYVAYHCPYAQR	69	100	Carbamidomethyl (C)[7]		Mascot

Calc. Mass	Obsrv. Mass	± da	± ppm	Start Seq.	End Seq.	Sequence	Ion Score	C. I. %	Modification	Rank	Result Type
1798.9222	1798.8801	-0.0421	-23	215	229	FIEEVNKIDAYTQTK					Mascot
1937.9127	1937.8662	-0.0465	-24	106	123	YIDSNFDGPALLPDDSAK					Mascot
2138.0752	2138.0918	0.0166	8	38	54	LYVAYHCPYAQRAWIAR			Carbamidomethyl (C)[7]		Mascot
2356.1667	2356.1624	-0.0043	-2	16	37	ENLPPSLTSTSEPPPLFD GTTR					Mascot
2356.1667	2356.1624	-0.0043	-2	16	37	ENLPPSLTSTSEPPPLFD GTTR	122	100			Mascot

3 hypothetical protein TRIUR3_35101 [Triticum urartu] gi|474033766 38149.2 8.5 10 48 36.08 12.211

Peptide Information

Calc. Mass	Obsrv. Mass	± da	± ppm	Start Seq.	End Seq.	Sequence	Ion Score	C. I. %	Modification	Rank	Result Type
825.4366	825.3926	-0.044	-53	13	18	SFRYPR					Mascot
844.4523	844.4527	0.0004	0	150	157	EGVEIAAR					Mascot
1388.6879	1388.5784	-0.1095	-79	311	322	TIPYPNGCPELK			Carbamidomethyl (C)[8]		Mascot
1651.8075	1651.838	0.0305	18	16	29	YPRADYDLESGIPR					Mascot
1742.7803	1742.8527	0.0724	42	285	299	SQFQEDPYGCVGSLR			Carbamidomethyl (C)[10]		Mascot
1757.9082	1757.8328	-0.0754	-43	246	260	FAQLHRSALGFPEER					Mascot
1948.0685	1947.9958	-0.0727	-37	33	51	KPKASHLDAPAPLGSAL MK			Oxidation (M)[18]		Mascot
2075.0332	2075.0427	0.0095	5	225	242	ELTGTYPQNITVVSYDFK					Mascot
2192.978	2193.0747	0.0967	44	323	341	GLFSYCGMVPYSGNLPW TQ			Carbamidomethyl (C)[6], Oxidation (M)[8]		Mascot
2192.978	2193.0747	0.0967	44	323	341	GLFSYCGMVPYSGNLPW TQ			Carbamidomethyl (C)[6], Oxidation (M)[8]		Mascot
3336.6577	3336.6396	-0.0181	-5	90	122	SGGNEVGAHPLPGLRNL VMVAGHSIYTSASCGK			Carbamidomethyl (C)[31]		Mascot

4 hypothetical protein TRIUR3_24984 [Triticum urartu] gi|474043561 13585.9 7.03 7 46 0 12.158

Peptide Information

Calc. Mass	Obsrv. Mass	± da	± ppm	Start Seq.	End Seq.	Sequence	Ion Score	C. I. %	Modification	Rank	Result Type
800.4161	800.3898	-0.0263	-33	47	52	ADPWRR					Mascot
962.4261	962.4642	0.0381	40	1	9	MQNGGGGWR					Mascot
978.421	978.4762	0.0552	56	1	9	MQNGGGGWR			Oxidation (M)[1]		Mascot
1001.5316	1001.4518	-0.0798	-80	83	89	LWFHDKR					Mascot
1034.5015	1034.4584	-0.0431	-42	58	65	EETRSAWR					Mascot
1725.8239	1725.8477	0.0238	14	1	16	MQNGGGGWRLQGPDPR					Mascot
2031.1545	2030.9945	-0.16	-79	66	82	SALRPRMLLLHPHHQPK					Mascot
2193.1538	2193.0747	-0.0791	-36	72	88	MLLLHPHHQPKLWFHDK			Oxidation (M)[1]		Mascot
2193.1538	2193.0747	-0.0791	-36	72	88	MLLLHPHHQPKLWFHDK			Oxidation (M)[1]		Mascot

5 hypothetical protein TRIUR3_05475 [Triticum urartu] gi|474451639 62486.4 7 13 45 0 4.6

Peptide Information

Calc. Mass	Obsrv. Mass	± da	± ppm	Start Seq.	End Seq.	Sequence	Ion Score	C. I. %	Modification	Rank	Result Type
800.4413	800.3898	-0.0515	-64	211	217	WPGSVVR					Mascot
965.4938	965.4653	-0.0285	-30	563	571	ELSFDVAGK					Mascot
1180.6208	1180.597	-0.0238	-20	513	523	SPPADIQDIPK					Mascot
1180.6208	1180.597	-0.0238	-20	513	523	SPPADIQDIPK					Mascot
1201.563	1201.6466	0.0836	70	398	408	MPDSVTNNAPR					Mascot
1290.7305	1290.6243	-0.1062	-82	386	397	TISPVVISSPYK					Mascot
1468.6775	1468.7197	0.0422	29	100	111	SRTPDLHEDDQR					Mascot
1675.8875	1675.8369	-0.0506	-30	346	361	HAAVSVHPKSSQTAQK					Mascot
1684.9091	1684.8153	-0.0938	-56	4	18	LDPLLLLPMFAGDNR					Mascot
1742.7109	1742.8527	0.1418	81	19	33	MGQYESMDVAHEASR			Oxidation (M)[1,7]		Mascot
1851.8754	1851.8801	0.0047	3	286	303	MTSTDGVLGVQPSTSSE R					Mascot
1927.0283	1926.8624	-0.1659	-86	185	202	ALSHGVEIENSASKIFPK					Mascot
1958.9203	1958.8541	-0.0662	-34	102	118	TPDLHEDDQRATQSFAK					Mascot
2031.0765	2030.9945	-0.082	-40	1	18	MSKLDPLLLLPMFAGDN R					Mascot

6	TPR repeat-containing thioredoxin TTL1 [Triticum urartu]	gi|473889643	56074.1	8.26	14	44	0	6.924	

Peptide Information

Calc. Mass	Obsrv. Mass	± da	± ppm	Start Seq.	End Seq.	Sequence	Ion Score	C. I. %	Modification	Rank	Result Type
806.4519	806.3834	-0.0685	-85	74	80	RFGDAIK					Mascot
809.3788	809.4021	0.0233	29	31	37	AGNEQYK					Mascot
965.4799	965.4653	-0.0146	-15	30	37	RAGNEQYK					Mascot
1086.6055	1086.59	-0.0155	-14	147	155	VGDWKNVLR					Mascot
1180.6797	1180.597	-0.0827	-70	131	140	LQTVEKHLGR					Mascot
1180.6797	1180.597	-0.0827	-70	131	140	LQTVEKHLGR					Mascot
1299.6553	1299.5959	-0.0594	-46	88	98	IDPSYGRAHQR					Mascot
1333.6416	1333.7086	0.067	50	19	30	AMSSADPEELKR					Mascot
1508.7744	1508.7002	-0.0742	-49	347	358	WAESLKDYEVLR					Mascot
1692.8109	1692.8411	0.0302	18	182	197	LNQLDEADMAISSASK					Mascot
1725.8878	1725.8477	-0.0401	-23	448	463	VDVNESPAVARAENVR					Mascot
1868.8881	1868.8864	-0.0017	-1	479	493	EMICPSQQLLEYSVR			Carbamidomethyl (C)[4], Oxidation (M)[2]		Mascot
1937.9447	1937.8662	-0.0785	-41	292	307	HHPVNPVLHCNRAACR			Carbamidomethyl (C)[10,15]		Mascot

Calc. Mass	Obsrv. Mass	± da	± ppm	Start Seq.	End Seq.	Sequence	Ion Score	C. I. %	Modification	Rank	Result Type
1979.9491	1979.9742	0.0251	13	156	175	ECDAAIAAGADSSALLFAAR			Carbamidomethyl (C)[2]		Mascot
2188.092	2188.0457	-0.0463	-21	360	379	ELPGDTEVAEAYFHAQVALK					Mascot

7 Protein CYPRO4 [Triticum urartu] gi|474008622 61443.9 5.79 13 44 0 15.467

Peptide Information

Calc. Mass	Obsrv. Mass	± da	± ppm	Start Seq.	End Seq.	Sequence	Ion Score	C. I. %	Modification	Rank	Result Type
851.437	851.4188	-0.0182	-21	422	429	EKTGFGGR					Mascot
891.3989	891.4105	0.0116	13	275	282	DGADINMR					Mascot
1001.5309	1001.4518	-0.0791	-79	430	438	MGNRIAAPR			Oxidation (M)[1]		Mascot
1038.5116	1038.5021	-0.0095	-9	204	212	NFEHGIHGK					Mascot
1068.5004	1068.5437	0.0433	41	424	433	TGFGGRMGNR			Oxidation (M)[7]		Mascot
1476.7019	1476.7216	0.0197	13	83	95	FLHADGYGHFNAK					Mascot
1507.7396	1507.7366	-0.003	-2	156	167	SPMLRPLMEDFR			Oxidation (M)[3]		Mascot
1508.7969	1508.7002	-0.0967	-64	204	217	NFEHGIHGKGVSVK					Mascot
1743.7867	1743.8324	0.0457	26	492	505	NSHHECYKNQEGLK			Carbamidomethyl (C)[6]		Mascot
1743.7867	1743.8324	0.0457	26	492	505	NSHHECYKNQEGLK			Carbamidomethyl (C)[6]		Mascot
1926.8499	1926.8624	0.0125	6	290	307	GAQMDASESTFLGLDDNR					Mascot
2075.0444	2075.0427	-0.0017	-1	379	398	TAFPGLGSPITHVDVTYDGK					Mascot
2188.0825	2188.0457	-0.0368	-17	149	167	GSPMPARSPMLRPLMEDFR					Mascot
2356.0657	2356.1624	0.0967	41	290	310	GAQMDASESTFLGLDDNRLCR			Carbamidomethyl (C)[20]		Mascot
2356.0657	2356.1624	0.0967	41	290	310	GAQMDASESTFLGLDDNRLCR			Carbamidomethyl (C)[20]		Mascot

8 hypothetical protein TRIUR3_20923 [Triticum urartu] gi|474402934 71417.2 8.27 13 40 0 18.668

Peptide Information

Calc. Mass	Obsrv. Mass	± da	± ppm	Start Seq.	End Seq.	Sequence	Ion Score	C. I. %	Modification	Rank	Result Type
806.4254	806.3834	-0.042	-52	559	565	EKLSSDK					Mascot
816.4097	816.3838	-0.0259	-32	531	537	DSQPELK					Mascot
891.4604	891.4105	-0.0499	-56	1	9	MAVATAAEK					Mascot
995.5408	995.4653	-0.0755	-76	257	265	EGVTVIYSK					Mascot
1086.6266	1086.59	-0.0366	-34	2	12	AVATAAEKAVR					Mascot
1340.6553	1340.6887	0.0334	25	395	406	NNRATSVNSYSK					Mascot
1652.908	1652.8206	-0.0874	-53	40	56	SSRETAPAAVPGVGVVR					Mascot
1742.8068	1742.8527	0.0459	26	480	492	LWLMYEHSQHENR					Mascot

1798.8649	1798.8801	0.0152	8	13	27	CLGLGFDMTCDLRLK			Carbamidomethyl (C)[1,10]		Mascot
1897.9299	1897.9169	-0.013	-7	337	353	MWAPVLGELPLGPCSDR			Carbamidomethyl (C)[14]		Mascot
2149.053	2149.0576	0.0046	2	167	186	YGTHVVVGLSMGGQDVV CVR			Carbamidomethyl (C)[18], Oxidation (M)[11]		Mascot
2150.0925	2150.0847	-0.0078	-4	205	224	LGDQLFTGACAVPPPHA RSR			Carbamidomethyl (C)[10]		Mascot
2193.0356	2193.0747	0.0391	18	121	138	CLAMDGYFISLFDLQLDR			Carbamidomethyl (C)[1], Oxidation (M)[4]		Mascot
2193.0356	2193.0747	0.0391	18	121	138	CLAMDGYFISLFDLQLDR			Carbamidomethyl (C)[1], Oxidation (M)[4]		Mascot

9 ABC transporter A family member 5 [Triticum urartu] gi|473803927 42836.6 6.28 11 40 0 8.822

Peptide Information

Calc. Mass	Obsrv. Mass	± da	± ppm	Start Seq.	End Seq.	Sequence	Ion Score	C. I. %	Modification	Rank	Result Type
916.4985	916.502	0.0035	4	304	311	EVEDLALK					Mascot
979.4699	979.4631	-0.0068	-7	266	273	LCIMVDGR			Carbamidomethyl (C)[2], Oxidation (M)[4]		Mascot
979.4699	979.4631	-0.0068	-7	266	273	LCIMVDGR			Carbamidomethyl (C)[2], Oxidation (M)[4]		Mascot
1290.6953	1290.6243	-0.071	-55	156	165	EHLQFYGRLK					Mascot
1340.6151	1340.6887	0.0736	55	54	65	NSGYAVVCDDLK			Carbamidomethyl (C)[8]		Mascot
1468.7101	1468.7197	0.0096	7	54	66	NSGYAVVCDDLKK			Carbamidomethyl (C)[8]		Mascot
1521.8094	1521.8235	0.0141	9	337	349	IADVFMAVENLKR			Oxidation (M)[6]		Mascot
1540.8806	1540.7316	-0.149	-97	182	196	SVNLLLGGAADKQVR					Mascot
1540.8806	1540.7316	-0.149	-97	182	196	SVNLLLGGAADKQVR					Mascot
1646.8596	1646.8737	0.0141	9	166	181	SLSGSALDLAVEESLR					Mascot
1652.8643	1652.8206	-0.0437	-26	319	332	VYHLSGTQKYELSK					Mascot
2138.0476	2138.0918	0.0442	21	351	369	VEVQAWGLADTTMEDVF VK					Mascot
2356.2449	2356.1624	-0.0825	-35	25	43	HHLLLDYVLTLLWYGESQ R					Mascot
2356.2449	2356.1624	-0.0825	-35	25	43	HHLLLDYVLTLLWYGESQ R					Mascot

10 [Protein-PII] uridylyltransferase [Triticum urartu] gi|473882697 36124.1 5.19 9 40 0 18.108

Peptide Information

Calc. Mass	Obsrv. Mass	± da	± ppm	Start Seq.	End Seq.	Sequence	Ion Score	C. I. %	Modification	Rank	Result Type
868.4159	868.4333	0.0174	20	107	114	AYISSDGR					Mascot
1162.5634	1162.5836	0.0202	17	191	200	SEAEMQRGVR					Mascot
1458.7799	1458.7119	-0.068	-47	221	234	ENGLLVAQAEVSTK					Mascot
1468.6678	1468.7197	0.0519	35	115	125	WFMDVFHVTDR			Oxidation (M)[3]		Mascot
1699.7816	1699.8292	0.0476	28	183	197	HADGSPIRSEAEMQR			Oxidation (M)[13]		Mascot
1727.7581	1727.8372	0.0791	46	43	56	QAAAEMEWLNEYEK			Oxidation (M)[6]		Mascot

2149.0593	2149.0576	-0.0017	-1	130	147	LTDDSVITYIQQCPDRPK	Carbamidomethyl (C)[13]	Mascot
2166.2493	2166.0786	-0.1707	-79	87	106	RGVLLEAVQVLADLDLSINK		Mascot
2166.2493	2166.0786	-0.1707	-79	87	106	RGVLLEAVQVLADLDLSINK		Mascot
2193.1008	2193.0747	-0.0261	-12	43	60	QAAAEMEWLNEYEKLVIR		Mascot
2193.1008	2193.0747	-0.0261	-12	43	60	QAAAEMEWLNEYEKLVIR		Mascot

Gel Idx/Pos	183/H10	Instr./Gel Origin	BA2151/Sample Project 20140814	Process Status	Analysis Succeeded
Plate [#] Name	[1] Sample Project 20140814	Instrument Sample Name		Spectra	11

Rank	Protein Name	Accession No.	Protein MW	Protein PI	Pep. Count	Protein Score	Protein Score C. I. %	Intensity Matched	Total Ion Score	Total Ion C. I. %	Confirmed
1	Chitinase 2 [Triticum urartu]	gi|474441224	24930.5	4.95	5	144	100	4.64	124	100	

Peptide Information

Calc. Mass	Obsrv. Mass	± da	± ppm	Start Seq.	End Seq.	Sequence	Ion Score	C. I. %	Modification	Rank	Result Type
816.3886	816.3824	-0.0062	-8	212	217	FTYETR					Mascot
875.5171	875.4537	-0.0634	-72	1	7	MAVKWLK					Mascot
996.4897	996.4843	-0.0054	-5	151	159	QTGFYPGAR					Mascot
996.4897	996.4843	-0.0054	-5	151	159	QTGFYPGAR	15	0			Mascot
1869.8865	1869.8718	-0.0147	-8	55	70	LINEYGLDGVDVDYER					Mascot
1869.8865	1869.8718	-0.0147	-8	55	70	LINEYGLDGVDVDYER	31	94.36			Mascot
2193.0823	2193.0718	-0.0105	-5	93	112	AAFPNITTSIAPFEDDTVQR					Mascot
2193.0823	2193.0718	-0.0105	-5	93	112	AAFPNITTSIAPFEDDTVQR	78	100			Mascot

Rank	Protein Name	Accession No.	Protein MW	Protein PI	Pep. Count	Protein Score	Protein Score C. I. %	Intensity Matched	Total Ion Score	Total Ion C. I. %	Confirmed
2	hypothetical protein TRIUR3_26563 [Triticum urartu]	gi|474400087	13679.8	6.29	5	57	91.766	3.057	33	95.905	

Peptide Information

Calc. Mass	Obsrv. Mass	± da	± ppm	Start Seq.	End Seq.	Sequence	Ion Score	C. I. %	Modification	Rank	Result Type
1201.6536	1201.6447	-0.0089	-7	86	97	EDAVAVATAAKR					Mascot
1211.5652	1211.566	0.0008	1	45	55	VTETDEPHAGR					Mascot
1367.6663	1367.6545	-0.0118	-9	45	56	VTETDEPHAGRR					Mascot
1527.7188	1527.7136	-0.0052	-3	10	24	TGDVYPPSAAAHDAR					Mascot
1527.7188	1527.7136	-0.0052	-3	10	24	TGDVYPPSAAAHDAR	33	95.905			Mascot
1609.7928	1609.7908	-0.002	-1	42	55	ELRVTETDEPHAGR					Mascot

Rank	Protein Name	Accession No.	Protein MW	Protein PI	Pep. Count	Protein Score	Protein Score C. I. %	Intensity Matched	Total Ion Score	Total Ion C. I. %	Confirmed
3	hypothetical protein TRIUR3_04849 [Triticum urartu]	gi|474111620	15661.8	5.92	9	55	86.949	3.35			

Peptide Information

Calc. Mass	Obsrv. Mass	± da	± ppm	Start Seq.	End Seq.	Sequence	Ion Score	C. I. %	Modification	Rank	Result Type
829.3794	829.4313	0.0519	63	1	7	MSSLMTK			Oxidation (M)[1,5]		Mascot
850.3763	850.418	0.0417	49	133	140	GPVCPSFS			Carbamidomethyl (C)[4]		Mascot
852.425	852.4203	-0.0047	-6	45	50	FWDEKK					Mascot

893.4476	893.4225	-0.0251	-28	19	26	FGVNDSVR						Mascot
925.4845	925.4169	-0.0676	-73	1	8	MSSLMTKK						Mascot
1167.6117	1167.5276	-0.0841	-72	9	18	EIGQTHDVLR						Mascot
1353.6039	1353.5756	-0.0283	-21	117	127	IPDMHHGMEVR				Oxidation (M)[4,8]		Mascot
1568.8544	1568.7797	-0.0747	-48	27	41	ADIAPAHPVQATIHK						Mascot
1927.0397	1926.8668	-0.1729	-90	27	44	ADIAPAHPVQATIHKETK						Mascot

4 Protein SRG1 [Triticum urartu] gi|474119852 35662.2 8.41 12 51 64.874 5.555

Peptide Information

Calc. Mass	Obsrv. Mass	± da	± ppm	Start Seq.	End Seq.	Sequence	Ion Score	C. I. %	Modification	Rank	Result Type
820.3981	820.4152	0.0171	21	90	96	TLEAACR			Carbamidomethyl (C)[6]		Mascot
841.3872	841.4304	0.0432	51	135	141	YMSADVR					Mascot
856.4999	856.5184	0.0185	22	78	84	LRVPSER					Mascot
904.5211	904.4572	-0.0639	-71	61	69	SVSAGTRVK					Mascot
919.4672	919.434	-0.0332	-36	317	324	LAFADVHF					Mascot
969.5112	969.47	-0.0412	-42	37	45	HLSDAGITR					Mascot
1068.5255	1068.4873	-0.0382	-36	133	141	ARYMSADVR					Mascot
1075.5684	1075.5291	-0.0393	-37	316	324	RLAFADVHF					Mascot
1084.5204	1084.4639	-0.0565	-52	133	141	ARYMSADVR			Oxidation (M)[4]		Mascot
1180.5593	1180.5895	0.0302	26	188	198	EVAAEYASANR					Mascot
1280.6416	1280.6161	-0.0255	-20	135	145	YMSADVRAPVR			Oxidation (M)[2]		Mascot
1507.8591	1507.7126	-0.1465	-97	2	16	AIVGLSNAGDRLPPK					Mascot
1553.7595	1553.7684	0.0089	6	174	187	DVVPSWPDSPADLR					Mascot
1553.7595	1553.7684	0.0089	6	174	187	DVVPSWPDSPADLR					Mascot

5 Protein BUD31-like protein 1 [Triticum urartu] gi|474287922 74105.9 5.44 15 47 13.775 6.148

Peptide Information

Calc. Mass	Obsrv. Mass	± da	± ppm	Start Seq.	End Seq.	Sequence	Ion Score	C. I. %	Modification	Rank	Result Type
808.4576	808.3929	-0.0647	-80	661	666	LRVHHF					Mascot
856.4635	856.5184	0.0549	64	157	163	EDLRPAR					Mascot
866.3971	866.4357	0.0386	45	207	213	MGQSCRK			Carbamidomethyl (C)[5]		Mascot
883.4857	883.4626	-0.0231	-26	52	58	ISHQRSR					Mascot
904.4774	904.4572	-0.0202	-22	565	572	LPTDSFPK					Mascot
1064.5259	1064.5117	-0.0142	-13	195	204	YALSSDPSPK					Mascot
1075.5266	1075.5291	0.0025	2	397	407	APSESSPSVSK					Mascot

1229.5216	1229.624	0.1024	83	259	270	SSGDGGGQYVMR			Oxidation (M)[11]		Mascot
1280.6674	1280.6161	-0.0513	-40	59	67	YIYDLFYKR					Mascot
1367.6995	1367.6545	-0.045	-33	57	66	SRYIYDLFYK					Mascot
1492.6584	1492.7629	0.1045	70	272	284	DDGDGEMERVLEK					Mascot
1507.7275	1507.7126	-0.0149	-10	285	297	QAELIGQYEAEEK					Mascot
1722.6726	1722.8147	0.1421	82	469	483	TDLYNDDDDGSSSYR					Mascot
2166.0649	2166.0857	0.0208	10	624	644	CNVPPGSGFLSGIPGLPE DPR			Carbamidomethyl (C)[1]		Mascot
2166.0649	2166.0857	0.0208	10	624	644	CNVPPGSGFLSGIPGLPE DPR			Carbamidomethyl (C)[1]		Mascot
2385.178	2385.0212	-0.1568	-66	383	407	YTTSASSSGLGAVKAPSE SSPSVSK					Mascot

6 Kinesin-4 [Triticum urartu] gi|473723820 93391.2 9.03 19 47 5.456 11.373

Peptide Information

Calc. Mass	Obsrv. Mass	± da	± ppm	Start Seq.	End Seq.	Sequence	Ion Score	C. I. %	Modification	Rank	Result Type
804.4032	804.4244	0.0212	26	816	822	EQIMAGR					Mascot
820.3981	820.4152	0.0171	21	816	822	EQIMAGR			Oxidation (M)[4]		Mascot
847.4705	847.4567	-0.0138	-16	516	522	IKQMAEK					Mascot
849.4828	849.4452	-0.0376	-44	424	430	EAKFINK					Mascot
850.4529	850.418	-0.0349	-41	498	505	AIEHGPAR					Mascot
863.4655	863.4581	-0.0074	-9	516	522	IKQMAEK			Oxidation (M)[4]		Mascot
870.458	870.5117	0.0537	62	446	452	NAHIPYR					Mascot
893.3781	893.4225	0.0444	50	560	566	ETEQACR			Carbamidomethyl (C)[6]		Mascot
948.5005	948.4896	-0.0109	-11	661	668	VSLVPMMR			Oxidation (M)[6]		Mascot
979.5281	979.4592	-0.0689	-70	690	697	MSIFPEKK					Mascot
1021.5359	1021.4922	-0.0437	-43	174	181	GNIRVFCR			Carbamidomethyl (C)[7]		Mascot
1105.6226	1105.5255	-0.0971	-88	496	505	VRAIEHGPAR					Mascot
1280.594	1280.6161	0.0221	17	265	275	TFTMEGVPENR					Mascot
1316.6692	1316.6222	-0.047	-36	574	584	ELENELANEKK					Mascot
1490.752	1490.6664	-0.0856	-57	641	653	ENIPTMNKTTVDK					Mascot
1490.752	1490.6664	-0.0856	-57	641	653	ENIPTMNKTTVDK					Mascot
1506.7468	1506.6841	-0.0627	-42	641	653	ENIPTMNKTTVDK			Oxidation (M)[6]		Mascot
1743.8595	1743.8315	-0.028	-16	397	411	SRSHMWLVDLAGSER					Mascot
1743.8848	1743.8315	-0.0533	-31	109	125	GIATPDCPAALQFLGGR			Carbamidomethyl (C)[7]		Mascot
1869.8912	1869.8718	-0.0194	-10	265	280	TFTMEGVPENRGVNYR					Mascot
1869.8912	1869.8718	-0.0194	-10	265	280	TFTMEGVPENRGVNYR					Mascot
2035.025	2034.9753	-0.0497	-24	612	629	QRPPSNNMPQPSGPSRL R			Oxidation (M)[8]		Mascot

Calc. Mass	Obsrv. Mass	± da	± ppm	Start Seq.	End Seq.	Sequence	Ion Score	C. I. %	Modification	Rank	Result Type
2193.2073	2193.0718	-0.1355	-62	592	611	SAKPSFAAPVRQRPPLAPMR			Oxidation (M)[19]		Mascot
2193.2073	2193.0718	-0.1355	-62	592	611	SAKPSFAAPVRQRPPLAPMR	2	0	Oxidation (M)[19]		Mascot
2440.196	2440.0295	-0.1665	-68	800	822	TTSMTSGTGIFDPALREQIMAGR					Mascot

7	hypothetical protein TRIUR3_21199 [Triticum urartu]	gi|474256322	80007.4	5.61	17	46	3.254	7.116

Peptide Information

Calc. Mass	Obsrv. Mass	± da	± ppm	Start Seq.	End Seq.	Sequence	Ion Score	C. I. %	Modification	Rank	Result Type
805.3872	805.4028	0.0156	19	192	197	MEQELR					Mascot
847.4567	847.4567	0	0	650	656	ASMVQRR					Mascot
850.5005	850.418	-0.0825	-97	220	226	LHTPARR					Mascot
853.3686	853.4183	0.0497	58	17	23	SDYEPSR					Mascot
863.4515	863.4581	0.0066	8	650	656	ASMVQRR			Oxidation (M)[3]		Mascot
883.4302	883.4626	0.0324	37	62	69	MAKSSTSR			Oxidation (M)[1]		Mascot
948.4567	948.4896	0.0329	35	57	64	ENSPRMAK			Oxidation (M)[6]		Mascot
1021.5676	1021.4922	-0.0754	-74	530	539	IGAAEAKSFK					Mascot
1037.5415	1037.5133	-0.0282	-27	354	361	IESWIFSR					Mascot
1075.5015	1075.5291	0.0276	26	5	13	ENGEVRDEK					Mascot
1105.5493	1105.5255	-0.0238	-22	438	446	LVMEQCVAR			Carbamidomethyl (C)[6]		Mascot
1280.6958	1280.6161	-0.0797	-62	170	180	IVDDDKAVLHR					Mascot
1474.7107	1474.7167	0.006	4	316	328	SMPVPWKNNSNGK			Oxidation (M)[2]		Mascot
1486.8186	1486.6873	-0.1313	-88	540	552	LLNELSDLLMTPK					Mascot
1568.8829	1568.7797	-0.1032	-66	562	575	KEICPSVGLPLVTR			Carbamidomethyl (C)[4]		Mascot
1676.8326	1676.8383	0.0057	3	410	423	AAFHDAYSRLCPLR			Carbamidomethyl (C)[11]		Mascot
2035.1587	2034.9753	-0.1834	-90	268	284	LTYWLSNTVVLREIISK					Mascot
2193.1274	2193.0718	-0.0556	-25	362	379	IVETVWWQALTPHMQTPR					Mascot
2193.1274	2193.0718	-0.0556	-25	362	379	IVETVWWQALTPHMQTPR					Mascot

8	resistance protein RGA2 [Triticum urartu var. pubescentiurartu]	gi|195975990	105639.7	5.85	21	46	3.254	9.63

Protein Group

resistance protein RGA2 [Triticum urartu var. urartu]	gi|195975994	105639.7	5.8499999046 3257

Peptide Information

Calc. Mass	Obsrv. Mass	± da	± ppm	Start Seq.	End Seq.	Sequence	Ion Score	C. I. %	Modification	Rank	Result Type

806.444	806.4187	-0.0253	-31	707	713	LVSSLCK			Carbamidomethyl (C)[6]		Mascot
841.4202	841.4304	0.0102	12	215	220	INFEYR					Mascot
854.3825	854.4212	0.0387	45	67	73	SCADFVR			Carbamidomethyl (C)[2]		Mascot
863.4258	863.4581	0.0323	37	151	157	TEFVDPR					Mascot
866.4955	866.4357	-0.0598	-69	115	121	NRHQIAK					Mascot
922.4628	922.4572	-0.0056	-6	208	214	QVYDELR					Mascot
948.5625	948.4896	-0.0729	-77	525	532	RLSLFANK					Mascot
991.5128	991.5018	-0.011	-11	228	236	SPDMATILK			Oxidation (M)[4]		Mascot
996.4884	996.4843	-0.0041	-4	22	29	TILDDEYK					Mascot
996.4884	996.4843	-0.0041	-4	22	29	TILDDEYK					Mascot
1064.5128	1064.5117	-0.0011	-1	754	761	GRMCWINK			Carbamidomethyl (C)[4]		Mascot
1068.5433	1068.4873	-0.056	-52	128	136	SQILEAHDR					Mascot
1182.63	1182.5619	-0.0681	-58	397	406	HKKPDIDAMK					Mascot
1182.6729	1182.5619	-0.111	-94	345	355	GVPPELKEVSK					Mascot
1265.6736	1265.6163	-0.0573	-45	20	29	LRTILDDEYK					Mascot
1507.7024	1507.7126	0.0102	7	453	465	YQDGEDLVQVGER					Mascot
1553.8646	1553.7684	-0.0962	-62	547	562	SLGASGHLGQLISSVK					Mascot
1553.8646	1553.7684	-0.0962	-62	547	562	SLGASGHLGQLISSVK					Mascot
1722.8181	1722.8147	-0.0034	-2	772	786	LYISGGDEIEQDDLR					Mascot
1958.8915	1958.8525	-0.039	-20	288	304	CALCKNSCGSVIMTTTR			Carbamidomethyl (C)[1,4,8]		Mascot
2011.076	2010.9916	-0.0844	-42	730	747	EKDGFIGHPFLPALNSIR					Mascot
2035.0504	2034.9753	-0.0751	-37	756	771	MCWINKWLLSLANLEK			Carbamidomethyl (C)[2], Oxidation (M)[1]		Mascot
2385.0737	2385.0212	-0.0525	-22	47	66	AMQSFLLMVADTEEHEQ FDK			Oxidation (M)[2]		Mascot

9 hypothetical protein TRIUR3_30438 [Triticum urartu] gi|473944792 22130 10.47 10 43 0 3.876

Peptide Information

Calc. Mass	Obsrv. Mass	± da	± ppm	Start Seq.	End Seq.	Sequence	Ion Score	C. I. %	Modification	Rank	Result Type
827.4846	827.4335	-0.0511	-62	159	165	QALPSRR					Mascot
837.4828	837.402	-0.0808	-96	63	69	IVYSLSR					Mascot
919.4706	919.434	-0.0366	-40	32	38	VNIMEWK					Mascot
950.4941	950.4682	-0.0259	-27	2	9	SPVTVYER					Mascot
1167.613	1167.5276	-0.0854	-73	165	174	RGIHGWTGQR					Mascot
1229.6056	1229.624	0.0184	15	109	119	ADPPLARSCSR			Carbamidomethyl (C)[9]		Mascot
1367.7291	1367.6545	-0.0746	-55	166	177	GIHGWTGQRVTR					Mascot
1926.9413	1926.8668	-0.0745	-39	1	17	MSPVTVYERVVNSAACK			Carbamidomethyl (C)[16], Oxidation (M)[1]		Mascot

1942.7188	1942.8545	0.1357	70	95	108	CSDYFDMCRCLDER			Carbamidomethyl (C)[1,8,10], Oxidation (M)[7]		Mascot
1958.9066	1958.8525	-0.0541	-28	126	142	MPPGYYTGSPQLEEHPR					Mascot

10	Pre-mRNA-processing factor 6 [Triticum urartu]	gi|473728804	120118.8	8.42	20	43	0	8.736	

Peptide Information

Calc. Mass	Obsrv. Mass	± da	± ppm	Start Seq.	End Seq.	Sequence	Ion Score	C. I. %	Modification	Rank	Result Type
806.4155	806.4187	0.0032	4	90	96	WISSTGR					Mascot
829.493	829.4313	-0.0617	-74	531	537	LWLQAAK					Mascot
841.4236	841.4304	0.0068	8	879	885	AFLTMSR			Oxidation (M)[5]		Mascot
853.3686	853.4183	0.0497	58	246	252	GYDENQK					Mascot
875.4662	875.4537	-0.0125	-14	819	825	LFSSFFK					Mascot
889.485	889.4669	-0.0181	-20	709	716	RGSIETAR					Mascot
967.4731	967.4658	-0.0073	-8	599	606	LETYDQAK					Mascot
969.5186	969.47	-0.0486	-50	879	886	AFLTMSRK			Oxidation (M)[5]		Mascot
1064.5623	1064.5117	-0.0506	-48	313	321	ITEQFADLK					Mascot
1068.5619	1068.4873	-0.0746	-70	877	885	SRAFLTMSR					Mascot
1075.5796	1075.5291	-0.0505	-47	464	473	HPPGWIAAAR					Mascot
1084.5569	1084.4639	-0.093	-86	877	885	SRAFLTMSR			Oxidation (M)[7]		Mascot
1201.6899	1201.6447	-0.0452	-38	193	203	ISLAEGLTNRK					Mascot
1211.6281	1211.566	-0.0621	-51	1023	1032	HGERWQAISK					Mascot
1229.696	1229.624	-0.072	-59	192	202	RISLAEGLTNR					Mascot
1537.7778	1537.8041	0.0263	17	435	448	SMKITSDAEISDIK					Mascot
1537.7778	1537.8041	0.0263	17	435	448	SMKITSDAEISDIK					Mascot
1549.8486	1549.762	-0.0866	-56	552	564	GLEHIPDSVRLWK					Mascot
1553.7727	1553.7684	-0.0043	-3	435	448	SMKITSDAEISDIK			Oxidation (M)[2]		Mascot
1553.7727	1553.7684	-0.0043	-3	435	448	SMKITSDAEISDIK			Oxidation (M)[2]		Mascot
1676.9078	1676.8383	-0.0695	-41	1033	1047	AVENSHQPVDAILRK					Mascot
1942.995	1942.8545	-0.1405	-72	982	998	AVTLAPDIGDFWALYYK					Mascot
2104.155	2103.9575	-0.1975	-94	710	729	GSIETARAIYAHALSVFVAK					Mascot
2385.1682	2385.0212	-0.147	-62	380	403	AAGGTETPWAQTPVTDLTAVGEGR					Mascot

Gel Idx/Pos	184/H11	Instr./Gel Origin	BA2151/Sample Project 20140814	Process Status	Analysis Succeeded
Plate [#] Name	[1] Sample Project 20140814	Instrument Sample Name		Spectra	11

Rank	Protein Name	Accession No.	Protein MW	Protein PI	Pep. Count	Protein Score	Protein Score C. I. %	Intensity Matched	Total Ion Score	Total Ion C. I. %	Confirmed
1	Chitinase 2 [Triticum urartu]	gi|474441224	24930.5	4.95	5	74	99.843	2.344	55	99.975	

Peptide Information

Calc. Mass	Obsrv. Mass	± da	± ppm	Start Seq.	End Seq.	Sequence	Ion Score	C. I. %	Modification	Rank	Result Type
816.3886	816.393	0.0044	5	212	217	FTYETR					Mascot
881.4475	881.3793	-0.0682	-77	218	225	AQEIVANH					Mascot
996.4897	996.4911	0.0014	1	151	159	QTGFYPGAR					Mascot
1869.8865	1869.8965	0.01	5	55	70	LINEYGLDGVDVDYER					Mascot
1869.8865	1869.8965	0.01	5	55	70	LINEYGLDGVDVDYER	55	99.975			Mascot
2193.0823	2193.0918	0.0095	4	93	112	AAFPNITTSIAPFEDDTVQR					Mascot

Rank	Protein Name	Accession No.	Protein MW	Protein PI	Pep. Count	Protein Score	Protein Score C. I. %	Intensity Matched	Total Ion Score	Total Ion C. I. %	Confirmed
2	Peroxidase 2 [Triticum urartu]	gi|474449493	36227.3	6.8	11	49	49.227	4.929			

Peptide Information

Calc. Mass	Obsrv. Mass	± da	± ppm	Start Seq.	End Seq.	Sequence	Ion Score	C. I. %	Modification	Rank	Result Type
805.3839	805.3314	-0.0525	-65	278	283	SDWELR					Mascot
853.3985	853.4216	0.0231	27	293	298	DMREFR					Mascot
881.4185	881.3793	-0.0392	-44	235	241	DMELAFR					Mascot
884.4472	884.4672	0.02	23	38	45	HDVADTVK					Mascot
897.4135	897.4288	0.0153	17	235	241	DMELAFR			Oxidation (M)[2]		Mascot
905.5124	905.4269	-0.0855	-94	47	54	EVAMSIKK					Mascot
921.5073	921.5189	0.0116	13	47	54	EVAMSIKK			Oxidation (M)[4]		Mascot
921.5073	921.5189	0.0116	13	46	53	KEVAMSIK			Oxidation (M)[5]		Mascot
1320.7059	1320.5967	-0.1092	-83	274	283	VLFRSDWELR					Mascot
1372.62	1372.6571	0.0371	27	242	255	NASGHNASGVDTSR					Mascot
1475.7522	1475.7523	0.0001	0	311	324	AMAKLSEIPAEGSR			Oxidation (M)[2]		Mascot
1659.8735	1659.8273	-0.0462	-28	113	128	LGESVSCADIVVLAAR			Carbamidomethyl (C)[7]		Mascot
1844.9899	1844.9404	-0.0495	-27	111	128	GKLGESVSCADIVVLAAR			Carbamidomethyl (C)[9]		Mascot

Rank	Protein Name	Accession No.	Protein MW	Protein PI	Pep. Count	Protein Score	Protein Score C. I. %	Intensity Matched	Total Ion Score	Total Ion C. I. %	Confirmed
3	Coatomer subunit epsilon-1 [Triticum urartu]	gi|472900321	25914.1	5.41	6	48	28.281	2.891	18	0	

Peptide Information

Calc. Mass	Obsrv. Mass	± da	± ppm	Start	End	Sequence	Ion	C. I. %	Modification	Rank	Result Type

Calc. Mass	Obsrv. Mass	± da	± ppm	Start Seq.	End Seq.	Sequence	Ion Score	C. I. %	Modification	Rank	Result Type
1270.5547	1270.5626	0.0079	6	217	227	TTTSAEDNFER					Mascot
1270.5547	1270.5626	0.0079	6	217	227	TTTSAEDNFER	18	0			Mascot
1473.7261	1473.7142	-0.0119	-8	131	142	EAYLIFQDFAEK					Mascot
1643.8387	1643.842	0.0033	2	37	51	EWLSDSAIGSNPVLR					Mascot
1865.9525	1865.8778	-0.0747	-40	1	18	MVISEIDSSAATSLQAVK			Oxidation (M)[1]		Mascot
2215.1982	2215.1887	-0.0095	-4	70	89	HTHTGGTLDLHALNVQIF LK					Mascot
2955.4517	2955.4148	-0.0369	-12	102	128	IMQQTDEDHTLTQLANA WLDIAVGGSK					Mascot
2955.4517	2955.4148	-0.0369	-12	102	128	IMQQTDEDHTLTQLANA WLDIAVGGSK					Mascot

4	hypothetical protein TRIUR3_21997 [Triticum urartu]	gi|474210228	27628.5	9.64	10	47	5.456	3.021	

Peptide Information

Calc. Mass	Obsrv. Mass	± da	± ppm	Start Seq.	End Seq.	Sequence	Ion Score	C. I. %	Modification	Rank	Result Type
941.4839	941.4062	-0.0777	-83	178	186	STLFGGFGR					Mascot
982.5026	982.4441	-0.0585	-60	156	163	HEDMPLLK					Mascot
996.4421	996.4911	0.049	49	17	24	DGNWFTEK					Mascot
1081.4796	1081.5175	0.0379	35	200	209	AEGTSSSEWK					Mascot
1167.619	1167.5627	-0.0563	-48	154	163	GKHEDMPLLK					Mascot
1184.6172	1184.5742	-0.043	-36	178	188	STLFGGFGRSR					Mascot
1309.7362	1309.6417	-0.0945	-72	232	244	FKLSSVASVTLSA					Mascot
1479.853	1479.7594	-0.0936	-63	28	40	KEVIPNPVLEVSR					Mascot
1556.8584	1556.7396	-0.1188	-76	173	186	FVIQKSTLFGGFGR					Mascot
1556.8584	1556.7396	-0.1188	-76	173	186	FVIQKSTLFGGFGR					Mascot
1572.8282	1572.7468	-0.0814	-52	99	113	GGLSTWIKGAFHGNK					Mascot

5	hypothetical protein TRIUR3_30879 [Triticum urartu]	gi|472909785	11663.1	9.09	8	44	0	3.217	

Peptide Information

Calc. Mass	Obsrv. Mass	± da	± ppm	Start Seq.	End Seq.	Sequence	Ion Score	C. I. %	Modification	Rank	Result Type
807.4108	807.3985	-0.0123	-15	92	97	NRDQFK					Mascot
963.5146	963.4689	-0.0457	-47	94	101	DQFKVVDL					Mascot
1081.5499	1081.5175	-0.0324	-30	1	10	MAAGGVWVFK			Oxidation (M)[1]		Mascot
1178.5875	1178.5804	-0.0071	-6	82	91	STHMYDVVVK					Mascot
1182.6953	1182.5792	-0.1161	-98	65	75	RGGVDLISIPR					Mascot
1182.6953	1182.5792	-0.1161	-98	65	75	RGGVDLISIPR					Mascot

1479.6744	1479.7594	0.085	57	11	23	NGVMELEQEATSR			Oxidation (M)[4]	Mascot
1491.7988	1491.733	-0.0658	-44	24	36	KALVYVPANETMR				Mascot
1507.7937	1507.7452	-0.0485	-32	24	36	KALVYVPANETMR			Oxidation (M)[12]	Mascot
1643.7812	1643.842	0.0608	37	45	57	LGSLGWERYYEDR				Mascot

6	Cis-zeatin O-glucosyltransferase 1 [Triticum urartu]	gi|473967644	25576.6	5.39	9	44	0	3.724			

Peptide Information

Calc. Mass	Obsrv. Mass	± da	± ppm	Start Seq.	End Seq.	Sequence	Ion Score	C. I. %	Modification	Rank	Result Type
852.4283	852.3857	-0.0426	-50	2	8	TMSISWK					Mascot
865.4488	865.3809	-0.0679	-78	84	90	MLPEFTK					Mascot
881.4437	881.3793	-0.0644	-73	84	90	MLPEFTK			Oxidation (M)[1]		Mascot
884.4836	884.4672	-0.0164	-19	168	175	HSEVITAK					Mascot
996.4051	996.4911	0.086	86	206	215	ASMEDGGSSR					Mascot
1140.495	1140.5684	0.0734	64	206	216	ASMEDGGSSRK			Oxidation (M)[3]		Mascot
1141.6437	1141.5553	-0.0884	-77	195	205	QGARVIGDAVR					Mascot
1259.6453	1259.6344	-0.0109	-9	81	90	HEKMLPEFTK					Mascot
1527.7368	1527.722	-0.0148	-10	1	13	MTMSISWKMDLGK					Mascot
1657.8503	1657.8033	-0.047	-28	43	57	DEQIEELAAALRGSR					Mascot

7	ent-kaurene synthase like 3 [Triticum aestivum]	gi|363987140	98115.3	5.87	14	42	0	5.187	10	0	

Peptide Information

Calc. Mass	Obsrv. Mass	± da	± ppm	Start Seq.	End Seq.	Sequence	Ion Score	C. I. %	Modification	Rank	Result Type
801.3525	801.3423	-0.0102	-13	617	622	WDEHSK					Mascot
807.4141	807.3985	-0.0156	-19	779	785	SVASCRK			Carbamidomethyl (C)[5]		Mascot
818.4519	818.371	-0.0809	-99	545	550	WVKETR					Mascot
893.4761	893.4317	-0.0444	-50	83	91	TMATSALAK					Mascot
897.374	897.4288	0.0548	61	730	736	LMGTCCR			Carbamidomethyl (C)[5,6]		Mascot
909.4709	909.4251	-0.0458	-50	83	91	TMATSALAK			Oxidation (M)[2]		Mascot
918.4639	918.3832	-0.0807	-88	258	266	LAGDKSDGR					Mascot
979.4666	979.4709	0.0043	4	71	79	APPYSMSAR					Mascot
1037.566	1037.5251	-0.0409	-39	83	92	TMATSALAKK			Oxidation (M)[2]		Mascot
1107.5695	1107.5435	-0.026	-23	187	195	WNVGPEHIR					Mascot
1182.6702	1182.5792	-0.091	-77	39	49	RNQPLGLASAR					Mascot
1182.6702	1182.5792	-0.091	-77	39	49	RNQPLGLASAR	10	0			Mascot
1259.7219	1259.6344	-0.0875	-69	551	560	LDQLLFARQR					Mascot

Calc. Mass	Obsrv. Mass	± da	± ppm	Start Seq.	End Seq.	Sequence	Ion Score	C. I. %	Modification	Rank	Result Type
1926.8505	1926.8954	0.0449	23	617	631	WDEHSKDEFYSEQVK					Mascot
2215.1956	2215.1887	-0.0069	-3	19	38	LRPAAVSPLVSNLSHSHH HR					Mascot
2633.2488	2633.2781	0.0293	11	559	581	QRTTFCYLAAAATMFPPE LSDAR			Carbamidomethyl (C)[6], Oxidation (M)[14]		Mascot

8 hypothetical protein TRIUR3_09072 [Triticum urartu] gi|474223938 53746.5 5.38 12 41 0 3.252

Peptide Information

Calc. Mass	Obsrv. Mass	± da	± ppm	Start Seq.	End Seq.	Sequence	Ion Score	C. I. %	Modification	Rank	Result Type
806.3389	806.3951	0.0562	70	134	139	YYDAMK			Oxidation (M)[5]		Mascot
807.4471	807.3985	-0.0486	-60	86	91	FVTRER					Mascot
837.4321	837.3568	-0.0753	-90	1	7	MITGLMR			Oxidation (M)[1]		Mascot
853.427	853.4216	-0.0054	-6	1	7	MITGLMR			Oxidation (M)[1,6]		Mascot
854.4479	854.3849	-0.063	-74	140	147	AEHGITAR					Mascot
891.4141	891.4144	0.0003	0	230	237	WDGAGMVR					Mascot
963.4377	963.4689	0.0312	32	347	355	EAIASSDDR					Mascot
989.4509	989.4658	0.0149	15	298	305	DDGWMLPR					Mascot
1265.5355	1265.6313	0.0958	76	16	26	EVFDGMPDPDK			Oxidation (M)[6]		Mascot
1320.6552	1320.5967	-0.0585	-44	230	241	WDGAGMVRGLMK					Mascot
1605.8563	1605.7428	-0.1135	-71	1	15	MITGLMRNGLVAAAR			Oxidation (M)[1,6]		Mascot
1643.8137	1643.842	0.0283	17	306	319	LRDDGDHVSLFVDR					Mascot
1675.8585	1675.8267	-0.0318	-19	27	41	VSWTALIDGCVKNGR			Carbamidomethyl (C)[10]		Mascot

9 Armadillo repeat-containing kinesin-like protein 2 [Triticum urartu] gi|474450999 94481.4 6.27 19 41 0 6.11 8 0

Peptide Information

Calc. Mass	Obsrv. Mass	± da	± ppm	Start Seq.	End Seq.	Sequence	Ion Score	C. I. %	Modification	Rank	Result Type
806.4003	806.3951	-0.0052	-6	234	240	LNTESSR					Mascot
810.3741	810.3497	-0.0244	-30	348	355	DSFGGTAR					Mascot
845.4614	845.3984	-0.063	-75	404	410	LDIELDK					Mascot
881.4549	881.3793	-0.0756	-86	215	221	SFMDLLR					Mascot
893.4258	893.4317	0.0059	7	809	815	ISRDCSR			Carbamidomethyl (C)[5]		Mascot
897.4498	897.4288	-0.021	-23	215	221	SFMDLLR			Oxidation (M)[3]		Mascot
912.4686	912.4173	-0.0513	-56	515	521	NQLSHWK					Mascot
951.4894	951.4586	-0.0308	-32	429	436	ITAEAQYR					Mascot
1064.5591	1064.4967	-0.0624	-59	382	390	AMKVQNMVK			Oxidation (M)[2]		Mascot
1107.5714	1107.5435	-0.0279	-25	446	454	ISLENEKMK			Oxidation (M)[8]		Mascot

1178.5834	1178.5804	-0.003	-3	536	545	RMLDTEASQK					Mascot
1182.5936	1182.5792	-0.0144	-12	816	824	EDIRMLAYR			Oxidation (M)[5]		Mascot
1182.5936	1182.5792	-0.0144	-12	816	824	EDIRMLAYR			Oxidation (M)[5]		Mascot
1193.5831	1193.6221	0.039	33	523	533	MEATATAEVVR			Oxidation (M)[1]		Mascot
1265.6042	1265.6313	0.0271	21	537	547	MLDTEASQKDK					Mascot
1372.7795	1372.6571	-0.1224	-89	285	297	SKLVVVDLAGSER					Mascot
1475.7312	1475.7523	0.0211	14	582	594	IFPGLDSLMSHSR	8	0	Oxidation (M)[9]		Mascot
1585.9346	1585.8348	-0.0998	-63	622	636	IVEAGGLTSLLMLLR					Mascot
1657.8037	1657.8033	-0.0004	0	370	384	GETTSTIMFGQRAMK					Mascot
1838.0535	1837.9287	-0.1248	-68	751	767	VGRSLLVDDGALPWIVK					Mascot
1927.0178	1926.8954	-0.1224	-64	234	250	LNTESSRSHAILMVNVR					Mascot
1943.0127	1942.8795	-0.1332	-69	234	250	LNTESSRSHAILMVNVR			Oxidation (M)[13]		Mascot

10	Myosin-J heavy chain [Triticum urartu]	gi|474114531	236907.2	6.35	29	40	0	20.949	

Peptide Information

Calc. Mass	Obsrv. Mass	± da	± ppm	Start Seq.	End Seq.	Sequence	Ion Score	C. I. %	Modification	Rank	Result Type
845.4111	845.3984	-0.0127	-15	1563	1569	QENDAIR					Mascot
889.4625	889.3993	-0.0632	-71	1507	1513	QEKDELK					Mascot
891.4166	891.4144	-0.0022	-2	1284	1291	QNQDGTTK					Mascot
893.441	893.4317	-0.0093	-10	1954	1959	QMRTWR			Oxidation (M)[2]		Mascot
905.4873	905.4269	-0.0604	-67	1261	1267	LMENVKR			Oxidation (M)[2]		Mascot
909.4577	909.4251	-0.0326	-36	767	773	YHTYVAR					Mascot
912.4131	912.4173	0.0042	5	1579	1585	NDELFMK			Oxidation (M)[6]		Mascot
932.5047	932.4513	-0.0534	-57	1116	1123	QESEAIKK					Mascot
996.4857	996.4911	0.0054	5	1067	1075	GELGEAHQR					Mascot
1037.5739	1037.5251	-0.0488	-47	258	265	TYLLERSR					Mascot
1167.6117	1167.5627	-0.049	-42	1593	1602	AHQLQDTVQK					Mascot
1192.6433	1192.5551	-0.0882	-74	1820	1830	TARTAASVPYR					Mascot
1201.6688	1201.6628	-0.006	-5	829	839	WSAVTLQAGLR					Mascot
1201.6688	1201.6628	-0.006	-5	829	839	WSAVTLQAGLR					Mascot
1217.6056	1217.6083	0.0027	2	742	752	AGQMAELDARR					Mascot
1223.6201	1223.6138	-0.0063	-5	420	429	DGLAKQIYCR			Carbamidomethyl (C)[9]		Mascot
1320.6583	1320.5967	-0.0616	-47	21	31	DLAWIDGEVFR					Mascot
1323.7379	1323.6639	-0.074	-56	1425	1435	HSLQLQLTVER					Mascot
1323.7379	1323.6639	-0.074	-56	1425	1435	HSLQLQLTVER					Mascot

1372.7067	1372.6571	-0.0496	-36	973	984	AVEEALAQEREK		Mascot
1490.781	1490.6857	-0.0953	-64	169	184	GKSNSILVSGESGAGK		Mascot
1491.7537	1491.733	-0.0207	-14	1244	1256	NSELLTKVDESEK		Mascot
1527.6479	1527.722	0.0741	49	1300	1312	NEELMKSSEDSDK	Oxidation (M)[5]	Mascot
1556.6897	1556.7396	0.0499	32	312	325	VDGMSDAEEYLATR		Mascot
1556.6897	1556.7396	0.0499	32	312	325	VDGMSDAEEYLATR		Mascot
1572.7864	1572.7468	-0.0396	-25	1669	1681	NEDLLDRNDDLIK		Mascot
1837.984	1837.9287	-0.0553	-30	1894	1908	KELNPLLELCIQDPR	Carbamidomethyl (C)[10]	Mascot
1838.8879	1838.9227	0.0348	19	1195	1210	FTDANRTNDTLQDSLK		Mascot
1844.8484	1844.9404	0.092	50	465	479	TNSFEQLCINFTNEK	Carbamidomethyl (C)[8]	Mascot
1865.9451	1865.8778	-0.0673	-36	171	189	SNSILVSGESGAGKTETT K		Mascot
2150.9312	2151.0779	0.1467	68	275	291	NYHCFYFLCSAPSEDIK	Carbamidomethyl (C)[4,9]	Mascot
2215.1968	2215.1887	-0.0081	-4	1725	1743	VLLSEGYQQPQDDQKLL LK		Mascot

Gel Idx/Pos	185/H12	Instr./Gel Origin	BA2151/Sample Project 20140814	Process Status	Analysis Succeeded
Plate [#] Name	[1] Sample Project 20140814	Instrument Sample Name		Spectra	11

Rank	Protein Name	Accession No.	Protein MW	Protein PI	Pep. Count	Protein Score	Protein Score C. I. %	Intensity Matched	Total Ion Score	Total Ion C. I. %	Confirmed
1	hypothetical protein TRIUR3_05104 [Triticum urartu]	gi|474427757	29228.5	5.54	6	275	100	27.561	250	100	

Peptide Information

Calc. Mass	Obsrv. Mass	± da	± ppm	Start Seq.	End Seq.	Sequence	Ion Score	C. I. %	Modification	Rank	Result Type
1187.5957	1187.5911	-0.0046	-4	168	176	TVHFWQVDR					Mascot
1187.5957	1187.5911	-0.0046	-4	168	176	TVHFWQVDR	72	100			Mascot
1213.6172	1213.6096	-0.0076	-6	247	258	EVDLPAANTGAR					Mascot
1513.8196	1513.8158	-0.0038	-3	141	155	GGVLFMPGVPGVVER					Mascot
1529.8146	1529.7975	-0.0171	-11	141	155	GGVLFMPGVPGVVER			Oxidation (M)[6]		Mascot
1529.8146	1529.7975	-0.0171	-11	141	155	GGVLFMPGVPGVVER	58	99.986	Oxidation (M)[6]		Mascot
1812.9711	1812.901	-0.0701	-39	177	193	GDALPLGLPQIMMALTR			Oxidation (M)[12]		Mascot
1828.9659	1828.9332	-0.0327	-18	177	193	GDALPLGLPQIMMALTR			Oxidation (M)[12,13]		Mascot
1828.9659	1828.9332	-0.0327	-18	177	193	GDALPLGLPQIMMALTR			Oxidation (M)[12,13]		Mascot
1878.928	1878.8839	-0.0441	-23	221	239	AYMSGPAHGIHPLANAA GK			Oxidation (M)[3]		Mascot
1961.9036	1961.8901	-0.0135	-7	80	95	QVEAHHFCAHLNEDVR			Carbamidomethyl (C)[8]		Mascot
1961.9036	1961.8901	-0.0135	-7	80	95	QVEAHHFCAHLNEDVR	120	100	Carbamidomethyl (C)[8]		Mascot

Rank	Protein Name	Accession No.	Protein MW	Protein PI	Pep. Count	Protein Score	Protein Score C. I. %	Intensity Matched	Total Ion Score	Total Ion C. I. %	Confirmed
2	hypothetical protein TRIUR3_17666 [Triticum urartu]	gi|474387985	26438.1	5.82	3	66	99.033	12.514	58	99.986	

Peptide Information

Calc. Mass	Obsrv. Mass	± da	± ppm	Start Seq.	End Seq.	Sequence	Ion Score	C. I. %	Modification	Rank	Result Type
1127.5845	1127.5596	-0.0249	-22	184	192	FSVSFQKER					Mascot
1213.6172	1213.6096	-0.0076	-6	222	233	EVDLPAANTGAR					Mascot
1513.8196	1513.8158	-0.0038	-3	116	130	GGVLFMPGVPGVVER					Mascot
1529.8146	1529.7975	-0.0171	-11	116	130	GGVLFMPGVPGVVER			Oxidation (M)[6]		Mascot
1529.8146	1529.7975	-0.0171	-11	116	130	GGVLFMPGVPGVVER	58	99.986	Oxidation (M)[6]		Mascot

Rank	Protein Name	Accession No.	Protein MW	Protein PI	Pep. Count	Protein Score	Protein Score C. I. %	Intensity Matched	Total Ion Score	Total Ion C. I. %	Confirmed
3	Ankyrin-2 [Triticum urartu]	gi|474347049	58755.6	6.69	15	64	98.157	18.984			

Peptide Information

Calc. Mass	Obsrv. Mass	± da	± ppm	Start Seq.	End Seq.	Sequence	Ion Score	C. I. %	Modification	Rank	Result Type

800.3897	800.3456	-0.0441	-55	510	516	HEGSDKK					Mascot
820.3618	820.3426	-0.0192	-23	253	258	QEDCLR			Carbamidomethyl (C)[4]		Mascot
858.4427	858.4106	-0.0321	-37	530	538	QAAAAAEAR					Mascot
1032.5321	1032.4746	-0.0575	-56	300	309	SGTIPLSSDR					Mascot
1187.6572	1187.5911	-0.0661	-56	4	14	LGFAAPWVQAK					Mascot
1187.6572	1187.5911	-0.0661	-56	4	14	LGFAAPWVQAK	2	0			Mascot
1191.662	1191.5863	-0.0757	-64	94	104	GFVDVVDTLVK					Mascot
1194.6953	1194.5979	-0.0974	-82	82	93	VAVHALVSAATR					Mascot
1219.6178	1219.5808	-0.037	-30	25	36	GYPATAAAREGR					Mascot
1219.6178	1219.5808	-0.037	-30	25	36	GYPATAAAREGR	5	0			Mascot
1231.6504	1231.5842	-0.0662	-54	540	550	GVPFKPGICEK			Carbamidomethyl (C)[9]		Mascot
1259.7219	1259.5978	-0.1241	-99	288	299	AGFERAVLGAIR					Mascot
1359.7454	1359.6335	-0.1119	-82	540	551	GVPFKPGICEKK			Carbamidomethyl (C)[9]		Mascot
1444.7948	1444.6819	-0.1129	-78	2	14	EKLGFAAPWVQAK					Mascot
1591.8302	1591.8108	-0.0194	-12	1	14	MEKLGFAAPWVQAK			Oxidation (M)[1]		Mascot
1661.7919	1661.7654	-0.0265	-16	115	129	LGAWSWDAATGEELR					Mascot
1828.9705	1828.9332	-0.0373	-20	4	21	LGFAAPWVQAKGADVNG K					Mascot
1828.9705	1828.9332	-0.0373	-20	4	21	LGFAAPWVQAKGADVNG K					Mascot

4 hypothetical protein TRIUR3_12297 [Triticum urartu] gi|473734608 16078.9 4.72 8 53 76.792 6.149

Peptide Information

Calc. Mass	Obsrv. Mass	± da	± ppm	Start Seq.	End Seq.	Sequence	Ion Score	C. I. %	Modification	Rank	Result Type
950.4425	950.4304	-0.0121	-13	85	94	ASEGADSVSK					Mascot
1191.5615	1191.5863	0.0248	21	53	61	MYVTFNFNR					Mascot
1249.5696	1249.5618	-0.0078	-6	123	133	QATEEAWDATK					Mascot
1373.6553	1373.6248	-0.0305	-22	23	33	FMLSKSTICDR			Carbamidomethyl (C)[9], Oxidation (M)[2]		Mascot
1375.5973	1375.6134	0.0161	12	34	46	VEHTETGDDTSGK					Mascot
1591.7559	1591.8108	0.0549	34	85	102	ASEGADSVSKAAGDAAG K					Mascot
1828.9287	1828.9332	0.0045	2	103	120	VQEAVEGAVEGAKDLGE K					Mascot
1828.9287	1828.9332	0.0045	2	103	120	VQEAVEGAVEGAKDLGE K					Mascot
1844.984	1844.9418	-0.0422	-23	47	61	LISLARMYVTFNFNR					Mascot

5 DNA cross-link repair 1A protein [Triticum urartu] gi|474425680 76924.2 5.46 14 45 0 10.015

Peptide Information

Calc. Mass	Obsrv. Mass	± da	± ppm	Start Seq.	End Seq.	Sequence	Ion Score	C. I. %	Modification	Rank	Result Type

810.3563	810.3467	-0.0096	-12	93	98	YTHSMR			Oxidation (M)[5]		Mascot
1185.6838	1185.5754	-0.1084	-91	308	318	LDGKEAIALQK					Mascot
1235.6226	1235.5739	-0.0487	-39	475	487	ATAAASKSESANK					Mascot
1241.6195	1241.5906	-0.0289	-23	234	243	EIMVERGYTK			Oxidation (M)[3]		Mascot
1244.5585	1244.6057	0.0472	38	652	661	TTAMLCNMFR			Carbamidomethyl (C)[6]		Mascot
1260.6179	1260.651	0.0331	26	141	152	QVKEESGAAGER					Mascot
1416.6545	1416.6532	-0.0013	-1	652	662	TTAMLCNMFRR			Carbamidomethyl (C)[6], Oxidation (M)[4]		Mascot
1416.6545	1416.6532	-0.0013	-1	652	662	TTAMLCNMFRR			Carbamidomethyl (C)[6], Oxidation (M)[4]		Mascot
1432.6494	1432.6614	0.012	8	652	662	TTAMLCNMFRR			Carbamidomethyl (C)[6], Oxidation (M)[4,8]		Mascot
1444.6777	1444.6819	0.0042	3	539	551	GDVNVAVDMFYSK					Mascot
1460.6726	1460.7471	0.0745	51	539	551	GDVNVAVDMFYSK			Oxidation (M)[9]		Mascot
1464.7441	1464.7979	0.0538	37	341	354	SAHASLSHEDVLAK					Mascot
1491.7286	1491.7179	-0.0107	-7	449	464	TSDLGSSADTKAGGPK					Mascot
1944.9451	1944.8665	-0.0786	-40	271	286	IHLVPYSEHSSYDELR					Mascot
1944.9451	1944.8665	-0.0786	-40	271	286	IHLVPYSEHSSYDELR					Mascot
2002.02	2001.9182	-0.1018	-51	8	26	STANSDIEPTTAELAAVR R					Mascot
2010.9152	2011.0291	0.1139	57	427	443	ERDFFEEANGSNNEKPK					Mascot
2322.272	2322.1433	-0.1287	-55	663	681	FSNVYHFIVYLSMLLLIIH					Mascot

6	Kinesin-4 [Triticum urartu]	gi|473723820	93391.2	9.03	19	44	0	19.063	

Peptide Information

Calc. Mass	Obsrv. Mass	± da	± ppm	Start Seq.	End Seq.	Sequence	Ion Score	C. I. %	Modification	Rank	Result Type
819.3843	819.3278	-0.0565	-69	415	421	TEVEGER					Mascot
820.3981	820.3426	-0.0555	-68	816	822	EQIMAGR			Oxidation (M)[4]		Mascot
870.458	870.5204	0.0624	72	446	452	NAHIPYR					Mascot
893.3781	893.4068	0.0287	32	560	566	ETEQACR			Carbamidomethyl (C)[6]		Mascot
896.4809	896.3973	-0.0836	-93	567	573	THQQRAR					Mascot
1127.515	1127.5596	0.0446	40	154	162	KYTAECAER			Carbamidomethyl (C)[6]		Mascot
1155.5211	1155.5795	0.0584	51	155	163	YTAECAERR			Carbamidomethyl (C)[5]		Mascot
1191.5714	1191.5863	0.0149	13	126	134	YNSLMEKYK			Oxidation (M)[5]		Mascot
1235.6161	1235.5739	-0.0422	-34	770	781	LRMQSGSGNASK					Mascot
1249.6423	1249.5618	-0.0805	-64	4	14	VFLSAGEEELR					Mascot
1322.6547	1322.5936	-0.0611	-46	365	377	SVGSTSVNELSSR					Mascot
1373.6179	1373.6248	0.0069	5	314	325	DLLDDNSEHTSK					Mascot
1415.7125	1415.6722	-0.0403	-28	572	583	ARELENELANEK					Mascot

Calc. Mass	Obsrv. Mass	± da	± ppm	Start Seq.	End Seq.	Sequence	Ion Score	C. I. %	Modification	Rank	Result Type
1491.7802	1491.7179	-0.0623	-42	281	292	ALEELFRISEER					Mascot
1529.7191	1529.7975	0.0784	51	314	326	DLLDDNSEHTSKR					Mascot
1529.7191	1529.7975	0.0784	51	314	326	DLLDDNSEHTSKR					Mascot
1743.8595	1743.834	-0.0255	-15	397	411	SRSHMWLVDLAGSER					Mascot
1812.9425	1812.901	-0.0415	-23	399	414	SHMWLVDLAGSERLAK					Mascot
1828.9375	1828.9332	-0.0043	-2	399	414	SHMWLVDLAGSERLAK			Oxidation (M)[3]		Mascot
1828.9375	1828.9332	-0.0043	-2	399	414	SHMWLVDLAGSERLAK			Oxidation (M)[3]		Mascot
2002.0312	2001.9182	-0.113	-56	365	383	SVGSTSVNELSSRSHSLV R					Mascot
2019.0302	2018.906	-0.1242	-62	612	629	QRPPSNNMPQPSGPSRL R					Mascot

7	26.8kDa heat-shock protein [Triticum turgidum subsp. dicoccon]	gi|147225076	26804.5	6.86	10	43	0	13.698			

Peptide Information

Calc. Mass	Obsrv. Mass	± da	± ppm	Start Seq.	End Seq.	Sequence	Ion Score	C. I. %	Modification	Rank	Result Type
1032.5143	1032.4746	-0.0397	-38	122	131	SLAAASEMPR					Mascot
1127.5441	1127.5596	0.0155	14	53	62	DNSVDVHVSR					Mascot
1187.6532	1187.5911	-0.0621	-52	2	13	AAANAPFAIVSR					Mascot
1187.6532	1187.5911	-0.0621	-52	2	13	AAANAPFAIVSR					Mascot
1201.661	1201.5857	-0.0753	-63	159	169	VMVEGDALVIR					Mascot
1209.5868	1209.579	-0.0078	-6	145	154	MRFDMPGLSR					Mascot
1217.6559	1217.5725	-0.0834	-68	159	169	VMVEGDALVIR			Oxidation (M)[2]		Mascot
1241.5765	1241.5906	0.0141	11	145	154	MRFDMPGLSR			Oxidation (M)[1,5]		Mascot
1397.6334	1397.6453	0.0119	9	98	108	TMRQMLDTMDR					Mascot
1464.7594	1464.7979	0.0385	26	109	121	LFDDAVGFPTARR					Mascot
1780.891	1780.942	0.051	29	63	78	QDGGNQQGNAVQRRPR					Mascot
1961.8624	1961.8901	0.0277	14	174	192	KEAGEGQGEAAEGGDG WWK					Mascot
1961.8624	1961.8901	0.0277	14	174	192	KEAGEGQGEAAEGGDG WWK					Mascot
2001.8893	2001.9182	0.0289	14	195	211	SVSSYDMRLALPDECDK			Carbamidomethyl (C)[15], Oxidation (M)[7]		Mascot

8	hypothetical protein TRIUR3_34298 [Triticum urartu]	gi|474054244	221550.5	6.49	23	42	0	22.186	17	0	

Peptide Information

Calc. Mass	Obsrv. Mass	± da	± ppm	Start Seq.	End Seq.	Sequence	Ion Score	C. I. %	Modification	Rank	Result Type
852.392	852.3701	-0.0219	-26	150	156	LPDGYCK			Carbamidomethyl (C)[6]		Mascot
864.4244	864.3787	-0.0457	-53	837	843	RVDAEMK			Oxidation (M)[6]		Mascot

896.4445	896.3973	-0.0472	-53	1088	1094	HEQNGRR					Mascot
1145.6161	1145.5809	-0.0352	-31	889	900	TAKPGASDAVTK					Mascot
1187.4965	1187.5911	0.0946	80	2004	2013	EPEGSWDPDR					Mascot
1187.4965	1187.5911	0.0946	80	2004	2013	EPEGSWDPDR	17	0			Mascot
1191.5488	1191.5863	0.0375	31	769	780	SGEGGSPKETDK					Mascot
1219.5801	1219.5808	0.0007	1	432	442	SGDELDSLQQK					Mascot
1219.5801	1219.5808	0.0007	1	432	442	SGDELDSLQQK					Mascot
1244.6091	1244.6057	-0.0034	-3	862	874	GGAAEVSNGGNRR					Mascot
1249.651	1249.5618	-0.0892	-71	351	359	YIMWKHLSR			Oxidation (M)[3]		Mascot
1340.7573	1340.6508	-0.1065	-79	1467	1479	LVAFPADVPNGIK					Mascot
1353.6831	1353.7025	0.0194	14	453	462	MERLDELLYR			Oxidation (M)[1]		Mascot
1397.63	1397.6453	0.0153	11	463	472	EEMMWLQRSR			Oxidation (M)[3,4]		Mascot
1416.7078	1416.6532	-0.0546	-39	875	888	SASSEASPKNPVSR					Mascot
1416.7078	1416.6532	-0.0546	-39	875	888	SASSEASPKNPVSR					Mascot
1431.7325	1431.7122	-0.0203	-14	930	943	SAGGSELPTVKEEK					Mascot
1432.7101	1432.6614	-0.0487	-34	1733	1747	SLPGGLPSTSMNGSK					Mascot
1491.7625	1491.7179	-0.0446	-30	562	575	GVELPAGISSFVCR			Carbamidomethyl (C)[13]		Mascot
1545.8132	1545.7981	-0.0151	-10	1618	1632	GSAATSAFRPAEPRK					Mascot
1557.7268	1557.8049	0.0781	50	335	346	SWRCTFVYGEPR			Carbamidomethyl (C)[4]		Mascot
1591.7745	1591.8108	0.0363	23	636	650	SGLDDGQNCILSKGK			Carbamidomethyl (C)[9]		Mascot
1743.8218	1743.834	0.0122	7	1021	1036	SLFQEKSSQSGMASEK					Mascot
1844.9501	1844.9418	-0.0083	-4	844	861	SNEAKPLVSGQAVSWSG K					Mascot
1960.9281	1960.8771	-0.051	-26	1244	1261	VECDIAPSQLQGSSDVQ K			Carbamidomethyl (C)[3]		Mascot
2322.0632	2322.1433	0.0801	34	1748	1768	NFFDLNNGPSLDEASTEP AQR					Mascot

9 Pyruvate kinase isozyme G, chloroplastic [Triticum urartu] gi|474060293 65805.1 5.63 13 41 0 7.905

Peptide Information

Calc. Mass	Obsrv. Mass	± da	± ppm	Start Seq.	End Seq.	Sequence	Ion Score	C. I. %	Modification	Rank	Result Type
802.5185	802.5666	0.0481	60	423	429	FPLKAVK					Mascot
849.4213	849.3477	-0.0736	-87	43	50	WSGSVASR					Mascot
869.379	869.3855	0.0065	7	369	375	MCRSMGK			Carbamidomethyl (C)[2]		Mascot
893.4575	893.4068	-0.0507	-57	240	247	SKTEDSVK					Mascot
1032.5573	1032.4746	-0.0827	-80	268	277	SATLPSITDK					Mascot
1182.6477	1182.5507	-0.097	-82	313	323	SSNADIHVIVK					Mascot
1217.6736	1217.5725	-0.1011	-83	266	277	GKSATLPSITDK					Mascot

1241.6776	1241.5906	-0.087	-70	533	543	TFADALSYLLK					Mascot
1273.6383	1273.5939	-0.0444	-35	549	560	EGEEVALVQSGR					Mascot
1461.6652	1461.7361	0.0709	49	139	151	LNMSHGDHASHQK					Mascot
1756.9263	1756.8777	-0.0486	-28	165	180	DNVIALMVDTKGPEVR					Mascot
1962.0179	1961.8901	-0.1278	-65	438	456	TEATIIGGETPSNLGQVF K					Mascot
1962.0179	1961.8901	-0.1278	-65	438	456	TEATIIGGETPSNLGQVF K					Mascot
2166.189	2166.179	-0.01	-5	89	108	AVQQLATAANGVWSKPN VRR					Mascot

10	1-phosphatidylinositol-3-phosphate 5-kinase fab1 [Triticum urartu]	gi|473824589	177878.2	6.14	20	40	0	21.314	

Peptide Information

Calc. Mass	Obsrv. Mass	± da	± ppm	Start Seq.	End Seq.	Sequence	Ion Score	C. I. %	Modification	Rank	Result Type
858.4138	858.4106	-0.0032	-4	1169	1175	DPMPNIR			Oxidation (M)[3]		Mascot
1145.5909	1145.5809	-0.01	-9	562	571	LKQVQGDDSR					Mascot
1185.5093	1185.5754	0.0661	56	303	313	GGSMDPTDYVK			Oxidation (M)[4]		Mascot
1194.5824	1194.5979	0.0155	13	572	581	MFEEISVSPR					Mascot
1219.6066	1219.5808	-0.0258	-21	1127	1137	IHSFDSTVASR					Mascot
1219.6066	1219.5808	-0.0258	-21	1127	1137	IHSFDSTVASR	5	0			Mascot
1244.5502	1244.6057	0.0555	45	93	104	RADGEENDGPGK					Mascot
1273.5704	1273.5939	0.0235	18	473	482	TLMFFEGCPR			Carbamidomethyl (C)[8], Oxidation (M)[3]		Mascot
1325.6631	1325.6569	-0.0062	-5	760	771	CEPKNGVPPATR			Carbamidomethyl (C)[1]		Mascot
1359.7366	1359.6335	-0.1031	-76	365	376	LASIDTILEQEK					Mascot
1373.6948	1373.6248	-0.07	-51	1156	1168	SAEYFGGLTSITK					Mascot
1433.7537	1433.678	-0.0757	-53	457	468	FLEFPPGKQTNR					Mascot
1433.7537	1433.678	-0.0757	-53	457	468	FLEFPPGKQTNR					Mascot
1463.7927	1463.7635	-0.0292	-20	1	14	MGVVEFSVLGAVQK					Mascot
1487.6777	1487.7373	0.0596	40	815	826	FYGYGNMVAFFR			Oxidation (M)[7]		Mascot
1491.7512	1491.7179	-0.0333	-22	1542	1554	NEAPTIISPMQYK					Mascot
1507.7461	1507.7184	-0.0277	-18	1542	1554	NEAPTIISPMQYK			Oxidation (M)[10]		Mascot
1743.8292	1743.834	0.0048	3	1383	1398	YLMESLASGSPTCLAK			Carbamidomethyl (C)[13], Oxidation (M)[3]		Mascot
1828.9011	1828.9332	0.0321	18	1058	1073	EWPSTRAAIGMEPVER					Mascot
1828.9011	1828.9332	0.0321	18	1058	1073	EWPSTRAAIGMEPVER					Mascot
1844.896	1844.9418	0.0458	25	1058	1073	EWPSTRAAIGMEPVER			Oxidation (M)[11]		Mascot
1904.8807	1904.8768	-0.0039	-2	582	597	SLCLNEEGESVVFEHR			Carbamidomethyl (C)[3]		Mascot
1960.9835	1960.8771	-0.1064	-54	921	937	DSNHHVQASIDILELNR					Mascot

2018.9636	2018.906	-0.0576	-29	1415	1430	EVKMDLMVMENLFFEK	Oxidation (M)[4]	Mascot
2166.0562	2166.179	0.1228	57	1030	1048	KLQIDLVDHPGDDTEDLD K		Mascot

Gel Idx/Pos	186/H13	Instr./Gel Origin	BA2151/Sample Project 20140814	Process Status	Analysis Succeeded
Plate [#] Name	[1] Sample Project 20140814	Instrument Sample Name		Spectra	11

Rank	Protein Name	Accession No.	Protein MW	Protein PI	Pep. Count	Protein Score	Protein Score C. I. %	Intensity Matched	Total Ion Score	Total Ion C. I. %	Confirmed
1	hypothetical protein TRIUR3_05104 [Triticum urartu]	gi|474427757	29228.5	5.54	8	320	100	34.243	277	100	

Peptide Information

Calc. Mass	Obsrv. Mass	± da	± ppm	Start Seq.	End Seq.	Sequence	Ion Score	C. I. %	Modification	Rank	Result Type
1187.5957	1187.5876	-0.0081	-7	168	176	TVHFWQVDR					Mascot
1187.5957	1187.5876	-0.0081	-7	168	176	TVHFWQVDR	62	99.995			Mascot
1213.6172	1213.6056	-0.0116	-10	247	258	EVDLPAANTGAR					Mascot
1405.653	1405.6421	-0.0109	-8	96	108	QCLVFDGPDAGAR			Carbamidomethyl (C)[2]		Mascot
1405.653	1405.6421	-0.0109	-8	96	108	QCLVFDGPDAGAR	106	100	Carbamidomethyl (C)[2]		Mascot
1513.8196	1513.7732	-0.0464	-31	141	155	GGVLFMPGVPGVVER					Mascot
1513.8196	1513.7732	-0.0464	-31	141	155	GGVLFMPGVPGVVER					Mascot
1529.8146	1529.7788	-0.0358	-23	141	155	GGVLFMPGVPGVVER			Oxidation (M)[6]		Mascot
1660.7959	1660.7781	-0.0178	-11	194	207	EGQLRQDLADCVEK			Carbamidomethyl (C)[11]		Mascot
1796.9762	1796.9534	-0.0228	-13	177	193	GDALPLGLPQIMMALTR					Mascot
1812.9711	1812.9301	-0.041	-23	177	193	GDALPLGLPQIMMALTR			Oxidation (M)[12]		Mascot
1828.9659	1828.9254	-0.0405	-22	177	193	GDALPLGLPQIMMALTR			Oxidation (M)[12,13]		Mascot
1828.9659	1828.9254	-0.0405	-22	177	193	GDALPLGLPQIMMALTR	3	0	Oxidation (M)[12,13]		Mascot
1878.928	1878.8932	-0.0348	-19	221	239	AYMSGPAHGIHPLANAAGK			Oxidation (M)[3]		Mascot
1961.9036	1961.8876	-0.016	-8	80	95	QVEAHHFCAHLNEDVR			Carbamidomethyl (C)[8]		Mascot
1961.9036	1961.8876	-0.016	-8	80	95	QVEAHHFCAHLNEDVR	109	100	Carbamidomethyl (C)[8]		Mascot

Rank	Protein Name	Accession No.	Protein MW	Protein PI	Pep. Count	Protein Score	Protein Score C. I. %	Intensity Matched	Total Ion Score	Total Ion C. I. %	Confirmed
2	hypothetical protein TRIUR3_11501 [Triticum urartu]	gi|474005353	37933.4	6.71	10	49	50.383	7.755	9	0	

Peptide Information

Calc. Mass	Obsrv. Mass	± da	± ppm	Start Seq.	End Seq.	Sequence	Ion Score	C. I. %	Modification	Rank	Result Type
817.489	817.471	-0.018	-22	215	221	IRTATQK					Mascot
893.4145	893.4263	0.0118	13	298	305	ISNGQCSK			Carbamidomethyl (C)[6]		Mascot
1229.6749	1229.6085	-0.0664	-54	24	33	FHRATSIELR					Mascot
1276.6797	1276.5894	-0.0903	-71	197	208	SVGHVFGSFALR					Mascot
1517.7781	1517.7479	-0.0302	-20	98	110	WTGRIDVDAPMLK			Oxidation (M)[11]		Mascot
1557.8173	1557.7686	-0.0487	-31	15	26	EFLDIDLPRFHR					Mascot

1561.7693	1561.7587	-0.0106	-7	1	12	MDNFFLHTRQPR					Mascot
1577.7642	1577.7471	-0.0171	-11	1	12	MDNFFLHTRQPR			Oxidation (M)[1]		Mascot
1577.7642	1577.7471	-0.0171	-11	1	12	MDNFFLHTRQPR	9	0	Oxidation (M)[1]		Mascot
1700.8425	1700.894	0.0515	30	298	311	ISNGQCSKIYDIFR			Carbamidomethyl (C)[6]		Mascot
1903.8895	1903.8823	-0.0072	-4	312	327	AYPYVECNVDPTYGKK			Carbamidomethyl (C)[7]		Mascot
1984.0698	1983.9065	-0.1633	-82	197	214	SVGHVFGSFALRLLGMHR					Mascot

3 R1R2R3-MYB protein [Triticum aestivum] gi|359950740 95869.9 5.79 14 48 36.08 19.582 15 0

Peptide Information

Calc. Mass	Obsrv. Mass	± da	± ppm	Start Seq.	End Seq.	Sequence	Ion Score	C. I. %	Modification	Rank	Result Type
1184.4672	1184.5667	0.0995	84	595	605	DCGSSAMPSTR			Carbamidomethyl (C)[2], Oxidation (M)[7]		Mascot
1219.5549	1219.578	0.0231	19	305	316	ANDAASTSPETR					Mascot
1219.5549	1219.578	0.0231	19	305	316	ANDAASTSPETR	15	0			Mascot
1231.6793	1231.5624	-0.1169	-95	182	192	FLPGRTDNAIK					Mascot
1276.5804	1276.5894	0.009	7	155	164	DAWTQEEEIR					Mascot
1314.6874	1314.6304	-0.057	-43	34	46	GQLSNGRTTGPAR					Mascot
1348.6665	1348.6271	-0.0394	-29	108	118	EEDDIIIQMVK			Oxidation (M)[9]		Mascot
1353.7889	1353.7039	-0.085	-63	92	102	WQKVLNPELVK					Mascot
1397.7019	1397.6353	-0.0666	-48	12	26	AAEGSPSALAVPDGR					Mascot
1487.8329	1487.7291	-0.1038	-70	125	138	WSTIAQALSGRIGK					Mascot
1517.7555	1517.7479	-0.0076	-5	635	647	TEDNANINTLEKR					Mascot
1700.8247	1700.894	0.0693	41	528	541	QFMRPSMSFSTPLR			Oxidation (M)[3]		Mascot
1788.8611	1788.8773	0.0162	9	612	627	DATRDDSLDLIPENSK					Mascot
1815.0222	1814.9299	-0.0923	-51	690	705	DIISSRSKPTELLVEK					Mascot
1903.947	1903.8823	-0.0647	-34	103	118	GPWSKEEDDIIIQMVK			Oxidation (M)[14]		Mascot

4 hypothetical protein TRIUR3_21162 [Triticum urartu] gi|474383469 95133.7 5.39 17 44 0 3.188

Peptide Information

Calc. Mass	Obsrv. Mass	± da	± ppm	Start Seq.	End Seq.	Sequence	Ion Score	C. I. %	Modification	Rank	Result Type
805.3331	805.4	0.0669	83	289	294	EMNHMK			Oxidation (M)[2]		Mascot
856.4271	856.4987	0.0716	84	659	665	IQHSESR					Mascot
909.4788	909.5074	0.0286	31	510	517	RAVDSFSK					Mascot
1107.6157	1107.5214	-0.0943	-85	511	520	AVDSFSKVVR					Mascot
1126.5963	1126.5515	-0.0448	-40	4	12	TEKQQLHSR					Mascot
1209.578	1209.5538	-0.0242	-20	492	502	SCSEAVSTLQK			Carbamidomethyl (C)[2]		Mascot

1229.6637	1229.6085	-0.0552	-45	775	785	GWISEPSGKLR					Mascot
1314.6552	1314.6304	-0.0248	-19	831	840	YSMIFLHPYK			Oxidation (M)[3]		Mascot
1348.6414	1348.6271	-0.0143	-11	415	426	IGMLESDIEDAR					Mascot
1397.5824	1397.6353	0.0529	38	451	461	LHGNEMEYNMK			Oxidation (M)[6,10]		Mascot
1416.6965	1416.6388	-0.0577	-41	162	174	DAEQEIGQQLASK					Mascot
1427.7393	1427.6283	-0.111	-78	831	841	YSMIFLHPYKL			Oxidation (M)[3]		Mascot
1465.8274	1465.7864	-0.041	-28	36	49	RPLFPAPTTAAAPR					Mascot
1517.7893	1517.7479	-0.0414	-27	659	670	IQHSESRFCLLK			Carbamidomethyl (C)[9]		Mascot
1533.8523	1533.7327	-0.1196	-78	300	312	VVQEKTEEIFALK					Mascot
1592.8207	1592.7347	-0.086	-54	262	275	VPPEEVFGDLSYLK					Mascot
1901.0161	1900.8373	-0.1788	-94	339	355	IGEVMARLDGLLLENNK			Oxidation (M)[5]		Mascot

5	1-phosphatidylinositol-3-phosphate 5-kinase fab1 [Triticum urartu]	gi|473824589	177878.2	6.14	21	43	0	27.202	7	0

Peptide Information

Calc. Mass	Obsrv. Mass	± da	± ppm	Start Seq.	End Seq.	Sequence	Ion Score	C. I. %	Modification	Rank	Result Type
811.4421	811.4283	-0.0138	-17	764	771	NGVPPATR					Mascot
842.4189	842.4792	0.0603	72	1169	1175	DPMPNIR					Mascot
856.4312	856.4987	0.0675	79	1348	1354	SNVYFAR					Mascot
1185.5093	1185.5723	0.063	53	303	313	GGSMDPTDYVK			Oxidation (M)[4]		Mascot
1219.6066	1219.578	-0.0286	-23	1127	1137	IHSFDSTVASR					Mascot
1219.6066	1219.578	-0.0286	-23	1127	1137	IHSFDSTVASR	7	0			Mascot
1233.6111	1233.5664	-0.0447	-36	156	166	FSLDSLEHGTK					Mascot
1244.5502	1244.6056	0.0554	45	93	104	RADGEENDGPGK					Mascot
1276.6644	1276.5894	-0.075	-59	32	44	QASAPPSPATPPR					Mascot
1297.6094	1297.6224	0.013	10	302	313	KGGSMDPTDYVK					Mascot
1433.7537	1433.6738	-0.0799	-56	457	468	FLEFPPGKQTNR					Mascot
1487.6777	1487.7291	0.0514	35	815	826	FYGYGNMVAFFR			Oxidation (M)[7]		Mascot
1497.7407	1497.7743	0.0336	22	773	786	VIMSDAAWGLSFGK			Oxidation (M)[3]		Mascot
1507.7461	1507.7123	-0.0338	-22	1542	1554	NEAPTIISPMQYK			Oxidation (M)[10]		Mascot
1512.8533	1512.7388	-0.1145	-76	1114	1126	LLDNPVYKNAPIR					Mascot
1517.7443	1517.7479	0.0036	2	17	31	SLITGSTPAADEEAR					Mascot
1763.7793	1763.8586	0.0793	45	1016	1030	EVCFSDDEYSISGKK			Carbamidomethyl (C)[3]		Mascot
1814.9006	1814.9299	0.0293	16	864	877	MKSLHWEISDFLHR			Oxidation (M)[1]		Mascot
1828.9011	1828.9254	0.0243	13	1058	1073	EWPSTRAAIGMEPVER					Mascot
1828.9011	1828.9254	0.0243	13	1058	1073	EWPSTRAAIGMEPVER					Mascot

Calc. Mass	Obsrv. Mass	± da	± ppm	Start Seq.	End Seq.	Sequence	Ion Score	C. I. %	Modification	Rank	Result Type
1844.896	1844.9261	0.0301	16	1058	1073	EWPSTRAAIGMEPVER			Oxidation (M)[11]		Mascot
1900.9983	1900.8373	-0.161	-85	906	920	MERNECEILLLPVIR			Carbamidomethyl (C)[6], Oxidation (M)[1]		Mascot
2018.9636	2018.9503	-0.0133	-7	1415	1430	EVKMDLMVMENLFFEK			Oxidation (M)[4]		Mascot
2166.0562	2166.1816	0.1254	58	1030	1048	KLQIDLVDHPGDDTEDLDK					Mascot

6	Cyclopropane-fatty-acyl-phospholipid synthase [Triticum urartu]	gi|474379868	88285.1	7.89	13	42	0	17.468	4	0	

Peptide Information

Calc. Mass	Obsrv. Mass	± da	± ppm	Start Seq.	End Seq.	Sequence	Ion Score	C. I. %	Modification	Rank	Result Type
809.4152	809.3976	-0.0176	-22	133	139	SQSFVNK					Mascot
1198.5699	1198.5801	0.0102	9	159	170	SVSSLDGSAGYR					Mascot
1209.528	1209.5538	0.0258	21	455	464	SMTYSCAVFK			Carbamidomethyl (C)[6], Oxidation (M)[2]		Mascot
1261.7052	1261.6027	-0.1025	-81	47	56	TNILKPSFWR					Mascot
1276.6355	1276.5894	-0.0461	-36	350	360	MIPSWTEAVAR			Oxidation (M)[1]		Mascot
1372.7267	1372.6777	-0.049	-36	207	217	RILGACQYVHR			Carbamidomethyl (C)[6]		Mascot
1388.7355	1388.6151	-0.1204	-87	349	360	KMIPSWTEAVAR					Mascot
1388.7355	1388.6151	-0.1204	-87	349	360	KMIPSWTEAVAR					Mascot
1405.6416	1405.6421	0.0005	0	749	759	SDMKTYDYINR					Mascot
1405.6416	1405.6421	0.0005	0	749	759	SDMKTYDYINR	6	0			Mascot
1447.6886	1447.6998	0.0112	8	2	13	EWLEGLGVEMER					Mascot
1533.7325	1533.7327	0.0002	0	465	477	MENESLEAAQQRK					Mascot
1813.0153	1812.9301	-0.0852	-47	350	365	MIPSWTEAVARLLVAR					Mascot
1828.8436	1828.9254	0.0818	45	231	246	NMSAWSAWNFLGTTSR					Mascot
1829.0103	1828.9254	-0.0849	-46	350	365	MIPSWTEAVARLLVAR	6	0	Oxidation (M)[1]		Mascot
1844.8385	1844.9261	0.0876	47	231	246	NMSAWSAWNFLGTTSR			Oxidation (M)[2]		Mascot
2018.9349	2018.9503	0.0154	8	26	46	GGGGGCEWGNGNGISSLLAQK			Carbamidomethyl (C)[6]		Mascot

7	SWI/SNF complex subunit SWI3D [Triticum urartu]	gi|474312027	84979.3	4.6	15	42	0	14.462	

Peptide Information

Calc. Mass	Obsrv. Mass	± da	± ppm	Start Seq.	End Seq.	Sequence	Ion Score	C. I. %	Modification	Rank	Result Type
805.46	805.4	-0.06	-74	727	732	KLLMER			Oxidation (M)[4]		Mascot
842.5345	842.4792	-0.0553	-66	688	695	LAALVVEK					Mascot
856.441	856.4987	0.0577	67	251	258	GTPEVPEK					Mascot
1195.5511	1195.6024	0.0513	43	259	268	METEEKVEGK			Oxidation (M)[1]		Mascot
1201.6899	1201.5734	-0.1165	-97	662	675	AAATAVSAAAAKAK					Mascot

Calc. Mass	Obsrv. Mass	± da	± ppm	Start Seq.	End Seq.	Sequence	Ion Score	C. I. %	Modification	Rank	Result Type
1233.6144	1233.5664	-0.048	-39	65	74	NEEKACLIEK			Carbamidomethyl (C)[6]		Mascot
1235.5751	1235.5642	-0.0109	-9	327	337	ENSVNLDTSEK					Mascot
1241.626	1241.5496	-0.0764	-62	523	533	ETDDPIPLVDK					Mascot
1405.6805	1405.6421	-0.0384	-27	288	300	TEEGSVVENKDAK					Mascot
1405.6805	1405.6421	-0.0384	-27	288	300	TEEGSVVENKDAK					Mascot
1487.7676	1487.7291	-0.0385	-26	704	716	MSLFADVEHVALR					Mascot
1513.8009	1513.7732	-0.0277	-18	201	213	WTEIAEHVATKTK					Mascot
1513.8009	1513.7732	-0.0277	-18	201	213	WTEIAEHVATKTK					Mascot
1559.7371	1559.7472	0.0101	6	504	517	NVSCDDEAPTVKPK			Carbamidomethyl (C)[4]		Mascot
1561.7704	1561.7587	-0.0117	-7	473	486	DINGKEENTVSVEK					Mascot
1900.8771	1900.8373	-0.0398	-21	583	600	DKPSSEVEPVDDSPSQG K					Mascot
1949.9031	1949.9172	0.0141	7	214	228	TQCMLHFLQMQIEDR			Carbamidomethyl (C)[3]		Mascot
1981.8929	1981.9564	0.0635	32	214	228	TQCMLHFLQMQIEDR			Carbamidomethyl (C)[3], Oxidation (M)[4,10]		Mascot

8	DEAD-box ATP-dependent RNA helicase 14 [Triticum urartu]	gi|474169041	62005.3	9.52	13	41	0	19.885

Peptide Information

Calc. Mass	Obsrv. Mass	± da	± ppm	Start Seq.	End Seq.	Sequence	Ion Score	C. I. %	Modification	Rank	Result Type
830.3792	830.4315	0.0523	63	543	549	FYSGNSR					Mascot
871.4017	871.425	0.0233	27	3	10	HTESAGNR					Mascot
925.3866	925.411	0.0244	26	336	342	MCDQLSR			Carbamidomethyl (C)[2], Oxidation (M)[1]		Mascot
1126.5641	1126.5515	-0.0126	-11	11	20	VQNAPFADHK					Mascot
1195.6252	1195.6024	-0.0228	-19	63	72	HEITIICPGR			Carbamidomethyl (C)[7]		Mascot
1219.5814	1219.578	-0.0034	-3	21	30	ARNDPSFEQR					Mascot
1219.5814	1219.578	-0.0034	-3	21	30	ARNDPSFEQR					Mascot
1353.7023	1353.7039	0.0016	1	11	22	VQNAPFADHKAR					Mascot
1487.8251	1487.7291	-0.096	-65	212	224	LNDILEMGKVSLR					Mascot
1507.7838	1507.7123	-0.0715	-47	257	268	RQTLMFTATWPR					Mascot
1599.8635	1599.7145	-0.149	-93	371	385	TGRCPILVATDVAAR			Carbamidomethyl (C)[4]		Mascot
1605.8119	1605.7603	-0.0516	-32	297	310	SITQHVEVTTSFEK					Mascot
1748.9403	1748.9541	0.0138	8	146	161	HNSRDGPTVLVLSPTR					Mascot
2165.9922	2166.1816	0.1894	87	414	433	TGRAGATGIAYTFFCDQD SK			Carbamidomethyl (C)[15]		Mascot

9	ATP-dependent Clp protease ATP-binding subunit ClpX [Triticum urartu]	gi|474317249	54682.3	6.77	12	40	0	6.323

Peptide Information

Calc. Mass	Obsrv. Mass	± da	± ppm	Start Seq.	End Seq.	Sequence	Ion Score	C. I. %	Modification	Rank	Result Type
856.4886	856.4987	0.0101	12	332	339	EPKNAIGK					Mascot
1235.6453	1235.5642	-0.0811	-66	213	223	MFEGTVISVPR					Mascot
1244.6635	1244.6056	-0.0579	-47	70	79	EADQVKQLWK					Mascot
1513.7322	1513.7732	0.041	27	64	75	YEAFWKEADQVK					Mascot
1513.8374	1513.7732	-0.0642	-42	4	16	NKQFVVQPPTVEK					Mascot
1517.7959	1517.7479	-0.048	-32	164	177	LLVAADFNVEAAER					Mascot
1557.8108	1557.7686	-0.0422	-27	261	273	LHRCPFGFGTPIR			Carbamidomethyl (C)[4]		Mascot
1748.8789	1748.9541	0.0752	43	23	36	QLFYTRIASSCQFK			Carbamidomethyl (C)[11]		Mascot
1796.9987	1796.9534	-0.0453	-25	443	457	VPGLIQRPKCSIICR			Carbamidomethyl (C)[10,14]		Mascot
1812.8763	1812.9301	0.0538	30	428	442	DDGTLELYVYQNNIR					Mascot
1871.017	1870.8809	-0.1361	-73	37	52	MKLGFLLDLTNFIMSK					Mascot
1982.0385	1981.9564	-0.0821	-41	452	468	CSIICRLCLLVAFSAAK			Carbamidomethyl (C)[1,5,8]		Mascot

10 DnaJ homolog subfamily C member 2 [Triticum urartu] gi|474262957 100684.4 9.15 16 38 0 25.29

Peptide Information

Calc. Mass	Obsrv. Mass	± da	± ppm	Start Seq.	End Seq.	Sequence	Ion Score	C. I. %	Modification	Rank	Result Type
830.473	830.4315	-0.0415	-50	50	56	LRGLDEK					Mascot
856.5039	856.4987	-0.0052	-6	489	495	AIHKYPK					Mascot
871.4995	871.425	-0.0745	-85	343	351	IAAAAADIR					Mascot
1191.594	1191.5807	-0.0133	-11	123	132	DMALKHHPDK					Mascot
1198.6791	1198.5801	-0.099	-83	544	555	AAPSIVSPLSTR					Mascot
1209.6223	1209.5538	-0.0685	-57	439	450	IHANGVADSTPK					Mascot
1213.7151	1213.6056	-0.1095	-90	523	533	TILLQKPDSAK					Mascot
1219.6542	1219.578	-0.0762	-62	283	292	VRNLVDNAYR					Mascot
1219.6542	1219.578	-0.0762	-62	283	292	VRNLVDNAYR	1	0			Mascot
1387.7216	1387.6543	-0.0673	-48	477	487	EWGKEEVELLR					Mascot
1513.8948	1513.7732	-0.1216	-80	520	533	ATKTILLQKPDSAK					Mascot
1513.8948	1513.7732	-0.1216	-80	520	533	ATKTILLQKPDSAK					Mascot
1557.8782	1557.7686	-0.1096	-70	822	835	MPRGNANILTTILK			Oxidation (M)[1]		Mascot
1593.7239	1593.7478	0.0239	15	556	572	DETAGGSTVGTGTEPSK					Mascot
1763.8997	1763.8586	-0.0411	-23	159	173	AIQEAYEVLMDPTKR					Mascot
1812.9392	1812.9301	-0.0091	-5	33	49	ASKYEPAGHSFHAVALK					Mascot
2001.9957	2001.8895	-0.1062	-53	798	814	TICRIVACISLDSSNHR			Carbamidomethyl (C)[3,8]		Mascot
2644.45	2644.2983	-0.1517	-57	738	760	VMQFLINPCKPMILALSR			Carbamidomethyl (C)[9], Oxidation (M)[2]		Mascot

PGSKK

Gel Idx/Pos	187/H14	Instr./Gel Origin	BA2151/Sample Project 20140814	Process Status	Analysis Succeeded
Plate [#] Name	[1] Sample Project 20140814	Instrument Sample Name		Spectra	11

Rank	Protein Name	Accession No.	Protein MW	Protein PI	Pep. Count	Protein Score	Protein Score C. I. %	Intensity Matched	Total Ion Score	Total Ion C. I. %	Confirmed
1	Triosephosphate isomerase, cytosolic [Triticum urartu]	gi|473927006	16951.5	5.08	4	350	100	19.054	330	100	

Peptide Information

Calc. Mass	Obsrv. Mass	± da	± ppm	Start Seq.	End Seq.	Sequence	Ion Score	C. I. %	Modification	Rank	Result Type
1577.8031	1577.8138	0.0107	7	55	69	VASPAQAQEVHDGLR					Mascot
1577.8031	1577.8138	0.0107	7	55	69	VASPAQAQEVHDGLR	99	100			Mascot
1607.8289	1607.8387	0.0098	6	71	85	WLHANVGPAVAESTR					Mascot
1607.8289	1607.8387	0.0098	6	71	85	WLHANVGPAVAESTR	138	100			Mascot
1705.8981	1705.9072	0.0091	5	55	70	VASPAQAQEVHDGLRK					Mascot
1705.8981	1705.9072	0.0091	5	55	70	VASPAQAQEVHDGLRK	94	100			Mascot
1735.9238	1735.9298	0.006	3	70	85	KWLHANVGPAVAESTR					Mascot

Rank	Protein Name	Accession No.	Protein MW	Protein PI	Pep. Count	Protein Score	Protein Score C. I. %	Intensity Matched	Total Ion Score	Total Ion C. I. %	Confirmed
2	unnamed protein product [Triticum aestivum]	gi|259662377	26526.7	5.2	7	134	100	13.668	101	100	

Protein Group

unnamed protein product [Triticum aestivum]	gi|300568722	26526.7	5.1999998092 6514

Peptide Information

Calc. Mass	Obsrv. Mass	± da	± ppm	Start Seq.	End Seq.	Sequence	Ion Score	C. I. %	Modification	Rank	Result Type
954.4832	954.4868	0.0036	4	6	13	FFVGGNWK					Mascot
1033.6041	1033.5706	-0.0335	-32	115	124	VAYALAQGLK					Mascot
1082.5782	1082.5771	-0.0011	-1	5	13	KFFVGGNWK					Mascot
1388.7202	1388.7308	0.0106	8	125	136	VIACIGETLEQR			Carbamidomethyl (C)[4]		Mascot
1388.7202	1388.7308	0.0106	8	125	136	VIACIGETLEQR	101	100	Carbamidomethyl (C)[4]		Mascot
1726.9236	1726.8116	-0.112	-65	183	198	KWLQANVGPAVAESTR					Mascot
1964.0005	1963.9949	-0.0056	-3	137	155	EAGTTMEVVAAQTKAIAEK			Oxidation (M)[6]		Mascot
2842.5237	2842.5278	0.0041	1	212	238	ELAAQPDLDGFLVGGASLKPEFVDIIK					Mascot

Rank	Protein Name	Accession No.	Protein MW	Protein PI	Pep. Count	Protein Score	Protein Score C. I. %	Intensity Matched	Total Ion Score	Total Ion C. I. %	Confirmed
3	Elicitor-responsive protein 1 [Triticum urartu]	gi|474213532	29523.9	8.3	5	106	100	6.45	90	100	

Peptide Information

Calc. Mass	Obsrv. Mass	± da	± ppm	Start Seq.	End Seq.	Sequence	Ion Score	C. I. %	Modification	Rank	Result Type
954.4832	954.4868	0.0036	4	6	13	FFVGGNWK					Mascot
1082.5782	1082.5771	-0.0011	-1	5	13	KFFVGGNWK					Mascot
1590.7363	1590.8282	0.0919	58	117	132	GGACSGLQLDPATMGR			Carbamidomethyl (C)[4]		Mascot
1605.7405	1605.8469	0.1064	66	174	186	DEGRNPSWNEVFR					Mascot
1973.0175	1973.0276	0.0101	5	55	71	GLLRPDFAVAAQNCWVR			Carbamidomethyl (C)[14]		Mascot
1973.0175	1973.0276	0.0101	5	55	71	GLLRPDFAVAAQNCWVR	90	100	Carbamidomethyl (C)[14]		Mascot

4 hypothetical protein TRIUR3_30538 [Triticum urartu] gi|473882607 197169.7 4.65 31 73 99.798 19.497

Peptide Information

Calc. Mass	Obsrv. Mass	± da	± ppm	Start Seq.	End Seq.	Sequence	Ion Score	C. I. %	Modification	Rank	Result Type
871.4156	871.4444	0.0288	33	341	347	DEHETLK					Mascot
954.5002	954.4868	-0.0134	-14	169	177	SLHAAQEAK					Mascot
1106.5623	1106.5262	-0.0361	-33	357	365	LSERDSMLR					Mascot
1114.6256	1114.5746	-0.051	-46	1376	1384	FDEVVHKLK					Mascot
1316.7056	1316.5984	-0.1072	-81	1241	1252	EKLVSLEAENGK					Mascot
1323.6678	1323.6578	-0.01	-8	640	650	LEELTAEYEVK					Mascot
1340.6078	1340.6927	0.0849	63	2	13	EAEQAAQVHDDK					Mascot
1353.7122	1353.6589	-0.0533	-39	1076	1087	EDVVTKLAEHGR					Mascot
1357.7434	1357.7043	-0.0391	-29	955	966	KLQQVGDNLSQK					Mascot
1370.7274	1370.7581	0.0307	22	1571	1581	DQLEQQLLEVR					Mascot
1404.7329	1404.6815	-0.0514	-37	348	360	GTLVDAESKLSER					Mascot
1487.6431	1487.7531	0.11	74	1	13	MEAEQAAQVHDDK			Oxidation (M)[1]		Mascot
1559.7119	1559.7791	0.0672	43	361	373	DSMLRQAEEEHAK			Oxidation (M)[3]		Mascot
1591.8075	1591.8215	0.014	9	1688	1701	SREFSLDSLAPQNK					Mascot
1605.7755	1605.8469	0.0714	44	595	608	VSHLSDDLEAYQTK					Mascot
1607.7946	1607.8387	0.0441	27	609	624	SASLEAVMEAASGKEK					Mascot
1607.7946	1607.8387	0.0441	27	609	624	SASLEAVMEAASGKEK					Mascot
1617.8517	1617.8246	-0.0271	-17	1425	1439	LNELQMTLAAAVAEK			Oxidation (M)[6]		Mascot
1623.7894	1623.832	0.0426	26	609	624	SASLEAVMEAASGKEK			Oxidation (M)[8]		Mascot
1660.8025	1660.8295	0.027	16	847	861	EDELSGKLAQANEEK					Mascot
1662.8618	1662.8446	-0.0172	-10	625	638	DLMDSLIQITEEKK					Mascot
1687.8749	1687.866	-0.0089	-5	283	297	LETELATVNEELQAK					Mascot
1726.8317	1726.8116	-0.0201	-12	1481	1494	YQSTLEEKQMLNDK					Mascot
1731.8218	1731.8478	0.026	15	195	208	EMENQINNLQEEIK					Mascot

1747.8167	1747.9216	0.1049	60	195	208	EMENQINNLQEEIK			Oxidation (M)[2]		Mascot
1838.9858	1838.9265	-0.0593	-32	1327	1342	LHEALETITQKEAEVK					Mascot
1867.8962	1867.91	0.0138	7	179	194	AAELDKMLEMAQLNMK			Oxidation (M)[7,10]		Mascot
1886.0229	1886.0022	-0.0207	-11	1648	1664	VAEESEKLALVNTQLNK					Mascot
1926.9767	1926.8969	-0.0798	-41	1309	1326	NLELHSAAESKIGETEAK					Mascot
1955.0518	1955.0406	-0.0112	-6	298	313	LLSLQEMEIKLEEHVK			Oxidation (M)[7]		Mascot
1977.0652	1977.0271	-0.0381	-19	1187	1203	LAVHEETIKHLTEELSK					Mascot
1988.9845	1989.0219	0.0374	19	1501	1517	ELDEAVAKLEEQMNLEK					Mascot
1988.9845	1989.0219	0.0374	19	1501	1517	ELDEAVAKLEEQMNLEK					Mascot
2004.9794	2005.015	0.0356	18	1501	1517	ELDEAVAKLEEQMNLEK			Oxidation (M)[13]		Mascot
2004.9794	2005.015	0.0356	18	1501	1517	ELDEAVAKLEEQMNLEK			Oxidation (M)[13]		Mascot
2011.0706	2011.0284	-0.0422	-21	1320	1337	IGETEAKLHEALETITQK					Mascot
2021.0802	2020.9664	-0.1138	-56	1163	1180	LVIVDELQEKFVSASSEK					Mascot

5 Cyclopropane-fatty-acyl-phospholipid synthase [Triticum urartu] gi|474443835 152981.1 8.43 17 62 97.078 26.782 31 93.9

Peptide Information

Calc. Mass	Obsrv. Mass	± da	± ppm	Start Seq.	End Seq.	Sequence	Ion Score	C. I. %	Modification	Rank	Result Type
1193.533	1193.6252	0.0922	77	455	464	SMTYSCAVFK			Carbamidomethyl (C)[6]		Mascot
1289.6962	1289.6503	-0.0459	-36	161	173	GLNNATIAVGGFR					Mascot
1316.6304	1316.5984	-0.032	-24	377	388	GSVFTFGKACDK			Carbamidomethyl (C)[10]		Mascot
1323.709	1323.6578	-0.0512	-39	1007	1017	SAYRVQIDMLK					Mascot
1388.7355	1388.7308	-0.0047	-3	343	354	KMIPSWTEAVAR					Mascot
1388.7355	1388.7308	-0.0047	-3	343	354	KMIPSWTEAVAR	31	93.9			Mascot
1404.7305	1404.6815	-0.049	-35	343	354	KMIPSWTEAVAR			Oxidation (M)[2]		Mascot
1416.7417	1416.7568	0.0151	11	1102	1112	VWRVCDLEAIR			Carbamidomethyl (C)[5]		Mascot
1491.7551	1491.7511	-0.004	-3	695	708	ALGDYQGSGQIINR					Mascot
1603.801	1603.8354	0.0344	21	912	925	EGMVWRVGTGENIR					Mascot
1605.7843	1605.8469	0.0626	39	1085	1097	LPEDGSHLFFRCK			Carbamidomethyl (C)[12]		Mascot
1611.8999	1611.8346	-0.0653	-41	865	878	LIQAPDSLCARVLR			Carbamidomethyl (C)[9]		Mascot
1619.7959	1619.8365	0.0406	25	912	925	EGMVWRVGTGENIR			Oxidation (M)[3]		Mascot
1619.7959	1619.8365	0.0406	25	912	925	EGMVWRVGTGENIR			Oxidation (M)[3]		Mascot
1674.7727	1674.8303	0.0576	34	221	233	DIYLHCDQNLMPR			Carbamidomethyl (C)[6]		Mascot
1689.8602	1689.8467	-0.0135	-8	1057	1070	VAHNSHPMLRNIER			Oxidation (M)[8]		Mascot
1743.7386	1743.864	0.1254	72	1	14	MMDWLEGLGVEMER			Oxidation (M)[1,2,12]		Mascot
1838.9912	1838.9265	-0.0647	-35	1157	1171	QNRAKPEGFYFLLQK					Mascot

1970.9714	1971.0166	0.0452	23	787	804	TVAQAIPAYAMACFDLTK			Carbamidomethyl (C)[13]		Mascot
1973.0175	1973.0276	0.0101	5	849	864	DLHAFNIAMLSRQVWR			Oxidation (M)[9]		Mascot
1973.0175	1973.0276	0.0101	5	849	864	DLHAFNIAMLSRQVWR			Oxidation (M)[9]		Mascot
1986.9664	1987.0134	0.047	24	787	804	TVAQAIPAYAMACFDLTK			Carbamidomethyl (C)[13], Oxidation (M)[11]		Mascot
1988.9243	1989.0219	0.0976	49	27	47	GGGGGCEWGNGNGISG LLAQK			Carbamidomethyl (C)[6]		Mascot
1988.9243	1989.0219	0.0976	49	27	47	GGGGGCEWGNGNGISG LLAQK			Carbamidomethyl (C)[6]		Mascot

6 Disease resistance protein RPP13 [Triticum urartu] gi|473786130 115619.3 8.15 22 62 97.01 5.646

Peptide Information

Calc. Mass	Obsrv. Mass	± da	± ppm	Start Seq.	End Seq.	Sequence	Ion Score	C. I. %	Modification	Rank	Result Type
807.3526	807.4046	0.052	64	508	513	NRSDCR			Carbamidomethyl (C)[5]		Mascot
954.4172	954.4868	0.0696	73	209	216	AWATMACK			Carbamidomethyl (C)[7], Oxidation (M)[5]		Mascot
1037.5011	1037.5319	0.0308	30	448	456	SAQQVAYDR					Mascot
1106.6205	1106.5262	-0.0943	-85	177	188	VVSIVGFGGSGK					Mascot
1182.6552	1182.572	-0.0832	-70	915	926	VVAPAEAMAPVK					Mascot
1193.6022	1193.6252	0.023	19	447	456	RSAQQVAYDR					Mascot
1357.7369	1357.7043	-0.0326	-24	946	956	LQRSCQVTPLR			Carbamidomethyl (C)[5]		Mascot
1374.6934	1374.7111	0.0177	13	1011	1023	TVPSIMPNGSKEV			Oxidation (M)[6]		Mascot
1491.8026	1491.7511	-0.0515	-35	464	476	NIIRPIDAHNNSK					Mascot
1615.8075	1615.8154	0.0079	5	494	507	SRSLNFISTSFNDK					Mascot
1617.6971	1617.8246	0.1275	79	204	216	EFSCRAWATMACK			Carbamidomethyl (C)[4,12]		Mascot
1633.692	1633.8154	0.1234	76	204	216	EFSCRAWATMACK			Carbamidomethyl (C)[4,12], Oxidation (M)[10]		Mascot
1660.7782	1660.8295	0.0513	31	973	987	NGLALLSCCEAPEAR			Carbamidomethyl (C)[8,9]		Mascot
1691.8719	1691.851	-0.0209	-12	482	495	THGIMNQLMLYKSR					Mascot
1731.8218	1731.8478	0.026	15	43	58	ELGMISASSEDQISHK					Mascot
1739.8997	1739.9189	0.0192	11	588	603	YLTLGSSVSRPLDGMK			Oxidation (M)[15]		Mascot
1747.8168	1747.9216	0.1048	60	43	58	ELGMISASSEDQISHK			Oxidation (M)[4]		Mascot
1749.8629	1749.8872	0.0243	14	189	203	TALAWEVYNCPQVAK			Carbamidomethyl (C)[10]		Mascot
1751.8857	1751.9213	0.0356	20	949	963	SCQVTPLREQSPPPR			Carbamidomethyl (C)[2]		Mascot
1867.9946	1867.91	-0.0846	-45	588	604	YLTLGSSVSRPLDGMKK			Oxidation (M)[15]		Mascot
1881.9132	1882.0166	0.1034	55	479	493	TCRTHGIMNQLMLYK			Carbamidomethyl (C)[2], Oxidation (M)[8]		Mascot
2011.0317	2011.0284	-0.0033	-2	328	345	LSVHGCSLEVEWGTPIVK			Carbamidomethyl (C)[6]		Mascot
2023.0502	2023.0468	-0.0034	-2	460	476	LMDRNIIRPIDAHNNSK			Oxidation (M)[2]		Mascot
2353.2068	2353.1724	-0.0344	-15	541	562	SLTVFGSAGEAVSELKSC ELLR			Carbamidomethyl (C)[18]		Mascot

7 hypothetical protein TRIUR3_27643 [Triticum urartu] gi|474329855 307031.8 4.73 35 58 93.896 22.83

Peptide Information

Calc. Mass	Obsrv. Mass	± da	± ppm	Start Seq.	End Seq.	Sequence	Ion Score	C. I. %	Modification	Rank	Result Type
832.3948	832.3271	-0.0677	-81	1464	1469	YDELHR					Mascot
905.4033	905.4636	0.0603	67	1510	1516	MLEPEDR			Oxidation (M)[1]		Mascot
977.5513	977.5466	-0.0047	-5	995	1003	LSTSLSEIK					Mascot
1033.516	1033.5706	0.0546	53	1266	1274	LDEALETSR					Mascot
1082.5477	1082.5771	0.0294	27	1347	1355	LKSYTEADR					Mascot
1107.4888	1107.5504	0.0616	56	2703	2712	IDGMWASGDR					Mascot
1193.5645	1193.6252	0.0607	51	2230	2239	EKEEESSSLR					Mascot
1323.621	1323.6578	0.0368	28	747	758	DSSNCTALQSLK			Carbamidomethyl (C)[5]		Mascot
1340.6376	1340.6927	0.0551	41	430	440	VAAHEEIQQCR			Carbamidomethyl (C)[10]		Mascot
1402.6598	1402.733	0.0732	52	2181	2192	NIDEHGFSIDQK					Mascot
1404.7039	1404.6815	-0.0224	-16	1900	1911	QTIEEMNAVIEK					Mascot
1410.7125	1410.6998	-0.0127	-9	33	44	GAQTTFNLQQFR					Mascot
1491.7472	1491.7511	0.0039	3	471	482	CEQLEIELRSSK			Carbamidomethyl (C)[1]		Mascot
1507.7794	1507.7521	-0.0273	-18	816	828	KLTQGMLCCLVGK			Carbamidomethyl (C)[8,9]		Mascot
1559.854	1559.7791	-0.0749	-48	759	772	GHLEVAKGELHELK					Mascot
1577.7227	1577.8138	0.0911	58	2095	2107	DVDLISYMESFMK					Mascot
1577.7227	1577.8138	0.0911	58	2095	2107	DVDLISYMESFMK					Mascot
1591.7744	1591.8215	0.0471	30	2193	2206	ATELAEVMSNLQNR			Oxidation (M)[8]		Mascot
1603.865	1603.8354	-0.0296	-18	930	944	ERASLSGLLSEVTNK					Mascot
1611.7795	1611.8346	0.0551	34	2655	2668	VNNDQVAIDVEMHK					Mascot
1627.7745	1627.8253	0.0508	31	2655	2668	VNNDQVAIDVEMHK			Oxidation (M)[12]		Mascot
1704.8837	1704.8618	-0.0219	-13	1086	1099	LLSKCEEVEEISLR			Carbamidomethyl (C)[5]		Mascot
1705.8868	1705.9072	0.0204	12	1524	1537	LLEVEHERDTLHSK					Mascot
1705.8868	1705.9072	0.0204	12	1524	1537	LLEVEHERDTLHSK					Mascot
1726.8494	1726.8116	-0.0378	-22	1281	1295	SSELEQSEQKLSSFK					Mascot
1739.8745	1739.9189	0.0444	26	2654	2668	KVNNDQVAIDVEMHK					Mascot
1743.8661	1743.864	-0.0021	-1	614	627	LKEDNVFLHTENER					Mascot
1838.9719	1838.9265	-0.0454	-25	2063	2078	QIDHLKELNSTSGQLR					Mascot
1885.9978	1886.0022	0.0044	2	1998	2013	RATELLLTELNEAQER					Mascot
1908.9047	1908.8147	-0.09	-47	707	723	HEHSLASSEITVENAER					Mascot
1954.9426	1955.0406	0.098	50	2430	2445	SNDLEQMLQEKEFSLK			Oxidation (M)[7]		Mascot
1985.8977	1986.0027	0.105	53	1090	1105	CEEVEEISLRCSSLMK			Carbamidomethyl (C)[1,11], Oxidation (M)[15]		Mascot
2005.0574	2005.015	-0.0424	-21	15	32	TPSLQQNRHLPTASTPTR					Mascot

Calc. Mass	Obsrv. Mass	± da	± ppm	Start Seq.	End Seq.	Sequence	Ion Score	C. I. %	Modification	Rank	Result Type
2005.0574	2005.015	-0.0424	-21	15	32	TPSLQQNRHLPTASTPTR					Mascot
2020.938	2020.9664	0.0284	14	2405	2421	KDQEIEGLMQALDEEEK			Oxidation (M)[9]		Mascot
2369.1555	2369.0232	-0.1323	-56	1470	1488	RFYELAEQNNMLEQSLVER					Mascot
2450.325	2450.2573	-0.0677	-28	909	929	LTNQAELLHNDITERISILEK					Mascot
2831.3503	2831.2188	-0.1315	-46	2406	2429	DQEIEGLMQALDEEEKELEVLENK					Mascot
3052.4417	3052.6523	0.2106	69	1532	1557	DTLHSKIEHLEDSSEMLITDLEESHK			Oxidation (M)[16]		Mascot

8 hypothetical protein TRIUR3_20753 [Triticum urartu] gi|473968637 66592.2 4.54 15 53 76.252 15.92

Peptide Information

Calc. Mass	Obsrv. Mass	± da	± ppm	Start Seq.	End Seq.	Sequence	Ion Score	C. I. %	Modification	Rank	Result Type
832.4047	832.3271	-0.0776	-93	80	87	EDDVGAVK					Mascot
1106.5398	1106.5262	-0.0136	-12	237	246	MVIVEDADAK			Oxidation (M)[1]		Mascot
1193.5355	1193.6252	0.0897	75	412	421	EEMDVNTEVK					Mascot
1316.6328	1316.5984	-0.0344	-26	517	527	NVEENKEETPK					Mascot
1507.6759	1507.7521	0.0762	51	503	516	DGKVTEEEASEADK					Mascot
1577.7363	1577.8138	0.0775	49	237	250	MVIVEDADAKEDDK					Mascot
1577.7363	1577.8138	0.0775	49	142	155	MVIVEDADAKEDDK					Mascot
1619.7548	1619.8365	0.0817	50	331	344	EDYGTKVAEHEDVK					Mascot
1619.7548	1619.8365	0.0817	50	331	344	EDYGTKVAEHEDVK					Mascot
1633.8392	1633.8154	-0.0238	-15	14	30	AVANGGTSTEVVASDKK					Mascot
1735.8055	1735.9298	0.1243	72	167	182	TVGIEDVVAMEDDNTK					Mascot
1751.8004	1751.9213	0.1209	69	167	182	TVGIEDVVAMEDDNTK			Oxidation (M)[10]		Mascot
1761.8324	1761.9474	0.115	65	191	206	MVIVEDANANEDDKAK					Mascot
1908.8491	1908.8147	-0.0344	-18	188	204	DSKMVIVEDANANEDDK			Oxidation (M)[4]		Mascot
1959.8965	1960.0547	0.1582	81	337	354	VAEHEDVKMGAVEDTDAK			Oxidation (M)[9]		Mascot
1988.9923	1989.0219	0.0296	15	215	233	AAGIEYTVAKEDAVSAEHK					Mascot
1988.9923	1989.0219	0.0296	15	215	233	AAGIEYTVAKEDAVSAEHK					Mascot
1996.8917	1996.9937	0.102	51	289	306	AEEFKDGNMNTVDNADVK					Mascot
2005.0172	2005.015	-0.0022	-1	361	378	EAEHQDIIMGAVKHADLK					Mascot
2005.0172	2005.015	-0.0022	-1	361	378	EAEHQDIIMGAVKHADLK					Mascot
2021.0121	2020.9664	-0.0457	-23	361	378	EAEHQDIIMGAVKHADLK			Oxidation (M)[9]		Mascot

9 hypothetical protein TRIUR3_29388 [Triticum urartu] gi|474319500 38387 8.88 5 51 65.673 12.356 37 98.567

Peptide Information

Calc. Mass	Obsrv. Mass	± da	± ppm	Start Seq.	End Seq.	Sequence	Ion Score	C. I. %	Modification	Rank	Result Type
1388.7678	1388.7308	-0.037	-27	171	182	RLMNIVTEIAGR			Oxidation (M)[3]		Mascot
1388.7678	1388.7308	-0.037	-27	171	182	RLMNIVTEIAGR	37	98.567	Oxidation (M)[3]		Mascot
1404.792	1404.6815	-0.1105	-79	116	127	VPTYKVTPMQLK					Mascot
1674.9479	1674.8303	-0.1176	-70	158	170	VIQRPWYAIWKSK					Mascot
1739.7561	1739.9189	0.1628	94	137	149	QFNGHEPENYWYR					Mascot
1908.9338	1908.8147	-0.1191	-62	85	100	ESVSLVEEGDFLKDWR					Mascot

10 Glyceraldehyde-3-phosphate dehydrogenase, cytosolic [Triticum urartu] gi|474166439 44425.5 8.54 12 50 52.616 8.093

Peptide Information

Calc. Mass	Obsrv. Mass	± da	± ppm	Start Seq.	End Seq.	Sequence	Ion Score	C. I. %	Modification	Rank	Result Type
977.5448	977.5466	0.0018	2	1	9	MASLSLSLR					Mascot
1308.6794	1308.6594	-0.02	-15	327	338	NASYEDVKAAIK					Mascot
1370.7386	1370.7581	0.0195	14	47	59	ATSSPARSIEPVR					Mascot
1371.7227	1371.7534	0.0307	22	276	290	GAGQNIIPSSTGAAK					Mascot
1577.8469	1577.8138	-0.0331	-21	21	35	AAAPIKVSFTASCVR			Carbamidomethyl (C)[13]		Mascot
1577.8469	1577.8138	-0.0331	-21	21	35	AAAPIKVSFTASCVR			Carbamidomethyl (C)[13]		Mascot
1603.8989	1603.8354	-0.0635	-40	295	309	VLPALNGKLTGMAFR			Oxidation (M)[12]		Mascot
1689.8906	1689.8467	-0.0439	-26	132	147	GTITVLDESTLEINGK					Mascot
1726.9447	1726.8116	-0.1331	-77	276	294	GAGQNIIPSSTGAAKAVG K					Mascot
1881.8358	1882.0166	0.1808	96	367	384	SSIFDANAGMGLSSSFM K			Oxidation (M)[10,17]		Mascot
1927.0317	1926.8969	-0.1348	-70	310	326	VPTPNVSVVDLTCRLEK			Carbamidomethyl (C)[13]		Mascot
1968.0306	1967.988	-0.0426	-22	399	415	FLQQCCVALPHAGVVLR			Carbamidomethyl (C)[5,6]		Mascot
1985.8925	1986.0027	0.1102	55	116	131	YMAYMFKYDSTHGPFK					Mascot

Gel Idx/Pos	188/H15	Instr./Gel Origin	BA2151/Sample Project 20140814	Process Status	Analysis Succeeded
Plate [#] Name	[1] Sample Project 20140814	Instrument Sample Name		Spectra	11

Rank	Protein Name	Accession No.	Protein MW	Protein PI	Pep. Count	Protein Score	Protein Score C. I. %	Intensity Matched	Total Ion Score	Total Ion C. I. %	Confirmed
1	hypothetical protein TRIUR3_31593 [Triticum urartu]	gi|473979984	19793.1	5.63	12	372	100	20.517	305	100	

Peptide Information

Calc. Mass	Obsrv. Mass	± da	± ppm	Start Seq.	End Seq.	Sequence	Ion Score	C. I. %	Modification	Rank	Result Type
807.3645	807.3835	0.019	24	86	91	GDHWHR					Mascot
888.4421	888.4516	0.0095	11	63	69	VEIEENR					Mascot
926.5669	926.5472	-0.0197	-21	129	136	KLAPEQIK					Mascot
1108.6473	1108.6488	0.0015	1	130	139	LAPEQIKGPR					Mascot
1203.5601	1203.5669	0.0068	6	155	167	TIGDAGAAGGEER					Mascot
1203.5601	1203.5669	0.0068	6	155	167	TIGDAGAAGGEER	33	95.893			Mascot
1247.6453	1247.6178	-0.0275	-22	6	16	MDVALLADPFR					Mascot
1329.6951	1329.6992	0.0041	3	17	27	ILEHVPFGFDR	20	26.452			Mascot
1331.655	1331.6841	0.0291	22	154	167	KTIGDAGAAGGEER					Mascot
1373.6907	1373.6976	0.0069	5	59	69	EDLKVEIEENR					Mascot
1770.8328	1770.8367	0.0039	2	42	57	ETSDSHEIVVDVPGMR					Mascot
1786.8276	1786.8215	-0.0061	-3	42	57	ETSDSHEIVVDVPGMR			Oxidation (M)[15]		Mascot
2226.1614	2226.1665	0.0051	2	105	126	LPDNADLDSIAASLDAGV LTVR					Mascot
2226.1614	2226.1665	0.0051	2	105	126	LPDNADLDSIAASLDAGV LTVR	241	100			Mascot
2421.1689	2421.1802	0.0113	5	17	37	ILEHVPFGFDRDDVAMVS MAR			Oxidation (M)[16]		Mascot
2437.1638	2437.1423	-0.0215	-9	17	37	ILEHVPFGFDRDDVAMVS MAR			Oxidation (M)[16,19]		Mascot
2437.1638	2437.1423	-0.0215	-9	17	37	ILEHVPFGFDRDDVAMVS MAR	12	0	Oxidation (M)[16,19]		Mascot

Rank	Protein Name	Accession No.	Protein MW	Protein PI	Pep. Count	Protein Score	Protein Score C. I. %	Intensity Matched	Total Ion Score	Total Ion C. I. %	Confirmed
2	HMW glutenin subunit 1ByX [Triticum durum]	gi|228015434	57657.7	13.28	16	53	78.341	10.093	2	0	

Peptide Information

Calc. Mass	Obsrv. Mass	± da	± ppm	Start Seq.	End Seq.	Sequence	Ion Score	C. I. %	Modification	Rank	Result Type
934.5065	934.4159	-0.0906	-97	397	405	ATTRTGATR					Mascot
1089.6051	1089.5977	-0.0074	-7	11	20	VLPNFSASVR					Mascot
1233.6699	1233.6416	-0.0283	-23	260	271	ALPSFSAAGRTR					Mascot
1233.6699	1233.6416	-0.0283	-23	260	271	ALPSFSAAGRTR	4	0			Mascot

1247.6855	1247.6178	-0.0677	-54	368	379	ALPSFSAAARTR					Mascot
1302.7278	1302.6642	-0.0636	-49	140	151	VLPNFSAAARTR					Mascot
1304.707	1304.6792	-0.0278	-21	257	269	ATRALPSFSAAGR					Mascot
1304.707	1304.6792	-0.0278	-21	152	164	ATRAVPSFSAAAR					Mascot
1317.7023	1317.6649	-0.0374	-28	245	256	ALHSFSAATRTR					Mascot
1318.7227	1318.66	-0.0627	-48	365	377	ATRALPSFSAAAR					Mascot
1331.7794	1331.6841	-0.0953	-72	26	37	VLPIFSAATRTR					Mascot
1332.7383	1332.6824	-0.0559	-42	427	438	VLPNFSATARTR					Mascot
1346.7539	1346.6832	-0.0707	-52	11	22	VLPNFSASVRTR					Mascot
1361.7245	1361.6946	-0.0299	-22	464	475	TRPTNRTSATTR					Mascot
1364.7281	1364.6674	-0.0607	-44	197	209	ATRAVPSFSTTAR					Mascot
1373.7648	1373.6976	-0.0672	-49	137	149	ATRVLPNFSAAAR					Mascot
1393.7546	1393.6891	-0.0655	-47	110	122	ATRVLSNFSTAAR					Mascot

3 G-type lectin S-receptor-like serine/threonine-protein kinase SRK [Triticum urartu] gi|473912990 45855.4 8.65 12 49 50.383 23.619

Peptide Information

Calc. Mass	Obsrv. Mass	± da	± ppm	Start Seq.	End Seq.	Sequence	Ion Score	C. I. %	Modification	Rank	Result Type
807.3995	807.3835	-0.016	-20	267	272	EFENLR					Mascot
814.3763	814.457	0.0807	99	146	151	KSYECK			Carbamidomethyl (C)[5]		Mascot
849.4677	849.4256	-0.0421	-50	395	403	KTISSVGTG					Mascot
942.3986	942.4316	0.033	35	255	262	NMTGFDNK			Oxidation (M)[2]		Mascot
1318.6307	1318.66	0.0293	22	240	251	GICENGEEIAVK			Carbamidomethyl (C)[3]		Mascot
1348.6644	1348.6592	-0.0052	-4	263	272	EFHKEFENLR					Mascot
1378.7379	1378.699	-0.0389	-28	33	43	VAYHIHSWLPR					Mascot
1381.6846	1381.6381	-0.0465	-34	288	299	ESEKVVVEYDGK					Mascot
1782.8336	1782.9763	0.1427	80	2	17	AMWMSAITLAPATSCR			Carbamidomethyl (C)[15], Oxidation (M)[2]		Mascot
1897.8792	1897.9833	0.1041	55	1	17	MAMWMSAITLAPATSCR			Carbamidomethyl (C)[16]		Mascot
1945.8639	1946.0093	0.1454	75	1	17	MAMWMSAITLAPATSCR			Carbamidomethyl (C)[16], Oxidation (M)[1,3,5]		Mascot
1996.9731	1997.0109	0.0378	19	306	322	MHTALCFEYVRNGSLAK			Carbamidomethyl (C)[6]		Mascot
2012.9681	2013.0306	0.0625	31	306	322	MHTALCFEYVRNGSLAK			Carbamidomethyl (C)[6], Oxidation (M)[1]		Mascot
2012.9681	2013.0306	0.0625	31	306	322	MHTALCFEYVRNGSLAK			Carbamidomethyl (C)[6], Oxidation (M)[1]		Mascot
2295.0852	2295.2002	0.115	50	44	63	EVLVPIMAELQMMCEGA VSR			Carbamidomethyl (C)[14], Oxidation (M)[7,12]		Mascot

4 Calnexin-like protein [Triticum urartu] gi|474105773 8765.4 5 6 48 38.957 13.85

Peptide Information

Calc. Mass	Obsrv. Mass	± da	± ppm	Start Seq.	End Seq.	Sequence	Ion Score	C. I. %	Modification	Rank	Result Type
923.4291	923.3699	-0.0592	-64	1	7	MEQSVWK			Oxidation (M)[1]		Mascot
1001.5513	1001.5844	0.0331	33	33	41	ELDSPVTLK					Mascot
1301.6307	1301.6265	-0.0042	-3	1	10	MEQSVWKHEK					Mascot
1317.6256	1317.6649	0.0393	30	1	10	MEQSVWKHEK			Oxidation (M)[1]		Mascot
1336.693	1336.6696	-0.0234	-18	42	52	DVTVVLQFEMR					Mascot
1336.693	1336.6696	-0.0234	-18	42	52	DVTVVLQFEMR					Mascot
1782.8293	1782.9763	0.147	82	11	26	SDGHEYYGLLVSESAR					Mascot
2335.2214	2335.158	-0.0634	-27	33	52	ELDSPVTLKDVTVVLQFE MR			Oxidation (M)[19]		Mascot

5 Kinesin-4 [Triticum urartu] gi|473942625 114264.5 9.09 20 47 13.775 24.841

Peptide Information

Calc. Mass	Obsrv. Mass	± da	± ppm	Start Seq.	End Seq.	Sequence	Ion Score	C. I. %	Modification	Rank	Result Type
807.3843	807.3835	-0.0008	-1	270	276	SEVESTR					Mascot
816.4825	816.4616	-0.0209	-26	650	656	EEIGKLK					Mascot
849.4386	849.4256	-0.013	-15	484	490	DVLDLMK			Oxidation (M)[6]		Mascot
867.3513	867.4164	0.0651	75	217	224	DDSGVCSK			Carbamidomethyl (C)[6]		Mascot
948.4421	948.4637	0.0216	23	279	285	WEEDLTR					Mascot
1001.5374	1001.5844	0.047	47	497	506	AVGATALNER					Mascot
1152.5929	1152.5472	-0.0457	-40	769	779	SGGTVMKTVEK			Oxidation (M)[6]		Mascot
1259.7294	1259.6282	-0.1012	-80	321	330	VYCRVKPLPK			Carbamidomethyl (C)[3]		Mascot
1291.7118	1291.6179	-0.0939	-73	510	521	SHSVLTVHVQGK					Mascot
1304.7178	1304.6792	-0.0386	-30	1	11	MALRNGLILCK			Carbamidomethyl (C)[10], Oxidation (M)[1]		Mascot
1304.7178	1304.6792	-0.0386	-30	1	11	MALRNGLILCK			Carbamidomethyl (C)[10], Oxidation (M)[1]		Mascot
1331.7026	1331.6841	-0.0185	-14	497	509	AVGATALNERSSR					Mascot
1346.691	1346.6832	-0.0078	-6	919	931	ASVREAEAVTEGK					Mascot
1373.673	1373.6976	0.0246	18	780	791	AESIIECTPTPR			Carbamidomethyl (C)[7]		Mascot
1768.9011	1768.8287	-0.0724	-41	531	546	GCLHLVDLAGSERVDK			Carbamidomethyl (C)[2]		Mascot
1770.9708	1770.8367	-0.1341	-76	585	601	NSKLTQVLQDALGGQAK					Mascot
1786.9407	1786.8215	-0.1192	-67	804	819	SRNNTPASILTEQSLR					Mascot
1966.9902	1966.946	-0.0442	-22	780	797	AESIIECTPTPRAEPPAK			Carbamidomethyl (C)[7]		Mascot
2226.0457	2226.1665	0.1208	54	698	716	FKPEARQDSSVDTCTSEI R			Carbamidomethyl (C)[14]		Mascot
2226.0457	2226.1665	0.1208	54	698	716	FKPEARQDSSVDTCTSEI R			Carbamidomethyl (C)[14]		Mascot
2248.241	2248.1287	-0.1123	-50	510	530	SHSVLTVHVQGKEIISGS					Mascot

Calc. Mass	Obsrv. Mass	± da	± ppm	Start Seq.	End Seq.	Sequence	Ion Score	C. I. %	Modification	Rank	Result Type
2404.2402	2404.197	-0.0432	-18	462	483	TLR NNSHVNGLNIPDANLVPV KCTK			Carbamidomethyl (C)[20]		Mascot

6	1-phosphatidylinositol-3-phosphate 5-kinase fab1 [Triticum urartu]	gi|473824589	177878.2	6.14	24	46	0	7.208

Peptide Information

Calc. Mass	Obsrv. Mass	± da	± ppm	Start Seq.	End Seq.	Sequence	Ion Score	C. I. %	Modification	Rank	Result Type
807.3488	807.3835	0.0347	43	1322	1327	DKCCPK			Carbamidomethyl (C)[3,4]		Mascot
856.4312	856.386	-0.0452	-53	1348	1354	SNVYFAR					Mascot
934.4485	934.4159	-0.0326	-35	757	763	CLKCEPK			Carbamidomethyl (C)[1,4]		Mascot
967.5432	967.4514	-0.0918	-95	764	772	NGVPPATRR					Mascot
979.5458	979.4996	-0.0462	-47	855	863	TVAVEIYGK					Mascot
1219.6066	1219.6005	-0.0061	-5	1127	1137	IHSFDSTVASR					Mascot
1233.6111	1233.6416	0.0305	25	156	166	FSLDSLEHGTK					Mascot
1233.6111	1233.6416	0.0305	25	156	166	FSLDSLEHGTK					Mascot
1237.6536	1237.6591	0.0055	4	1328	1337	DIDYIRSLSR					Mascot
1303.6423	1303.6538	0.0115	9	1177	1188	ACSQRSPGAIEK			Carbamidomethyl (C)[2]		Mascot
1318.6421	1318.66	0.0179	14	314	325	VKCIASGDPTDR			Carbamidomethyl (C)[3]		Mascot
1329.6708	1329.6992	0.0284	21	751	759	IWMWHRCLK			Carbamidomethyl (C)[7]		Mascot
1332.7522	1332.6824	-0.0698	-52	2	14	GVVEFSVLGAVQK					Mascot
1334.7103	1334.6627	-0.0476	-36	941	951	GLILDAYLWDR					Mascot
1335.6943	1335.6426	-0.0517	-39	628	638	ISPYFLDLDPR					Mascot
1349.7246	1349.644	-0.0806	-60	446	456	LGQCEIFKVQK			Carbamidomethyl (C)[4]		Mascot
1373.6948	1373.6976	0.0028	2	1156	1168	SAEYFGGLTSITK					Mascot
1393.707	1393.6891	-0.0179	-13	45	59	SGGVSPVDSPPPAAR					Mascot
1722.7211	1722.8527	0.1316	76	545	561	GDTDCFAGSAGVGMPHK			Carbamidomethyl (C)[5], Oxidation (M)[14]		Mascot
1770.9246	1770.8367	-0.0879	-50	1122	1137	NAPIRIHSFDSTVASR					Mascot
1782.9572	1782.9763	0.0191	11	1	16	MGVVEFSVLGAVQKFR			Oxidation (M)[1]		Mascot
1822.8424	1822.9637	0.1213	67	1418	1431	MDLMVMENLFFEKK			Oxidation (M)[1,4,6]		Mascot
1897.9556	1897.9833	0.0277	15	866	880	SLHWEISDFLHRTEK					Mascot
2034.9585	2034.9897	0.0312	15	1415	1430	EVKMDLMVMENLFFEK			Oxidation (M)[4,7]		Mascot
2166.0562	2166.1382	0.082	38	1030	1048	KLQIDLVDHPGDDTEDLDK					Mascot

7	ABC transporter F family member 4 [Triticum urartu]	gi|474393400	66356.1	5.96	15	45	0	3.25

Peptide Information

Calc. Mass	Obsrv. Mass	± da	± ppm	Start Seq.	End Seq.	Sequence	Ion Score	C. I. %	Modification	Rank	Result Type

Calc. Mass	Obsrv. Mass	± da	± ppm	Start Seq.	End Seq.	Sequence	Ion Score	C. I. %	Modification	Rank	Result Type
814.3981	814.457	0.0589	72	291	296	FEVYEK					Mascot
816.4686	816.4616	-0.007	-9	495	502	LSGGQKAR					Mascot
942.493	942.4316	-0.0614	-65	290	296	KFEVYEK					Mascot
948.4706	948.4637	-0.0069	-7	586	593	DELMAEIK					Mascot
1108.5457	1108.6488	0.1031	93	74	84	YGLVGPNGMGK			Oxidation (M)[9]		Mascot
1264.6467	1264.6171	-0.0296	-23	73	84	RYGLVGPNGMGK			Oxidation (M)[10]		Mascot
1332.5551	1332.6824	0.1273	96	139	150	LEASNDPDDNDK					Mascot
1335.6903	1335.6426	-0.0477	-36	45	56	DIVLDNFSVSAR					Mascot
1361.6591	1361.6946	0.0355	26	158	169	LNLCDSDAARAR			Carbamidomethyl (C)[4]		Mascot
1393.6628	1393.6891	0.0263	19	388	400	LSDVDVGIDMGTR			Oxidation (M)[10]		Mascot
1822.948	1822.9637	0.0157	9	173	189	ILAGLGFDQAMQARSTK			Oxidation (M)[11]		Mascot
1826.9495	1826.769	-0.1805	-99	101	116	NIDVLLVEQEIVGDDR					Mascot
1966.9539	1966.946	-0.0079	-4	151	167	LAEVYEKLNLCDSDAAR			Carbamidomethyl (C)[11]		Mascot
2341.218	2341.2322	0.0142	6	388	411	LSDVDVGIDMGTRVAIVG PNGAGK					Mascot
2357.2129	2357.2146	0.0017	1	388	411	LSDVDVGIDMGTRVAIVG PNGAGK			Oxidation (M)[10]		Mascot
2453.2632	2453.1562	-0.107	-44	19	44	DAISVVIGARVPGSAAEG DAADGNIK					Mascot

8 Guanylate kinase [Triticum urartu] gi|474404653 46350 6.58 11 44 0 3.018

Peptide Information

Calc. Mass	Obsrv. Mass	± da	± ppm	Start Seq.	End Seq.	Sequence	Ion Score	C. I. %	Modification	Rank	Result Type
1182.6477	1182.5615	-0.0862	-73	334	343	KLLSLNDDHK					Mascot
1219.6682	1219.6005	-0.0677	-56	138	148	IKGSEVVSWSK					Mascot
1259.6412	1259.6282	-0.013	-10	249	259	CILDIDVQGAR			Carbamidomethyl (C)[1]		Mascot
1318.6598	1318.66	0.0002	0	289	299	GTETEEQIQKR					Mascot
1332.7747	1332.6824	-0.0923	-69	2	14	VATVVGASFLGRR					Mascot
1346.637	1346.6832	0.0462	34	46	57	DCQQNAIVVGDK			Carbamidomethyl (C)[2]		Mascot
1768.9017	1768.8287	-0.073	-41	178	192	EYPLKFGFSVSHTTR					Mascot
1897.9518	1897.9833	0.0315	17	270	284	FIFVCPPSFEELEKR			Carbamidomethyl (C)[5]		Mascot
1946.0012	1946.0093	0.0081	4	40	57	ITLGSKDCQQNAIVVGDK			Carbamidomethyl (C)[8]		Mascot
1968.0624	1967.9647	-0.0977	-50	80	97	LTQTWIMPTVLGAQPPSK					Mascot
2437.3523	2437.1423	-0.21	-86	76	97	ILDKLTQTWIMPTVLGAQ PPSK					Mascot
2437.3523	2437.1423	-0.21	-86	76	97	ILDKLTQTWIMPTVLGAQ PPSK					Mascot
2453.3474	2453.1562	-0.1912	-78	76	97	ILDKLTQTWIMPTVLGAQ PPSK			Oxidation (M)[11]		Mascot

9	Disease resistance protein RPP13 [Triticum urartu]	gi|473954621	142534.1	9.2	19	42	0	13.564

Peptide Information

Calc. Mass	Obsrv. Mass	± da	± ppm	Start Seq.	End Seq.	Sequence	Ion Score	C. I. %	Modification	Rank	Result Type
916.5112	916.4604	-0.0508	-55	97	104	TGAGWLRR					Mascot
1152.5718	1152.5472	-0.0246	-21	851	860	DIFAALSEMR					Mascot
1233.6838	1233.6416	-0.0422	-34	667	676	LQFLETLDVR					Mascot
1233.6838	1233.6416	-0.0422	-34	667	676	LQFLETLDVR					Mascot
1290.6511	1290.6478	-0.0033	-3	426	437	FLQSAGNPTPMK					Mascot
1303.7621	1303.6538	-0.1083	-83	650	662	YLSLGAAVTALPK					Mascot
1304.7322	1304.6792	-0.053	-41	321	332	LVVKNQNGTFGK					Mascot
1304.7322	1304.6792	-0.053	-41	321	332	LVVKNQNGTFGK					Mascot
1317.6443	1317.6649	0.0206	16	1125	1134	IVEGMRYMYR					Mascot
1335.7056	1335.6426	-0.063	-47	212	222	TLLANHVYNYK					Mascot
1349.634	1349.644	0.01	7	1125	1134	IVEGMRYMYR			Oxidation (M)[5,8]		Mascot
1361.7788	1361.6946	-0.0842	-62	666	676	KLQFLETLDVR					Mascot
1364.7202	1364.6674	-0.0528	-39	711	723	MSKLQASLSANSK					Mascot
1378.7213	1378.699	-0.0223	-16	724	736	LETVAGFVVDSNK					Mascot
1381.6998	1381.6381	-0.0617	-45	1158	1169	SLWSAYLEVADK					Mascot
2012.1111	2012.0647	-0.0464	-23	194	211	QQQQLKVITIVGFHGMGK					Mascot
2028.9331	2029.0111	0.078	38	623	639	VLDLEEYDHQMSDGHLK					Mascot
2341.1494	2341.2322	0.0828	35	40	59	FIKDEFAMISAVIQDEQNSR					Mascot
2357.1443	2357.2146	0.0703	30	40	59	FIKDEFAMISAVIQDEQNSR			Oxidation (M)[8]		Mascot
2373.1345	2373.1953	0.0608	26	391	411	EIFQDDELPPDVEELGSEALK					Mascot
2404.1489	2404.197	0.0481	20	438	457	WAKLCTDLGTYLESDELFSR			Carbamidomethyl (C)[5]		Mascot
2437.1665	2437.1423	-0.0242	-10	1010	1032	SVPGLGSSYSGRDCGPELEIQTK			Carbamidomethyl (C)[14]		Mascot
2437.1665	2437.1423	-0.0242	-10	1010	1032	SVPGLGSSYSGRDCGPELEIQTK			Carbamidomethyl (C)[14]		Mascot
2501.2295	2501.2498	0.0203	8	391	412	EIFQDDELPPDVEELGSEALKK					Mascot

10	Putative serpin-Z12 [Triticum urartu]	gi|474116890	68690.1	7.24	13	41	0	8.115

Peptide Information

Calc. Mass	Obsrv. Mass	± da	± ppm	Start Seq.	End Seq.	Sequence	Ion Score	C. I. %	Modification	Rank	Result Type
856.3869	856.386	-0.0009	-1	200	206	TVFSCDK			Carbamidomethyl (C)[5]		Mascot
910.4563	910.4223	-0.034	-37	340	346	REFMATR					Mascot

942.4944	942.4316	-0.0628	-67	45	51	SPHFWLR		Mascot
1291.6615	1291.6179	-0.0436	-34	546	557	DGHGHGRLSISR		Mascot
1304.7322	1304.6792	-0.053	-41	368	379	LRVNAFVADATK		Mascot
1304.7322	1304.6792	-0.053	-41	368	379	LRVNAFVADATK		Mascot
1318.562	1318.66	0.098	74	516	526	FEFEASSDMQK		Mascot
1329.6951	1329.6992	0.0041	3	153	164	RFLGFGYAPSSK		Mascot
1334.6951	1334.6627	-0.0324	-24	115	127	SSLDGVVFNPATK		Mascot
1348.7385	1348.6592	-0.0793	-59	45	54	SPHFWLRHLR		Mascot
1357.657	1357.7228	0.0658	48	532	545	AFKGGDFSGMVGGK		Mascot
1373.6519	1373.6976	0.0457	33	532	545	AFKGGDFSGMVGGK	Oxidation (M)[10]	Mascot
2035.0026	2034.9897	-0.0129	-6	318	335	LNGLPQTSFACDVSVDR R	Carbamidomethyl (C)[11]	Mascot
2208.1184	2208.1582	0.0398	18	132	152	LSVPSLPPPAAPEDDDG YVLR		Mascot
2421.2913	2421.1802	-0.1111	-46	384	406	DILPPGSIDSSTTVVLANA LYFK		Mascot

Gel Idx/Pos	189/H16	Instr./Gel Origin	BA2151/Sample Project 20140814	Process Status	Analysis Succeeded
Plate [#] Name	[1] Sample Project 20140814	Instrument Sample Name		Spectra	11

Rank	Protein Name	Accession No.	Protein MW	Protein PI	Pep. Count	Protein Score	Protein Score C. I. %	Intensity Matched	Total Ion Score	Total Ion C. I. %	Confirmed
1	hypothetical protein TRIUR3_31593 [Triticum urartu]	gi|473979984	19793.1	5.63	6	245	100	4.387	223	100	

Peptide Information

Calc. Mass	Obsrv. Mass	± da	± ppm	Start Seq.	End Seq.	Sequence	Ion Score	C. I. %	Modification	Rank	Result Type
807.3645	807.4138	0.0493	61	86	91	GDHWHR					Mascot
926.5669	926.511	-0.0559	-60	129	136	KLAPEQIK					Mascot
1108.6473	1108.6591	0.0118	11	130	139	LAPEQIKGPR					Mascot
1203.5601	1203.5775	0.0174	14	155	167	TIGDAGAAGGEER					Mascot
1786.8276	1786.8252	-0.0024	-1	42	57	ETSDSHEIVVDVPGMR			Oxidation (M)[15]		Mascot
2226.1614	2226.1792	0.0178	8	105	126	LPDNADLDSIAASLDAGV LTVR					Mascot
2226.1614	2226.1792	0.0178	8	105	126	LPDNADLDSIAASLDAGV LTVR	223	100			Mascot

Rank	Protein Name	Accession No.	Protein MW	Protein PI	Pep. Count	Protein Score	Protein Score C. I. %	Intensity Matched	Total Ion Score	Total Ion C. I. %	Confirmed
2	unnamed protein product [Triticum aestivum]	gi|259439696	22043.6	5.73	5	234	100	4.681	203	100	

Protein Group

cold shock domain protein 3 [Triticum aestivum]	gi|42391858	22043.6	5.7300 000190 7349
unnamed protein product [Triticum aestivum]	gi|300645711	22043.6	5.7300 000190 7349

Peptide Information

Calc. Mass	Obsrv. Mass	± da	± ppm	Start Seq.	End Seq.	Sequence	Ion Score	C. I. %	Modification	Rank	Result Type
1465.5874	1465.6221	0.0347	24	188	204	DCPQGGGGGGYGGGG GR			Carbamidomethyl (C)[2]		Mascot
1886.7294	1886.7604	0.031	16	205	224	GGGGGGGGCFSCGESG HFSR			Carbamidomethyl (C)[9,12]		Mascot
2027.801	2027.8228	0.0218	11	150	174	DCPQGGGGGGGYGGG GYGGGGGGGR			Carbamidomethyl (C)[2]		Mascot
2027.801	2027.8228	0.0218	11	150	174	DCPQGGGGGGGYGGG GYGGGGGGGR	71	100	Carbamidomethyl (C)[2]		Mascot
2093.9622	2093.9812	0.019	9	44	62	SLNENDAVEFEIITGDDG R					Mascot
2093.9622	2093.9812	0.019	9	44	62	SLNENDAVEFEIITGDDG R	133	100			Mascot

Calc. Mass	Obsrv. Mass	± da	± ppm	Start Seq.	End Seq.	Sequence	Ion Score	C. I. %	Modification	Rank	Result Type
3263.2932	3263.3474	0.0542	17	93	136	GGYGGGGGGYGGGGG GYGGGGGGYGGGGGG YGGGGYGGGGGGGR					Mascot

3	unnamed protein product [Triticum aestivum]	gi|259439834	21883.5	5.74	5	194	100	4.389	164	100		

Protein Group

cold shock protein-1 [Triticum aestivum]	gi|21322752	21883.5	5.7399 997711 1816
unnamed protein product [Triticum aestivum]	gi|300645849	21883.5	5.7399 997711 1816

Peptide Information

Calc. Mass	Obsrv. Mass	± da	± ppm	Start Seq.	End Seq.	Sequence	Ion Score	C. I. %	Modification	Rank	Result Type
1693.6733	1693.7076	0.0343	20	182	202	DCPQGGGGGGGGGGY GGGGGR			Carbamidomethyl (C)[2]		Mascot
1886.7294	1886.7604	0.031	16	203	222	GGGGGGGGCFSCGESG HFSR			Carbamidomethyl (C)[9,12]		Mascot
2027.801	2027.8228	0.0218	11	144	168	DCPQGGGGGGGYGGG GYGGGGGGGR			Carbamidomethyl (C)[2]		Mascot
2027.801	2027.8228	0.0218	11	144	168	DCPQGGGGGGGYGGG GYGGGGGGGR	71	100	Carbamidomethyl (C)[2]		Mascot
2109.957	2109.9753	0.0183	9	44	62	SLNENDTVEFEVITGDDG R					Mascot
2109.957	2109.9753	0.0183	9	44	62	SLNENDTVEFEVITGDDG R	93	100			Mascot
2815.1226	2815.1584	0.0358	13	93	130	GGYGGGGYGGGGGGG GYGGGGGGYGGGGGG YGGGGGGR					Mascot

4	Glycine-rich protein 2 [Triticum urartu]	gi|474091493	15630.7	4.66	1	98	100	1.041	93	100		

Peptide Information

Calc. Mass	Obsrv. Mass	± da	± ppm	Start Seq.	End Seq.	Sequence	Ion Score	C. I. %	Modification	Rank	Result Type
2109.957	2109.9753	0.0183	9	44	62	SLNENDTVEFEVITGDDG R					Mascot
2109.957	2109.9753	0.0183	9	44	62	SLNENDTVEFEVITGDDG R	93	100			Mascot

5	Vicilin-like antimicrobial peptides 2-2 [Triticum urartu]	gi|473890163	75298.3	5.79	12	90	99.996	6.306	66	99.998		

Peptide Information

Calc. Mass	Obsrv. Mass	± da	± ppm	Start Seq.	End Seq.	Sequence	Ion Score	C. I. %	Modification	Rank	Result Type
807.4359	807.4138	-0.0221	-27	660	667	KGAVFQSA					Mascot
847.3904	847.4501	0.0597	70	607	612	EEEERR					Mascot

	849.4941	849.432	-0.0621	-73	360	366	NSVFRVK					Mascot
	1231.5186	1231.6066	0.088	71	602	611	GDDPREEEER					Mascot
	1232.5753	1232.6047	0.0294	24	458	468	KAEQEEQEGGK					Mascot
	1233.5342	1233.6423	0.1081	88	551	559	EQEEEEERR					Mascot
	1490.7485	1490.7582	0.0097	7	202	214	SPQSIITYNPDQK					Mascot
	1875.8403	1875.9401	0.0998	53	348	364	GGDHGQEGVECKNSVFR			Carbamidomethyl (C)[11]		Mascot
	1982.9666	1982.9745	0.0079	4	102	119	VTYIQEGGSETSSLEVQR					Mascot
	2021.1251	2021.0497	-0.0754	-37	315	334	ATEIAIVTHGSGIVQVGGRR					Mascot
	2027.9194	2027.8228	-0.0966	-48	441	458	SGSTIMACVSCAEELERK			Carbamidomethyl (C)[8,11]		Mascot
	2027.9194	2027.8228	-0.0966	-48	441	458	SGSTIMACVSCAEELERK			Carbamidomethyl (C)[8,11]		Mascot
	2501.2307	2501.2637	0.033	13	120	141	GDVYNLEQGSILYIQSYPNATR					Mascot
	2501.2307	2501.2637	0.033	13	120	141	GDVYNLEQGSILYIQSYPNATR	66	99.998			Mascot

6	Aldehyde dehydrogenase family 2 member C4 [Triticum urartu]	gi|474376937	80767.3	8.38	16	55	85.357	6.025

Peptide Information

Calc. Mass	Obsrv. Mass	± da	± ppm	Start Seq.	End Seq.	Sequence	Ion Score	C. I. %	Modification	Rank	Result Type
864.4574	864.4284	-0.029	-34	264	271	ALFAGETR					Mascot
906.4713	906.4753	0.004	4	497	504	LIMEASAR			Oxidation (M)[3]		Mascot
1090.5059	1090.593	0.0871	80	1	10	MAEAENRGGR					Mascot
1106.5007	1106.5249	0.0242	22	1	10	MAEAENRGGR			Oxidation (M)[1]		Mascot
1182.6589	1182.5759	-0.083	-70	414	424	IHGESLRVSGK					Mascot
1319.7206	1319.6652	-0.0554	-42	156	167	ITASSLQPFVEK					Mascot
1323.6515	1323.6796	0.0281	21	172	182	LSSWWASMLSR					Mascot
1334.6627	1334.65	-0.0127	-10	722	733	SVITAVPDSPWY					Mascot
1348.7253	1348.7158	-0.0095	-7	497	508	LIMEASARSNLK			Oxidation (M)[3]		Mascot
1371.7665	1371.7228	-0.0437	-32	394	405	IIDIPSAVQMLR			Oxidation (M)[10]		Mascot
1415.7754	1415.656	-0.1194	-84	117	128	SKNHGVITYQLR					Mascot
1859.9539	1859.9205	-0.0334	-18	295	311	YTKLFINGEFVDAASGK					Mascot
1982.8636	1982.9745	0.1109	56	344	359	EAFEHGMWPRMSGYER					Mascot
2182.0593	2182.1174	0.0581	27	623	641	EDMKIAQDEIFGPVMSLMK					Mascot
2310.1282	2310.1892	0.061	26	366	387	LADLMEQHIEELAALDGADAGK					Mascot
2313.1577	2313.1821	0.0244	11	272	294	IAMGSNEGGGCDGKPVVVVPEIK			Carbamidomethyl (C)[11]		Mascot
2322.053	2322.1763	0.1233	53	43	64	EEDVVAGSPVMPGPFGWSGMTR			Oxidation (M)[11]		Mascot

7	hypothetical protein TRIUR3_03512 [Triticum urartu]	gi|474370952	37722.6	6.09	10	48	23.152	7.054

Peptide Information

Calc. Mass	Obsrv. Mass	± da	± ppm	Start Seq.	End Seq.	Sequence	Ion Score	C. I. %	Modification	Rank	Result Type
834.4138	834.3316	-0.0822	-99	1	7	MVVGQER			Oxidation (M)[1]		Mascot
988.517	988.4916	-0.0254	-26	19	26	DQKVQTNR					Mascot
1201.5491	1201.5886	0.0395	33	301	311	MRNTADGHGAR			Oxidation (M)[1]		Mascot
1203.6633	1203.5775	-0.0858	-71	173	182	VVSYKAHFPR					Mascot
1323.6633	1323.6796	0.0163	12	118	126	YVWHTLYWR					Mascot
1838.892	1838.9313	0.0393	21	157	171	HNEDHLLEDLAYEIK					Mascot
1886.8372	1886.7604	-0.0768	-41	132	147	NSKEALDAMMNYNPTR			Oxidation (M)[9,10]		Mascot
1966.9868	1966.9479	-0.0389	-20	157	172	HNEDHLLEDLAYEIKK					Mascot
1996.9467	1997.0073	0.0606	30	135	151	EALDAMMNYNPTREVVK			Oxidation (M)[6]		Mascot
2012.9417	2013.0465	0.1048	52	135	151	EALDAMMNYNPTREVVK			Oxidation (M)[6,7]		Mascot
2012.9417	2013.0465	0.1048	52	135	151	EALDAMMNYNPTREVVK	3	0	Oxidation (M)[6,7]		Mascot
2021.1073	2021.0497	-0.0576	-28	1	18	MVVGQERGPLVPVRPTNR			Oxidation (M)[1]		Mascot

8	hypothetical protein TRIUR3_34539 [Triticum urartu]	gi|474392058	19949.9	7.74	8	43	0	2.692

Peptide Information

Calc. Mass	Obsrv. Mass	± da	± ppm	Start Seq.	End Seq.	Sequence	Ion Score	C. I. %	Modification	Rank	Result Type
816.4396	816.4059	-0.0337	-41	16	23	LPAMSAAR					Mascot
864.4396	864.4284	-0.0112	-13	157	163	TSWALMR					Mascot
896.4402	896.4395	-0.0007	-1	1	8	MAMAMLTK					Mascot
928.43	928.4812	0.0512	55	1	8	MAMAMLTK			Oxidation (M)[1,3]		Mascot
944.4249	944.4505	0.0256	27	1	8	MAMAMLTK			Oxidation (M)[1,3,5]		Mascot
988.5356	988.4916	-0.044	-45	15	23	RLPAMSAAR			Oxidation (M)[5]		Mascot
1068.599	1068.526	-0.073	-68	111	118	WTILPWPR					Mascot
1127.5514	1127.5261	-0.0253	-22	148	156	HLMELESPR			Oxidation (M)[3]		Mascot
1284.6014	1284.5867	-0.0147	-11	24	33	SWPAHNTCRR			Carbamidomethyl (C)[8]		Mascot
1956.9783	1956.9739	-0.0044	-2	148	163	HLMELESPRTSWALMR					Mascot

9	Putative U-box domain-containing protein 42 [Triticum urartu]	gi|474186070	109352.1	6.13	17	42	0	8.837

Peptide Information

Calc. Mass	Obsrv. Mass	± da	± ppm	Start Seq.	End Seq.	Sequence	Ion Score	C. I. %	Modification	Rank	Result Type

870.468	870.553	0.085	98	563	570	EGHVITSK					Mascot
925.485	925.4399	-0.0451	-49	84	91	NIGHELSR					Mascot
1027.4626	1027.4832	0.0206	20	147	155	ALHDGDMPR			Oxidation (M)[7]		Mascot
1033.579	1033.5269	-0.0521	-50	92	101	IPASAFGSKR					Mascot
1106.5663	1106.5249	-0.0414	-37	254	262	STIAEWIMR					Mascot
1108.6949	1108.6591	-0.0358	-32	659	668	VPGQLGRLIR					Mascot
1127.6354	1127.5261	-0.1093	-97	656	665	LCKVPGQLGR			Carbamidomethyl (C)[2]		Mascot
1145.5546	1145.5854	0.0308	27	910	920	NDEQGSGAVLR					Mascot
1203.5609	1203.5775	0.0166	14	483	493	MVHSGNASICK			Carbamidomethyl (C)[10]		Mascot
1301.6556	1301.6255	-0.0301	-23	910	921	NDEQGSGAVLRR					Mascot
1501.6998	1501.8094	0.1096	73	714	726	IEEMQHGGMRASR					Mascot
1522.644	1522.6498	0.0058	4	976	988	MPDYSATYMSVEL			Oxidation (M)[1]		Mascot
1693.8326	1693.7076	-0.125	-74	254	267	STIAEWIMRNEATR			Oxidation (M)[8]		Mascot
1951.1045	1950.9423	-0.1622	-83	280	296	TEAMILEAIHELKLLAR					Mascot
1956.9021	1956.9739	0.0718	37	921	936	RCFWALESFLENGGDR			Carbamidomethyl (C)[2]		Mascot
1967.0994	1966.9479	-0.1515	-77	280	296	TEAMILEAIHELKLLAR			Oxidation (M)[4]		Mascot
2110.0962	2109.9753	-0.1209	-57	551	570	SGMNPEAIVVNKEGHVIT SK					Mascot
2110.0962	2109.9753	-0.1209	-57	551	570	SGMNPEAIVVNKEGHVIT SK					Mascot
2226.2163	2226.1792	-0.0371	-17	273	292	TALSLARTEAMILEAIHEL K			Oxidation (M)[11]		Mascot
2226.2163	2226.1792	-0.0371	-17	273	292	TALSLARTEAMILEAIHEL K			Oxidation (M)[11]		Mascot

10 hypothetical protein TRIUR3_25993 [Triticum urartu] gi|474447249 12715.7 7.01 6 41 0 5.629

Peptide Information

Calc. Mass	Obsrv. Mass	± da	± ppm	Start Seq.	End Seq.	Sequence	Ion Score	C. I. %	Modification	Rank	Result Type
828.4111	828.4328	0.0217	26	25	30	KDSWHR					Mascot
1108.5933	1108.6591	0.0658	59	16	25	VAAFCGSLRK			Carbamidomethyl (C)[5]		Mascot
1406.691	1406.6732	-0.0178	-13	77	89	FDGEGNLIDAVTR					Mascot
1592.8313	1592.7451	-0.0862	-54	1	15	MDSVTVSTAAKPTLR			Oxidation (M)[1]		Mascot
1592.8313	1592.7451	-0.0862	-54	1	15	MDSVTVSTAAKPTLR			Oxidation (M)[1]		Mascot
1860.0967	1859.9205	-0.1762	-95	95	110	VLLSLHAFALRLQHNK					Mascot
2013.0433	2013.0465	0.0032	2	31	48	GLIRAAEELCEESIPGLR			Carbamidomethyl (C)[10]		Mascot
2013.0433	2013.0465	0.0032	2	31	48	GLIRAAEELCEESIPGLR			Carbamidomethyl (C)[10]		Mascot

Gel Idx/Pos	190/H17	Instr./Gel Origin	BA2151/Sample Project 20140814	Process Status	Analysis Succeeded
Plate [#] Name	[1] Sample Project 20140814	Instrument Sample Name		Spectra	11

Rank	Protein Name	Accession No.	Protein MW	Protein PI	Pep. Count	Protein Score	Protein Score C. I. %	Intensity Matched	Total Ion Score	Total Ion C. I. %	Confirmed
1	serpin-N3.2 [Triticum aestivum]	gi|379060943	43026.4	5.18	5	105	100	8.133	93	100	

Protein Group

RecName: Full=Serpin-Z2B; AltName: Full=TriaeZ2b; AltName: Full=WSZ2b; AltName: Full=WZS3	gi|75279909	43011.4	5.1799 998283 3862
serpin [Triticum aestivum]	gi|1885346	43011.4	5.1799 998283 3862

Peptide Information

Calc. Mass	Obsrv. Mass	± da	± ppm	Start Seq.	End Seq.	Sequence	Ion Score	C. I. %	Modification	Rank	Result Type
925.5214	925.5336	0.0122	13	11	18	LSIAHQTR					Mascot
925.5214	925.5336	0.0122	13	11	18	LSIAHQTR					Mascot
1005.5363	1005.5119	-0.0244	-24	230	238	LPYKQGGDK					Mascot
1223.5903	1223.6508	0.0605	49	127	137	AEAQSVDFQTK					Mascot
1258.7253	1258.7123	-0.013	-10	289	300	ISLGIEASDLLK					Mascot
1665.8595	1665.8937	0.0342	21	261	274	LSAEPEFLEQHIPR					Mascot
1665.8595	1665.8937	0.0342	21	261	274	LSAEPEFLEQHIPR	93	100			Mascot

Rank	Protein Name	Accession No.	Protein MW	Protein PI	Pep. Count	Protein Score	Protein Score C. I. %	Intensity Matched	Total Ion Score	Total Ion C. I. %	Confirmed
2	Serpin-Z2B [Triticum urartu]	gi|473793747	45225.7	6.03	4	102	100	7.758	93	100	

Peptide Information

Calc. Mass	Obsrv. Mass	± da	± ppm	Start Seq.	End Seq.	Sequence	Ion Score	C. I. %	Modification	Rank	Result Type
925.5214	925.5336	0.0122	13	11	18	LSIAHQTR					Mascot
925.5214	925.5336	0.0122	13	11	18	LSIAHQTR					Mascot
1005.5363	1005.5119	-0.0244	-24	247	255	LPYKQGGDK					Mascot
1223.5903	1223.6508	0.0605	49	127	137	AEAQSVDFQTK					Mascot
1665.8595	1665.8937	0.0342	21	278	291	LSAEPEFLEQHIPR					Mascot
1665.8595	1665.8937	0.0342	21	278	291	LSAEPEFLEQHIPR	93	100			Mascot

Rank	Protein Name	Accession No.	Protein MW	Protein PI	Pep. Count	Protein Score	Protein Score C. I. %	Intensity Matched	Total Ion Score	Total Ion C. I. %	Confirmed
3	4-hydroxy-3-methylbut-2-en-1-yl diphosphate synthase [Triticum urartu]	gi|474125335	73992.9	5.71	17	56	90.325	12.169	5	0	

Peptide Information

Calc. Mass	Obsrv. Mass	± da	± ppm	Start Seq.	End Seq.	Sequence	Ion Score	C. I. %	Modification	Rank	Result Type
807.4611	807.4244	-0.0367	-45	626	632	IDLYVGK					Mascot
870.5043	870.5579	0.0536	62	347	354	SGQLPLQK					Mascot
897.4676	897.4619	-0.0057	-6	168	174	ELEHIEK					Mascot
972.4309	972.4764	0.0455	47	658	665	WVDPPTEE					Mascot
989.4799	989.5588	0.0789	80	143	151	VNPGNFADR					Mascot
989.4799	989.5588	0.0789	80	143	151	VNPGNFADR					Mascot
1026.6055	1026.5063	-0.0992	-97	346	354	RSGQLPLQK					Mascot
1145.5811	1145.5726	-0.0085	-7	143	152	VNPGNFADRR					Mascot
1193.5369	1193.6523	0.1154	97	32	40	YCESIHQTR			Carbamidomethyl (C)[2]		Mascot
1258.6652	1258.7123	0.0471	37	141	151	IRVNPGNFADR					Mascot
1349.6379	1349.7034	0.0655	49	32	41	YCESIHQTRR			Carbamidomethyl (C)[2]		Mascot
1507.7673	1507.7797	0.0124	8	368	381	DGSVLMSVSLDQLK			Oxidation (M)[6]		Mascot
1665.8741	1665.8937	0.0196	12	46	61	TVMVGNVALGSDHPIR	6	0			Mascot
1665.8927	1665.8937	0.001	1	1	16	MATGMAPAPLSHVKVR					Mascot
1673.9142	1673.7948	-0.1194	-71	638	653	GIAMEGATEALIQLIK			Oxidation (M)[4]		Mascot
1818.963	1818.9581	-0.0049	-3	282	299	SAIGIGTLLMDGLGDTIR			Oxidation (M)[10]		Mascot
1926.9226	1926.903	-0.0196	-10	300	315	VSLTEPPEEEIDPCRR			Carbamidomethyl (C)[14]		Mascot
2334.2373	2334.1328	-0.1045	-45	368	388	DGSVLMSVSLDQLKAPE LLYR					Mascot

4 hypothetical protein TRIUR3_30168 [Triticum urartu] gi|473888425 75325.4 5.21 24 54 83.944 9.678

Peptide Information

Calc. Mass	Obsrv. Mass	± da	± ppm	Start Seq.	End Seq.	Sequence	Ion Score	C. I. %	Modification	Rank	Result Type
831.4359	831.4246	-0.0113	-14	53	58	VWELER					Mascot
832.3795	832.3348	-0.0447	-54	533	540	EAAEEAGR					Mascot
836.4148	836.4257	0.0109	13	375	381	GNLEEFK					Mascot
844.5138	844.5013	-0.0125	-15	549	555	LKDDLLK					Mascot
846.4567	846.4658	0.0091	11	451	457	VEEIQTK					Mascot
848.5101	848.4423	-0.0678	-80	2	8	AVIAYRR					Mascot
872.52	872.4808	-0.0392	-45	443	449	DLEVLKR					Mascot
905.4509	905.5015	0.0506	56	295	301	EMDNLKR					Mascot
943.5458	943.4969	-0.0489	-52	155	162	LEEIQAIK					Mascot
974.5516	974.515	-0.0366	-38	451	458	VEEIQTKK					Mascot

1013.5989	1013.5285	-0.0704	-69	269	278	QVVGALVEAK					Mascot
1015.5782	1015.5372	-0.041	-40	382	390	VNKDLLEGK					Mascot
1021.5173	1021.5103	-0.007	-7	8	16	RAGYDVGQR					Mascot
1088.5695	1088.587	0.0175	16	112	120	SEAEVERLR					Mascot
1117.61	1117.6404	0.0304	27	70	78	LEKQLEETK					Mascot
1126.5813	1126.5544	-0.0269	-24	514	522	FMKEVESLK			Oxidation (M)[2]		Mascot
1189.6609	1189.6564	-0.0045	-4	60	69	ITQLICEKGK			Carbamidomethyl (C)[6]		Mascot
1193.5468	1193.6523	0.1055	88	577	586	LNDEMNSVQK			Oxidation (M)[5]		Mascot
1258.6288	1258.7123	0.0835	66	9	20	AGYDVGQRHGAK					Mascot
1263.6077	1263.6604	0.0527	42	136	145	EFNDERANIR					Mascot
1308.6794	1308.699	0.0196	15	246	256	LRSEVFTAEEK					Mascot
1349.6803	1349.7034	0.0231	17	290	300	VEIMKEMDNLK					Mascot
1455.6971	1455.7954	0.0983	68	653	665	CSILSFLSPCGSK			Carbamidomethyl (C)[1,10]		Mascot
1739.8558	1739.9204	0.0646	37	525	540	IEEIHASKEAAEEAGR					Mascot

5 hypothetical protein TRIUR3_29935 [Triticum urartu] gi|473990946 76571.9 4.97 19 54 83.57 5.407

Peptide Information

Calc. Mass	Obsrv. Mass	± da	± ppm	Start Seq.	End Seq.	Sequence	Ion Score	C. I. %	Modification	Rank	Result Type
806.389	806.4419	0.0529	66	288	294	SDAEEKK					Mascot
897.4498	897.4619	0.0121	13	615	621	GEMLYLR			Oxidation (M)[3]		Mascot
905.4574	905.5015	0.0441	49	315	322	DSELLSNK					Mascot
922.4993	922.4858	-0.0135	-15	16	24	AGPPQPEVK					Mascot
1003.5306	1003.5573	0.0267	27	606	614	LEEELATAK					Mascot
1009.5564	1009.5217	-0.0347	-34	476	483	ELLDTYKK					Mascot
1015.4955	1015.5372	0.0417	41	183	190	HAFEENLR					Mascot
1033.491	1033.5393	0.0483	47	511	519	QSQVESEAR					Mascot
1171.6681	1171.5927	-0.0754	-64	115	124	LLDEQVAQKK					Mascot
1184.6093	1184.5907	-0.0186	-16	581	590	NMVDAQHLIK			Oxidation (M)[2]		Mascot
1189.592	1189.6564	0.0644	54	511	520	QSQVESEARR					Mascot
1205.6195	1205.6394	0.0199	17	359	368	TMLLNEVETR					Mascot
1258.6174	1258.7123	0.0949	75	183	192	HAFEENLRDK					Mascot
1302.7263	1302.686	-0.0403	-31	562	573	EVLSAALSEQKK					Mascot
1304.6879	1304.691	0.0031	2	146	156	TVKNLNEEVMK					Mascot
1318.6671	1318.6818	0.0147	11	97	108	LAEDEAAMSLLR					Mascot
1340.7103	1340.6893	-0.021	-16	581	591	NMVDAQHLIKR			Oxidation (M)[2]		Mascot
1349.7094	1349.7034	-0.006	-4	359	369	TMLLNEVETRK			Oxidation (M)[2]		Mascot

1738.7258	1738.8406	0.1148	66	2	15	DIQMSEEMQPEETR			Oxidation (M)[4]		Mascot

6	50S ribosomal protein L17 [Triticum urartu]	gi|473912988	15328	10.23	10	54	82.796	3.795

Peptide Information

Calc. Mass	Obsrv. Mass	± da	± ppm	Start Seq.	End Seq.	Sequence	Ion Score	C. I. %	Modification	Rank	Result Type
806.4631	806.4419	-0.0212	-26	62	68	RASAFVR					Mascot
905.5125	905.5015	-0.011	-12	20	27	TMVSQLVK					Mascot
972.4897	972.4764	-0.0133	-14	117	125	ASQQWAGPK					Mascot
974.5013	974.515	0.0137	14	54	62	DGTLDAARR					Mascot
989.5084	989.5588	0.0504	51	45	53	ADQMVQLGK					Mascot
989.5084	989.5588	0.0504	51	45	53	ADQMVQLGK					Mascot
1005.5034	1005.5119	0.0085	8	45	53	ADQMVQLGK			Oxidation (M)[4]		Mascot
1117.6034	1117.6404	0.037	33	44	53	KADQMVQLGK					Mascot
1193.6321	1193.6523	0.0202	17	10	19	HAAHRVSMLR			Oxidation (M)[8]		Mascot
1400.7281	1400.6951	-0.033	-24	63	75	ASAFVRGDDVVHK					Mascot
1507.8335	1507.7797	-0.0538	-36	15	27	VSMLRTMVSQLVK			Oxidation (M)[3]		Mascot
2444.239	2444.1562	-0.0828	-34	95	116	EAKPATPPPPQCAPLDP WTKSR			Carbamidomethyl (C)[12]		Mascot

7	hypothetical protein TRIUR3_16515 [Triticum urartu]	gi|474399185	127477.6	6.71	22	50	51.512	7.564

Peptide Information

Calc. Mass	Obsrv. Mass	± da	± ppm	Start Seq.	End Seq.	Sequence	Ion Score	C. I. %	Modification	Rank	Result Type
800.4625	800.4326	-0.0299	-37	1101	1108	LAEAALGR					Mascot
802.4166	802.4518	0.0352	44	574	580	DGERLGR					Mascot
807.4247	807.4244	-0.0003	0	960	966	SVPETFK					Mascot
814.4781	814.4571	-0.021	-26	319	326	ILPSSAAR					Mascot
844.4635	844.5013	0.0378	45	217	223	RAALEER					Mascot
847.4342	847.47	0.0358	42	503	509	QVMVDQK					Mascot
888.5049	888.4644	-0.0405	-46	343	349	LPRYSPR					Mascot
897.4386	897.4619	0.0233	26	815	821	FTMEELK					Mascot
925.4699	925.5336	0.0637	69	385	391	EFELLMK			Oxidation (M)[6]		Mascot
925.4699	925.5336	0.0637	69	385	391	EFELLMK			Oxidation (M)[6]		Mascot
974.5629	974.515	-0.0479	-49	1109	1117	IGATKLTDR					Mascot
1003.4803	1003.5573	0.077	77	569	577	DNLGKDGER					Mascot
1016.4829	1016.5201	0.0372	37	720	728	DALHQMASK			Oxidation (M)[6]		Mascot

1036.4979	1036.5175	0.0196	19	994	1002	QVLMSEDSK					Mascot
1041.6163	1041.554	-0.0623	-60	319	328	ILPSSAARAR					Mascot
1065.5245	1065.5699	0.0454	43	527	535	MELALSETR			Oxidation (M)[1]		Mascot
1103.6936	1103.594	-0.0996	-90	1058	1066	QILSRVFLK					Mascot
1107.5463	1107.5663	0.02	18	683	691	AQVRETMEK			Oxidation (M)[7]		Mascot
1223.6453	1223.6508	0.0055	4	812	821	GLRFTMEELK					Mascot
1245.578	1245.6302	0.0522	42	777	786	EEEMKISHDK					Mascot
1323.6804	1323.6802	-0.0002	0	515	526	VWHLGGEAGIER					Mascot
1393.6603	1393.6858	0.0255	18	113	123	IMQFHETLSCK			Carbamidomethyl (C)[10]		Mascot
1605.798	1605.7922	-0.0058	-4	787	800	LLNELIGHSDSHDR					Mascot

8	hypothetical protein TRIUR3_18987 [Triticum urartu]	gi|474353780	59488.2	5.99	14	49	50.383	3.648	

Peptide Information

Calc. Mass	Obsrv. Mass	± da	± ppm	Start Seq.	End Seq.	Sequence	Ion Score	C. I. %	Modification	Rank	Result Type
807.4512	807.4244	-0.0268	-33	192	197	QPLFFR					Mascot
814.5145	814.4571	-0.0574	-70	93	100	LVQAGKAK					Mascot
852.4396	852.4548	0.0152	18	11	17	LCIFSGR			Carbamidomethyl (C)[2]		Mascot
916.5098	916.4991	-0.0107	-12	404	411	LAIDDITR					Mascot
922.488	922.4858	-0.0022	-2	135	142	YEGDLVVK					Mascot
982.4662	982.4998	0.0336	34	430	437	LAFDEMTR					Mascot
1016.5206	1016.5201	-0.0005	0	18	24	RCFHQLR			Carbamidomethyl (C)[2]		Mascot
1119.5582	1119.5428	-0.0154	-14	126	134	IGSWEWVSR					Mascot
1189.6357	1189.6564	0.0207	17	458	467	VNSLCSLIQR			Carbamidomethyl (C)[5]		Mascot
1302.7528	1302.686	-0.0668	-51	114	125	LKASNFPGSILR					Mascot
1475.7563	1475.7739	0.0176	12	163	174	IEIQWSDICALK			Carbamidomethyl (C)[9]		Mascot
1493.8475	1493.7631	-0.0844	-56	148	160	HKLVWEVLDGGLK					Mascot
1605.7448	1605.7922	0.0474	30	225	237	RHFLQCAPGMMNK			Carbamidomethyl (C)[6], Oxidation (M)[10]		Mascot
1926.9136	1926.903	-0.0106	-6	226	241	HFLQCAPGMMNKHVEK			Carbamidomethyl (C)[5]		Mascot

9	Serine/threonine-protein phosphatase 7 long form-like protein [Triticum urartu]	gi|474387690	171909.1	7.55	24	49	41.705	10.251	

Peptide Information

Calc. Mass	Obsrv. Mass	± da	± ppm	Start Seq.	End Seq.	Sequence	Ion Score	C. I. %	Modification	Rank	Result Type
802.4094	802.4518	0.0424	53	861	867	WVPTDGK					Mascot
808.4563	808.439	-0.0173	-21	297	303	LYTGLNK					Mascot

Calc. Mass	Obsrv. Mass	± da	± ppm	Start Seq.	End Seq.	Sequence	Ion Score	C. I. %	Modification	Rank	Result Type
814.4781	814.4571	-0.021	-26	1493	1499	LEGINLR					Mascot
832.406	832.3348	-0.0712	-86	137	142	TNWQQR					Mascot
872.52	872.4808	-0.0392	-45	1486	1492	LQENIKK					Mascot
886.449	886.4823	0.0333	38	510	517	TQDRPGGR					Mascot
888.4574	888.4644	0.007	8	1075	1082	HFAVGTEK					Mascot
905.4323	905.5015	0.0692	76	235	242	SLDDATQR					Mascot
911.4767	911.4898	0.0131	14	659	666	RGSAMFVK			Oxidation (M)[5]		Mascot
989.5448	989.5588	0.014	14	1459	1466	QVDQIKMK					Mascot
989.5448	989.5588	0.014	14	1459	1466	QVDQIKMK					Mascot
1005.5397	1005.5119	-0.0278	-28	1459	1466	QVDQIKMK			Oxidation (M)[7]		Mascot
1109.6174	1109.6252	0.0078	7	675	685	KQHSGGIGGLR					Mascot
1109.6174	1109.6252	0.0078	7	675	685	KQHSGGIGGLR					Mascot
1136.6245	1136.5609	-0.0636	-56	628	637	IQPMPSAHKK					Mascot
1193.5872	1193.6523	0.0651	55	764	773	DGVMTLWDIK			Oxidation (M)[4]		Mascot
1347.709	1347.7191	0.0101	7	1198	1209	LFSLSHAIQGMK			Oxidation (M)[11]		Mascot
1393.6271	1393.6858	0.0587	42	985	996	TATANEAYFYDK					Mascot
1412.7533	1412.6868	-0.0665	-47	493	504	DLLSPDPFQRPK					Mascot
1455.6863	1455.7954	0.1091	75	397	408	NQSIIDWGEEHK					Mascot
1475.804	1475.7739	-0.0301	-20	1198	1210	LFSLSHAIQGMKK			Oxidation (M)[11]		Mascot
1738.9236	1738.8406	-0.083	-48	158	172	TSGVPLTWLSEHRQK					Mascot
1739.8567	1739.9204	0.0637	37	34	47	FIENGEMLQALRMR			Oxidation (M)[7,13]		Mascot
1818.9821	1818.9581	-0.024	-13	2	18	APKSETRPPLADNIGPR					Mascot
1828.7603	1828.8724	0.1121	61	46	60	MRGHGVHGHMDYDER			Oxidation (M)[1,10]		Mascot
1828.9148	1828.8724	-0.0424	-23	426	443	KATQDLIGSSSHAPSSSR					Mascot
1926.9167	1926.903	-0.0137	-7	640	658	GVDPGSPASGFFVGFCL GR			Carbamidomethyl (C)[16]		Mascot

10 Pattern formation protein EMB30 [Triticum urartu] gi|473732124 144008.4 5.68 22 48 36.08 6.584

Peptide Information

Calc. Mass	Obsrv. Mass	± da	± ppm	Start Seq.	End Seq.	Sequence	Ion Score	C. I. %	Modification	Rank	Result Type
808.41	808.439	0.029	36	708	713	YGHYIR					Mascot
824.4005	824.4363	0.0358	43	1179	1184	MEKCIK			Carbamidomethyl (C)[4], Oxidation (M)[1]		Mascot
831.4934	831.4246	-0.0688	-83	807	813	TLQTIQK					Mascot
835.4454	835.4909	0.0455	54	773	780	KSSGLMGR					Mascot
852.476	852.4548	-0.0212	-25	1	7	MPIVQHK					Mascot
925.5142	925.5336	0.0194	21	473	479	LFLETFR					Mascot
925.5142	925.5336	0.0194	21	473	479	LFLETFR					Mascot

943.5723	943.4969	-0.0754	-80	1171	1178	LWLGVLSR		Mascot
1009.52	1009.5217	0.0017	2	816	824	IDGIFTESK		Mascot
1016.5847	1016.5201	-0.0646	-64	1022	1030	VGLTDRSIR		Mascot
1021.5564	1021.5103	-0.0461	-45	31	39	YEDAGIIIK		Mascot
1036.5356	1036.5175	-0.0181	-17	73	80	HVMHELVR	Oxidation (M)[3]	Mascot
1117.7205	1117.6404	-0.0801	-72	726	735	LHKLGLLPAR		Mascot
1140.5605	1140.5725	0.012	11	534	542	KMTEEDFIK		Mascot
1155.6481	1155.6353	-0.0128	-11	480	489	LPGESQKIQR		Mascot
1189.5782	1189.6564	0.0782	66	390	399	LMIGADHFNR	Oxidation (M)[2]	Mascot
1308.6597	1308.699	0.0393	30	708	717	YGHYIRTGWR		Mascot
1318.7366	1318.6818	-0.0548	-42	199	209	VLSLVQDELFR		Mascot
1347.7454	1347.7191	-0.0263	-20	1171	1181	LWLGVLSRMEK	Oxidation (M)[9]	Mascot
1412.6475	1412.6868	0.0393	28	535	545	MTEEDFIKNNR	Oxidation (M)[1]	Mascot
1493.7456	1493.7631	0.0175	12	794	806	SQPTEQQLAAHQR		Mascot
1664.9006	1664.8496	-0.051	-31	473	486	LFLETFRLPGESQK		Mascot
1738.8065	1738.8406	0.0341	20	940	954	VADAYCENITQEVAR	Carbamidomethyl (C)[6]	Mascot

Gel Idx/Pos	191/H18	Instr./Gel Origin	BA2151/Sample Project 20140814	Process Status	Analysis Succeeded
Plate [#] Name	[1] Sample Project 20140814	Instrument Sample Name		Spectra	11

Rank	Protein Name	Accession No.	Protein MW	Protein PI	Pep. Count	Protein Score	Protein Score C. I. %	Intensity Matched	Total Ion Score	Total Ion C. I. %	Confirmed
1	Vicilin-like antimicrobial peptides 2-2 [Triticum urartu]	gi|474404647	43446.1	6.74	4	123	100	8.332	110	100	

Peptide Information

Calc. Mass	Obsrv. Mass	± da	± ppm	Start Seq.	End Seq.	Sequence	Ion Score	C. I. %	Modification	Rank	Result Type
869.386	869.4692	0.0832	96	144	151	EHGQGGER					Mascot
2166.1958	2166.2502	0.0544	25	5	22	TLFVPQYIDSNLILFVQR					Mascot
2166.1958	2166.2502	0.0544	25	5	22	TLFVPQYIDSNLILFVQR	110	100			Mascot
2565.4077	2565.2175	-0.1902	-74	5	26	TLFVPQYIDSNLILFVQRG DVK					Mascot
2931.3982	2931.4702	0.072	25	65	92	LHIICSIDASDSAGFAPYQ SFYLGGGGK			Carbamidomethyl (C)[5]		Mascot

Rank	Protein Name	Accession No.	Protein MW	Protein PI	Pep. Count	Protein Score	Protein Score C. I. %	Intensity Matched	Total Ion Score	Total Ion C. I. %	Confirmed
2	Alanyl-tRNA synthetase [Triticum urartu]	gi|474423501	208125.5	8.47	31	63	97.625	15.39			

Peptide Information

Calc. Mass	Obsrv. Mass	± da	± ppm	Start Seq.	End Seq.	Sequence	Ion Score	C. I. %	Modification	Rank	Result Type
800.4485	800.3987	-0.0498	-62	1716	1724	RGGAGGVAR					Mascot
809.4264	809.4132	-0.0132	-16	634	639	VDYTRR					Mascot
810.3596	810.4244	0.0648	80	1277	1283	MISCAGR			Carbamidomethyl (C)[4], Oxidation (M)[1]		Mascot
813.5053	813.4472	-0.0581	-71	1015	1021	AIANRLR					Mascot
818.3613	818.433	0.0717	88	1328	1333	NSMHWK			Oxidation (M)[3]		Mascot
850.4893	850.4501	-0.0392	-46	1160	1166	GIRVSYR					Mascot
860.4724	860.4551	-0.0173	-20	832	839	LEGATLEK					Mascot
881.4298	881.4735	0.0437	50	1343	1350	EKGGMGFR					Mascot
884.4083	884.4766	0.0683	77	1397	1403	FSSAMWR					Mascot
897.4247	897.459	0.0343	38	1343	1350	EKGGMGFR			Oxidation (M)[5]		Mascot
963.4781	963.4902	0.0121	13	1425	1432	SISIWDDK					Mascot
1026.5844	1026.4967	-0.0877	-85	1753	1761	NGVLFAHLR					Mascot
1033.5459	1033.5256	-0.0203	-20	921	929	EAVVKAMNR			Oxidation (M)[7]		Mascot
1048.584	1048.5896	0.0056	5	1552	1558	VRVFWWR					Mascot
1068.5619	1068.5385	-0.0234	-22	1510	1518	SAYRALMTR					Mascot
1161.6263	1161.6694	0.0431	37	274	285	LAGAGIQPYSGK					Mascot
1210.6613	1210.6152	-0.0461	-38	1104	1113	CVTSIKFSIR			Carbamidomethyl (C)[1]		Mascot

1210.6613	1210.6152	-0.0461	-38	1104	1113	CVTSIKFSIR			Carbamidomethyl (C)[1]		Mascot
1353.6168	1353.746	0.1292	95	380	391	DEEASFENTLAK					Mascot
1353.6168	1353.746	0.1292	95	380	391	DEEASFENTLAK					Mascot
1368.6292	1368.5964	-0.0328	-24	1490	1502	GGGDDVVAWAHER					Mascot
1375.7944	1375.6714	-0.123	-89	703	713	KIEYIVNQQIK					Mascot
1391.7543	1391.7126	-0.0417	-30	1616	1626	RPELHPLTWSR					Mascot
1474.7723	1474.7936	0.0213	14	1433	1445	WIPGTLSMTPTVR			Oxidation (M)[8]		Mascot
1480.7135	1480.8369	0.1234	83	804	817	ITAVTAECASQAMK			Carbamidomethyl (C)[8]		Mascot
1656.9166	1656.8723	-0.0443	-27	887	903	AVKIAIDAAEAALSEGK					Mascot
1748.033	1747.9246	-0.1084	-62	1783	1797	HNVRAIVAPILFEIR					Mascot
1757.8615	1757.9246	0.0631	36	1328	1342	NSMHWKAWMALASPK					Mascot
1959.0599	1959.0446	-0.0153	-8	1798	1813	EHVIYFKSFVIQHVGR					Mascot
2158.2424	2158.0427	-0.1997	-93	1787	1804	AIVAPILFEIREHVIYFK					Mascot
2266.1697	2266.1055	-0.0642	-28	639	657	RTLIAPNHTCTHMLNFAL R			Carbamidomethyl (C)[10]		Mascot
2266.1697	2266.1055	-0.0642	-28	639	657	RTLIAPNHTCTHMLNFAL R			Carbamidomethyl (C)[10]		Mascot
2380.1892	2380.1516	-0.0376	-16	307	328	TLSFAIADGSQPGNEGRE YVLR					Mascot
2380.1892	2380.1516	-0.0376	-16	307	328	TLSFAIADGSQPGNEGRE YVLR					Mascot
2701.4277	2701.3364	-0.0913	-34	1559	1582	VLHGILPAESTLMHRHITT IGTCK			Carbamidomethyl (C)[23], Oxidation (M)[13]		Mascot
2709.4797	2709.2837	-0.196	-72	992	1014	FPQSITQYRPISLCPVLYK IASK			Carbamidomethyl (C)[14]		Mascot

3 hypothetical protein TRIUR3_33234 [Triticum urartu] gi|474386845 15580.5 8.28 8 54 84.667 3.763

Peptide Information

Calc. Mass	Obsrv. Mass	± da	± ppm	Start Seq.	End Seq.	Sequence	Ion Score	C. I. %	Modification	Rank	Result Type
854.3131	854.3173	0.0042	5	1	7	MEACGDR			Carbamidomethyl (C)[4], Oxidation (M)[1]		Mascot
860.52	860.4551	-0.0649	-75	35	43	AVGSVTKAK					Mascot
881.4839	881.4735	-0.0104	-12	8	16	LAAGEPAPR					Mascot
1429.7356	1429.7078	-0.0278	-19	42	55	AKEELIGAPAMDGK					Mascot
1618.7537	1618.791	0.0373	23	87	102	GHCSRASSAAAMTGVR			Carbamidomethyl (C)[3]		Mascot
1634.7487	1634.7892	0.0405	25	87	102	GHCSRASSAAAMTGVR			Carbamidomethyl (C)[3], Oxidation (M)[12]		Mascot
1634.7487	1634.7892	0.0405	25	87	102	GHCSRASSAAAMTGVR			Carbamidomethyl (C)[3], Oxidation (M)[12]		Mascot
2266.0149	2266.1055	0.0906	40	67	86	KDCTSEAEMPEQAPAMI VSR			Carbamidomethyl (C)[3], Oxidation (M)[9]		Mascot
2266.0149	2266.1055	0.0906	40	67	86	KDCTSEAEMPEQAPAMI VSR			Carbamidomethyl (C)[3], Oxidation (M)[9]		Mascot
2512.1919	2512.2041	0.0122	5	44	66	EELIGAPAMDGKCVHGE LQSAQR			Carbamidomethyl (C)[13], Oxidation (M)[9]		Mascot
2717.1533	2717.3303	0.177	65	114	137	EGCPDVTEAWDAEDVGE			Carbamidomethyl (C)[3]		Mascot

EGGWPRR

4	hypothetical protein TRIUR3_13852 [Triticum urartu]	gi|474387534	50487.5	8.69	13	47	11.766	9.894

Peptide Information

Calc. Mass	Obsrv. Mass	± da	± ppm	Start Seq.	End Seq.	Sequence	Ion Score	C. I. %	Modification	Rank	Result Type
813.3811	813.4472	0.0661	81	275	281	YGDSLMK					Mascot
829.376	829.4493	0.0733	88	275	281	YGDSLMK			Oxidation (M)[6]		Mascot
832.4159	832.3384	-0.0775	-93	374	380	TLQEEGR					Mascot
850.5032	850.4501	-0.0531	-62	233	239	QVYSIIK					Mascot
1177.567	1177.6122	0.0452	38	39	48	CEYGVPSLPR			Carbamidomethyl (C)[1]		Mascot
1381.7046	1381.76	0.0554	40	364	373	WRSLFLQMER			Oxidation (M)[8]		Mascot
1391.7166	1391.7126	-0.004	-3	26	38	KDIPQFSAEGATK					Mascot
1480.7867	1480.8369	0.0502	34	374	385	TLQEEGRHLEIR					Mascot
1741.8433	1741.9788	0.1355	78	116	131	VGSSDAAELNPFFPYK					Mascot
1757.8599	1757.9246	0.0647	37	100	115	GENRMSDAPLGLDLNR					Mascot
1958.9714	1959.0446	0.0732	37	402	418	QVTCEAPLLGPMAELWK			Carbamidomethyl (C)[4], Oxidation (M)[12]		Mascot
2158.0371	2158.0427	0.0056	3	244	264	SEELENASQPGLAGQTG ERGK					Mascot
2184.1555	2184.0283	-0.1272	-58	402	420	QVTCEAPLLGPMAELWK LK			Carbamidomethyl (C)[4]		Mascot
2422.1707	2422.1443	-0.0264	-11	27	48	DIPQFSAEGATKCEYGVP SLPR			Carbamidomethyl (C)[13]		Mascot

5	hypothetical protein TRIUR3_27519 [Triticum urartu]	gi|474390988	71745.7	5.57	15	46	0	4.711

Peptide Information

Calc. Mass	Obsrv. Mass	± da	± ppm	Start Seq.	End Seq.	Sequence	Ion Score	C. I. %	Modification	Rank	Result Type
807.4359	807.4217	-0.0142	-18	557	565	GGFSIGAAK					Mascot
829.4315	829.4493	0.0178	21	150	156	GVDFVHR					Mascot
834.4105	834.343	-0.0675	-81	540	546	TYGTHQK					Mascot
838.369	838.4225	0.0535	64	613	619	DEGQSFR					Mascot
860.4584	860.4551	-0.0033	-4	599	605	VNNETKR					Mascot
876.4057	876.3741	-0.0316	-36	249	256	VEDNQGSK					Mascot
897.4498	897.459	0.0092	10	312	319	SSMLNAFK					Mascot
921.5516	921.538	-0.0136	-15	214	221	SYLAKALR					Mascot
963.537	963.4902	-0.0468	-49	557	566	GGFSIGAAKR					Mascot
1132.6222	1132.5575	-0.0647	-57	627	636	QRIGVFNNGK					Mascot
1177.6576	1177.6122	-0.0454	-39	554	565	LEKGGFSIGAAK					Mascot
1556.8027	1556.7747	-0.028	-18	219	233	ALRDLEVESGGSAPR					Mascot

Calc. Mass	Obsrv. Mass	± da	± ppm	Start Seq.	End Seq.	Sequence	Ion Score	C. I. %	Modification	Rank	Result Type
1888.0586	1887.8765	-0.1821	-96	191	206	QPLCASRPDHIVIILR			Carbamidomethyl (C)[4]		Mascot
1986.9226	1986.8711	-0.0515	-26	1	18	MPFDRYSALDSVGGSQEK					Mascot
2002.9175	2002.9275	0.01	5	1	18	MPFDRYSALDSVGGSQEK			Oxidation (M)[1]		Mascot
2266.0876	2266.1055	0.0179	8	288	306	VCNACIENEIVSEISIKCK			Carbamidomethyl (C)[2,5,18]		Mascot
2266.0876	2266.1055	0.0179	8	288	306	VCNACIENEIVSEISIKCK			Carbamidomethyl (C)[2,5,18]		Mascot

6	Cysteine-rich receptor-like protein kinase 26 [Triticum urartu]	gi|474325169	21669.1	7.64	7	42	0	6.826	14	0		

Peptide Information

Calc. Mass	Obsrv. Mass	± da	± ppm	Start Seq.	End Seq.	Sequence	Ion Score	C. I. %	Modification	Rank	Result Type
875.437	875.4569	0.0199	23	62	68	GLEWNTR					Mascot
909.4676	909.446	-0.0216	-24	55	61	YITDELR					Mascot
1353.6943	1353.746	0.0517	38	1	11	MLNIEHENVVR					Mascot
1353.6943	1353.746	0.0517	38	1	11	MLNIEHENVVR	14	0			Mascot
1556.8431	1556.7747	-0.0684	-44	26	38	LEGLKEHIYAEVR					Mascot
1741.8765	1741.9788	0.1023	59	157	171	LILMMVSSHGSFFTR			Oxidation (M)[4]		Mascot
1757.8713	1757.9246	0.0533	30	157	171	LILMMVSSHGSFFTR			Oxidation (M)[4,5]		Mascot
2266.0884	2266.1055	0.0171	8	105	124	LGTLGYCAPEYLSQGKMSFK			Carbamidomethyl (C)[7], Oxidation (M)[17]		Mascot
2266.0884	2266.1055	0.0171	8	105	124	LGTLGYCAPEYLSQGKMSFK			Carbamidomethyl (C)[7], Oxidation (M)[17]		Mascot
2474.2922	2474.2229	-0.0693	-28	121	142	MSFKTDMYSLGIIIVELVTGEK					Mascot
2506.282	2506.1956	-0.0864	-34	121	142	MSFKTDMYSLGIIIVELVTGEK			Oxidation (M)[1,7]		Mascot

7	Methylglutaconyl-CoA hydratase, mitochondrial [Triticum urartu]	gi|474367514	71275.7	7.18	14	42	0	8.672	

Peptide Information

Calc. Mass	Obsrv. Mass	± da	± ppm	Start Seq.	End Seq.	Sequence	Ion Score	C. I. %	Modification	Rank	Result Type
818.4254	818.433	0.0076	9	66	73	IEADATAK					Mascot
870.4791	870.5201	0.041	47	434	440	LERPEAR					Mascot
878.473	878.4639	-0.0091	-10	480	486	EFVNTLR					Mascot
893.4509	893.438	-0.0129	-14	450	457	GLRSAMDK			Oxidation (M)[6]		Mascot
904.4557	904.4666	0.0109	12	472	479	LMGPSEVR			Oxidation (M)[2]		Mascot
909.4901	909.446	-0.0441	-48	336	343	HQSPTLAR					Mascot
928.5397	928.4925	-0.0472	-51	212	220	GPLGVRMAK					Mascot
1048.5674	1048.5896	0.0222	21	642	651	LEGLAAFAEK					Mascot

1068.5394	1068.5385	-0.0009	-1	160	169	VPISFSSGMK			Oxidation (M)[9]		Mascot
1161.6296	1161.6694	0.0398	34	201	210	ALELAREMTK					Mascot
1177.6245	1177.6122	-0.0123	-10	201	210	ALELAREMTK			Oxidation (M)[8]		Mascot
1353.7598	1353.746	-0.0138	-10	434	445	LERPEARNAIGK					Mascot
1353.7598	1353.746	-0.0138	-10	434	445	LERPEARNAIGK					Mascot
1747.916	1747.9246	0.0086	5	472	486	LMGPSEVREFVNTLR					Mascot
2174.1638	2174.0112	-0.1526	-70	74	94	VVLVASSVPGVFCAGAD LKER			Carbamidomethyl (C)[13]		Mascot
2219.0398	2219.2107	0.1709	77	234	252	SALAVEGECYEQLLHTQ DR			Carbamidomethyl (C)[9]		Mascot

8 Disease resistance protein RPP8 [Triticum urartu] gi|474240937 90551.8 8.12 15 41 0 4.739

Peptide Information

Calc. Mass	Obsrv. Mass	± da	± ppm	Start Seq.	End Seq.	Sequence	Ion Score	C. I. %	Modification	Rank	Result Type
806.3679	806.4398	0.0719	89	784	790	SFESHLS					Mascot
876.4421	876.3741	-0.068	-78	125	131	VEEESKR					Mascot
878.4036	878.4639	0.0603	69	161	168	DLVGMDGR			Oxidation (M)[5]		Mascot
906.5407	906.4791	-0.0616	-68	105	111	IKTIFER					Mascot
1047.5867	1047.5476	-0.0391	-37	635	644	GMASLTNILK					Mascot
1048.5786	1048.5896	0.011	10	34	41	KDIEFLQR					Mascot
1132.578	1132.5575	-0.0205	-18	158	168	AAKDLVGMDGR					Mascot
1429.8049	1429.7078	-0.0971	-68	427	438	EILPLVDFQSLR					Mascot
1536.7992	1536.6725	-0.1267	-82	700	714	FGAIQFAAGAMPALR			Oxidation (M)[11]		Mascot
1536.7992	1536.6725	-0.1267	-82	700	714	FGAIQFAAGAMPALR			Oxidation (M)[11]		Mascot
1906.077	1905.9642	-0.1128	-59	767	783	AVALHPNLHTLQLHVSR					Mascot
2219.0908	2219.2107	0.1199	54	658	675	NILMGMPYLTYLQLDVMR			Oxidation (M)[4,6,17]		Mascot
2512.1946	2512.2041	0.0095	4	46	67	MQTLVSTLADMEGLDEV AKDWK			Oxidation (M)[1,11]		Mascot
2594.4011	2594.2253	-0.1758	-68	1	27	MAGVGVGTGAFGSVLGK LATLLGNEFK					Mascot
2687.2739	2687.3103	0.0364	14	197	221	TTLAMEAFGMIGGQFGC KAHVSVSR			Carbamidomethyl (C)[17], Oxidation (M)[5,10]		Mascot
2717.2764	2717.3303	0.0539	20	676	699	SAPDVMNVSSEGFNLLE VFDYEVR					Mascot

9 hypothetical protein TRIUR3_05231 [Triticum urartu] gi|473786240 11213.6 4.85 6 40 0 2.221

Peptide Information

Calc. Mass	Obsrv. Mass	± da	± ppm	Start Seq.	End Seq.	Sequence	Ion Score	C. I. %	Modification	Rank	Result Type
807.4029	807.4217	0.0188	23	72	78	VVSAEMR			Oxidation (M)[6]		Mascot
928.521	928.4925	-0.0285	-31	58	66	LPADKGATR					Mascot

1033.5459	1033.5256	-0.0203	-20	72	80	VVSAEMRNK					Mascot
1391.6583	1391.7126	0.0543	39	67	78	EDAERVVSAEMR					Mascot
1536.6595	1536.6725	0.013	8	42	55	DAAMLQSAENEDAR			Oxidation (M)[4]		Mascot
1536.6595	1536.6725	0.013	8	42	55	DAAMLQSAENEDAR			Oxidation (M)[4]		Mascot
2581.3582	2581.2151	-0.1431	-55	81	105	LDLTTTPGGVAEAVTTAA RLNQERP					Mascot

10	Disease resistance protein RGA2 [Triticum urartu]	gi|474345500	205228.7	6.62	15	39	0	8.791	27	89.278

Peptide Information

Calc. Mass	Obsrv. Mass	± da	± ppm	Start Seq.	End Seq.	Sequence	Ion Score	C. I. %	Modification	Rank	Result Type
818.4916	818.433	-0.0586	-72	1040	1046	LARMSIK					Mascot
821.4628	821.4166	-0.0462	-56	1125	1130	RVYIDR					Mascot
824.4638	824.429	-0.0348	-42	425	430	HWRAVR					Mascot
860.4584	860.4551	-0.0033	-4	498	504	LEETGRR					Mascot
870.5519	870.5201	-0.0318	-37	300	306	NLLKNIR					Mascot
875.4621	875.4569	-0.0052	-6	1777	1783	LPENFQK					Mascot
1177.5995	1177.6122	0.0127	11	177	187	GLCTIGSSVQR			Carbamidomethyl (C)[3]		Mascot
1353.7195	1353.746	0.0265	20	960	970	ELELMDLHVVR					Mascot
1353.7195	1353.746	0.0265	20	960	970	ELELMDLHVVR	27	89.278			Mascot
1368.639	1368.5964	-0.0426	-31	1786	1797	SQTTSWDSTSRI					Mascot
1568.8254	1568.8074	-0.018	-11	509	521	LVDLCFFQKVGSR			Carbamidomethyl (C)[5]		Mascot
1905.9222	1905.9642	0.042	22	148	164	REEEGPAQSTMLTSEIK					Mascot
1958.9575	1959.0446	0.0871	44	518	534	VGSRYVMHDLMHELAGK			Oxidation (M)[7]		Mascot
2146.1655	2146.0002	-0.1653	-77	1137	1154	WLSQLLSHSPPLEELQLR					Mascot
2183.9326	2184.0283	0.0957	44	77	93	ELLYDSEDVMDELDYYR			Oxidation (M)[10]		Mascot
2266.1714	2266.1055	-0.0659	-29	1757	1775	AKELHPEFIIDEELIGEQR					Mascot
2266.1714	2266.1055	-0.0659	-29	1757	1775	AKELHPEFIIDEELIGEQR					Mascot

Gel Idx/Pos	192/H19	Instr./Gel Origin	BA2151/Sample Project 20140814	Process Status	Analysis Succeeded
Plate [#] Name	[1] Sample Project 20140814	Instrument Sample Name		Spectra	11

Rank	Protein Name	Accession No.	Protein MW	Protein PI	Pep. Count	Protein Score	Protein Score C. I. %	Intensity Matched	Total Ion Score	Total Ion C. I. %	Confirmed
1	unnamed protein product [Triticum aestivum]	gi|300586307	27013.9	5.38	12	386	100	39.275	306	100	

Protein Group

Protein Name	Accession No.	Protein MW	Protein PI
triosephosphat-isomerase [Triticum aestivum]	gi|11124572	27013.9	5.3800001144092
unnamed protein product [Triticum aestivum]	gi|300567607	27013.9	5.3800001144092
unnamed protein product [Triticum aestivum]	gi|259703023	27013.9	5.3800001144092
unnamed protein product [Triticum aestivum]	gi|300659112	27013.9	5.3800001144092
unnamed protein product [Triticum aestivum]	gi|259656593	27013.9	5.3800001144092
unnamed protein product [Triticum aestivum]	gi|259654835	27013.9	5.3800001144092

Peptide Information

Calc. Mass	Obsrv. Mass	± da	± ppm	Start Seq.	End Seq.	Sequence	Ion Score	C. I. %	Modification	Rank	Result Type
954.4832	954.5059	0.0227	24	5	12	FFVGGNWK					Mascot
954.4832	954.5059	0.0227	24	5	12	FFVGGNWK	63	99.997			Mascot
1033.6041	1033.6255	0.0214	21	114	123	VAYALAQGLK					Mascot
1289.6332	1289.6741	0.0409	32	195	206	TNVSPEVAESTR					Mascot
1289.6332	1289.6741	0.0409	32	195	206	TNVSPEVAESTR	53	99.966			Mascot
1312.6566	1312.6898	0.0332	25	207	219	IIYGGSVTGASCK			Carbamidomethyl (C)[12]		Mascot

Calc. Mass	Obsrv. Mass	± da	± ppm	Start Seq.	End Seq.	Sequence	Ion Score	C. I. %	Modification	Rank	Result Type
1374.7046	1374.7457	0.0411	30	124	135	VIACVGETLEQR			Carbamidomethyl (C)[4]		Mascot
1374.7046	1374.7457	0.0411	30	124	135	VIACVGETLEQR	94	100	Carbamidomethyl (C)[4]		Mascot
1415.6359	1415.6653	0.0294	21	101	113	SLMGESSEFVGEK			Oxidation (M)[3]		Mascot
1571.7371	1571.7865	0.0494	31	100	113	RSLMGESSEFVGEK			Oxidation (M)[4]		Mascot
1811.9585	1812.0146	0.0561	31	56	70	LRPEIQVAAQNCWVK			Carbamidomethyl (C)[12]		Mascot
1811.9585	1812.0146	0.0561	31	56	70	LRPEIQVAAQNCWVK	95	100	Carbamidomethyl (C)[12]		Mascot
1997.075	1997.0447	-0.0303	-15	54	70	GKLRPEIQVAAQNCWVK			Carbamidomethyl (C)[14]		Mascot
1997.075	1997.0447	-0.0303	-15	54	70	GKLRPEIQVAAQNCWVK			Carbamidomethyl (C)[14]		Mascot
2346.2493	2346.2766	0.0273	12	155	175	IKDWTNVVVAYEPVWAIG TGK					Mascot
2925.4929	2925.5884	0.0955	33	72	99	GGAFTGEVSAEMLVNLG VPWVILGHSER					Mascot
2941.4878	2941.5771	0.0893	30	72	99	GGAFTGEVSAEMLVNLG VPWVILGHSER			Oxidation (M)[12]		Mascot
3053.5876	3053.6904	0.1028	34	71	99	KGGAFTGEVSAEMLVNL GVPWVILGHSER					Mascot
3069.5828	3069.6748	0.092	30	71	99	KGGAFTGEVSAEMLVNL GVPWVILGHSER			Oxidation (M)[13]		Mascot

2 Triosephosphate isomerase, cytosolic [Triticum urartu] gi|474302100 17829.2 5.71 6 251 100 28.905 212 100

Peptide Information

Calc. Mass	Obsrv. Mass	± da	± ppm	Start Seq.	End Seq.	Sequence	Ion Score	C. I. %	Modification	Rank	Result Type
954.4832	954.5059	0.0227	24	5	12	FFVGGNWK					Mascot
954.4832	954.5059	0.0227	24	5	12	FFVGGNWK	63	99.997			Mascot
1289.6332	1289.6741	0.0409	32	119	130	TNVSPEVAESTR					Mascot
1289.6332	1289.6741	0.0409	32	119	130	TNVSPEVAESTR	53	99.966			Mascot
1811.9585	1812.0146	0.0561	31	56	70	LRPEIQVAAQNCWVK			Carbamidomethyl (C)[12]		Mascot
1811.9585	1812.0146	0.0561	31	56	70	LRPEIQVAAQNCWVK	95	100	Carbamidomethyl (C)[12]		Mascot
1997.075	1997.0447	-0.0303	-15	54	70	GKLRPEIQVAAQNCWVK			Carbamidomethyl (C)[14]		Mascot
1997.075	1997.0447	-0.0303	-15	54	70	GKLRPEIQVAAQNCWVK			Carbamidomethyl (C)[14]		Mascot
2925.4929	2925.5884	0.0955	33	72	99	GGAFTGEVSAEMLVNLG VPWVILGHSER					Mascot
2941.4878	2941.5771	0.0893	30	72	99	GGAFTGEVSAEMLVNLG VPWVILGHSER			Oxidation (M)[12]		Mascot
3053.5876	3053.6904	0.1028	34	71	99	KGGAFTGEVSAEMLVNL GVPWVILGHSER					Mascot
3069.5828	3069.6748	0.092	30	71	99	KGGAFTGEVSAEMLVNL GVPWVILGHSER			Oxidation (M)[13]		Mascot

3 unnamed protein product [Triticum aestivum] gi|259662377 26526.7 5.2 3 70 99.606 2.442 63 99.997

Protein Group

unnamed protein product [Triticum aestivum] gi|300568722 26526.7 5.1999 998092 6514

Peptide Information

Calc. Mass	Obsrv. Mass	± da	± ppm	Start Seq.	End Seq.	Sequence	Ion Score	C. I. %	Modification	Rank	Result Type
954.4832	954.5059	0.0227	24	6	13	FFVGGNWK					Mascot
954.4832	954.5059	0.0227	24	6	13	FFVGGNWK	63	99.997			Mascot
1033.6041	1033.6255	0.0214	21	115	124	VAYALAQGLK					Mascot
1388.7202	1388.7347	0.0145	10	125	136	VIACIGETLEQR			Carbamidomethyl (C)[4]		Mascot

4	Elicitor-responsive protein 1 [Triticum urartu]	gi|474213532	29523.9	8.3	2	68	99.315	2.174	63	99.997	

Peptide Information

Calc. Mass	Obsrv. Mass	± da	± ppm	Start Seq.	End Seq.	Sequence	Ion Score	C. I. %	Modification	Rank	Result Type
954.4832	954.5059	0.0227	24	6	13	FFVGGNWK					Mascot
954.4832	954.5059	0.0227	24	6	13	FFVGGNWK	63	99.997			Mascot
1765.9232	1765.9767	0.0535	30	154	167	IDPYVIVQYRSQER					Mascot

5	hypothetical protein TRIUR3_06862 [Triticum urartu]	gi|474201925	53725.7	10.09	4	52	69.406	9.44	45	99.752	

Peptide Information

Calc. Mass	Obsrv. Mass	± da	± ppm	Start Seq.	End Seq.	Sequence	Ion Score	C. I. %	Modification	Rank	Result Type
931.4955	931.5103	0.0148	16	161	168	ETLEGARR					Mascot
1373.8489	1373.7323	-0.1166	-85	16	27	QILRPLGRHVGK					Mascot
1374.7336	1374.7457	0.0121	9	155	167	ATGITRETLEGAR					Mascot
1374.7336	1374.7457	0.0121	9	155	167	ATGITRETLEGAR	45	99.752			Mascot
2006.0376	2006.0277	-0.0099	-5	442	460	YKPTVPGGAVEVTVQSM AR			Oxidation (M)[17]		Mascot

6	Phenylalanyl-tRNA synthetase, chloroplastic/mitochondrial [Triticum urartu]	gi|473940057	51441.7	6.41	12	45	0	4.915			

Peptide Information

Calc. Mass	Obsrv. Mass	± da	± ppm	Start Seq.	End Seq.	Sequence	Ion Score	C. I. %	Modification	Rank	Result Type
992.5267	992.4642	-0.0625	-63	13	20	CLMTLNIK			Carbamidomethyl (C)[1]		Mascot
1008.5216	1008.4674	-0.0542	-54	13	20	CLMTLNIK			Carbamidomethyl (C)[1], Oxidation (M)[3]		Mascot
1388.6852	1388.7347	0.0495	36	29	39	YQSMHRPALNR			Oxidation (M)[4]		Mascot
1396.744	1396.7214	-0.0226	-16	13	23	CLMTLNIKTFR			Carbamidomethyl (C)[1]		Mascot
1402.7764	1402.7765	0.0001	0	296	307	LAMVLFDIPDIR					Mascot

1494.7911	1494.8499	0.0588	39	361	374	GIAGDLVEEVHISR					Mascot
1571.7965	1571.7865	-0.01	-6	282	295	TDHVAWAFGLGLER					Mascot
1587.8173	1587.8773	0.06	38	27	39	SKYQSMHRPALNR					Mascot
1798.9348	1798.9899	0.0551	31	167	181	EGHTHFLVIGDVYRR					Mascot
1823.8447	1823.9821	0.1374	75	140	154	SYNDTYYVDSQTVLR					Mascot
1850.0131	1849.9739	-0.0392	-21	361	377	GIAGDLVEEVHISRNLK					Mascot
1865.8335	1865.9346	0.1011	54	412	426	SLTDEEINDMQWNVR			Oxidation (M)[10]		Mascot
2322.072	2322.2231	0.1511	65	181	199	RDSIDSTHYPVFHQMEG FR					Mascot
2322.072	2322.2231	0.1511	65	181	199	RDSIDSTHYPVFHQMEG FR					Mascot

7	hypothetical protein TRIUR3_02076 [Triticum urartu]	gi|474249336	48655.5	6.14	11	44	0	33.345

Peptide Information

Calc. Mass	Obsrv. Mass	± da	± ppm	Start Seq.	End Seq.	Sequence	Ion Score	C. I. %	Modification	Rank	Result Type
1237.686	1237.7399	0.0539	44	60	69	AQILELMLYK			Oxidation (M)[7]		Mascot
1374.774	1374.7457	-0.0283	-21	106	119	ITAFLGGLDLAAGR					Mascot
1374.774	1374.7457	-0.0283	-21	106	119	ITAFLGGLDLAAGR	1	0			Mascot
1559.7887	1559.8802	0.0915	59	424	437	VLGSTNKFPDHLTM					Mascot
1682.9033	1682.929	0.0257	15	56	69	SETKAQILELMLYK			Oxidation (M)[11]		Mascot
1798.9811	1798.9899	0.0088	5	267	283	SLAVEQSIHAAYVAAIR					Mascot
1809.913	1809.9985	0.0855	47	164	179	LDGAAAYDVLKNFEQR					Mascot
1811.936	1812.0146	0.0786	43	344	359	MSLWEEHLGGLAEVLK					Mascot
1811.936	1812.0146	0.0786	43	344	359	MSLWEEHLGGLAEVLK					Mascot
1827.9309	1828.0045	0.0736	40	344	359	MSLWEEHLGGLAEVLK			Oxidation (M)[1]		Mascot
1857.9851	1857.9862	0.0011	1	14	31	GTVLPSNVAALNLSCVSR			Carbamidomethyl (C)[15]		Mascot
1982.8834	1983.0376	0.1542	78	381	397	YTDEGEVVETMQGHLMK			Oxidation (M)[11]		Mascot
2024.976	2025.0769	0.1009	50	223	238	VLPEDDPRCWHAQVFR			Carbamidomethyl (C)[9]		Mascot
2368.1326	2368.2983	0.1657	70	155	174	LPWHDMHCRLDGAAAY DVLK			Carbamidomethyl (C)[8]		Mascot
2384.1274	2384.2849	0.1575	66	155	174	LPWHDMHCRLDGAAAY DVLK			Carbamidomethyl (C)[8], Oxidation (M)[6]		Mascot

8	hypothetical protein TRIUR3_28731 [Triticum urartu]	gi|473822664	16549.5	9.25	6	41	0	11.579

Peptide Information

Calc. Mass	Obsrv. Mass	± da	± ppm	Start Seq.	End Seq.	Sequence	Ion Score	C. I. %	Modification	Rank	Result Type
1374.6835	1374.7457	0.0622	45	1	11	MEYTINPKNHK					Mascot
1374.6835	1374.7457	0.0622	45	1	11	MEYTINPKNHK					Mascot

Calc. Mass	Obsrv. Mass	± da	± ppm	Start Seq.	End Seq.	Sequence	Ion Score	C. I. %	Modification	Rank	Result Type
1751.9738	1752.0167	0.0429	24	46	59	SRNLHVWCLVNLLK			Carbamidomethyl (C)[8]		Mascot
1809.968	1809.9985	0.0305	17	112	127	KPLYSPSYAGAKVICR			Carbamidomethyl (C)[15]		Mascot
2163.1169	2163.1426	0.0257	12	65	82	LYVDMNLFLGFGREHVP R					Mascot
2179.1118	2179.1619	0.0501	23	65	82	LYVDMNLFLGFGREHVP R			Oxidation (M)[5]		Mascot
2893.4214	2893.5686	0.1472	51	15	39	IVYLEIQVSIDRNLHFGSE SDDSEK					Mascot
3069.5654	3069.6748	0.1094	36	87	111	TENHVYLHVVQWQKPNR NEPENPLK					Mascot

9 peroxisomal ascorbate peroxidase [Triticum aestivum] gi|148250118 31802.5 7.77 10 41 0 18.516

Peptide Information

Calc. Mass	Obsrv. Mass	± da	± ppm	Start Seq.	End Seq.	Sequence	Ion Score	C. I. %	Modification	Rank	Result Type
976.4629	976.4907	0.0278	28	118	125	RDSSVCPR			Carbamidomethyl (C)[6]		Mascot
1231.6429	1231.6635	0.0206	17	207	216	ALLDDPEFRR					Mascot
1374.6722	1374.7457	0.0735	53	141	151	DIFYRMGLTDK			Oxidation (M)[6]		Mascot
1374.6722	1374.7457	0.0735	53	141	151	DIFYRMGLTDK			Oxidation (M)[6]		Mascot
1388.7097	1388.7347	0.025	18	183	193	FDNSYFLELLK					Mascot
1586.7458	1586.844	0.0982	62	165	178	AHPERSGFDGAWTR					Mascot
1618.7609	1618.8558	0.0949	59	37	50	LAWHDAGTYDVNTR					Mascot
1734.9021	1734.9626	0.0605	35	239	255	LSELGFTPRSSGPASTK					Mascot
1765.8684	1765.9767	0.1083	61	217	230	YVELYAKDEDVFFK					Mascot
1841.9684	1842.0173	0.0489	27	179	193	DPLKFDNSYFLELLK					Mascot
1843.9774	1843.9996	0.0222	12	152	169	DIVALSGGHSLGKAHPER					Mascot
1843.9774	1843.9996	0.0222	12	152	169	DIVALSGGHSLGKAHPER					Mascot

10 Aldo-keto reductase family 4 member C9 [Triticum urartu] gi|474378832 74710.9 6.97 12 39 0 6.298

Peptide Information

Calc. Mass	Obsrv. Mass	± da	± ppm	Start Seq.	End Seq.	Sequence	Ion Score	C. I. %	Modification	Rank	Result Type
1033.5347	1033.6255	0.0908	88	524	532	MEDLLAVAR			Oxidation (M)[1]		Mascot
1237.6357	1237.7399	0.1042	84	364	375	MAGSFILNTGAR					Mascot
1373.7246	1373.7323	0.0077	6	242	253	WGLQMGQSILPK			Oxidation (M)[5]		Mascot
1505.7628	1505.8064	0.0436	29	274	287	DLMAELSGKVAESR					Mascot
1751.9003	1752.0167	0.1164	66	260	273	IKENFDIFDWSIPK					Mascot
1997.0227	1997.0447	0.022	11	425	441	LFEDGVIEREDLFVTSK					Mascot
1997.0227	1997.0447	0.022	11	425	441	LFEDGVIEREDLFVTSK					Mascot

2025.1168	2025.0769	-0.0399	-20	99	115	DLQLDFLDLFLIHGPIR		Mascot
2145.0547	2145.1814	0.1267	59	533	550	VPPAVNQVECHPIWQQD K	Carbamidomethyl (C)[10]	Mascot
2163.1128	2163.1426	0.0298	14	405	423	HIDCAPAYHNQKQVGLAL K	Carbamidomethyl (C)[4]	Mascot
2973.3896	2973.563	0.1734	58	619	643	SIDEAMIKENYDIFDWSIP EDLMAK		Mascot
3069.6409	3069.6748	0.0339	11	376	404	IPPIGLGTWQIEPDVVGD AIYAAVKAGYR		Mascot
3085.6721	3085.6816	0.0095	3	13	41	IPLIGLGTWQIEPDVVGD AIYAAVKAGYR		Mascot

Rank	Protein Name	Accession No.	Protein MW	Protein PI	Pep. Count	Protein Score	Protein Score C. I. %	Intensity Matched	Total Ion Score	Total Ion C. I. %	Confirmed
1	Disease resistance protein RPP13 [Triticum urartu]	gi|473786130	115619.3	8.15	24	69	99.417	4.106			

Peptide Information

Calc. Mass	Obsrv. Mass	± da	± ppm	Start Seq.	End Seq.	Sequence	Ion Score	C. I. %	Modification	Rank	Result Type
807.3526	807.4201	0.0675	84	508	513	NRSDCR			Carbamidomethyl (C)[5]		Mascot
810.4104	810.4314	0.021	26	957	963	EQSPPPR					Mascot
906.4791	906.4875	0.0084	9	105	111	FRDEIAR					Mascot
954.4172	954.484	0.0668	70	209	216	AWATMACK			Carbamidomethyl (C)[7], Oxidation (M)[5]		Mascot
960.4931	960.5721	0.079	82	949	956	SCQVTPLR			Carbamidomethyl (C)[2]		Mascot
1036.5786	1036.5469	-0.0317	-31	434	442	RLSAEGYIK					Mascot
1043.5343	1043.5565	0.0222	21	273	280	MELWHAIK			Oxidation (M)[1]		Mascot
1106.6205	1106.5507	-0.0698	-63	177	188	VVSIVGFGGSGK					Mascot
1141.5969	1141.5421	-0.0548	-48	675	683	LMVHMNQLR					Mascot
1182.6552	1182.6012	-0.054	-46	915	926	VVAPAEAMAPVK					Mascot
1193.6022	1193.6482	0.046	39	447	456	RSAQQVAYDR					Mascot
1210.5708	1210.6221	0.0513	42	209	218	AWATMACKQK			Carbamidomethyl (C)[7], Oxidation (M)[5]		Mascot
1338.7012	1338.707	0.0058	4	250	260	ELEDNISHLLR					Mascot
1357.7369	1357.7451	0.0082	6	946	956	LQRSCQVTPLR			Carbamidomethyl (C)[5]		Mascot
1374.6934	1374.729	0.0356	26	1011	1023	TVPSIMPNGSKEV			Oxidation (M)[6]		Mascot
1444.7941	1444.7816	-0.0125	-9	261	272	TNRCLVVIDNIK			Carbamidomethyl (C)[4]		Mascot
1455.7366	1455.7355	-0.0011	-1	754	766	DDPTFLGYLSSLK					Mascot
1491.8026	1491.7732	-0.0294	-20	464	476	NIIRPIDAHNNSK					Mascot
1633.692	1633.845	0.153	94	204	216	EFSCRAWATMACK			Carbamidomethyl (C)[4,12], Oxidation (M)[10]		Mascot
2011.0317	2011.0259	-0.0058	-3	328	345	LSVHGCSLEVEWGTPIVK			Carbamidomethyl (C)[6]		Mascot
2064.0066	2064.2024	0.1958	95	147	164	TYEACPAVGIEQAKEELR			Carbamidomethyl (C)[5]		Mascot
2278.3469	2278.2344	-0.1125	-49	835	854	LCLVVQKPIFPTIRQGALP K			Carbamidomethyl (C)[2]		Mascot
2344.3057	2344.1826	-0.1231	-53	583	603	LLHIKYLTLGSSVSRPLD GMK			Oxidation (M)[20]		Mascot
2353.2068	2353.2222	0.0154	7	541	562	SLTVFGSAGEAVSELKSC ELLR			Carbamidomethyl (C)[18]		Mascot

Rank	Protein Name	Accession No.	Protein MW	Protein PI	Pep. Count	Protein Score	Protein Score C. I. %	Intensity Matched	Total Ion Score	Total Ion C. I. %	Confirmed
2	Vicilin-like antimicrobial peptides 2-2 [Triticum urartu]	gi|473890163	75298.3	5.79	4	63	97.732	1.283	58	99.989	

Peptide Information

Calc. Mass	Obsrv. Mass	± da	± ppm	Start Seq.	End Seq.	Sequence	Ion Score	C. I. %	Modification	Rank	Result Type
807.4359	807.4201	-0.0158	-20	660	667	KGAVFQSA					Mascot
847.3904	847.4603	0.0699	82	607	612	EEEERR					Mascot
1085.595	1085.6261	0.0311	29	31	42	AGAAVGGQVVEK					Mascot
1982.9666	1983.0228	0.0562	28	102	119	VTYIQEGGSETSSLEVQR					Mascot
1982.9666	1983.0228	0.0562	28	102	119	VTYIQEGGSETSSLEVQR	58	99.989			Mascot

3	Xyloglucan galactosyltransferase KATAMARI1-like protein [Triticum urartu]	gi|474371812	65845.6	6.9	15	59	94.56	12.475	

Peptide Information

Calc. Mass	Obsrv. Mass	± da	± ppm	Start Seq.	End Seq.	Sequence	Ion Score	C. I. %	Modification	Rank	Result Type
807.4148	807.4201	0.0053	7	112	117	IAWDFR					Mascot
906.5117	906.4875	-0.0242	-27	130	137	LLFMPAAK			Oxidation (M)[4]		Mascot
1201.5895	1201.6433	0.0538	45	94	103	RPEWNVMGGR					Mascot
1320.6041	1320.627	0.0229	17	71	80	YLWGYNTTMR			Oxidation (M)[9]		Mascot
1325.6995	1325.7107	0.0112	8	310	320	SIHPDVVKQMR			Oxidation (M)[10]		Mascot
1325.6995	1325.7107	0.0112	8	310	320	SIHPDVVKQMR			Oxidation (M)[10]		Mascot
1351.6158	1351.7251	0.1093	81	509	521	CTSADELGQSGVK			Carbamidomethyl (C)[1]		Mascot
1633.8406	1633.845	0.0044	3	486	500	VPKASHVHLSDSSNR					Mascot
1688.8656	1688.8695	0.0039	2	104	117	DHFLVGGRIAWDFR					Mascot
1699.7891	1699.8739	0.0848	50	202	215	GQLIDQCRTSSFCK			Carbamidomethyl (C)[7,13]		Mascot
1873.8671	1874.0049	0.1378	74	210	225	TSSFCKLLECDLGESK			Carbamidomethyl (C)[5,10]		Mascot
2011.0437	2011.0259	-0.0178	-9	390	406	TIGPHEWDPFFSKPKPK					Mascot
2072.0554	2072.0522	-0.0032	-2	540	560	QSDGGVASKQGIQSVPI MGGR					Mascot
2181.0532	2181.1062	0.053	24	119	137	LTDEESDWGNKLLFMPA AK			Oxidation (M)[15]		Mascot
2321.0803	2321.1953	0.115	50	235	253	MFQSSLFCLQPQGDSYT RR			Carbamidomethyl (C)[8]		Mascot
3442.6389	3442.7466	0.1077	31	254	283	SAFDSMLAGCIPVFFHPG SAYVQYTWHLPK			Carbamidomethyl (C)[10], Oxidation (M)[6]		Mascot

4	hypothetical protein TRIUR3_19508 [Triticum urartu]	gi|474413405	35530.8	9.03	14	59	94.303	2.156	

Peptide Information

Calc. Mass	Obsrv. Mass	± da	± ppm	Start Seq.	End Seq.	Sequence	Ion Score	C. I. %	Modification	Rank	Result Type
804.4396	804.3704	-0.0692	-86	1	7	MNAALLR			Oxidation (M)[1]		Mascot
807.4319	807.4201	-0.0118	-15	148	155	STRSTAGK					Mascot

960.5261	960.5721	0.046	48	173	180	TWGQKNVK					Mascot
993.4611	993.5272	0.0661	67	262	268	IWWSNCK			Carbamidomethyl (C)[6]		Mascot
1193.6017	1193.6482	0.0465	39	178	187	NVKIDLMCGK			Carbamidomethyl (C)[8], Oxidation (M)[7]		Mascot
1259.7583	1259.6898	-0.0685	-54	270	279	SLQLQLRFVR					Mascot
1308.6902	1308.6979	0.0077	6	292	302	LMQMELAIISK			Oxidation (M)[2,4]		Mascot
1336.6791	1336.7051	0.026	19	140	150	GLTCWLSRSTR			Carbamidomethyl (C)[4]		Mascot
1338.7311	1338.707	-0.0241	-18	159	169	KPQHERAMTLK					Mascot
1407.7222	1407.7445	0.0223	16	292	303	LMQMELAIISKD			Oxidation (M)[2]		Mascot
1756.8977	1756.939	0.0413	24	2	16	NAALLRVAEDDLDWR					Mascot
1979.0526	1979.025	-0.0276	-14	123	139	IYKLMLPLLPCGNATMK			Carbamidomethyl (C)[11], Oxidation (M)[5]		Mascot
2011.0537	2011.0259	-0.0278	-14	74	90	LFLKQALNMEMAIVMAR			Oxidation (M)[9,11]		Mascot
2185.0046	2185.0598	0.0552	25	78	96	QALNMEMAIVMARESSA ER			Oxidation (M)[5,7,11]		Mascot

5 Cytochrome P450 71D7 [Triticum urartu] gi|473954864 84793.5 7.25 18 58 93.459 3.567

Peptide Information

Calc. Mass	Obsrv. Mass	± da	± ppm	Start Seq.	End Seq.	Sequence	Ion Score	C. I. %	Modification	Rank	Result Type
906.5295	906.4875	-0.042	-46	635	642	VLGYDVLK					Mascot
929.4938	929.4902	-0.0036	-4	268	276	GGEESILPK					Mascot
954.4648	954.484	0.0192	20	205	211	ACCKFLR			Carbamidomethyl (C)[2,3]		Mascot
1033.6154	1033.5406	-0.0748	-72	609	616	LVIRETFR					Mascot
1126.6038	1126.5538	-0.05	-44	65	76	KPVAGAAAGPCK			Carbamidomethyl (C)[11]		Mascot
1253.6848	1253.5927	-0.0921	-73	268	279	GGEESILPKAPR					Mascot
1259.6968	1259.6898	-0.007	-6	517	526	GIIHEHLERR					Mascot
1357.7257	1357.7451	0.0194	14	575	587	NPAAMVKATAEVR					Mascot
1363.7581	1363.7045	-0.0536	-39	441	453	ALLSALISDGTFR					Mascot
1373.7206	1373.7097	-0.0109	-8	575	587	NPAAMVKATAEVR			Oxidation (M)[5]		Mascot
1379.6954	1379.7577	0.0623	45	684	696	GTDFELLPFGAGR					Mascot
1475.8403	1475.7908	-0.0495	-34	601	612	LGELPYMRLVIR			Oxidation (M)[7]		Mascot
1487.6584	1487.7484	0.09	60	218	230	AVVSTNGFDDFCR			Carbamidomethyl (C)[12]		Mascot
2029.0502	2029.0277	-0.0225	-11	478	496	LATGLNTADLWPSSWLA GR					Mascot
2054.2485	2054.1418	-0.1067	-52	747	765	ANLLLRPSLRVPLPTSPP L					Mascot
2321.0359	2321.1953	0.1594	69	218	237	AVVSTNGFDDFCRSCPT LMK			Carbamidomethyl (C)[12,15], Oxidation (M)[19]		Mascot
2533.2942	2533.2239	-0.0703	-28	397	419	LTATMGVLTYGGRDMIFA PYAIR			Oxidation (M)[5]		Mascot
3525.6951	3525.7358	0.0407	12	543	574	IHKDGVIDMDIFSAGSETS ATTLEWVMAELMK					Mascot
3874.8257	3874.905	0.0793	20	546	581	DGVIDMDIFSAGSETSAT			Oxidation (M)[6]		Mascot

						TLEWVMAELMKNPAAMV K						
3874.8257	3874.905	0.0793	20	546	581	DGVIDMDIFSAGSETSAT TLEWVMAELMKNPAAMV K			Oxidation (M)[6]		Mascot

6 Anaphase-promoting complex subunit 7 [Triticum urartu] gi|474342493 64971 6.28 16 50 56.785 3.002

Peptide Information

Calc. Mass	Obsrv. Mass	± da	± ppm	Start Seq.	End Seq.	Sequence	Ion Score	C. I. %	Modification	Rank	Result Type
807.4835	807.4201	-0.0634	-79	104	109	LYRISR					Mascot
906.5043	906.4875	-0.0168	-19	157	164	FPTSSVLR					Mascot
942.5618	942.531	-0.0308	-33	482	489	EAVLLLER					Mascot
993.5112	993.5272	0.016	16	526	534	INPHNEAAK					Mascot
1193.6381	1193.6482	0.0101	8	94	103	NLKMNLMLGK			Oxidation (M)[4,7]		Mascot
1259.6235	1259.6898	0.0663	53	420	430	EAMKVMHQSAK					Mascot
1291.6133	1291.6307	0.0174	13	420	430	EAMKVMHQSAK			Oxidation (M)[3,6]		Mascot
1347.6461	1347.6825	0.0364	27	80	91	EALQEMEGIPSK			Oxidation (M)[6]		Mascot
1357.6859	1357.7451	0.0592	44	173	185	GGKPPGDFLDAQR					Mascot
1374.8104	1374.729	-0.0814	-59	482	492	EAVLLLERYLR					Mascot
1407.7301	1407.7445	0.0144	10	204	215	GGLDIYLELMQR					Mascot
1507.8632	1507.7864	-0.0768	-51	216	228	FPNNVHILLEIAK					Mascot
1743.7466	1743.9038	0.1572	90	190	203	YVEAQCCIASHDYK			Carbamidomethyl (C)[6,7]		Mascot
1839.0164	1838.9741	-0.0423	-23	388	403	SYQGSLIWILFSGLVR					Mascot
2034.0291	2034.0125	-0.0166	-8	43	60	AESLVLHGDALYGEKEFR					Mascot
2185.0669	2185.0598	-0.0071	-3	124	143	QCPYVFEAIAALAEMGLS SK			Carbamidomethyl (C)[2]		Mascot
2353.2295	2353.2222	-0.0073	-3	246	265	ARLIDPNIMAYMDEYAILL K					Mascot

7 hypothetical protein TRIUR3_34298 [Triticum urartu] gi|474054244 221550.5 6.49 28 50 55.778 23.618

Peptide Information

Calc. Mass	Obsrv. Mass	± da	± ppm	Start Seq.	End Seq.	Sequence	Ion Score	C. I. %	Modification	Rank	Result Type
804.4097	804.3704	-0.0393	-49	1513	1520	LSDDVGAK					Mascot
929.4219	929.4902	0.0683	73	425	431	YMLMSPR			Oxidation (M)[2,4]		Mascot
948.4608	948.4719	0.0111	12	829	835	CLVENWK			Carbamidomethyl (C)[1]		Mascot
960.5546	960.5721	0.0175	18	279	286	TLLCLNVK			Carbamidomethyl (C)[4]		Mascot
993.5211	993.5272	0.0061	6	901	910	SNLLTSGSSK					Mascot
1022.5265	1022.5522	0.0257	25	576	583	RVYDIDNK					Mascot

Calc. Mass	Obsrv. Mass	± da	± ppm	Start Seq.	End Seq.	Sequence	Ion Score	C. I. %	Modification	Rank	Result Type
1085.5586	1085.6261	0.0675	62	655	664	VEQGIDPATR					Mascot
1107.5793	1107.5734	-0.0059	-5	700	710	GGLPHAEAVEK					Mascot
1122.5071	1122.6133	0.1062	95	463	470	EEMMWLQR					Mascot
1126.5527	1126.5538	0.0011	1	2014	2022	SSYKQLSWQ					Mascot
1141.6113	1141.5421	-0.0692	-61	78	87	AAWNPAEKVR					Mascot
1220.6708	1220.6515	-0.0193	-16	782	791	VEEFLMALLR					Mascot
1263.6288	1263.7169	0.0881	70	1009	1020	EATAGRSTSLDR					Mascot
1320.6881	1320.627	-0.0611	-46	109	118	IMERGPWIFR			Oxidation (M)[2]		Mascot
1323.7056	1323.6747	-0.0309	-23	512	522	VSWLYRSADVK					Mascot
1353.6831	1353.7358	0.0527	39	453	462	MERLDELLYR			Oxidation (M)[1]		Mascot
1491.7625	1491.7732	0.0107	7	562	575	GVELPAGISSFVCR			Carbamidomethyl (C)[13]		Mascot
1627.8837	1627.9191	0.0354	22	796	810	LPINLNALQSCSIGK			Carbamidomethyl (C)[11]		Mascot
1633.7816	1633.845	0.0634	39	1820	1834	GEQPYPIETASGTQR					Mascot
1699.7705	1699.8739	0.1034	61	1	16	MASHPDEQSPTSVGTR					Mascot
1702.7338	1702.8925	0.1587	93	1835	1851	MIASAADSSQFGSDSGR			Oxidation (M)[1]		Mascot
1741.9331	1741.9064	-0.0267	-15	1633	1651	VVGASLTAPDIVGTDAAG K					Mascot
1743.8218	1743.9038	0.082	47	1021	1036	SLFQEKSSQSGMASEK					Mascot
1844.9501	1844.978	0.0279	15	844	861	SNEAKPLVSGQAVSWSG K					Mascot
2024.9933	2025.0688	0.0755	37	456	470	LDELLYREEMMWLQR					Mascot
2024.9933	2025.0688	0.0755	37	456	470	LDELLYREEMMWLQR					Mascot
2321.2764	2321.1953	-0.0811	-35	748	766	FVQLRGLPILNEWLQETH K					Mascot
2322.0632	2322.2219	0.1587	68	1748	1768	NFFDLNNGPSLDEASTEP AQR					Mascot
2322.0632	2322.2219	0.1587	68	1748	1768	NFFDLNNGPSLDEASTEP AQR					Mascot
3442.6018	3442.7466	0.1448	42	1789	1819	MNSTEINNMSPWFASAN PYAPVAMQSFLPAR					Mascot

8 SKP1-like protein 3 [Triticum urartu] gi|474367027 28826.9 5.35 9 48 37.535 11.001

Peptide Information

Calc. Mass	Obsrv. Mass	± da	± ppm	Start Seq.	End Seq.	Sequence	Ion Score	C. I. %	Modification	Rank	Result Type
906.4899	906.4875	-0.0024	-3	9	15	EKMVMLR					Mascot
1085.6313	1085.6261	-0.0052	-5	67	76	LPVRGDTLSK					Mascot
1325.6948	1325.7107	0.0159	12	71	82	GDTLSKVIDYSK					Mascot
1325.6948	1325.7107	0.0159	12	71	82	GDTLSKVIDYSK	4	0			Mascot
1389.7406	1389.7164	-0.0242	-17	124	136	GLIDLACQTIASK			Carbamidomethyl (C)[7]		Mascot
1604.7996	1604.8911	0.0915	57	225	237	FDIFNLAFFDLDK					Mascot
1688.8677	1688.8695	0.0018	1	153	167	SVFPPELDGEMIAKR					Mascot

2184.9351	2185.0598	0.1247	57	16	35	SEDGVDFVLSESEAEAQ CGR			Carbamidomethyl (C)[18]		Mascot
2831.2644	2831.2942	0.0298	11	11	35	MVMLRSEDGVDFVLSES EAEAQCGR			Carbamidomethyl (C)[23], Oxidation (M)[1]		Mascot
3670.5903	3670.8557	0.2654	72	168	197	LQYSTTCSSDKELPCSET QCLMQELEFEEK			Carbamidomethyl (C)[7,15,20]		Mascot

9	DNA topoisomerase 6 subunit A [Triticum urartu]	gi|474413071	48509.4	9.27	13	47	15.737	19.056

Peptide Information

Calc. Mass	Obsrv. Mass	± da	± ppm	Start Seq.	End Seq.	Sequence	Ion Score	C. I. %	Modification	Rank	Result Type
810.4944	810.4314	-0.063	-78	174	180	GIHVTKR					Mascot
1085.5297	1085.6261	0.0964	89	374	382	LPMTDHDIK			Oxidation (M)[3]		Mascot
1107.5364	1107.5734	0.037	33	366	373	YRVPEQCR			Carbamidomethyl (C)[7]		Mascot
1353.7195	1353.7358	0.0163	12	374	385	LPMTDHDIKVGK					Mascot
1389.7234	1389.7164	-0.007	-5	143	155	ESARPFANVATAR					Mascot
1444.7504	1444.7816	0.0312	22	8	23	AGADAAAGGSTSKKPR					Mascot
1517.8184	1517.7731	-0.0453	-30	143	156	ESARPFANVATARK					Mascot
1604.8544	1604.8911	0.0367	23	355	367	WLGVRPSDLDKYR					Mascot
1633.8768	1633.845	-0.0318	-19	21	36	KPRGASAAASYAQSLR					Mascot
1838.8888	1838.9741	0.0853	46	368	382	VPEQCRLPMTDHDIK			Carbamidomethyl (C)[5]		Mascot
1996.9321	1997.0375	0.1054	53	386	401	EMLEEDFVKQNEGWVK			Oxidation (M)[2]		Mascot
1996.9321	1997.0375	0.1054	53	386	401	EMLEEDFVKQNEGWVK			Oxidation (M)[2]		Mascot
2011.1658	2011.0259	-0.1399	-70	39	58	LRPDASILASLRALASAAS K					Mascot
2072.1499	2072.0522	-0.0977	-47	124	141	ASSNQVYLPDLDRIVLLR					Mascot

10	Protein HIRA [Triticum urartu]	gi|474156140	110180.9	7.04	17	46	0	9.505

Peptide Information

Calc. Mass	Obsrv. Mass	± da	± ppm	Start Seq.	End Seq.	Sequence	Ion Score	C. I. %	Modification	Rank	Result Type
954.425	954.484	0.059	62	269	275	FNHSMFR			Oxidation (M)[5]		Mascot
972.5836	972.5209	-0.0627	-64	473	481	VTRVSSPVK					Mascot
1036.5092	1036.5469	0.0377	36	638	645	ETEIRCTK			Carbamidomethyl (C)[6]		Mascot
1066.5251	1066.5424	0.0173	16	269	276	FNHSMFRK					Mascot
1193.5909	1193.6482	0.0573	48	866	875	FLAREADESR					Mascot
1201.6787	1201.6433	-0.0354	-29	557	567	ANISESLVIQK					Mascot
1291.7191	1291.6307	-0.0884	-68	812	822	LQIDIGKFMAR					Mascot
1374.7046	1374.729	0.0244	18	918	929	EDILPSMATNRK					Mascot
1390.6995	1390.7231	0.0236	17	918	929	EDILPSMATNRK			Oxidation (M)[7]		Mascot

1427.6836	1427.8096	0.126	88	623	637	SAGDMIGLGGSFSTK		Mascot
1517.7708	1517.7731	0.0023	2	286	301	TAPAGWANGASKTSAK		Mascot
1743.9137	1743.9038	-0.0099	-6	823	837	KPVWSRVTDDGVQTR		Mascot
1873.8546	1874.0049	0.1503	80	773	786	HAFLYDMSMKCWLR	Carbamidomethyl (C)[11], Oxidation (M)[7]	Mascot
1890.8723	1891.0214	0.1491	79	48	63	DNQNDDSNQRLLATMR		Mascot
1890.8723	1891.0214	0.1491	79	48	63	DNQNDDSNQRLLATMR		Mascot
2029.0098	2029.0277	0.0179	9	277	297	NLATGQDAKTAPAGWAN GASK		Mascot
2071.9966	2072.0522	0.0556	27	623	642	SAGDMIGLGGSFSTKETE IR	Oxidation (M)[5]	Mascot
2166.9939	2167.0752	0.0813	38	96	116	KAGSGTSEFGSGEPADIE NWK		Mascot
3671.698	3671.8105	0.1125	31	933	965	LLNEFMDLLSEYESAAEE NVDKMDVTPPAADAK	Oxidation (M)[6]	Mascot
3671.698	3671.8105	0.1125	31	933	965	LLNEFMDLLSEYESAAEE NVDKMDVTPPAADAK	Oxidation (M)[6]	Mascot
3687.6929	3687.8135	0.1206	33	933	965	LLNEFMDLLSEYESAAEE NVDKMDVTPPAADAK	Oxidation (M)[6,23]	Mascot

Gel Idx/Pos	194/H21	Instr./Gel Origin	BA2151/Sample Project 20140814	Process Status	Analysis Succeeded
Plate [#] Name	[1] Sample Project 20140814	Instrument Sample Name		Spectra	11

Rank	Protein Name	Accession No.	Protein MW	Protein PI	Pep. Count	Protein Score	Protein Score C. I. %	Intensity Matched	Total Ion Score	Total Ion C. I. %	Confirmed
1	Vicilin-like antimicrobial peptides 2-2 [Triticum urartu]	gi|473890163	75298.3	5.79	7	193	100	25.518	178	100	

Peptide Information

Calc. Mass	Obsrv. Mass	± da	± ppm	Start Seq.	End Seq.	Sequence	Ion Score	C. I. %	Modification	Rank	Result Type
1085.595	1085.6288	0.0338	31	31	42	AGAAVGGQVVEK					Mascot
1370.7386	1370.7841	0.0455	33	31	44	AGAAVGGQVVEKER					Mascot
1844.0389	1844.0031	-0.0358	-19	182	198	ILRQGFGVSAEVVEAIR					Mascot
1982.9666	1983.0311	0.0645	33	102	119	VTYIQEGGSETSSLEVQR					Mascot
1982.9666	1983.0311	0.0645	33	102	119	VTYIQEGGSETSSLEVQR	178	100			Mascot
2168.083	2168.1536	0.0706	33	100	119	GKVTYIQEGGSETSSLEV QR					Mascot
2351.0828	2351.2314	0.1486	63	219	238	SNWTEEIFDALWGDESP LNK					Mascot
3258.677	3258.8091	0.1321	41	71	99	LQFITMDPGALFLPVQLH ADAVFYVHSGR			Oxidation (M)[6]		Mascot

Rank	Protein Name	Accession No.	Protein MW	Protein PI	Pep. Count	Protein Score	Protein Score C. I. %	Intensity Matched	Total Ion Score	Total Ion C. I. %	Confirmed
2	Cell division control protein 48-like protein B [Triticum urartu]	gi|473886645	61577.5	8.92	13	47	9.711	22.963			

Peptide Information

Calc. Mass	Obsrv. Mass	± da	± ppm	Start Seq.	End Seq.	Sequence	Ion Score	C. I. %	Modification	Rank	Result Type
986.5993	986.507	-0.0923	-94	76	84	TSLVQAIVR					Mascot
1033.5823	1033.6332	0.0509	49	305	313	SQIKASMIR					Mascot
1107.5398	1107.5854	0.0456	41	1	10	MGEVMAARSR					Mascot
1221.6409	1221.6392	-0.0017	-1	361	372	GVLLHGPPGCSK			Carbamidomethyl (C)[10]		Mascot
1415.7013	1415.6531	-0.0482	-34	516	528	LGEDVDLGEIAER					Mascot
1471.6885	1471.7883	0.0998	68	9	23	SRGGANGEDGEPVAR					Mascot
1720.8364	1720.9299	0.0935	54	489	503	FDMVLYVPPPDAQGR			Oxidation (M)[3]		Mascot
1754.9589	1754.9846	0.0257	15	42	55	ELVIYPFLYARQSR					Mascot
1843.9436	1844.0031	0.0595	32	318	334	EAPTVSWDDIGGLKDLK					Mascot
1966.0658	1966.057	-0.0088	-4	337	353	LQQAVEWPIKHATAFAR					Mascot
2167.2097	2167.1758	-0.0339	-16	35	52	MALQALRELVIYPFLYAR					Mascot
2353.2122	2353.2297	0.0175	7	489	508	FDMVLYVPPPDAQGRHE ILR					Mascot
2353.2122	2353.2297	0.0175	7	489	508	FDMVLYVPPPDAQGRHE					Mascot

	3670.8323	3670.8213	-0.011	-3	135	166	ILR FLREAFSEAYSQASQGR PAIIFIDELDAICPR			Carbamidomethyl (C)[30]		Mascot

3	Pantothenate kinase 2 [Triticum urartu]	gi|474259602	91498.7	5.76	14	45	0	6.321

Peptide Information

Calc. Mass	Obsrv. Mass	± da	± ppm	Start Seq.	End Seq.	Sequence	Ion Score	C. I. %	Modification	Rank	Result Type
929.5567	929.5223	-0.0344	-37	15	22	SIWVIVGR					Mascot
1004.5636	1004.6089	0.0453	45	107	115	RSYPVLGGR					Mascot
1022.5451	1022.5771	0.032	31	380	388	GEAKAMFLR					Mascot
1145.6677	1145.6146	-0.0531	-46	584	593	FGLANLLELR					Mascot
1289.6848	1289.6774	-0.0074	-6	430	441	IHGPPLGDLNEK					Mascot
1325.6406	1325.713	0.0724	55	126	136	LNECLDFISSK			Carbamidomethyl (C)[4]		Mascot
1997.0549	1997.0426	-0.0123	-6	296	315	IGLSASTIASSFGKTISDN K					Mascot
2309.2031	2309.2227	0.0196	8	772	792	DADLLILEGMGRSLHTNL NAR					Mascot
2375.1675	2375.2063	0.0388	16	384	404	AMFLRHEGFLGALGAFM SYEK					Mascot
2394.2275	2394.3521	0.1246	52	419	441	FPMGAPYVGGKIHGPPL GDLNEK					Mascot
2499.1763	2499.2859	0.1096	44	362	383	GHAYTMDTISFAVHFWS KGEAK			Oxidation (M)[6]		Mascot
3352.6167	3352.7761	0.1594	48	389	418	HEGFLGALGAFMSYEKH GLDDLSAHHLVER			Oxidation (M)[12]		Mascot
3470.8894	3470.7981	-0.0913	-26	479	511	GDILRSDASAALNVGVLH LVPSLDVFPLLEDPK					Mascot
3509.9001	3509.7595	-0.1406	-40	50	81	NPTILLPNQSDDISHLALD IGGSLIKLVYFSR					Mascot
3509.9001	3509.7595	-0.1406	-40	50	81	NPTILLPNQSDDISHLALD IGGSLIKLVYFSR					Mascot

4	DNA-binding protein [Triticum aestivum]	gi|6958202	69254.2	7.47	16	43	0	2.7

Peptide Information

Calc. Mass	Obsrv. Mass	± da	± ppm	Start Seq.	End Seq.	Sequence	Ion Score	C. I. %	Modification	Rank	Result Type
1085.6677	1085.6288	-0.0389	-36	319	328	LLVDTKGAIR					Mascot
1145.6776	1145.6146	-0.063	-55	484	494	LGISTIDSLVK					Mascot
1289.5872	1289.6774	0.0902	70	438	447	VFEDMFATWK			Oxidation (M)[5]		Mascot
1308.6332	1308.709	0.0758	58	166	176	KWLDGGNTFDR					Mascot
1325.71	1325.713	0.003	2	308	318	ENVKPSLFTYK					Mascot
1370.7137	1370.7841	0.0704	51	177	187	SDIFYVIMNLR					Mascot
1415.5968	1415.6531	0.0563	40	37	48	NAEQHSEQGCQK			Carbamidomethyl (C)[10]		Mascot
1713.8881	1713.8516	-0.0365	-21	305	318	MEKENVKPSLFTYK					Mascot

1719.8469	1719.8934	0.0465	27	366	381	EKAEAILESMEGGDIK					Mascot
1720.9818	1720.9299	-0.0519	-30	385	399	NACKILLPLYAFLGK			Carbamidomethyl (C)[3]		Mascot
1964.9939	1965.0131	0.0192	10	453	468	FYNALMKVYADQNLFK					Mascot
2024.9628	2025.0753	0.1125	56	477	494	MDEDGCRLGISTIDSLVK			Carbamidomethyl (C)[6], Oxidation (M)[1]		Mascot
2164.2417	2164.0935	-0.1482	-68	273	291	DLGFPVTVFAINQLLLLYK					Mascot
2196.1079	2196.1731	0.0652	30	518	535	MKPQYSSYLMLLDTYSK K					Mascot
2375.2163	2375.2063	-0.01	-4	336	357	VVESMQAEGVEPDLLFQ ATIAK					Mascot
2381.2764	2381.2573	-0.0191	-8	559	578	QYQLLLNAYVHAKTPVY GFR					Mascot

5 Disease resistance protein RPM1 [Triticum urartu] gi|473968240 69201.6 9.02 15 42 0 12.438

Peptide Information

Calc. Mass	Obsrv. Mass	± da	± ppm	Start Seq.	End Seq.	Sequence	Ion Score	C. I. %	Modification	Rank	Result Type
929.476	929.5223	0.0463	50	429	436	KAEEMPPK					Mascot
946.5833	946.5666	-0.0167	-18	588	595	RPKFITGK					Mascot
1035.4775	1035.5721	0.0946	91	104	112	DMSVEELGR					Mascot
1107.6521	1107.5854	-0.0667	-60	591	600	FITGKSIVSR					Mascot
1270.6387	1270.7268	0.0881	69	41	51	KHLGSELESDR					Mascot
1289.6882	1289.6774	-0.0108	-8	472	482	QVDVEVMGKLR			Oxidation (M)[7]		Mascot
1308.6682	1308.709	0.0408	31	264	274	FLETLDVQDTK					Mascot
1379.677	1379.7474	0.0704	51	1	11	MMEQASRILER			Oxidation (M)[1]		Mascot
1415.7465	1415.6531	-0.0934	-66	447	458	LGNLPGWMDRLK			Oxidation (M)[8]		Mascot
1505.774	1505.8254	0.0514	34	185	197	NEKMASLNLSQVR			Oxidation (M)[4]		Mascot
1590.9438	1590.8871	-0.0567	-36	359	373	NLQVLGVVHIARGSK					Mascot
1713.9659	1713.8516	-0.1143	-67	235	248	HIGELHHLRYLGLR					Mascot
1845.015	1844.9824	-0.0326	-18	465	480	LIQTQLKQVDVEVMGK			Oxidation (M)[14]		Mascot
1845.015	1844.9824	-0.0326	-18	465	480	LIQTQLKQVDVEVMGK			Oxidation (M)[14]		Mascot
3670.9031	3670.8213	-0.0818	-22	541	573	CVNLDDSKEGLSLVLVLQ NLNELVLTSCGDKPK			Carbamidomethyl (C)[1,28]		Mascot
3671.8025	3671.8333	0.0308	8	56	85	VITSSYDGLPYHLKCCFL YLSIFPENHEIR			Carbamidomethyl (C)[15,16]		Mascot
3671.8025	3671.8333	0.0308	8	56	85	VITSSYDGLPYHLKCCFL YLSIFPENHEIR			Carbamidomethyl (C)[15,16]		Mascot

6 BEL1-like homeodomain protein 1 [Triticum urartu] gi|474189237 43807.9 9.45 10 42 0 2.129

Peptide Information

Calc. Mass	Obsrv. Mass	± da	± ppm	Start Seq.	End Seq.	Sequence	Ion Score	C. I. %	Modification	Rank	Result Type
1231.7191	1231.6735	-0.0456	-37	337	347	IMLAKQTGLTR					Mascot

Calc. Mass	Obsrv. Mass	± da	± ppm	Start Seq.	End Seq.	Sequence	Ion Score	C. I. %	Modification	Rank	Result Type
1289.5791	1289.6774	0.0983	76	182	194	GGAPAPEMSTAER			Oxidation (M)[8]		Mascot
1323.7491	1323.7091	-0.04	-30	60	72	IGVDGVNGRAPIR					Mascot
1691.729	1691.8612	0.1322	78	258	274	DAIASQDCDAEGLGGGR			Carbamidomethyl (C)[8]		Mascot
1730.937	1730.8875	-0.0495	-29	17	31	IPYSSNPLRILCAAR			Carbamidomethyl (C)[12]		Mascot
1964.9495	1965.0131	0.0636	32	109	127	YHLGASTAASQGQVVMNSK			Oxidation (M)[16]		Mascot
2164.1584	2164.0935	-0.0649	-30	41	59	LYALPLFVLRSGVDTCPSR			Carbamidomethyl (C)[16]		Mascot
2381.2031	2381.2573	0.0542	23	106	127	FLRYHLGASTAASQGQVVMNSK			Oxidation (M)[19]		Mascot
2835.2971	2835.3923	0.0952	34	370	396	GRPGGSGAAGYDMNMQTTKSFAAQLMR			Oxidation (M)[13,15]		Mascot
3258.5959	3258.8091	0.2132	65	217	247	QYHQQMASVSSSFEAVAGAGSARTYTALALR					Mascot

7	2-succinylbenzoate--CoA ligase [Triticum urartu]	gi|474012066	61656.3	6.95	11	40	0	3.672

Peptide Information

Calc. Mass	Obsrv. Mass	± da	± ppm	Start Seq.	End Seq.	Sequence	Ion Score	C. I. %	Modification	Rank	Result Type
954.4713	954.5046	0.0333	35	394	401	SYGEIVMR					Mascot
970.4662	970.5124	0.0462	48	394	401	SYGEIVMR			Oxidation (M)[7]		Mascot
1379.7893	1379.7474	-0.0419	-30	262	274	QVSTKAIYTGIAK					Mascot
1415.6954	1415.6531	-0.0423	-30	174	185	TGDPEFNWKPPK					Mascot
1619.6868	1619.8414	0.1546	95	1	17	MGSEGPGHGFGSGAAER			Oxidation (M)[1]		Mascot
1720.7806	1720.9299	0.1493	87	496	512	EGAGGSDEVALANDIMR			Oxidation (M)[16]		Mascot
1965.9773	1966.057	0.0797	41	384	401	TMVPVPADGKSYGEIVMR			Oxidation (M)[2]		Mascot
1981.9722	1982.0155	0.0433	22	384	401	TMVPVPADGKSYGEIVMR			Oxidation (M)[2,17]		Mascot
1997.1152	1997.0426	-0.0726	-36	41	59	AALAQPDRASVVHGPVRPR					Mascot
2164.2522	2164.0935	-0.1587	-73	305	326	VVNVMLAGAAPTPSLLAALSIR					Mascot
2167.9858	2168.1536	0.1678	77	496	515	EGAGGSDEVALANDIMRFCR			Carbamidomethyl (C)[19]		Mascot
2322.2639	2322.2332	-0.0307	-13	136	155	KQNFRPPILIVIGDPTCEPK			Carbamidomethyl (C)[17]		Mascot
3538.8826	3538.7793	-0.1033	-29	447	479	DIIISGGENISTLEVEKVVYMHPAVLEASVVAR					Mascot

8	hypothetical protein TRIUR3_16063 [Triticum urartu]	gi|474415566	201802	4.61	20	39	0	37.72

Peptide Information

Calc. Mass	Obsrv. Mass	± da	± ppm	Start Seq.	End Seq.	Sequence	Ion Score	C. I. %	Modification	Rank	Result Type
1035.4954	1035.5721	0.0767	74	1107	1115	AKDTEENTK					Mascot
1109.595	1109.6063	0.0113	10	1712	1722	HGAVEPVSVSK					Mascot

1221.6475	1221.6392	-0.0083	-7	1674	1684	THSSSISVYLK					Mascot
1370.6886	1370.7841	0.0955	70	18	29	FVLPGHPESTMR					Mascot
1505.7628	1505.8254	0.0626	42	390	404	GDDGNLMAKSLVGTK					Mascot
1619.8066	1619.8414	0.0348	21	127	140	MNPMMIREVLNSGK					Mascot
1705.8215	1705.8915	0.07	41	232	247	DVAVFTPCSEPGVTAR			Carbamidomethyl (C)[8]		Mascot
1714.001	1713.8516	-0.1494	-87	45	60	VALGPAVLASLYRDLR					Mascot
1719.7677	1719.8934	0.1257	73	900	914	MKGTHKPMEEDTDGK			Oxidation (M)[1]		Mascot
1841.9908	1841.8971	-0.0937	-51	2	17	DAVIEHASFLALWLTR					Mascot
1844.9106	1844.9824	0.0718	39	1795	1810	QVMNMNHVGEVISRSK			Oxidation (M)[3]		Mascot
1844.9106	1844.9824	0.0718	39	1795	1810	QVMNMNHVGEVISRSK			Oxidation (M)[3]		Mascot
1938.9451	1938.9795	0.0344	18	1544	1560	VSSQNQQKCVPLGDHNK			Carbamidomethyl (C)[9]		Mascot
1979.0127	1979.0253	0.0126	6	1467	1483	ISLQNQQKCVPLGDHNK			Carbamidomethyl (C)[9]		Mascot
1982.8767	1983.0311	0.1544	78	215	231	ANEDWGVAWETYDLEG K					Mascot
1982.8767	1983.0311	0.1544	78	215	231	ANEDWGVAWETYDLEG K					Mascot
2131.9812	2132.1311	0.1499	70	949	968	SIGIDGTHGPMEESTDDK VK			Oxidation (M)[11]		Mascot
2499.2378	2499.2859	0.0481	19	1787	1808	AQIASMQRQVMNMNHV GEVISR					Mascot
2531.2275	2531.3113	0.0838	33	1787	1808	AQIASMQRQVMNMNHV GEVISR			Oxidation (M)[6,11]		Mascot
3352.5532	3352.7761	0.2229	66	1216	1245	HCLQDVGLINERESLSDG AAMEIQGVDDHK			Carbamidomethyl (C)[2], Oxidation (M)[21]		Mascot
3538.4895	3538.7793	0.2898	82	1606	1637	ENDEASEQEQTYTESASI APSGMDHRGENNNK					Mascot
3654.6096	3654.8074	0.1978	54	1599	1631	TELSIGRENDEASEQEQT YTESASIAPSGMDHR			Oxidation (M)[30]		Mascot
3671.7388	3671.8333	0.0945	26	973	1005	VNAEAEQVNGTMMGND RPDSILQIDLPARDETR			Oxidation (M)[12]		Mascot
3671.7388	3671.8333	0.0945	26	973	1005	VNAEAEQVNGTMMGND RPDSILQIDLPARDETR			Oxidation (M)[12]		Mascot

9	Transcription factor MYC4 [Triticum urartu]	gi|474117031	40826.7	7.23	9	38	0	4.785	

Peptide Information

Calc. Mass	Obsrv. Mass	± da	± ppm	Start Seq.	End Seq.	Sequence	Ion Score	C. I. %	Modification	Rank	Result Type
1268.6844	1268.6333	-0.0511	-40	253	263	ETLHSQIEALK					Mascot
1619.8608	1619.8414	-0.0194	-12	335	348	DIMIQQVAVKMATR			Oxidation (M)[3]		Mascot
1819.9912	1819.9508	-0.0404	-22	228	244	ASLLGDAISYINELRGK					Mascot
1819.9912	1819.9508	-0.0404	-22	228	244	ASLLGDAISYINELRGK					Mascot
1844.863	1844.9824	0.1194	65	2	20	AMVVDGAGPAGLASAPC ER			Carbamidomethyl (C)[17], Oxidation (M)[2]		Mascot
1844.863	1844.9824	0.1194	65	2	20	AMVVDGAGPAGLASAPC ER			Carbamidomethyl (C)[17], Oxidation (M)[2]		Mascot
1959.9086	1960.0253	0.1167	60	1	20	MAMVVDGAGPAGLASAP			Carbamidomethyl (C)[18]		Mascot

						CER						
2010.9159	2011.0569	0.141	70	265	283	ERDARPAAPSSGMHDN GAR			Oxidation (M)[13]		Mascot	
2025.0321	2025.0753	0.0432	21	225	242	MDKASLLGDAISYINELR			Oxidation (M)[1]		Mascot	
2056.0063	2056.1357	0.1294	63	2	22	AMVVDGAGPAGLASAPC ERAR			Carbamidomethyl (C)[17]		Mascot	
2375.2639	2375.2063	-0.0576	-24	314	334	LMTALRELDLDVYHASVS VVK			Oxidation (M)[2]		Mascot	

10	hypothetical protein TRIUR3_07605 [Triticum urartu]	gi|474443810	36454.2	6.43	9	38	0	2.067

Peptide Information

Calc. Mass	Obsrv. Mass	± da	± ppm	Start Seq.	End Seq.	Sequence	Ion Score	C. I. %	Modification	Rank	Result Type
1107.5793	1107.5854	0.0061	6	240	250	LSAAAFLDSGR					Mascot
1351.7039	1351.7256	0.0217	16	262	273	MFASISNLAELR					Mascot
1491.7108	1491.7885	0.0777	52	168	181	DAVAGKEDAMESLR					Mascot
1505.7516	1505.8254	0.0738	49	138	150	ELEQAKMALEEAK			Oxidation (M)[7]		Mascot
1523.7999	1523.8282	0.0283	19	261	273	RMFASISNLAELR			Oxidation (M)[2]		Mascot
1720.9242	1720.9299	0.0057	3	2	17	AIGSGQEFHPGRLVPR					Mascot
1752.9048	1753.0168	0.112	64	123	137	QTETKMLESLISQTK			Oxidation (M)[6]		Mascot
2322.1011	2322.2332	0.1321	57	27	47	VVGRWAEDSSGFQFQS HASAR					Mascot
3670.6213	3670.8213	0.2	54	262	294	MFASISNLAELRSAAAAA AMDDYYEFDHFDDGR					Mascot
3686.6162	3686.8279	0.2117	57	262	294	MFASISNLAELRSAAAAA AMDDYYEFDHFDDGR			Oxidation (M)[1]		Mascot

Gel Idx/Pos	195/H22	Instr./Gel Origin	BA2151/Sample Project 20140814	Process Status	Analysis Succeeded
Plate [#] Name	[1] Sample Project 20140814	Instrument Sample Name		Spectra	11

Rank	Protein Name	Accession No.	Protein MW	Protein PI	Pep. Count	Protein Score	Protein Score C. I. %	Intensity Matched	Total Ion Score	Total Ion C. I. %	Confirmed
1	dehydroascorbate reductase [Triticum aestivum]	gi|28192421	23457.2	5.88	14	533	100	30.02	431	100	

Peptide Information

Calc. Mass	Obsrv. Mass	± da	± ppm	Start Seq.	End Seq.	Sequence	Ion Score	C. I. %	Modification	Rank	Result Type
866.411	866.447	0.036	42	1	7	MTEVCVK			Carbamidomethyl (C)[5]		Mascot
909.4862	909.5072	0.021	23	33	39	KVPYQMK			Oxidation (M)[6]		Mascot
944.5662	944.5848	0.0186	20	25	32	VLLTLEEK					Mascot
1005.5	1005.5252	0.0252	25	59	68	VPVYNGGDGK					Mascot
1023.4589	1023.4998	0.0409	40	112	121	SKDASDGSEK					Mascot
1098.5942	1098.6393	0.0451	41	200	209	ENLIAGWAPK					Mascot
1202.682	1202.7219	0.0399	33	102	111	IFSTFVTFLK					Mascot
1202.682	1202.7219	0.0399	33	102	111	IFSTFVTFLK	78	100			Mascot
1497.8213	1497.8668	0.0455	30	157	168	LYHLQVALEHFK					Mascot
1497.8213	1497.8668	0.0455	30	157	168	LYHLQVALEHFK	90	100			Mascot
1574.8578	1574.9065	0.0487	31	40	52	LIDVSNKPDWFLK					Mascot
1607.8639	1607.9105	0.0466	29	122	135	ALVDELQALEEHLK					Mascot
1827.8444	1827.9144	0.07	38	8	24	AAVGHPDTLGDCPFSQR			Carbamidomethyl (C)[12]		Mascot
1827.8444	1827.9144	0.07	38	8	24	AAVGHPDTLGDCPFSQR	137	100	Carbamidomethyl (C)[12]		Mascot
1866.9484	1866.9868	0.0384	21	84	101	YPTPSLVTPAEYASVGSK					Mascot
2021.0338	2021.1149	0.0811	40	172	189	VPETLTSVHAYTEALFSR					Mascot
2021.0338	2021.1149	0.0811	40	172	189	VPETLTSVHAYTEALFSR	126	100			Mascot
2108.1135	2108.1692	0.0557	26	136	156	AHGPYINGANISAVDLSL APK					Mascot

Rank	Protein Name	Accession No.	Protein MW	Protein PI	Pep. Count	Protein Score	Protein Score C. I. %	Intensity Matched	Total Ion Score	Total Ion C. I. %	Confirmed
2	dehydroascorbate reductase [Triticum aestivum]	gi|259017810	23457.2	5.88	13	522	100	29.369	431	100	

Peptide Information

Calc. Mass	Obsrv. Mass	± da	± ppm	Start Seq.	End Seq.	Sequence	Ion Score	C. I. %	Modification	Rank	Result Type
866.411	866.447	0.036	42	1	7	MTEVCVK			Carbamidomethyl (C)[5]		Mascot
909.4862	909.5072	0.021	23	33	39	KVPYQMK			Oxidation (M)[6]		Mascot
944.5662	944.5848	0.0186	20	25	32	VLLTLEEK					Mascot
1005.5	1005.5252	0.0252	25	59	68	VPVYNGGDGK					Mascot

1023.4589	1023.4998	0.0409	40	112	121	SKDASDGSEK					Mascot
1098.5942	1098.6393	0.0451	41	200	209	ENLIAGWAPK					Mascot
1202.682	1202.7219	0.0399	33	102	111	IFSTFVTFLK					Mascot
1202.682	1202.7219	0.0399	33	102	111	IFSTFVTFLK	78	100			Mascot
1497.8213	1497.8668	0.0455	30	157	168	LYHLQVALEHFK					Mascot
1497.8213	1497.8668	0.0455	30	157	168	LYHLQVALEHFK	90	100			Mascot
1607.8639	1607.9105	0.0466	29	122	135	ALVDELQALEEHLK					Mascot
1827.8444	1827.9144	0.07	38	8	24	AAVGHPDTLGDCPFSQR			Carbamidomethyl (C)[12]		Mascot
1827.8444	1827.9144	0.07	38	8	24	AAVGHPDTLGDCPFSQR	137	100	Carbamidomethyl (C)[12]		Mascot
1892.9641	1893.01	0.0459	24	84	101	YPTPSLVTPPEYASVGSK					Mascot
2021.0338	2021.1149	0.0811	40	172	189	VPETLTSVHAYTEALFSR					Mascot
2021.0338	2021.1149	0.0811	40	172	189	VPETLTSVHAYTEALFSR	126	100			Mascot
2108.1135	2108.1692	0.0557	26	136	156	AHGPYINGANISAVDLSL APK					Mascot

3 Glutathione S-transferase DHAR2 [Triticum urartu] gi|474023258 45260 8.3 15 503 100 30.216 431 100

Peptide Information

Calc. Mass	Obsrv. Mass	± da	± ppm	Start Seq.	End Seq.	Sequence	Ion Score	C. I. %	Modification	Rank	Result Type
909.4862	909.5072	0.021	23	78	84	KVPYQMK			Oxidation (M)[6]		Mascot
944.5662	944.5848	0.0186	20	70	77	VLLTLEEK					Mascot
951.4683	951.5133	0.045	47	339	346	AYDAAVWR					Mascot
1005.5	1005.5252	0.0252	25	104	113	VPVYNGGDGK					Mascot
1023.4589	1023.4998	0.0409	40	157	166	SKDASDGSEK					Mascot
1098.5942	1098.6393	0.0451	41	245	254	ENLIAGWAPK					Mascot
1128.5909	1128.6136	0.0227	20	2	10	GSWPQRELR					Mascot
1202.682	1202.7219	0.0399	33	147	156	IFSTFVTFLK					Mascot
1202.682	1202.7219	0.0399	33	147	156	IFSTFVTFLK	78	100			Mascot
1497.8213	1497.8668	0.0455	30	202	213	LYHLQVALEHFK					Mascot
1497.8213	1497.8668	0.0455	30	202	213	LYHLQVALEHFK	90	100			Mascot
1574.8578	1574.9065	0.0487	31	85	97	LIDVSNKPDWFLK					Mascot
1607.8639	1607.9105	0.0466	29	167	180	ALVDELQALEEHLK					Mascot
1827.8444	1827.9144	0.07	38	53	69	AAVGHPDTLGDCPFSQR			Carbamidomethyl (C)[12]		Mascot
1827.8444	1827.9144	0.07	38	53	69	AAVGHPDTLGDCPFSQR	137	100	Carbamidomethyl (C)[12]		Mascot
1866.9484	1866.9868	0.0384	21	129	146	YPTPSLVTPAEYASVGSK					Mascot
2021.0338	2021.1149	0.0811	40	217	234	VPETLTSVHAYTEALFSR					Mascot
2021.0338	2021.1149	0.0811	40	217	234	VPETLTSVHAYTEALFSR	126	100			Mascot
2108.1135	2108.1692	0.0557	26	181	201	AHGPYINGANISAVDLSL					Mascot

APK

4	hypothetical protein TRIUR3_28410 [Triticum urartu]	gi|474060617	28387.9	5.53	7	302	100	24.585	273	100

Peptide Information

Calc. Mass	Obsrv. Mass	± da	± ppm	Start Seq.	End Seq.	Sequence	Ion Score	C. I. %	Modification	Rank	Result Type
930.4389	930.4752	0.0363	39	194	200	FEAYICK			Carbamidomethyl (C)[6]		Mascot
971.5673	971.6036	0.0363	37	51	58	FVTNHLLK					Mascot
971.5673	971.6036	0.0363	37	51	58	FVTNHLLK	34	96.781			Mascot
1078.535	1078.5786	0.0436	40	207	215	GYPLLEACR			Carbamidomethyl (C)[8]		Mascot
1078.535	1078.5786	0.0436	40	207	215	GYPLLEACR	55	99.975	Carbamidomethyl (C)[8]		Mascot
1145.5698	1145.615	0.0452	39	156	165	GHNLSLEYGR					Mascot
1145.5698	1145.615	0.0452	39	156	165	GHNLSLEYGR	85	100			Mascot
1406.6271	1406.6857	0.0586	42	130	139	HREWESCFQK			Carbamidomethyl (C)[7]		Mascot
1796.9075	1796.9415	0.034	19	113	127	VHLGFIYCVSDLVMK			Carbamidomethyl (C)[8], Oxidation (M)[14]		Mascot
1927.9371	1928.0159	0.0788	41	35	50	VHVAIYYESLCPYSAR			Carbamidomethyl (C)[11]		Mascot
1927.9371	1928.0159	0.0788	41	35	50	VHVAIYYESLCPYSAR	99	100	Carbamidomethyl (C)[11]		Mascot

5	DRP5 protein [Triticum durum]	gi|85857188	17705.2	5.57	10	239	100	12.572	168	100

Peptide Information

Calc. Mass	Obsrv. Mass	± da	± ppm	Start Seq.	End Seq.	Sequence	Ion Score	C. I. %	Modification	Rank	Result Type
909.4862	909.5072	0.021	23	15	21	KVPYQMK			Oxidation (M)[6]		Mascot
944.5662	944.5848	0.0186	20	7	14	VLLTLEEK					Mascot
1005.5	1005.5252	0.0252	25	41	50	VPVYNGGDGK					Mascot
1023.4589	1023.4998	0.0409	40	94	103	SKDASDGSEK					Mascot
1202.682	1202.7219	0.0399	33	84	93	IFSTFVTFLK					Mascot
1202.682	1202.7219	0.0399	33	84	93	IFSTFVTFLK	78	100			Mascot
1497.8213	1497.8668	0.0455	30	139	150	LYHLQVALEHFK					Mascot
1497.8213	1497.8668	0.0455	30	139	150	LYHLQVALEHFK	90	100			Mascot
1574.8578	1574.9065	0.0487	31	22	34	LIDVSNKPDWFLK					Mascot
1607.8639	1607.9105	0.0466	29	104	117	ALVDELQALEEHLK					Mascot
1866.9484	1866.9868	0.0384	21	66	83	YPTPSLVTPAEYASVGSK					Mascot
2108.1135	2108.1692	0.0557	26	118	138	AHGPYINGANISAVDLSL APK					Mascot

6	27K protein [Triticum aestivum]	gi|30793446	23271.5	6.06	3	149	100	19.206	140	100

Protein Group

Tri a Bd 27K, partial [Triticum aestivum]	gi|283480515	23214.5	6.0599

Peptide Information

Calc. Mass	Obsrv. Mass	± da	± ppm	Start Seq.	End Seq.	Sequence	Ion Score	C. I. %	Modification	Rank	Result Type
1078.535	1078.5786	0.0436	40	181	189	GYPLLEACR			Carbamidomethyl (C)[8]		Mascot
1078.535	1078.5786	0.0436	40	181	189	GYPLLEACR	55	99.975	Carbamidomethyl (C)[8]		Mascot
1145.5698	1145.615	0.0452	39	133	142	GHNLSLEYGR					Mascot
1145.5698	1145.615	0.0452	39	133	142	GHNLSLEYGR	85	100			Mascot
1406.6271	1406.6857	0.0586	42	107	116	HREWESCFQK			Carbamidomethyl (C)[7]		Mascot

7	27k protein [Triticum aestivum]	gi|290350670	24399.1	6.06	3	148	100	19.206	140	100	

Peptide Information

Calc. Mass	Obsrv. Mass	± da	± ppm	Start Seq.	End Seq.	Sequence	Ion Score	C. I. %	Modification	Rank	Result Type
1078.535	1078.5786	0.0436	40	193	201	GYPLLEACR			Carbamidomethyl (C)[8]		Mascot
1078.535	1078.5786	0.0436	40	193	201	GYPLLEACR	55	99.975	Carbamidomethyl (C)[8]		Mascot
1145.5698	1145.615	0.0452	39	145	154	GHNLSLEYGR					Mascot
1145.5698	1145.615	0.0452	39	145	154	GHNLSLEYGR	85	100			Mascot
1406.6271	1406.6857	0.0586	42	119	128	HREWESCFQK			Carbamidomethyl (C)[7]		Mascot

8	Ras-related protein RGP1 [Triticum urartu]	gi|474381041	23292.9	6.75	11	52	74.553	9.101			

Peptide Information

Calc. Mass	Obsrv. Mass	± da	± ppm	Start Seq.	End Seq.	Sequence	Ion Score	C. I. %	Modification	Rank	Result Type
930.468	930.4752	0.0072	8	65	72	AVTSAYYR					Mascot
1057.6252	1057.5444	-0.0808	-76	11	21	VVLIGDSAVGK					Mascot
1097.599	1097.6506	0.0516	47	2	10	GQKIDYVFK					Mascot
1114.6653	1114.6252	-0.0401	-36	106	115	NIVIMLIGNK					Mascot
1130.6602	1130.6245	-0.0357	-32	106	115	NIVIMLIGNK			Oxidation (M)[5]		Mascot
1130.6602	1130.6245	-0.0357	-32	106	115	NIVIMLIGNK			Oxidation (M)[5]		Mascot
1187.6743	1187.6726	-0.0017	-1	42	51	TLQIDNRTVK					Mascot
1228.6395	1228.5991	-0.0404	-33	1	10	MGQKIDYVFK					Mascot
1572.8229	1572.8794	0.0565	36	116	130	SDLGTLRAVPTEDAK					Mascot
1602.9214	1602.8824	-0.039	-24	188	203	GTKVIIPGQEPAPPAK					Mascot
1784.0277	1783.8997	-0.128	-72	11	27	VVLIGDSAVGKSQLLER					Mascot

1823.0314	1823.0471	0.0157	9	5	21	IDYVFKVVLIGDSAVGK					Mascot
1833.8701	1833.9481	0.078	43	87	100	QSFDHMARWLEELR			Oxidation (M)[6]		Mascot

9 ribulose-1,5-bisphosphate carboxylase/oxygenase small subunit [Triticum urartu] gi|4038713 18853.5 8.82 8 46 3.254 2.291

Protein Group

ribulose-1,5-bisphosphate carboxylase/oxygenase small subunit [Triticum timopheevii subsp. armeniacum] gi|4038715 18804.4 8.8299999237 0605

Peptide Information

Calc. Mass	Obsrv. Mass	± da	± ppm	Start Seq.	End Seq.	Sequence	Ion Score	C. I. %	Modification	Rank	Result Type
906.5043	906.4809	-0.0234	-26	71	77	QVDYLIR					Mascot
930.4178	930.4752	0.0574	62	107	112	YWTMWK			Oxidation (M)[4]		Mascot
1165.571	1165.6284	0.0574	49	80	88	WVPCLEFSK			Carbamidomethyl (C)[4]		Mascot
1320.6615	1320.6687	0.0072	5	26	39	RSSGSLGSVSNGGR					Mascot
1380.6981	1380.7648	0.0667	48	78	88	SKWVPCLEFSK			Carbamidomethyl (C)[6]		Mascot
1607.8462	1607.9105	0.0643	40	1	16	VMASSATTVAPFQGLK					Mascot
1629.8604	1629.884	0.0236	14	40	52	IRCMQVWPIEGIK			Carbamidomethyl (C)[3]		Mascot
1926.9708	1926.9629	-0.0079	-4	134	149	EYPDAYVRVIGFDNLR					Mascot

10 putative galacturonosyltransferase 4 [Triticum urartu] gi|474127664 78174.2 8.72 15 46 1 6.744

Peptide Information

Calc. Mass	Obsrv. Mass	± da	± ppm	Start Seq.	End Seq.	Sequence	Ion Score	C. I. %	Modification	Rank	Result Type
906.4276	906.4809	0.0533	59	219	226	ENTDGRSK					Mascot
1096.5051	1096.6034	0.0983	90	225	234	SKNMASSDTR					Mascot
1114.6005	1114.6252	0.0247	22	411	419	QVFHIVTDR					Mascot
1126.6216	1126.6205	-0.0011	-1	28	37	LTVAINPNER					Mascot
1128.6987	1128.6136	-0.0851	-75	292	301	VKALEQTLVK					Mascot
1136.5477	1136.6084	0.0607	53	227	236	NMASSDTRVR					Mascot
1152.5426	1152.606	0.0634	55	227	236	NMASSDTRVR			Oxidation (M)[2]		Mascot
1169.6241	1169.564	-0.0601	-51	498	506	FYLPEIYPK					Mascot
1187.6631	1187.6726	0.0095	8	270	280	EVQKVLGEASK					Mascot
1224.6624	1224.7079	0.0455	37	552	562	YLNFSSPVVAK					Mascot
1278.6399	1278.6633	0.0234	18	522	532	DITGLWSIDMK					Mascot
1406.7349	1406.6857	-0.0492	-35	521	532	KDITGLWSIDMK					Mascot

1422.7297	1422.6721	-0.0576	-40	521	532	KDITGLWSIDMK	Oxidation (M)[11]	Mascot
1606.8013	1606.9036	0.1023	64	586	597	QNITEIYHFWQK		Mascot
1607.7523	1607.9105	0.1582	98	460	472	QLGSQSMIDYYFR		Mascot
1613.8282	1613.8188	-0.0094	-6	38	52	KDFPAEIASQGHSVK		Mascot
3419.7847	3419.8794	0.0947	28	376	406	LDDPKQYHYALFSDNILA TAVVVNSTVLNAK		Mascot

Gel Idx/Pos	196/H23	Instr./Gel Origin	BA2151/Sample Project 20140814	Process Status	Analysis Succeeded
Plate [#] Name	[1] Sample Project 20140814	Instrument Sample Name		Spectra	11

Rank	Protein Name	Accession No.	Protein MW	Protein PI	Pep. Count	Protein Score	Protein Score C. I. %	Intensity Matched	Total Ion Score	Total Ion C. I. %	Confirmed
1	hypothetical protein TRIUR3_31593 [Triticum urartu]	gi|473979984	19793.1	5.63	13	289	100	13.744	220	100	

Peptide Information

Calc. Mass	Obsrv. Mass	± da	± ppm	Start Seq.	End Seq.	Sequence	Ion Score	C. I. %	Modification	Rank	Result Type
817.4162	817.4504	0.0342	42	79	84	REVEER					Mascot
888.4421	888.4931	0.051	57	63	69	VEIEENR					Mascot
926.5669	926.6085	0.0416	45	129	136	KLAPEQIK					Mascot
935.4595	935.5031	0.0436	47	85	91	KGDHWHR					Mascot
939.4617	939.5032	0.0415	44	99	104	FWRQMR			Oxidation (M)[5]		Mascot
943.4785	943.5114	0.0329	35	95	101	SYGKFWR					Mascot
1108.6473	1108.7036	0.0563	51	130	139	LAPEQIKGPR					Mascot
1329.6951	1329.762	0.0669	50	17	27	ILEHVPFGFDR					Mascot
1373.6907	1373.7559	0.0652	47	59	69	EDLKVEIEENR					Mascot
1770.8328	1770.8926	0.0598	34	42	57	ETSDSHEIVVDVPGMR					Mascot
2002.9626	2003.1459	0.1833	92	1	16	MEHWRMDVALLADPFR			Oxidation (M)[1]		Mascot
2226.1614	2226.271	0.1096	49	105	126	LPDNADLDSIAASLDAGV LTVR					Mascot
2226.1614	2226.271	0.1096	49	105	126	LPDNADLDSIAASLDAGV LTVR	220	100			Mascot
2421.1689	2421.2732	0.1043	43	17	37	ILEHVPFGFDRDDVAMVS MAR			Oxidation (M)[16]		Mascot
2437.1638	2437.2385	0.0747	31	17	37	ILEHVPFGFDRDDVAMVS MAR			Oxidation (M)[16,19]		Mascot

Rank	Protein Name	Accession No.	Protein MW	Protein PI	Pep. Count	Protein Score	Protein Score C. I. %	Intensity Matched	Total Ion Score	Total Ion C. I. %	Confirmed
2	Proteasome subunit beta type-6 [Triticum urartu]	gi|474432171	24640.6	6.06	9	209	100	9.35	161	100	

Peptide Information

Calc. Mass	Obsrv. Mass	± da	± ppm	Start Seq.	End Seq.	Sequence	Ion Score	C. I. %	Modification	Rank	Result Type
817.4163	817.4504	0.0341	42	144	152	DGASGGVVR					Mascot
828.5302	828.579	0.0488	59	136	143	VVSLAIAR					Mascot
849.4828	849.4609	-0.0219	-26	62	68	LLAYQNK					Mascot
943.4744	943.5114	0.037	39	163	170	RSFHPGDK					Mascot
1245.6798	1245.7362	0.0564	45	153	163	TVTINEEGVKR					Mascot
1467.7261	1467.7992	0.0731	50	11	22	ISQLTDNVYVCR			Carbamidomethyl (C)[11]		Mascot

Calc. Mass	Obsrv. Mass	± da	± ppm	Start Seq.	End Seq.	Sequence	Ion Score	C. I. %	Modification	Rank	Result Type
1467.7261	1467.7992	0.0731	50	11	22	ISQLTDNVYVCR	43	99.702	Carbamidomethyl (C)[11]		Mascot
1582.7708	1582.8483	0.0775	49	23	37	SGSAADTQIISDYVR					Mascot
1822.9698	1823.0623	0.0925	51	83	99	YEGGQIYSVPLGGTILR					Mascot
1822.9698	1823.0623	0.0925	51	83	99	YEGGQIYSVPLGGTILR	118	100			Mascot
1981.0654	1981.1412	0.0758	38	38	54	YFLHQHTIQLGQPATVK					Mascot

3	Proteasome subunit beta type-6 [Triticum urartu]	gi|473930301	26187	5.32	8	199	100	9.11	161	100	

Peptide Information

Calc. Mass	Obsrv. Mass	± da	± ppm	Start Seq.	End Seq.	Sequence	Ion Score	C. I. %	Modification	Rank	Result Type
817.4163	817.4504	0.0341	42	190	198	DGASGGVVR					Mascot
828.5302	828.579	0.0488	59	182	189	VVSLAIAR					Mascot
849.4828	849.4609	-0.0219	-26	108	114	LLAYQNK					Mascot
943.4744	943.5114	0.037	39	209	216	RSFHPGDK					Mascot
1467.7261	1467.7992	0.0731	50	57	68	ISQLTDNVYVCR			Carbamidomethyl (C)[11]		Mascot
1467.7261	1467.7992	0.0731	50	57	68	ISQLTDNVYVCR	43	99.702	Carbamidomethyl (C)[11]		Mascot
1582.7708	1582.8483	0.0775	49	69	83	SGSAADTQIISDYVR					Mascot
1822.9698	1823.0623	0.0925	51	129	145	YEGGQIYSVPLGGTILR					Mascot
1822.9698	1823.0623	0.0925	51	129	145	YEGGQIYSVPLGGTILR	118	100			Mascot
1981.0654	1981.1412	0.0758	38	84	100	YFLHQHTIQLGQPATVK					Mascot

4	hypothetical protein TRIUR3_02653 [Triticum urartu]	gi|473895929	88455.4	5.43	23	62	97.21	18.749	

Peptide Information

Calc. Mass	Obsrv. Mass	± da	± ppm	Start Seq.	End Seq.	Sequence	Ion Score	C. I. %	Modification	Rank	Result Type
805.4124	805.4517	0.0393	49	648	654	VMEAVEK					Mascot
828.5302	828.579	0.0488	59	232	238	RIIEGLK					Mascot
830.4618	830.5016	0.0398	48	12	18	VEELNVK					Mascot
832.441	832.3593	-0.0817	-98	483	489	ADLEKEK					Mascot
888.4785	888.4931	0.0146	16	690	697	VITAQDNK					Mascot
906.4349	906.4817	0.0468	52	455	461	KELDDMR					Mascot
935.4985	935.5031	0.0046	5	182	189	FGGILDWK					Mascot
963.4564	963.5076	0.0512	53	552	559	LACDEVTR			Carbamidomethyl (C)[3]		Mascot
1120.5997	1120.6395	0.0398	36	212	220	RFIAEEVEK					Mascot
1179.6256	1179.6649	0.0393	33	213	222	FIAEEVEKSK					Mascot
1196.6127	1196.6122	-0.0005	0	440	449	VRSMVSVQMK			Oxidation (M)[4,9]		Mascot
1228.6	1228.6025	0.0025	2	776	785	SIAMMFMNRK					Mascot

1253.6593	1253.6095	-0.0498	-40	442	452	SMVSVQMKLAK			Oxidation (M)[2,7]		Mascot
1259.6478	1259.6926	0.0448	36	755	767	ADTIATTAAAEPK					Mascot
1320.6941	1320.7295	0.0354	27	221	231	SKVLQELCSTR			Carbamidomethyl (C)[8]		Mascot
1347.705	1347.7017	-0.0033	-2	516	526	TMRELSVVQER					Mascot
1358.7349	1358.731	-0.0039	-3	527	538	TEAAKMPIELQK					Mascot
1582.7681	1582.8483	0.0802	51	132	144	EQQQISRHNSAER					Mascot
1754.8014	1754.92	0.1186	68	102	116	NFSEDIGSLTINECR			Carbamidomethyl (C)[14]		Mascot
1770.8076	1770.8926	0.085	48	117	131	ANKVEENCHDQLEGK			Carbamidomethyl (C)[8]		Mascot
1927.9971	1928.0287	0.0316	16	2	18	EELNVEGAQKVEELNVK					Mascot
2012.9304	2013.1245	0.1941	96	618	635	GSDKSMMVPLEDYDALNK					Mascot
2012.9304	2013.1245	0.1941	96	618	635	GSDKSMMVPLEDYDALNK					Mascot
2035.0568	2035.0688	0.012	6	497	514	DKVHHATVSVSSLQEELR					Mascot

5 Sorting nexin-1 [Triticum urartu] gi|474048334 106281.9 8.45 18 54 83.187 9.146

Peptide Information

Calc. Mass	Obsrv. Mass	± da	± ppm	Start Seq.	End Seq.	Sequence	Ion Score	C. I. %	Modification	Rank	Result Type
888.4323	888.4931	0.0608	68	4	10	HDFVSQR					Mascot
926.6033	926.6085	0.0052	6	783	792	VAAVIVGGLK					Mascot
939.4795	939.5032	0.0237	25	670	676	FQGFRER					Mascot
971.5632	971.5494	-0.0138	-14	561	570	GTGVPARVSK					Mascot
1200.71	1200.6013	-0.1087	-91	677	686	LLADPKFLQR					Mascot
1291.7006	1291.6893	-0.0113	-9	576	586	DHVVVLLDPER					Mascot
1302.7086	1302.7197	0.0111	9	137	148	TTTAHLGMTLIK			Oxidation (M)[8]		Mascot
1303.6324	1303.7183	0.0859	66	1	10	MQRHDFVSQR					Mascot
1359.7267	1359.7513	0.0246	18	758	770	GLLGSLPDNAFQK					Mascot
1467.7551	1467.7992	0.0441	30	495	507	SGRSFIENITSTR					Mascot
1467.7551	1467.7992	0.0441	30	495	507	SGRSFIENITSTR					Mascot
1706.9225	1706.9257	0.0032	2	53	68	HWTTTVNAAIPTVPAK					Mascot
1794.9061	1794.8594	-0.0467	-26	592	607	EGQIPVADFLWYSAAK					Mascot
1875.96	1876.0045	0.0445	24	35	52	TFLTEPGAIAAFQGEAPR					Mascot
1951.0072	1951.0365	0.0293	15	591	607	REGQIPVADFLWYSAAK					Mascot
1966.9613	1967.0161	0.0548	28	276	292	EYESIKIPEGGLCLCAK			Carbamidomethyl (C)[13,15]		Mascot
1966.9613	1967.0161	0.0548	28	276	292	EYESIKIPEGGLCLCAK			Carbamidomethyl (C)[13,15]		Mascot
1994.0601	1994.0789	0.0188	9	622	639	VMNAAGRRPEALPQDLQK					Mascot
2035.1335	2035.0688	-0.0647	-32	541	560	DPPGILIVVAGASHLIYGS					Mascot

Calc. Mass	Obsrv. Mass	± da	± ppm	Start Seq.	End Seq.	Sequence	Ion Score	C. I. %	Modification	Rank	Result Type
						R					
3373.8591	3373.8052	-0.0539	-16	793	828	LAGVGFISGVGAGVSSDLLYAARGLLKPSASVGAGR					Mascot

6 hypothetical protein TRIUR3_15152 [Triticum urartu] gi\|474074286 24206.7 9.61 9 46 0 3.028

Peptide Information

Calc. Mass	Obsrv. Mass	± da	± ppm	Start Seq.	End Seq.	Sequence	Ion Score	C. I. %	Modification	Rank	Result Type
805.4566	805.4517	-0.0049	-6	40	45	IEQIFR					Mascot
1145.679	1145.6306	-0.0484	-42	40	48	IEQIFRAIR					Mascot
1196.6462	1196.6122	-0.034	-28	160	168	SFLEFVLWR					Mascot
1332.6696	1332.7328	0.0632	47	16	26	FKTDFSHPTPR					Mascot
1359.7955	1359.7513	-0.0442	-33	2	14	STSRVTVIGVVNK					Mascot
1381.6931	1381.7048	0.0117	8	169	182	LHDAARAGGDATAR					Mascot
1490.8359	1490.8264	-0.0095	-6	1	14	MSTSRVTVIGVVNK					Mascot
1859.9916	1859.9945	0.0029	2	160	174	SFLEFVLWRLHDAAR					Mascot
2209.2017	2209.3037	0.102	46	46	65	AIRSWVEVFTATAVGVFIDK					Mascot

7 hypothetical protein TRIUR3_26078 [Triticum urartu] gi\|474369513 42322.3 6.1 11 46 0 13.64

Peptide Information

Calc. Mass	Obsrv. Mass	± da	± ppm	Start Seq.	End Seq.	Sequence	Ion Score	C. I. %	Modification	Rank	Result Type
800.3944	800.3726	-0.0218	-27	112	117	GRQMHR			Oxidation (M)[4]		Mascot
817.4315	817.4504	0.0189	23	300	305	RWQEAK					Mascot
963.4927	963.5076	0.0149	15	310	317	VMEERGVK			Oxidation (M)[2]		Mascot
971.4727	971.5494	0.0767	79	36	43	CHDLLSAR			Carbamidomethyl (C)[1]		Mascot
1291.7004	1291.6893	-0.0111	-9	232	243	AGRLEEAYGVVK					Mascot
1376.7103	1376.7657	0.0554	40	2	13	YAACALPDLARR			Carbamidomethyl (C)[4]		Mascot
1507.7509	1507.799	0.0481	32	1	13	MYAACALPDLARR			Carbamidomethyl (C)[5]		Mascot
1582.7241	1582.8483	0.1242	78	53	66	DVVSWSAMIDGCVK			Carbamidomethyl (C)[12], Oxidation (M)[8]		Mascot
1909.0537	1909.0807	0.027	14	346	361	EIYALLNLITVEMKMK					Mascot
1949.9426	1950.0818	0.1392	71	360	375	MKDDVAIPEYFLHTDR					Mascot
2012.9417	2013.1245	0.1828	91	72	90	EALALFEMMETTGAGNGVR			Oxidation (M)[8]		Mascot
2012.9417	2013.1245	0.1828	91	72	90	EALALFEMMETTGAGNGVR			Oxidation (M)[8]		Mascot

8 Disease resistance protein RPM1 [Triticum urartu] gi\|473894777 103193.5 6.68 18 44 0 6.255

Peptide Information

Calc. Mass	Obsrv. Mass	± da	± ppm	Start Seq.	End Seq.	Sequence	Ion Score	C. I. %	Modification	Rank	Result Type
828.5189	828.579	0.0601	73	629	636	LLLVDGAK					Mascot
830.528	830.5016	-0.0264	-32	673	679	MRILGLK					Mascot
834.4502	834.4919	0.0417	50	212	218	SMLSQIR					Mascot
835.4924	835.4278	-0.0646	-77	567	573	LEIGYLK					Mascot
935.5309	935.5031	-0.0278	-30	710	717	LFVKTDGR					Mascot
1120.6361	1120.6395	0.0034	3	166	177	VVSLIGFGGSGK					Mascot
1179.662	1179.6649	0.0029	2	564	573	SEKLEIGYLK					Mascot
1233.7202	1233.6908	-0.0294	-24	438	449	VVGGKILFDTGK					Mascot
1291.7621	1291.6893	-0.0728	-56	567	577	LEIGYLKNITK					Mascot
1329.7009	1329.762	0.0611	46	26	37	LAEAEALDTQLR					Mascot
1349.6617	1349.7072	0.0455	34	14	25	DELSSMNALLEK					Mascot
1376.7897	1376.7657	-0.024	-17	702	713	LGSSQLERLFVK					Mascot
1406.7638	1406.7023	-0.0615	-44	514	525	LSIDYRGLENVK					Mascot
1794.9783	1794.8594	-0.1189	-66	331	345	CPLHLNEISTEIIKK			Carbamidomethyl (C)[1]		Mascot
1927.9219	1928.0287	0.1068	55	227	242	SSDEAWLINSMRDFLK			Oxidation (M)[11]		Mascot
1949.9896	1950.0818	0.0922	47	273	289	CALLENSCGSRIIVTTR			Carbamidomethyl (C)[1,8]		Mascot
1956.1562	1956.0511	-0.1051	-54	345	363	KCGGLPLAIITIGSLLTTK			Carbamidomethyl (C)[2]		Mascot
1994.9753	1995.0762	0.1009	51	191	207	LFNCQAFVSVSQNPDIR			Carbamidomethyl (C)[4]		Mascot

9 gamma gliadin [Triticum aestivum] gi|133741924 16537.3 8.88 2 43 0 3.943 36 98.251

Peptide Information

Calc. Mass	Obsrv. Mass	± da	± ppm	Start Seq.	End Seq.	Sequence	Ion Score	C. I. %	Modification	Rank	Result Type
856.416	856.4077	-0.0083	-10	138	145	STTTRSGF					Mascot
1950.9413	1951.0365	0.0952	49	60	76	DALLQQCSPVADMSFLR			Carbamidomethyl (C)[7]		Mascot
1966.9362	1967.0161	0.0799	41	60	76	DALLQQCSPVADMSFLR			Carbamidomethyl (C)[7], Oxidation (M)[13]		Mascot
1966.9362	1967.0161	0.0799	41	60	76	DALLQQCSPVADMSFLR	36	98.251	Carbamidomethyl (C)[7], Oxidation (M)[13]		Mascot

10 Acid phosphatase 1 [Triticum urartu] gi|474391855 30920.3 8.55 9 41 0 6.432

Peptide Information

Calc. Mass	Obsrv. Mass	± da	± ppm	Start Seq.	End Seq.	Sequence	Ion Score	C. I. %	Modification	Rank	Result Type
839.4258	839.4061	-0.0197	-23	233	240	GQSSSVFK					Mascot
1107.5793	1107.6079	0.0286	26	245	253	KQLVDEGYR					Mascot
1196.5914	1196.6122	0.0208	17	224	232	LMMRTPEYR					Mascot

1228.5813	1228.6025	0.0212	17	224	232	LMMRTPEYR	Oxidation (M)[2,3]	Mascot
1302.63	1302.7197	0.0897	69	156	166	RFGAYDPMAFK		Mascot
1318.6249	1318.7152	0.0903	68	156	166	RFGAYDPMAFK	Oxidation (M)[8]	Mascot
1329.6945	1329.762	0.0675	51	1	14	MRDVVLGGGSGPGK		Mascot
1467.7705	1467.7992	0.0287	20	274	285	VFKIPNPMYFVP	Oxidation (M)[8]	Mascot
1467.7705	1467.7992	0.0287	20	274	285	VFKIPNPMYFVP	Oxidation (M)[8]	Mascot
1501.7581	1501.8647	0.1066	71	44	56	GKQSMQWPASLPR	Oxidation (M)[5]	Mascot
1753.9166	1753.9247	0.0081	5	46	60	QSMQWPASLPRSIPR		Mascot
1902.9313	1903.0477	0.1164	61	15	31	GKQSMQWPASLPQSAM R		Mascot
1902.9313	1903.0477	0.1164	61	15	31	GKQSMQWPASLPQSAM R		Mascot

Gel Idx/Pos	197/H24	Instr./Gel Origin	BA2151/Sample Project 20140814	Process Status	Analysis Succeeded
Plate [#] Name	[1] Sample Project 20140814	Instrument Sample Name		Spectra	11

Rank	Protein Name	Accession No.	Protein MW	Protein PI	Pep. Count	Protein Score	Protein Score C. I. %	Intensity Matched	Total Ion Score	Total Ion C. I. %	Confirmed
1	gliadin/avenin-like seed protein [Triticum aestivum]	gi|281335538	22813.2	6.2	4	174	100	18.147	152	100	

Peptide Information

Calc. Mass	Obsrv. Mass	± da	± ppm	Start Seq.	End Seq.	Sequence	Ion Score	C. I. %	Modification	Rank	Result Type
1950.9413	1951.0499	0.1086	56	60	76	DALLQQCSPVADMSFLR			Carbamidomethyl (C)[7]		Mascot
1966.9362	1967.0344	0.0982	50	60	76	DALLQQCSPVADMSFLR			Carbamidomethyl (C)[7], Oxidation (M)[13]		Mascot
1966.9362	1967.0344	0.0982	50	60	76	DALLQQCSPVADMSFLR	111	100	Carbamidomethyl (C)[7], Oxidation (M)[13]		Mascot
2635.1885	2635.3235	0.135	51	77	97	SQVVQHSSCLVMWEQC CQQLK			Carbamidomethyl (C)[9,16,17]		Mascot
2651.1833	2651.3323	0.149	56	77	97	SQVVQHSSCLVMWEQC CQQLK			Carbamidomethyl (C)[9,16,17], Oxidation (M)[12]		Mascot
3060.4521	3060.6226	0.1705	56	77	101	SQVVQHSSCLVMWEQC CQQLKAIPK			Carbamidomethyl (C)[9,16,17], Oxidation (M)[12]		Mascot
3217.5198	3217.7222	0.2024	63	27	54	SAWEPQHPSSPEHQPTP QPQEHPVPHQK					Mascot
3217.5198	3217.7222	0.2024	63	27	54	SAWEPQHPSSPEHQPTP QPQEHPVPHQK	41	99.568			Mascot

Rank	Protein Name	Accession No.	Protein MW	Protein PI	Pep. Count	Protein Score	Protein Score C. I. %	Intensity Matched	Total Ion Score	Total Ion C. I. %	Confirmed
2	Avenin-3 [Triticum urartu]	gi|474329936	22657.1	6.35	4	132	100	17.231	111	100	

Peptide Information

Calc. Mass	Obsrv. Mass	± da	± ppm	Start Seq.	End Seq.	Sequence	Ion Score	C. I. %	Modification	Rank	Result Type
1950.9413	1951.0499	0.1086	56	60	76	DALLQQCSPVADMSFLR			Carbamidomethyl (C)[7]		Mascot
1966.9362	1967.0344	0.0982	50	60	76	DALLQQCSPVADMSFLR			Carbamidomethyl (C)[7], Oxidation (M)[13]		Mascot
1966.9362	1967.0344	0.0982	50	60	76	DALLQQCSPVADMSFLR	111	100	Carbamidomethyl (C)[7], Oxidation (M)[13]		Mascot
2635.1885	2635.3235	0.135	51	77	97	SQVVQHSSCLVMWEQC CQQLK			Carbamidomethyl (C)[9,16,17]		Mascot
2651.1833	2651.3323	0.149	56	77	97	SQVVQHSSCLVMWEQC CQQLK			Carbamidomethyl (C)[9,16,17], Oxidation (M)[12]		Mascot
3060.4521	3060.6226	0.1705	56	77	101	SQVVQHSSCLVMWEQC CQQLKAIPK			Carbamidomethyl (C)[9,16,17], Oxidation (M)[12]		Mascot
3222.5352	3222.7131	0.1779	55	27	54	TAWEPHHPSSPEQQPTP QPQEQPVPHQK					Mascot

Rank	Protein Name	Accession No.	Protein MW	Protein PI	Pep. Count	Protein Score	Protein Score C. I. %	Intensity Matched	Total Ion Score	Total Ion C. I. %	Confirmed
3	gamma gliadin [Triticum aestivum]	gi|133741924	16537.3	8.88	2	118	100	16.792	111	100	

Peptide Information

Calc. Mass	Obsrv. Mass	± da	± ppm	Start Seq.	End Seq.	Sequence	Ion Score	C. I. %	Modification	Rank	Result Type
856.416	856.4152	-0.0008	-1	138	145	STTTRSGF					Mascot

1950.9413	1951.0499	0.1086	56	60	76	DALLQQCSPVADMSFLR			Carbamidomethyl (C)[7]		Mascot
1966.9362	1967.0344	0.0982	50	60	76	DALLQQCSPVADMSFLR			Carbamidomethyl (C)[7], Oxidation (M)[13]		Mascot
1966.9362	1967.0344	0.0982	50	60	76	DALLQQCSPVADMSFLR	111	100	Carbamidomethyl (C)[7], Oxidation (M)[13]		Mascot

4	putative methyltransferase PMT26 [Triticum urartu]	gi|474134322	109681.1	6.45	18	47	11.766	22.724	

Peptide Information

Calc. Mass	Obsrv. Mass	± da	± ppm	Start Seq.	End Seq.	Sequence	Ion Score	C. I. %	Modification	Rank	Result Type
883.5148	883.4744	-0.0404	-46	498	504	SPIRWPK					Mascot
906.4163	906.4916	0.0753	83	358	365	QQADSTEK					Mascot
1142.551	1142.6293	0.0783	69	21	29	DLKPMEEHK			Oxidation (M)[5]		Mascot
1144.6395	1144.6077	-0.0318	-28	613	624	GIPAISAVMGTK					Mascot
1182.5209	1182.6144	0.0935	79	944	953	TMWRPNTSSS			Oxidation (M)[2]		Mascot
1187.6572	1187.6923	0.0351	30	758	766	VERPPYWLK					Mascot
1297.7151	1297.6373	-0.0778	-60	308	318	LFNGLTKDVYK					Mascot
1490.7784	1490.8168	0.0384	26	693	705	VGVAIFQKPMDNR			Oxidation (M)[10]		Mascot
1493.7794	1493.8135	0.0341	23	123	134	HGIQHMKPFVQR			Oxidation (M)[6]		Mascot
1794.8125	1794.8651	0.0526	29	251	265	FHNMMVFEAMHVGTK			Oxidation (M)[4]		Mascot
1794.8125	1794.8651	0.0526	29	251	265	FHNMMVFEAMHVGTK			Oxidation (M)[4]		Mascot
1810.8074	1810.8536	0.0462	26	251	265	FHNMMVFEAMHVGTK			Oxidation (M)[4,5]		Mascot
1826.8024	1826.8625	0.0601	33	251	265	FHNMMVFEAMHVGTK			Oxidation (M)[4,5,10]		Mascot
1859.9143	1860.0336	0.1193	64	809	825	NIMDMKAVYGGFAAALR			Oxidation (M)[3,5]		Mascot
1909.98	1910.0164	0.0364	19	291	307	KMILEHDLADDNAAVVR					Mascot
1950.9738	1951.0499	0.0761	39	135	150	DMLMVENQLPLMFLEK					Mascot
1966.9686	1967.0344	0.0658	33	135	150	DMLMVENQLPLMFLEK			Oxidation (M)[2]		Mascot
1966.9686	1967.0344	0.0658	33	135	150	DMLMVENQLPLMFLEK			Oxidation (M)[2]		Mascot
1982.9636	1983.0267	0.0631	32	135	150	DMLMVENQLPLMFLEK			Oxidation (M)[2,4]		Mascot
1982.9636	1983.0267	0.0631	32	135	150	DMLMVENQLPLMFLEK			Oxidation (M)[2,4]		Mascot
1996.9797	1997.0676	0.0879	44	791	808	VISNSYMEGLGIDWSAVR					Mascot
2322.1001	2322.283	0.1829	79	78	96	GKFLEMMITDGCFLLEVMR			Carbamidomethyl (C)[12], Oxidation (M)[6,7]		Mascot
2651.3228	2651.3323	0.0095	4	524	547	GHQNWVNVSGEHLVFPGGGTQFKR					Mascot
3060.478	3060.6226	0.1446	47	251	276	FHNMMVFEAMHVGTKNDVTAYVLFVK			Oxidation (M)[4,5]		Mascot
3419.804	3419.8896	0.0856	25	659	687	LLRPGGYFVWSATPVYQKLPEDVEIWEVK					Mascot

5	hypothetical protein TRIUR3_18929 [Triticum urartu]	gi|474195889	44166.9	10.11	11	45	0	3.013	

Peptide Information

Calc. Mass	Obsrv. Mass	± da	± ppm	Start Seq.	End Seq.	Sequence	Ion Score	C. I. %	Modification	Rank	Result Type
1092.5209	1092.6106	0.0897	82	299	308	EGDTEAPFVK					Mascot
1142.6165	1142.6293	0.0128	11	165	176	DGSAPKPASGKK					Mascot
1219.629	1219.6055	-0.0235	-19	53	64	GRSPGVPAAHDR					Mascot
1235.6453	1235.5841	-0.0612	-50	176	187	KFVSGSPSAMPK					Mascot
1493.8435	1493.8135	-0.03	-20	250	264	DGGEAPAAKRPLLAK					Mascot
1810.9011	1810.8536	-0.0475	-26	384	398	QFLLNFYDEVPNSPK					Mascot
1811.8156	1811.973	0.1574	87	214	230	DSTEAPSATDKNHGEPR					Mascot
1811.8156	1811.973	0.1574	87	214	230	DSTEAPSATDKNHGEPR	1	0			Mascot
1822.8488	1822.8593	0.0105	6	318	335	SEPAKSTCGSPSTSIADK			Carbamidomethyl (C)[8]		Mascot
1827.0222	1826.8625	-0.1597	-87	231	249	GLALVIVNDGSGAALVTE K					Mascot
1928.9382	1929.0305	0.0923	48	354	371	KPSLMAAADDEITPNEAR					Mascot
1972.9434	1973.0416	0.0982	50	137	156	DDSQAAAVAVSAWPPMT GAK					Mascot
1988.9382	1989.0048	0.0666	33	137	156	DDSQAAAVAVSAWPPMT GAK			Oxidation (M)[16]		Mascot

6 hypothetical protein TRIUR3_21898 [Triticum urartu] gi|474418703 35926.1 5.85 9 42 0 7.654

Peptide Information

Calc. Mass	Obsrv. Mass	± da	± ppm	Start Seq.	End Seq.	Sequence	Ion Score	C. I. %	Modification	Rank	Result Type
1179.5939	1179.6605	0.0666	56	226	235	AGRFMEAIER					Mascot
1182.6453	1182.6144	-0.0309	-26	27	35	VFFERMVVR					Mascot
1219.5446	1219.6055	0.0609	50	301	311	MGEVMHDAGIK			Oxidation (M)[1,5]		Mascot
1297.6859	1297.6373	-0.0486	-37	270	281	NLVALGPQQSDR					Mascot
1406.775	1406.7052	-0.0698	-50	257	269	LHGNVELVAEIGR					Mascot
1859.9724	1860.0336	0.0612	33	239	256	MPFLPDASVWGALLGAS K					Mascot
1875.9674	1876.0226	0.0552	29	239	256	MPFLPDASVWGALLGAS K			Oxidation (M)[1]		Mascot
1875.9674	1876.0226	0.0552	29	239	256	MPFLPDASVWGALLGAS K			Oxidation (M)[1]		Mascot
1918.9337	1919.051	0.1173	61	209	225	VTPMMAHYGCVVDLLGR			Carbamidomethyl (C)[10]		Mascot
1950.9235	1951.0499	0.1264	65	209	225	VTPMMAHYGCVVDLLGR			Carbamidomethyl (C)[10], Oxidation (M)[4,5]		Mascot
2635.2524	2635.3235	0.0711	27	1	26	MASPDLASSNAMLDVLC LAGDVAAAR			Carbamidomethyl (C)[17], Oxidation (M)[1]		Mascot
2651.2473	2651.3323	0.085	32	1	26	MASPDLASSNAMLDVLC LAGDVAAAR			Carbamidomethyl (C)[17], Oxidation (M)[1,12]		Mascot
3247.7297	3247.7231	-0.0066	-2	239	269	MPFLPDASVWGALLGAS KLHGNVELVAEIGR					Mascot

7 Sorting nexin-13 [Triticum urartu] gi|474446083 115810 7.39 16 42 0 19.756

Peptide Information

Calc. Mass	Obsrv. Mass	± da	± ppm	Start Seq.	End Seq.	Sequence	Ion Score	C. I. %	Modification	Rank	Result Type
938.5305	938.516	-0.0145	-15	1010	1017	LIEGIHEK					Mascot
1097.595	1097.6626	0.0676	62	345	354	SKVLVDDHGK					Mascot
1187.6565	1187.6923	0.0358	30	396	405	NMVKVERPSK					Mascot
1219.6351	1219.6055	-0.0296	-24	156	165	EMNLVDLLTR			Oxidation (M)[2]		Mascot
1228.6144	1228.5996	-0.0148	-12	542	551	CRVVGAYFEK			Carbamidomethyl (C)[1]		Mascot
1297.7151	1297.6373	-0.0778	-60	544	555	VVGAYFEKLGSK					Mascot
1493.7191	1493.8135	0.0944	63	504	517	SNQTALSSESLNSR					Mascot
1557.8384	1557.953	0.1146	74	360	373	QADWALVLDAATKR					Mascot
1851.9429	1852.0175	0.0746	40	888	902	WIQDTLWPNGVFFTK					Mascot
1860.1205	1860.0336	-0.0869	-47	952	969	KLLLGGTPSTLVSIIGYK					Mascot
1944.0007	1944.0503	0.0496	26	524	541	GSVPSSHGEVLMYAPKIR			Oxidation (M)[12]		Mascot
1949.979	1950.0786	0.0996	51	974	989	SARDIYYFLQSTVCVK			Carbamidomethyl (C)[14]		Mascot
1950.9073	1951.0499	0.1426	73	181	198	NQSEIGVDVMGTLSSEER					Mascot
1966.9022	1967.0344	0.1322	67	181	198	NQSEIGVDVMGTLSSEER			Oxidation (M)[10]		Mascot
1966.9022	1967.0344	0.1322	67	181	198	NQSEIGVDVMGTLSSEER	5	0	Oxidation (M)[10]		Mascot
1983.0658	1983.0267	-0.0391	-20	355	372	TVQPRQADWALVLDAATK					Mascot
1983.0658	1983.0267	-0.0391	-20	355	372	TVQPRQADWALVLDAATK					Mascot
3268.7656	3268.7007	-0.0649	-20	225	253	VLQELVGGIMALVLRPQDAQSPLVRCFSR			Carbamidomethyl (C)[26], Oxidation (M)[10]		Mascot
3419.6333	3419.8896	0.2563	75	888	917	WIQDTLWPNGVFFTKLDGYQGNAGSSQFDK					Mascot

8 hypothetical protein TRIUR3_04057 [Triticum urartu] gi|474150269 26394.9 5.03 8 42 0 2.195

Peptide Information

Calc. Mass	Obsrv. Mass	± da	± ppm	Start Seq.	End Seq.	Sequence	Ion Score	C. I. %	Modification	Rank	Result Type
865.4373	865.4634	0.0261	30	203	210	TEASSKSR					Mascot
871.4421	871.5052	0.0631	72	2	8	GKDHTWK					Mascot
1144.5957	1144.6077	0.012	10	91	101	QKPADGSADKK					Mascot
1145.5797	1145.6301	0.0504	44	114	124	DSNAAPVESKK					Mascot
1631.7959	1631.8483	0.0524	32	20	33	LQENEGMVGKFSHR					Mascot
1647.7908	1647.8419	0.0511	31	20	33	LQENEGMVGKFSHR			Oxidation (M)[7]		Mascot
1892.9601	1893.0314	0.0713	38	211	228	STKAQPPAVESSSLSEFK					Mascot
1988.9772	1989.0048	0.0276	14	164	183	ASETSVAQQEPSAVASGIEK					Mascot

3218.5408	3218.7151	0.1743	54	150	183	EVSSSGQGGANGAKASE TSVAQQEPSAVASGIEK						Mascot

9 WW domain-binding protein 4 [Triticum urartu] gi|473797577 30998.9 9.75 9 39 0 2.583

Peptide Information

Calc. Mass	Obsrv. Mass	± da	± ppm	Start Seq.	End Seq.	Sequence	Ion Score	C. I. %	Modification	Rank	Result Type
871.4818	871.5052	0.0234	27	215	221	KPLNPMR			Oxidation (M)[6]		Mascot
1078.5966	1078.5875	-0.0091	-8	268	276	EKPLMGLYK					Mascot
1135.5531	1135.4916	-0.0615	-54	36	45	GPYWVSQGNK					Mascot
1647.8239	1647.8419	0.018	11	31	45	ADLGRGPYWVSQGNK					Mascot
1855.8861	1856.0358	0.1497	81	166	180	WVTQEEAYKWAETSK					Mascot
1902.0808	1902.057	-0.0238	-13	193	214	TPSGGGPVAGIKGGPAP GVVVK					Mascot
1949.9563	1950.0786	0.1223	63	175	192	WAETSKTNVAQSSTSQP K					Mascot
1973.0603	1973.0416	-0.0187	-9	52	68	IFISNNPFSIRTHELGK					Mascot
1989.0739	1989.0048	-0.0691	-35	1	17	MTEVLPHLPPPFEIRGR					Mascot
2005.0688	2004.9845	-0.0843	-42	1	17	MTEVLPHLPPPFEIRGR			Oxidation (M)[1]		Mascot

10 39S ribosomal protein L28, mitochondrial [Triticum urartu] gi|474019718 35173.7 9.22 10 39 0 3.105

Peptide Information

Calc. Mass	Obsrv. Mass	± da	± ppm	Start Seq.	End Seq.	Sequence	Ion Score	C. I. %	Modification	Rank	Result Type
866.4036	866.452	0.0484	56	130	136	GKMETER			Oxidation (M)[3]		Mascot
1078.5779	1078.5875	0.0096	9	202	211	AGGIDEYLLK					Mascot
1120.5634	1120.6555	0.0921	82	252	260	IEQGFEEIR					Mascot
1142.5953	1142.6293	0.034	30	168	176	NWKPNVQEK					Mascot
1380.665	1380.7871	0.1221	88	217	228	MDTEMGIAWKAK					Mascot
1406.7274	1406.7052	-0.0222	-16	252	263	IEQGFEEIRSAK					Mascot
1631.7483	1631.8483	0.1	61	98	113	WGGGSATCPAEDKAPK			Carbamidomethyl (C)[8]		Mascot
1931.8918	1932.0483	0.1565	81	232	248	MYSELAGMEVGFFPPEK					Mascot
3060.5085	3060.6226	0.1141	37	70	95	RLVLGFHVHGLFSSDAW GCLYMVSHR			Carbamidomethyl (C)[19], Oxidation (M)[22]		Mascot
3249.4468	3249.7166	0.2698	83	138	166	YENGNGNLQNGNGNGIF YAETETKTDWSR					Mascot
3249.4468	3249.7166	0.2698	83	138	166	YENGNGNLQNGNGNGIF YAETETKTDWSR					Mascot

Gel Idx/Pos	201/I1	Instr./Gel Origin	BA2151/Sample Project 20140814	Process Status	Analysis Succeeded
Plate [#] Name	[1] Sample Project 20140814	Instrument Sample Name		Spectra	11

Rank	Protein Name	Accession No.	Protein MW	Protein PI	Pep. Count	Protein Score	Protein Score C. I. %	Intensity Matched	Total Ion Score	Total Ion C. I. %	Confirmed
1	Avenin-3 [Triticum urartu]	gi|474329936	22657.1	6.35	4	136	100	18.408	115	100	

Peptide Information

Calc. Mass	Obsrv. Mass	± da	± ppm	Start Seq.	End Seq.	Sequence	Ion Score	C. I. %	Modification	Rank	Result Type
1950.9413	1950.929	-0.0123	-6	60	76	DALLQQCSPVADMSFLR			Carbamidomethyl (C)[7]		Mascot
1966.9362	1966.9025	-0.0337	-17	60	76	DALLQQCSPVADMSFLR			Carbamidomethyl (C)[7], Oxidation (M)[13]		Mascot
1966.9362	1966.9025	-0.0337	-17	60	76	DALLQQCSPVADMSFLR	115	100	Carbamidomethyl (C)[7], Oxidation (M)[13]		Mascot
2591.2527	2591.2151	-0.0376	-15	55	76	LNPCRDALLQQCSPVAD MSFLR			Carbamidomethyl (C)[4,12]		Mascot
2635.1885	2635.1636	-0.0249	-9	77	97	SQVVQHSSCLVMWEQC CQQLK			Carbamidomethyl (C)[9,16,17]		Mascot
2651.1833	2651.147	-0.0363	-14	77	97	SQVVQHSSCLVMWEQC CQQLK			Carbamidomethyl (C)[9,16,17], Oxidation (M)[12]		Mascot
2651.1833	2651.147	-0.0363	-14	77	97	SQVVQHSSCLVMWEQC CQQLK			Carbamidomethyl (C)[9,16,17], Oxidation (M)[12]		Mascot
3222.5352	3222.5293	-0.0059	-2	27	54	TAWEPHHPSSPEQQPTP QPQEQPVPHQK					Mascot

Rank	Protein Name	Accession No.	Protein MW	Protein PI	Pep. Count	Protein Score	Protein Score C. I. %	Intensity Matched	Total Ion Score	Total Ion C. I. %	Confirmed
2	gliadin/avenin-like seed protein [Triticum aestivum]	gi|281335538	22813.2	6.2	4	136	100	18.353	115	100	

Peptide Information

Calc. Mass	Obsrv. Mass	± da	± ppm	Start Seq.	End Seq.	Sequence	Ion Score	C. I. %	Modification	Rank	Result Type
1950.9413	1950.929	-0.0123	-6	60	76	DALLQQCSPVADMSFLR			Carbamidomethyl (C)[7]		Mascot
1966.9362	1966.9025	-0.0337	-17	60	76	DALLQQCSPVADMSFLR			Carbamidomethyl (C)[7], Oxidation (M)[13]		Mascot
1966.9362	1966.9025	-0.0337	-17	60	76	DALLQQCSPVADMSFLR	115	100	Carbamidomethyl (C)[7], Oxidation (M)[13]		Mascot
2591.2527	2591.2151	-0.0376	-15	55	76	LNPCRDALLQQCSPVAD MSFLR			Carbamidomethyl (C)[4,12]		Mascot
2635.1885	2635.1636	-0.0249	-9	77	97	SQVVQHSSCLVMWEQC CQQLK			Carbamidomethyl (C)[9,16,17]		Mascot
2651.1833	2651.147	-0.0363	-14	77	97	SQVVQHSSCLVMWEQC CQQLK			Carbamidomethyl (C)[9,16,17], Oxidation (M)[12]		Mascot
2651.1833	2651.147	-0.0363	-14	77	97	SQVVQHSSCLVMWEQC CQQLK			Carbamidomethyl (C)[9,16,17], Oxidation (M)[12]		Mascot
3217.5198	3217.5295	0.0097	3	27	54	SAWEPQHPSSPEHQPTP QPQEHPVPHQK					Mascot

Rank	Protein Name	Accession No.	Protein MW	Protein PI	Pep. Count	Protein Score	Protein Score C. I. %	Intensity Matched	Total Ion Score	Total Ion C. I. %	Confirmed
3	gamma gliadin [Triticum aestivum]	gi|133741924	16537.3	8.88	3	129	100	17.871	115	100	

Peptide Information

Calc. Mass	Obsrv. Mass	± da	± ppm	Start Seq.	End Seq.	Sequence	Ion Score	C. I. %	Modification	Rank	Result Type

856.416	856.3511	-0.0649	-76	138	145	STTTRSGF					Mascot
1950.9413	1950.929	-0.0123	-6	60	76	DALLQQCSPVADMSFLR			Carbamidomethyl (C)[7]		Mascot
1966.9362	1966.9025	-0.0337	-17	60	76	DALLQQCSPVADMSFLR			Carbamidomethyl (C)[7], Oxidation (M)[13]		Mascot
1966.9362	1966.9025	-0.0337	-17	60	76	DALLQQCSPVADMSFLR	115	100	Carbamidomethyl (C)[7], Oxidation (M)[13]		Mascot
2591.2527	2591.2151	-0.0376	-15	55	76	LNPCRDALLQQCSPVAD MSFLR			Carbamidomethyl (C)[4,12]		Mascot

4 hypothetical protein TRIUR3_19596 [Triticum urartu] gi|473887867 16404.2 8.88 6 41 0 5.128

Peptide Information

Calc. Mass	Obsrv. Mass	± da	± ppm	Start Seq.	End Seq.	Sequence	Ion Score	C. I. %	Modification	Rank	Result Type
849.4465	849.4175	-0.029	-34	80	86	VEEGFIR					Mascot
1097.5739	1097.5914	0.0175	16	28	37	FYVRAGEGAK					Mascot
1811.7874	1811.9285	0.1411	78	131	144	FSQQKDLMHTECCK			Carbamidomethyl (C)[12,13]		Mascot
1811.7874	1811.9285	0.1411	78	131	144	FSQQKDLMHTECCK			Carbamidomethyl (C)[12,13]		Mascot
1851.9019	1851.8625	-0.0394	-21	62	79	SLKSWAASTGVSCDGAV R			Carbamidomethyl (C)[13]		Mascot
3233.5586	3233.5259	-0.0327	-10	100	130	HHAFDVVDAPDGVVATG CPPAGGIFSAYHIR			Carbamidomethyl (C)[18]		Mascot
3389.6597	3389.6235	-0.0362	-11	99	130	RHHAFDVVDAPDGVVAT GCPPAGGIFSAYHIR			Carbamidomethyl (C)[19]		Mascot

5 3-deoxy-manno-octulosonate cytidylyltransferase [Triticum urartu] gi|474449980 24529.4 6.07 7 41 0 1.347

Peptide Information

Calc. Mass	Obsrv. Mass	± da	± ppm	Start Seq.	End Seq.	Sequence	Ion Score	C. I. %	Modification	Rank	Result Type
1060.5244	1060.5123	-0.0121	-11	184	192	ACQSWLAPK			Carbamidomethyl (C)[2]		Mascot
1097.566	1097.5914	0.0254	23	175	183	VLENGYKMK			Oxidation (M)[8]		Mascot
1335.6548	1335.6204	-0.0344	-26	182	192	MKACQSWLAPK			Carbamidomethyl (C)[4], Oxidation (M)[1]		Mascot
1918.948	1918.9167	-0.0313	-16	107	122	VKCVVDNQGYAIYFSR			Carbamidomethyl (C)[3]		Mascot
2635.3362	2635.1636	-0.1726	-65	84	108	APDAVFSTAATALKPEDA FDTNRVK					Mascot
3277.6772	3277.5459	-0.1313	-40	55	83	HYDIVVNIQGDEPLIEPDII DGVVMALQR			Oxidation (M)[25]		Mascot
3389.7773	3389.6235	-0.1538	-45	54	83	KHYDIVVNIQGDEPLIEPD IIDGVVMALQR					Mascot

6 Cysteine proteinase inhibitor 3 [Triticum urartu] gi|473777552 14557.3 6.92 6 40 0 1.019

Peptide Information

Calc. Mass	Obsrv. Mass	± da	± ppm	Start Seq.	End Seq.	Sequence	Ion Score	C. I. %	Modification	Rank	Result Type

Calc. Mass	Obsrv. Mass	± da	± ppm	Start Seq.	End Seq.	Sequence	Ion Score	C. I. %	Modification	Rank	Result Type
832.3795	832.3098	-0.0697	-84	9	16	AEAEDAAR					Mascot
1117.5232	1117.5082	-0.015	-13	7	16	ERAEAEDAAR					Mascot
1924.7826	1924.931	0.1484	77	116	133	MHSNENTGLSVDSSSSQ E			Oxidation (M)[1]		Mascot
1997.0372	1996.9279	-0.1093	-55	41	59	QVVAGTLHDLMVEVVDS GK					Mascot
2993.3462	2993.3516	0.0054	2	106	133	LSLQTDMAPKMHSNENT GLSVDSSSSQE					Mascot
3406.6431	3406.6287	-0.0144	-4	83	115	HAGDFQSESSVASDAST GQAILKLSLQTDMAPK			Oxidation (M)[30]		Mascot

7	auxin-responsive Aux/IAA family protein [Triticum monococcum]	gi|207175053	16116.2	5.22	6	38	0	1.051	

Peptide Information

Calc. Mass	Obsrv. Mass	± da	± ppm	Start Seq.	End Seq.	Sequence	Ion Score	C. I. %	Modification	Rank	Result Type
1298.5681	1298.5768	0.0087	7	40	50	VDLAACDSYER			Carbamidomethyl (C)[6]		Mascot
1679.8599	1679.7333	-0.1266	-75	1	16	ASKQPPEPQIGEAEAK					Mascot
1909.9689	1909.8794	-0.0895	-47	40	56	VDLAACDSYERLSLAVK			Carbamidomethyl (C)[6]		Mascot
1919.0209	1918.9167	-0.1042	-54	106	122	MLVGDVPWNVFVSTAKR					Mascot
1924.9359	1924.931	-0.0049	-3	128	146	SSELSHDLIGATPERAAN G					Mascot
2593.1941	2593.1467	-0.0474	-18	83	105	IFSQLLDGSGEYTLVYED SEGDR					Mascot

8	KDEL-tailed cysteine endopeptidase CEP1 [Triticum urartu]	gi|474157044	39269.3	6.45	8	36	0	3.106	

Peptide Information

Calc. Mass	Obsrv. Mass	± da	± ppm	Start Seq.	End Seq.	Sequence	Ion Score	C. I. %	Modification	Rank	Result Type
1060.5092	1060.5123	0.0031	3	104	113	CSSPLPNSAK			Carbamidomethyl (C)[1]		Mascot
1097.5739	1097.5914	0.0175	16	62	70	FSVFKENAR					Mascot
1520.7527	1520.7272	-0.0255	-17	194	208	ESGGCKGGNPVLAFK			Carbamidomethyl (C)[5]		Mascot
1664.7816	1664.7518	-0.0298	-18	317	330	NSWGPKWGEDGYIR					Mascot
3217.4531	3217.5295	0.0764	24	75	103	FNHGGAPYKLSLNLFGD MTDEEVDDGYGR					Mascot
3233.448	3233.5259	0.0779	24	75	103	FNHGGAPYKLSLNLFGD MTDEEVDDGYGR			Oxidation (M)[18]		Mascot
3234.4185	3234.5254	0.1069	33	209	236	YMIDHGGISTEADYPYFA DDHGYCLVPK			Carbamidomethyl (C)[24]		Mascot
3250.4133	3250.5164	0.1031	32	209	236	YMIDHGGISTEADYPYFA DDHGYCLVPK			Carbamidomethyl (C)[24], Oxidation (M)[2]		Mascot
3389.8159	3389.6235	-0.1924	-57	4	36	AIATILLMAIVLVAAMQAV SSVDITDTDLASEK			Oxidation (M)[8]		Mascot
3406.5144	3406.6287	0.1143	34	209	237	YMIDHGGISTEADYPYFA DDHGYCLVPKR			Carbamidomethyl (C)[24], Oxidation (M)[2]		Mascot

9	hypothetical protein TRIUR3_21898 [Triticum urartu]	gi|474418703	35926.1	5.85	8	36	0	6.349	

Peptide Information

Calc. Mass	Obsrv. Mass	± da	± ppm	Start Seq.	End Seq.	Sequence	Ion Score	C. I. %	Modification	Rank	Result Type
895.4341	895.4161	-0.018	-20	229	235	FMEAIER					Mascot
1182.6453	1182.5458	-0.0995	-84	27	35	VFFERMVVR					Mascot
1187.5548	1187.6215	0.0667	56	301	311	MGEVMHDAGIK					Mascot
1347.6395	1347.599	-0.0405	-30	301	312	MGEVMHDAGIKK			Oxidation (M)[1,5]		Mascot
1875.9674	1875.8912	-0.0762	-41	239	256	MPFLPDASVWGALLGAS K			Oxidation (M)[1]		Mascot
1875.9674	1875.8912	-0.0762	-41	239	256	MPFLPDASVWGALLGAS K			Oxidation (M)[1]		Mascot
1918.9337	1918.9167	-0.017	-9	209	225	VTPMMAHYGCVVDLLGR			Carbamidomethyl (C)[10]		Mascot
1950.9235	1950.929	0.0055	3	209	225	VTPMMAHYGCVVDLLGR			Carbamidomethyl (C)[10], Oxidation (M)[4,5]		Mascot
2619.2576	2619.1821	-0.0755	-29	1	26	MASPDLASSNAMLDVLC LAGDVAAAR			Carbamidomethyl (C)[17]		Mascot
2635.2524	2635.1636	-0.0888	-34	1	26	MASPDLASSNAMLDVLC LAGDVAAAR			Carbamidomethyl (C)[17], Oxidation (M)[1]		Mascot
2651.2473	2651.147	-0.1003	-38	1	26	MASPDLASSNAMLDVLC LAGDVAAAR			Carbamidomethyl (C)[17], Oxidation (M)[1,12]		Mascot
2651.2473	2651.147	-0.1003	-38	1	26	MASPDLASSNAMLDVLC LAGDVAAAR			Carbamidomethyl (C)[17], Oxidation (M)[1,12]		Mascot
3247.7297	3247.5312	-0.1985	-61	239	269	MPFLPDASVWGALLGAS KLHGNVELVAEIGR					Mascot

10	hypothetical protein TRIUR3_08605 [Triticum urartu]	gi|474248787	15287.8	9.23	6	36	0	1.124	

Peptide Information

Calc. Mass	Obsrv. Mass	± da	± ppm	Start Seq.	End Seq.	Sequence	Ion Score	C. I. %	Modification	Rank	Result Type
812.4447	812.4184	-0.0263	-32	1	8	MAAPLPGR					Mascot
1097.6249	1097.5914	-0.0335	-31	1	10	MAAPLPGRLR			Oxidation (M)[1]		Mascot
1145.5626	1145.559	-0.0036	-3	96	105	ADAFIGNFYK					Mascot
1851.8344	1851.8625	0.0281	15	118	131	LQDLCSYHHHHNYK			Carbamidomethyl (C)[5]		Mascot
1921.9324	1921.9042	-0.0282	-15	79	95	VDASSHDLMSKQELYAK					Mascot
3389.5881	3389.6235	0.0354	10	42	74	ASIFFIGSADPHDAGEIVD ATSTLQDNQAGEGR					Mascot

Gel Idx/Pos	202/I2	Instr./Gel Origin	BA2151/Sample Project 20140814	Process Status	Analysis Succeeded
Plate [#] Name	[1] Sample Project 20140814	Instrument Sample Name		Spectra	11

Rank	Protein Name	Accession No.	Protein MW	Protein PI	Pep. Count	Protein Score	Protein Score C. I. %	Intensity Matched	Total Ion Score	Total Ion C. I. %	Confirmed
1	glutathione S-transferase [Triticum aestivum]	gi|5923877	23609.1	5.79	4	264	100	36.388	247	100	

Protein Group

Protein Name	Accession No.	Protein MW	Protein PI
glutathione S-transferase [Triticum aestivum]	gi|14538027	23609.1	5.7899999618 5303
unnamed protein product [Triticum aestivum]	gi|257737764	23609.1	5.7899999618 5303
unnamed protein product [Triticum aestivum]	gi|257343711	23609.1	5.7899999618 5303
unnamed protein product [Triticum aestivum]	gi|257293238	23609.1	5.7899999618 5303
unnamed protein product [Triticum aestivum]	gi|219899599	23609.1	5.7899999618 5303
unnamed protein product [Triticum aestivum]	gi|219744934	23609.1	5.7899999618 5303
unnamed protein product [Triticum aestivum]	gi|219740288	23609.1	5.7899999618 5303

Peptide Information

Calc. Mass	Obsrv. Mass	± da	± ppm	Start Seq.	End Seq.	Sequence	Ion Score	C. I. %	Modification	Rank	Result Type
1343.7318	1343.7368	0.005	4	141	152	NVLAVYEAHLSK					Mascot
1380.7271	1380.7369	0.0098	7	6	17	LYGATLSWNVTR					Mascot
1380.7271	1380.7369	0.0098	7	6	17	LYGATLSWNVTR	68	99.999			Mascot

2252.0984	2252.1233	0.0249	11	50	69	NPFGQVPALQDGDLYVF ESR					Mascot
2252.0984	2252.1233	0.0249	11	50	69	NPFGQVPALQDGDLYVF ESR	179	100			Mascot
2732.3237	2732.3357	0.012	4	18	42	CVAALEEAGVEYELVPIN FGTGEHK			Carbamidomethyl (C)[1]		Mascot

2	unnamed protein product [Triticum aestivum]	gi|257343673	23668.2	5.96	4	264	100	35.916	247	100	

Protein Group

glutathione transferase F1 [Triticum aestivum]	gi|23504749	23668.2	5.9600 000381 4697
unnamed protein product [Triticum aestivum]	gi|257293200	23668.2	5.9600 000381 4697
unnamed protein product [Triticum aestivum]	gi|219899559	23668.2	5.9600 000381 4697
unnamed protein product [Triticum aestivum]	gi|257737348	23668.2	5.9600 000381 4697
unnamed protein product [Triticum aestivum]	gi|219744894	23668.2	5.9600 000381 4697
unnamed protein product [Triticum aestivum]	gi|219740248	23668.2	5.9600 000381 4697

Peptide Information

Calc. Mass	Obsrv. Mass	± da	± ppm	Start Seq.	End Seq.	Sequence	Ion Score	C. I. %	Modification	Rank	Result Type
1343.7318	1343.7368	0.005	4	141	152	NVLAVYEAHLSK					Mascot
1380.7271	1380.7369	0.0098	7	6	17	LYGATLSWNVTR					Mascot
1380.7271	1380.7369	0.0098	7	6	17	LYGATLSWNVTR	68	99.999			Mascot
2252.0984	2252.1233	0.0249	11	50	69	NPFGQVPALQDGDLYVF ESR					Mascot
2252.0984	2252.1233	0.0249	11	50	69	NPFGQVPALQDGDLYVF ESR	179	100			Mascot
2731.3396	2731.304	-0.0356	-13	18	42	CVAALEEAGVQYEIVPIN FGTGEHK			Carbamidomethyl (C)[1]		Mascot

3	unnamed protein product [Triticum aestivum]	gi|257659932	23669.1	5.79	4	264	100	36.388	247	100

Peptide Information

Calc. Mass	Obsrv. Mass	± da	± ppm	Start Seq.	End Seq.	Sequence	Ion Score	C. I. %	Modification	Rank	Result Type
1343.7318	1343.7368	0.005	4	141	152	NVLAVYEAHLSK					Mascot
1380.7271	1380.7369	0.0098	7	6	17	LYGATLSWNVTR					Mascot
1380.7271	1380.7369	0.0098	7	6	17	LYGATLSWNVTR	68	99.999			Mascot
2252.0984	2252.1233	0.0249	11	50	69	NPFGQVPALQDGDLYVF ESR					Mascot
2252.0984	2252.1233	0.0249	11	50	69	NPFGQVPALQDGDLYVF ESR	179	100			Mascot
2732.3237	2732.3357	0.012	4	18	42	CVAALEEAGVEYEIVPINF GTGEHK			Carbamidomethyl (C)[1]		Mascot

4 Glutathione S-transferase 1 [Triticum urartu] gi|474049299 26837.7 5.8 3 78 99.936 8.566 68 99.999

Peptide Information

Calc. Mass	Obsrv. Mass	± da	± ppm	Start Seq.	End Seq.	Sequence	Ion Score	C. I. %	Modification	Rank	Result Type
1343.7318	1343.7368	0.005	4	170	181	NVLAVYEAHLSK					Mascot
1380.7271	1380.7369	0.0098	7	6	17	LYGATLSWNVTR					Mascot
1380.7271	1380.7369	0.0098	7	6	17	LYGATLSWNVTR	68	99.999			Mascot
2732.3237	2732.3357	0.012	4	18	42	CVAALEEAGVEYELVPIN FGTGEHK			Carbamidomethyl (C)[1]		Mascot

5 hypothetical protein TRIUR3_32347 [Triticum urartu] gi|474449898 101677.7 4.96 16 58 93.307 9.032 26 77.416

Peptide Information

Calc. Mass	Obsrv. Mass	± da	± ppm	Start Seq.	End Seq.	Sequence	Ion Score	C. I. %	Modification	Rank	Result Type
887.4944	887.4387	-0.0557	-63	486	492	LEQTQLR					Mascot
1079.4972	1079.552	0.0548	51	298	306	ECEGLRGMK			Carbamidomethyl (C)[2]		Mascot
1085.4971	1085.5514	0.0543	50	477	485	QENHDISSR					Mascot
1088.6171	1088.5459	-0.0712	-65	97	105	RTLQSQLSR					Mascot
1120.519	1120.5863	0.0673	60	366	374	DLDEMLEQK					Mascot
1131.5641	1131.6042	0.0401	35	238	247	GAEDDVEKLR					Mascot
1131.5641	1131.6042	0.0401	35	238	247	GAEDDVEKLR	26	77.416			Mascot
1136.514	1136.5918	0.0778	68	366	374	DLDEMLEQK			Oxidation (M)[5]		Mascot
1145.6637	1145.5773	-0.0864	-75	246	255	LRSEIGTLTR					Mascot
1211.554	1211.5697	0.0157	13	652	661	SKSDEFENQK					Mascot
1236.5161	1236.571	0.0549	44	106	115	CEDEEAEKAR			Carbamidomethyl (C)[1]		Mascot
1366.6631	1366.6991	0.036	26	278	289	GQDLSKEMSSLR			Oxidation (M)[8]		Mascot

1406.6581	1406.6692	0.0111	8	366	376	DLDEMLEQKNR			Oxidation (M)[5]		Mascot
1430.7373	1430.6981	-0.0392	-27	668	679	LNDLSEEIEQLK					Mascot
1592.722	1592.7312	0.0092	6	696	709	NEQKDMELAANGEK			Oxidation (M)[6]		Mascot
1836.8876	1836.932	0.0444	24	559	573	WNNATTAERLQEEFK					Mascot
1865.0201	1864.9832	-0.0369	-20	758	773	LQITIGSLKLQYDNMK					Mascot
2250.1836	2250.0808	-0.1028	-46	574	594	SLSSQVSSAFSANERLLV QAR					Mascot

6 starch branching enzyme I [Triticum aestivum] gi|1935006 93916.7 6.27 18 55 86.016 4.6

Protein Group

starch branching enzyme 1 [Triticum aestivum] gi|11037530 93916.7 6.2699 999809 2651

Peptide Information

Calc. Mass	Obsrv. Mass	± da	± ppm	Start Seq.	End Seq.	Sequence	Ion Score	C. I. %	Modification	Rank	Result Type
1060.5422	1060.5676	0.0254	24	271	279	EFADNVLPR					Mascot
1079.5884	1079.552	-0.0364	-34	499	506	WIDYLKNK					Mascot
1085.537	1085.5514	0.0144	13	545	553	TMAFLLMDK			Oxidation (M)[2]		Mascot
1136.5769	1136.5918	0.0149	13	694	703	VGCDLPGKYK			Carbamidomethyl (C)[3]		Mascot
1161.58	1161.5846	0.0046	4	373	381	GYHKLWDSR					Mascot
1192.5514	1192.5831	0.0317	27	658	667	QIVSDMNEEK					Mascot
1199.6797	1199.5828	-0.0969	-81	237	245	YVFKHPRPR					Mascot
1236.6624	1236.571	-0.0914	-74	817	827	GINFVFLSPDK					Mascot
1320.6464	1320.6163	-0.0301	-23	658	668	QIVSDMNEEKK					Mascot
1336.6414	1336.6594	0.018	13	658	668	QIVSDMNEEKK			Oxidation (M)[6]		Mascot
1371.7743	1371.7085	-0.0658	-48	324	335	YLVDKAHSLGLR					Mascot
1424.7322	1424.7264	-0.0058	-4	382	392	LFNYANWEVLR					Mascot
1428.5961	1428.7225	0.1264	88	610	620	EGNNWSYDKCR			Carbamidomethyl (C)[10]		Mascot
1469.7206	1469.7185	-0.0021	-1	164	175	MTKDNYGVWSIR					Mascot
1836.9061	1836.932	0.0259	14	702	718	YKVALDSDALMFGGHGR					Mascot
2234.0918	2234.1318	0.04	18	675	693	GDLVFVFNFHPSKTYDG YK					Mascot
2234.0918	2234.1318	0.04	18	675	693	GDLVFVFNFHPSKTYDG YK					Mascot
2264.2227	2264.1145	-0.1082	-48	669	687	IIVFERGDLVFVFNFHPSK					Mascot
2268.2288	2268.1216	-0.1072	-47	382	399	LFNYANWEVLRFLLSNLR					Mascot
2274.1772	2274.0916	-0.0856	-38	329	349	AHSLGLRVLMDVVHSHA SSNK			Oxidation (M)[10]		Mascot

7 hypothetical protein TRIUR3_18298 [Triticum urartu] gi|474057860 302316.3 4.98 36 55 85.016 24.988

Peptide Information

Calc. Mass	Obsrv. Mass	± da	± ppm	Start Seq.	End Seq.	Sequence	Ion Score	C. I. %	Modification	Rank	Result Type
887.404	887.4387	0.0347	39	1963	1969	ETCNHVK			Carbamidomethyl (C)[3]		Mascot
1060.5283	1060.5676	0.0393	37	117	125	SVDGRFHSR					Mascot
1076.5582	1076.5511	-0.0071	-7	1746	1754	LKDSEELSR					Mascot
1120.5919	1120.5863	-0.0056	-5	980	989	VEAETLLMSK					Mascot
1136.5868	1136.5918	0.005	4	980	989	VEAETLLMSK			Oxidation (M)[8]		Mascot
1151.5912	1151.5947	0.0035	3	2204	2213	GMLMKEISSR					Mascot
1167.5861	1167.5881	0.002	2	2204	2213	GMLMKEISSR			Oxidation (M)[2]		Mascot
1177.6133	1177.5775	-0.0358	-30	469	478	DIISCLISEK			Carbamidomethyl (C)[5]		Mascot
1183.5809	1183.5747	-0.0062	-5	2204	2213	GMLMKEISSR			Oxidation (M)[2,4]		Mascot
1195.5511	1195.5868	0.0357	30	781	790	NTILEMDSEK			Oxidation (M)[6]		Mascot
1195.5511	1195.5868	0.0357	30	781	790	NTILEMDSEK			Oxidation (M)[6]		Mascot
1199.554	1199.5828	0.0288	24	2066	2075	DDELHKADEK					Mascot
1207.6173	1207.5801	-0.0372	-31	727	736	CMLAETTLLR			Carbamidomethyl (C)[1]		Mascot
1236.6327	1236.571	-0.0617	-50	1658	1667	LSDECLMLKK			Carbamidomethyl (C)[5]		Mascot
1336.6855	1336.6594	-0.0261	-20	666	676	RLAQDLEYSNK					Mascot
1343.757	1343.7368	-0.0202	-15	355	367	DALGVSPPLFTVK					Mascot
1349.6431	1349.6985	0.0554	41	935	946	SEVSETQLEAEK					Mascot
1351.7073	1351.6703	-0.037	-27	726	736	KCMLAETTLLR			Carbamidomethyl (C)[2], Oxidation (M)[3]		Mascot
1365.7737	1365.7122	-0.0615	-45	823	833	EKVHLLEQELK					Mascot
1366.5692	1366.6991	0.1299	95	2343	2352	HKEQYMEDDR			Oxidation (M)[6]		Mascot
1380.7958	1380.7369	-0.0589	-43	1896	1908	AAHSAEVVISRLK					Mascot
1380.7958	1380.7369	-0.0589	-43	1896	1908	AAHSAEVVISRLK					Mascot
1392.6675	1392.6808	0.0133	10	1424	1435	LNEVENMSSELK					Mascot
1394.5853	1394.7161	0.1308	94	457	468	ADNSENECQSLK			Carbamidomethyl (C)[8]		Mascot
1406.7023	1406.6692	-0.0331	-24	256	267	LINEDADSFARR					Mascot
1408.7318	1408.7352	0.0034	2	1668	1680	SFSNAIVETEALK					Mascot
1412.7202	1412.7311	0.0109	8	2001	2011	DLHLQKCELEK			Carbamidomethyl (C)[7]		Mascot
1412.7202	1412.7311	0.0109	8	2001	2011	DLHLQKCELEK			Carbamidomethyl (C)[7]		Mascot
1424.7268	1424.7264	-0.0004	0	1081	1092	KEEFDSLQTSLK					Mascot
1425.7849	1425.7273	-0.0576	-40	31	42	LVVSYSEGFRIR					Mascot
1428.6788	1428.7225	0.0437	31	2502	2514	GKSPMSSEYSSIR					Mascot
1443.7186	1443.6909	-0.0277	-19	2266	2277	EGNLELVQDDRR					Mascot
1452.6577	1452.7034	0.0457	31	636	646	LQDEHFNYMQK					Mascot

1469.698	1469.7185	0.0205	14	369	382	NGTHPDEIGSLSSR						Mascot
1505.72	1505.6841	-0.0359	-24	541	552	IMMQQQELAQNR			Oxidation (M)[2]			Mascot
1818.8617	1818.8566	-0.0051	-3	837	852	EVVDGLHNNLEEEGHK						Mascot
1836.8708	1836.932	0.0612	33	935	950	SEVSETQLEAEKTEEK						Mascot
2040.9906	2041.0183	0.0277	14	1726	1743	SMLEISFSNVNSEIGELR			Oxidation (M)[2]			Mascot
2235.1292	2235.1545	0.0253	11	804	822	LSYLEAHLFDVQSDLDKN K						Mascot
2268.281	2268.1216	-0.1594	-70	2475	2495	LATDAQGLLIIQASIEEVK QK						Mascot
2274.1936	2274.0916	-0.102	-45	1274	1293	DTTLLQQNQSLVRVSDLE SK						Mascot
2288.0579	2288.0762	0.0183	8	2538	2557	AENYPAFEVNADLEGYS SRR						Mascot

8 hypothetical protein TRIUR3_31082 [Triticum urartu] gi|473961885 62552.5 7.88 7 51 64.055 6.037 38 98.75

Peptide Information

Calc. Mass	Obsrv. Mass	± da	± ppm	Start Seq.	End Seq.	Sequence	Ion Score	C. I. %	Modification	Rank	Result Type
1058.559	1058.5356	-0.0234	-22	74	83	ALEEQKAGGR					Mascot
1079.5341	1079.552	0.0179	17	240	248	NYSAQGQRR					Mascot
1129.6576	1129.5697	-0.0879	-78	99	108	ASVNKLQELK					Mascot
1131.6005	1131.6042	0.0037	3	109	119	DAAVSTLAEVR					Mascot
1131.6005	1131.6042	0.0037	3	109	119	DAAVSTLAEVR	38	98.75			Mascot
1320.6768	1320.6163	-0.0605	-46	462	473	RPSGSANRNYAK					Mascot
1366.7339	1366.6991	-0.0348	-25	496	507	RPAAPVGTWQQR					Mascot
1410.7773	1410.7175	-0.0598	-42	439	450	LQKECPLGPLQK			Carbamidomethyl (C)[5]		Mascot

9 hypothetical protein TRIUR3_20519 [Triticum urartu] gi|473929633 122927 5.87 25 51 64.055 18.484 12 0

Peptide Information

Calc. Mass	Obsrv. Mass	± da	± ppm	Start Seq.	End Seq.	Sequence	Ion Score	C. I. %	Modification	Rank	Result Type
832.3795	832.3247	-0.0548	-66	827	834	AGEEEAAR					Mascot
907.4666	907.4711	0.0045	5	279	286	LKSEMGSR					Mascot
1076.5405	1076.5511	0.0106	10	170	178	LEESLAMQR					Mascot
1078.5198	1078.5381	0.0183	17	867	875	DNMVQLQSK			Oxidation (M)[3]		Mascot
1088.5695	1088.5459	-0.0236	-22	490	499	QVSAEAREAK					Mascot
1131.5892	1131.6042	0.015	13	287	296	EKGAEEIIDK					Mascot
1131.5892	1131.6042	0.015	13	287	296	EKGAEEIIDK	2	0			Mascot
1135.5664	1135.5942	0.0278	24	543	552	KAEMLESEAK					Mascot
1143.6368	1143.5775	-0.0593	-52	289	298	GAEEIIDKLR					Mascot

Calc. Mass	Obsrv. Mass	± da	± ppm	Start Seq.	End Seq.	Sequence	Ion Score	C. I. %	Modification	Rank	Result Type
1151.5613	1151.5947	0.0334	29	543	552	KAEMLESEAK			Oxidation (M)[4]		Mascot
1187.5764	1187.5979	0.0215	18	947	958	GGNQSAGRSPEK					Mascot
1192.6064	1192.5831	-0.0233	-20	269	278	MEILMAEVTR					Mascot
1207.6252	1207.5801	-0.0451	-37	590	599	EVGFAEMLRR					Mascot
1247.5862	1247.6451	0.0589	47	827	838	AGEEEAARSAEK					Mascot
1320.6464	1320.6163	-0.0301	-23	544	555	AEMLESEAKNAK					Mascot
1336.6412	1336.6594	0.0182	14	544	555	AEMLESEAKNAK			Oxidation (M)[3]		Mascot
1351.6501	1351.6703	0.0202	15	1071	1081	SSFKQEHHSHK					Mascot
1365.7195	1365.7122	-0.0073	-5	587	598	VSKEVGFAEMLR					Mascot
1380.6974	1380.7369	0.0395	29	268	278	RMEILMAEVTR			Oxidation (M)[2,6]		Mascot
1380.6974	1380.7369	0.0395	29	268	278	RMEILMAEVTR	12	0	Oxidation (M)[2,6]		Mascot
1392.6788	1392.6808	0.002	1	864	875	DAKDNMVQLQSK			Oxidation (M)[6]		Mascot
1410.77	1410.7175	-0.0525	-37	220	232	HAQDVNSLAKVTK					Mascot
1424.7241	1424.7264	0.0023	2	96	108	ASPHRTSTTPDVR					Mascot
1425.6106	1425.7273	0.1167	82	965	976	KMMCSPMENAAR			Carbamidomethyl (C)[4]		Mascot
1432.6737	1432.696	0.0223	16	712	724	AEQSMVAESSHLK			Oxidation (M)[5]		Mascot
1452.6061	1452.7034	0.0973	67	338	350	AEMDADQSAQGWK			Oxidation (M)[3]		Mascot
1467.6744	1467.7151	0.0407	28	698	711	ADEASKLMDAASSR			Oxidation (M)[8]		Mascot
1592.7122	1592.7312	0.019	12	337	350	RAEMDADQSAQGWK					Mascot
2264.0645	2264.1145	0.05	22	704	724	LMDAASSRAEQSMVAES SHLK			Oxidation (M)[2]		Mascot
2280.0596	2280.1387	0.0791	35	704	724	LMDAASSRAEQSMVAES SHLK			Oxidation (M)[2,13]		Mascot

10 LEA1 protein [Triticum aestivum] gi|25989705 23195.5 8.81 11 49 46.834 9.117

Protein Group

RecName: Full=Late embryogenesis abundant protein, group 3; Short=LEA; AltName: Full=PMA2005	gi|7387828	23224.5	8.8100 004196 167
embryonic abundant protein, group 3 - common wheat x Sanduri wheat	gi|100829	23224.5	8.8100 004196 167
group 3 late embryogenesis abundant protein (LEA) [Triticum aestivum]	gi|21637	23224.5	8.8100 004196 167

Peptide Information

Calc. Mass	Obsrv. Mass	± da	± ppm	Start Seq.	End Seq.	Sequence	Ion Score	C. I. %	Modification	Rank	Result Type

906.4527	906.4601	0.0074	8	34	42	AGQTTEATK		Mascot
907.4367	907.4711	0.0344	38	45	53	AGETTEATK		Mascot
1135.5776	1135.5942	0.0166	15	23	33	TGQVMGATKDK		Mascot
1147.5953	1147.5962	0.0009	1	56	66	AAETTEAAKQK		Mascot
1147.5953	1147.5962	0.0009	1	54	64	QKAAETTEAAK		Mascot
1151.5725	1151.5947	0.0222	19	23	33	TGQVMGATKDK	Oxidation (M)[5]	Mascot
1163.5903	1163.5946	0.0043	4	45	55	AGETTEATKQK		Mascot
1163.5903	1163.5946	0.0043	4	43	53	QKAGETTEATK		Mascot
1175.6014	1175.588	-0.0134	-11	129	139	AAETTEAARQK		Mascot
1283.6227	1283.6488	0.0261	20	96	106	TYETAQSAKER		Mascot
1392.6788	1392.6808	0.002	1	19	31	NEEKTGQVMGATK		Mascot
1408.6737	1408.7352	0.0615	44	19	31	NEEKTGQVMGATK	Oxidation (M)[9]	Mascot
1928.0448	1927.9574	-0.0874	-45	169	188	TGSVLQQAGETVVNAVVGAK		Mascot

Gel Idx/Pos	203/I3	Instr./Gel Origin	BA2151/Sample Project 20140814	Process Status	Analysis Succeeded
Plate [#] Name	[1] Sample Project 20140814	Instrument Sample Name		Spectra	11

Rank	Protein Name	Accession No.	Protein MW	Protein PI	Pep. Count	Protein Score	Protein Score C. I. %	Intensity Matched	Total Ion Score	Total Ion C. I. %	Confirmed
1	hypothetical protein TRIUR3_28410 [Triticum urartu]	gi|474060617	28387.9	5.53	9	250	100	17.641	202	100	

Peptide Information

Calc. Mass	Obsrv. Mass	± da	± ppm	Start Seq.	End Seq.	Sequence	Ion Score	C. I. %	Modification	Rank	Result Type
930.4389	930.4361	-0.0028	-3	194	200	FEAYICK			Carbamidomethyl (C)[6]		Mascot
971.5673	971.5276	-0.0397	-41	51	58	FVTNHLLK					Mascot
1078.535	1078.5316	-0.0034	-3	207	215	GYPLLEACR			Carbamidomethyl (C)[8]		Mascot
1078.535	1078.5316	-0.0034	-3	207	215	GYPLLEACR	55	99.98	Carbamidomethyl (C)[8]		Mascot
1145.5698	1145.5648	-0.005	-4	156	165	GHNLSLEYGR					Mascot
1145.5698	1145.5648	-0.005	-4	156	165	GHNLSLEYGR	79	100			Mascot
1406.6271	1406.6199	-0.0072	-5	130	139	HREWESCFQK			Carbamidomethyl (C)[7]		Mascot
1406.6271	1406.6199	-0.0072	-5	130	139	HREWESCFQK			Carbamidomethyl (C)[7]		Mascot
1796.9075	1796.84	-0.0675	-38	113	127	VHLGFIYCVSDLVMK			Carbamidomethyl (C)[8], Oxidation (M)[14]		Mascot
1845.9594	1845.8855	-0.0739	-40	62	79	DGLLDAADLTLVPYGNAK					Mascot
1927.9371	1927.9288	-0.0083	-4	35	50	VHVAIYYESLCPYSAR			Carbamidomethyl (C)[11]		Mascot
1927.9371	1927.9288	-0.0083	-4	35	50	VHVAIYYESLCPYSAR	68	99.999	Carbamidomethyl (C)[11]		Mascot
3402.8162	3402.6677	-0.1485	-44	1	34	MARGLHHLLLLAALLQQL SATSAGDVATGSGGEK			Oxidation (M)[1]		Mascot

Rank	Protein Name	Accession No.	Protein MW	Protein PI	Pep. Count	Protein Score	Protein Score C. I. %	Intensity Matched	Total Ion Score	Total Ion C. I. %	Confirmed
2	27K protein [Triticum aestivum]	gi|30793446	23271.5	6.06	4	148	100	11.048	134	100	

Protein Group

Tri a Bd 27K, partial [Triticum aestivum]	gi|283480515	23214.5	6.0599 999427 7954

Peptide Information

Calc. Mass	Obsrv. Mass	± da	± ppm	Start Seq.	End Seq.	Sequence	Ion Score	C. I. %	Modification	Rank	Result Type
1078.535	1078.5316	-0.0034	-3	181	189	GYPLLEACR			Carbamidomethyl (C)[8]		Mascot
1078.535	1078.5316	-0.0034	-3	181	189	GYPLLEACR	55	99.98	Carbamidomethyl (C)[8]		Mascot
1145.5698	1145.5648	-0.005	-4	133	142	GHNLSLEYGR					Mascot
1145.5698	1145.5648	-0.005	-4	133	142	GHNLSLEYGR	79	100			Mascot
1406.6271	1406.6199	-0.0072	-5	107	116	HREWESCFQK			Carbamidomethyl (C)[7]		Mascot

1406.6271	1406.6199	-0.0072	-5	107	116	HREWESCFQK			Carbamidomethyl (C)[7]		Mascot
1955.9684	1955.9403	-0.0281	-14	12	27	VHVAIYYESLCPYSVR			Carbamidomethyl (C)[11]		Mascot

3 27k protein [Triticum aestivum] gi|290350670 24399.1 6.06 4 147 100 11.048 134 100

Peptide Information

Calc. Mass	Obsrv. Mass	± da	± ppm	Start Seq.	End Seq.	Sequence	Ion Score	C. I. %	Modification	Rank	Result Type
1078.535	1078.5316	-0.0034	-3	193	201	GYPLLEACR			Carbamidomethyl (C)[8]		Mascot
1078.535	1078.5316	-0.0034	-3	193	201	GYPLLEACR	55	99.98	Carbamidomethyl (C)[8]		Mascot
1145.5698	1145.5648	-0.005	-4	145	154	GHNLSLEYGR					Mascot
1145.5698	1145.5648	-0.005	-4	145	154	GHNLSLEYGR	79	100			Mascot
1406.6271	1406.6199	-0.0072	-5	119	128	HREWESCFQK			Carbamidomethyl (C)[7]		Mascot
1406.6271	1406.6199	-0.0072	-5	119	128	HREWESCFQK			Carbamidomethyl (C)[7]		Mascot
1955.9684	1955.9403	-0.0281	-14	24	39	VHVAIYYESLCPYSVR			Carbamidomethyl (C)[11]		Mascot

4 Avenin-3 [Triticum urartu] gi|474329936 22657.1 6.35 3 116 100 18.253 102 100

Peptide Information

Calc. Mass	Obsrv. Mass	± da	± ppm	Start Seq.	End Seq.	Sequence	Ion Score	C. I. %	Modification	Rank	Result Type
1950.9413	1950.9283	-0.013	-7	60	76	DALLQQCSPVADMSFLR			Carbamidomethyl (C)[7]		Mascot
1966.9362	1966.8981	-0.0381	-19	60	76	DALLQQCSPVADMSFLR			Carbamidomethyl (C)[7], Oxidation (M)[13]		Mascot
1966.9362	1966.8981	-0.0381	-19	60	76	DALLQQCSPVADMSFLR	102	100	Carbamidomethyl (C)[7], Oxidation (M)[13]		Mascot
2635.1885	2635.1633	-0.0252	-10	77	97	SQVVQHSSCLVMWEQC CQQLK			Carbamidomethyl (C)[9,16,17]		Mascot
2651.1833	2651.1372	-0.0461	-17	77	97	SQVVQHSSCLVMWEQC CQQLK			Carbamidomethyl (C)[9,16,17], Oxidation (M)[12]		Mascot
3222.5352	3222.5425	0.0073	2	27	54	TAWEPHHPSSPEQQPTP QPQEQPVPHQK					Mascot

5 gamma gliadin [Triticum aestivum] gi|133741924 16537.3 8.88 3 114 100 18.272 102 100

Peptide Information

Calc. Mass	Obsrv. Mass	± da	± ppm	Start Seq.	End Seq.	Sequence	Ion Score	C. I. %	Modification	Rank	Result Type
856.416	856.3607	-0.0553	-65	138	145	STTTRSGF					Mascot
1885.8064	1885.9266	0.1202	64	84	97	SCLVMWEQCCQQLK			Carbamidomethyl (C)[2,9,10], Oxidation (M)[5]		Mascot
1950.9413	1950.9283	-0.013	-7	60	76	DALLQQCSPVADMSFLR			Carbamidomethyl (C)[7]		Mascot
1966.9362	1966.8981	-0.0381	-19	60	76	DALLQQCSPVADMSFLR			Carbamidomethyl (C)[7], Oxidation (M)[13]		Mascot
1966.9362	1966.8981	-0.0381	-19	60	76	DALLQQCSPVADMSFLR	102	100	Carbamidomethyl (C)[7], Oxidation (M)[13]		Mascot

6 gliadin/avenin-like seed protein [Triticum aestivum] gi|281335538 22813.2 6.2 2 109 100 18.089 102 100

Peptide Information

Calc. Mass	Obsrv. Mass	± da	± ppm	Start Seq.	End Seq.	Sequence	Ion Score	C. I. %	Modification	Rank	Result Type
1950.9413	1950.9283	-0.013	-7	60	76	DALLQQCSPVADMSFLR			Carbamidomethyl (C)[7]		Mascot
1966.9362	1966.8981	-0.0381	-19	60	76	DALLQQCSPVADMSFLR			Carbamidomethyl (C)[7], Oxidation (M)[13]		Mascot
1966.9362	1966.8981	-0.0381	-19	60	76	DALLQQCSPVADMSFLR	102	100	Carbamidomethyl (C)[7], Oxidation (M)[13]		Mascot
2635.1885	2635.1633	-0.0252	-10	77	97	SQVVQHSSCLVMWEQC CQQLK			Carbamidomethyl (C)[9,16,17]		Mascot
2651.1833	2651.1372	-0.0461	-17	77	97	SQVVQHSSCLVMWEQC CQQLK			Carbamidomethyl (C)[9,16,17], Oxidation (M)[12]		Mascot

7 DNA repair protein rhp54 [Triticum urartu] gi|474247395 115707.3 5.93 17 47 21.362 10.126

Peptide Information

Calc. Mass	Obsrv. Mass	± da	± ppm	Start Seq.	End Seq.	Sequence	Ion Score	C. I. %	Modification	Rank	Result Type
865.3807	865.4037	0.023	27	941	946	MVHCYR			Carbamidomethyl (C)[4]		Mascot
889.4162	889.4243	0.0081	9	886	893	FNHSADAK					Mascot
987.5833	987.5199	-0.0634	-64	811	820	SGIDIKVGAK					Mascot
1046.559	1046.5054	-0.0536	-51	558	566	ANTRSEQLK					Mascot
1163.4885	1163.5836	0.0951	82	251	260	DSMDIDNEPK					Mascot
1187.6208	1187.6111	-0.0097	-8	536	544	TEFVHWQIK					Mascot
1507.7136	1507.6714	-0.0422	-28	278	290	QGQNDINIYAESR					Mascot
1517.7756	1517.7177	-0.0579	-38	858	869	MKGWKPEVHMFK					Mascot
1647.8346	1647.7654	-0.0692	-42	775	788	KTACSAVYLHPCLK			Carbamidomethyl (C)[4,12]		Mascot
1901.9685	1901.934	-0.0345	-18	396	411	DDLGIVCRVCGLIQQR			Carbamidomethyl (C)[7,10]		Mascot
1902.8386	1902.957	0.1184	62	947	964	LVAADSPEEEDHMTASG K			Oxidation (M)[13]		Mascot
1902.8386	1902.957	0.1184	62	947	964	LVAADSPEEEDHMTASG K			Oxidation (M)[13]		Mascot
1926.9994	1926.8879	-0.1115	-58	821	837	AKFIYNLLCLSEAAGEK			Carbamidomethyl (C)[9]		Mascot
1959.8324	1959.8823	0.0499	25	380	395	NNSEVVDDCHHDFLMK			Carbamidomethyl (C)[9]		Mascot
2840.4934	2840.3169	-0.1765	-62	901	927	ACGEGISLVGASRVVILE VQENPSVTR			Carbamidomethyl (C)[2]		Mascot
3218.4922	3218.5381	0.0459	14	2	30	WVGMGNPCNEEDLNFFY ASTTIIIGNGAK			Carbamidomethyl (C)[8]		Mascot
3222.6238	3222.5425	-0.0813	-25	737	764	GELLEELPGLVDFTVFLN MSTEQENILR			Oxidation (M)[19]		Mascot
3373.6658	3373.6201	-0.0457	-14	261	290	EIALFDGHSTSEPQLIKQ GQNDINIYAESR					Mascot

8 VIL2 protein [Triticum monococcum subsp. aegilopoides] gi|116562951 84613.7 8.15 16 47 21.362 5.858

Protein Group

VIL2 protein [Triticum monococcum subsp. monococcum]	gi|116563031	84613.7	8.1499 996185 3027		

Peptide Information

Calc. Mass	Obsrv. Mass	± da	± ppm	Start Seq.	End Seq.	Sequence	Ion Score	C. I. %	Modification	Rank	Result Type
823.456	823.4077	-0.0483	-59	263	269	VLFSTEK					Mascot
832.3795	832.3134	-0.0661	-79	640	647	EPGNSSNK					Mascot
906.46	906.4385	-0.0215	-24	149	156	AILSMEDK					Mascot
1060.5457	1060.5466	0.0009	1	207	216	TGILPSGQCK			Carbamidomethyl (C)[9]		Mascot
1173.528	1173.5994	0.0714	61	339	348	SNMMPSSFIK			Oxidation (M)[3,4]		Mascot
1182.6378	1182.547	-0.0908	-77	230	238	QHDLLRSWK					Mascot
1380.6498	1380.7098	0.06	43	314	325	LCAEALDVMQSK			Carbamidomethyl (C)[2], Oxidation (M)[9]		Mascot
1438.7397	1438.6079	-0.1318	-92	446	460	GLAPGGAGLVDQNNR					Mascot
1517.8105	1517.7177	-0.0928	-61	300	313	GIVSRLTCGAEVQK			Carbamidomethyl (C)[8]		Mascot
1550.8578	1550.7809	-0.0769	-50	263	275	VLFSTEKYSVLHK					Mascot
1647.9039	1647.7654	-0.1385	-84	250	262	LDILCYRIFLGHK			Carbamidomethyl (C)[5]		Mascot
1811.856	1811.9325	0.0765	42	94	109	EPTLDPNTSNHQYPAK					Mascot
1828.8971	1828.8572	-0.0399	-22	282	299	QKLEAEVGSVAGHGSMG R			Oxidation (M)[16]		Mascot
1836.8983	1836.912	0.0137	7	598	614	FVGSMPPTAPRVMETGK			Oxidation (M)[5,13]		Mascot
1901.8521	1901.934	0.0819	43	174	189	DPTIWLSCSSDHPMQK			Carbamidomethyl (C)[8]		Mascot
3419.6926	3419.707	0.0144	4	705	735	IVSVYVDTLINDPASLCG QLTDTFSEAIYSK			Carbamidomethyl (C)[17]		Mascot
3419.6926	3419.707	0.0144	4	705	735	IVSVYVDTLINDPASLCG QLTDTFSEAIYSK			Carbamidomethyl (C)[17]		Mascot

9	hypothetical protein TRIUR3_22196 [Triticum urartu]	gi|474074592	41219.9	8.73	10	45	0	4.04	

Peptide Information

Calc. Mass	Obsrv. Mass	± da	± ppm	Start Seq.	End Seq.	Sequence	Ion Score	C. I. %	Modification	Rank	Result Type
889.4924	889.4243	-0.0681	-77	1	8	MPATAVKR			Oxidation (M)[1]		Mascot
987.5105	987.5199	0.0094	10	308	316	EGLEEALAR					Mascot
1281.5239	1281.5493	0.0254	20	204	214	GCMGTYSFNTK			Carbamidomethyl (C)[2], Oxidation (M)[3]		Mascot
1412.7241	1412.7141	-0.01	-7	337	348	GELQITDHRSTR					Mascot
1680.8197	1680.7667	-0.053	-32	284	298	KPHMGVSLSYVGMSR			Oxidation (M)[4,13]		Mascot
1885.8103	1885.9266	0.1163	62	9	25	GRGGSSICTSCWMTGPR			Carbamidomethyl (C)[8,11], Oxidation (M)[13]		Mascot
1908.8368	1908.8411	0.0043	2	204	219	GCMGTYSFNTKDSVWR			Carbamidomethyl (C)[2]		Mascot
1951.9478	1951.9329	-0.0149	-8	282	298	DRKPHMGVSLSYVGMSR			Oxidation (M)[6,15]		Mascot

1951.9478	1951.9329	-0.0149	-8	282	298	DRKPHMGVSLSYVGMSR			Oxidation (M)[6,15]		Mascot
2684.2542	2684.1558	-0.0984	-37	215	236	DSVWRWHGEWALPFSG QAHFDR					Mascot
3222.5535	3222.5425	-0.011	-3	64	92	LECPVGTVPHPGMSFSA LGTQIFTFMNQR			Carbamidomethyl (C)[3]		Mascot
3238.5483	3238.552	0.0037	1	64	92	LECPVGTVPHPGMSFSA LGTQIFTFMNQR			Carbamidomethyl (C)[3], Oxidation (M)[13]		Mascot
3254.5432	3254.533	-0.0102	-3	64	92	LECPVGTVPHPGMSFSA LGTQIFTFMNQR			Carbamidomethyl (C)[3], Oxidation (M)[13,26]		Mascot
3254.5432	3254.533	-0.0102	-3	64	92	LECPVGTVPHPGMSFSA LGTQIFTFMNQR			Carbamidomethyl (C)[3], Oxidation (M)[13,26]		Mascot

10	Disease resistance protein RPM1 [Triticum urartu]	gi|473982211	115263.9	8.51	16	42	0	4.796

Peptide Information

Calc. Mass	Obsrv. Mass	± da	± ppm	Start Seq.	End Seq.	Sequence	Ion Score	C. I. %	Modification	Rank	Result Type
969.4168	969.4487	0.0319	33	873	879	MEWCSLK			Carbamidomethyl (C)[4], Oxidation (M)[1]		Mascot
987.5833	987.5199	-0.0634	-64	558	566	DISTIGLLR					Mascot
1380.7158	1380.7098	-0.006	-4	400	410	SIFPEDFEIKR					Mascot
1421.6941	1421.6545	-0.0396	-28	600	612	NGNMTSLPTEITK			Oxidation (M)[4]		Mascot
1631.8785	1631.7623	-0.1162	-71	586	599	TIGKLQDLQTLDMR					Mascot
1647.8735	1647.7654	-0.1081	-66	586	599	TIGKLQDLQTLDMR			Oxidation (M)[13]		Mascot
1676.9482	1676.7921	-0.1561	-93	544	557	ILDLHDAHFVIKQK					Mascot
1812.0413	1811.9325	-0.1088	-60	368	385	SGQLPLAILTIGGMLATR					Mascot
1837.0154	1836.912	-0.1034	-56	541	555	MLRILDLHDAHFVIK			Oxidation (M)[1]		Mascot
1851.9357	1851.8595	-0.0762	-41	659	673	HPEIMKLIHMAWTGR			Oxidation (M)[5,10]		Mascot
1857.9568	1857.9412	-0.0156	-8	386	399	MVNEWETYFLRILK			Oxidation (M)[1]		Mascot
1956.1311	1955.9403	-0.1908	-98	367	385	KSGQLPLAILTIGGMLATR			Oxidation (M)[15]		Mascot
2635.3066	2635.1633	-0.1433	-54	590	612	LQDLQTLDMRNGNMTSL PTEITK			Oxidation (M)[9]		Mascot
2651.3015	2651.1372	-0.1643	-62	590	612	LQDLQTLDMRNGNMTSL PTEITK			Oxidation (M)[9,14]		Mascot
2684.2671	2684.1558	-0.1113	-41	207	228	VYESMKSFSCCAWIIVSQ SFVR			Carbamidomethyl (C)[10,11]		Mascot
3254.6846	3254.533	-0.1516	-47	417	445	WIAEGFIIARSGVSLEDV GNSYFIDLINR					Mascot
3254.6846	3254.533	-0.1516	-47	417	445	WIAEGFIIARSGVSLEDV GNSYFIDLINR					Mascot
3402.6194	3402.6677	0.0483	14	2	35	AETVMSMARSMLGGAIS TAASAAADEMSLLMGVR			Oxidation (M)[5]		Mascot
3418.6143	3418.6348	0.0205	6	2	35	AETVMSMARSMLGGAIS TAASAAADEMSLLMGVR			Oxidation (M)[5,7]		Mascot
3448.8093	3448.7388	-0.0705	-20	174	205	LIDHLHVHANNGPPWVV CVVGMGGLGKTTLTR			Carbamidomethyl (C)[18]		Mascot

Gel Idx/Pos	204/I4	Instr./Gel Origin	BA2151/Sample Project 20140814	Process Status	Analysis Succeeded
Plate [#] Name	[1] Sample Project 20140814	Instrument Sample Name		Spectra	11

Rank	Protein Name	Accession No.	Protein MW	Protein PI	Pep. Count	Protein Score	Protein Score C. I. %	Intensity Matched	Total Ion Score	Total Ion C. I. %	Confirmed
1	L-ascorbate peroxidase 1, cytosolic [Triticum urartu]	gi|474311703	27561.1	5.85	12	417	100	29.858	330	100	

Peptide Information

Calc. Mass	Obsrv. Mass	± da	± ppm	Start Seq.	End Seq.	Sequence	Ion Score	C. I. %	Modification	Rank	Result Type
895.4342	895.4674	0.0332	37	53	61	TGGPFGTMK					Mascot
911.4291	911.429	-0.0001	0	53	61	TGGPFGTMK			Oxidation (M)[8]		Mascot
919.4196	919.4172	-0.0024	-3	230	236	AFFEDYK					Mascot
1006.4808	1006.4757	-0.0051	-5	31	38	NCSPLMLR			Carbamidomethyl (C)[2], Oxidation (M)[6]		Mascot
1036.4847	1036.4836	-0.0011	-1	173	181	SGFEGPWTR					Mascot
1036.4847	1036.4836	-0.0011	-1	173	181	SGFEGPWTR	25	64.002			Mascot
1249.6172	1249.6107	-0.0065	-5	120	130	EDKPQPPPEGR					Mascot
1249.6172	1249.6107	-0.0065	-5	120	130	EDKPQPPPEGR	58	99.984			Mascot
1505.7384	1505.7271	-0.0113	-8	39	52	LAWHSAGTFDVSSK					Mascot
1525.7434	1525.7363	-0.0071	-5	230	241	AFFEDYKEAHLR					Mascot
1525.7434	1525.7363	-0.0071	-5	230	241	AFFEDYKEAHLR	44	99.547			Mascot
1627.9418	1627.9352	-0.0066	-4	210	223	TLLTDPVFRPLVEK					Mascot
1627.9418	1627.9352	-0.0066	-4	210	223	TLLTDPVFRPLVEK	52	99.937			Mascot
1712.8378	1712.8055	-0.0323	-19	4	18	TYPVVSAEYQEAVEK					Mascot
1831.9773	1831.9725	-0.0048	-3	62	79	KPAEQAHAANAGLDIAVR					Mascot
1831.9773	1831.9725	-0.0048	-3	62	79	KPAEQAHAANAGLDIAVR	152	100			Mascot
2071.0237	2071.0107	-0.013	-6	148	167	QMGLSDQDIVALSGGHT LGR			Oxidation (M)[2]		Mascot
2559.3711	2559.2598	-0.1113	-43	62	85	KPAEQAHAANAGLDIAVR MLEPIK			Oxidation (M)[19]		Mascot

Rank	Protein Name	Accession No.	Protein MW	Protein PI	Pep. Count	Protein Score	Protein Score C. I. %	Intensity Matched	Total Ion Score	Total Ion C. I. %	Confirmed
2	ascorbate peroxidase [Triticum aestivum]	gi|226897533	26776.6	5.54	6	162	100	21.527	134	100	

Peptide Information

Calc. Mass	Obsrv. Mass	± da	± ppm	Start Seq.	End Seq.	Sequence	Ion Score	C. I. %	Modification	Rank	Result Type
895.4342	895.4674	0.0332	37	46	54	TGGPFGTMK					Mascot
911.4291	911.429	-0.0001	0	46	54	TGGPFGTMK			Oxidation (M)[8]		Mascot
919.4196	919.4172	-0.0024	-3	223	229	AFFEDYK					Mascot
1036.4847	1036.4836	-0.0011	-1	166	174	SGFEGPWTR					Mascot

1036.4847	1036.4836	-0.0011	-1	166	174	SGFEGPWTR	25	64.002			Mascot
1249.6172	1249.6107	-0.0065	-5	113	123	QDKPEPPPEGR					Mascot
1249.6172	1249.6107	-0.0065	-5	113	123	QDKPEPPPEGR	14	0			Mascot
1525.7434	1525.7363	-0.0071	-5	223	234	AFFEDYKEAHLR					Mascot
1525.7434	1525.7363	-0.0071	-5	223	234	AFFEDYKEAHLR	44	99.547			Mascot
1627.9418	1627.9352	-0.0066	-4	203	216	TLLTDPVFRPLVEK					Mascot
1627.9418	1627.9352	-0.0066	-4	203	216	TLLTDPVFRPLVEK	52	99.937			Mascot

3 hypothetical protein TRIUR3_15565 [Triticum urartu] gi|473990913 43642.9 6.09 10 44 0 16.003

Peptide Information

Calc. Mass	Obsrv. Mass	± da	± ppm	Start Seq.	End Seq.	Sequence	Ion Score	C. I. %	Modification	Rank	Result Type
976.4669	976.47	0.0031	3	162	169	WNDVGMVR					Mascot
1021.4441	1021.5009	0.0568	56	233	241	MVAGMDHTK			Oxidation (M)[1,5]		Mascot
1036.5343	1036.4836	-0.0507	-49	171	179	LMSDEGLKK			Oxidation (M)[2]		Mascot
1036.5343	1036.4836	-0.0507	-49	171	179	LMSDEGLKK			Oxidation (M)[2]		Mascot
1049.5262	1049.4971	-0.0291	-28	320	328	SKLADWSDK					Mascot
1064.5405	1064.5017	-0.0388	-36	170	178	RLMSDEGLK			Oxidation (M)[3]		Mascot
1109.6174	1109.5332	-0.0842	-76	242	251	GAALRLHSER					Mascot
1353.5967	1353.6576	0.0609	45	64	73	YFQMMVSEYR					Mascot
1353.5967	1353.6576	0.0609	45	64	73	YFQMMVSEYR					Mascot
1525.6926	1525.7363	0.0437	29	63	73	RYFQMMVSEYR			Oxidation (M)[5]		Mascot
1525.6926	1525.7363	0.0437	29	63	73	RYFQMMVSEYR			Oxidation (M)[5]		Mascot
1847.9296	1847.952	0.0224	12	64	77	YFQMMVSEYRIKPR					Mascot
2071.094	2071.0107	-0.0833	-40	74	90	IKPRIEHYGCMVDILAR			Carbamidomethyl (C)[10]		Mascot
2087.0889	2086.9973	-0.0916	-44	74	90	IKPRIEHYGCMVDILAR			Carbamidomethyl (C)[10], Oxidation (M)[11]		Mascot

4 porphobilinogen deaminase [Triticum aestivum] gi|19849545 21636.6 9.16 7 43 0 13.085

Peptide Information

Calc. Mass	Obsrv. Mass	± da	± ppm	Start Seq.	End Seq.	Sequence	Ion Score	C. I. %	Modification	Rank	Result Type
943.5432	943.478	-0.0652	-69	191	198	GNVQTRLR					Mascot
1035.5946	1035.4946	-0.1	-97	173	180	RQSQILYK					Mascot
1182.6477	1182.5508	-0.0969	-82	28	39	AAVAVQAEAQPK					Mascot
1537.7679	1537.7174	-0.0505	-33	143	155	EDVRDAFICLTAK			Carbamidomethyl (C)[9]		Mascot
1627.9014	1627.9352	0.0338	21	156	172	TLGELPAGSVIGSASLR					Mascot
1627.9014	1627.9352	0.0338	21	156	172	TLGELPAGSVIGSASLR					Mascot

Calc. Mass	Obsrv. Mass	± da	± ppm	Start Seq.	End Seq.	Sequence	Ion Score	C. I. %	Modification	Rank	Result Type
1760.9099	1760.8677	-0.0422	-24	84	100	TTGDMILDKPLADIGGK			Oxidation (M)[5]		Mascot
2542.28	2542.2664	-0.0136	-5	1	22	MATLRCTNHTLLGSPTCL ARPR			Carbamidomethyl (C)[6,17], Oxidation (M)[1]		Mascot

5	hypothetical protein TRIUR3_33372 [Triticum urartu]	gi|473848470	21525.9	8.17	7	40	0	3.364		

Peptide Information

Calc. Mass	Obsrv. Mass	± da	± ppm	Start Seq.	End Seq.	Sequence	Ion Score	C. I. %	Modification	Rank	Result Type
870.5229	870.51	-0.0129	-15	160	166	LMRPPLK			Oxidation (M)[2]		Mascot
919.4124	919.4172	0.0048	5	132	138	EMLPNCR			Carbamidomethyl (C)[6]		Mascot
1050.5038	1050.4817	-0.0221	-21	152	159	SLESCWIR			Carbamidomethyl (C)[5]		Mascot
1108.5853	1108.5126	-0.0727	-66	2	10	RTLPMTMVK			Oxidation (M)[5,7]		Mascot
1126.5198	1126.5605	0.0407	36	82	91	FAVESMDATR					Mascot
1537.7792	1537.7174	-0.0618	-40	82	95	FAVESMDATRLAAR					Mascot
1886.0139	1885.8928	-0.1211	-64	152	166	SLESCWIRLMRPPLK			Carbamidomethyl (C)[5]		Mascot

6	hypothetical protein TRIUR3_21372 [Triticum urartu]	gi|474266694	67269.7	5.44	12	39	0	14.238	9	0

Peptide Information

Calc. Mass	Obsrv. Mass	± da	± ppm	Start Seq.	End Seq.	Sequence	Ion Score	C. I. %	Modification	Rank	Result Type
915.5006	915.4647	-0.0359	-39	300	307	RAEEAALR					Mascot
1007.552	1007.5112	-0.0408	-40	371	379	LLTTAAFDR					Mascot
1007.552	1007.5112	-0.0408	-40	371	379	LLTTAAFDR					Mascot
1049.4382	1049.4971	0.0589	56	411	419	DDDESELAR					Mascot
1050.3859	1050.4817	0.0958	91	402	410	ENEEDDDGK					Mascot
1067.5044	1067.4625	-0.0419	-39	390	397	DEDWLVYK					Mascot
1090.5011	1090.4667	-0.0344	-32	133	141	NNKEEAEEK					Mascot
1108.5997	1108.5126	-0.0871	-79	328	336	ARYLELSEK					Mascot
1231.7004	1231.6003	-0.1001	-81	231	241	SDILKATQSLR					Mascot
1386.5414	1386.6509	0.1095	79	2	12	TECECNPSFSR			Carbamidomethyl (C)[3,5]		Mascot
1525.7169	1525.7363	0.0194	13	316	327	TENPELYFDELR					Mascot
1525.7169	1525.7363	0.0194	13	316	327	TENPELYFDELR	9	0			Mascot
1760.715	1760.8677	0.1527	87	1	14	MTECECNPSFSRAR			Carbamidomethyl (C)[4,6], Oxidation (M)[1]		Mascot
2583.2839	2583.2676	-0.0163	-6	424	446	IQEVDPTFVNKHDAVEPA PDPHK					Mascot

7	Peroxisomal 2,4-dienoyl-CoA reductase [Triticum urartu]	gi|474413719	26291.7	8.52	7	38	0	4.757		

Peptide Information

Calc. Mass	Obsrv. Mass	± da	± ppm	Start Seq.	End Seq.	Sequence	Ion Score	C. I. %	Modification	Rank	Result Type
895.4416	895.4674	0.0258	29	171	177	EMMPLFK					Mascot
906.4349	906.4397	0.0048	5	1	8	MATESPVR			Oxidation (M)[1]		Mascot
911.4365	911.429	-0.0075	-8	171	177	EMMPLFK			Oxidation (M)[2]		Mascot
1051.5427	1051.4642	-0.0785	-75	170	177	REMMPLFK					Mascot
1061.5297	1061.5869	0.0572	54	160	169	LAPDEMGKGK			Oxidation (M)[6]		Mascot
1067.5376	1067.4625	-0.0751	-70	170	177	REMMPLFK			Oxidation (M)[3]		Mascot
1525.7567	1525.7363	-0.0204	-13	183	197	DIAMAALYLASDAGK			Oxidation (M)[4]		Mascot
1525.7567	1525.7363	-0.0204	-13	183	197	DIAMAALYLASDAGK			Oxidation (M)[4]		Mascot
2559.3831	2559.2598	-0.1233	-48	198	220	YVNGTTLVVDGGLWLSH PRHIPK					Mascot
2583.2471	2583.2676	0.0205	8	61	82	GFRTVLEIDTVGTYTMCY EALK			Carbamidomethyl (C)[17], Oxidation (M)[16]		Mascot

8 Myosin-2 heavy chain [Triticum urartu] gi|474125466 128134.6 7.77 15 37 0 19.392

Peptide Information

Calc. Mass	Obsrv. Mass	± da	± ppm	Start Seq.	End Seq.	Sequence	Ion Score	C. I. %	Modification	Rank	Result Type
915.5258	915.4647	-0.0611	-67	920	927	AEALLRDK					Mascot
1036.4767	1036.4836	0.0069	7	381	389	TSRNDNSSR					Mascot
1036.4767	1036.4836	0.0069	7	381	389	TSRNDNSSR					Mascot
1078.5198	1078.5327	0.0129	12	976	985	SLAADDMVTR					Mascot
1084.5535	1084.4686	-0.0849	-78	596	605	QHLSGNPGFK					Mascot
1084.5535	1084.6349	0.0814	75	596	605	QHLSGNPGFK					Mascot
1096.5018	1096.5054	0.0036	3	384	392	NDNSSRFEK					Mascot
1125.5181	1125.5319	0.0138	12	730	738	CCGVFEVVR			Carbamidomethyl (C)[1,2]		Mascot
1126.6442	1126.5605	-0.0837	-74	186	194	VSFIVKFMR					Mascot
1145.5797	1145.563	-0.0167	-15	104	114	EIGGQEAKEGK					Mascot
1182.5902	1182.5508	-0.0394	-33	269	277	YSRDHIYTK					Mascot
1642.8105	1642.9016	0.0911	55	82	95	KPEAVNENKPEMEK					Mascot
1655.85	1655.9353	0.0853	52	1036	1049	INAVNHLAKEFEDR					Mascot
1760.8232	1760.8677	0.0445	25	1065	1080	SGQIGSNMNPDDELRK					Mascot
1831.8717	1831.9725	0.1008	55	192	205	FMRFILLSCEQDEK			Carbamidomethyl (C)[9], Oxidation (M)[2]		Mascot
1831.8717	1831.9725	0.1008	55	192	205	FMRFILLSCEQDEK			Carbamidomethyl (C)[9], Oxidation (M)[2]		Mascot
1955.9491	1955.9791	0.03	15	654	671	DFASVMIADSQNKSSLSR					Mascot
2559.3025	2559.2598	-0.0427	-17	304	325	QKLTNDPHVYAIADIAFN EMLR					Mascot

9 hypothetical protein TRIUR3_16487 [Triticum urartu] gi|474196041 25608.9 5.29 7 36 0 14.997

Peptide Information

Calc. Mass	Obsrv. Mass	± da	± ppm	Start Seq.	End Seq.	Sequence	Ion Score	C. I. %	Modification	Rank	Result Type
906.4713	906.4397	-0.0316	-35	1	7	MEVERVK			Oxidation (M)[1]		Mascot
976.4694	976.47	0.0006	1	12	19	EEEKAQSR					Mascot
1036.5786	1036.4836	-0.095	-92	76	85	AVHLVDAPSK					Mascot
1036.5786	1036.4836	-0.095	-92	76	85	AVHLVDAPSK					Mascot
1231.61	1231.6003	-0.0097	-8	32	43	AIGLTSPADACR			Carbamidomethyl (C)[11]		Mascot
1370.7349	1370.6801	-0.0548	-40	20	31	IIQGIPEECLAK			Carbamidomethyl (C)[9]		Mascot
1370.7349	1370.6801	-0.0548	-40	20	31	IIQGIPEECLAK			Carbamidomethyl (C)[9]		Mascot
2071.0706	2071.0107	-0.0599	-29	86	103	KELFIDLSNEHVVLDDGK					Mascot
2583.3269	2583.2676	-0.0593	-23	20	43	IIQGIPEECLAKAIGLTSPA DACR			Carbamidomethyl (C)[9,23]		Mascot

10	glycosyltransferase, HGA-like,putative,expressed [Triticum aestivum]	gi|383100754	67949.7	5.85	11	36	0	14.258

Peptide Information

Calc. Mass	Obsrv. Mass	± da	± ppm	Start Seq.	End Seq.	Sequence	Ion Score	C. I. %	Modification	Rank	Result Type
870.4403	870.51	0.0697	80	583	589	GFGFMRR					Mascot
906.3887	906.4397	0.051	56	392	398	GETHCFR			Carbamidomethyl (C)[5]		Mascot
915.5522	915.4647	-0.0875	-96	279	285	VRPYPRK					Mascot
1035.543	1035.4946	-0.0484	-47	67	76	TVKSSSSSPR					Mascot
1052.4612	1052.4778	0.0166	16	285	293	KGDQTCMGR			Carbamidomethyl (C)[6]		Mascot
1052.4612	1052.4778	0.0166	16	285	293	KGDQTCMGR			Carbamidomethyl (C)[6]		Mascot
1068.4562	1068.4725	0.0163	15	285	293	KGDQTCMGR			Carbamidomethyl (C)[6], Oxidation (M)[7]		Mascot
1068.4562	1068.4725	0.0163	15	285	293	KGDQTCMGR			Carbamidomethyl (C)[6], Oxidation (M)[7]		Mascot
1090.5599	1090.4667	-0.0932	-85	70	80	SSSSSPRGLGR					Mascot
1145.665	1145.563	-0.102	-89	399	408	HAVVSLRAHR					Mascot
1386.774	1386.6509	-0.1231	-89	77	89	GLGRDFLVDVAPK					Mascot
1633.902	1633.8083	-0.0937	-57	376	391	ELSRHAPLDLAAAAAK					Mascot
1643.8163	1643.9189	0.1026	62	550	563	YVQYEIGVEESSLK					Mascot
1655.8347	1655.9353	0.1006	61	90	105	LGDALGRQPEQSAGEK					Mascot

Gel Idx/Pos	205/I5	Instr./Gel Origin	BA2151/Sample Project 20140814	Process Status	Analysis Succeeded
Plate [#] Name	[1] Sample Project 20140814	Instrument Sample Name		Spectra	11

Rank	Protein Name	Accession No.	Protein MW	Protein PI	Pep. Count	Protein Score	Protein Score C. I. %	Intensity Matched	Total Ion Score	Total Ion C. I. %	Confirmed
1	gamma gliadin [Triticum aestivum]	gi|133741924	16537.3	8.88	3	82	99.971	15.537	69	100	

Peptide Information

Calc. Mass	Obsrv. Mass	± da	± ppm	Start Seq.	End Seq.	Sequence	Ion Score	C. I. %	Modification	Rank	Result Type
856.416	856.3726	-0.0434	-51	138	145	STTTRSGF					Mascot
1885.8064	1885.9272	0.1208	64	84	97	SCLVMWEQCCQQLK			Carbamidomethyl (C)[2,9,10], Oxidation (M)[5]		Mascot
1966.9362	1966.9166	-0.0196	-10	60	76	DALLQQCSPVADMSFLR			Carbamidomethyl (C)[7], Oxidation (M)[13]		Mascot
1966.9362	1966.9166	-0.0196	-10	60	76	DALLQQCSPVADMSFLR	69	100	Carbamidomethyl (C)[7], Oxidation (M)[13]		Mascot

Rank	Protein Name	Accession No.	Protein MW	Protein PI	Pep. Count	Protein Score	Protein Score C. I. %	Intensity Matched	Total Ion Score	Total Ion C. I. %	Confirmed
2	gliadin/avenin-like seed protein [Triticum aestivum]	gi|281335538	22813.2	6.2	1	69	99.48	14.6	69	100	

Protein Group

Avenin-3 [Triticum urartu]	gi|474329936	22657.1	6.3499 999046 3257

Peptide Information

Calc. Mass	Obsrv. Mass	± da	± ppm	Start Seq.	End Seq.	Sequence	Ion Score	C. I. %	Modification	Rank	Result Type
1966.9362	1966.9166	-0.0196	-10	60	76	DALLQQCSPVADMSFLR			Carbamidomethyl (C)[7], Oxidation (M)[13]		Mascot
1966.9362	1966.9166	-0.0196	-10	60	76	DALLQQCSPVADMSFLR	69	100	Carbamidomethyl (C)[7], Oxidation (M)[13]		Mascot

Rank	Protein Name	Accession No.	Protein MW	Protein PI	Pep. Count	Protein Score	Protein Score C. I. %	Intensity Matched	Total Ion Score	Total Ion C. I. %	Confirmed
3	Zinc finger protein 7 [Triticum urartu]	gi|474142506	12280.2	9.18	6	50	54.748	4.279			

Peptide Information

Calc. Mass	Obsrv. Mass	± da	± ppm	Start Seq.	End Seq.	Sequence	Ion Score	C. I. %	Modification	Rank	Result Type
1097.5586	1097.6028	0.0442	40	101	109	DHELDLSLR					Mascot
1210.6427	1210.552	-0.0907	-75	101	110	DHELDLSLRL					Mascot
1637.8256	1637.806	-0.0196	-12	35	49	FRSSQALGGHQNAHK					Mascot
1925.9062	1925.8889	-0.0173	-9	73	92	AAGAVWVSEESAAAGG MAYK					Mascot
1941.9011	1941.9039	0.0028	1	73	92	AAGAVWVSEESAAAGG MAYK			Oxidation (M)[17]		Mascot
3417.6282	3417.6863	0.0581	17	59	92	ETAVATTHAWNPGRAAG AVWVSEESAAAGGMAYK					Mascot

Calc. Mass	Obsrv. Mass	± da	± ppm	Start Seq.	End Seq.	Sequence	Ion Score	C. I. %	Modification	Rank	Result Type
3433.623	3433.6763	0.0533	16	59	92	ETAVATTHAWNPGRAAG AVWVSEESAAAGGMAYK			Oxidation (M)[31]		Mascot
3477.7183	3477.6982	-0.0201	-6	2	32	EHAQDELSLELTLATAVV APAPGFFLCVYCR			Carbamidomethyl (C)[27,30]		Mascot

4 O-methyltransferase ZRP4 [Triticum urartu] gi|473849432 39910.2 5.4 9 44 0 11.493

Peptide Information

Calc. Mass	Obsrv. Mass	± da	± ppm	Start Seq.	End Seq.	Sequence	Ion Score	C. I. %	Modification	Rank	Result Type
834.3628	834.3173	-0.0455	-55	330	335	DEQEWK					Mascot
1637.8932	1637.806	-0.0872	-53	294	309	VIIVDMVVGAGPQDPK					Mascot
1749.829	1749.933	0.104	59	92	108	DDGVEGEAVYALTPASR					Mascot
1903.047	1902.928	-0.119	-63	294	311	VIIVDMVVGAGPQDPKHK					Mascot
1903.047	1902.928	-0.119	-63	294	311	VIIVDMVVGAGPQDPKHK					Mascot
1919.042	1918.9171	-0.1249	-65	294	311	VIIVDMVVGAGPQDPKHK			Oxidation (M)[6]		Mascot
1937.0452	1936.897	-0.1482	-77	49	68	QGGAATLSQIAATATLHP TK					Mascot
2221.0669	2221.0715	0.0046	2	312	329	ETQVMFDLFIMFINGVER			Oxidation (M)[5,11]		Mascot
3402.7034	3402.6343	-0.0691	-20	92	123	DDGVEGEAVYALTPASR LLVCSASVNMVHITK			Carbamidomethyl (C)[21]		Mascot
3427.7031	3427.6877	-0.0154	-4	124	152	MLLHTNLVSPFSDLGTW FQHELPEPDLFK			Oxidation (M)[1]		Mascot
3445.7651	3445.6343	-0.1308	-38	2	30	APTREQHILDQGLLDAQL ELWHHTFSYVK					Mascot

5 Protein Rf1, mitochondrial [Triticum urartu] gi|474436132 56266.2 8.88 13 44 0 4.008

Peptide Information

Calc. Mass	Obsrv. Mass	± da	± ppm	Start Seq.	End Seq.	Sequence	Ion Score	C. I. %	Modification	Rank	Result Type
839.3273	839.3144	-0.0129	-15	384	390	MDDAMEK					Mascot
1089.6415	1089.561	-0.0805	-74	8	18	ALSGLFAALAR					Mascot
1147.614	1147.6825	0.0685	60	208	217	AMDKAEVVLR			Oxidation (M)[2]		Mascot
1199.4854	1199.5419	0.0565	47	381	390	MGRMDDAMEK			Oxidation (M)[1]		Mascot
1298.5868	1298.5831	-0.0037	-3	177	186	ACDLFQEMIR			Carbamidomethyl (C)[2], Oxidation (M)[8]		Mascot
1550.719	1550.7864	0.0674	43	311	323	EGSLIDMIDLCER			Carbamidomethyl (C)[11]		Mascot
1637.7261	1637.806	0.0799	49	349	361	MDVAMLFFEDMLK			Oxidation (M)[1,5,11]		Mascot
1733.8312	1733.9216	0.0904	52	349	362	MDVAMLFFEDMLKK			Oxidation (M)[1]		Mascot
1749.826	1749.933	0.107	61	349	362	MDVAMLFFEDMLKK			Oxidation (M)[1,5]		Mascot
1765.8209	1765.9094	0.0885	50	349	362	MDVAMLFFEDMLKK			Oxidation (M)[1,5,11]		Mascot
1908.8976	1908.891	-0.0066	-3	311	326	EGSLIDMIDLCERMAR			Carbamidomethyl (C)[11]		Mascot
1965.9271	1965.8792	-0.0479	-24	346	361	YGRMDVAMLFFEDMLK					Mascot
2875.2915	2875.3521	0.0606	21	151	176	GADHSPDVMSYNMVIDG			Oxidation (M)[9]		Mascot

Calc. Mass	Obsrv. Mass	± da	± ppm	Start Seq.	End Seq.	Sequence	Ion Score	C. I. %	Modification	Rank	Result Type
						FFKEGEVSK					
3378.6716	3378.627	-0.0446	-13	281	310	EAVEFFYSMAVNGQKPD VISYSVLLHGYAK			Oxidation (M)[9]		Mascot
3463.7275	3463.6655	-0.062	-18	218	249	SMVHNGAQPDAVTYNSL IQGYSTLGQLKEVAR			Oxidation (M)[2]		Mascot

6 Zinc finger protein 7 [Triticum urartu] gi|474443748 12722.5 9.75 5 40 0 1.865

Peptide Information

Calc. Mass	Obsrv. Mass	± da	± ppm	Start Seq.	End Seq.	Sequence	Ion Score	C. I. %	Modification	Rank	Result Type
1067.5593	1067.608	0.0487	46	87	96	HAAAPDTRTK					Mascot
1228.5441	1228.5308	-0.0133	-11	98	107	LDDDDDKHQK					Mascot
1228.5441	1228.5308	-0.0133	-11	98	107	LDDDDDKHQK					Mascot
1845.8524	1845.8774	0.025	14	38	52	GGARSWLFSCLFCER			Carbamidomethyl (C)[10,13]		Mascot
3438.7759	3438.6631	-0.1128	-33	1	35	MAAQAPPAAAAAPLDLSL TLSPMYNLAVLPSGGSR			Oxidation (M)[1]		Mascot
3463.8367	3463.6655	-0.1712	-49	2	36	AAQAPPAAAAAPLDLSLT LSPMYNLAVLPSGGSRR			Oxidation (M)[22]		Mascot

7 Acyl carrier protein, mitochondrial [Triticum urartu] gi|474168731 9921 4.6 5 38 0 2.016

Peptide Information

Calc. Mass	Obsrv. Mass	± da	± ppm	Start Seq.	End Seq.	Sequence	Ion Score	C. I. %	Modification	Rank	Result Type
1067.5303	1067.608	0.0777	73	23	31	CHPKVDPSK			Carbamidomethyl (C)[1]		Mascot
1180.5529	1180.533	-0.0199	-17	1	10	MPSHDAHLTR			Oxidation (M)[1]		Mascot
1208.682	1208.5667	-0.1153	-95	17	26	VLDVLKCHPK			Carbamidomethyl (C)[7]		Mascot
1925.979	1925.8889	-0.0901	-47	72	89	INSLPLAIEYVANHPMAG			Oxidation (M)[16]		Mascot
3406.6611	3406.6189	-0.0422	-12	32	61	VTPEAHFEKDLGLDSLDT VEVVMAIEEEFK			Oxidation (M)[23]		Mascot

8 putative adenylate kinase 1, chloroplastic [Triticum urartu] gi|474438700 28399.3 5.08 8 37 0 2.641

Peptide Information

Calc. Mass	Obsrv. Mass	± da	± ppm	Start Seq.	End Seq.	Sequence	Ion Score	C. I. %	Modification	Rank	Result Type
1067.5844	1067.608	0.0236	22	68	77	ALSEIVNHGK					Mascot
1199.6089	1199.5419	-0.067	-56	1	11	MATSLLGTFSR			Oxidation (M)[1]		Mascot
1200.5902	1200.5245	-0.0657	-55	57	67	TGGREVHASMR					Mascot
1893.96	1893.9132	-0.0468	-25	61	77	EVHASMRALSEIVNHGK			Oxidation (M)[6]		Mascot
1983.0586	1982.9031	-0.1555	-78	218	235	GKLLEFDLPGGIPESWPK					Mascot
1983.1008	1982.9031	-0.1977	-100	114	131	QAEILEGVTDIDLVINLK					Mascot
1990.8958	1990.8903	-0.0055	-3	1	18	MATSLLGTFSRDGECSS			Carbamidomethyl (C)[15], Oxidation (M)[1]		Mascot

1998.0292	1998.1549	0.1257	63	22	40	R EKDSPPGTSELVGWLAV GR						Mascot

9 hypothetical protein TRIUR3_11930 [Triticum urartu] gi|474096673 12591.4 5.3 5 36 0 16.414

Peptide Information

Calc. Mass	Obsrv. Mass	± da	± ppm	Start Seq.	End Seq.	Sequence	Ion Score	C. I. %	Modification	Rank	Result Type
867.5159	867.4315	-0.0844	-97	47	54	GRPVPVSR					Mascot
1637.833	1637.806	-0.027	-16	47	60	GRPVPVSRGCSFYR			Carbamidomethyl (C)[10]		Mascot
1859.8998	1859.9421	0.0423	23	7	23	VVGFFGEFMGSVSPDLR			Oxidation (M)[9]		Mascot
1967.0631	1966.9166	-0.1465	-74	78	95	DCVEPATKPGELALVVIR			Carbamidomethyl (C)[2]		Mascot
1967.0631	1966.9166	-0.1465	-74	78	95	DCVEPATKPGELALVVIR			Carbamidomethyl (C)[2]		Mascot
3448.7649	3448.6882	-0.0767	-22	24	54	FVIDDISGEDPSAVGVTW HLEWKGRPVPVSR					Mascot
3448.7649	3448.6882	-0.0767	-22	24	54	FVIDDISGEDPSAVGVTW HLEWKGRPVPVSR					Mascot

10 GA2ox4-3 protein [Triticum aestivum] gi|440577526 36833.8 7.79 8 35 0 3.491

Peptide Information

Calc. Mass	Obsrv. Mass	± da	± ppm	Start Seq.	End Seq.	Sequence	Ion Score	C. I. %	Modification	Rank	Result Type
1224.5677	1224.5448	-0.0229	-19	132	142	DAINDYVGAMR					Mascot
1637.8064	1637.806	-0.0004	0	132	146	DAINDYVGAMRGLAR			Oxidation (M)[10]		Mascot
1810.9092	1810.8914	-0.0178	-10	271	286	SRMSMIYFGGPPLAQR					Mascot
1935.1062	1934.9248	-0.1814	-94	287	304	IAPLPQLPAGTQSLPLYR					Mascot
1951.9873	1951.9303	-0.057	-29	238	255	DAFFVNVGDSLQVLTNG R					Mascot
1955.0743	1954.9728	-0.1015	-52	143	161	GLARTVLEMVAEGLGVS PR					Mascot
2221.1433	2221.0715	-0.0718	-32	98	116	IGRNGDMGWVEYLLLAIE R			Oxidation (M)[7]		Mascot
3378.667	3378.627	-0.04	-12	147	179	TVLEMVAEGLGVSPRGA LADMVTGDAASDQVFR			Oxidation (M)[5]		Mascot

Gel Idx/Pos	206/I6	Instr./Gel Origin	BA2151/Sample Project 20140814	Process Status	Analysis Succeeded
Plate [#] Name	[1] Sample Project 20140814	Instrument Sample Name		Spectra	11

Rank	Protein Name	Accession No.	Protein MW	Protein PI	Pep. Count	Protein Score	Protein Score C. I. %	Intensity Matched	Total Ion Score	Total Ion C. I. %	Confirmed
1	hypothetical protein TRIUR3_28410 [Triticum urartu]	gi|474060617	28387.9	5.53	7	86	99.989	10.984	52	99.962	

Peptide Information

Calc. Mass	Obsrv. Mass	± da	± ppm	Start Seq.	End Seq.	Sequence	Ion Score	C. I. %	Modification	Rank	Result Type
971.5673	971.5522	-0.0151	-16	51	58	FVTNHLLK					Mascot
1078.535	1078.5322	-0.0028	-3	207	215	GYPLLEACR			Carbamidomethyl (C)[8]		Mascot
1145.5698	1145.5636	-0.0062	-5	156	165	GHNLSLEYGR					Mascot
1145.5698	1145.5636	-0.0062	-5	156	165	GHNLSLEYGR	24	77.688			Mascot
1406.6271	1406.6254	-0.0017	-1	130	139	HREWESCFQK			Carbamidomethyl (C)[7]		Mascot
1534.7571	1534.7842	0.0271	18	140	152	QGLDPKPVTECYK			Carbamidomethyl (C)[11]		Mascot
1927.9371	1927.9271	-0.01	-5	35	50	VHVAIYYESLCPYSAR			Carbamidomethyl (C)[11]		Mascot
1927.9371	1927.9271	-0.01	-5	35	50	VHVAIYYESLCPYSAR	28	89.66	Carbamidomethyl (C)[11]		Mascot
3402.8162	3402.6047	-0.2115	-62	1	34	MARGLHHLLLLAALLQQL SATSAGDVATGSGGEK			Oxidation (M)[1]		Mascot

Rank	Protein Name	Accession No.	Protein MW	Protein PI	Pep. Count	Protein Score	Protein Score C. I. %	Intensity Matched	Total Ion Score	Total Ion C. I. %	Confirmed
2	gamma gliadin [Triticum aestivum]	gi|133741924	16537.3	8.88	2	77	99.92	18.909	68	100	

Peptide Information

Calc. Mass	Obsrv. Mass	± da	± ppm	Start Seq.	End Seq.	Sequence	Ion Score	C. I. %	Modification	Rank	Result Type
1950.9413	1950.9211	-0.0202	-10	60	76	DALLQQCSPVADMSFLR			Carbamidomethyl (C)[7]		Mascot
1966.9362	1966.9214	-0.0148	-8	60	76	DALLQQCSPVADMSFLR			Carbamidomethyl (C)[7], Oxidation (M)[13]		Mascot
1966.9362	1966.9214	-0.0148	-8	60	76	DALLQQCSPVADMSFLR	68	100	Carbamidomethyl (C)[7], Oxidation (M)[13]		Mascot
2652.2151	2652.1565	-0.0586	-22	77	97	SQAVQPRSCLVMWEQC CQQLK			Carbamidomethyl (C)[9,16,17], Oxidation (M)[12]		Mascot

Rank	Protein Name	Accession No.	Protein MW	Protein PI	Pep. Count	Protein Score	Protein Score C. I. %	Intensity Matched	Total Ion Score	Total Ion C. I. %	Confirmed
3	gliadin/avenin-like seed protein [Triticum aestivum]	gi|281335538	22813.2	6.2	1	68	99.283	18.83	68	100	

Protein Group

Avenin-3 [Triticum urartu]	gi|474329936	22657.1	6.3499 999046 3257

Peptide Information

Calc. Mass	Obsrv. Mass	± da	± ppm	Start Seq.	End Seq.	Sequence	Ion Score	C. I. %	Modification	Rank	Result Type

Calc. Mass	Obsrv. Mass	± da	± ppm	Start Seq.	End Seq.	Sequence	Ion Score	C. I. %	Modification	Rank	Result Type
1950.9413	1950.9211	-0.0202	-10	60	76	DALLQQCSPVADMSFLR			Carbamidomethyl (C)[7]		Mascot
1966.9362	1966.9214	-0.0148	-8	60	76	DALLQQCSPVADMSFLR			Carbamidomethyl (C)[7], Oxidation (M)[13]		Mascot
1966.9362	1966.9214	-0.0148	-8	60	76	DALLQQCSPVADMSFLR	68	100	Carbamidomethyl (C)[7], Oxidation (M)[13]		Mascot

4	Tri a Bd 27K, partial [Triticum aestivum]	gi|283480515	23214.5	6.06	4	38	0	6.869	24	77.688	

Peptide Information

Calc. Mass	Obsrv. Mass	± da	± ppm	Start Seq.	End Seq.	Sequence	Ion Score	C. I. %	Modification	Rank	Result Type
1078.535	1078.5322	-0.0028	-3	180	188	GYPLLEACR			Carbamidomethyl (C)[8]		Mascot
1145.5698	1145.5636	-0.0062	-5	132	141	GHNLSLEYGR					Mascot
1145.5698	1145.5636	-0.0062	-5	132	141	GHNLSLEYGR	24	77.688			Mascot
1406.6271	1406.6254	-0.0017	-1	106	115	HREWESCFQK			Carbamidomethyl (C)[7]		Mascot
1534.7571	1534.7842	0.0271	18	116	128	QGLDPKPVTECYK			Carbamidomethyl (C)[11]		Mascot

5	27K protein [Triticum aestivum]	gi|30793446	23271.5	6.06	4	38	0	6.869	24	77.688	

Peptide Information

Calc. Mass	Obsrv. Mass	± da	± ppm	Start Seq.	End Seq.	Sequence	Ion Score	C. I. %	Modification	Rank	Result Type
1078.535	1078.5322	-0.0028	-3	181	189	GYPLLEACR			Carbamidomethyl (C)[8]		Mascot
1145.5698	1145.5636	-0.0062	-5	133	142	GHNLSLEYGR					Mascot
1145.5698	1145.5636	-0.0062	-5	133	142	GHNLSLEYGR	24	77.688			Mascot
1406.6271	1406.6254	-0.0017	-1	107	116	HREWESCFQK			Carbamidomethyl (C)[7]		Mascot
1534.7571	1534.7842	0.0271	18	117	129	QGLDPKPVTECYK			Carbamidomethyl (C)[11]		Mascot

6	27k protein [Triticum aestivum]	gi|290350670	24399.1	6.06	4	37	0	6.869	24	77.688	

Peptide Information

Calc. Mass	Obsrv. Mass	± da	± ppm	Start Seq.	End Seq.	Sequence	Ion Score	C. I. %	Modification	Rank	Result Type
1078.535	1078.5322	-0.0028	-3	193	201	GYPLLEACR			Carbamidomethyl (C)[8]		Mascot
1145.5698	1145.5636	-0.0062	-5	145	154	GHNLSLEYGR					Mascot
1145.5698	1145.5636	-0.0062	-5	145	154	GHNLSLEYGR	24	77.688			Mascot
1406.6271	1406.6254	-0.0017	-1	119	128	HREWESCFQK			Carbamidomethyl (C)[7]		Mascot
1534.7571	1534.7842	0.0271	18	129	141	QGLDPKPVTECYK			Carbamidomethyl (C)[11]		Mascot

7	Kinesin-like protein KIFC3 [Triticum urartu]	gi|473859842	47769.5	9.82	9	35	0	6.959			

Peptide Information

Calc. Mass	Obsrv. Mass	± da	± ppm	Start Seq.	End Seq.	Sequence	Ion Score	C. I. %	Modification	Rank	Result Type

Calc. Mass	Obsrv. Mass	± da	± ppm	Start Seq.	End Seq.	Sequence	Ion Score	C. I. %	Modification	Rank	Result Type
1170.6589	1170.5618	-0.0971	-83	410	419	ESLVSRPVQR					Mascot
1263.644	1263.6754	0.0314	25	367	378	ARSVAFSSGEPR					Mascot
1517.8044	1517.7848	-0.0196	-13	329	342	QRHGAASHSVSKPR					Mascot
1518.801	1518.798	-0.003	-2	218	230	LIESSRELSEDLK					Mascot
1617.7213	1617.7544	0.0331	20	1	14	MAYAYEVEVDGAQR			Oxidation (M)[1]		Mascot
1950.988	1950.9211	-0.0669	-34	288	306	SILVAGHIDAADSPQATE R					Mascot
1957.9647	1957.9929	0.0282	14	243	259	EICDAEQELGVLNAQIR			Carbamidomethyl (C)[3]		Mascot
3394.624	3394.6272	0.0032	1	2	31	AYAYEVEVDGAQRWTNH LPWSYVEGGAGLR					Mascot
3408.7268	3408.6533	-0.0735	-22	41	73	SSDVEFSNLSILATTSGS VQVEGLTDVTVPDLK					Mascot

8 Programmed cell death protein 4 [Triticum urartu] gi|473951553 71006.1 4.88 12 35 0 8.954

Peptide Information

Calc. Mass	Obsrv. Mass	± da	± ppm	Start Seq.	End Seq.	Sequence	Ion Score	C. I. %	Modification	Rank	Result Type
808.4047	808.3493	-0.0554	-69	179	186	GSLTESSK					Mascot
1037.5626	1037.5201	-0.0425	-41	318	326	SQFQTLVSK					Mascot
1060.6184	1060.5651	-0.0533	-50	397	405	LITIAMDRK					Mascot
1422.6213	1422.6089	-0.0124	-9	237	248	NGDTAEACRCIR			Carbamidomethyl (C)[8,10]		Mascot
1518.8203	1518.798	-0.0223	-15	383	395	ELGLPEYNPVFIK					Mascot
1810.8534	1810.8898	0.0364	20	483	499	CSGAETLNMARSLASAR			Carbamidomethyl (C)[1], Oxidation (M)[9]		Mascot
1861.047	1860.9288	-0.1182	-64	380	395	TLKELGLPEYNPVFIK					Mascot
1875.9171	1875.9167	-0.0004	0	505	521	LLRCWGGGTGWAVEDA K			Carbamidomethyl (C)[4]		Mascot
1875.9171	1875.9167	-0.0004	0	505	521	LLRCWGGGTGWAVEDA K			Carbamidomethyl (C)[4]		Mascot
1902.0365	1901.9048	-0.1317	-69	262	279	RAVTLGMESPAAETLIVK			Oxidation (M)[7]		Mascot
1949.026	1948.9332	-0.0928	-48	280	297	LLKEASEEGLISSSQMVK					Mascot
3155.5144	3155.47	-0.0444	-14	570	596	ECMLALLHECFGEGIITIN QMTKGFSR			Carbamidomethyl (C)[2,10]		Mascot
3256.4692	3256.5	0.0308	9	34	61	NDPNYDSDEEPYELIEAP ATTPLEDYKK					Mascot

9 putative gag-pol polyprotein [Triticum monococcum subsp. aegilopoides] gi|115392332 196089.4 8.17 24 35 0 10.195

Peptide Information

Calc. Mass	Obsrv. Mass	± da	± ppm	Start Seq.	End Seq.	Sequence	Ion Score	C. I. %	Modification	Rank	Result Type
987.5621	987.5455	-0.0166	-17	612	619	NLYTLVHK					Mascot
991.5319	991.5373	0.0054	5	1274	1281	DVERFVAR					Mascot

1144.535	1144.5614	0.0264	23	1678	1687	NMHGVDGMRK					Mascot
1193.6749	1193.6127	-0.0622	-52	86	95	LPTHRETALR					Mascot
1308.6543	1308.6357	-0.0186	-14	1688	1699	GIKNGVTSDDFR					Mascot
1315.7151	1315.6089	-0.1062	-81	1226	1237	ANKLCIPASSVR			Carbamidomethyl (C)[5]		Mascot
1343.7179	1343.7344	0.0165	12	851	861	VDLRSGYHQIR					Mascot
1347.6573	1347.6012	-0.0561	-42	770	780	TNPEETKEIMR					Mascot
1491.693	1491.7422	0.0492	33	289	301	CGIVEENEAMLAR			Carbamidomethyl (C)[1]		Mascot
1507.688	1507.7377	0.0497	33	289	301	CGIVEENEAMLAR			Carbamidomethyl (C)[1], Oxidation (M)[10]		Mascot
1517.7992	1517.7848	-0.0144	-9	777	788	EIMRQVQELLDK			Oxidation (M)[3]		Mascot
1518.7345	1518.798	0.0635	42	1336	1347	FSKMAHFIPCHK			Carbamidomethyl (C)[10], Oxidation (M)[4]		Mascot
1631.9155	1631.8994	-0.0161	-10	1043	1056	LTYAPLLQLPNFNK					Mascot
1700.8795	1700.816	-0.0635	-37	252	264	WVPPYYQHELLQK					Mascot
1813.0139	1812.9358	-0.0781	-43	620	635	DKNITLLPMTPESILK					Mascot
1875.0375	1874.9512	-0.0863	-46	1041	1056	DKLTYAPLLQLPNFNK					Mascot
1891.9524	1891.9485	-0.0039	-2	1258	1272	TEDILATHFFWPKMR					Mascot
1927.7797	1927.9271	0.1474	76	1	17	MAGSQADETDWENMTN K					Mascot
1927.9946	1927.9271	-0.0675	-35	302	317	FMGGLNREIQTILEYK			Oxidation (M)[2]		Mascot
1936.0367	1935.9364	-0.1003	-52	1145	1159	WVEFIETFPYVIKHK					Mascot
1943.7747	1943.8976	0.1229	63	1	17	MAGSQADETDWENMTN K			Oxidation (M)[1]		Mascot
1958.0283	1957.9929	-0.0354	-18	250	264	TRWVPPYYQHELLQK					Mascot
1959.9117	1959.8427	-0.069	-35	273	288	NSVEEYYQELQTGMIR					Mascot
1980.0186	1979.9556	-0.063	-32	1348	1364	SDDAVNVADLFFREIIR					Mascot
2668.3784	2668.1592	-0.2192	-82	540	561	LSLITRPHPHPYYIQWFN NSGK					Mascot
3405.7375	3405.6074	-0.1301	-38	1292	1321	LNPHGLYMPLPVPSVPW EDISMDFVLGLPR			Oxidation (M)[8]		Mascot

10 Putative cysteine-rich receptor-like protein kinase 12 [Triticum urartu] gi|474201971 66220.6 6.4 11 34 0 6.525

Peptide Information

Calc. Mass	Obsrv. Mass	± da	± ppm	Start Seq.	End Seq.	Sequence	Ion Score	C. I. %	Modification	Rank	Result Type
1106.6312	1106.5375	-0.0937	-85	3	11	KIVLMVEMK			Oxidation (M)[5]		Mascot
1438.6996	1438.6182	-0.0814	-57	429	441	DMEAKIGDFGLSR					Mascot
1518.8428	1518.798	-0.0448	-29	299	312	KNIIGHGGYGIVYK					Mascot
1600.804	1600.7502	-0.0538	-34	164	176	TSLYMPPEYISKR			Oxidation (M)[5]		Mascot
1844.9364	1844.9073	-0.0291	-16	325	338	KLHQMLWIEDEQFK					Mascot
1858.944	1858.8917	-0.0523	-28	517	532	VRLQATMSSHVTEEVR			Oxidation (M)[7]		Mascot

1860.9313	1860.9288	-0.0025	-1	325	338	KLHQMLWIEDEQFK	Oxidation (M)[5]	Mascot
1950.9114	1950.9211	0.0097	5	442	457	LFDSTQTYMTESRDIK	Oxidation (M)[9]	Mascot
1991.9432	1991.897	-0.0462	-23	494	509	EGYFNCPHTPPREFIK	Carbamidomethyl (C)[6]	Mascot
2440.145	2440.2778	0.1328	54	434	454	IGDFGLSRLFDSTQTYMT ESR	Oxidation (M)[17]	Mascot
3256.533	3256.5	-0.033	-10	204	230	CADIPPEEFIEPVYEFWV DRMQGTISK	Carbamidomethyl (C)[1]	Mascot
3410.6997	3410.6309	-0.0688	-20	87	115	VKHQNTIPLVGYCHHTAQ GICEGLHYLHK	Carbamidomethyl (C)[13,21]	Mascot

Gel Idx/Pos	207/I7	Instr./Gel Origin	BA2151/Sample Project 20140814	Process Status	Analysis Succeeded
Plate [#] Name	[1] Sample Project 20140814	Instrument Sample Name		Spectra	11

Rank	Protein Name	Accession No.	Protein MW	Protein PI	Pep. Count	Protein Score	Protein Score C. I. %	Intensity Matched	Total Ion Score	Total Ion C. I. %	Confirmed
1	27k protein [Triticum aestivum]	gi|290350670	24399.1	6.06	5	215	100	39.017	194	100	

Peptide Information

Calc. Mass	Obsrv. Mass	± da	± ppm	Start Seq.	End Seq.	Sequence	Ion Score	C. I. %	Modification	Rank	Result Type
1078.535	1078.5276	-0.0074	-7	193	201	GYPLLEACR			Carbamidomethyl (C)[8]		Mascot
1078.535	1078.5276	-0.0074	-7	193	201	GYPLLEACR	66	99.998	Carbamidomethyl (C)[8]		Mascot
1113.467	1113.4602	-0.0068	-6	121	128	EWESCFQK			Carbamidomethyl (C)[5]		Mascot
1406.6271	1406.6171	-0.01	-7	119	128	HREWESCFQK			Carbamidomethyl (C)[7]		Mascot
1406.6271	1406.6171	-0.01	-7	119	128	HREWESCFQK			Carbamidomethyl (C)[7]		Mascot
1955.9684	1955.9551	-0.0133	-7	24	39	VHVAIYYESLCPYSVR			Carbamidomethyl (C)[11]		Mascot
1955.9684	1955.9551	-0.0133	-7	24	39	VHVAIYYESLCPYSVR	128	100	Carbamidomethyl (C)[11]		Mascot
2204.1519	2204.1172	-0.0347	-16	1	23	LQLLSATSAATAGDVATG RGSEK					Mascot

Rank	Protein Name	Accession No.	Protein MW	Protein PI	Pep. Count	Protein Score	Protein Score C. I. %	Intensity Matched	Total Ion Score	Total Ion C. I. %	Confirmed
2	27K protein [Triticum aestivum]	gi|30793446	23271.5	6.06	4	208	100	38.844	194	100	

Protein Group

Tri a Bd 27K, partial [Triticum aestivum]	gi|283480515	23214.5	6.0599 999427 7954

Peptide Information

Calc. Mass	Obsrv. Mass	± da	± ppm	Start Seq.	End Seq.	Sequence	Ion Score	C. I. %	Modification	Rank	Result Type
1078.535	1078.5276	-0.0074	-7	181	189	GYPLLEACR			Carbamidomethyl (C)[8]		Mascot
1078.535	1078.5276	-0.0074	-7	181	189	GYPLLEACR	66	99.998	Carbamidomethyl (C)[8]		Mascot
1113.467	1113.4602	-0.0068	-6	109	116	EWESCFQK			Carbamidomethyl (C)[5]		Mascot
1406.6271	1406.6171	-0.01	-7	107	116	HREWESCFQK			Carbamidomethyl (C)[7]		Mascot
1406.6271	1406.6171	-0.01	-7	107	116	HREWESCFQK			Carbamidomethyl (C)[7]		Mascot
1955.9684	1955.9551	-0.0133	-7	12	27	VHVAIYYESLCPYSVR			Carbamidomethyl (C)[11]		Mascot
1955.9684	1955.9551	-0.0133	-7	12	27	VHVAIYYESLCPYSVR	128	100	Carbamidomethyl (C)[11]		Mascot

Rank	Protein Name	Accession No.	Protein MW	Protein PI	Pep. Count	Protein Score	Protein Score C. I. %	Intensity Matched	Total Ion Score	Total Ion C. I. %	Confirmed
3	hypothetical protein TRIUR3_28410 [Triticum urartu]	gi|474060617	28387.9	5.53	8	207	100	18.142	168	100	

Peptide Information

Calc. Mass	Obsrv. Mass	± da	± ppm	Start Seq.	End Seq.	Sequence	Ion Score	C. I. %	Modification	Rank	Result Type
1074.5062	1074.5544	0.0482	45	232	241	ADGDAIDEIR					Mascot
1078.535	1078.5276	-0.0074	-7	207	215	GYPLLEACR			Carbamidomethyl (C)[8]		Mascot
1078.535	1078.5276	-0.0074	-7	207	215	GYPLLEACR	66	99.998	Carbamidomethyl (C)[8]		Mascot
1113.467	1113.4602	-0.0068	-6	132	139	EWESCFQK			Carbamidomethyl (C)[5]		Mascot
1406.6271	1406.6171	-0.01	-7	130	139	HREWESCFQK			Carbamidomethyl (C)[7]		Mascot
1406.6271	1406.6171	-0.01	-7	130	139	HREWESCFQK			Carbamidomethyl (C)[7]		Mascot
1796.9075	1796.8546	-0.0529	-29	113	127	VHLGFIYCVSDLVMK			Carbamidomethyl (C)[8], Oxidation (M)[14]		Mascot
1796.9075	1796.8546	-0.0529	-29	113	127	VHLGFIYCVSDLVMK	84	100	Carbamidomethyl (C)[8], Oxidation (M)[14]		Mascot
1845.9594	1845.8875	-0.0719	-39	62	79	DGLLDAADLTLVPYGNAK					Mascot
1899.7782	1899.9275	0.1493	79	216	231	RPGMEAENMCTADFDR			Carbamidomethyl (C)[10]		Mascot
2236.1609	2236.1377	-0.0232	-10	59	79	AYRDGLLDAADLTLVPYG NAK					Mascot
2236.1609	2236.1377	-0.0232	-10	59	79	AYRDGLLDAADLTLVPYG NAK	17	0			Mascot

4	Uncharacterized protein ycf45 [Triticum urartu]	gi|473741424	53468.6	7.19	14	53	76.792	28.456

Peptide Information

Calc. Mass	Obsrv. Mass	± da	± ppm	Start Seq.	End Seq.	Sequence	Ion Score	C. I. %	Modification	Rank	Result Type
1021.4697	1021.5018	0.0321	31	187	196	AVGEFGGDNR					Mascot
1092.583	1092.5381	-0.0449	-41	255	263	TTVMREIAR			Oxidation (M)[4]		Mascot
1113.547	1113.4602	-0.0868	-78	226	235	AVNGHVDMVR			Oxidation (M)[8]		Mascot
1252.5892	1252.5615	-0.0277	-22	418	426	MHFYNLQQR			Oxidation (M)[1]		Mascot
1377.7267	1377.6057	-0.121	-88	84	96	ASIRSVCASSALR			Carbamidomethyl (C)[7]		Mascot
1385.7206	1385.6241	-0.0965	-70	449	460	TERSVDMLLHGK					Mascot
1409.743	1409.6029	-0.1401	-99	223	235	VGRAVNGHVDMVR					Mascot
1434.745	1434.6283	-0.1167	-81	396	407	APPTFPFLIEMR			Oxidation (M)[11]		Mascot
1511.7206	1511.7238	0.0032	2	298	309	RMQVPEPSMQHR			Oxidation (M)[2]		Mascot
1562.84	1562.7413	-0.0987	-63	395	407	KAPPTFPFLIEMR			Oxidation (M)[12]		Mascot
1796.9436	1796.8546	-0.089	-50	339	355	SIAERGVMLIGTAHGER					Mascot
1796.9436	1796.8546	-0.089	-50	339	355	SIAERGVMLIGTAHGER					Mascot
1955.9683	1955.9551	-0.0132	-7	187	205	AVGEFGGDNRAGIEGTL HR					Mascot
1955.9683	1955.9551	-0.0132	-7	187	205	AVGEFGGDNRAGIEGTL HR					Mascot
1976.0634	1975.9426	-0.1208	-61	97	117	MELVLASAAAAFVGLGLG GGR			Oxidation (M)[1]		Mascot
2242.0298	2242.1096	0.0798	36	470	488	DNEFEVIIERWATYDGDG L					Mascot

5	PERQ amino acid-rich with GYF domain-containing protein 2 [Triticum urartu]	gi|474146978	156052	5.9	22	52	72.733	7.228	

Peptide Information

Calc. Mass	Obsrv. Mass	± da	± ppm	Start Seq.	End Seq.	Sequence	Ion Score	C. I. %	Modification	Rank	Result Type
847.407	847.4771	0.0701	83	852	858	FGHGHHR					Mascot
1053.5436	1053.5063	-0.0373	-35	974	983	GGQGIHSLER					Mascot
1117.6252	1117.5453	-0.0799	-71	1395	1404	QIDPSLLGFK					Mascot
1145.5546	1145.4414	-0.1132	-99	87	95	EVDREVENR					Mascot
1145.5546	1145.4414	-0.1132	-99	87	95	EVDREVENR					Mascot
1159.5953	1159.5585	-0.0368	-32	176	185	LLPESDSRDK					Mascot
1252.6168	1252.5615	-0.0553	-44	645	655	ADFQLTSLDSR					Mascot
1317.6587	1317.6372	-0.0215	-16	1127	1139	GFPDVPAPASFGR					Mascot
1377.625	1377.6057	-0.0193	-14	780	790	EQLQQEQMMGR					Mascot
1385.6002	1385.6241	0.0239	17	158	169	MDAEKDEPHGEK					Mascot
1409.6149	1409.6029	-0.012	-9	780	790	EQLQQEQMMGR			Oxidation (M)[8,9]		Mascot
1420.718	1420.6033	-0.1147	-81	275	286	QQKVVQSFEDGR					Mascot
1422.6284	1422.6168	-0.0116	-8	63	74	NAFDAGWLDEER					Mascot
1434.7224	1434.6283	-0.0941	-66	341	353	NKDLEAFGIDANK					Mascot
1450.7384	1450.6208	-0.1176	-81	325	338	VTSSEATNTTALQK					Mascot
1511.7158	1511.7238	0.008	5	1105	1117	NPEAELSDMIHQK					Mascot
1549.7209	1549.7284	0.0075	5	779	790	REQLQQEQMMGR			Oxidation (M)[9]		Mascot
1562.822	1562.7413	-0.0807	-52	721	734	HGPLSRMNEAPVVR					Mascot
1926.978	1926.8663	-0.1117	-58	394	411	KPTEENAPSHPHGLSGIR					Mascot
1953.96	1953.9353	-0.0247	-13	727	743	MNEAPVVRDEWPANLG R					Mascot
1969.955	1969.9392	-0.0158	-8	727	743	MNEAPVVRDEWPANLG R			Oxidation (M)[1]		Mascot
2226.1221	2226.1218	-0.0003	0	1280	1301	RTANPHAATSQAASTDL SSAIR					Mascot
2234.0935	2234.1152	0.0217	10	354	375	DKSAEAFSGLSHEGSAA LISEK					Mascot
2680.3181	2680.1682	-0.1499	-56	1177	1200	MGHLGQDGPTTLDILPN NIEMNRK			Oxidation (M)[1]		Mascot
3187.5437	3187.5171	-0.0266	-8	1052	1079	NWSDANGQLQNSLMEA SRINQLQIEAEK					Mascot

6	hypothetical protein TRIUR3_08384 [Triticum urartu]	gi|474206074	87381.7	8.06	17	51	66.454	4.539	

Peptide Information

Calc. Mass	Obsrv. Mass	± da	± ppm	Start Seq.	End Seq.	Sequence	Ion Score	C. I. %	Modification	Rank	Result Type

816.4323	816.4512	0.0189	23	470	477	VGVTGNNR					Mascot
922.4815	922.4555	-0.026	-28	252	259	ELMGGFIR					Mascot
1092.5103	1092.5381	0.0278	25	276	284	CAKQATEER			Carbamidomethyl (C)[1]		Mascot
1139.5765	1139.5443	-0.0322	-28	757	766	ALYKNIEMAS					Mascot
1145.4351	1145.4414	0.0063	6	232	240	MCNSDFSER			Carbamidomethyl (C)[2]		Mascot
1145.4351	1145.4414	0.0063	6	232	240	MCNSDFSER			Carbamidomethyl (C)[2]		Mascot
1182.6339	1182.549	-0.0849	-72	467	477	HTKVGVTGNNR					Mascot
1330.6686	1330.6598	-0.0088	-7	713	724	CPAAGPTFTRPR			Carbamidomethyl (C)[1]		Mascot
1402.6671	1402.7367	0.0696	50	79	90	VCEFIYNATASK			Carbamidomethyl (C)[2]		Mascot
1450.7512	1450.6208	-0.1304	-90	609	621	CLFFADAVAAPLR			Carbamidomethyl (C)[1]		Mascot
1477.8679	1477.7775	-0.0904	-61	492	504	AVGLGVWHWLVLK					Mascot
1632.6815	1632.8257	0.1442	88	228	240	EMVKMCNSDFSER			Carbamidomethyl (C)[6]		Mascot
1845.9467	1845.8875	-0.0592	-32	323	339	LALSSYGANHHSHAPRK					Mascot
1910.0898	1909.9011	-0.1887	-99	57	73	VWKEFAVIVPGLPGELR					Mascot
1939.9192	1939.937	0.0178	9	260	275	CELTFVHREANEAAHR			Carbamidomethyl (C)[1]		Mascot
2012.9899	2012.9529	-0.037	-18	74	90	FTHPKVCEFIYNATASK			Carbamidomethyl (C)[7]		Mascot
2234.1274	2234.1152	-0.0122	-5	38	56	KAQIYDIGFITQISYMNGR			Oxidation (M)[16]		Mascot
2250.0425	2250.0649	0.0224	10	1	18	MDPFSVEFDELFQLYNQK					Mascot

7 Protein TRANSPARENT TESTA 1 [Triticum urartu] gi|474069866 29913.8 9.59 10 44 0 4.384

Peptide Information

Calc. Mass	Obsrv. Mass	± da	± ppm	Start Seq.	End Seq.	Sequence	Ion Score	C. I. %	Modification	Rank	Result Type
1046.5742	1046.4946	-0.0796	-76	150	157	TLQTHYKR					Mascot
1230.6624	1230.5654	-0.097	-79	2	14	ALGVGCASLGIGR			Carbamidomethyl (C)[6]		Mascot
1230.6624	1230.5654	-0.097	-79	2	14	ALGVGCASLGIGR			Carbamidomethyl (C)[6]		Mascot
1273.6656	1273.5621	-0.1035	-81	159	168	HGIKPFMCRK			Carbamidomethyl (C)[8]		Mascot
1377.6978	1377.6057	-0.0921	-67	1	14	MALGVGCASLGIGR			Carbamidomethyl (C)[7], Oxidation (M)[1]		Mascot
1406.5868	1406.6171	0.0303	22	189	199	LWYCACGSDFK			Carbamidomethyl (C)[4,6]		Mascot
1406.5868	1406.6171	0.0303	22	189	199	LWYCACGSDFK			Carbamidomethyl (C)[4,6]		Mascot
1511.6908	1511.7238	0.033	22	47	59	YNNMQVSGLDAQR			Oxidation (M)[4]		Mascot
1651.797	1651.7041	-0.0929	-56	47	60	YNNMQVSGLDAQRR					Mascot
1865.7769	1865.9438	0.1669	89	185	199	NCGKLWYCACGSDFK			Carbamidomethyl (C)[2,8,10]		Mascot
1977.7703	1977.9188	0.1485	75	214	230	GHAPCGIDCFDLDDDDR			Carbamidomethyl (C)[5,9]		Mascot
2219.9314	2220.1172	0.1858	84	124	141	LPCYCCAAGCRNNVDHPR			Carbamidomethyl (C)[3,5,6,10]		Mascot

8 putative beta-1,3-galactosyltransferase 6 [Triticum urartu] gi|474183439 40536.8 6.22 11 43 0 4.209

Peptide Information

Calc. Mass	Obsrv. Mass	± da	± ppm	Start Seq.	End Seq.	Sequence	Ion Score	C. I. %	Modification	Rank	Result Type
922.4628	922.4555	-0.0073	-8	160	166	EYNDILR					Mascot
1021.5095	1021.5018	-0.0077	-8	39	47	AMLSQAQTR			Oxidation (M)[2]		Mascot
1053.5371	1053.5063	-0.0308	-29	1	9	MAASRPQHR					Mascot
1074.5837	1074.5544	-0.0293	-27	64	73	LAAARAAQMR			Oxidation (M)[9]		Mascot
1347.6475	1347.5969	-0.0506	-38	115	125	TWMPEGEGLRR			Oxidation (M)[3]		Mascot
1927.0283	1926.8663	-0.162	-84	253	269	QLYAITRDLATYVSANK					Mascot
1939.8716	1939.937	0.0654	34	341	356	QCWEHREAALPQAQES			Carbamidomethyl (C)[2]		Mascot
1975.8887	1975.9426	0.0539	27	74	92	SQGVSPSDSAADQGNM RPR			Oxidation (M)[16]		Mascot
2017.8767	2017.8745	-0.0022	-1	141	159	SANPGPDSEVERAMDAE DK					Mascot
2236.0996	2236.1377	0.0381	17	180	198	IQMFLSTALSTWDADFYV K					Mascot
2236.0996	2236.1377	0.0381	17	180	198	IQMFLSTALSTWDADFYV K					Mascot
2680.2527	2680.1682	-0.0845	-32	275	297	YTNEDVSFGSWLIGLDVE HVDER					Mascot

9	E3 ubiquitin-protein ligase ATL9 [Triticum urartu]	gi|474451402	25995.7	5.22	8	40	0	4.407

Peptide Information

Calc. Mass	Obsrv. Mass	± da	± ppm	Start Seq.	End Seq.	Sequence	Ion Score	C. I. %	Modification	Rank	Result Type
872.4221	872.4965	0.0744	85	210	217	EHVGSTSR					Mascot
1060.4906	1060.5416	0.051	48	226	234	EQVLDDGER					Mascot
1145.4827	1145.4414	-0.0413	-36	186	194	EHSTCPVCR			Carbamidomethyl (C)[5,8]		Mascot
1145.4827	1145.4414	-0.0413	-36	186	194	EHSTCPVCR			Carbamidomethyl (C)[5,8]		Mascot
1187.6433	1187.6162	-0.0271	-23	89	97	WYLVHRSAR					Mascot
1865.9352	1865.9438	0.0086	5	218	234	AVTSPPPREQVLDDGER					Mascot
1905.8495	1905.8704	0.0209	11	98	116	QQEEEATAAATDAAPGM AK			Oxidation (M)[17]		Mascot
2017.9307	2017.8745	-0.0562	-28	171	185	CMHVFHRSCVDVWLR			Carbamidomethyl (C)[1,9], Oxidation (M)[2]		Mascot
2204.0249	2204.1172	0.0923	42	95	116	SARQQEEEATAAATDAA PGMAK					Mascot
2220.0198	2220.1172	0.0974	44	95	116	SARQQEEEATAAATDAA PGMAK			Oxidation (M)[20]		Mascot

10	Putative disease resistance RPP13-like protein 1 [Triticum urartu]	gi|474219021	59003.2	9.36	13	40	0	3.882

Peptide Information

Calc. Mass	Obsrv. Mass	± da	± ppm	Start	End	Sequence	Ion	C. I. %	Modification	Rank	Result Type

				Seq.	Seq.		Score		
1054.5284	1054.4314	-0.097	-92	68	75	MLRQGMYR			Mascot
1060.611	1060.5416	-0.0694	-65	306	314	IIITSRSDR			Mascot
1100.5518	1100.4924	-0.0594	-54	218	226	STLVEHVCR		Carbamidomethyl (C)[8]	Mascot
1116.583	1116.5048	-0.0782	-70	488	497	MQAPQLTVGR		Oxidation (M)[1]	Mascot
1409.6652	1409.6029	-0.0623	-44	142	154	MVDTLEDTVAGMK			Mascot
1420.693	1420.6033	-0.0897	-63	462	473	LPCGEFEALAWK		Carbamidomethyl (C)[3]	Mascot
1441.6549	1441.6571	0.0022	2	142	154	MVDTLEDTVAGMK		Oxidation (M)[1,12]	Mascot
1477.7753	1477.7775	0.0022	1	354	366	LATMAMEIALELR		Oxidation (M)[4]	Mascot
1507.729	1507.7239	-0.0051	-3	155	165	EFLFFMELYPR		Oxidation (M)[6]	Mascot
1511.7192	1511.7238	0.0046	3	291	305	LKSSTTGMTPCGGSK		Carbamidomethyl (C)[11]	Mascot
1590.8091	1590.7792	-0.0299	-19	484	497	VSCKMQAPQLTVGR		Carbamidomethyl (C)[3], Oxidation (M)[5]	Mascot
1953.9733	1953.9353	-0.038	-19	293	311	SSTTGMTPCGGSKIIITSR		Carbamidomethyl (C)[9]	Mascot
1955.0028	1954.9359	-0.0669	-34	400	415	QTNLNMFGQHPNLRLR		Oxidation (M)[6]	Mascot
1969.9681	1969.9392	-0.0289	-15	293	311	SSTTGMTPCGGSKIIITSR		Carbamidomethyl (C)[9], Oxidation (M)[6]	Mascot
1977.902	1977.9188	0.0168	8	169	184	QPYGMYLLLDNCMFGR		Carbamidomethyl (C)[12]	Mascot
2009.8918	2009.8527	-0.0391	-19	169	184	QPYGMYLLLDNCMFGR		Carbamidomethyl (C)[12], Oxidation (M)[5,13]	Mascot

Gel Idx/Pos	208/I8	Instr./Gel Origin	BA2151/Sample Project 20140814	Process Status	Analysis Succeeded
Plate [#] Name	[1] Sample Project 20140814	Instrument Sample Name		Spectra	11

Rank	Protein Name	Accession No.	Protein MW	Protein PI	Pep. Count	Protein Score	Protein Score C. I. %	Intensity Matched	Total Ion Score	Total Ion C. I. %	Confirmed
1	27K protein [Triticum aestivum]	gi|30793446	23271.5	6.06	13	516	100	62.403	416	100	

Peptide Information

Calc. Mass	Obsrv. Mass	± da	± ppm	Start Seq.	End Seq.	Sequence	Ion Score	C. I. %	Modification	Rank	Result Type
975.5411	975.5275	-0.0136	-14	28	35	FVANHLFK					Mascot
1076.5331	1076.55	0.0169	16	1	11	GDVATGRGSEK					Mascot
1078.535	1078.5328	-0.0022	-2	181	189	GYPLLEACR			Carbamidomethyl (C)[8]		Mascot
1078.535	1078.5328	-0.0022	-2	181	189	GYPLLEACR	71	100	Carbamidomethyl (C)[8]		Mascot
1113.467	1113.4681	0.0011	1	109	116	EWESCFQK			Carbamidomethyl (C)[5]		Mascot
1145.5698	1145.5669	-0.0029	-3	133	142	GHNLSLEYGR					Mascot
1145.5698	1145.5669	-0.0029	-3	133	142	GHNLSLEYGR	83	100			Mascot
1406.6271	1406.6235	-0.0036	-3	107	116	HREWESCFQK			Carbamidomethyl (C)[7]		Mascot
1406.6271	1406.6235	-0.0036	-3	107	116	HREWESCFQK	53	99.968	Carbamidomethyl (C)[7]		Mascot
1534.7571	1534.7111	-0.046	-30	117	129	QGLDPKPVTECYK			Carbamidomethyl (C)[11]		Mascot
1762.9561	1762.9292	-0.0269	-15	90	104	VHLGFIYCVSDLVLK			Carbamidomethyl (C)[8]		Mascot
1876.9222	1876.9094	-0.0128	-7	117	132	QGLDPKPVTECYKGER			Carbamidomethyl (C)[11]		Mascot
1876.9222	1876.9094	-0.0128	-7	117	132	QGLDPKPVTECYKGER	67	99.999	Carbamidomethyl (C)[11]		Mascot
1955.9684	1955.9619	-0.0065	-3	12	27	VHVAIYYESLCPYSVR			Carbamidomethyl (C)[11]		Mascot
1955.9684	1955.9619	-0.0065	-3	12	27	VHVAIYYESLCPYSVR	142	100	Carbamidomethyl (C)[11]		Mascot
2357.1594	2357.1438	-0.0156	-7	8	27	GSEKVHVAIYYESLCPYS VR			Carbamidomethyl (C)[15]		Mascot
3457.7581	3457.7588	0.0007	0	143	172	QTAELVPPHQFVPWVVV DGKPLYNDYGNFK					Mascot
3499.5564	3499.6301	0.0737	21	59	89	NDGTISCQHGPEECLLNT VEACAIDAWPDVK			Carbamidomethyl (C)[7,14,22]		Mascot

Rank	Protein Name	Accession No.	Protein MW	Protein PI	Pep. Count	Protein Score	Protein Score C. I. %	Intensity Matched	Total Ion Score	Total Ion C. I. %	Confirmed
2	Tri a Bd 27K, partial [Triticum aestivum]	gi|283480515	23214.5	6.06	12	505	100	62.324	416	100	

Peptide Information

Calc. Mass	Obsrv. Mass	± da	± ppm	Start Seq.	End Seq.	Sequence	Ion Score	C. I. %	Modification	Rank	Result Type
975.5411	975.5275	-0.0136	-14	27	34	FVANHLFK					Mascot
1078.535	1078.5328	-0.0022	-2	180	188	GYPLLEACR			Carbamidomethyl (C)[8]		Mascot
1078.535	1078.5328	-0.0022	-2	180	188	GYPLLEACR	71	100	Carbamidomethyl (C)[8]		Mascot
1113.467	1113.4681	0.0011	1	108	115	EWESCFQK			Carbamidomethyl (C)[5]		Mascot

Calc. Mass	Obsrv. Mass	± da	± ppm	Start Seq.	End Seq.	Sequence	Ion Score	C. I. %	Modification	Rank	Result Type
1145.5698	1145.5669	-0.0029	-3	132	141	GHNLSLEYGR					Mascot
1145.5698	1145.5669	-0.0029	-3	132	141	GHNLSLEYGR	83	100			Mascot
1406.6271	1406.6235	-0.0036	-3	106	115	HREWESCFQK			Carbamidomethyl (C)[7]		Mascot
1406.6271	1406.6235	-0.0036	-3	106	115	HREWESCFQK	53	99.968	Carbamidomethyl (C)[7]		Mascot
1534.7571	1534.7111	-0.046	-30	116	128	QGLDPKPVTECYK			Carbamidomethyl (C)[11]		Mascot
1762.9561	1762.9292	-0.0269	-15	89	103	VHLGFIYCVSDLVLK			Carbamidomethyl (C)[8]		Mascot
1876.9222	1876.9094	-0.0128	-7	116	131	QGLDPKPVTECYKGER			Carbamidomethyl (C)[11]		Mascot
1876.9222	1876.9094	-0.0128	-7	116	131	QGLDPKPVTECYKGER	67	99.999	Carbamidomethyl (C)[11]		Mascot
1955.9684	1955.9619	-0.0065	-3	11	26	VHVAIYYESLCPYSVR			Carbamidomethyl (C)[11]		Mascot
1955.9684	1955.9619	-0.0065	-3	11	26	VHVAIYYESLCPYSVR	142	100	Carbamidomethyl (C)[11]		Mascot
2357.1594	2357.1438	-0.0156	-7	7	26	GSEKVHVAIYYESLCPYS VR			Carbamidomethyl (C)[15]		Mascot
3457.7581	3457.7588	0.0007	0	142	171	QTAELVPPHQFVPWVVV DGKPLYNDYGNFK					Mascot
3499.5564	3499.6301	0.0737	21	58	88	NDGTISCQHGPEECLLNT VEACAIDAWPDVDK			Carbamidomethyl (C)[7,14,22]		Mascot

3 27k protein [Triticum aestivum] gi|290350670 24399.1 6.06 12 502 100 62.324 416 100

Peptide Information

Calc. Mass	Obsrv. Mass	± da	± ppm	Start Seq.	End Seq.	Sequence	Ion Score	C. I. %	Modification	Rank	Result Type
975.5411	975.5275	-0.0136	-14	40	47	FVANHLFK					Mascot
1078.535	1078.5328	-0.0022	-2	193	201	GYPLLEACR			Carbamidomethyl (C)[8]		Mascot
1078.535	1078.5328	-0.0022	-2	193	201	GYPLLEACR	71	100	Carbamidomethyl (C)[8]		Mascot
1113.467	1113.4681	0.0011	1	121	128	EWESCFQK			Carbamidomethyl (C)[5]		Mascot
1145.5698	1145.5669	-0.0029	-3	145	154	GHNLSLEYGR					Mascot
1145.5698	1145.5669	-0.0029	-3	145	154	GHNLSLEYGR	83	100			Mascot
1406.6271	1406.6235	-0.0036	-3	119	128	HREWESCFQK			Carbamidomethyl (C)[7]		Mascot
1406.6271	1406.6235	-0.0036	-3	119	128	HREWESCFQK	53	99.968	Carbamidomethyl (C)[7]		Mascot
1534.7571	1534.7111	-0.046	-30	129	141	QGLDPKPVTECYK			Carbamidomethyl (C)[11]		Mascot
1762.9561	1762.9292	-0.0269	-15	102	116	VHLGFIYCVSDLVLK			Carbamidomethyl (C)[8]		Mascot
1876.9222	1876.9094	-0.0128	-7	129	144	QGLDPKPVTECYKGER			Carbamidomethyl (C)[11]		Mascot
1876.9222	1876.9094	-0.0128	-7	129	144	QGLDPKPVTECYKGER	67	99.999	Carbamidomethyl (C)[11]		Mascot
1955.9684	1955.9619	-0.0065	-3	24	39	VHVAIYYESLCPYSVR			Carbamidomethyl (C)[11]		Mascot
1955.9684	1955.9619	-0.0065	-3	24	39	VHVAIYYESLCPYSVR	142	100	Carbamidomethyl (C)[11]		Mascot
2357.1594	2357.1438	-0.0156	-7	20	39	GSEKVHVAIYYESLCPYS VR			Carbamidomethyl (C)[15]		Mascot
3457.7581	3457.7588	0.0007	0	155	184	QTAELVPPHQFVPWVVV DGKPLYNDYGNFK					Mascot
3499.5564	3499.6301	0.0737	21	71	101	NDGTISCQHGPEECLLNT VEACAIDAWPDVDK			Carbamidomethyl (C)[7,14,22]		Mascot

4	hypothetical protein TRIUR3_28410 [Triticum urartu]	gi|474060617	28387.9	5.53	6	297	100	36.334	274	100	

Peptide Information

Calc. Mass	Obsrv. Mass	± da	± ppm	Start Seq.	End Seq.	Sequence	Ion Score	C. I. %	Modification	Rank	Result Type
1078.535	1078.5328	-0.0022	-2	207	215	GYPLLEACR			Carbamidomethyl (C)[8]		Mascot
1078.535	1078.5328	-0.0022	-2	207	215	GYPLLEACR	71	100	Carbamidomethyl (C)[8]		Mascot
1113.467	1113.4681	0.0011	1	132	139	EWESCFQK			Carbamidomethyl (C)[5]		Mascot
1145.5698	1145.5669	-0.0029	-3	156	165	GHNLSLEYGR					Mascot
1145.5698	1145.5669	-0.0029	-3	156	165	GHNLSLEYGR	83	100			Mascot
1406.6271	1406.6235	-0.0036	-3	130	139	HREWESCFQK			Carbamidomethyl (C)[7]		Mascot
1406.6271	1406.6235	-0.0036	-3	130	139	HREWESCFQK	53	99.968	Carbamidomethyl (C)[7]		Mascot
1534.7571	1534.7111	-0.046	-30	140	152	QGLDPKPVTECYK			Carbamidomethyl (C)[11]		Mascot
1876.9222	1876.9094	-0.0128	-7	140	155	QGLDPKPVTECYKGER			Carbamidomethyl (C)[11]		Mascot
1876.9222	1876.9094	-0.0128	-7	140	155	QGLDPKPVTECYKGER	67	99.999	Carbamidomethyl (C)[11]		Mascot

5	hypothetical protein TRIUR3_22316 [Triticum urartu]	gi|474365597	46281.9	6.15	13	52	75.132	2.981	

Peptide Information

Calc. Mass	Obsrv. Mass	± da	± ppm	Start Seq.	End Seq.	Sequence	Ion Score	C. I. %	Modification	Rank	Result Type
987.5218	987.5245	0.0027	3	119	127	VLKGDGDQR					Mascot
1106.5762	1106.5667	-0.0095	-9	101	110	SMLEVAVTEK					Mascot
1229.6637	1229.627	-0.0367	-30	2	11	DHQTKFVVQK					Mascot
1301.692	1301.5677	-0.1243	-95	328	339	LADSRSAGLQQR					Mascot
1410.6351	1410.6219	-0.0132	-9	30	41	ASEEKAEMISCR			Carbamidomethyl (C)[11]		Mascot
1488.8281	1488.6923	-0.1358	-91	224	235	IIKHESHIQDLR					Mascot
1490.7308	1490.7308	0	0	262	274	WKEACALEVEAGK			Carbamidomethyl (C)[5]		Mascot
1559.8098	1559.7373	-0.0725	-46	264	278	EACALEVEAGKAAIK			Carbamidomethyl (C)[3]		Mascot
1876.9255	1876.9094	-0.0161	-9	309	327	EKLAAGAMAAQAAADAC LK			Carbamidomethyl (C)[17], Oxidation (M)[8]		Mascot
1876.9255	1876.9094	-0.0161	-9	309	327	EKLAAGAMAAQAAADAC LK			Carbamidomethyl (C)[17], Oxidation (M)[8]		Mascot
1940.0812	1939.9827	-0.0985	-51	236	251	EREILLLQSVEELNVR					Mascot
1954.0757	1953.9396	-0.1361	-70	12	29	IVVSLHSAGEIFENILGR					Mascot
1967.9781	1967.9226	-0.0555	-28	340	355	IEELTRQIEQEDTHAR					Mascot
3095.3569	3095.4785	0.1216	39	142	172	VGFGFIMEVISGESEGDE MSSSSASAASNGR					Mascot

6	DEAD-box ATP-dependent RNA helicase 17 [Triticum urartu]	gi|474072519	67679.7	9.58	13	50	51.512	24.128	

Peptide Information

Calc. Mass	Obsrv. Mass	± da	± ppm	Start Seq.	End Seq.	Sequence	Ion Score	C. I. %	Modification	Rank	Result Type
978.4673	978.5208	0.0535	55	213	220	SKTDPMQR			Oxidation (M)[6]		Mascot
1145.6062	1145.5669	-0.0393	-34	530	539	AYTAHRGELK					Mascot
1145.6062	1145.5669	-0.0393	-34	530	539	AYTAHRGELK					Mascot
1230.5168	1230.5765	0.0597	49	374	383	LHGNMDQEDR			Oxidation (M)[5]		Mascot
1343.7529	1343.6383	-0.1146	-85	221	232	QNLLLSATLNEK					Mascot
1448.6991	1448.7418	0.0427	29	9	20	HMVVEVGHFSYK			Oxidation (M)[2]		Mascot
1856.9535	1856.9032	-0.0503	-27	243	258	NPVMIGLDEQKKPSER			Oxidation (M)[4]		Mascot
1899.0369	1898.926	-0.1109	-58	395	412	SAILVCTDVAARGLDIPK			Carbamidomethyl (C)[6]		Mascot
1938.9847	1938.9674	-0.0173	-9	176	192	WIVFDEADSILELGFGK					Mascot
1983.0944	1982.9535	-0.1409	-71	236	253	LANISLKNPVMIGLDEQK					Mascot
2072.0806	2072.0723	-0.0083	-4	215	232	TDPMQRQNLLLSATLNE K					Mascot
2323.1111	2323.1287	0.0176	8	9	28	HMVVEVGHFSYKMGFQ GPTR			Oxidation (M)[2]		Mascot
3369.7729	3369.6255	-0.1474	-44	458	487	DLELHGVSLTEYPFQKVL DGFPVNGEKPLK					Mascot
3457.7783	3457.7588	-0.0195	-6	489	518	KPISLDMHPWILSVQRTL ENYVASEATTNK			Oxidation (M)[7]		Mascot

7 Ribosomal protein S3, mitochondrial [Triticum urartu] gi|474387796 89207.9 10.28 16 49 49.227 5.506

Peptide Information

Calc. Mass	Obsrv. Mass	± da	± ppm	Start Seq.	End Seq.	Sequence	Ion Score	C. I. %	Modification	Rank	Result Type
847.4421	847.4274	-0.0147	-17	479	485	NNPISFR					Mascot
975.5192	975.5275	0.0083	9	627	633	NCKIWVR			Carbamidomethyl (C)[2]		Mascot
978.4832	978.5208	0.0376	38	113	120	GWWPTFGK					Mascot
997.5214	997.5127	-0.0087	-9	2	9	GDYLARFR					Mascot
1128.5619	1128.5417	-0.0202	-18	1	9	MGDYLARFR					Mascot
1144.5569	1144.5374	-0.0195	-17	1	9	MGDYLARFR			Oxidation (M)[1]		Mascot
1390.7743	1390.6548	-0.1195	-86	83	93	RTFIHFFLPGR					Mascot
1461.8168	1461.7588	-0.058	-40	412	423	GIEMMIEIILRK			Oxidation (M)[4]		Mascot
1477.8116	1477.7103	-0.1013	-69	412	423	GIEMMIEIILRK			Oxidation (M)[4,5]		Mascot
1706.8544	1706.7568	-0.0976	-57	611	625	ATIGQFHRAMSGQFR					Mascot
1856.8444	1856.9032	0.0588	32	121	137	VGPIGCLHSSEGTEEER			Carbamidomethyl (C)[6]		Mascot
1876.9763	1876.9094	-0.0669	-36	539	556	IDYAPAEVSTRNGISGVK					Mascot
1876.9763	1876.9094	-0.0669	-36	539	556	IDYAPAEVSTRNGISGVK					Mascot
1937.9323	1937.9778	0.0455	23	691	706	FLMVMFVAFERPSMGK			Oxidation (M)[3,5,14]		Mascot

Calc. Mass	Obsrv. Mass	± da	± ppm	Start Seq.	End Seq.	Sequence	Ion Score	C. I. %	Modification	Rank	Result Type
1939.0793	1938.9674	-0.1119	-58	634	651	VLADLPITGKPAEVRMGR			Oxidation (M)[16]		Mascot
1940.0753	1939.9827	-0.0926	-48	55	71	SYFSSIRPPTKLALGFR					Mascot
3079.5493	3079.48	-0.0693	-23	263	291	NKMHFDPVLVLNHFVAP GVAEPSTMGGAK			Oxidation (M)[3]		Mascot
3079.6396	3079.48	-0.1596	-52	189	217	VSGAFKRPQYAGVVNDI PFLMGNAASLLK			Oxidation (M)[21]		Mascot
3095.5442	3095.4785	-0.0657	-21	263	291	NKMHFDPVLVLNHFVAP GVAEPSTMGGAK			Oxidation (M)[3,25]		Mascot
3469.6196	3469.71	0.0904	26	712	742	AWFERFSSVVQAAGFSS SEHDPALFTHTSDR					Mascot

8 ADP-ribosylation factor 1 [Triticum urartu] gi|473888229 54713 5.98 12 48 29.914 35.541

Peptide Information

Calc. Mass	Obsrv. Mass	± da	± ppm	Start Seq.	End Seq.	Sequence	Ion Score	C. I. %	Modification	Rank	Result Type
918.4778	918.4587	-0.0191	-21	399	406	LDETIEAK					Mascot
1046.5663	1046.5059	-0.0604	-58	231	240	VIDAATMLGR					Mascot
1078.4834	1078.5328	0.0494	46	344	353	DDLSGMDVAR					Mascot
1078.4834	1078.5328	0.0494	46	344	353	DDLSGMDVAR					Mascot
1088.6462	1088.5469	-0.0993	-91	104	113	NAVLLVFANK					Mascot
1128.5573	1128.5417	-0.0156	-14	77	85	FNVETVEYK					Mascot
1145.6412	1145.5669	-0.0743	-65	399	408	LDETIEAKVK					Mascot
1145.6412	1145.5669	-0.0743	-65	399	408	LDETIEAKVK					Mascot
1206.5784	1206.606	0.0276	23	344	354	DDLSGMDVARK					Mascot
1418.7274	1418.625	-0.1024	-72	331	343	TAKALGYTEPDPR					Mascot
1559.8291	1559.7373	-0.0918	-59	1	15	MGLSFGTLFGALFAK					Mascot
3095.5659	3095.4785	-0.0874	-28	471	498	MTTLIVQHDNFFFGWAA NLEALGAIKST					Mascot
3241.4453	3241.5405	0.0952	29	296	323	FESTCFTNNNTIHSFTLG YVMSELEDGK			Carbamidomethyl (C)[5]		Mascot
3241.4453	3241.5405	0.0952	29	296	323	FESTCFTNNNTIHSFTLG YVMSELEDGK			Carbamidomethyl (C)[5]		Mascot
3369.5403	3369.6255	0.0852	25	296	324	FESTCFTNNNTIHSFTLG YVMSELEDGKK			Carbamidomethyl (C)[5]		Mascot

9 NADH-dependent glutamate synthase [Triticum turgidum] gi|530452549 237135.1 6.26 29 47 9.711 17.675

Peptide Information

Calc. Mass	Obsrv. Mass	± da	± ppm	Start Seq.	End Seq.	Sequence	Ion Score	C. I. %	Modification	Rank	Result Type
975.5469	975.5275	-0.0194	-20	1623	1632	VANGVSVTTK					Mascot
997.5676	997.5127	-0.0549	-55	1572	1580	LHTESVLAK					Mascot
1021.565	1021.5082	-0.0568	-56	11	20	HAAPTGVGRR					Mascot

	1046.5663	1046.5059	-0.0604	-58	1282	1290	EIMAQLGLR				Oxidation (M)[3]	Mascot
	1060.5634	1060.5507	-0.0127	-12	702	710	KGLEETLDR					Mascot
	1076.5657	1076.55	-0.0157	-15	1604	1612	VLEEMKAEK					Mascot
	1078.5496	1078.5328	-0.0168	-16	954	962	ACNLRGMLK				Carbamidomethyl (C)[2], Oxidation (M)[7]	Mascot
	1078.5496	1078.5328	-0.0168	-16	954	962	ACNLRGMLK				Carbamidomethyl (C)[2], Oxidation (M)[7]	Mascot
	1207.6039	1207.6091	0.0052	4	24	35	SHAASAHGRSAR					Mascot
	1238.5988	1238.5806	-0.0182	-15	1825	1834	MGHFVTVFER				Oxidation (M)[1]	Mascot
	1273.6971	1273.5808	-0.1163	-91	1645	1656	SSSRPTRVANAK					Mascot
	1315.6022	1315.6135	0.0113	9	1026	1037	MEPLPDGSMNPK					Mascot
	1331.597	1331.6117	0.0147	11	1026	1037	MEPLPDGSMNPK				Oxidation (M)[1]	Mascot
	1347.5919	1347.6084	0.0165	12	1026	1037	MEPLPDGSMNPK				Oxidation (M)[1,9]	Mascot
	1410.6942	1410.6219	-0.0723	-51	1838	1850	IGGLMMYGVPNMK					Mascot
	1497.8346	1497.7245	-0.1101	-74	830	843	VLAKMGISTLASYK				Oxidation (M)[5]	Mascot
	1517.7168	1517.7039	-0.0129	-8	818	829	KYFYASIYGMMK				Oxidation (M)[10]	Mascot
	1768.8544	1768.8678	0.0134	8	1835	1850	SDRIGGLMMYGVPNMK					Mascot
	1784.9114	1784.9188	0.0074	4	819	833	YFYASIYGMMKVLAK					Mascot
	1818.879	1818.8286	-0.0504	-28	1299	1314	SDMLEVDPEVVKSNEK					Mascot
	1856.947	1856.9032	-0.0438	-24	112	127	ATVVDAIQMLERMAHR				Oxidation (M)[9]	Mascot
	1876.9758	1876.9094	-0.0664	-35	663	679	LALKGPLVSMDEMESIK				Oxidation (M)[10]	Mascot
	1876.9758	1876.9094	-0.0664	-35	663	679	LALKGPLVSMDEMESIK				Oxidation (M)[10]	Mascot
	1898.9872	1898.926	-0.0612	-32	135	152	NTGDGAGILVALPHNFFR					Mascot
	1938.9888	1938.9674	-0.0214	-11	1565	1580	MMIEQHRLHTESVLAK				Oxidation (M)[1]	Mascot
	1954.9838	1954.9324	-0.0514	-26	1565	1580	MMIEQHRLHTESVLAK				Oxidation (M)[1,2]	Mascot
	1970.0601	1969.9535	-0.1066	-54	1888	1905	LRSENNAVILACGATKPR				Carbamidomethyl (C)[12]	Mascot
	1983.058	1982.9535	-0.1045	-53	467	485	VVMGSEVGVVDIPAQDV LR					Mascot
	2011.0643	2010.9703	-0.094	-47	573	590	AFGYTLEALEMLLLPMAK					Mascot
	2356.241	2356.1257	-0.1153	-49	135	156	NTGDGAGILVALPHNFFR EVTK					Mascot
	3079.5518	3079.48	-0.0718	-23	1860	1887	RVNLMAEEGVTFVVNAN VGSDPLYSIER					Mascot
	3079.5518	3079.48	-0.0718	-23	1860	1887	RVNLMAEEGVTFVVNAN VGSDPLYSIER					Mascot
	3095.5466	3095.4785	-0.0681	-22	1860	1887	RVNLMAEEGVTFVVNAN VGSDPLYSIER				Oxidation (M)[5]	Mascot
	3109.3733	3109.4917	0.1184	38	391	417	GGRSLPEAVMMMIPEAW QNDVNMEPDK				Oxidation (M)[10,11,12,23]	Mascot
	3457.697	3457.7588	0.0618	18	205	236	QVPTDNSDLGQDALDTE PAIEQVFLTKSPNSK					Mascot
	3469.8359	3469.71	-0.1259	-36	1311	1341	SNEKLENIDLSLILKPAAEI RPGAAQYCVEK				Carbamidomethyl (C)[28]	Mascot
10	Putative receptor protein kinase ZmPK1 [Triticum urartu]	gi|474373778	89054.3	5.58	15	46	0	30.25				

Peptide Information

Calc. Mass	Obsrv. Mass	± da	± ppm	Start Seq.	End Seq.	Sequence	Ion Score	C. I. %	Modification	Rank	Result Type
951.5081	951.4813	-0.0268	-28	491	498	LVMGTQFR					Mascot
978.5843	978.5208	-0.0635	-65	560	567	INHINLVR					Mascot
1144.5957	1144.5374	-0.0583	-51	253	262	LTIEQDGNVR					Mascot
1207.5702	1207.6091	0.0389	32	508	518	ATGNFNEELGR					Mascot
1229.7465	1229.627	-0.1195	-97	527	538	GVLDKTTVVAVK					Mascot
1418.7234	1418.625	-0.0984	-69	712	723	ITEQTTVDRAER					Mascot
1434.7192	1434.6251	-0.0941	-66	662	674	KAVADGMQLSQMR					Mascot
1530.8123	1530.7861	-0.0262	-17	735	749	QVVASGDVVSLVDSR					Mascot
1768.9229	1768.8678	-0.0551	-31	579	592	LLVYEYVENRSLDR					Mascot
1818.7852	1818.8286	0.0434	24	785	800	ALIACDDEDEHPAYLS			Carbamidomethyl (C)[5]		Mascot
1955.9822	1955.9619	-0.0203	-10	123	140	AASNNYFTVSLLDTGNLR					Mascot
1955.9822	1955.9619	-0.0203	-10	123	140	AASNNYFTVSLLDTGNLR					Mascot
1982.9679	1982.9535	-0.0144	-7	508	526	ATGNFNEELGRGGSGVV YR					Mascot
2323.1565	2323.1287	-0.0278	-12	100	122	VSFGADGKLALADANGT TVWDSK					Mascot
2356.0908	2356.1257	0.0349	15	319	340	KGCNPTFSLPTTTPTNCS TSGK			Carbamidomethyl (C)[3,17]		Mascot
3241.6528	3241.5405	-0.1123	-35	141	169	VVDPSTGRPVWQSFDW PTDTLLPSQALTK					Mascot
3241.6528	3241.5405	-0.1123	-35	141	169	VVDPSTGRPVWQSFDW PTDTLLPSQALTK					Mascot

Gel Idx/Pos	209/I9	Instr./Gel Origin	BA2151/Sample Project 20140814	Process Status	Analysis Succeeded
Plate [#] Name	[1] Sample Project 20140814	Instrument Sample Name		Spectra	11

Rank	Protein Name	Accession No.	Protein MW	Protein PI	Pep. Count	Protein Score	Protein Score C. I. %	Intensity Matched	Total Ion Score	Total Ion C. I. %	Confirmed
1	27K protein [Triticum aestivum]	gi|30793446	23271.5	6.06	4	270	100	42.971	256	100	

Protein Group

Tri a Bd 27K, partial [Triticum aestivum]	gi|283480515	23214.5	6.0599 999427 7954

Peptide Information

Calc. Mass	Obsrv. Mass	± da	± ppm	Start Seq.	End Seq.	Sequence	Ion Score	C. I. %	Modification	Rank	Result Type
1078.535	1078.5236	-0.0114	-11	181	189	GYPLLEACR			Carbamidomethyl (C)[8]		Mascot
1078.535	1078.5236	-0.0114	-11	181	189	GYPLLEACR	71	100	Carbamidomethyl (C)[8]		Mascot
1113.467	1113.45	-0.017	-15	109	116	EWESCFQK			Carbamidomethyl (C)[5]		Mascot
1406.6271	1406.6101	-0.017	-12	107	116	HREWESCFQK			Carbamidomethyl (C)[7]		Mascot
1406.6271	1406.6101	-0.017	-12	107	116	HREWESCFQK	38	98.881	Carbamidomethyl (C)[7]		Mascot
1955.9684	1955.949	-0.0194	-10	12	27	VHVAIYYESLCPYSVR			Carbamidomethyl (C)[11]		Mascot
1955.9684	1955.949	-0.0194	-10	12	27	VHVAIYYESLCPYSVR	146	100	Carbamidomethyl (C)[11]		Mascot

Rank	Protein Name	Accession No.	Protein MW	Protein PI	Pep. Count	Protein Score	Protein Score C. I. %	Intensity Matched	Total Ion Score	Total Ion C. I. %	Confirmed
2	27k protein [Triticum aestivum]	gi|290350670	24399.1	6.06	4	269	100	42.971	256	100	

Peptide Information

Calc. Mass	Obsrv. Mass	± da	± ppm	Start Seq.	End Seq.	Sequence	Ion Score	C. I. %	Modification	Rank	Result Type
1078.535	1078.5236	-0.0114	-11	193	201	GYPLLEACR			Carbamidomethyl (C)[8]		Mascot
1078.535	1078.5236	-0.0114	-11	193	201	GYPLLEACR	71	100	Carbamidomethyl (C)[8]		Mascot
1113.467	1113.45	-0.017	-15	121	128	EWESCFQK			Carbamidomethyl (C)[5]		Mascot
1406.6271	1406.6101	-0.017	-12	119	128	HREWESCFQK			Carbamidomethyl (C)[7]		Mascot
1406.6271	1406.6101	-0.017	-12	119	128	HREWESCFQK	38	98.881	Carbamidomethyl (C)[7]		Mascot
1955.9684	1955.949	-0.0194	-10	24	39	VHVAIYYESLCPYSVR			Carbamidomethyl (C)[11]		Mascot
1955.9684	1955.949	-0.0194	-10	24	39	VHVAIYYESLCPYSVR	146	100	Carbamidomethyl (C)[11]		Mascot

Rank	Protein Name	Accession No.	Protein MW	Protein PI	Pep. Count	Protein Score	Protein Score C. I. %	Intensity Matched	Total Ion Score	Total Ion C. I. %	Confirmed
3	hypothetical protein TRIUR3_28410 [Triticum urartu]	gi|474060617	28387.9	5.53	6	266	100	21.334	241	100	

Peptide Information

Calc. Mass	Obsrv. Mass	± da	± ppm	Start Seq.	End Seq.	Sequence	Ion Score	C. I. %	Modification	Rank	Result Type

Calc. Mass	Obsrv. Mass	± da	± ppm	Start Seq.	End Seq.	Sequence	Ion Score	C. I. %	Modification	Rank	Result Type
1078.535	1078.5236	-0.0114	-11	207	215	GYPLLEACR			Carbamidomethyl (C)[8]		Mascot
1078.535	1078.5236	-0.0114	-11	207	215	GYPLLEACR	71	100	Carbamidomethyl (C)[8]		Mascot
1113.467	1113.45	-0.017	-15	132	139	EWESCFQK			Carbamidomethyl (C)[5]		Mascot
1406.6271	1406.6101	-0.017	-12	130	139	HREWESCFQK			Carbamidomethyl (C)[7]		Mascot
1406.6271	1406.6101	-0.017	-12	130	139	HREWESCFQK	38	98.881	Carbamidomethyl (C)[7]		Mascot
1796.9075	1796.8391	-0.0684	-38	113	127	VHLGFIYCVSDLVMK			Carbamidomethyl (C)[8], Oxidation (M)[14]		Mascot
1796.9075	1796.8391	-0.0684	-38	113	127	VHLGFIYCVSDLVMK	84	100	Carbamidomethyl (C)[8], Oxidation (M)[14]		Mascot
1845.9594	1845.8745	-0.0849	-46	62	79	DGLLDAADLTLVPYGNAK					Mascot
2236.1609	2236.1265	-0.0344	-15	59	79	AYRDGLLDAADLTLVPYG NAK					Mascot
2236.1609	2236.1265	-0.0344	-15	59	79	AYRDGLLDAADLTLVPYG NAK	48	99.884			Mascot

4	hypothetical protein TRIUR3_08536 [Triticum urartu]	gi|474407060	49568.3	5.63	14	53	78.834	20.556

Peptide Information

Calc. Mass	Obsrv. Mass	± da	± ppm	Start Seq.	End Seq.	Sequence	Ion Score	C. I. %	Modification	Rank	Result Type
1054.447	1054.4301	-0.0169	-16	330	338	DTTEQTMGR			Oxidation (M)[7]		Mascot
1078.5388	1078.5236	-0.0152	-14	33	42	GHGTGHKEQK					Mascot
1078.5388	1078.5236	-0.0152	-14	33	42	GHGTGHKEQK					Mascot
1141.575	1141.5007	-0.0743	-65	2	11	AAWHVDTVSR					Mascot
1193.5468	1193.5795	0.0327	27	377	389	DSVMGAAGGAVDK			Oxidation (M)[4]		Mascot
1249.6033	1249.564	-0.0393	-31	23	32	QHHTKQEEGR					Mascot
1312.5984	1312.6602	0.0618	47	266	276	TRDMTAQTMNK			Oxidation (M)[4]		Mascot
1410.6383	1410.604	-0.0343	-24	244	256	AEEPSFDTSTQAK					Mascot
1422.6278	1422.608	-0.0198	-14	315	329	DSVMGAAGGTADNTR					Mascot
1422.6278	1422.608	-0.0198	-14	315	329	DSVMGAAGGTADNTR					Mascot
1434.7006	1434.649	-0.0516	-36	377	391	DSVMGAAGGAVDKTR					Mascot
1438.6228	1438.5996	-0.0232	-16	315	329	DSVMGAAGGTADNTR			Oxidation (M)[4]		Mascot
1438.6228	1438.5996	-0.0232	-16	315	329	DSVMGAAGGTADNTR			Oxidation (M)[4]		Mascot
1450.6956	1450.6421	-0.0535	-37	377	391	DSVMGAAGGAVDKTR			Oxidation (M)[4]		Mascot
1651.832	1651.7448	-0.0872	-53	408	425	VVETGKSATGGGMTGTA K					Mascot
1705.8425	1705.821	-0.0215	-13	66	82	DAAMQALGITGDQTVAK			Oxidation (M)[4]		Mascot
1779.829	1779.8074	-0.0216	-12	341	358	EATGDAGNRTGSMAAQV K			Oxidation (M)[13]		Mascot
1796.9276	1796.8391	-0.0885	-49	464	481	GIVAGEEELIPVEGEAGK					Mascot
1796.9276	1796.8391	-0.0885	-49	464	481	GIVAGEEELIPVEGEAGK					Mascot
1969.9713	1969.9294	-0.0419	-21	143	162	DVTVSTGGTAAEYAKTA					Mascot

AEK

5	hypothetical protein TRIUR3_30538 [Triticum urartu]	gi|473882607	197169.7	4.65	28	51	65.673	8.82

Peptide Information

Calc. Mass	Obsrv. Mass	± da	± ppm	Start Seq.	End Seq.	Sequence	Ion Score	C. I. %	Modification	Rank	Result Type
856.4523	856.4855	0.0332	39	930	937	ALEHATSK					Mascot
987.5356	987.5131	-0.0225	-23	736	743	EELELNLK					Mascot
1036.5463	1036.4789	-0.0674	-65	1602	1609	EIKWDAFK					Mascot
1094.559	1094.5156	-0.0434	-40	726	735	SLLSDAHSHK					Mascot
1106.5623	1106.5458	-0.0165	-15	357	365	LSERDSMLR					Mascot
1110.6014	1110.5044	-0.097	-87	169	178	SLHAAQEAKR					Mascot
1139.5579	1139.5282	-0.0297	-26	862	870	EKYEELSNK					Mascot
1147.6028	1147.5582	-0.0446	-39	233	242	MVQEALELSK					Mascot
1208.5836	1208.5681	-0.0155	-13	185	194	MLEMAQLNMK					Mascot
1238.5569	1238.5681	0.0112	9	991	1002	AAGATEEVESMK			Oxidation (M)[11]		Mascot
1238.5569	1238.5681	0.0112	9	991	1002	AAGATEEVESMK			Oxidation (M)[11]		Mascot
1242.5709	1242.558	-0.0129	-10	919	929	SAEEQLEHSGR					Mascot
1265.6008	1265.5752	-0.0256	-20	583	592	EVEAELEQYR					Mascot
1269.578	1269.5499	-0.0281	-22	1268	1277	VAMYDELQER			Oxidation (M)[3]		Mascot
1323.6678	1323.6306	-0.0372	-28	640	650	LEELTAEYEVK					Mascot
1337.6583	1337.6232	-0.0351	-26	979	990	LAEEQVASYEAK					Mascot
1340.6078	1340.6639	0.0561	42	2	13	EAEQAAQVHDDK					Mascot
1345.6707	1345.6273	-0.0434	-32	1590	1601	TIANQEEQASVR					Mascot
1404.7329	1404.6033	-0.1296	-92	348	360	GTLVDAESKLSER					Mascot
1511.7224	1511.6993	-0.0231	-15	1173	1186	FVSASSEKEEAAEK					Mascot
1559.7119	1559.7186	0.0067	4	361	373	DSMLRQAEEEHAK			Oxidation (M)[3]		Mascot
1562.8094	1562.7217	-0.0877	-56	76	88	VLSMRIEELESEK					Mascot
1618.7742	1618.7343	-0.0399	-25	896	909	MEEVENDLQALGVR			Oxidation (M)[1]		Mascot
1678.8568	1678.7368	-0.12	-71	625	638	DLMDSLIQITEEKK			Oxidation (M)[3]		Mascot
1838.9858	1838.8784	-0.1074	-58	1327	1342	LHEALETITQKEAEVK					Mascot
1845.9188	1845.8745	-0.0443	-24	687	703	ESGENTIADLNLQLSNK					Mascot
1867.8962	1867.9077	0.0115	6	179	194	AAELDKMLEMAQLNMK			Oxidation (M)[7,10]		Mascot
1883.8911	1883.8615	-0.0296	-16	179	194	AAELDKMLEMAQLNMK			Oxidation (M)[7,10,15]		Mascot
1926.9767	1926.8483	-0.1284	-67	1309	1326	NLELHSAAESKIGETEAK					Mascot
1955.0518	1954.9196	-0.1322	-68	298	313	LLSLQEMEIKLEEHVK			Oxidation (M)[7]		Mascot

6	PERQ amino acid-rich with GYF domain-containing	gi|474146978	156052	5.9	22	50	52.616	7.502

protein 2 [Triticum urartu]

Peptide Information

Calc. Mass	Obsrv. Mass	± da	± ppm	Start Seq.	End Seq.	Sequence	Ion Score	C. I. %	Modification	Rank	Result Type
1036.5059	1036.4789	-0.027	-26	278	286	VVQSFEDGR					Mascot
1117.6252	1117.5424	-0.0828	-74	1395	1404	QIDPSLLGFK					Mascot
1117.6252	1117.5424	-0.0828	-74	1395	1404	QIDPSLLGFK					Mascot
1145.5546	1145.4415	-0.1131	-99	87	95	EVDREVENR					Mascot
1242.5453	1242.558	0.0127	10	1205	1215	SSSATMLDMQR			Oxidation (M)[6]		Mascot
1249.6212	1249.564	-0.0572	-46	590	600	TGWFPSVEAQK					Mascot
1252.6168	1252.5513	-0.0655	-52	645	655	ADFQLTSLDSR					Mascot
1265.6532	1265.5752	-0.078	-62	964	973	SLSMHERLHR					Mascot
1418.6407	1418.6205	-0.0202	-14	952	963	SSSVEHHDHLDR					Mascot
1420.718	1420.6115	-0.1065	-75	275	286	QQKVVQSFEDGR					Mascot
1422.6284	1422.608	-0.0204	-14	63	74	NAFDAGWLDEER					Mascot
1422.6284	1422.608	-0.0204	-14	63	74	NAFDAGWLDEER					Mascot
1434.7224	1434.649	-0.0734	-51	341	353	NKDLEAFGIDANK					Mascot
1450.7384	1450.6421	-0.0963	-66	325	338	VTSSEATNTTALQK					Mascot
1511.7158	1511.6993	-0.0165	-11	1105	1117	NPEAELSDMIHQK					Mascot
1549.7209	1549.7122	-0.0087	-6	779	790	REQLQQEQMMGR			Oxidation (M)[9]		Mascot
1562.822	1562.7217	-0.1003	-64	721	734	HGPLSRMNEAPVVR					Mascot
1698.8446	1698.7634	-0.0812	-48	1141	1156	DPSSLFAQPAADNPLR					Mascot
1887.8583	1887.8469	-0.0114	-6	376	393	DAFYNEGMFAGGITGPP K			Oxidation (M)[8]		Mascot
1904.9396	1904.8774	-0.0622	-33	1405	1420	VHSNRILMGEIHRPDD			Oxidation (M)[8]		Mascot
1926.978	1926.8483	-0.1297	-67	394	411	KPTEENAPSHPHGLSGIR					Mascot
1953.96	1953.9296	-0.0304	-16	727	743	MNEAPVVRDEWPANLG R					Mascot
1969.955	1969.9294	-0.0256	-13	727	743	MNEAPVVRDEWPANLG R			Oxidation (M)[1]		Mascot
2036.9091	2036.9698	0.0607	30	1052	1069	NWSDANGQLQNSLMEA SR			Oxidation (M)[14]		Mascot
3187.5437	3187.5303	-0.0134	-4	1052	1079	NWSDANGQLQNSLMEA SRINQLQIEAEK					Mascot

7 hypothetical protein TRIUR3_22710 [Triticum urartu] gi|474191119 23930.7 5.54 8 46 0 2.157

Peptide Information

Calc. Mass	Obsrv. Mass	± da	± ppm	Start Seq.	End Seq.	Sequence	Ion Score	C. I. %	Modification	Rank	Result Type
1046.5299	1046.4967	-0.0332	-32	66	75	STPMDGAVLR					Mascot

Calc. Mass	Obsrv. Mass	± da	± ppm	Start Seq.	End Seq.	Sequence	Ion Score	C. I. %	Modification	Rank	Result Type
1205.6749	1205.5809	-0.094	-78	30	40	HGLVLISHSSR					Mascot
1315.7019	1315.6025	-0.0994	-76	180	190	GWRFGHTLSVR					Mascot
1418.6846	1418.6205	-0.0641	-45	191	201	IHGRVMEEEYR					Mascot
1434.6794	1434.649	-0.0304	-21	191	201	IHGRVMEEEYR			Oxidation (M)[6]		Mascot
1635.6865	1635.7322	0.0457	28	202	214	LRWCCFMGAGTNY			Carbamidomethyl (C)[4,5]		Mascot
1651.6815	1651.7448	0.0633	38	202	214	LRWCCFMGAGTNY			Carbamidomethyl (C)[4,5], Oxidation (M)[7]		Mascot
1883.9069	1883.8615	-0.0454	-24	163	179	VPTNMFAEGRLDGYASR					Mascot
2037.0084	2036.9698	-0.0386	-19	66	84	STPMDGAVLRVAGDAHH FR					Mascot
3382.7126	3382.7234	0.0108	3	12	40	FSVHLHDAGYHFHLLSC RHGLVLISHSSR			Carbamidomethyl (C)[17]		Mascot

8 hypothetical protein TRIUR3_35091 [Triticum urartu] gi|474277421 70803.8 6.31 15 41 0 4.625

Peptide Information

Calc. Mass	Obsrv. Mass	± da	± ppm	Start Seq.	End Seq.	Sequence	Ion Score	C. I. %	Modification	Rank	Result Type
856.4788	856.4855	0.0067	8	217	224	HPPLGHAK					Mascot
1147.5994	1147.5582	-0.0412	-36	499	509	FVGSAVVDEPK					Mascot
1187.515	1187.5753	0.0603	51	130	138	FQDAEQMYR					Mascot
1208.5953	1208.5681	-0.0272	-23	410	419	CPRPLHDSAR			Carbamidomethyl (C)[1]		Mascot
1312.6428	1312.6602	0.0174	13	86	95	VWIQMMDYVK					Mascot
1320.6589	1320.6083	-0.0506	-38	454	464	INSRSMHPHNK					Mascot
1420.743	1420.6115	-0.1315	-93	194	205	NYTELKSNPVQK					Mascot
1545.6863	1545.6985	0.0122	8	1	14	MAADADHQHGHIDK					Mascot
1573.6959	1573.7382	0.0423	27	397	409	GNDQNVMCHKNTR			Carbamidomethyl (C)[8]		Mascot
1619.745	1619.7397	-0.0053	-3	114	126	AAFYMAYAIYYEK			Oxidation (M)[5]		Mascot
1705.8644	1705.821	-0.0434	-25	560	576	AAPVSGFSILADDDISK					Mascot
1796.8273	1796.8391	0.0118	7	63	76	FLQKCAEEFQDEPR			Carbamidomethyl (C)[5]		Mascot
1796.8273	1796.8391	0.0118	7	63	76	FLQKCAEEFQDEPR			Carbamidomethyl (C)[5]		Mascot
1818.8188	1818.8351	0.0163	9	1	16	MAADADHQHGHIDKEK			Oxidation (M)[1]		Mascot
1898.7494	1898.9174	0.168	88	605	620	DVMSEINDMFGMSLDF			Oxidation (M)[3,9,12]		Mascot
2188.1133	2188.1082	-0.0051	-2	560	581	AAPVSGFSILADDDISKD PAAK					Mascot

9 hypothetical protein TRIUR3_22661 [Triticum urartu] gi|474428008 12867.6 9.1 7 41 0 6.063

Peptide Information

Calc. Mass	Obsrv. Mass	± da	± ppm	Start Seq.	End Seq.	Sequence	Ion Score	C. I. %	Modification	Rank	Result Type
1187.6354	1187.5753	-0.0601	-51	79	87	LVCVHYQIR			Carbamidomethyl (C)[3]		Mascot

1250.5544	1250.563	0.0086	7	68	77	CMPEEALSWK			Carbamidomethyl (C)[1]		Mascot
1406.6555	1406.6101	-0.0454	-32	68	78	CMPEEALSWKR			Carbamidomethyl (C)[1]		Mascot
1406.6555	1406.6101	-0.0454	-32	68	78	CMPEEALSWKR			Carbamidomethyl (C)[1]		Mascot
1418.7539	1418.6205	-0.1334	-94	2	12	ELHVFREVFSR					Mascot
1422.6504	1422.608	-0.0424	-30	68	78	CMPEEALSWKR			Carbamidomethyl (C)[1], Oxidation (M)[2]		Mascot
1422.6504	1422.608	-0.0424	-30	68	78	CMPEEALSWKR			Carbamidomethyl (C)[1], Oxidation (M)[2]		Mascot
1549.7944	1549.7122	-0.0822	-53	1	12	MELHVFREVFSR					Mascot
1618.782	1618.7343	-0.0477	-29	88	101	QSRNNGDPFDLDIK					Mascot
1977.8616	1977.9203	0.0587	30	62	77	GDHMVRCMPEEALSWK			Carbamidomethyl (C)[7], Oxidation (M)[4,8]		Mascot

10	hypothetical protein TRIUR3_01759 [Triticum urartu]	gi|474404033	5902.9	6.05	5	41	0	14.573

Peptide Information

Calc. Mass	Obsrv. Mass	± da	± ppm	Start Seq.	End Seq.	Sequence	Ion Score	C. I. %	Modification	Rank	Result Type
1078.5316	1078.5236	-0.008	-7	41	48	EDVLHYFR					Mascot
1078.5316	1078.5236	-0.008	-7	41	48	EDVLHYFR					Mascot
1450.7253	1450.6421	-0.0832	-57	21	31	FKEFEFSEFIK					Mascot
1484.7758	1484.817	0.0412	28	41	51	EDVLHYFRIHR					Mascot
1856.9575	1856.8613	-0.0962	-52	5	22	IGPAPEMVNEATGPAKFK					Mascot
1926.9457	1926.8483	-0.0974	-51	32	48	AYSAAAASREDVLHYFR					Mascot

Gel Idx/Pos	210/I10	Instr./Gel Origin	BA2151/Sample Project 20140814	Process Status	Analysis Succeeded
Plate [#] Name	[1] Sample Project 20140814	Instrument Sample Name		Spectra	11

Rank	Protein Name	Accession No.	Protein MW	Protein PI	Pep. Count	Protein Score	Protein Score C. I. %	Intensity Matched	Total Ion Score	Total Ion C. I. %	Confirmed
1	1-Cys peroxiredoxin [Triticum durum]	gi|12247762	24106.2	6.3	13	195	100	20.187	112	100	

Protein Group

Protein Name	Accession No.	Protein MW	Protein PI
1-Cys-peroxiredoxine [Triticum aestivum]	gi|34539782	24178.2	6.0799999237 0605
RecName: Full=1-Cys peroxiredoxin PER1; AltName: Full=Rehydrin homolog; AltName: Full=Thioredoxin peroxidase	gi|75324900	24178.2	6.0799999237 0605
unnamed protein product [Triticum turgidum]	gi|114209484	24106.2	6.3000001907 3486

Peptide Information

Calc. Mass	Obsrv. Mass	± da	± ppm	Start Seq.	End Seq.	Sequence	Ion Score	C. I. %	Modification	Rank	Result Type
878.4036	878.4022	-0.0014	-2	151	157	NMDEVVR			Oxidation (M)[2]		Mascot
972.4745	972.4695	-0.005	-5	119	127	DAEGQLPSR					Mascot
979.5571	979.5389	-0.0182	-19	128	136	TLHIVGPDK					Mascot
988.5673	988.5319	-0.0354	-36	158	167	AVDSLLTAAK					Mascot
1107.5681	1107.5558	-0.0123	-11	84	93	DIEAYKPGSK					Mascot
1253.7212	1253.6019	-0.1193	-95	158	169	AVDSLLTAAKHK					Mascot
1277.6195	1277.6052	-0.0143	-11	94	104	VTYPIMADPDR					Mascot
1293.6144	1293.5979	-0.0165	-13	94	104	VTYPIMADPDR	24	68.193	Oxidation (M)[6]		Mascot
1300.6355	1300.6293	-0.0062	-5	140	150	LSFLYPSCTGR			Carbamidomethyl (C)[8]		Mascot
1300.6355	1300.6293	-0.0062	-5	140	150	LSFLYPSCTGR	88	100	Carbamidomethyl (C)[8]		Mascot
1471.7209	1471.7075	-0.0134	-9	67	79	LLGISCDDVQSHK			Carbamidomethyl (C)[6]		Mascot
1583.7411	1583.6924	-0.0487	-31	196	209	MFPQGFETADLPSK			Oxidation (M)[1]		Mascot
1711.8361	1711.7836	-0.0525	-31	196	210	MFPQGFETADLPSKK			Oxidation (M)[1]		Mascot
2064.0608	2064.0044	-0.0564	-27	2	21	PGLTIGDTVPNLELDSTHGK					Mascot
2144.0264	2143.9248	-0.1016	-47	140	157	LSFLYPSCTGRNMDEVVR			Carbamidomethyl (C)[8]		Mascot

Rank	Protein Name	Accession No.	Protein MW	Protein PI	Pep. Count	Protein Score	Protein Score C. I. %	Intensity Matched	Total Ion Score	Total Ion C. I. %	Confirmed
2	1-Cys peroxiredoxin PER1 [Triticum urartu]	gi|474094006	24248.3	6.08	12	188	100	19.711	112	100	

Peptide Information

Calc. Mass	Obsrv. Mass	± da	± ppm	Start Seq.	End Seq.	Sequence	Ion Score	C. I. %	Modification	Rank	Result Type
958.4589	958.4544	-0.0045	-5	119	127	DGEGQLPSR					Mascot
979.5571	979.5389	-0.0182	-19	128	136	TLHIVGPDK					Mascot
988.5673	988.5319	-0.0354	-36	159	168	AVDSLLTAAK					Mascot
1107.5681	1107.5558	-0.0123	-11	84	93	DIEAYKPGSK					Mascot
1253.7212	1253.6019	-0.1193	-95	159	170	AVDSLLTAAKHK					Mascot
1277.6195	1277.6052	-0.0143	-11	94	104	VTYPIMADPDR					Mascot
1293.6144	1293.5979	-0.0165	-13	94	104	VTYPIMADPDR	24	68.193	Oxidation (M)[6]		Mascot
1300.6355	1300.6293	-0.0062	-5	140	150	LSFLYPSCTGR			Carbamidomethyl (C)[8]		Mascot
1300.6355	1300.6293	-0.0062	-5	140	150	LSFLYPSCTGR	88	100	Carbamidomethyl (C)[8]		Mascot
1471.7209	1471.7075	-0.0134	-9	67	79	LLGISCDDVQSHK			Carbamidomethyl (C)[6]		Mascot
1583.7411	1583.6924	-0.0487	-31	197	210	MFPQGFETADLPSK			Oxidation (M)[1]		Mascot
1711.8361	1711.7836	-0.0525	-31	197	211	MFPQGFETADLPSKK			Oxidation (M)[1]		Mascot
2064.0608	2064.0044	-0.0564	-27	2	21	PGLTIGDTVPNLELDSTHGK					Mascot
2143.9924	2143.9248	-0.0676	-32	109	127	QLNMVDPDEKDGEGQLPSR			Oxidation (M)[4]		Mascot

3	peroxiredoxin [Triticum aestivum]	gi|190684059	24190.2	6.31	12	186	100	19.786	112	100	

Peptide Information

Calc. Mass	Obsrv. Mass	± da	± ppm	Start Seq.	End Seq.	Sequence	Ion Score	C. I. %	Modification	Rank	Result Type
878.4036	878.4022	-0.0014	-2	151	157	NMDEVVR			Oxidation (M)[2]		Mascot
957.4749	957.4382	-0.0367	-38	119	127	DGQGQLPSR					Mascot
979.5571	979.5389	-0.0182	-19	128	136	TLHIVGPDK					Mascot
988.5673	988.5319	-0.0354	-36	158	167	AVDSLLTAAK					Mascot
1253.7212	1253.6019	-0.1193	-95	158	169	AVDSLLTAAKHK					Mascot
1277.6195	1277.6052	-0.0143	-11	94	104	VTYPIMADPDR					Mascot
1293.6144	1293.5979	-0.0165	-13	94	104	VTYPIMADPDR	24	68.193	Oxidation (M)[6]		Mascot
1300.6355	1300.6293	-0.0062	-5	140	150	LSFLYPSCTGR			Carbamidomethyl (C)[8]		Mascot
1300.6355	1300.6293	-0.0062	-5	140	150	LSFLYPSCTGR	88	100	Carbamidomethyl (C)[8]		Mascot
1471.7209	1471.7075	-0.0134	-9	67	79	LLGISCDDVQSHK			Carbamidomethyl (C)[6]		Mascot
1583.7411	1583.6924	-0.0487	-31	196	209	MFPQGFETADLPSK			Oxidation (M)[1]		Mascot
1711.8361	1711.7836	-0.0525	-31	196	210	MFPQGFETADLPSKK			Oxidation (M)[1]		Mascot
2064.0608	2064.0044	-0.0564	-27	2	21	PGLTIGDTVPNLELDSTHGK					Mascot

2144.0264	2143.9248	-0.1016	-47	140	157	LSFLYPSCTGRNMDEVV R			Carbamidomethyl (C)[8]		Mascot

4 27K protein [Triticum aestivum] gi|30793446 23271.5 6.06 6 169 100 7.276 142 100

Peptide Information

Calc. Mass	Obsrv. Mass	± da	± ppm	Start Seq.	End Seq.	Sequence	Ion Score	C. I. %	Modification	Rank	Result Type
975.5411	975.5283	-0.0128	-13	28	35	FVANHLFK					Mascot
1076.5331	1076.5466	0.0135	13	1	11	GDVATGRGSEK					Mascot
1078.535	1078.5311	-0.0039	-4	181	189	GYPLLEACR			Carbamidomethyl (C)[8]		Mascot
1078.535	1078.5311	-0.0039	-4	181	189	GYPLLEACR	42	99.478	Carbamidomethyl (C)[8]		Mascot
1145.5698	1145.5634	-0.0064	-6	133	142	GHNLSLEYGR					Mascot
1145.5698	1145.5634	-0.0064	-6	133	142	GHNLSLEYGR	68	99.999			Mascot
1406.6271	1406.6166	-0.0105	-7	107	116	HREWESCFQK			Carbamidomethyl (C)[7]		Mascot
1955.9684	1955.9583	-0.0101	-5	12	27	VHVAIYYESLCPYSVR			Carbamidomethyl (C)[11]		Mascot
1955.9684	1955.9583	-0.0101	-5	12	27	VHVAIYYESLCPYSVR	31	93.683	Carbamidomethyl (C)[11]		Mascot

5 Tri a Bd 27K, partial [Triticum aestivum] gi|283480515 23214.5 6.06 5 162 100 6.916 142 100

Peptide Information

Calc. Mass	Obsrv. Mass	± da	± ppm	Start Seq.	End Seq.	Sequence	Ion Score	C. I. %	Modification	Rank	Result Type
975.5411	975.5283	-0.0128	-13	27	34	FVANHLFK					Mascot
1078.535	1078.5311	-0.0039	-4	180	188	GYPLLEACR			Carbamidomethyl (C)[8]		Mascot
1078.535	1078.5311	-0.0039	-4	180	188	GYPLLEACR	42	99.478	Carbamidomethyl (C)[8]		Mascot
1145.5698	1145.5634	-0.0064	-6	132	141	GHNLSLEYGR					Mascot
1145.5698	1145.5634	-0.0064	-6	132	141	GHNLSLEYGR	68	99.999			Mascot
1406.6271	1406.6166	-0.0105	-7	106	115	HREWESCFQK			Carbamidomethyl (C)[7]		Mascot
1955.9684	1955.9583	-0.0101	-5	11	26	VHVAIYYESLCPYSVR			Carbamidomethyl (C)[11]		Mascot
1955.9684	1955.9583	-0.0101	-5	11	26	VHVAIYYESLCPYSVR	31	93.683	Carbamidomethyl (C)[11]		Mascot

6 27k protein [Triticum aestivum] gi|290350670 24399.1 6.06 5 161 100 6.916 142 100

Peptide Information

Calc. Mass	Obsrv. Mass	± da	± ppm	Start Seq.	End Seq.	Sequence	Ion Score	C. I. %	Modification	Rank	Result Type
975.5411	975.5283	-0.0128	-13	40	47	FVANHLFK					Mascot
1078.535	1078.5311	-0.0039	-4	193	201	GYPLLEACR			Carbamidomethyl (C)[8]		Mascot
1078.535	1078.5311	-0.0039	-4	193	201	GYPLLEACR	42	99.478	Carbamidomethyl (C)[8]		Mascot
1145.5698	1145.5634	-0.0064	-6	145	154	GHNLSLEYGR					Mascot

Calc. Mass	Obsrv. Mass	± da	± ppm	Start Seq.	End Seq.	Sequence	Ion Score	C. I. %	Modification	Rank	Result Type
1145.5698	1145.5634	-0.0064	-6	145	154	GHNLSLEYGR	68	99.999			Mascot
1406.6271	1406.6166	-0.0105	-7	119	128	HREWESCFQK			Carbamidomethyl (C)[7]		Mascot
1955.9684	1955.9583	-0.0101	-5	24	39	VHVAIYYESLCPYSVR			Carbamidomethyl (C)[11]		Mascot
1955.9684	1955.9583	-0.0101	-5	24	39	VHVAIYYESLCPYSVR	31	93.683	Carbamidomethyl (C)[11]		Mascot

7	hypothetical protein TRIUR3_28410 [Triticum urartu]	gi|474060617	28387.9	5.53	3	118	100	4.641	110	100

Peptide Information

Calc. Mass	Obsrv. Mass	± da	± ppm	Start Seq.	End Seq.	Sequence	Ion Score	C. I. %	Modification	Rank	Result Type
1078.535	1078.5311	-0.0039	-4	207	215	GYPLLEACR			Carbamidomethyl (C)[8]		Mascot
1078.535	1078.5311	-0.0039	-4	207	215	GYPLLEACR	42	99.478	Carbamidomethyl (C)[8]		Mascot
1145.5698	1145.5634	-0.0064	-6	156	165	GHNLSLEYGR					Mascot
1145.5698	1145.5634	-0.0064	-6	156	165	GHNLSLEYGR	68	99.999			Mascot
1406.6271	1406.6166	-0.0105	-7	130	139	HREWESCFQK			Carbamidomethyl (C)[7]		Mascot

8	Vicilin-like antimicrobial peptides 2-2 [Triticum urartu]	gi|474404647	43446.1	6.74	4	76	99.891	7.034	67	99.998

Peptide Information

Calc. Mass	Obsrv. Mass	± da	± ppm	Start Seq.	End Seq.	Sequence	Ion Score	C. I. %	Modification	Rank	Result Type
1060.5575	1060.5504	-0.0071	-7	294	302	AGDVFWIPR					Mascot
1490.7428	1490.736	-0.0068	-5	313	326	GGPFVFFGFTTSAR					Mascot
1490.7428	1490.736	-0.0068	-5	313	326	GGPFVFFGFTTSAR	67	99.998			Mascot
1557.8748	1557.8629	-0.0119	-8	328	341	NKPQFLTGPTSVLR					Mascot
1818.8446	1818.834	-0.0106	-6	207	221	APEPYNLFDHEPSFR					Mascot

9	hypothetical protein TRIUR3_03511 [Triticum urartu]	gi|474370951	11104.9	7.42	7	52	75.132	1.693		

Peptide Information

Calc. Mass	Obsrv. Mass	± da	± ppm	Start Seq.	End Seq.	Sequence	Ion Score	C. I. %	Modification	Rank	Result Type
808.3869	808.3878	0.0009	1	15	21	CATDITK			Carbamidomethyl (C)[1]		Mascot
1106.5623	1106.5503	-0.012	-11	12	21	AARCATDITK			Carbamidomethyl (C)[4]		Mascot
1135.4296	1135.4246	-0.005	-4	40	48	FGGDTYCCR			Carbamidomethyl (C)[7,8]		Mascot
1253.5402	1253.6019	0.0617	49	1	11	MNFEPVGGACR			Carbamidomethyl (C)[10], Oxidation (M)[1]		Mascot
1259.5725	1259.5912	0.0187	15	66	76	GKCPDAYSYAK			Carbamidomethyl (C)[3]		Mascot
1535.7206	1535.8141	0.0935	61	1	14	MNFEPVGGACRAAR			Carbamidomethyl (C)[10]		Mascot
1706.734	1706.7325	-0.0015	-1	49	62	GQFEHNCPPTNYSR			Carbamidomethyl (C)[7]		Mascot

10	hypothetical protein TRIUR3_03844 [Triticum urartu]	gi|474366583	16503.3	9.19	4	48	37.535	5.669	30	91.899

Peptide Information

Calc. Mass	Obsrv. Mass	± da	± ppm	Start Seq.	End Seq.	Sequence	Ion Score	C. I. %	Modification	Rank	Result Type
957.5265	957.4382	-0.0883	-92	14	22	WQGIGAIGR					Mascot
1085.6215	1085.5709	-0.0506	-47	13	22	KWQGIGAIGR					Mascot
1107.6045	1107.5558	-0.0487	-44	64	72	TTVFEELLR					Mascot
1490.6217	1490.736	0.1143	77	73	85	MSQEEFGFTSDGR					Mascot
1490.6217	1490.736	0.1143	77	73	85	MSQEEFGFTSDGR	30	91.988			Mascot

Gel Idx/Pos	211/I11	Instr./Gel Origin	BA2151/Sample Project 20140814	Process Status	Analysis Succeeded
Plate [#] Name	[1] Sample Project 20140814	Instrument Sample Name		Spectra	11

Rank	Protein Name	Accession No.	Protein MW	Protein PI	Pep. Count	Protein Score	Protein Score C. I. %	Intensity Matched	Total Ion Score	Total Ion C. I. %	Confirmed
1	1-Cys peroxiredoxin [Triticum durum]	gi|12247762	24106.2	6.3	10	320	100	23.538	261	100	

Protein Group

1-Cys-peroxiredoxine [Triticum aestivum]	gi|34539782	24178.2	6.0799 999237 0605
RecName: Full=1-Cys peroxiredoxin PER1; AltName: Full=Rehydrin homolog; AltName: Full=Thioredoxin peroxidase	gi|75324900	24178.2	6.0799 999237 0605
unnamed protein product [Triticum turgidum]	gi|114209484	24106.2	6.3000 001907 3486

Peptide Information

Calc. Mass	Obsrv. Mass	± da	± ppm	Start Seq.	End Seq.	Sequence	Ion Score	C. I. %	Modification	Rank	Result Type
862.4087	862.4234	0.0147	17	151	157	NMDEVVR					Mascot
878.4036	878.4014	-0.0022	-3	151	157	NMDEVVR			Oxidation (M)[2]		Mascot
972.4745	972.4718	-0.0027	-3	119	127	DAEGQLPSR					Mascot
1277.6195	1277.613	-0.0065	-5	94	104	VTYPIMADPDR					Mascot
1293.6144	1293.5973	-0.0171	-13	94	104	VTYPIMADPDR	15	0	Oxidation (M)[6]		Mascot
1300.6355	1300.6281	-0.0074	-6	140	150	LSFLYPSCTGR			Carbamidomethyl (C)[8]		Mascot
1300.6355	1300.6281	-0.0074	-6	140	150	LSFLYPSCTGR	77	100	Carbamidomethyl (C)[8]		Mascot
1471.7209	1471.7009	-0.02	-14	67	79	LLGISCDDVQSHK			Carbamidomethyl (C)[6]		Mascot
1583.7411	1583.7004	-0.0407	-26	196	209	MFPQGFETADLPSK			Oxidation (M)[1]		Mascot
1711.8361	1711.7919	-0.0442	-26	196	210	MFPQGFETADLPSKK			Oxidation (M)[1]		Mascot
2064.0608	2064.0266	-0.0342	-17	2	21	PGLTIGDTVPNLELDSTH GK					Mascot
2064.0608	2064.0266	-0.0342	-17	2	21	PGLTIGDTVPNLELDSTH GK	80	100			Mascot
2144.0264	2143.95	-0.0764	-36	140	157	LSFLYPSCTGRNMDEVV R			Carbamidomethyl (C)[8]		Mascot
2158.0081	2157.9651	-0.043	-20	109	127	QLNMVDPDEKDAEGQLP SR			Oxidation (M)[4]		Mascot
2158.0081	2157.9651	-0.043	-20	109	127	QLNMVDPDEKDAEGQLP SR	89	100	Oxidation (M)[4]		Mascot

2	peroxiredoxin [Triticum aestivum]	gi|190684059	24190.2	6.31	9	222	100	21.624	172	100

Peptide Information

Calc. Mass	Obsrv. Mass	± da	± ppm	Start Seq.	End Seq.	Sequence	Ion Score	C. I. %	Modification	Rank	Result Type
862.4087	862.4234	0.0147	17	151	157	NMDEVVR					Mascot
878.4036	878.4014	-0.0022	-3	151	157	NMDEVVR			Oxidation (M)[2]		Mascot
1277.6195	1277.613	-0.0065	-5	94	104	VTYPIMADPDR					Mascot
1293.6144	1293.5973	-0.0171	-13	94	104	VTYPIMADPDR	15	0	Oxidation (M)[6]		Mascot
1300.6355	1300.6281	-0.0074	-6	140	150	LSFLYPSCTGR			Carbamidomethyl (C)[8]		Mascot
1300.6355	1300.6281	-0.0074	-6	140	150	LSFLYPSCTGR	77	100	Carbamidomethyl (C)[8]		Mascot
1471.7209	1471.7009	-0.02	-14	67	79	LLGISCDDVQSHK			Carbamidomethyl (C)[6]		Mascot
1583.7411	1583.7004	-0.0407	-26	196	209	MFPQGFETADLPSK			Oxidation (M)[1]		Mascot
1711.8361	1711.7919	-0.0442	-26	196	210	MFPQGFETADLPSKK			Oxidation (M)[1]		Mascot
2064.0608	2064.0266	-0.0342	-17	2	21	PGLTIGDTVPNLELDSTH GK					Mascot
2064.0608	2064.0266	-0.0342	-17	2	21	PGLTIGDTVPNLELDSTH GK	80	100			Mascot
2127.0137	2126.957	-0.0567	-27	109	127	QLNMVDPDEKDGQGQL PSR					Mascot
2144.0264	2143.95	-0.0764	-36	140	157	LSFLYPSCTGRNMDEVV R			Carbamidomethyl (C)[8]		Mascot

3	1-Cys peroxiredoxin PER1 [Triticum urartu]	gi|474094006	24248.3	6.08	8	217	100	20.702	172	100

Peptide Information

Calc. Mass	Obsrv. Mass	± da	± ppm	Start Seq.	End Seq.	Sequence	Ion Score	C. I. %	Modification	Rank	Result Type
1277.6195	1277.613	-0.0065	-5	94	104	VTYPIMADPDR					Mascot
1293.6144	1293.5973	-0.0171	-13	94	104	VTYPIMADPDR	15	0	Oxidation (M)[6]		Mascot
1300.6355	1300.6281	-0.0074	-6	140	150	LSFLYPSCTGR			Carbamidomethyl (C)[8]		Mascot
1300.6355	1300.6281	-0.0074	-6	140	150	LSFLYPSCTGR	77	100	Carbamidomethyl (C)[8]		Mascot
1471.7209	1471.7009	-0.02	-14	67	79	LLGISCDDVQSHK			Carbamidomethyl (C)[6]		Mascot
1583.7411	1583.7004	-0.0407	-26	197	210	MFPQGFETADLPSK			Oxidation (M)[1]		Mascot
1711.8361	1711.7919	-0.0442	-26	197	211	MFPQGFETADLPSKK			Oxidation (M)[1]		Mascot
2064.0608	2064.0266	-0.0342	-17	2	21	PGLTIGDTVPNLELDSTH GK					Mascot
2064.0608	2064.0266	-0.0342	-17	2	21	PGLTIGDTVPNLELDSTH GK	80	100			Mascot
2143.9924	2143.95	-0.0424	-20	109	127	QLNMVDPDEKDGEGQLP SR			Oxidation (M)[4]		Mascot
2610.2869	2610.2419	-0.045	-17	171	195	VATPANWKPGECVVIAP GVSDDEAK			Carbamidomethyl (C)[12]		Mascot

4	27K protein [Triticum aestivum]	gi|30793446	23271.5	6.06	6	191	100	10.359	163	100

Peptide Information

Calc. Mass	Obsrv. Mass	± da	± ppm	Start Seq.	End Seq.	Sequence	Ion Score	C. I. %	Modification	Rank	Result Type
1076.5331	1076.5358	0.0027	3	1	11	GDVATGRGSEK					Mascot
1078.535	1078.5282	-0.0068	-6	181	189	GYPLLEACR			Carbamidomethyl (C)[8]		Mascot
1078.535	1078.5282	-0.0068	-6	181	189	GYPLLEACR	54	99.969	Carbamidomethyl (C)[8]		Mascot
1145.5698	1145.5577	-0.0121	-11	133	142	GHNLSLEYGR					Mascot
1145.5698	1145.5577	-0.0121	-11	133	142	GHNLSLEYGR	14	0			Mascot
1406.6271	1406.6256	-0.0015	-1	107	116	HREWESCFQK			Carbamidomethyl (C)[7]		Mascot
1955.9684	1955.9543	-0.0141	-7	12	27	VHVAIYYESLCPYSVR			Carbamidomethyl (C)[11]		Mascot
1955.9684	1955.9543	-0.0141	-7	12	27	VHVAIYYESLCPYSVR	94	100	Carbamidomethyl (C)[11]		Mascot
2071.1182	2071.0967	-0.0215	-10	39	58	DGLLDAANLTLVPYGNAV VR					Mascot

5	Tri a Bd 27K, partial [Triticum aestivum]	gi|283480515	23214.5	6.06	5	185	100	10.127	163	100		

Peptide Information

Calc. Mass	Obsrv. Mass	± da	± ppm	Start Seq.	End Seq.	Sequence	Ion Score	C. I. %	Modification	Rank	Result Type
1078.535	1078.5282	-0.0068	-6	180	188	GYPLLEACR			Carbamidomethyl (C)[8]		Mascot
1078.535	1078.5282	-0.0068	-6	180	188	GYPLLEACR	54	99.969	Carbamidomethyl (C)[8]		Mascot
1145.5698	1145.5577	-0.0121	-11	132	141	GHNLSLEYGR					Mascot
1145.5698	1145.5577	-0.0121	-11	132	141	GHNLSLEYGR	14	0			Mascot
1406.6271	1406.6256	-0.0015	-1	106	115	HREWESCFQK			Carbamidomethyl (C)[7]		Mascot
1955.9684	1955.9543	-0.0141	-7	11	26	VHVAIYYESLCPYSVR			Carbamidomethyl (C)[11]		Mascot
1955.9684	1955.9543	-0.0141	-7	11	26	VHVAIYYESLCPYSVR	94	100	Carbamidomethyl (C)[11]		Mascot
2071.1182	2071.0967	-0.0215	-10	38	57	DGLLDAANLTLVPYGNAV VR					Mascot

6	27k protein [Triticum aestivum]	gi|290350670	24399.1	6.06	5	184	100	10.127	163	100		

Peptide Information

Calc. Mass	Obsrv. Mass	± da	± ppm	Start Seq.	End Seq.	Sequence	Ion Score	C. I. %	Modification	Rank	Result Type
1078.535	1078.5282	-0.0068	-6	193	201	GYPLLEACR			Carbamidomethyl (C)[8]		Mascot
1078.535	1078.5282	-0.0068	-6	193	201	GYPLLEACR	54	99.969	Carbamidomethyl (C)[8]		Mascot
1145.5698	1145.5577	-0.0121	-11	145	154	GHNLSLEYGR					Mascot
1145.5698	1145.5577	-0.0121	-11	145	154	GHNLSLEYGR	14	0			Mascot
1406.6271	1406.6256	-0.0015	-1	119	128	HREWESCFQK			Carbamidomethyl (C)[7]		Mascot
1955.9684	1955.9543	-0.0141	-7	24	39	VHVAIYYESLCPYSVR			Carbamidomethyl (C)[11]		Mascot

1955.9684	1955.9543	-0.0141	-7	24	39	VHVAIYYESLCPYSVR	94	100	Carbamidomethyl (C)[11]		Mascot
2071.1182	2071.0967	-0.0215	-10	51	70	DGLLDAANLTLVPYGNAV VR					Mascot

7	hypothetical protein TRIUR3_28410 [Triticum urartu]	gi|474060617	28387.9	5.53	5	87	99.991	6.292	69	99.999	

Peptide Information

Calc. Mass	Obsrv. Mass	± da	± ppm	Start Seq.	End Seq.	Sequence	Ion Score	C. I. %	Modification	Rank	Result Type
1078.535	1078.5282	-0.0068	-6	207	215	GYPLLEACR			Carbamidomethyl (C)[8]		Mascot
1078.535	1078.5282	-0.0068	-6	207	215	GYPLLEACR	54	99.969	Carbamidomethyl (C)[8]		Mascot
1145.5698	1145.5577	-0.0121	-11	156	165	GHNLSLEYGR					Mascot
1145.5698	1145.5577	-0.0121	-11	156	165	GHNLSLEYGR	14	0			Mascot
1406.6271	1406.6256	-0.0015	-1	130	139	HREWESCFQK			Carbamidomethyl (C)[7]		Mascot
1796.9075	1796.8629	-0.0446	-25	113	127	VHLGFIYCVSDLVMK			Carbamidomethyl (C)[8], Oxidation (M)[14]		Mascot
2236.1609	2236.1199	-0.041	-18	59	79	AYRDGLLDAADLTLVPYG NAK					Mascot

8	ATP-dependent Clp protease ATP-binding subunit clpA-like protein CD4B, chloroplastic [Triticum urartu]	gi|473787307	98387.6	5.91	23	69	99.492	8.806	

Peptide Information

Calc. Mass	Obsrv. Mass	± da	± ppm	Start Seq.	End Seq.	Sequence	Ion Score	C. I. %	Modification	Rank	Result Type
848.4771	848.4555	-0.0216	-25	26	32	TRATMLR					Mascot
856.4635	856.499	0.0355	41	384	390	DPALERR					Mascot
948.507	948.5135	0.0065	7	787	794	EIADIMLK			Oxidation (M)[6]		Mascot
964.4993	964.5176	0.0183	19	76	84	GSGSRMVVR			Oxidation (M)[6]		Mascot
972.536	972.4718	-0.0642	-66	573	581	IIGQDEAVK					Mascot
1173.4915	1173.5924	0.1009	86	635	643	LDMSEFMER			Oxidation (M)[3]		Mascot
1277.696	1277.613	-0.083	-65	461	471	LRHAQLPDEAK					Mascot
1315.7151	1315.6162	-0.0989	-75	97	107	VIMLAQEEARR					Mascot
1399.7904	1399.6564	-0.134	-96	573	585	IIGQDEAVKAISR					Mascot
1406.6282	1406.6256	-0.0026	-2	854	867	EGDSAIVDVDSDGK					Mascot
1421.7748	1421.682	-0.0928	-65	49	60	QTNFLDTRSVIK					Mascot
1435.6733	1435.705	0.0317	22	496	507	AGELRDEEMELK			Oxidation (M)[9]		Mascot
1471.8301	1471.7009	-0.1292	-88	94	106	AIKVIMLAQEEAR					Mascot
1583.821	1583.7004	-0.1206	-76	257	271	TKNNPCLIGEPGVGK			Carbamidomethyl (C)[6]		Mascot
1609.8796	1609.783	-0.0966	-60	396	409	VPEPTVEESIQILR					Mascot
1864.8706	1864.946	0.0754	40	196	216	MVGESTEAVGAGVGGG SSGQK					Mascot
1916.0753	1915.9664	-0.1089	-57	151	168	IIGRGSGFVAVEIPFTPR					Mascot

2064.0259	2064.0266	0.0007	0	815	832	DRVVDEGYNPSYGARPL R					Mascot
2064.0259	2064.0266	0.0007	0	815	832	DRVVDEGYNPSYGARPL R					Mascot
2092.1067	2092.0098	-0.0969	-46	714	733	NTLLIMTSNVGSSVIEKG GR			Oxidation (M)[6]		Mascot
2124.053	2124.0225	-0.0305	-14	217	235	MPTLEEYGTNLTKLAEEG K					Mascot
2135.0173	2135.0557	0.0384	18	847	867	MLAGEVKEGDSAIVDVD SDGK					Mascot
2236.0955	2236.1199	0.0244	11	615	634	SELAKALASYYFGSEEAM IR					Mascot
2264.1558	2264.1147	-0.0411	-18	649	670	LIGSPPGYVGYTEGGQLT EAVR					Mascot

9 hypothetical protein [Triticum durum] gi|188038091 121493.3 5.4 22 65 98.569 11.677

Peptide Information

Calc. Mass	Obsrv. Mass	± da	± ppm	Start Seq.	End Seq.	Sequence	Ion Score	C. I. %	Modification	Rank	Result Type
929.4938	929.4634	-0.0304	-33	923	930	TIIDDPQK					Mascot
948.4421	948.5135	0.0714	75	1002	1008	TWDELER					Mascot
1037.5262	1037.5084	-0.0178	-17	524	532	NSNELVAYK					Mascot
1048.5344	1048.599	0.0646	62	189	197	DATEIICVK			Carbamidomethyl (C)[7]		Mascot
1100.5446	1100.5155	-0.0291	-26	869	877	NFDMAIVFK			Oxidation (M)[4]		Mascot
1106.551	1106.5609	0.0099	9	429	438	AAEILTNCSK			Carbamidomethyl (C)[8]		Mascot
1154.6165	1154.7151	0.0986	85	621	630	HSSEVVQQIK					Mascot
1173.572	1173.5924	0.0204	17	507	519	RLAGGGSGNGDGR					Mascot
1208.6494	1208.5988	-0.0506	-42	491	500	QHQAELARQK					Mascot
1209.6838	1209.6177	-0.0661	-55	353	363	NAPELLPNLTK					Mascot
1209.6838	1209.6177	-0.0661	-55	353	363	NAPELLPNLTK					Mascot
1385.6598	1385.6602	0.0004	0	796	806	VNDHWSQPQFK					Mascot
1490.7349	1490.7244	-0.0105	-7	869	880	NFDMAIVFKDFK			Oxidation (M)[4]		Mascot
1506.7772	1506.7382	-0.039	-26	1051	1065	HAPLPQRAGSSSGNK					Mascot
1530.6918	1530.777	0.0852	56	765	777	SALDPDEIEEEQR					Mascot
1628.7778	1628.7738	-0.004	-2	586	599	IFFNVPGMPFSNDK			Oxidation (M)[8]		Mascot
1647.8953	1647.8063	-0.089	-54	414	428	QFSLLLADTALVNDK					Mascot
1818.8545	1818.8328	-0.0217	-12	899	912	EWLDTTDLKYYESR					Mascot
2092.0315	2092.0098	-0.0217	-10	325	344	AQEAALAACKPGNQMSA VFK			Carbamidomethyl (C)[9]		Mascot
2124.0583	2124.0225	-0.0358	-17	705	722	VDIMYGNIKHAFFQPAEK			Oxidation (M)[4]		Mascot
2127.1003	2126.957	-0.1433	-67	225	243	VSHSSLMDDTEKVILDPL K					Mascot
2135.0286	2135.0557	0.0271	13	122	141	NGDGIDLMDDILRAVSAE SK			Oxidation (M)[8]		Mascot

2144.033	2143.95	-0.083	-39	745	763	DVQFYVEVMDVVQTVGG SR			Oxidation (M)[9]		Mascot

10 Disease resistance protein RPM1 [Triticum urartu] gi|474368921 159248.7 6.36 28 65 98.569 9.267

Peptide Information

Calc. Mass	Obsrv. Mass	± da	± ppm	Start Seq.	End Seq.	Sequence	Ion Score	C. I. %	Modification	Rank	Result Type
848.4553	848.4555	0.0002	0	1237	1242	YLYVYK					Mascot
862.4265	862.4234	-0.0031	-4	1302	1308	EEKTEAR					Mascot
941.5527	941.5426	-0.0101	-11	536	543	RLSLHTSK					Mascot
967.438	967.4795	0.0415	43	382	389	HDSAHWSK					Mascot
987.6196	987.5217	-0.0979	-99	105	113	SLNLLTKAK					Mascot
1004.5563	1004.5391	-0.0172	-17	1237	1243	YLYVYKR					Mascot
1021.5346	1021.5037	-0.0309	-30	476	484	SLIQTMNSK					Mascot
1037.5297	1037.5084	-0.0213	-21	476	484	SLIQTMNSK			Oxidation (M)[6]		Mascot
1060.4663	1060.5459	0.0796	75	799	806	HLNMDVCR			Carbamidomethyl (C)[7], Oxidation (M)[4]		Mascot
1106.551	1106.5609	0.0099	9	175	183	SNELVEMLR			Oxidation (M)[7]		Mascot
1226.5624	1226.6398	0.0774	63	95	104	SHTFMGFIDR			Oxidation (M)[5]		Mascot
1230.5797	1230.6077	0.028	23	1190	1200	GSKAMANHWGR			Oxidation (M)[5]		Mascot
1274.6344	1274.6309	-0.0035	-3	549	559	VLSTMNMSHVR					Mascot
1319.7352	1319.6462	-0.089	-67	1320	1330	LDLLRTNVTMK			Oxidation (M)[10]		Mascot
1372.6227	1372.6962	0.0735	54	71	82	DLSHDLEDSVDK					Mascot
1385.6709	1385.6602	-0.0107	-8	382	392	HDSAHWSKVYR					Mascot
1421.7352	1421.682	-0.0532	-37	975	987	VAMGMNALTGLRR			Oxidation (M)[3,5]		Mascot
1435.7073	1435.705	-0.0023	-2	697	707	CLDMKVIEWNK			Carbamidomethyl (C)[1]		Mascot
1436.6873	1436.7708	0.0835	58	892	904	MGEMEAAEATLRK					Mascot
1448.7852	1448.7361	-0.0491	-34	1	13	MAQLVMTATLLPK			Oxidation (M)[1,6]		Mascot
1471.7573	1471.7009	-0.0564	-38	905	917	ALDMNPNKPTLNK			Oxidation (M)[4]		Mascot
1562.8115	1562.7469	-0.0646	-41	813	826	QAWPVIGATGYPFR					Mascot
1583.8574	1583.7004	-0.157	-99	904	917	KALDMNPNKPTLNK					Mascot
1620.8593	1620.7449	-0.1144	-71	49	63	VSQAPIDQPPDVQVK					Mascot
1839.8695	1839.8969	0.0274	15	89	104	TDGLDKSHTFMGFIDR					Mascot
1864.9844	1864.946	-0.0384	-21	277	291	YIIVIDDIWDKSAWK					Mascot
1926.9001	1926.866	-0.0341	-18	318	334	EDGDVYEMKPLSITDSK					Mascot
2157.9626	2157.9651	0.0025	1	590	606	QVDDHHSMLICSMFHLR			Carbamidomethyl (C)[11], Oxidation (M)[8,13]		Mascot
2157.9626	2157.9651	0.0025	1	590	606	QVDDHHSMLICSMFHLR			Carbamidomethyl (C)[11], Oxidation (M)[8,13]		Mascot
2610.2869	2610.2419	-0.045	-17	1148	1169	VVCLDPTTGTNQSIETYW ERIK			Carbamidomethyl (C)[3]		Mascot

Gel Idx/Pos	212/I12	Instr./Gel Origin	BA2151/Sample Project 20140814	Process Status	Analysis Succeeded
Plate [#] Name	[1] Sample Project 20140814	Instrument Sample Name		Spectra	11

Rank	Protein Name	Accession No.	Protein MW	Protein PI	Pep. Count	Protein Score	Protein Score C. I. %	Intensity Matched	Total Ion Score	Total Ion C. I. %	Confirmed
1	L-ascorbate peroxidase 1, cytosolic [Triticum urartu]	gi|474311703	27561.1	5.85	15	673	100	47.949	562	100	

Peptide Information

Calc. Mass	Obsrv. Mass	± da	± ppm	Start Seq.	End Seq.	Sequence	Ion Score	C. I. %	Modification	Rank	Result Type
911.4291	911.4224	-0.0067	-7	53	61	TGGPFGTMK			Oxidation (M)[8]		Mascot
919.4196	919.4149	-0.0047	-5	230	236	AFFEDYK					Mascot
990.4859	990.4738	-0.0121	-12	31	38	NCSPLMLR			Carbamidomethyl (C)[2]		Mascot
1006.4808	1006.4717	-0.0091	-9	31	38	NCSPLMLR			Carbamidomethyl (C)[2], Oxidation (M)[6]		Mascot
1036.4847	1036.4802	-0.0045	-4	173	181	SGFEGPWTR					Mascot
1036.4847	1036.4802	-0.0045	-4	173	181	SGFEGPWTR	50	99.924			Mascot
1249.6172	1249.6124	-0.0048	-4	120	130	EDKPQPPPEGR					Mascot
1249.6172	1249.6124	-0.0048	-4	120	130	EDKPQPPPEGR	70	100			Mascot
1505.7384	1505.7155	-0.0229	-15	39	52	LAWHSAGTFDVSSK					Mascot
1525.7434	1525.7373	-0.0061	-4	230	241	AFFEDYKEAHLR					Mascot
1525.7434	1525.7373	-0.0061	-4	230	241	AFFEDYKEAHLR	88	100			Mascot
1627.9418	1627.9331	-0.0087	-5	210	223	TLLTDPVFRPLVEK					Mascot
1627.9418	1627.9331	-0.0087	-5	210	223	TLLTDPVFRPLVEK	69	100			Mascot
1635.7538	1635.7223	-0.0315	-19	186	199	FDNSYFTELLSGDK					Mascot
1712.8378	1712.7953	-0.0425	-25	4	18	TYPVVSAEYQEAVEK					Mascot
1831.9773	1831.9717	-0.0056	-3	62	79	KPAEQAHAANAGLDIAVR					Mascot
1831.9773	1831.9717	-0.0056	-3	62	79	KPAEQAHAANAGLDIAVR	153	100			Mascot
1939.976	1939.9512	-0.0248	-13	4	20	TYPVVSAEYQEAVEKAR					Mascot
2071.0237	2070.9912	-0.0325	-16	148	167	QMGLSDQDIVALSGGHT LGR			Oxidation (M)[2]		Mascot
2071.0237	2070.9912	-0.0325	-16	148	167	QMGLSDQDIVALSGGHT LGR	132	100	Oxidation (M)[2]		Mascot
2716.3352	2716.3118	-0.0234	-9	186	209	FDNSYFTELLSGDKEGLL QLPSDK					Mascot
3580.8687	3580.8896	0.0209	6	86	119	EEIPTISYADLYQLAGVVA VEVSGGPVIPFHPGR					Mascot

Rank	Protein Name	Accession No.	Protein MW	Protein PI	Pep. Count	Protein Score	Protein Score C. I. %	Intensity Matched	Total Ion Score	Total Ion C. I. %	Confirmed
2	ascorbate peroxidase [Triticum aestivum]	gi|226897533	26776.6	5.54	9	277	100	35.462	230	100	

Peptide Information

Calc. Mass	Obsrv. Mass	± da	± ppm	Start Seq.	End Seq.	Sequence	Ion Score	C. I. %	Modification	Rank	Result Type

Calc. Mass	Obsrv. Mass	± da	± ppm	Start Seq.	End Seq.	Sequence	Ion Score	C. I. %	Modification	Rank	Result Type
911.4291	911.4224	-0.0067	-7	46	54	TGGPFGTMK			Oxidation (M)[8]		Mascot
919.4196	919.4149	-0.0047	-5	223	229	AFFEDYK					Mascot
990.4859	990.4738	-0.0121	-12	24	31	NCAPLMLR			Carbamidomethyl (C)[2], Oxidation (M)[6]		Mascot
1036.4847	1036.4802	-0.0045	-4	166	174	SGFEGPWTR					Mascot
1036.4847	1036.4802	-0.0045	-4	166	174	SGFEGPWTR	50	99.924			Mascot
1249.6172	1249.6124	-0.0048	-4	113	123	QDKPEPPPEGR					Mascot
1249.6172	1249.6124	-0.0048	-4	113	123	QDKPEPPPEGR	23	65.072			Mascot
1525.7434	1525.7373	-0.0061	-4	223	234	AFFEDYKEAHLR					Mascot
1525.7434	1525.7373	-0.0061	-4	223	234	AFFEDYKEAHLR	88	100			Mascot
1627.9418	1627.9331	-0.0087	-5	203	216	TLLTDPVFRPLVEK					Mascot
1627.9418	1627.9331	-0.0087	-5	203	216	TLLTDPVFRPLVEK	69	100			Mascot
1635.7538	1635.7223	-0.0315	-19	179	192	FDNSYFTELLSGDK					Mascot
2716.3352	2716.3118	-0.0234	-9	179	202	FDNSYFTELLSGDKEGLL QLPSDK					Mascot

3 hypothetical protein TRIUR3_23412 [Triticum urartu] gi|474122276 44908.9 5.5 18 80 99.956 10.355

Peptide Information

Calc. Mass	Obsrv. Mass	± da	± ppm	Start Seq.	End Seq.	Sequence	Ion Score	C. I. %	Modification	Rank	Result Type
811.4019	811.3736	-0.0283	-35	407	413	LAEGFMK			Oxidation (M)[6]		Mascot
875.4257	875.4254	-0.0003	0	91	97	AEDYLHK					Mascot
911.4694	911.4224	-0.047	-52	300	309	KPSGGSHGGK					Mascot
924.4646	924.4166	-0.048	-52	362	370	HGGRPESGK					Mascot
981.4973	981.4382	-0.0591	-60	267	276	RPSAGSHGGR					Mascot
1022.5013	1022.4612	-0.0401	-39	186	196	KPSSGGYGGGR					Mascot
1036.4066	1036.4802	0.0736	71	351	359	EENEEDSGK					Mascot
1036.4066	1036.4802	0.0736	71	351	359	EENEEDSGK					Mascot
1051.5167	1051.4648	-0.0519	-49	98	109	FGAKEGGSGGGK					Mascot
1052.5596	1052.4727	-0.0869	-83	361	370	KHGGRPESGK					Mascot
1052.5596	1052.4727	-0.0869	-83	361	370	KHGGRPESGK					Mascot
1090.4211	1090.4438	0.0227	21	259	266	YEEDDEYK					Mascot
1118.4272	1118.5089	0.0817	73	155	162	YEEDDEYR					Mascot
1132.443	1132.4781	0.0351	31	177	184	YEEEDEYR					Mascot
1204.4641	1204.5304	0.0663	55	139	147	YEQDDDEYK					Mascot
1541.6901	1541.7266	0.0365	24	391	406	KDGEGGSGAGMGDYLK					Mascot
1888.8383	1888.9642	0.1259	67	55	72	GDVSIDYGGACGDFTNL K			Carbamidomethyl (C)[11]		Mascot

	1994.8839	1994.9786	0.0947	47	249	266	KPTGGHGGGRYEEDDE YK					Mascot
	2052.9006	2053.0679	0.1673	81	259	276	YEEDDEYKRPSAGSHGG R					Mascot
	2323.0256	2323.1377	0.1121	48	362	383	HGGRPESGKDESEGSG MADYLK			Oxidation (M)[17]		Mascot

4	hypothetical protein TRIUR3_34620 [Triticum urartu]	gi|474175186	56855.9	7.73	14	60	95.151	13.341	

Peptide Information

Calc. Mass	Obsrv. Mass	± da	± ppm	Start Seq.	End Seq.	Sequence	Ion Score	C. I. %	Modification	Rank	Result Type
981.4055	981.4382	0.0327	33	1	11	MADASSGGGGR			Oxidation (M)[1]		Mascot
1050.4586	1050.4625	0.0039	4	413	421	KEEEDADSK					Mascot
1078.4946	1078.5057	0.0111	10	400	408	VSCQNSEVR			Carbamidomethyl (C)[3]		Mascot
1204.5858	1204.5304	-0.0554	-46	153	163	FATAWHSATGR					Mascot
1231.6715	1231.6027	-0.0688	-56	327	338	NLSAGVLEMLGK					Mascot
1247.6664	1247.6113	-0.0551	-44	327	338	NLSAGVLEMLGK			Oxidation (M)[9]		Mascot
1646.7994	1646.8035	0.0041	2	238	250	EQQQQQHDHILSR					Mascot
1649.7991	1649.8871	0.088	53	251	267	TPPDANASANAHGITGR					Mascot
1734.8951	1734.8104	-0.0847	-49	327	342	NLSAGVLEMLGKMPMK			Oxidation (M)[9]		Mascot
1814.9032	1814.9757	0.0725	40	102	117	SSDPRIAQPLDPYAER					Mascot
1831.8505	1831.9717	0.1212	66	164	178	EVHDVFGAMQQNRER			Oxidation (M)[9]		Mascot
1831.8505	1831.9717	0.1212	66	164	178	EVHDVFGAMQQNRER			Oxidation (M)[9]		Mascot
1845.9607	1845.9391	-0.0216	-12	107	123	IAQPLDPYAERVGGGFR					Mascot
1988.9342	1989.0907	0.1565	79	343	360	EKHQVTAPSSSSVDMDV R			Oxidation (M)[15]		Mascot
2054.0601	2053.9731	-0.087	-42	320	338	WQGHASRNLSAGVLEML GK					Mascot
2054.0601	2053.9731	-0.087	-42	320	338	WQGHASRNLSAGVLEML GK					Mascot
2070.0549	2069.9834	-0.0715	-35	320	338	WQGHASRNLSAGVLEML GK			Oxidation (M)[16]		Mascot
2716.2798	2716.3118	0.032	12	153	176	FATAWHSATGREVHDVF GAMQQNR					Mascot

5	hypothetical protein TRIUR3_19781 [Triticum urartu]	gi|473930259	38500.4	8.44	11	52	75.698	12.412	

Peptide Information

Calc. Mass	Obsrv. Mass	± da	± ppm	Start Seq.	End Seq.	Sequence	Ion Score	C. I. %	Modification	Rank	Result Type
875.4655	875.4254	-0.0401	-46	83	90	LDAICGVK			Carbamidomethyl (C)[5]		Mascot
1048.5092	1048.4572	-0.052	-50	1	11	MDLSAAGQAGK					Mascot
1064.5042	1064.4897	-0.0145	-14	1	11	MDLSAAGQAGK			Oxidation (M)[1]		Mascot
1132.4689	1132.4781	0.0092	8	290	298	DETCDHLSR			Carbamidomethyl (C)[4]		Mascot

Calc. Mass	Obsrv. Mass	± da	± ppm	Start Seq.	End Seq.	Sequence	Ion Score	C. I. %	Modification	Rank	Result Type
1262.6223	1262.6104	-0.0119	-9	250	261	TSASEQQSSPLK					Mascot
1583.8315	1583.8131	-0.0184	-12	40	53	GYVISDAVLEQVYK					Mascot
1635.8391	1635.7223	-0.1168	-71	201	215	IAHPPFGNHIGANYK					Mascot
1703.8381	1703.8724	0.0343	20	132	147	SGPNTSVSLVTQPCTR			Carbamidomethyl (C)[14]		Mascot
1831.9331	1831.9717	0.0386	21	132	148	SGPNTSVSLVTQPCTRK			Carbamidomethyl (C)[14]		Mascot
1831.9331	1831.9717	0.0386	21	132	148	SGPNTSVSLVTQPCTRK			Carbamidomethyl (C)[14]		Mascot
1859.9393	1859.9668	0.0275	15	131	147	RSGPNTSVSLVTQPCTR			Carbamidomethyl (C)[15]		Mascot
2021.9359	2022.0011	0.0652	32	290	305	DETCDHLSRAQFLHHR			Carbamidomethyl (C)[4]		Mascot
2071.0496	2070.9912	-0.0584	-28	263	280	SSGYVIQVPQFPSNYEKK					Mascot
2071.0496	2070.9912	-0.0584	-28	263	280	SSGYVIQVPQFPSNYEKK					Mascot

6 ascorbate peroxidase [Triticum aestivum] gi|21666264 15417.5 4.7 7 50 54.748 1.434

Peptide Information

Calc. Mass	Obsrv. Mass	± da	± ppm	Start Seq.	End Seq.	Sequence	Ion Score	C. I. %	Modification	Rank	Result Type
1247.5579	1247.6113	0.0534	43	119	128	YAEDQEAFFK					Mascot
1541.8547	1541.7266	-0.1281	-83	9	22	LPDAGPRLPAEHLR					Mascot
1814.8345	1814.9757	0.1412	78	64	80	DGPGEPGGQSWTAEWLK					Mascot
1837.8644	1838.0114	0.147	80	114	128	VYAEKYAEDQEAFFK					Mascot
1983.0328	1982.9631	-0.0697	-35	28	46	MGLDDKEIVALSGAHTLGR					Mascot
2004.8611	2004.9878	0.1267	63	119	135	YAEDQEAFFKDYAEAHA					Mascot
2716.2314	2716.3118	0.0804	30	64	87	DGPGEPGGQSWTAEWLKFDNSYFK					Mascot

7 Disease resistance protein RPM1 [Triticum urartu] gi|474256457 143409.9 8.38 24 50 52.616 24.407

Peptide Information

Calc. Mass	Obsrv. Mass	± da	± ppm	Start Seq.	End Seq.	Sequence	Ion Score	C. I. %	Modification	Rank	Result Type
911.4417	911.4224	-0.0193	-21	144	149	RCPHWR			Carbamidomethyl (C)[2]		Mascot
1022.5087	1022.4612	-0.0475	-46	670	677	CFEALINR			Carbamidomethyl (C)[1]		Mascot
1048.5463	1048.4572	-0.0891	-85	29	37	WYGVEVPAK					Mascot
1050.5619	1050.4625	-0.0994	-95	298	305	FTHEYIIK					Mascot
1132.4575	1132.4781	0.0206	18	313	322	GHMEEDADTK					Mascot
1145.6565	1145.557	-0.0995	-87	337	346	EVFPPVTTKK					Mascot
1204.6473	1204.5304	-0.1169	-97	28	37	RWYGVEVPAK					Mascot
1262.6959	1262.6104	-0.0855	-68	778	787	DICRTMLLLK			Carbamidomethyl (C)[3]		Mascot
1508.7996	1508.7277	-0.0719	-48	1157	1168	LEKIIWSFTEDK					Mascot

Calc. Mass	Obsrv. Mass	± da	± ppm	Start Seq.	End Seq.	Sequence	Ion Score	C. I. %	Modification	Rank	Result Type
1525.7065	1525.7373	0.0308	20	611	622	KMFMFSYSDLPK			Oxidation (M)[2,4]		Mascot
1525.822	1525.7373	-0.0847	-56	398	411	KPGPAELQEDKSVK					Mascot
1587.8047	1587.6609	-0.1438	-91	395	408	TMKKPGPAELQEDK			Oxidation (M)[2]		Mascot
1626.881	1626.8962	0.0152	9	794	807	TDVKQLPSEINNLR					Mascot
1813.9113	1813.9677	0.0564	31	382	397	SVHKPGAELQEDKTMK			Oxidation (M)[15]		Mascot
1814.9899	1814.9757	-0.0142	-8	1181	1196	LKELEFNGDLIPGELK					Mascot
1831.9623	1831.9717	0.0094	5	209	223	WKLLDEVTEQIQAMK					Mascot
1831.9623	1831.9717	0.0094	5	209	223	WKLLDEVTEQIQAMK					Mascot
1837.8307	1838.0114	0.1807	98	452	467	GIMDKIEDCLTGQDDK			Carbamidomethyl (C)[9]		Mascot
1845.8186	1845.9391	0.1205	65	612	625	MFMFSYSDLPKEYR			Oxidation (M)[1,3]		Mascot
1853.8256	1853.9221	0.0965	52	452	467	GIMDKIEDCLTGQDDK			Carbamidomethyl (C)[9], Oxidation (M)[3]		Mascot
1988.0613	1988.0076	-0.0537	-27	718	733	LSHYLARHFSIFNNLR					Mascot
1990.0161	1990.0204	0.0043	2	211	227	LLDEVTEQIQAMKVDNK			Oxidation (M)[12]		Mascot
1994.9537	1994.9786	0.0249	12	487	504	NAFSMFGCIAGALMLTFK			Carbamidomethyl (C)[8], Oxidation (M)[5]		Mascot
2037.1273	2037.0439	-0.0834	-41	982	998	QDNLKVLNQLPNIVCLR			Carbamidomethyl (C)[15]		Mascot
2071.0527	2070.9912	-0.0615	-30	539	555	NHMLEDNPKIYLDILDK					Mascot
2071.0527	2070.9912	-0.0615	-30	539	555	NHMLEDNPKIYLDILDK					Mascot
2087.0479	2087.0125	-0.0354	-17	539	555	NHMLEDNPKIYLDILDK			Oxidation (M)[3]		Mascot
2323.1284	2323.1377	0.0093	4	484	504	DTKNAFSMFGCIAGALML TFK			Carbamidomethyl (C)[11]		Mascot

8 Protein furry homolog-like protein [Triticum urartu] gi|474346744 243185.5 6.08 28 48 26.61 17.26

Peptide Information

Calc. Mass	Obsrv. Mass	± da	± ppm	Start Seq.	End Seq.	Sequence	Ion Score	C. I. %	Modification	Rank	Result Type
911.4404	911.4224	-0.018	-20	577	583	DMQSFKR					Mascot
916.4846	916.4686	-0.016	-17	2108	2115	DVALQNTR					Mascot
981.4999	981.4382	-0.0617	-63	600	607	SKDISEFR					Mascot
1040.4288	1040.4735	0.0447	43	1670	1677	WAMECTSR			Carbamidomethyl (C)[5]		Mascot
1050.5653	1050.4625	-0.1028	-98	2065	2073	MLLPQSSFK					Mascot
1052.5524	1052.4727	-0.0797	-76	151	158	FFIELNNR					Mascot
1052.5524	1052.4727	-0.0797	-76	151	158	FFIELNNR					Mascot
1066.5602	1066.4674	-0.0928	-87	2065	2073	MLLPQSSFK			Oxidation (M)[1]		Mascot
1078.5487	1078.5057	-0.043	-40	1808	1816	LESRTTSER					Mascot
1118.5082	1118.5089	0.0007	1	311	319	SMALDCLHR			Carbamidomethyl (C)[6], Oxidation (M)[2]		Mascot
1204.6321	1204.5304	-0.1017	-84	1258	1267	ESGWSERLLK					Mascot
1231.698	1231.6027	-0.0953	-77	558	566	LVELMRLWR			Oxidation (M)[5]		Mascot
1247.6412	1247.6113	-0.0299	-24	167	177	SETLNIINGMR					Mascot

1262.6814	1262.6104	-0.071	-56	2	12	VEMLGISWVTK					Mascot
1300.6678	1300.7076	0.0398	31	1134	1143	QEIPKCEIQR			Carbamidomethyl (C)[6]		Mascot
1490.7122	1490.7324	0.0202	14	1591	1602	HLELYEVESSER					Mascot
1525.7111	1525.7373	0.0262	17	308	319	NHRSMALDCLHR	4	0	Carbamidomethyl (C)[9], Oxidation (M)[5]		Mascot
1525.7502	1525.7373	-0.0129	-8	68	80	LAVECIFSSACIR			Carbamidomethyl (C)[5,11]		Mascot
1547.8615	1547.7114	-0.1501	-97	2061	2073	VLERMLLPQSSFK					Mascot
1618.8218	1618.8385	0.0167	10	1780	1793	DRTTENVLLSSMPR					Mascot
1643.9016	1643.8926	-0.009	-5	795	808	AARDIFHTIFPSLR					Mascot
1646.7843	1646.8035	0.0192	12	1620	1633	GSLMWENEDPTLVR					Mascot
1734.793	1734.8104	0.0174	10	51	66	ESESPKGAHDASTYQK					Mascot
1814.0317	1813.9677	-0.064	-35	1689	1704	ALRPSVKSDSCVLLLR			Carbamidomethyl (C)[11]		Mascot
1818.8804	1818.8752	-0.0052	-3	1619	1633	RGSLMWENEDPTLVR			Oxidation (M)[5]		Mascot
1885.9034	1885.87	-0.0334	-18	567	582	ACLSEELLAKDMQSFK			Carbamidomethyl (C)[2], Oxidation (M)[12]		Mascot
1988.0126	1988.0076	-0.005	-3	1455	1473	SGQLLPALMNMSGPLMG VR			Oxidation (M)[9]		Mascot
2038.8958	2039.0055	0.1097	54	1212	1227	DHPELSEHLCEEIMQR			Carbamidomethyl (C)[10], Oxidation (M)[14]		Mascot
2054.1069	2053.9731	-0.1338	-65	921	938	YALASVIRYLAPEFVDAR					Mascot
2054.1069	2053.9731	-0.1338	-65	921	938	YALASVIRYLAPEFVDAR					Mascot
2070.0549	2069.9834	-0.0715	-35	725	742	YAAELCPASVHVARQEL R			Carbamidomethyl (C)[6]		Mascot

9	Ubiquitin carboxyl-terminal hydrolase 18 [Triticum urartu]	gi|473990377	48353.8	5.91	12	44	0	3.171	

Peptide Information

Calc. Mass	Obsrv. Mass	± da	± ppm	Start Seq.	End Seq.	Sequence	Ion Score	C. I. %	Modification	Rank	Result Type
924.5084	924.4166	-0.0918	-99	73	80	KPMGHLNK					Mascot
990.4131	990.4738	0.0607	61	250	257	CEGCSLHK			Carbamidomethyl (C)[1,4]		Mascot
1118.5082	1118.5089	0.0007	1	250	258	CEGCSLHKK			Carbamidomethyl (C)[1,4]		Mascot
1132.4471	1132.4781	0.031	27	235	243	ECMDAFTMK			Carbamidomethyl (C)[2]		Mascot
1249.6497	1249.6124	-0.0373	-30	214	223	DFQMLEIPIK			Oxidation (M)[4]		Mascot
1249.6497	1249.6124	-0.0373	-30	214	223	DFQMLEIPIK			Oxidation (M)[4]		Mascot
1587.671	1587.6609	-0.0101	-6	338	349	YNGEWYEINDER					Mascot
1669.8037	1669.8751	0.0714	43	81	94	SLNCYANSVLQCLK			Carbamidomethyl (C)[4,12]		Mascot
1740.7633	1740.833	0.0697	40	44	58	MENGEDDLVFGELEK			Oxidation (M)[1]		Mascot
1813.8505	1813.9677	0.1172	65	19	33	DLHSDLHNWAIYDSK					Mascot
1845.9396	1845.9391	-0.0005	0	314	328	YDLYGVIHHRQYGPK					Mascot
1859.8448	1859.9668	0.122	66	235	249	ECMDAFTMKKPLNSR			Carbamidomethyl (C)[2], Oxidation (M)[3,8]		Mascot

Calc. Mass	Obsrv. Mass	± da	± ppm	Start Seq.	End Seq.	Sequence	Ion Score	C. I. %	Modification	Rank	Result Type
1885.9364	1885.87	-0.0664	-35	159	175	LGSQFSADEFLCAILSK			Carbamidomethyl (C)[12]		Mascot

10	Expansin-B9 [Triticum urartu]	gi|474118794	27694.9	9.07	9	44	0	1.649	

Peptide Information

Calc. Mass	Obsrv. Mass	± da	± ppm	Start Seq.	End Seq.	Sequence	Ion Score	C. I. %	Modification	Rank	Result Type
919.4955	919.4149	-0.0806	-88	215	222	LTTESGRR					Mascot
924.4244	924.4166	-0.0078	-8	141	148	CQYAPGTK			Carbamidomethyl (C)[1]		Mascot
1010.4611	1010.3917	-0.0694	-69	113	122	SFGAMAEPGK			Oxidation (M)[5]		Mascot
1060.6183	1060.5332	-0.0851	-80	1	9	MALEKSLLR					Mascot
1064.4248	1064.4897	0.0649	61	79	87	CTNHSACSK			Carbamidomethyl (C)[1,7]		Mascot
1084.4663	1084.4668	0.0005	0	70	78	GCGSCFQIR			Carbamidomethyl (C)[2,5]		Mascot
1247.6783	1247.6113	-0.067	-54	204	214	VEPLVGPFSFR					Mascot
1649.7952	1649.8871	0.0919	56	113	127	SFGAMAEPGKEQELR					Mascot
1669.8003	1669.8751	0.0748	45	107	122	FDLSGRSFGAMAEPGK					Mascot

Gel Idx/Pos	213/I13	Instr./Gel Origin	BA2151/Sample Project 20140814	Process Status	Analysis Succeeded
Plate [#] Name	[1] Sample Project 20140814	Instrument Sample Name		Spectra	11

Rank	Protein Name	Accession No.	Protein MW	Protein PI	Pep. Count	Protein Score	Protein Score C. I. %	Intensity Matched	Total Ion Score	Total Ion C. I. %	Confirmed
1	Sucrose synthase 2 [Triticum urartu]	gi|474169938	114503.8	6.95	20	53	76.252	14.323			

Peptide Information

Calc. Mass	Obsrv. Mass	± da	± ppm	Start Seq.	End Seq.	Sequence	Ion Score	C. I. %	Modification	Rank	Result Type
858.4791	858.444	-0.0351	-41	9	15	TPSIRER					Mascot
960.4883	960.4428	-0.0455	-47	803	810	EEIAEIEK					Mascot
975.5006	975.538	0.0374	38	826	833	WISAQTNR					Mascot
978.5115	978.5276	0.0161	16	834	841	ARNGELYR					Mascot
985.5676	985.5165	-0.0511	-52	310	318	KQGLDVTPK					Mascot
1072.5027	1072.6053	0.1026	96	176	184	GHVMMLNDR					Mascot
1072.5027	1072.6053	0.1026	96	176	184	GHVMMLNDR					Mascot
1145.5739	1145.5726	-0.0013	-1	817	825	TYNLFGQFR					Mascot
1145.5739	1145.5726	-0.0013	-1	817	825	TYNLFGQFR					Mascot
1188.6776	1188.6024	-0.0752	-63	346	355	NSLALIWPFK					Mascot
1196.5986	1196.5959	-0.0027	-2	518	526	FDVWPYLEK					Mascot
1202.6389	1202.6088	-0.0301	-25	826	835	WISAQTNRAR					Mascot
1213.6432	1213.6439	0.0007	1	551	559	ICTIYIMKR			Carbamidomethyl (C)[2], Oxidation (M)[7]		Mascot
1220.5801	1220.6034	0.0233	19	481	490	GTSCNQRLER			Carbamidomethyl (C)[4]		Mascot
1242.7065	1242.6113	-0.0952	-77	150	159	HLSSILFRNR					Mascot
1357.6239	1357.6716	0.0477	35	461	471	TPSCVESFMRK			Carbamidomethyl (C)[4], Oxidation (M)[9]		Mascot
1369.6464	1369.6947	0.0483	35	174	184	HKGHVMMLNDR			Oxidation (M)[6,7]		Mascot
1375.7443	1375.7534	0.0091	7	977	986	RYLEMFYILK					Mascot
1390.7035	1390.6556	-0.0479	-34	160	170	DCLEPLLDFLR			Carbamidomethyl (C)[2]		Mascot
1455.7512	1455.6522	-0.099	-68	765	777	VKNMTGLVEAYSK			Oxidation (M)[4]		Mascot
1738.8951	1738.8336	-0.0615	-35	514	526	WISRFDVWPYLEK					Mascot
1985.0549	1985.0256	-0.0293	-15	25	42	NELVALLSKSSDPLDEVR					Mascot

Rank	Protein Name	Accession No.	Protein MW	Protein PI	Pep. Count	Protein Score	Protein Score C. I. %	Intensity Matched	Total Ion Score	Total Ion C. I. %	Confirmed
2	TPR repeat-containing thioredoxin TTL1 [Triticum urartu]	gi|473951385	58817	8.74	16	52	73.96	7.61			

Peptide Information

Calc. Mass	Obsrv. Mass	± da	± ppm	Start Seq.	End Seq.	Sequence	Ion Score	C. I. %	Modification	Rank	Result Type

Calc. Mass	Obsrv. Mass	± da	± ppm	Start Seq.	End Seq.	Sequence	Ion Score	C. I. %	Modification	Rank	Result Type
805.493	805.4406	-0.0524	-65	108	114	LAGLYLR					Mascot
858.4138	858.444	0.0302	35	364	370	RAMSYSK			Oxidation (M)[3]		Mascot
906.468	906.4659	-0.0021	-2	295	302	AQGNELFK					Mascot
960.4931	960.4428	-0.0503	-52	235	243	AQVDMALGR					Mascot
1037.5415	1037.5117	-0.0298	-29	40	47	LGNEWYKK					Mascot
1070.5299	1070.5306	0.0007	1	365	373	AMSYSKLDR					Mascot
1086.5248	1086.5631	0.0383	35	365	373	AMSYSKLDR			Oxidation (M)[2]		Mascot
1168.6909	1168.6206	-0.0703	-60	72	83	GNRAAALIGLGR					Mascot
1213.5994	1213.6439	0.0445	37	142	151	LQDVEMHLGR			Oxidation (M)[6]		Mascot
1255.6066	1255.5323	-0.0743	-59	517	527	YSVRHYAVSSS					Mascot
1357.6635	1357.6716	0.0081	6	306	318	FSDASIAYGEGLK					Mascot
1419.7577	1419.7521	-0.0056	-4	196	208	LEEADSTLTSLLK					Mascot
1444.739	1444.7241	-0.0149	-10	84	96	LADALRDSEEAVR					Mascot
1743.8442	1743.8684	0.0242	14	142	156	LQDVEMHLGRSTDAR			Oxidation (M)[6]		Mascot
1955.9491	1955.9672	0.0181	9	20	39	SPASSPVAPPSGGSLQE MTR					Mascot
2086.0886	2086.0217	-0.0669	-32	163	183	SALREADAAIAAGADSSQ LLR					Mascot
2264.2544	2264.1421	-0.1123	-50	272	292	LIDPGNAEVGMILNNVRL VAR					Mascot

3 hypothetical protein TRIUR3_06254 [Triticum urartu] gi|474356981 10405.1 6.71 7 51 67.218 11.287

Peptide Information

Calc. Mass	Obsrv. Mass	± da	± ppm	Start Seq.	End Seq.	Sequence	Ion Score	C. I. %	Modification	Rank	Result Type
832.4199	832.3696	-0.0503	-60	29	35	SWVSPEK					Mascot
887.4846	887.4902	0.0056	6	67	73	QSRFPPR					Mascot
960.5149	960.4428	-0.0721	-75	29	36	SWVSPEKK					Mascot
1037.4786	1037.5117	0.0331	32	85	93	FDLAEEGEK					Mascot
1094.5001	1094.5256	0.0255	23	85	94	FDLAEEGEKG					Mascot
1094.5001	1094.5256	0.0255	23	85	94	FDLAEEGEKG					Mascot
1214.6376	1214.5305	-0.1071	-88	74	84	EEGLEPRASVK					Mascot
1422.7111	1422.7657	0.0546	38	81	93	ASVKFDLAEEGEK					Mascot
1422.7111	1422.7657	0.0546	38	81	93	ASVKFDLAEEGEK					Mascot

4 hypothetical protein TRIUR3_33368 [Triticum urartu] gi|474005303 10109.3 11.36 7 51 64.055 2.713

Peptide Information

Calc. Mass	Obsrv. Mass	± da	± ppm	Start Seq.	End Seq.	Sequence	Ion Score	C. I. %	Modification	Rank	Result Type

Calc. Mass	Obsrv. Mass	± da	± ppm	Start Seq.	End Seq.	Sequence	Ion Score	C. I. %	Modification	Rank	Result Type
960.4857	960.4428	-0.0429	-45	54	62	SSRSSPPSR					Mascot
1254.6624	1254.5894	-0.073	-58	1	13	MAPPALNLAGSGR					Mascot
1272.6543	1272.5577	-0.0966	-76	18	30	SAENGSIPAISAR					Mascot
1444.7906	1444.7241	-0.0665	-46	31	43	IRPGLLPYDASSR					Mascot
1462.8489	1462.7073	-0.1416	-97	71	82	KLHQILQEAQVR					Mascot
1490.855	1490.7338	-0.1212	-81	72	83	LHQILQEAQVRR					Mascot
1743.9071	1743.8684	-0.0387	-22	1	17	MAPPALNLAGSGRHHAK			Oxidation (M)[1]		Mascot

5 hypothetical protein TRIUR3_06020 [Triticum urartu] gi|474060146 49092.5 8.21 13 46 1 9.885

Peptide Information

Calc. Mass	Obsrv. Mass	± da	± ppm	Start Seq.	End Seq.	Sequence	Ion Score	C. I. %	Modification	Rank	Result Type
836.4737	836.4016	-0.0721	-86	433	438	YTIQRR					Mascot
870.4792	870.5242	0.045	52	127	134	TAKTHGQK					Mascot
900.5261	900.4498	-0.0763	-85	426	432	QVKEQLR					Mascot
913.4156	913.4791	0.0635	70	57	64	SRSMSSSR			Oxidation (M)[4]		Mascot
1019.6108	1019.5519	-0.0589	-58	73	80	LEHRPLVR					Mascot
1206.6589	1206.6058	-0.0531	-44	429	437	EQLRYTIQR					Mascot
1247.6024	1247.6299	0.0275	22	39	50	MMGGAPGVAWVR			Oxidation (M)[1]		Mascot
1255.5203	1255.5323	0.012	10	1	11	MACGMAAWAMR			Carbamidomethyl (C)[3]		Mascot
1271.5153	1271.5457	0.0304	24	1	11	MACGMAAWAMR			Carbamidomethyl (C)[3], Oxidation (M)[1]		Mascot
1272.6543	1272.5577	-0.0966	-76	175	186	AAKEEADQLAAR					Mascot
1404.6398	1404.7189	0.0791	56	142	157	VRGNGGGGEGGAGGMR			Oxidation (M)[15]		Mascot
1490.7356	1490.7338	-0.0018	-1	37	50	SRMMGGAPGVAWVR			Oxidation (M)[3]		Mascot
1926.8442	1926.8766	0.0324	17	39	55	MMGGAPGVAWVRNCCT K			Carbamidomethyl (C)[14,15], Oxidation (M)[1,2]		Mascot
3079.6206	3079.4966	-0.124	-40	396	425	DGLAMVLADPQPRATSV GGNDVQSVLLALR			Oxidation (M)[5]		Mascot

6 Phosphoenolpyruvate carboxylase, housekeeping isozyme [Triticum urartu] gi|474167022 121290.5 6.03 19 46 0 15.446

Peptide Information

Calc. Mass	Obsrv. Mass	± da	± ppm	Start Seq.	End Seq.	Sequence	Ion Score	C. I. %	Modification	Rank	Result Type
807.3519	807.4277	0.0758	94	475	480	EWSEEK					Mascot
860.4301	860.4647	0.0346	40	733	738	FVEYFR					Mascot
975.5258	975.538	0.0122	13	725	732	SIVFQEPR					Mascot
989.5197	989.5119	-0.0078	-8	749	757	MNIGSRPSK					Mascot
1025.5487	1025.4946	-0.0541	-53	338	346	ADQLHRASK					Mascot

1070.6317	1070.5306	-0.1011	-94	800	808	DIRNLQALK					Mascot
1072.5422	1072.6053	0.0631	59	359	367	QVPPSEPYR					Mascot
1072.5422	1072.6053	0.0631	59	359	367	QVPPSEPYR					Mascot
1099.5571	1099.6479	0.0908	83	606	614	FSAGWQLYK					Mascot
1106.6681	1106.5641	-0.104	-94	864	873	QLLLQVAGHK					Mascot
1139.6572	1139.5858	-0.0714	-63	493	502	RPLFGPDLPK					Mascot
1145.5909	1145.5726	-0.0183	-16	448	456	LDIRQESER					Mascot
1145.5909	1145.5726	-0.0183	-16	448	456	LDIRQESER					Mascot
1172.6357	1172.613	-0.0227	-19	1051	1059	FVHRMVDIR					Mascot
1188.6306	1188.6024	-0.0282	-24	1051	1059	FVHRMVDIR			Oxidation (M)[5]		Mascot
1206.6842	1206.6058	-0.0784	-65	437	447	QVSTFGLSLVR					Mascot
1313.6042	1313.714	0.1098	84	590	601	QEVMIGYSDSGK					Mascot
1369.7012	1369.6947	-0.0065	-5	576	585	LFSVEWYRNR					Mascot
1404.7166	1404.7189	0.0023	2	630	642	LTMFHGRGGTVGR			Oxidation (M)[3]		Mascot
1703.9803	1703.9159	-0.0644	-38	437	451	QVSTFGLSLVRLDIR					Mascot
1703.9803	1703.9159	-0.0644	-38	437	451	QVSTFGLSLVRLDIR					Mascot
1743.7928	1743.8684	0.0756	43	710	724	ALMDEMAVVATEEYR			Oxidation (M)[3]		Mascot
2086.0215	2086.0217	0.0002	0	803	818	NLQALKEMYNEWPFFR					Mascot

7 hypothetical protein TRIUR3_09054 [Triticum urartu] gi|474315691 36924.6 9.04 12 46 0 9.172

Peptide Information

Calc. Mass	Obsrv. Mass	± da	± ppm	Start Seq.	End Seq.	Sequence	Ion Score	C. I. %	Modification	Rank	Result Type
805.3695	805.4406	0.0711	88	120	125	HMLCTK			Carbamidomethyl (C)[4], Oxidation (M)[2]		Mascot
836.4009	836.4016	0.0007	1	143	148	EFDNRR					Mascot
870.4427	870.5242	0.0815	94	292	298	QENNPIR					Mascot
949.485	949.5055	0.0205	22	141	147	IREFDNR					Mascot
1139.6281	1139.5858	-0.0423	-37	292	300	QENNPIRIR					Mascot
1172.6998	1172.613	-0.0868	-74	108	119	KLLSGNAAVATK					Mascot
1198.6038	1198.6031	-0.0007	-1	177	185	YNVLFLCNR			Carbamidomethyl (C)[7]		Mascot
1259.6276	1259.6913	0.0637	51	99	108	LSFFCVACKK			Carbamidomethyl (C)[5,8]		Mascot
1313.6056	1313.714	0.1084	83	218	228	AGRYFPACTDR			Carbamidomethyl (C)[8]		Mascot
1365.7314	1365.7449	0.0135	10	82	93	HPVWEGFAVPVK					Mascot
1743.9363	1743.8684	-0.0679	-39	173	185	LLYRYNVLFLCNR			Carbamidomethyl (C)[11]		Mascot
1985.0426	1985.0256	-0.017	-9	259	275	FKATVEAMFSIVHHVPR			Oxidation (M)[8]		Mascot

8 RING finger and CHY zinc finger domain-containing protein 1 [Triticum urartu] gi|474441132 30109.9 6.16 9 45 0 3.067

Peptide Information

Calc. Mass	Obsrv. Mass	± da	± ppm	Start Seq.	End Seq.	Sequence	Ion Score	C. I. %	Modification	Rank	Result Type
864.3668	864.4263	0.0595	69	97	102	EQYHCK			Carbamidomethyl (C)[5]		Mascot
913.405	913.4791	0.0741	81	70	76	FFDDDVR					Mascot
978.4825	978.5276	0.0451	46	227	235	VYPGMAGQR					Mascot
1106.5775	1106.5641	-0.0134	-12	227	236	VYPGMAGQRK					Mascot
1202.5293	1202.6088	0.0795	66	121	130	CGSCYSVTLR			Carbamidomethyl (C)[1,4]		Mascot
1272.5679	1272.5577	-0.0102	-8	67	76	ACKFFDDDVR			Carbamidomethyl (C)[2]		Mascot
1375.7579	1375.7534	-0.0045	-3	196	207	FLRALDAEIEAK					Mascot
2263.9492	2264.1421	0.1929	85	178	195	HDKFTCPMCSVSIFDMEK			Carbamidomethyl (C)[6,9], Oxidation (M)[8,16]		Mascot
3079.3418	3079.4966	0.1548	50	133	156	HVCIEDSMKNNCPICYEY LFDSLR			Carbamidomethyl (C)[3,12,15], Oxidation (M)[8]		Mascot

9 hypothetical protein TRIUR3_27818 [Triticum urartu] gi|473923671 34692.6 8.92 9 44 0 12.316 13 0

Peptide Information

Calc. Mass	Obsrv. Mass	± da	± ppm	Start Seq.	End Seq.	Sequence	Ion Score	C. I. %	Modification	Rank	Result Type
985.5023	985.5165	0.0142	14	279	286	KSFSEMIK			Oxidation (M)[6]		Mascot
1072.5496	1072.6053	0.0557	52	155	163	LAFGTCYLK			Carbamidomethyl (C)[6]		Mascot
1072.5496	1072.6053	0.0557	52	155	163	LAFGTCYLK			Carbamidomethyl (C)[6]		Mascot
1078.5674	1078.535	-0.0324	-30	19	28	GGTLSCISRK			Carbamidomethyl (C)[6]		Mascot
1078.5674	1078.535	-0.0324	-30	18	27	KGGTLSCISR	15	0	Carbamidomethyl (C)[7]		Mascot
1178.5988	1178.5955	-0.0033	-3	296	305	SRPLLCGFQT			Carbamidomethyl (C)[6]		Mascot
1202.6562	1202.6088	-0.0474	-39	180	190	MASGLIQLQNK					Mascot
1255.5922	1255.5323	-0.0599	-48	112	121	LRQEGYCMAK			Carbamidomethyl (C)[7]		Mascot
1271.5872	1271.5457	-0.0415	-33	112	121	LRQEGYCMAK			Carbamidomethyl (C)[7], Oxidation (M)[8]		Mascot
1365.6508	1365.7449	0.0941	69	94	105	LATWGGMPYPEK			Oxidation (M)[7]		Mascot
1703.7694	1703.9159	0.1465	86	1	16	MEEAATGAGEPWVAER					Mascot
1703.7694	1703.9159	0.1465	86	1	16	MEEAATGAGEPWVAER					Mascot

10 hypothetical protein TRIUR3_20587 [Triticum urartu] gi|474154164 31473.2 5.73 9 44 0 13.172

Peptide Information

Calc. Mass	Obsrv. Mass	± da	± ppm	Start Seq.	End Seq.	Sequence	Ion Score	C. I. %	Modification	Rank	Result Type
1106.5146	1106.5641	0.0495	45	38	47	GTEEAMSLPR			Oxidation (M)[6]		Mascot
1172.6746	1172.613	-0.0616	-53	98	108	ATLRSANLQAK					Mascot

1193.5004	1193.6086	0.1082	91	183	192	MAEGEYQGHR	Oxidation (M)[1]	Mascot
1214.6488	1214.5305	-0.1183	-97	109	119	VNIGKAAQEER		Mascot
1242.7164	1242.6113	-0.1051	-85	102	113	SANLQAKVNIGK		Mascot
1422.6716	1422.7657	0.0941	66	35	47	MGKGTEEAMSLPR	Oxidation (M)[1]	Mascot
1422.6716	1422.7657	0.0941	66	35	47	MGKGTEEAMSLPR	Oxidation (M)[1]	Mascot
1438.6665	1438.6818	0.0153	11	35	47	MGKGTEEAMSLPR	Oxidation (M)[1,9]	Mascot
1743.96	1743.8684	-0.0916	-53	254	271	LADAIRSGSVSAGDILAK		Mascot
1985.0233	1985.0256	0.0023	1	48	65	LNGSAQDALQLLNALQC R	Carbamidomethyl (C)[17]	Mascot
2058.0461	2058.0051	-0.041	-20	114	132	AAQEERELLLGGGEESTI R		Mascot
2058.0461	2058.0051	-0.041	-20	114	132	AAQEERELLLGGGEESTI R		Mascot

Gel Idx/Pos	214/I14	Instr./Gel Origin	BA2151/Sample Project 20140814	Process Status	Analysis Succeeded
Plate [#] Name	[1] Sample Project 20140814	Instrument Sample Name		Spectra	11

Rank	Protein Name	Accession No.	Protein MW	Protein PI	Pep. Count	Protein Score	Protein Score C. I. %	Intensity Matched	Total Ion Score	Total Ion C. I. %	Confirmed
1	hypothetical protein TRIUR3_22532 [Triticum urartu]	gi|473797438	36043.2	5.13	10	350	100	44.449	303	100	

Peptide Information

Calc. Mass	Obsrv. Mass	± da	± ppm	Start Seq.	End Seq.	Sequence	Ion Score	C. I. %	Modification	Rank	Result Type
892.4022	892.4059	0.0037	4	113	118	EFMFYR					Mascot
908.3971	908.395	-0.0021	-2	113	118	EFMFYR			Oxidation (M)[3]		Mascot
908.3971	908.395	-0.0021	-2	113	118	EFMFYR	25	73.488	Oxidation (M)[3]		Mascot
963.5873	963.5838	-0.0035	-4	99	107	TALAFVTLK					Mascot
965.4761	965.4636	-0.0125	-13	191	198	DGIMSIWK			Oxidation (M)[4]		Mascot
1209.674	1209.6798	0.0058	5	180	190	LPLWPSAQAAR					Mascot
1209.674	1209.6798	0.0058	5	180	190	LPLWPSAQAAR	99	100			Mascot
1451.6948	1451.6985	0.0037	3	167	179	SAGILCSYDPNVR			Carbamidomethyl (C)[6]		Mascot
1451.6948	1451.6985	0.0037	3	167	179	SAGILCSYDPNVR	57	99.984	Carbamidomethyl (C)[6]		Mascot
1792.9052	1792.9064	0.0012	1	141	155	IFHYGSISLITEPCR			Carbamidomethyl (C)[14]		Mascot
1792.9052	1792.9064	0.0012	1	141	155	IFHYGSISLITEPCR	85	100	Carbamidomethyl (C)[14]		Mascot
1798.8463	1798.806	-0.0403	-22	295	310	EVLQFSNACGAICTTK			Carbamidomethyl (C)[9,13]		Mascot
1895.1212	1895.0588	-0.0624	-33	311	329	KGAIPALPTTATALELISK					Mascot
2068.0315	2068.0317	0.0002	0	293	310	LREVLQFSNACGAICTTK			Carbamidomethyl (C)[11,15]		Mascot
2068.0315	2068.0317	0.0002	0	293	310	LREVLQFSNACGAICTTK	39	99.017	Carbamidomethyl (C)[11,15]		Mascot
2128.0955	2128.0962	0.0007	0	119	137	NPSADMLLTEAELNLDLIR					Mascot
2144.0903	2144.0696	-0.0207	-10	119	137	NPSADMLLTEAELNLDLIR			Oxidation (M)[6]		Mascot

Rank	Protein Name	Accession No.	Protein MW	Protein PI	Pep. Count	Protein Score	Protein Score C. I. %	Intensity Matched	Total Ion Score	Total Ion C. I. %	Confirmed
2	Fructokinase-2 [Triticum urartu]	gi|474190636	42253.2	4.78	11	212	100	27.743	164	100	

Peptide Information

Calc. Mass	Obsrv. Mass	± da	± ppm	Start Seq.	End Seq.	Sequence	Ion Score	C. I. %	Modification	Rank	Result Type
842.4301	842.4733	0.0432	51	251	258	SAHVAAMR					Mascot
858.425	858.4254	0.0004	0	251	258	SAHVAAMR			Oxidation (M)[7]		Mascot
892.4022	892.4059	0.0037	4	208	213	EFMFYR					Mascot
908.3971	908.395	-0.0021	-2	208	213	EFMFYR			Oxidation (M)[3]		Mascot
908.3971	908.395	-0.0021	-2	208	213	EFMFYR	25	73.488	Oxidation (M)[3]		Mascot

Calc. Mass	Obsrv. Mass	± da	± ppm	Start Seq.	End Seq.	Sequence	Ion Score	C. I. %	Modification	Rank	Result Type
963.5873	963.5838	-0.0035	-4	194	202	TALAFVTLK					Mascot
965.4761	965.4636	-0.0125	-13	286	293	DGIMSIWK			Oxidation (M)[4]		Mascot
1305.7202	1305.7113	-0.0089	-7	318	328	NVLSLWFEGLK					Mascot
1451.6948	1451.6985	0.0037	3	262	274	SAGILCSYDPNVR			Carbamidomethyl (C)[6]		Mascot
1451.6948	1451.6985	0.0037	3	262	274	SAGILCSYDPNVR	57	99.984	Carbamidomethyl (C)[6]		Mascot
1735.8361	1735.871	0.0349	20	163	177	FGDDEFGHMLVDILK					Mascot
1771.8392	1771.8171	-0.0221	-12	64	80	GNVEVIHGEGAQMSATR			Oxidation (M)[13]		Mascot
1792.9052	1792.9064	0.0012	1	236	250	IFHYGSISLITEPCR			Carbamidomethyl (C)[14]		Mascot
1792.9052	1792.9064	0.0012	1	236	250	IFHYGSISLITEPCR	85	100	Carbamidomethyl (C)[14]		Mascot
2127.04	2127.0386	-0.0014	-1	61	80	WGKGNVEVIHGEGAQM SATR					Mascot
2128.0955	2128.0962	0.0007	0	214	232	NPSADMLLTEAELNLDLI R					Mascot
2144.0903	2144.0696	-0.0207	-10	214	232	NPSADMLLTEAELNLDLI R			Oxidation (M)[6]		Mascot

3 hypothetical protein TRIUR3_24487 [Triticum urartu] gi|474171341 22815.6 5.27 11 195 100 25.276 124 100

Peptide Information

Calc. Mass	Obsrv. Mass	± da	± ppm	Start Seq.	End Seq.	Sequence	Ion Score	C. I. %	Modification	Rank	Result Type
842.4301	842.4733	0.0432	51	33	40	SAHVAAMR					Mascot
858.425	858.4254	0.0004	0	33	40	SAHVAAMR			Oxidation (M)[7]		Mascot
965.4761	965.4636	-0.0125	-13	68	75	DGIMSIWK			Oxidation (M)[4]		Mascot
987.5721	987.5706	-0.0015	-2	111	119	LLIVTDGEK					Mascot
1201.5372	1201.5342	-0.003	-2	160	169	DDSIFYNEAK					Mascot
1305.7202	1305.7113	-0.0089	-7	100	110	NVLSLWFEGLK					Mascot
1360.7253	1360.687	-0.0383	-28	111	122	LLIVTDGEKGCR			Carbamidomethyl (C)[11]		Mascot
1792.9052	1792.9064	0.0012	1	18	32	IFHYGSISLITEPCR			Carbamidomethyl (C)[14]		Mascot
1792.9052	1792.9064	0.0012	1	18	32	IFHYGSISLITEPCR	85	100	Carbamidomethyl (C)[14]		Mascot
1798.8463	1798.806	-0.0403	-22	172	187	EVLQFSNACGAICTTK			Carbamidomethyl (C)[9,13]		Mascot
1815.9998	1815.8248	-0.175	-96	1	15	MLLTEAELNLDLIRR			Oxidation (M)[1]		Mascot
1895.1212	1895.0588	-0.0624	-33	188	206	KGAIPALPTTATALELISK					Mascot
2068.0315	2068.0317	0.0002	0	170	187	LREVLQFSNACGAICTTK			Carbamidomethyl (C)[11,15]		Mascot
2068.0315	2068.0317	0.0002	0	170	187	LREVLQFSNACGAICTTK	39	99.017	Carbamidomethyl (C)[11,15]		Mascot

4 Protein strawberry notch-like protein 1 [Triticum urartu] gi|474429003 142165 6.59 20 53 78.834 26.428 12 0

Peptide Information

Calc. Mass	Obsrv. Mass	± da	± ppm	Start Seq.	End Seq.	Sequence	Ion Score	C. I. %	Modification	Rank	Result Type

Calc. Mass	Obsrv. Mass	± da	± ppm	Start Seq.	End Seq.	Sequence	Ion Score	C. I. %	Modification	Rank	Result Type
844.3691	844.4085	0.0394	47	495	501	MTNMYGK					Mascot
860.3641	860.4041	0.04	46	495	501	MTNMYGK			Oxidation (M)[1]		Mascot
868.4345	868.3714	-0.0631	-73	425	431	NLGYMVR			Oxidation (M)[5]		Mascot
873.5152	873.4371	-0.0781	-89	626	632	VTEIQRK					Mascot
911.3928	911.4236	0.0308	34	847	853	QQFMDDK					Mascot
987.5556	987.5706	0.015	15	934	940	IWKPKCR			Carbamidomethyl (C)[6]		Mascot
1009.5611	1009.5046	-0.0565	-56	989	996	RALTMVYR					Mascot
1182.6127	1182.6022	-0.0105	-9	222	231	HLHHLPTGDR					Mascot
1209.6951	1209.6798	-0.0153	-13	1054	1064	LLGLAPDIQNR					Mascot
1209.6951	1209.6798	-0.0153	-13	1054	1064	LLGLAPDIQNR	12	0			Mascot
1213.6549	1213.6744	0.0195	16	895	904	AIQQFGRTHR					Mascot
1231.6179	1231.6525	0.0346	28	634	644	HSAQDVSFKGR					Mascot
1241.5944	1241.6672	0.0728	59	1039	1049	IVDADMHDVAR					Mascot
1241.5944	1241.6672	0.0728	59	1039	1049	IVDADMHDVAR					Mascot
1390.7069	1390.6998	-0.0071	-5	454	467	GGVGALELVAMDMK					Mascot
1394.7177	1394.6755	-0.0422	-30	232	246	AGFFVGDGAGVGKGR					Mascot
1407.7413	1407.6786	-0.0627	-45	939	950	CRLLFTNLGGEK			Carbamidomethyl (C)[1]		Mascot
1473.7375	1473.6735	-0.064	-43	544	556	HMCMSAKVPAVVK			Carbamidomethyl (C)[3], Oxidation (M)[2]		Mascot
1747.9337	1747.8489	-0.0848	-49	905	919	SNQTSAPQYRILIEK					Mascot
1771.8909	1771.8171	-0.0738	-42	1039	1053	IVDADMHDVARFLNR					Mascot
1806.8182	1806.8787	0.0605	33	832	846	NAKEVSMEMINMHEK			Oxidation (M)[7]		Mascot
1838.8081	1838.8496	0.0415	23	832	846	NAKEVSMEMINMHEK			Oxidation (M)[7,9,12]		Mascot
1919.8779	1919.9467	0.0688	36	495	510	MTNMYGKAAEFWADLR			Oxidation (M)[1]		Mascot
1954.9725	1954.9463	-0.0262	-13	206	221	TLSCLQIETIVYACQR			Carbamidomethyl (C)[4,14]		Mascot

5 hypothetical protein TRIUR3_30538 [Triticum urartu] gi|473882607 197169.7 4.65 27 50 54.748 14.014

Peptide Information

Calc. Mass	Obsrv. Mass	± da	± ppm	Start Seq.	End Seq.	Sequence	Ion Score	C. I. %	Modification	Rank	Result Type
873.4465	873.4371	-0.0094	-11	1376	1382	FDEVVHK					Mascot
874.4265	874.4091	-0.0174	-20	404	412	AVAEDANGK					Mascot
890.4366	890.3925	-0.0441	-50	1278	1285	FSATHAEK					Mascot
904.4622	904.5242	0.062	69	1233	1240	EAEVTDLK					Mascot
937.3931	937.4059	0.0128	14	94	101	DMTEAENK					Mascot
987.5356	987.5706	0.035	35	736	743	EELELNLK					Mascot
997.4836	997.4595	-0.0241	-24	1481	1488	YQSTLEEK					Mascot
1009.604	1009.5046	-0.0994	-98	158	165	LHKLEELK					Mascot

1233.6208	1233.6298	0.009	7	81	90	IEELESEKEK					Mascot
1237.5365	1237.6586	0.1221	99	967	976	ETDCQQLSEK			Carbamidomethyl (C)[4]		Mascot
1337.6583	1337.6886	0.0303	23	979	990	LAEEQVASYEAK					Mascot
1451.7628	1451.6985	-0.0643	-44	639	650	KLEELTAEYEVK					Mascot
1451.7965	1451.6985	-0.098	-68	14	26	EKLVNGNSNLHVK					Mascot
1464.7363	1464.7437	0.0074	5	1665	1677	LTQNVSKSAEMEK					Mascot
1701.9017	1701.8953	-0.0064	-4	831	846	EKSASLEALLAEANQK					Mascot
1747.8167	1747.8489	0.0322	18	195	208	EMENQINNLQEEIK			Oxidation (M)[2]		Mascot
1771.8708	1771.8171	-0.0537	-30	341	356	DEHETLKGTLVDAESK					Mascot
1791.9044	1791.8419	-0.0625	-35	623	637	EKDLMDSLIQITEEK					Mascot
1803.8906	1803.8069	-0.0837	-46	894	909	GKMEEVENDLQALGVR			Oxidation (M)[3]		Mascot
1813.9655	1813.8052	-0.1603	-88	325	340	QQSQILNLQSGLDELK					Mascot
1820.9025	1820.8286	-0.0739	-41	593	608	SKVSHLSDDLEAYQTK					Mascot
1838.9858	1838.8496	-0.1362	-74	1327	1342	LHEALETITQKEAEVK					Mascot
1955.0518	1954.9463	-0.1055	-54	298	313	LLSLQEMEIKLEEHVK			Oxidation (M)[7]		Mascot
1977.0652	1977.0205	-0.0447	-23	1187	1203	LAVHEETIKHLTEELSK					Mascot
1977.0652	1977.0205	-0.0447	-23	1187	1203	LAVHEETIKHLTEELSK					Mascot
2080.0054	2080.093	0.0876	42	919	937	SAEEQLEHSGRALEHAT SK					Mascot
2090.0249	2090.0027	-0.0222	-11	243	261	SQVDDLEQKLASQDADIS K					Mascot
2128.0955	2128.0962	0.0007	0	1138	1156	LIAVQSETESLMHVNEAL K			Oxidation (M)[12]		Mascot

6 Long chain acyl-CoA synthetase 3 [Triticum urartu] gi|474097070 70628.4 5.74 13 40 0 2.358

Peptide Information

Calc. Mass	Obsrv. Mass	± da	± ppm	Start Seq.	End Seq.	Sequence	Ion Score	C. I. %	Modification	Rank	Result Type
868.428	868.3714	-0.0566	-65	347	352	QFRMMR					Mascot
965.4833	965.4636	-0.0197	-20	61	69	SCGLTKGSR			Carbamidomethyl (C)[2]		Mascot
1009.5829	1009.5046	-0.0783	-78	578	585	LKGFEFIR					Mascot
1237.5994	1237.6586	0.0592	48	1	10	MAVEKYPNNR			Oxidation (M)[1]		Mascot
1305.6943	1305.7113	0.017	13	55	66	IGNSIRSCGLTK			Carbamidomethyl (C)[8]		Mascot
1327.6893	1327.7024	0.0131	10	44	54	TYKEVFDIANK					Mascot
1337.7311	1337.6886	-0.0425	-32	563	574	EYILGELSKTGK					Mascot
1360.6492	1360.687	0.0378	28	215	226	AGDQEFDLPVNR					Mascot
1464.8058	1464.7437	-0.0621	-42	141	153	TIVSFGKVTQEQK					Mascot
1863.9674	1863.9353	-0.0321	-17	328	344	ISAGGFIKSTMFNLAYK			Oxidation (M)[11]		Mascot
1894.8851	1895.0588	0.1737	92	443	459	LESVPDMNYDALASTPR			Oxidation (M)[7]		Mascot

1955.1324	1954.9463	-0.1861	-95	27	43	LQLISPENIKAAEFLLR					Mascot
2440.2119	2440.208	-0.0039	-2	276	296	VVEELFMFHGASIGFWR GDVK			Oxidation (M)[7]		Mascot

7 hypothetical protein TRIUR3_21551 [Triticum urartu] gi|474385881 61851.9 5.83 12 40 0 5.184

Peptide Information

Calc. Mass	Obsrv. Mass	± da	± ppm	Start Seq.	End Seq.	Sequence	Ion Score	C. I. %	Modification	Rank	Result Type
987.537	987.5706	0.0336	34	270	278	QLSIGGAWR					Mascot
1009.452	1009.5046	0.0526	52	285	292	TVSSNCWR			Carbamidomethyl (C)[6]		Mascot
1213.5776	1213.6744	0.0968	80	54	62	YTSEYFLYK					Mascot
1225.5995	1225.6718	0.0723	59	132	142	TLPNPCPGVDR			Carbamidomethyl (C)[6]		Mascot
1225.5995	1225.6718	0.0723	59	132	142	TLPNPCPGVDR			Carbamidomethyl (C)[6]		Mascot
1394.6522	1394.6755	0.0233	17	530	540	HYGPYNLDLMR			Oxidation (M)[10]		Mascot
1701.9185	1701.8953	-0.0232	-14	270	284	QLSIGGAWRAVTWTR					Mascot
1723.8334	1723.8422	0.0088	5	279	292	AVTWTRTVSSNCWR			Carbamidomethyl (C)[12]		Mascot
1725.8364	1725.8115	-0.0249	-14	247	261	GDVIYDSDLIMSLER					Mascot
1729.8361	1729.8168	-0.0193	-11	399	413	HLDMTPGIEVSACRK			Carbamidomethyl (C)[13], Oxidation (M)[4]		Mascot
1791.9423	1791.8419	-0.1004	-56	132	147	TLPNPCPGVDRIIPDK			Carbamidomethyl (C)[6]		Mascot
1814.861	1814.8474	-0.0136	-7	208	221	KAEDESHPNLWWFR					Mascot
2128.092	2128.0962	0.0042	2	435	451	VLELEQEIEQELERGWK					Mascot

8 hypothetical protein TRIUR3_16642 [Triticum urartu] gi|474247535 24459.1 10.98 8 39 0 5.656

Peptide Information

Calc. Mass	Obsrv. Mass	± da	± ppm	Start Seq.	End Seq.	Sequence	Ion Score	C. I. %	Modification	Rank	Result Type
983.4905	983.4583	-0.0322	-33	2	10	GSSTKNSFR					Mascot
1237.592	1237.6586	0.0666	54	143	155	VGAPSGHAEEGAR					Mascot
1464.7302	1464.7437	0.0135	9	143	157	VGAPSGHAEEGARAR					Mascot
1481.7802	1481.7051	-0.0751	-51	112	123	GVVVGHLWWWSR					Mascot
1481.7802	1481.7051	-0.0751	-51	112	123	GVVVGHLWWWSR					Mascot
1771.8868	1771.8171	-0.0697	-39	175	192	RGHEVATASGGVMVAAS R			Oxidation (M)[13]		Mascot
1788.9677	1789.0099	0.0422	24	12	27	ILIGFNPMNQTINIGK			Oxidation (M)[8]		Mascot
1917.0626	1917.0919	0.0293	15	11	27	KILIGFNPMNQTINIGK			Oxidation (M)[9]		Mascot
2127.1995	2127.0386	-0.1609	-76	12	30	ILIGFNPMNQTINIGKIPK			Oxidation (M)[8]		Mascot

9 ABI3-interacting protein 2-1 protein [Triticum aestivum] gi|223703116 36622.3 5.1 9 39 0 8.606

Protein Group

ABI3-interacting protein 2-1 [Triticum aestivum]	gi|223470525	36622.3	5.0999 999046 3257
ABI3-interacting protein 2-1 protein [Triticum aestivum]	gi|223703118	36594.2	5.0999 999046 3257
ABI3-interacting protein 2-2 [Triticum aestivum]	gi|223470527	36594.2	5.0999 999046 3257

Peptide Information

Calc. Mass	Obsrv. Mass	± da	± ppm	Start Seq.	End Seq.	Sequence	Ion Score	C. I. %	Modification	Rank	Result Type
832.3546	832.3198	-0.0348	-42	317	323	GGEFMYI			Oxidation (M)[5]		Mascot
921.4168	921.426	0.0092	10	256	262	MQELPCK			Carbamidomethyl (C)[6], Oxidation (M)[1]		Mascot
997.489	997.4595	-0.0295	-30	69	76	YTAPGFWR					Mascot
1394.6217	1394.6755	0.0538	39	1	14	MSATAGDEAAVAER			Oxidation (M)[1]		Mascot
1798.9447	1798.806	-0.1387	-77	25	40	KQHFEEAVADLAATLR					Mascot
1820.7716	1820.8286	0.057	31	113	127	ENEESMPTNREPDTR			Oxidation (M)[6]		Mascot
1866.0021	1865.8585	-0.1436	-77	61	76	VATVLQTRYTAPGFWR					Mascot
1938.0907	1937.9644	-0.1263	-65	219	236	EVVANLPVVTVTEEVIAR					Mascot
1976.9706	1977.0205	0.0499	25	1	19	MSATAGDEAAVAERLEA LR			Oxidation (M)[1]		Mascot
1976.9706	1977.0205	0.0499	25	1	19	MSATAGDEAAVAERLEA LR			Oxidation (M)[1]		Mascot

10	hypothetical protein TRIUR3_18298 [Triticum urartu]	gi|474057860	302316.3	4.98	33	38	0	12.195

Peptide Information

Calc. Mass	Obsrv. Mass	± da	± ppm	Start Seq.	End Seq.	Sequence	Ion Score	C. I. %	Modification	Rank	Result Type
804.4461	804.4033	-0.0428	-53	553	559	KDLVDSK					Mascot
858.493	858.4254	-0.0676	-79	2573	2579	LDLELQK					Mascot
873.3883	873.4371	0.0488	56	509	515	LSDHMDR					Mascot
904.5251	904.5242	-0.0009	-1	103	109	LVQEFIR					Mascot
937.4295	937.4059	-0.0236	-25	1543	1550	LNEMESSK					Mascot
983.4905	983.4583	-0.0322	-33	627	635	EEAGAIHTR					Mascot
997.4585	997.4595	0.001	1	2355	2363	YDADNSKGK					Mascot
1156.5997	1156.5503	-0.0494	-43	31	40	LVVSYSEGFR					Mascot

1201.5848	1201.5342	-0.0506	-42	2436	2445	SQYTSFEIAR		Mascot
1207.6173	1207.6512	0.0339	28	727	736	CMLAETTLLR	Carbamidomethyl (C)[1]	Mascot
1213.599	1213.6744	0.0754	62	2199	2208	MITEKGMLMK	Oxidation (M)[1,7]	Mascot
1223.6123	1223.6556	0.0433	35	727	736	CMLAETTLLR	Carbamidomethyl (C)[1], Oxidation (M)[2]	Mascot
1237.5518	1237.6586	0.1068	86	1695	1704	YDVCLHSSEK	Carbamidomethyl (C)[4]	Mascot
1281.6798	1281.6918	0.012	9	481	491	ALVQYSESTKR		Mascot
1327.7693	1327.7024	-0.0669	-50	770	780	LNLQNISRELK		Mascot
1332.6907	1332.6498	-0.0409	-31	560	570	SKFESEIHSLR		Mascot
1360.6954	1360.687	-0.0084	-6	1338	1349	LNEVENVSSELK		Mascot
1394.5853	1394.6755	0.0902	65	457	468	ADNSENECQSLK	Carbamidomethyl (C)[8]	Mascot
1407.6785	1407.6786	0.0001	0	1539	1550	LNDKLNEMESSK		Mascot
1464.8281	1464.7437	-0.0844	-58	1446	1458	EAALLQHKQSLAR		Mascot
1481.7404	1481.7051	-0.0353	-24	2143	2155	EESLVSTLEMVTK	Oxidation (M)[10]	Mascot
1481.7404	1481.7051	-0.0353	-24	2143	2155	EESLVSTLEMVTK	Oxidation (M)[10]	Mascot
1729.9517	1729.8168	-0.1349	-78	695	709	IASKAQILEQELVCK	Carbamidomethyl (C)[14]	Mascot
1749.8741	1749.8501	-0.024	-14	275	287	RPELMKHVEEFYR	Oxidation (M)[5]	Mascot
1765.7632	1765.8038	0.0406	23	1626	1639	QYCSLEHEMEAVNR	Carbamidomethyl (C)[3]	Mascot
1773.8436	1773.8701	0.0265	15	1551	1565	LDLESMISKHAEDNR	Oxidation (M)[6]	Mascot
1780.8568	1780.8276	-0.0292	-16	2224	2240	IAAMGAETAELKAEMGR	Oxidation (M)[4,15]	Mascot
1820.8331	1820.8286	-0.0045	-2	423	438	QDASDDIMNLQNEISK		Mascot
1836.8708	1836.8644	-0.0064	-3	935	950	SEVSETQLEAEKTEEK		Mascot
1849.9114	1849.895	-0.0164	-9	84	102	GTGAGGDCGIVNLSLYA PK	Carbamidomethyl (C)[8]	Mascot
1937.9637	1937.9644	0.0007	0	962	978	NVMVDFLQSSLQDEGKK		Mascot
1952.9607	1952.9612	0.0005	0	2025	2040	NENHKLIELNEQMGQR		Mascot
2128.0227	2128.0962	0.0735	35	647	665	EAALLAMEDLHSQSQEE VK		Mascot
2144.0176	2144.0696	0.052	24	647	665	EAALLAMEDLHSQSQEE VK	Oxidation (M)[7]	Mascot
2197.0525	2197.1462	0.0937	43	238	255	WLQENLTDMDMMVKAMI K		Mascot
2341.134	2341.05	-0.084	-36	581	600	DVALLQHQQCIEEVSDLE SK	Carbamidomethyl (C)[10]	Mascot

Gel Idx/Pos	215/I15	Instr./Gel Origin	BA2151/Sample Project 20140814	Process Status	Analysis Succeeded
Plate [#] Name	[1] Sample Project 20140814	Instrument Sample Name		Spectra	11

Rank	Protein Name	Accession No.	Protein MW	Protein PI	Pep. Count	Protein Score	Protein Score C. I. %	Intensity Matched	Total Ion Score	Total Ion C. I. %	Confirmed
1	Late embryogenesis abundant protein Lea14-A [Triticum urartu]	gi|474359227	38327.9	5.03	14	270	100	25.559	192	100	

Peptide Information

Calc. Mass	Obsrv. Mass	± da	± ppm	Start Seq.	End Seq.	Sequence	Ion Score	C. I. %	Modification	Rank	Result Type
1178.6906	1178.6841	-0.0065	-6	77	86	IHVPHIGLHR					Mascot
1252.5879	1252.5829	-0.005	-4	296	306	IDVDTPFGNMK			Oxidation (M)[10]		Mascot
1262.6199	1262.6775	0.0576	46	274	284	DLGSAVWDMIR					Mascot
1262.6199	1262.6775	0.0576	46	274	284	DLGSAVWDMIR	18	0			Mascot
1278.6147	1278.603	-0.0117	-9	274	284	DLGSAVWDMIR			Oxidation (M)[9]		Mascot
1280.682	1280.6505	-0.0315	-25	264	273	MQIPFTFRPK			Oxidation (M)[1]		Mascot
1295.7682	1295.7664	-0.0018	-1	119	131	LVAGLIPDAGTIR					Mascot
1295.7682	1295.7664	-0.0018	-1	119	131	LVAGLIPDAGTIR	72	100			Mascot
1300.6896	1300.6146	-0.075	-58	50	60	VKDFIHDIGEK					Mascot
1303.6893	1303.6859	-0.0034	-3	140	150	VPITLDFDDIR					Mascot
1303.6893	1303.6859	-0.0034	-3	140	150	VPITLDFDDIR	38	98.725			Mascot
1360.7988	1360.7285	-0.0703	-52	168	179	VIFLVDVPVFGR					Mascot
1360.7988	1360.7285	-0.0703	-52	168	179	VIFLVDVPVFGR	64	99.997			Mascot
1423.8632	1423.8552	-0.008	-6	118	131	KLVAGLIPDAGTIR					Mascot
1645.8545	1645.8457	-0.0088	-5	61	76	IEEAVGFGKPSADVAR					Mascot
1907.0637	1907.0546	-0.0091	-5	151	167	STYADIKPGSIIPYLLR					Mascot
2013.0903	2013.0117	-0.0786	-39	187	204	SGEIPIPYKPDVDVDKIK					Mascot
2322.1978	2322.1709	-0.0269	-12	97	117	NPNPVPIPLVDIDYLIDSD GR					Mascot
2450.2925	2450.2693	-0.0232	-9	97	118	NPNPVPIPLVDIDYLIDSD GRK					Mascot

Rank	Protein Name	Accession No.	Protein MW	Protein PI	Pep. Count	Protein Score	Protein Score C. I. %	Intensity Matched	Total Ion Score	Total Ion C. I. %	Confirmed
2	Transaldolase [Triticum urartu]	gi|473926683	30855.8	5.24	6	265	100	6.778	242	100	

Peptide Information

Calc. Mass	Obsrv. Mass	± da	± ppm	Start Seq.	End Seq.	Sequence	Ion Score	C. I. %	Modification	Rank	Result Type
890.4578	890.4591	0.0013	1	140	147	VSTEIDAR					Mascot
1328.6958	1328.6915	-0.0043	-3	180	191	IPATWQGIEASR					Mascot

1328.6958	1328.6915	-0.0043	-3	180	191	IPATWQGIEASR	87	100			Mascot
1424.6653	1424.7144	0.0491	34	235	247	NHSGDPEIDEALK					Mascot
1588.8555	1588.8538	-0.0017	-1	124	139	ALGNVGAELAHQVPGR					Mascot
1588.8555	1588.8538	-0.0017	-1	124	139	ALGNVGAELAHQVPGR	90	100			Mascot
2092.0459	2092.043	-0.0029	-1	159	175	VHELLQIYNEHDIPSER					Mascot
2092.0459	2092.043	-0.0029	-1	159	175	VHELLQIYNEHDIPSER	65	99.998			Mascot
2230.2329	2230.1558	-0.0771	-35	71	93	FAPTAATVSSSLLLGIAGL PDTK					Mascot

3	high molecular weight glutenin subunit Dx5 [Triticum aestivum]	gi|343174543	13442.9	5.12	3	125	100	4.485	110	100	

Peptide Information

Calc. Mass	Obsrv. Mass	± da	± ppm	Start Seq.	End Seq.	Sequence	Ion Score	C. I. %	Modification	Rank	Result Type
1044.532	1044.5123	-0.0197	-19	34	41	ELQELQER					Mascot
1392.6359	1392.6281	-0.0078	-6	45	55	ACQQVMDQQLR			Carbamidomethyl (C)[2], Oxidation (M)[6]		Mascot
1990.9618	1990.96	-0.0018	-1	83	100	GGSFYPGETTPPQQLQQ R					Mascot
1990.9618	1990.96	-0.0018	-1	83	100	GGSFYPGETTPPQQLQQ R	110	100			Mascot

4	HMW glutenin subunit, partial [Triticum aestivum]	gi|45533903	20974.4	5.15	3	121	100	4.485	110	100

Protein Group

HMW glutenin subunit, partial [Triticum aestivum]	gi|45533901	20984.4	5.1500 000953 6743
HMW glutenin subunit, partial [Triticum aestivum]	gi|45533897	20974.4	5.1500 000953 6743
HMW glutenin subunit, partial [Triticum aestivum]	gi|45533895	20984.4	5.1500 000953 6743
HMW glutenin subunit, partial [Triticum aestivum]	gi|45533893	20901.4	5.1500 000953 6743
HMW glutenin subunit, partial [Triticum aestivum]	gi|45533905	20901.4	5.1500 000953 6743

Peptide Information

Calc. Mass	Obsrv. Mass	± da	± ppm	Start Seq.	End Seq.	Sequence	Ion Score	C. I. %	Modification	Rank	Result Type
1044.532	1044.5123	-0.0197	-19	34	41	ELQELQER					Mascot
1392.6359	1392.6281	-0.0078	-6	45	55	ACQQVMDQQLR			Carbamidomethyl (C)[2], Oxidation (M)[6]		Mascot
1990.9618	1990.96	-0.0018	-1	83	100	GGSFYPGETTPPQQLQQR					Mascot
1990.9618	1990.96	-0.0018	-1	83	100	GGSFYPGETTPPQQLQQR	110	100			Mascot

5	high molecular weight glutenin subunit 1Dx [Triticum spelta]	gi|469469524	89470.2	6.1	4	110	100	4.685	110	100	

Protein Group

HMW glutenin subunit 1Dx5' [Triticum aestivum]	gi|296280726	89058.1	6.42999982833862
HMW glutenin subunit [Triticum aestivum]	gi|26515125	103218.4	5.61000013351440
HMW subunit of glutenin 1Dx2.2* precursor [Triticum aestivum]	gi|61966513	108927.1	5.73999977111816
RecName: Full=Glutenin, high molecular weight subunit DX5; Flags: Precursor	gi|300669719	90523.6	6.42999982833862
RecName: Full=Glutenin, high molecular weight subunit PW212; Flags: Precursor	gi|121453	89348.1	5.82000017166138
TPA_inf: high-molecular-weight glutenin Dx2 [Triticum aestivum]	gi|262205150	89116	5.82000017166138
X-type HMW glutenin [Triticum aestivum]	gi|110341804	90523.6	6.42999982833862
glutenin [Triticum aestivum]	gi|736319	89348.1	5.82000017166138

high molecular weight glutenin subunit 1Dx [Triticum spelta]	gi|469469506	89435.1	5.8200 001716 6138
high molecular weight glutenin subunit 1Dx [Triticum spelta]	gi|469469502	90566.7	6.4299 998283 3862
high molecular weight glutenin subunit 1Dx [Triticum spelta]	gi|469469498	90574.7	7.5500 001907 3486
high molecular weight glutenin subunit 1Dx [Triticum spelta]	gi|469469496	90647.8	6.8800 001144 4092
high molecular weight glutenin subunit 1Dx [Triticum spelta]	gi|469469494	90722.8	8.0299 997329 7119
high molecular weight glutenin subunit 1Dx [Triticum spelta]	gi|469469526	89530.2	6.8800 001144 4092
high molecular weight glutenin subunit 1Dx [Triticum spelta]	gi|469469492	90506.6	6.4200 000762 9395
high molecular weight glutenin subunit 1Dx [Triticum spelta]	gi|469469490	90537.7	6.8800 001144 4092
high molecular weight glutenin subunit 1Dx [Triticum spelta]	gi|469469488	90521.6	6.4299 998283 3862
high molecular weight glutenin subunit 2.8 [Triticum aestivum]	gi|255957523	80320	6.0900 001525 8789
high molecular weight glutenin subunit 5 [Triticum aestivum]	gi|288860106	90523.6	6.4299 998283 3862

high molecular weight glutenin subunit [Triticum aestivum]	gi|294719862	88098.5	5.5900 001525 8789
high molecular weight glutenin subunit [Triticum aestivum]	gi|162415985	89512.2	6.4299 998283 3862
high-molecular-weight glutenin 1Dx2.2 [Triticum aestivum]	gi|399933061	103170.4	5.6100 001335 144
high-molecular-weight glutenin subunit 1Dx2.1 [Triticum aestivum]	gi|50512298	89149	5.8400 001525 8789
high-molecular-weight glutenin subunit 2.6 [Triticum aestivum]	gi|194077448	109047.2	5.4099 998474 1211
high-molecular-weight glutenin subunit [Triticum aestivum]	gi|478428127	103169.4	5.75
high-molecular-weight glutenin subunit x5 [Triticum aestivum]	gi|226088593	90567.7	8.0600 004196 167

Peptide Information

Calc. Mass	Obsrv. Mass	± da	± ppm	Start Seq.	End Seq.	Sequence	Ion Score	C. I. %	Modification	Rank	Result Type
1044.532	1044.5123	-0.0197	-19	34	41	ELQELQER					Mascot
1392.6359	1392.6281	-0.0078	-6	45	55	ACQQVMDQQLR			Carbamidomethyl (C)[2], Oxidation (M)[6]		Mascot
1473.7301	1473.7666	0.0365	25	816	828	AQQLAAQLPAMCR			Carbamidomethyl (C)[12], Oxidation (M)[11]		Mascot
1990.9618	1990.96	-0.0018	-1	83	100	GGSFYPGETTPPQQLQQ R					Mascot
1990.9618	1990.96	-0.0018	-1	83	100	GGSFYPGETTPPQQLQQ R	110	100			Mascot

6	high molecular weight glutenin subunit 1Dx [Triticum spelta]	gi|469469500	90547.7	6.88	5	110	100	4.918	110	100

Peptide Information

Calc. Mass	Obsrv. Mass	± da	± ppm	Start Seq.	End Seq.	Sequence	Ion Score	C. I. %	Modification	Rank	Result Type

Calc. Mass	Obsrv. Mass	± da	± ppm	Start Seq.	End Seq.	Sequence	Ion Score	C. I. %	Modification	Rank	Result Type
1044.532	1044.5123	-0.0197	-19	34	41	ELQELQER					Mascot
1392.6359	1392.6281	-0.0078	-6	45	55	ACQQVMDQQLR			Carbamidomethyl (C)[2], Oxidation (M)[6]		Mascot
1473.7301	1473.7666	0.0365	25	825	837	AQQLAAQLPAMCR			Carbamidomethyl (C)[12], Oxidation (M)[11]		Mascot
1524.7441	1524.7439	-0.0002	0	168	180	YYPTSPQQSGQLR					Mascot
1990.9618	1990.96	-0.0018	-1	83	100	GGSFYPGETTPPQQLQQR					Mascot
1990.9618	1990.96	-0.0018	-1	83	100	GGSFYPGETTPPQQLQQR	110	100			Mascot

7	HMW-glutenin subunit Glu-1Stx1 [Triticum aestivum]	gi|430801112	54146.5	6.42	3	110	100	18.085	110	100	

Peptide Information

Calc. Mass	Obsrv. Mass	± da	± ppm	Start Seq.	End Seq.	Sequence	Ion Score	C. I. %	Modification	Rank	Result Type
1360.6638	1360.7285	0.0647	48	34	44	GLQERELEACR			Carbamidomethyl (C)[10]		Mascot
1360.6638	1360.7285	0.0647	48	34	44	GLQERELEACR			Carbamidomethyl (C)[10]		Mascot
1473.7301	1473.7666	0.0365	25	482	494	AQQLAAQLPAMCR			Carbamidomethyl (C)[12], Oxidation (M)[11]		Mascot
1990.9618	1990.96	-0.0018	-1	80	97	GGSFYPGETTPPQQLQQR					Mascot
1990.9618	1990.96	-0.0018	-1	80	97	GGSFYPGETTPPQQLQQR	110	100			Mascot

8	HMW glutenin protein Dx5 [Triticum aestivum]	gi|77864615	66765.6	5.21	3	110	100	4.485	110	100	

Protein Group

TPA_inf: high-molecular-weight glutenin Dx5 [Triticum aestivum]	gi|262205115	90715.5	6.42999982833862

Peptide Information

Calc. Mass	Obsrv. Mass	± da	± ppm	Start Seq.	End Seq.	Sequence	Ion Score	C. I. %	Modification	Rank	Result Type
1044.532	1044.5123	-0.0197	-19	34	41	ELQELQER					Mascot
1392.6359	1392.6281	-0.0078	-6	45	55	ACQQVMDQQLR			Carbamidomethyl (C)[2], Oxidation (M)[6]		Mascot
1990.9618	1990.96	-0.0018	-1	83	100	GGSFYPGETTPPQQLQQR					Mascot
1990.9618	1990.96	-0.0018	-1	83	100	GGSFYPGETTPPQQLQQR	110	100			Mascot

9	hypothetical protein TRIUR3_27643 [Triticum urartu]	gi|474329855	307031.8	4.73	36	48	36.08	13.66	

Peptide Information

Calc. Mass	Obsrv. Mass	± da	± ppm	Start Seq.	End Seq.	Sequence	Ion Score	C. I. %	Modification	Rank	Result Type
820.4233	820.4283	0.005	6	1135	1141	CLSIAEK			Carbamidomethyl (C)[1]		Mascot

832.3948	832.3207	-0.0741	-89	1464	1469	YDELHR		Mascot
892.4556	892.4098	-0.0458	-51	2589	2595	MEELSRK		Mascot
894.5156	894.45	-0.0656	-73	648	654	QLELHVR		Mascot
905.4033	905.4487	0.0454	50	1510	1516	MLEPEDR	Oxidation (M)[1]	Mascot
908.4506	908.3867	-0.0639	-70	2589	2595	MEELSRK	Oxidation (M)[1]	Mascot
991.5319	991.5569	0.025	25	2695	2702	FTRPISDR		Mascot
993.5033	993.5036	0.0003	0	2587	2594	TKMEELSR		Mascot
1082.5477	1082.554	0.0063	6	1347	1355	LKSYTEADR		Mascot
1128.6736	1128.6434	-0.0302	-27	2284	2294	SVVERLGAAVK		Mascot
1131.6555	1131.6167	-0.0388	-34	2685	2694	SLTMSRIVPK		Mascot
1203.658	1203.6522	-0.0058	-5	2395	2404	IKELTDELSR		Mascot
1300.6968	1300.6146	-0.0822	-63	2036	2047	NVIESARADAVR		Mascot
1310.6587	1310.5969	-0.0618	-47	2441	2451	EFSLKSLEDSR		Mascot
1310.6587	1310.5969	-0.0618	-47	2441	2451	EFSLKSLEDSR		Mascot
1328.6328	1328.6915	0.0587	44	1917	1927	EQHIESLESEK		Mascot
1328.6482	1328.6915	0.0433	33	2516	2526	QYSSQINDFVK		Mascot
1346.7162	1346.7379	0.0217	16	2561	2572	IVSLISESDDLR		Mascot
1404.7039	1404.7207	0.0168	12	1900	1911	QTIEEMNAVIEK		Mascot
1415.6689	1415.725	0.0561	40	1571	1582	AEKDFFSESLDK		Mascot
1424.6726	1424.7144	0.0418	29	1415	1428	MAVGASFTLPDGDK	Oxidation (M)[1]	Mascot
1428.8322	1428.7058	-0.1264	-88	2691	2702	IVPKFTRPISDR		Mascot
1451.686	1451.6829	-0.0031	-2	2503	2515	STNELLTTEDSNK		Mascot
1456.6989	1456.7053	0.0064	4	874	885	MDELSEKLSNYK		Mascot
1456.6989	1456.7053	0.0064	4	874	885	MDELSEKLSNYK		Mascot
1507.7794	1507.7323	-0.0471	-31	816	828	KLTQGMLCCLVGK	Carbamidomethyl (C)[8,9]	Mascot
1544.7626	1544.6699	-0.0927	-60	1229	1241	EEIVPQVCDLQSK	Carbamidomethyl (C)[8]	Mascot
1603.865	1603.8112	-0.0538	-34	930	944	ERASLSGLLSEVTNK		Mascot
1621.7426	1621.8403	0.0977	60	1005	1018	AHSDVQDRNEHASR		Mascot
1627.7745	1627.8575	0.083	51	2655	2668	VNNDQVAIDVEMHK	Oxidation (M)[12]	Mascot
1645.8829	1645.8457	-0.0372	-23	1900	1913	QTIEEMNAVIEKLK		Mascot
1838.9719	1838.8809	-0.091	-49	2063	2078	QIDHLKELNSTSGQLR		Mascot
1885.9978	1885.9521	-0.0457	-24	1998	2013	RATELLLTELNEAQER		Mascot
2083.9709	2083.9597	-0.0112	-5	1106	1122	NMDDMCLLNEELKLVSK	Carbamidomethyl (C)[6], Oxidation (M)[2,5]	Mascot
2092.0315	2092.043	0.0115	5	2355	2371	MQVHNLEEHVEMLLNQK		Mascot
2092.0996	2092.043	-0.0566	-27	1409	1428	IELLSKMAVGASFTLPDG DK		Mascot
2153.0479	2153.1294	0.0815	38	648	664	QLELHVRDMETHMEQLK	Oxidation (M)[9]	Mascot
2213.0542	2213.0586	0.0044	2	1471	1488	FYELAEQNNMLEQSLVE		Mascot

2450.325	2450.2693	-0.0557	-23	909	929	R LTNQAELLHNDITERISILE K	Mascot

10 hypothetical protein TRIUR3_23901 [Triticum urartu] gi\|474125413 27090.1 9.43 9 48 34.591 2.25

Peptide Information

Calc. Mass	Obsrv. Mass	± da	± ppm	Start Seq.	End Seq.	Sequence	Ion Score	C. I. %	Modification	Rank	Result Type
890.5054	890.4591	-0.0463	-52	46	54	SVASAATKR					Mascot
892.4379	892.4098	-0.0281	-31	126	132	AMLECLR			Carbamidomethyl (C)[5]		Mascot
908.4328	908.3867	-0.0461	-51	126	132	AMLECLR			Carbamidomethyl (C)[5], Oxidation (M)[2]		Mascot
1415.8118	1415.725	-0.0868	-61	79	90	DLLQLFVRAQGR					Mascot
1451.8104	1451.6829	-0.1275	-88	162	174	SHELVLGLDVLEK					Mascot
1473.7849	1473.7666	-0.0183	-12	55	67	SVLAWPQYSPAVR					Mascot
1610.9113	1610.8207	-0.0906	-56	178	191	VTELLLQKAPEDVR					Mascot
1907.075	1907.0546	-0.0204	-11	162	177	SHELVLGLDVLEKWLR					Mascot
2213.1458	2213.0586	-0.0872	-39	126	143	AMLECLRFLLATDVEIYR			Carbamidomethyl (C)[5]		Mascot
2450.1704	2450.2693	0.0989	40	24	45	GGLLVSHGDYPPDHQGV LLCCR			Carbamidomethyl (C)[20,21]		Mascot

Gel Idx/Pos	216/I16	Instr./Gel Origin	BA2151/Sample Project 20140814	Process Status	Analysis Succeeded
Plate [#] Name	[1] Sample Project 20140814	Instrument Sample Name		Spectra	11

Rank	Protein Name	Accession No.	Protein MW	Protein PI	Pep. Count	Protein Score	Protein Score C. I. %	Intensity Matched	Total Ion Score	Total Ion C. I. %	Confirmed
1	Adenosine kinase 2 [Triticum urartu]	gi|474049015	37679.9	5.01	8	235	100	6.086	199	100	

Peptide Information

Calc. Mass	Obsrv. Mass	± da	± ppm	Start Seq.	End Seq.	Sequence	Ion Score	C. I. %	Modification	Rank	Result Type
1335.625	1335.6327	0.0077	6	46	56	HLPMYDELSSK			Oxidation (M)[4]		Mascot
1424.7202	1424.714	-0.0062	-4	133	145	SLIANLSAANCYK			Carbamidomethyl (C)[11]		Mascot
1772.9177	1772.9188	0.0011	1	232	246	VRGWETENVEEIALK					Mascot
1781.9644	1781.9213	-0.0431	-24	260	277	IAVITQGADPVVVAEDGK					Mascot
1938.0656	1938.037	-0.0286	-15	259	277	RIAVITQGADPVVVAEDG K					Mascot
1990.9918	1990.9954	0.0036	2	191	206	VFLMNLSAPFICEFFR			Carbamidomethyl (C)[12]		Mascot
2006.9867	2006.991	0.0043	2	191	206	VFLMNLSAPFICEFFR			Carbamidomethyl (C)[12], Oxidation (M)[4]		Mascot
2703.2178	2703.2292	0.0114	4	321	344	AGCYAANVIIQQSGCTYP EKPDFN			Carbamidomethyl (C)[3,15]		Mascot
2911.2986	2911.3374	0.0388	13	105	132	AAGVTAHYYEDEAAPTG TCAVCVVGGER			Carbamidomethyl (C)[19,22]		Mascot
2911.2986	2911.3374	0.0388	13	105	132	AAGVTAHYYEDEAAPTG TCAVCVVGGER	199	100	Carbamidomethyl (C)[19,22]		Mascot

Rank	Protein Name	Accession No.	Protein MW	Protein PI	Pep. Count	Protein Score	Protein Score C. I. %	Intensity Matched	Total Ion Score	Total Ion C. I. %	Confirmed
2	sedoheptulose-1,7-bisphosphatase, chloroplast precursor, expressed [Triticum aestivum]	gi|300681420	42580.3	6.17	16	133	100	16.781	52	99.953	

Peptide Information

Calc. Mass	Obsrv. Mass	± da	± ppm	Start Seq.	End Seq.	Sequence	Ion Score	C. I. %	Modification	Rank	Result Type
853.4414	853.4326	-0.0088	-10	360	367	TQVAYGSK					Mascot
921.4524	921.455	0.0026	3	250	258	DTTTIGEGK					Mascot
930.5255	930.532	0.0065	7	352	359	VISVLDER					Mascot
937.456	937.4582	0.0022	2	259	266	MFSPGNLR			Oxidation (M)[1]		Mascot
1008.5109	1008.4807	-0.0302	-30	2	11	ETVAAAGYAR					Mascot
1185.5059	1185.5493	0.0434	37	267	276	ATFDNPDYDK					Mascot
1188.5532	1188.5646	0.0114	10	373	382	FEETLYGSSR					Mascot
1225.5737	1225.5691	-0.0046	-4	95	104	LLICMGEAMR			Carbamidomethyl (C)[4], Oxidation (M)[5,9]		Mascot
1235.6631	1235.6394	-0.0237	-19	307	318	GIFTNVTSPTAK					Mascot
1588.9349	1588.8877	-0.0472	-30	323	336	LLFEVAPLGFLIEK					Mascot
1608.8091	1608.8002	-0.0089	-6	138	150	LLFEALEYSHVCK			Carbamidomethyl (C)[12]		Mascot

1649.8568	1649.8385	-0.0183	-11	290	304	YTGGMVPDVNQIIVK			Oxidation (M)[5]		Mascot
1649.8568	1649.8385	-0.0183	-11	290	304	YTGGMVPDVNQIIVK			Oxidation (M)[5]		Mascot
1730.8054	1730.8131	0.0077	4	230	244	DCPGTHEFLLLDEGK			Carbamidomethyl (C)[2]		Mascot
1906.9944	1907.0732	0.0788	41	290	306	YTGGMVPDVNQIIVKEK			Oxidation (M)[5]		Mascot
1933.9801	1934.0062	0.0261	13	202	221	LTGVTGGDQVAAAMGIY GPR					Mascot
1949.975	1949.9709	-0.0041	-2	202	221	LTGVTGGDQVAAAMGIY GPR			Oxidation (M)[14]		Mascot
1949.975	1949.9709	-0.0041	-2	202	221	LTGVTGGDQVAAAMGIY GPR	52	99.953	Oxidation (M)[14]		Mascot
2064.9915	2064.97	-0.0215	-10	267	283	ATFDNPDYDKLVNYYVK					Mascot

3	unnamed protein product [Triticum aestivum]	gi|257336984	42547.2	6.04	15	126	100	16.784	52	99.953

Protein Group

RecName: Full=Sedoheptulose-1,7-bisphosphatase, chloroplastic; AltName: Full=SED(1,7)P2ase; AltName: Full=Sedoheptulose bisphosphatase; Short=SBPase; Flags: Precursor	gi|1173347	42547.2	6.0399 999618 5303
sedoheptulose-1,7-bisphosphatase [Triticum aestivum]	gi|14265	42547.2	6.0399 999618 5303
unnamed protein product [Triticum aestivum]	gi|219741570	42547.2	6.0399 999618 5303

Peptide Information

Calc. Mass	Obsrv. Mass	± da	± ppm	Start Seq.	End Seq.	Sequence	Ion Score	C. I. %	Modification	Rank	Result Type
853.4414	853.4326	-0.0088	-10	360	367	TQVAYGSK					Mascot
921.4611	921.455	-0.0061	-7	259	266	MFSPGNLR					Mascot
930.5255	930.532	0.0065	7	352	359	VISVLDER					Mascot
937.456	937.4582	0.0022	2	259	266	MFSPGNLR			Oxidation (M)[1]		Mascot
1185.5059	1185.5493	0.0434	37	267	276	ATFDNPDYDK					Mascot
1188.5532	1188.5646	0.0114	10	373	382	FEETLYGSSR					Mascot
1225.5737	1225.5691	-0.0046	-4	95	104	LLICMGEAMR			Carbamidomethyl (C)[4], Oxidation (M)[5,9]		Mascot
1235.6631	1235.6394	-0.0237	-19	307	318	GIFTNVTSPTAK					Mascot
1585.797	1585.8354	0.0384	24	245	258	WQHVKDTTSIGEGK					Mascot
1588.9349	1588.8877	-0.0472	-30	323	336	LLFEVAPLGFLIEK					Mascot
1608.8091	1608.8002	-0.0089	-6	138	150	LLFEALEYSHVCK			Carbamidomethyl (C)[12]		Mascot
1649.8568	1649.8385	-0.0183	-11	290	304	YTGGMVPDVNQIIVK			Oxidation (M)[5]		Mascot

1649.8568	1649.8385	-0.0183	-11	290	304	YTGGMVPDVNQIIVK			Oxidation (M)[5]		Mascot
1730.8054	1730.8131	0.0077	4	230	244	DCPGTHEFLLLDEGK			Carbamidomethyl (C)[2]		Mascot
1906.9944	1907.0732	0.0788	41	290	306	YTGGMVPDVNQIIVKEK			Oxidation (M)[5]		Mascot
1933.9801	1934.0062	0.0261	13	202	221	LTGVTGGDQVAAAMGIY GPR					Mascot
1949.975	1949.9709	-0.0041	-2	202	221	LTGVTGGDQVAAAMGIY GPR			Oxidation (M)[14]		Mascot
1949.975	1949.9709	-0.0041	-2	202	221	LTGVTGGDQVAAAMGIY GPR	52	99.953	Oxidation (M)[14]		Mascot
2064.9915	2064.97	-0.0215	-10	267	283	ATFDNPDYDKLVNYYVK					Mascot

4 Sedoheptulose-1,7-bisphosphatase, chloroplastic [Triticum urartu] gi|474425491 40102.1 5.74 14 123 100 16.016 52 99.953

Peptide Information

Calc. Mass	Obsrv. Mass	± da	± ppm	Start Seq.	End Seq.	Sequence	Ion Score	C. I. %	Modification	Rank	Result Type
853.4414	853.4326	-0.0088	-10	336	343	TQVAYGSK					Mascot
921.4611	921.455	-0.0061	-7	236	243	MFSPGNLR					Mascot
930.5255	930.532	0.0065	7	328	335	VISVLDER					Mascot
937.456	937.4582	0.0022	2	236	243	MFSPGNLR			Oxidation (M)[1]		Mascot
1185.5059	1185.5493	0.0434	37	244	253	ATFDNPDYDK					Mascot
1188.5532	1188.5646	0.0114	10	349	358	FEETLYGSSR					Mascot
1225.5737	1225.5691	-0.0046	-4	72	81	LLICMGEAMR			Carbamidomethyl (C)[4], Oxidation (M)[5,9]		Mascot
1235.6631	1235.6394	-0.0237	-19	285	296	GIFTNVTSPTAK					Mascot
1585.797	1585.8354	0.0384	24	222	235	WQHVKDTTSIGEGK					Mascot
1588.9349	1588.8877	-0.0472	-30	299	312	LLFEVAPLGFLIEK					Mascot
1608.8091	1608.8002	-0.0089	-6	115	127	LLFEALEYSHVCK			Carbamidomethyl (C)[12]		Mascot
1649.8568	1649.8385	-0.0183	-11	268	282	YTGGMVPDVNQIIVK			Oxidation (M)[5]		Mascot
1649.8568	1649.8385	-0.0183	-11	268	282	YTGGMVPDVNQIIVK			Oxidation (M)[5]		Mascot
1730.8054	1730.8131	0.0077	4	207	221	DCPGTHEFLLLDEGK			Carbamidomethyl (C)[2]		Mascot
1906.9944	1907.0732	0.0788	41	268	284	YTGGMVPDVNQIIVKEK			Oxidation (M)[5]		Mascot
1933.9801	1934.0062	0.0261	13	179	198	LTGVTGGDQVAAAMGIY GPR					Mascot
1949.975	1949.9709	-0.0041	-2	179	198	LTGVTGGDQVAAAMGIY GPR			Oxidation (M)[14]		Mascot
1949.975	1949.9709	-0.0041	-2	179	198	LTGVTGGDQVAAAMGIY GPR	52	99.953	Oxidation (M)[14]		Mascot

5 Pyruvate dehydrogenase E1 component subunit beta, mitochondrial [Triticum urartu] gi|474369502 100638.9 6.03 15 73 99.768 13.984 40 99.228

Peptide Information

Calc. Mass	Obsrv. Mass	± da	± ppm	Start Seq.	End Seq.	Sequence	Ion Score	C. I. %	Modification	Rank	Result Type

Calc. Mass	Obsrv. Mass	± da	± ppm	Start Seq.	End Seq.	Sequence	Ion Score	C. I. %	Modification	Rank	Result Type
801.4577	801.4723	0.0146	18	231	238	AAINASVR					Mascot
856.4999	856.5052	0.0053	6	224	230	SIRPLDR					Mascot
937.423	937.4582	0.0352	38	405	411	ELCLCSR			Carbamidomethyl (C)[3,5]		Mascot
959.552	959.5163	-0.0357	-37	691	699	LSNAQSVLK					Mascot
1200.6583	1200.6696	0.0113	9	213	223	EGISAEVINLR					Mascot
1207.5953	1207.6014	0.0061	5	128	138	VLAPYSSEDAR					Mascot
1225.6536	1225.5691	-0.0845	-69	905	916	QPLPAAAESVSR					Mascot
1262.5868	1262.645	0.0582	46	294	304	LAVPQDMADMR			Oxidation (M)[7]		Mascot
1273.7012	1273.6152	-0.086	-68	736	747	GSFSGPIPLRSR					Mascot
1278.5818	1278.6135	0.0317	25	294	304	LAVPQDMADMR			Oxidation (M)[7,10]		Mascot
1295.6842	1295.7776	0.0934	72	187	198	EGKDVTITAFSK					Mascot
1303.7257	1303.7012	-0.0245	-19	435	445	ELFEVLLNAQK					Mascot
1326.7013	1326.6162	-0.0851	-64	905	917	QPLPAAAESVSRT					Mascot
1687.8473	1687.8282	-0.0191	-11	278	293	IAGADVPMPYAANLER					Mascot
1703.8422	1703.8239	-0.0183	-11	278	293	IAGADVPMPYAANLER			Oxidation (M)[8]		Mascot
1795.916	1795.9297	0.0137	8	84	99	SNYMSAGQINVPIVFR					Mascot
1811.9109	1811.9072	-0.0037	-2	84	99	SNYMSAGQINVPIVFR			Oxidation (M)[4]		Mascot
1811.9109	1811.9072	-0.0037	-2	84	99	SNYMSAGQINVPIVFR	41	99.443	Oxidation (M)[4]		Mascot
1827.8794	1827.9229	0.0435	24	889	904	MSLSSLPSTQTFLEDR			Oxidation (M)[1]		Mascot

6	Late embryogenesis abundant protein Lea14-A [Triticum urartu]	gi|474359227	38327.9	5.03	13	66	99.01	6.038	

Peptide Information

Calc. Mass	Obsrv. Mass	± da	± ppm	Start Seq.	End Seq.	Sequence	Ion Score	C. I. %	Modification	Rank	Result Type
853.4778	853.4326	-0.0452	-53	15	22	GLFSKSSK					Mascot
1178.6906	1178.6509	-0.0397	-34	77	86	IHVPHIGLHR					Mascot
1262.6199	1262.645	0.0251	20	274	284	DLGSAVWDMIR					Mascot
1278.6147	1278.6135	-0.0012	-1	274	284	DLGSAVWDMIR			Oxidation (M)[9]		Mascot
1280.682	1280.6726	-0.0094	-7	264	273	MQIPFTFRPK			Oxidation (M)[1]		Mascot
1295.7682	1295.7776	0.0094	7	119	131	LVAGLIPDAGTIR					Mascot
1300.6896	1300.7095	0.0199	15	50	60	VKDFIHDIGEK					Mascot
1303.6893	1303.7012	0.0119	9	140	150	VPITLDFDDIR					Mascot
1360.7988	1360.743	-0.0558	-41	168	179	VIFLVDVPVFGR					Mascot
1423.8632	1423.8723	0.0091	6	118	131	KLVAGLIPDAGTIR					Mascot
1645.8545	1645.8647	0.0102	6	61	76	IEEAVGFGKPSADVAR					Mascot
1907.0637	1907.0732	0.0095	5	151	167	STYADIKPGSIIPYLLR					Mascot

2013.0903	2013.0487	-0.0416	-21	187	204	SGEIPIPYKPDVDVDKIK					Mascot
2450.2925	2450.3176	0.0251	10	97	118	NPNPVPIPLVDIDYLIDSD GRK					Mascot

7 Protein strawberry notch-like protein 1 [Triticum urartu] gi|474429003 142165 6.59 25 62 97.145 18.59

Peptide Information

Calc. Mass	Obsrv. Mass	± da	± ppm	Start Seq.	End Seq.	Sequence	Ion Score	C. I. %	Modification	Rank	Result Type
801.4828	801.4723	-0.0105	-13	1095	1101	GKNIELK					Mascot
832.4047	832.3243	-0.0804	-97	279	286	DLDDVGAK					Mascot
853.46	853.4326	-0.0274	-32	990	996	ALTMVYR					Mascot
873.5152	873.4637	-0.0515	-59	626	632	VTEIQRK					Mascot
959.5057	959.5163	0.0106	11	525	532	GVSNQIWR					Mascot
1067.6095	1067.5153	-0.0942	-88	1097	1105	NIELKEPPK					Mascot
1182.6127	1182.5994	-0.0133	-11	222	231	HLHHLPTGDR					Mascot
1259.6591	1259.5955	-0.0636	-50	960	971	LESLGALTQGDR					Mascot
1268.6594	1268.5988	-0.0606	-48	384	395	NLIPDAGSQPTR					Mascot
1273.6635	1273.6152	-0.0483	-38	1083	1094	EGQLDSGIVDIK					Mascot
1295.7471	1295.7776	0.0305	24	318	328	VHPLNKLPYSK					Mascot
1326.5995	1326.6162	0.0167	13	307	317	TYDPKNDMSQK					Mascot
1569.8207	1569.8461	0.0254	16	1	15	MAAASHLFLHGITGK			Oxidation (M)[1]		Mascot
1627.7599	1627.8702	0.1103	68	1209	1222	VSKGWQDEYDVSSK					Mascot
1649.835	1649.8385	0.0035	2	454	469	GGVGALELVAMDMKAR			Oxidation (M)[11,13]		Mascot
1649.835	1649.8385	0.0035	2	454	469	GGVGALELVAMDMKAR			Oxidation (M)[11,13]		Mascot
1671.8602	1671.7992	-0.061	-36	247	260	TIAGLIWENWQQGR					Mascot
1677.7755	1677.8925	0.117	70	973	988	AGPSLSAFNYDSTYGK					Mascot
1747.9337	1747.9344	0.0007	0	905	919	SNQTSAPQYRILIEK					Mascot
1747.9337	1747.9344	0.0007	0	905	919	SNQTSAPQYRILIEK					Mascot
1795.8783	1795.9297	0.0514	29	997	1012	GIMEQASIEEFITEAK					Mascot
1811.8732	1811.9072	0.034	19	997	1012	GIMEQASIEEFITEAK			Oxidation (M)[3]		Mascot
1811.8732	1811.9072	0.034	19	997	1012	GIMEQASIEEFITEAK			Oxidation (M)[3]		Mascot
1838.8081	1838.9103	0.1022	56	832	846	NAKEVSMEMINMHEK			Oxidation (M)[7,9,12]		Mascot
1880.0236	1879.8922	-0.1314	-70	855	873	LIAIISEAGSAGVSLHADR					Mascot
2047.939	2047.9648	0.0258	13	728	745	KIEGQEMEHDANVADFS K					Mascot
2082.0576	2081.937	-0.1206	-58	587	604	YGVEMEDFVSGPRELLL K					Mascot
2406.0588	2406.2886	0.2298	96	481	501	GADFDTVEAPLEERMTN MYGK			Oxidation (M)[15,18]		Mascot
3042.3254	3042.4875	0.1621	53	704	728	VHPSCLTPPWTGMLTDD			Carbamidomethyl (C)[5,20,23], Oxidation		Mascot

		WSCYSCKK					(M)[13]		
8	hypothetical protein TRIUR3_25712 [Triticum urartu]	gi|474317317	47918.6	9.36	14	56	87.82	5.141	

Peptide Information

Calc. Mass	Obsrv. Mass	± da	± ppm	Start Seq.	End Seq.	Sequence	Ion Score	C. I. %	Modification	Rank	Result Type
921.5403	921.455	-0.0853	-93	343	350	LFGKLESK					Mascot
959.517	959.5163	-0.0007	-1	398	405	RHFSIGSR					Mascot
1188.677	1188.5646	-0.1124	-95	378	388	VPRLGTSISMK					Mascot
1268.6039	1268.5988	-0.0051	-4	128	139	SLDMVSVASDTK			Oxidation (M)[4]		Mascot
1280.6859	1280.6726	-0.0133	-10	389	398	DSFSRPFLRR					Mascot
1322.5464	1322.6655	0.1191	90	161	172	GHSLGMDTDSMR			Oxidation (M)[6]		Mascot
1627.8075	1627.8702	0.0627	39	177	190	SPGYQLYRQVSDSK					Mascot
1677.8	1677.8925	0.0925	55	355	370	MPTNVILDGDLDGSSK			Oxidation (M)[1]		Mascot
1788.8657	1788.9181	0.0524	29	111	126	DRPTASNSSPEAKLCR			Carbamidomethyl (C)[15]		Mascot
1873.8345	1873.9147	0.0802	43	266	282	SNASNHTTVSPDQEVCK			Carbamidomethyl (C)[16]		Mascot
1895.9531	1895.8658	-0.0873	-46	249	265	WSIDSELLGSVSSKMTR					Mascot
2006.8655	2006.991	0.1255	63	154	172	ADPSSSRGHSLGMDTDS MR					Mascot
2022.8605	2022.9879	0.1274	63	154	172	ADPSSSRGHSLGMDTDS MR			Oxidation (M)[13]		Mascot
2047.8671	2047.9648	0.0977	48	326	342	YDPPCPVCTHGEQCTVK			Carbamidomethyl (C)[5,8,14]		Mascot
2939.4568	2939.3745	-0.0823	-28	42	67	THIEDIMEIPTFLSNHSSG SIRPETK					Mascot

9	Kinesin-like calmodulin-binding protein [Triticum urartu]	gi|474241655	90038.4	7.79	18	49	40.347	8.225

Peptide Information

Calc. Mass	Obsrv. Mass	± da	± ppm	Start Seq.	End Seq.	Sequence	Ion Score	C. I. %	Modification	Rank	Result Type
921.5265	921.455	-0.0715	-78	273	280	RLYGVGTR					Mascot
1188.6471	1188.5646	-0.0825	-69	2	12	QSLPDTLSSLK					Mascot
1225.6801	1225.5691	-0.111	-91	364	373	KPHVPYRNSK					Mascot
1235.6267	1235.6394	0.0127	10	302	312	ISLTSFDAPER					Mascot
1250.6488	1250.6459	-0.0029	-2	543	553	RPSSVYAESVR					Mascot
1273.647	1273.6152	-0.0318	-25	511	521	FMKPTAASQHR					Mascot
1276.6355	1276.5928	-0.0427	-33	319	329	IWMIDLGGSER					Mascot
1300.5798	1300.7095	0.1297	100	283	295	STASTMANSTSSR					Mascot
1310.7175	1310.6073	-0.1102	-84	522	533	IGLNNNVPSINR					Mascot
1335.6825	1335.6327	-0.0498	-37	1	12	MQSLPDTLSSLK			Oxidation (M)[1]		Mascot
1569.765	1569.8461	0.0811	52	281	295	LRSTASTMANSTSSR					Mascot

1585.7599	1585.8354	0.0755	48	281	295	LRSTASTMANSTSSR			Oxidation (M)[8]		Mascot
1632.8778	1632.8032	-0.0746	-46	319	332	IWMIDLGGSERLVK			Oxidation (M)[3]		Mascot
1639.7281	1639.8578	0.1297	79	102	114	MRPFNHEESYSSR					Mascot
1687.9048	1687.8282	-0.0766	-45	64	78	IQDEMVSLGAQLKQK					Mascot
1703.8997	1703.8239	-0.0758	-44	64	78	IQDEMVSLGAQLKQK			Oxidation (M)[5]		Mascot
1772.9581	1772.9188	-0.0393	-22	17	31	YLTPSWIESVSHILK					Mascot
1781.9103	1781.9213	0.011	6	1	16	MQSLPDTLSSLKGFNK			Oxidation (M)[1]		Mascot
1804.9626	1804.9218	-0.0408	-23	181	196	TYTMEGKPKDLGVIPR					Mascot
1996.9659	1996.9857	0.0198	10	212	227	FQFTFSMLEIYMGNLR					Mascot
2012.9609	2013.0487	0.0878	44	212	227	FQFTFSMLEIYMGNLR			Oxidation (M)[7]		Mascot

10	BTB/POZ and MATH domain-containing protein 2 [Triticum urartu]	gi|473781595	39746.7	5.4	11	48	28.281	11.617

Peptide Information

Calc. Mass	Obsrv. Mass	± da	± ppm	Start Seq.	End Seq.	Sequence	Ion Score	C. I. %	Modification	Rank	Result Type
856.4887	856.5052	0.0165	19	103	110	SIVVHSSK					Mascot
1026.4418	1026.4851	0.0433	42	276	283	ICEGMLCK			Carbamidomethyl (C)[2,7], Oxidation (M)[5]		Mascot
1182.5428	1182.5994	0.0566	48	275	283	RICEGMLCK			Carbamidomethyl (C)[3,8], Oxidation (M)[6]		Mascot
1252.6532	1252.7351	0.0819	65	24	35	ATVAVEISGYSR					Mascot
1252.6532	1252.7351	0.0819	65	24	35	ATVAVEISGYSR					Mascot
1295.7405	1295.7776	0.0371	29	89	98	VLHKWMLVNR					Mascot
1303.7118	1303.7012	-0.0106	-8	99	110	FSGRSIVVHSSK					Mascot
1346.7097	1346.7938	0.0841	62	2	14	AVSQTSIAMVPSR					Mascot
1346.7097	1346.7938	0.0841	62	2	14	AVSQTSIAMVPSR					Mascot
1677.8014	1677.8925	0.0911	54	319	333	MDDGIASQGYAHLKR			Oxidation (M)[1]		Mascot
1788.9202	1788.9181	-0.0021	-1	217	232	GVQDIIIDDMQPAVFK					Mascot
1804.915	1804.9218	0.0068	4	217	232	GVQDIIIDDMQPAVFK			Oxidation (M)[10]		Mascot
1933.0178	1932.9972	-0.0206	-11	178	195	IGADVTFKVQGEVFSAHK					Mascot
2406.1501	2406.2886	0.1385	58	2	23	AVSQTSIAMVPSRCTSET HTAR			Carbamidomethyl (C)[14], Oxidation (M)[9]		Mascot

Gel Idx/Pos	217/I17	Instr./Gel Origin	BA2151/Sample Project 20140814	Process Status	Analysis Succeeded
Plate [#] Name	[1] Sample Project 20140814	Instrument Sample Name		Spectra	11

Rank	Protein Name	Accession No.	Protein MW	Protein PI	Pep. Count	Protein Score	Protein Score C. I. %	Intensity Matched	Total Ion Score	Total Ion C. I. %	Confirmed
1	Adenosine kinase 2 [Triticum urartu]	gi|474049015	37679.9	5.01	12	462	100	19.39	395	100	

Peptide Information

Calc. Mass	Obsrv. Mass	± da	± ppm	Start Seq.	End Seq.	Sequence	Ion Score	C. I. %	Modification	Rank	Result Type
1027.655	1027.6213	-0.0337	-33	280	288	TFPVILLPK					Mascot
1213.6576	1213.661	0.0034	3	151	160	KPENWALVEK					Mascot
1424.7202	1424.7365	0.0163	11	133	145	SLIANLSAANCYK			Carbamidomethyl (C)[11]		Mascot
1517.7483	1517.7593	0.011	7	234	246	GWETENVEEIALK					Mascot
1707.8297	1707.8503	0.0206	12	57	72	DNVEYIAGGATQNSIR	125	100			Mascot
1772.9177	1772.938	0.0203	11	232	246	VRGWETENVEEIALK					Mascot
1772.9177	1772.938	0.0203	11	232	246	VRGWETENVEEIALK					Mascot
1938.0656	1938.075	0.0094	5	259	277	RIAVITQGADPVVVAEDG K					Mascot
1990.9918	1991.0117	0.0199	10	191	206	VFLMNLSAPFICEFFR			Carbamidomethyl (C)[12]		Mascot
2006.9867	2006.9976	0.0109	5	191	206	VFLMNLSAPFICEFFR			Carbamidomethyl (C)[12], Oxidation (M)[4]		Mascot
2006.9867	2006.9976	0.0109	5	191	206	VFLMNLSAPFICEFFR	70	100	Carbamidomethyl (C)[12], Oxidation (M)[4]		Mascot
2293.1824	2293.1672	-0.0152	-7	291	313	LVDTNGAGDAFVGGFLS QLVQGK					Mascot
2703.2178	2703.2261	0.0083	3	321	344	AGCYAANVIIQQSGCTYP EKPDFN			Carbamidomethyl (C)[3,15]		Mascot
2911.2986	2911.344	0.0454	16	105	132	AAGVTAHYYEDEAAPTG TCAVCVVGGER			Carbamidomethyl (C)[19,22]		Mascot
2911.2986	2911.344	0.0454	16	105	132	AAGVTAHYYEDEAAPTG TCAVCVVGGER	199	100	Carbamidomethyl (C)[19,22]		Mascot
3024.4368	3024.4753	0.0385	13	46	72	HLPMYDELSSKDNVEYIA GGATQNSIR			Oxidation (M)[4]		Mascot

Rank	Protein Name	Accession No.	Protein MW	Protein PI	Pep. Count	Protein Score	Protein Score C. I. %	Intensity Matched	Total Ion Score	Total Ion C. I. %	Confirmed
2	Protein kinase G11A [Triticum urartu]	gi|474393627	45786	8.82	11	51	66.454	2.406			

Peptide Information

Calc. Mass	Obsrv. Mass	± da	± ppm	Start Seq.	End Seq.	Sequence	Ion Score	C. I. %	Modification	Rank	Result Type
1182.6841	1182.5792	-0.1049	-89	146	155	DLKPENVLVR					Mascot
1735.7957	1735.8698	0.0741	43	156	170	DDGHVMLSDFDLSLR			Oxidation (M)[6]		Mascot
1758.9021	1758.8623	-0.0398	-23	304	320	FPEGGPPTSSASKDLIR					Mascot
1800.866	1800.9573	0.0913	51	22	39	APTVYAGVASAMAFMGP K			Oxidation (M)[12,15]		Mascot
1902.0305	1901.9435	-0.087	-46	287	303	GQTNRATLFNVVGQQLR					Mascot

Calc. Mass	Obsrv. Mass	± da	± ppm	Start Seq.	End Seq.	Sequence	Ion Score	C. I. %	Modification	Rank	Result Type
1937.9678	1938.075	0.1072	55	242	258	SMSFVGTHEYLAPEIIK			Oxidation (M)[2]		Mascot
1940.9722	1941.0311	0.0589	30	22	40	APTVYAGVASAMAFMGPKR			Oxidation (M)[12]		Mascot
2084.0078	2083.9565	-0.0513	-25	222	241	SGEQQQGAMPELVVEPTGAR					Mascot
2293.1831	2293.1672	-0.0159	-7	356	377	CSTPPGVPRPVEPIAFAAMPAK			Carbamidomethyl (C)[1]		Mascot
2315.1296	2315.1794	0.0498	22	220	241	SKSGEQQQGAMPELVVEPTGAR			Oxidation (M)[11]		Mascot
2894.3699	2894.3984	0.0285	10	11	39	QPDDGGLPEGKAPTVYAGVASAMAFMGPK			Oxidation (M)[23,26]		Mascot

3 ferritin 2 [Triticum aestivum] gi|126583394 28396.4 6.01 10 50 51.512 2.544

Peptide Information

Calc. Mass	Obsrv. Mass	± da	± ppm	Start Seq.	End Seq.	Sequence	Ion Score	C. I. %	Modification	Rank	Result Type
1027.5782	1027.6213	0.0431	42	71	80	GELSLVPQGK					Mascot
1081.5459	1081.4679	-0.078	-72	146	153	LMEYQNKR					Mascot
1273.5696	1273.6179	0.0483	38	131	140	FFKESSDEER					Mascot
1730.9661	1730.8745	-0.0916	-53	190	204	LVNEKLHNLHSVATR					Mascot
1838.8883	1838.9241	0.0358	19	174	189	KAMPCMAMELALALEK			Carbamidomethyl (C)[5], Oxidation (M)[3,6]		Mascot
1839.8483	1839.9252	0.0769	42	242	257	GHGVWHFDQMLLEEAA					Mascot
1855.8433	1855.9155	0.0722	39	242	257	GHGVWHFDQMLLEEAA			Oxidation (M)[10]		Mascot
1873.0793	1872.901	-0.1783	-95	159	174	LQSIVTPLTEFDILRK					Mascot
1938.0042	1938.075	0.0708	37	54	70	GKEVLSGVMFQPFEELK					Mascot
2294.1262	2294.1538	0.0276	12	175	194	AMPCMAMELALALEKLVNEK			Carbamidomethyl (C)[4], Oxidation (M)[2,5]		Mascot
2725.2661	2725.2405	-0.0256	-9	205	227	CNDPQLTDFVESEFLQEQVDAIK			Carbamidomethyl (C)[1]		Mascot

4 hypothetical protein TRIUR3_13145 [Triticum urartu] gi|473964953 11885.7 8.61 3 49 50.383 3.085 32 96.324

Peptide Information

Calc. Mass	Obsrv. Mass	± da	± ppm	Start Seq.	End Seq.	Sequence	Ion Score	C. I. %	Modification	Rank	Result Type
1721.8164	1721.8636	0.0472	27	1	16	MDAVQSSSPYSPPSLR					Mascot
1721.8164	1721.8636	0.0472	27	1	16	MDAVQSSSPYSPPSLR	32	96.324			Mascot
1855.9297	1855.9155	-0.0142	-8	2	18	DAVQSSSPYSPPSLRHK					Mascot
2002.9651	2002.9561	-0.009	-4	1	18	MDAVQSSSPYSPPSLRHK			Oxidation (M)[1]		Mascot

5 unnamed protein product [Triticum aestivum] gi|194239079 22590.7 4.96 8 48 38.957 4.306

Peptide Information

Calc. Mass	Obsrv. Mass	± da	± ppm	Start Seq.	End Seq.	Sequence	Ion Score	C. I. %	Modification	Rank	Result Type

1310.5287	1310.6139	0.0852	65	1	13	MGPAACGSSGEMR			Carbamidomethyl (C)[6]		Mascot
1424.6765	1424.7365	0.06	42	151	163	LTEPDENGIHSGR					Mascot
1515.6567	1515.7552	0.0985	65	76	87	CLDFLTSQECSR			Carbamidomethyl (C)[1,10]		Mascot
1788.832	1788.9294	0.0974	54	41	56	LLGENVDDEQDMSVPK					Mascot
1804.827	1804.9277	0.1007	56	41	56	LLGENVDDEQDMSVPK			Oxidation (M)[12]		Mascot
1804.827	1804.9277	0.1007	56	41	56	LLGENVDDEQDMSVPK			Oxidation (M)[12]		Mascot
1864.9309	1865.0181	0.0872	47	60	75	WLHDHGGAMMIKLEAR					Mascot
1899.9236	1899.9515	0.0279	15	164	179	DAVLWQFEVHGVNEEK					Mascot
2083.9099	2083.9565	0.0466	22	180	195	DYELCVENTQYCYAKK			Carbamidomethyl (C)[5,12]		Mascot
2894.3672	2894.3984	0.0312	11	14	40	NMDGGWGIHTEGGSSM LRSALNYTALR					Mascot
2910.3621	2910.3157	-0.0464	-16	14	40	NMDGGWGIHTEGGSSM LRSALNYTALR			Oxidation (M)[2]		Mascot

6 3-ketoacyl-CoA synthase 6 [Triticum urartu] gi|473777652 45096.8 8.96 11 48 26.61 5.696

Peptide Information

Calc. Mass	Obsrv. Mass	± da	± ppm	Start Seq.	End Seq.	Sequence	Ion Score	C. I. %	Modification	Rank	Result Type
1273.6576	1273.6179	-0.0397	-31	6	16	AVYLVDYAGFR					Mascot
1295.6995	1295.7493	0.0498	38	388	398	YPLDVPDVLKH					Mascot
1346.6713	1346.8013	0.13	97	376	387	NAHGPWATSIHR					Mascot
1603.8302	1603.795	-0.0352	-22	259	271	EQLMFAFSFIARK			Oxidation (M)[4]		Mascot
1795.9966	1795.9189	-0.0777	-43	2	16	LRPRAVYLVDYAGFR					Mascot
1839.7717	1839.9252	0.1535	83	133	148	SDVRSMHLSGMGCCSR			Carbamidomethyl (C)[13,14]		Mascot
1855.7667	1855.9155	0.1488	80	133	148	SDVRSMHLSGMGCCSR			Carbamidomethyl (C)[13,14], Oxidation (M)[6]		Mascot
1871.7616	1871.9022	0.1406	75	133	148	SDVRSMHLSGMGCCSR			Carbamidomethyl (C)[13,14], Oxidation (M)[6,11]		Mascot
1927.0371	1926.8949	-0.1422	-74	1	16	MLRPRAVYLVDYAGFR					Mascot
1943.032	1943.0565	0.0245	13	1	16	MLRPRAVYLVDYAGFR			Oxidation (M)[1]		Mascot
1943.032	1943.0565	0.0245	13	1	16	MLRPRAVYLVDYAGFR			Oxidation (M)[1]		Mascot
1988.9647	1988.996	0.0313	16	6	22	AVYLVDYAGFRTPSNCR			Carbamidomethyl (C)[16]		Mascot
2002.9917	2002.9561	-0.0356	-18	17	33	TPSNCRVPFATFLEHAR			Carbamidomethyl (C)[5]		Mascot
2820.3662	2820.3445	-0.0217	-8	320	343	MTLHRFGNTSSSSLWYE LAYIEAK			Oxidation (M)[1]		Mascot
2894.542	2894.3984	-0.1436	-50	245	270	ANITAMGPLVLPTREQLM FAFSFIAR					Mascot
2910.5369	2910.3157	-0.2212	-76	245	270	ANITAMGPLVLPTREQLM FAFSFIAR			Oxidation (M)[6]		Mascot

7 Activator of 90 kDa heat shock protein ATPase-like protein 1 [Triticum urartu] gi|474417943 45508.5 8.48 11 48 23.152 1.691

Peptide Information

Calc. Mass	Obsrv. Mass	± da	± ppm	Start Seq.	End Seq.	Sequence	Ion Score	C. I. %	Modification	Rank	Result Type
1081.5095	1081.4679	-0.0416	-38	1	10	MAKFGEGDAR					Mascot
1328.6403	1328.6826	0.0423	32	219	229	EGFKTIEMTEK			Oxidation (M)[8]		Mascot
1549.7792	1549.7339	-0.0453	-29	155	169	IRDYVAAMANGGPAK			Oxidation (M)[8]		Mascot
1730.9436	1730.8745	-0.0691	-40	335	351	GAKLPPPLTEDHGLSAK					Mascot
1733.9044	1733.8594	-0.045	-26	353	368	HANPMLAPAPYEVPVK					Mascot
1758.8593	1758.8623	0.003	2	304	318	ALSMEFDPAKHQTQR					Mascot
1800.9491	1800.9573	0.0082	5	60	75	TPPLDKLDGEAYVNIR					Mascot
1890.0055	1889.9321	-0.0734	-39	352	368	RHANPMLAPAPYEVPVK					Mascot
1899.843	1899.9515	0.1085	57	399	414	QLEESKETTWGFPCTS			Carbamidomethyl (C)[14]		Mascot
2178.322	2178.1689	-0.1531	-70	38	59	ALLSKLLAGLPVLSGEGG LTLR					Mascot
2854.4106	2854.3103	-0.1003	-35	257	283	ISKDVGGQFSLFDGSITG VNEELQEGK					Mascot

8 hypothetical protein TRIUR3_14840 [Triticum urartu] gi|474389623 61462.5 5.68 12 45 0 22.969

Peptide Information

Calc. Mass	Obsrv. Mass	± da	± ppm	Start Seq.	End Seq.	Sequence	Ion Score	C. I. %	Modification	Rank	Result Type
1500.7727	1500.7501	-0.0226	-15	226	238	DGPLEELVVKNCK			Carbamidomethyl (C)[12]		Mascot
1588.8475	1588.8275	-0.02	-13	50	63	QSLGLLEQAQKMSR					Mascot
1603.7091	1603.795	0.0859	54	518	531	SVQYSSECSNALMK			Carbamidomethyl (C)[8]		Mascot
1703.9122	1703.8556	-0.0566	-33	61	75	MSRRPNIAPVGPQHK			Oxidation (M)[1]		Mascot
1747.799	1747.9017	0.1027	59	518	532	SVQYSSECSNALMKK			Carbamidomethyl (C)[8], Oxidation (M)[13]		Mascot
1873.9167	1873.9156	-0.0011	-1	239	254	GINHHDFLKFGSGWMK					Mascot
1873.9167	1873.9156	-0.0011	-1	239	254	GINHHDFLKFGSGWMK					Mascot
1889.9116	1889.9321	0.0205	11	239	254	GINHHDFLKFGSGWMK			Oxidation (M)[15]		Mascot
1901.9095	1901.9435	0.034	18	515	531	VAKSVQYSSECSNALMK			Carbamidomethyl (C)[11]		Mascot
1958.9786	1958.962	-0.0166	-8	98	115	VGSHLCIATEALTSLCAR			Carbamidomethyl (C)[6,16]		Mascot
1991.0094	1991.0117	0.0023	1	32	49	GVEEWHLSAQQAHAATK K					Mascot
2084.0085	2083.9565	-0.052	-25	464	480	FIADTPCLSNLTLRMCR			Carbamidomethyl (C)[7,16], Oxidation (M)[15]		Mascot
2583.2695	2583.2178	-0.0517	-20	4	24	WRQELLMSTLANMVDNL ETFR			Oxidation (M)[7]		Mascot
2725.3423	2725.2405	-0.1018	-37	439	463	ALSSSPYLDTLELILCEAV TDAGMR			Carbamidomethyl (C)[16]		Mascot

9 Protein TOC75, chloroplastic [Triticum urartu] gi|474326689 55823.1 8.01 11 43 0 1.892

Peptide Information

Calc. Mass	Obsrv. Mass	± da	± ppm	Start Seq.	End Seq.	Sequence	Ion Score	C. I. %	Modification	Rank	Result Type
1186.498	1186.5852	0.0872	73	408	418	GYGMGELGACR			Carbamidomethyl (C)[10], Oxidation (M)[4]		Mascot
1346.7274	1346.8013	0.0739	55	250	261	ANITENLTKQSK					Mascot
1549.7792	1549.7339	-0.0453	-29	334	347	CIFQVDQGLGIGSR			Carbamidomethyl (C)[1]		Mascot
1707.8926	1707.8503	-0.0423	-25	466	482	AGQGSSYGVGVRFGPLR					Mascot
1812.0239	1811.9083	-0.1156	-64	367	384	QEKGAGKPPPAVLVAHG R					Mascot
1840.8613	1840.9182	0.0569	31	483	499	AEYAVDHNAGTGSVFFR					Mascot
1933.9039	1933.9926	0.0887	46	436	453	NTHVYAFAEHGTDLGSS K					Mascot
2213.072	2213.0493	-0.0227	-10	175	195	SLAGSFSSSNLLNPQDD VSFK					Mascot
2226.0344	2226.1304	0.096	43	287	309	TTATGALGMDGPPTTFS GTGVDR			Oxidation (M)[9]		Mascot
2332.0742	2332.0894	0.0152	7	483	503	AEYAVDHNAGTGSVFFR YGDR					Mascot
2925.4048	2925.3499	-0.0549	-19	262	286	FTYGLVLEEITTRDESND VCTHGLR			Carbamidomethyl (C)[20]		Mascot

10	Tryptophanyl-tRNA synthetase, cytoplasmic [Triticum urartu]	gi|474250777	74886.1	8.28	12	42	0	23.101	

Peptide Information

Calc. Mass	Obsrv. Mass	± da	± ppm	Start Seq.	End Seq.	Sequence	Ion Score	C. I. %	Modification	Rank	Result Type
1721.9585	1721.8636	-0.0949	-55	311	325	VSFRVLLSLNPFDSK					Mascot
1721.9585	1721.8636	-0.0949	-55	311	325	VSFRVLLSLNPFDSK					Mascot
1786.8164	1786.8899	0.0735	41	331	347	MSASDPNSAIYVTDSTK					Mascot
1789.0483	1788.9294	-0.1189	-66	370	385	KLGANLEVHPFPVVLR					Mascot
1829.8552	1829.9071	0.0519	28	12	27	EEEEQVVNPWEVSAGK					Mascot
1874.0006	1873.9156	-0.085	-45	276	290	MPEAWRHFIHPALIR					Mascot
1874.0006	1873.9156	-0.085	-45	276	290	MPEAWRHFIHPALIR					Mascot
1889.9956	1889.9321	-0.0635	-34	276	290	MPEAWRHFIHPALIR			Oxidation (M)[1]		Mascot
1940.9495	1941.0311	0.0816	42	581	598	RCGLLSENPAVPNSPSD K			Carbamidomethyl (C)[2]		Mascot
1957.9502	1957.9976	0.0474	24	11	27	KEEEEQVVNPWEVSAGK					Mascot
2084.0635	2083.9565	-0.107	-51	95	113	GPSSEALHLGHLVPFMFT K			Oxidation (M)[16]		Mascot
2294.083	2294.1538	0.0708	31	582	602	CGLLSENPAVPNSPSDK HSER			Carbamidomethyl (C)[1]		Mascot
2332.061	2332.0894	0.0284	12	326	347	GENTKMSASDPNSAIYVT DSTK			Oxidation (M)[6]		Mascot
2933.502	2933.3538	-0.1482	-51	95	120	GPSSEALHLGHLVPFMFT KYLQDAFK					Mascot
2968.5178	2968.3787	-0.1391	-47	88	113	FYLYTGRGPSSEALHLG HLVPFMFTK					Mascot

Gel Idx/Pos	218/I18	Instr./Gel Origin	BA2151/Sample Project 20140814	Process Status	Analysis Succeeded
Plate [#] Name	[1] Sample Project 20140814	Instrument Sample Name		Spectra	11

Rank	Protein Name	Accession No.	Protein MW	Protein PI	Pep. Count	Protein Score	Protein Score C. I. %	Intensity Matched	Total Ion Score	Total Ion C. I. %	Confirmed
1	40S ribosomal protein SA [Triticum urartu]	gi|474222337	33930.8	4.97	14	458	100	55.328	374	100	

Peptide Information

Calc. Mass	Obsrv. Mass	± da	± ppm	Start Seq.	End Seq.	Sequence	Ion Score	C. I. %	Modification	Rank	Result Type
809.4008	809.4124	0.0116	14	190	195	MVLQMR			Oxidation (M)[1,5]		Mascot
822.4832	822.4948	0.0116	14	196	203	GTILPGHK					Mascot
838.4417	838.4663	0.0246	29	138	144	TDHQPIK					Mascot
940.5826	940.6027	0.0201	21	130	137	LLILTDPR					Mascot
940.5826	940.6027	0.0201	21	130	137	LLILTDPR	61	99.994			Mascot
1099.4296	1099.4526	0.023	21	38	45	NCDFQMER			Carbamidomethyl (C)[2]		Mascot
1099.4296	1099.4526	0.023	21	38	45	NCDFQMER	29	92.17	Carbamidomethyl (C)[2]		Mascot
1115.4244	1115.4442	0.0198	18	38	45	NCDFQMER			Carbamidomethyl (C)[2], Oxidation (M)[6]		Mascot
1115.4244	1115.4442	0.0198	18	38	45	NCDFQMER	12	0	Carbamidomethyl (C)[2], Oxidation (M)[6]		Mascot
1192.6572	1192.6821	0.0249	21	52	62	SDGIYIINLGK					Mascot
1203.6368	1203.655	0.0182	15	165	175	YVDIGIPANNK					Mascot
1313.6709	1313.7035	0.0326	25	99	111	FAQHTGANAIAGR					Mascot
1313.6709	1313.7035	0.0326	25	99	111	FAQHTGANAIAGR	117	100			Mascot
1348.7583	1348.7883	0.03	22	51	62	RSDGIYIINLGK					Mascot
1348.7583	1348.7883	0.03	22	51	62	RSDGIYIINLGK	67	99.999			Mascot
1465.7621	1465.7946	0.0325	22	178	189	NSIGCLYWLLAR			Carbamidomethyl (C)[5]		Mascot
1465.7621	1465.7946	0.0325	22	178	189	NSIGCLYWLLAR	55	99.977	Carbamidomethyl (C)[5]		Mascot
1915.9253	1915.9432	0.0179	9	21	37	EQDIQMMLAADVHLGTK			Oxidation (M)[6]		Mascot
1931.9202	1931.9381	0.0179	9	21	37	EQDIQMMLAADVHLGTK			Oxidation (M)[6,7]		Mascot
2065.9397	2065.9922	0.0525	25	112	129	HTPGTFTNQMQTSFSEPR					Mascot
2065.9397	2065.9922	0.0525	25	112	129	HTPGTFTNQMQTSFSEPR					Mascot
2081.9346	2081.9744	0.0398	19	112	129	HTPGTFTNQMQTSFSEPR			Oxidation (M)[10]		Mascot
2081.9346	2081.9744	0.0398	19	112	129	HTPGTFTNQMQTSFSEPR	44	99.757	Oxidation (M)[10]		Mascot
2195.0107	2195.0593	0.0486	22	145	164	ESALGNIPTIAFCDTDSPMR			Carbamidomethyl (C)[13]		Mascot
2466.3464	2466.4006	0.0542	22	73	94	VIVAIENPQDIIVQSARPYGQR					Mascot

Rank	Protein Name	Accession No.	Protein MW	Protein PI	Pep. Count	Protein Score	Protein Score C. I. %	Intensity Matched	Total Ion Score	Total Ion C. I. %	Confirmed
2	40S ribosomal protein SA [Triticum urartu]	gi|474262479	27262.4	4.76	8	174	100	31.221	134	100	

Peptide Information

Calc. Mass	Obsrv. Mass	± da	± ppm	Start Seq.	End Seq.	Sequence	Ion Score	C. I. %	Modification	Rank	Result Type
822.4832	822.4948	0.0116	14	138	145	GTILPGHK					Mascot
847.4091	847.4722	0.0631	74	1	9	MAAEGGGVR					Mascot
940.5826	940.6027	0.0201	21	124	131	LLILTDPR					Mascot
940.5826	940.6027	0.0201	21	124	131	LLILTDPR	61	99.994			Mascot
1099.4296	1099.4526	0.023	21	32	39	NCDFQMER			Carbamidomethyl (C)[2]		Mascot
1099.4296	1099.4526	0.023	21	32	39	NCDFQMER	29	92.17	Carbamidomethyl (C)[2]		Mascot
1115.4244	1115.4442	0.0198	18	32	39	NCDFQMER			Carbamidomethyl (C)[2], Oxidation (M)[6]		Mascot
1115.4244	1115.4442	0.0198	18	32	39	NCDFQMER	12	0	Carbamidomethyl (C)[2], Oxidation (M)[6]		Mascot
1339.6754	1339.7114	0.036	27	93	105	FAQYTGANAIAGR					Mascot
1915.9253	1915.9432	0.0179	9	15	31	EQDIQMMLAADVHLGTK			Oxidation (M)[6]		Mascot
1931.9202	1931.9381	0.0179	9	15	31	EQDIQMMLAADVHLGTK			Oxidation (M)[6,7]		Mascot
2065.9397	2065.9922	0.0525	25	106	123	HTPGTFTNQMQTSFSEPR					Mascot
2065.9397	2065.9922	0.0525	25	106	123	HTPGTFTNQMQTSFSEPR					Mascot
2081.9346	2081.9744	0.0398	19	106	123	HTPGTFTNQMQTSFSEPR			Oxidation (M)[10]		Mascot
2081.9346	2081.9744	0.0398	19	106	123	HTPGTFTNQMQTSFSEPR	44	99.757	Oxidation (M)[10]		Mascot
2466.3464	2466.4006	0.0542	22	67	88	VIVAIENPQDIIVQSARPYGQR					Mascot

3	Chain A, Localization Of The Small Subunit Ribosomal Proteins Into A 5.5 A Cryo-Em Map Of Triticum Aestivum Translating 80s Ribosome	gi|313103629	33122.5	4.97	9	131	100	24.741	90	100	

Peptide Information

Calc. Mass	Obsrv. Mass	± da	± ppm	Start Seq.	End Seq.	Sequence	Ion Score	C. I. %	Modification	Rank	Result Type
809.4008	809.4124	0.0116	14	185	190	MVLQMR			Oxidation (M)[1,5]		Mascot
822.4832	822.4948	0.0116	14	191	198	GTILPGHK					Mascot
838.4417	838.4663	0.0246	29	133	139	TDHQPIK					Mascot
940.5826	940.6027	0.0201	21	125	132	LLILTDPR					Mascot
940.5826	940.6027	0.0201	21	125	132	LLILTDPR	61	99.994			Mascot
1099.4296	1099.4526	0.023	21	33	40	NCDFQMER			Carbamidomethyl (C)[2]		Mascot
1099.4296	1099.4526	0.023	21	33	40	NCDFQMER	29	92.17	Carbamidomethyl (C)[2]		Mascot
1115.4244	1115.4442	0.0198	18	33	40	NCDFQMER			Carbamidomethyl (C)[2], Oxidation (M)[6]		Mascot
1115.4244	1115.4442	0.0198	18	33	40	NCDFQMER	12	0	Carbamidomethyl (C)[2], Oxidation (M)[6]		Mascot
1203.6368	1203.655	0.0182	15	160	170	YVDIGIPANNK					Mascot

1915.9253	1915.9432	0.0179	9	16	32	EQDIQMMLAADVHLGTK			Oxidation (M)[6]		Mascot
1931.9202	1931.9381	0.0179	9	16	32	EQDIQMMLAADVHLGTK			Oxidation (M)[6,7]		Mascot
2195.0107	2195.0593	0.0486	22	140	159	ESALGNIPTIAFCDTDSP MR			Carbamidomethyl (C)[13]		Mascot
2466.3464	2466.4006	0.0542	22	68	89	VIVAIENPQDIIVQSARPY GQR					Mascot

4 hypothetical protein TRIUR3_01307 [Triticum urartu] gi|473888829 12399.3 9.93 7 48 31.509 1.554

Peptide Information

Calc. Mass	Obsrv. Mass	± da	± ppm	Start Seq.	End Seq.	Sequence	Ion Score	C. I. %	Modification	Rank	Result Type
962.5054	962.5842	0.0788	82	79	87	SGKGNSVWK					Mascot
1049.4833	1049.489	0.0057	5	70	78	WIDMSNAGR					Mascot
1131.4954	1131.4368	-0.0586	-52	49	59	SFGGTADDFSK					Mascot
1337.6267	1337.6847	0.058	43	70	81	WIDMSNAGRSGK			Oxidation (M)[4]		Mascot
1473.806	1473.799	-0.007	-5	82	95	GNSVWKAVSSTPLK					Mascot
2195.2104	2195.0593	-0.1511	-69	2	23	ASFSSASVLALTIMTVLVT GAR					Mascot
2284.1458	2284.252	0.1062	46	37	59	GSTPTNLIVAAKSFGGTA DDFSK					Mascot

5 hypothetical protein TRIUR3_11018 [Triticum urartu] gi|473764713 13035.6 6.3 9 47 17.656 3.211

Peptide Information

Calc. Mass	Obsrv. Mass	± da	± ppm	Start Seq.	End Seq.	Sequence	Ion Score	C. I. %	Modification	Rank	Result Type
821.4702	821.4848	0.0146	18	100	106	VVIMFGR					Mascot
837.4651	837.4676	0.0025	3	100	106	VVIMFGR			Oxidation (M)[4]		Mascot
968.5999	968.6313	0.0314	32	25	33	ARAALLTPR					Mascot
977.5713	977.4871	-0.0842	-86	99	106	RVVIMFGR					Mascot
993.5662	993.5045	-0.0617	-62	99	106	RVVIMFGR			Oxidation (M)[5]		Mascot
1051.4473	1051.449	0.0017	2	11	19	SMEDGNINR			Oxidation (M)[2]		Mascot
1191.5535	1191.6454	0.0919	77	10	19	RSMEDGNINR					Mascot
1337.6188	1337.6847	0.0659	49	34	45	SASVQMDEALMR					Mascot
1412.6515	1412.7524	0.1009	71	88	98	KHMFAEEFETK			Oxidation (M)[3]		Mascot
1440.6577	1440.7563	0.0986	68	89	99	HMFAEEFETKR			Oxidation (M)[2]		Mascot
1465.67	1465.7946	0.1246	85	11	24	SMEDGNINRSGGTK					Mascot
1465.67	1465.7946	0.1246	85	11	24	SMEDGNINRSGGTK					Mascot
1481.6649	1481.79	0.1251	84	11	24	SMEDGNINRSGGTK			Oxidation (M)[2]		Mascot

6 Kinesin-like protein KIF19 [Triticum urartu] gi|474383745 86606 8.83 17 45 0 3.878

Peptide Information

Calc. Mass	Obsrv. Mass	± da	± ppm	Start Seq.	End Seq.	Sequence	Ion Score	C. I. %	Modification	Rank	Result Type
822.4468	822.4948	0.048	58	270	276	YGSQVLR					Mascot
905.4145	905.4918	0.0773	85	723	730	SPCSGELR			Carbamidomethyl (C)[3]		Mascot
968.5635	968.6313	0.0678	70	2	10	PTIRAPASR					Mascot
1067.4388	1067.4417	0.0029	3	519	528	DNDEAGAGYR					Mascot
1126.6692	1126.6113	-0.0579	-51	510	518	RQVILDNIR					Mascot
1145.6677	1145.655	-0.0127	-11	756	765	LHSIILELAH					Mascot
1170.6365	1170.6051	-0.0314	-27	43	53	NVAVLDPDISK					Mascot
1193.6049	1193.6666	0.0617	52	468	477	ALFELEETNK					Mascot
1213.5848	1213.6422	0.0574	47	731	741	TPYSGIEGFSR					Mascot
1284.6947	1284.7228	0.0281	22	556	565	TYLQILSQYR					Mascot
1295.6954	1295.7073	0.0119	9	429	439	EHQLSVKPTEK					Mascot
1320.7271	1320.6189	-0.1082	-82	54	64	GYLDLIQNRTK					Mascot
1406.775	1406.7751	0.0001	0	499	510	DYALLQALTSRR					Mascot
1473.7003	1473.799	0.0987	67	161	174	SPSSGKTYTMVGSR			Oxidation (M)[10]		Mascot
1497.7445	1497.7837	0.0392	26	731	743	TPYSGIEGFSRQR					Mascot
1561.8445	1561.728	-0.1165	-75	229	242	EDPEHGIIVAGLRR					Mascot
1789.9695	1789.9362	-0.0333	-19	463	477	INLQKALFELEETNK					Mascot

7 Sucrose synthase 2 [Triticum urartu] gi|474393599 93127.3 6.01 16 43 0 23.182

Peptide Information

Calc. Mass	Obsrv. Mass	± da	± ppm	Start Seq.	End Seq.	Sequence	Ion Score	C. I. %	Modification	Rank	Result Type
896.506	896.585	0.079	88	13	19	IHSVRER					Mascot
1078.6078	1078.5864	-0.0214	-20	575	583	KPIIFSMAR			Oxidation (M)[7]		Mascot
1225.6576	1225.6497	-0.0079	-6	513	523	VVHGIDVFDPK					Mascot
1284.7747	1284.7228	-0.0519	-40	203	214	IRSLGTLQGALR					Mascot
1299.7129	1299.6346	-0.0783	-60	788	797	RYLEMLYALK					Mascot
1313.6525	1313.7035	0.051	39	448	457	YPNSDLYWKK					Mascot
1313.6525	1313.7035	0.051	39	448	457	YPNSDLYWKK	0	0			Mascot
1320.7158	1320.6189	-0.0969	-73	67	77	LKDTAFEDLLR					Mascot
1337.6487	1337.6847	0.036	27	194	204	GMTMMLNDRIR					Mascot
1357.7434	1357.735	-0.0084	-6	744	755	ISQGGLQRIEEK					Mascot
1417.7185	1417.7758	0.0573	40	765	776	LMTLSGVYGFWK			Oxidation (M)[2]		Mascot
1497.7552	1497.7837	0.0285	19	587	599	VKNMTGLVEMYGR					Mascot

1513.7502	1513.7456	-0.0046	-3	587	599	VKNMTGLVEMYGR			Oxidation (M)[4]		Mascot
1561.7137	1561.728	0.0143	9	657	668	NGELYRYICDMK			Carbamidomethyl (C)[9]		Mascot
1915.9873	1915.9432	-0.0441	-23	20	37	IGDSLSAHTNELVAVFSR					Mascot
2066.0415	2065.9922	-0.0493	-24	760	776	LYSERLMTLSGVYGFWK			Oxidation (M)[7]		Mascot
2066.0415	2065.9922	-0.0493	-24	760	776	LYSERLMTLSGVYGFWK			Oxidation (M)[7]		Mascot
2094.1377	2093.9805	-0.1572	-75	603	621	LQELVNLVVVCGDHGKVSK			Carbamidomethyl (C)[11]		Mascot
2147.1392	2147.0608	-0.0784	-37	600	618	NPRLQELVNLVVVCGDHGK			Carbamidomethyl (C)[14]		Mascot

8 hypothetical protein TRIUR3_05161 [Triticum urartu] gi|474022902 45146 4.95 12 43 0 2.816

Peptide Information

Calc. Mass	Obsrv. Mass	± da	± ppm	Start Seq.	End Seq.	Sequence	Ion Score	C. I. %	Modification	Rank	Result Type
809.3458	809.4124	0.0666	82	255	260	MREDDK			Oxidation (M)[1]		Mascot
844.4886	844.501	0.0124	15	30	38	AALGQLSGK					Mascot
1024.4429	1024.4341	-0.0088	-9	312	321	SGETDTASEK					Mascot
1214.6813	1214.6683	-0.013	-11	198	208	MASPLLEPKTK					Mascot
1299.5999	1299.6346	0.0347	27	39	50	SALYCSDGSIAR			Carbamidomethyl (C)[5]		Mascot
1308.6166	1308.6809	0.0643	49	351	361	EEETETVSEKK					Mascot
1339.5835	1339.7114	0.1279	95	83	94	WDEISGEAMTGK			Oxidation (M)[9]		Mascot
1406.6394	1406.7751	0.1357	96	388	403	GEGIASAGKSGSSSDP					Mascot
1770.8593	1770.9772	0.1179	67	242	256	SPVTFNINDYAARMR			Oxidation (M)[14]		Mascot
1915.9695	1915.9432	-0.0263	-14	39	55	SALYCSDGSIARYLIAR			Carbamidomethyl (C)[5]		Mascot
2125.9619	2126.0396	0.0777	37	342	360	GENTTESVKEEETETVSEK					Mascot
2128.9678	2129.0708	0.103	48	224	241	IMEDLFNLDELECAFGGR			Carbamidomethyl (C)[13]		Mascot

9 Putative disease resistance RPP13-like protein 2 [Triticum urartu] gi|474060631 82914.2 8.56 15 43 0 25.551

Peptide Information

Calc. Mass	Obsrv. Mass	± da	± ppm	Start Seq.	End Seq.	Sequence	Ion Score	C. I. %	Modification	Rank	Result Type
838.5145	838.4663	-0.0482	-57	560	567	LPATPALR					Mascot
940.5251	940.6027	0.0776	83	445	452	FELPHGIK					Mascot
940.5251	940.6027	0.0776	83	445	452	FELPHGIK					Mascot
962.5054	962.5842	0.0788	82	720	728	QPQPTIHAA					Mascot
1118.6066	1118.5	-0.1066	-95	719	728	RQPQPTIHAA					Mascot
1214.6025	1214.6683	0.0658	54	601	611	LAEDGHGFRGR					Mascot

1320.6213	1320.6189	-0.0024	-2	1	13	MSATNDDAGILGR					Mascot
1348.7294	1348.7883	0.0589	44	541	552	FLQAPCAITSIK			Carbamidomethyl (C)[6]		Mascot
1348.7294	1348.7883	0.0589	44	541	552	FLQAPCAITSIK	3	0	Carbamidomethyl (C)[6]		Mascot
1357.7509	1357.735	-0.0159	-12	633	644	LPQVKIEQGSMK					Mascot
1390.7438	1390.7084	-0.0354	-25	399	410	GTDVDHLHQKIK					Mascot
1473.8159	1473.799	-0.0169	-11	523	536	ALQILSIDSSDLAK					Mascot
1481.8574	1481.79	-0.0674	-45	422	434	DTLVEVVPIEVIR					Mascot
1770.9393	1770.9772	0.0379	21	279	292	CKVHSIMLEFIIHK			Carbamidomethyl (C)[1], Oxidation (M)[7]		Mascot
1789.963	1789.9362	-0.0268	-15	21	36	LCQLSVVLGPTYGNLR			Carbamidomethyl (C)[2]		Mascot
1838.8451	1838.9626	0.1175	64	159	175	GQTENHVAGGLTTEHCK			Carbamidomethyl (C)[16]		Mascot
2017.9972	2017.9926	-0.0046	-2	330	348	EGVDDAVCSARGIDLSVV R			Carbamidomethyl (C)[8]		Mascot
2017.9972	2017.9926	-0.0046	-2	330	348	EGVDDAVCSARGIDLSVV R			Carbamidomethyl (C)[8]		Mascot

10	Fatty acyl-CoA reductase 1 [Triticum urartu]	gi|473954707	54704.9	8.6	13	42	0	19.524

Peptide Information

Calc. Mass	Obsrv. Mass	± da	± ppm	Start Seq.	End Seq.	Sequence	Ion Score	C. I. %	Modification	Rank	Result Type
807.4028	807.4128	0.01	12	433	439	KAMESNK					Mascot
827.441	827.5119	0.0709	86	359	365	FFSTVAR					Mascot
837.4101	837.4676	0.0575	69	396	402	YNELNGK					Mascot
870.4791	870.5576	0.0785	90	346	353	GKNGEPIR					Mascot
896.5353	896.585	0.0497	55	462	469	VHFPGVLK					Mascot
993.5112	993.5045	-0.0067	-7	395	402	RYNELNGK					Mascot
1156.5746	1156.6448	0.0702	61	396	404	YNELNGKYR					Mascot
1238.6528	1238.7329	0.0801	65	222	232	DPLPGWIEGVR					Mascot
1296.6252	1296.6962	0.071	55	423	432	FDDMNLEKLR			Oxidation (M)[4]		Mascot
1308.5671	1308.6809	0.1138	87	155	165	MELNANCATER			Carbamidomethyl (C)[7]		Mascot
1313.6743	1313.7035	0.0292	22	348	358	NGEPIRLNNMR					Mascot
1313.6743	1313.7035	0.0292	22	348	358	NGEPIRLNNMR					Mascot
1412.7057	1412.7524	0.0467	33	105	117	YDVAFDTNVLGAK					Mascot
1723.8704	1723.9309	0.0605	35	179	192	ARHFGWPNTYVFTK					Mascot

Rank	Protein Name	Accession No.	Protein MW	Protein PI	Pep. Count	Protein Score	Protein Score C. I. %	Intensity Matched	Total Ion Score	Total Ion C. I. %	Confirmed
1	Glucose-1-phosphate adenylyltransferase large subunit 1, chloroplastic/amyloplastic [Triticum urartu]	gi|474056532	51530	8.53	13	75	99.863	12.192	28	90.88	

Peptide Information

Calc. Mass	Obsrv. Mass	± da	± ppm	Start Seq.	End Seq.	Sequence	Ion Score	C. I. %	Modification	Rank	Result Type
876.4897	876.5085	0.0188	21	450	458	ARAGSISSK					Mascot
925.5214	925.5168	-0.0046	-5	17	26	AAPGGQGVLR					Mascot
952.4887	952.5057	0.017	18	260	267	TPFFTSPR					Mascot
952.4887	952.5057	0.017	18	260	267	TPFFTSPR	25	82.622			Mascot
1023.5945	1023.6124	0.0179	17	295	303	IEHSIIGVR					Mascot
1266.7278	1266.6205	-0.1073	-85	295	305	IEHSIIGVRSR					Mascot
1302.6624	1302.6932	0.0308	24	281	291	EAIISHGCFLR			Carbamidomethyl (C)[8]		Mascot
1302.6624	1302.6932	0.0308	24	281	291	EAIISHGCFLR			Carbamidomethyl (C)[8]		Mascot
1306.6243	1306.662	0.0377	29	348	358	ISNCIIDMNAR			Carbamidomethyl (C)[4]		Mascot
1322.6191	1322.6451	0.026	20	348	358	ISNCIIDMNAR			Carbamidomethyl (C)[4], Oxidation (M)[8]		Mascot
1543.8413	1543.8767	0.0354	23	279	291	IKEAIISHGCFLR			Carbamidomethyl (C)[10]		Mascot
1646.8174	1646.8459	0.0285	17	201	214	YAELHDFGSEILPR					Mascot
1646.8174	1646.8459	0.0285	17	201	214	YAELHDFGSEILPR	3	0			Mascot
1756.9592	1756.9988	0.0396	23	157	171	SIEHILILSGDQLYR					Mascot
1838.8776	1838.976	0.0984	54	72	87	LIDIPMSNCFNSGINK			Carbamidomethyl (C)[9], Oxidation (M)[6]		Mascot
1910.8879	1910.9208	0.0329	17	369	384	EGVQEADRPEEGYYIR					Mascot
2763.3162	2763.3708	0.0546	20	215	237	ALHDHNVQAYVFTDYWE DIGTIR					Mascot

Rank	Protein Name	Accession No.	Protein MW	Protein PI	Pep. Count	Protein Score	Protein Score C. I. %	Intensity Matched	Total Ion Score	Total Ion C. I. %	Confirmed
2	ADP-glucose pyrophosphorylase large subunit [Triticum aestivum]	gi|110729318	58399.1	6.12	13	70	99.568	12.368	28	90.88	

Protein Group

Protein Name	Accession No.	Protein MW	Protein PI
ADP-glucose pyrophosphorylase large subunit [Triticum aestivum]	gi|995746	58399.1	6.1199 998855 5908
RecName: Full=Glucose-1-phosphate adenylyltransferase large subunit, chloroplastic/amyloplastic; AltName: Full=ADP-glucose pyrophosphorylase; AltName: Full=ADP-glucose	gi|1707930	58399.1	6.1199 998855 5908

synthase; AltName: Full=AGPase S; AltName: Full=Alpha-D-glucose-1-phosphate adeny			
unnamed protein product [Triticum aestivum]	gi|300634107	58399.1	6.1199998855 5908
unnamed protein product [Triticum aestivum]	gi|300544593	58399.1	6.1199998855 5908
unnamed protein product [Triticum aestivum]	gi|259471795	58399.1	6.1199998855 5908
unnamed protein product [Triticum aestivum]	gi|259439208	58399.1	6.1199998855 5908
unnamed protein product [Triticum aestivum]	gi|257671708	58399.1	6.1199998855 5908
unnamed protein product [Triticum aestivum]	gi|257306977	58399.1	6.1199998855 5908
unnamed protein product [Triticum aestivum]	gi|257308981	58399.1	6.1199998855 5908
unnamed protein product [Triticum aestivum]	gi|219764742	58399.1	6.1199998855 5908
unnamed protein product [Triticum aestivum]	gi|219752155	58399.1	6.1199998855 5908

Peptide Information

Calc. Mass	Obsrv. Mass	± da	± ppm	Start Seq.	End Seq.	Sequence	Ion Score	C. I. %	Modification	Rank	Result Type
866.4618	866.447	-0.0148	-17	251	258	ASEYGLVK					Mascot
952.4887	952.5057	0.017	18	380	387	TPFFTSPR					Mascot
952.4887	952.5057	0.017	18	380	387	TPFFTSPR	25	82.622			Mascot

1023.5945	1023.6124	0.0179	17	415	423	IEHSIIGVR					Mascot
1266.7278	1266.6205	-0.1073	-85	415	425	IEHSIIGVRSR					Mascot
1302.6624	1302.6932	0.0308	24	401	411	EAIISHGCFLR			Carbamidomethyl (C)[8]		Mascot
1302.6624	1302.6932	0.0308	24	401	411	EAIISHGCFLR			Carbamidomethyl (C)[8]		Mascot
1306.6243	1306.662	0.0377	29	468	478	ISNCIIDMNAR			Carbamidomethyl (C)[4]		Mascot
1322.6191	1322.6451	0.026	20	468	478	ISNCIIDMNAR			Carbamidomethyl (C)[4], Oxidation (M)[8]		Mascot
1543.8413	1543.8767	0.0354	23	399	411	IKEAIISHGCFLR			Carbamidomethyl (C)[10]		Mascot
1646.8174	1646.8459	0.0285	17	321	334	YAELHDFGSEILPR					Mascot
1646.8174	1646.8459	0.0285	17	321	334	YAELHDFGSEILPR	3	0			Mascot
1756.9592	1756.9988	0.0396	23	208	222	SIEHILILSGDQLYR					Mascot
1838.8776	1838.976	0.0984	54	123	138	LIDIPMSNCFNSGINK			Carbamidomethyl (C)[9], Oxidation (M)[6]		Mascot
1910.8879	1910.9208	0.0329	17	489	504	EGVQEADRPEEGYYIR					Mascot
2055.9399	2055.9924	0.0525	26	232	250	HVDDNADITLSCAPVGES R			Carbamidomethyl (C)[12]		Mascot
2763.3162	2763.3708	0.0546	20	335	357	ALHDHNVQAYVFTDYWE DIGTIR					Mascot

3 ADP-glucose-pyrophosphorylase large subunit [Triticum aestivum] gi|2583072 32915.3 5.21 10 68 99.331 10.747 28 90.88

Peptide Information

Calc. Mass	Obsrv. Mass	± da	± ppm	Start Seq.	End Seq.	Sequence	Ion Score	C. I. %	Modification	Rank	Result Type
866.4618	866.447	-0.0148	-17	19	26	ASEYGLVK					Mascot
952.4887	952.5057	0.017	18	148	155	TPFFTSPR					Mascot
952.4887	952.5057	0.017	18	148	155	TPFFTSPR	25	82.622			Mascot
1023.5945	1023.6124	0.0179	17	183	191	IEHSIIGVR					Mascot
1266.7278	1266.6205	-0.1073	-85	183	193	IEHSIIGVRSR					Mascot
1302.6624	1302.6932	0.0308	24	169	179	EAIISHGCFLR			Carbamidomethyl (C)[8]		Mascot
1302.6624	1302.6932	0.0308	24	169	179	EAIISHGCFLR			Carbamidomethyl (C)[8]		Mascot
1306.6243	1306.662	0.0377	29	236	246	ISNCIIDMNAR			Carbamidomethyl (C)[4]		Mascot
1322.6191	1322.6451	0.026	20	236	246	ISNCIIDMNAR			Carbamidomethyl (C)[4], Oxidation (M)[8]		Mascot
1543.8413	1543.8767	0.0354	23	167	179	IKEAIISHGCFLR			Carbamidomethyl (C)[10]		Mascot
1646.8174	1646.8459	0.0285	17	89	102	YAELHDFGSEILPR					Mascot
1646.8174	1646.8459	0.0285	17	89	102	YAELHDFGSEILPR	3	0			Mascot
1910.8879	1910.9208	0.0329	17	257	272	EGVQEADRPEEGYYIR					Mascot
2763.3162	2763.3708	0.0546	20	103	125	ALHDHNVQAYVFTDYWE DIGTIR					Mascot

4 ADP-glucose pyrophosophorylase (1 is 2nd base in codon) [Triticum aestivum] gi|829293 33580.7 5.13 9 67 99.076 7.81 28 90.88

Protein Group

ADP glucose pyrophosphatase AGA.3	gi|226874	33580.7	5.1300 001144 4092

Peptide Information

Calc. Mass	Obsrv. Mass	± da	± ppm	Start Seq.	End Seq.	Sequence	Ion Score	C. I. %	Modification	Rank	Result Type
866.4618	866.447	-0.0148	-17	25	32	ASEYGLVK					Mascot
952.4887	952.5057	0.017	18	154	161	TPFFTSPR					Mascot
952.4887	952.5057	0.017	18	154	161	TPFFTSPR	25	82.622			Mascot
1306.6243	1306.662	0.0377	29	242	252	ISNCIIDMNAR			Carbamidomethyl (C)[4]		Mascot
1322.6191	1322.6451	0.026	20	242	252	ISNCIIDMNAR			Carbamidomethyl (C)[4], Oxidation (M)[8]		Mascot
1646.8174	1646.8459	0.0285	17	95	108	YAELHDFGSEILPR					Mascot
1646.8174	1646.8459	0.0285	17	95	108	YAELHDFGSEILPR	3	0			Mascot
1745.8826	1745.9778	0.0952	55	175	188	EAIILHGCFLRECK			Carbamidomethyl (C)[8,13]		Mascot
1786.7776	1786.9343	0.1567	88	132	146	SFFDANMSLCEQPPK			Carbamidomethyl (C)[10], Oxidation (M)[7]		Mascot
1910.8879	1910.9208	0.0329	17	263	278	EGVQEADRPEEGYYIR					Mascot
2055.9399	2055.9924	0.0525	26	6	24	HVDDNADITLSCAPVGES R			Carbamidomethyl (C)[12]		Mascot
2763.3162	2763.3708	0.0546	20	109	131	ALHDHNVQAYVFTDYWE DIGTIR					Mascot

5	ADP-glucose pyrophosophorylase preprotein [Triticum aestivum]	gi|21680	56037.9	6.61	11	64	98.07	9.844	28	90.88	

Protein Group

ADP glucose pyrophosphatase AGA.7	gi|226875	56037.9	6.6100 001335 144
RecName: Full=Glucose-1-phosphate adenylyltransferase large subunit, chloroplastic/amyloplastic; AltName: Full=ADP-glucose pyrophosphorylase; AltName: Full=ADP-glucose synthase; AltName: Full=AGPase S; AltName: Full=Alpha-D-glucose-1-phosphate adeny	gi|121293	56037.9	6.6100 001335 144

Peptide Information

Calc. Mass	Obsrv. Mass	± da	± ppm	Start Seq.	End Seq.	Sequence	Ion Score	C. I. %	Modification	Rank	Result Type
866.4618	866.447	-0.0148	-17	232	239	ASEYGLVK					Mascot
908.5424	908.486	-0.0564	-62	10	17	APLRAPQR					Mascot
952.4887	952.5057	0.017	18	361	368	TPFFTSPR					Mascot

	952.4887	952.5057	0.017	18	361	368	TPFFTSPR	25	82.622			Mascot
	1306.6243	1306.662	0.0377	29	446	456	ISNCIIDMNAR			Carbamidomethyl (C)[4]		Mascot
	1322.6191	1322.6451	0.026	20	446	456	ISNCIIDMNAR			Carbamidomethyl (C)[4], Oxidation (M)[8]		Mascot
	1646.8174	1646.8459	0.0285	17	302	315	YAELHDFGSEILPR					Mascot
	1646.8174	1646.8459	0.0285	17	302	315	YAELHDFGSEILPR	3	0			Mascot
	1745.8826	1745.9778	0.0952	55	382	395	EAIILHGCFLRECK			Carbamidomethyl (C)[8,13]		Mascot
	1756.9592	1756.9988	0.0396	23	189	203	SIEHILILSGDQLYR					Mascot
	1838.8776	1838.976	0.0984	54	104	119	LIDIPMSNCFNSGINK			Carbamidomethyl (C)[9], Oxidation (M)[6]		Mascot
	1910.8879	1910.9208	0.0329	17	467	482	EGVQEADRPEEGYYIR					Mascot
	2055.9399	2055.9924	0.0525	26	213	231	HVDDNADITLSCAPVGES R			Carbamidomethyl (C)[12]		Mascot
	2763.3162	2763.3708	0.0546	20	316	338	ALHDHNVQAYVFTDYWE DIGTIR					Mascot

6	unnamed protein product [Triticum aestivum]	gi|257304087	58353.9	5.89	12	63	97.883	11.991	28	90.88	

Protein Group

ADP-glucose pyrophosphorylase large subunit [Triticum aestivum]	gi|32812836	58353.9	5.8899 998664 856
unnamed protein product [Triticum aestivum]	gi|257308933	58353.9	5.8899 998664 856
unnamed protein product [Triticum aestivum]	gi|219764692	58353.9	5.8899 998664 856
unnamed protein product [Triticum aestivum]	gi|257671660	58353.9	5.8899 998664 856
unnamed protein product [Triticum aestivum]	gi|219752105	58353.9	5.8899 998664 856

Peptide Information

Calc. Mass	Obsrv. Mass	± da	± ppm	Start Seq.	End Seq.	Sequence	Ion Score	C. I. %	Modification	Rank	Result Type

866.4618	866.447	-0.0148	-17	251	258	ASEYGLVK					Mascot
952.4887	952.5057	0.017	18	380	387	TPFFTSPR					Mascot
952.4887	952.5057	0.017	18	380	387	TPFFTSPR	25	82.622			Mascot
1023.5945	1023.6124	0.0179	17	415	423	IEHSIIGVR					Mascot
1266.7278	1266.6205	-0.1073	-85	415	425	IEHSIIGVRSR					Mascot
1302.6624	1302.6932	0.0308	24	401	411	EAIISHGCFLR			Carbamidomethyl (C)[8]		Mascot
1302.6624	1302.6932	0.0308	24	401	411	EAIISHGCFLR			Carbamidomethyl (C)[8]		Mascot
1306.6243	1306.662	0.0377	29	468	478	ISNCIIDMNAR			Carbamidomethyl (C)[4]		Mascot
1322.6191	1322.6451	0.026	20	468	478	ISNCIIDMNAR			Carbamidomethyl (C)[4], Oxidation (M)[8]		Mascot
1543.8413	1543.8767	0.0354	23	399	411	IKEAIISHGCFLR			Carbamidomethyl (C)[10]		Mascot
1646.8174	1646.8459	0.0285	17	321	334	YAELHDFGSEILPR					Mascot
1646.8174	1646.8459	0.0285	17	321	334	YAELHDFGSEILPR	3	0			Mascot
1756.9592	1756.9988	0.0396	23	208	222	SIEHILILSGDQLYR					Mascot
1838.8776	1838.976	0.0984	54	123	138	LIDIPMSNCFNSGINK			Carbamidomethyl (C)[9], Oxidation (M)[6]		Mascot
1910.8879	1910.9208	0.0329	17	489	504	EGVQEADRPEEGYYIR					Mascot
2055.9399	2055.9924	0.0525	26	232	250	HVDDNADITLSCAPVGES R			Carbamidomethyl (C)[12]		Mascot

7	ADP-glucose pyrophosphorylase [Triticum aestivum]	gi|1432141	21415.7	6.58	7	59	94.303	8.817	28	90.88	

Protein Group

ADP-glucose pyrophosphorylase, partial [Triticum aestivum]	gi|1432143	21415.7	6.5799 999237 0605

Peptide Information

Calc. Mass	Obsrv. Mass	± da	± ppm	Start Seq.	End Seq.	Sequence	Ion Score	C. I. %	Modification	Rank	Result Type
952.4887	952.5057	0.017	18	85	92	TPFFTSPR					Mascot
952.4887	952.5057	0.017	18	85	92	TPFFTSPR	25	82.622			Mascot
1023.5945	1023.6124	0.0179	17	120	128	IEHSIIGVR					Mascot
1266.7278	1266.6205	-0.1073	-85	120	130	IEHSIIGVRSR					Mascot
1302.6624	1302.6932	0.0308	24	106	116	EAIISHGCFLR			Carbamidomethyl (C)[8]		Mascot
1302.6624	1302.6932	0.0308	24	106	116	EAIISHGCFLR			Carbamidomethyl (C)[8]		Mascot
1543.8413	1543.8767	0.0354	23	104	116	IKEAIISHGCFLR			Carbamidomethyl (C)[10]		Mascot
1646.8174	1646.8459	0.0285	17	26	39	YAELHDFGSEILPR					Mascot
1646.8174	1646.8459	0.0285	17	26	39	YAELHDFGSEILPR	3	0			Mascot
2763.3162	2763.3708	0.0546	20	40	62	ALHDHNVQAYVFTDYWE DIGTIR					Mascot

8	ADP-glucose pyrophosphorylase [Triticum monococcum subsp. aegilopoides]	gi|148529550	17493.5	5.21	6	55	86.016	7.001	25	82.622

Protein Group

ADP-glucose pyrophosphorylase [Triticum monococcum subsp. aegilopoides]	gi|148529546	17467.4	5.2100 000381 4697
ADP-glucose pyrophosphorylase [Triticum monococcum subsp. aegilopoides]	gi|148529544	17467.4	5.2100 000381 4697
ADP-glucose pyrophosphorylase [Triticum monococcum subsp. aegilopoides]	gi|148529534	17467.4	5.2100 000381 4697
ADP-glucose pyrophosphorylase [Triticum monococcum subsp. aegilopoides]	gi|148529528	17467.4	5.2100 000381 4697
ADP-glucose pyrophosphorylase [Triticum monococcum subsp. aegilopoides]	gi|148529530	17467.4	5.2100 000381 4697
ADP-glucose pyrophosphorylase [Triticum monococcum subsp. aegilopoides]	gi|148529532	17467.4	5.2100 000381 4697
ADP-glucose pyrophosphorylase [Triticum monococcum subsp. aegilopoides]	gi|148529526	17467.4	5.2100 000381 4697
ADP-glucose pyrophosphorylase [Triticum monococcum subsp. aegilopoides]	gi|148529522	17467.4	5.2100 000381 4697
ADP-glucose pyrophosphorylase [Triticum monococcum subsp. aegilopoides]	gi|148529520	17467.4	5.2100 000381 4697

ADP-glucose pyrophosphorylase [Triticum monococcum subsp. aegilopoides]	gi|148529552	17467.4	5.2100 000381 4697
ADP-glucose pyrophosphorylase [Triticum monococcum subsp. aegilopoides]	gi|148529524	17467.4	5.2100 000381 4697
ADP-glucose pyrophosphorylase [Triticum monococcum]	gi|148529548	17467.4	5.2100 000381 4697
ADP-glucose pyrophosphorylase [Triticum monococcum]	gi|148529516	17467.4	5.2100 000381 4697
ADP-glucose pyrophosphorylase [Triticum monococcum]	gi|148529518	17467.4	5.2100 000381 4697
ADP-glucose pyrophosphorylase [Triticum urartu]	gi|148529542	17467.4	5.2100 000381 4697
ADP-glucose pyrophosphorylase [Triticum urartu]	gi|148529540	17467.4	5.2100 000381 4697
ADP-glucose pyrophosphorylase [Triticum urartu]	gi|148529538	17467.4	5.2100 000381 4697
ADP-glucose pyrophosphorylase [Triticum urartu]	gi|148529536	17467.4	5.2100 000381 4697

Peptide Information

Calc. Mass	Obsrv. Mass	± da	± ppm	Start Seq.	End Seq.	Sequence	Ion Score	C. I. %	Modification	Rank	Result Type
952.4887	952.5057	0.017	18	55	62	TPFFTSPR					Mascot
952.4887	952.5057	0.017	18	55	62	TPFFTSPR	25	82.622			Mascot
1023.5945	1023.6124	0.0179	17	90	98	IEHSIIGVR					Mascot
1266.7278	1266.6205	-0.1073	-85	90	100	IEHSIIGVRSR					Mascot
1302.6624	1302.6932	0.0308	24	76	86	EAIISHGCFLR			Carbamidomethyl (C)[8]		Mascot
1302.6624	1302.6932	0.0308	24	76	86	EAIISHGCFLR			Carbamidomethyl (C)[8]		Mascot
1543.8413	1543.8767	0.0354	23	74	86	IKEAIISHGCFLR			Carbamidomethyl (C)[10]		Mascot
2763.3162	2763.3708	0.0546	20	10	32	ALHDHNVQAYVFTDYWE DIGTIR					Mascot

9	glutamine synthetase isoform GS1 [Triticum aestivum]	gi|321531577	39400.7	5.41	7	47	7.608	10.029	22	61.363	

Protein Group

Glutamine synthetase cytosolic isozyme 1-1 [Triticum urartu]	gi|473888366	39400.7	5.4099 998474 1211
unnamed protein product [Triticum aestivum]	gi|227478291	39400.7	5.4099 998474 1211
unnamed protein product [Triticum aestivum]	gi|227478289	39416.7	5.4099 998474 1211

Peptide Information

Calc. Mass	Obsrv. Mass	± da	± ppm	Start Seq.	End Seq.	Sequence	Ion Score	C. I. %	Modification	Rank	Result Type
872.5087	872.5273	0.0186	21	265	272	VIVDAVEK					Mascot
1345.6132	1345.646	0.0328	24	279	290	EHIAAYGEGNER					Mascot
1345.6132	1345.646	0.0328	24	279	290	EHIAAYGEGNER					Mascot
1501.7142	1501.728	0.0138	9	279	291	EHIAAYGEGNERR					Mascot
1501.7142	1501.728	0.0138	9	279	291	EHIAAYGEGNERR					Mascot
1610.767	1610.807	0.04	25	277	290	HKEHIAAYGEGNER					Mascot
1793.9255	1793.9658	0.0403	22	19	34	IIAEYIWIGGSGMDLR					Mascot

1809.9204	1809.9457	0.0253	14	19	34	IIAEYIWIGGSGMDLR			Oxidation (M)[13]		Mascot
1817.8566	1817.8987	0.0421	23	296	311	HETADINTFSWGVANR					Mascot
1817.8566	1817.8987	0.0421	23	296	311	HETADINTFSWGVANR	22	61.363			Mascot
2288.1167	2288.1177	0.001	0	296	316	HETADINTFSWGVANRG ASVR					Mascot

10	unnamed protein product [Triticum aestivum]	gi|227478287	39444.7	5.41	1	0	22	61.363	

Protein Group

glutamine synthetase isoform GS1a [Triticum aestivum]	gi|71361900	39400.7	5.4099 998474 1211
glutamine synthetase isoform GS1b [Triticum aestivum]	gi|71361902	39416.7	5.4099 998474 1211
glutamine synthetase isoform GS1c [Triticum aestivum]	gi|71361904	39444.7	5.4099 998474 1211
unnamed protein product [Triticum aestivum]	gi|227477545	39400.7	5.4099 998474 1211
unnamed protein product [Triticum aestivum]	gi|227477543	39416.7	5.4099 998474 1211
unnamed protein product [Triticum aestivum]	gi|227477541	39444.7	5.4099 998474 1211

Peptide Information

Calc. Mass	Obsrv. Mass	± da	± ppm	Start Seq.	End Seq.	Sequence	Ion Score	C. I. %	Modification	Rank	Result Type
1817.8566	1817.8987	0.0421	23	296	311	HETADINTFSWGVANR	22	61.363		1	Mascot

Gel Idx/Pos	220/I20	Instr./Gel Origin	BA2151/Sample Project 20140814	Process Status	Analysis Succeeded
Plate [#] Name	[1] Sample Project 20140814	Instrument Sample Name		Spectra	11

Rank	Protein Name	Accession No.	Protein MW	Protein PI	Pep. Count	Protein Score	Protein Score C. I. %	Intensity Matched	Total Ion Score	Total Ion C. I. %	Confirmed
1	unnamed protein product [Triticum aestivum]	gi|227478321	38990.4	5.35	11	210	100	17.285	152	100	

Protein Group

Protein Name	Accession No.	Protein MW	Protein PI
glutamine synthetase isoform GSr1 [Triticum aestivum]	gi|40317416	38990.4	5.3499999046 3257
unnamed protein product [Triticum aestivum]	gi|227477575	38990.4	5.3499999046 3257

Peptide Information

Calc. Mass	Obsrv. Mass	± da	± ppm	Start Seq.	End Seq.	Sequence	Ion Score	C. I. %	Modification	Rank	Result Type
1173.6124	1173.6902	0.0778	66	306	316	WGVADRGASVR					Mascot
1440.7693	1440.8202	0.0509	35	39	52	TVNGPITDASQLPK					Mascot
1552.7251	1552.7704	0.0453	29	277	290	HAQHIAAYGEGNER					Mascot
1552.7251	1552.7704	0.0453	29	277	290	HAQHIAAYGEGNER	129	100			Mascot
1708.8263	1708.8668	0.0405	24	277	291	HAQHIAAYGEGNERR					Mascot
1739.8824	1739.9308	0.0484	28	291	305	RLTGHHETADINTFK					Mascot
1739.8824	1739.9308	0.0484	28	291	305	RLTGHHETADINTFK	4	0			Mascot
1789.9847	1790.0315	0.0468	26	19	34	IIVEYLWVGGSGIDIR					Mascot
1789.9847	1790.0315	0.0468	26	19	34	IIVEYLWVGGSGIDIR	19	15.692			Mascot
1849.8848	1849.8967	0.0119	6	2	18	ASLADLVNLDLSDCTDK			Carbamidomethyl (C)[14]		Mascot
2271.1179	2271.1506	0.0327	14	119	137	VAAEETWYGIEQEYTLLQK					Mascot
2438.2307	2438.2896	0.0589	24	333	354	RPASNMDPYVVTSMIAETTLLL			Oxidation (M)[6]		Mascot
2454.2256	2454.2661	0.0405	17	333	354	RPASNMDPYVVTSMIAETTLLL			Oxidation (M)[6,14]		Mascot
2648.2629	2648.3406	0.0777	29	85	107	GDNLLVMCDCYTPQGVPIPTNKR			Carbamidomethyl (C)[8,10]		Mascot
2664.2578	2664.3223	0.0645	24	85	107	GDNLLVMCDCYTPQGVPIPTNKR			Carbamidomethyl (C)[8,10], Oxidation (M)[7]		Mascot
2972.3948	2972.4504	0.0556	19	53	79	WNYDGSSTGQAPGEDSEVILYPQAIFK					Mascot

Rank	Protein Name	Accession No.	Protein MW	Protein PI	Pep. Count	Protein Score	Protein Score C. I. %	Intensity Matched	Total Ion Score	Total Ion C. I. %	Confirmed
2	unnamed protein product [Triticum aestivum]	gi|298556780	38918.3	5.34	9	195	100	16.5	152	100	

Protein Group

glutamine synthetase isoform GSr2 [Triticum aestivum]	gi|40317418	38918.3	5.3400 001525 8789
unnamed protein product [Triticum aestivum]	gi|227478319	38918.3	5.3400 001525 8789
unnamed protein product [Triticum aestivum]	gi|227477573	38918.3	5.3400 001525 8789

Peptide Information

Calc. Mass	Obsrv. Mass	± da	± ppm	Start Seq.	End Seq.	Sequence	Ion Score	C. I. %	Modification	Rank	Result Type
1440.7693	1440.8202	0.0509	35	39	52	TVNGPITDASQLPK					Mascot
1552.7251	1552.7704	0.0453	29	277	290	HAQHIAAYGEGNER					Mascot
1552.7251	1552.7704	0.0453	29	277	290	HAQHIAAYGEGNER	129	100			Mascot
1708.8263	1708.8668	0.0405	24	277	291	HAQHIAAYGEGNERR					Mascot
1739.8824	1739.9308	0.0484	28	291	305	RLTGHHETADINTFK					Mascot
1739.8824	1739.9308	0.0484	28	291	305	RLTGHHETADINTFK	4	0			Mascot
1789.9847	1790.0315	0.0468	26	19	34	IIVEYLWVGGSGIDIR					Mascot
1789.9847	1790.0315	0.0468	26	19	34	IIVEYLWVGGSGIDIR	19	15.692			Mascot
1849.8848	1849.8967	0.0119	6	2	18	ASLADLVNLDLSDCTDK			Carbamidomethyl (C)[14]		Mascot
2438.2307	2438.2896	0.0589	24	333	354	RPASNMDPYVVTSMIAET TLLL			Oxidation (M)[6]		Mascot
2454.2256	2454.2661	0.0405	17	333	354	RPASNMDPYVVTSMIAET TLLL			Oxidation (M)[6,14]		Mascot
2648.2629	2648.3406	0.0777	29	85	107	GDNLLVMCDCYTPQGVP IPTNKR			Carbamidomethyl (C)[8,10]		Mascot
2664.2578	2664.3223	0.0645	24	85	107	GDNLLVMCDCYTPQGVP IPTNKR			Carbamidomethyl (C)[8,10], Oxidation (M)[7]		Mascot
2972.3948	2972.4504	0.0556	19	53	79	WNYDGSSTGQAPGEDS EVILYPQAIFK					Mascot

3	Glutamine synthetase cytosolic isozyme 1-2 [Triticum urartu]	gi|473798921	42771.6	8.08	10	179	100	15.442	133	100

Peptide Information

Calc. Mass	Obsrv. Mass	± da	± ppm	Start Seq.	End Seq.	Sequence	Ion Score	C. I. %	Modification	Rank	Result Type

Calc. Mass	Obsrv. Mass	± da	± ppm	Start Seq.	End Seq.	Sequence	Ion Score	C. I. %	Modification	Rank	Result Type
1132.5382	1132.6221	0.0839	74	146	155	SYQNGLADHK					Mascot
1370.6562	1370.7211	0.0649	47	132	141	CFKFIYYSSR			Carbamidomethyl (C)[1]		Mascot
1440.7693	1440.8202	0.0509	35	39	52	TVNGPITDASQLPK					Mascot
1552.7251	1552.7704	0.0453	29	304	317	HAQHIAAYGEGNER					Mascot
1552.7251	1552.7704	0.0453	29	304	317	HAQHIAAYGEGNER	129	100			Mascot
1708.8263	1708.8668	0.0405	24	304	318	HAQHIAAYGEGNERR					Mascot
1739.8824	1739.9308	0.0484	28	318	332	RLTGHHETADINTFK					Mascot
1739.8824	1739.9308	0.0484	28	318	332	RLTGHHETADINTFK	4	0			Mascot
1849.8848	1849.8967	0.0119	6	2	18	ASLADLVNLDLSDCTDK			Carbamidomethyl (C)[14]		Mascot
2438.2307	2438.2896	0.0589	24	360	381	RPASNMDPYVVTSMIAET TLLL			Oxidation (M)[6]		Mascot
2454.2256	2454.2661	0.0405	17	360	381	RPASNMDPYVVTSMIAET TLLL			Oxidation (M)[6,14]		Mascot
2648.2629	2648.3406	0.0777	29	85	107	GDNLLVMCDCYTPQGVP IPTNKR			Carbamidomethyl (C)[8,10]		Mascot
2664.2578	2664.3223	0.0645	24	85	107	GDNLLVMCDCYTPQGVP IPTNKR			Carbamidomethyl (C)[8,10], Oxidation (M)[7]		Mascot
2972.3948	2972.4504	0.0556	19	53	79	WNYDGSSTGQAPGEDS EVILYPQAIFK					Mascot

4 unnamed protein product [Triticum aestivum] gi|227478731 38946.4 5.46 9 176 100 15.367 133 100

Protein Group

unnamed protein product [Triticum aestivum] gi|227479475 38946.4 5.4600 000381 4697

Peptide Information

Calc. Mass	Obsrv. Mass	± da	± ppm	Start Seq.	End Seq.	Sequence	Ion Score	C. I. %	Modification	Rank	Result Type
1440.7693	1440.8202	0.0509	35	39	52	TVNGPITDASQLPK					Mascot
1552.7251	1552.7704	0.0453	29	277	290	HAQHIAAYGEGNER					Mascot
1552.7251	1552.7704	0.0453	29	277	290	HAQHIAAYGEGNER	129	100			Mascot
1708.8263	1708.8668	0.0405	24	277	291	HAQHIAAYGEGNERR					Mascot
1739.8824	1739.9308	0.0484	28	291	305	RLTGHHETADINTFK					Mascot
1739.8824	1739.9308	0.0484	28	291	305	RLTGHHETADINTFK	4	0			Mascot
1849.8848	1849.8967	0.0119	6	2	18	ASLADLVNLDLSDCTDK			Carbamidomethyl (C)[14]		Mascot
2271.1179	2271.1506	0.0327	14	119	137	VAAEETWYGIEQEYTLLQ K					Mascot
2438.2307	2438.2896	0.0589	24	333	354	RPASNMDPYVVTSMIAET TLLL			Oxidation (M)[6]		Mascot
2454.2256	2454.2661	0.0405	17	333	354	RPASNMDPYVVTSMIAET TLLL			Oxidation (M)[6,14]		Mascot
2648.2629	2648.3406	0.0777	29	85	107	GDNLLVMCDCYTPQGVP			Carbamidomethyl (C)[8,10]		Mascot

						IPTNKR					
2664.2578	2664.3223	0.0645	24	85	107	GDNLLVMCDCYTPQGVP IPTNKR			Carbamidomethyl (C)[8,10], Oxidation (M)[7]		Mascot
2972.3948	2972.4504	0.0556	19	53	79	WNYDGSSTGQAPGEDS EVILYPQAIFK					Mascot

5	ADP-glucose pyrophosophorylase (1 is 2nd base in codon) [Triticum aestivum]	gi|829293	33580.7	5.13	9	56	89.869	3.737	18	0	

Protein Group

ADP glucose pyrophosphatase AGA.3	gi|226874	33580.7	5.1300 001144 4092

Peptide Information

Calc. Mass	Obsrv. Mass	± da	± ppm	Start Seq.	End Seq.	Sequence	Ion Score	C. I. %	Modification	Rank	Result Type
866.4618	866.468	0.0062	7	25	32	ASEYGLVK					Mascot
927.5873	927.5176	-0.0697	-75	85	92	DVLLNLLK					Mascot
952.4887	952.5087	0.02	21	154	161	TPFFTSPR					Mascot
1370.8367	1370.7211	-0.1156	-84	279	291	SGIVVIQKNATIK					Mascot
1646.8174	1646.8601	0.0427	26	95	108	YAELHDFGSEILPR					Mascot
1646.8174	1646.8601	0.0427	26	95	108	YAELHDFGSEILPR	18	0			Mascot
1710.881	1710.8741	-0.0069	-4	33	47	FDSSGRVVQFSEKPK					Mascot
1745.8826	1745.9574	0.0748	43	175	188	EAIILHGCFLRECK			Carbamidomethyl (C)[8,13]		Mascot
1910.8879	1910.9399	0.052	27	263	278	EGVQEADRPEEGYYIR					Mascot
2055.9399	2056.0344	0.0945	46	6	24	HVDDNADITLSCAPVGES R			Carbamidomethyl (C)[12]		Mascot

6	unnamed protein product [Triticum aestivum]	gi|257304087	58353.9	5.89	12	56	88.633	4.868	18	0	

Protein Group

ADP-glucose pyrophosphorylase large subunit [Triticum aestivum]	gi|32812836	58353.9	5.8899 998664 856
unnamed protein product [Triticum aestivum]	gi|257308933	58353.9	5.8899 998664 856
unnamed protein product [Triticum aestivum]	gi|219764692	58353.9	5.8899 998664 856

unnamed protein product [Triticum aestivum]	gi|257671660	58353.9	5.8899 998664 856
unnamed protein product [Triticum aestivum]	gi|219752105	58353.9	5.8899 998664 856

Peptide Information

Calc. Mass	Obsrv. Mass	± da	± ppm	Start Seq.	End Seq.	Sequence	Ion Score	C. I. %	Modification	Rank	Result Type
866.4618	866.468	0.0062	7	251	258	ASEYGLVK					Mascot
927.5873	927.5176	-0.0697	-75	311	318	DVLLNLLK					Mascot
952.4887	952.5087	0.02	21	380	387	TPFFTSPR					Mascot
1302.6624	1302.6931	0.0307	24	401	411	EAIISHGCFLR			Carbamidomethyl (C)[8]		Mascot
1370.8367	1370.7211	-0.1156	-84	505	517	SGIVVIQKNATIK					Mascot
1440.7628	1440.8202	0.0574	40	412	423	ECKIEHSIIGVR			Carbamidomethyl (C)[2]		Mascot
1617.8312	1617.87	0.0388	24	196	207	FIWVLEDYYKNK					Mascot
1646.8174	1646.8601	0.0427	26	321	334	YAELHDFGSEILPR					Mascot
1646.8174	1646.8601	0.0427	26	321	334	YAELHDFGSEILPR	18	0			Mascot
1710.881	1710.8741	-0.0069	-4	259	273	FDSSGRVVQFSEKPK					Mascot
1756.9592	1757.0048	0.0456	26	208	222	SIEHILILSGDQLYR					Mascot
1910.8879	1910.9399	0.052	27	489	504	EGVQEADRPEEGYYIR					Mascot
2055.9399	2056.0344	0.0945	46	232	250	HVDDNADITLSCAPVGES R			Carbamidomethyl (C)[12]		Mascot

7	ADP-glucose pyrophosphorylase large subunit [Triticum aestivum]	gi|110729318	58399.1	6.12	12	54	83.944	4.868	18	0	

Protein Group

ADP-glucose pyrophosphorylase large subunit [Triticum aestivum]	gi|995746	58399.1	6.1199 998855 5908
RecName: Full=Glucose-1-phosphate adenylyltransferase large subunit, chloroplastic/amyloplastic; AltName: Full=ADP-glucose pyrophosphorylase; AltName: Full=ADP-glucose synthase; AltName: Full=AGPase S; AltName: Full=Alpha-D-glucose-1-phosphate adeny	gi|1707930	58399.1	6.1199 998855 5908

unnamed protein product [Triticum aestivum]	gi|300634107	58399.1	6.1199 998855 5908
unnamed protein product [Triticum aestivum]	gi|300544593	58399.1	6.1199 998855 5908
unnamed protein product [Triticum aestivum]	gi|259471795	58399.1	6.1199 998855 5908
unnamed protein product [Triticum aestivum]	gi|259439208	58399.1	6.1199 998855 5908
unnamed protein product [Triticum aestivum]	gi|257671708	58399.1	6.1199 998855 5908
unnamed protein product [Triticum aestivum]	gi|257306977	58399.1	6.1199 998855 5908
unnamed protein product [Triticum aestivum]	gi|257308981	58399.1	6.1199 998855 5908
unnamed protein product [Triticum aestivum]	gi|219764742	58399.1	6.1199 998855 5908
unnamed protein product [Triticum aestivum]	gi|219752155	58399.1	6.1199 998855 5908

Peptide Information

Calc. Mass	Obsrv. Mass	± da	± ppm	Start	End	Sequence	Ion	C. I. %	Modification	Rank	Result Type

Calc. Mass	Obsrv. Mass	± da	± ppm	Start Seq.	End Seq.	Sequence	Ion Score	C. I. %	Modification	Rank	Result Type
866.4618	866.468	0.0062	7	251	258	ASEYGLVK					Mascot
927.5873	927.5176	-0.0697	-75	311	318	DVLLNLLK					Mascot
952.4887	952.5087	0.02	21	380	387	TPFFTSPR					Mascot
1302.6624	1302.6931	0.0307	24	401	411	EAIISHGCFLR			Carbamidomethyl (C)[8]		Mascot
1370.8367	1370.7211	-0.1156	-84	505	517	SGIVVIQKNATIK					Mascot
1440.7628	1440.8202	0.0574	40	412	423	ECKIEHSIIGVR			Carbamidomethyl (C)[2]		Mascot
1617.8312	1617.87	0.0388	24	196	207	FIWVLEDYYKNK					Mascot
1646.8174	1646.8601	0.0427	26	321	334	YAELHDFGSEILPR					Mascot
1646.8174	1646.8601	0.0427	26	321	334	YAELHDFGSEILPR	18	0			Mascot
1710.881	1710.8741	-0.0069	-4	259	273	FDSSGRVVQFSEKPK					Mascot
1756.9592	1757.0048	0.0456	26	208	222	SIEHILILSGDQLYR					Mascot
1910.8879	1910.9399	0.052	27	489	504	EGVQEADRPEEGYYIR					Mascot
2055.9399	2056.0344	0.0945	46	232	250	HVDDNADITLSCAPVGES R			Carbamidomethyl (C)[12]		Mascot

8	ADP-glucose-pyrophosphorylase large subunit [Triticum aestivum]	gi|2583072	32915.3	5.21	9	53	77.32	3.77	18	0		

Peptide Information

Calc. Mass	Obsrv. Mass	± da	± ppm	Start Seq.	End Seq.	Sequence	Ion Score	C. I. %	Modification	Rank	Result Type
866.4618	866.468	0.0062	7	19	26	ASEYGLVK					Mascot
927.5873	927.5176	-0.0697	-75	79	86	DVLLNLLK					Mascot
952.4887	952.5087	0.02	21	148	155	TPFFTSPR					Mascot
1302.6624	1302.6931	0.0307	24	169	179	EAIISHGCFLR			Carbamidomethyl (C)[8]		Mascot
1370.8367	1370.7211	-0.1156	-84	273	285	SGIVVIQKNATIK					Mascot
1440.7628	1440.8202	0.0574	40	180	191	ECKIEHSIIGVR			Carbamidomethyl (C)[2]		Mascot
1646.8174	1646.8601	0.0427	26	89	102	YAELHDFGSEILPR					Mascot
1646.8174	1646.8601	0.0427	26	89	102	YAELHDFGSEILPR	18	0			Mascot
1710.881	1710.8741	-0.0069	-4	27	41	FDSSGRVVQFSEKPK					Mascot
1910.8879	1910.9399	0.052	27	257	272	EGVQEADRPEEGYYIR					Mascot

9	pre-sequence wheat 33K [Triticum aestivum]	gi|579235	7845	9.84	6	50	58.73	1.609				

Peptide Information

Calc. Mass	Obsrv. Mass	± da	± ppm	Start Seq.	End Seq.	Sequence	Ion Score	C. I. %	Modification	Rank	Result Type
1149.5569	1149.6318	0.0749	65	51	61	EVASKCADAAK			Carbamidomethyl (C)[6]		Mascot
1192.5991	1192.5765	-0.0226	-19	41	50	ITCSLQSDIR			Carbamidomethyl (C)[3]		Mascot

1343.7351	1343.7076	-0.0275	-20	2	15	AASLQAAATLMPAK						Mascot
1608.8678	1608.8066	-0.0612	-38	16	31	IGGRASSARPSSHVAR						Mascot
1742.9581	1742.943	-0.0151	-9	2	19	AASLQAAATLMPAKIGGR			Oxidation (M)[11]			Mascot
1873.9987	1874.0134	0.0147	8	1	19	MAASLQAAATLMPAKIGG R			Oxidation (M)[1]			Mascot

10	plastid acetyl co-enzyme A carboxylase, partial [Triticum aestivum]	gi|379997141	42349.5	5.29	11	50	52.616	3.966	

Peptide Information

Calc. Mass	Obsrv. Mass	± da	± ppm	Start Seq.	End Seq.	Sequence	Ion Score	C. I. %	Modification	Rank	Result Type
925.5465	925.5417	-0.0048	-5	30	38	ISASVIAHK					Mascot
927.457	927.5176	0.0606	65	370	376	IEFYAER					Mascot
1182.5573	1182.5966	0.0393	33	291	300	TAQAMLDFNR			Oxidation (M)[5]		Mascot
1415.6478	1415.7003	0.0525	37	223	234	DSFVETFEGWAK					Mascot
1440.7522	1440.8202	0.068	47	338	349	TYNQPAFVYIPK					Mascot
1617.8846	1617.87	-0.0146	-9	110	124	TDQPIILTGFSALNK					Mascot
1745.897	1745.9574	0.0604	35	275	290	SVPRAGQVWFPDSATK					Mascot
1826.0211	1825.9365	-0.0846	-46	166	182	WLSYVPANIGGPLPITK					Mascot
2454.187	2454.2661	0.0791	32	279	300	AGQVWFPDSATKTAQAM LDFNR					Mascot
2648.3467	2648.3406	-0.0061	-2	16	38	GFQYIYLTEEDHARISAS VIAHK					Mascot
2664.374	2664.3223	-0.0517	-19	313	337	GFSGGQRDLFEGILQAG STIVENLR					Mascot

Gel Idx/Pos	221/I21	Instr./Gel Origin	BA2151/Sample Project 20140814	Process Status	Analysis Succeeded
Plate [#] Name	[1] Sample Project 20140814	Instrument Sample Name		Spectra	11

Rank	Protein Name	Accession No.	Protein MW	Protein PI	Pep. Count	Protein Score	Protein Score C. I. %	Intensity Matched	Total Ion Score	Total Ion C. I. %	Confirmed
1	Fructose-bisphosphate aldolase, chloroplastic [Triticum urartu]	gi|473848356	42204.5	5.94	14	144	100	26.182	75	100	

Peptide Information

Calc. Mass	Obsrv. Mass	± da	± ppm	Start Seq.	End Seq.	Sequence	Ion Score	C. I. %	Modification	Rank	Result Type
822.4355	822.4597	0.0242	29	229	235	TFEVAQK					Mascot
873.4577	873.501	0.0433	50	193	200	EAAWGLAR					Mascot
901.5101	901.4962	-0.0139	-15	359	367	ANSLAQLGK					Mascot
947.4979	947.5263	0.0284	30	330	337	ALQNTCLK			Carbamidomethyl (C)[6]		Mascot
1156.5382	1156.5863	0.0481	42	163	172	EAAYYQQGAR					Mascot
1156.5382	1156.5863	0.0481	42	163	172	EAAYYQQGAR	75	100			Mascot
1308.6682	1308.7185	0.0503	38	271	282	ATPEEVASYTLK					Mascot
1387.7175	1387.7709	0.0534	38	73	85	LASIGLENTEANR					Mascot
1510.8475	1510.8711	0.0236	16	178	192	TVVSIPNGPSELAVK					Mascot
1543.8187	1543.9049	0.0862	56	72	85	RLASIGLENTEANR					Mascot
1569.8782	1569.8785	0.0003	0	23	37	QAAAPKPVTVSMVVR			Oxidation (M)[12]		Mascot
1592.9622	1592.9935	0.0313	20	122	136	IVDILVEQGIVPGIK					Mascot
1721.0571	1721.0935	0.0364	21	121	136	KIVDILVEQGIVPGIK					Mascot
2052.0986	2052.1704	0.0718	35	338	356	TWGGRPENVAAAQEALLLR					Mascot
2430.1719	2430.2747	0.1028	42	140	162	GLVPLVGSNDESWCQGLDGLASR			Carbamidomethyl (C)[14]		Mascot

Rank	Protein Name	Accession No.	Protein MW	Protein PI	Pep. Count	Protein Score	Protein Score C. I. %	Intensity Matched	Total Ion Score	Total Ion C. I. %	Confirmed
2	chloroplast fructose-bisphosphate aldolase [Triticum aestivum]	gi|223018643	42216.6	5.94	13	137	100	26.041	75	100	

Peptide Information

Calc. Mass	Obsrv. Mass	± da	± ppm	Start Seq.	End Seq.	Sequence	Ion Score	C. I. %	Modification	Rank	Result Type
822.4355	822.4597	0.0242	29	229	235	TFEVAQK					Mascot
873.4577	873.501	0.0433	50	193	200	EAAWGLAR					Mascot
947.4979	947.5263	0.0284	30	330	337	ALQNTCLK			Carbamidomethyl (C)[6]		Mascot
1156.5382	1156.5863	0.0481	42	163	172	EAAYYQQGAR					Mascot
1156.5382	1156.5863	0.0481	42	163	172	EAAYYQQGAR	75	100			Mascot
1308.6682	1308.7185	0.0503	38	271	282	ATPEEVASYTLK					Mascot

Calc. Mass	Obsrv. Mass	± da	± ppm	Start Seq.	End Seq.	Sequence	Ion Score	C. I. %	Modification	Rank	Result Type
1387.7175	1387.7709	0.0534	38	73	85	LASIGLENTEANR					Mascot
1510.8475	1510.8711	0.0236	16	178	192	TVVSIPNGPSELAVK					Mascot
1543.8187	1543.9049	0.0862	56	72	85	RLASIGLENTEANR					Mascot
1569.8782	1569.8785	0.0003	0	23	37	QAAAPKPVTVSMVVR			Oxidation (M)[12]		Mascot
1592.9622	1592.9935	0.0313	20	122	136	IVDILVEQGIVPGIK					Mascot
1721.0571	1721.0935	0.0364	21	121	136	KIVDILVEQGIVPGIK					Mascot
2052.0986	2052.1704	0.0718	35	338	356	TWGGRPENVAAAQEALL LR					Mascot
2430.1719	2430.2747	0.1028	42	140	162	GLVPLVGSNDESWCQGL DGLASR			Carbamidomethyl (C)[14]		Mascot

3 ATP synthase beta subunit [Triticum aestivum] gi|525291 59325.9 5.56 13 50 56.785 4.322

Protein Group

unnamed protein product [Triticum aestivum]	gi|227303166	59325.9	5.5599 999427 7954

Peptide Information

Calc. Mass	Obsrv. Mass	± da	± ppm	Start Seq.	End Seq.	Sequence	Ion Score	C. I. %	Modification	Rank	Result Type
1135.6007	1135.6478	0.0471	41	183	191	THDFLPIHR					Mascot
1173.6627	1173.6998	0.0371	32	212	221	VVDLLAPYQR					Mascot
1347.7631	1347.804	0.0409	30	167	178	IINVIGEPIDHK					Mascot
1390.6863	1390.7452	0.0589	42	249	262	AHGGFSVFAGVGER					Mascot
1399.7693	1399.8275	0.0582	42	307	319	VGLTGLTVAEHFR					Mascot
1409.8112	1409.8662	0.055	39	148	161	VLNTGSPITVPVGR					Mascot
1492.7755	1492.8411	0.0656	44	336	349	FTQANSEVSALLGR					Mascot
1675.949	1676.0194	0.0704	42	118	132	LVLEVAQHLGENVVR					Mascot
1864.944	1865.0157	0.0717	38	320	335	DAEGQDVLLFIDNIFR					Mascot
1908.1025	1908.0735	-0.029	-15	148	166	VLNTGSPITVPVGRATLG R					Mascot
2186.1453	2186.2415	0.0962	44	350	370	IPSAVGYQPTLATDLGGL QER					Mascot
2430.3789	2430.2747	-0.1042	-43	225	248	IGLFGGAGVGKTVLIMELI NNVAK			Oxidation (M)[16]		Mascot
2591.3135	2591.4238	0.1103	43	73	97	ITDEFTGAGSIGQVCQVI GAVVDVR			Carbamidomethyl (C)[15]		Mascot

4 Long chain acyl-CoA synthetase 9, chloroplastic [Triticum urartu] gi|474094741 122821.7 7.86 21 50 53.694 6.779

Peptide Information

Calc. Mass	Obsrv. Mass	± da	± ppm	Start Seq.	End Seq.	Sequence	Ion Score	C. I. %	Modification	Rank	Result Type

Calc. Mass	Obsrv. Mass	± da	± ppm	Start Seq.	End Seq.	Sequence	Ion Score	C. I. %	Modification	Rank	Result Type
855.5522	855.4846	-0.0676	-79	818	825	AILGGRIR					Mascot
871.456	871.4879	0.0319	37	513	519	TYIEAFK					Mascot
887.5197	887.4814	-0.0383	-43	1037	1044	EVLQSLAK					Mascot
889.4567	889.4929	0.0362	41	195	201	YGVFTFR					Mascot
947.4428	947.5263	0.0835	88	491	499	ETEVAADGR					Mascot
1143.6732	1143.6388	-0.0344	-30	1037	1047	EVLQSLAKAGK					Mascot
1143.6732	1143.6388	-0.0344	-30	302	313	GKLAIGLDGATK					Mascot
1178.6239	1178.5887	-0.0352	-30	876	886	VGAPLSCSFIK			Carbamidomethyl (C)[7]		Mascot
1251.6741	1251.7062	0.0321	26	249	257	CILLMIFWR			Carbamidomethyl (C)[1]		Mascot
1292.6594	1292.6969	0.0375	29	536	546	DERVAIFADTR					Mascot
1342.7577	1342.7056	-0.0521	-39	597	608	LIDINGQLDTLK					Mascot
1387.6774	1387.7709	0.0935	67	398	409	MNYITDSVTISK			Oxidation (M)[1]		Mascot
1492.7366	1492.8411	0.1045	70	547	558	AEWQIALQACFR			Carbamidomethyl (C)[10]		Mascot
1532.8068	1532.8721	0.0653	43	826	840	FILSGGAPLSGETQR					Mascot
1566.8169	1566.8159	-0.001	-1	1	14	MAAPKPKNHVSNTR			Oxidation (M)[1]		Mascot
1600.8087	1600.9432	0.1345	84	360	372	IIFSACRALFSCR			Carbamidomethyl (C)[6,12]		Mascot
1630.8105	1630.9734	0.1629	100	398	411	MNYITDSVTISKSR			Oxidation (M)[1]		Mascot
1665.8271	1665.9258	0.0987	59	500	512	SFEKLHLGEYEWK					Mascot
2051.9744	2052.1704	0.196	96	887	904	LIDWVEGGYLTSDSPMP R			Oxidation (M)[16]		Mascot
2084.1169	2084.1321	0.0152	7	747	766	GDASALRPTLMTAVPAIL DR			Oxidation (M)[11]		Mascot
2430.197	2430.2747	0.0777	32	114	134	EMKPDLGFLVTDGTTYLR DSR			Oxidation (M)[2]		Mascot

5 hypothetical protein TRIUR3_25094 [Triticum urartu] gi|474448549 64241.6 6.13 6 47 17.656 5.63 35 97.851

Peptide Information

Calc. Mass	Obsrv. Mass	± da	± ppm	Start Seq.	End Seq.	Sequence	Ion Score	C. I. %	Modification	Rank	Result Type
984.5472	984.6262	0.079	80	238	246	AAQISIEPR					Mascot
984.5472	984.6262	0.079	80	238	246	AAQISIEPR	35	97.851			Mascot
1155.535	1155.5908	0.0558	48	153	162	SPSYNIESMK					Mascot
1171.53	1171.6323	0.1023	87	153	162	SPSYNIESMK			Oxidation (M)[9]		Mascot
1328.7169	1328.7007	-0.0162	-12	235	246	SEKAAQISIEPR					Mascot
1569.7955	1569.8785	0.083	53	127	140	MLNGFARGQHDVPK					Mascot
1983.8107	1983.9323	0.1216	61	302	318	HTCFTQCEASTGNMPSR			Carbamidomethyl (C)[3,7]		Mascot
2185.1936	2185.2559	0.0623	29	247	266	NQSALNLIQVNGSRSILE TK					Mascot

6 Isocitrate dehydrogenase [NADP] [Triticum urartu] gi|473892099 38929.5 5.54 11 43 0 25.605

Peptide Information

Calc. Mass	Obsrv. Mass	± da	± ppm	Start Seq.	End Seq.	Sequence	Ion Score	C. I. %	Modification	Rank	Result Type
966.5268	966.5223	-0.0045	-5	272	278	HFRLHEK					Mascot
1054.5891	1054.5814	-0.0077	-7	332	341	DLALLSHGTK					Mascot
1135.651	1135.6478	-0.0032	-3	161	169	KWPLYLSTK					Mascot
1156.5092	1156.5863	0.0771	67	218	227	SEGGYVWACK			Carbamidomethyl (C)[9]		Mascot
1156.5092	1156.5863	0.0771	67	218	227	SEGGYVWACK			Carbamidomethyl (C)[9]		Mascot
1335.7559	1335.7206	-0.0353	-26	18	28	LIFPYLDLDVK					Mascot
1355.6914	1355.7061	0.0147	11	259	271	TLEAEAAHGTVTR					Mascot
1390.6996	1390.7452	0.0456	33	84	95	CATITPDETRVK			Carbamidomethyl (C)[1]		Mascot
1487.7233	1487.8141	0.0908	61	148	161	AFAESSMAMALSKK			Oxidation (M)[7]		Mascot
1578.8778	1578.9756	0.0978	62	16	28	DKLIFPYLDLDVK					Mascot
2341.2585	2341.2424	-0.0161	-7	51	71	WITSGSRMTNLITSILYAL GK			Oxidation (M)[8]		Mascot
3048.4475	3048.7109	0.2634	86	132	160	GPGVALAMYNVDESIRAF AESSMAMALSK			Oxidation (M)[8,23]		Mascot

7 hypothetical protein TRIUR3_09113 [Triticum urartu] gi|474039660 225607 5.03 30 42 0 12.272

Peptide Information

Calc. Mass	Obsrv. Mass	± da	± ppm	Start Seq.	End Seq.	Sequence	Ion Score	C. I. %	Modification	Rank	Result Type
877.4526	877.4959	0.0433	49	1892	1899	EGGFPSKR					Mascot
887.5673	887.4814	-0.0859	-97	1104	1111	TLLKLSGR					Mascot
901.5287	901.4962	-0.0325	-36	63	69	KRPELMK					Mascot
1081.4644	1081.5247	0.0603	56	1655	1664	DEESTNSTAK					Mascot
1135.6106	1135.6478	0.0372	33	987	995	DLVYDQVRK					Mascot
1138.5562	1138.58	0.0238	21	921	929	AEFEDMRIK					Mascot
1147.6205	1147.6328	0.0123	11	471	480	LTQDLDTTIK					Mascot
1155.5826	1155.5908	0.0082	7	740	749	DLHCELGALK			Carbamidomethyl (C)[4]		Mascot
1197.5504	1197.6063	0.0559	47	1966	1975	SPFCGCVRSK			Carbamidomethyl (C)[4,6]		Mascot
1285.7474	1285.7229	-0.0245	-19	1086	1096	ILISQLEDKAR					Mascot
1292.6403	1292.6969	0.0566	44	219	229	LTDEMALEVQK			Oxidation (M)[5]		Mascot
1328.6047	1328.7007	0.096	72	1521	1531	EMALQFMMEAK					Mascot
1335.6638	1335.7206	0.0568	43	524	535	VSDLESQLSETK					Mascot
1355.71	1355.7061	-0.0039	-3	597	607	LHREIEMLNGK			Oxidation (M)[7]		Mascot
1357.7686	1357.7185	-0.0501	-37	298	308	EEVQRLTIEIK					Mascot
1418.756	1418.7769	0.0209	15	830	841	ALLSEKLQEMEK					Mascot

1431.7261	1431.7681	0.042	29	1065	1076	DENVALLNKCQK			Carbamidomethyl (C)[10]		Mascot
1431.7261	1431.7681	0.042	29	1065	1076	DENVALLNKCQK			Carbamidomethyl (C)[10]		Mascot
1492.7424	1492.8411	0.0987	66	630	642	DATLLKNQESSMR					Mascot
1511.7999	1511.8571	0.0572	38	1548	1560	ISMRDAHIEALQK					Mascot
1630.8376	1630.9734	0.1358	83	11	23	QYSWLWVSHISPK					Mascot
1665.8373	1665.9258	0.0885	53	1665	1678	MMAVNMEIQELKTK					Mascot
1766.9218	1767.0519	0.1301	74	740	754	DLHCELGALKELNVR			Carbamidomethyl (C)[4]		Mascot
1785.9089	1785.9917	0.0828	46	161	177	SLKQSNDLSLGGENAPR					Mascot
1907.9491	1908.0735	0.1244	65	368	384	NEIQSIRGTMSQLSAEK			Oxidation (M)[10]		Mascot
1974.9325	1975.1183	0.1858	94	1401	1417	DDELLEAEENVQLMQAK					Mascot
2084.1233	2084.1321	0.0088	4	799	817	DSAAANQLLIEELQITNLK					Mascot
2430.1895	2430.2747	0.0852	35	1273	1293	EHQSLIEKYNALEDENGTALR					Mascot
2591.3306	2591.4238	0.0932	36	545	567	MQLLELEIGQMSESVNSLELALK			Oxidation (M)[1]		Mascot
3047.564	3047.7188	0.1548	51	541	567	SEQKMQLLELEIGQMSESVNSLELALK					Mascot
3047.564	3047.7188	0.1548	51	541	567	SEQKMQLLELEIGQMSESVNSLELALK					Mascot
3075.5588	3075.7417	0.1829	59	545	572	MQLLELEIGQMSESVNSLELALKDEAGK					Mascot

8 Alpha-soluble NSF attachment protein [Triticum urartu] gi|474212649 42706.3 6.36 11 41 0 4.284

Peptide Information

Calc. Mass	Obsrv. Mass	± da	± ppm	Start Seq.	End Seq.	Sequence	Ion Score	C. I. %	Modification	Rank	Result Type
855.4505	855.4846	0.0341	40	148	154	IANCHLK			Carbamidomethyl (C)[4]		Mascot
1147.6218	1147.6328	0.011	10	48	57	NYGKPNSVIR					Mascot
1178.6052	1178.5887	-0.0165	-14	260	269	ATEIFEEIAR					Mascot
1292.6304	1292.6969	0.0665	51	95	107	SGFETPPGMGGKK					Mascot
1308.6254	1308.7185	0.0931	71	95	107	SGFETPPGMGGKK			Oxidation (M)[9]		Mascot
1355.8046	1355.7061	-0.0985	-73	352	362	LDPWKTTLLLR					Mascot
1413.6646	1413.7448	0.0802	57	308	319	YQEIDPTFSGTR					Mascot
1459.7792	1459.7859	0.0067	5	247	259	VAEIAAQLEQYPK					Mascot
1487.7821	1487.8141	0.032	22	283	295	GILLNAGICQLCR			Carbamidomethyl (C)[9,12]		Mascot
1587.8676	1587.8683	0.0007	0	141	154	AASVYIKIANCHLK			Carbamidomethyl (C)[11]		Mascot
1587.8676	1587.8683	0.0007	0	141	154	AASVYIKIANCHLK			Carbamidomethyl (C)[11]		Mascot
1665.7704	1665.9258	0.1554	93	110	122	QYFGSPMHWRGQR			Oxidation (M)[7]		Mascot
3046.5779	3046.7024	0.1245	41	175	202	FSPQEAAQALDQAVNLFLEIGRLSMAAR					Mascot

9 Tetratricopeptide repeat protein 35-A [Triticum urartu] gi|474100421 23154 8.18 7 41 0 9.232

Peptide Information

Calc. Mass	Obsrv. Mass	± da	± ppm	Start Seq.	End Seq.	Sequence	Ion Score	C. I. %	Modification	Rank	Result Type
905.4761	905.488	0.0119	13	11	18	DCIGILSK			Carbamidomethyl (C)[2]		Mascot
905.4761	905.488	0.0119	13	11	18	DCIGILSK	5	0	Carbamidomethyl (C)[2]		Mascot
1074.5803	1074.5715	-0.0088	-8	19	28	QFPGSARVGR					Mascot
1251.582	1251.7062	0.1242	99	1	10	MDCQRLDVAK			Carbamidomethyl (C)[3], Oxidation (M)[1]		Mascot
1431.7876	1431.7681	-0.0195	-14	6	18	LDVAKDCIGILSK			Carbamidomethyl (C)[7]		Mascot
1431.7876	1431.7681	-0.0195	-14	6	18	LDVAKDCIGILSK			Carbamidomethyl (C)[7]		Mascot
1864.8844	1865.0157	0.1313	70	167	183	EEESSELQSLAAEALMK					Mascot
1947.9811	1948.0806	0.0995	51	29	45	LEALLFEAKGDWAEAER					Mascot
2052.073	2052.1704	0.0974	47	146	164	ALFGVCLCSAAISQLTKG R			Carbamidomethyl (C)[6,8]		Mascot

10 coronatine insensitive 2-like protein [Triticum aestivum] gi|301318118 67460.6 6.58 8 40 0 16.196 23 65.47

Peptide Information

Calc. Mass	Obsrv. Mass	± da	± ppm	Start Seq.	End Seq.	Sequence	Ion Score	C. I. %	Modification	Rank	Result Type
905.4509	905.488	0.0371	41	38	45	EAASLVCR			Carbamidomethyl (C)[7]		Mascot
905.4509	905.488	0.0371	41	38	45	EAASLVCR	23	65.47	Carbamidomethyl (C)[7]		Mascot
984.62	984.6262	0.0062	6	411	418	LVLLDRQK					Mascot
984.62	984.6262	0.0062	6	411	418	LVLLDRQK					Mascot
1143.5576	1143.6388	0.0812	71	1	10	MGGEVPEPRR			Oxidation (M)[1]		Mascot
1143.5576	1143.6388	0.0812	71	1	10	MGGEVPEPRR			Oxidation (M)[1]		Mascot
1173.699	1173.6998	0.0008	1	75	84	FPRLESLALK					Mascot
1357.7509	1357.7185	-0.0324	-24	526	538	ASPAGLELLLMAR			Oxidation (M)[11]		Mascot
1569.8669	1569.8785	0.0116	7	209	222	VVPADLELLAKNCK			Carbamidomethyl (C)[13]		Mascot
1785.918	1785.9917	0.0737	41	269	283	FPPRVCFLGLTFMGK			Carbamidomethyl (C)[6], Oxidation (M)[13]		Mascot
2373.1909	2373.2463	0.0554	23	14	35	ALSFGVPDEALHLVMGY VDAPR			Oxidation (M)[15]		Mascot

Gel Idx/Pos	222/I22	Instr./Gel Origin	BA2151/Sample Project 20140814	Process Status	Analysis Succeeded
Plate [#] Name	[1] Sample Project 20140814	Instrument Sample Name		Spectra	11

Rank	Protein Name	Accession No.	Protein MW	Protein PI	Pep. Count	Protein Score	Protein Score C. I. %	Intensity Matched	Total Ion Score	Total Ion C. I. %	Confirmed
1	chloroplast fructose-bisphosphate aldolase [Triticum aestivum]	gi|223018643	42216.6	5.94	18	602	100	40.062	489	100	

Peptide Information

Calc. Mass	Obsrv. Mass	± da	± ppm	Start Seq.	End Seq.	Sequence	Ion Score	C. I. %	Modification	Rank	Result Type
822.4355	822.4487	0.0132	16	229	235	TFEVAQK					Mascot
873.4577	873.4947	0.037	42	193	200	EAAWGLAR					Mascot
947.4979	947.5132	0.0153	16	330	337	ALQNTCLK			Carbamidomethyl (C)[6]		Mascot
1156.5382	1156.5806	0.0424	37	163	172	EAAYYQQGAR					Mascot
1156.5382	1156.5806	0.0424	37	163	172	EAAYYQQGAR	74	100			Mascot
1308.6682	1308.6915	0.0233	18	271	282	ATPEEVASYTLK					Mascot
1387.7175	1387.769	0.0515	37	73	85	LASIGLENTEANR					Mascot
1387.7175	1387.769	0.0515	37	73	85	LASIGLENTEANR	112	100			Mascot
1510.8475	1510.8488	0.0013	1	178	192	TVVSIPNGPSELAVK					Mascot
1543.8187	1543.8806	0.0619	40	72	85	RLASIGLENTEANR					Mascot
1579.7963	1579.8947	0.0984	62	269	282	DRATPEEVASYTLK					Mascot
1592.9622	1592.9685	0.0063	4	122	136	IVDILVEQGIVPGIK					Mascot
1592.9622	1592.9685	0.0063	4	122	136	IVDILVEQGIVPGIK	68	100			Mascot
1622.7626	1622.8186	0.056	35	58	72	GILAMDESNATCGKR			Carbamidomethyl (C)[12]		Mascot
1721.0571	1721.0658	0.0087	5	121	136	KIVDILVEQGIVPGIK					Mascot
1818.996	1819.0166	0.0206	11	271	286	ATPEEVASYTLKLLQR					Mascot
1864.7905	1864.8185	0.028	15	368	384	YTSDGEAAEASENMFVK			Oxidation (M)[14]		Mascot
2052.0986	2052.1692	0.0706	34	338	356	TWGGRPENVAAAQEALLLR					Mascot
2052.0986	2052.1692	0.0706	34	338	356	TWGGRPENVAAAQEALLLR	124	100			Mascot
2355.2603	2355.2554	-0.0049	-2	16	37	AEWGTTRQAAAPKPVTVSMVVR					Mascot
2371.2551	2371.2515	-0.0036	-2	16	37	AEWGTTRQAAAPKPVTVSMVVR			Oxidation (M)[19]		Mascot
2430.1719	2430.2661	0.0942	39	140	162	GLVPLVGSNDESWCQGLDGLASR			Carbamidomethyl (C)[14]		Mascot
2430.1719	2430.2661	0.0942	39	140	162	GLVPLVGSNDESWCQGLDGLASR	112	100	Carbamidomethyl (C)[14]		Mascot
3081.5198	3081.6509	0.1311	43	201	228	YAAISQDNGLVPIVEPEIMLDGEHGIER			Oxidation (M)[19]		Mascot

Rank	Protein Name	Accession No.	Protein MW	Protein PI	Pep. Count	Protein Score	Protein Score C. I. %	Intensity Matched	Total Ion Score	Total Ion C. I. %	Confirmed
2	Fructose-bisphosphate aldolase, chloroplastic [Triticum	gi|473848356	42204.5	5.94	18	600	100	39.912	489	100	

urartu]

Peptide Information

Calc. Mass	Obsrv. Mass	± da	± ppm	Start Seq.	End Seq.	Sequence	Ion Score	C. I. %	Modification	Rank	Result Type
822.4355	822.4487	0.0132	16	229	235	TFEVAQK					Mascot
873.4577	873.4947	0.037	42	193	200	EAAWGLAR					Mascot
901.5101	901.495	-0.0151	-17	359	367	ANSLAQLGK					Mascot
947.4979	947.5132	0.0153	16	330	337	ALQNTCLK			Carbamidomethyl (C)[6]		Mascot
1156.5382	1156.5806	0.0424	37	163	172	EAAYYQQGAR					Mascot
1156.5382	1156.5806	0.0424	37	163	172	EAAYYQQGAR	74	100			Mascot
1308.6682	1308.6915	0.0233	18	271	282	ATPEEVASYTLK					Mascot
1387.7175	1387.769	0.0515	37	73	85	LASIGLENTEANR					Mascot
1387.7175	1387.769	0.0515	37	73	85	LASIGLENTEANR	112	100			Mascot
1510.8475	1510.8488	0.0013	1	178	192	TVVSIPNGPSELAVK					Mascot
1543.8187	1543.8806	0.0619	40	72	85	RLASIGLENTEANR					Mascot
1579.7963	1579.8947	0.0984	62	269	282	DRATPEEVASYTLK					Mascot
1592.9622	1592.9685	0.0063	4	122	136	IVDILVEQGIVPGIK					Mascot
1592.9622	1592.9685	0.0063	4	122	136	IVDILVEQGIVPGIK	68	100			Mascot
1622.7626	1622.8186	0.056	35	58	72	GILAMDESNATCGKR			Carbamidomethyl (C)[12]		Mascot
1721.0571	1721.0658	0.0087	5	121	136	KIVDILVEQGIVPGIK					Mascot
1818.996	1819.0166	0.0206	11	271	286	ATPEEVASYTLKLLQR					Mascot
1864.7905	1864.8185	0.028	15	368	384	YTSDGEAAEASENMFVK			Oxidation (M)[14]		Mascot
2052.0986	2052.1692	0.0706	34	338	356	TWGGRPENVAAAQEALL LR					Mascot
2052.0986	2052.1692	0.0706	34	338	356	TWGGRPENVAAAQEALL LR	124	100			Mascot
2430.1719	2430.2661	0.0942	39	140	162	GLVPLVGSNDESWCQGL DGLASR			Carbamidomethyl (C)[14]		Mascot
2430.1719	2430.2661	0.0942	39	140	162	GLVPLVGSNDESWCQGL DGLASR	112	100	Carbamidomethyl (C)[14]		Mascot
3081.5198	3081.6509	0.1311	43	201	228	YAAISQDNGLVPIVEPEIM LDGEHGIER			Oxidation (M)[19]		Mascot

3	enolase [Triticum aestivum]	gi|461744058	48459.5	5.49	8	103	100	6.352	78	100	

Peptide Information

Calc. Mass	Obsrv. Mass	± da	± ppm	Start Seq.	End Seq.	Sequence	Ion Score	C. I. %	Modification	Rank	Result Type
1138.6102	1138.5708	-0.0394	-35	235	245	TAIEKAGYTGK					Mascot
1138.6102	1138.5708	-0.0394	-35	235	245	TAIEKAGYTGK					Mascot
1143.5681	1143.6222	0.0541	47	262	270	DKTYDLNFK					Mascot

1175.6565	1175.6224	-0.0341	-29	1	11	MAATIQSVKAR					Mascot
1579.9166	1579.8947	-0.0219	-14	67	82	AVNNVNSIIAPALVGK					Mascot
1790.9283	1790.99	0.0617	34	36	53	AAVPSGASTGVYEALELR					Mascot
1790.9283	1790.99	0.0617	34	36	53	AAVPSGASTGVYEALELR	78	100			Mascot
1884.9598	1885.0559	0.0961	51	169	185	LAMQEFMILPTGATSFK					Mascot
1951.1335	1951.0858	-0.0477	-24	63	82	GVSKAVNNVNSIIAPALV GK					Mascot
1983.8536	1983.9185	0.0649	33	18	35	GNPTVEVDVCCSDGTFA R			Carbamidomethyl (C)[10,11]		Mascot

4	enolase [Triticum aestivum]	gi|461744056	48416.5	5.59	5	88	99.994	5.867	78	100	

Protein Group

enolase [Triticum aestivum]	gi|461744078	48416.5	5.5900 001525 8789

Peptide Information

Calc. Mass	Obsrv. Mass	± da	± ppm	Start Seq.	End Seq.	Sequence	Ion Score	C. I. %	Modification	Rank	Result Type
1138.6102	1138.5708	-0.0394	-35	235	245	TAIEKAGYTGK					Mascot
1138.6102	1138.5708	-0.0394	-35	235	245	TAIEKAGYTGK					Mascot
1143.5681	1143.6222	0.0541	47	262	270	DKTYDLNFK					Mascot
1175.6453	1175.6224	-0.0229	-19	345	354	EKSCNALLLK			Carbamidomethyl (C)[4]		Mascot
1790.9283	1790.99	0.0617	34	36	53	AAVPSGASTGVYEALELR					Mascot
1790.9283	1790.99	0.0617	34	36	53	AAVPSGASTGVYEALELR	78	100			Mascot
1983.8536	1983.9185	0.0649	33	18	35	GNPTVEVDVCCSDGTFA R			Carbamidomethyl (C)[10,11]		Mascot

5	Enolase [Triticum urartu]	gi|474188401	43643.1	5.54	4	86	99.989	5.316	78	100

Peptide Information

Calc. Mass	Obsrv. Mass	± da	± ppm	Start Seq.	End Seq.	Sequence	Ion Score	C. I. %	Modification	Rank	Result Type
1138.6102	1138.5708	-0.0394	-35	182	192	TAIEKAGYTGK					Mascot
1138.6102	1138.5708	-0.0394	-35	182	192	TAIEKAGYTGK					Mascot
1143.5681	1143.6222	0.0541	47	209	217	DKTYDLNFK					Mascot
1175.6453	1175.6224	-0.0229	-19	292	301	EKSCNALLLK			Carbamidomethyl (C)[4]		Mascot
1790.9283	1790.99	0.0617	34	34	51	AAVPSGASTGVYEALELR					Mascot
1790.9283	1790.99	0.0617	34	34	51	AAVPSGASTGVYEALELR	78	100			Mascot

6	Enolase 1 [Triticum urartu]	gi|474407144	48212.4	5.21	6	51	67.964	2.565	36	98.179

Peptide Information

Calc. Mass	Obsrv. Mass	± da	± ppm	Start Seq.	End Seq.	Sequence	Ion Score	C. I. %	Modification	Rank	Result Type
932.5233	932.4723	-0.051	-55	350	357	TCNALLLK			Carbamidomethyl (C)[2]		Mascot
1143.5317	1143.6222	0.0905	79	265	273	DQTYDLNFK					Mascot
1308.6794	1308.6915	0.0121	9	57	69	DGGSDYLGKGVLK					Mascot
1790.9283	1790.99	0.0617	34	39	56	GAVPSGASTGIYEALELR					Mascot
1790.9283	1790.99	0.0617	34	39	56	GAVPSGASTGIYEALELR	36	98.179			Mascot
2035.0488	2035.2089	0.1601	79	358	376	VNQIGSVTESIEAVSMSK R					Mascot
2051.0437	2051.1526	0.1089	53	358	376	VNQIGSVTESIEAVSMSK R			Oxidation (M)[16]		Mascot
2057.1609	2057.168	0.0071	3	115	135	LGANAILAVSLAVCKAGA MVK			Carbamidomethyl (C)[14]		Mascot

7 Tubulin beta-2 chain [Triticum urartu] gi|474369340 70289.9 5.67 14 47 21.362 20.317

Peptide Information

Calc. Mass	Obsrv. Mass	± da	± ppm	Start Seq.	End Seq.	Sequence	Ion Score	C. I. %	Modification	Rank	Result Type
855.4683	855.48	0.0117	14	2	8	PRPETQK					Mascot
905.455	905.4818	0.0268	30	620	626	CLQWVIS			Carbamidomethyl (C)[1]		Mascot
905.455	905.4818	0.0268	30	620	626	CLQWVIS			Carbamidomethyl (C)[1]		Mascot
1081.4224	1081.5139	0.0915	85	427	435	NMMCAADPR			Carbamidomethyl (C)[4], Oxidation (M)[2]		Mascot
1143.6481	1143.6222	-0.0259	-23	208	219	GVGSERLGLGAK					Mascot
1215.5828	1215.6906	0.1078	89	510	519	VSEQFTAMFR					Mascot
1274.6852	1274.6641	-0.0211	-17	371	381	FPGQLNSDLRK					Mascot
1387.6787	1387.769	0.0903	65	510	520	VSEQFTAMFRR			Oxidation (M)[8]		Mascot
1387.6787	1387.769	0.0903	65	509	519	RVSEQFTAMFR			Oxidation (M)[9]		Mascot
1431.6322	1431.751	0.1188	83	266	277	VNVYYNEASCGR			Carbamidomethyl (C)[10]		Mascot
1622.8512	1622.8186	-0.0326	-20	392	405	LHFFMVGFAPLTSR					Mascot
1993.0172	1993.1018	0.0846	42	186	204	EGIGQEMEIGPHLAALVG R			Oxidation (M)[7]		Mascot
2056.9944	2057.168	0.1736	84	14	32	WTPPGKTLAGATCPMPA ER			Carbamidomethyl (C)[13], Oxidation (M)[15]		Mascot
2126.0593	2126.115	0.0557	26	220	238	MREILHIQGGQCGNQIGA K			Carbamidomethyl (C)[12], Oxidation (M)[1]		Mascot
2446.1943	2446.2585	0.0642	26	592	617	SAAATTTALDTGALSAAE QIGPADDK					Mascot

8 U3 small nucleolar RNA-associated protein 6-like protein [Triticum urartu] gi|474404716 78692 6.31 15 45 0 10.948

Peptide Information

Calc. Mass	Obsrv. Mass	± da	± ppm	Start	End	Sequence	Ion	C. I. %	Modification	Rank	Result Type

				Seq.	Seq.		Score				
887.408	887.4738	0.0658	74	139	144	YLEFCR			Carbamidomethyl (C)[5]		Mascot
932.4982	932.4723	-0.0259	-28	153	160	QVMAQAIR			Oxidation (M)[3]		Mascot
1027.5128	1027.5494	0.0366	36	210	217	MELTYLNK			Oxidation (M)[1]		Mascot
1042.6003	1042.5782	-0.0221	-21	28	36	AELAEVVRR					Mascot
1143.5753	1143.6222	0.0469	41	451	460	LGRSEEAPER					Mascot
1175.6388	1175.6224	-0.0164	-14	151	160	MKQVMAQAIR					Mascot
1300.7471	1300.634	-0.1131	-87	223	234	VALGEDVKTLQK					Mascot
1387.6699	1387.769	0.0991	71	454	465	SEEAPERLEEAK					Mascot
1387.6699	1387.769	0.0991	71	454	465	SEEAPERLEEAK					Mascot
1534.8224	1534.8323	0.0099	6	367	379	LNKSIQVYDEAVR					Mascot
1884.9425	1885.0559	0.1134	60	527	542	AEGLWHTAMKLFSHEK					Mascot
1951.0494	1951.0858	0.0364	19	306	322	ILDILNSVELAHSEELR					Mascot
2067.9998	2068.1685	0.1687	82	386	402	MYSLYANFWLGVVFSDR					Mascot
2083.9946	2084.1597	0.1651	79	386	402	MYSLYANFWLGVVFSDR			Oxidation (M)[1]		Mascot
2083.9946	2084.1597	0.1651	79	386	402	MYSLYANFWLGVVFSDR			Oxidation (M)[1]		Mascot
2095.9487	2096.1001	0.1514	72	244	261	EENKELFMSLDEQGDSPK					Mascot
2355.2239	2355.2554	0.0315	13	466	486	NLAEKLCSGPLSQAANLWNLR			Carbamidomethyl (C)[7]		Mascot
2371.0547	2371.2515	0.1968	83	646	665	YYDMELTAGTSETSNAIYWR					Mascot

9 N-alpha-acetyltransferase 40, NatD catalytic subunit [Triticum urartu] gi|473878239 35086.8 9.18 10 43 0 1.878

Peptide Information

Calc. Mass	Obsrv. Mass	± da	± ppm	Start Seq.	End Seq.	Sequence	Ion Score	C. I. %	Modification	Rank	Result Type
1008.5182	1008.473	-0.0452	-45	274	281	LEFIEAMR					Mascot
1042.5752	1042.5782	0.003	3	2	10	ATAENKRPR					Mascot
1274.7117	1274.6641	-0.0476	-37	157	167	GGRLLGFVHYR					Mascot
1494.8059	1494.8651	0.0592	40	196	207	FLMQLIELIACK			Carbamidomethyl (C)[11], Oxidation (M)[3]		Mascot
1510.7207	1510.8488	0.1281	85	220	233	SNTDAMVSTTIWGK					Mascot
1518.6425	1518.7659	0.1234	81	144	156	QDSGVECAHVTCR			Carbamidomethyl (C)[7,12]		Mascot
1622.9009	1622.8186	-0.0823	-51	195	207	KFLMQLIELIACK			Carbamidomethyl (C)[12], Oxidation (M)[4]		Mascot
1630.7972	1630.9464	0.1492	91	261	273	TRYSYVDSFISHR					Mascot
2371.1797	2371.2515	0.0718	30	286	304	HLNLKPSANWRPMMYERVE					Mascot
3081.6143	3081.6509	0.0366	12	168	194	FIVEEDVPVLYVYELQLESSAQGKGLR					Mascot

10 hypothetical protein TRIUR3_19286 [Triticum urartu] gi|473887298 66948.3 5.21 14 43 0 4.348

Peptide Information

Calc. Mass	Obsrv. Mass	± da	± ppm	Start Seq.	End Seq.	Sequence	Ion Score	C. I. %	Modification	Rank	Result Type
873.4723	873.4947	0.0224	26	430	436	RNTMPVR					Mascot
889.4672	889.4843	0.0171	19	430	436	RNTMPVR			Oxidation (M)[4]		Mascot
904.4094	904.4595	0.0501	55	122	128	YGPMHQR			Oxidation (M)[4]		Mascot
927.4795	927.4596	-0.0199	-21	497	503	RGYTFQR					Mascot
932.4796	932.4723	-0.0073	-8	388	396	ASDVGVQTR					Mascot
947.4792	947.5132	0.034	36	320	327	ESKEQQAK					Mascot
1008.5472	1008.473	-0.0742	-74	72	80	TSAFEKAVR					Mascot
1308.6444	1308.6915	0.0471	36	140	152	HNAIDAGPPGFGR					Mascot
1347.6759	1347.8099	0.134	99	520	531	SSMPPKPMLATR			Oxidation (M)[3,8]		Mascot
1415.6947	1415.7927	0.098	69	18	30	AQILPCSEAAAER			Carbamidomethyl (C)[6]		Mascot
1543.7897	1543.8806	0.0909	59	18	31	AQILPCSEAAAERK			Carbamidomethyl (C)[6]		Mascot
1617.8199	1617.8737	0.0538	33	520	533	SSMPPKPMLATRNR			Oxidation (M)[3,8]		Mascot
2047.0964	2047.1085	0.0121	6	534	551	LNSLNVRITSTACLTDIR			Carbamidomethyl (C)[13]		Mascot
2050.9675	2051.1526	0.1851	90	241	257	ELDEEYVIEEASRQANR					Mascot
2063.9426	2064.0559	0.1133	55	504	519	TTSELCLRFSFCSNWR			Carbamidomethyl (C)[6,12]		Mascot

Gel Idx/Pos	223/I23	Instr./Gel Origin	BA2151/Sample Project 20140814	Process Status	Analysis Succeeded
Plate [#] Name	[1] Sample Project 20140814	Instrument Sample Name		Spectra	11

Rank	Protein Name	Accession No.	Protein MW	Protein PI	Pep. Count	Protein Score	Protein Score C. I. %	Intensity Matched	Total Ion Score	Total Ion C. I. %	Confirmed
1	actin [Triticum aestivum]	gi|58533119	41929	5.23	16	637	100	65.141	542	100	

Protein Group

Protein Name	Accession No.	Protein MW	Protein PI
Actin-97 [Triticum urartu]	gi|474287474	41929	5.2300 000190 7349
actin [Triticum turgidum]	gi|58533114	41929	5.2300 000190 7349

Peptide Information

Calc. Mass	Obsrv. Mass	± da	± ppm	Start Seq.	End Seq.	Sequence	Ion Score	C. I. %	Modification	Rank	Result Type
895.5359	895.569	0.0331	37	331	338	VVAPPERK					Mascot
976.4483	976.486	0.0377	39	21	30	AGFAGDDAPR					Mascot
1118.5114	1118.5577	0.0463	41	199	208	GYSFTTSAER					Mascot
1118.5114	1118.5577	0.0463	41	199	208	GYSFTTSAER	22	53.779			Mascot
1176.55	1176.6389	0.0889	76	42	52	HTGVMVGMGQK			Oxidation (M)[5,8]		Mascot
1182.5273	1182.587	0.0597	50	53	63	DAYVGDEAQSK					Mascot
1198.7056	1198.7498	0.0442	37	31	41	AVFPSIVGRPR					Mascot
1198.7056	1198.7498	0.0442	37	31	41	AVFPSIVGRPR	43	99.665			Mascot
1487.6761	1487.7434	0.0673	45	362	374	DEYDESGPAIVHR					Mascot
1487.6761	1487.7434	0.0673	45	362	374	DEYDESGPAIVHR	118	100			Mascot
1515.7491	1515.8101	0.061	40	87	97	IWHHTFYNELR					Mascot
1515.7491	1515.8101	0.061	40	87	97	IWHHTFYNELR	75	100			Mascot
1547.8098	1547.8048	-0.005	-3	180	193	LDLAGRDLTDSLMK					Mascot
1547.8098	1547.8048	-0.005	-3	180	193	LDLAGRDLTDSLMK					Mascot
1563.8047	1563.7969	-0.0078	-5	180	193	LDLAGRDLTDSLMK			Oxidation (M)[13]		Mascot
1747.8861	1747.9542	0.0681	39	241	256	SYELPDGQVITIGAER					Mascot
1747.8861	1747.9542	0.0681	39	241	256	SYELPDGQVITIGAER	135	100			Mascot
1855.9324	1855.9874	0.055	30	218	233	LAYVALDYEQELETAK					Mascot

1954.0645	1954.1345	0.07	36	98	115	VAPEEHPVLLTEAPLNPK					Mascot
1954.0645	1954.1345	0.07	36	98	115	VAPEEHPVLLTEAPLNPK	97	100			Mascot
2149.9563	2149.8726	-0.0837	-39	1	20	MADGEDIQPLVCDNGTGMVK			Carbamidomethyl (C)[12]		Mascot
2197.0959	2197.1804	0.0845	38	294	314	DLYGNIVLSGGTTMFPGIADR					Mascot
2213.0908	2213.1753	0.0845	38	294	314	DLYGNIVLSGGTTMFPGIADR			Oxidation (M)[14]		Mascot
2325.1909	2325.2661	0.0752	32	293	314	KDLYGNIVLSGGTTMFPGIADR					Mascot
2341.1858	2341.2668	0.081	35	293	314	KDLYGNIVLSGGTTMFPGIADR			Oxidation (M)[15]		Mascot
3151.6423	3151.7881	0.1458	46	150	179	TTGIVLDSGDGVSHTVPIYEGYALPHAILR					Mascot
3151.6423	3151.7881	0.1458	46	150	179	TTGIVLDSGDGVSHTVPIYEGYALPHAILR	52	99.951			Mascot

2	Actin-3 [Triticum urartu]	gi|474259583	44624.2	5.26	13	570	100	53.609	508	100

Peptide Information

Calc. Mass	Obsrv. Mass	± da	± ppm	Start Seq.	End Seq.	Sequence	Ion Score	C. I. %	Modification	Rank	Result Type
895.5359	895.569	0.0331	37	354	361	VVAPPERK					Mascot
976.4483	976.486	0.0377	39	44	53	AGFAGDDAPR					Mascot
1132.527	1132.5756	0.0486	43	222	231	GYSFTTTAER					Mascot
1176.55	1176.6389	0.0889	76	65	75	HTGVMVGMGQK			Oxidation (M)[5,8]		Mascot
1182.5273	1182.587	0.0597	50	76	86	DAYVGDEAQSK					Mascot
1198.7056	1198.7498	0.0442	37	54	64	AVFPSIVGRPR					Mascot
1198.7056	1198.7498	0.0442	37	54	64	AVFPSIVGRPR	43	99.665			Mascot
1429.6707	1429.7329	0.0622	44	385	397	GEYDESGPAIVHR					Mascot
1429.6707	1429.7329	0.0622	44	385	397	GEYDESGPAIVHR	106	100			Mascot
1515.7491	1515.8101	0.061	40	110	120	IWHHTFYNELR					Mascot
1515.7491	1515.8101	0.061	40	110	120	IWHHTFYNELR	75	100			Mascot
1747.8861	1747.9542	0.0681	39	264	279	SYELPDGQVITIGAER					Mascot
1747.8861	1747.9542	0.0681	39	264	279	SYELPDGQVITIGAER	135	100			Mascot
1868.9276	1869.0087	0.0811	43	241	256	LAYVALDYEQELENAK					Mascot
1954.0645	1954.1345	0.07	36	121	138	VAPEEHPVLLTEAPLNPK					Mascot
1954.0645	1954.1345	0.07	36	121	138	VAPEEHPVLLTEAPLNPK	97	100			Mascot
2183.0801	2183.1589	0.0788	36	317	337	DLYGNIVLSGGSTMFPGIADR					Mascot
2199.0752	2199.1707	0.0955	43	317	337	DLYGNIVLSGGSTMFPGIADR			Oxidation (M)[14]		Mascot
3151.6423	3151.7881	0.1458	46	173	202	TTGIVLDSGDGVSHTVPIYEGYALPHAILR					Mascot
3151.6423	3151.7881	0.1458	46	173	202	TTGIVLDSGDGVSHTVPIYEGYALPHAILR	52	99.951			Mascot

3	Actin-2 [Triticum urartu]	gi|474372381	41863.1	5.31	14	478	100	57.332	402	100	

Peptide Information

Calc. Mass	Obsrv. Mass	± da	± ppm	Start Seq.	End Seq.	Sequence	Ion Score	C. I. %	Modification	Rank	Result Type
895.5359	895.569	0.0331	37	331	338	VVAPPERK					Mascot
976.4483	976.486	0.0377	39	21	30	AGFAGDDAPR					Mascot
1132.527	1132.5756	0.0486	43	199	208	GYSFTTTAER					Mascot
1176.55	1176.6389	0.0889	76	42	52	HTGVMVGMGQK			Oxidation (M)[5,8]		Mascot
1182.5273	1182.587	0.0597	50	53	63	DAYVGDEAQSK					Mascot
1198.7056	1198.7498	0.0442	37	31	41	AVFPSIVGRPR					Mascot
1198.7056	1198.7498	0.0442	37	31	41	AVFPSIVGRPR	43	99.665			Mascot
1457.7019	1457.7529	0.051	35	362	374	AEYEESGPAIVHR					Mascot
1515.7491	1515.8101	0.061	40	87	97	IWHHTFYNELR					Mascot
1515.7491	1515.8101	0.061	40	87	97	IWHHTFYNELR	75	100			Mascot
1547.8098	1547.8048	-0.005	-3	180	193	LDLAGRDLTDSLMK					Mascot
1547.8098	1547.8048	-0.005	-3	180	193	LDLAGRDLTDSLMK					Mascot
1563.8047	1563.7969	-0.0078	-5	180	193	LDLAGRDLTDSLMK			Oxidation (M)[13]		Mascot
1747.8861	1747.9542	0.0681	39	241	256	SYELPDGQVITIGAER					Mascot
1747.8861	1747.9542	0.0681	39	241	256	SYELPDGQVITIGAER	135	100			Mascot
1855.9324	1855.9874	0.055	30	218	233	LAYVALDYEQELETAK					Mascot
1954.0645	1954.1345	0.07	36	98	115	VAPEEHPVLLTEAPLNPK					Mascot
1954.0645	1954.1345	0.07	36	98	115	VAPEEHPVLLTEAPLNPK	97	100			Mascot
2183.0801	2183.1589	0.0788	36	294	314	DLYGNIVLSGGSTMFPGI ADR					Mascot
2199.0752	2199.1707	0.0955	43	294	314	DLYGNIVLSGGSTMFPGI ADR			Oxidation (M)[14]		Mascot
3151.6423	3151.7881	0.1458	46	150	179	TTGIVLDSGDGVSHTVPI YEGYALPHAILR					Mascot
3151.6423	3151.7881	0.1458	46	150	179	TTGIVLDSGDGVSHTVPI YEGYALPHAILR	52	99.951			Mascot

4	actin [Triticum aestivum]	gi|255684860	28232.3	5.34	8	403	100	43.513	359	100	

Peptide Information

Calc. Mass	Obsrv. Mass	± da	± ppm	Start Seq.	End Seq.	Sequence	Ion Score	C. I. %	Modification	Rank	Result Type
895.5359	895.569	0.0331	37	245	252	VVAPPERK					Mascot
1132.527	1132.5756	0.0486	43	113	122	GYSFTTTAER					Mascot
1515.7491	1515.8101	0.061	40	1	11	IWHHTFYNELR					Mascot
1515.7491	1515.8101	0.061	40	1	11	IWHHTFYNELR	75	100			Mascot

Calc. Mass	Obsrv. Mass	± da	± ppm	Start Seq.	End Seq.	Sequence	Ion Score	C. I. %	Modification	Rank	Result Type
1747.8861	1747.9542	0.0681	39	155	170	SYELPDGQVITIGAER					Mascot
1747.8861	1747.9542	0.0681	39	155	170	SYELPDGQVITIGAER	135	100			Mascot
1868.9276	1869.0087	0.0811	43	132	147	LAYVALDYEQELENAK					Mascot
1954.0645	1954.1345	0.07	36	12	29	VAPEEHPVLLTEAPLNPK					Mascot
1954.0645	1954.1345	0.07	36	12	29	VAPEEHPVLLTEAPLNPK	97	100			Mascot
2183.0801	2183.1589	0.0788	36	208	228	DLYGNIVLSGGSTMFPGIADR					Mascot
2199.0752	2199.1707	0.0955	43	208	228	DLYGNIVLSGGSTMFPGIADR			Oxidation (M)[14]		Mascot
3151.6423	3151.7881	0.1458	46	64	93	TTGIVLDSGDGVSHTVPIYEGYALPHAILR					Mascot
3151.6423	3151.7881	0.1458	46	64	93	TTGIVLDSGDGVSHTVPIYEGYALPHAILR	52	99.951			Mascot

5	actin [Triticum monococcum]	gi|15088544	20822.4	5.15	8	315	100	30.004	268	100	

Peptide Information

Calc. Mass	Obsrv. Mass	± da	± ppm	Start Seq.	End Seq.	Sequence	Ion Score	C. I. %	Modification	Rank	Result Type
976.4483	976.486	0.0377	39	21	30	AGFAGDDAPR					Mascot
1176.55	1176.6389	0.0889	76	42	52	HTGVMVGMGQK			Oxidation (M)[5,8]		Mascot
1182.5273	1182.587	0.0597	50	53	63	DAYVGDEAQSK					Mascot
1198.7056	1198.7498	0.0442	37	31	41	AVFPSIVGRPR					Mascot
1198.7056	1198.7498	0.0442	37	31	41	AVFPSIVGRPR	43	99.665			Mascot
1515.7491	1515.8101	0.061	40	87	97	IWHHTFYNELR					Mascot
1515.7491	1515.8101	0.061	40	87	97	IWHHTFYNELR	75	100			Mascot
1954.0645	1954.1345	0.07	36	98	115	VAPEEHPVLLTEAPLNPK					Mascot
1954.0645	1954.1345	0.07	36	98	115	VAPEEHPVLLTEAPLNPK	97	100			Mascot
2149.9563	2149.8726	-0.0837	-39	1	20	MADGEDIQPLVCDNGTGMVK			Carbamidomethyl (C)[12]		Mascot
3151.6423	3151.7881	0.1458	46	150	179	TTGIVLDSGDGVSHTVPIYEGYALPHAILR					Mascot
3151.6423	3151.7881	0.1458	46	150	179	TTGIVLDSGDGVSHTVPIYEGYALPHAILR	52	99.951			Mascot

6	actin-like protein [Triticum aestivum]	gi|37694729	21516.8	4.84	6	218	100	30.368	186	100	

Peptide Information

Calc. Mass	Obsrv. Mass	± da	± ppm	Start Seq.	End Seq.	Sequence	Ion Score	C. I. %	Modification	Rank	Result Type
1132.527	1132.5756	0.0486	43	83	92	GYSFTTTAER					Mascot
1547.8098	1547.8048	-0.005	-3	64	77	LDLAGRDLTDSLMK					Mascot
1547.8098	1547.8048	-0.005	-3	64	77	LDLAGRDLTDSLMK					Mascot
1563.8047	1563.7969	-0.0078	-5	64	77	LDLAGRDLTDSLMK			Oxidation (M)[13]		Mascot
1747.8861	1747.9542	0.0681	39	125	140	SYELPDGQVITIGAER					Mascot

1747.8861	1747.9542	0.0681	39	125	140	SYELPDGQVITIGAER	135	100			Mascot
1812.9202	1813.0503	0.1301	72	177	193	KDLYGNIVLSGGTTMFP					Mascot
1855.9324	1855.9874	0.055	30	102	117	LAYVALDYEQELETAK					Mascot
3151.6423	3151.7881	0.1458	46	34	63	TTGIVLDSGDGVSHTVPI YEGYALPHAILR					Mascot
3151.6423	3151.7881	0.1458	46	34	63	TTGIVLDSGDGVSHTVPI YEGYALPHAILR	52	99.951			Mascot

7	Actin-7 [Triticum urartu]	gi|474317121	41833.9	5.29	9	154	100	24.494	119	100

Peptide Information

Calc. Mass	Obsrv. Mass	± da	± ppm	Start Seq.	End Seq.	Sequence	Ion Score	C. I. %	Modification	Rank	Result Type
976.4483	976.486	0.0377	39	20	29	AGFAGDDAPR					Mascot
1098.5426	1098.5544	0.0118	11	198	207	GYSLTTTAER					Mascot
1176.55	1176.6389	0.0889	76	41	51	HTGVMVGMGQK			Oxidation (M)[5,8]		Mascot
1198.7056	1198.7498	0.0442	37	30	40	AVFPSIVGRPR					Mascot
1198.7056	1198.7498	0.0442	37	30	40	AVFPSIVGRPR	43	99.665			Mascot
1515.7491	1515.8101	0.061	40	86	96	IWHHTFYNELR					Mascot
1515.7491	1515.8101	0.061	40	86	96	IWHHTFYNELR	75	100			Mascot
1781.8375	1781.9031	0.0656	37	240	255	SYEMPDGQVITIGSER					Mascot
1797.8324	1797.9056	0.0732	41	240	255	SYEMPDGQVITIGSER			Oxidation (M)[4]		Mascot
1883.9385	1884.0074	0.0689	37	217	232	LAYVALDYEQELETAR					Mascot
2169.0645	2169.1455	0.081	37	293	313	DLYGNVVLSGGSTMFPGI ADR					Mascot
3181.6528	3181.7896	0.1368	43	149	178	TTGIVLDSGDGVSHTVPI YEGYTLPHAILR					Mascot

8	Actin-7 [Triticum urartu]	gi|474086769	21834.8	5.31	5	137	100	23.14	119	100

Peptide Information

Calc. Mass	Obsrv. Mass	± da	± ppm	Start Seq.	End Seq.	Sequence	Ion Score	C. I. %	Modification	Rank	Result Type
976.4483	976.486	0.0377	39	20	29	AGFAGDDAPR					Mascot
1176.55	1176.6389	0.0889	76	41	51	HTGVMVGMGQK			Oxidation (M)[5,8]		Mascot
1182.5273	1182.587	0.0597	50	52	62	DAYVGDEAQSK					Mascot
1198.7056	1198.7498	0.0442	37	30	40	AVFPSIVGRPR					Mascot
1198.7056	1198.7498	0.0442	37	30	40	AVFPSIVGRPR	43	99.665			Mascot
1515.7491	1515.8101	0.061	40	86	96	IWHHTFYNELR					Mascot
1515.7491	1515.8101	0.061	40	86	96	IWHHTFYNELR	75	100			Mascot

9	actin [Triticum aestivum]	gi|48927618	33601.8	5.33	6	97	100	17.201	75	100

Peptide Information

Calc. Mass	Obsrv. Mass	± da	± ppm	Start Seq.	End Seq.	Sequence	Ion Score	C. I. %	Modification	Rank	Result Type
1098.5426	1098.5544	0.0118	11	121	130	GYSLTTTAER					Mascot
1515.7491	1515.8101	0.061	40	9	19	IWHHTFYNELR					Mascot
1515.7491	1515.8101	0.061	40	9	19	IWHHTFYNELR	75	100			Mascot
1781.8375	1781.9031	0.0656	37	163	178	SYEMPDGQVITIGSER					Mascot
1797.8324	1797.9056	0.0732	41	163	178	SYEMPDGQVITIGSER			Oxidation (M)[4]		Mascot
1883.9385	1884.0074	0.0689	37	140	155	LAYVALDYEQELETAR					Mascot
2169.0645	2169.1455	0.081	37	216	236	DLYGNVVLSGGSTMFPGI ADR					Mascot
3181.6528	3181.7896	0.1368	43	72	101	TTGIVLDSGDGVSHTVPI YEGYTLPHAILR					Mascot

10	Translocase of chloroplast 34, chloroplastic [Triticum urartu]	gi|472900218	38216.2	9.22	10	45	0	10.153	

Peptide Information

Calc. Mass	Obsrv. Mass	± da	± ppm	Start Seq.	End Seq.	Sequence	Ion Score	C. I. %	Modification	Rank	Result Type
874.4741	874.5458	0.0717	82	336	343	VSSSRNPK					Mascot
1002.5367	1002.6156	0.0789	79	204	213	YIHSGAGIGK					Mascot
1547.7812	1547.8048	0.0236	15	322	335	YRDLVGSGNPVDQK					Mascot
1547.7812	1547.8048	0.0236	15	322	335	YRDLVGSGNPVDQK					Mascot
1703.8752	1703.8879	0.0127	7	8	22	EWVGLQQFPAATQTK					Mascot
1774.9983	1774.958	-0.0403	-23	30	45	LKEENVSTLTILVMGK					Mascot
1813.0663	1813.0503	-0.016	-9	286	300	YIPLILAVQYFFVVK					Mascot
1883.9167	1884.0074	0.0907	48	145	159	LDTYRMDTLDEQVIR			Oxidation (M)[6]		Mascot
1954.0604	1954.1345	0.0741	38	255	272	EITIVVSNGSKSIHVDQK					Mascot
1954.0604	1954.1345	0.0741	38	255	272	EITIVVSNGSKSIHVDQK					Mascot
1983.9519	1984.0651	0.1132	57	305	321	AIHSDISNGKLDDWEQR					Mascot
2325.1907	2325.2661	0.0754	32	234	254	TNENGAKILPDGTPWIPN LMK			Oxidation (M)[20]		Mascot
