# Supplementary material for: Proteomic analysis of middle and late stages of bread wheat (Triticum aestivum L.) grain development
Source: Front Plant Sci. 2015 Sep 15;6:735. doi: 10.3389/fpls.2015.00735 (PMC4569854; doi:10.3389/fpls.2015.00735)
Supplement: Supplementary file 4 [file DataSheet3.PDF]

Analysis Information

Report Type	Protein-Peptide Summary by Spot	Analysis Type	Combined (MS+MS/MS)
Sample Set Name	Sample set_20140814	Database	triticum
Analysis Name	R14026-4-T2	Creation Date	09/30/2014 08:41:36
Reported By	09/30/2014 14:53:43 - admin	Last Modified	09/30/2014 08:59:29

MS Acq. : Proc. Methods	(Unspecified) : (Unspecified)
Interpretation Method	(Unspecified)

**Gel Idx/Pos**	240/J16	**Instr./Gel Origin**	BA2151/Sample Project 20140814	**Process Status**	Analysis Succeeded
**Plate [#] Name**	[1] Sample Project 20140814	**Instrument Sample Name**		**Spectra**	11

Rank	Protein Name	Accession No.	Protein MW	Protein PI	Pep. Count	Protein Score	Protein Score C. I. %	Intensity Matched	Total Ion Score	Total Ion C. I. %	Confirmed
1	unnamed protein product [Triticum aestivum]	gi|296511811	46517.8	5.23	11	573	100	25.571	527	100	

Protein Group

unnamed protein product [Triticum aestivum]	gi|296511991	46517.8	5.2300000190 7349

Peptide Information

Calc. Mass	Obsrv. Mass	± da	± ppm	Start Seq.	End Seq.	Sequence	Ion Score	C. I. %	Modification	Rank	Result Type
809.3788	809.3802	0.0014	2	306	311	IYNDER					Mascot
978.4752	978.4617	-0.0135	-14	313	320	VEHVDHSR					Mascot
1149.6051	1149.6075	0.0024	2	54	63	AYLFPQSPAR					Mascot
1149.6051	1149.6075	0.0024	2	54	63	AYLFPQSPAR	63	99.996			Mascot
1430.7638	1430.762	-0.0018	-1	41	53	EAVAPYERPALSK					Mascot
1439.7642	1439.7705	0.0063	4	9	23	YIVLGGGVSGGYAAR					Mascot
1439.7642	1439.7705	0.0063	4	9	23	YIVLGGGVSGGYAAR	96	100			Mascot
1471.7112	1471.7186	0.0074	5	64	77	LPGFHVCVGSGGER			Carbamidomethyl (C)[7]		Mascot
1471.7112	1471.7186	0.0074	5	64	77	LPGFHVCVGSGGER	72	100	Carbamidomethyl (C)[7]		Mascot
1905.9011	1905.9282	0.0271	14	392	410	CVGAFLEGGSPDENAAIAK			Carbamidomethyl (C)[1]		Mascot
1983.9771	1983.9939	0.0168	8	130	147	LSDFGTQGADSNNILYLR					Mascot
1983.9771	1983.9939	0.0168	8	130	147	LSDFGTQGADSNNILYLR	146	100			Mascot
2158.9604	2158.9907	0.0303	14	334	351	EAGSAVEEYDYLPYFYS					Mascot

Calc. Mass	Obsrv. Mass	± da	± ppm	Start Seq.	End Seq.	Sequence	Ion Score	C. I. %	Modification	Rank	Result Type
						R					
2158.9604	2158.9907	0.0303	14	334	351	EAGSAVEEYDYLPYFYS R	150	100			Mascot
2442.3604	2442.3828	0.0224	9	247	270	DGSVLEADIVVVGVGGR PLTTLFK					Mascot
2683.5393	2683.572	0.0327	12	245	270	LKDGSVLEADIVVVGVGG RPLTTLFK					Mascot

2 Monodehydroascorbate reductase [Triticum urartu] gi|474291016 47953.5 5.24 9 397 100 19.728 368 100

Peptide Information

Calc. Mass	Obsrv. Mass	± da	± ppm	Start Seq.	End Seq.	Sequence	Ion Score	C. I. %	Modification	Rank	Result Type
809.3788	809.3802	0.0014	2	319	324	IYNDER					Mascot
978.4752	978.4617	-0.0135	-14	326	333	VEHVDHSR					Mascot
1176.6161	1176.594	-0.0221	-19	54	63	AYLFPQNPAR					Mascot
1425.7485	1425.746	-0.0025	-2	9	23	YVVLGGGVSGGYAAR					Mascot
1430.7638	1430.762	-0.0018	-1	41	53	EAVAPYERPALSK					Mascot
1471.7112	1471.7186	0.0074	5	64	77	LPGFHVCVGSGGER			Carbamidomethyl (C)[7]		Mascot
1471.7112	1471.7186	0.0074	5	64	77	LPGFHVCVGSGGER	72	100	Carbamidomethyl (C)[7]		Mascot
1837.9708	1838.0157	0.0449	24	6	23	HFKYVVLGGGVSGGYAA R					Mascot
1983.9771	1983.9939	0.0168	8	130	147	LSDFGTQGADSNNILYLR					Mascot
1983.9771	1983.9939	0.0168	8	130	147	LSDFGTQGADSNNILYLR	146	100			Mascot
2158.9604	2158.9907	0.0303	14	347	364	EAGSAVEEYDYLPYFYS R					Mascot
2158.9604	2158.9907	0.0303	14	347	364	EAGSAVEEYDYLPYFYS R	150	100			Mascot

3 Actin-7 [Triticum urartu] gi|474317121 41833.9 5.29 15 244 100 13.967 164 100

Peptide Information

Calc. Mass	Obsrv. Mass	± da	± ppm	Start Seq.	End Seq.	Sequence	Ion Score	C. I. %	Modification	Rank	Result Type
800.5352	800.4624	-0.0728	-91	63	69	RGILTLK					Mascot
976.4483	976.4434	-0.0049	-5	20	29	AGFAGDDAPR					Mascot
1098.5426	1098.516	-0.0266	-24	198	207	GYSLTTTAER					Mascot
1176.55	1176.594	0.044	37	41	51	HTGVMVGMGQK			Oxidation (M)[5,8]		Mascot
1198.7056	1198.6981	-0.0075	-6	30	40	AVFPSIVGRPR					Mascot
1515.7491	1515.7528	0.0037	2	86	96	IWHHTFYNELR					Mascot
1797.8324	1797.8264	-0.006	-3	240	255	SYEMPDGQVITIGSER			Oxidation (M)[4]		Mascot
1883.9385	1883.9622	0.0237	13	217	232	LAYVALDYEQELETAR					Mascot
1883.9385	1883.9622	0.0237	13	217	232	LAYVALDYEQELETAR	109	100			Mascot
1940.0488	1940.0363	-0.0125	-6	97	114	VAPEDHPVLLTEAPLNPK					Mascot

1963.8737	1964.0367	0.163	83	2	19	ADEDVQPIVCDNGTGMV K			Carbamidomethyl (C)[10], Oxidation (M)[16]		Mascot
1975.8855	1976.0479	0.1624	82	70	85	YPIEHGIVNNWDDMEK			Oxidation (M)[14]		Mascot
2141.0762	2140.9949	-0.0813	-38	215	232	EKLAYVALDYEQELETAR					Mascot
2169.0645	2169.092	0.0275	13	293	313	DLYGNVVLSGGSTMFPGI ADR					Mascot
2185.0596	2185.071	0.0114	5	293	313	DLYGNVVLSGGSTMFPGI ADR			Oxidation (M)[14]		Mascot
2313.1545	2313.1672	0.0127	5	292	313	KDLYGNVVLSGGSTMFP GIADR			Oxidation (M)[15]		Mascot
3181.6528	3181.7122	0.0594	19	149	178	TTGIVLDSGDGVSHTVPI YEGYTLPHAILR					Mascot
3181.6528	3181.7122	0.0594	19	149	178	TTGIVLDSGDGVSHTVPI YEGYTLPHAILR	55	99.977			Mascot

4 actin [Triticum aestivum] gi|48927618 33601.8 5.33 9 206 100 11.692 164 100

Peptide Information

Calc. Mass	Obsrv. Mass	± da	± ppm	Start Seq.	End Seq.	Sequence	Ion Score	C. I. %	Modification	Rank	Result Type
1098.5426	1098.516	-0.0266	-24	121	130	GYSLTTTAER					Mascot
1515.7491	1515.7528	0.0037	2	9	19	IWHHTFYNELR					Mascot
1797.8324	1797.8264	-0.006	-3	163	178	SYEMPDGQVITIGSER			Oxidation (M)[4]		Mascot
1883.9385	1883.9622	0.0237	13	140	155	LAYVALDYEQELETAR					Mascot
1883.9385	1883.9622	0.0237	13	140	155	LAYVALDYEQELETAR	109	100			Mascot
1940.0488	1940.0363	-0.0125	-6	20	37	VAPEDHPVLLTEAPLNPK					Mascot
2141.0762	2140.9949	-0.0813	-38	138	155	EKLAYVALDYEQELETAR					Mascot
2169.0645	2169.092	0.0275	13	216	236	DLYGNVVLSGGSTMFPGI ADR					Mascot
2185.0596	2185.071	0.0114	5	216	236	DLYGNVVLSGGSTMFPGI ADR			Oxidation (M)[14]		Mascot
2313.1545	2313.1672	0.0127	5	215	236	KDLYGNVVLSGGSTMFP GIADR			Oxidation (M)[15]		Mascot
3181.6528	3181.7122	0.0594	19	72	101	TTGIVLDSGDGVSHTVPI YEGYTLPHAILR					Mascot
3181.6528	3181.7122	0.0594	19	72	101	TTGIVLDSGDGVSHTVPI YEGYTLPHAILR	55	99.977			Mascot

5 Actin-2 [Triticum urartu] gi|474372381 41863.1 5.31 12 56 90.325 7.29

Peptide Information

Calc. Mass	Obsrv. Mass	± da	± ppm	Start Seq.	End Seq.	Sequence	Ion Score	C. I. %	Modification	Rank	Result Type
800.5352	800.4624	-0.0728	-91	64	70	RGILTLK					Mascot
976.4483	976.4434	-0.0049	-5	21	30	AGFAGDDAPR					Mascot
1132.527	1132.5295	0.0025	2	199	208	GYSFTTTAER					Mascot
1176.55	1176.594	0.044	37	42	52	HTGVMVGMGQK			Oxidation (M)[5,8]		Mascot
1198.7056	1198.6981	-0.0075	-6	31	41	AVFPSIVGRPR					Mascot

Calc. Mass	Obsrv. Mass	± da	± ppm	Start Seq.	End Seq.	Sequence	Ion Score	C. I. %	Modification	Rank	Result Type
1515.7491	1515.7528	0.0037	2	87	97	IWHHTFYNELR					Mascot
1547.8098	1547.7478	-0.062	-40	180	193	LDLAGRDLTDSLMK					Mascot
1547.8098	1547.7478	-0.062	-40	180	193	LDLAGRDLTDSLMK					Mascot
1747.8861	1747.8982	0.0121	7	241	256	SYELPDGQVITIGAER					Mascot
1954.0645	1954.0579	-0.0066	-3	98	115	VAPEEHPVLLTEAPLNPK					Mascot
2199.0752	2199.0608	-0.0144	-7	294	314	DLYGNIVLSGGSTMFPGI ADR			Oxidation (M)[14]		Mascot
2774.4333	2774.4192	-0.0141	-5	338	361	KYSVWIGGSILASLSTFQ QMWISR			Oxidation (M)[20]		Mascot
3151.6423	3151.7039	0.0616	20	150	179	TTGIVLDSGDGVSHTVPI YEGYALPHAILR					Mascot

6	Actin-3 [Triticum urartu]	gi|474259583	44624.2	5.26	12	51	63.218	4.547

Peptide Information

Calc. Mass	Obsrv. Mass	± da	± ppm	Start Seq.	End Seq.	Sequence	Ion Score	C. I. %	Modification	Rank	Result Type
800.5352	800.4624	-0.0728	-91	87	93	RGILTLK					Mascot
976.4483	976.4434	-0.0049	-5	44	53	AGFAGDDAPR					Mascot
1132.527	1132.5295	0.0025	2	222	231	GYSFTTTAER					Mascot
1176.55	1176.594	0.044	37	65	75	HTGVMVGMGQK			Oxidation (M)[5,8]		Mascot
1198.7056	1198.6981	-0.0075	-6	54	64	AVFPSIVGRPR					Mascot
1429.6707	1429.6914	0.0207	14	385	397	GEYDESGPAIVHR					Mascot
1515.7491	1515.7528	0.0037	2	110	120	IWHHTFYNELR					Mascot
1623.8081	1623.776	-0.0321	-20	209	221	DLTDCLMKILTER			Carbamidomethyl (C)[5], Oxidation (M)[7]		Mascot
1747.8861	1747.8982	0.0121	7	264	279	SYELPDGQVITIGAER					Mascot
1954.0645	1954.0579	-0.0066	-3	121	138	VAPEEHPVLLTEAPLNPK					Mascot
2199.0752	2199.0608	-0.0144	-7	317	337	DLYGNIVLSGGSTMFPGI ADR			Oxidation (M)[14]		Mascot
3151.6423	3151.7039	0.0616	20	173	202	TTGIVLDSGDGVSHTVPI YEGYALPHAILR					Mascot

7	Disease resistance protein RPM1 [Triticum urartu]	gi|474330240	108709.4	7.05	18	48	28.281	13.787

Peptide Information

Calc. Mass	Obsrv. Mass	± da	± ppm	Start Seq.	End Seq.	Sequence	Ion Score	C. I. %	Modification	Rank	Result Type
800.4777	800.4624	-0.0153	-19	258	263	QFLKHK					Mascot
832.4709	832.4478	-0.0231	-28	717	723	LGMLNLR			Oxidation (M)[3]		Mascot
918.4825	918.4247	-0.0578	-63	653	660	IGNMQALR			Oxidation (M)[4]		Mascot
934.5468	934.4917	-0.0551	-59	595	601	YLRLDVR					Mascot
1102.5626	1102.616	0.0534	48	892	901	EVEALEDAVK					Mascot
1396.7869	1396.6571	-0.1298	-93	487	498	VHDMILDLIISK					Mascot

1412.7817	1412.7198	-0.0619	-44	487	498	VHDMILDLIISK			Oxidation (M)[4]		Mascot
1430.6654	1430.762	0.0966	68	705	716	AYTDNLVMSLCK			Carbamidomethyl (C)[11], Oxidation (M)[8]		Mascot
1536.7428	1536.7622	0.0194	13	42	54	KISEFEDPDELSK					Mascot
1547.7748	1547.7478	-0.027	-17	333	346	RAFGSEGGCPLQLR			Carbamidomethyl (C)[9]		Mascot
1547.7748	1547.7478	-0.027	-17	333	346	RAFGSEGGCPLQLR			Carbamidomethyl (C)[9]		Mascot
1600.7854	1600.7819	-0.0035	-2	499	512	SIEENFVTFSGDKK					Mascot
1641.7758	1641.8778	0.102	62	91	104	GLLSRCMDLMTNSK			Carbamidomethyl (C)[6], Oxidation (M)[7]		Mascot
1782.7997	1782.8594	0.0597	33	902	917	NAAGCLSDELTLEMSR			Carbamidomethyl (C)[5], Oxidation (M)[14]		Mascot
1881.9528	1882.0273	0.0745	40	142	157	TSIDPRLPAFFTEMTR					Mascot
1953.9447	1954.0579	0.1132	58	283	299	CAFPENNSSSRILTTTR			Carbamidomethyl (C)[1]		Mascot
1982.0277	1981.9966	-0.0311	-16	687	704	ILGLNLNWHISNTNGGMK					Mascot
1984.0498	1983.9939	-0.0559	-28	570	585	VLDLENREVLEHNYLK					Mascot
1984.0498	1983.9939	-0.0559	-28	570	585	VLDLENREVLEHNYLK					Mascot
1995.9369	1996.0089	0.072	36	43	58	ISEFEDPDELSKCWIK			Carbamidomethyl (C)[13]		Mascot
2313.1763	2313.1672	-0.0091	-4	207	226	LEGQFEYQAFVSVSQKPDLK					Mascot

8	actin [Triticum aestivum]	gi|58533119	41929	5.23	11	48	24.901	7.328

Protein Group

Actin-97 [Triticum urartu]	gi|474287474	41929	5.23000001907349
actin [Triticum turgidum]	gi|58533114	41929	5.23000001907349

Peptide Information

Calc. Mass	Obsrv. Mass	± da	± ppm	Start Seq.	End Seq.	Sequence	Ion Score	C. I. %	Modification	Rank	Result Type
800.5352	800.4624	-0.0728	-91	64	70	RGILTLK					Mascot
976.4483	976.4434	-0.0049	-5	21	30	AGFAGDDAPR					Mascot
1176.55	1176.594	0.044	37	42	52	HTGVMVGMGQK			Oxidation (M)[5,8]		Mascot
1198.7056	1198.6981	-0.0075	-6	31	41	AVFPSIVGRPR					Mascot
1487.6761	1487.6979	0.0218	15	362	374	DEYDESGPAIVHR					Mascot
1515.7491	1515.7528	0.0037	2	87	97	IWHHTFYNELR					Mascot
1547.8098	1547.7478	-0.062	-40	180	193	LDLAGRDLTDSLMK					Mascot
1547.8098	1547.7478	-0.062	-40	180	193	LDLAGRDLTDSLMK					Mascot
1747.8861	1747.8982	0.0121	7	241	256	SYELPDGQVITIGAER					Mascot
1954.0645	1954.0579	-0.0066	-3	98	115	VAPEEHPVLLTEAPLNPK					Mascot
2197.0959	2197.0081	-0.0878	-40	294	314	DLYGNIVLSGGTTMFPGIADR					Mascot

Calc. Mass	Obsrv. Mass	± da	± ppm	Start Seq.	End Seq.	Sequence	Ion Score	C. I. %	Modification	Rank	Result Type
2213.0908	2213.0962	0.0054	2	294	314	DLYGNIVLSGGTTMFPGI ADR			Oxidation (M)[14]		Mascot
3151.6423	3151.7039	0.0616	20	150	179	TTGIVLDSGDGVSHTVPI YEGYALPHAILR					Mascot

9	Myosin-J heavy chain [Triticum urartu]	gi|474219098	176351.8	9.12	24	45	0	13.403

Peptide Information

Calc. Mass	Obsrv. Mass	± da	± ppm	Start Seq.	End Seq.	Sequence	Ion Score	C. I. %	Modification	Rank	Result Type
832.441	832.4478	0.0068	8	1039	1045	ADELEKK					Mascot
925.4738	925.5115	0.0377	41	1255	1263	SSSATLFGR					Mascot
927.4934	927.5099	0.0165	18	542	548	LYQTFQK					Mascot
945.5363	945.5457	0.0094	10	926	934	ETGALKQAK					Mascot
945.5363	945.5457	0.0094	10	926	934	ETGALKQAK					Mascot
976.4815	976.4434	-0.0381	-39	763	769	MRTHIMR			Oxidation (M)[1,6]		Mascot
1196.6093	1196.5663	-0.043	-36	788	797	GTLACKLYDR			Carbamidomethyl (C)[5]		Mascot
1198.6678	1198.6981	0.0303	25	1128	1139	ITPESAVVASPK					Mascot
1234.5957	1234.6034	0.0077	6	1242	1253	ASGSTGMAPQRR			Oxidation (M)[7]		Mascot
1420.7213	1420.6106	-0.1107	-78	1239	1252	TLKASGSTGMAPQR			Oxidation (M)[10]		Mascot
1425.7386	1425.746	0.0074	5	475	485	LQQHFNQHVFK					Mascot
1471.7177	1471.7186	0.0009	1	1193	1204	SFEVERTSVFDR					Mascot
1471.7388	1471.7186	-0.0202	-14	30	43	INGEEAEIQVANGK					Mascot
1780.048	1780.0416	-0.0064	-4	339	355	VVAAILHIGNIEFAKGK					Mascot
1837.8361	1838.0157	0.1796	98	125	138	LQHLYDSHMMQQYK			Oxidation (M)[9]		Mascot
1865.9451	1865.9912	0.0461	25	166	184	SNSILVSGESGAGKTETT K					Mascot
1880.812	1880.9396	0.1276	68	460	474	SNSFEQFCINYTNEK			Carbamidomethyl (C)[8]		Mascot
1881.9375	1882.0273	0.0898	48	963	979	AQELSKFQSSMDALQAK					Mascot
1912.0579	1912.0156	-0.0423	-22	1298	1313	YPALLFKQQLTAYVEK					Mascot
1943.8726	1943.9467	0.0741	38	52	69	LYPKDMEAAAGGVDDMT K			Oxidation (M)[6,16]		Mascot
1975.998	1976.0479	0.0499	25	180	196	TETTKMLMQYLAYLGGR					Mascot
1979.979	1980.0222	0.0432	22	670	687	CGGVLEAIRISCAGYPTR			Carbamidomethyl (C)[1,12]		Mascot
2100.0503	2100.1575	0.1072	51	159	179	AMIHEGRSNSILVSGESG AGK					Mascot
2179.074	2179.1211	0.0471	22	599	618	CSFIAGLFPPPPEETSKS SK			Carbamidomethyl (C)[1]		Mascot
2313.2231	2313.1672	-0.0559	-24	1270	1292	GTPQGVNLALINGSMVS GVETLR					Mascot

10	hypothetical protein TRIUR3_27266 [Triticum urartu]	gi|474369292	77343.8	4.94	14	45	0	12.417

Peptide Information

Calc. Mass	Obsrv. Mass	± da	± ppm	Start	End	Sequence	Ion	C. I. %	Modification	Rank	Result Type

				Seq.	Seq.		Score		
800.3859	800.4624	0.0765	96	133	138	IYMETK		Oxidation (M)[3]	Mascot
925.5002	925.5115	0.0113	12	346	352	NLWVHTR			Mascot
934.4629	934.4917	0.0288	31	337	344	GFTELDPR			Mascot
1453.7543	1453.7487	-0.0056	-4	386	398	CMVSFAVNVISVK		Carbamidomethyl (C)[1]	Mascot
1456.7101	1456.7161	0.006	4	573	584	TQMIVYDSRDTK			Mascot
1515.7108	1515.7528	0.042	28	259	272	EEQAPMDIAASPTR			Mascot
1531.7057	1531.7411	0.0354	23	259	272	EEQAPMDIAASPTR		Oxidation (M)[6]	Mascot
1547.6948	1547.7478	0.053	34	353	365	FCSFNIGGFDLDR		Carbamidomethyl (C)[2]	Mascot
1547.6948	1547.7478	0.053	34	353	365	FCSFNIGGFDLDR		Carbamidomethyl (C)[2]	Mascot
1780.9303	1781.0354	0.1051	59	139	154	MYPDVLFLGGELVTAR			Mascot
1791.8695	1791.9362	0.0667	37	555	572	GAAPFSGKICAGTSENPK		Carbamidomethyl (C)[10]	Mascot
1837.9663	1838.0157	0.0494	27	383	398	LDRCMVSFAVNVISVK		Carbamidomethyl (C)[4]	Mascot
2141.073	2140.9949	-0.0781	-36	386	404	CMVSFAVNVISVKVTESDR		Carbamidomethyl (C)[1]	Mascot
2159.1133	2158.9907	-0.1226	-57	405	423	GYPISVYGTLVARDTIDYR			Mascot
2159.1133	2158.9907	-0.1226	-57	405	423	GYPISVYGTLVARDTIDYR			Mascot
2170.0269	2170.0986	0.0717	33	563	581	ICAGTSENPKTQMIVYDSR		Carbamidomethyl (C)[2]	Mascot
2179.158	2179.1211	-0.0369	-17	139	157	MYPDVLFLGGELVTARELR			Mascot

Gel Idx/Pos	241/J17	Instr./Gel Origin	BA2151/Sample Project 20140814	Process Status	Analysis Succeeded
Plate [#] Name	[1] Sample Project 20140814	Instrument Sample Name		Spectra	11

Rank	Protein Name	Accession No.	Protein MW	Protein PI	Pep. Count	Protein Score	Protein Score C. I. %	Intensity Matched	Total Ion Score	Total Ion C. I. %	Confirmed
1	glutathione synthetase [Triticum aestivum]	gi|33352233	53262.1	5.57	8	23	0	2.306			

Peptide Information

Calc. Mass	Obsrv. Mass	± da	± ppm	Start Seq.	End Seq.	Sequence	Ion Score	C. I. %	Modification	Rank	Result Type
815.3538	815.3464	-0.0074	-9	444	449	CGYLMR			Carbamidomethyl (C)[1], Oxidation (M)[5]		Mascot
1149.5205	1149.5764	0.0559	49	8	18	ADAAAVEEMAR			Oxidation (M)[9]		Mascot
1439.6625	1439.7367	0.0742	52	335	346	QCFAGLWSLDDK			Carbamidomethyl (C)[2]		Mascot
1665.9646	1665.8687	-0.0959	-58	309	322	KIQQELAKPNVLER					Mascot
1736.812	1736.7794	-0.0326	-19	2	18	STTEGKADAAAVEEMAR					Mascot
1746.8051	1746.9088	0.1037	59	19	34	SATAWCAMHGLVVGDR			Carbamidomethyl (C)[6], Oxidation (M)[8]		Mascot
1883.8474	1883.9219	0.0745	40	1	18	MSTTEGKADAAAVEEMA R			Oxidation (M)[1]		Mascot
2186.0229	2186.0127	-0.0102	-5	19	38	SATAWCAMHGLVVGDR ADPR			Carbamidomethyl (C)[6], Oxidation (M)[8]		Mascot

Rank	Protein Name	Accession No.	Protein MW	Protein PI	Pep. Count	Protein Score	Protein Score C. I. %	Intensity Matched	Total Ion Score	Total Ion C. I. %	Confirmed
2	Homeobox protein knotted-1-like 4 [Triticum urartu]	gi|474308816	11527.7	5.44	4	22	0	1.66			

Peptide Information

Calc. Mass	Obsrv. Mass	± da	± ppm	Start Seq.	End Seq.	Sequence	Ion Score	C. I. %	Modification	Rank	Result Type
809.4012	809.3536	-0.0476	-59	31	36	SNYNRR					Mascot
838.4166	838.4088	-0.0078	-9	29	35	GKSNYNR					Mascot
856.4675	856.5344	0.0669	78	16	23	APPATPFR					Mascot
1471.7024	1471.6855	-0.0169	-11	2	15	DQHAASTSTSPLEK					Mascot

Rank	Protein Name	Accession No.	Protein MW	Protein PI	Pep. Count	Protein Score	Protein Score C. I. %	Intensity Matched	Total Ion Score	Total Ion C. I. %	Confirmed
3	unnamed protein product [Triticum aestivum]	gi|296511811	46517.8	5.23	7	21	0	2.356			

Protein Group

unnamed protein product [Triticum aestivum]	gi|296511991	46517.8	5.2300 000190 7349

Peptide Information

Calc. Mass	Obsrv. Mass	± da	± ppm	Start Seq.	End Seq.	Sequence	Ion Score	C. I. %	Modification	Rank	Result Type
809.3788	809.3536	-0.0252	-31	306	311	IYNDER					Mascot
860.4836	860.4083	-0.0753	-88	321	328	KSAEQAVK					Mascot

Calc. Mass	Obsrv. Mass	± da	± ppm	Start Seq.	End Seq.	Sequence	Ion Score	C. I. %	Modification	Rank	Result Type
1149.6051	1149.5764	-0.0287	-25	54	63	AYLFPQSPAR					Mascot
1439.7642	1439.7367	-0.0275	-19	9	23	YIVLGGGVSGGYAAR					Mascot
1471.7112	1471.6855	-0.0257	-17	64	77	LPGFHVCVGSGGER			Carbamidomethyl (C)[7]		Mascot
1983.9771	1983.955	-0.0221	-11	130	147	LSDFGTQGADSNNILYLR					Mascot
2158.9604	2158.9312	-0.0292	-14	334	351	EAGSAVEEYDYLPYFYS R					Mascot

4	60S ribosomal protein L30 [Triticum urartu]	gi|474051608	10722.5	9.74	5	21	0	1.055

Peptide Information

Calc. Mass	Obsrv. Mass	± da	± ppm	Start Seq.	End Seq.	Sequence	Ion Score	C. I. %	Modification	Rank	Result Type
856.5251	856.5344	0.0093	11	42	49	GISPVQKK					Mascot
989.5812	989.5205	-0.0607	-61	58	65	NKMQLVIK			Oxidation (M)[3]		Mascot
1016.5411	1016.5295	-0.0116	-11	66	74	SGKYTLGYK					Mascot
1050.4487	1050.5026	0.0539	51	11	18	EDTQEWSR					Mascot
1627.7559	1627.8523	0.0964	59	19	32	ASSLQHDELDEDLR					Mascot

5	hypothetical protein TRIUR3_18195 [Triticum urartu]	gi|474072490	16069.4	5.83	4	20	0	1.371

Peptide Information

Calc. Mass	Obsrv. Mass	± da	± ppm	Start Seq.	End Seq.	Sequence	Ion Score	C. I. %	Modification	Rank	Result Type
831.4319	831.3587	-0.0732	-88	123	129	DKNEAVR					Mascot
856.4635	856.5344	0.0709	83	24	30	DPALERR					Mascot
1983.9878	1983.955	-0.0328	-17	1	18	MALVASLCIGATTLDEYR			Carbamidomethyl (C)[8]		Mascot
2186.124	2186.0127	-0.1113	-51	55	73	YELHHKLQYTDDALVAAA K					Mascot

6	putative NADH dehydrogenase [ubiquinone] 1 alpha subcomplex subunit 5, mitochondrial [Triticum urartu]	gi|473878444	19607.7	4.63	5	20	0	1.244

Peptide Information

Calc. Mass	Obsrv. Mass	± da	± ppm	Start Seq.	End Seq.	Sequence	Ion Score	C. I. %	Modification	Rank	Result Type
809.4152	809.3536	-0.0616	-76	98	104	AVESFTR					Mascot
1118.5988	1118.5861	-0.0127	-11	40	51	ILSGGGGLAAMR			Oxidation (M)[11]		Mascot
1471.6006	1471.6855	0.0849	58	26	38	ESEEEDGGGMFLR			Oxidation (M)[10]		Mascot
1627.7017	1627.8523	0.1506	93	26	39	ESEEEDGGGMFLRR			Oxidation (M)[10]		Mascot
1665.8047	1665.8687	0.064	38	1	18	MTGLGLAAGCVSSAAAG R			Carbamidomethyl (C)[10], Oxidation (M)[1]		Mascot

7	hypothetical protein TRIUR3_04821 [Triticum urartu]	gi|474447708	9549.8	9.58	3	20	0	.829

Peptide Information

Calc. Mass	Obsrv. Mass	± da	± ppm	Start Seq.	End Seq.	Sequence	Ion Score	C. I. %	Modification	Rank	Result Type
1016.5371	1016.5295	-0.0076	-7	69	79	GGEPSGLKSGK					Mascot
1471.7573	1471.6855	-0.0718	-49	62	76	TAPQMAKGGEPSGLK					Mascot
1746.8262	1746.9088	0.0826	47	53	68	QQHMAASSKTAPQMAK			Oxidation (M)[4,14]		Mascot

8	hypothetical protein TRIUR3_22266 [Triticum urartu]	gi|473824728	17014.4	5.05	4	20	0	1.289

Peptide Information

Calc. Mass	Obsrv. Mass	± da	± ppm	Start Seq.	End Seq.	Sequence	Ion Score	C. I. %	Modification	Rank	Result Type
802.4053	802.3508	-0.0545	-68	27	33	TDSPNLR					Mascot
847.3904	847.4102	0.0198	23	10	15	EEEERR					Mascot
1471.67	1471.6855	0.0155	11	34	46	SFTQQFPGTSEDK					Mascot
2159.0696	2158.9312	-0.1384	-64	131	147	LSDNFFQVWIDIEELYK					Mascot

9	hypothetical protein TRIUR3_14163 [Triticum urartu]	gi|474323280	25626.4	5.09	5	19	0	1.918

Peptide Information

Calc. Mass	Obsrv. Mass	± da	± ppm	Start Seq.	End Seq.	Sequence	Ion Score	C. I. %	Modification	Rank	Result Type
1265.7001	1265.6566	-0.0435	-34	182	192	LFPSKQGSVFR					Mascot
1439.7927	1439.7367	-0.056	-39	209	221	ESPLHIALTAIMK			Oxidation (M)[12]		Mascot
1570.8007	1570.7826	-0.0181	-12	2	15	DICSLVIHGSLSNR			Carbamidomethyl (C)[3]		Mascot
1883.7634	1883.9219	0.1585	84	85	101	TTMEADDAQTQMEDPGK			Oxidation (M)[3]		Mascot
2203.1475	2203.1479	0.0004	0	2	20	DICSLVIHGSLSNRVMFV R			Carbamidomethyl (C)[3]		Mascot

10	hypothetical protein TRIUR3_34183 [Triticum urartu]	gi|474046378	7852.9	5.82	3	19	0	4.057

Peptide Information

Calc. Mass	Obsrv. Mass	± da	± ppm	Start Seq.	End Seq.	Sequence	Ion Score	C. I. %	Modification	Rank	Result Type
815.3947	815.3464	-0.0483	-59	31	36	WHPYGR					Mascot
889.401	889.3234	-0.0776	-87	39	47	EDEVAGGGR					Mascot
1471.8016	1471.6855	-0.1161	-79	52	64	VGISRGEWEVALR					Mascot

Gel Idx/Pos	242/J18	Instr./Gel Origin	BA2151/Sample Project 20140814	Process Status	Analysis Succeeded
Plate [#] Name	[1] Sample Project 20140814	Instrument Sample Name		Spectra	11

Rank	Protein Name	Accession No.	Protein MW	Protein PI	Pep. Count	Protein Score	Protein Score C. I. %	Intensity Matched	Total Ion Score	Total Ion C. I. %	Confirmed
1	serpin-N3.2 [Triticum aestivum]	gi|379060943	43026.4	5.18	15	577	100	41.952	494	100	

Protein Group

RecName: Full=Serpin-Z2B; AltName: Full=TriaeZ2b; AltName: Full=WSZ2b; AltName: Full=WZS3	gi|75279909	43011.4	5.1799 998283 3862
serpin [Triticum aestivum]	gi|1885346	43011.4	5.1799 998283 3862

Peptide Information

Calc. Mass	Obsrv. Mass	± da	± ppm	Start Seq.	End Seq.	Sequence	Ion Score	C. I. %	Modification	Rank	Result Type
860.5604	860.4865	-0.0739	-86	227	233	VLKLPYK					Mascot
925.5214	925.5234	0.002	2	11	18	LSIAHQTR					Mascot
925.5214	925.5234	0.002	2	11	18	LSIAHQTR	60	99.992			Mascot
947.5156	947.5029	-0.0127	-13	2	10	ATTLATDVR					Mascot
1078.5562	1078.6249	0.0687	64	1	10	MATTLATDVR					Mascot
1137.6667	1137.6506	-0.0161	-14	172	181	LVLGNALYFK					Mascot
1192.5382	1192.5416	0.0034	3	182	191	GAWTDQFDPR					Mascot
1192.5382	1192.5416	0.0034	3	182	191	GAWTDQFDPR	44	99.708			Mascot
1223.5903	1223.5654	-0.0249	-20	127	137	AEAQSVDFQTK					Mascot
1258.7253	1258.7069	-0.0184	-15	289	300	ISLGIEASDLLK					Mascot
1372.7068	1372.7174	0.0106	8	159	171	DILPAGSIDNTTR					Mascot
1372.7068	1372.7174	0.0106	8	159	171	DILPAGSIDNTTR	99	100			Mascot
1514.7485	1514.7397	-0.0088	-6	125	137	YKAEAQSVDFQTK					Mascot
1665.8595	1665.8872	0.0277	17	261	274	LSAEPEFLEQHIPR					Mascot
1665.8595	1665.8872	0.0277	17	261	274	LSAEPEFLEQHIPR	118	100			Mascot
1922.9706	1922.9829	0.0123	6	335	353	AFVEVNETGTEAAATTIA K					Mascot
2083.1072	2083.1067	-0.0005	0	379	398	EDTSGVVLFIGHVVNPLL SS					Mascot
2838.4858	2838.5356	0.0498	18	99	124	VAFANGVFVDASLQLKPS FQELAVCK			Carbamidomethyl (C)[25]		Mascot
3751.9614	3752.0615	0.1001	27	23	61	LASAISSNPESTVNNAAF SPVSLHVALSLITAGAGG ATR					Mascot
3751.9614	3752.0615	0.1001	27	23	61	LASAISSNPESTVNNAAF SPVSLHVALSLITAGAGG ATR	174	100			Mascot

2	serpin 1 [Triticum aestivum]	gi|224589266	43261.1	5.44	15	421	100	16.635	336	100

Protein Group

serpin 3 [Triticum aestivum]	gi|224589270	43227.1	5.5599 999427 7954

Peptide Information

Calc. Mass	Obsrv. Mass	± da	± ppm	Start Seq.	End Seq.	Sequence	Ion Score	C. I. %	Modification	Rank	Result Type
806.4744	806.4302	-0.0442	-55	272	277	HIPRQR					Mascot
860.5604	860.4865	-0.0739	-86	228	234	VLKLPYK					Mascot
925.5214	925.5234	0.002	2	11	18	LSIAHQTR					Mascot
925.5214	925.5234	0.002	2	11	18	LSIAHQTR	60	99.992			Mascot
947.5156	947.5029	-0.0127	-13	2	10	ATTLATDVR					Mascot
1078.5562	1078.6249	0.0687	64	1	10	MATTLATDVR					Mascot
1151.6824	1151.661	-0.0214	-19	172	181	LVLANALYFK					Mascot
1176.5896	1176.5983	0.0087	7	262	271	LSAEPDFLER					Mascot
1176.5896	1176.5983	0.0087	7	262	271	LSAEPDFLER	82	100			Mascot
1292.7097	1292.6886	-0.0211	-16	290	301	ISFGIEASDLLK					Mascot
1345.6958	1345.7329	0.0371	28	159	171	NILPSGSVDNTTK					Mascot
1544.7592	1544.7599	0.0007	0	125	137	YKAETQSVDFQTK					Mascot
1561.7856	1561.7687	-0.0169	-11	138	151	AAEVTTQVNSWVEK					Mascot
2685.3955	2685.448	0.0525	20	33	61	SAASNAAFSPVSLHSALS LLAAGAGSATR					Mascot
2685.3955	2685.448	0.0525	20	33	61	SAASNAAFSPVSLHSALS LLAAGAGSATR	195	100			Mascot
2720.3525	2720.3938	0.0413	15	329	354	VSSVFHQAFVEVNEQGT EAAASTAIK					Mascot
3071.3796	3071.4233	0.0437	14	302	328	CLGLQLPFSDEADFSEM VDSPMPQGLR			Carbamidomethyl (C)[1], Oxidation (M)[17,22]		Mascot
3705.9082	3705.9927	0.0845	23	62	98	DQLVATLGTGEVEGLHAL AEQVVQFVLADASSAGG PR					Mascot

3	serpin [Triticum aestivum]	gi|1885350	43119.9	5.44	14	408	100	16.349	336	100

Protein Group

RecName: Full=Serpin-Z1B; AltName: Full=TriaeZ1b; AltName: Full=WSZ1b; AltName: Full=WZS2	gi|75279910	43119.9	5.4400 000572 2046

Peptide Information

Calc. Mass	Obsrv. Mass	± da	± ppm	Start Seq.	End Seq.	Sequence	Ion Score	C. I. %	Modification	Rank	Result Type
806.4744	806.4302	-0.0442	-55	272	277	HIPRQR					Mascot
860.5604	860.4865	-0.0739	-86	228	234	VLKLPYK					Mascot

925.5214	925.5234	0.002	2	11	18	LSIAHQTR					Mascot
925.5214	925.5234	0.002	2	11	18	LSIAHQTR	60	99.992			Mascot
947.5156	947.5029	-0.0127	-13	2	10	ATTLATDVR					Mascot
1078.5562	1078.6249	0.0687	64	1	10	MATTLATDVR					Mascot
1151.6824	1151.661	-0.0214	-19	172	181	LVLANALYFK					Mascot
1176.5896	1176.5983	0.0087	7	262	271	LSAEPDFLER					Mascot
1176.5896	1176.5983	0.0087	7	262	271	LSAEPDFLER	82	100			Mascot
1345.6958	1345.7329	0.0371	28	159	171	NILPSGSVDNTTK					Mascot
1544.7592	1544.7599	0.0007	0	125	137	YKAETQSVDFQTK					Mascot
1561.7856	1561.7687	-0.0169	-11	138	151	AAEVTTQVNSWVEK					Mascot
1585.8295	1585.7592	-0.0703	-44	288	301	FKISFGMEASDLLK					Mascot
2685.3955	2685.448	0.0525	20	33	61	SAASNAAFSPVSLHSALS LLAAGAGSATR					Mascot
2685.3955	2685.448	0.0525	20	33	61	SAASNAAFSPVSLHSALS LLAAGAGSATR	195	100			Mascot
2720.3525	2720.3938	0.0413	15	329	354	VSSVFHQAFVEVNEQGT EAAASTAIK					Mascot
3071.3796	3071.4233	0.0437	14	302	328	CLGLQLPFSDEADFSEM VDSPMPQGLR			Carbamidomethyl (C)[1], Oxidation (M)[17,22]		Mascot

4	Serpin-Z2B [Triticum urartu]	gi|473793747	45225.7	6.03	14	290	100	36.307	221	100	

Peptide Information

Calc. Mass	Obsrv. Mass	± da	± ppm	Start Seq.	End Seq.	Sequence	Ion Score	C. I. %	Modification	Rank	Result Type
860.5604	860.4865	-0.0739	-86	244	250	VLKLPYK					Mascot
925.5214	925.5234	0.002	2	11	18	LSIAHQTR					Mascot
925.5214	925.5234	0.002	2	11	18	LSIAHQTR	60	99.992			Mascot
947.5156	947.5029	-0.0127	-13	2	10	ATTLATDVR					Mascot
1078.5562	1078.6249	0.0687	64	1	10	MATTLATDVR					Mascot
1137.6667	1137.6506	-0.0161	-14	189	198	LVLGNALYFK					Mascot
1192.5382	1192.5416	0.0034	3	199	208	GAWTDQFDPR					Mascot
1192.5382	1192.5416	0.0034	3	199	208	GAWTDQFDPR	44	99.708			Mascot
1223.5903	1223.5654	-0.0249	-20	127	137	AEAQSVDFQTK					Mascot
1292.7097	1292.6886	-0.0211	-16	306	317	ISFGIEASDLLK					Mascot
1514.7485	1514.7397	-0.0088	-6	125	137	YKAEAQSVDFQTK					Mascot
1665.8595	1665.8872	0.0277	17	278	291	LSAEPEFLEQHIPR					Mascot
1665.8595	1665.8872	0.0277	17	278	291	LSAEPEFLEQHIPR	118	100			Mascot
1922.9706	1922.9829	0.0123	6	352	370	AFVEVNETGTEAAATTIA K					Mascot
2083.1072	2083.1067	-0.0005	0	396	415	EDTSGVVLFIGHVVNPLL SS					Mascot

Calc. Mass	Obsrv. Mass	± da	± ppm	Start Seq.	End Seq.	Sequence	Ion Score	C. I. %	Modification	Rank	Result Type
2667.385	2667.5034	0.1184	44	146	168	YVMQLFLPKAAEVTAQV NSWVEK			Oxidation (M)[3]		Mascot
2838.4858	2838.5356	0.0498	18	99	124	VAFANGVFVDASLQLKPS FQELAVCK			Carbamidomethyl (C)[25]		Mascot

5 serpin [Triticum aestivum] gi|871551 43262.2 5.6 11 185 100 11.72 141 100

Protein Group

RecName: Full=Serpin-Z1A; AltName: Full=TriaeZ1a; AltName: Full=WSZ1a; Short=WSZ1; AltName: Full=WSZCI gi|75282265 43262.2 5.5999999046 3257

Peptide Information

Calc. Mass	Obsrv. Mass	± da	± ppm	Start Seq.	End Seq.	Sequence	Ion Score	C. I. %	Modification	Rank	Result Type
806.4744	806.4302	-0.0442	-55	271	276	HIPRQR					Mascot
860.5604	860.4865	-0.0739	-86	227	233	VLKLPYK					Mascot
925.5214	925.5234	0.002	2	11	18	LSIAHQTR					Mascot
925.5214	925.5234	0.002	2	11	18	LSIAHQTR	60	99.992			Mascot
947.5156	947.5029	-0.0127	-13	2	10	ATTLATDVR					Mascot
1078.5562	1078.6249	0.0687	64	1	10	MATTLATDVR					Mascot
1151.6824	1151.661	-0.0214	-19	172	181	LVLANALYFK					Mascot
1176.5896	1176.5983	0.0087	7	261	270	LSAEPDFLER					Mascot
1176.5896	1176.5983	0.0087	7	261	270	LSAEPDFLER	82	100			Mascot
1292.7097	1292.6886	-0.0211	-16	289	300	ISFGIEASDLLK					Mascot
1544.7592	1544.7599	0.0007	0	125	137	YKAETQSVDFQTK					Mascot
1561.7856	1561.7687	-0.0169	-11	138	151	AAEVTTQVNSWVEK					Mascot
2720.3525	2720.3938	0.0413	15	328	353	VSSVFHQAFVEVNEQGT EAAASTAIK					Mascot

6 serpin-N3.7 [Triticum aestivum] gi|379060945 42639.8 5.52 10 181 100 11.581 141 100

Peptide Information

Calc. Mass	Obsrv. Mass	± da	± ppm	Start Seq.	End Seq.	Sequence	Ion Score	C. I. %	Modification	Rank	Result Type
806.4744	806.4302	-0.0442	-55	271	276	HIPRQR					Mascot
860.5604	860.4865	-0.0739	-86	227	233	VLKLPYK					Mascot
925.5214	925.5234	0.002	2	11	18	LSIAHQTR					Mascot
925.5214	925.5234	0.002	2	11	18	LSIAHQTR	60	99.992			Mascot
947.5156	947.5029	-0.0127	-13	2	10	ATTLATDVR					Mascot
1078.5562	1078.6249	0.0687	64	1	10	MATTLATDVR					Mascot
1151.6824	1151.661	-0.0214	-19	172	181	LVLANALYFK					Mascot
1176.5896	1176.5983	0.0087	7	261	270	LSAEPDFLER					Mascot
1176.5896	1176.5983	0.0087	7	261	270	LSAEPDFLER	82	100			Mascot

1292.7097	1292.6886	-0.0211	-16	289	300	ISFGIEASDLLK					Mascot
2720.3525	2720.3938	0.0413	15	328	353	VSSVFHQAFVEVNEQGT EAAASTAIK					Mascot
3071.3796	3071.4233	0.0437	14	301	327	CLGLQLPFSDEADFSEM VDSPMPQGLR			Carbamidomethyl (C)[1], Oxidation (M)[17,22]		Mascot

7	Serpin-Z1C [Triticum urartu]	gi|474075261	42956	5.62	9	172	100	11.326	141	100

Peptide Information

Calc. Mass	Obsrv. Mass	± da	± ppm	Start Seq.	End Seq.	Sequence	Ion Score	C. I. %	Modification	Rank	Result Type
806.4744	806.4302	-0.0442	-55	271	276	HIPRQR					Mascot
860.5604	860.4865	-0.0739	-86	227	233	VLKLPYK					Mascot
925.5214	925.5234	0.002	2	11	18	LSIAHQTR					Mascot
925.5214	925.5234	0.002	2	11	18	LSIAHQTR	60	99.992			Mascot
947.5156	947.5029	-0.0127	-13	2	10	ATTLATDVR					Mascot
1078.5562	1078.6249	0.0687	64	1	10	MATTLATDVR					Mascot
1151.6824	1151.661	-0.0214	-19	172	181	LVLANALYFK					Mascot
1176.5896	1176.5983	0.0087	7	261	270	LSAEPDFLER					Mascot
1176.5896	1176.5983	0.0087	7	261	270	LSAEPDFLER	82	100			Mascot
1544.7592	1544.7599	0.0007	0	125	137	YKAETQSVDFQTK					Mascot
2720.3525	2720.3938	0.0413	15	328	353	VSSVFHQAFVEVNEQGT EAAASTAIK					Mascot

8	serpin [Triticum aestivum]	gi|5734504	42969	5.62	8	166	100	11.179	141	100

Protein Group

RecName: Full=Serpin-Z1C; AltName: Full=TriaeZ1c; AltName: Full=WSZ1c	gi|75313848	42969	5.6199 998855 5908

Peptide Information

Calc. Mass	Obsrv. Mass	± da	± ppm	Start Seq.	End Seq.	Sequence	Ion Score	C. I. %	Modification	Rank	Result Type
806.4744	806.4302	-0.0442	-55	271	276	HIPRQR					Mascot
860.5604	860.4865	-0.0739	-86	227	233	VLKLPYK					Mascot
925.5214	925.5234	0.002	2	11	18	LSIAHQTR					Mascot
925.5214	925.5234	0.002	2	11	18	LSIAHQTR	60	99.992			Mascot
947.5156	947.5029	-0.0127	-13	2	10	ATTLATDVR					Mascot
1078.5562	1078.6249	0.0687	64	1	10	MATTLATDVR					Mascot
1151.6824	1151.661	-0.0214	-19	172	181	LVLANALYFK					Mascot
1176.5896	1176.5983	0.0087	7	261	270	LSAEPDFLER					Mascot
1176.5896	1176.5983	0.0087	7	261	270	LSAEPDFLER	82	100			Mascot

2720.3525	2720.3938	0.0413	15	328	353	VSSVFHQAFVEVNEQGT EAAASTAIK						Mascot

9	serpin [Triticum aestivum]	gi|5734506	43341.5	5.46	9	89	99.995	7.267	60	99.992	

Protein Group

RecName: Full=Serpin-Z2A; AltName: Full=TriaeZ2a; AltName: Full=WSZ2a	gi|75313847	43341.5	5.4600 000381 4697

Peptide Information

Calc. Mass	Obsrv. Mass	± da	± ppm	Start Seq.	End Seq.	Sequence	Ion Score	C. I. %	Modification	Rank	Result Type
860.5604	860.4865	-0.0739	-86	227	233	VLKLPYK					Mascot
925.5214	925.5234	0.002	2	11	18	LSIAHQTR					Mascot
925.5214	925.5234	0.002	2	11	18	LSIAHQTR	60	99.992			Mascot
947.5156	947.5029	-0.0127	-13	2	10	ATTLATDVR					Mascot
1078.5562	1078.6249	0.0687	64	1	10	MATTLATDVR					Mascot
1137.6667	1137.6506	-0.0161	-14	172	181	LVLGNALYFK					Mascot
1223.5903	1223.5654	-0.0249	-20	127	137	AEAQSVDFQTK					Mascot
1292.7097	1292.6886	-0.0211	-16	289	300	ISFGIEASDLLK					Mascot
1514.7485	1514.7397	-0.0088	-6	125	137	YKAEAQSVDFQTK					Mascot
1922.9706	1922.9829	0.0123	6	335	353	TFVEVNETGTEAAAATIA K					Mascot

10	Cullin-3A [Triticum urartu]	gi|474304968	93558.8	8.49	24	61	96.236	11.445	

Peptide Information

Calc. Mass	Obsrv. Mass	± da	± ppm	Start Seq.	End Seq.	Sequence	Ion Score	C. I. %	Modification	Rank	Result Type
812.4157	812.4317	0.016	20	479	484	VMMLFR			Oxidation (M)[2]		Mascot
832.3909	832.3279	-0.063	-76	362	367	MYDLFK			Oxidation (M)[1]		Mascot
866.3859	866.4454	0.0595	69	240	246	MGEMINR			Oxidation (M)[1]		Mascot
907.5247	907.4628	-0.0619	-68	195	202	TFIPSSKK					Mascot
925.5002	925.5234	0.0232	25	174	180	RWADHIK					Mascot
925.5002	925.5234	0.0232	25	174	180	RWADHIK					Mascot
945.4901	945.5374	0.0473	50	136	143	HGGRLYDK					Mascot
972.4971	972.4874	-0.0097	-10	362	368	MYDLFKR					Mascot
989.501	989.5266	0.0256	26	303	310	RLAEESER					Mascot
1078.5385	1078.6249	0.0864	80	668	676	CLQSLACVK			Carbamidomethyl (C)[1,7]		Mascot
1137.6124	1137.6506	0.0382	34	368	377	RVPDGHSTIR					Mascot
1206.6729	1206.5811	-0.0918	-76	140	149	LYDKLAENLK					Mascot
1220.5253	1220.5529	0.0276	23	524	533	TECGYQFTSK			Carbamidomethyl (C)[3]		Mascot

1223.6307	1223.5654	-0.0653	-53	18	27	VEVDPKFFDK		Mascot
1232.5753	1232.6323	0.057	46	719	728	ESEPEKQETR		Mascot
1263.6548	1263.7106	0.0558	44	668	678	CLQSLACVKGK	Carbamidomethyl (C)[1,7]	Mascot
1355.6229	1355.6924	0.0695	51	240	250	MGEMINRGLMR	Oxidation (M)[1,4,10]	Mascot
1491.7261	1491.6368	-0.0893	-60	608	620	LTWQTNMGNADIK		Mascot
1507.7209	1507.6311	-0.0898	-60	608	620	LTWQTNMGNADIK	Oxidation (M)[7]	Mascot
1514.806	1514.7397	-0.0663	-44	711	724	IGTVVAQKESEPEK		Mascot
1647.8271	1647.8423	0.0152	9	607	620	RLTWQTNMGNADIK		Mascot
1663.8221	1663.87	0.0479	29	607	620	RLTWQTNMGNADIK	Oxidation (M)[8]	Mascot
1681.8796	1681.869	-0.0106	-6	447	460	SPEFISLYVDDKLR		Mascot
1722.9385	1722.9036	-0.0349	-20	653	667	EIEQNTAIPPVDLKR		Mascot
2066.1182	2066.1067	-0.0115	-6	203	219	TPVFEHGLELWRDIVVR		Mascot
2082.9072	2083.1067	0.1995	96	684	701	EPMSKDISDSDAFHFNDK		Mascot
2734.354	2734.4077	0.0537	20	339	361	LFLMENSGLVNMLINDKH EDLTR	Oxidation (M)[4,12]	Mascot

Gel Idx/Pos	243/J19	Instr./Gel Origin	BA2151/Sample Project 20140814	Process Status	Analysis Succeeded
Plate [#] Name	[1] Sample Project 20140814	Instrument Sample Name		Spectra	11

Rank	Protein Name	Accession No.	Protein MW	Protein PI	Pep. Count	Protein Score	Protein Score C. I. %	Intensity Matched	Total Ion Score	Total Ion C. I. %	Confirmed
1	Vicilin-like antimicrobial peptides 2-2 [Triticum urartu]	gi|473890163	75298.3	5.79	12	131	100	9.187	117	100	

Peptide Information

Calc. Mass	Obsrv. Mass	± da	± ppm	Start Seq.	End Seq.	Sequence	Ion Score	C. I. %	Modification	Rank	Result Type
807.4359	807.3883	-0.0476	-59	660	667	KGAVFQSA					Mascot
819.3478	819.3619	0.0141	17	519	524	EEEEQR					Mascot
847.3904	847.3969	0.0065	8	607	612	EEEERR					Mascot
849.4941	849.4177	-0.0764	-90	360	366	NSVFRVK					Mascot
947.5673	947.4882	-0.0791	-83	177	184	GFDVKILR					Mascot
989.4646	989.5185	0.0539	54	543	550	EEEEAARR					Mascot
989.4646	989.5185	0.0539	54	543	550	EEEEAARR					Mascot
1017.5364	1017.5435	0.0071	7	367	375	EGDVFVVPR					Mascot
1017.5364	1017.5435	0.0071	7	367	375	EGDVFVVPR	45	99.748			Mascot
1117.5232	1117.5873	0.0641	57	541	549	QREEEEAAR					Mascot
1161.4767	1161.5367	0.06	52	590	601	EEEEGGGGQGGR					Mascot
1232.5753	1232.6104	0.0351	28	458	468	KAEQEEQEGGK					Mascot
1244.6998	1244.7128	0.013	10	365	375	VKEGDVFVVPR					Mascot
1244.6998	1244.7128	0.013	10	365	375	VKEGDVFVVPR	71	100			Mascot
1899.8246	1899.9703	0.1457	77	441	457	SGSTIMACVSCAEELER			Carbamidomethyl (C)[8,11]		Mascot
1915.8195	1915.8275	0.008	4	441	457	SGSTIMACVSCAEELER			Carbamidomethyl (C)[8,11], Oxidation (M)[6]		Mascot

Rank	Protein Name	Accession No.	Protein MW	Protein PI	Pep. Count	Protein Score	Protein Score C. I. %	Intensity Matched	Total Ion Score	Total Ion C. I. %	Confirmed
2	serpin-N3.2 [Triticum aestivum]	gi|379060943	43026.4	5.18	7	107	100	4.227	86	100	

Protein Group

Protein Name	Accession No.	Protein MW	Protein PI
RecName: Full=Serpin-Z2B; AltName: Full=TriaeZ2b; AltName: Full=WSZ2b; AltName: Full=WZS3	gi|75279909	43011.4	5.1799998283862
serpin [Triticum aestivum]	gi|1885346	43011.4	5.1799998283862

Peptide Information

Calc. Mass	Obsrv. Mass	± da	± ppm	Start Seq.	End Seq.	Sequence	Ion Score	C. I. %	Modification	Rank	Result Type
925.5214	925.5213	-0.0001	0	11	18	LSIAHQTR					Mascot

Calc. Mass	Obsrv. Mass	± da	± ppm	Start Seq.	End Seq.	Sequence	Ion Score	C. I. %	Modification	Rank	Result Type
925.5214	925.5213	-0.0001	0	11	18	LSIAHQTR	6	0			Mascot
947.5156	947.4882	-0.0274	-29	2	10	ATTLATDVR					Mascot
1137.6667	1137.6163	-0.0504	-44	172	181	LVLGNALYFK					Mascot
1192.5382	1192.6343	0.0961	81	182	191	GAWTDQFDPR					Mascot
1372.7068	1372.7135	0.0067	5	159	171	DILPAGSIDNTTR					Mascot
1372.7068	1372.7135	0.0067	5	159	171	DILPAGSIDNTTR	22	44.148			Mascot
1514.7485	1514.751	0.0025	2	125	137	YKAEAQSVDFQTK					Mascot
1665.8595	1665.8821	0.0226	14	261	274	LSAEPEFLEQHIPR					Mascot
1665.8595	1665.8821	0.0226	14	261	274	LSAEPEFLEQHIPR	58	99.987			Mascot

3	Serpin-Z2B [Triticum urartu]	gi|473793747	45225.7	6.03	7	84	99.981	3.72	64	99.997

Peptide Information

Calc. Mass	Obsrv. Mass	± da	± ppm	Start Seq.	End Seq.	Sequence	Ion Score	C. I. %	Modification	Rank	Result Type
925.5214	925.5213	-0.0001	0	11	18	LSIAHQTR					Mascot
925.5214	925.5213	-0.0001	0	11	18	LSIAHQTR	6	0			Mascot
947.5156	947.4882	-0.0274	-29	2	10	ATTLATDVR					Mascot
1137.6667	1137.6163	-0.0504	-44	189	198	LVLGNALYFK					Mascot
1192.5382	1192.6343	0.0961	81	199	208	GAWTDQFDPR					Mascot
1437.7948	1437.7842	-0.0106	-7	234	246	QYISSSDGLKVLK					Mascot
1514.7485	1514.751	0.0025	2	125	137	YKAEAQSVDFQTK					Mascot
1665.8595	1665.8821	0.0226	14	278	291	LSAEPEFLEQHIPR					Mascot
1665.8595	1665.8821	0.0226	14	278	291	LSAEPEFLEQHIPR	58	99.987			Mascot

4	hypothetical protein TRIUR3_15243 [Triticum urartu]	gi|473989782	52754.8	10.12	18	61	96.322	5.874		

Peptide Information

Calc. Mass	Obsrv. Mass	± da	± ppm	Start Seq.	End Seq.	Sequence	Ion Score	C. I. %	Modification	Rank	Result Type
807.4393	807.3883	-0.051	-63	147	154	IAGMGKSK			Oxidation (M)[4]		Mascot
810.341	810.3917	0.0507	63	31	37	MTGNESR			Oxidation (M)[1]		Mascot
819.3955	819.3619	-0.0336	-41	473	479	TDGKNER					Mascot
830.4366	830.403	-0.0336	-40	427	433	ELQVDAR					Mascot
831.4505	831.4928	0.0423	51	464	471	TAPAKGMR					Mascot
841.4638	841.4263	-0.0375	-45	416	423	RPPSSAAR					Mascot
847.4454	847.3969	-0.0485	-57	464	471	TAPAKGMR			Oxidation (M)[7]		Mascot
904.4482	904.5279	0.0797	88	21	28	SKDQSSPR					Mascot
904.4482	904.5279	0.0797	88	21	28	SKDQSSPR					Mascot

932.4544	932.4841	0.0297	32	23	30	DQSSPRSR					Mascot
936.4996	936.4963	-0.0033	-4	283	291	TEATKTSAK					Mascot
960.421	960.4867	0.0657	68	220	226	HDYDWPK					Mascot
1023.4701	1023.519	0.0489	48	331	341	ANTGTSSNSGK					Mascot
1068.6022	1068.522	-0.0802	-75	414	423	ARRPPSSAAR					Mascot
1116.6008	1116.5566	-0.0442	-40	424	433	ASKELQVDAR					Mascot
1192.6685	1192.6343	-0.0342	-29	379	389	NLLTTGSIFAR					Mascot
1207.5736	1207.6359	0.0623	52	126	135	CETSSVLQQR			Carbamidomethyl (C)[1]		Mascot
1243.7079	1243.681	-0.0269	-22	210	219	RDLLMPIIEK			Oxidation (M)[5]		Mascot
1320.692	1320.6121	-0.0799	-60	95	106	GPTFRAPGHPQR					Mascot
1733.8236	1733.8721	0.0485	28	441	458	GKAVAGTGSEPVCNSSGR			Carbamidomethyl (C)[13]		Mascot

5 Ferredoxin--NADP reductase, leaf isozyme, chloroplastic [Triticum urartu] gi|474066121 27862 5.38 13 60 95.678 3.254

Peptide Information

Calc. Mass	Obsrv. Mass	± da	± ppm	Start Seq.	End Seq.	Sequence	Ion Score	C. I. %	Modification	Rank	Result Type
806.444	806.4159	-0.0281	-35	91	97	TVSLCVK			Carbamidomethyl (C)[5]		Mascot
807.4359	807.3883	-0.0476	-59	163	169	LDFAVSR					Mascot
811.413	811.405	-0.008	-10	179	184	MYIQTR					Mascot
827.408	827.4014	-0.0066	-8	179	184	MYIQTR			Oxidation (M)[1]		Mascot
947.4428	947.4882	0.0454	48	170	178	EQTNAAGEK					Mascot
949.569	949.4977	-0.0713	-75	67	74	NGKPHKLR					Mascot
989.4898	989.5185	0.0287	29	26	34	QDEGVVTNK					Mascot
989.4898	989.5185	0.0287	29	26	34	QDEGVVTNK					Mascot
1077.5286	1077.5514	0.0228	21	190	197	EELWEMLK					Mascot
1117.5848	1117.5873	0.0025	2	25	34	KQDEGVVTNK					Mascot
1137.5211	1137.6163	0.0952	84	240	248	AEQWNVEVY					Mascot
1205.6235	1205.6381	0.0146	12	190	198	EELWEMLKK					Mascot
1221.6184	1221.6074	-0.011	-9	190	198	EELWEMLKK			Oxidation (M)[6]		Mascot
1423.6774	1423.7733	0.0959	67	141	152	DPNATIIMEFEK			Oxidation (M)[8]		Mascot
1507.692	1507.7479	0.0559	37	198	209	KDNTYVYMCGLK			Carbamidomethyl (C)[9], Oxidation (M)[8]		Mascot
1899.9811	1899.9703	-0.0108	-6	73	90	LRLYSIASSAIGDFGDSK					Mascot

6 Sucrose synthase 2 [Triticum urartu] gi|474393599 93127.3 6.01 17 50 59.669 4.893

Peptide Information

Calc. Mass	Obsrv. Mass	± da	± ppm	Start Seq.	End Seq.	Sequence	Ion Score	C. I. %	Modification	Rank	Result Type

Calc. Mass	Obsrv. Mass	± da	± ppm	Start Seq.	End Seq.	Sequence	Ion Score	C. I. %	Modification	Rank	Result Type
829.3583	829.3928	0.0345	42	663	668	YICDMK			Carbamidomethyl (C)[3]		Mascot
850.3723	850.4424	0.0701	82	1	8	MGETAGER					Mascot
866.4366	866.4255	-0.0111	-13	777	783	YVSNLDR					Mascot
963.5145	963.5167	0.0022	2	236	243	FQELGLEK					Mascot
999.5469	999.5292	-0.0177	-18	328	336	QQGLDITPK					Mascot
999.5469	999.5292	-0.0177	-18	328	336	QQGLDITPK					Mascot
1021.4883	1021.5261	0.0378	37	647	654	WISAQMNR			Oxidation (M)[6]		Mascot
1068.4635	1068.522	0.0585	55	194	202	GMTMMLNDR					Mascot
1096.5674	1096.5728	0.0054	5	752	759	IEEKYTWK					Mascot
1100.4534	1100.536	0.0826	75	194	202	GMTMMLNDR			Oxidation (M)[2,4]		Mascot
1116.4482	1116.5566	0.1084	97	194	202	GMTMMLNDR			Oxidation (M)[2,4,5]		Mascot
1146.5862	1146.5868	0.0006	1	2	12	GETAGERALSR					Mascot
1190.7078	1190.606	-0.1018	-85	574	583	KKPIIFSMAR					Mascot
1240.726	1240.7271	0.0011	1	326	336	IKQQGLDITPK					Mascot
1313.6525	1313.6639	0.0114	9	448	457	YPNSDLYWKK					Mascot
1320.7158	1320.6121	-0.1037	-79	67	77	LKDTAFEDLLR					Mascot
1746.8943	1746.9242	0.0299	17	800	816	KMAETVPLAVEGETSGK					Mascot
1747.0015	1746.9242	-0.0773	-44	359	373	VLGTEHTHILRVPFK					Mascot
1851.9097	1851.9326	0.0229	12	736	751	EDPSHWNKISQGGLQR					Mascot
1915.9873	1915.8275	-0.1598	-83	20	37	IGDSLSAHTNELVAVFSR					Mascot

7 DNA repair protein Rad50 [Triticum turgidum] gi|157863728 152759.1 6.45 28 50 55.778 7.333

Peptide Information

Calc. Mass	Obsrv. Mass	± da	± ppm	Start Seq.	End Seq.	Sequence	Ion Score	C. I. %	Modification	Rank	Result Type
806.4189	806.4159	-0.003	-4	1262	1267	IMENRK			Oxidation (M)[2]		Mascot
810.3661	810.3917	0.0256	32	1128	1134	TTEMANK			Oxidation (M)[4]		Mascot
817.4162	817.4156	-0.0006	-1	480	485	IDERER					Mascot
818.4366	818.401	-0.0356	-43	1041	1047	TKAEVDR					Mascot
829.4566	829.3928	-0.0638	-77	1296	1301	YYRISK					Mascot
831.4207	831.4928	0.0721	87	199	205	DQAQEIK					Mascot
834.4203	834.3426	-0.0777	-93	71	78	VAGETETK					Mascot
840.4283	840.4257	-0.0026	-3	105	111	ASKMEFK					Mascot
921.4901	921.4808	-0.0093	-10	219	225	DQAYRLR					Mascot
932.4836	932.4841	0.0005	1	557	563	KIYDEHK					Mascot
999.4604	999.5292	0.0688	69	680	687	QMYEPFGK					Mascot

999.4604	999.5292	0.0688	69	680	687	QMYEPFGK					Mascot
1016.537	1016.5453	0.0083	8	247	255	TNIQAVENK					Mascot
1023.5582	1023.519	-0.0392	-38	486	493	HLQIEVER					Mascot
1051.5089	1051.576	0.0671	64	259	267	TETSMVDLR					Mascot
1060.527	1060.5253	-0.0017	-2	602	610	SQEAEQQLK					Mascot
1068.5585	1068.522	-0.0365	-34	881	888	WHALREEK					Mascot
1117.6285	1117.5873	-0.0412	-37	1155	1163	MEEINKIIK					Mascot
1137.6263	1137.6163	-0.01	-9	98	107	SFQLTQKASK					Mascot
1197.6218	1197.5466	-0.0752	-63	1	10	MSTVDKMLIK			Oxidation (M)[1,7]		Mascot
1199.6056	1199.6254	0.0198	17	176	185	KFDDIFSATR					Mascot
1207.6027	1207.6359	0.0332	27	714	724	QRTTGTSTAER					Mascot
1224.5896	1224.5382	-0.0514	-42	307	315	EWQTKFEEK					Mascot
1224.5896	1224.5382	-0.0514	-42	307	315	EWQTKFEEK					Mascot
1320.741	1320.6121	-0.1289	-98	312	322	FEEKIALLETK					Mascot
1423.6709	1423.7733	0.1024	72	1150	1160	FHTMKMEEINK			Oxidation (M)[4]		Mascot
1693.8505	1693.8749	0.0244	14	433	447	VNARYSEVDGQIQSK					Mascot
1899.991	1899.9703	-0.0207	-11	758	774	ETIPLAEKDLEQLSADK					Mascot
1988.0547	1987.9363	-0.1184	-60	775	792	SEKEQISDDLVSVLAQVK					Mascot
2407.1824	2407.3208	0.1384	57	379	400	HNLGPVPDAPFTNDIAMN LTNR					Mascot

8	DNA repair protein Rad50 [Triticum turgidum]	gi|157863720	152748.1	6.49	28	49	43.032	7.333

Peptide Information

Calc. Mass	Obsrv. Mass	± da	± ppm	Start Seq.	End Seq.	Sequence	Ion Score	C. I. %	Modification	Rank	Result Type
806.4189	806.4159	-0.003	-4	1262	1267	IMENRK			Oxidation (M)[2]		Mascot
810.3661	810.3917	0.0256	32	1128	1134	TTEMANK			Oxidation (M)[4]		Mascot
817.4162	817.4156	-0.0006	-1	480	485	IDERER					Mascot
818.4366	818.401	-0.0356	-43	1041	1047	TKAEVDR					Mascot
829.4566	829.3928	-0.0638	-77	1296	1301	YYRISK					Mascot
831.4207	831.4928	0.0721	87	199	205	DQAQEIK					Mascot
834.4203	834.3426	-0.0777	-93	71	78	VAGETETK					Mascot
840.4283	840.4257	-0.0026	-3	105	111	ASKMEFK					Mascot
921.4901	921.4808	-0.0093	-10	219	225	DQAYRLR					Mascot
932.4836	932.4841	0.0005	1	557	563	KIYDEHK					Mascot
999.4604	999.5292	0.0688	69	680	687	QMYEPFGK					Mascot
999.4604	999.5292	0.0688	69	680	687	QMYEPFGK					Mascot

1016.537	1016.5453	0.0083	8	247	255	TNIQAVENK					Mascot
1023.5582	1023.519	-0.0392	-38	486	493	HLQIEVER					Mascot
1051.5089	1051.576	0.0671	64	259	267	TETSMVDLR					Mascot
1060.527	1060.5253	-0.0017	-2	602	610	SQEAEQQLK					Mascot
1068.5585	1068.522	-0.0365	-34	881	888	WHALREEK					Mascot
1117.6285	1117.5873	-0.0412	-37	1155	1163	MEEINKIIK					Mascot
1137.6263	1137.6163	-0.01	-9	98	107	SFQLTQKASK					Mascot
1197.6218	1197.5466	-0.0752	-63	1	10	MSTVDKMLIK			Oxidation (M)[1,7]		Mascot
1199.6056	1199.6254	0.0198	17	176	185	KFDDIFSATR					Mascot
1207.6027	1207.6359	0.0332	27	714	724	QRTTGTSTAER					Mascot
1224.5896	1224.5382	-0.0514	-42	307	315	EWQTKFEEK					Mascot
1224.5896	1224.5382	-0.0514	-42	307	315	EWQTKFEEK					Mascot
1320.741	1320.6121	-0.1289	-98	312	322	FEEKIALLETK					Mascot
1423.6709	1423.7733	0.1024	72	1150	1160	FHTMKMEEINK			Oxidation (M)[4]		Mascot
1693.8505	1693.8749	0.0244	14	433	447	VNARYSEVDGQIQSK					Mascot
1899.991	1899.9703	-0.0207	-11	758	774	ETIPLAEKDLEQLSADK					Mascot
1988.0547	1987.9363	-0.1184	-60	775	792	SEKEQISDDLVSVLAQVK					Mascot
2407.1824	2407.3208	0.1384	57	379	400	HNLGPVPDAPFTNDIAMN LTNR					Mascot

9 hypothetical protein TRIUR3_19555 [Triticum urartu] gi|474424891 48621.3 8.33 15 49 41.705 4.427

Peptide Information

Calc. Mass	Obsrv. Mass	± da	± ppm	Start Seq.	End Seq.	Sequence	Ion Score	C. I. %	Modification	Rank	Result Type
810.4216	810.3917	-0.0299	-37	407	413	LEGQHAR					Mascot
850.4855	850.4424	-0.0431	-51	1	7	MKPAFLK			Oxidation (M)[1]		Mascot
872.4849	872.4377	-0.0472	-54	98	104	SFLHGRR					Mascot
1023.5231	1023.519	-0.0041	-4	277	284	KPHDHHPR					Mascot
1029.5	1029.5114	0.0114	11	105	113	SSNFYGINK					Mascot
1040.4895	1040.5435	0.054	52	50	57	EYEQKTDK					Mascot
1060.5422	1060.5253	-0.0169	-16	47	54	HVKEYEQK					Mascot
1132.6912	1132.6008	-0.0904	-80	1	10	MKPAFLKAVK					Mascot
1171.6582	1171.6182	-0.04	-34	332	341	GLRSPSWQLK					Mascot
1243.6542	1243.681	0.0268	22	335	344	SPSWQLKNQR					Mascot
1278.6688	1278.6514	-0.0174	-14	35	44	NYEEEVRVLK					Mascot
1373.7019	1373.7117	0.0098	7	222	234	AQLNTSAPEISSR					Mascot
1514.7081	1514.751	0.0429	28	376	389	SSLDHEISESSPAR					Mascot
1665.798	1665.8821	0.0841	50	105	119	SSNFYGINKHAGNEK					Mascot

1665.798	1665.8821	0.0841	50	105	119	SSNFYGINKHAGNEK					Mascot
1988.027	1987.9363	-0.0907	-46	18	34	APRLLEDLCDEFALGIR			Carbamidomethyl (C)[9]		Mascot

10	DNA repair protein Rad50 [Triticum monococcum]	gi|157863716	152880.2	6.38	28	48	29.914	11.226	

Peptide Information

Calc. Mass	Obsrv. Mass	± da	± ppm	Start Seq.	End Seq.	Sequence	Ion Score	C. I. %	Modification	Rank	Result Type
810.3661	810.3917	0.0256	32	1128	1134	TTEMANK			Oxidation (M)[4]		Mascot
817.4162	817.4156	-0.0006	-1	480	485	IDERER					Mascot
818.4366	818.401	-0.0356	-43	1041	1047	TKAEVDR					Mascot
829.4566	829.3928	-0.0638	-77	1296	1301	YYRISK					Mascot
831.4207	831.4928	0.0721	87	199	205	DQAQEIK					Mascot
834.4203	834.3426	-0.0777	-93	71	78	VAGETETK					Mascot
840.4283	840.4257	-0.0026	-3	105	111	ASKMEFK					Mascot
921.4901	921.4808	-0.0093	-10	219	225	DQAYRLR					Mascot
932.4836	932.4841	0.0005	1	557	563	KIYDEHK					Mascot
1016.537	1016.5453	0.0083	8	247	255	TNIQAVENK					Mascot
1023.5582	1023.519	-0.0392	-38	486	493	HLQIEVER					Mascot
1051.5089	1051.576	0.0671	64	259	267	TETSMVDLR					Mascot
1060.527	1060.5253	-0.0017	-2	602	610	SQEAEQQLK					Mascot
1068.5585	1068.522	-0.0365	-34	881	888	WHALREEK					Mascot
1117.6285	1117.5873	-0.0412	-37	1155	1163	MEEINKIIK					Mascot
1137.6263	1137.6163	-0.01	-9	98	107	SFQLTQKASK					Mascot
1197.6218	1197.5466	-0.0752	-63	1	10	MSTVDKMLIK			Oxidation (M)[1,7]		Mascot
1199.6056	1199.6254	0.0198	17	176	185	KFDDIFSATR					Mascot
1207.6027	1207.6359	0.0332	27	714	724	QRTTGTSTAER					Mascot
1224.5896	1224.5382	-0.0514	-42	307	315	EWQTKFEEK					Mascot
1224.5896	1224.5382	-0.0514	-42	307	315	EWQTKFEEK					Mascot
1244.6594	1244.7128	0.0534	43	224	234	LRGSIAQDQEK					Mascot
1244.6594	1244.7128	0.0534	43	224	234	LRGSIAQDQEK					Mascot
1320.741	1320.6121	-0.1289	-98	312	322	FEEKIALLETK					Mascot
1324.6855	1324.6562	-0.0293	-22	1091	1102	GTISVYQSNISR					Mascot
1423.6709	1423.7733	0.1024	72	1150	1160	FHTMKMEEINK			Oxidation (M)[4]		Mascot
1693.8505	1693.8749	0.0244	14	433	447	VNARYSEVDGQIQSK					Mascot
1899.991	1899.9703	-0.0207	-11	758	774	ETIPLAEKDLEQLSADK					Mascot
1988.0547	1987.9363	-0.1184	-60	775	792	SEKEQISDDLVSVLAQVK					Mascot

2407.1824	2407.3208	0.1384	57	379	400	HNLGPVPDAPFTNDIAMN LTNR	Mascot

Gel Idx/Pos	244/J20	Instr./Gel Origin	BA2151/Sample Project 20140814	Process Status	Analysis Succeeded
Plate [#] Name	[1] Sample Project 20140814	Instrument Sample Name		Spectra	11

Rank	Protein Name	Accession No.	Protein MW	Protein PI	Pep. Count	Protein Score	Protein Score C. I. %	Intensity Matched	Total Ion Score	Total Ion C. I. %	Confirmed
1	Glutaminyl-tRNA synthetase [Triticum urartu]	gi|474021464	90535.6	6.65	25	77	99.921	10.168			

Peptide Information

Calc. Mass	Obsrv. Mass	± da	± ppm	Start Seq.	End Seq.	Sequence	Ion Score	C. I. %	Modification	Rank	Result Type
804.421	804.3451	-0.0759	-94	550	556	SDNSLIR					Mascot
807.3454	807.3951	0.0497	62	374	379	MDSPWR			Oxidation (M)[1]		Mascot
809.3676	809.3757	0.0081	10	673	679	AEYDPSK					Mascot
813.3923	813.4012	0.0089	11	627	632	TDFRMK			Oxidation (M)[5]		Mascot
830.4519	830.3871	-0.0648	-78	560	565	LEYHIR					Mascot
856.5615	856.5229	-0.0386	-45	522	529	LLTLAGLR					Mascot
887.5197	887.4554	-0.0643	-72	594	601	VIDLDGKK					Mascot
935.4404	935.4622	0.0218	23	373	379	KMDSPWR			Oxidation (M)[2]		Mascot
947.5043	947.4869	-0.0174	-18	165	172	ATKEEIEK					Mascot
960.5434	960.4643	-0.0791	-82	1	8	MEEVIVLK					Mascot
1001.5812	1001.5284	-0.0528	-53	25	32	QMLLLDLR					Mascot
1096.4921	1096.5713	0.0792	72	325	332	WMGWEPYK					Mascot
1106.5378	1106.5371	-0.0007	-1	428	437	FTPHPHAGDK					Mascot
1182.6477	1182.6322	-0.0155	-13	247	256	AHNTKEILEK					Mascot
1193.5653	1193.611	0.0457	38	493	502	LNISHTMMSK			Oxidation (M)[7,8]		Mascot
1204.5641	1204.6403	0.0762	63	294	302	ERNGHCYLR			Carbamidomethyl (C)[6]		Mascot
1227.7168	1227.6271	-0.0897	-73	395	406	RGLIAEGAATLR					Mascot
1232.594	1232.6223	0.0283	23	133	142	ENMEAILEQR					Mascot
1251.5457	1251.663	0.1173	94	407	416	MKQDMQNDNK					Mascot
1346.7461	1346.6886	-0.0575	-43	396	408	GLIAEGAATLRMK			Oxidation (M)[12]		Mascot
1347.7168	1347.7183	0.0015	1	426	437	IKFTPHPHAGDK					Mascot
1514.8213	1514.7424	-0.0789	-52	313	324	KEYIDHIQEIVK					Mascot
1676.8544	1676.9122	0.0578	34	269	283	FPPEPNGYLHIGHAK					Mascot
1828.0062	1827.9441	-0.0621	-34	584	600	VVITNLEDGKVIDLDGK					Mascot
1990.8708	1990.9856	0.1148	58	33	49	FTHMMLTFEAGITGCDK			Carbamidomethyl (C)[15], Oxidation (M)[4,5]		Mascot

Rank	Protein Name	Accession No.	Protein MW	Protein PI	Pep. Count	Protein Score	Protein Score C. I. %	Intensity Matched	Total Ion Score	Total Ion C. I. %	Confirmed
2	unnamed protein product [Triticum aestivum]	gi|218381978	90487.6	6.22	21	52	69.406	8.818			

Protein Group

unnamed protein product [Triticum aestivum]	gi|218401616	90487.6	6.2199 997901 9165

Peptide Information

Calc. Mass	Obsrv. Mass	± da	± ppm	Start Seq.	End Seq.	Sequence	Ion Score	C. I. %	Modification	Rank	Result Type
804.421	804.3451	-0.0759	-94	556	562	SDNSLIR					Mascot
807.3454	807.3951	0.0497	62	380	385	MDSPWR			Oxidation (M)[1]		Mascot
809.3676	809.3757	0.0081	10	679	685	AEYDPSK					Mascot
830.4519	830.3871	-0.0648	-78	566	571	LEYHIR					Mascot
856.5615	856.5229	-0.0386	-45	528	535	LLTLAGLR					Mascot
887.5197	887.4554	-0.0643	-72	600	607	VIDLDGKK					Mascot
935.4404	935.4622	0.0218	23	379	385	KMDSPWR			Oxidation (M)[2]		Mascot
947.5043	947.4869	-0.0174	-18	171	178	ATKEEIEK					Mascot
1096.4921	1096.5713	0.0792	72	331	338	WMGWEPYK					Mascot
1106.5378	1106.5371	-0.0007	-1	434	443	FTPHPHAGDK					Mascot
1182.6477	1182.6322	-0.0155	-13	253	262	AHNTKEILEK					Mascot
1193.5653	1193.611	0.0457	38	499	508	LNISHTMMSK			Oxidation (M)[7,8]		Mascot
1204.5641	1204.6403	0.0762	63	300	308	ERNGHCYLR			Carbamidomethyl (C)[6]		Mascot
1227.7168	1227.6271	-0.0897	-73	401	412	RGLIAEGAATLR					Mascot
1232.594	1232.6223	0.0283	23	139	148	ENMEAILEQR					Mascot
1251.5457	1251.663	0.1173	94	413	422	MKQDMQNDNK					Mascot
1346.7461	1346.6886	-0.0575	-43	402	414	GLIAEGAATLRMK			Oxidation (M)[12]		Mascot
1347.7168	1347.7183	0.0015	1	432	443	IKFTPHPHAGDK					Mascot
1514.8213	1514.7424	-0.0789	-52	319	330	KEYIDHIQEIVK					Mascot
1676.8544	1676.9122	0.0578	34	275	289	FPPEPNGYLHIGHAK					Mascot
1828.0062	1827.9441	-0.0621	-34	590	606	VVITNLEDGKVIDLDGK					Mascot

3	Cell division control protein 45-like protein [Triticum urartu]	gi|473962627	68147.2	6.08	16	50	52.616	6.489	

Peptide Information

Calc. Mass	Obsrv. Mass	± da	± ppm	Start Seq.	End Seq.	Sequence	Ion Score	C. I. %	Modification	Rank	Result Type
818.4519	818.3856	-0.0663	-81	408	414	VHGYKSK					Mascot
819.4505	819.3804	-0.0701	-86	1	6	MVRELR			Oxidation (M)[1]		Mascot
830.4114	830.3871	-0.0243	-29	125	132	NLAAGNDR					Mascot
837.3585	837.3818	0.0233	28	432	439	SAESNDSK					Mascot

968.4254	968.4797	0.0543	56	384	390	MRDEFDR					Mascot
1067.5667	1067.5404	-0.0263	-25	510	518	LCHPQALTK			Carbamidomethyl (C)[2]		Mascot
1106.6106	1106.5371	-0.0735	-66	404	412	SFLRVHGYK					Mascot
1182.5282	1182.6322	0.104	88	362	371	MGFPLADCQK			Carbamidomethyl (C)[8], Oxidation (M)[1]		Mascot
1227.6117	1227.6271	0.0154	13	4	13	ELRADSFYAR					Mascot
1244.7322	1244.6871	-0.0451	-36	475	486	AILRQGSSAITK					Mascot
1251.5385	1251.663	0.1245	99	372	381	GFQYMSMEVK			Oxidation (M)[5,7]		Mascot
1263.658	1263.7039	0.0459	36	133	143	VVVLFTADDER					Mascot
1320.6586	1320.5986	-0.06	-45	534	544	MKPLVCACLGR			Carbamidomethyl (C)[6,8], Oxidation (M)[1]		Mascot
1692.9796	1692.8248	-0.1548	-91	545	559	EPEKVLVVGVWGKPR					Mascot
2399.054	2399.0337	-0.0203	-8	362	381	MGFPLADCQKGFQYMS MEVK			Carbamidomethyl (C)[8], Oxidation (M)[1,15]		Mascot
2664.2983	2664.3176	0.0193	7	322	343	LMLLREWSLFDSMVCSS YVATR			Carbamidomethyl (C)[15]		Mascot

4 Kinesin-like protein KIF22-B [Triticum urartu] gi|474410318 60637.5 7.06 15 48 23.152 6.088

Peptide Information

Calc. Mass	Obsrv. Mass	± da	± ppm	Start Seq.	End Seq.	Sequence	Ion Score	C. I. %	Modification	Rank	Result Type
819.3777	819.3804	0.0027	3	193	199	ACNEGIR			Carbamidomethyl (C)[2]		Mascot
838.3546	838.4029	0.0483	58	1	7	MEACGVR			Carbamidomethyl (C)[4], Oxidation (M)[1]		Mascot
887.4832	887.4554	-0.0278	-31	483	489	EELQQLK					Mascot
935.4866	935.4622	-0.0244	-26	290	297	VKVDMEAK			Oxidation (M)[5]		Mascot
947.5156	947.4869	-0.0287	-30	170	178	VSNDTVKGK					Mascot
963.5258	963.4664	-0.0594	-62	458	466	FNALGSNLK					Mascot
1006.5567	1006.4957	-0.061	-61	496	503	AEYILELR					Mascot
1149.582	1149.5952	0.0132	11	108	117	AKEIMALDDK			Oxidation (M)[5]		Mascot
1224.6406	1224.554	-0.0866	-71	438	448	TPIGACHIVEK			Carbamidomethyl (C)[6]		Mascot
1227.6077	1227.6271	0.0194	16	150	161	AAHTGLNDVSSR					Mascot
1265.6096	1265.6576	0.048	38	314	324	MNGLFSPTGWR					Mascot
1265.6096	1265.6576	0.048	38	314	324	MNGLFSPTGWR					Mascot
1320.6035	1320.5986	-0.0049	-4	272	284	SGHMVTNMASASK					Mascot
1625.8428	1625.8792	0.0364	22	325	339	TPSSMSHVKQPLSAR					Mascot
1676.9581	1676.9122	-0.0459	-27	206	221	INSSLFALSNVISALK					Mascot
1909.0066	1908.9042	-0.1024	-54	467	482	ESLIQQYLEFLNVANK					Mascot

5 3'-N-debenzoyl-2'-deoxytaxol N-benzoyltransferase [Triticum urartu] gi|473759202 38706.3 9.07 6 46 0 4.987 28 86.295

Peptide Information

Calc. Mass	Obsrv. Mass	± da	± ppm	Start Seq.	End Seq.	Sequence	Ion Score	C. I. %	Modification	Rank	Result Type
1117.5823	1117.6324	0.0501	45	1	9	MHIFSTLPR			Oxidation (M)[1]		Mascot
1117.5823	1117.6324	0.0501	45	1	9	MHIFSTLPR			Oxidation (M)[1]		Mascot
1205.6677	1205.6748	0.0071	6	183	193	ALVPYYPVAGR					Mascot
1205.6677	1205.6748	0.0071	6	183	193	ALVPYYPVAGR	28	86.295			Mascot
1370.7791	1370.7018	-0.0773	-56	118	131	SPPALIPPAGPTPR					Mascot
1379.7352	1379.7412	0.006	4	247	258	LDGLIFMVQATR			Oxidation (M)[7]		Mascot
1395.7744	1395.7	-0.0744	-53	293	305	GLKAPSVAPVWDR					Mascot
1676.8538	1676.9122	0.0584	35	166	182	DLGAAAAVAAMREGFAR					Mascot
1692.8486	1692.8248	-0.0238	-14	166	182	DLGAAAAVAAMREGFAR			Oxidation (M)[11]		Mascot

6 hypothetical protein TRIUR3_18392 [Triticum urartu] gi|473741048 40617.8 5.07 11 45 0 5.108

Peptide Information

Calc. Mass	Obsrv. Mass	± da	± ppm	Start Seq.	End Seq.	Sequence	Ion Score	C. I. %	Modification	Rank	Result Type
816.4322	816.4474	0.0152	19	248	254	DADLRAR					Mascot
819.3665	819.3804	0.0139	17	145	151	MDQAPNK			Oxidation (M)[1]		Mascot
935.4792	935.4622	-0.017	-18	152	160	GNKSSTDVK					Mascot
937.4625	937.4332	-0.0293	-31	261	268	VDINDAYK					Mascot
1003.553	1003.5351	-0.0179	-18	317	325	QLTESKAAR					Mascot
1058.559	1058.5045	-0.0545	-51	135	144	EASANKPSVR					Mascot
1104.5908	1104.5818	-0.009	-8	60	70	HHEAKAAAAAK					Mascot
1149.5244	1149.5952	0.0708	62	110	120	RQSDGTGSEGR					Mascot
1265.7324	1265.6576	-0.0748	-59	65	78	AAAAAKVTPAAAPR					Mascot
1265.7324	1265.6576	-0.0748	-59	65	78	AAAAAKVTPAAAPR					Mascot
1374.6383	1374.6686	0.0303	22	285	296	LEEEEIDAAAER					Mascot
1885.8927	1885.9563	0.0636	34	297	310	EEYLTLEQEEYRQR					Mascot
1885.8927	1885.9563	0.0636	34	297	310	EEYLTLEQEEYRQR					Mascot

7 NADH-ubiquinone oxidoreductase 75 kDa subunit, mitochondrial [Triticum urartu] gi|473891076 76594.8 6.33 16 45 0 6.356

Peptide Information

Calc. Mass	Obsrv. Mass	± da	± ppm	Start Seq.	End Seq.	Sequence	Ion Score	C. I. %	Modification	Rank	Result Type
813.3923	813.4012	0.0089	11	147	152	FTDMKR			Oxidation (M)[4]		Mascot
837.3705	837.3818	0.0113	13	170	175	CIQCTR			Carbamidomethyl (C)[1,4]		Mascot

874.4451	874.4691	0.024	27	296	302	LNDPMIR			Oxidation (M)[5]		Mascot
890.4512	890.421	-0.0302	-34	63	70	LSIAGNCR			Carbamidomethyl (C)[7]		Mascot
902.4077	902.4413	0.0336	37	286	292	FCYDGLK			Carbamidomethyl (C)[2]		Mascot
960.4931	960.4643	-0.0288	-30	401	409	VEAAMVNAR					Mascot
1001.5237	1001.5284	0.0047	5	452	459	HPFCSILK			Carbamidomethyl (C)[4]		Mascot
1023.4849	1023.5413	0.0564	55	71	78	MCLVEVEK			Carbamidomethyl (C)[2], Oxidation (M)[1]		Mascot
1058.5088	1058.5045	-0.0043	-4	286	293	FCYDGLKR			Carbamidomethyl (C)[2]		Mascot
1227.5596	1227.6271	0.0675	55	339	349	LSDAESMMALK			Oxidation (M)[7,8]		Mascot
1251.6436	1251.663	0.0194	15	686	696	IMAQCSATLLK			Carbamidomethyl (C)[5], Oxidation (M)[2]		Mascot
1315.7369	1315.7001	-0.0368	-28	389	400	ADVFLLVGTQPR					Mascot
1379.7385	1379.7412	0.0027	2	686	697	IMAQCSATLLKK			Carbamidomethyl (C)[5], Oxidation (M)[2]		Mascot
1665.8265	1665.8916	0.0651	39	179	194	FASEVAGVQDLGMLGR			Oxidation (M)[13]		Mascot
1886.0018	1885.9563	-0.0455	-24	611	629	ALSEVAGAPLPYDSVAAV R					Mascot
1886.0018	1885.9563	-0.0455	-24	611	629	ALSEVAGAPLPYDSVAAV R					Mascot
2399.2466	2399.0337	-0.2129	-89	15	36	APRLPPNPEDALEVFVD GHAVR					Mascot

8 leucine-rich repeat protein [Triticum aestivum] gi|148250116 29927.1 5.87 7 44 0 3.207 15 0

Peptide Information

Calc. Mass	Obsrv. Mass	± da	± ppm	Start Seq.	End Seq.	Sequence	Ion Score	C. I. %	Modification	Rank	Result Type
802.4417	802.4266	-0.0151	-19	155	161	KPLDSSR					Mascot
818.4366	818.3856	-0.051	-62	164	170	SEQSLVR					Mascot
874.4741	874.4691	-0.005	-6	1	7	DDLSRLR					Mascot
1428.6866	1428.7281	0.0415	29	239	250	RQSGEELGFSYR					Mascot
1865.9248	1865.8721	-0.0527	-28	185	201	MVDPALNGMYPAKSLSR			Oxidation (M)[1]		Mascot
1885.9729	1885.9563	-0.0166	-9	138	154	SDVYSFGVVMLELLTGR					Mascot
1885.9729	1885.9563	-0.0166	-9	138	154	SDVYSFGVVMLELLTGR	15	0			Mascot
1990.9917	1990.9856	-0.0061	-3	8	24	HPNIVPLTGYCVEHAQR			Carbamidomethyl (C)[11]		Mascot

9 hypothetical protein TRIUR3_15243 [Triticum urartu] gi|473989782 52754.8 10.12 15 44 0 6.86

Peptide Information

Calc. Mass	Obsrv. Mass	± da	± ppm	Start Seq.	End Seq.	Sequence	Ion Score	C. I. %	Modification	Rank	Result Type
804.421	804.3451	-0.0759	-94	324	330	QQKSDAK					Mascot
807.4393	807.3951	-0.0442	-55	147	154	IAGMGKSK			Oxidation (M)[4]		Mascot
819.3955	819.3804	-0.0151	-18	473	479	TDGKNER					Mascot

820.4312	820.3632	-0.068	-83	83	89	DLPHPNK					Mascot
830.4366	830.3871	-0.0495	-60	427	433	ELQVDAR					Mascot
841.4638	841.4668	0.003	4	416	423	RPPSSAAR					Mascot
847.4454	847.4008	-0.0446	-53	464	471	TAPAKGMR			Oxidation (M)[7]		Mascot
904.4482	904.4779	0.0297	33	21	28	SKDQSSPR					Mascot
960.421	960.4643	0.0433	45	220	226	HDYDWPK					Mascot
1023.4701	1023.5413	0.0712	70	331	341	ANTGTSSNSGK					Mascot
1227.713	1227.6271	-0.0859	-70	210	219	RDLLMPIIEK					Mascot
1255.6318	1255.6177	-0.0141	-11	163	172	DKDESIVFFR					Mascot
1320.692	1320.5986	-0.0934	-71	95	106	GPTFRAPGHPQR					Mascot
1370.6006	1370.7018	0.1012	74	31	42	MTGNESRDDAFK					Mascot
1480.7239	1480.7672	0.0433	29	288	303	TSAKTPAGSSSSNSAK					Mascot

10 Glycogenin-1 [Triticum urartu] gi|473869868 44791.4 7.96 11 43 0 7.843

Peptide Information

Calc. Mass	Obsrv. Mass	± da	± ppm	Start Seq.	End Seq.	Sequence	Ion Score	C. I. %	Modification	Rank	Result Type
802.4166	802.4266	0.01	12	340	346	TTPRGDR					Mascot
807.3705	807.3951	0.0246	30	158	163	WMVDEK					Mascot
823.3655	823.3993	0.0338	41	158	163	WMVDEK			Oxidation (M)[2]		Mascot
837.4352	837.3818	-0.0534	-64	58	64	SIEDVFK					Mascot
963.4564	963.4664	0.01	10	94	101	DMISQVDR					Mascot
1003.4917	1003.5351	0.0434	43	43	50	IFNMTSYK					Mascot
1049.5739	1049.5192	-0.0547	-52	211	219	NPHDQLVVK					Mascot
1182.5824	1182.6322	0.0498	42	58	67	SIEDVFKCGK			Carbamidomethyl (C)[8]		Mascot
1205.5984	1205.6748	0.0764	63	158	166	WMVDEKELR					Mascot
1205.5984	1205.6748	0.0764	63	158	166	WMVDEKELR					Mascot
1244.6708	1244.6871	0.0163	13	41	50	LKIFNMTSYK					Mascot
1428.7441	1428.7281	-0.016	-11	197	210	QKLEESLPGTGGGR					Mascot
1885.9728	1885.9563	-0.0165	-9	141	157	LSTLYNADVGLYMLANK					Mascot
1885.9728	1885.9563	-0.0165	-9	141	157	LSTLYNADVGLYMLANK					Mascot

Gel Idx/Pos	245/J21	Instr./Gel Origin	BA2151/Sample Project 20140814	Process Status	Analysis Succeeded
Plate [#] Name	[1] Sample Project 20140814	Instrument Sample Name		Spectra	11

Rank	Protein Name	Accession No.	Protein MW	Protein PI	Pep. Count	Protein Score	Protein Score C. I. %	Intensity Matched	Total Ion Score	Total Ion C. I. %	Confirmed
1	hypothetical protein TRIUR3_09741 [Triticum urartu]	gi|473781913	7289	10.02	5	40	0	3.138			

Peptide Information

Calc. Mass	Obsrv. Mass	± da	± ppm	Start Seq.	End Seq.	Sequence	Ion Score	C. I. %	Modification	Rank	Result Type
848.4229	848.4524	0.0295	35	1	6	MCRPLR			Carbamidomethyl (C)[2], Oxidation (M)[1]		Mascot
1104.6095	1104.6295	0.02	18	2	9	CRPLRQFK			Carbamidomethyl (C)[1]		Mascot
1251.6449	1251.6506	0.0057	5	1	9	MCRPLRQFK			Carbamidomethyl (C)[2], Oxidation (M)[1]		Mascot
1251.6449	1251.6506	0.0057	5	1	9	MCRPLRQFK			Carbamidomethyl (C)[2], Oxidation (M)[1]		Mascot
1367.8005	1367.8048	0.0043	3	18	29	VEVLPDATLRVR					Mascot
2723.4768	2723.3367	-0.1401	-51	30	55	LVSGTAGIFGTELPPEGW LTIPPRSK					Mascot

Rank	Protein Name	Accession No.	Protein MW	Protein PI	Pep. Count	Protein Score	Protein Score C. I. %	Intensity Matched	Total Ion Score	Total Ion C. I. %	Confirmed
2	60S ribosomal protein L5-1 [Triticum urartu]	gi|474062146	15487.8	9.66	7	38	0	2.688			

Peptide Information

Calc. Mass	Obsrv. Mass	± da	± ppm	Start Seq.	End Seq.	Sequence	Ion Score	C. I. %	Modification	Rank	Result Type
887.4468	887.4655	0.0187	21	81	88	SIADEQPK					Mascot
923.5057	923.4854	-0.0203	-22	60	67	QLGAEIHR					Mascot
1015.5418	1015.5254	-0.0164	-16	81	89	SIADEQPKK					Mascot
1180.6433	1180.6313	-0.012	-10	14	26	NPLLPGTGGAGAR					Mascot
1827.9382	1827.9742	0.036	20	33	50	ITMGALDGGLGISHSDKR					Mascot
1991.0016	1991.006	0.0044	2	31	49	FRITMGALDGGLGISHSD K			Oxidation (M)[5]		Mascot
2695.2419	2695.2893	0.0474	18	1	26	MGADTCSLCTMVRNPLL PGTGGAGAR			Carbamidomethyl (C)[6,9], Oxidation (M)[1,11]		Mascot

Rank	Protein Name	Accession No.	Protein MW	Protein PI	Pep. Count	Protein Score	Protein Score C. I. %	Intensity Matched	Total Ion Score	Total Ion C. I. %	Confirmed
3	hypothetical protein TRIUR3_29935 [Triticum urartu]	gi|473990946	76571.9	4.97	15	35	0	6.778			

Peptide Information

Calc. Mass	Obsrv. Mass	± da	± ppm	Start Seq.	End Seq.	Sequence	Ion Score	C. I. %	Modification	Rank	Result Type
806.389	806.4201	0.0311	39	288	294	SDAEEKK					Mascot
815.5097	815.4651	-0.0446	-55	497	503	INATLKR					Mascot
838.4053	838.4112	0.0059	7	109	114	ENYEKR					Mascot
881.4549	881.4303	-0.0246	-28	615	621	GEMLYLR					Mascot

Calc. Mass	Obsrv. Mass	± da	± ppm	Start Seq.	End Seq.	Sequence	Ion Score	C. I. %	Modification	Rank	Result Type
897.4498	897.4319	-0.0179	-20	615	621	GEMLYLR			Oxidation (M)[3]		Mascot
1015.4955	1015.5254	0.0299	29	183	190	HAFEENLR					Mascot
1022.5265	1022.5748	0.0483	47	384	391	ESRNELFK					Mascot
1105.5736	1105.6094	0.0358	32	427	436	KVEEAESVSK					Mascot
1205.6195	1205.6827	0.0632	52	359	368	TMLLNEVETR					Mascot
1233.6144	1233.6294	0.015	12	149	158	NLNEEVMKEK					Mascot
1244.6482	1244.6997	0.0515	41	203	213	VNLLSQDVNDK					Mascot
1319.7053	1319.6517	-0.0536	-41	603	614	STKLEEELATAK					Mascot
1324.7155	1324.6554	-0.0601	-45	581	591	NMVDAQHLIKR					Mascot
1381.7686	1381.7231	-0.0455	-33	191	202	DKLETLDILHGK					Mascot
1865.9677	1865.8956	-0.0721	-39	606	621	LEEELATAKGEMLYLR					Mascot
1991.0179	1991.006	-0.0119	-6	428	446	VEEAESVSKALSDELASV K					Mascot

4	RuBisCO large subunit-binding protein subunit beta, chloroplastic [Triticum urartu]	gi|474137978	66766.1	7.46	13	34	0	8.348

Peptide Information

Calc. Mass	Obsrv. Mass	± da	± ppm	Start Seq.	End Seq.	Sequence	Ion Score	C. I. %	Modification	Rank	Result Type
806.4189	806.4201	0.0012	1	62	68	DGLAMRK			Oxidation (M)[5]		Mascot
807.4611	807.3878	-0.0733	-91	519	525	SLSYPLK					Mascot
813.4141	813.425	0.0109	13	56	61	ELYFNK					Mascot
815.4621	815.4651	0.003	4	101	107	NVVLENK					Mascot
856.5363	856.5277	-0.0086	-10	137	144	LVRQAAAK					Mascot
871.5359	871.4689	-0.067	-77	511	518	VGAEIVRK					Mascot
963.5509	963.529	-0.0219	-23	190	197	ALVYELQK					Mascot
963.5509	963.529	-0.0219	-23	190	197	ALVYELQK					Mascot
1015.5782	1015.5254	-0.0528	-52	113	122	IVNDGVTVAK					Mascot
1068.5685	1068.558	-0.0105	-10	235	244	HGVVTLEEGK					Mascot
1245.5715	1245.6625	0.091	73	576	586	CCLEHAASVAK			Carbamidomethyl (C)[1,2]		Mascot
1263.7307	1263.7278	-0.0029	-2	187	197	TAKALVYELQK					Mascot
1263.7307	1263.7278	-0.0029	-2	187	197	TAKALVYELQK					Mascot
1470.6893	1470.7897	0.1004	68	245	257	SSENSLYVVEGMR					Mascot
1625.9585	1625.8925	-0.066	-41	526	541	LIAKNAGINGSVVIEK					Mascot

5	hypothetical protein TRIUR3_32614 [Triticum urartu]	gi|474296787	22731	4.95	7	34	0	2.751

Peptide Information

Calc. Mass	Obsrv. Mass	± da	± ppm	Start	End	Sequence	Ion	C. I. %	Modification	Rank	Result Type

				Seq.	Seq.		Score				
897.5192	897.4319	-0.0873	-97	159	165	IYYIGLR					Mascot
903.4642	903.4348	-0.0294	-33	166	173	GEATQNKR					Mascot
923.4879	923.4854	-0.0025	-3	96	102	MRAFINR			Oxidation (M)[1]		Mascot
1106.475	1106.5505	0.0755	68	194	204	SETGGGFSHVE					Mascot
1335.6176	1335.6156	-0.002	-1	192	204	TKSETGGGFSHVE					Mascot
1335.6176	1335.6156	-0.002	-1	192	204	TKSETGGGFSHVE					Mascot
1373.702	1373.7078	0.0058	4	81	95	SIAVVGGADGTSPSR					Mascot
1625.8646	1625.8925	0.0279	17	159	172	IYYIGLRGEATQNK					Mascot

6 Protein SRG1 [Triticum urartu] gi|474119852 35662.2 8.41 9 32 0 4.706

Peptide Information

Calc. Mass	Obsrv. Mass	± da	± ppm	Start Seq.	End Seq.	Sequence	Ion Score	C. I. %	Modification	Rank	Result Type
820.3981	820.3737	-0.0244	-30	90	96	TLEAACR			Carbamidomethyl (C)[6]		Mascot
841.3872	841.4519	0.0647	77	135	141	YMSADVR					Mascot
856.4999	856.5277	0.0278	32	78	84	LRVPSER					Mascot
1016.5636	1016.5561	-0.0075	-7	308	315	HKTFLQSR					Mascot
1016.5636	1016.5561	-0.0075	-7	308	315	HKTFLQSR					Mascot
1068.5255	1068.558	0.0325	30	133	141	ARYMSADVR					Mascot
1180.5593	1180.6313	0.072	61	188	198	EVAAEYASANR					Mascot
1324.722	1324.6554	-0.0666	-50	270	282	VVGPAAELLDQGR					Mascot
1507.8591	1507.7622	-0.0969	-64	2	16	AIVGLSNAGDRLPPK					Mascot
1625.9122	1625.8925	-0.0197	-12	46	60	LPGKYVLPASDRPGR					Mascot

7 hypothetical protein TRIUR3_20409 [Triticum urartu] gi|474054174 72798.3 5.58 16 31 0 7.89

Peptide Information

Calc. Mass	Obsrv. Mass	± da	± ppm	Start Seq.	End Seq.	Sequence	Ion Score	C. I. %	Modification	Rank	Result Type
807.4471	807.3878	-0.0593	-73	142	147	FRSEIR					Mascot
887.4693	887.4655	-0.0038	-4	402	408	AELDRQR					Mascot
921.5074	921.4561	-0.0513	-56	419	426	VKEVMTAK			Oxidation (M)[5]		Mascot
947.523	947.4752	-0.0478	-50	89	96	MAEIEKVK					Mascot
963.5179	963.529	0.0111	12	89	96	MAEIEKVK			Oxidation (M)[1]		Mascot
963.5179	963.529	0.0111	12	89	96	MAEIEKVK			Oxidation (M)[1]		Mascot
1052.4684	1052.5562	0.0878	83	123	130	AEFQDWEK					Mascot

1068.5433	1068.558	0.0147	14	409	417	EAVVTEHQR					Mascot
1106.5265	1106.5505	0.024	22	587	594	EWEYSHKK					Mascot
1116.6008	1116.5586	-0.0422	-38	64	73	IDRAVTEGQK					Mascot
1133.5375	1133.6034	0.0659	58	481	489	VHTGYEWNK					Mascot
1170.6729	1170.6189	-0.054	-46	427	436	AKKPDELELK					Mascot
1180.5634	1180.6313	0.0679	58	123	131	AEFQDWEKK					Mascot
1251.6885	1251.6506	-0.0379	-30	603	612	GILHLYFNFK					Mascot
1251.6885	1251.6506	-0.0379	-30	603	612	GILHLYFNFK					Mascot
1317.7083	1317.6106	-0.0977	-74	429	439	KPDELELKAMK			Oxidation (M)[10]		Mascot
1326.6284	1326.6749	0.0465	35	239	249	GEEPPREVEER					Mascot
1373.7635	1373.7078	-0.0557	-41	67	79	AVTEGQKVDISVK					Mascot
1838.9429	1838.947	0.0041	2	103	118	AIEKAQHEEEMALLAR					Mascot

8 hypothetical protein TRIUR3_11914 [Triticum urartu] gi|473999168 47340.6 6.68 10 31 0 4.615

Peptide Information

Calc. Mass	Obsrv. Mass	± da	± ppm	Start Seq.	End Seq.	Sequence	Ion Score	C. I. %	Modification	Rank	Result Type
807.4247	807.3878	-0.0369	-46	333	339	FAAIEEK					Mascot
820.4094	820.3737	-0.0357	-44	50	55	RCLSER			Carbamidomethyl (C)[2]		Mascot
832.377	832.3358	-0.0412	-49	92	97	WSHQMK			Oxidation (M)[5]		Mascot
834.4104	834.3618	-0.0486	-58	114	120	TQNAWSK					Mascot
1015.5142	1015.5254	0.0112	11	90	97	AKWSHQMK					Mascot
1105.5525	1105.6094	0.0569	51	341	349	LEDPYNINK					Mascot
1180.662	1180.6313	-0.0307	-26	1	11	MAGRPPGLLPR			Oxidation (M)[1]		Mascot
1233.6473	1233.6294	-0.0179	-15	340	349	KLEDPYNINK					Mascot
1326.6689	1326.6749	0.006	5	134	144	FDQSFTLNQVK					Mascot
1507.754	1507.7622	0.0082	5	160	172	LLDVSGFGWDKDR					Mascot

9 truncated hemoglobin Hb2a [Triticum aestivum] gi|198385790 19619.6 5.97 6 30 0 1.517

Peptide Information

Calc. Mass	Obsrv. Mass	± da	± ppm	Start Seq.	End Seq.	Sequence	Ion Score	C. I. %	Modification	Rank	Result Type
840.421	840.4349	0.0139	17	164	171	HATSKPAE					Mascot
923.4944	923.4854	-0.009	-10	59	66	EIFSGSRK					Mascot
1105.5533	1105.6094	0.0561	51	133	140	TKMMIFFR			Oxidation (M)[3,4]		Mascot
1245.6521	1245.6625	0.0104	8	81	91	MGGPPLFSQRR					Mascot
1625.774	1625.8925	0.1185	73	141	154	HTAYFLVAGNEMTR			Oxidation (M)[12]		Mascot

1865.9175	1865.8956	-0.0219	-12	155	171	QTQSVPPCKHATSKPAE			Carbamidomethyl (C)[8]		Mascot

10	Carbonic anhydrase, chloroplastic [Triticum urartu]	gi|474340346	16367.1	7.53	6	30	0	2.589

Peptide Information

Calc. Mass	Obsrv. Mass	± da	± ppm	Start Seq.	End Seq.	Sequence	Ion Score	C. I. %	Modification	Rank	Result Type
856.4635	856.5277	0.0642	75	39	46	NKPSAPSR					Mascot
864.4356	864.4142	-0.0214	-25	47	54	SSRCGGIK			Carbamidomethyl (C)[4]		Mascot
1172.682	1172.6251	-0.0569	-49	50	60	CGGIKALLNVK			Carbamidomethyl (C)[1]		Mascot
1326.6003	1326.6749	0.0746	56	28	38	NIAAMVPCYCK			Carbamidomethyl (C)[8,10]		Mascot
1838.7981	1838.947	0.1489	81	61	75	DSADDTFHFVEDWVR					Mascot
1909.0066	1908.9491	-0.0575	-30	103	119	EAVNVSLQNLSTYPFVK					Mascot

Gel Idx/Pos	246/J22	Instr./Gel Origin	BA2151/Sample Project 20140814	Process Status	Analysis Succeeded
Plate [#] Name	[1] Sample Project 20140814	Instrument Sample Name		Spectra	11

Rank	Protein Name	Accession No.	Protein MW	Protein PI	Pep. Count	Protein Score	Protein Score C. I. %	Intensity Matched	Total Ion Score	Total Ion C. I. %	Confirmed
1	ATP synthase subunit beta, mitochondrial [Triticum urartu]	gi|473798701	57827	5.25	25	933	100	47.459	763	100	

Peptide Information

Calc. Mass	Obsrv. Mass	± da	± ppm	Start Seq.	End Seq.	Sequence	Ion Score	C. I. %	Modification	Rank	Result Type
832.4597	832.3896	-0.0701	-84	497	504	IMGVLDGK					Mascot
866.4003	866.4259	0.0256	30	219	225	EGNDLYR					Mascot
1100.5847	1100.6163	0.0316	29	137	145	TNSYLPIHR					Mascot
1173.6627	1173.6957	0.033	28	166	175	VVDLLAPYQR					Mascot
1173.6627	1173.6957	0.033	28	166	175	VVDLLAPYQR	47	99.862			Mascot
1278.6359	1278.6624	0.0265	21	87	98	TIAMDGTEGLVR			Oxidation (M)[4]		Mascot
1390.6863	1390.7303	0.044	32	203	216	AHGGFSVFAGVGER					Mascot
1390.6863	1390.7303	0.044	32	203	216	AHGGFSVFAGVGER	109	100			Mascot
1399.7693	1399.8136	0.0443	32	261	273	VGLTGLTVAEHFR					Mascot
1399.7693	1399.8136	0.0443	32	261	273	VGLTGLTVAEHFR	45	99.773			Mascot
1409.8112	1409.854	0.0428	30	102	115	VLNTGSPITVPVGR					Mascot
1473.8346	1473.8148	-0.0198	-13	190	202	TVLIMELINNVAK			Oxidation (M)[5]		Mascot
1492.7755	1492.8224	0.0469	31	290	303	FTQANSEVSALLGR					Mascot
1492.7755	1492.8224	0.0469	31	290	303	FTQANSEVSALLGR	71	100			Mascot
1513.8121	1513.8231	0.011	7	133	145	GDIKTNSYLPIHR					Mascot
1675.949	1676.0051	0.0561	33	72	86	LVLEVAQHLGENVVR					Mascot
1675.949	1676.0051	0.0561	33	72	86	LVLEVAQHLGENVVR	101	100			Mascot
1678.7676	1678.7964	0.0288	17	244	258	CALVYGQMNEPPGAR			Carbamidomethyl (C)[1], Oxidation (M)[8]		Mascot
1811.8857	1811.936	0.0503	28	386	401	MLSPHVLGEAHYNTAR			Oxidation (M)[1]		Mascot
1852.9109	1853.0294	0.1185	64	219	234	EGNDLYREMIESGVIK					Mascot
1864.944	1865.0048	0.0608	33	274	289	DAEGQDVLLFIDNIFR					Mascot
1864.944	1865.0048	0.0608	33	274	289	DAEGQDVLLFIDNIFR	117	100			Mascot
1868.9059	1869.0281	0.1222	65	219	234	EGNDLYREMIESGVIK			Oxidation (M)[9]		Mascot
2061.0498	2061.1155	0.0657	32	367	385	QISELGIYPAVDPLDSTSR					Mascot
2172.1548	2172.2068	0.052	24	146	165	EAPAFVEQATEQQILVTG IK					Mascot
2186.1453	2186.2266	0.0813	37	304	324	IPSAVGYQPTLATDLGGL QER					Mascot

Calc. Mass	Obsrv. Mass	± da	± ppm	Start Seq.	End Seq.	Sequence	Ion Score	C. I. %	Modification	Rank	Result Type
2186.1453	2186.2266	0.0813	37	304	324	IPSAVGYQPTLATDLGGL QER	206	100			Mascot
2208.1343	2208.1892	0.0549	25	386	405	MLSPHVLGEAHYNTARG VQK					Mascot
2212.186	2212.2603	0.0743	34	52	71	FDEGLPPILTALEVLDNSI R					Mascot
2212.186	2212.2603	0.0743	34	52	71	FDEGLPPILTALEVLDNSI R	68	99.999			Mascot
2548.2712	2548.3699	0.0987	39	27	51	ITDDFTGAGAVGEVCQVI GAVVDVR			Carbamidomethyl (C)[15]		Mascot
2688.376	2688.4717	0.0957	36	412	435	NLQDIIAILGMDELSEDDK LTVAR			Oxidation (M)[11]		Mascot
3714.8862	3715.0525	0.1663	45	331	366	GSITSVQAIYVPADDLTD PAPATTFAHLDATTVLSR					Mascot
3842.9812	3843.1782	0.197	51	330	366	KGSITSVQAIYVPADDLT DPAPATTFAHLDATTVLS R					Mascot

2	ATP synthase beta subunit [Triticum aestivum]	gi|525291	59325.9	5.56	20	882	100	46.063	765	100	

Protein Group

unnamed protein product [Triticum aestivum]	gi|227303166	59325.9	5.5599 999427 7954

Peptide Information

Calc. Mass	Obsrv. Mass	± da	± ppm	Start Seq.	End Seq.	Sequence	Ion Score	C. I. %	Modification	Rank	Result Type
866.4003	866.4259	0.0256	30	265	271	EGNDLYR					Mascot
1173.6627	1173.6957	0.033	28	212	221	VVDLLAPYQR					Mascot
1173.6627	1173.6957	0.033	28	212	221	VVDLLAPYQR	47	99.862			Mascot
1278.6359	1278.6624	0.0265	21	133	144	TIAMDGTEGLVR			Oxidation (M)[4]		Mascot
1390.6863	1390.7303	0.044	32	249	262	AHGGFSVFAGVGER					Mascot
1390.6863	1390.7303	0.044	32	249	262	AHGGFSVFAGVGER	109	100			Mascot
1399.7693	1399.8136	0.0443	32	307	319	VGLTGLTVAEHFR					Mascot
1399.7693	1399.8136	0.0443	32	307	319	VGLTGLTVAEHFR	45	99.773			Mascot
1409.8112	1409.854	0.0428	30	148	161	VLNTGSPITVPVGR					Mascot
1473.8346	1473.8148	-0.0198	-13	236	248	TVLIMELINNVAK			Oxidation (M)[5]		Mascot
1490.7771	1490.7906	0.0135	9	272	284	EMIESGVIKLDEK					Mascot
1492.7755	1492.8224	0.0469	31	336	349	FTQANSEVSALLGR					Mascot
1492.7755	1492.8224	0.0469	31	336	349	FTQANSEVSALLGR	71	100			Mascot
1675.949	1676.0051	0.0561	33	118	132	LVLEVAQHLGENVVR					Mascot
1675.949	1676.0051	0.0561	33	118	132	LVLEVAQHLGENVVR	101	100			Mascot
1678.7676	1678.7964	0.0288	17	290	304	CALVYGQMNEPPGAR			Carbamidomethyl (C)[1], Oxidation (M)[8]		Mascot
1852.9109	1853.0294	0.1185	64	265	280	EGNDLYREMIESGVIK					Mascot
1864.944	1865.0048	0.0608	33	320	335	DAEGQDVLLFIDNIFR					Mascot
1864.944	1865.0048	0.0608	33	320	335	DAEGQDVLLFIDNIFR	117	100			Mascot

1868.9059	1869.0281	0.1222	65	265	280	EGNDLYREMIESGVIK			Oxidation (M)[9]		Mascot
2061.0498	2061.1155	0.0657	32	413	431	QISELGIYPAVDPLDSTSR					Mascot
2172.1548	2172.2068	0.052	24	192	211	EAPAFVEQATEQQILVTG IK					Mascot
2186.1453	2186.2266	0.0813	37	350	370	IPSAVGYQPTLATDLGGL QER					Mascot
2186.1453	2186.2266	0.0813	37	350	370	IPSAVGYQPTLATDLGGL QER	206	100			Mascot
2212.186	2212.2603	0.0743	34	98	117	FDEGLPPILTALEVLDNSI R					Mascot
2212.186	2212.2603	0.0743	34	98	117	FDEGLPPILTALEVLDNSI R	68	99.999			Mascot
2591.3135	2591.3718	0.0583	22	73	97	ITDEFTGAGSIGQVCQVI GAVVDVR			Carbamidomethyl (C)[15]		Mascot
3714.8862	3715.0525	0.1663	45	377	412	GSITSVQAIYVPADDLTD PAPATTFAHLDATTVLSR					Mascot
3842.9812	3843.1782	0.197	51	376	412	KGSITSVQAIYVPADDLT DPAPATTFAHLDATTVLS R					Mascot

3 ATP synthase subunit beta, mitochondrial [Triticum urartu] gi|473990219 58081.1 5.13 19 874 100 44.222 765 100

Peptide Information

Calc. Mass	Obsrv. Mass	± da	± ppm	Start Seq.	End Seq.	Sequence	Ion Score	C. I. %	Modification	Rank	Result Type
866.4003	866.4259	0.0256	30	253	259	EGNDLYR					Mascot
1173.6627	1173.6957	0.033	28	200	209	VVDLLAPYQR					Mascot
1173.6627	1173.6957	0.033	28	200	209	VVDLLAPYQR	47	99.862			Mascot
1278.6359	1278.6624	0.0265	21	92	103	TIAMDGTEGLVR			Oxidation (M)[4]		Mascot
1390.6863	1390.7303	0.044	32	237	250	AHGGFSVFAGVGER					Mascot
1390.6863	1390.7303	0.044	32	237	250	AHGGFSVFAGVGER	109	100			Mascot
1399.7693	1399.8136	0.0443	32	295	307	VGLTGLTVAEHFR					Mascot
1399.7693	1399.8136	0.0443	32	295	307	VGLTGLTVAEHFR	45	99.773			Mascot
1473.8346	1473.8148	-0.0198	-13	224	236	TVLIMELINNVAK			Oxidation (M)[5]		Mascot
1490.7771	1490.7906	0.0135	9	260	272	EMIESGVIKLDEK					Mascot
1492.7755	1492.8224	0.0469	31	324	337	FTQANSEVSALLGR					Mascot
1492.7755	1492.8224	0.0469	31	324	337	FTQANSEVSALLGR	71	100			Mascot
1675.949	1676.0051	0.0561	33	77	91	LVLEVAQHLGENVVR					Mascot
1675.949	1676.0051	0.0561	33	77	91	LVLEVAQHLGENVVR	101	100			Mascot
1678.7676	1678.7964	0.0288	17	278	292	CALVYGQMNEPPGAR			Carbamidomethyl (C)[1], Oxidation (M)[8]		Mascot
1852.9109	1853.0294	0.1185	64	253	268	EGNDLYREMIESGVIK					Mascot
1864.944	1865.0048	0.0608	33	308	323	DAEGQDVLLFIDNIFR					Mascot
1864.944	1865.0048	0.0608	33	308	323	DAEGQDVLLFIDNIFR	117	100			Mascot
1868.9059	1869.0281	0.1222	65	253	268	EGNDLYREMIESGVIK			Oxidation (M)[9]		Mascot

Calc. Mass	Obsrv. Mass	± da	± ppm	Start Seq.	End Seq.	Sequence	Ion Score	C. I. %	Modification	Rank	Result Type
2061.0498	2061.1155	0.0657	32	401	419	QISELGIYPAVDPLDSTSR					Mascot
2172.1548	2172.2068	0.052	24	180	199	EAPAFVEQATEQQILVTGIK					Mascot
2186.1453	2186.2266	0.0813	37	338	358	IPSAVGYQPTLATDLGGLQER					Mascot
2186.1453	2186.2266	0.0813	37	338	358	IPSAVGYQPTLATDLGGLQER	206	100			Mascot
2212.186	2212.2603	0.0743	34	57	76	FDEGLPPILTALEVLDNSIR					Mascot
2212.186	2212.2603	0.0743	34	57	76	FDEGLPPILTALEVLDNSIR	68	99.999			Mascot
2591.3135	2591.3718	0.0583	22	32	56	ITDEFTGAGSIGQVCQVIGAVVDVR			Carbamidomethyl (C)[15]		Mascot
3714.8862	3715.0525	0.1663	45	365	400	GSITSVQAIYVPADDLTDPAPATTFAHLDATTVLSR					Mascot
3842.9812	3843.1782	0.197	51	364	400	KGSITSVQAIYVPADDLTDPAPATTFAHLDATTVLSR					Mascot

4	enolase [Triticum aestivum]	gi|461744056	48416.5	5.59	9	169	100	9.365	134	100		

Protein Group

enolase [Triticum aestivum]	gi|461744078	48416.5	5.5900001525 8789

Peptide Information

Calc. Mass	Obsrv. Mass	± da	± ppm	Start Seq.	End Seq.	Sequence	Ion Score	C. I. %	Modification	Rank	Result Type
806.4519	806.4757	0.0238	30	418	423	YNQLLR					Mascot
978.5043	978.5253	0.021	21	439	446	FRAPVEPY					Mascot
1499.8615	1499.7412	-0.1203	-80	112	126	LGANAILAVSLAVCK			Carbamidomethyl (C)[14]		Mascot
1790.9283	1790.9888	0.0605	34	36	53	AAVPSGASTGVYEALELR					Mascot
1790.9283	1790.9888	0.0605	34	36	53	AAVPSGASTGVYEALELR	134	100			Mascot
1886.9391	1886.9723	0.0332	18	169	185	LAMQEFMILPTGAASFK			Oxidation (M)[3,7]		Mascot
1983.8536	1983.9176	0.064	32	18	35	GNPTVEVDVCCSDGTFAR			Carbamidomethyl (C)[10,11]		Mascot
2016.9584	2017.0199	0.0615	30	246	263	VVIGMDVAASEFYNDKDK			Oxidation (M)[5]		Mascot
2591.2329	2591.3718	0.1389	54	316	338	MTEECGVEVQIVGDDLLVTNPTR			Carbamidomethyl (C)[5], Oxidation (M)[1]		Mascot
3842.948	3843.1782	0.2302	60	67	102	AVDNVNSIIAPALIGKDPTAQTELDNYMVQQLDGTK					Mascot

5	enolase [Triticum aestivum]	gi|461744058	48459.5	5.49	8	159	100	9.13	134	100		

Peptide Information

Calc. Mass	Obsrv. Mass	± da	± ppm	Start Seq.	End Seq.	Sequence	Ion Score	C. I. %	Modification	Rank	Result Type
806.4519	806.4757	0.0238	30	418	423	YNQLLR					Mascot
978.5043	978.5253	0.021	21	439	446	FRAPVEPY					Mascot

1499.8615	1499.7412	-0.1203	-80	112	126	LGANAILAVSLAVCK			Carbamidomethyl (C)[14]		Mascot
1577.8058	1577.8263	0.0205	13	424	438	IEEELGDAAVYAGLK					Mascot
1790.9283	1790.9888	0.0605	34	36	53	AAVPSGASTGVYEALELR					Mascot
1790.9283	1790.9888	0.0605	34	36	53	AAVPSGASTGVYEALELR	134	100			Mascot
1983.8536	1983.9176	0.064	32	18	35	GNPTVEVDVCCSDGTFAR			Carbamidomethyl (C)[10,11]		Mascot
2016.9584	2017.0199	0.0615	30	246	263	VVIGMDVAASEFYNDKDK			Oxidation (M)[5]		Mascot
2591.2329	2591.3718	0.1389	54	316	338	MTEECGVEVQIVGDDLLVTNPTR			Carbamidomethyl (C)[5], Oxidation (M)[1]		Mascot

6	Enolase [Triticum urartu]	gi|474188401	43643.1	5.54	4	144	100	5.208	134	100

Peptide Information

Calc. Mass	Obsrv. Mass	± da	± ppm	Start Seq.	End Seq.	Sequence	Ion Score	C. I. %	Modification	Rank	Result Type
921.4901	921.4991	0.009	10	12	18	QIFDSRR					Mascot
1790.9283	1790.9888	0.0605	34	34	51	AAVPSGASTGVYEALELR					Mascot
1790.9283	1790.9888	0.0605	34	34	51	AAVPSGASTGVYEALELR	134	100			Mascot
2016.9584	2017.0199	0.0615	30	193	210	VVIGMDVAASEFYNDKDK			Oxidation (M)[5]		Mascot
2591.2329	2591.3718	0.1389	54	263	285	MTEECGVEVQIVGDDLLVTNPTR			Carbamidomethyl (C)[5], Oxidation (M)[1]		Mascot

7	putative alanine aminotransferase [Triticum aestivum]	gi|40641599	12310.1	5.89	8	125	100	5.2	61	99.994

Peptide Information

Calc. Mass	Obsrv. Mass	± da	± ppm	Start Seq.	End Seq.	Sequence	Ion Score	C. I. %	Modification	Rank	Result Type
860.3607	860.3792	0.0185	22	90	95	FMDEFR			Oxidation (M)[2]		Mascot
1170.5613	1170.5811	0.0198	17	7	16	AEGAMYLFPR			Oxidation (M)[5]		Mascot
1530.6682	1530.809	0.1408	92	85	95	EFHEKFMDEFR			Oxidation (M)[7]		Mascot
1820.929	1820.9922	0.0632	35	23	41	AIGAAQAAGAAPDAYYALR					Mascot
1820.929	1820.9922	0.0632	35	23	41	AIGAAQAAGAAPDAYYALR	61	99.994			Mascot
1854.0154	1854.0549	0.0395	21	67	82	CTILPQEDKIPAIISR			Carbamidomethyl (C)[1]		Mascot
1861.8394	1861.9919	0.1525	82	1	16	GMTCNKAEGAMYLFPR			Carbamidomethyl (C)[4], Oxidation (M)[2]		Mascot
1886.9946	1886.9723	-0.0223	-12	7	22	AEGAMYLFPRLHLPQK			Oxidation (M)[5]		Mascot
2572.3672	2572.4668	0.0996	39	42	66	LLQATGIVVVPGSGFGQAPGTYHFR					Mascot

8	Alanine aminotransferase 2 [Triticum urartu]	gi|474343747	59649.9	5.3	13	108	100	6.554	61	99.994

Peptide Information

Calc. Mass	Obsrv. Mass	± da	± ppm	Start Seq.	End Seq.	Sequence	Ion Score	C. I. %	Modification	Rank	Result Type

Calc. Mass	Obsrv. Mass	± da	± ppm	Start Seq.	End Seq.	Sequence	Ion Score	C. I. %	Modification	Rank	Result Type
860.3607	860.3792	0.0185	22	513	518	FMDEFR			Oxidation (M)[2]		Mascot
921.4941	921.4991	0.005	5	308	314	QFHSFKK					Mascot
931.5207	931.5323	0.0116	12	402	410	DGIISSLAR					Mascot
1170.5613	1170.5811	0.0198	17	430	439	AEGAMYLFPR			Oxidation (M)[5]		Mascot
1312.7332	1312.7657	0.0325	25	147	159	GLRDEIAAGIAAR					Mascot
1530.6682	1530.809	0.1408	92	508	518	EFHEKFMDEFR			Oxidation (M)[7]		Mascot
1531.6846	1531.7164	0.0318	21	346	359	GGYMEVTGFNADVR			Oxidation (M)[4]		Mascot
1563.6632	1563.6967	0.0335	21	389	401	VGDESYESFMVER			Oxidation (M)[10]		Mascot
1578.7395	1578.7994	0.0599	38	110	123	SETHALYSSDAIER					Mascot
1820.929	1820.9922	0.0632	35	446	464	AIGAAQAAGAAPDAYYAL R					Mascot
1820.929	1820.9922	0.0632	35	446	464	AIGAAQAAGAAPDAYYAL R	61	99.994			Mascot
1854.0154	1854.0549	0.0395	21	490	505	CTILPQEDKIPAIISR			Carbamidomethyl (C)[1]		Mascot
1886.9946	1886.9723	-0.0223	-12	430	445	AEGAMYLFPRLHLPQK			Oxidation (M)[5]		Mascot
2572.3672	2572.4668	0.0996	39	465	489	LLQATGIVVVPGSGFGQA PGTYHFR					Mascot

9	Eukaryotic translation initiation factor 3 subunit A [Triticum urartu]	gi|474111197	117354.8	9.4	25	68	99.331	16.551

Peptide Information

Calc. Mass	Obsrv. Mass	± da	± ppm	Start Seq.	End Seq.	Sequence	Ion Score	C. I. %	Modification	Rank	Result Type
800.376	800.4019	0.0259	32	182	187	AFQFCK			Carbamidomethyl (C)[5]		Mascot
803.4257	803.4128	-0.0129	-16	708	713	RLEEEK					Mascot
1016.5986	1016.5812	-0.0174	-17	375	384	ATLLSELAAK					Mascot
1100.5518	1100.6163	0.0645	59	685	693	TMDHLERAK					Mascot
1270.6023	1270.6082	0.0059	5	973	985	QPDGAAPAASTER					Mascot
1279.7118	1279.7102	-0.0016	-1	13	23	RAEELIHVGQK					Mascot
1384.7332	1384.735	0.0018	1	744	754	LLEHKNAFQER					Mascot
1389.6646	1389.7036	0.039	28	337	348	YGASHLELENEK					Mascot
1427.7563	1427.8065	0.0502	35	253	265	SVEDIHGLMSLVK					Mascot
1473.6865	1473.8148	0.1283	87	791	801	LMYFLNMEEQR					Mascot
1490.8186	1490.7906	-0.028	-19	931	943	QDAPPVRDARPIR					Mascot
1492.7941	1492.8224	0.0283	19	1	13	MATFAKPENALKR			Oxidation (M)[1]		Mascot
1492.7941	1492.8224	0.0283	19	1	13	MATFAKPENALKR			Oxidation (M)[1]		Mascot
1499.7604	1499.7412	-0.0192	-13	473	484	MIPFFEFNVVEK					Mascot
1531.7639	1531.7164	-0.0475	-31	695	707	QEEAPLIEEAFQK					Mascot
1563.8165	1563.6967	-0.1198	-77	240	252	IATELSLWQEAFR					Mascot

Calc. Mass	Obsrv. Mass	± da	± ppm	Start Seq.	End Seq.	Sequence	Ion Score	C. I. %	Modification	Rank	Result Type
1583.8574	1583.7832	-0.0742	-47	253	266	SVEDIHGLMSLVKR					Mascot
1623.8047	1623.8525	0.0478	29	124	137	RPEDLMLSYVSGEK					Mascot
1659.8562	1659.9407	0.0845	51	957	972	QEAPPAARQEAPPAAR					Mascot
1786.007	1785.9702	-0.0368	-21	421	438	ISTIGGKLSAASSVPEIR					Mascot
1861.9403	1861.9919	0.0516	28	723	738	EIELSKQHHAGDLQEK					Mascot
1865.0127	1865.0048	-0.0079	-4	714	728	ILHEQEQLREIELSK					Mascot
1865.0127	1865.0048	-0.0079	-4	714	728	ILHEQEQLREIELSK					Mascot
1886.9252	1886.9723	0.0471	25	791	804	LMYFLNMEEQRIQR			Oxidation (M)[2]		Mascot
2086.9775	2087.1243	0.1468	70	105	123	NQAQALEDALDVEDLEA DK					Mascot
2212.1724	2212.2603	0.0879	40	473	491	MIPFFEFNVVEKIAVDAVK			Oxidation (M)[1]		Mascot
2212.1724	2212.2603	0.0879	40	473	491	MIPFFEFNVVEKIAVDAVK			Oxidation (M)[1]		Mascot
2548.301	2548.3699	0.0689	27	649	670	IHIEGDMTKQHAMEVVLN QQVK					Mascot

10	eukaryotic translation initiation factor 3 subunit A, putative, expressed [Triticum aestivum]	gi|383100786	117173.6	9.38	25	67	99.214	16.551

Peptide Information

Calc. Mass	Obsrv. Mass	± da	± ppm	Start Seq.	End Seq.	Sequence	Ion Score	C. I. %	Modification	Rank	Result Type
800.376	800.4019	0.0259	32	182	187	AFQFCK			Carbamidomethyl (C)[5]		Mascot
803.4257	803.4128	-0.0129	-16	708	713	RLEEEK					Mascot
1016.5986	1016.5812	-0.0174	-17	375	384	ATLLSELAAK					Mascot
1100.5518	1100.6163	0.0645	59	685	693	TMDHLERAK					Mascot
1270.6023	1270.6082	0.0059	5	978	990	QPDGAAPAASTER					Mascot
1279.7118	1279.7102	-0.0016	-1	13	23	RAEELIHVGQK					Mascot
1384.7332	1384.735	0.0018	1	744	754	LLEHKNAFQER					Mascot
1389.6646	1389.7036	0.039	28	337	348	YGASHLELENEK					Mascot
1427.7563	1427.8065	0.0502	35	253	265	SVEDIHGLMSLVK					Mascot
1473.6865	1473.8148	0.1283	87	791	801	LMYFLNMEEQR					Mascot
1490.8186	1490.7906	-0.028	-19	936	948	QDAPPVRDARPIR					Mascot
1492.7941	1492.8224	0.0283	19	1	13	MATFAKPENALKR			Oxidation (M)[1]		Mascot
1492.7941	1492.8224	0.0283	19	1	13	MATFAKPENALKR			Oxidation (M)[1]		Mascot
1499.7604	1499.7412	-0.0192	-13	473	484	MIPFFEFNVVEK					Mascot
1531.7639	1531.7164	-0.0475	-31	695	707	QEEAPLIEEAFQK					Mascot
1563.8165	1563.6967	-0.1198	-77	240	252	IATELSLWQEAFR					Mascot
1583.8574	1583.7832	-0.0742	-47	253	266	SVEDIHGLMSLVKR					Mascot
1623.8047	1623.8525	0.0478	29	124	137	RPEDLMLSYVSGEK					Mascot

1659.8562	1659.9407	0.0845	51	962	977	QEAPPAARQEAPPAAR		Mascot
1786.007	1785.9702	-0.0368	-21	421	438	ISTIGGKLSAASSVPEIR		Mascot
1861.9403	1861.9919	0.0516	28	723	738	EIELSKQHHAGDLQEK		Mascot
1865.0127	1865.0048	-0.0079	-4	714	728	ILHEQEQLREIELSK		Mascot
1865.0127	1865.0048	-0.0079	-4	714	728	ILHEQEQLREIELSK		Mascot
1886.9252	1886.9723	0.0471	25	791	804	LMYFLNMEEQRIQR	Oxidation (M)[2]	Mascot
2086.9775	2087.1243	0.1468	70	105	123	NQAQALEDALDVEDLEA DK		Mascot
2212.1724	2212.2603	0.0879	40	473	491	MIPFFEFNVVEKIAVDAVK	Oxidation (M)[1]	Mascot
2212.1724	2212.2603	0.0879	40	473	491	MIPFFEFNVVEKIAVDAVK	Oxidation (M)[1]	Mascot
2548.301	2548.3699	0.0689	27	649	670	IHIEGDMTKQHAMEVVLN QQVK		Mascot

Gel Idx/Pos	247/J23	Instr./Gel Origin	BA2151/Sample Project 20140814	Process Status	Analysis Succeeded
Plate [#] Name	[1] Sample Project 20140814	Instrument Sample Name		Spectra	11

Rank	Protein Name	Accession No.	Protein MW	Protein PI	Pep. Count	Protein Score	Protein Score C. I. %	Intensity Matched	Total Ion Score	Total Ion C. I. %	Confirmed
1	UTP--glucose-1-phosphate uridylyltransferase [Triticum urartu]	gi|473993048	51082.9	5.76	18	516	100	17.784	420	100	

Peptide Information

Calc. Mass	Obsrv. Mass	± da	± ppm	Start Seq.	End Seq.	Sequence	Ion Score	C. I. %	Modification	Rank	Result Type
839.5098	839.5275	0.0177	21	334	341	AIGINVPR					Mascot
918.5519	918.5631	0.0112	12	390	397	KVANFLAR					Mascot
949.5465	949.5652	0.0187	20	27	35	AGFISLVSR					Mascot
949.5465	949.5652	0.0187	20	27	35	AGFISLVSR	62	99.996			Mascot
1014.5942	1014.6111	0.0169	17	294	302	RLVDAEALK					Mascot
1052.5371	1052.5609	0.0238	23	245	254	GGTLISYEGR					Mascot
1052.5371	1052.5609	0.0238	23	245	254	GGTLISYEGR	64	99.997			Mascot
1114.6216	1114.548	-0.0736	-66	8	17	IDNLRDAVAK					Mascot
1173.6222	1173.6803	0.0581	50	2	12	AAADSKIDNLR					Mascot
1300.7358	1300.7156	-0.0202	-16	400	411	SIPSIVELDSLK					Mascot
1312.7583	1312.7961	0.0378	29	317	329	VLQLETAAGAAIR					Mascot
1312.7583	1312.7961	0.0378	29	317	329	VLQLETAAGAAIR	110	100			Mascot
1350.7264	1350.6886	-0.0378	-28	412	425	VSGDVSFGSGVVLK					Mascot
1358.7566	1358.7612	0.0046	3	185	196	IVTEDFLPLPSK					Mascot
1390.7842	1390.6754	-0.1088	-78	330	341	FFEKAIGINVPR					Mascot
1390.7842	1390.6754	-0.1088	-78	330	341	FFEKAIGINVPR					Mascot
1539.7325	1539.7522	0.0197	13	36	48	YLSGEAEQIEWSK					Mascot
1641.8846	1641.9037	0.0191	12	375	389	VKPSNPSIELGPEFK					Mascot
1940.0812	1940.1102	0.029	15	311	329	EVDGVKVLQLETAAGAAIR					Mascot
2108.1023	2108.1689	0.0666	32	255	272	VQLLEIAQVPDEHVNEFK					Mascot
2108.1023	2108.1689	0.0666	32	255	272	VQLLEIAQVPDEHVNEFK	81	100			Mascot
2143.9832	2144.0635	0.0803	37	202	221	DGWYPPGHGDVFPSLNNSGK					Mascot
2454.3127	2454.4099	0.0972	40	349	370	ATSDLLLVQSDLYTLVDGYVIR					Mascot
2454.3127	2454.4099	0.0972	40	349	370	ATSDLLLVQSDLYTLVDGYVIR	104	100			Mascot

Rank	Protein Name	Accession No.	Protein MW	Protein PI	Pep. Count	Protein Score	Protein Score C. I. %	Intensity Matched	Total Ion Score	Total Ion C. I. %	Confirmed
2	hypothetical protein TRIUR3_05034 [Triticum urartu]	gi|473889078	66517.6	9.96	13	51	65.673	8.31			

Peptide Information

Calc. Mass	Obsrv. Mass	± da	± ppm	Start Seq.	End Seq.	Sequence	Ion Score	C. I. %	Modification	Rank	Result Type
1128.6194	1128.5408	-0.0786	-70	60	69	KACLPPVTSR			Carbamidomethyl (C)[3]		Mascot
1312.6855	1312.7961	0.1106	84	304	316	SSPSTPARTSPPK					Mascot
1312.6855	1312.7961	0.1106	84	304	316	SSPSTPARTSPPK	7	0			Mascot
1342.7074	1342.8049	0.0975	73	448	458	ESRPEKEKPSR					Mascot
1342.7074	1342.8049	0.0975	73	448	458	ESRPEKEKPSR					Mascot
1350.7125	1350.6886	-0.0239	-18	319	331	SSPPTPARTSPPR					Mascot
1513.8081	1513.8071	-0.001	-1	266	280	NSGRPLSQNGSAVVK					Mascot
1545.714	1545.8044	0.0904	58	440	453	DNSPDSGKESRPEK					Mascot
1641.9031	1641.9037	0.0006	0	266	281	NSGRPLSQNGSAVVKK					Mascot
1801.865	1801.9606	0.0956	53	33	50	EAAGLASHVGCGAFIGE R			Carbamidomethyl (C)[11]		Mascot
2019.058	2019.0635	0.0055	3	354	370	RVTETVSWDTLPTCLIK			Carbamidomethyl (C)[14]		Mascot
2108.1804	2108.1689	-0.0115	-5	424	439	FFQLNRLIIQQNIFWK					Mascot
2108.1804	2108.1689	-0.0115	-5	424	439	FFQLNRLIIQQNIFWK					Mascot
2159.0881	2159.1079	0.0198	9	2	21	TTALAPPFPSWTSRPSAT DR					Mascot
2197.2551	2197.1072	-0.1479	-67	380	401	TIALIVAAEAQREATAAAS LVK					Mascot
2255.1335	2255.1072	-0.0263	-12	586	606	QKAYSCLLGTVETAASAL ESR			Carbamidomethyl (C)[6]		Mascot

3 putative serine acetyltransferase 5 [Triticum urartu] gi|474393091 45231 9.37 11 48 37.535 2.069

Peptide Information

Calc. Mass	Obsrv. Mass	± da	± ppm	Start Seq.	End Seq.	Sequence	Ion Score	C. I. %	Modification	Rank	Result Type
1016.5523	1016.5781	0.0258	25	187	195	SLSFHLANK					Mascot
1161.6627	1161.6145	-0.0482	-41	41	51	DVLFLAAGLSR					Mascot
1380.6979	1380.7655	0.0676	49	126	140	VGAFGTTRASGSGGR					Mascot
1675.8585	1675.9844	0.1259	75	2	14	EFQQQLLCIQVNR			Carbamidomethyl (C)[8]		Mascot
1801.9807	1801.9606	-0.0201	-11	41	57	DVLFLAAGLSRVPDNSK					Mascot
1967.0709	1967.0576	-0.0133	-7	23	40	KEVLVLFAGGDGNLLEH R					Mascot
2168.2107	2168.1306	-0.0801	-37	339	362	IGDGVLIGAGATILGNVMI GAGAK					Mascot
2187.2046	2187.2407	0.0361	17	250	268	GFLAIQAHRVAHVLWAQ NR					Mascot
2220.2458	2220.0837	-0.1621	-73	363	385	IGAGSVVLIDVPARSTAV GNPAR					Mascot
2299.3259	2299.1716	-0.1543	-67	259	278	VAHVLWAQNRRPLALAL QSR					Mascot
2382.9812	2383.1287	0.1475	62	392	412	GESDKDEDMPGESMDH					Mascot

TSFIR

4	Alanine--glyoxylate aminotransferase 2-like protein 3, mitochondrial [Triticum urartu]	gi|474093999	48521	8.72	11	46	0	2.949

Peptide Information

Calc. Mass	Obsrv. Mass	± da	± ppm	Start Seq.	End Seq.	Sequence	Ion Score	C. I. %	Modification	Rank	Result Type
1016.4948	1016.5781	0.0833	82	408	416	GGFYGNVFR					Mascot
1342.7227	1342.8049	0.0822	61	10	20	LELPWRSAQSR					Mascot
1342.7227	1342.8049	0.0822	61	10	20	LELPWRSAQSR					Mascot
1480.7465	1480.7419	-0.0046	-3	368	381	GAGFMLGVELVTDR			Oxidation (M)[5]		Mascot
1492.8231	1492.8119	-0.0112	-8	355	367	GLQEKHQIIGDVR					Mascot
1790.9219	1790.9744	0.0525	29	248	264	KAGGLCIADEVQVGFAR			Carbamidomethyl (C)[6]		Mascot
1833.9891	1833.9451	-0.044	-24	368	384	GAGFMLGVELVTDRQLK					Mascot
2122.9724	2123.1272	0.1548	73	154	173	NSYHGNASGTMGATAQK NWK					Mascot
2187.0903	2187.2407	0.1504	69	311	331	SYFNTFGGNPLCTAGGL AVLK			Carbamidomethyl (C)[12]		Mascot
2206.9963	2207.1404	0.1441	65	389	407	DEICQAMEHMKDMGVLV GK			Carbamidomethyl (C)[4], Oxidation (M)[7]		Mascot
2383.2551	2383.1287	-0.1264	-53	360	381	HQIIGDVRGAGFMLGVEL VTDR					Mascot
3239.5752	3239.8269	0.2517	78	112	141	YLDAFAGIATVVFFTNSG TEANELAIMMAR			Oxidation (M)[27]		Mascot

5	DNA repair and recombination protein RAD54-like protein [Triticum urartu]	gi|474174849	95723	5.98	16	43	0	11.657

Peptide Information

Calc. Mass	Obsrv. Mass	± da	± ppm	Start Seq.	End Seq.	Sequence	Ion Score	C. I. %	Modification	Rank	Result Type
1052.5259	1052.5609	0.035	33	16	24	SEIFIDNSK					Mascot
1052.5259	1052.5609	0.035	33	16	24	SEIFIDNSK					Mascot
1216.6355	1216.6571	0.0216	18	2	11	SINPKLMEER					Mascot
1312.7583	1312.7961	0.0378	29	288	299	KLAGGIDIEQLR					Mascot
1312.7583	1312.7961	0.0378	29	288	299	KLAGGIDIEQLR					Mascot
1358.7825	1358.7612	-0.0213	-16	698	709	EILLMSGNVRVK					Mascot
1492.8846	1492.8119	-0.0727	-49	581	593	ESVVILNPLPRQK					Mascot
1529.7483	1529.8113	0.063	41	12	24	YADKSEIFIDNSK					Mascot
1553.7443	1553.8059	0.0616	40	244	259	SVGYEGASNVALGSDK					Mascot
1701.8517	1701.9419	0.0902	53	834	847	EELKDMFVQILPSH			Oxidation (M)[6]		Mascot
1802.0323	1801.9606	-0.0717	-40	32	48	FYQVPALALALLQAAGR					Mascot
2108.0303	2108.1689	0.1386	66	204	220	CDYIEIEIRHVFPSMAK			Carbamidomethyl (C)[1]		Mascot

2108.0999	2108.1689	0.069	33	645	661	LNPCEGVKTWFVFEIIR			Carbamidomethyl (C)[4]		Mascot
2130.0173	2130.1709	0.1536	72	226	243	EPAAEHDRLDMFVDDILK			Oxidation (M)[11]		Mascot
2155.9463	2156.1594	0.2131	99	168	186	DAESDEHEIPAHGGTFCK R			Carbamidomethyl (C)[17]		Mascot
2173.9219	2174.1304	0.2085	96	272	287	EDMFPHQREGFEFMWR			Oxidation (M)[3,14]		Mascot
2186.2444	2186.2288	-0.0156	-7	27	48	GAGAKFYQVPALALALLQ AAGR					Mascot
2264.0461	2264.1724	0.1263	56	594	613	EIIAMMEESAGKGFLDAE YK			Oxidation (M)[5,6]		Mascot

6 unnamed protein product [Triticum aestivum] gi|257633015 39942.3 7.14 10 42 0 4.283

Peptide Information

Calc. Mass	Obsrv. Mass	± da	± ppm	Start Seq.	End Seq.	Sequence	Ion Score	C. I. %	Modification	Rank	Result Type
1114.5906	1114.548	-0.0426	-38	25	33	HFASFPPRR					Mascot
1371.6903	1371.7751	0.0848	62	326	337	FPKDTSASYSLR					Mascot
1396.6743	1396.7513	0.077	55	292	303	SDVFPIYIGDDR					Mascot
1614.936	1614.8572	-0.0788	-49	311	325	VLRNMGQGIGILVTK			Oxidation (M)[5]		Mascot
1790.8378	1790.9744	0.1366	76	233	247	CVEEAEWDALGREVK			Carbamidomethyl (C)[1]		Mascot
1833.8225	1833.9451	0.1226	67	69	84	HAMPPAGADHEEWIEK			Oxidation (M)[3]		Mascot
1939.9537	1940.1102	0.1565	81	45	63	MDLATSGVLMAGSLLDS MK					Mascot
2264.1418	2264.1724	0.0306	14	2	24	TNHAAFAAEDAVTAVAAP VQPGR					Mascot
2408.2346	2408.2825	0.0479	20	40	63	AALGRMDLATSGVLMAG SLLDSMK					Mascot
2454.2036	2454.4099	0.2063	84	45	68	MDLATSGVLMAGSLLDS MKASSPR			Oxidation (M)[1]		Mascot
2454.2036	2454.4099	0.2063	84	45	68	MDLATSGVLMAGSLLDS MKASSPR			Oxidation (M)[1]		Mascot

7 SWI/SNF complex component SNF12-like protein [Triticum urartu] gi|473957856 46899.6 9.43 12 41 0 3.377

Peptide Information

Calc. Mass	Obsrv. Mass	± da	± ppm	Start Seq.	End Seq.	Sequence	Ion Score	C. I. %	Modification	Rank	Result Type
847.4645	847.3915	-0.073	-86	336	341	IHEHRR					Mascot
918.4832	918.5631	0.0799	87	139	145	FSSFFKR					Mascot
1114.5527	1114.548	-0.0047	-4	102	111	NGDPPTWSLK					Mascot
1428.6827	1428.7368	0.0541	38	191	201	LEMNYNPEKFK			Oxidation (M)[3]		Mascot
1553.8435	1553.8059	-0.0376	-24	102	115	NGDPPTWSLKIIGR					Mascot
1790.9912	1790.9744	-0.0168	-9	266	281	ISQHLAPPPPINLEHK					Mascot
1801.9365	1801.9606	0.0241	13	202	217	LSQPLMEVLGVEVDTR			Oxidation (M)[6]		Mascot
1833.9453	1833.9451	-0.0002	0	2	20	AAAYKAANSGSVDLTPA					Mascot

						AR				
1996.9796	1997.1179	0.1383	69	184	199	EFLANIRLEMNYNPEK			Oxidation (M)[10]	Mascot
2061.105	2061.0715	-0.0335	-16	200	217	FKLSQPLMEVLGVEVDTR				Mascot
2168.0151	2168.1306	0.1155	53	233	250	LQNPSDPSYFMCDPQLKK			Carbamidomethyl (C)[12]	Mascot
2174.1758	2174.1304	-0.0454	-21	83	101	IYVFNTFANQAPRTIPPPK				Mascot

8	Putative disease resistance protein RGA4 [Triticum urartu]	gi|473865684	229627.6	7.58	25	41	0	9.816

Peptide Information

Calc. Mass	Obsrv. Mass	± da	± ppm	Start Seq.	End Seq.	Sequence	Ion Score	C. I. %	Modification	Rank	Result Type
847.4672	847.3915	-0.0757	-89	592	598	YLGELPR					Mascot
949.5254	949.5652	0.0398	42	744	750	LQWLFSR					Mascot
949.5254	949.5652	0.0398	42	744	750	LQWLFSR					Mascot
1064.5558	1064.5604	0.0046	4	796	803	LFILNCER			Carbamidomethyl (C)[6]		Mascot
1074.5327	1074.5496	0.0169	16	88	96	EAQNKGHYK					Mascot
1140.4661	1140.5739	0.1078	95	24	32	VMEGMEEQR			Oxidation (M)[2,5]		Mascot
1173.5754	1173.6803	0.1049	89	1459	1467	QMKYMTALR			Oxidation (M)[2,5]		Mascot
1216.7048	1216.6571	-0.0477	-39	592	601	YLGELPRQLK					Mascot
1371.6614	1371.7751	0.1137	83	366	376	LSYNDLPQYMK					Mascot
1396.7795	1396.7513	-0.0282	-20	1271	1283	AVEELPAAVRDVK					Mascot
1513.797	1513.8071	0.0101	7	214	227	TPLDSLQNVVSGQR					Mascot
1545.8934	1545.8044	-0.089	-58	1979	1992	SRLFLSIVPVGAMR					Mascot
1593.8635	1593.7589	-0.1046	-66	429	442	SFFQDVKPVQATVK					Mascot
1614.7615	1614.8572	0.0957	59	1004	1018	MDISGCGVLASIYGR			Carbamidomethyl (C)[6], Oxidation (M)[1]		Mascot
1641.892	1641.9037	0.0117	7	213	227	KTPLDSLQNVVSGQR					Mascot
1739.8456	1739.8984	0.0528	30	458	472	IHDLMHDVALSVMDK			Oxidation (M)[5]		Mascot
2061.0498	2061.0715	0.0217	11	191	209	SIVEAALEKHYGEEAVTSK					Mascot
2159.0405	2159.1079	0.0674	31	1302	1319	TTCKIHDLMHDVALSVMR			Carbamidomethyl (C)[3], Oxidation (M)[9,17]		Mascot
2164.1108	2164.1487	0.0379	18	836	854	LSVLTVEGNEAELFMWVAR					Mascot
2176.1033	2176.0674	-0.0359	-16	1757	1775	ENGNDQDFPVEVLVLRGFK					Mascot
2180.1057	2180.1731	0.0674	31	836	854	LSVLTVEGNEAELFMWVAR			Oxidation (M)[15]		Mascot
2181.1333	2181.1753	0.042	19	1512	1530	NLKLGGQLELNHLENMTEK					Mascot
2181.1333	2181.1753	0.042	19	1512	1530	NLKLGGQLELNHLENMTEK					Mascot
2186.1484	2186.2288	0.0804	37	302	320	KEEERPAVLVSMVGEIVER			Oxidation (M)[12]		Mascot
2197.1282	2197.1072	-0.021	-10	1512	1530	NLKLGGQLELNHLENMT			Oxidation (M)[16]		Mascot

Calc. Mass	Obsrv. Mass	± da	± ppm	Start Seq.	End Seq.	Sequence	Ion Score	C. I. %	Modification	Rank	Result Type
						EK					
2214.0715	2214.0752	0.0037	2	454	472	TTCKIHDLMHDVALSVMDK			Carbamidomethyl (C)[3]		Mascot
2255.9849	2256.1047	0.1198	53	1643	1659	WWEIDNGMDREEIMFPR			Oxidation (M)[8,14]		Mascot
2724.3853	2724.4475	0.0622	23	1229	1256	SPGRIGGGEGHGDPAPVPPSFSPPTVVR					Mascot
3015.5054	3015.6877	0.1823	60	1429	1454	ITALPEDMSILYNLQTLNLSGCEYLK			Carbamidomethyl (C)[22], Oxidation (M)[8]		Mascot

9 Bifunctional dihydroflavonol 4-reductase/flavanone 4-reductase [Triticum urartu] gi|474089461 49554.8 7.03 9 41 0 4.893 16 0

Peptide Information

Calc. Mass	Obsrv. Mass	± da	± ppm	Start Seq.	End Seq.	Sequence	Ion Score	C. I. %	Modification	Rank	Result Type
1016.5384	1016.5781	0.0397	39	38	46	GYHVRGTAR					Mascot
1312.708	1312.7961	0.0881	67	112	124	NVVNAAADAGVRR					Mascot
1312.708	1312.7961	0.0881	67	112	124	NVVNAAADAGVRR	16	0			Mascot
1366.6671	1366.6021	-0.065	-48	299	310	LGDLGLEFTDMR					Mascot
1492.8231	1492.8119	-0.0112	-8	52	65	KNVHLLALDGAGER					Mascot
1513.7396	1513.8071	0.0675	45	429	441	SPEFQLPAFSLFM					Mascot
1529.7345	1529.8113	0.0768	50	429	441	SPEFQLPAFSLFM			Oxidation (M)[13]		Mascot
1545.7592	1545.8044	0.0452	29	71	84	ADVMHGDSLRAAFR					Mascot
1648.9309	1648.8499	-0.081	-49	267	280	LLKDLYPQYPVTAK					Mascot
2089.9539	2090.0583	0.1044	50	159	176	QTGNLYCCAKMMAEVVAK			Carbamidomethyl (C)[7,8], Oxidation (M)[11]		Mascot
2197.0747	2197.1072	0.0325	15	343	360	KWVGSFHTTELVVMEYDR					Mascot

10 hypothetical protein TRIUR3_30361 [Triticum urartu] gi|474448357 34615.7 9.44 9 40 0 2.514

Peptide Information

Calc. Mass	Obsrv. Mass	± da	± ppm	Start Seq.	End Seq.	Sequence	Ion Score	C. I. %	Modification	Rank	Result Type
918.5659	918.5631	-0.0028	-3	14	21	LTFPISLK					Mascot
1358.6774	1358.7612	0.0838	62	275	285	IGKDLFTMDYR					Mascot
1396.802	1396.7513	-0.0507	-36	122	134	SARVIGLNQVSPR					Mascot
1898.8293	1898.968	0.1387	73	160	177	MNCVMSSIPASAVEEGGK			Carbamidomethyl (C)[3], Oxidation (M)[1,5]		Mascot
2054.9304	2055.0527	0.1223	60	159	177	RMNCVMSSIPASAVEEGGK			Carbamidomethyl (C)[4], Oxidation (M)[2,6]		Mascot
2089.9597	2090.0583	0.0986	47	227	241	WHEQLQCWCLNFRGR			Carbamidomethyl (C)[7,9]		Mascot
2154.0134	2154.1321	0.1187	55	68	85	KPTYTDYLIYLDMGDMSK					Mascot
2236.1021	2236.0493	-0.0528	-24	39	58	TTQTYYLYIGLTEALADDGK					Mascot
3239.5063	3239.8269	0.3206	99	278	304	DLFTMDYRYPISAFQSFAICLSSFDTK			Carbamidomethyl (C)[20], Oxidation (M)[5]		Mascot

Gel Idx/Pos	248/J24	Instr./Gel Origin	BA2151/Sample Project 20140814	Process Status	Analysis Succeeded
Plate [#] Name	[1] Sample Project 20140814	Instrument Sample Name		Spectra	11

Rank	Protein Name	Accession No.	Protein MW	Protein PI	Pep. Count	Protein Score	Protein Score C. I. %	Intensity Matched	Total Ion Score	Total Ion C. I. %	Confirmed
1	ATP synthase beta subunit [Triticum aestivum]	gi|525291	59325.9	5.56	22	1,310	100	73.952	1171	100	

Protein Group

unnamed protein product [Triticum aestivum]	gi|227303166	59325.9	5.5599999427 7954

Peptide Information

Calc. Mass	Obsrv. Mass	± da	± ppm	Start Seq.	End Seq.	Sequence	Ion Score	C. I. %	Modification	Rank	Result Type
866.4003	866.4203	0.02	23	265	271	EGNDLYR					Mascot
1135.6007	1135.6233	0.0226	20	183	191	THDFLPIHR					Mascot
1173.6627	1173.6893	0.0266	23	212	221	VVDLLAPYQR					Mascot
1173.6627	1173.6893	0.0266	23	212	221	VVDLLAPYQR	72	100			Mascot
1278.6359	1278.6506	0.0147	11	133	144	TIAMDGTEGLVR			Oxidation (M)[4]		Mascot
1347.7631	1347.7711	0.008	6	167	178	IINVIGEPIDHK					Mascot
1390.6863	1390.7252	0.0389	28	249	262	AHGGFSVFAGVGER					Mascot
1390.6863	1390.7252	0.0389	28	249	262	AHGGFSVFAGVGER	130	100			Mascot
1399.7693	1399.8077	0.0384	27	307	319	VGLTGLTVAEHFR					Mascot
1399.7693	1399.8077	0.0384	27	307	319	VGLTGLTVAEHFR	37	98.495			Mascot
1409.8112	1409.847	0.0358	25	148	161	VLNTGSPITVPVGR					Mascot
1473.8346	1473.8335	-0.0011	-1	236	248	TVLIMELINNVAK			Oxidation (M)[5]		Mascot
1492.7755	1492.8168	0.0413	28	336	349	FTQANSEVSALLGR					Mascot
1492.7755	1492.8168	0.0413	28	336	349	FTQANSEVSALLGR	123	100			Mascot
1675.949	1676.0007	0.0517	31	118	132	LVLEVAQHLGENVVR					Mascot
1675.949	1676.0007	0.0517	31	118	132	LVLEVAQHLGENVVR	100	100			Mascot
1678.7676	1678.7976	0.03	18	290	304	CALVYGQMNEPPGAR			Carbamidomethyl (C)[1], Oxidation (M)[8]		Mascot
1825.9014	1825.9404	0.039	21	432	447	MLSPHVLGVDHYNTAR			Oxidation (M)[1]		Mascot
1864.944	1865.0068	0.0628	34	320	335	DAEGQDVLLFIDNIFR					Mascot
1864.944	1865.0068	0.0628	34	320	335	DAEGQDVLLFIDNIFR	142	100			Mascot
2061.0498	2061.1125	0.0627	30	413	431	QISELGIYPAVDPLDSTSR					Mascot
2061.0498	2061.1125	0.0627	30	413	431	QISELGIYPAVDPLDSTSR	93	100			Mascot
2172.1548	2172.2222	0.0674	31	192	211	EAPAFVEQATEQQILVTG IK					Mascot
2186.1453	2186.2305	0.0852	39	350	370	IPSAVGYQPTLATDLGGL					Mascot

Calc. Mass	Obsrv. Mass	± da	± ppm	Start Seq.	End Seq.	Sequence	Ion Score	C. I. %	Modification	Rank	Result Type
						QER					
2186.1453	2186.2305	0.0852	39	350	370	IPSAVGYQPTLATDLGGL QER	207	100			Mascot
2212.186	2212.2642	0.0782	35	98	117	FDEGLPPILTALEVLDNSI R					Mascot
2212.186	2212.2642	0.0782	35	98	117	FDEGLPPILTALEVLDNSI R	74	100			Mascot
2591.3135	2591.4268	0.1133	44	73	97	ITDEFTGAGSIGQVCQVI GAVVDVR			Carbamidomethyl (C)[15]		Mascot
2591.3135	2591.4268	0.1133	44	73	97	ITDEFTGAGSIGQVCQVI GAVVDVR	194	100	Carbamidomethyl (C)[15]		Mascot
2722.3274	2722.426	0.0986	36	458	481	NLQDIIAILGMDELSEDDK MTVAR			Oxidation (M)[11,20]		Mascot
3714.8862	3715.0779	0.1917	52	377	412	GSITSVQAIYVPADDLTD PAPATTFAHLDATTVLSR					Mascot
3842.9812	3843.1807	0.1995	52	376	412	KGSITSVQAIYVPADDLT DPAPATTFAHLDATTVLS R					Mascot

2	ATP synthase subunit beta, mitochondrial [Triticum urartu]	gi|473990219	58081.1	5.13	21	1,300	100	72.497	1171	100	

Peptide Information

Calc. Mass	Obsrv. Mass	± da	± ppm	Start Seq.	End Seq.	Sequence	Ion Score	C. I. %	Modification	Rank	Result Type
866.4003	866.4203	0.02	23	253	259	EGNDLYR					Mascot
1135.6007	1135.6233	0.0226	20	171	179	THDFLPIHR					Mascot
1173.6627	1173.6893	0.0266	23	200	209	VVDLLAPYQR					Mascot
1173.6627	1173.6893	0.0266	23	200	209	VVDLLAPYQR	72	100			Mascot
1278.6359	1278.6506	0.0147	11	92	103	TIAMDGTEGLVR			Oxidation (M)[4]		Mascot
1347.7631	1347.7711	0.008	6	155	166	IINVIGEPIDHK					Mascot
1390.6863	1390.7252	0.0389	28	237	250	AHGGFSVFAGVGER					Mascot
1390.6863	1390.7252	0.0389	28	237	250	AHGGFSVFAGVGER	130	100			Mascot
1399.7693	1399.8077	0.0384	27	295	307	VGLTGLTVAEHFR					Mascot
1399.7693	1399.8077	0.0384	27	295	307	VGLTGLTVAEHFR	37	98.495			Mascot
1473.8346	1473.8335	-0.0011	-1	224	236	TVLIMELINNVAK			Oxidation (M)[5]		Mascot
1492.7755	1492.8168	0.0413	28	324	337	FTQANSEVSALLGR					Mascot
1492.7755	1492.8168	0.0413	28	324	337	FTQANSEVSALLGR	123	100			Mascot
1675.949	1676.0007	0.0517	31	77	91	LVLEVAQHLGENVVR					Mascot
1675.949	1676.0007	0.0517	31	77	91	LVLEVAQHLGENVVR	100	100			Mascot
1678.7676	1678.7976	0.03	18	278	292	CALVYGQMNEPPGAR			Carbamidomethyl (C)[1], Oxidation (M)[8]		Mascot
1825.9014	1825.9404	0.039	21	420	435	MLSPHVLGVDHYNTAR			Oxidation (M)[1]		Mascot
1864.944	1865.0068	0.0628	34	308	323	DAEGQDVLLFIDNIFR					Mascot
1864.944	1865.0068	0.0628	34	308	323	DAEGQDVLLFIDNIFR	142	100			Mascot
2061.0498	2061.1125	0.0627	30	401	419	QISELGIYPAVDPLDSTSR					Mascot

2061.0498	2061.1125	0.0627	30	401	419	QISELGIYPAVDPLDSTSR	93	100			Mascot
2172.1548	2172.2222	0.0674	31	180	199	EAPAFVEQATEQQILVTGIK					Mascot
2186.1453	2186.2305	0.0852	39	338	358	IPSAVGYQPTLATDLGGLQER					Mascot
2186.1453	2186.2305	0.0852	39	338	358	IPSAVGYQPTLATDLGGLQER	207	100			Mascot
2212.186	2212.2642	0.0782	35	57	76	FDEGLPPILTALEVLDNSIR					Mascot
2212.186	2212.2642	0.0782	35	57	76	FDEGLPPILTALEVLDNSIR	74	100			Mascot
2591.3135	2591.4268	0.1133	44	32	56	ITDEFTGAGSIGQVCQVIGAVVDVR			Carbamidomethyl (C)[15]		Mascot
2591.3135	2591.4268	0.1133	44	32	56	ITDEFTGAGSIGQVCQVIGAVVDVR	194	100	Carbamidomethyl (C)[15]		Mascot
2722.3274	2722.426	0.0986	36	446	469	NLQDIIAILGMDELSEDDKMTVAR			Oxidation (M)[11,20]		Mascot
3714.8862	3715.0779	0.1917	52	365	400	GSITSVQAIYVPADDLTDPAPATTFAHLDATTVLSR					Mascot
3842.9812	3843.1807	0.1995	52	364	400	KGSITSVQAIYVPADDLTDPAPATTFAHLDATTVLSR					Mascot

3	ATP synthase subunit beta, mitochondrial [Triticum urartu]	gi|473798701	57827	5.25	21	1,110	100	63.625	976	100	

Peptide Information

Calc. Mass	Obsrv. Mass	± da	± ppm	Start Seq.	End Seq.	Sequence	Ion Score	C. I. %	Modification	Rank	Result Type
866.4003	866.4203	0.02	23	219	225	EGNDLYR					Mascot
1173.6627	1173.6893	0.0266	23	166	175	VVDLLAPYQR					Mascot
1173.6627	1173.6893	0.0266	23	166	175	VVDLLAPYQR	72	100			Mascot
1278.6359	1278.6506	0.0147	11	87	98	TIAMDGTEGLVR			Oxidation (M)[4]		Mascot
1347.7631	1347.7711	0.008	6	121	132	IINVIGEPIDHK					Mascot
1390.6863	1390.7252	0.0389	28	203	216	AHGGFSVFAGVGER					Mascot
1390.6863	1390.7252	0.0389	28	203	216	AHGGFSVFAGVGER	130	100			Mascot
1399.7693	1399.8077	0.0384	27	261	273	VGLTGLTVAEHFR					Mascot
1399.7693	1399.8077	0.0384	27	261	273	VGLTGLTVAEHFR	37	98.495			Mascot
1409.8112	1409.847	0.0358	25	102	115	VLNTGSPITVPVGR					Mascot
1473.8346	1473.8335	-0.0011	-1	190	202	TVLIMELINNVAK			Oxidation (M)[5]		Mascot
1492.7755	1492.8168	0.0413	28	290	303	FTQANSEVSALLGR					Mascot
1492.7755	1492.8168	0.0413	28	290	303	FTQANSEVSALLGR	123	100			Mascot
1675.949	1676.0007	0.0517	31	72	86	LVLEVAQHLGENVVR					Mascot
1675.949	1676.0007	0.0517	31	72	86	LVLEVAQHLGENVVR	100	100			Mascot
1678.7676	1678.7976	0.03	18	244	258	CALVYGQMNEPPGAR			Carbamidomethyl (C)[1], Oxidation (M)[8]		Mascot
1864.944	1865.0068	0.0628	34	274	289	DAEGQDVLLFIDNIFR					Mascot
1864.944	1865.0068	0.0628	34	274	289	DAEGQDVLLFIDNIFR	142	100			Mascot

Calc. Mass	Obsrv. Mass	± da	± ppm	Start Seq.	End Seq.	Sequence	Ion Score	C. I. %	Modification	Rank	Result Type
2061.0498	2061.1125	0.0627	30	367	385	QISELGIYPAVDPLDSTSR					Mascot
2061.0498	2061.1125	0.0627	30	367	385	QISELGIYPAVDPLDSTSR	93	100			Mascot
2172.1548	2172.2222	0.0674	31	146	165	EAPAFVEQATEQQILVTGIK					Mascot
2186.1453	2186.2305	0.0852	39	304	324	IPSAVGYQPTLATDLGGLQER					Mascot
2186.1453	2186.2305	0.0852	39	304	324	IPSAVGYQPTLATDLGGLQER	207	100			Mascot
2208.1343	2208.1965	0.0622	28	386	405	MLSPHVLGEAHYNTARGVQK					Mascot
2212.186	2212.2642	0.0782	35	52	71	FDEGLPPILTALEVLDNSIR					Mascot
2212.186	2212.2642	0.0782	35	52	71	FDEGLPPILTALEVLDNSIR	74	100			Mascot
2234.1663	2234.2341	0.0678	30	5	26	LSLSEAAPVTPPPTSDKHTGTK					Mascot
2548.2712	2548.3926	0.1214	48	27	51	ITDDFTGAGAVGEVCQVIGAVVDVR			Carbamidomethyl (C)[15]		Mascot
3714.8862	3715.0779	0.1917	52	331	366	GSITSVQAIYVPADDLTDPAPATTFAHLDATTVLSR					Mascot
3842.9812	3843.1807	0.1995	52	330	366	KGSITSVQAIYVPADDLTDPAPATTFAHLDATTVLSR					Mascot

4 hypothetical protein TRIUR3_08343 [Triticum urartu] gi|474423666 70251.9 4.9 14 52 74.553 11.134

Peptide Information

Calc. Mass	Obsrv. Mass	± da	± ppm	Start Seq.	End Seq.	Sequence	Ion Score	C. I. %	Modification	Rank	Result Type
832.4159	832.3357	-0.0802	-96	398	404	TNAELER					Mascot
1237.694	1237.6478	-0.0462	-37	412	421	LTKYHLSTFK					Mascot
1347.6937	1347.7711	0.0774	57	362	374	TIASCNVEAGVVK			Carbamidomethyl (C)[5]		Mascot
1390.74	1390.7252	-0.0148	-11	476	487	IVIAFSITDHMK			Oxidation (M)[11]		Mascot
1390.74	1390.7252	-0.0148	-11	476	487	IVIAFSITDHMK			Oxidation (M)[11]		Mascot
1427.7021	1427.817	0.1149	80	213	223	FCSRMEDLLLK			Carbamidomethyl (C)[2], Oxidation (M)[5]		Mascot
1492.7179	1492.8168	0.0989	66	489	500	LFYSEQGYSSRR					Mascot
1492.7179	1492.8168	0.0989	66	489	500	LFYSEQGYSSRR					Mascot
1514.7697	1514.7892	0.0195	13	542	556	IESSSSPVQAPSTPK					Mascot
1801.8578	1801.9672	0.1094	61	57	72	VLFYDADIGGAPMNFR			Oxidation (M)[13]		Mascot
1819.9912	1819.9908	-0.0004	0	142	157	EELLQFTQSAISGLKR					Mascot
2208.0918	2208.1965	0.1047	47	569	588	SEQTLDLGSELAELELEFGK					Mascot
2214.0132	2213.8374	-0.1758	-79	336	353	EERDQFDEANNQMIFSLK					Mascot
2324.1855	2324.1865	0.001	0	52	72	TPLARVLFYDADIGGAPMNFR					Mascot
2619.3401	2619.45	0.1099	42	565	588	SPVKSEQTLDLGSELAELELEFGK					Mascot
3714.7559	3715.0779	0.322	87	375	404	TWINFLEDTWQLQSSYD					Mascot

EQIEQKTNAELER

5	hypothetical protein TRIUR3_33895 [Triticum urartu]	gi|474185893	15997.7	8.04	8	50	58.73	6.271

Peptide Information

Calc. Mass	Obsrv. Mass	± da	± ppm	Start Seq.	End Seq.	Sequence	Ion Score	C. I. %	Modification	Rank	Result Type
921.4822	921.4911	0.0089	10	36	43	TLGMVQTR			Oxidation (M)[4]		Mascot
1244.6117	1244.6693	0.0576	46	87	97	LLQQNDGEAEK					Mascot
1390.6433	1390.7252	0.0819	59	23	34	VPMWHGNGEHAR					Mascot
1390.6433	1390.7252	0.0819	59	23	34	VPMWHGNGEHAR					Mascot
1424.6699	1424.772	0.1021	72	2	13	PVENNKCAEHAR			Carbamidomethyl (C)[7]		Mascot
1520.7235	1520.8304	0.1069	70	98	113	GTAGSGGGSEVAMQRR					Mascot
1801.8577	1801.9672	0.1095	61	114	132	SSEAGTWGPAGGTRPGT GR					Mascot
1892.9661	1893.0204	0.0543	29	36	51	TLGMVQTRAFHTTHHR					Mascot
2590.2163	2590.457	0.2407	93	87	112	LLQQNDGEAEKGTAGSG GGSEVAMQR					Mascot

6	Topless-related protein 2 [Triticum urartu]	gi|474140346	88404.5	6.71	16	50	51.512	14.659

Peptide Information

Calc. Mass	Obsrv. Mass	± da	± ppm	Start Seq.	End Seq.	Sequence	Ion Score	C. I. %	Modification	Rank	Result Type
1173.6263	1173.6893	0.063	54	337	346	SDKGKPWDLK					Mascot
1173.6263	1173.6893	0.063	54	337	346	SDKGKPWDLK					Mascot
1214.559	1214.67	0.111	91	116	125	AWFYDNVGSR					Mascot
1381.7158	1381.8042	0.0884	64	552	562	TKSTVHVHMWR					Mascot
1473.6943	1473.8335	0.1392	94	114	125	MKAWFYDNVGSR					Mascot
1550.8062	1550.837	0.0308	20	238	252	EGNLLAVTTVDSGFK					Mascot
1665.8364	1665.8971	0.0607	36	356	370	LDTMLETDQASKVSK					Mascot
1697.9432	1697.9658	0.0226	13	371	384	VLDDDDLLREIIIR					Mascot
1703.944	1704.0222	0.0782	46	180	195	KTSGVVQGVVQFDIAR					Mascot
1801.8037	1801.9672	0.1635	91	695	709	HVGDYAEFMFLEMGR					Mascot
1819.955	1819.9908	0.0358	20	570	584	TYLTLDTDQLLDPRR					Mascot
1846.9164	1847.0297	0.1133	61	758	772	FAFWPSDDLYIAFVR					Mascot
1915.9557	1916.0688	0.1131	59	742	757	MIWPPMFPALKDNPAR			Oxidation (M)[1,6]		Mascot
2042.9827	2043.1368	0.1541	75	693	709	IKHVGDYAEFMFLEMGR					Mascot
2212.1682	2212.2642	0.096	43	723	741	KVYIMAEEEVQLGDIHPL K					Mascot
2212.1682	2212.2642	0.096	43	723	741	KVYIMAEEEVQLGDIHPL K					Mascot
2458.0837	2458.1516	0.0679	28	134	154	WCTTMLYSADGTRLFSC			Carbamidomethyl (C)[2,17], Oxidation (M)[5]		Mascot

	2658.2327	2658.4358	0.2031	76	671	692	GTSK EMVANMWIPGYRVEDES YAPLR			Oxidation (M)[2,6]	Mascot

7	Disease resistance protein RPM1 [Triticum urartu]	gi|474426983	174445.3	8.79	25	48	24.901	19.737

Peptide Information

Calc. Mass	Obsrv. Mass	± da	± ppm	Start Seq.	End Seq.	Sequence	Ion Score	C. I. %	Modification	Rank	Result Type
1201.5742	1201.6842	0.11	92	158	167	SENIARHCSK			Carbamidomethyl (C)[8]		Mascot
1224.538	1224.5602	0.0222	18	87	96	QLDAEDYENK					Mascot
1278.6987	1278.6506	-0.0481	-38	815	824	ARLVVYICER			Carbamidomethyl (C)[8]		Mascot
1312.7181	1312.7727	0.0546	42	1290	1300	LKTLLETGMYK			Oxidation (M)[9]		Mascot
1326.6947	1326.8053	0.1106	83	1304	1314	GHQTLCDVLKR			Carbamidomethyl (C)[6]		Mascot
1399.7501	1399.8077	0.0576	41	1292	1303	TLLETGMYKSLK			Oxidation (M)[7]		Mascot
1399.7501	1399.8077	0.0576	41	1292	1303	TLLETGMYKSLK			Oxidation (M)[7]		Mascot
1412.6951	1412.6931	-0.002	-1	343	354	GIDSCQVHDLIR			Carbamidomethyl (C)[5]		Mascot
1421.7053	1421.7644	0.0591	42	590	603	ALDTMGVVNVSGSR			Oxidation (M)[5]		Mascot
1514.7395	1514.7892	0.0497	33	416	426	WRPFFMSEKMR					Mascot
1520.8108	1520.8304	0.0196	13	1341	1353	AEQYPKTTWVAVK					Mascot
1527.7043	1527.8323	0.128	84	1395	1407	YIGTNCRNGPTMK			Carbamidomethyl (C)[6], Oxidation (M)[12]		Mascot
1552.8887	1552.9175	0.0288	19	682	694	LPEWIAALWNLVK					Mascot
1562.8479	1562.9657	0.1178	75	973	984	VVKHYPEQFFIR					Mascot
1597.9498	1597.8131	-0.1367	-86	1515	1528	LIASKPPLKMWVAK			Oxidation (M)[10]		Mascot
1733.001	1733.0186	0.0176	10	857	872	RPAFAAPAFKPPPIPR					Mascot
1769.8494	1769.9623	0.1129	64	1332	1346	INADGSYWKAEQYPK					Mascot
1801.9946	1801.9672	-0.0274	-15	740	756	DAAFPSLAVLELSLLDK					Mascot
1881.0804	1881.0056	-0.0748	-40	955	972	KVADEIVAALGVRPTDVK					Mascot
1898.9569	1898.9681	0.0112	6	175	190	LQILGYEDGLNLFMEK			Oxidation (M)[14]		Mascot
1983.8505	1983.9176	0.0671	34	557	573	DVCTACCCNKLPSAATR			Carbamidomethyl (C)[3,6,7,8]		Mascot
2043.911	2044.0897	0.1787	87	243	259	LNDHMSAELEMNPELER			Oxidation (M)[5]		Mascot
2061.0977	2061.1125	0.0148	7	430	449	VLDLEGTSGLVDHHLTGI GK					Mascot
2061.0977	2061.1125	0.0148	7	430	449	VLDLEGTSGLVDHHLTGI GK					Mascot
2212.2158	2212.2642	0.0484	22	215	235	KCDGLPLAIVTIGGFLAN QPK			Carbamidomethyl (C)[2]		Mascot
2212.2158	2212.2642	0.0484	22	215	235	KCDGLPLAIVTIGGFLAN QPK			Carbamidomethyl (C)[2]		Mascot
2635.2668	2635.4866	0.2198	83	1222	1244	ENESLRAEQISSAQEGFE PLCLK			Carbamidomethyl (C)[21]		Mascot
2658.3484	2658.4358	0.0874	33	169	190	QENIYKLQILGYEDGLNLF MEK					Mascot

8	beta-amylase [Triticum aestivum]	gi|1771782	56860.2	5.24	12	44	0	1.665

Protein Group

RecName: Full=Beta-amylase; AltName: Full=1,4-alpha-D-glucan maltohydrolase	gi|3334120	56860.2	5.2399 997711 1816

Peptide Information

Calc. Mass	Obsrv. Mass	± da	± ppm	Start Seq.	End Seq.	Sequence	Ion Score	C. I. %	Modification	Rank	Result Type
1016.5564	1016.5719	0.0155	15	411	418	LFGFTYLR					Mascot
1300.6532	1300.6714	0.0182	14	384	394	YDATAYNTILR					Mascot
1355.7318	1355.782	0.0502	37	72	83	QVFDLVHEAGLK					Mascot
1447.7535	1447.7524	-0.0011	-1	458	469	SKPEMPIEMILK			Oxidation (M)[5,9]		Mascot
1769.9181	1769.9623	0.0442	25	355	370	SAPEELVQQVLSAGWR					Mascot
1870.0487	1870.0319	-0.0168	-9	288	302	VQLAIKISGIHWWYR					Mascot
2055.0076	2055.0437	0.0361	18	439	457	MHANLGHDPSVDPVAPL ER					Mascot
2087.0557	2087.124	0.0683	33	129	146	NIEYLTLGVDDQPLFHGR					Mascot
2176.0955	2176.21	0.1145	53	166	186	FLDAGTIVDIEVGLGPAG EMR			Oxidation (M)[20]		Mascot
2187.9507	2187.7874	-0.1633	-75	147	164	TAVQMYADYMASFRENM K			Oxidation (M)[5,10]		Mascot
2458.2473	2458.1516	-0.0957	-39	125	146	GGTRNIEYLTLGVDDQPL FHGR					Mascot
2572.3118	2572.4675	0.1557	61	40	63	LTEAGVDGVMIDVWWGL VEGKGPK			Oxidation (M)[10]		Mascot

9	Mono- and diacylglycerol lipase [Triticum urartu]	gi|474144640	49703.4	8.3	11	44	0	18.311	

Peptide Information

Calc. Mass	Obsrv. Mass	± da	± ppm	Start Seq.	End Seq.	Sequence	Ion Score	C. I. %	Modification	Rank	Result Type
1300.7155	1300.6714	-0.0441	-34	99	109	LIRNAMQGELR					Mascot
1421.743	1421.7644	0.0214	15	102	113	NAMQGELRIPHR					Mascot
1552.8595	1552.9175	0.058	37	208	221	ALGLQRNAIGWPEK					Mascot
1675.8591	1676.0007	0.1416	84	315	327	YFRFVYSNDIVPR					Mascot
1675.8591	1676.0007	0.1416	84	315	327	YFRFVYSNDIVPR					Mascot
1825.996	1825.9404	-0.0556	-30	222	236	IEAIKERPFAYYAVR					Mascot
1858.9923	1859.0514	0.0591	32	277	292	DLLARLHGVYTYGQPR					Mascot
1863.9884	1863.9734	-0.015	-8	135	152	IKPEDGSYLAALGIMASK					Mascot
2054.9966	2055.0437	0.0471	23	6	23	EEFSGDFMVLRPDKGGV R			Oxidation (M)[8]		Mascot
2591.3003	2591.4268	0.1265	49	282	304	LHGVYTYGQPRVGDAQL GEFVER					Mascot
2591.3003	2591.4268	0.1265	49	282	304	LHGVYTYGQPRVGDAQL GEFVER					Mascot

2619.3962	2619.45	0.0538	21	78	101	LGSAVEYWMNLITDNGG GVLKLIR						Mascot
2635.3914	2635.4866	0.0952	36	78	101	LGSAVEYWMNLITDNGG GVLKLIR			Oxidation (M)[9]			Mascot
2658.5354	2658.4358	-0.0996	-37	249	274	ARFAVTGHSLGGALAVLF PAILALHR						Mascot

10	Glutathione S-transferase theta-1 [Triticum urartu]	gi|472900065	26340	9.04	8	43	0	1.071

Peptide Information

Calc. Mass	Obsrv. Mass	± da	± ppm	Start Seq.	End Seq.	Sequence	Ion Score	C. I. %	Modification	Rank	Result Type
1195.6504	1195.6599	0.0095	8	1	10	MALLKVYADR			Oxidation (M)[1]		Mascot
1716.0055	1715.9706	-0.0349	-20	140	154	LLSRSLGTIETVWLK					Mascot
1761.9143	1761.9744	0.0601	34	97	110	LESVLDWHHSNLRR					Mascot
1769.8857	1769.9623	0.0766	43	208	222	ATSPHFDEVHELIFK					Mascot
1801.963	1801.9672	0.0042	2	49	64	INPMAQVPTIVDGRFK			Oxidation (M)[4]		Mascot
1869.9818	1870.0319	0.0501	27	27	42	IDFQELTVDLAKGQHR					Mascot
2184.1482	2184.2227	0.0745	34	43	62	APEFTKINPMAQVPTIVD GR					Mascot
2619.4253	2619.45	0.0247	9	110	135	RGAATYVLHTALGPALGL TPNPETAK					Mascot

Gel Idx/Pos	249/K1	Instr./Gel Origin	BA2151/Sample Project 20140814	Process Status	Analysis Succeeded
Plate [#] Name	[1] Sample Project 20140814	Instrument Sample Name		Spectra	11

Rank	Protein Name	Accession No.	Protein MW	Protein PI	Pep. Count	Protein Score	Protein Score C. I. %	Intensity Matched	Total Ion Score	Total Ion C. I. %	Confirmed
1	UTP--glucose-1-phosphate uridylyltransferase [Triticum urartu]	gi|473993048	51082.9	5.76	23	511	100	35.188	368	100	

Peptide Information

Calc. Mass	Obsrv. Mass	± da	± ppm	Start Seq.	End Seq.	Sequence	Ion Score	C. I. %	Modification	Rank	Result Type
839.5098	839.5059	-0.0039	-5	334	341	AIGINVPR					Mascot
858.493	858.4778	-0.0152	-18	295	302	LVDAEALK					Mascot
918.5519	918.5441	-0.0078	-8	390	397	KVANFLAR					Mascot
918.5519	918.5441	-0.0078	-8	390	397	KVANFLAR					Mascot
949.5465	949.542	-0.0045	-5	27	35	AGFISLVSR					Mascot
949.5465	949.542	-0.0045	-5	27	35	AGFISLVSR	36	97.711			Mascot
957.5073	957.4853	-0.022	-23	303	310	MEIIPNPK			Oxidation (M)[1]		Mascot
1014.5942	1014.5879	-0.0063	-6	294	302	RLVDAEALK					Mascot
1018.5051	1018.5077	0.0026	3	18	26	LGEISENEK					Mascot
1052.5371	1052.5386	0.0015	1	245	254	GGTLISYEGR					Mascot
1052.5371	1052.5386	0.0015	1	245	254	GGTLISYEGR	75	100			Mascot
1111.745	1111.6816	-0.0634	-57	75	84	ALLDKLVVLK					Mascot
1114.6216	1114.5669	-0.0547	-49	8	17	IDNLRDAVAK					Mascot
1173.6222	1173.6573	0.0351	30	2	12	AAADSKIDNLR					Mascot
1297.6998	1297.6887	-0.0111	-9	438	449	LEIPDGAVLENK					Mascot
1300.7358	1300.7202	-0.0156	-12	400	411	SIPSIVELDSLK					Mascot
1312.7583	1312.7721	0.0138	11	317	329	VLQLETAAGAAIR					Mascot
1312.7583	1312.7721	0.0138	11	317	329	VLQLETAAGAAIR	101	100			Mascot
1350.7264	1350.7135	-0.0129	-10	412	425	VSGDVSFGSGVVLK					Mascot
1358.7566	1358.7418	-0.0148	-11	185	196	IVTEDFLPLPSK					Mascot
1358.7566	1358.7418	-0.0148	-11	185	196	IVTEDFLPLPSK	24	66.692			Mascot
1390.7842	1390.6921	-0.0921	-66	330	341	FFEKAIGINVPR					Mascot
1390.7842	1390.6921	-0.0921	-66	330	341	FFEKAIGINVPR					Mascot
1641.8846	1641.8799	-0.0047	-3	375	389	VKPSNPSIELGPEFK					Mascot
1679.8964	1679.8489	-0.0475	-28	239	254	TLADVKGGTLISYEGR					Mascot
1769.9796	1769.975	-0.0046	-3	375	390	VKPSNPSIELGPEFKK					Mascot

Calc. Mass	Obsrv. Mass	± da	± ppm	Start Seq.	End Seq.	Sequence	Ion Score	C. I. %	Modification	Rank	Result Type
2108.1023	2108.1201	0.0178	8	255	272	VQLLEIAQVPDEHVNEFK					Mascot
2108.1023	2108.1201	0.0178	8	255	272	VQLLEIAQVPDEHVNEFK	133	100			Mascot
2143.9832	2144.0171	0.0339	16	202	221	DGWYPPGHGDVFPSLNNSGK					Mascot
2454.3127	2454.3491	0.0364	15	349	370	ATSDLLLVQSDLYTLVDGYVIR					Mascot

2	DNA repair and recombination protein RAD54-like protein [Triticum urartu]	gi|474174849	95723	5.98	20	55	86.016	24.009		

Peptide Information

Calc. Mass	Obsrv. Mass	± da	± ppm	Start Seq.	End Seq.	Sequence	Ion Score	C. I. %	Modification	Rank	Result Type
816.4573	816.4169	-0.0404	-49	429	436	NLAKGEGK					Mascot
892.4483	892.4319	-0.0164	-18	518	526	GVASTSQSR					Mascot
911.5421	911.4834	-0.0587	-64	69	76	SSLVPPRR					Mascot
916.4557	916.4633	0.0076	8	645	652	LNPCEGVK			Carbamidomethyl (C)[4]		Mascot
947.4978	947.4996	0.0018	2	830	837	MAAREELK					Mascot
955.4414	955.4649	0.0235	25	160	167	VSSHTCHK			Carbamidomethyl (C)[6]		Mascot
964.521	964.451	-0.07	-73	766	773	AYRIGQEK					Mascot
1018.5349	1018.5077	-0.0272	-27	662	669	LCEALKER			Carbamidomethyl (C)[2]		Mascot
1052.5259	1052.5386	0.0127	12	16	24	SEIFIDNSK					Mascot
1052.5259	1052.5386	0.0127	12	16	24	SEIFIDNSK					Mascot
1213.6357	1213.6454	0.0097	8	388	398	DHGLGQKLAMK			Oxidation (M)[10]		Mascot
1312.7583	1312.7721	0.0138	11	288	299	KLAGGIDIEQLR					Mascot
1312.7583	1312.7721	0.0138	11	288	299	KLAGGIDIEQLR					Mascot
1358.7825	1358.7418	-0.0407	-30	698	709	EILLMSGNVRVK					Mascot
1358.7825	1358.7418	-0.0407	-30	698	709	EILLMSGNVRVK					Mascot
1492.8846	1492.7826	-0.102	-68	581	593	ESVVILNPLPRQK					Mascot
1529.7483	1529.7804	0.0321	21	12	24	YADKSEIFIDNSK					Mascot
1553.7443	1553.7727	0.0284	18	244	259	SVGYEGASNVALGSDK					Mascot
1701.8517	1701.9033	0.0516	30	834	847	EELKDMFVQILPSH			Oxidation (M)[6]		Mascot
2108.0303	2108.1201	0.0898	43	204	220	CDYIEIEIRHVFPSMAK			Carbamidomethyl (C)[1]		Mascot
2108.0999	2108.1201	0.0202	10	645	661	LNPCEGVKTWFVFEIIR			Carbamidomethyl (C)[4]		Mascot
2130.0173	2130.1218	0.1045	49	226	243	EPAAEHDRLDMFVDDILK			Oxidation (M)[11]		Mascot
2186.2444	2186.1707	-0.0737	-34	27	48	GAGAKFYQVPALALALLQAAGR					Mascot

3	ATP synthase subunit beta, mitochondrial [Triticum urartu]	gi|473798701	57827	5.25	11	52	70.103	3.741	16	0

Peptide Information

Calc. Mass	Obsrv. Mass	± da	± ppm	Start Seq.	End Seq.	Sequence	Ion Score	C. I. %	Modification	Rank	Result Type
832.4597	832.3926	-0.0671	-81	497	504	IMGVLDGK					Mascot
866.4003	866.4043	0.004	5	219	225	EGNDLYR					Mascot
1173.6627	1173.6573	-0.0054	-5	166	175	VVDLLAPYQR					Mascot
1390.6863	1390.6921	0.0058	4	203	216	AHGGFSVFAGVGER					Mascot
1390.6863	1390.6921	0.0058	4	203	216	AHGGFSVFAGVGER	16	0			Mascot
1399.7693	1399.7716	0.0023	2	261	273	VGLTGLTVAEHFR					Mascot
1409.8112	1409.8132	0.002	1	102	115	VLNTGSPITVPVGR					Mascot
1492.7755	1492.7826	0.0071	5	290	303	FTQANSEVSALLGR					Mascot
1513.8121	1513.7909	-0.0212	-14	133	145	GDIKTNSYLPIHR					Mascot
1675.949	1675.9578	0.0088	5	72	86	LVLEVAQHLGENVVR					Mascot
1864.944	1864.9424	-0.0016	-1	274	289	DAEGQDVLLFIDNIFR					Mascot
2186.1453	2186.1707	0.0254	12	304	324	IPSAVGYQPTLATDLGGL QER					Mascot

4 hypothetical protein TRIUR3_28636 [Triticum urartu] gi|473799591 169958.7 5.01 27 48 26.61 19.219

Peptide Information

Calc. Mass	Obsrv. Mass	± da	± ppm	Start Seq.	End Seq.	Sequence	Ion Score	C. I. %	Modification	Rank	Result Type
832.3658	832.3926	0.0268	32	23	28	AEMYYR					Mascot
892.4444	892.4319	-0.0125	-14	1409	1415	LEVEMQK			Oxidation (M)[5]		Mascot
949.5214	949.542	0.0206	22	43	50	AYRALAER					Mascot
949.5214	949.542	0.0206	22	43	50	AYRALAER					Mascot
1016.537	1016.5568	0.0198	19	853	861	VDDLRAEAK					Mascot
1029.4558	1029.5254	0.0696	68	750	757	YESLDQMK			Oxidation (M)[7]		Mascot
1057.5636	1057.504	-0.0596	-56	1099	1107	LNAVEIENR					Mascot
1102.6102	1102.6478	0.0376	34	1079	1087	EVLKEDITR					Mascot
1140.5645	1140.5513	-0.0132	-12	242	250	QKHDELNEK					Mascot
1173.6222	1173.6573	0.0351	30	812	821	NTLKQDQQAK					Mascot
1213.6648	1213.6454	-0.0194	-16	1099	1108	LNAVEIENRR					Mascot
1218.6511	1218.6379	-0.0132	-11	1406	1415	IGRLEVEMQK			Oxidation (M)[8]		Mascot
1297.7111	1297.6887	-0.0224	-17	1313	1323	ELSIVNQELPR					Mascot
1300.6855	1300.7202	0.0347	27	1097	1107	DKLNAVEIENR					Mascot
1312.6743	1312.7721	0.0978	75	257	267	LNISTEEEHLK					Mascot
1312.6743	1312.7721	0.0978	75	257	267	LNISTEEEHLK					Mascot
1326.7012	1326.6827	-0.0185	-14	928	938	SLHEDRSLELK					Mascot

Calc. Mass	Obsrv. Mass	± da	± ppm	Start Seq.	End Seq.	Sequence	Ion Score	C. I. %	Modification	Rank	Result Type
1358.6547	1358.7418	0.0871	64	225	235	DNQDLQLEVER					Mascot
1358.6547	1358.7418	0.0871	64	225	235	DNQDLQLEVER					Mascot
1405.6458	1405.7507	0.1049	75	100	111	YFLSFMNSGDPK					Mascot
1491.7803	1491.7806	0.0003	0	862	874	FLVGQLSELQDSR					Mascot
1525.7059	1525.7748	0.0689	45	268	280	CMQAEMVSLSLEK			Carbamidomethyl (C)[1]		Mascot
1675.8762	1675.9578	0.0816	49	361	375	NLTSQYHSSSAVIIR					Mascot
1679.8823	1679.8489	-0.0334	-20	463	476	TEALHAENLRQLER					Mascot
2108.1094	2108.1201	0.0107	5	884	901	LIQQNSLLANELHDSREK					Mascot
2108.1094	2108.1201	0.0107	5	884	901	LIQQNSLLANELHDSREK					Mascot
2129.9841	2130.1218	0.1377	65	693	711	ECLGDMASANSDYLAKLQK			Carbamidomethyl (C)[2], Oxidation (M)[6]		Mascot
2143.9124	2144.0171	0.1047	49	833	849	QELAEISDEFWEEMESR			Oxidation (M)[14]		Mascot
2180.0574	2180.0825	0.0251	12	141	159	ITSVLEQSNCAESEVLCLK			Carbamidomethyl (C)[10,17]		Mascot
2181.0054	2181.1042	0.0988	45	1371	1390	AEEFTSADGLDGDNIDLRSR					Mascot
2226.1396	2226.0798	-0.0598	-27	1060	1078	IIELIVTCESYEISAMVQK			Carbamidomethyl (C)[8]		Mascot

5	putative DEAD-box ATP-dependent RNA helicase [Triticum aestivum]	gi|310656783	85847.7	8.92	17	46	0	22.033	

Peptide Information

Calc. Mass	Obsrv. Mass	± da	± ppm	Start Seq.	End Seq.	Sequence	Ion Score	C. I. %	Modification	Rank	Result Type
858.4427	858.4778	0.0351	41	29	36	QAAVADQR					Mascot
866.4036	866.4043	0.0007	1	568	574	ESEKMSR					Mascot
885.5264	885.4975	-0.0289	-33	21	27	QRLALER					Mascot
892.4556	892.4319	-0.0237	-27	156	162	LEKMAER			Oxidation (M)[4]		Mascot
918.4713	918.5441	0.0728	79	458	464	LLDCLER			Carbamidomethyl (C)[4]		Mascot
918.4713	918.5441	0.0728	79	458	464	LLDCLER			Carbamidomethyl (C)[4]		Mascot
964.4404	964.451	0.0106	11	291	298	GIEEMTER					Mascot
1014.5439	1014.5879	0.044	43	29	37	QAAVADQRR					Mascot
1074.5725	1074.5149	-0.0576	-54	458	465	LLDCLERR			Carbamidomethyl (C)[4]		Mascot
1146.6001	1146.5894	-0.0107	-9	360	371	DVIGIAETGSGK					Mascot
1213.6801	1213.6454	-0.0347	-29	607	618	AGFRVTALHGGK					Mascot
1396.7292	1396.7217	-0.0075	-5	611	623	VTALHGGKSQDQR					Mascot
1507.8003	1507.7792	-0.0211	-14	167	179	ELEAIKEQYLGSK					Mascot
1555.8374	1555.7319	-0.1055	-68	444	457	IRQGCEVVIATPGR			Carbamidomethyl (C)[5]		Mascot
1701.7975	1701.9033	0.1058	62	521	535	TTYMFSATMPPAVER					Mascot
2186.1057	2186.1707	0.065	30	446	464	QGCEVVIATPGRLLDCLER			Carbamidomethyl (C)[3,16]		Mascot

Calc. Mass	Obsrv. Mass	± da	± ppm	Start Seq.	End Seq.	Sequence	Ion Score	C. I. %	Modification	Rank	Result Type
2198.0659	2198.0654	-0.0005	0	465	482	RYAVLNQCNYVVLDEAD R			Carbamidomethyl (C)[8]		Mascot
2198.0659	2198.0654	-0.0005	0	465	482	RYAVLNQCNYVVLDEAD R			Carbamidomethyl (C)[8]		Mascot
2210.0481	2210.0691	0.021	10	207	224	DMNMLYQAPHEARLLYG R			Oxidation (M)[2,4]		Mascot

6 ATP synthase beta subunit [Triticum aestivum] gi|525291 59325.9 5.56 10 45 0 3.619 16 0

Protein Group

unnamed protein product [Triticum aestivum] gi|227303166 59325.9 5.5599 999427 7954

Peptide Information

Calc. Mass	Obsrv. Mass	± da	± ppm	Start Seq.	End Seq.	Sequence	Ion Score	C. I. %	Modification	Rank	Result Type
866.4003	866.4043	0.004	5	265	271	EGNDLYR					Mascot
1135.6007	1135.5879	-0.0128	-11	183	191	THDFLPIHR					Mascot
1173.6627	1173.6573	-0.0054	-5	212	221	VVDLLAPYQR					Mascot
1390.6863	1390.6921	0.0058	4	249	262	AHGGFSVFAGVGER					Mascot
1390.6863	1390.6921	0.0058	4	249	262	AHGGFSVFAGVGER	16	0			Mascot
1399.7693	1399.7716	0.0023	2	307	319	VGLTGLTVAEHFR					Mascot
1409.8112	1409.8132	0.002	1	148	161	VLNTGSPITVPVGR					Mascot
1492.7755	1492.7826	0.0071	5	336	349	FTQANSEVSALLGR					Mascot
1675.949	1675.9578	0.0088	5	118	132	LVLEVAQHLGENVVR					Mascot
1864.944	1864.9424	-0.0016	-1	320	335	DAEGQDVLLFIDNIFR					Mascot
2186.1453	2186.1707	0.0254	12	350	370	IPSAVGYQPTLATDLGGL QER					Mascot

7 Serine/arginine-rich splicing factor 4 [Triticum urartu] gi|474386528 36654.5 9.33 11 45 0 23.039

Peptide Information

Calc. Mass	Obsrv. Mass	± da	± ppm	Start Seq.	End Seq.	Sequence	Ion Score	C. I. %	Modification	Rank	Result Type
845.3682	845.4409	0.0727	86	149	156	CATHGGSR			Carbamidomethyl (C)[1]		Mascot
847.3727	847.3715	-0.0012	-1	204	210	NCQNSPK			Carbamidomethyl (C)[2]		Mascot
955.4955	955.4649	-0.0306	-32	229	236	SLSRSYSR					Mascot
1057.5095	1057.504	-0.0055	-5	195	203	CGEKGHIEK			Carbamidomethyl (C)[1]		Mascot
1342.6863	1342.7803	0.094	70	157	169	EYLGRGPPPGSGR					Mascot
1342.6863	1342.7803	0.094	70	157	169	EYLGRGPPPGSGR					Mascot
1396.6564	1396.7217	0.0653	47	285	297	EQAEVNGSHNGVR					Mascot
1553.7161	1553.7727	0.0566	36	130	142	ALMEGDWMMNLVK			Oxidation (M)[3]		Mascot
1571.7561	1571.7231	-0.033	-21	298	311	GNSLSPARDSRPWE					Mascot

2179.0598	2179.0078	-0.052	-24	285	305	EQAEVNGSHNGVRGNSL SPAR						Mascot
2197.9768	2198.0654	0.0886	40	162	181	GPPPGSGRCFNCGIDGH WAR			Carbamidomethyl (C)[9,12]			Mascot
2197.9768	2198.0654	0.0886	40	162	181	GPPPGSGRCFNCGIDGH WAR			Carbamidomethyl (C)[9,12]			Mascot
2210.1089	2210.0691	-0.0398	-18	106	124	YELDGQEIDGSRIIVEFAR						Mascot

8 DEAD-box ATP-dependent RNA helicase 21 [Triticum urartu] gi|474238397 75728.6 8.65 14 44 0 21.202

Peptide Information

Calc. Mass	Obsrv. Mass	± da	± ppm	Start Seq.	End Seq.	Sequence	Ion Score	C. I. %	Modification	Rank	Result Type
866.4036	866.4043	0.0007	1	482	488	ESEKMSR					Mascot
892.4556	892.4319	-0.0237	-27	70	76	LEKMAER			Oxidation (M)[4]		Mascot
918.4713	918.5441	0.0728	79	372	378	LLDCLER			Carbamidomethyl (C)[4]		Mascot
918.4713	918.5441	0.0728	79	372	378	LLDCLER			Carbamidomethyl (C)[4]		Mascot
964.4404	964.451	0.0106	11	205	212	GIEEMTER					Mascot
1074.5725	1074.5149	-0.0576	-54	372	379	LLDCLERR			Carbamidomethyl (C)[4]		Mascot
1146.6001	1146.5894	-0.0107	-9	274	285	DVIGIAETGSGK					Mascot
1213.6801	1213.6454	-0.0347	-29	521	532	AGFRVTALHGGK					Mascot
1396.7292	1396.7217	-0.0075	-5	525	537	VTALHGGKSQDQR					Mascot
1507.8003	1507.7792	-0.0211	-14	81	93	ELEAIKEQYLGSK					Mascot
1555.8374	1555.7319	-0.1055	-68	358	371	IRQGCEVVIATPGR			Carbamidomethyl (C)[5]		Mascot
1701.7975	1701.9033	0.1058	62	435	449	TTYMFSATMPPAVER					Mascot
2186.1057	2186.1707	0.065	30	360	378	QGCEVVIATPGRLLDCLE R			Carbamidomethyl (C)[3,16]		Mascot
2198.0659	2198.0654	-0.0005	0	379	396	RYAVLNQCNYVVLDEAD R			Carbamidomethyl (C)[8]		Mascot
2198.0659	2198.0654	-0.0005	0	379	396	RYAVLNQCNYVVLDEAD R			Carbamidomethyl (C)[8]		Mascot
2210.0481	2210.0691	0.021	10	121	138	DMNMLYQAPHEARLLYG R			Oxidation (M)[2,4]		Mascot

9 Bifunctional aspartokinase/homoserine dehydrogenase 2, chloroplastic [Triticum urartu] gi|474369356 109971.5 7.56 18 42 0 15.284

Peptide Information

Calc. Mass	Obsrv. Mass	± da	± ppm	Start Seq.	End Seq.	Sequence	Ion Score	C. I. %	Modification	Rank	Result Type
810.3637	810.386	0.0223	28	35	40	CMIGWK			Carbamidomethyl (C)[1], Oxidation (M)[2]		Mascot
832.441	832.3926	-0.0484	-58	794	801	GLLETGDK					Mascot
878.4618	878.4689	0.0071	8	987	995	LASYLGAPS					Mascot
957.5112	957.4853	-0.0259	-27	753	761	KANSGPLDR					Mascot

965.5203	965.5365	0.0162	17	340	346	LEKWFSR					Mascot
965.5203	965.5365	0.0162	17	340	346	LEKWFSR					Mascot
978.4713	978.4984	0.0271	28	693	699	WKEEMQK					Mascot
1029.5438	1029.5254	-0.0184	-18	56	63	KTFDLFMK					Mascot
1052.5259	1052.5386	0.0127	12	825	834	SFSDVVAEAK					Mascot
1052.5259	1052.5386	0.0127	12	825	834	SFSDVVAEAK					Mascot
1146.5685	1146.5894	0.0209	18	616	624	QEDCVRALR			Carbamidomethyl (C)[4]		Mascot
1322.6488	1322.705	0.0562	42	712	722	HLSEDHVFPNK					Mascot
1507.7751	1507.7792	0.0041	3	372	386	DGSDFSAAIIGSLVR					Mascot
1525.8597	1525.7748	-0.0849	-56	537	550	EVAAVSAALHVRFR					Mascot
1529.8071	1529.7804	-0.0267	-17	255	267	FLSQLHSDISNLR					Mascot
1571.7346	1571.7231	-0.0115	-7	57	69	TFDLFMKCHGSTK			Carbamidomethyl (C)[8]		Mascot
1663.8762	1663.8755	-0.0007	0	371	386	RDGSDFSAAIIGSLVR					Mascot
1691.9037	1691.8555	-0.0482	-28	577	593	MASTPGVSAILFDALAK					Mascot
2186.1526	2186.1707	0.0181	8	16	34	ILPSYESMVEHPVSLLSIR			Oxidation (M)[8]		Mascot
2333.1633	2333.1367	-0.0266	-11	938	958	RDHPFAQLSGSDNIIAFTTSR					Mascot

10 Protein kinase 2B, chloroplastic [Triticum urartu] gi|474374929 51195.2 9.39 11 42 0 10.084

Peptide Information

Calc. Mass	Obsrv. Mass	± da	± ppm	Start Seq.	End Seq.	Sequence	Ion Score	C. I. %	Modification	Rank	Result Type
856.4271	856.5117	0.0846	99	96	102	DRSEPPR					Mascot
870.5771	870.5146	-0.0625	-72	237	245	LKVAIGAAK					Mascot
902.5054	902.4512	-0.0542	-60	427	434	RTSNTVPK					Mascot
1057.483	1057.504	0.021	20	71	81	TSMSSATSASK					Mascot
1128.5579	1128.5319	-0.026	-23	435	444	SPMRGQPSPR			Oxidation (M)[3]		Mascot
1140.6161	1140.5513	-0.0648	-57	168	177	LKPEGFQGHK					Mascot
1312.7372	1312.7721	0.0349	27	227	238	GADPLSWGIRLK					Mascot
1312.7372	1312.7721	0.0349	27	227	238	GADPLSWGIRLK	3	0			Mascot
1433.6512	1433.7853	0.1341	94	11	23	VDHSMNTSAACLK			Carbamidomethyl (C)[11]		Mascot
1472.7261	1472.7577	0.0316	21	67	81	VTSKTSMSSATSASK					Mascot
1864.864	1864.9424	0.0784	42	7	23	STARVDHSMNTSAACLK			Carbamidomethyl (C)[15], Oxidation (M)[9]		Mascot
2226.1653	2226.0798	-0.0855	-38	103	122	TEGEILSSSNLKAFLFNDLK					Mascot

Gel Idx/Pos	250/K2	Instr./Gel Origin	BA2151/Sample Project 20140814	Process Status	Analysis Succeeded
Plate [#] Name	[1] Sample Project 20140814	Instrument Sample Name		Spectra	11

Rank	Protein Name	Accession No.	Protein MW	Protein PI	Pep. Count	Protein Score	Protein Score C. I. %	Intensity Matched	Total Ion Score	Total Ion C. I. %	Confirmed
1	ATP synthase CF1 beta subunit [Triticum aestivum]	gi|14017579	53880.9	5.06	25	1,160	100	53.676	977	100	

Protein Group

ATP synthase CF1 beta subunit (chloroplast) [Triticum aestivum]	gi|521301494	53937.9	5.0599999427 7954
ATP synthase beta subunit [Triticum aestivum]	gi|473690	53880.9	5.0599999427 7954
ATPase beta subunit [Triticum aestivum]	gi|13928212	53880.9	5.0599999427 7954
RecName: Full=ATP synthase subunit beta, chloroplastic; AltName: Full=ATP synthase F1 sector subunit beta; AltName: Full=F-ATPase subunit beta	gi|114574	53880.9	5.0599999427 7954
unnamed protein product [Triticum aestivum]	gi|227303890	53880.9	5.0599999427 7954

Peptide Information

Calc. Mass	Obsrv. Mass	± da	± ppm	Start Seq.	End Seq.	Sequence	Ion Score	C. I. %	Modification	Rank	Result Type
873.5152	873.5121	-0.0031	-4	391	397	VKETLQR					Mascot
1007.5771	1007.562	-0.0151	-15	146	154	LSIFETGIK					Mascot
1201.7052	1201.7001	-0.0051	-4	155	164	VVDLLAPYRR					Mascot
1254.5818	1254.5714	-0.0104	-8	76	87	AVAMSATDGLMR			Oxidation (M)[4,11]		Mascot
1328.6707	1328.6815	0.0108	8	192	205	AHGGVSVFGGVGER					Mascot
1328.6707	1328.6815	0.0108	8	192	205	AHGGVSVFGGVGER	116	100			Mascot
1416.6866	1416.6992	0.0126	9	379	390	IVGNEHYETAQR					Mascot
1416.6866	1416.6992	0.0126	9	379	390	IVGNEHYETAQR	91	100			Mascot
1433.7748	1433.7893	0.0145	10	278	291	FVQAGSEVSALLGR					Mascot
1433.7748	1433.7893	0.0145	10	278	291	FVQAGSEVSALLGR	113	100			Mascot
1471.7614	1471.7592	-0.0022	-1	249	261	VGLTALTMAEYFR					Mascot
1487.7563	1487.7515	-0.0048	-3	249	261	VGLTALTMAEYFR			Oxidation (M)[8]		Mascot
1487.7563	1487.7515	-0.0048	-3	249	261	VGLTALTMAEYFR	30	90.704	Oxidation (M)[8]		Mascot
1492.8159	1492.816	0.0001	0	266	277	QDVLLFIDNIFR					Mascot
1518.6564	1518.7524	0.096	63	206	217	TREGNDLYMEMK			Oxidation (M)[9,11]		Mascot
1535.858	1535.8685	0.0105	7	40	52	LPYIYNALVVQSR					Mascot

1535.858	1535.8685	0.0105	7	40	52	LPYIYNALVVQSR	102	100		Mascot
1601.8104	1601.818	0.0076	5	232	246	VALVYGQMNEPPGAR				Mascot
1617.8054	1617.8005	-0.0049	-3	232	246	VALVYGQMNEPPGAR			Oxidation (M)[8]	Mascot
1617.8054	1617.8005	-0.0049	-3	232	246	VALVYGQMNEPPGAR	49	99.891	Oxidation (M)[8]	Mascot
1790.8928	1790.9406	0.0478	27	247	261	MRVGLTALTMAEYFR			Oxidation (M)[1,10]	Mascot
1809.0157	1808.9768	-0.0389	-22	23	39	IDQIIGPVLDVTFPPGK				Mascot
1885.955	1885.9711	0.0161	9	58	73	QINVTCEVQQLLGNNR			Carbamidomethyl (C)[6]	Mascot
1949.0491	1949.0708	0.0217	11	262	277	DVNKQDVLLFIDNIFR				Mascot
1949.0491	1949.0708	0.0217	11	262	277	DVNKQDVLLFIDNIFR	118	100		Mascot
2030.9989	2031.0533	0.0544	27	3	22	TNPTTSPPGASTIEEKSTGR				Mascot
2061.0322	2061.0659	0.0337	16	360	378	GIYPAVDPLDSTSTMLQPR				Mascot
2077.0271	2077.0442	0.0171	8	360	378	GIYPAVDPLDSTSTMLQPR			Oxidation (M)[15]	Mascot
2097.1008	2097.1152	0.0144	7	88	109	GMEVIDTGAPLSVPVGGATLGR				Mascot
2113.0959	2113.1067	0.0108	5	88	109	GMEVIDTGAPLSVPVGGATLGR			Oxidation (M)[2]	Mascot
2314.0691	2314.0925	0.0234	10	292	312	MPSAVGYQPTLSTEMGSLQER			Oxidation (M)[1,15]	Mascot
2460.1785	2460.217	0.0385	16	53	73	DTDDKQINVTCEVQQLLGNNR			Carbamidomethyl (C)[11]	Mascot
2460.1785	2460.217	0.0385	16	53	73	DTDDKQINVTCEVQQLLGNNR	190	100	Carbamidomethyl (C)[11]	Mascot
2694.3887	2694.4514	0.0627	23	110	134	IFNVLGEPVDNLGPVDSSATFPIHR				Mascot
2694.3887	2694.4514	0.0627	23	110	134	IFNVLGEPVDNLGPVDSSATFPIHR	170	100		Mascot
3325.856	3325.9202	0.0642	19	23	52	IDQIIGPVLDVTFPPGKLPYIYNALVVQSR				Mascot
3714.8862	3714.9929	0.1067	29	319	354	GSITSIQAVYVPADDLTDPAPATTFAHLDATTVLSR				Mascot
3842.9812	3843.0984	0.1172	30	318	354	KGSITSIQAVYVPADDLTDPAPATTFAHLDATTVLSR				Mascot

2	ATP synthase CF1 beta subunit (chloroplast) [Triticum monococcum]	gi|525778512	53865	5.17	23	945	100	50.025	787	100

Protein Group

ATP synthase CF1 beta subunit (chloroplast) [Triticum monococcum subsp. aegilopoides]	gi|521301354	53865	5.17000007629395
ATP synthase CF1 beta subunit (chloroplast) [Triticum monococcum]	gi|521301197	53865	5.17000007629395
ATP synthase CF1 beta subunit (chloroplast) [Triticum urartu]	gi|521301433	53865	5.17000007629395
ATP synthase CF1 beta subunit (chloroplast) [Triticum urartu]	gi|525782302	53865	5.17000007629395

Peptide Information

Calc. Mass	Obsrv. Mass	± da	± ppm	Start Seq.	End Seq.	Sequence	Ion Score	C. I. %	Modification	Rank	Result Type
873.5152	873.5121	-0.0031	-4	391	397	VKETLQR					Mascot
1007.5771	1007.562	-0.0151	-15	146	154	LSIFETGIK					Mascot
1201.7052	1201.7001	-0.0051	-4	155	164	VVDLLAPYRR					Mascot
1254.5818	1254.5714	-0.0104	-8	76	87	AVAMSATDGLMR			Oxidation (M)[4,11]		Mascot
1328.6707	1328.6815	0.0108	8	192	205	AHGGVSVFGGVGER					Mascot
1328.6707	1328.6815	0.0108	8	192	205	AHGGVSVFGGVGER	116	100			Mascot
1416.6866	1416.6992	0.0126	9	379	390	IVGNEHYETAQR					Mascot
1416.6866	1416.6992	0.0126	9	379	390	IVGNEHYETAQR	91	100			Mascot
1433.7748	1433.7893	0.0145	10	278	291	FVQAGSEVSALLGR					Mascot
1433.7748	1433.7893	0.0145	10	278	291	FVQAGSEVSALLGR	113	100			Mascot
1471.7614	1471.7592	-0.0022	-1	249	261	VGLTALTMAEYFR					Mascot
1487.7563	1487.7515	-0.0048	-3	249	261	VGLTALTMAEYFR			Oxidation (M)[8]		Mascot
1487.7563	1487.7515	-0.0048	-3	249	261	VGLTALTMAEYFR	30	90.704	Oxidation (M)[8]		Mascot
1492.8159	1492.816	0.0001	0	266	277	QDVLLFIDNIFR					Mascot
1518.6564	1518.7524	0.096	63	206	217	TREGNDLYMEMK			Oxidation (M)[9,11]		Mascot
1535.858	1535.8685	0.0105	7	40	52	LPYIYNALVVQSR					Mascot
1535.858	1535.8685	0.0105	7	40	52	LPYIYNALVVQSR	102	100			Mascot
1601.8104	1601.818	0.0076	5	232	246	VALVYGQMNEPPGAR					Mascot
1617.8054	1617.8005	-0.0049	-3	232	246	VALVYGQMNEPPGAR			Oxidation (M)[8]		Mascot
1617.8054	1617.8005	-0.0049	-3	232	246	VALVYGQMNEPPGAR	49	99.891	Oxidation (M)[8]		Mascot
1790.8928	1790.9406	0.0478	27	247	261	MRVGLTALTMAEYFR			Oxidation (M)[1,10]		Mascot
1809.0157	1808.9768	-0.0389	-22	23	39	IDQIIGPVLDVTFPPGK					Mascot
1885.955	1885.9711	0.0161	9	58	73	QINVTCEVQQLLGNNR			Carbamidomethyl (C)[6]		Mascot
1949.0491	1949.0708	0.0217	11	262	277	DVNKQDVLLFIDNIFR					Mascot
1949.0491	1949.0708	0.0217	11	262	277	DVNKQDVLLFIDNIFR	118	100			Mascot
2061.0322	2061.0659	0.0337	16	360	378	GIYPAVDPLDSTSTMLQP R					Mascot
2077.0271	2077.0442	0.0171	8	360	378	GIYPAVDPLDSTSTMLQP R			Oxidation (M)[15]		Mascot
2097.1008	2097.1152	0.0144	7	88	109	GMEVIDTGAPLSVPVGG ATLGR					Mascot
2113.0959	2113.1067	0.0108	5	88	109	GMEVIDTGAPLSVPVGG ATLGR			Oxidation (M)[2]		Mascot
2314.0691	2314.0925	0.0234	10	292	312	MPSAVGYQPTLSTEMGS LQER			Oxidation (M)[1,15]		Mascot
2694.3887	2694.4514	0.0627	23	110	134	IFNVLGEPVDNLGPVDSS ATFPIHR					Mascot
2694.3887	2694.4514	0.0627	23	110	134	IFNVLGEPVDNLGPVDSS ATFPIHR	170	100			Mascot

Calc. Mass	Obsrv. Mass	± da	± ppm	Start Seq.	End Seq.	Sequence	Ion Score	C. I. %	Modification	Rank	Result Type
3325.856	3325.9202	0.0642	19	23	52	IDQIIGPVLDVTFPPGKLP YIYNALVVQSR					Mascot
3714.8862	3714.9929	0.1067	29	319	354	GSITSIQAVYVPADDLTD PAPATTFAHLDATTVLSR					Mascot
3842.9812	3843.0984	0.1172	30	318	354	KGSITSIQAVYVPADDLT DPAPATTFAHLDATTVLS R					Mascot

3	ATP synthase subunit beta, chloroplastic [Triticum urartu]	gi|474022890	36113.7	5.21	18	818	100	40.107	686	100	

Peptide Information

Calc. Mass	Obsrv. Mass	± da	± ppm	Start Seq.	End Seq.	Sequence	Ion Score	C. I. %	Modification	Rank	Result Type
873.5152	873.5121	-0.0031	-4	303	309	VKETLQR					Mascot
1007.5771	1007.562	-0.0151	-15	58	66	LSIFETGIK					Mascot
1201.7052	1201.7001	-0.0051	-4	67	76	VVDLLAPYRR					Mascot
1328.6707	1328.6815	0.0108	8	104	117	AHGGVSVFGGVGER					Mascot
1328.6707	1328.6815	0.0108	8	104	117	AHGGVSVFGGVGER	116	100			Mascot
1416.6866	1416.6992	0.0126	9	291	302	IVGNEHYETAQR					Mascot
1416.6866	1416.6992	0.0126	9	291	302	IVGNEHYETAQR	91	100			Mascot
1433.7748	1433.7893	0.0145	10	190	203	FVQAGSEVSALLGR					Mascot
1433.7748	1433.7893	0.0145	10	190	203	FVQAGSEVSALLGR	113	100			Mascot
1471.7614	1471.7592	-0.0022	-1	161	173	VGLTALTMAEYFR					Mascot
1487.7563	1487.7515	-0.0048	-3	161	173	VGLTALTMAEYFR			Oxidation (M)[8]		Mascot
1487.7563	1487.7515	-0.0048	-3	161	173	VGLTALTMAEYFR	30	90.704	Oxidation (M)[8]		Mascot
1492.8159	1492.816	0.0001	0	178	189	QDVLLFIDNIFR					Mascot
1518.6564	1518.7524	0.096	63	118	129	TREGNDLYMEMK			Oxidation (M)[9,11]		Mascot
1601.8104	1601.818	0.0076	5	144	158	VALVYGQMNEPPGAR					Mascot
1617.8054	1617.8005	-0.0049	-3	144	158	VALVYGQMNEPPGAR			Oxidation (M)[8]		Mascot
1617.8054	1617.8005	-0.0049	-3	144	158	VALVYGQMNEPPGAR	49	99.891	Oxidation (M)[8]		Mascot
1790.8928	1790.9406	0.0478	27	159	173	MRVGLTALTMAEYFR			Oxidation (M)[1,10]		Mascot
1909.0389	1909.0364	-0.0025	-1	2	21	EVIDTGAPLSVPVGGATL GR					Mascot
1949.0491	1949.0708	0.0217	11	174	189	DVNKQDVLLFIDNIFR					Mascot
1949.0491	1949.0708	0.0217	11	174	189	DVNKQDVLLFIDNIFR	118	100			Mascot
2061.0322	2061.0659	0.0337	16	272	290	GIYPAVDPLDSTSTMLQP R					Mascot
2077.0271	2077.0442	0.0171	8	272	290	GIYPAVDPLDSTSTMLQP R			Oxidation (M)[15]		Mascot
2314.0691	2314.0925	0.0234	10	204	224	MPSAVGYQPTLSTEMGS LQER			Oxidation (M)[1,15]		Mascot
2694.3887	2694.4514	0.0627	23	22	46	IFNVLGEPVDNLGPVDSS ATFPIHR					Mascot
2694.3887	2694.4514	0.0627	23	22	46	IFNVLGEPVDNLGPVDSS	170	100			Mascot

						ATFPIHR					
3714.8862	3714.9929	0.1067	29	231	266	GSITSIQAVYVPADDLTDPAPATTFAHLDATTVLSR					Mascot
3842.9812	3843.0984	0.1172	30	230	266	KGSITSIQAVYVPADDLTDPAPATTFAHLDATTVLSR					Mascot

4	ATP synthase subunit beta, chloroplastic [Triticum urartu]	gi|474124791	50614.5	5.09	18	795	100	40.107	686	100

Peptide Information

Calc. Mass	Obsrv. Mass	± da	± ppm	Start Seq.	End Seq.	Sequence	Ion Score	C. I. %	Modification	Rank	Result Type
873.5152	873.5121	-0.0031	-4	303	309	VKETLQR					Mascot
1007.5771	1007.562	-0.0151	-15	58	66	LSIFETGIK					Mascot
1201.7052	1201.7001	-0.0051	-4	67	76	VVDLLAPYRR					Mascot
1328.6707	1328.6815	0.0108	8	104	117	AHGGVSVFGGVGER					Mascot
1328.6707	1328.6815	0.0108	8	104	117	AHGGVSVFGGVGER	116	100			Mascot
1416.6866	1416.6992	0.0126	9	291	302	IVGNEHYETAQR					Mascot
1416.6866	1416.6992	0.0126	9	291	302	IVGNEHYETAQR	91	100			Mascot
1433.7748	1433.7893	0.0145	10	190	203	FVQAGSEVSALLGR					Mascot
1433.7748	1433.7893	0.0145	10	190	203	FVQAGSEVSALLGR	113	100			Mascot
1471.7614	1471.7592	-0.0022	-1	161	173	VGLTALTMAEYFR					Mascot
1487.7563	1487.7515	-0.0048	-3	161	173	VGLTALTMAEYFR			Oxidation (M)[8]		Mascot
1487.7563	1487.7515	-0.0048	-3	161	173	VGLTALTMAEYFR	30	90.704	Oxidation (M)[8]		Mascot
1492.8159	1492.816	0.0001	0	178	189	QDVLLFIDNIFR					Mascot
1518.6564	1518.7524	0.096	63	118	129	TREGNDLYMEMK			Oxidation (M)[9,11]		Mascot
1601.8104	1601.818	0.0076	5	144	158	VALVYGQMNEPPGAR					Mascot
1617.8054	1617.8005	-0.0049	-3	144	158	VALVYGQMNEPPGAR			Oxidation (M)[8]		Mascot
1617.8054	1617.8005	-0.0049	-3	144	158	VALVYGQMNEPPGAR	49	99.891	Oxidation (M)[8]		Mascot
1790.8928	1790.9406	0.0478	27	159	173	MRVGLTALTMAEYFR			Oxidation (M)[1,10]		Mascot
1909.0389	1909.0364	-0.0025	-1	2	21	EVIDTGAPLSVPVGGATLGR					Mascot
1949.0491	1949.0708	0.0217	11	174	189	DVNKQDVLLFIDNIFR					Mascot
1949.0491	1949.0708	0.0217	11	174	189	DVNKQDVLLFIDNIFR	118	100			Mascot
2061.0322	2061.0659	0.0337	16	272	290	GIYPAVDPLDSTSTMLQPR					Mascot
2077.0271	2077.0442	0.0171	8	272	290	GIYPAVDPLDSTSTMLQPR			Oxidation (M)[15]		Mascot
2314.0691	2314.0925	0.0234	10	204	224	MPSAVGYQPTLSTEMGSLQER			Oxidation (M)[1,15]		Mascot
2694.3887	2694.4514	0.0627	23	22	46	IFNVLGEPVDNLGPVDSSATFPIHR					Mascot
2694.3887	2694.4514	0.0627	23	22	46	IFNVLGEPVDNLGPVDSSATFPIHR	170	100			Mascot

Calc. Mass	Obsrv. Mass	± da	± ppm	Start Seq.	End Seq.	Sequence	Ion Score	C. I. %	Modification	Rank	Result Type
3714.8862	3714.9929	0.1067	29	231	266	GSITSIQAVYVPADDLTDPAPATTFAHLDATTVLSR					Mascot
3842.9812	3843.0984	0.1172	30	230	266	KGSITSIQAVYVPADDLTDPAPATTFAHLDATTVLSR					Mascot

5	ATP synthase beta subunit [Triticum aestivum]	gi|525291	59325.9	5.56	17	88	99.993	5.786

Protein Group

unnamed protein product [Triticum aestivum]	gi|227303166	59325.9	5.55999994277954

Peptide Information

Calc. Mass	Obsrv. Mass	± da	± ppm	Start Seq.	End Seq.	Sequence	Ion Score	C. I. %	Modification	Rank	Result Type
1173.6627	1173.6654	0.0027	2	212	221	VVDLLAPYQR					Mascot
1390.6863	1390.6973	0.011	8	249	262	AHGGFSVFAGVGER					Mascot
1399.7693	1399.7745	0.0052	4	307	319	VGLTGLTVAEHFR					Mascot
1409.8112	1409.8087	-0.0025	-2	148	161	VLNTGSPITVPVGR					Mascot
1415.8005	1415.7726	-0.0279	-20	212	224	VVDLLAPYQRGGK					Mascot
1457.8396	1457.7507	-0.0889	-61	236	248	TVLIMELINNVAK					Mascot
1492.7755	1492.816	0.0405	27	336	349	FTQANSEVSALLGR					Mascot
1675.949	1675.9551	0.0061	4	118	132	LVLEVAQHLGENVVR					Mascot
1864.944	1864.9534	0.0094	5	320	335	DAEGQDVLLFIDNIFR					Mascot
1868.9059	1868.9799	0.074	40	265	280	EGNDLYREMIESGVIK			Oxidation (M)[9]		Mascot
2061.0498	2061.0659	0.0161	8	413	431	QISELGIYPAVDPLDSTSR					Mascot
2186.1453	2186.1687	0.0234	11	350	370	IPSAVGYQPTLATDLGGLQER					Mascot
2212.186	2212.1946	0.0086	4	98	117	FDEGLPPILTALEVLDNSIR					Mascot
2716.281	2716.4258	0.1448	53	524	547	YDDISEHAFYMVGGIDEVIAKAEK			Oxidation (M)[11]		Mascot
2722.3274	2722.4614	0.134	49	458	481	NLQDIIAILGMDELSEDDKMTVAR			Oxidation (M)[11,20]		Mascot
3714.8862	3714.9929	0.1067	29	377	412	GSITSVQAIYVPADDLTDPAPATTFAHLDATTVLSR					Mascot
3842.9812	3843.0984	0.1172	30	376	412	KGSITSVQAIYVPADDLTDPAPATTFAHLDATTVLSR					Mascot

6	ATP synthase subunit beta, mitochondrial [Triticum urartu]	gi|473990219	58081.1	5.13	16	81	99.969	5.67

Peptide Information

Calc. Mass	Obsrv. Mass	± da	± ppm	Start Seq.	End Seq.	Sequence	Ion Score	C. I. %	Modification	Rank	Result Type
1173.6627	1173.6654	0.0027	2	200	209	VVDLLAPYQR					Mascot
1390.6863	1390.6973	0.011	8	237	250	AHGGFSVFAGVGER					Mascot

Calc. Mass	Obsrv. Mass	± da	± ppm	Start Seq.	End Seq.	Sequence	Ion Score	C. I. %	Modification	Rank	Result Type
1399.7693	1399.7745	0.0052	4	295	307	VGLTGLTVAEHFR					Mascot
1415.8005	1415.7726	-0.0279	-20	200	212	VVDLLAPYQRGGK					Mascot
1457.8396	1457.7507	-0.0889	-61	224	236	TVLIMELINNVAK					Mascot
1492.7755	1492.816	0.0405	27	324	337	FTQANSEVSALLGR					Mascot
1675.949	1675.9551	0.0061	4	77	91	LVLEVAQHLGENVVR					Mascot
1864.944	1864.9534	0.0094	5	308	323	DAEGQDVLLFIDNIFR					Mascot
1868.9059	1868.9799	0.074	40	253	268	EGNDLYREMIESGVIK			Oxidation (M)[9]		Mascot
2061.0498	2061.0659	0.0161	8	401	419	QISELGIYPAVDPLDSTSR					Mascot
2186.1453	2186.1687	0.0234	11	338	358	IPSAVGYQPTLATDLGGL QER					Mascot
2212.186	2212.1946	0.0086	4	57	76	FDEGLPPILTALEVLDNSI R					Mascot
2716.281	2716.4258	0.1448	53	512	535	YDDISEHAFYMVGGIDEV IAKAEK			Oxidation (M)[11]		Mascot
2722.3274	2722.4614	0.134	49	446	469	NLQDIIAILGMDELSEDDK MTVAR			Oxidation (M)[11,20]		Mascot
3714.8862	3714.9929	0.1067	29	365	400	GSITSVQAIYVPADDLTD PAPATTFAHLDATTVLSR					Mascot
3842.9812	3843.0984	0.1172	30	364	400	KGSITSVQAIYVPADDLT DPAPATTFAHLDATTVLS R					Mascot

7 ATP synthase subunit beta, mitochondrial [Triticum urartu] gi|473798701 57827 5.25 16 80 99.955 5.573

Peptide Information

Calc. Mass	Obsrv. Mass	± da	± ppm	Start Seq.	End Seq.	Sequence	Ion Score	C. I. %	Modification	Rank	Result Type
1173.6627	1173.6654	0.0027	2	166	175	VVDLLAPYQR					Mascot
1390.6863	1390.6973	0.011	8	203	216	AHGGFSVFAGVGER					Mascot
1399.7693	1399.7745	0.0052	4	261	273	VGLTGLTVAEHFR					Mascot
1409.8112	1409.8087	-0.0025	-2	102	115	VLNTGSPITVPVGR					Mascot
1415.8005	1415.7726	-0.0279	-20	166	178	VVDLLAPYQRGGK					Mascot
1457.8396	1457.7507	-0.0889	-61	190	202	TVLIMELINNVAK					Mascot
1492.7755	1492.816	0.0405	27	290	303	FTQANSEVSALLGR					Mascot
1675.949	1675.9551	0.0061	4	72	86	LVLEVAQHLGENVVR					Mascot
1864.944	1864.9534	0.0094	5	274	289	DAEGQDVLLFIDNIFR					Mascot
1868.9059	1868.9799	0.074	40	219	234	EGNDLYREMIESGVIK			Oxidation (M)[9]		Mascot
2061.0498	2061.0659	0.0161	8	367	385	QISELGIYPAVDPLDSTSR					Mascot
2139.1116	2139.0647	-0.0469	-22	2	21	ICRLSLSEAAPVTPPPTS DK			Carbamidomethyl (C)[2]		Mascot
2186.1453	2186.1687	0.0234	11	304	324	IPSAVGYQPTLATDLGGL QER					Mascot
2212.186	2212.1946	0.0086	4	52	71	FDEGLPPILTALEVLDNSI R					Mascot

3714.8862	3714.9929	0.1067	29	331	366	GSITSVQAIYVPADDLTDPAPATTFAHLDATTVLSR					Mascot
3842.9812	3843.0984	0.1172	30	330	366	KGSITSVQAIYVPADDLTDPAPATTFAHLDATTVLSR					Mascot

8	Disease resistance protein RPM1 [Triticum urartu]	gi|474426983	174445.3	8.79	25	56	89.145	11.762

Peptide Information

Calc. Mass	Obsrv. Mass	± da	± ppm	Start Seq.	End Seq.	Sequence	Ion Score	C. I. %	Modification	Rank	Result Type
920.4142	920.4473	0.0331	36	1090	1097	MAEADDIR					Mascot
1379.7067	1379.741	0.0343	25	300	310	WIAEGYSRELR					Mascot
1399.7501	1399.7745	0.0244	17	1292	1303	TLLETGMYKSLK			Oxidation (M)[7]		Mascot
1421.7053	1421.7694	0.0641	45	590	603	ALDTMGVVNVSGSR			Oxidation (M)[5]		Mascot
1422.7489	1422.7961	0.0472	33	1383	1394	LFKNQNGEVFAR					Mascot
1455.8066	1455.749	-0.0576	-40	380	392	GTIRHLAISSSWK					Mascot
1457.7859	1457.7507	-0.0352	-24	723	734	LLRHSFASEELR					Mascot
1503.7349	1503.7604	0.0255	17	874	884	LFGRCCQFLFR			Carbamidomethyl (C)[5,6]		Mascot
1552.8887	1552.8545	-0.0342	-22	682	694	LPEWIAALWNLVK					Mascot
1553.7443	1553.8235	0.0792	51	1199	1211	HETLSTTHEELEK					Mascot
1553.7443	1553.8235	0.0792	51	1199	1211	HETLSTTHEELEK					Mascot
1581.955	1581.8412	-0.1138	-72	1515	1528	LIASKPPLKMWVAK					Mascot
1590.8269	1590.8767	0.0498	31	590	605	ALDTMGVVNVSGSRGK					Mascot
1812.9347	1812.9279	-0.0068	-4	400	415	SIVDMSCIRSLTVFGK			Carbamidomethyl (C)[7]		Mascot
1930.9188	1931.0276	0.1088	56	839	856	EADATGGWQVVMSRNGPR					Mascot
2043.911	2044.0477	0.1367	67	243	259	LNDHMSAELEMNPELER			Oxidation (M)[5]		Mascot
2061.0977	2061.0659	-0.0318	-15	430	449	VLDLEGTSGLVDHHLTGIGK					Mascot
2092.9832	2093.0483	0.0651	31	277	293	SCMLYFSIFPQDDKVSR			Carbamidomethyl (C)[2]		Mascot
2098.1519	2098.1343	-0.0176	-8	682	698	LPEWIAALWNLVKMELR			Oxidation (M)[14]		Mascot
2212.2158	2212.1946	-0.0212	-10	215	235	KCDGLPLAIVTIGGFLANQPK			Carbamidomethyl (C)[2]		Mascot
2235.0215	2235.2056	0.1841	82	1482	1498	TKNYSAYFYEYYCPPLR			Carbamidomethyl (C)[13]		Mascot
2340.3479	2340.2817	-0.0662	-28	675	694	LYGNLVKLPEWIAALWNLVK					Mascot
2443.3667	2443.2559	-0.1108	-45	427	449	LLRVLDLEGTSGLVDHHLTGIGK					Mascot
2460.1526	2460.217	0.0644	26	1192	1211	YEELESRHETLSTTHEELEK					Mascot
2460.1526	2460.217	0.0644	26	1192	1211	YEELESRHETLSTTHEELEK					Mascot
2693.5098	2693.5212	0.0114	4	430	454	VLDLEGTSGLVDHHLTGIGKLLHLR					Mascot
3002.5503	3002.6616	0.1113	37	19	46	SDIIKLISNEDSQQLDVISVWGMGGLGK					Mascot

9	Bifunctional dihydroflavonol 4-reductase/flavanone 4-reductase [Triticum urartu]	gi|474086534	37922	6.88	10	45	0	6.003

Peptide Information

Calc. Mass	Obsrv. Mass	± da	± ppm	Start Seq.	End Seq.	Sequence	Ion Score	C. I. %	Modification	Rank	Result Type
920.506	920.4473	-0.0587	-64	297	303	FSNQRLR					Mascot
1422.6682	1422.7961	0.1279	90	285	296	EDDGKPMAKPYR			Oxidation (M)[7]		Mascot
1423.6635	1423.7823	0.1188	83	224	236	TCPNAVAGYTDVR			Carbamidomethyl (C)[2]		Mascot
1492.7795	1492.816	0.0365	24	273	284	DLFPQYPITDKR					Mascot
1551.7584	1551.8368	0.0784	51	223	236	KTCPNAVAGYTDVR			Carbamidomethyl (C)[3]		Mascot
1864.8971	1864.9534	0.0563	30	224	240	TCPNAVAGYTDVRDVAR			Carbamidomethyl (C)[2]		Mascot
2065.0972	2065.1108	0.0136	7	56	73	NAHLLTLEGAQERLALCR			Carbamidomethyl (C)[17]		Mascot
2138.9226	2139.0647	0.1421	66	163	180	ETGNMYCCSKIMAEITAK			Carbamidomethyl (C)[7,8], Oxidation (M)[5,12]		Mascot
2198.1838	2198.0789	-0.1049	-48	255	272	YLCMGAVLHRAHFLQLL R			Carbamidomethyl (C)[3]		Mascot
2676.439	2676.5054	0.0664	25	181	206	EEAANRGLDLAVVVPSM TIGPALQPK					Mascot

10	hypothetical protein TRIUR3_13290 [Triticum urartu]	gi|473890931	59042.1	6.31	12	44	0	10.872

Peptide Information

Calc. Mass	Obsrv. Mass	± da	± ppm	Start Seq.	End Seq.	Sequence	Ion Score	C. I. %	Modification	Rank	Result Type
827.3936	827.4746	0.081	98	421	426	VRMMMK			Oxidation (M)[3,4]		Mascot
1173.6449	1173.6654	0.0205	17	81	89	FMLLVELHR			Oxidation (M)[2]		Mascot
1472.8042	1472.7803	-0.0239	-16	78	89	GNKFMLLVELHR			Oxidation (M)[5]		Mascot
1535.771	1535.8685	0.0975	63	178	189	VEPCLQVFQLMR			Carbamidomethyl (C)[4], Oxidation (M)[11]		Mascot
1535.771	1535.8685	0.0975	63	178	189	VEPCLQVFQLMR			Carbamidomethyl (C)[4], Oxidation (M)[11]		Mascot
1777.8901	1777.9419	0.0518	29	429	444	GLKTNTGCSWIEIGNK			Carbamidomethyl (C)[8]		Mascot
1990.9282	1990.9961	0.0679	34	91	109	GMSADISILASAMSFCAV K			Carbamidomethyl (C)[16], Oxidation (M)[2,13]		Mascot
2128.9614	2129.1074	0.146	69	144	160	CYQLENACQVFQNMPVK			Carbamidomethyl (C)[1,8]		Mascot
2198.0264	2198.0789	0.0525	24	20	37	RLINDCMHDIMGVQPEN R			Carbamidomethyl (C)[6]		Mascot
2235.0244	2235.2056	0.1812	81	327	345	LGMEPELDHYSCMVDLL GR			Carbamidomethyl (C)[12]		Mascot
2443.2141	2443.2559	0.0418	17	91	113	GMSADISILASAMSFCAV KQSIR			Carbamidomethyl (C)[16]		Mascot
2460.125	2460.217	0.092	37	21	40	LINDCMHDIMGVQPENRV MR			Carbamidomethyl (C)[5], Oxidation (M)[6,10]		Mascot
2460.125	2460.217	0.092	37	21	40	LINDCMHDIMGVQPENRV MR			Carbamidomethyl (C)[5], Oxidation (M)[6,10]		Mascot
2722.282	2722.4614	0.1794	66	323	345	AMIRLGMEPELDHYSCM VDLLGR			Carbamidomethyl (C)[16], Oxidation (M)[2]		Mascot

Gel Idx/Pos	251/K3	Instr./Gel Origin	BA2151/Sample Project 20140814	Process Status	Analysis Succeeded
Plate [#] Name	[1] Sample Project 20140814	Instrument Sample Name		Spectra	11

Rank	Protein Name	Accession No.	Protein MW	Protein PI	Pep. Count	Protein Score	Protein Score C. I. %	Intensity Matched	Total Ion Score	Total Ion C. I. %	Confirmed
1	Tubulin alpha chain [Triticum urartu]	gi|474224323	50367.6	4.89	16	470	100	22.494	386	100	

Protein Group

RecName: Full=Tubulin alpha chain	gi|8928408	50395.6	4.8899998664856
Tubulin alpha-3 chain [Triticum urartu]	gi|473887549	50381.6	4.8899998664856
alpha tubulin-2A [Triticum aestivum]	gi|90289596	50367.6	4.8899998664856
alpha tubulin-2B [Triticum aestivum]	gi|90289600	50367.6	4.8899998664856
alpha tubulin-2D [Triticum aestivum]	gi|90289598	50367.6	4.8899998664856
alpha tubulin-3A [Triticum aestivum]	gi|90289604	50381.6	4.8899998664856
alpha tubulin-3B [Triticum aestivum]	gi|90289602	50381.6	4.8899998664856
alpha tubulin-3D [Triticum aestivum]	gi|90289606	50381.6	4.8899998664856
alpha-tubulin [Triticum aestivum]	gi|4098272	50395.6	4.8899998664856

Peptide Information

Calc. Mass	Obsrv. Mass	± da	± ppm	Start Seq.	End Seq.	Sequence	Ion Score	C. I. %	Modification	Rank	Result Type
1007.454	1007.4469	-0.0071	-7	97	105	EDAANNFAR					Mascot
1132.5668	1132.5575	-0.0093	-8	113	121	EIVDLCLDR			Carbamidomethyl (C)[6]		Mascot
1299.4989	1299.472	-0.0269	-21	312	320	YMACCLMYR			Carbamidomethyl (C)[4,5], Oxidation (M)[2,7]		Mascot
1396.693	1396.7186	0.0256	18	391	401	IDHKFDLMYAK			Oxidation (M)[8]		Mascot
1473.8635	1473.854	-0.0095	-6	230	243	LVSQVISSLTASLR					Mascot
1473.8635	1473.854	-0.0095	-6	230	243	LVSQVISSLTASLR	102	100			Mascot
1589.6844	1589.6995	0.0151	9	309	320	HGKYMACCLMYR			Carbamidomethyl (C)[7,8]		Mascot
1691.8711	1691.8533	-0.0178	-11	216	229	SLDIERPTYTNLNR					Mascot

1691.8711	1691.8533	-0.0178	-11	216	229	SLDIERPTYTNLNR	67	99.998			Mascot
1701.9059	1701.8979	-0.008	-5	65	79	AVFVDLEPTVIDEVR					Mascot
1701.9059	1701.8979	-0.008	-5	65	79	AVFVDLEPTVIDEVR	71	100			Mascot
1808.9252	1808.8848	-0.0404	-22	265	280	IHFMLSSYAPVISAEK			Oxidation (M)[4]		Mascot
1847.9723	1847.8995	-0.0728	-39	215	229	RSLDIERPTYTNLNR					Mascot
1885.9147	1885.9192	0.0045	2	374	390	AVCMISNSTSVVEVFSR			Carbamidomethyl (C)[3]		Mascot
1901.9097	1901.9373	0.0276	15	374	390	AVCMISNSTSVVEVFSR			Carbamidomethyl (C)[3], Oxidation (M)[4]		Mascot
1977.8826	1977.8553	-0.0273	-14	41	60	TVGGGDDAFNTFFSETG AGK					Mascot
2346.0132	2346.0142	0.001	0	403	422	AFVHWYVGEGMEEGEF SEAR			Oxidation (M)[11]		Mascot
2385.1946	2385.2019	0.0073	3	85	105	QLFHPEQLISGKEDAANN FAR					Mascot
2395.1746	2395.2053	0.0307	13	374	394	AVCMISNSTSVVEVFSRI DHK			Carbamidomethyl (C)[3], Oxidation (M)[4]		Mascot
2408.1882	2408.2029	0.0147	6	244	264	FDGALNVDVNEFQTNLV PYPR					Mascot
2408.1882	2408.2029	0.0147	6	244	264	FDGALNVDVNEFQTNLV PYPR	145	100			Mascot

2 protein disulfide isomerase [Triticum durum] gi|12056121 41818.6 5.29 20 319 100 16.487 191 100

Protein Group

protein disulfide isomerase [Triticum durum] gi|12056117 41818.6 5.2899 999618 5303

Peptide Information

Calc. Mass	Obsrv. Mass	± da	± ppm	Start Seq.	End Seq.	Sequence	Ion Score	C. I. %	Modification	Rank	Result Type
938.473	938.4479	-0.0251	-27	270	277	FFQSNAPK					Mascot
1023.4993	1023.4863	-0.013	-13	236	244	DFDVSALEK					Mascot
1150.5851	1150.5656	-0.0195	-17	124	133	NGGKNIQEYK					Mascot
1181.6201	1181.5886	-0.0315	-27	111	120	YEVQGFPTLK					Mascot
1182.6589	1182.6346	-0.0243	-21	212	222	GDAAVERPLVR					Mascot
1182.6589	1182.6346	-0.0243	-21	212	222	GDAAVERPLVR	46	99.774			Mascot
1210.6216	1210.5975	-0.0241	-20	260	269	NPDNHPYLLK					Mascot
1278.694	1278.6486	-0.0454	-36	137	147	EAEGIVEYLKK					Mascot
1308.5955	1308.6018	0.0063	5	158	169	APEDATYLEDGK					Mascot
1320.6107	1320.578	-0.0327	-25	295	306	AYYGAVEEFSGK					Mascot
1358.674	1358.6945	0.0205	15	361	371	DYFDGKLTPFR					Mascot
1536.8308	1536.7975	-0.0333	-22	223	235	LFKPFDELVVDSK					Mascot
1647.8951	1647.7646	-0.1305	-79	73	87	SLAPEYEKAAQLLSK					Mascot
1647.8951	1647.7646	-0.1305	-79	73	87	SLAPEYEKAAQLLSK					Mascot
1671.8436	1671.7244	-0.1192	-71	329	343	EDQAPLILIQDSDSK					Mascot

1674.9901	1674.916	-0.0741	-44	81	96	AAQLLSKHDPAIVLAK					Mascot
1799.9386	1799.9027	-0.0359	-20	329	344	EDQAPLILIQDSDSKK					Mascot
1835.9037	1835.9165	0.0128	7	278	293	AMLFLNFSTGPFESFK					Mascot
1865.8678	1865.8667	-0.0011	-1	196	211	SDYDFGHTVHANHLPR					Mascot
1865.8678	1865.8667	-0.0011	-1	196	211	SDYDFGHTVHANHLPR	105	100			Mascot
1899.9963	1899.9719	-0.0244	-13	254	269	VVTFDKNPDNHPYLLK					Mascot
1899.9963	1899.9719	-0.0244	-13	254	269	VVTFDKNPDNHPYLLK	41	99.345			Mascot
2104.075	2104.0588	-0.0162	-8	310	328	FLIGDIEASQGAFQYFGL K					Mascot
2135.053	2135.0745	0.0215	10	194	211	LRSDYDFGHTVHANHLP R					Mascot

3 protein disulfide isomerase [Triticum aestivum] gi|222446342 56662.9 5.03 22 315 100 16.942 191 100

Protein Group

protein disulfide isomerase 2 precursor [Triticum aestivum] gi|13925726 56690.9 5.0300 002098 0835

protein disulfide isomerase precursor [Triticum aestivum] gi|67508767 56690.9 5.0300 002098 0835

protein disulfide isomerase precursor [Triticum aestivum] gi|67508773 56690.9 5.0300 002098 0835

Peptide Information

Calc. Mass	Obsrv. Mass	± da	± ppm	Start Seq.	End Seq.	Sequence	Ion Score	C. I. %	Modification	Rank	Result Type
938.473	938.4479	-0.0251	-27	270	277	FFQSNAPK					Mascot
1023.4993	1023.4863	-0.013	-13	236	244	DFDVSALEK					Mascot
1150.5851	1150.5656	-0.0195	-17	124	133	NGGKNIQEYK					Mascot
1181.6201	1181.5886	-0.0315	-27	111	120	YEVQGFPTLK					Mascot
1182.6589	1182.6346	-0.0243	-21	212	222	GDAAVERPLVR					Mascot
1182.6589	1182.6346	-0.0243	-21	212	222	GDAAVERPLVR	46	99.774			Mascot
1210.6216	1210.5975	-0.0241	-20	260	269	NPDNHPYLLK					Mascot
1278.694	1278.6486	-0.0454	-36	137	147	EAEGIVEYLKK					Mascot
1308.5955	1308.6018	0.0063	5	158	169	APEDATYLEDGK					Mascot
1320.6107	1320.578	-0.0327	-25	295	306	AYYGAVEEFSGK					Mascot
1358.674	1358.6945	0.0205	15	361	371	DYFDGKLTPFR					Mascot
1423.7064	1423.6896	-0.0168	-12	373	385	SEPIPEANNEPVK					Mascot
1440.7847	1440.7448	-0.0399	-28	386	398	VVVADNVHDVVFK					Mascot
1536.8308	1536.7975	-0.0333	-22	223	235	LFKPFDELVVDSK					Mascot
1647.8951	1647.7646	-0.1305	-79	73	87	SLAPEYEKAAQLLSK					Mascot

Calc. Mass	Obsrv. Mass	± da	± ppm	Start Seq.	End Seq.	Sequence	Ion Score	C. I. %	Modification	Rank	Result Type
1647.8951	1647.7646	-0.1305	-79	73	87	SLAPEYEKAAQLLSK					Mascot
1671.8436	1671.7244	-0.1192	-71	329	343	EDQAPLILIQDSDSK					Mascot
1674.9901	1674.916	-0.0741	-44	81	96	AAQLLSKHDPAIVLAK					Mascot
1799.9386	1799.9027	-0.0359	-20	329	344	EDQAPLILIQDSDSKK					Mascot
1835.9037	1835.9165	0.0128	7	278	293	AMLFLNFSTGPFESFK					Mascot
1865.8678	1865.8667	-0.0011	-1	196	211	SDYDFGHTVHANHLPR					Mascot
1865.8678	1865.8667	-0.0011	-1	196	211	SDYDFGHTVHANHLPR	105	100			Mascot
1899.9963	1899.9719	-0.0244	-13	254	269	VVTFDKNPDNHPYLLK					Mascot
1899.9963	1899.9719	-0.0244	-13	254	269	VVTFDKNPDNHPYLLK	41	99.345			Mascot
2104.075	2104.0588	-0.0162	-8	310	328	FLIGDIEASQGAFQYFGLK					Mascot
2135.053	2135.0745	0.0215	10	194	211	LRSDYDFGHTVHANHLPR					Mascot

4	protein disulfide isomerase [Triticum aestivum]	gi|335906217	56649.8	4.96	21	306	100	16.811	191	100	

Protein Group

protein disulfide isomerase [Triticum aestivum]	gi|335906219	56618.9	5.0599 999427 7954

Peptide Information

Calc. Mass	Obsrv. Mass	± da	± ppm	Start Seq.	End Seq.	Sequence	Ion Score	C. I. %	Modification	Rank	Result Type
938.473	938.4479	-0.0251	-27	270	277	FFQSNAPK					Mascot
1023.4993	1023.4863	-0.013	-13	236	244	DFDVSALEK					Mascot
1150.5851	1150.5656	-0.0195	-17	124	133	NGGKNIQEYK					Mascot
1181.6201	1181.5886	-0.0315	-27	111	120	YEVQGFPTLK					Mascot
1182.6589	1182.6346	-0.0243	-21	212	222	GDAAVERPLVR					Mascot
1182.6589	1182.6346	-0.0243	-21	212	222	GDAAVERPLVR	46	99.774			Mascot
1210.6216	1210.5975	-0.0241	-20	260	269	NPDNHPYLLK					Mascot
1308.5955	1308.6018	0.0063	5	158	169	APEDATYLEDGK					Mascot
1320.6107	1320.578	-0.0327	-25	295	306	AYYGAVEEFSGK					Mascot
1358.674	1358.6945	0.0205	15	361	371	DYFDGKLTPFR					Mascot
1423.7064	1423.6896	-0.0168	-12	373	385	SEPIPEANNEPVK					Mascot
1440.7847	1440.7448	-0.0399	-28	386	398	VVVADNVHDVVFK					Mascot
1536.8308	1536.7975	-0.0333	-22	223	235	LFKPFDELVVDSK					Mascot
1647.8951	1647.7646	-0.1305	-79	73	87	SLAPEYEKAAQLLSK					Mascot
1647.8951	1647.7646	-0.1305	-79	73	87	SLAPEYEKAAQLLSK					Mascot
1671.8436	1671.7244	-0.1192	-71	329	343	EDQAPLILIQDSDSK					Mascot
1674.9901	1674.916	-0.0741	-44	81	96	AAQLLSKHDPAIVLAK					Mascot
1799.9386	1799.9027	-0.0359	-20	329	344	EDQAPLILIQDSDSKK					Mascot

Calc. Mass	Obsrv. Mass	± da	± ppm	Start Seq.	End Seq.	Sequence	Ion Score	C. I. %	Modification	Rank	Result Type
1835.9037	1835.9165	0.0128	7	278	293	AMLFLNFSTGPFESFK					Mascot
1865.8678	1865.8667	-0.0011	-1	196	211	SDYDFGHTVHANHLPR					Mascot
1865.8678	1865.8667	-0.0011	-1	196	211	SDYDFGHTVHANHLPR	105	100			Mascot
1899.9963	1899.9719	-0.0244	-13	254	269	VVTFDKNPDNHPYLLK					Mascot
1899.9963	1899.9719	-0.0244	-13	254	269	VVTFDKNPDNHPYLLK	41	99.345			Mascot
2104.075	2104.0588	-0.0162	-8	310	328	FLIGDIEASQGAFQYFGL K					Mascot
2135.053	2135.0745	0.0215	10	194	211	LRSDYDFGHTVHANHLP R					Mascot

5 protein disulfide isomerase [Triticum aestivum] gi|222446340 56921 4.96 21 306 100 16.41 191 100

Protein Group

protein disulfide isomerase 3 precursor [Triticum aestivum]	gi|13925728	56879	4.9600 000381 4697
protein disulfide isomerase precursor [Triticum aestivum]	gi|67508769	56879	4.9600 000381 4697
protein disulfide isomerase precursor [Triticum aestivum]	gi|67508775	56879	4.9600 000381 4697

Peptide Information

Calc. Mass	Obsrv. Mass	± da	± ppm	Start Seq.	End Seq.	Sequence	Ion Score	C. I. %	Modification	Rank	Result Type
1023.4993	1023.4863	-0.013	-13	236	244	DFDVSALEK					Mascot
1150.5851	1150.5656	-0.0195	-17	124	133	NGGKNIQEYK					Mascot
1181.6201	1181.5886	-0.0315	-27	111	120	YEVQGFPTLK					Mascot
1182.6589	1182.6346	-0.0243	-21	212	222	GDAAVERPLVR					Mascot
1182.6589	1182.6346	-0.0243	-21	212	222	GDAAVERPLVR	46	99.774			Mascot
1210.6216	1210.5975	-0.0241	-20	260	269	NPDNHPYLLK					Mascot
1278.694	1278.6486	-0.0454	-36	137	147	EAEGIVEYLKK					Mascot
1308.5955	1308.6018	0.0063	5	158	169	APEDATYLEDGK					Mascot
1358.674	1358.6945	0.0205	15	361	371	DYFDGKLTPFR					Mascot
1407.6427	1407.6097	-0.033	-23	294	306	SAYYGAVEEFSGK					Mascot
1423.7064	1423.6896	-0.0168	-12	373	385	SEPIPEANNEPVK					Mascot
1440.7847	1440.7448	-0.0399	-28	386	398	VVVADNVHDVVFK					Mascot
1536.8308	1536.7975	-0.0333	-22	223	235	LFKPFDELVVDSK					Mascot
1647.8951	1647.7646	-0.1305	-79	73	87	SLAPEYEKAAQLLSK					Mascot
1647.8951	1647.7646	-0.1305	-79	73	87	SLAPEYEKAAQLLSK					Mascot
1671.8436	1671.7244	-0.1192	-71	329	343	EDQAPLILIQDSDSK					Mascot
1674.9901	1674.916	-0.0741	-44	81	96	AAQLLSKHDPAIVLAK					Mascot

Calc. Mass	Obsrv. Mass	± da	± ppm	Start Seq.	End Seq.	Sequence	Ion Score	C. I. %	Modification	Rank	Result Type
1799.9386	1799.9027	-0.0359	-20	329	344	EDQAPLILIQDSDSKK					Mascot
1835.9037	1835.9165	0.0128	7	278	293	AMLFLNFSTGPFESFK					Mascot
1865.8678	1865.8667	-0.0011	-1	196	211	SDYDFGHTVHANHLPR					Mascot
1865.8678	1865.8667	-0.0011	-1	196	211	SDYDFGHTVHANHLPR	105	100			Mascot
1899.9963	1899.9719	-0.0244	-13	254	269	VVTFDKNPDNHPYLLK					Mascot
1899.9963	1899.9719	-0.0244	-13	254	269	VVTFDKNPDNHPYLLK	41	99.345			Mascot
2104.075	2104.0588	-0.0162	-8	310	328	FLIGDIEASQGAFQYFGL K					Mascot
2135.053	2135.0745	0.0215	10	194	211	LRSDYDFGHTVHANHLP R					Mascot

6	protein disulfide isomerase [Triticum durum]	gi|12056115	56772	4.99	21	306	100	16.495	191	100	

Protein Group

protein disulfide isomerase 1 proprotein [Triticum aestivum]	gi|13925723	56772	4.9899 997711 1816
protein disulfide isomerase [Triticum durum]	gi|12056119	56772	4.9899 997711 1816

Peptide Information

Calc. Mass	Obsrv. Mass	± da	± ppm	Start Seq.	End Seq.	Sequence	Ion Score	C. I. %	Modification	Rank	Result Type
1023.4993	1023.4863	-0.013	-13	236	244	DFDVSALEK					Mascot
1150.5851	1150.5656	-0.0195	-17	124	133	NGGKNIQEYK					Mascot
1181.6201	1181.5886	-0.0315	-27	111	120	YEVQGFPTLK					Mascot
1182.6589	1182.6346	-0.0243	-21	212	222	GDAAVERPLVR					Mascot
1182.6589	1182.6346	-0.0243	-21	212	222	GDAAVERPLVR	46	99.774			Mascot
1210.6216	1210.5975	-0.0241	-20	260	269	NPDNHPYLLK					Mascot
1218.5961	1218.5593	-0.0368	-30	489	501	ETAGQAAAAATEK					Mascot
1278.694	1278.6486	-0.0454	-36	137	147	EAEGIVEYLKK					Mascot
1308.5955	1308.6018	0.0063	5	158	169	APEDATYLEDGK					Mascot
1358.674	1358.6945	0.0205	15	361	371	DYFDGKLTPFR					Mascot
1407.6427	1407.6097	-0.033	-23	294	306	SAYYGAVEEFSGK					Mascot
1423.7064	1423.6896	-0.0168	-12	373	385	SEPIPEANNEPVK					Mascot
1536.8308	1536.7975	-0.0333	-22	223	235	LFKPFDELVVDSK					Mascot
1647.8951	1647.7646	-0.1305	-79	73	87	SLAPEYEKAAQLLSK					Mascot
1647.8951	1647.7646	-0.1305	-79	73	87	SLAPEYEKAAQLLSK					Mascot
1671.8436	1671.7244	-0.1192	-71	329	343	EDQAPLILIQDSDSK					Mascot
1674.9901	1674.916	-0.0741	-44	81	96	AAQLLSKHDPAIVLAK					Mascot
1799.9386	1799.9027	-0.0359	-20	329	344	EDQAPLILIQDSDSKK					Mascot
1835.9037	1835.9165	0.0128	7	278	293	AMLFLNFSTGPFESFK					Mascot

1865.8678	1865.8667	-0.0011	-1	196	211	SDYDFGHTVHANHLPR					Mascot
1865.8678	1865.8667	-0.0011	-1	196	211	SDYDFGHTVHANHLPR	105	100			Mascot
1899.9963	1899.9719	-0.0244	-13	254	269	VVTFDKNPDNHPYLLK					Mascot
1899.9963	1899.9719	-0.0244	-13	254	269	VVTFDKNPDNHPYLLK	41	99.345			Mascot
2104.075	2104.0588	-0.0162	-8	310	328	FLIGDIEASQGAFQYFGLK					Mascot
2135.053	2135.0745	0.0215	10	194	211	LRSDYDFGHTVHANHLPR					Mascot

7 Protein disulfide-isomerase [Triticum urartu] gi|474046595 56482 5.92 20 297 100 16.138 191 100

Peptide Information

Calc. Mass	Obsrv. Mass	± da	± ppm	Start Seq.	End Seq.	Sequence	Ion Score	C. I. %	Modification	Rank	Result Type
1023.4993	1023.4863	-0.013	-13	241	249	DFDVSALEK					Mascot
1150.5851	1150.5656	-0.0195	-17	129	138	NGGKNIQEYK					Mascot
1181.6201	1181.5886	-0.0315	-27	116	125	YEVQGFPTLK					Mascot
1182.6589	1182.6346	-0.0243	-21	217	227	GDAAVERPLVR					Mascot
1182.6589	1182.6346	-0.0243	-21	217	227	GDAAVERPLVR	46	99.774			Mascot
1210.6216	1210.5975	-0.0241	-20	265	274	NPDNHPYLLK					Mascot
1278.694	1278.6486	-0.0454	-36	142	152	EAEGIVEYLKK					Mascot
1308.5955	1308.6018	0.0063	5	163	174	APEDATYLEDGK					Mascot
1358.674	1358.6945	0.0205	15	366	376	DYFDGKLTPFR					Mascot
1407.6427	1407.6097	-0.033	-23	299	311	SAYYGAVEEFSGK					Mascot
1423.7064	1423.6896	-0.0168	-12	378	390	SEPIPEANNEPVK					Mascot
1536.8308	1536.7975	-0.0333	-22	228	240	LFKPFDELVVDSK					Mascot
1647.8951	1647.7646	-0.1305	-79	78	92	SLAPEYEKAAQLLSK					Mascot
1647.8951	1647.7646	-0.1305	-79	78	92	SLAPEYEKAAQLLSK					Mascot
1671.8436	1671.7244	-0.1192	-71	334	348	EDQAPLILIQDSDSK					Mascot
1674.9901	1674.916	-0.0741	-44	86	101	AAQLLSKHDPAIVLAK					Mascot
1799.9386	1799.9027	-0.0359	-20	334	349	EDQAPLILIQDSDSKK					Mascot
1835.9037	1835.9165	0.0128	7	283	298	AMLFLNFSTGPFESFK					Mascot
1865.8678	1865.8667	-0.0011	-1	201	216	SDYDFGHTVHANHLPR					Mascot
1865.8678	1865.8667	-0.0011	-1	201	216	SDYDFGHTVHANHLPR	105	100			Mascot
1899.9963	1899.9719	-0.0244	-13	259	274	VVTFDKNPDNHPYLLK					Mascot
1899.9963	1899.9719	-0.0244	-13	259	274	VVTFDKNPDNHPYLLK	41	99.345			Mascot
2104.075	2104.0588	-0.0162	-8	315	333	FLIGDIEASQGAFQYFGLK					Mascot
2135.053	2135.0745	0.0215	10	199	216	LRSDYDFGHTVHANHLPR					Mascot

8	protein disulfide isomerase [Triticum aestivum]	gi|222446344	56921	4.93	20	296	100	16.044	191	100	

Peptide Information

Calc. Mass	Obsrv. Mass	± da	± ppm	Start Seq.	End Seq.	Sequence	Ion Score	C. I. %	Modification	Rank	Result Type
1023.4993	1023.4863	-0.013	-13	236	244	DFDVSALEK					Mascot
1150.5851	1150.5656	-0.0195	-17	124	133	NGGKNIQEYK					Mascot
1181.6201	1181.5886	-0.0315	-27	111	120	YEVQGFPTLK					Mascot
1182.6589	1182.6346	-0.0243	-21	212	222	GDAAVERPLVR					Mascot
1182.6589	1182.6346	-0.0243	-21	212	222	GDAAVERPLVR	46	99.774			Mascot
1210.6216	1210.5975	-0.0241	-20	260	269	NPDNHPYLLK					Mascot
1278.694	1278.6486	-0.0454	-36	137	147	EAEGIVEYLKK					Mascot
1308.5955	1308.6018	0.0063	5	158	169	APEDATYLEDGK					Mascot
1320.6107	1320.578	-0.0327	-25	295	306	AYYGAVEEFSGK					Mascot
1358.674	1358.6945	0.0205	15	361	371	DYFDGKLTPFR					Mascot
1423.7064	1423.6896	-0.0168	-12	373	385	SEPIPEANNEPVK					Mascot
1440.7847	1440.7448	-0.0399	-28	386	398	VVVADNVHDVVFK					Mascot
1536.8308	1536.7975	-0.0333	-22	223	235	LFKPFDELVVDSK					Mascot
1647.8951	1647.7646	-0.1305	-79	73	87	SLAPEYEKAAQLLSK					Mascot
1647.8951	1647.7646	-0.1305	-79	73	87	SLAPEYEKAAQLLSK					Mascot
1671.8436	1671.7244	-0.1192	-71	329	343	EDQAPLILIQDSDSK					Mascot
1799.9386	1799.9027	-0.0359	-20	329	344	EDQAPLILIQDSDSKK					Mascot
1835.9037	1835.9165	0.0128	7	278	293	AMLFLNFSTGPFESFK					Mascot
1865.8678	1865.8667	-0.0011	-1	196	211	SDYDFGHTVHANHLPR					Mascot
1865.8678	1865.8667	-0.0011	-1	196	211	SDYDFGHTVHANHLPR	105	100			Mascot
1899.9963	1899.9719	-0.0244	-13	254	269	VVTFDKNPDNHPYLLK					Mascot
1899.9963	1899.9719	-0.0244	-13	254	269	VVTFDKNPDNHPYLLK	41	99.345			Mascot
2104.075	2104.0588	-0.0162	-8	310	328	FLIGDIEASQGAFQYFGL K					Mascot
2135.053	2135.0745	0.0215	10	194	211	LRSDYDFGHTVHANHLP R					Mascot

9	protein disulfide isomerase [Triticum aestivum]	gi|335906213	56496.9	5.06	20	296	100	16.36	191	100	

Peptide Information

Calc. Mass	Obsrv. Mass	± da	± ppm	Start Seq.	End Seq.	Sequence	Ion Score	C. I. %	Modification	Rank	Result Type
938.473	938.4479	-0.0251	-27	270	277	FFQSNAPK					Mascot
1023.4993	1023.4863	-0.013	-13	236	244	DFDVSALEK					Mascot
1150.5851	1150.5656	-0.0195	-17	124	133	NGGKNIQEYK					Mascot

Calc. Mass	Obsrv. Mass	± da	± ppm	Start Seq.	End Seq.	Sequence	Ion Score	C. I. %	Modification	Rank	Result Type
1181.6201	1181.5886	-0.0315	-27	111	120	YEVQGFPTLK					Mascot
1182.6589	1182.6346	-0.0243	-21	212	222	GDAAVERPLVR					Mascot
1182.6589	1182.6346	-0.0243	-21	212	222	GDAAVERPLVR	46	99.774			Mascot
1210.6216	1210.5975	-0.0241	-20	260	269	NPDNHPYLLK					Mascot
1308.5955	1308.6018	0.0063	5	158	169	APEDATYLEDGK					Mascot
1320.6107	1320.578	-0.0327	-25	295	306	AYYGAVEEFSGK					Mascot
1358.674	1358.6945	0.0205	15	361	371	DYFDGKLTPFR					Mascot
1423.7064	1423.6896	-0.0168	-12	373	385	SEPIPEANNEPVK					Mascot
1440.7847	1440.7448	-0.0399	-28	386	398	VVVADNVHDVVFK					Mascot
1536.8308	1536.7975	-0.0333	-22	223	235	LFKPFDELVVDSK					Mascot
1647.8951	1647.7646	-0.1305	-79	73	87	SLAPEYEKAAQLLSK					Mascot
1647.8951	1647.7646	-0.1305	-79	73	87	SLAPEYEKAAQLLSK					Mascot
1671.8436	1671.7244	-0.1192	-71	329	343	EDQAPLILIQDSDSK					Mascot
1674.9901	1674.916	-0.0741	-44	81	96	AAQLLSKHDPAIVLAK					Mascot
1799.9386	1799.9027	-0.0359	-20	329	344	EDQAPLILIQDSDSKK					Mascot
1835.9037	1835.9165	0.0128	7	278	293	AMLFLNFSTGPFESFK					Mascot
1865.8678	1865.8667	-0.0011	-1	196	211	SDYDFGHTVHANHLPR					Mascot
1865.8678	1865.8667	-0.0011	-1	196	211	SDYDFGHTVHANHLPR	105	100			Mascot
1899.9963	1899.9719	-0.0244	-13	254	269	VVTFDKNPDNHPYLLK					Mascot
1899.9963	1899.9719	-0.0244	-13	254	269	VVTFDKNPDNHPYLLK	41	99.345			Mascot
2135.053	2135.0745	0.0215	10	194	211	LRSDYDFGHTVHANHLPR					Mascot

10	protein disulfide isomerase [Triticum aestivum]	gi|508975	56726	4.99	20	296	100	16.044	191	100

Protein Group

RecName: Full=Protein disulfide-isomerase; Short=PDI; Flags: Precursor	gi|1709620	56726	4.9899997711816
protein disulfide isomerase	gi|1094851	56726	4.9899997711816

Peptide Information

Calc. Mass	Obsrv. Mass	± da	± ppm	Start Seq.	End Seq.	Sequence	Ion Score	C. I. %	Modification	Rank	Result Type
1023.4993	1023.4863	-0.013	-13	236	244	DFDVSALEK					Mascot
1150.599	1150.5656	-0.0334	-29	137	146	EAEGIVEYLK					Mascot
1181.6201	1181.5886	-0.0315	-27	111	120	YEVQGFPTLK					Mascot
1182.6589	1182.6346	-0.0243	-21	212	222	GDAAVERPLVR					Mascot
1182.6589	1182.6346	-0.0243	-21	212	222	GDAAVERPLVR	46	99.774			Mascot

1210.6216	1210.5975	-0.0241	-20	260	269	NPDNHPYLLK			Mascot
1218.5961	1218.5593	-0.0368	-30	489	501	ETAGQAAAAATEK			Mascot
1278.694	1278.6486	-0.0454	-36	137	147	EAEGIVEYLKK			Mascot
1308.5955	1308.6018	0.0063	5	158	169	APEDATYLEDGK			Mascot
1358.674	1358.6945	0.0205	15	361	371	DYFDGKLTPFR			Mascot
1407.6427	1407.6097	-0.033	-23	294	306	SAYYGAVEEFSGK			Mascot
1423.7064	1423.6896	-0.0168	-12	373	385	SEPIPEANNEPVK			Mascot
1536.8308	1536.7975	-0.0333	-22	223	235	LFKPFDELVVDSK			Mascot
1647.8951	1647.7646	-0.1305	-79	73	87	SLAPEYEKAAQLLSK			Mascot
1647.8951	1647.7646	-0.1305	-79	73	87	SLAPEYEKAAQLLSK			Mascot
1671.8436	1671.7244	-0.1192	-71	329	343	EDQAPLILIQDSDSK			Mascot
1674.9901	1674.916	-0.0741	-44	81	96	AAQLLSKHDPAIVLAK			Mascot
1799.9386	1799.9027	-0.0359	-20	329	344	EDQAPLILIQDSDSKK			Mascot
1835.9037	1835.9165	0.0128	7	278	293	AMLFLNFSTGPFESFK			Mascot
1865.8678	1865.8667	-0.0011	-1	196	211	SDYDFGHTVHANHLPR			Mascot
1865.8678	1865.8667	-0.0011	-1	196	211	SDYDFGHTVHANHLPR	105	100	Mascot
1899.9963	1899.9719	-0.0244	-13	254	269	VVTFDKNPDNHPYLLK			Mascot
1899.9963	1899.9719	-0.0244	-13	254	269	VVTFDKNPDNHPYLLK	41	99.324	Mascot
2135.053	2135.0745	0.0215	10	194	211	LRSDYDFGHTVHANHLP R			Mascot

Gel Idx/Pos	252/K4	Instr./Gel Origin	BA2151/Sample Project 20140814	Process Status	Analysis Succeeded
Plate [#] Name	[1] Sample Project 20140814	Instrument Sample Name		Spectra	11

Rank	Protein Name	Accession No.	Protein MW	Protein PI	Pep. Count	Protein Score	Protein Score C. I. %	Intensity Matched	Total Ion Score	Total Ion C. I. %	Confirmed
1	Beta-amylase [Triticum urartu]	gi|474451266	58995	5.34	13	166	100	20.227	116	100	

Peptide Information

Calc. Mass	Obsrv. Mass	± da	± ppm	Start Seq.	End Seq.	Sequence	Ion Score	C. I. %	Modification	Rank	Result Type
802.4305	802.4324	0.0019	2	276	282	ILDEANK					Mascot
947.5057	947.4904	-0.0153	-16	322	329	DGYRPIAR					Mascot
947.5057	947.4904	-0.0153	-16	322	329	DGYRPIAR	23	60.739			Mascot
1016.5564	1016.5534	-0.003	-3	412	419	LFGFTYLR					Mascot
1016.5564	1016.5534	-0.003	-3	412	419	LFGFTYLR	48	99.865			Mascot
1326.6688	1326.6642	-0.0046	-3	385	395	YDPTAYNTILR					Mascot
1326.6688	1326.6642	-0.0046	-3	385	395	YDPTAYNTILR	45	99.733			Mascot
1335.7202	1335.677	-0.0432	-32	322	332	DGYRPIARMLK			Oxidation (M)[9]		Mascot
1474.6777	1474.6841	0.0064	4	372	384	EGLNMACENALPR			Carbamidomethyl (C)[7]		Mascot
1490.6726	1490.6832	0.0106	7	372	384	EGLNMACENALPR			Carbamidomethyl (C)[7], Oxidation (M)[5]		Mascot
1646.781	1646.7871	0.0061	4	246	259	FFVDNGTYLTEQGR					Mascot
1668.7952	1668.7889	-0.0063	-4	218	232	AAAAMVGHPEWEFPR					Mascot
1684.79	1684.7834	-0.0066	-4	218	232	AAAAMVGHPEWEFPR			Oxidation (M)[5]		Mascot
1701.7247	1701.8429	0.1182	69	148	161	SAVQMYTDYMASFR			Oxidation (M)[5,10]		Mascot
1752.8916	1752.866	-0.0256	-15	420	434	LSNQLVEGQNYVNFK					Mascot
1841.9678	1841.9316	-0.0362	-20	459	475	SGPELTIEMILQAAQPK			Oxidation (M)[9]		Mascot
2087.0557	2087.0625	0.0068	3	130	147	NIEYLTLGVDDQPLFHGR					Mascot
2183.0486	2183.0332	-0.0154	-7	440	458	MHANLPHDPCVDPVAPL QR			Carbamidomethyl (C)[10], Oxidation (M)[1]		Mascot

Rank	Protein Name	Accession No.	Protein MW	Protein PI	Pep. Count	Protein Score	Protein Score C. I. %	Intensity Matched	Total Ion Score	Total Ion C. I. %	Confirmed
2	beta amylase [Triticum aestivum]	gi|32400764	31099.9	8.6	8	98	100	8.419	65	99.998	

Peptide Information

Calc. Mass	Obsrv. Mass	± da	± ppm	Start Seq.	End Seq.	Sequence	Ion Score	C. I. %	Modification	Rank	Result Type
802.4305	802.4324	0.0019	2	91	97	ILDEANK					Mascot
947.5057	947.4904	-0.0153	-16	137	144	DGYRPIAR					Mascot
947.5057	947.4904	-0.0153	-16	137	144	DGYRPIAR	23	60.739			Mascot
1326.6688	1326.6642	-0.0046	-3	200	210	YDPTAYNTILR					Mascot

Calc. Mass	Obsrv. Mass	± da	± ppm	Start Seq.	End Seq.	Sequence	Ion Score	C. I. %	Modification	Rank	Result Type
1326.6688	1326.6642	-0.0046	-3	200	210	YDPTAYNTILR	45	99.733			Mascot
1335.7202	1335.677	-0.0432	-32	137	147	DGYRPIARMLK			Oxidation (M)[9]		Mascot
1474.6777	1474.6841	0.0064	4	187	199	EGLNMACENALPR			Carbamidomethyl (C)[7]		Mascot
1490.6726	1490.6832	0.0106	7	187	199	EGLNMACENALPR			Carbamidomethyl (C)[7], Oxidation (M)[5]		Mascot
1646.781	1646.7871	0.0061	4	61	74	FFVDNGTYLTEQGR					Mascot
1668.7952	1668.7889	-0.0063	-4	33	47	AAAAMVGHPEWEFPR					Mascot
1684.79	1684.7834	-0.0066	-4	33	47	AAAAMVGHPEWEFPR			Oxidation (M)[5]		Mascot
1842.0425	1841.9316	-0.1109	-60	104	118	VQLAIKISGIHWWYK					Mascot

3 beta-amylase [Triticum aestivum] gi|1771782 56860.2 5.24 9 69 99.492 12.111 48 99.865

Protein Group

RecName: Full=Beta-amylase; AltName: Full=1,4-alpha-D-glucan maltohydrolase gi|3334120 56860.2 5.2399997111816

Peptide Information

Calc. Mass	Obsrv. Mass	± da	± ppm	Start Seq.	End Seq.	Sequence	Ion Score	C. I. %	Modification	Rank	Result Type
802.4305	802.4324	0.0019	2	275	281	ILDEANK					Mascot
1016.5564	1016.5534	-0.003	-3	411	418	LFGFTYLR					Mascot
1016.5564	1016.5534	-0.003	-3	411	418	LFGFTYLR	48	99.865			Mascot
1285.6212	1285.6085	-0.0127	-10	61	71	GPKAYDWSAYK					Mascot
1297.6787	1297.6343	-0.0444	-34	210	221	YLEADFKAAAAK					Mascot
1425.6903	1425.6925	0.0022	2	371	383	EGLHVACENALGR			Carbamidomethyl (C)[7]		Mascot
1487.7893	1487.7458	-0.0435	-29	470	482	AAQPKLEPFPFDK					Mascot
1607.6512	1607.7291	0.0779	48	333	345	HHASMNFTCAEMR			Carbamidomethyl (C)[9], Oxidation (M)[5]		Mascot
1623.6461	1623.769	0.1229	76	333	345	HHASMNFTCAEMR			Carbamidomethyl (C)[9], Oxidation (M)[5,12]		Mascot
1691.8275	1691.8262	-0.0013	-1	243	256	TQFFKDNGTYLTEK					Mascot
2087.0557	2087.0625	0.0068	3	129	146	NIEYLTLGVDDQPLFHGR					Mascot

4 Putative disease resistance protein RGA4 [Triticum urartu] gi|473781055 115939 5.83 21 63 98.025 6.899

Peptide Information

Calc. Mass	Obsrv. Mass	± da	± ppm	Start Seq.	End Seq.	Sequence	Ion Score	C. I. %	Modification	Rank	Result Type
800.4083	800.3776	-0.0307	-38	490	495	MHDLLR			Oxidation (M)[1]		Mascot
815.5098	815.4643	-0.0455	-56	524	530	RLSVVNK					Mascot
846.4567	846.4782	0.0215	25	266	273	LAETVEGK					Mascot
847.4883	847.4324	-0.0559	-66	372	380	VTASALASK					Mascot
930.5077	930.4921	-0.0156	-17	894	901	LKVEGCPK			Carbamidomethyl (C)[6]		Mascot

1193.6525	1193.614	-0.0385	-32	89	99	LLPDHPLSSSK					Mascot
1256.6846	1256.6787	-0.0059	-5	162	173	SSNLVEPNLVGK					Mascot
1326.7198	1326.6642	-0.0556	-42	896	907	VEGCPKLSALPR			Carbamidomethyl (C)[4]		Mascot
1326.7198	1326.6642	-0.0556	-42	896	907	VEGCPKLSALPR			Carbamidomethyl (C)[4]		Mascot
1384.7189	1384.6818	-0.0371	-27	490	500	MHDLLRQLACK			Carbamidomethyl (C)[10]		Mascot
1425.6315	1425.6925	0.061	43	504	515	DECFIGDAETLR			Carbamidomethyl (C)[3]		Mascot
1543.6992	1543.7452	0.046	30	105	118	GLSVSSCFCNIGSR			Carbamidomethyl (C)[7,9]		Mascot
1547.8104	1547.7762	-0.0342	-22	826	839	VGEPISNEFIAFPK					Mascot
1607.8131	1607.7291	-0.084	-52	1	15	MATILESLLGSCANK			Carbamidomethyl (C)[12]		Mascot
1620.8812	1620.8102	-0.071	-44	534	547	LVLTSMDNMVVKVR			Oxidation (M)[6]		Mascot
1623.8081	1623.769	-0.0391	-24	1	15	MATILESLLGSCANK			Carbamidomethyl (C)[12], Oxidation (M)[1]		Mascot
1668.8083	1668.7889	-0.0194	-12	41	53	VELIRCCIYDAEK			Carbamidomethyl (C)[6,7]		Mascot
1698.9385	1698.8132	-0.1253	-74	902	917	LSALPRQLGEDTASLK					Mascot
1729.912	1729.8627	-0.0493	-28	89	104	LLPDHPLSSSKSASYK					Mascot
1890.9556	1890.9586	0.003	2	372	388	VTASALASKDLTENEWR					Mascot
1908.9927	1908.8231	-0.1696	-89	702	717	LERTAHFHTNVVLEDK					Mascot
2087.1497	2087.0625	-0.0872	-42	594	613	LLDLGHTGISHLPESIGSL K					Mascot
2408.312	2408.2168	-0.0952	-40	787	807	NLQSCVDLPPVGQLPNL KFLR			Carbamidomethyl (C)[5]		Mascot

5 beta-amylase, partial [Triticum aestivum] gi|451798942 15408.7 5.21 3 62 97.21 11.111 48 99.865

Peptide Information

Calc. Mass	Obsrv. Mass	± da	± ppm	Start Seq.	End Seq.	Sequence	Ion Score	C. I. %	Modification	Rank	Result Type
1016.5564	1016.5534	-0.003	-3	29	36	LFGFTYLR					Mascot
1016.5564	1016.5534	-0.003	-3	29	36	LFGFTYLR	48	99.865			Mascot
1841.9678	1841.9316	-0.0362	-20	76	92	SGPELTIEMILQAAQPK			Oxidation (M)[9]		Mascot
2183.0486	2183.0332	-0.0154	-7	57	75	MHANLPHDPCVDPVAPL QR			Carbamidomethyl (C)[10], Oxidation (M)[1]		Mascot

6 hypothetical protein TRIUR3_29779 [Triticum urartu] gi|474262217 35184.4 9.62 13 60 95.369 5.145

Peptide Information

Calc. Mass	Obsrv. Mass	± da	± ppm	Start Seq.	End Seq.	Sequence	Ion Score	C. I. %	Modification	Rank	Result Type
802.4529	802.4324	-0.0205	-26	131	136	ELQRTR					Mascot
1060.582	1060.5542	-0.0278	-26	69	77	DKLVAEVMR					Mascot
1141.5419	1141.5322	-0.0097	-8	83	92	THQNSGCPIK			Carbamidomethyl (C)[7]		Mascot
1197.6951	1197.6552	-0.0399	-33	228	237	QALELLVQQR					Mascot

1297.6431	1297.6343	-0.0088	-7	83	93	THQNSGCPIKR			Carbamidomethyl (C)[7]		Mascot
1390.7954	1390.6838	-0.1116	-80	53	63	KVFDVFIRPNR					Mascot
1507.8115	1507.7383	-0.0732	-49	94	105	EELTQIITKNYR					Mascot
1507.8115	1507.7383	-0.0732	-49	94	105	EELTQIITKNYR					Mascot
1541.7972	1541.7408	-0.0564	-37	18	30	SWIAWRPATNSPR					Mascot
1589.8507	1589.6962	-0.1545	-97	2	16	AAVASLPPATRNHER					Mascot
1684.8475	1684.7834	-0.0641	-38	121	134	LAATFGYEMRELQR					Mascot
1700.8425	1700.8146	-0.0279	-16	121	134	LAATFGYEMRELQR			Oxidation (M)[9]		Mascot
1701.7538	1701.8429	0.0891	52	37	52	YATPCSSPFPSSSSAR			Carbamidomethyl (C)[5]		Mascot
1716.801	1716.7914	-0.0096	-6	116	130	ETGDRLAATFGYEMR					Mascot
1732.7959	1732.8326	0.0367	21	116	130	ETGDRLAATFGYEMR			Oxidation (M)[14]		Mascot
2119.0122	2119.135	0.1228	58	137	156	STSTRSGRPSSQQPSNQ DAK					Mascot

7 hypothetical protein TRIUR3_27843 [Triticum urartu] gi|474375451 9920.8 6.29 8 59 94.17 4.514

Peptide Information

Calc. Mass	Obsrv. Mass	± da	± ppm	Start Seq.	End Seq.	Sequence	Ion Score	C. I. %	Modification	Rank	Result Type
832.402	832.3227	-0.0793	-95	50	57	GSDGQGRR					Mascot
962.5265	962.4447	-0.0818	-85	30	38	LEATTSGRK					Mascot
1032.5182	1032.5344	0.0162	16	39	49	TGDAAAGTGRR					Mascot
1235.6161	1235.6245	0.0084	7	26	37	GMGRLEATTSGR					Mascot
1251.6111	1251.642	0.0309	25	26	37	GMGRLEATTSGR			Oxidation (M)[2]		Mascot
1544.7412	1544.7347	-0.0065	-4	2	14	QEAGSERDLQQQR					Mascot
1652.8351	1652.8069	-0.0282	-17	79	96	GGDQEGLLGRLGQAAG GP					Mascot
1675.7816	1675.7504	-0.0312	-19	1	14	MQEAGSERDLQQQR					Mascot
1691.7766	1691.8262	0.0496	29	1	14	MQEAGSERDLQQQR			Oxidation (M)[1]		Mascot
2118.9534	2119.135	0.1816	86	68	88	SGEEASEQGDKGGDQE GLLGR					Mascot

8 hypothetical protein TRIUR3_26886 [Triticum urartu] gi|474169710 18643.6 9.74 8 53 78.341 5.769

Peptide Information

Calc. Mass	Obsrv. Mass	± da	± ppm	Start Seq.	End Seq.	Sequence	Ion Score	C. I. %	Modification	Rank	Result Type
1256.6416	1256.6787	0.0371	30	134	144	LIPTHSMSTNR					Mascot
1308.546	1308.6633	0.1173	90	76	86	MGCWPGGERDK			Carbamidomethyl (C)[3], Oxidation (M)[1]		Mascot
1384.7366	1384.6818	-0.0548	-40	133	144	KLIPTHSMSTNR					Mascot
1838.9219	1838.9227	0.0008	0	99	115	ATCPAWLEISGPGPNLR			Carbamidomethyl (C)[3]		Mascot

1909.0001	1908.8231	-0.177	-93	154	169	IWSYLNRSCVGIVVDK			Carbamidomethyl (C)[9]		Mascot
1999.9767	1999.9746	-0.0021	-1	51	70	ANRPASQGAAAEGACVV WGK			Carbamidomethyl (C)[15]		Mascot
1999.9767	1999.9746	-0.0021	-1	51	70	ANRPASQGAAAEGACVV WGK			Carbamidomethyl (C)[15]		Mascot
2183.1489	2183.0332	-0.1157	-53	134	153	LIPTHSMSTNRLALSTDT PK					Mascot
2369.1892	2369.03	-0.1592	-67	47	70	AAARANRPASQGAAAEG ACVVWGK			Carbamidomethyl (C)[19]		Mascot

9 hypothetical protein TRIUR3_00606 [Triticum urartu] gi|473868751 34805.2 4.64 11 52 72.733 5.531

Peptide Information

Calc. Mass	Obsrv. Mass	± da	± ppm	Start Seq.	End Seq.	Sequence	Ion Score	C. I. %	Modification	Rank	Result Type
962.4975	962.4447	-0.0528	-55	56	63	EDSLMLVR					Mascot
963.5146	963.5081	-0.0065	-7	287	295	LDFSSPLGK					Mascot
1118.6099	1118.5227	-0.0872	-78	164	174	ARSLAVSMAGR					Mascot
1370.8002	1370.6747	-0.1255	-92	225	236	LQLEALLAEKSR					Mascot
1487.8112	1487.7458	-0.0654	-44	166	180	SLAVSMAGRAANLAR					Mascot
1629.7827	1629.8932	0.1105	68	126	141	VVNESSDNGAADAKPR					Mascot
1647.7821	1647.778	-0.0041	-2	2	18	DSAAASSLVVSPASDDR					Mascot
1647.7821	1647.778	-0.0041	-2	2	18	DSAAASSLVVSPASDDR					Mascot
1675.781	1675.7504	-0.0306	-18	296	312	FDGEGELDGVPATPGSK					Mascot
1698.8115	1698.8132	0.0017	1	111	125	RCTASTEFASLEAQK			Carbamidomethyl (C)[2]		Mascot
1773.9388	1773.8314	-0.1074	-61	36	53	LVASAASTCAAASGRPK R			Carbamidomethyl (C)[9]		Mascot
2620.2778	2620.2808	0.003	1	287	312	LDFSSPLGKFDGEGELD GVPATPGSK					Mascot

10 hypothetical protein TRIUR3_11352 [Triticum urartu] gi|474189085 15304.3 6.29 9 51 68.694 2.409

Peptide Information

Calc. Mass	Obsrv. Mass	± da	± ppm	Start Seq.	End Seq.	Sequence	Ion Score	C. I. %	Modification	Rank	Result Type
846.4315	846.4782	0.0467	55	22	29	VEAETAAR					Mascot
962.4975	962.4447	-0.0528	-55	107	114	ECLDKLGK			Carbamidomethyl (C)[2]		Mascot
1139.5917	1139.538	-0.0537	-47	20	29	HRVEAETAAR					Mascot
1237.5618	1237.5525	-0.0093	-8	128	138	LEDTDVSVSIC			Carbamidomethyl (C)[11]		Mascot
1259.6313	1259.6554	0.0241	19	7	18	FAVGQALGGHMR			Oxidation (M)[11]		Mascot
1323.6184	1323.6493	0.0309	23	118	127	YLQLDPCCVR			Carbamidomethyl (C)[7,8]		Mascot
1379.739	1379.7136	-0.0254	-18	67	81	NVAGLGAATGQGVHK					Mascot
1399.7375	1399.6593	-0.0782	-56	7	19	FAVGQALGGHMRR					Mascot
1607.7782	1607.7291	-0.0491	-31	116	127	QRYLQLDPCCVR			Carbamidomethyl (C)[9,10]		Mascot

Gel Idx/Pos	253/K5	Instr./Gel Origin	BA2151/Sample Project 20140814	Process Status	Analysis Succeeded
Plate [#] Name	[1] Sample Project 20140814	Instrument Sample Name		Spectra	11

Rank	Protein Name	Accession No.	Protein MW	Protein PI	Pep. Count	Protein Score	Protein Score C. I. %	Intensity Matched	Total Ion Score	Total Ion C. I. %	Confirmed
1	Tubulin alpha chain [Triticum urartu]	gi|474224323	50367.6	4.89	10	350	100	15.351	313	100	

Protein Group

RecName: Full=Tubulin alpha chain	gi|8928408	50395.6	4.8899998664856
Tubulin alpha-3 chain [Triticum urartu]	gi|473887549	50381.6	4.8899998664856
alpha tubulin-2A [Triticum aestivum]	gi|90289596	50367.6	4.8899998664856
alpha tubulin-2B [Triticum aestivum]	gi|90289600	50367.6	4.8899998664856
alpha tubulin-2D [Triticum aestivum]	gi|90289598	50367.6	4.8899998664856
alpha tubulin-3A [Triticum aestivum]	gi|90289604	50381.6	4.8899998664856
alpha tubulin-3B [Triticum aestivum]	gi|90289602	50381.6	4.8899998664856
alpha tubulin-3D [Triticum aestivum]	gi|90289606	50381.6	4.8899998664856
alpha-tubulin [Triticum aestivum]	gi|4098272	50395.6	4.8899998664856

Peptide Information

Calc. Mass	Obsrv. Mass	± da	± ppm	Start Seq.	End Seq.	Sequence	Ion Score	C. I. %	Modification	Rank	Result Type
1007.454	1007.4495	-0.0045	-4	97	105	EDAANNFAR					Mascot
1132.5668	1132.5371	-0.0297	-26	113	121	EIVDLCLDR			Carbamidomethyl (C)[6]		Mascot
1396.693	1396.6779	-0.0151	-11	391	401	IDHKFDLMYAK			Oxidation (M)[8]		Mascot
1473.8635	1473.8313	-0.0322	-22	230	243	LVSQVISSLTASLR					Mascot
1473.8635	1473.8313	-0.0322	-22	230	243	LVSQVISSLTASLR	71	100			Mascot
1589.6844	1589.6282	-0.0562	-35	309	320	HGKYMACCLMYR			Carbamidomethyl (C)[7,8]		Mascot
1691.8711	1691.8196	-0.0515	-30	216	229	SLDIERPTYTNLNR					Mascot
1691.8711	1691.8196	-0.0515	-30	216	229	SLDIERPTYTNLNR	76	100			Mascot

1701.9059	1701.8625	-0.0434	-26	65	79	AVFVDLEPTVIDEVR					Mascot
1701.9059	1701.8625	-0.0434	-26	65	79	AVFVDLEPTVIDEVR	76	100			Mascot
1808.9252	1808.8646	-0.0606	-34	265	280	IHFMLSSYAPVISAEK			Oxidation (M)[4]		Mascot
2385.1946	2385.1514	-0.0432	-18	85	105	QLFHPEQLISGKEDAANN FAR					Mascot
2408.1882	2408.1445	-0.0437	-18	244	264	FDGALNVDVNEFQTNLV PYPR					Mascot
2408.1882	2408.1445	-0.0437	-18	244	264	FDGALNVDVNEFQTNLV PYPR	90	100			Mascot

2	protein disulfide isomerase [Triticum aestivum]	gi|222446340	56921	4.96	20	308	100	31.36	203	100	

Protein Group

protein disulfide isomerase 3 precursor [Triticum aestivum]	gi|13925728	56879	4.9600 000381 4697
protein disulfide isomerase precursor [Triticum aestivum]	gi|67508769	56879	4.9600 000381 4697
protein disulfide isomerase precursor [Triticum aestivum]	gi|67508775	56879	4.9600 000381 4697

Peptide Information

Calc. Mass	Obsrv. Mass	± da	± ppm	Start Seq.	End Seq.	Sequence	Ion Score	C. I. %	Modification	Rank	Result Type
952.4887	952.4437	-0.045	-47	270	277	FFQTNAPK					Mascot
1023.4993	1023.471	-0.0283	-28	236	244	DFDVSALEK					Mascot
1104.5797	1104.5514	-0.0283	-26	128	136	NIQEYKGPR					Mascot
1150.5851	1150.5203	-0.0648	-56	124	133	NGGKNIQEYK					Mascot
1181.6201	1181.5607	-0.0594	-50	111	120	YEVQGFPTLK					Mascot
1182.6589	1182.6149	-0.044	-37	212	222	GDAAVERPLVR					Mascot
1182.6589	1182.6149	-0.044	-37	212	222	GDAAVERPLVR	31	94.005			Mascot
1210.6216	1210.5952	-0.0264	-22	260	269	NPDNHPYLLK					Mascot
1278.694	1278.6205	-0.0735	-57	137	147	EAEGIVEYLKK					Mascot
1407.6427	1407.5763	-0.0664	-47	294	306	SAYYGAVEEFSGK					Mascot
1440.7847	1440.7064	-0.0783	-54	386	398	VVVADNVHDVVFK					Mascot
1454.7373	1454.7169	-0.0204	-14	502	515	AAEPAATEPLKDEL					Mascot
1536.8308	1536.7449	-0.0859	-56	223	235	LFKPFDELVVDSK					Mascot
1647.8951	1647.7367	-0.1584	-96	73	87	SLAPEYEKAAQLLSK					Mascot
1647.8951	1647.7367	-0.1584	-96	73	87	SLAPEYEKAAQLLSK					Mascot
1654.8688	1654.7891	-0.0797	-48	245	259	FIDASSTPKVVTFDK					Mascot
1671.8436	1671.6882	-0.1554	-93	329	343	EDQAPLILIQDSDSK					Mascot
1674.9901	1674.8242	-0.1659	-99	81	96	AAQLLSKHDPAIVLAK					Mascot

1799.9386	1799.8619	-0.0767	-43	329	344	EDQAPLILIQDSDSKK					Mascot
1865.8678	1865.8357	-0.0321	-17	196	211	SDYDFGHTVHANHLPR					Mascot
1865.8678	1865.8357	-0.0321	-17	196	211	SDYDFGHTVHANHLPR	115	100			Mascot
1899.9963	1899.9279	-0.0684	-36	254	269	VVTFDKNPDNHPYLLK					Mascot
1899.9963	1899.9279	-0.0684	-36	254	269	VVTFDKNPDNHPYLLK	57	99.984			Mascot
2135.053	2135.0029	-0.0501	-23	194	211	LRSDYDFGHTVHANHLPR					Mascot

3 Protein disulfide-isomerase [Triticum urartu] gi|474046595 56482 5.92 19 300 100 30.92 203 100

Peptide Information

Calc. Mass	Obsrv. Mass	± da	± ppm	Start Seq.	End Seq.	Sequence	Ion Score	C. I. %	Modification	Rank	Result Type
1023.4993	1023.471	-0.0283	-28	241	249	DFDVSALEK					Mascot
1104.5797	1104.5514	-0.0283	-26	133	141	NIQEYKGPR					Mascot
1150.5851	1150.5203	-0.0648	-56	129	138	NGGKNIQEYK					Mascot
1181.6201	1181.5607	-0.0594	-50	116	125	YEVQGFPTLK					Mascot
1182.6589	1182.6149	-0.044	-37	217	227	GDAAVERPLVR					Mascot
1182.6589	1182.6149	-0.044	-37	217	227	GDAAVERPLVR	31	94.005			Mascot
1210.6216	1210.5952	-0.0264	-22	265	274	NPDNHPYLLK					Mascot
1278.694	1278.6205	-0.0735	-57	142	152	EAEGIVEYLKK					Mascot
1407.6427	1407.5763	-0.0664	-47	299	311	SAYYGAVEEFSGK					Mascot
1454.8003	1454.7169	-0.0834	-57	391	403	VVVADNIHDVVFK					Mascot
1480.714	1480.7242	0.0102	7	51	65	GRFGGVSDASVTGDR					Mascot
1536.8308	1536.7449	-0.0859	-56	228	240	LFKPFDELVVDSK					Mascot
1647.8951	1647.7367	-0.1584	-96	78	92	SLAPEYEKAAQLLSK					Mascot
1647.8951	1647.7367	-0.1584	-96	78	92	SLAPEYEKAAQLLSK					Mascot
1654.8688	1654.7891	-0.0797	-48	250	264	FIDASSTPKVVTFDK					Mascot
1671.8436	1671.6882	-0.1554	-93	334	348	EDQAPLILIQDSDSK					Mascot
1674.9901	1674.8242	-0.1659	-99	86	101	AAQLLSKHDPAIVLAK					Mascot
1799.9386	1799.8619	-0.0767	-43	334	349	EDQAPLILIQDSDSKK					Mascot
1865.8678	1865.8357	-0.0321	-17	201	216	SDYDFGHTVHANHLPR					Mascot
1865.8678	1865.8357	-0.0321	-17	201	216	SDYDFGHTVHANHLPR	115	100			Mascot
1899.9963	1899.9279	-0.0684	-36	259	274	VVTFDKNPDNHPYLLK					Mascot
1899.9963	1899.9279	-0.0684	-36	259	274	VVTFDKNPDNHPYLLK	57	99.984			Mascot
2135.053	2135.0029	-0.0501	-23	199	216	LRSDYDFGHTVHANHLPR					Mascot

4 protein disulfide isomerase [Triticum aestivum] gi|222446344 56921 4.93 19 299 100 29.64 203 100

Peptide Information

Calc. Mass	Obsrv. Mass	± da	± ppm	Start Seq.	End Seq.	Sequence	Ion Score	C. I. %	Modification	Rank	Result Type
952.4887	952.4437	-0.045	-47	270	277	FFQTNAPK					Mascot
1023.4993	1023.471	-0.0283	-28	236	244	DFDVSALEK					Mascot
1104.5797	1104.5514	-0.0283	-26	128	136	NIQEYKGPR					Mascot
1150.5851	1150.5203	-0.0648	-56	124	133	NGGKNIQEYK					Mascot
1181.6201	1181.5607	-0.0594	-50	111	120	YEVQGFPTLK					Mascot
1182.6589	1182.6149	-0.044	-37	212	222	GDAAVERPLVR					Mascot
1182.6589	1182.6149	-0.044	-37	212	222	GDAAVERPLVR	31	94.005			Mascot
1210.6216	1210.5952	-0.0264	-22	260	269	NPDNHPYLLK					Mascot
1278.694	1278.6205	-0.0735	-57	137	147	EAEGIVEYLKK					Mascot
1320.6107	1320.5748	-0.0359	-27	295	306	AYYGAVEEFSGK					Mascot
1440.7847	1440.7064	-0.0783	-54	386	398	VVVADNVHDVVFK					Mascot
1454.7373	1454.7169	-0.0204	-14	502	515	AAEPAATEPLKDEL					Mascot
1536.8308	1536.7449	-0.0859	-56	223	235	LFKPFDELVVDSK					Mascot
1647.8951	1647.7367	-0.1584	-96	73	87	SLAPEYEKAAQLLSK					Mascot
1647.8951	1647.7367	-0.1584	-96	73	87	SLAPEYEKAAQLLSK					Mascot
1654.8688	1654.7891	-0.0797	-48	245	259	FIDASSTPKVVTFDK					Mascot
1671.8436	1671.6882	-0.1554	-93	329	343	EDQAPLILIQDSDSK					Mascot
1799.9386	1799.8619	-0.0767	-43	329	344	EDQAPLILIQDSDSKK					Mascot
1865.8678	1865.8357	-0.0321	-17	196	211	SDYDFGHTVHANHLPR					Mascot
1865.8678	1865.8357	-0.0321	-17	196	211	SDYDFGHTVHANHLPR	115	100			Mascot
1899.9963	1899.9279	-0.0684	-36	254	269	VVTFDKNPDNHPYLLK					Mascot
1899.9963	1899.9279	-0.0684	-36	254	269	VVTFDKNPDNHPYLLK	57	99.984			Mascot
2135.053	2135.0029	-0.0501	-23	194	211	LRSDYDFGHTVHANHLPR					Mascot

5 protein disulfide isomerase [Triticum durum] gi|12056121 41818.6 5.29 16 292 100 28.722 203 100

Protein Group

protein disulfide isomerase [Triticum durum] gi|12056117 41818.6 5.28999996185303

Peptide Information

Calc. Mass	Obsrv. Mass	± da	± ppm	Start Seq.	End Seq.	Sequence	Ion Score	C. I. %	Modification	Rank	Result Type
1023.4993	1023.471	-0.0283	-28	236	244	DFDVSALEK					Mascot
1104.5797	1104.5514	-0.0283	-26	128	136	NIQEYKGPR					Mascot
1150.5851	1150.5203	-0.0648	-56	124	133	NGGKNIQEYK					Mascot

1181.6201	1181.5607	-0.0594	-50	111	120	YEVQGFPTLK					Mascot
1182.6589	1182.6149	-0.044	-37	212	222	GDAAVERPLVR					Mascot
1182.6589	1182.6149	-0.044	-37	212	222	GDAAVERPLVR	31	94.005			Mascot
1210.6216	1210.5952	-0.0264	-22	260	269	NPDNHPYLLK					Mascot
1278.694	1278.6205	-0.0735	-57	137	147	EAEGIVEYLKK					Mascot
1320.6107	1320.5748	-0.0359	-27	295	306	AYYGAVEEFSGK					Mascot
1536.8308	1536.7449	-0.0859	-56	223	235	LFKPFDELVVDSK					Mascot
1647.8951	1647.7367	-0.1584	-96	73	87	SLAPEYEKAAQLLSK					Mascot
1647.8951	1647.7367	-0.1584	-96	73	87	SLAPEYEKAAQLLSK					Mascot
1671.8436	1671.6882	-0.1554	-93	329	343	EDQAPLILIQDSDSK					Mascot
1674.9901	1674.8242	-0.1659	-99	81	96	AAQLLSKHDPAIVLAK					Mascot
1799.9386	1799.8619	-0.0767	-43	329	344	EDQAPLILIQDSDSKK					Mascot
1865.8678	1865.8357	-0.0321	-17	196	211	SDYDFGHTVHANHLPR					Mascot
1865.8678	1865.8357	-0.0321	-17	196	211	SDYDFGHTVHANHLPR	115	100			Mascot
1899.9963	1899.9279	-0.0684	-36	254	269	VVTFDKNPDNHPYLLK					Mascot
1899.9963	1899.9279	-0.0684	-36	254	269	VVTFDKNPDNHPYLLK	57	99.984			Mascot
2135.053	2135.0029	-0.0501	-23	194	211	LRSDYDFGHTVHANHLPR					Mascot

6 protein disulfide isomerase [Triticum aestivum] gi|335906217 56649.8 4.96 18 292 100 30.052 203 100

Peptide Information

Calc. Mass	Obsrv. Mass	± da	± ppm	Start Seq.	End Seq.	Sequence	Ion Score	C. I. %	Modification	Rank	Result Type
1023.4993	1023.471	-0.0283	-28	236	244	DFDVSALEK					Mascot
1104.5797	1104.5514	-0.0283	-26	128	136	NIQEYKGPR					Mascot
1150.5851	1150.5203	-0.0648	-56	124	133	NGGKNIQEYK					Mascot
1181.6201	1181.5607	-0.0594	-50	111	120	YEVQGFPTLK					Mascot
1182.6589	1182.6149	-0.044	-37	212	222	GDAAVERPLVR					Mascot
1182.6589	1182.6149	-0.044	-37	212	222	GDAAVERPLVR	31	94.005			Mascot
1210.6216	1210.5952	-0.0264	-22	260	269	NPDNHPYLLK					Mascot
1320.6107	1320.5748	-0.0359	-27	295	306	AYYGAVEEFSGK					Mascot
1440.7847	1440.7064	-0.0783	-54	386	398	VVVADNVHDVVFK					Mascot
1454.7373	1454.7169	-0.0204	-14	499	512	AAEPAATEPLKDEL					Mascot
1536.8308	1536.7449	-0.0859	-56	223	235	LFKPFDELVVDSK					Mascot
1647.8951	1647.7367	-0.1584	-96	73	87	SLAPEYEKAAQLLSK					Mascot
1647.8951	1647.7367	-0.1584	-96	73	87	SLAPEYEKAAQLLSK					Mascot
1654.8688	1654.7891	-0.0797	-48	245	259	FIDASSTPKVVTFDK					Mascot
1671.8436	1671.6882	-0.1554	-93	329	343	EDQAPLILIQDSDSK					Mascot

1674.9901	1674.8242	-0.1659	-99	81	96	AAQLLSKHDPAIVLAK						Mascot
1799.9386	1799.8619	-0.0767	-43	329	344	EDQAPLILIQDSDSKK						Mascot
1865.8678	1865.8357	-0.0321	-17	196	211	SDYDFGHTVHANHLPR						Mascot
1865.8678	1865.8357	-0.0321	-17	196	211	SDYDFGHTVHANHLPR	115	100				Mascot
1899.9963	1899.9279	-0.0684	-36	254	269	VVTFDKNPDNHPYLLK						Mascot
1899.9963	1899.9279	-0.0684	-36	254	269	VVTFDKNPDNHPYLLK	57	99.984				Mascot
2135.053	2135.0029	-0.0501	-23	194	211	LRSDYDFGHTVHANHLPR						Mascot

7	protein disulfide isomerase [Triticum aestivum]	gi|222446342	56662.9	5.03	18	291	100	30.079	203	100		

Protein Group

protein disulfide isomerase 2 precursor [Triticum aestivum]	gi|13925726	56690.9	5.03000020980835
protein disulfide isomerase precursor [Triticum aestivum]	gi|67508767	56690.9	5.03000020980835
protein disulfide isomerase precursor [Triticum aestivum]	gi|67508773	56690.9	5.03000020980835

Peptide Information

Calc. Mass	Obsrv. Mass	± da	± ppm	Start Seq.	End Seq.	Sequence	Ion Score	C. I. %	Modification	Rank	Result Type
1023.4993	1023.471	-0.0283	-28	236	244	DFDVSALEK					Mascot
1104.5797	1104.5514	-0.0283	-26	128	136	NIQEYKGPR					Mascot
1150.5851	1150.5203	-0.0648	-56	124	133	NGGKNIQEYK					Mascot
1181.6201	1181.5607	-0.0594	-50	111	120	YEVQGFPTLK					Mascot
1182.6589	1182.6149	-0.044	-37	212	222	GDAAVERPLVR					Mascot
1182.6589	1182.6149	-0.044	-37	212	222	GDAAVERPLVR	31	94.005			Mascot
1210.6216	1210.5952	-0.0264	-22	260	269	NPDNHPYLLK					Mascot
1278.694	1278.6205	-0.0735	-57	137	147	EAEGIVEYLKK					Mascot
1320.6107	1320.5748	-0.0359	-27	295	306	AYYGAVEEFSGK					Mascot
1440.7847	1440.7064	-0.0783	-54	386	398	VVVADNVHDVVFK					Mascot
1454.7373	1454.7169	-0.0204	-14	499	512	AAEPAATEPLKDEL					Mascot
1536.8308	1536.7449	-0.0859	-56	223	235	LFKPFDELVVDSK					Mascot
1647.8951	1647.7367	-0.1584	-96	73	87	SLAPEYEKAAQLLSK					Mascot
1647.8951	1647.7367	-0.1584	-96	73	87	SLAPEYEKAAQLLSK					Mascot
1671.8436	1671.6882	-0.1554	-93	329	343	EDQAPLILIQDSDSK					Mascot
1674.9901	1674.8242	-0.1659	-99	81	96	AAQLLSKHDPAIVLAK					Mascot
1799.9386	1799.8619	-0.0767	-43	329	344	EDQAPLILIQDSDSKK					Mascot

1865.8678	1865.8357	-0.0321	-17	196	211	SDYDFGHTVHANHLPR					Mascot
1865.8678	1865.8357	-0.0321	-17	196	211	SDYDFGHTVHANHLPR	115	100			Mascot
1899.9963	1899.9279	-0.0684	-36	254	269	VVTFDKNPDNHPYLLK					Mascot
1899.9963	1899.9279	-0.0684	-36	254	269	VVTFDKNPDNHPYLLK	57	99.984			Mascot
2135.053	2135.0029	-0.0501	-23	194	211	LRSDYDFGHTVHANHLP R					Mascot

8	protein disulfide isomerase [Triticum aestivum]	gi|508975	56726	4.99	18	291	100	30.747	203	100	

Protein Group

RecName: Full=Protein disulfide-isomerase; Short=PDI; Flags: Precursor	gi|1709620	56726	4.9899 997711 1816
protein disulfide isomerase	gi|1094851	56726	4.9899 997711 1816

Peptide Information

Calc. Mass	Obsrv. Mass	± da	± ppm	Start Seq.	End Seq.	Sequence	Ion Score	C. I. %	Modification	Rank	Result Type
1023.4993	1023.471	-0.0283	-28	236	244	DFDVSALEK					Mascot
1104.5797	1104.5514	-0.0283	-26	128	136	NIQEYKGPR					Mascot
1150.599	1150.5203	-0.0787	-68	137	146	EAEGIVEYLK					Mascot
1181.6201	1181.5607	-0.0594	-50	111	120	YEVQGFPTLK					Mascot
1182.6589	1182.6149	-0.044	-37	212	222	GDAAVERPLVR					Mascot
1182.6589	1182.6149	-0.044	-37	212	222	GDAAVERPLVR	31	94.005			Mascot
1210.6216	1210.5952	-0.0264	-22	260	269	NPDNHPYLLK					Mascot
1278.694	1278.6205	-0.0735	-57	137	147	EAEGIVEYLKK					Mascot
1407.6427	1407.5763	-0.0664	-47	294	306	SAYYGAVEEFSGK					Mascot
1454.7373	1454.7169	-0.0204	-14	502	515	AAEPAATEPLKDEL					Mascot
1536.8308	1536.7449	-0.0859	-56	223	235	LFKPFDELVVDSK					Mascot
1647.8951	1647.7367	-0.1584	-96	73	87	SLAPEYEKAAQLLSK					Mascot
1647.8951	1647.7367	-0.1584	-96	73	87	SLAPEYEKAAQLLSK					Mascot
1654.8688	1654.7891	-0.0797	-48	245	259	FIDASSTPKVVTFDK					Mascot
1671.8436	1671.6882	-0.1554	-93	329	343	EDQAPLILIQDSDSK					Mascot
1674.9901	1674.8242	-0.1659	-99	81	96	AAQLLSKHDPAIVLAK					Mascot
1799.9386	1799.8619	-0.0767	-43	329	344	EDQAPLILIQDSDSKK					Mascot
1865.8678	1865.8357	-0.0321	-17	196	211	SDYDFGHTVHANHLPR					Mascot
1865.8678	1865.8357	-0.0321	-17	196	211	SDYDFGHTVHANHLPR	115	100			Mascot
1899.9963	1899.9279	-0.0684	-36	254	269	VVTFDKNPDNHPYLLK					Mascot
1899.9963	1899.9279	-0.0684	-36	254	269	VVTFDKNPDNHPYLLK	57	99.984			Mascot
2135.053	2135.0029	-0.0501	-23	194	211	LRSDYDFGHTVHANHLP					Mascot

R

9	protein disulfide isomerase [Triticum durum]	gi|12056115	56772	4.99	18	291	100	30.747	203	100

Protein Group

protein disulfide isomerase 1 proprotein [Triticum aestivum]	gi|13925723	56772	4.9899 997711 1816
protein disulfide isomerase [Triticum durum]	gi|12056119	56772	4.9899 997711 1816

Peptide Information

Calc. Mass	Obsrv. Mass	± da	± ppm	Start Seq.	End Seq.	Sequence	Ion Score	C. I. %	Modification	Rank	Result Type
1023.4993	1023.471	-0.0283	-28	236	244	DFDVSALEK					Mascot
1104.5797	1104.5514	-0.0283	-26	128	136	NIQEYKGPR					Mascot
1150.5851	1150.5203	-0.0648	-56	124	133	NGGKNIQEYK					Mascot
1181.6201	1181.5607	-0.0594	-50	111	120	YEVQGFPTLK					Mascot
1182.6589	1182.6149	-0.044	-37	212	222	GDAAVERPLVR					Mascot
1182.6589	1182.6149	-0.044	-37	212	222	GDAAVERPLVR	31	94.005			Mascot
1210.6216	1210.5952	-0.0264	-22	260	269	NPDNHPYLLK					Mascot
1278.694	1278.6205	-0.0735	-57	137	147	EAEGIVEYLKK					Mascot
1407.6427	1407.5763	-0.0664	-47	294	306	SAYYGAVEEFSGK					Mascot
1454.7373	1454.7169	-0.0204	-14	502	515	AAEPAATEPLKDEL					Mascot
1536.8308	1536.7449	-0.0859	-56	223	235	LFKPFDELVVDSK					Mascot
1647.8951	1647.7367	-0.1584	-96	73	87	SLAPEYEKAAQLLSK					Mascot
1647.8951	1647.7367	-0.1584	-96	73	87	SLAPEYEKAAQLLSK					Mascot
1654.8688	1654.7891	-0.0797	-48	245	259	FIDASSTPKVVTFDK					Mascot
1671.8436	1671.6882	-0.1554	-93	329	343	EDQAPLILIQDSDSK					Mascot
1674.9901	1674.8242	-0.1659	-99	81	96	AAQLLSKHDPAIVLAK					Mascot
1799.9386	1799.8619	-0.0767	-43	329	344	EDQAPLILIQDSDSKK					Mascot
1865.8678	1865.8357	-0.0321	-17	196	211	SDYDFGHTVHANHLPR					Mascot
1865.8678	1865.8357	-0.0321	-17	196	211	SDYDFGHTVHANHLPR	115	100			Mascot
1899.9963	1899.9279	-0.0684	-36	254	269	VVTFDKNPDNHPYLLK					Mascot
1899.9963	1899.9279	-0.0684	-36	254	269	VVTFDKNPDNHPYLLK	57	99.984			Mascot
2135.053	2135.0029	-0.0501	-23	194	211	LRSDYDFGHTVHANHLP R					Mascot

10	protein disulfide isomerase [Triticum aestivum]	gi|335906215	56840	4.93	18	291	100	29.989	203	100

Peptide Information

Calc. Mass	Obsrv. Mass	± da	± ppm	Start Seq.	End Seq.	Sequence	Ion Score	C. I. %	Modification	Rank	Result Type

952.4887	952.4437	-0.045	-47	270	277	FFQTNAPK			Mascot
1023.4993	1023.471	-0.0283	-28	236	244	DFDVSALEK			Mascot
1104.5797	1104.5514	-0.0283	-26	128	136	NIQEYKGPR			Mascot
1150.5851	1150.5203	-0.0648	-56	124	133	NGGKNIQEYK			Mascot
1181.6201	1181.5607	-0.0594	-50	111	120	YEVQGFPTLK			Mascot
1182.6589	1182.6149	-0.044	-37	212	222	GDAAVERPLVR			Mascot
1182.6589	1182.6149	-0.044	-37	212	222	GDAAVERPLVR	31	95.26	Mascot
1210.6216	1210.5952	-0.0264	-22	260	269	NPDNHPYLLK			Mascot
1278.694	1278.6205	-0.0735	-57	137	147	EAEGIVEYLKK			Mascot
1440.7847	1440.7064	-0.0783	-54	386	398	VVVADNVHDVVFK			Mascot
1454.7373	1454.7169	-0.0204	-14	502	515	AAEPAATEPLKDEL			Mascot
1536.8308	1536.7449	-0.0859	-56	223	235	LFKPFDELVVDSK			Mascot
1647.8951	1647.7367	-0.1584	-96	73	87	SLAPEYEKAAQLLSK			Mascot
1647.8951	1647.7367	-0.1584	-96	73	87	SLAPEYEKAAQLLSK			Mascot
1671.8436	1671.6882	-0.1554	-93	329	343	EDQAPLILIQDSDSK			Mascot
1674.9901	1674.8242	-0.1659	-99	81	96	AAQLLSKHDPAIVLAK			Mascot
1799.9386	1799.8619	-0.0767	-43	329	344	EDQAPLILIQDSDSKK			Mascot
1865.8678	1865.8357	-0.0321	-17	196	211	SDYDFGHTVHANHLPR			Mascot
1865.8678	1865.8357	-0.0321	-17	196	211	SDYDFGHTVHANHLPR	115	100	Mascot
1899.9963	1899.9279	-0.0684	-36	254	269	VVTFDKNPDNHPYLLK			Mascot
1899.9963	1899.9279	-0.0684	-36	254	269	VVTFDKNPDNHPYLLK	57	99.987	Mascot
2135.053	2135.0029	-0.0501	-23	194	211	LRSDYDFGHTVHANHLP R			Mascot

Gel Idx/Pos	254/K6	Instr./Gel Origin	BA2151/Sample Project 20140814	Process Status	Analysis Succeeded
Plate [#] Name	[1] Sample Project 20140814	Instrument Sample Name		Spectra	11

Rank	Protein Name	Accession No.	Protein MW	Protein PI	Pep. Count	Protein Score	Protein Score C. I. %	Intensity Matched	Total Ion Score	Total Ion C. I. %	Confirmed
1	Beta-amylase [Triticum urartu]	gi|474451266	58995	5.34	19	720	100	46.233	612	100	

Peptide Information

Calc. Mass	Obsrv. Mass	± da	± ppm	Start Seq.	End Seq.	Sequence	Ion Score	C. I. %	Modification	Rank	Result Type
947.5057	947.4756	-0.0301	-32	322	329	DGYRPIAR					Mascot
1016.5564	1016.5394	-0.017	-17	412	419	LFGFTYLR					Mascot
1016.5564	1016.5394	-0.017	-17	412	419	LFGFTYLR	56	99.983			Mascot
1315.5769	1315.5422	-0.0347	-26	336	346	ASLNFTCAEMR			Carbamidomethyl (C)[7], Oxidation (M)[10]		Mascot
1326.6688	1326.6444	-0.0244	-18	385	395	YDPTAYNTILR					Mascot
1326.6688	1326.6444	-0.0244	-18	385	395	YDPTAYNTILR	82	100			Mascot
1382.7791	1382.7256	-0.0535	-39	73	84	QLFQLVHEAGLK					Mascot
1490.6726	1490.6362	-0.0364	-24	372	384	EGLNMACENALPR			Carbamidomethyl (C)[7], Oxidation (M)[5]		Mascot
1646.781	1646.7638	-0.0172	-10	246	259	FFVDNGTYLTEQGR					Mascot
1668.7952	1668.7675	-0.0277	-17	218	232	AAAAMVGHPEWEFPR					Mascot
1684.79	1684.7556	-0.0344	-20	218	232	AAAAMVGHPEWEFPR			Oxidation (M)[5]		Mascot
1685.7299	1685.7437	0.0138	8	148	161	SAVQMYTDYMASFR			Oxidation (M)[5]		Mascot
1701.7247	1701.7063	-0.0184	-11	148	161	SAVQMYTDYMASFR			Oxidation (M)[5,10]		Mascot
1701.7247	1701.7063	-0.0184	-11	148	161	SAVQMYTDYMASFR	1	0	Oxidation (M)[5,10]		Mascot
1752.8916	1752.8329	-0.0587	-33	420	434	LSNQLVEGQNYVNFK					Mascot
1841.9678	1841.8951	-0.0727	-39	459	475	SGPELTIEMILQAAQPK			Oxidation (M)[9]		Mascot
1992.9047	1992.8866	-0.0181	-9	112	129	NVGASDPDIFYTDQHGT R					Mascot
1992.9047	1992.8866	-0.0181	-9	112	129	NVGASDPDIFYTDQHGT R	128	100			Mascot
2013.9778	2013.9647	-0.0131	-7	304	321	VPSHAAEITAGYYNLHDR					Mascot
2013.9778	2013.9647	-0.0131	-7	304	321	VPSHAAEITAGYYNLHDR	112	100			Mascot
2087.0557	2087.0425	-0.0132	-6	130	147	NIEYLTLGVDDQPLFHGR					Mascot
2087.0557	2087.0425	-0.0132	-6	130	147	NIEYLTLGVDDQPLFHGR	150	100			Mascot
2183.0486	2183.0105	-0.0381	-17	440	458	MHANLPHDPCVDPVAPL QR			Carbamidomethyl (C)[10], Oxidation (M)[1]		Mascot
2183.0486	2183.0105	-0.0381	-17	440	458	MHANLPHDPCVDPVAPL QR	85	100	Carbamidomethyl (C)[10], Oxidation (M)[1]		Mascot
2269.2075	2269.1899	-0.0176	-8	166	187	EFLDAGVIVDIEVGLGPA GELR					Mascot
2733.2786	2733.2993	0.0207	8	347	371	DSEQSSQAMSAPEELVQ					Mascot

Calc. Mass	Obsrv. Mass	± da	± ppm	Start Seq.	End Seq.	Sequence	Ion Score	C. I. %	Modification	Rank	Result Type
						QVLSAGWR					
2749.2734	2749.2756	0.0022	1	347	371	DSEQSSQAMSAPEELVQ QVLSAGWR			Oxidation (M)[9]		Mascot
2773.4077	2773.3901	-0.0176	-6	162	187	DNMKEFLDAGVIVDIEVG LGPAGELR			Oxidation (M)[3]		Mascot
2794.3916	2794.3499	-0.0417	-15	87	111	AIMSFHQCGGNVGDVVN IPIPQWVR			Carbamidomethyl (C)[8]		Mascot
2810.3865	2810.3384	-0.0481	-17	87	111	AIMSFHQCGGNVGDVVN IPIPQWVR			Carbamidomethyl (C)[8], Oxidation (M)[3]		Mascot

2	beta amylase [Triticum aestivum]	gi|32400764	31099.9	8.6	9	233	100	17.309	190	100

Peptide Information

Calc. Mass	Obsrv. Mass	± da	± ppm	Start Seq.	End Seq.	Sequence	Ion Score	C. I. %	Modification	Rank	Result Type
947.5057	947.4756	-0.0301	-32	137	144	DGYRPIAR					Mascot
1315.5769	1315.5422	-0.0347	-26	151	161	ASLNFTCAEMR			Carbamidomethyl (C)[7], Oxidation (M)[10]		Mascot
1326.6688	1326.6444	-0.0244	-18	200	210	YDPTAYNTILR					Mascot
1326.6688	1326.6444	-0.0244	-18	200	210	YDPTAYNTILR	82	100			Mascot
1490.6726	1490.6362	-0.0364	-24	187	199	EGLNMACENALPR			Carbamidomethyl (C)[7], Oxidation (M)[5]		Mascot
1646.781	1646.7638	-0.0172	-10	61	74	FFVDNGTYLTEQGR					Mascot
1668.7952	1668.7675	-0.0277	-17	33	47	AAAAMVGHPEWEFPR					Mascot
1684.79	1684.7556	-0.0344	-20	33	47	AAAAMVGHPEWEFPR			Oxidation (M)[5]		Mascot
1842.0425	1841.8951	-0.1474	-80	104	118	VQLAIKISGIHWWYK					Mascot
2013.9778	2013.9647	-0.0131	-7	119	136	VPSHAAEITAGYYNLHDR					Mascot
2013.9778	2013.9647	-0.0131	-7	119	136	VPSHAAEITAGYYNLHDR	112	100			Mascot
2733.2786	2733.2993	0.0207	8	162	186	DSEQSSQAMSAPEELVQ QVLSAGWR					Mascot
2749.2734	2749.2756	0.0022	1	162	186	DSEQSSQAMSAPEELVQ QVLSAGWR			Oxidation (M)[9]		Mascot

3	beta-amylase [Triticum aestivum]	gi|1771782	56860.2	5.24	5	217	100	19.612	206	100

Protein Group

RecName: Full=Beta-amylase; AltName: Full=1,4-alpha-D-glucan maltohydrolase	gi|3334120	56860.2	5.2399 997711 1816

Peptide Information

Calc. Mass	Obsrv. Mass	± da	± ppm	Start Seq.	End Seq.	Sequence	Ion Score	C. I. %	Modification	Rank	Result Type
1016.5564	1016.5394	-0.017	-17	411	418	LFGFTYLR					Mascot
1016.5564	1016.5394	-0.017	-17	411	418	LFGFTYLR	56	99.983			Mascot
1285.6212	1285.5829	-0.0383	-30	61	71	GPKAYDWSAYK					Mascot
1685.7299	1685.7437	0.0138	8	147	160	TAVQMYADYMASFR			Oxidation (M)[5,10]		Mascot
2087.0557	2087.0425	-0.0132	-6	129	146	NIEYLTLGVDDQPLFHGR					Mascot

2087.0557	2087.0425	-0.0132	-6	129	146	NIEYLTLGVDDQPLFHGR	150	100			Mascot
2773.3274	2773.3901	0.0627	23	346	370	DSEQSEEAKSAPEELVQ QVLSAGWR					Mascot

4	Beta-amylase [Triticum urartu]	gi|474019719	63864.1	5.29	7	166	100	9.951	150	100	

Peptide Information

Calc. Mass	Obsrv. Mass	± da	± ppm	Start Seq.	End Seq.	Sequence	Ion Score	C. I. %	Modification	Rank	Result Type
1285.6212	1285.5829	-0.0383	-30	61	71	GPKAYDWSAYK					Mascot
1449.7307	1449.6591	-0.0716	-49	487	497	KQWPYVMNDLR					Mascot
1571.858	1571.7524	-0.1056	-67	456	469	FYLQPLPPAEAAVR					Mascot
1683.7506	1683.8013	0.0507	30	147	160	TAVQMYADYMTSFR					Mascot
1842.0021	1841.8951	-0.107	-58	454	469	NRFYLQPLPPAEAAVR					Mascot
2087.0557	2087.0425	-0.0132	-6	129	146	NIEYLTLGVDDQPLFHGR					Mascot
2087.0557	2087.0425	-0.0132	-6	129	146	NIEYLTLGVDDQPLFHGR	150	100			Mascot
2701.3792	2701.3049	-0.0743	-28	403	428	NTDLPVKDHTDVGDEVV HAGTVAAIK					Mascot

5	beta amylase, partial [Triticum monococcum subsp. aegilopoides]	gi|56130898	25218.4	5.35	3	162	100	9.371	150	100	

Peptide Information

Calc. Mass	Obsrv. Mass	± da	± ppm	Start Seq.	End Seq.	Sequence	Ion Score	C. I. %	Modification	Rank	Result Type
1683.7506	1683.8013	0.0507	30	61	74	TAVQMYADYMTSFR					Mascot
2087.0557	2087.0425	-0.0132	-6	43	60	NIEYLTLGVDDQPLFHGR					Mascot
2087.0557	2087.0425	-0.0132	-6	43	60	NIEYLTLGVDDQPLFHGR	150	100			Mascot
2751.3857	2751.3047	-0.081	-29	1	24	IMSFHQCGGNVADIVNIPI PQWVR			Carbamidomethyl (C)[7]		Mascot
2767.3809	2767.2705	-0.1104	-40	1	24	IMSFHQCGGNVADIVNIPI PQWVR			Carbamidomethyl (C)[7], Oxidation (M)[2]		Mascot

6	beta amylase [Triticum urartu]	gi|260060415	25204.3	5.35	2	157	100	9.153	150	100	

Protein Group

beta amylase, partial [Triticum monococcum]	gi|56130900	23475.6	5.3800 001144 4092

Peptide Information

Calc. Mass	Obsrv. Mass	± da	± ppm	Start Seq.	End Seq.	Sequence	Ion Score	C. I. %	Modification	Rank	Result Type
1683.7506	1683.8013	0.0507	30	62	75	TAVQMYADYMTSFR					Mascot
2087.0557	2087.0425	-0.0132	-6	44	61	NIEYLTLGVDDQPLFHGR					Mascot
2087.0557	2087.0425	-0.0132	-6	44	61	NIEYLTLGVDDQPLFHGR	150	100			Mascot

7	beta-amylase, partial [Triticum aestivum]	gi|451798942	15408.7	5.21	3	155	100	11.626	141	100

Peptide Information

Calc. Mass	Obsrv. Mass	± da	± ppm	Start Seq.	End Seq.	Sequence	Ion Score	C. I. %	Modification	Rank	Result Type
1016.5564	1016.5394	-0.017	-17	29	36	LFGFTYLR					Mascot
1016.5564	1016.5394	-0.017	-17	29	36	LFGFTYLR	56	99.983			Mascot
1841.9678	1841.8951	-0.0727	-39	76	92	SGPELTIEMILQAAQPK			Oxidation (M)[9]		Mascot
2183.0486	2183.0105	-0.0381	-17	57	75	MHANLPHDPCVDPVAPL QR			Carbamidomethyl (C)[10], Oxidation (M)[1]		Mascot
2183.0486	2183.0105	-0.0381	-17	57	75	MHANLPHDPCVDPVAPL QR	85	100	Carbamidomethyl (C)[10], Oxidation (M)[1]		Mascot

8	hypothetical protein TRIUR3_22966 [Triticum urartu]	gi|474350833	48251.5	4.99	11	46	0	13.468

Peptide Information

Calc. Mass	Obsrv. Mass	± da	± ppm	Start Seq.	End Seq.	Sequence	Ion Score	C. I. %	Modification	Rank	Result Type
1352.6918	1352.7415	0.0497	37	343	356	TGNAAVPAPQSPSR					Mascot
1571.6793	1571.7524	0.0731	47	103	119	GPARGDDGGEQDAGGG R					Mascot
1574.7744	1574.755	-0.0194	-12	89	102	FDMGGHIKNALEAR			Oxidation (M)[3]		Mascot
1620.7058	1620.7842	0.0784	48	1	15	MLYTSTSSATNDDSK					Mascot
1629.765	1629.7559	-0.0091	-6	396	411	STDNTAIGVAGSPMHR			Oxidation (M)[14]		Mascot
1636.7007	1636.7894	0.0887	54	1	15	MLYTSTSSATNDDSK			Oxidation (M)[1]		Mascot
1646.7993	1646.7638	-0.0355	-22	234	248	AGNHDHAANEDLVRK					Mascot
1670.7518	1670.7533	0.0015	1	137	152	APEPGHPGTHVADDDR					Mascot
1685.809	1685.7437	-0.0653	-39	412	426	SPKEDEGSQQTNPIR					Mascot
1688.9	1688.7451	-0.1549	-92	186	202	AASAVDQVANVMAAKVK			Oxidation (M)[12]		Mascot
2013.9976	2013.9647	-0.0329	-16	2	20	LYTSTSSATNDDSKLSPV K					Mascot
2013.9976	2013.9647	-0.0329	-16	2	20	LYTSTSSATNDDSKLSPV K					Mascot
2685.3301	2685.3147	-0.0154	-6	26	50	QGLWGLLAQQAKAMLDE SAPAEDAR			Oxidation (M)[14]		Mascot

9	Spastin [Triticum urartu]	gi|473923330	102782	7.29	15	40	0	16.366

Peptide Information

Calc. Mass	Obsrv. Mass	± da	± ppm	Start Seq.	End Seq.	Sequence	Ion Score	C. I. %	Modification	Rank	Result Type
929.388	929.47	0.082	88	604	612	ESMASSSSK			Oxidation (M)[3]		Mascot
1016.5483	1016.5394	-0.0089	-9	97	107	LAAAATGSQAR					Mascot
1016.5483	1016.5394	-0.0089	-9	97	107	LAAAATGSQAR					Mascot

1048.5382	1048.5602	0.022	21	475	484	GSSGSKLQER						Mascot
1237.5696	1237.5747	0.0051	4	617	626	DVVTENEFER						Mascot
1251.6692	1251.6128	-0.0564	-45	241	251	VSQTFTDGLKR						Mascot
1326.6318	1326.6444	0.0126	9	601	612	IQRESMASSSSK			Oxidation (M)[6]			Mascot
1326.6318	1326.6444	0.0126	9	601	612	IQRESMASSSSK			Oxidation (M)[6]			Mascot
1572.834	1572.7811	-0.0529	-34	61	77	APVAAAAAAEKATTSSR						Mascot
1688.8048	1688.7451	-0.0597	-35	307	321	IMLSSSSGTEIYQEK			Oxidation (M)[2]			Mascot
1712.0217	1711.9052	-0.1165	-68	665	679	RPELFSKGQLLKPAK						Mascot
1800.9603	1800.8634	-0.0969	-54	22	39	APSSSPPQPPSPASPIKR						Mascot
2115.0098	2115.0557	0.0459	22	697	717	AVATESGASFMNVSMSSI TPK						Mascot
2118.1301	2118.0449	-0.0852	-40	510	527	AQLSYLNQQIRLDAESLR						Mascot
2717.4944	2717.2849	-0.2095	-77	854	878	EKSVAIAEGRPEPALLTA DDIRPLR						Mascot
2717.4944	2717.6816	0.1872	69	854	878	EKSVAIAEGRPEPALLTA DDIRPLR						Mascot
2797.4441	2797.3223	-0.1218	-44	627	652	SLLSNVIAPHEIGVTFDDI GALECVK			Carbamidomethyl (C)[24]			Mascot
2810.4458	2810.3384	-0.1074	-38	411	435	QFGIDELNQVISEESQSS TLIVFVK						Mascot

10 Double-stranded RNA-binding protein 6 [Triticum urartu] gi|474383954 45809 7.66 10 40 0 12.181

Peptide Information

Calc. Mass	Obsrv. Mass	± da	± ppm	Start Seq.	End Seq.	Sequence	Ion Score	C. I. %	Modification	Rank	Result Type
1048.5244	1048.5602	0.0358	34	124	131	KFPMQPER			Oxidation (M)[4]		Mascot
1120.5997	1120.5054	-0.0943	-84	334	345	DAPPAATGPPVK					Mascot
1615.826	1615.7502	-0.0758	-47	110	124	IAMANNPNASPFPKK			Oxidation (M)[3]		Mascot
1615.826	1615.7502	-0.0758	-47	110	124	IAMANNPNASPFPKK			Oxidation (M)[3]		Mascot
1622.8385	1622.782	-0.0565	-35	319	333	DVQPQPAKEPLSEGK					Mascot
1643.9149	1643.7832	-0.1317	-80	284	298	AIRESMSSVIPVQVK					Mascot
1646.9225	1646.7638	-0.1587	-96	232	246	TQQLPPLLSNPPSVR					Mascot
1705.9272	1705.7739	-0.1533	-90	215	231	SSIPVFSAPPLPPPSGR					Mascot
1744.8687	1744.77	-0.0987	-57	108	123	EKIAMANNPNASPFPK			Oxidation (M)[5]		Mascot
2733.2017	2733.2993	0.0976	36	74	96	QLAREEATTTNEPENND EQEQMR					Mascot
2749.1965	2749.2756	0.0791	29	74	96	QLAREEATTTNEPENND EQEQMR			Oxidation (M)[22]		Mascot
2842.5081	2842.3542	-0.1539	-54	253	278	IRPASPHFAPSGPAQRPR PVMSVQMK					Mascot

Gel Idx/Pos	255/K7	Instr./Gel Origin	BA2151/Sample Project 20140814	Process Status	Analysis Succeeded
Plate [#] Name	[1] Sample Project 20140814	Instrument Sample Name		Spectra	11

Rank	Protein Name	Accession No.	Protein MW	Protein PI	Pep. Count	Protein Score	Protein Score C. I. %	Intensity Matched	Total Ion Score	Total Ion C. I. %	Confirmed
1	unnamed protein product [Triticum aestivum]	gi|257726669	45131.2	5.31	23	1,140	100	51.671	1000	100	

Peptide Information

Calc. Mass	Obsrv. Mass	± da	± ppm	Start Seq.	End Seq.	Sequence	Ion Score	C. I. %	Modification	Rank	Result Type
907.4818	907.4484	-0.0334	-37	235	241	FMNKPVR			Oxidation (M)[2]		Mascot
910.4702	910.4323	-0.0379	-42	373	379	MLFDIQK			Oxidation (M)[1]		Mascot
935.5197	935.483	-0.0367	-39	131	139	ALGDYLGVK					Mascot
952.5033	952.4671	-0.0362	-38	170	176	VFDMLRR			Oxidation (M)[4]		Mascot
976.5574	976.5386	-0.0188	-19	360	368	GVAINFVTR					Mascot
1035.5768	1035.5374	-0.0394	-38	234	241	KFMNKPVR			Oxidation (M)[3]		Mascot
1070.5953	1070.5756	-0.0197	-18	177	185	QSLRPDNIK					Mascot
1104.6525	1104.6372	-0.0153	-14	359	368	KGVAINFVTR					Mascot
1104.6525	1104.6372	-0.0153	-14	359	368	KGVAINFVTR	75	100			Mascot
1114.6831	1114.6646	-0.0185	-17	315	324	VLITTDLLAR					Mascot
1114.6831	1114.6646	-0.0185	-17	315	324	VLITTDLLAR	74	100			Mascot
1142.5736	1142.5586	-0.015	-13	140	150	VHACVGGTSVR			Carbamidomethyl (C)[4]		Mascot
1142.5736	1142.5586	-0.015	-13	140	150	VHACVGGTSVR	71	100	Carbamidomethyl (C)[4]		Mascot
1173.6475	1173.6292	-0.0183	-16	246	255	RDELTLEGIK					Mascot
1226.6964	1226.6692	-0.0272	-22	176	185	RQSLRPDNIK					Mascot
1401.7333	1401.6835	-0.0498	-36	78	91	GLDVIQQAQSGTGK					Mascot
1461.8538	1461.8395	-0.0143	-10	155	169	ILASGVHVVVGTPGR					Mascot
1461.8538	1461.8395	-0.0143	-10	155	169	ILASGVHVVVGTPGR	102	100			Mascot
1571.708	1571.6785	-0.0295	-19	186	198	MFVLDEADEMLSR			Oxidation (M)[1]		Mascot
1579.8367	1579.7844	-0.0523	-33	202	214	DQIYDIFQLLPSK					Mascot
1587.703	1587.6549	-0.0481	-30	186	198	MFVLDEADEMLSR			Oxidation (M)[1,10]		Mascot
1587.703	1587.6549	-0.0481	-30	186	198	MFVLDEADEMLSR	26	84.129	Oxidation (M)[1,10]		Mascot
1827.9388	1827.933	-0.0058	-3	55	70	GIYAYGFEKPSAIQQR					Mascot
1827.9388	1827.933	-0.0058	-3	55	70	GIYAYGFEKPSAIQQR	138	100			Mascot
1912.0215	1911.9354	-0.0861	-45	199	214	GFKDQIYDIFQLLPSK					Mascot
2011.0892	2011.105	0.0158	8	215	233	IQVGVVSATMPPEALEIT R					Mascot
2011.0892	2011.105	0.0158	8	215	233	IQVGVVSATMPPEALEIT	93	100			Mascot

Calc. Mass	Obsrv. Mass	± da	± ppm	Start Seq.	End Seq.	Sequence	Ion Score	C. I. %	Modification	Rank	Result Type
						R					
2027.0842	2027.068	-0.0162	-8	215	233	IQVGVVSATMPPEALEIT R			Oxidation (M)[10]		Mascot
2059.0754	2059.0776	0.0022	1	131	150	ALGDYLGVKVHACVGGT SVR			Carbamidomethyl (C)[13]		Mascot
2685.3804	2685.3704	-0.01	-4	268	290	LDTLCDLYETLAITQSVIF VNTR			Carbamidomethyl (C)[5]		Mascot
2911.4949	2911.4919	-0.003	-1	325	349	GIDVQQVSLVINYDLPTQ PENYLHR					Mascot
2911.4949	2911.4919	-0.003	-1	325	349	GIDVQQVSLVINYDLPTQ PENYLHR	183	100			Mascot
3124.5806	3124.575	-0.0056	-2	92	119	TATFCSGILQQLDYGLVE CQALVLAPTR			Carbamidomethyl (C)[5,19]		Mascot
3124.5806	3124.575	-0.0056	-2	92	119	TATFCSGILQQLDYGLVE CQALVLAPTR	237	100	Carbamidomethyl (C)[5,19]		Mascot

2	Eukaryotic initiation factor 4A-1 [Triticum urartu]	gi|474441074	47158	5.38	24	1,120	100	54.095	972	100	

Peptide Information

Calc. Mass	Obsrv. Mass	± da	± ppm	Start Seq.	End Seq.	Sequence	Ion Score	C. I. %	Modification	Rank	Result Type
907.4818	907.4484	-0.0334	-37	235	241	FMNKPVR			Oxidation (M)[2]		Mascot
910.4702	910.4323	-0.0379	-42	391	397	MLFDIQK			Oxidation (M)[1]		Mascot
935.5197	935.483	-0.0367	-39	131	139	ALGDYLGVK					Mascot
952.5033	952.4671	-0.0362	-38	170	176	VFDMLRR			Oxidation (M)[4]		Mascot
976.5574	976.5386	-0.0188	-19	378	386	GVAINFVTR					Mascot
1035.5768	1035.5374	-0.0394	-38	234	241	KFMNKPVR			Oxidation (M)[3]		Mascot
1070.5953	1070.5756	-0.0197	-18	177	185	QSLRPDNIK					Mascot
1104.6525	1104.6372	-0.0153	-14	377	386	KGVAINFVTR					Mascot
1104.6525	1104.6372	-0.0153	-14	377	386	KGVAINFVTR	75	100			Mascot
1114.6831	1114.6646	-0.0185	-17	333	342	VLITTDLLAR					Mascot
1114.6831	1114.6646	-0.0185	-17	333	342	VLITTDLLAR	74	100			Mascot
1142.5736	1142.5586	-0.015	-13	140	150	VHACVGGTSVR			Carbamidomethyl (C)[4]		Mascot
1142.5736	1142.5586	-0.015	-13	140	150	VHACVGGTSVR	71	100	Carbamidomethyl (C)[4]		Mascot
1173.6475	1173.6292	-0.0183	-16	246	255	RDELTLEGIK					Mascot
1226.6964	1226.6692	-0.0272	-22	176	185	RQSLRPDNIK					Mascot
1401.7333	1401.6835	-0.0498	-36	78	91	GLDVIQQAQSGTGK					Mascot
1461.8538	1461.8395	-0.0143	-10	155	169	ILASGVHVVVGTPGR					Mascot
1461.8538	1461.8395	-0.0143	-10	155	169	ILASGVHVVVGTPGR	102	100			Mascot
1571.708	1571.6785	-0.0295	-19	186	198	MFVLDEADEMLSR			Oxidation (M)[1]		Mascot
1579.8367	1579.7844	-0.0523	-33	202	214	DQIYDIFQLLPSK					Mascot
1587.703	1587.6549	-0.0481	-30	186	198	MFVLDEADEMLSR			Oxidation (M)[1,10]		Mascot
1587.703	1587.6549	-0.0481	-30	186	198	MFVLDEADEMLSR	26	84.129	Oxidation (M)[1,10]		Mascot
1800.7566	1800.7279	-0.0287	-16	304	319	DHTVSATHGDMDQNTR			Oxidation (M)[11]		Mascot

1827.9388	1827.933	-0.0058	-3	55	70	GIYAYGFEKPSAIQQR					Mascot
1827.9388	1827.933	-0.0058	-3	55	70	GIYAYGFEKPSAIQQR	138	100			Mascot
1912.0215	1911.9354	-0.0861	-45	199	214	GFKDQIYDIFQLLPSK					Mascot
2059.0754	2059.0776	0.0022	1	131	150	ALGDYLGVKVHACVGGTSVR			Carbamidomethyl (C)[13]		Mascot
2075.0842	2075.0544	-0.0298	-14	215	233	IQVGVFSATMPPEALEITR			Oxidation (M)[10]		Mascot
2075.0842	2075.0544	-0.0298	-14	215	233	IQVGVFSATMPPEALEITR	65	99.998	Oxidation (M)[10]		Mascot
2685.3804	2685.3704	-0.01	-4	268	290	LDTLCDLYETLAITQSVIFVNTR			Carbamidomethyl (C)[5]		Mascot
2911.4949	2911.4919	-0.003	-1	343	367	GIDVQQVSLVINYDLPTQPENYLHR					Mascot
2911.4949	2911.4919	-0.003	-1	343	367	GIDVQQVSLVINYDLPTQPENYLHR	183	100			Mascot
3124.5806	3124.575	-0.0056	-2	92	119	TATFCSGILQQLDYGLVECQALVLAPTR			Carbamidomethyl (C)[5,19]		Mascot
3124.5806	3124.575	-0.0056	-2	92	119	TATFCSGILQQLDYGLVECQALVLAPTR	237	100	Carbamidomethyl (C)[5,19]		Mascot

3	unnamed protein product [Triticum aestivum]	gi|257726671	47227.1	5.31	22	1,100	100	53.614	972	100	

Peptide Information

Calc. Mass	Obsrv. Mass	± da	± ppm	Start Seq.	End Seq.	Sequence	Ion Score	C. I. %	Modification	Rank	Result Type
907.4818	907.4484	-0.0334	-37	235	241	FMNKPVR			Oxidation (M)[2]		Mascot
910.4702	910.4323	-0.0379	-42	391	397	MLFDIQK			Oxidation (M)[1]		Mascot
935.5197	935.483	-0.0367	-39	131	139	ALGDYLGVK					Mascot
952.5033	952.4671	-0.0362	-38	170	176	VFDMLRR			Oxidation (M)[4]		Mascot
976.5574	976.5386	-0.0188	-19	378	386	GVAINFVTR					Mascot
1035.5768	1035.5374	-0.0394	-38	234	241	KFMNKPVR			Oxidation (M)[3]		Mascot
1070.5953	1070.5756	-0.0197	-18	177	185	QSLRPDNIK					Mascot
1104.6525	1104.6372	-0.0153	-14	377	386	KGVAINFVTR					Mascot
1104.6525	1104.6372	-0.0153	-14	377	386	KGVAINFVTR	75	100			Mascot
1114.6831	1114.6646	-0.0185	-17	333	342	VLITTDLLAR					Mascot
1114.6831	1114.6646	-0.0185	-17	333	342	VLITTDLLAR	74	100			Mascot
1142.5736	1142.5586	-0.015	-13	140	150	VHACVGGTSVR			Carbamidomethyl (C)[4]		Mascot
1142.5736	1142.5586	-0.015	-13	140	150	VHACVGGTSVR	71	100	Carbamidomethyl (C)[4]		Mascot
1173.6475	1173.6292	-0.0183	-16	246	255	RDELTLEGIK					Mascot
1226.6964	1226.6692	-0.0272	-22	176	185	RQSLRPDNIK					Mascot
1401.7333	1401.6835	-0.0498	-36	78	91	GLDVIQQAQSGTGK					Mascot
1461.8538	1461.8395	-0.0143	-10	155	169	ILASGVHVVVGTPGR					Mascot
1461.8538	1461.8395	-0.0143	-10	155	169	ILASGVHVVVGTPGR	102	100			Mascot
1571.708	1571.6785	-0.0295	-19	186	198	MFVLDEADEMLSR			Oxidation (M)[1]		Mascot

1587.703	1587.6549	-0.0481	-30	186	198	MFVLDEADEMLSR			Oxidation (M)[1,10]		Mascot
1587.703	1587.6549	-0.0481	-30	186	198	MFVLDEADEMLSR	26	84.129	Oxidation (M)[1,10]		Mascot
1800.7566	1800.7279	-0.0287	-16	304	319	DHTVSATHGDMDQNTR			Oxidation (M)[11]		Mascot
1827.9388	1827.933	-0.0058	-3	55	70	GIYAYGFEKPSAIQQR					Mascot
1827.9388	1827.933	-0.0058	-3	55	70	GIYAYGFEKPSAIQQR	138	100			Mascot
2059.0754	2059.0776	0.0022	1	131	150	ALGDYLGVKVHACVGGT SVR			Carbamidomethyl (C)[13]		Mascot
2075.0842	2075.0544	-0.0298	-14	215	233	IQVGVFSATMPPEALEIT R			Oxidation (M)[10]		Mascot
2075.0842	2075.0544	-0.0298	-14	215	233	IQVGVFSATMPPEALEIT R	65	99.998	Oxidation (M)[10]		Mascot
2685.3804	2685.3704	-0.01	-4	268	290	LDTLCDLYETLAITQSVIF VNTR			Carbamidomethyl (C)[5]		Mascot
2911.4949	2911.4919	-0.003	-1	343	367	GIDVQQVSLVINYDLPTQ PENYLHR					Mascot
2911.4949	2911.4919	-0.003	-1	343	367	GIDVQQVSLVINYDLPTQ PENYLHR	183	100			Mascot
3124.5806	3124.575	-0.0056	-2	92	119	TATFCSGILQQLDYGLVE CQALVLAPTR			Carbamidomethyl (C)[5,19]		Mascot
3124.5806	3124.575	-0.0056	-2	92	119	TATFCSGILQQLDYGLVE CQALVLAPTR	237	100	Carbamidomethyl (C)[5,19]		Mascot

4	unnamed protein product [Triticum aestivum]	gi|257721534	47183.1	5.31	20	898	100	50.013	790	100

Protein Group

RecName: Full=Eukaryotic initiation factor 4A; Short=eIF-4A; AltName: Full=ATP-dependent RNA helicase eIF4A	gi|1170509	47183.1	5.3099 999427 7954

Peptide Information

Calc. Mass	Obsrv. Mass	± da	± ppm	Start Seq.	End Seq.	Sequence	Ion Score	C. I. %	Modification	Rank	Result Type
907.4818	907.4484	-0.0334	-37	235	241	FMNKPVR			Oxidation (M)[2]		Mascot
910.4702	910.4323	-0.0379	-42	391	397	MLFDIQK			Oxidation (M)[1]		Mascot
935.5197	935.483	-0.0367	-39	131	139	ALGDYLGVK					Mascot
976.5574	976.5386	-0.0188	-19	378	386	GVAINFVTR					Mascot
1035.5768	1035.5374	-0.0394	-38	234	241	KFMNKPVR			Oxidation (M)[3]		Mascot
1070.5953	1070.5756	-0.0197	-18	177	185	QSLRPDNIK					Mascot
1104.6525	1104.6372	-0.0153	-14	377	386	KGVAINFVTR					Mascot
1104.6525	1104.6372	-0.0153	-14	377	386	KGVAINFVTR	75	100			Mascot
1114.6831	1114.6646	-0.0185	-17	333	342	VLITTDLLAR					Mascot
1114.6831	1114.6646	-0.0185	-17	333	342	VLITTDLLAR	74	100			Mascot
1142.5736	1142.5586	-0.015	-13	140	150	VHACVGGTSVR			Carbamidomethyl (C)[4]		Mascot
1142.5736	1142.5586	-0.015	-13	140	150	VHACVGGTSVR	71	100	Carbamidomethyl (C)[4]		Mascot
1173.6475	1173.6292	-0.0183	-16	246	255	RDELTLEGIK					Mascot
1226.6964	1226.6692	-0.0272	-22	176	185	RQSLRPDNIK					Mascot

Calc. Mass	Obsrv. Mass	± da	± ppm	Start Seq.	End Seq.	Sequence	Ion Score	C. I. %	Modification	Rank	Result Type
1401.7333	1401.6835	-0.0498	-36	78	91	GLDVIQQAQSGTGK					Mascot
1461.8538	1461.8395	-0.0143	-10	155	169	ILASGVHVVVGTPGR					Mascot
1461.8538	1461.8395	-0.0143	-10	155	169	ILASGVHVVVGTPGR	102	100			Mascot
1571.708	1571.6785	-0.0295	-19	186	198	MFVLDEADEMLSR			Oxidation (M)[1]		Mascot
1587.703	1587.6549	-0.0481	-30	186	198	MFVLDEADEMLSR			Oxidation (M)[1,10]		Mascot
1587.703	1587.6549	-0.0481	-30	186	198	MFVLDEADEMLSR	26	84.129	Oxidation (M)[1,10]		Mascot
1800.7566	1800.7279	-0.0287	-16	304	319	DHTVSATHGDMDQNTR			Oxidation (M)[11]		Mascot
1827.9388	1827.933	-0.0058	-3	55	70	GIYAYGFEKPSAIQQR					Mascot
1827.9388	1827.933	-0.0058	-3	55	70	GIYAYGFEKPSAIQQR	138	100			Mascot
2059.0754	2059.0776	0.0022	1	131	150	ALGDYLGVKVHACVGGTSVR			Carbamidomethyl (C)[13]		Mascot
2075.0842	2075.0544	-0.0298	-14	215	233	IQVGVFSATMPPEALEITR			Oxidation (M)[10]		Mascot
2075.0842	2075.0544	-0.0298	-14	215	233	IQVGVFSATMPPEALEITR	65	99.998	Oxidation (M)[10]		Mascot
2685.3804	2685.3704	-0.01	-4	268	290	LDTLCDLYETLAITQSVIFVNTR			Carbamidomethyl (C)[5]		Mascot
3124.5806	3124.575	-0.0056	-2	92	119	TATFCSGILQQLDYGLVECQALVLAPTR			Carbamidomethyl (C)[5,19]		Mascot
3124.5806	3124.575	-0.0056	-2	92	119	TATFCSGILQQLDYGLVECQALVLAPTR	237	100	Carbamidomethyl (C)[5,19]		Mascot

5	eukaryotic initiation factor 4A-like protein, partial [Triticum aestivum]	gi|451798948	32137.8	8.72	17	639	100	24.802	522	100		

Peptide Information

Calc. Mass	Obsrv. Mass	± da	± ppm	Start Seq.	End Seq.	Sequence	Ion Score	C. I. %	Modification	Rank	Result Type
935.5197	935.483	-0.0367	-39	27	35	ALGDYLGVK					Mascot
952.5033	952.4671	-0.0362	-38	66	72	VFDMLRR			Oxidation (M)[4]		Mascot
1070.5953	1070.5756	-0.0197	-18	73	81	QSLRPDNIK					Mascot
1114.6831	1114.6646	-0.0185	-17	229	238	VLITTDLLAR					Mascot
1114.6831	1114.6646	-0.0185	-17	229	238	VLITTDLLAR	74	100			Mascot
1142.5736	1142.5586	-0.015	-13	36	46	VHACVGGTSVR			Carbamidomethyl (C)[4]		Mascot
1142.5736	1142.5586	-0.015	-13	36	46	VHACVGGTSVR	71	100	Carbamidomethyl (C)[4]		Mascot
1173.6475	1173.6292	-0.0183	-16	142	151	RDELTLEGIK					Mascot
1226.6964	1226.6692	-0.0272	-22	72	81	RQSLRPDNIK					Mascot
1461.8538	1461.8395	-0.0143	-10	51	65	ILASGVHVVVGTPGR					Mascot
1461.8538	1461.8395	-0.0143	-10	51	65	ILASGVHVVVGTPGR	102	100			Mascot
1571.708	1571.6785	-0.0295	-19	82	94	MFVLDEADEMLSR			Oxidation (M)[1]		Mascot
1579.8367	1579.7844	-0.0523	-33	98	110	DQIYDIFQLLPSK					Mascot
1587.703	1587.6549	-0.0481	-30	82	94	MFVLDEADEMLSR			Oxidation (M)[1,10]		Mascot
1587.703	1587.6549	-0.0481	-30	82	94	MFVLDEADEMLSR	26	84.129	Oxidation (M)[1,10]		Mascot

1689.8993	1689.832	-0.0673	-40	1	15	YGLVECQALVLAPTR			Carbamidomethyl (C)[6]		Mascot
1800.7566	1800.7279	-0.0287	-16	200	215	DHTVSATHGDMDQNTR			Oxidation (M)[11]		Mascot
1912.0215	1911.9354	-0.0861	-45	95	110	GFKDQIYDIFQLLPSK					Mascot
2059.0754	2059.0776	0.0022	1	27	46	ALGDYLGVKVHACVGGT SVR			Carbamidomethyl (C)[13]		Mascot
2075.0842	2075.0544	-0.0298	-14	111	129	IQVGVFSATMPPEALEIT R			Oxidation (M)[10]		Mascot
2075.0842	2075.0544	-0.0298	-14	111	129	IQVGVFSATMPPEALEIT R	65	99.998	Oxidation (M)[10]		Mascot
2685.3804	2685.3704	-0.01	-4	164	186	LDTLCDLYETLAITQSVIF VNTR			Carbamidomethyl (C)[5]		Mascot
2911.4949	2911.4919	-0.003	-1	239	263	GIDVQQVSLVINYDLPTQ PENYLHR					Mascot
2911.4949	2911.4919	-0.003	-1	239	263	GIDVQQVSLVINYDLPTQ PENYLHR	183	100			Mascot

6	group 3 late embryogenesis abundant protein, partial [Triticum aestivum]	gi|170692	33404.2	5.01	13	58	93.896	10.464

Peptide Information

Calc. Mass	Obsrv. Mass	± da	± ppm	Start Seq.	End Seq.	Sequence	Ion Score	C. I. %	Modification	Rank	Result Type
800.3495	800.4261	0.0766	96	126	131	EYMVDK			Oxidation (M)[3]		Mascot
1104.5531	1104.6372	0.0841	76	66	76	AAETKDAAAEK					Mascot
1104.5531	1104.6372	0.0841	76	66	76	AAETKDAAAEK					Mascot
1192.5701	1192.6097	0.0396	33	207	216	MTMPTDVIER					Mascot
1306.642	1306.6725	0.0305	23	77	89	ASGAGEMVTEKAR					Mascot
1366.6454	1366.5525	-0.0929	-68	260	272	MGEEGTGMKAALR			Oxidation (M)[1]		Mascot
1539.6744	1539.6932	0.0188	12	245	259	EADQMTGAGFNDVGK					Mascot
1603.7744	1603.693	-0.0814	-51	269	282	AALRADDEEDVMLR					Mascot
1664.7797	1664.86	0.0803	48	71	87	DAAAEKASGAGEMVTEK					Mascot
1782.8328	1782.8729	0.0401	22	243	259	VKEADQMTGAGFNDVGK			Oxidation (M)[7]		Mascot
1826.8477	1826.9088	0.0611	33	115	131	DAALDTAEGAKEYMVDK					Mascot
2008.8625	2009.0594	0.1969	98	145	162	DSKGETNESACQQGQD VR			Carbamidomethyl (C)[11]		Mascot
2052.0608	2052.0508	-0.01	-5	185	204	SKSATENILGSAQGLTEA FK					Mascot
2058.9873	2059.0776	0.0903	44	223	242	GTPTDAGRGEALNADDV MLR					Mascot
2074.9822	2075.0544	0.0722	35	223	242	GTPTDAGRGEALNADDV MLR			Oxidation (M)[18]		Mascot
2074.9822	2075.0544	0.0722	35	223	242	GTPTDAGRGEALNADDV MLR	3	0	Oxidation (M)[18]		Mascot

7	eukaryotic translation initiation factor 3 subunit A, putative, expressed [Triticum aestivum]	gi|383100786	117173.6	9.38	23	48	23.152	6.239

Peptide Information

Calc. Mass	Obsrv. Mass	± da	± ppm	Start Seq.	End Seq.	Sequence	Ion Score	C. I. %	Modification	Rank	Result Type
800.376	800.4261	0.0501	63	182	187	AFQFCK			Carbamidomethyl (C)[5]		Mascot
834.3951	834.316	-0.0791	-95	599	605	KSADEER					Mascot
846.429	846.4315	0.0025	3	492	498	HNFVAMK					Mascot
864.4574	864.4236	-0.0338	-39	68	74	DGLIQYR					Mascot
942.5982	942.526	-0.0722	-77	829	837	LKLDAIAAK					Mascot
1016.5986	1016.5222	-0.0764	-75	375	384	ATLLSELAAK					Mascot
1036.484	1036.5051	0.0211	20	367	374	ENREMVSR			Oxidation (M)[5]		Mascot
1053.5404	1053.5509	0.0105	10	145	152	EFVTPWFK					Mascot
1082.5477	1082.5944	0.0467	43	760	768	ESEFSSLRK					Mascot
1226.7216	1226.6692	-0.0524	-43	831	841	LDAIAAKQLQR					Mascot
1260.6808	1260.6487	-0.0321	-25	749	758	NAFQERIVQR					Mascot
1320.698	1320.6298	-0.0682	-52	1	12	MATFAKPENALK					Mascot
1384.7332	1384.6855	-0.0477	-34	744	754	LLEHKNAFQER					Mascot
1443.7512	1443.8027	0.0515	36	253	265	SVEDIHGLMSLVK			Oxidation (M)[9]		Mascot
1508.8431	1508.7566	-0.0865	-57	24	37	QAALQALHDLITSK					Mascot
1523.7999	1523.6869	-0.113	-74	658	670	QHAMEVVLNQQVK					Mascot
1539.7948	1539.6932	-0.1016	-66	658	670	QHAMEVVLNQQVK			Oxidation (M)[4]		Mascot
1551.8312	1551.7512	-0.08	-52	454	466	VLQQASRIFQSMK			Oxidation (M)[12]		Mascot
1601.7815	1601.7963	0.0148	9	790	801	KLMYFLNMEEQR					Mascot
1664.8602	1664.86	-0.0002	0	880	899	EPAAAAPAAAAAATGAA PSK					Mascot
1689.8013	1689.832	0.0307	18	91	104	HFMQLSNEKAEEAR					Mascot
1808.9211	1808.8812	-0.0399	-22	124	139	RPEDLMLSYVSGEKGK					Mascot
2009.99	2010.0741	0.0841	42	978	996	QPDGAAPAASTERWRP GSR					Mascot
2091.0215	2091.0576	0.0361	17	970	990	QEAPPAARQPDGAAPAA STER					Mascot

8	eukaryotic translation initiation factor,putative, expressed [Triticum aestivum]	gi|300681560	117424.6	9.36	23	46	0	6.239	

Peptide Information

Calc. Mass	Obsrv. Mass	± da	± ppm	Start Seq.	End Seq.	Sequence	Ion Score	C. I. %	Modification	Rank	Result Type
800.376	800.4261	0.0501	63	182	187	AFQFCK			Carbamidomethyl (C)[5]		Mascot
834.3951	834.316	-0.0791	-95	599	605	KSADEER					Mascot
846.429	846.4315	0.0025	3	492	498	HNFVAMK					Mascot
864.4574	864.4236	-0.0338	-39	68	74	DGLIQYR					Mascot

942.5982	942.526	-0.0722	-77	829	837	LKLDAIAAK					Mascot
1016.5986	1016.5222	-0.0764	-75	375	384	ATLLSELAAK					Mascot
1036.484	1036.5051	0.0211	20	367	374	ENREMVSR			Oxidation (M)[5]		Mascot
1053.5404	1053.5509	0.0105	10	145	152	EFVTPWFK					Mascot
1082.5477	1082.5944	0.0467	43	760	768	ESEFSSLRK					Mascot
1226.7216	1226.6692	-0.0524	-43	831	841	LDAIAAKQLQR					Mascot
1260.6808	1260.6487	-0.0321	-25	749	758	NAFQERIVQR					Mascot
1320.698	1320.6298	-0.0682	-52	1	12	MATFAKPENALK					Mascot
1384.7332	1384.6855	-0.0477	-34	744	754	LLEHKNAFQER					Mascot
1443.7512	1443.8027	0.0515	36	253	265	SVEDIHGLMSLVK			Oxidation (M)[9]		Mascot
1508.8431	1508.7566	-0.0865	-57	24	37	QAALQALHDLITSK					Mascot
1523.7999	1523.6869	-0.113	-74	658	670	QHAMEVVLNQQVK					Mascot
1539.7948	1539.6932	-0.1016	-66	658	670	QHAMEVVLNQQVK			Oxidation (M)[4]		Mascot
1551.8312	1551.7512	-0.08	-52	454	466	VLQQASRIFQSMK			Oxidation (M)[12]		Mascot
1601.7815	1601.7963	0.0148	9	790	801	KLMYFLNMEEQR					Mascot
1664.8602	1664.86	-0.0002	0	880	899	EPAAAAPAAAAAATGAA PSK					Mascot
1689.8013	1689.832	0.0307	18	91	104	HFMQLSNEKAEEAR					Mascot
1808.9211	1808.8812	-0.0399	-22	124	139	RPEDLMLSYVSGEKGK					Mascot
2009.99	2010.0741	0.0841	42	978	996	QPDGAAPAASTERWRP GSR					Mascot
2091.0215	2091.0576	0.0361	17	970	990	QEAPPAARQPDGAAPAA STER					Mascot

9	Putative disease resistance RPP13-like protein 1 [Triticum urartu]	gi|474219021	59003.2	9.36	14	46	0	9.326

Peptide Information

Calc. Mass	Obsrv. Mass	± da	± ppm	Start Seq.	End Seq.	Sequence	Ion Score	C. I. %	Modification	Rank	Result Type
1070.5234	1070.5756	0.0522	49	68	75	MLRQGMYR			Oxidation (M)[1]		Mascot
1082.5338	1082.5944	0.0606	56	264	272	YRNSASQTR					Mascot
1085.5521	1085.5814	0.0293	27	56	64	HITNQGMLR			Oxidation (M)[7]		Mascot
1384.7366	1384.6855	-0.0511	-37	215	226	VGKSTLVEHVCR			Carbamidomethyl (C)[11]		Mascot
1457.786	1457.7389	-0.0471	-32	367	380	QGFTSAHIASGILR					Mascot
1461.7804	1461.8395	0.0591	40	354	366	LATMAMEIALELR					Mascot
1461.7804	1461.8395	0.0591	40	354	366	LATMAMEIALELR					Mascot
1477.7753	1477.7294	-0.0459	-31	354	366	LATMAMEIALELR			Oxidation (M)[4]		Mascot
1507.729	1507.7181	-0.0109	-7	155	165	EFLFFMELYPR			Oxidation (M)[6]		Mascot
1601.8932	1601.7963	-0.0969	-60	1	15	MDILVSAIVGDLISR					Mascot
1782.8997	1782.8729	-0.0268	-15	232	246	SHFSMILFFPQGSLR			Oxidation (M)[5]		Mascot

1790.9548	1790.911	-0.0438	-24	24	37	YFQQQPDINKILQR					Mascot
1809.8999	1809.9155	0.0156	9	326	339	LDFLPPEAYWHFFK					Mascot
1953.9733	1953.9196	-0.0537	-27	293	311	SSTTGMTPCGGSKIIITSR			Carbamidomethyl (C)[9]		Mascot
2009.8918	2010.0741	0.1823	91	169	184	QPYGMYLLLDNCMFGR			Carbamidomethyl (C)[12], Oxidation (M)[5,13]		Mascot
2051.9451	2052.0508	0.1057	52	85	103	FQAARGEEEMSHSSSALSK					Mascot

10 Eukaryotic translation initiation factor 3 subunit A [Triticum urartu] gi|474111197 117354.8 9.4 23 46 0 6.239

Peptide Information

Calc. Mass	Obsrv. Mass	± da	± ppm	Start Seq.	End Seq.	Sequence	Ion Score	C. I. %	Modification	Rank	Result Type
800.376	800.4261	0.0501	63	182	187	AFQFCK			Carbamidomethyl (C)[5]		Mascot
834.3951	834.316	-0.0791	-95	599	605	KSADEER					Mascot
846.429	846.4315	0.0025	3	492	498	HNFVAMK					Mascot
864.4574	864.4236	-0.0338	-39	68	74	DGLIQYR					Mascot
942.5982	942.526	-0.0722	-77	846	854	LKLDAIAAK					Mascot
1016.5986	1016.5222	-0.0764	-75	375	384	ATLLSELAAK					Mascot
1036.484	1036.5051	0.0211	20	367	374	ENREMVSR			Oxidation (M)[5]		Mascot
1053.5404	1053.5509	0.0105	10	145	152	EFVTPWFK					Mascot
1082.5477	1082.5944	0.0467	43	760	768	ESEFSSLRK					Mascot
1226.7216	1226.6692	-0.0524	-43	848	858	LDAIAAKQLQR					Mascot
1260.6808	1260.6487	-0.0321	-25	749	758	NAFQERIVQR					Mascot
1320.698	1320.6298	-0.0682	-52	1	12	MATFAKPENALK					Mascot
1384.7332	1384.6855	-0.0477	-34	744	754	LLEHKNAFQER					Mascot
1443.7512	1443.8027	0.0515	36	253	265	SVEDIHGLMSLVK			Oxidation (M)[9]		Mascot
1508.8431	1508.7566	-0.0865	-57	24	37	QAALQALHDLITSK					Mascot
1523.7999	1523.6869	-0.113	-74	658	670	QHAMEVVLNQQVK					Mascot
1539.7948	1539.6932	-0.1016	-66	658	670	QHAMEVVLNQQVK			Oxidation (M)[4]		Mascot
1551.8312	1551.7512	-0.08	-52	454	466	VLQQASRIFQSMK			Oxidation (M)[12]		Mascot
1601.7815	1601.7963	0.0148	9	790	801	KLMYFLNMEEQR					Mascot
1664.9442	1664.86	-0.0842	-51	24	38	QAALQALHDLITSKR					Mascot
1689.8013	1689.832	0.0307	18	91	104	HFMQLSNEKAEEAR					Mascot
1808.9211	1808.8812	-0.0399	-22	124	139	RPEDLMLSYVSGEKGK					Mascot
2009.99	2010.0741	0.0841	42	973	991	QPDGAAPAASTERWRPGSR					Mascot
2091.0215	2091.0576	0.0361	17	965	985	QEAPPAARQPDGAAPAASTER					Mascot

Gel Idx/Pos	256/K8	Instr./Gel Origin	BA2151/Sample Project 20140814	Process Status	Analysis Succeeded
Plate [#] Name	[1] Sample Project 20140814	Instrument Sample Name		Spectra	11

Rank	Protein Name	Accession No.	Protein MW	Protein PI	Pep. Count	Protein Score	Protein Score C. I. %	Intensity Matched	Total Ion Score	Total Ion C. I. %	Confirmed
1	unnamed protein product [Triticum aestivum]	gi|257726671	47227.1	5.31	25	895	100	49.505	739	100	

Peptide Information

Calc. Mass	Obsrv. Mass	± da	± ppm	Start Seq.	End Seq.	Sequence	Ion Score	C. I. %	Modification	Rank	Result Type
907.4818	907.4412	-0.0406	-45	235	241	FMNKPVR			Oxidation (M)[2]		Mascot
910.4702	910.4266	-0.0436	-48	391	397	MLFDIQK			Oxidation (M)[1]		Mascot
935.5197	935.4751	-0.0446	-48	131	139	ALGDYLGVK					Mascot
952.5033	952.4612	-0.0421	-44	170	176	VFDMLRR			Oxidation (M)[4]		Mascot
976.5574	976.5323	-0.0251	-26	378	386	GVAINFVTR					Mascot
1035.5768	1035.5188	-0.058	-56	234	241	KFMNKPVR			Oxidation (M)[3]		Mascot
1070.5953	1070.5665	-0.0288	-27	177	185	QSLRPDNIK					Mascot
1104.6525	1104.6274	-0.0251	-23	377	386	KGVAINFVTR					Mascot
1104.6525	1104.6274	-0.0251	-23	377	386	KGVAINFVTR	63	99.996			Mascot
1114.6831	1114.6573	-0.0258	-23	333	342	VLITTDLLAR					Mascot
1114.6831	1114.6573	-0.0258	-23	333	342	VLITTDLLAR	77	100			Mascot
1142.5736	1142.5491	-0.0245	-21	140	150	VHACVGGTSVR			Carbamidomethyl (C)[4]		Mascot
1142.5736	1142.5491	-0.0245	-21	140	150	VHACVGGTSVR	70	100	Carbamidomethyl (C)[4]		Mascot
1173.6475	1173.6195	-0.028	-24	246	255	RDELTLEGIK					Mascot
1226.6964	1226.662	-0.0344	-28	176	185	RQSLRPDNIK					Mascot
1401.7333	1401.6686	-0.0647	-46	78	91	GLDVIQQAQSGTGK					Mascot
1461.8538	1461.8269	-0.0269	-18	155	169	ILASGVHVVVGTPGR					Mascot
1461.8538	1461.8269	-0.0269	-18	155	169	ILASGVHVVVGTPGR	108	100			Mascot
1549.8262	1549.7515	-0.0747	-48	202	214	DQIYDIFQLLPGK					Mascot
1555.7131	1555.6875	-0.0256	-16	186	198	MFVLDEADEMLSR					Mascot
1571.708	1571.6927	-0.0153	-10	186	198	MFVLDEADEMLSR			Oxidation (M)[1]		Mascot
1587.703	1587.6492	-0.0538	-34	186	198	MFVLDEADEMLSR			Oxidation (M)[1,10]		Mascot
1598.7849	1598.726	-0.0589	-37	256	267	QFYVNVEKEEWK					Mascot
1827.9388	1827.9164	-0.0224	-12	55	70	GIYAYGFEKPSAIQQR					Mascot
1827.9388	1827.9164	-0.0224	-12	55	70	GIYAYGFEKPSAIQQR	141	100			Mascot
1882.011	1881.8552	-0.1558	-83	199	214	GFKDQIYDIFQLLPGK					Mascot
1903.8928	1903.9167	0.0239	13	186	201	MFVLDEADEMLSRGFK			Oxidation (M)[1]		Mascot

1919.8878	1919.9137	0.0259	13	186	201	MFVLDEADEMLSRGFK			Oxidation (M)[1,10]		Mascot
2059.0754	2059.063	-0.0124	-6	131	150	ALGDYLGVKVHACVGGT SVR			Carbamidomethyl (C)[13]		Mascot
2059.0894	2059.063	-0.0264	-13	215	233	IQVGVFSATMPPEALEIT R	84	100			Mascot
2075.0842	2075.033	-0.0512	-25	215	233	IQVGVFSATMPPEALEIT R			Oxidation (M)[10]		Mascot
2075.0842	2075.033	-0.0512	-25	215	233	IQVGVFSATMPPEALEIT R	66	99.998	Oxidation (M)[10]		Mascot
2685.3804	2685.3508	-0.0296	-11	268	290	LDTLCDLYETLAITQSVIF VNTR			Carbamidomethyl (C)[5]		Mascot
2911.4949	2911.4683	-0.0266	-9	343	367	GIDVQQVSLVINYDLPTQ PENYLHR					Mascot
2911.4949	2911.4683	-0.0266	-9	343	367	GIDVQQVSLVINYDLPTQ PENYLHR	197	100			Mascot
3124.5806	3124.5498	-0.0308	-10	92	119	TATFCSGILQQLDYGLVE CQALVLAPTR			Carbamidomethyl (C)[5,19]		Mascot

2 Eukaryotic initiation factor 4A-1 [Triticum urartu] gi|474441074 47158 5.38 23 875 100 48.804 739 100

Peptide Information

Calc. Mass	Obsrv. Mass	± da	± ppm	Start Seq.	End Seq.	Sequence	Ion Score	C. I. %	Modification	Rank	Result Type
907.4818	907.4412	-0.0406	-45	235	241	FMNKPVR			Oxidation (M)[2]		Mascot
910.4702	910.4266	-0.0436	-48	391	397	MLFDIQK			Oxidation (M)[1]		Mascot
935.5197	935.4751	-0.0446	-48	131	139	ALGDYLGVK					Mascot
952.5033	952.4612	-0.0421	-44	170	176	VFDMLRR			Oxidation (M)[4]		Mascot
976.5574	976.5323	-0.0251	-26	378	386	GVAINFVTR					Mascot
1035.5768	1035.5188	-0.058	-56	234	241	KFMNKPVR			Oxidation (M)[3]		Mascot
1070.5953	1070.5665	-0.0288	-27	177	185	QSLRPDNIK					Mascot
1104.6525	1104.6274	-0.0251	-23	377	386	KGVAINFVTR					Mascot
1104.6525	1104.6274	-0.0251	-23	377	386	KGVAINFVTR	63	99.996			Mascot
1114.6831	1114.6573	-0.0258	-23	333	342	VLITTDLLAR					Mascot
1114.6831	1114.6573	-0.0258	-23	333	342	VLITTDLLAR	77	100			Mascot
1142.5736	1142.5491	-0.0245	-21	140	150	VHACVGGTSVR			Carbamidomethyl (C)[4]		Mascot
1142.5736	1142.5491	-0.0245	-21	140	150	VHACVGGTSVR	70	100	Carbamidomethyl (C)[4]		Mascot
1173.6475	1173.6195	-0.028	-24	246	255	RDELTLEGIK					Mascot
1226.6964	1226.662	-0.0344	-28	176	185	RQSLRPDNIK					Mascot
1401.7333	1401.6686	-0.0647	-46	78	91	GLDVIQQAQSGTGK					Mascot
1461.8538	1461.8269	-0.0269	-18	155	169	ILASGVHVVVGTPGR					Mascot
1461.8538	1461.8269	-0.0269	-18	155	169	ILASGVHVVVGTPGR	108	100			Mascot
1555.7131	1555.6875	-0.0256	-16	186	198	MFVLDEADEMLSR					Mascot
1571.708	1571.6927	-0.0153	-10	186	198	MFVLDEADEMLSR			Oxidation (M)[1]		Mascot
1587.703	1587.6492	-0.0538	-34	186	198	MFVLDEADEMLSR			Oxidation (M)[1,10]		Mascot

1598.7849	1598.726	-0.0589	-37	256	267	QFYVNVEKEEWK					Mascot
1827.9388	1827.9164	-0.0224	-12	55	70	GIYAYGFEKPSAIQQR					Mascot
1827.9388	1827.9164	-0.0224	-12	55	70	GIYAYGFEKPSAIQQR	141	100			Mascot
1903.8928	1903.9167	0.0239	13	186	201	MFVLDEADEMLSRGFK			Oxidation (M)[1]		Mascot
1919.8878	1919.9137	0.0259	13	186	201	MFVLDEADEMLSRGFK			Oxidation (M)[1,10]		Mascot
2059.0754	2059.063	-0.0124	-6	131	150	ALGDYLGVKVHACVGGT SVR			Carbamidomethyl (C)[13]		Mascot
2059.0894	2059.063	-0.0264	-13	215	233	IQVGVFSATMPPEALEIT R	84	100			Mascot
2075.0842	2075.033	-0.0512	-25	215	233	IQVGVFSATMPPEALEIT R			Oxidation (M)[10]		Mascot
2075.0842	2075.033	-0.0512	-25	215	233	IQVGVFSATMPPEALEIT R	66	99.998	Oxidation (M)[10]		Mascot
2685.3804	2685.3508	-0.0296	-11	268	290	LDTLCDLYETLAITQSVIF VNTR			Carbamidomethyl (C)[5]		Mascot
2911.4949	2911.4683	-0.0266	-9	343	367	GIDVQQVSLVINYDLPTQ PENYLHR					Mascot
2911.4949	2911.4683	-0.0266	-9	343	367	GIDVQQVSLVINYDLPTQ PENYLHR	197	100			Mascot
3124.5806	3124.5498	-0.0308	-10	92	119	TATFCSGILQQLDYGLVE CQALVLAPTR			Carbamidomethyl (C)[5,19]		Mascot

3	unnamed protein product [Triticum aestivum]	gi|257726669	45131.2	5.31	23	864	100	47.539	723	100		

Peptide Information

Calc. Mass	Obsrv. Mass	± da	± ppm	Start Seq.	End Seq.	Sequence	Ion Score	C. I. %	Modification	Rank	Result Type
907.4818	907.4412	-0.0406	-45	235	241	FMNKPVR			Oxidation (M)[2]		Mascot
910.4702	910.4266	-0.0436	-48	373	379	MLFDIQK			Oxidation (M)[1]		Mascot
935.5197	935.4751	-0.0446	-48	131	139	ALGDYLGVK					Mascot
952.5033	952.4612	-0.0421	-44	170	176	VFDMLRR			Oxidation (M)[4]		Mascot
976.5574	976.5323	-0.0251	-26	360	368	GVAINFVTR					Mascot
1035.5768	1035.5188	-0.058	-56	234	241	KFMNKPVR			Oxidation (M)[3]		Mascot
1070.5953	1070.5665	-0.0288	-27	177	185	QSLRPDNIK					Mascot
1104.6525	1104.6274	-0.0251	-23	359	368	KGVAINFVTR					Mascot
1104.6525	1104.6274	-0.0251	-23	359	368	KGVAINFVTR	63	99.996			Mascot
1114.6831	1114.6573	-0.0258	-23	315	324	VLITTDLLAR					Mascot
1114.6831	1114.6573	-0.0258	-23	315	324	VLITTDLLAR	77	100			Mascot
1142.5736	1142.5491	-0.0245	-21	140	150	VHACVGGTSVR			Carbamidomethyl (C)[4]		Mascot
1142.5736	1142.5491	-0.0245	-21	140	150	VHACVGGTSVR	70	100	Carbamidomethyl (C)[4]		Mascot
1173.6475	1173.6195	-0.028	-24	246	255	RDELTLEGIK					Mascot
1226.6964	1226.662	-0.0344	-28	176	185	RQSLRPDNIK					Mascot
1401.7333	1401.6686	-0.0647	-46	78	91	GLDVIQQAQSGTGK					Mascot
1461.8538	1461.8269	-0.0269	-18	155	169	ILASGVHVVVGTPGR					Mascot

1461.8538	1461.8269	-0.0269	-18	155	169	ILASGVHVVVGTPGR	108	100			Mascot
1555.7131	1555.6875	-0.0256	-16	186	198	MFVLDEADEMLSR					Mascot
1571.708	1571.6927	-0.0153	-10	186	198	MFVLDEADEMLSR			Oxidation (M)[1]		Mascot
1587.703	1587.6492	-0.0538	-34	186	198	MFVLDEADEMLSR			Oxidation (M)[1,10]		Mascot
1598.7849	1598.726	-0.0589	-37	256	267	QFYVNVEKEEWK					Mascot
1827.9388	1827.9164	-0.0224	-12	55	70	GIYAYGFEKPSAIQQR					Mascot
1827.9388	1827.9164	-0.0224	-12	55	70	GIYAYGFEKPSAIQQR	141	100			Mascot
1903.8928	1903.9167	0.0239	13	186	201	MFVLDEADEMLSRGFK			Oxidation (M)[1]		Mascot
1919.8878	1919.9137	0.0259	13	186	201	MFVLDEADEMLSRGFK			Oxidation (M)[1,10]		Mascot
2011.0892	2011.1105	0.0213	11	215	233	IQVGVVSATMPPEALEIT R					Mascot
2011.0892	2011.1105	0.0213	11	215	233	IQVGVVSATMPPEALEIT R	67	99.999			Mascot
2027.0842	2027.056	-0.0282	-14	215	233	IQVGVVSATMPPEALEIT R			Oxidation (M)[10]		Mascot
2059.0754	2059.063	-0.0124	-6	131	150	ALGDYLGVKVHACVGGT SVR			Carbamidomethyl (C)[13]		Mascot
2059.0754	2059.063	-0.0124	-6	131	150	ALGDYLGVKVHACVGGT SVR			Carbamidomethyl (C)[13]		Mascot
2685.3804	2685.3508	-0.0296	-11	268	290	LDTLCDLYETLAITQSVIF VNTR			Carbamidomethyl (C)[5]		Mascot
2911.4949	2911.4683	-0.0266	-9	325	349	GIDVQQVSLVINYDLPTQ PENYLHR					Mascot
2911.4949	2911.4683	-0.0266	-9	325	349	GIDVQQVSLVINYDLPTQ PENYLHR	197	100			Mascot
3124.5806	3124.5498	-0.0308	-10	92	119	TATFCSGILQQLDYGLVE CQALVLAPTR			Carbamidomethyl (C)[5,19]		Mascot

4 unnamed protein product [Triticum aestivum] gi|257721534 47183.1 5.31 23 678 100 45.591 542 100

Protein Group

RecName: Full=Eukaryotic initiation factor 4A; Short=eIF-4A; AltName: Full=ATP-dependent RNA helicase eIF4A	gi|1170509	47183.1	5.3099999427 7954

Peptide Information

Calc. Mass	Obsrv. Mass	± da	± ppm	Start Seq.	End Seq.	Sequence	Ion Score	C. I. %	Modification	Rank	Result Type
907.4818	907.4412	-0.0406	-45	235	241	FMNKPVR			Oxidation (M)[2]		Mascot
910.4702	910.4266	-0.0436	-48	391	397	MLFDIQK			Oxidation (M)[1]		Mascot
935.5197	935.4751	-0.0446	-48	131	139	ALGDYLGVK					Mascot
976.5574	976.5323	-0.0251	-26	378	386	GVAINFVTR					Mascot
1035.5768	1035.5188	-0.058	-56	234	241	KFMNKPVR			Oxidation (M)[3]		Mascot
1070.5953	1070.5665	-0.0288	-27	177	185	QSLRPDNIK					Mascot
1104.6525	1104.6274	-0.0251	-23	377	386	KGVAINFVTR					Mascot
1104.6525	1104.6274	-0.0251	-23	377	386	KGVAINFVTR	63	99.996			Mascot
1114.6831	1114.6573	-0.0258	-23	333	342	VLITTDLLAR					Mascot

1114.6831	1114.6573	-0.0258	-23	333	342	VLITTDLLAR	77	100			Mascot
1142.5736	1142.5491	-0.0245	-21	140	150	VHACVGGTSVR			Carbamidomethyl (C)[4]		Mascot
1142.5736	1142.5491	-0.0245	-21	140	150	VHACVGGTSVR	70	100	Carbamidomethyl (C)[4]		Mascot
1173.6475	1173.6195	-0.028	-24	246	255	RDELTLEGIK					Mascot
1226.6964	1226.662	-0.0344	-28	176	185	RQSLRPDNIK					Mascot
1401.7333	1401.6686	-0.0647	-46	78	91	GLDVIQQAQSGTGK					Mascot
1461.8538	1461.8269	-0.0269	-18	155	169	ILASGVHVVVGTPGR					Mascot
1461.8538	1461.8269	-0.0269	-18	155	169	ILASGVHVVVGTPGR	108	100			Mascot
1549.8262	1549.7515	-0.0747	-48	202	214	DQIYDIFQLLPGK					Mascot
1555.7131	1555.6875	-0.0256	-16	186	198	MFVLDEADEMLSR					Mascot
1571.708	1571.6927	-0.0153	-10	186	198	MFVLDEADEMLSR			Oxidation (M)[1]		Mascot
1587.703	1587.6492	-0.0538	-34	186	198	MFVLDEADEMLSR			Oxidation (M)[1,10]		Mascot
1598.7849	1598.726	-0.0589	-37	256	267	QFYVNVEKEEWK					Mascot
1827.9388	1827.9164	-0.0224	-12	55	70	GIYAYGFEKPSAIQQR					Mascot
1827.9388	1827.9164	-0.0224	-12	55	70	GIYAYGFEKPSAIQQR	141	100			Mascot
1882.011	1881.8552	-0.1558	-83	199	214	GFKDQIYDIFQLLPGK					Mascot
1903.8928	1903.9167	0.0239	13	186	201	MFVLDEADEMLSRGFK			Oxidation (M)[1]		Mascot
1919.8878	1919.9137	0.0259	13	186	201	MFVLDEADEMLSRGFK			Oxidation (M)[1,10]		Mascot
2059.0754	2059.063	-0.0124	-6	131	150	ALGDYLGVKVHACVGGT SVR			Carbamidomethyl (C)[13]		Mascot
2059.0894	2059.063	-0.0264	-13	215	233	IQVGVFSATMPPEALEIT R	84	100			Mascot
2075.0842	2075.033	-0.0512	-25	215	233	IQVGVFSATMPPEALEIT R			Oxidation (M)[10]		Mascot
2075.0842	2075.033	-0.0512	-25	215	233	IQVGVFSATMPPEALEIT R	66	99.998	Oxidation (M)[10]		Mascot
2685.3804	2685.3508	-0.0296	-11	268	290	LDTLCDLYETLAITQSVIF VNTR			Carbamidomethyl (C)[5]		Mascot
3124.5806	3124.5498	-0.0308	-10	92	119	TATFCSGILQQLDYGLVE CQALVLAPTR			Carbamidomethyl (C)[5,19]		Mascot

5	eukaryotic initiation factor 4A-like protein, partial [Triticum aestivum]	gi|451798948	32137.8	8.72	15	631	100	21.672	535	100	

Peptide Information

Calc. Mass	Obsrv. Mass	± da	± ppm	Start Seq.	End Seq.	Sequence	Ion Score	C. I. %	Modification	Rank	Result Type
935.5197	935.4751	-0.0446	-48	27	35	ALGDYLGVK					Mascot
952.5033	952.4612	-0.0421	-44	66	72	VFDMLRR			Oxidation (M)[4]		Mascot
1070.5953	1070.5665	-0.0288	-27	73	81	QSLRPDNIK					Mascot
1114.6831	1114.6573	-0.0258	-23	229	238	VLITTDLLAR					Mascot
1114.6831	1114.6573	-0.0258	-23	229	238	VLITTDLLAR	77	100			Mascot
1142.5736	1142.5491	-0.0245	-21	36	46	VHACVGGTSVR			Carbamidomethyl (C)[4]		Mascot

Calc. Mass	Obsrv. Mass	± da	± ppm	Start Seq.	End Seq.	Sequence	Ion Score	C. I. %	Modification	Rank	Result Type
1142.5736	1142.5491	-0.0245	-21	36	46	VHACVGGTSVR	70	100	Carbamidomethyl (C)[4]		Mascot
1173.6475	1173.6195	-0.028	-24	142	151	RDELTLEGIK					Mascot
1226.6964	1226.662	-0.0344	-28	72	81	RQSLRPDNIK					Mascot
1461.8538	1461.8269	-0.0269	-18	51	65	ILASGVHVVVGTPGR					Mascot
1461.8538	1461.8269	-0.0269	-18	51	65	ILASGVHVVVGTPGR	108	100			Mascot
1555.7131	1555.6875	-0.0256	-16	82	94	MFVLDEADEMLSR					Mascot
1571.708	1571.6927	-0.0153	-10	82	94	MFVLDEADEMLSR			Oxidation (M)[1]		Mascot
1587.703	1587.6492	-0.0538	-34	82	94	MFVLDEADEMLSR			Oxidation (M)[1,10]		Mascot
1598.7849	1598.726	-0.0589	-37	152	163	QFYVNVEKEEWK					Mascot
1903.8928	1903.9167	0.0239	13	82	97	MFVLDEADEMLSRGFK			Oxidation (M)[1]		Mascot
1919.8878	1919.9137	0.0259	13	82	97	MFVLDEADEMLSRGFK			Oxidation (M)[1,10]		Mascot
2059.0754	2059.063	-0.0124	-6	27	46	ALGDYLGVKVHACVGGT SVR			Carbamidomethyl (C)[13]		Mascot
2059.0894	2059.063	-0.0264	-13	111	129	IQVGVFSATMPPEALEIT R	84	100			Mascot
2075.0842	2075.033	-0.0512	-25	111	129	IQVGVFSATMPPEALEIT R			Oxidation (M)[10]		Mascot
2075.0842	2075.033	-0.0512	-25	111	129	IQVGVFSATMPPEALEIT R	66	99.998	Oxidation (M)[10]		Mascot
2685.3804	2685.3508	-0.0296	-11	164	186	LDTLCDLYETLAITQSVIF VNTR			Carbamidomethyl (C)[5]		Mascot
2911.4949	2911.4683	-0.0266	-9	239	263	GIDVQQVSLVINYDLPTQ PENYLHR					Mascot
2911.4949	2911.4683	-0.0266	-9	239	263	GIDVQQVSLVINYDLPTQ PENYLHR	197	100			Mascot

6 Beta-amylase [Triticum urartu] gi|474451266 58995 5.34 8 61 96.487 3.88 41 99.348

Peptide Information

Calc. Mass	Obsrv. Mass	± da	± ppm	Start Seq.	End Seq.	Sequence	Ion Score	C. I. %	Modification	Rank	Result Type
947.5057	947.4658	-0.0399	-42	322	329	DGYRPIAR					Mascot
1016.5564	1016.5276	-0.0288	-28	412	419	LFGFTYLR					Mascot
1016.5564	1016.5276	-0.0288	-28	412	419	LFGFTYLR	41	99.348			Mascot
1326.6688	1326.6377	-0.0311	-23	385	395	YDPTAYNTILR					Mascot
1646.781	1646.7588	-0.0222	-13	246	259	FFVDNGTYLTEQGR					Mascot
1752.8916	1752.8147	-0.0769	-44	420	434	LSNQLVEGQNYVNFK					Mascot
1825.9728	1825.8894	-0.0834	-46	459	475	SGPELTIEMILQAAQPK					Mascot
1841.9678	1841.8945	-0.0733	-40	459	475	SGPELTIEMILQAAQPK			Oxidation (M)[9]		Mascot
1903.9298	1903.9167	-0.0131	-7	244	259	TRFFVDNGTYLTEQGR					Mascot
2087.0557	2087.0337	-0.022	-11	130	147	NIEYLTLGVDDQPLFHGR					Mascot

7 beta-amylase [Triticum aestivum] gi|1771782 56860.2 5.24 6 53 79.316 2.451 41 99.348

Protein Group

RecName: Full=Beta-amylase; AltName: Full=1,4-alpha-D-glucan maltohydrolase	gi|3334120	56860.2	5.2399 997711 1816

Peptide Information

Calc. Mass	Obsrv. Mass	± da	± ppm	Start Seq.	End Seq.	Sequence	Ion Score	C. I. %	Modification	Rank	Result Type
885.4352	885.4784	0.0432	49	210	216	YLEADFK					Mascot
1016.5564	1016.5276	-0.0288	-28	411	418	LFGFTYLR					Mascot
1016.5564	1016.5276	-0.0288	-28	411	418	LFGFTYLR	41	99.348			Mascot
1431.7585	1431.6772	-0.0813	-57	458	469	SKPEMPIEMILK			Oxidation (M)[5]		Mascot
1769.9181	1769.8894	-0.0287	-16	355	370	SAPEELVQQVLSAGWR					Mascot
1870.0487	1869.9163	-0.1324	-71	288	302	VQLAIKISGIHWWYR					Mascot
2087.0557	2087.0337	-0.022	-11	129	146	NIEYLTLGVDDQPLFHGR					Mascot

8	Disease resistance protein RPM1 [Triticum urartu]	gi|474086298	129568	6.87	20	48	38.957	9.581	

Peptide Information

Calc. Mass	Obsrv. Mass	± da	± ppm	Start Seq.	End Seq.	Sequence	Ion Score	C. I. %	Modification	Rank	Result Type
846.4178	846.4166	-0.0012	-1	381	386	MLFYTR			Oxidation (M)[1]		Mascot
908.4472	908.4121	-0.0351	-39	1012	1018	YAEQELR					Mascot
976.5608	976.5323	-0.0285	-29	511	518	LVNKSMIR			Oxidation (M)[6]		Mascot
983.5308	983.5084	-0.0224	-23	960	967	EAGRFIYK					Mascot
1035.5504	1035.5188	-0.0316	-31	693	702	GTVMKGEIGK			Oxidation (M)[4]		Mascot
1142.6317	1142.5491	-0.0826	-72	895	904	KDAASLVPWR					Mascot
1142.6317	1142.5491	-0.0826	-72	895	904	KDAASLVPWR					Mascot
1173.611	1173.6195	0.0085	7	927	936	EVKEAEEALR					Mascot
1192.646	1192.6027	-0.0433	-36	15	24	LGELLVEEYK					Mascot
1226.6562	1226.662	0.0058	5	533	542	VHDMVLDLIR			Oxidation (M)[4]		Mascot
1549.8584	1549.7515	-0.1069	-69	647	660	TAVKIPEEIGHDLK					Mascot
1598.7343	1598.726	-0.0083	-5	305	316	TMLVSFKCYYDR			Carbamidomethyl (C)[8], Oxidation (M)[2]		Mascot
1770.0194	1769.8894	-0.13	-73	404	421	CGGVPLAITTIASLLVGK			Carbamidomethyl (C)[1]		Mascot
1802.0463	1801.8799	-0.1664	-92	835	849	LPSLLFLLLWSKDEK					Mascot
1810.0143	1809.9073	-0.107	-59	871	887	IEIAVGEGALPMLEVLR					Mascot
1826.0092	1825.8894	-0.1198	-66	871	887	IEIAVGEGALPMLEVLR			Oxidation (M)[12]		Mascot
1884.9136	1884.9072	-0.0064	-3	1104	1119	GFWLGPAENKMYLCAK			Carbamidomethyl (C)[14]		Mascot
1903.9946	1903.9167	-0.0779	-41	251	266	ATETMAEWQLINQLKK					Mascot
1919.9895	1919.9137	-0.0758	-39	251	266	ATETMAEWQLINQLKK			Oxidation (M)[5]		Mascot

Calc. Mass	Obsrv. Mass	± da	± ppm	Start Seq.	End Seq.	Sequence	Ion Score	C. I. %	Modification	Rank	Result Type
1955.9895	1955.9517	-0.0378	-19	730	746	LRVLNIFFGEMEESAGK			Oxidation (M)[11]		Mascot
1985.9031	1985.9178	0.0147	7	1114	1129	MYLCAKAESCFHVDVR			Carbamidomethyl (C)[4,10]		Mascot
2059.1084	2059.063	-0.0454	-22	287	304	NLSQSWLAVSKSYHVAL R					Mascot
2059.1084	2059.063	-0.0454	-22	287	304	NLSQSWLAVSKSYHVAL R					Mascot
3033.5527	3033.5342	-0.0185	-6	964	991	FIYKLEEVLGGLDRPGEE GITAGEGDIR					Mascot

9	beta-amylase, partial [Triticum aestivum]	gi|451798942	15408.7	5.21	2	48	38.957	2.178	41	99.348	

Peptide Information

Calc. Mass	Obsrv. Mass	± da	± ppm	Start Seq.	End Seq.	Sequence	Ion Score	C. I. %	Modification	Rank	Result Type
1016.5564	1016.5276	-0.0288	-28	29	36	LFGFTYLR					Mascot
1016.5564	1016.5276	-0.0288	-28	29	36	LFGFTYLR	41	99.348			Mascot
1825.9728	1825.8894	-0.0834	-46	76	92	SGPELTIEMILQAAQPK					Mascot
1841.9678	1841.8945	-0.0733	-40	76	92	SGPELTIEMILQAAQPK			Oxidation (M)[9]		Mascot

10	hypothetical protein TRIUR3_01716 [Triticum urartu]	gi|473996123	34800.3	6.27	10	45	0	2.99	

Peptide Information

Calc. Mass	Obsrv. Mass	± da	± ppm	Start Seq.	End Seq.	Sequence	Ion Score	C. I. %	Modification	Rank	Result Type
881.4033	881.4152	0.0119	14	248	255	MESTQSAK					Mascot
908.3825	908.4121	0.0296	33	54	61	CRGGNGCK			Carbamidomethyl (C)[1,7]		Mascot
947.5421	947.4658	-0.0763	-81	258	265	RSFTLPAR					Mascot
1053.4994	1053.5422	0.0428	41	247	255	RMESTQSAK			Oxidation (M)[2]		Mascot
1156.5667	1156.5646	-0.0021	-2	248	257	MESTQSAKFK					Mascot
1431.6719	1431.6772	0.0053	4	20	32	EAAGMMALHEALR			Oxidation (M)[5,6]		Mascot
1630.804	1630.7186	-0.0854	-52	18	32	SKEAAGMMALHEALR			Oxidation (M)[7]		Mascot
1644.7507	1644.7849	0.0342	21	1	19	MVGSGAAGGGGGGDHA RSK			Oxidation (M)[1]		Mascot
1646.799	1646.7588	-0.0402	-24	18	32	SKEAAGMMALHEALR			Oxidation (M)[7,8]		Mascot
1826.7241	1826.8981	0.174	95	82	96	MAECLEEMEGEDPVR			Carbamidomethyl (C)[4], Oxidation (M)[1,8]		Mascot
1955.8691	1955.9517	0.0826	42	299	315	AQHEGSVYSDLMETFLE					Mascot

Gel Idx/Pos	257/K9	Instr./Gel Origin	BA2151/Sample Project 20140814	Process Status	Analysis Succeeded
Plate [#] Name	[1] Sample Project 20140814	Instrument Sample Name		Spectra	11

Rank	Protein Name	Accession No.	Protein MW	Protein PI	Pep. Count	Protein Score	Protein Score C. I. %	Intensity Matched	Total Ion Score	Total Ion C. I. %	Confirmed
1	unnamed protein product [Triticum aestivum]	gi|257726671	47227.1	5.31	16	180	100	15.504	106	100	

Peptide Information

Calc. Mass	Obsrv. Mass	± da	± ppm	Start Seq.	End Seq.	Sequence	Ion Score	C. I. %	Modification	Rank	Result Type
976.5574	976.53	-0.0274	-28	378	386	GVAINFVTR					Mascot
1070.5953	1070.5638	-0.0315	-29	177	185	QSLRPDNIK					Mascot
1104.6525	1104.6251	-0.0274	-25	377	386	KGVAINFVTR					Mascot
1114.6831	1114.6538	-0.0293	-26	333	342	VLITTDLLAR					Mascot
1142.5736	1142.5458	-0.0278	-24	140	150	VHACVGGTSVR			Carbamidomethyl (C)[4]		Mascot
1173.6475	1173.6141	-0.0334	-28	246	255	RDELTLEGIK					Mascot
1461.8538	1461.8214	-0.0324	-22	155	169	ILASGVHVVVGTPGR					Mascot
1571.708	1571.6755	-0.0325	-21	186	198	MFVLDEADEMLSR			Oxidation (M)[1]		Mascot
1587.703	1587.6487	-0.0543	-34	186	198	MFVLDEADEMLSR			Oxidation (M)[1,10]		Mascot
1800.7566	1800.8596	0.103	57	304	319	DHTVSATHGDMDQNTR			Oxidation (M)[11]		Mascot
1827.9388	1827.9044	-0.0344	-19	55	70	GIYAYGFEKPSAIQQR					Mascot
1827.9388	1827.9044	-0.0344	-19	55	70	GIYAYGFEKPSAIQQR	106	100			Mascot
1882.011	1881.8931	-0.1179	-63	199	214	GFKDQIYDIFQLLPGK					Mascot
2013.8792	2013.9648	0.0856	43	302	319	GRDHTVSATHGDMDQNTR			Oxidation (M)[13]		Mascot
2075.0842	2075.022	-0.0622	-30	215	233	IQVGVFSATMPPEALEITR			Oxidation (M)[10]		Mascot
2685.3804	2685.3381	-0.0423	-16	268	290	LDTLCDLYETLAITQSVIFVNTR			Carbamidomethyl (C)[5]		Mascot
2911.4949	2911.4631	-0.0318	-11	343	367	GIDVQQVSLVINYDLPTQPENYLHR					Mascot
3124.5806	3124.5454	-0.0352	-11	92	119	TATFCSGILQQLDYGLVECQALVLAPTR			Carbamidomethyl (C)[5,19]		Mascot

Rank	Protein Name	Accession No.	Protein MW	Protein PI	Pep. Count	Protein Score	Protein Score C. I. %	Intensity Matched	Total Ion Score	Total Ion C. I. %	Confirmed
2	Beta-amylase [Triticum urartu]	gi|474451266	58995	5.34	11	177	100	22.988	139	100	

Peptide Information

Calc. Mass	Obsrv. Mass	± da	± ppm	Start Seq.	End Seq.	Sequence	Ion Score	C. I. %	Modification	Rank	Result Type
947.5057	947.4587	-0.047	-50	322	329	DGYRPIAR					Mascot
1016.5564	1016.526	-0.0304	-30	412	419	LFGFTYLR					Mascot
1016.5564	1016.526	-0.0304	-30	412	419	LFGFTYLR	60	99.993			Mascot

1326.6688	1326.6288	-0.04	-30	385	395	YDPTAYNTILR					Mascot
1326.6688	1326.6288	-0.04	-30	385	395	YDPTAYNTILR	79	100			Mascot
1646.781	1646.7448	-0.0362	-22	246	259	FFVDNGTYLTEQGR					Mascot
1669.7349	1669.7363	0.0014	1	148	161	SAVQMYTDYMASFR					Mascot
1752.8916	1752.8192	-0.0724	-41	420	434	LSNQLVEGQNYVNFK					Mascot
1841.9678	1841.882	-0.0858	-47	459	475	SGPELTIEMILQAAQPK			Oxidation (M)[9]		Mascot
2013.9778	2013.9648	-0.013	-6	304	321	VPSHAAEITAGYYNLHDR					Mascot
2087.0557	2087.0181	-0.0376	-18	130	147	NIEYLTLGVDDQPLFHGR					Mascot
2183.0486	2182.9871	-0.0615	-28	440	458	MHANLPHDPCVDPVAPLQR			Carbamidomethyl (C)[10], Oxidation (M)[1]		Mascot
2269.2075	2269.1658	-0.0417	-18	166	187	EFLDAGVIVDIEVGLGPAGELR					Mascot

3	Eukaryotic initiation factor 4A-1 [Triticum urartu]	gi|474441074	47158	5.38	15	173	100	15.39	106	100

Peptide Information

Calc. Mass	Obsrv. Mass	± da	± ppm	Start Seq.	End Seq.	Sequence	Ion Score	C. I. %	Modification	Rank	Result Type
976.5574	976.53	-0.0274	-28	378	386	GVAINFVTR					Mascot
1070.5953	1070.5638	-0.0315	-29	177	185	QSLRPDNIK					Mascot
1104.6525	1104.6251	-0.0274	-25	377	386	KGVAINFVTR					Mascot
1114.6831	1114.6538	-0.0293	-26	333	342	VLITTDLLAR					Mascot
1142.5736	1142.5458	-0.0278	-24	140	150	VHACVGGTSVR			Carbamidomethyl (C)[4]		Mascot
1173.6475	1173.6141	-0.0334	-28	246	255	RDELTLEGIK					Mascot
1461.8538	1461.8214	-0.0324	-22	155	169	ILASGVHVVVGTPGR					Mascot
1571.708	1571.6755	-0.0325	-21	186	198	MFVLDEADEMLSR			Oxidation (M)[1]		Mascot
1587.703	1587.6487	-0.0543	-34	186	198	MFVLDEADEMLSR			Oxidation (M)[1,10]		Mascot
1800.7566	1800.8596	0.103	57	304	319	DHTVSATHGDMDQNTR			Oxidation (M)[11]		Mascot
1827.9388	1827.9044	-0.0344	-19	55	70	GIYAYGFEKPSAIQQR					Mascot
1827.9388	1827.9044	-0.0344	-19	55	70	GIYAYGFEKPSAIQQR	106	100			Mascot
2013.8792	2013.9648	0.0856	43	302	319	GRDHTVSATHGDMDQNTR			Oxidation (M)[13]		Mascot
2075.0842	2075.022	-0.0622	-30	215	233	IQVGVFSATMPPEALEITR			Oxidation (M)[10]		Mascot
2685.3804	2685.3381	-0.0423	-16	268	290	LDTLCDLYETLAITQSVIFVNTR			Carbamidomethyl (C)[5]		Mascot
2911.4949	2911.4631	-0.0318	-11	343	367	GIDVQQVSLVINYDLPTQPENYLHR					Mascot
3124.5806	3124.5454	-0.0352	-11	92	119	TATFCSGILQQLDYGLVECQALVLAPTR			Carbamidomethyl (C)[5,19]		Mascot

4	unnamed protein product [Triticum aestivum]	gi|257721534	47183.1	5.31	15	171	100	14.134	106	100

Protein Group

RecName: Full=Eukaryotic initiation factor 4A;	gi|1170509	47183.1	5.3099

	Short=eIF-4A; AltName: Full=ATP-dependent RNA helicase eIF4A		999427 7954										

Peptide Information

Calc. Mass	Obsrv. Mass	± da	± ppm	Start Seq.	End Seq.	Sequence	Ion Score	C. I. %	Modification	Rank	Result Type
976.5574	976.53	-0.0274	-28	378	386	GVAINFVTR					Mascot
1070.5953	1070.5638	-0.0315	-29	177	185	QSLRPDNIK					Mascot
1104.6525	1104.6251	-0.0274	-25	377	386	KGVAINFVTR					Mascot
1114.6831	1114.6538	-0.0293	-26	333	342	VLITTDLLAR					Mascot
1142.5736	1142.5458	-0.0278	-24	140	150	VHACVGGTSVR			Carbamidomethyl (C)[4]		Mascot
1173.6475	1173.6141	-0.0334	-28	246	255	RDELTLEGIK					Mascot
1461.8538	1461.8214	-0.0324	-22	155	169	ILASGVHVVVGTPGR					Mascot
1571.708	1571.6755	-0.0325	-21	186	198	MFVLDEADEMLSR			Oxidation (M)[1]		Mascot
1587.703	1587.6487	-0.0543	-34	186	198	MFVLDEADEMLSR			Oxidation (M)[1,10]		Mascot
1800.7566	1800.8596	0.103	57	304	319	DHTVSATHGDMDQNTR			Oxidation (M)[11]		Mascot
1827.9388	1827.9044	-0.0344	-19	55	70	GIYAYGFEKPSAIQQR					Mascot
1827.9388	1827.9044	-0.0344	-19	55	70	GIYAYGFEKPSAIQQR	106	100			Mascot
1882.011	1881.8931	-0.1179	-63	199	214	GFKDQIYDIFQLLPGK					Mascot
2013.8792	2013.9648	0.0856	43	302	319	GRDHTVSATHGDMDQNTR			Oxidation (M)[13]		Mascot
2075.0842	2075.022	-0.0622	-30	215	233	IQVGVFSATMPPEALEITR			Oxidation (M)[10]		Mascot
2685.3804	2685.3381	-0.0423	-16	268	290	LDTLCDLYETLAITQSVIFVNTR			Carbamidomethyl (C)[5]		Mascot
3124.5806	3124.5454	-0.0352	-11	92	119	TATFCSGILQQLDYGLVECQALVLAPTR			Carbamidomethyl (C)[5,19]		Mascot

5	unnamed protein product [Triticum aestivum]	gi|257726669	45131.2	5.31	13	159	100	14.372	106	100

Peptide Information

Calc. Mass	Obsrv. Mass	± da	± ppm	Start Seq.	End Seq.	Sequence	Ion Score	C. I. %	Modification	Rank	Result Type
976.5574	976.53	-0.0274	-28	360	368	GVAINFVTR					Mascot
1070.5953	1070.5638	-0.0315	-29	177	185	QSLRPDNIK					Mascot
1104.6525	1104.6251	-0.0274	-25	359	368	KGVAINFVTR					Mascot
1114.6831	1114.6538	-0.0293	-26	315	324	VLITTDLLAR					Mascot
1142.5736	1142.5458	-0.0278	-24	140	150	VHACVGGTSVR			Carbamidomethyl (C)[4]		Mascot
1173.6475	1173.6141	-0.0334	-28	246	255	RDELTLEGIK					Mascot
1461.8538	1461.8214	-0.0324	-22	155	169	ILASGVHVVVGTPGR					Mascot
1571.708	1571.6755	-0.0325	-21	186	198	MFVLDEADEMLSR			Oxidation (M)[1]		Mascot
1587.703	1587.6487	-0.0543	-34	186	198	MFVLDEADEMLSR			Oxidation (M)[1,10]		Mascot

1827.9388	1827.9044	-0.0344	-19	55	70	GIYAYGFEKPSAIQQR					Mascot
1827.9388	1827.9044	-0.0344	-19	55	70	GIYAYGFEKPSAIQQR	106	100			Mascot
2011.0892	2011.0721	-0.0171	-9	215	233	IQVGVVSATMPPEALEIT R					Mascot
2685.3804	2685.3381	-0.0423	-16	268	290	LDTLCDLYETLAITQSVIF VNTR			Carbamidomethyl (C)[5]		Mascot
2911.4949	2911.4631	-0.0318	-11	325	349	GIDVQQVSLVINYDLPTQ PENYLHR					Mascot
3124.5806	3124.5454	-0.0352	-11	92	119	TATFCSGILQQLDYGLVE CQALVLAPTR			Carbamidomethyl (C)[5,19]		Mascot

6	beta amylase [Triticum aestivum]	gi|32400764	31099.9	8.6	5	93	99.998	9.531	76	100	

Peptide Information

Calc. Mass	Obsrv. Mass	± da	± ppm	Start Seq.	End Seq.	Sequence	Ion Score	C. I. %	Modification	Rank	Result Type
947.5057	947.4587	-0.047	-50	137	144	DGYRPIAR					Mascot
1326.6688	1326.6288	-0.04	-30	200	210	YDPTAYNTILR					Mascot
1326.6688	1326.6288	-0.04	-30	200	210	YDPTAYNTILR	79	100			Mascot
1646.781	1646.7448	-0.0362	-22	61	74	FFVDNGTYLTEQGR					Mascot
1842.0425	1841.882	-0.1605	-87	104	118	VQLAIKISGIHWWYK					Mascot
2013.9778	2013.9648	-0.013	-6	119	136	VPSHAAEITAGYYNLHDR					Mascot

7	beta-amylase, partial [Triticum aestivum]	gi|451798942	15408.7	5.21	3	74	99.828	12.414	60	99.993	

Peptide Information

Calc. Mass	Obsrv. Mass	± da	± ppm	Start Seq.	End Seq.	Sequence	Ion Score	C. I. %	Modification	Rank	Result Type
1016.5564	1016.526	-0.0304	-30	29	36	LFGFTYLR					Mascot
1016.5564	1016.526	-0.0304	-30	29	36	LFGFTYLR	60	99.993			Mascot
1841.9678	1841.882	-0.0858	-47	76	92	SGPELTIEMILQAAQPK			Oxidation (M)[9]		Mascot
2183.0486	2182.9871	-0.0615	-28	57	75	MHANLPHDPCVDPVAPL QR			Carbamidomethyl (C)[10], Oxidation (M)[1]		Mascot

8	beta-amylase [Triticum aestivum]	gi|1771782	56860.2	5.24	6	73	99.778	13.938	60	99.993	

Protein Group

RecName: Full=Beta-amylase; AltName: Full=1,4-alpha-D-glucan maltohydrolase	gi|3334120	56860.2	5.2399 997711 1816

Peptide Information

Calc. Mass	Obsrv. Mass	± da	± ppm	Start Seq.	End Seq.	Sequence	Ion Score	C. I. %	Modification	Rank	Result Type
1016.5564	1016.526	-0.0304	-30	411	418	LFGFTYLR					Mascot
1016.5564	1016.526	-0.0304	-30	411	418	LFGFTYLR	60	99.993			Mascot

1297.6787	1297.6023	-0.0764	-59	210	221	YLEADFKAAAAK					Mascot
1607.6512	1607.7827	0.1315	82	333	345	HHASMNFTCAEMR			Carbamidomethyl (C)[9], Oxidation (M)[5]		Mascot
1623.6461	1623.6915	0.0454	28	333	345	HHASMNFTCAEMR			Carbamidomethyl (C)[9], Oxidation (M)[5,12]		Mascot
1669.7349	1669.7363	0.0014	1	147	160	TAVQMYADYMASFR			Oxidation (M)[5]		Mascot
1769.9181	1769.8868	-0.0313	-18	355	370	SAPEELVQQVLSAGWR					Mascot
2087.0557	2087.0181	-0.0376	-18	129	146	NIEYLTLGVDDQPLFHGR					Mascot

9 hypothetical protein TRIUR3_23974 [Triticum urartu] gi|473996411 25499.2 9.46 12 61 96.796 9.884

Peptide Information

Calc. Mass	Obsrv. Mass	± da	± ppm	Start Seq.	End Seq.	Sequence	Ion Score	C. I. %	Modification	Rank	Result Type
846.4315	846.4894	0.0579	68	30	37	EALGTAER					Mascot
976.5284	976.53	0.0016	2	96	102	EKWCILK			Carbamidomethyl (C)[4]		Mascot
1104.5579	1104.6251	0.0672	61	2	12	ATRSPMAGSAR					Mascot
1156.6572	1156.5728	-0.0844	-73	18	28	ATESPIQIGIK					Mascot
1251.5933	1251.6223	0.029	23	1	12	MATRSPMAGSAR			Oxidation (M)[1]		Mascot
1326.6073	1326.6288	0.0215	16	51	62	GGGGSTWYEERK					Mascot
1326.6073	1326.6288	0.0215	16	51	62	GGGGSTWYEERK					Mascot
1442.7737	1442.6742	-0.0995	-69	144	157	SEPSLLEDLAVAAK					Mascot
1442.7737	1442.6742	-0.0995	-69	144	157	SEPSLLEDLAVAAK					Mascot
1544.8068	1544.7675	-0.0393	-25	115	127	DIGPELRPNDYKK					Mascot
1607.8098	1607.7827	-0.0271	-17	170	183	IYMTRASTYTSAVK			Oxidation (M)[3]		Mascot
1797.9858	1797.8824	-0.1034	-58	13	28	WGELRATESPIQIGIK					Mascot
2042.9917	2042.9392	-0.0525	-26	184	200	NYVETYQEGLKDVLDEK					Mascot
2075.0696	2075.022	-0.0476	-23	69	87	YDLKEIAFPSSLPDPPGTK					Mascot

10 eukaryotic initiation factor 4A-like protein, partial [Triticum aestivum] gi|451798948 32137.8 8.72 11 57 91.953 5.576

Peptide Information

Calc. Mass	Obsrv. Mass	± da	± ppm	Start Seq.	End Seq.	Sequence	Ion Score	C. I. %	Modification	Rank	Result Type
1070.5953	1070.5638	-0.0315	-29	73	81	QSLRPDNIK					Mascot
1114.6831	1114.6538	-0.0293	-26	229	238	VLITTDLLAR					Mascot
1142.5736	1142.5458	-0.0278	-24	36	46	VHACVGGTSVR			Carbamidomethyl (C)[4]		Mascot
1173.6475	1173.6141	-0.0334	-28	142	151	RDELTLEGIK					Mascot
1461.8538	1461.8214	-0.0324	-22	51	65	ILASGVHVVVGTPGR					Mascot
1571.708	1571.6755	-0.0325	-21	82	94	MFVLDEADEMLSR			Oxidation (M)[1]		Mascot

1587.703	1587.6487	-0.0543	-34	82	94	MFVLDEADEMLSR	Oxidation (M)[1,10]	Mascot
1800.7566	1800.8596	0.103	57	200	215	DHTVSATHGDMDQNTR	Oxidation (M)[11]	Mascot
2013.8792	2013.9648	0.0856	43	198	215	GRDHTVSATHGDMDQNT R	Oxidation (M)[13]	Mascot
2075.0842	2075.022	-0.0622	-30	111	129	IQVGVFSATMPPEALEIT R	Oxidation (M)[10]	Mascot
2685.3804	2685.3381	-0.0423	-16	164	186	LDTLCDLYETLAITQSVIF VNTR	Carbamidomethyl (C)[5]	Mascot
2911.4949	2911.4631	-0.0318	-11	239	263	GIDVQQVSLVINYDLPTQ PENYLHR		Mascot

Gel Idx/Pos	258/K10	Instr./Gel Origin	BA2151/Sample Project 20140814	Process Status	Analysis Succeeded
Plate [#] Name	[1] Sample Project 20140814	Instrument Sample Name		Spectra	11

Rank	Protein Name	Accession No.	Protein MW	Protein PI	Pep. Count	Protein Score	Protein Score C. I. %	Intensity Matched	Total Ion Score	Total Ion C. I. %	Confirmed
1	enolase [Triticum aestivum]	gi|461744058	48459.5	5.49	17	627	100	54.224	531	100	

Peptide Information

Calc. Mass	Obsrv. Mass	± da	± ppm	Start Seq.	End Seq.	Sequence	Ion Score	C. I. %	Modification	Rank	Result Type
806.4519	806.4372	-0.0147	-18	418	423	YNQLLR					Mascot
918.5077	918.4749	-0.0328	-36	347	354	SCNALLLK			Carbamidomethyl (C)[2]		Mascot
978.5043	978.4792	-0.0251	-26	439	446	FRAPVEPY					Mascot
978.5043	978.4792	-0.0251	-26	439	446	FRAPVEPY	39	99.317			Mascot
1189.6034	1189.614	0.0106	9	190	199	MGVEVYHNLK					Mascot
1205.5984	1205.5643	-0.0341	-28	190	199	MGVEVYHNLK			Oxidation (M)[1]		Mascot
1238.5848	1238.552	-0.0328	-26	373	383	HAGWGVMTSHR					Mascot
1254.5797	1254.5375	-0.0422	-34	373	383	HAGWGVMTSHR			Oxidation (M)[7]		Mascot
1551.8643	1551.8042	-0.0601	-39	134	147	IPLYQHIANLAGNK					Mascot
1573.8433	1573.7914	-0.0519	-33	355	369	VNQIGSVTESIEAVK					Mascot
1577.8058	1577.7406	-0.0652	-41	424	438	IEEELGDAAVYAGLK					Mascot
1790.9283	1790.9041	-0.0242	-14	36	53	AAVPSGASTGVYEALELR					Mascot
1790.9283	1790.9041	-0.0242	-14	36	53	AAVPSGASTGVYEALELR	155	100			Mascot
1901.8512	1901.8148	-0.0364	-19	264	279	TYDLNFKEENNDGSQK					Mascot
1916.9497	1916.8655	-0.0842	-44	169	185	LAMQEFMILPTGATSFK			Oxidation (M)[3,7]		Mascot
1983.8536	1983.8239	-0.0297	-15	18	35	GNPTVEVDVCCSDGTFA R			Carbamidomethyl (C)[10,11]		Mascot
1983.8536	1983.8239	-0.0297	-15	18	35	GNPTVEVDVCCSDGTFA R	157	100	Carbamidomethyl (C)[10,11]		Mascot
2016.9584	2016.8817	-0.0767	-38	246	263	VVIGMDVAASEFYNDKD K			Oxidation (M)[5]		Mascot
2132.1611	2132.0872	-0.0739	-35	148	168	QLVLPVPAFNVINGGSHA GNK					Mascot
2251.0547	2251.0205	-0.0342	-15	83	102	DPTAQTELDNFMVQQLD GTK					Mascot
2252.1294	2252.0425	-0.0869	-39	384	405	SGETEDTFIADLAVGLST GQIK					Mascot
2267.0496	2266.9934	-0.0562	-25	83	102	DPTAQTELDNFMVQQLD GTK			Oxidation (M)[12]		Mascot
2575.2378	2575.2151	-0.0227	-9	316	338	MTEECGVEVQIVGDDLLV TNPTR			Carbamidomethyl (C)[5]		Mascot
2575.2378	2575.2151	-0.0227	-9	316	338	MTEECGVEVQIVGDDLLV TNPTR	171	100	Carbamidomethyl (C)[5]		Mascot
2591.2329	2591.1909	-0.042	-16	316	338	MTEECGVEVQIVGDDLLV TNPTR			Carbamidomethyl (C)[5], Oxidation (M)[1]		Mascot

2591.2329	2591.1909	-0.042	-16	316	338	MTEECGVEVQIVGDDLLV TNPTR	180	100	Carbamidomethyl (C)[5], Oxidation (M)[1]		Mascot

2	enolase [Triticum aestivum]	gi|461744056	48416.5	5.59	17	625	100	54.29	531	100	

Protein Group

enolase [Triticum aestivum]	gi|461744078	48416.5	5.5900 001525 8789

Peptide Information

Calc. Mass	Obsrv. Mass	± da	± ppm	Start Seq.	End Seq.	Sequence	Ion Score	C. I. %	Modification	Rank	Result Type
806.4519	806.4372	-0.0147	-18	418	423	YNQLLR					Mascot
918.5077	918.4749	-0.0328	-36	347	354	SCNALLLK			Carbamidomethyl (C)[2]		Mascot
978.5043	978.4792	-0.0251	-26	439	446	FRAPVEPY					Mascot
978.5043	978.4792	-0.0251	-26	439	446	FRAPVEPY	39	99.317			Mascot
1189.6034	1189.614	0.0106	9	190	199	MGVEVYHNLK					Mascot
1205.5984	1205.5643	-0.0341	-28	190	199	MGVEVYHNLK			Oxidation (M)[1]		Mascot
1238.5848	1238.552	-0.0328	-26	373	383	HAGWGVMTSHR					Mascot
1254.5797	1254.5375	-0.0422	-34	373	383	HAGWGVMTSHR			Oxidation (M)[7]		Mascot
1551.8643	1551.8042	-0.0601	-39	134	147	IPLYQHIANLAGNK					Mascot
1573.8433	1573.7914	-0.0519	-33	355	369	VNQIGSVTESIEAVK					Mascot
1790.9283	1790.9041	-0.0242	-14	36	53	AAVPSGASTGVYEALELR					Mascot
1790.9283	1790.9041	-0.0242	-14	36	53	AAVPSGASTGVYEALELR	155	100			Mascot
1886.9391	1886.8562	-0.0829	-44	169	185	LAMQEFMILPTGAASFK			Oxidation (M)[3,7]		Mascot
1901.8512	1901.8148	-0.0364	-19	264	279	TYDLNFKEENNDGSQK					Mascot
1983.8536	1983.8239	-0.0297	-15	18	35	GNPTVEVDVCCSDGTFA R			Carbamidomethyl (C)[10,11]		Mascot
1983.8536	1983.8239	-0.0297	-15	18	35	GNPTVEVDVCCSDGTFA R	157	100	Carbamidomethyl (C)[10,11]		Mascot
2016.9584	2016.8817	-0.0767	-38	246	263	VVIGMDVAASEFYNDKD K			Oxidation (M)[5]		Mascot
2132.1611	2132.0872	-0.0739	-35	148	168	QLVLPVPAFNVINGGSHA GNK					Mascot
2252.1294	2252.0425	-0.0869	-39	384	405	SGETEDTFIADLAVGLST GQIK					Mascot
2267.0496	2266.9934	-0.0562	-25	83	102	DPTAQTELDNYMVQQLD GTK					Mascot
2346.1543	2345.9802	-0.1741	-74	169	189	LAMQEFMILPTGAASFKE AMK			Oxidation (M)[3,7]		Mascot
2575.2378	2575.2151	-0.0227	-9	316	338	MTEECGVEVQIVGDDLLV TNPTR			Carbamidomethyl (C)[5]		Mascot
2575.2378	2575.2151	-0.0227	-9	316	338	MTEECGVEVQIVGDDLLV TNPTR	171	100	Carbamidomethyl (C)[5]		Mascot
2591.2329	2591.1909	-0.042	-16	316	338	MTEECGVEVQIVGDDLLV TNPTR			Carbamidomethyl (C)[5], Oxidation (M)[1]		Mascot
2591.2329	2591.1909	-0.042	-16	316	338	MTEECGVEVQIVGDDLLV TNPTR	180	100	Carbamidomethyl (C)[5], Oxidation (M)[1]		Mascot

3	Enolase [Triticum urartu]	gi|474188401	43643.1	5.54	12	394	100	22.623	335	100

Peptide Information

Calc. Mass	Obsrv. Mass	± da	± ppm	Start Seq.	End Seq.	Sequence	Ion Score	C. I. %	Modification	Rank	Result Type
918.5077	918.4749	-0.0328	-36	294	301	SCNALLLK			Carbamidomethyl (C)[2]		Mascot
1189.6034	1189.614	0.0106	9	137	146	MGVEVYHNLK					Mascot
1205.5984	1205.5643	-0.0341	-28	137	146	MGVEVYHNLK			Oxidation (M)[1]		Mascot
1238.5848	1238.552	-0.0328	-26	320	330	HAGWGVMTSHR					Mascot
1254.5797	1254.5375	-0.0422	-34	320	330	HAGWGVMTSHR			Oxidation (M)[7]		Mascot
1573.8433	1573.7914	-0.0519	-33	302	316	VNQIGSVTESIEAVK					Mascot
1790.9283	1790.9041	-0.0242	-14	34	51	AAVPSGASTGVYEALELR					Mascot
1790.9283	1790.9041	-0.0242	-14	34	51	AAVPSGASTGVYEALELR	155	100			Mascot
1901.8512	1901.8148	-0.0364	-19	211	226	TYDLNFKEENNDGSQK					Mascot
2016.9584	2016.8817	-0.0767	-38	193	210	VVIGMDVAASEFYNDKD K			Oxidation (M)[5]		Mascot
2154.1667	2154.0757	-0.091	-42	114	132	KIPLYQEFMILPTGAASFK					Mascot
2252.1294	2252.0425	-0.0869	-39	331	352	SGETEDTFIADLAVGLST GQIK					Mascot
2324.0425	2323.9866	-0.0559	-24	153	174	YGQDATNVGDEGGFAPN IQENK					Mascot
2452.1375	2452.0972	-0.0403	-16	152	174	KYGQDATNVGDEGGFAP NIQENK					Mascot
2575.2378	2575.2151	-0.0227	-9	263	285	MTEECGVEVQIVGDDLLV TNPTR			Carbamidomethyl (C)[5]		Mascot
2575.2378	2575.2151	-0.0227	-9	263	285	MTEECGVEVQIVGDDLLV TNPTR	171	100	Carbamidomethyl (C)[5]		Mascot
2591.2329	2591.1909	-0.042	-16	263	285	MTEECGVEVQIVGDDLLV TNPTR			Carbamidomethyl (C)[5], Oxidation (M)[1]		Mascot
2591.2329	2591.1909	-0.042	-16	263	285	MTEECGVEVQIVGDDLLV TNPTR	180	100	Carbamidomethyl (C)[5], Oxidation (M)[1]		Mascot

4	Enolase 2 [Triticum urartu]	gi|474377047	71283.3	5.4	14	232	100	5.881	180	100

Peptide Information

Calc. Mass	Obsrv. Mass	± da	± ppm	Start Seq.	End Seq.	Sequence	Ion Score	C. I. %	Modification	Rank	Result Type
918.5077	918.4749	-0.0328	-36	573	580	SCNALLLK			Carbamidomethyl (C)[2]		Mascot
932.5121	932.4705	-0.0416	-45	1	8	MEEVAILK					Mascot
1189.6034	1189.614	0.0106	9	416	425	MGVEVYHNLK					Mascot
1205.5984	1205.5643	-0.0341	-28	416	425	MGVEVYHNLK			Oxidation (M)[1]		Mascot
1238.5848	1238.552	-0.0328	-26	599	609	HAGWGVMTSHR					Mascot
1254.5797	1254.5375	-0.0422	-34	599	609	HAGWGVMTSHR			Oxidation (M)[7]		Mascot
1507.7244	1507.7058	-0.0186	-12	1	13	MEEVAILKGSDCR			Carbamidomethyl (C)[12]		Mascot

Calc. Mass	Obsrv. Mass	± da	± ppm	Start Seq.	End Seq.	Sequence	Ion Score	C. I. %	Modification	Rank	Result Type
1573.8433	1573.7914	-0.0519	-33	581	595	VNQIGSVTESIEAVK					Mascot
1901.8512	1901.8148	-0.0364	-19	490	505	TYDLNFKEENNDGSQK					Mascot
2016.9584	2016.8817	-0.0767	-38	472	489	VVIGMDVAASEFYNDKD K			Oxidation (M)[5]		Mascot
2154.1667	2154.0757	-0.091	-42	393	411	KIPLYQEFMILPTGAASFK					Mascot
2251.0911	2251.0205	-0.0706	-31	343	362	DPTAQTELDNFMVQKLD GTK					Mascot
2252.1294	2252.0425	-0.0869	-39	610	631	SGETEDTFIADLAVGLST GQIK					Mascot
2267.0859	2266.9934	-0.0925	-41	343	362	DPTAQTELDNFMVQKLD GTK			Oxidation (M)[12]		Mascot
2324.0425	2323.9866	-0.0559	-24	432	453	YGQDATNVGDEGGFAPN IQENK					Mascot
2452.1375	2452.0972	-0.0403	-16	431	453	KYGQDATNVGDEGGFAP NIQENK					Mascot
2575.2378	2575.2151	-0.0227	-9	542	564	MTEECGVEVQIVGDDLLV TNPTR			Carbamidomethyl (C)[5]		Mascot
2575.2378	2575.2151	-0.0227	-9	542	564	MTEECGVEVQIVGDDLLV TNPTR	171	100	Carbamidomethyl (C)[5]		Mascot
2591.2329	2591.1909	-0.042	-16	542	564	MTEECGVEVQIVGDDLLV TNPTR			Carbamidomethyl (C)[5], Oxidation (M)[1]		Mascot
2591.2329	2591.1909	-0.042	-16	542	564	MTEECGVEVQIVGDDLLV TNPTR	180	100	Carbamidomethyl (C)[5], Oxidation (M)[1]		Mascot

5	plastid ADP-glucose pyrophosphorylase small subunit [Triticum aestivum]	gi|224021585	56652.9	6.4	13	210	100	2.831	158	100

Peptide Information

Calc. Mass	Obsrv. Mass	± da	± ppm	Start Seq.	End Seq.	Sequence	Ion Score	C. I. %	Modification	Rank	Result Type
1017.5952	1017.5392	-0.056	-55	407	415	IHHSVVGLR					Mascot
1025.5925	1025.5387	-0.0538	-52	301	308	HVMLQLLR			Oxidation (M)[3]		Mascot
1032.5472	1032.5209	-0.0263	-25	371	379	SAPIYTQPR					Mascot
1256.7109	1256.597	-0.1139	-91	105	116	AKPAVPLGANYR					Mascot
1384.6896	1384.6577	-0.0319	-23	360	370	KPIPDFSFYDR					Mascot
1384.6896	1384.6577	-0.0319	-23	360	370	KPIPDFSFYDR	75	100			Mascot
1573.6912	1573.7914	0.1002	64	19	34	ASAATAAASTSCDSFR			Carbamidomethyl (C)[12]		Mascot
1625.8646	1625.8336	-0.031	-19	133	146	IYVLTQFNSASLNR	83	100			Mascot
1746.9684	1746.8699	-0.0985	-56	2	18	AMAAAASPSKILIPPHR			Oxidation (M)[2]		Mascot
1769.9684	1769.8848	-0.0836	-47	489	504	ETDGYFIKSGIVTVIK					Mascot
1852.9143	1852.8784	-0.0359	-19	269	285	AMMVDTTILGLDDARAK			Oxidation (M)[2,3]		Mascot
1862.0139	1861.8931	-0.1208	-65	1	18	MAMAAAASPSKILIPPHR					Mascot
1916.9861	1916.8655	-0.1206	-63	284	300	AKEMPYIASMGIYVISK			Oxidation (M)[4]		Mascot
3061.4287	3061.4038	-0.0249	-8	161	188	NEGFVEVLAAQQSPDNP DWFQGTADAVR					Mascot

6	small subunit ADP glucose pyrophosphorylase [Triticum aestivum]	gi|20127139	52313.6	5.53	12	207	100	2.811	158	100

Protein Group

cytosolic small subunit ADP glucose pyrophosphorylase [Triticum aestivum]	gi|125976023	52277.6	5.5300 002098 0835
small subunit ADP glucose pyrophosphorylase [Triticum aestivum]	gi|7340287	52313.6	5.5300 002098 0835
unnamed protein product [Triticum aestivum]	gi|257671690	52313.6	5.5300 002098 0835
unnamed protein product [Triticum aestivum]	gi|257304117	52313.6	5.5300 002098 0835
unnamed protein product [Triticum aestivum]	gi|257308963	52313.6	5.5300 002098 0835
unnamed protein product [Triticum aestivum]	gi|219764724	52313.6	5.5300 002098 0835
unnamed protein product [Triticum aestivum]	gi|219752137	52313.6	5.5300 002098 0835

Peptide Information

Calc. Mass	Obsrv. Mass	± da	± ppm	Start Seq.	End Seq.	Sequence	Ion Score	C. I. %	Modification	Rank	Result Type
1017.5952	1017.5392	-0.056	-55	366	374	IHHSVVGLR					Mascot
1025.5925	1025.5387	-0.0538	-52	260	267	HVMLQLLR			Oxidation (M)[3]		Mascot
1032.5472	1032.5209	-0.0263	-25	330	338	SAPIYTQPR					Mascot
1256.7109	1256.597	-0.1139	-91	64	75	AKPAVPLGANYR					Mascot
1384.6896	1384.6577	-0.0319	-23	319	329	KPIPDFSFYDR					Mascot
1384.6896	1384.6577	-0.0319	-23	319	329	KPIPDFSFYDR	75	100			Mascot
1625.8646	1625.8336	-0.031	-19	92	105	IYVLTQFNSASLNR	83	100			Mascot
1769.9684	1769.8848	-0.0836	-47	448	463	ETDGYFIKSGIVTVIK					Mascot
1788.92	1788.8937	-0.0263	-15	1	17	MDVPLASKTFPSPSPSK					Mascot
1852.9143	1852.8784	-0.0359	-19	228	244	AMMVDTTILGLDDARAK			Oxidation (M)[2,3]		Mascot
1916.9861	1916.8655	-0.1206	-63	243	259	AKEMPYIASMGIYVISK			Oxidation (M)[4]		Mascot
2355.2163	2355.1826	-0.0337	-14	32	55	HADLNPHANDSVLGIILG GGAGTR					Mascot
3061.4287	3061.4038	-0.0249	-8	120	147	NEGFVEVLAAQQSPDNP DWFQGTADAVR					Mascot

7	Glucose-1-phosphate adenylyltransferase small subunit, chloroplastic/amyloplastic [Triticum urartu]	gi|474108293	65309.1	7.9	11	192	100	2.42	158	100	

Peptide Information

Calc. Mass	Obsrv. Mass	± da	± ppm	Start Seq.	End Seq.	Sequence	Ion Score	C. I. %	Modification	Rank	Result Type

Calc. Mass	Obsrv. Mass	± da	± ppm	Start Seq.	End Seq.	Sequence	Ion Score	C. I. %	Modification	Rank	Result Type
1017.5952	1017.5392	-0.056	-55	407	415	IHHSVVGLR					Mascot
1025.5925	1025.5387	-0.0538	-52	301	308	HVMLQLLR			Oxidation (M)[3]		Mascot
1032.5472	1032.5209	-0.0263	-25	371	379	SAPIYTQPR					Mascot
1256.7109	1256.597	-0.1139	-91	105	116	AKPAVPLGANYR					Mascot
1384.6896	1384.6577	-0.0319	-23	360	370	KPIPDFSFYDR					Mascot
1384.6896	1384.6577	-0.0319	-23	360	370	KPIPDFSFYDR	75	100			Mascot
1625.8646	1625.8336	-0.031	-19	133	146	IYVLTQFNSASLNR	83	100			Mascot
1746.9684	1746.8699	-0.0985	-56	2	18	AMAAAASPSKILIPPHR			Oxidation (M)[2]		Mascot
1852.9143	1852.8784	-0.0359	-19	269	285	AMMVDTTILGLDDARAK			Oxidation (M)[2,3]		Mascot
1862.0139	1861.8931	-0.1208	-65	1	18	MAMAAAASPSKILIPPHR					Mascot
1916.9861	1916.8655	-0.1206	-63	284	300	AKEMPYIASMGIYVISK			Oxidation (M)[4]		Mascot
3061.4287	3061.4038	-0.0249	-8	161	188	NEGFVEVLAAQQSPDNP DWFQGTADAVR					Mascot

8	plastid ADP-glucose pyrophosphorylase small subunit [Triticum aestivum]	gi|182894563	52263.6	5.48	9	184	100	2.103	158	100	

Peptide Information

Calc. Mass	Obsrv. Mass	± da	± ppm	Start Seq.	End Seq.	Sequence	Ion Score	C. I. %	Modification	Rank	Result Type
1017.5952	1017.5392	-0.056	-55	368	376	IHHSVVGLR					Mascot
1025.5925	1025.5387	-0.0538	-52	262	269	HVMLQLLR			Oxidation (M)[3]		Mascot
1032.5472	1032.5209	-0.0263	-25	332	340	SAPIYTQPR					Mascot
1256.7109	1256.597	-0.1139	-91	66	77	AKPAVPLGANYR					Mascot
1384.6896	1384.6577	-0.0319	-23	321	331	KPIPDFSFYDR					Mascot
1384.6896	1384.6577	-0.0319	-23	321	331	KPIPDFSFYDR	75	100			Mascot
1625.8646	1625.8336	-0.031	-19	94	107	IYVLTQFNSASLNR	83	100			Mascot
1769.9684	1769.8848	-0.0836	-47	450	465	ETDGYFIKSGIVTVIK					Mascot
1852.9143	1852.8784	-0.0359	-19	230	246	AMMVDTTILGLDDARAK			Oxidation (M)[2,3]		Mascot
1916.9861	1916.8655	-0.1206	-63	245	261	AKEMPYIASMGIYVISK			Oxidation (M)[4]		Mascot

9	ADP glucose pyrophosphorylase [Triticum aestivum]	gi|469952290	52399.6	5.54	10	110	100	2.4	75	100	

Protein Group

ADP-glucose pyrophosphorylase [Triticum aestivum]	gi|21687	52399.6	5.5399 999618 5303
RecName: Full=Glucose-1-phosphate adenylyltransferase small subunit, chloroplastic/amyloplastic; AltName: Full=ADP-glucose pyrophosphorylase; AltName: Full=ADP-glucose synthase; AltName: Full=AGPase B; AltName:	gi|232172	52399.6	5.5399 999618 5303

Full=Alpha-D-glucose-1-phosphate adeny			
unnamed protein product [Triticum aestivum]	gi|300634091	52399.6	5.5399 999618 5303
unnamed protein product [Triticum aestivum]	gi|300544577	52399.6	5.5399 999618 5303
unnamed protein product [Triticum aestivum]	gi|259471779	52399.6	5.5399 999618 5303
unnamed protein product [Triticum aestivum]	gi|259439192	52399.6	5.5399 999618 5303
unnamed protein product [Triticum aestivum]	gi|257671726	52399.6	5.5399 999618 5303
unnamed protein product [Triticum aestivum]	gi|257306995	52399.6	5.5399 999618 5303
unnamed protein product [Triticum aestivum]	gi|257308999	52399.6	5.5399 999618 5303
unnamed protein product [Triticum aestivum]	gi|219764760	52399.6	5.5399 999618 5303
unnamed protein product [Triticum aestivum]	gi|219752173	52399.6	5.5399 999618 5303

Peptide Information

Calc. Mass	Obsrv. Mass	± da	± ppm	Start Seq.	End Seq.	Sequence	Ion Score	C. I. %	Modification	Rank	Result Type
1017.5952	1017.5392	-0.056	-55	366	374	IHHSVVGLR					Mascot
1025.5925	1025.5387	-0.0538	-52	260	267	HVMLQLLR			Oxidation (M)[3]		Mascot
1032.5472	1032.5209	-0.0263	-25	330	338	SAPIYTQPR					Mascot
1256.5801	1256.597	0.0169	13	18	27	REQCNIDGHK			Carbamidomethyl (C)[4]		Mascot
1384.6896	1384.6577	-0.0319	-23	319	329	KPIPDFSFYDR					Mascot
1384.6896	1384.6577	-0.0319	-23	319	329	KPIPDFSFYDR	75	100			Mascot
1769.9684	1769.8848	-0.0836	-47	448	463	ETDGYFIKSGIVTVIK					Mascot
1788.92	1788.8937	-0.0263	-15	1	17	MDVPLASKTFPSPSPSK					Mascot
1852.9143	1852.8784	-0.0359	-19	228	244	AMMVDTTILGLDDARAK			Oxidation (M)[2,3]		Mascot
1916.9861	1916.8655	-0.1206	-63	243	259	AKEMPYIASMGIYVISK			Oxidation (M)[4]		Mascot
3061.4287	3061.4038	-0.0249	-8	120	147	NEGFVEVLAAQQSPDNP DWFQGTADAVR					Mascot

10	Glucose-1-phosphate adenylyltransferase small subunit, chloroplastic/amyloplastic [Triticum urartu]	gi|474444719	55553.8	5.84	7	100	100	2.722	83	100	

Peptide Information

Calc. Mass	Obsrv. Mass	± da	± ppm	Start Seq.	End Seq.	Sequence	Ion Score	C. I. %	Modification	Rank	Result Type
959.5342	959.5381	0.0039	4	324	331	DAMLQLLR					Mascot
1256.7109	1256.597	-0.1139	-91	128	139	AKPAVPLGANYR					Mascot
1625.8646	1625.8336	-0.031	-19	156	169	IYVLTQFNSASLNR	83	100			Mascot
1772.9211	1772.9235	0.0024	1	140	155	LIDIPVSNCLNSNVSK			Carbamidomethyl (C)[9]		Mascot
1892.9821	1892.8184	-0.1637	-86	290	306	LKAMMVDTTILGLDSER					Mascot
1892.9821	1892.8184	-0.1637	-86	290	306	LKAMMVDTTILGLDSER					Mascot
1908.9769	1908.8273	-0.1496	-78	290	306	LKAMMVDTTILGLDSER			Oxidation (M)[4]		Mascot
1917.019	1916.8655	-0.1535	-80	307	323	AKELPYIASMGIYVFSK					Mascot
2155.0486	2155.0837	0.0351	16	77	94	EVVISDDNEEQQRPRGQ R					Mascot

Gel Idx/Pos	259/K11	Instr./Gel Origin	BA2151/Sample Project 20140814	Process Status	Analysis Succeeded
Plate [#] Name	[1] Sample Project 20140814	Instrument Sample Name		Spectra	11

Rank	Protein Name	Accession No.	Protein MW	Protein PI	Pep. Count	Protein Score	Protein Score C. I. %	Intensity Matched	Total Ion Score	Total Ion C. I. %	Confirmed
1	serpin 1 [Triticum aestivum]	gi|224589266	43261.1	5.44	13	649	100	65.417	574	100	

Peptide Information

Calc. Mass	Obsrv. Mass	± da	± ppm	Start Seq.	End Seq.	Sequence	Ion Score	C. I. %	Modification	Rank	Result Type
925.5214	925.5015	-0.0199	-22	11	18	LSIAHQTR					Mascot
925.5214	925.5015	-0.0199	-22	11	18	LSIAHQTR	31	94.044			Mascot
1151.6824	1151.6327	-0.0497	-43	172	181	LVLANALYFK					Mascot
1176.5896	1176.572	-0.0176	-15	262	271	LSAEPDFLER					Mascot
1176.5896	1176.572	-0.0176	-15	262	271	LSAEPDFLER	76	100			Mascot
1292.7097	1292.6455	-0.0642	-50	290	301	ISFGIEASDLLK					Mascot
1345.6958	1345.7191	0.0233	17	159	171	NILPSGSVDNTTK					Mascot
1475.6438	1475.641	-0.0028	-2	182	194	GAWTDQFDSYGTK					Mascot
2062.0564	2061.9919	-0.0645	-31	138	156	AAEVTTQVNSWVEKVTS GR					Mascot
2113.0999	2113.0837	-0.0162	-8	380	399	EDISGVVLFMGHVVNPLL SS					Mascot
2129.0947	2128.9883	-0.1064	-50	380	399	EDISGVVLFMGHVVNPLL SS			Oxidation (M)[10]		Mascot
2685.3955	2685.3792	-0.0163	-6	33	61	SAASNAAFSPVSLHSALS LLAAGAGSATR					Mascot
2685.3955	2685.3792	-0.0163	-6	33	61	SAASNAAFSPVSLHSALS LLAAGAGSATR	240	100			Mascot
2720.3525	2720.3101	-0.0424	-16	329	354	VSSVFHQAFVEVNEQGT EAAASTAIK					Mascot
2720.3525	2720.3101	-0.0424	-16	329	354	VSSVFHQAFVEVNEQGT EAAASTAIK	39	99.218			Mascot
2941.5215	2941.5994	0.0779	26	355	379	MVPQQARPPSVMDFIAD HPFLFLLR			Oxidation (M)[1]		Mascot
2957.5166	2957.4834	-0.0332	-11	355	379	MVPQQARPPSVMDFIAD HPFLFLLR			Oxidation (M)[1,12]		Mascot
3055.3845	3055.3315	-0.053	-17	302	328	CLGLQLPFSDEADFSEM VDSPMPQGLR			Carbamidomethyl (C)[1], Oxidation (M)[17]		Mascot
3071.3796	3071.325	-0.0546	-18	302	328	CLGLQLPFSDEADFSEM VDSPMPQGLR			Carbamidomethyl (C)[1], Oxidation (M)[17,22]		Mascot
3705.9082	3705.8901	-0.0181	-5	62	98	DQLVATLGTGEVEGLHAL AEQVVQFVLADASSAGG PR					Mascot
3705.9082	3705.8901	-0.0181	-5	62	98	DQLVATLGTGEVEGLHAL AEQVVQFVLADASSAGG PR	189	100			Mascot

Rank	Protein Name	Accession No.	Protein MW	Protein PI	Pep. Count	Protein Score	Protein Score C. I. %	Intensity Matched	Total Ion Score	Total Ion C. I. %	Confirmed
2	serpin 3 [Triticum aestivum]	gi|224589270	43227.1	5.56	12	640	100	65.017	574	100	

Peptide Information

Calc. Mass	Obsrv. Mass	± da	± ppm	Start Seq.	End Seq.	Sequence	Ion Score	C. I. %	Modification	Rank	Result Type
925.5214	925.5015	-0.0199	-22	11	18	LSIAHQTR					Mascot
925.5214	925.5015	-0.0199	-22	11	18	LSIAHQTR	31	94.044			Mascot
1151.6824	1151.6327	-0.0497	-43	172	181	LVLANALYFK					Mascot
1176.5896	1176.572	-0.0176	-15	262	271	LSAEPDFLER					Mascot
1176.5896	1176.572	-0.0176	-15	262	271	LSAEPDFLER	76	100			Mascot
1292.7097	1292.6455	-0.0642	-50	290	301	ISFGIEASDLLK					Mascot
1345.6958	1345.7191	0.0233	17	159	171	NILPSGSVDNTTK					Mascot
1475.6438	1475.641	-0.0028	-2	182	194	GAWTDQFDSYGTK					Mascot
2062.0564	2061.9919	-0.0645	-31	138	156	AAEVTTQVNSWVEKVTS GR					Mascot
2685.3955	2685.3792	-0.0163	-6	33	61	SAASNAAFSPVSLHSALS LLAAGAGSATR					Mascot
2685.3955	2685.3792	-0.0163	-6	33	61	SAASNAAFSPVSLHSALS LLAAGAGSATR	240	100			Mascot
2720.3525	2720.3101	-0.0424	-16	329	354	VSSVFHQAFVEVNEQGT EAAASTAIK					Mascot
2720.3525	2720.3101	-0.0424	-16	329	354	VSSVFHQAFVEVNEQGT EAAASTAIK	39	99.218			Mascot
2941.5215	2941.5994	0.0779	26	355	379	MVPQQARPPSVMDFIAD HPFLFLLR			Oxidation (M)[1]		Mascot
2957.5166	2957.4834	-0.0332	-11	355	379	MVPQQARPPSVMDFIAD HPFLFLLR			Oxidation (M)[1,12]		Mascot
3055.3845	3055.3315	-0.053	-17	302	328	CLGLQLPFSDEADFSEM VDSPMPQGLR			Carbamidomethyl (C)[1], Oxidation (M)[17]		Mascot
3071.3796	3071.325	-0.0546	-18	302	328	CLGLQLPFSDEADFSEM VDSPMPQGLR			Carbamidomethyl (C)[1], Oxidation (M)[17,22]		Mascot
3705.9082	3705.8901	-0.0181	-5	62	98	DQLVATLGTGEVEGLHAL AEQVVQFVLADASSAGG PR					Mascot
3705.9082	3705.8901	-0.0181	-5	62	98	DQLVATLGTGEVEGLHAL AEQVVQFVLADASSAGG PR	189	100			Mascot

3	serpin [Triticum aestivum]	gi|1885350	43119.9	5.44	11	440	100	63.593	385	100	

Protein Group

RecName: Full=Serpin-Z1B; AltName: Full=TriaeZ1b; AltName: Full=WSZ1b; AltName: Full=WZS2	gi|75279910	43119.9	5.4400 000572 2046

Peptide Information

Calc. Mass	Obsrv. Mass	± da	± ppm	Start Seq.	End Seq.	Sequence	Ion Score	C. I. %	Modification	Rank	Result Type
925.5214	925.5015	-0.0199	-22	11	18	LSIAHQTR					Mascot
925.5214	925.5015	-0.0199	-22	11	18	LSIAHQTR	31	94.044			Mascot
1151.6824	1151.6327	-0.0497	-43	172	181	LVLANALYFK					Mascot

Calc. Mass	Obsrv. Mass	± da	± ppm	Start Seq.	End Seq.	Sequence	Ion Score	C. I. %	Modification	Rank	Result Type
1176.5896	1176.572	-0.0176	-15	262	271	LSAEPDFLER					Mascot
1176.5896	1176.572	-0.0176	-15	262	271	LSAEPDFLER	76	100			Mascot
1345.6958	1345.7191	0.0233	17	159	171	NILPSGSVDNTTK					Mascot
1475.6438	1475.641	-0.0028	-2	182	194	GAWTDQFDSYGTK					Mascot
2062.0564	2061.9919	-0.0645	-31	138	156	AAEVTTQVNSWVEKVTS GR					Mascot
2113.0999	2113.0837	-0.0162	-8	380	399	EDISGVVLFMGHVVNPLL SS					Mascot
2129.0947	2128.9883	-0.1064	-50	380	399	EDISGVVLFMGHVVNPLL SS			Oxidation (M)[10]		Mascot
2685.3955	2685.3792	-0.0163	-6	33	61	SAASNAAFSPVSLHSALS LLAAGAGSATR					Mascot
2685.3955	2685.3792	-0.0163	-6	33	61	SAASNAAFSPVSLHSALS LLAAGAGSATR	240	100			Mascot
2720.3525	2720.3101	-0.0424	-16	329	354	VSSVFHQAFVEVNEQGT EAAASTAIK					Mascot
2720.3525	2720.3101	-0.0424	-16	329	354	VSSVFHQAFVEVNEQGT EAAASTAIK	39	99.218			Mascot
2941.5215	2941.5994	0.0779	26	355	379	MVPQQARPPSVMDFIAD HPFLFLLR			Oxidation (M)[1]		Mascot
2957.5166	2957.4834	-0.0332	-11	355	379	MVPQQARPPSVMDFIAD HPFLFLLR			Oxidation (M)[1,12]		Mascot
3055.3845	3055.3315	-0.053	-17	302	328	CLGLQLPFSDEADFSEM VDSPMPQGLR			Carbamidomethyl (C)[1], Oxidation (M)[17]		Mascot
3071.3796	3071.325	-0.0546	-18	302	328	CLGLQLPFSDEADFSEM VDSPMPQGLR			Carbamidomethyl (C)[1], Oxidation (M)[17,22]		Mascot

4 serpin-N3.7 [Triticum aestivum] gi|379060945 42639.8 5.52 9 185 100 16.967 145 100

Peptide Information

Calc. Mass	Obsrv. Mass	± da	± ppm	Start Seq.	End Seq.	Sequence	Ion Score	C. I. %	Modification	Rank	Result Type
925.5214	925.5015	-0.0199	-22	11	18	LSIAHQTR					Mascot
925.5214	925.5015	-0.0199	-22	11	18	LSIAHQTR	31	94.044			Mascot
1151.6824	1151.6327	-0.0497	-43	172	181	LVLANALYFK					Mascot
1176.5896	1176.572	-0.0176	-15	261	270	LSAEPDFLER					Mascot
1176.5896	1176.572	-0.0176	-15	261	270	LSAEPDFLER	76	100			Mascot
1239.5852	1239.6814	0.0962	78	127	137	ADTQSVDFQTK					Mascot
1292.7097	1292.6455	-0.0642	-50	289	300	ISFGIEASDLLK					Mascot
2720.3525	2720.3101	-0.0424	-16	328	353	VSSVFHQAFVEVNEQGT EAAASTAIK					Mascot
2720.3525	2720.3101	-0.0424	-16	328	353	VSSVFHQAFVEVNEQGT EAAASTAIK	39	99.218			Mascot
2725.4631	2725.325	-0.1381	-51	33	61	SAASNAVFSPVSLHVALS LLAAGAGSATR					Mascot
2941.5215	2941.5994	0.0779	26	354	378	MVPQQARPPSVMDFIAD HPFLFLLR			Oxidation (M)[1]		Mascot
2957.5166	2957.4834	-0.0332	-11	354	378	MVPQQARPPSVMDFIAD HPFLFLLR			Oxidation (M)[1,12]		Mascot
3055.3845	3055.3315	-0.053	-17	301	327	CLGLQLPFSDEADFSEM VDSPMPQGLR			Carbamidomethyl (C)[1], Oxidation (M)[17]		Mascot

Calc. Mass	Obsrv. Mass	± da	± ppm	Start Seq.	End Seq.	Sequence	Ion Score	C. I. %	Modification	Rank	Result Type
3071.3796	3071.325	-0.0546	-18	301	327	CLGLQLPFSDEADFSEM VDSPMPQGLR			Carbamidomethyl (C)[1], Oxidation (M)[17,22]		Mascot

5	serpin [Triticum aestivum]	gi|871551	43262.2	5.6	9	181	100	17.136	145	100	

Protein Group

RecName: Full=Serpin-Z1A; AltName: Full=TriaeZ1a; AltName: Full=WSZ1a; Short=WSZ1; AltName: Full=WSZCI	gi|75282265	43262.2	5.5999999046 3257

Peptide Information

Calc. Mass	Obsrv. Mass	± da	± ppm	Start Seq.	End Seq.	Sequence	Ion Score	C. I. %	Modification	Rank	Result Type
925.5214	925.5015	-0.0199	-22	11	18	LSIAHQTR					Mascot
925.5214	925.5015	-0.0199	-22	11	18	LSIAHQTR	31	94.044			Mascot
1151.6824	1151.6327	-0.0497	-43	172	181	LVLANALYFK					Mascot
1176.5896	1176.572	-0.0176	-15	261	270	LSAEPDFLER					Mascot
1176.5896	1176.572	-0.0176	-15	261	270	LSAEPDFLER	76	100			Mascot
1292.7097	1292.6455	-0.0642	-50	289	300	ISFGIEASDLLK					Mascot
1611.8953	1611.766	-0.1293	-80	157	171	IKDILPPGSIDNTTK					Mascot
2062.0564	2061.9919	-0.0645	-31	138	156	AAEVTTQVNSWVEKVTS GR					Mascot
2113.0999	2113.0837	-0.0162	-8	379	398	EDISGVVLFMGHVVNPLL SS					Mascot
2129.0947	2128.9883	-0.1064	-50	379	398	EDISGVVLFMGHVVNPLL SS			Oxidation (M)[10]		Mascot
2720.3525	2720.3101	-0.0424	-16	328	353	VSSVFHQAFVEVNEQGT EAAASTAIK					Mascot
2720.3525	2720.3101	-0.0424	-16	328	353	VSSVFHQAFVEVNEQGT EAAASTAIK	39	99.218			Mascot
2943.5374	2943.7	0.1626	55	354	378	MVLQQARPPSVMDFIAD HPFLFLVR			Oxidation (M)[1]		Mascot
2943.5374	2943.7	0.1626	55	354	378	MVLQQARPPSVMDFIAD HPFLFLVR			Oxidation (M)[1]		Mascot

6	serpin [Triticum aestivum]	gi|5734504	42969	5.62	7	170	100	15.837	145	100	

Protein Group

RecName: Full=Serpin-Z1C; AltName: Full=TriaeZ1c; AltName: Full=WSZ1c	gi|75313848	42969	5.6199998855 5908

Peptide Information

Calc. Mass	Obsrv. Mass	± da	± ppm	Start Seq.	End Seq.	Sequence	Ion Score	C. I. %	Modification	Rank	Result Type
925.5214	925.5015	-0.0199	-22	11	18	LSIAHQTR					Mascot
925.5214	925.5015	-0.0199	-22	11	18	LSIAHQTR	31	94.044			Mascot
1151.6824	1151.6327	-0.0497	-43	172	181	LVLANALYFK					Mascot
1176.5896	1176.572	-0.0176	-15	261	270	LSAEPDFLER					Mascot
1176.5896	1176.572	-0.0176	-15	261	270	LSAEPDFLER	76	100			Mascot

Calc. Mass	Obsrv. Mass	± da	± ppm	Start Seq.	End Seq.	Sequence	Ion Score	C. I. %	Modification	Rank	Result Type
1239.5852	1239.6814	0.0962	78	127	137	ADTQSVDFQTK					Mascot
2113.0999	2113.0837	-0.0162	-8	379	398	EDISGVVLFMGHVVNPLL SS					Mascot
2129.0947	2128.9883	-0.1064	-50	379	398	EDISGVVLFMGHVVNPLL SS			Oxidation (M)[10]		Mascot
2720.3525	2720.3101	-0.0424	-16	328	353	VSSVFHQAFVEVNEQGT EAAASTAIK					Mascot
2720.3525	2720.3101	-0.0424	-16	328	353	VSSVFHQAFVEVNEQGT EAAASTAIK	39	99.218			Mascot
2725.4631	2725.325	-0.1381	-51	33	61	SAASNAVFSPVSLHVALS LLAAGAGSATR					Mascot

7	Serpin-Z1C [Triticum urartu]	gi|474075261	42956	5.62	6	165	100	15.724	145	100	

Peptide Information

Calc. Mass	Obsrv. Mass	± da	± ppm	Start Seq.	End Seq.	Sequence	Ion Score	C. I. %	Modification	Rank	Result Type
925.5214	925.5015	-0.0199	-22	11	18	LSIAHQTR					Mascot
925.5214	925.5015	-0.0199	-22	11	18	LSIAHQTR	31	94.044			Mascot
1151.6824	1151.6327	-0.0497	-43	172	181	LVLANALYFK					Mascot
1176.5896	1176.572	-0.0176	-15	261	270	LSAEPDFLER					Mascot
1176.5896	1176.572	-0.0176	-15	261	270	LSAEPDFLER	76	100			Mascot
2113.0999	2113.0837	-0.0162	-8	379	398	EDISGVVLFMGHVVNPLL SS					Mascot
2129.0947	2128.9883	-0.1064	-50	379	398	EDISGVVLFMGHVVNPLL SS			Oxidation (M)[10]		Mascot
2720.3525	2720.3101	-0.0424	-16	328	353	VSSVFHQAFVEVNEQGT EAAASTAIK					Mascot
2720.3525	2720.3101	-0.0424	-16	328	353	VSSVFHQAFVEVNEQGT EAAASTAIK	39	99.218			Mascot
2725.4631	2725.325	-0.1381	-51	33	61	SAASNAVFSPVSLHVALS LLAAGAGSATR					Mascot

8	ADP-glucose brittle-1 transporter precursor [Triticum aestivum]	gi|261286811	46104.2	9.69	12	53	80.247	1.816	

Peptide Information

Calc. Mass	Obsrv. Mass	± da	± ppm	Start Seq.	End Seq.	Sequence	Ion Score	C. I. %	Modification	Rank	Result Type
836.4261	836.4518	0.0257	31	364	371	EGAAGLYR					Mascot
1100.6423	1100.5343	-0.108	-98	126	137	LVSGAIAGAVSR					Mascot
1175.5942	1175.5756	-0.0186	-16	204	214	YLTPEAGEPAK					Mascot
1198.5807	1198.5554	-0.0253	-21	2	13	AAAMAATTMVTK			Oxidation (M)[4,9]		Mascot
1345.6161	1345.7191	0.103	77	1	13	MAAAMAATTMVTK			Oxidation (M)[1,5,10]		Mascot
1648.8417	1648.7787	-0.063	-38	166	178	WIMRTEGWPGLFR					Mascot
1801.996	1801.8665	-0.1295	-72	250	264	DVYDNLLHAFVKIVR					Mascot
2075.063	2074.9597	-0.1033	-50	77	97	ARPADDVAHQLAAAGEA					Mascot

Calc. Mass	Obsrv. Mass	± da	± ppm	Start Seq.	End Seq.	Sequence	Ion Score	C. I. %	Modification	Rank	Result Type
						GVQK					
2641.3076	2641.3579	0.0503	19	72	97	EHDGKARPADDVAHQLA AAGEAGVQK					Mascot
2683.4143	2683.3301	-0.0842	-31	24	45	KNWFLRPVPEVAFPWSS QPEIR					Mascot
2758.2034	2758.2551	0.0517	19	403	429	EDEPQEETETGQAGGQA APKSSSGDRP					Mascot
2982.4812	2982.437	-0.0442	-15	138	165	TFVAPLETIRTHLMVGSS GADSMAGVFR			Oxidation (M)[14,23]		Mascot

9	Thiosulfate sulfurtransferase [Triticum urartu]	gi|473951518	59109.2	8.52	12	45	0	4.89	

Peptide Information

Calc. Mass	Obsrv. Mass	± da	± ppm	Start Seq.	End Seq.	Sequence	Ion Score	C. I. %	Modification	Rank	Result Type
887.5461	887.5195	-0.0266	-30	162	168	INLFPKR					Mascot
1100.6422	1100.5343	-0.1079	-98	170	178	SLLLKNEQR					Mascot
1204.6078	1204.6011	-0.0067	-6	91	99	WMLCPRVAR			Carbamidomethyl (C)[4], Oxidation (M)[2]		Mascot
1204.6078	1204.6011	-0.0067	-6	91	99	WMLCPRVAR			Carbamidomethyl (C)[4], Oxidation (M)[2]		Mascot
1281.6984	1281.5765	-0.1219	-95	131	141	RSASILQFMTK					Mascot
1611.7526	1611.766	0.0134	8	246	257	VWWMFRAYGHDK			Oxidation (M)[4]		Mascot
1687.7957	1687.7643	-0.0314	-19	346	360	CEEEAVSLDGQVVPR			Carbamidomethyl (C)[1]		Mascot
1815.8906	1815.8557	-0.0349	-19	346	361	CEEEAVSLDGQVVPRK			Carbamidomethyl (C)[1]		Mascot
1887.8993	1887.8213	-0.078	-41	387	402	AGWMKWHQASGILCDK			Carbamidomethyl (C)[14]		Mascot
1903.8943	1903.8177	-0.0766	-40	387	402	AGWMKWHQASGILCDK			Carbamidomethyl (C)[14], Oxidation (M)[4]		Mascot
2075.1294	2074.9597	-0.1697	-82	81	96	TILFILNELRWMLCPR			Carbamidomethyl (C)[14]		Mascot
2683.2053	2683.3301	0.1248	47	362	384	DTFRYLGSMLQEDGGID EDVNHR			Oxidation (M)[9]		Mascot
2748.4026	2748.2622	-0.1404	-51	204	229	ASNLPHMLPSEKAFSAAV SSLGIYNK			Oxidation (M)[7]		Mascot
2943.416	2943.7	0.284	96	498	525	LGKTDVAVYDGSWTEW GAHPDTPVATAV					Mascot
2943.416	2943.7	0.284	96	498	525	LGKTDVAVYDGSWTEW GAHPDTPVATAV					Mascot

10	SWI/SNF complex subunit SWI3A [Triticum urartu]	gi|473869925	90574.7	6.32	15	43	0	2.728	

Peptide Information

Calc. Mass	Obsrv. Mass	± da	± ppm	Start Seq.	End Seq.	Sequence	Ion Score	C. I. %	Modification	Rank	Result Type
837.4213	837.4443	0.023	27	585	591	GHELPER					Mascot
1151.6896	1151.6327	-0.0569	-49	704	714	KAAVVNPSLPR					Mascot
1173.674	1173.6484	-0.0256	-22	604	614	LALIDHGVAHK					Mascot
1189.5696	1189.6082	0.0386	32	675	685	VEGEQSNEVAK					Mascot
1198.6216	1198.5554	-0.0662	-55	474	483	NFISTAYQVR					Mascot
1341.6104	1341.74	0.1296	97	458	469	EAEMHGQTVPDK					Mascot

1415.6077	1415.6708	0.0631	45	236	247	VCSTCCKTNSAK	Carbamidomethyl (C)[2,5,6]	Mascot
1593.7252	1593.7354	0.0102	6	326	339	SDSRFHTNQTTDGK		Mascot
1646.8795	1646.7543	-0.1252	-76	1	17	MSPAAPGAASPPAPLRR		Mascot
1648.8363	1648.7787	-0.0576	-35	549	561	ESLMKEWQTVLER		Mascot
1801.9556	1801.8665	-0.0891	-49	562	577	AFQTGAPLQRDEVLTR		Mascot
2087.1562	2087.0217	-0.1345	-64	43	59	AWPPHQLRVLRPAGWF R		Mascot
2456.2351	2456.2378	0.0027	1	308	329	LIQLPFGEHMLGNINNGK SDSR	Oxidation (M)[10]	Mascot
2725.3914	2725.325	-0.0664	-24	302	325	TECIARLIQLPFGEHMLG NINNGK	Carbamidomethyl (C)[3]	Mascot
3071.1702	3071.325	0.1548	50	746	773	WESSPGKDDIAEDEGAS DDEDDDEMADK		Mascot
3087.165	3087.3323	0.1673	54	746	773	WESSPGKDDIAEDEGAS DDEDDDEMADK	Oxidation (M)[25]	Mascot

Gel Idx/Pos	260/K12	Instr./Gel Origin	BA2151/Sample Project 20140814	Process Status	Analysis Succeeded
Plate [#] Name	[1] Sample Project 20140814	Instrument Sample Name		Spectra	11

Rank	Protein Name	Accession No.	Protein MW	Protein PI	Pep. Count	Protein Score	Protein Score C. I. %	Intensity Matched	Total Ion Score	Total Ion C. I. %	Confirmed
1	Bifunctional polymyxin resistance protein ArnA [Triticum urartu]	gi|474224464	43507.3	7.53	16	307	100	20.033	220	100	

Peptide Information

Calc. Mass	Obsrv. Mass	± da	± ppm	Start Seq.	End Seq.	Sequence	Ion Score	C. I. %	Modification	Rank	Result Type
842.4519	842.4771	0.0252	30	345	351	QLGWNPK					Mascot
862.5032	862.4528	-0.0504	-58	140	147	TIGSFLPK					Mascot
933.3908	933.4069	0.0161	17	40	48	EGEEAGDAR					Mascot
960.4745	960.4437	-0.0308	-32	246	254	LVDGGESQR					Mascot
960.4745	960.4437	-0.0308	-32	246	254	LVDGGESQR	50	99.917			Mascot
1216.6719	1216.6477	-0.0242	-20	335	344	RIPDMTLINK			Oxidation (M)[5]		Mascot
1306.6936	1306.6619	-0.0317	-24	230	240	VLACFSNNLLR			Carbamidomethyl (C)[4]		Mascot
1306.6936	1306.6619	-0.0317	-24	230	240	VLACFSNNLLR	87	100	Carbamidomethyl (C)[4]		Mascot
1427.7489	1427.6968	-0.0521	-36	242	254	EPLKLVDGGESQR					Mascot
1453.7145	1453.6626	-0.0519	-36	128	139	LIHFSTCEVYGK			Carbamidomethyl (C)[7]		Mascot
1461.7584	1461.6963	-0.0621	-42	356	367	DLLETTLTYQHK					Mascot
1524.7628	1524.6855	-0.0773	-51	177	188	WSYACAKQLIER			Carbamidomethyl (C)[5]		Mascot
1580.65	1580.6161	-0.0339	-21	323	335	EFYGEGYDDSDKR					Mascot
1654.8833	1654.8354	-0.0479	-29	261	275	DAIEAVLLMIENPAR					Mascot
1670.8782	1670.8124	-0.0658	-39	261	275	DAIEAVLLMIENPAR			Oxidation (M)[9]		Mascot
1673.7362	1673.7976	0.0614	37	31	48	ASPGGDAGKEGEEAGDAR					Mascot
1688.7949	1688.7306	-0.0643	-38	214	229	MDFIPGVDGPSEGVPR			Oxidation (M)[1]		Mascot
1688.7949	1688.7306	-0.0643	-38	214	229	MDFIPGVDGPSEGVPR	83	100	Oxidation (M)[1]		Mascot
1765.9219	1765.8307	-0.0912	-52	306	322	VSGEPPLEEPVIDVSAK					Mascot
1952.9574	1952.915	-0.0424	-22	276	293	ANGHIFNVGNPDNEVTVR					Mascot

Rank	Protein Name	Accession No.	Protein MW	Protein PI	Pep. Count	Protein Score	Protein Score C. I. %	Intensity Matched	Total Ion Score	Total Ion C. I. %	Confirmed
2	hypothetical protein [Triticum monococcum subsp. aegilopoides]	gi|355389675	35263	5.08	13	292	100	19.122	220	100	

Peptide Information

Calc. Mass	Obsrv. Mass	± da	± ppm	Start Seq.	End Seq.	Sequence	Ion Score	C. I. %	Modification	Rank	Result Type
842.4519	842.4771	0.0252	30	293	299	QLGWNPK					Mascot

862.5032	862.4528	-0.0504	-58	88	95	TIGSFLPK					Mascot
960.4745	960.4437	-0.0308	-32	194	202	LVDGGESQR					Mascot
960.4745	960.4437	-0.0308	-32	194	202	LVDGGESQR	50	99.917			Mascot
1216.6719	1216.6477	-0.0242	-20	283	292	RIPDMTLINK			Oxidation (M)[5]		Mascot
1306.6936	1306.6619	-0.0317	-24	178	188	VLACFSNNLLR			Carbamidomethyl (C)[4]		Mascot
1306.6936	1306.6619	-0.0317	-24	178	188	VLACFSNNLLR	87	100	Carbamidomethyl (C)[4]		Mascot
1427.7489	1427.6968	-0.0521	-36	190	202	EPLKLVDGGESQR					Mascot
1453.7145	1453.6626	-0.0519	-36	76	87	LIHFSTCEVYGK			Carbamidomethyl (C)[7]		Mascot
1524.7628	1524.6855	-0.0773	-51	125	136	WSYACAKQLIER			Carbamidomethyl (C)[5]		Mascot
1580.65	1580.6161	-0.0339	-21	271	283	EFYGEGYDDSDKR					Mascot
1654.8833	1654.8354	-0.0479	-29	209	223	DAIEAVLLMIENPAR					Mascot
1670.8782	1670.8124	-0.0658	-39	209	223	DAIEAVLLMIENPAR			Oxidation (M)[9]		Mascot
1688.7949	1688.7306	-0.0643	-38	162	177	MDFIPGVDGPSEGVPR			Oxidation (M)[1]		Mascot
1688.7949	1688.7306	-0.0643	-38	162	177	MDFIPGVDGPSEGVPR	83	100	Oxidation (M)[1]		Mascot
1765.9219	1765.8307	-0.0912	-52	254	270	VSGEPPLEEPVIDVSAK					Mascot
1952.9574	1952.915	-0.0424	-22	224	241	ANGHIFNVGNPDNEVTV R					Mascot

3	hypothetical protein TRIUR3_09763 [Triticum urartu]	gi|474119782	17354.6	4.9	7	126	100	4.181	83	100	

Peptide Information

Calc. Mass	Obsrv. Mass	± da	± ppm	Start Seq.	End Seq.	Sequence	Ion Score	C. I. %	Modification	Rank	Result Type
1216.6719	1216.6477	-0.0242	-20	88	97	RIPDMTLINK			Oxidation (M)[5]		Mascot
1377.658	1377.588	-0.07	-51	17	28	VLACFSNENPAR			Carbamidomethyl (C)[4]		Mascot
1541.7595	1541.719	-0.0405	-26	2	16	DFIPGVDGPSEGVPR					Mascot
1580.65	1580.6161	-0.0339	-21	76	88	EFYGEGYDDSDKR					Mascot
1688.7949	1688.7306	-0.0643	-38	1	16	MDFIPGVDGPSEGVPR			Oxidation (M)[1]		Mascot
1688.7949	1688.7306	-0.0643	-38	1	16	MDFIPGVDGPSEGVPR	83	100	Oxidation (M)[1]		Mascot
1780.8964	1780.8284	-0.068	-38	59	75	VSGEPPQEEPVIDVSAK					Mascot
1952.9574	1952.915	-0.0424	-22	29	46	ANGHIFNVGNPDNEVTV R					Mascot

4	auxin amidohydrolase [Triticum aestivum]	gi|51538213	47455	5.47	11	115	100	6.947	71	100	

Peptide Information

Calc. Mass	Obsrv. Mass	± da	± ppm	Start Seq.	End Seq.	Sequence	Ion Score	C. I. %	Modification	Rank	Result Type
857.4363	857.3925	-0.0438	-51	431	437	VEPRDEL					Mascot
1164.5718	1164.5533	-0.0185	-16	71	80	ELDAMGIPYR					Mascot

Calc. Mass	Obsrv. Mass	± da	± ppm	Start Seq.	End Seq.	Sequence	Ion Score	C. I. %	Modification	Rank	Result Type
1180.5667	1180.5294	-0.0373	-32	71	80	ELDAMGIPYR			Oxidation (M)[5]		Mascot
1280.6958	1280.6528	-0.043	-34	145	154	ILQEHRDELK					Mascot
1336.6678	1336.6178	-0.05	-37	70	80	RELDAMGIPYR			Oxidation (M)[6]		Mascot
1341.7162	1341.6488	-0.0674	-50	419	430	YLLEHQPATAAK					Mascot
1377.625	1377.588	-0.037	-27	343	354	VCSEMVGPNNVR			Carbamidomethyl (C)[2], Oxidation (M)[5]		Mascot
1471.8115	1471.7726	-0.0389	-26	300	312	IEEVIVTQASVQR					Mascot
1471.8115	1471.7726	-0.0389	-26	300	312	IEEVIVTQASVQR	71	100			Mascot
1701.8806	1701.7955	-0.0851	-50	155	172	GTVALLFQPAEEGGGGA K					Mascot
1755.9712	1755.9146	-0.0566	-32	298	312	QRIEEVIVTQASVQR					Mascot
2140.0823	2140.0449	-0.0374	-17	266	286	FQGGGAFNVIPDSVTIGG TFR					Mascot
2250.2395	2250.2	-0.0395	-18	81	103	HPFAVTGVVATVGTGGP PFVALR					Mascot

5	Eukaryotic translation initiation factor 3 subunit E [Triticum urartu]	gi|473882632	49699.2	5.53	14	105	100	11.427	47	99.831

Peptide Information

Calc. Mass	Obsrv. Mass	± da	± ppm	Start Seq.	End Seq.	Sequence	Ion Score	C. I. %	Modification	Rank	Result Type
874.4741	874.4027	-0.0714	-82	62	68	RSEVVER					Mascot
1071.4742	1071.4359	-0.0383	-36	285	293	YDFDGAQQK					Mascot
1164.5896	1164.5533	-0.0363	-31	17	26	GVYADEEILR					Mascot
1248.6082	1248.5784	-0.0298	-24	331	339	LFIFETYCR			Carbamidomethyl (C)[8]		Mascot
1347.6475	1347.5894	-0.0581	-43	97	106	QYNLHMLQER			Oxidation (M)[6]		Mascot
1427.72	1427.6968	-0.0232	-16	391	402	VDVHEQIIESMK					Mascot
1443.7148	1443.6593	-0.0555	-38	391	402	VDVHEQIIESMK			Oxidation (M)[11]		Mascot
1477.7506	1477.7043	-0.0463	-31	414	428	SVVDPGHAAAQQAAR					Mascot
1537.7891	1537.7323	-0.0568	-37	377	390	IDSVTGTLIMTTNR			Oxidation (M)[10]		Mascot
1599.7432	1599.73	-0.0132	-8	47	61	SLHGTDDVPADMVAR			Oxidation (M)[12]		Mascot
1623.8966	1623.8596	-0.037	-23	227	240	YLNAIQTNAPHLLR					Mascot
1623.8966	1623.8596	-0.037	-23	227	240	YLNAIQTNAPHLLR	47	99.831			Mascot
1654.8523	1654.8354	-0.0169	-10	331	342	LFIFETYCRIHR			Carbamidomethyl (C)[8]		Mascot
1755.8444	1755.9146	0.0702	40	47	62	SLHGTDDVPADMVARR			Oxidation (M)[12]		Mascot
1873.9443	1873.8832	-0.0611	-33	107	122	HQIGPDQIEALYQYAK					Mascot
1936.0361	1935.8962	-0.1399	-72	1	16	MDPHLVLPVLEFLQER					Mascot
1936.0361	1935.8962	-0.1399	-72	1	16	MDPHLVLPVLEFLQER					Mascot
1952.031	1951.8915	-0.1395	-71	1	16	MDPHLVLPVLEFLQER			Oxidation (M)[1]		Mascot

6	Alanyl-tRNA synthetase [Triticum urartu]	gi|474423501	208125.5	8.47	31	64	98.467	14.563		

Peptide Information

Calc. Mass	Obsrv. Mass	± da	± ppm	Start Seq.	End Seq.	Sequence	Ion Score	C. I. %	Modification	Rank	Result Type
800.4485	800.3928	-0.0557	-70	1716	1724	RGGAGGVAR					Mascot
840.4461	840.4304	-0.0157	-19	1311	1318	SLTSSFAK					Mascot
842.4982	842.4771	-0.0211	-25	668	675	GSIVLPEK					Mascot
860.4724	860.3868	-0.0856	-99	832	839	LEGATLEK					Mascot
941.4509	941.5041	0.0532	57	632	638	CKVDYTR			Carbamidomethyl (C)[1]		Mascot
941.4509	941.5041	0.0532	57	632	638	CKVDYTR			Carbamidomethyl (C)[1]		Mascot
963.4781	963.4682	-0.0099	-10	1425	1432	SISIWDDK					Mascot
988.4945	988.4675	-0.027	-27	146	155	AGLAPDTESK					Mascot
1012.4694	1012.4534	-0.016	-16	84	92	HNDLDDVGK					Mascot
1030.535	1030.4806	-0.0544	-53	1574	1582	HITTIGTCK			Carbamidomethyl (C)[8]		Mascot
1071.5293	1071.4359	-0.0934	-87	1091	1099	LGFGMEFVR			Oxidation (M)[5]		Mascot
1079.5858	1079.5215	-0.0643	-60	333	341	RAVHFGHQK					Mascot
1111.6833	1111.5736	-0.1097	-99	668	677	GSIVLPEKLR					Mascot
1139.5692	1139.5908	0.0216	19	658	667	EVLGDHVDQK					Mascot
1164.5103	1164.5533	0.043	37	238	247	HVDTGMGFER			Oxidation (M)[6]		Mascot
1204.6243	1204.6057	-0.0186	-15	1662	1671	ELDPIQSMKK			Oxidation (M)[8]		Mascot
1247.6453	1247.5989	-0.0464	-37	1334	1344	AWMALASPKEK			Oxidation (M)[3]		Mascot
1275.5812	1275.5959	0.0147	12	527	538	NQGLDSTNDSPK					Mascot
1305.6467	1305.6398	-0.0069	-5	1704	1715	INTDVSVAMEAR					Mascot
1362.7053	1362.6494	-0.0559	-41	736	747	AVFGEIYPDPVR					Mascot
1461.7333	1461.6963	-0.037	-25	1420	1432	IGDGRSISIWDDK					Mascot
1477.7428	1477.7043	-0.0385	-26	1704	1716	INTDVSVAMEARR			Oxidation (M)[9]		Mascot
1507.743	1507.6327	-0.1103	-73	1061	1074	GGNAVCAIKLDMMK			Carbamidomethyl (C)[6]		Mascot
1533.7479	1533.6355	-0.1124	-73	1	13	MEVPSDWTAARVR			Oxidation (M)[1]		Mascot
1537.7209	1537.7323	0.0114	7	1270	1283	LQGGSERMISCAGR			Carbamidomethyl (C)[11], Oxidation (M)[8]		Mascot
1624.7887	1624.8463	0.0576	35	932	946	GLPIMVFSTDEASNK			Oxidation (M)[5]		Mascot
1642.8799	1642.7435	-0.1364	-83	146	160	AGLAPDTESKNIWLK					Mascot
1673.9156	1673.7976	-0.118	-70	1559	1573	VLHGILPAESTLMHR					Mascot
1773.8563	1773.8767	0.0204	12	1328	1342	NSMHWKAWMALASPK			Oxidation (M)[3]		Mascot
1869.0129	1869.0179	0.005	3	1351	1366	NLELFNTALLGKHGWR					Mascot
1954.0215	1953.9014	-0.1201	-61	1345	1362	GGMGFRNLELFNTALLG K			Oxidation (M)[3]		Mascot
1954.0215	1953.9014	-0.1201	-61	1345	1362	GGMGFRNLELFNTALLG K			Oxidation (M)[3]		Mascot
2685.4329	2685.3525	-0.0804	-30	1559	1582	VLHGILPAESTLMHRHITT IGTCK			Carbamidomethyl (C)[23]		Mascot

7	putative bifunctional polymyxin resistance arnA protein [Triticum monococcum]	gi|210077765	12688.5	6.95	2	63	97.784	4.63	54	99.968

Protein Group

putative bifunctional polymyxin resistance arnA protein [Triticum urartu]	gi|210077767	12688.5	6.9499 998092 6514

Peptide Information

Calc. Mass	Obsrv. Mass	± da	± ppm	Start Seq.	End Seq.	Sequence	Ion Score	C. I. %	Modification	Rank	Result Type
1405.7699	1405.7205	-0.0494	-35	20	32	HLVDPPPPHLAGR					Mascot
1405.7699	1405.7205	-0.0494	-35	20	32	HLVDPPPPHLAGR	54	99.968			Mascot
1453.7145	1453.6626	-0.0519	-36	97	108	LIHFSTCEVYGK			Carbamidomethyl (C)[7]		Mascot

8	hypothetical protein TRIUR3_28460 [Triticum urartu]	gi|473999752	14237	4.7	9	56	87.82	1.647	

Peptide Information

Calc. Mass	Obsrv. Mass	± da	± ppm	Start Seq.	End Seq.	Sequence	Ion Score	C. I. %	Modification	Rank	Result Type
846.454	846.4209	-0.0331	-39	8	14	ASTQQRR					Mascot
913.4374	913.4075	-0.0299	-33	97	104	ADDDKPPR					Mascot
933.416	933.4069	-0.0091	-10	27	33	EEEEELR					Mascot
1347.6978	1347.5894	-0.1084	-80	69	79	AELEWLMAQLK			Oxidation (M)[7]		Mascot
1417.7217	1417.6949	-0.0268	-19	85	96	LEDVLRQMGNAR			Oxidation (M)[8]		Mascot
1507.7104	1507.6327	-0.0777	-52	1	13	MGNCLNKASTQQR			Carbamidomethyl (C)[4]		Mascot
1570.7391	1570.7356	-0.0035	-2	91	104	QMGNARADDDKPPR					Mascot
1584.7864	1584.844	0.0576	36	21	33	VAPEVREEEEELR					Mascot
1765.8729	1765.8307	-0.0422	-24	97	111	ADDDKPPRADAWRPR					Mascot

9	hypothetical protein TRIUR3_29514 [Triticum urartu]	gi|473940046	36122.5	9.81	13	55	87.537	2.976	

Peptide Information

Calc. Mass	Obsrv. Mass	± da	± ppm	Start Seq.	End Seq.	Sequence	Ion Score	C. I. %	Modification	Rank	Result Type
840.4825	840.4304	-0.0521	-62	86	93	SPPPLTTK					Mascot
1164.6147	1164.5533	-0.0614	-53	113	122	YPTSDLVEIK					Mascot
1225.6106	1225.5708	-0.0398	-32	123	132	HQMEINALNR					Mascot
1232.6018	1232.6259	0.0241	20	268	278	SGYGVNKNEHK					Mascot
1453.5682	1453.6626	0.0944	65	217	229	DVAAACGMQEDDR			Carbamidomethyl (C)[6], Oxidation (M)[8]		Mascot
1477.6411	1477.7043	0.0632	43	1	14	MGGCPPTTTTSPDR			Carbamidomethyl (C)[4]		Mascot
1499.8251	1499.7408	-0.0843	-56	230	242	VPLAEVVLDCTKR			Carbamidomethyl (C)[10]		Mascot

Calc. Mass	Obsrv. Mass	± da	± ppm	Start Seq.	End Seq.	Sequence	Ion Score	C. I. %	Modification	Rank	Result Type
1524.7554	1524.6855	-0.0699	-46	39	51	SHYPRTTSVDHPK					Mascot
1537.7899	1537.7323	-0.0576	-37	279	291	GTMLVTQIQMMLR			Oxidation (M)[3]		Mascot
1563.7584	1563.7241	-0.0343	-22	26	38	DDCSRNPLQFLAK			Carbamidomethyl (C)[3]		Mascot
1570.8489	1570.7356	-0.1133	-72	31	43	NPLQFLAKSHYPR					Mascot
1665.8849	1665.8066	-0.0783	-47	279	292	GTMLVTQIQMMLRK			Oxidation (M)[3]		Mascot
1981.9583	1981.9203	-0.038	-19	250	267	EARAGDAAMQVLVGQMYR			Oxidation (M)[9]		Mascot

10	hypothetical protein TRIUR3_26594 [Triticum urartu]	gi|474446112	90423.5	7.27	18	53	78.341	6.275

Peptide Information

Calc. Mass	Obsrv. Mass	± da	± ppm	Start Seq.	End Seq.	Sequence	Ion Score	C. I. %	Modification	Rank	Result Type
826.3334	826.3394	0.006	7	781	786	FPCSCR			Carbamidomethyl (C)[3,5]		Mascot
840.3589	840.4304	0.0715	85	470	476	LDMSACK			Carbamidomethyl (C)[6], Oxidation (M)[3]		Mascot
863.5209	863.4437	-0.0772	-89	269	275	KHNVLPR					Mascot
874.4669	874.4027	-0.0642	-73	748	754	DEIIAWK					Mascot
913.4019	913.4075	0.0056	6	433	439	CICYAAR			Carbamidomethyl (C)[1,3]		Mascot
963.5621	963.4682	-0.0939	-97	382	389	TRYPSLVK					Mascot
1016.6099	1016.5134	-0.0965	-95	95	103	RAIVLSETK					Mascot
1151.6354	1151.5886	-0.0468	-41	603	612	FLRITGAAMR			Oxidation (M)[9]		Mascot
1362.6869	1362.6494	-0.0375	-28	465	476	IPGVRLDMSACK			Carbamidomethyl (C)[11], Oxidation (M)[8]		Mascot
1427.6698	1427.6968	0.027	19	727	737	TMFVIEPMYHK			Oxidation (M)[2,8]		Mascot
1541.8336	1541.719	-0.1146	-74	594	605	NFLVHENPRFLR					Mascot
1570.7999	1570.7356	-0.0643	-41	45	57	KAVEVIETFDEYK					Mascot
1606.8774	1606.8546	-0.0228	-14	58	71	RWLVTEIGFGGMLK					Mascot
1622.8723	1622.8184	-0.0539	-33	58	71	RWLVTEIGFGGMLK			Oxidation (M)[12]		Mascot
1665.7407	1665.8066	0.0659	40	304	316	CMIRQGHDICYGR			Carbamidomethyl (C)[1,10]		Mascot
1701.8953	1701.7955	-0.0998	-59	406	420	EINTARVNVQADMIK					Mascot
1755.8966	1755.9146	0.018	10	551	564	YVFRAGFQTDPWIR					Mascot
1952.0349	1951.8915	-0.1434	-73	739	754	SRPLTTHERDEIIAWK					Mascot
2250.1562	2250.2	0.0438	19	175	193	DCFQIAFIIFVMGHVLAPR			Carbamidomethyl (C)[2], Oxidation (M)[12]		Mascot

Gel Idx/Pos	261/K13	Instr./Gel Origin	BA2151/Sample Project 20140814	Process Status	Analysis Succeeded
Plate [#] Name	[1] Sample Project 20140814	Instrument Sample Name		Spectra	11

Rank	Protein Name	Accession No.	Protein MW	Protein PI	Pep. Count	Protein Score	Protein Score C. I. %	Intensity Matched	Total Ion Score	Total Ion C. I. %	Confirmed
1	cytosolic malate dehydrogenase [Triticum aestivum]	gi|49343245	35805.3	5.75	13	486	100	39.609	409	100	

Protein Group

cytosolic malate dehydrogenase [Triticum aestivum]	gi|229358240	35831.3	5.75

Peptide Information

Calc. Mass	Obsrv. Mass	± da	± ppm	Start Seq.	End Seq.	Sequence	Ion Score	C. I. %	Modification	Rank	Result Type
863.4404	863.4255	-0.0149	-17	153	159	NISCLTR			Carbamidomethyl (C)[4]		Mascot
873.4788	873.4628	-0.016	-18	165	172	ALGQISER					Mascot
970.5316	970.508	-0.0236	-24	204	212	TPSGEKPVR					Mascot
1360.7545	1360.6942	-0.0603	-44	57	68	MELIDAAFPLLK					Mascot
1376.7494	1376.6934	-0.056	-41	57	68	MELIDAAFPLLK			Oxidation (M)[1]		Mascot
1508.7928	1508.6887	-0.1041	-69	160	172	LDHNRALGQISER					Mascot
1645.7963	1645.7635	-0.0328	-20	242	257	LSSALSAASSACDHIR			Carbamidomethyl (C)[12]		Mascot
1649.9949	1649.85	-0.1449	-88	128	143	VLVVANPANTNALILK					Mascot
1773.8912	1773.8575	-0.0337	-19	241	257	KLSSALSAASSACDHIR			Carbamidomethyl (C)[13]		Mascot
2000.1361	2000.0997	-0.0364	-18	9	28	VLVTGAAGGQIGYALVPMI AR					Mascot
2000.1361	2000.0997	-0.0364	-18	9	28	VLVTGAAGGQIGYALVPMI AR	44	99.758			Mascot
2016.1311	2016.0746	-0.0565	-28	9	28	VLVTGAAGGQIGYALVPMI AR			Oxidation (M)[17]		Mascot
2016.1311	2016.0746	-0.0565	-28	9	28	VLVTGAAGGQIGYALVPMI AR	96	100	Oxidation (M)[17]		Mascot
2390.1624	2390.1448	-0.0176	-7	213	232	ELVQDDEWLNGEFIATV QQR					Mascot
2390.1624	2390.1448	-0.0176	-7	213	232	ELVQDDEWLNGEFIATV QQR	187	100			Mascot
2438.1848	2438.1343	-0.0505	-21	182	203	NAIIWGNHSSSQYPDVN HATVK					Mascot
2438.1848	2438.1343	-0.0505	-21	182	203	NAIIWGNHSSSQYPDVN HATVK					Mascot
2605.3113	2605.2822	-0.0291	-11	69	94	GVVATTDVVEACTGVNV AVMVGGFPR			Carbamidomethyl (C)[12]		Mascot
2605.3113	2605.2822	-0.0291	-11	69	94	GVVATTDVVEACTGVNV AVMVGGFPR	126	100	Carbamidomethyl (C)[12]		Mascot
2621.3064	2621.2654	-0.041	-16	69	94	GVVATTDVVEACTGVNV AVMVGGFPR			Carbamidomethyl (C)[12], Oxidation (M)[20]		Mascot
2621.3064	2621.2654	-0.041	-16	69	94	GVVATTDVVEACTGVNV AVMVGGFPR	87	100	Carbamidomethyl (C)[12], Oxidation (M)[20]		Mascot
2648.5022	2648.2666	-0.2356	-89	128	152	VLVVANPANTNALILKEFA					Mascot

PSIPEK

2	unnamed protein product [Triticum aestivum]	gi|257700508	24559.7	6.6	13	409	100	30.684	313	100

Protein Group

cytosolic malate dehydrogenase [Triticum aestivum]	gi|37928995	24559.7	6.5999999046 3257
unnamed protein product [Triticum aestivum]	gi|257645258	24559.7	6.5999999046 3257
unnamed protein product [Triticum aestivum]	gi|219913686	24559.7	6.5999999046 3257
unnamed protein product [Triticum aestivum]	gi|219789621	24559.7	6.5999999046 3257

Peptide Information

Calc. Mass	Obsrv. Mass	± da	± ppm	Start Seq.	End Seq.	Sequence	Ion Score	C. I. %	Modification	Rank	Result Type
863.4404	863.4255	-0.0149	-17	123	129	NISCLTR			Carbamidomethyl (C)[4]		Mascot
873.4788	873.4628	-0.016	-18	135	142	ALGQISER					Mascot
970.5316	970.508	-0.0236	-24	174	182	TPSGEKPVR					Mascot
1360.7545	1360.6942	-0.0603	-44	27	38	MELIDAAFPLLK					Mascot
1376.7494	1376.6934	-0.056	-41	27	38	MELIDAAFPLLK			Oxidation (M)[1]		Mascot
1508.7928	1508.6887	-0.1041	-69	130	142	LDHNRALGQISER					Mascot
1645.7963	1645.7635	-0.0328	-20	212	227	LSSALSAASSACDHIR			Carbamidomethyl (C)[12]		Mascot
1649.9949	1649.85	-0.1449	-88	98	113	VLVVANPANTNALILK					Mascot
1773.8912	1773.8575	-0.0337	-19	211	227	KLSSALSAASSACDHIR			Carbamidomethyl (C)[13]		Mascot
2390.1624	2390.1448	-0.0176	-7	183	202	ELVQDDEWLNGEFIATVQQR					Mascot
2390.1624	2390.1448	-0.0176	-7	183	202	ELVQDDEWLNGEFIATVQQR	187	100			Mascot
2438.1848	2438.1343	-0.0505	-21	152	173	NAIIWGNHSSSQYPDVNHATVK					Mascot
2438.1848	2438.1343	-0.0505	-21	152	173	NAIIWGNHSSSQYPDVNHATVK					Mascot
2557.3042	2557.3127	0.0085	3	1	23	MLGADQPVILHMLDIEFAAEALK			Oxidation (M)[1,12]		Mascot
2557.3042	2557.3127	0.0085	3	1	23	MLGADQPVILHMLDIEFAAEALK			Oxidation (M)[1,12]		Mascot
2605.3113	2605.2822	-0.0291	-11	39	64	GVVATTDVVEACTGVNVAVMVGGFPR			Carbamidomethyl (C)[12]		Mascot
2605.3113	2605.2822	-0.0291	-11	39	64	GVVATTDVVEACTGVNVAVMVGGFPR	126	100	Carbamidomethyl (C)[12]		Mascot
2621.3064	2621.2654	-0.041	-16	39	64	GVVATTDVVEACTGVNVAVMVGGFPR			Carbamidomethyl (C)[12], Oxidation (M)[20]		Mascot
2621.3064	2621.2654	-0.041	-16	39	64	GVVATTDVVEACTGVNVAVMVGGFPR	87	100	Carbamidomethyl (C)[12], Oxidation (M)[20]		Mascot
2648.5022	2648.2666	-0.2356	-89	98	122	VLVVANPANTNALILKEFAPSIPEK					Mascot

3	hypothetical protein TRIUR3_33832 [Triticum urartu]	gi|474157193	40178.6	9.02	10	44	0	1.735

Peptide Information

Calc. Mass	Obsrv. Mass	± da	± ppm	Start Seq.	End Seq.	Sequence	Ion Score	C. I. %	Modification	Rank	Result Type
1317.6719	1317.6321	-0.0398	-30	189	200	MTLLEDPTAQAK					Mascot
1473.7729	1473.7206	-0.0523	-35	189	201	MTLLEDPTAQAKR					Mascot
1476.7377	1476.7167	-0.021	-14	72	84	GKDQRPAAFSPMR			Oxidation (M)[12]		Mascot
1489.7679	1489.6906	-0.0773	-52	189	201	MTLLEDPTAQAKR			Oxidation (M)[1]		Mascot
2332.2681	2332.0686	-0.1995	-86	242	263	GGKVVEADLVTLTEALMS ELLK			Oxidation (M)[17]		Mascot
2403.198	2403.1375	-0.0605	-25	130	150	YAGVYHEVYINSQASFGE LKK					Mascot
2517.3733	2517.2581	-0.1152	-46	245	267	VVEADLVTLTEALMSELL KLDAK			Oxidation (M)[14]		Mascot
2533.3118	2533.2451	-0.0667	-26	15	41	VAGEGGHPSVAVSTLDL SSGAVGPGRR					Mascot
2573.2124	2573.2739	0.0615	24	342	364	LIPYNDLLHRCMVAVDGD GDGDK			Carbamidomethyl (C)[11]		Mascot
2602.4548	2602.2651	-0.1897	-73	264	286	LDAKAARPPLRPRPAPA QQHQQR					Mascot
2714.3931	2714.2739	-0.1192	-44	85	108	ESVAAAVQEEVWEVRPS GMLVQKR			Oxidation (M)[19]		Mascot

4	Serine-threonine kinase receptor-associated protein [Triticum urartu]	gi|473898062	35043	5.65	9	43	0	9.511

Peptide Information

Calc. Mass	Obsrv. Mass	± da	± ppm	Start Seq.	End Seq.	Sequence	Ion Score	C. I. %	Modification	Rank	Result Type
1072.615	1072.6005	-0.0145	-14	306	314	KIEGFHITK					Mascot
1316.6442	1316.6512	0.007	5	166	178	APVTSAEVSQDGR					Mascot
1596.7476	1596.8741	0.1265	79	11	24	GYNLGYPDSNPMLR					Mascot
1842.8505	1842.8567	0.0062	3	256	273	FAPVGESYASGSEDGTIR					Mascot
1981.8994	1982.0679	0.1685	85	85	102	ACAFSEDTHMLLTGGVE K			Carbamidomethyl (C)[2], Oxidation (M)[10]		Mascot
2373.1833	2373.1409	-0.0424	-18	274	295	IWPLNPANTEDPEAPNAN GKPK					Mascot
2402.2363	2402.1431	-0.0932	-39	65	84	IWDALTGNELHSFEHKHI VR					Mascot
2406.1177	2406.1262	0.0085	4	128	149	TVAWLHSDQSILSSCSD MGGVR			Carbamidomethyl (C)[15]		Mascot
2422.1125	2422.1191	0.0066	3	128	149	TVAWLHSDQSILSSCSD MGGVR			Carbamidomethyl (C)[15], Oxidation (M)[18]		Mascot
2422.1125	2422.1191	0.0066	3	128	149	TVAWLHSDQSILSSCSD MGGVR			Carbamidomethyl (C)[15], Oxidation (M)[18]		Mascot
2423.2048	2423.1118	-0.093	-38	166	189	APVTSAEVSQDGRFITTA DGSSVK					Mascot

5	TBC1 domain family member 8 [Triticum urartu]	gi|474190209	94417.5	4.69	14	38	0	5.98

Peptide Information

Calc. Mass	Obsrv. Mass	± da	± ppm	Start Seq.	End Seq.	Sequence	Ion Score	C. I. %	Modification	Rank	Result Type
1316.6991	1316.6512	-0.0479	-36	569	578	VELCRLLEER			Carbamidomethyl (C)[4]		Mascot
1411.6602	1411.7876	0.1274	90	265	277	TFPGHPALDEDGR					Mascot
1473.718	1473.7206	0.0026	2	765	777	LNNNTEQSVETPK					Mascot
1508.6786	1508.6887	0.0101	7	809	822	MDGDVPTVETTTDK					Mascot
1842.9564	1842.8567	-0.0997	-54	181	196	EELECLVRGGLPMALR			Carbamidomethyl (C)[5]		Mascot
1862.9243	1862.8969	-0.0274	-15	645	661	VTEDARIFAEQDAAAQK					Mascot
2022.0247	2022.0005	-0.0242	-12	580	597	SAVLRADELETALMEMV K			Oxidation (M)[14]		Mascot
2038.0195	2038.0443	0.0248	12	580	597	SAVLRADELETALMEMV K			Oxidation (M)[14,16]		Mascot
2346.1428	2346.123	-0.0198	-8	626	644	EEQEQAMLQVLMRVEQE QK					Mascot
2404.1516	2404.1299	-0.0217	-9	213	236	GYYESLLGVVDGGGDSK GSDSLTK					Mascot
2423.2864	2423.1118	-0.1746	-72	96	116	IQTWSEIRPSLGHIGELM SLR					Mascot
2438.355	2438.1343	-0.2207	-91	412	433	VMLFRTALALMELYGPAL VTTK					Mascot
2438.355	2438.1343	-0.2207	-91	412	433	VMLFRTALALMELYGPAL VTTK					Mascot
2445.1389	2445.1235	-0.0154	-6	716	737	TPTRDASPGQVNQDSSQ EFQPR					Mascot
2470.3447	2470.113	-0.2317	-94	412	433	VMLFRTALALMELYGPAL VTTK			Oxidation (M)[2,11]		Mascot
2509.1499	2509.1311	-0.0188	-7	800	822	DEPTLETAKMDGDVPTV ETTTDK			Oxidation (M)[10]		Mascot
2714.2549	2714.2739	0.019	7	662	684	FASHVLQEKYDEAMASL TQMENR			Oxidation (M)[14]		Mascot

6 Callose synthase 11 [Triticum urartu] gi|473945522 184478.2 9.23 21 38 0 6.211

Peptide Information

Calc. Mass	Obsrv. Mass	± da	± ppm	Start Seq.	End Seq.	Sequence	Ion Score	C. I. %	Modification	Rank	Result Type
1134.6418	1134.5875	-0.0543	-48	1293	1301	LYARSHFIK					Mascot
1148.5959	1148.5892	-0.0067	-6	1141	1149	LGHRTDFFR					Mascot
1316.6157	1316.6512	0.0355	27	656	666	FPGENDLSFYK					Mascot
1373.6056	1373.7098	0.1042	76	1054	1064	MHYGHPDVFDR					Mascot
1476.7019	1476.7167	0.0148	10	109	119	SFWNVYRSFDR					Mascot
1649.7642	1649.85	0.0858	52	334	345	RNWFEFMPHTER					Mascot
1681.8446	1681.8541	0.0095	6	1279	1292	GFVVQHKSFAENYR					Mascot
1862.9041	1862.8969	-0.0072	-4	693	707	RIAFFSNSLFMNMPR			Oxidation (M)[11,13]		Mascot
1895.9471	1896.0557	0.1086	57	45	61	AEVEASRNGTKPHSAWR					Mascot

Calc. Mass	Obsrv. Mass	± da	± ppm	Start Seq.	End Seq.	Sequence	Ion Score	C. I. %	Modification	Rank	Result Type
2031.9475	2032.0758	0.1283	63	2	19	ALDLSHVMDCSIDIETGR			Carbamidomethyl (C)[10]		Mascot
2038.0275	2038.0443	0.0168	8	859	878	GASTVSQLFKGQEDGAA LMK					Mascot
2186.1909	2186.1008	-0.0901	-41	268	284	ILYVLTWWFQTRTFVGR					Mascot
2332.1465	2332.0686	-0.0779	-33	694	713	IAFFSNSLFMNMPRAPTV EK			Oxidation (M)[10,12]		Mascot
2372.3201	2372.1453	-0.1748	-74	192	212	MVLKAFVAAGWTITFSVL YVR					Mascot
2372.3201	2372.1453	-0.1748	-74	192	212	MVLKAFVAAGWTITFSVL YVR					Mascot
2388.3149	2388.1274	-0.1875	-79	192	212	MVLKAFVAAGWTITFSVL YVR			Oxidation (M)[1]		Mascot
2462.1155	2462.1294	0.0139	6	397	416	FQFFASAMQFNLMPEEH LDK			Oxidation (M)[8,13]		Mascot
2466.3015	2466.2424	-0.0591	-24	468	488	EEDIVSDKEVELLELPPV VWK					Mascot
2492.3044	2492.1184	-0.186	-75	996	1016	NLLQQYNYYHGSQKPTL LGVR					Mascot
2533.333	2533.2451	-0.0879	-35	532	552	CAVIEAYDSIRQLLLEIIEE R			Carbamidomethyl (C)[1]		Mascot
2588.1755	2588.2944	0.1189	46	974	995	GDAVQTIDMNQDNYFEE ALKMR					Mascot
2602.2	2602.2651	0.0651	25	1356	1376	TVYDFEDFMTWIWFPGGI FSK			Oxidation (M)[9]		Mascot
2604.1704	2604.2747	0.1043	40	974	995	GDAVQTIDMNQDNYFEE ALKMR			Oxidation (M)[9]		Mascot
2620.1655	2620.2605	0.095	36	974	995	GDAVQTIDMNQDNYFEE ALKMR			Oxidation (M)[9,21]		Mascot
2633.3335	2633.3242	-0.0093	-4	196	217	AFVAAGWTITFSVLYVRM WDQR			Oxidation (M)[18]		Mascot

7	putative serine/threonine-protein kinase WNK5 [Triticum urartu]	gi|474224677	45025.5	7.4	9	37	0	9.996

Peptide Information

Calc. Mass	Obsrv. Mass	± da	± ppm	Start Seq.	End Seq.	Sequence	Ion Score	C. I. %	Modification	Rank	Result Type
1649.7555	1649.85	0.0945	57	144	157	GFDQEEGIEVAWNR					Mascot
1842.9875	1842.8567	-0.1308	-71	65	79	VFQLHLNFGHAFTRR					Mascot
1949.9135	1950.1018	0.1883	97	2	19	TPSAPDHEGERHASSML K					Mascot
1951.9542	1952.1282	0.174	89	200	217	DAGVLNFITEVCNSGSLR			Carbamidomethyl (C)[12]		Mascot
1951.9542	1952.1282	0.174	89	200	217	DAGVLNFITEVCNSGSLR			Carbamidomethyl (C)[12]		Mascot
2400.1614	2400.1523	-0.0091	-4	200	220	DAGVLNFITEVCNSGSLR EYR			Carbamidomethyl (C)[12]		Mascot
2466.1982	2466.2424	0.0442	18	107	128	IHGGGFFMNNLEEVDLTG RFGR					Mascot
2509.1711	2509.1311	-0.04	-16	104	125	CARIHGGGFFMNNLEEV DLTGR			Carbamidomethyl (C)[1], Oxidation (M)[11]		Mascot
2604.2307	2604.2747	0.044	17	79	102	RQMGDAAEHLGFDGQM GVVHGHVR					Mascot
2620.2256	2620.2605	0.0349	13	79	102	RQMGDAAEHLGFDGQM GVVHGHVR			Oxidation (M)[3]		Mascot
2621.3142	2621.2654	-0.0488	-19	195	217	VWLDRDAGVLNFITEVCN			Carbamidomethyl (C)[17]		Mascot

2621.3142	2621.2654	-0.0488	-19	195	217	SGSLR VWLDRDAGVLNFITEVCN SGSLR			Carbamidomethyl (C)[17]		Mascot

8 26S proteasome non-ATPase regulatory subunit 2 1B [Triticum urartu] gi|474393501 95591.6 6.62 13 36 0 9.921

Peptide Information

Calc. Mass	Obsrv. Mass	± da	± ppm	Start Seq.	End Seq.	Sequence	Ion Score	C. I. %	Modification	Rank	Result Type
1681.8214	1681.8541	0.0327	19	630	643	SLEQLLQYGDCSIR			Carbamidomethyl (C)[11]		Mascot
1763.0175	1762.9231	-0.0944	-54	209	224	DLGSALRIVLLVNDHK					Mascot
1842.9645	1842.8567	-0.1078	-58	768	782	YPYMLYILALAMQPR					Mascot
1950.0035	1950.1018	0.0983	50	44	61	KEVCAATSSMTSVPKPLK			Carbamidomethyl (C)[4], Oxidation (M)[10]		Mascot
1989.04	1988.9559	-0.0841	-42	417	435	DHALALISEYSKGAASVT R					Mascot
1991.0995	1990.9529	-0.1466	-74	783	799	MLLTVDEDLKPLHVPVR			Oxidation (M)[1]		Mascot
2000.0117	2000.0997	0.088	44	85	103	HMADILSVLALTASAEGE R			Oxidation (M)[2]		Mascot
2000.0117	2000.0997	0.088	44	85	103	HMADILSVLALTASAEGE R			Oxidation (M)[2]		Mascot
2394.9976	2395.1372	0.1396	58	108	126	YCMMGSLVDICSWGHEY VR			Carbamidomethyl (C)[2,11], Oxidation (M)[3,4]		Mascot
2400.2817	2400.1523	-0.1294	-54	718	739	IAQGLVHLGKGLLTLDPC HSDR			Carbamidomethyl (C)[18]		Mascot
2548.3406	2548.241	-0.0996	-39	389	413	YLHSNDTNVVAGTFLGIG IVSSGVK					Mascot
2602.2932	2602.2651	-0.0281	-11	185	206	ILYLSTPDHKATLDIAYDM YMK					Mascot
2604.1865	2604.2747	0.0882	34	108	128	YCMMGSLVDICSWGHEY VRLK			Carbamidomethyl (C)[2,11]		Mascot
2620.1816	2620.2605	0.0789	30	108	128	YCMMGSLVDICSWGHEY VRLK			Carbamidomethyl (C)[2,11], Oxidation (M)[3]		Mascot
2633.2739	2633.3242	0.0503	19	365	388	ASVVASLGMIHMWDPDS GFPKLDK			Oxidation (M)[9,12]		Mascot

9 DNA topoisomerase 1 [Triticum urartu] gi|474103406 92304.6 9.26 12 35 0 15.253

Peptide Information

Calc. Mass	Obsrv. Mass	± da	± ppm	Start Seq.	End Seq.	Sequence	Ion Score	C. I. %	Modification	Rank	Result Type
1317.6798	1317.6321	-0.0477	-36	607	618	GGHVFDKLDTTK					Mascot
1952.0334	1952.1282	0.0948	49	637	653	TYNASITLDKILNEQTK					Mascot
1952.0334	1952.1282	0.0948	49	637	653	TYNASITLDKILNEQTK					Mascot
2014.0538	2013.9919	-0.0619	-31	135	152	FSPVARNSVDMPIKPNNK					Mascot
2373.2866	2373.1409	-0.1457	-61	271	291	WSTLVHNGVIFPPPYKPH GVK					Mascot
2412.23	2412.1206	-0.1094	-45	141	163	NSVDMPIKPNNKALNTSA SSAPR					Mascot
2423.074	2423.1118	0.0378	16	185	207	CTDSSASAISEDDGSDNV			Carbamidomethyl (C)[1]		Mascot

						PLARR					
2443.1089	2443.1365	0.0276	11	317	336	DTEYASKETFINNFFTDW SK					Mascot
2466.105	2466.2424	0.1374	56	183	206	AKCTDSSASAISEDDGSD NVPLAR			Carbamidomethyl (C)[3]		Mascot
2470.1038	2470.113	0.0092	4	85	106	IINTCSADADYSEDDEKP LSAR			Carbamidomethyl (C)[5]		Mascot
2544.0508	2544.2454	0.1946	76	1	23	MPAAVHTFDSDDDDEFD GPTSFK					Mascot
2556.269	2556.3577	0.0887	35	583	604	YLNTVEVEPPVYEAIKEF CAGK			Carbamidomethyl (C)[19]		Mascot
2605.2834	2605.2822	-0.0012	0	457	477	HDNTVTWLAFWNDLINQ KDFK					Mascot
2605.2834	2605.2822	-0.0012	0	457	477	HDNTVTWLAFWNDLINQ KDFK					Mascot

10	cystatin [Triticum aestivum]	gi|71535090	8532.4	9.52	5	34	0	10.634	

Protein Group

cystatin [Triticum aestivum]	gi|78173103	8532.4	9.5200 004577 6367

Peptide Information

Calc. Mass	Obsrv. Mass	± da	± ppm	Start Seq.	End Seq.	Sequence	Ion Score	C. I. %	Modification	Rank	Result Type
1148.5881	1148.5892	0.0011	1	60	68	LMEFRPAER					Mascot
2015.9507	2016.0746	0.1239	61	44	58	MYEAKVWERPWMDFK					Mascot
2015.9507	2016.0746	0.1239	61	44	58	MYEAKVWERPWMDFK					Mascot
2031.9456	2032.0758	0.1302	64	44	58	MYEAKVWERPWMDFK			Oxidation (M)[1]		Mascot
2332.1311	2332.0686	-0.0625	-27	22	42	QQVVAGMMYYITIQVNE GGAK			Oxidation (M)[7,8]		Mascot
2460.2261	2460.1162	-0.1099	-45	22	43	QQVVAGMMYYITIQVNE GGAKK			Oxidation (M)[7,8]		Mascot
2557.3154	2557.3127	-0.0027	-1	20	42	LKQQVVAGMMYYITIQVN EGGAK			Oxidation (M)[9]		Mascot
2557.3154	2557.3127	-0.0027	-1	20	42	LKQQVVAGMMYYITIQVN EGGAK			Oxidation (M)[9]		Mascot
2573.3103	2573.2739	-0.0364	-14	20	42	LKQQVVAGMMYYITIQVN EGGAK			Oxidation (M)[9,10]		Mascot

Gel Idx/Pos	262/K14	Instr./Gel Origin	BA2151/Sample Project 20140814	Process Status	Analysis Succeeded
Plate [#] Name	[1] Sample Project 20140814	Instrument Sample Name		Spectra	11

Rank	Protein Name	Accession No.	Protein MW	Protein PI	Pep. Count	Protein Score	Protein Score C. I. %	Intensity Matched	Total Ion Score	Total Ion C. I. %	Confirmed
1	Beta-amylase [Triticum urartu]	gi|474451266	58995	5.34	17	549	100	26.762	469	100	

Peptide Information

Calc. Mass	Obsrv. Mass	± da	± ppm	Start Seq.	End Seq.	Sequence	Ion Score	C. I. %	Modification	Rank	Result Type
947.5057	947.4637	-0.042	-44	322	329	DGYRPIAR					Mascot
993.4999	993.4631	-0.0368	-37	28	35	FEKGDELR					Mascot
1239.6328	1239.5414	-0.0914	-74	272	282	HGDKILDEANK					Mascot
1299.582	1299.552	-0.03	-23	336	346	ASLNFTCAEMR			Carbamidomethyl (C)[7]		Mascot
1313.6273	1313.6583	0.031	24	62	72	GPRAYDWSAYK					Mascot
1315.5769	1315.5256	-0.0513	-39	336	346	ASLNFTCAEMR			Carbamidomethyl (C)[7], Oxidation (M)[10]		Mascot
1335.7202	1335.6029	-0.1173	-88	322	332	DGYRPIARMLK			Oxidation (M)[9]		Mascot
1382.7791	1382.7128	-0.0663	-48	73	84	QLFQLVHEAGLK					Mascot
1515.7994	1515.6556	-0.1438	-95	260	271	FFLAWYSNNLIK					Mascot
1592.7421	1592.6691	-0.073	-46	334	346	HRASLNFTCAEMR			Carbamidomethyl (C)[9]		Mascot
1623.9581	1623.8064	-0.1517	-93	73	86	QLFQLVHEAGLKLK					Mascot
1646.781	1646.7518	-0.0292	-18	246	259	FFVDNGTYLTEQGR					Mascot
1646.781	1646.7518	-0.0292	-18	246	259	FFVDNGTYLTEQGR	84	100			Mascot
1668.7952	1668.7633	-0.0319	-19	218	232	AAAAMVGHPEWEFPR					Mascot
1669.7349	1669.7563	0.0214	13	148	161	SAVQMYTDYMASFR					Mascot
1684.79	1684.7444	-0.0456	-27	218	232	AAAAMVGHPEWEFPR			Oxidation (M)[5]		Mascot
1701.7247	1701.6964	-0.0283	-17	148	161	SAVQMYTDYMASFR			Oxidation (M)[5,10]		Mascot
1992.9047	1992.8723	-0.0324	-16	112	129	NVGASDPDIFYTDQHGTR					Mascot
1992.9047	1992.8723	-0.0324	-16	112	129	NVGASDPDIFYTDQHGTR	146	100			Mascot
2013.9778	2013.9503	-0.0275	-14	304	321	VPSHAAEITAGYYNLHDR					Mascot
2013.9778	2013.9503	-0.0275	-14	304	321	VPSHAAEITAGYYNLHDR	118	100			Mascot
2087.0557	2087.0286	-0.0271	-13	130	147	NIEYLTLGVDDQPLFHGR					Mascot
2087.0557	2087.0286	-0.0271	-13	130	147	NIEYLTLGVDDQPLFHGR	121	100			Mascot
2269.2075	2269.1716	-0.0359	-16	166	187	EFLDAGVIVDIEVGLGPAGELR					Mascot

Rank	Protein Name	Accession No.	Protein MW	Protein PI	Pep. Count	Protein Score	Protein Score C. I. %	Intensity Matched	Total Ion Score	Total Ion C. I. %	Confirmed
2	beta amylase [Triticum aestivum]	gi|32400764	31099.9	8.6	9	242	100	13.75	202	100	

Peptide Information

Calc. Mass	Obsrv. Mass	± da	± ppm	Start Seq.	End Seq.	Sequence	Ion Score	C. I. %	Modification	Rank	Result Type
947.5057	947.4637	-0.042	-44	137	144	DGYRPIAR					Mascot
1239.6328	1239.5414	-0.0914	-74	87	97	HGDKILDEANK					Mascot
1299.582	1299.552	-0.03	-23	151	161	ASLNFTCAEMR			Carbamidomethyl (C)[7]		Mascot
1315.5769	1315.5256	-0.0513	-39	151	161	ASLNFTCAEMR			Carbamidomethyl (C)[7], Oxidation (M)[10]		Mascot
1335.7202	1335.6029	-0.1173	-88	137	147	DGYRPIARMLK			Oxidation (M)[9]		Mascot
1515.7994	1515.6556	-0.1438	-95	75	86	FFLAWYSNNLIK					Mascot
1592.7421	1592.6691	-0.073	-46	149	161	HRASLNFTCAEMR			Carbamidomethyl (C)[9]		Mascot
1646.781	1646.7518	-0.0292	-18	61	74	FFVDNGTYLTEQGR					Mascot
1646.781	1646.7518	-0.0292	-18	61	74	FFVDNGTYLTEQGR	84	100			Mascot
1668.7952	1668.7633	-0.0319	-19	33	47	AAAAMVGHPEWEFPR					Mascot
1684.79	1684.7444	-0.0456	-27	33	47	AAAAMVGHPEWEFPR			Oxidation (M)[5]		Mascot
2013.9778	2013.9503	-0.0275	-14	119	136	VPSHAAEITAGYYNLHDR					Mascot
2013.9778	2013.9503	-0.0275	-14	119	136	VPSHAAEITAGYYNLHDR	118	100			Mascot

3	reversibly glycosylated polypeptide [Triticum aestivum]	gi|4158232	41985	5.82	12	231	100	22.746	175	100	

Protein Group

glycosyltransferase 75 [Triticum aestivum]	gi|301072492	41985	5.8200 001716 6138

Peptide Information

Calc. Mass	Obsrv. Mass	± da	± ppm	Start Seq.	End Seq.	Sequence	Ion Score	C. I. %	Modification	Rank	Result Type
839.441	839.4164	-0.0246	-29	159	165	GYPFSLR					Mascot
1180.6321	1180.5765	-0.0556	-47	123	132	DINALEQHIK					Mascot
1201.6365	1201.5807	-0.0558	-46	266	275	TGLPYLWHSK					Mascot
1283.7206	1283.6833	-0.0373	-29	20	30	DELDIVIPTIR					Mascot
1401.625	1401.5948	-0.0302	-22	83	94	ASCISFKDSACR			Carbamidomethyl (C)[3,11]		Mascot
1483.7461	1483.6969	-0.0492	-33	315	326	CYISLSEQVKEK			Carbamidomethyl (C)[1]		Mascot
1501.6958	1501.6659	-0.0299	-20	61	72	VPEGFDYELYNR					Mascot
1501.6958	1501.6659	-0.0299	-20	61	72	VPEGFDYELYNR	99	100			Mascot
1592.7302	1592.6691	-0.0611	-38	105	117	YVFTIDDDCFVAK			Carbamidomethyl (C)[9]		Mascot
1745.7809	1745.7543	-0.0266	-15	209	223	GTLFPMCGMNLAFDR			Carbamidomethyl (C)[7], Oxidation (M)[6]		Mascot
1761.7758	1761.7183	-0.0575	-33	209	223	GTLFPMCGMNLAFDR			Carbamidomethyl (C)[7], Oxidation (M)[6,9]		Mascot
2086.9785	2087.0286	0.0501	24	308	324	ECDTVQKCYISLSEQVK			Carbamidomethyl (C)[2,8]		Mascot
2086.9785	2087.0286	0.0501	24	308	324	ECDTVQKCYISLSEQVK			Carbamidomethyl (C)[2,8]		Mascot
2137.0569	2136.9932	-0.0637	-30	224	243	QLIGPAMYFGLMGDGQPI			Oxidation (M)[7]		Mascot

Calc. Mass	Obsrv. Mass	± da	± ppm	Start Seq.	End Seq.	Sequence	Ion Score	C. I. %	Modification	Rank	Result Type
						GR					
2153.0518	2152.9929	-0.0589	-27	224	243	QLIGPAMYFGLMGDGQPIGR			Oxidation (M)[7,12]		Mascot
2292.1335	2292.1003	-0.0332	-14	133	151	NLLSPSTPFFFNTLYDPYR					Mascot
2292.1335	2292.1003	-0.0332	-14	133	151	NLLSPSTPFFFNTLYDPYR	76	100			Mascot

4	Alpha-1,4-glucan-protein synthase [UDP-forming] 1 [Triticum urartu]	gi|474042704	63955.1	5.55	13	217	100	16.945	175	100	

Peptide Information

Calc. Mass	Obsrv. Mass	± da	± ppm	Start Seq.	End Seq.	Sequence	Ion Score	C. I. %	Modification	Rank	Result Type
839.441	839.4164	-0.0246	-29	150	156	GYPFSLR					Mascot
989.5414	989.4857	-0.0557	-56	267	275	ASNPFVNLK					Mascot
1180.6321	1180.5765	-0.0556	-47	114	123	DINALEQHIK					Mascot
1201.6365	1201.5807	-0.0558	-46	257	266	TGLPYIWHSK					Mascot
1271.5275	1271.5276	0.0001	0	550	560	YVDLTSDTNDE					Mascot
1283.7206	1283.6833	-0.0373	-29	11	21	DELDIVIPTIR					Mascot
1401.625	1401.5948	-0.0302	-22	74	85	ASCISFKDSACR			Carbamidomethyl (C)[3,11]		Mascot
1501.6958	1501.6659	-0.0299	-20	52	63	VPEGFDYELYNR					Mascot
1501.6958	1501.6659	-0.0299	-20	52	63	VPEGFDYELYNR	99	100			Mascot
1574.8285	1574.7452	-0.0833	-53	374	388	AGLETSAQSRAIGWK					Mascot
1745.7809	1745.7543	-0.0266	-15	200	214	GTLFPMCGMNLAFDR			Carbamidomethyl (C)[7], Oxidation (M)[6]		Mascot
1761.7758	1761.7183	-0.0575	-33	200	214	GTLFPMCGMNLAFDR			Carbamidomethyl (C)[7], Oxidation (M)[6,9]		Mascot
1789.777	1789.8464	0.0694	39	429	447	LSCGSSSAGSSFAGASNSR			Carbamidomethyl (C)[3]		Mascot
2136.0278	2135.9727	-0.0551	-26	514	531	EEEVNDVLFEEGLARVCK			Carbamidomethyl (C)[17]		Mascot
2292.1335	2292.1003	-0.0332	-14	124	142	NLLSPSTPFFFNTLYDPYR					Mascot
2292.1335	2292.1003	-0.0332	-14	124	142	NLLSPSTPFFFNTLYDPYR	76	100			Mascot

5	Beta-amylase [Triticum urartu]	gi|474019719	63864.1	5.29	8	139	100	8.455	121	100	

Peptide Information

Calc. Mass	Obsrv. Mass	± da	± ppm	Start Seq.	End Seq.	Sequence	Ion Score	C. I. %	Modification	Rank	Result Type
1285.6212	1285.5837	-0.0375	-29	61	71	GPKAYDWSAYK					Mascot
1449.7307	1449.6445	-0.0862	-59	487	497	KQWPYVMNDLR					Mascot
1571.858	1571.7612	-0.0968	-62	456	469	FYLQPLPPAEAAVR					Mascot
1606.8159	1606.7373	-0.0786	-49	488	499	QWPYVMNDLRLR			Oxidation (M)[6]		Mascot
1623.6461	1623.8064	0.1603	99	333	345	HHASMNFTCAEMR			Carbamidomethyl (C)[9], Oxidation (M)[5,12]		Mascot

1686.8949	1686.7264	-0.1685	-100	552	566	YYGETKTVLSDVLAK					Mascot
1696.948	1696.8121	-0.1359	-80	472	486	ESAQDILNLKPLIDK					Mascot
2087.0557	2087.0286	-0.0271	-13	129	146	NIEYLTLGVDDQPLFHGR					Mascot
2087.0557	2087.0286	-0.0271	-13	129	146	NIEYLTLGVDDQPLFHGR	121	100			Mascot

6	beta-amylase [Triticum aestivum]	gi|1771782	56860.2	5.24	6	132	100	7.41	121	100	

Protein Group

RecName: Full=Beta-amylase; AltName: Full=1,4-alpha-D-glucan maltohydrolase	gi|3334120	56860.2	5.2399 997711 1816

Peptide Information

Calc. Mass	Obsrv. Mass	± da	± ppm	Start Seq.	End Seq.	Sequence	Ion Score	C. I. %	Modification	Rank	Result Type
993.4999	993.4631	-0.0368	-37	27	34	FEKGDEIR					Mascot
1239.6328	1239.5414	-0.0914	-74	271	281	HGDKILDEANK					Mascot
1285.6212	1285.5837	-0.0375	-29	61	71	GPKAYDWSAYK					Mascot
1623.6461	1623.8064	0.1603	99	333	345	HHASMNFTCAEMR			Carbamidomethyl (C)[9], Oxidation (M)[5,12]		Mascot
1669.7349	1669.7563	0.0214	13	147	160	TAVQMYADYMASFR			Oxidation (M)[5]		Mascot
2087.0557	2087.0286	-0.0271	-13	129	146	NIEYLTLGVDDQPLFHGR					Mascot
2087.0557	2087.0286	-0.0271	-13	129	146	NIEYLTLGVDDQPLFHGR	121	100			Mascot

7	beta amylase, partial [Triticum monococcum subsp. aegilopoides]	gi|56130898	25218.4	5.35	1	121	100	6.488	121	100	

Protein Group

beta amylase [Triticum urartu]	gi|260060415	25204.3	5.3499 999046 3257
beta amylase, partial [Triticum monococcum]	gi|56130900	23475.6	5.3800 001144 4092

Peptide Information

Calc. Mass	Obsrv. Mass	± da	± ppm	Start Seq.	End Seq.	Sequence	Ion Score	C. I. %	Modification	Rank	Result Type
2087.0557	2087.0286	-0.0271	-13	43	60	NIEYLTLGVDDQPLFHGR					Mascot
2087.0557	2087.0286	-0.0271	-13	43	60	NIEYLTLGVDDQPLFHGR	121	100			Mascot

8	hypothetical protein TRIUR3_35144 [Triticum urartu]	gi|474320863	25811.9	9	8	44	0	16.061	

Peptide Information

Calc. Mass	Obsrv. Mass	± da	± ppm	Start Seq.	End Seq.	Sequence	Ion Score	C. I. %	Modification	Rank	Result Type
930.4904	930.4849	-0.0055	-6	71	78	SHGFAQRK					Mascot

Calc. Mass	Obsrv. Mass	± da	± ppm	Start Seq.	End Seq.	Sequence	Ion Score	C. I. %	Modification	Rank	Result Type
1501.7645	1501.6659	-0.0986	-66	82	94	VVPEAPSYLENQR					Mascot
1501.7645	1501.6659	-0.0986	-66	82	94	VVPEAPSYLENQR	1	0			Mascot
1574.738	1574.7452	0.0072	5	173	186	GSMDPQVGYLSQHR					Mascot
1684.7571	1684.7444	-0.0127	-8	188	201	DWSLMTMPPEQGHR					Mascot
1700.752	1700.7507	-0.0013	-1	188	201	DWSLMTMPPEQGHR			Oxidation (M)[5]		Mascot
1714.8396	1714.8383	-0.0013	-1	43	59	GPELEGAGNTVFGDVPR					Mascot
1716.7469	1716.7482	0.0013	1	188	201	DWSLMTMPPEQGHR			Oxidation (M)[5,7]		Mascot
1716.7469	1716.7482	0.0013	1	188	201	DWSLMTMPPEQGHR			Oxidation (M)[5,7]		Mascot
1728.9028	1728.8491	-0.0537	-31	80	94	ARVVPEAPSYLENQR					Mascot
1730.8391	1730.8306	-0.0085	-5	173	187	GSMDPQVGYLSQHRR					Mascot
3015.5391	3015.6692	0.1301	43	120	146	MINAAGPPQDLLSQLYR QGMEIDAVLR			Oxidation (M)[1]		Mascot

9	hypothetical protein TRIUR3_29779 [Triticum urartu]	gi|474262217	35184.4	9.62	10	44	0	7.053	

Peptide Information

Calc. Mass	Obsrv. Mass	± da	± ppm	Start Seq.	End Seq.	Sequence	Ion Score	C. I. %	Modification	Rank	Result Type
1382.8002	1382.7128	-0.0874	-63	108	120	ALPALVIKETGDR					Mascot
1416.7482	1416.7468	-0.0014	-1	199	209	ISEEDLWRQLK					Mascot
1507.8115	1507.6997	-0.1118	-74	94	105	EELTQIITKNYR					Mascot
1553.7694	1553.7124	-0.057	-37	298	312	EYISQVVGVSTATAE					Mascot
1684.8475	1684.7444	-0.1031	-61	121	134	LAATFGYEMRELQR					Mascot
1697.8983	1697.7742	-0.1241	-73	17	30	RSWIAWRPATNSPR					Mascot
1700.8425	1700.7507	-0.0918	-54	121	134	LAATFGYEMRELQR			Oxidation (M)[9]		Mascot
1701.7538	1701.6964	-0.0574	-34	37	52	YATPCSSPFPSSSSAR			Carbamidomethyl (C)[5]		Mascot
1716.801	1716.7482	-0.0528	-31	116	130	ETGDRLAATFGYEMR					Mascot
1716.801	1716.7482	-0.0528	-31	116	130	ETGDRLAATFGYEMR					Mascot
1732.7959	1732.7701	-0.0258	-15	116	130	ETGDRLAATFGYEMR			Oxidation (M)[14]		Mascot
1758.8479	1758.8601	0.0122	7	271	286	IAGPEGHSMVYELAER					Mascot
2070.0627	2070.0596	-0.0031	-1	18	35	SWIAWRPATNSPRSTSP R					Mascot

10	Zinc finger CCCH domain-containing protein 8 [Triticum urartu]	gi|474341013	69792.4	8.87	13	42	0	11.131	

Peptide Information

Calc. Mass	Obsrv. Mass	± da	± ppm	Start Seq.	End Seq.	Sequence	Ion Score	C. I. %	Modification	Rank	Result Type
930.4614	930.4849	0.0235	25	318	324	CKFNHPK			Carbamidomethyl (C)[1]		Mascot
947.371	947.4637	0.0927	98	457	464	TGSCMYGR			Carbamidomethyl (C)[4], Oxidation (M)[5]		Mascot

1206.5579	1206.6012	0.0433	36	2	11	SGYPFYPYGR		Mascot
1233.5504	1233.5753	0.0249	20	128	136	ECAFYMRTR	Carbamidomethyl (C)[2]	Mascot
1255.5923	1255.5529	-0.0394	-31	581	591	TGACKFGVQCK	Carbamidomethyl (C)[4,10]	Mascot
1507.6669	1507.6997	0.0328	22	170	183	AGGDDAWVPMGMLR	Oxidation (M)[10,12]	Mascot
1647.7731	1647.7443	-0.0288	-17	170	184	AGGDDAWVPMGMLRR	Oxidation (M)[10]	Mascot
1728.9869	1728.8491	-0.1378	-80	229	242	WKMDLHVGLIYLLK		Mascot
1789.9629	1789.8464	-0.1165	-65	474	490	HIQASVVPGEMLNPAVK		Mascot
2014.0215	2013.9503	-0.0712	-35	586	603	FGVQCKFDHPPLGEAIAK	Carbamidomethyl (C)[5]	Mascot
2014.0215	2013.9503	-0.0712	-35	586	603	FGVQCKFDHPPLGEAIAK	Carbamidomethyl (C)[5]	Mascot
2069.0928	2069.0164	-0.0764	-37	552	569	WEPSQQPVTLTVAGFPR R		Mascot
2114.8872	2114.9851	0.0979	46	12	31	GGVGDDASAMSDYPFHP YGR	Oxidation (M)[10]	Mascot
2269.1208	2269.1716	0.0508	22	32	53	GGVGDNAAARYSSYEID LIAAR		Mascot

Gel Idx/Pos	263/K15	Instr./Gel Origin	BA2151/Sample Project 20140814	Process Status	Analysis Succeeded
Plate [#] Name	[1] Sample Project 20140814	Instrument Sample Name		Spectra	11

Rank	Protein Name	Accession No.	Protein MW	Protein PI	Pep. Count	Protein Score	Protein Score C. I. %	Intensity Matched	Total Ion Score	Total Ion C. I. %	Confirmed
1	Glutelin type-A 1 [Triticum urartu]	gi|474023259	33443.4	6.23	16	627	100	53.945	521	100	

Peptide Information

Calc. Mass	Obsrv. Mass	± da	± ppm	Start Seq.	End Seq.	Sequence	Ion Score	C. I. %	Modification	Rank	Result Type
877.5142	877.4645	-0.0497	-57	12	19	VAYVLQGK					Mascot
981.5364	981.5081	-0.0283	-29	132	140	LPVPVDADR					Mascot
981.5364	981.5081	-0.0283	-29	132	140	LPVPVDADR	56	99.975			Mascot
1028.5735	1028.5447	-0.0288	-28	175	184	EVGLGADLVR					Mascot
1028.5735	1028.5447	-0.0288	-28	175	184	EVGLGADLVR	85	100			Mascot
1054.6005	1054.5669	-0.0336	-32	213	222	VQVVGPDGKR					Mascot
1055.5481	1055.5266	-0.0215	-20	152	161	LDVDIPNGGR					Mascot
1109.6313	1109.5995	-0.0318	-29	132	141	LPVPVDADRK					Mascot
1109.6313	1109.5995	-0.0318	-29	132	141	LPVPVDADRK	44	99.591			Mascot
1184.6886	1184.616	-0.0726	-61	20	31	GTVGIVLPEATK					Mascot
1185.6838	1185.6259	-0.0579	-49	114	125	LVSSQPASGIVK					Mascot
1187.6783	1187.6514	-0.0269	-23	228	238	IEGGSLFIVPR					Mascot
1187.6783	1187.6514	-0.0269	-23	228	238	IEGGSLFIVPR	101	100			Mascot
1291.6497	1291.6804	0.0307	24	141	151	KDMALNCLQAK			Carbamidomethyl (C)[7]		Mascot
1291.6497	1291.6804	0.0307	24	141	151	KDMALNCLQAK			Carbamidomethyl (C)[7]		Mascot
1308.6583	1308.626	-0.0323	-25	300	310	RLDSEIFFAPN					Mascot
1308.6583	1308.626	-0.0323	-25	300	310	RLDSEIFFAPN	90	100			Mascot
1379.8621	1379.7728	-0.0893	-65	162	174	VVVLNTANLPLVK					Mascot
2109.0056	2108.918	-0.0876	-42	276	294	AISPEVLEASFNTTPEME K			Oxidation (M)[17]		Mascot
2694.3333	2694.3499	0.0166	6	271	294	TSVWKAISPEVLEASFNT TPEMEK					Mascot
2764.2166	2764.1853	-0.0313	-11	185	208	IDAHSMCSPGFSCDSAY QVTYIVR			Carbamidomethyl (C)[7,13]		Mascot
2780.2114	2780.1611	-0.0503	-18	185	208	IDAHSMCSPGFSCDSAY QVTYIVR			Carbamidomethyl (C)[7,13], Oxidation (M)[6]		Mascot
2780.2114	2780.1611	-0.0503	-18	185	208	IDAHSMCSPGFSCDSAY QVTYIVR	145	100	Carbamidomethyl (C)[7,13], Oxidation (M)[6]		Mascot
3116.5337	3116.499	-0.0347	-11	74	102	GHRPGQFTNFQLTGASG IFTGFSTEFVGR					Mascot

Rank	Protein Name	Accession No.	Protein MW	Protein PI	Pep. Count	Protein Score	Protein Score C. I. %	Intensity Matched	Total Ion Score	Total Ion C. I. %	Confirmed
2	11S globulin seed storage protein 2 [Triticum urartu]	gi|473838597	35786.7	6.46	11	244	100	44.071	190	100	

Peptide Information

Calc. Mass	Obsrv. Mass	± da	± ppm	Start Seq.	End Seq.	Sequence	Ion Score	C. I. %	Modification	Rank	Result Type
877.5142	877.4645	-0.0497	-57	71	78	VAYVLQGK					Mascot
1072.6514	1072.5752	-0.0762	-71	3	12	LVFPLLSAGR					Mascot
1180.5337	1180.5558	0.0221	19	201	210	DMALNCLEAK			Carbamidomethyl (C)[6], Oxidation (M)[2]		Mascot
1185.6838	1185.6259	-0.0579	-49	173	184	LVSSQPASGIVK					Mascot
1187.6783	1187.6514	-0.0269	-23	255	265	IEGGSLFIVPR					Mascot
1187.6783	1187.6514	-0.0269	-23	255	265	IEGGSLFIVPR	101	100			Mascot
1308.6287	1308.626	-0.0027	-2	200	210	KDMALNCLEAK			Carbamidomethyl (C)[7], Oxidation (M)[3]		Mascot
1308.6583	1308.626	-0.0323	-25	327	337	RLDSEIFFAPN	90	100			Mascot
1379.8621	1379.7728	-0.0893	-65	221	233	VVVLNTANLPLVK					Mascot
2109.0056	2108.918	-0.0876	-42	303	321	AISPEVLEASFNTTPEME K			Oxidation (M)[17]		Mascot
2694.3333	2694.3499	0.0166	6	298	321	TSVWKAISPEVLEASFNT TPEMEK					Mascot
3116.5337	3116.499	-0.0347	-11	133	161	GHRPGQFTNFQLTGASG IFTGFSTEFVGR					Mascot

3	Glutelin type-A 1 [Triticum urartu]	gi|474374792	38789.6	5.36	4	100	100	16.835	90	100

Peptide Information

Calc. Mass	Obsrv. Mass	± da	± ppm	Start Seq.	End Seq.	Sequence	Ion Score	C. I. %	Modification	Rank	Result Type
1185.6838	1185.6259	-0.0579	-49	172	183	IVSTQPGSGIVK					Mascot
1308.6583	1308.626	-0.0323	-25	354	364	RLDSEIFFAPN					Mascot
1308.6583	1308.626	-0.0323	-25	354	364	RLDSEIFFAPN	90	100			Mascot
1908.9154	1908.8682	-0.0472	-25	2	18	VQRTSNAEVMSMDLSPK			Oxidation (M)[10]		Mascot
2344.0044	2343.9456	-0.0588	-25	247	266	SMCSPGFSCDSAYQVTY IVR			Carbamidomethyl (C)[3,9], Oxidation (M)[2]		Mascot

4	12S seed storage globulin 1 [Triticum urartu]	gi|474247136	33055.9	5.48	3	93	99.998	4.007	85	100

Peptide Information

Calc. Mass	Obsrv. Mass	± da	± ppm	Start Seq.	End Seq.	Sequence	Ion Score	C. I. %	Modification	Rank	Result Type
1028.5735	1028.5447	-0.0288	-28	174	183	EVGLGADLVR					Mascot
1028.5735	1028.5447	-0.0288	-28	174	183	EVGLGADLVR	85	100			Mascot
1384.7656	1384.65	-0.1156	-83	208	221	GGGRVQVLGIDGTR					Mascot
2344.0044	2343.9456	-0.0588	-25	188	207	SMCSPGFSCDSAYQVTY IVR			Carbamidomethyl (C)[3,9], Oxidation (M)[2]		Mascot

5	unnamed protein product [Triticum aestivum]	gi|257700508	24559.7	6.6	10	60	95.262	1.9

Protein Group

cytosolic malate dehydrogenase [Triticum aestivum]	gi|37928995	24559.7	6.5999 999046 3257
unnamed protein product [Triticum aestivum]	gi|257645258	24559.7	6.5999 999046 3257
unnamed protein product [Triticum aestivum]	gi|219913686	24559.7	6.5999 999046 3257
unnamed protein product [Triticum aestivum]	gi|219789621	24559.7	6.5999 999046 3257

Peptide Information

Calc. Mass	Obsrv. Mass	± da	± ppm	Start Seq.	End Seq.	Sequence	Ion Score	C. I. %	Modification	Rank	Result Type
863.4404	863.4173	-0.0231	-27	123	129	NISCLTR			Carbamidomethyl (C)[4]		Mascot
873.4788	873.457	-0.0218	-25	135	142	ALGQISER					Mascot
944.5411	944.4796	-0.0615	-65	143	151	LGVQVSDVK					Mascot
970.5316	970.4947	-0.0369	-38	174	182	TPSGEKPVR					Mascot
1017.5251	1017.5208	-0.0043	-4	114	122	EFAPSIPEK					Mascot
1376.7494	1376.6771	-0.0723	-53	27	38	MELIDAAFPLLK			Oxidation (M)[1]		Mascot
1645.7963	1645.7552	-0.0411	-25	212	227	LSSALSAASSACDHIR			Carbamidomethyl (C)[12]		Mascot
1773.8912	1773.8527	-0.0385	-22	211	227	KLSSALSAASSACDHIR			Carbamidomethyl (C)[13]		Mascot
2621.3064	2621.2588	-0.0476	-18	39	64	GVVATTDVVEACTGVNV AVMVGGFPR			Carbamidomethyl (C)[12], Oxidation (M)[20]		Mascot
2694.4536	2694.3499	-0.1037	-38	2	26	LGADQPVILHMLDIEFAA EALKGVK			Oxidation (M)[11]		Mascot

6	cytochrome P450, putative, expressed [Triticum aestivum]	gi|300681513	57250	8.95	7	51	62.361	29.134	35	97.233	

Peptide Information

Calc. Mass	Obsrv. Mass	± da	± ppm	Start Seq.	End Seq.	Sequence	Ion Score	C. I. %	Modification	Rank	Result Type
804.3886	804.4453	0.0567	70	431	437	WIGEDGK					Mascot
863.4404	863.4173	-0.0231	-27	508	514	RQVMNSL			Oxidation (M)[4]		Mascot
919.4917	919.4357	-0.056	-61	460	467	DMAVVQLK			Oxidation (M)[2]		Mascot
1187.6565	1187.6514	-0.0051	-4	130	140	AKAQLLMSGPR			Oxidation (M)[7]		Mascot
1187.6565	1187.6514	-0.0051	-4	130	140	AKAQLLMSGPR	35	97.233	Oxidation (M)[7]		Mascot
1291.694	1291.6804	-0.0136	-11	132	142	AQLLMSGPRFR			Oxidation (M)[5]		Mascot
1291.694	1291.6804	-0.0136	-11	132	142	AQLLMSGPRFR			Oxidation (M)[5]		Mascot
1330.7035	1330.6212	-0.0823	-62	210	221	AIDDAMDVLLVR					Mascot
2625.4585	2625.2393	-0.2192	-83	29	52	QNPALPLDWPLVGMLPA			Oxidation (M)[14]		Mascot

Calc. Mass	Obsrv. Mass	± da	± ppm	Start Seq.	End Seq.	Sequence	Ion Score	C. I. %	Modification	Rank	Result Type
						LLANLPR					
2625.4585	2625.6243	0.1658	63	29	52	QNPALPLDWPLVGMLPA LLANLPR			Oxidation (M)[14]		Mascot

7	hypothetical protein TRIUR3_05553 [Triticum urartu]	gi|474417641	66942.7	11.62	8	50	60.587	6.084	35	97.214	

Peptide Information

Calc. Mass	Obsrv. Mass	± da	± ppm	Start Seq.	End Seq.	Sequence	Ion Score	C. I. %	Modification	Rank	Result Type
1028.5959	1028.5447	-0.0512	-50	514	523	SSAGKARPVR					Mascot
1028.5959	1028.5447	-0.0512	-50	514	523	SSAGKARPVR	35	97.214			Mascot
1109.5909	1109.5995	0.0086	8	45	54	TLTRSSSTTR					Mascot
1109.5909	1109.5995	0.0086	8	45	54	TLTRSSSTTR					Mascot
1199.6492	1199.6232	-0.026	-22	251	261	SSTPTRQPAVR					Mascot
1233.6031	1233.5961	-0.007	-6	1	11	MEDLLDAEIGK					Mascot
1336.7141	1336.6443	-0.0698	-52	480	491	TISKNSLDMAIK			Oxidation (M)[9]		Mascot
1467.7261	1467.7562	0.0301	21	592	603	YEAMLLRGEDVR			Oxidation (M)[4]		Mascot
1507.6343	1507.7054	0.0711	47	524	537	MSDPGHPTSNGDHR					Mascot
2020.067	2019.8905	-0.1765	-87	96	115	TSVLSASISSVSSRPTTP SR					Mascot

8	cytochrome P450, putative, expressed [Triticum aestivum]	gi|300681510	53915.9	8.86	7	50	58.73	28.629	35	97.233	

Peptide Information

Calc. Mass	Obsrv. Mass	± da	± ppm	Start Seq.	End Seq.	Sequence	Ion Score	C. I. %	Modification	Rank	Result Type
944.5233	944.4796	-0.0437	-46	119	126	DMLPLLAR			Oxidation (M)[2]		Mascot
970.5754	970.4947	-0.0807	-83	456	463	ISIILHMK			Oxidation (M)[7]		Mascot
1187.6565	1187.6514	-0.0051	-4	93	103	AKAQLLMSGPR			Oxidation (M)[7]		Mascot
1187.6565	1187.6514	-0.0051	-4	93	103	AKAQLLMSGPR	35	97.233	Oxidation (M)[7]		Mascot
1291.694	1291.6804	-0.0136	-11	95	105	AQLLMSGPRFR			Oxidation (M)[5]		Mascot
1291.694	1291.6804	-0.0136	-11	95	105	AQLLMSGPRFR			Oxidation (M)[5]		Mascot
1328.7355	1328.6558	-0.0797	-60	116	126	VERDMLPLLAR			Oxidation (M)[5]		Mascot
1330.7035	1330.6212	-0.0823	-62	173	184	AIDDAMDVLLVR					Mascot
1400.8082	1400.6851	-0.1231	-88	456	467	ISIILHMKNGFK					Mascot

9	cyclin-dependent kinase-like D2 [Triticum aestivum]	gi|226359371	38229.5	8.03	11	50	52.616	19.279			

Peptide Information

Calc. Mass	Obsrv. Mass	± da	± ppm	Start Seq.	End Seq.	Sequence	Ion Score	C. I. %	Modification	Rank	Result Type
905.4146	905.4305	0.0159	18	16	24	VDMSGGAPR			Oxidation (M)[3]		Mascot

Calc. Mass	Obsrv. Mass	± da	± ppm	Start Seq.	End Seq.	Sequence	Ion Score	C. I. %	Modification	Rank	Result Type
1109.6677	1109.5995	-0.0682	-61	284	293	LVTPPQVISR					Mascot
1109.6677	1109.5995	-0.0682	-61	284	293	LVTPPQVISR					Mascot
1175.6201	1175.6349	0.0148	13	315	324	GLLSCNIDKR			Carbamidomethyl (C)[5]		Mascot
1184.569	1184.616	0.047	40	213	222	YMAPEMLLGK			Oxidation (M)[2,6]		Mascot
1225.6688	1225.5853	-0.0835	-68	58	68	HLTNGWTVAVK					Mascot
1233.6331	1233.5961	-0.037	-30	151	161	CIMEQLLGAAK			Carbamidomethyl (C)[1]		Mascot
1308.6803	1308.626	-0.0543	-41	212	222	RYMAPEMLLGK					Mascot
1308.6803	1308.626	-0.0543	-41	212	222	RYMAPEMLLGK					Mascot
1314.6591	1314.646	-0.0131	-10	5	15	RVFPGDHPDFK					Mascot
1324.6752	1324.6145	-0.0607	-46	212	222	RYMAPEMLLGK			Oxidation (M)[3]		Mascot
1340.6702	1340.7201	0.0499	37	212	222	RYMAPEMLLGK			Oxidation (M)[3,7]		Mascot
1515.7849	1515.7006	-0.0843	-56	89	101	TLREAAFLAACHR			Carbamidomethyl (C)[11]		Mascot
2016.0437	2016.0641	0.0204	10	298	314	QHFPEDLLSKEGFEVLK					Mascot
2044.9546	2044.9443	-0.0103	-5	6	24	VFPGDHPDFKVDMSGGAPR			Oxidation (M)[13]		Mascot

10	putative E3 ubiquitin-protein ligase ARI7 [Triticum urartu]	gi|473903712	102944	5.41	18	49	43.032	5.249

Peptide Information

Calc. Mass	Obsrv. Mass	± da	± ppm	Start Seq.	End Seq.	Sequence	Ion Score	C. I. %	Modification	Rank	Result Type
813.3526	813.4188	0.0662	81	752	757	DFNDFR					Mascot
871.4996	871.504	0.0044	5	812	820	NKAPVGASK					Mascot
919.438	919.4357	-0.0023	-3	644	651	WAANQSSR					Mascot
970.4628	970.4947	0.0319	33	399	406	SYNWSVSK					Mascot
1017.4418	1017.5208	0.079	78	428	435	QNEMPNER					Mascot
1054.5316	1054.5669	0.0353	33	769	776	NYFENLVR					Mascot
1169.5289	1169.6375	0.1086	93	246	254	MMQSKDIER			Oxidation (M)[1,2]		Mascot
1175.5917	1175.6349	0.0432	37	644	653	WAANQSSRQK					Mascot
1184.5795	1184.616	0.0365	31	140	150	QVIDYSGSTSK					Mascot
1314.6033	1314.646	0.0427	32	16	28	QDNGALSGQPDGR					Mascot
1501.6741	1501.6655	-0.0086	-6	2	15	GSWSTPSSPEHMAK					Mascot
1630.7609	1630.7665	0.0056	3	632	643	NSLERYTHYYER					Mascot
1632.7145	1632.7684	0.0539	33	1	15	MGSWSTPSSPEHMAK					Mascot
1671.8912	1671.7748	-0.1164	-70	654	668	ALGDLQSLQNDKLEK					Mascot
1957.9033	1957.9441	0.0408	21	777	796	ALESGLNDVGAHSGQGACSK			Carbamidomethyl (C)[18]		Mascot
2044.9723	2044.9443	-0.028	-14	715	731	RQFFEYVQGEAESGLER					Mascot

2280.0457	2279.9924	-0.0533	-23	204	224	VLFNCGGPLEAEMAAMD EGLR	Carbamidomethyl (C)[5]	Mascot
2764.259	2764.1853	-0.0737	-27	359	381	QQQNYSVLSETDIKQHQ ADDMNR	Oxidation (M)[21]	Mascot

Gel Idx/Pos	264/K16	Instr./Gel Origin	BA2151/Sample Project 20140814	Process Status	Analysis Succeeded
Plate [#] Name	[1] Sample Project 20140814	Instrument Sample Name		Spectra	11

Rank	Protein Name	Accession No.	Protein MW	Protein PI	Pep. Count	Protein Score	Protein Score C. I. %	Intensity Matched	Total Ion Score	Total Ion C. I. %	Confirmed
1	Serpin-Z2B [Triticum urartu]	gi|473793747	45225.7	6.03	12	318	100	26.106	267	100	

Peptide Information

Calc. Mass	Obsrv. Mass	± da	± ppm	Start Seq.	End Seq.	Sequence	Ion Score	C. I. %	Modification	Rank	Result Type
925.5214	925.4976	-0.0238	-26	11	18	LSIAHQTR					Mascot
925.5214	925.4976	-0.0238	-26	11	18	LSIAHQTR	37	98.6			Mascot
1137.6667	1137.6052	-0.0615	-54	189	198	LVLGNALYFK					Mascot
1192.5382	1192.5138	-0.0244	-20	199	208	GAWTDQFDPR					Mascot
1223.5903	1223.5265	-0.0638	-52	127	137	AEAQSVDFQTK					Mascot
1292.7097	1292.651	-0.0587	-45	306	317	ISFGIEASDLLK					Mascot
1385.7019	1385.6683	-0.0336	-24	176	188	DILPAGSIDNNTR					Mascot
1385.7019	1385.6683	-0.0336	-24	176	188	DILPAGSIDNNTR	84	100			Mascot
1446.7965	1446.6721	-0.1244	-86	11	22	LSIAHQTRFAFR					Mascot
1483.7614	1483.7064	-0.055	-37	257	268	QFSMYILLPEAR			Oxidation (M)[4]		Mascot
1483.7614	1483.7064	-0.055	-37	257	268	QFSMYILLPEAR	36	98.229	Oxidation (M)[4]		Mascot
1514.7485	1514.6949	-0.0536	-35	125	137	YKAEAQSVDFQTK					Mascot
1665.8595	1665.8331	-0.0264	-16	278	291	LSAEPEFLEQHIPR					Mascot
1665.8595	1665.8331	-0.0264	-16	278	291	LSAEPEFLEQHIPR	109	100			Mascot
1922.9706	1922.9031	-0.0675	-35	352	370	AFVEVNETGTEAAATTIA K					Mascot
2083.1072	2083.0286	-0.0786	-38	396	415	EDTSGVVLFIGHVVNPLL SS					Mascot

Rank	Protein Name	Accession No.	Protein MW	Protein PI	Pep. Count	Protein Score	Protein Score C. I. %	Intensity Matched	Total Ion Score	Total Ion C. I. %	Confirmed
2	serpin-N3.2 [Triticum aestivum]	gi|379060943	43026.4	5.18	9	178	100	20.611	146	100	

Protein Group

RecName: Full=Serpin-Z2B; AltName: Full=TriaeZ2b; AltName: Full=WSZ2b; AltName: Full=WZS3	gi|75279909	43011.4	5.1799 998283 3862
serpin [Triticum aestivum]	gi|1885346	43011.4	5.1799 998283 3862

Peptide Information

Calc. Mass	Obsrv. Mass	± da	± ppm	Start Seq.	End Seq.	Sequence	Ion Score	C. I. %	Modification	Rank	Result Type
925.5214	925.4976	-0.0238	-26	11	18	LSIAHQTR					Mascot

Calc. Mass	Obsrv. Mass	± da	± ppm	Start Seq.	End Seq.	Sequence	Ion Score	C. I. %	Modification	Rank	Result Type
925.5214	925.4976	-0.0238	-26	11	18	LSIAHQTR	37	98.6			Mascot
1137.6667	1137.6052	-0.0615	-54	172	181	LVLGNALYFK					Mascot
1192.5382	1192.5138	-0.0244	-20	182	191	GAWTDQFDPR					Mascot
1223.5903	1223.5265	-0.0638	-52	127	137	AEAQSVDFQTK					Mascot
1446.7965	1446.6721	-0.1244	-86	11	22	LSIAHQTRFAFR					Mascot
1514.7485	1514.6949	-0.0536	-35	125	137	YKAEAQSVDFQTK					Mascot
1665.8595	1665.8331	-0.0264	-16	261	274	LSAEPEFLEQHIPR					Mascot
1665.8595	1665.8331	-0.0264	-16	261	274	LSAEPEFLEQHIPR	109	100			Mascot
1922.9706	1922.9031	-0.0675	-35	335	353	AFVEVNETGTEAAATTIAK					Mascot
2083.1072	2083.0286	-0.0786	-38	379	398	EDTSGVVLFIGHVVNPLLSS					Mascot

3 hypothetical protein TRIUR3_07605 [Triticum urartu] gi|474443810 36454.2 6.43 13 59 94.683 2.714

Peptide Information

Calc. Mass	Obsrv. Mass	± da	± ppm	Start Seq.	End Seq.	Sequence	Ion Score	C. I. %	Modification	Rank	Result Type
807.3917	807.3633	-0.0284	-35	144	150	MALEEAK			Oxidation (M)[1]		Mascot
833.4661	833.3911	-0.075	-90	251	258	VMAAASRK					Mascot
1133.5408	1133.5016	-0.0392	-35	295	304	QYGGLEHAMK					Mascot
1205.6372	1205.566	-0.0712	-59	117	127	EVATAKQTETK					Mascot
1402.6532	1402.7021	0.0489	35	1	13	MAIGSGQEFHPGR			Oxidation (M)[1]		Mascot
1417.7006	1417.7142	0.0136	10	295	306	QYGGLEHAMKQR					Mascot
1507.7057	1507.703	-0.0027	-2	168	181	DAVAGKEDAMESLR			Oxidation (M)[10]		Mascot
1544.8027	1544.7765	-0.0262	-17	151	165	LEAATLRQAGGENSK					Mascot
1557.7577	1557.7008	-0.0569	-37	85	97	EDALQLQDLMEPR					Mascot
1561.8254	1561.7313	-0.0941	-60	144	157	MALEEAKLEAATLR			Oxidation (M)[1]		Mascot
1720.9242	1720.951	0.0268	16	2	17	AIGSGQEFHPGRLVPR					Mascot
1763.848	1763.8708	0.0228	13	174	188	EDAMESLRLENESLK					Mascot
2014.0353	2014.0059	-0.0294	-15	98	116	SDGATPTSPASARVWLLER					Mascot

4 reversibly glycosylated polypeptide [Triticum aestivum] gi|4158232 41985 5.82 6 59 94.17 3.59 42 99.463

Protein Group

glycosyltransferase 75 [Triticum aestivum] gi|301072492 41985 5.8200 001716 6138

Peptide Information

Calc. Mass	Obsrv. Mass	± da	± ppm	Start Seq.	End Seq.	Sequence	Ion Score	C. I. %	Modification	Rank	Result Type

839.441	839.4224	-0.0186	-22	159	165	GYPFSLR					Mascot
1446.5818	1446.6721	0.0903	62	244	254	YDDMWAGWCVK			Carbamidomethyl (C)[9], Oxidation (M)[4]		Mascot
1483.7461	1483.7064	-0.0397	-27	315	326	CYISLSEQVKEK			Carbamidomethyl (C)[1]		Mascot
1483.7461	1483.7064	-0.0397	-27	315	326	CYISLSEQVKEK			Carbamidomethyl (C)[1]		Mascot
1501.6958	1501.6715	-0.0243	-16	61	72	VPEGFDYELYNR					Mascot
1501.6958	1501.6715	-0.0243	-16	61	72	VPEGFDYELYNR	42	99.463			Mascot
1720.8252	1720.951	0.1258	73	104	117	KYVFTIDDDCFVAK			Carbamidomethyl (C)[10]		Mascot
2292.1335	2292.0925	-0.041	-18	133	151	NLLSPSTPFFFNTLYDPY R					Mascot

5 serpin [Triticum aestivum] gi|5734506 43341.5 5.46 7 58 92.49 4.536 37 98.6

Protein Group

RecName: Full=Serpin-Z2A; AltName: Full=TriaeZ2a; AltName: Full=WSZ2a	gi|75313847	43341.5	5.4600 000381 4697

Peptide Information

Calc. Mass	Obsrv. Mass	± da	± ppm	Start Seq.	End Seq.	Sequence	Ion Score	C. I. %	Modification	Rank	Result Type
925.5214	925.4976	-0.0238	-26	11	18	LSIAHQTR					Mascot
925.5214	925.4976	-0.0238	-26	11	18	LSIAHQTR	37	98.6			Mascot
1137.6667	1137.6052	-0.0615	-54	172	181	LVLGNALYFK					Mascot
1223.5903	1223.5265	-0.0638	-52	127	137	AEAQSVDFQTK					Mascot
1292.7097	1292.651	-0.0587	-45	289	300	ISFGIEASDLLK					Mascot
1510.7285	1510.7201	-0.0084	-6	182	194	GAWTDQFDSRVTK					Mascot
1514.7485	1514.6949	-0.0536	-35	125	137	YKAEAQSVDFQTK					Mascot
1922.9706	1922.9031	-0.0675	-35	335	353	TFVEVNETGTEAAAATIA K					Mascot

6 hypothetical protein TRIUR3_29177 [Triticum urartu] gi|474234225 25562.7 11.72 13 56 89.392 3.337

Peptide Information

Calc. Mass	Obsrv. Mass	± da	± ppm	Start Seq.	End Seq.	Sequence	Ion Score	C. I. %	Modification	Rank	Result Type
800.4485	800.4001	-0.0484	-60	139	146	GQLRGGGR					Mascot
834.425	834.4103	-0.0147	-18	28	33	MEQVRR			Oxidation (M)[1]		Mascot
837.3705	837.4459	0.0754	90	102	108	LGCGMQR			Carbamidomethyl (C)[3], Oxidation (M)[5]		Mascot
972.5221	972.5	-0.0221	-23	36	44	SLSDPAARR					Mascot
1133.5334	1133.5016	-0.0318	-28	222	232	TGRWGSGQGEV					Mascot
1137.5074	1137.6052	0.0978	86	102	110	LGCGMQRCR			Carbamidomethyl (C)[3,8]		Mascot
1196.5403	1196.5106	-0.0297	-25	45	56	HTGSSHGGEATR					Mascot
1205.5182	1205.566	0.0478	40	147	158	DAEGEGGGTWAR					Mascot

1402.6493	1402.7021	0.0528	38	14	26	HSSSAMVGSQQQR					Mascot
1417.619	1417.7142	0.0952	67	163	177	GGGGAEDGEDDVIAR					Mascot
1483.7554	1483.7064	-0.049	-33	188	200	GDGAWLRAPWQAR					Mascot
1483.7554	1483.7064	-0.049	-33	188	200	GDGAWLRAPWQAR					Mascot
1557.7405	1557.7008	-0.0397	-25	147	161	DAEGEGGGTWARVPR					Mascot
2013.9043	2014.0059	0.1016	50	67	86	EEHLQQGDGAGVASGG AMQR			Oxidation (M)[18]		Mascot

7	Alpha-1,4-glucan-protein synthase [UDP-forming] 1 [Triticum urartu]	gi|474042704	63955.1	5.55	5	50	60.587	1.862	42	99.463	

Peptide Information

Calc. Mass	Obsrv. Mass	± da	± ppm	Start Seq.	End Seq.	Sequence	Ion Score	C. I. %	Modification	Rank	Result Type
839.441	839.4224	-0.0186	-22	150	156	GYPFSLR					Mascot
989.5414	989.491	-0.0504	-51	267	275	ASNPFVNLK					Mascot
1501.6958	1501.6715	-0.0243	-16	52	63	VPEGFDYELYNR					Mascot
1501.6958	1501.6715	-0.0243	-16	52	63	VPEGFDYELYNR	42	99.463			Mascot
1585.6864	1585.7167	0.0303	19	547	560	DAKYVDLTSDTNDE					Mascot
2292.1335	2292.0925	-0.041	-18	124	142	NLLSPSTPFFFNTLYDPY R					Mascot

8	Homeobox-leucine zipper protein HOX33 [Triticum urartu]	gi|474291991	101472.2	7.25	18	50	58.73	9.109	

Peptide Information

Calc. Mass	Obsrv. Mass	± da	± ppm	Start Seq.	End Seq.	Sequence	Ion Score	C. I. %	Modification	Rank	Result Type
813.5053	813.4486	-0.0567	-70	121	126	RQQIIR					Mascot
834.4138	834.4103	-0.0035	-4	732	739	ESVAAMAR					Mascot
837.4254	837.4459	0.0205	24	351	356	DFWTLR					Mascot
877.456	877.4555	-0.0005	-1	744	751	AVMASVQR			Oxidation (M)[3]		Mascot
925.4448	925.4976	0.0528	57	862	869	ALCAEFSK			Carbamidomethyl (C)[3]		Mascot
925.4448	925.4976	0.0528	57	862	869	ALCAEFSK			Carbamidomethyl (C)[3]		Mascot
1022.5516	1022.4725	-0.0791	-77	187	195	LVYENASVK					Mascot
1022.5516	1022.4725	-0.0791	-77	187	195	LVYENASVK					Mascot
1056.5731	1056.5651	-0.008	-8	55	63	VACVRPAQR			Carbamidomethyl (C)[3]		Mascot
1115.5439	1115.502	-0.0419	-38	22	31	TPARDEEAAR					Mascot
1174.6136	1174.5302	-0.0834	-71	291	301	ACGLVSLEPTK			Carbamidomethyl (C)[2]		Mascot
1223.5797	1223.5265	-0.0532	-43	155	164	ESSRMQTVNR			Oxidation (M)[5]		Mascot
1557.8207	1557.7008	-0.1199	-77	40	54	GAGTLFAGHMIVIDR					Mascot

1608.9142	1608.7598	-0.1544	-96	539	553	ASMLVQDVPPALLVR						Mascot
1612.8905	1612.7401	-0.1504	-93	672	686	VIPLDTKTDVPSATR						Mascot
1693.8248	1693.8617	0.0369	22	166	179	LTAMNKLLMEENDR			Oxidation (M)[4]			Mascot
1693.8248	1693.8617	0.0369	22	166	179	LTAMNKLLMEENDR			Oxidation (M)[4]			Mascot
1727.882	1727.7576	-0.1244	-72	127	140	DCPILCNIEPKQIK			Carbamidomethyl (C)[2,6]			Mascot
1828.0249	1827.8425	-0.1824	-100	291	307	ACGLVSLEPTKVAEILK			Carbamidomethyl (C)[2]			Mascot
1873.9403	1873.8807	-0.0596	-32	372	390	SLTQSTGGPSGPNTPGFI R						Mascot
2078.0527	2077.896	-0.1567	-75	448	466	HIRQIAHESSGEIPYGAG R						Mascot

9 Cullin-3A [Triticum urartu] gi|474304968 93558.8 8.49 19 48 33.068 7.378

Peptide Information

Calc. Mass	Obsrv. Mass	± da	± ppm	Start Seq.	End Seq.	Sequence	Ion Score	C. I. %	Modification	Rank	Result Type
832.3909	832.3566	-0.0343	-41	362	367	MYDLFK			Oxidation (M)[1]		Mascot
833.3999	833.3911	-0.0088	-11	304	310	LAEESER					Mascot
925.5002	925.4976	-0.0026	-3	174	180	RWADHIK					Mascot
925.5002	925.4976	-0.0026	-3	174	180	RWADHIK					Mascot
972.4971	972.5	0.0029	3	362	368	MYDLFKR					Mascot
989.501	989.491	-0.01	-10	303	310	RLAEESER					Mascot
1044.4125	1044.4735	0.061	58	291	298	CDCGEYLK			Carbamidomethyl (C)[1,3]		Mascot
1056.4852	1056.5651	0.0799	76	187	194	DILMYMDR					Mascot
1083.5391	1083.5453	0.0062	6	534	542	LESMFTDLK					Mascot
1088.4751	1088.46	-0.0151	-14	187	194	DILMYMDR			Oxidation (M)[4,6]		Mascot
1137.6124	1137.6052	-0.0072	-6	368	377	RVPDGHSTIR					Mascot
1223.6307	1223.5265	-0.1042	-85	18	27	VEVDPKFFDK					Mascot
1357.7322	1357.7001	-0.0321	-24	386	397	ETGKILVTDPER					Mascot
1474.6921	1474.7527	0.0606	41	84	97	NENDIHAGSLGYGK					Mascot
1483.7587	1483.7064	-0.0523	-35	127	139	TAYNMVLHKHGGR					Mascot
1483.7587	1483.7064	-0.0523	-35	127	139	TAYNMVLHKHGGR					Mascot
1507.7209	1507.703	-0.0179	-12	608	620	LTWQTNMGNADIK			Oxidation (M)[7]		Mascot
1510.6445	1510.7201	0.0756	50	689	701	DISDSDAFHFNDK					Mascot
1514.806	1514.6949	-0.1111	-73	711	724	IGTVVAQKESEPEK					Mascot
1647.8271	1647.8115	-0.0156	-9	607	620	RLTWQTNMGNADIK					Mascot
2014.0939	2014.0059	-0.088	-44	98	114	SKMTSMLVLWGMILLFK			Oxidation (M)[3]		Mascot
2082.9072	2083.0286	0.1214	58	684	701	EPMSKDISDSDAFHFNDK					Mascot

10 hypothetical protein TRIUR3_19712 [Triticum urartu] gi|474222453 35520.2 8.14 11 46 0 4.937

Peptide Information

Calc. Mass	Obsrv. Mass	± da	± ppm	Start Seq.	End Seq.	Sequence	Ion Score	C. I. %	Modification	Rank	Result Type
877.427	877.4555	0.0285	32	154	160	IMHSMVK			Oxidation (M)[2,5]		Mascot
1079.636	1079.5386	-0.0974	-90	65	72	HLLWLIER					Mascot
1133.5408	1133.5016	-0.0392	-35	99	107	LLNDADMWR					Mascot
1246.5885	1246.5029	-0.0856	-69	233	243	DIAQPMHSGYK					Mascot
1262.5835	1262.5427	-0.0408	-32	233	243	DIAQPMHSGYK			Oxidation (M)[6]		Mascot
1313.5717	1313.656	0.0843	64	298	310	ASRDPQGPQGGED					Mascot
1402.6896	1402.7021	0.0125	9	232	243	RDIAQPMHSGYK					Mascot
1483.8043	1483.7064	-0.0979	-66	178	190	FSLVSEVVIFESK					Mascot
1483.8043	1483.7064	-0.0979	-66	178	190	FSLVSEVVIFESK					Mascot
1727.9084	1727.7576	-0.1508	-87	65	77	HLLWLIERMVDMR			Oxidation (M)[9]		Mascot
1845.9496	1845.8678	-0.0818	-44	108	123	NMAPGLPMLVTRCTLR			Carbamidomethyl (C)[13], Oxidation (M)[2]		Mascot
1861.9446	1861.8522	-0.0924	-50	108	123	NMAPGLPMLVTRCTLR			Carbamidomethyl (C)[13], Oxidation (M)[2,8]		Mascot
1861.9446	1861.8522	-0.0924	-50	108	123	NMAPGLPMLVTRCTLR			Carbamidomethyl (C)[13], Oxidation (M)[2,8]		Mascot
2014.1188	2014.0059	-0.1129	-56	143	160	LLLQISVGSNKIMHSMVK			Oxidation (M)[13]		Mascot
2069.0994	2069.0168	-0.0826	-40	120	137	CTLRLAHSVMIGETLVPR			Carbamidomethyl (C)[1], Oxidation (M)[10]		Mascot

Gel Idx/Pos	265/K17	Instr./Gel Origin	BA2151/Sample Project 20140814	Process Status	Analysis Succeeded
Plate [#] Name	[1] Sample Project 20140814	Instrument Sample Name		Spectra	11

Rank	Protein Name	Accession No.	Protein MW	Protein PI	Pep. Count	Protein Score	Protein Score C. I. %	Intensity Matched	Total Ion Score	Total Ion C. I. %	Confirmed
1	serpin [Triticum aestivum]	gi|5734504	42969	5.62	10	148	100	10.664	105	100	

Protein Group

RecName: Full=Serpin-Z1C; AltName: Full=TriaeZ1c; AltName: Full=WSZ1c	gi|75313848	42969	5.6199 998855 5908

Peptide Information

Calc. Mass	Obsrv. Mass	± da	± ppm	Start Seq.	End Seq.	Sequence	Ion Score	C. I. %	Modification	Rank	Result Type
925.5214	925.5001	-0.0213	-23	11	18	LSIAHQTR					Mascot
925.5214	925.5001	-0.0213	-23	11	18	LSIAHQTR	35	97.639			Mascot
947.5156	947.4678	-0.0478	-50	2	10	ATTLATDVR					Mascot
1151.6824	1151.5846	-0.0978	-85	172	181	LVLANALYFK					Mascot
1176.5896	1176.5691	-0.0205	-17	261	270	LSAEPDFLER					Mascot
1176.5896	1176.5691	-0.0205	-17	261	270	LSAEPDFLER	70	100			Mascot
1239.5852	1239.6824	0.0972	78	127	137	ADTQSVDFQTK					Mascot
1352.6945	1352.6407	-0.0538	-40	289	300	ISFETEASDLLK					Mascot
1399.6125	1399.6244	0.0119	9	182	194	GAWTDQFDSSGTK					Mascot
2129.0947	2129.0059	-0.0888	-42	379	398	EDISGVVLFMGHVVNPLL SS			Oxidation (M)[10]		Mascot
2720.3525	2720.302	-0.0505	-19	328	353	VSSVFHQAFVEVNEQGT EAAASTAIK					Mascot
2725.4631	2725.4294	-0.0337	-12	33	61	SAASNAVFSPVSLHVALS LLAAGAGSATR					Mascot

Rank	Protein Name	Accession No.	Protein MW	Protein PI	Pep. Count	Protein Score	Protein Score C. I. %	Intensity Matched	Total Ion Score	Total Ion C. I. %	Confirmed
2	Serpin-Z1C [Triticum urartu]	gi|474075261	42956	5.62	9	141	100	10.43	105	100	

Peptide Information

Calc. Mass	Obsrv. Mass	± da	± ppm	Start Seq.	End Seq.	Sequence	Ion Score	C. I. %	Modification	Rank	Result Type
925.5214	925.5001	-0.0213	-23	11	18	LSIAHQTR					Mascot
925.5214	925.5001	-0.0213	-23	11	18	LSIAHQTR	35	97.639			Mascot
947.5156	947.4678	-0.0478	-50	2	10	ATTLATDVR					Mascot
1151.6824	1151.5846	-0.0978	-85	172	181	LVLANALYFK					Mascot
1176.5896	1176.5691	-0.0205	-17	261	270	LSAEPDFLER					Mascot
1176.5896	1176.5691	-0.0205	-17	261	270	LSAEPDFLER	70	100			Mascot
1352.6945	1352.6407	-0.0538	-40	289	300	ISFETEASDLLK					Mascot

Calc. Mass	Obsrv. Mass	± da	± ppm	Start Seq.	End Seq.	Sequence	Ion Score	C. I. %	Modification	Rank	Result Type
1399.6125	1399.6244	0.0119	9	182	194	GAWTDQFDSSGTK					Mascot
2129.0947	2129.0059	-0.0888	-42	379	398	EDISGVVLFMGHVVNPLLSS			Oxidation (M)[10]		Mascot
2720.3525	2720.302	-0.0505	-19	328	353	VSSVFHQAFVEVNEQGTEAAASTAIK					Mascot
2725.4631	2725.4294	-0.0337	-12	33	61	SAASNAVFSPVSLHVALSLLAAGAGSATR					Mascot

3	serpin-N3.7 [Triticum aestivum]	gi|379060945	42639.8	5.52	8	135	100	10.377	105	100

Peptide Information

Calc. Mass	Obsrv. Mass	± da	± ppm	Start Seq.	End Seq.	Sequence	Ion Score	C. I. %	Modification	Rank	Result Type
925.5214	925.5001	-0.0213	-23	11	18	LSIAHQTR					Mascot
925.5214	925.5001	-0.0213	-23	11	18	LSIAHQTR	35	97.639			Mascot
947.5156	947.4678	-0.0478	-50	2	10	ATTLATDVR					Mascot
1151.6824	1151.5846	-0.0978	-85	172	181	LVLANALYFK					Mascot
1176.5896	1176.5691	-0.0205	-17	261	270	LSAEPDFLER					Mascot
1176.5896	1176.5691	-0.0205	-17	261	270	LSAEPDFLER	70	100			Mascot
1239.5852	1239.6824	0.0972	78	127	137	ADTQSVDFQTK					Mascot
1399.6125	1399.6244	0.0119	9	182	194	GAWTDQFDSSGTK					Mascot
2720.3525	2720.302	-0.0505	-19	328	353	VSSVFHQAFVEVNEQGTEAAASTAIK					Mascot
2725.4631	2725.4294	-0.0337	-12	33	61	SAASNAVFSPVSLHVALSLLAAGAGSATR					Mascot

4	serpin [Triticum aestivum]	gi|1885350	43119.9	5.44	7	128	100	9.708	105	100

Protein Group

RecName: Full=Serpin-Z1B; AltName: Full=TriaeZ1b; AltName: Full=WSZ1b; AltName: Full=WZS2	gi|75279910	43119.9	5.4400000572046

Peptide Information

Calc. Mass	Obsrv. Mass	± da	± ppm	Start Seq.	End Seq.	Sequence	Ion Score	C. I. %	Modification	Rank	Result Type
925.5214	925.5001	-0.0213	-23	11	18	LSIAHQTR					Mascot
925.5214	925.5001	-0.0213	-23	11	18	LSIAHQTR	35	97.639			Mascot
947.5156	947.4678	-0.0478	-50	2	10	ATTLATDVR					Mascot
1151.6824	1151.5846	-0.0978	-85	172	181	LVLANALYFK					Mascot
1176.5896	1176.5691	-0.0205	-17	262	271	LSAEPDFLER					Mascot
1176.5896	1176.5691	-0.0205	-17	262	271	LSAEPDFLER	70	100			Mascot
1585.8295	1585.7083	-0.1212	-76	288	301	FKISFGMEASDLLK					Mascot
1601.8243	1601.7025	-0.1218	-76	288	301	FKISFGMEASDLLK			Oxidation (M)[7]		Mascot
2129.0947	2129.0059	-0.0888	-42	380	399	EDISGVVLFMGHVVNPLL			Oxidation (M)[10]		Mascot

Calc. Mass	Obsrv. Mass	± da	± ppm	Start Seq.	End Seq.	Sequence	Ion Score	C. I. %	Modification	Rank	Result Type
						SS					
2720.3525	2720.302	-0.0505	-19	329	354	VSSVFHQAFVEVNEQGT EAAASTAIK					Mascot

5	serpin [Triticum aestivum]	gi|871551	43262.2	5.6	6	123	100	9.412	105	100	

Protein Group

RecName: Full=Serpin-Z1A; AltName: Full=TriaeZ1a; AltName: Full=WSZ1a; Short=WSZ1; AltName: Full=WSZCI	gi|75282265	43262.2	5.5999 999046 3257
serpin 1 [Triticum aestivum]	gi|224589266	43261.1	5.4400 000572 2046

Peptide Information

Calc. Mass	Obsrv. Mass	± da	± ppm	Start Seq.	End Seq.	Sequence	Ion Score	C. I. %	Modification	Rank	Result Type
925.5214	925.5001	-0.0213	-23	11	18	LSIAHQTR					Mascot
925.5214	925.5001	-0.0213	-23	11	18	LSIAHQTR	35	97.639			Mascot
947.5156	947.4678	-0.0478	-50	2	10	ATTLATDVR					Mascot
1151.6824	1151.5846	-0.0978	-85	172	181	LVLANALYFK					Mascot
1176.5896	1176.5691	-0.0205	-17	261	270	LSAEPDFLER					Mascot
1176.5896	1176.5691	-0.0205	-17	261	270	LSAEPDFLER	70	100			Mascot
2129.0947	2129.0059	-0.0888	-42	379	398	EDISGVVLFMGHVVNPLL SS			Oxidation (M)[10]		Mascot
2720.3525	2720.302	-0.0505	-19	328	353	VSSVFHQAFVEVNEQGT EAAASTAIK					Mascot

6	serpin 3 [Triticum aestivum]	gi|224589270	43227.1	5.56	5	118	100	9.286	105	100	

Peptide Information

Calc. Mass	Obsrv. Mass	± da	± ppm	Start Seq.	End Seq.	Sequence	Ion Score	C. I. %	Modification	Rank	Result Type
925.5214	925.5001	-0.0213	-23	11	18	LSIAHQTR					Mascot
925.5214	925.5001	-0.0213	-23	11	18	LSIAHQTR	35	97.639			Mascot
947.5156	947.4678	-0.0478	-50	2	10	ATTLATDVR					Mascot
1151.6824	1151.5846	-0.0978	-85	172	181	LVLANALYFK					Mascot
1176.5896	1176.5691	-0.0205	-17	262	271	LSAEPDFLER					Mascot
1176.5896	1176.5691	-0.0205	-17	262	271	LSAEPDFLER	70	100			Mascot
2720.3525	2720.302	-0.0505	-19	329	354	VSSVFHQAFVEVNEQGT EAAASTAIK					Mascot

7	Sec14 cytosolic factor [Triticum urartu]	gi|474102547	76258.1	8.33	17	52	72.733	3.938			

Peptide Information

Calc. Mass	Obsrv. Mass	± da	± ppm	Start Seq.	End Seq.	Sequence	Ion Score	C. I. %	Modification	Rank	Result Type

Calc. Mass	Obsrv. Mass	± da	± ppm	Start Seq.	End Seq.	Sequence	Ion Score	C. I. %	Modification	Rank	Result Type
823.4421	823.4119	-0.0302	-37	237	243	NFSKTAR					Mascot
843.5046	843.4642	-0.0404	-48	531	537	LQKLEGR					Mascot
870.5043	870.4689	-0.0354	-41	177	184	LGKVDPNK					Mascot
889.5175	889.4562	-0.0613	-69	576	583	VLQATVMK					Mascot
993.5146	993.4438	-0.0708	-71	241	248	TAREMLTR			Oxidation (M)[5]		Mascot
1033.6074	1033.5057	-0.1017	-98	575	583	KVLQATVMK			Oxidation (M)[8]		Mascot
1068.5177	1068.5057	-0.012	-11	244	251	EMLTRMQK			Oxidation (M)[2,6]		Mascot
1151.642	1151.5846	-0.0574	-50	599	608	LHVIEGNELK					Mascot
1232.6635	1232.5883	-0.0752	-61	555	564	SLLDSWDRIK					Mascot
1507.8591	1507.7097	-0.1494	-99	599	611	LHVIEGNELKISR					Mascot
1516.8523	1516.8213	-0.031	-20	275	287	LLWNSVKGFLDPK					Mascot
1657.8069	1657.7853	-0.0216	-13	7	20	SESTEGLFLFDERK					Mascot
2004.8645	2005.0156	0.1511	75	397	413	ESAAYYSCDDHFVVVDK			Carbamidomethyl (C)[8]		Mascot
2209.1433	2209.1145	-0.0288	-13	85	102	AVFTFQQELLNRNLLCDK			Carbamidomethyl (C)[16]		Mascot
2232.0027	2232.1465	0.1438	64	395	413	ARESAAYYSCDDHFVVVDK			Carbamidomethyl (C)[10]		Mascot
2253.0781	2253.0725	-0.0056	-2	79	96	DEEEERAVFTFQQELLNR					Mascot
2392.3323	2392.1055	-0.2268	-95	643	661	LKEINPQQWLGCWILLRPK			Carbamidomethyl (C)[12]		Mascot

8 puroindoline b-like protein 2v5 [Triticum turgidum] gi|307099831 17240.4 8.76 9 49 48.044 1.931

Peptide Information

Calc. Mass	Obsrv. Mass	± da	± ppm	Start Seq.	End Seq.	Sequence	Ion Score	C. I. %	Modification	Rank	Result Type
809.3895	809.3904	0.0009	1	127	133	AMMLPSK			Oxidation (M)[2,3]		Mascot
812.3607	812.3884	0.0277	34	52	57	DYVMER					Mascot
834.3926	834.3884	-0.0042	-5	98	103	CEAIWR			Carbamidomethyl (C)[1]		Mascot
851.4291	851.4117	-0.0174	-20	45	51	TKLDSCK			Carbamidomethyl (C)[6]		Mascot
947.4257	947.4678	0.0421	44	142	149	FVANNGYY					Mascot
1150.5245	1150.4816	-0.0429	-37	96	103	CRCEAIWR			Carbamidomethyl (C)[1,3]		Mascot
1399.6709	1399.6244	-0.0465	-33	52	62	DYVMERCLAVK			Carbamidomethyl (C)[7], Oxidation (M)[4]		Mascot
1431.6243	1431.5555	-0.0688	-48	47	57	LDSCKDYVMER			Carbamidomethyl (C)[4], Oxidation (M)[9]		Mascot
1664.7374	1664.7168	-0.0206	-12	136	149	LDSNCKFVANNGYY			Carbamidomethyl (C)[5]		Mascot

9 hypothetical protein TRIUR3_10997 [Triticum urartu] gi|474397164 31545.5 5.9 10 48 29.914 1.776

Peptide Information

Calc. Mass	Obsrv. Mass	± da	± ppm	Start Seq.	End Seq.	Sequence	Ion Score	C. I. %	Modification	Rank	Result Type

822.4719	822.4172	-0.0547	-67	150	156	SFKLAEK					Mascot
860.4519	860.4457	-0.0062	-7	1	8	MAVGGNRR					Mascot
947.4866	947.4678	-0.0188	-20	110	117	EGLILCDK			Carbamidomethyl (C)[6]		Mascot
1151.5183	1151.5846	0.0663	58	237	245	NIECEKACK			Carbamidomethyl (C)[4,8]		Mascot
1232.6304	1232.5883	-0.0421	-34	110	119	EGLILCDKER			Carbamidomethyl (C)[6]		Mascot
1399.6998	1399.6244	-0.0754	-54	138	149	LGRDMEEIGVHK			Oxidation (M)[5]		Mascot
1434.7145	1434.6682	-0.0463	-32	213	225	SSMLTPENSDLIK					Mascot
2005.0648	2005.0156	-0.0492	-25	122	140	GQQGVSLDAMVGIFTRL GR					Mascot
2742.4634	2742.3042	-0.1592	-58	213	236	SSMLTPENSDLIKQSIFTL IFTLK			Oxidation (M)[3]		Mascot
2758.2778	2758.2742	-0.0036	-1	246	269	FLTCYFQSISSDISGFTGD EATRR			Carbamidomethyl (C)[4]		Mascot

10	Adaptin ear-binding coat-associated protein 1 [Triticum urartu]	gi|474159928	30124	4.93	10	47	19.53	2.365

Peptide Information

Calc. Mass	Obsrv. Mass	± da	± ppm	Start Seq.	End Seq.	Sequence	Ion Score	C. I. %	Modification	Rank	Result Type
834.4025	834.3884	-0.0141	-17	72	78	GEECVIK			Carbamidomethyl (C)[4]		Mascot
885.4974	885.4501	-0.0473	-53	1	8	MAPLGRPK			Oxidation (M)[1]		Mascot
963.4377	963.4927	0.055	57	43	51	DAEEGVTSR					Mascot
1140.5685	1140.541	-0.0275	-24	155	164	SAFFEQGLNK					Mascot
1151.6056	1151.5846	-0.021	-18	165	174	LSFNEKANTK					Mascot
1381.6958	1381.6832	-0.0126	-9	79	90	LEDKSTGELYAR					Mascot
1601.7601	1601.7025	-0.0576	-36	9	21	GYPRPDPVDNRCR			Carbamidomethyl (C)[12]		Mascot
1918.903	1918.9313	0.0283	15	226	242	AEFPSQEEALDDIVEAR					Mascot
1918.903	1918.9313	0.0283	15	226	242	AEFPSQEEALDDIVEAR					Mascot
2005.0726	2005.0156	-0.057	-28	2	19	APLGRPKGYPRPDPVDN R					Mascot
2219.0251	2219.0793	0.0542	24	204	225	AEFPSQEQPGTGDAGDV AAPFK					Mascot

Gel Idx/Pos	266/K18	Instr./Gel Origin	BA2151/Sample Project 20140814	Process Status	Analysis Succeeded
Plate [#] Name	[1] Sample Project 20140814	Instrument Sample Name		Spectra	11

Rank	Protein Name	Accession No.	Protein MW	Protein PI	Pep. Count	Protein Score	Protein Score C. I. %	Intensity Matched	Total Ion Score	Total Ion C. I. %	Confirmed
1	unnamed protein product [Triticum aestivum]	gi|298541521	42152.7	5.64	19	651	100	57.398	530	100	

Protein Group

RecName: Full=Phosphoglycerate kinase, cytosolic	gi|129916	42152.7	5.6399998664856
unnamed protein product [Triticum aestivum]	gi|21835	42152.7	5.6399998664856

Peptide Information

Calc. Mass	Obsrv. Mass	± da	± ppm	Start Seq.	End Seq.	Sequence	Ion Score	C. I. %	Modification	Rank	Result Type
1030.6409	1030.6362	-0.0047	-5	75	83	FSLKPLVAR					Mascot
1030.6409	1030.6362	-0.0047	-5	75	83	FSLKPLVAR	46	99.82			Mascot
1089.5786	1089.5455	-0.0331	-30	6	16	SVGTLGEADLK					Mascot
1096.5521	1096.5519	-0.0002	0	307	316	TFAEALDTTK					Mascot
1245.6798	1245.6925	0.0127	10	5	16	RSVGTLGEADLK					Mascot
1274.6951	1274.6445	-0.0506	-40	6	18	SVGTLGEADLKGK					Mascot
1298.6587	1298.6355	-0.0232	-18	24	35	ADLNVPLDDAQK					Mascot
1375.8196	1375.7859	-0.0337	-24	44	55	ASIPTIKYLLEK					Mascot
1388.7421	1388.708	-0.0341	-25	179	191	ELDYLVGAVANPK					Mascot
1443.8782	1443.7881	-0.0901	-62	203	216	VSSKIGVIESLLAK					Mascot
1493.8297	1493.8295	-0.0002	0	166	178	FLRPSVAGFLMQK					Mascot
1509.8247	1509.8046	-0.0201	-13	166	178	FLRPSVAGFLMQK			Oxidation (M)[11]		Mascot
1509.8247	1509.8046	-0.0201	-13	166	178	FLRPSVAGFLMQK	25	76.441	Oxidation (M)[11]		Mascot
1573.8433	1573.7906	-0.0527	-33	350	366	GVTTIIGGGDSVAAVEK					Mascot
1720.9956	1721.0099	0.0143	8	106	122	LAAALPDGGVLLLENVR					Mascot
1720.9956	1721.0099	0.0143	8	106	122	LAAALPDGGVLLLENVR	152	100			Mascot
1769.8568	1769.8281	-0.0287	-16	317	331	TVIWNGPMGVFEFEK			Oxidation (M)[8]		Mascot
1919.9611	1919.9753	0.0142	7	138	155	LASVADLYVNDAFGTAHR					Mascot
1919.9611	1919.9753	0.0142	7	138	155	LASVADLYVNDAFGTAHR	151	100			Mascot
2048.0559	2048.0669	0.011	5	137	155	KLASVADLYVNDAFGTAHR					Mascot
2048.0559	2048.0669	0.011	5	137	155	KLASVADLYVNDAFGTAHR	155	100			Mascot

Calc. Mass	Obsrv. Mass	± da	± ppm	Start Seq.	End Seq.	Sequence	Ion Score	C. I. %	Modification	Rank	Result Type
2089.1274	2089.0889	-0.0385	-18	241	259	SLVEEDKLELATSLIETAK					Mascot
2159.2224	2159.1467	-0.0757	-35	106	125	LAAALPDGGVLLLENVRFYK					Mascot
2446.2092	2446.1875	-0.0217	-9	84	105	LSELLGLEVVMAPDCIGEEVEK			Carbamidomethyl (C)[15], Oxidation (M)[11]		Mascot
2878.4866	2878.4949	0.0083	3	373	401	MSHISTGGGASLELLEGKPLPGVLALDEA			Oxidation (M)[1]		Mascot

2	Phosphoglycerate kinase, cytosolic [Triticum urartu]	gi|473781647	45286.1	5.9	18	642	100	57.478	540	100	

Peptide Information

Calc. Mass	Obsrv. Mass	± da	± ppm	Start Seq.	End Seq.	Sequence	Ion Score	C. I. %	Modification	Rank	Result Type
1056.6565	1056.6521	-0.0044	-4	75	83	FSLKPLVPR					Mascot
1056.6565	1056.6521	-0.0044	-4	75	83	FSLKPLVPR	56	99.983			Mascot
1089.5786	1089.5455	-0.0331	-30	6	16	SVGTLGEADLK					Mascot
1096.5521	1096.5519	-0.0002	0	307	316	TFAEALDTTK					Mascot
1245.6798	1245.6925	0.0127	10	5	16	RSVGTLGEADLK					Mascot
1274.6951	1274.6445	-0.0506	-40	6	18	SVGTLGEADLKGK					Mascot
1298.6587	1298.6355	-0.0232	-18	24	35	ADLNVPLDDAQK					Mascot
1375.8196	1375.7859	-0.0337	-24	44	55	ASIPTIKYLLEK					Mascot
1388.7421	1388.708	-0.0341	-25	179	191	ELDYLVGAVANPK					Mascot
1443.8782	1443.7881	-0.0901	-62	203	216	VSSKIGVIESLLAK					Mascot
1493.8297	1493.8295	-0.0002	0	166	178	FLRPSVAGFLMQK					Mascot
1509.8247	1509.8046	-0.0201	-13	166	178	FLRPSVAGFLMQK			Oxidation (M)[11]		Mascot
1509.8247	1509.8046	-0.0201	-13	166	178	FLRPSVAGFLMQK	25	76.441	Oxidation (M)[11]		Mascot
1573.8433	1573.7906	-0.0527	-33	362	378	GVTTIIGGGDSVAAVEK					Mascot
1720.9956	1721.0099	0.0143	8	106	122	LAAALPDGGVLLLENVR					Mascot
1720.9956	1721.0099	0.0143	8	106	122	LAAALPDGGVLLLENVR	152	100			Mascot
1769.8568	1769.8281	-0.0287	-16	317	331	TVIWNGPMGVFEFEK			Oxidation (M)[8]		Mascot
1919.9611	1919.9753	0.0142	7	138	155	LASVADLYVNDAFGTAHR					Mascot
1919.9611	1919.9753	0.0142	7	138	155	LASVADLYVNDAFGTAHR	151	100			Mascot
2048.0559	2048.0669	0.011	5	137	155	KLASVADLYVNDAFGTAHR					Mascot
2048.0559	2048.0669	0.011	5	137	155	KLASVADLYVNDAFGTAHR	155	100			Mascot
2089.1274	2089.0889	-0.0385	-18	241	259	SLVEEDKLELATSLIETAK					Mascot
2159.2224	2159.1467	-0.0757	-35	106	125	LAAALPDGGVLLLENVRFYK					Mascot
2446.2092	2446.1875	-0.0217	-9	84	105	LSELLGLEVVMAPDCIGEEVEK			Carbamidomethyl (C)[15], Oxidation (M)[11]		Mascot

3	cytosolic 3-phosphoglycerate kinase [Triticum urartu]	gi|28172905	31371.7	4.98	13	620	100	56.993	540	100	

Protein Group

cytosolic 3-phosphoglycerate kinase [Triticum aestivum] gi|28172911 31371.7 4.9800 000190 7349

Peptide Information

Calc. Mass	Obsrv. Mass	± da	± ppm	Start Seq.	End Seq.	Sequence	Ion Score	C. I. %	Modification	Rank	Result Type
1056.6565	1056.6521	-0.0044	-4	1	9	FSLKPLVPR					Mascot
1056.6565	1056.6521	-0.0044	-4	1	9	FSLKPLVPR	56	99.983			Mascot
1096.5521	1096.5519	-0.0002	0	233	242	TFAEALDTTK					Mascot
1388.7421	1388.708	-0.0341	-25	105	117	ELDYLVGAVANPK					Mascot
1443.8782	1443.7881	-0.0901	-62	129	142	VSSKIGVIESLLAK					Mascot
1493.8297	1493.8295	-0.0002	0	92	104	FLRPSVAGFLMQK					Mascot
1509.8247	1509.8046	-0.0201	-13	92	104	FLRPSVAGFLMQK			Oxidation (M)[11]		Mascot
1509.8247	1509.8046	-0.0201	-13	92	104	FLRPSVAGFLMQK	25	76.441	Oxidation (M)[11]		Mascot
1573.8433	1573.7906	-0.0527	-33	276	292	GVTTIIGGGDSVAAVEK					Mascot
1720.9956	1721.0099	0.0143	8	32	48	LAAALPDGGVLLLENVR					Mascot
1720.9956	1721.0099	0.0143	8	32	48	LAAALPDGGVLLLENVR	152	100			Mascot
1769.8568	1769.8281	-0.0287	-16	243	257	TVIWNGPMGVFEFEK			Oxidation (M)[8]		Mascot
1919.9611	1919.9753	0.0142	7	64	81	LASVADLYVNDAFGTAH R					Mascot
1919.9611	1919.9753	0.0142	7	64	81	LASVADLYVNDAFGTAH R	151	100			Mascot
2048.0559	2048.0669	0.011	5	63	81	KLASVADLYVNDAFGTAH R					Mascot
2048.0559	2048.0669	0.011	5	63	81	KLASVADLYVNDAFGTAH R	155	100			Mascot
2089.1274	2089.0889	-0.0385	-18	167	185	SLVEEDKLELATSLIETAK					Mascot
2159.2224	2159.1467	-0.0757	-35	32	51	LAAALPDGGVLLLENVRF YK					Mascot
2446.2092	2446.1875	-0.0217	-9	10	31	LSELLGLEVVMAPDCIGE EVEK			Carbamidomethyl (C)[15], Oxidation (M)[11]		Mascot

4 cytosolic 3-phosphoglycerate kinase [Triticum aestivum] gi|28172909 31320.7 4.91 12 585 100 49.021 515 100

Peptide Information

Calc. Mass	Obsrv. Mass	± da	± ppm	Start Seq.	End Seq.	Sequence	Ion Score	C. I. %	Modification	Rank	Result Type
1056.6565	1056.6521	-0.0044	-4	1	9	FSLKPLVPR					Mascot
1056.6565	1056.6521	-0.0044	-4	1	9	FSLKPLVPR	56	99.983			Mascot
1096.5521	1096.5519	-0.0002	0	233	242	TFAEALDTTK					Mascot
1388.7421	1388.708	-0.0341	-25	105	117	ELDYLVGAVANPK					Mascot
1443.8782	1443.7881	-0.0901	-62	129	142	VSSKIGVIESLLAK					Mascot
1573.8433	1573.7906	-0.0527	-33	276	292	GVTTIIGGGDSVAAVEK					Mascot

1720.9956	1721.0099	0.0143	8	32	48	LAAALPDGGVLLLENVR					Mascot
1720.9956	1721.0099	0.0143	8	32	48	LAAALPDGGVLLLENVR	152	100			Mascot
1769.8568	1769.8281	-0.0287	-16	243	257	TVIWNGPMGVFEFEK			Oxidation (M)[8]		Mascot
1919.9611	1919.9753	0.0142	7	64	81	LASVADLYVNDAFGTAHR					Mascot
1919.9611	1919.9753	0.0142	7	64	81	LASVADLYVNDAFGTAHR	151	100			Mascot
2048.0559	2048.0669	0.011	5	63	81	KLASVADLYVNDAFGTAHR					Mascot
2048.0559	2048.0669	0.011	5	63	81	KLASVADLYVNDAFGTAHR	155	100			Mascot
2089.1274	2089.0889	-0.0385	-18	167	185	SLVEEDKLELATSLIETAK					Mascot
2159.2224	2159.1467	-0.0757	-35	32	51	LAAALPDGGVLLLENVRFYK					Mascot
2446.2092	2446.1875	-0.0217	-9	10	31	LSELLGLEVVMAPDCIGEEVEK			Carbamidomethyl (C)[15], Oxidation (M)[11]		Mascot

5	Phosphoglycerate kinase, cytosolic [Triticum urartu]	gi|473995124	55052.6	5.45	9	505	100	54.801	475	100	

Peptide Information

Calc. Mass	Obsrv. Mass	± da	± ppm	Start Seq.	End Seq.	Sequence	Ion Score	C. I. %	Modification	Rank	Result Type
1493.8297	1493.8295	-0.0002	0	209	221	YLRPAVAGFLMQK					Mascot
1509.8247	1509.8046	-0.0201	-13	209	221	YLRPAVAGFLMQK			Oxidation (M)[11]		Mascot
1509.8247	1509.8046	-0.0201	-13	209	221	YLRPAVAGFLMQK	16	0	Oxidation (M)[11]		Mascot
1536.8269	1536.8265	-0.0004	0	506	520	VVFLNSQLVSAATTS					Mascot
1573.8433	1573.7906	-0.0527	-33	393	409	GVTTIIGGGDSVAAVEK					Mascot
1720.9956	1721.0099	0.0143	8	149	165	LAAALPDGGVLLLENVR					Mascot
1720.9956	1721.0099	0.0143	8	149	165	LAAALPDGGVLLLENVR	152	100			Mascot
1769.8568	1769.8281	-0.0287	-16	360	374	TVIWNGPMGVFEFEK			Oxidation (M)[8]		Mascot
1919.9611	1919.9753	0.0142	7	181	198	LASVADLYVNDAFGTAHR					Mascot
1919.9611	1919.9753	0.0142	7	181	198	LASVADLYVNDAFGTAHR	151	100			Mascot
2048.0559	2048.0669	0.011	5	180	198	KLASVADLYVNDAFGTAHR					Mascot
2048.0559	2048.0669	0.011	5	180	198	KLASVADLYVNDAFGTAHR	155	100			Mascot
2159.2224	2159.1467	-0.0757	-35	149	168	LAAALPDGGVLLLENVRFYK					Mascot
2382.2373	2382.208	-0.0293	-12	328	350	IVPASAIPDGWMGLDVGPDSIKK			Oxidation (M)[12]		Mascot

6	resistance protein RGA2 [Triticum urartu]	gi|195975992	105314.6	5.85	21	61	96.869	22.17	

Peptide Information

Calc. Mass	Obsrv. Mass	± da	± ppm	Start Seq.	End Seq.	Sequence	Ion Score	C. I. %	Modification	Rank	Result Type

1068.5433	1068.5649	0.0216	20	128	136	SQILEAHDR					Mascot
1069.5677	1069.6381	0.0704	66	466	473	YLYELVNR					Mascot
1106.6357	1106.6294	-0.0063	-6	176	183	YELVKWLR					Mascot
1156.6508	1156.5665	-0.0843	-73	648	657	LPDLIGNMKR					Mascot
1245.7162	1245.6925	-0.0237	-19	708	719	LVSSLSKLDAGR					Mascot
1274.6124	1274.6445	0.0321	25	181	190	WLRNGEDEQK					Mascot
1362.7781	1362.728	-0.0501	-37	842	852	LDLHVYLSKFK					Mascot
1363.7104	1363.6987	-0.0117	-9	474	486	SLIESVGVPYDGK					Mascot
1388.7573	1388.708	-0.0493	-35	720	730	LSNLYVTFYLR					Mascot
1436.7744	1436.694	-0.0804	-56	203	214	TTLAKQVYDELR					Mascot
1507.7024	1507.754	0.0516	34	453	465	YQDGEDLVQVGER					Mascot
1573.8009	1573.7906	-0.0103	-7	215	227	INFEYQAFVSISR					Mascot
1702.8184	1702.9664	0.148	87	382	396	EEWGHVGLSSVFAER					Mascot
1769.8132	1769.8281	0.0149	8	83	97	IDNSMSLMLHHACPK			Carbamidomethyl (C)[13], Oxidation (M)[5]		Mascot
1920.0146	1919.9753	-0.0393	-20	640	656	LFVSDETKLPDLIGNMK					Mascot
1920.0146	1919.9753	-0.0393	-20	640	656	LFVSDETKLPDLIGNMK					Mascot
1941.908	1941.9265	0.0185	10	1	19	MAPCLVSASTGAMGSLQ TK			Carbamidomethyl (C)[4], Oxidation (M)[1,13]		Mascot
2048.0627	2048.0669	0.0042	2	2	21	APCLVSASTGAMGSLQT KLR			Carbamidomethyl (C)[3]		Mascot
2048.0627	2048.0669	0.0042	2	2	21	APCLVSASTGAMGSLQT KLR			Carbamidomethyl (C)[3]		Mascot
2069.0017	2069.0693	0.0676	33	757	772	MCWMNKWLLSLANLEK			Carbamidomethyl (C)[2], Oxidation (M)[1,4]		Mascot
2094.9995	2095.0801	0.0806	38	894	911	SMVNAHPNPNRPMLEMT R					Mascot
2110.9944	2111.105	0.1106	52	894	911	SMVNAHPNPNRPMLEMT R			Oxidation (M)[2]		Mascot
2300.1672	2300.1282	-0.039	-17	270	287	YFVIIDDIWDMKTWDVLK					Mascot
2878.3057	2878.4949	0.1892	66	74	97	ELSYDIEDKIDNSMSLML HHACPK			Carbamidomethyl (C)[22], Oxidation (M)[14,17]		Mascot

7 Disease resistance protein RPM1 [Triticum urartu] gi|474393435 105112.6 6 21 60 95.578 22.17

Peptide Information

Calc. Mass	Obsrv. Mass	± da	± ppm	Start Seq.	End Seq.	Sequence	Ion Score	C. I. %	Modification	Rank	Result Type
1068.5433	1068.5649	0.0216	20	126	134	SQILEAHDR					Mascot
1069.5677	1069.6381	0.0704	66	464	471	YLYELVNR					Mascot
1106.6357	1106.6294	-0.0063	-6	174	181	YELVKWLR					Mascot
1156.6508	1156.5665	-0.0843	-73	646	655	LPDLIGNMKR					Mascot
1245.7162	1245.6925	-0.0237	-19	706	717	LVSSLSKLDAGR					Mascot
1274.6124	1274.6445	0.0321	25	179	188	WLRNGEDEQK					Mascot
1362.7781	1362.728	-0.0501	-37	840	850	LDLHVYLSKFK					Mascot

1363.7104	1363.6987	-0.0117	-9	472	484	SLIESVGVPYDGK					Mascot
1388.7573	1388.708	-0.0493	-35	718	728	LSNLYVTFYLR					Mascot
1436.7744	1436.694	-0.0804	-56	201	212	TTLAKQVYDELR					Mascot
1507.7024	1507.754	0.0516	34	451	463	YQDGEDLVQVGER					Mascot
1573.7607	1573.7906	0.0299	19	268	279	YFVIIDDIWDMK			Oxidation (M)[11]		Mascot
1702.8184	1702.9664	0.148	87	380	394	EEWGHVGLSSVFAER					Mascot
1769.8132	1769.8281	0.0149	8	81	95	IDNSMSLMLHHACPK			Carbamidomethyl (C)[13], Oxidation (M)[5]		Mascot
1920.0146	1919.9753	-0.0393	-20	638	654	LFVSDETKLPDLIGNMK					Mascot
1920.0146	1919.9753	-0.0393	-20	638	654	LFVSDETKLPDLIGNMK					Mascot
1941.908	1941.9265	0.0185	10	1	19	MAPCLVSASTGAMGSLQ TK			Carbamidomethyl (C)[4], Oxidation (M)[1,13]		Mascot
2048.0627	2048.0669	0.0042	2	2	21	APCLVSASTGAMGSLQT KLR			Carbamidomethyl (C)[3]		Mascot
2048.0627	2048.0669	0.0042	2	2	21	APCLVSASTGAMGSLQT KLR			Carbamidomethyl (C)[3]		Mascot
2069.0017	2069.0693	0.0676	33	755	770	MCWMNKWLLSLANLEK			Carbamidomethyl (C)[2], Oxidation (M)[1,4]		Mascot
2094.9995	2095.0801	0.0806	38	892	909	SMVNAHPNPNRPMLEMT R					Mascot
2110.9944	2111.105	0.1106	52	892	909	SMVNAHPNPNRPMLEMT R			Oxidation (M)[2]		Mascot
2300.1672	2300.1282	-0.039	-17	268	285	YFVIIDDIWDMKTWDVLK					Mascot
2878.3057	2878.4949	0.1892	66	72	95	ELSYDIEDKIDNSMSLML HHACPK			Carbamidomethyl (C)[22], Oxidation (M)[14,17]		Mascot

8	Disease resistance protein RPM1 [Triticum urartu]	gi|473895841	113356.9	8.99	19	50	61.485	11.726

Peptide Information

Calc. Mass	Obsrv. Mass	± da	± ppm	Start Seq.	End Seq.	Sequence	Ion Score	C. I. %	Modification	Rank	Result Type
1055.5554	1055.5972	0.0418	40	216	224	TTIAMALYR			Oxidation (M)[5]		Mascot
1060.6262	1060.6387	0.0125	12	700	708	AFGQLKQLR					Mascot
1096.5416	1096.5519	0.0103	9	591	600	RLSMQSSGSK			Oxidation (M)[4]		Mascot
1118.5801	1118.5406	-0.0395	-35	403	412	QSISESPSRK					Mascot
1233.6871	1233.6084	-0.0787	-64	281	291	NLLVDMKSSVK					Mascot
1443.6606	1443.7881	0.1275	88	1	13	MEFVVGASEATMR			Oxidation (M)[1]		Mascot
1445.7305	1445.8466	0.1161	80	176	188	TENPVGVKVDMEK					Mascot
1445.7305	1445.8466	0.1161	80	176	188	TENPVGVKVDMEK					Mascot
1461.7255	1461.8082	0.0827	57	176	188	TENPVGVKVDMEK			Oxidation (M)[11]		Mascot
1483.8124	1483.7872	-0.0252	-17	651	662	HLNDICKMLVLK			Carbamidomethyl (C)[6]		Mascot
1491.7295	1491.8365	0.107	72	925	936	EVMPALERIDMR			Oxidation (M)[3,11]		Mascot
1493.7708	1493.8295	0.0587	39	399	411	SLFRQSISESPSR					Mascot
1747.9412	1747.8895	-0.0517	-30	917	932	LSFAVTGKEVMPALER					Mascot

1759.0225	1758.9252	-0.0973	-55	19	34	LGGLLAQEYTLIRGVR					Mascot
1801.9404	1801.8566	-0.0838	-47	552	566	SFKVNDMILEYIVSK			Oxidation (M)[7]		Mascot
1864.9698	1865.0361	0.0663	36	364	380	VIVTTRFQAVGAACSER			Carbamidomethyl (C)[14]		Mascot
1876.0902	1876.0261	-0.0641	-34	835	850	ELNNLYKLTLSVTVLR					Mascot
1941.9773	1941.9265	-0.0508	-26	1	18	MEFVVGASEATMRSLLGK			Oxidation (M)[1]		Mascot
1957.9722	1957.9142	-0.058	-30	1	18	MEFVVGASEATMRSLLGK			Oxidation (M)[1,12]		Mascot
2446.1709	2446.1875	0.0166	7	567	588	SSEENFITVVGGHWLMPTPSNK			Oxidation (M)[16]		Mascot
2878.5632	2878.4949	-0.0683	-24	734	760	ALRILSGIEIDEESSAVASLHQLTGLR					Mascot
2891.5222	2891.4795	-0.0427	-15	250	276	DVLGQIKPANSQIKPKEEGQEGNNIGK					Mascot

9 Peroxidase 1 [Triticum urartu] gi|474256573 27025.7 5.7 9 49 49.227 1.015

Peptide Information

Calc. Mass	Obsrv. Mass	± da	± ppm	Start Seq.	End Seq.	Sequence	Ion Score	C. I. %	Modification	Rank	Result Type
1465.772	1465.8077	0.0357	24	81	93	KEMLFAAVNLDAK			Oxidation (M)[3]		Mascot
1507.6774	1507.754	0.0766	51	205	217	DEFFADFAASMIK			Oxidation (M)[11]		Mascot
1573.7891	1573.7906	0.0015	1	67	80	LLAESCPSVEDVVR			Carbamidomethyl (C)[6]		Mascot
1701.884	1701.8932	0.0092	5	67	81	LLAESCPSVEDVVRK			Carbamidomethyl (C)[6]		Mascot
1746.881	1746.957	0.076	44	177	192	GLFHSDGALLTDPFTR					Mascot
1902.9822	1903.0122	0.03	16	176	192	RGLFHSDGALLTDPFTR					Mascot
2204.0481	2204.1465	0.0984	45	198	217	HATGAFKDEFFADFAASMIK					Mascot
2878.2732	2878.4949	0.2217	77	116	139	LYNFTGMENPSDIDPTLEPQYMMR			Oxidation (M)[7]		Mascot
3017.4131	3017.6877	0.2746	91	205	232	DEFFADFAASMIKMGNANPLTGSQGEIR					Mascot

10 hypothetical protein TRIUR3_30538 [Triticum urartu] gi|473882607 197169.7 4.65 27 49 41.705 7.708

Peptide Information

Calc. Mass	Obsrv. Mass	± da	± ppm	Start Seq.	End Seq.	Sequence	Ion Score	C. I. %	Modification	Rank	Result Type
874.4265	874.4257	-0.0008	-1	404	412	AVAEDANGK					Mascot
993.5251	993.5469	0.0218	22	1397	1404	VELFQTEK					Mascot
993.5251	993.5469	0.0218	22	1397	1404	VELFQTEK					Mascot
1018.5779	1018.5896	0.0117	11	910	918	ESSVLDKLK					Mascot
1030.5527	1030.6362	0.0835	81	1500	1508	RELDEAVAK					Mascot
1030.5527	1030.6362	0.0835	81	1500	1508	RELDEAVAK					Mascot
1106.5623	1106.6294	0.0671	61	357	365	LSERDSMLR					Mascot

1224.5785	1224.5216	-0.0569	-46	185	194	MLEMAQLNMK	Oxidation (M)[1]	Mascot
1233.6208	1233.6084	-0.0124	-10	81	90	IEELESEKEK		Mascot
1238.5569	1238.634	0.0771	62	991	1002	AAGATEEVESMK	Oxidation (M)[11]	Mascot
1253.5831	1253.6404	0.0573	46	1268	1277	VAMYDELQER		Mascot
1298.7314	1298.6355	-0.0959	-74	546	557	SIELASLLHTSK		Mascot
1370.7274	1370.6874	-0.04	-29	1571	1581	DQLEQQLLEVR		Mascot
1465.6765	1465.8077	0.1312	90	61	73	TSESATLNQETER		Mascot
1478.7156	1478.782	0.0664	45	967	978	ETDCQQLSEKLK	Carbamidomethyl (C)[4]	Mascot
1483.75	1483.7872	0.0372	25	917	929	LKSAEEQLEHSGR		Mascot
1493.7152	1493.8295	0.1143	77	1509	1520	LEEQMNLEKSEK	Oxidation (M)[5]	Mascot
1498.8224	1498.8066	-0.0158	-11	1571	1582	DQLEQQLLEVRK		Mascot
1662.8618	1662.8624	0.0006	0	625	638	DLMDSLIQITEEKK		Mascot
1699.865	1699.8444	-0.0206	-12	1253	1267	LADKNEHLEGEVFAK		Mascot
1701.9017	1701.8932	-0.0085	-5	831	846	EKSASLEALLAEANQK		Mascot
1706.8556	1706.9015	0.0459	27	61	75	TSESATLNQETERLK		Mascot
1742.9786	1742.9556	-0.023	-13	672	686	VLDLEELLAKLTEEK		Mascot
1747.8167	1747.8895	0.0728	42	195	208	EMENQINNLQEEIK	Oxidation (M)[2]	Mascot
1769.8341	1769.8281	-0.006	-3	489	503	QFEEAVNNAEAYKEK		Mascot
1875.9116	1876.0261	0.1145	61	1033	1051	AEEALAESTAMGAANQA LK		Mascot
1977.0652	1976.9766	-0.0886	-45	1187	1203	LAVHEETIKHLTEELSK		Mascot
2095.0237	2095.0801	0.0564	27	1553	1570	NEALTSKMQEHANLVHE K	Oxidation (M)[8]	Mascot
2446.2307	2446.1875	-0.0432	-18	682	703	LTEEKESGENTIADLNLQ LSNK		Mascot

Gel Idx/Pos	267/K19	Instr./Gel Origin	BA2151/Sample Project 20140814	Process Status	Analysis Succeeded
Plate [#] Name	[1] Sample Project 20140814	Instrument Sample Name		Spectra	11

Rank	Protein Name	Accession No.	Protein MW	Protein PI	Pep. Count	Protein Score	Protein Score C. I. %	Intensity Matched	Total Ion Score	Total Ion C. I. %	Confirmed
1	serpin [Triticum aestivum]	gi|5734504	42969	5.62	14	345	100	60.817	269	100	

Protein Group

RecName: Full=Serpin-Z1C; AltName: Full=TriaeZ1c; AltName: Full=WSZ1c	gi|75313848	42969	5.6199998855 5908

Peptide Information

Calc. Mass	Obsrv. Mass	± da	± ppm	Start Seq.	End Seq.	Sequence	Ion Score	C. I. %	Modification	Rank	Result Type
806.4744	806.4313	-0.0431	-53	271	276	HIPRQR					Mascot
925.5214	925.5156	-0.0058	-6	11	18	LSIAHQTR					Mascot
925.5214	925.5156	-0.0058	-6	11	18	LSIAHQTR	43	99.606			Mascot
947.5156	947.4764	-0.0392	-41	2	10	ATTLATDVR					Mascot
1078.5562	1078.6202	0.064	59	1	10	MATTLATDVR					Mascot
1151.6824	1151.6451	-0.0373	-32	172	181	LVLANALYFK					Mascot
1176.5896	1176.5881	-0.0015	-1	261	270	LSAEPDFLER					Mascot
1176.5896	1176.5881	-0.0015	-1	261	270	LSAEPDFLER	69	100			Mascot
1352.6945	1352.6516	-0.0429	-32	289	300	ISFETEASDLLK					Mascot
1399.6125	1399.5831	-0.0294	-21	182	194	GAWTDQFDSSGTK					Mascot
1530.7435	1530.7631	0.0196	13	125	137	YKADTQSVDFQTK					Mascot
2129.0947	2129.0332	-0.0615	-29	379	398	EDISGVVLFMGHVVNPLLSS			Oxidation (M)[10]		Mascot
2720.3525	2720.3455	-0.007	-3	328	353	VSSVFHQAFVEVNEQGTEAAASTAIK					Mascot
2725.4631	2725.4707	0.0076	3	33	61	SAASNAVFSPVSLHVALSLLAAGAGSATR					Mascot
2725.4631	2725.4707	0.0076	3	33	61	SAASNAVFSPVSLHVALSLLAAGAGSATR	120	100			Mascot
2930.542	2930.5276	-0.0144	-5	354	378	MALLQARPPSVMDFIADHPFLFLLR			Oxidation (M)[1,12]		Mascot
3053.3801	3053.3704	-0.0097	-3	301	327	CLGLQLPFSNEADFSEMVDSPMAHGLR			Carbamidomethyl (C)[1], Oxidation (M)[17,22]		Mascot
3053.3801	3053.3704	-0.0097	-3	301	327	CLGLQLPFSNEADFSEMVDSPMAHGLR	37	98.639	Carbamidomethyl (C)[1], Oxidation (M)[17,22]		Mascot

Rank	Protein Name	Accession No.	Protein MW	Protein PI	Pep. Count	Protein Score	Protein Score C. I. %	Intensity Matched	Total Ion Score	Total Ion C. I. %	Confirmed
2	Serpin-Z1C [Triticum urartu]	gi|474075261	42956	5.62	13	337	100	60.708	269	100	

Peptide Information

Calc. Mass	Obsrv. Mass	± da	± ppm	Start Seq.	End Seq.	Sequence	Ion Score	C. I. %	Modification	Rank	Result Type

Calc. Mass	Obsrv. Mass	± da	± ppm	Start Seq.	End Seq.	Sequence	Ion Score	C. I. %	Modification	Rank	Result Type
806.4744	806.4313	-0.0431	-53	271	276	HIPRQR					Mascot
925.5214	925.5156	-0.0058	-6	11	18	LSIAHQTR					Mascot
925.5214	925.5156	-0.0058	-6	11	18	LSIAHQTR	43	99.606			Mascot
947.5156	947.4764	-0.0392	-41	2	10	ATTLATDVR					Mascot
1078.5562	1078.6202	0.064	59	1	10	MATTLATDVR					Mascot
1151.6824	1151.6451	-0.0373	-32	172	181	LVLANALYFK					Mascot
1176.5896	1176.5881	-0.0015	-1	261	270	LSAEPDFLER					Mascot
1176.5896	1176.5881	-0.0015	-1	261	270	LSAEPDFLER	69	100			Mascot
1352.6945	1352.6516	-0.0429	-32	289	300	ISFETEASDLLK					Mascot
1399.6125	1399.5831	-0.0294	-21	182	194	GAWTDQFDSSGTK					Mascot
2129.0947	2129.0332	-0.0615	-29	379	398	EDISGVVLFMGHVVNPLL SS			Oxidation (M)[10]		Mascot
2720.3525	2720.3455	-0.007	-3	328	353	VSSVFHQAFVEVNEQGT EAAASTAIK					Mascot
2725.4631	2725.4707	0.0076	3	33	61	SAASNAVFSPVSLHVALS LLAAGAGSATR					Mascot
2725.4631	2725.4707	0.0076	3	33	61	SAASNAVFSPVSLHVALS LLAAGAGSATR	120	100			Mascot
2930.542	2930.5276	-0.0144	-5	354	378	MALLQARPPSVMDFIADH PFLFLLR			Oxidation (M)[1,12]		Mascot
3053.3801	3053.3704	-0.0097	-3	301	327	CLGLQLPFSNEADFSEM VDSPMAHGLR			Carbamidomethyl (C)[1], Oxidation (M)[17,22]		Mascot
3053.3801	3053.3704	-0.0097	-3	301	327	CLGLQLPFSNEADFSEM VDSPMAHGLR	37	98.639	Carbamidomethyl (C)[1], Oxidation (M)[17,22]		Mascot

3	serpin-N3.7 [Triticum aestivum]	gi|379060945	42639.8	5.52	10	272	100	59.038	232	100	

Peptide Information

Calc. Mass	Obsrv. Mass	± da	± ppm	Start Seq.	End Seq.	Sequence	Ion Score	C. I. %	Modification	Rank	Result Type
806.4744	806.4313	-0.0431	-53	271	276	HIPRQR					Mascot
925.5214	925.5156	-0.0058	-6	11	18	LSIAHQTR					Mascot
925.5214	925.5156	-0.0058	-6	11	18	LSIAHQTR	43	99.606			Mascot
947.5156	947.4764	-0.0392	-41	2	10	ATTLATDVR					Mascot
1078.5562	1078.6202	0.064	59	1	10	MATTLATDVR					Mascot
1151.6824	1151.6451	-0.0373	-32	172	181	LVLANALYFK					Mascot
1176.5896	1176.5881	-0.0015	-1	261	270	LSAEPDFLER					Mascot
1176.5896	1176.5881	-0.0015	-1	261	270	LSAEPDFLER	69	100			Mascot
1399.6125	1399.5831	-0.0294	-21	182	194	GAWTDQFDSSGTK					Mascot
1530.7435	1530.7631	0.0196	13	125	137	YKADTQSVDFQTK					Mascot
2720.3525	2720.3455	-0.007	-3	328	353	VSSVFHQAFVEVNEQGT EAAASTAIK					Mascot
2725.4631	2725.4707	0.0076	3	33	61	SAASNAVFSPVSLHVALS LLAAGAGSATR					Mascot
2725.4631	2725.4707	0.0076	3	33	61	SAASNAVFSPVSLHVALS	120	100			Mascot

LLAAGAGSATR

4	serpin [Triticum aestivum]	gi|1885350	43119.9	5.44	10	151	100	26.805	112	100

Protein Group

RecName: Full=Serpin-Z1B; AltName: Full=TriaeZ1b; AltName: Full=WSZ1b; AltName: Full=WZS2	gi|75279910	43119.9	5.4400000572 2046

Peptide Information

Calc. Mass	Obsrv. Mass	± da	± ppm	Start Seq.	End Seq.	Sequence	Ion Score	C. I. %	Modification	Rank	Result Type
806.4744	806.4313	-0.0431	-53	272	277	HIPRQR					Mascot
925.5214	925.5156	-0.0058	-6	11	18	LSIAHQTR					Mascot
925.5214	925.5156	-0.0058	-6	11	18	LSIAHQTR	43	99.606			Mascot
947.5156	947.4764	-0.0392	-41	2	10	ATTLATDVR					Mascot
1078.5562	1078.6202	0.064	59	1	10	MATTLATDVR					Mascot
1151.6824	1151.6451	-0.0373	-32	172	181	LVLANALYFK					Mascot
1176.5896	1176.5881	-0.0015	-1	262	271	LSAEPDFLER					Mascot
1176.5896	1176.5881	-0.0015	-1	262	271	LSAEPDFLER	69	100			Mascot
1585.8295	1585.7332	-0.0963	-61	288	301	FKISFGMEASDLLK					Mascot
1601.8243	1601.7195	-0.1048	-65	288	301	FKISFGMEASDLLK			Oxidation (M)[7]		Mascot
2062.0564	2061.9585	-0.0979	-47	138	156	AAEVTTQVNSWVEKVTSGR					Mascot
2129.0947	2129.0332	-0.0615	-29	380	399	EDISGVVLFMGHVVNPLLSS			Oxidation (M)[10]		Mascot
2720.3525	2720.3455	-0.007	-3	329	354	VSSVFHQAFVEVNEQGTEAAASTAIK					Mascot

5	serpin [Triticum aestivum]	gi|871551	43262.2	5.6	9	145	100	26.392	112	100

Protein Group

RecName: Full=Serpin-Z1A; AltName: Full=TriaeZ1a; AltName: Full=WSZ1a; Short=WSZ1; AltName: Full=WSZCI	gi|75282265	43262.2	5.5999999046 3257
serpin 1 [Triticum aestivum]	gi|224589266	43261.1	5.4400000572 2046

Peptide Information

Calc. Mass	Obsrv. Mass	± da	± ppm	Start Seq.	End Seq.	Sequence	Ion Score	C. I. %	Modification	Rank	Result Type
806.4744	806.4313	-0.0431	-53	271	276	HIPRQR					Mascot
925.5214	925.5156	-0.0058	-6	11	18	LSIAHQTR					Mascot
925.5214	925.5156	-0.0058	-6	11	18	LSIAHQTR	43	99.606			Mascot
947.5156	947.4764	-0.0392	-41	2	10	ATTLATDVR					Mascot
1078.5562	1078.6202	0.064	59	1	10	MATTLATDVR					Mascot

Calc. Mass	Obsrv. Mass	± da	± ppm	Start Seq.	End Seq.	Sequence	Ion Score	C. I. %	Modification	Rank	Result Type
1151.6824	1151.6451	-0.0373	-32	172	181	LVLANALYFK					Mascot
1176.5896	1176.5881	-0.0015	-1	261	270	LSAEPDFLER					Mascot
1176.5896	1176.5881	-0.0015	-1	261	270	LSAEPDFLER	69	100			Mascot
2062.0564	2061.9585	-0.0979	-47	138	156	AAEVTTQVNSWVEKVTS GR					Mascot
2129.0947	2129.0332	-0.0615	-29	379	398	EDISGVVLFMGHVVNPLL SS			Oxidation (M)[10]		Mascot
2720.3525	2720.3455	-0.007	-3	328	353	VSSVFHQAFVEVNEQGT EAAASTAIK					Mascot

6	serpin 3 [Triticum aestivum]	gi|224589270	43227.1	5.56	8	138	100	26.013	112	100	

Peptide Information

Calc. Mass	Obsrv. Mass	± da	± ppm	Start Seq.	End Seq.	Sequence	Ion Score	C. I. %	Modification	Rank	Result Type
806.4744	806.4313	-0.0431	-53	272	277	HIPRQR					Mascot
925.5214	925.5156	-0.0058	-6	11	18	LSIAHQTR					Mascot
925.5214	925.5156	-0.0058	-6	11	18	LSIAHQTR	43	99.606			Mascot
947.5156	947.4764	-0.0392	-41	2	10	ATTLATDVR					Mascot
1078.5562	1078.6202	0.064	59	1	10	MATTLATDVR					Mascot
1151.6824	1151.6451	-0.0373	-32	172	181	LVLANALYFK					Mascot
1176.5896	1176.5881	-0.0015	-1	262	271	LSAEPDFLER					Mascot
1176.5896	1176.5881	-0.0015	-1	262	271	LSAEPDFLER	69	100			Mascot
2062.0564	2061.9585	-0.0979	-47	138	156	AAEVTTQVNSWVEKVTS GR					Mascot
2720.3525	2720.3455	-0.007	-3	329	354	VSSVFHQAFVEVNEQGT EAAASTAIK					Mascot

7	zeta-carotene desaturase [Triticum aestivum]	gi|336185123	63061.2	7.09	15	55	85.357	4.881	

Protein Group

zeta-carotene desaturase [Triticum aestivum]	gi|336185125	63061.2	7.0900 001525 8789

Peptide Information

Calc. Mass	Obsrv. Mass	± da	± ppm	Start Seq.	End Seq.	Sequence	Ion Score	C. I. %	Modification	Rank	Result Type
806.4077	806.4313	0.0236	29	562	568	VQMLQTS					Mascot
836.4625	836.4458	-0.0167	-20	482	488	IGQSLYR					Mascot
842.4744	842.4411	-0.0333	-40	296	302	GGRFHLR					Mascot
848.4724	848.4305	-0.0419	-49	328	335	ATSSEIIK					Mascot
900.4938	900.4388	-0.055	-61	351	357	LLPSEWR					Mascot
1042.5891	1042.611	0.0219	21	133	142	VGADNNLLVK					Mascot
1204.6572	1204.6191	-0.0381	-32	471	481	GLEVTWSSVVK					Mascot

Calc. Mass	Obsrv. Mass	± da	± ppm	Start Seq.	End Seq.	Sequence	Ion Score	C. I. %	Modification	Rank	Result Type
1204.6572	1204.6191	-0.0381	-32	471	481	GLEVTWSSVVK					Mascot
1232.6522	1232.6344	-0.0178	-14	278	289	GSPDVYLSGPIK					Mascot
1341.6587	1341.7524	0.0937	70	49	59	GLFPPEPEHYR					Mascot
1621.8401	1621.847	0.0069	4	1	16	MAATSCALVSALVVGR			Carbamidomethyl (C)[6], Oxidation (M)[1]		Mascot
1623.8279	1623.9089	0.081	50	49	62	GLFPPEPEHYRGPK					Mascot
1642.7378	1642.83	0.0922	56	516	530	QDYIDSMEGATLSGR					Mascot
1733.7477	1733.8416	0.0939	54	213	226	DLDDVSFTDWFMSR					Mascot
2331.2708	2331.2705	-0.0003	0	461	481	QVLDLFPSARGLEVTWS SVVK					Mascot
2331.2708	2331.2705	-0.0003	0	461	481	QVLDLFPSARGLEVTWS SVVK					Mascot
2930.3899	2930.5276	0.1377	47	108	131	QGNHIEMGLHVFFGCYS NLFRLMK			Carbamidomethyl (C)[15], Oxidation (M)[7,23]		Mascot

8	Serpin-Z2B [Triticum urartu]	gi|473793747	45225.7	6.03	4	49	49.227	6.464	43	99.606	

Peptide Information

Calc. Mass	Obsrv. Mass	± da	± ppm	Start Seq.	End Seq.	Sequence	Ion Score	C. I. %	Modification	Rank	Result Type
925.5214	925.5156	-0.0058	-6	11	18	LSIAHQTR					Mascot
925.5214	925.5156	-0.0058	-6	11	18	LSIAHQTR	43	99.606			Mascot
947.5156	947.4764	-0.0392	-41	2	10	ATTLATDVR					Mascot
1078.5562	1078.6202	0.064	59	1	10	MATTLATDVR					Mascot
1623.8676	1623.9089	0.0413	25	256	268	RQFSMYILLPEAR					Mascot

9	serpin [Triticum aestivum]	gi|5734506	43341.5	5.46	4	49	49.227	6.301	43	99.606	

Protein Group

RecName: Full=Serpin-Z2A; AltName: Full=TriaeZ2a; AltName: Full=WSZ2a	gi|75313847	43341.5	5.4600 000381 4697

Peptide Information

Calc. Mass	Obsrv. Mass	± da	± ppm	Start Seq.	End Seq.	Sequence	Ion Score	C. I. %	Modification	Rank	Result Type
925.5214	925.5156	-0.0058	-6	11	18	LSIAHQTR					Mascot
925.5214	925.5156	-0.0058	-6	11	18	LSIAHQTR	43	99.606			Mascot
947.5156	947.4764	-0.0392	-41	2	10	ATTLATDVR					Mascot
1078.5562	1078.6202	0.064	59	1	10	MATTLATDVR					Mascot
1156.6208	1156.5862	-0.0346	-30	261	270	LSAEPELLER					Mascot

10	zeta-carotene desaturase [Triticum aestivum]	gi|336185127	63071.2	6.81	14	48	23.152	4.317			

Protein Group

zeta-carotene desaturase [Triticum aestivum]	gi|205371883	63071.2	6.8099

			999427<br>7954
zeta-carotene desaturase [Triticum aestivum]	gi|336185129	63071.2	6.8099<br>999427<br>7954
zeta-carotene desaturase enzyme [Triticum aestivum]	gi|231274761	63071.2	6.8099<br>999427<br>7954

Peptide Information

Calc. Mass	Obsrv. Mass	± da	± ppm	Start Seq.	End Seq.	Sequence	Ion Score	C. I. %	Modification	Rank	Result Type
836.4625	836.4458	-0.0167	-20	482	488	IGQSLYR					Mascot
848.4724	848.4305	-0.0419	-49	328	335	ATSSEIIK					Mascot
860.4155	860.4617	0.0462	54	18	24	RGPSCQR			Carbamidomethyl (C)[5]		Mascot
900.4938	900.4388	-0.055	-61	351	357	LLPSEWR					Mascot
1042.5891	1042.611	0.0219	21	133	142	VGADNNLLVK					Mascot
1204.6572	1204.6191	-0.0381	-32	471	481	GLEVTWSSVVK					Mascot
1204.6572	1204.6191	-0.0381	-32	471	481	GLEVTWSSVVK					Mascot
1232.6522	1232.6344	-0.0178	-14	278	289	GSPDVYLSGPIK					Mascot
1341.6587	1341.7524	0.0937	70	49	59	GLFPPEPEHYR					Mascot
1399.7223	1399.5831	-0.1392	-99	19	32	GPSCQRAAAAGVVR			Carbamidomethyl (C)[4]		Mascot
1621.8401	1621.847	0.0069	4	1	16	MAATSCALVSALVVGR			Carbamidomethyl (C)[6], Oxidation (M)[1]		Mascot
1623.8279	1623.9089	0.081	50	49	62	GLFPPEPEHYRGPK					Mascot
1642.7378	1642.83	0.0922	56	516	530	QDYIDSMEGATLSGR					Mascot
1733.7477	1733.8416	0.0939	54	213	226	DLDDVSFTDWFMSR					Mascot
2331.2708	2331.2705	-0.0003	0	461	481	QVLDLFPSARGLEVTWS SVVK					Mascot
2331.2708	2331.2705	-0.0003	0	461	481	QVLDLFPSARGLEVTWS SVVK					Mascot

Gel Idx/Pos	268/K20	Instr./Gel Origin	BA2151/Sample Project 20140814	Process Status	Analysis Succeeded
Plate [#] Name	[1] Sample Project 20140814	Instrument Sample Name		Spectra	11

Rank	Protein Name	Accession No.	Protein MW	Protein PI	Pep. Count	Protein Score	Protein Score C. I. %	Intensity Matched	Total Ion Score	Total Ion C. I. %	Confirmed
1	Beta-amylase [Triticum urartu]	gi|474451266	58995	5.34	17	445	100	26.36	362	100	

Peptide Information

Calc. Mass	Obsrv. Mass	± da	± ppm	Start Seq.	End Seq.	Sequence	Ion Score	C. I. %	Modification	Rank	Result Type
947.5057	947.4819	-0.0238	-25	322	329	DGYRPIAR					Mascot
1016.5564	1016.5454	-0.011	-11	412	419	LFGFTYLR					Mascot
1016.5564	1016.5454	-0.011	-11	412	419	LFGFTYLR	50	99.926			Mascot
1189.6154	1189.5902	-0.0252	-21	295	303	ISGIHWWYK					Mascot
1299.582	1299.5822	0.0002	0	336	346	ASLNFTCAEMR			Carbamidomethyl (C)[7]		Mascot
1315.5769	1315.552	-0.0249	-19	336	346	ASLNFTCAEMR			Carbamidomethyl (C)[7], Oxidation (M)[10]		Mascot
1326.6688	1326.6537	-0.0151	-11	385	395	YDPTAYNTILR					Mascot
1326.6688	1326.6537	-0.0151	-11	385	395	YDPTAYNTILR	66	99.998			Mascot
1474.6777	1474.698	0.0203	14	372	384	EGLNMACENALPR			Carbamidomethyl (C)[7]		Mascot
1490.6726	1490.6471	-0.0255	-17	372	384	EGLNMACENALPR			Carbamidomethyl (C)[7], Oxidation (M)[5]		Mascot
1511.8329	1511.7593	-0.0736	-49	276	288	ILDEANKVFLGHR					Mascot
1623.9581	1623.8678	-0.0903	-56	73	86	QLFQLVHEAGLKLK					Mascot
1646.781	1646.777	-0.004	-2	246	259	FFVDNGTYLTEQGR					Mascot
1646.781	1646.777	-0.004	-2	246	259	FFVDNGTYLTEQGR	95	100			Mascot
1668.7952	1668.797	0.0018	1	218	232	AAAAMVGHPEWEFPR					Mascot
1669.7349	1669.7777	0.0428	26	148	161	SAVQMYTDYMASFR					Mascot
1684.79	1684.7666	-0.0234	-14	218	232	AAAAMVGHPEWEFPR			Oxidation (M)[5]		Mascot
1685.7299	1685.7469	0.017	10	148	161	SAVQMYTDYMASFR			Oxidation (M)[5]		Mascot
1701.7247	1701.7129	-0.0118	-7	148	161	SAVQMYTDYMASFR			Oxidation (M)[5,10]		Mascot
1738.9095	1738.8765	-0.033	-19	396	411	NARPHGINKSGPPEHK					Mascot
1752.8916	1752.8417	-0.0499	-28	420	434	LSNQLVEGQNYVNFK					Mascot
2013.9778	2013.9774	-0.0004	0	304	321	VPSHAAEITAGYYNLHDR					Mascot
2013.9778	2013.9774	-0.0004	0	304	321	VPSHAAEITAGYYNLHDR	150	100			Mascot
2087.0557	2087.041	-0.0147	-7	130	147	NIEYLTLGVDDQPLFHGR					Mascot
2167.0537	2167.0452	-0.0085	-4	440	458	MHANLPHDPCVDPVAPL QR			Carbamidomethyl (C)[10]		Mascot
2183.0486	2183.0181	-0.0305	-14	440	458	MHANLPHDPCVDPVAPL QR			Carbamidomethyl (C)[10], Oxidation (M)[1]		Mascot
2269.2075	2269.1758	-0.0317	-14	166	187	EFLDAGVIVDIEVGLGPA					Mascot

GELR

2	beta amylase [Triticum aestivum]	gi|32400764	31099.9	8.6	9	350	100	14.654	309	100	

Peptide Information

Calc. Mass	Obsrv. Mass	± da	± ppm	Start Seq.	End Seq.	Sequence	Ion Score	C. I. %	Modification	Rank	Result Type
947.5057	947.4819	-0.0238	-25	137	144	DGYRPIAR					Mascot
1189.6154	1189.5902	-0.0252	-21	110	118	ISGIHWWYK					Mascot
1299.582	1299.5822	0.0002	0	151	161	ASLNFTCAEMR			Carbamidomethyl (C)[7]		Mascot
1315.5769	1315.552	-0.0249	-19	151	161	ASLNFTCAEMR			Carbamidomethyl (C)[7], Oxidation (M)[10]		Mascot
1326.6688	1326.6537	-0.0151	-11	200	210	YDPTAYNTILR					Mascot
1326.6688	1326.6537	-0.0151	-11	200	210	YDPTAYNTILR	66	99.998			Mascot
1474.6777	1474.698	0.0203	14	187	199	EGLNMACENALPR			Carbamidomethyl (C)[7]		Mascot
1490.6726	1490.6471	-0.0255	-17	187	199	EGLNMACENALPR			Carbamidomethyl (C)[7], Oxidation (M)[5]		Mascot
1511.8329	1511.7593	-0.0736	-49	91	103	ILDEANKVFLGHR					Mascot
1646.781	1646.777	-0.004	-2	61	74	FFVDNGTYLTEQGR					Mascot
1646.781	1646.777	-0.004	-2	61	74	FFVDNGTYLTEQGR	95	100			Mascot
1668.7952	1668.797	0.0018	1	33	47	AAAAMVGHPEWEFPR					Mascot
1684.79	1684.7666	-0.0234	-14	33	47	AAAAMVGHPEWEFPR			Oxidation (M)[5]		Mascot
2013.9778	2013.9774	-0.0004	0	119	136	VPSHAAEITAGYYNLHDR					Mascot
2013.9778	2013.9774	-0.0004	0	119	136	VPSHAAEITAGYYNLHDR	150	100			Mascot

3	putative cinnamyl alcohol dehydrogenase [Triticum urartu]	gi|474360235	39042.5	5.62	9	192	100	5.769	156	100	

Peptide Information

Calc. Mass	Obsrv. Mass	± da	± ppm	Start Seq.	End Seq.	Sequence	Ion Score	C. I. %	Modification	Rank	Result Type
1128.5983	1128.5863	-0.012	-11	175	184	HFGLMTPGLR					Mascot
1144.5933	1144.5651	-0.0282	-25	175	184	HFGLMTPGLR			Oxidation (M)[5]		Mascot
1253.5944	1253.5831	-0.0113	-9	109	118	ANVEQYCNKK			Carbamidomethyl (C)[7]		Mascot
1272.5677	1272.5724	0.0047	4	328	337	MDYVNQAFER					Mascot
1288.5627	1288.5341	-0.0286	-22	328	337	MDYVNQAFER			Oxidation (M)[1]		Mascot
1517.7563	1517.7496	-0.0067	-4	88	102	AGDVVGVGVIVGCCR			Carbamidomethyl (C)[13,14]		Mascot
1517.7563	1517.7496	-0.0067	-4	88	102	AGDVVGVGVIVGCCR	85	100	Carbamidomethyl (C)[13,14]		Mascot
1594.786	1594.7784	-0.0076	-5	18	31	DATGHLSPYTYTLR					Mascot
1594.786	1594.7784	-0.0076	-5	18	31	DATGHLSPYTYTLR	71	100			Mascot
1611.8271	1611.7799	-0.0472	-29	203	217	SMGHHVTVISSSNKK					Mascot
1665.9469	1665.8182	-0.1287	-77	185	202	GGILGLGGVGHMGVKVA			Oxidation (M)[12]		Mascot

Calc. Mass	Obsrv. Mass	± da	± ppm	Start Seq.	End Seq.	Sequence	Ion Score	C. I. %	Modification	Rank	Result Type
						K					
1738.8065	1738.8765	0.07	40	1	17	MGSVDASETTVTGWAAR					Mascot
1797.9276	1797.9135	-0.0141	-8	200	216	VAKSMGHHVTVISSSNK			Oxidation (M)[5]		Mascot

4	unnamed protein product [Triticum aestivum]	gi|257656445	39113.6	5.75	9	121	100	5.33	85	100	

Protein Group

cinnamyl alcohol dehydrogenase [Triticum aestivum]	gi|298162735	39113.6	5.75
unnamed protein product [Triticum aestivum]	gi|219971024	39113.6	5.75

Peptide Information

Calc. Mass	Obsrv. Mass	± da	± ppm	Start Seq.	End Seq.	Sequence	Ion Score	C. I. %	Modification	Rank	Result Type
1128.5983	1128.5863	-0.012	-11	175	184	HFGLMTPGLR					Mascot
1144.5933	1144.5651	-0.0282	-25	175	184	HFGLMTPGLR			Oxidation (M)[5]		Mascot
1253.5944	1253.5831	-0.0113	-9	109	118	ANVEQYCNKK			Carbamidomethyl (C)[7]		Mascot
1272.5677	1272.5724	0.0047	4	328	337	MDYVNQAFER					Mascot
1288.5627	1288.5341	-0.0286	-22	328	337	MDYVNQAFER			Oxidation (M)[1]		Mascot
1517.7563	1517.7496	-0.0067	-4	88	102	AGDVVGVGVIVGCCR			Carbamidomethyl (C)[13,14]		Mascot
1517.7563	1517.7496	-0.0067	-4	88	102	AGDVVGVGVIVGCCR	85	100	Carbamidomethyl (C)[13,14]		Mascot
1611.8271	1611.7799	-0.0472	-29	203	217	SMGHHVTVISSSNKK					Mascot
1649.8395	1649.772	-0.0675	-41	18	31	DATGHLSPYRYTLR					Mascot
1665.9469	1665.8182	-0.1287	-77	185	202	GGILGLGGVGHMGVKVA<br>K			Oxidation (M)[12]		Mascot
1738.8065	1738.8765	0.07	40	1	17	MGSVDASETTVTGWAAR					Mascot
1797.9276	1797.9135	-0.0141	-8	200	216	VAKSMGHHVTVISSSNK			Oxidation (M)[5]		Mascot

5	putative acyl transferase 3 [Triticum aestivum]	gi|151175355	48417.3	5.73	14	88	99.993	10.901	30	92.301	

Peptide Information

Calc. Mass	Obsrv. Mass	± da	± ppm	Start Seq.	End Seq.	Sequence	Ion Score	C. I. %	Modification	Rank	Result Type
925.4481	925.5078	0.0597	65	412	419	MLASCVTK			Carbamidomethyl (C)[5], Oxidation (M)[1]		Mascot
939.5258	939.513	-0.0128	-14	403	411	APLPLDGTR					Mascot
999.5081	999.5031	-0.005	-5	285	292	LCFFASVR			Carbamidomethyl (C)[2]		Mascot
1188.6194	1188.6051	-0.0143	-12	55	64	GLVESMHIFR					Mascot
1204.6144	1204.6002	-0.0142	-12	55	64	GLVESMHIFR			Oxidation (M)[6]		Mascot
1225.6729	1225.6733	0.0004	0	81	91	ALAFFYPLAGR					Mascot
1225.6729	1225.6733	0.0004	0	81	91	ALAFFYPLAGR	31	94.448			Mascot

Calc. Mass	Obsrv. Mass	± da	± ppm	Start Seq.	End Seq.	Sequence	Ion Score	C. I. %	Modification	Rank	Result Type
1244.5916	1244.585	-0.0066	-5	163	173	FTCGGFVMGLR			Carbamidomethyl (C)[3]		Mascot
1253.5903	1253.5831	-0.0072	-6	1	11	MSTTEAQSRAR			Oxidation (M)[1]		Mascot
1260.5864	1260.5579	-0.0285	-23	163	173	FTCGGFVMGLR			Carbamidomethyl (C)[3], Oxidation (M)[8]		Mascot
1311.6283	1311.6132	-0.0151	-12	321	331	SSVMEVIDMIR			Oxidation (M)[4,9]		Mascot
1399.6998	1399.609	-0.0908	-65	12	25	AGAAMASSSFKVTR			Oxidation (M)[5]		Mascot
1463.8005	1463.7959	-0.0046	-3	197	209	GLPEPAVKPVWDR					Mascot
1517.757	1517.7496	-0.0074	-5	335	346	QRMAVEFFQFAK			Oxidation (M)[3]		Mascot
1517.757	1517.7496	-0.0074	-5	335	346	QRMAVEFFQFAK			Oxidation (M)[3]		Mascot
1543.7792	1543.7543	-0.0249	-16	297	310	LDAGYYGNSIFPVK					Mascot
1623.8081	1623.8678	0.0597	37	321	334	SSVMEVIDMIREAK			Oxidation (M)[4]		Mascot
1680.7799	1680.8047	0.0248	15	174	189	FNHASADGMGAAQFIK			Oxidation (M)[9]		Mascot

6 putative acyl transferase 4 [Triticum aestivum] gi|151175357 46848.5 5.72 11 71 99.68 11.766 30 92.301

Peptide Information

Calc. Mass	Obsrv. Mass	± da	± ppm	Start Seq.	End Seq.	Sequence	Ion Score	C. I. %	Modification	Rank	Result Type
925.4481	925.5078	0.0597	65	397	404	MLASCVTK			Carbamidomethyl (C)[5], Oxidation (M)[1]		Mascot
939.5258	939.513	-0.0128	-14	388	396	APLPLDGTR					Mascot
999.5081	999.5031	-0.005	-5	270	277	LCFFASVR			Carbamidomethyl (C)[2]		Mascot
1188.6194	1188.6051	-0.0143	-12	40	49	GLVESMHIFR					Mascot
1204.6144	1204.6002	-0.0142	-12	40	49	GLVESMHIFR			Oxidation (M)[6]		Mascot
1225.6729	1225.6733	0.0004	0	66	76	ALAFFYPLAGR					Mascot
1225.6729	1225.6733	0.0004	0	66	76	ALAFFYPLAGR	31	94.448			Mascot
1244.5916	1244.585	-0.0066	-5	148	158	FTCGGFVMGLR			Carbamidomethyl (C)[3]		Mascot
1260.5864	1260.5579	-0.0285	-23	148	158	FTCGGFVMGLR			Carbamidomethyl (C)[3], Oxidation (M)[8]		Mascot
1479.7955	1479.7738	-0.0217	-15	182	194	GLPEPSVKPVWDR					Mascot
1517.757	1517.7496	-0.0074	-5	320	331	QRMAVEFFQFAK			Oxidation (M)[3]		Mascot
1517.757	1517.7496	-0.0074	-5	320	331	QRMAVEFFQFAK			Oxidation (M)[3]		Mascot
1543.7792	1543.7543	-0.0249	-16	282	295	LDAGYYGNSIFPVK					Mascot
1620.8336	1620.7993	-0.0343	-21	303	316	VLESSVMEVIDMIR					Mascot
1636.8285	1636.8065	-0.022	-13	303	316	VLESSVMEVIDMIR			Oxidation (M)[7]		Mascot
1652.8234	1652.7933	-0.0301	-18	303	316	VLESSVMEVIDMIR			Oxidation (M)[7,12]		Mascot
1680.7799	1680.8047	0.0248	15	159	174	FNHASADGMGAAQFIK			Oxidation (M)[9]		Mascot

7 beta-amylase, partial [Triticum aestivum] gi|451798942 15408.7 5.21 3 64 98.319 8.806 50 99.926

Peptide Information

Calc. Mass	Obsrv. Mass	± da	± ppm	Start Seq.	End Seq.	Sequence	Ion Score	C. I. %	Modification	Rank	Result Type

Calc. Mass	Obsrv. Mass	± da	± ppm	Start Seq.	End Seq.	Sequence	Ion Score	C. I. %	Modification	Rank	Result Type
1016.5564	1016.5454	-0.011	-11	29	36	LFGFTYLR					Mascot
1016.5564	1016.5454	-0.011	-11	29	36	LFGFTYLR	50	99.926			Mascot
1738.9095	1738.8765	-0.033	-19	13	28	NARPHGINKSGPPEHK					Mascot
2167.0537	2167.0452	-0.0085	-4	57	75	MHANLPHDPCVDPVAPLQR			Carbamidomethyl (C)[10]		Mascot
2183.0486	2183.0181	-0.0305	-14	57	75	MHANLPHDPCVDPVAPLQR			Carbamidomethyl (C)[10], Oxidation (M)[1]		Mascot

8 beta-amylase [Triticum aestivum] gi|1771782 56860.2 5.24 6 64 98.28 12.558 50 99.926

Protein Group

RecName: Full=Beta-amylase; AltName: Full=1,4-alpha-D-glucan maltohydrolase gi|3334120 56860.2 5.2399997711816

Peptide Information

Calc. Mass	Obsrv. Mass	± da	± ppm	Start Seq.	End Seq.	Sequence	Ion Score	C. I. %	Modification	Rank	Result Type
1016.5564	1016.5454	-0.011	-11	411	418	LFGFTYLR					Mascot
1016.5564	1016.5454	-0.011	-11	411	418	LFGFTYLR	50	99.926			Mascot
1225.606	1225.6733	0.0673	55	248	258	DNGTYLTEKGK					Mascot
1225.606	1225.6733	0.0673	55	248	258	DNGTYLTEKGK					Mascot
1297.6787	1297.6333	-0.0454	-35	210	221	YLEADFKAAAAK					Mascot
1669.7349	1669.7777	0.0428	26	147	160	TAVQMYADYMASFR			Oxidation (M)[5]		Mascot
1685.7299	1685.7469	0.017	10	147	160	TAVQMYADYMASFR			Oxidation (M)[5,10]		Mascot
2087.0557	2087.041	-0.0147	-7	129	146	NIEYLTLGVDDQPLFHGR					Mascot
2233.1348	2233.0786	-0.0562	-25	483	503	NTDLPVKDHTDVGDEVLVAPV					Mascot

9 10-deacetylbaccatin III 10-O-acetyltransferase [Triticum urartu] gi|474330353 51608.9 8.63 9 53 80.247 10.626 30 92.301

Peptide Information

Calc. Mass	Obsrv. Mass	± da	± ppm	Start Seq.	End Seq.	Sequence	Ion Score	C. I. %	Modification	Rank	Result Type
999.5081	999.5031	-0.005	-5	332	339	LCFFASVR			Carbamidomethyl (C)[2]		Mascot
1188.6194	1188.6051	-0.0143	-12	40	49	GLVESMHIFR					Mascot
1204.6144	1204.6002	-0.0142	-12	40	49	GLVESMHIFR			Oxidation (M)[6]		Mascot
1225.6729	1225.6733	0.0004	0	66	76	ALAFFYPLAGR					Mascot
1225.6729	1225.6733	0.0004	0	66	76	ALAFFYPLAGR	31	94.448			Mascot
1244.5916	1244.585	-0.0066	-5	210	220	FTCGGFVMGLR			Carbamidomethyl (C)[3]		Mascot
1260.5864	1260.5579	-0.0285	-23	210	220	FTCGGFVMGLR			Carbamidomethyl (C)[3], Oxidation (M)[8]		Mascot
1479.7955	1479.7738	-0.0217	-15	244	256	GLPEPSVKPVWDR					Mascot

1517.7272	1517.7496	0.0224	15	344	357	LDAGYYGNSSFPVK					Mascot
1517.757	1517.7496	-0.0074	-5	382	393	QRMAVEFFQFAK			Oxidation (M)[3]		Mascot
1620.8336	1620.7993	-0.0343	-21	365	378	VLESSVMEVIDMIR					Mascot
1636.8285	1636.8065	-0.022	-13	365	378	VLESSVMEVIDMIR			Oxidation (M)[7]		Mascot
1652.8234	1652.7933	-0.0301	-18	365	378	VLESSVMEVIDMIR			Oxidation (M)[7,12]		Mascot
1680.7799	1680.8047	0.0248	15	221	236	FNHASADGMGAAQFIK			Oxidation (M)[9]		Mascot

10	Beta-amylase 1, chloroplastic [Triticum urartu]	gi|474105092	31764	4.78	7	48	36.08	10.376	21	35.669

Peptide Information

Calc. Mass	Obsrv. Mass	± da	± ppm	Start Seq.	End Seq.	Sequence	Ion Score	C. I. %	Modification	Rank	Result Type
1016.5234	1016.5454	0.022	22	251	258	MVAFTYLR			Oxidation (M)[1]		Mascot
1016.5234	1016.5454	0.022	22	251	258	MVAFTYLR	21	35.669	Oxidation (M)[1]		Mascot
1224.6696	1224.5577	-0.1119	-91	221	233	AGVGLAGENALPR					Mascot
1263.5924	1263.6122	0.0198	16	2	15	AESAAAAGQSSSAR					Mascot
1426.7625	1426.7197	-0.0428	-30	272	283	FALFVTRMSQAR					Mascot
1647.7697	1647.7715	0.0018	1	259	271	MGPDLFQPDNWRR			Oxidation (M)[1]		Mascot
1649.8641	1649.772	-0.0921	-56	17	31	SGVPLFVMMPLDTVK			Oxidation (M)[8]		Mascot
1665.8591	1665.8182	-0.0409	-25	17	31	SGVPLFVMMPLDTVK			Oxidation (M)[8,9]		Mascot
1669.7235	1669.7777	0.0542	32	127	139	DQDLAYTDQCERR			Carbamidomethyl (C)[10]		Mascot

Gel Idx/Pos	269/K21	Instr./Gel Origin	BA2151/Sample Project 20140814	Process Status	Analysis Succeeded
Plate [#] Name	[1] Sample Project 20140814	Instrument Sample Name		Spectra	11

Rank	Protein Name	Accession No.	Protein MW	Protein PI	Pep. Count	Protein Score	Protein Score C. I. %	Intensity Matched	Total Ion Score	Total Ion C. I. %	Confirmed
1	putative NADP-dependent oxidoreductase P1 [Triticum urartu]	gi|473799043	38359.4	5.53	12	348	100	19.046	287	100	

Peptide Information

Calc. Mass	Obsrv. Mass	± da	± ppm	Start Seq.	End Seq.	Sequence	Ion Score	C. I. %	Modification	Rank	Result Type
885.5152	885.502	-0.0132	-15	339	346	QLVAVARE					Mascot
1008.5546	1008.5253	-0.0293	-29	271	278	NLFCIITK			Carbamidomethyl (C)[4]		Mascot
1188.6219	1188.6544	0.0325	27	212	221	EQDLDATLKR					Mascot
1223.5369	1223.5138	-0.0231	-19	201	210	FGFDDAFNYK					Mascot
1360.6454	1360.6564	0.011	8	296	306	KFEEEMAGYLK			Oxidation (M)[6]		Mascot
1413.6831	1413.6804	-0.0027	-2	49	59	NLYLSCDPYLR			Carbamidomethyl (C)[6]		Mascot
1413.6831	1413.6804	-0.0027	-2	49	59	NLYLSCDPYLR	65	99.998	Carbamidomethyl (C)[6]		Mascot
1528.6948	1528.6642	-0.0306	-20	179	193	ISGCYVVGSAGSDEK			Carbamidomethyl (C)[4]		Mascot
1656.8163	1656.7595	-0.0568	-34	49	61	NLYLSCDPYLRSR			Carbamidomethyl (C)[6]		Mascot
1688.7738	1688.8044	0.0306	18	282	295	MEGFIVTDHYGTYR					Mascot
1704.7687	1704.7454	-0.0233	-14	282	295	MEGFIVTDHYGTYR			Oxidation (M)[1]		Mascot
1704.7687	1704.7454	-0.0233	-14	282	295	MEGFIVTDHYGTYR	75	100	Oxidation (M)[1]		Mascot
2113.9746	2113.9597	-0.0149	-7	17	35	YVTGFPSEDDMELVPATAR			Oxidation (M)[11]		Mascot
2138.0547	2138.0662	0.0115	5	252	270	VSVCGLISQYNLEQSEGVR			Carbamidomethyl (C)[4]		Mascot
2138.0547	2138.0662	0.0115	5	252	270	VSVCGLISQYNLEQSEGVR	147	100	Carbamidomethyl (C)[4]		Mascot
2279.2395	2279.2256	-0.0139	-6	156	178	KGEYVFVSAASGAVGQLVGQLAK					Mascot

Rank	Protein Name	Accession No.	Protein MW	Protein PI	Pep. Count	Protein Score	Protein Score C. I. %	Intensity Matched	Total Ion Score	Total Ion C. I. %	Confirmed
2	putative NADP-dependent oxidoreductase P1 [Triticum urartu]	gi|474246662	38736.6	5.75	6	82	99.975	12.735	65	99.998	

Peptide Information

Calc. Mass	Obsrv. Mass	± da	± ppm	Start Seq.	End Seq.	Sequence	Ion Score	C. I. %	Modification	Rank	Result Type
885.5152	885.502	-0.0132	-15	342	349	QLVAVARE					Mascot
1223.5369	1223.5138	-0.0231	-19	204	213	FGFDDAFNYK					Mascot
1360.6454	1360.6564	0.011	8	299	309	KFEEEMAGYLK			Oxidation (M)[6]		Mascot
1413.6831	1413.6804	-0.0027	-2	52	62	NLYLSCDPYLR			Carbamidomethyl (C)[6]		Mascot

1413.6831	1413.6804	-0.0027	-2	52	62	NLYLSCDPYLR	65	99.998	Carbamidomethyl (C)[6]		Mascot
1528.6948	1528.6642	-0.0306	-20	182	196	ISGCYVVGSAGSDEK			Carbamidomethyl (C)[4]		Mascot
2046.9524	2047.0533	0.1009	49	283	298	IRMEGFIVMDHYSNYR			Oxidation (M)[3]		Mascot

3 unnamed protein product [Triticum aestivum] gi|257710966 38195.3 5.66 6 82 99.974 12.056 65 99.998

Peptide Information

Calc. Mass	Obsrv. Mass	± da	± ppm	Start Seq.	End Seq.	Sequence	Ion Score	C. I. %	Modification	Rank	Result Type
1008.5546	1008.5253	-0.0293	-29	268	275	NLFCIITK			Carbamidomethyl (C)[4]		Mascot
1223.5369	1223.5138	-0.0231	-19	198	207	FGFDDAFNYK					Mascot
1360.6454	1360.6564	0.011	8	293	303	KFEEEMAGYLK			Oxidation (M)[6]		Mascot
1413.6831	1413.6804	-0.0027	-2	46	56	NLYLSCDPYLR			Carbamidomethyl (C)[6]		Mascot
1413.6831	1413.6804	-0.0027	-2	46	56	NLYLSCDPYLR	65	99.998	Carbamidomethyl (C)[6]		Mascot
1528.6948	1528.6642	-0.0306	-20	176	190	ISGCYVVGSAGSDEK			Carbamidomethyl (C)[4]		Mascot
1656.8163	1656.7595	-0.0568	-34	46	58	NLYLSCDPYLRSR			Carbamidomethyl (C)[6]		Mascot

4 hypothetical protein TRIUR3_08615 [Triticum urartu] gi|474346849 84352.6 5.51 17 55 86.645 12.133

Peptide Information

Calc. Mass	Obsrv. Mass	± da	± ppm	Start Seq.	End Seq.	Sequence	Ion Score	C. I. %	Modification	Rank	Result Type
804.4614	804.4032	-0.0582	-72	467	473	NSVFLPK					Mascot
825.473	825.4481	-0.0249	-30	596	601	YHHKLK					Mascot
870.4832	870.5237	0.0405	47	736	742	NIILFNH					Mascot
890.4618	890.4373	-0.0245	-28	588	595	DASPIYPK					Mascot
993.5363	993.4908	-0.0455	-46	458	466	DTNVKAFAK					Mascot
998.4724	998.5615	0.0891	89	42	50	YMQSLGAGR			Oxidation (M)[2]		Mascot
1004.4571	1004.4809	0.0238	24	68	75	YYESDLSK					Mascot
1018.5164	1018.5104	-0.006	-6	701	709	EQSLSGIER					Mascot
1106.5585	1106.5085	-0.05	-45	498	507	EMLSTVAMPK					Mascot
1122.5533	1122.5563	0.003	3	498	507	EMLSTVAMPK			Oxidation (M)[2]		Mascot
1187.6631	1187.6718	0.0087	7	448	457	LQSLETLDIR					Mascot
1187.6631	1187.6718	0.0087	7	448	457	LQSLETLDIR					Mascot
1253.6372	1253.6788	0.0416	33	145	155	EEEVIGQPQPK					Mascot
1322.7614	1322.6727	-0.0887	-67	218	228	MLIAQLQQHLK					Mascot
1322.7614	1322.6727	-0.0887	-67	218	228	MLIAQLQQHLK					Mascot
1443.6421	1443.6581	0.016	11	650	661	QESCNESTLTFK			Carbamidomethyl (C)[4]		Mascot
1507.8777	1507.7378	-0.1399	-93	218	230	MLIAQLQQHLKGK					Mascot

Calc. Mass	Obsrv. Mass	± da	± ppm	Start Seq.	End Seq.	Sequence	Ion Score	C. I. %	Modification	Rank	Result Type
1622.7921	1622.849	0.0569	35	347	360	LSVHYEGQPHQAEK					Mascot
1704.8916	1704.7454	-0.1462	-86	76	91	LLIDVGEDHGGQPNLK					Mascot
1704.8916	1704.7454	-0.1462	-86	76	91	LLIDVGEDHGGQPNLK					Mascot
2021.0338	2021.0325	-0.0013	-1	92	109	AWIGLREESTEAAAFLEK					Mascot
2021.0338	2021.0325	-0.0013	-1	92	109	AWIGLREESTEAAAFLEK					Mascot

5 hypothetical protein TRIUR3_12046 [Triticum urartu] gi|474373380 94319.1 5.93 18 53 78.834 10.221

Peptide Information

Calc. Mass	Obsrv. Mass	± da	± ppm	Start Seq.	End Seq.	Sequence	Ion Score	C. I. %	Modification	Rank	Result Type
819.3478	819.3983	0.0505	62	734	739	EEQEER					Mascot
847.4706	847.4264	-0.0442	-52	424	431	VMGDKLGK					Mascot
857.4039	857.4737	0.0698	81	860	866	YDEGFVK					Mascot
863.4655	863.397	-0.0685	-79	424	431	VMGDKLGK			Oxidation (M)[2]		Mascot
870.5043	870.5237	0.0194	22	455	461	VEEPLRK					Mascot
960.5472	960.4789	-0.0683	-71	69	79	AVGGVSGAKSK					Mascot
1028.5194	1028.5176	-0.0018	-2	441	448	MIVDEHLR			Oxidation (M)[1]		Mascot
1134.5824	1134.601	0.0186	16	243	252	TNTPMLEKGK			Oxidation (M)[5]		Mascot
1320.6577	1320.595	-0.0627	-47	538	549	GGQTLSMVVQER			Oxidation (M)[7]		Mascot
1372.7155	1372.6547	-0.0608	-44	331	341	MVVNELRGPWR			Oxidation (M)[1]		Mascot
1375.6709	1375.759	0.0881	64	693	704	VMPQNVAEACIK			Carbamidomethyl (C)[10], Oxidation (M)[2]		Mascot
1443.7394	1443.6581	-0.0813	-56	369	380	SHVLHAGPWHFR					Mascot
1476.7588	1476.7444	-0.0144	-10	537	549	RGGQTLSMVVQER			Oxidation (M)[8]		Mascot
1507.7574	1507.7378	-0.0196	-13	784	796	RMPDGQDLVVSFK			Oxidation (M)[2]		Mascot
1551.7571	1551.7861	0.029	19	462	475	VIDTVETMLDGNTK			Oxidation (M)[8]		Mascot
1692.8334	1692.8475	0.0141	8	538	552	GGQTLSMVVQERESR			Oxidation (M)[7]		Mascot
1972.0573	1972.0367	-0.0206	-10	321	337	VLSPYPVDPKMVVNELR			Oxidation (M)[11]		Mascot
2016.2076	2016.1077	-0.0999	-50	296	314	APNKPIVINLEAALRAVAR					Mascot
2138.0813	2138.0662	-0.0151	-7	1	22	MEPAVAAPTPGWAQGK AAGGVR			Oxidation (M)[1]		Mascot
2138.0813	2138.0662	-0.0151	-7	1	22	MEPAVAAPTPGWAQGK AAGGVR			Oxidation (M)[1]		Mascot

6 hypothetical protein TRIUR3_13384 [Triticum urartu] gi|474396601 13366.8 5.48 8 51 64.055 13.438

Peptide Information

Calc. Mass	Obsrv. Mass	± da	± ppm	Start Seq.	End Seq.	Sequence	Ion Score	C. I. %	Modification	Rank	Result Type
807.4359	807.3952	-0.0407	-50	110	116	VADALYR					Mascot
857.5091	857.4737	-0.0354	-41	67	73	LVEILDR					Mascot

1187.6743	1187.6718	-0.0025	-2	67	76	LVEILDRSSR					Mascot
1187.6743	1187.6718	-0.0025	-2	67	76	LVEILDRSSR					Mascot
1372.6857	1372.6547	-0.031	-23	37	49	ITDEPPPPQGGHK					Mascot
1413.6501	1413.6804	0.0303	21	1	12	MAGVKQSCFEEK			Carbamidomethyl (C)[8]		Mascot
1413.6501	1413.6804	0.0303	21	1	12	MAGVKQSCFEEK			Carbamidomethyl (C)[8]		Mascot
1429.645	1429.6732	0.0282	20	1	12	MAGVKQSCFEEK			Carbamidomethyl (C)[8], Oxidation (M)[1]		Mascot
1507.6871	1507.7378	0.0507	34	14	28	TEAPTQGSSEQGTSK					Mascot
1528.7867	1528.6642	-0.1225	-80	37	50	ITDEPPPPQGGHKR					Mascot
1692.8036	1692.8475	0.0439	26	14	30	TEAPTQGSSEQGTSKGK					Mascot

7 Centromere-associated protein E [Triticum urartu] gi|474097328 99338.5 5.26 21 47 13.775 19.678

Peptide Information

Calc. Mass	Obsrv. Mass	± da	± ppm	Start Seq.	End Seq.	Sequence	Ion Score	C. I. %	Modification	Rank	Result Type
810.3992	810.415	0.0158	19	204	210	EGSYINK					Mascot
847.4705	847.4264	-0.0441	-52	839	845	AKIQEMK					Mascot
857.4628	857.4737	0.0109	13	227	233	ATHIPYR					Mascot
863.4655	863.397	-0.0685	-79	839	845	AKIQEMK			Oxidation (M)[6]		Mascot
873.4788	873.473	-0.0058	-7	287	293	NRLVDEK					Mascot
904.4258	904.4449	0.0191	21	848	855	ETDPLSDK					Mascot
905.476	905.4454	-0.0306	-34	841	847	IQEMKEK					Mascot
963.4564	963.4813	0.0249	26	539	546	LTAQCNEK			Carbamidomethyl (C)[5]		Mascot
981.5112	981.5152	0.004	4	631	638	DLINQNHK					Mascot
993.5475	993.4908	-0.0567	-57	279	286	RVEIYASR					Mascot
1058.5477	1058.5524	0.0047	4	331	339	QQLEEGQVK					Mascot
1058.5477	1058.5524	0.0047	4	331	339	QQLEEGQVK					Mascot
1107.5906	1107.5645	-0.0261	-24	280	288	VEIYASRNR					Mascot
1187.6168	1187.6718	0.055	46	227	236	ATHIPYRDSK					Mascot
1187.6168	1187.6718	0.055	46	227	236	ATHIPYRDSK					Mascot
1413.7155	1413.6804	-0.0351	-25	677	688	ELLIAQEMAHSR			Oxidation (M)[8]		Mascot
1413.7155	1413.6804	-0.0351	-25	677	688	ELLIAQEMAHSR			Oxidation (M)[8]		Mascot
1424.7454	1424.6902	-0.0552	-39	465	478	MLAGEIAFGTSSLK					Mascot
1429.707	1429.6732	-0.0338	-24	620	630	YQHLEVIEENR					Mascot
1551.7068	1551.7861	0.0793	51	158	170	VREDAQMIESSDR			Oxidation (M)[7]		Mascot
1622.8132	1622.849	0.0358	22	493	505	NQIENLEHEIQQK					Mascot
1656.8665	1656.7595	-0.107	-65	554	567	SADNRVLQEQLQQK					Mascot

1760.9146	1761.0294	0.1148	65	315	330	RGMLGGASQEEIMILR						Mascot
1972.0433	1972.0367	-0.0066	-3	2	19	GLCRGAVASIPTDLSWIR			Carbamidomethyl (C)[3]			Mascot
2016.0835	2016.1077	0.0242	12	752	769	KELSLENDLAGMWVLVAK						Mascot

8 hypothetical protein TRIUR3_18216 [Triticum urartu] gi|474403174 102897.7 9.04 24 47 7.608 18.269

Peptide Information

Calc. Mass	Obsrv. Mass	± da	± ppm	Start Seq.	End Seq.	Sequence	Ion Score	C. I. %	Modification	Rank	Result Type
813.3777	813.4261	0.0484	60	664	669	WSYTEK					Mascot
815.4873	815.4124	-0.0749	-92	43	49	DIIDVIK					Mascot
832.4523	832.4285	-0.0238	-29	590	596	KEAITDR					Mascot
855.5298	855.4562	-0.0736	-86	191	198	APIEGLKK					Mascot
866.411	866.4102	-0.0008	-1	325	331	LMQMESK					Mascot
868.4444	868.4597	0.0153	18	201	208	TTITSSMK					Mascot
902.4578	902.4393	-0.0185	-20	124	131	ALNAEQEK					Mascot
944.5159	944.493	-0.0229	-24	638	645	LAKLNDDR					Mascot
970.5833	970.5104	-0.0729	-75	804	811	GKWLPTLR					Mascot
1031.5116	1031.5057	-0.0059	-6	308	315	EQQLTQER					Mascot
1127.4708	1127.5574	0.0866	77	795	803	CCSEIETTK			Carbamidomethyl (C)[1,2]		Mascot
1193.5872	1193.6071	0.0199	17	680	688	IWEMEKDVK			Oxidation (M)[4]		Mascot
1321.6271	1321.5924	-0.0347	-26	163	173	AQFLEAQEEEK					Mascot
1322.6335	1322.6727	0.0392	30	693	704	DVAGAAKEYENR					Mascot
1322.6335	1322.6727	0.0392	30	693	704	DVAGAAKEYENR					Mascot
1375.67	1375.759	0.089	65	223	234	EVTDDEIQLSAR					Mascot
1413.7557	1413.6804	-0.0753	-53	210	221	IANQINQNTNKR					Mascot
1413.7836	1413.6804	-0.1032	-73	73	84	LTPIQLLEETEK					Mascot
1474.7148	1474.7662	0.0514	35	856	867	SGQFLPFCISYR			Carbamidomethyl (C)[8]		Mascot
1476.7792	1476.7444	-0.0348	-24	777	789	EIVSISTKLEDDK					Mascot
1501.6952	1501.7302	0.035	23	281	293	DEMTQLTNDIGHK					Mascot
1633.8102	1633.8066	-0.0036	-2	561	574	NVEGMEEALKELQK			Oxidation (M)[5]		Mascot
1703.905	1703.7974	-0.1076	-63	868	881	SKILQTAHFGWLMR			Oxidation (M)[13]		Mascot
2114.0547	2113.9597	-0.095	-45	281	299	DEMTQLTNDIGHKISGINK					Mascot
2279.1853	2279.2256	0.0403	18	359	378	NMFRGEVYGPVLLEVNVQSK					Mascot

9 hypothetical protein TRIUR3_09611 [Triticum urartu] gi|474097349 8900.5 5.7 8 44 0 2.098

Peptide Information

Calc. Mass	Obsrv. Mass	± da	± ppm	Start	End	Sequence	Ion	C. I. %	Modification	Rank	Result Type

				Seq.	Seq.		Score				
832.4523	832.4285	-0.0238	-29	2	9	AEVSSALR					Mascot
902.4955	902.4393	-0.0562	-62	59	65	QQPHHKK					Mascot
963.4927	963.4813	-0.0114	-12	1	9	MAEVSSALR					Mascot
1106.5762	1106.5085	-0.0677	-61	23	31	LELSLQMEK			Oxidation (M)[7]		Mascot
1122.4984	1122.5563	0.0579	52	68	78	LVSSAAAMEED					Mascot
1262.6774	1262.6157	-0.0617	-49	22	31	RLELSLQMEK			Oxidation (M)[8]		Mascot
1321.6304	1321.5924	-0.038	-29	66	78	AKLVSSAAAMEED					Mascot
1375.725	1375.759	0.034	25	23	33	LELSLQMEKER					Mascot

10 hypothetical protein TRIUR3_07605 [Triticum urartu] gi|474443810 36454.2 6.43 11 43 0 3.291

Peptide Information

Calc. Mass	Obsrv. Mass	± da	± ppm	Start Seq.	End Seq.	Sequence	Ion Score	C. I. %	Modification	Rank	Result Type
807.3917	807.3952	0.0035	4	144	150	MALEEAK			Oxidation (M)[1]		Mascot
815.4774	815.4124	-0.065	-80	111	116	VWLLER					Mascot
832.441	832.4285	-0.0125	-15	182	188	LENESLK					Mascot
849.4611	849.4046	-0.0565	-67	251	258	VMAAASRK			Oxidation (M)[2]		Mascot
944.4737	944.493	0.0193	20	315	321	FGDFFRR					Mascot
1107.5793	1107.5645	-0.0148	-13	240	250	LSAAAFLDSGR					Mascot
1205.6372	1205.6465	0.0093	8	117	127	EVATAKQTETK					Mascot
1360.693	1360.6564	-0.0366	-27	222	233	ACAAGLLLEEWK			Carbamidomethyl (C)[2]		Mascot
1507.7057	1507.7378	0.0321	21	168	181	DAVAGKEDAMESLR			Oxidation (M)[10]		Mascot
1720.9242	1720.7665	-0.1577	-92	2	17	AIGSGQEFHPGRLVPR					Mascot
2113.9819	2113.9597	-0.0222	-11	48	66	AQHVLATSTTTYSMQSSER			Oxidation (M)[14]		Mascot

Gel Idx/Pos	270/K22	Instr./Gel Origin	BA2151/Sample Project 20140814	Process Status	Analysis Succeeded
Plate [#] Name	[1] Sample Project 20140814	Instrument Sample Name		Spectra	11

Rank	Protein Name	Accession No.	Protein MW	Protein PI	Pep. Count	Protein Score	Protein Score C. I. %	Intensity Matched	Total Ion Score	Total Ion C. I. %	Confirmed
1	cytosolic 3-phosphoglycerate kinase [Triticum urartu]	gi|28172905	31371.7	4.98	16	650	100	55.763	539	100	

Protein Group

cytosolic 3-phosphoglycerate kinase [Triticum aestivum]	gi|28172911	31371.7	4.9800 000190 7349

Peptide Information

Calc. Mass	Obsrv. Mass	± da	± ppm	Start Seq.	End Seq.	Sequence	Ion Score	C. I. %	Modification	Rank	Result Type
1056.6565	1056.6548	-0.0017	-2	1	9	FSLKPLVPR					Mascot
1056.6565	1056.6548	-0.0017	-2	1	9	FSLKPLVPR	60	99.993			Mascot
1074.6306	1074.6031	-0.0275	-26	118	128	KPFAAIVGGSK					Mascot
1335.6063	1335.6732	0.0669	50	52	62	EEEKNDPEFAK					Mascot
1388.7421	1388.7196	-0.0225	-16	105	117	ELDYLVGAVANPK					Mascot
1509.8247	1509.8083	-0.0164	-11	92	104	FLRPSVAGFLMQK			Oxidation (M)[11]		Mascot
1509.8247	1509.8083	-0.0164	-11	92	104	FLRPSVAGFLMQK	16	0	Oxidation (M)[11]		Mascot
1573.8433	1573.8103	-0.033	-21	276	292	GVTTIIGGGDSVAAVEK					Mascot
1720.9956	1721.0206	0.025	15	32	48	LAAALPDGGVLLLENVR					Mascot
1720.9956	1721.0206	0.025	15	32	48	LAAALPDGGVLLLENVR	152	100			Mascot
1769.8568	1769.8505	-0.0063	-4	243	257	TVIWNGPMGVFEFEK			Oxidation (M)[8]		Mascot
1802.9762	1802.8641	-0.1121	-62	143	158	VDILILGGGMIFTFYK			Oxidation (M)[10]		Mascot
1919.9611	1919.9871	0.026	14	64	81	LASVADLYVNDAFGTAH R					Mascot
1919.9611	1919.9871	0.026	14	64	81	LASVADLYVNDAFGTAH R	148	100			Mascot
2048.0559	2048.0833	0.0274	13	63	81	KLASVADLYVNDAFGTAH R					Mascot
2048.0559	2048.0833	0.0274	13	63	81	KLASVADLYVNDAFGTAH R	163	100			Mascot
2089.1274	2089.1179	-0.0095	-5	167	185	SLVEEDKLELATSLIETAK					Mascot
2102.1379	2102.135	-0.0029	-1	191	210	LLLPTDVVVADKFAADAE SK					Mascot
2159.2224	2159.1672	-0.0552	-26	32	51	LAAALPDGGVLLLENVRF YK					Mascot
2268.1582	2268.1665	0.0083	4	211	232	IVPATAIPDGWMGLDVGP DSIK			Oxidation (M)[12]		Mascot
2446.2092	2446.2239	0.0147	6	10	31	LSELLGLEVVMAPDCIGE EVEK			Carbamidomethyl (C)[15], Oxidation (M)[11]		Mascot

Rank	Protein Name	Accession No.	Protein MW	Protein PI	Pep. Count	Protein Score	Protein Score C. I. %	Intensity Matched	Total Ion Score	Total Ion C. I. %	Confirmed
2	Phosphoglycerate kinase, cytosolic [Triticum urartu]	gi|473781647	45286.1	5.9	18	646	100	56.039	539	100	

Peptide Information

Calc. Mass	Obsrv. Mass	± da	± ppm	Start Seq.	End Seq.	Sequence	Ion Score	C. I. %	Modification	Rank	Result Type
1056.6565	1056.6548	-0.0017	-2	75	83	FSLKPLVPR					Mascot
1056.6565	1056.6548	-0.0017	-2	75	83	FSLKPLVPR	60	99.993			Mascot
1074.6306	1074.6031	-0.0275	-26	192	202	KPFAAIVGGSK					Mascot
1089.5786	1089.5549	-0.0237	-22	6	16	SVGTLGEADLK					Mascot
1298.6587	1298.6323	-0.0264	-20	24	35	ADLNVPLDDAQK					Mascot
1335.6063	1335.6732	0.0669	50	126	136	EEEKNDPEFAK					Mascot
1388.7421	1388.7196	-0.0225	-16	179	191	ELDYLVGAVANPK					Mascot
1509.8247	1509.8083	-0.0164	-11	166	178	FLRPSVAGFLMQK			Oxidation (M)[11]		Mascot
1509.8247	1509.8083	-0.0164	-11	166	178	FLRPSVAGFLMQK	16	0	Oxidation (M)[11]		Mascot
1573.8433	1573.8103	-0.033	-21	362	378	GVTTIIGGGDSVAAVEK					Mascot
1720.9956	1721.0206	0.025	15	106	122	LAAALPDGGVLLLENVR					Mascot
1720.9956	1721.0206	0.025	15	106	122	LAAALPDGGVLLLENVR	152	100			Mascot
1769.8568	1769.8505	-0.0063	-4	317	331	TVIWNGPMGVFEFEK			Oxidation (M)[8]		Mascot
1802.9762	1802.8641	-0.1121	-62	217	232	VDILILGGGMIFTFYK			Oxidation (M)[10]		Mascot
1919.9611	1919.9871	0.026	14	138	155	LASVADLYVNDAFGTAH R					Mascot
1919.9611	1919.9871	0.026	14	138	155	LASVADLYVNDAFGTAH R	148	100			Mascot
2048.0559	2048.0833	0.0274	13	137	155	KLASVADLYVNDAFGTAH R					Mascot
2048.0559	2048.0833	0.0274	13	137	155	KLASVADLYVNDAFGTAH R	163	100			Mascot
2089.1274	2089.1179	-0.0095	-5	241	259	SLVEEDKLELATSLIETAK					Mascot
2102.1379	2102.135	-0.0029	-1	265	284	LLLPTDVVVADKFAADAE SK					Mascot
2159.2224	2159.1672	-0.0552	-26	106	125	LAAALPDGGVLLLENVRF YK					Mascot
2268.1582	2268.1665	0.0083	4	285	306	IVPATAIPDGWMGLDVGP DSIK			Oxidation (M)[12]		Mascot
2446.2092	2446.2239	0.0147	6	84	105	LSELLGLEVVMAPDCIGE EVEK			Carbamidomethyl (C)[15], Oxidation (M)[11]		Mascot

3	cytosolic 3-phosphoglycerate kinase [Triticum aestivum]	gi|28172909	31320.7	4.91	16	634	100	55.451	524	100

Peptide Information

Calc. Mass	Obsrv. Mass	± da	± ppm	Start Seq.	End Seq.	Sequence	Ion Score	C. I. %	Modification	Rank	Result Type
1056.6565	1056.6548	-0.0017	-2	1	9	FSLKPLVPR					Mascot
1056.6565	1056.6548	-0.0017	-2	1	9	FSLKPLVPR	60	99.993			Mascot
1074.6306	1074.6031	-0.0275	-26	118	128	KPFAAIVGGSK					Mascot
1335.6063	1335.6732	0.0669	50	52	62	EEEKNDPEFAK					Mascot

Calc. Mass	Obsrv. Mass	± da	± ppm	Start Seq.	End Seq.	Sequence	Ion Score	C. I. %	Modification	Rank	Result Type
1388.7421	1388.7196	-0.0225	-16	105	117	ELDYLVGAVANPK					Mascot
1500.759	1500.7413	-0.0177	-12	92	104	FLMPSVAGFLMQK			Oxidation (M)[3,11]		Mascot
1573.8433	1573.8103	-0.033	-21	276	292	GVTTIIGGGDSVAAVEK					Mascot
1720.9956	1721.0206	0.025	15	32	48	LAAALPDGGVLLLENVR					Mascot
1720.9956	1721.0206	0.025	15	32	48	LAAALPDGGVLLLENVR	152	100			Mascot
1769.8568	1769.8505	-0.0063	-4	243	257	TVIWNGPMGVFEFEK			Oxidation (M)[8]		Mascot
1802.9762	1802.8641	-0.1121	-62	143	158	VDILILGGGMIFTFYK			Oxidation (M)[10]		Mascot
1919.9611	1919.9871	0.026	14	64	81	LASVADLYVNDAFGTAHR					Mascot
1919.9611	1919.9871	0.026	14	64	81	LASVADLYVNDAFGTAHR	148	100			Mascot
2048.0559	2048.0833	0.0274	13	63	81	KLASVADLYVNDAFGTAHR					Mascot
2048.0559	2048.0833	0.0274	13	63	81	KLASVADLYVNDAFGTAHR	163	100			Mascot
2089.1274	2089.1179	-0.0095	-5	167	185	SLVEEDKLELATSLIETAK					Mascot
2102.1379	2102.135	-0.0029	-1	191	210	LLLPTDVVVADKFAADAESK					Mascot
2159.2224	2159.1672	-0.0552	-26	32	51	LAAALPDGGVLLLENVRFYK					Mascot
2268.1582	2268.1665	0.0083	4	211	232	IVPATAIPDGWMGLDVGPDSIK			Oxidation (M)[12]		Mascot
2446.2092	2446.2239	0.0147	6	10	31	LSELLGLEVVMAPDCIGEEVEK			Carbamidomethyl (C)[15], Oxidation (M)[11]		Mascot

4	unnamed protein product [Triticum aestivum]	gi|298541521	42152.7	5.64	19	602	100	52.168	479	100

Protein Group

RecName: Full=Phosphoglycerate kinase, cytosolic	gi|129916	42152.7	5.6399998664856
unnamed protein product [Triticum aestivum]	gi|21835	42152.7	5.6399998664856

Peptide Information

Calc. Mass	Obsrv. Mass	± da	± ppm	Start Seq.	End Seq.	Sequence	Ion Score	C. I. %	Modification	Rank	Result Type
1030.6409	1030.6354	-0.0055	-5	75	83	FSLKPLVAR					Mascot
1074.6306	1074.6031	-0.0275	-26	192	202	KPFAAIVGGSK					Mascot
1089.5786	1089.5549	-0.0237	-22	6	16	SVGTLGEADLK					Mascot
1298.6587	1298.6323	-0.0264	-20	24	35	ADLNVPLDDAQK					Mascot
1335.6063	1335.6732	0.0669	50	126	136	EEEKNDPEFAK					Mascot
1388.7421	1388.7196	-0.0225	-16	179	191	ELDYLVGAVANPK					Mascot
1509.8247	1509.8083	-0.0164	-11	166	178	FLRPSVAGFLMQK			Oxidation (M)[11]		Mascot
1509.8247	1509.8083	-0.0164	-11	166	178	FLRPSVAGFLMQK	16	0	Oxidation (M)[11]		Mascot
1573.8433	1573.8103	-0.033	-21	350	366	GVTTIIGGGDSVAAVEK					Mascot

1720.9956	1721.0206	0.025	15	106	122	LAAALPDGGVLLLENVR					Mascot
1720.9956	1721.0206	0.025	15	106	122	LAAALPDGGVLLLENVR	152	100			Mascot
1769.8568	1769.8505	-0.0063	-4	317	331	TVIWNGPMGVFEFEK			Oxidation (M)[8]		Mascot
1802.9762	1802.8641	-0.1121	-62	217	232	VDILILGGGMIFTFYK			Oxidation (M)[10]		Mascot
1919.9611	1919.9871	0.026	14	138	155	LASVADLYVNDAFGTAH R					Mascot
1919.9611	1919.9871	0.026	14	138	155	LASVADLYVNDAFGTAH R	148	100			Mascot
2048.0559	2048.0833	0.0274	13	137	155	KLASVADLYVNDAFGTAH R					Mascot
2048.0559	2048.0833	0.0274	13	137	155	KLASVADLYVNDAFGTAH R	163	100			Mascot
2089.1274	2089.1179	-0.0095	-5	241	259	SLVEEDKLELATSLIETAK					Mascot
2102.1379	2102.135	-0.0029	-1	265	284	LLLPTDVVVADKFAADAE SK					Mascot
2159.2224	2159.1672	-0.0552	-26	106	125	LAAALPDGGVLLLENVRF YK					Mascot
2268.1582	2268.1665	0.0083	4	285	306	IVPATAIPDGWMGLDVGP DSIK			Oxidation (M)[12]		Mascot
2446.2092	2446.2239	0.0147	6	84	105	LSELLGLEVVMAPDCIGE EVEK			Carbamidomethyl (C)[15], Oxidation (M)[11]		Mascot
2878.4866	2878.5269	0.0403	14	373	401	MSHISTGGGASLELLEGK PLPGVLALDEA			Oxidation (M)[1]		Mascot

5	Phosphoglycerate kinase, cytosolic [Triticum urartu]	gi|473995124	55052.6	5.45	12	519	100	50.49	472	100	

Peptide Information

Calc. Mass	Obsrv. Mass	± da	± ppm	Start Seq.	End Seq.	Sequence	Ion Score	C. I. %	Modification	Rank	Result Type
1074.5765	1074.6031	0.0266	25	463	472	APSPIVMFGR					Mascot
1176.7212	1176.6947	-0.0265	-23	102	112	VVLASHLGRPK					Mascot
1335.6063	1335.6732	0.0669	50	169	179	EEEKNDPEFAK					Mascot
1509.8247	1509.8083	-0.0164	-11	209	221	YLRPAVAGFLMQK			Oxidation (M)[11]		Mascot
1509.8247	1509.8083	-0.0164	-11	209	221	YLRPAVAGFLMQK	9	0	Oxidation (M)[11]		Mascot
1573.8433	1573.8103	-0.033	-21	393	409	GVTTIIGGGDSVAAVEK					Mascot
1720.9956	1721.0206	0.025	15	149	165	LAAALPDGGVLLLENVR					Mascot
1720.9956	1721.0206	0.025	15	149	165	LAAALPDGGVLLLENVR	152	100			Mascot
1769.8568	1769.8505	-0.0063	-4	360	374	TVIWNGPMGVFEFEK			Oxidation (M)[8]		Mascot
1802.9762	1802.8641	-0.1121	-62	260	275	VDILILGGGMIYTFYK					Mascot
1919.9611	1919.9871	0.026	14	181	198	LASVADLYVNDAFGTAH R					Mascot
1919.9611	1919.9871	0.026	14	181	198	LASVADLYVNDAFGTAH R	148	100			Mascot
2048.0559	2048.0833	0.0274	13	180	198	KLASVADLYVNDAFGTAH R					Mascot
2048.0559	2048.0833	0.0274	13	180	198	KLASVADLYVNDAFGTAH R	163	100			Mascot
2159.2224	2159.1672	-0.0552	-26	149	168	LAAALPDGGVLLLENVRF					Mascot

2382.2373	2382.2368	-0.0005	0	328	350	YK IVPASAIPDGWMGLDVGP DSIKK			Oxidation (M)[12]		Mascot

6	reversibly glycosylated polypeptide [Triticum aestivum]	gi|4158232	41985	5.82	9	70	99.606	2.995	36	98.174

Protein Group

glycosyltransferase 75 [Triticum aestivum]	gi|301072492	41985	5.8200 001716 6138

Peptide Information

Calc. Mass	Obsrv. Mass	± da	± ppm	Start Seq.	End Seq.	Sequence	Ion Score	C. I. %	Modification	Rank	Result Type
1118.6456	1118.6246	-0.021	-19	199	208	YVDAVLTIPK					Mascot
1201.6365	1201.6372	0.0007	1	266	275	TGLPYLWHSK					Mascot
1320.7345	1320.6322	-0.1023	-77	78	89	ILGPKASCISFK			Carbamidomethyl (C)[8]		Mascot
1483.7461	1483.7449	-0.0012	-1	315	326	CYISLSEQVKEK			Carbamidomethyl (C)[1]		Mascot
1501.6958	1501.712	0.0162	11	61	72	VPEGFDYELYNR					Mascot
1501.6958	1501.712	0.0162	11	61	72	VPEGFDYELYNR	36	98.174			Mascot
1580.6655	1580.7913	0.1258	80	90	102	DSACRCFGYMVSK			Carbamidomethyl (C)[4,6]		Mascot
1596.6604	1596.7736	0.1132	71	90	102	DSACRCFGYMVSK			Carbamidomethyl (C)[4,6], Oxidation (M)[10]		Mascot
1710.9637	1710.8643	-0.0994	-58	2	19	AGTVTVPGSSVPSTPLLK					Mascot
2086.9785	2087.0811	0.1026	49	308	324	ECDTVQKCYISLSEQVK			Carbamidomethyl (C)[2,8]		Mascot
2292.1335	2292.1704	0.0369	16	133	151	NLLSPSTPFFFNTLYDPY R					Mascot

7	Protein SRG1 [Triticum urartu]	gi|473874548	41717.2	5.05	14	70	99.537	3.633

Peptide Information

Calc. Mass	Obsrv. Mass	± da	± ppm	Start Seq.	End Seq.	Sequence	Ion Score	C. I. %	Modification	Rank	Result Type
963.5258	963.517	-0.0088	-9	343	350	TVDFLNVR					Mascot
1110.6881	1110.6849	-0.0032	-3	43	52	LVIPIVDVSR					Mascot
1198.5488	1198.5521	0.0033	3	117	126	FQNEGYGIDR					Mascot
1201.6477	1201.6372	-0.0105	-9	152	160	LQFWPQNLR					Mascot
1217.6307	1217.6416	0.0109	9	106	116	QKCGNVIDGVK			Carbamidomethyl (C)[3]		Mascot
1238.6483	1238.679	0.0307	25	173	183	VTMDVLKAMAK			Oxidation (M)[3,9]		Mascot
1287.7096	1287.6227	-0.0869	-67	96	105	KFFHLPLEEK					Mascot
1335.6791	1335.6732	-0.0059	-4	85	95	EFLDEILEATR					Mascot
1426.7325	1426.7292	-0.0033	-2	140	150	LWLQLQPEDER					Mascot
1463.774	1463.7686	-0.0054	-4	85	96	EFLDEILEATRK					Mascot
1580.7777	1580.7913	0.0136	9	292	304	LSLAMFYQPEPER					Mascot

Calc. Mass	Obsrv. Mass	± da	± ppm	Start Seq.	End Seq.	Sequence	Ion Score	C. I. %	Modification	Rank	Result Type
1596.7727	1596.7736	0.0009	1	292	304	LSLAMFYQPEPER			Oxidation (M)[5]		Mascot
1738.952	1738.8728	-0.0792	-46	9	25	LVQMVAADVGLVAPPSR			Oxidation (M)[4]		Mascot
1895.932	1895.9723	0.0403	21	184	199	LLNQEEGFFINMVGER					Mascot
1911.927	1911.9336	0.0066	3	184	199	LLNQEEGFFINMVGER			Oxidation (M)[12]		Mascot
2382.2585	2382.2368	-0.0217	-9	43	64	LVIPIVDVSRVAMPDDVE EAAK			Oxidation (M)[13]		Mascot

8	Serpin-Z2B [Triticum urartu]	gi|473793747	45225.7	6.03	5	64	98.394	1.919	54	99.973

Peptide Information

Calc. Mass	Obsrv. Mass	± da	± ppm	Start Seq.	End Seq.	Sequence	Ion Score	C. I. %	Modification	Rank	Result Type
925.5214	925.5118	-0.0096	-10	11	18	LSIAHQTR					Mascot
1192.5382	1192.5385	0.0003	0	199	208	GAWTDQFDPR					Mascot
1385.7019	1385.7043	0.0024	2	176	188	DILPAGSIDNNTR					Mascot
1483.7614	1483.7449	-0.0165	-11	257	268	QFSMYILLPEAR			Oxidation (M)[4]		Mascot
1665.8595	1665.8763	0.0168	10	278	291	LSAEPEFLEQHIPR					Mascot
1665.8595	1665.8763	0.0168	10	278	291	LSAEPEFLEQHIPR	54	99.973			Mascot

9	Allene oxide synthase-lipoxygenase protein [Triticum urartu]	gi|474213407	49109.9	5.61	14	64	98.114	3.767	

Peptide Information

Calc. Mass	Obsrv. Mass	± da	± ppm	Start Seq.	End Seq.	Sequence	Ion Score	C. I. %	Modification	Rank	Result Type
1217.5508	1217.6416	0.0908	75	180	189	FDEGKEMYAK					Mascot
1224.618	1224.5302	-0.0878	-72	56	65	MYEDIPSLLK			Oxidation (M)[1]		Mascot
1233.5457	1233.6392	0.0935	76	180	189	FDEGKEMYAK			Oxidation (M)[7]		Mascot
1233.5457	1233.6392	0.0935	76	180	189	FDEGKEMYAK			Oxidation (M)[7]		Mascot
1287.556	1287.6227	0.0667	52	81	92	HSNSLSADDDAR					Mascot
1436.7678	1436.746	-0.0218	-15	331	341	SLIYEVCQRLR			Carbamidomethyl (C)[7]		Mascot
1463.7866	1463.7686	-0.018	-12	148	160	APHVRDAVWGSLR					Mascot
1596.7761	1596.7736	-0.0025	-2	413	427	VEDGGYLGALCLAMK			Carbamidomethyl (C)[11]		Mascot
1612.771	1612.79	0.019	12	413	427	VEDGGYLGALCLAMK			Carbamidomethyl (C)[11], Oxidation (M)[14]		Mascot
1742.8053	1742.9684	0.1631	94	81	96	HSNSLSADDDARLDAR					Mascot
1758.9783	1758.9395	-0.0388	-22	23	39	IVGNLSANLAITTMNVK					Mascot
1903.0681	1903.0101	-0.058	-30	22	39	KIVGNLSANLAITTMNVK			Oxidation (M)[15]		Mascot
1909.9291	1909.9153	-0.0138	-7	222	237	DESDTRPLLFLADDFR					Mascot
1918.0546	1917.9778	-0.0768	-40	240	255	VEVPDGVRYVFQLQLR					Mascot
2013.1115	2013.045	-0.0665	-33	102	120	ILSDDGEVPLLDLTLKSG K					Mascot

2382.1865	2382.2368	0.0503	21	405	427	AVAMPGVKVEDGGYLGA LCLAMK			Carbamidomethyl (C)[19], Oxidation (M)[4,22]		Mascot
2413.2334	2413.3105	0.0771	32	126	146	TIADFATWLVCGLPAREE HVK			Carbamidomethyl (C)[11]		Mascot

10	serpin-N3.2 [Triticum aestivum]	gi|379060943	43026.4	5.18	4	62	97.078	1.654	54	99.973	

Protein Group

RecName: Full=Serpin-Z2B; AltName: Full=TriaeZ2b; AltName: Full=WSZ2b; AltName: Full=WZS3	gi|75279909	43011.4	5.1799 998283 3862
serpin [Triticum aestivum]	gi|1885346	43011.4	5.1799 998283 3862

Peptide Information

Calc. Mass	Obsrv. Mass	± da	± ppm	Start Seq.	End Seq.	Sequence	Ion Score	C. I. %	Modification	Rank	Result Type
925.5214	925.5118	-0.0096	-10	11	18	LSIAHQTR					Mascot
1192.5382	1192.5385	0.0003	0	182	191	GAWTDQFDPR					Mascot
1372.7068	1372.7031	-0.0037	-3	159	171	DILPAGSIDNTTR					Mascot
1665.8595	1665.8763	0.0168	10	261	274	LSAEPEFLEQHIPR					Mascot
1665.8595	1665.8763	0.0168	10	261	274	LSAEPEFLEQHIPR	54	99.973			Mascot

Gel Idx/Pos	271/K23	Instr./Gel Origin	BA2151/Sample Project 20140814	Process Status	Analysis Succeeded
Plate [#] Name	[1] Sample Project 20140814	Instrument Sample Name		Spectra	11

Rank	Protein Name	Accession No.	Protein MW	Protein PI	Pep. Count	Protein Score	Protein Score C. I. %	Intensity Matched	Total Ion Score	Total Ion C. I. %	Confirmed
1	Beta-amylase [Triticum urartu]	gi|474451266	58995	5.34	10	276	100	23.259	247	100	

Peptide Information

Calc. Mass	Obsrv. Mass	± da	± ppm	Start Seq.	End Seq.	Sequence	Ion Score	C. I. %	Modification	Rank	Result Type
884.4512	884.4348	-0.0164	-19	211	217	YLQADFK					Mascot
947.5057	947.4902	-0.0155	-16	322	329	DGYRPIAR					Mascot
947.5057	947.4902	-0.0155	-16	322	329	DGYRPIAR	26	82.903			Mascot
993.4999	993.4896	-0.0103	-10	28	35	FEKGDELR					Mascot
1335.7202	1335.637	-0.0832	-62	322	332	DGYRPIARMLK			Oxidation (M)[9]		Mascot
1623.9581	1623.9006	-0.0575	-35	73	86	QLFQLVHEAGLKLK					Mascot
1646.781	1646.7966	0.0156	9	246	259	FFVDNGTYLTEQGR					Mascot
1646.781	1646.7966	0.0156	9	246	259	FFVDNGTYLTEQGR	88	100			Mascot
1668.7952	1668.7944	-0.0008	0	218	232	AAAAMVGHPEWEFPR					Mascot
1684.79	1684.7838	-0.0062	-4	218	232	AAAAMVGHPEWEFPR			Oxidation (M)[5]		Mascot
1701.7247	1701.8016	0.0769	45	148	161	SAVQMYTDYMASFR			Oxidation (M)[5,10]		Mascot
2087.0557	2087.0823	0.0266	13	130	147	NIEYLTLGVDDQPLFHGR					Mascot
2087.0557	2087.0823	0.0266	13	130	147	NIEYLTLGVDDQPLFHGR	132	100			Mascot
2173.9351	2174.0701	0.135	62	148	165	SAVQMYTDYMASFRDNM K			Oxidation (M)[5]		Mascot

Rank	Protein Name	Accession No.	Protein MW	Protein PI	Pep. Count	Protein Score	Protein Score C. I. %	Intensity Matched	Total Ion Score	Total Ion C. I. %	Confirmed
2	Beta-amylase [Triticum urartu]	gi|474019719	63864.1	5.29	7	147	100	13.115	132	100	

Peptide Information

Calc. Mass	Obsrv. Mass	± da	± ppm	Start Seq.	End Seq.	Sequence	Ion Score	C. I. %	Modification	Rank	Result Type
1285.6212	1285.6163	-0.0049	-4	61	71	GPKAYDWSAYK					Mascot
1449.7307	1449.6873	-0.0434	-30	487	497	KQWPYVMNDLR					Mascot
1607.6512	1607.7347	0.0835	52	333	345	HHASMNFTCAEMR			Carbamidomethyl (C)[9], Oxidation (M)[5]		Mascot
1686.8949	1686.7914	-0.1035	-61	552	566	YYGETKTVLSDVLAK					Mascot
1696.948	1696.7971	-0.1509	-89	472	486	ESAQDILNLKPLIDK					Mascot
2087.0557	2087.0823	0.0266	13	129	146	NIEYLTLGVDDQPLFHGR					Mascot
2087.0557	2087.0823	0.0266	13	129	146	NIEYLTLGVDDQPLFHGR	132	100			Mascot
2124.9194	2125.0215	0.1021	48	329	345	MLTRHHASMNFTCAEMR			Carbamidomethyl (C)[13], Oxidation (M)[1,9]		Mascot

3	beta-amylase [Triticum aestivum]	gi|1771782	56860.2	5.24	5	142	100	12.516	132	100

Protein Group

RecName: Full=Beta-amylase; AltName: Full=1,4-alpha-D-glucan maltohydrolase	gi|3334120	56860.2	5.2399 997711 1816

Peptide Information

Calc. Mass	Obsrv. Mass	± da	± ppm	Start Seq.	End Seq.	Sequence	Ion Score	C. I. %	Modification	Rank	Result Type
993.4999	993.4896	-0.0103	-10	27	34	FEKGDEIR					Mascot
1285.6212	1285.6163	-0.0049	-4	61	71	GPKAYDWSAYK					Mascot
1607.6512	1607.7347	0.0835	52	333	345	HHASMNFTCAEMR			Carbamidomethyl (C)[9], Oxidation (M)[5]		Mascot
2087.0557	2087.0823	0.0266	13	129	146	NIEYLTLGVDDQPLFHGR					Mascot
2087.0557	2087.0823	0.0266	13	129	146	NIEYLTLGVDDQPLFHGR	132	100			Mascot
2124.9194	2125.0215	0.1021	48	329	345	MLTRHHASMNFTCAEMR			Carbamidomethyl (C)[13], Oxidation (M)[1,9]		Mascot

4	beta amylase, partial [Triticum monococcum subsp. aegilopoides]	gi|56130898	25218.4	5.35	1	132	100	10.856	132	100

Protein Group

beta amylase [Triticum urartu]	gi|260060415	25204.3	5.3499 999046 3257
beta amylase, partial [Triticum monococcum]	gi|56130900	23475.6	5.3800 001144 4092

Peptide Information

Calc. Mass	Obsrv. Mass	± da	± ppm	Start Seq.	End Seq.	Sequence	Ion Score	C. I. %	Modification	Rank	Result Type
2087.0557	2087.0823	0.0266	13	43	60	NIEYLTLGVDDQPLFHGR					Mascot
2087.0557	2087.0823	0.0266	13	43	60	NIEYLTLGVDDQPLFHGR	132	100			Mascot

5	beta amylase [Triticum aestivum]	gi|32400764	31099.9	8.6	5	130	100	10.571	115	100

Peptide Information

Calc. Mass	Obsrv. Mass	± da	± ppm	Start Seq.	End Seq.	Sequence	Ion Score	C. I. %	Modification	Rank	Result Type
884.4512	884.4348	-0.0164	-19	26	32	YLQADFK					Mascot
947.5057	947.4902	-0.0155	-16	137	144	DGYRPIAR					Mascot
947.5057	947.4902	-0.0155	-16	137	144	DGYRPIAR	26	82.903			Mascot
1335.7202	1335.637	-0.0832	-62	137	147	DGYRPIARMLK			Oxidation (M)[9]		Mascot
1646.781	1646.7966	0.0156	9	61	74	FFVDNGTYLTEQGR					Mascot
1646.781	1646.7966	0.0156	9	61	74	FFVDNGTYLTEQGR	88	100			Mascot

1668.7952	1668.7944	-0.0008	0	33	47	AAAAMVGHPEWEFPR					Mascot
1684.79	1684.7838	-0.0062	-4	33	47	AAAAMVGHPEWEFPR			Oxidation (M)[5]		Mascot

6	reversibly glycosylated polypeptide [Triticum aestivum]	gi|4158232	41985	5.82	8	113	100	13.594	83	100	

Protein Group

glycosyltransferase 75 [Triticum aestivum]	gi|301072492	41985	5.8200 001716 6138

Peptide Information

Calc. Mass	Obsrv. Mass	± da	± ppm	Start Seq.	End Seq.	Sequence	Ion Score	C. I. %	Modification	Rank	Result Type
839.441	839.4368	-0.0042	-5	159	165	GYPFSLR					Mascot
1180.6321	1180.6431	0.011	9	123	132	DINALEQHIK					Mascot
1501.6958	1501.7019	0.0061	4	61	72	VPEGFDYELYNR					Mascot
1501.6958	1501.7019	0.0061	4	61	72	VPEGFDYELYNR	83	100			Mascot
1761.7758	1761.7963	0.0205	12	209	223	GTLFPMCGMNLAFDR			Carbamidomethyl (C)[7], Oxidation (M)[6,9]		Mascot
2086.9785	2087.0823	0.1038	50	308	324	ECDTVQKCYISLSEQVK			Carbamidomethyl (C)[2,8]		Mascot
2086.9785	2087.0823	0.1038	50	308	324	ECDTVQKCYISLSEQVK			Carbamidomethyl (C)[2,8]		Mascot
2153.0518	2153.0459	-0.0059	-3	224	243	QLIGPAMYFGLMGDGQPI GR			Oxidation (M)[7,12]		Mascot
2240.1387	2240.1995	0.0608	27	289	307	GIFWQEDIIPFFQNASLSK					Mascot
2292.1335	2292.1665	0.033	14	133	151	NLLSPSTPFFFNTLYDPY R					Mascot

7	Alpha-1,4-glucan-protein synthase [UDP-forming] 1 [Triticum urartu]	gi|474042704	63955.1	5.55	8	99	100	2.835	83	100	

Peptide Information

Calc. Mass	Obsrv. Mass	± da	± ppm	Start Seq.	End Seq.	Sequence	Ion Score	C. I. %	Modification	Rank	Result Type
839.441	839.4368	-0.0042	-5	150	156	GYPFSLR					Mascot
884.4771	884.4348	-0.0423	-48	366	373	AAVAHMLR			Oxidation (M)[6]		Mascot
989.5414	989.5106	-0.0308	-31	267	275	ASNPFVNLK					Mascot
1180.6321	1180.6431	0.011	9	114	123	DINALEQHIK					Mascot
1271.5275	1271.571	0.0435	34	550	560	YVDLTSDTNDE					Mascot
1501.6958	1501.7019	0.0061	4	52	63	VPEGFDYELYNR					Mascot
1501.6958	1501.7019	0.0061	4	52	63	VPEGFDYELYNR	83	100			Mascot
1761.7758	1761.7963	0.0205	12	200	214	GTLFPMCGMNLAFDR			Carbamidomethyl (C)[7], Oxidation (M)[6,9]		Mascot
2292.1335	2292.1665	0.033	14	124	142	NLLSPSTPFFFNTLYDPY R					Mascot

8	hypothetical protein TRIUR3_30538 [Triticum urartu]	gi|473882607	197169.7	4.65	26	50	51.512	19.782			

Peptide Information

Calc. Mass	Obsrv. Mass	± da	± ppm	Start Seq.	End Seq.	Sequence	Ion Score	C. I. %	Modification	Rank	Result Type
854.4254	854.4709	0.0455	53	1173	1180	FVSASSEK					Mascot
993.5251	993.4896	-0.0355	-36	1397	1404	VELFQTEK					Mascot
1063.4836	1063.4554	-0.0282	-27	1110	1118	NAEVEAQMR			Oxidation (M)[8]		Mascot
1063.4836	1063.4554	-0.0282	-27	1110	1118	NAEVEAQMR			Oxidation (M)[8]		Mascot
1124.6422	1124.6307	-0.0115	-10	1521	1529	ELHISKLER					Mascot
1229.6484	1229.674	0.0256	21	956	966	LQQVGDNLSQK					Mascot
1237.5365	1237.5508	0.0143	12	967	976	ETDCQQLSEK			Carbamidomethyl (C)[4]		Mascot
1237.5365	1237.5508	0.0143	12	967	976	ETDCQQLSEK			Carbamidomethyl (C)[4]		Mascot
1253.5831	1253.5743	-0.0088	-7	1268	1277	VAMYDELQER					Mascot
1335.6475	1335.637	-0.0105	-8	1560	1570	MQEHANLVHEK					Mascot
1366.6519	1366.724	0.0721	53	609	622	SASLEAVMEAASGK			Oxidation (M)[8]		Mascot
1498.8224	1498.6951	-0.1273	-85	1571	1582	DQLEQQLLEVRK					Mascot
1543.7169	1543.7749	0.058	38	361	373	DSMLRQAEEEHAK					Mascot
1559.7119	1559.7656	0.0537	34	361	373	DSMLRQAEEEHAK			Oxidation (M)[3]		Mascot
1602.7792	1602.771	-0.0082	-5	896	909	MEEVENDLQALGVR					Mascot
1605.7755	1605.729	-0.0465	-29	595	608	VSHLSDDLEAYQTK					Mascot
1607.7946	1607.7347	-0.0599	-37	609	624	SASLEAVMEAASGKEK					Mascot
1623.7894	1623.9006	0.1112	68	609	624	SASLEAVMEAASGKEK			Oxidation (M)[8]		Mascot
1632.834	1632.8073	-0.0267	-16	1690	1703	EFSLDSLAPQNKQR					Mascot
1662.8618	1662.8118	-0.05	-30	625	638	DLMDSLIQITEEKK					Mascot
1671.837	1671.6985	-0.1385	-83	432	446	SHLEEASMNVKVAEK					Mascot
1671.837	1671.6985	-0.1385	-83	432	446	SHLEEASMNVKVAEK					Mascot
1687.832	1687.7386	-0.0934	-55	432	446	SHLEEASMNVKVAEK			Oxidation (M)[8]		Mascot
1696.8752	1696.7971	-0.0781	-46	1181	1195	EEAAEKLAVHEETIK					Mascot
1701.9017	1701.8016	-0.1001	-59	831	846	EKSASLEALLAEANQK					Mascot
1728.7858	1728.8325	0.0467	27	1	15	MEAEQAAQVHDDKEK					Mascot
1744.7806	1744.8055	0.0249	14	1	15	MEAEQAAQVHDDKEK			Oxidation (M)[1]		Mascot
1851.9012	1851.9681	0.0669	36	179	194	AAELDKMLEMAQLNMK			Oxidation (M)[7]		Mascot
1861.8848	1861.9032	0.0184	10	262	277	LTEELSLHCSSEESLK			Carbamidomethyl (C)[9]		Mascot
2021.0802	2020.953	-0.1272	-63	1163	1180	LVIVDELQEKFVSASSEK					Mascot
2021.0802	2020.953	-0.1272	-63	1163	1180	LVIVDELQEKFVSASSEK					Mascot
2077.9819	2077.9719	-0.01	-5	744	762	SLSDLHTESKTAAESAM QR			Oxidation (M)[17]		Mascot
2125.002	2125.0215	0.0195	9	1268	1285	VAMYDELQERFSATHAE K					Mascot

2297.0825	2297.2	0.1175	51	195	213	EMENQINNLQEEIKGHQD K						Mascot

9 hypothetical protein TRIUR3_07884 [Triticum urartu] gi\|474186540 5672.9 10.02 5 43 0 .892

Peptide Information

Calc. Mass	Obsrv. Mass	± da	± ppm	Start Seq.	End Seq.	Sequence	Ion Score	C. I. %	Modification	Rank	Result Type
925.5287	925.5121	-0.0166	-18	1	8	MAPPRPLK			Oxidation (M)[1]		Mascot
1419.6903	1419.6417	-0.0486	-34	16	28	DPGQEPFAFNIGK					Mascot
1593.7512	1593.755	0.0038	2	29	42	GSMIKGDPLCSWAR			Carbamidomethyl (C)[10], Oxidation (M)[3]		Mascot
1981.941	1982.0659	0.1249	63	34	51	GDPLCSWARNGSAAHNL R			Carbamidomethyl (C)[5]		Mascot
2078.0554	2077.9719	-0.0835	-40	9	28	TTVAGTKDPGQEPFAFNI GK					Mascot

10 Poly [ADP-ribose] polymerase 2-A [Triticum urartu] gi\|474201429 93834.6 6.31 17 42 0 8.544

Peptide Information

Calc. Mass	Obsrv. Mass	± da	± ppm	Start Seq.	End Seq.	Sequence	Ion Score	C. I. %	Modification	Rank	Result Type
801.4478	801.4733	0.0255	32	305	311	WGRVGAR					Mascot
837.3658	837.4368	0.071	85	827	833	DDLEKMS					Mascot
854.4189	854.4709	0.052	61	92	98	LQSMGYR					Mascot
989.5626	989.5106	-0.052	-53	374	382	KGSITDQIK					Mascot
993.4748	993.4896	0.0148	15	337	344	TKNSWSDR					Mascot
1180.5344	1180.6431	0.1087	92	594	603	GVYFADMFSK			Oxidation (M)[7]		Mascot
1543.7461	1543.7749	0.0288	19	484	495	LEMEDPLYARYK			Oxidation (M)[3]		Mascot
1553.9122	1553.7815	-0.1307	-84	17	31	RGLDASGTKPALVLR					Mascot
1589.7542	1589.6837	-0.0705	-44	140	153	EVEEADDGKVEELK					Mascot
1593.7982	1593.755	-0.0432	-27	579	593	IAPPEAPVTGYMFGK			Oxidation (M)[12]		Mascot
1607.8574	1607.7347	-0.1227	-76	92	105	LQSMGYRELQALAK					Mascot
1607.8574	1607.9587	0.1013	63	92	105	LQSMGYRELQALAK					Mascot
1620.8229	1620.8291	0.0062	4	790	804	QLSGAADVVSEFELR					Mascot
1623.8523	1623.9006	0.0483	30	92	105	LQSMGYRELQALAK			Oxidation (M)[4]		Mascot
1671.7384	1671.6985	-0.0399	-24	323	336	DEAIDEFEGKFEDK					Mascot
1671.7384	1671.6985	-0.0399	-24	323	336	DEAIDEFEGKFEDK					Mascot
2012.978	2013.0109	0.0329	16	157	174	IVTDVMLDAAQLQGMGY R			Oxidation (M)[6,15]		Mascot
2125.0781	2125.0215	-0.0566	-27	156	174	KIVTDVMLDAAQLQGMG YR			Oxidation (M)[7]		Mascot
2297.0908	2297.2	0.1092	48	285	304	YYIIQALESDAGGSFMVF NR			Oxidation (M)[16]		Mascot
2685.3342	2685.4417	0.1075	40	516	538	TYLTNTHGKTHSGYTIDIL			Oxidation (M)[21]		Mascot

QMFK

Gel Idx/Pos	272/K24	Instr./Gel Origin	BA2151/Sample Project 20140814	Process Status	Analysis Succeeded
Plate [#] Name	[1] Sample Project 20140814	Instrument Sample Name		Spectra	11

Rank	Protein Name	Accession No.	Protein MW	Protein PI	Pep. Count	Protein Score	Protein Score C. I. %	Intensity Matched	Total Ion Score	Total Ion C. I. %	Confirmed
1	putative NADP-dependent oxidoreductase P1 [Triticum urartu]	gi|473799043	38359.4	5.53	15	430	100	36.195	340	100	

Peptide Information

Calc. Mass	Obsrv. Mass	± da	± ppm	Start Seq.	End Seq.	Sequence	Ion Score	C. I. %	Modification	Rank	Result Type
860.4472	860.4519	0.0047	5	2	10	AAAAEVSNK					Mascot
1008.5546	1008.5222	-0.0324	-32	271	278	NLFCIITK			Carbamidomethyl (C)[4]		Mascot
1032.5209	1032.5051	-0.0158	-15	212	220	EQDLDATLK					Mascot
1188.6219	1188.6204	-0.0015	-1	212	221	EQDLDATLKR					Mascot
1223.5369	1223.5114	-0.0255	-21	201	210	FGFDDAFNYK					Mascot
1223.5369	1223.5114	-0.0255	-21	201	210	FGFDDAFNYK	58	99.987			Mascot
1232.5504	1232.6123	0.0619	50	297	306	FEEEMAGYLK			Oxidation (M)[5]		Mascot
1360.6454	1360.6233	-0.0221	-16	296	306	KFEEEMAGYLK			Oxidation (M)[6]		Mascot
1413.6831	1413.6869	0.0038	3	49	59	NLYLSCDPYLR			Carbamidomethyl (C)[6]		Mascot
1413.6831	1413.6869	0.0038	3	49	59	NLYLSCDPYLR	85	100	Carbamidomethyl (C)[6]		Mascot
1528.6948	1528.6652	-0.0296	-19	179	193	ISGCYVVGSAGSDEK			Carbamidomethyl (C)[4]		Mascot
1704.7687	1704.751	-0.0177	-10	282	295	MEGFIVTDHYGTYR			Oxidation (M)[1]		Mascot
2097.9797	2097.9773	-0.0024	-1	17	35	YVTGFPSEDDMELVPAT AR					Mascot
2097.9797	2097.9773	-0.0024	-1	17	35	YVTGFPSEDDMELVPAT AR	28	87.94			Mascot
2113.9746	2113.9873	0.0127	6	17	35	YVTGFPSEDDMELVPAT AR			Oxidation (M)[11]		Mascot
2138.0547	2138.0818	0.0271	13	252	270	VSVCGLISQYNLEQSEGV R			Carbamidomethyl (C)[4]		Mascot
2138.0547	2138.0818	0.0271	13	252	270	VSVCGLISQYNLEQSEGV R	170	100	Carbamidomethyl (C)[4]		Mascot
2279.2395	2279.2351	-0.0044	-2	156	178	KGEYVFVSAASGAVGQL VGQLAK					Mascot
2759.3193	2759.342	0.0227	8	62	87	MSGNDEPSHVPDFVQGE VLTTLGVSK			Oxidation (M)[1]		Mascot
2760.4131	2760.4214	0.0083	3	310	334	ITYVEDVAEGIESFPTALI GLFYGR					Mascot

Rank	Protein Name	Accession No.	Protein MW	Protein PI	Pep. Count	Protein Score	Protein Score C. I. %	Intensity Matched	Total Ion Score	Total Ion C. I. %	Confirmed
2	unnamed protein product [Triticum aestivum]	gi|257710966	38195.3	5.66	9	230	100	23.705	197	100	

Peptide Information

Calc. Mass	Obsrv. Mass	± da	± ppm	Start Seq.	End Seq.	Sequence	Ion Score	C. I. %	Modification	Rank	Result Type

Calc. Mass	Obsrv. Mass	± da	± ppm	Start Seq.	End Seq.	Sequence	Ion Score	C. I. %	Modification	Rank	Result Type
911.5309	911.4791	-0.0518	-57	336	343	QLVPVARE					Mascot
1008.5546	1008.5222	-0.0324	-32	268	275	NLFCIITK			Carbamidomethyl (C)[4]		Mascot
1223.5369	1223.5114	-0.0255	-21	198	207	FGFDDAFNYK					Mascot
1223.5369	1223.5114	-0.0255	-21	198	207	FGFDDAFNYK	58	99.987			Mascot
1232.5504	1232.6123	0.0619	50	294	303	FEEEMAGYLK			Oxidation (M)[5]		Mascot
1360.6454	1360.6233	-0.0221	-16	293	303	KFEEEMAGYLK			Oxidation (M)[6]		Mascot
1413.6831	1413.6869	0.0038	3	46	56	NLYLSCDPYLR			Carbamidomethyl (C)[6]		Mascot
1413.6831	1413.6869	0.0038	3	46	56	NLYLSCDPYLR	85	100	Carbamidomethyl (C)[6]		Mascot
1528.6948	1528.6652	-0.0296	-19	176	190	ISGCYVVGSAGSDEK			Carbamidomethyl (C)[4]		Mascot
1674.7581	1674.774	0.0159	9	279	292	MEGFIVTDHYGSYR					Mascot
1690.7531	1690.7365	-0.0166	-10	279	292	MEGFIVTDHYGSYR			Oxidation (M)[1]		Mascot
1690.7531	1690.7365	-0.0166	-10	279	292	MEGFIVTDHYGSYR	55	99.978	Oxidation (M)[1]		Mascot
2759.3193	2759.342	0.0227	8	59	84	MSGNDEPSHVPDFVQGE VLTTLGVSK			Oxidation (M)[1]		Mascot

3	putative NADP-dependent oxidoreductase P1 [Triticum urartu]	gi|474246662	38736.6	5.75	6	160	100	18.834	142	100	

Peptide Information

Calc. Mass	Obsrv. Mass	± da	± ppm	Start Seq.	End Seq.	Sequence	Ion Score	C. I. %	Modification	Rank	Result Type
1223.5369	1223.5114	-0.0255	-21	204	213	FGFDDAFNYK					Mascot
1223.5369	1223.5114	-0.0255	-21	204	213	FGFDDAFNYK	58	99.987			Mascot
1232.5504	1232.6123	0.0619	50	300	309	FEEEMAGYLK			Oxidation (M)[5]		Mascot
1360.6454	1360.6233	-0.0221	-16	299	309	KFEEEMAGYLK			Oxidation (M)[6]		Mascot
1413.6831	1413.6869	0.0038	3	52	62	NLYLSCDPYLR			Carbamidomethyl (C)[6]		Mascot
1413.6831	1413.6869	0.0038	3	52	62	NLYLSCDPYLR	85	100	Carbamidomethyl (C)[6]		Mascot
1528.6948	1528.6652	-0.0296	-19	182	196	ISGCYVVGSAGSDEK			Carbamidomethyl (C)[4]		Mascot
2046.9524	2047.0742	0.1218	60	283	298	IRMEGFIVMDHYSNYR			Oxidation (M)[3]		Mascot
2046.9524	2047.0742	0.1218	60	283	298	IRMEGFIVMDHYSNYR			Oxidation (M)[3]		Mascot

4	unnamed protein product [Triticum aestivum]	gi|257710968	38536.3	5.66	5	71	99.68	7.601	58	99.987	

Peptide Information

Calc. Mass	Obsrv. Mass	± da	± ppm	Start Seq.	End Seq.	Sequence	Ion Score	C. I. %	Modification	Rank	Result Type
1223.5369	1223.5114	-0.0255	-21	202	211	FGFDDAFNYK					Mascot
1223.5369	1223.5114	-0.0255	-21	202	211	FGFDDAFNYK	58	99.987			Mascot
1232.5504	1232.6123	0.0619	50	298	307	FEEEMAGYLK			Oxidation (M)[5]		Mascot
1360.6454	1360.6233	-0.0221	-16	297	307	KFEEEMAGYLK			Oxidation (M)[6]		Mascot

Calc. Mass	Obsrv. Mass	± da	± ppm	Start Seq.	End Seq.	Sequence	Ion Score	C. I. %	Modification	Rank	Result Type
1674.7727	1674.774	0.0013	1	50	62	NLYLSCDPYMRSR			Carbamidomethyl (C)[6]		Mascot
1690.7676	1690.7365	-0.0311	-18	50	62	NLYLSCDPYMRSR			Carbamidomethyl (C)[6], Oxidation (M)[10]		Mascot
1690.7676	1690.7365	-0.0311	-18	50	62	NLYLSCDPYMRSR			Carbamidomethyl (C)[6], Oxidation (M)[10]		Mascot
2166.0317	2166.1021	0.0704	33	253	271	VAVCGMISQYNLEQPEG VR			Carbamidomethyl (C)[4], Oxidation (M)[6]		Mascot

5	putative NADP-dependent oxidoreductase P1 [Triticum urartu]	gi|474172228	38292.4	6.21	2	58	92.315	1.373	58	99.987

Peptide Information

Calc. Mass	Obsrv. Mass	± da	± ppm	Start Seq.	End Seq.	Sequence	Ion Score	C. I. %	Modification	Rank	Result Type
847.4155	847.4285	0.013	15	2	9	AEAEVSNK					Mascot
1223.5369	1223.5114	-0.0255	-21	199	208	FGFDDAFNYK					Mascot
1223.5369	1223.5114	-0.0255	-21	199	208	FGFDDAFNYK	58	99.987			Mascot

6	putative NADP-dependent oxidoreductase P1 [Triticum urartu]	gi|474145110	22793.5	5.31	1	58	92.315	1.143	58	99.987

Peptide Information

Calc. Mass	Obsrv. Mass	± da	± ppm	Start Seq.	End Seq.	Sequence	Ion Score	C. I. %	Modification	Rank	Result Type
1223.5369	1223.5114	-0.0255	-21	63	72	FGFDDAFNYK					Mascot
1223.5369	1223.5114	-0.0255	-21	63	72	FGFDDAFNYK	58	99.987			Mascot

7	beta-glucosidase 4, partial [Triticum aestivum]	gi|359828768	56731.2	8.58	14	52	73.354	8.857		

Peptide Information

Calc. Mass	Obsrv. Mass	± da	± ppm	Start Seq.	End Seq.	Sequence	Ion Score	C. I. %	Modification	Rank	Result Type
800.405	800.3974	-0.0076	-9	251	256	YQPHQK					Mascot
807.3917	807.4159	0.0242	30	93	99	EDVGIMK			Oxidation (M)[6]		Mascot
809.4628	809.3958	-0.067	-83	367	374	NGVPIGPR					Mascot
819.3414	819.4123	0.0709	87	219	226	CPAGGDSR			Carbamidomethyl (C)[1]		Mascot
820.4676	820.4104	-0.0572	-70	184	190	VFGDRVK					Mascot
825.4175	825.4537	0.0362	44	301	307	YPSSMLK					Mascot
844.4635	844.4587	-0.0048	-6	440	447	KAIDNGAR					Mascot
849.4498	849.4354	-0.0144	-17	493	499	NMLSEKK					Mascot
866.4407	866.4147	-0.026	-30	486	492	DSALWFK					Mascot
882.4468	882.449	0.0022	2	108	114	FSISWSR					Mascot
1009.5564	1009.5527	-0.0037	-4	433	440	DYITELKK					Mascot

1107.543	1107.582	0.039	35	313	322	LPGFSTNESR					Mascot
1210.5641	1210.5837	0.0196	16	191	199	NWFTFNEPR					Mascot
2760.3311	2760.4214	0.0903	33	34	60	QGFPAGFVFGTAASAYQ VEGMARQGGR					Mascot

8 hypothetical protein TRIUR3_09442 [Triticum urartu] gi|474400632 105615.1 5.39 19 50 57.769 28.277

Peptide Information

Calc. Mass	Obsrv. Mass	± da	± ppm	Start Seq.	End Seq.	Sequence	Ion Score	C. I. %	Modification	Rank	Result Type
800.4512	800.3974	-0.0538	-67	134	140	IDPNTIK					Mascot
872.4836	872.423	-0.0606	-69	47	54	EGLGQQLK					Mascot
923.5673	923.4753	-0.092	-100	432	439	VHEIGKLK					Mascot
1031.4761	1031.5171	0.041	40	454	461	DPMPEMRR					Mascot
1210.5708	1210.5837	0.0129	11	21	30	QAFVCLMDAR			Carbamidomethyl (C)[5]		Mascot
1250.6814	1250.6512	-0.0302	-24	755	765	KDYSMVPIIGK					Mascot
1360.6539	1360.6233	-0.0306	-22	799	809	IHGGNMFRQER			Oxidation (M)[6]		Mascot
1413.7155	1413.6869	-0.0286	-20	513	525	SNSLNHMLIGEAK					Mascot
1413.7155	1413.6869	-0.0286	-20	513	525	SNSLNHMLIGEAK					Mascot
1528.6697	1528.6652	-0.0045	-3	96	107	HQCVEDIDLDER			Carbamidomethyl (C)[3]		Mascot
1561.7904	1561.7512	-0.0392	-25	793	806	TSGQLKIHGGNMFR			Oxidation (M)[12]		Mascot
1622.8273	1622.869	0.0417	26	118	131	ASDDLLYEEVLTVR					Mascot
1672.7926	1672.7924	-0.0002	0	592	606	SQPESGTHIDEVFAR					Mascot
1674.8502	1674.774	-0.0762	-45	5	17	QRPMWGLRACMLR			Carbamidomethyl (C)[10]		Mascot
1675.7963	1675.786	-0.0103	-6	620	633	EKYLFADDSTPSFR					Mascot
1690.8451	1690.7365	-0.1086	-64	5	17	QRPMWGLRACMLR			Carbamidomethyl (C)[10], Oxidation (M)[4]		Mascot
1690.8451	1690.7365	-0.1086	-64	5	17	QRPMWGLRACMLR			Carbamidomethyl (C)[10], Oxidation (M)[4]		Mascot
1706.84	1706.7487	-0.0913	-53	5	17	QRPMWGLRACMLR			Carbamidomethyl (C)[10], Oxidation (M)[4,11]		Mascot
2033.8804	2034.0166	0.1362	67	851	867	SSPYDCSERGPIMPHER			Carbamidomethyl (C)[6], Oxidation (M)[13]		Mascot
2033.8804	2034.0166	0.1362	67	851	867	SSPYDCSERGPIMPHER			Carbamidomethyl (C)[6], Oxidation (M)[13]		Mascot
2097.0144	2097.0562	0.0418	20	251	268	CLDFTDFGSIQIPSLMPR			Carbamidomethyl (C)[1]		Mascot
2113.9204	2113.9873	0.0669	32	91	107	NEEGRHQCVEDIDLDER			Carbamidomethyl (C)[8]		Mascot
2301.0671	2301.2361	0.169	73	233	250	LMNEEMSDDQIIRCFLVR			Carbamidomethyl (C)[14], Oxidation (M)[2,6]		Mascot
2760.3701	2760.4214	0.0513	19	810	833	STLVNRTDDFYNTNLSLG RPSSFR					Mascot

9 hypothetical protein TRIUR3_07605 [Triticum urartu] gi|474443810 36454.2 6.43 12 50 56.785 7.248

Peptide Information

Calc. Mass	Obsrv. Mass	± da	± ppm	Start Seq.	End Seq.	Sequence	Ion Score	C. I. %	Modification	Rank	Result Type

Calc. Mass	Obsrv. Mass	± da	± ppm	Start Seq.	End Seq.	Sequence	Ion Score	C. I. %	Modification	Rank	Result Type
807.3917	807.4159	0.0242	30	144	150	MALEEAK			Oxidation (M)[1]		Mascot
829.489	829.4282	-0.0608	-73	166	173	LRDAVAGK					Mascot
832.441	832.3732	-0.0678	-81	182	188	LENESLK					Mascot
849.4611	849.4354	-0.0257	-30	251	258	VMAAASRK			Oxidation (M)[2]		Mascot
860.422	860.4519	0.0299	35	189	197	ASEAAAQGR					Mascot
1107.5793	1107.582	0.0027	2	240	250	LSAAAFLDSGR					Mascot
1205.6372	1205.6243	-0.0129	-11	117	127	EVATAKQTETK					Mascot
1360.693	1360.6233	-0.0697	-51	222	233	ACAAGLLLEEWK			Carbamidomethyl (C)[2]		Mascot
1426.7802	1426.7145	-0.0657	-46	323	335	SLHKSDFGPVLAR					Mascot
1507.7057	1507.7505	0.0448	30	168	181	DAVAGKEDAMESLR			Oxidation (M)[10]		Mascot
1561.8254	1561.7512	-0.0742	-48	144	157	MALEEAKLEAATLR			Oxidation (M)[1]		Mascot
2097.9871	2097.9773	-0.0098	-5	48	66	AQHVLATSTTTYSMQSS ER					Mascot
2097.9871	2097.9773	-0.0098	-5	48	66	AQHVLATSTTTYSMQSS ER					Mascot
2113.9819	2113.9873	0.0054	3	48	66	AQHVLATSTTTYSMQSS ER			Oxidation (M)[14]		Mascot

10 hypothetical protein TRIUR3_03956 [Triticum urartu] gi|473905046 31223.1 9.17 11 48 33.068 3.861

Peptide Information

Calc. Mass	Obsrv. Mass	± da	± ppm	Start Seq.	End Seq.	Sequence	Ion Score	C. I. %	Modification	Rank	Result Type
807.4723	807.4159	-0.0564	-70	39	45	LTLTGFR					Mascot
818.4002	818.4371	0.0369	45	208	214	GEEERAK					Mascot
872.4723	872.423	-0.0493	-57	161	167	EKELEPK					Mascot
1232.6416	1232.6123	-0.0293	-24	213	225	AKAAAAATAMEAR					Mascot
1253.6484	1253.693	0.0446	36	168	180	APQAAAAAQTPEK					Mascot
1322.7025	1322.6819	-0.0206	-16	89	100	FEAGVLTLTMPK			Oxidation (M)[10]		Mascot
1322.7025	1322.6819	-0.0206	-16	89	100	FEAGVLTLTMPK	1	0	Oxidation (M)[10]		Mascot
1323.6283	1323.6782	0.0499	38	244	255	MTEGLGQLTDMK					Mascot
1435.6925	1435.6493	-0.0432	-30	46	58	TDDFRVQVDGAGR					Mascot
1441.7434	1441.7936	0.0502	35	39	50	LTLTGFRTDDFR					Mascot
1675.865	1675.786	-0.079	-47	73	88	VFQLPSSASLDDIAGR					Mascot
2279.1262	2279.2351	0.1089	48	168	190	APQAAAAAQTPEKQAPN PTSSDK					Mascot

Gel Idx/Pos	273/L1	Instr./Gel Origin	BA2151/Sample Project 20140814	Process Status	Analysis Succeeded
Plate [#] Name	[1] Sample Project 20140814	Instrument Sample Name		Spectra	11

Rank	Protein Name	Accession No.	Protein MW	Protein PI	Pep. Count	Protein Score	Protein Score C. I. %	Intensity Matched	Total Ion Score	Total Ion C. I. %	Confirmed
1	globulin-3A [Triticum aestivum]	gi|390979705	66626.8	8.48	14	325	100	9.473	278	100	

Peptide Information

Calc. Mass	Obsrv. Mass	± da	± ppm	Start Seq.	End Seq.	Sequence	Ion Score	C. I. %	Modification	Rank	Result Type
823.4645	823.4	-0.0645	-78	350	356	IANRHGR					Mascot
837.4101	837.4008	-0.0093	-11	357	363	LYEADAR					Mascot
906.468	906.4623	-0.0057	-6	535	541	EVQEVFR					Mascot
1091.509	1091.5485	0.0395	36	82	91	GHGRHGEGER					Mascot
1187.5917	1187.6055	0.0138	12	354	363	HGRLYEADAR					Mascot
1280.6495	1280.6497	0.0002	0	364	374	SFHALAQHDVR					Mascot
1280.6495	1280.6497	0.0002	0	364	374	SFHALAQHDVR	51	99.935			Mascot
1360.7219	1360.7036	-0.0183	-13	339	349	DTFNLLEQRPK					Mascot
1360.7219	1360.7036	-0.0183	-13	339	349	DTFNLLEQRPK	29	89.635			Mascot
1381.7488	1381.6913	-0.0575	-42	132	142	RPYVFGPRSFR					Mascot
1565.631	1565.6531	0.0221	14	446	459	GSGSESEEEQDQQR					Mascot
1655.85	1655.8416	-0.0084	-5	520	534	LDDPAQELAFGRPAR					Mascot
1822.8752	1822.8926	0.0174	10	489	504	GSSNLQVVCFEINAER			Carbamidomethyl (C)[9]		Mascot
1822.8752	1822.8926	0.0174	10	489	504	GSSNLQVVCFEINAER	110	100	Carbamidomethyl (C)[9]		Mascot
1906.0182	1906.0371	0.0189	10	470	488	GSAFVVPPGHPVVEIASS R					Mascot
1906.0182	1906.0371	0.0189	10	470	488	GSAFVVPPGHPVVEIASS R	87	100			Mascot
2176.1431	2176.1775	0.0344	16	397	416	LAVVLEGEGEVEIVCPHL GR			Carbamidomethyl (C)[15]		Mascot
2426.0967	2426.1196	0.0229	9	542	562	AKDQQDEGFVAGPEQQ QEHER					Mascot

Rank	Protein Name	Accession No.	Protein MW	Protein PI	Pep. Count	Protein Score	Protein Score C. I. %	Intensity Matched	Total Ion Score	Total Ion C. I. %	Confirmed
2	globulin 3 [Triticum aestivum]	gi|215398470	66651.7	7.78	13	319	100	9.242	278	100	

Peptide Information

Calc. Mass	Obsrv. Mass	± da	± ppm	Start Seq.	End Seq.	Sequence	Ion Score	C. I. %	Modification	Rank	Result Type
823.4645	823.4	-0.0645	-78	350	356	IANRHGR					Mascot
837.4101	837.4008	-0.0093	-11	357	363	LYEADAR					Mascot
906.468	906.4623	-0.0057	-6	535	541	EVQEVFR					Mascot

1091.509	1091.5485	0.0395	36	82	91	GHGRHGEGER					Mascot
1187.5917	1187.6055	0.0138	12	354	363	HGRLYEADAR					Mascot
1280.6495	1280.6497	0.0002	0	364	374	SFHALAQHDVR					Mascot
1280.6495	1280.6497	0.0002	0	364	374	SFHALAQHDVR	51	99.935			Mascot
1360.7219	1360.7036	-0.0183	-13	339	349	DTFNLLEQRPK					Mascot
1360.7219	1360.7036	-0.0183	-13	339	349	DTFNLLEQRPK	29	89.635			Mascot
1381.7488	1381.6913	-0.0575	-42	132	142	RPYVFGPRSFR					Mascot
1565.631	1565.6531	0.0221	14	446	459	GSGSESEEEQDQQR					Mascot
1822.8752	1822.8926	0.0174	10	489	504	GSSNLQVVCFEINAER			Carbamidomethyl (C)[9]		Mascot
1822.8752	1822.8926	0.0174	10	489	504	GSSNLQVVCFEINAER	110	100	Carbamidomethyl (C)[9]		Mascot
1906.0182	1906.0371	0.0189	10	470	488	GSAFVVPPGHPVVEIASSR					Mascot
1906.0182	1906.0371	0.0189	10	470	488	GSAFVVPPGHPVVEIASSR	87	100			Mascot
2176.1431	2176.1775	0.0344	16	397	416	LAVVLEGEGEVEIVCPHLGR			Carbamidomethyl (C)[15]		Mascot
2426.0967	2426.1196	0.0229	9	542	562	AKDQQDEGFVAGPEQQQEHER					Mascot

3 Globulin-1 S allele [Triticum urartu] gi|474411419 57108.4 9.1 12 284 100 6.944 248 100

Peptide Information

Calc. Mass	Obsrv. Mass	± da	± ppm	Start Seq.	End Seq.	Sequence	Ion Score	C. I. %	Modification	Rank	Result Type
818.4003	818.4104	0.0101	12	226	232	ASEEQVR					Mascot
823.4645	823.4	-0.0645	-78	269	275	IANRHGR					Mascot
837.4101	837.4008	-0.0093	-11	276	282	LYEADAR					Mascot
906.468	906.4623	-0.0057	-6	457	463	EVQEVFR					Mascot
990.5214	990.4847	-0.0367	-37	195	203	AALKTSDER					Mascot
1187.5917	1187.6055	0.0138	12	273	282	HGRLYEADAR					Mascot
1280.6495	1280.6497	0.0002	0	283	293	SFHALAQHDVR					Mascot
1280.6495	1280.6497	0.0002	0	283	293	SFHALAQHDVR	51	99.935			Mascot
1320.5452	1320.6154	0.0702	53	349	358	WGEEEEDDRR					Mascot
1791.8984	1791.7439	-0.1545	-86	254	268	GDSRDTYNLLEQRPK					Mascot
1822.8752	1822.8926	0.0174	10	411	426	GSSNLQVVCFEINAER			Carbamidomethyl (C)[9]		Mascot
1822.8752	1822.8926	0.0174	10	411	426	GSSNLQVVCFEINAER	110	100	Carbamidomethyl (C)[9]		Mascot
1906.0182	1906.0371	0.0189	10	392	410	GSAFVVPPGHPVVEIASSR					Mascot
1906.0182	1906.0371	0.0189	10	392	410	GSAFVVPPGHPVVEIASSR	87	100			Mascot
2176.1431	2176.1775	0.0344	16	316	335	LAVVLEGEGEVEIVCPHLGR			Carbamidomethyl (C)[15]		Mascot

4 globulin 3B [Triticum aestivum] gi|215398472 57067.8 7.36 10 75 99.85 4.06 51 99.935

Peptide Information

Calc. Mass	Obsrv. Mass	± da	± ppm	Start Seq.	End Seq.	Sequence	Ion Score	C. I. %	Modification	Rank	Result Type
823.4645	823.4	-0.0645	-78	308	314	IANRHGR					Mascot
837.4101	837.4008	-0.0093	-11	315	321	LYEADAR					Mascot
906.468	906.4623	-0.0057	-6	452	458	EVQEVFR					Mascot
944.5272	944.4589	-0.0683	-72	381	389	QGKQGSALR					Mascot
1187.5917	1187.6055	0.0138	12	312	321	HGRLYEADAR					Mascot
1280.6495	1280.6497	0.0002	0	322	332	SFHALAQHDVR					Mascot
1280.6495	1280.6497	0.0002	0	322	332	SFHALAQHDVR	51	99.935			Mascot
1340.5939	1340.6417	0.0478	36	61	72	HGEGGREEEQGR					Mascot
1381.7488	1381.6913	-0.0575	-42	125	135	RPYVFGPRSFR					Mascot
1430.6156	1430.6456	0.03	21	49	60	HEQEEQGHSHGR					Mascot
1791.8984	1791.7439	-0.1545	-86	293	307	GDSRDTYNLLEQRPK					Mascot

5	RecName: Full=Avenin-like b4; Flags: Precursor	gi|338817624	33700.4	8.29	4	62	97.513	4.047	52	99.942	

Protein Group

RecName: Full=Avenin-like b5; Flags: Precursor	gi|338817625	33763.4	8.2799 997329 7119
avenin-like protein [Triticum aestivum]	gi|145321072	33700.4	8.2899 999618 5303
avenin-like type-B [Triticum aestivum]	gi|156630230	33763.4	8.2799 997329 7119

Peptide Information

Calc. Mass	Obsrv. Mass	± da	± ppm	Start Seq.	End Seq.	Sequence	Ion Score	C. I. %	Modification	Rank	Result Type
818.4553	818.4104	-0.0449	-55	248	254	MSLQALR					Mascot
834.4502	834.3798	-0.0704	-84	248	254	MSLQALR			Oxidation (M)[1]		Mascot
1245.6587	1245.6598	0.0011	1	202	211	QLSQIPEQFR					Mascot
1245.6587	1245.6598	0.0011	1	202	211	QLSQIPEQFR	52	99.942			Mascot
1280.6455	1280.6497	0.0042	3	115	124	QERQQQAQHK					Mascot
1280.6455	1280.6497	0.0042	3	115	124	QERQQQAQHK					Mascot
1381.7006	1381.6913	-0.0093	-7	212	223	CQAIHNVAEAIR			Carbamidomethyl (C)[1]		Mascot

6	avenin-like b [Triticum aestivum]	gi|295853633	33381.3	8.12	4	62	97.455	3.192	52	99.942	

Protein Group

RecName: Full=Avenin-like b10; Flags: Precursor	gi|338817630	33381.3	8.1199

998855
5908

Peptide Information

Calc. Mass	Obsrv. Mass	± da	± ppm	Start Seq.	End Seq.	Sequence	Ion Score	C. I. %	Modification	Rank	Result Type
818.4553	818.4104	-0.0449	-55	248	254	MSLQALR					Mascot
834.4502	834.3798	-0.0704	-84	248	254	MSLQALR			Oxidation (M)[1]		Mascot
1192.6289	1192.5261	-0.1028	-86	248	257	MSLQALRSMR					Mascot
1245.6587	1245.6598	0.0011	1	202	211	QLSQIPEQFR					Mascot
1245.6587	1245.6598	0.0011	1	202	211	QLSQIPEQFR	52	99.942			Mascot
1381.7006	1381.6913	-0.0093	-7	212	223	CQAIHNVAEAIR			Carbamidomethyl (C)[1]		Mascot

7	RecName: Full=Avenin-like b9; Flags: Precursor	gi|338817629	33411.3	7.82	3	59	94.683	2.903	52	99.942	

Protein Group

RecName: Full=Avenin-like b1; Short=TaAvlike-b1; Flags: Precursor	gi|122232330	33788.2	8.0799 999237 0605
RecName: Full=Avenin-like b2; Flags: Precursor	gi|338817622	33575.2	7.8200 001716 6138
RecName: Full=Avenin-like b3; Flags: Precursor	gi|338817623	33442.3	7.8299 999237 0605
RecName: Full=Avenin-like b6; AltName: Full=Avenin-like protein s1; Flags: Precursor	gi|338817626	33385.3	7.8299 999237 0605
RecName: Full=Avenin-like b7; AltName: Full=Avenin-like protein s2; Flags: Precursor	gi|338817627	33357.2	7.8299 999237 0605
RecName: Full=Avenin-like b8; Flags: Precursor	gi|338817628	33355.3	7.8299 999237 0605
avenin-like b [Triticum aestivum]	gi|295853631	33385.3	7.8299 999237 0605
avenin-like b [Triticum aestivum]	gi|295853629	33385.3	7.8299 999237 0605
avenin-like b [Triticum aestivum]	gi|295853627	33355.3	7.8299 999237 0605
avenin-like b [Triticum aestivum]	gi|295853625	33411.3	7.8200 001716 6138
avenin-like protein [Triticum aestivum]	gi|363981082	33385.3	7.8299 999237 0605
avenin-like protein [Triticum aestivum]	gi|363981080	33371.3	7.8299 999237

			0605
avenin-like protein [Triticum aestivum]	gi|363981078	33413.3	8.0900 001525 8789
avenin-like protein [Triticum aestivum]	gi|363981076	33385.3	7.8299 999237 0605
avenin-like protein [Triticum aestivum]	gi|363981074	33385.3	7.8299 999237 0605
avenin-like protein [Triticum aestivum]	gi|363981070	33385.3	7.8299 999237 0605
avenin-like protein [Triticum aestivum]	gi|363981068	33454.3	8.0900 001525 8789
avenin-like protein [Triticum aestivum]	gi|363981064	33385.3	7.8299 999237 0605
avenin-like protein [Triticum aestivum]	gi|363981062	33415.3	7.8299 999237 0605
avenin-like protein [Triticum aestivum]	gi|363981060	33360.3	7.8499 999046 3257
avenin-like protein [Triticum aestivum]	gi|363981058	33385.3	7.8299 999237 0605
avenin-like protein [Triticum aestivum]	gi|363981056	33413.3	8.0900 001525 8789
avenin-like protein [Triticum aestivum]	gi|363981054	33385.3	7.8299 999237 0605
avenin-like protein [Triticum aestivum]	gi|363981051	33385.3	7.8299 999237 0605
avenin-like protein [Triticum aestivum]	gi|145321070	33385.3	7.8299 999237 0605
avenin-like protein [Triticum aestivum]	gi|363981047	33385.3	7.8299 999237 0605
avenin-like protein [Triticum aestivum]	gi|363981045	33357.3	7.8299 999237 0605
avenin-like protein [Triticum aestivum]	gi|363981043	33401.3	7.8299 999237 0605
avenin-like protein s1 [Triticum aestivum]	gi|260600231	33385.3	7.8299 999237 0605
avenin-like protein s2 [Triticum aestivum]	gi|260600233	33357.2	7.8299 999237 0605

	putative avenin-like b precursor [Triticum aestivum]	gi|89143122	33788.2	8.0799 999237 0605		

Peptide Information

Calc. Mass	Obsrv. Mass	± da	± ppm	Start Seq.	End Seq.	Sequence	Ion Score	C. I. %	Modification	Rank	Result Type
818.4553	818.4104	-0.0449	-55	248	254	MSLQALR					Mascot
834.4502	834.3798	-0.0704	-84	248	254	MSLQALR			Oxidation (M)[1]		Mascot
1245.6587	1245.6598	0.0011	1	202	211	QLSQIPEQFR					Mascot
1245.6587	1245.6598	0.0011	1	202	211	QLSQIPEQFR	52	99.942			Mascot
1381.7006	1381.6913	-0.0093	-7	212	223	CQAIHNVAEAIR			Carbamidomethyl (C)[1]		Mascot

8	putative protein [Triticum aestivum]	gi|283099379	58569.9	5.26	15	58	93.459	4.215		

Peptide Information

Calc. Mass	Obsrv. Mass	± da	± ppm	Start Seq.	End Seq.	Sequence	Ion Score	C. I. %	Modification	Rank	Result Type
803.3417	803.4163	0.0746	93	524	530	IPEDSEN					Mascot
813.3777	813.4298	0.0521	64	138	143	WEYTSK					Mascot
851.3853	851.438	0.0527	62	114	122	SGTSSAGER					Mascot
863.4291	863.4218	-0.0073	-8	239	246	CPTTSLGK			Carbamidomethyl (C)[1]		Mascot
944.4683	944.4589	-0.0094	-10	451	459	DTAPTSQPK					Mascot
990.511	990.4847	-0.0263	-27	1	9	MVQLPAMGK			Oxidation (M)[1]		Mascot
1033.5677	1033.5012	-0.0665	-64	125	134	ASNFPASVLK					Mascot
1057.4586	1057.5293	0.0707	67	321	329	NDTNDFFGK					Mascot
1111.5782	1111.5903	0.0121	11	135	143	IGKWEYTSK					Mascot
1331.7682	1331.6564	-0.1118	-84	125	137	ASNFPASVLKIGK					Mascot
1381.6781	1381.6913	0.0132	10	239	250	CPTTSLGKNFEK			Carbamidomethyl (C)[1]		Mascot
1404.6324	1404.7167	0.0843	60	289	299	CHDFANLKDER			Carbamidomethyl (C)[1]		Mascot
1507.8632	1507.745	-0.1182	-78	157	169	HKLVWEVLEGGLK					Mascot
1714.8582	1714.8402	-0.018	-10	11	27	QHPEAAEPPMAPAAAVK					Mascot
2116.9163	2117.0945	0.1782	84	300	320	EDLPGFSGSVSPCAGSS MSTK			Carbamidomethyl (C)[13], Oxidation (M)[18]		Mascot

9	avenin-like protein [Triticum aestivum]	gi|363981072	33415.2	7.41	2	57	91.953	2.247	52	99.942

Peptide Information

Calc. Mass	Obsrv. Mass	± da	± ppm	Start Seq.	End Seq.	Sequence	Ion Score	C. I. %	Modification	Rank	Result Type
1245.6587	1245.6598	0.0011	1	202	211	QLSQIPEQFR					Mascot
1245.6587	1245.6598	0.0011	1	202	211	QLSQIPEQFR	52	99.942			Mascot

1381.7006	1381.6913	-0.0093	-7	212	223	CQAIHNVAEAIR			Carbamidomethyl (C)[1]		Mascot

10	Anaphase-promoting complex subunit 7 [Triticum urartu]	gi|474342493	64971	6.28	18	57	91.766	6.43

Peptide Information

Calc. Mass	Obsrv. Mass	± da	± ppm	Start Seq.	End Seq.	Sequence	Ion Score	C. I. %	Modification	Rank	Result Type
800.4083	800.4175	0.0092	11	424	430	VMHQSAK					Mascot
806.4263	806.3817	-0.0446	-55	97	103	MNLMLGK					Mascot
807.4835	807.4081	-0.0754	-93	104	109	LYRISR					Mascot
816.4033	816.4218	0.0185	23	424	430	VMHQSAK			Oxidation (M)[2]		Mascot
854.4618	854.4067	-0.0551	-64	266	272	LKSDYTK					Mascot
906.5043	906.4623	-0.042	-46	157	164	FPTSSVLR					Mascot
919.4631	919.4503	-0.0128	-14	165	172	VEYAAPNR					Mascot
1053.5762	1053.4933	-0.0829	-79	404	412	AYLALSKCK			Carbamidomethyl (C)[8]		Mascot
1081.5459	1081.5199	-0.026	-24	411	419	CKDALFTAR			Carbamidomethyl (C)[1]		Mascot
1177.6432	1177.5564	-0.0868	-74	94	103	NLKMNLMLGK			Oxidation (M)[4]		Mascot
1212.6008	1212.574	-0.0268	-22	493	502	QWADDSLHIK					Mascot
1212.6008	1212.574	-0.0268	-22	493	502	QWADDSLHIK					Mascot
1240.5514	1240.6106	0.0592	48	236	245	NEEAIMNFEK			Oxidation (M)[6]		Mascot
1259.6235	1259.6522	0.0287	23	420	430	EAMKVMHQSAK					Mascot
1331.6511	1331.6564	0.0053	4	80	91	EALQEMEGIPSK					Mascot
1447.7362	1447.689	-0.0472	-33	316	327	VDDRHITGYIMK					Mascot
1507.8632	1507.745	-0.1182	-78	216	228	FPNNVHILLEIAK					Mascot
1554.8235	1554.7832	-0.0403	-26	434	448	LVGDVHAISSSGREK					Mascot
1684.8588	1684.8519	-0.0069	-4	66	79	SKIAICHSALHEYR			Carbamidomethyl (C)[6]		Mascot
2120.1499	2120.0562	-0.0937	-44	459	479	LEPGFLGAALALADLHVAEGR					Mascot

Rank	Protein Name	Accession No.	Protein MW	Protein PI	Pep. Count	Protein Score	Protein Score C. I. %	Intensity Matched	Total Ion Score	Total Ion C. I. %	Confirmed
1	Guanine nucleotide-binding protein subunit beta-like protein A [Triticum urartu]	gi|473957859	27532.7	6.29	9	706	100	32.98	652	100	

Peptide Information

Calc. Mass	Obsrv. Mass	± da	± ppm	Start Seq.	End Seq.	Sequence	Ion Score	C. I. %	Modification	Rank	Result Type
1455.6937	1455.6965	0.0028	2	211	222	YWLCAATQDSIK			Carbamidomethyl (C)[4]		Mascot
1602.8486	1602.8632	0.0146	9	178	191	DGVTLLWDLTEGKR					Mascot
1717.9596	1717.9836	0.024	14	230	244	HIVQDLRPEVPVSTK					Mascot
1717.9596	1717.9836	0.024	14	230	244	HIVQDLRPEVPVSTK	65	99.997			Mascot
1894.9083	1894.9457	0.0374	20	121	137	FSPNNFAPTIVSGSWDR					Mascot
1894.9083	1894.9457	0.0374	20	121	137	FSPNNFAPTIVSGSWDR	131	100			Mascot
2127.054	2127.1038	0.0498	23	192	210	LYSLDAGSIINSLCFSPNR			Carbamidomethyl (C)[14]		Mascot
2127.054	2127.1038	0.0498	23	192	210	LYSLDAGSIINSLCFSPNR	125	100	Carbamidomethyl (C)[14]		Mascot
2178.0034	2178.0469	0.0435	20	100	120	YTIGGDLGGGEGHTGWVSCVR			Carbamidomethyl (C)[19]		Mascot
2178.0034	2178.0469	0.0435	20	100	120	YTIGGDLGGGEGHTGWVSCVR	160	100	Carbamidomethyl (C)[19]		Mascot
2209.1038	2209.0549	-0.0489	-22	121	140	FSPNNFAPTIVSGSWDRSVK					Mascot
2497.2683	2497.333	0.0647	26	16	39	GHNDVVTAIATPIDNSPFIVSSSR					Mascot
2497.2683	2497.333	0.0647	26	16	39	GHNDVVTAIATPIDNSPFIVSSSR	172	100			Mascot
2923.4473	2923.5444	0.0971	33	42	67	SLLVWDLTNPIQATQDSSSEYGVPFR					Mascot

Rank	Protein Name	Accession No.	Protein MW	Protein PI	Pep. Count	Protein Score	Protein Score C. I. %	Intensity Matched	Total Ion Score	Total Ion C. I. %	Confirmed
2	unnamed protein product [Triticum aestivum]	gi|218389324	53505.2	5.29	12	49	46.834	3.286			

Peptide Information

Calc. Mass	Obsrv. Mass	± da	± ppm	Start Seq.	End Seq.	Sequence	Ion Score	C. I. %	Modification	Rank	Result Type
1093.6729	1093.5804	-0.0925	-85	316	324	VVHILKQEK					Mascot
1320.6754	1320.689	0.0136	10	141	153	QNGSSAAESTLKK					Mascot
1455.7587	1455.6965	-0.0622	-43	397	409	ALISSSPVMLFMK			Oxidation (M)[9,12]		Mascot
1898.9131	1898.9443	0.0312	16	429	446	GAGISFGSFDILSDEEVR					Mascot
1906.9287	1906.9453	0.0166	9	135	152	VQEMARQNGSSAAESTLK					Mascot
1910.9818	1910.9414	-0.0404	-21	86	104	EGKPVDTLEGANPASLANK					Mascot

1922.9237	1922.9436	0.0199	10	135	152	VQEMARQNGSSAAESTLK			Oxidation (M)[4]		Mascot
1924.0837	1923.9412	-0.1425	-74	154	169	RLEQLVNSHPVILFMK					Mascot
1949.0525	1948.9265	-0.126	-65	286	302	ARLESLTNSNPVMIFIK			Oxidation (M)[13]		Mascot
2132.0364	2132.1035	0.0671	31	358	377	GELVGGSDIVMEMHKSGELK			Oxidation (M)[11]		Mascot
2166.1077	2166.041	-0.0667	-31	325	343	IPFSSFDILSDDEVRQGLK					Mascot
2190.1045	2190.1123	0.0078	4	397	416	ALISSSPVMLFMKGNPDNPR			Oxidation (M)[9]		Mascot
2206.0996	2206.0479	-0.0517	-23	397	416	ALISSSPVMLFMKGNPDNPR			Oxidation (M)[9,12]		Mascot
2209.1824	2209.0549	-0.1275	-58	86	107	EGKPVDTLEGANPASLANKVAK					Mascot

3	Transcriptional regulator ATRX [Triticum urartu]	gi|473759274	164310.8	5.49	24	49	40.347	19.324

Peptide Information

Calc. Mass	Obsrv. Mass	± da	± ppm	Start Seq.	End Seq.	Sequence	Ion Score	C. I. %	Modification	Rank	Result Type
872.5312	872.4738	-0.0574	-66	952	958	LSQLQRK					Mascot
922.4199	922.4426	0.0227	25	373	379	RTDGWCK			Carbamidomethyl (C)[6]		Mascot
1075.5266	1075.561	0.0344	32	116	125	AAEAQESLEK					Mascot
1248.6252	1248.6611	0.0359	29	575	585	NLGEKCISEAK			Carbamidomethyl (C)[6]		Mascot
1279.6865	1279.6604	-0.0261	-20	1170	1182	AGSLGVNLHAANR					Mascot
1302.6899	1302.6322	-0.0577	-44	126	136	ESLEKIEAEVR					Mascot
1396.6149	1396.6738	0.0589	42	644	655	IMDDTELGEETK			Oxidation (M)[2]		Mascot
1443.7802	1443.7639	-0.0163	-11	131	142	IEAEVRLELSER					Mascot
1455.7084	1455.6965	-0.0119	-8	926	937	GFVQRMDMNVVK			Oxidation (M)[6,8]		Mascot
1474.7294	1474.7556	0.0262	18	754	767	GFGCILAHNMGLGK			Carbamidomethyl (C)[4]		Mascot
1494.7408	1494.7688	0.028	19	1378	1390	QAQQPKSHSNNQK					Mascot
1535.735	1535.7836	0.0486	32	880	892	EGFLGSSHEFRNR					Mascot
1630.7959	1630.8601	0.0642	39	102	115	VEELVAEFHDVESK					Mascot
1673.8354	1673.8966	0.0612	37	1147	1160	QNLVERFNDPANTR					Mascot
1717.9385	1717.9836	0.0451	26	917	930	SHILYEQLKGFVQR					Mascot
1717.9385	1717.9836	0.0451	26	917	930	SHILYEQLKGFVQR					Mascot
1743.9164	1743.9794	0.063	36	476	491	GVDIIDLDLFPSQSPK					Mascot
1877.8997	1877.9895	0.0898	48	750	767	SGDKGFGCILAHNMGLGK			Carbamidomethyl (C)[8], Oxidation (M)[14]		Mascot
1898.9324	1898.9443	0.0119	6	1392	1407	CTNLNHMLTLRSHGTK			Carbamidomethyl (C)[1], Oxidation (M)[7]		Mascot
1909.0654	1908.9429	-0.1225	-64	840	857	GGVLLIGYSSFRNLSLGR					Mascot
1923.9117	1923.9412	0.0295	15	863	879	DTADEISNALQMVDFVR					Mascot
1967.0228	1966.968	-0.0548	-28	1081	1097	MVLLLDILATCYELGDK			Carbamidomethyl (C)[11]		Mascot

Calc. Mass	Obsrv. Mass	± da	± ppm	Start Seq.	End Seq.	Sequence	Ion Score	C. I. %	Modification	Rank	Result Type
2118.9873	2119.0366	0.0493	23	194	211	SIESQAPNVCETEAWKNR			Carbamidomethyl (C)[10]		Mascot
2149.1335	2149.0676	-0.0659	-31	990	1007	LAQIWNHPGLLQMAKEQR			Oxidation (M)[13]		Mascot
2497.1414	2497.333	0.1916	77	1332	1352	EEQDMAWSNFKQSQQLDAVAR			Oxidation (M)[5]		Mascot
2497.1414	2497.333	0.1916	77	1332	1352	EEQDMAWSNFKQSQQLDAVAR			Oxidation (M)[5]		Mascot

4 hypothetical protein TRIUR3_05572 [Triticum urartu] gi|474378111 36423.6 9.36 12 47 15.737 13.412

Peptide Information

Calc. Mass	Obsrv. Mass	± da	± ppm	Start Seq.	End Seq.	Sequence	Ion Score	C. I. %	Modification	Rank	Result Type
1066.5966	1066.4919	-0.1047	-98	183	191	IAAKMEYLK					Mascot
1075.6187	1075.561	-0.0577	-54	256	263	YYVVKYLK					Mascot
1262.6959	1262.6654	-0.0305	-24	203	213	IALSKSPMMLR			Oxidation (M)[8]		Mascot
1279.5848	1279.6604	0.0756	59	1	11	MGAGRESQNWK			Oxidation (M)[1]		Mascot
1509.6903	1509.7313	0.041	27	170	182	HAMHAVAFHSEEK			Oxidation (M)[3]		Mascot
1509.6903	1509.7313	0.041	27	170	182	HAMHAVAFHSEEK			Oxidation (M)[3]		Mascot
1537.7428	1537.7664	0.0236	15	156	169	ADGLGVSRESPMFR			Oxidation (M)[12]		Mascot
1618.88	1618.853	-0.027	-17	21	35	QDHVPLLPPSVTTSK					Mascot
1745.8467	1745.9937	0.147	84	6	19	ESQNWKGYHSTRPR					Mascot
1892.9436	1892.9376	-0.006	-3	170	186	HAMHAVAFHSEEKIAAK			Oxidation (M)[3]		Mascot
1908.8936	1908.9429	0.0493	26	273	288	DYYSALTPSEMVFLEK			Oxidation (M)[11]		Mascot
2192.1638	2192.0759	-0.0879	-40	82	100	LQYYLTLFGSSEAFLQALK					Mascot
2497.2869	2497.333	0.0461	18	101	122	FNCNLLTHSIESAVEPNVALLR			Carbamidomethyl (C)[3]		Mascot
2497.2869	2497.333	0.0461	18	101	122	FNCNLLTHSIESAVEPNVALLR			Carbamidomethyl (C)[3]		Mascot

5 ATP-dependent DNA helicase 2 subunit KU80 [Triticum urartu] gi|474055978 85956.1 8.84 15 47 15.737 5.906

Peptide Information

Calc. Mass	Obsrv. Mass	± da	± ppm	Start Seq.	End Seq.	Sequence	Ion Score	C. I. %	Modification	Rank	Result Type
1075.6259	1075.561	-0.0649	-60	361	370	GVKLLGFADR					Mascot
1403.7498	1403.7765	0.0267	19	404	415	AMQEMNKVAILR					Mascot
1494.7621	1494.7688	0.0067	4	612	624	ARAIEVMFPSTEK			Oxidation (M)[7]		Mascot
1535.8138	1535.7836	-0.0302	-20	614	627	AIEVMFPSTEKVGK					Mascot
1745.9769	1745.9937	0.0168	10	90	105	TVSQSIPPRPPPSRAR					Mascot
1850.8235	1850.9246	0.1011	55	46	60	GVCDDFMICRFSLSK			Carbamidomethyl (C)[3,9], Oxidation (M)[7]		Mascot
1870.9805	1871.1074	0.1269	68	28	45	RLSGNVCSVGGVVPIDN			Carbamidomethyl (C)[7]		Mascot

						K					
1878.0266	1877.9895	-0.0371	-20	136	152	NKEGLVLLLDVGPSMHR					Mascot
1910.1322	1909.9454	-0.1868	-98	238	256	SVAKVVQVDTPTALLGAL K					Mascot
2165.0398	2165.041	0.0012	1	659	676	KYTTNLVENSLQGDYYE K					Mascot
2189.085	2189.1606	0.0756	35	169	188	ASGTLFPSGTLCWFFIGA TR			Carbamidomethyl (C)[12]		Mascot
2193.1226	2193.0671	-0.0555	-25	336	355	GYLYGPQVVPISSAEWE AVK					Mascot
2210.04	2210.0381	-0.0019	-1	301	319	APPSDQFASHEVKVDYE YK					Mascot
2226.0747	2226.0239	-0.0508	-23	686	703	KACIIEQEPEEYNQFVTK			Carbamidomethyl (C)[3]		Mascot
2923.4805	2923.5444	0.0639	22	486	510	MLDLAPPGREEILRPDFT PNPMLER			Oxidation (M)[1]		Mascot
2939.4753	2939.5347	0.0594	20	486	510	MLDLAPPGREEILRPDFT PNPMLER			Oxidation (M)[1,22]		Mascot

6 Aldehyde dehydrogenase family 3 member H1 [Triticum urartu] gi|474427784 36522.1 8.88 10 46 0 2.221

Peptide Information

Calc. Mass	Obsrv. Mass	± da	± ppm	Start Seq.	End Seq.	Sequence	Ion Score	C. I. %	Modification	Rank	Result Type
1262.7467	1262.6654	-0.0813	-64	58	69	HLTPVVLELGGK					Mascot
1294.6321	1294.6664	0.0343	26	287	298	AVMNRGFAGEAR			Oxidation (M)[3]		Mascot
1430.7825	1430.7469	-0.0356	-25	320	332	LGDMIQAILGFPR					Mascot
1455.759	1455.6965	-0.0625	-43	27	38	ETTALLNHKWDK					Mascot
1494.7179	1494.7688	0.0509	34	150	162	LKGLMDEEMVSDK					Mascot
1615.8989	1615.8309	-0.068	-42	320	334	LGDMIQAILGFPRGK					Mascot
1893.0011	1892.9376	-0.0635	-34	70	86	CPVVVDSNVDLHIAAKR			Carbamidomethyl (C)[1]		Mascot
2035.9576	2036.0845	0.1269	62	152	169	GLMDEEMVSDKIVFGGH R			Oxidation (M)[3]		Mascot
2166.1299	2166.041	-0.0889	-41	1	19	MSSLLAELLPRFVDGTCI K			Carbamidomethyl (C)[17], Oxidation (M)[1]		Mascot
2479.3167	2479.364	0.0473	19	208	229	IHESFGLINSMAKPLAAYL FTR					Mascot

7 Ku80 [Triticum aestivum] gi|308052434 80415.4 5.91 15 46 0 6.286

Peptide Information

Calc. Mass	Obsrv. Mass	± da	± ppm	Start Seq.	End Seq.	Sequence	Ion Score	C. I. %	Modification	Rank	Result Type
1075.6259	1075.561	-0.0649	-60	311	320	GVKLLGFADR					Mascot
1403.7498	1403.7765	0.0267	19	354	365	AMQEMNKVAILR					Mascot
1433.7029	1433.7699	0.067	47	154	164	HDIKMDCIVFR			Carbamidomethyl (C)[7]		Mascot
1494.7621	1494.7688	0.0067	4	562	574	ARAIEVMFPSTEK			Oxidation (M)[7]		Mascot
1535.8138	1535.7836	-0.0302	-20	564	577	AIEVMFPSTEKVGK					Mascot

Calc. Mass	Obsrv. Mass	± da	± ppm	Start Seq.	End Seq.	Sequence	Ion Score	C. I. %	Modification	Rank	Result Type
1602.7871	1602.8632	0.0761	47	54	67	ETHNDLARELGGYK					Mascot
1878.0266	1877.9895	-0.0371	-20	4	20	NKEGLVLLLDVGPSMHR					Mascot
1910.1322	1909.9454	-0.1868	-98	188	206	SVAKVVQVDTPTALLGAL K					Mascot
2131.9812	2132.1035	0.1223	57	134	152	DPPQGTKEDQVDTIADQ MK			Oxidation (M)[18]		Mascot
2165.0398	2165.041	0.0012	1	609	626	KYTTNLVENSLQGDYYE K					Mascot
2176.1543	2176.0364	-0.1179	-54	122	140	LCLITDAQHLLRDPPQGT K			Carbamidomethyl (C)[2]		Mascot
2193.1226	2193.0671	-0.0555	-25	286	305	GYLYGPQVVPISSAEWE AVK					Mascot
2210.04	2210.0381	-0.0019	-1	251	269	APPSDQFASHEVKVDYE YK					Mascot
2226.0747	2226.0239	-0.0508	-23	636	653	KACIIEQEPEEYNQFVTK			Carbamidomethyl (C)[3]		Mascot
2923.4805	2923.5444	0.0639	22	436	460	MLDLAPPGREEILRPDFT PNPMLER			Oxidation (M)[1]		Mascot
2939.4753	2939.5347	0.0594	20	436	460	MLDLAPPGREEILRPDFT PNPMLER			Oxidation (M)[1,22]		Mascot

8 hypothetical protein TRIUR3_13495 [Triticum urartu] gi|474389127 30891.4 7.78 9 45 0 5.479

Peptide Information

Calc. Mass	Obsrv. Mass	± da	± ppm	Start Seq.	End Seq.	Sequence	Ion Score	C. I. %	Modification	Rank	Result Type
1248.6121	1248.6611	0.049	39	16	26	FYAAQLGHADR					Mascot
1320.7158	1320.689	-0.0268	-20	265	275	EILAIYQTKNQ					Mascot
1443.7816	1443.7639	-0.0177	-12	125	138	EAHLQVLLGGGGHR					Mascot
1461.806	1461.7517	-0.0543	-37	108	121	FAVPGSQSLSLLSR					Mascot
1630.8007	1630.8601	0.0594	36	1	14	MDSEHWISRLAAAK			Oxidation (M)[1]		Mascot
1871.0134	1871.1074	0.094	50	243	259	GEKSTANQAFVLIISHR					Mascot
2109.218	2109.1035	-0.1145	-54	246	264	STANQAFVLIISHRLLPTK					Mascot
2134.032	2134.0605	0.0285	13	86	102	DMLNHITMQHGYLFKNR			Oxidation (M)[2]		Mascot
2178.0835	2178.0469	-0.0366	-17	83	100	VSKDMLNHITMQHGYLF K			Oxidation (M)[5]		Mascot
2178.0835	2178.0469	-0.0366	-17	83	100	VSKDMLNHITMQHGYLF K			Oxidation (M)[5]		Mascot
2194.0784	2194.0432	-0.0352	-16	83	100	VSKDMLNHITMQHGYLF K			Oxidation (M)[5,11]		Mascot

9 putative NAC transcription factor [Triticum aestivum] gi|296044562 38870.9 7.24 9 43 0 6.766

Peptide Information

Calc. Mass	Obsrv. Mass	± da	± ppm	Start Seq.	End Seq.	Sequence	Ion Score	C. I. %	Modification	Rank	Result Type
1403.8158	1403.7765	-0.0393	-28	132	143	KALVFYQGRPPK					Mascot
1892.9514	1892.9376	-0.0138	-7	93	109	YPNGVRPNRAAGSGYW K					Mascot

1978.8607	1978.9783	0.1176	59	74	89	AVFEGDNDEWYFFSPR					Mascot
2118.9468	2119.0366	0.0898	42	305	324	QATADSNNDGEMSSLHASKR					Mascot
2149.1433	2149.0676	-0.0757	-35	1	20	MTIELQTRPSLPAPAGTPPR			Oxidation (M)[1]		Mascot
2165.1362	2165.041	-0.0952	-44	156	175	LADAHAAAAAHTYRPPTRFK					Mascot
2249.9888	2250.0776	0.0888	39	74	91	AVFEGDNDEWYFFSPRDR					Mascot
2923.5022	2923.5444	0.0422	14	47	73	AAAAPCPAAVIAEVDIYKLDPWELPSR			Carbamidomethyl (C)[6]		Mascot
2955.6165	2955.5305	-0.086	-29	2	30	TIELQTRPSLPAPAGTPPRAGTGLPPGFR					Mascot

10 CNL2 [Triticum monococcum subsp. monococcum] gi|521311581 106129.4 5.85 17 43 0 13.322

Peptide Information

Calc. Mass	Obsrv. Mass	± da	± ppm	Start Seq.	End Seq.	Sequence	Ion Score	C. I. %	Modification	Rank	Result Type
1401.722	1401.7839	0.0619	44	120	132	ISDALEEAVQQAK					Mascot
1422.6417	1422.6573	0.0156	11	403	414	GLTEDQNLEEMK			Oxidation (M)[11]		Mascot
1433.6438	1433.7699	0.1261	88	87	99	MENGGEPTNSKNR					Mascot
1444.8312	1444.7787	-0.0525	-36	855	866	LDFVLPAKWISR					Mascot
1487.7754	1487.7015	-0.0739	-50	391	402	HQWYTLLNSIGR					Mascot
1489.7581	1489.7595	0.0014	1	863	874	WISRENIDLGMR					Mascot
1535.7134	1535.7836	0.0702	46	749	760	RLAFMSSCSWFK			Carbamidomethyl (C)[8], Oxidation (M)[5]		Mascot
1743.851	1743.9794	0.1284	74	71	84	ELSYQMEEIIDVFK					Mascot
1922.993	1922.9436	-0.0494	-26	116	132	DLHRISDALEEAVQQAK					Mascot
1924.13	1923.9412	-0.1888	-98	32	49	GIVSLVTELKLMHAVLGK			Oxidation (M)[12]		Mascot
1926.8651	1926.9362	0.0711	37	219	235	GQFNCDAFISVSQNPDK			Carbamidomethyl (C)[5]		Mascot
1926.8651	1926.9362	0.0711	37	219	235	GQFNCDAFISVSQNPDK			Carbamidomethyl (C)[5]		Mascot
1967.0597	1966.968	-0.0917	-47	53	70	VPVDQLDEGVKIWAGNVK					Mascot
1999.0205	1998.9618	-0.0587	-29	71	86	ELSYQMEEIIDVFKVR					Mascot
2087.0037	2087.0476	0.0439	21	158	175	MMALYTDVTELVGIEETR			Oxidation (M)[1]		Mascot
2102.9985	2103.04	0.0415	20	158	175	MMALYTDVTELVGIEETR			Oxidation (M)[1,2]		Mascot
2119.0601	2119.0366	-0.0235	-11	344	361	RVFSQENGCPQELLQVSK			Carbamidomethyl (C)[9]		Mascot
2164.9929	2165.041	0.0481	22	139	157	QRYEQEMPDTSVGASVDPR					Mascot
2249.072	2249.0774	0.0054	2	464	482	QETSLYELGNSYFSELVNR					Mascot

Gel Idx/Pos	275/L3	Instr./Gel Origin	BA2151/Sample Project 20140814	Process Status	Analysis Succeeded
Plate [#] Name	[1] Sample Project 20140814	Instrument Sample Name		Spectra	11

Rank	Protein Name	Accession No.	Protein MW	Protein PI	Pep. Count	Protein Score	Protein Score C. I. %	Intensity Matched	Total Ion Score	Total Ion C. I. %	Confirmed
1	apt1-2 [Triticum aestivum]	gi|291498596	55556.8	5.7	21	759	100	55.254	641	100	

Protein Group

ATP synthase subunit alpha [Triticum durum x Triticosecale sp.]	gi|1405781	55556.8	5.6999998092 6514
apt1 [Triticum aestivum]	gi|169649046	55556.8	5.6999998092 6514
apt1-1 [Triticum aestivum]	gi|291498595	55556.8	5.6999998092 6514
atp1 [Triticum aestivum]	gi|78675233	55556.8	5.6999998092 6514
atp1 [Triticum aestivum]	gi|81176509	55556.8	5.6999998092 6514
unnamed protein product [Triticum aestivum]	gi|13725	55556.8	5.6999998092 6514

Peptide Information

Calc. Mass	Obsrv. Mass	± da	± ppm	Start Seq.	End Seq.	Sequence	Ion Score	C. I. %	Modification	Rank	Result Type
815.4621	815.4589	-0.0032	-4	167	173	ELIIGDR					Mascot
860.5022	860.4783	-0.0239	-28	283	289	QMSLLLR					Mascot
876.4971	876.4769	-0.0202	-23	283	289	QMSLLLR			Oxidation (M)[2]		Mascot
884.4221	884.4244	0.0023	3	121	128	GALSDHER					Mascot
892.4886	892.4926	0.004	4	395	401	LELAQYR					Mascot
972.5473	972.5458	-0.0015	-2	33	42	VVSVGDGIAR					Mascot
1026.5942	1026.593	-0.0012	-1	154	163	AVDSLVPIGR					Mascot
1026.5942	1026.593	-0.0012	-1	154	163	AVDSLVPIGR	70	100			Mascot
1203.658	1203.6637	0.0057	5	7	17	AAELTTLLESR					Mascot
1242.6147	1242.5919	-0.0228	-18	143	153	SVHEPMQTGLK			Oxidation (M)[6]		Mascot
1300.7471	1300.7046	-0.0425	-33	178	189	TAIAIDTILNQK					Mascot
1326.7627	1326.7194	-0.0433	-33	470	481	AILSTINPELQK					Mascot
1341.705	1341.667	-0.038	-28	433	443	QPQYEPLPIEK					Mascot
1438.8489	1438.8525	0.0036	3	363	376	GIRPAINVGLSVSR					Mascot

1438.8489	1438.8525	0.0036	3	363	376	GIRPAINVGLSVSR	54	99.971			Mascot
1537.7434	1537.7633	0.0199	13	295	307	EAFPGDVFYLHSR					Mascot
1537.7434	1537.7633	0.0199	13	295	307	EAFPGDVFYLHSR	109	100			Mascot
1638.8268	1638.778	-0.0488	-30	388	401	QVCGSSKLELAQYR			Carbamidomethyl (C)[3]		Mascot
1704.8262	1704.8168	-0.0094	-6	262	276	DNGMHALIIYDDLSK					Mascot
1724.8789	1724.9037	0.0248	14	444	458	QIVVIYAAVNGFCDR			Carbamidomethyl (C)[13]		Mascot
1834.8429	1834.8728	0.0299	16	18	32	MTNFYTNFQVDEIGR					Mascot
1834.8429	1834.8728	0.0299	16	18	32	MTNFYTNFQVDEIGR	130	100			Mascot
1850.8378	1850.8463	0.0085	5	18	32	MTNFYTNFQVDEIGR			Oxidation (M)[1]		Mascot
1850.8378	1850.8463	0.0085	5	18	32	MTNFYTNFQVDEIGR	167	100	Oxidation (M)[1]		Mascot
2031.9692	2031.9708	0.0016	1	195	212	GTNESETLYCVYVAIGQK			Carbamidomethyl (C)[10]		Mascot
2141.0583	2141.0762	0.0179	8	43	62	VYGLNEIQAGEMVEFAS GVK					Mascot
2157.0532	2157.0676	0.0144	7	43	62	VYGLNEIQAGEMVEFAS GVK			Oxidation (M)[12]		Mascot
2308.1567	2308.2153	0.0586	25	402	423	EVAAFAQFGSDLDAATQ ALLNR					Mascot
2308.1567	2308.2153	0.0586	25	402	423	EVAAFAQFGSDLDAATQ ALLNR	241	100			Mascot
2373.2661	2373.2607	-0.0054	-2	63	85	GIALNLENENVGIVVFGS DTAIK					Mascot

2	ATP synthase subunit alpha, mitochondrial [Triticum urartu]	gi|474033641	44894.3	5.54	16	564	100	31.126	478	100

Peptide Information

Calc. Mass	Obsrv. Mass	± da	± ppm	Start Seq.	End Seq.	Sequence	Ion Score	C. I. %	Modification	Rank	Result Type
815.4621	815.4589	-0.0032	-4	150	156	ELIIGDR					Mascot
884.4221	884.4244	0.0023	3	104	111	GALSDHER					Mascot
892.4886	892.4926	0.004	4	297	303	LELAQYR					Mascot
972.5473	972.5458	-0.0015	-2	16	25	VVSVGDGIAR					Mascot
1026.5942	1026.593	-0.0012	-1	137	146	AVDSLVPIGR					Mascot
1026.5942	1026.593	-0.0012	-1	137	146	AVDSLVPIGR	70	100			Mascot
1242.6147	1242.5919	-0.0228	-18	126	136	SVHEPMQTGLK			Oxidation (M)[6]		Mascot
1300.7471	1300.7046	-0.0425	-33	161	172	TAIAIDTILNQK					Mascot
1341.705	1341.667	-0.038	-28	335	345	QPQYEPLPIEK					Mascot
1638.8268	1638.778	-0.0488	-30	290	303	QVCGSSKLELAQYR			Carbamidomethyl (C)[3]		Mascot
1646.9225	1646.8326	-0.0899	-55	16	31	VVSVGDGIARVYGLNK					Mascot
1703.8025	1703.8191	0.0166	10	2	15	TNFYTNFQVDEIGR					Mascot
1704.8262	1704.8168	-0.0094	-6	245	259	DNGMHALIIYDDLSK					Mascot
1834.8429	1834.8728	0.0299	16	1	15	MTNFYTNFQVDEIGR					Mascot

1834.8429	1834.8728	0.0299	16	1	15	MTNFYTNFQVDEIGR	130	100			Mascot
1850.8378	1850.8463	0.0085	5	1	15	MTNFYTNFQVDEIGR			Oxidation (M)[1]		Mascot
1850.8378	1850.8463	0.0085	5	1	15	MTNFYTNFQVDEIGR	167	100	Oxidation (M)[1]		Mascot
2031.9692	2031.9708	0.0016	1	178	195	GTNESETLYCVYVAIGQK			Carbamidomethyl (C)[10]		Mascot
2308.1567	2308.2153	0.0586	25	304	325	EVAAFAQFGSDLDAATQ ALLNR					Mascot
2308.1567	2308.2153	0.0586	25	304	325	EVAAFAQFGSDLDAATQ ALLNR	241	100			Mascot
2373.2661	2373.2607	-0.0054	-2	46	68	GIALNLENENVGIVVFGS DTAIK					Mascot

3 RecName: Full=ATP synthase subunit alpha, mitochondrial | gi\|114419 | 55514.8 | 5.7 | 20 | 507 | 100 | 38.614 | 400 | 100

Peptide Information

Calc. Mass	Obsrv. Mass	± da	± ppm	Start Seq.	End Seq.	Sequence	Ion Score	C. I. %	Modification	Rank	Result Type
815.4621	815.4589	-0.0032	-4	167	173	ELIIGDR					Mascot
860.5022	860.4783	-0.0239	-28	283	289	QMSLLLR					Mascot
876.4971	876.4769	-0.0202	-23	283	289	QMSLLLR			Oxidation (M)[2]		Mascot
884.4221	884.4244	0.0023	3	121	128	GALSDHER					Mascot
892.4886	892.4926	0.004	4	395	401	LELAQYR					Mascot
972.5473	972.5458	-0.0015	-2	33	42	VVSVGDGIAR					Mascot
1026.5942	1026.593	-0.0012	-1	154	163	AVDSLVPIGR					Mascot
1026.5942	1026.593	-0.0012	-1	154	163	AVDSLVPIGR	70	100			Mascot
1203.658	1203.6637	0.0057	5	7	17	AAELTTLLESR					Mascot
1242.6147	1242.5919	-0.0228	-18	143	153	SVHEPMQTGLK			Oxidation (M)[6]		Mascot
1300.7471	1300.7046	-0.0425	-33	178	189	TAIAIDTILNQK					Mascot
1326.7627	1326.7194	-0.0433	-33	470	481	AILSTINPELQK					Mascot
1341.705	1341.667	-0.038	-28	433	443	QPQYEPLPIEK					Mascot
1438.8489	1438.8525	0.0036	3	363	376	GIRPAINVGLSVSR					Mascot
1438.8489	1438.8525	0.0036	3	363	376	GIRPAINVGLSVSR	54	99.971			Mascot
1537.7434	1537.7633	0.0199	13	295	307	EAFPGDVFYLHSR					Mascot
1537.7434	1537.7633	0.0199	13	295	307	EAFPGDVFYLHSR	109	100			Mascot
1638.8268	1638.778	-0.0488	-30	388	401	QVCGSSKLELAQYR			Carbamidomethyl (C)[3]		Mascot
1704.8262	1704.8168	-0.0094	-6	262	276	DNGMHALIIYDDLSK					Mascot
1724.8789	1724.9037	0.0248	14	444	458	QIVVIYAAVNGFCDR			Carbamidomethyl (C)[13]		Mascot
1834.8429	1834.8728	0.0299	16	18	32	MTNFYTNFQVDEIGR					Mascot
1834.8429	1834.8728	0.0299	16	18	32	MTNFYTNFQVDEIGR	130	100			Mascot
1850.8378	1850.8463	0.0085	5	18	32	MTNFYTNFQVDEIGR			Oxidation (M)[1]		Mascot
1850.8378	1850.8463	0.0085	5	18	32	MTNFYTNFQVDEIGR	167	100	Oxidation (M)[1]		Mascot

Calc. Mass	Obsrv. Mass	± da	± ppm	Start Seq.	End Seq.	Sequence	Ion Score	C. I. %	Modification	Rank	Result Type
2031.9692	2031.9708	0.0016	1	195	212	GTNESETLYCVYVAIGQK			Carbamidomethyl (C)[10]		Mascot
2141.0583	2141.0762	0.0179	8	43	62	VYGLNEIQAGEMVEFAS GVK					Mascot
2157.0532	2157.0676	0.0144	7	43	62	VYGLNEIQAGEMVEFAS GVK			Oxidation (M)[12]		Mascot
2373.2661	2373.2607	-0.0054	-2	63	85	GIALNLENENVGIVVFGS DTAIK					Mascot

4	ATP synthase subunit alpha, mitochondrial [Triticum urartu]	gi|474046276	47623.9	9.18	16	441	100	26.719	365	100

Peptide Information

Calc. Mass	Obsrv. Mass	± da	± ppm	Start Seq.	End Seq.	Sequence	Ion Score	C. I. %	Modification	Rank	Result Type
815.4621	815.4589	-0.0032	-4	148	154	ELIIGDR					Mascot
860.5022	860.4783	-0.0239	-28	238	244	QMSLLLR					Mascot
876.4971	876.4769	-0.0202	-23	238	244	QMSLLLR			Oxidation (M)[2]		Mascot
884.4221	884.4244	0.0023	3	102	109	GALSDHER					Mascot
892.4886	892.4926	0.004	4	350	356	LELAQYR					Mascot
1026.5942	1026.593	-0.0012	-1	135	144	AVDSLVPIGR					Mascot
1026.5942	1026.593	-0.0012	-1	135	144	AVDSLVPIGR	70	100			Mascot
1242.6147	1242.5919	-0.0228	-18	124	134	SVHEPMQTGLK			Oxidation (M)[6]		Mascot
1259.6777	1259.603	-0.0747	-59	73	84	RTGSIVDVPMGK					Mascot
1300.7471	1300.7046	-0.0425	-33	159	170	TAIAIDTILNQK					Mascot
1341.705	1341.667	-0.038	-28	388	398	QPQYEPLPIEK					Mascot
1438.8489	1438.8525	0.0036	3	318	331	GIRPAINVGLSVSR					Mascot
1438.8489	1438.8525	0.0036	3	318	331	GIRPAINVGLSVSR	54	99.971			Mascot
1638.8268	1638.778	-0.0488	-30	343	356	QVCGSSKLELAQYR			Carbamidomethyl (C)[3]		Mascot
1704.8262	1704.8168	-0.0094	-6	217	231	DNGMHALIIYDDLSK					Mascot
1707.9099	1707.8719	-0.038	-22	85	101	AMLGHVVDALGVPIDGK			Oxidation (M)[2]		Mascot
1724.8789	1724.9037	0.0248	14	399	413	QIVVIYAAVNGFCDR			Carbamidomethyl (C)[13]		Mascot
1784.7771	1784.9336	0.1565	88	202	216	FLAPYSGCAMGEYFR			Carbamidomethyl (C)[8], Oxidation (M)[10]		Mascot
2308.1567	2308.2153	0.0586	25	357	378	EVAAFAQFGSDLDAATQ ALLNR					Mascot
2308.1567	2308.2153	0.0586	25	357	378	EVAAFAQFGSDLDAATQ ALLNR	241	100			Mascot

5	ATP synthase subunit alpha, mitochondrial [Triticum urartu]	gi|474247591	36637.9	5.92	14	425	100	32.271	346	100

Peptide Information

Calc. Mass	Obsrv. Mass	± da	± ppm	Start Seq.	End Seq.	Sequence	Ion Score	C. I. %	Modification	Rank	Result Type

Calc. Mass	Obsrv. Mass	± da	± ppm	Start Seq.	End Seq.	Sequence	Ion Score	C. I. %	Modification	Rank	Result Type
815.4621	815.4589	-0.0032	-4	150	156	ELIIGDR					Mascot
860.5022	860.4783	-0.0239	-28	266	272	QMSLLLR					Mascot
876.4971	876.4769	-0.0202	-23	266	272	QMSLLLR			Oxidation (M)[2]		Mascot
884.4221	884.4244	0.0023	3	104	111	GALSDHER					Mascot
972.5473	972.5458	-0.0015	-2	16	25	VVSVGDGIAR					Mascot
1026.5942	1026.593	-0.0012	-1	137	146	AVDSLVPIGR					Mascot
1026.5942	1026.593	-0.0012	-1	137	146	AVDSLVPIGR	70	100			Mascot
1242.6147	1242.5919	-0.0228	-18	126	136	SVHEPMQTGLK			Oxidation (M)[6]		Mascot
1300.7471	1300.7046	-0.0425	-33	161	172	TAIAIDTILNQK					Mascot
1537.7434	1537.7633	0.0199	13	278	290	EAFPGDVFYLHSR					Mascot
1537.7434	1537.7633	0.0199	13	278	290	EAFPGDVFYLHSR	109	100			Mascot
1703.8025	1703.8191	0.0166	10	2	15	TNFYTNFQVDEIGR					Mascot
1704.8262	1704.8168	-0.0094	-6	245	259	DNGMHALIIYDDLSK					Mascot
1834.8429	1834.8728	0.0299	16	1	15	MTNFYTNFQVDEIGR					Mascot
1834.8429	1834.8728	0.0299	16	1	15	MTNFYTNFQVDEIGR	130	100			Mascot
1850.8378	1850.8463	0.0085	5	1	15	MTNFYTNFQVDEIGR			Oxidation (M)[1]		Mascot
1850.8378	1850.8463	0.0085	5	1	15	MTNFYTNFQVDEIGR	167	100	Oxidation (M)[1]		Mascot
2031.9692	2031.9708	0.0016	1	178	195	GTNESETLYCVYVAIGQK			Carbamidomethyl (C)[10]		Mascot
2141.0583	2141.0762	0.0179	8	26	45	VYGLNEIQAGEMVEFAS GVK					Mascot
2157.0532	2157.0676	0.0144	7	26	45	VYGLNEIQAGEMVEFAS GVK			Oxidation (M)[12]		Mascot
2373.2661	2373.2607	-0.0054	-2	46	68	GIALNLENENVGIVVFGS DTAIK					Mascot

6	ATP synthase subunit alpha, mitochondrial [Triticum urartu]	gi|474015734	16963	5.25	6	197	100	9.337	167	100	

Peptide Information

Calc. Mass	Obsrv. Mass	± da	± ppm	Start Seq.	End Seq.	Sequence	Ion Score	C. I. %	Modification	Rank	Result Type
815.4621	815.4589	-0.0032	-4	152	158	ELIIGDR					Mascot
884.4221	884.4244	0.0023	3	106	113	GALSDHER					Mascot
972.5473	972.5458	-0.0015	-2	16	25	VVSVGDGIAR					Mascot
1703.8025	1703.8191	0.0166	10	2	15	TNFYTNFQVDEIGR					Mascot
1834.8429	1834.8728	0.0299	16	1	15	MTNFYTNFQVDEIGR					Mascot
1834.8429	1834.8728	0.0299	16	1	15	MTNFYTNFQVDEIGR	130	100			Mascot
1850.8378	1850.8463	0.0085	5	1	15	MTNFYTNFQVDEIGR			Oxidation (M)[1]		Mascot
1850.8378	1850.8463	0.0085	5	1	15	MTNFYTNFQVDEIGR	167	100	Oxidation (M)[1]		Mascot
2373.2661	2373.2607	-0.0054	-2	46	68	GIALNLENENVGIVVFGS DTAIK					Mascot

7	ATP synthase subunit alpha, mitochondrial [Triticum urartu]	gi|474015735	23605.2	5.96	5	128	100	20.692	109	100	

Peptide Information

Calc. Mass	Obsrv. Mass	± da	± ppm	Start Seq.	End Seq.	Sequence	Ion Score	C. I. %	Modification	Rank	Result Type
1276.7307	1276.6971	-0.0336	-26	1	11	MSLLLHRPPGR					Mascot
1326.7627	1326.7194	-0.0433	-33	175	186	AILSTINPELQK					Mascot
1341.705	1341.667	-0.038	-28	138	148	QPQYEPLPIEK					Mascot
1537.7434	1537.7633	0.0199	13	12	24	EAFPGDVFYLHSR					Mascot
1537.7434	1537.7633	0.0199	13	12	24	EAFPGDVFYLHSR	109	100			Mascot
1724.8789	1724.9037	0.0248	14	149	163	QIVVIYAAVNGFCDR			Carbamidomethyl (C)[13]		Mascot

8	Exocyst complex component 7 [Triticum urartu]	gi|474090276	39671.3	6.03	9	51	66.454	19.239	14	0	

Peptide Information

Calc. Mass	Obsrv. Mass	± da	± ppm	Start Seq.	End Seq.	Sequence	Ion Score	C. I. %	Modification	Rank	Result Type
859.5108	859.4566	-0.0542	-63	233	239	QLSTRVR					Mascot
1535.7734	1535.7847	0.0113	7	88	102	AEVSAVCNTLGSSIK			Carbamidomethyl (C)[7]		Mascot
1790.9105	1790.9232	0.0127	7	306	322	AAVEDSVMPAYAALINR					Mascot
2032.0896	2031.9708	-0.1188	-58	306	324	AAVEDSVMPAYAALINRL K					Mascot
2227.2446	2227.1453	-0.0993	-45	248	270	STWGKVTTVLQIGGSGV GALPAK					Mascot
2239.2844	2239.1904	-0.094	-42	253	275	VTTVLQIGGSGVGALPAK AMLQK					Mascot
2241.1406	2241.1792	0.0386	17	198	216	DPSLACIFLMNNGKYIIQK			Carbamidomethyl (C)[6], Oxidation (M)[10]		Mascot
2264.0396	2264.1965	0.1569	69	139	160	AACGSRQTLEEVMEGDL GAGGR			Carbamidomethyl (C)[3]		Mascot
2308.1423	2308.2153	0.073	32	302	322	MDVRAAVEDSVMPAYAA LINR			Oxidation (M)[1]		Mascot
2308.1423	2308.2153	0.073	32	302	322	MDVRAAVEDSVMPAYAA LINR	14	0	Oxidation (M)[1]		Mascot

9	Diaminopimelate epimerase, chloroplastic [Triticum urartu]	gi|474357580	40520.2	6.47	11	47	17.656	4.747			

Peptide Information

Calc. Mass	Obsrv. Mass	± da	± ppm	Start Seq.	End Seq.	Sequence	Ion Score	C. I. %	Modification	Rank	Result Type
859.4454	859.4566	0.0112	13	1	9	MTPAAAAAR					Mascot
876.4509	876.4769	0.026	30	300	305	MRVWER					Mascot
892.4457	892.4926	0.0469	53	300	305	MRVWER			Oxidation (M)[1]		Mascot
1185.6838	1185.6628	-0.021	-18	218	228	LPSTKNEAVVK					Mascot

1203.6515	1203.6637	0.0122	10	41	52	RAVASMAVSAPK			Oxidation (M)[6]		Mascot
1300.5892	1300.7046	0.1154	89	275	284	FEHHEMFPAR					Mascot
1638.9122	1638.778	-0.1342	-82	29	41	LLRPFCGNPRPRR			Carbamidomethyl (C)[6]		Mascot
1657.7639	1657.8154	0.0515	31	74	87	YQGLGNDFIMVDNR			Oxidation (M)[10]		Mascot
1746.8619	1746.9115	0.0496	28	202	217	VDMGEPILYGPDVPTK			Oxidation (M)[3]		Mascot
1759.9337	1759.8188	-0.1149	-65	285	299	TNTEFVEVLSRSHLK					Mascot
1910.9358	1910.9065	-0.0293	-15	184	199	AYTTIVFHSYYSIMSK					Mascot
2239.0813	2239.1904	0.1091	49	74	93	YQGLGNDFIMVDNRDSAVPK					Mascot

10 Protein kinase APK1A, chloroplastic [Triticum urartu] gi|474411736 41841.2 9.14 12 44 0 3.708

Peptide Information

Calc. Mass	Obsrv. Mass	± da	± ppm	Start Seq.	End Seq.	Sequence	Ion Score	C. I. %	Modification	Rank	Result Type
921.5152	921.4993	-0.0159	-17	335	342	LKGQFNSK					Mascot
957.5298	957.5562	0.0264	28	327	334	VARLMDPR					Mascot
1072.5786	1072.5801	0.0015	1	212	221	GLAFLHASEK					Mascot
1300.7261	1300.7046	-0.0215	-17	139	149	FLTVHLQSDIK					Mascot
1326.7053	1326.7194	0.0141	11	80	90	FTFLEIQTATR					Mascot
1638.8446	1638.778	-0.0666	-41	243	258	LSDYGLAKLGSTGNSR					Mascot
1657.8188	1657.8154	-0.0034	-2	125	138	MAVAIMHGKEEWQK					Mascot
1708.9164	1708.8625	-0.0539	-32	343	357	QALQVVHVALSCLDR			Carbamidomethyl (C)[12]		Mascot
1724.9806	1724.9037	-0.0769	-45	205	221	IAIGAARGLAFLHASEK					Mascot
1801.9443	1801.9081	-0.0362	-20	63	78	QGSFLEGQILETPNLR					Mascot
2031.9773	2031.9708	-0.0065	-3	115	133	TMNPTTSGPRMAVAIMHGK			Oxidation (M)[2,11]		Mascot
2054.095	2053.99	-0.105	-51	358	375	DPSCRPSIKVVLEALEQI			Carbamidomethyl (C)[4]		Mascot

Gel Idx/Pos	276/L4	Instr./Gel Origin	BA2151/Sample Project 20140814	Process Status	Analysis Succeeded
Plate [#] Name	[1] Sample Project 20140814	Instrument Sample Name		Spectra	11

Rank	Protein Name	Accession No.	Protein MW	Protein PI	Pep. Count	Protein Score	Protein Score C. I. %	Intensity Matched	Total Ion Score	Total Ion C. I. %	Confirmed
1	Beta-amylase [Triticum urartu]	gi|474451266	58995	5.34	15	449	100	34.98	384	100	

Peptide Information

Calc. Mass	Obsrv. Mass	± da	± ppm	Start Seq.	End Seq.	Sequence	Ion Score	C. I. %	Modification	Rank	Result Type
947.5057	947.4861	-0.0196	-21	322	329	DGYRPIAR					Mascot
1016.5564	1016.5525	-0.0039	-4	412	419	LFGFTYLR					Mascot
1016.5564	1016.5525	-0.0039	-4	412	419	LFGFTYLR	59	99.99			Mascot
1234.5448	1234.5884	0.0436	35	233	243	DAGQYNDAPQR					Mascot
1234.5448	1234.5884	0.0436	35	233	243	DAGQYNDAPQR					Mascot
1315.5769	1315.5629	-0.014	-11	336	346	ASLNFTCAEMR			Carbamidomethyl (C)[7], Oxidation (M)[10]		Mascot
1326.6688	1326.6639	-0.0049	-4	385	395	YDPTAYNTILR					Mascot
1326.6688	1326.6639	-0.0049	-4	385	395	YDPTAYNTILR	81	100			Mascot
1382.7791	1382.7528	-0.0263	-19	73	84	QLFQLVHEAGLK					Mascot
1490.6726	1490.6649	-0.0077	-5	372	384	EGLNMACENALPR			Carbamidomethyl (C)[7], Oxidation (M)[5]		Mascot
1646.781	1646.793	0.012	7	246	259	FFVDNGTYLTEQGR					Mascot
1668.7952	1668.7999	0.0047	3	218	232	AAAAMVGHPEWEFPR					Mascot
1684.79	1684.7843	-0.0057	-3	218	232	AAAAMVGHPEWEFPR			Oxidation (M)[5]		Mascot
1701.7247	1701.7289	0.0042	2	148	161	SAVQMYTDYMASFR			Oxidation (M)[5,10]		Mascot
1752.8916	1752.8677	-0.0239	-14	420	434	LSNQLVEGQNYVNFK					Mascot
1992.9047	1992.9198	0.0151	8	112	129	NVGASDPDIFYTDQHGT R					Mascot
1992.9047	1992.9198	0.0151	8	112	129	NVGASDPDIFYTDQHGT R	117	100			Mascot
2013.9778	2013.9973	0.0195	10	304	321	VPSHAAEITAGYYNLHDR					Mascot
2013.9778	2013.9973	0.0195	10	304	321	VPSHAAEITAGYYNLHDR	127	100			Mascot
2087.0557	2087.0767	0.021	10	130	147	NIEYLTLGVDDQPLFHGR					Mascot
2167.0537	2167.0732	0.0195	9	440	458	MHANLPHDPCVDPVAPL QR			Carbamidomethyl (C)[10]		Mascot
2183.0486	2183.0481	-0.0005	0	440	458	MHANLPHDPCVDPVAPL QR			Carbamidomethyl (C)[10], Oxidation (M)[1]		Mascot

Rank	Protein Name	Accession No.	Protein MW	Protein PI	Pep. Count	Protein Score	Protein Score C. I. %	Intensity Matched	Total Ion Score	Total Ion C. I. %	Confirmed
2	Aldehyde dehydrogenase family 2 member B7, mitochondrial [Triticum urartu]	gi|473987280	59912.1	5.84	13	290	100	9.32	243	100	

Protein Group

unnamed protein product [Triticum aestivum]	gi|291047856	59581.9	6.4000

Peptide Information

Calc. Mass	Obsrv. Mass	± da	± ppm	Start Seq.	End Seq.	Sequence	Ion Score	C. I. %	Modification	Rank	Result Type
900.5513	900.5527	0.0014	2	301	308	VILELSAR					Mascot
917.5203	917.5028	-0.0175	-19	379	386	VVGDPFRK					Mascot
932.4836	932.4709	-0.0127	-14	86	93	TFPTVDPR					Mascot
1028.4935	1028.5205	0.027	26	363	370	VYDEFVEK					Mascot
1074.5004	1074.5442	0.0438	41	119	127	AFDEGPWPR					Mascot
1202.5953	1202.5991	0.0038	3	118	127	KAFDEGPWPR					Mascot
1247.6743	1247.6477	-0.0266	-21	459	468	FNDLNEVIKR					Mascot
1507.8003	1507.7488	-0.0515	-34	242	255	TAEQTPLSALYVSK					Mascot
1589.7443	1589.7802	0.0359	23	387	400	GVEQGPQIDDEQFK					Mascot
1705.916	1705.8896	-0.0264	-15	444	458	IAQEEIFGPVQSIFK					Mascot
1977.9263	1977.917	-0.0093	-5	427	443	GYYIQPTIFSDVQDGMK			Oxidation (M)[16]		Mascot
2583.2798	2583.3201	0.0403	16	469	493	ANASQYGLAAGVFTNNL DTANTLTR					Mascot
2583.2798	2583.3201	0.0403	16	469	493	ANASQYGLAAGVFTNNL DTANTLTR	243	100			Mascot
2739.3809	2739.4189	0.038	14	468	493	RANASQYGLAAGVFTNN LDTANTLTR					Mascot

3	beta amylase [Triticum aestivum]	gi|32400764	31099.9	8.6	8	239	100	18.934	206	100		

Peptide Information

Calc. Mass	Obsrv. Mass	± da	± ppm	Start Seq.	End Seq.	Sequence	Ion Score	C. I. %	Modification	Rank	Result Type
947.5057	947.4861	-0.0196	-21	137	144	DGYRPIAR					Mascot
1234.5448	1234.5884	0.0436	35	48	58	DAGQYNDAPQR					Mascot
1234.5448	1234.5884	0.0436	35	48	58	DAGQYNDAPQR					Mascot
1315.5769	1315.5629	-0.014	-11	151	161	ASLNFTCAEMR			Carbamidomethyl (C)[7], Oxidation (M)[10]		Mascot
1326.6688	1326.6639	-0.0049	-4	200	210	YDPTAYNTILR					Mascot
1326.6688	1326.6639	-0.0049	-4	200	210	YDPTAYNTILR	81	100			Mascot
1490.6726	1490.6649	-0.0077	-5	187	199	EGLNMACENALPR			Carbamidomethyl (C)[7], Oxidation (M)[5]		Mascot
1646.781	1646.793	0.012	7	61	74	FFVDNGTYLTEQGR					Mascot
1668.7952	1668.7999	0.0047	3	33	47	AAAAMVGHPEWEFPR					Mascot
1684.79	1684.7843	-0.0057	-3	33	47	AAAAMVGHPEWEFPR			Oxidation (M)[5]		Mascot
2013.9778	2013.9973	0.0195	10	119	136	VPSHAAEITAGYYNLHDR					Mascot
2013.9778	2013.9973	0.0195	10	119	136	VPSHAAEITAGYYNLHDR	127	100			Mascot

4	beta-amylase [Triticum aestivum]	gi|1771782	56860.2	5.24	6	71	99.649	11.117	59	99.99

Protein Group

RecName: Full=Beta-amylase; AltName: Full=1,4-alpha-D-glucan maltohydrolase	gi|3334120	56860.2	5.2399 997711 1816

Peptide Information

Calc. Mass	Obsrv. Mass	± da	± ppm	Start Seq.	End Seq.	Sequence	Ion Score	C. I. %	Modification	Rank	Result Type
1016.5564	1016.5525	-0.0039	-4	411	418	LFGFTYLR					Mascot
1016.5564	1016.5525	-0.0039	-4	411	418	LFGFTYLR	59	99.99			Mascot
1285.6212	1285.5966	-0.0246	-19	61	71	GPKAYDWSAYK					Mascot
1431.7585	1431.6398	-0.1187	-83	458	469	SKPEMPIEMILK			Oxidation (M)[5]		Mascot
1591.6562	1591.7605	0.1043	66	333	345	HHASMNFTCAEMR			Carbamidomethyl (C)[9]		Mascot
1775.9229	1775.828	-0.0949	-53	404	418	NGPPEHKLFGFTYLR					Mascot
2087.0557	2087.0767	0.021	10	129	146	NIEYLTLGVDDQPLFHGR					Mascot

5	beta-amylase, partial [Triticum aestivum]	gi|451798942	15408.7	5.21	2	67	99.055	9.878	59	99.99

Peptide Information

Calc. Mass	Obsrv. Mass	± da	± ppm	Start Seq.	End Seq.	Sequence	Ion Score	C. I. %	Modification	Rank	Result Type
1016.5564	1016.5525	-0.0039	-4	29	36	LFGFTYLR					Mascot
1016.5564	1016.5525	-0.0039	-4	29	36	LFGFTYLR	59	99.99			Mascot
2167.0537	2167.0732	0.0195	9	57	75	MHANLPHDPCVDPVAPL QR			Carbamidomethyl (C)[10]		Mascot
2183.0486	2183.0481	-0.0005	0	57	75	MHANLPHDPCVDPVAPL QR			Carbamidomethyl (C)[10], Oxidation (M)[1]		Mascot

6	hypothetical protein TRIUR3_29779 [Triticum urartu]	gi|474262217	35184.4	9.62	12	56	88.633	5.161		

Peptide Information

Calc. Mass	Obsrv. Mass	± da	± ppm	Start Seq.	End Seq.	Sequence	Ion Score	C. I. %	Modification	Rank	Result Type
1074.6041	1074.5442	-0.0599	-56	94	102	EELTQIITK					Mascot
1197.6951	1197.6605	-0.0346	-29	228	237	QALELLVQQR					Mascot
1262.7004	1262.6199	-0.0805	-64	54	63	VFDVFIRPNR					Mascot
1382.8002	1382.7528	-0.0474	-34	108	120	ALPALVIKETGDR					Mascot
1507.8115	1507.7488	-0.0627	-42	94	105	EELTQIITKNYR					Mascot
1589.8507	1589.7802	-0.0705	-44	2	16	AAVASLPPATRNHER					Mascot
1684.8475	1684.7843	-0.0632	-38	121	134	LAATFGYEMRELQR					Mascot
1700.8425	1700.7959	-0.0466	-27	121	134	LAATFGYEMRELQR			Oxidation (M)[9]		Mascot

Calc. Mass	Obsrv. Mass	± da	± ppm	Start Seq.	End Seq.	Sequence	Ion Score	C. I. %	Modification	Rank	Result Type
1701.7538	1701.7289	-0.0249	-15	37	52	YATPCSSPFPSSSSAR			Carbamidomethyl (C)[5]		Mascot
1716.801	1716.775	-0.026	-15	116	130	ETGDRLAATFGYEMR					Mascot
1732.7959	1732.792	-0.0039	-2	116	130	ETGDRLAATFGYEMR			Oxidation (M)[14]		Mascot
1774.8429	1774.8851	0.0422	24	271	286	IAGPEGHSMVYELAER			Oxidation (M)[9]		Mascot
1832.0177	1831.8981	-0.1196	-65	54	68	VFDVFIRPNRALQEK					Mascot
2119.0122	2119.1121	0.0999	47	137	156	STSTRSGRPSSQQPSNQ DAK					Mascot

7	3-hydroxybenzoate 6-hydroxylase 1 [Triticum urartu]	gi|474387897	44459.4	5.99	12	50	60.587	9.014

Peptide Information

Calc. Mass	Obsrv. Mass	± da	± ppm	Start Seq.	End Seq.	Sequence	Ion Score	C. I. %	Modification	Rank	Result Type
1146.4811	1146.585	0.1039	91	340	349	GFGHEEGEER					Mascot
1197.6475	1197.6605	0.013	11	262	272	VGPEVLEAVER					Mascot
1205.6749	1205.619	-0.0559	-46	100	110	VNGKLGPHEVR					Mascot
1253.6848	1253.5848	-0.1	-80	147	157	ILHLADDSTLR					Mascot
1262.7355	1262.6199	-0.1156	-92	287	298	SPLSLLFASISK					Mascot
1591.8262	1591.7605	-0.0657	-41	273	286	SEMSDVLAAPLRFR					Mascot
1643.9149	1643.8148	-0.1001	-61	158	173	AKVLIGCDGINSVVAK			Carbamidomethyl (C)[7]		Mascot
1652.7856	1652.8204	0.0348	21	48	61	TSGFAFFTWTNAFR					Mascot
1652.7856	1652.8204	0.0348	21	48	61	TSGFAFFTWTNAFR					Mascot
1680.8374	1680.8463	0.0089	5	242	256	EDRSAAAMNQFVLTK					Mascot
1729.9344	1729.874	-0.0604	-35	174	189	WLGLAKPSESGRTATR					Mascot
1752.9102	1752.8677	-0.0425	-24	369	382	EVECHLPVFPIEKR			Carbamidomethyl (C)[4]		Mascot
1992.8223	1992.9198	0.0975	49	353	368	HTSCHCLMDSDGFNRR			Carbamidomethyl (C)[4,6]		Mascot
1992.8223	1992.9198	0.0975	49	353	368	HTSCHCLMDSDGFNRR			Carbamidomethyl (C)[4,6]		Mascot

8	hypothetical protein TRIUR3_13181 [Triticum urartu]	gi|474069655	32404.3	4.89	10	49	50.383	5.49

Peptide Information

Calc. Mass	Obsrv. Mass	± da	± ppm	Start Seq.	End Seq.	Sequence	Ion Score	C. I. %	Modification	Rank	Result Type
1123.6544	1123.6426	-0.0118	-11	139	148	KLGIIGTFMK			Oxidation (M)[9]		Mascot
1431.705	1431.6398	-0.0652	-46	271	283	TGGSWVRGPELCI			Carbamidomethyl (C)[12]		Mascot
1449.7633	1449.6862	-0.0771	-53	1	11	MTLLVPWMIWK			Oxidation (M)[1,8]		Mascot
1507.8124	1507.7488	-0.0636	-42	149	161	GCILTKLPAMFTR			Carbamidomethyl (C)[2]		Mascot
1591.7184	1591.7605	0.0421	26	12	24	YRNDCVFNGGHPR			Carbamidomethyl (C)[5]		Mascot
1605.8975	1605.7754	-0.1221	-76	2	13	TLLVPWMIWKYR					Mascot
1651.9275	1651.8462	-0.0813	-49	140	154	LGIIGTFMKGCILTK			Carbamidomethyl (C)[11]		Mascot

1652.9105	1652.8204	-0.0901	-55	124	138	ESYVKLSLESLSGIK					Mascot
1652.9105	1652.8204	-0.0901	-55	124	138	ESYVKLSLESLSGIK					Mascot
1727.7946	1727.8981	0.1035	60	162	175	LEDIGFMINFEDER					Mascot
1743.7894	1743.8541	0.0647	37	162	175	LEDIGFMINFEDER			Oxidation (M)[7]		Mascot
1752.9329	1752.8677	-0.0652	-37	1	13	MTLLVPWMIWKYR			Oxidation (M)[1]		Mascot

9	NADH-dependent glutamate synthase [Triticum turgidum]	gi|530452549	237135.1	6.26	30	47	7.608	29.032	

Peptide Information

Calc. Mass	Obsrv. Mass	± da	± ppm	Start Seq.	End Seq.	Sequence	Ion Score	C. I. %	Modification	Rank	Result Type
900.5261	900.5527	0.0266	30	947	953	RIQELNK					Mascot
932.4683	932.4709	0.0026	3	703	710	GLEETLDR					Mascot
946.4476	946.4897	0.0421	44	2143	2151	DEQNVAGLT					Mascot
947.4873	947.4861	-0.0012	-1	614	620	LTFEYFK					Mascot
1016.5847	1016.5525	-0.0322	-32	1039	1048	SAIKQVASGR					Mascot
1016.5847	1016.5525	-0.0322	-32	1039	1048	SAIKQVASGR					Mascot
1035.5219	1035.4246	-0.0973	-94	2025	2033	RFIGDENGK					Mascot
1035.5219	1035.4246	-0.0973	-94	2025	2033	RFIGDENGK					Mascot
1072.5997	1072.5723	-0.0274	-26	1199	1208	AVLQTDGQLK					Mascot
1078.5496	1078.484	-0.0656	-61	954	962	ACNLRGMLK			Carbamidomethyl (C)[2], Oxidation (M)[7]		Mascot
1218.5961	1218.5922	-0.0039	-3	1930	1940	SLLDSNLEDGR					Mascot
1222.6038	1222.5835	-0.0203	-17	1825	1834	MGHFVTVFER					Mascot
1234.5699	1234.5884	0.0185	15	2001	2011	VDYGHQEASTK					Mascot
1234.5699	1234.5884	0.0185	15	2001	2011	VDYGHQEASTK					Mascot
1251.6732	1251.6166	-0.0566	-45	237	246	ADFEQLFILR					Mascot
1253.6195	1253.5848	-0.0347	-28	869	879	IEGATFEMLAR			Oxidation (M)[8]		Mascot
1259.6115	1259.5924	-0.0191	-15	533	543	DIVESVPETDR					Mascot
1285.7223	1285.5966	-0.1257	-98	1197	1208	GRAVLQTDGQLK					Mascot
1315.5736	1315.5629	-0.0107	-8	1473	1484	GEAYFNGMAAER					Mascot
1426.6892	1426.7069	0.0177	12	1838	1850	IGGLMMYGVPNMK			Oxidation (M)[5]		Mascot
1573.7566	1573.8138	0.0572	36	1010	1025	LGGKSNTGEGGEQPSR					Mascot
1636.8079	1636.8292	0.0213	13	1998	2011	VFRVDYGHQEASTK					Mascot
1680.7104	1680.8463	0.1359	81	262	275	GGERDFYMCSLSSR			Carbamidomethyl (C)[9], Oxidation (M)[8]		Mascot
1684.9116	1684.7843	-0.1273	-76	970	984	ISLDEVEPASEIVKR					Mascot
1700.8749	1700.7959	-0.079	-46	1890	1905	SENNAVILACGATKPR			Carbamidomethyl (C)[10]		Mascot
1716.9042	1716.775	-0.1292	-75	452	466	NGLRPGRFYVTHSGR					Mascot

1727.9036	1727.8981	-0.0055	-3	1376	1391	NTNRAVGTTLSHEVTK					Mascot
2026.0386	2026.0259	-0.0127	-6	1342	1359	QDHGLDMALDNKLIALSR			Oxidation (M)[7]		Mascot
2043.0541	2043.0416	-0.0125	-6	573	590	AFGYTLEALEMLLLPMAK			Oxidation (M)[11,16]		Mascot
2167.0747	2167.0732	-0.0015	-1	1241	1259	KCHTNTCPVGIATQDPVLR			Carbamidomethyl (C)[2,7]		Mascot
2183.0522	2183.0481	-0.0041	-2	33	54	SARQAHGAMSLEGGGFLGGAHR			Oxidation (M)[9]		Mascot
2255.0413	2255.1514	0.1101	49	2097	2117	AQFGHFGTSVDGVFAAGDCRR			Carbamidomethyl (C)[19]		Mascot
2277.1101	2277.1443	0.0342	15	1291	1310	TINEMVGRSDMLEVDPEVVK			Oxidation (M)[5]		Mascot

10	Disease resistance protein RGA2 [Triticum urartu]	gi|473970852	187258.6	5.81	24	46	1	24.73	

Peptide Information

Calc. Mass	Obsrv. Mass	± da	± ppm	Start Seq.	End Seq.	Sequence	Ion Score	C. I. %	Modification	Rank	Result Type
900.472	900.5527	0.0807	90	249	256	ILQGCGPR			Carbamidomethyl (C)[5]		Mascot
1016.6138	1016.5525	-0.0613	-60	1163	1170	LSKLEIWK					Mascot
1016.6138	1016.5525	-0.0613	-60	1163	1170	LSKLEIWK					Mascot
1026.5038	1026.4773	-0.0265	-26	226	233	VDFSQRMK			Oxidation (M)[7]		Mascot
1052.516	1052.5461	0.0301	29	1631	1639	TFQNWSAAK					Mascot
1078.5779	1078.484	-0.0939	-87	819	827	ELGIYNLEK					Mascot
1106.5623	1106.493	-0.0693	-63	511	520	QTNGIMSALR			Oxidation (M)[6]		Mascot
1106.5623	1106.493	-0.0693	-63	511	520	QTNGIMSALR			Oxidation (M)[6]		Mascot
1123.6582	1123.6426	-0.0156	-14	810	818	QLEHLSKLR					Mascot
1146.6881	1146.585	-0.1031	-90	790	798	LLEELKVFR					Mascot
1197.6296	1197.6605	0.0309	26	731	740	EMHLPLNISK			Oxidation (M)[2]		Mascot
1247.5984	1247.6477	0.0493	40	108	118	GCVCNLALNAR			Carbamidomethyl (C)[2,4]		Mascot
1264.5746	1264.5583	-0.0163	-13	1334	1343	FFADWDPNPR					Mascot
1275.644	1275.5962	-0.0478	-37	847	856	RLSLNWDSER					Mascot
1326.7126	1326.6639	-0.0487	-37	1602	1613	FGEGFVLMVLAK			Oxidation (M)[8]		Mascot
1326.7126	1326.6639	-0.0487	-37	1602	1613	FGEGFVLMVLAK			Oxidation (M)[8]		Mascot
1342.6572	1342.6393	-0.0179	-13	938	947	DFIIERSFCR			Carbamidomethyl (C)[9]		Mascot
1549.7349	1549.7682	0.0333	21	59	71	EICGTAMEELLQR			Carbamidomethyl (C)[3]		Mascot
1573.8043	1573.8138	0.0095	6	1	15	MAEAALGAAQWVVEK					Mascot
1589.7992	1589.7802	-0.019	-12	1	15	MAEAALGAAQWVVEK			Oxidation (M)[1]		Mascot
1606.8687	1606.8096	-0.0591	-37	819	832	ELGIYNLEKIDTAK					Mascot
1615.7864	1615.778	-0.0084	-5	1052	1065	QFWYSKSSGGAELR					Mascot
1652.9442	1652.8204	-0.1238	-75	257	271	TIPDIAQSRPITKGR					Mascot
1652.9442	1652.8204	-0.1238	-75	257	271	TIPDIAQSRPITKGR					Mascot

1752.9967	1752.8677	-0.129	-74	1428	1443	DVQQLPAGLRNLTSLK		Mascot
1775.9109	1775.828	-0.0829	-47	1310	1325	CQGLWSLLTTGGQLNK	Carbamidomethyl (C)[1]	Mascot
2012.9098	2013.0154	0.1056	52	91	107	IHDELYHTYDAADQHGK		Mascot
2119.104	2119.1121	0.0081	4	294	314	GQYCTKGLTVLPVVGPG GMGK	Carbamidomethyl (C)[4]	Mascot
2154.9622	2155.0588	0.0966	45	127	147	QLSSLSAYCFGANTGGG GDHR	Carbamidomethyl (C)[9]	Mascot

Gel Idx/Pos	277/L5	Instr./Gel Origin	BA2151/Sample Project 20140814	Process Status	Analysis Succeeded
Plate [#] Name	[1] Sample Project 20140814	Instrument Sample Name		Spectra	11

Rank	Protein Name	Accession No.	Protein MW	Protein PI	Pep. Count	Protein Score	Protein Score C. I. %	Intensity Matched	Total Ion Score	Total Ion C. I. %	Confirmed
1	Adenosylhomocysteinase [Triticum urartu]	gi|474154141	46127.7	6.48	16	354	100	20.902	280	100	

Peptide Information

Calc. Mass	Obsrv. Mass	± da	± ppm	Start Seq.	End Seq.	Sequence	Ion Score	C. I. %	Modification	Rank	Result Type
827.5713	827.5648	-0.0065	-8	107	113	IVLTIIR					Mascot
1008.4745	1008.4741	-0.0004	0	18	26	DLSQADFGR					Mascot
1008.4745	1008.4741	-0.0004	0	18	26	DLSQADFGR	75	100			Mascot
1028.6099	1028.6066	-0.0033	-3	316	325	TGIIVLAEGR					Mascot
1028.6099	1028.6066	-0.0033	-3	316	325	TGIIVLAEGR	13	0			Mascot
1041.5146	1041.5033	-0.0113	-11	177	185	HSLPDGLMR			Oxidation (M)[8]		Mascot
1044.4568	1044.4551	-0.0017	-2	169	176	FDNLYGCR			Carbamidomethyl (C)[7]		Mascot
1044.4568	1044.4551	-0.0017	-2	169	176	FDNLYGCR	30	93.958	Carbamidomethyl (C)[7]		Mascot
1071.6157	1071.6138	-0.0019	-2	300	308	ITIKPQTDR					Mascot
1137.5576	1137.5415	-0.0161	-14	44	53	TEFGPSQPFK					Mascot
1161.5392	1161.538	-0.0012	-1	260	268	DIIMVDHMR			Oxidation (M)[4,8]		Mascot
1235.6379	1235.6345	-0.0034	-3	16	26	VKDLSQADFGR					Mascot
1259.5837	1259.5884	0.0047	4	167	176	SKFDNLYGCR			Carbamidomethyl (C)[9]		Mascot
1259.5837	1259.5884	0.0047	4	167	176	SKFDNLYGCR	44	99.751	Carbamidomethyl (C)[9]		Mascot
1916.0641	1916.0217	-0.0424	-22	309	325	WVFPETKTGIIVLAEGR					Mascot
1978.9282	1978.9274	-0.0008	0	27	43	LELDLAEVEMPGLMACR			Carbamidomethyl (C)[16], Oxidation (M)[10,14]		Mascot
2241.1187	2241.1587	0.04	18	396	415	SQSDYISIPVEGPYKPAAYR					Mascot
2241.1187	2241.1587	0.04	18	396	415	SQSDYISIPVEGPYKPAAYR	116	100			Mascot
2583.3455	2583.3264	-0.0191	-7	393	415	LTKSQSDYISIPVEGPYKPAAYR					Mascot
2584.2964	2584.322	0.0256	10	144	166	LYQMQESGTLLFPAINVNDSVTK			Oxidation (M)[4]		Mascot
2740.3975	2740.4712	0.0737	27	143	166	RLYQMQESGTLLFPAINVNDSVTK			Oxidation (M)[5]		Mascot

Rank	Protein Name	Accession No.	Protein MW	Protein PI	Pep. Count	Protein Score	Protein Score C. I. %	Intensity Matched	Total Ion Score	Total Ion C. I. %	Confirmed
2	Adenosylhomocysteinase [Triticum urartu]	gi|474160132	83795.8	6.45	18	289	100	19.279	232	100	

Peptide Information

Calc. Mass	Obsrv. Mass	± da	± ppm	Start Seq.	End Seq.	Sequence	Ion Score	C. I. %	Modification	Rank	Result Type

827.5713	827.5648	-0.0065	-8	443	449	IVLTIIR					Mascot
909.3948	909.4017	0.0069	8	420	426	AEEEFER					Mascot
1008.4745	1008.4741	-0.0004	0	327	335	DLSQADFGR					Mascot
1008.4745	1008.4741	-0.0004	0	327	335	DLSQADFGR	75	100			Mascot
1041.5146	1041.5033	-0.0113	-11	513	521	HSLPDGLMR			Oxidation (M)[8]		Mascot
1044.4568	1044.4551	-0.0017	-2	505	512	FDNLYGCR			Carbamidomethyl (C)[7]		Mascot
1044.4568	1044.4551	-0.0017	-2	505	512	FDNLYGCR	30	93.958	Carbamidomethyl (C)[7]		Mascot
1071.6157	1071.6138	-0.0019	-2	636	644	ITIKPQTDR					Mascot
1107.547	1107.5271	-0.0199	-18	353	362	AEFGPSQPFK					Mascot
1161.5392	1161.538	-0.0012	-1	596	604	DIIMVDHMR			Oxidation (M)[4,8]		Mascot
1235.6379	1235.6345	-0.0034	-3	325	335	VKDLSQADFGR					Mascot
1259.5837	1259.5884	0.0047	4	503	512	SKFDNLYGCR			Carbamidomethyl (C)[9]		Mascot
1259.5837	1259.5884	0.0047	4	503	512	SKFDNLYGCR	44	99.751	Carbamidomethyl (C)[9]		Mascot
1806.797	1806.7625	-0.0345	-19	65	78	AKDWDTFYFEAEER					Mascot
1902.0121	1902.0322	0.0201	11	645	661	WVFPETNTGIIVLAEGR					Mascot
1902.0121	1902.0322	0.0201	11	645	661	WVFPETNTGIIVLAEGR	81	100			Mascot
1916.9647	1917.0282	0.0635	33	239	254	VKEINMVHYQGDALQR			Oxidation (M)[6]		Mascot
1992.9438	1992.9331	-0.0107	-5	336	352	LELELAEVEMPGLMACR			Carbamidomethyl (C)[16], Oxidation (M)[10,14]		Mascot
2179.0205	2179.0144	-0.0061	-3	241	258	EINMVHYQGDALQRMMAR			Oxidation (M)[4]		Mascot
2179.0205	2179.0144	-0.0061	-3	241	258	EINMVHYQGDALQRMMAR			Oxidation (M)[4]		Mascot
2225.1238	2225.1555	0.0317	14	732	751	AQSEYISIPVDGPYKPAAYR					Mascot
2584.2964	2584.322	0.0256	10	480	502	LYQMQESGTLLFPAINVNDSVTK			Oxidation (M)[4]		Mascot
2740.3975	2740.4712	0.0737	27	479	502	RLYQMQESGTLLFPAINVNDSVTK			Oxidation (M)[5]		Mascot

3	S-adenosyl-L-homocysteine hydrolase [Triticum aestivum]	gi|170773	54086.4	5.65	13	214	100	12.763	167	100		

Protein Group

RecName: Full=Adenosylhomocysteinase; Short=AdoHcyase; AltName: Full=S-adenosyl-L-homocysteine hydrolase	gi|417745	54086.4	5.6500000953 6743

Peptide Information

Calc. Mass	Obsrv. Mass	± da	± ppm	Start Seq.	End Seq.	Sequence	Ion Score	C. I. %	Modification	Rank	Result Type
827.5713	827.5648	-0.0065	-8	176	182	IVLTIIR					Mascot
1028.6099	1028.6066	-0.0033	-3	385	394	TGIIVLAEGR					Mascot
1028.6099	1028.6066	-0.0033	-3	385	394	TGIIVLAEGR	13	0			Mascot
1041.5146	1041.5033	-0.0113	-11	246	254	HSLPDGLMR			Oxidation (M)[8]		Mascot

1044.4568	1044.4551	-0.0017	-2	238	245	FDNLYGCR			Carbamidomethyl (C)[7]		Mascot
1044.4568	1044.4551	-0.0017	-2	238	245	FDNLYGCR	30	93.958	Carbamidomethyl (C)[7]		Mascot
1071.6157	1071.6138	-0.0019	-2	369	377	ITIKPQTDR					Mascot
1137.5576	1137.5415	-0.0161	-14	44	53	TEFGPSQPFK					Mascot
1161.5392	1161.538	-0.0012	-1	329	337	DIIMVDHMR			Oxidation (M)[4,8]		Mascot
1259.5837	1259.5884	0.0047	4	236	245	SKFDNLYGCR			Carbamidomethyl (C)[9]		Mascot
1259.5837	1259.5884	0.0047	4	236	245	SKFDNLYGCR	44	99.751	Carbamidomethyl (C)[9]		Mascot
1758.7428	1758.7628	0.02	11	111	123	GETLEEYWWCTER			Carbamidomethyl (C)[10]		Mascot
1758.7428	1758.7628	0.02	11	111	123	GETLEEYWWCTER	79	100	Carbamidomethyl (C)[10]		Mascot
1916.0641	1916.0217	-0.0424	-22	378	394	WVFPETKTGIIVLAEGR					Mascot
1992.9438	1992.9331	-0.0107	-5	27	43	LELELAEVEMPGLMACR			Carbamidomethyl (C)[16], Oxidation (M)[10,14]		Mascot
2584.2964	2584.322	0.0256	10	213	235	LYQMQESGTLLFPAINVN DSVTK			Oxidation (M)[4]		Mascot
2740.4915	2740.4712	-0.0203	-7	57	82	ISGSLHMTIQTAVLIETLT ALGAEVR			Oxidation (M)[7]		Mascot

4 S-adenosylhomocysteine hydrolase [Triticum monococcum] gi|115589748 4646.5 9.46 2 135 100 7.476 116 100

Peptide Information

Calc. Mass	Obsrv. Mass	± da	± ppm	Start Seq.	End Seq.	Sequence	Ion Score	C. I. %	Modification	Rank	Result Type
2241.1187	2241.1587	0.04	18	22	41	SQSDYISIPVEGPYKPAA YR					Mascot
2241.1187	2241.1587	0.04	18	22	41	SQSDYISIPVEGPYKPAA YR	116	100			Mascot
2583.3455	2583.3264	-0.0191	-7	19	41	LTKSQSDYISIPVEGPYKP AAYR					Mascot

5 sucrose:fructan 6-fructosyltransferase [Triticum urartu] gi|10180903 11302.6 6.38 7 56 88.633 5.167 9 0

Peptide Information

Calc. Mass	Obsrv. Mass	± da	± ppm	Start Seq.	End Seq.	Sequence	Ion Score	C. I. %	Modification	Rank	Result Type
1016.4717	1016.5544	0.0827	81	95	103	LVVHEMDSA			Oxidation (M)[6]		Mascot
1353.6144	1353.6483	0.0339	25	68	78	VYPMEAYHEAK			Oxidation (M)[4]		Mascot
1701.8741	1701.744	-0.1301	-76	46	61	VLVDHSIVQGFAMGGR			Oxidation (M)[13]		Mascot
1758.848	1758.7628	-0.0852	-48	79	94	VYLFNNATGASVMAER			Oxidation (M)[13]		Mascot
1758.848	1758.7628	-0.0852	-48	79	94	VYLFNNATGASVMAER	9	0	Oxidation (M)[13]		Mascot
1804.8284	1804.9176	0.0892	49	3	18	GLDGGLHTSFCQDELR			Carbamidomethyl (C)[11]		Mascot
1934.0165	1934.0212	0.0047	2	28	45	RVIGSTVPVLDGEAFSMR					Mascot
2740.3069	2740.4712	0.1643	60	79	103	VYLFNNATGASVMAERL VVHEMDSA			Oxidation (M)[13]		Mascot

6 initiation factor (iso)4f p82 subunit [Triticum aestivum] gi|951453 87096 7.57 16 51 66.454 10.96

Protein Group

RecName: Full=Eukaryotic translation initiation factor isoform 4G-2; Short=eIF(iso)-4G-2; Short=eIF(iso)4G-2; AltName: Full=Eukaryotic initiation factor iso-4F subunit p82-16; Short=eIF-(iso)4F p82-16 subunit	gi|75102610	87096	7.5700 001716 6138

Peptide Information

Calc. Mass	Obsrv. Mass	± da	± ppm	Start Seq.	End Seq.	Sequence	Ion Score	C. I. %	Modification	Rank	Result Type
866.4982	866.4177	-0.0805	-93	753	760	AIFTSVTK					Mascot
947.4469	947.4871	0.0402	42	278	286	FPSEEPGGK					Mascot
1016.5775	1016.5544	-0.0231	-23	680	687	LLEHLYTK					Mascot
1028.5385	1028.6066	0.0681	66	433	440	SNNWVPRR					Mascot
1028.5385	1028.6066	0.0681	66	433	440	SNNWVPRR					Mascot
1044.5255	1044.4551	-0.0704	-67	523	531	SMPRGDPLR			Oxidation (M)[2]		Mascot
1044.5255	1044.4551	-0.0704	-67	523	531	SMPRGDPLR			Oxidation (M)[2]		Mascot
1146.5902	1146.5802	-0.01	-9	149	157	SWDNIREVK					Mascot
1167.7096	1167.5931	-0.1165	-100	553	564	LLPQGTGALIGK					Mascot
1646.7942	1646.8029	0.0087	5	310	324	VEIASLTGPDQEMEK					Mascot
1664.7963	1664.9005	0.1042	63	50	62	ALQDWCLHLSHER			Carbamidomethyl (C)[6]		Mascot
1818.8903	1818.8462	-0.0441	-24	310	325	VEIASLTGPDQEMEKR			Oxidation (M)[13]		Mascot
1952.9972	1952.9818	-0.0154	-8	1	20	MTTDQPVISLRPGGGGG GPR					Mascot
1973.0968	1973.0117	-0.0851	-43	181	199	AQVGPPPALIKADVPWS AR					Mascot
1979.0557	1978.9274	-0.1283	-65	173	191	QDQSSSQKAQVGPPPAL IK					Mascot
2223.1411	2223.1384	-0.0027	-1	1	23	MTTDQPVISLRPGGGGG GPRGGR					Mascot
2239.136	2239.1709	0.0349	16	1	23	MTTDQPVISLRPGGGGG GPRGGR			Oxidation (M)[1]		Mascot
2263.2517	2263.116	-0.1357	-60	532	552	NQGSLINKVSSINKPSPIN PR					Mascot
2496.3357	2496.231	-0.1047	-42	400	420	INDTYFVHLRELVANPQL TPR					Mascot

7	initiation factor (iso)4f p82 subunit [Triticum aestivum]	gi|452440	86754.9	8.2	16	51	65.673	11.771

Protein Group

RecName: Full=Eukaryotic translation initiation factor isoform 4G-1; Short=eIF(iso)-4G-1; Short=eIF(iso)4G-1; AltName: Full=Eukaryotic initiation factor iso-4F subunit p82-34; Short=eIF-(iso)4F p82-34 subunit	gi|1170504	86754.9	8.1999 998092 6514

Peptide Information

Calc. Mass	Obsrv. Mass	± da	± ppm	Start Seq.	End Seq.	Sequence	Ion Score	C. I. %	Modification	Rank	Result Type
866.4982	866.4177	-0.0805	-93	754	761	AIFTSVTK					Mascot

977.5302	977.5073	-0.0229	-23	736	744	FEAAEGILK					Mascot
1016.5775	1016.5544	-0.0231	-23	681	688	LLEHLYTK					Mascot
1028.5385	1028.6066	0.0681	66	434	441	SNNWVPRR					Mascot
1028.5385	1028.6066	0.0681	66	434	441	SNNWVPRR					Mascot
1044.5255	1044.4551	-0.0704	-67	524	532	SMPRGDPLR			Oxidation (M)[2]		Mascot
1044.5255	1044.4551	-0.0704	-67	524	532	SMPRGDPLR			Oxidation (M)[2]		Mascot
1146.5902	1146.5802	-0.01	-9	150	158	SWDNIREVK					Mascot
1167.7096	1167.5931	-0.1165	-100	554	565	LLPQGTGALIGK					Mascot
1646.7942	1646.8029	0.0087	5	311	325	VEIASLTGPDQEMEK					Mascot
1818.8903	1818.8462	-0.0441	-24	311	326	VEIASLTGPDQEMEKR			Oxidation (M)[13]		Mascot
1952.9972	1952.9818	-0.0154	-8	1	20	MTTDQPVISLRPGGGGG GPR					Mascot
1973.0968	1973.0117	-0.0851	-43	182	200	AQVGPPPALIKADVPWS AR					Mascot
1974.963	1975.0582	0.0952	48	736	752	FEAAEGILKAMEDTFFR					Mascot
1979.0557	1978.9274	-0.1283	-65	174	192	QDQSSSQKAQVGPPPAL IK					Mascot
2087.1431	2087.0798	-0.0633	-30	717	735	APTQFGEVVARLILSCGL R			Carbamidomethyl (C)[16]		Mascot
2223.1411	2223.1384	-0.0027	-1	1	23	MTTDQPVISLRPGGGGG GPRGGR					Mascot
2239.136	2239.1709	0.0349	16	1	23	MTTDQPVISLRPGGGGG GPRGGR			Oxidation (M)[1]		Mascot
2267.1123	2267.1194	0.0071	3	105	124	NDSNVQLQTQAQPQVQA QNR					Mascot

8	sucrose:fructan 6-fructosyltransferase [Triticum aestivum]	gi|7637954	11293.6	6.25	6	47	13.775	4.816	9	0

Peptide Information

Calc. Mass	Obsrv. Mass	± da	± ppm	Start Seq.	End Seq.	Sequence	Ion Score	C. I. %	Modification	Rank	Result Type
1016.4717	1016.5544	0.0827	81	95	103	LVVHEMDSA			Oxidation (M)[6]		Mascot
1701.8741	1701.744	-0.1301	-76	46	61	VLVDHSIVQGFAMGGR			Oxidation (M)[13]		Mascot
1758.848	1758.7628	-0.0852	-48	79	94	VYLFNNATGASVMAER			Oxidation (M)[13]		Mascot
1758.848	1758.7628	-0.0852	-48	79	94	VYLFNNATGASVMAER	9	0	Oxidation (M)[13]		Mascot
1804.8284	1804.9176	0.0892	49	3	18	GLDGGLHTSFCQDELR			Carbamidomethyl (C)[11]		Mascot
1934.0165	1934.0212	0.0047	2	28	45	RVIGSTVPVLDGEAFSMR					Mascot
2740.3069	2740.4712	0.1643	60	79	103	VYLFNNATGASVMAERL VVHEMDSA			Oxidation (M)[13]		Mascot

9	hypothetical protein TRIUR3_04705 [Triticum urartu]	gi|474360334	7546.8	6.52	4	45	0	4.543	15	0

Peptide Information

Calc. Mass	Obsrv. Mass	± da	± ppm	Start Seq.	End Seq.	Sequence	Ion Score	C. I. %	Modification	Rank	Result Type

1008.4745	1008.4741	-0.0004	0	45	54	LAGSADDFGR					Mascot
1008.4745	1008.4741	-0.0004	0	45	54	LAGSADDFGR	15	0			Mascot
1044.511	1044.4551	-0.0559	-54	22	31	APTGDSPFPR					Mascot
1044.511	1044.4551	-0.0559	-54	22	31	APTGDSPFPR					Mascot
1107.6198	1107.5271	-0.0927	-84	2	10	EGLLPFLYR					Mascot
2253.1775	2253.1028	-0.0747	-33	11	31	AILLHYIDGGRAPTGDSPFPR					Mascot

10 lipoxygenase 2 [Triticum turgidum] gi|90903443 17795.2 9.43 7 44 0 2.156

Peptide Information

Calc. Mass	Obsrv. Mass	± da	± ppm	Start Seq.	End Seq.	Sequence	Ion Score	C. I. %	Modification	Rank	Result Type
1161.5793	1161.538	-0.0413	-36	85	94	DTMNINARAR					Mascot
1443.8054	1443.7437	-0.0617	-43	1	14	LSSPLIQGDLTTAK					Mascot
1786.9884	1786.8365	-0.1519	-85	95	111	GLLINAGGVIEMTVFPR					Mascot
1850.9442	1850.871	-0.0732	-40	78	92	LLHPHYRDTMNINAR					Mascot
1915.0834	1915.0067	-0.0767	-40	95	112	GLLINAGGVIEMTVFPRK					Mascot
2014.1267	2013.998	-0.1287	-64	93	111	ARGLLINAGGVIEMTVFPR					Mascot
2088.1501	2088.0635	-0.0866	-41	68	84	QLSVTHPVYKLLHPHYR					Mascot

Gel Idx/Pos	278/L6	Instr./Gel Origin	BA2151/Sample Project 20140814	Process Status	Analysis Succeeded
Plate [#] Name	[1] Sample Project 20140814	Instrument Sample Name		Spectra	11

Rank	Protein Name	Accession No.	Protein MW	Protein PI	Pep. Count	Protein Score	Protein Score C. I. %	Intensity Matched	Total Ion Score	Total Ion C. I. %	Confirmed
1	apt1-2 [Triticum aestivum]	gi|291498596	55556.8	5.7	19	837	100	47.484	739	100	

Protein Group

Protein Name	Accession No.	Protein MW	Protein PI
ATP synthase subunit alpha [Triticum durum x Triticosecale sp.]	gi|1405781	55556.8	5.6999998092 6514
apt1 [Triticum aestivum]	gi|169649046	55556.8	5.6999998092 6514
apt1-1 [Triticum aestivum]	gi|291498595	55556.8	5.6999998092 6514
atp1 [Triticum aestivum]	gi|78675233	55556.8	5.6999998092 6514
atp1 [Triticum aestivum]	gi|81176509	55556.8	5.6999998092 6514
unnamed protein product [Triticum aestivum]	gi|13725	55556.8	5.6999998092 6514

Peptide Information

Calc. Mass	Obsrv. Mass	± da	± ppm	Start Seq.	End Seq.	Sequence	Ion Score	C. I. %	Modification	Rank	Result Type
815.4621	815.4484	-0.0137	-17	167	173	ELIIGDR					Mascot
860.5022	860.4489	-0.0533	-62	283	289	QMSLLLR					Mascot
874.4741	874.4857	0.0116	13	487	494	GGLTNERK					Mascot
876.4971	876.4717	-0.0254	-29	283	289	QMSLLLR			Oxidation (M)[2]		Mascot
884.4221	884.4128	-0.0093	-11	121	128	GALSDHER					Mascot
892.4886	892.4791	-0.0095	-11	395	401	LELAQYR					Mascot
972.5473	972.5329	-0.0144	-15	33	42	VVSVGDGIAR					Mascot
972.5473	972.5329	-0.0144	-15	33	42	VVSVGDGIAR	72	100			Mascot
1026.5942	1026.5762	-0.018	-18	154	163	AVDSLVPIGR					Mascot
1026.5942	1026.5762	-0.018	-18	154	163	AVDSLVPIGR	73	100			Mascot

1203.658	1203.6455	-0.0125	-10	7	17	AAELTTLLESR					Mascot
1203.658	1203.6455	-0.0125	-10	7	17	AAELTTLLESR	74	100			Mascot
1242.6147	1242.5756	-0.0391	-31	143	153	SVHEPMQTGLK			Oxidation (M)[6]		Mascot
1300.7471	1300.6886	-0.0585	-45	178	189	TAIAIDTILNQK					Mascot
1326.7627	1326.7087	-0.054	-41	470	481	AILSTINPELQK					Mascot
1341.705	1341.649	-0.056	-42	433	443	QPQYEPLPIEK					Mascot
1438.8489	1438.8287	-0.0202	-14	363	376	GIRPAINVGLSVSR					Mascot
1438.8489	1438.8287	-0.0202	-14	363	376	GIRPAINVGLSVSR	45	99.744			Mascot
1537.7434	1537.7349	-0.0085	-6	295	307	EAFPGDVFYLHSR					Mascot
1537.7434	1537.7349	-0.0085	-6	295	307	EAFPGDVFYLHSR	108	100			Mascot
1720.821	1720.9739	0.1529	89	262	276	DNGMHALIIYDDLSK			Oxidation (M)[4]		Mascot
1724.8789	1724.8748	-0.0041	-2	444	458	QIVVIYAAVNGFCDR			Carbamidomethyl (C)[13]		Mascot
1834.8429	1834.851	0.0081	4	18	32	MTNFYTNFQVDEIGR					Mascot
1850.8378	1850.8162	-0.0216	-12	18	32	MTNFYTNFQVDEIGR			Oxidation (M)[1]		Mascot
1850.8378	1850.8162	-0.0216	-12	18	32	MTNFYTNFQVDEIGR	133	100	Oxidation (M)[1]		Mascot
2157.0532	2157.0288	-0.0244	-11	43	62	VYGLNEIQAGEMVEFAS GVK			Oxidation (M)[12]		Mascot
2308.1567	2308.1646	0.0079	3	402	423	EVAAFAQFGSDLDAATQ ALLNR					Mascot
2308.1567	2308.1646	0.0079	3	402	423	EVAAFAQFGSDLDAATQ ALLNR	233	100			Mascot

2	RecName: Full=ATP synthase subunit alpha, mitochondrial	gi|114419	55514.8	5.7	18	593	100	42.72	505	100	

Peptide Information

Calc. Mass	Obsrv. Mass	± da	± ppm	Start Seq.	End Seq.	Sequence	Ion Score	C. I. %	Modification	Rank	Result Type
815.4621	815.4484	-0.0137	-17	167	173	ELIIGDR					Mascot
860.5022	860.4489	-0.0533	-62	283	289	QMSLLLR					Mascot
874.4741	874.4857	0.0116	13	487	494	GGLTNERK					Mascot
876.4971	876.4717	-0.0254	-29	283	289	QMSLLLR			Oxidation (M)[2]		Mascot
884.4221	884.4128	-0.0093	-11	121	128	GALSDHER					Mascot
892.4886	892.4791	-0.0095	-11	395	401	LELAQYR					Mascot
972.5473	972.5329	-0.0144	-15	33	42	VVSVGDGIAR					Mascot
972.5473	972.5329	-0.0144	-15	33	42	VVSVGDGIAR	72	100			Mascot
1026.5942	1026.5762	-0.018	-18	154	163	AVDSLVPIGR					Mascot
1026.5942	1026.5762	-0.018	-18	154	163	AVDSLVPIGR	73	100			Mascot
1203.658	1203.6455	-0.0125	-10	7	17	AAELTTLLESR					Mascot
1203.658	1203.6455	-0.0125	-10	7	17	AAELTTLLESR	74	100			Mascot
1242.6147	1242.5756	-0.0391	-31	143	153	SVHEPMQTGLK			Oxidation (M)[6]		Mascot

1300.7471	1300.6886	-0.0585	-45	178	189	TAIAIDTILNQK					Mascot
1326.7627	1326.7087	-0.054	-41	470	481	AILSTINPELQK					Mascot
1341.705	1341.649	-0.056	-42	433	443	QPQYEPLPIEK					Mascot
1438.8489	1438.8287	-0.0202	-14	363	376	GIRPAINVGLSVSR					Mascot
1438.8489	1438.8287	-0.0202	-14	363	376	GIRPAINVGLSVSR	45	99.744			Mascot
1537.7434	1537.7349	-0.0085	-6	295	307	EAFPGDVFYLHSR					Mascot
1537.7434	1537.7349	-0.0085	-6	295	307	EAFPGDVFYLHSR	108	100			Mascot
1720.821	1720.9739	0.1529	89	262	276	DNGMHALIIYDDLSK			Oxidation (M)[4]		Mascot
1724.8789	1724.8748	-0.0041	-2	444	458	QIVVIYAAVNGFCDR			Carbamidomethyl (C)[13]		Mascot
1834.8429	1834.851	0.0081	4	18	32	MTNFYTNFQVDEIGR					Mascot
1850.8378	1850.8162	-0.0216	-12	18	32	MTNFYTNFQVDEIGR			Oxidation (M)[1]		Mascot
1850.8378	1850.8162	-0.0216	-12	18	32	MTNFYTNFQVDEIGR	133	100	Oxidation (M)[1]		Mascot
2157.0532	2157.0288	-0.0244	-11	43	62	VYGLNEIQAGEMVEFAS GVK			Oxidation (M)[12]		Mascot

3	ATP synthase subunit alpha, mitochondrial [Triticum urartu]	gi|474033641	44894.3	5.54	14	578	100	21.668	511	100	

Peptide Information

Calc. Mass	Obsrv. Mass	± da	± ppm	Start Seq.	End Seq.	Sequence	Ion Score	C. I. %	Modification	Rank	Result Type
815.4621	815.4484	-0.0137	-17	150	156	ELIIGDR					Mascot
874.4741	874.4857	0.0116	13	389	396	GGLTNERK					Mascot
884.4221	884.4128	-0.0093	-11	104	111	GALSDHER					Mascot
892.4886	892.4791	-0.0095	-11	297	303	LELAQYR					Mascot
972.5473	972.5329	-0.0144	-15	16	25	VVSVGDGIAR					Mascot
972.5473	972.5329	-0.0144	-15	16	25	VVSVGDGIAR	72	100			Mascot
1026.5942	1026.5762	-0.018	-18	137	146	AVDSLVPIGR					Mascot
1026.5942	1026.5762	-0.018	-18	137	146	AVDSLVPIGR	73	100			Mascot
1242.6147	1242.5756	-0.0391	-31	126	136	SVHEPMQTGLK			Oxidation (M)[6]		Mascot
1300.7471	1300.6886	-0.0585	-45	161	172	TAIAIDTILNQK					Mascot
1341.705	1341.649	-0.056	-42	335	345	QPQYEPLPIEK					Mascot
1646.9225	1646.8026	-0.1199	-73	16	31	VVSVGDGIARVYGLNK					Mascot
1703.8025	1703.7719	-0.0306	-18	2	15	TNFYTNFQVDEIGR					Mascot
1720.821	1720.9739	0.1529	89	245	259	DNGMHALIIYDDLSK			Oxidation (M)[4]		Mascot
1834.8429	1834.851	0.0081	4	1	15	MTNFYTNFQVDEIGR					Mascot
1850.8378	1850.8162	-0.0216	-12	1	15	MTNFYTNFQVDEIGR			Oxidation (M)[1]		Mascot
1850.8378	1850.8162	-0.0216	-12	1	15	MTNFYTNFQVDEIGR	133	100	Oxidation (M)[1]		Mascot
2308.1567	2308.1646	0.0079	3	304	325	EVAAFAQFGSDLDAATQ					Mascot

2308.1567	2308.1646	0.0079	3	304	325	ALLNR EVAAFAQFGSDLDAATQ ALLNR	233	100			Mascot

4	ATP synthase subunit alpha, mitochondrial [Triticum urartu]	gi|474247591	36637.9	5.92	13	453	100	30.5	386	100

Peptide Information

Calc. Mass	Obsrv. Mass	± da	± ppm	Start Seq.	End Seq.	Sequence	Ion Score	C. I. %	Modification	Rank	Result Type
815.4621	815.4484	-0.0137	-17	150	156	ELIIGDR					Mascot
860.5022	860.4489	-0.0533	-62	266	272	QMSLLLR					Mascot
876.4971	876.4717	-0.0254	-29	266	272	QMSLLLR			Oxidation (M)[2]		Mascot
884.4221	884.4128	-0.0093	-11	104	111	GALSDHER					Mascot
952.5098	952.4523	-0.0575	-60	332	339	TRELASIY					Mascot
972.5473	972.5329	-0.0144	-15	16	25	VVSVGDGIAR					Mascot
972.5473	972.5329	-0.0144	-15	16	25	VVSVGDGIAR	72	100			Mascot
1026.5942	1026.5762	-0.018	-18	137	146	AVDSLVPIGR					Mascot
1026.5942	1026.5762	-0.018	-18	137	146	AVDSLVPIGR	73	100			Mascot
1242.6147	1242.5756	-0.0391	-31	126	136	SVHEPMQTGLK			Oxidation (M)[6]		Mascot
1300.7471	1300.6886	-0.0585	-45	161	172	TAIAIDTILNQK					Mascot
1537.7434	1537.7349	-0.0085	-6	278	290	EAFPGDVFYLHSR					Mascot
1537.7434	1537.7349	-0.0085	-6	278	290	EAFPGDVFYLHSR	108	100			Mascot
1703.8025	1703.7719	-0.0306	-18	2	15	TNFYTNFQVDEIGR					Mascot
1720.821	1720.9739	0.1529	89	245	259	DNGMHALIIYDDLSK			Oxidation (M)[4]		Mascot
1834.8429	1834.851	0.0081	4	1	15	MTNFYTNFQVDEIGR					Mascot
1850.8378	1850.8162	-0.0216	-12	1	15	MTNFYTNFQVDEIGR			Oxidation (M)[1]		Mascot
1850.8378	1850.8162	-0.0216	-12	1	15	MTNFYTNFQVDEIGR	133	100	Oxidation (M)[1]		Mascot
2157.0532	2157.0288	-0.0244	-11	26	45	VYGLNEIQAGEMVEFAS GVK			Oxidation (M)[12]		Mascot

5	ATP synthase subunit alpha, mitochondrial [Triticum urartu]	gi|474046276	47623.9	9.18	13	405	100	23.069	352	100

Peptide Information

Calc. Mass	Obsrv. Mass	± da	± ppm	Start Seq.	End Seq.	Sequence	Ion Score	C. I. %	Modification	Rank	Result Type
815.4621	815.4484	-0.0137	-17	148	154	ELIIGDR					Mascot
860.5022	860.4489	-0.0533	-62	238	244	QMSLLLR					Mascot
876.4971	876.4717	-0.0254	-29	238	244	QMSLLLR			Oxidation (M)[2]		Mascot
884.4221	884.4128	-0.0093	-11	102	109	GALSDHER					Mascot
892.4886	892.4791	-0.0095	-11	350	356	LELAQYR					Mascot

Calc. Mass	Obsrv. Mass	± da	± ppm	Start Seq.	End Seq.	Sequence	Ion Score	C. I. %	Modification	Rank	Result Type
1026.5942	1026.5762	-0.018	-18	135	144	AVDSLVPIGR					Mascot
1026.5942	1026.5762	-0.018	-18	135	144	AVDSLVPIGR	73	100			Mascot
1242.6147	1242.5756	-0.0391	-31	124	134	SVHEPMQTGLK			Oxidation (M)[6]		Mascot
1259.6777	1259.5688	-0.1089	-86	73	84	RTGSIVDVPMGK					Mascot
1300.7471	1300.6886	-0.0585	-45	159	170	TAIAIDTILNQK					Mascot
1341.705	1341.649	-0.056	-42	388	398	QPQYEPLPIEK					Mascot
1438.8489	1438.8287	-0.0202	-14	318	331	GIRPAINVGLSVSR					Mascot
1438.8489	1438.8287	-0.0202	-14	318	331	GIRPAINVGLSVSR	45	99.744			Mascot
1720.821	1720.9739	0.1529	89	217	231	DNGMHALIIYDDLSK			Oxidation (M)[4]		Mascot
1724.8789	1724.8748	-0.0041	-2	399	413	QIVVIYAAVNGFCDR			Carbamidomethyl (C)[13]		Mascot
2308.1567	2308.1646	0.0079	3	357	378	EVAAFAQFGSDLDAATQ ALLNR					Mascot
2308.1567	2308.1646	0.0079	3	357	378	EVAAFAQFGSDLDAATQ ALLNR	233	100			Mascot

6	ATP synthase subunit alpha, mitochondrial [Triticum urartu]	gi|474015734	16963	5.25	6	233	100	6.155	204	100	

Peptide Information

Calc. Mass	Obsrv. Mass	± da	± ppm	Start Seq.	End Seq.	Sequence	Ion Score	C. I. %	Modification	Rank	Result Type
815.4621	815.4484	-0.0137	-17	152	158	ELIIGDR					Mascot
884.4221	884.4128	-0.0093	-11	106	113	GALSDHER					Mascot
972.5473	972.5329	-0.0144	-15	16	25	VVSVGDGIAR					Mascot
972.5473	972.5329	-0.0144	-15	16	25	VVSVGDGIAR	72	100			Mascot
1703.8025	1703.7719	-0.0306	-18	2	15	TNFYTNFQVDEIGR					Mascot
1728.9061	1728.8871	-0.019	-11	75	91	RTGSIVDVPAGEAMLGR					Mascot
1834.8429	1834.851	0.0081	4	1	15	MTNFYTNFQVDEIGR					Mascot
1850.8378	1850.8162	-0.0216	-12	1	15	MTNFYTNFQVDEIGR			Oxidation (M)[1]		Mascot
1850.8378	1850.8162	-0.0216	-12	1	15	MTNFYTNFQVDEIGR	133	100	Oxidation (M)[1]		Mascot

7	ATP synthase subunit alpha, mitochondrial [Triticum urartu]	gi|474015735	23605.2	5.96	5	128	100	20.053	108	100	

Peptide Information

Calc. Mass	Obsrv. Mass	± da	± ppm	Start Seq.	End Seq.	Sequence	Ion Score	C. I. %	Modification	Rank	Result Type
874.4741	874.4857	0.0116	13	192	199	GGLTNERK					Mascot
1326.7627	1326.7087	-0.054	-41	175	186	AILSTINPELQK					Mascot
1341.705	1341.649	-0.056	-42	138	148	QPQYEPLPIEK					Mascot
1537.7434	1537.7349	-0.0085	-6	12	24	EAFPGDVFYLHSR					Mascot

Calc. Mass	Obsrv. Mass	± da	± ppm	Start Seq.	End Seq.	Sequence	Ion Score	C. I. %	Modification	Rank	Result Type
1537.7434	1537.7349	-0.0085	-6	12	24	EAFPGDVFYLHSR	108	100			Mascot
1724.8789	1724.8748	-0.0041	-2	149	163	QIVVIYAAVNGFCDR			Carbamidomethyl (C)[13]		Mascot

8 Adenosylhomocysteinase [Triticum urartu] gi|474154141 46127.7 6.48 12 92 99.997 4.604 52 99.946

Peptide Information

Calc. Mass	Obsrv. Mass	± da	± ppm	Start Seq.	End Seq.	Sequence	Ion Score	C. I. %	Modification	Rank	Result Type
827.5713	827.549	-0.0223	-27	107	113	IVLTIIR					Mascot
1008.4745	1008.4617	-0.0128	-13	18	26	DLSQADFGR					Mascot
1008.4745	1008.4617	-0.0128	-13	18	26	DLSQADFGR	52	99.946			Mascot
1041.5146	1041.4823	-0.0323	-31	177	185	HSLPDGLMR			Oxidation (M)[8]		Mascot
1044.4568	1044.4391	-0.0177	-17	169	176	FDNLYGCR			Carbamidomethyl (C)[7]		Mascot
1071.6157	1071.5541	-0.0616	-57	300	308	ITIKPQTDR					Mascot
1129.5493	1129.4744	-0.0749	-66	260	268	DIIMVDHMR					Mascot
1137.5576	1137.5282	-0.0294	-26	44	53	TEFGPSQPFK					Mascot
1235.6379	1235.6002	-0.0377	-31	16	26	VKDLSQADFGR					Mascot
1259.5837	1259.5688	-0.0149	-12	167	176	SKFDNLYGCR			Carbamidomethyl (C)[9]		Mascot
1281.6467	1281.5728	-0.0739	-58	1	12	MALSVEKTSSGR			Oxidation (M)[1]		Mascot
2241.1187	2241.136	0.0173	8	396	415	SQSDYISIPVEGPYKPAAYR					Mascot
2583.3455	2583.2893	-0.0562	-22	393	415	LTKSQSDYISIPVEGPYKPAAYR					Mascot

9 Adenosylhomocysteinase [Triticum urartu] gi|474160132 83795.8 6.45 12 74 99.832 4.164 52 99.946

Peptide Information

Calc. Mass	Obsrv. Mass	± da	± ppm	Start Seq.	End Seq.	Sequence	Ion Score	C. I. %	Modification	Rank	Result Type
827.5713	827.549	-0.0223	-27	443	449	IVLTIIR					Mascot
1008.4745	1008.4617	-0.0128	-13	327	335	DLSQADFGR					Mascot
1008.4745	1008.4617	-0.0128	-13	327	335	DLSQADFGR	52	99.946			Mascot
1037.5747	1037.4928	-0.0819	-79	255	262	MMARLLFR					Mascot
1041.5146	1041.4823	-0.0323	-31	513	521	HSLPDGLMR			Oxidation (M)[8]		Mascot
1044.4568	1044.4391	-0.0177	-17	505	512	FDNLYGCR			Carbamidomethyl (C)[7]		Mascot
1071.6157	1071.5541	-0.0616	-57	636	644	ITIKPQTDR					Mascot
1129.5493	1129.4744	-0.0749	-66	596	604	DIIMVDHMR					Mascot
1211.6313	1211.6774	0.0461	38	1	12	MASPSPAARAPR					Mascot
1235.6379	1235.6002	-0.0377	-31	325	335	VKDLSQADFGR					Mascot
1259.5837	1259.5688	-0.0149	-12	503	512	SKFDNLYGCR			Carbamidomethyl (C)[9]		Mascot
1720.915	1720.9739	0.0589	34	301	316	IQSTNLLSSMALSVEK					Mascot

2225.1238	2225.1238	0	0	732	751	AQSEYISIPVDGPYKPAAYR						Mascot

10	hypothetical protein TRIUR3_17642 [Triticum urartu]	gi|473817258	12524.3	8.09	9	58	93.896	5.429

Peptide Information

Calc. Mass	Obsrv. Mass	± da	± ppm	Start Seq.	End Seq.	Sequence	Ion Score	C. I. %	Modification	Rank	Result Type
925.4639	925.5013	0.0374	40	54	61	LGAHGWER					Mascot
1137.551	1137.5282	-0.0228	-20	1	10	MADGGVWVFR					Mascot
1178.5875	1178.5682	-0.0193	-16	92	101	STHMYDVVVK					Mascot
1185.6475	1185.611	-0.0365	-31	75	85	DGSLDLISLPR					Mascot
1281.6409	1281.5728	-0.0681	-53	1	11	MADGGVWVFRK			Oxidation (M)[1]		Mascot
1341.7485	1341.649	-0.0995	-74	74	85	RDGSLDLISLPR					Mascot
1363.7039	1363.6704	-0.0335	-25	34	45	ALVYVPANETMR					Mascot
1724.812	1724.8748	0.0628	36	11	27	KDGVMELESAAGSTSSR					Mascot
1850.962	1850.8162	-0.1458	-79	46	61	SLQALEQRLGAHGWER					Mascot
1850.962	1850.8162	-0.1458	-79	46	61	SLQALEQRLGAHGWER					Mascot

Gel Idx/Pos	279/L7	Instr./Gel Origin	BA2151/Sample Project 20140814	Process Status	Analysis Succeeded
Plate [#] Name	[1] Sample Project 20140814	Instrument Sample Name		Spectra	11

Rank	Protein Name	Accession No.	Protein MW	Protein PI	Pep. Count	Protein Score	Protein Score C. I. %	Intensity Matched	Total Ion Score	Total Ion C. I. %	Confirmed
1	6-phosphogluconate dehydrogenase, decarboxylating [Triticum urartu]	gi|474156904	53712.4	9.45	19	500	100	33.803	409	100	

Peptide Information

Calc. Mass	Obsrv. Mass	± da	± ppm	Start Seq.	End Seq.	Sequence	Ion Score	C. I. %	Modification	Rank	Result Type
846.4316	846.4282	-0.0034	-4	140	146	VDETVQR					Mascot
943.5207	943.5165	-0.0042	-4	321	328	AQLIEDVR					Mascot
1052.5524	1052.5509	-0.0015	-1	127	135	GFPISVYNR					Mascot
1052.5524	1052.5509	-0.0015	-1	127	135	GFPISVYNR	70	100			Mascot
1064.5735	1064.5676	-0.0059	-6	293	301	FLSGLKDER					Mascot
1064.5735	1064.5676	-0.0059	-6	293	301	FLSGLKDER	43	99.541			Mascot
1071.6157	1071.6086	-0.0071	-7	321	329	AQLIEDVRK					Mascot
1075.5742	1075.5322	-0.042	-39	140	148	VDETVQRTK					Mascot
1109.6426	1109.641	-0.0016	-1	442	451	LPANLVQAQR					Mascot
1185.6376	1185.6371	-0.0005	0	354	364	GWGLNLGELAR					Mascot
1258.5488	1258.5519	0.0031	2	452	461	DYFGAHTYER					Mascot
1258.5488	1258.5519	0.0031	2	452	461	DYFGAHTYER	79	100			Mascot
1263.6539	1263.6343	-0.0196	-16	136	146	TTSKVDETVQR					Mascot
1380.7706	1380.7672	-0.0034	-2	440	451	DRLPANLVQAQR					Mascot
1380.7706	1380.7672	-0.0034	-2	440	451	DRLPANLVQAQR	66	99.998			Mascot
1439.6848	1439.724	0.0392	27	24	34	MQWIRGENYSR					Mascot
1709.9255	1709.822	-0.1035	-61	91	106	VKLGLASSAYEMALTR					Mascot
1777.9232	1777.8411	-0.0821	-46	62	77	RSDPPLELPAGHFVDK					Mascot
2012.1208	2012.077	-0.0438	-22	107	126	IGLAGLAVMGQNLALNIAEK			Oxidation (M)[9]		Mascot
2304.1177	2304.1191	0.0014	1	387	406	NGELANLLIDPEFAQEIMDR			Oxidation (M)[18]		Mascot
2470.2097	2470.2358	0.0261	11	307	328	IFQGDYSSGETVDKAQLIEDVR					Mascot
2699.394	2699.4307	0.0367	14	149	172	LEGNLPLYGFHDPASFVNSIQKPR					Mascot
2699.394	2699.4307	0.0367	14	149	172	LEGNLPLYGFHDPASFVNSIQKPR	152	100			Mascot
2809.3462	2809.3469	0.0007	0	383	406	AYDRNGELANLLIDPEFAQEIMDR			Oxidation (M)[22]		Mascot

Rank	Protein Name	Accession No.	Protein MW	Protein PI	Pep. Count	Protein Score	Protein Score C. I. %	Intensity Matched	Total Ion Score	Total Ion C. I. %	Confirmed
2	6-phosphogluconate dehydrogenase, decarboxylating	gi|474379872	81632	8.56	20	498	100	36.703	406	100	

[Triticum urartu]

Peptide Information

Calc. Mass	Obsrv. Mass	± da	± ppm	Start Seq.	End Seq.	Sequence	Ion Score	C. I. %	Modification	Rank	Result Type
846.4316	846.4282	-0.0034	-4	481	487	VDETVQR					Mascot
908.4142	908.4077	-0.0065	-7	115	121	EKAMEER			Oxidation (M)[4]		Mascot
943.5207	943.5165	-0.0042	-4	578	585	AQLIEDVR					Mascot
1052.5524	1052.5509	-0.0015	-1	468	476	GFPISVYNR					Mascot
1052.5524	1052.5509	-0.0015	-1	26	34	GFPISVYNR	70	100			Mascot
1064.5735	1064.5676	-0.0059	-6	271	279	FLSGLKDER					Mascot
1064.5735	1064.5676	-0.0059	-6	271	279	FLSGLKDER	43	99.541			Mascot
1071.6157	1071.6086	-0.0071	-7	578	586	AQLIEDVRK					Mascot
1109.6426	1109.641	-0.0016	-1	699	708	LPANLVQAQR					Mascot
1185.6376	1185.6371	-0.0005	0	611	621	GWGLNLGELAR					Mascot
1258.5488	1258.5519	0.0031	2	709	718	DYFGAHTYER					Mascot
1258.5488	1258.5519	0.0031	2	709	718	DYFGAHTYER	79	100			Mascot
1263.6539	1263.6343	-0.0196	-16	477	487	TTSKVDETVQR					Mascot
1380.7706	1380.7672	-0.0034	-2	697	708	DRLPANLVQAQR					Mascot
1380.7706	1380.7672	-0.0034	-2	697	708	DRLPANLVQAQR	66	99.998			Mascot
2012.1208	2012.077	-0.0438	-22	448	467	IGLAGLAVMGQNLALNIA EK			Oxidation (M)[9]		Mascot
2304.1177	2304.1191	0.0014	1	644	663	NGELANLLIDPEFAQEIM DR			Oxidation (M)[18]		Mascot
2443.2463	2443.2715	0.0252	10	248	270	WTVQQAAELSVAAPTIEA SLDSR					Mascot
2470.2097	2470.2358	0.0261	11	564	585	IFQGDYSSGETVDKAQLI EDVR					Mascot
2596.2998	2596.3823	0.0825	32	117	140	AMEERGLLYLGMGVSGG EEDILLK			Oxidation (M)[2]		Mascot
2699.394	2699.4307	0.0367	14	490	513	LEGNLPLYGFHDPASFVN SIQKPR					Mascot
2699.394	2699.4307	0.0367	14	48	71	LEGNLPLYGFHDPASFVN SIQKPR	152	100			Mascot
2809.3462	2809.3469	0.0007	0	640	663	AYDRNGELANLLIDPEFA QEIMDR			Oxidation (M)[22]		Mascot
2880.427	2880.4761	0.0491	17	166	191	MVHNGIEYGDMQLIAEAY DVLKSVGK					Mascot
2898.5261	2898.5608	0.0347	12	488	513	AKLEGNLPLYGFHDPASF VNSIQKPR					Mascot

3	Argininosuccinate synthase, chloroplastic [Triticum urartu]	gi|474097032	48277.5	5.75	10	142	100	4.314	117	100	

Peptide Information

Calc. Mass	Obsrv. Mass	± da	± ppm	Start Seq.	End Seq.	Sequence	Ion Score	C. I. %	Modification	Rank	Result Type

Calc. Mass	Obsrv. Mass	± da	± ppm	Start Seq.	End Seq.	Sequence	Ion Score	C. I. %	Modification	Rank	Result Type
819.4723	819.4624	-0.0099	-12	422	428	LYGLPTR					Mascot
833.4304	833.4158	-0.0146	-18	354	359	WFDPLR					Mascot
846.4866	846.4282	-0.0584	-69	429	435	VRAMLEK					Mascot
885.4465	885.4439	-0.0026	-3	393	399	SPYSLYR					Mascot
1013.5414	1013.5345	-0.0069	-7	392	399	KSPYSLYR					Mascot
1041.5364	1041.5333	-0.0031	-3	345	353	YAELVYAGR					Mascot
1075.563	1075.5322	-0.0308	-29	324	332	ELESLTLDR					Mascot
1608.8051	1608.7914	-0.0137	-9	308	323	GVYETPGGTIMAAAVR			Oxidation (M)[11]		Mascot
2003.9419	2003.9576	0.0157	8	100	115	DLTEEFVGEYIYPCLR			Carbamidomethyl (C)[14]		Mascot
2003.9419	2003.9576	0.0157	8	100	115	DLTEEFVGEYIYPCLR	117	100	Carbamidomethyl (C)[14]		Mascot
2505.1052	2505.1401	0.0349	14	400	421	EDISSFENGEIYDQADAE GFIR					Mascot

4	SHAGGY-like kinase [Triticum aestivum]	gi|480578280	45822.3	7.62	11	45	0	4.574

Protein Group

SHAGGY-like kinase [Triticum aestivum]	gi|480578282	45822.3	7.6199 998855 5908

Peptide Information

Calc. Mass	Obsrv. Mass	± da	± ppm	Start Seq.	End Seq.	Sequence	Ion Score	C. I. %	Modification	Rank	Result Type
897.4135	897.4235	0.01	11	205	212	ICDFGSAK			Carbamidomethyl (C)[2]		Mascot
1064.5809	1064.5676	-0.0133	-12	157	165	QGMPLIYVK			Oxidation (M)[3]		Mascot
1064.5809	1064.5676	-0.0133	-12	157	165	QGMPLIYVK			Oxidation (M)[3]		Mascot
1075.5677	1075.5322	-0.0355	-33	102	109	NRELQLMR			Oxidation (M)[7]		Mascot
1199.6089	1199.6263	0.0174	15	316	326	MPPEAIDLASR					Mascot
1213.6437	1213.6333	-0.0104	-9	391	402	RQAGLAFVHAGS					Mascot
1286.6906	1286.578	-0.1126	-88	305	314	AHPWHKVFHK					Mascot
1343.6987	1343.7355	0.0368	27	315	326	KMPPEAIDLASR			Oxidation (M)[2]		Mascot
1394.7018	1394.7758	0.074	53	82	93	CLETGEMVAIKK			Carbamidomethyl (C)[1], Oxidation (M)[7]		Mascot
1409.7788	1409.7181	-0.0607	-43	68	81	VVGTGSFGIVFQAK					Mascot
2505.2808	2505.1401	-0.1407	-56	59	81	QTISYMAERVVGTGSFGI VFQAK			Oxidation (M)[6]		Mascot
2808.4714	2808.3503	-0.1211	-43	188	212	DVKPQNVLVDPLTHQVKI CDFGSAK			Carbamidomethyl (C)[19]		Mascot

5	unnamed protein product [Triticum aestivum]	gi|257626285	37835.4	6.34	10	42	0	10.734

Peptide Information

Calc. Mass	Obsrv. Mass	± da	± ppm	Start	End	Sequence	Ion	C. I. %	Modification	Rank	Result Type

Calc. Mass	Obsrv. Mass	± da	± ppm	Start Seq.	End Seq.	Sequence	Ion Score	C. I. %	Modification	Rank	Result Type
1034.4789	1034.4993	0.0204	20	98	106	FNEEPAEAK					Mascot
1075.5929	1075.5322	-0.0607	-56	1	10	MASDRLAALK					Mascot
1216.5966	1216.625	0.0284	23	300	309	MSVACFFRAK			Carbamidomethyl (C)[5]		Mascot
1224.6517	1224.6736	0.0219	18	166	175	YTAQIRMLGR			Oxidation (M)[7]		Mascot
1258.5256	1258.5519	0.0263	21	136	145	DTIYMDDVDR			Oxidation (M)[5]		Mascot
1258.5256	1258.5519	0.0263	21	136	145	DTIYMDDVDR			Oxidation (M)[5]		Mascot
1289.6307	1289.6516	0.0209	16	36	46	HPPDPLPQCTK			Carbamidomethyl (C)[9]		Mascot
1350.7377	1350.708	-0.0297	-22	327	337	YRTVTAEELLR					Mascot
1580.785	1580.7152	-0.0698	-44	310	326	GATVCAPVVADGGGPPR			Carbamidomethyl (C)[5]		Mascot
1912.9521	1912.9417	-0.0104	-5	291	307	VVANSVGPRMSVACFFR			Carbamidomethyl (C)[14], Oxidation (M)[10]		Mascot
2880.3027	2880.4761	0.1734	60	136	160	DTIYMDDVDRLAAGEEEE VLPPACR			Carbamidomethyl (C)[24], Oxidation (M)[5]		Mascot

6	hypothetical protein TRIUR3_24112 [Triticum urartu]	gi|474055143	15160.6	10.09	7	42	0	4.099	

Peptide Information

Calc. Mass	Obsrv. Mass	± da	± ppm	Start Seq.	End Seq.	Sequence	Ion Score	C. I. %	Modification	Rank	Result Type
885.4788	885.4439	-0.0349	-39	116	123	EVSPAINR					Mascot
890.4407	890.425	-0.0157	-18	124	131	FFSGFSAK					Mascot
908.4373	908.4077	-0.0296	-33	101	107	YSQHAFR					Mascot
1041.58	1041.5333	-0.0467	-45	115	123	REVSPAINR					Mascot
1064.5385	1064.5676	0.0291	27	101	108	YSQHAFRR					Mascot
1064.5385	1064.5676	0.0291	27	58	65	YSQHAFRR					Mascot
1073.5739	1073.4934	-0.0805	-75	30	38	NVFLNPDVR					Mascot
1756.9017	1756.9557	0.054	31	116	131	EVSPAINRFFSGFSAK					Mascot

7	ubiquitin-activating enzyme E1 [Triticum aestivum]	gi|170780	117846.4	5.16	17	42	0	5.503	

Protein Group

RecName: Full=Ubiquitin-activating enzyme E1 1	gi|136632	117846.4	5.1599 998474 1211
ubiquitin-protein ligase (EC 6.3.2.19) E1 - wheat	gi|100841	117846.4	5.1599 998474 1211

Peptide Information

Calc. Mass	Obsrv. Mass	± da	± ppm	Start Seq.	End Seq.	Sequence	Ion Score	C. I. %	Modification	Rank	Result Type

897.4135	897.4235	0.01	11	1018	1024	MEVPSYR			Oxidation (M)[1]		Mascot
908.4948	908.4077	-0.0871	-96	336	342	FRTELSR					Mascot
1064.5259	1064.5676	0.0417	39	874	882	NYSIPEVDK					Mascot
1064.5259	1064.5676	0.0417	39	874	882	NYSIPEVDK					Mascot
1075.5491	1075.5322	-0.0169	-16	20	29	TRAGEGEVTR					Mascot
1256.6844	1256.6576	-0.0268	-21	781	792	TPNKLAAEAVDK					Mascot
1284.5815	1284.5996	0.0181	14	40	50	GNEIDEDLHSR					Mascot
1315.6754	1315.6133	-0.0621	-47	914	925	ALAGGHKVEDYR					Mascot
1457.7847	1457.6823	-0.1024	-70	7	19	EIVAGEVEDLQKK					Mascot
1644.8125	1644.684	-0.1285	-78	465	479	IFMVGSGALGCEFLK			Carbamidomethyl (C)[11], Oxidation (M)[3]		Mascot
1697.8857	1697.8199	-0.0658	-39	445	459	YDAQISVFGSKLQNK					Mascot
1756.7959	1756.9557	0.1598	91	693	706	DKCETFQDSITWAR			Carbamidomethyl (C)[3]		Mascot
1805.8082	1805.9734	0.1652	91	22	39	AGEGEVTREEGDAAMAG R					Mascot
1840.0215	1839.8864	-0.1351	-73	785	801	LAAEAVDKVIVPDFQPK					Mascot
1911.0045	1910.9023	-0.1022	-53	582	598	CVYFQKPLLESGTLGAK			Carbamidomethyl (C)[1]		Mascot
2077.0964	2077.1428	0.0464	22	147	164	EHLSKFQAVVFTDISLDK					Mascot
2598.3213	2598.3579	0.0366	14	949	969	HQELSWTVWDRWTVTG NITLR					Mascot
2880.5725	2880.4761	-0.0964	-33	887	913	FIAGRIIPAIATSTAMATGL VCLELYK			Carbamidomethyl (C)[22]		Mascot

8 Ankyrin-3 [Triticum urartu] gi|474308451 132023.4 7.49 18 41 0 21.954

Peptide Information

Calc. Mass	Obsrv. Mass	± da	± ppm	Start Seq.	End Seq.	Sequence	Ion Score	C. I. %	Modification	Rank	Result Type
819.4108	819.4624	0.0516	63	612	617	HDYTRK					Mascot
833.3748	833.4158	0.041	49	115	121	QDNDVSR					Mascot
1052.5735	1052.5509	-0.0226	-21	982	992	ADLVQPGPAGK					Mascot
1052.5735	1052.5509	-0.0226	-21	982	992	ADLVQPGPAGK					Mascot
1201.6973	1201.6334	-0.0639	-53	235	245	LVALEVALMSR					Mascot
1212.6583	1212.6384	-0.0199	-16	955	965	RPDLVESAPTK					Mascot
1217.6923	1217.6348	-0.0575	-47	235	245	LVALEVALMSR			Oxidation (M)[9]		Mascot
1217.6923	1217.6348	-0.0575	-47	235	245	LVALEVALMSR			Oxidation (M)[9]		Mascot
1241.7212	1241.6105	-0.1107	-89	268	278	DLNAVRLLAEK					Mascot
1310.7175	1310.6392	-0.0783	-60	1015	1025	IDLNQPRPTTR					Mascot
1328.606	1328.6818	0.0758	57	1047	1056	FSFFHSQWDK					Mascot
1343.7576	1343.7355	-0.0221	-16	214	224	CIQLLLSRNAR			Carbamidomethyl (C)[1]		Mascot
1544.8717	1544.8306	-0.0411	-27	295	308	VAALAMLLLVTEER			Oxidation (M)[6]		Mascot

1567.772	1567.7902	0.0182	12	798	810	LSDFMRSLPGICR			Carbamidomethyl (C)[12], Oxidation (M)[5]		Mascot
1644.7543	1644.684	-0.0703	-43	1072	1085	SCEAAMELVPCLHK			Carbamidomethyl (C)[2,11]		Mascot
1680.7867	1680.8358	0.0491	29	1116	1130	CQLMVVCSGADAVVR			Carbamidomethyl (C)[1,7], Oxidation (M)[4]		Mascot
2003.9678	2003.9576	-0.0102	-5	528	545	FLLNSANVVPMNHGSMEK			Oxidation (M)[11]		Mascot
2003.9678	2003.9576	-0.0102	-5	528	545	FLLNSANVVPMNHGSMEK			Oxidation (M)[11]		Mascot
2470.2759	2470.2358	-0.0401	-16	458	479	GNYKALQSLLLHAQDCVDIPSK			Carbamidomethyl (C)[16]		Mascot
2506.1814	2506.1213	-0.0601	-24	1026	1046	FVDRPVERPAITPSSCENEMR			Carbamidomethyl (C)[16], Oxidation (M)[20]		Mascot
2598.4373	2598.3579	-0.0794	-31	811	834	MRVVPVGNGPATHMVLLPPLSRPK			Oxidation (M)[1,14]		Mascot
2809.2681	2809.3469	0.0788	28	899	925	SNGESLPDGDSTSNCSLLDEVTVSTPK			Carbamidomethyl (C)[15]		Mascot

9 SHAGGY-like kinase [Triticum aestivum] gi|483171662 45794.3 7.62 10 39 0 3.886

Protein Group

SHAGGY-like kinase [Triticum aestivum] gi|480578271 45822.3 7.23000001907349

SHAGGY-like kinase [Triticum aestivum] gi|480578267 45822.3 7.23000001907349

SHAGGY-like kinase [Triticum aestivum] gi|483171680 45794.3 7.61999988555908

Peptide Information

Calc. Mass	Obsrv. Mass	± da	± ppm	Start Seq.	End Seq.	Sequence	Ion Score	C. I. %	Modification	Rank	Result Type
897.4135	897.4235	0.01	11	205	212	ICDFGSAK			Carbamidomethyl (C)[2]		Mascot
1064.5809	1064.5676	-0.0133	-12	157	165	QGMPLIYVK			Oxidation (M)[3]		Mascot
1064.5809	1064.5676	-0.0133	-12	157	165	QGMPLIYVK			Oxidation (M)[3]		Mascot
1075.5677	1075.5322	-0.0355	-33	102	109	NRELQLMR			Oxidation (M)[7]		Mascot
1199.6089	1199.6263	0.0174	15	316	326	MPPEAIDLASR					Mascot
1213.6437	1213.6333	-0.0104	-9	391	402	RQAGLAFVHAGS					Mascot
1286.6906	1286.578	-0.1126	-88	305	314	AHPWHKVFHK					Mascot
1343.6987	1343.7355	0.0368	27	315	326	KMPPEAIDLASR			Oxidation (M)[2]		Mascot
1409.7788	1409.7181	-0.0607	-43	68	81	VVGTGSFGIVFQAK					Mascot
2505.2808	2505.1401	-0.1407	-56	59	81	QTISYMAERVVGTGSFGIVFQAK			Oxidation (M)[6]		Mascot
2808.4714	2808.3503	-0.1211	-43	188	212	DVKPQNVLVDPLTHQVKICDFGSAK			Carbamidomethyl (C)[19]		Mascot

10 Anthranilate N-benzoyltransferase protein 2 [Triticum gi|474111564 37853.3 8.27 10 38 0 3.295

urartu]

Peptide Information

Calc. Mass	Obsrv. Mass	± da	± ppm	Start Seq.	End Seq.	Sequence	Ion Score	C. I. %	Modification	Rank	Result Type
833.4913	833.4158	-0.0755	-91	81	88	MGAVLSKK					Mascot
865.4084	865.4156	0.0072	8	270	276	MAETELR			Oxidation (M)[1]		Mascot
890.4149	890.425	0.0101	11	171	179	LGMGGQDGR					Mascot
1041.5211	1041.5333	0.0122	12	50	58	TGYDQKTTK					Mascot
1240.611	1240.6108	-0.0002	0	132	141	GHTPTVYFYR					Mascot
1544.7738	1544.8306	0.0568	37	102	116	VVESGMVAPSAQTPR			Oxidation (M)[6]		Mascot
1646.8684	1646.8103	-0.0581	-35	180	195	AVIDCAGQGALFVVAR			Carbamidomethyl (C)[5]		Mascot
2011.972	2012.077	0.105	52	196	213	SDLAVDDFSGFRPSTELR					Mascot
2077.1023	2077.1428	0.0405	19	61	80	KLDISSSLELTSAQSAGEI K					Mascot
2458.2473	2458.2971	0.0498	20	2	22	RSHITTDLAVVYTNEPWS VNR					Mascot

Gel Idx/Pos	280/L8	Instr./Gel Origin	BA2151/Sample Project 20140814	Process Status	Analysis Succeeded
Plate [#] Name	[1] Sample Project 20140814	Instrument Sample Name		Spectra	11

Rank	Protein Name	Accession No.	Protein MW	Protein PI	Pep. Count	Protein Score	Protein Score C. I. %	Intensity Matched	Total Ion Score	Total Ion C. I. %	Confirmed
1	waxy A1, partial [Triticum spelta]	gi|334086816	64047.7	8.73	23	665	100	28.717	558	100	

Peptide Information

Calc. Mass	Obsrv. Mass	± da	± ppm	Start Seq.	End Seq.	Sequence	Ion Score	C. I. %	Modification	Rank	Result Type
817.46	817.4323	-0.0277	-34	110	116	VMVISPR			Oxidation (M)[2]		Mascot
821.3723	821.3473	-0.025	-30	510	516	TGFHMGR			Oxidation (M)[5]		Mascot
908.5173	908.4616	-0.0557	-61	50	56	QSRKPHR					Mascot
917.3974	917.3704	-0.027	-29	143	148	YFHCYK			Carbamidomethyl (C)[4]		Mascot
971.6036	971.574	-0.0296	-30	391	399	VPLVAFIGR					Mascot
1099.6986	1099.6765	-0.0221	-20	390	399	KVPLVAFIGR					Mascot
1123.4871	1123.4641	-0.023	-20	327	335	GCELDNIMR			Carbamidomethyl (C)[2], Oxidation (M)[8]		Mascot
1346.6774	1346.636	-0.0414	-31	542	553	VVGTPAYHEMVK			Oxidation (M)[10]		Mascot
1390.6825	1390.6514	-0.0311	-22	154	164	VFVDHPCFLEK			Carbamidomethyl (C)[7]		Mascot
1396.7432	1396.7229	-0.0203	-15	377	389	EALQAEVGLPVDR					Mascot
1503.7988	1503.7808	-0.018	-12	296	308	INWMKAGILQADK			Oxidation (M)[4]		Mascot
1503.7988	1503.7808	-0.018	-12	189	201	FSLLCQAALEVPR	88	100	Carbamidomethyl (C)[5]		Mascot
1524.838	1524.7905	-0.0475	-31	377	390	EALQAEVGLPVDRK					Mascot
1564.7611	1564.7469	-0.0142	-9	476	488	FEPCGLIQLQGMR			Carbamidomethyl (C)[4], Oxidation (M)[12]		Mascot
1564.7689	1564.7469	-0.022	-14	250	262	VAFCIHNISYQGR	67	99.998	Carbamidomethyl (C)[4]		Mascot
1684.7966	1684.7849	-0.0117	-7	263	276	FSFDDFAQLNLPDR					Mascot
1684.7966	1684.7849	-0.0117	-7	263	276	FSFDDFAQLNLPDR	119	100			Mascot
1831.8497	1831.8289	-0.0208	-11	279	294	SSFDFIDGYDKPVEGR					Mascot
1831.8497	1831.8289	-0.0208	-11	279	294	SSFDFIDGYDKPVEGR	113	100			Mascot
1841.7937	1841.7733	-0.0204	-11	173	188	IYGPDAGTDYEDNQQR					Mascot
1841.7937	1841.7733	-0.0204	-11	173	188	IYGPDAGTDYEDNQQR	68	99.999			Mascot
1913.9855	1913.9971	0.0116	6	356	372	DKFLTVNYDVTTALEGK					Mascot
1997.0226	1997.0088	-0.0138	-7	309	326	VLTVSPYYAEELISGEAR					Mascot
1997.0226	1997.0088	-0.0138	-7	309	326	VLTVSPYYAEELISGEAR	106	100			Mascot
2014.0399	2013.9863	-0.0536	-27	22	40	AGFQGLRPRNPADAALG MR			Oxidation (M)[18]		Mascot
2098.9312	2098.9155	-0.0157	-7	171	188	EKIYGPDAGTDYEDNQQ R					Mascot
2232.0901	2232.0498	-0.0403	-18	455	475	FNAPLAHQMMAGADVLA			Oxidation (M)[9,10]		Mascot

			VTSR								
2	waxy D1, partial [Triticum spelta]	gi|310619520		64035.6	8.62	23	663	100	28.621	558	100

Protein Group

waxy D1, partial [Triticum spelta]	gi|310619514	64035.6	8.6199 998855 5908
waxy D1, partial [Triticum spelta]	gi|310619508	64035.6	8.6199 998855 5908
waxy D1, partial [Triticum spelta]	gi|310619502	64035.6	8.6199 998855 5908
waxy D1, partial [Triticum spelta]	gi|310619526	64035.6	8.6199 998855 5908

Peptide Information

Calc. Mass	Obsrv. Mass	± da	± ppm	Start Seq.	End Seq.	Sequence	Ion Score	C. I. %	Modification	Rank	Result Type
817.46	817.4323	-0.0277	-34	110	116	VMVISPR			Oxidation (M)[2]		Mascot
821.3723	821.3473	-0.025	-30	510	516	TGFHMGR			Oxidation (M)[5]		Mascot
908.4836	908.4616	-0.022	-24	134	140	VVDKYER					Mascot
917.3974	917.3704	-0.027	-29	143	148	YFHCYK			Carbamidomethyl (C)[4]		Mascot
971.6036	971.574	-0.0296	-30	391	399	VPLVAFIGR					Mascot
1099.6986	1099.6765	-0.0221	-20	390	399	KVPLVAFIGR					Mascot
1123.4871	1123.4641	-0.023	-20	327	335	GCELDNIMR			Carbamidomethyl (C)[2], Oxidation (M)[8]		Mascot
1273.6859	1273.6185	-0.0674	-53	41	53	TVGASAAPTQSRK					Mascot
1346.6774	1346.636	-0.0414	-31	542	553	VVGTPAYHEMVK			Oxidation (M)[10]		Mascot
1390.6825	1390.6514	-0.0311	-22	154	164	VFVDHPCFLEK			Carbamidomethyl (C)[7]		Mascot
1396.7432	1396.7229	-0.0203	-15	377	389	EALQAEVGLPVDR					Mascot
1503.7988	1503.7808	-0.018	-12	296	308	INWMKAGILQADK			Oxidation (M)[4]		Mascot
1503.7988	1503.7808	-0.018	-12	189	201	FSLLCQAALEVPR	88	100	Carbamidomethyl (C)[5]		Mascot
1524.838	1524.7905	-0.0475	-31	377	390	EALQAEVGLPVDRK					Mascot
1564.7611	1564.7469	-0.0142	-9	476	488	FEPCGLIQLQGMR			Carbamidomethyl (C)[4], Oxidation (M)[12]		Mascot
1564.7689	1564.7469	-0.022	-14	250	262	VAFCIHNISYQGR	67	99.998	Carbamidomethyl (C)[4]		Mascot
1654.8687	1654.8173	-0.0514	-31	358	372	FLAVNYDITTALEGK					Mascot
1684.7966	1684.7849	-0.0117	-7	263	276	FSFDDFAQLNLPDR					Mascot
1684.7966	1684.7849	-0.0117	-7	263	276	FSFDDFAQLNLPDR	119	100			Mascot
1831.8497	1831.8289	-0.0208	-11	279	294	SSFDFIDGYDKPVEGR					Mascot

1831.8497	1831.8289	-0.0208	-11	279	294	SSFDFIDGYDKPVEGR	113	100			Mascot
1841.7937	1841.7733	-0.0204	-11	173	188	IYGPDAGTDYEDNQQR					Mascot
1841.7937	1841.7733	-0.0204	-11	173	188	IYGPDAGTDYEDNQQR	68	99.999			Mascot
1997.0226	1997.0088	-0.0138	-7	309	326	VLTVSPYYAEELISGEAR					Mascot
1997.0226	1997.0088	-0.0138	-7	309	326	VLTVSPYYAEELISGEAR	106	100			Mascot
2098.9312	2098.9155	-0.0157	-7	171	188	EKIYGPDAGTDYEDNQQR					Mascot
2232.0901	2232.0498	-0.0403	-18	455	475	FNAPLAHQMMAGADVLAVTSR			Oxidation (M)[9,10]		Mascot

3	unnamed protein product [Triticum aestivum]	gi|298543905	63967.6	8.84	23	662	100	28.621	558	100		

Protein Group

granule bound starch synthase, partial [Triticum spelta]	gi|378939984	64251.7	8.60999965667725
granule bound starch synthase, partial [Triticum spelta]	gi|378939990	64308.8	8.60999965667725
granule bound starch synthase, partial [Triticum spelta]	gi|378939980	64308.8	8.60999965667725
granule-bound starch synthase precursor [Triticum aestivum]	gi|4588609	63967.6	8.84000015258789
unnamed protein product [Triticum aestivum]	gi|298545815	63967.6	8.84000015258789

Peptide Information

Calc. Mass	Obsrv. Mass	± da	± ppm	Start Seq.	End Seq.	Sequence	Ion Score	C. I. %	Modification	Rank	Result Type
817.46	817.4323	-0.0277	-34	113	119	VMVISPR			Oxidation (M)[2]		Mascot
821.3723	821.3473	-0.025	-30	513	519	TGFHMGR			Oxidation (M)[5]		Mascot
908.4836	908.4616	-0.022	-24	137	143	VVDKYER					Mascot
917.3974	917.3704	-0.027	-29	146	151	YFHCYK			Carbamidomethyl (C)[4]		Mascot
971.6036	971.574	-0.0296	-30	394	402	VPLVAFIGR					Mascot
1099.6986	1099.6765	-0.0221	-20	393	402	KVPLVAFIGR					Mascot
1123.4871	1123.4641	-0.023	-20	330	338	GCELDNIMR			Carbamidomethyl (C)[2], Oxidation (M)[8]		Mascot
1273.6859	1273.6185	-0.0674	-53	44	56	TVGASAAPTQSRK					Mascot
1346.6774	1346.636	-0.0414	-31	545	556	VVGTPAYHEMVK			Oxidation (M)[10]		Mascot
1390.6825	1390.6514	-0.0311	-22	157	167	VFVDHPCFLEK			Carbamidomethyl (C)[7]		Mascot

1396.7432	1396.7229	-0.0203	-15	380	392	EALQAEVGLPVDR					Mascot
1503.7988	1503.7808	-0.018	-12	299	311	INWMKAGILQADK			Oxidation (M)[4]		Mascot
1503.7988	1503.7808	-0.018	-12	192	204	FSLLCQAALEVPR	88	100	Carbamidomethyl (C)[5]		Mascot
1524.838	1524.7905	-0.0475	-31	380	393	EALQAEVGLPVDRK					Mascot
1564.7611	1564.7469	-0.0142	-9	479	491	FEPCGLIQLQGMR			Carbamidomethyl (C)[4], Oxidation (M)[12]		Mascot
1564.7689	1564.7469	-0.022	-14	253	265	VAFCIHNISYQGR	67	99.998	Carbamidomethyl (C)[4]		Mascot
1654.8687	1654.8173	-0.0514	-31	361	375	FLAVNYDITTALEGK					Mascot
1684.7966	1684.7849	-0.0117	-7	266	279	FSFDDFAQLNLPDR					Mascot
1684.7966	1684.7849	-0.0117	-7	266	279	FSFDDFAQLNLPDR	119	100			Mascot
1831.8497	1831.8289	-0.0208	-11	282	297	SSFDFIDGYDKPVEGR					Mascot
1831.8497	1831.8289	-0.0208	-11	282	297	SSFDFIDGYDKPVEGR	113	100			Mascot
1841.7937	1841.7733	-0.0204	-11	176	191	IYGPDAGTDYEDNQQR					Mascot
1841.7937	1841.7733	-0.0204	-11	176	191	IYGPDAGTDYEDNQQR	68	99.999			Mascot
1997.0226	1997.0088	-0.0138	-7	312	329	VLTVSPYYAEELISGEAR					Mascot
1997.0226	1997.0088	-0.0138	-7	312	329	VLTVSPYYAEELISGEAR	106	100			Mascot
2098.9312	2098.9155	-0.0157	-7	174	191	EKIYGPDAGTDYEDNQQR					Mascot
2232.0901	2232.0498	-0.0403	-18	458	478	FNAPLAHQMMAGADVLAVTSR			Oxidation (M)[9,10]		Mascot

4 granule-bound starch synthase precursor [Triticum aestivum] gi|4588607 63388.4 7.86 22 660 100 28.567 558 100

Peptide Information

Calc. Mass	Obsrv. Mass	± da	± ppm	Start Seq.	End Seq.	Sequence	Ion Score	C. I. %	Modification	Rank	Result Type
817.46	817.4323	-0.0277	-34	74	80	VMVISPR			Oxidation (M)[2]		Mascot
821.3723	821.3473	-0.025	-30	474	480	TGFHMGR			Oxidation (M)[5]		Mascot
908.5173	908.4616	-0.0557	-61	53	59	QSRKPHR					Mascot
917.3974	917.3704	-0.027	-29	107	112	YFHCYK			Carbamidomethyl (C)[4]		Mascot
971.6036	971.574	-0.0296	-30	355	363	VPLVAFIGR					Mascot
1099.6986	1099.6765	-0.0221	-20	354	363	KVPLVAFIGR					Mascot
1123.4871	1123.4641	-0.023	-20	291	299	GCELDNIMR			Carbamidomethyl (C)[2], Oxidation (M)[8]		Mascot
1346.6774	1346.636	-0.0414	-31	506	517	VVGTPAYHEMVK			Oxidation (M)[10]		Mascot
1390.6825	1390.6514	-0.0311	-22	118	128	VFVDHPCFLEK			Carbamidomethyl (C)[7]		Mascot
1396.7432	1396.7229	-0.0203	-15	341	353	EALQAEVGLPVDR					Mascot
1503.7988	1503.7808	-0.018	-12	260	272	INWMKAGILQADK			Oxidation (M)[4]		Mascot
1503.7988	1503.7808	-0.018	-12	153	165	FSLLCQAALEVPR	88	100	Carbamidomethyl (C)[5]		Mascot
1524.838	1524.7905	-0.0475	-31	341	354	EALQAEVGLPVDRK					Mascot

1564.7611	1564.7469	-0.0142	-9	440	452	FEPCGLIQLQGMR			Carbamidomethyl (C)[4], Oxidation (M)[12]		Mascot
1564.7689	1564.7469	-0.022	-14	214	226	VAFCIHNISYQGR	67	99.998	Carbamidomethyl (C)[4]		Mascot
1684.7966	1684.7849	-0.0117	-7	227	240	FSFDDFAQLNLPDR					Mascot
1684.7966	1684.7849	-0.0117	-7	227	240	FSFDDFAQLNLPDR	119	100			Mascot
1831.8497	1831.8289	-0.0208	-11	243	258	SSFDFIDGYDKPVEGR					Mascot
1831.8497	1831.8289	-0.0208	-11	243	258	SSFDFIDGYDKPVEGR	113	100			Mascot
1841.7937	1841.7733	-0.0204	-11	137	152	IYGPDAGTDYEDNQQR					Mascot
1841.7937	1841.7733	-0.0204	-11	137	152	IYGPDAGTDYEDNQQR	68	99.999			Mascot
1913.9855	1913.9971	0.0116	6	320	336	DKFLTVNYDVTTALEGK					Mascot
1997.0226	1997.0088	-0.0138	-7	273	290	VLTVSPYYAEELISGEAR					Mascot
1997.0226	1997.0088	-0.0138	-7	273	290	VLTVSPYYAEELISGEAR	106	100			Mascot
2098.9312	2098.9155	-0.0157	-7	135	152	EKIYGPDAGTDYEDNQQ R					Mascot
2232.0901	2232.0498	-0.0403	-18	419	439	FNAPLAHQMMAGADVLA VTSR			Oxidation (M)[9,10]		Mascot

5	waxy protein [Triticum aestivum]	gi|399153338	67223.3	8.1	23	660	100	28.707	558	100

Peptide Information

Calc. Mass	Obsrv. Mass	± da	± ppm	Start Seq.	End Seq.	Sequence	Ion Score	C. I. %	Modification	Rank	Result Type
817.46	817.4323	-0.0277	-34	113	119	VMVISPR			Oxidation (M)[2]		Mascot
821.3723	821.3473	-0.025	-30	513	519	TGFHMGR			Oxidation (M)[5]		Mascot
908.5173	908.4616	-0.0557	-61	53	59	QSRKPHR					Mascot
917.3974	917.3704	-0.027	-29	146	151	YFHCYK			Carbamidomethyl (C)[4]		Mascot
971.6036	971.574	-0.0296	-30	394	402	VPLVAFIGR					Mascot
1099.6986	1099.6765	-0.0221	-20	393	402	KVPLVAFIGR					Mascot
1123.4871	1123.4641	-0.023	-20	330	338	GCELDNIMR			Carbamidomethyl (C)[2], Oxidation (M)[8]		Mascot
1346.6774	1346.636	-0.0414	-31	545	556	VVGTPAYHEMVK			Oxidation (M)[10]		Mascot
1362.7264	1362.6519	-0.0745	-55	125	136	DAWDTSVISKIK					Mascot
1390.6825	1390.6514	-0.0311	-22	157	167	VFVDHPCFLEK			Carbamidomethyl (C)[7]		Mascot
1396.7432	1396.7229	-0.0203	-15	380	392	EALQAEVGLPVDR					Mascot
1503.7988	1503.7808	-0.018	-12	299	311	INWMKAGILQADK			Oxidation (M)[4]		Mascot
1503.7988	1503.7808	-0.018	-12	192	204	FSLLCQAALEVPR	88	100	Carbamidomethyl (C)[5]		Mascot
1524.838	1524.7905	-0.0475	-31	380	393	EALQAEVGLPVDRK					Mascot
1564.7611	1564.7469	-0.0142	-9	479	491	FEPCGLIQLQGMR			Carbamidomethyl (C)[4], Oxidation (M)[12]		Mascot
1564.7689	1564.7469	-0.022	-14	253	265	VAFCIHNISYQGR	67	99.998	Carbamidomethyl (C)[4]		Mascot
1684.7966	1684.7849	-0.0117	-7	266	279	FSFDDFAQLNLPDR					Mascot
1684.7966	1684.7849	-0.0117	-7	266	279	FSFDDFAQLNLPDR	119	100			Mascot

Calc. Mass	Obsrv. Mass	± da	± ppm	Start Seq.	End Seq.	Sequence	Ion Score	C. I. %	Modification	Rank	Result Type
1831.8497	1831.8289	-0.0208	-11	282	297	SSFDFIDGYDKPVEGR					Mascot
1831.8497	1831.8289	-0.0208	-11	282	297	SSFDFIDGYDKPVEGR	113	100			Mascot
1841.7937	1841.7733	-0.0204	-11	176	191	IYGPDAGTDYEDNQQR					Mascot
1841.7937	1841.7733	-0.0204	-11	176	191	IYGPDAGTDYEDNQQR	68	99.999			Mascot
1913.9855	1913.9971	0.0116	6	359	375	DKFLTVNYDVTTALEGK					Mascot
1997.0226	1997.0088	-0.0138	-7	312	329	VLTVSPYYAEELISGEAR					Mascot
1997.0226	1997.0088	-0.0138	-7	312	329	VLTVSPYYAEELISGEAR	106	100			Mascot
2098.9312	2098.9155	-0.0157	-7	174	191	EKIYGPDAGTDYEDNQQR					Mascot
2232.0901	2232.0498	-0.0403	-18	458	478	FNAPLAHQMMAGADVLAVTSR			Oxidation (M)[9,10]		Mascot

6	waxy protein [Triticum dicoccoides]	gi|399153332	67165.2	7.85	23	660	100	29.323	558	100	

Peptide Information

Calc. Mass	Obsrv. Mass	± da	± ppm	Start Seq.	End Seq.	Sequence	Ion Score	C. I. %	Modification	Rank	Result Type
817.46	817.4323	-0.0277	-34	113	119	VMVISPR			Oxidation (M)[2]		Mascot
821.3723	821.3473	-0.025	-30	513	519	TGFHMGR			Oxidation (M)[5]		Mascot
908.5173	908.4616	-0.0557	-61	53	59	QSRKPHR					Mascot
917.3974	917.3704	-0.027	-29	146	151	YFHCYK			Carbamidomethyl (C)[4]		Mascot
971.6036	971.574	-0.0296	-30	394	402	VPLVAFIGR					Mascot
1099.6986	1099.6765	-0.0221	-20	393	402	KVPLVAFIGR					Mascot
1123.4871	1123.4641	-0.023	-20	330	338	GCELDNIMR			Carbamidomethyl (C)[2], Oxidation (M)[8]		Mascot
1346.6774	1346.636	-0.0414	-31	545	556	VVGTPAYHEMVK			Oxidation (M)[10]		Mascot
1390.6825	1390.6514	-0.0311	-22	157	167	VFVDHPCFLEK			Carbamidomethyl (C)[7]		Mascot
1396.7432	1396.7229	-0.0203	-15	380	392	EALQAEVGLPVDR					Mascot
1500.78	1500.7559	-0.0241	-16	408	421	GPDVMIAAIPEIMK			Oxidation (M)[5]		Mascot
1503.7988	1503.7808	-0.018	-12	299	311	INWMKAGILQADK			Oxidation (M)[4]		Mascot
1503.7988	1503.7808	-0.018	-12	192	204	FSLLCQAALEVPR	88	100	Carbamidomethyl (C)[5]		Mascot
1524.838	1524.7905	-0.0475	-31	380	393	EALQAEVGLPVDRK					Mascot
1564.7611	1564.7469	-0.0142	-9	479	491	FEPCGLIQLQGMR			Carbamidomethyl (C)[4], Oxidation (M)[12]		Mascot
1564.7689	1564.7469	-0.022	-14	253	265	VAFCIHNISYQGR	67	99.998	Carbamidomethyl (C)[4]		Mascot
1684.7966	1684.7849	-0.0117	-7	266	279	FSFDDFAQLNLPDR					Mascot
1684.7966	1684.7849	-0.0117	-7	266	279	FSFDDFAQLNLPDR	119	100			Mascot
1831.8497	1831.8289	-0.0208	-11	282	297	SSFDFIDGYDKPVEGR					Mascot
1831.8497	1831.8289	-0.0208	-11	282	297	SSFDFIDGYDKPVEGR	113	100			Mascot
1841.7937	1841.7733	-0.0204	-11	176	191	IYGPDAGTDYEDNQQR					Mascot
1841.7937	1841.7733	-0.0204	-11	176	191	IYGPDAGTDYEDNQQR	68	99.999			Mascot

1913.9855	1913.9971	0.0116	6	359	375	DKFLTVNYDVTTALEGK					Mascot
1997.0226	1997.0088	-0.0138	-7	312	329	VLTVSPYYAEELISGEAR					Mascot
1997.0226	1997.0088	-0.0138	-7	312	329	VLTVSPYYAEELISGEAR	106	100			Mascot
2098.9312	2098.9155	-0.0157	-7	174	191	EKIYGPDAGTDYEDNQQ R					Mascot
2232.0901	2232.0498	-0.0403	-18	458	478	FNAPLAHQMMAGADVLA VTSR			Oxidation (M)[9,10]		Mascot

7	granule-bound starch synthase I [Triticum aestivum]	gi|6969978	66968.1	7.49	23	658	100	28.621	558	100		

Protein Group

Wx [Triticum aestivum]	gi|190366235	66968.1	7.4899 997711 1816
glycogen (starch) synthase [Triticum aestivum]	gi|29788056	66977.1	7.5
starch synthase (GBSSI) [Triticum aestivum]	gi|4760584	66968.1	7.4899 997711 1816

Peptide Information

Calc. Mass	Obsrv. Mass	± da	± ppm	Start Seq.	End Seq.	Sequence	Ion Score	C. I. %	Modification	Rank	Result Type
817.46	817.4323	-0.0277	-34	113	119	VMVISPR			Oxidation (M)[2]		Mascot
821.3723	821.3473	-0.025	-30	513	519	TGFHMGR			Oxidation (M)[5]		Mascot
908.4836	908.4616	-0.022	-24	137	143	VVDKYER					Mascot
917.3974	917.3704	-0.027	-29	146	151	YFHCYK			Carbamidomethyl (C)[4]		Mascot
971.6036	971.574	-0.0296	-30	394	402	VPLVAFIGR					Mascot
1099.6986	1099.6765	-0.0221	-20	393	402	KVPLVAFIGR					Mascot
1123.4871	1123.4641	-0.023	-20	330	338	GCELDNIMR			Carbamidomethyl (C)[2], Oxidation (M)[8]		Mascot
1273.6859	1273.6185	-0.0674	-53	44	56	TVGASAAPTQSRK					Mascot
1346.6774	1346.636	-0.0414	-31	545	556	VVGTPAYHEMVK			Oxidation (M)[10]		Mascot
1390.6825	1390.6514	-0.0311	-22	157	167	VFVDHPCFLEK			Carbamidomethyl (C)[7]		Mascot
1396.7432	1396.7229	-0.0203	-15	380	392	EALQAEVGLPVDR					Mascot
1503.7988	1503.7808	-0.018	-12	299	311	INWMKAGILQADK			Oxidation (M)[4]		Mascot
1503.7988	1503.7808	-0.018	-12	192	204	FSLLCQAALEVPR	88	100	Carbamidomethyl (C)[5]		Mascot
1524.838	1524.7905	-0.0475	-31	380	393	EALQAEVGLPVDRK					Mascot
1564.7611	1564.7469	-0.0142	-9	479	491	FEPCGLIQLQGMR			Carbamidomethyl (C)[4], Oxidation (M)[12]		Mascot
1564.7689	1564.7469	-0.022	-14	253	265	VAFCIHNISYQGR	67	99.998	Carbamidomethyl (C)[4]		Mascot
1654.8687	1654.8173	-0.0514	-31	361	375	FLAVNYDITTALEGK					Mascot
1684.7966	1684.7849	-0.0117	-7	266	279	FSFDDFAQLNLPDR					Mascot

1684.7966	1684.7849	-0.0117	-7	266	279	FSFDDFAQLNLPDR	119	100			Mascot
1831.8497	1831.8289	-0.0208	-11	282	297	SSFDFIDGYDKPVEGR					Mascot
1831.8497	1831.8289	-0.0208	-11	282	297	SSFDFIDGYDKPVEGR	113	100			Mascot
1841.7937	1841.7733	-0.0204	-11	176	191	IYGPDAGTDYEDNQQR					Mascot
1841.7937	1841.7733	-0.0204	-11	176	191	IYGPDAGTDYEDNQQR	68	99.999			Mascot
1997.0226	1997.0088	-0.0138	-7	312	329	VLTVSPYYAEELISGEAR					Mascot
1997.0226	1997.0088	-0.0138	-7	312	329	VLTVSPYYAEELISGEAR	106	100			Mascot
2098.9312	2098.9155	-0.0157	-7	174	191	EKIYGPDAGTDYEDNQQ R					Mascot
2232.0901	2232.0498	-0.0403	-18	458	478	FNAPLAHQMMAGADVLA VTSR			Oxidation (M)[9,10]		Mascot

8	waxy A1, partial [Triticum spelta]	gi|310619522	64218.8	8.7	22	657	100	28.567	558	100

Protein Group

waxy A1, partial [Triticum spelta]	gi|310619516	64218.8	8.6999 998092 6514
waxy A1, partial [Triticum spelta]	gi|310619510	64218.8	8.6999 998092 6514
waxy A1, partial [Triticum spelta]	gi|310619504	64218.8	8.6999 998092 6514
waxy A1, partial [Triticum spelta]	gi|310619498	64218.8	8.6999 998092 6514
waxy A1, partial [Triticum spelta]	gi|334086818	64089.7	8.7299 995422 3633

Peptide Information

Calc. Mass	Obsrv. Mass	± da	± ppm	Start Seq.	End Seq.	Sequence	Ion Score	C. I. %	Modification	Rank	Result Type
817.46	817.4323	-0.0277	-34	110	116	VMVISPR			Oxidation (M)[2]		Mascot
821.3723	821.3473	-0.025	-30	510	516	TGFHMGR			Oxidation (M)[5]		Mascot
908.5173	908.4616	-0.0557	-61	50	56	QSRKPHR					Mascot
917.3974	917.3704	-0.027	-29	143	148	YFHCYK			Carbamidomethyl (C)[4]		Mascot
971.6036	971.574	-0.0296	-30	391	399	VPLVAFIGR					Mascot
1099.6986	1099.6765	-0.0221	-20	390	399	KVPLVAFIGR					Mascot
1123.4871	1123.4641	-0.023	-20	327	335	GCELDNIMR			Carbamidomethyl (C)[2], Oxidation (M)[8]		Mascot
1346.6774	1346.636	-0.0414	-31	542	553	VVGTPAYHEMVK			Oxidation (M)[10]		Mascot

1390.6825	1390.6514	-0.0311	-22	154	164	VFVDHPCFLEK			Carbamidomethyl (C)[7]		Mascot
1396.7432	1396.7229	-0.0203	-15	377	389	EALQAEVGLPVDR					Mascot
1503.7988	1503.7808	-0.018	-12	296	308	INWMKAGILQADK			Oxidation (M)[4]		Mascot
1503.7988	1503.7808	-0.018	-12	189	201	FSLLCQAALEVPR	88	100	Carbamidomethyl (C)[5]		Mascot
1524.838	1524.7905	-0.0475	-31	377	390	EALQAEVGLPVDRK					Mascot
1564.7611	1564.7469	-0.0142	-9	476	488	FEPCGLIQLQGMR			Carbamidomethyl (C)[4], Oxidation (M)[12]		Mascot
1564.7689	1564.7469	-0.022	-14	250	262	VAFCIHNISYQGR	67	99.998	Carbamidomethyl (C)[4]		Mascot
1684.7966	1684.7849	-0.0117	-7	263	276	FSFDDFAQLNLPDR					Mascot
1684.7966	1684.7849	-0.0117	-7	263	276	FSFDDFAQLNLPDR	119	100			Mascot
1831.8497	1831.8289	-0.0208	-11	279	294	SSFDFIDGYDKPVEGR					Mascot
1831.8497	1831.8289	-0.0208	-11	279	294	SSFDFIDGYDKPVEGR	113	100			Mascot
1841.7937	1841.7733	-0.0204	-11	173	188	IYGPDAGTDYEDNQQR					Mascot
1841.7937	1841.7733	-0.0204	-11	173	188	IYGPDAGTDYEDNQQR	68	99.999			Mascot
1913.9855	1913.9971	0.0116	6	356	372	DKFLTVNYDVTTALEGK					Mascot
1997.0226	1997.0088	-0.0138	-7	309	326	VLTVSPYYAEELISGEAR					Mascot
1997.0226	1997.0088	-0.0138	-7	309	326	VLTVSPYYAEELISGEAR	106	100			Mascot
2098.9312	2098.9155	-0.0157	-7	171	188	EKIYGPDAGTDYEDNQQ R					Mascot
2232.0901	2232.0498	-0.0403	-18	455	475	FNAPLAHQMMAGADVLA VTSR			Oxidation (M)[9,10]		Mascot

9 granule bound starch synthase, partial [Triticum spelta] gi|378939978 64546.9 8.67 22 656 100 28.567 558 100

Protein Group

granule bound starch synthase [Triticum turgidum subsp. dicoccon] gi|308229780 64617 8.6199998855 5908

granule bound starch synthase, partial [Triticum turgidum subsp. dicoccon] gi|378939996 64546.9 8.6700000762 9395

Peptide Information

Calc. Mass	Obsrv. Mass	± da	± ppm	Start Seq.	End Seq.	Sequence	Ion Score	C. I. %	Modification	Rank	Result Type
817.46	817.4323	-0.0277	-34	113	119	VMVISPR			Oxidation (M)[2]		Mascot
821.3723	821.3473	-0.025	-30	513	519	TGFHMGR			Oxidation (M)[5]		Mascot
908.5173	908.4616	-0.0557	-61	53	59	QSRKPHR					Mascot
917.3974	917.3704	-0.027	-29	146	151	YFHCYK			Carbamidomethyl (C)[4]		Mascot
971.6036	971.574	-0.0296	-30	394	402	VPLVAFIGR					Mascot
1099.6986	1099.6765	-0.0221	-20	393	402	KVPLVAFIGR					Mascot
1123.4871	1123.4641	-0.023	-20	330	338	GCELDNIMR			Carbamidomethyl (C)[2], Oxidation (M)[8]		Mascot

Calc. Mass	Obsrv. Mass	± da	± ppm	Start Seq.	End Seq.	Sequence	Ion Score	C. I. %	Modification	Rank	Result Type
1346.6774	1346.636	-0.0414	-31	545	556	VVGTPAYHEMVK			Oxidation (M)[10]		Mascot
1390.6825	1390.6514	-0.0311	-22	157	167	VFVDHPCFLEK			Carbamidomethyl (C)[7]		Mascot
1396.7432	1396.7229	-0.0203	-15	380	392	EALQAEVGLPVDR					Mascot
1503.7988	1503.7808	-0.018	-12	299	311	INWMKAGILQADK			Oxidation (M)[4]		Mascot
1503.7988	1503.7808	-0.018	-12	192	204	FSLLCQAALEVPR	88	100	Carbamidomethyl (C)[5]		Mascot
1524.838	1524.7905	-0.0475	-31	380	393	EALQAEVGLPVDRK					Mascot
1564.7611	1564.7469	-0.0142	-9	479	491	FEPCGLIQLQGMR			Carbamidomethyl (C)[4], Oxidation (M)[12]		Mascot
1564.7689	1564.7469	-0.022	-14	253	265	VAFCIHNISYQGR	67	99.998	Carbamidomethyl (C)[4]		Mascot
1684.7966	1684.7849	-0.0117	-7	266	279	FSFDDFAQLNLPDR					Mascot
1684.7966	1684.7849	-0.0117	-7	266	279	FSFDDFAQLNLPDR	119	100			Mascot
1831.8497	1831.8289	-0.0208	-11	282	297	SSFDFIDGYDKPVEGR					Mascot
1831.8497	1831.8289	-0.0208	-11	282	297	SSFDFIDGYDKPVEGR	113	100			Mascot
1841.7937	1841.7733	-0.0204	-11	176	191	IYGPDAGTDYEDNQQR					Mascot
1841.7937	1841.7733	-0.0204	-11	176	191	IYGPDAGTDYEDNQQR	68	99.999			Mascot
1913.9855	1913.9971	0.0116	6	359	375	DKFLTVNYDVTTALEGK					Mascot
1997.0226	1997.0088	-0.0138	-7	312	329	VLTVSPYYAEELISGEAR					Mascot
1997.0226	1997.0088	-0.0138	-7	312	329	VLTVSPYYAEELISGEAR	106	100			Mascot
2098.9312	2098.9155	-0.0157	-7	174	191	EKIYGPDAGTDYEDNQQ R					Mascot
2232.0901	2232.0498	-0.0403	-18	458	478	FNAPLAHQMMAGADVLA VTSR			Oxidation (M)[9,10]		Mascot

10	granule bound starch synthase, partial [Triticum spelta]	gi|378939988	64491.9	8.7	22	655	100	28.567	558	100	

Protein Group

granule bound starch synthase, partial [Triticum spelta]	gi|378939992	64491.9	8.6999998092 6514
starch synthase (GBSSI) [Triticum aestivum]	gi|4760580	67254.2	7.1100001335 144

Peptide Information

Calc. Mass	Obsrv. Mass	± da	± ppm	Start Seq.	End Seq.	Sequence	Ion Score	C. I. %	Modification	Rank	Result Type
817.46	817.4323	-0.0277	-34	113	119	VMVISPR			Oxidation (M)[2]		Mascot
821.3723	821.3473	-0.025	-30	513	519	TGFHMGR			Oxidation (M)[5]		Mascot
908.5173	908.4616	-0.0557	-61	53	59	QSRKPHR					Mascot
917.3974	917.3704	-0.027	-29	146	151	YFHCYK			Carbamidomethyl (C)[4]		Mascot
971.6036	971.574	-0.0296	-30	394	402	VPLVAFIGR					Mascot
1099.6986	1099.6765	-0.0221	-20	393	402	KVPLVAFIGR					Mascot

1123.4871	1123.4641	-0.023	-20	330	338	GCELDNIMR			Carbamidomethyl (C)[2], Oxidation (M)[8]	Mascot
1346.6774	1346.636	-0.0414	-31	545	556	VVGTPAYHEMVK			Oxidation (M)[10]	Mascot
1390.6825	1390.6514	-0.0311	-22	157	167	VFVDHPCFLEK			Carbamidomethyl (C)[7]	Mascot
1396.7432	1396.7229	-0.0203	-15	380	392	EALQAEVGLPVDR				Mascot
1503.7988	1503.7808	-0.018	-12	299	311	INWMKAGILQADK			Oxidation (M)[4]	Mascot
1503.7988	1503.7808	-0.018	-12	192	204	FSLLCQAALEVPR	88	100	Carbamidomethyl (C)[5]	Mascot
1524.838	1524.7905	-0.0475	-31	380	393	EALQAEVGLPVDRK				Mascot
1564.7611	1564.7469	-0.0142	-9	479	491	FEPCGLIQLQGMR			Carbamidomethyl (C)[4], Oxidation (M)[12]	Mascot
1564.7689	1564.7469	-0.022	-14	253	265	VAFCIHNISYQGR	67	99.998	Carbamidomethyl (C)[4]	Mascot
1684.7966	1684.7849	-0.0117	-7	266	279	FSFDDFAQLNLPDR				Mascot
1684.7966	1684.7849	-0.0117	-7	266	279	FSFDDFAQLNLPDR	119	100		Mascot
1831.8497	1831.8289	-0.0208	-11	282	297	SSFDFIDGYDKPVEGR				Mascot
1831.8497	1831.8289	-0.0208	-11	282	297	SSFDFIDGYDKPVEGR	113	100		Mascot
1841.7937	1841.7733	-0.0204	-11	176	191	IYGPDAGTDYEDNQQR				Mascot
1841.7937	1841.7733	-0.0204	-11	176	191	IYGPDAGTDYEDNQQR	68	99.999		Mascot
1913.9855	1913.9971	0.0116	6	359	375	DKFLTVNYDVTTALEGK				Mascot
1997.0226	1997.0088	-0.0138	-7	312	329	VLTVSPYYAEELISGEAR				Mascot
1997.0226	1997.0088	-0.0138	-7	312	329	VLTVSPYYAEELISGEAR	106	100		Mascot
2098.9312	2098.9155	-0.0157	-7	174	191	EKIYGPDAGTDYEDNQQ R				Mascot
2232.0901	2232.0498	-0.0403	-18	458	478	FNAPLAHQMMAGADVLA VTSR			Oxidation (M)[9,10]	Mascot

Gel Idx/Pos	281/L9	Instr./Gel Origin	BA2151/Sample Project 20140814	Process Status	Analysis Succeeded
Plate [#] Name	[1] Sample Project 20140814	Instrument Sample Name		Spectra	11

Rank	Protein Name	Accession No.	Protein MW	Protein PI	Pep. Count	Protein Score	Protein Score C. I. %	Intensity Matched	Total Ion Score	Total Ion C. I. %	Confirmed
1	granule bound starch synthase [Triticum turgidum subsp. dicoccon]	gi|262385348	64477.8	8.42	16	99	100	5.302	43	99.586	

Peptide Information

Calc. Mass	Obsrv. Mass	± da	± ppm	Start Seq.	End Seq.	Sequence	Ion Score	C. I. %	Modification	Rank	Result Type
881.3999	881.4545	0.0546	62	135	141	VADEYER					Mascot
1099.6986	1099.6649	-0.0337	-31	391	400	KVPLVAFIGR					Mascot
1310.5868	1310.675	0.0882	67	555	564	NCMIQDLSWK			Carbamidomethyl (C)[2], Oxidation (M)[3]		Mascot
1390.6825	1390.6663	-0.0162	-12	155	165	VFVDHPCFLEK			Carbamidomethyl (C)[7]		Mascot
1396.7432	1396.699	-0.0442	-32	378	390	EALQAEVGLPVDR					Mascot
1400.7632	1400.7122	-0.051	-36	420	432	EEDVQIVLLGTGK					Mascot
1487.804	1487.7142	-0.0898	-60	297	309	INWMKAGILQADK					Mascot
1503.7988	1503.7736	-0.0252	-17	297	309	INWMKAGILQADK			Oxidation (M)[4]		Mascot
1544.7626	1544.6357	-0.1269	-82	518	531	LSVDCNVVEPADVK			Carbamidomethyl (C)[5]		Mascot
1544.7626	1544.6357	-0.1269	-82	518	531	LSVDCNVVEPADVK			Carbamidomethyl (C)[5]		Mascot
1564.7611	1564.7455	-0.0156	-10	477	489	FEPCGLIQLQGMR			Carbamidomethyl (C)[4], Oxidation (M)[12]		Mascot
1628.8829	1628.8304	-0.0525	-32	540	554	AVKVVGTPAYHEMVK					Mascot
1672.8575	1672.8046	-0.0529	-32	518	532	LSVDCNVVEPADVKK			Carbamidomethyl (C)[5]		Mascot
1684.7966	1684.7802	-0.0164	-10	264	277	FSFDDFAQLNLPDR					Mascot
1684.7966	1684.7802	-0.0164	-10	264	277	FSFDDFAQLNLPDR	43	99.586			Mascot
1798.9116	1798.8046	-0.107	-59	31	49	SPADAPLGMRTTGASAA PK					Mascot
1831.8497	1831.8252	-0.0245	-13	280	295	SSFDFIDGYDKPVEGR					Mascot
1976.9971	1977.0291	0.032	16	90	110	TGGLGDVLGGLPPAMAA NGHR			Oxidation (M)[15]		Mascot
2039.0808	2038.9558	-0.125	-61	359	377	FLAANYDVTTALEGKALN K					Mascot

Rank	Protein Name	Accession No.	Protein MW	Protein PI	Pep. Count	Protein Score	Protein Score C. I. %	Intensity Matched	Total Ion Score	Total Ion C. I. %	Confirmed
2	granule bound starch synthase, partial [Triticum durum]	gi|262385352	64458.8	8.29	16	99	100	5.302	43	99.586	

Peptide Information

Calc. Mass	Obsrv. Mass	± da	± ppm	Start Seq.	End Seq.	Sequence	Ion Score	C. I. %	Modification	Rank	Result Type
881.3999	881.4545	0.0546	62	135	141	VADEYER					Mascot
1099.6986	1099.6649	-0.0337	-31	391	400	KVPLVAFIGR					Mascot

1310.5868	1310.675	0.0882	67	555	564	NCMIQDLSWK			Carbamidomethyl (C)[2], Oxidation (M)[3]		Mascot
1390.6825	1390.6663	-0.0162	-12	155	165	VFVDHPCFLEK			Carbamidomethyl (C)[7]		Mascot
1396.7432	1396.699	-0.0442	-32	378	390	EALQAEVGLPVDR					Mascot
1400.7632	1400.7122	-0.051	-36	420	432	EEDVQIVLLGTGK					Mascot
1487.804	1487.7142	-0.0898	-60	297	309	INWMKAGILQADK					Mascot
1503.7988	1503.7736	-0.0252	-17	297	309	INWMKAGILQADK			Oxidation (M)[4]		Mascot
1544.7626	1544.6357	-0.1269	-82	518	531	LSVDCNVVEPADVK			Carbamidomethyl (C)[5]		Mascot
1544.7626	1544.6357	-0.1269	-82	518	531	LSVDCNVVEPADVK			Carbamidomethyl (C)[5]		Mascot
1564.7611	1564.7455	-0.0156	-10	477	489	FEPCGLIQLQGMR			Carbamidomethyl (C)[4], Oxidation (M)[12]		Mascot
1628.8829	1628.8304	-0.0525	-32	540	554	AVKVVGTPAYHEMVK					Mascot
1672.8575	1672.8046	-0.0529	-32	518	532	LSVDCNVVEPADVKK			Carbamidomethyl (C)[5]		Mascot
1684.7966	1684.7802	-0.0164	-10	264	277	FSFDDFAQLNLPDR					Mascot
1684.7966	1684.7802	-0.0164	-10	264	277	FSFDDFAQLNLPDR	43	99.586			Mascot
1798.9116	1798.8046	-0.107	-59	31	49	SPADAPLGMRTTGASAAPK					Mascot
1831.8497	1831.8252	-0.0245	-13	280	295	SSFDFIDGYDKPVEGR					Mascot
1976.9971	1977.0291	0.032	16	90	110	TGGLGDVLGGLPPAMAANGHR			Oxidation (M)[15]		Mascot
2039.0808	2038.9558	-0.125	-61	359	377	FLAANYDVTTALEGKALNK					Mascot

3	starch synthase (GBSSI) [Triticum dicoccoides]	gi|6624283	66982	7.49	16	98	100	5.302	43	99.586	

Peptide Information

Calc. Mass	Obsrv. Mass	± da	± ppm	Start Seq.	End Seq.	Sequence	Ion Score	C. I. %	Modification	Rank	Result Type
881.3999	881.4545	0.0546	62	138	144	VADEYER					Mascot
1099.6986	1099.6649	-0.0337	-31	394	403	KVPLVAFIGR					Mascot
1310.5868	1310.675	0.0882	67	558	567	NCMIQDLSWK			Carbamidomethyl (C)[2], Oxidation (M)[3]		Mascot
1390.6825	1390.6663	-0.0162	-12	158	168	VFVDHPCFLEK			Carbamidomethyl (C)[7]		Mascot
1396.7432	1396.699	-0.0442	-32	381	393	EALQAEVGLPVDR					Mascot
1400.7632	1400.7122	-0.051	-36	423	435	EEDVQIVLLGTGK					Mascot
1487.804	1487.7142	-0.0898	-60	300	312	INWMKAGILQADK					Mascot
1503.7988	1503.7736	-0.0252	-17	300	312	INWMKAGILQADK			Oxidation (M)[4]		Mascot
1544.7626	1544.6357	-0.1269	-82	521	534	LSVDCNVVEPADVK			Carbamidomethyl (C)[5]		Mascot
1544.7626	1544.6357	-0.1269	-82	521	534	LSVDCNVVEPADVK			Carbamidomethyl (C)[5]		Mascot
1564.7611	1564.7455	-0.0156	-10	480	492	FEPCGLIQLQGMR			Carbamidomethyl (C)[4], Oxidation (M)[12]		Mascot
1628.8829	1628.8304	-0.0525	-32	543	557	AVKVVGTPAYHEMVK					Mascot
1672.8575	1672.8046	-0.0529	-32	521	535	LSVDCNVVEPADVKK			Carbamidomethyl (C)[5]		Mascot
1684.7966	1684.7802	-0.0164	-10	267	280	FSFDDFAQLNLPDR					Mascot

1684.7966	1684.7802	-0.0164	-10	267	280	FSFDDFAQLNLPDR	43	99.586			Mascot
1798.9116	1798.8046	-0.107	-59	34	52	SPADAPLGMRTTGASAA PK					Mascot
1831.8497	1831.8252	-0.0245	-13	283	298	SSFDFIDGYDKPVEGR					Mascot
1976.9971	1977.0291	0.032	16	93	113	TGGLGDVLGGLPPAMAA NGHR			Oxidation (M)[15]		Mascot
2039.0808	2038.9558	-0.125	-61	362	380	FLAANYDVTTALEGKALN K					Mascot

4 unnamed protein product [Triticum aestivum] gi|218035594 67044 7.08 16 97 100 5.302 43 99.586

Protein Group

starch synthase (GBSSI) [Triticum aestivum]	gi|4760582	67044	7.0799 999237 0605
unnamed protein product [Triticum aestivum]	gi|218049817	67044	7.0799 999237 0605

Peptide Information

Calc. Mass	Obsrv. Mass	± da	± ppm	Start Seq.	End Seq.	Sequence	Ion Score	C. I. %	Modification	Rank	Result Type
881.3999	881.4545	0.0546	62	138	144	VADEYER					Mascot
1099.6986	1099.6649	-0.0337	-31	394	403	KVPLVAFIGR					Mascot
1310.5868	1310.675	0.0882	67	558	567	NCMIQDLSWK			Carbamidomethyl (C)[2], Oxidation (M)[3]		Mascot
1390.6825	1390.6663	-0.0162	-12	158	168	VFVDHPCFLEK			Carbamidomethyl (C)[7]		Mascot
1396.7432	1396.699	-0.0442	-32	381	393	EALQAEVGLPVDR					Mascot
1400.7632	1400.7122	-0.051	-36	423	435	EEDVQIVLLGTGK					Mascot
1487.804	1487.7142	-0.0898	-60	300	312	INWMKAGILQADK					Mascot
1503.7988	1503.7736	-0.0252	-17	300	312	INWMKAGILQADK			Oxidation (M)[4]		Mascot
1544.7626	1544.6357	-0.1269	-82	521	534	LSVDCNVVEPADVK			Carbamidomethyl (C)[5]		Mascot
1544.7626	1544.6357	-0.1269	-82	521	534	LSVDCNVVEPADVK			Carbamidomethyl (C)[5]		Mascot
1564.7611	1564.7455	-0.0156	-10	480	492	FEPCGLIQLQGMR			Carbamidomethyl (C)[4], Oxidation (M)[12]		Mascot
1628.8829	1628.8304	-0.0525	-32	543	557	AVKVVGTPAYHEMVK					Mascot
1672.8575	1672.8046	-0.0529	-32	521	535	LSVDCNVVEPADVKK			Carbamidomethyl (C)[5]		Mascot
1684.7966	1684.7802	-0.0164	-10	267	280	FSFDDFAQLNLPDR					Mascot
1684.7966	1684.7802	-0.0164	-10	267	280	FSFDDFAQLNLPDR	43	99.586			Mascot
1798.9116	1798.8046	-0.107	-59	34	52	SPADAPLGMRTTGASAA PK					Mascot
1831.8497	1831.8252	-0.0245	-13	283	298	SSFDFIDGYDKPVEGR					Mascot
1976.9971	1977.0291	0.032	16	93	113	TGGLGDVLGGLPPAMAA NGHR			Oxidation (M)[15]		Mascot
2039.0808	2038.9558	-0.125	-61	362	380	FLAANYDVTTALEGKALN K					Mascot

5 mutant granule bound starch synthase I [Triticum aestivum] gi|17736918 59580.1 5.6 14 94 99.998 4.464 43 99.586

Peptide Information

Calc. Mass	Obsrv. Mass	± da	± ppm	Start Seq.	End Seq.	Sequence	Ion Score	C. I. %	Modification	Rank	Result Type
1099.6986	1099.6649	-0.0337	-31	323	332	KVPLVAFIGR					Mascot
1310.5868	1310.675	0.0882	67	487	496	NCMIQDLSWK			Carbamidomethyl (C)[2], Oxidation (M)[3]		Mascot
1390.6825	1390.6663	-0.0162	-12	87	97	VFVDHPCFLEK			Carbamidomethyl (C)[7]		Mascot
1396.7432	1396.699	-0.0442	-32	310	322	EALQAEVGLPVDR					Mascot
1400.7632	1400.7122	-0.051	-36	352	364	EEDVQIVLLGTGK					Mascot
1487.804	1487.7142	-0.0898	-60	229	241	INWMKAGILQADK					Mascot
1503.7988	1503.7736	-0.0252	-17	229	241	INWMKAGILQADK			Oxidation (M)[4]		Mascot
1544.7626	1544.6357	-0.1269	-82	450	463	LSVDCNVVEPADVK			Carbamidomethyl (C)[5]		Mascot
1544.7626	1544.6357	-0.1269	-82	450	463	LSVDCNVVEPADVK			Carbamidomethyl (C)[5]		Mascot
1564.7611	1564.7455	-0.0156	-10	409	421	FEPCGLIQLQGMR			Carbamidomethyl (C)[4], Oxidation (M)[12]		Mascot
1628.8829	1628.8304	-0.0525	-32	472	486	AVKVVGTPAYHEMVK					Mascot
1672.8575	1672.8046	-0.0529	-32	450	464	LSVDCNVVEPADVKK			Carbamidomethyl (C)[5]		Mascot
1684.7966	1684.7802	-0.0164	-10	196	209	FSFDDFAQLNLPDR					Mascot
1684.7966	1684.7802	-0.0164	-10	196	209	FSFDDFAQLNLPDR	43	99.586			Mascot
1831.8497	1831.8252	-0.0245	-13	212	227	SSFDFIDGYDKPVEGR					Mascot
1841.7937	1841.7708	-0.0229	-12	106	121	IYGPDAGTDYEDNQQR					Mascot
1976.9971	1977.0291	0.032	16	22	42	TGGLGDVLGGLPPAMAA NGHR			Oxidation (M)[15]		Mascot

6 waxy protein [Triticum aestivum] gi|399153330 67152.2 7.85 15 89 99.994 4.652 43 99.586

Peptide Information

Calc. Mass	Obsrv. Mass	± da	± ppm	Start Seq.	End Seq.	Sequence	Ion Score	C. I. %	Modification	Rank	Result Type
1099.6986	1099.6649	-0.0337	-31	393	402	KVPLVAFIGR					Mascot
1310.5868	1310.675	0.0882	67	557	566	NCMIQDLSWK			Carbamidomethyl (C)[2], Oxidation (M)[3]		Mascot
1363.6741	1363.7131	0.039	29	125	136	DAWDTSVISEIK					Mascot
1390.6825	1390.6663	-0.0162	-12	157	167	VFVDHPCFLEK			Carbamidomethyl (C)[7]		Mascot
1396.7432	1396.699	-0.0442	-32	380	392	EALQAEVGLPVDR					Mascot
1400.7632	1400.7122	-0.051	-36	422	434	EEDVQIVLLGTGK					Mascot
1487.804	1487.7142	-0.0898	-60	299	311	INWMKAGILQADK					Mascot
1503.7988	1503.7736	-0.0252	-17	299	311	INWMKAGILQADK			Oxidation (M)[4]		Mascot
1544.7626	1544.6357	-0.1269	-82	520	533	LSVDCNVVEPADVK			Carbamidomethyl (C)[5]		Mascot

Calc. Mass	Obsrv. Mass	± da	± ppm	Start Seq.	End Seq.	Sequence	Ion Score	C. I. %	Modification	Rank	Result Type
1544.7626	1544.6357	-0.1269	-82	520	533	LSVDCNVVEPADVK			Carbamidomethyl (C)[5]		Mascot
1564.7611	1564.7455	-0.0156	-10	479	491	FEPCGLIQLQGMR			Carbamidomethyl (C)[4], Oxidation (M)[12]		Mascot
1628.8829	1628.8304	-0.0525	-32	542	556	AVKVVGTPAYHEMVK					Mascot
1672.8575	1672.8046	-0.0529	-32	520	534	LSVDCNVVEPADVKK			Carbamidomethyl (C)[5]		Mascot
1684.7966	1684.7802	-0.0164	-10	266	279	FSFDDFAQLNLPDR					Mascot
1684.7966	1684.7802	-0.0164	-10	266	279	FSFDDFAQLNLPDR	43	99.586			Mascot
1831.8497	1831.8252	-0.0245	-13	282	297	SSFDFIDGYDKPVEGR					Mascot
1841.7937	1841.7708	-0.0229	-12	176	191	IYGPDAGTDYEDNQQR					Mascot
2008.1147	2008.0212	-0.0935	-47	403	421	LEGQKGPDVMIAAIPEIVK					Mascot

7	granule-bound starch synthase precursor [Triticum aestivum]	gi|4588607	63388.4	7.86	14	88	99.992	4.52	43	99.586	

Peptide Information

Calc. Mass	Obsrv. Mass	± da	± ppm	Start Seq.	End Seq.	Sequence	Ion Score	C. I. %	Modification	Rank	Result Type
1099.6986	1099.6649	-0.0337	-31	354	363	KVPLVAFIGR					Mascot
1310.5868	1310.675	0.0882	67	518	527	NCMIQDLSWK			Carbamidomethyl (C)[2], Oxidation (M)[3]		Mascot
1363.6741	1363.7131	0.039	29	86	97	DAWDTSVISEIK					Mascot
1390.6825	1390.6663	-0.0162	-12	118	128	VFVDHPCFLEK			Carbamidomethyl (C)[7]		Mascot
1396.7432	1396.699	-0.0442	-32	341	353	EALQAEVGLPVDR					Mascot
1400.7632	1400.7122	-0.051	-36	383	395	EEDVQIVLLGTGK					Mascot
1487.804	1487.7142	-0.0898	-60	260	272	INWMKAGILQADK					Mascot
1503.7988	1503.7736	-0.0252	-17	260	272	INWMKAGILQADK			Oxidation (M)[4]		Mascot
1544.7626	1544.6357	-0.1269	-82	481	494	LSVDCNVVEPADVK			Carbamidomethyl (C)[5]		Mascot
1544.7626	1544.6357	-0.1269	-82	481	494	LSVDCNVVEPADVK			Carbamidomethyl (C)[5]		Mascot
1564.7611	1564.7455	-0.0156	-10	440	452	FEPCGLIQLQGMR			Carbamidomethyl (C)[4], Oxidation (M)[12]		Mascot
1628.8829	1628.8304	-0.0525	-32	503	517	AVKVVGTPAYHEMVK					Mascot
1672.8575	1672.8046	-0.0529	-32	481	495	LSVDCNVVEPADVKK			Carbamidomethyl (C)[5]		Mascot
1684.7966	1684.7802	-0.0164	-10	227	240	FSFDDFAQLNLPDR					Mascot
1684.7966	1684.7802	-0.0164	-10	227	240	FSFDDFAQLNLPDR	43	99.586			Mascot
1831.8497	1831.8252	-0.0245	-13	243	258	SSFDFIDGYDKPVEGR					Mascot
1841.7937	1841.7708	-0.0229	-12	137	152	IYGPDAGTDYEDNQQR					Mascot

8	waxy protein [Triticum dicoccoides]	gi|399153332	67165.2	7.85	15	87	99.991	4.63	43	99.586	

Peptide Information

Calc. Mass	Obsrv. Mass	± da	± ppm	Start Seq.	End Seq.	Sequence	Ion Score	C. I. %	Modification	Rank	Result Type

Calc. Mass	Obsrv. Mass	± da	± ppm	Start Seq.	End Seq.	Sequence	Ion Score	C. I. %	Modification	Rank	Result Type
1099.6986	1099.6649	-0.0337	-31	393	402	KVPLVAFIGR					Mascot
1310.5868	1310.675	0.0882	67	557	566	NCMIQDLSWK			Carbamidomethyl (C)[2], Oxidation (M)[3]		Mascot
1363.6741	1363.7131	0.039	29	125	136	DAWDTSVISEIK					Mascot
1390.6825	1390.6663	-0.0162	-12	157	167	VFVDHPCFLEK			Carbamidomethyl (C)[7]		Mascot
1396.7432	1396.699	-0.0442	-32	380	392	EALQAEVGLPVDR					Mascot
1400.7632	1400.7122	-0.051	-36	422	434	EEDVQIVLLGTGK					Mascot
1487.804	1487.7142	-0.0898	-60	299	311	INWMKAGILQADK					Mascot
1500.78	1500.7604	-0.0196	-13	408	421	GPDVMIAAIPEIMK			Oxidation (M)[5]		Mascot
1503.7988	1503.7736	-0.0252	-17	299	311	INWMKAGILQADK			Oxidation (M)[4]		Mascot
1544.7626	1544.6357	-0.1269	-82	520	533	LSVDCNVVEPADVK			Carbamidomethyl (C)[5]		Mascot
1544.7626	1544.6357	-0.1269	-82	520	533	LSVDCNVVEPADVK			Carbamidomethyl (C)[5]		Mascot
1564.7611	1564.7455	-0.0156	-10	479	491	FEPCGLIQLQGMR			Carbamidomethyl (C)[4], Oxidation (M)[12]		Mascot
1628.8829	1628.8304	-0.0525	-32	542	556	AVKVVGTPAYHEMVK					Mascot
1672.8575	1672.8046	-0.0529	-32	520	534	LSVDCNVVEPADVKK			Carbamidomethyl (C)[5]		Mascot
1684.7966	1684.7802	-0.0164	-10	266	279	FSFDDFAQLNLPDR					Mascot
1684.7966	1684.7802	-0.0164	-10	266	279	FSFDDFAQLNLPDR	43	99.586			Mascot
1831.8497	1831.8252	-0.0245	-13	282	297	SSFDFIDGYDKPVEGR					Mascot
1841.7937	1841.7708	-0.0229	-12	176	191	IYGPDAGTDYEDNQQR					Mascot

9 granule bound starch synthase, partial [Triticum spelta] gi|378939986 64365.6 8.41 14 87 99.991 4.194 43 99.586

Protein Group

granule bound starch synthase, partial [Triticum spelta]	gi|378939982	64365.6	8.4099998474 1211
granule bound starch synthase, partial [Triticum spelta]	gi|378939994	64365.6	8.4099998474 1211

Peptide Information

Calc. Mass	Obsrv. Mass	± da	± ppm	Start Seq.	End Seq.	Sequence	Ion Score	C. I. %	Modification	Rank	Result Type
881.3999	881.4545	0.0546	62	138	144	VADEYER					Mascot
1099.6986	1099.6649	-0.0337	-31	394	403	KVPLVAFIGR					Mascot
1310.5868	1310.675	0.0882	67	558	567	NCMIQDLSWK			Carbamidomethyl (C)[2], Oxidation (M)[3]		Mascot
1390.6825	1390.6663	-0.0162	-12	158	168	VFVDHPCFLEK			Carbamidomethyl (C)[7]		Mascot
1396.7432	1396.699	-0.0442	-32	381	393	EALQAEVGLPVDR					Mascot
1400.7632	1400.7122	-0.051	-36	423	435	EEDVQIVLLGTGK					Mascot
1487.804	1487.7142	-0.0898	-60	300	312	INWMKAGILQADK					Mascot
1503.7988	1503.7736	-0.0252	-17	300	312	INWMKAGILQADK			Oxidation (M)[4]		Mascot

1564.7611	1564.7455	-0.0156	-10	480	492	FEPCGLIQLQGMR			Carbamidomethyl (C)[4], Oxidation (M)[12]		Mascot
1628.8829	1628.8304	-0.0525	-32	543	557	AVKVVGTPAYHEMVK					Mascot
1684.7966	1684.7802	-0.0164	-10	267	280	FSFDDFAQLNLPDR					Mascot
1684.7966	1684.7802	-0.0164	-10	267	280	FSFDDFAQLNLPDR	43	99.586			Mascot
1798.9116	1798.8046	-0.107	-59	34	52	SPADAPLGMRTTGASAA PK					Mascot
1831.8497	1831.8252	-0.0245	-13	283	298	SSFDFIDGYDKPVEGR					Mascot
1976.9971	1977.0291	0.032	16	93	113	TGGLGDVLGGLPPAMAA NGHR			Oxidation (M)[15]		Mascot
2039.0808	2038.9558	-0.125	-61	362	380	FLAANYDVTTALEGKALN K					Mascot

10	waxy B1, partial [Triticum spelta]	gi|310619512	64078.5	8.42	14	87	99.991	4.194	43	99.586

Protein Group

granule bound starch synthase, partial [Triticum turgidum subsp. dicoccon]	gi|378939998	64092.5	8.4200 000762 9395
waxy B1 [Triticum spelta]	gi|310619506	64092.5	8.4200 000762 9395
waxy B1 [Triticum spelta]	gi|310619500	64092.5	8.4200 000762 9395

Peptide Information

Calc. Mass	Obsrv. Mass	± da	± ppm	Start Seq.	End Seq.	Sequence	Ion Score	C. I. %	Modification	Rank	Result Type
881.3999	881.4545	0.0546	62	135	141	VADEYER					Mascot
1099.6986	1099.6649	-0.0337	-31	391	400	KVPLVAFIGR					Mascot
1310.5868	1310.675	0.0882	67	555	564	NCMIQDLSWK			Carbamidomethyl (C)[2], Oxidation (M)[3]		Mascot
1390.6825	1390.6663	-0.0162	-12	155	165	VFVDHPCFLEK			Carbamidomethyl (C)[7]		Mascot
1396.7432	1396.699	-0.0442	-32	378	390	EALQAEVGLPVDR					Mascot
1400.7632	1400.7122	-0.051	-36	420	432	EEDVQIVLLGTGK					Mascot
1487.804	1487.7142	-0.0898	-60	297	309	INWMKAGILQADK					Mascot
1503.7988	1503.7736	-0.0252	-17	297	309	INWMKAGILQADK			Oxidation (M)[4]		Mascot
1564.7611	1564.7455	-0.0156	-10	477	489	FEPCGLIQLQGMR			Carbamidomethyl (C)[4], Oxidation (M)[12]		Mascot
1628.8829	1628.8304	-0.0525	-32	540	554	AVKVVGTPAYHEMVK					Mascot
1684.7966	1684.7802	-0.0164	-10	264	277	FSFDDFAQLNLPDR					Mascot
1684.7966	1684.7802	-0.0164	-10	264	277	FSFDDFAQLNLPDR	43	99.586			Mascot
1798.9116	1798.8046	-0.107	-59	31	49	SPADAPLGMRTTGASAA PK					Mascot
1831.8497	1831.8252	-0.0245	-13	280	295	SSFDFIDGYDKPVEGR					Mascot

1976.9971	1977.0291	0.032	16	90	110	TGGLGDVLGGLPPAMAA NGHR	Oxidation (M)[15]	Mascot
2039.0808	2038.9558	-0.125	-61	359	377	FLAANYDVTTALEGKALN K		Mascot

Gel Idx/Pos	282/L10	Instr./Gel Origin	BA2151/Sample Project 20140814	Process Status	Analysis Succeeded
Plate [#] Name	[1] Sample Project 20140814	Instrument Sample Name		Spectra	11

Rank	Protein Name	Accession No.	Protein MW	Protein PI	Pep. Count	Protein Score	Protein Score C. I. %	Intensity Matched	Total Ion Score	Total Ion C. I. %	Confirmed
1	Betaine aldehyde dehydrogenase 1, chloroplastic [Triticum urartu]	gi|474447706	46073	4.97	13	362	100	11.653	297	100	

Peptide Information

Calc. Mass	Obsrv. Mass	± da	± ppm	Start Seq.	End Seq.	Sequence	Ion Score	C. I. %	Modification	Rank	Result Type
823.4164	823.3699	-0.0465	-56	1	6	MMIERK			Oxidation (M)[1]		Mascot
1161.5205	1161.4916	-0.0289	-25	239	248	VSDPLEEGCR			Carbamidomethyl (C)[9]		Mascot
1229.6161	1229.5562	-0.0599	-49	150	161	VAFTGSYATGQK					Mascot
1276.6606	1276.6068	-0.0538	-42	308	318	EEVFGPVLCVK			Carbamidomethyl (C)[9]		Mascot
1305.6686	1305.6112	-0.0574	-44	249	260	LGPVVSEGQYEK					Mascot
1385.7748	1385.7404	-0.0344	-25	270	283	SEGATILTGGVRPK					Mascot
1385.7748	1385.7404	-0.0344	-25	270	283	SEGATILTGGVRPK	29	90.964			Mascot
1457.7859	1457.7318	-0.0541	-37	48	60	RQNAAVALPENFK					Mascot
1461.7472	1461.6838	-0.0634	-43	181	193	SPIVVFDDVDIDK					Mascot
1507.7639	1507.7036	-0.0603	-40	386	399	ELGEGGIDNYLSIK					Mascot
1507.7639	1507.7036	-0.0603	-40	386	399	ELGEGGIDNYLSIK					Mascot
1911.0984	1911.0066	-0.0918	-48	162	180	IMVAAAPTVKPVTLELGG K			Oxidation (M)[2]		Mascot
2012.0084	2012.0045	-0.0039	-2	381	399	SGFGRELGEGGIDNYLSI K					Mascot
2994.3962	2994.3801	-0.0161	-5	319	346	EFSTEEEAIELANDTHYG LAGAVISGDR					Mascot
2994.3962	2994.3801	-0.0161	-5	319	346	EFSTEEEAIELANDTHYG LAGAVISGDR	268	100			Mascot
3056.5898	3056.5515	-0.0383	-13	119	149	EIGLPSGVLNIVTGLGNE AGAPLSSHPDVDK					Mascot

Rank	Protein Name	Accession No.	Protein MW	Protein PI	Pep. Count	Protein Score	Protein Score C. I. %	Intensity Matched	Total Ion Score	Total Ion C. I. %	Confirmed
2	betaine-aldehyde dehydrogenase [Triticum aestivum]	gi|21747870	55228	5.44	12	344	100	12.362	297	100	

Protein Group

unnamed protein product [Triticum aestivum]	gi|291047652	55228	5.4400 000572 2046

Peptide Information

Calc. Mass	Obsrv. Mass	± da	± ppm	Start Seq.	End Seq.	Sequence	Ion Score	C. I. %	Modification	Rank	Result Type
1149.5687	1149.5292	-0.0395	-34	11	19	QLFIDGDWR					Mascot

1161.5205	1161.4916	-0.0289	-25	324	333	VSDPLEEGCR			Carbamidomethyl (C)[9]	Mascot
1229.6161	1229.5562	-0.0599	-49	235	246	VAFTGSYATGQK				Mascot
1276.6606	1276.6068	-0.0538	-42	393	403	EEVFGPVLCVK			Carbamidomethyl (C)[9]	Mascot
1305.6686	1305.6112	-0.0574	-44	334	345	LGPVVSEGQYEK				Mascot
1385.7748	1385.7404	-0.0344	-25	355	368	SEGATILTGGVRPK				Mascot
1385.7748	1385.7404	-0.0344	-25	355	368	SEGATILTGGVRPK	29	90.964		Mascot
1457.7859	1457.7318	-0.0541	-37	133	145	RQNAAVALPENFK				Mascot
1461.7472	1461.6838	-0.0634	-43	266	278	SPIVVFDDVDIDK				Mascot
1507.7639	1507.7036	-0.0603	-40	471	484	ELGEGGIDNYLSIK				Mascot
1507.7639	1507.7036	-0.0603	-40	471	484	ELGEGGIDNYLSIK				Mascot
1911.0984	1911.0066	-0.0918	-48	247	265	IMVAAAPTVKPVTLELGGK			Oxidation (M)[2]	Mascot
2012.0084	2012.0045	-0.0039	-2	466	484	SGFGRELGEGGIDNYLSIK				Mascot
2994.3962	2994.3801	-0.0161	-5	404	431	EFSTEEEAIELANDTHYGLAGAVISGDR				Mascot
2994.3962	2994.3801	-0.0161	-5	404	431	EFSTEEEAIELANDTHYGLAGAVISGDR	268	100		Mascot

3	granule-bound starch synthase 1 [Triticum durum]	gi|306518235	29663	4.84	11	214	100	7.984	153	100

Protein Group

granule-bound starch synthase 1 [Triticum durum]	gi|306518233	29663	4.84000015258789
granule-bound starch synthase 1 [Triticum durum]	gi|306518229	29663	4.84000015258789
granule-bound starch synthase 1 [Triticum durum]	gi|306518219	29663	4.84000015258789
granule-bound starch synthase 1 [Triticum durum]	gi|306518217	29663	4.84000015258789
granule-bound starch synthase 1 [Triticum durum]	gi|306518203	29648.9	4.84000015258789
granule-bound starch synthase 1 [Triticum durum]	gi|306518199	29663	4.84000015258789
granule-bound starch synthase 1 [Triticum durum]	gi|306518195	29663	4.84000015258789
granule-bound starch synthase 1 [Triticum durum]	gi|306518191	29663	4.8400

			001525<br>8789
granule-bound starch synthase 1 [Triticum durum]	gi|306518187	29663	4.8400<br>001525<br>8789
granule-bound starch synthase 1 [Triticum durum]	gi|306518237	29663	4.8400<br>001525<br>8789
granule-bound starch synthase 1 [Triticum durum]	gi|306518177	29663	4.8400<br>001525<br>8789
granule-bound starch synthase 1 [Triticum durum]	gi|306518179	29663	4.8400<br>001525<br>8789
granule-bound starch synthase 1 [Triticum durum]	gi|306518181	29663	4.8400<br>001525<br>8789
granule-bound starch synthase 1 [Triticum durum]	gi|306518183	29663	4.8400<br>001525<br>8789
granule-bound starch synthase 1 [Triticum durum]	gi|306518185	29663	4.8400<br>001525<br>8789
granule-bound starch synthase 1 [Triticum durum]	gi|306518189	29663	4.8400<br>001525<br>8789
granule-bound starch synthase 1 [Triticum durum]	gi|306518193	29663	4.8400<br>001525<br>8789
granule-bound starch synthase 1 [Triticum durum]	gi|306518197	29663	4.8400<br>001525<br>8789
granule-bound starch synthase 1 [Triticum durum]	gi|306518201	29663	4.8400<br>001525<br>8789
granule-bound starch synthase 1 [Triticum durum]	gi|306518205	29663	4.8400<br>001525<br>8789
granule-bound starch synthase 1 [Triticum durum]	gi|306518225	29663	4.8400<br>001525<br>8789

granule-bound starch synthase 1 [Triticum durum]	gi|306518215	29663	4.8400 001525 8789
granule-bound starch synthase 1 [Triticum polonicum]	gi|306518155	29663	4.8400 001525 8789
granule-bound starch synthase 1 [Triticum polonicum]	gi|306518153	29663	4.8400 001525 8789
granule-bound starch synthase 1 [Triticum turgidum subsp. dicoccon]	gi|306518231	29663	4.8400 001525 8789
granule-bound starch synthase 1 [Triticum turgidum subsp. dicoccon]	gi|306518159	29663	4.8400 001525 8789
granule-bound starch synthase 1 [Triticum turgidum subsp. dicoccon]	gi|306518161	29663	4.8400 001525 8789
granule-bound starch synthase 1 [Triticum turgidum subsp. dicoccon]	gi|306518163	29663	4.8400 001525 8789
granule-bound starch synthase 1 [Triticum turgidum subsp. dicoccon]	gi|306518165	29648.9	4.8299 999237 0605
granule-bound starch synthase 1 [Triticum turgidum subsp. dicoccon]	gi|306518167	29663	4.8400 001525 8789
granule-bound starch synthase 1 [Triticum turgidum subsp. dicoccon]	gi|306518169	29663	4.8400 001525 8789
granule-bound starch synthase 1 [Triticum turgidum subsp. dicoccon]	gi|306518171	29663	4.8400 001525 8789
granule-bound starch synthase 1 [Triticum turgidum subsp. dicoccon]	gi|306518173	29663	4.8400 001525 8789
granule-bound starch synthase 1 [Triticum turgidum subsp. dicoccon]	gi|306518175	29663	4.8400 001525 8789
granule-bound starch synthase 1 [Triticum turgidum subsp. dicoccon]	gi|306518207	29663	4.8400 001525 8789

granule-bound starch synthase 1 [Triticum turgidum subsp. dicoccon]	gi|306518209	29663	4.8400 001525 8789
granule-bound starch synthase 1 [Triticum turgidum subsp. dicoccon]	gi|306518227	29663	4.8400 001525 8789
granule-bound starch synthase 1 [Triticum turgidum subsp. dicoccon]	gi|306518223	29663	4.8400 001525 8789
granule-bound starch synthase 1 [Triticum turgidum subsp. dicoccon]	gi|306518211	29663	4.8400 001525 8789
granule-bound starch synthase 1 [Triticum turgidum subsp. dicoccon]	gi|306518213	29663	4.8400 001525 8789
granule-bound starch synthase 1 [Triticum turgidum subsp. paleocolchicum]	gi|306518221	29663	4.8400 001525 8789
granule-bound starch synthase 1 [Triticum turgidum subsp. pyramidale]	gi|306518157	29663	4.8400 001525 8789
granule-bound starch synthase 1 [Triticum turgidum]	gi|306518151	29663	4.8400 001525 8789
granule-bound starch synthase 1 [Triticum turgidum]	gi|306518149	29663	4.8400 001525 8789
granule-bound starch synthase 1 [Triticum turgidum]	gi|306518147	29663	4.8400 001525 8789
granule-bound starch synthase 1 [Triticum turgidum]	gi|306518145	29663	4.8400 001525 8789

Peptide Information

Calc. Mass	Obsrv. Mass	± da	± ppm	Start Seq.	End Seq.	Sequence	Ion Score	C. I. %	Modification	Rank	Result Type
971.6036	971.5504	-0.0532	-55	226	234	VPLVAFIGR					Mascot
1099.6986	1099.6608	-0.0378	-34	225	234	KVPLVAFIGR					Mascot
1107.4922	1107.5118	0.0196	18	162	170	GCELDNIMR			Carbamidomethyl (C)[2]		Mascot
1201.5597	1201.5629	0.0032	3	72	81	SNYQSNGIYR					Mascot

1396.7432	1396.7061	-0.0371	-27	212	224	EALQAEVGLPVDR					Mascot
1503.7988	1503.7635	-0.0353	-23	131	143	INWMKAGILQADK				Oxidation (M)[4]	Mascot
1564.7689	1564.7291	-0.0398	-25	85	97	VAFCIHNISYQGR				Carbamidomethyl (C)[4]	Mascot
1684.7966	1684.7695	-0.0271	-16	98	111	FSFDDFAQLNLPDR					Mascot
1684.7966	1684.7695	-0.0271	-16	98	111	FSFDDFAQLNLPDR		91	100		Mascot
1831.8497	1831.8109	-0.0388	-21	114	129	SSFDFIDGYDKPVEGR					Mascot
1841.7937	1841.7545	-0.0392	-21	8	23	IYGPDAGTDYEDNQQR					Mascot
1997.0226	1996.9845	-0.0381	-19	144	161	VLTVSPYYAEELISGEAR					Mascot
1997.0226	1996.9845	-0.0381	-19	144	161	VLTVSPYYAEELISGEAR		63	99.996		Mascot

4	granule-bound starch synthase, partial [Triticum aestivum]	gi|417381857	12867.4	6.75	2		0	153	100

Protein Group

GBSS [Triticum aestivum]	gi|90018524	12867.4	6.75
RecName: Full=Granule-bound starch synthase 1, chloroplastic/amyloplastic; AltName: Full=Granule-bound starch synthase I; Short=GBSS-I; Flags: Precursor	gi|136765	68506	8.25
Wx [Triticum aestivum]	gi|190366235	66968.1	7.4899 997711 1816
Wx [Triticum aestivum]	gi|190366232	67025	6.8099 999427 7954
Wx [Triticum aestivum]	gi|190366229	67224.3	7.5
Wx-A1, partial [Triticum durum]	gi|239509211	13811.8	6.0999 999046 3257
Wx-B1, partial [Triticum durum]	gi|239509214	13784.8	6.0999 999046 3257
glycogen (starch) synthase [Triticum aestivum]	gi|21902	68506	8.25
glycogen (starch) synthase [Triticum aestivum]	gi|29788056	66977.1	7.5

granule bound starch synthase I [Triticum aestivum]	gi|11037536	67025	6.80999994277954
granule bound starch synthase [Triticum turgidum subsp. dicoccon]	gi|308229780	64617	8.61999988555908
granule bound starch synthase [Triticum turgidum subsp. dicoccon]	gi|262385350	64391.7	8.28999996185303
granule bound starch synthase [Triticum turgidum subsp. dicoccon]	gi|262385348	64477.8	8.42000007629395
granule bound starch synthase [Triticum turgidum subsp. dicoccon]	gi|262385346	64490.7	8.28999996185303
granule bound starch synthase, partial [Triticum durum]	gi|262385352	64458.8	8.28999996185303
granule bound starch synthase, partial [Triticum spelta]	gi|378939994	64365.6	8.40999984741211
granule bound starch synthase, partial [Triticum spelta]	gi|378939992	64491.9	8.69999980926514
granule bound starch synthase, partial [Triticum spelta]	gi|378939990	64308.8	8.60999965667725
granule bound starch synthase, partial [Triticum spelta]	gi|378939984	64251.7	8.60999965667725
granule bound starch synthase, partial [Triticum spelta]	gi|378939986	64365.6	8.40999984741211
granule bound starch synthase, partial [Triticum spelta]	gi|378939988	64491.9	8.69999980926514
granule bound starch synthase, partial [Triticum spelta]	gi|378939982	64365.6	8.40999984741211
granule bound starch synthase, partial [Triticum spelta]	gi|378939978	64546.9	8.67000007629395

granule bound starch synthase, partial [Triticum spelta]	gi|378939980	64308.8	8.6099996566 7725
granule bound starch synthase, partial [Triticum turgidum subsp. dicoccon]	gi|378939998	64092.5	8.4200000762 9395
granule bound starch synthase, partial [Triticum turgidum subsp. dicoccon]	gi|378939996	64546.9	8.6700000762 9395
granule-bound starch synthase 1 [Triticum durum]	gi|306518413	17279.4	4.8299999237 0605
granule-bound starch synthase 1 [Triticum durum]	gi|306518409	17279.4	4.8299999237 0605
granule-bound starch synthase 1 [Triticum durum]	gi|306518405	17279.4	4.8299999237 0605
granule-bound starch synthase 1 [Triticum durum]	gi|306518401	17279.4	4.8299999237 0605
granule-bound starch synthase 1 [Triticum durum]	gi|306518353	17279.4	4.8299999237 0605
granule-bound starch synthase 1 [Triticum durum]	gi|306518355	17279.4	4.8299999237 0605
granule-bound starch synthase 1 [Triticum durum]	gi|306518357	17279.4	4.8299999237 0605
granule-bound starch synthase 1 [Triticum durum]	gi|306518359	17279.4	4.8299999237 0605
granule-bound starch synthase 1 [Triticum durum]	gi|306518361	17279.4	4.8299999237 0605
granule-bound starch synthase 1 [Triticum durum]	gi|306518363	17243.3	4.8299999237 0605
granule-bound starch synthase 1 [Triticum durum]	gi|306518365	17279.4	4.8299999237

			0605
granule-bound starch synthase 1 [Triticum durum]	gi|306518367	17279.4	4.8299 999237 0605
granule-bound starch synthase 1 [Triticum durum]	gi|306518369	17279.4	4.8299 999237 0605
granule-bound starch synthase 1 [Triticum durum]	gi|306518371	17279.4	4.8299 999237 0605
granule-bound starch synthase 1 [Triticum durum]	gi|306518373	17279.4	4.8299 999237 0605
granule-bound starch synthase 1 [Triticum durum]	gi|306518375	17279.4	4.8299 999237 0605
granule-bound starch synthase 1 [Triticum durum]	gi|306518377	17279.4	4.8299 999237 0605
granule-bound starch synthase 1 [Triticum durum]	gi|306518379	17279.4	4.8299 999237 0605
granule-bound starch synthase 1 [Triticum durum]	gi|306518381	17279.4	4.8299 999237 0605
granule-bound starch synthase 1 [Triticum durum]	gi|306518255	17279.4	4.8299 999237 0605
granule-bound starch synthase 1 [Triticum durum]	gi|306518257	17279.4	4.8299 999237 0605
granule-bound starch synthase 1 [Triticum durum]	gi|306518259	17279.4	4.8299 999237 0605
granule-bound starch synthase 1 [Triticum durum]	gi|306518261	17279.4	4.8299 999237 0605
granule-bound starch synthase 1 [Triticum durum]	gi|306518263	17279.4	4.8299 999237 0605
granule-bound starch synthase 1 [Triticum durum]	gi|306518265	17279.4	4.8299

			999237 0605
granule-bound starch synthase 1 [Triticum durum]	gi|306518267	17279.4	4.8299 999237 0605
granule-bound starch synthase 1 [Triticum durum]	gi|306518269	17279.4	4.8299 999237 0605
granule-bound starch synthase 1 [Triticum durum]	gi|306518271	17279.4	4.8299 999237 0605
granule-bound starch synthase 1 [Triticum durum]	gi|306518273	17279.4	4.8299 999237 0605
granule-bound starch synthase 1 [Triticum durum]	gi|306518275	17279.4	4.8299 999237 0605
granule-bound starch synthase 1 [Triticum durum]	gi|306518277	17279.4	4.8299 999237 0605
granule-bound starch synthase 1 [Triticum durum]	gi|306518079	29663	4.8400 001525 8789
granule-bound starch synthase 1 [Triticum durum]	gi|306518081	29663	4.8400 001525 8789
granule-bound starch synthase 1 [Triticum durum]	gi|306518083	29663	4.8400 001525 8789
granule-bound starch synthase 1 [Triticum durum]	gi|306518085	29663	4.8400 001525 8789
granule-bound starch synthase 1 [Triticum durum]	gi|306518087	29663	4.8400 001525 8789
granule-bound starch synthase 1 [Triticum durum]	gi|306518089	29663	4.8400 001525 8789
granule-bound starch synthase 1 [Triticum durum]	gi|306518091	29663	4.8400 001525 8789

granule-bound starch synthase 1 [Triticum durum]	gi|306518093	29663	4.8400 001525 8789
granule-bound starch synthase 1 [Triticum durum]	gi|306518095	29663	4.8400 001525 8789
granule-bound starch synthase 1 [Triticum durum]	gi|306518097	29663	4.8400 001525 8789
granule-bound starch synthase 1 [Triticum durum]	gi|306518099	29663	4.8400 001525 8789
granule-bound starch synthase 1 [Triticum durum]	gi|306518101	29663	4.8400 001525 8789
granule-bound starch synthase 1 [Triticum durum]	gi|306518103	29663	4.8400 001525 8789
granule-bound starch synthase 1 [Triticum durum]	gi|306518105	29663	4.8400 001525 8789
granule-bound starch synthase 1 [Triticum durum]	gi|306518107	29663	4.8400 001525 8789
granule-bound starch synthase 1 [Triticum durum]	gi|306518109	29663	4.8400 001525 8789
granule-bound starch synthase 1 [Triticum durum]	gi|306518111	29663	4.8400 001525 8789
granule-bound starch synthase 1 [Triticum durum]	gi|306518113	29663	4.8400 001525 8789
granule-bound starch synthase 1 [Triticum durum]	gi|306518279	17279.4	4.8299 999237 0605
granule-bound starch synthase 1 [Triticum durum]	gi|306518281	17279.4	4.8299 999237 0605
granule-bound starch synthase 1 [Triticum durum]	gi|306518283	17279.4	4.8299 999237 0605

granule-bound starch synthase 1 [Triticum durum]	gi|306518285	17279.4	4.8299 999237 0605
granule-bound starch synthase 1 [Triticum durum]	gi|306518287	17279.4	4.8299 999237 0605
granule-bound starch synthase 1 [Triticum durum]	gi|306518289	17279.4	4.8299 999237 0605
granule-bound starch synthase 1 [Triticum durum]	gi|306518391	17279.4	4.8299 999237 0605
granule-bound starch synthase 1 [Triticum durum]	gi|306518393	17279.4	4.8299 999237 0605
granule-bound starch synthase 1 [Triticum durum]	gi|306518395	17279.4	4.8299 999237 0605
granule-bound starch synthase 1 [Triticum durum]	gi|306518411	17279.4	4.8299 999237 0605
granule-bound starch synthase 1 [Triticum polonicum]	gi|306518331	17279.4	4.8299 999237 0605
granule-bound starch synthase 1 [Triticum polonicum]	gi|306518329	17279.4	4.8299 999237 0605
granule-bound starch synthase 1 [Triticum turgidum subsp. dicoccon]	gi|306518407	17249.4	4.8299 999237 0605
granule-bound starch synthase 1 [Triticum turgidum subsp. dicoccon]	gi|306518399	17279.4	4.8299 999237 0605
granule-bound starch synthase 1 [Triticum turgidum subsp. dicoccon]	gi|306518389	17279.4	4.8299 999237 0605
granule-bound starch synthase 1 [Triticum turgidum subsp. dicoccon]	gi|306518385	17279.4	4.8299 999237 0605
granule-bound starch synthase 1 [Triticum turgidum subsp. dicoccon]	gi|306518063	29663	4.8400 001525

			8789
granule-bound starch synthase 1 [Triticum turgidum subsp. dicoccon]	gi|306518065	29663	4.8400 001525 8789
granule-bound starch synthase 1 [Triticum turgidum subsp. dicoccon]	gi|306518067	29663	4.8400 001525 8789
granule-bound starch synthase 1 [Triticum turgidum subsp. dicoccon]	gi|306518069	29663	4.8400 001525 8789
granule-bound starch synthase 1 [Triticum turgidum subsp. dicoccon]	gi|306518071	29663	4.8400 001525 8789
granule-bound starch synthase 1 [Triticum turgidum subsp. dicoccon]	gi|306518073	29663	4.8400 001525 8789
granule-bound starch synthase 1 [Triticum turgidum subsp. dicoccon]	gi|306518075	29663	4.8400 001525 8789
granule-bound starch synthase 1 [Triticum turgidum subsp. dicoccon]	gi|306518077	29663	4.8400 001525 8789
granule-bound starch synthase 1 [Triticum turgidum subsp. dicoccon]	gi|306518241	17279.4	4.8299 999237 0605
granule-bound starch synthase 1 [Triticum turgidum subsp. dicoccon]	gi|306518243	17279.4	4.8299 999237 0605
granule-bound starch synthase 1 [Triticum turgidum subsp. dicoccon]	gi|306518245	17279.4	4.8299 999237 0605
granule-bound starch synthase 1 [Triticum turgidum subsp. dicoccon]	gi|306518247	17279.4	4.8299 999237 0605
granule-bound starch synthase 1 [Triticum turgidum subsp. dicoccon]	gi|306518249	17279.4	4.8299 999237 0605
granule-bound starch synthase 1 [Triticum turgidum subsp. dicoccon]	gi|306518251	17279.4	4.8299 999237 0605
granule-bound starch synthase 1 [Triticum turgidum	gi|306518253	17279.4	4.8299

subsp. dicoccon]			999237 0605
granule-bound starch synthase 1 [Triticum turgidum subsp. dicoccon]	gi|306518335	17279.4	4.8299 999237 0605
granule-bound starch synthase 1 [Triticum turgidum subsp. dicoccon]	gi|306518337	17279.4	4.8299 999237 0605
granule-bound starch synthase 1 [Triticum turgidum subsp. dicoccon]	gi|306518239	17279.4	4.8299 999237 0605
granule-bound starch synthase 1 [Triticum turgidum subsp. dicoccon]	gi|306518339	17279.4	4.8299 999237 0605
granule-bound starch synthase 1 [Triticum turgidum subsp. dicoccon]	gi|306518341	17279.4	4.8299 999237 0605
granule-bound starch synthase 1 [Triticum turgidum subsp. dicoccon]	gi|306518343	17227.3	4.8299 999237 0605
granule-bound starch synthase 1 [Triticum turgidum subsp. dicoccon]	gi|306518345	17279.4	4.8299 999237 0605
granule-bound starch synthase 1 [Triticum turgidum subsp. dicoccon]	gi|306518347	17279.4	4.8299 999237 0605
granule-bound starch synthase 1 [Triticum turgidum subsp. dicoccon]	gi|306518349	17279.4	4.8299 999237 0605
granule-bound starch synthase 1 [Triticum turgidum subsp. dicoccon]	gi|306518351	17279.4	4.8299 999237 0605
granule-bound starch synthase 1 [Triticum turgidum subsp. dicoccon]	gi|306518383	17279.4	4.8299 999237 0605
granule-bound starch synthase 1 [Triticum turgidum subsp. dicoccon]	gi|306518387	17279.4	4.8299 999237 0605
granule-bound starch synthase 1 [Triticum turgidum subsp. dicoccon]	gi|306518403	17279.4	4.8299 999237 0605

granule-bound starch synthase 1 [Triticum turgidum subsp. paleocolchicum]	gi|306518397	17279.4	4.8299999237 0605
granule-bound starch synthase 1 [Triticum turgidum subsp. pyramidale]	gi|306518333	17279.4	4.8299999237 0605
granule-bound starch synthase 1 [Triticum turgidum subsp. turgidum]	gi|306518309	17279.4	4.8299999237 0605
granule-bound starch synthase 1 [Triticum turgidum subsp. turgidum]	gi|306518303	17279.4	4.8299999237 0605
granule-bound starch synthase 1 [Triticum turgidum subsp. turgidum]	gi|306518299	17279.4	4.8299999237 0605
granule-bound starch synthase 1 [Triticum turgidum subsp. turgidum]	gi|306518295	17279.4	4.8299999237 0605
granule-bound starch synthase 1 [Triticum turgidum subsp. turgidum]	gi|306518115	29663	4.8400001525 8789
granule-bound starch synthase 1 [Triticum turgidum subsp. turgidum]	gi|306518117	29663	4.8400001525 8789
granule-bound starch synthase 1 [Triticum turgidum subsp. turgidum]	gi|306518119	29663	4.8400001525 8789
granule-bound starch synthase 1 [Triticum turgidum subsp. turgidum]	gi|306518121	29663	4.8400001525 8789
granule-bound starch synthase 1 [Triticum turgidum subsp. turgidum]	gi|306518123	29663	4.8400001525 8789
granule-bound starch synthase 1 [Triticum turgidum subsp. turgidum]	gi|306518125	29663	4.8400001525 8789
granule-bound starch synthase 1 [Triticum turgidum subsp. turgidum]	gi|306518127	29663	4.8400001525 8789
granule-bound starch synthase 1 [Triticum turgidum subsp. turgidum]	gi|306518129	29663	4.8400001525 8789

granule-bound starch synthase 1 [Triticum turgidum subsp. turgidum]	gi|306518131	29663	4.8400 001525 8789
granule-bound starch synthase 1 [Triticum turgidum subsp. turgidum]	gi|306518133	29663	4.8400 001525 8789
granule-bound starch synthase 1 [Triticum turgidum subsp. turgidum]	gi|306518291	17279.4	4.8299 999237 0605
granule-bound starch synthase 1 [Triticum turgidum subsp. turgidum]	gi|306518293	17279.4	4.8299 999237 0605
granule-bound starch synthase 1 [Triticum turgidum subsp. turgidum]	gi|306518297	17279.4	4.8299 999237 0605
granule-bound starch synthase 1 [Triticum turgidum subsp. turgidum]	gi|306518301	17279.4	4.8299 999237 0605
granule-bound starch synthase 1 [Triticum turgidum subsp. turgidum]	gi|306518305	17279.4	4.8299 999237 0605
granule-bound starch synthase 1 [Triticum turgidum subsp. turgidum]	gi|306518307	17279.4	4.8299 999237 0605
granule-bound starch synthase 1 [Triticum turgidum]	gi|306518327	17279.4	4.8299 999237 0605
granule-bound starch synthase 1 [Triticum turgidum]	gi|306518323	17279.4	4.8299 999237 0605
granule-bound starch synthase 1 [Triticum turgidum]	gi|306518319	17279.4	4.8299 999237 0605
granule-bound starch synthase 1 [Triticum turgidum]	gi|306518315	17279.4	4.8299 999237 0605
granule-bound starch synthase 1 [Triticum turgidum]	gi|306518135	29663	4.8400 001525 8789
granule-bound starch synthase 1 [Triticum turgidum]	gi|306518137	29663	4.8400 001525

			8789
granule-bound starch synthase 1 [Triticum turgidum]	gi|306518139	29663	4.8400 001525 8789
granule-bound starch synthase 1 [Triticum turgidum]	gi|306518141	29663	4.8400 001525 8789
granule-bound starch synthase 1 [Triticum turgidum]	gi|306518143	29663	4.8400 001525 8789
granule-bound starch synthase 1 [Triticum turgidum]	gi|306518311	17279.4	4.8299 999237 0605
granule-bound starch synthase 1 [Triticum turgidum]	gi|306518313	17279.4	4.8299 999237 0605
granule-bound starch synthase 1 [Triticum turgidum]	gi|306518317	17279.4	4.8299 999237 0605
granule-bound starch synthase 1 [Triticum turgidum]	gi|306518321	17279.4	4.8299 999237 0605
granule-bound starch synthase 1 [Triticum turgidum]	gi|306518325	17279.4	4.8299 999237 0605
granule-bound starch synthase GBSS [Triticum aestivum]	gi|63054377	12842.3	5.7600 002288 8184
granule-bound starch synthase I [Triticum aestivum]	gi|6969978	66968.1	7.4899 997711 1816
granule-bound starch synthase WX-TmA protein [Triticum monococcum]	gi|6318538	67056.1	7.5100 002288 8184
granule-bound starch synthase [Triticum aestivum]	gi|21628918	68506	8.25
granule-bound starch synthase precursor [Triticum aestivum]	gi|4588609	63967.6	8.8400 001525 8789
granule-bound starch synthase precursor [Triticum	gi|4588607	63388.4	7.8600

aestivum]			001335 144
granule-bound starch synthase precursor [Triticum monococcum]	gi|33321030	67071.2	7.5
granule-bound starch synthase, GBSSI=waxy protein {EC 2.4.1.21} [Triticum aestivum=wheat, cv. Chinese Spring, hexaploid, Peptide Chloroplast, 615 aa]	gi|386023	68506	8.25
granule-bound starch synthase, partial [Triticum aestivum]	gi|417381855	12867.4	6.75
granule-bound starch synthase, partial [Triticum aestivum]	gi|417381851	12867.4	6.75
granule-bound starch synthase, partial [Triticum aestivum]	gi|417381843	12867.4	6.75
starch synthase (GBSSI) [Triticum aestivum]	gi|4760584	66968.1	7.4899 997711 1816
starch synthase (GBSSI) [Triticum aestivum]	gi|4760580	67254.2	7.1100 001335 144
starch synthase (GBSSI) [Triticum aestivum]	gi|4760582	67044	7.0799 999237 0605
starch synthase (GBSSI) [Triticum dicoccoides]	gi|6624283	66982	7.4899 997711 1816
starch synthase (GBSSI) [Triticum dicoccoides]	gi|6624281	67152.2	7.8499 999046 3257
starch synthase (GBSSI) [Triticum durum]	gi|6624287	66926	7.0799 999237 0605
starch synthase (GBSSI) [Triticum durum]	gi|6624285	67133.2	7.8499 999046 3257
unnamed protein product [Triticum aestivum]	gi|298545815	63967.6	8.8400 001525 8789

unnamed protein product [Triticum aestivum]	gi|218049817	67044	7.0799<br>999237<br>0605
unnamed protein product [Triticum aestivum]	gi|298543905	63967.6	8.8400<br>001525<br>8789
unnamed protein product [Triticum aestivum]	gi|218035594	67044	7.0799<br>999237<br>0605
unnamed protein product [Triticum monococcum]	gi|298545807	67056.1	7.5100<br>002288<br>8184
unnamed protein product [Triticum monococcum]	gi|298543897	67056.1	7.5100<br>002288<br>8184
unnamed protein product [Triticum turgidum]	gi|298545801	66926	7.0799<br>999237<br>0605
unnamed protein product [Triticum turgidum]	gi|298543891	66926	7.0799<br>999237<br>0605
waxy A1, partial [Triticum spelta]	gi|334086818	64089.7	8.7299<br>995422<br>3633
waxy A1, partial [Triticum spelta]	gi|310619498	64218.8	8.6999<br>998092<br>6514
waxy A1, partial [Triticum spelta]	gi|310619504	64218.8	8.6999<br>998092<br>6514
waxy A1, partial [Triticum spelta]	gi|310619510	64218.8	8.6999<br>998092<br>6514
waxy A1, partial [Triticum spelta]	gi|310619516	64218.8	8.6999<br>998092<br>6514
waxy A1, partial [Triticum spelta]	gi|310619522	64218.8	8.6999<br>998092<br>6514
waxy A1, partial [Triticum spelta]	gi|334086816	64047.7	8.7299<br>995422<br>3633

waxy B1 [Triticum spelta]	gi|310619506	64092.5	8.4200<br>000762<br>9395
waxy B1 [Triticum spelta]	gi|310619500	64092.5	8.4200<br>000762<br>9395
waxy B1, partial [Triticum spelta]	gi|310619524	63993.6	8.5200<br>004577<br>6367
waxy B1, partial [Triticum spelta]	gi|310619518	63993.6	8.5200<br>004577<br>6367
waxy B1, partial [Triticum spelta]	gi|310619512	64078.5	8.4200<br>000762<br>9395
waxy D1, partial [Triticum spelta]	gi|310619526	64035.6	8.6199<br>998855<br>5908
waxy D1, partial [Triticum spelta]	gi|310619520	64035.6	8.6199<br>998855<br>5908
waxy D1, partial [Triticum spelta]	gi|310619502	64035.6	8.6199<br>998855<br>5908
waxy D1, partial [Triticum spelta]	gi|310619508	64035.6	8.6199<br>998855<br>5908
waxy D1, partial [Triticum spelta]	gi|310619514	64035.6	8.6199<br>998855<br>5908
waxy protein [Triticum aestivum]	gi|399153338	67223.3	8.1000<br>003814<br>6973
waxy protein [Triticum aestivum]	gi|399153330	67152.2	7.8499<br>999046<br>3257
waxy protein [Triticum dicoccoides]	gi|399153332	67165.2	7.8499<br>999046<br>3257
waxy protein [Triticum durum]	gi|399153336	67133.2	7.8499<br>999046

			3257
waxy protein [Triticum durum]	gi|399153334	67132.3	8.2700 004577 6367
waxy protein [Triticum urartu]	gi|378406303	66969.1	7.5

Peptide Information

Calc. Mass	Obsrv. Mass	± da	± ppm	Start Seq.	End Seq.	Sequence	Ion Score	C. I. %	Modification	Rank	Result Type
1684.7966	1684.7695	-0.0271	-16	35	48	FSFDDFAQLNLPDR	91	100		1	Mascot
1997.0226	1996.9845	-0.0381	-19	81	98	VLTVSPYYAEELISGEAR	63	99.996		1	Mascot

5	Lysosomal alpha-mannosidase [Triticum urartu]	gi|474367528	139761.5	5.83	1	0	69	100

Protein Group

Lysosomal alpha-mannosidase [Triticum urartu]	gi|474443198	25690	5.4000 000953 6743

Peptide Information

Calc. Mass	Obsrv. Mass	± da	± ppm	Start Seq.	End Seq.	Sequence	Ion Score	C. I. %	Modification	Rank	Result Type
1447.7369	1447.7146	-0.0223	-15	28	38	FIYVEQAFFQR	69	100		1	Mascot

6	Coatomer subunit beta'-2 [Triticum urartu]	gi|474014221	138162.4	5.3	1	0	10	0

Peptide Information

Calc. Mass	Obsrv. Mass	± da	± ppm	Start Seq.	End Seq.	Sequence	Ion Score	C. I. %	Modification	Rank	Result Type
1372.7617	1372.6837	-0.078	-57	450	461	EKGNMVQQVIVK	10	0		1	Mascot

7	hypothetical protein TRIUR3_02638 [Triticum urartu]	gi|474419357	24232.3	8.47	1	0	9	0

Peptide Information

Calc. Mass	Obsrv. Mass	± da	± ppm	Start Seq.	End Seq.	Sequence	Ion Score	C. I. %	Modification	Rank	Result Type
1181.6273	1181.531	-0.0963	-81	173	183	KNYIGSSSAVR	9	0		1	Mascot

8	MATE efflux family protein 3, chloroplastic [Triticum urartu]	gi|473740428	78435.7	8.69	1	0	9	0

Peptide Information

Calc. Mass	Obsrv. Mass	± da	± ppm	Start	End	Sequence	Ion	C. I. %	Modification	Rank	Result Type

Calc. Mass	Obsrv. Mass	± da	± ppm	Start Seq.	End Seq.	Sequence	Ion Score	C. I. %	Modification	Rank	Result Type
1372.6855	1372.6837	-0.0018	-1	589	600	AINEVFPDSPQR	9	0		2	Mascot

9	putative RNA-dependent RNA polymerase SHL2 [Triticum urartu]	gi|473782429	117919.8	7.74	1		0	9	0

Peptide Information

Calc. Mass	Obsrv. Mass	± da	± ppm	Start Seq.	End Seq.	Sequence	Ion Score	C. I. %	Modification	Rank	Result Type
1164.5579	1164.509	-0.0489	-42	649	658	NPCLHPGDVR	9	0	Carbamidomethyl (C)[3]	1	Mascot

10	Bifunctional protein FolD [Triticum urartu]	gi|474143071	20740.8	6.99	1		0	9	0

Peptide Information

Calc. Mass	Obsrv. Mass	± da	± ppm	Start Seq.	End Seq.	Sequence	Ion Score	C. I. %	Modification	Rank	Result Type
1372.7333	1372.6837	-0.0496	-36	115	126	GDSQSHVRFIVK	9	0		3	Mascot

Gel Idx/Pos	283/L11	Instr./Gel Origin	BA2151/Sample Project 20140814	Process Status	Analysis Succeeded
Plate [#] Name	[1] Sample Project 20140814	Instrument Sample Name		Spectra	11

Rank	Protein Name	Accession No.	Protein MW	Protein PI	Pep. Count	Protein Score	Protein Score C. I. %	Intensity Matched	Total Ion Score	Total Ion C. I. %	Confirmed
1	hypothetical protein TRIUR3_12214 [Triticum urartu]	gi|473926526	87847.5	6.54	19	63	97.732	8.608			

Peptide Information

Calc. Mass	Obsrv. Mass	± da	± ppm	Start Seq.	End Seq.	Sequence	Ion Score	C. I. %	Modification	Rank	Result Type
808.3981	808.3535	-0.0446	-55	286	292	ARAMSEK			Oxidation (M)[4]		Mascot
962.4955	962.4772	-0.0183	-19	413	419	FHSYPRR					Mascot
976.4815	976.486	0.0045	5	426	432	QRCPLMR			Carbamidomethyl (C)[3], Oxidation (M)[6]		Mascot
1161.5358	1161.5178	-0.018	-15	726	735	GIMFDPNDPR					Mascot
1164.5466	1164.5353	-0.0113	-10	489	498	SPHLAMEHDK					Mascot
1177.5306	1177.531	0.0004	0	726	735	GIMFDPNDPR			Oxidation (M)[3]		Mascot
1453.7542	1453.6378	-0.1164	-80	553	564	MLLLDGCYILSR			Carbamidomethyl (C)[7]		Mascot
1508.7778	1508.7253	-0.0525	-35	513	525	RPGMTVEVDVYVK			Oxidation (M)[4]		Mascot
1508.8142	1508.7253	-0.0889	-59	89	101	ATRPTMTVEVYLK					Mascot
1528.7543	1528.6823	-0.072	-47	271	283	WRSATEYNFAGVK					Mascot
1551.8234	1551.744	-0.0794	-51	499	512	LMSLDMVITAAKAR			Oxidation (M)[2,6]		Mascot
1564.7827	1564.741	-0.0417	-27	704	718	GVIVHGQGNNDEVAR					Mascot
1735.916	1735.7894	-0.1266	-73	511	525	ARRPGMTVEVDVYVK			Oxidation (M)[6]		Mascot
1831.8491	1831.8303	-0.0188	-10	617	633	IGELTGLDDMSETGHTR					Mascot
1891.0106	1890.9558	-0.0548	-29	513	528	RPGMTVEVDVYVKELR					Mascot
1984.0345	1984.0321	-0.0024	-1	2	19	DQLAHELESKLATLNSSK					Mascot
1992.1133	1991.9901	-0.1232	-62	129	145	MLLLDGCYILSRLVGLR			Carbamidomethyl (C)[7]		Mascot
2008.1083	2008.0262	-0.0821	-41	129	145	MLLLDGCYILSRLVGLR			Carbamidomethyl (C)[7], Oxidation (M)[1]		Mascot
2037.9191	2037.9758	0.0567	28	719	735	CLADLCKGIMFDPNDPR			Carbamidomethyl (C)[1,6], Oxidation (M)[10]		Mascot
2083.8484	2084.0059	0.1575	76	112	128	NCYANTFDDMSSEQFVR			Carbamidomethyl (C)[2]		Mascot
2576.2332	2576.3042	0.071	28	617	639	IGELTGLDDMSETGHTRC ILDVK			Carbamidomethyl (C)[18], Oxidation (M)[10]		Mascot

Rank	Protein Name	Accession No.	Protein MW	Protein PI	Pep. Count	Protein Score	Protein Score C. I. %	Intensity Matched	Total Ion Score	Total Ion C. I. %	Confirmed
2	hypothetical protein TRIUR3_08536 [Triticum urartu]	gi|474407060	49568.3	5.63	14	53	78.834	3.549			

Peptide Information

Calc. Mass	Obsrv. Mass	± da	± ppm	Start Seq.	End Seq.	Sequence	Ion Score	C. I. %	Modification	Rank	Result Type
808.3505	808.3535	0.003	4	217	223	EMAEQGK			Oxidation (M)[2]		Mascot

Calc. Mass	Obsrv. Mass	± da	± ppm	Start Seq.	End Seq.	Sequence	Ion Score	C. I. %	Modification	Rank	Result Type
947.4905	947.4896	-0.0009	-1	258	267	SAAGAADKTR					Mascot
1177.5519	1177.531	-0.0209	-18	377	389	DSVMGAAGGAVDK					Mascot
1288.6104	1288.6733	0.0629	49	1	11	MAAWHVDTVSR			Oxidation (M)[1]		Mascot
1328.5934	1328.6801	0.0867	65	266	276	TRDMTAQTMNK			Oxidation (M)[4,9]		Mascot
1410.6383	1410.6685	0.0302	21	244	256	AEEPSFDTSTQAK					Mascot
1525.7856	1525.7972	0.0116	8	173	186	TTADYTQQAAVKTK					Mascot
1705.8425	1705.7919	-0.0506	-30	66	82	DAAMQALGITGDQTVAK			Oxidation (M)[4]		Mascot
1798.864	1798.8063	-0.0577	-32	359	376	DATGAMAQKAGDTAAYI K			Oxidation (M)[6]		Mascot
1960.9658	1960.9724	0.0066	3	1	18	MAAWHVDTVSRATVASS R			Oxidation (M)[1]		Mascot
1976.0117	1976.0081	-0.0036	-2	63	82	SAKDAAMQALGITGDQT VAK					Mascot
1992.0067	1991.9901	-0.0166	-8	63	82	SAKDAAMQALGITGDQT VAK			Oxidation (M)[7]		Mascot
2024.0295	2024.006	-0.0235	-12	165	184	DTALAAGKTTADYTQQA AVK					Mascot
2068.0017	2067.9839	-0.0178	-9	368	389	AGDTAAYIKDSVMGAAG GAVDK					Mascot
2083.9966	2084.0059	0.0093	4	368	389	AGDTAAYIKDSVMGAAG GAVDK			Oxidation (M)[13]		Mascot
2085.8811	2085.9619	0.0808	39	268	287	DMTAQTMNKAEDATGGT GDR			Oxidation (M)[2]		Mascot

3	wheat kinase-START domain protein splice variant WKS1.2 [Triticum dicoccoides]	gi|194131660	71393.9	8.68	14	46	0	10.166

Peptide Information

Calc. Mass	Obsrv. Mass	± da	± ppm	Start Seq.	End Seq.	Sequence	Ion Score	C. I. %	Modification	Rank	Result Type
1267.6827	1267.5898	-0.0929	-73	568	577	TRTQVQYLMK					Mascot
1283.6776	1283.561	-0.1166	-91	568	577	TRTQVQYLMK			Oxidation (M)[9]		Mascot
1328.6805	1328.6801	-0.0004	0	8	19	LADLNQGNENLK					Mascot
1394.7322	1394.6827	-0.0495	-35	304	314	KRPEMNHVVER					Mascot
1410.7271	1410.6685	-0.0586	-42	304	314	KRPEMNHVVER			Oxidation (M)[5]		Mascot
1480.6519	1480.681	0.0291	20	234	247	AMAAQNGTCNDLAK			Carbamidomethyl (C)[9], Oxidation (M)[2]		Mascot
1480.6519	1480.681	0.0291	20	234	247	AMAAQNGTCNDLAK			Carbamidomethyl (C)[9], Oxidation (M)[2]		Mascot
1508.7428	1508.7253	-0.0175	-12	536	548	EHPKCGPQPGFVR			Carbamidomethyl (C)[5]		Mascot
1508.7428	1508.7253	-0.0175	-12	536	548	EHPKCGPQPGFVR			Carbamidomethyl (C)[5]		Mascot
1551.8173	1551.744	-0.0733	-47	305	316	RPEMNHVVERLR			Oxidation (M)[4]		Mascot
1592.752	1592.6254	-0.1266	-79	234	248	AMAAQNGTCNDLAKK			Carbamidomethyl (C)[9]		Mascot
1705.8505	1705.7919	-0.0586	-34	373	387	GVEAVDEENQHPLLR					Mascot
1960.9512	1960.9724	0.0212	11	523	539	NDDGGYVVLFQSREHPK					Mascot
1996.011	1996.016	0.005	3	540	557	CGPQPGFVRAYIEIGGFK			Carbamidomethyl (C)[1]		Mascot
2000.0059	2000.0134	0.0075	4	607	623	TLGSVIIFCTTFARYHN			Carbamidomethyl (C)[9]		Mascot

2068.0498	2067.9839	-0.0659	-32	50	69	GAFGEVFRGFLDDGSPV AVK						Mascot
2084.0408	2084.0059	-0.0349	-17	118	136	GNLKDLLHGSDDPISFEA R						Mascot
2085.9468	2085.9619	0.0151	7	272	289	EMFDTQIANASNMEVLEK			Oxidation (M)[2]			Mascot

4 DNA replication licensing factor MCM8 [Triticum urartu] gi|474193997 100327.2 6.41 17 44 0 14.566

Peptide Information

Calc. Mass	Obsrv. Mass	± da	± ppm	Start Seq.	End Seq.	Sequence	Ion Score	C. I. %	Modification		Rank	Result Type
860.4472	860.4469	-0.0003	0	814	820	QSELQQK						Mascot
877.4349	877.4656	0.0307	35	94	100	WQLMSGR						Mascot
891.4716	891.4403	-0.0313	-35	609	616	MNAALLSR			Oxidation (M)[1]			Mascot
1288.7625	1288.6733	-0.0892	-69	452	464	AGITLALFGAVQK						Mascot
1384.7906	1384.6716	-0.119	-86	743	754	QLESLVRLAEAR						Mascot
1429.7429	1429.7172	-0.0257	-18	866	877	IKMVSSLTPYMK			Oxidation (M)[3,11]			Mascot
1473.6752	1473.7283	0.0531	36	729	742	AHSTCSDGTPITAR			Carbamidomethyl (C)[5]			Mascot
1507.7462	1507.7208	-0.0254	-17	40	52	ILDDVMAWSQSVK			Oxidation (M)[6]			Mascot
1508.7275	1508.7253	-0.0022	-1	635	647	VSDHIMALHTNDR						Mascot
1508.7275	1508.7253	-0.0022	-1	635	647	VSDHIMALHTNDR						Mascot
1615.8248	1615.7275	-0.0973	-60	835	848	ISLQVPDLDDIMEK						Mascot
1664.8286	1664.9684	0.1398	84	634	647	RVSDHIMALHTNDR						Mascot
1978.0062	1978.0176	0.0114	6	656	673	LRTVPQFNGSMGIGIDEK			Oxidation (M)[11]			Mascot
1980.0405	1980.0363	-0.0042	-2	227	244	ENPKEALLCMGVAVHLA K			Carbamidomethyl (C)[9]			Mascot
1980.0405	1980.0363	-0.0042	-2	227	244	ENPKEALLCMGVAVHLA K			Carbamidomethyl (C)[9]			Mascot
1996.0354	1996.016	-0.0194	-10	227	244	ENPKEALLCMGVAVHLA K			Carbamidomethyl (C)[9], Oxidation (M)[10]			Mascot
2028.0372	2028.0066	-0.0306	-15	435	451	QILHSFCPSIYGHELVK			Carbamidomethyl (C)[7]			Mascot
2038.0638	2037.9758	-0.088	-43	504	523	GIYVCGNTTTNAGLTVAV VK			Carbamidomethyl (C)[5]			Mascot
2067.9883	2067.9839	-0.0044	-2	158	175	YFPEETEHPVPAGDPRA R						Mascot
2165.0339	2165.106	0.0721	33	129	147	MYADTQKGRPGCRPVD ASR			Carbamidomethyl (C)[12]			Mascot

5 hypothetical protein TRIUR3_25626 [Triticum urartu] gi|473808691 9131.7 8.34 6 43 0 .661

Peptide Information

Calc. Mass	Obsrv. Mass	± da	± ppm	Start Seq.	End Seq.	Sequence	Ion Score	C. I. %	Modification		Rank	Result Type
962.4876	962.4772	-0.0104	-11	5	12	CAVSALWR			Carbamidomethyl (C)[1]			Mascot
976.4153	976.486	0.0707	72	16	25	NSPAEGGGCK			Carbamidomethyl (C)[9]			Mascot

1161.6263	1161.5178	-0.1085	-93	26	36	AEALQVGAFQK						Mascot
1328.7645	1328.6801	-0.0844	-64	53	64	ASELLRILNGSR						Mascot
1363.6974	1363.7046	0.0072	5	5	15	CAVSALWRLCK			Carbamidomethyl (C)[1,10]			Mascot
2023.9753	2024.006	0.0307	15	59	76	ILNGSRDGAECIDTLDFK			Carbamidomethyl (C)[11]			Mascot

6	Peroxidase 5 [Triticum urartu]	gi|474249697	70673.3	7.58	15	41	0	14.889	

Peptide Information

Calc. Mass	Obsrv. Mass	± da	± ppm	Start Seq.	End Seq.	Sequence	Ion Score	C. I. %	Modification	Rank	Result Type
1197.6335	1197.5975	-0.036	-30	463	474	VPGGRPDGTVSR					Mascot
1267.61	1267.5898	-0.0202	-16	475	486	ASMANSDLPPHK					Mascot
1283.6049	1283.561	-0.0439	-34	475	486	ASMANSDLPPHK			Oxidation (M)[3]		Mascot
1363.7693	1363.7046	-0.0647	-47	161	172	HQLTLVLHSSTK					Mascot
1371.6686	1371.6715	0.0029	2	206	216	EMRELNLGEHK			Oxidation (M)[2]		Mascot
1372.7067	1372.7041	-0.0026	-2	579	591	TVLESDAALNSPR					Mascot
1372.7067	1372.7041	-0.0026	-2	579	591	TVLESDAALNSPR					Mascot
1385.7212	1385.701	-0.0202	-15	489	499	NVDLLAQYFFR					Mascot
1447.8011	1447.7117	-0.0894	-62	310	322	LTVAVILETMAMR					Mascot
1473.7333	1473.7283	-0.005	-3	224	236	VAEAIWGKEDAER					Mascot
1490.8036	1490.7202	-0.0834	-56	124	136	CYAAITVVLPDLR			Carbamidomethyl (C)[1]		Mascot
1490.8036	1490.7202	-0.0834	-56	124	136	CYAAITVVLPDLR			Carbamidomethyl (C)[1]		Mascot
1551.7697	1551.744	-0.0257	-17	475	488	ASMANSDLPPHKQR					Mascot
1646.9047	1646.7592	-0.1455	-88	124	137	CYAAITVVLPDLRR			Carbamidomethyl (C)[1]		Mascot
1664.8378	1664.9684	0.1306	78	173	186	IKTENPSIIEDDYK					Mascot
1735.7415	1735.7894	0.0479	28	521	534	CGTFDYRLMSDQDK			Carbamidomethyl (C)[1]		Mascot
1975.812	1976.0081	0.1961	99	192	208	CESSATSESSQWGKEM R			Carbamidomethyl (C)[1], Oxidation (M)[16]		Mascot
1991.9822	1991.9901	0.0079	4	359	375	AESIVFEEVQKAWNADR					Mascot

7	Auxilin-related protein 2 [Triticum urartu]	gi|474056531	186931.2	5.59	22	41	0	25.421	

Peptide Information

Calc. Mass	Obsrv. Mass	± da	± ppm	Start Seq.	End Seq.	Sequence	Ion Score	C. I. %	Modification	Rank	Result Type
860.4584	860.4469	-0.0115	-13	1424	1431	QRASAEAK					Mascot
947.4978	947.4896	-0.0082	-9	1411	1418	AEKMALER					Mascot
1149.5068	1149.5471	0.0403	35	182	190	SFVMNFNMK			Oxidation (M)[4,8]		Mascot
1229.6121	1229.5475	-0.0646	-53	1243	1252	EAERELPTER					Mascot

1354.642	1354.6958	0.0538	40	494	504	EAMEYAEARLR			Oxidation (M)[3]		Mascot
1384.6339	1384.6716	0.0377	27	991	1002	EAEKPHSENSEK					Mascot
1394.6151	1394.6827	0.0676	48	336	348	MDNGIMANGDRGK			Oxidation (M)[1]		Mascot
1410.6101	1410.6685	0.0584	41	336	348	MDNGIMANGDRGK			Oxidation (M)[1,6]		Mascot
1473.7656	1473.7283	-0.0373	-25	1120	1133	SANDGPTRLTVNTK					Mascot
1525.7493	1525.7972	0.0479	31	661	673	VTTQGSDLERYEK					Mascot
1551.806	1551.744	-0.062	-40	1625	1637	ATLCVHPDKVQQR			Carbamidomethyl (C)[4]		Mascot
1583.7635	1583.8403	0.0768	48	237	249	WMSSTDVLFGRER					Mascot
1615.8845	1615.7275	-0.157	-97	584	596	MVLPLKKPQQMMR			Oxidation (M)[1]		Mascot
1649.7258	1649.8036	0.0778	47	597	610	THTMPCQTSSNIEK			Carbamidomethyl (C)[6], Oxidation (M)[4]		Mascot
1771.8909	1771.8419	-0.049	-28	1274	1288	ISMAEPKHQHSHLEK					Mascot
1890.8861	1890.9558	0.0697	37	474	493	ADAALSNTEANPSSAAAA MK					Mascot
1977.0361	1977.005	-0.0311	-16	195	211	TIHEVYSMLKSAEVEIK					Mascot
1993.0311	1992.9772	-0.0539	-27	195	211	TIHEVYSMLKSAEVEIK			Oxidation (M)[8]		Mascot
2007.8998	2008.0262	0.1264	63	1134	1151	DEPTSCSEMSTGLQQLP K			Carbamidomethyl (C)[6]		Mascot
2011.939	2012.0264	0.0874	43	1181	1198	ELATTFEENGSVNRMEG K					Mascot
2011.939	2012.0264	0.0874	43	1181	1198	ELATTFEENGSVNRMEG K					Mascot
2017.9244	2018.0063	0.0819	41	639	656	EDNCGRSKPVTEPSEGQ K			Carbamidomethyl (C)[4]		Mascot
2023.8948	2024.006	0.1112	55	1134	1151	DEPTSCSEMSTGLQQLP K			Carbamidomethyl (C)[6], Oxidation (M)[9]		Mascot
2027.9338	2028.0066	0.0728	36	1181	1198	ELATTFEENGSVNRMEG K			Oxidation (M)[15]		Mascot
2056.9612	2056.9448	-0.0164	-8	871	887	EKFSFVGESYLHNENEK					Mascot
2056.9612	2056.9448	-0.0164	-8	871	887	EKFSFVGESYLHNENEK					Mascot
2068.0669	2067.9839	-0.083	-40	957	975	GDLDQEIQGSLGPIEVKN R					Mascot
2084.0254	2084.0059	-0.0195	-9	819	838	TSQEPPEVANVGNSQAS QIK					Mascot

8	SIR2-family protein HDAC1 [Triticum aestivum]	gi|109716216	40318.9	8.89	11	41	0	1.775	

Peptide Information

Calc. Mass	Obsrv. Mass	± da	± ppm	Start Seq.	End Seq.	Sequence	Ion Score	C. I. %	Modification	Rank	Result Type
1055.548	1055.5889	0.0409	39	130	139	LAELHGNSSK					Mascot
1161.5457	1161.5178	-0.0279	-24	13	23	EDVGTVGMPEK					Mascot
1164.6147	1164.5353	-0.0794	-68	152	161	DFEIETIGLK					Mascot
1177.5405	1177.531	-0.0095	-8	13	23	EDVGTVGMPEK			Oxidation (M)[8]		Mascot
1390.761	1390.6732	-0.0878	-63	34	45	IEELAVMVQKSK			Oxidation (M)[7]		Mascot
1583.9269	1583.8403	-0.0866	-55	274	286	TDFIQLLLRHTVK					Mascot

1698.9823	1698.8505	-0.1318	-78	29	43	LLQGKIEELAVMVQK						Mascot
1733.87	1733.7957	-0.0743	-43	337	351	ETSMTSIFSMLLTLK				Oxidation (M)[4,10]		Mascot
1735.8208	1735.7894	-0.0314	-18	13	28	EDVGTVGMPEKFDSPK						Mascot
2010.1495	2010.0133	-0.1362	-68	105	122	AGLLKFVISQNVDSLHLR						Mascot
2134.2053	2134.1006	-0.1047	-49	90	109	AAPTLTHMALVELERAGL LK						Mascot
2576.1831	2576.3042	0.1211	47	337	359	ETSMTSIFSMLLTLKFSD GCGNH				Carbamidomethyl (C)[20]		Mascot

9 hypothetical protein TRIUR3_29169 [Triticum urartu] gi|473827401 12535.7 10.12 6 41 0 1.762

Peptide Information

Calc. Mass	Obsrv. Mass	± da	± ppm	Start Seq.	End Seq.	Sequence	Ion Score	C. I. %	Modification	Rank	Result Type
851.437	851.4162	-0.0208	-24	55	62	THPGPNTK					Mascot
877.5141	877.4656	-0.0485	-55	40	46	YLNLNLK					Mascot
1473.6929	1473.7283	0.0354	24	10	25	AAEEVAEAKGGDGGGR					Mascot
1798.9811	1798.8063	-0.1748	-97	47	62	YTLIISTRTHPGPNTK					Mascot
1979.1396	1979.0122	-0.1274	-64	19	38	GGDGGGRVGLLAIVINIEI R					Mascot
2007.9553	2008.0262	0.0709	35	68	85	SEPQQAISAMEQPVAEH R					Mascot
2023.9502	2024.006	0.0558	28	68	85	SEPQQAISAMEQPVAEH R			Oxidation (M)[10]		Mascot

10 Serine/threonine-protein kinase PBS1 [Triticum urartu] gi|474016289 67060.6 6.46 14 40 0 8.876

Peptide Information

Calc. Mass	Obsrv. Mass	± da	± ppm	Start Seq.	End Seq.	Sequence	Ion Score	C. I. %	Modification	Rank	Result Type
860.5022	860.4469	-0.0553	-64	342	350	MKIAAGAAK					Mascot
863.433	863.4956	0.0626	73	539	546	SRVSDSGR					Mascot
947.4177	947.4896	0.0719	76	561	568	SDRDDSPR					Mascot
1009.5248	1009.5166	-0.0082	-8	113	121	GVFMQRGSK					Mascot
1149.5859	1149.5471	-0.0388	-34	170	182	TVSAASSSGVGAR					Mascot
1177.6437	1177.531	-0.1127	-96	233	242	QLTAATRNFR					Mascot
1310.6488	1310.6962	0.0474	36	87	96	NQSIDTYWKR					Mascot
1507.7145	1507.7208	0.0063	4	472	484	MADPGLQGRYPMR			Oxidation (M)[1]		Mascot
1508.7349	1508.7253	-0.0096	-6	581	593	MVAEAKMWGANLR			Oxidation (M)[1,7]		Mascot
1508.7349	1508.7253	-0.0096	-6	581	593	MVAEAKMWGANLR			Oxidation (M)[1,7]		Mascot
1551.7988	1551.744	-0.0548	-35	106	118	LVDPDFKGVFMQR					Mascot
1735.8367	1735.7894	-0.0473	-27	122	136	AMANRWGLIQSACNK			Carbamidomethyl (C)[13], Oxidation (M)[2]		Mascot
1996.1115	1996.016	-0.0955	-48	425	442	SDVYSFGVVLLELITGRK					Mascot

2257.1355	2257.1133	-0.0222	-10	170	191	TVSAASSSGVGAREERP MVPPR	Oxidation (M)[18]	Mascot
3512.7666	3512.7153	-0.0513	-15	281	310	EFLVEVLMLSLLHHQNLV NLVGYCADGEQR	Carbamidomethyl (C)[24], Oxidation (M)[8]	Mascot

Gel Idx/Pos	284/L12	Instr./Gel Origin	BA2151/Sample Project 20140814	Process Status	Analysis Succeeded
Plate [#] Name	[1] Sample Project 20140814	Instrument Sample Name		Spectra	11

Rank	Protein Name	Accession No.	Protein MW	Protein PI	Pep. Count	Protein Score	Protein Score C. I. %	Intensity Matched	Total Ion Score	Total Ion C. I. %	Confirmed
1	small subunit ADP glucose pyrophosphorylase [Triticum aestivum]	gi|7340287	52313.6	5.53	22	954	100	41.718	814	100	

Protein Group

Protein Name	Accession No.	Protein MW	Protein PI
small subunit ADP glucose pyrophosphorylase [Triticum aestivum]	gi|20127139	52313.6	5.5300002098 0835
unnamed protein product [Triticum aestivum]	gi|257671690	52313.6	5.5300002098 0835
unnamed protein product [Triticum aestivum]	gi|257304117	52313.6	5.5300002098 0835
unnamed protein product [Triticum aestivum]	gi|257308963	52313.6	5.5300002098 0835
unnamed protein product [Triticum aestivum]	gi|219764724	52313.6	5.5300002098 0835
unnamed protein product [Triticum aestivum]	gi|219752137	52313.6	5.5300002098 0835

Peptide Information

Calc. Mass	Obsrv. Mass	± da	± ppm	Start Seq.	End Seq.	Sequence	Ion Score	C. I. %	Modification	Rank	Result Type
854.444	854.4305	-0.0135	-16	200	207	ATAFGLMK			Oxidation (M)[7]		Mascot
947.4833	947.4744	-0.0089	-9	9	17	TFPSPSPSK					Mascot
972.4673	972.4487	-0.0186	-19	448	455	ETDGYFIK					Mascot
1009.5975	1009.5825	-0.015	-15	260	267	HVMLQLLR					Mascot
1017.5952	1017.5868	-0.0084	-8	366	374	IHHSVVGLR					Mascot
1025.5925	1025.5709	-0.0216	-21	260	267	HVMLQLLR			Oxidation (M)[3]		Mascot
1029.5	1029.5046	0.0046	4	110	119	AYGSNIGGYK					Mascot
1032.5472	1032.5397	-0.0075	-7	330	338	SAPIYTQPR					Mascot
1032.5472	1032.5397	-0.0075	-7	330	338	SAPIYTQPR	45	99.784			Mascot
1074.6194	1074.5847	-0.0347	-32	214	222	IIEFAEKPK					Mascot

1256.7109	1256.7004	-0.0105	-8	64	75	AKPAVPLGANYR					Mascot
1256.7109	1256.7004	-0.0105	-8	64	75	AKPAVPLGANYR	29	91.339			Mascot
1384.6896	1384.6841	-0.0055	-4	319	329	KPIPDFSFYDR					Mascot
1384.6896	1384.6841	-0.0055	-4	319	329	KPIPDFSFYDR	88	100			Mascot
1412.8121	1412.6833	-0.1288	-91	63	75	RAKPAVPLGANYR					Mascot
1621.7925	1621.7832	-0.0093	-6	228	242	AMMVDTTILGLDDAR					Mascot
1625.8646	1625.8612	-0.0034	-2	92	105	IYVLTQFNSASLNR	114	100			Mascot
1637.7874	1637.786	-0.0014	-1	228	242	AMMVDTTILGLDDAR			Oxidation (M)[2]		Mascot
1653.7822	1653.77	-0.0122	-7	228	242	AMMVDTTILGLDDAR			Oxidation (M)[2,3]		Mascot
1733.8489	1733.8354	-0.0135	-8	245	259	EMPYIASMGIYVISK			Oxidation (M)[2,8]		Mascot
1786.9368	1786.9095	-0.0273	-15	76	91	LIDIPVSNCLNSNISK			Carbamidomethyl (C)[9]		Mascot
1804.915	1804.9409	0.0259	14	1	17	MDVPLASKTFPSPSPSK			Oxidation (M)[1]		Mascot
1875.864	1875.8551	-0.0089	-5	183	199	ETDADITVAALPMDEER					Mascot
1889.9525	1889.9269	-0.0256	-14	345	362	VLDADVTDSVIGEGCVIK			Carbamidomethyl (C)[15]		Mascot
1891.859	1891.8402	-0.0188	-10	183	199	ETDADITVAALPMDEER			Oxidation (M)[13]		Mascot
1970.9965	1970.9867	-0.0098	-5	430	447	IGDNVMIINVDNVQEAAR					Mascot
1970.9965	1970.9867	-0.0098	-5	430	447	IGDNVMIINVDNVQEAAR	164	100			Mascot
1986.9913	1986.968	-0.0233	-12	430	447	IGDNVMIINVDNVQEAAR			Oxidation (M)[6]		Mascot
2355.2163	2355.2185	0.0022	1	32	55	HADLNPHANDSVLGIILG GGAGTR					Mascot
2355.2163	2355.2185	0.0022	1	32	55	HADLNPHANDSVLGIILG GGAGTR	153	100			Mascot
2368.0874	2368.0959	0.0085	4	268	290	EQFPGANDFGSEVIPGAT STGMR					Mascot
3061.4287	3061.4373	0.0086	3	120	147	NEGFVEVLAAQQSPDNP DWFQGTADAVR					Mascot
3061.4287	3061.4373	0.0086	3	120	147	NEGFVEVLAAQQSPDNP DWFQGTADAVR	221	100			Mascot

2 cytosolic small subunit ADP glucose pyrophosphorylase [Triticum aestivum] gi|125976023 52277.6 5.53 20 932 100 40.623 814 100

Peptide Information

Calc. Mass	Obsrv. Mass	± da	± ppm	Start Seq.	End Seq.	Sequence	Ion Score	C. I. %	Modification	Rank	Result Type
854.444	854.4305	-0.0135	-16	200	207	ATAFGLMK			Oxidation (M)[7]		Mascot
947.4833	947.4744	-0.0089	-9	9	17	TFPSPSPSK					Mascot
972.4673	972.4487	-0.0186	-19	448	455	ETDGYFIK					Mascot
1009.5975	1009.5825	-0.015	-15	260	267	HVMLQLLR					Mascot
1017.5952	1017.5868	-0.0084	-8	366	374	IHHSVVGLR					Mascot
1025.5925	1025.5709	-0.0216	-21	260	267	HVMLQLLR			Oxidation (M)[3]		Mascot
1029.5	1029.5046	0.0046	4	110	119	AYGSNIGGYK					Mascot

Calc. Mass	Obsrv. Mass	± da	± ppm	Start Seq.	End Seq.	Sequence	Ion Score	C. I. %	Modification	Rank	Result Type
1032.5472	1032.5397	-0.0075	-7	330	338	SAPIYTQPR					Mascot
1032.5472	1032.5397	-0.0075	-7	330	338	SAPIYTQPR	45	99.784			Mascot
1074.6194	1074.5847	-0.0347	-32	214	222	IIEFAEKPK					Mascot
1256.7109	1256.7004	-0.0105	-8	64	75	AKPAVPLGANYR					Mascot
1256.7109	1256.7004	-0.0105	-8	64	75	AKPAVPLGANYR	29	91.339			Mascot
1384.6896	1384.6841	-0.0055	-4	319	329	KPIPDFSFYDR					Mascot
1384.6896	1384.6841	-0.0055	-4	319	329	KPIPDFSFYDR	88	100			Mascot
1412.8121	1412.6833	-0.1288	-91	63	75	RAKPAVPLGANYR					Mascot
1621.7925	1621.7832	-0.0093	-6	228	242	AMMVDTTILGLDDAR					Mascot
1625.8646	1625.8612	-0.0034	-2	92	105	IYVLTQFNSASLNR	114	100			Mascot
1637.7874	1637.786	-0.0014	-1	228	242	AMMVDTTILGLDDAR			Oxidation (M)[2]		Mascot
1653.7822	1653.77	-0.0122	-7	228	242	AMMVDTTILGLDDAR			Oxidation (M)[2,3]		Mascot
1733.8489	1733.8354	-0.0135	-8	245	259	EMPYIASMGIYVISK			Oxidation (M)[2,8]		Mascot
1804.915	1804.9409	0.0259	14	1	17	MDVPLASKTFPSPSPSK			Oxidation (M)[1]		Mascot
1889.9525	1889.9269	-0.0256	-14	345	362	VLDADVTDSVIGEGCVIK			Carbamidomethyl (C)[15]		Mascot
1970.9965	1970.9867	-0.0098	-5	430	447	IGDNVMIINVDNVQEAAR					Mascot
1970.9965	1970.9867	-0.0098	-5	430	447	IGDNVMIINVDNVQEAAR	164	100			Mascot
1986.9913	1986.968	-0.0233	-12	430	447	IGDNVMIINVDNVQEAAR			Oxidation (M)[6]		Mascot
2355.2163	2355.2185	0.0022	1	32	55	HADLNPHANDSVLGIILG GGAGTR					Mascot
2355.2163	2355.2185	0.0022	1	32	55	HADLNPHANDSVLGIILG GGAGTR	153	100			Mascot
2368.0874	2368.0959	0.0085	4	268	290	EQFPGANDFGSEVIPGAT STGMR					Mascot
3061.4287	3061.4373	0.0086	3	120	147	NEGFVEVLAAQQSPDNP DWFQGTADAVR					Mascot
3061.4287	3061.4373	0.0086	3	120	147	NEGFVEVLAAQQSPDNP DWFQGTADAVR	221	100			Mascot

3 plastid ADP-glucose pyrophosphorylase small subunit [Triticum aestivum] gi|224021585 56652.9 6.4 21 783 100 35.843 661 100

Peptide Information

Calc. Mass	Obsrv. Mass	± da	± ppm	Start Seq.	End Seq.	Sequence	Ion Score	C. I. %	Modification	Rank	Result Type
854.444	854.4305	-0.0135	-16	241	248	ATAFGLMK			Oxidation (M)[7]		Mascot
972.4673	972.4487	-0.0186	-19	489	496	ETDGYFIK					Mascot
1009.5975	1009.5825	-0.015	-15	301	308	HVMLQLLR					Mascot
1017.5952	1017.5868	-0.0084	-8	407	415	IHHSVVGLR					Mascot
1025.5925	1025.5709	-0.0216	-21	301	308	HVMLQLLR			Oxidation (M)[3]		Mascot
1029.5	1029.5046	0.0046	4	151	160	AYGSNIGGYK					Mascot
1032.5472	1032.5397	-0.0075	-7	371	379	SAPIYTQPR					Mascot

1032.5472	1032.5397	-0.0075	-7	371	379	SAPIYTQPR	45	99.784		Mascot
1074.6194	1074.5847	-0.0347	-32	255	263	IIEFAEKPK				Mascot
1256.7109	1256.7004	-0.0105	-8	105	116	AKPAVPLGANYR				Mascot
1256.7109	1256.7004	-0.0105	-8	105	116	AKPAVPLGANYR	29	91.339		Mascot
1384.6896	1384.6841	-0.0055	-4	360	370	KPIPDFSFYDR				Mascot
1384.6896	1384.6841	-0.0055	-4	360	370	KPIPDFSFYDR	88	100		Mascot
1412.8121	1412.6833	-0.1288	-91	104	116	RAKPAVPLGANYR				Mascot
1573.6912	1573.8218	0.1306	83	19	34	ASAATAAASTSCDSFR			Carbamidomethyl (C)[12]	Mascot
1621.7925	1621.7832	-0.0093	-6	269	283	AMMVDTTILGLDDAR				Mascot
1625.8646	1625.8612	-0.0034	-2	133	146	IYVLTQFNSASLNR	114	100		Mascot
1637.7874	1637.786	-0.0014	-1	269	283	AMMVDTTILGLDDAR			Oxidation (M)[2]	Mascot
1653.7822	1653.77	-0.0122	-7	269	283	AMMVDTTILGLDDAR			Oxidation (M)[2,3]	Mascot
1733.8489	1733.8354	-0.0135	-8	286	300	EMPYIASMGIYVISK			Oxidation (M)[2,8]	Mascot
1786.9368	1786.9095	-0.0273	-15	117	132	LIDIPVSNCLNSNISK			Carbamidomethyl (C)[9]	Mascot
1875.864	1875.8551	-0.0089	-5	224	240	ETDADITVAALPMDEER				Mascot
1889.9525	1889.9269	-0.0256	-14	386	403	VLDADVTDSVIGEGCVIK			Carbamidomethyl (C)[15]	Mascot
1891.859	1891.8402	-0.0188	-10	224	240	ETDADITVAALPMDEER			Oxidation (M)[13]	Mascot
1970.9965	1970.9867	-0.0098	-5	471	488	IGDNVMIINVDNVQEAAR				Mascot
1970.9965	1970.9867	-0.0098	-5	471	488	IGDNVMIINVDNVQEAAR	164	100		Mascot
1986.9913	1986.968	-0.0233	-12	471	488	IGDNVMIINVDNVQEAAR			Oxidation (M)[6]	Mascot
2368.0874	2368.0959	0.0085	4	309	331	EQFPGANDFGSEVIPGAT STGMR				Mascot
2400.209	2400.0647	-0.1443	-60	12	34	ILIPPHRASAATAAASTSC DSFR			Carbamidomethyl (C)[19]	Mascot
3061.4287	3061.4373	0.0086	3	161	188	NEGFVEVLAAQQSPDNP DWFQGTADAVR				Mascot
3061.4287	3061.4373	0.0086	3	161	188	NEGFVEVLAAQQSPDNP DWFQGTADAVR	221	100		Mascot

4	ADP glucose pyrophosphorylase [Triticum aestivum]	gi|469952290	52399.6	5.54	21	673	100	35.931	546	100

Protein Group

ADP-glucose pyrophosphorylase [Triticum aestivum]	gi|21687	52399.6	5.5399 999618 5303
RecName: Full=Glucose-1-phosphate adenylyltransferase small subunit, chloroplastic/amyloplastic; AltName: Full=ADP-glucose pyrophosphorylase; AltName: Full=ADP-glucose synthase; AltName: Full=AGPase B; AltName: Full=Alpha-D-glucose-1-phosphate adeny	gi|232172	52399.6	5.5399 999618 5303
unnamed protein product [Triticum aestivum]	gi|300634091	52399.6	5.5399 999618 5303

unnamed protein product [Triticum aestivum]	gi|300544577	52399.6	5.5399 999618 5303
unnamed protein product [Triticum aestivum]	gi|259471779	52399.6	5.5399 999618 5303
unnamed protein product [Triticum aestivum]	gi|259439192	52399.6	5.5399 999618 5303
unnamed protein product [Triticum aestivum]	gi|257671726	52399.6	5.5399 999618 5303
unnamed protein product [Triticum aestivum]	gi|257306995	52399.6	5.5399 999618 5303
unnamed protein product [Triticum aestivum]	gi|257308999	52399.6	5.5399 999618 5303
unnamed protein product [Triticum aestivum]	gi|219764760	52399.6	5.5399 999618 5303
unnamed protein product [Triticum aestivum]	gi|219752173	52399.6	5.5399 999618 5303

Peptide Information

Calc. Mass	Obsrv. Mass	± da	± ppm	Start Seq.	End Seq.	Sequence	Ion Score	C. I. %	Modification	Rank	Result Type
854.444	854.4305	-0.0135	-16	200	207	ATAFGLMK			Oxidation (M)[7]		Mascot
947.4833	947.4744	-0.0089	-9	9	17	TFPSPSPSK					Mascot
972.4673	972.4487	-0.0186	-19	448	455	ETDGYFIK					Mascot
1009.5975	1009.5825	-0.015	-15	260	267	HVMLQLLR					Mascot
1017.5952	1017.5868	-0.0084	-8	366	374	IHHSVVGLR					Mascot
1025.5925	1025.5709	-0.0216	-21	260	267	HVMLQLLR			Oxidation (M)[3]		Mascot
1029.5	1029.5046	0.0046	4	110	119	AYGSNIGGYK					Mascot
1032.5472	1032.5397	-0.0075	-7	330	338	SAPIYTQPR					Mascot
1032.5472	1032.5397	-0.0075	-7	330	338	SAPIYTQPR	45	99.784			Mascot
1074.6194	1074.5847	-0.0347	-32	214	222	IIEFAEKPK					Mascot
1256.7109	1256.7004	-0.0105	-8	64	75	AKPAVPLGANYR					Mascot
1256.7109	1256.7004	-0.0105	-8	64	75	AKPAVPLGANYR	29	91.339			Mascot

1384.6896	1384.6841	-0.0055	-4	319	329	KPIPDFSFYDR					Mascot
1384.6896	1384.6841	-0.0055	-4	319	329	KPIPDFSFYDR	88	100			Mascot
1412.8121	1412.6833	-0.1288	-91	63	75	RAKPAVPLGANYR					Mascot
1621.7925	1621.7832	-0.0093	-6	228	242	AMMVDTTILGLDDAR					Mascot
1637.7874	1637.786	-0.0014	-1	228	242	AMMVDTTILGLDDAR			Oxidation (M)[2]		Mascot
1653.7822	1653.77	-0.0122	-7	228	242	AMMVDTTILGLDDAR			Oxidation (M)[2,3]		Mascot
1733.8489	1733.8354	-0.0135	-8	245	259	EMPYIASMGIYVISK			Oxidation (M)[2,8]		Mascot
1786.9368	1786.9095	-0.0273	-15	76	91	LIDIPVSNCLNSNISK			Carbamidomethyl (C)[9]		Mascot
1804.915	1804.9409	0.0259	14	1	17	MDVPLASKTFPSPSPSK			Oxidation (M)[1]		Mascot
1875.864	1875.8551	-0.0089	-5	183	199	ETDADITVAALPMDEER					Mascot
1889.9525	1889.9269	-0.0256	-14	345	362	VLDADVTDSVIGEGCVIK			Carbamidomethyl (C)[15]		Mascot
1891.859	1891.8402	-0.0188	-10	183	199	ETDADITVAALPMDEER			Oxidation (M)[13]		Mascot
1970.9965	1970.9867	-0.0098	-5	430	447	IGDNVMIINVDNVQEAAR					Mascot
1970.9965	1970.9867	-0.0098	-5	430	447	IGDNVMIINVDNVQEAAR	164	100			Mascot
1986.9913	1986.968	-0.0233	-12	430	447	IGDNVMIINVDNVQEAAR			Oxidation (M)[6]		Mascot
2318.2537	2318.1902	-0.0635	-27	76	95	LIDIPVSNCLNSNISKIYVR			Carbamidomethyl (C)[9]		Mascot
2368.0874	2368.0959	0.0085	4	268	290	EQFPGANDFGSEVIPGAT STGMR					Mascot
3061.4287	3061.4373	0.0086	3	120	147	NEGFVEVLAAQQSPDNP DWFQGTADAVR					Mascot
3061.4287	3061.4373	0.0086	3	120	147	NEGFVEVLAAQQSPDNP DWFQGTADAVR	221	100			Mascot

5	Glucose-1-phosphate adenylyltransferase small subunit, chloroplastic/amyloplastic [Triticum urartu]	gi|474108293	65309.1	7.9	18	582	100	32.514	497	100	

Peptide Information

Calc. Mass	Obsrv. Mass	± da	± ppm	Start Seq.	End Seq.	Sequence	Ion Score	C. I. %	Modification	Rank	Result Type
854.444	854.4305	-0.0135	-16	241	248	ATAFGLMK			Oxidation (M)[7]		Mascot
1009.5975	1009.5825	-0.015	-15	301	308	HVMLQLLR					Mascot
1017.5952	1017.5868	-0.0084	-8	407	415	IHHSVVGLR					Mascot
1025.5925	1025.5709	-0.0216	-21	301	308	HVMLQLLR			Oxidation (M)[3]		Mascot
1029.5	1029.5046	0.0046	4	151	160	AYGSNIGGYK					Mascot
1032.5472	1032.5397	-0.0075	-7	371	379	SAPIYTQPR					Mascot
1032.5472	1032.5397	-0.0075	-7	371	379	SAPIYTQPR	45	99.784			Mascot
1074.6194	1074.5847	-0.0347	-32	255	263	IIEFAEKPK					Mascot
1256.7109	1256.7004	-0.0105	-8	105	116	AKPAVPLGANYR					Mascot
1256.7109	1256.7004	-0.0105	-8	105	116	AKPAVPLGANYR	29	91.339			Mascot
1384.6896	1384.6841	-0.0055	-4	360	370	KPIPDFSFYDR					Mascot

1384.6896	1384.6841	-0.0055	-4	360	370	KPIPDFSFYDR	88	100			Mascot
1412.8121	1412.6833	-0.1288	-91	104	116	RAKPAVPLGANYR					Mascot
1621.7925	1621.7832	-0.0093	-6	269	283	AMMVDTTILGLDDAR					Mascot
1625.8646	1625.8612	-0.0034	-2	133	146	IYVLTQFNSASLNR	114	100			Mascot
1637.7874	1637.786	-0.0014	-1	269	283	AMMVDTTILGLDDAR			Oxidation (M)[2]		Mascot
1653.7822	1653.77	-0.0122	-7	269	283	AMMVDTTILGLDDAR			Oxidation (M)[2,3]		Mascot
1733.8489	1733.8354	-0.0135	-8	286	300	EMPYIASMGIYVISK			Oxidation (M)[2,8]		Mascot
1786.9368	1786.9095	-0.0273	-15	117	132	LIDIPVSNCLNSNISK			Carbamidomethyl (C)[9]		Mascot
1875.864	1875.8551	-0.0089	-5	224	240	ETDADITVAALPMDEER					Mascot
1889.9525	1889.9269	-0.0256	-14	386	403	VLDADVTDSVIGEGCVIK			Carbamidomethyl (C)[15]		Mascot
1891.859	1891.8402	-0.0188	-10	224	240	ETDADITVAALPMDEER			Oxidation (M)[13]		Mascot
2366.2246	2366.0994	-0.1252	-53	12	34	ILIPPHRASAATAAASTSC DSLR			Carbamidomethyl (C)[19]		Mascot
2368.0874	2368.0959	0.0085	4	309	331	EQFPGANDFGSEVIPGAT STGMR					Mascot
3061.4287	3061.4373	0.0086	3	161	188	NEGFVEVLAAQQSPDNP DWFQGTADAVR					Mascot
3061.4287	3061.4373	0.0086	3	161	188	NEGFVEVLAAQQSPDNP DWFQGTADAVR	221	100			Mascot

6	plastid ADP-glucose pyrophosphorylase small subunit [Triticum aestivum]	gi|182894563	52263.6	5.48	16	519	100	31.018	440	100	

Peptide Information

Calc. Mass	Obsrv. Mass	± da	± ppm	Start Seq.	End Seq.	Sequence	Ion Score	C. I. %	Modification	Rank	Result Type
972.4673	972.4487	-0.0186	-19	450	457	ETDGYFIK					Mascot
1009.5975	1009.5825	-0.015	-15	262	269	HVMLQLLR					Mascot
1017.5952	1017.5868	-0.0084	-8	368	376	IHHSVVGLR					Mascot
1025.5925	1025.5709	-0.0216	-21	262	269	HVMLQLLR			Oxidation (M)[3]		Mascot
1029.5	1029.5046	0.0046	4	112	121	AYGSNIGGYK					Mascot
1032.5472	1032.5397	-0.0075	-7	332	340	SAPIYTQPR					Mascot
1032.5472	1032.5397	-0.0075	-7	332	340	SAPIYTQPR	45	99.784			Mascot
1256.7109	1256.7004	-0.0105	-8	66	77	AKPAVPLGANYR					Mascot
1256.7109	1256.7004	-0.0105	-8	66	77	AKPAVPLGANYR	29	91.339			Mascot
1384.6896	1384.6841	-0.0055	-4	321	331	KPIPDFSFYDR					Mascot
1384.6896	1384.6841	-0.0055	-4	321	331	KPIPDFSFYDR	88	100			Mascot
1412.8121	1412.6833	-0.1288	-91	65	77	RAKPAVPLGANYR					Mascot
1621.7925	1621.7832	-0.0093	-6	230	244	AMMVDTTILGLDDAR					Mascot
1625.8646	1625.8612	-0.0034	-2	94	107	IYVLTQFNSASLNR	114	100			Mascot
1637.7874	1637.786	-0.0014	-1	230	244	AMMVDTTILGLDDAR			Oxidation (M)[2]		Mascot

Calc. Mass	Obsrv. Mass	± da	± ppm	Start Seq.	End Seq.	Sequence	Ion Score	C. I. %	Modification	Rank	Result Type
1653.7822	1653.77	-0.0122	-7	230	244	AMMVDTTILGLDDAR			Oxidation (M)[2,3]		Mascot
1733.8489	1733.8354	-0.0135	-8	247	261	EMPYIASMGIYVISK			Oxidation (M)[2,8]		Mascot
1786.9368	1786.9095	-0.0273	-15	78	93	LIDIPVSNCLNSNISK			Carbamidomethyl (C)[9]		Mascot
1875.864	1875.8551	-0.0089	-5	185	201	ETDADITVAALPMDEER					Mascot
1889.9525	1889.9269	-0.0256	-14	347	364	VLDADVTDSVIGEGCVIK			Carbamidomethyl (C)[15]		Mascot
1891.859	1891.8402	-0.0188	-10	185	201	ETDADITVAALPMDEER			Oxidation (M)[13]		Mascot
1970.9965	1970.9867	-0.0098	-5	432	449	IGDNVMIINVDNVQEAAR					Mascot
1970.9965	1970.9867	-0.0098	-5	432	449	IGDNVMIINVDNVQEAAR	164	100			Mascot
1986.9913	1986.968	-0.0233	-12	432	449	IGDNVMIINVDNVQEAAR			Oxidation (M)[6]		Mascot
2368.0874	2368.0959	0.0085	4	270	292	EQFPGANDFGSEVIPGAT STGMR					Mascot

7 ADP-glucose pyrophosphorylase small subunit, partial [Triticum aestivum] gi|377657086 19687.8 5.31 7 407 100 7.528 364 100

Peptide Information

Calc. Mass	Obsrv. Mass	± da	± ppm	Start Seq.	End Seq.	Sequence	Ion Score	C. I. %	Modification	Rank	Result Type
1029.5	1029.5046	0.0046	4	86	95	AYGSNIGGYK					Mascot
1256.7109	1256.7004	-0.0105	-8	40	51	AKPAVPLGANYR					Mascot
1256.7109	1256.7004	-0.0105	-8	40	51	AKPAVPLGANYR	29	91.339			Mascot
1412.8121	1412.6833	-0.1288	-91	39	51	RAKPAVPLGANYR					Mascot
1625.8646	1625.8612	-0.0034	-2	68	81	IYVLTQFNSASLNR	114	100			Mascot
1786.9368	1786.9095	-0.0273	-15	52	67	LIDIPVSNCLNSNISK			Carbamidomethyl (C)[9]		Mascot
1875.864	1875.8551	-0.0089	-5	159	175	ETDADITVAALPMDEER					Mascot
1891.859	1891.8402	-0.0188	-10	159	175	ETDADITVAALPMDEER			Oxidation (M)[13]		Mascot
3061.4287	3061.4373	0.0086	3	96	123	NEGFVEVLAAQQSPDNP DWFQGTADAVR					Mascot
3061.4287	3061.4373	0.0086	3	96	123	NEGFVEVLAAQQSPDNP DWFQGTADAVR	221	100			Mascot

8 unnamed protein product [Triticum aestivum] gi|257671648 54622.7 5.67 8 166 100 5.01 143 100

Protein Group

ADP-glucose pyrophosphorylase small subunit [Triticum aestivum] gi|52430025 54622.7 5.6700 000762 9395

unnamed protein product [Triticum aestivum] gi|257304075 54622.7 5.6700 000762 9395

unnamed protein product [Triticum aestivum] gi|257308921 54622.7 5.6700 000762 9395

unnamed protein product [Triticum aestivum]	gi|219764680	54622.7	5.6700 000762 9395
unnamed protein product [Triticum aestivum]	gi|219752093	54622.7	5.6700 000762 9395

Peptide Information

Calc. Mass	Obsrv. Mass	± da	± ppm	Start Seq.	End Seq.	Sequence	Ion Score	C. I. %	Modification	Rank	Result Type
1256.7109	1256.7004	-0.0105	-8	89	100	AKPAVPLGANYR					Mascot
1256.7109	1256.7004	-0.0105	-8	89	100	AKPAVPLGANYR	29	91.339			Mascot
1412.8121	1412.6833	-0.1288	-91	88	100	RAKPAVPLGANYR					Mascot
1436.7103	1436.7429	0.0326	23	197	207	MDYQKFIQAHR					Mascot
1625.8646	1625.8612	-0.0034	-2	117	130	IYVLTQFNSASLNR	114	100			Mascot
1733.882	1733.8354	-0.0466	-27	270	284	ELPYIASMGIYVFSK			Oxidation (M)[8]		Mascot
1875.864	1875.8551	-0.0089	-5	208	224	ETDADITVAALPMDEER					Mascot
1891.859	1891.8402	-0.0188	-10	208	224	ETDADITVAALPMDEER			Oxidation (M)[13]		Mascot
2320.1716	2320.1497	-0.0219	-9	270	289	ELPYIASMGIYVFSKDAM LR			Oxidation (M)[8]		Mascot
2320.1716	2320.1497	-0.0219	-9	270	289	ELPYIASMGIYVFSKDAM LR			Oxidation (M)[8]		Mascot
2406.1946	2406.1035	-0.0911	-38	2	26	AMATAMAATYGAPITAPA PAAFSPR					Mascot

9	Glucose-1-phosphate adenylyltransferase small subunit, chloroplastic/amyloplastic [Triticum urartu]	gi|474444719	55553.8	5.84	8	163	100	3.48	143	100

Peptide Information

Calc. Mass	Obsrv. Mass	± da	± ppm	Start Seq.	End Seq.	Sequence	Ion Score	C. I. %	Modification	Rank	Result Type
854.444	854.4305	-0.0135	-16	264	271	ATAFGLMK			Oxidation (M)[7]		Mascot
1256.7109	1256.7004	-0.0105	-8	128	139	AKPAVPLGANYR					Mascot
1256.7109	1256.7004	-0.0105	-8	128	139	AKPAVPLGANYR	29	91.339			Mascot
1270.7478	1270.7382	-0.0096	-8	106	119	SVLGIILGGGAGTR					Mascot
1412.8121	1412.6833	-0.1288	-91	127	139	RAKPAVPLGANYR					Mascot
1436.7103	1436.7429	0.0326	23	236	246	MDYQKFIQAHR					Mascot
1625.8646	1625.8612	-0.0034	-2	156	169	IYVLTQFNSASLNR	114	100			Mascot
1733.882	1733.8354	-0.0466	-27	309	323	ELPYIASMGIYVFSK			Oxidation (M)[8]		Mascot
1875.864	1875.8551	-0.0089	-5	247	263	ETDADITVAALPMDEER					Mascot
1891.859	1891.8402	-0.0188	-10	247	263	ETDADITVAALPMDEER			Oxidation (M)[13]		Mascot

10	cytosolic ADP glucose pyrophosphorylase [Triticum	gi|25271998	9141.8	9.34	4	54	81.985	2.338	27	87.989

aestivum]

Peptide Information

Calc. Mass	Obsrv. Mass	± da	± ppm	Start Seq.	End Seq.	Sequence	Ion Score	C. I. %	Modification	Rank	Result Type
947.4833	947.4744	-0.0089	-9	9	17	TFPSPSPSK					Mascot
1256.7109	1256.7004	-0.0105	-8	64	75	AKPAVPLGANYR					Mascot
1256.7109	1256.7004	-0.0105	-8	64	75	AKPAVPLGANYR	29	90.805			Mascot
1412.8121	1412.6833	-0.1288	-91	63	75	RAKPAVPLGANYR					Mascot
1804.915	1804.9409	0.0259	14	1	17	MDVPLASKTFPSPSPSK			Oxidation (M)[1]		Mascot

Gel Idx/Pos	285/L13	Instr./Gel Origin	BA2151/Sample Project 20140814	Process Status	Analysis Succeeded
Plate [#] Name	[1] Sample Project 20140814	Instrument Sample Name		Spectra	11

Rank	Protein Name	Accession No.	Protein MW	Protein PI	Pep. Count	Protein Score	Protein Score C. I. %	Intensity Matched	Total Ion Score	Total Ion C. I. %	Confirmed
1	2,3-bisphosphoglycerate-independent phosphoglycerate mutase [Triticum urartu]	gi|473886714	57764.3	5.28	15	657	100	21.43	600	100	

Peptide Information

Calc. Mass	Obsrv. Mass	± da	± ppm	Start Seq.	End Seq.	Sequence	Ion Score	C. I. %	Modification	Rank	Result Type
801.4464	801.4352	-0.0112	-14	374	380	ALEIAER					Mascot
924.5261	924.4982	-0.0279	-30	161	168	LHILTDGR					Mascot
974.4214	974.399	-0.0224	-23	345	352	SGYFDETR					Mascot
1403.6511	1403.5999	-0.0512	-36	313	324	YAGMLQYDGELK			Oxidation (M)[4]		Mascot
1407.7015	1407.6813	-0.0202	-14	222	234	GWDAQVLGEAPHK					Mascot
1481.7074	1481.6864	-0.021	-14	333	344	FGHVTFFWNGNR					Mascot
1499.7166	1499.6884	-0.0282	-19	294	305	ALEFPDFDKFDR					Mascot
1499.7166	1499.6884	-0.0282	-19	294	305	ALEFPDFDKFDR	82	100			Mascot
1513.725	1513.6738	-0.0512	-34	198	211	IASGGGRMYVTMDR					Mascot
1563.8027	1563.7573	-0.0454	-29	221	234	RGWDAQVLGEAPHK					Mascot
1731.9501	1731.9226	-0.0275	-16	124	140	GTLHLIGLLSDGGVHSR					Mascot
1731.9501	1731.9226	-0.0275	-16	124	140	GTLHLIGLLSDGGVHSR	117	100			Mascot
1792.9229	1792.8931	-0.0298	-17	268	284	SVGPIVDGDAVVTFNFR					Mascot
1792.9229	1792.8931	-0.0298	-17	268	284	SVGPIVDGDAVVTFNFR	121	100			Mascot
2350.1729	2350.0681	-0.1048	-45	325	344	LPSNETVKFGHVTFFWN GNR					Mascot
2721.2283	2721.1909	-0.0374	-14	63	90	AHGTAVGLPSDDDMGNS EVGHNALGAGR			Oxidation (M)[14]		Mascot
2888.6106	2888.5979	-0.0127	-4	463	491	SGSIQILTSHTLQPVPVAI GGPGLHPGVR					Mascot
2888.6106	2888.5979	-0.0127	-4	463	491	SGSIQILTSHTLQPVPVAI GGPGLHPGVR	186	100			Mascot
3137.4221	3137.4116	-0.0105	-3	345	371	SGYFDETREEYVEIPSDS GITFNEQPK					Mascot
3137.4221	3137.4116	-0.0105	-3	345	371	SGYFDETREEYVEIPSDS GITFNEQPK	94	100			Mascot

Rank	Protein Name	Accession No.	Protein MW	Protein PI	Pep. Count	Protein Score	Protein Score C. I. %	Intensity Matched	Total Ion Score	Total Ion C. I. %	Confirmed
2	phosphoglycerate mutase, partial [Triticum aestivum]	gi|32400802	29615.1	5.43	13	416	100	21.994	344	100	

Peptide Information

Calc. Mass	Obsrv. Mass	± da	± ppm	Start Seq.	End Seq.	Sequence	Ion Score	C. I. %	Modification	Rank	Result Type

924.5261	924.4982	-0.0279	-30	7	14	LHILTDGR				Mascot
974.4214	974.399	-0.0224	-23	218	225	SGYFDETR				Mascot
1186.6831	1186.6573	-0.0258	-22	175	184	YLVSPPLIER				Mascot
1186.6831	1186.6573	-0.0258	-22	175	184	YLVSPPLIER	46	99.839		Mascot
1306.7954	1306.7146	-0.0808	-62	4	14	IIRLHILTDGR				Mascot
1403.6511	1403.5999	-0.0512	-36	159	170	YAGMLQYDGELK			Oxidation (M)[4]	Mascot
1407.7015	1407.6813	-0.0202	-14	68	80	GWDAQVLGEAPHK				Mascot
1481.7074	1481.6864	-0.021	-14	206	217	FGHVTFFWNGNR				Mascot
1499.7166	1499.6884	-0.0282	-19	140	151	ALEFPDFDKFDR				Mascot
1499.7166	1499.6884	-0.0282	-19	140	151	ALEFPDFDKFDR	82	100		Mascot
1513.725	1513.6738	-0.0512	-34	44	57	IASGGGRMYVTMDR				Mascot
1563.8027	1563.7573	-0.0454	-29	67	80	RGWDAQVLGEAPHK				Mascot
1792.9229	1792.8931	-0.0298	-17	114	130	SVGPIVDGDAVVTFNFR				Mascot
1792.9229	1792.8931	-0.0298	-17	114	130	SVGPIVDGDAVVTFNFR	121	100		Mascot
2064.1377	2064.1187	-0.019	-9	175	192	YLVSPPLIERTSGEYLVK				Mascot
3137.4221	3137.4116	-0.0105	-3	218	244	SGYFDETREEYVEIPSDS GITFNEQPK				Mascot
3137.4221	3137.4116	-0.0105	-3	218	244	SGYFDETREEYVEIPSDS GITFNEQPK	94	100		Mascot

3	Bp2A protein, partial [Triticum aestivum]	gi|133872550	25773.1	5.86	11	401	100	21.174	344	100

Protein Group

Bp2A protein, partial [Triticum aestivum]	gi|133872548	25773.1	5.8600 001335 144
Bp2A protein, partial [Triticum aestivum]	gi|133872546	25773.1	5.8600 001335 144
Bp2A protein, partial [Triticum aestivum]	gi|133872544	25773.1	5.8600 001335 144
Bp2A protein, partial [Triticum aestivum]	gi|133872538	25773.1	5.8600 001335 144
Bp2A protein, partial [Triticum aestivum]	gi|133872534	25773.1	5.8600 001335 144
Bp2A protein, partial [Triticum aestivum]	gi|133872530	25773.1	5.8600 001335 144
Bp2A protein, partial [Triticum aestivum]	gi|133872526	25773.1	5.8600

			001335 144
Bp2A protein, partial [Triticum aestivum]	gi|133872522	25773.1	5.8600 001335 144
Bp2A protein, partial [Triticum aestivum]	gi|133872518	25773.1	5.8600 001335 144
Bp2A protein, partial [Triticum aestivum]	gi|133872500	25773.1	5.8600 001335 144
Bp2A protein, partial [Triticum aestivum]	gi|133872502	25773.1	5.8600 001335 144
Bp2A protein, partial [Triticum aestivum]	gi|133872506	25773.1	5.8600 001335 144
Bp2A protein, partial [Triticum aestivum]	gi|133872508	25773.1	5.8600 001335 144
Bp2A protein, partial [Triticum aestivum]	gi|133872512	25773.1	5.8600 001335 144
Bp2A protein, partial [Triticum aestivum]	gi|133872516	25773.1	5.8600 001335 144
Bp2A protein, partial [Triticum aestivum]	gi|133872520	25773.1	5.8600 001335 144
Bp2A protein, partial [Triticum aestivum]	gi|133872524	25773.1	5.8600 001335 144
Bp2A protein, partial [Triticum aestivum]	gi|133872528	25773.1	5.8600 001335 144
Bp2A protein, partial [Triticum aestivum]	gi|133872480	25773.1	5.8600 001335 144
Bp2A protein, partial [Triticum aestivum]	gi|133872482	25773.1	5.8600 001335 144

Bp2A protein, partial [Triticum aestivum]	gi|133872484	25773.1	5.8600<br>001335<br>144
Bp2A protein, partial [Triticum aestivum]	gi|133872486	25773.1	5.8600<br>001335<br>144
Bp2A protein, partial [Triticum aestivum]	gi|133872488	25773.1	5.8600<br>001335<br>144
Bp2A protein, partial [Triticum aestivum]	gi|133872490	25773.1	5.8600<br>001335<br>144
Bp2A protein, partial [Triticum aestivum]	gi|133872492	25773.1	5.8600<br>001335<br>144
Bp2A protein, partial [Triticum aestivum]	gi|133872494	25773.1	5.8600<br>001335<br>144
Bp2A protein, partial [Triticum aestivum]	gi|133872496	25773.1	5.8600<br>001335<br>144
Bp2A protein, partial [Triticum aestivum]	gi|133872498	25773.1	5.8600<br>001335<br>144
Bp2A protein, partial [Triticum aestivum]	gi|133872552	25773.1	5.8600<br>001335<br>144
Bp2A protein, partial [Triticum aestivum]	gi|133872532	25773.1	5.8600<br>001335<br>144
Bp2A protein, partial [Triticum aestivum]	gi|133872536	25773.1	5.8600<br>001335<br>144
Bp2A protein, partial [Triticum aestivum]	gi|133872540	25773.1	5.8600<br>001335<br>144
Bp2A protein, partial [Triticum dicoccoides]	gi|133872470	25773.1	5.8600<br>001335<br>144
Bp2A protein, partial [Triticum dicoccoides]	gi|133872460	25773.1	5.8600<br>001335<br>144

Bp2A protein, partial [Triticum dicoccoides]	gi|133872458	25773.1	5.8600 001335 144
Bp2A protein, partial [Triticum dicoccoides]	gi|133872462	25773.1	5.8600 001335 144
Bp2A protein, partial [Triticum dicoccoides]	gi|133872468	25773.1	5.8600 001335 144
Bp2A protein, partial [Triticum dicoccoides]	gi|133872464	25773.1	5.8600 001335 144
Bp2A protein, partial [Triticum dicoccoides]	gi|133872466	25773.1	5.8600 001335 144
Bp2A protein, partial [Triticum durum]	gi|133872542	25773.1	5.8600 001335 144
Bp2A protein, partial [Triticum durum]	gi|133872504	25773.1	5.8600 001335 144
Bp2A protein, partial [Triticum durum]	gi|133872510	25773.1	5.8600 001335 144
Bp2A protein, partial [Triticum timopheevii subsp. armeniacum]	gi|133872478	25773.1	5.8600 001335 144
Bp2A protein, partial [Triticum timopheevii subsp. armeniacum]	gi|133872476	25773.1	5.8600 001335 144
Bp2A protein, partial [Triticum timopheevii subsp. armeniacum]	gi|133872472	25773.1	5.8600 001335 144
Bp2A protein, partial [Triticum timopheevii subsp. armeniacum]	gi|133872474	25773.1	5.8600 001335 144
Bp2A protein, partial [Triticum turgidum subsp. dicoccon]	gi|133872514	25773.1	5.8600 001335 144

Peptide Information

Calc. Mass	Obsrv. Mass	± da	± ppm	Start	End	Sequence	Ion	C. I. %	Modification	Rank	Result Type

				Seq.	Seq.		Score			
801.4464	801.4352	-0.0112	-14	201	207	ALEIAER				Mascot
974.4214	974.399	-0.0224	-23	172	179	SGYFDETR				Mascot
1186.6831	1186.6573	-0.0258	-22	129	138	YLVSPPLIER				Mascot
1186.6831	1186.6573	-0.0258	-22	129	138	YLVSPPLIER	46	99.839		Mascot
1403.6511	1403.5999	-0.0512	-36	113	124	YAGMLQYDGELK			Oxidation (M)[4]	Mascot
1407.7015	1407.6813	-0.0202	-14	22	34	GWDAQVLGEAPHK				Mascot
1481.7074	1481.6864	-0.021	-14	160	171	FGHVTFFWNGNR				Mascot
1499.7166	1499.6884	-0.0282	-19	94	105	ALEFPDFDKFDR				Mascot
1499.7166	1499.6884	-0.0282	-19	94	105	ALEFPDFDKFDR	82	100		Mascot
1563.8027	1563.7573	-0.0454	-29	21	34	RGWDAQVLGEAPHK				Mascot
1792.9229	1792.8931	-0.0298	-17	68	84	SVGPIVDGDAVVTFNFR				Mascot
1792.9229	1792.8931	-0.0298	-17	68	84	SVGPIVDGDAVVTFNFR	121	100		Mascot
2064.1377	2064.1187	-0.019	-9	129	146	YLVSPPLIERTSGEYLVK				Mascot
3137.4221	3137.4116	-0.0105	-3	172	198	SGYFDETREEYVEIPSDS GITFNEQPK				Mascot
3137.4221	3137.4116	-0.0105	-3	172	198	SGYFDETREEYVEIPSDS GITFNEQPK	94	100		Mascot

4 Bp2A protein, partial [Triticum dicoccoides] gi|133872456 25773.1 5.86 4 0 344 100

Protein Group

Bp2A protein, partial [Triticum dicoccoides] gi|133872454 25773.1 5.8600 001335 144

Bp2A protein, partial [Triticum dicoccoides] gi|133872452 25773.1 5.8600 001335 144

Bp2A protein, partial [Triticum dicoccoides] gi|133872450 25773.1 5.8600 001335 144

Bp2A protein, partial [Triticum dicoccoides] gi|133872448 25773.1 5.8600 001335 144

Bp2A protein, partial [Triticum dicoccoides] gi|133872446 25773.1 5.8600 001335 144

Bp2A protein, partial [Triticum dicoccoides] gi|133872444 25773.1 5.8600 001335 144

Bp2A protein, partial [Triticum dicoccoides] gi|133872442 25773.1 5.8600

			001335<br>144
Bp2A protein, partial [Triticum dicoccoides]	gi|133872440	25773.1	5.8600<br>001335<br>144
Bp2A protein, partial [Triticum dicoccoides]	gi|133872438	25773.1	5.8600<br>001335<br>144
Bp2A protein, partial [Triticum dicoccoides]	gi|133872382	25773.1	5.8600<br>001335<br>144
Bp2A protein, partial [Triticum dicoccoides]	gi|133872384	25773.1	5.8600<br>001335<br>144
Bp2A protein, partial [Triticum dicoccoides]	gi|133872386	25773.1	5.8600<br>001335<br>144
Bp2A protein, partial [Triticum dicoccoides]	gi|133872388	25773.1	5.8600<br>001335<br>144
Bp2A protein, partial [Triticum dicoccoides]	gi|133872390	25773.1	5.8600<br>001335<br>144
Bp2A protein, partial [Triticum dicoccoides]	gi|133872392	25773.1	5.8600<br>001335<br>144
Bp2A protein, partial [Triticum dicoccoides]	gi|133872394	25773.1	5.8600<br>001335<br>144
Bp2A protein, partial [Triticum dicoccoides]	gi|133872398	25773.1	5.8600<br>001335<br>144
Bp2A protein, partial [Triticum dicoccoides]	gi|133872436	25759	5.8600<br>001335<br>144
Bp2A protein, partial [Triticum durum]	gi|133872434	25773.1	5.8600<br>001335<br>144
Bp2A protein, partial [Triticum durum]	gi|133872432	25773.1	5.8600<br>001335<br>144

Bp2A protein, partial [Triticum durum]	gi|133872430	25773.1	5.8600 001335 144
Bp2A protein, partial [Triticum durum]	gi|133872428	25773.1	5.8600 001335 144
Bp2A protein, partial [Triticum durum]	gi|133872426	25773.1	5.8600 001335 144
Bp2A protein, partial [Triticum durum]	gi|133872424	25773.1	5.8600 001335 144
Bp2A protein, partial [Triticum durum]	gi|133872422	25773.1	5.8600 001335 144
Bp2A protein, partial [Triticum durum]	gi|133872420	25773.1	5.8600 001335 144
Bp2A protein, partial [Triticum durum]	gi|133872418	25773.1	5.8600 001335 144
Bp2A protein, partial [Triticum durum]	gi|133872416	25773.1	5.8600 001335 144
Bp2A protein, partial [Triticum durum]	gi|133872414	25773.1	5.8600 001335 144
Bp2A protein, partial [Triticum durum]	gi|133872412	25773.1	5.8600 001335 144
Bp2A protein, partial [Triticum durum]	gi|133872410	25773.1	5.8600 001335 144
Bp2A protein, partial [Triticum durum]	gi|133872408	25773.1	5.8600 001335 144
Bp2A protein, partial [Triticum durum]	gi|133872406	25773.1	5.8600 001335 144
Bp2A protein, partial [Triticum durum]	gi|133872404	25773.1	5.8600 001335 144

Bp2A protein, partial [Triticum durum]	gi|133872402	25773.1	5.8600 001335 144
Bp2A protein, partial [Triticum timopheevii subsp. armeniacum]	gi|133872400	25773.1	5.8600 001335 144
Bp2A protein, partial [Triticum turgidum subsp. dicoccon]	gi|133872380	25773.1	5.8600 001335 144
Bp2A protein, partial [Triticum turgidum subsp. dicoccon]	gi|133872378	25773.1	5.8600 001335 144
Bp2A protein, partial [Triticum turgidum subsp. dicoccon]	gi|133872376	25773.1	5.8600 001335 144
Bp2A protein, partial [Triticum turgidum subsp. dicoccon]	gi|133872374	25773.1	5.8600 001335 144
Bp2A protein, partial [Triticum turgidum subsp. dicoccon]	gi|133872372	25773.1	5.8600 001335 144
Bp2A protein, partial [Triticum turgidum subsp. dicoccon]	gi|133872370	25773.1	5.8600 001335 144
Bp2A protein, partial [Triticum turgidum subsp. dicoccon]	gi|133872368	25773.1	5.8600 001335 144
Bp2A protein, partial [Triticum turgidum subsp. dicoccon]	gi|133872366	25773.1	5.8600 001335 144
Bp2A protein, partial [Triticum turgidum subsp. dicoccon]	gi|133872364	25773.1	5.8600 001335 144
Bp2A protein, partial [Triticum turgidum subsp. dicoccon]	gi|133872362	25773.1	5.8600 001335 144
Bp2A protein, partial [Triticum turgidum subsp. dicoccon]	gi|133872360	25773.1	5.8600 001335 144

Peptide Information

Calc. Mass	Obsrv. Mass	± da	± ppm	Start	End	Sequence	Ion	C. I. %	Modification	Rank	Result Type

				Seq.	Seq.		Score				
1186.6831	1186.6573	-0.0258	-22	129	138	YLVSPPLIER	46	99.839		1	Mascot
1499.7166	1499.6884	-0.0282	-19	94	105	ALEFPDFDKFDR	82	100		1	Mascot
1792.9229	1792.8931	-0.0298	-17	68	84	SVGPIVDGDAVVTFNFR	121	100		1	Mascot
3137.4221	3137.4116	-0.0105	-3	172	198	SGYFDETREEYVEIPSDS GITFNEQPK	94	100		1	Mascot

5	hypothetical protein TRIUR3_15619 [Triticum urartu]	gi|474057801	20820.6	5.11	1	0	24	74.559

Peptide Information

Calc. Mass	Obsrv. Mass	± da	± ppm	Start Seq.	End Seq.	Sequence	Ion Score	C. I. %	Modification	Rank	Result Type
1068.5698	1068.4869	-0.0829	-78	167	174	RIDSWHVR	24	74.559		1	Mascot

6	Receptor-like serine/threonine-protein kinase SD1-6 [Triticum urartu]	gi|474299006	76920	6.76	1	0	18	0

Peptide Information

Calc. Mass	Obsrv. Mass	± da	± ppm	Start Seq.	End Seq.	Sequence	Ion Score	C. I. %	Modification	Rank	Result Type
1068.5658	1068.4869	-0.0789	-74	453	461	RIHASSNQR	18	0		2	Mascot

7	arginase [Triticum aestivum]	gi|345447290	37052.1	5.62	1	0	16	0

Protein Group

Arginase [Triticum urartu]	gi|474404660	37139.1	5.6199 998855 5908

Peptide Information

Calc. Mass	Obsrv. Mass	± da	± ppm	Start Seq.	End Seq.	Sequence	Ion Score	C. I. %	Modification	Rank	Result Type
1068.5255	1068.4869	-0.0386	-36	206	214	IMEGGYARR	16	0	Oxidation (M)[2]	3	Mascot

8	hypothetical protein TRIUR3_12931 [Triticum urartu]	gi|474345800	85517.4	9.3	1	0	16	0

Peptide Information

Calc. Mass	Obsrv. Mass	± da	± ppm	Start Seq.	End Seq.	Sequence	Ion Score	C. I. %	Modification	Rank	Result Type
1068.587	1068.4869	-0.1001	-94	765	773	ISFLSMAKR	16	0	Oxidation (M)[6]	4	Mascot

9	putative protein phosphatase 2C 39 [Triticum urartu]	gi|474048284	69246.5	5.2	1	0	14	0

Peptide Information

Calc. Mass	Obsrv. Mass	± da	± ppm	Start	End	Sequence	Ion	C. I. %	Modification	Rank	Result Type

				Seq.	Seq.		Score				
1068.5546	1068.4869	-0.0677	-63	520	528	REVESVHGR	14	0		5	Mascot

10	Disease resistance protein RPM1 [Triticum urartu]	gi|473886552	104338.5	7.77	1		0	13	0

Peptide Information

Calc. Mass	Obsrv. Mass	± da	± ppm	Start Seq.	End Seq.	Sequence	Ion Score	C. I. %	Modification	Rank	Result Type
1499.7952	1499.6884	-0.1068	-71	120	132	IAEEVIEIEGEIR	13	0		2	Mascot

Gel Idx/Pos	286/L14	Instr./Gel Origin	BA2151/Sample Project 20140814	Process Status	Analysis Succeeded
Plate [#] Name	[1] Sample Project 20140814	Instrument Sample Name		Spectra	11

Rank	Protein Name	Accession No.	Protein MW	Protein PI	Pep. Count	Protein Score	Protein Score C. I. %	Intensity Matched	Total Ion Score	Total Ion C. I. %	Confirmed
1	2,3-bisphosphoglycerate-independent phosphoglycerate mutase [Triticum urartu]	gi|473886714	57764.3	5.28	21	1,020	100	38.67	920	100	

Peptide Information

Calc. Mass	Obsrv. Mass	± da	± ppm	Start Seq.	End Seq.	Sequence	Ion Score	C. I. %	Modification	Rank	Result Type
801.4464	801.4344	-0.012	-15	374	380	ALEIAER					Mascot
924.5261	924.4944	-0.0317	-34	161	168	LHILTDGR					Mascot
947.3961	947.4342	0.0381	40	205	211	MYVTMDR			Oxidation (M)[1,5]		Mascot
974.4214	974.3896	-0.0318	-33	345	352	SGYFDETR					Mascot
974.4214	974.3896	-0.0318	-33	345	352	SGYFDETR	48	99.898			Mascot
1019.552	1019.5002	-0.0518	-51	235	243	FQNALEAVK					Mascot
1139.5369	1139.5072	-0.0297	-26	212	220	YENDWSVVK					Mascot
1403.6511	1403.5919	-0.0592	-42	313	324	YAGMLQYDGELK			Oxidation (M)[4]		Mascot
1407.7015	1407.6548	-0.0467	-33	222	234	GWDAQVLGEAPHK					Mascot
1413.6686	1413.6185	-0.0501	-35	108	118	IWEDEGFNYIK					Mascot
1481.7074	1481.6821	-0.0253	-17	333	344	FGHVTFFWNGNR					Mascot
1499.7166	1499.6847	-0.0319	-21	294	305	ALEFPDFDKFDR					Mascot
1499.7166	1499.6847	-0.0319	-21	294	305	ALEFPDFDKFDR	103	100			Mascot
1513.725	1513.6747	-0.0503	-33	198	211	IASGGGRMYVTMDR					Mascot
1563.8027	1563.7623	-0.0404	-26	221	234	RGWDAQVLGEAPHK					Mascot
1731.9501	1731.92	-0.0301	-17	124	140	GTLHLIGLLSDGGVHSR					Mascot
1731.9501	1731.92	-0.0301	-17	124	140	GTLHLIGLLSDGGVHSR	124	100			Mascot
1792.9229	1792.894	-0.0289	-16	268	284	SVGPIVDGDAVVTFNFR					Mascot
1792.9229	1792.894	-0.0289	-16	268	284	SVGPIVDGDAVVTFNFR	137	100			Mascot
1891.9436	1891.8815	-0.0621	-33	251	267	ANDQYLPPFVIVDESGK					Mascot
2035.9253	2035.931	0.0057	3	205	220	MYVTMDRYENDWSVVK					Mascot
2377.1882	2377.1641	-0.0241	-10	169	190	DVLDGSSVGFVETIENDL AQLR					Mascot
2377.1882	2377.1641	-0.0241	-10	169	190	DVLDGSSVGFVETIENDL AQLR	191	100			Mascot
2721.2283	2721.1895	-0.0388	-14	63	90	AHGTAVGLPSDDDMGNS EVGHNALGAGR			Oxidation (M)[14]		Mascot
2888.6106	2888.5957	-0.0149	-5	463	491	SGSIQILTSHTLQPVPVAI GGPGLHPGVR					Mascot
2888.6106	2888.5957	-0.0149	-5	463	491	SGSIQILTSHTLQPVPVAI	191	100			Mascot

Calc. Mass	Obsrv. Mass	± da	± ppm	Start Seq.	End Seq.	Sequence	Ion Score	C. I. %	Modification	Rank	Result Type
						GGPGLHPGVR					
3137.4221	3137.4119	-0.0102	-3	345	371	SGYFDETREEYVEIPSDS GITFNEQPK					Mascot
3137.4221	3137.4119	-0.0102	-3	345	371	SGYFDETREEYVEIPSDS GITFNEQPK	125	100			Mascot

2	phosphoglycerate mutase, partial [Triticum aestivum]	gi|32400802	29615.1	5.43	21	825	100	41.97	674	100

Peptide Information

Calc. Mass	Obsrv. Mass	± da	± ppm	Start Seq.	End Seq.	Sequence	Ion Score	C. I. %	Modification	Rank	Result Type
896.4724	896.4312	-0.0412	-46	185	192	TSGEYLVK					Mascot
924.5261	924.4944	-0.0317	-34	7	14	LHILTDGR					Mascot
947.3961	947.4342	0.0381	40	51	57	MYVTMDR			Oxidation (M)[1,5]		Mascot
974.4214	974.3896	-0.0318	-33	218	225	SGYFDETR					Mascot
974.4214	974.3896	-0.0318	-33	218	225	SGYFDETR	48	99.898			Mascot
1019.552	1019.5002	-0.0518	-51	81	89	FQNALEAVK					Mascot
1042.4874	1042.4586	-0.0288	-28	197	205	TFACSETVK			Carbamidomethyl (C)[4]		Mascot
1139.5369	1139.5072	-0.0297	-26	58	66	YENDWSVVK					Mascot
1186.6831	1186.6544	-0.0287	-24	175	184	YLVSPPLIER					Mascot
1186.6831	1186.6544	-0.0287	-24	175	184	YLVSPPLIER	69	100			Mascot
1322.7063	1322.6094	-0.0969	-73	185	196	TSGEYLVKNGVR					Mascot
1403.6511	1403.5919	-0.0592	-42	159	170	YAGMLQYDGELK			Oxidation (M)[4]		Mascot
1407.7015	1407.6548	-0.0467	-33	68	80	GWDAQVLGEAPHK					Mascot
1481.7074	1481.6821	-0.0253	-17	206	217	FGHVTFFWNGNR					Mascot
1499.7166	1499.6847	-0.0319	-21	140	151	ALEFPDFDKFDR					Mascot
1499.7166	1499.6847	-0.0319	-21	140	151	ALEFPDFDKFDR	103	100			Mascot
1513.725	1513.6747	-0.0503	-33	44	57	IASGGGRMYVTMDR					Mascot
1563.8027	1563.7623	-0.0404	-26	67	80	RGWDAQVLGEAPHK					Mascot
1611.9469	1611.788	-0.1589	-99	171	184	LPSKYLVSPPLIER					Mascot
1792.9229	1792.894	-0.0289	-16	114	130	SVGPIVDGDAVVTFNFR					Mascot
1792.9229	1792.894	-0.0289	-16	114	130	SVGPIVDGDAVVTFNFR	137	100			Mascot
1891.9436	1891.8815	-0.0621	-33	97	113	ANDQYLPPFVIVDESGK					Mascot
2035.9253	2035.931	0.0057	3	51	66	MYVTMDRYENDWSVVK					Mascot
2377.1882	2377.1641	-0.0241	-10	15	36	DVLDGSSVGFVETIENDL AQLR					Mascot
2377.1882	2377.1641	-0.0241	-10	15	36	DVLDGSSVGFVETIENDL AQLR	191	100			Mascot
3137.4221	3137.4119	-0.0102	-3	218	244	SGYFDETREEYVEIPSDS GITFNEQPK					Mascot
3137.4221	3137.4119	-0.0102	-3	218	244	SGYFDETREEYVEIPSDS GITFNEQPK	125	100			Mascot

3	Bp2A protein, partial [Triticum aestivum]	gi|133872550	25773.1	5.86	19	611	100	38.412	483	100

Protein Group

Bp2A protein, partial [Triticum aestivum]	gi|133872548	25773.1	5.8600<br>001335<br>144
Bp2A protein, partial [Triticum aestivum]	gi|133872546	25773.1	5.8600<br>001335<br>144
Bp2A protein, partial [Triticum aestivum]	gi|133872544	25773.1	5.8600<br>001335<br>144
Bp2A protein, partial [Triticum aestivum]	gi|133872538	25773.1	5.8600<br>001335<br>144
Bp2A protein, partial [Triticum aestivum]	gi|133872534	25773.1	5.8600<br>001335<br>144
Bp2A protein, partial [Triticum aestivum]	gi|133872530	25773.1	5.8600<br>001335<br>144
Bp2A protein, partial [Triticum aestivum]	gi|133872526	25773.1	5.8600<br>001335<br>144
Bp2A protein, partial [Triticum aestivum]	gi|133872522	25773.1	5.8600<br>001335<br>144
Bp2A protein, partial [Triticum aestivum]	gi|133872518	25773.1	5.8600<br>001335<br>144
Bp2A protein, partial [Triticum aestivum]	gi|133872500	25773.1	5.8600<br>001335<br>144
Bp2A protein, partial [Triticum aestivum]	gi|133872502	25773.1	5.8600<br>001335<br>144
Bp2A protein, partial [Triticum aestivum]	gi|133872506	25773.1	5.8600<br>001335<br>144
Bp2A protein, partial [Triticum aestivum]	gi|133872508	25773.1	5.8600<br>001335<br>144
Bp2A protein, partial [Triticum aestivum]	gi|133872512	25773.1	5.8600

			001335<br>144
Bp2A protein, partial [Triticum aestivum]	gi|133872516	25773.1	5.8600<br>001335<br>144
Bp2A protein, partial [Triticum aestivum]	gi|133872520	25773.1	5.8600<br>001335<br>144
Bp2A protein, partial [Triticum aestivum]	gi|133872524	25773.1	5.8600<br>001335<br>144
Bp2A protein, partial [Triticum aestivum]	gi|133872528	25773.1	5.8600<br>001335<br>144
Bp2A protein, partial [Triticum aestivum]	gi|133872480	25773.1	5.8600<br>001335<br>144
Bp2A protein, partial [Triticum aestivum]	gi|133872482	25773.1	5.8600<br>001335<br>144
Bp2A protein, partial [Triticum aestivum]	gi|133872484	25773.1	5.8600<br>001335<br>144
Bp2A protein, partial [Triticum aestivum]	gi|133872486	25773.1	5.8600<br>001335<br>144
Bp2A protein, partial [Triticum aestivum]	gi|133872488	25773.1	5.8600<br>001335<br>144
Bp2A protein, partial [Triticum aestivum]	gi|133872490	25773.1	5.8600<br>001335<br>144
Bp2A protein, partial [Triticum aestivum]	gi|133872492	25773.1	5.8600<br>001335<br>144
Bp2A protein, partial [Triticum aestivum]	gi|133872494	25773.1	5.8600<br>001335<br>144
Bp2A protein, partial [Triticum aestivum]	gi|133872496	25773.1	5.8600<br>001335<br>144

Bp2A protein, partial [Triticum aestivum]	gi|133872498	25773.1	5.8600 001335 144
Bp2A protein, partial [Triticum aestivum]	gi|133872552	25773.1	5.8600 001335 144
Bp2A protein, partial [Triticum aestivum]	gi|133872532	25773.1	5.8600 001335 144
Bp2A protein, partial [Triticum aestivum]	gi|133872536	25773.1	5.8600 001335 144
Bp2A protein, partial [Triticum aestivum]	gi|133872540	25773.1	5.8600 001335 144
Bp2A protein, partial [Triticum dicoccoides]	gi|133872470	25773.1	5.8600 001335 144
Bp2A protein, partial [Triticum dicoccoides]	gi|133872460	25773.1	5.8600 001335 144
Bp2A protein, partial [Triticum dicoccoides]	gi|133872458	25773.1	5.8600 001335 144
Bp2A protein, partial [Triticum dicoccoides]	gi|133872462	25773.1	5.8600 001335 144
Bp2A protein, partial [Triticum dicoccoides]	gi|133872468	25773.1	5.8600 001335 144
Bp2A protein, partial [Triticum dicoccoides]	gi|133872464	25773.1	5.8600 001335 144
Bp2A protein, partial [Triticum dicoccoides]	gi|133872466	25773.1	5.8600 001335 144
Bp2A protein, partial [Triticum durum]	gi|133872542	25773.1	5.8600 001335 144
Bp2A protein, partial [Triticum durum]	gi|133872504	25773.1	5.8600 001335 144

Bp2A protein, partial [Triticum durum]	gi|133872510	25773.1	5.8600 001335 144
Bp2A protein, partial [Triticum timopheevii subsp. armeniacum]	gi|133872478	25773.1	5.8600 001335 144
Bp2A protein, partial [Triticum timopheevii subsp. armeniacum]	gi|133872476	25773.1	5.8600 001335 144
Bp2A protein, partial [Triticum timopheevii subsp. armeniacum]	gi|133872472	25773.1	5.8600 001335 144
Bp2A protein, partial [Triticum timopheevii subsp. armeniacum]	gi|133872474	25773.1	5.8600 001335 144
Bp2A protein, partial [Triticum turgidum subsp. dicoccon]	gi|133872514	25773.1	5.8600 001335 144

Peptide Information

Calc. Mass	Obsrv. Mass	± da	± ppm	Start Seq.	End Seq.	Sequence	Ion Score	C. I. %	Modification	Rank	Result Type
801.4464	801.4344	-0.012	-15	201	207	ALEIAER					Mascot
896.4724	896.4312	-0.0412	-46	139	146	TSGEYLVK					Mascot
947.3961	947.4342	0.0381	40	5	11	MYVTMDR			Oxidation (M)[1,5]		Mascot
974.4214	974.3896	-0.0318	-33	172	179	SGYFDETR					Mascot
974.4214	974.3896	-0.0318	-33	172	179	SGYFDETR	48	99.898			Mascot
1019.552	1019.5002	-0.0518	-51	35	43	FQNALEAVK					Mascot
1042.4874	1042.4586	-0.0288	-28	151	159	TFACSETVK			Carbamidomethyl (C)[4]		Mascot
1139.5369	1139.5072	-0.0297	-26	12	20	YENDWSVVK					Mascot
1186.6831	1186.6544	-0.0287	-24	129	138	YLVSPPLIER					Mascot
1186.6831	1186.6544	-0.0287	-24	129	138	YLVSPPLIER	69	100			Mascot
1322.7063	1322.6094	-0.0969	-73	139	150	TSGEYLVKNGVR					Mascot
1403.6511	1403.5919	-0.0592	-42	113	124	YAGMLQYDGELK			Oxidation (M)[4]		Mascot
1407.7015	1407.6548	-0.0467	-33	22	34	GWDAQVLGEAPHK					Mascot
1481.7074	1481.6821	-0.0253	-17	160	171	FGHVTFFWNGNR					Mascot
1499.7166	1499.6847	-0.0319	-21	94	105	ALEFPDFDKFDR					Mascot
1499.7166	1499.6847	-0.0319	-21	94	105	ALEFPDFDKFDR	103	100			Mascot
1563.8027	1563.7623	-0.0404	-26	21	34	RGWDAQVLGEAPHK					Mascot

1611.9469	1611.788	-0.1589	-99	125	138	LPSKYLVSPPLIER					Mascot
1792.9229	1792.894	-0.0289	-16	68	84	SVGPIVDGDAVVTFNFR					Mascot
1792.9229	1792.894	-0.0289	-16	68	84	SVGPIVDGDAVVTFNFR	137	100			Mascot
1891.9436	1891.8815	-0.0621	-33	51	67	ANDQYLPPFVIVDESGK					Mascot
2035.9253	2035.931	0.0057	3	5	20	MYVTMDRYENDWSVVK					Mascot
3137.4221	3137.4119	-0.0102	-3	172	198	SGYFDETREEYVEIPSDS GITFNEQPK					Mascot
3137.4221	3137.4119	-0.0102	-3	172	198	SGYFDETREEYVEIPSDS GITFNEQPK	125	100			Mascot

4	Disease resistance protein RPM1 [Triticum urartu]	gi|473886552	104338.5	7.77	1	0	14	0

Peptide Information

Calc. Mass	Obsrv. Mass	± da	± ppm	Start Seq.	End Seq.	Sequence	Ion Score	C. I. %	Modification	Rank	Result Type
1499.7952	1499.6847	-0.1105	-74	120	132	IAEEVIEIEGEIR	14	0		2	Mascot

5	hypothetical protein TRIUR3_13456 [Triticum urartu]	gi|474040344	98262.6	5.43	1	0	13	0

Peptide Information

Calc. Mass	Obsrv. Mass	± da	± ppm	Start Seq.	End Seq.	Sequence	Ion Score	C. I. %	Modification	Rank	Result Type
1499.6649	1499.6847	0.0198	13	133	145	TDNFDPDAYVQSK	13	0		3	Mascot

6	hypothetical protein TRIUR3_19735 [Triticum urartu]	gi|473747916	25933.2	5.59	1	0	11	0

Peptide Information

Calc. Mass	Obsrv. Mass	± da	± ppm	Start Seq.	End Seq.	Sequence	Ion Score	C. I. %	Modification	Rank	Result Type
1186.6289	1186.6544	0.0255	21	29	37	YIMTKLFDR	11	0		2	Mascot

7	hypothetical protein TRIUR3_15279 [Triticum urartu]	gi|474019004	74664.7	6.39	1	0	11	0

Peptide Information

Calc. Mass	Obsrv. Mass	± da	± ppm	Start Seq.	End Seq.	Sequence	Ion Score	C. I. %	Modification	Rank	Result Type
1186.574	1186.6544	0.0804	68	284	293	SSVSFFDIER	11	0		3	Mascot

8	Sucrose synthase 2 [Triticum urartu]	gi|474169938	114504	6.95	1	0	10	0

Peptide Information

Calc. Mass	Obsrv. Mass	± da	± ppm	Start Seq.	End Seq.	Sequence	Ion Score	C. I. %	Modification	Rank	Result Type
1186.5415	1186.6544	0.1129	95	626	634	YPDSDIYWK	10	0		4	Mascot

9	putative beta-1,3-galactosyltransferase 18 [Triticum urartu]	gi|474224937	52008.8	6.65	1	0	10	0

Peptide Information

Calc. Mass	Obsrv. Mass	± da	± ppm	Start Seq.	End Seq.	Sequence	Ion Score	C. I. %	Modification	Rank	Result Type
974.4222	974.3896	-0.0326	-33	222	228	SWMMYTR	10	0		2	Mascot

10	Helicase domino [Triticum urartu]	gi|473885833	232239.9	5.03	2	0	9	0

Peptide Information

Calc. Mass	Obsrv. Mass	± da	± ppm	Start Seq.	End Seq.	Sequence	Ion Score	C. I. %	Modification	Rank	Result Type
1499.7965	1499.6847	-0.1118	-75	1844	1855	HLDLVLDFRTDR	8	0		6	Mascot
1731.9752	1731.92	-0.0552	-32	1879	1894	TVVPVEQSLLSGLPHR	2	0		10	Mascot

Gel Idx/Pos	287/L15	Instr./Gel Origin	BA2151/Sample Project 20140814	Process Status	Analysis Succeeded
Plate [#] Name	[1] Sample Project 20140814	Instrument Sample Name		Spectra	11

Rank	Protein Name	Accession No.	Protein MW	Protein PI	Pep. Count	Protein Score	Protein Score C. I. %	Intensity Matched	Total Ion Score	Total Ion C. I. %	Confirmed
1	2,3-bisphosphoglycerate-independent phosphoglycerate mutase [Triticum urartu]	gi|473886714	57764.3	5.28	18	744	100	38.98	664	100	

Peptide Information

Calc. Mass	Obsrv. Mass	± da	± ppm	Start Seq.	End Seq.	Sequence	Ion Score	C. I. %	Modification	Rank	Result Type
801.4464	801.4226	-0.0238	-30	374	380	ALEIAER					Mascot
924.5261	924.5121	-0.014	-15	161	168	LHILTDGR					Mascot
947.3961	947.4465	0.0504	53	205	211	MYVTMDR			Oxidation (M)[1,5]		Mascot
974.4214	974.4093	-0.0121	-12	345	352	SGYFDETR					Mascot
974.4214	974.4093	-0.0121	-12	345	352	SGYFDETR	27	85.01			Mascot
1139.5369	1139.5653	0.0284	25	212	220	YENDWSVVK					Mascot
1403.6511	1403.6001	-0.051	-36	313	324	YAGMLQYDGELK			Oxidation (M)[4]		Mascot
1413.6686	1413.6373	-0.0313	-22	108	118	IWEDEGFNYIK					Mascot
1481.7074	1481.6935	-0.0139	-9	333	344	FGHVTFFWNGNR					Mascot
1499.7166	1499.7074	-0.0092	-6	294	305	ALEFPDFDKFDR					Mascot
1499.7166	1499.7074	-0.0092	-6	294	305	ALEFPDFDKFDR	100	100			Mascot
1513.725	1513.6871	-0.0379	-25	198	211	IASGGGRMYVTMDR					Mascot
1731.9501	1731.9423	-0.0078	-5	124	140	GTLHLIGLLSDGGVHSR					Mascot
1731.9501	1731.9423	-0.0078	-5	124	140	GTLHLIGLLSDGGVHSR	126	100			Mascot
1792.9229	1792.9136	-0.0093	-5	268	284	SVGPIVDGDAVVTFNFR					Mascot
1792.9229	1792.9136	-0.0093	-5	268	284	SVGPIVDGDAVVTFNFR	136	100			Mascot
1891.9436	1891.907	-0.0366	-19	251	267	ANDQYLPPFVIVDESGK					Mascot
2035.9253	2035.9226	-0.0027	-1	205	220	MYVTMDRYENDWSVVK					Mascot
2377.1882	2377.1819	-0.0063	-3	169	190	DVLDGSSVGFVETIENDL AQLR					Mascot
2721.2283	2721.22	-0.0083	-3	63	90	AHGTAVGLPSDDDMGNS EVGHNALGAGR			Oxidation (M)[14]		Mascot
2888.6106	2888.6157	0.0051	2	463	491	SGSIQILTSHTLQPVPVAI GGPGLHPGVR					Mascot
2888.6106	2888.6157	0.0051	2	463	491	SGSIQILTSHTLQPVPVAI GGPGLHPGVR	169	100			Mascot
3137.4221	3137.4365	0.0144	5	345	371	SGYFDETREEYVEIPSDS GITFNEQPK					Mascot
3137.4221	3137.4365	0.0144	5	345	371	SGYFDETREEYVEIPSDS GITFNEQPK	106	100			Mascot

Rank	Protein Name	Accession No.	Protein MW	Protein PI	Pep. Count	Protein Score	Protein Score C. I. %	Intensity Matched	Total Ion Score	Total Ion C. I. %	Confirmed
2	phosphoglycerate mutase, partial [Triticum aestivum]	gi|32400802	29615.1	5.43	16	535	100	43.009	437	100	

Peptide Information

Calc. Mass	Obsrv. Mass	± da	± ppm	Start Seq.	End Seq.	Sequence	Ion Score	C. I. %	Modification	Rank	Result Type
896.4724	896.4455	-0.0269	-30	185	192	TSGEYLVK					Mascot
924.5261	924.5121	-0.014	-15	7	14	LHILTDGR					Mascot
947.3961	947.4465	0.0504	53	51	57	MYVTMDR			Oxidation (M)[1,5]		Mascot
974.4214	974.4093	-0.0121	-12	218	225	SGYFDETR					Mascot
974.4214	974.4093	-0.0121	-12	218	225	SGYFDETR	27	85.01			Mascot
1042.4874	1042.4751	-0.0123	-12	197	205	TFACSETVK			Carbamidomethyl (C)[4]		Mascot
1139.5369	1139.5653	0.0284	25	58	66	YENDWSVVK					Mascot
1186.6831	1186.6746	-0.0085	-7	175	184	YLVSPPLIER					Mascot
1186.6831	1186.6746	-0.0085	-7	175	184	YLVSPPLIER	68	99.999			Mascot
1403.6511	1403.6001	-0.051	-36	159	170	YAGMLQYDGELK			Oxidation (M)[4]		Mascot
1481.7074	1481.6935	-0.0139	-9	206	217	FGHVTFFWNGNR					Mascot
1499.7166	1499.7074	-0.0092	-6	140	151	ALEFPDFDKFDR					Mascot
1499.7166	1499.7074	-0.0092	-6	140	151	ALEFPDFDKFDR	100	100			Mascot
1513.725	1513.6871	-0.0379	-25	44	57	IASGGGRMYVTMDR					Mascot
1792.9229	1792.9136	-0.0093	-5	114	130	SVGPIVDGDAVVTFNFR					Mascot
1792.9229	1792.9136	-0.0093	-5	114	130	SVGPIVDGDAVVTFNFR	136	100			Mascot
1891.9436	1891.907	-0.0366	-19	97	113	ANDQYLPPFVIVDESGK					Mascot
2035.9253	2035.9226	-0.0027	-1	51	66	MYVTMDRYENDWSVVK					Mascot
2377.1882	2377.1819	-0.0063	-3	15	36	DVLDGSSVGFVETIENDL AQLR					Mascot
3137.4221	3137.4365	0.0144	5	218	244	SGYFDETREEYVEIPSDS GITFNEQPK					Mascot
3137.4221	3137.4365	0.0144	5	218	244	SGYFDETREEYVEIPSDS GITFNEQPK	106	100			Mascot

3	Bp2A protein, partial [Triticum aestivum]	gi|133872550	25773.1	5.86	14	516	100	41.841	437	100	

Protein Group

Bp2A protein, partial [Triticum aestivum]	gi|133872548	25773.1	5.8600 001335 144
Bp2A protein, partial [Triticum aestivum]	gi|133872546	25773.1	5.8600 001335 144
Bp2A protein, partial [Triticum aestivum]	gi|133872544	25773.1	5.8600 001335 144
Bp2A protein, partial [Triticum aestivum]	gi|133872538	25773.1	5.8600

			001335 144
Bp2A protein, partial [Triticum aestivum]	gi|133872534	25773.1	5.8600 001335 144
Bp2A protein, partial [Triticum aestivum]	gi|133872530	25773.1	5.8600 001335 144
Bp2A protein, partial [Triticum aestivum]	gi|133872526	25773.1	5.8600 001335 144
Bp2A protein, partial [Triticum aestivum]	gi|133872522	25773.1	5.8600 001335 144
Bp2A protein, partial [Triticum aestivum]	gi|133872518	25773.1	5.8600 001335 144
Bp2A protein, partial [Triticum aestivum]	gi|133872500	25773.1	5.8600 001335 144
Bp2A protein, partial [Triticum aestivum]	gi|133872502	25773.1	5.8600 001335 144
Bp2A protein, partial [Triticum aestivum]	gi|133872506	25773.1	5.8600 001335 144
Bp2A protein, partial [Triticum aestivum]	gi|133872508	25773.1	5.8600 001335 144
Bp2A protein, partial [Triticum aestivum]	gi|133872512	25773.1	5.8600 001335 144
Bp2A protein, partial [Triticum aestivum]	gi|133872516	25773.1	5.8600 001335 144
Bp2A protein, partial [Triticum aestivum]	gi|133872520	25773.1	5.8600 001335 144
Bp2A protein, partial [Triticum aestivum]	gi|133872524	25773.1	5.8600 001335 144

Bp2A protein, partial [Triticum aestivum]	gi|133872528	25773.1	5.8600<br>001335<br>144
Bp2A protein, partial [Triticum aestivum]	gi|133872480	25773.1	5.8600<br>001335<br>144
Bp2A protein, partial [Triticum aestivum]	gi|133872482	25773.1	5.8600<br>001335<br>144
Bp2A protein, partial [Triticum aestivum]	gi|133872484	25773.1	5.8600<br>001335<br>144
Bp2A protein, partial [Triticum aestivum]	gi|133872486	25773.1	5.8600<br>001335<br>144
Bp2A protein, partial [Triticum aestivum]	gi|133872488	25773.1	5.8600<br>001335<br>144
Bp2A protein, partial [Triticum aestivum]	gi|133872490	25773.1	5.8600<br>001335<br>144
Bp2A protein, partial [Triticum aestivum]	gi|133872492	25773.1	5.8600<br>001335<br>144
Bp2A protein, partial [Triticum aestivum]	gi|133872494	25773.1	5.8600<br>001335<br>144
Bp2A protein, partial [Triticum aestivum]	gi|133872496	25773.1	5.8600<br>001335<br>144
Bp2A protein, partial [Triticum aestivum]	gi|133872498	25773.1	5.8600<br>001335<br>144
Bp2A protein, partial [Triticum aestivum]	gi|133872552	25773.1	5.8600<br>001335<br>144
Bp2A protein, partial [Triticum aestivum]	gi|133872532	25773.1	5.8600<br>001335<br>144
Bp2A protein, partial [Triticum aestivum]	gi|133872536	25773.1	5.8600<br>001335<br>144

Bp2A protein, partial [Triticum aestivum]	gi|133872540	25773.1	5.8600<br>001335<br>144
Bp2A protein, partial [Triticum dicoccoides]	gi|133872470	25773.1	5.8600<br>001335<br>144
Bp2A protein, partial [Triticum dicoccoides]	gi|133872460	25773.1	5.8600<br>001335<br>144
Bp2A protein, partial [Triticum dicoccoides]	gi|133872458	25773.1	5.8600<br>001335<br>144
Bp2A protein, partial [Triticum dicoccoides]	gi|133872462	25773.1	5.8600<br>001335<br>144
Bp2A protein, partial [Triticum dicoccoides]	gi|133872468	25773.1	5.8600<br>001335<br>144
Bp2A protein, partial [Triticum dicoccoides]	gi|133872464	25773.1	5.8600<br>001335<br>144
Bp2A protein, partial [Triticum dicoccoides]	gi|133872466	25773.1	5.8600<br>001335<br>144
Bp2A protein, partial [Triticum durum]	gi|133872542	25773.1	5.8600<br>001335<br>144
Bp2A protein, partial [Triticum durum]	gi|133872504	25773.1	5.8600<br>001335<br>144
Bp2A protein, partial [Triticum durum]	gi|133872510	25773.1	5.8600<br>001335<br>144
Bp2A protein, partial [Triticum timopheevii subsp. armeniacum]	gi|133872478	25773.1	5.8600<br>001335<br>144
Bp2A protein, partial [Triticum timopheevii subsp. armeniacum]	gi|133872476	25773.1	5.8600<br>001335<br>144
Bp2A protein, partial [Triticum timopheevii subsp. armeniacum]	gi|133872472	25773.1	5.8600<br>001335

			144
Bp2A protein, partial [Triticum timopheevii subsp. armeniacum]	gi|133872474	25773.1	5.8600 001335 144
Bp2A protein, partial [Triticum turgidum subsp. dicoccon]	gi|133872514	25773.1	5.8600 001335 144

Peptide Information

Calc. Mass	Obsrv. Mass	± da	± ppm	Start Seq.	End Seq.	Sequence	Ion Score	C. I. %	Modification	Rank	Result Type
801.4464	801.4226	-0.0238	-30	201	207	ALEIAER					Mascot
896.4724	896.4455	-0.0269	-30	139	146	TSGEYLVK					Mascot
947.3961	947.4465	0.0504	53	5	11	MYVTMDR			Oxidation (M)[1,5]		Mascot
974.4214	974.4093	-0.0121	-12	172	179	SGYFDETR					Mascot
974.4214	974.4093	-0.0121	-12	172	179	SGYFDETR	27	85.01			Mascot
1042.4874	1042.4751	-0.0123	-12	151	159	TFACSETVK			Carbamidomethyl (C)[4]		Mascot
1139.5369	1139.5653	0.0284	25	12	20	YENDWSVVK					Mascot
1186.6831	1186.6746	-0.0085	-7	129	138	YLVSPPLIER					Mascot
1186.6831	1186.6746	-0.0085	-7	129	138	YLVSPPLIER	68	99.999			Mascot
1403.6511	1403.6001	-0.051	-36	113	124	YAGMLQYDGELK			Oxidation (M)[4]		Mascot
1481.7074	1481.6935	-0.0139	-9	160	171	FGHVTFFWNGNR					Mascot
1499.7166	1499.7074	-0.0092	-6	94	105	ALEFPDFDKFDR					Mascot
1499.7166	1499.7074	-0.0092	-6	94	105	ALEFPDFDKFDR	100	100			Mascot
1792.9229	1792.9136	-0.0093	-5	68	84	SVGPIVDGDAVVTFNFR					Mascot
1792.9229	1792.9136	-0.0093	-5	68	84	SVGPIVDGDAVVTFNFR	136	100			Mascot
1891.9436	1891.907	-0.0366	-19	51	67	ANDQYLPPFVIVDESGK					Mascot
2035.9253	2035.9226	-0.0027	-1	5	20	MYVTMDRYENDWSVVK					Mascot
3137.4221	3137.4365	0.0144	5	172	198	SGYFDETREEYVEIPSDS GITFNEQPK					Mascot
3137.4221	3137.4365	0.0144	5	172	198	SGYFDETREEYVEIPSDS GITFNEQPK	106	100			Mascot

4	Bp2A protein, partial [Triticum dicoccoides]	gi|133872456	25773.1	5.86	5	0	437	100	

Protein Group

Bp2A protein, partial [Triticum dicoccoides]	gi|133872454	25773.1	5.8600 001335 144
Bp2A protein, partial [Triticum dicoccoides]	gi|133872452	25773.1	5.8600 001335 144

Bp2A protein, partial [Triticum dicoccoides]	gi|133872450	25773.1	5.8600 001335 144
Bp2A protein, partial [Triticum dicoccoides]	gi|133872448	25773.1	5.8600 001335 144
Bp2A protein, partial [Triticum dicoccoides]	gi|133872446	25773.1	5.8600 001335 144
Bp2A protein, partial [Triticum dicoccoides]	gi|133872444	25773.1	5.8600 001335 144
Bp2A protein, partial [Triticum dicoccoides]	gi|133872442	25773.1	5.8600 001335 144
Bp2A protein, partial [Triticum dicoccoides]	gi|133872440	25773.1	5.8600 001335 144
Bp2A protein, partial [Triticum dicoccoides]	gi|133872438	25773.1	5.8600 001335 144
Bp2A protein, partial [Triticum dicoccoides]	gi|133872382	25773.1	5.8600 001335 144
Bp2A protein, partial [Triticum dicoccoides]	gi|133872384	25773.1	5.8600 001335 144
Bp2A protein, partial [Triticum dicoccoides]	gi|133872386	25773.1	5.8600 001335 144
Bp2A protein, partial [Triticum dicoccoides]	gi|133872388	25773.1	5.8600 001335 144
Bp2A protein, partial [Triticum dicoccoides]	gi|133872390	25773.1	5.8600 001335 144
Bp2A protein, partial [Triticum dicoccoides]	gi|133872392	25773.1	5.8600 001335 144
Bp2A protein, partial [Triticum dicoccoides]	gi|133872394	25773.1	5.8600 001335

			144
Bp2A protein, partial [Triticum dicoccoides]	gi|133872398	25773.1	5.8600 001335 144
Bp2A protein, partial [Triticum dicoccoides]	gi|133872436	25759	5.8600 001335 144
Bp2A protein, partial [Triticum durum]	gi|133872434	25773.1	5.8600 001335 144
Bp2A protein, partial [Triticum durum]	gi|133872432	25773.1	5.8600 001335 144
Bp2A protein, partial [Triticum durum]	gi|133872430	25773.1	5.8600 001335 144
Bp2A protein, partial [Triticum durum]	gi|133872428	25773.1	5.8600 001335 144
Bp2A protein, partial [Triticum durum]	gi|133872426	25773.1	5.8600 001335 144
Bp2A protein, partial [Triticum durum]	gi|133872424	25773.1	5.8600 001335 144
Bp2A protein, partial [Triticum durum]	gi|133872422	25773.1	5.8600 001335 144
Bp2A protein, partial [Triticum durum]	gi|133872420	25773.1	5.8600 001335 144
Bp2A protein, partial [Triticum durum]	gi|133872418	25773.1	5.8600 001335 144
Bp2A protein, partial [Triticum durum]	gi|133872416	25773.1	5.8600 001335 144
Bp2A protein, partial [Triticum durum]	gi|133872414	25773.1	5.8600 001335 144
Bp2A protein, partial [Triticum durum]	gi|133872412	25773.1	5.8600

			001335 144
Bp2A protein, partial [Triticum durum]	gi|133872410	25773.1	5.8600 001335 144
Bp2A protein, partial [Triticum durum]	gi|133872408	25773.1	5.8600 001335 144
Bp2A protein, partial [Triticum durum]	gi|133872406	25773.1	5.8600 001335 144
Bp2A protein, partial [Triticum durum]	gi|133872404	25773.1	5.8600 001335 144
Bp2A protein, partial [Triticum durum]	gi|133872402	25773.1	5.8600 001335 144
Bp2A protein, partial [Triticum timopheevii subsp. armeniacum]	gi|133872400	25773.1	5.8600 001335 144
Bp2A protein, partial [Triticum turgidum subsp. dicoccon]	gi|133872380	25773.1	5.8600 001335 144
Bp2A protein, partial [Triticum turgidum subsp. dicoccon]	gi|133872378	25773.1	5.8600 001335 144
Bp2A protein, partial [Triticum turgidum subsp. dicoccon]	gi|133872376	25773.1	5.8600 001335 144
Bp2A protein, partial [Triticum turgidum subsp. dicoccon]	gi|133872374	25773.1	5.8600 001335 144
Bp2A protein, partial [Triticum turgidum subsp. dicoccon]	gi|133872372	25773.1	5.8600 001335 144
Bp2A protein, partial [Triticum turgidum subsp. dicoccon]	gi|133872370	25773.1	5.8600 001335 144
Bp2A protein, partial [Triticum turgidum subsp. dicoccon]	gi|133872368	25773.1	5.8600 001335 144

Bp2A protein, partial [Triticum turgidum subsp. dicoccon]	gi|133872366	25773.1	5.8600 001335 144
Bp2A protein, partial [Triticum turgidum subsp. dicoccon]	gi|133872364	25773.1	5.8600 001335 144
Bp2A protein, partial [Triticum turgidum subsp. dicoccon]	gi|133872362	25773.1	5.8600 001335 144
Bp2A protein, partial [Triticum turgidum subsp. dicoccon]	gi|133872360	25773.1	5.8600 001335 144

Peptide Information

Calc. Mass	Obsrv. Mass	± da	± ppm	Start Seq.	End Seq.	Sequence	Ion Score	C. I. %	Modification	Rank	Result Type
974.4214	974.4093	-0.0121	-12	172	179	SGYFDETR	27	85.01		1	Mascot
1186.6831	1186.6746	-0.0085	-7	129	138	YLVSPPLIER	68	99.999		1	Mascot
1499.7166	1499.7074	-0.0092	-6	94	105	ALEFPDFDKFDR	100	100		1	Mascot
1792.9229	1792.9136	-0.0093	-5	68	84	SVGPIVDGDAVVTFNFR	136	100		1	Mascot
3137.4221	3137.4365	0.0144	5	172	198	SGYFDETREEYVEIPSDS GITFNEQPK	106	100		1	Mascot

5	zeta-carotene desaturase [Triticum aestivum]	gi|336185127	63071.2	6.81	1		0	14	0

Protein Group

zeta-carotene desaturase [Triticum aestivum]	gi|336185125	63061.2	7.0900 001525 8789
zeta-carotene desaturase [Triticum aestivum]	gi|336185123	63061.2	7.0900 001525 8789
zeta-carotene desaturase [Triticum aestivum]	gi|205371883	63071.2	6.8099 999427 7954
zeta-carotene desaturase [Triticum aestivum]	gi|336185129	63071.2	6.8099 999427 7954
zeta-carotene desaturase enzyme [Triticum aestivum]	gi|231274761	63071.2	6.8099 999427 7954

Peptide Information

Calc. Mass	Obsrv. Mass	± da	± ppm	Start	End	Sequence	Ion	C. I. %	Modification	Rank	Result Type

				Seq.	Seq.		Score				
1621.8401	1621.7865	-0.0536	-33	1	16	MAATSCALVSALVVGR	14	0	Carbamidomethyl (C)[6], Oxidation (M)[1]	1	Mascot

6	Disease resistance protein RPM1 [Triticum urartu]	gi|473886552	104338.5	7.77	1	0	13	0	

Peptide Information

Calc. Mass	Obsrv. Mass	± da	± ppm	Start Seq.	End Seq.	Sequence	Ion Score	C. I. %	Modification	Rank	Result Type
1499.7952	1499.7074	-0.0878	-59	120	132	IAEEVIEIEGEIR	13	0		2	Mascot

7	Helicase domino [Triticum urartu]	gi|473885833	232239.9	5.03	2	0	12	0

Peptide Information

Calc. Mass	Obsrv. Mass	± da	± ppm	Start Seq.	End Seq.	Sequence	Ion Score	C. I. %	Modification	Rank	Result Type
1499.7965	1499.7074	-0.0891	-59	1844	1855	HLDLVLDFRTDR	10	0		6	Mascot
1731.9752	1731.9423	-0.0329	-19	1879	1894	TVVPVEQSLLSGLPHR	2	0		10	Mascot

8	hypothetical protein TRIUR3_13456 [Triticum urartu]	gi|474040344	98262.6	5.43	1	0	12	0

Peptide Information

Calc. Mass	Obsrv. Mass	± da	± ppm	Start Seq.	End Seq.	Sequence	Ion Score	C. I. %	Modification	Rank	Result Type
1499.6649	1499.7074	0.0425	28	133	145	TDNFDPDAYVQSK	12	0		3	Mascot

9	putative protein phosphatase 2C 12 [Triticum urartu]	gi|474236789	58903.4	5.34	1	0	12	0

Peptide Information

Calc. Mass	Obsrv. Mass	± da	± ppm	Start Seq.	End Seq.	Sequence	Ion Score	C. I. %	Modification	Rank	Result Type
1499.8176	1499.7074	-0.1102	-73	386	399	QGKDLIIANLGDSR	12	0		4	Mascot

10	hypothetical protein TRIUR3_32964 [Triticum urartu]	gi|474203701	25148.4	9.78	1	0	11	0

Peptide Information

Calc. Mass	Obsrv. Mass	± da	± ppm	Start Seq.	End Seq.	Sequence	Ion Score	C. I. %	Modification	Rank	Result Type
1499.7826	1499.7074	-0.0752	-50	67	79	ASLNGQHFSNIRR	11	0		5	Mascot

Gel Idx/Pos	288/L16	Instr./Gel Origin	BA2151/Sample Project 20140814	Process Status	Analysis Succeeded
Plate [#] Name	[1] Sample Project 20140814	Instrument Sample Name		Spectra	11

Rank	Protein Name	Accession No.	Protein MW	Protein PI	Pep. Count	Protein Score	Protein Score C. I. %	Intensity Matched	Total Ion Score	Total Ion C. I. %	Confirmed
1	Alanine aminotransferase 2 [Triticum urartu]	gi|473789790	57750.7	6.77	20	437	100	36.801	327	100	

Peptide Information

Calc. Mass	Obsrv. Mass	± da	± ppm	Start Seq.	End Seq.	Sequence	Ion Score	C. I. %	Modification	Rank	Result Type
915.5258	915.5275	0.0017	2	402	410	DGILASLAR					Mascot
960.5109	960.5087	-0.0022	-2	134	143	DAIASGIASR					Mascot
980.6251	980.6287	0.0036	4	110	118	QILALIPGR					Mascot
1022.5741	1022.5752	0.0011	1	34	42	GEIVIHAQR					Mascot
1023.5258	1023.5353	0.0095	9	456	464	APDAFYALR					Mascot
1023.5258	1023.5353	0.0095	9	456	464	APDAFYALR	64	99.997			Mascot
1089.4783	1089.488	0.0097	9	337	345	GYYGECGKR			Carbamidomethyl (C)[6]		Mascot
1096.5634	1096.5681	0.0047	4	98	107	TLFSADSISR					Mascot
1243.7004	1243.7025	0.0021	2	399	410	AEKDGILASLAR					Mascot
1407.7379	1407.7445	0.0066	5	452	464	AANKAPDAFYALR					Mascot
1484.7203	1484.7324	0.0121	8	346	359	GGYMEITGFSAPVR					Mascot
1500.7152	1500.7115	-0.0037	-2	346	359	GGYMEITGFSAPVR			Oxidation (M)[4]		Mascot
1500.7152	1500.7115	-0.0037	-2	346	359	GGYMEITGFSAPVR	74	100	Oxidation (M)[4]		Mascot
1522.7935	1522.7615	-0.032	-21	80	92	EVLALCDHPDLLK			Carbamidomethyl (C)[6]		Mascot
1640.8214	1640.8143	-0.0071	-4	345	359	RGGYMEITGFSAPVR					Mascot
1650.8884	1650.7999	-0.0885	-54	80	93	EVLALCDHPDLLKK			Carbamidomethyl (C)[6]		Mascot
1656.8163	1656.8071	-0.0092	-6	345	359	RGGYMEITGFSAPVR			Oxidation (M)[5]		Mascot
1677.7731	1677.8005	0.0274	16	506	518	FTVFHETFMAEYR					Mascot
1693.7679	1693.7909	0.023	14	506	518	FTVFHETFMAEYR			Oxidation (M)[9]		Mascot
1792.8	1792.8221	0.0221	12	506	519	FTVFHETFMAEYRD					Mascot
1792.8	1792.8221	0.0221	12	506	519	FTVFHETFMAEYRD	55	99.976			Mascot
1808.7949	1808.8041	0.0092	5	506	519	FTVFHETFMAEYRD			Oxidation (M)[9]		Mascot
1808.7949	1808.8041	0.0092	5	506	519	FTVFHETFMAEYRD	36	98.095	Oxidation (M)[9]		Mascot
1868.031	1867.9723	-0.0587	-31	490	505	CTILPQEEKIPAIISR			Carbamidomethyl (C)[1]		Mascot
1881.9238	1881.8962	-0.0276	-15	430	445	AEGAMYLFPQICLPQK			Carbamidomethyl (C)[12], Oxidation (M)[5]		Mascot
2538.2722	2538.3054	0.0332	13	287	308	NEGLVLLADEVYQENIYA DNKK					Mascot
2640.3933	2640.4429	0.0496	19	465	489	LLESTGIVVVPGSGFGQV					Mascot

						PGTWHFR					
2640.3933	2640.4429	0.0496	19	465	489	LLESTGIVVVPGSGFGQV PGTWHFR	134	100			Mascot
3146.5286	3146.6016	0.073	23	144	172	DGFPANADDIFLTDGASP GVHLMMQLLIR			Oxidation (M)[23,24]		Mascot

2	hypothetical protein TRIUR3_10172 [Triticum urartu]	gi|474163113	90775.5	6.85	18	60	95.777	24.947	

Peptide Information

Calc. Mass	Obsrv. Mass	± da	± ppm	Start Seq.	End Seq.	Sequence	Ion Score	C. I. %	Modification	Rank	Result Type
838.4088	838.3538	-0.055	-66	67	74	STVTGSMR					Mascot
1096.6473	1096.5681	-0.0792	-72	712	722	VANLPIGSVAR					Mascot
1146.5725	1146.5254	-0.0471	-41	579	587	ISSFFSMRR			Oxidation (M)[7]		Mascot
1225.5477	1225.5969	0.0492	40	76	87	SSSDRSGGTPMK			Oxidation (M)[11]		Mascot
1350.6537	1350.7031	0.0494	37	805	816	FADDDAVVLETR					Mascot
1351.6853	1351.7388	0.0535	40	534	546	SVPVSSSAFDSLR					Mascot
1516.7754	1516.7238	-0.0516	-34	453	465	WPSASSNEIIREK					Mascot
1629.7533	1629.7914	0.0381	23	471	485	SLSTLGEMLAMSDMK			Oxidation (M)[8]		Mascot
1656.809	1656.8071	-0.0019	-1	250	264	QSGEFHSAPTSPQRK					Mascot
1691.9061	1691.8809	-0.0252	-15	648	663	ESEALSSSKALISLEK					Mascot
1714.8218	1714.823	0.0012	1	20	33	CSRQLQGVYDDFVK			Carbamidomethyl (C)[1]		Mascot
1744.9453	1744.8315	-0.1138	-65	706	722	QGYSGRVANLPIGSVAR					Mascot
1744.9453	1744.8315	-0.1138	-65	706	722	QGYSGRVANLPIGSVAR					Mascot
1786.8566	1786.837	-0.0196	-11	694	711	SASSSESAITAKQGYSGR					Mascot
1792.8746	1792.8221	-0.0525	-29	88	103	TLIAQEMAKEGDTNQK			Oxidation (M)[7]		Mascot
1792.8746	1792.8221	-0.0525	-29	88	103	TLIAQEMAKEGDTNQK			Oxidation (M)[7]		Mascot
1801.8492	1801.863	0.0138	8	470	485	RSLSTLGEMLAMSDMK			Oxidation (M)[9,12]		Mascot
1817.8442	1817.9269	0.0827	45	470	485	RSLSTLGEMLAMSDMK			Oxidation (M)[9,12,15]		Mascot
2385.0598	2385.2009	0.1411	59	144	163	VNNQMSFDKHAGCVENV EYK			Carbamidomethyl (C)[13], Oxidation (M)[5]		Mascot
2640.2148	2640.4429	0.2281	86	153	174	HAGCVENVEYKDVYEVG YQPQR			Carbamidomethyl (C)[4]		Mascot
2640.2148	2640.4429	0.2281	86	153	174	HAGCVENVEYKDVYEVG YQPQR			Carbamidomethyl (C)[4]		Mascot
2665.2119	2665.2549	0.043	16	508	533	VAVPSNCSVDGAEGESS SNNNISRSK			Carbamidomethyl (C)[7]		Mascot

3	hypothetical protein TRIUR3_03956 [Triticum urartu]	gi|473905046	31223.1	9.17	11	56	87.82	21.025	

Peptide Information

Calc. Mass	Obsrv. Mass	± da	± ppm	Start Seq.	End Seq.	Sequence	Ion Score	C. I. %	Modification	Rank	Result Type
1023.5694	1023.5353	-0.0341	-33	63	72	GTRPGASLHK					Mascot

Calc. Mass	Obsrv. Mass	± da	± ppm	Start Seq.	End Seq.	Sequence	Ion Score	C. I. %	Modification	Rank	Result Type
1023.5694	1023.5353	-0.0341	-33	63	72	GTRPGASLHK					Mascot
1351.6423	1351.7388	0.0965	71	256	266	WTESAVEMARR			Oxidation (M)[8]		Mascot
1435.6925	1435.7279	0.0354	25	46	58	TDDFRVQVDGAGR					Mascot
1618.7853	1618.8442	0.0589	36	215	230	AAAAATAMEARAEQEK					Mascot
1640.7618	1640.8143	0.0525	32	242	255	ERMTEGLGQLTDMK			Oxidation (M)[3,13]		Mascot
1691.8203	1691.8809	0.0606	36	191	205	AMAAVDRECLAETVR			Carbamidomethyl (C)[9]		Mascot
1744.913	1744.8315	-0.0815	-47	9	25	QHPHPAPPPGATVVDPK					Mascot
1744.913	1744.8315	-0.0815	-47	9	25	QHPHPAPPPGATVVDPK					Mascot
1758.844	1758.8611	0.0171	10	181	197	QAPNPTSSDKAMAAVDR					Mascot
1849.9655	1849.8336	-0.1319	-71	163	180	ELEPKAPQAAAAAQTPE K					Mascot
2063.1133	2062.9578	-0.1555	-75	129	148	KVPKPAPSGDAAPTSIEEI R					Mascot
2385.333	2385.2009	-0.1321	-55	269	290	EVVAVGVAAFTLGFFVSQ RLFK					Mascot

4	Putative disease resistance protein RGA4 [Triticum urartu]	gi|473781055	115939	5.83	17	48	28.281	27.6	

Peptide Information

Calc. Mass	Obsrv. Mass	± da	± ppm	Start Seq.	End Seq.	Sequence	Ion Score	C. I. %	Modification	Rank	Result Type
1005.5727	1005.5178	-0.0549	-55	214	221	IFNDQKIK					Mascot
1023.5258	1023.5353	0.0095	9	817	825	VGPEFVGYR					Mascot
1023.5258	1023.5353	0.0095	9	817	825	VGPEFVGYR					Mascot
1170.7092	1170.6429	-0.0663	-57	918	928	ELLLIGANSLK					Mascot
1229.6354	1229.5897	-0.0457	-37	548	557	TFLAFYGPWK					Mascot
1465.8009	1465.7679	-0.033	-23	86	99	GSKLLPDHPLSSSK					Mascot
1484.8049	1484.7324	-0.0725	-49	546	557	VRTFLAFYGPWK					Mascot
1592.8386	1592.8442	0.0056	4	532	545	DKLVLTSMDNMVVK					Mascot
1743.9388	1743.8402	-0.0986	-57	294	309	TPFHTTNSGVILVTTR					Mascot
1874.9178	1875.0422	0.1244	66	474	489	NLLQPDGTIFNQAQCR			Carbamidomethyl (C)[15]		Mascot
1890.9556	1890.9266	-0.029	-15	372	388	VTASALASKDLTENEWR					Mascot
1908.9927	1908.9388	-0.0539	-28	702	717	LERTAHFHTNVVLEDK					Mascot
2408.312	2408.2119	-0.1001	-42	787	807	NLQSCVDLPPVGQLPNL KFLR			Carbamidomethyl (C)[5]		Mascot
2581.1897	2581.3735	0.1838	71	840	858	LEWFFVKDMPNWEEWS FFK			Oxidation (M)[9]		Mascot
2640.3135	2640.4429	0.1294	49	474	495	NLLQPDGTIFNQAQCRM HDLLR			Carbamidomethyl (C)[15]		Mascot
2640.3135	2640.4429	0.1294	49	474	495	NLLQPDGTIFNQAQCRM HDLLR			Carbamidomethyl (C)[15]		Mascot
2652.5237	2652.4846	-0.0391	-15	571	593	VLALNYSIVQNIPDYIGKLI HLR					Mascot
2656.3083	2656.4346	0.1263	48	474	495	NLLQPDGTIFNQAQCRM			Carbamidomethyl (C)[15], Oxidation (M)[17]		Mascot

						HDLLR					
2656.3083	2656.4346	0.1263	48	474	495	NLLQPDGTIFNQAQCRM HDLLR			Carbamidomethyl (C)[15], Oxidation (M)[17]		Mascot
2662.5332	2662.4246	-0.1086	-41	566	588	FLLLRVLALNYSIVQNIPD YIGK					Mascot
2719.5618	2719.3882	-0.1736	-64	589	613	LIHLRLLDLGHTGISHLPE SIGSLK					Mascot

5 Beta-glucosidase 12 [Triticum urartu] gi|474093165 60671.1 9.48 12 44 0 4.187

Peptide Information

Calc. Mass	Obsrv. Mass	± da	± ppm	Start Seq.	End Seq.	Sequence	Ion Score	C. I. %	Modification	Rank	Result Type
806.3502	806.3668	0.0166	21	215	220	CSPWEK			Carbamidomethyl (C)[1]		Mascot
870.3661	870.3408	-0.0253	-29	302	309	GDYPGSMK			Oxidation (M)[7]		Mascot
1247.6631	1247.5634	-0.0997	-80	403	413	NPLVYITENGK					Mascot
1456.7642	1456.6592	-0.105	-72	449	461	SLPLEEALKDDAR					Mascot
1484.7632	1484.7324	-0.0308	-21	129	140	YYNNLIDELLSK					Mascot
1522.7319	1522.7615	0.0296	19	302	315	GDYPGSMKELVGNR					Mascot
1691.8389	1691.8809	0.042	25	40	55	GFLFGTATSSYQVSAR					Mascot
1778.9298	1778.8208	-0.109	-61	287	301	AIEFMLGWFLDPLAR					Mascot
1835.8204	1835.8195	-0.0009	0	97	111	DMGMDAYRFSISWTR					Mascot
1851.8153	1851.957	0.1417	77	97	111	DMGMDAYRFSISWTR			Oxidation (M)[2]		Mascot
1867.8102	1867.9723	0.1621	87	97	111	DMGMDAYRFSISWTR			Oxidation (M)[2,4]		Mascot
1875.0487	1875.0422	-0.0065	-3	260	276	GKIGISLVSNWFTPLSR					Mascot
2646.2732	2646.4424	0.1692	64	287	309	AIEFMLGWFLDPLARGDY PGSMK			Oxidation (M)[5,22]		Mascot
2688.5671	2688.4185	-0.1486	-55	2	29	AARAVAAAVLPLLLLVVA TSAYGGAHGR					Mascot

6 RuBisCO large subunit-binding protein subunit beta, chloroplastic [Triticum urartu] gi|474137978 66766.1 7.46 13 43 0 11.749

Peptide Information

Calc. Mass	Obsrv. Mass	± da	± ppm	Start Seq.	End Seq.	Sequence	Ion Score	C. I. %	Modification	Rank	Result Type
806.4189	806.3668	-0.0521	-65	62	68	DGLAMRK			Oxidation (M)[5]		Mascot
960.4996	960.5087	0.0091	9	467	475	VEDALNATK					Mascot
963.5509	963.6027	0.0518	54	190	197	ALVYELQK					Mascot
963.5509	963.6027	0.0518	54	190	197	ALVYELQK					Mascot
1229.6848	1229.5897	-0.0951	-77	465	475	LRVEDALNATK					Mascot
1309.7185	1309.6528	-0.0657	-50	190	200	ALVYELQKMSK					Mascot
1435.7792	1435.7279	-0.0513	-36	587	599	TFITSDAVVVDIR					Mascot
1456.7253	1456.6592	-0.0661	-45	56	67	ELYFNKDGLAMR					Mascot

1700.9	1700.8652	-0.0348	-20	476	492	AAVEEGIVVGGGCTLIR			Carbamidomethyl (C)[13]		Mascot
1743.8549	1743.8402	-0.0147	-8	542	558	VLAIDNSSYGYNAATGK					Mascot
1890.9994	1890.9266	-0.0728	-38	559	575	YEDLMAAGIIDPTKVVR					Mascot
2538.3298	2538.3054	-0.0244	-10	113	136	IVNDGVTVAKEVELEDPV ENIGAK					Mascot
2642.3818	2642.1409	-0.2409	-91	467	492	VEDALNATKAAVEEGIVV GGGCTLIR			Carbamidomethyl (C)[22]		Mascot
2662.3328	2662.4246	0.0918	34	576	599	CCLEHAASVAKTFITSDA VVVDIR			Carbamidomethyl (C)[1,2]		Mascot

7 31 kDa ribonucleoprotein, chloroplastic [Triticum urartu] gi|474097120 55079.2 6.12 11 43 0 9.647

Peptide Information

Calc. Mass	Obsrv. Mass	± da	± ppm	Start Seq.	End Seq.	Sequence	Ion Score	C. I. %	Modification	Rank	Result Type
915.5046	915.5275	0.0229	25	432	439	ELFGKHGK					Mascot
1170.6704	1170.6429	-0.0275	-23	386	395	LFGMPLIVHK			Oxidation (M)[4]		Mascot
1452.8508	1452.7223	-0.1285	-88	386	398	LFGMPLIVHKAAR					Mascot
1691.9943	1691.8809	-0.1134	-67	23	37	LIGLSQSTYLDKILK					Mascot
1790.9647	1790.9417	-0.023	-13	139	155	RASLGGSLDEVFIVAEK					Mascot
1791.8695	1791.8958	0.0263	15	356	372	GTGQSRGYGFVTMSTVK			Oxidation (M)[13]		Mascot
1849.9695	1849.8336	-0.1359	-73	416	431	IYVGNLSLQVDDSWLK					Mascot
1890.8691	1890.9266	0.0575	30	451	468	GGTWSSQGFGFVTMATE K					Mascot
1973.0015	1973.001	-0.0005	0	339	355	LFEQAGVVEFSEVIYSR					Mascot
2656.2673	2656.4346	0.1673	63	362	385	GYGFVTMSTVKEAELAV EMFDGFK					Mascot
2656.2673	2656.4346	0.1673	63	362	385	GYGFVTMSTVKEAELAV EMFDGFK					Mascot
2672.2622	2672.4204	0.1582	59	362	385	GYGFVTMSTVKEAELAV EMFDGFK			Oxidation (M)[7]		Mascot
2672.2622	2672.4204	0.1582	59	362	385	GYGFVTMSTVKEAELAV EMFDGFK			Oxidation (M)[7]		Mascot
2685.2373	2685.4236	0.1863	69	60	87	GAAAPDVVVISSDEEEEE GDGSAPVAGK					Mascot
2688.2573	2688.4185	0.1612	60	362	385	GYGFVTMSTVKEAELAV EMFDGFK			Oxidation (M)[7,19]		Mascot

8 putative replication factor C subunit 3 [Triticum urartu] gi|473936914 40601.6 5.64 10 43 0 14.35

Peptide Information

Calc. Mass	Obsrv. Mass	± da	± ppm	Start Seq.	End Seq.	Sequence	Ion Score	C. I. %	Modification	Rank	Result Type
1005.5687	1005.5178	-0.0509	-51	339	347	SLITKNSSR					Mascot
1023.5582	1023.5353	-0.0229	-22	108	116	VIVVHDADR					Mascot
1023.5582	1023.5353	-0.0229	-22	108	116	VIVVHDADR					Mascot
1408.7294	1408.7418	0.0124	9	333	343	FMSFYRSLITK			Oxidation (M)[2]		Mascot

1484.8292	1484.7324	-0.0968	-65	181	194	IAASASNNLRQAIR					Mascot
1500.8241	1500.7115	-0.1126	-75	176	190	GLASRIAASASNNLR					Mascot
1500.8241	1500.7115	-0.1126	-75	176	190	GLASRIAASASNNLR					Mascot
1640.9734	1640.8143	-0.1591	-97	147	160	VVRLKPPSFDEIIK					Mascot
1700.9218	1700.8652	-0.0566	-33	161	175	VLEFIATQEGIDLPR					Mascot
2622.2188	2622.4661	0.2473	94	2	24	MDGQVTERQCNHFIFEG AQAVGK			Carbamidomethyl (C)[10]		Mascot
2638.2139	2638.4333	0.2194	83	2	24	MDGQVTERQCNHFIFEG AQAVGK			Carbamidomethyl (C)[10], Oxidation (M)[1]		Mascot
2646.332	2646.4424	0.1104	42	246	266	MIEHNVSPYFIFCHLVNEL KR			Carbamidomethyl (C)[13]		Mascot
2662.3293	2662.4246	0.0953	36	302	325	RAINIEGFAVEGPDQGEA IQCFIK			Carbamidomethyl (C)[21]		Mascot

9 hypothetical protein TRIUR3_05398 [Triticum urartu] gi|474374693 7143.6 8.85 4 43 0 11.88 13 0

Peptide Information

Calc. Mass	Obsrv. Mass	± da	± ppm	Start Seq.	End Seq.	Sequence	Ion Score	C. I. %	Modification	Rank	Result Type
806.4268	806.3668	-0.06	-74	17	23	SPNVHPR					Mascot
1677.853	1677.8005	-0.0525	-31	2	16	VTAFRAGIGAWPMER			Oxidation (M)[13]		Mascot
1792.8986	1792.8221	-0.0765	-43	1	16	MVTAFRAGIGAWPMER					Mascot
1792.8986	1792.8221	-0.0765	-43	1	16	MVTAFRAGIGAWPMER					Mascot
1808.8934	1808.8041	-0.0893	-49	1	16	MVTAFRAGIGAWPMER			Oxidation (M)[1]		Mascot
1808.8934	1808.8041	-0.0893	-49	1	16	MVTAFRAGIGAWPMER	13	0	Oxidation (M)[14]		Mascot
1824.8884	1824.8032	-0.0852	-47	1	16	MVTAFRAGIGAWPMER			Oxidation (M)[1,14]		Mascot
1874.9443	1875.0422	0.0979	52	7	23	AGIGAWPMERSPNVHPR					Mascot
1890.9392	1890.9266	-0.0126	-7	7	23	AGIGAWPMERSPNVHPR			Oxidation (M)[8]		Mascot

10 Zinc finger A20 and AN1 domain-containing stress-associated protein 8 [Triticum urartu] gi|474443429 30470.2 9.11 8 43 0 29.032 11 0

Peptide Information

Calc. Mass	Obsrv. Mass	± da	± ppm	Start Seq.	End Seq.	Sequence	Ion Score	C. I. %	Modification	Rank	Result Type
963.5179	963.6027	0.0848	88	12	19	LEMKEAVK			Oxidation (M)[3]		Mascot
963.5179	963.6027	0.0848	88	12	19	LEMKEAVK	11	0	Oxidation (M)[3]		Mascot
1023.504	1023.5353	0.0313	31	221	229	VGLTGFNCR			Carbamidomethyl (C)[8]		Mascot
1023.504	1023.5353	0.0313	31	221	229	VGLTGFNCR			Carbamidomethyl (C)[8]		Mascot
1225.5552	1225.5969	0.0417	34	143	152	EMAMKQEQAK			Oxidation (M)[2,4]		Mascot
1622.8021	1622.7867	-0.0154	-9	153	169	LAASSFDSIVNGGDAAK					Mascot
1674.8268	1674.8202	-0.0066	-4	95	108	NRDEGFLLSVMEHK					Mascot

1744.7531	1744.8315	0.0784	45	230	243	CGNLYCATHRYSDK	Carbamidomethyl (C)[1,6]	Mascot
1744.7531	1744.8315	0.0784	45	230	243	CGNLYCATHRYSDK	Carbamidomethyl (C)[1,6]	Mascot
2622.3191	2622.4661	0.147	56	188	213	TLITAQPADIAGPSEAAM ENPKGPSR		Mascot
2638.3142	2638.4333	0.1191	45	188	213	TLITAQPADIAGPSEAAM ENPKGPSR	Oxidation (M)[18]	Mascot
2665.1335	2665.2549	0.1214	46	116	139	EGPILCVNNCGFFGSAAT MNMCSK	Carbamidomethyl (C)[6,10,22]	Mascot

Gel Idx/Pos	289/L17	Instr./Gel Origin	BA2151/Sample Project 20140814	Process Status	Analysis Succeeded
Plate [#] Name	[1] Sample Project 20140814	Instrument Sample Name		Spectra	11

Rank	Protein Name	Accession No.	Protein MW	Protein PI	Pep. Count	Protein Score	Protein Score C. I. %	Intensity Matched	Total Ion Score	Total Ion C. I. %	Confirmed
1	RecName: Full=Ribulose bisphosphate carboxylase large chain; Short=RuBisCO large subunit; Flags: Precursor	gi|132065	53444.6	6.22	28	858	100	58.535	661	100	

Protein Group

RuBisCO large subunit [Triticum aestivum]	gi|13928213	53444.6	6.2199 997901 9165
ribulose-1,5-bisphosphate carboxylase/oxygenase large subunit (H-type) [Triticum aestivum]	gi|344053	53444.6	6.2199 997901 9165
ribulose-1,5-bisphosphate carboxylase/oxygenase large subunit (chloroplast) [Triticum aestivum]	gi|521301495	53444.6	6.2199 997901 9165
ribulose-1,5-bisphosphate carboxylase/oxygenase large subunit [Triticum aestivum]	gi|14017580	53444.6	6.2199 997901 9165
ribulose-1,5-bisphosphate carboxylase/oxygenase large subunit [Triticum aestivum]	gi|169666638	53444.6	6.2199 997901 9165

Peptide Information

Calc. Mass	Obsrv. Mass	± da	± ppm	Start Seq.	End Seq.	Sequence	Ion Score	C. I. %	Modification	Rank	Result Type
830.4366	830.4283	-0.0083	-10	440	446	EGNEIIR					Mascot
898.41	898.4123	0.0023	3	306	312	NHGMHFR					Mascot
912.472	912.4646	-0.0074	-8	296	303	AMHAVIDR					Mascot
914.405	914.3882	-0.0168	-18	306	312	NHGMHFR			Oxidation (M)[4]		Mascot
928.4669	928.441	-0.0259	-28	296	303	AMHAVIDR			Oxidation (M)[2]		Mascot
971.4073	971.4023	-0.005	-5	188	194	ACYECLR			Carbamidomethyl (C)[2,5]		Mascot
971.4073	971.4023	-0.005	-5	188	194	ACYECLR	24	71.301	Carbamidomethyl (C)[2,5]		Mascot
1021.5312	1021.5198	-0.0114	-11	33	41	DTDILAAFR					Mascot
1021.5312	1021.5198	-0.0114	-11	33	41	DTDILAAFR	56	99.983			Mascot
1037.4899	1037.4851	-0.0048	-5	351	358	DDFIEKDR					Mascot
1116.583	1116.5812	-0.0018	-2	422	431	VALEACVQAR			Carbamidomethyl (C)[6]		Mascot
1170.5586	1170.6375	0.0789	67	304	312	QKNHGMHFR			Oxidation (M)[6]		Mascot

1170.5586	1170.6375	0.0789	67	304	312	QKNHGMHFR			Oxidation (M)[6]	Mascot
1187.6644	1187.6611	-0.0033	-3	286	295	DNGLLLHIHR				Mascot
1247.6129	1247.5988	-0.0141	-11	218	227	FVFCAEAIYK			Carbamidomethyl (C)[4]	Mascot
1285.6859	1285.5776	-0.1083	-84	436	446	DLAREGNEIIR				Mascot
1293.6871	1293.6863	-0.0008	-1	340	350	EMTLGFVDLLR	90	100		Mascot
1309.6821	1309.6604	-0.0217	-17	340	350	EMTLGFVDLLR			Oxidation (M)[2]	Mascot
1325.6896	1325.6711	-0.0185	-14	306	316	NHGMHFRVLAK			Oxidation (M)[4]	Mascot
1339.6417	1339.6206	-0.0211	-16	467	477	FEFEPVDTIDK				Mascot
1407.6678	1407.6458	-0.022	-16	22	32	LTYYTPEYETK				Mascot
1451.6219	1451.6394	0.0175	12	202	213	DDENVNSQPFMR				Mascot
1465.7546	1465.765	0.0104	7	147	159	TFQGPPHGIQVER				Mascot
1465.7546	1465.765	0.0104	7	147	159	TFQGPPHGIQVER	102	100		Mascot
1502.8512	1502.849	-0.0022	-1	165	177	YGRPLLGCTIKPK			Carbamidomethyl (C)[8]	Mascot
1502.8512	1502.849	-0.0022	-1	165	177	YGRPLLGCTIKPK	6	0	Carbamidomethyl (C)[8]	Mascot
1518.741	1518.7408	-0.0002	0	216	227	DRFVFCAEAIYK			Carbamidomethyl (C)[6]	Mascot
1546.736	1546.7114	-0.0246	-16	451	463	WSPELAAACEVWK			Carbamidomethyl (C)[9]	Mascot
1651.8578	1651.8324	-0.0254	-15	464	477	AIKFEFEPVDTIDK				Mascot
1893.9739	1893.9507	-0.0232	-12	335	350	LEGEREMTLGFVDLLR			Oxidation (M)[7]	Mascot
1950.916	1950.9291	0.0131	7	237	253	GHYLNATAGTCEEMIKR			Carbamidomethyl (C)[11]	Mascot
1966.911	1966.9098	-0.0012	-1	237	253	GHYLNATAGTCEEMIKR			Carbamidomethyl (C)[11], Oxidation (M)[14]	Mascot
2041.0311	2041.0352	0.0041	2	340	356	EMTLGFVDLLRDDFIEK				Mascot
2057.0261	2057.0132	-0.0129	-6	340	356	EMTLGFVDLLRDDFIEK			Oxidation (M)[2]	Mascot
2169.9871	2169.999	0.0119	5	195	213	GGLDFTKDDENVNSQPFMR				Mascot
2169.9871	2169.999	0.0119	5	195	213	GGLDFTKDDENVNSQPFMR	93	100		Mascot
2185.9819	2185.9814	-0.0005	0	195	213	GGLDFTKDDENVNSQPFMR			Oxidation (M)[18]	Mascot
2252.1824	2252.1777	-0.0047	-2	140	159	IPPTYSKTFQGPPHGIQVER				Mascot
3036.4707	3036.5229	0.0522	17	259	285	ELGVPIVMHDYLTGGFTANTTLAHYCR			Carbamidomethyl (C)[26]	Mascot
3036.4707	3036.5229	0.0522	17	259	285	ELGVPIVMHDYLTGGFTANTTLAHYCR	159	100	Carbamidomethyl (C)[26]	Mascot
3052.4656	3052.5239	0.0583	19	259	285	ELGVPIVMHDYLTGGFTANTTLAHYCR			Carbamidomethyl (C)[26], Oxidation (M)[8]	Mascot
3840.8564	3840.968	0.1116	29	42	79	VSPQPGVPPEEAGAAVAAESSTGTWTTVWTDGLTSLDR				Mascot
3840.8564	3840.968	0.1116	29	42	79	VSPQPGVPPEEAGAAVAAESSTGTWTTVWTDGLTSLDR	131	100		Mascot

2	ribulose-1,5-bisphosphate carboxylase/oxygenase large subunit (chloroplast) [Triticum monococcum]	gi|525778513	53343.5	6.04	27	850	100	58.301	661	100

Protein Group

ribulose-1,5-bisphosphate carboxylase/oxygenase large subunit (chloroplast) [Triticum monococcum subsp. aegilopoides]	gi|521301355	53343.5	6.0399 999618 5303
ribulose-1,5-bisphosphate carboxylase/oxygenase large subunit (chloroplast) [Triticum monococcum]	gi|521301198	53343.5	6.0399 999618 5303
ribulose-1,5-bisphosphate carboxylase/oxygenase large subunit (chloroplast) [Triticum urartu]	gi|521301434	53343.5	6.0399 999618 5303
ribulose-1,5-bisphosphate carboxylase/oxygenase large subunit (chloroplast) [Triticum urartu]	gi|525782303	53343.5	6.0399 999618 5303
ribulose-1,5-bisphosphate carboxylase/oxygenase large subunit [Triticum monococcum]	gi|61378618	53343.5	6.0399 999618 5303

Peptide Information

Calc. Mass	Obsrv. Mass	± da	± ppm	Start Seq.	End Seq.	Sequence	Ion Score	C. I. %	Modification	Rank	Result Type
830.4366	830.4283	-0.0083	-10	440	446	EGNEIIR					Mascot
898.41	898.4123	0.0023	3	306	312	NHGMHFR					Mascot
912.472	912.4646	-0.0074	-8	296	303	AMHAVIDR					Mascot
914.405	914.3882	-0.0168	-18	306	312	NHGMHFR			Oxidation (M)[4]		Mascot
928.4669	928.441	-0.0259	-28	296	303	AMHAVIDR			Oxidation (M)[2]		Mascot
971.4073	971.4023	-0.005	-5	188	194	ACYECLR			Carbamidomethyl (C)[2,5]		Mascot
971.4073	971.4023	-0.005	-5	188	194	ACYECLR	24	71.301	Carbamidomethyl (C)[2,5]		Mascot
1021.5312	1021.5198	-0.0114	-11	33	41	DTDILAAFR					Mascot
1021.5312	1021.5198	-0.0114	-11	33	41	DTDILAAFR	56	99.983			Mascot
1037.4899	1037.4851	-0.0048	-5	351	358	DDFIEKDR					Mascot
1116.583	1116.5812	-0.0018	-2	422	431	VALEACVQAR			Carbamidomethyl (C)[6]		Mascot
1170.5586	1170.6375	0.0789	67	304	312	QKNHGMHFR			Oxidation (M)[6]		Mascot
1170.5586	1170.6375	0.0789	67	304	312	QKNHGMHFR			Oxidation (M)[6]		Mascot
1187.6644	1187.6611	-0.0033	-3	286	295	DNGLLLHIHR					Mascot
1247.6129	1247.5988	-0.0141	-11	218	227	FVFCAEAIYK			Carbamidomethyl (C)[4]		Mascot
1285.6859	1285.5776	-0.1083	-84	436	446	DLAREGNEIIR					Mascot
1293.6871	1293.6863	-0.0008	-1	340	350	EMTLGFVDLLR	90	100			Mascot
1309.6821	1309.6604	-0.0217	-17	340	350	EMTLGFVDLLR			Oxidation (M)[2]		Mascot
1325.6896	1325.6711	-0.0185	-14	306	316	NHGMHFRVLAK			Oxidation (M)[4]		Mascot

Calc. Mass	Obsrv. Mass	± da	± ppm	Start Seq.	End Seq.	Sequence	Ion Score	C. I. %	Modification	Rank	Result Type
1339.7006	1339.6206	-0.08	-60	9	21	AGVGFQAGVKDYK					Mascot
1407.6678	1407.6458	-0.022	-16	22	32	LTYYTPEYETK					Mascot
1451.6219	1451.6394	0.0175	12	202	213	DDENVNSQPFMR					Mascot
1465.7546	1465.765	0.0104	7	147	159	TFQGPPHGIQVER					Mascot
1465.7546	1465.765	0.0104	7	147	159	TFQGPPHGIQVER	102	100			Mascot
1502.8512	1502.849	-0.0022	-1	165	177	YGRPLLGCTIKPK			Carbamidomethyl (C)[8]		Mascot
1502.8512	1502.849	-0.0022	-1	165	177	YGRPLLGCTIKPK	6	0	Carbamidomethyl (C)[8]		Mascot
1518.741	1518.7408	-0.0002	0	216	227	DRFVFCAEAIYK			Carbamidomethyl (C)[6]		Mascot
1546.736	1546.7114	-0.0246	-16	451	463	WSPELAAACEVWK			Carbamidomethyl (C)[9]		Mascot
1893.9739	1893.9507	-0.0232	-12	335	350	LEGEREMTLGFVDLLR			Oxidation (M)[7]		Mascot
1950.916	1950.9291	0.0131	7	237	253	GHYLNATAGTCEEMIKR			Carbamidomethyl (C)[11]		Mascot
1966.911	1966.9098	-0.0012	-1	237	253	GHYLNATAGTCEEMIKR			Carbamidomethyl (C)[11], Oxidation (M)[14]		Mascot
2041.0311	2041.0352	0.0041	2	340	356	EMTLGFVDLLRDDFIEK					Mascot
2057.0261	2057.0132	-0.0129	-6	340	356	EMTLGFVDLLRDDFIEK			Oxidation (M)[2]		Mascot
2169.9871	2169.999	0.0119	5	195	213	GGLDFTKDDENVNSQPF MR					Mascot
2169.9871	2169.999	0.0119	5	195	213	GGLDFTKDDENVNSQPF MR	93	100			Mascot
2185.9819	2185.9814	-0.0005	0	195	213	GGLDFTKDDENVNSQPF MR			Oxidation (M)[18]		Mascot
2252.1824	2252.1777	-0.0047	-2	140	159	IPPTYSKTFQGPPHGIQV ER					Mascot
3036.4707	3036.5229	0.0522	17	259	285	ELGVPIVMHDYLTGGFTA NTTLAHYCR			Carbamidomethyl (C)[26]		Mascot
3036.4707	3036.5229	0.0522	17	259	285	ELGVPIVMHDYLTGGFTA NTTLAHYCR	159	100	Carbamidomethyl (C)[26]		Mascot
3052.4656	3052.5239	0.0583	19	259	285	ELGVPIVMHDYLTGGFTA NTTLAHYCR			Carbamidomethyl (C)[26], Oxidation (M)[8]		Mascot
3840.8564	3840.968	0.1116	29	42	79	VSPQPGVPPEEAGAAVA AESSTGTWTTVWTDGLT SLDR					Mascot
3840.8564	3840.968	0.1116	29	42	79	VSPQPGVPPEEAGAAVA AESSTGTWTTVWTDGLT SLDR	131	100			Mascot

3	ribulose-1,5-bisphosphate carboxylase/oxygenase large subunit [Triticum aestivum]	gi|32966580	53441.6	6.22	27	845	100	58.422	661	100	

Peptide Information

Calc. Mass	Obsrv. Mass	± da	± ppm	Start Seq.	End Seq.	Sequence	Ion Score	C. I. %	Modification	Rank	Result Type
830.4366	830.4283	-0.0083	-10	440	446	EGNEIIR					Mascot
898.41	898.4123	0.0023	3	306	312	NHGMHFR					Mascot
912.472	912.4646	-0.0074	-8	296	303	AMHAVIDR					Mascot
914.405	914.3882	-0.0168	-18	306	312	NHGMHFR			Oxidation (M)[4]		Mascot
928.4669	928.441	-0.0259	-28	296	303	AMHAVIDR			Oxidation (M)[2]		Mascot

971.4073	971.4023	-0.005	-5	188	194	ACYECLR			Carbamidomethyl (C)[2,5]	Mascot
971.4073	971.4023	-0.005	-5	188	194	ACYECLR	24	71.301	Carbamidomethyl (C)[2,5]	Mascot
1021.5312	1021.5198	-0.0114	-11	33	41	DTDILAAFR				Mascot
1021.5312	1021.5198	-0.0114	-11	33	41	DTDILAAFR	56	99.983		Mascot
1037.4899	1037.4851	-0.0048	-5	351	358	DDFIEKDR				Mascot
1116.583	1116.5812	-0.0018	-2	422	431	VALEACVQAR			Carbamidomethyl (C)[6]	Mascot
1170.5586	1170.6375	0.0789	67	304	312	QKNHGMHFR			Oxidation (M)[6]	Mascot
1170.5586	1170.6375	0.0789	67	304	312	QKNHGMHFR			Oxidation (M)[6]	Mascot
1187.6644	1187.6611	-0.0033	-3	286	295	DNGLLLHIHR				Mascot
1247.6129	1247.5988	-0.0141	-11	218	227	FVFCAEAIYK			Carbamidomethyl (C)[4]	Mascot
1285.6859	1285.5776	-0.1083	-84	436	446	DLAREGNEIIR				Mascot
1293.6871	1293.6863	-0.0008	-1	340	350	EMTLGFVDLLR	90	100		Mascot
1309.6821	1309.6604	-0.0217	-17	340	350	EMTLGFVDLLR			Oxidation (M)[2]	Mascot
1325.6896	1325.6711	-0.0185	-14	306	316	NHGMHFRVLAK			Oxidation (M)[4]	Mascot
1339.6417	1339.6206	-0.0211	-16	467	477	FEFEPVDTIDK				Mascot
1407.6678	1407.6458	-0.022	-16	22	32	LTYYTPEYETK				Mascot
1451.6219	1451.6394	0.0175	12	202	213	DDENVNSQPFMR				Mascot
1465.7546	1465.765	0.0104	7	147	159	TFQGPPHGIQVER				Mascot
1465.7546	1465.765	0.0104	7	147	159	TFQGPPHGIQVER	102	100		Mascot
1502.8512	1502.849	-0.0022	-1	165	177	YGRPLLGCTIKPK			Carbamidomethyl (C)[8]	Mascot
1502.8512	1502.849	-0.0022	-1	165	177	YGRPLLGCTIKPK	6	0	Carbamidomethyl (C)[8]	Mascot
1518.741	1518.7408	-0.0002	0	216	227	DRFVFCAEAIYK			Carbamidomethyl (C)[6]	Mascot
1546.736	1546.7114	-0.0246	-16	451	463	WSPELAAACEVWK			Carbamidomethyl (C)[9]	Mascot
1651.8578	1651.8324	-0.0254	-15	464	477	AIKFEFEPVDTIDK				Mascot
1893.9739	1893.9507	-0.0232	-12	335	350	LEGEREMTLGFVDLLR			Oxidation (M)[7]	Mascot
1950.916	1950.9291	0.0131	7	237	253	GHYLNATAGTCEEMIKR			Carbamidomethyl (C)[11]	Mascot
1966.911	1966.9098	-0.0012	-1	237	253	GHYLNATAGTCEEMIKR			Carbamidomethyl (C)[11], Oxidation (M)[14]	Mascot
2041.0311	2041.0352	0.0041	2	340	356	EMTLGFVDLLRDDFIEK				Mascot
2057.0261	2057.0132	-0.0129	-6	340	356	EMTLGFVDLLRDDFIEK			Oxidation (M)[2]	Mascot
2169.9871	2169.999	0.0119	5	195	213	GGLDFTKDDENVNSQPF MR				Mascot
2169.9871	2169.999	0.0119	5	195	213	GGLDFTKDDENVNSQPF MR	93	100		Mascot
2185.9819	2185.9814	-0.0005	0	195	213	GGLDFTKDDENVNSQPF MR			Oxidation (M)[18]	Mascot
3036.4707	3036.5229	0.0522	17	259	285	ELGVPIVMHDYLTGGFTA NTTLAHYCR			Carbamidomethyl (C)[26]	Mascot
3036.4707	3036.5229	0.0522	17	259	285	ELGVPIVMHDYLTGGFTA NTTLAHYCR	159	100	Carbamidomethyl (C)[26]	Mascot
3052.4656	3052.5239	0.0583	19	259	285	ELGVPIVMHDYLTGGFTA NTTLAHYCR			Carbamidomethyl (C)[26], Oxidation (M)[8]	Mascot

3840.8564	3840.968	0.1116	29	42	79	VSPQPGVPPEEAGAAVA AESSTGTWTTVWTDGLT SLDR					Mascot
3840.8564	3840.968	0.1116	29	42	79	VSPQPGVPPEEAGAAVA AESSTGTWTTVWTDGLT SLDR	131	100			Mascot

4	Ribulose bisphosphate carboxylase large chain [Triticum urartu]	gi|473930390	41662	8.32	23	614	100	52.992	474	100

Peptide Information

Calc. Mass	Obsrv. Mass	± da	± ppm	Start Seq.	End Seq.	Sequence	Ion Score	C. I. %	Modification	Rank	Result Type
830.4366	830.4283	-0.0083	-10	334	340	EGNEIIR					Mascot
898.41	898.4123	0.0023	3	200	206	NHGMHFR					Mascot
912.472	912.4646	-0.0074	-8	190	197	AMHAVIDR					Mascot
914.405	914.3882	-0.0168	-18	200	206	NHGMHFR			Oxidation (M)[4]		Mascot
928.4669	928.441	-0.0259	-28	190	197	AMHAVIDR			Oxidation (M)[2]		Mascot
971.4073	971.4023	-0.005	-5	82	88	ACYECLR			Carbamidomethyl (C)[2,5]		Mascot
971.4073	971.4023	-0.005	-5	82	88	ACYECLR	24	71.301	Carbamidomethyl (C)[2,5]		Mascot
1037.4899	1037.4851	-0.0048	-5	245	252	DDFIEKDR					Mascot
1116.583	1116.5812	-0.0018	-2	316	325	VALEACVQAR			Carbamidomethyl (C)[6]		Mascot
1170.5586	1170.6375	0.0789	67	198	206	QKNHGMHFR			Oxidation (M)[6]		Mascot
1170.5586	1170.6375	0.0789	67	198	206	QKNHGMHFR			Oxidation (M)[6]		Mascot
1187.6644	1187.6611	-0.0033	-3	180	189	DNGLLLHIHR					Mascot
1247.6129	1247.5988	-0.0141	-11	112	121	FVFCAEAIYK			Carbamidomethyl (C)[4]		Mascot
1285.6859	1285.5776	-0.1083	-84	330	340	DLAREGNEIIR					Mascot
1293.6871	1293.6863	-0.0008	-1	234	244	EMTLGFVDLLR	90	100			Mascot
1309.6821	1309.6604	-0.0217	-17	234	244	EMTLGFVDLLR			Oxidation (M)[2]		Mascot
1325.6896	1325.6711	-0.0185	-14	200	210	NHGMHFRVLAK			Oxidation (M)[4]		Mascot
1451.6219	1451.6394	0.0175	12	96	107	DDENVNSQPFMR					Mascot
1465.7546	1465.765	0.0104	7	41	53	TFQGPPHGIQVER					Mascot
1465.7546	1465.765	0.0104	7	41	53	TFQGPPHGIQVER	102	100			Mascot
1502.8512	1502.849	-0.0022	-1	59	71	YGRPLLGCTIKPK			Carbamidomethyl (C)[8]		Mascot
1502.8512	1502.849	-0.0022	-1	59	71	YGRPLLGCTIKPK	6	0	Carbamidomethyl (C)[8]		Mascot
1518.741	1518.7408	-0.0002	0	110	121	DRFVFCAEAIYK			Carbamidomethyl (C)[6]		Mascot
1546.736	1546.7114	-0.0246	-16	345	357	WSPELAAACEVWK			Carbamidomethyl (C)[9]		Mascot
1893.9739	1893.9507	-0.0232	-12	229	244	LEGEREMTLGFVDLLR			Oxidation (M)[7]		Mascot
1950.916	1950.9291	0.0131	7	131	147	GHYLNATAGTCEEMIKR			Carbamidomethyl (C)[11]		Mascot
1966.911	1966.9098	-0.0012	-1	131	147	GHYLNATAGTCEEMIKR			Carbamidomethyl (C)[11], Oxidation (M)[14]		Mascot
2041.0311	2041.0352	0.0041	2	234	250	EMTLGFVDLLRDDFIEK					Mascot

2057.0261	2057.0132	-0.0129	-6	234	250	EMTLGFVDLLRDDFIEK			Oxidation (M)[2]		Mascot
2169.9871	2169.999	0.0119	5	89	107	GGLDFTKDDENVNSQPF MR					Mascot
2169.9871	2169.999	0.0119	5	89	107	GGLDFTKDDENVNSQPF MR	93	100			Mascot
2185.9819	2185.9814	-0.0005	0	89	107	GGLDFTKDDENVNSQPF MR			Oxidation (M)[18]		Mascot
2252.1824	2252.1777	-0.0047	-2	34	53	IPPTYSKTFQGPPHGIQV ER					Mascot
3036.4707	3036.5229	0.0522	17	153	179	ELGVPIVMHDYLTGGFTA NTTLAHYCR			Carbamidomethyl (C)[26]		Mascot
3036.4707	3036.5229	0.0522	17	153	179	ELGVPIVMHDYLTGGFTA NTTLAHYCR	159	100	Carbamidomethyl (C)[26]		Mascot
3052.4656	3052.5239	0.0583	19	153	179	ELGVPIVMHDYLTGGFTA NTTLAHYCR			Carbamidomethyl (C)[26], Oxidation (M)[8]		Mascot

5 rbcL [Triticum aestivum] gi|12344 47599.8 6.6 24 469 100 48.987 314 100

Peptide Information

Calc. Mass	Obsrv. Mass	± da	± ppm	Start Seq.	End Seq.	Sequence	Ion Score	C. I. %	Modification	Rank	Result Type
830.4366	830.4283	-0.0083	-10	385	391	EGNEIIR					Mascot
898.41	898.4123	0.0023	3	251	257	NHGMHFR					Mascot
912.472	912.4646	-0.0074	-8	241	248	AMHAVIDR					Mascot
914.405	914.3882	-0.0168	-18	251	257	NHGMHFR			Oxidation (M)[4]		Mascot
928.4669	928.441	-0.0259	-28	241	248	AMHAVIDR			Oxidation (M)[2]		Mascot
971.4073	971.4023	-0.005	-5	133	139	ACYECLR			Carbamidomethyl (C)[2,5]		Mascot
971.4073	971.4023	-0.005	-5	133	139	ACYECLR	24	71.301	Carbamidomethyl (C)[2,5]		Mascot
1037.4899	1037.4851	-0.0048	-5	296	303	DDFIEKDR					Mascot
1116.583	1116.5812	-0.0018	-2	367	376	VALEACVQAR			Carbamidomethyl (C)[6]		Mascot
1170.5586	1170.6375	0.0789	67	249	257	QKNHGMHFR			Oxidation (M)[6]		Mascot
1170.5586	1170.6375	0.0789	67	249	257	QKNHGMHFR			Oxidation (M)[6]		Mascot
1187.6644	1187.6611	-0.0033	-3	231	240	DNGLLLHIHR					Mascot
1247.6129	1247.5988	-0.0141	-11	163	172	FVFCAEAIYK			Carbamidomethyl (C)[4]		Mascot
1285.6859	1285.5776	-0.1083	-84	381	391	DLAREGNEIIR					Mascot
1293.6871	1293.6863	-0.0008	-1	285	295	EMTLGFVDLLR	90	100			Mascot
1309.6821	1309.6604	-0.0217	-17	285	295	EMTLGFVDLLR			Oxidation (M)[2]		Mascot
1325.6896	1325.6711	-0.0185	-14	251	261	NHGMHFRVLAK			Oxidation (M)[4]		Mascot
1339.6417	1339.6206	-0.0211	-16	412	422	FEFEPVDTIDK					Mascot
1451.6219	1451.6394	0.0175	12	147	158	DDENVNSQPFMR					Mascot
1465.7546	1465.765	0.0104	7	92	104	TFQGPPHGIQVER					Mascot
1465.7546	1465.765	0.0104	7	92	104	TFQGPPHGIQVER	102	100			Mascot
1502.8512	1502.849	-0.0022	-1	110	122	YGRPLLGCTIKPK			Carbamidomethyl (C)[8]		Mascot

Calc. Mass	Obsrv. Mass	± da	± ppm	Start Seq.	End Seq.	Sequence	Ion Score	C. I. %	Modification	Rank	Result Type
1502.8512	1502.849	-0.0022	-1	110	122	YGRPLLGCTIKPK	6	0	Carbamidomethyl (C)[8]		Mascot
1518.741	1518.7408	-0.0002	0	161	172	DRFVFCAEAIYK			Carbamidomethyl (C)[6]		Mascot
1546.736	1546.7114	-0.0246	-16	396	408	WSPELAAACEVWK			Carbamidomethyl (C)[9]		Mascot
1651.8578	1651.8324	-0.0254	-15	409	422	AIKFEFEPVDTIDK					Mascot
1893.9739	1893.9507	-0.0232	-12	280	295	LEGEREMTLGFVDLLR			Oxidation (M)[7]		Mascot
1950.916	1950.9291	0.0131	7	182	198	GHYLNATAGTCEEMIKR			Carbamidomethyl (C)[11]		Mascot
1966.911	1966.9098	-0.0012	-1	182	198	GHYLNATAGTCEEMIKR			Carbamidomethyl (C)[11], Oxidation (M)[14]		Mascot
2041.0311	2041.0352	0.0041	2	285	301	EMTLGFVDLLRDDFIEK					Mascot
2057.0261	2057.0132	-0.0129	-6	285	301	EMTLGFVDLLRDDFIEK			Oxidation (M)[2]		Mascot
2169.9871	2169.999	0.0119	5	140	158	GGLDFTKDDENVNSQPF MR					Mascot
2169.9871	2169.999	0.0119	5	140	158	GGLDFTKDDENVNSQPF MR	93	100			Mascot
2185.9819	2185.9814	-0.0005	0	140	158	GGLDFTKDDENVNSQPF MR			Oxidation (M)[18]		Mascot
2252.1824	2252.1777	-0.0047	-2	85	104	IPPTYSKTFQGPPHGIQV ER					Mascot

6	ribulose-1,5-bisphosphate carboxylase/oxygenase large subunit, partial [Triticum aestivum]	gi|313664567	22653.3	5.78	7	360	100	35.462	319	100	

Protein Group

ribulose-1,5-bisphosphate carboxylase/oxygenase large subunit [Triticum aestivum]	gi|339742637	22185.1	7.5700 001716 6138

Peptide Information

Calc. Mass	Obsrv. Mass	± da	± ppm	Start Seq.	End Seq.	Sequence	Ion Score	C. I. %	Modification	Rank	Result Type
971.4073	971.4023	-0.005	-5	179	185	ACYECLR			Carbamidomethyl (C)[2,5]		Mascot
971.4073	971.4023	-0.005	-5	179	185	ACYECLR	24	71.301	Carbamidomethyl (C)[2,5]		Mascot
1021.5312	1021.5198	-0.0114	-11	24	32	DTDILAAFR					Mascot
1021.5312	1021.5198	-0.0114	-11	24	32	DTDILAAFR	56	99.983			Mascot
1407.6678	1407.6458	-0.022	-16	13	23	LTYYTPEYETK					Mascot
1465.7546	1465.765	0.0104	7	138	150	TFQGPPHGIQVER					Mascot
1465.7546	1465.765	0.0104	7	138	150	TFQGPPHGIQVER	102	100			Mascot
1502.8512	1502.849	-0.0022	-1	156	168	YGRPLLGCTIKPK			Carbamidomethyl (C)[8]		Mascot
1502.8512	1502.849	-0.0022	-1	156	168	YGRPLLGCTIKPK	6	0	Carbamidomethyl (C)[8]		Mascot
2252.1824	2252.1777	-0.0047	-2	131	150	IPPTYSKTFQGPPHGIQV ER					Mascot
3840.8564	3840.968	0.1116	29	33	70	VSPQPGVPPEEAGAAVA AESSTGTWTTVWTDGLT SLDR					Mascot
3840.8564	3840.968	0.1116	29	33	70	VSPQPGVPPEEAGAAVA AESSTGTWTTVWTDGLT	131	100			Mascot

SLDR

7	Ribulose bisphosphate carboxylase large chain [Triticum urartu]	gi|474087389	46868.7	5.78	16	336	100	41.708	257	100	

Peptide Information

Calc. Mass	Obsrv. Mass	± da	± ppm	Start Seq.	End Seq.	Sequence	Ion Score	C. I. %	Modification	Rank	Result Type
830.4366	830.4283	-0.0083	-10	385	391	EGNEIIR					Mascot
880.4536	880.4	-0.0536	-61	251	257	NHGIHFR					Mascot
963.5622	963.5828	0.0206	21	9	18	ASVGFKAGVK					Mascot
1021.5312	1021.5198	-0.0114	-11	33	41	DTDILAAFR					Mascot
1021.5312	1021.5198	-0.0114	-11	33	41	DTDILAAFR	56	99.983			Mascot
1116.583	1116.5812	-0.0018	-2	367	376	VALEACVQAR			Carbamidomethyl (C)[6]		Mascot
1245.6337	1245.6863	0.0526	42	218	227	FLFCAEAIFK			Carbamidomethyl (C)[4]		Mascot
1245.6433	1245.6863	0.043	35	385	395	EGNEIIREASK					Mascot
1275.7307	1275.6725	-0.0582	-46	285	295	EITLGFVDLLR					Mascot
1285.6859	1285.5776	-0.1083	-84	381	391	DLAREGNEIIR					Mascot
1291.7383	1291.6506	-0.0877	-68	251	261	NHGIHFRVLAK					Mascot
1407.6678	1407.6458	-0.022	-16	22	32	LTYYTPEYETK					Mascot
1451.6219	1451.6394	0.0175	12	202	213	DDENVNSQPFMR					Mascot
1465.7546	1465.765	0.0104	7	147	159	TFQGPPHGIQVER					Mascot
1465.7546	1465.765	0.0104	7	147	159	TFQGPPHGIQVER	102	100			Mascot
1502.8512	1502.849	-0.0022	-1	165	177	YGRPLLGCTIKPK			Carbamidomethyl (C)[8]		Mascot
1502.8512	1502.849	-0.0022	-1	165	177	YGRPLLGCTIKPK	6	0	Carbamidomethyl (C)[8]		Mascot
1546.736	1546.7114	-0.0246	-16	396	408	WSPELAAACEVWK			Carbamidomethyl (C)[9]		Mascot
2169.9871	2169.999	0.0119	5	195	213	GGLDFTKDDENVNSQPF MR					Mascot
2169.9871	2169.999	0.0119	5	195	213	GGLDFTKDDENVNSQPF MR	93	100			Mascot
2185.9819	2185.9814	-0.0005	0	195	213	GGLDFTKDDENVNSQPF MR			Oxidation (M)[18]		Mascot

8	ribulose-1,5-bisphosphate carboxylase/oxygenase large subunit [Triticum monococcum]	gi|355344487	20660.3	6.18	6	331	100	32.891	295	100	

Protein Group

ribulose-1,5-bisphosphate carboxylase/oxygenase large subunit [Triticum spelta]	gi|355344485	20633.4	6.9200 000762 9395
ribulose-1,5-bisphosphate carboxylase/oxygenase large subunit [Triticum turgidum subsp. dicoccon]	gi|355344489	20633.4	6.9200 000762 9395

Peptide Information

Calc. Mass	Obsrv. Mass	± da	± ppm	Start Seq.	End Seq.	Sequence	Ion Score	C. I. %	Modification	Rank	Result Type
1021.5312	1021.5198	-0.0114	-11	24	32	DTDILAAFR					Mascot
1021.5312	1021.5198	-0.0114	-11	24	32	DTDILAAFR	56	99.983			Mascot
1407.6678	1407.6458	-0.022	-16	13	23	LTYYTPEYETK					Mascot
1465.7546	1465.765	0.0104	7	138	150	TFQGPPHGIQVER					Mascot
1465.7546	1465.765	0.0104	7	138	150	TFQGPPHGIQVER	102	100			Mascot
1502.8512	1502.849	-0.0022	-1	156	168	YGRPLLGCTIKPK			Carbamidomethyl (C)[8]		Mascot
1502.8512	1502.849	-0.0022	-1	156	168	YGRPLLGCTIKPK	6	0	Carbamidomethyl (C)[8]		Mascot
2252.1824	2252.1777	-0.0047	-2	131	150	IPPTYSKTFQGPPHGIQVER					Mascot
3840.8564	3840.968	0.1116	29	33	70	VSPQPGVPPEEAGAAVAAESSTGTWTTVWTDGLTSLDR					Mascot
3840.8564	3840.968	0.1116	29	33	70	VSPQPGVPPEEAGAAVAAESSTGTWTTVWTDGLTSLDR	131	100			Mascot

9	Ribulose bisphosphate carboxylase large chain [Triticum urartu]	gi|474173630	30533.1	6.39	9	262	100	37.124	225	100		

Peptide Information

Calc. Mass	Obsrv. Mass	± da	± ppm	Start Seq.	End Seq.	Sequence	Ion Score	C. I. %	Modification	Rank	Result Type
971.4073	971.4023	-0.005	-5	179	185	ACYECLR			Carbamidomethyl (C)[2,5]		Mascot
971.4073	971.4023	-0.005	-5	179	185	ACYECLR	24	71.301	Carbamidomethyl (C)[2,5]		Mascot
1247.6129	1247.5988	-0.0141	-11	209	218	FVFCAEAIYK			Carbamidomethyl (C)[4]		Mascot
1451.6219	1451.6394	0.0175	12	193	204	DDENVNSQPFMR					Mascot
1465.7546	1465.765	0.0104	7	138	150	TFQGPPHGIQVER					Mascot
1465.7546	1465.765	0.0104	7	138	150	TFQGPPHGIQVER	102	100			Mascot
1502.8512	1502.849	-0.0022	-1	156	168	YGRPLLGCTIKPK			Carbamidomethyl (C)[8]		Mascot
1502.8512	1502.849	-0.0022	-1	156	168	YGRPLLGCTIKPK	6	0	Carbamidomethyl (C)[8]		Mascot
1518.741	1518.7408	-0.0002	0	207	218	DRFVFCAEAIYK			Carbamidomethyl (C)[6]		Mascot
1950.916	1950.9291	0.0131	7	228	244	GHYLNATAGTCEEMIKR			Carbamidomethyl (C)[11]		Mascot
1966.911	1966.9098	-0.0012	-1	228	244	GHYLNATAGTCEEMIKR			Carbamidomethyl (C)[11], Oxidation (M)[14]		Mascot
2169.9871	2169.999	0.0119	5	186	204	GGLDFTKDDENVNSQPFMR					Mascot
2169.9871	2169.999	0.0119	5	186	204	GGLDFTKDDENVNSQPFMR	93	100			Mascot
2185.9819	2185.9814	-0.0005	0	186	204	GGLDFTKDDENVNSQPFMR			Oxidation (M)[18]		Mascot
2252.1824	2252.1777	-0.0047	-2	131	150	IPPTYSKTFQGPPHGIQVER					Mascot

10 Superkiller viralicidic activity 2-like 2 [Triticum urartu] gi|474423721 118282.3 6.05 20 51 62.361 9.915

Peptide Information

Calc. Mass	Obsrv. Mass	± da	± ppm	Start Seq.	End Seq.	Sequence	Ion Score	C. I. %	Modification	Rank	Result Type
1021.5095	1021.5198	0.0103	10	841	848	MRDSQLQK			Oxidation (M)[1]		Mascot
1021.5425	1021.5198	-0.0227	-22	843	850	DSQLQKFR	4	0			Mascot
1032.5983	1032.5159	-0.0824	-80	1	9	MLGVRALTR			Oxidation (M)[1]		Mascot
1037.5482	1037.4851	-0.0631	-61	370	377	IVKMIMDR			Oxidation (M)[4,6]		Mascot
1116.6147	1116.5812	-0.0335	-30	624	633	LGLDISELEK					Mascot
1244.7097	1244.6775	-0.0322	-26	624	634	LGLDISELEKK					Mascot
1261.6392	1261.6537	0.0145	11	635	644	IMSEMIRPER					Mascot
1275.7056	1275.6725	-0.0331	-26	1002	1011	RLDEFLNQLK					Mascot
1293.629	1293.6863	0.0573	44	635	644	IMSEMIRPER	5	0	Oxidation (M)[2,5]		Mascot
1340.7362	1340.6538	-0.0824	-61	379	389	FQPVIIFSFSR					Mascot
1421.724	1421.748	0.024	17	634	644	KIMSEMIRPER			Oxidation (M)[3,6]		Mascot
1464.8169	1464.7468	-0.0701	-48	746	758	ISIPTDLRPPEAR					Mascot
1515.8643	1515.7655	-0.0988	-65	759	771	QNILFAVQELGKR					Mascot
1518.6246	1518.7408	0.1162	77	390	401	RECEHHAMSMSK			Carbamidomethyl (C)[3], Oxidation (M)[8]		Mascot
1555.8149	1555.8782	0.0633	41	862	875	MLGHIDTDGVLQLK			Oxidation (M)[1]		Mascot
1561.8948	1561.7406	-0.1542	-99	169	182	VIYTSPLKALSNQK					Mascot
1568.7955	1568.7291	-0.0664	-42	324	336	EDNFLKLQDTFAK					Mascot
1723.8176	1723.8146	-0.003	-2	521	535	GMDARGICVIMIDEK			Carbamidomethyl (C)[8], Oxidation (M)[2]		Mascot
2003.9127	2004.01	0.0973	49	100	118	DEAVHGTLSNPAFNGEM AK			Oxidation (M)[17]		Mascot
2328.0925	2328.1528	0.0603	26	411	429	DNIEQVFRSAIFCLSEED R			Carbamidomethyl (C)[13]		Mascot
3068.6125	3068.498	-0.1145	-37	772	798	YPQGLPKLHPITDMGIEE PELVDLVHK					Mascot

Gel Idx/Pos	290/L18	Instr./Gel Origin	BA2151/Sample Project 20140814	Process Status	Analysis Succeeded
Plate [#] Name	[1] Sample Project 20140814	Instrument Sample Name		Spectra	11

Rank	Protein Name	Accession No.	Protein MW	Protein PI	Pep. Count	Protein Score	Protein Score C. I. %	Intensity Matched	Total Ion Score	Total Ion C. I. %	Confirmed
1	ADP-glucose pyrophosphorylase large subunit [Triticum aestivum]	gi|110729318	58399.1	6.12	29	1,140	100	63.89	955	100	

Protein Group

Protein Name	Accession No.	Protein MW	Protein PI
ADP-glucose pyrophosphorylase large subunit [Triticum aestivum]	gi|995746	58399.1	6.1199998855 5908
RecName: Full=Glucose-1-phosphate adenylyltransferase large subunit, chloroplastic/amyloplastic; AltName: Full=ADP-glucose pyrophosphorylase; AltName: Full=ADP-glucose synthase; AltName: Full=AGPase S; AltName: Full=Alpha-D-glucose-1-phosphate adeny	gi|1707930	58399.1	6.1199998855 5908
unnamed protein product [Triticum aestivum]	gi|300634107	58399.1	6.1199998855 5908
unnamed protein product [Triticum aestivum]	gi|300544593	58399.1	6.1199998855 5908
unnamed protein product [Triticum aestivum]	gi|259471795	58399.1	6.1199998855 5908
unnamed protein product [Triticum aestivum]	gi|259439208	58399.1	6.1199998855 5908
unnamed protein product [Triticum aestivum]	gi|257671708	58399.1	6.1199998855 5908
unnamed protein product [Triticum aestivum]	gi|257306977	58399.1	6.1199998855 5908
unnamed protein product [Triticum aestivum]	gi|257308981	58399.1	6.1199998855 5908
unnamed protein product [Triticum aestivum]	gi|219764742	58399.1	6.1199998855 5908
unnamed protein product [Triticum aestivum]	gi|219752155	58399.1	6.1199

Peptide Information

Calc. Mass	Obsrv. Mass	± da	± ppm	Start Seq.	End Seq.	Sequence	Ion Score	C. I. %	Modification	Rank	Result Type
802.424	802.4251	0.0011	1	16	22	ACISPVR			Carbamidomethyl (C)[2]		Mascot
820.4159	820.4044	-0.0115	-14	33	40	VGDSSSIR					Mascot
847.452	847.4308	-0.0212	-25	426	433	LNSGSELK					Mascot
866.4618	866.4388	-0.023	-27	251	258	ASEYGLVK					Mascot
927.5873	927.5493	-0.038	-41	311	318	DVLLNLLK					Mascot
945.4352	945.4153	-0.0199	-21	373	379	FEFYDPK					Mascot
952.4887	952.4767	-0.012	-13	380	387	TPFFTSPR					Mascot
952.4887	952.4767	-0.012	-13	380	387	TPFFTSPR	51	99.936			Mascot
1013.5626	1013.5337	-0.0289	-29	458	467	VPIGVGENTK					Mascot
1023.5945	1023.5873	-0.0072	-7	415	423	IEHSIIGVR					Mascot
1023.5945	1023.5873	-0.0072	-7	415	423	IEHSIIGVR	53	99.956			Mascot
1061.595	1061.5651	-0.0299	-28	31	40	LKVGDSSSIR					Mascot
1170.7205	1170.6298	-0.0907	-77	311	320	DVLLNLLKSR					Mascot
1261.6359	1261.6375	0.0016	1	111	122	ATPAVPIGGCYR			Carbamidomethyl (C)[10]		Mascot
1261.6359	1261.6375	0.0016	1	111	122	ATPAVPIGGCYR	76	100	Carbamidomethyl (C)[10]		Mascot
1302.6624	1302.6641	0.0017	1	401	411	EAIISHGCFLR			Carbamidomethyl (C)[8]		Mascot
1302.6624	1302.6641	0.0017	1	401	411	EAIISHGCFLR	83	100	Carbamidomethyl (C)[8]		Mascot
1322.6191	1322.5989	-0.0202	-15	468	478	ISNCIIDMNAR			Carbamidomethyl (C)[4], Oxidation (M)[8]		Mascot
1322.6191	1322.5989	-0.0202	-15	468	478	ISNCIIDMNAR	37	98.398	Carbamidomethyl (C)[4], Oxidation (M)[8]		Mascot
1503.7882	1503.7552	-0.033	-22	195	205	KFIWVLEDYYK					Mascot
1553.7709	1553.7338	-0.0371	-24	297	309	YPYIASMGVYVFK			Oxidation (M)[7]		Mascot
1643.821	1643.8031	-0.0179	-11	139	152	IFVMTQFNSASLNR			Oxidation (M)[4]		Mascot
1646.8174	1646.8174	0	0	321	334	YAELHDFGSEILPR					Mascot
1646.8174	1646.8174	0	0	321	334	YAELHDFGSEILPR	108	100			Mascot
1652.8167	1652.7986	-0.0181	-11	282	296	VDTSFLNFAIDDPAK					Mascot
1674.8732	1674.8409	-0.0323	-19	452	467	LMSEGKVPIGVGENTK			Oxidation (M)[2]		Mascot
1709.8719	1709.8036	-0.0683	-40	297	310	YPYIASMGVYVFKR			Oxidation (M)[7]		Mascot
1756.9592	1756.9644	0.0052	3	208	222	SIEHILILSGDQLYR					Mascot
1756.9592	1756.9644	0.0052	3	208	222	SIEHILILSGDQLYR	110	100			Mascot
1770.7826	1770.7554	-0.0272	-15	358	372	SFFDANMALCEQPPK			Carbamidomethyl (C)[10], Oxidation (M)[7]		Mascot
1838.8776	1838.8567	-0.0209	-11	123	138	LIDIPMSNCFNSGINK			Carbamidomethyl (C)[9], Oxidation (M)[6]		Mascot
1910.8879	1910.8832	-0.0047	-2	489	504	EGVQEADRPEEGYYIR					Mascot

2055.9399	2055.9438	0.0039	2	232	250	HVDDNADITLSCAPVGES R			Carbamidomethyl (C)[12]		Mascot
2371.1924	2371.2029	0.0105	4	54	77	GPAATGAQCVLTSDASP ADTLVLR			Carbamidomethyl (C)[9]		Mascot
2371.1924	2371.2029	0.0105	4	54	77	GPAATGAQCVLTSDASP ADTLVLR	198	100	Carbamidomethyl (C)[9]		Mascot
2763.3162	2763.3372	0.021	8	335	357	ALHDHNVQAYVFTDYWE DIGTIR					Mascot
2862.4634	2862.4819	0.0185	6	83	110	NYADPNEVAAVILGGGT GTQLFPLTSTR					Mascot
2862.4634	2862.4819	0.0185	6	83	110	NYADPNEVAAVILGGGT GTQLFPLTSTR	239	100			Mascot

2	unnamed protein product [Triticum aestivum]	gi|257304087	58353.9	5.89	27	1,120	100	63.388	955	100

Protein Group

ADP-glucose pyrophosphorylase large subunit [Triticum aestivum]	gi|32812836	58353.9	5.8899998664856
unnamed protein product [Triticum aestivum]	gi|257308933	58353.9	5.8899998664856
unnamed protein product [Triticum aestivum]	gi|219764692	58353.9	5.8899998664856
unnamed protein product [Triticum aestivum]	gi|257671660	58353.9	5.8899998664856
unnamed protein product [Triticum aestivum]	gi|219752105	58353.9	5.8899998664856

Peptide Information

Calc. Mass	Obsrv. Mass	± da	± ppm	Start Seq.	End Seq.	Sequence	Ion Score	C. I. %	Modification	Rank	Result Type
808.3254	808.3248	-0.0006	-1	24	30	EGSACER			Carbamidomethyl (C)[5]		Mascot
820.4159	820.4044	-0.0115	-14	33	40	VGDSSSIR					Mascot
847.452	847.4308	-0.0212	-25	426	433	LNSGSELK					Mascot
866.4618	866.4388	-0.023	-27	251	258	ASEYGLVK					Mascot
927.5873	927.5493	-0.038	-41	311	318	DVLLNLLK					Mascot
945.4352	945.4153	-0.0199	-21	373	379	FEFYDPK					Mascot
952.4887	952.4767	-0.012	-13	380	387	TPFFTSPR					Mascot
952.4887	952.4767	-0.012	-13	380	387	TPFFTSPR	51	99.936			Mascot
1013.5626	1013.5337	-0.0289	-29	458	467	VPIGVGENTK					Mascot
1023.5945	1023.5873	-0.0072	-7	415	423	IEHSIIGVR					Mascot

Calc. Mass	Obsrv. Mass	± da	± ppm	Start Seq.	End Seq.	Sequence	Ion Score	C. I. %	Modification	Rank	Result Type
1023.5945	1023.5873	-0.0072	-7	415	423	IEHSIIGVR	53	99.956			Mascot
1061.595	1061.5651	-0.0299	-28	31	40	LKVGDSSSIR					Mascot
1170.7205	1170.6298	-0.0907	-77	311	320	DVLLNLLKSR					Mascot
1261.6359	1261.6375	0.0016	1	111	122	ATPAVPIGGCYR			Carbamidomethyl (C)[10]		Mascot
1261.6359	1261.6375	0.0016	1	111	122	ATPAVPIGGCYR	76	100	Carbamidomethyl (C)[10]		Mascot
1302.6624	1302.6641	0.0017	1	401	411	EAIISHGCFLR			Carbamidomethyl (C)[8]		Mascot
1302.6624	1302.6641	0.0017	1	401	411	EAIISHGCFLR	83	100	Carbamidomethyl (C)[8]		Mascot
1322.6191	1322.5989	-0.0202	-15	468	478	ISNCIIDMNAR			Carbamidomethyl (C)[4], Oxidation (M)[8]		Mascot
1322.6191	1322.5989	-0.0202	-15	468	478	ISNCIIDMNAR	37	98.398	Carbamidomethyl (C)[4], Oxidation (M)[8]		Mascot
1503.7882	1503.7552	-0.033	-22	195	205	KFIWVLEDYYK					Mascot
1553.7709	1553.7338	-0.0371	-24	297	309	YPYIASMGVYVFK			Oxidation (M)[7]		Mascot
1643.821	1643.8031	-0.0179	-11	139	152	IFVMTQFNSASLNR			Oxidation (M)[4]		Mascot
1646.8174	1646.8174	0	0	321	334	YAELHDFGSEILPR					Mascot
1646.8174	1646.8174	0	0	321	334	YAELHDFGSEILPR	108	100			Mascot
1652.8167	1652.7986	-0.0181	-11	282	296	VDTSFLNFAIDDPAK					Mascot
1674.8732	1674.8409	-0.0323	-19	452	467	LMSEGKVPIGVGENTK			Oxidation (M)[2]		Mascot
1709.8719	1709.8036	-0.0683	-40	297	310	YPYIASMGVYVFKR			Oxidation (M)[7]		Mascot
1756.9592	1756.9644	0.0052	3	208	222	SIEHILILSGDQLYR					Mascot
1756.9592	1756.9644	0.0052	3	208	222	SIEHILILSGDQLYR	110	100			Mascot
1838.8776	1838.8567	-0.0209	-11	123	138	LIDIPMSNCFNSGINK			Carbamidomethyl (C)[9], Oxidation (M)[6]		Mascot
1910.8879	1910.8832	-0.0047	-2	489	504	EGVQEADRPEEGYYIR					Mascot
2055.9399	2055.9438	0.0039	2	232	250	HVDDNADITLSCAPVGES R			Carbamidomethyl (C)[12]		Mascot
2371.1924	2371.2029	0.0105	4	54	77	GPAATGAQCVLTSDASP ADTLVLR			Carbamidomethyl (C)[9]		Mascot
2371.1924	2371.2029	0.0105	4	54	77	GPAATGAQCVLTSDASP ADTLVLR	198	100	Carbamidomethyl (C)[9]		Mascot
2862.4634	2862.4819	0.0185	6	83	110	NYADPNEVAAVILGGGT GTQLFPLTSTR					Mascot
2862.4634	2862.4819	0.0185	6	83	110	NYADPNEVAAVILGGGT GTQLFPLTSTR	239	100			Mascot

3 Glucose-1-phosphate adenylyltransferase large subunit 1, chloroplastic/amyloplastic [Triticum urartu] gi|474056532 51530 8.53 18 851 100 55.153 758 100

Peptide Information

Calc. Mass	Obsrv. Mass	± da	± ppm	Start Seq.	End Seq.	Sequence	Ion Score	C. I. %	Modification	Rank	Result Type
847.452	847.4308	-0.0212	-25	306	313	LNSGSELK					Mascot
945.4352	945.4153	-0.0199	-21	253	259	FEFYDPK					Mascot
952.4887	952.4767	-0.012	-13	260	267	TPFFTSPR					Mascot
952.4887	952.4767	-0.012	-13	260	267	TPFFTSPR	51	99.936			Mascot

1013.5626	1013.5337	-0.0289	-29	338	347	VPIGVGENTK					Mascot
1023.5945	1023.5873	-0.0072	-7	295	303	IEHSIIGVR					Mascot
1023.5945	1023.5873	-0.0072	-7	295	303	IEHSIIGVR	53	99.956			Mascot
1261.6359	1261.6375	0.0016	1	60	71	ATPAVPIGGCYR			Carbamidomethyl (C)[10]		Mascot
1261.6359	1261.6375	0.0016	1	60	71	ATPAVPIGGCYR	76	100	Carbamidomethyl (C)[10]		Mascot
1302.6624	1302.6641	0.0017	1	281	291	EAIISHGCFLR			Carbamidomethyl (C)[8]		Mascot
1302.6624	1302.6641	0.0017	1	281	291	EAIISHGCFLR	83	100	Carbamidomethyl (C)[8]		Mascot
1322.6191	1322.5989	-0.0202	-15	348	358	ISNCIIDMNAR			Carbamidomethyl (C)[4], Oxidation (M)[8]		Mascot
1322.6191	1322.5989	-0.0202	-15	348	358	ISNCIIDMNAR	37	98.398	Carbamidomethyl (C)[4], Oxidation (M)[8]		Mascot
1503.7882	1503.7552	-0.033	-22	144	154	KFIWVLEDYYK					Mascot
1643.821	1643.8031	-0.0179	-11	88	101	IFVMTQFNSASLNR			Oxidation (M)[4]		Mascot
1646.8174	1646.8174	0	0	201	214	YAELHDFGSEILPR					Mascot
1646.8174	1646.8174	0	0	201	214	YAELHDFGSEILPR	108	100			Mascot
1674.8732	1674.8409	-0.0323	-19	332	347	LMSEGKVPIGVGENTK			Oxidation (M)[2]		Mascot
1756.9592	1756.9644	0.0052	3	157	171	SIEHILILSGDQLYR					Mascot
1756.9592	1756.9644	0.0052	3	157	171	SIEHILILSGDQLYR	110	100			Mascot
1770.7826	1770.7554	-0.0272	-15	238	252	SFFDANMALCEQPPK			Carbamidomethyl (C)[10], Oxidation (M)[7]		Mascot
1838.8776	1838.8567	-0.0209	-11	72	87	LIDIPMSNCFNSGINK			Carbamidomethyl (C)[9], Oxidation (M)[6]		Mascot
1910.8879	1910.8832	-0.0047	-2	369	384	EGVQEADRPEEGYYIR					Mascot
2763.3162	2763.3372	0.021	8	215	237	ALHDHNVQAYVFTDYWE DIGTIR					Mascot
2862.4634	2862.4819	0.0185	6	32	59	NYADPNEVAAVILGGGT GTQLFPLTSTR					Mascot
2862.4634	2862.4819	0.0185	6	32	59	NYADPNEVAAVILGGGT GTQLFPLTSTR	239	100			Mascot

4	ADP-glucose pyrophosophorylase preprotein [Triticum aestivum]	gi|21680	56037.9	6.61	22	496	100	47.287	383	100	

Protein Group

ADP glucose pyrophosphatase AGA.7	gi|226875	56037.9	6.6100 001335 144
RecName: Full=Glucose-1-phosphate adenylyltransferase large subunit, chloroplastic/amyloplastic; AltName: Full=ADP-glucose pyrophosphorylase; AltName: Full=ADP-glucose synthase; AltName: Full=AGPase S; AltName: Full=Alpha-D-glucose-1-phosphate adeny	gi|121293	56037.9	6.6100 001335 144

Peptide Information

Calc. Mass	Obsrv. Mass	± da	± ppm	Start Seq.	End Seq.	Sequence	Ion Score	C. I. %	Modification	Rank	Result Type
847.452	847.4308	-0.0212	-25	404	411	LNSGSELK					Mascot

866.4618	866.4388	-0.023	-27	232	239	ASEYGLVK					Mascot
908.5424	908.4543	-0.0881	-97	10	17	APLRAPQR					Mascot
927.5873	927.5493	-0.038	-41	292	299	DVLLNLLK					Mascot
945.4352	945.4153	-0.0199	-21	354	360	FEFYDPK					Mascot
952.4887	952.4767	-0.012	-13	361	368	TPFFTSPR					Mascot
952.4887	952.4767	-0.012	-13	361	368	TPFFTSPR	51	99.936			Mascot
1013.5626	1013.5337	-0.0289	-29	436	445	VPIGVGENTK					Mascot
1061.5626	1061.5651	0.0025	2	246	254	VVQFSEQPK					Mascot
1170.7205	1170.6298	-0.0907	-77	292	301	DVLLNLLKSR					Mascot
1261.6359	1261.6375	0.0016	1	92	103	ATPAVPIGGCYR			Carbamidomethyl (C)[10]		Mascot
1261.6359	1261.6375	0.0016	1	92	103	ATPAVPIGGCYR	76	100	Carbamidomethyl (C)[10]		Mascot
1322.6191	1322.5989	-0.0202	-15	446	456	ISNCIIDMNAR			Carbamidomethyl (C)[4], Oxidation (M)[8]		Mascot
1322.6191	1322.5989	-0.0202	-15	446	456	ISNCIIDMNAR	37	98.398	Carbamidomethyl (C)[4], Oxidation (M)[8]		Mascot
1553.7709	1553.7338	-0.0371	-24	278	290	YPYIASMGVYVFK			Oxidation (M)[7]		Mascot
1643.821	1643.8031	-0.0179	-11	120	133	IFVMTQFNSASLNR			Oxidation (M)[4]		Mascot
1646.8174	1646.8174	0	0	302	315	YAELHDFGSEILPR					Mascot
1646.8174	1646.8174	0	0	302	315	YAELHDFGSEILPR	108	100			Mascot
1652.8167	1652.7986	-0.0181	-11	263	277	VDTSFLNFAIDDPAK					Mascot
1674.8732	1674.8409	-0.0323	-19	430	445	LMSEGKVPIGVGENTK			Oxidation (M)[2]		Mascot
1709.8719	1709.8036	-0.0683	-40	278	291	YPYIASMGVYVFKR			Oxidation (M)[7]		Mascot
1756.9592	1756.9644	0.0052	3	189	203	SIEHILILSGDQLYR					Mascot
1756.9592	1756.9644	0.0052	3	189	203	SIEHILILSGDQLYR	110	100			Mascot
1838.8776	1838.8567	-0.0209	-11	104	119	LIDIPMSNCFNSGINK			Carbamidomethyl (C)[9], Oxidation (M)[6]		Mascot
1910.8879	1910.8832	-0.0047	-2	467	482	EGVQEADRPEEGYYIR					Mascot
2055.9399	2055.9438	0.0039	2	213	231	HVDDNADITLSCAPVGES R			Carbamidomethyl (C)[12]		Mascot
2763.3162	2763.3372	0.021	8	316	338	ALHDHNVQAYVFTDYWE DIGTIR					Mascot

5 ADP-glucose-pyrophosphorylase large subunit [Triticum aestivum] gi|2583072 32915.3 5.21 19 456 100 34.933 332 100

Peptide Information

Calc. Mass	Obsrv. Mass	± da	± ppm	Start Seq.	End Seq.	Sequence	Ion Score	C. I. %	Modification	Rank	Result Type
847.452	847.4308	-0.0212	-25	194	201	LNSGSELK					Mascot
866.4618	866.4388	-0.023	-27	19	26	ASEYGLVK					Mascot
927.5873	927.5493	-0.038	-41	79	86	DVLLNLLK					Mascot
945.4352	945.4153	-0.0199	-21	141	147	FEFYDPK					Mascot
952.4887	952.4767	-0.012	-13	148	155	TPFFTSPR					Mascot

952.4887	952.4767	-0.012	-13	148	155	TPFFTSPR	51	99.936			Mascot
1013.5626	1013.5337	-0.0289	-29	226	235	VPIGVGENTK					Mascot
1023.5945	1023.5873	-0.0072	-7	183	191	IEHSIIGVR					Mascot
1023.5945	1023.5873	-0.0072	-7	183	191	IEHSIIGVR	53	99.956			Mascot
1061.599	1061.5651	-0.0339	-32	33	41	VVQFSEKPK					Mascot
1170.7205	1170.6298	-0.0907	-77	79	88	DVLLNLLKSR					Mascot
1302.6624	1302.6641	0.0017	1	169	179	EAIISHGCFLR			Carbamidomethyl (C)[8]		Mascot
1302.6624	1302.6641	0.0017	1	169	179	EAIISHGCFLR	83	100	Carbamidomethyl (C)[8]		Mascot
1322.6191	1322.5989	-0.0202	-15	236	246	ISNCIIDMNAR			Carbamidomethyl (C)[4], Oxidation (M)[8]		Mascot
1322.6191	1322.5989	-0.0202	-15	236	246	ISNCIIDMNAR	37	98.398	Carbamidomethyl (C)[4], Oxidation (M)[8]		Mascot
1553.7709	1553.7338	-0.0371	-24	65	77	YPYIASMGVYVFK			Oxidation (M)[7]		Mascot
1646.8174	1646.8174	0	0	89	102	YAELHDFGSEILPR					Mascot
1646.8174	1646.8174	0	0	89	102	YAELHDFGSEILPR	108	100			Mascot
1652.8167	1652.7986	-0.0181	-11	50	64	VDTSFLNFAIDDPAK					Mascot
1674.8732	1674.8409	-0.0323	-19	220	235	LMSEGKVPIGVGENTK			Oxidation (M)[2]		Mascot
1709.8719	1709.8036	-0.0683	-40	65	78	YPYIASMGVYVFKR			Oxidation (M)[7]		Mascot
1770.7826	1770.7554	-0.0272	-15	126	140	SFFDANMALCEQPPK			Carbamidomethyl (C)[10], Oxidation (M)[7]		Mascot
1910.8879	1910.8832	-0.0047	-2	257	272	EGVQEADRPEEGYYIR					Mascot
2763.3162	2763.3372	0.021	8	103	125	ALHDHNVQAYVFTDYWE DIGTIR					Mascot

6	ADP-glucose pyrophosphorylase [Triticum aestivum]	gi|1432141	21415.7	6.58	13	373	100	30.894	295	100	

Protein Group

ADP-glucose pyrophosphorylase, partial [Triticum aestivum]	gi|1432143	21415.7	6.5799 999237 0605

Peptide Information

Calc. Mass	Obsrv. Mass	± da	± ppm	Start Seq.	End Seq.	Sequence	Ion Score	C. I. %	Modification	Rank	Result Type
847.452	847.4308	-0.0212	-25	131	138	LNSGSELK					Mascot
927.5873	927.5493	-0.038	-41	16	23	DVLLNLLK					Mascot
945.4352	945.4153	-0.0199	-21	78	84	FEFYDPK					Mascot
952.4887	952.4767	-0.012	-13	85	92	TPFFTSPR					Mascot
952.4887	952.4767	-0.012	-13	85	92	TPFFTSPR	51	99.936			Mascot
962.4975	962.4715	-0.026	-27	173	180	ISNCIIDK			Carbamidomethyl (C)[4]		Mascot
1013.5626	1013.5337	-0.0289	-29	163	172	VPIGVGENTK					Mascot

1023.5945	1023.5873	-0.0072	-7	120	128	IEHSIIGVR					Mascot
1023.5945	1023.5873	-0.0072	-7	120	128	IEHSIIGVR	53	99.956			Mascot
1170.7205	1170.6298	-0.0907	-77	16	25	DVLLNLLKSR					Mascot
1302.6624	1302.6641	0.0017	1	106	116	EAIISHGCFLR			Carbamidomethyl (C)[8]		Mascot
1302.6624	1302.6641	0.0017	1	106	116	EAIISHGCFLR	83	100	Carbamidomethyl (C)[8]		Mascot
1646.8174	1646.8174	0	0	26	39	YAELHDFGSEILPR					Mascot
1646.8174	1646.8174	0	0	26	39	YAELHDFGSEILPR	108	100			Mascot
1674.8732	1674.8409	-0.0323	-19	157	172	LMSEGKVPIGVGENTK			Oxidation (M)[2]		Mascot
1770.7826	1770.7554	-0.0272	-15	63	77	SFFDANMALCEQPPK			Carbamidomethyl (C)[10], Oxidation (M)[7]		Mascot
2763.3162	2763.3372	0.021	8	40	62	ALHDHNVQAYVFTDYWE DIGTIR					Mascot

7	ADP-glucose pyrophosophorylase (1 is 2nd base in codon) [Triticum aestivum]	gi|829293	33580.7	5.13	18	314	100	25.77	196	100		

Protein Group

ADP glucose pyrophosphatase AGA.3	gi|226874	33580.7	5.1300 001144 4092

Peptide Information

Calc. Mass	Obsrv. Mass	± da	± ppm	Start Seq.	End Seq.	Sequence	Ion Score	C. I. %	Modification	Rank	Result Type
847.452	847.4308	-0.0212	-25	200	207	LNSGSELK					Mascot
866.4618	866.4388	-0.023	-27	25	32	ASEYGLVK					Mascot
927.5873	927.5493	-0.038	-41	85	92	DVLLNLLK					Mascot
945.4352	945.4153	-0.0199	-21	147	153	FEFYDPK					Mascot
952.4887	952.4767	-0.012	-13	154	161	TPFFTSPR					Mascot
952.4887	952.4767	-0.012	-13	154	161	TPFFTSPR	51	99.936			Mascot
1013.5626	1013.5337	-0.0289	-29	232	241	VPIGVGENTK					Mascot
1061.599	1061.5651	-0.0339	-32	39	47	VVQFSEKPK					Mascot
1170.7205	1170.6298	-0.0907	-77	85	94	DVLLNLLKSR					Mascot
1322.6191	1322.5989	-0.0202	-15	242	252	ISNCIIDMNAR			Carbamidomethyl (C)[4], Oxidation (M)[8]		Mascot
1322.6191	1322.5989	-0.0202	-15	242	252	ISNCIIDMNAR	37	98.398	Carbamidomethyl (C)[4], Oxidation (M)[8]		Mascot
1553.7709	1553.7338	-0.0371	-24	71	83	YPYIASMGVYVFK			Oxidation (M)[7]		Mascot
1646.8174	1646.8174	0	0	95	108	YAELHDFGSEILPR					Mascot
1646.8174	1646.8174	0	0	95	108	YAELHDFGSEILPR	108	100			Mascot
1652.8167	1652.7986	-0.0181	-11	56	70	VDTSFLNFAIDDPAK					Mascot
1674.8732	1674.8409	-0.0323	-19	226	241	LMSEGKVPIGVGENTK			Oxidation (M)[2]		Mascot

1709.8719	1709.8036	-0.0683	-40	71	84	YPYIASMGVYVFKR		Oxidation (M)[7]	Mascot
1770.7826	1770.7554	-0.0272	-15	132	146	SFFDANMSLCEQPPK		Carbamidomethyl (C)[10]	Mascot
1910.8879	1910.8832	-0.0047	-2	263	278	EGVQEADRPEEGYYIR			Mascot
2055.9399	2055.9438	0.0039	2	6	24	HVDDNADITLSCAPVGES R		Carbamidomethyl (C)[12]	Mascot
2763.3162	2763.3372	0.021	8	109	131	ALHDHNVQAYVFTDYWE DIGTIR			Mascot

8	ADP-glucose pyrophosphorylase [Triticum monococcum subsp. aegilopoides]	gi|148529550	17493.5	5.21	9	242	100	21.525	187	100	

Protein Group

ADP-glucose pyrophosphorylase [Triticum monococcum subsp. aegilopoides]	gi|148529546	17467.4	5.2100 000381 4697
ADP-glucose pyrophosphorylase [Triticum monococcum subsp. aegilopoides]	gi|148529544	17467.4	5.2100 000381 4697
ADP-glucose pyrophosphorylase [Triticum monococcum subsp. aegilopoides]	gi|148529534	17467.4	5.2100 000381 4697
ADP-glucose pyrophosphorylase [Triticum monococcum subsp. aegilopoides]	gi|148529528	17467.4	5.2100 000381 4697
ADP-glucose pyrophosphorylase [Triticum monococcum subsp. aegilopoides]	gi|148529530	17467.4	5.2100 000381 4697
ADP-glucose pyrophosphorylase [Triticum monococcum subsp. aegilopoides]	gi|148529532	17467.4	5.2100 000381 4697
ADP-glucose pyrophosphorylase [Triticum monococcum subsp. aegilopoides]	gi|148529526	17467.4	5.2100 000381 4697
ADP-glucose pyrophosphorylase [Triticum	gi|148529522	17467.4	5.2100

monococcum subsp. aegilopoides]			000381 4697
ADP-glucose pyrophosphorylase [Triticum monococcum subsp. aegilopoides]	gi|148529520	17467.4	5.2100 000381 4697
ADP-glucose pyrophosphorylase [Triticum monococcum subsp. aegilopoides]	gi|148529552	17467.4	5.2100 000381 4697
ADP-glucose pyrophosphorylase [Triticum monococcum subsp. aegilopoides]	gi|148529524	17467.4	5.2100 000381 4697
ADP-glucose pyrophosphorylase [Triticum monococcum]	gi|148529548	17467.4	5.2100 000381 4697
ADP-glucose pyrophosphorylase [Triticum monococcum]	gi|148529516	17467.4	5.2100 000381 4697
ADP-glucose pyrophosphorylase [Triticum monococcum]	gi|148529518	17467.4	5.2100 000381 4697
ADP-glucose pyrophosphorylase [Triticum urartu]	gi|148529542	17467.4	5.2100 000381 4697
ADP-glucose pyrophosphorylase [Triticum urartu]	gi|148529540	17467.4	5.2100 000381 4697
ADP-glucose pyrophosphorylase [Triticum urartu]	gi|148529538	17467.4	5.2100 000381 4697

ADP-glucose pyrophosphorylase [Triticum urartu]	gi|148529536	17467.4	5.2100 000381 4697

Peptide Information

Calc. Mass	Obsrv. Mass	± da	± ppm	Start Seq.	End Seq.	Sequence	Ion Score	C. I. %	Modification	Rank	Result Type
847.452	847.4308	-0.0212	-25	101	108	LNSGSELK					Mascot
945.4352	945.4153	-0.0199	-21	48	54	FEFYDPK					Mascot
952.4887	952.4767	-0.012	-13	55	62	TPFFTSPR					Mascot
952.4887	952.4767	-0.012	-13	55	62	TPFFTSPR	51	99.936			Mascot
1013.5626	1013.5337	-0.0289	-29	133	142	VPIGVGENTK					Mascot
1023.5945	1023.5873	-0.0072	-7	90	98	IEHSIIGVR					Mascot
1023.5945	1023.5873	-0.0072	-7	90	98	IEHSIIGVR	53	99.956			Mascot
1302.6624	1302.6641	0.0017	1	76	86	EAIISHGCFLR			Carbamidomethyl (C)[8]		Mascot
1302.6624	1302.6641	0.0017	1	76	86	EAIISHGCFLR	83	100	Carbamidomethyl (C)[8]		Mascot
1674.8732	1674.8409	-0.0323	-19	127	142	LMSEGKVPIGVGENTK			Oxidation (M)[2]		Mascot
1770.7826	1770.7554	-0.0272	-15	33	47	SFFDANMALCEQPPK			Carbamidomethyl (C)[10], Oxidation (M)[7]		Mascot
2763.3162	2763.3372	0.021	8	10	32	ALHDHNVQAYVFTDYWE DIGTIR					Mascot

9	chloroplast putative glucose-1-phosphate adenylyltransferase large subunit 1 precursor [Triticum monococcum]	gi|210063654	12054.9	5.21	7	214	100	13.719	173	100	

Protein Group

chloroplast putative glucose-1-phosphate adenylyltransferase large subunit 1 precursor [Triticum urartu]	gi|210063658	12054.9	5.2100 000381 4697

Peptide Information

Calc. Mass	Obsrv. Mass	± da	± ppm	Start Seq.	End Seq.	Sequence	Ion Score	C. I. %	Modification	Rank	Result Type
847.452	847.4308	-0.0212	-25	28	35	LNSGSELK					Mascot
1013.5626	1013.5337	-0.0289	-29	60	69	VPIGVGENTK					Mascot
1023.5945	1023.5873	-0.0072	-7	17	25	IEHSIIGVR					Mascot
1023.5945	1023.5873	-0.0072	-7	17	25	IEHSIIGVR	53	99.956			Mascot

1302.6624	1302.6641	0.0017	1	3	13	EAIISHGCFLR			Carbamidomethyl (C)[8]		Mascot
1302.6624	1302.6641	0.0017	1	3	13	EAIISHGCFLR	83	100	Carbamidomethyl (C)[8]		Mascot
1322.6191	1322.5989	-0.0202	-15	70	80	ISNCIIDMNAR			Carbamidomethyl (C)[4], Oxidation (M)[8]		Mascot
1322.6191	1322.5989	-0.0202	-15	70	80	ISNCIIDMNAR	37	98.398	Carbamidomethyl (C)[4], Oxidation (M)[8]		Mascot
1674.8732	1674.8409	-0.0323	-19	54	69	LMSEGKVPIGVGENTK			Oxidation (M)[2]		Mascot
1910.8879	1910.8832	-0.0047	-2	91	106	EGVQEADRPEEGYYIR					Mascot

10	chloroplast putative glucose-1-phosphate adenylyltransferase large subunit 1 precursor [Triticum urartu]	gi|210063887	21209.7	5.84	12	193	100	16.832	110	100	

Peptide Information

Calc. Mass	Obsrv. Mass	± da	± ppm	Start Seq.	End Seq.	Sequence	Ion Score	C. I. %	Modification	Rank	Result Type
866.4618	866.4388	-0.023	-27	116	123	ASEYGLVK					Mascot
927.5873	927.5493	-0.038	-41	176	183	DVLLNLLK					Mascot
1061.599	1061.5651	-0.0339	-32	130	138	VVQFSEKPK					Mascot
1170.7205	1170.6298	-0.0907	-77	176	185	DVLLNLLKSR					Mascot
1503.7882	1503.7552	-0.033	-22	60	70	KFIWVLEDYYK					Mascot
1553.7709	1553.7338	-0.0371	-24	162	174	YPYIASMGVYVFK			Oxidation (M)[7]		Mascot
1643.821	1643.8031	-0.0179	-11	4	17	IFVMTQFNSASLNR			Oxidation (M)[4]		Mascot
1652.8167	1652.7986	-0.0181	-11	147	161	VDTSFLNFAIDDPAK					Mascot
1709.8719	1709.8036	-0.0683	-40	162	175	YPYIASMGVYVFKR			Oxidation (M)[7]		Mascot
1756.9592	1756.9644	0.0052	3	73	87	SIEHILILSGDQLYR					Mascot
1756.9592	1756.9644	0.0052	3	73	87	SIEHILILSGDQLYR	110	100			Mascot
1892.9786	1892.9161	-0.0625	-33	130	146	VVQFSEKPKGADLEAMK			Oxidation (M)[16]		Mascot
2055.9399	2055.9438	0.0039	2	97	115	HVDDNADITLSCAPVGESR			Carbamidomethyl (C)[12]		Mascot

Gel Idx/Pos	291/L19	Instr./Gel Origin	BA2151/Sample Project 20140814	Process Status	Analysis Succeeded
Plate [#] Name	[1] Sample Project 20140814	Instrument Sample Name		Spectra	11

Rank	Protein Name	Accession No.	Protein MW	Protein PI	Pep. Count	Protein Score	Protein Score C. I. %	Intensity Matched	Total Ion Score	Total Ion C. I. %	Confirmed
1	ATP synthase CF1 alpha subunit (chloroplast) [Triticum aestivum]	gi|521301484	55318.3	6.11	27	932	100	63.84	754	100	

Protein Group

Protein Name	Accession No.	Protein MW	Protein PI
ATP synthase CF1 alpha subunit (chloroplast) [Triticum monococcum subsp. aegilopoides]	gi|521301345	55318.3	6.1100 001335 144
ATP synthase CF1 alpha subunit (chloroplast) [Triticum urartu]	gi|521301423	55318.3	6.1100 001335 144
ATP synthase CF1 alpha subunit (chloroplast) [Triticum urartu]	gi|525782292	55318.3	6.1100 001335 144
ATP synthase CF1 alpha subunit [Triticum aestivum]	gi|14017569	55318.3	6.1100 001335 144
ATPase alpha subunit [Triticum aestivum]	gi|13928202	55318.3	6.1100 001335 144
RecName: Full=ATP synthase subunit alpha, chloroplastic; AltName: Full=ATP synthase F1 sector subunit alpha; AltName: Full=F-ATPase subunit alpha	gi|17371040	55318.3	6.1100 001335 144

Peptide Information

Calc. Mass	Obsrv. Mass	± da	± ppm	Start Seq.	End Seq.	Sequence	Ion Score	C. I. %	Modification	Rank	Result Type
815.4621	815.4515	-0.0106	-13	166	172	ELIIGDR					Mascot
822.4104	822.4001	-0.0103	-13	17	22	IEQYNR					Mascot

857.4839	857.4806	-0.0033	-4	24	31	VGIENIGR					Mascot
876.4971	876.4763	-0.0208	-24	273	279	QMSLLLR				Oxidation (M)[2]	Mascot
917.4799	917.4731	-0.0068	-7	406	413	TSQNQLAR					Mascot
920.52	920.5038	-0.0162	-18	457	463	FLDELRK					Mascot
961.4949	961.4859	-0.009	-9	120	128	GEIIASESR					Mascot
985.5789	985.571	-0.0079	-8	23	31	KVGIENIGR					Mascot
1013.5739	1013.5659	-0.008	-8	32	41	VVQVGDGIAR					Mascot
1107.5542	1107.5417	-0.0125	-11	15	22	ERIEQYNR					Mascot
1115.5691	1115.5665	-0.0026	-2	492	500	EAIQEQLER					Mascot
1115.5691	1115.5665	-0.0026	-2	492	500	EAIQEQLER		52	99.954		Mascot
1274.6951	1274.6492	-0.0459	-36	177	188	TAVATDTILNQK					Mascot
1282.7366	1282.7344	-0.0022	-2	129	140	LIESPAPSIISR					Mascot
1282.7366	1282.7344	-0.0022	-2	129	140	LIESPAPSIISR		72	100		Mascot
1292.7097	1292.6729	-0.0368	-28	481	491	TFTEQAEILLK					Mascot
1317.705	1317.6711	-0.0339	-26	256	266	HTLIIYDDLSK					Mascot
1416.7845	1416.7905	0.006	4	95	107	IAQIPVSEAYLGR					Mascot
1416.7845	1416.7905	0.006	4	95	107	IAQIPVSEAYLGR		124	100		Mascot
1438.8376	1438.7535	-0.0841	-58	129	141	LIESPAPSIISRR					Mascot
1507.7639	1507.7301	-0.0338	-22	444	456	GYLDSLEIEQVNK					Mascot
1507.7639	1507.7301	-0.0338	-22	444	456	GYLDSLEIEQVNK		66	99.998		Mascot
1519.8051	1519.8083	0.0032	2	189	202	GQGVICVYVAIGQR				Carbamidomethyl (C)[6]	Mascot
1519.8051	1519.8083	0.0032	2	189	202	GQGVICVYVAIGQR		55	99.976	Carbamidomethyl (C)[6]	Mascot
1553.7384	1553.7443	0.0059	4	285	297	EAYPGDVFYLHSR					Mascot
1553.7384	1553.7443	0.0059	4	285	297	EAYPGDVFYLHSR		103	100		Mascot
1593.853	1593.7144	-0.1386	-87	267	279	QAQAYRQMSLLLR				Oxidation (M)[8]	Mascot
1607.8275	1607.8008	-0.0267	-17	467	480	DTKPQFQEIISSSK					Mascot
2121.0896	2121.1057	0.0161	8	42	61	IIGLGEIMSGELVEFAEGT R					Mascot
2137.0845	2137.0872	0.0027	1	42	61	IIGLGEIMSGELVEFAEGT R				Oxidation (M)[8]	Mascot
2137.0845	2137.0872	0.0027	1	42	61	IIGLGEIMSGELVEFAEGT R		132	100	Oxidation (M)[8]	Mascot
2225.2136	2225.2644	0.0508	23	120	140	GEIIASESRLIESPAPSIIS R					Mascot
2289.2158	2289.2336	0.0178	8	142	162	SVYEPLQTGLIAIDSMIPI GR				Oxidation (M)[16]	Mascot
2316.1831	2316.2073	0.0242	10	423	443	QSQANPLPVEEQIATIYT GTR					Mascot
2316.1831	2316.2073	0.0242	10	423	443	QSQANPLPVEEQIATIYT GTR		148	100		Mascot
2445.3171	2445.3088	-0.0083	-3	141	162	RSVYEPLQTGLIAIDSMIPI GR				Oxidation (M)[17]	Mascot

2	ATP synthase CF1 alpha subunit (chloroplast) [Triticum	gi|521301187	55318.3	6.11	27	932	100	63.84	754	100	

monococcum]

Protein Group

ATP synthase CF1 alpha subunit (chloroplast) [Triticum monococcum]	gi|525778502	55318.3	6.1100 001335 144

Peptide Information

Calc. Mass	Obsrv. Mass	± da	± ppm	Start Seq.	End Seq.	Sequence	Ion Score	C. I. %	Modification	Rank	Result Type
815.4621	815.4515	-0.0106	-13	166	172	ELIIGDR					Mascot
822.4104	822.4001	-0.0103	-13	17	22	IEQYNR					Mascot
857.4839	857.4806	-0.0033	-4	24	31	VGIENIGR					Mascot
876.4971	876.4763	-0.0208	-24	273	279	QMSLLLR			Oxidation (M)[2]		Mascot
917.4799	917.4731	-0.0068	-7	406	413	TSQNQLAR					Mascot
920.52	920.5038	-0.0162	-18	457	463	FLDELRK					Mascot
961.4949	961.4859	-0.009	-9	120	128	GEILASESR					Mascot
985.5789	985.571	-0.0079	-8	23	31	KVGIENIGR					Mascot
1013.5739	1013.5659	-0.008	-8	32	41	VVQVGDGIAR					Mascot
1107.5542	1107.5417	-0.0125	-11	15	22	ERIEQYNR					Mascot
1115.5691	1115.5665	-0.0026	-2	492	500	EAIQEQLER					Mascot
1115.5691	1115.5665	-0.0026	-2	492	500	EAIQEQLER	52	99.954			Mascot
1274.6951	1274.6492	-0.0459	-36	177	188	TAVATDTILNQK					Mascot
1282.7366	1282.7344	-0.0022	-2	129	140	LIESPAPSIISR					Mascot
1282.7366	1282.7344	-0.0022	-2	129	140	LIESPAPSIISR	72	100			Mascot
1292.7097	1292.6729	-0.0368	-28	481	491	TFTEQAEILLK					Mascot
1317.705	1317.6711	-0.0339	-26	256	266	HTLIIYDDLSK					Mascot
1416.7845	1416.7905	0.006	4	95	107	IAQIPVSEAYLGR					Mascot
1416.7845	1416.7905	0.006	4	95	107	IAQIPVSEAYLGR	124	100			Mascot
1438.8376	1438.7535	-0.0841	-58	129	141	LIESPAPSIISRR					Mascot
1507.7639	1507.7301	-0.0338	-22	444	456	GYLDSLEIEQVNK					Mascot
1507.7639	1507.7301	-0.0338	-22	444	456	GYLDSLEIEQVNK	66	99.998			Mascot
1519.8051	1519.8083	0.0032	2	189	202	GQGVICVYVAIGQR			Carbamidomethyl (C)[6]		Mascot
1519.8051	1519.8083	0.0032	2	189	202	GQGVICVYVAIGQR	55	99.976	Carbamidomethyl (C)[6]		Mascot
1553.7384	1553.7443	0.0059	4	285	297	EAYPGDVFYLHSR					Mascot
1553.7384	1553.7443	0.0059	4	285	297	EAYPGDVFYLHSR	103	100			Mascot
1593.853	1593.7144	-0.1386	-87	267	279	QAQAYRQMSLLLR			Oxidation (M)[8]		Mascot

1607.8275	1607.8008	-0.0267	-17	467	480	DTKPQFQEIISSSK					Mascot
2121.0896	2121.1057	0.0161	8	42	61	IIGLGEIMSGELVEFAEGT R					Mascot
2137.0845	2137.0872	0.0027	1	42	61	IIGLGEIMSGELVEFAEGT R			Oxidation (M)[8]		Mascot
2137.0845	2137.0872	0.0027	1	42	61	IIGLGEIMSGELVEFAEGT R	132	100	Oxidation (M)[8]		Mascot
2225.2136	2225.2644	0.0508	23	120	140	GEILASESRLIESPAPSIIS R					Mascot
2289.2158	2289.2336	0.0178	8	142	162	SVYEPLQTGLIAIDSMIPI GR			Oxidation (M)[16]		Mascot
2316.1831	2316.2073	0.0242	10	423	443	QSQANPLPVEEQIATIYT GTR					Mascot
2316.1831	2316.2073	0.0242	10	423	443	QSQANPLPVEEQIATIYT GTR	148	100			Mascot
2445.3171	2445.3088	-0.0083	-3	141	162	RSVYEPLQTGLIAIDSMIPI GR			Oxidation (M)[17]		Mascot

3	ATP synthase subunit alpha, chloroplastic [Triticum urartu]	gi|473750243	58399.8	6.03	27	927	100	63.84	754	100	

Peptide Information

Calc. Mass	Obsrv. Mass	± da	± ppm	Start Seq.	End Seq.	Sequence	Ion Score	C. I. %	Modification	Rank	Result Type
815.4621	815.4515	-0.0106	-13	166	172	ELIIGDR					Mascot
822.4104	822.4001	-0.0103	-13	17	22	IEQYNR					Mascot
857.4839	857.4806	-0.0033	-4	24	31	VGIENIGR					Mascot
876.4971	876.4763	-0.0208	-24	298	304	QMSLLLR			Oxidation (M)[2]		Mascot
917.4799	917.4731	-0.0068	-7	431	438	TSQNQLAR					Mascot
920.52	920.5038	-0.0162	-18	482	488	FLDELRK					Mascot
961.4949	961.4859	-0.009	-9	120	128	GEIIASESR					Mascot
985.5789	985.571	-0.0079	-8	23	31	KVGIENIGR					Mascot
1013.5739	1013.5659	-0.008	-8	32	41	VVQVGDGIAR					Mascot
1107.5542	1107.5417	-0.0125	-11	15	22	ERIEQYNR					Mascot
1115.5691	1115.5665	-0.0026	-2	517	525	EAIQEQLER					Mascot
1115.5691	1115.5665	-0.0026	-2	517	525	EAIQEQLER	52	99.954			Mascot
1274.6951	1274.6492	-0.0459	-36	177	188	TAVATDTILNQK					Mascot
1282.7366	1282.7344	-0.0022	-2	129	140	LIESPAPSIISR					Mascot
1282.7366	1282.7344	-0.0022	-2	129	140	LIESPAPSIISR	72	100			Mascot
1292.7097	1292.6729	-0.0368	-28	506	516	TFTEQAEILLK					Mascot
1317.705	1317.6711	-0.0339	-26	281	291	HTLIIYDDLSK					Mascot
1416.7845	1416.7905	0.006	4	95	107	IAQIPVSEAYLGR					Mascot
1416.7845	1416.7905	0.006	4	95	107	IAQIPVSEAYLGR	124	100			Mascot
1438.8376	1438.7535	-0.0841	-58	129	141	LIESPAPSIISRR					Mascot
1507.7639	1507.7301	-0.0338	-22	469	481	GYLDSLEIEQVNK					Mascot

1507.7639	1507.7301	-0.0338	-22	469	481	GYLDSLEIEQVNK	66	99.998			Mascot
1519.8051	1519.8083	0.0032	2	189	202	GQGVICVYVAIGQR			Carbamidomethyl (C)[6]		Mascot
1519.8051	1519.8083	0.0032	2	189	202	GQGVICVYVAIGQR	55	99.976	Carbamidomethyl (C)[6]		Mascot
1553.7384	1553.7443	0.0059	4	310	322	EAYPGDVFYLHSR					Mascot
1553.7384	1553.7443	0.0059	4	310	322	EAYPGDVFYLHSR	103	100			Mascot
1593.853	1593.7144	-0.1386	-87	292	304	QAQAYRQMSLLLR			Oxidation (M)[8]		Mascot
1607.8275	1607.8008	-0.0267	-17	492	505	DTKPQFQEIISSSK					Mascot
2121.0896	2121.1057	0.0161	8	42	61	IIGLGEIMSGELVEFAEGT R					Mascot
2137.0845	2137.0872	0.0027	1	42	61	IIGLGEIMSGELVEFAEGT R			Oxidation (M)[8]		Mascot
2137.0845	2137.0872	0.0027	1	42	61	IIGLGEIMSGELVEFAEGT R	132	100	Oxidation (M)[8]		Mascot
2225.2136	2225.2644	0.0508	23	120	140	GEIIASESRLIESPAPSIIS R					Mascot
2289.2158	2289.2336	0.0178	8	142	162	SVYEPLQTGLIAIDSMIPI GR			Oxidation (M)[16]		Mascot
2316.1831	2316.2073	0.0242	10	448	468	QSQANPLPVEEQIATIYT GTR					Mascot
2316.1831	2316.2073	0.0242	10	448	468	QSQANPLPVEEQIATIYT GTR	148	100			Mascot
2445.3171	2445.3088	-0.0083	-3	141	162	RSVYEPLQTGLIAIDSMIPI GR			Oxidation (M)[17]		Mascot

4	ATP synthase subunit alpha, chloroplastic [Triticum urartu]	gi|473826750	61894.6	6.2	27	920	100	63.84	754	100

Protein Group

ATP synthase subunit alpha, chloroplastic [Triticum urartu]	gi|474384779	61894.6	6.1999 998092 6514

Peptide Information

Calc. Mass	Obsrv. Mass	± da	± ppm	Start Seq.	End Seq.	Sequence	Ion Score	C. I. %	Modification	Rank	Result Type
815.4621	815.4515	-0.0106	-13	230	236	ELIIGDR					Mascot
822.4104	822.4001	-0.0103	-13	81	86	IEQYNR					Mascot
857.4839	857.4806	-0.0033	-4	88	95	VGIENIGR					Mascot
876.4971	876.4763	-0.0208	-24	337	343	QMSLLLR			Oxidation (M)[2]		Mascot
917.4799	917.4731	-0.0068	-7	470	477	TSQNQLAR					Mascot
920.52	920.5038	-0.0162	-18	521	527	FLDELRK					Mascot
961.4949	961.4859	-0.009	-9	184	192	GEIIASESR					Mascot
985.5789	985.571	-0.0079	-8	87	95	KVGIENIGR					Mascot
1013.5739	1013.5659	-0.008	-8	96	105	VVQVGDGIAR					Mascot

1107.5542	1107.5417	-0.0125	-11	79	86	ERIEQYNR					Mascot
1115.5691	1115.5665	-0.0026	-2	556	564	EAIQEQLER					Mascot
1115.5691	1115.5665	-0.0026	-2	556	564	EAIQEQLER	52	99.954			Mascot
1274.6951	1274.6492	-0.0459	-36	241	252	TAVATDTILNQK					Mascot
1282.7366	1282.7344	-0.0022	-2	193	204	LIESPAPSIISR					Mascot
1282.7366	1282.7344	-0.0022	-2	193	204	LIESPAPSIISR	72	100			Mascot
1292.7097	1292.6729	-0.0368	-28	545	555	TFTEQAEILLK					Mascot
1317.705	1317.6711	-0.0339	-26	320	330	HTLIIYDDLSK					Mascot
1416.7845	1416.7905	0.006	4	159	171	IAQIPVSEAYLGR					Mascot
1416.7845	1416.7905	0.006	4	159	171	IAQIPVSEAYLGR	124	100			Mascot
1438.8376	1438.7535	-0.0841	-58	193	205	LIESPAPSIISRR					Mascot
1507.7639	1507.7301	-0.0338	-22	508	520	GYLDSLEIEQVNK					Mascot
1507.7639	1507.7301	-0.0338	-22	508	520	GYLDSLEIEQVNK	66	99.998			Mascot
1519.8051	1519.8083	0.0032	2	253	266	GQGVICVYVAIGQR			Carbamidomethyl (C)[6]		Mascot
1519.8051	1519.8083	0.0032	2	253	266	GQGVICVYVAIGQR	55	99.976	Carbamidomethyl (C)[6]		Mascot
1553.7384	1553.7443	0.0059	4	349	361	EAYPGDVFYLHSR					Mascot
1553.7384	1553.7443	0.0059	4	349	361	EAYPGDVFYLHSR	103	100			Mascot
1593.853	1593.7144	-0.1386	-87	331	343	QAQAYRQMSLLLR			Oxidation (M)[8]		Mascot
1607.8275	1607.8008	-0.0267	-17	531	544	DTKPQFQEIISSSK					Mascot
2121.0896	2121.1057	0.0161	8	106	125	IIGLGEIMSGELVEFAEGTR					Mascot
2137.0845	2137.0872	0.0027	1	106	125	IIGLGEIMSGELVEFAEGTR			Oxidation (M)[8]		Mascot
2137.0845	2137.0872	0.0027	1	106	125	IIGLGEIMSGELVEFAEGTR	132	100	Oxidation (M)[8]		Mascot
2225.2136	2225.2644	0.0508	23	184	204	GEIIASESRLIESPAPSIISR					Mascot
2289.2158	2289.2336	0.0178	8	206	226	SVYEPLQTGLIAIDSMIPIGR			Oxidation (M)[16]		Mascot
2316.1831	2316.2073	0.0242	10	487	507	QSQANPLPVEEQIATIYTGTR					Mascot
2316.1831	2316.2073	0.0242	10	487	507	QSQANPLPVEEQIATIYTGTR	148	100			Mascot
2445.3171	2445.3088	-0.0083	-3	205	226	RSVYEPLQTGLIAIDSMIPIGR			Oxidation (M)[17]		Mascot

5 ATP synthase alpha subunit [Triticum aestivum] gi|552976 55287.2 5.94 25 787 100 45.51 629 100

Peptide Information

Calc. Mass	Obsrv. Mass	± da	± ppm	Start Seq.	End Seq.	Sequence	Ion Score	C. I. %	Modification	Rank	Result Type
815.4621	815.4515	-0.0106	-13	166	172	ELIIGDR					Mascot
857.4839	857.4806	-0.0033	-4	24	31	VGIENIGR					Mascot
876.4971	876.4763	-0.0208	-24	273	279	QMSLLLR			Oxidation (M)[2]		Mascot

Calc. Mass	Obsrv. Mass	± da	± ppm	Start Seq.	End Seq.	Sequence	Ion Score	C. I. %	Modification	Rank	Result Type
917.4799	917.4731	-0.0068	-7	406	413	TSQNQLAR					Mascot
920.52	920.5038	-0.0162	-18	457	463	FLDELRK					Mascot
961.4949	961.4859	-0.009	-9	120	128	GEIIASESR					Mascot
985.5789	985.571	-0.0079	-8	23	31	KVGIENIGR					Mascot
1013.5739	1013.5659	-0.008	-8	32	41	VVQVGDGIAR					Mascot
1115.5691	1115.5665	-0.0026	-2	492	500	EAIQEQLER					Mascot
1115.5691	1115.5665	-0.0026	-2	492	500	EAIQEQLER	52	99.954			Mascot
1274.6951	1274.6492	-0.0459	-36	177	188	TAVATDTILNQK					Mascot
1282.7366	1282.7344	-0.0022	-2	129	140	LIESPAPSIISR					Mascot
1282.7366	1282.7344	-0.0022	-2	129	140	LIESPAPSIISR	72	100			Mascot
1292.7097	1292.6729	-0.0368	-28	481	491	TFTEQAEILLK					Mascot
1317.705	1317.6711	-0.0339	-26	256	266	HTLIIYDDLSK					Mascot
1438.8376	1438.7535	-0.0841	-58	129	141	LIESPAPSIISRR					Mascot
1442.8002	1442.7566	-0.0436	-30	95	107	IPQIPVSEAYLGR					Mascot
1507.7639	1507.7301	-0.0338	-22	444	456	GYLDSLEIEQVNK					Mascot
1507.7639	1507.7301	-0.0338	-22	444	456	GYLDSLEIEQVNK	66	99.998			Mascot
1519.8051	1519.8083	0.0032	2	189	202	GQGVICVYVAIGQR			Carbamidomethyl (C)[6]		Mascot
1519.8051	1519.8083	0.0032	2	189	202	GQGVICVYVAIGQR	55	99.976	Carbamidomethyl (C)[6]		Mascot
1553.7384	1553.7443	0.0059	4	285	297	EAYPGDVFYLHSR					Mascot
1553.7384	1553.7443	0.0059	4	285	297	EAYPGDVFYLHSR	103	100			Mascot
1593.853	1593.7144	-0.1386	-87	267	279	QAQAYRQMSLLLR			Oxidation (M)[8]		Mascot
1607.8275	1607.8008	-0.0267	-17	467	480	DTKPQFQEIISSSK					Mascot
2121.0896	2121.1057	0.0161	8	42	61	IIGLGEIMSGELVEFAEGTR					Mascot
2137.0845	2137.0872	0.0027	1	42	61	IIGLGEIMSGELVEFAEGTR			Oxidation (M)[8]		Mascot
2137.0845	2137.0872	0.0027	1	42	61	IIGLGEIMSGELVEFAEGTR	132	100	Oxidation (M)[8]		Mascot
2225.2136	2225.2644	0.0508	23	120	140	GEIIASESRLIESPAPSIISR					Mascot
2289.2158	2289.2336	0.0178	8	142	162	SVYEPLQTGLIAIDSMIPIGR			Oxidation (M)[16]		Mascot
2316.1831	2316.2073	0.0242	10	423	443	QSQANPLPVEEQIATIYTGTR					Mascot
2316.1831	2316.2073	0.0242	10	423	443	QSQANPLPVEEQIATIYTGTR	148	100			Mascot
2445.3171	2445.3088	-0.0083	-3	141	162	RSVYEPLQTGLIAIDSMIPIGR			Oxidation (M)[17]		Mascot

6	ATP synthase subunit alpha, chloroplastic [Triticum urartu]	gi|474391517	31843.3	6.03	7	185	100	16.437	159	100	

Peptide Information

Calc. Mass	Obsrv. Mass	± da	± ppm	Start Seq.	End Seq.	Sequence	Ion Score	C. I. %	Modification	Rank	Result Type

Calc. Mass	Obsrv. Mass	± da	± ppm	Start Seq.	End Seq.	Sequence	Ion Score	C. I. %	Modification	Rank	Result Type
876.4971	876.4763	-0.0208	-24	171	177	QMSLLLR			Oxidation (M)[2]		Mascot
920.4724	920.5038	0.0314	34	283	290	LELAQFAE					Mascot
1317.705	1317.6711	-0.0339	-26	154	164	HTLIIYDDLSK					Mascot
1519.8051	1519.8083	0.0032	2	87	100	GQGVICVYVAIGQR			Carbamidomethyl (C)[6]		Mascot
1519.8051	1519.8083	0.0032	2	87	100	GQGVICVYVAIGQR	55	99.976	Carbamidomethyl (C)[6]		Mascot
1553.7384	1553.7443	0.0059	4	183	195	EAYPGDVFYLHSR					Mascot
1553.7384	1553.7443	0.0059	4	183	195	EAYPGDVFYLHSR	103	100			Mascot
1593.853	1593.7144	-0.1386	-87	165	177	QAQAYRQMSLLLR			Oxidation (M)[8]		Mascot
2298.0999	2298.2869	0.187	81	16	34	MEESLTVFSLNYSNFLFE K					Mascot

7	hypothetical protein TRIUR3_14033 [Triticum urartu]	gi|474367026	31559.3	8.67	11	50	60.587	17.419	

Peptide Information

Calc. Mass	Obsrv. Mass	± da	± ppm	Start Seq.	End Seq.	Sequence	Ion Score	C. I. %	Modification	Rank	Result Type
857.4338	857.4806	0.0468	55	57	62	CVYLFR			Carbamidomethyl (C)[1]		Mascot
1013.5349	1013.5659	0.031	31	57	63	CVYLFRR			Carbamidomethyl (C)[1]		Mascot
1097.6578	1097.5565	-0.1013	-92	255	263	LVWTAVPRR					Mascot
1384.743	1384.6361	-0.1069	-77	190	203	AAAEIEAGTVPLSR					Mascot
1471.7209	1471.7582	0.0373	25	128	140	QTMGVSLNSYLSR			Oxidation (M)[3]		Mascot
1519.7687	1519.8083	0.0396	26	163	176	GPCSVSRVAAWTTK			Carbamidomethyl (C)[3]		Mascot
1519.7687	1519.8083	0.0396	26	163	176	GPCSVSRVAAWTTK			Carbamidomethyl (C)[3]		Mascot
1553.6577	1553.7443	0.0866	56	177	189	NYEYSMDLYNGGK					Mascot
1553.6577	1553.7443	0.0866	56	177	189	NYEYSMDLYNGGK					Mascot
1569.6526	1569.7413	0.0887	57	177	189	NYEYSMDLYNGGK			Oxidation (M)[6]		Mascot
1593.7499	1593.7144	-0.0355	-22	266	279	QDMIKDVGDESLLM					Mascot
1713.8776	1713.9271	0.0495	29	78	92	LTLMDPCRGLVPADR			Carbamidomethyl (C)[7]		Mascot
2327.0649	2327.1997	0.1348	58	170	189	VAAWTTKNYEYSMDLYN GGK			Oxidation (M)[13]		Mascot
2787.4434	2787.4216	-0.0218	-8	10	33	NFVFCNVPQQHVGSCVN VITLKVK			Carbamidomethyl (C)[5,15]		Mascot

8	unnamed protein product [Triticum aestivum]	gi|298543883	87139.7	6.27	16	48	31.509	17.303	

Protein Group

starch synthase II-B [Triticum aestivum]	gi|82943646	87139.7	6.2699 999809 2651
starch synthase IIa-3 [Triticum aestivum]	gi|8953573	87139.7	6.2699 999809

			2651	
unnamed protein product [Triticum aestivum]	gi|298545793	87139.7	6.2699 999809 2651	

Peptide Information

Calc. Mass	Obsrv. Mass	± da	± ppm	Start Seq.	End Seq.	Sequence	Ion Score	C. I. %	Modification	Rank	Result Type
811.4205	811.4323	0.0118	15	413	418	MILFCK			Carbamidomethyl (C)[5]		Mascot
815.4808	815.4515	-0.0293	-36	343	349	VMVVVPR			Oxidation (M)[2]		Mascot
820.4345	820.4316	-0.0029	-4	407	412	QEIMKR			Oxidation (M)[4]		Mascot
920.4697	920.5038	0.0341	37	664	670	EHHDKVR					Mascot
1143.6521	1143.5878	-0.0643	-56	610	620	GDVPLLGFIGR					Mascot
1281.6361	1281.678	0.0419	33	267	277	YIGFEEPVEAK					Mascot
1292.623	1292.6729	0.0499	39	101	114	DAAEGGAPSPPAPR					Mascot
1317.6295	1317.6711	0.0416	32	396	406	HRQEDIYGGSR					Mascot
1384.7598	1384.6361	-0.1237	-89	671	682	GWVGFSVRLAHR					Mascot
1452.719	1452.7124	-0.0066	-5	65	78	DAGIDDAAPARQPR					Mascot
1519.688	1519.8083	0.1203	79	120	133	LPSMNGMPVNGENK			Oxidation (M)[4,7]		Mascot
1519.688	1519.8083	0.1203	79	120	133	LPSMNGMPVNGENK			Oxidation (M)[4,7]		Mascot
1535.6649	1535.7393	0.0744	48	350	362	YGDYEEAYDVGVR					Mascot
1535.6649	1535.7393	0.0744	48	350	362	YGDYEEAYDVGVR					Mascot
1967.9855	1968.004	0.0185	9	1	21	MSSAVASAASFLALASAS PGR			Oxidation (M)[1]		Mascot
2118.9543	2119.1565	0.2022	95	115	133	QEDARLPSMNGMPVNG ENK			Oxidation (M)[9,12]		Mascot
2305.0674	2305.2258	0.1584	69	723	742	DTVPPFDPFNHSGLGWT FDR					Mascot
2316.1331	2316.2073	0.0742	32	343	362	VMVVVPRYGDYEEAYDV GVR					Mascot
2316.1331	2316.2073	0.0742	32	343	362	VMVVVPRYGDYEEAYDV GVR					Mascot

9	T-complex protein 1 subunit eta [Triticum urartu]	gi|474157243	63092.4	6.03	13	47	9.711	28.186	

Peptide Information

Calc. Mass	Obsrv. Mass	± da	± ppm	Start Seq.	End Seq.	Sequence	Ion Score	C. I. %	Modification	Rank	Result Type
989.4898	989.487	-0.0028	-3	339	346	VTEEDLQR					Mascot
1170.63	1170.6118	-0.0182	-16	413	422	SLHDAIMIVR			Oxidation (M)[7]		Mascot

1196.6311	1196.7262	0.0951	79	232	242	DSFLVNGVAFK					Mascot
1310.7362	1310.7318	-0.0044	-3	413	423	SLHDAIMIVRR					Mascot
1317.7308	1317.6711	-0.0597	-45	306	317	CVQSGAKIVLSR			Carbamidomethyl (C)[1]		Mascot
1321.6019	1321.611	0.0091	7	401	412	GGADQFIEEAER					Mascot
1519.8044	1519.8083	0.0039	3	1	13	MSSMMQPQIILLK					Mascot
1519.8044	1519.8083	0.0039	3	1	13	MSSMMQPQIILLK					Mascot
1535.7994	1535.7393	-0.0601	-39	1	13	MSSMMQPQIILLK			Oxidation (M)[1]		Mascot
1535.7994	1535.7393	-0.0601	-39	1	13	MSSMMQPQIILLK			Oxidation (M)[1]		Mascot
1551.7943	1551.7449	-0.0494	-32	1	13	MSSMMQPQIILLK			Oxidation (M)[1,4]		Mascot
1553.7959	1553.7443	-0.0516	-33	318	331	LAIGDLATQYFADR					Mascot
1553.7959	1553.7443	-0.0516	-33	318	331	LAIGDLATQYFADR					Mascot
1646.7551	1646.8112	0.0561	34	548	564	NPKSESAQGDAAAMGG R					Mascot
1910.9794	1910.8906	-0.0888	-46	225	242	VPGGTMRDSFLVNGVAF K			Oxidation (M)[6]		Mascot
1968.0009	1968.004	0.0031	2	383	400	FNIFSGCPSGQTATIVLR			Carbamidomethyl (C)[7]		Mascot
2122.1655	2122.1187	-0.0468	-22	313	331	IVLSRLAIGDLATQYFADR					Mascot
2316.2117	2316.2073	-0.0044	-2	526	547	INAINAATEAACLILSVDE TVK			Carbamidomethyl (C)[12]		Mascot
2316.2117	2316.2073	-0.0044	-2	526	547	INAINAATEAACLILSVDE TVK			Carbamidomethyl (C)[12]		Mascot

10	E3 ubiquitin-protein ligase CHIP [Triticum urartu]	gi|474196219	47783.4	7.98	12	47	5.456	6.741

Peptide Information

Calc. Mass	Obsrv. Mass	± da	± ppm	Start Seq.	End Seq.	Sequence	Ion Score	C. I. %	Modification	Rank	Result Type
815.4621	815.4515	-0.0106	-13	144	151	QELAGGIK					Mascot
820.4716	820.4316	-0.04	-49	174	180	LVFGAWK					Mascot
857.5203	857.4806	-0.0397	-46	13	19	RQAELLK					Mascot
876.3998	876.4763	0.0765	87	294	300	DHGWAYK					Mascot
920.5087	920.5038	-0.0049	-5	286	293	EAVDVFLK					Mascot
1013.5738	1013.5659	-0.0079	-8	303	312	AEGRPGTVVK					Mascot
1108.4939	1108.5254	0.0315	28	1	12	MSPAADSASGSK					Mascot
1282.759	1282.7344	-0.0246	-19	301	312	IRAEGRPGTVVK					Mascot
1282.759	1282.7344	-0.0246	-19	301	312	IRAEGRPGTVVK					Mascot
1339.6125	1339.6807	0.0682	51	409	419	FGREEETQDTK					Mascot
1371.7566	1371.7211	-0.0355	-26	132	143	AHYMLGLALLNR					Mascot
1408.6162	1408.6421	0.0259	18	110	120	SEWAKVEEDCR			Carbamidomethyl (C)[10]		Mascot
2338.241	2338.1621	-0.0789	-34	74	93	MHTYAIDLPIPLMRNIPNT K					Mascot

Gel Idx/Pos	292/L20	Instr./Gel Origin	BA2151/Sample Project 20140814	Process Status	Analysis Succeeded
Plate [#] Name	[1] Sample Project 20140814	Instrument Sample Name		Spectra	11

Rank	Protein Name	Accession No.	Protein MW	Protein PI	Pep. Count	Protein Score	Protein Score C. I. %	Intensity Matched	Total Ion Score	Total Ion C. I. %	Confirmed
1	triticin [Triticum aestivum]	gi|171027826	65294.9	6.43	10	507	100	72.448	478	100	

Peptide Information

Calc. Mass	Obsrv. Mass	± da	± ppm	Start Seq.	End Seq.	Sequence	Ion Score	C. I. %	Modification	Rank	Result Type
826.3512	826.3635	0.0123	15	44	49	ECTFNR			Carbamidomethyl (C)[2]		Mascot
885.5152	885.5341	0.0189	21	50	57	LQASTPLR					Mascot
923.4767	923.4944	0.0177	19	78	85	CTGVFAIR			Carbamidomethyl (C)[1]		Mascot
1116.5472	1116.5741	0.0269	24	202	210	EFLFAGNYR					Mascot
1116.5472	1116.5741	0.0269	24	202	210	EFLFAGNYR	67	99.999			Mascot
1263.7208	1263.7505	0.0297	24	259	268	FLKPVFTQQR					Mascot
1263.7208	1263.7505	0.0297	24	259	268	FLKPVFTQQR	56	99.985			Mascot
1381.7012	1381.7372	0.036	26	200	210	HKEFLFAGNYR					Mascot
1381.7012	1381.7372	0.036	26	200	210	HKEFLFAGNYR	65	99.998			Mascot
1908.9199	1908.9667	0.0468	25	211	227	SSQLHSSQNIFSGFDVR					Mascot
1908.9199	1908.9667	0.0468	25	211	227	SSQLHSSQNIFSGFDVR	137	100			Mascot
2062.8989	2062.9482	0.0493	24	61	77	SQAGLTEYFDEENEQFR					Mascot
2062.8989	2062.9482	0.0493	24	61	77	SQAGLTEYFDEENEQFR	152	100			Mascot
2107.9204	2107.9399	0.0195	9	269	286	EPESYPHTQYEEGQSQAK					Mascot
3353.7634	3353.6753	-0.0881	-26	508	540	TNANSMVSHIAGKNSILGALPVDVIANAYGISR					Mascot

Rank	Protein Name	Accession No.	Protein MW	Protein PI	Pep. Count	Protein Score	Protein Score C. I. %	Intensity Matched	Total Ion Score	Total Ion C. I. %	Confirmed
2	triticin [Triticum aestivum]	gi|171027813	65240.8	6.37	9	498	100	72.252	478	100	

Protein Group

Protein Name	Accession No.	Protein MW	Protein PI
triticin [Triticum aestivum]	gi|171027837	65240.8	6.36999988555908

Peptide Information

Calc. Mass	Obsrv. Mass	± da	± ppm	Start Seq.	End Seq.	Sequence	Ion Score	C. I. %	Modification	Rank	Result Type
826.3512	826.3635	0.0123	15	44	49	ECTFNR			Carbamidomethyl (C)[2]		Mascot
885.5152	885.5341	0.0189	21	50	57	LQASTPLR					Mascot

923.4767	923.4944	0.0177	19	78	85	CTGVFAIR			Carbamidomethyl (C)[1]		Mascot
1116.5472	1116.5741	0.0269	24	202	210	EFLFAGNYR					Mascot
1116.5472	1116.5741	0.0269	24	202	210	EFLFAGNYR	67	99.999			Mascot
1263.7208	1263.7505	0.0297	24	259	268	FLKPVFTQQR					Mascot
1263.7208	1263.7505	0.0297	24	259	268	FLKPVFTQQR	56	99.985			Mascot
1381.7012	1381.7372	0.036	26	200	210	HKEFLFAGNYR					Mascot
1381.7012	1381.7372	0.036	26	200	210	HKEFLFAGNYR	65	99.998			Mascot
1908.9199	1908.9667	0.0468	25	211	227	SSQLHSSQNIFSGFDVR					Mascot
1908.9199	1908.9667	0.0468	25	211	227	SSQLHSSQNIFSGFDVR	137	100			Mascot
2062.8989	2062.9482	0.0493	24	61	77	SQAGLTEYFDEENEQFR					Mascot
2062.8989	2062.9482	0.0493	24	61	77	SQAGLTEYFDEENEQFR	152	100			Mascot
2107.9204	2107.9399	0.0195	9	269	286	EPESYPHTQYEEGQSQAK					Mascot

3 12S seed storage globulin 1 [Triticum urartu] gi|474246751 64229.5 6.62 9 498 100 71.519 476 100

Peptide Information

Calc. Mass	Obsrv. Mass	± da	± ppm	Start Seq.	End Seq.	Sequence	Ion Score	C. I. %	Modification	Rank	Result Type
826.3512	826.3635	0.0123	15	44	49	ECTFNR			Carbamidomethyl (C)[2]		Mascot
885.5152	885.5341	0.0189	21	50	57	LQASTPLR					Mascot
1116.5472	1116.5741	0.0269	24	202	210	EFLFAGNYR					Mascot
1116.5472	1116.5741	0.0269	24	202	210	EFLFAGNYR	67	99.999			Mascot
1263.7208	1263.7505	0.0297	24	259	268	FLKPVFTQQR					Mascot
1263.7208	1263.7505	0.0297	24	259	268	FLKPVFTQQR	56	99.985			Mascot
1381.7012	1381.7372	0.036	26	200	210	HKEFLFAGNYR					Mascot
1381.7012	1381.7372	0.036	26	200	210	HKEFLFAGNYR	65	99.998			Mascot
1666.7966	1666.7762	-0.0204	-12	312	326	HIQGEQPQMGQSQAK					Mascot
1908.9199	1908.9667	0.0468	25	211	227	SSQLHSSQNIFSGFDVR					Mascot
1908.9199	1908.9667	0.0468	25	211	227	SSQLHSSQNIFSGFDVR	137	100			Mascot
2062.8989	2062.9482	0.0493	24	61	77	SQAGLTEYFDEENEQFR					Mascot
2062.8989	2062.9482	0.0493	24	61	77	SQAGLTEYFDEENEQFR	152	100			Mascot
2107.9204	2107.9399	0.0195	9	269	286	EPESYPHTQYEEGQSQAK					Mascot

4 triticin precursor [Triticum aestivum] gi|7548844 57317.5 9.37 8 496 100 72.103 478 100

Peptide Information

Calc. Mass	Obsrv. Mass	± da	± ppm	Start Seq.	End Seq.	Sequence	Ion Score	C. I. %	Modification	Rank	Result Type
826.3512	826.3635	0.0123	15	44	49	ECTFNR			Carbamidomethyl (C)[2]		Mascot

885.5152	885.5341	0.0189	21	50	57	LQASTPLR					Mascot
923.4767	923.4944	0.0177	19	78	85	CTGVFAIR			Carbamidomethyl (C)[1]		Mascot
1116.5472	1116.5741	0.0269	24	202	210	EFLFAGNYR					Mascot
1116.5472	1116.5741	0.0269	24	202	210	EFLFAGNYR	67	99.999			Mascot
1263.7208	1263.7505	0.0297	24	259	268	FLKPVFTQQR					Mascot
1263.7208	1263.7505	0.0297	24	259	268	FLKPVFTQQR	56	99.985			Mascot
1381.7012	1381.7372	0.036	26	200	210	HKEFLFAGNYR					Mascot
1381.7012	1381.7372	0.036	26	200	210	HKEFLFAGNYR	65	99.998			Mascot
1908.9199	1908.9667	0.0468	25	211	227	SSQLHSSQNIFSGFDVR					Mascot
1908.9199	1908.9667	0.0468	25	211	227	SSQLHSSQNIFSGFDVR	137	100			Mascot
2062.8989	2062.9482	0.0493	24	61	77	SQAGLTEYFDEENEQFR					Mascot
2062.8989	2062.9482	0.0493	24	61	77	SQAGLTEYFDEENEQFR	152	100			Mascot

5 triticin [Triticum aestivum] gi|147744620 24404.1 9.37 5 346 100 44.953 325 100

Peptide Information

Calc. Mass	Obsrv. Mass	± da	± ppm	Start Seq.	End Seq.	Sequence	Ion Score	C. I. %	Modification	Rank	Result Type
1116.5472	1116.5741	0.0269	24	75	83	EFLFAGNYR					Mascot
1116.5472	1116.5741	0.0269	24	75	83	EFLFAGNYR	67	99.999			Mascot
1263.7208	1263.7505	0.0297	24	132	141	FLKPVFTQQR					Mascot
1263.7208	1263.7505	0.0297	24	132	141	FLKPVFTQQR	56	99.985			Mascot
1381.7012	1381.7372	0.036	26	73	83	HKEFLFAGNYR					Mascot
1381.7012	1381.7372	0.036	26	73	83	HKEFLFAGNYR	65	99.998			Mascot
1908.9199	1908.9667	0.0468	25	84	100	SSQLHSSQNIFSGFDVR					Mascot
1908.9199	1908.9667	0.0468	25	84	100	SSQLHSSQNIFSGFDVR	137	100			Mascot
2107.9204	2107.9399	0.0195	9	142	159	EPESYPHTQYEEGQSQAK					Mascot

6 hypothetical protein TRIUR3_30538 [Triticum urartu] gi|473882607 197169.7 4.65 24 44 0 33.359

Peptide Information

Calc. Mass	Obsrv. Mass	± da	± ppm	Start Seq.	End Seq.	Sequence	Ion Score	C. I. %	Modification	Rank	Result Type
822.4025	822.3866	-0.0159	-19	1672	1678	SAEMEKK					Mascot
979.4917	979.4737	-0.018	-18	1723	1731	EPSGIMAFK					Mascot
1116.5532	1116.5741	0.0209	19	744	753	SLSDLHTESK					Mascot
1116.5532	1116.5741	0.0209	19	744	753	SLSDLHTESK					Mascot
1212.495	1212.5336	0.0386	32	807	816	DVTDECNGFR			Carbamidomethyl (C)[6]		Mascot

1219.6603	1219.7335	0.0732	60	298	307	LLSLQEMEIK			Oxidation (M)[7]		Mascot
1269.578	1269.6855	0.1075	85	1268	1277	VAMYDELQER			Oxidation (M)[3]		Mascot
1340.6078	1340.655	0.0472	35	2	13	EAEQAAQVHDDK					Mascot
1534.767	1534.8468	0.0798	52	625	637	DLMDSLIQITEEK					Mascot
1600.8905	1600.7362	-0.1543	-96	1383	1396	LKSLEVQLAETQNK					Mascot
1632.834	1632.7489	-0.0851	-52	1690	1703	EFSLDSLAPQNKQR					Mascot
1678.8568	1678.8494	-0.0074	-4	625	638	DLMDSLIQITEEKK			Oxidation (M)[3]		Mascot
1696.8752	1696.7806	-0.0946	-56	1181	1195	EEAAEKLAVHEETIK					Mascot
2046.0237	2046.0138	-0.0099	-5	763	781	TAELETQVQELSAAEEGLK					Mascot
2061.9871	2061.988	0.0009	0	744	762	SLSDLHTESKTAAESAMQR					Mascot
2063.0767	2062.9482	-0.1285	-62	726	743	SLLSDAHSHKEELELNLK					Mascot
2063.0767	2062.9482	-0.1285	-62	726	743	SLLSDAHSHKEELELNLK					Mascot
2079.0288	2078.9392	-0.0896	-43	1553	1570	NEALTSKMQEHANLVHEK					Mascot
2085.071	2084.9121	-0.1589	-76	736	753	EELELNLKSLSDLHTESK					Mascot
2090.0249	2089.9927	-0.0322	-15	243	261	SQVDDLEQKLASQDADISK					Mascot
2112.1006	2111.9329	-0.1677	-79	1138	1156	LIAVQSETESLMHVNEALK					Mascot
2119.0225	2119.031	0.0085	4	262	279	LTEELSLHCSSEESLKEK			Carbamidomethyl (C)[9]		Mascot
2178.0786	2178.0657	-0.0129	-6	1583	1601	ELEAAYRTIANQEEQASVR					Mascot
2192.0288	2192.1018	0.073	33	214	232	AVDHQQVEESLNTTMSQFK					Mascot
2208.0237	2208.0264	0.0027	1	214	232	AVDHQQVEESLNTTMSQFK			Oxidation (M)[15]		Mascot
2232.1619	2232.0066	-0.1553	-70	366	385	QAEEEHAKAQLLLSEALSHK					Mascot
2265.0615	2265.0088	-0.0527	-23	1461	1480	EELESQVSSTLEEQEELASK					Mascot

7 hypothetical protein TRIUR3_03528 [Triticum urartu] gi|474065809 47163.6 7.66 11 43 0 16.542

Peptide Information

Calc. Mass	Obsrv. Mass	± da	± ppm	Start Seq.	End Seq.	Sequence	Ion Score	C. I. %	Modification	Rank	Result Type
875.4694	875.4527	-0.0167	-19	91	99	ASNGSKGVR					Mascot
1003.5492	1003.4968	-0.0524	-52	372	380	LAMLVEEAK					Mascot
1058.4935	1058.5083	0.0148	14	287	297	GMGEAPAPNSK					Mascot
1285.6608	1285.724	0.0632	49	419	431	QGGPISPSSSGRR					Mascot
1403.6583	1403.7128	0.0545	39	283	297	GGSKGMGEAPAPNSK			Oxidation (M)[6]		Mascot
1760.8596	1760.7662	-0.0934	-53	396	413	AMPGGTTSGITSPRGQDK					Mascot
1908.9558	1908.9667	0.0109	6	349	364	LMEYCLSPAITVLQDR			Carbamidomethyl (C)[5]		Mascot
1908.9558	1908.9667	0.0109	6	349	364	LMEYCLSPAITVLQDR			Carbamidomethyl (C)[5]		Mascot

Calc. Mass	Obsrv. Mass	± da	± ppm	Start Seq.	End Seq.	Sequence	Ion Score	C. I. %	Modification	Rank	Result Type
1965.8607	1965.986	0.1253	64	132	148	SQDMDLYPSLDTNHSAR			Oxidation (M)[4]		Mascot
2152.114	2152.0068	-0.1072	-50	349	366	LMEYCLSPAITVLQDRVK			Carbamidomethyl (C)[5], Oxidation (M)[2]		Mascot
2216.0046	2216.0227	0.0181	8	330	348	MHPLTSMHHTSTYNASLCK			Carbamidomethyl (C)[18]		Mascot
2231.9995	2232.0066	0.0071	3	330	348	MHPLTSMHHTSTYNASLCK			Carbamidomethyl (C)[18], Oxidation (M)[1]		Mascot
2247.9944	2247.9897	-0.0047	-2	330	348	MHPLTSMHHTSTYNASLCK			Carbamidomethyl (C)[18], Oxidation (M)[1,7]		Mascot
3647.7683	3647.6433	-0.125	-34	100	131	GVDYHSIIVFFPTGWSMFKAGAYFAHANVWGR			Oxidation (M)[17]		Mascot

8	hypothetical protein TRIUR3_31919 [Triticum urartu]	gi|474428178	20980.8	9.17	8	41	0	8.268	

Peptide Information

Calc. Mass	Obsrv. Mass	± da	± ppm	Start Seq.	End Seq.	Sequence	Ion Score	C. I. %	Modification	Rank	Result Type
842.4665	842.4449	-0.0216	-26	162	168	ALKMHSR					Mascot
885.5305	885.5341	0.0036	4	46	53	VLAAAVWR					Mascot
906.4713	906.4699	-0.0014	-2	63	70	MNSANILK			Oxidation (M)[1]		Mascot
951.4564	951.4329	-0.0235	-25	2	9	TVSENVMR			Oxidation (M)[7]		Mascot
1098.4918	1098.5603	0.0685	62	1	9	MTVSENVMR			Oxidation (M)[1,8]		Mascot
1116.5619	1116.5741	0.0122	11	54	62	SWLCQVPAR			Carbamidomethyl (C)[4]		Mascot
1116.5619	1116.5741	0.0122	11	54	62	SWLCQVPAR			Carbamidomethyl (C)[4]		Mascot
1906.9592	1907.0146	0.0554	29	165	180	MHSRGFNILEIFETGR					Mascot
1922.9542	1922.9498	-0.0044	-2	165	180	MHSRGFNILEIFETGR			Oxidation (M)[1]		Mascot
1965.9634	1965.986	0.0226	11	1	16	MTVSENVMRNLSWINR			Oxidation (M)[1]		Mascot

9	Cyclopropane-fatty-acyl-phospholipid synthase [Triticum urartu]	gi|474379868	88285.1	7.89	14	40	0	21.376	

Peptide Information

Calc. Mass	Obsrv. Mass	± da	± ppm	Start Seq.	End Seq.	Sequence	Ion Score	C. I. %	Modification	Rank	Result Type
977.5336	977.4518	-0.0818	-84	723	731	SALMVEALK			Oxidation (M)[4]		Mascot
1204.615	1204.566	-0.049	-41	403	411	VHDPLFYWK					Mascot
1261.7052	1261.7255	0.0203	16	47	56	TNILKPSFWR					Mascot
1263.658	1263.7505	0.0925	73	297	307	AYLDLDQIQGK					Mascot
1263.658	1263.7505	0.0925	73	297	307	AYLDLDQIQGK					Mascot
1291.6893	1291.7385	0.0492	38	666	676	DQVLKLGFDEK					Mascot
1403.7502	1403.7128	-0.0374	-27	196	207	VLGVEATHHERR					Mascot
1438.6996	1438.7573	0.0577	40	659	670	DNFMANKDQVLK			Oxidation (M)[4]		Mascot
1953.0665	1952.9351	-0.1314	-67	123	139	HSQLPTVKPRSQSFVNK					Mascot

2018.9349	2018.9517	0.0168	8	26	46	GGGGGCEWGNGNGISS LLAQK			Carbamidomethyl (C)[6]		Mascot
2045.0928	2045.01	-0.0828	-40	490	509	GHHVLDIGSGWGSLAIQA VK					Mascot
2135.1504	2134.9883	-0.1621	-76	47	63	TNILKPSFWRMVCEILK			Carbamidomethyl (C)[13]		Mascot
2178.9814	2179.175	0.1936	89	309	327	GIWFCGAYQGHGFHEDG LK			Carbamidomethyl (C)[5]		Mascot
2193.991	2194.0408	0.0498	23	732	748	ATYQENSEVLCFWYLDR			Carbamidomethyl (C)[11]		Mascot
2193.991	2194.0408	0.0498	23	732	748	ATYQENSEVLCFWYLDR			Carbamidomethyl (C)[11]		Mascot
2216.1309	2216.0227	-0.1082	-49	436	454	EGLLNLMLSNDFFSLFLD K					Mascot
2232.1257	2232.0066	-0.1191	-53	436	454	EGLLNLMLSNDFFSLFLD K			Oxidation (M)[7]		Mascot

10 hypothetical protein TRIUR3_29349 [Triticum urartu] gi|474074465 171410.4 5.77 27 39 0 6.946

Peptide Information

Calc. Mass	Obsrv. Mass	± da	± ppm	Start Seq.	End Seq.	Sequence	Ion Score	C. I. %	Modification	Rank	Result Type
822.4138	822.3866	-0.0272	-33	1411	1417	MSEKVGR			Oxidation (M)[1]		Mascot
875.5349	875.4527	-0.0822	-94	1425	1431	FQQVLLK					Mascot
923.4833	923.4944	0.0111	12	334	341	FKDVEASK					Mascot
929.4186	929.4495	0.0309	33	128	134	DMTPFFR			Oxidation (M)[2]		Mascot
1057.5095	1057.5122	0.0027	3	618	626	NMVHNEAVK			Oxidation (M)[2]		Mascot
1058.6093	1058.5083	-0.101	-95	372	380	LQDEIISLK					Mascot
1245.6686	1245.7174	0.0488	39	444	454	DGNVELKETIK					Mascot
1261.6821	1261.7255	0.0434	34	336	346	DVEASKLMLQK					Mascot
1284.6504	1284.6344	-0.016	-12	746	756	YGSLDLMKDVK			Oxidation (M)[7]		Mascot
1364.8009	1364.7655	-0.0354	-26	1291	1302	DIRLALVQPPSR					Mascot
1380.7482	1380.7087	-0.0395	-29	1248	1259	DIQLDLVQPPSR					Mascot
1393.707	1393.7296	0.0226	16	1211	1222	LDFSSNLQDARK					Mascot
1403.7749	1403.7128	-0.0621	-44	342	352	LMLQKELEMIR					Mascot
1416.8396	1416.8582	0.0186	13	1279	1290	MLALWSVARLLK			Oxidation (M)[1]		Mascot
1664.8677	1664.807	-0.0607	-36	62	74	RPELMSLLEELYR			Oxidation (M)[5]		Mascot
1878.8691	1879.0239	0.1548	82	128	143	DMTPFFRSFINTGDSK			Oxidation (M)[2]		Mascot
1922.8583	1922.9498	0.0915	48	690	707	CLGDMAEANSDVSGQLQ K			Carbamidomethyl (C)[1]		Mascot
1930.9869	1930.9395	-0.0474	-25	215	230	LQNLKSEISHTQEEFK					Mascot
1947.0732	1946.9208	-0.1524	-78	1047	1064	LLANMQIATANAALFKEK					Mascot
1966.0101	1965.986	-0.0241	-12	563	579	DLEESAQALRNHNSLLR					Mascot
2061.9983	2061.988	-0.0103	-5	202	219	EAAVSQCQQSSDRLQNL K			Carbamidomethyl (C)[7]		Mascot
2073.9871	2074.0391	0.052	25	1029	1046	NNEQPVIDVRSCDAEISK			Carbamidomethyl (C)[12]		Mascot

2091.0273	2090.9734	-0.0539	-26	1357	1375	AQMIESEEAITQLIDTNGK		Mascot
2091.0273	2090.9734	-0.0539	-26	1357	1375	AQMIESEEAITQLIDTNGK		Mascot
2107.0225	2106.9263	-0.0962	-46	1357	1375	AQMIESEEAITQLIDTNGK	Oxidation (M)[3]	Mascot
2129.0808	2128.9453	-0.1355	-64	455	472	NHDGVKALYVENLMQLE R		Mascot
2135.0398	2134.9883	-0.0515	-24	951	970	ELSRTLSICDGSSPEIGSA R	Carbamidomethyl (C)[9]	Mascot
2146.1311	2146.085	-0.0461	-21	1065	1083	VLELIVACESSEISEIVQK	Carbamidomethyl (C)[8]	Mascot
2146.1311	2146.085	-0.0461	-21	1065	1083	VLELIVACESSEISEIVQK	Carbamidomethyl (C)[8]	Mascot
2192.105	2192.1018	-0.0032	-1	288	306	LSISIQEEQLKSMQAEMA R		Mascot
2208.0999	2208.0264	-0.0735	-33	288	306	LSISIQEEQLKSMQAEMA R	Oxidation (M)[13]	Mascot

Gel Idx/Pos	293/L21	Instr./Gel Origin	BA2151/Sample Project 20140814	Process Status	Analysis Succeeded
Plate [#] Name	[1] Sample Project 20140814	Instrument Sample Name		Spectra	11

Rank	Protein Name	Accession No.	Protein MW	Protein PI	Pep. Count	Protein Score	Protein Score C. I. %	Intensity Matched	Total Ion Score	Total Ion C. I. %	Confirmed
1	triticin [Triticum aestivum]	gi|171027826	65294.9	6.43	12	570	100	72.035	529	100	

Peptide Information

Calc. Mass	Obsrv. Mass	± da	± ppm	Start Seq.	End Seq.	Sequence	Ion Score	C. I. %	Modification	Rank	Result Type
826.3512	826.3522	0.001	1	44	49	ECTFNR			Carbamidomethyl (C)[2]		Mascot
885.5152	885.5197	0.0045	5	50	57	LQASTPLR					Mascot
923.4767	923.4773	0.0006	1	78	85	CTGVFAIR			Carbamidomethyl (C)[1]		Mascot
1116.5472	1116.5555	0.0083	7	202	210	EFLFAGNYR					Mascot
1116.5472	1116.5555	0.0083	7	202	210	EFLFAGNYR	61	99.994			Mascot
1263.7208	1263.7356	0.0148	12	259	268	FLKPVFTQQR					Mascot
1263.7208	1263.7356	0.0148	12	259	268	FLKPVFTQQR	67	99.999			Mascot
1381.7012	1381.718	0.0168	12	200	210	HKEFLFAGNYR					Mascot
1381.7012	1381.718	0.0168	12	200	210	HKEFLFAGNYR	91	100			Mascot
1639.7671	1639.7712	0.0041	3	132	146	YGQSQSVQGQSQSQK					Mascot
1728.7759	1728.7894	0.0135	8	322	336	HTQGEQPQMGWSQAK			Oxidation (M)[9]		Mascot
1908.9199	1908.9552	0.0353	18	211	227	SSQLHSSQNIFSGFDVR					Mascot
1908.9199	1908.9552	0.0353	18	211	227	SSQLHSSQNIFSGFDVR	142	100			Mascot
2062.8989	2062.9451	0.0462	22	61	77	SQAGLTEYFDEENEQFR					Mascot
2062.8989	2062.9451	0.0462	22	61	77	SQAGLTEYFDEENEQFR	146	100			Mascot
2107.9204	2107.9377	0.0173	8	269	286	EPESYPHTQYEEGQSQA K					Mascot
2107.9204	2107.9377	0.0173	8	269	286	EPESYPHTQYEEGQSQA K	23	63.717			Mascot
3353.7634	3353.7017	-0.0617	-18	508	540	TNANSMVSHIAGKNSILG ALPVDVIANAYGISR					Mascot

Rank	Protein Name	Accession No.	Protein MW	Protein PI	Pep. Count	Protein Score	Protein Score C. I. %	Intensity Matched	Total Ion Score	Total Ion C. I. %	Confirmed
2	triticin [Triticum aestivum]	gi|171027813	65240.8	6.37	11	561	100	71.66	529	100	

Protein Group

triticin [Triticum aestivum]	gi|171027837	65240.8	6.3699 998855 5908

Peptide Information

Calc. Mass	Obsrv. Mass	± da	± ppm	Start	End	Sequence	Ion	C. I. %	Modification	Rank	Result Type

				Seq.	Seq.		Score				
826.3512	826.3522	0.001	1	44	49	ECTFNR			Carbamidomethyl (C)[2]		Mascot
885.5152	885.5197	0.0045	5	50	57	LQASTPLR					Mascot
923.4767	923.4773	0.0006	1	78	85	CTGVFAIR			Carbamidomethyl (C)[1]		Mascot
1116.5472	1116.5555	0.0083	7	202	210	EFLFAGNYR					Mascot
1116.5472	1116.5555	0.0083	7	202	210	EFLFAGNYR	61	99.994			Mascot
1263.7208	1263.7356	0.0148	12	259	268	FLKPVFTQQR					Mascot
1263.7208	1263.7356	0.0148	12	259	268	FLKPVFTQQR	67	99.999			Mascot
1381.7012	1381.718	0.0168	12	200	210	HKEFLFAGNYR					Mascot
1381.7012	1381.718	0.0168	12	200	210	HKEFLFAGNYR	91	100			Mascot
1639.7671	1639.7712	0.0041	3	132	146	YGQSQSVQGQSQSQK					Mascot
1728.7759	1728.7894	0.0135	8	322	336	HTQGEQPQMGWSQAK			Oxidation (M)[9]		Mascot
1908.9199	1908.9552	0.0353	18	211	227	SSQLHSSQNIFSGFDVR					Mascot
1908.9199	1908.9552	0.0353	18	211	227	SSQLHSSQNIFSGFDVR	142	100			Mascot
2062.8989	2062.9451	0.0462	22	61	77	SQAGLTEYFDEENEQFR					Mascot
2062.8989	2062.9451	0.0462	22	61	77	SQAGLTEYFDEENEQFR	146	100			Mascot
2107.9204	2107.9377	0.0173	8	269	286	EPESYPHTQYEEGQSQA K					Mascot
2107.9204	2107.9377	0.0173	8	269	286	EPESYPHTQYEEGQSQA K	23	63.717			Mascot

3 12S seed storage globulin 1 [Triticum urartu] gi|474246751 64229.5 6.62 9 550 100 71.136 528 100

Peptide Information

Calc. Mass	Obsrv. Mass	± da	± ppm	Start Seq.	End Seq.	Sequence	Ion Score	C. I. %	Modification	Rank	Result Type
826.3512	826.3522	0.001	1	44	49	ECTFNR			Carbamidomethyl (C)[2]		Mascot
885.5152	885.5197	0.0045	5	50	57	LQASTPLR					Mascot
1116.5472	1116.5555	0.0083	7	202	210	EFLFAGNYR					Mascot
1116.5472	1116.5555	0.0083	7	202	210	EFLFAGNYR	61	99.994			Mascot
1263.7208	1263.7356	0.0148	12	259	268	FLKPVFTQQR					Mascot
1263.7208	1263.7356	0.0148	12	259	268	FLKPVFTQQR	67	99.999			Mascot
1381.7012	1381.718	0.0168	12	200	210	HKEFLFAGNYR					Mascot
1381.7012	1381.718	0.0168	12	200	210	HKEFLFAGNYR	91	100			Mascot
1666.7966	1666.7633	-0.0333	-20	312	326	HIQGEQPQMGQSQAK					Mascot
1908.9199	1908.9552	0.0353	18	211	227	SSQLHSSQNIFSGFDVR					Mascot
1908.9199	1908.9552	0.0353	18	211	227	SSQLHSSQNIFSGFDVR	142	100			Mascot
2062.8989	2062.9451	0.0462	22	61	77	SQAGLTEYFDEENEQFR					Mascot
2062.8989	2062.9451	0.0462	22	61	77	SQAGLTEYFDEENEQFR	146	100			Mascot

Calc. Mass	Obsrv. Mass	± da	± ppm	Start Seq.	End Seq.	Sequence	Ion Score	C. I. %	Modification	Rank	Result Type
2107.9204	2107.9377	0.0173	8	269	286	EPESYPHTQYEEGQSQA K					Mascot
2107.9204	2107.9377	0.0173	8	269	286	EPESYPHTQYEEGQSQA K	23	63.717			Mascot

4	triticin precursor [Triticum aestivum]	gi|7548844	57317.5	9.37	9	531	100	70.749	507	100

Peptide Information

Calc. Mass	Obsrv. Mass	± da	± ppm	Start Seq.	End Seq.	Sequence	Ion Score	C. I. %	Modification	Rank	Result Type
826.3512	826.3522	0.001	1	44	49	ECTFNR			Carbamidomethyl (C)[2]		Mascot
885.5152	885.5197	0.0045	5	50	57	LQASTPLR					Mascot
923.4767	923.4773	0.0006	1	78	85	CTGVFAIR			Carbamidomethyl (C)[1]		Mascot
1116.5472	1116.5555	0.0083	7	202	210	EFLFAGNYR					Mascot
1116.5472	1116.5555	0.0083	7	202	210	EFLFAGNYR	61	99.994			Mascot
1263.7208	1263.7356	0.0148	12	259	268	FLKPVFTQQR					Mascot
1263.7208	1263.7356	0.0148	12	259	268	FLKPVFTQQR	67	99.999			Mascot
1381.7012	1381.718	0.0168	12	200	210	HKEFLFAGNYR					Mascot
1381.7012	1381.718	0.0168	12	200	210	HKEFLFAGNYR	91	100			Mascot
1639.7671	1639.7712	0.0041	3	132	146	YGQSQSVQGQSQSQK					Mascot
1908.9199	1908.9552	0.0353	18	211	227	SSQLHSSQNIFSGFDVR					Mascot
1908.9199	1908.9552	0.0353	18	211	227	SSQLHSSQNIFSGFDVR	142	100			Mascot
2062.8989	2062.9451	0.0462	22	61	77	SQAGLTEYFDEENEQFR					Mascot
2062.8989	2062.9451	0.0462	22	61	77	SQAGLTEYFDEENEQFR	146	100			Mascot

5	triticin [Triticum aestivum]	gi|147744620	24404.1	9.37	6	411	100	50.413	383	100

Peptide Information

Calc. Mass	Obsrv. Mass	± da	± ppm	Start Seq.	End Seq.	Sequence	Ion Score	C. I. %	Modification	Rank	Result Type
1116.5472	1116.5555	0.0083	7	75	83	EFLFAGNYR					Mascot
1116.5472	1116.5555	0.0083	7	75	83	EFLFAGNYR	61	99.994			Mascot
1263.7208	1263.7356	0.0148	12	132	141	FLKPVFTQQR					Mascot
1263.7208	1263.7356	0.0148	12	132	141	FLKPVFTQQR	67	99.999			Mascot
1381.7012	1381.718	0.0168	12	73	83	HKEFLFAGNYR					Mascot
1381.7012	1381.718	0.0168	12	73	83	HKEFLFAGNYR	91	100			Mascot
1639.7671	1639.7712	0.0041	3	5	19	YGQSQSVQGQSQSQK					Mascot
1908.9199	1908.9552	0.0353	18	84	100	SSQLHSSQNIFSGFDVR					Mascot
1908.9199	1908.9552	0.0353	18	84	100	SSQLHSSQNIFSGFDVR	142	100			Mascot
2107.9204	2107.9377	0.0173	8	142	159	EPESYPHTQYEEGQSQA K					Mascot

2107.9204	2107.9377	0.0173	8	142	159	EPESYPHTQYEEGQSQAK	23	63.717			Mascot

6	hypothetical protein TRIUR3_34298 [Triticum urartu]	gi|474054244	221550.5	6.49	27	50	52.616	70.676

Peptide Information

Calc. Mass	Obsrv. Mass	± da	± ppm	Start Seq.	End Seq.	Sequence	Ion Score	C. I. %	Modification	Rank	Result Type
823.446	823.4287	-0.0173	-21	512	517	VSWLYR					Mascot
875.4833	875.459	-0.0243	-28	1420	1427	DGSKLDLK					Mascot
929.4219	929.4438	0.0219	24	425	431	YMLMSPR			Oxidation (M)[2,4]		Mascot
1116.5474	1116.5555	0.0081	7	66	74	NFNQTAFFK					Mascot
1116.5474	1116.5555	0.0081	7	66	74	NFNQTAFFK					Mascot
1244.6091	1244.6582	0.0491	39	862	874	GGAAEVSNGGNRR					Mascot
1261.7086	1261.6919	-0.0167	-13	1992	2002	MYQLPGVGIKR					Mascot
1262.5859	1262.6959	0.11	87	1455	1466	EQPSSTSADVNK					Mascot
1263.6288	1263.7356	0.1068	85	1009	1020	EATAGRSTSLDR					Mascot
1263.6288	1263.7356	0.1068	85	1009	1020	EATAGRSTSLDR					Mascot
1301.6519	1301.6593	0.0074	6	1270	1280	ADPCLIAKEER			Carbamidomethyl (C)[4]		Mascot
1318.5854	1318.6046	0.0192	15	197	206	LACFCKFCGR			Carbamidomethyl (C)[3,5,8]		Mascot
1320.6881	1320.7546	0.0665	50	109	118	IMERGPWIFR			Oxidation (M)[2]		Mascot
1376.7719	1376.693	-0.0789	-57	781	791	RVEEFLMALLR					Mascot
1381.6351	1381.718	0.0829	60	463	472	EEMMWLQRSR			Oxidation (M)[3]		Mascot
1381.6351	1381.718	0.0829	60	463	472	EEMMWLQRSR			Oxidation (M)[3]		Mascot
1432.7101	1432.6985	-0.0116	-8	1733	1747	SLPGGLPSTSMNGSK					Mascot
1534.7108	1534.8329	0.1221	80	66	77	NFNQTAFFKDMR			Oxidation (M)[11]		Mascot
1547.7887	1547.7727	-0.016	-10	1173	1186	GNSFNPMNALIEIK					Mascot
1664.7722	1664.7537	-0.0185	-11	432	446	SGDELDSLQQKSSDR					Mascot
1696.8442	1696.7863	-0.0579	-34	61	74	VHTDKNFNQTAFFK					Mascot
1715.7654	1715.7756	0.0102	6	1	16	MASHPDEQSPTSVGTR			Oxidation (M)[1]		Mascot
1728.8302	1728.7894	-0.0408	-24	119	132	NWAVLLTPYDDFMK			Oxidation (M)[13]		Mascot
1908.9271	1908.9552	0.0281	15	1037	1054	GGDTPSDNNSNGHRLIVR					Mascot
1908.9271	1908.9552	0.0281	15	1037	1054	GGDTPSDNNSNGHRLIVR					Mascot
1936.8917	1936.9377	0.046	24	1226	1243	NSSANEVGCEGDSIEKLK			Carbamidomethyl (C)[9]		Mascot
2063.0339	2062.9451	-0.0888	-43	911	929	LQHMQPANVATNSKDPPSK					Mascot
2063.0339	2062.9451	-0.0888	-43	911	929	LQHMQPANVATNSKDPPSK					Mascot
2129.9841	2129.9331	-0.051	-24	425	442	YMLMSPRSGDELDSLQQK			Oxidation (M)[2,4]		Mascot
2194.0159	2194.0378	0.0219	10	1926	1945	QYAINRPEGSSSDGLDG					Mascot

						NWK					
2224.1543	2224.0454	-0.1089	-49	700	719	GGLPHAEAVEKLVQFMQ VDR					Mascot
3528.5449	3528.6387	0.0938	27	1293	1326	TTTSTGLSPQNGTDCNA VESSAKTENQAEGCANK			Carbamidomethyl (C)[15,31]		Mascot

7 porphobilinogen deaminase [Triticum aestivum] gi|19849543 33568.2 5.5 8 43 0 3.712

Peptide Information

Calc. Mass	Obsrv. Mass	± da	± ppm	Start Seq.	End Seq.	Sequence	Ion Score	C. I. %	Modification	Rank	Result Type
1098.4634	1098.5452	0.0818	74	251	259	DKDGNCSFR			Carbamidomethyl (C)[6]		Mascot
1507.8479	1507.7385	-0.1094	-73	159	173	EGDVHATLLALAGLK					Mascot
1664.849	1664.7537	-0.0953	-57	260	275	GLLASPDGSIVYETSR					Mascot
1760.9099	1760.764	-0.1459	-83	41	57	TTGDMILDKPLADIGGK			Oxidation (M)[5]		Mascot
2085.0508	2084.9087	-0.1421	-68	82	99	DVPTYLPEGMILPCNLPR			Carbamidomethyl (C)[14]		Mascot
2101.0457	2100.8921	-0.1536	-73	82	99	DVPTYLPEGMILPCNLPR			Carbamidomethyl (C)[14], Oxidation (M)[10]		Mascot
2241.0129	2241.0149	0.002	1	276	296	SGTYSFDDMVALGQDAG HELK					Mascot
3618.9299	3618.656	-0.2739	-76	23	57	AAHTELAEDGAIEIVIIKTT GDMILDKPLADIGGK					Mascot
3618.9299	3618.656	-0.2739	-76	23	57	AAHTELAEDGAIEIVIIKTT GDMILDKPLADIGGK					Mascot
3646.7761	3646.6821	-0.094	-26	175	209	LGMPETATSVLSVDEML PAVAQGAIGITCRSNDDK			Carbamidomethyl (C)[29]		Mascot

8 hypothetical protein TRIUR3_30538 [Triticum urartu] gi|473882607 197169.7 4.65 23 42 0 27.015

Peptide Information

Calc. Mass	Obsrv. Mass	± da	± ppm	Start Seq.	End Seq.	Sequence	Ion Score	C. I. %	Modification	Rank	Result Type
904.4622	904.4637	0.0015	2	1233	1240	EAEVTDLK					Mascot
979.4917	979.4662	-0.0255	-26	1723	1731	EPSGIMAFK					Mascot
1116.5532	1116.5555	0.0023	2	744	753	SLSDLHTESK					Mascot
1116.5532	1116.5555	0.0023	2	744	753	SLSDLHTESK					Mascot
1219.6603	1219.7087	0.0484	40	298	307	LLSLQEMEIK			Oxidation (M)[7]		Mascot
1244.594	1244.6582	0.0642	52	432	442	SHLEEASMNVK					Mascot
1269.578	1269.6744	0.0964	76	1268	1277	VAMYDELQER			Oxidation (M)[3]		Mascot
1534.767	1534.8329	0.0659	43	625	637	DLMDSLIQITEEK					Mascot
1678.8568	1678.8386	-0.0182	-11	625	638	DLMDSLIQITEEKK			Oxidation (M)[3]		Mascot
1696.8752	1696.7863	-0.0889	-52	1181	1195	EEAAEKLAVHEETIK					Mascot
1715.8922	1715.7756	-0.1166	-68	1590	1604	TIANQEEQASVREIK					Mascot
1728.7858	1728.7894	0.0036	2	1	15	MEAEQAAQVHDDKEK					Mascot
1875.9116	1875.9746	0.063	34	1033	1051	AEEALAESTAMGAANQA LK					Mascot

Calc. Mass	Obsrv. Mass	± da	± ppm	Start Seq.	End Seq.	Sequence	Ion Score	C. I. %	Modification	Rank	Result Type
1955.0518	1954.9762	-0.0756	-39	298	313	LLSLQEMEIKLEEHVK			Oxidation (M)[7]		Mascot
2004.9794	2004.9366	-0.0428	-21	1501	1517	ELDEAVAKLEEQMNLEK			Oxidation (M)[13]		Mascot
2046.0237	2046.0022	-0.0215	-11	763	781	TAELETQVQELSAAEEGL K					Mascot
2061.9871	2062.0437	0.0566	27	744	762	SLSDLHTESKTAAESAM QR					Mascot
2063.0767	2062.9451	-0.1316	-64	726	743	SLLSDAHSHKEELELNLK					Mascot
2063.0767	2062.9451	-0.1316	-64	726	743	SLLSDAHSHKEELELNLK					Mascot
2085.071	2084.9087	-0.1623	-78	736	753	EELELNLKSLSDLHTESK					Mascot
2090.0249	2089.9851	-0.0398	-19	243	261	SQVDDLEQKLASQDADIS K					Mascot
2119.0225	2119.0391	0.0166	8	262	279	LTEELSLHCSSEESLKEK			Carbamidomethyl (C)[9]		Mascot
2178.0786	2178.0786	0	0	1583	1601	ELEAAYRTIANQEEQASV R					Mascot
2192.0288	2192.0518	0.023	10	214	232	AVDHQQVEESLNTTMSQ FK					Mascot
2195.1052	2195.0439	-0.0613	-28	546	566	SIELASLLHTSKSDADGA HSR					Mascot
2208.0237	2208.0034	-0.0203	-9	214	232	AVDHQQVEESLNTTMSQ FK			Oxidation (M)[15]		Mascot

9	RGA2 [Triticum aestivum]	gi|226427700	37906.4	8.25	8	41	0	1.741	9	0

Peptide Information

Calc. Mass	Obsrv. Mass	± da	± ppm	Start Seq.	End Seq.	Sequence	Ion Score	C. I. %	Modification	Rank	Result Type
1246.658	1246.7515	0.0935	75	2	12	WIAEGYPGVVR					Mascot
1248.6848	1248.7161	0.0313	25	202	212	LQHLHAGFPTK					Mascot
1416.8131	1416.8495	0.0364	26	186	198	GTSIVMLPQTIIK			Oxidation (M)[6]		Mascot
1432.7465	1432.6985	-0.048	-34	293	305	LGVMGINEKNDVK			Oxidation (M)[4]		Mascot
1661.8892	1661.7749	-0.1143	-69	57	71	EIGISKSMEGNLVLR			Oxidation (M)[8]		Mascot
2119.0012	2119.0391	0.0379	18	13	30	NKSTEEVAESYFMDLISR					Mascot
2210.2041	2210.0188	-0.1853	-84	271	291	GVHVAYGDAVIQEIGRLS GLR					Mascot
2210.2041	2210.0188	-0.1853	-84	271	291	GVHVAYGDAVIQEIGRLS GLR	9	0			Mascot
2241.1179	2241.0149	-0.103	-46	306	325	FCSAISNLSRLESLSVQS DK			Carbamidomethyl (C)[2]		Mascot

10	ribosomal protein L14 (chloroplast) [Triticum monococcum]	gi|525778541	13711.3	8.52	7	40	0	1.627

Protein Group

RecName: Full=50S ribosomal protein L14, chloroplastic	gi|45477200	13639.3	8.9099 998474 1211
ribosomal protein L14 (chloroplast) [Triticum aestivum]	gi|521301523	13639.3	8.9099

			998474 1211
ribosomal protein L14 (chloroplast) [Triticum monococcum subsp. aegilopoides]	gi|521301383	13711.3	8.5200 004577 6367
ribosomal protein L14 (chloroplast) [Triticum monococcum]	gi|521301226	13711.3	8.5200 004577 6367
ribosomal protein L14 (chloroplast) [Triticum urartu]	gi|521301444	13711.3	8.5200 004577 6367
ribosomal protein L14 (chloroplast) [Triticum urartu]	gi|525782313	13711.3	8.5200 004577 6367
ribosomal protein L14 [Triticum aestivum]	gi|14017608	13639.3	8.9099 998474 1211
ribosomal protein L14 [Triticum aestivum]	gi|13928241	13639.3	8.9099 998474 1211

Peptide Information

Calc. Mass	Obsrv. Mass	± da	± ppm	Start Seq.	End Seq.	Sequence	Ion Score	C. I. %	Modification	Rank	Result Type
885.4901	885.5197	0.0296	33	24	32	VIGAAGNQR					Mascot
940.5714	940.485	-0.0864	-92	115	123	IVSLAPEVL					Mascot
1275.6917	1275.7289	0.0372	29	24	35	VIGAAGNQRYAR					Mascot
1364.6693	1364.7455	0.0762	56	80	91	YDDNAAVIIDQK					Mascot
1416.8573	1416.8495	-0.0078	-6	33	45	YARIGDVIVAVIK					Mascot
1696.8977	1696.7863	-0.1114	-66	2	17	IQPQTLLNVADNSGAR					Mascot
1760.8813	1760.764	-0.1173	-67	80	95	YDDNAAVIIDQKGNPK					Mascot

Gel Idx/Pos	294/L22	Instr./Gel Origin	BA2151/Sample Project 20140814	Process Status	Analysis Succeeded
Plate [#] Name	[1] Sample Project 20140814	Instrument Sample Name		Spectra	11

Rank	Protein Name	Accession No.	Protein MW	Protein PI	Pep. Count	Protein Score	Protein Score C. I. %	Intensity Matched	Total Ion Score	Total Ion C. I. %	Confirmed
1	hypothetical protein TRIUR3_30168 [Triticum urartu]	gi|473888425	75325.4	5.21	26	64	98.198	9.817			

Peptide Information

Calc. Mass	Obsrv. Mass	± da	± ppm	Start Seq.	End Seq.	Sequence	Ion Score	C. I. %	Modification	Rank	Result Type
819.3843	819.433	0.0487	59	112	118	SEAEVER					Mascot
823.4494	823.4265	-0.0229	-28	1	7	MAVIAYR					Mascot
831.4359	831.471	0.0351	42	53	58	VWELER					Mascot
832.441	832.4409	-0.0001	0	146	152	LESENLK					Mascot
839.4443	839.431	-0.0133	-16	1	7	MAVIAYR			Oxidation (M)[1]		Mascot
846.4567	846.5141	0.0574	68	451	457	VEEIQTK					Mascot
921.4458	921.4852	0.0394	43	295	301	EMDNLKR			Oxidation (M)[2]		Mascot
946.4588	946.5076	0.0488	52	541	548	NKDVEADR					Mascot
960.4897	960.5273	0.0376	39	90	97	SFKAENHK					Mascot
988.5421	988.5034	-0.0387	-39	146	153	LESENLKR					Mascot
991.4942	991.5438	0.0496	50	100	108	SEVSIAEEK					Mascot
995.5455	995.6255	0.08	80	1	8	MAVIAYRR			Oxidation (M)[1]		Mascot
995.5455	995.6255	0.08	80	1	8	MAVIAYRR			Oxidation (M)[1]		Mascot
1004.5445	1004.5174	-0.0271	-27	60	67	ITQLICEK			Carbamidomethyl (C)[6]		Mascot
1021.5173	1021.5029	-0.0144	-14	8	16	RAGYDVGQR					Mascot
1023.5105	1023.606	0.0955	93	85	92	EEVERSFK					Mascot
1049.5626	1049.5669	0.0043	4	205	214	EAAAKAFDVK					Mascot
1106.5259	1106.5123	-0.0136	-12	257	265	CRQSEAEVK			Carbamidomethyl (C)[1]		Mascot
1118.6125	1118.5353	-0.0772	-69	417	426	MELVALVEAK			Oxidation (M)[1]		Mascot
1164.5895	1164.5743	-0.0152	-13	279	289	EAAAKAFEAEK					Mascot
1189.6609	1189.5776	-0.0833	-70	60	69	ITQLICEKGK			Carbamidomethyl (C)[6]		Mascot
1193.5468	1193.6187	0.0719	60	577	586	LNDEMNSVQK			Oxidation (M)[5]		Mascot
1199.6201	1199.646	0.0259	22	479	488	HAMSELEVKR					Mascot
1260.6794	1260.6345	-0.0449	-36	98	108	LRSEVSIAEEK					Mascot
1289.7134	1289.6633	-0.0501	-39	489	500	LKMDLAALAEAK			Oxidation (M)[3]		Mascot
1455.6971	1455.7073	0.0102	7	653	665	CSILSFLSPCGSK			Carbamidomethyl (C)[1,10]		Mascot
1463.7158	1463.7532	0.0374	26	183	194	QNMSEAEIERLK			Oxidation (M)[3]		Mascot

1479.6744	1479.7737	0.0993	67	181	192	EKQNMSEAEIER			Oxidation (M)[5]		Mascot

2	Signal recognition particle 54 kDa protein 3 [Triticum urartu]	gi|474414481	54042.9	9.38	18	58	93.307	7.237	2	0	

Peptide Information

Calc. Mass	Obsrv. Mass	± da	± ppm	Start Seq.	End Seq.	Sequence	Ion Score	C. I. %	Modification	Rank	Result Type
804.421	804.4183	-0.0027	-3	364	370	GQEKQSK					Mascot
835.4164	835.4343	0.0179	21	485	493	MLAGMKGGA					Mascot
849.4465	849.4623	0.0158	19	142	149	AGAFDQLK					Mascot
851.4113	851.4108	-0.0005	-1	485	493	MLAGMKGGA			Oxidation (M)[1]		Mascot
858.493	858.5036	0.0106	12	171	178	IAVEGLEK					Mascot
863.4807	863.4628	-0.0179	-21	425	431	LAKMWSK					Mascot
864.4825	864.4678	-0.0147	-17	468	475	SAFEALVK					Mascot
1004.4829	1004.5174	0.0345	34	30	37	ECLNEIAR			Carbamidomethyl (C)[2]		Mascot
1101.6263	1101.5842	-0.0421	-38	2	12	VLAELGGSISR					Mascot
1118.635	1118.5353	-0.0997	-89	395	403	LMTESRILR					Mascot
1127.5258	1127.5483	0.0225	20	481	490	DMSKMLAGMK			Oxidation (M)[2]		Mascot
1189.6423	1189.5776	-0.0647	-54	184	194	SDLIIIDTSGR					Mascot
1232.6667	1232.623	-0.0437	-35	1	12	MVLAELGGSISR					Mascot
1366.7035	1366.6744	-0.0291	-21	332	342	LMSEQFQNLLK			Oxidation (M)[2]		Mascot
1366.8053	1366.6744	-0.1309	-96	294	306	LLGRGDLPGLLDK					Mascot
1415.7853	1415.7048	-0.0805	-57	58	71	KSVNLEALAAGTNK					Mascot
1482.7444	1482.7701	0.0257	17	46	57	FKMVCDLQANIK			Carbamidomethyl (C)[5], Oxidation (M)[3]		Mascot
1507.6669	1507.7626	0.0957	63	195	206	HMQEAALFEEMR			Oxidation (M)[2]		Mascot
1507.6669	1507.7626	0.0957	63	195	206	HMQEAALFEEMR	2	0	Oxidation (M)[2]		Mascot
1838.7856	1838.9429	0.1573	86	376	391	YMTIMDSMTAAELDSK			Oxidation (M)[2,5]		Mascot

3	Protein strawberry notch [Triticum urartu]	gi|474424519	138678.5	5.96	25	54	81.985	10.181			

Peptide Information

Calc. Mass	Obsrv. Mass	± da	± ppm	Start Seq.	End Seq.	Sequence	Ion Score	C. I. %	Modification	Rank	Result Type
800.4736	800.4361	-0.0375	-47	1090	1096	ALNKQAR					Mascot
807.4108	807.4246	0.0138	17	735	741	ATQQFGR					Mascot
817.405	817.4589	0.0539	66	424	430	EALAEER					Mascot
825.4689	825.4743	0.0054	7	859	866	HGGKLTGR					Mascot
831.4584	831.471	0.0126	15	1153	1158	NLSRWR					Mascot

832.4047	832.4409	0.0362	43	171	178	DLDDVGAK					Mascot
868.4345	868.4468	0.0123	14	289	295	NLGYMVR			Oxidation (M)[5]		Mascot
891.541	891.4639	-0.0771	-86	766	773	FASIVAKR					Mascot
988.5058	988.5034	-0.0024	-2	170	178	RDLDDVGAK					Mascot
995.5706	995.6255	0.0549	55	803	810	KALTLMYR					Mascot
995.5706	995.6255	0.0549	55	803	810	KALTLMYR					Mascot
1004.5193	1004.5174	-0.0019	-2	963	970	AMLEERQK					Mascot
1011.5655	1011.624	0.0585	58	803	810	KALTLMYR			Oxidation (M)[6]		Mascot
1011.5655	1011.624	0.0585	58	803	810	KALTLMYR			Oxidation (M)[6]		Mascot
1021.4263	1021.5029	0.0766	75	359	365	MMNMYRK			Oxidation (M)[1,2,4]		Mascot
1027.4764	1027.5166	0.0402	39	600	608	DAYMAELSK					Mascot
1158.6729	1158.6189	-0.054	-47	914	924	LDSGIVDIKAK					Mascot
1189.5023	1189.5776	0.0753	63	1051	1059	QCMHGPKCR			Carbamidomethyl (C)[2,8], Oxidation (M)[3]		Mascot
1199.5725	1199.646	0.0735	61	600	609	DAYMAELSKR			Oxidation (M)[4]		Mascot
1312.6201	1312.634	0.0139	11	598	608	ERDAYMAELSK					Mascot
1415.7601	1415.7048	-0.0553	-39	774	786	LESLGALTQGDRR					Mascot
1473.7375	1473.7614	0.0239	16	408	420	HMCMSAKVPAVVK			Carbamidomethyl (C)[3], Oxidation (M)[2]		Mascot
1516.7616	1516.7252	-0.0364	-24	742	754	THRSNQTSAPVYR					Mascot
1707.9065	1707.7964	-0.1101	-64	155	169	ALWISIGSDLKYDAR					Mascot
1818.8724	1818.9351	0.0627	34	672	686	NTKEIALDMINMHEK			Oxidation (M)[9,12]		Mascot
1839.0885	1838.9429	-0.1456	-79	841	858	AALVAVGIIRDAVICIGK			Carbamidomethyl (C)[15]		Mascot
2246.1484	2246.2185	0.0701	31	1007	1027	VTRPAVGEASKEMPFAE LEGK					Mascot
2839.3179	2839.3608	0.0429	15	223	245	LRQLVEWCGSEYDGLVV FDECHK			Carbamidomethyl (C)[8,21]		Mascot
2839.3179	2839.3608	0.0429	15	223	245	LRQLVEWCGSEYDGLVV FDECHK			Carbamidomethyl (C)[8,21]		Mascot

4	putative rhamnose biosynthetic enzyme 1 [Triticum urartu]	gi|473775808	76545.8	6.38	16	52	72.098	6.697	

Peptide Information

Calc. Mass	Obsrv. Mass	± da	± ppm	Start Seq.	End Seq.	Sequence	Ion Score	C. I. %	Modification	Rank	Result Type
829.5002	829.4321	-0.0681	-82	120	126	VTGQIRR					Mascot
835.511	835.4343	-0.0767	-92	203	209	FILLAMK					Mascot
839.3967	839.431	0.0343	41	1	7	MATYEPK					Mascot
922.488	922.4679	-0.0201	-22	268	275	LFGLDTEK					Mascot
932.5121	932.4739	-0.0382	-41	524	531	AMVEELLK					Mascot
960.4996	960.5273	0.0277	29	356	365	SLASSPAEAK					Mascot
963.4781	963.4975	0.0194	20	306	313	TTWEEGLK					Mascot

1001.5778	1001.5231	-0.0547	-55	396	405	TGWIGGLLGK						Mascot
1033.517	1033.5222	0.0052	5	339	347	MLMTPGVER						Mascot
1049.5118	1049.5669	0.0551	53	339	347	MLMTPGVER			Oxidation (M)[1]			Mascot
1106.6205	1106.5123	-0.1082	-98	176	185	SYGLPVITTR						Mascot
1155.51	1155.6075	0.0975	84	532	540	DYDNVCTLR			Carbamidomethyl (C)[6]			Mascot
1216.5991	1216.5809	-0.0182	-15	543	553	MPISSDLSNPR						Mascot
1216.5991	1216.5809	-0.0182	-15	543	553	MPISSDLSNPR						Mascot
1232.594	1232.623	0.029	24	543	553	MPISSDLSNPR			Oxidation (M)[1]			Mascot
1366.6638	1366.6744	0.0106	8	621	631	WTNFTLEEQAK						Mascot
1366.6638	1366.6744	0.0106	8	621	631	WTNFTLEEQAK						Mascot
1372.722	1372.6788	-0.0432	-31	242	254	GEVGHVYNIGTVK						Mascot
1461.8101	1461.7346	-0.0755	-52	29	40	SYPHYKIVVLDK						Mascot
1463.6914	1463.7532	0.0618	42	186	198	GNNVYGPNQFPEK						Mascot

5	asynapsis 1 [Triticum aestivum]	gi|148970484	66664	5.36	19	50	54.748	7.109	

Peptide Information

Calc. Mass	Obsrv. Mass	± da	± ppm	Start Seq.	End Seq.	Sequence	Ion Score	C. I. %	Modification	Rank	Result Type
819.4756	819.433	-0.0426	-52	157	163	TLVSLMR					Mascot
821.4549	821.4269	-0.028	-34	2	8	VMAQKTK			Oxidation (M)[2]		Mascot
835.4706	835.4343	-0.0363	-43	157	163	TLVSLMR			Oxidation (M)[6]		Mascot
890.4651	890.4818	0.0167	19	48	55	SVPALEMK			Oxidation (M)[7]		Mascot
893.4727	893.4639	-0.0088	-10	76	83	GVYDALQK					Mascot
993.4669	993.5377	0.0708	71	213	221	MEVGNVNSK			Oxidation (M)[1]		Mascot
1021.5676	1021.5029	-0.0647	-63	76	84	GVYDALQKK					Mascot
1049.4755	1049.5669	0.0914	87	59	67	LMPMDAESR					Mascot
1106.5663	1106.5123	-0.054	-49	68	75	RLIDWMEK			Oxidation (M)[6]		Mascot
1109.5586	1109.5552	-0.0034	-3	506	515	FQSVGSDLTR					Mascot
1115.6493	1115.6151	-0.0342	-31	48	57	SVPALEMKIK					Mascot
1118.5146	1118.5353	0.0207	19	164	172	TLDQMPEER					Mascot
1147.5742	1147.5923	0.0181	16	272	282	YVIAPNDGNGK					Mascot
1189.5808	1189.5776	-0.0032	-3	415	425	LDGEANQSTVR					Mascot
1193.5653	1193.6187	0.0534	45	58	67	KLMPMDAESR			Oxidation (M)[3]		Mascot
1320.6794	1320.6062	-0.0732	-55	354	365	DGYAVNKITDPK					Mascot
1320.6794	1320.6062	-0.0732	-55	354	365	DGYAVNKITDPK					Mascot
1357.6787	1357.6873	0.0086	6	37	47	GLFPEKYFNDK					Mascot

1366.7074	1366.6744	-0.033	-24	506	517	FQSVGSDLTRTR	Mascot
1366.7074	1366.6744	-0.033	-24	506	517	FQSVGSDLTRTR	Mascot
1433.7231	1433.7382	0.0151	11	538	551	ESATSLESGVLGQR	Mascot
1628.7585	1628.8672	0.1087	67	371	385	EEVAMHNVSPTEGTK	Mascot

6	unnamed protein product [Triticum aestivum]	gi|257695339	41711.4	9.87	13	47	19.53	5.205

Protein Group

chimeric SDH2-RPS14 protein [Triticum aestivum]	gi|28611030	41711.4	9.8699 998855 5908
unnamed protein product [Triticum aestivum]	gi|257661236	41711.4	9.8699 998855 5908
unnamed protein product [Triticum aestivum]	gi|257319684	41711.4	9.8699 998855 5908
unnamed protein product [Triticum aestivum]	gi|257289356	41711.4	9.8699 998855 5908
unnamed protein product [Triticum aestivum]	gi|257289100	41711.4	9.8699 998855 5908
unnamed protein product [Triticum aestivum]	gi|219984044	41711.4	9.8699 998855 5908
unnamed protein product [Triticum aestivum]	gi|257700384	41711.4	9.8699 998855 5908
unnamed protein product [Triticum aestivum]	gi|219925569	41711.4	9.8699 998855 5908

unnamed protein product [Triticum aestivum]	gi|219788309	41711.4	9.8699<br>998855<br>5908
unnamed protein product [Triticum aestivum]	gi|219786134	41711.4	9.8699<br>998855<br>5908
unnamed protein product [Triticum aestivum]	gi|219760709	41711.4	9.8699<br>998855<br>5908
unnamed protein product [Triticum aestivum]	gi|219759160	41711.4	9.8699<br>998855<br>5908

Peptide Information

Calc. Mass	Obsrv. Mass	± da	± ppm	Start Seq.	End Seq.	Sequence	Ion Score	C. I. %	Modification	Rank	Result Type
832.4709	832.4409	-0.03	-36	1	8	MAAAALLR			Oxidation (M)[1]		Mascot
837.4577	837.4022	-0.0555	-66	335	341	SRGVYQK					Mascot
858.4719	858.5036	0.0317	37	160	166	SVEPWLK					Mascot
908.5134	908.4781	-0.0353	-39	344	350	MSRIVFR					Mascot
988.572	988.5034	-0.0686	-69	1	9	MAAAALLRR			Oxidation (M)[1]		Mascot
991.5101	991.5438	0.0337	34	319	326	NSNMTRLR					Mascot
1014.5731	1014.5282	-0.0449	-44	160	167	SVEPWLKR					Mascot
1023.5153	1023.606	0.0907	89	327	334	NRCIFTGR			Carbamidomethyl (C)[3]		Mascot
1109.5586	1109.5552	-0.0034	-3	177	185	EIYQSKADR					Mascot
1260.6981	1260.6345	-0.0636	-50	351	362	TLANKGELMGVK					Mascot
1302.5995	1302.6438	0.0443	34	298	308	DPELPSDMRDK					Mascot
1496.8544	1496.7445	-0.1099	-73	10	24	SPAARALLSPALSSR					Mascot
1507.754	1507.7626	0.0086	6	55	67	WDPDSPSTKPHLK					Mascot
1507.754	1507.7626	0.0086	6	55	67	WDPDSPSTKPHLK					Mascot

7	hypothetical protein TRIUR3_27544 [Triticum urartu]	gi|473892699	74264.7	9.73	16	44	0	5.973

Peptide Information

Calc. Mass	Obsrv. Mass	± da	± ppm	Start Seq.	End Seq.	Sequence	Ion Score	C. I. %	Modification	Rank	Result Type
819.4393	819.433	-0.0063	-8	163	170	IAAAMQAK			Oxidation (M)[5]		Mascot
827.437	827.4335	-0.0035	-4	235	242	APTSQPAR					Mascot
831.4108	831.471	0.0602	72	146	152	TNGFHQK					Mascot
846.4427	846.5141	0.0714	84	430	436	QLQSSQR					Mascot
858.454	858.5036	0.0496	58	507	513	RVQANDR					Mascot
963.5258	963.4975	-0.0283	-29	300	308	AASQKFPSK					Mascot
1033.5459	1033.5222	-0.0237	-23	270	277	EMLLSRER					Mascot
1049.5408	1049.5669	0.0261	25	270	277	EMLLSRER			Oxidation (M)[2]		Mascot
1087.5643	1087.604	0.0397	37	146	154	TNGFHQKQK					Mascot
1107.5793	1107.5498	-0.0295	-27	259	267	RPESKFESK					Mascot
1199.624	1199.646	0.022	18	520	530	QPSRQVSANGR					Mascot
1302.6835	1302.6438	-0.0397	-30	110	120	SMKEIQNTVPR					Mascot
1340.6852	1340.7075	0.0223	17	278	289	AVDNGRMHSVVR					Mascot
1461.7631	1461.7346	-0.0285	-19	121	132	ERRPPGVTYMQK					Mascot
1516.6196	1516.7252	0.1056	70	210	222	SAESADGWFCCVK			Carbamidomethyl (C)[10,11]		Mascot
1661.8025	1661.824	0.0215	13	284	299	MHSVVRNGSSQATGSK			Oxidation (M)[1]		Mascot
1838.9369	1838.9429	0.006	3	493	507	QRPLSSQGHYPEQRR					Mascot

8 hypothetical protein TRIUR3_13415 [Triticum urartu] gi|474021942 109224.8 6.41 19 44 0 8.056

Peptide Information

Calc. Mass	Obsrv. Mass	± da	± ppm	Start Seq.	End Seq.	Sequence	Ion Score	C. I. %	Modification	Rank	Result Type
804.4726	804.4183	-0.0543	-67	354	360	ANFAIIR					Mascot
819.3843	819.433	0.0487	59	7	13	VDVDESR					Mascot
821.4403	821.4269	-0.0134	-16	564	570	EIVDFAK					Mascot
825.3923	825.4743	0.082	99	67	73	CFTGVNK			Carbamidomethyl (C)[1]		Mascot
832.4159	832.4409	0.025	30	347	353	EQSELAR					Mascot
845.4614	845.4951	0.0337	40	756	762	EVDEIIK					Mascot
846.4315	846.5141	0.0826	98	748	754	DLNELSR					Mascot
847.4892	847.481	-0.0082	-10	283	289	LIMAIMR					Mascot
863.4841	863.4628	-0.0213	-25	283	289	LIMAIMR			Oxidation (M)[3]		Mascot
1001.5626	1001.5231	-0.0395	-39	45	52	LTIIEEQR					Mascot
1014.485	1014.5282	0.0432	43	403	412	DAIHGSETGK					Mascot
1023.5159	1023.606	0.0901	88	166	172	WAWRYNK					Mascot
1101.6011	1101.5842	-0.0169	-15	746	754	VRDLNELSR					Mascot

1189.694	1189.5776	-0.1164	-98	561	570	IVREIVDFAK					Mascot
1407.6863	1407.715	0.0287	20	582	594	ENISGANAFSLER					Mascot
1455.7413	1455.7073	-0.034	-23	154	165	GELKESFHHMLK					Mascot
1473.7795	1473.7614	-0.0181	-12	844	856	EVNIQTSIELAEK					Mascot
1699.858	1699.8628	0.0048	3	229	243	MAYILTNVALTCMAK			Carbamidomethyl (C)[12]		Mascot
1839.0159	1838.9429	-0.073	-40	888	902	EMTVWIILILAVYMK			Oxidation (M)[2]		Mascot
2839.4407	2839.3608	-0.0799	-28	173	197	NLEEQAAQLHMLTSWSQ IVEVAVSR					Mascot
2839.4407	2839.3608	-0.0799	-28	173	197	NLEEQAAQLHMLTSWSQ IVEVAVSR					Mascot

9	Sec14 cytosolic factor [Triticum urartu]	gi|474102547	76258.1	8.33	18	44	0	6.661

Peptide Information

Calc. Mass	Obsrv. Mass	± da	± ppm	Start Seq.	End Seq.	Sequence	Ion Score	C. I. %	Modification	Rank	Result Type
818.473	818.4578	-0.0152	-19	45	52	KALNASSK					Mascot
823.4421	823.4265	-0.0156	-19	237	243	NFSKTAR					Mascot
870.5043	870.5388	0.0345	40	177	184	LGKVDPNK					Mascot
991.4843	991.5438	0.0595	60	555	562	SLLDSWDR					Mascot
993.5146	993.5377	0.0231	23	241	248	TAREMLTR			Oxidation (M)[5]		Mascot
1021.4619	1021.5029	0.041	40	565	572	CIESDLER			Carbamidomethyl (C)[1]		Mascot
1033.6074	1033.5222	-0.0852	-82	575	583	KVLQATVMK			Oxidation (M)[8]		Mascot
1066.4768	1066.55	0.0732	69	389	396	EEMQMRAR			Oxidation (M)[3]		Mascot
1107.5219	1107.5498	0.0279	25	197	204	YHVQEFER					Mascot
1189.5782	1189.5776	-0.0006	-1	103	111	QNDYHMLLR					Mascot
1232.6635	1232.623	-0.0405	-33	555	564	SLLDSWDRIK					Mascot
1316.7103	1316.5812	-0.1291	-98	377	388	STLGCSRLAPVR			Carbamidomethyl (C)[5]		Mascot
1415.8118	1415.7048	-0.107	-76	168	179	QGRPVYIERLGK					Mascot
1482.7985	1482.7701	-0.0284	-19	185	196	LMNITTVDRYIK			Oxidation (M)[2]		Mascot
1507.8591	1507.7626	-0.0965	-64	599	611	LHVIEGNELKISR					Mascot
1507.8591	1507.7626	-0.0965	-64	599	611	LHVIEGNELKISR					Mascot
1516.7213	1516.7252	0.0039	3	331	343	SNKGPWNDPNIMK			Oxidation (M)[12]		Mascot
1699.856	1699.8628	0.0068	4	123	135	AIHMWAEMLQWRK					Mascot
2224.0315	2224.0476	0.0161	7	118	134	FDTEKAIHMWAEMLQWR			Oxidation (M)[9,13]		Mascot

10	hypothetical protein TRIUR3_25683 [Triticum urartu]	gi|474075206	68030.7	4.94	14	43	0	4.814

Peptide Information

Calc. Mass	Obsrv. Mass	± da	± ppm	Start Seq.	End Seq.	Sequence	Ion Score	C. I. %	Modification	Rank	Result Type

809.4192	809.4311	0.0119	15	398	403	SFELWK		Mascot
817.4203	817.4589	0.0386	47	595	601	VSSQFHL		Mascot
819.393	819.433	0.04	49	278	283	HCFLSR	Carbamidomethyl (C)[2]	Mascot
908.4836	908.4781	-0.0055	-6	141	147	IYVDRDK		Mascot
960.4971	960.5273	0.0302	31	90	97	CPLQFPAK	Carbamidomethyl (C)[1]	Mascot
991.524	991.5438	0.0198	20	183	191	ATEMLALAR	Oxidation (M)[4]	Mascot
1004.5371	1004.5174	-0.0197	-20	533	541	TVLSDISNR		Mascot
1033.5021	1033.5222	0.0201	19	489	497	EENSTARAR		Mascot
1155.6157	1155.6075	-0.0082	-7	136	145	AFGSKIYVDR		Mascot
1193.6525	1193.6187	-0.0338	-28	510	520	FTAETIGKQAK		Mascot
1199.6096	1199.646	0.0364	30	398	406	SFELWKDFK		Mascot
1479.7625	1479.7737	0.0112	8	498	509	FSEICKPRESVK	Carbamidomethyl (C)[5]	Mascot
1496.6752	1496.7445	0.0693	46	374	386	DEYESVQVAEEAK		Mascot
1516.7941	1516.7252	-0.0689	-45	90	102	CPLQFPAKEASIR	Carbamidomethyl (C)[1]	Mascot

Gel Idx/Pos	295/L23	Instr./Gel Origin	BA2151/Sample Project 20140814	Process Status	Analysis Succeeded
Plate [#] Name	[1] Sample Project 20140814	Instrument Sample Name		Spectra	11

Rank	Protein Name	Accession No.	Protein MW	Protein PI	Pep. Count	Protein Score	Protein Score C. I. %	Intensity Matched	Total Ion Score	Total Ion C. I. %	Confirmed
1	Cleavage stimulation factor subunit 1 [Triticum urartu]	gi|474365350	70971.2	9.27	19	68	99.299	7.25			

Peptide Information

Calc. Mass	Obsrv. Mass	± da	± ppm	Start Seq.	End Seq.	Sequence	Ion Score	C. I. %	Modification	Rank	Result Type
816.4686	816.4243	-0.0443	-54	271	277	ASEVVRR					Mascot
822.462	822.4008	-0.0612	-74	638	643	SVRFWK					Mascot
826.4893	826.4271	-0.0622	-75	290	297	GLGPRQAK					Mascot
849.4424	849.4384	-0.004	-5	2	9	DTSTRAAK					Mascot
861.4828	861.4442	-0.0386	-45	322	329	GPSKTFPK					Mascot
1015.5319	1015.516	-0.0159	-16	326	333	TFPKHETR					Mascot
1068.6161	1068.5432	-0.0729	-68	262	270	LQVNQNKPK					Mascot
1081.575	1081.5223	-0.0527	-49	435	443	VIQDTHNVR					Mascot
1095.4922	1095.5701	0.0779	71	54	63	MQVASGEAMR			Oxidation (M)[1]		Mascot
1106.6205	1106.5417	-0.0788	-71	166	175	YKGGIDVVQK					Mascot
1187.6816	1187.6211	-0.0605	-51	168	178	GGIDVVQKIMK					Mascot
1232.7362	1232.6223	-0.1139	-92	65	75	NALATFKNILK					Mascot
1235.5984	1235.6146	0.0162	13	54	64	MQVASGEAMRR					Mascot
1259.7107	1259.6743	-0.0364	-29	85	97	GFGTVITGAIPAR					Mascot
1262.6311	1262.642	0.0109	9	155	165	LMVQGYSGHVR			Oxidation (M)[2]		Mascot
1325.7648	1325.6694	-0.0954	-72	260	270	TRLQVNQNKPK					Mascot
1367.6149	1367.7141	0.0992	73	491	503	YSGTGSMYVTASK			Oxidation (M)[7]		Mascot
1384.7042	1384.7765	0.0723	52	213	224	MIWSALGHLNDK					Mascot
1384.7042	1384.7765	0.0723	52	213	224	MIWSALGHLNDK					Mascot
1443.8319	1443.7413	-0.0906	-63	72	84	NILKVDGVPGLYR					Mascot

Rank	Protein Name	Accession No.	Protein MW	Protein PI	Pep. Count	Protein Score	Protein Score C. I. %	Intensity Matched	Total Ion Score	Total Ion C. I. %	Confirmed
2	putative mitochondrial carrier protein [Triticum monococcum]	gi|18496651	35130.2	9.55	13	66	98.963	5.014			

Peptide Information

Calc. Mass	Obsrv. Mass	± da	± ppm	Start Seq.	End Seq.	Sequence	Ion Score	C. I. %	Modification	Rank	Result Type
816.4686	816.4243	-0.0443	-54	271	277	ASEVVRR					Mascot
849.4424	849.4384	-0.004	-5	2	9	DTSTRAAK					Mascot

1068.6161	1068.5432	-0.0729	-68	262	270	LQVNQNKPK					Mascot
1095.4922	1095.5701	0.0779	71	54	63	MQVASGEAMR			Oxidation (M)[1]		Mascot
1106.6205	1106.5417	-0.0788	-71	166	175	YKGGIDVVQK					Mascot
1187.6816	1187.6211	-0.0605	-51	168	178	GGIDVVQKIMK					Mascot
1232.7362	1232.6223	-0.1139	-92	65	75	NALATFKNILK					Mascot
1235.5984	1235.6146	0.0162	13	54	64	MQVASGEAMRR					Mascot
1259.7107	1259.6743	-0.0364	-29	85	97	GFGTVITGAIPAR					Mascot
1262.6311	1262.642	0.0109	9	155	165	LMVQGYSGHVR			Oxidation (M)[2]		Mascot
1325.7648	1325.6694	-0.0954	-72	260	270	TRLQVNQNKPK					Mascot
1384.7042	1384.7765	0.0723	52	213	224	MIWSALGHLNDK					Mascot
1384.7042	1384.7765	0.0723	52	213	224	MIWSALGHLNDK					Mascot
1443.8319	1443.7413	-0.0906	-63	72	84	NILKVDGVPGLYR					Mascot

3 hypothetical protein TRIUR3_22357 [Triticum urartu] gi|474210352 46810.1 8.75 15 53 79.787 6.375

Peptide Information

Calc. Mass	Obsrv. Mass	± da	± ppm	Start Seq.	End Seq.	Sequence	Ion Score	C. I. %	Modification	Rank	Result Type
813.4828	813.408	-0.0748	-92	117	123	LELPVSR					Mascot
826.4166	826.4271	0.0105	13	188	194	EPRDPGR					Mascot
861.4617	861.4442	-0.0175	-20	405	410	FYKFTR					Mascot
865.4461	865.4575	0.0114	13	52	58	FGQMRAR					Mascot
870.5229	870.5538	0.0309	35	396	402	LVMVKHK			Oxidation (M)[3]		Mascot
891.4128	891.4423	0.0295	33	411	417	DEIECIL			Carbamidomethyl (C)[5]		Mascot
1032.5143	1032.5116	-0.0027	-3	146	154	ILCVDAEGR			Carbamidomethyl (C)[3]		Mascot
1068.6095	1068.5432	-0.0663	-62	1	9	MGRTLVHVR					Mascot
1114.6514	1114.5632	-0.0882	-79	59	67	QLVNVVMRR					Mascot
1199.6096	1199.6605	0.0509	42	128	136	LDFLPFYER					Mascot
1201.6835	1201.6339	-0.0496	-41	57	66	ARQLVNVVMR			Oxidation (M)[9]		Mascot
1259.7008	1259.6743	-0.0265	-21	282	292	AGRWTLPFVGR					Mascot
1290.6147	1290.6887	0.074	57	68	79	YGMGSSLYAVSR					Mascot
1316.682	1316.5952	-0.0868	-66	361	370	FCVAKIFEFR			Carbamidomethyl (C)[2]		Mascot
1323.7321	1323.6731	-0.059	-45	227	237	WHQLPPPPVPR					Mascot
1323.7321	1323.6731	-0.059	-45	227	237	WHQLPPPPVPR					Mascot

4 DNA repair protein Rad50 [Triticum turgidum] gi|157863720 152748.1 6.49 31 52 75.132 8.936

Peptide Information

Calc. Mass	Obsrv. Mass	± da	± ppm	Start	End	Sequence	Ion	C. I. %	Modification	Rank	Result Type

				Seq.	Seq.		Score		
806.4189	806.4059	-0.013	-16	1262	1267	IMENRK		Oxidation (M)[2]	Mascot
810.3661	810.4192	0.0531	66	1128	1134	TTEMANK		Oxidation (M)[4]	Mascot
820.4424	820.4149	-0.0275	-34	572	578	GRPPHEK			Mascot
831.4207	831.4301	0.0094	11	199	205	DQAQEIK			Mascot
836.3784	836.4198	0.0414	49	14	20	SFDPENK			Mascot
864.4131	864.4208	0.0077	9	240	246	TQMEDLK			Mascot
946.5203	946.478	-0.0423	-45	269	276	LQEQISTK			Mascot
999.4604	999.4961	0.0357	36	680	687	QMYEPFGK			Mascot
1015.4553	1015.516	0.0607	60	680	687	QMYEPFGK		Oxidation (M)[2]	Mascot
1016.537	1016.5214	-0.0156	-15	247	255	TNIQAVENK			Mascot
1023.5582	1023.628	0.0698	68	486	493	HLQIEVER			Mascot
1033.4796	1033.5253	0.0457	44	226	234	DSIAQDQEK			Mascot
1037.5626	1037.5352	-0.0274	-26	975	983	IKGYLDSNK			Mascot
1060.5708	1060.5697	-0.0011	-1	140	149	EIPALMGVSK		Oxidation (M)[6]	Mascot
1064.635	1064.5533	-0.0817	-77	186	194	YTKALEVIK			Mascot
1067.5038	1067.5596	0.0558	52	259	267	TETSMVDLR		Oxidation (M)[5]	Mascot
1068.5585	1068.5432	-0.0153	-14	881	888	WHALREEK			Mascot
1106.551	1106.5417	-0.0093	-8	657	665	ILKDAMDER		Oxidation (M)[6]	Mascot
1199.6056	1199.6605	0.0549	46	176	185	KFDDIFSATR			Mascot
1235.6379	1235.6146	-0.0233	-19	199	208	DQAQEIKTFR			Mascot
1302.6648	1302.6768	0.012	9	224	234	LRDSIAQDQEK			Mascot
1308.7019	1308.6779	-0.024	-18	484	493	ERHLQIEVER			Mascot
1320.741	1320.6177	-0.1233	-93	312	322	FEEKIALLETK			Mascot
1325.6671	1325.6694	0.0023	2	680	690	QMYEPFGKVAR			Mascot
1381.6958	1381.7578	0.062	45	437	448	YSEVDGQIQSKK			Mascot
1407.6759	1407.7302	0.0543	39	1150	1160	FHTMKMEEINK			Mascot
1463.7166	1463.802	0.0854	58	177	188	FDDIFSATRYTK			Mascot
1493.7384	1493.7603	0.0219	15	418	429	TNETQLEFLWGR			Mascot
1519.6982	1519.7815	0.0833	55	462	474	ENERDAAETELSR			Mascot
1584.7687	1584.7711	0.0024	2	1089	1102	CQGTISVYQSNISK		Carbamidomethyl (C)[1]	Mascot
1621.8334	1621.8745	0.0411	25	417	429	KTNETQLEFLWGR			Mascot
1812.924	1812.8986	-0.0254	-14	1268	1282	GQENFQLIIITHDER			Mascot

5	Signal recognition particle 54 kDa protein 3 [Triticum urartu]	gi|474414481	54042.9	9.38	17	52	73.354	6.912

Peptide Information

Calc. Mass	Obsrv. Mass	± da	± ppm	Start Seq.	End Seq.	Sequence	Ion Score	C. I. %	Modification	Rank	Result Type
816.4825	816.4243	-0.0582	-71	74	81	VIETAVGK					Mascot
835.4164	835.4055	-0.0109	-13	485	493	MLAGMKGGA					Mascot
849.4465	849.4384	-0.0081	-10	142	149	AGAFDQLK					Mascot
864.4825	864.4208	-0.0617	-71	468	475	SAFEALVK					Mascot
927.5146	927.4841	-0.0305	-33	298	306	GDLPGLLDK					Mascot
1013.439	1013.5228	0.0838	83	476	484	QMGGKDMSK			Oxidation (M)[2,7]		Mascot
1101.6263	1101.616	-0.0103	-9	2	12	VLAELGGSISR					Mascot
1127.5258	1127.5436	0.0178	16	481	490	DMSKMLAGMK			Oxidation (M)[2]		Mascot
1232.6667	1232.6223	-0.0444	-36	1	12	MVLAELGGSISR					Mascot
1308.665	1308.6779	0.0129	10	444	454	MSDQAIQKMLK			Oxidation (M)[1]		Mascot
1366.7035	1366.6907	-0.0128	-9	332	342	LMSEQFQNLLK			Oxidation (M)[2]		Mascot
1366.8053	1366.6907	-0.1146	-84	294	306	LLGRGDLPGLLDK					Mascot
1381.7145	1381.7578	0.0433	31	468	480	SAFEALVKQMGGK			Oxidation (M)[10]		Mascot
1443.7914	1443.7413	-0.0501	-35	59	72	SVNLEALAAGTNKR					Mascot
1507.6669	1507.7723	0.1054	70	195	206	HMQEAALFEEMR			Oxidation (M)[2]		Mascot
1507.6669	1507.7723	0.1054	70	195	206	HMQEAALFEEMR			Oxidation (M)[2]		Mascot
1657.8326	1657.8401	0.0075	5	250	267	MDGHAKGGGALSAVAAT K			Oxidation (M)[1]		Mascot
1812.9637	1812.8986	-0.0651	-36	238	255	QSASVGAVIITKMDGHAK					Mascot

6 DNA repair protein Rad50 [Triticum turgidum] gi|157863728 152759.1 6.45 30 51 68.694 8.592

Peptide Information

Calc. Mass	Obsrv. Mass	± da	± ppm	Start Seq.	End Seq.	Sequence	Ion Score	C. I. %	Modification	Rank	Result Type
806.4189	806.4059	-0.013	-16	1262	1267	IMENRK			Oxidation (M)[2]		Mascot
810.3661	810.4192	0.0531	66	1128	1134	TTEMANK			Oxidation (M)[4]		Mascot
831.4207	831.4301	0.0094	11	199	205	DQAQEIK					Mascot
836.3784	836.4198	0.0414	49	14	20	SFDPENK					Mascot
864.4131	864.4208	0.0077	9	240	246	TQMEDLK					Mascot
946.5203	946.478	-0.0423	-45	269	276	LQEQISTK					Mascot
999.4604	999.4961	0.0357	36	680	687	QMYEPFGK					Mascot
1015.4553	1015.516	0.0607	60	680	687	QMYEPFGK			Oxidation (M)[2]		Mascot
1016.537	1016.5214	-0.0156	-15	247	255	TNIQAVENK					Mascot
1023.5582	1023.628	0.0698	68	486	493	HLQIEVER					Mascot
1033.4796	1033.5253	0.0457	44	226	234	DSIAQDQEK					Mascot

Calc. Mass	Obsrv. Mass	± da	± ppm	Start Seq.	End Seq.	Sequence	Ion Score	C. I. %	Modification	Rank	Result Type
1037.5626	1037.5352	-0.0274	-26	975	983	IKGYLDSNK					Mascot
1060.5708	1060.5697	-0.0011	-1	140	149	EIPALMGVSK			Oxidation (M)[6]		Mascot
1064.635	1064.5533	-0.0817	-77	186	194	YTKALEVIK					Mascot
1067.5038	1067.5596	0.0558	52	259	267	TETSMVDLR			Oxidation (M)[5]		Mascot
1068.5585	1068.5432	-0.0153	-14	881	888	WHALREEK					Mascot
1106.551	1106.5417	-0.0093	-8	657	665	ILKDAMDER			Oxidation (M)[6]		Mascot
1199.6056	1199.6605	0.0549	46	176	185	KFDDIFSATR					Mascot
1235.6379	1235.6146	-0.0233	-19	199	208	DQAQEIKTFR					Mascot
1302.6648	1302.6768	0.012	9	224	234	LRDSIAQDQEK					Mascot
1308.7019	1308.6779	-0.024	-18	484	493	ERHLQIEVER					Mascot
1320.741	1320.6177	-0.1233	-93	312	322	FEEKIALLETK					Mascot
1325.6671	1325.6694	0.0023	2	680	690	QMYEPFGKVAR					Mascot
1381.6958	1381.7578	0.062	45	437	448	YSEVDGQIQSKK					Mascot
1407.6759	1407.7302	0.0543	39	1150	1160	FHTMKMEEINK					Mascot
1463.7166	1463.802	0.0854	58	177	188	FDDIFSATRYTK					Mascot
1493.7384	1493.7603	0.0219	15	418	429	TNETQLEFLWGR					Mascot
1519.6982	1519.7815	0.0833	55	462	474	ENERDAAETELSR					Mascot
1584.7687	1584.7711	0.0024	2	1089	1102	CQGTISVYQSNISK			Carbamidomethyl (C)[1]		Mascot
1621.8334	1621.8745	0.0411	25	417	429	KTNETQLEFLWGR					Mascot
1812.924	1812.8986	-0.0254	-14	1268	1282	GQENFQLIIITHDER					Mascot

7 Serine/threonine-protein kinase svkA [Triticum urartu] gi|473790097 74991.3 8.93 15 48 34.591 5.958

Peptide Information

Calc. Mass	Obsrv. Mass	± da	± ppm	Start Seq.	End Seq.	Sequence	Ion Score	C. I. %	Modification	Rank	Result Type
806.4003	806.4059	0.0056	7	365	372	SEGTGTVR					Mascot
856.525	856.5396	0.0146	17	297	303	TPKLLER					Mascot
865.4526	865.4575	0.0049	6	58	65	LGQPDHAK					Mascot
891.512	891.4423	-0.0697	-78	244	250	VLFMIPR			Oxidation (M)[4]		Mascot
913.4122	913.4289	0.0167	18	426	433	EAQTDHGR					Mascot
1023.5292	1023.628	0.0988	97	266	273	EFVSLCLR			Carbamidomethyl (C)[6]		Mascot
1037.564	1037.5352	-0.0288	-28	71	79	AGVQLAHWR					Mascot
1193.665	1193.6241	-0.0409	-34	71	80	AGVQLAHWRR					Mascot
1212.5902	1212.6089	0.0187	15	147	156	CPQANQNRPK			Carbamidomethyl (C)[1]		Mascot
1277.6195	1277.7323	0.1128	88	388	398	FDMPQNPSTLK					Mascot
1308.5955	1308.6779	0.0824	63	35	46	VTDSPFVDEGDK					Mascot

1325.692	1325.6694	-0.0226	-17	343	355	HAASPSSQGTVRK						Mascot
1605.769	1605.7699	0.0009	1	385	398	DGRFDMPQNPSTLK						Mascot
1621.7639	1621.8745	0.1106	68	385	398	DGRFDMPQNPSTLK			Oxidation (M)[6]			Mascot
1812.8585	1812.8986	0.0401	22	251	265	ENPPQLDEHFSKPMK			Oxidation (M)[14]			Mascot
3133.5161	3133.6973	0.1812	58	458	485	ASQVYPAGPNHSSKMHI YNALEQYTQAK						Mascot

8 MORC family CW-type zinc finger protein 4 [Triticum urartu] gi|474210267 159639.2 9.01 26 47 11.766 8.913

Peptide Information

Calc. Mass	Obsrv. Mass	± da	± ppm	Start Seq.	End Seq.	Sequence	Ion Score	C. I. %	Modification	Rank	Result Type
810.458	810.4192	-0.0388	-48	378	383	IHREQK					Mascot
816.4574	816.4243	-0.0331	-41	1443	1449	SVNDLLR					Mascot
823.4308	823.4351	0.0043	5	1151	1157	SYPKSNK					Mascot
833.4475	833.372	-0.0755	-91	401	408	SSAGVKER					Mascot
847.4706	847.4644	-0.0062	-7	175	181	KKPTDMK					Mascot
861.4677	861.4442	-0.0235	-27	1426	1434	GVDGLSSVK					Mascot
864.4309	864.4208	-0.0101	-12	667	674	SVDVSTEK					Mascot
865.4639	865.4575	-0.0064	-7	1388	1394	NQPHLTR					Mascot
877.4737	877.4401	-0.0336	-38	477	484	REISSSAK					Mascot
891.424	891.4423	0.0183	21	1158	1164	QDMEKPK			Oxidation (M)[3]		Mascot
909.4709	909.4614	-0.0095	-10	351	358	SMEKVTSK					Mascot
909.4709	909.4614	-0.0095	-10	351	358	SMEKVTSK					Mascot
927.424	927.4841	0.0601	65	187	194	NDVSAFMK			Oxidation (M)[7]		Mascot
999.5582	999.4961	-0.0621	-62	1120	1128	KTAAPIQDR					Mascot
1037.5812	1037.5352	-0.046	-44	1302	1311	KMAAAALAYK					Mascot
1067.4786	1067.5596	0.081	76	24	32	ERCESTASK			Carbamidomethyl (C)[3]		Mascot
1095.5099	1095.5701	0.0602	55	468	477	DSDMLGASKR			Oxidation (M)[4]		Mascot
1127.5229	1127.5436	0.0207	18	1081	1090	GYNHLHSGDK					Mascot
1139.5579	1139.5697	0.0118	10	359	368	EASLYDTNVK					Mascot
1171.6793	1171.5842	-0.0951	-81	57	67	IKVGSDNVLAR					Mascot
1259.5638	1259.6743	0.1105	88	859	869	SEELYSTTDSK					Mascot
1302.6649	1302.6768	0.0119	9	1204	1216	VAKQGTSNPTDGK					Mascot
1316.6693	1316.5952	-0.0741	-56	195	206	NEIDVETLAGQK					Mascot
1320.5776	1320.6177	0.0401	30	706	716	EMDYGEYKASK					Mascot
1487.7628	1487.7836	0.0208	14	533	545	LLPYESDPDTLPK					Mascot
1812.9226	1812.8986	-0.024	-13	854	869	SKPIKSEELYSTTDSK					Mascot

3133.4929	3133.6973	0.2044	65	982	1010	DGPMNANPGTMPSSVKY LNTEVDIVDNVR					Mascot

9	hypothetical protein TRIUR3_20788 [Triticum urartu]	gi|474346268	11622.5	4.19	3	47	5.456	2.248	32	94.688	

Peptide Information

Calc. Mass	Obsrv. Mass	± da	± ppm	Start Seq.	End Seq.	Sequence	Ion Score	C. I. %	Modification	Rank	Result Type
1101.6012	1101.616	0.0148	13	93	105	AGGVGAAVTTAAR					Mascot
1139.6281	1139.5697	-0.0584	-51	2	13	AAPSRAAAAPTR					Mascot
1366.6598	1366.6907	0.0309	23	78	90	VVQAETYSDAGAR					Mascot
1366.6598	1366.6907	0.0309	23	78	90	VVQAETYSDAGAR	32	94.688			Mascot

10	hypothetical protein TRIUR3_09582 [Triticum urartu]	gi|473822602	43982.1	8.75	14	47	5.456	5.652	

Peptide Information

Calc. Mass	Obsrv. Mass	± da	± ppm	Start Seq.	End Seq.	Sequence	Ion Score	C. I. %	Modification	Rank	Result Type
812.3528	812.4122	0.0594	73	121	126	EMMELK			Oxidation (M)[2,3]		Mascot
836.3857	836.4198	0.0341	41	341	349	GGSSASNTR					Mascot
877.4526	877.4401	-0.0125	-14	384	390	NLTTSWR					Mascot
1010.4901	1010.5079	0.0178	18	283	290	YENLSQTR					Mascot
1023.5555	1023.628	0.0725	71	103	112	GGRPPRGNGR					Mascot
1060.6361	1060.5697	-0.0664	-63	127	135	TLVTRTLEK					Mascot
1171.6543	1171.5842	-0.0701	-60	29	39	TSLTRGGPNLR					Mascot
1212.5618	1212.6089	0.0471	39	45	55	GGHSGYGREHR					Mascot
1232.6344	1232.6223	-0.0121	-10	236	245	VYLPECNLPK			Carbamidomethyl (C)[6]		Mascot
1302.6219	1302.6768	0.0549	42	330	340	QMPPSQTSDRR					Mascot
1360.7067	1360.7316	0.0249	18	369	380	TSTALENIQLDR					Mascot
1366.7069	1366.6907	-0.0162	-12	121	131	EMMELKTLVTR			Oxidation (M)[2]		Mascot
1366.7069	1366.6907	-0.0162	-12	121	131	EMMELKTLVTR			Oxidation (M)[2]		Mascot
1621.9021	1621.8745	-0.0276	-17	61	76	TGPLQPQGARVEGALK					Mascot
1837.9252	1837.955	0.0298	16	266	282	SAESGPMLLDVLEGYLK			Oxidation (M)[7]		Mascot

Gel Idx/Pos	296/L24	Instr./Gel Origin	BA2151/Sample Project 20140814	Process Status	Analysis Succeeded
Plate [#] Name	[1] Sample Project 20140814	Instrument Sample Name		Spectra	11

Rank	Protein Name	Accession No.	Protein MW	Protein PI	Pep. Count	Protein Score	Protein Score C. I. %	Intensity Matched	Total Ion Score	Total Ion C. I. %	Confirmed
1	5-methyltetrahydropteroyltriglutamate--homocysteine methyltransferase [Triticum urartu]	gi|473993302	84841.4	5.74	20	1,120	100	30.702	1030	100	

Peptide Information

Calc. Mass	Obsrv. Mass	± da	± ppm	Start Seq.	End Seq.	Sequence	Ion Score	C. I. %	Modification	Rank	Result Type
1096.5786	1096.6073	0.0287	26	287	296	YLFAGVVDGR					Mascot
1096.5786	1096.6073	0.0287	26	287	296	YLFAGVVDGR	86	100			Mascot
1199.5732	1199.6453	0.0721	60	19	28	FALESFWDGK					Mascot
1373.7019	1373.7046	0.0027	2	389	400	SSPRVTNEEVQK					Mascot
1456.7505	1456.765	0.0145	10	577	588	FETCYQIALAIK			Carbamidomethyl (C)[4]		Mascot
1540.739	1540.7914	0.0524	34	373	387	DEAYFAANAAALASR					Mascot
1540.739	1540.7914	0.0524	34	373	387	DEAYFAANAAALASR	58	99.992			Mascot
1790.9623	1790.9556	-0.0067	-4	556	571	GMLTGPVTILNWSFVR					Mascot
1806.9572	1807.0002	0.043	24	556	571	GMLTGPVTILNWSFVR			Oxidation (M)[2]		Mascot
1859.9386	1860.0032	0.0646	35	254	270	TLTSLSSVTAYGFDLER					Mascot
1859.9386	1860.0032	0.0646	35	254	270	TLTSLSSVTAYGFDLER	130	100			Mascot
1864.8766	1864.9762	0.0996	53	618	632	SEHAFYLDWAVHSFR					Mascot
1991.0081	1991.0825	0.0744	37	477	493	IQEELDIDVLVHGEPER					Mascot
1991.0081	1991.0825	0.0744	37	477	493	IQEELDIDVLVHGEPER	123	100			Mascot
2043.0295	2043.1112	0.0817	40	685	704	EGVVYGAGIGPGVYDIHS PR					Mascot
2043.0295	2043.1112	0.0817	40	685	704	EGVVYGAGIGPGVYDIHS PR	167	100			Mascot
2067.0327	2067.125	0.0923	45	572	588	NDQPRFETCYQIALAIK			Carbamidomethyl (C)[9]		Mascot
2098.9539	2099.0269	0.073	35	82	99	YSWTGGEINLSTYFSMA R			Oxidation (M)[16]		Mascot
2109.0723	2109.1504	0.0781	37	367	387	ALAGQKDEAYFAANAAA LASR					Mascot
2109.0723	2109.1504	0.0781	37	367	387	ALAGQKDEAYFAANAAA LASR	91	100			Mascot
2165.0703	2165.1357	0.0654	30	112	129	WFDTNYHFIVPELAPSTK					Mascot
2268.1719	2268.2661	0.0942	42	590	610	EVEDLEAGGIQVIQIDEAA LR					Mascot
2268.1719	2268.2661	0.0942	42	590	610	EVEDLEAGGIQVIQIDEAA LR	232	100			Mascot
2296.2913	2296.3796	0.0883	38	427	447	LNLPVLPTTTIGSFPQTVE LR					Mascot
2296.2913	2296.3796	0.0883	38	427	447	LNLPVLPTTTIGSFPQTVE	23	70.093			Mascot

						LR					
2396.2668	2396.3687	0.1019	43	589	610	KEVEDLEAGGIQVIQIDEA ALR					Mascot
2424.3862	2424.4856	0.0994	41	426	447	KLNLPVLPTTTIGSFPQTV ELR					Mascot
2424.3862	2424.4856	0.0994	41	426	447	KLNLPVLPTTTIGSFPQTV ELR	119	100			Mascot
2707.3735	2707.4761	0.1026	38	522	544	CVKPPIIYGDVSRPNPMT VFWSK			Carbamidomethyl (C)[1], Oxidation (M)[17]		Mascot
2937.3975	2937.5366	0.1391	47	56	81	YIPSNTFSYYDQVLDTTA MLGAVPDR					Mascot
2953.3926	2953.5283	0.1357	46	56	81	YIPSNTFSYYDQVLDTTA MLGAVPDR			Oxidation (M)[19]		Mascot

2	unnamed protein product [Triticum aestivum]	gi|257676183	84754.4	5.89	18	513	100	14.749	440	100	

Peptide Information

Calc. Mass	Obsrv. Mass	± da	± ppm	Start Seq.	End Seq.	Sequence	Ion Score	C. I. %	Modification	Rank	Result Type
857.4952	857.4821	-0.0131	-15	413	420	RATPVSAR					Mascot
1096.5786	1096.6073	0.0287	26	288	297	YLFAGVVDGR	86	100			Mascot
1096.5858	1096.6073	0.0215	20	402	412	AAAALKGSDHR					Mascot
1199.5732	1199.6453	0.0721	60	19	28	FALESFWDGK					Mascot
1456.7505	1456.765	0.0145	10	578	589	FETCYQIALAIK			Carbamidomethyl (C)[4]		Mascot
1503.7649	1503.8326	0.0677	45	29	42	SSAEDLEKVAADLR					Mascot
1516.743	1516.7673	0.0243	16	130	142	FSYASHKAVSEYK					Mascot
1790.9623	1790.9556	-0.0067	-4	557	572	GMLTGPVTILNWSFVR					Mascot
1806.9572	1807.0002	0.043	24	557	572	GMLTGPVTILNWSFVR			Oxidation (M)[2]		Mascot
1864.8766	1864.9762	0.0996	53	619	633	SEHAFYLDWAVHSFR					Mascot
1991.0081	1991.0825	0.0744	37	478	494	IQEELDIDVLVHGEPER					Mascot
1991.0081	1991.0825	0.0744	37	478	494	IQEELDIDVLVHGEPER	123	100			Mascot
2067.0327	2067.125	0.0923	45	573	589	NDQPRFETCYQIALAIK			Carbamidomethyl (C)[9]		Mascot
2109.0723	2109.1504	0.0781	37	368	388	ALAGLKDEAYFAANAAA QASR					Mascot
2109.0723	2109.1504	0.0781	37	368	388	ALAGLKDEAYFAANAAA QASR					Mascot
2165.0703	2165.1357	0.0654	30	112	129	WFDTNYHFIVPELSPATK					Mascot
2268.1719	2268.2661	0.0942	42	591	611	EVEDLEAGGIQVIQIDEAA LR					Mascot
2268.1719	2268.2661	0.0942	42	591	611	EVEDLEAGGIQVIQIDEAA LR	232	100			Mascot
2396.2668	2396.3687	0.1019	43	590	611	KEVEDLEAGGIQVIQIDEA ALR					Mascot
2707.3735	2707.4761	0.1026	38	523	545	CVKPPIIYGDVSRPNPMT VFWSK			Carbamidomethyl (C)[1], Oxidation (M)[17]		Mascot
2937.3975	2937.5366	0.1391	47	56	81	YIPSNTFSYYDQVLDTTA MLGAVPDR					Mascot
2953.3926	2953.5283	0.1357	46	56	81	YIPSNTFSYYDQVLDTTA MLGAVPDR			Oxidation (M)[19]		Mascot

Calc. Mass	Obsrv. Mass	± da	± ppm	Start Seq.	End Seq.	Sequence	Ion Score	C. I. %	Modification	Rank	Result Type
3312.4758	3312.4648	-0.011	-3	82	111	YSWTGGEIGHSTYFSMA RGNATVPAMEMTK			Oxidation (M)[16,26]		Mascot

3	5-methyltetrahydropteroyltriglutamate--homocysteine methyltransferase [Triticum urartu]	gi|473954466	82012.1	6.17	17	510	100	14.749	441	100

Peptide Information

Calc. Mass	Obsrv. Mass	± da	± ppm	Start Seq.	End Seq.	Sequence	Ion Score	C. I. %	Modification	Rank	Result Type
857.4952	857.4821	-0.0131	-15	387	394	RATPVSAR					Mascot
1096.5786	1096.6073	0.0287	26	256	265	YLFAGVVDGR					Mascot
1096.5786	1096.6073	0.0287	26	256	265	YLFAGVVDGR	86	100			Mascot
1199.5732	1199.6453	0.0721	60	19	28	FALESFWDGK					Mascot
1456.7505	1456.765	0.0145	10	552	563	FETCYQIALAIK			Carbamidomethyl (C)[4]		Mascot
1503.7649	1503.8326	0.0677	45	29	42	SSAEDLEKVAADLR					Mascot
1516.743	1516.7673	0.0243	16	130	142	FSYASHKAVSEYK					Mascot
1790.9623	1790.9556	-0.0067	-4	531	546	GMLTGPVTILNWSFVR					Mascot
1806.9572	1807.0002	0.043	24	531	546	GMLTGPVTILNWSFVR			Oxidation (M)[2]		Mascot
1864.8766	1864.9762	0.0996	53	593	607	SEHAFYLDWAVHSFR					Mascot
1991.0081	1991.0825	0.0744	37	452	468	IQEELDIDVLVHGEPER					Mascot
1991.0081	1991.0825	0.0744	37	452	468	IQEELDIDVLVHGEPER	123	100			Mascot
2067.0327	2067.125	0.0923	45	547	563	NDQPRFETCYQIALAIK			Carbamidomethyl (C)[9]		Mascot
2109.0723	2109.1504	0.0781	37	336	356	ALAGLKDEAYFAANAAA QASR					Mascot
2109.0723	2109.1504	0.0781	37	336	356	ALAGLKDEAYFAANAAA QASR					Mascot
2165.0703	2165.1357	0.0654	30	112	129	WFDTNYHFIVPELSPATK					Mascot
2268.1719	2268.2661	0.0942	42	565	585	EVEDLEAGGIQVIQIDEAA LR					Mascot
2268.1719	2268.2661	0.0942	42	565	585	EVEDLEAGGIQVIQIDEAA LR	232	100			Mascot
2396.2668	2396.3687	0.1019	43	564	585	KEVEDLEAGGIQVIQIDEA ALR					Mascot
2707.3735	2707.4761	0.1026	38	497	519	CVKPPIIYGDVSRPNPMT VFWSK			Carbamidomethyl (C)[1], Oxidation (M)[17]		Mascot
2937.3975	2937.5366	0.1391	47	56	81	YIPSNTFSYYDQVLDTTA MLGAVPDR					Mascot
2953.3926	2953.5283	0.1357	46	56	81	YIPSNTFSYYDQVLDTTA MLGAVPDR			Oxidation (M)[19]		Mascot
3312.4758	3312.4648	-0.011	-3	82	111	YSWTGGEIGHSTYFSMA RGNATVPAMEMTK			Oxidation (M)[16,26]		Mascot

4	cobalamin-independent methionine synthase [Triticum monococcum]	gi|115589740	26374.4	6.1	8	444	100	13.172	399	100

Peptide Information

Calc. Mass	Obsrv. Mass	± da	± ppm	Start Seq.	End Seq.	Sequence	Ion Score	C. I. %	Modification	Rank	Result Type

Calc. Mass	Obsrv. Mass	± da	± ppm	Start Seq.	End Seq.	Sequence	Ion Score	C. I. %	Modification	Rank	Result Type
1132.5786	1132.5739	-0.0047	-4	3	11	HEVTVFWSK					Mascot
1456.7505	1456.765	0.0145	10	44	55	FETCYQIALAIK			Carbamidomethyl (C)[4]		Mascot
1790.9623	1790.9556	-0.0067	-4	23	38	GMLTGPVTILNWSFVR					Mascot
1806.9572	1807.0002	0.043	24	23	38	GMLTGPVTILNWSFVR			Oxidation (M)[2]		Mascot
1864.8766	1864.9762	0.0996	53	85	99	SEHAFYLDWAVHSFR					Mascot
2043.0295	2043.1112	0.0817	40	152	171	EGVVYGAGIGPGVYDIHSPR					Mascot
2043.0295	2043.1112	0.0817	40	152	171	EGVVYGAGIGPGVYDIHSPR	167	100			Mascot
2067.0327	2067.125	0.0923	45	39	55	NDQPRFETCYQIALAIK			Carbamidomethyl (C)[9]		Mascot
2268.1719	2268.2661	0.0942	42	57	77	EVEDLEAGGIQVIQIDEAALR					Mascot
2268.1719	2268.2661	0.0942	42	57	77	EVEDLEAGGIQVIQIDEAALR	232	100			Mascot
2396.2668	2396.3687	0.1019	43	56	77	KEVEDLEAGGIQVIQIDEAALR					Mascot

5 hypothetical protein TRIUR3_18498 [Triticum urartu] gi|474394976 47927.6 9.9 14 62 97.21 2.518

Peptide Information

Calc. Mass	Obsrv. Mass	± da	± ppm	Start Seq.	End Seq.	Sequence	Ion Score	C. I. %	Modification	Rank	Result Type
944.5121	944.5458	0.0337	36	423	430	LVEPLMDK					Mascot
1068.4667	1068.543	0.0763	71	408	415	EFDEELMR					Mascot
1092.5428	1092.5673	0.0245	22	91	100	TPSPTALMMK			Oxidation (M)[8]		Mascot
1163.5692	1163.641	0.0718	62	297	306	ISEPSPYQSR					Mascot
1197.5647	1197.6593	0.0946	79	371	381	AGFPGQQYSSR					Mascot
1308.5864	1308.7032	0.1168	89	130	138	VYWKSYMCR			Carbamidomethyl (C)[8], Oxidation (M)[7]		Mascot
1399.7441	1399.7325	-0.0116	-8	307	317	VNEPQIYQPRR					Mascot
1487.8832	1487.7833	-0.0999	-67	101	114	FPLKSTLPSVASLK					Mascot
1582.8475	1582.8448	-0.0027	-2	139	152	VIYKFQSDAEAALK					Mascot
1786.8678	1786.9355	0.0677	38	234	251	SNDEGGLAAASALRDSPR					Mascot
1822.9519	1823.0042	0.0523	29	2	18	RPSDPTSAGVKRPPSDR					Mascot
1953.9923	1954.0707	0.0784	40	1	18	MRPSDPTSAGVKRPPSDR					Mascot
2031.9342	2032.1113	0.1771	87	334	350	QSDAPQPFNMQPQYQPR					Mascot
2066.9348	2067.125	0.1902	92	371	389	AGFPGQQYSSRSDDMPPAR					Mascot

6 Disease resistance protein RPM1 [Triticum urartu] gi|473968240 69201.6 9.02 19 62 97.078 3.641

Peptide Information

Calc. Mass	Obsrv. Mass	± da	± ppm	Start Seq.	End Seq.	Sequence	Ion Score	C. I. %	Modification	Rank	Result Type

Calc. Mass	Obsrv. Mass	± da	± ppm	Start Seq.	End Seq.	Sequence	Ion Score	C. I. %	Modification	Rank	Result Type
807.4723	807.4216	-0.0507	-63	581	587	QISGFKK					Mascot
993.4822	993.515	0.0328	33	35	41	EWMDLRK			Oxidation (M)[3]		Mascot
1107.6521	1107.568	-0.0841	-76	591	600	FITGKSIVSR					Mascot
1111.6119	1111.5896	-0.0223	-20	235	243	HIGELHHLR					Mascot
1118.5986	1118.5522	-0.0464	-41	188	197	MASLNLSQVR					Mascot
1132.5415	1132.5739	0.0324	29	122	131	SMIQPSENAR					Mascot
1263.7532	1263.7069	-0.0463	-37	408	417	LVHLERLEVR					Mascot
1308.6682	1308.7032	0.035	27	264	274	FLETLDVQDTK					Mascot
1379.677	1379.7571	0.0801	58	1	11	MMEQASRILER			Oxidation (M)[1]		Mascot
1399.7515	1399.7325	-0.019	-14	447	458	LGNLPGWMDRLK					Mascot
1425.8676	1425.7517	-0.1159	-81	275	287	VTQLPVGITKLEK					Mascot
1487.77	1487.7833	0.0133	9	386	399	RLGVDVDATEEVGK					Mascot
1764.9677	1764.9193	-0.0484	-27	198	213	SLTIFGRCPVSLISSK			Carbamidomethyl (C)[8]		Mascot
1775.0902	1775.0265	-0.0637	-36	14	31	GLPLAIATIGGLLANRPK					Mascot
1873.9277	1874.02	0.0923	49	219	234	VLDLEDTLELENDDLK					Mascot
1908.8903	1908.8901	-0.0002	0	122	138	SMIQPSENARASMAVER			Oxidation (M)[2,13]		Mascot
2187.0498	2187.1741	0.1243	57	114	131	YYNELISRSMIQPSENAR			Oxidation (M)[10]		Mascot
2250.0918	2250.259	0.1672	74	387	407	LGVDVDATEEVGKDLCS SISR			Carbamidomethyl (C)[16]		Mascot
2350.186	2350.1877	0.0017	1	93	112	WIAEGYITKPRDMSVEEL GR					Mascot

7 DNA-directed RNA polymerase 3, chloroplastic [Triticum urartu] gi|474213641 106067.8 8.65 20 56 90.325 6.126

Peptide Information

Calc. Mass	Obsrv. Mass	± da	± ppm	Start Seq.	End Seq.	Sequence	Ion Score	C. I. %	Modification	Rank	Result Type
944.5047	944.5458	0.0411	44	672	679	VLVDEVDR					Mascot
1122.6782	1122.6044	-0.0738	-66	525	533	WLKIHLANK					Mascot
1201.6974	1201.6487	-0.0487	-41	39	49	QQTLVMALAVK					Mascot
1262.6991	1262.6431	-0.056	-44	384	395	VFEALDILGGTK					Mascot
1340.6958	1340.7307	0.0349	26	402	412	VHDVVETIWSR					Mascot
1390.7941	1390.7449	-0.0492	-35	383	395	KVFEALDILGGTK					Mascot
1399.7693	1399.7325	-0.0368	-26	528	540	IHLANKYGGGIEK					Mascot
1404.7118	1404.7596	0.0478	34	534	546	YGGGIEKLSHESK					Mascot
1425.7996	1425.7517	-0.0479	-34	728	740	RQGHAVTVVSLMK					Mascot
1485.7375	1485.7977	0.0602	41	120	131	TCCVILHNMIPK			Carbamidomethyl (C)[2,3]		Mascot
1516.7577	1516.7673	0.0096	6	717	728	LLYDVSCYATRR			Carbamidomethyl (C)[7]		Mascot

1657.9675	1657.8494	-0.1181	-71	164	177	HVYAKYLLLLPAEK					Mascot
1754.9041	1754.9259	0.0218	12	793	806	NYMIRTSLQCLALR			Carbamidomethyl (C)[10], Oxidation (M)[3]		Mascot
1790.8477	1790.9556	0.1079	60	655	671	EDSMKDPATDPSVSLAK					Mascot
1806.8427	1807.0002	0.1575	87	655	671	EDSMKDPATDPSVSLAK			Oxidation (M)[4]		Mascot
1891.0037	1891.0414	0.0377	20	178	194	VAVIVMHKMMGLLMSSK			Oxidation (M)[6]		Mascot
1990.9375	1991.0825	0.145	73	491	507	AYPMHPHLSHLGSDLCR			Carbamidomethyl (C)[16]		Mascot
1990.9375	1991.0825	0.145	73	491	507	AYPMHPHLSHLGSDLCR			Carbamidomethyl (C)[16]		Mascot
2031.923	2032.1113	0.1883	93	132	147	DERGMNLEFFYDNPLR			Oxidation (M)[5]		Mascot
2064.135	2064.2173	0.0823	40	330	346	HVEIPYLPMLVPPTKWK			Oxidation (M)[9]		Mascot
2114.9878	2115.0454	0.0576	27	903	921	LWDIKLSIAEESSHADDA D					Mascot
2165.1865	2165.1357	-0.0508	-23	771	789	NQPVRWTSPVGLPVVQP YK					Mascot

8 Tetratricopeptide repeat protein 37 [Triticum urartu] gi|473913094 113426.3 8.76 19 50 59.669 6.607

Peptide Information

Calc. Mass	Obsrv. Mass	± da	± ppm	Start Seq.	End Seq.	Sequence	Ion Score	C. I. %	Modification	Rank	Result Type
807.3553	807.4216	0.0663	82	472	477	ECEELK			Carbamidomethyl (C)[2]		Mascot
857.4008	857.4821	0.0813	95	433	439	FALGMMR			Oxidation (M)[5,6]		Mascot
1027.5167	1027.5306	0.0139	14	500	508	HDEALSISR					Mascot
1053.4564	1053.5461	0.0897	85	1	9	MTEGSGRCR			Carbamidomethyl (C)[8]		Mascot
1308.6543	1308.7032	0.0489	37	823	834	AYVVQGDLSNSR					Mascot
1379.7318	1379.7571	0.0253	18	447	458	YPFVDVSVNLAR					Mascot
1404.7225	1404.7596	0.0371	26	256	268	MSLGGLMLEPVNK			Oxidation (M)[1]		Mascot
1492.7292	1492.7697	0.0405	27	717	728	LVHQEPWNQDAR					Mascot
1499.8251	1499.7708	-0.0543	-36	382	395	SGELLSPQVLMAVR					Mascot
1540.7867	1540.7914	0.0047	3	23	35	WSDAIQSLQHSIR					Mascot
1540.7867	1540.7914	0.0047	3	23	35	WSDAIQSLQHSIR					Mascot
1582.7496	1582.8448	0.0952	60	419	432	SDFQSAITSYQHAK					Mascot
1742.9377	1742.9983	0.0606	35	988	1001	NLRLEWFSWPPGLK					Mascot
1822.8905	1823.0042	0.1137	62	184	198	GMDEQNFKASILQWR					Mascot
1838.8854	1839.0028	0.1174	64	184	198	GMDEQNFKASILQWR			Oxidation (M)[2]		Mascot
1953.8859	1954.0707	0.1848	95	903	920	DEDFASAEKALAQACAE K			Carbamidomethyl (C)[15]		Mascot
1991.0127	1991.0825	0.0698	35	192	209	ASILQWRNTCLSAANSAK			Carbamidomethyl (C)[10]		Mascot
1991.0127	1991.0825	0.0698	35	192	209	ASILQWRNTCLSAANSAK			Carbamidomethyl (C)[10]		Mascot
2019.0043	2019.1102	0.1059	52	401	418	APHYPESHNINGLVSEVR					Mascot
2082.1489	2082.0764	-0.0725	-35	382	400	SGELLSPQVLMAVRQAV QR					Mascot

Calc. Mass	Obsrv. Mass	± da	± ppm	Start Seq.	End Seq.	Sequence	Ion Score	C. I. %	Modification	Rank	Result Type
2102.1646	2102.0679	-0.0967	-46	478	496	TQGLLNVDGLQVYALALWK					Mascot
2888.2947	2888.5527	0.258	89	670	694	GLRSPHQIQACATMSCYATCTSYPK			Carbamidomethyl (C)[11,16,20]		Mascot

9 Xyloglucan galactosyltransferase KATAMARI1-like protein [Triticum urartu] gi|474371812 65845.6 6.9 14 50 52.616 2.492

Peptide Information

Calc. Mass	Obsrv. Mass	± da	± ppm	Start Seq.	End Seq.	Sequence	Ion Score	C. I. %	Modification	Rank	Result Type
807.4148	807.4216	0.0068	8	112	117	IAWDFR					Mascot
857.5567	857.4821	-0.0746	-87	444	450	LLLNTRK					Mascot
963.5159	963.5042	-0.0117	-12	112	118	IAWDFRR					Mascot
989.4833	989.5598	0.0765	77	202	209	GQLIDQCR			Carbamidomethyl (C)[7]		Mascot
1163.5613	1163.641	0.0797	68	216	225	LLECDLGESK			Carbamidomethyl (C)[4]		Mascot
1201.5895	1201.6487	0.0592	49	94	103	RPEWNVMGGR					Mascot
1325.6995	1325.6797	-0.0198	-15	310	320	SIHPDVVKQMR			Oxidation (M)[10]		Mascot
1485.8094	1485.7977	-0.0117	-8	318	329	QMREEVINLIPK			Oxidation (M)[2]		Mascot
1699.7891	1699.8865	0.0974	57	202	215	GQLIDQCRTSSFCK			Carbamidomethyl (C)[7,13]		Mascot
1873.8671	1874.02	0.1529	82	210	225	TSSFCKLLECDLGESK			Carbamidomethyl (C)[5,10]		Mascot
1941.9158	1942.0283	0.1125	58	420	436	QQDEHTCGGLMILQSPK			Carbamidomethyl (C)[7]		Mascot
2099.0352	2099.0269	-0.0083	-4	94	111	RPEWNVMGGRDHFLVGGR			Oxidation (M)[7]		Mascot
2165.0583	2165.1357	0.0774	36	119	137	LTDEESDWGNKLLFMPAAK					Mascot
3312.5493	3312.4648	-0.0845	-26	138	166	NMSMLVVESSPWNANDFAVPYPTYFHPAK					Mascot

10 Replication factor C subunit 2 [Triticum urartu] gi|474262868 39962.7 9.3 11 44 0 5.235

Peptide Information

Calc. Mass	Obsrv. Mass	± da	± ppm	Start Seq.	End Seq.	Sequence	Ion Score	C. I. %	Modification	Rank	Result Type
989.5699	989.5598	-0.0101	-10	165	173	LMIVVAAEK			Oxidation (M)[2]		Mascot
1106.5874	1106.559	-0.0284	-26	267	276	SMVKNVLDGK			Oxidation (M)[2]		Mascot
1231.5624	1231.5936	0.0312	25	120	129	TMEIYSNTTR			Oxidation (M)[2]		Mascot
1486.761	1486.8109	0.0499	34	304	315	VIKNYDMAEFLK			Oxidation (M)[7]		Mascot
1742.9033	1742.9983	0.095	55	307	320	NYDMAEFLKLELLK			Oxidation (M)[4]		Mascot
1774.9092	1775.0265	0.1173	66	256	270	VCDQPHPLHVKSMVK			Carbamidomethyl (C)[2]		Mascot
1790.9041	1790.9556	0.0515	29	256	270	VCDQPHPLHVKSMVK			Carbamidomethyl (C)[2], Oxidation (M)[13]		Mascot
1859.9684	1860.0032	0.0348	19	46	62	TTSILALAHEMLGPSYR					Mascot
1859.9684	1860.0032	0.0348	19	46	62	TTSILALAHEMLGPSYR					Mascot

2032.038	2032.1113	0.0733	36	100	118	IVILDEADSMTTGAQQAL R		Mascot
2035.0641	2035.0667	0.0026	1	130	147	FALACNTSSKIIEPIQSR	Carbamidomethyl (C)[5]	Mascot
2707.3696	2707.4761	0.1065	39	321	345	ETGFAHMRICDGVGSFL QLSGLLAK	Carbamidomethyl (C)[10]	Mascot
2937.4551	2937.5366	0.0815	28	206	232	LTGLSQEVPYVPEGLEAII FTADGDMR	Oxidation (M)[26]	Mascot

Gel Idx/Pos	299/M1	Instr./Gel Origin	BA2151/Sample Project 20140814	Process Status	Analysis Succeeded
Plate [#] Name	[1] Sample Project 20140814	Instrument Sample Name		Spectra	11

Rank	Protein Name	Accession No.	Protein MW	Protein PI	Pep. Count	Protein Score	Protein Score C. I. %	Intensity Matched	Total Ion Score	Total Ion C. I. %	Confirmed
1	Elongation factor 2 [Triticum urartu]	gi|474416088	97317.3	5.96	23	611	100	24.977	528	100	

Peptide Information

Calc. Mass	Obsrv. Mass	± da	± ppm	Start Seq.	End Seq.	Sequence	Ion Score	C. I. %	Modification	Rank	Result Type
865.4414	865.4394	-0.002	-2	4	10	FTAEELR					Mascot
890.5095	890.5068	-0.0027	-3	504	511	FSVSPVVR					Mascot
955.532	955.5226	-0.0094	-10	723	732	GGGQVIPTAR					Mascot
959.4615	959.4772	0.0157	16	493	500	EVDACPIR			Carbamidomethyl (C)[5]		Mascot
1039.6622	1039.6492	-0.013	-13	172	180	IRPVLTVNK					Mascot
1135.5049	1135.5085	0.0036	3	695	704	EGALAEENMR			Oxidation (M)[9]		Mascot
1188.6194	1188.5979	-0.0215	-18	342	351	VMQTWLPASR					Mascot
1204.6144	1204.6097	-0.0047	-4	342	351	VMQTWLPASR			Oxidation (M)[2]		Mascot
1220.682	1220.6019	-0.0801	-66	501	511	AMKFSVSPVVR					Mascot
1236.677	1236.5984	-0.0786	-64	501	511	AMKFSVSPVVR			Oxidation (M)[2]		Mascot
1315.7191	1315.6771	-0.042	-32	432	443	IMGPNFVPGQKK					Mascot
1346.7791	1346.7725	-0.0066	-5	734	745	VIYASQLTAKPR					Mascot
1543.7727	1543.7751	0.0024	2	290	301	RGFVQFCYEPIK			Carbamidomethyl (C)[7]		Mascot
1745.8965	1745.9255	0.029	17	352	366	ALLEMMIFHLPSPSK			Oxidation (M)[5,6]		Mascot
1745.8965	1745.9255	0.029	17	352	366	ALLEMMIFHLPSPSK			Oxidation (M)[5,6]		Mascot
1786.9375	1786.9601	0.0226	13	792	807	AYLPVIESFGFSSTLR					Mascot
1786.9375	1786.9601	0.0226	13	792	807	AYLPVIESFGFSSTLR	107	100			Mascot
1830.9015	1830.9303	0.0288	16	485	500	NATLTNEKEVDACPIR			Carbamidomethyl (C)[13]		Mascot
2065.0237	2065.0598	0.0361	17	372	389	VENLYEGPLDDIYANAIR					Mascot
2065.0237	2065.0598	0.0361	17	372	389	VENLYEGPLDDIYANAIR	112	100			Mascot
2132.0593	2132.0872	0.0279	13	774	791	GHVFEEMQRPGTPLYNI K			Oxidation (M)[7]		Mascot
2206.0122	2206.0483	0.0361	16	184	201	CFLELQVEGEEAYQTFS R			Carbamidomethyl (C)[1]		Mascot
2206.0122	2206.0483	0.0361	16	184	201	CFLELQVEGEEAYQTFS R	92	100	Carbamidomethyl (C)[1]		Mascot
2257.2034	2257.2478	0.0444	20	54	76	STLTDSLVAAAGIIAQEVA GDVR					Mascot
2257.2034	2257.2478	0.0444	20	54	76	STLTDSLVAAAGIIAQEVA GDVR	136	100			Mascot
2264.0864	2264.1226	0.0362	16	608	627	LYMEARPLEEGLAEAIDD			Oxidation (M)[3]		Mascot

Calc. Mass	Obsrv. Mass	± da	± ppm	Start Seq.	End Seq.	Sequence	Ion Score	C. I. %	Modification	Rank	Result Type
						GR					
2384.188	2384.2336	0.0456	19	370	389	YRVENLYEGPLDDIYANA IR					Mascot
2384.188	2384.2336	0.0456	19	370	389	YRVENLYEGPLDDIYANA IR	82	100			Mascot
2406.2009	2406.2437	0.0428	18	565	586	DLQEDFMGGAEIIVSPPV VSFR					Mascot
2422.196	2422.2366	0.0406	17	565	586	DLQEDFMGGAEIIVSPPV VSFR			Oxidation (M)[7]		Mascot
2646.3479	2646.4104	0.0625	24	142	165	ITDGALVVVDCIEGVCVQ TETVLR			Carbamidomethyl (C)[11,16]		Mascot
2933.4163	2933.4939	0.0776	26	115	141	DGDEYLINLIDSPGHVDF SSEVTAALR					Mascot

2	Elongation factor 2 [Triticum urartu]	gi|473786548	94753.9	5.85	23	607	100	24.537	528	100

Peptide Information

Calc. Mass	Obsrv. Mass	± da	± ppm	Start Seq.	End Seq.	Sequence	Ion Score	C. I. %	Modification	Rank	Result Type
865.4414	865.4394	-0.002	-2	4	10	FTAEELR					Mascot
890.5095	890.5068	-0.0027	-3	483	490	FSVSPVVR					Mascot
955.532	955.5226	-0.0094	-10	702	711	GGGQVIPTAR					Mascot
959.4615	959.4772	0.0157	16	472	479	EVDACPIR			Carbamidomethyl (C)[5]		Mascot
1039.6622	1039.6492	-0.013	-13	151	159	IRPVLTVNK					Mascot
1135.5049	1135.5085	0.0036	3	674	683	EGALAEENMR			Oxidation (M)[9]		Mascot
1148.5154	1148.5072	-0.0082	-7	753	761	GHVFEEMQR			Oxidation (M)[7]		Mascot
1188.6194	1188.5979	-0.0215	-18	321	330	VMQTWLPASR					Mascot
1204.6144	1204.6097	-0.0047	-4	321	330	VMQTWLPASR			Oxidation (M)[2]		Mascot
1220.682	1220.6019	-0.0801	-66	480	490	AMKFSVSPVVR					Mascot
1236.677	1236.5984	-0.0786	-64	480	490	AMKFSVSPVVR			Oxidation (M)[2]		Mascot
1315.7191	1315.6771	-0.042	-32	411	422	IMGPNFVPGQKK					Mascot
1346.7791	1346.7725	-0.0066	-5	713	724	VIYASQLTAKPR					Mascot
1543.7727	1543.7751	0.0024	2	269	280	RGFVQFCYEPIK			Carbamidomethyl (C)[7]		Mascot
1745.8965	1745.9255	0.029	17	331	345	ALLEMMIFHLPSPSK			Oxidation (M)[5,6]		Mascot
1745.8965	1745.9255	0.029	17	331	345	ALLEMMIFHLPSPSK			Oxidation (M)[5,6]		Mascot
1786.9375	1786.9601	0.0226	13	771	786	AYLPVIESFGFSSTLR					Mascot
1786.9375	1786.9601	0.0226	13	771	786	AYLPVIESFGFSSTLR	107	100			Mascot
1830.9015	1830.9303	0.0288	16	464	479	NATLTNEKEVDACPIR			Carbamidomethyl (C)[13]		Mascot
2065.0237	2065.0598	0.0361	17	351	368	VENLYEGPLDDIYANAIR					Mascot
2065.0237	2065.0598	0.0361	17	351	368	VENLYEGPLDDIYANAIR	112	100			Mascot
2206.0122	2206.0483	0.0361	16	163	180	CFLELQVEGEEAYQTFS R			Carbamidomethyl (C)[1]		Mascot
2206.0122	2206.0483	0.0361	16	163	180	CFLELQVEGEEAYQTFS R	92	100	Carbamidomethyl (C)[1]		Mascot
2257.2034	2257.2478	0.0444	20	33	55	STLTDSLVAAAGIIAQEVA					Mascot

						GDVR					
2257.2034	2257.2478	0.0444	20	33	55	STLTDSLVAAAGIIAQEVAGDVR	136	100			Mascot
2264.0864	2264.1226	0.0362	16	587	606	LYMEARPLEEGLAEAIDDGR			Oxidation (M)[3]		Mascot
2384.188	2384.2336	0.0456	19	349	368	YRVENLYEGPLDDIYANAIR					Mascot
2384.188	2384.2336	0.0456	19	349	368	YRVENLYEGPLDDIYANAIR	82	100			Mascot
2408.1804	2408.2285	0.0481	20	544	565	DLQDDFMGGAEIIVSPPVVSFR			Oxidation (M)[7]		Mascot
2646.3479	2646.4104	0.0625	24	121	144	ITDGALVVVDCIEGVCVQTETVLR			Carbamidomethyl (C)[11,16]		Mascot
2933.4163	2933.4939	0.0776	26	94	120	DGDEYLINLIDSPGHVDFSSEVTAALR					Mascot

3 Elongation factor 2 [Triticum urartu] gi|474224670 94757.9 5.85 22 601 100 24.364 528 100

Peptide Information

Calc. Mass	Obsrv. Mass	± da	± ppm	Start Seq.	End Seq.	Sequence	Ion Score	C. I. %	Modification	Rank	Result Type
865.4414	865.4394	-0.002	-2	4	10	FTAEELR					Mascot
890.5095	890.5068	-0.0027	-3	483	490	FSVSPVVR					Mascot
955.532	955.5226	-0.0094	-10	702	711	GGGQVIPTAR					Mascot
959.4615	959.4772	0.0157	16	472	479	EVDACPIR			Carbamidomethyl (C)[5]		Mascot
1039.6622	1039.6492	-0.013	-13	151	159	IRPVLTVNK					Mascot
1135.5049	1135.5085	0.0036	3	674	683	EGALAEENMR			Oxidation (M)[9]		Mascot
1148.5154	1148.5072	-0.0082	-7	753	761	GHVFEEMQR			Oxidation (M)[7]		Mascot
1188.6194	1188.5979	-0.0215	-18	321	330	VMQTWLPASR					Mascot
1204.6144	1204.6097	-0.0047	-4	321	330	VMQTWLPASR			Oxidation (M)[2]		Mascot
1220.682	1220.6019	-0.0801	-66	480	490	AMKFSVSPVVR					Mascot
1236.677	1236.5984	-0.0786	-64	480	490	AMKFSVSPVVR			Oxidation (M)[2]		Mascot
1315.7191	1315.6771	-0.042	-32	411	422	IMGPNFVPGQKK					Mascot
1346.7791	1346.7725	-0.0066	-5	713	724	VIYASQLTAKPR					Mascot
1543.7727	1543.7751	0.0024	2	269	280	RGFVQFCYEPIK			Carbamidomethyl (C)[7]		Mascot
1745.8965	1745.9255	0.029	17	331	345	ALLEMMIFHLPSPSK			Oxidation (M)[5,6]		Mascot
1745.8965	1745.9255	0.029	17	331	345	ALLEMMIFHLPSPSK			Oxidation (M)[5,6]		Mascot
1786.9375	1786.9601	0.0226	13	771	786	AYLPVIESFGFSSTLR					Mascot
1786.9375	1786.9601	0.0226	13	771	786	AYLPVIESFGFSSTLR	107	100			Mascot
2065.0237	2065.0598	0.0361	17	351	368	VENLYEGPLDDIYANAIR					Mascot
2065.0237	2065.0598	0.0361	17	351	368	VENLYEGPLDDIYANAIR	112	100			Mascot
2206.0122	2206.0483	0.0361	16	163	180	CFLELQVEGEEAYQTFSR			Carbamidomethyl (C)[1]		Mascot
2206.0122	2206.0483	0.0361	16	163	180	CFLELQVEGEEAYQTFSR	92	100	Carbamidomethyl (C)[1]		Mascot

2257.2034	2257.2478	0.0444	20	33	55	STLTDSLVAAAGIIAQEVA GDVR				Mascot
2257.2034	2257.2478	0.0444	20	33	55	STLTDSLVAAAGIIAQEVA GDVR	136	100		Mascot
2264.0864	2264.1226	0.0362	16	587	606	LYMEARPLEEGLAEAIDD GR			Oxidation (M)[3]	Mascot
2384.188	2384.2336	0.0456	19	349	368	YRVENLYEGPLDDIYANA IR				Mascot
2384.188	2384.2336	0.0456	19	349	368	YRVENLYEGPLDDIYANA IR	82	100		Mascot
2408.1804	2408.2285	0.0481	20	544	565	DLQDDFMGGAEIIVSPPV VSFR			Oxidation (M)[7]	Mascot
2646.3479	2646.4104	0.0625	24	121	144	ITDGALVVVDCIEGVCVQ TETVLR			Carbamidomethyl (C)[11,16]	Mascot
2933.4163	2933.4939	0.0776	26	94	120	DGDEYLINLIDSPGHVDF SSEVTAALR				Mascot

4	Elongation factor 2 [Triticum urartu]	gi|474416089	94980	5.85	21	499	100	22.937	436	100	

Peptide Information

Calc. Mass	Obsrv. Mass	± da	± ppm	Start Seq.	End Seq.	Sequence	Ion Score	C. I. %	Modification	Rank	Result Type
865.4414	865.4394	-0.002	-2	4	10	FTAEELR					Mascot
890.5095	890.5068	-0.0027	-3	483	490	FSVSPVVR					Mascot
955.532	955.5226	-0.0094	-10	702	711	GGGQVIPTAR					Mascot
959.4615	959.4772	0.0157	16	472	479	EVDACPIR			Carbamidomethyl (C)[5]		Mascot
1039.6622	1039.6492	-0.013	-13	151	159	IRPVLTVNK					Mascot
1148.5154	1148.5072	-0.0082	-7	753	761	GHVFEEMQR			Oxidation (M)[7]		Mascot
1188.6194	1188.5979	-0.0215	-18	321	330	VMQTWLPASR					Mascot
1204.6144	1204.6097	-0.0047	-4	321	330	VMQTWLPASR			Oxidation (M)[2]		Mascot
1220.682	1220.6019	-0.0801	-66	480	490	AMKFSVSPVVR					Mascot
1236.677	1236.5984	-0.0786	-64	480	490	AMKFSVSPVVR			Oxidation (M)[2]		Mascot
1315.7191	1315.6771	-0.042	-32	411	422	IMGPNFVPGQKK					Mascot
1346.7791	1346.7725	-0.0066	-5	713	724	VIYASQLTAKPR					Mascot
1543.7727	1543.7751	0.0024	2	269	280	RGFVQFCYEPIK			Carbamidomethyl (C)[7]		Mascot
1745.8965	1745.9255	0.029	17	331	345	ALLEMMIFHLPSPSK			Oxidation (M)[5,6]		Mascot
1745.8965	1745.9255	0.029	17	331	345	ALLEMMIFHLPSPSK			Oxidation (M)[5,6]		Mascot
1786.9375	1786.9601	0.0226	13	771	786	AYLPVIESFGFSSTLR					Mascot
1786.9375	1786.9601	0.0226	13	771	786	AYLPVIESFGFSSTLR	107	100			Mascot
1830.9015	1830.9303	0.0288	16	464	479	NATLTNEKEVDACPIR			Carbamidomethyl (C)[13]		Mascot
2065.0237	2065.0598	0.0361	17	351	368	VENLYEGPLDDIYANAIR					Mascot
2065.0237	2065.0598	0.0361	17	351	368	VENLYEGPLDDIYANAIR	112	100			Mascot
2257.2034	2257.2478	0.0444	20	33	55	STLTDSLVAAAGIIAQEVA GDVR					Mascot
2257.2034	2257.2478	0.0444	20	33	55	STLTDSLVAAAGIIAQEVA	136	100			Mascot

						GDVR					
2264.0864	2264.1226	0.0362	16	587	606	LYMEARPLEEGLAEAIDDGR			Oxidation (M)[3]		Mascot
2384.188	2384.2336	0.0456	19	349	368	YRVENLYEGPLDDIYANAIR					Mascot
2384.188	2384.2336	0.0456	19	349	368	YRVENLYEGPLDDIYANAIR	82	100			Mascot
2408.1804	2408.2285	0.0481	20	544	565	DLQDDFMGGAEIIVSPPVVSFR			Oxidation (M)[7]		Mascot
2646.3479	2646.4104	0.0625	24	121	144	ITDGALVVVDCIEGVCVQTETVLR			Carbamidomethyl (C)[11,16]		Mascot
2933.4163	2933.4939	0.0776	26	94	120	DGDEYLINLIDSPGHVDFSSEVTAALR					Mascot

5	unnamed protein product [Triticum turgidum]	gi|257623547	101230.2	5.89	31	333	100	17.938	199	100	

Protein Group

heat shock protein 101 [Triticum durum]	gi|110623253	101230.2	5.8899998664856
unnamed protein product [Triticum turgidum]	gi|296529764	101230.2	5.8899998664856

Peptide Information

Calc. Mass	Obsrv. Mass	± da	± ppm	Start Seq.	End Seq.	Sequence	Ion Score	C. I. %	Modification	Rank	Result Type
842.5206	842.4762	-0.0444	-53	443	449	ARLVEVR					Mascot
929.5203	929.4902	-0.0301	-32	546	553	WTGIPVTR					Mascot
954.454	954.4502	-0.0038	-4	361	368	YEGHHGVR					Mascot
959.4905	959.4772	-0.0133	-14	554	561	LGQNDKER					Mascot
1005.5034	1005.4828	-0.0206	-20	743	750	DLVMQEVR			Oxidation (M)[4]		Mascot
1043.5845	1043.5747	-0.0098	-9	217	226	TAVVEGLAQR					Mascot
1085.5011	1085.5	-0.0011	-1	255	262	YRGEFEER					Mascot
1098.663	1098.6564	-0.0066	-6	373	383	AIVVAAQLSAR					Mascot
1181.6538	1181.6431	-0.0107	-9	752	760	HFRPELLNR					Mascot
1184.6271	1184.6323	0.0052	4	230	240	GDVPSNLLDVR					Mascot
1188.5968	1188.5979	0.0011	1	865	876	SGGLVNAETGQR					Mascot
1315.7151	1315.6771	-0.038	-29	740	750	VARDLVMQEVR					Mascot
1440.7152	1440.7169	0.0017	1	314	325	CIGATTLEEYRK			Carbamidomethyl (C)[1]		Mascot
1445.7053	1445.7189	0.0136	9	394	406	AIDLVDEACANVR			Carbamidomethyl (C)[9]		Mascot
1492.7278	1492.74	0.0122	8	484	495	EELQFTLQEAER					Mascot

1594.8475	1594.8578	0.0103	6	672	684	RPYSVILFDEVEK					Mascot
1648.829	1648.8375	0.0085	5	484	496	EELQFTLQEAERR					Mascot
1725.9495	1725.9689	0.0194	11	573	589	VVGQTEAVNAVAEAVLR					Mascot
1734.9684	1734.8088	-0.1596	-92	373	388	AIVVAAQLSARYIMGR			Oxidation (M)[14]		Mascot
1745.9069	1745.9255	0.0186	11	620	634	ALAEQLFDDENLLVR					Mascot
1745.9069	1745.9255	0.0186	11	620	634	ALAEQLFDDENLLVR	75	100			Mascot
1750.9486	1750.9218	-0.0268	-15	671	684	RRPYSVILFDEVEK					Mascot
1765.0371	1765.0576	0.0205	12	277	292	VILFIDEIHLVLGAGR					Mascot
1784.8661	1784.8901	0.024	13	407	421	VQLDSQPEEIDNLER					Mascot
1868.0026	1868.0242	0.0216	12	685	701	AHVAVFNTLLQVLDDGR					Mascot
1910.0018	1910.0229	0.0211	11	761	776	LDEIVVFDPLSHEQLR					Mascot
1910.0018	1910.0229	0.0211	11	761	776	LDEIVVFDPLSHEQLR	86	100			Mascot
2136.1335	2136.1714	0.0379	18	337	355	FQQVYVAEPSVADTISILR					Mascot
2136.1335	2136.1714	0.0379	18	337	355	FQQVYVAEPSVADTISILR	8	0			Mascot
2285.2402	2285.2891	0.0489	21	592	614	AGLGRPQQPTGSFLFLG PTGVGK					Mascot
2292.2346	2292.2676	0.033	14	336	355	RFQQVYVAEPSVADTISIL R					Mascot
2306.1743	2306.1892	0.0149	6	293	313	TEGSMDAANLFKPMLAR GQLR					Mascot
2307.1729	2307.2207	0.0478	21	649	670	LIGAPPGYVGHEEGGQL TEQVR					Mascot
2307.1729	2307.2207	0.0478	21	649	670	LIGAPPGYVGHEEGGQL TEQVR	30	94.374			Mascot
2817.3823	2817.4485	0.0662	23	520	545	LEGETGENLMLTETVGP EQIAEVVSR			Oxidation (M)[10]		Mascot

6	heat shock protein 101 [Triticum aestivum]	gi|4558484	101288.3	5.95	31	332	100	17.938	199	100	

Protein Group

unnamed protein product [Triticum aestivum]	gi|257623561	101288.3	5.9499 998092 6514
unnamed protein product [Triticum aestivum]	gi|296529810	101288.3	5.9499 998092 6514

Peptide Information

Calc. Mass	Obsrv. Mass	± da	± ppm	Start Seq.	End Seq.	Sequence	Ion Score	C. I. %	Modification	Rank	Result Type
842.5206	842.4762	-0.0444	-53	443	449	ARLVEVR					Mascot
929.5203	929.4902	-0.0301	-32	546	553	WTGIPVTR					Mascot

954.454	954.4502	-0.0038	-4	361	368	YEGHHGVR				Mascot
959.4905	959.4772	-0.0133	-14	554	561	LGQNDKER				Mascot
1005.5034	1005.4828	-0.0206	-20	743	750	DLVMQEVR			Oxidation (M)[4]	Mascot
1043.5845	1043.5747	-0.0098	-9	217	226	TAVVEGLAQR				Mascot
1085.5011	1085.5	-0.0011	-1	255	262	YRGEFEER				Mascot
1098.663	1098.6564	-0.0066	-6	373	383	AIVVAAQLSAR				Mascot
1181.6538	1181.6431	-0.0107	-9	752	760	HFRPELLNR				Mascot
1184.6271	1184.6323	0.0052	4	230	240	GDVPSNLLDVR				Mascot
1188.5968	1188.5979	0.0011	1	865	876	SGGLVNAETGQR				Mascot
1315.7151	1315.6771	-0.038	-29	740	750	VARDLVMQEVR				Mascot
1440.7152	1440.7169	0.0017	1	314	325	CIGATTLEEYRK			Carbamidomethyl (C)[1]	Mascot
1445.7053	1445.7189	0.0136	9	394	406	AIDLVDEACANVR			Carbamidomethyl (C)[9]	Mascot
1492.7278	1492.74	0.0122	8	484	495	EELQFTLQEAER				Mascot
1594.8475	1594.8578	0.0103	6	672	684	RPYSVILFDEVEK				Mascot
1648.829	1648.8375	0.0085	5	484	496	EELQFTLQEAERR				Mascot
1725.9495	1725.9689	0.0194	11	573	589	VVGQTEAVNAVAEAVLR				Mascot
1734.9684	1734.8088	-0.1596	-92	373	388	AIVVAAQLSARYIMGR			Oxidation (M)[14]	Mascot
1745.9069	1745.9255	0.0186	11	620	634	ALAEQLFDDENLLVR				Mascot
1745.9069	1745.9255	0.0186	11	620	634	ALAEQLFDDENLLVR	75	100		Mascot
1750.9486	1750.9218	-0.0268	-15	671	684	RRPYSVILFDEVEK				Mascot
1765.0371	1765.0576	0.0205	12	277	292	VILFIDEIHLVLGAGR				Mascot
1784.8661	1784.8901	0.024	13	407	421	VQLDSQPEEIDNLER				Mascot
1868.0026	1868.0242	0.0216	12	685	701	AHVAVFNTLLQVLDDGR				Mascot
1910.0018	1910.0229	0.0211	11	761	776	LDEIVVFDPLSHEQLR				Mascot
1910.0018	1910.0229	0.0211	11	761	776	LDEIVVFDPLSHEQLR	86	100		Mascot
2136.1335	2136.1714	0.0379	18	337	355	FQQVYVAEPSVADTISILR				Mascot
2136.1335	2136.1714	0.0379	18	337	355	FQQVYVAEPSVADTISILR	8	0		Mascot
2285.2402	2285.2891	0.0489	21	592	614	AGLGRPQQPTGSFLFLG PTGVGK				Mascot
2292.2346	2292.2676	0.033	14	336	355	RFQQVYVAEPSVADTISIL R				Mascot
2306.1743	2306.1892	0.0149	6	293	313	TEGSMDAANLFKPMLAR GQLR				Mascot
2307.1729	2307.2207	0.0478	21	649	670	LIGAPPGYVGHEEGGQL TEQVR				Mascot
2307.1729	2307.2207	0.0478	21	649	670	LIGAPPGYVGHEEGGQL TEQVR	30	94.374		Mascot
2817.3823	2817.4485	0.0662	23	520	545	LEGETGENLMLTETVGP EQIAEVVSR			Oxidation (M)[10]	Mascot

7	unnamed protein product [Triticum aestivum]	gi|257623571	101163.2	5.84	29	307	100	16.222	191	100	

Protein Group

unnamed protein product [Triticum aestivum] gi|296530054 101163.2 5.8400 001525 8789

Peptide Information

Calc. Mass	Obsrv. Mass	± da	± ppm	Start Seq.	End Seq.	Sequence	Ion Score	C. I. %	Modification	Rank	Result Type
842.5206	842.4762	-0.0444	-53	443	449	ARLVEVR					Mascot
929.5203	929.4902	-0.0301	-32	546	553	WTGIPVTR					Mascot
954.454	954.4502	-0.0038	-4	361	368	YEGHHGVR					Mascot
959.4905	959.4772	-0.0133	-14	554	561	LGQNDKER					Mascot
1005.5034	1005.4828	-0.0206	-20	743	750	DLVMQEVR			Oxidation (M)[4]		Mascot
1043.5845	1043.5747	-0.0098	-9	217	226	TAVVEGLAQR					Mascot
1085.5011	1085.5	-0.0011	-1	255	262	YRGEFEER					Mascot
1098.663	1098.6564	-0.0066	-6	373	383	AIVVAAQLSAR					Mascot
1181.6538	1181.6431	-0.0107	-9	752	760	HFRPELLNR					Mascot
1184.6271	1184.6323	0.0052	4	230	240	GDVPSNLLDVR					Mascot
1188.5968	1188.5979	0.0011	1	865	876	SGGLVNAETGQR					Mascot
1315.7151	1315.6771	-0.038	-29	740	750	VARDLVMQEVR					Mascot
1445.7053	1445.7189	0.0136	9	394	406	AIDLVDEACANVR			Carbamidomethyl (C)[9]		Mascot
1492.7278	1492.74	0.0122	8	484	495	EELQFTLQEAER					Mascot
1594.8475	1594.8578	0.0103	6	672	684	RPYSVILFDEVEK					Mascot
1648.829	1648.8375	0.0085	5	484	496	EELQFTLQEAERR					Mascot
1725.9495	1725.9689	0.0194	11	573	589	VVGQTEAVNAVAEAVLR					Mascot
1734.9684	1734.8088	-0.1596	-92	373	388	AIVVAAQLSARYIMGR			Oxidation (M)[14]		Mascot
1745.9069	1745.9255	0.0186	11	620	634	ALAEQLFDDENLLVR					Mascot
1745.9069	1745.9255	0.0186	11	620	634	ALAEQLFDDENLLVR	75	100			Mascot
1750.9486	1750.9218	-0.0268	-15	671	684	RRPYSVILFDEVEK					Mascot
1765.0371	1765.0576	0.0205	12	277	292	VILFIDEIHLVLGAGR					Mascot
1784.8661	1784.8901	0.024	13	407	421	VQLDSQPEEIDNLER					Mascot
1868.0026	1868.0242	0.0216	12	685	701	AHVAVFNTLLQVLDDGR					Mascot
1910.0018	1910.0229	0.0211	11	761	776	LDEIVVFDPLSHEQLR					Mascot
1910.0018	1910.0229	0.0211	11	761	776	LDEIVVFDPLSHEQLR	86	100			Mascot
2276.2397	2276.1965	-0.0432	-19	336	355	RFQQVFVAEPSVADTISIL R					Mascot
2285.2402	2285.2891	0.0489	21	592	614	AGLGRPQQPTGSFLFLG PTGVGK					Mascot

2306.1743	2306.1892	0.0149	6	293	313	TEGSMDAANLFKPMLAR GQLR					Mascot
2307.1729	2307.2207	0.0478	21	649	670	LIGAPPGYVGHEEGGQL TEQVR					Mascot
2307.1729	2307.2207	0.0478	21	649	670	LIGAPPGYVGHEEGGQL TEQVR	30	94.374			Mascot
2817.3823	2817.4485	0.0662	23	520	545	LEGETGENLMLTETVGP EQIAEVVSR			Oxidation (M)[10]		Mascot

8	unnamed protein product [Triticum turgidum]	gi|257623549	100617	6.07	30	237	100	16.53	113	100	

Protein Group

heat shock protein 101 [Triticum durum]	gi|110623251	100617	6.0700 001716 6138
unnamed protein product [Triticum turgidum]	gi|296529766	100617	6.0700 001716 6138

Peptide Information

Calc. Mass	Obsrv. Mass	± da	± ppm	Start Seq.	End Seq.	Sequence	Ion Score	C. I. %	Modification	Rank	Result Type
842.5206	842.4762	-0.0444	-53	443	449	ARLVEVR					Mascot
929.5203	929.4902	-0.0301	-32	546	553	WTGIPVTR					Mascot
954.454	954.4502	-0.0038	-4	361	368	YEGHHGVR					Mascot
959.4905	959.4772	-0.0133	-14	554	561	LGQNDKER					Mascot
1005.5034	1005.4828	-0.0206	-20	743	750	DLVMQEVR			Oxidation (M)[4]		Mascot
1043.5845	1043.5747	-0.0098	-9	217	226	TAVVEGLAQR					Mascot
1085.5011	1085.5	-0.0011	-1	255	262	YRGEFEER					Mascot
1098.663	1098.6564	-0.0066	-6	373	383	AIVVAAQLSAR					Mascot
1181.6538	1181.6431	-0.0107	-9	752	760	HFRPELLNR					Mascot
1184.6271	1184.6323	0.0052	4	230	240	GDVPSNLLDVR					Mascot
1188.5968	1188.5979	0.0011	1	865	876	SGGLVNAETGQR					Mascot
1315.7151	1315.6771	-0.038	-29	740	750	VARDLVMQEVR					Mascot
1440.7152	1440.7169	0.0017	1	314	325	CIGATTLEEYRK			Carbamidomethyl (C)[1]		Mascot
1445.7053	1445.7189	0.0136	9	394	406	AIDLVDEACANVR			Carbamidomethyl (C)[9]		Mascot
1492.7278	1492.74	0.0122	8	484	495	EELQFTLQEAER					Mascot
1594.8475	1594.8578	0.0103	6	672	684	RPYSVILFDEVEK					Mascot
1648.829	1648.8375	0.0085	5	484	496	EELQFTLQEAERR					Mascot
1725.9495	1725.9689	0.0194	11	573	589	VVGQTEAVNAVAEAVLR					Mascot

1734.9684	1734.8088	-0.1596	-92	373	388	AIVVAAQLSARYIMGR			Oxidation (M)[14]		Mascot
1745.9069	1745.9255	0.0186	11	620	634	ALAEQLFDDENLLVR					Mascot
1745.9069	1745.9255	0.0186	11	620	634	ALAEQLFDDENLLVR	75	100			Mascot
1750.9486	1750.9218	-0.0268	-15	671	684	RRPYSVILFDEVEK					Mascot
1765.0371	1765.0576	0.0205	12	277	292	VILFIDEIHLVLGAGR					Mascot
1784.8661	1784.8901	0.024	13	407	421	VQLDSQPEEIDNLER					Mascot
1868.0026	1868.0242	0.0216	12	685	701	AHVAVFNTLLQVLDDGR					Mascot
2136.1335	2136.1714	0.0379	18	337	355	FQQVYVAEPSVADTISILR					Mascot
2136.1335	2136.1714	0.0379	18	337	355	FQQVYVAEPSVADTISILR	8	0			Mascot
2285.2402	2285.2891	0.0489	21	592	614	AGLGRPQQPTGSFLFLG PTGVGK					Mascot
2292.2346	2292.2676	0.033	14	336	355	RFQQVYVAEPSVADTISIL R					Mascot
2306.1743	2306.1892	0.0149	6	293	313	TEGSMDAANLFKPMLAR GQLR					Mascot
2307.1729	2307.2207	0.0478	21	649	670	LIGAPPGYVGHEEGGQL TEQVR					Mascot
2307.1729	2307.2207	0.0478	21	649	670	LIGAPPGYVGHEEGGQL TEQVR	30	94.374			Mascot
2817.3823	2817.4485	0.0662	23	520	545	LEGETGENLMLTETVGP EQIAEVVSR			Oxidation (M)[10]		Mascot

9	101 kDa heat shock protein [Triticum aestivum]	gi|11561808	101281.2	5.84	30	235	100	16.53	113	100

Protein Group

unnamed protein product [Triticum aestivum]	gi|257623563	101281.2	5.8400 001525 8789
unnamed protein product [Triticum aestivum]	gi|296529812	101281.2	5.8400 001525 8789

Peptide Information

Calc. Mass	Obsrv. Mass	± da	± ppm	Start Seq.	End Seq.	Sequence	Ion Score	C. I. %	Modification	Rank	Result Type
842.5206	842.4762	-0.0444	-53	443	449	ARLVEVR					Mascot
929.5203	929.4902	-0.0301	-32	546	553	WTGIPVTR					Mascot
954.454	954.4502	-0.0038	-4	361	368	YEGHHGVR					Mascot
959.4905	959.4772	-0.0133	-14	554	561	LGQNDKER					Mascot
1005.5034	1005.4828	-0.0206	-20	743	750	DLVMQEVR			Oxidation (M)[4]		Mascot
1043.5845	1043.5747	-0.0098	-9	217	226	TAVVEGLAQR					Mascot
1085.5011	1085.5	-0.0011	-1	255	262	YRGEFEER					Mascot

1098.663	1098.6564	-0.0066	-6	373	383	AIVVAAQLSAR				Mascot
1181.6538	1181.6431	-0.0107	-9	752	760	HFRPELLNR				Mascot
1184.6271	1184.6323	0.0052	4	230	240	GDVPSNLLDVR				Mascot
1188.5968	1188.5979	0.0011	1	865	876	SGGLVNAETGQR				Mascot
1315.7151	1315.6771	-0.038	-29	740	750	VARDLVMQEVR				Mascot
1440.7152	1440.7169	0.0017	1	314	325	CIGATTLEEYRK			Carbamidomethyl (C)[1]	Mascot
1445.7053	1445.7189	0.0136	9	394	406	AIDLVDEACANVR			Carbamidomethyl (C)[9]	Mascot
1492.7278	1492.74	0.0122	8	484	495	EELQFTLQEAER				Mascot
1594.8475	1594.8578	0.0103	6	672	684	RPYSVILFDEVEK				Mascot
1648.829	1648.8375	0.0085	5	484	496	EELQFTLQEAERR				Mascot
1725.9495	1725.9689	0.0194	11	573	589	VVGQTEAVNAVAEAVLR				Mascot
1734.9684	1734.8088	-0.1596	-92	373	388	AIVVAAQLSARYIMGR			Oxidation (M)[14]	Mascot
1745.9069	1745.9255	0.0186	11	620	634	ALAEQLFDDENLLVR				Mascot
1745.9069	1745.9255	0.0186	11	620	634	ALAEQLFDDENLLVR	75	100		Mascot
1750.9486	1750.9218	-0.0268	-15	671	684	RRPYSVILFDEVEK				Mascot
1765.0371	1765.0576	0.0205	12	277	292	VILFIDEIHLVLGAGR				Mascot
1784.8661	1784.8901	0.024	13	407	421	VQLDSQPEEIDNLER				Mascot
1868.0026	1868.0242	0.0216	12	685	701	AHVAVFNTLLQVLDDGR				Mascot
2136.1335	2136.1714	0.0379	18	337	355	FQQVYVAEPSVADTISILR				Mascot
2136.1335	2136.1714	0.0379	18	337	355	FQQVYVAEPSVADTISILR	8	0		Mascot
2285.2402	2285.2891	0.0489	21	592	614	AGLGRPQQPTGSFLFLGPTGVGK				Mascot
2292.2346	2292.2676	0.033	14	336	355	RFQQVYVAEPSVADTISILR				Mascot
2306.1743	2306.1892	0.0149	6	293	313	TEGSMDAANLFKPMLARGQLR				Mascot
2307.1729	2307.2207	0.0478	21	649	670	LIGAPPGYVGHEEGGQLTEQVR				Mascot
2307.1729	2307.2207	0.0478	21	649	670	LIGAPPGYVGHEEGGQLTEQVR	30	94.374		Mascot
2817.3823	2817.4485	0.0662	23	520	545	LEGETGENLMLTETVGPEQIAEVVSR			Oxidation (M)[10]	Mascot

10	heat shock protein 101 [Triticum aestivum]	gi|6013196	100949.1	5.95	24	183	100	13.301	105	100

Protein Group

unnamed protein product [Triticum aestivum]	gi|257623559	100949.1	5.94999980926514
unnamed protein product [Triticum aestivum]	gi|296529806	100949.1	5.94999980926514

Peptide Information

Calc. Mass	Obsrv. Mass	± da	± ppm	Start Seq.	End Seq.	Sequence	Ion Score	C. I. %	Modification	Rank	Result Type
842.557	842.4762	-0.0808	-96	193	199	VVRILSR					Mascot
929.5203	929.4902	-0.0301	-32	545	552	WTGIPVTR					Mascot
954.454	954.4502	-0.0038	-4	360	367	YEGHHGVR					Mascot
1001.5738	1001.5527	-0.0211	-21	131	140	EAGISAARVK					Mascot
1005.5034	1005.4828	-0.0206	-20	741	748	DLVMQEVR			Oxidation (M)[4]		Mascot
1043.5845	1043.5747	-0.0098	-9	216	225	TAVVEGLAQR					Mascot
1085.5011	1085.5	-0.0011	-1	254	261	YRGEFEER					Mascot
1181.6538	1181.6431	-0.0107	-9	750	758	HFRPELLNR					Mascot
1184.6271	1184.6323	0.0052	4	229	239	GDVPSNLLDVR					Mascot
1315.7151	1315.6771	-0.038	-29	738	748	VARDLVMQEVR					Mascot
1440.7152	1440.7169	0.0017	1	313	324	CIGATTLEEYRK			Carbamidomethyl (C)[1]		Mascot
1445.7053	1445.7189	0.0136	9	393	405	AIDLVDEACANVR			Carbamidomethyl (C)[9]		Mascot
1492.7278	1492.74	0.0122	8	483	494	EELQFTLQEAER					Mascot
1594.8475	1594.8578	0.0103	6	671	683	RPYSVILFDEVEK					Mascot
1648.829	1648.8375	0.0085	5	483	495	EELQFTLQEAERR					Mascot
1745.9069	1745.9255	0.0186	11	619	633	ALAEQLFDDENLLVR					Mascot
1745.9069	1745.9255	0.0186	11	619	633	ALAEQLFDDENLLVR	75	100			Mascot
1750.9486	1750.9218	-0.0268	-15	670	683	RRPYSVILFDEVEK					Mascot
1765.0371	1765.0576	0.0205	12	276	291	VILFIDEIHLVLGAGR					Mascot
1784.8661	1784.8901	0.024	13	406	420	VQLDSQPEEIDNLER					Mascot
1868.0026	1868.0242	0.0216	12	684	700	AHVAVFNTLLQVLDDGR					Mascot
2132.1387	2132.0872	-0.0515	-24	336	354	FQQVFVAEPSVPDTVSIL R					Mascot
2285.2402	2285.2891	0.0489	21	591	613	AGLGRPQQPTGSFLFLG PTGVGK					Mascot
2306.1743	2306.1892	0.0149	6	292	312	TEGSMDAANLFKPMLAR GQLR					Mascot
2307.1729	2307.2207	0.0478	21	648	669	LIGAPPGYVGHEEGGQL TEQVR					Mascot
2307.1729	2307.2207	0.0478	21	648	669	LIGAPPGYVGHEEGGQL TEQVR	30	93.485			Mascot

Gel Idx/Pos	300/M2	Instr./Gel Origin	BA2151/Sample Project 20140814	Process Status	Analysis Succeeded
Plate [#] Name	[1] Sample Project 20140814	Instrument Sample Name		Spectra	11

Rank	Protein Name	Accession No.	Protein MW	Protein PI	Pep. Count	Protein Score	Protein Score C. I. %	Intensity Matched	Total Ion Score	Total Ion C. I. %	Confirmed
1	Chlorophyll a-b binding protein 1, chloroplastic [Triticum urartu]	gi|473965828	30439.1	5.25	6	149	100	8.616	129	100	

Peptide Information

Calc. Mass	Obsrv. Mass	± da	± ppm	Start Seq.	End Seq.	Sequence	Ion Score	C. I. %	Modification	Rank	Result Type
804.3998	804.4075	0.0077	10	23	30	AAPFGEGR					Mascot
983.4985	983.468	-0.0305	-31	125	132	FGEAVWFK					Mascot
1055.5303	1055.5291	-0.0012	-1	96	103	ELEVIHCR			Carbamidomethyl (C)[7]		Mascot
1055.5303	1055.5291	-0.0012	-1	96	103	ELEVIHCR	42	99.56	Carbamidomethyl (C)[7]		Mascot
1325.6743	1325.6754	0.0011	1	94	103	NRELEVIHCR			Carbamidomethyl (C)[9]		Mascot
1382.6335	1382.6498	0.0163	12	42	54	VAASSSPWYGSDR					Mascot
1382.6335	1382.6498	0.0163	12	42	54	VAASSSPWYGSDR	87	100			Mascot
1919.9871	1920.0138	0.0267	14	104	120	WAMLGALGCVFPELLAR			Carbamidomethyl (C)[9], Oxidation (M)[3]		Mascot

Rank	Protein Name	Accession No.	Protein MW	Protein PI	Pep. Count	Protein Score	Protein Score C. I. %	Intensity Matched	Total Ion Score	Total Ion C. I. %	Confirmed
2	unnamed protein product [Triticum aestivum]	gi|219746360	26622.7	5.01	6	118	100	3.327	95	100	

Protein Group

glutathione transferase [Triticum aestivum]	gi|20067423	26622.7	5.0100 002288 8184
unnamed protein product [Triticum aestivum]	gi|257333174	26622.7	5.0100 002288 8184

Peptide Information

Calc. Mass	Obsrv. Mass	± da	± ppm	Start Seq.	End Seq.	Sequence	Ion Score	C. I. %	Modification	Rank	Result Type
816.4362	816.4263	-0.0099	-12	201	207	LAAWAER					Mascot
1051.5089	1051.4957	-0.0132	-13	134	142	MEETLSGLR			Oxidation (M)[1]		Mascot
1135.618	1135.5906	-0.0274	-24	114	123	LFPSCTGILK			Carbamidomethyl (C)[5]		Mascot
1192.7048	1192.7067	0.0019	2	186	197	LAGLGPIIDPAR					Mascot
1192.7048	1192.7067	0.0019	2	186	197	LAGLGPIIDPAR	68	99.999			Mascot

1327.6165	1327.635	0.0185	14	32	42	GLSYDYLPEDR					Mascot
1327.6165	1327.635	0.0185	14	32	42	GLSYDYLPEDR	27	84.336			Mascot
1465.8083	1465.6833	-0.125	-85	114	126	LFPSCTGILKTTK			Carbamidomethyl (C)[5]		Mascot

3	unnamed protein product [Triticum aestivum]	gi|219746300	26555.9	5.26	3	76	99.901	1.788	68	99.999	

Protein Group

unnamed protein product [Triticum aestivum]	gi|257333114	26555.9	5.2600 002288 8184

Peptide Information

Calc. Mass	Obsrv. Mass	± da	± ppm	Start Seq.	End Seq.	Sequence	Ion Score	C. I. %	Modification	Rank	Result Type
816.4362	816.4263	-0.0099	-12	202	208	LAAWAER					Mascot
1192.7048	1192.7067	0.0019	2	187	198	LAGLGPLIDPAR					Mascot
1192.7048	1192.7067	0.0019	2	187	198	LAGLGPLIDPAR	68	99.999			Mascot
1259.6954	1259.6858	-0.0096	-8	132	143	AGKVEETLSGLR					Mascot

4	unnamed protein product [Triticum aestivum]	gi|332617595	28328.2	5.17	7	72	99.74	6.232	42	99.56	

Protein Group

unnamed protein product [Triticum aestivum]	gi|330730540	28328.2	5.1700 000762 9395
unnamed protein product [Triticum aestivum]	gi|330728755	28328.2	5.1700 000762 9395
unnamed protein product [Triticum aestivum]	gi|332628256	28328.2	5.1700 000762 9395

Peptide Information

Calc. Mass	Obsrv. Mass	± da	± ppm	Start Seq.	End Seq.	Sequence	Ion Score	C. I. %	Modification	Rank	Result Type
983.4985	983.468	-0.0305	-31	125	132	FGEAVWFK					Mascot
1055.5303	1055.5291	-0.0012	-1	96	103	ELEVIHCR			Carbamidomethyl (C)[7]		Mascot

Calc. Mass	Obsrv. Mass	± da	± ppm	Start Seq.	End Seq.	Sequence	Ion Score	C. I. %	Modification	Rank	Result Type
1055.5303	1055.5291	-0.0012	-1	96	103	ELEVIHCR	42	99.56	Carbamidomethyl (C)[7]		Mascot
1325.6743	1325.6754	0.0011	1	94	103	NRELEVIHCR			Carbamidomethyl (C)[9]		Mascot
1409.7094	1409.6514	-0.058	-41	2	16	AASTMALSSPAFAGK					Mascot
1575.849	1575.8531	0.0041	3	17	32	AVKLSPGASEVFGTGR					Mascot
1870.9402	1871.043	0.1028	55	1	19	MAASTMALSSPAFAGKA VK			Oxidation (M)[1,6]		Mascot
1919.9871	1920.0138	0.0267	14	104	120	WAMLGALGCVFPELLAR			Carbamidomethyl (C)[9], Oxidation (M)[3]		Mascot

5	Chlorophyll a-b binding protein, chloroplastic [Triticum urartu]	gi|474413347	28531.3	5.29	7	70	99.597	6.502	42	99.56

Peptide Information

Calc. Mass	Obsrv. Mass	± da	± ppm	Start Seq.	End Seq.	Sequence	Ion Score	C. I. %	Modification	Rank	Result Type
983.4985	983.468	-0.0305	-31	126	133	FGEAVWFK					Mascot
1055.5303	1055.5291	-0.0012	-1	97	104	ELEVIHCR			Carbamidomethyl (C)[7]		Mascot
1055.5303	1055.5291	-0.0012	-1	97	104	ELEVIHCR	42	99.56	Carbamidomethyl (C)[7]		Mascot
1325.6743	1325.6754	0.0011	1	95	104	NRELEVIHCR			Carbamidomethyl (C)[9]		Mascot
1439.72	1439.6439	-0.0761	-53	2	16	AATTMSLSSPAFAGK					Mascot
1455.7148	1455.6631	-0.0517	-36	2	16	AATTMSLSSPAFAGK			Oxidation (M)[5]		Mascot
1469.6656	1469.6794	0.0138	9	43	55	QVTSSSPWYGSDR					Mascot
1575.849	1575.8531	0.0041	3	17	31	AVKNLPSSSLFGEAR					Mascot
1919.9871	1920.0138	0.0267	14	105	121	WAMLGALGCVFPELLAR			Carbamidomethyl (C)[9], Oxidation (M)[3]		Mascot

6	chloroplast light-harvesting chlorophyll a/b binding protein [Triticum aestivum]	gi|302566696	28367.2	5.14	6	65	98.602	6.269	42	99.56

Peptide Information

Calc. Mass	Obsrv. Mass	± da	± ppm	Start Seq.	End Seq.	Sequence	Ion Score	C. I. %	Modification	Rank	Result Type
983.4985	983.468	-0.0305	-31	126	133	FGEAVWFK					Mascot
1055.5303	1055.5291	-0.0012	-1	97	104	ELEVIHCR			Carbamidomethyl (C)[7]		Mascot
1055.5303	1055.5291	-0.0012	-1	97	104	ELEVIHCR	42	99.56	Carbamidomethyl (C)[7]		Mascot
1325.6743	1325.6754	0.0011	1	95	104	NRELEVIHCR			Carbamidomethyl (C)[9]		Mascot
1439.72	1439.6439	-0.0761	-53	2	16	ATTAMALSPSSFAGK					Mascot
1455.7148	1455.6631	-0.0517	-36	2	16	ATTAMALSPSSFAGK			Oxidation (M)[5]		Mascot
1593.7656	1593.8019	0.0363	23	41	55	AKPVSSGSPWYGSDR					Mascot
1919.9871	1920.0138	0.0267	14	105	121	WAMLGALGCVFPELLAR			Carbamidomethyl (C)[9], Oxidation (M)[3]		Mascot

7	Chlorophyll a-b binding protein 3C, chloroplastic [Triticum urartu]	gi|474060351	28204.2	5.27	6	65	98.602	5.971	42	99.56

Peptide Information

Calc. Mass	Obsrv. Mass	± da	± ppm	Start Seq.	End Seq.	Sequence	Ion Score	C. I. %	Modification	Rank	Result Type
983.4985	983.468	-0.0305	-31	125	132	FGEAVWFK					Mascot
1055.5303	1055.5291	-0.0012	-1	96	103	ELEVIHCR			Carbamidomethyl (C)[7]		Mascot
1055.5303	1055.5291	-0.0012	-1	96	103	ELEVIHCR	42	99.56	Carbamidomethyl (C)[7]		Mascot
1325.6743	1325.6754	0.0011	1	94	103	NRELEVIHCR			Carbamidomethyl (C)[9]		Mascot
1463.7601	1463.714	-0.0461	-31	17	30	QAKIASSNVFGEGR					Mascot
1568.7659	1568.7498	-0.0161	-10	1	16	MASSTMALSSPALAAK			Oxidation (M)[1,6]		Mascot
1919.9871	1920.0138	0.0267	14	104	120	WAMLGALGCVFPELLAR			Carbamidomethyl (C)[9], Oxidation (M)[3]		Mascot

8 Chlorophyll a-b binding protein, chloroplastic [Triticum urartu] gi|473952980 28455.3 5.15 6 64 98.319 6.176 42 99.56

Peptide Information

Calc. Mass	Obsrv. Mass	± da	± ppm	Start Seq.	End Seq.	Sequence	Ion Score	C. I. %	Modification	Rank	Result Type
983.4985	983.468	-0.0305	-31	126	133	FGEAVWFK					Mascot
1055.5303	1055.5291	-0.0012	-1	97	104	ELEVIHCR			Carbamidomethyl (C)[7]		Mascot
1055.5303	1055.5291	-0.0012	-1	97	104	ELEVIHCR	42	99.56	Carbamidomethyl (C)[7]		Mascot
1325.6743	1325.6754	0.0011	1	95	104	NRELEVIHCR			Carbamidomethyl (C)[9]		Mascot
1409.6445	1409.6514	0.0069	5	43	55	QVSSGSPWYGADR					Mascot
1439.7318	1439.6439	-0.0879	-61	122	133	NDVKFGEAVWFK					Mascot
1919.9871	1920.0138	0.0267	14	105	121	WAMLGALGCVFPELLAR			Carbamidomethyl (C)[9], Oxidation (M)[3]		Mascot

9 Chlorophyll a-b binding protein, chloroplastic [Triticum urartu] gi|474413348 28483.3 5.28 6 62 97.57 6.3 42 99.56

Peptide Information

Calc. Mass	Obsrv. Mass	± da	± ppm	Start Seq.	End Seq.	Sequence	Ion Score	C. I. %	Modification	Rank	Result Type
983.4985	983.468	-0.0305	-31	126	133	FGEAVWFK					Mascot
1055.5303	1055.5291	-0.0012	-1	97	104	ELEVIHCR			Carbamidomethyl (C)[7]		Mascot
1055.5303	1055.5291	-0.0012	-1	97	104	ELEVIHCR	42	99.56	Carbamidomethyl (C)[7]		Mascot
1325.6743	1325.6754	0.0011	1	95	104	NRELEVIHCR			Carbamidomethyl (C)[9]		Mascot
1409.6809	1409.6514	-0.0295	-21	43	55	KVASGSPWYGSDR					Mascot
1439.72	1439.6439	-0.0761	-53	2	16	AATTMSLSSPAFAGK					Mascot
1455.7148	1455.6631	-0.0517	-36	2	16	AATTMSLSSPAFAGK			Oxidation (M)[5]		Mascot
1919.9871	1920.0138	0.0267	14	105	121	WAMLGALGCVFPELLAR			Carbamidomethyl (C)[9], Oxidation (M)[3]		Mascot

10 putative chlorophyll a/b binding protein [Triticum durum] gi|62176932 26752.4 5.13 5 60 95.262 5.685 42 99.56

Peptide Information

Calc. Mass	Obsrv. Mass	± da	± ppm	Start Seq.	End Seq.	Sequence	Ion Score	C. I. %	Modification	Rank	Result Type
1013.501	1013.4961	-0.0049	-5	2	11	SRSSSSFAGK					Mascot
1055.5303	1055.5291	-0.0012	-1	92	99	ELEVIHCR			Carbamidomethyl (C)[7]		Mascot
1055.5303	1055.5291	-0.0012	-1	92	99	ELEVIHCR	42	99.577	Carbamidomethyl (C)[7]		Mascot
1325.6743	1325.6754	0.0011	1	90	99	NRELEVIHCR			Carbamidomethyl (C)[9]		Mascot
1455.6499	1455.6631	0.0132	9	38	50	QVSSSSPWYGSDR					Mascot
1919.9871	1920.0138	0.0267	14	100	116	WAMLGALGCVFPELLAR			Carbamidomethyl (C)[9], Oxidation (M)[3]		Mascot

Gel Idx/Pos	301/M3	Instr./Gel Origin	BA2151/Sample Project 20140814	Process Status	Analysis Succeeded
Plate [#] Name	[1] Sample Project 20140814	Instrument Sample Name		Spectra	11

Rank	Protein Name	Accession No.	Protein MW	Protein PI	Pep. Count	Protein Score	Protein Score C. I. %	Intensity Matched	Total Ion Score	Total Ion C. I. %	Confirmed
1	20 kDa chaperonin, chloroplastic [Triticum urartu]	gi|474407512	29805.7	6.77	8	389	100	6.672	348	100	

Peptide Information

Calc. Mass	Obsrv. Mass	± da	± ppm	Start Seq.	End Seq.	Sequence	Ion Score	C. I. %	Modification	Rank	Result Type
943.5207	943.5082	-0.0125	-13	142	149	DLKPLSDR					Mascot
1122.579	1122.5712	-0.0078	-7	257	266	GSEGEYIVLR					Mascot
1122.579	1122.5712	-0.0078	-7	257	266	GSEGEYIVLR	70	100			Mascot
1135.6106	1135.5966	-0.014	-12	42	51	YTSVKPLGDR					Mascot
1569.8846	1569.8328	-0.0518	-33	90	105	SLGSNIVEISVPVGAK					Mascot
1904.9388	1905.0001	0.0613	32	250	266	YAGSEFKGSEGEYIVLR					Mascot
2094.0828	2094.0974	0.0146	7	213	234	EKPSVGTVVAVGPGPLG DDGSR					Mascot
2094.0828	2094.0974	0.0146	7	213	234	EKPSVGTVVAVGPGPLG DDGSR	175	100			Mascot
2618.467	2618.5288	0.0618	24	1	25	MAKPALADGLRIPTSSSA RPLRPGR					Mascot
2635.4163	2635.4487	0.0324	12	63	89	TAGGILLPTTAQSRPQAG EVVAVGEGR					Mascot
2635.4163	2635.4487	0.0324	12	63	89	TAGGILLPTTAQSRPQAG EVVAVGEGR	103	100			Mascot

Rank	Protein Name	Accession No.	Protein MW	Protein PI	Pep. Count	Protein Score	Protein Score C. I. %	Intensity Matched	Total Ion Score	Total Ion C. I. %	Confirmed
2	chloroplast light-harvesting chlorophyll a/b binding protein [Triticum aestivum]	gi|302566696	28367.2	5.14	6	214	100	15.318	191	100	

Peptide Information

Calc. Mass	Obsrv. Mass	± da	± ppm	Start Seq.	End Seq.	Sequence	Ion Score	C. I. %	Modification	Rank	Result Type
983.4985	983.4614	-0.0371	-38	126	133	FGEAVWFK					Mascot
1055.5303	1055.5221	-0.0082	-8	97	104	ELEVIHCR			Carbamidomethyl (C)[7]		Mascot
1055.5303	1055.5221	-0.0082	-8	97	104	ELEVIHCR	58	99.99	Carbamidomethyl (C)[7]		Mascot
1325.6743	1325.6655	-0.0088	-7	95	104	NRELEVIHCR			Carbamidomethyl (C)[9]		Mascot
1325.6743	1325.6655	-0.0088	-7	95	104	NRELEVIHCR	64	99.997	Carbamidomethyl (C)[9]		Mascot
1439.72	1439.6511	-0.0689	-48	2	16	ATTAMALSPSSFAGK					Mascot
1593.7656	1593.7772	0.0116	7	41	55	AKPVSSGSPWYGSDR					Mascot
1593.7656	1593.7772	0.0116	7	41	55	AKPVSSGSPWYGSDR	69	100			Mascot
1903.9922	1904.0045	0.0123	6	105	121	WAMLGALGCVFPELLAR			Carbamidomethyl (C)[9]		Mascot
1919.9871	1919.9934	0.0063	3	105	121	WAMLGALGCVFPELLAR			Carbamidomethyl (C)[9], Oxidation (M)[3]		Mascot

3	Chlorophyll a-b binding protein, chloroplastic [Triticum urartu]	gi|473952980	28455.3	5.15	7	151	100	10.215	122	100	

Peptide Information

Calc. Mass	Obsrv. Mass	± da	± ppm	Start Seq.	End Seq.	Sequence	Ion Score	C. I. %	Modification	Rank	Result Type
983.4985	983.4614	-0.0371	-38	126	133	FGEAVWFK					Mascot
1055.5303	1055.5221	-0.0082	-8	97	104	ELEVIHCR			Carbamidomethyl (C)[7]		Mascot
1055.5303	1055.5221	-0.0082	-8	97	104	ELEVIHCR	58	99.99	Carbamidomethyl (C)[7]		Mascot
1325.6743	1325.6655	-0.0088	-7	95	104	NRELEVIHCR			Carbamidomethyl (C)[9]		Mascot
1325.6743	1325.6655	-0.0088	-7	95	104	NRELEVIHCR	64	99.997	Carbamidomethyl (C)[9]		Mascot
1439.7318	1439.6511	-0.0807	-56	122	133	NDVKFGEAVWFK					Mascot
1592.7295	1592.7844	0.0549	34	1	16	MAAATMSLSSSTFAGK			Oxidation (M)[1,6]		Mascot
1608.7765	1608.7917	0.0152	9	41	55	AKQVSSGSPWYGADR					Mascot
1903.9922	1904.0045	0.0123	6	105	121	WAMLGALGCVFPELLAR			Carbamidomethyl (C)[9]		Mascot
1919.9871	1919.9934	0.0063	3	105	121	WAMLGALGCVFPELLAR			Carbamidomethyl (C)[9], Oxidation (M)[3]		Mascot

4	unnamed protein product [Triticum aestivum]	gi|332617595	28328.2	5.17	6	146	100	9.939	122	100	

Protein Group

unnamed protein product [Triticum aestivum]	gi|330730540	28328.2	5.1700 000762 9395
unnamed protein product [Triticum aestivum]	gi|330728755	28328.2	5.1700 000762 9395
unnamed protein product [Triticum aestivum]	gi|332628256	28328.2	5.1700 000762 9395

Peptide Information

Calc. Mass	Obsrv. Mass	± da	± ppm	Start Seq.	End Seq.	Sequence	Ion Score	C. I. %	Modification	Rank	Result Type
983.4985	983.4614	-0.0371	-38	125	132	FGEAVWFK					Mascot
1055.5303	1055.5221	-0.0082	-8	96	103	ELEVIHCR			Carbamidomethyl (C)[7]		Mascot
1055.5303	1055.5221	-0.0082	-8	96	103	ELEVIHCR	58	99.99	Carbamidomethyl (C)[7]		Mascot

1325.6743	1325.6655	-0.0088	-7	94	103	NRELEVIHCR			Carbamidomethyl (C)[9]		Mascot
1325.6743	1325.6655	-0.0088	-7	94	103	NRELEVIHCR	64	99.997	Carbamidomethyl (C)[9]		Mascot
1575.849	1575.8107	-0.0383	-24	17	32	AVKLSPGASEVFGTGR					Mascot
1870.9402	1871.0072	0.067	36	1	19	MAASTMALSSPAFAGKAVK			Oxidation (M)[1,6]		Mascot
1903.9922	1904.0045	0.0123	6	104	120	WAMLGALGCVFPELLAR			Carbamidomethyl (C)[9]		Mascot
1919.9871	1919.9934	0.0063	3	104	120	WAMLGALGCVFPELLAR			Carbamidomethyl (C)[9], Oxidation (M)[3]		Mascot

5	Chlorophyll a-b binding protein 3C, chloroplastic [Triticum urartu]	gi|474060351	28204.2	5.27	6	145	100	10.645	122	100

Peptide Information

Calc. Mass	Obsrv. Mass	± da	± ppm	Start Seq.	End Seq.	Sequence	Ion Score	C. I. %	Modification	Rank	Result Type
983.4985	983.4614	-0.0371	-38	125	132	FGEAVWFK					Mascot
1055.5303	1055.5221	-0.0082	-8	96	103	ELEVIHCR			Carbamidomethyl (C)[7]		Mascot
1055.5303	1055.5221	-0.0082	-8	96	103	ELEVIHCR	58	99.99	Carbamidomethyl (C)[7]		Mascot
1325.6743	1325.6655	-0.0088	-7	94	103	NRELEVIHCR			Carbamidomethyl (C)[9]		Mascot
1325.6743	1325.6655	-0.0088	-7	94	103	NRELEVIHCR	64	99.997	Carbamidomethyl (C)[9]		Mascot
1589.7708	1589.7832	0.0124	8	40	54	AKPAASSSPWYGPDR					Mascot
1637.8428	1637.7593	-0.0835	-51	20	34	IASSNVFGEGRITMR					Mascot
1653.8378	1653.7649	-0.0729	-44	20	34	IASSNVFGEGRITMR			Oxidation (M)[14]		Mascot
1903.9922	1904.0045	0.0123	6	104	120	WAMLGALGCVFPELLAR			Carbamidomethyl (C)[9]		Mascot
1919.9871	1919.9934	0.0063	3	104	120	WAMLGALGCVFPELLAR			Carbamidomethyl (C)[9], Oxidation (M)[3]		Mascot

6	unnamed protein product [Triticum aestivum]	gi|332617637	28377.3	5.3	6	145	100	10.022	122	100

Protein Group

chlorophyll a/b-binding protein WCAB precursor [Triticum aestivum]	gi|1657859	28377.3	5.30000019073486
unnamed protein product [Triticum aestivum]	gi|330730582	28377.3	5.30000019073486
unnamed protein product [Triticum aestivum]	gi|332628299	28377.3	5.30000019073486
unnamed protein product [Triticum aestivum]	gi|330728797	28377.3	5.3000

Peptide Information

Calc. Mass	Obsrv. Mass	± da	± ppm	Start Seq.	End Seq.	Sequence	Ion Score	C. I. %	Modification	Rank	Result Type
983.4985	983.4614	-0.0371	-38	126	133	FGEAVWFK					Mascot
1055.5303	1055.5221	-0.0082	-8	97	104	ELEVIHCR			Carbamidomethyl (C)[7]		Mascot
1055.5303	1055.5221	-0.0082	-8	97	104	ELEVIHCR	58	99.99	Carbamidomethyl (C)[7]		Mascot
1325.6743	1325.6655	-0.0088	-7	95	104	NRELEVIHCR			Carbamidomethyl (C)[9]		Mascot
1325.6743	1325.6655	-0.0088	-7	95	104	NRELEVIHCR	64	99.997	Carbamidomethyl (C)[9]		Mascot
1592.7295	1592.7844	0.0549	34	1	16	MAAATMSLSSSTFAGK			Oxidation (M)[1,6]		Mascot
1608.7765	1608.7917	0.0152	9	41	55	AKQVSSGSPWYGADR					Mascot
1903.9922	1904.0045	0.0123	6	105	121	WAMLGALGCVFPELLAR			Carbamidomethyl (C)[9]		Mascot
1919.9871	1919.9934	0.0063	3	105	121	WAMLGALGCVFPELLAR			Carbamidomethyl (C)[9], Oxidation (M)[3]		Mascot

7	Chlorophyll a-b binding protein, chloroplastic [Triticum urartu]	gi|474413347	28531.3	5.29	6	144	100	10.031	122	100	

Peptide Information

Calc. Mass	Obsrv. Mass	± da	± ppm	Start Seq.	End Seq.	Sequence	Ion Score	C. I. %	Modification	Rank	Result Type
983.4985	983.4614	-0.0371	-38	126	133	FGEAVWFK					Mascot
1055.5303	1055.5221	-0.0082	-8	97	104	ELEVIHCR			Carbamidomethyl (C)[7]		Mascot
1055.5303	1055.5221	-0.0082	-8	97	104	ELEVIHCR	58	99.99	Carbamidomethyl (C)[7]		Mascot
1325.6743	1325.6655	-0.0088	-7	95	104	NRELEVIHCR			Carbamidomethyl (C)[9]		Mascot
1325.6743	1325.6655	-0.0088	-7	95	104	NRELEVIHCR	64	99.997	Carbamidomethyl (C)[9]		Mascot
1439.72	1439.6511	-0.0689	-48	2	16	AATTMSLSSPAFAGK					Mascot
1575.849	1575.8107	-0.0383	-24	17	31	AVKNLPSSSLFGEAR					Mascot
1903.9922	1904.0045	0.0123	6	105	121	WAMLGALGCVFPELLAR			Carbamidomethyl (C)[9]		Mascot
1919.9871	1919.9934	0.0063	3	105	121	WAMLGALGCVFPELLAR			Carbamidomethyl (C)[9], Oxidation (M)[3]		Mascot

8	Chlorophyll a-b binding protein, chloroplastic [Triticum urartu]	gi|474238080	34864.4	5.48	6	141	100	9.934	122	100	

Peptide Information

Calc. Mass	Obsrv. Mass	± da	± ppm	Start Seq.	End Seq.	Sequence	Ion Score	C. I. %	Modification	Rank	Result Type
983.4985	983.4614	-0.0371	-38	126	133	FGEAVWFK					Mascot

1055.5303	1055.5221	-0.0082	-8	97	104	ELEVIHCR			Carbamidomethyl (C)[7]		Mascot
1055.5303	1055.5221	-0.0082	-8	97	104	ELEVIHCR	58	99.99	Carbamidomethyl (C)[7]		Mascot
1325.6743	1325.6655	-0.0088	-7	95	104	NRELEVIHCR			Carbamidomethyl (C)[9]		Mascot
1325.6743	1325.6655	-0.0088	-7	95	104	NRELEVIHCR	64	99.997	Carbamidomethyl (C)[9]		Mascot
1789.9	1789.8918	-0.0082	-5	2	19	AATTMSLSSSSFASKAVK			Oxidation (M)[5]		Mascot
1903.9922	1904.0045	0.0123	6	105	121	WAMIGALGCVFPELLAR			Carbamidomethyl (C)[9]		Mascot
1904.9457	1905.0001	0.0544	29	1	19	MAATTMSLSSSSFASKAVK					Mascot
1919.9871	1919.9934	0.0063	3	105	121	WAMIGALGCVFPELLAR			Carbamidomethyl (C)[9], Oxidation (M)[3]		Mascot

9	Chlorophyll a-b binding protein, chloroplastic [Triticum urartu]	gi|473952982	31970	5.75	6	140	100	9.28	122	100	

Peptide Information

Calc. Mass	Obsrv. Mass	± da	± ppm	Start Seq.	End Seq.	Sequence	Ion Score	C. I. %	Modification	Rank	Result Type
983.4985	983.4614	-0.0371	-38	126	133	FGEAVWFK					Mascot
1055.5303	1055.5221	-0.0082	-8	97	104	ELEVIHCR			Carbamidomethyl (C)[7]		Mascot
1055.5303	1055.5221	-0.0082	-8	97	104	ELEVIHCR	58	99.99	Carbamidomethyl (C)[7]		Mascot
1308.6543	1308.704	0.0497	38	20	31	NVQSSTLFGEAR					Mascot
1325.6743	1325.6655	-0.0088	-7	95	104	NRELEVIHCR			Carbamidomethyl (C)[9]		Mascot
1325.6743	1325.6655	-0.0088	-7	95	104	NRELEVIHCR	64	99.997	Carbamidomethyl (C)[9]		Mascot
1696.7921	1696.7897	-0.0024	-1	1	16	MATTTMSLSFSTFAGK			Oxidation (M)[1]		Mascot
1929.9851	1929.9065	-0.0786	-41	264	280	EAELAEGQGKLMFVHVR			Oxidation (M)[12]		Mascot

10	unnamed protein product [Triticum aestivum]	gi|332617597	27867	5.17	5	139	100	9.839	122	100	

Protein Group

unnamed protein product [Triticum aestivum]	gi|330730542	27867	5.1700000762 9395
unnamed protein product [Triticum aestivum]	gi|330728757	27867	5.1700000762 9395
unnamed protein product [Triticum aestivum]	gi|332628258	27867	5.1700000762 9395

Peptide Information

Calc. Mass	Obsrv. Mass	± da	± ppm	Start Seq.	End Seq.	Sequence	Ion Score	C. I. %	Modification	Rank	Result Type
983.4985	983.4614	-0.0371	-38	120	127	FGEAVWFK					Mascot
1055.5303	1055.5221	-0.0082	-8	91	98	ELEVIHCR			Carbamidomethyl (C)[7]		Mascot
1055.5303	1055.5221	-0.0082	-8	91	98	ELEVIHCR	58	99.987	Carbamidomethyl (C)[7]		Mascot
1325.6743	1325.6655	-0.0088	-7	89	98	NRELEVIHCR			Carbamidomethyl (C)[9]		Mascot
1325.6743	1325.6655	-0.0088	-7	89	98	NRELEVIHCR	64	99.996	Carbamidomethyl (C)[9]		Mascot
1575.849	1575.8107	-0.0383	-24	12	27	AVKLSPGASEVFGTGR					Mascot
1903.9922	1904.0045	0.0123	6	99	115	WAMLGALGCVFPELLAR			Carbamidomethyl (C)[9]		Mascot
1919.9871	1919.9934	0.0063	3	99	115	WAMLGALGCVFPELLAR			Carbamidomethyl (C)[9], Oxidation (M)[3]		Mascot

Gel Idx/Pos	302/M4	Instr./Gel Origin	BA2151/Sample Project 20140814	Process Status	Analysis Succeeded
Plate [#] Name	[1] Sample Project 20140814	Instrument Sample Name		Spectra	11

Rank	Protein Name	Accession No.	Protein MW	Protein PI	Pep. Count	Protein Score	Protein Score C. I. %	Intensity Matched	Total Ion Score	Total Ion C. I. %	Confirmed
1	Chlorophyll a-b binding protein, chloroplastic [Triticum urartu]	gi|473952980	28455.3	5.15	7	102	100	19.083	73	100	

Peptide Information

Calc. Mass	Obsrv. Mass	± da	± ppm	Start Seq.	End Seq.	Sequence	Ion Score	C. I. %	Modification	Rank	Result Type
983.4985	983.463	-0.0355	-36	126	133	FGEAVWFK					Mascot
983.4985	983.463	-0.0355	-36	126	133	FGEAVWFK	25	73.662			Mascot
1055.5303	1055.5171	-0.0132	-13	97	104	ELEVIHCR			Carbamidomethyl (C)[7]		Mascot
1055.5303	1055.5171	-0.0132	-13	97	104	ELEVIHCR	48	99.843	Carbamidomethyl (C)[7]		Mascot
1273.6899	1273.6294	-0.0605	-47	20	31	NVPSLALFGEAR					Mascot
1325.6743	1325.652	-0.0223	-17	95	104	NRELEVIHCR			Carbamidomethyl (C)[9]		Mascot
1439.7318	1439.6124	-0.1194	-83	122	133	NDVKFGEAVWFK					Mascot
1439.7318	1439.6124	-0.1194	-83	122	133	NDVKFGEAVWFK					Mascot
1576.7346	1576.7382	0.0036	2	1	16	MAAATMSLSSSTFAGK			Oxidation (M)[1]		Mascot
1919.9871	1919.9635	-0.0236	-12	105	121	WAMLGALGCVFPELLAR			Carbamidomethyl (C)[9], Oxidation (M)[3]		Mascot

Rank	Protein Name	Accession No.	Protein MW	Protein PI	Pep. Count	Protein Score	Protein Score C. I. %	Intensity Matched	Total Ion Score	Total Ion C. I. %	Confirmed
2	chloroplast light-harvesting chlorophyll a/b binding protein [Triticum aestivum]	gi|302566696	28367.2	5.14	6	96	99.999	19.092	73	100	

Peptide Information

Calc. Mass	Obsrv. Mass	± da	± ppm	Start Seq.	End Seq.	Sequence	Ion Score	C. I. %	Modification	Rank	Result Type
983.4985	983.463	-0.0355	-36	126	133	FGEAVWFK					Mascot
983.4985	983.463	-0.0355	-36	126	133	FGEAVWFK	25	73.662			Mascot
1055.5303	1055.5171	-0.0132	-13	97	104	ELEVIHCR			Carbamidomethyl (C)[7]		Mascot
1055.5303	1055.5171	-0.0132	-13	97	104	ELEVIHCR	48	99.843	Carbamidomethyl (C)[7]		Mascot
1325.6743	1325.652	-0.0223	-17	95	104	NRELEVIHCR			Carbamidomethyl (C)[9]		Mascot
1439.72	1439.6124	-0.1076	-75	2	16	ATTAMALSPSSFAGK					Mascot
1439.72	1439.6124	-0.1076	-75	2	16	ATTAMALSPSSFAGK					Mascot
1455.7148	1455.6071	-0.1077	-74	2	16	ATTAMALSPSSFAGK			Oxidation (M)[5]		Mascot
1593.7656	1593.7512	-0.0144	-9	41	55	AKPVSSGSPWYGSDR					Mascot
1919.9871	1919.9635	-0.0236	-12	105	121	WAMLGALGCVFPELLAR			Carbamidomethyl (C)[9], Oxidation (M)[3]		Mascot

Rank	Protein Name	Accession No.	Protein MW	Protein PI	Pep. Count	Protein Score	Protein Score C. I. %	Intensity Matched	Total Ion Score	Total Ion C. I. %	Confirmed
3	Chlorophyll a-b binding protein, chloroplastic [Triticum	gi|474413347	28531.3	5.29	6	96	99.999	19.332	73	100	

urartu]

Peptide Information

Calc. Mass	Obsrv. Mass	± da	± ppm	Start Seq.	End Seq.	Sequence	Ion Score	C. I. %	Modification	Rank	Result Type
983.4985	983.463	-0.0355	-36	126	133	FGEAVWFK					Mascot
983.4985	983.463	-0.0355	-36	126	133	FGEAVWFK	25	73.662			Mascot
1055.5303	1055.5171	-0.0132	-13	97	104	ELEVIHCR			Carbamidomethyl (C)[7]		Mascot
1055.5303	1055.5171	-0.0132	-13	97	104	ELEVIHCR	48	99.843	Carbamidomethyl (C)[7]		Mascot
1325.6743	1325.652	-0.0223	-17	95	104	NRELEVIHCR			Carbamidomethyl (C)[9]		Mascot
1411.7369	1411.6213	-0.1156	-82	122	133	NSVKFGEAVWFK					Mascot
1439.72	1439.6124	-0.1076	-75	2	16	AATTMSLSSPAFAGK					Mascot
1439.72	1439.6124	-0.1076	-75	2	16	AATTMSLSSPAFAGK					Mascot
1455.7148	1455.6071	-0.1077	-74	2	16	AATTMSLSSPAFAGK			Oxidation (M)[5]		Mascot
1919.9871	1919.9635	-0.0236	-12	105	121	WAMLGALGCVFPELLAR			Carbamidomethyl (C)[9], Oxidation (M)[3]		Mascot

4 Chlorophyll a-b binding protein, chloroplastic [Triticum urartu] gi|474413348 28483.3 5.28 6 93 99.998 19.14 73 100

Peptide Information

Calc. Mass	Obsrv. Mass	± da	± ppm	Start Seq.	End Seq.	Sequence	Ion Score	C. I. %	Modification	Rank	Result Type
983.4985	983.463	-0.0355	-36	126	133	FGEAVWFK					Mascot
983.4985	983.463	-0.0355	-36	126	133	FGEAVWFK	25	73.662			Mascot
1055.5303	1055.5171	-0.0132	-13	97	104	ELEVIHCR			Carbamidomethyl (C)[7]		Mascot
1055.5303	1055.5171	-0.0132	-13	97	104	ELEVIHCR	48	99.843	Carbamidomethyl (C)[7]		Mascot
1281.5859	1281.5435	-0.0424	-33	44	55	VASGSPWYGSDR					Mascot
1325.6743	1325.652	-0.0223	-17	95	104	NRELEVIHCR			Carbamidomethyl (C)[9]		Mascot
1439.72	1439.6124	-0.1076	-75	2	16	AATTMSLSSPAFAGK					Mascot
1439.72	1439.6124	-0.1076	-75	2	16	AATTMSLSSPAFAGK					Mascot
1455.7148	1455.6071	-0.1077	-74	2	16	AATTMSLSSPAFAGK			Oxidation (M)[5]		Mascot
1919.9871	1919.9635	-0.0236	-12	105	121	WAMLGALGCVFPELLAR			Carbamidomethyl (C)[9], Oxidation (M)[3]		Mascot

5 Chlorophyll a-b binding protein 3C, chloroplastic [Triticum urartu] gi|474060351 28204.2 5.27 5 90 99.996 10.114 73 100

Peptide Information

Calc. Mass	Obsrv. Mass	± da	± ppm	Start Seq.	End Seq.	Sequence	Ion Score	C. I. %	Modification	Rank	Result Type
983.4985	983.463	-0.0355	-36	125	132	FGEAVWFK					Mascot

983.4985	983.463	-0.0355	-36	125	132	FGEAVWFK	25	73.662			Mascot
1055.5303	1055.5171	-0.0132	-13	96	103	ELEVIHCR			Carbamidomethyl (C)[7]		Mascot
1055.5303	1055.5171	-0.0132	-13	96	103	ELEVIHCR	48	99.843	Carbamidomethyl (C)[7]		Mascot
1325.6743	1325.652	-0.0223	-17	94	103	NRELEVIHCR			Carbamidomethyl (C)[9]		Mascot
1405.7356	1405.621	-0.1146	-82	2	16	ASSTMALSSPALAAK					Mascot
1421.7305	1421.6143	-0.1162	-82	2	16	ASSTMALSSPALAAK			Oxidation (M)[5]		Mascot
1919.9871	1919.9635	-0.0236	-12	104	120	WAMLGALGCVFPELLAR			Carbamidomethyl (C)[9], Oxidation (M)[3]		Mascot

6	Chlorophyll a-b binding protein, chloroplastic [Triticum urartu]	gi|474169245	28454.3	5.46	5	90	99.996	9.714	73	100

Peptide Information

Calc. Mass	Obsrv. Mass	± da	± ppm	Start Seq.	End Seq.	Sequence	Ion Score	C. I. %	Modification	Rank	Result Type
983.4985	983.463	-0.0355	-36	126	133	FGEAVWFK					Mascot
983.4985	983.463	-0.0355	-36	126	133	FGEAVWFK	25	73.662			Mascot
1055.5303	1055.5171	-0.0132	-13	97	104	ELEVIHCR			Carbamidomethyl (C)[7]		Mascot
1055.5303	1055.5171	-0.0132	-13	97	104	ELEVIHCR	48	99.843	Carbamidomethyl (C)[7]		Mascot
1325.6743	1325.652	-0.0223	-17	95	104	NRELEVIHCR			Carbamidomethyl (C)[9]		Mascot
1576.7523	1576.7382	-0.0141	-9	1	16	MAATTTSLSSSTFAGK			Oxidation (M)[1]		Mascot
1919.9871	1919.9635	-0.0236	-12	105	121	WAMLGALGCVFPELLAR			Carbamidomethyl (C)[9], Oxidation (M)[3]		Mascot

7	unnamed protein product [Triticum aestivum]	gi|332617637	28377.3	5.3	5	90	99.996	9.714	73	100

Protein Group

chlorophyll a/b-binding protein WCAB precursor [Triticum aestivum]	gi|1657859	28377.3	5.3000 001907 3486
unnamed protein product [Triticum aestivum]	gi|330730582	28377.3	5.3000 001907 3486
unnamed protein product [Triticum aestivum]	gi|332628299	28377.3	5.3000 001907 3486
unnamed protein product [Triticum aestivum]	gi|330728797	28377.3	5.3000 001907 3486

Peptide Information

Calc. Mass	Obsrv. Mass	± da	± ppm	Start Seq.	End Seq.	Sequence	Ion Score	C. I. %	Modification	Rank	Result Type
983.4985	983.463	-0.0355	-36	126	133	FGEAVWFK					Mascot
983.4985	983.463	-0.0355	-36	126	133	FGEAVWFK	25	73.662			Mascot
1055.5303	1055.5171	-0.0132	-13	97	104	ELEVIHCR			Carbamidomethyl (C)[7]		Mascot
1055.5303	1055.5171	-0.0132	-13	97	104	ELEVIHCR	48	99.843	Carbamidomethyl (C)[7]		Mascot
1325.6743	1325.652	-0.0223	-17	95	104	NRELEVIHCR			Carbamidomethyl (C)[9]		Mascot
1576.7346	1576.7382	0.0036	2	1	16	MAAATMSLSSSTFAGK			Oxidation (M)[1]		Mascot
1919.9871	1919.9635	-0.0236	-12	105	121	WAMLGALGCVFPELLAR			Carbamidomethyl (C)[9], Oxidation (M)[3]		Mascot

8 Chlorophyll a-b binding protein 1, chloroplastic [Triticum urartu] gi|473965828 30439.1 5.25 5 89 99.994 9.852 73 100

Peptide Information

Calc. Mass	Obsrv. Mass	± da	± ppm	Start Seq.	End Seq.	Sequence	Ion Score	C. I. %	Modification	Rank	Result Type
983.4985	983.463	-0.0355	-36	125	132	FGEAVWFK					Mascot
983.4985	983.463	-0.0355	-36	125	132	FGEAVWFK	25	73.662			Mascot
1055.5303	1055.5171	-0.0132	-13	96	103	ELEVIHCR			Carbamidomethyl (C)[7]		Mascot
1055.5303	1055.5171	-0.0132	-13	96	103	ELEVIHCR	48	99.843	Carbamidomethyl (C)[7]		Mascot
1325.6743	1325.652	-0.0223	-17	94	103	NRELEVIHCR			Carbamidomethyl (C)[9]		Mascot
1382.6335	1382.626	-0.0075	-5	42	54	VAASSSPWYGSDR					Mascot
1919.9871	1919.9635	-0.0236	-12	104	120	WAMLGALGCVFPELLAR			Carbamidomethyl (C)[9], Oxidation (M)[3]		Mascot

9 Chlorophyll a-b binding protein, chloroplastic [Triticum urartu] gi|474238080 34864.4 5.48 5 87 99.991 9.689 73 100

Peptide Information

Calc. Mass	Obsrv. Mass	± da	± ppm	Start Seq.	End Seq.	Sequence	Ion Score	C. I. %	Modification	Rank	Result Type
983.4985	983.463	-0.0355	-36	126	133	FGEAVWFK					Mascot
983.4985	983.463	-0.0355	-36	126	133	FGEAVWFK	25	73.662			Mascot
1055.5303	1055.5171	-0.0132	-13	97	104	ELEVIHCR			Carbamidomethyl (C)[7]		Mascot
1055.5303	1055.5171	-0.0132	-13	97	104	ELEVIHCR	48	99.843	Carbamidomethyl (C)[7]		Mascot
1325.6743	1325.652	-0.0223	-17	95	104	NRELEVIHCR			Carbamidomethyl (C)[9]		Mascot
1455.6499	1455.6071	-0.0428	-29	43	55	QVSSSSPWYGSDR					Mascot
1919.9871	1919.9635	-0.0236	-12	105	121	WAMIGALGCVFPELLAR			Carbamidomethyl (C)[9], Oxidation (M)[3]		Mascot

10	unnamed protein product [Triticum aestivum]	gi|332617599	25049.5	4.92	4	86	99.99	9.512	73	100

Protein Group

unnamed protein product [Triticum aestivum]	gi|330730544	25049.5	4.9200 000762 9395
unnamed protein product [Triticum aestivum]	gi|330728759	25049.5	4.9200 000762 9395
unnamed protein product [Triticum aestivum]	gi|332628260	25049.5	4.9200 000762 9395

Peptide Information

Calc. Mass	Obsrv. Mass	± da	± ppm	Start Seq.	End Seq.	Sequence	Ion Score	C. I. %	Modification	Rank	Result Type
983.4985	983.463	-0.0355	-36	91	98	FGEAVWFK					Mascot
983.4985	983.463	-0.0355	-36	91	98	FGEAVWFK	25	84.92			Mascot
1055.5303	1055.5171	-0.0132	-13	62	69	ELEVIHCR			Carbamidomethyl (C)[7]		Mascot
1055.5303	1055.5171	-0.0132	-13	62	69	ELEVIHCR	48	99.91	Carbamidomethyl (C)[7]		Mascot
1325.6743	1325.652	-0.0223	-17	60	69	NRELEVIHCR			Carbamidomethyl (C)[9]		Mascot
1919.9611	1919.9635	0.0024	1	3	20	KTVKPTGPSGSPWYGSD R					Mascot

Gel Idx/Pos	303/M5	Instr./Gel Origin	BA2151/Sample Project 20140814	Process Status	Analysis Succeeded
Plate [#] Name	[1] Sample Project 20140814	Instrument Sample Name		Spectra	11

Rank	Protein Name	Accession No.	Protein MW	Protein PI	Pep. Count	Protein Score	Protein Score C. I. %	Intensity Matched	Total Ion Score	Total Ion C. I. %	Confirmed
1	gamma gliadin [Triticum aestivum]	gi|133741924	16537.3	8.88	2	95	99.999	22.896	87	100	

Peptide Information

Calc. Mass	Obsrv. Mass	± da	± ppm	Start Seq.	End Seq.	Sequence	Ion Score	C. I. %	Modification	Rank	Result Type
1885.8064	1885.9667	0.1603	85	84	97	SCLVMWEQCCQQLK			Carbamidomethyl (C)[2,9,10], Oxidation (M)[5]		Mascot
1950.9413	1950.9327	-0.0086	-4	60	76	DALLQQCSPVADMSFLR			Carbamidomethyl (C)[7]		Mascot
1950.9413	1950.9327	-0.0086	-4	60	76	DALLQQCSPVADMSFLR	87	100	Carbamidomethyl (C)[7]		Mascot
1966.9362	1966.9016	-0.0346	-18	60	76	DALLQQCSPVADMSFLR			Carbamidomethyl (C)[7], Oxidation (M)[13]		Mascot
1966.9362	1966.9016	-0.0346	-18	60	76	DALLQQCSPVADMSFLR	81	100	Carbamidomethyl (C)[7], Oxidation (M)[13]		Mascot

Rank	Protein Name	Accession No.	Protein MW	Protein PI	Pep. Count	Protein Score	Protein Score C. I. %	Intensity Matched	Total Ion Score	Total Ion C. I. %	Confirmed
2	gliadin/avenin-like seed protein [Triticum aestivum]	gi|281335538	22813.2	6.2	2	94	99.998	23.294	87	100	

Protein Group

Avenin-3 [Triticum urartu]	gi|474329936	22657.1	6.3499 999046 3257

Peptide Information

Calc. Mass	Obsrv. Mass	± da	± ppm	Start Seq.	End Seq.	Sequence	Ion Score	C. I. %	Modification	Rank	Result Type
1950.9413	1950.9327	-0.0086	-4	60	76	DALLQQCSPVADMSFLR			Carbamidomethyl (C)[7]		Mascot
1950.9413	1950.9327	-0.0086	-4	60	76	DALLQQCSPVADMSFLR	87	100	Carbamidomethyl (C)[7]		Mascot
1966.9362	1966.9016	-0.0346	-18	60	76	DALLQQCSPVADMSFLR			Carbamidomethyl (C)[7], Oxidation (M)[13]		Mascot
1966.9362	1966.9016	-0.0346	-18	60	76	DALLQQCSPVADMSFLR	81	100	Carbamidomethyl (C)[7], Oxidation (M)[13]		Mascot
2635.1885	2635.1743	-0.0142	-5	77	97	SQVVQHSSCLVMWEQCCQQLK			Carbamidomethyl (C)[9,16,17]		Mascot
2651.1833	2651.157	-0.0263	-10	77	97	SQVVQHSSCLVMWEQCCQQLK			Carbamidomethyl (C)[9,16,17], Oxidation (M)[12]		Mascot

Rank	Protein Name	Accession No.	Protein MW	Protein PI	Pep. Count	Protein Score	Protein Score C. I. %	Intensity Matched	Total Ion Score	Total Ion C. I. %	Confirmed
3	hypothetical protein TRIUR3_33673 [Triticum urartu]	gi|474071653	44186.3	8.98	9	42	0	19.394			

Peptide Information

Calc. Mass	Obsrv. Mass	± da	± ppm	Start Seq.	End Seq.	Sequence	Ion Score	C. I. %	Modification	Rank	Result Type
945.5264	945.4924	-0.034	-36	362	369	ARPPYSVR					Mascot

Calc. Mass	Obsrv. Mass	± da	± ppm	Start Seq.	End Seq.	Sequence	Ion Score	C. I. %	Modification	Rank	Result Type
1786.9712	1786.8433	-0.1279	-72	273	287	VIYRWQQAVSNPGLR					Mascot
1851.986	1851.8918	-0.0942	-51	8	24	IPFFGCNAGALMVLTLK			Carbamidomethyl (C)[6]		Mascot
1916.8755	1916.9415	0.066	34	129	142	FSEPLYYEWGFWHR					Mascot
1988.9131	1988.8542	-0.0589	-30	339	356	EGDIEGDSVPGVGKQWC R			Carbamidomethyl (C)[17]		Mascot
3375.8787	3375.6782	-0.2005	-59	191	221	AINVDILGVELAGSAAQLP LKWNIQVSTWLR					Mascot
3381.5615	3381.6865	0.125	37	129	154	FSEPLYYEWGFWHRLFF QYMSGFTAR			Oxidation (M)[20]		Mascot
3396.5925	3396.6963	0.1038	31	52	80	LAKLPSLVEYFGYCLCCG SHFAGPVYEMK			Carbamidomethyl (C)[14,16,17]		Mascot
3419.7234	3419.7341	0.0107	3	8	38	IPFFGCNAGALMVLTLKVI SCSMNYSDGLLK			Carbamidomethyl (C)[6,21]		Mascot
3419.7234	3419.7341	0.0107	3	8	38	IPFFGCNAGALMVLTLKVI SCSMNYSDGLLK			Carbamidomethyl (C)[6,21]		Mascot
3435.7183	3435.7095	-0.0088	-3	8	38	IPFFGCNAGALMVLTLKVI SCSMNYSDGLLK			Carbamidomethyl (C)[6,21], Oxidation (M)[12]		Mascot

4	putative cadmium/zinc-transporting ATPase [Triticum durum]	gi|400621213	86726.8	6.29	12	42	0	4.21

Peptide Information

Calc. Mass	Obsrv. Mass	± da	± ppm	Start Seq.	End Seq.	Sequence	Ion Score	C. I. %	Modification	Rank	Result Type
1298.53	1298.5414	0.0114	9	762	771	GCHCCHKPSR			Carbamidomethyl (C)[2,4,5]		Mascot
1507.7317	1507.702	-0.0297	-20	200	214	ASAGMSSLMSMIPPK					Mascot
1683.8041	1683.7494	-0.0547	-32	291	305	TMALAENSTVAKMER			Oxidation (M)[2,13]		Mascot
1875.9811	1875.8892	-0.0919	-49	394	411	GGDVLESLGEIRAVAFDK					Mascot
1875.9811	1875.8892	-0.0919	-49	394	411	GGDVLESLGEIRAVAFDK					Mascot
1924.8324	1924.9072	0.0748	39	755	771	TGGDAPKGCHCCHKPSR			Carbamidomethyl (C)[9,11,12]		Mascot
1935.0659	1934.9537	-0.1122	-58	65	82	TVVVEHDPAAVSQSRIVK					Mascot
3265.6733	3265.5474	-0.1259	-39	228	259	DIGVGVVIAVRAGEMVPV DGVVVDGQSEVDER					Mascot
3271.7979	3271.5298	-0.2681	-82	357	387	LALVLLVSACPCALVLST PVATFCALLTAAR			Carbamidomethyl (C)[10,12,24]		Mascot
3372.6199	3372.6665	0.0466	14	518	549	GVSIGYVICDGDLVGVFS LSDDCRTGAAEAIR			Carbamidomethyl (C)[9,23]		Mascot
3375.7219	3375.6782	-0.0437	-13	260	290	SLTGESYPVPKQPQSEV WAGTLNLDGYIAVR					Mascot
3411.7173	3411.6733	-0.044	-13	542	575	TGAAEAIRELASMGISSV LLTGDSAEAAVHAQER					Mascot
3433.7419	3433.7078	-0.0341	-10	271	302	QPQSEVWAGTLNLDGYI AVRTMALAENSTVAK					Mascot

5	hypothetical protein TRIUR3_09989 [Triticum urartu]	gi|473996362	20828.6	8.32	7	40	0	.748

Peptide Information

Calc. Mass	Obsrv. Mass	± da	± ppm	Start Seq.	End Seq.	Sequence	Ion Score	C. I. %	Modification	Rank	Result Type
836.4512	836.3863	-0.0649	-78	2	8	SFLNEVK					Mascot

868.4635	868.468	0.0045	5	177	183	ERHGLEK					Mascot
1138.5562	1138.5187	-0.0375	-33	74	84	AYGGVAAECLK			Carbamidomethyl (C)[9]		Mascot
1351.6675	1351.6569	-0.0106	-8	1	11	MSFLNEVKDPR			Oxidation (M)[1]		Mascot
1858.9658	1858.9884	0.0226	12	139	157	VTPYPDSRGPVAAVGSA SK					Mascot
1892.0059	1891.9006	-0.1053	-56	66	84	LGTGGLVRAYGGVAAEC LK			Carbamidomethyl (C)[17]		Mascot
3458.6819	3458.6831	0.0012	0	27	59	YNDDGEPSSTAGKPIYSA ISSSGIDMVMVVVIR					Mascot

6 dimeric alpha-amylase inhibitor [Triticum dicoccoides] gi|227808841 15899.7 8.33 5 38 0 1.929

Peptide Information

Calc. Mass	Obsrv. Mass	± da	± ppm	Start Seq.	End Seq.	Sequence	Ion Score	C. I. %	Modification	Rank	Result Type
1928.8781	1928.9218	0.0437	23	84	101	EHGAQEGQAGTGAFPR CR			Carbamidomethyl (C)[17]		Mascot
3233.4084	3233.54	0.1316	41	71	99	CDALYNMLDSMYKEHGA QEGQAGTGAFPR			Carbamidomethyl (C)[1], Oxidation (M)[7]		Mascot
3249.5537	3249.533	-0.0207	-6	2	30	LVATPVAAKYDAWSVNS GPWMCYPGYAFK			Carbamidomethyl (C)[22]		Mascot
3249.5537	3249.533	-0.0207	-6	2	30	LVATPVAAKYDAWSVNS GPWMCYPGYAFK			Carbamidomethyl (C)[22]		Mascot
3265.5486	3265.5474	-0.0012	0	2	30	LVATPVAAKYDAWSVNS GPWMCYPGYAFK			Carbamidomethyl (C)[22], Oxidation (M)[21]		Mascot
3396.5891	3396.6963	0.1072	32	1	30	MLVATPVAAKYDAWSVN SGPWMCYPGYAFK			Carbamidomethyl (C)[23], Oxidation (M)[1]		Mascot
3404.5352	3404.7297	0.1945	57	43	70	LQCNGSQVPEAVVRDCC QQLANINEWCR			Carbamidomethyl (C)[3,16,17,27]		Mascot

7 hypothetical protein TRIUR3_34788 [Triticum urartu] gi|474411787 22001.5 9.3 7 38 0 .903

Peptide Information

Calc. Mass	Obsrv. Mass	± da	± ppm	Start Seq.	End Seq.	Sequence	Ion Score	C. I. %	Modification	Rank	Result Type
818.4366	818.3765	-0.0601	-73	88	94	SDELKAR					Mascot
820.3981	820.3776	-0.0205	-25	100	105	EMQERK					Mascot
836.3931	836.3863	-0.0068	-8	100	105	EMQERK			Oxidation (M)[2]		Mascot
1138.4768	1138.5187	0.0419	37	55	63	AIWCADCSR			Carbamidomethyl (C)[4,7]		Mascot
1263.6539	1263.6095	-0.0444	-35	186	197	SSSKELSSSVPR					Mascot
1857.974	1857.9189	-0.0551	-30	1	19	MSAGTATPGLVVTATDPI R					Mascot
1873.9689	1873.9329	-0.036	-19	1	19	MSAGTATPGLVVTATDPI R			Oxidation (M)[1]		Mascot
3277.751	3277.5522	-0.1988	-61	154	185	ALFSNSPVMNAAGGILGV VGGMLMETVLFIIR					Mascot
3445.7446	3445.6912	-0.0534	-15	2	36	SAGTATPGLVVTATDPIR SFLSSAAGSPDLSDDLR					Mascot

8 dimeric alpha-amylase inhibitor [Triticum dicoccoides] gi|227808878 15959.7 6.54 5 37 0 1.972

Peptide Information

Calc. Mass	Obsrv. Mass	± da	± ppm	Start Seq.	End Seq.	Sequence	Ion Score	C. I. %	Modification	Rank	Result Type
1928.8781	1928.9218	0.0437	23	84	101	EHGAQEGQAGTGAFPR CR			Carbamidomethyl (C)[17]		Mascot
3233.4084	3233.54	0.1316	41	71	99	CDALYNMLDSMYKEHGA QEGQAGTGAFPR			Carbamidomethyl (C)[1], Oxidation (M)[7]		Mascot
3249.4033	3249.533	0.1297	40	71	99	CDALYNMLDSMYKEHGA QEGQAGTGAFPR			Carbamidomethyl (C)[1], Oxidation (M)[7,11]		Mascot
3249.4033	3249.533	0.1297	40	71	99	CDALYNMLDSMYKEHGA QEGQAGTGAFPR			Carbamidomethyl (C)[1], Oxidation (M)[7,11]		Mascot
3278.5325	3278.5632	0.0307	9	2	30	LVATPIAAEYDAWSINSG PWMCYPGYAFK			Carbamidomethyl (C)[22]		Mascot
3441.563	3441.7024	0.1394	41	1	30	MLVATPIAAEYDAWSINS GPWMCYPGYAFK			Carbamidomethyl (C)[23], Oxidation (M)[1,22]		Mascot
3476.4143	3476.7288	0.3145	90	57	83	DCCQQLANISEWCRCDA LYNMLDSMYK			Carbamidomethyl (C)[2,3,13,15], Oxidation (M)[21,25]		Mascot

9	dimeric alpha-amylase inhibitor [Triticum dicoccoides]	gi|227809049	15739.6	8.33	5	37	0	1.86

Peptide Information

Calc. Mass	Obsrv. Mass	± da	± ppm	Start Seq.	End Seq.	Sequence	Ion Score	C. I. %	Modification	Rank	Result Type
1928.8781	1928.9218	0.0437	23	84	101	EHGAQEGQAGTGAFPR CR			Carbamidomethyl (C)[17]		Mascot
3233.4084	3233.54	0.1316	41	71	99	CDALYNMLDSMYKEHGA QEGQAGTGAFPR			Carbamidomethyl (C)[1], Oxidation (M)[7]		Mascot
3249.5537	3249.533	-0.0207	-6	2	30	LVATPVAAKYDAWSVNS GPWMCYPGYAFK			Carbamidomethyl (C)[22]		Mascot
3249.5537	3249.533	-0.0207	-6	2	30	LVATPVAAKYDAWSVNS GPWMCYPGYAFK			Carbamidomethyl (C)[22]		Mascot
3265.5486	3265.5474	-0.0012	0	2	30	LVATPVAAKYDAWSVNS GPWMCYPGYAFK			Carbamidomethyl (C)[22], Oxidation (M)[21]		Mascot
3396.5891	3396.6963	0.1072	32	1	30	MLVATPVAAKYDAWSVN SGPWMCYPGYAFK			Carbamidomethyl (C)[23], Oxidation (M)[1]		Mascot
3476.4143	3476.7288	0.3145	90	57	83	DCCQQLANISEWCRCDA LYNMLDSMYK			Carbamidomethyl (C)[2,3,13,15], Oxidation (M)[21,25]		Mascot

10	hypothetical protein TRIUR3_27022 [Triticum urartu]	gi|473723623	7577.8	6.7	5	37	0	.631

Peptide Information

Calc. Mass	Obsrv. Mass	± da	± ppm	Start Seq.	End Seq.	Sequence	Ion Score	C. I. %	Modification	Rank	Result Type
836.4261	836.3863	-0.0398	-48	18	24	DVNVFSR					Mascot
1105.4653	1105.525	0.0597	54	42	51	VCGDMPPSDK			Carbamidomethyl (C)[2]		Mascot
1584.7727	1584.7068	-0.0659	-42	25	38	MEVYAITSVFGDPR					Mascot
1857.9164	1857.9189	0.0025	1	25	40	MEVYAITSVFGDPRTR			Oxidation (M)[1]		Mascot
1925.0637	1924.9072	-0.1565	-81	1	17	MAHRVLELTLVSGQNLK			Oxidation (M)[1]		Mascot

Gel Idx/Pos	304/M6	Instr./Gel Origin	BA2151/Sample Project 20140814	Process Status	Analysis Succeeded
Plate [#] Name	[1] Sample Project 20140814	Instrument Sample Name		Spectra	11

Rank	Protein Name	Accession No.	Protein MW	Protein PI	Pep. Count	Protein Score	Protein Score C. I. %	Intensity Matched	Total Ion Score	Total Ion C. I. %	Confirmed
1	dehydroascorbate reductase [Triticum aestivum]	gi|259017810	23457.2	5.88	7	233	100	29.282	199	100	

Peptide Information

Calc. Mass	Obsrv. Mass	± da	± ppm	Start Seq.	End Seq.	Sequence	Ion Score	C. I. %	Modification	Rank	Result Type
909.4862	909.4286	-0.0576	-63	33	39	KVPYQMK			Oxidation (M)[6]		Mascot
944.5662	944.4809	-0.0853	-90	25	32	VLLTLEEK					Mascot
1202.682	1202.6294	-0.0526	-44	102	111	IFSTFVTFLK					Mascot
1497.8213	1497.7782	-0.0431	-29	157	168	LYHLQVALEHFK					Mascot
1607.8639	1607.8101	-0.0538	-33	122	135	ALVDELQALEEHLK					Mascot
1827.8444	1827.8322	-0.0122	-7	8	24	AAVGHPDTLGDCPFSQR			Carbamidomethyl (C)[12]		Mascot
1827.8444	1827.8322	-0.0122	-7	8	24	AAVGHPDTLGDCPFSQR	147	100	Carbamidomethyl (C)[12]		Mascot
1892.9641	1892.8965	-0.0676	-36	84	101	YPTPSLVTPPEYASVGSK					Mascot
1892.9641	1892.8965	-0.0676	-36	84	101	YPTPSLVTPPEYASVGSK	52	99.954			Mascot

Rank	Protein Name	Accession No.	Protein MW	Protein PI	Pep. Count	Protein Score	Protein Score C. I. %	Intensity Matched	Total Ion Score	Total Ion C. I. %	Confirmed
2	dehydroascorbate reductase [Triticum aestivum]	gi|28192421	23457.2	5.88	7	180	100	28.255	147	100	

Peptide Information

Calc. Mass	Obsrv. Mass	± da	± ppm	Start Seq.	End Seq.	Sequence	Ion Score	C. I. %	Modification	Rank	Result Type
909.4862	909.4286	-0.0576	-63	33	39	KVPYQMK			Oxidation (M)[6]		Mascot
944.5662	944.4809	-0.0853	-90	25	32	VLLTLEEK					Mascot
1202.682	1202.6294	-0.0526	-44	102	111	IFSTFVTFLK					Mascot
1497.8213	1497.7782	-0.0431	-29	157	168	LYHLQVALEHFK					Mascot
1574.8578	1574.7878	-0.07	-44	40	52	LIDVSNKPDWFLK					Mascot
1607.8639	1607.8101	-0.0538	-33	122	135	ALVDELQALEEHLK					Mascot
1827.8444	1827.8322	-0.0122	-7	8	24	AAVGHPDTLGDCPFSQR			Carbamidomethyl (C)[12]		Mascot
1827.8444	1827.8322	-0.0122	-7	8	24	AAVGHPDTLGDCPFSQR	147	100	Carbamidomethyl (C)[12]		Mascot

Rank	Protein Name	Accession No.	Protein MW	Protein PI	Pep. Count	Protein Score	Protein Score C. I. %	Intensity Matched	Total Ion Score	Total Ion C. I. %	Confirmed
3	Glutathione S-transferase DHAR2 [Triticum urartu]	gi|474023258	45260	8.3	9	173	100	28.695	147	100	

Peptide Information

Calc. Mass	Obsrv. Mass	± da	± ppm	Start Seq.	End Seq.	Sequence	Ion Score	C. I. %	Modification	Rank	Result Type

Calc. Mass	Obsrv. Mass	± da	± ppm	Start Seq.	End Seq.	Sequence	Ion Score	C. I. %	Modification	Rank	Result Type
909.4862	909.4286	-0.0576	-63	78	84	KVPYQMK			Oxidation (M)[6]		Mascot
943.4744	943.4584	-0.016	-17	297	304	ARSSFGYR					Mascot
944.5662	944.4809	-0.0853	-90	70	77	VLLTLEEK					Mascot
1202.682	1202.6294	-0.0526	-44	147	156	IFSTFVTFLK					Mascot
1497.8213	1497.7782	-0.0431	-29	202	213	LYHLQVALEHFK					Mascot
1574.781	1574.7878	0.0068	4	325	338	LSLGTFETVHEADR					Mascot
1607.8639	1607.8101	-0.0538	-33	167	180	ALVDELQALEEHLK					Mascot
1827.8444	1827.8322	-0.0122	-7	53	69	AAVGHPDTLGDCPFSQR			Carbamidomethyl (C)[12]		Mascot
1827.8444	1827.8322	-0.0122	-7	53	69	AAVGHPDTLGDCPFSQR	147	100	Carbamidomethyl (C)[12]		Mascot
2038.9242	2038.9896	0.0654	32	386	402	RQCCLLITEADELAMAE			Carbamidomethyl (C)[3,4], Oxidation (M)[15]		Mascot

4	hypothetical protein TRIUR3_12273 [Triticum urartu]	gi|474139724	93416.6	5.26	19	48	23.152	9.845	

Peptide Information

Calc. Mass	Obsrv. Mass	± da	± ppm	Start Seq.	End Seq.	Sequence	Ion Score	C. I. %	Modification	Rank	Result Type
932.4618	932.4376	-0.0242	-26	257	263	QLNECLR			Carbamidomethyl (C)[5]		Mascot
933.4999	933.4335	-0.0664	-71	334	341	RAETELSK					Mascot
943.5458	943.4584	-0.0874	-93	581	588	LQAIEELK					Mascot
974.5265	974.4619	-0.0646	-66	166	173	LNKEIDSR					Mascot
976.5057	976.4539	-0.0518	-53	424	432	AESASREVK					Mascot
982.4146	982.4176	0.003	3	462	470	DGDDMSSKK					Mascot
998.4095	998.4162	0.0067	7	462	470	DGDDMSSKK			Oxidation (M)[5]		Mascot
1078.5415	1078.5182	-0.0233	-22	720	728	EYPLAETQK					Mascot
1102.4946	1102.5439	0.0493	45	451	459	DHNMLSNQK			Oxidation (M)[4]		Mascot
1202.6124	1202.6294	0.017	14	519	528	SLEREGDQLR					Mascot
1232.6191	1232.5913	-0.0278	-23	246	255	MLNEQLEDLK					Mascot
1320.6808	1320.576	-0.1048	-79	754	764	TNPIRSSFWGR					Mascot
1507.7891	1507.7115	-0.0776	-51	398	410	ELEVTLEFAEISK					Mascot
1588.8428	1588.7958	-0.047	-30	335	348	AETELSKLQEIEAK					Mascot
1590.8235	1590.8219	-0.0016	-1	538	552	LGHGDYSASSTKVLR					Mascot
1644.7751	1644.7634	-0.0117	-7	661	674	FTLQSVYAQTDDEK					Mascot
1810.9255	1810.8842	-0.0413	-23	75	90	QMVKSEFVVALDTAEK			Oxidation (M)[2]		Mascot
2037.0176	2037.0267	0.0091	4	711	728	YDALPVDGKEYPLAETQK					Mascot
2055.0393	2054.9504	-0.0889	-43	79	96	SEFVVALDTAEKAVQEYR					Mascot
2055.0393	2054.9504	-0.0889	-43	79	96	SEFVVALDTAEKAVQEYR					Mascot
2080.9993	2081.0181	0.0188	9	169	188	EIDSRISAESSSASAQESVK					Mascot

2080.9993	2081.0181	0.0188	9	169	188	EIDSRISAESSSASAQESVK					Mascot

5 NBS-LRR resistance protein CIN14 [Triticum aestivum] gi|302583873 100033.9 6.1 17 48 23.152 9.18

Peptide Information

Calc. Mass	Obsrv. Mass	± da	± ppm	Start Seq.	End Seq.	Sequence	Ion Score	C. I. %	Modification	Rank	Result Type
822.4654	822.3881	-0.0773	-94	45	50	LKFQMR					Mascot
837.4651	837.4003	-0.0648	-77	612	618	TLMLFGR					Mascot
1057.5889	1057.4988	-0.0901	-85	95	103	LEQQIIEGK					Mascot
1102.5626	1102.5439	-0.0187	-17	35	44	EIGLAEDVEK					Mascot
1232.6382	1232.5913	-0.0469	-38	406	416	EHHPSLQSIGK					Mascot
1235.6599	1235.5559	-0.104	-84	357	367	VKGCMVLATTR			Carbamidomethyl (C)[4]		Mascot
1302.6147	1302.6364	0.0217	17	766	776	EEAWAAMLTHK			Oxidation (M)[7]		Mascot
1497.8384	1497.7782	-0.0602	-40	316	327	LQEILQENIRNK					Mascot
1507.7097	1507.7115	0.0018	1	210	222	DHYMTTSVPIESK					Mascot
1629.824	1629.8071	-0.0169	-10	47	60	FQMRYGQMVLAAAK			Oxidation (M)[3]		Mascot
1768.8344	1768.8688	0.0344	19	374	389	MIGTMDEITLSGLDEK			Oxidation (M)[1]		Mascot
1826.0018	1825.8855	-0.1163	-64	240	259	GGSSDLNVLPVVGSIGGIGK					Mascot
1826.8452	1826.8615	0.0163	9	329	342	FLLVMDDMWEEKDR					Mascot
1838.933	1838.9059	-0.0271	-15	847	861	EWQILSTHGMHSLRK			Oxidation (M)[10]		Mascot
1892.9059	1892.8965	-0.0094	-5	29	44	MEAWTREIGLAEDVEK			Oxidation (M)[1]		Mascot
1892.9059	1892.8965	-0.0094	-5	29	44	MEAWTREIGLAEDVEK			Oxidation (M)[1]		Mascot
1930.9441	1930.8867	-0.0574	-30	129	145	LVCSATSQITDWISHGR			Carbamidomethyl (C)[3]		Mascot
2039.0049	2038.9896	-0.0153	-8	149	165	REEEGLAHCNMLPLEIK			Carbamidomethyl (C)[9]		Mascot
2054.9998	2054.9504	-0.0494	-24	149	165	REEEGLAHCNMLPLEIK			Carbamidomethyl (C)[9], Oxidation (M)[11]		Mascot
2054.9998	2054.9504	-0.0494	-24	149	165	REEEGLAHCNMLPLEIK			Carbamidomethyl (C)[9], Oxidation (M)[11]		Mascot

6 hypothetical protein TRIUR3_31101 [Triticum urartu] gi|473999512 76855.9 9.13 16 45 0 15.204

Peptide Information

Calc. Mass	Obsrv. Mass	± da	± ppm	Start Seq.	End Seq.	Sequence	Ion Score	C. I. %	Modification	Rank	Result Type
940.5251	940.4492	-0.0759	-81	420	427	AFYVGQKK					Mascot
974.5095	974.4619	-0.0476	-49	81	88	TFFFSAVR					Mascot
976.4768	976.4539	-0.0229	-23	227	235	CELDAAAVK			Carbamidomethyl (C)[1]		Mascot
988.5462	988.4509	-0.0953	-96	110	117	ILPNFQEK					Mascot
1130.6066	1130.5435	-0.0631	-56	596	605	WSTGTGPRIR					Mascot
1130.6066	1130.5435	-0.0631	-56	596	605	WSTGTGPRIR	3	0			Mascot

1135.5565	1135.5653	0.0088	8	141	149	THKFDMVNK			Oxidation (M)[6]		Mascot
1145.4714	1145.5424	0.071	62	92	100	TYDCMKGDR			Carbamidomethyl (C)[4]		Mascot
1320.6074	1320.576	-0.0314	-24	202	212	QSMVNGNQDRR			Oxidation (M)[3]		Mascot
1507.7639	1507.7115	-0.0524	-35	478	490	FLQENDVSLTDVK					Mascot
1590.821	1590.8219	0.0009	1	175	187	ATLMHSVSFKQWR					Mascot
1606.8159	1606.801	-0.0149	-9	175	187	ATLMHSVSFKQWR			Oxidation (M)[4]		Mascot
1606.8159	1606.801	-0.0149	-9	175	187	ATLMHSVSFKQWR			Oxidation (M)[4]		Mascot
1635.8589	1635.8245	-0.0344	-21	478	491	FLQENDVSLTDVKK					Mascot
1657.7612	1657.7754	0.0142	9	590	603	NHQTCRWSTGTGPR			Carbamidomethyl (C)[5]		Mascot
1838.9283	1838.9059	-0.0224	-12	27	43	IFSDARSIGGDVPVDYK					Mascot
1892.925	1892.8965	-0.0285	-15	271	288	HSSISFFNGGKPETAASR					Mascot
1892.925	1892.8965	-0.0285	-15	271	288	HSSISFFNGGKPETAASR					Mascot
1915.0695	1914.9117	-0.1578	-82	636	653	RAPVPSPRPSPGMILSPR					Mascot
1931.0645	1930.8867	-0.1778	-92	636	653	RAPVPSPRPSPGMILSPR			Oxidation (M)[13]		Mascot
2039.0444	2038.9896	-0.0548	-27	474	490	DFIRFLQENDVSLTDVK					Mascot

7	uncoupling protein [Triticum aestivum]	gi|10716672	30876	9.33	9	44	0	7.77

Peptide Information

Calc. Mass	Obsrv. Mass	± da	± ppm	Start Seq.	End Seq.	Sequence	Ion Score	C. I. %	Modification	Rank	Result Type
904.5138	904.5524	0.0386	43	86	93	VGLYEPVK					Mascot
960.3914	960.4383	0.0469	49	231	238	MMGDSTYR					Mascot
960.3914	960.4383	0.0469	49	231	238	MMGDSTYR					Mascot
976.3863	976.4539	0.0676	69	231	238	MMGDSTYR			Oxidation (M)[1]		Mascot
998.4941	998.4162	-0.0779	-78	250	258	NDGLAAFYK					Mascot
1235.5144	1235.5559	0.0415	34	229	238	SRMMGDSTYR			Oxidation (M)[3,4]		Mascot
1535.7965	1535.7308	-0.0657	-43	144	157	HYSGALNAYATIVR					Mascot
1786.8905	1786.8097	-0.0808	-45	158	174	QEGIGALWTGLGPNMAR			Oxidation (M)[15]		Mascot
1843.9749	1843.7957	-0.1792	-97	70	85	GIIPGFHRQCLYGGLR			Carbamidomethyl (C)[10]		Mascot
1892.9391	1892.8965	-0.0426	-23	41	60	TAAGPAGTVGMLGTMMS IAR					Mascot
1892.9391	1892.8965	-0.0426	-23	41	60	TAAGPAGTVGMLGTMMS IAR					Mascot
1908.9341	1908.8068	-0.1273	-67	41	60	TAAGPAGTVGMLGTMMS IAR			Oxidation (M)[11]		Mascot
2037.0291	2037.0267	-0.0024	-1	40	60	KTAAGPAGTVGMLGTMM SIAR			Oxidation (M)[12]		Mascot

8	Dihydroflavonol-4-reductase [Triticum urartu]	gi|473776250	31108.8	6.05	9	43	0	5.523

Peptide Information

Calc. Mass	Obsrv. Mass	± da	± ppm	Start Seq.	End Seq.	Sequence	Ion Score	C. I. %	Modification	Rank	Result Type
870.4355	870.5151	0.0796	91	136	143	EAFAYAAK					Mascot
968.4869	968.4633	-0.0236	-24	78	85	ACYEAKVK			Carbamidomethyl (C)[2]		Mascot
972.4857	972.4381	-0.0476	-49	2	13	AAAAAAADGGAR					Mascot
974.5265	974.4619	-0.0646	-66	181	188	DTVENKLR					Mascot
1081.571	1081.5464	-0.0246	-23	213	221	YICSSKPVK			Carbamidomethyl (C)[3]		Mascot
1786.7225	1786.8097	0.0872	49	107	120	LFDEDSWSDEDLCR			Carbamidomethyl (C)[13]		Mascot
1914.9742	1914.9117	-0.0625	-33	87	104	VVMVSSVAAVSNNPNWP K			Oxidation (M)[3]		Mascot
2055.0803	2054.9504	-0.1299	-63	86	104	RVVMVSSVAAVSNNPNW PK					Mascot
2055.0803	2054.9504	-0.1299	-63	86	104	RVVMVSSVAAVSNNPNW PK					Mascot
2071.0754	2070.9636	-0.1118	-54	86	104	RVVMVSSVAAVSNNPNW PK			Oxidation (M)[4]		Mascot
2586.3159	2586.197	-0.1189	-46	189	212	NVVDVRDVADALLLAYEN PEASGR					Mascot

9 hypothetical protein TRIUR3_23027 [Triticum urartu] gi|474188442 113296.8 9.17 18 42 0 4.643

Peptide Information

Calc. Mass	Obsrv. Mass	± da	± ppm	Start Seq.	End Seq.	Sequence	Ion Score	C. I. %	Modification	Rank	Result Type
837.4326	837.4003	-0.0323	-39	36	42	SFGQRSR					Mascot
905.5064	905.5419	0.0355	39	220	226	SHQRHLK					Mascot
940.3863	940.4492	0.0629	67	378	385	CVSEGCTK			Carbamidomethyl (C)[1,6]		Mascot
952.5211	952.4574	-0.0637	-67	850	859	VPAGPVEGAR					Mascot
954.4639	954.4504	-0.0135	-14	638	646	ATHSGEQPK					Mascot
982.4523	982.4176	-0.0347	-35	230	238	FNGCSKGGR			Carbamidomethyl (C)[4]		Mascot
1057.4078	1057.4988	0.091	86	353	360	CQYPDCSK			Carbamidomethyl (C)[1,6]		Mascot
1068.5837	1068.4933	-0.0904	-85	712	721	HGPPLGYLSK					Mascot
1126.4253	1126.5062	0.0809	72	595	604	EGCTAEGSCR			Carbamidomethyl (C)[3,9]		Mascot
1145.5559	1145.5424	-0.0135	-12	702	711	RAHHGDAEPR					Mascot
1487.804	1487.7212	-0.0828	-56	821	833	VVHVPCSISKAYK			Carbamidomethyl (C)[6]		Mascot
1535.7595	1535.7308	-0.0287	-19	579	593	EVVVAGTRASGSSCR			Carbamidomethyl (C)[14]		Mascot
1578.8275	1578.7988	-0.0287	-18	140	153	KSGDYQLSPLFAPR					Mascot
1588.7611	1588.7958	0.0347	22	1	13	MDLNMTNLWLHGK			Oxidation (M)[1]		Mascot
1604.756	1604.8212	0.0652	41	1	13	MDLNMTNLWLHGK			Oxidation (M)[1,5]		Mascot
1607.7231	1607.8101	0.087	54	464	479	SAQGSTDFCKAHGGGK			Carbamidomethyl (C)[9]		Mascot
1635.7882	1635.8245	0.0363	22	236	252	GGRGASGLCISHGGGHR			Carbamidomethyl (C)[9]		Mascot

1657.6759	1657.7754	0.0995	60	605	618	CSEQDTWEATSTSR			Carbamidomethyl (C)[1]		Mascot
2039.0206	2038.9896	-0.031	-15	703	721	AHHGDAEPRHGPPLGYL SK					Mascot

10	Glutamyl-tRNA reductase 3, chloroplastic [Triticum urartu]	gi|473777000	62312.6	6.88	14	41	0	7.23	

Peptide Information

Calc. Mass	Obsrv. Mass	± da	± ppm	Start Seq.	End Seq.	Sequence	Ion Score	C. I. %	Modification	Rank	Result Type
822.3774	822.3881	0.0107	13	195	201	SECLSAR			Carbamidomethyl (C)[3]		Mascot
940.4444	940.4492	0.0048	5	444	451	MFGLDTEK					Mascot
974.5516	974.4619	-0.0897	-92	389	397	IGEDALTKK					Mascot
1057.6001	1057.4988	-0.1013	-96	91	100	KSGIPASELR					Mascot
1081.571	1081.5464	-0.0246	-23	156	165	MFKDAITAGK					Mascot
1135.5817	1135.5653	-0.0164	-14	83	91	EIVDWMSKK					Mascot
1535.7056	1535.7308	0.0252	16	4	15	CIISFCGYMKEK			Carbamidomethyl (C)[1,6]		Mascot
1590.8407	1590.8219	-0.0188	-12	339	352	KAMEAQTIISEELK					Mascot
1605.8451	1605.7703	-0.0748	-47	195	209	SECLSARMLLIGAGK			Carbamidomethyl (C)[3]		Mascot
1606.7643	1606.801	0.0367	23	101	113	EHLFMLRDSDATR			Oxidation (M)[5]		Mascot
1606.8357	1606.801	-0.0347	-22	339	352	KAMEAQTIISEELK			Oxidation (M)[3]		Mascot
1809.884	1809.8578	-0.0262	-14	354	368	FEAWRDSMETVPTIK					Mascot
1825.8789	1825.8855	0.0066	4	354	368	FEAWRDSMETVPTIK			Oxidation (M)[8]		Mascot
1855.9847	1855.8387	-0.146	-79	92	107	SGIPASELREHLFMLR					Mascot
2119.0676	2118.9736	-0.094	-44	460	478	TKGACVFPIPTNQSLNPM K			Carbamidomethyl (C)[5], Oxidation (M)[18]		Mascot
2340.1421	2340.272	0.1299	56	169	191	SETNISCGAVSVSSAAVE LALMK			Carbamidomethyl (C)[7], Oxidation (M)[22]		Mascot

Gel Idx/Pos	305/M7	Instr./Gel Origin	BA2151/Sample Project 20140814	Process Status	Analysis Succeeded
Plate [#] Name	[1] Sample Project 20140814	Instrument Sample Name		Spectra	11

Rank	Protein Name	Accession No.	Protein MW	Protein PI	Pep. Count	Protein Score	Protein Score C. I. %	Intensity Matched	Total Ion Score	Total Ion C. I. %	Confirmed
1	dehydroascorbate reductase [Triticum aestivum]	gi|28192421	23457.2	5.88	9	435	100	48.738	382	100	

Peptide Information

Calc. Mass	Obsrv. Mass	± da	± ppm	Start Seq.	End Seq.	Sequence	Ion Score	C. I. %	Modification	Rank	Result Type
909.4862	909.4279	-0.0583	-64	33	39	KVPYQMK			Oxidation (M)[6]		Mascot
944.5662	944.5034	-0.0628	-66	25	32	VLLTLEEK					Mascot
1202.682	1202.6251	-0.0569	-47	102	111	IFSTFVTFLK					Mascot
1497.8213	1497.7751	-0.0462	-31	157	168	LYHLQVALEHFK					Mascot
1497.8213	1497.7751	-0.0462	-31	157	168	LYHLQVALEHFK	82	100			Mascot
1607.8639	1607.8123	-0.0516	-32	122	135	ALVDELQALEEHLK					Mascot
1827.8444	1827.8257	-0.0187	-10	8	24	AAVGHPDTLGDCPFSQR			Carbamidomethyl (C)[12]		Mascot
1827.8444	1827.8257	-0.0187	-10	8	24	AAVGHPDTLGDCPFSQR	148	100	Carbamidomethyl (C)[12]		Mascot
1866.9484	1866.8588	-0.0896	-48	84	101	YPTPSLVTPAEYASVGSK					Mascot
2021.0338	2021.0204	-0.0134	-7	172	189	VPETLTSVHAYTEALFSR					Mascot
2021.0338	2021.0204	-0.0134	-7	172	189	VPETLTSVHAYTEALFSR	151	100			Mascot
2108.1135	2108.0623	-0.0512	-24	136	156	AHGPYINGANISAVDLSL APK					Mascot

Rank	Protein Name	Accession No.	Protein MW	Protein PI	Pep. Count	Protein Score	Protein Score C. I. %	Intensity Matched	Total Ion Score	Total Ion C. I. %	Confirmed
2	dehydroascorbate reductase [Triticum aestivum]	gi|259017810	23457.2	5.88	9	435	100	48.926	382	100	

Peptide Information

Calc. Mass	Obsrv. Mass	± da	± ppm	Start Seq.	End Seq.	Sequence	Ion Score	C. I. %	Modification	Rank	Result Type
909.4862	909.4279	-0.0583	-64	33	39	KVPYQMK			Oxidation (M)[6]		Mascot
944.5662	944.5034	-0.0628	-66	25	32	VLLTLEEK					Mascot
1202.682	1202.6251	-0.0569	-47	102	111	IFSTFVTFLK					Mascot
1497.8213	1497.7751	-0.0462	-31	157	168	LYHLQVALEHFK					Mascot
1497.8213	1497.7751	-0.0462	-31	157	168	LYHLQVALEHFK	82	100			Mascot
1607.8639	1607.8123	-0.0516	-32	122	135	ALVDELQALEEHLK					Mascot
1827.8444	1827.8257	-0.0187	-10	8	24	AAVGHPDTLGDCPFSQR			Carbamidomethyl (C)[12]		Mascot
1827.8444	1827.8257	-0.0187	-10	8	24	AAVGHPDTLGDCPFSQR	148	100	Carbamidomethyl (C)[12]		Mascot
1892.9641	1892.8944	-0.0697	-37	84	101	YPTPSLVTPPEYASVGSK					Mascot
2021.0338	2021.0204	-0.0134	-7	172	189	VPETLTSVHAYTEALFSR					Mascot

2021.0338	2021.0204	-0.0134	-7	172	189	VPETLTSVHAYTEALFSR	151	100			Mascot
2108.1135	2108.0623	-0.0512	-24	136	156	AHGPYINGANISAVDLSL APK					Mascot

3	Glutathione S-transferase DHAR2 [Triticum urartu]	gi|474023258	45260	8.3	11	425	100	49.034	382	100	

Peptide Information

Calc. Mass	Obsrv. Mass	± da	± ppm	Start Seq.	End Seq.	Sequence	Ion Score	C. I. %	Modification	Rank	Result Type
909.4862	909.4279	-0.0583	-64	78	84	KVPYQMK			Oxidation (M)[6]		Mascot
944.5662	944.5034	-0.0628	-66	70	77	VLLTLEEK					Mascot
1202.682	1202.6251	-0.0569	-47	147	156	IFSTFVTFLK					Mascot
1497.8213	1497.7751	-0.0462	-31	202	213	LYHLQVALEHFK					Mascot
1497.8213	1497.7751	-0.0462	-31	202	213	LYHLQVALEHFK	82	100			Mascot
1607.8639	1607.8123	-0.0516	-32	167	180	ALVDELQALEEHLK					Mascot
1645.8881	1645.7777	-0.1104	-67	21	34	EHASPQIDRARPLR					Mascot
1722.8381	1722.7915	-0.0466	-27	347	360	LFRTSLHMNFDDAR					Mascot
1827.8444	1827.8257	-0.0187	-10	53	69	AAVGHPDTLGDCPFSQR			Carbamidomethyl (C)[12]		Mascot
1827.8444	1827.8257	-0.0187	-10	53	69	AAVGHPDTLGDCPFSQR	148	100	Carbamidomethyl (C)[12]		Mascot
1866.8282	1866.8588	0.0306	16	387	402	QCCLLITEADELAMAE			Carbamidomethyl (C)[2,3]		Mascot
2021.0338	2021.0204	-0.0134	-7	217	234	VPETLTSVHAYTEALFSR					Mascot
2021.0338	2021.0204	-0.0134	-7	217	234	VPETLTSVHAYTEALFSR	151	100			Mascot
2108.1135	2108.0623	-0.0512	-24	181	201	AHGPYINGANISAVDLSL APK					Mascot

4	glutathione S-transferase [Triticum aestivum]	gi|5923877	23609.1	5.79	1	176	100	1.966	176	100

Protein Group

glutathione S-transferase [Triticum aestivum]	gi|14538027	23609.1	5.7899 999618 5303
glutathione transferase F1 [Triticum aestivum]	gi|23504749	23668.2	5.9600 000381 4697
unnamed protein product [Triticum aestivum]	gi|257737764	23609.1	5.7899 999618 5303
unnamed protein product [Triticum aestivum]	gi|257737348	23668.2	5.9600

			000381<br>4697
unnamed protein product [Triticum aestivum]	gi|257659932	23669.1	5.7899<br>999618<br>5303
unnamed protein product [Triticum aestivum]	gi|257343711	23609.1	5.7899<br>999618<br>5303
unnamed protein product [Triticum aestivum]	gi|257343673	23668.2	5.9600<br>000381<br>4697
unnamed protein product [Triticum aestivum]	gi|257293238	23609.1	5.7899<br>999618<br>5303
unnamed protein product [Triticum aestivum]	gi|257293200	23668.2	5.9600<br>000381<br>4697
unnamed protein product [Triticum aestivum]	gi|219899599	23609.1	5.7899<br>999618<br>5303
unnamed protein product [Triticum aestivum]	gi|219899559	23668.2	5.9600<br>000381<br>4697
unnamed protein product [Triticum aestivum]	gi|219744934	23609.1	5.7899<br>999618<br>5303
unnamed protein product [Triticum aestivum]	gi|219744894	23668.2	5.9600<br>000381<br>4697

unnamed protein product [Triticum aestivum]	gi|219740288	23609.1	5.7899 999618 5303
unnamed protein product [Triticum aestivum]	gi|219740248	23668.2	5.9600 000381 4697

Peptide Information

Calc. Mass	Obsrv. Mass	± da	± ppm	Start Seq.	End Seq.	Sequence	Ion Score	C. I. %	Modification	Rank	Result Type
2252.0984	2252.085	-0.0134	-6	50	69	NPFGQVPALQDGDLYVF ESR					Mascot
2252.0984	2252.085	-0.0134	-6	50	69	NPFGQVPALQDGDLYVF ESR	176	100			Mascot

5	DRP5 protein [Triticum durum]	gi|85857188	17705.2	5.57	7	124	100	5.205	82	100	

Peptide Information

Calc. Mass	Obsrv. Mass	± da	± ppm	Start Seq.	End Seq.	Sequence	Ion Score	C. I. %	Modification	Rank	Result Type
909.4862	909.4279	-0.0583	-64	15	21	KVPYQMK			Oxidation (M)[6]		Mascot
944.5662	944.5034	-0.0628	-66	7	14	VLLTLEEK					Mascot
1202.682	1202.6251	-0.0569	-47	84	93	IFSTFVTFLK					Mascot
1497.8213	1497.7751	-0.0462	-31	139	150	LYHLQVALEHFK					Mascot
1497.8213	1497.7751	-0.0462	-31	139	150	LYHLQVALEHFK	82	100			Mascot
1607.8639	1607.8123	-0.0516	-32	104	117	ALVDELQALEEHLK					Mascot
1866.9484	1866.8588	-0.0896	-48	66	83	YPTPSLVTPAEYASVGSK					Mascot
2108.1135	2108.0623	-0.0512	-24	118	138	AHGPYINGANISAVDLSL APK					Mascot

6	NBS-LRR resistance protein CIN14 [Triticum aestivum]	gi|302583873	100033.9	6.1	19	56	89.869	5.719

Peptide Information

Calc. Mass	Obsrv. Mass	± da	± ppm	Start Seq.	End Seq.	Sequence	Ion Score	C. I. %	Modification	Rank	Result Type
837.4651	837.4064	-0.0587	-70	612	618	TLMLFGR					Mascot
1008.4965	1008.4695	-0.027	-27	359	367	GCMVLATTR			Carbamidomethyl (C)[2]		Mascot
1057.5889	1057.4866	-0.1023	-97	95	103	LEQQIIEGK					Mascot

1102.5626	1102.5476	-0.015	-14	35	44	EIGLAEDVEK					Mascot
1347.6427	1347.6158	-0.0269	-20	518	528	LEETGHEYLEK					Mascot
1497.8384	1497.7751	-0.0633	-42	316	327	LQEILQENIRNK					Mascot
1497.8384	1497.7751	-0.0633	-42	316	327	LQEILQENIRNK					Mascot
1507.7097	1507.7048	-0.0049	-3	210	222	DHYMTTSVPIESK					Mascot
1587.707	1587.7635	0.0565	36	329	340	FLLVMDDMWEEK			Oxidation (M)[5,8]		Mascot
1589.8422	1589.8545	0.0123	8	455	468	SLQGYDDDILPILK					Mascot
1629.824	1629.8051	-0.0189	-12	47	60	FQMRYGQMVLAAAK			Oxidation (M)[3]		Mascot
1645.819	1645.7777	-0.0413	-25	47	60	FQMRYGQMVLAAAK			Oxidation (M)[3,8]		Mascot
1694.8431	1694.7759	-0.0672	-40	847	860	EWQILSTHGMHSLR					Mascot
1768.8344	1768.8822	0.0478	27	374	389	MIGTMDEITLSGLDEK			Oxidation (M)[1]		Mascot
1822.9381	1822.9509	0.0128	7	847	861	EWQILSTHGMHSLRK					Mascot
1826.0018	1825.8992	-0.1026	-56	240	259	GGSSDLNVLPVVGSIGGI GK					Mascot
1826.8452	1826.8519	0.0067	4	329	342	FLLVMDDMWEEKDR					Mascot
1892.9059	1892.8944	-0.0115	-6	29	44	MEAWTREIGLAEDVEK			Oxidation (M)[1]		Mascot
1930.9441	1930.8986	-0.0455	-24	129	145	LVCSATSQITDWISHGR			Carbamidomethyl (C)[3]		Mascot
2059.0391	2058.9429	-0.0962	-47	129	146	LVCSATSQITDWISHGRK			Carbamidomethyl (C)[3]		Mascot
2108.123	2108.0623	-0.0607	-29	862	880	LTLIGMLNLMELSVLDLST			Oxidation (M)[6,10]		Mascot

7	1-phosphatidylinositol-3-phosphate 5-kinase fab1 [Triticum urartu]	gi|473824589	177878.2	6.14	24	51	64.055	8.967

Peptide Information

Calc. Mass	Obsrv. Mass	± da	± ppm	Start Seq.	End Seq.	Sequence	Ion Score	C. I. %	Modification	Rank	Result Type
856.4312	856.3597	-0.0715	-83	1348	1354	SNVYFAR					Mascot
944.4559	944.5034	0.0475	50	751	756	IWMWHR			Oxidation (M)[3]		Mascot
979.5458	979.4794	-0.0664	-68	855	863	TVAVEIYGK					Mascot
1112.5735	1112.5459	-0.0276	-25	787	795	FLELSFSNR					Mascot
1145.5909	1145.5505	-0.0404	-35	562	571	LKQVQGDDSR					Mascot
1396.6777	1396.6927	0.015	11	303	315	GGSMDPTDYVKVK					Mascot
1412.6726	1412.703	0.0304	22	303	315	GGSMDPTDYVKVK			Oxidation (M)[4]		Mascot
1412.6726	1412.703	0.0304	22	303	315	GGSMDPTDYVKVK			Oxidation (M)[4]		Mascot
1481.7457	1481.7145	-0.0312	-21	773	786	VIMSDAAWGLSFGK					Mascot
1497.6362	1497.7751	0.1389	93	80	92	CYPVSGTGFHGCR			Carbamidomethyl (C)[1,12]		Mascot
1497.7407	1497.7751	0.0344	23	773	786	VIMSDAAWGLSFGK			Oxidation (M)[3]		Mascot
1507.7461	1507.7048	-0.0413	-27	1542	1554	NEAPTIISPMQYK			Oxidation (M)[10]		Mascot
1589.7451	1589.8545	0.1094	69	470	482	STKTLMFFEGCPR			Carbamidomethyl (C)[11], Oxidation (M)[6]		Mascot

1635.8411	1635.8202	-0.0209	-13	1542	1555	NEAPTIISPMQYKK	Oxidation (M)[10]	Mascot
1645.9207	1645.7777	-0.143	-87	674	686	SLVCERPQLFRIK	Carbamidomethyl (C)[4]	Mascot
1677.8555	1677.8026	-0.0529	-32	881	895	NIPSEDDPVKTGIHR		Mascot
1694.7474	1694.7759	0.0285	17	1418	1430	MDLMVMENLFFEK	Oxidation (M)[1,4,6]	Mascot
1706.7262	1706.7218	-0.0044	-3	545	561	GDTDCFAGSAGVGMPHK	Carbamidomethyl (C)[5]	Mascot
1722.7211	1722.7915	0.0704	41	545	561	GDTDCFAGSAGVGMPHK	Carbamidomethyl (C)[5], Oxidation (M)[14]	Mascot
1770.9246	1770.8192	-0.1054	-60	1122	1137	NAPIRIHSFDSTVASR		Mascot
1782.9572	1782.8263	-0.1309	-73	1	16	MGVVEFSVLGAVQKFR	Oxidation (M)[1]	Mascot
1783.8029	1783.8257	0.0228	13	1196	1212	SPTHISSASHMVDDGAR	Oxidation (M)[11]	Mascot
1785.88	1785.843	-0.037	-21	234	250	TVGADMITDIAHAEGLR	Oxidation (M)[6]	Mascot
1817.9644	1817.8241	-0.1403	-77	1106	1121	IDENGSLKLLDNPVYK		Mascot
1822.8424	1822.9509	0.1085	60	1418	1431	MDLMVMENLFFEKK	Oxidation (M)[1,4,6]	Mascot
1885.0034	1884.8418	-0.1616	-86	906	920	MERNECEILLLPVIR	Carbamidomethyl (C)[6]	Mascot
1904.8807	1904.8815	0.0008	0	582	597	SLCLNEEGESVVFEHR	Carbamidomethyl (C)[3]	Mascot
2002.9686	2003.072	0.1034	52	1415	1430	EVKMDLMVMENLFFEK		Mascot
2034.9585	2034.975	0.0165	8	1415	1430	EVKMDLMVMENLFFEK	Oxidation (M)[4,7]	Mascot

8	RNA polymerase beta' subunit (chloroplast) [Triticum urartu]	gi|521301418	170735.3	6.6	22	49	44.328	29.37

Protein Group

RNA polymerase beta' subunit (chloroplast) [Triticum urartu] gi|525782287 170735.3 6.5999999046 3257

Peptide Information

Calc. Mass	Obsrv. Mass	± da	± ppm	Start Seq.	End Seq.	Sequence	Ion Score	C. I. %	Modification	Rank	Result Type
909.504	909.4279	-0.0761	-84	762	769	GTKEFSLK					Mascot
944.5524	944.5034	-0.049	-52	187	195	KGVVDTAVR					Mascot
1006.4986	1006.4622	-0.0364	-36	13	21	EIDGTGMKR					Mascot
1008.5585	1008.4695	-0.089	-88	1060	1068	NSKHPNAIK					Mascot
1129.5525	1129.5466	-0.0059	-5	1360	1368	ALDESIYYR					Mascot
1347.6573	1347.6158	-0.0415	-31	225	237	GISVSPQNGMTEK					Mascot
1396.7114	1396.6927	-0.0187	-13	138	150	GNASQVHQLVGMR					Mascot
1412.7064	1412.703	-0.0034	-2	138	150	GNASQVHQLVGMR			Oxidation (M)[12]		Mascot
1412.7064	1412.703	-0.0034	-2	138	150	GNASQVHQLVGMR	2	0	Oxidation (M)[12]		Mascot
1481.7635	1481.7145	-0.049	-33	1435	1446	SPQDKNLYFEIK					Mascot

1589.7927	1589.8545	0.0618	39	476	488	TSHLWILSVSMCR		Carbamidomethyl (C)[12]	Mascot
1605.7876	1605.774	-0.0136	-8	476	488	TSHLWILSVSMCR		Carbamidomethyl (C)[12], Oxidation (M)[11]	Mascot
1622.8472	1622.7886	-0.0586	-36	285	297	AQPIYIRTPFTCR		Carbamidomethyl (C)[12]	Mascot
1628.9218	1628.8	-0.1218	-75	795	809	ILDNSIIGVNTQLTK			Mascot
1677.802	1677.8026	0.0006	0	584	596	NTIFAYFDDPRYR			Mascot
1705.8809	1705.7797	-0.1012	-59	85	98	HYYYGAVHAVEKLR			Mascot
1735.8763	1735.8351	-0.0412	-24	342	358	TFHTGGVFTGGTADLVR			Mascot
1783.8102	1783.8257	0.0155	9	1040	1054	EYHSLMILSASNCSR		Carbamidomethyl (C)[13], Oxidation (M)[6]	Mascot
1785.9606	1785.843	-0.1176	-66	922	936	LVNFISHENSKLTQR			Mascot
1827.8131	1827.8257	0.0126	7	683	698	TLEKDSEEEYGSPESK			Mascot
1827.8131	1827.8257	0.0126	7	683	698	TLEKDSEEEYGSPESK			Mascot
1928.0245	1927.9122	-0.1123	-58	473	488	LPKTSHLWILSVSMCR		Carbamidomethyl (C)[15]	Mascot
2018.0553	2017.9011	-0.1542	-76	1239	1256	SSDITQGLPKVEQIFEAR			Mascot
2034.959	2034.975	0.016	8	885	901	YFVSVRPTVAYEMDEGR		Oxidation (M)[13]	Mascot
2131.1968	2131.0413	-0.1555	-73	790	809	SSSLKILDNSIIGVNTQLTK			Mascot
2147.1604	2147.0247	-0.1357	-63	564	582	ELISCFGISIEIPLMGVLR		Carbamidomethyl (C)[5]	Mascot

9	RNA polymerase beta' subunit (chloroplast) [Triticum monococcum subsp. aegilopoides]	gi|521301340	170721.3	6.6	21	44	0	29.285

Protein Group

RNA polymerase beta' subunit (chloroplast) [Triticum monococcum]	gi|521301182	170721.3	6.5999999046 3257
RNA polymerase beta' subunit (chloroplast) [Triticum monococcum]	gi|525778497	170721.3	6.5999999046 3257

Peptide Information

Calc. Mass	Obsrv. Mass	± da	± ppm	Start Seq.	End Seq.	Sequence	Ion Score	C. I. %	Modification	Rank	Result Type
909.504	909.4279	-0.0761	-84	762	769	GTKEFSLK					Mascot
944.5524	944.5034	-0.049	-52	187	195	KGVVDTAVR					Mascot
1006.4986	1006.4622	-0.0364	-36	13	21	EIDGTGMKR					Mascot
1008.5585	1008.4695	-0.089	-88	1060	1068	NSKHPNAIK					Mascot
1129.5525	1129.5466	-0.0059	-5	1360	1368	ALDESIYYR					Mascot
1347.6573	1347.6158	-0.0415	-31	225	237	GISVSPQNGMTEK					Mascot

Calc. Mass	Obsrv. Mass	± da	± ppm	Start Seq.	End Seq.	Sequence	Ion Score	C. I. %	Modification	Rank	Result Type
1396.7114	1396.6927	-0.0187	-13	138	150	GNASQVHQLVGMR					Mascot
1412.7064	1412.703	-0.0034	-2	138	150	GNASQVHQLVGMR			Oxidation (M)[12]		Mascot
1412.7064	1412.703	-0.0034	-2	138	150	GNASQVHQLVGMR	2	0	Oxidation (M)[12]		Mascot
1481.7635	1481.7145	-0.049	-33	1435	1446	SPQDKNLYFEIK					Mascot
1589.7927	1589.8545	0.0618	39	476	488	TSHLWILSVSMCR			Carbamidomethyl (C)[12]		Mascot
1605.7876	1605.774	-0.0136	-8	476	488	TSHLWILSVSMCR			Carbamidomethyl (C)[12], Oxidation (M)[11]		Mascot
1628.9218	1628.8	-0.1218	-75	795	809	ILDNSIIGVNTQLTK					Mascot
1677.802	1677.8026	0.0006	0	584	596	NTIFAYFDDPRYR					Mascot
1705.8809	1705.7797	-0.1012	-59	85	98	HYYYGAVHAVEKLR					Mascot
1735.8763	1735.8351	-0.0412	-24	342	358	TFHTGGVFTGGTADLVR					Mascot
1783.8102	1783.8257	0.0155	9	1040	1054	EYHSLMILSASNCSR			Carbamidomethyl (C)[13], Oxidation (M)[6]		Mascot
1785.9606	1785.843	-0.1176	-66	922	936	LVNFISHENSKLTQR					Mascot
1827.8131	1827.8257	0.0126	7	683	698	TLEKDSEEEYGSPESK					Mascot
1827.8131	1827.8257	0.0126	7	683	698	TLEKDSEEEYGSPESK					Mascot
1928.0245	1927.9122	-0.1123	-58	473	488	LPKTSHLWILSVSMCR			Carbamidomethyl (C)[15]		Mascot
2018.0553	2017.9011	-0.1542	-76	1239	1256	SSDITQGLPKVEQIFEAR					Mascot
2034.959	2034.975	0.016	8	885	901	YFVSVRPTVAYEMDEGR			Oxidation (M)[13]		Mascot
2131.1968	2131.0413	-0.1555	-73	790	809	SSSLKILDNSIIGVNTQLTK					Mascot
2147.1604	2147.0247	-0.1357	-63	564	582	ELISCFGISIEIPLMGVLR			Carbamidomethyl (C)[5]		Mascot

10 hypothetical protein TRIUR3_23380 [Triticum urartu] gi|474308957 62167.9 9.11 16 44 0 7.778

Peptide Information

Calc. Mass	Obsrv. Mass	± da	± ppm	Start Seq.	End Seq.	Sequence	Ion Score	C. I. %	Modification	Rank	Result Type
944.5087	944.5034	-0.0053	-6	247	254	DEFPPVIK					Mascot
968.52	968.4636	-0.0564	-58	53	60	FYSSIVPR					Mascot
979.5393	979.4794	-0.0599	-61	186	193	MFAVLLNR			Oxidation (M)[1]		Mascot
1057.5023	1057.4866	-0.0157	-15	310	317	LFTSMWEK			Oxidation (M)[5]		Mascot
1078.5562	1078.5234	-0.0328	-30	517	526	SPSMTGKNLK			Oxidation (M)[4]		Mascot
1243.7157	1243.6611	-0.0546	-44	274	283	RVWNEIIVSK					Mascot
1347.6937	1347.6158	-0.0779	-58	90	102	VLKSCAADADLGK			Carbamidomethyl (C)[5]		Mascot
1603.8802	1603.7729	-0.1073	-67	38	52	QNALEVLPDAALPPR					Mascot
1606.8523	1606.8026	-0.0497	-31	186	199	MFAVLLNRYAGAHK			Oxidation (M)[1]		Mascot
1606.8523	1606.8026	-0.0497	-31	186	199	MFAVLLNRYAGAHK			Oxidation (M)[1]		Mascot
1622.6963	1622.7886	0.0923	57	509	523	DGPETEDRSPSMTGK			Oxidation (M)[12]		Mascot
1628.7725	1628.8	0.0275	17	374	386	TEKVYELLDDMEK			Oxidation (M)[11]		Mascot
1677.7611	1677.8026	0.0415	25	388	401	GCSPNNMTYTYILK			Carbamidomethyl (C)[2], Oxidation (M)[7]		Mascot

1781.9697	1781.8245	-0.1452	-81	53	68	FYSSIVPRGFVIGSPR		Mascot
1810.8429	1810.8756	0.0327	18	440	453	YEKGVQQVWEEMER		Mascot
1826.8378	1826.8519	0.0141	8	440	453	YEKGVQQVWEEMER	Oxidation (M)[12]	Mascot
1866.9749	1866.8588	-0.1161	-62	194	209	YAGAHKVQEAIEIFYK		Mascot
2018.0151	2017.9011	-0.114	-56	423	439	LDSDTYNLILNLYVSMK	Oxidation (M)[16]	Mascot

Gel Idx/Pos	306/M8	Instr./Gel Origin	BA2151/Sample Project 20140814	Process Status	Analysis Succeeded
Plate [#] Name	[1] Sample Project 20140814	Instrument Sample Name		Spectra	11

Rank	Protein Name	Accession No.	Protein MW	Protein PI	Pep. Count	Protein Score	Protein Score C. I. %	Intensity Matched	Total Ion Score	Total Ion C. I. %	Confirmed
1	23kDa oxygen evolving protein of photosystem II [Triticum aestivum]	gi|21837	27423.9	8.84	15	525	100	50.016	411	100	

Protein Group

RecName: Full=Oxygen-evolving enhancer protein 2, chloroplastic; Short=OEE2; AltName: Full=23 kDa subunit of oxygen evolving system of photosystem II; AltName: Full=23 kDa thylakoid membrane protein; AltName: Full=OEC 23 kDa subunit; Flags: Precurso	gi|131394	27423.9	8.8400001525 8789

Peptide Information

Calc. Mass	Obsrv. Mass	± da	± ppm	Start Seq.	End Seq.	Sequence	Ion Score	C. I. %	Modification	Rank	Result Type
849.3585	849.3956	0.0371	44	208	216	TADGDEGGK					Mascot
895.4706	895.4012	-0.0694	-78	28	34	TQLFVCK			Carbamidomethyl (C)[6]		Mascot
945.5152	945.4871	-0.0281	-30	114	121	EFPGQVLR					Mascot
945.5152	945.4871	-0.0281	-30	114	121	EFPGQVLR	59	99.991			Mascot
1230.6589	1230.6284	-0.0305	-25	112	121	EREFPGQVLR					Mascot
1243.6682	1243.6417	-0.0265	-21	198	207	QYYSITVLTR					Mascot
1243.6682	1243.6417	-0.0265	-21	198	207	QYYSITVLTR	67	99.999			Mascot
1253.6848	1253.6121	-0.0727	-58	217	228	HQLITATVADGK					Mascot
1326.6688	1326.6298	-0.039	-29	246	258	KFVENAAGSFSVA					Mascot
1434.6537	1434.5995	-0.0542	-38	88	100	NTDFVAYSGEGFK					Mascot
1434.6537	1434.5995	-0.0542	-38	88	100	NTDFVAYSGEGFK	91	100			Mascot
1562.7487	1562.6932	-0.0555	-36	87	100	KNTDFVAYSGEGFK					Mascot
1562.7487	1562.6932	-0.0555	-36	87	100	KNTDFVAYSGEGFK	82	100			Mascot
2084.0254	2083.97	-0.0554	-27	208	228	TADGDEGGKHQLITATVADGK					Mascot
2242.0874	2242.0212	-0.0662	-30	122	141	YEDNFDATSNLSVIINPTTK					Mascot
2242.0874	2242.0212	-0.0662	-30	122	141	YEDNFDATSNLSVIINPTTK	112	100			Mascot
2752.2795	2752.23	-0.0495	-18	170	197	TDSEGGFESDAVATANVLESSAPVVDGK					Mascot
2921.4204	2921.3787	-0.0417	-14	143	169	TITDYGSPEEFLSQVGFLLGQQSYGGK					Mascot
2943.6301	2943.3691	-0.261	-89	53	84	AALSLLAGAAAIAVKVSPAAAAYGEAANVFGK					Mascot
3049.5154	3049.4673	-0.0481	-16	142	169	KTITDYGSPEEFLSQVGFLLGQQSYGGK					Mascot

2	Oxygen-evolving enhancer protein 2, chloroplastic [Triticum urartu]	gi|474077556	25713.9	8.94	13	505	100	49.823	411	100

Peptide Information

Calc. Mass	Obsrv. Mass	± da	± ppm	Start Seq.	End Seq.	Sequence	Ion Score	C. I. %	Modification	Rank	Result Type
849.3585	849.3956	0.0371	44	187	195	TADGDEGGK					Mascot
945.5152	945.4871	-0.0281	-30	93	100	EFPGQVLR					Mascot
945.5152	945.4871	-0.0281	-30	93	100	EFPGQVLR	59	99.991			Mascot
1230.6589	1230.6284	-0.0305	-25	91	100	EREFPGQVLR					Mascot
1243.6682	1243.6417	-0.0265	-21	177	186	QYYSITVLTR					Mascot
1243.6682	1243.6417	-0.0265	-21	177	186	QYYSITVLTR	67	99.999			Mascot
1253.6848	1253.6121	-0.0727	-58	196	207	HQLITATVADGK					Mascot
1326.6688	1326.6298	-0.039	-29	226	238	KFVENAAGSFSVA					Mascot
1434.6537	1434.5995	-0.0542	-38	67	79	NTDFVAYSGEGFK					Mascot
1434.6537	1434.5995	-0.0542	-38	67	79	NTDFVAYSGEGFK	91	100			Mascot
1562.7487	1562.6932	-0.0555	-36	66	79	KNTDFVAYSGEGFK					Mascot
1562.7487	1562.6932	-0.0555	-36	66	79	KNTDFVAYSGEGFK	82	100			Mascot
2084.0254	2083.97	-0.0554	-27	187	207	TADGDEGGKHQLITATVA DGK					Mascot
2242.0874	2242.0212	-0.0662	-30	101	120	YEDNFDATSNLSVIINPTT K					Mascot
2242.0874	2242.0212	-0.0662	-30	101	120	YEDNFDATSNLSVIINPTT K	112	100			Mascot
2752.2795	2752.23	-0.0495	-18	149	176	TDSEGGFESDAVATANV LESSAPVVDGK					Mascot
2921.4204	2921.3787	-0.0417	-14	122	148	TITDYGSPEEFLSQVGFL LGQQSYGGK					Mascot
3049.5154	3049.4673	-0.0481	-16	121	148	KTITDYGSPEEFLSQVGF LLGQQSYGGK					Mascot

3	hypothetical protein TRIUR3_28410 [Triticum urartu]	gi|474060617	28387.9	5.53	4	79	99.944	2.321	67	99.998

Peptide Information

Calc. Mass	Obsrv. Mass	± da	± ppm	Start Seq.	End Seq.	Sequence	Ion Score	C. I. %	Modification	Rank	Result Type
971.5673	971.4952	-0.0721	-74	51	58	FVTNHLLK					Mascot
1078.535	1078.5118	-0.0232	-22	207	215	GYPLLEACR			Carbamidomethyl (C)[8]		Mascot
1145.5698	1145.5449	-0.0249	-22	156	165	GHNLSLEYGR					Mascot
1145.5698	1145.5449	-0.0249	-22	156	165	GHNLSLEYGR	67	99.998			Mascot
1927.9371	1927.9097	-0.0274	-14	35	50	VHVAIYYESLCPYSAR			Carbamidomethyl (C)[11]		Mascot

4	27K protein [Triticum aestivum]	gi|30793446	23271.5	6.06	2	73	99.763	1.676	67	99.998

Protein Group

Tri a Bd 27K, partial [Triticum aestivum]	gi|283480515	23214.5	6.0599<br>999427<br>7954

Peptide Information

Calc. Mass	Obsrv. Mass	± da	± ppm	Start Seq.	End Seq.	Sequence	Ion Score	C. I. %	Modification	Rank	Result Type
1078.535	1078.5118	-0.0232	-22	181	189	GYPLLEACR			Carbamidomethyl (C)[8]		Mascot
1145.5698	1145.5449	-0.0249	-22	133	142	GHNLSLEYGR					Mascot
1145.5698	1145.5449	-0.0249	-22	133	142	GHNLSLEYGR	67	99.998			Mascot

5	27k protein [Triticum aestivum]	gi|290350670	24399.1	6.06	2	72	99.751	1.676	67	99.998

Peptide Information

Calc. Mass	Obsrv. Mass	± da	± ppm	Start Seq.	End Seq.	Sequence	Ion Score	C. I. %	Modification	Rank	Result Type
1078.535	1078.5118	-0.0232	-22	193	201	GYPLLEACR			Carbamidomethyl (C)[8]		Mascot
1145.5698	1145.5449	-0.0249	-22	145	154	GHNLSLEYGR					Mascot
1145.5698	1145.5449	-0.0249	-22	145	154	GHNLSLEYGR	67	99.998			Mascot

6	unnamed protein product [Triticum aestivum]	gi|227248194	23269.6	5.78	10	51	62.361	1.832	

Peptide Information

Calc. Mass	Obsrv. Mass	± da	± ppm	Start Seq.	End Seq.	Sequence	Ion Score	C. I. %	Modification	Rank	Result Type
809.3709	809.3794	0.0085	11	49	55	TLEMDGK			Oxidation (M)[4]		Mascot
817.4526	817.4125	-0.0401	-49	172	179	SKAGGLER					Mascot
824.3897	824.3775	-0.0122	-15	109	115	YANDSVR					Mascot
906.4713	906.4324	-0.0389	-43	181	188	ASNLVQMK			Oxidation (M)[7]		Mascot
957.3764	957.459	0.0826	86	199	206	QKSSCCST			Carbamidomethyl (C)[5,6]		Mascot
1078.5562	1078.5118	-0.0444	-41	47	55	IRTLEMDGK			Oxidation (M)[6]		Mascot
1279.5664	1279.6042	0.0378	30	1	10	MGNEFDYLFK			Oxidation (M)[1]		Mascot
1474.7683	1474.7225	-0.0458	-31	117	129	LLVGNKCDLAESR			Carbamidomethyl (C)[7]		Mascot
1822.9255	1822.9395	0.014	8	154	170	ESINVEEAFLAMSAAIK					Mascot
1967.0154	1966.8835	-0.1319	-67	154	171	ESINVEEAFLAMSAAIKK			Oxidation (M)[12]		Mascot

7	Nudix hydrolase 23, chloroplastic [Triticum urartu]	gi|473993049	34120.8	4.86	11	50	57.769	5.321

Peptide Information

Calc. Mass	Obsrv. Mass	± da	± ppm	Start	End	Sequence	Ion	C. I. %	Modification	Rank	Result Type

				Seq.	Seq.		Score				
816.4872	816.4324	-0.0548	-67	135	140	VLLCRR			Carbamidomethyl (C)[4]		Mascot
899.4001	899.4482	0.0481	53	76	82	YMDMVPK			Oxidation (M)[2]		Mascot
943.4731	943.4584	-0.0147	-16	97	105	LAVPDGDEK					Mascot
1126.5385	1126.4995	-0.039	-35	76	84	YMDMVPKSR					Mascot
1126.5385	1126.4995	-0.039	-35	74	82	ARYMDMVPK			Oxidation (M)[4]		Mascot
1199.5078	1199.6095	0.1017	85	106	115	MRAVCSSCGR			Carbamidomethyl (C)[5,8], Oxidation (M)[1]		Mascot
1230.6147	1230.6284	0.0137	11	97	107	LAVPDGDEKMR					Mascot
1238.6099	1238.5933	-0.0166	-13	280	288	FHYCTINKR			Carbamidomethyl (C)[4]		Mascot
1265.6083	1265.587	-0.0213	-17	8	17	SLWDELMLDK			Oxidation (M)[7]		Mascot
1396.7141	1396.7544	0.0403	29	268	279	MYIEDVKAGNIK			Oxidation (M)[1]		Mascot
1997.0088	1996.9503	-0.0585	-29	289	307	IGAGPSDLRSFDIDNHLAV					Mascot

8	ATP-dependent zinc metalloprotease FTSH 5, mitochondrial [Triticum urartu]	gi|473987342	73431.1	6.15	15	46	0	19.95		

Peptide Information

Calc. Mass	Obsrv. Mass	± da	± ppm	Start Seq.	End Seq.	Sequence	Ion Score	C. I. %	Modification	Rank	Result Type
809.3934	809.3794	-0.014	-17	558	564	TKNMSGR			Oxidation (M)[4]		Mascot
961.5135	961.4619	-0.0516	-54	371	379	GVDVMTIAR					Mascot
967.4699	967.4511	-0.0188	-19	420	427	IMMGSERK			Oxidation (M)[2]		Mascot
983.4648	983.4235	-0.0413	-42	420	427	IMMGSERK			Oxidation (M)[2,3]		Mascot
1098.55	1098.6064	0.0564	51	357	365	QILEAYMSK			Oxidation (M)[7]		Mascot
1126.498	1126.4995	0.0015	1	418	426	DRIMMGSER			Oxidation (M)[4,5]		Mascot
1126.498	1126.4995	0.0015	1	418	426	DRIMMGSER			Oxidation (M)[4,5]		Mascot
1145.6208	1145.5449	-0.0759	-66	88	98	MLNRGISGAAR					Mascot
1145.6208	1145.5449	-0.0759	-66	88	98	MLNRGISGAAR					Mascot
1148.5681	1148.5115	-0.0566	-49	565	574	TSELVDEEVK					Mascot
1220.6093	1220.5409	-0.0684	-56	62	72	CVHYGVASLSK			Carbamidomethyl (C)[1]		Mascot
1226.6199	1226.618	-0.0019	-2	428	437	SMVISDQYRK					Mascot
1226.6199	1226.618	-0.0019	-2	427	436	KSMVISDQYR					Mascot
1238.6561	1238.5933	-0.0628	-51	356	365	RQILEAYMSK					Mascot
1242.6147	1242.6104	-0.0043	-3	428	437	SMVISDQYRK			Oxidation (M)[2]		Mascot
1568.8353	1568.7262	-0.1091	-70	357	370	QILEAYMSKVSTAK					Mascot
1584.8302	1584.7068	-0.1234	-78	357	370	QILEAYMSKVSTAK			Oxidation (M)[7]		Mascot
1592.7706	1592.7015	-0.0691	-43	489	502	KQMLANLDVCMGGR			Carbamidomethyl (C)[10]		Mascot

1945.8055	1945.9672	0.1617	83	400	417	ASMDGANAVGMDHFEY AK			Oxidation (M)[3,11]		Mascot
2084.1035	2083.97	-0.1335	-64	25	45	SFPPFRAPAANAPSGAG SLLR					Mascot

9 hypothetical protein TRIUR3_33505 [Triticum urartu] gi|474378462 12441.4 9.82 7 44 0 29.985

Peptide Information

Calc. Mass	Obsrv. Mass	± da	± ppm	Start Seq.	End Seq.	Sequence	Ion Score	C. I. %	Modification	Rank	Result Type
805.3556	805.376	0.0204	25	31	36	GCRCPR			Carbamidomethyl (C)[2,4]		Mascot
817.405	817.4125	0.0075	9	19	27	AAEDAAAAK					Mascot
912.4938	912.4209	-0.0729	-80	110	118	HLPTPPPPG					Mascot
945.4999	945.4871	-0.0128	-14	18	27	KAAEDAAAAK					Mascot
945.4999	945.4871	-0.0128	-14	18	27	KAAEDAAAAK	11	0			Mascot
1195.5996	1195.5626	-0.037	-31	88	96	ICKNIMLCK			Carbamidomethyl (C)[2,8], Oxidation (M)[6]		Mascot
1230.6511	1230.6284	-0.0227	-18	97	107	NMAVQSPEVKK					Mascot
1413.7168	1413.6572	-0.0596	-42	37	48	GPPYRCRPSAPR			Carbamidomethyl (C)[6]		Mascot

10 hypothetical protein TRIUR3_13626 [Triticum urartu] gi|474378477 506914.5 5.1 44 44 0 49.119

Peptide Information

Calc. Mass	Obsrv. Mass	± da	± ppm	Start Seq.	End Seq.	Sequence	Ion Score	C. I. %	Modification	Rank	Result Type
805.3798	805.376	-0.0038	-5	806	813	QAASEGSR					Mascot
816.4574	816.4324	-0.025	-31	718	724	KDVVEAR					Mascot
817.3686	817.4125	0.0439	54	1377	1383	EATEDPR					Mascot
822.4216	822.3802	-0.0414	-50	249	255	QHPPTSR					Mascot
830.389	830.3892	0.0002	0	3722	3729	EPPSSDAK					Mascot
835.4341	835.399	-0.0351	-42	1685	1691	ANEMVKK			Oxidation (M)[4]		Mascot
856.4886	856.4432	-0.0454	-53	3260	3267	SLPNQVAK					Mascot
866.3924	866.3989	0.0065	8	2722	2729	GASEMVEK			Oxidation (M)[5]		Mascot
872.4472	872.4561	0.0089	10	2603	2609	QQDPKEK					Mascot
899.4404	899.4482	0.0078	9	214	220	VMHEVER					Mascot
901.4738	901.4521	-0.0217	-24	1422	1430	VADSAPSVR					Mascot
910.4741	910.4152	-0.0589	-65	489	495	SVRYTER					Mascot
912.4897	912.4209	-0.0688	-75	2950	2958	DRAPAPGTK					Mascot
944.4796	944.4563	-0.0233	-25	1352	1360	KEAADAPSR					Mascot
945.4636	945.4871	0.0235	25	1376	1383	KEATEDPR	9	0			Mascot
945.5073	945.4871	-0.0202	-21	3747	3754	SEKPLPMK			Oxidation (M)[7]		Mascot
960.5109	960.4432	-0.0677	-70	4017	4025	TNISEAGLR					Mascot

961.481	961.4619	-0.0191	-20	806	814	QAASEGSRR		Mascot
1080.532	1080.5211	-0.0109	-10	1266	1274	YPRAEETSK		Mascot
1098.6154	1098.6064	-0.009	-8	1908	1918	KLAPAAPESSK		Mascot
1115.5579	1115.5532	-0.0047	-4	3128	3137	SDEVALPEQK		Mascot
1148.5542	1148.5115	-0.0427	-37	4234	4245	TSGDGPATSSLR		Mascot
1154.4961	1154.6008	0.1047	91	1753	1762	DTSDQEFANK		Mascot
1182.6477	1182.5923	-0.0554	-47	3864	3873	DIQPSPLKER		Mascot
1188.6121	1188.6014	-0.0107	-9	1763	1772	SDAQRWNALK		Mascot
1199.6855	1199.6095	-0.076	-63	2555	2564	DTQVSRRPIK		Mascot
1213.5332	1213.6421	0.1089	90	959	968	RYTDDEDTAK		Mascot
1220.6118	1220.5409	-0.0709	-58	3931	3942	TSGDVPTTSSLR		Mascot
1243.6066	1243.6417	0.0351	28	1107	1117	DASFPKHAQDK		Mascot
1243.6066	1243.6417	0.0351	28	1107	1117	DASFPKHAQDK		Mascot
1248.5717	1248.5892	0.0175	14	1946	1956	QVPSSDAHHDR		Mascot
1255.6827	1255.6243	-0.0584	-47	211	220	VLKVMHEVER	Oxidation (M)[5]	Mascot
1279.5947	1279.6042	0.0095	7	2722	2732	GASEMVEKEQR	Oxidation (M)[5]	Mascot
1291.6642	1291.5746	-0.0896	-69	1144	1155	TTTPQDAVHPPK		Mascot
1306.6638	1306.5751	-0.0887	-68	3517	3529	VVPASASDTQFGK		Mascot
1348.5396	1348.6049	0.0653	48	909	919	MMSQDAFEETK	Oxidation (M)[1,2]	Mascot
1389.7413	1389.668	-0.0733	-53	3483	3494	KPPPFETPVYSK		Mascot
1434.686	1434.5995	-0.0865	-60	2867	2879	AQLSEQNFAPSDK		Mascot
1434.686	1434.5995	-0.0865	-60	2867	2879	AQLSEQNFAPSDK		Mascot
1461.718	1461.7522	0.0342	23	3060	3073	KSAPSDQDSIQSAK		Mascot
1472.6509	1472.5912	-0.0597	-41	908	919	RMMSQDAFEETK		Mascot
1592.7915	1592.7015	-0.09	-57	1569	1582	HQQDPVVPSISEEK		Mascot
1786.9043	1786.8795	-0.0248	-14	3705	3721	KDVANADQTNVSQAGLR		Mascot
1928.0461	1927.9097	-0.1364	-71	1275	1292	QPRAPVSIPTHIAAQDAR		Mascot
2839.4255	2839.2737	-0.1518	-53	2300	2327	AARPTSTEGMTPTAGPA PTSKAQLPEQK	Oxidation (M)[10]	Mascot
3049.3904	3049.4673	0.0769	25	4312	4340	DIANTDQTDVSPAGLRET TSSDAMPPSEK	Oxidation (M)[24]	Mascot

Gel Idx/Pos	307/M9	Instr./Gel Origin	BA2151/Sample Project 20140814	Process Status	Analysis Succeeded
Plate [#] Name	[1] Sample Project 20140814	Instrument Sample Name		Spectra	11

Rank	Protein Name	Accession No.	Protein MW	Protein PI	Pep. Count	Protein Score	Protein Score C. I. %	Intensity Matched	Total Ion Score	Total Ion C. I. %	Confirmed
1	unnamed protein product [Triticum aestivum]	gi|296520469	28254.3	5.58	6	78	99.932	1.698	56	99.987	

Peptide Information

Calc. Mass	Obsrv. Mass	± da	± ppm	Start Seq.	End Seq.	Sequence	Ion Score	C. I. %	Modification	Rank	Result Type
805.3985	805.3553	-0.0432	-54	1	8	MAAAGVNR			Oxidation (M)[1]		Mascot
878.4189	878.4049	-0.014	-16	59	64	RWEEMK					Mascot
1112.6423	1112.5942	-0.0481	-43	95	104	LDILVNNVGR					Mascot
1458.7435	1458.7021	-0.0414	-28	209	221	SVDPEIVENTLSR					Mascot
1590.8962	1590.8569	-0.0393	-25	27	42	GIGHAIVEELVGLGAR					Mascot
1590.8962	1590.8569	-0.0393	-25	27	42	GIGHAIVEELVGLGAR	56	99.987			Mascot
1796.0024	1795.8834	-0.119	-66	192	208	IRVNSVAPGGINTELQK					Mascot

Rank	Protein Name	Accession No.	Protein MW	Protein PI	Pep. Count	Protein Score	Protein Score C. I. %	Intensity Matched	Total Ion Score	Total Ion C. I. %	Confirmed
2	Tropinone reductase 1 [Triticum urartu]	gi|473901715	26007.3	5.54	3	64	98.431	1.65	56	99.987	

Peptide Information

Calc. Mass	Obsrv. Mass	± da	± ppm	Start Seq.	End Seq.	Sequence	Ion Score	C. I. %	Modification	Rank	Result Type
1112.6423	1112.5942	-0.0481	-43	85	94	LDILVNNVGR					Mascot
1384.6777	1384.6011	-0.0766	-55	73	84	LMETAKQTFDGK			Oxidation (M)[2]		Mascot
1590.8962	1590.8569	-0.0393	-25	27	42	GIGHAIVEELVGLGAR					Mascot
1590.8962	1590.8569	-0.0393	-25	27	42	GIGHAIVEELVGLGAR	56	99.987			Mascot

Rank	Protein Name	Accession No.	Protein MW	Protein PI	Pep. Count	Protein Score	Protein Score C. I. %	Intensity Matched	Total Ion Score	Total Ion C. I. %	Confirmed
3	Pre-mRNA-splicing factor SF2 [Triticum urartu]	gi|474319872	39635.6	10.08	12	55	85.016	2.244			

Peptide Information

Calc. Mass	Obsrv. Mass	± da	± ppm	Start Seq.	End Seq.	Sequence	Ion Score	C. I. %	Modification	Rank	Result Type
822.4216	822.3672	-0.0544	-66	156	163	VEPAHGGR					Mascot
869.386	869.3177	-0.0683	-79	164	172	GSGGPSHDR					Mascot
885.3995	885.3408	-0.0587	-66	263	269	CGRSYSR			Carbamidomethyl (C)[1]		Mascot
1177.5696	1177.5364	-0.0332	-28	342	352	SSGKPEETQSK					Mascot
1210.5197	1210.5563	0.0366	30	173	188	SSSFGGGGGGGGGGGR					Mascot
1286.5835	1286.5712	-0.0123	-10	206	216	AGDVCFAEVYR			Carbamidomethyl (C)[5]		Mascot

1450.5951	1450.7036	0.1085	75	194	204	HTDYRGMDHMR			Oxidation (M)[7,10]		Mascot
1672.7899	1672.782	-0.0079	-5	156	172	VEPAHGGRGSGGPSHD R					Mascot
1795.828	1795.8834	0.0554	31	337	352	WCSGKSSGKPEETQSK			Carbamidomethyl (C)[2]		Mascot
1947.8641	1947.9255	0.0614	32	217	234	EGGGTIGIVDYTNYDDMK					Mascot
2002.7944	2002.8453	0.0509	25	136	153	DAEDACAGRDGYNFDGN R			Carbamidomethyl (C)[6]		Mascot
2839.4448	2839.2639	-0.1809	-64	312	336	SPSSCLWNFLQITSKIQIP NSFSGK			Carbamidomethyl (C)[5]		Mascot

4 hypothetical protein TRIUR3_10241 [Triticum urartu] gi|473750146 32718.3 8.53 11 49 46.834 2.188

Peptide Information

Calc. Mass	Obsrv. Mass	± da	± ppm	Start Seq.	End Seq.	Sequence	Ion Score	C. I. %	Modification	Rank	Result Type
941.4839	941.4282	-0.0557	-59	33	40	YIAFNSAR					Mascot
993.5112	993.4419	-0.0693	-70	223	230	GLESFRER					Mascot
1068.4667	1068.4587	-0.008	-7	139	146	FLENECEK			Carbamidomethyl (C)[6]		Mascot
1184.6058	1184.5255	-0.0803	-68	33	42	YIAFNSARDK					Mascot
1219.5413	1219.5609	0.0196	16	149	159	GFASESTMAYR					Mascot
1302.7389	1302.6205	-0.1184	-91	2	12	HNIKNIHTGLR					Mascot
1433.7794	1433.6365	-0.1429	-100	1	12	MHNIKNIHTGLR					Mascot
1446.7046	1446.5999	-0.1047	-72	147	159	VKGFASESTMAYR					Mascot
1491.7261	1491.649	-0.0771	-52	127	138	EVSEHFKESIMR					Mascot
1507.7209	1507.693	-0.0279	-19	127	138	EVSEHFKESIMR			Oxidation (M)[11]		Mascot
1684.767	1684.756	-0.011	-7	134	146	ESIMRFLENECEK			Carbamidomethyl (C)[11]		Mascot
2361.9973	2362.1111	0.1138	48	258	277	NNKNNNNNNNNNNEAF CPK			Carbamidomethyl (C)[18]		Mascot

5 unnamed protein product [Triticum aestivum] gi|227248194 23269.6 5.78 9 45 0 1.834

Peptide Information

Calc. Mass	Obsrv. Mass	± da	± ppm	Start Seq.	End Seq.	Sequence	Ion Score	C. I. %	Modification	Rank	Result Type
809.3709	809.3481	-0.0228	-28	49	55	TLEMDGK			Oxidation (M)[4]		Mascot
817.4526	817.3801	-0.0725	-89	172	179	SKAGGLER					Mascot
824.3897	824.4399	0.0502	61	109	115	YANDSVR					Mascot
906.4713	906.4189	-0.0524	-58	181	188	ASNLVQMK			Oxidation (M)[7]		Mascot
952.4846	952.437	-0.0476	-50	109	116	YANDSVRK					Mascot
1447.7765	1447.666	-0.1105	-76	189	200	GQPIQQQHKQK					Mascot
1838.9204	1838.8755	-0.0449	-24	154	170	ESINVEEAFLAMSAAIK			Oxidation (M)[12]		Mascot
2352.1541	2352.0896	-0.0645	-27	80	100	GAHGIIIVYDITDMESFNN VK			Oxidation (M)[14]		Mascot

2362.2	2362.1111	-0.0889	-38	1	21	MGNEFDYLFKLLLIGDSS VGK			Oxidation (M)[1]		Mascot

6	hypothetical protein TRIUR3_23949 [Triticum urartu]	gi|474201759	9062.5	9.43	8	42	0	1.399

Peptide Information

Calc. Mass	Obsrv. Mass	± da	± ppm	Start Seq.	End Seq.	Sequence	Ion Score	C. I. %	Modification	Rank	Result Type
807.3851	807.3825	-0.0026	-3	57	63	GALCCVK			Carbamidomethyl (C)[4,5]		Mascot
822.3563	822.3672	0.0109	13	34	39	WCETAR			Carbamidomethyl (C)[2]		Mascot
836.3931	836.381	-0.0121	-14	43	49	RVDGDMK			Oxidation (M)[6]		Mascot
870.5519	870.5071	-0.0448	-51	50	56	EARLLLR					Mascot
1238.5405	1238.4939	-0.0466	-38	34	42	WCETAREMR			Carbamidomethyl (C)[2]		Mascot
1302.7385	1302.6205	-0.118	-91	53	63	LLLRGALCCVK			Carbamidomethyl (C)[8,9]		Mascot
1446.7305	1446.5999	-0.1306	-90	1	13	MGMAVLGEAQRQR					Mascot
1631.7371	1631.7579	0.0208	13	64	77	DYASVYRTEICPSA			Carbamidomethyl (C)[11]		Mascot

7	hypothetical protein TRIUR3_19508 [Triticum urartu]	gi|474413405	35530.8	9.03	11	38	0	2.74

Peptide Information

Calc. Mass	Obsrv. Mass	± da	± ppm	Start Seq.	End Seq.	Sequence	Ion Score	C. I. %	Modification	Rank	Result Type
807.4319	807.3825	-0.0494	-61	148	155	STRSTAGK					Mascot
825.4539	825.3993	-0.0546	-66	229	235	IISMYAK					Mascot
836.4005	836.381	-0.0195	-23	181	187	IDLMCGK			Carbamidomethyl (C)[5]		Mascot
993.4611	993.4419	-0.0192	-19	262	268	IWWSNCK			Carbamidomethyl (C)[6]		Mascot
1149.5623	1149.5146	-0.0477	-41	262	269	IWWSNCKR			Carbamidomethyl (C)[6]		Mascot
1177.6068	1177.5364	-0.0704	-60	178	187	NVKIDLMCGK			Carbamidomethyl (C)[8]		Mascot
1259.7583	1259.6461	-0.1122	-89	270	279	SLQLQLRFVR					Mascot
1590.8053	1590.8569	0.0516	32	126	139	LMLPLLPCGNATMK			Carbamidomethyl (C)[8], Oxidation (M)[2,13]		Mascot
1590.8053	1590.8569	0.0516	32	126	139	LMLPLLPCGNATMK			Carbamidomethyl (C)[8], Oxidation (M)[2,13]		Mascot
1756.8977	1756.835	-0.0627	-36	2	16	NAALLRVAEDDLDWR					Mascot
2011.0537	2011.001	-0.0527	-26	74	90	LFLKQALNMEMAIVMAR			Oxidation (M)[9,11]		Mascot
2483.188	2483.2815	0.0935	38	229	249	IISMYAKQPASWVCAPCL SER			Carbamidomethyl (C)[14,17], Oxidation (M)[4]		Mascot

8	hypothetical protein TRIUR3_05046 [Triticum urartu]	gi|474105814	133904.5	6.6	19	38	0	4.351

Peptide Information

Calc. Mass	Obsrv. Mass	± da	± ppm	Start Seq.	End Seq.	Sequence	Ion Score	C. I. %	Modification	Rank	Result Type
807.4359	807.3825	-0.0534	-66	249	255	SVALFDR					Mascot

822.4369	822.3672	-0.0697	-85	145	150	FWRASR					Mascot
870.5771	870.5071	-0.07	-80	1081	1087	KILDLLR					Mascot
1029.4976	1029.4346	-0.063	-61	175	181	FLWNMYR					Mascot
1030.4551	1030.3969	-0.0582	-56	635	642	EMPYTFDK					Mascot
1102.4987	1102.4987	0	0	110	118	CSFIFDTGR			Carbamidomethyl (C)[1]		Mascot
1112.6133	1112.5942	-0.0191	-17	869	877	VHELLKDMK					Mascot
1177.5518	1177.5364	-0.0154	-13	875	885	DMKGNGISPDK			Oxidation (M)[2]		Mascot
1271.5792	1271.6239	0.0447	35	838	847	CKDLVQSMMK			Carbamidomethyl (C)[1], Oxidation (M)[8,9]		Mascot
1445.7383	1445.6007	-0.1376	-95	1022	1034	LPSEASYSSVIHR					Mascot
1517.7781	1517.7074	-0.0707	-47	457	468	HAKYILEDMIER					Mascot
1642.8622	1642.7039	-0.1583	-96	105	118	LIVSKCSFIFDTGR			Carbamidomethyl (C)[6]		Mascot
1646.9324	1646.7739	-0.1585	-96	330	345	SLNIEISKGSLSTVAK					Mascot
1783.8142	1783.8173	0.0031	2	351	364	KDIGDMMNFLEEWR					Mascot
1795.9198	1795.8834	-0.0364	-20	1019	1034	HGRLPSEASYSSVIHR					Mascot
1947.9626	1947.9255	-0.0371	-19	904	921	SVTMLDACIAQGLTPTNR			Carbamidomethyl (C)[8]		Mascot
2003.0056	2002.8453	-0.1603	-80	439	456	VCAYNAIIGGVFTEGLYR			Carbamidomethyl (C)[2]		Mascot
2447.2817	2447.1404	-0.1413	-58	2	23	ALLFLLDTNFISFSTTSSSNLR					Mascot
2483.2268	2483.2815	0.0547	22	763	785	TGYTNVGCAMLEALLEKGVDVSR			Carbamidomethyl (C)[8]		Mascot

9	hypothetical protein TRIUR3_27530 [Triticum urartu]	gi|474188825	63772.9	5.48	12	37	0	5.737	

Peptide Information

Calc. Mass	Obsrv. Mass	± da	± ppm	Start Seq.	End Seq.	Sequence	Ion Score	C. I. %	Modification	Rank	Result Type
843.5159	843.488	-0.0279	-33	112	118	VSRQVVR					Mascot
906.5043	906.4189	-0.0854	-94	22	29	SVDGFLLR					Mascot
945.4537	945.5183	0.0646	68	414	420	HNYNELR					Mascot
950.4625	950.3837	-0.0788	-83	175	181	MQSWRSR					Mascot
1149.5933	1149.5146	-0.0787	-68	313	321	EQELSRMLK			Oxidation (M)[7]		Mascot
1210.6249	1210.5563	-0.0686	-57	539	548	LTDMVAFKNR			Oxidation (M)[4]		Mascot
1425.6467	1425.5509	-0.0958	-67	488	499	SSCFSFTHEPVK			Carbamidomethyl (C)[3]		Mascot
1425.6467	1425.5509	-0.0958	-67	488	499	SSCFSFTHEPVK			Carbamidomethyl (C)[3]		Mascot
1433.6002	1433.6365	0.0363	25	522	533	SNYCADDYAVQK			Carbamidomethyl (C)[4]		Mascot
1453.7223	1453.7041	-0.0182	-13	98	111	APAFPPSPWGGDVR					Mascot
1684.8344	1684.756	-0.0784	-47	69	82	AHAVHTSFHEFVFR					Mascot
1795.924	1795.8834	-0.0406	-23	98	114	APAFPPSPWGGDVRVSR					Mascot
2018.8873	2018.7576	-0.1297	-64	522	538	SNYCADDYAVQKVSENR			Carbamidomethyl (C)[4]		Mascot

	2018.8873	2018.7576	-0.1297	-64	522	538	SNYCADDYAVQKVSENR			Carbamidomethyl (C)[4]		Mascot

10	Splicing factor U2af small subunit B [Triticum urartu]	gi|473993293	30289.5	7.11	8	35	0	1.813

Peptide Information

Calc. Mass	Obsrv. Mass	± da	± ppm	Start Seq.	End Seq.	Sequence	Ion Score	C. I. %	Modification	Rank	Result Type
817.4163	817.3801	-0.0362	-44	244	252	GGGDISVGR					Mascot
822.3853	822.3672	-0.0181	-22	209	215	DRGGDFR					Mascot
1074.5078	1074.5298	0.022	20	16	23	VNCPFYFK			Carbamidomethyl (C)[3]		Mascot
1302.6688	1302.6205	-0.0483	-37	2	13	AEHLASIFGTEK					Mascot
1433.7094	1433.6365	-0.0729	-51	1	13	MAEHLASIFGTEK					Mascot
1631.7821	1631.7579	-0.0242	-15	16	28	VNCPFYFKIGACR			Carbamidomethyl (C)[3,12]		Mascot
1682.7891	1682.7833	-0.0058	-3	165	178	GGYCNFMHVKQIGR			Carbamidomethyl (C)[4], Oxidation (M)[7]		Mascot
2352.0012	2352.0896	0.0884	38	74	91	MQEHFEDFYEDIYEELSK					Mascot

Gel Idx/Pos	308/M10	Instr./Gel Origin	BA2151/Sample Project 20140814	Process Status	Analysis Succeeded
Plate [#] Name	[1] Sample Project 20140814	Instrument Sample Name		Spectra	11

Rank	Protein Name	Accession No.	Protein MW	Protein PI	Pep. Count	Protein Score	Protein Score C. I. %	Intensity Matched	Total Ion Score	Total Ion C. I. %	Confirmed
1	unnamed protein product [Triticum aestivum]	gi|259439698	19555.7	5.63	4	216	100	6.059	190	100	

Protein Group

Protein Name	Accession No.	Protein MW	Protein PI
cold shock domain protein 2 [Triticum aestivum]	gi|42391853	19555.7	5.6300001144 4092
putative glycine-rich protein [Triticum aestivum]	gi|40363759	19555.7	5.6300001144 4092
unnamed protein product [Triticum aestivum]	gi|300645713	19555.7	5.6300001144 4092

Peptide Information

Calc. Mass	Obsrv. Mass	± da	± ppm	Start Seq.	End Seq.	Sequence	Ion Score	C. I. %	Modification	Rank	Result Type
1579.6304	1579.5938	-0.0366	-23	160	178	DCPQGGGGGGGGYGGGGGR			Carbamidomethyl (C)[2]		Mascot
2107.9778	2107.9207	-0.0571	-27	44	62	SLNENDVVEFEVITGDDGR					Mascot
2107.9778	2107.9207	-0.0571	-27	44	62	SLNENDVVEFEVITGDDGR	190	100			Mascot
2380.1458	2380.0764	-0.0694	-29	17	38	GFGFISPEDGSEDLFVHQSAIK					Mascot
3350.3252	3350.2742	-0.051	-15	93	137	GGGGYGGGGYGGGGGGYGGGGGGYGGGGGSYGGGGGYGGGGGGGR					Mascot

Rank	Protein Name	Accession No.	Protein MW	Protein PI	Pep. Count	Protein Score	Protein Score C. I. %	Intensity Matched	Total Ion Score	Total Ion C. I. %	Confirmed
2	hypothetical protein TRIUR3_32194 [Triticum urartu]	gi|474207867	19544.8	9.83	8	42	0	3.101			

Peptide Information

Calc. Mass	Obsrv. Mass	± da	± ppm	Start Seq.	End Seq.	Sequence	Ion Score	C. I. %	Modification	Rank	Result Type
808.3505	808.3643	0.0138	17	115	121	TSEPSCK			Carbamidomethyl (C)[6]		Mascot
1042.5428	1042.46	-0.0828	-79	19	27	SGYLYRGAR					Mascot
1276.6016	1276.5819	-0.0197	-15	60	71	DGDDNGTVKIDK					Mascot

Calc. Mass	Obsrv. Mass	± da	± ppm	Start Seq.	End Seq.	Sequence	Ion Score	C. I. %	Modification	Rank	Result Type
1323.6692	1323.6052	-0.064	-48	14	24	NEPPKSGYLYR					Mascot
1487.7067	1487.6934	-0.0133	-9	152	163	FDFSKHPYAYGR					Mascot
1490.7961	1490.7118	-0.0843	-57	46	59	LAVADEIHTHVASK					Mascot
1837.8837	1837.8822	-0.0015	-1	137	151	CKCFSPHISGQTFLR			Carbamidomethyl (C)[1,3]		Mascot
1908.9379	1908.7632	-0.1747	-92	28	45	RPAGLSCSGTTTIRCGSK			Carbamidomethyl (C)[7,15]		Mascot

3	hypothetical protein TRIUR3_30879 [Triticum urartu]	gi|472909785	11663.1	9.09	7	41	0	3.611			

Peptide Information

Calc. Mass	Obsrv. Mass	± da	± ppm	Start Seq.	End Seq.	Sequence	Ion Score	C. I. %	Modification	Rank	Result Type
807.4108	807.3381	-0.0727	-90	92	97	NRDQFK					Mascot
1065.5551	1065.4583	-0.0968	-91	1	10	MAAGGVWVFK					Mascot
1194.5824	1194.5653	-0.0171	-14	82	91	STHMYDVVVK			Oxidation (M)[4]		Mascot
1379.6987	1379.6514	-0.0473	-34	25	36	ALVYVPANETMR			Oxidation (M)[11]		Mascot
1479.6744	1479.6942	0.0198	13	11	23	NGVMELEQEATSR			Oxidation (M)[4]		Mascot
1491.7988	1491.6946	-0.1042	-70	24	36	KALVYVPANETMR					Mascot
1507.7937	1507.6915	-0.1022	-68	24	36	KALVYVPANETMR			Oxidation (M)[12]		Mascot
1507.7937	1507.6915	-0.1022	-68	24	36	KALVYVPANETMR			Oxidation (M)[12]		Mascot
2510.2168	2510.0449	-0.1719	-68	1	23	MAAGGVWVFKNGVMEL EQEATSR					Mascot

4	hypothetical protein TRIUR3_08300 [Triticum urartu]	gi|474193236	20749.5	10.11	6	35	0	1.45	8	0	

Peptide Information

Calc. Mass	Obsrv. Mass	± da	± ppm	Start Seq.	End Seq.	Sequence	Ion Score	C. I. %	Modification	Rank	Result Type
1037.556	1037.4777	-0.0783	-75	47	55	ATVNRFMAK					Mascot
1141.5823	1141.504	-0.0783	-69	52	61	FMAKGTPAYR					Mascot
1232.6165	1232.5566	-0.0599	-49	36	46	TGRDAQIAMNR					Mascot
1475.7384	1475.7074	-0.031	-21	39	51	DAQIAMNRATVNR	8	0	Oxidation (M)[6]		Mascot
1490.7711	1490.7118	-0.0593	-40	106	119	TGSSFIRPASPTNR					Mascot
2138.9707	2138.9399	-0.0308	-14	146	164	THMADSSSAYRSMASLV HR			Oxidation (M)[3,13]		Mascot

5	hypothetical protein TRIUR3_19950 [Triticum urartu]	gi|473732842	117587	9.66	10	33	0	2.983	20	34.786	

Peptide Information

Calc. Mass	Obsrv. Mass	± da	± ppm	Start Seq.	End Seq.	Sequence	Ion Score	C. I. %	Modification	Rank	Result Type
842.4843	842.4788	-0.0055	-7	668	674	LTPAERR					Mascot
1060.5786	1060.511	-0.0676	-64	496	504	LYAETKHAK					Mascot

Calc. Mass	Obsrv. Mass	± da	± ppm	Start Seq.	End Seq.	Sequence	Ion Score	C. I. %	Modification	Rank	Result Type
1127.548	1127.4773	-0.0707	-63	903	911	NYDLYLGNR					Mascot
1194.663	1194.5653	-0.0977	-82	754	763	GYNFLKTVPR					Mascot
1277.6016	1277.6653	0.0637	50	639	649	QLSRGGNMDQR			Oxidation (M)[8]		Mascot
1339.6389	1339.6162	-0.0227	-17	393	403	IGLEEGWNEHR					Mascot
1434.7886	1434.7098	-0.0788	-55	349	361	GRLALLMAPSSYR	20	34.786			Mascot
1490.7573	1490.7118	-0.0455	-31	246	258	AAMVHTWGGYLIR			Oxidation (M)[3]		Mascot
1491.818	1491.6946	-0.1234	-83	864	875	GNEIFTVRRPFR					Mascot
2136.0415	2135.9487	-0.0928	-43	546	566	DEVGTSSIQGSGLTVSG REEK					Mascot

6 hypothetical protein TRIUR3_09957 [Triticum urartu] gi|474355150 11617.8 5.5 5 32 0 1.541

Peptide Information

Calc. Mass	Obsrv. Mass	± da	± ppm	Start Seq.	End Seq.	Sequence	Ion Score	C. I. %	Modification	Rank	Result Type
998.4036	998.435	0.0314	31	40	46	MWEDFDR					Mascot
1014.3986	1014.4041	0.0055	5	40	46	MWEDFDR			Oxidation (M)[1]		Mascot
1125.5093	1125.4442	-0.0651	-58	1	11	MGSLGSSDVEK			Oxidation (M)[1]		Mascot
1265.6155	1265.583	-0.0325	-26	1	12	MGSLGSSDVEKR					Mascot
1487.7642	1487.6934	-0.0708	-48	54	66	NVPYYLSVNPHGK					Mascot
2136.1099	2135.9487	-0.1612	-75	47	66	GHVAGARNVPYYLSVNP HGK					Mascot

7 hypothetical protein TRIUR3_16366 [Triticum urartu] gi|473726607 11451.7 5.07 6 32 0 2.308

Peptide Information

Calc. Mass	Obsrv. Mass	± da	± ppm	Start Seq.	End Seq.	Sequence	Ion Score	C. I. %	Modification	Rank	Result Type
810.3232	810.2953	-0.0279	-34	17	23	CAEMGSR			Carbamidomethyl (C)[1]		Mascot
826.3182	826.3212	0.003	4	17	23	CAEMGSR			Carbamidomethyl (C)[1], Oxidation (M)[4]		Mascot
982.4193	982.403	-0.0163	-17	17	24	CAEMGSRR			Carbamidomethyl (C)[1], Oxidation (M)[4]		Mascot
1232.6093	1232.5566	-0.0527	-43	2	12	AAEIDGMALWR					Mascot
1235.5984	1235.562	-0.0364	-29	13	23	LGVRCAEMGSR			Carbamidomethyl (C)[5]		Mascot
1379.6447	1379.6514	0.0067	5	1	12	MAAEIDGMALWR			Oxidation (M)[1]		Mascot
1657.8843	1657.7584	-0.1259	-76	2	16	AAEIDGMALWRLGVR					Mascot

8 hypothetical protein TRIUR3_12298 [Triticum urartu] gi|473734609 9794.7 4.47 5 32 0 1.31

Peptide Information

Calc. Mass	Obsrv. Mass	± da	± ppm	Start Seq.	End Seq.	Sequence	Ion Score	C. I. %	Modification	Rank	Result Type

993.4999	993.4412	-0.0587	-59	59	67	AKDFGEQAK					Mascot
1033.516	1033.4742	-0.0418	-40	51	60	GASEVDEKAK					Mascot
1339.576	1339.6162	0.0402	30	2	14	GQAYNDAADATDK					Mascot
1390.6445	1390.6479	0.0034	2	29	43	GVAEAAAEAGSGNTEK					Mascot
1490.6969	1490.7118	0.0149	10	44	58	AAEEAGKGASEVDEK					Mascot

9 unnamed protein product [Triticum aestivum] gi|227248200 23205.5 5.28 7 31 0 1.833

Peptide Information

Calc. Mass	Obsrv. Mass	± da	± ppm	Start Seq.	End Seq.	Sequence	Ion Score	C. I. %	Modification	Rank	Result Type
1006.4622	1006.3874	-0.0748	-74	174	182	MASQPTAER			Oxidation (M)[1]		Mascot
1118.5623	1118.4744	-0.0879	-79	174	183	MASQPTAERK					Mascot
1232.6052	1232.5566	-0.0486	-39	172	182	NKMASQPTAER					Mascot
1252.5732	1252.5413	-0.0319	-25	2	11	STSEYDYLFK					Mascot
1383.6138	1383.6151	0.0013	1	1	11	MSTSEYDYLFK					Mascot
1460.7778	1460.704	-0.0738	-51	118	130	LLVGNKCDLVDSK			Carbamidomethyl (C)[7]		Mascot
1707.8107	1707.7307	-0.08	-47	124	138	CDLVDSKVVDTEEAK			Carbamidomethyl (C)[1]		Mascot

10 Mitochondrial import inner membrane translocase subunit Tim8 [Triticum urartu] gi|474250473 12948.1 6.56 6 31 0 3.809

Peptide Information

Calc. Mass	Obsrv. Mass	± da	± ppm	Start Seq.	End Seq.	Sequence	Ion Score	C. I. %	Modification	Rank	Result Type
948.4316	948.3737	-0.0579	-61	100	106	EDNRQMR					Mascot
1193.5586	1193.5719	0.0133	11	90	99	GSWEDFSLPR					Mascot
1308.7345	1308.6113	-0.1232	-94	59	69	YLDMSVIIAKR					Mascot
1491.7413	1491.6946	-0.0467	-31	104	116	QMRVGVDFAPQPF					Mascot
1507.7363	1507.6915	-0.0448	-30	104	116	QMRVGVDFAPQPF			Oxidation (M)[2]		Mascot
1507.7363	1507.6915	-0.0448	-30	104	116	QMRVGVDFAPQPF			Oxidation (M)[2]		Mascot
1707.7722	1707.7307	-0.0415	-24	90	103	GSWEDFSLPREDNR					Mascot
2135.9302	2135.9487	0.0185	9	18	34	MMMSEMVTKLTNVCWDK			Carbamidomethyl (C)[14], Oxidation (M)[1,2]		Mascot

Gel Idx/Pos	309/M11	Instr./Gel Origin	BA2151/Sample Project 20140814	Process Status	Analysis Succeeded
Plate [#] Name	[1] Sample Project 20140814	Instrument Sample Name		Spectra	11

Rank	Protein Name	Accession No.	Protein MW	Protein PI	Pep. Count	Protein Score	Protein Score C. I. %	Intensity Matched	Total Ion Score	Total Ion C. I. %	Confirmed
1	small heat shock protein Hsp23.5 [Triticum aestivum]	gi|4138869	23448	6.22	6	224	100	5.515	203	100	

Peptide Information

Calc. Mass	Obsrv. Mass	± da	± ppm	Start Seq.	End Seq.	Sequence	Ion Score	C. I. %	Modification	Rank	Result Type
1033.5459	1033.4652	-0.0807	-78	186	194	AEMKNGVLR			Oxidation (M)[3]		Mascot
1035.547	1035.4945	-0.0525	-51	172	180	LELAGDVYR					Mascot
1035.547	1035.4945	-0.0525	-51	172	180	LELAGDVYR	28	90.255			Mascot
1174.5852	1174.5254	-0.0598	-51	80	90	DPFSAPQSLGR					Mascot
1174.5852	1174.5254	-0.0598	-51	80	90	DPFSAPQSLGR	78	100			Mascot
1485.7373	1485.6854	-0.0519	-35	67	79	DIGVPSFFSDVFR					Mascot
1485.7373	1485.6854	-0.0519	-35	67	79	DIGVPSFFSDVFR	98	100			Mascot
1770.881	1770.8145	-0.0665	-38	65	79	ERDIGVPSFFSDVFR					Mascot
1788.8796	1788.7855	-0.0941	-53	91	108	LLSMLDDVAAASPDGAAR			Oxidation (M)[4]		Mascot

Rank	Protein Name	Accession No.	Protein MW	Protein PI	Pep. Count	Protein Score	Protein Score C. I. %	Intensity Matched	Total Ion Score	Total Ion C. I. %	Confirmed
2	alpha purothionin, partial [Triticum aestivum]	gi|402485682	12724.6	5.04	4	204	100	6.815	183	100	

Peptide Information

Calc. Mass	Obsrv. Mass	± da	± ppm	Start Seq.	End Seq.	Sequence	Ion Score	C. I. %	Modification	Rank	Result Type
835.3913	835.3531	-0.0382	-46	25	31	LCAGVCR			Carbamidomethyl (C)[2,6]		Mascot
841.3477	841.3763	0.0286	34	1	6	MKSCCR			Carbamidomethyl (C)[4,5]		Mascot
999.4135	999.3727	-0.0408	-41	12	18	NCYNLCR			Carbamidomethyl (C)[2,6]		Mascot
999.4135	999.3727	-0.0408	-41	12	18	NCYNLCR	41	99.486	Carbamidomethyl (C)[2,6]		Mascot
2456.0706	2455.999	-0.0716	-29	47	67	LALESNSDEPDTIEYCNLGCR			Carbamidomethyl (C)[16,20]		Mascot
2456.0706	2455.999	-0.0716	-29	47	67	LALESNSDEPDTIEYCNLGCR	142	100	Carbamidomethyl (C)[16,20]		Mascot

Rank	Protein Name	Accession No.	Protein MW	Protein PI	Pep. Count	Protein Score	Protein Score C. I. %	Intensity Matched	Total Ion Score	Total Ion C. I. %	Confirmed
3	alpha2 purothionin [Triticum aestivum]	gi|21848	14371.6	4.76	3	197	100	6.536	183	100	

Protein Group

RecName: Full=Alpha-1-purothionin; AltName: Full=Purothionin A-II; Contains: RecName: Full=Alpha-1-purothionin; Contains: RecName: Full=Acidic protein; Flags: Precursor	gi|401182	14371.6	4.76000022888184

alpha purothionin [Triticum aestivum]	gi|4007850	15475.2	5.1300 001144 4092

Peptide Information

Calc. Mass	Obsrv. Mass	± da	± ppm	Start Seq.	End Seq.	Sequence	Ion Score	C. I. %	Modification	Rank	Result Type
835.3913	835.3531	-0.0382	-46	40	46	LCAGVCR			Carbamidomethyl (C)[2,6]		Mascot
999.4135	999.3727	-0.0408	-41	27	33	NCYNLCR			Carbamidomethyl (C)[2,6]		Mascot
999.4135	999.3727	-0.0408	-41	27	33	NCYNLCR	41	99.486	Carbamidomethyl (C)[2,6]		Mascot
2456.0706	2455.999	-0.0716	-29	62	82	LALESNSDEPDTIEYCNL GCR			Carbamidomethyl (C)[16,20]		Mascot
2456.0706	2455.999	-0.0716	-29	62	82	LALESNSDEPDTIEYCNL GCR	142	100	Carbamidomethyl (C)[16,20]		Mascot

4	alpha 1 purothionin precursor [Triticum aestivum]	gi|241995021	15403.1	5.13	2	192	100	5.567	183	100

Protein Group

R59-thionin precursor [Triticum aestivum]	gi|197111876	15403.1	5.1300 001144 4092
RecName: Full=Alpha-2-purothionin; Contains: RecName: Full=Alpha-2-purothionin; Contains: RecName: Full=Acidic protein; Flags: Precursor	gi|401184	15403.1	5.1300 001144 4092
alpha purothionin [Triticum aestivum]	gi|4007848	15403.1	5.1300 001144 4092
alpha-1 purothionin [Triticum aestivum]	gi|1304262	15387.1	5.1300 001144 4092
alpha1 purothionin [Triticum aestivum]	gi|21846	15403.1	5.1300 001144 4092

Peptide Information

Calc. Mass	Obsrv. Mass	± da	± ppm	Start	End	Sequence	Ion	C. I. %	Modification	Rank	Result Type

				Seq.	Seq.		Score				
999.4135	999.3727	-0.0408	-41	38	44	NCYNLCR			Carbamidomethyl (C)[2,6]		Mascot
999.4135	999.3727	-0.0408	-41	38	44	NCYNLCR	41	99.486	Carbamidomethyl (C)[2,6]		Mascot
2456.0706	2455.999	-0.0716	-29	73	93	LALESNSDEPDTIEYCNL GCR			Carbamidomethyl (C)[16,20]		Mascot
2456.0706	2455.999	-0.0716	-29	73	93	LALESNSDEPDTIEYCNL GCR	142	100	Carbamidomethyl (C)[16,20]		Mascot

5	alpha purothionin [Triticum aestivum]	gi|5912388	10093.4	4.24	3	160	100	2.893	142	100

Peptide Information

Calc. Mass	Obsrv. Mass	± da	± ppm	Start Seq.	End Seq.	Sequence	Ion Score	C. I. %	Modification	Rank	Result Type
835.3913	835.3531	-0.0382	-46	4	10	LCAGVCR			Carbamidomethyl (C)[2,6]		Mascot
932.4869	932.4337	-0.0532	-57	13	21	IASGLSCPK			Carbamidomethyl (C)[7]		Mascot
2456.0706	2455.999	-0.0716	-29	26	46	LALESNSDEPDTIEYCNL GCR			Carbamidomethyl (C)[16,20]		Mascot
2456.0706	2455.999	-0.0716	-29	26	46	LALESNSDEPDTIEYCNL GCR	142	100	Carbamidomethyl (C)[16,20]		Mascot

6	purothionin beta	gi|229588	5379.6	9.54	2	54	83.187	4.205	41	99.486

Protein Group

Chain A, Structure Of Beta-Purothionin At Room Temperature And 1.7 Angstroms Resolution	gi|157830318	5379.6	9.5399 999618 5303

Peptide Information

Calc. Mass	Obsrv. Mass	± da	± ppm	Start Seq.	End Seq.	Sequence	Ion Score	C. I. %	Modification	Rank	Result Type
999.4135	999.3727	-0.0408	-41	11	17	NCYNLCR			Carbamidomethyl (C)[2,6]		Mascot
999.4135	999.3727	-0.0408	-41	11	17	NCYNLCR	41	99.486	Carbamidomethyl (C)[2,6]		Mascot
1068.4926	1068.4742	-0.0184	-17	2	10	SCCKSTLGR			Carbamidomethyl (C)[2,3]		Mascot

7	purothionin AII	gi|350008	5278.5	9.75	2	54	82.395	4.864	41	99.486

Protein Group

Chain A, Structure Of Alpha-1-Purothionin At Room Temperature And 2.8 Angstroms Resolution	gi|157835697	5278.5	9.75

Peptide Information

Calc. Mass	Obsrv. Mass	± da	± ppm	Start Seq.	End Seq.	Sequence	Ion Score	C. I. %	Modification	Rank	Result Type

Calc. Mass	Obsrv. Mass	± da	± ppm	Start Seq.	End Seq.	Sequence	Ion Score	C. I. %	Modification	Rank	Result Type
835.3913	835.3531	-0.0382	-46	24	30	LCAGVCR			Carbamidomethyl (C)[2,6]		Mascot
999.4135	999.3727	-0.0408	-41	11	17	NCYNLCR			Carbamidomethyl (C)[2,6]		Mascot
999.4135	999.3727	-0.0408	-41	11	17	NCYNLCR	41	99.486	Carbamidomethyl (C)[2,6]		Mascot

8	beta purothionin [Triticum monococcum subsp. monococcum]	gi|218767014	15458.1	4.94	3	53	78.834	4.457	41	99.486	

Peptide Information

Calc. Mass	Obsrv. Mass	± da	± ppm	Start Seq.	End Seq.	Sequence	Ion Score	C. I. %	Modification	Rank	Result Type
932.487	932.4337	-0.0533	-57	60	68	LTSGLGCPK			Carbamidomethyl (C)[7]		Mascot
999.4135	999.3727	-0.0408	-41	38	44	NCYNLCR			Carbamidomethyl (C)[2,6]		Mascot
999.4135	999.3727	-0.0408	-41	38	44	NCYNLCR	41	99.486	Carbamidomethyl (C)[2,6]		Mascot
1068.4926	1068.4742	-0.0184	-17	29	37	SCCKSTLGR			Carbamidomethyl (C)[2,3]		Mascot

9	hypothetical protein TRIUR3_16366 [Triticum urartu]	gi|473726607	11451.7	5.07	8	50	56.785	3.495			

Peptide Information

Calc. Mass	Obsrv. Mass	± da	± ppm	Start Seq.	End Seq.	Sequence	Ion Score	C. I. %	Modification	Rank	Result Type
810.3232	810.3514	0.0282	35	17	23	CAEMGSR			Carbamidomethyl (C)[1]		Mascot
982.4193	982.396	-0.0233	-24	17	24	CAEMGSRR			Carbamidomethyl (C)[1], Oxidation (M)[4]		Mascot
1232.6093	1232.553	-0.0563	-46	2	12	AAEIDGMALWR					Mascot
1235.5984	1235.4944	-0.104	-84	13	23	LGVRCAEMGSR			Carbamidomethyl (C)[5]		Mascot
1235.5984	1235.4944	-0.104	-84	13	23	LGVRCAEMGSR			Carbamidomethyl (C)[5]		Mascot
1379.6447	1379.6552	0.0105	8	1	12	MAAEIDGMALWR			Oxidation (M)[1]		Mascot
1657.8843	1657.7355	-0.1488	-90	2	16	AAEIDGMALWRLGVR					Mascot
1770.9425	1770.8145	-0.128	-72	80	93	IILNFARYVEEFEK					Mascot
1788.9248	1788.7855	-0.1393	-78	1	16	MAAEIDGMALWRLGVR					Mascot

10	Purothionin A-1 [Triticum urartu]	gi|474271605	15491.1	4.78	2	48	34.591	4.205	41	99.486	

Protein Group

RecName: Full=Purothionin A-1; AltName: Full=Beta-purothionin; AltName: Full=Purothionin A-I; Contains: RecName: Full=Purothionin A-1; Contains: RecName: Full=Acidic protein; Flags: Precursor	gi|2851607	15470.2	4.9400 000572 2046
beta purothionin [Triticum monococcum subsp. aegilopoides]	gi|218767004	15484.2	5.1500 000953 6743

beta purothionin [Triticum monococcum subsp. aegilopoides]	gi|218767002	15488.1	4.9400 000572 2046
beta purothionin [Triticum monococcum subsp. aegilopoides]	gi|218767000	15416.1	5.1100 001335 144
beta purothionin [Triticum monococcum subsp. aegilopoides]	gi|218766998	15488.1	4.9400 000572 2046
beta purothionin [Triticum monococcum subsp. aegilopoides]	gi|218766988	15488.1	4.9400 000572 2046
beta purothionin [Triticum monococcum subsp. aegilopoides]	gi|218766981	15514.2	4.9400 000572 2046
beta purothionin [Triticum monococcum subsp. aegilopoides]	gi|218766977	15474.1	4.9400 000572 2046
beta purothionin [Triticum monococcum subsp. aegilopoides]	gi|218766975	15488.1	4.9400 000572 2046
beta purothionin [Triticum monococcum subsp. aegilopoides]	gi|218766979	15488.1	4.9400 000572 2046
beta purothionin [Triticum monococcum subsp. aegilopoides]	gi|218766986	15488.1	4.9400 000572 2046

beta purothionin [Triticum monococcum subsp. aegilopoides]	gi|218766992	15488.1	4.9400 000572 2046
beta purothionin [Triticum monococcum subsp. aegilopoides]	gi|218766994	15488.1	4.9400 000572 2046
beta purothionin [Triticum monococcum subsp. aegilopoides]	gi|218766996	15488.1	4.9400 000572 2046
beta purothionin [Triticum monococcum subsp. monococcum]	gi|218767024	15488.1	4.9400 000572 2046
beta purothionin [Triticum monococcum subsp. monococcum]	gi|218767022	15502.1	4.8299 999237 0605
beta purothionin [Triticum monococcum subsp. monococcum]	gi|218767020	15488.1	4.9400 000572 2046
beta purothionin [Triticum monococcum subsp. monococcum]	gi|218767016	15488.1	4.9400 000572 2046
beta purothionin [Triticum monococcum subsp. monococcum]	gi|218767008	15454.2	4.9400 000572 2046
beta purothionin [Triticum monococcum subsp. monococcum]	gi|218767010	15488.1	4.9400 000572 2046
beta purothionin [Triticum monococcum subsp. monococcum]	gi|218767012	15488.1	4.9400 000572

			2046
beta purothionin [Triticum monococcum subsp. monococcum]	gi|218767018	15488.1	4.9400 000572 2046
beta purothionin [Triticum monococcum subsp. monococcum]	gi|218767006	15488.1	4.9400 000572 2046
beta purothionin [Triticum urartu]	gi|218767043	15488.1	4.9400 000572 2046
beta purothionin [Triticum urartu]	gi|218767039	15557.2	5.1500 000953 6743
beta purothionin [Triticum urartu]	gi|218767037	15488.1	4.9400 000572 2046
beta purothionin [Triticum urartu]	gi|218767035	15488.1	4.9400 000572 2046
beta purothionin [Triticum urartu]	gi|218767031	15488.1	4.9400 000572 2046
beta purothionin [Triticum urartu]	gi|218767029	15502.2	5.1500 000953 6743
beta purothionin [Triticum urartu]	gi|218767026	15488.1	4.9400 000572 2046

Peptide Information

Calc. Mass	Obsrv. Mass	± da	± ppm	Start Seq.	End Seq.	Sequence	Ion Score	C. I. %	Modification	Rank	Result Type
999.4135	999.3727	-0.0408	-41	38	44	NCYNLCR			Carbamidomethyl (C)[2,6]		Mascot
999.4135	999.3727	-0.0408	-41	38	44	NCYNLCR	41	99.522	Carbamidomethyl (C)[2,6]		Mascot
1068.4926	1068.4742	-0.0184	-17	29	37	SCCKSTLGR			Carbamidomethyl (C)[2,3]		Mascot

Gel Idx/Pos	310/M12	Instr./Gel Origin	BA2151/Sample Project 20140814	Process Status	Analysis Succeeded
Plate [#] Name	[1] Sample Project 20140814	Instrument Sample Name		Spectra	11

Rank	Protein Name	Accession No.	Protein MW	Protein PI	Pep. Count	Protein Score	Protein Score C. I. %	Intensity Matched	Total Ion Score	Total Ion C. I. %	Confirmed
1	CM2 protein [Triticum durum]	gi|21920	16019.7	6.86	5	312	100	31.26	282	100	

Protein Group

Protein Name	Accession No.	Protein MW	Protein PI
CM2 protein - durum wheat	gi|100833	16019.7	6.8600001335144
RecName: Full=Alpha-amylase/trypsin inhibitor CM2; AltName: Full=Chloroform/methanol-soluble protein CM2; Flags: Precursor	gi|123956	16019.7	6.8600001335144

Peptide Information

Calc. Mass	Obsrv. Mass	± da	± ppm	Start Seq.	End Seq.	Sequence	Ion Score	C. I. %	Modification	Rank	Result Type
814.3876	814.3585	-0.0291	-36	107	113	DLPGCPR			Carbamidomethyl (C)[5]		Mascot
1017.5211	1017.4822	-0.0389	-38	97	106	TSDPNSGVLK					Mascot
1278.5532	1278.514	-0.0392	-31	77	86	ELYDASQHCR			Carbamidomethyl (C)[9]		Mascot
1278.5532	1278.514	-0.0392	-31	77	86	ELYDASQHCR	83	100	Carbamidomethyl (C)[9]		Mascot
2703.3044	2703.2476	-0.0568	-21	46	71	EYVAQQTCGVGIVGSPV STEPGNTPR			Carbamidomethyl (C)[8]		Mascot
2703.3044	2703.2476	-0.0568	-21	46	71	EYVAQQTCGVGIVGSPV STEPGNTPR	199	100	Carbamidomethyl (C)[8]		Mascot
2726.3101	2726.219	-0.0911	-33	122	145	VLVTPGHCNVMTVHNTP YCLGLDI			Carbamidomethyl (C)[8,19], Oxidation (M)[11]		Mascot

Rank	Protein Name	Accession No.	Protein MW	Protein PI	Pep. Count	Protein Score	Protein Score C. I. %	Intensity Matched	Total Ion Score	Total Ion C. I. %	Confirmed
2	putative alpha-amylase inhibitor CM2, partial [Triticum aestivum]	gi|283465827	13566.3	5.83	5	293	100	15.541	258	100	

Peptide Information

Calc. Mass	Obsrv. Mass	± da	± ppm	Start Seq.	End Seq.	Sequence	Ion Score	C. I. %	Modification	Rank	Result Type
814.3876	814.3585	-0.0291	-36	82	88	DLPGCPR			Carbamidomethyl (C)[5]		Mascot
1017.5211	1017.4822	-0.0389	-38	72	81	TSDPNSGVLK					Mascot
2241.9727	2241.9055	-0.0672	-30	1	20	TGPYCYPGMGLPSNPLE GCR			Carbamidomethyl (C)[5,19], Oxidation (M)[9]		Mascot
2241.9727	2241.9055	-0.0672	-30	1	20	TGPYCYPGMGLPSNPLE GCR	59	99.993	Carbamidomethyl (C)[5,19], Oxidation (M)[9]		Mascot
2703.3044	2703.2476	-0.0568	-21	21	46	EYVAQQTCGVGIVGSPV			Carbamidomethyl (C)[8]		Mascot

						STEPGNTPR					
2703.3044	2703.2476	-0.0568	-21	21	46	EYVAQQTCGVGIVGSPV STEPGNTPR	199	100	Carbamidomethyl (C)[8]		Mascot
2726.3101	2726.219	-0.0911	-33	97	120	VLVTPGHCNVMTVHNTP YCLGLDI			Carbamidomethyl (C)[8,19], Oxidation (M)[11]		Mascot

3 alpha amylase inhibitor CM1, partial [Triticum aestivum] gi|253783731 13657.4 6.73 7 173 100 26.411 122 100

Peptide Information

Calc. Mass	Obsrv. Mass	± da	± ppm	Start Seq.	End Seq.	Sequence	Ion Score	C. I. %	Modification	Rank	Result Type
811.4573	811.4189	-0.0384	-47	67	72	YFIGRR					Mascot
814.3876	814.3585	-0.0291	-36	82	88	DLPGCPR			Carbamidomethyl (C)[5]		Mascot
1102.5852	1102.5223	-0.0629	-57	72	81	RSDPNSSVLK					Mascot
1278.5532	1278.514	-0.0392	-31	52	61	ELYDASQHCR			Carbamidomethyl (C)[9]		Mascot
1278.5532	1278.514	-0.0392	-31	52	61	ELYDASQHCR	83	100	Carbamidomethyl (C)[9]		Mascot
1741.8538	1741.7435	-0.1103	-63	73	88	SDPNSSVLKDLPGCPR			Carbamidomethyl (C)[14]		Mascot
2242.009	2241.9055	-0.1035	-46	1	20	TGPYCYAGMGLPINPLE GCR			Carbamidomethyl (C)[5,19], Oxidation (M)[9]		Mascot
2242.009	2241.9055	-0.1035	-46	1	20	TGPYCYAGMGLPINPLE GCR	39	99.182	Carbamidomethyl (C)[5,19], Oxidation (M)[9]		Mascot
2709.2786	2709.2214	-0.0572	-21	21	46	EYVAQQTCGISISGSAVS TEPGNTPR			Carbamidomethyl (C)[8]		Mascot

4 dimeric alpha-amylase inhibitor [Triticum dicoccoides] gi|114215776 13742.5 6.49 4 169 100 3.115 146 100

Protein Group

0.19 dimeric alpha-amylase inhibitor [Triticum aestivum]	gi|54778511	13756.6	6.4899 997711 1816
0.19 dimeric alpha-amylase inhibitor [Triticum aestivum]	gi|54778507	13814.6	5.7300 000190 7349
dimeric alpha-amylase inhibitor [Triticum dicoccoides]	gi|114215772	13742.5	6.4899 997711 1816
dimeric alpha-amylase inhibitor [Triticum dicoccoides]	gi|114215790	13742.5	6.4899 997711 1816
dimeric alpha-amylase inhibitor [Triticum dicoccoides]	gi|114215768	13742.5	6.4899

			997711<br>1816
dimeric alpha-amylase inhibitor [Triticum dicoccoides]	gi|114215762	13742.5	6.4899<br>997711<br>1816

Peptide Information

Calc. Mass	Obsrv. Mass	± da	± ppm	Start Seq.	End Seq.	Sequence	Ion Score	C. I. %	Modification	Rank	Result Type
1306.7665	1306.7229	-0.0436	-33	14	25	VPALPGCRPVLK			Carbamidomethyl (C)[7]		Mascot
1570.8007	1570.7483	-0.0524	-33	26	39	LQCNGSQVPEAVLR			Carbamidomethyl (C)[3]		Mascot
1677.8517	1677.7589	-0.0928	-55	101	116	LPIVIDASGDGAYVCK			Carbamidomethyl (C)[15]		Mascot
1887.8403	1887.7843	-0.056	-30	67	84	EHGVQEGQAGTGAFPSC R			Carbamidomethyl (C)[17]		Mascot
1887.8403	1887.7843	-0.056	-30	67	84	EHGVQEGQAGTGAFPSC R	146	100	Carbamidomethyl (C)[17]		Mascot

5	dimeric alpha-amylase inhibitor [Triticum timopheevii subsp. armeniacum]	gi|227809294	15588.4	5.28	4	167	100	3.115	146	100

Protein Group

dimeric alpha-amylase inhibitor [Triticum dicoccoides]	gi|227809240	15588.5	5.2800<br>002098<br>0835
dimeric alpha-amylase inhibitor [Triticum dicoccoides]	gi|227809226	15574.4	5.2800<br>002098<br>0835
dimeric alpha-amylase inhibitor [Triticum dicoccoides]	gi|227809216	15574.4	5.2800<br>002098<br>0835
dimeric alpha-amylase inhibitor [Triticum dicoccoides]	gi|227809212	15574.4	5.2800<br>002098<br>0835
dimeric alpha-amylase inhibitor [Triticum dicoccoides]	gi|227809196	15574.4	5.2800<br>002098<br>0835

dimeric alpha-amylase inhibitor [Triticum dicoccoides]	gi|227809192	15604.4	5.2800 002098 0835
dimeric alpha-amylase inhibitor [Triticum dicoccoides]	gi|227809174	15574.4	5.2800 002098 0835
dimeric alpha-amylase inhibitor [Triticum dicoccoides]	gi|227809172	15548.4	5.5799 999237 0605
dimeric alpha-amylase inhibitor [Triticum dicoccoides]	gi|227809154	15574.4	5.2800 002098 0835
dimeric alpha-amylase inhibitor [Triticum dicoccoides]	gi|227809140	15574.4	5.2800 002098 0835
dimeric alpha-amylase inhibitor [Triticum dicoccoides]	gi|227809092	15574.4	5.2800 002098 0835
dimeric alpha-amylase inhibitor [Triticum dicoccoides]	gi|227809084	15574.4	5.2800 002098 0835
dimeric alpha-amylase inhibitor [Triticum dicoccoides]	gi|227809082	15574.4	5.2800 002098 0835
dimeric alpha-amylase inhibitor [Triticum dicoccoides]	gi|227809055	15558.5	5.7399 997711 1816

dimeric alpha-amylase inhibitor [Triticum dicoccoides]	gi|227809047	15574.4	5.2800<br>002098<br>0835
dimeric alpha-amylase inhibitor [Triticum dicoccoides]	gi|227809039	15574.4	5.2800<br>002098<br>0835
dimeric alpha-amylase inhibitor [Triticum dicoccoides]	gi|227809037	15574.4	5.2800<br>002098<br>0835
dimeric alpha-amylase inhibitor [Triticum dicoccoides]	gi|227809033	15574.4	5.2800<br>002098<br>0835
dimeric alpha-amylase inhibitor [Triticum dicoccoides]	gi|227809025	15574.4	5.2800<br>002098<br>0835
dimeric alpha-amylase inhibitor [Triticum dicoccoides]	gi|227809003	15574.4	5.2800<br>002098<br>0835
dimeric alpha-amylase inhibitor [Triticum dicoccoides]	gi|227808981	15574.4	5.2800<br>002098<br>0835
dimeric alpha-amylase inhibitor [Triticum dicoccoides]	gi|227808979	15574.4	5.2800<br>002098<br>0835
dimeric alpha-amylase inhibitor [Triticum dicoccoides]	gi|227808972	15574.4	5.2800<br>002098<br>0835
dimeric alpha-amylase inhibitor [Triticum dicoccoides]	gi|227808962	15574.4	5.2800

			002098<br>0835
dimeric alpha-amylase inhibitor [Triticum dicoccoides]	gi|227808960	15574.4	5.2800<br>002098<br>0835
dimeric alpha-amylase inhibitor [Triticum dicoccoides]	gi|227808948	15574.4	5.2800<br>002098<br>0835
dimeric alpha-amylase inhibitor [Triticum dicoccoides]	gi|227808942	15574.4	5.2800<br>002098<br>0835
dimeric alpha-amylase inhibitor [Triticum dicoccoides]	gi|227808940	15574.4	5.2800<br>002098<br>0835
dimeric alpha-amylase inhibitor [Triticum dicoccoides]	gi|227808932	15574.4	5.2800<br>002098<br>0835
dimeric alpha-amylase inhibitor [Triticum dicoccoides]	gi|227808926	15574.4	5.2800<br>002098<br>0835
dimeric alpha-amylase inhibitor [Triticum dicoccoides]	gi|227808924	15574.4	5.2800<br>002098<br>0835
dimeric alpha-amylase inhibitor [Triticum dicoccoides]	gi|227808908	15574.4	5.2800<br>002098<br>0835
dimeric alpha-amylase inhibitor [Triticum dicoccoides]	gi|227808906	15574.4	5.2800<br>002098<br>0835

dimeric alpha-amylase inhibitor [Triticum dicoccoides]	gi|227808896	15588.5	5.2800 002098 0835
dimeric alpha-amylase inhibitor [Triticum dicoccoides]	gi|227808889	15588.5	5.2800 002098 0835
dimeric alpha-amylase inhibitor [Triticum dicoccoides]	gi|227808887	15588.5	5.2800 002098 0835
dimeric alpha-amylase inhibitor [Triticum timopheevii subsp. armeniacum]	gi|227809296	15574.4	5.2800 002098 0835

Peptide Information

Calc. Mass	Obsrv. Mass	± da	± ppm	Start Seq.	End Seq.	Sequence	Ion Score	C. I. %	Modification	Rank	Result Type
1306.7665	1306.7229	-0.0436	-33	31	42	VPALPGCRPVLK			Carbamidomethyl (C)[7]		Mascot
1570.8007	1570.7483	-0.0524	-33	43	56	LQCNGSQVPEAVLR			Carbamidomethyl (C)[3]		Mascot
1677.8517	1677.7589	-0.0928	-55	118	133	LPIVIDASGDGAYVCK			Carbamidomethyl (C)[15]		Mascot
1887.8403	1887.7843	-0.056	-30	84	101	EHGVQEGQAGTGAFPSCR			Carbamidomethyl (C)[17]		Mascot
1887.8403	1887.7843	-0.056	-30	84	101	EHGVQEGQAGTGAFPSCR	146	100	Carbamidomethyl (C)[17]		Mascot

6	dimeric alpha-amylase inhibitor [Triticum timopheevii subsp. armeniacum]	gi|227809292	15571.4	4.81	1	0	146	100	

Protein Group

dimeric alpha-amylase inhibitor [Triticum aestivum]	gi|65993941	15631.5	5.7699 999809 2651
dimeric alpha-amylase inhibitor [Triticum aestivum]	gi|65993852	15558.5	5.3000 001907 3486

dimeric alpha-amylase inhibitor [Triticum aestivum]	gi|65993807	15588.5	5.3000<br>001907<br>3486
dimeric alpha-amylase inhibitor [Triticum aestivum]	gi|65993756	15820.6	6.5399<br>999618<br>5303
dimeric alpha-amylase inhibitor [Triticum dicoccoides]	gi|227809222	15602.5	5.75
dimeric alpha-amylase inhibitor [Triticum dicoccoides]	gi|227809204	15564.4	5.2800<br>002098<br>0835
dimeric alpha-amylase inhibitor [Triticum dicoccoides]	gi|227809132	15575.4	5.2600<br>002288<br>8184
dimeric alpha-amylase inhibitor [Triticum dicoccoides]	gi|227809021	15804.6	6.5399<br>999618<br>5303
dimeric alpha-amylase inhibitor [Triticum dicoccoides]	gi|227809015	15575.4	5.2600<br>002288<br>8184
dimeric alpha-amylase inhibitor [Triticum dicoccoides]	gi|227809045	15558.4	5.2800<br>002098<br>0835
dimeric alpha-amylase inhibitor [Triticum dicoccoides]	gi|227809112	15602.5	5.2800<br>002098<br>0835
dimeric alpha-amylase inhibitor [Triticum dicoccoides]	gi|227809001	15573.5	5.7399

			997711<br>1816
dimeric alpha-amylase inhibitor [Triticum dicoccoides]	gi|227808884	15588.5	5.2800<br>002098<br>0835
dimeric alpha-amylase inhibitor [Triticum dicoccoides]	gi|227808839	15588.5	5.2800<br>002098<br>0835
dimeric alpha-amylase inhibitor [Triticum dicoccoides]	gi|227808843	15588.5	5.2800<br>002098<br>0835
dimeric alpha-amylase inhibitor [Triticum dicoccoides]	gi|227808849	15588.5	5.2800<br>002098<br>0835
dimeric alpha-amylase inhibitor [Triticum dicoccoides]	gi|227808855	15588.5	5.2800<br>002098<br>0835
dimeric alpha-amylase inhibitor [Triticum dicoccoides]	gi|227808857	15562.4	5.5799<br>999237<br>0605
dimeric alpha-amylase inhibitor [Triticum dicoccoides]	gi|227808859	15588.5	5.2800<br>002098<br>0835
dimeric alpha-amylase inhibitor [Triticum dicoccoides]	gi|227808863	15588.5	5.2800<br>002098<br>0835
dimeric alpha-amylase inhibitor [Triticum dicoccoides]	gi|227808876	15588.5	5.2800<br>002098<br>0835

dimeric alpha-amylase inhibitor [Triticum dicoccoides]	gi|227808880	15588.5	5.2800<br>002098<br>0835
dimeric alpha-amylase inhibitor [Triticum dicoccoides]	gi|114215784	13770.6	6.4899<br>997711<br>1816
dimeric alpha-amylase inhibitor [Triticum dicoccoides]	gi|227808997	15575.4	5.2600<br>002288<br>8184
dimeric alpha-amylase inhibitor [Triticum timopheevii subsp. armeniacum]	gi|227809290	15571.4	4.8099<br>999427<br>7954
dimeric alpha-amylase inhibitor [Triticum timopheevii subsp. armeniacum]	gi|227809286	15513.4	4.9899<br>997711<br>1816
dimeric alpha-amylase inhibitor [Triticum timopheevii subsp. armeniacum]	gi|227809284	15588.5	5.2800<br>002098<br>0835
dimeric alpha-amylase inhibitor [Triticum timopheevii subsp. armeniacum]	gi|227809282	15606.4	5.2800<br>002098<br>0835
dimeric alpha-amylase inhibitor [Triticum timopheevii subsp. armeniacum]	gi|227809280	15588.5	5.2800<br>002098<br>0835
dimeric alpha-amylase inhibitor [Triticum timopheevii subsp. armeniacum]	gi|227809278	15588.5	5.2800<br>002098<br>0835

dimeric alpha-amylase inhibitor, partial [Triticum durum]	gi|386877064	14502.8	4.7800 002098 0835

Peptide Information

Calc. Mass	Obsrv. Mass	± da	± ppm	Start Seq.	End Seq.	Sequence	Ion Score	C. I. %	Modification	Rank	Result Type
1887.8403	1887.7843	-0.056	-30	84	101	EHGVQEGQAGTGAFPSC R	146	100	Carbamidomethyl (C)[17]	1	Mascot

7	WCI proteinase inhibitor [Triticum aestivum]	gi|20798981	13505.5	7.42	1	0	129	100	

Protein Group

RecName: Full=Chymotrypsin inhibitor WCI; AltName: Full=Chloroform/methanol-soluble protein WCI	gi|75107149	13505.5	7.4200 000762 9395

Peptide Information

Calc. Mass	Obsrv. Mass	± da	± ppm	Start Seq.	End Seq.	Sequence	Ion Score	C. I. %	Modification	Rank	Result Type
1986.0193	1985.9745	-0.0448	-23	67	86	AFPPSQSQGGGPPQPPL APR	129	100		1	Mascot

8	RecName: Full=Trypsin/alpha-amylase inhibitor CMX2; AltName: Full=ITRL-2; Flags: Precursor	gi|3913903	14279.3	9.08	1	0	67	99.999

Protein Group

CMx [Triticum aestivum]	gi|640019	14279.3	9.0799 999237 0605
CMx [Triticum aestivum]	gi|640015	14221.3	9.2299 995422 3633
CMx [Triticum aestivum]	gi|640013	14221.3	9.2299 995422 3633
RecName: Full=Trypsin/alpha-amylase inhibitor CMX1/CMX3; AltName: Full=ITRL-1/ITRL-3; Flags: Precursor	gi|3913904	14221.3	9.2299 995422 3633

Trypsin/alpha-amylase inhibitor CMX1/CMX3 [Triticum urartu]	gi|474202113	16973.5	8.5900 001525 8789
trypsin inhibitor CMx precursor - wheat	gi|2130114	17243.6	8.5900 001525 8789

Peptide Information

Calc. Mass	Obsrv. Mass	± da	± ppm	Start Seq.	End Seq.	Sequence	Ion Score	C. I. %	Modification	Rank	Result Type
961.5465	961.514	-0.0325	-34	112	120	EFIAGIVGR	67	99.999		1	Mascot

9	putative replication factor C subunit 3 [Triticum urartu]	gi|473936914	40601.7	5.64	1	0	25	78.587

Peptide Information

Calc. Mass	Obsrv. Mass	± da	± ppm	Start Seq.	End Seq.	Sequence	Ion Score	C. I. %	Modification	Rank	Result Type
961.5941	961.514	-0.0801	-83	235	241	QLYLIRR	25	78.587		2	Mascot

10	putative cyclic nucleotide-gated ion channel 20, chloroplastic [Triticum urartu]	gi|473932349	64124.3	9.32	1	0	22	60.945

Peptide Information

Calc. Mass	Obsrv. Mass	± da	± ppm	Start Seq.	End Seq.	Sequence	Ion Score	C. I. %	Modification	Rank	Result Type
961.5247	961.514	-0.0107	-11	288	294	LEMQLRR	22	60.945	Oxidation (M)[3]	3	Mascot

Gel Idx/Pos	311/M13	Instr./Gel Origin	BA2151/Sample Project 20140814	Process Status	Analysis Succeeded
Plate [#] Name	[1] Sample Project 20140814	Instrument Sample Name		Spectra	11

Rank	Protein Name	Accession No.	Protein MW	Protein PI	Pep. Count	Protein Score	Protein Score C. I. %	Intensity Matched	Total Ion Score	Total Ion C. I. %	Confirmed
1	CM2 protein [Triticum durum]	gi|21920	16019.7	6.86	4	302	100	17.919	282	100	

Protein Group

CM2 protein - durum wheat	gi|100833	16019.7	6.8600001335144
RecName: Full=Alpha-amylase/trypsin inhibitor CM2; AltName: Full=Chloroform/methanol-soluble protein CM2; Flags: Precursor	gi|123956	16019.7	6.8600001335144

Peptide Information

Calc. Mass	Obsrv. Mass	± da	± ppm	Start Seq.	End Seq.	Sequence	Ion Score	C. I. %	Modification	Rank	Result Type
814.3876	814.3555	-0.0321	-39	107	113	DLPGCPR			Carbamidomethyl (C)[5]		Mascot
1017.5211	1017.4763	-0.0448	-44	97	106	TSDPNSGVLK					Mascot
1278.5532	1278.5017	-0.0515	-40	77	86	ELYDASQHCR			Carbamidomethyl (C)[9]		Mascot
1278.5532	1278.5017	-0.0515	-40	77	86	ELYDASQHCR	78	100	Carbamidomethyl (C)[9]		Mascot
2703.3044	2703.2388	-0.0656	-24	46	71	EYVAQQTCGVGIVGSPVSTEPGNTPR			Carbamidomethyl (C)[8]		Mascot
2703.3044	2703.2388	-0.0656	-24	46	71	EYVAQQTCGVGIVGSPVSTEPGNTPR	204	100	Carbamidomethyl (C)[8]		Mascot

Rank	Protein Name	Accession No.	Protein MW	Protein PI	Pep. Count	Protein Score	Protein Score C. I. %	Intensity Matched	Total Ion Score	Total Ion C. I. %	Confirmed
2	putative alpha-amylase inhibitor CM2, partial [Triticum aestivum]	gi|283465827	13566.3	5.83	4	228	100	9.635	204	100	

Peptide Information

Calc. Mass	Obsrv. Mass	± da	± ppm	Start Seq.	End Seq.	Sequence	Ion Score	C. I. %	Modification	Rank	Result Type
814.3876	814.3555	-0.0321	-39	82	88	DLPGCPR			Carbamidomethyl (C)[5]		Mascot
1017.5211	1017.4763	-0.0448	-44	72	81	TSDPNSGVLK					Mascot
2241.9727	2241.8916	-0.0811	-36	1	20	TGPYCYPGMGLPSNPLEGCR			Carbamidomethyl (C)[5,19], Oxidation (M)[9]		Mascot
2703.3044	2703.2388	-0.0656	-24	21	46	EYVAQQTCGVGIVGSPVSTEPGNTPR			Carbamidomethyl (C)[8]		Mascot
2703.3044	2703.2388	-0.0656	-24	21	46	EYVAQQTCGVGIVGSPVSTEPGNTPR	204	100	Carbamidomethyl (C)[8]		Mascot

Rank	Protein Name	Accession No.	Protein MW	Protein PI	Pep. Count	Protein Score	Protein Score C. I. %	Intensity Matched	Total Ion Score	Total Ion C. I. %	Confirmed
3	dimeric alpha-amylase inhibitor [Triticum aestivum]	gi|65993852	15558.5	5.3	5	170	100	4.475	142	100	

Peptide Information

Calc. Mass	Obsrv. Mass	± da	± ppm	Start Seq.	End Seq.	Sequence	Ion Score	C. I. %	Modification	Rank	Result Type
1306.7665	1306.7191	-0.0474	-36	31	42	VPALPGCRPVLK			Carbamidomethyl (C)[7]		Mascot
1306.7665	1306.7191	-0.0474	-36	31	42	VPALPGCRPVLK	9	0	Carbamidomethyl (C)[7]		Mascot
1524.6709	1524.6825	0.0116	8	71	83	CGALYSTLDSMYK			Carbamidomethyl (C)[1], Oxidation (M)[11]		Mascot
1570.8007	1570.7379	-0.0628	-40	43	56	LQCNGSQVPEAVLR			Carbamidomethyl (C)[3]		Mascot
1677.8517	1677.755	-0.0967	-58	118	133	LPIVIDASGDGAYVCK			Carbamidomethyl (C)[15]		Mascot
1887.8403	1887.771	-0.0693	-37	84	101	EHGVQEGQAGTGAFPSC R			Carbamidomethyl (C)[17]		Mascot
1887.8403	1887.771	-0.0693	-37	84	101	EHGVQEGQAGTGAFPSC R	133	100	Carbamidomethyl (C)[17]		Mascot

4	dimeric alpha-amylase inhibitor [Triticum dicoccoides]	gi|114215776	13742.5	6.49	4	164	100	4.327	142	100	

Protein Group

0.19 dimeric alpha-amylase inhibitor [Triticum aestivum]	gi|54778511	13756.6	6.4899 997711 1816
0.19 dimeric alpha-amylase inhibitor [Triticum aestivum]	gi|54778507	13814.6	5.7300 000190 7349
dimeric alpha-amylase inhibitor [Triticum dicoccoides]	gi|114215772	13742.5	6.4899 997711 1816
dimeric alpha-amylase inhibitor [Triticum dicoccoides]	gi|114215790	13742.5	6.4899 997711 1816
dimeric alpha-amylase inhibitor [Triticum dicoccoides]	gi|114215768	13742.5	6.4899 997711 1816
dimeric alpha-amylase inhibitor [Triticum dicoccoides]	gi|114215762	13742.5	6.4899 997711 1816

Peptide Information

Calc. Mass	Obsrv. Mass	± da	± ppm	Start Seq.	End Seq.	Sequence	Ion Score	C. I. %	Modification	Rank	Result Type
1306.7665	1306.7191	-0.0474	-36	14	25	VPALPGCRPVLK			Carbamidomethyl (C)[7]		Mascot
1306.7665	1306.7191	-0.0474	-36	14	25	VPALPGCRPVLK	9	0	Carbamidomethyl (C)[7]		Mascot
1570.8007	1570.7379	-0.0628	-40	26	39	LQCNGSQVPEAVLR			Carbamidomethyl (C)[3]		Mascot
1677.8517	1677.755	-0.0967	-58	101	116	LPIVIDASGDGAYVCK			Carbamidomethyl (C)[15]		Mascot
1887.8403	1887.771	-0.0693	-37	67	84	EHGVQEGQAGTGAFPSC R			Carbamidomethyl (C)[17]		Mascot
1887.8403	1887.771	-0.0693	-37	67	84	EHGVQEGQAGTGAFPSC R	133	100	Carbamidomethyl (C)[17]		Mascot

5	dimeric alpha-amylase inhibitor [Triticum timopheevii subsp. armeniacum]	gi|227809294	15588.4	5.28	4	163	100	4.327	142	100	

Protein Group

dimeric alpha-amylase inhibitor [Triticum dicoccoides]	gi|227809240	15588.5	5.2800 002098 0835
dimeric alpha-amylase inhibitor [Triticum dicoccoides]	gi|227809226	15574.4	5.2800 002098 0835
dimeric alpha-amylase inhibitor [Triticum dicoccoides]	gi|227809216	15574.4	5.2800 002098 0835
dimeric alpha-amylase inhibitor [Triticum dicoccoides]	gi|227809212	15574.4	5.2800 002098 0835
dimeric alpha-amylase inhibitor [Triticum dicoccoides]	gi|227809196	15574.4	5.2800 002098 0835
dimeric alpha-amylase inhibitor [Triticum dicoccoides]	gi|227809192	15604.4	5.2800 002098 0835

dimeric alpha-amylase inhibitor [Triticum dicoccoides]	gi|227809174	15574.4	5.2800<br>002098<br>0835
dimeric alpha-amylase inhibitor [Triticum dicoccoides]	gi|227809172	15548.4	5.5799<br>999237<br>0605
dimeric alpha-amylase inhibitor [Triticum dicoccoides]	gi|227809154	15574.4	5.2800<br>002098<br>0835
dimeric alpha-amylase inhibitor [Triticum dicoccoides]	gi|227809140	15574.4	5.2800<br>002098<br>0835
dimeric alpha-amylase inhibitor [Triticum dicoccoides]	gi|227809092	15574.4	5.2800<br>002098<br>0835
dimeric alpha-amylase inhibitor [Triticum dicoccoides]	gi|227809084	15574.4	5.2800<br>002098<br>0835
dimeric alpha-amylase inhibitor [Triticum dicoccoides]	gi|227809082	15574.4	5.2800<br>002098<br>0835
dimeric alpha-amylase inhibitor [Triticum dicoccoides]	gi|227809055	15558.5	5.7399<br>997711<br>1816
dimeric alpha-amylase inhibitor [Triticum dicoccoides]	gi|227809047	15574.4	5.2800<br>002098<br>0835
dimeric alpha-amylase inhibitor [Triticum dicoccoides]	gi|227809039	15574.4	5.2800

			002098<br>0835
dimeric alpha-amylase inhibitor [Triticum dicoccoides]	gi|227809037	15574.4	5.2800<br>002098<br>0835
dimeric alpha-amylase inhibitor [Triticum dicoccoides]	gi|227809033	15574.4	5.2800<br>002098<br>0835
dimeric alpha-amylase inhibitor [Triticum dicoccoides]	gi|227809025	15574.4	5.2800<br>002098<br>0835
dimeric alpha-amylase inhibitor [Triticum dicoccoides]	gi|227809003	15574.4	5.2800<br>002098<br>0835
dimeric alpha-amylase inhibitor [Triticum dicoccoides]	gi|227808981	15574.4	5.2800<br>002098<br>0835
dimeric alpha-amylase inhibitor [Triticum dicoccoides]	gi|227808979	15574.4	5.2800<br>002098<br>0835
dimeric alpha-amylase inhibitor [Triticum dicoccoides]	gi|227808972	15574.4	5.2800<br>002098<br>0835
dimeric alpha-amylase inhibitor [Triticum dicoccoides]	gi|227808962	15574.4	5.2800<br>002098<br>0835
dimeric alpha-amylase inhibitor [Triticum dicoccoides]	gi|227808960	15574.4	5.2800<br>002098<br>0835

dimeric alpha-amylase inhibitor [Triticum dicoccoides]	gi|227808948	15574.4	5.2800 002098 0835
dimeric alpha-amylase inhibitor [Triticum dicoccoides]	gi|227808942	15574.4	5.2800 002098 0835
dimeric alpha-amylase inhibitor [Triticum dicoccoides]	gi|227808940	15574.4	5.2800 002098 0835
dimeric alpha-amylase inhibitor [Triticum dicoccoides]	gi|227808932	15574.4	5.2800 002098 0835
dimeric alpha-amylase inhibitor [Triticum dicoccoides]	gi|227808926	15574.4	5.2800 002098 0835
dimeric alpha-amylase inhibitor [Triticum dicoccoides]	gi|227808924	15574.4	5.2800 002098 0835
dimeric alpha-amylase inhibitor [Triticum dicoccoides]	gi|227808908	15574.4	5.2800 002098 0835
dimeric alpha-amylase inhibitor [Triticum dicoccoides]	gi|227808906	15574.4	5.2800 002098 0835
dimeric alpha-amylase inhibitor [Triticum dicoccoides]	gi|227808896	15588.5	5.2800 002098 0835

dimeric alpha-amylase inhibitor [Triticum dicoccoides]	gi|227808889	15588.5	5.2800 002098 0835
dimeric alpha-amylase inhibitor [Triticum dicoccoides]	gi|227808887	15588.5	5.2800 002098 0835
dimeric alpha-amylase inhibitor [Triticum timopheevii subsp. armeniacum]	gi|227809296	15574.4	5.2800 002098 0835

Peptide Information

Calc. Mass	Obsrv. Mass	± da	± ppm	Start Seq.	End Seq.	Sequence	Ion Score	C. I. %	Modification	Rank	Result Type
1306.7665	1306.7191	-0.0474	-36	31	42	VPALPGCRPVLK			Carbamidomethyl (C)[7]		Mascot
1306.7665	1306.7191	-0.0474	-36	31	42	VPALPGCRPVLK	9	0	Carbamidomethyl (C)[7]		Mascot
1570.8007	1570.7379	-0.0628	-40	43	56	LQCNGSQVPEAVLR			Carbamidomethyl (C)[3]		Mascot
1677.8517	1677.755	-0.0967	-58	118	133	LPIVIDASGDGAYVCK			Carbamidomethyl (C)[15]		Mascot
1887.8403	1887.771	-0.0693	-37	84	101	EHGVQEGQAGTGAFPSCR			Carbamidomethyl (C)[17]		Mascot
1887.8403	1887.771	-0.0693	-37	84	101	EHGVQEGQAGTGAFPSCR	133	100	Carbamidomethyl (C)[17]		Mascot

6	dimeric alpha-amylase inhibitor [Triticum timopheevii subsp. armeniacum]	gi|227809284	15588.5	5.28	2	0	142	100	

Protein Group

dimeric alpha-amylase inhibitor [Triticum aestivum]	gi|65993941	15631.5	5.7699 999809 2651
dimeric alpha-amylase inhibitor [Triticum aestivum]	gi|65993807	15588.5	5.3000 001907 3486
dimeric alpha-amylase inhibitor [Triticum aestivum]	gi|65993756	15820.6	6.5399 999618 5303

dimeric alpha-amylase inhibitor [Triticum dicoccoides]	gi|227809222	15602.5	5.75
dimeric alpha-amylase inhibitor [Triticum dicoccoides]	gi|227809112	15602.5	5.2800 002098 0835
dimeric alpha-amylase inhibitor [Triticum dicoccoides]	gi|227809001	15573.5	5.7399 997711 1816
dimeric alpha-amylase inhibitor [Triticum dicoccoides]	gi|227809045	15558.4	5.2800 002098 0835
dimeric alpha-amylase inhibitor [Triticum dicoccoides]	gi|227808884	15588.5	5.2800 002098 0835
dimeric alpha-amylase inhibitor [Triticum dicoccoides]	gi|227808876	15588.5	5.2800 002098 0835
dimeric alpha-amylase inhibitor [Triticum dicoccoides]	gi|227808859	15588.5	5.2800 002098 0835
dimeric alpha-amylase inhibitor [Triticum dicoccoides]	gi|227808855	15588.5	5.2800 002098 0835
dimeric alpha-amylase inhibitor [Triticum dicoccoides]	gi|227808843	15588.5	5.2800 002098 0835
dimeric alpha-amylase inhibitor [Triticum dicoccoides]	gi|114215784	13770.6	6.4899

			997711 1816
dimeric alpha-amylase inhibitor [Triticum dicoccoides]	gi|227808839	15588.5	5.2800 002098 0835
dimeric alpha-amylase inhibitor [Triticum dicoccoides]	gi|227808849	15588.5	5.2800 002098 0835
dimeric alpha-amylase inhibitor [Triticum dicoccoides]	gi|227808857	15562.4	5.5799 999237 0605
dimeric alpha-amylase inhibitor [Triticum dicoccoides]	gi|227808863	15588.5	5.2800 002098 0835
dimeric alpha-amylase inhibitor [Triticum dicoccoides]	gi|227808880	15588.5	5.2800 002098 0835
dimeric alpha-amylase inhibitor [Triticum timopheevii subsp. armeniacum]	gi|227809282	15606.4	5.2800 002098 0835
dimeric alpha-amylase inhibitor [Triticum timopheevii subsp. armeniacum]	gi|227809280	15588.5	5.2800 002098 0835
dimeric alpha-amylase inhibitor [Triticum timopheevii subsp. armeniacum]	gi|227809278	15588.5	5.2800 002098 0835

Peptide Information

Calc. Mass	Obsrv. Mass	± da	± ppm	Start Seq.	End Seq.	Sequence	Ion Score	C. I. %	Modification	Rank	Result Type

1306.7665	1306.7191	-0.0474	-36	31	42	VPALPGCRPVLK	9	0	Carbamidomethyl (C)[7]	2	Mascot
1887.8403	1887.771	-0.0693	-37	84	101	EHGVQEGQAGTGAFPSCR	133	100	Carbamidomethyl (C)[17]	1	Mascot

7 WCI proteinase inhibitor [Triticum aestivum] gi|20798981 13505.5 7.42 1 0 90 100

Protein Group

RecName: Full=Chymotrypsin inhibitor WCI; AltName: Full=Chloroform/methanol-soluble protein WCI	gi|75107149	13505.5	7.4200000762 9395

Peptide Information

Calc. Mass	Obsrv. Mass	± da	± ppm	Start Seq.	End Seq.	Sequence	Ion Score	C. I. %	Modification	Rank	Result Type
1986.0193	1985.9674	-0.0519	-26	67	86	AFPPSQSQGGGPPQPPLAPR	90	100		1	Mascot

8 monomeric alpha-amylase inhibitor [Triticum dicoccoides] gi|229615151 17176.2 5.82 1 0 26 81.976

Peptide Information

Calc. Mass	Obsrv. Mass	± da	± ppm	Start Seq.	End Seq.	Sequence	Ion Score	C. I. %	Modification	Rank	Result Type
1050.5579	1050.5188	-0.0391	-37	95	103	SVYQEIGVR	26	81.976		1	Mascot

9 monomeric alpha-amylase inhibitor [Triticum dicoccoides] gi|229615664 17201.3 7.45 1 0 26 81.976

Protein Group

RecName: Full=Alpha-amylase inhibitor 0.28; AltName: Full=CIII; AltName: Full=WMAI-1; Flags: Precursor	gi|110282974	17358.3	7.4499998092 6514
monomeric alpha-amylase inhibitor [Triticum aestivum]	gi|134034649	13658.5	7.4000000953 6743
monomeric alpha-amylase inhibitor [Triticum aestivum]	gi|134034647	13617.5	5.3699998855 5908
monomeric alpha-amylase inhibitor [Triticum aestivum]	gi|134034645	13658.5	7.4000000953

			6743
monomeric alpha-amylase inhibitor [Triticum aestivum]	gi|134034643	13658.5	7.4000<br>000953<br>6743
monomeric alpha-amylase inhibitor [Triticum aestivum]	gi|134034635	13658.5	7.4000<br>000953<br>6743
monomeric alpha-amylase inhibitor [Triticum aestivum]	gi|134034631	13716.5	6.1799<br>998283<br>3862
monomeric alpha-amylase inhibitor [Triticum aestivum]	gi|134034627	13658.5	7.4000<br>000953<br>6743
monomeric alpha-amylase inhibitor [Triticum aestivum]	gi|134034623	13716.5	6.1799<br>998283<br>3862
monomeric alpha-amylase inhibitor [Triticum aestivum]	gi|134034619	13716.5	6.1799<br>998283<br>3862
monomeric alpha-amylase inhibitor [Triticum aestivum]	gi|134034601	13716.5	6.1799<br>998283<br>3862
monomeric alpha-amylase inhibitor [Triticum aestivum]	gi|134034603	13716.5	6.1799<br>998283<br>3862
monomeric alpha-amylase inhibitor [Triticum aestivum]	gi|134034605	13716.5	6.1799<br>998283<br>3862

monomeric alpha-amylase inhibitor [Triticum aestivum]	gi|134034607	13716.5	6.1799 998283 3862
monomeric alpha-amylase inhibitor [Triticum aestivum]	gi|134034609	13716.5	6.1799 998283 3862
monomeric alpha-amylase inhibitor [Triticum aestivum]	gi|134034611	13716.5	6.1799 998283 3862
monomeric alpha-amylase inhibitor [Triticum aestivum]	gi|134034613	13637.5	7.4099 998474 1211
monomeric alpha-amylase inhibitor [Triticum aestivum]	gi|134034615	13617.5	5.3699 998855 5908
monomeric alpha-amylase inhibitor [Triticum aestivum]	gi|134034617	13716.5	6.1799 998283 3862
monomeric alpha-amylase inhibitor [Triticum aestivum]	gi|2894148	17187.3	7.4499 998092 6514
monomeric alpha-amylase inhibitor [Triticum aestivum]	gi|134034573	13716.5	6.1799 998283 3862
monomeric alpha-amylase inhibitor [Triticum aestivum]	gi|134034575	13716.5	6.1799 998283 3862

monomeric alpha-amylase inhibitor [Triticum aestivum]	gi|134034577	13667.5	6.1799 998283 3862
monomeric alpha-amylase inhibitor [Triticum aestivum]	gi|134034579	13716.5	6.1799 998283 3862
monomeric alpha-amylase inhibitor [Triticum aestivum]	gi|134034581	13716.5	6.1799 998283 3862
monomeric alpha-amylase inhibitor [Triticum aestivum]	gi|134034583	13716.5	6.1799 998283 3862
monomeric alpha-amylase inhibitor [Triticum aestivum]	gi|134034585	13716.5	6.1799 998283 3862
monomeric alpha-amylase inhibitor [Triticum aestivum]	gi|134034587	13716.5	6.1799 998283 3862
monomeric alpha-amylase inhibitor [Triticum aestivum]	gi|134034589	13716.5	6.1799 998283 3862
monomeric alpha-amylase inhibitor [Triticum aestivum]	gi|134034591	13716.5	6.1799 998283 3862
monomeric alpha-amylase inhibitor [Triticum aestivum]	gi|134034593	13716.5	6.1799 998283 3862
monomeric alpha-amylase inhibitor [Triticum aestivum]	gi|134034595	13716.5	6.1799 998283

			3862
monomeric alpha-amylase inhibitor [Triticum aestivum]	gi|134034597	13716.5	6.1799 998283 3862
monomeric alpha-amylase inhibitor [Triticum aestivum]	gi|134034599	13716.5	6.1799 998283 3862
monomeric alpha-amylase inhibitor [Triticum aestivum]	gi|134034621	13716.5	6.1799 998283 3862
monomeric alpha-amylase inhibitor [Triticum aestivum]	gi|134034625	13716.5	6.1799 998283 3862
monomeric alpha-amylase inhibitor [Triticum aestivum]	gi|134034629	13716.5	6.1799 998283 3862
monomeric alpha-amylase inhibitor [Triticum aestivum]	gi|134034633	13658.5	7.4000 000953 6743
monomeric alpha-amylase inhibitor [Triticum aestivum]	gi|134034637	13716.5	6.1799 998283 3862
monomeric alpha-amylase inhibitor [Triticum aestivum]	gi|134034639	13658.5	7.4000 000953 6743
monomeric alpha-amylase inhibitor [Triticum aestivum]	gi|134034641	13587.5	6.1799 998283 3862

monomeric alpha-amylase inhibitor [Triticum dicoccoides]	gi|229615662	17173.2	7.4499 998092 6514
monomeric alpha-amylase inhibitor [Triticum dicoccoides]	gi|229615660	17102.2	6.5500 001907 3486
monomeric alpha-amylase inhibitor [Triticum dicoccoides]	gi|229615658	17173.3	8.0299 997329 7119
monomeric alpha-amylase inhibitor [Triticum dicoccoides]	gi|229615656	17102.2	6.5500 001907 3486
monomeric alpha-amylase inhibitor [Triticum dicoccoides]	gi|229615654	17173.2	7.4499 998092 6514
monomeric alpha-amylase inhibitor [Triticum dicoccoides]	gi|229615652	17114.3	8.3800 001144 4092
monomeric alpha-amylase inhibitor [Triticum dicoccoides]	gi|229615650	17102.2	6.5500 001907 3486
monomeric alpha-amylase inhibitor [Triticum dicoccoides]	gi|229615648	17102.2	6.5500 001907 3486
monomeric alpha-amylase inhibitor [Triticum dicoccoides]	gi|229615646	17102.2	6.5500 001907 3486

monomeric alpha-amylase inhibitor [Triticum dicoccoides]	gi|229615644	17102.2	6.5500 001907 3486
monomeric alpha-amylase inhibitor [Triticum dicoccoides]	gi|229615642	17074.2	6.5500 001907 3486
monomeric alpha-amylase inhibitor [Triticum dicoccoides]	gi|229615640	17102.2	6.5500 001907 3486
monomeric alpha-amylase inhibitor [Triticum dicoccoides]	gi|229615638	17102.2	6.5500 001907 3486
monomeric alpha-amylase inhibitor [Triticum dicoccoides]	gi|229615636	17102.2	6.5500 001907 3486
monomeric alpha-amylase inhibitor [Triticum dicoccoides]	gi|229615634	17102.2	6.5500 001907 3486
monomeric alpha-amylase inhibitor [Triticum dicoccoides]	gi|229615632	17102.2	6.5500 001907 3486
monomeric alpha-amylase inhibitor [Triticum dicoccoides]	gi|229615630	17102.2	6.5500 001907 3486
monomeric alpha-amylase inhibitor [Triticum dicoccoides]	gi|229615592	17102.2	6.5500 001907 3486
monomeric alpha-amylase inhibitor [Triticum dicoccoides]	gi|229615594	17188.3	6.5500 001907

			3486
monomeric alpha-amylase inhibitor [Triticum dicoccoides]	gi|229615596	17188.3	6.5500 001907 3486
monomeric alpha-amylase inhibitor [Triticum dicoccoides]	gi|229615598	17188.3	6.5500 001907 3486
monomeric alpha-amylase inhibitor [Triticum dicoccoides]	gi|229615602	17188.3	6.5500 001907 3486
monomeric alpha-amylase inhibitor [Triticum dicoccoides]	gi|229615604	17188.3	6.5500 001907 3486
monomeric alpha-amylase inhibitor [Triticum dicoccoides]	gi|229615606	17070.2	7.4600 000381 4697
monomeric alpha-amylase inhibitor [Triticum dicoccoides]	gi|229615608	17104.2	6.5500 001907 3486
monomeric alpha-amylase inhibitor [Triticum dicoccoides]	gi|229615610	17100.2	7.4600 000381 4697
monomeric alpha-amylase inhibitor [Triticum dicoccoides]	gi|229615558	17104.2	6.5500 001907 3486
monomeric alpha-amylase inhibitor [Triticum dicoccoides]	gi|229615560	17173.2	7.4499 998092 6514

monomeric alpha-amylase inhibitor [Triticum dicoccoides]	gi|229615562	17104.2	7.4600 000381 4697
monomeric alpha-amylase inhibitor [Triticum dicoccoides]	gi|229615564	17203.2	7.4499 998092 6514
monomeric alpha-amylase inhibitor [Triticum dicoccoides]	gi|229615566	17173.2	7.4499 998092 6514
monomeric alpha-amylase inhibitor [Triticum dicoccoides]	gi|229615568	17102.2	6.5500 001907 3486
monomeric alpha-amylase inhibitor [Triticum dicoccoides]	gi|229615570	17102.2	6.5500 001907 3486
monomeric alpha-amylase inhibitor [Triticum dicoccoides]	gi|229615572	17102.2	6.5500 001907 3486
monomeric alpha-amylase inhibitor [Triticum dicoccoides]	gi|229615574	17101.2	8.0299 997329 7119
monomeric alpha-amylase inhibitor [Triticum dicoccoides]	gi|229615524	17203.2	7.4499 998092 6514
monomeric alpha-amylase inhibitor [Triticum dicoccoides]	gi|229615526	17173.3	7.4499 998092 6514

monomeric alpha-amylase inhibitor [Triticum dicoccoides]	gi|229615528	17187.3	7.4499 998092 6514
monomeric alpha-amylase inhibitor [Triticum dicoccoides]	gi|229615530	17215.3	7.4499 998092 6514
monomeric alpha-amylase inhibitor [Triticum dicoccoides]	gi|229615532	17187.3	7.4499 998092 6514
monomeric alpha-amylase inhibitor [Triticum dicoccoides]	gi|229615534	17187.3	7.4499 998092 6514
monomeric alpha-amylase inhibitor [Triticum dicoccoides]	gi|229615536	17187.3	7.4499 998092 6514
monomeric alpha-amylase inhibitor [Triticum dicoccoides]	gi|229615538	17203.2	7.4499 998092 6514
monomeric alpha-amylase inhibitor [Triticum dicoccoides]	gi|229615540	17233.3	7.4499 998092 6514
monomeric alpha-amylase inhibitor [Triticum dicoccoides]	gi|229615490	17102.2	6.5500 001907 3486
monomeric alpha-amylase inhibitor [Triticum dicoccoides]	gi|229615492	17102.2	6.5500 001907 3486
monomeric alpha-amylase inhibitor [Triticum dicoccoides]	gi|229615494	17187.3	7.4499 998092

			6514
monomeric alpha-amylase inhibitor [Triticum dicoccoides]	gi|229615496	17187.3	7.4499 998092 6514
monomeric alpha-amylase inhibitor [Triticum dicoccoides]	gi|229615498	17187.3	7.4499 998092 6514
monomeric alpha-amylase inhibitor [Triticum dicoccoides]	gi|229615500	17187.3	7.4499 998092 6514
monomeric alpha-amylase inhibitor [Triticum dicoccoides]	gi|229615502	17187.3	7.4499 998092 6514
monomeric alpha-amylase inhibitor [Triticum dicoccoides]	gi|229615504	17187.3	7.4499 998092 6514
monomeric alpha-amylase inhibitor [Triticum dicoccoides]	gi|229615506	17187.3	7.4499 998092 6514
monomeric alpha-amylase inhibitor [Triticum dicoccoides]	gi|229615456	17187.3	7.4499 998092 6514
monomeric alpha-amylase inhibitor [Triticum dicoccoides]	gi|229615458	17187.3	7.4499 998092 6514
monomeric alpha-amylase inhibitor [Triticum dicoccoides]	gi|229615460	17187.3	7.4499 998092 6514

monomeric alpha-amylase inhibitor [Triticum dicoccoides]	gi|229615462	17187.3	7.4499998092 6514
monomeric alpha-amylase inhibitor [Triticum dicoccoides]	gi|229615464	17187.3	7.4499998092 6514
monomeric alpha-amylase inhibitor [Triticum dicoccoides]	gi|229615466	17203.2	7.4499998092 6514
monomeric alpha-amylase inhibitor [Triticum dicoccoides]	gi|229615468	17173.2	7.4499998092 6514
monomeric alpha-amylase inhibitor [Triticum dicoccoides]	gi|229615470	17072.2	6.5500001907 3486
monomeric alpha-amylase inhibitor [Triticum dicoccoides]	gi|229615472	17203.2	7.4499998092 6514
monomeric alpha-amylase inhibitor [Triticum dicoccoides]	gi|229615422	17102.2	6.5500001907 3486
monomeric alpha-amylase inhibitor [Triticum dicoccoides]	gi|229615424	17102.2	6.5500001907 3486
monomeric alpha-amylase inhibitor [Triticum dicoccoides]	gi|229615426	17098.3	7.4600000381 4697

monomeric alpha-amylase inhibitor [Triticum dicoccoides]	gi|229615428	17157.3	8.0299 997329 7119
monomeric alpha-amylase inhibitor [Triticum dicoccoides]	gi|229615430	17159.2	7.4499 998092 6514
monomeric alpha-amylase inhibitor [Triticum dicoccoides]	gi|229615432	17187.3	7.4499 998092 6514
monomeric alpha-amylase inhibitor [Triticum dicoccoides]	gi|229615434	17187.3	7.4499 998092 6514
monomeric alpha-amylase inhibitor [Triticum dicoccoides]	gi|229615436	17187.3	7.4499 998092 6514
monomeric alpha-amylase inhibitor [Triticum dicoccoides]	gi|229615438	17187.3	7.4499 998092 6514
monomeric alpha-amylase inhibitor [Triticum dicoccoides]	gi|229615386	17102.2	6.5500 001907 3486
monomeric alpha-amylase inhibitor [Triticum dicoccoides]	gi|229615388	17102.2	6.5500 001907 3486
monomeric alpha-amylase inhibitor [Triticum dicoccoides]	gi|229615390	17102.2	6.5500 001907 3486
monomeric alpha-amylase inhibitor [Triticum dicoccoides]	gi|229615392	17046.1	6.5500 001907

			3486
monomeric alpha-amylase inhibitor [Triticum dicoccoides]	gi|229615394	17102.2	6.5500 001907 3486
monomeric alpha-amylase inhibitor [Triticum dicoccoides]	gi|229615396	17102.2	6.5500 001907 3486
monomeric alpha-amylase inhibitor [Triticum dicoccoides]	gi|229615398	17070.1	6.5500 001907 3486
monomeric alpha-amylase inhibitor [Triticum dicoccoides]	gi|229615400	17102.2	6.5500 001907 3486
monomeric alpha-amylase inhibitor [Triticum dicoccoides]	gi|229615402	17102.2	6.5500 001907 3486
monomeric alpha-amylase inhibitor [Triticum dicoccoides]	gi|229615221	17159.2	7.4499 998092 6514
monomeric alpha-amylase inhibitor [Triticum dicoccoides]	gi|229615223	17104.2	6.5500 001907 3486
monomeric alpha-amylase inhibitor [Triticum dicoccoides]	gi|229615225	17102.2	6.5500 001907 3486
monomeric alpha-amylase inhibitor [Triticum dicoccoides]	gi|229615227	17102.2	6.5500 001907 3486

monomeric alpha-amylase inhibitor [Triticum dicoccoides]	gi|229615229	17102.2	6.5500 001907 3486
monomeric alpha-amylase inhibitor [Triticum dicoccoides]	gi|229615231	17102.2	6.5500 001907 3486
monomeric alpha-amylase inhibitor [Triticum dicoccoides]	gi|229615233	17072.2	6.5500 001907 3486
monomeric alpha-amylase inhibitor [Triticum dicoccoides]	gi|229615235	17102.2	6.5500 001907 3486
monomeric alpha-amylase inhibitor [Triticum dicoccoides]	gi|229615237	17102.2	6.5500 001907 3486
monomeric alpha-amylase inhibitor [Triticum dicoccoides]	gi|229615239	17102.2	6.5500 001907 3486
monomeric alpha-amylase inhibitor [Triticum dicoccoides]	gi|229615241	17102.2	6.5500 001907 3486
monomeric alpha-amylase inhibitor [Triticum dicoccoides]	gi|229615243	17102.2	6.5500 001907 3486
monomeric alpha-amylase inhibitor [Triticum dicoccoides]	gi|229615245	17102.2	6.5500 001907 3486

monomeric alpha-amylase inhibitor [Triticum dicoccoides]	gi|229615247	17102.2	6.5500 001907 3486
monomeric alpha-amylase inhibitor [Triticum dicoccoides]	gi|229615249	17102.2	6.5500 001907 3486
monomeric alpha-amylase inhibitor [Triticum dicoccoides]	gi|229615251	17102.2	6.5500 001907 3486
monomeric alpha-amylase inhibitor [Triticum dicoccoides]	gi|229615253	17003.1	5.8000 001907 3486
monomeric alpha-amylase inhibitor [Triticum dicoccoides]	gi|229615255	17102.2	6.5500 001907 3486
monomeric alpha-amylase inhibitor [Triticum dicoccoides]	gi|229615257	17119.2	8.0299 997329 7119
monomeric alpha-amylase inhibitor [Triticum dicoccoides]	gi|229615259	17132.2	6.5500 001907 3486
monomeric alpha-amylase inhibitor [Triticum dicoccoides]	gi|229615261	17104.2	6.5500 001907 3486
monomeric alpha-amylase inhibitor [Triticum dicoccoides]	gi|229615263	17104.2	6.5500 001907 3486
monomeric alpha-amylase inhibitor [Triticum dicoccoides]	gi|229615265	17102.2	6.5500 001907

			3486
monomeric alpha-amylase inhibitor [Triticum dicoccoides]	gi|229615267	17201.3	7.4499 998092 6514
monomeric alpha-amylase inhibitor [Triticum dicoccoides]	gi|229615269	17189.3	7.4499 998092 6514
monomeric alpha-amylase inhibitor [Triticum dicoccoides]	gi|229615271	17102.2	6.5500 001907 3486
monomeric alpha-amylase inhibitor [Triticum dicoccoides]	gi|229615273	17102.2	6.5500 001907 3486
monomeric alpha-amylase inhibitor [Triticum dicoccoides]	gi|229615275	17102.2	6.5500 001907 3486
monomeric alpha-amylase inhibitor [Triticum dicoccoides]	gi|229615278	17102.2	6.5500 001907 3486
monomeric alpha-amylase inhibitor [Triticum dicoccoides]	gi|229615280	17102.2	6.5500 001907 3486
monomeric alpha-amylase inhibitor [Triticum dicoccoides]	gi|229615282	17102.2	6.5500 001907 3486
monomeric alpha-amylase inhibitor [Triticum dicoccoides]	gi|229615284	17102.2	6.5500 001907 3486

monomeric alpha-amylase inhibitor [Triticum dicoccoides]	gi|229615286	17102.2	6.5500 001907 3486
monomeric alpha-amylase inhibitor [Triticum dicoccoides]	gi|229615288	17102.2	6.5500 001907 3486
monomeric alpha-amylase inhibitor [Triticum dicoccoides]	gi|229615290	17162.2	6.5500 001907 3486
monomeric alpha-amylase inhibitor [Triticum dicoccoides]	gi|229615292	17102.2	6.5500 001907 3486
monomeric alpha-amylase inhibitor [Triticum dicoccoides]	gi|229615294	17102.2	6.5500 001907 3486
monomeric alpha-amylase inhibitor [Triticum dicoccoides]	gi|229615296	17102.2	6.5500 001907 3486
monomeric alpha-amylase inhibitor [Triticum dicoccoides]	gi|229615298	17102.2	6.5500 001907 3486
monomeric alpha-amylase inhibitor [Triticum dicoccoides]	gi|229615300	17102.2	6.5500 001907 3486
monomeric alpha-amylase inhibitor [Triticum dicoccoides]	gi|229615302	17102.2	6.5500 001907 3486

monomeric alpha-amylase inhibitor [Triticum dicoccoides]	gi|229615304	17102.2	6.5500 001907 3486
monomeric alpha-amylase inhibitor [Triticum dicoccoides]	gi|229615306	17102.2	6.5500 001907 3486
monomeric alpha-amylase inhibitor [Triticum dicoccoides]	gi|229615308	17102.2	6.5500 001907 3486
monomeric alpha-amylase inhibitor [Triticum dicoccoides]	gi|229615310	17102.2	6.5500 001907 3486
monomeric alpha-amylase inhibitor [Triticum dicoccoides]	gi|229615312	17102.2	6.5500 001907 3486
monomeric alpha-amylase inhibitor [Triticum dicoccoides]	gi|229615314	17102.2	6.5500 001907 3486
monomeric alpha-amylase inhibitor [Triticum dicoccoides]	gi|229615316	17102.2	6.5500 001907 3486
monomeric alpha-amylase inhibitor [Triticum dicoccoides]	gi|229615318	17102.2	6.5500 001907 3486
monomeric alpha-amylase inhibitor [Triticum dicoccoides]	gi|229615320	17074.2	6.5500 001907 3486
monomeric alpha-amylase inhibitor [Triticum dicoccoides]	gi|229615322	17054.2	6.5500 001907

			3486
monomeric alpha-amylase inhibitor [Triticum dicoccoides]	gi|229615324	17102.2	6.5500 001907 3486
monomeric alpha-amylase inhibitor [Triticum dicoccoides]	gi|229615326	17102.2	6.5500 001907 3486
monomeric alpha-amylase inhibitor [Triticum dicoccoides]	gi|229615328	17086.2	6.5500 001907 3486
monomeric alpha-amylase inhibitor [Triticum dicoccoides]	gi|229615330	17102.2	6.5500 001907 3486
monomeric alpha-amylase inhibitor [Triticum dicoccoides]	gi|229615334	17074.2	6.5500 001907 3486
monomeric alpha-amylase inhibitor [Triticum dicoccoides]	gi|229615336	17108.2	6.5500 001907 3486
monomeric alpha-amylase inhibitor [Triticum dicoccoides]	gi|229615338	17173.2	7.4499 998092 6514
monomeric alpha-amylase inhibitor [Triticum dicoccoides]	gi|229615340	17102.2	6.5500 001907 3486
monomeric alpha-amylase inhibitor [Triticum dicoccoides]	gi|229615342	17134.2	6.5500 001907 3486

monomeric alpha-amylase inhibitor [Triticum dicoccoides]	gi|229615344	17102.2	6.5500 001907 3486
monomeric alpha-amylase inhibitor [Triticum dicoccoides]	gi|229615346	17102.2	6.5500 001907 3486
monomeric alpha-amylase inhibitor [Triticum dicoccoides]	gi|229615348	17102.2	6.5500 001907 3486
monomeric alpha-amylase inhibitor [Triticum dicoccoides]	gi|229615350	17102.2	6.5500 001907 3486
monomeric alpha-amylase inhibitor [Triticum dicoccoides]	gi|229615352	17102.2	6.5500 001907 3486
monomeric alpha-amylase inhibitor [Triticum dicoccoides]	gi|229615354	17102.2	6.5500 001907 3486
monomeric alpha-amylase inhibitor [Triticum dicoccoides]	gi|229615356	17102.2	6.5500 001907 3486
monomeric alpha-amylase inhibitor [Triticum dicoccoides]	gi|229615358	17102.2	6.5500 001907 3486
monomeric alpha-amylase inhibitor [Triticum dicoccoides]	gi|229615360	17102.2	6.5500 001907 3486

monomeric alpha-amylase inhibitor [Triticum dicoccoides]	gi|229615362	17102.2	6.5500 001907 3486
monomeric alpha-amylase inhibitor [Triticum dicoccoides]	gi|229615364	17102.2	6.5500 001907 3486
monomeric alpha-amylase inhibitor [Triticum dicoccoides]	gi|229615366	17187.3	7.4499 998092 6514
monomeric alpha-amylase inhibitor [Triticum dicoccoides]	gi|229615368	17173.2	7.4499 998092 6514
monomeric alpha-amylase inhibitor [Triticum dicoccoides]	gi|229614961	17104.2	6.5500 001907 3486
monomeric alpha-amylase inhibitor [Triticum dicoccoides]	gi|229614963	17074.2	6.5500 001907 3486
monomeric alpha-amylase inhibitor [Triticum dicoccoides]	gi|229614965	17074.2	6.5500 001907 3486
monomeric alpha-amylase inhibitor [Triticum dicoccoides]	gi|229614967	17074.2	6.5500 001907 3486
monomeric alpha-amylase inhibitor [Triticum dicoccoides]	gi|229614969	17104.2	6.5500 001907 3486
monomeric alpha-amylase inhibitor [Triticum dicoccoides]	gi|229614971	17074.2	6.5500 001907

			3486
monomeric alpha-amylase inhibitor [Triticum dicoccoides]	gi|229614973	17104.2	6.5500 001907 3486
monomeric alpha-amylase inhibitor [Triticum dicoccoides]	gi|229614975	17104.2	6.5500 001907 3486
monomeric alpha-amylase inhibitor [Triticum dicoccoides]	gi|229614977	17173.2	7.4499 998092 6514
monomeric alpha-amylase inhibitor [Triticum dicoccoides]	gi|229614979	17104.2	6.5500 001907 3486
monomeric alpha-amylase inhibitor [Triticum dicoccoides]	gi|229614981	17189.3	7.4499 998092 6514
monomeric alpha-amylase inhibitor [Triticum dicoccoides]	gi|229614983	17104.2	6.5500 001907 3486
monomeric alpha-amylase inhibitor [Triticum dicoccoides]	gi|229614985	17104.2	6.5500 001907 3486
monomeric alpha-amylase inhibitor [Triticum dicoccoides]	gi|229614987	17104.2	6.5500 001907 3486
monomeric alpha-amylase inhibitor [Triticum dicoccoides]	gi|229614989	17104.2	6.5500 001907 3486

monomeric alpha-amylase inhibitor [Triticum dicoccoides]	gi|229614991	17072.2	7.4499998092 6514
monomeric alpha-amylase inhibitor [Triticum dicoccoides]	gi|229614993	17102.2	6.5500001907 3486
monomeric alpha-amylase inhibitor [Triticum dicoccoides]	gi|229614995	17102.2	6.5500001907 3486
monomeric alpha-amylase inhibitor [Triticum dicoccoides]	gi|229614997	17046.1	6.5500001907 3486
monomeric alpha-amylase inhibitor [Triticum dicoccoides]	gi|229614999	17042.2	8.0299997329 7119
monomeric alpha-amylase inhibitor [Triticum dicoccoides]	gi|229615001	17102.2	6.5500001907 3486
monomeric alpha-amylase inhibitor [Triticum dicoccoides]	gi|229615003	17104.2	6.5500001907 3486
monomeric alpha-amylase inhibitor [Triticum dicoccoides]	gi|229615005	17104.2	6.5500001907 3486
monomeric alpha-amylase inhibitor [Triticum dicoccoides]	gi|229615007	17107.2	6.5500001907 3486

monomeric alpha-amylase inhibitor [Triticum dicoccoides]	gi|229615009	17104.2	6.5500 001907 3486
monomeric alpha-amylase inhibitor [Triticum dicoccoides]	gi|229615011	17104.2	6.5500 001907 3486
monomeric alpha-amylase inhibitor [Triticum dicoccoides]	gi|229615013	17102.2	6.5500 001907 3486
monomeric alpha-amylase inhibitor [Triticum dicoccoides]	gi|229615015	17102.2	6.5500 001907 3486
monomeric alpha-amylase inhibitor [Triticum dicoccoides]	gi|229615017	17102.2	6.5500 001907 3486
monomeric alpha-amylase inhibitor [Triticum dicoccoides]	gi|229615019	17102.2	6.5500 001907 3486
monomeric alpha-amylase inhibitor [Triticum dicoccoides]	gi|229615021	17102.2	6.5500 001907 3486
monomeric alpha-amylase inhibitor [Triticum dicoccoides]	gi|229615023	17102.2	6.5500 001907 3486
monomeric alpha-amylase inhibitor [Triticum dicoccoides]	gi|229615025	17102.2	6.5500 001907 3486
monomeric alpha-amylase inhibitor [Triticum dicoccoides]	gi|229615027	17094.2	6.5500 001907

			3486
monomeric alpha-amylase inhibitor [Triticum dicoccoides]	gi|229615029	17105.2	5.8000 001907 3486
monomeric alpha-amylase inhibitor [Triticum dicoccoides]	gi|229615031	17102.2	6.5500 001907 3486
monomeric alpha-amylase inhibitor [Triticum dicoccoides]	gi|229615033	17190.2	6.5500 001907 3486
monomeric alpha-amylase inhibitor [Triticum dicoccoides]	gi|229615035	17104.2	6.5500 001907 3486
monomeric alpha-amylase inhibitor [Triticum dicoccoides]	gi|229615037	17159.2	7.4499 998092 6514
monomeric alpha-amylase inhibitor [Triticum dicoccoides]	gi|229615039	17118.2	6.5500 001907 3486
monomeric alpha-amylase inhibitor [Triticum dicoccoides]	gi|229615041	17074.2	6.5500 001907 3486
monomeric alpha-amylase inhibitor [Triticum dicoccoides]	gi|229615043	17044.2	6.5500 001907 3486
monomeric alpha-amylase inhibitor [Triticum dicoccoides]	gi|229615045	17173.2	7.4499 998092 6514

monomeric alpha-amylase inhibitor [Triticum dicoccoides]	gi|229615047	17104.2	6.5500001907 3486
monomeric alpha-amylase inhibitor [Triticum dicoccoides]	gi|229615049	17102.2	6.5500001907 3486
monomeric alpha-amylase inhibitor [Triticum dicoccoides]	gi|229615051	17102.2	6.5500001907 3486
monomeric alpha-amylase inhibitor [Triticum dicoccoides]	gi|229615053	17103.2	6.5500001907 3486
monomeric alpha-amylase inhibitor [Triticum dicoccoides]	gi|229615055	17102.2	6.5500001907 3486
monomeric alpha-amylase inhibitor [Triticum dicoccoides]	gi|229615057	17074.2	6.5500001907 3486
monomeric alpha-amylase inhibitor [Triticum dicoccoides]	gi|229615059	17159.2	7.4499998092 6514
monomeric alpha-amylase inhibitor [Triticum dicoccoides]	gi|229615061	17104.2	6.5500001907 3486
monomeric alpha-amylase inhibitor [Triticum dicoccoides]	gi|229615063	17203.2	7.4499998092 6514

monomeric alpha-amylase inhibitor [Triticum dicoccoides]	gi|229615065	17216.3	7.4499998092 6514
monomeric alpha-amylase inhibitor [Triticum dicoccoides]	gi|229615067	17189.3	7.4499998092 6514
monomeric alpha-amylase inhibitor [Triticum dicoccoides]	gi|229615069	17104.2	6.5500001907 3486
monomeric alpha-amylase inhibitor [Triticum dicoccoides]	gi|229615071	17104.2	6.5500001907 3486
monomeric alpha-amylase inhibitor [Triticum dicoccoides]	gi|229615073	17102.2	6.5500001907 3486
monomeric alpha-amylase inhibitor [Triticum dicoccoides]	gi|229615075	17104.2	6.5500001907 3486
monomeric alpha-amylase inhibitor [Triticum dicoccoides]	gi|229615079	17102.2	6.5500001907 3486
monomeric alpha-amylase inhibitor [Triticum dicoccoides]	gi|229615081	17104.2	6.5500001907 3486
monomeric alpha-amylase inhibitor [Triticum dicoccoides]	gi|229615083	17076.1	6.5500001907 3486
monomeric alpha-amylase inhibitor [Triticum dicoccoides]	gi|229615085	17106.2	6.5500001907

			3486
monomeric alpha-amylase inhibitor [Triticum dicoccoides]	gi|229615087	17044.2	6.5500 001907 3486
monomeric alpha-amylase inhibitor [Triticum dicoccoides]	gi|229615089	17033.1	5.8000 001907 3486
monomeric alpha-amylase inhibitor [Triticum dicoccoides]	gi|229615091	17074.2	6.5500 001907 3486
monomeric alpha-amylase inhibitor [Triticum dicoccoides]	gi|229615093	17074.2	6.5500 001907 3486
monomeric alpha-amylase inhibitor [Triticum dicoccoides]	gi|229615095	17104.2	6.5500 001907 3486
monomeric alpha-amylase inhibitor [Triticum dicoccoides]	gi|229615097	17084.2	7.4600 000381 4697
monomeric alpha-amylase inhibitor [Triticum dicoccoides]	gi|229615099	17102.2	6.5500 001907 3486
monomeric alpha-amylase inhibitor [Triticum dicoccoides]	gi|229615101	17187.3	7.4499 998092 6514
monomeric alpha-amylase inhibitor [Triticum dicoccoides]	gi|229615103	17189.3	7.4499 998092 6514

monomeric alpha-amylase inhibitor [Triticum dicoccoides]	gi|229615105	17102.2	6.5500 001907 3486
monomeric alpha-amylase inhibitor [Triticum dicoccoides]	gi|229615107	17074.2	6.5500 001907 3486
monomeric alpha-amylase inhibitor [Triticum dicoccoides]	gi|229615109	17074.2	6.5500 001907 3486
monomeric alpha-amylase inhibitor [Triticum dicoccoides]	gi|229615111	17076.1	6.5500 001907 3486
monomeric alpha-amylase inhibitor [Triticum dicoccoides]	gi|229615113	17104.2	6.5500 001907 3486
monomeric alpha-amylase inhibitor [Triticum dicoccoides]	gi|229615115	17094.2	6.5500 001907 3486
monomeric alpha-amylase inhibitor [Triticum dicoccoides]	gi|229615117	17203.2	7.4499 998092 6514
monomeric alpha-amylase inhibitor [Triticum dicoccoides]	gi|229615119	17272.3	8.0299 997329 7119
monomeric alpha-amylase inhibitor [Triticum dicoccoides]	gi|229615121	17102.2	6.5500 001907 3486

monomeric alpha-amylase inhibitor [Triticum dicoccoides]	gi|229615123	17044.2	7.4499 998092 6514
monomeric alpha-amylase inhibitor [Triticum dicoccoides]	gi|229615125	17102.2	6.5500 001907 3486
monomeric alpha-amylase inhibitor [Triticum dicoccoides]	gi|229615127	17102.2	6.5500 001907 3486
monomeric alpha-amylase inhibitor [Triticum dicoccoides]	gi|229615129	17102.2	6.5500 001907 3486
monomeric alpha-amylase inhibitor [Triticum dicoccoides]	gi|229615131	17203.2	7.4499 998092 6514
monomeric alpha-amylase inhibitor [Triticum dicoccoides]	gi|229615133	17090.2	6.5399 999618 5303
monomeric alpha-amylase inhibitor [Triticum dicoccoides]	gi|229615135	17104.2	6.5500 001907 3486
monomeric alpha-amylase inhibitor [Triticum dicoccoides]	gi|229615137	17203.2	7.4499 998092 6514
monomeric alpha-amylase inhibitor [Triticum dicoccoides]	gi|229615139	17104.2	6.5500 001907 3486
monomeric alpha-amylase inhibitor [Triticum dicoccoides]	gi|229615141	17104.2	6.5500 001907

			3486
monomeric alpha-amylase inhibitor [Triticum dicoccoides]	gi|229615143	17104.2	6.5500 001907 3486
monomeric alpha-amylase inhibitor [Triticum dicoccoides]	gi|229615145	17173.2	7.4499 998092 6514
monomeric alpha-amylase inhibitor [Triticum dicoccoides]	gi|229615147	17030.2	7.4499 998092 6514
monomeric alpha-amylase inhibitor [Triticum dicoccoides]	gi|229615149	17104.2	6.5500 001907 3486
monomeric alpha-amylase inhibitor [Triticum dicoccoides]	gi|229615153	17102.2	6.5500 001907 3486
monomeric alpha-amylase inhibitor [Triticum dicoccoides]	gi|229615155	17104.2	6.5500 001907 3486
monomeric alpha-amylase inhibitor [Triticum dicoccoides]	gi|229615157	17173.2	7.4499 998092 6514
monomeric alpha-amylase inhibitor [Triticum dicoccoides]	gi|229615159	17187.3	7.4499 998092 6514
monomeric alpha-amylase inhibitor [Triticum dicoccoides]	gi|229615161	17187.3	7.4499 998092 6514

monomeric alpha-amylase inhibitor [Triticum dicoccoides]	gi|229615163	17190.2	6.5399 999618 5303
monomeric alpha-amylase inhibitor [Triticum dicoccoides]	gi|229615165	17187.3	7.4499 998092 6514
monomeric alpha-amylase inhibitor [Triticum dicoccoides]	gi|229615167	17187.3	7.4499 998092 6514
monomeric alpha-amylase inhibitor [Triticum dicoccoides]	gi|229615169	17102.2	6.5500 001907 3486
monomeric alpha-amylase inhibitor [Triticum dicoccoides]	gi|229615171	17056.2	6.5500 001907 3486
monomeric alpha-amylase inhibitor [Triticum dicoccoides]	gi|229615173	17130.3	7.4600 000381 4697
monomeric alpha-amylase inhibitor [Triticum dicoccoides]	gi|229615175	17102.2	6.5500 001907 3486
monomeric alpha-amylase inhibitor [Triticum dicoccoides]	gi|229615177	17102.2	6.5500 001907 3486
monomeric alpha-amylase inhibitor [Triticum dicoccoides]	gi|229615179	17130.2	6.5500 001907 3486

monomeric alpha-amylase inhibitor [Triticum dicoccoides]	gi|229615181	17102.2	6.5500 001907 3486
monomeric alpha-amylase inhibitor [Triticum dicoccoides]	gi|229615183	17130.2	6.5500 001907 3486
monomeric alpha-amylase inhibitor [Triticum dicoccoides]	gi|229615185	17102.2	6.5500 001907 3486
monomeric alpha-amylase inhibitor [Triticum dicoccoides]	gi|229615187	17187.3	7.4499 998092 6514
monomeric alpha-amylase inhibitor [Triticum dicoccoides]	gi|229615189	17187.3	7.4499 998092 6514
monomeric alpha-amylase inhibitor [Triticum dicoccoides]	gi|229615191	17187.3	7.4499 998092 6514
monomeric alpha-amylase inhibitor [Triticum dicoccoides]	gi|229615193	17217.3	7.4499 998092 6514
monomeric alpha-amylase inhibitor [Triticum dicoccoides]	gi|229615195	17187.3	7.4499 998092 6514
monomeric alpha-amylase inhibitor [Triticum dicoccoides]	gi|229615197	17103.3	7.4899 997711 1816
monomeric alpha-amylase inhibitor [Triticum dicoccoides]	gi|229615199	17231.3	7.4499 998092

			6514
monomeric alpha-amylase inhibitor [Triticum dicoccoides]	gi|229615201	17173.2	7.4499 998092 6514
monomeric alpha-amylase inhibitor [Triticum dicoccoides]	gi|229615203	17143.3	8.0299 997329 7119
monomeric alpha-amylase inhibitor [Triticum dicoccoides]	gi|229615205	17173.2	7.4499 998092 6514
monomeric alpha-amylase inhibitor [Triticum dicoccoides]	gi|229615207	17173.2	7.4499 998092 6514
monomeric alpha-amylase inhibitor [Triticum dicoccoides]	gi|229615209	17203.2	7.4499 998092 6514
monomeric alpha-amylase inhibitor [Triticum dicoccoides]	gi|229615211	17173.2	7.4499 998092 6514
monomeric alpha-amylase inhibitor [Triticum dicoccoides]	gi|229615213	17173.2	7.4499 998092 6514
monomeric alpha-amylase inhibitor [Triticum dicoccoides]	gi|229615215	17104.2	6.5500 001907 3486
monomeric alpha-amylase inhibitor [Triticum dicoccoides]	gi|229615217	17173.2	7.4499 998092 6514

monomeric alpha-amylase inhibitor [Triticum dicoccoides]	gi|229615219	17215.3	7.4499 998092 6514
monomeric alpha-amylase inhibitor [Triticum dicoccoides]	gi|229615370	17203.2	7.4499 998092 6514
monomeric alpha-amylase inhibitor [Triticum dicoccoides]	gi|229615372	17173.2	7.4499 998092 6514
monomeric alpha-amylase inhibitor [Triticum dicoccoides]	gi|229615374	17102.2	6.5500 001907 3486
monomeric alpha-amylase inhibitor [Triticum dicoccoides]	gi|229615376	17102.2	6.5500 001907 3486
monomeric alpha-amylase inhibitor [Triticum dicoccoides]	gi|229615378	17130.2	6.5500 001907 3486
monomeric alpha-amylase inhibitor [Triticum dicoccoides]	gi|229615380	17103.2	5.8000 001907 3486
monomeric alpha-amylase inhibitor [Triticum dicoccoides]	gi|229615382	17102.2	6.5500 001907 3486
monomeric alpha-amylase inhibitor [Triticum dicoccoides]	gi|229615384	17187.3	7.4499 998092 6514

monomeric alpha-amylase inhibitor [Triticum dicoccoides]	gi|229615404	17102.2	6.5500 001907 3486
monomeric alpha-amylase inhibitor [Triticum dicoccoides]	gi|229615406	17102.2	6.5500 001907 3486
monomeric alpha-amylase inhibitor [Triticum dicoccoides]	gi|229615408	17131.2	5.3800 001144 4092
monomeric alpha-amylase inhibitor [Triticum dicoccoides]	gi|229615412	17102.2	6.5500 001907 3486
monomeric alpha-amylase inhibitor [Triticum dicoccoides]	gi|229615414	17102.2	6.5500 001907 3486
monomeric alpha-amylase inhibitor [Triticum dicoccoides]	gi|229615416	17102.2	6.5500 001907 3486
monomeric alpha-amylase inhibitor [Triticum dicoccoides]	gi|229615418	17102.2	6.5500 001907 3486
monomeric alpha-amylase inhibitor [Triticum dicoccoides]	gi|229615420	17044.2	7.4499 998092 6514
monomeric alpha-amylase inhibitor [Triticum dicoccoides]	gi|229615440	17189.3	7.4800 000190 7349
monomeric alpha-amylase inhibitor [Triticum dicoccoides]	gi|229615442	17102.2	6.5500 001907

			3486
monomeric alpha-amylase inhibitor [Triticum dicoccoides]	gi|229615444	17102.2	6.5500 001907 3486
monomeric alpha-amylase inhibitor [Triticum dicoccoides]	gi|229615446	17102.2	6.5500 001907 3486
monomeric alpha-amylase inhibitor [Triticum dicoccoides]	gi|229615448	17047.2	6.5500 001907 3486
monomeric alpha-amylase inhibitor [Triticum dicoccoides]	gi|229615450	17074.2	6.5500 001907 3486
monomeric alpha-amylase inhibitor [Triticum dicoccoides]	gi|229615452	17104.2	6.5500 001907 3486
monomeric alpha-amylase inhibitor [Triticum dicoccoides]	gi|229615454	17187.3	7.4499 998092 6514
monomeric alpha-amylase inhibitor [Triticum dicoccoides]	gi|229615474	17206.3	7.4600 000381 4697
monomeric alpha-amylase inhibitor [Triticum dicoccoides]	gi|229615476	17177.2	7.4499 998092 6514
monomeric alpha-amylase inhibitor [Triticum dicoccoides]	gi|229615478	17217.3	7.4499 998092 6514

monomeric alpha-amylase inhibitor [Triticum dicoccoides]	gi|229615480	17104.2	6.5500 001907 3486
monomeric alpha-amylase inhibitor [Triticum dicoccoides]	gi|229615482	17173.2	7.4499 998092 6514
monomeric alpha-amylase inhibitor [Triticum dicoccoides]	gi|229615484	17187.3	7.4499 998092 6514
monomeric alpha-amylase inhibitor [Triticum dicoccoides]	gi|229615486	17102.2	6.5500 001907 3486
monomeric alpha-amylase inhibitor [Triticum dicoccoides]	gi|229615488	17134.2	6.5500 001907 3486
monomeric alpha-amylase inhibitor [Triticum dicoccoides]	gi|229615508	17187.3	7.4499 998092 6514
monomeric alpha-amylase inhibitor [Triticum dicoccoides]	gi|229615510	17187.3	7.4499 998092 6514
monomeric alpha-amylase inhibitor [Triticum dicoccoides]	gi|229615512	17187.3	7.4499 998092 6514
monomeric alpha-amylase inhibitor [Triticum dicoccoides]	gi|229615514	17187.3	7.4499 998092 6514

monomeric alpha-amylase inhibitor [Triticum dicoccoides]	gi|229615516	17187.3	7.4499 998092 6514
monomeric alpha-amylase inhibitor [Triticum dicoccoides]	gi|229615518	17187.3	7.4499 998092 6514
monomeric alpha-amylase inhibitor [Triticum dicoccoides]	gi|229615520	17102.2	6.5500 001907 3486
monomeric alpha-amylase inhibitor [Triticum dicoccoides]	gi|229615522	17102.2	6.5500 001907 3486
monomeric alpha-amylase inhibitor [Triticum dicoccoides]	gi|229615542	17044.2	7.4499 998092 6514
monomeric alpha-amylase inhibitor [Triticum dicoccoides]	gi|229615544	17102.2	6.5500 001907 3486
monomeric alpha-amylase inhibitor [Triticum dicoccoides]	gi|229615546	17187.3	7.4499 998092 6514
monomeric alpha-amylase inhibitor [Triticum dicoccoides]	gi|229615548	17187.3	7.4499 998092 6514
monomeric alpha-amylase inhibitor [Triticum dicoccoides]	gi|229615550	17157.3	7.4499 998092 6514
monomeric alpha-amylase inhibitor [Triticum dicoccoides]	gi|229615552	17187.3	7.4499 998092

			6514
monomeric alpha-amylase inhibitor [Triticum dicoccoides]	gi|229615554	17100.2	7.4600 000381 4697
monomeric alpha-amylase inhibitor [Triticum dicoccoides]	gi|229615556	17203.2	7.4499 998092 6514
monomeric alpha-amylase inhibitor [Triticum dicoccoides]	gi|229615576	17102.2	6.5500 001907 3486
monomeric alpha-amylase inhibitor [Triticum dicoccoides]	gi|229615578	17102.2	6.5500 001907 3486
monomeric alpha-amylase inhibitor [Triticum dicoccoides]	gi|229615580	17102.2	6.5500 001907 3486
monomeric alpha-amylase inhibitor [Triticum dicoccoides]	gi|229615582	17102.2	6.5500 001907 3486
monomeric alpha-amylase inhibitor [Triticum dicoccoides]	gi|229615584	17187.3	7.4499 998092 6514
monomeric alpha-amylase inhibitor [Triticum dicoccoides]	gi|229615586	17187.3	7.4499 998092 6514
monomeric alpha-amylase inhibitor [Triticum dicoccoides]	gi|229615588	17103.2	5.8000 001907 3486

monomeric alpha-amylase inhibitor [Triticum dicoccoides]	gi|229615590	17118.2	6.5500 001907 3486
monomeric alpha-amylase inhibitor [Triticum dicoccoides]	gi|229615612	17102.2	6.5500 001907 3486
monomeric alpha-amylase inhibitor [Triticum dicoccoides]	gi|229615614	17104.2	6.5500 001907 3486
monomeric alpha-amylase inhibitor [Triticum dicoccoides]	gi|229615616	17104.2	6.5500 001907 3486
monomeric alpha-amylase inhibitor [Triticum dicoccoides]	gi|229615624	17102.2	6.5500 001907 3486
monomeric alpha-amylase inhibitor [Triticum dicoccoides]	gi|229615622	17102.2	6.5500 001907 3486
monomeric alpha-amylase inhibitor [Triticum dicoccoides]	gi|229615620	17102.2	6.5500 001907 3486
monomeric alpha-amylase inhibitor [Triticum monococcum]	gi|134034543	13716.5	6.1799 998283 3862
monomeric alpha-amylase inhibitor [Triticum monococcum]	gi|134034541	13716.5	6.1799 998283 3862

monomeric alpha-amylase inhibitor [Triticum monococcum]	gi|134034506	13658.5	7.4000 000953 6743
monomeric alpha-amylase inhibitor [Triticum monococcum]	gi|134034508	13657.5	8.0299 997329 7119
monomeric alpha-amylase inhibitor [Triticum monococcum]	gi|134034510	13658.5	7.4000 000953 6743
monomeric alpha-amylase inhibitor [Triticum monococcum]	gi|134034512	13658.5	7.4000 000953 6743
monomeric alpha-amylase inhibitor [Triticum monococcum]	gi|134034514	13658.5	7.4000 000953 6743
monomeric alpha-amylase inhibitor [Triticum monococcum]	gi|134034516	13658.5	7.4000 000953 6743
monomeric alpha-amylase inhibitor [Triticum monococcum]	gi|134034518	13658.5	7.4000 000953 6743
monomeric alpha-amylase inhibitor [Triticum monococcum]	gi|134034521	13716.5	6.1799 998283 3862
monomeric alpha-amylase inhibitor [Triticum monococcum]	gi|134034523	13658.5	7.4000 000953 6743
monomeric alpha-amylase inhibitor [Triticum monococcum]	gi|134034525	13658.5	7.4000 000953

			6743
monomeric alpha-amylase inhibitor [Triticum monococcum]	gi|134034537	13699.5	5.3699 998855 5908
monomeric alpha-amylase inhibitor [Triticum monococcum]	gi|134034535	13658.5	7.4000 000953 6743
monomeric alpha-amylase inhibitor [Triticum monococcum]	gi|134034539	13642.5	7.4000 000953 6743
monomeric alpha-amylase inhibitor [Triticum urartu]	gi|134034533	13658.5	7.4000 000953 6743
monomeric alpha-amylase inhibitor [Triticum urartu]	gi|134034527	13658.5	7.4000 000953 6743
monomeric alpha-amylase inhibitor [Triticum urartu]	gi|134034531	13716.5	6.1799 998283 3862
monomeric alpha-amylase inhibitor [Triticum urartu]	gi|134034529	13716.5	6.1799 998283 3862
monomeric alpha-amylase inhibitor, partial [Triticum dicoccoides]	gi|229615618	17230.2	6.5500 001907 3486

Peptide Information

Calc. Mass	Obsrv. Mass	± da	± ppm	Start Seq.	End Seq.	Sequence	Ion Score	C. I. %	Modification	Rank	Result Type

1050.5579	1050.5188	-0.0391	-37	95	103	SVYQELGVR	26	81.976		1	Mascot

10	hypothetical protein TRIUR3_11548 [Triticum urartu]	gi|474069482	20430.8	6.51	1	0	16	0

Peptide Information

Calc. Mass	Obsrv. Mass	± da	± ppm	Start Seq.	End Seq.	Sequence	Ion Score	C. I. %	Modification	Rank	Result Type
1050.5402	1050.5188	-0.0214	-20	64	71	FNPLEKMR	16	0	Oxidation (M)[7]	3	Mascot

Gel Idx/Pos	312/M14	Instr./Gel Origin	BA2151/Sample Project 20140814	Process Status	Analysis Succeeded
Plate [#] Name	[1] Sample Project 20140814	Instrument Sample Name		Spectra	11

Rank	Protein Name	Accession No.	Protein MW	Protein PI	Pep. Count	Protein Score	Protein Score C. I. %	Intensity Matched	Total Ion Score	Total Ion C. I. %	Confirmed
1	0.19 dimeric alpha-amylase inhibitor [Triticum aestivum]	gi|54778507	13814.6	5.73	7	259	100	35.271	209	100	

Protein Group

0.19 dimeric alpha-amylase inhibitor [Triticum aestivum]	gi|54778511	13756.6	6.4899 997711 1816

Peptide Information

Calc. Mass	Obsrv. Mass	± da	± ppm	Start Seq.	End Seq.	Sequence	Ion Score	C. I. %	Modification	Rank	Result Type
1306.7665	1306.7219	-0.0446	-34	14	25	VPALPGCRPVLK			Carbamidomethyl (C)[7]		Mascot
1306.7665	1306.7219	-0.0446	-34	14	25	VPALPGCRPVLK	57	99.988	Carbamidomethyl (C)[7]		Mascot
1554.6637	1554.719	0.0553	36	54	66	CGALYSMLDSMYK			Carbamidomethyl (C)[1], Oxidation (M)[7]		Mascot
1570.8007	1570.7502	-0.0505	-32	26	39	LQCNGSQVPEAVLR			Carbamidomethyl (C)[3]		Mascot
1677.8517	1677.7567	-0.095	-57	101	116	LPIVIDASGDGAYVCK			Carbamidomethyl (C)[15]		Mascot
1854.7568	1854.7277	-0.0291	-16	40	53	ECCQQLADISEWCR			Carbamidomethyl (C)[2,3,13]		Mascot
1887.8403	1887.7791	-0.0612	-32	67	84	EHGVQEGQAGTGAFPSC R			Carbamidomethyl (C)[17]		Mascot
1887.8403	1887.7791	-0.0612	-32	67	84	EHGVQEGQAGTGAFPSC R	152	100	Carbamidomethyl (C)[17]		Mascot
2043.9414	2043.8724	-0.069	-34	67	85	EHGVQEGQAGTGAFPSC RR			Carbamidomethyl (C)[17]		Mascot

Rank	Protein Name	Accession No.	Protein MW	Protein PI	Pep. Count	Protein Score	Protein Score C. I. %	Intensity Matched	Total Ion Score	Total Ion C. I. %	Confirmed
2	dimeric alpha-amylase inhibitor [Triticum aestivum]	gi|65993807	15588.5	5.3	7	256	100	35.271	209	100	

Protein Group

dimeric alpha-amylase inhibitor [Triticum aestivum]	gi|65993941	15631.5	5.7699 999809 2651

Peptide Information

Calc. Mass	Obsrv. Mass	± da	± ppm	Start Seq.	End Seq.	Sequence	Ion Score	C. I. %	Modification	Rank	Result Type
1306.7665	1306.7219	-0.0446	-34	31	42	VPALPGCRPVLK			Carbamidomethyl (C)[7]		Mascot
1306.7665	1306.7219	-0.0446	-34	31	42	VPALPGCRPVLK	57	99.988	Carbamidomethyl (C)[7]		Mascot
1554.6637	1554.719	0.0553	36	71	83	CGALYSMLDSMYK			Carbamidomethyl (C)[1], Oxidation (M)[7]		Mascot

1570.8007	1570.7502	-0.0505	-32	43	56	LQCNGSQVPEAVLR			Carbamidomethyl (C)[3]		Mascot
1677.8517	1677.7567	-0.095	-57	118	133	LPIVIDASGDGAYVCK			Carbamidomethyl (C)[15]		Mascot
1854.7568	1854.7277	-0.0291	-16	57	70	ECCQQLADISEWCR			Carbamidomethyl (C)[2,3,13]		Mascot
1887.8403	1887.7791	-0.0612	-32	84	101	EHGVQEGQAGTGAFPSC R			Carbamidomethyl (C)[17]		Mascot
1887.8403	1887.7791	-0.0612	-32	84	101	EHGVQEGQAGTGAFPSC R	152	100	Carbamidomethyl (C)[17]		Mascot
2043.9414	2043.8724	-0.069	-34	84	102	EHGVQEGQAGTGAFPSC RR			Carbamidomethyl (C)[17]		Mascot

3	dimeric alpha-amylase inhibitor [Triticum dicoccoides]	gi|114215776	13742.5	6.49	6	249	100	35.11	209	100	

Protein Group

dimeric alpha-amylase inhibitor [Triticum dicoccoides]	gi|114215772	13742.5	6.4899 997711 1816
dimeric alpha-amylase inhibitor [Triticum dicoccoides]	gi|114215768	13742.5	6.4899 997711 1816
dimeric alpha-amylase inhibitor [Triticum dicoccoides]	gi|114215762	13742.5	6.4899 997711 1816
dimeric alpha-amylase inhibitor [Triticum dicoccoides]	gi|114215790	13742.5	6.4899 997711 1816

Peptide Information

Calc. Mass	Obsrv. Mass	± da	± ppm	Start Seq.	End Seq.	Sequence	Ion Score	C. I. %	Modification	Rank	Result Type
1306.7665	1306.7219	-0.0446	-34	14	25	VPALPGCRPVLK			Carbamidomethyl (C)[7]		Mascot
1306.7665	1306.7219	-0.0446	-34	14	25	VPALPGCRPVLK	57	99.988	Carbamidomethyl (C)[7]		Mascot
1554.6637	1554.719	0.0553	36	54	66	CGALYSMLDSMYK			Carbamidomethyl (C)[1], Oxidation (M)[7]		Mascot
1570.8007	1570.7502	-0.0505	-32	26	39	LQCNGSQVPEAVLR			Carbamidomethyl (C)[3]		Mascot
1677.8517	1677.7567	-0.095	-57	101	116	LPIVIDASGDGAYVCK			Carbamidomethyl (C)[15]		Mascot
1887.8403	1887.7791	-0.0612	-32	67	84	EHGVQEGQAGTGAFPSC R			Carbamidomethyl (C)[17]		Mascot
1887.8403	1887.7791	-0.0612	-32	67	84	EHGVQEGQAGTGAFPSC R	152	100	Carbamidomethyl (C)[17]		Mascot
2043.9414	2043.8724	-0.069	-34	67	85	EHGVQEGQAGTGAFPSC RR			Carbamidomethyl (C)[17]		Mascot

4	dimeric alpha-amylase inhibitor [Triticum aestivum]	gi|65993852	15558.5	5.3	6	247	100	35.074	209	100

Peptide Information

Calc. Mass	Obsrv. Mass	± da	± ppm	Start Seq.	End Seq.	Sequence	Ion Score	C. I. %	Modification	Rank	Result Type
1306.7665	1306.7219	-0.0446	-34	31	42	VPALPGCRPVLK			Carbamidomethyl (C)[7]		Mascot
1306.7665	1306.7219	-0.0446	-34	31	42	VPALPGCRPVLK	57	99.988	Carbamidomethyl (C)[7]		Mascot
1570.8007	1570.7502	-0.0505	-32	43	56	LQCNGSQVPEAVLR			Carbamidomethyl (C)[3]		Mascot
1677.8517	1677.7567	-0.095	-57	118	133	LPIVIDASGDGAYVCK			Carbamidomethyl (C)[15]		Mascot
1854.7568	1854.7277	-0.0291	-16	57	70	ECCQQLADISEWCR			Carbamidomethyl (C)[2,3,13]		Mascot
1887.8403	1887.7791	-0.0612	-32	84	101	EHGVQEGGQAGTGAFPSC R			Carbamidomethyl (C)[17]		Mascot
1887.8403	1887.7791	-0.0612	-32	84	101	EHGVQEGGQAGTGAFPSC R	152	100	Carbamidomethyl (C)[17]		Mascot
2043.9414	2043.8724	-0.069	-34	84	102	EHGVQEGGQAGTGAFPSC RR			Carbamidomethyl (C)[17]		Mascot

5	dimeric alpha-amylase inhibitor [Triticum timopheevii subsp. armeniacum]	gi|227809294	15588.4	5.28	6	246	100	35.11	209	100

Protein Group

dimeric alpha-amylase inhibitor [Triticum dicoccoides]	gi|227809240	15588.5	5.2800 002098 0835
dimeric alpha-amylase inhibitor [Triticum dicoccoides]	gi|227809226	15574.4	5.2800 002098 0835
dimeric alpha-amylase inhibitor [Triticum dicoccoides]	gi|227809216	15574.4	5.2800 002098 0835
dimeric alpha-amylase inhibitor [Triticum dicoccoides]	gi|227809212	15574.4	5.2800 002098 0835
dimeric alpha-amylase inhibitor [Triticum dicoccoides]	gi|227809196	15574.4	5.2800 002098 0835

dimeric alpha-amylase inhibitor [Triticum dicoccoides]	gi|227809192	15604.4	5.2800<br>002098<br>0835
dimeric alpha-amylase inhibitor [Triticum dicoccoides]	gi|227809174	15574.4	5.2800<br>002098<br>0835
dimeric alpha-amylase inhibitor [Triticum dicoccoides]	gi|227809172	15548.4	5.5799<br>999237<br>0605
dimeric alpha-amylase inhibitor [Triticum dicoccoides]	gi|227809154	15574.4	5.2800<br>002098<br>0835
dimeric alpha-amylase inhibitor [Triticum dicoccoides]	gi|227809140	15574.4	5.2800<br>002098<br>0835
dimeric alpha-amylase inhibitor [Triticum dicoccoides]	gi|227809092	15574.4	5.2800<br>002098<br>0835
dimeric alpha-amylase inhibitor [Triticum dicoccoides]	gi|227809084	15574.4	5.2800<br>002098<br>0835
dimeric alpha-amylase inhibitor [Triticum dicoccoides]	gi|227809082	15574.4	5.2800<br>002098<br>0835
dimeric alpha-amylase inhibitor [Triticum dicoccoides]	gi|227809055	15558.5	5.7399<br>997711<br>1816
dimeric alpha-amylase inhibitor [Triticum dicoccoides]	gi|227809047	15574.4	5.2800<br>002098

			0835
dimeric alpha-amylase inhibitor [Triticum dicoccoides]	gi|227809039	15574.4	5.2800 002098 0835
dimeric alpha-amylase inhibitor [Triticum dicoccoides]	gi|227809037	15574.4	5.2800 002098 0835
dimeric alpha-amylase inhibitor [Triticum dicoccoides]	gi|227809033	15574.4	5.2800 002098 0835
dimeric alpha-amylase inhibitor [Triticum dicoccoides]	gi|227809025	15574.4	5.2800 002098 0835
dimeric alpha-amylase inhibitor [Triticum dicoccoides]	gi|227809003	15574.4	5.2800 002098 0835
dimeric alpha-amylase inhibitor [Triticum dicoccoides]	gi|227808981	15574.4	5.2800 002098 0835
dimeric alpha-amylase inhibitor [Triticum dicoccoides]	gi|227808979	15574.4	5.2800 002098 0835
dimeric alpha-amylase inhibitor [Triticum dicoccoides]	gi|227808972	15574.4	5.2800 002098 0835
dimeric alpha-amylase inhibitor [Triticum dicoccoides]	gi|227808962	15574.4	5.2800 002098 0835

dimeric alpha-amylase inhibitor [Triticum dicoccoides]	gi|227808960	15574.4	5.2800<br>002098<br>0835
dimeric alpha-amylase inhibitor [Triticum dicoccoides]	gi|227808948	15574.4	5.2800<br>002098<br>0835
dimeric alpha-amylase inhibitor [Triticum dicoccoides]	gi|227808942	15574.4	5.2800<br>002098<br>0835
dimeric alpha-amylase inhibitor [Triticum dicoccoides]	gi|227808940	15574.4	5.2800<br>002098<br>0835
dimeric alpha-amylase inhibitor [Triticum dicoccoides]	gi|227808932	15574.4	5.2800<br>002098<br>0835
dimeric alpha-amylase inhibitor [Triticum dicoccoides]	gi|227808926	15574.4	5.2800<br>002098<br>0835
dimeric alpha-amylase inhibitor [Triticum dicoccoides]	gi|227808924	15574.4	5.2800<br>002098<br>0835
dimeric alpha-amylase inhibitor [Triticum dicoccoides]	gi|227808908	15574.4	5.2800<br>002098<br>0835
dimeric alpha-amylase inhibitor [Triticum dicoccoides]	gi|227808906	15574.4	5.2800<br>002098<br>0835

dimeric alpha-amylase inhibitor [Triticum dicoccoides]	gi|227808896	15588.5	5.2800 002098 0835
dimeric alpha-amylase inhibitor [Triticum dicoccoides]	gi|227808889	15588.5	5.2800 002098 0835
dimeric alpha-amylase inhibitor [Triticum dicoccoides]	gi|227808887	15588.5	5.2800 002098 0835
dimeric alpha-amylase inhibitor [Triticum dicoccoides]	gi|227808884	15588.5	5.2800 002098 0835
dimeric alpha-amylase inhibitor [Triticum timopheevii subsp. armeniacum]	gi|227809296	15574.4	5.2800 002098 0835

Peptide Information

Calc. Mass	Obsrv. Mass	± da	± ppm	Start Seq.	End Seq.	Sequence	Ion Score	C. I. %	Modification	Rank	Result Type
1306.7665	1306.7219	-0.0446	-34	31	42	VPALPGCRPVLK			Carbamidomethyl (C)[7]		Mascot
1306.7665	1306.7219	-0.0446	-34	31	42	VPALPGCRPVLK	57	99.988	Carbamidomethyl (C)[7]		Mascot
1554.6637	1554.719	0.0553	36	71	83	CGALYSMLDSMYK			Carbamidomethyl (C)[1], Oxidation (M)[7]		Mascot
1570.8007	1570.7502	-0.0505	-32	43	56	LQCNGSQVPEAVLR			Carbamidomethyl (C)[3]		Mascot
1677.8517	1677.7567	-0.095	-57	118	133	LPIVIDASGDGAYVCK			Carbamidomethyl (C)[15]		Mascot
1887.8403	1887.7791	-0.0612	-32	84	101	EHGVQEGQAGTGAFPSCR			Carbamidomethyl (C)[17]		Mascot
1887.8403	1887.7791	-0.0612	-32	84	101	EHGVQEGQAGTGAFPSCR	152	100	Carbamidomethyl (C)[17]		Mascot
2043.9414	2043.8724	-0.069	-34	84	102	EHGVQEGQAGTGAFPSCRR			Carbamidomethyl (C)[17]		Mascot

6	dimeric alpha-amylase inhibitor [Triticum dicoccoides]	gi|227809045	15558.4	5.28	6	246	100	35.11	209	100

Peptide Information

Calc. Mass	Obsrv. Mass	± da	± ppm	Start Seq.	End Seq.	Sequence	Ion Score	C. I. %	Modification	Rank	Result Type

1306.7665	1306.7219	-0.0446	-34	31	42	VPALPGCRPVLK			Carbamidomethyl (C)[7]	Mascot
1306.7665	1306.7219	-0.0446	-34	31	42	VPALPGCRPVLK	57	99.988	Carbamidomethyl (C)[7]	Mascot
1554.7694	1554.719	-0.0504	-32	43	56	LQCNGSQVPEAVPR			Carbamidomethyl (C)[3]	Mascot
1570.6587	1570.7502	0.0915	58	71	83	CGALYSMLDSMYK			Carbamidomethyl (C)[1], Oxidation (M)[7,11]	Mascot
1677.8517	1677.7567	-0.095	-57	118	133	LPIVIDASGDGAYVCK			Carbamidomethyl (C)[15]	Mascot
1887.8403	1887.7791	-0.0612	-32	84	101	EHGVQEGQAGTGAFPSC R			Carbamidomethyl (C)[17]	Mascot
1887.8403	1887.7791	-0.0612	-32	84	101	EHGVQEGQAGTGAFPSC R	152	100	Carbamidomethyl (C)[17]	Mascot
2043.9414	2043.8724	-0.069	-34	84	102	EHGVQEGQAGTGAFPSC RR			Carbamidomethyl (C)[17]	Mascot

7	dimeric alpha-amylase inhibitor [Triticum timopheevii subsp. armeniacum]	gi|227809284	15588.5	5.28	2	0	209	100

Protein Group

dimeric alpha-amylase inhibitor [Triticum aestivum]	gi|65993756	15820.6	6.5399 999618 5303
dimeric alpha-amylase inhibitor [Triticum dicoccoides]	gi|227809222	15602.5	5.75
dimeric alpha-amylase inhibitor [Triticum dicoccoides]	gi|227809112	15602.5	5.2800 002098 0835
dimeric alpha-amylase inhibitor [Triticum dicoccoides]	gi|227808880	15588.5	5.2800 002098 0835
dimeric alpha-amylase inhibitor [Triticum dicoccoides]	gi|227809001	15573.5	5.7399 997711 1816
dimeric alpha-amylase inhibitor [Triticum dicoccoides]	gi|227808876	15588.5	5.2800 002098 0835
dimeric alpha-amylase inhibitor [Triticum dicoccoides]	gi|227808859	15588.5	5.2800 002098

			0835
dimeric alpha-amylase inhibitor [Triticum dicoccoides]	gi|227808855	15588.5	5.2800 002098 0835
dimeric alpha-amylase inhibitor [Triticum dicoccoides]	gi|227808843	15588.5	5.2800 002098 0835
dimeric alpha-amylase inhibitor [Triticum dicoccoides]	gi|114215784	13770.6	6.4899 997711 1816
dimeric alpha-amylase inhibitor [Triticum dicoccoides]	gi|227808839	15588.5	5.2800 002098 0835
dimeric alpha-amylase inhibitor [Triticum dicoccoides]	gi|227808849	15588.5	5.2800 002098 0835
dimeric alpha-amylase inhibitor [Triticum dicoccoides]	gi|227808857	15562.4	5.5799 999237 0605
dimeric alpha-amylase inhibitor [Triticum dicoccoides]	gi|227808863	15588.5	5.2800 002098 0835
dimeric alpha-amylase inhibitor [Triticum timopheevii subsp. armeniacum]	gi|227809282	15606.4	5.2800 002098 0835
dimeric alpha-amylase inhibitor [Triticum timopheevii subsp. armeniacum]	gi|227809280	15588.5	5.2800 002098 0835

	dimeric alpha-amylase inhibitor [Triticum timopheevii subsp. armeniacum]	gi|227809278	15588.5	5.2800 002098 0835

Peptide Information

Calc. Mass	Obsrv. Mass	± da	± ppm	Start Seq.	End Seq.	Sequence	Ion Score	C. I. %	Modification	Rank	Result Type
1306.7665	1306.7219	-0.0446	-34	31	42	VPALPGCRPVLK	57	99.988	Carbamidomethyl (C)[7]	1	Mascot
1887.8403	1887.7791	-0.0612	-32	84	101	EHGVQEGQAGTGAFPSCR	152	100	Carbamidomethyl (C)[17]	1	Mascot

8	Glycerol-3-phosphate dehydrogenase SDP6, mitochondrial [Triticum urartu]	gi|474261841	58632.6	7.25	1	0	16	0

Peptide Information

Calc. Mass	Obsrv. Mass	± da	± ppm	Start Seq.	End Seq.	Sequence	Ion Score	C. I. %	Modification	Rank	Result Type
1480.7213	1480.7294	0.0081	5	180	193	VVVNASGAFCDSVR	16	0	Carbamidomethyl (C)[10]	1	Mascot

9	hypothetical protein TRIUR3_09519 [Triticum urartu]	gi|473894932	54375.6	9.57	1	0	12	0

Peptide Information

Calc. Mass	Obsrv. Mass	± da	± ppm	Start Seq.	End Seq.	Sequence	Ion Score	C. I. %	Modification	Rank	Result Type
1571.7772	1571.7378	-0.0394	-25	9	23	RLPSDDNVASDGGLR	13	0		1	Mascot

10	Expansin-A23 [Triticum urartu]	gi|473874952	28602.9	8.61	1	0	11	0

Peptide Information

Calc. Mass	Obsrv. Mass	± da	± ppm	Start Seq.	End Seq.	Sequence	Ion Score	C. I. %	Modification	Rank	Result Type
1306.7339	1306.7219	-0.012	-9	215	227	GARLLGGEALGHR	11	0		2	Mascot

Gel Idx/Pos	313/M15	Instr./Gel Origin	BA2151/Sample Project 20140814	Process Status	Analysis Succeeded
Plate [#] Name	[1] Sample Project 20140814	Instrument Sample Name		Spectra	11

Rank	Protein Name	Accession No.	Protein MW	Protein PI	Pep. Count	Protein Score	Protein Score C. I. %	Intensity Matched	Total Ion Score	Total Ion C. I. %	Confirmed
1	monomeric alpha-amylase inhibitor [Triticum aestivum]	gi|134034615	13617.5	5.37	8	375	100	58.489	317	100	

Protein Group

monomeric alpha-amylase inhibitor [Triticum aestivum]	gi|134034647	13617.5	5.3699998855 5908

Peptide Information

Calc. Mass	Obsrv. Mass	± da	± ppm	Start Seq.	End Seq.	Sequence	Ion Score	C. I. %	Modification	Rank	Result Type
830.4189	830.389	-0.0299	-36	77	83	EVLPGCR			Carbamidomethyl (C)[6]		Mascot
863.4404	863.4152	-0.0252	-29	15	22	VSALTGCR			Carbamidomethyl (C)[7]		Mascot
1050.5579	1050.522	-0.0359	-34	65	73	SVYQELGVR					Mascot
1050.5579	1050.522	-0.0359	-34	65	73	SVYQELGVR	72	100			Mascot
1054.4657	1054.4099	-0.0558	-53	56	64	CGDLSSMLR			Carbamidomethyl (C)[1], Oxidation (M)[7]		Mascot
1174.6136	1174.5415	-0.0721	-61	89	99	LTAASVPEVCK			Carbamidomethyl (C)[10]		Mascot
1555.8262	1555.7826	-0.0436	-28	27	40	LQCVGSQVPEAVLR			Carbamidomethyl (C)[3]		Mascot
1555.8262	1555.7826	-0.0436	-28	27	40	LQCVGSQVPEAVLR	113	100	Carbamidomethyl (C)[3]		Mascot
1611.6897	1611.6116	-0.0781	-48	1	14	SGPWSWCDPATGYK			Carbamidomethyl (C)[7]		Mascot
1981.795	1981.7411	-0.0539	-27	41	55	DCCQQLADINNEWCR			Carbamidomethyl (C)[2,3,14]		Mascot
1981.795	1981.7411	-0.0539	-27	41	55	DCCQQLADINNEWCR	132	100	Carbamidomethyl (C)[2,3,14]		Mascot

Rank	Protein Name	Accession No.	Protein MW	Protein PI	Pep. Count	Protein Score	Protein Score C. I. %	Intensity Matched	Total Ion Score	Total Ion C. I. %	Confirmed
2	monomeric alpha-amylase inhibitor [Triticum monococcum]	gi|134034508	13657.5	8.03	8	374	100	59.369	317	100	

Peptide Information

Calc. Mass	Obsrv. Mass	± da	± ppm	Start Seq.	End Seq.	Sequence	Ion Score	C. I. %	Modification	Rank	Result Type
830.4189	830.389	-0.0299	-36	77	83	EVLPGCR			Carbamidomethyl (C)[6]		Mascot
863.4404	863.4152	-0.0252	-29	15	22	VSALTGCR			Carbamidomethyl (C)[7]		Mascot
1037.4867	1037.3872	-0.0995	-96	56	64	CGNLSSMLR			Carbamidomethyl (C)[1]		Mascot
1037.4867	1037.3872	-0.0995	-96	56	64	CGNLSSMLR			Carbamidomethyl (C)[1]		Mascot
1050.5579	1050.522	-0.0359	-34	65	73	SVYQELGVR					Mascot
1050.5579	1050.522	-0.0359	-34	65	73	SVYQELGVR	72	100			Mascot

1174.6136	1174.5415	-0.0721	-61	89	99	LTAASVPEVCK			Carbamidomethyl (C)[10]	Mascot
1555.8262	1555.7826	-0.0436	-28	27	40	LQCVGSQVPEAVLR			Carbamidomethyl (C)[3]	Mascot
1555.8262	1555.7826	-0.0436	-28	27	40	LQCVGSQVPEAVLR	113	100	Carbamidomethyl (C)[3]	Mascot
1611.6897	1611.6116	-0.0781	-48	1	14	SGPWSWCDPATGYK			Carbamidomethyl (C)[7]	Mascot
1981.795	1981.7411	-0.0539	-27	41	55	DCCQQLADINNEWCR			Carbamidomethyl (C)[2,3,14]	Mascot
1981.795	1981.7411	-0.0539	-27	41	55	DCCQQLADINNEWCR	132	100	Carbamidomethyl (C)[2,3,14]	Mascot

3	monomeric alpha-amylase inhibitor [Triticum aestivum]	gi|134034645	13658.5	7.4	8	373	100	58.489	317	100

Protein Group

monomeric alpha-amylase inhibitor [Triticum aestivum]	gi|134034643	13658.5	7.4000<br>000953<br>6743
monomeric alpha-amylase inhibitor [Triticum aestivum]	gi|134034639	13658.5	7.4000<br>000953<br>6743
monomeric alpha-amylase inhibitor [Triticum aestivum]	gi|134034637	13716.5	6.1799<br>998283<br>3862
monomeric alpha-amylase inhibitor [Triticum aestivum]	gi|134034635	13658.5	7.4000<br>000953<br>6743
monomeric alpha-amylase inhibitor [Triticum aestivum]	gi|134034633	13658.5	7.4000<br>000953<br>6743
monomeric alpha-amylase inhibitor [Triticum aestivum]	gi|134034631	13716.5	6.1799<br>998283<br>3862
monomeric alpha-amylase inhibitor [Triticum aestivum]	gi|134034629	13716.5	6.1799<br>998283<br>3862

monomeric alpha-amylase inhibitor [Triticum aestivum]	gi|134034627	13658.5	7.4000 000953 6743
monomeric alpha-amylase inhibitor [Triticum aestivum]	gi|134034625	13716.5	6.1799 998283 3862
monomeric alpha-amylase inhibitor [Triticum aestivum]	gi|134034623	13716.5	6.1799 998283 3862
monomeric alpha-amylase inhibitor [Triticum aestivum]	gi|134034621	13716.5	6.1799 998283 3862
monomeric alpha-amylase inhibitor [Triticum aestivum]	gi|134034619	13716.5	6.1799 998283 3862
monomeric alpha-amylase inhibitor [Triticum aestivum]	gi|134034617	13716.5	6.1799 998283 3862
monomeric alpha-amylase inhibitor [Triticum aestivum]	gi|134034611	13716.5	6.1799 998283 3862
monomeric alpha-amylase inhibitor [Triticum aestivum]	gi|134034609	13716.5	6.1799 998283 3862
monomeric alpha-amylase inhibitor [Triticum aestivum]	gi|134034607	13716.5	6.1799 998283 3862
monomeric alpha-amylase inhibitor [Triticum aestivum]	gi|134034605	13716.5	6.1799 998283

			3862
monomeric alpha-amylase inhibitor [Triticum aestivum]	gi|134034649	13658.5	7.4000 000953 6743
monomeric alpha-amylase inhibitor [Triticum aestivum]	gi|134034603	13716.5	6.1799 998283 3862
monomeric alpha-amylase inhibitor [Triticum aestivum]	gi|134034601	13716.5	6.1799 998283 3862
monomeric alpha-amylase inhibitor [Triticum aestivum]	gi|134034599	13716.5	6.1799 998283 3862
monomeric alpha-amylase inhibitor [Triticum aestivum]	gi|134034597	13716.5	6.1799 998283 3862
monomeric alpha-amylase inhibitor [Triticum aestivum]	gi|134034595	13716.5	6.1799 998283 3862
monomeric alpha-amylase inhibitor [Triticum aestivum]	gi|134034593	13716.5	6.1799 998283 3862
monomeric alpha-amylase inhibitor [Triticum aestivum]	gi|134034591	13716.5	6.1799 998283 3862
monomeric alpha-amylase inhibitor [Triticum aestivum]	gi|134034589	13716.5	6.1799 998283 3862

monomeric alpha-amylase inhibitor [Triticum aestivum]	gi|134034587	13716.5	6.1799 998283 3862
monomeric alpha-amylase inhibitor [Triticum aestivum]	gi|134034585	13716.5	6.1799 998283 3862
monomeric alpha-amylase inhibitor [Triticum aestivum]	gi|134034583	13716.5	6.1799 998283 3862
monomeric alpha-amylase inhibitor [Triticum aestivum]	gi|134034581	13716.5	6.1799 998283 3862
monomeric alpha-amylase inhibitor [Triticum aestivum]	gi|134034579	13716.5	6.1799 998283 3862
monomeric alpha-amylase inhibitor [Triticum aestivum]	gi|134034575	13716.5	6.1799 998283 3862
monomeric alpha-amylase inhibitor [Triticum aestivum]	gi|134034573	13716.5	6.1799 998283 3862
monomeric alpha-amylase inhibitor [Triticum monococcum]	gi|134034543	13716.5	6.1799 998283 3862
monomeric alpha-amylase inhibitor [Triticum monococcum]	gi|134034514	13658.5	7.4000 000953 6743

monomeric alpha-amylase inhibitor [Triticum monococcum]	gi|134034516	13658.5	7.4000 000953 6743
monomeric alpha-amylase inhibitor [Triticum monococcum]	gi|134034518	13658.5	7.4000 000953 6743
monomeric alpha-amylase inhibitor [Triticum monococcum]	gi|134034521	13716.5	6.1799 998283 3862
monomeric alpha-amylase inhibitor [Triticum monococcum]	gi|134034523	13658.5	7.4000 000953 6743
monomeric alpha-amylase inhibitor [Triticum monococcum]	gi|134034525	13658.5	7.4000 000953 6743
monomeric alpha-amylase inhibitor [Triticum monococcum]	gi|134034541	13716.5	6.1799 998283 3862
monomeric alpha-amylase inhibitor [Triticum monococcum]	gi|134034535	13658.5	7.4000 000953 6743
monomeric alpha-amylase inhibitor [Triticum urartu]	gi|134034533	13658.5	7.4000 000953 6743
monomeric alpha-amylase inhibitor [Triticum urartu]	gi|134034531	13716.5	6.1799 998283 3862
monomeric alpha-amylase inhibitor [Triticum urartu]	gi|134034529	13716.5	6.1799 998283

			3862
monomeric alpha-amylase inhibitor [Triticum urartu]	gi|134034527	13658.5	7.4000000953 6743

Peptide Information

Calc. Mass	Obsrv. Mass	± da	± ppm	Start Seq.	End Seq.	Sequence	Ion Score	C. I. %	Modification	Rank	Result Type
830.4189	830.389	-0.0299	-36	77	83	EVLPGCR			Carbamidomethyl (C)[6]		Mascot
863.4404	863.4152	-0.0252	-29	15	22	VSALTGCR			Carbamidomethyl (C)[7]		Mascot
1050.5579	1050.522	-0.0359	-34	65	73	SVYQELGVR					Mascot
1050.5579	1050.522	-0.0359	-34	65	73	SVYQELGVR	72	100			Mascot
1054.4657	1054.4099	-0.0558	-53	56	64	CGDLSSMLR			Carbamidomethyl (C)[1], Oxidation (M)[7]		Mascot
1174.6136	1174.5415	-0.0721	-61	89	99	LTAASVPEVCK			Carbamidomethyl (C)[10]		Mascot
1555.8262	1555.7826	-0.0436	-28	27	40	LQCVGSQVPEAVLR			Carbamidomethyl (C)[3]		Mascot
1555.8262	1555.7826	-0.0436	-28	27	40	LQCVGSQVPEAVLR	113	100	Carbamidomethyl (C)[3]		Mascot
1611.6897	1611.6116	-0.0781	-48	1	14	SGPWSWCDPATGYK			Carbamidomethyl (C)[7]		Mascot
1981.795	1981.7411	-0.0539	-27	41	55	DCCQQLADINNEWCR			Carbamidomethyl (C)[2,3,14]		Mascot
1981.795	1981.7411	-0.0539	-27	41	55	DCCQQLADINNEWCR	132	100	Carbamidomethyl (C)[2,3,14]		Mascot

4	monomeric alpha-amylase inhibitor [Triticum dicoccoides]	gi|229615664	17201.3	7.45	3	0	317	100	

Protein Group

RecName: Full=Alpha-amylase inhibitor 0.28; AltName: Full=CIII; AltName: Full=WMAI-1; Flags: Precursor	gi|110282974	17358.3	7.4499998092 6514
monomeric alpha-amylase inhibitor [Triticum aestivum]	gi|134034613	13637.5	7.4099998474 1211
monomeric alpha-amylase inhibitor [Triticum aestivum]	gi|134034577	13667.5	6.1799998283 3862
monomeric alpha-amylase inhibitor [Triticum aestivum]	gi|2894148	17187.3	7.4499

			998092 6514
monomeric alpha-amylase inhibitor [Triticum dicoccoides]	gi|229615662	17173.2	7.4499 998092 6514
monomeric alpha-amylase inhibitor [Triticum dicoccoides]	gi|229615660	17102.2	6.5500 001907 3486
monomeric alpha-amylase inhibitor [Triticum dicoccoides]	gi|229615658	17173.3	8.0299 997329 7119
monomeric alpha-amylase inhibitor [Triticum dicoccoides]	gi|229615656	17102.2	6.5500 001907 3486
monomeric alpha-amylase inhibitor [Triticum dicoccoides]	gi|229615654	17173.2	7.4499 998092 6514
monomeric alpha-amylase inhibitor [Triticum dicoccoides]	gi|229615650	17102.2	6.5500 001907 3486
monomeric alpha-amylase inhibitor [Triticum dicoccoides]	gi|229615648	17102.2	6.5500 001907 3486
monomeric alpha-amylase inhibitor [Triticum dicoccoides]	gi|229615646	17102.2	6.5500 001907 3486
monomeric alpha-amylase inhibitor [Triticum dicoccoides]	gi|229615644	17102.2	6.5500 001907 3486

monomeric alpha-amylase inhibitor [Triticum dicoccoides]	gi|229615642	17074.2	6.5500 001907 3486
monomeric alpha-amylase inhibitor [Triticum dicoccoides]	gi|229615640	17102.2	6.5500 001907 3486
monomeric alpha-amylase inhibitor [Triticum dicoccoides]	gi|229615638	17102.2	6.5500 001907 3486
monomeric alpha-amylase inhibitor [Triticum dicoccoides]	gi|229615636	17102.2	6.5500 001907 3486
monomeric alpha-amylase inhibitor [Triticum dicoccoides]	gi|229615634	17102.2	6.5500 001907 3486
monomeric alpha-amylase inhibitor [Triticum dicoccoides]	gi|229615632	17102.2	6.5500 001907 3486
monomeric alpha-amylase inhibitor [Triticum dicoccoides]	gi|229615630	17102.2	6.5500 001907 3486
monomeric alpha-amylase inhibitor [Triticum dicoccoides]	gi|229615624	17102.2	6.5500 001907 3486
monomeric alpha-amylase inhibitor [Triticum dicoccoides]	gi|229615590	17118.2	6.5500 001907 3486

monomeric alpha-amylase inhibitor [Triticum dicoccoides]	gi|229615592	17102.2	6.5500 001907 3486
monomeric alpha-amylase inhibitor [Triticum dicoccoides]	gi|229615594	17188.3	6.5500 001907 3486
monomeric alpha-amylase inhibitor [Triticum dicoccoides]	gi|229615596	17188.3	6.5500 001907 3486
monomeric alpha-amylase inhibitor [Triticum dicoccoides]	gi|229615598	17188.3	6.5500 001907 3486
monomeric alpha-amylase inhibitor [Triticum dicoccoides]	gi|229615602	17188.3	6.5500 001907 3486
monomeric alpha-amylase inhibitor [Triticum dicoccoides]	gi|229615604	17188.3	6.5500 001907 3486
monomeric alpha-amylase inhibitor [Triticum dicoccoides]	gi|229615606	17070.2	7.4600 000381 4697
monomeric alpha-amylase inhibitor [Triticum dicoccoides]	gi|229615608	17104.2	6.5500 001907 3486
monomeric alpha-amylase inhibitor [Triticum dicoccoides]	gi|229615554	17100.2	7.4600 000381 4697
monomeric alpha-amylase inhibitor [Triticum	gi|229615556	17203.2	7.4499

dicoccoides]			998092 6514
monomeric alpha-amylase inhibitor [Triticum dicoccoides]	gi|229615558	17104.2	6.5500 001907 3486
monomeric alpha-amylase inhibitor [Triticum dicoccoides]	gi|229615560	17173.2	7.4499 998092 6514
monomeric alpha-amylase inhibitor [Triticum dicoccoides]	gi|229615562	17104.2	7.4600 000381 4697
monomeric alpha-amylase inhibitor [Triticum dicoccoides]	gi|229615564	17203.2	7.4499 998092 6514
monomeric alpha-amylase inhibitor [Triticum dicoccoides]	gi|229615566	17173.2	7.4499 998092 6514
monomeric alpha-amylase inhibitor [Triticum dicoccoides]	gi|229615568	17102.2	6.5500 001907 3486
monomeric alpha-amylase inhibitor [Triticum dicoccoides]	gi|229615570	17102.2	6.5500 001907 3486
monomeric alpha-amylase inhibitor [Triticum dicoccoides]	gi|229615472	17203.2	7.4499 998092 6514
monomeric alpha-amylase inhibitor [Triticum dicoccoides]	gi|229615474	17206.3	7.4600 000381 4697

monomeric alpha-amylase inhibitor [Triticum dicoccoides]	gi|229615476	17177.2	7.4499998092 6514
monomeric alpha-amylase inhibitor [Triticum dicoccoides]	gi|229615480	17104.2	6.5500001907 3486
monomeric alpha-amylase inhibitor [Triticum dicoccoides]	gi|229615482	17173.2	7.4499998092 6514
monomeric alpha-amylase inhibitor [Triticum dicoccoides]	gi|229615484	17187.3	7.4499998092 6514
monomeric alpha-amylase inhibitor [Triticum dicoccoides]	gi|229615486	17102.2	6.5500001907 3486
monomeric alpha-amylase inhibitor [Triticum dicoccoides]	gi|229615488	17134.2	6.5500001907 3486
monomeric alpha-amylase inhibitor [Triticum dicoccoides]	gi|229615490	17102.2	6.5500001907 3486
monomeric alpha-amylase inhibitor [Triticum dicoccoides]	gi|229615406	17102.2	6.5500001907 3486
monomeric alpha-amylase inhibitor [Triticum dicoccoides]	gi|229615408	17131.2	5.3800001144 4092

monomeric alpha-amylase inhibitor [Triticum dicoccoides]	gi|229615412	17102.2	6.5500 001907 3486
monomeric alpha-amylase inhibitor [Triticum dicoccoides]	gi|229615414	17102.2	6.5500 001907 3486
monomeric alpha-amylase inhibitor [Triticum dicoccoides]	gi|229615416	17102.2	6.5500 001907 3486
monomeric alpha-amylase inhibitor [Triticum dicoccoides]	gi|229615418	17102.2	6.5500 001907 3486
monomeric alpha-amylase inhibitor [Triticum dicoccoides]	gi|229615422	17102.2	6.5500 001907 3486
monomeric alpha-amylase inhibitor [Triticum dicoccoides]	gi|229615424	17102.2	6.5500 001907 3486
monomeric alpha-amylase inhibitor [Triticum dicoccoides]	gi|229615426	17098.3	7.4600 000381 4697
monomeric alpha-amylase inhibitor [Triticum dicoccoides]	gi|229615368	17173.2	7.4499 998092 6514
monomeric alpha-amylase inhibitor [Triticum dicoccoides]	gi|229615370	17203.2	7.4499 998092 6514
monomeric alpha-amylase inhibitor [Triticum	gi|229615372	17173.2	7.4499

dicoccoides]			998092 6514
monomeric alpha-amylase inhibitor [Triticum dicoccoides]	gi|229615374	17102.2	6.5500 001907 3486
monomeric alpha-amylase inhibitor [Triticum dicoccoides]	gi|229615376	17102.2	6.5500 001907 3486
monomeric alpha-amylase inhibitor [Triticum dicoccoides]	gi|229615378	17130.2	6.5500 001907 3486
monomeric alpha-amylase inhibitor [Triticum dicoccoides]	gi|229615380	17103.2	5.8000 001907 3486
monomeric alpha-amylase inhibitor [Triticum dicoccoides]	gi|229615382	17102.2	6.5500 001907 3486
monomeric alpha-amylase inhibitor [Triticum dicoccoides]	gi|229615386	17102.2	6.5500 001907 3486
monomeric alpha-amylase inhibitor [Triticum dicoccoides]	gi|229615330	17102.2	6.5500 001907 3486
monomeric alpha-amylase inhibitor [Triticum dicoccoides]	gi|229615334	17074.2	6.5500 001907 3486
monomeric alpha-amylase inhibitor [Triticum dicoccoides]	gi|229615336	17108.2	6.5500 001907 3486

monomeric alpha-amylase inhibitor [Triticum dicoccoides]	gi|229615338	17173.2	7.4499 998092 6514
monomeric alpha-amylase inhibitor [Triticum dicoccoides]	gi|229615340	17102.2	6.5500 001907 3486
monomeric alpha-amylase inhibitor [Triticum dicoccoides]	gi|229615344	17102.2	6.5500 001907 3486
monomeric alpha-amylase inhibitor [Triticum dicoccoides]	gi|229615346	17102.2	6.5500 001907 3486
monomeric alpha-amylase inhibitor [Triticum dicoccoides]	gi|229615348	17102.2	6.5500 001907 3486
monomeric alpha-amylase inhibitor [Triticum dicoccoides]	gi|229615350	17102.2	6.5500 001907 3486
monomeric alpha-amylase inhibitor [Triticum dicoccoides]	gi|229615294	17102.2	6.5500 001907 3486
monomeric alpha-amylase inhibitor [Triticum dicoccoides]	gi|229615296	17102.2	6.5500 001907 3486
monomeric alpha-amylase inhibitor [Triticum dicoccoides]	gi|229615298	17102.2	6.5500 001907 3486

monomeric alpha-amylase inhibitor [Triticum dicoccoides]	gi|229615300	17102.2	6.5500 001907 3486
monomeric alpha-amylase inhibitor [Triticum dicoccoides]	gi|229615302	17102.2	6.5500 001907 3486
monomeric alpha-amylase inhibitor [Triticum dicoccoides]	gi|229615304	17102.2	6.5500 001907 3486
monomeric alpha-amylase inhibitor [Triticum dicoccoides]	gi|229615306	17102.2	6.5500 001907 3486
monomeric alpha-amylase inhibitor [Triticum dicoccoides]	gi|229615308	17102.2	6.5500 001907 3486
monomeric alpha-amylase inhibitor [Triticum dicoccoides]	gi|229615310	17102.2	6.5500 001907 3486
monomeric alpha-amylase inhibitor [Triticum dicoccoides]	gi|229615093	17074.2	6.5500 001907 3486
monomeric alpha-amylase inhibitor [Triticum dicoccoides]	gi|229615095	17104.2	6.5500 001907 3486
monomeric alpha-amylase inhibitor [Triticum dicoccoides]	gi|229615099	17102.2	6.5500 001907 3486
monomeric alpha-amylase inhibitor [Triticum	gi|229615101	17187.3	7.4499

dicoccoides]			998092<br>6514
monomeric alpha-amylase inhibitor [Triticum dicoccoides]	gi|229615105	17102.2	6.5500<br>001907<br>3486
monomeric alpha-amylase inhibitor [Triticum dicoccoides]	gi|229615107	17074.2	6.5500<br>001907<br>3486
monomeric alpha-amylase inhibitor [Triticum dicoccoides]	gi|229615109	17074.2	6.5500<br>001907<br>3486
monomeric alpha-amylase inhibitor [Triticum dicoccoides]	gi|229615111	17076.1	6.5500<br>001907<br>3486
monomeric alpha-amylase inhibitor [Triticum dicoccoides]	gi|229615113	17104.2	6.5500<br>001907<br>3486
monomeric alpha-amylase inhibitor [Triticum dicoccoides]	gi|229615115	17094.2	6.5500<br>001907<br>3486
monomeric alpha-amylase inhibitor [Triticum dicoccoides]	gi|229615117	17203.2	7.4499<br>998092<br>6514
monomeric alpha-amylase inhibitor [Triticum dicoccoides]	gi|229615119	17272.3	8.0299<br>997329<br>7119
monomeric alpha-amylase inhibitor [Triticum dicoccoides]	gi|229615121	17102.2	6.5500<br>001907<br>3486

monomeric alpha-amylase inhibitor [Triticum dicoccoides]	gi|229615123	17044.2	7.4499 998092 6514
monomeric alpha-amylase inhibitor [Triticum dicoccoides]	gi|229615125	17102.2	6.5500 001907 3486
monomeric alpha-amylase inhibitor [Triticum dicoccoides]	gi|229615127	17102.2	6.5500 001907 3486
monomeric alpha-amylase inhibitor [Triticum dicoccoides]	gi|229615129	17102.2	6.5500 001907 3486
monomeric alpha-amylase inhibitor [Triticum dicoccoides]	gi|229615131	17203.2	7.4499 998092 6514
monomeric alpha-amylase inhibitor [Triticum dicoccoides]	gi|229615135	17104.2	6.5500 001907 3486
monomeric alpha-amylase inhibitor [Triticum dicoccoides]	gi|229615137	17203.2	7.4499 998092 6514
monomeric alpha-amylase inhibitor [Triticum dicoccoides]	gi|229615139	17104.2	6.5500 001907 3486
monomeric alpha-amylase inhibitor [Triticum dicoccoides]	gi|229615141	17104.2	6.5500 001907 3486

monomeric alpha-amylase inhibitor [Triticum dicoccoides]	gi|229615143	17104.2	6.5500 001907 3486
monomeric alpha-amylase inhibitor [Triticum dicoccoides]	gi|229615145	17173.2	7.4499 998092 6514
monomeric alpha-amylase inhibitor [Triticum dicoccoides]	gi|229615147	17030.2	7.4499 998092 6514
monomeric alpha-amylase inhibitor [Triticum dicoccoides]	gi|229615149	17104.2	6.5500 001907 3486
monomeric alpha-amylase inhibitor [Triticum dicoccoides]	gi|229615153	17102.2	6.5500 001907 3486
monomeric alpha-amylase inhibitor [Triticum dicoccoides]	gi|229615155	17104.2	6.5500 001907 3486
monomeric alpha-amylase inhibitor [Triticum dicoccoides]	gi|229615157	17173.2	7.4499 998092 6514
monomeric alpha-amylase inhibitor [Triticum dicoccoides]	gi|229615169	17102.2	6.5500 001907 3486
monomeric alpha-amylase inhibitor [Triticum dicoccoides]	gi|229615171	17056.2	6.5500 001907 3486
monomeric alpha-amylase inhibitor [Triticum	gi|229615173	17130.3	7.4600

dicoccoides]			000381 4697
monomeric alpha-amylase inhibitor [Triticum dicoccoides]	gi|229615175	17102.2	6.5500 001907 3486
monomeric alpha-amylase inhibitor [Triticum dicoccoides]	gi|229615177	17102.2	6.5500 001907 3486
monomeric alpha-amylase inhibitor [Triticum dicoccoides]	gi|229615179	17130.2	6.5500 001907 3486
monomeric alpha-amylase inhibitor [Triticum dicoccoides]	gi|229615181	17102.2	6.5500 001907 3486
monomeric alpha-amylase inhibitor [Triticum dicoccoides]	gi|229615183	17130.2	6.5500 001907 3486
monomeric alpha-amylase inhibitor [Triticum dicoccoides]	gi|229615185	17102.2	6.5500 001907 3486
monomeric alpha-amylase inhibitor [Triticum dicoccoides]	gi|229615197	17103.3	7.4899 997711 1816
monomeric alpha-amylase inhibitor [Triticum dicoccoides]	gi|229615199	17231.3	7.4499 998092 6514
monomeric alpha-amylase inhibitor [Triticum dicoccoides]	gi|229615201	17173.2	7.4499 998092 6514

monomeric alpha-amylase inhibitor [Triticum dicoccoides]	gi|229615203	17143.3	8.0299 997329 7119
monomeric alpha-amylase inhibitor [Triticum dicoccoides]	gi|229615205	17173.2	7.4499 998092 6514
monomeric alpha-amylase inhibitor [Triticum dicoccoides]	gi|229615207	17173.2	7.4499 998092 6514
monomeric alpha-amylase inhibitor [Triticum dicoccoides]	gi|229615209	17203.2	7.4499 998092 6514
monomeric alpha-amylase inhibitor [Triticum dicoccoides]	gi|229615211	17173.2	7.4499 998092 6514
monomeric alpha-amylase inhibitor [Triticum dicoccoides]	gi|229615213	17173.2	7.4499 998092 6514
monomeric alpha-amylase inhibitor [Triticum dicoccoides]	gi|229615215	17104.2	6.5500 001907 3486
monomeric alpha-amylase inhibitor [Triticum dicoccoides]	gi|229615217	17173.2	7.4499 998092 6514
monomeric alpha-amylase inhibitor [Triticum dicoccoides]	gi|229615223	17104.2	6.5500 001907 3486

monomeric alpha-amylase inhibitor [Triticum dicoccoides]	gi|229615225	17102.2	6.5500 001907 3486
monomeric alpha-amylase inhibitor [Triticum dicoccoides]	gi|229615227	17102.2	6.5500 001907 3486
monomeric alpha-amylase inhibitor [Triticum dicoccoides]	gi|229615229	17102.2	6.5500 001907 3486
monomeric alpha-amylase inhibitor [Triticum dicoccoides]	gi|229615231	17102.2	6.5500 001907 3486
monomeric alpha-amylase inhibitor [Triticum dicoccoides]	gi|229615233	17072.2	6.5500 001907 3486
monomeric alpha-amylase inhibitor [Triticum dicoccoides]	gi|229615235	17102.2	6.5500 001907 3486
monomeric alpha-amylase inhibitor [Triticum dicoccoides]	gi|229615237	17102.2	6.5500 001907 3486
monomeric alpha-amylase inhibitor [Triticum dicoccoides]	gi|229615239	17102.2	6.5500 001907 3486
monomeric alpha-amylase inhibitor [Triticum dicoccoides]	gi|229615241	17102.2	6.5500 001907 3486
monomeric alpha-amylase inhibitor [Triticum	gi|229615243	17102.2	6.5500

dicoccoides]			001907 3486
monomeric alpha-amylase inhibitor [Triticum dicoccoides]	gi|229615245	17102.2	6.5500 001907 3486
monomeric alpha-amylase inhibitor [Triticum dicoccoides]	gi|229615247	17102.2	6.5500 001907 3486
monomeric alpha-amylase inhibitor [Triticum dicoccoides]	gi|229615249	17102.2	6.5500 001907 3486
monomeric alpha-amylase inhibitor [Triticum dicoccoides]	gi|229615251	17102.2	6.5500 001907 3486
monomeric alpha-amylase inhibitor [Triticum dicoccoides]	gi|229615253	17003.1	5.8000 001907 3486
monomeric alpha-amylase inhibitor [Triticum dicoccoides]	gi|229615255	17102.2	6.5500 001907 3486
monomeric alpha-amylase inhibitor [Triticum dicoccoides]	gi|229615259	17132.2	6.5500 001907 3486
monomeric alpha-amylase inhibitor [Triticum dicoccoides]	gi|229615261	17104.2	6.5500 001907 3486
monomeric alpha-amylase inhibitor [Triticum dicoccoides]	gi|229615263	17104.2	6.5500 001907 3486

monomeric alpha-amylase inhibitor [Triticum dicoccoides]	gi|229615265	17102.2	6.5500 001907 3486
monomeric alpha-amylase inhibitor [Triticum dicoccoides]	gi|229615271	17102.2	6.5500 001907 3486
monomeric alpha-amylase inhibitor [Triticum dicoccoides]	gi|229615273	17102.2	6.5500 001907 3486
monomeric alpha-amylase inhibitor [Triticum dicoccoides]	gi|229615275	17102.2	6.5500 001907 3486
monomeric alpha-amylase inhibitor [Triticum dicoccoides]	gi|229614961	17104.2	6.5500 001907 3486
monomeric alpha-amylase inhibitor [Triticum dicoccoides]	gi|229614963	17074.2	6.5500 001907 3486
monomeric alpha-amylase inhibitor [Triticum dicoccoides]	gi|229614965	17074.2	6.5500 001907 3486
monomeric alpha-amylase inhibitor [Triticum dicoccoides]	gi|229614967	17074.2	6.5500 001907 3486
monomeric alpha-amylase inhibitor [Triticum dicoccoides]	gi|229614969	17104.2	6.5500 001907 3486

monomeric alpha-amylase inhibitor [Triticum dicoccoides]	gi|229614971	17074.2	6.5500 001907 3486
monomeric alpha-amylase inhibitor [Triticum dicoccoides]	gi|229614973	17104.2	6.5500 001907 3486
monomeric alpha-amylase inhibitor [Triticum dicoccoides]	gi|229614975	17104.2	6.5500 001907 3486
monomeric alpha-amylase inhibitor [Triticum dicoccoides]	gi|229614977	17173.2	7.4499 998092 6514
monomeric alpha-amylase inhibitor [Triticum dicoccoides]	gi|229614979	17104.2	6.5500 001907 3486
monomeric alpha-amylase inhibitor [Triticum dicoccoides]	gi|229614983	17104.2	6.5500 001907 3486
monomeric alpha-amylase inhibitor [Triticum dicoccoides]	gi|229614985	17104.2	6.5500 001907 3486
monomeric alpha-amylase inhibitor [Triticum dicoccoides]	gi|229614987	17104.2	6.5500 001907 3486
monomeric alpha-amylase inhibitor [Triticum dicoccoides]	gi|229614989	17104.2	6.5500 001907 3486
monomeric alpha-amylase inhibitor [Triticum	gi|229614991	17072.2	7.4499

dicoccoides]			998092 6514
monomeric alpha-amylase inhibitor [Triticum dicoccoides]	gi|229614993	17102.2	6.5500 001907 3486
monomeric alpha-amylase inhibitor [Triticum dicoccoides]	gi|229614995	17102.2	6.5500 001907 3486
monomeric alpha-amylase inhibitor [Triticum dicoccoides]	gi|229614997	17046.1	6.5500 001907 3486
monomeric alpha-amylase inhibitor [Triticum dicoccoides]	gi|229615001	17102.2	6.5500 001907 3486
monomeric alpha-amylase inhibitor [Triticum dicoccoides]	gi|229615003	17104.2	6.5500 001907 3486
monomeric alpha-amylase inhibitor [Triticum dicoccoides]	gi|229615005	17104.2	6.5500 001907 3486
monomeric alpha-amylase inhibitor [Triticum dicoccoides]	gi|229615007	17107.2	6.5500 001907 3486
monomeric alpha-amylase inhibitor [Triticum dicoccoides]	gi|229615009	17104.2	6.5500 001907 3486
monomeric alpha-amylase inhibitor [Triticum dicoccoides]	gi|229615011	17104.2	6.5500 001907 3486

monomeric alpha-amylase inhibitor [Triticum dicoccoides]	gi|229615013	17102.2	6.5500 001907 3486
monomeric alpha-amylase inhibitor [Triticum dicoccoides]	gi|229615015	17102.2	6.5500 001907 3486
monomeric alpha-amylase inhibitor [Triticum dicoccoides]	gi|229615017	17102.2	6.5500 001907 3486
monomeric alpha-amylase inhibitor [Triticum dicoccoides]	gi|229615019	17102.2	6.5500 001907 3486
monomeric alpha-amylase inhibitor [Triticum dicoccoides]	gi|229615021	17102.2	6.5500 001907 3486
monomeric alpha-amylase inhibitor [Triticum dicoccoides]	gi|229615023	17102.2	6.5500 001907 3486
monomeric alpha-amylase inhibitor [Triticum dicoccoides]	gi|229615025	17102.2	6.5500 001907 3486
monomeric alpha-amylase inhibitor [Triticum dicoccoides]	gi|229615027	17094.2	6.5500 001907 3486
monomeric alpha-amylase inhibitor [Triticum dicoccoides]	gi|229615031	17102.2	6.5500 001907 3486

monomeric alpha-amylase inhibitor [Triticum dicoccoides]	gi|229615035	17104.2	6.5500 001907 3486
monomeric alpha-amylase inhibitor [Triticum dicoccoides]	gi|229615039	17118.2	6.5500 001907 3486
monomeric alpha-amylase inhibitor [Triticum dicoccoides]	gi|229615041	17074.2	6.5500 001907 3486
monomeric alpha-amylase inhibitor [Triticum dicoccoides]	gi|229615043	17044.2	6.5500 001907 3486
monomeric alpha-amylase inhibitor [Triticum dicoccoides]	gi|229615045	17173.2	7.4499 998092 6514
monomeric alpha-amylase inhibitor [Triticum dicoccoides]	gi|229615047	17104.2	6.5500 001907 3486
monomeric alpha-amylase inhibitor [Triticum dicoccoides]	gi|229615049	17102.2	6.5500 001907 3486
monomeric alpha-amylase inhibitor [Triticum dicoccoides]	gi|229615051	17102.2	6.5500 001907 3486
monomeric alpha-amylase inhibitor [Triticum dicoccoides]	gi|229615055	17102.2	6.5500 001907 3486
monomeric alpha-amylase inhibitor [Triticum	gi|229615057	17074.2	6.5500

dicoccoides]			001907 3486
monomeric alpha-amylase inhibitor [Triticum dicoccoides]	gi|229615061	17104.2	6.5500 001907 3486
monomeric alpha-amylase inhibitor [Triticum dicoccoides]	gi|229615063	17203.2	7.4499 998092 6514
monomeric alpha-amylase inhibitor [Triticum dicoccoides]	gi|229615069	17104.2	6.5500 001907 3486
monomeric alpha-amylase inhibitor [Triticum dicoccoides]	gi|229615071	17104.2	6.5500 001907 3486
monomeric alpha-amylase inhibitor [Triticum dicoccoides]	gi|229615073	17102.2	6.5500 001907 3486
monomeric alpha-amylase inhibitor [Triticum dicoccoides]	gi|229615075	17104.2	6.5500 001907 3486
monomeric alpha-amylase inhibitor [Triticum dicoccoides]	gi|229615079	17102.2	6.5500 001907 3486
monomeric alpha-amylase inhibitor [Triticum dicoccoides]	gi|229615081	17104.2	6.5500 001907 3486
monomeric alpha-amylase inhibitor [Triticum dicoccoides]	gi|229615085	17106.2	6.5500 001907 3486

monomeric alpha-amylase inhibitor [Triticum dicoccoides]	gi|229615087	17044.2	6.5500 001907 3486
monomeric alpha-amylase inhibitor [Triticum dicoccoides]	gi|229615091	17074.2	6.5500 001907 3486
monomeric alpha-amylase inhibitor [Triticum dicoccoides]	gi|229615278	17102.2	6.5500 001907 3486
monomeric alpha-amylase inhibitor [Triticum dicoccoides]	gi|229615280	17102.2	6.5500 001907 3486
monomeric alpha-amylase inhibitor [Triticum dicoccoides]	gi|229615282	17102.2	6.5500 001907 3486
monomeric alpha-amylase inhibitor [Triticum dicoccoides]	gi|229615284	17102.2	6.5500 001907 3486
monomeric alpha-amylase inhibitor [Triticum dicoccoides]	gi|229615286	17102.2	6.5500 001907 3486
monomeric alpha-amylase inhibitor [Triticum dicoccoides]	gi|229615288	17102.2	6.5500 001907 3486
monomeric alpha-amylase inhibitor [Triticum dicoccoides]	gi|229615290	17162.2	6.5500 001907 3486

monomeric alpha-amylase inhibitor [Triticum dicoccoides]	gi|229615292	17102.2	6.5500 001907 3486
monomeric alpha-amylase inhibitor [Triticum dicoccoides]	gi|229615312	17102.2	6.5500 001907 3486
monomeric alpha-amylase inhibitor [Triticum dicoccoides]	gi|229615314	17102.2	6.5500 001907 3486
monomeric alpha-amylase inhibitor [Triticum dicoccoides]	gi|229615316	17102.2	6.5500 001907 3486
monomeric alpha-amylase inhibitor [Triticum dicoccoides]	gi|229615318	17102.2	6.5500 001907 3486
monomeric alpha-amylase inhibitor [Triticum dicoccoides]	gi|229615322	17054.2	6.5500 001907 3486
monomeric alpha-amylase inhibitor [Triticum dicoccoides]	gi|229615324	17102.2	6.5500 001907 3486
monomeric alpha-amylase inhibitor [Triticum dicoccoides]	gi|229615326	17102.2	6.5500 001907 3486
monomeric alpha-amylase inhibitor [Triticum dicoccoides]	gi|229615328	17086.2	6.5500 001907 3486
monomeric alpha-amylase inhibitor [Triticum	gi|229615352	17102.2	6.5500

dicoccoides]			001907<br>3486
monomeric alpha-amylase inhibitor [Triticum dicoccoides]	gi|229615354	17102.2	6.5500<br>001907<br>3486
monomeric alpha-amylase inhibitor [Triticum dicoccoides]	gi|229615356	17102.2	6.5500<br>001907<br>3486
monomeric alpha-amylase inhibitor [Triticum dicoccoides]	gi|229615358	17102.2	6.5500<br>001907<br>3486
monomeric alpha-amylase inhibitor [Triticum dicoccoides]	gi|229615360	17102.2	6.5500<br>001907<br>3486
monomeric alpha-amylase inhibitor [Triticum dicoccoides]	gi|229615362	17102.2	6.5500<br>001907<br>3486
monomeric alpha-amylase inhibitor [Triticum dicoccoides]	gi|229615364	17102.2	6.5500<br>001907<br>3486
monomeric alpha-amylase inhibitor [Triticum dicoccoides]	gi|229615366	17187.3	7.4499<br>998092<br>6514
monomeric alpha-amylase inhibitor [Triticum dicoccoides]	gi|229615388	17102.2	6.5500<br>001907<br>3486
monomeric alpha-amylase inhibitor [Triticum dicoccoides]	gi|229615390	17102.2	6.5500<br>001907<br>3486

monomeric alpha-amylase inhibitor [Triticum dicoccoides]	gi|229615394	17102.2	6.5500 001907 3486
monomeric alpha-amylase inhibitor [Triticum dicoccoides]	gi|229615396	17102.2	6.5500 001907 3486
monomeric alpha-amylase inhibitor [Triticum dicoccoides]	gi|229615398	17070.1	6.5500 001907 3486
monomeric alpha-amylase inhibitor [Triticum dicoccoides]	gi|229615400	17102.2	6.5500 001907 3486
monomeric alpha-amylase inhibitor [Triticum dicoccoides]	gi|229615402	17102.2	6.5500 001907 3486
monomeric alpha-amylase inhibitor [Triticum dicoccoides]	gi|229615404	17102.2	6.5500 001907 3486
monomeric alpha-amylase inhibitor [Triticum dicoccoides]	gi|229615442	17102.2	6.5500 001907 3486
monomeric alpha-amylase inhibitor [Triticum dicoccoides]	gi|229615444	17102.2	6.5500 001907 3486
monomeric alpha-amylase inhibitor [Triticum dicoccoides]	gi|229615446	17102.2	6.5500 001907 3486

monomeric alpha-amylase inhibitor [Triticum dicoccoides]	gi|229615450	17074.2	6.5500 001907 3486
monomeric alpha-amylase inhibitor [Triticum dicoccoides]	gi|229615452	17104.2	6.5500 001907 3486
monomeric alpha-amylase inhibitor [Triticum dicoccoides]	gi|229615466	17203.2	7.4499 998092 6514
monomeric alpha-amylase inhibitor [Triticum dicoccoides]	gi|229615468	17173.2	7.4499 998092 6514
monomeric alpha-amylase inhibitor [Triticum dicoccoides]	gi|229615470	17072.2	6.5500 001907 3486
monomeric alpha-amylase inhibitor [Triticum dicoccoides]	gi|229615492	17102.2	6.5500 001907 3486
monomeric alpha-amylase inhibitor [Triticum dicoccoides]	gi|229615520	17102.2	6.5500 001907 3486
monomeric alpha-amylase inhibitor [Triticum dicoccoides]	gi|229615522	17102.2	6.5500 001907 3486
monomeric alpha-amylase inhibitor [Triticum dicoccoides]	gi|229615524	17203.2	7.4499 998092 6514
monomeric alpha-amylase inhibitor [Triticum	gi|229615538	17203.2	7.4499

dicoccoides]			998092<br>6514
monomeric alpha-amylase inhibitor [Triticum dicoccoides]	gi|229615540	17233.3	7.4499<br>998092<br>6514
monomeric alpha-amylase inhibitor [Triticum dicoccoides]	gi|229615542	17044.2	7.4499<br>998092<br>6514
monomeric alpha-amylase inhibitor [Triticum dicoccoides]	gi|229615544	17102.2	6.5500<br>001907<br>3486
monomeric alpha-amylase inhibitor [Triticum dicoccoides]	gi|229615572	17102.2	6.5500<br>001907<br>3486
monomeric alpha-amylase inhibitor [Triticum dicoccoides]	gi|229615574	17101.2	8.0299<br>997329<br>7119
monomeric alpha-amylase inhibitor [Triticum dicoccoides]	gi|229615576	17102.2	6.5500<br>001907<br>3486
monomeric alpha-amylase inhibitor [Triticum dicoccoides]	gi|229615578	17102.2	6.5500<br>001907<br>3486
monomeric alpha-amylase inhibitor [Triticum dicoccoides]	gi|229615580	17102.2	6.5500<br>001907<br>3486
monomeric alpha-amylase inhibitor [Triticum dicoccoides]	gi|229615582	17102.2	6.5500<br>001907<br>3486

monomeric alpha-amylase inhibitor [Triticum dicoccoides]	gi|229615586	17187.3	7.4499998092 6514
monomeric alpha-amylase inhibitor [Triticum dicoccoides]	gi|229615588	17103.2	5.8000001907 3486
monomeric alpha-amylase inhibitor [Triticum dicoccoides]	gi|229615610	17100.2	7.4600000381 4697
monomeric alpha-amylase inhibitor [Triticum dicoccoides]	gi|229615612	17102.2	6.5500001907 3486
monomeric alpha-amylase inhibitor [Triticum dicoccoides]	gi|229615614	17104.2	6.5500001907 3486
monomeric alpha-amylase inhibitor [Triticum dicoccoides]	gi|229615616	17104.2	6.5500001907 3486
monomeric alpha-amylase inhibitor [Triticum dicoccoides]	gi|229615622	17102.2	6.5500001907 3486
monomeric alpha-amylase inhibitor [Triticum dicoccoides]	gi|229615620	17102.2	6.5500001907 3486
monomeric alpha-amylase inhibitor [Triticum monococcum]	gi|134034537	13699.5	5.3699998855 5908

monomeric alpha-amylase inhibitor [Triticum monococcum]	gi|134034512	13658.5	7.4000 000953 6743
monomeric alpha-amylase inhibitor [Triticum monococcum]	gi|134034506	13658.5	7.4000 000953 6743
monomeric alpha-amylase inhibitor [Triticum monococcum]	gi|134034510	13658.5	7.4000 000953 6743
monomeric alpha-amylase inhibitor, partial [Triticum dicoccoides]	gi|229615618	17230.2	6.5500 001907 3486

Peptide Information

Calc. Mass	Obsrv. Mass	± da	± ppm	Start Seq.	End Seq.	Sequence	Ion Score	C. I. %	Modification	Rank	Result Type
1050.5579	1050.522	-0.0359	-34	95	103	SVYQELGVR	72	100		1	Mascot
1555.8262	1555.7826	-0.0436	-28	57	70	LQCVGSQVPEAVLR	113	100	Carbamidomethyl (C)[3]	1	Mascot
1981.795	1981.7411	-0.0539	-27	71	85	DCCQQLADINNEWCR	132	100	Carbamidomethyl (C)[2,3,14]	1	Mascot

5	monomeric alpha-amylase inhibitor [Triticum dicoccoides]	gi|229615151	17176.2	5.82	3	0	317	100

Peptide Information

Calc. Mass	Obsrv. Mass	± da	± ppm	Start Seq.	End Seq.	Sequence	Ion Score	C. I. %	Modification	Rank	Result Type
1050.5579	1050.522	-0.0359	-34	95	103	SVYQEIGVR	72	100		1	Mascot
1555.8262	1555.7826	-0.0436	-28	57	70	LQCVGSQVPEAVLR	113	100	Carbamidomethyl (C)[3]	1	Mascot
1981.795	1981.7411	-0.0539	-27	71	85	DCCQQLADINNEWCR	132	100	Carbamidomethyl (C)[2,3,14]	1	Mascot

6	monomeric alpha-amylase inhibitor [Triticum dicoccoides]	gi|229615033	17190.2	6.55	3	0	279	100

Peptide Information

Calc. Mass	Obsrv. Mass	± da	± ppm	Start Seq.	End Seq.	Sequence	Ion Score	C. I. %	Modification	Rank	Result Type

1050.5579	1050.522	-0.0359	-34	95	103	SVYQELGVR	72	100		1	Mascot
1555.8262	1555.7826	-0.0436	-28	57	70	LQCVGSQVPEAVLR	113	100	Carbamidomethyl (C)[3]	1	Mascot
1981.795	1981.7411	-0.0539	-27	71	85	DCCQQLANIDNEWCR	94	100	Carbamidomethyl (C)[2,3,14]	2	Mascot

7	dimeric alpha-amylase inhibitor [Triticum timopheevii subsp. armeniacum]	gi|227809296	15574.4	5.28	1	0	115	100	

Protein Group

0.19 dimeric alpha-amylase inhibitor [Triticum aestivum]	gi|54778511	13756.6	6.4899 997711 1816
0.19 dimeric alpha-amylase inhibitor [Triticum aestivum]	gi|54778507	13814.6	5.7300 000190 7349
dimeric alpha-amylase inhibitor [Triticum aestivum]	gi|65993941	15631.5	5.7699 999809 2651
dimeric alpha-amylase inhibitor [Triticum aestivum]	gi|65993852	15558.5	5.3000 001907 3486
dimeric alpha-amylase inhibitor [Triticum aestivum]	gi|65993756	15820.6	6.5399 999618 5303
dimeric alpha-amylase inhibitor [Triticum aestivum]	gi|65993807	15588.5	5.3000 001907 3486
dimeric alpha-amylase inhibitor [Triticum dicoccoides]	gi|227809240	15588.5	5.2800 002098 0835
dimeric alpha-amylase inhibitor [Triticum dicoccoides]	gi|227809226	15574.4	5.2800 002098 0835

dimeric alpha-amylase inhibitor [Triticum dicoccoides]	gi|227809222	15602.5	5.75
dimeric alpha-amylase inhibitor [Triticum dicoccoides]	gi|227809216	15574.4	5.2800<br>002098<br>0835
dimeric alpha-amylase inhibitor [Triticum dicoccoides]	gi|227809192	15604.4	5.2800<br>002098<br>0835
dimeric alpha-amylase inhibitor [Triticum dicoccoides]	gi|227809172	15548.4	5.5799<br>999237<br>0605
dimeric alpha-amylase inhibitor [Triticum dicoccoides]	gi|227809140	15574.4	5.2800<br>002098<br>0835
dimeric alpha-amylase inhibitor [Triticum dicoccoides]	gi|227809112	15602.5	5.2800<br>002098<br>0835
dimeric alpha-amylase inhibitor [Triticum dicoccoides]	gi|227809084	15574.4	5.2800<br>002098<br>0835
dimeric alpha-amylase inhibitor [Triticum dicoccoides]	gi|227809021	15804.6	6.5399<br>999618<br>5303
dimeric alpha-amylase inhibitor [Triticum dicoccoides]	gi|227809025	15574.4	5.2800<br>002098<br>0835
dimeric alpha-amylase inhibitor [Triticum dicoccoides]	gi|227809033	15574.4	5.2800

			002098<br>0835
dimeric alpha-amylase inhibitor [Triticum dicoccoides]	gi|227809037	15574.4	5.2800<br>002098<br>0835
dimeric alpha-amylase inhibitor [Triticum dicoccoides]	gi|227809039	15574.4	5.2800<br>002098<br>0835
dimeric alpha-amylase inhibitor [Triticum dicoccoides]	gi|227809045	15558.4	5.2800<br>002098<br>0835
dimeric alpha-amylase inhibitor [Triticum dicoccoides]	gi|227809047	15574.4	5.2800<br>002098<br>0835
dimeric alpha-amylase inhibitor [Triticum dicoccoides]	gi|227809055	15558.5	5.7399<br>997711<br>1816
dimeric alpha-amylase inhibitor [Triticum dicoccoides]	gi|227809082	15574.4	5.2800<br>002098<br>0835
dimeric alpha-amylase inhibitor [Triticum dicoccoides]	gi|227808960	15574.4	5.2800<br>002098<br>0835
dimeric alpha-amylase inhibitor [Triticum dicoccoides]	gi|227808962	15574.4	5.2800<br>002098<br>0835
dimeric alpha-amylase inhibitor [Triticum dicoccoides]	gi|227808972	15574.4	5.2800<br>002098<br>0835

dimeric alpha-amylase inhibitor [Triticum dicoccoides]	gi|227808979	15574.4	5.2800 002098 0835
dimeric alpha-amylase inhibitor [Triticum dicoccoides]	gi|227808981	15574.4	5.2800 002098 0835
dimeric alpha-amylase inhibitor [Triticum dicoccoides]	gi|227808997	15575.4	5.2600 002288 8184
dimeric alpha-amylase inhibitor [Triticum dicoccoides]	gi|227809001	15573.5	5.7399 997711 1816
dimeric alpha-amylase inhibitor [Triticum dicoccoides]	gi|227809003	15574.4	5.2800 002098 0835
dimeric alpha-amylase inhibitor [Triticum dicoccoides]	gi|227809015	15575.4	5.2600 002288 8184
dimeric alpha-amylase inhibitor [Triticum dicoccoides]	gi|227808857	15562.4	5.5799 999237 0605
dimeric alpha-amylase inhibitor [Triticum dicoccoides]	gi|227808859	15588.5	5.2800 002098 0835
dimeric alpha-amylase inhibitor [Triticum dicoccoides]	gi|227808863	15588.5	5.2800 002098 0835

dimeric alpha-amylase inhibitor [Triticum dicoccoides]	gi|227808876	15588.5	5.2800<br>002098<br>0835
dimeric alpha-amylase inhibitor [Triticum dicoccoides]	gi|227808880	15588.5	5.2800<br>002098<br>0835
dimeric alpha-amylase inhibitor [Triticum dicoccoides]	gi|227808884	15588.5	5.2800<br>002098<br>0835
dimeric alpha-amylase inhibitor [Triticum dicoccoides]	gi|227808887	15588.5	5.2800<br>002098<br>0835
dimeric alpha-amylase inhibitor [Triticum dicoccoides]	gi|227808889	15588.5	5.2800<br>002098<br>0835
dimeric alpha-amylase inhibitor [Triticum dicoccoides]	gi|227808896	15588.5	5.2800<br>002098<br>0835
dimeric alpha-amylase inhibitor [Triticum dicoccoides]	gi|114215762	13742.5	6.4899<br>997711<br>1816
dimeric alpha-amylase inhibitor [Triticum dicoccoides]	gi|114215768	13742.5	6.4899<br>997711<br>1816
dimeric alpha-amylase inhibitor [Triticum dicoccoides]	gi|114215772	13742.5	6.4899<br>997711<br>1816
dimeric alpha-amylase inhibitor [Triticum dicoccoides]	gi|114215776	13742.5	6.4899

			997711<br>1816
dimeric alpha-amylase inhibitor [Triticum dicoccoides]	gi|114215784	13770.6	6.4899<br>997711<br>1816
dimeric alpha-amylase inhibitor [Triticum dicoccoides]	gi|114215790	13742.5	6.4899<br>997711<br>1816
dimeric alpha-amylase inhibitor [Triticum dicoccoides]	gi|227808839	15588.5	5.2800<br>002098<br>0835
dimeric alpha-amylase inhibitor [Triticum dicoccoides]	gi|227808843	15588.5	5.2800<br>002098<br>0835
dimeric alpha-amylase inhibitor [Triticum dicoccoides]	gi|227808849	15588.5	5.2800<br>002098<br>0835
dimeric alpha-amylase inhibitor [Triticum dicoccoides]	gi|227808855	15588.5	5.2800<br>002098<br>0835
dimeric alpha-amylase inhibitor [Triticum dicoccoides]	gi|227808906	15574.4	5.2800<br>002098<br>0835
dimeric alpha-amylase inhibitor [Triticum dicoccoides]	gi|227808908	15574.4	5.2800<br>002098<br>0835
dimeric alpha-amylase inhibitor [Triticum dicoccoides]	gi|227808924	15574.4	5.2800<br>002098<br>0835

dimeric alpha-amylase inhibitor [Triticum dicoccoides]	gi|227808926	15574.4	5.2800<br>002098<br>0835
dimeric alpha-amylase inhibitor [Triticum dicoccoides]	gi|227808932	15574.4	5.2800<br>002098<br>0835
dimeric alpha-amylase inhibitor [Triticum dicoccoides]	gi|227808940	15574.4	5.2800<br>002098<br>0835
dimeric alpha-amylase inhibitor [Triticum dicoccoides]	gi|227808942	15574.4	5.2800<br>002098<br>0835
dimeric alpha-amylase inhibitor [Triticum dicoccoides]	gi|227808948	15574.4	5.2800<br>002098<br>0835
dimeric alpha-amylase inhibitor [Triticum dicoccoides]	gi|227809092	15574.4	5.2800<br>002098<br>0835
dimeric alpha-amylase inhibitor [Triticum dicoccoides]	gi|227809132	15575.4	5.2600<br>002288<br>8184
dimeric alpha-amylase inhibitor [Triticum dicoccoides]	gi|227809154	15574.4	5.2800<br>002098<br>0835
dimeric alpha-amylase inhibitor [Triticum dicoccoides]	gi|227809174	15574.4	5.2800<br>002098<br>0835

dimeric alpha-amylase inhibitor [Triticum dicoccoides]	gi|227809196	15574.4	5.2800 002098 0835
dimeric alpha-amylase inhibitor [Triticum dicoccoides]	gi|227809204	15564.4	5.2800 002098 0835
dimeric alpha-amylase inhibitor [Triticum dicoccoides]	gi|227809212	15574.4	5.2800 002098 0835
dimeric alpha-amylase inhibitor [Triticum timopheevii subsp. armeniacum]	gi|227809294	15588.4	5.2800 002098 0835
dimeric alpha-amylase inhibitor [Triticum timopheevii subsp. armeniacum]	gi|227809292	15571.4	4.8099 999427 7954
dimeric alpha-amylase inhibitor [Triticum timopheevii subsp. armeniacum]	gi|227809290	15571.4	4.8099 999427 7954
dimeric alpha-amylase inhibitor [Triticum timopheevii subsp. armeniacum]	gi|227809286	15513.4	4.9899 997711 1816
dimeric alpha-amylase inhibitor [Triticum timopheevii subsp. armeniacum]	gi|227809284	15588.5	5.2800 002098 0835
dimeric alpha-amylase inhibitor [Triticum timopheevii subsp. armeniacum]	gi|227809282	15606.4	5.2800 002098 0835
dimeric alpha-amylase inhibitor [Triticum timopheevii	gi|227809280	15588.5	5.2800

subsp. armeniacum]			002098<br>0835
dimeric alpha-amylase inhibitor [Triticum timopheevii subsp. armeniacum]	gi|227809278	15588.5	5.2800<br>002098<br>0835
dimeric alpha-amylase inhibitor, partial [Triticum durum]	gi|386877064	14502.8	4.7800<br>002098<br>0835

Peptide Information

Calc. Mass	Obsrv. Mass	± da	± ppm	Start Seq.	End Seq.	Sequence	Ion Score	C. I. %	Modification	Rank	Result Type
1887.8403	1887.7812	-0.0591	-31	84	101	EHGVQEGQAGTGAFPSCR	115	100	Carbamidomethyl (C)[17]	1	Mascot

8	E3 ubiquitin-protein ligase RMA3 [Triticum urartu]	gi|473881667	49391.1	10.05	1	0	20	24.309

Peptide Information

Calc. Mass	Obsrv. Mass	± da	± ppm	Start Seq.	End Seq.	Sequence	Ion Score	C. I. %	Modification	Rank	Result Type
1037.468	1037.3872	-0.0808	-78	224	232	QQMESAATR	20	24.309	Oxidation (M)[3]	1	Mascot

9	hypothetical protein TRIUR3_17239 [Triticum urartu]	gi|474446971	29769.3	9.79	1	0	17	0

Peptide Information

Calc. Mass	Obsrv. Mass	± da	± ppm	Start Seq.	End Seq.	Sequence	Ion Score	C. I. %	Modification	Rank	Result Type
1037.4867	1037.3872	-0.0995	-96	58	66	MANLSIGCR	17	0	Carbamidomethyl (C)[8], Oxidation (M)[1]	2	Mascot

10	hypothetical protein TRIUR3_09869 [Triticum urartu]	gi|474051755	28022.1	9.51	1	0	15	0

Peptide Information

Calc. Mass	Obsrv. Mass	± da	± ppm	Start Seq.	End Seq.	Sequence	Ion Score	C. I. %	Modification	Rank	Result Type
1050.5361	1050.522	-0.0141	-13	181	189	TRSVADMVR	15	0	Oxidation (M)[7]	3	Mascot
