# Supplementary material for: Proteomic analysis of middle and late stages of bread wheat (Triticum aestivum L.) grain development
Source: Front Plant Sci. 2015 Sep 15;6:735. doi: 10.3389/fpls.2015.00735 (PMC4569854; doi:10.3389/fpls.2015.00735)
Supplement: Supplementary file 5 [file DataSheet4.PDF]

Applied Biosystems

Analysis Information

Report Type	Protein-Peptide Summary by Spot	Analysis Type	Combined (MS+MS/MS)
Sample Set Name	Sample set_20140814	Database	SwissProt
Analysis Name	BSA0919	Creation Date	09/19/2014 16:44:19
Reported By	09/30/2014 15:03:24 - admin	Last Modified	09/19/2014 17:01:20

MS Acq. : Proc. Methods	(Unspecified) : (Unspecified)
Interpretation Method	(Unspecified)

**Gel Idx/Pos**	110/E9	**Instr./Gel Origin**	BA2151/Sample Project 20140814	**Process Status**	Analysis Succeeded
**Plate [#] Name**	[1] Sample Project 20140814	**Instrument Sample Name**		**Spectra**	11

Rank	Protein Name	Accession No.	Protein MW	Protein PI	Pep. Count	Protein Score	Protein Score C. I. %	Intensity Matched	Total Ion Score	Total Ion C. I. %	Confirmed
1	Cysteine desulfurase OS=Aeromonas hydrophila subsp. hydrophila (strain ATCC 7966 / NCIB 9240) GN=iscS PE=3 SV=1	ISCS_AERHH	45061.9	5.85	7	23	0	1.759			

Peptide Information

Calc. Mass	Obsrv. Mass	± da	± ppm	Start Seq.	End Seq.	Sequence	Ion Score	C. I. %	Modification	Rank	Result Type
834.4138	834.3561	-0.0577	-69	106	112	AVLDTCR			Carbamidomethyl (C)[6]		Mascot
848.4988	848.4734	-0.0254	-30	212	219	GIGALYVR					Mascot
983.5771	983.4911	-0.086	-87	188	196	IPVDVEALK					Mascot
1015.5683	1015.5669	-0.0014	-1	275	282	QRLWDGIK					Mascot
1376.6838	1376.8007	0.1169	85	258	269	IAKEEMVSEGQR					Mascot
1816.9011	1816.9352	0.0341	19	241	257	SGTLPTHQIVGMGEAFR			Oxidation (M)[12]		Mascot
2724.3992	2724.4023	0.0031	1	68	93	EIVFTSGATESNNLAIKGV AHFYAGK					Mascot

Rank	Protein Name	Accession No.	Protein MW	Protein PI	Pep. Count	Protein Score	Protein Score C. I. %	Intensity Matched	Total Ion Score	Total Ion C. I. %	Confirmed
2	Protein THEM6 OS=Danio rerio GN=them6 PE=2 SV=1	THEM6_DANRE	23742.1	9.08	5	22	0	1.209			

Peptide Information

Calc. Mass	Obsrv. Mass	± da	± ppm	Start Seq.	End Seq.	Sequence	Ion Score	C. I. %	Modification	Rank	Result Type
834.3839	834.3561	-0.0278	-33	193	200	AESGLDDK					Mascot
942.468	942.4318	-0.0362	-38	126	132	SFYLEQR					Mascot
1799.8962	1799.9218	0.0256	14	119	132	IVSWDEKSFYLEQR					Mascot

Calc. Mass	Obsrv. Mass	± da	± ppm	Start Seq.	End Seq.	Sequence	Ion Score	C. I. %	Modification	Rank	Result Type
1816.9084	1816.9352	0.0268	15	133	148	FVSKSDGFISAVMLCR			Carbamidomethyl (C)[15]		Mascot
2840.4885	2840.448	-0.0405	-14	1	23	MLLWFLSGSLLLFGTFDV WYFLR			Oxidation (M)[1]		Mascot

3	Pseudin-1 OS=Pseudis paradoxa PE=1 SV=1	PS1_PSEPD	2713.5	9.71	2	22	0	.934

Peptide Information

Calc. Mass	Obsrv. Mass	± da	± ppm	Start Seq.	End Seq.	Sequence	Ion Score	C. I. %	Modification	Rank	Result Type
1269.7314	1269.6726	-0.0588	-46	7	17	KVFQGLHEAIK					Mascot
1960.0763	1960.1753	0.099	51	8	24	VFQGLHEAIKLINNHVQ					Mascot

4	Pseudin-2 OS=Pseudis paradoxa PE=1 SV=1	PS2_PSEPD	2683.5	9.71	2	22	0	.934

Peptide Information

Calc. Mass	Obsrv. Mass	± da	± ppm	Start Seq.	End Seq.	Sequence	Ion Score	C. I. %	Modification	Rank	Result Type
1269.7314	1269.6726	-0.0588	-46	7	17	KVFQGIHEAIK					Mascot
1960.0763	1960.1753	0.099	51	8	24	VFQGIHEAIKLINNHVQ					Mascot

5	Major surface protein MspTL OS=Treponema lecithinolyticum GN=mspTL PE=4 SV=1	MSP_TRELE	64849.3	6.99	7	22	0	2.185

Peptide Information

Calc. Mass	Obsrv. Mass	± da	± ppm	Start Seq.	End Seq.	Sequence	Ion Score	C. I. %	Modification	Rank	Result Type
818.3791	818.3299	-0.0492	-60	347	353	WNGANEK					Mascot
833.3748	833.363	-0.0118	-14	305	312	AVGDSDNR					Mascot
1376.8009	1376.8007	-0.0002	0	441	454	GLNVAALVHANVAK					Mascot
1440.8308	1440.7295	-0.1013	-70	195	207	VEGVILPKDTEIK					Mascot
1799.9579	1799.9218	-0.0361	-20	331	346	FVVNPVVTYTLYADAK					Mascot
1817.0531	1816.9352	-0.1179	-65	480	497	LIGAKGLQIGLAASYDVK					Mascot
2840.4434	2840.448	0.0046	2	498	523	LNDITIVPAAAMLWTHGM LKGEADTR			Oxidation (M)[12]		Mascot

6	Putative cytochrome c oxidase subunit II PS17 (Fragments) OS=Pinus strobus PE=1 SV=1	PS17_PINST	1707	9.63	2	21	0	3.12

Peptide Information

Calc. Mass	Obsrv. Mass	± da	± ppm	Start Seq.	End Seq.	Sequence	Ion Score	C. I. %	Modification	Rank	Result Type
856.5251	856.5733	0.0482	56	1	8	SPTVIALR					Mascot
870.5043	870.5853	0.081	93	9	16	VVEALSPR					Mascot

7	Peptide YY-like OS=Myoxocephalus scorpius PE=1 SV=1	PYY_MYOSC	4167	8.39	2	21	0	.153

Peptide Information

Calc. Mass	Obsrv. Mass	± da	± ppm	Start Seq.	End Seq.	Sequence	Ion Score	C. I. %	Modification	Rank	Result Type
1015.5683	1015.5669	-0.0014	-1	26	33	HYVNLITR					Mascot
2724.28	2724.4023	0.1223	45	1	25	YPPQPESPGGNASPED WAKYHAAVR					Mascot

8	50S ribosomal protein L29 OS=Streptococcus equi subsp. zooepidemicus (strain MGCS10565) GN=rpmC PE=3 SV=1	RL29_STREM	7899.3	9.34	3	20	0	.763

Protein Group

50S ribosomal protein L29 OS=Streptococcus equi subsp. equi (strain 4047) GN=rpmC PE=3 SV=1	RL29_STRE4	7899.3	9.3400 001525 8789
50S ribosomal protein L29 OS=Streptococcus equi subsp. zooepidemicus (strain H70) GN=rpmC PE=3 SV=1	RL29_STRS7	7899.3	9.3400 001525 8789
50S ribosomal protein L29 OS=Streptococcus uberis (strain ATCC BAA-854 / 0140J) GN=rpmC PE=3 SV=1	RL29_STRU0	7899.3	9.3400 001525 8789

Peptide Information

Calc. Mass	Obsrv. Mass	± da	± ppm	Start Seq.	End Seq.	Sequence	Ion Score	C. I. %	Modification	Rank	Result Type
822.4025	822.375	-0.0275	-33	62	68	TVQSEMK					Mascot
1376.6918	1376.8007	0.1089	79	37	49	FQAAAGQLDQTAR					Mascot
1960.0247	1960.1753	0.1506	77	37	54	FQAAAGQLDQTARLNEV K					Mascot

9	Tautomerase PptA OS=Erwinia carotovora subsp. atroseptica (strain SCRI 1043 / ATCC BAA-672) GN=pptA PE=3 SV=1	PPTA_ERWCT	8756.5	6.04	3	20	0	1.203

Peptide Information

Calc. Mass	Obsrv. Mass	± da	± ppm	Start Seq.	End Seq.	Sequence	Ion Score	C. I. %	Modification	Rank	Result Type
848.3995	848.4734	0.0739	87	12	18	NLSEEEK					Mascot
1269.7777	1269.6726	-0.1051	-83	20	31	IVAEDLAAVLKK					Mascot
1376.7256	1376.8007	0.0751	55	1	11	MPHIDVKHFPR					Mascot

10	Tautomerase PptA OS=Pectobacterium carotovorum subsp. carotovorum (strain PC1) GN=pptA PE=3 SV=1	PPTA_PECCP	8743.5	5.72	3	20	0	1.203

Peptide Information

Calc. Mass	Obsrv. Mass	± da	± ppm	Start	End	Sequence	Ion	C. I. %	Modification	Rank	Result Type

				Seq.	Seq.		Score	
848.3995	848.4734	0.0739	87	12	18	NLSEEEK		Mascot
1269.7777	1269.6726	-0.1051	-83	20	31	VIAEDLAAVLKK		Mascot
1376.7256	1376.8007	0.0751	55	1	11	MPHIDVKHFPR		Mascot

Gel Idx/Pos	111/E10	Instr./Gel Origin	BA2151/Sample Project 20140814	Process Status	Analysis Succeeded
Plate [#] Name	[1] Sample Project 20140814	Instrument Sample Name		Spectra	11

Rank	Protein Name	Accession No.	Protein MW	Protein PI	Pep. Count	Protein Score	Protein Score C. I. %	Intensity Matched	Total Ion Score	Total Ion C. I. %	Confirmed
1	Keratin, type II cytoskeletal 1 OS=Homo sapiens GN=KRT1 PE=1 SV=6	K2C1_HUMAN	66170.1	8.15	27	480	100	31.254	324	100	

Peptide Information

Calc. Mass	Obsrv. Mass	± da	± ppm	Start Seq.	End Seq.	Sequence	Ion Score	C. I. %	Modification	Rank	Result Type
802.4417	802.4954	0.0537	67	180	185	EREQIK					Mascot
832.4886	832.5322	0.0436	52	75	82	SISISVAR					Mascot
973.5312	973.5909	0.0597	61	396	403	IEISELNR					Mascot
973.5312	973.5909	0.0597	61	396	403	IEISELNR	32	42.753			Mascot
999.4966	999.4979	0.0013	1	387	395	HGDSVRNSK					Mascot
1006.4297	1006.492	0.0623	62	589	602	GGSGGGGGGSSGGR					Mascot
1033.516	1033.5781	0.0621	60	484	492	TLLEGEESR					Mascot
1065.5211	1065.5627	0.0416	39	356	364	AQYEDIAQK					Mascot
1066.5164	1066.5685	0.0521	49	270	277	YEDEINKR					Mascot
1092.5029	1092.5721	0.0692	63	603	616	GSGGGSSGGSIGGR					Mascot
1127.5402	1127.5874	0.0472	42	289	298	KDVDGAYMTK					Mascot
1141.5194	1141.6199	0.1005	88	464	472	DYQELMNTK					Mascot
1179.6005	1179.6729	0.0724	61	377	386	YEELQITAGR					Mascot
1265.6372	1265.688	0.0508	40	278	288	TNAENEFVTIK					Mascot
1277.71	1277.7864	0.0764	60	473	483	LALDLEIATYR					Mascot
1300.5297	1300.6179	0.0882	68	258	267	NMQDMVEDYR					Mascot
1332.5195	1332.6359	0.1164	87	258	267	NMQDMVEDYR			Oxidation (M)[2,5]		Mascot
1393.7322	1393.7693	0.0371	27	278	289	TNAENEFVTIKK					Mascot
1421.7383	1421.7313	-0.007	-5	277	288	RTNAENEFVTIK					Mascot
1475.7489	1475.8389	0.09	61	212	223	WELLQQVDTSTR	96	100			Mascot
1475.7853	1475.8389	0.0536	36	200	211	FLEQQNQVLQTK					Mascot
1638.8599	1638.9562	0.0963	59	186	199	SLNNQFASFIDKVR					Mascot
1657.793	1657.8982	0.1052	63	13	29	SGGGFSSGSAGIINYQR					Mascot
1657.793	1657.8982	0.1052	63	13	29	SGGGFSSGSAGIINYQR	24	0			Mascot
1716.8511	1716.9579	0.1068	62	418	432	QISNLQQSISDAEQR					Mascot
1716.8511	1716.9579	0.1068	62	418	432	QISNLQQSISDAEQR	79	99.999			Mascot

1813.894	1813.8425	-0.0515	-28	13	30	SGGGFSSGSAGIINYQRR					Mascot
1993.9767	1994.1018	0.1251	63	224	239	THNLEPYFESFINNLR	95	100			Mascot
1993.9827	1994.1018	0.1191	60	625	644	SSGGSSSVKFVSTTYSG VTR					Mascot
2383.9519	2384.1062	0.1543	65	519	549	GGGGGGYGSGGSSYGS GGGSYGSGGGGGGGR					Mascot
3312.3083	3312.5403	0.232	70	550	588	GSYGSGGSSYGSGGGS YGSGGGGGGHGSYGSG SSSGGYR					Mascot
3312.3083	3312.5403	0.232	70	550	588	GSYGSGGSSYGSGGGS YGSGGGGGGHGSYGSG SSSGGYR					Mascot

2	Keratin, type II cytoskeletal 1 OS=Pan troglodytes GN=KRT1 PE=1 SV=1	K2C1_PANTR	65620.8	7.62	25	461	100	30.716	324	100

Peptide Information

Calc. Mass	Obsrv. Mass	± da	± ppm	Start Seq.	End Seq.	Sequence	Ion Score	C. I. %	Modification	Rank	Result Type
802.4417	802.4954	0.0537	67	175	180	EREQIK					Mascot
832.4886	832.5322	0.0436	52	75	82	SISISVAR					Mascot
973.5312	973.5909	0.0597	61	391	398	IEISELNR					Mascot
973.5312	973.5909	0.0597	61	391	398	IEISELNR	32	42.753			Mascot
999.4966	999.4979	0.0013	1	382	390	HGDSVRNSK					Mascot
1033.516	1033.5781	0.0621	60	479	487	TLLEGEESR					Mascot
1065.5211	1065.5627	0.0416	39	351	359	AQYEDIAQK					Mascot
1066.5164	1066.5685	0.0521	49	265	272	YEDEINKR					Mascot
1092.5029	1092.5721	0.0692	63	596	609	GSGGGSSGGSIGGR					Mascot
1127.5402	1127.5874	0.0472	42	284	293	KDVDGAYMTK					Mascot
1141.5194	1141.6199	0.1005	88	459	467	DYQELMNTK					Mascot
1179.6005	1179.6729	0.0724	61	372	381	YEELQITAGR					Mascot
1265.6372	1265.688	0.0508	40	273	283	TNAENEFVTIK					Mascot
1277.71	1277.7864	0.0764	60	468	478	LALDLEIATYR					Mascot
1393.7322	1393.7693	0.0371	27	273	284	TNAENEFVTIKK					Mascot
1421.7383	1421.7313	-0.007	-5	272	283	RTNAENEFVTIK					Mascot
1475.7489	1475.8389	0.09	61	207	218	WELLQQVDTSTR	96	100			Mascot
1475.7853	1475.8389	0.0536	36	195	206	FLEQQNQVLQTK					Mascot
1638.8599	1638.9562	0.0963	59	181	194	SLNNQFASFIDKVR					Mascot
1657.793	1657.8982	0.1052	63	13	29	SGGGFSSGSAGIINYQR					Mascot
1657.793	1657.8982	0.1052	63	13	29	SGGGFSSGSAGIINYQR	24	0			Mascot
1716.8511	1716.9579	0.1068	62	413	427	QISNLQQSISDAEQR					Mascot
1716.8511	1716.9579	0.1068	62	413	427	QISNLQQSISDAEQR	79	99.999			Mascot

1813.894	1813.8425	-0.0515	-28	13	30	SGGGFSSGSAGIINYQRR					Mascot
1993.9767	1994.1018	0.1251	63	219	234	THNLEPYFESFINNLR	95	100			Mascot
1993.9827	1994.1018	0.1191	60	618	637	SSGGSSSVKFVSTTYSG VTR					Mascot
2383.9519	2384.1062	0.1543	65	514	544	GGGGGGYGSGGSSYGS GGGSYGSGGGGGGGR					Mascot
3312.3083	3312.5403	0.232	70	545	583	GSYGSGGSSYGSGGGS YGSGGGGGGHGSYGSG SSSGGYR					Mascot
3312.3083	3312.5403	0.232	70	545	583	GSYGSGGSSYGSGGGS YGSGGGGGGHGSYGSG SSSGGYR					Mascot

3	Keratin, type I cytoskeletal 9 OS=Homo sapiens GN=KRT9 PE=1 SV=3	K1C9_HUMAN	62254.9	5.14	17	417	100	18.943	337	100

Peptide Information

Calc. Mass	Obsrv. Mass	± da	± ppm	Start Seq.	End Seq.	Sequence	Ion Score	C. I. %	Modification	Rank	Result Type
897.4135	897.4689	0.0554	62	234	240	MTLDDFR					Mascot
913.4084	913.4479	0.0395	43	234	240	MTLDDFR			Oxidation (M)[1]		Mascot
982.4337	982.4957	0.062	63	35	46	FSSSGGGGGGGR					Mascot
1060.5634	1060.6218	0.0584	55	225	233	TLLDIDNTR					Mascot
1065.4994	1065.5627	0.0633	59	155	163	STMQELNSR					Mascot
1066.4987	1066.5685	0.0698	65	243	250	FEMEQNLR					Mascot
1074.5215	1074.5909	0.0694	65	5	13	QFSSSYLSR					Mascot
1081.4943	1081.5435	0.0492	45	155	163	STMQELNSR			Oxidation (M)[3]		Mascot
1082.4935	1082.5487	0.0552	51	243	250	FEMEQNLR			Oxidation (M)[3]		Mascot
1232.5979	1232.6776	0.0797	65	14	29	SGGGGGGGLGSGGSIR					Mascot
1235.5288	1235.6145	0.0857	69	47	59	FSSSSGYGGGSSR					Mascot
1307.6776	1307.7587	0.0811	62	241	250	IKFEMEQNLR					Mascot
1323.6726	1323.7312	0.0586	44	241	250	IKFEMEQNLR			Oxidation (M)[5]		Mascot
1323.6726	1323.7312	0.0586	44	241	250	IKFEMEQNLR	14	0	Oxidation (M)[5]		Mascot
1605.7432	1605.8174	0.0742	46	200	212	NYSPYYNTIDDLK					Mascot
1707.7504	1707.8805	0.1301	76	47	63	FSSSSGYGGGSSRVCGR			Carbamidomethyl (C)[15]		Mascot
1791.7278	1791.8446	0.1168	65	491	513	GGSGGSYGGGGSGGGY GGGSGSR					Mascot
1791.7278	1791.8446	0.1168	65	491	513	GGSGGSYGGGGSGGGY GGGSGSR	70	99.99			Mascot
1837.9655	1838.0459	0.0804	44	375	390	HGVQELEIELQSQLSK					Mascot
1851.9269	1852.033	0.1061	57	322	336	TLNDMRQEYEQLIAK					Mascot
1867.9219	1867.9961	0.0742	40	322	336	TLNDMRQEYEQLIAK			Oxidation (M)[5]		Mascot
2510.1318	2510.2524	0.1206	48	450	472	EIETYHNLLEGGQEDFES SGAGK					Mascot
2705.1611	2705.3398	0.1787	66	64	95	GGGGSFGYSYGGGSGG					Mascot

Calc. Mass	Obsrv. Mass	± da	± ppm	Start Seq.	End Seq.	Sequence	Ion Score	C. I. %	Modification	Rank	Result Type
						GFSASSLGGGFGGGSR					
2705.1611	2705.3398	0.1787	66	64	95	GGGGSFGYSYGGGSGG GFSASSLGGGFGGGSR	253	100			Mascot
3223.2817	3223.5027	0.221	69	580	619	GGSGGSHGGGSGFGGE SGGSYGGGEEASGSGG GYGGGSGK					Mascot

4	Keratin, type II cytoskeletal 1 OS=Rattus norvegicus GN=Krt1 PE=2 SV=1	K2C1_RAT	65190.2	8	17	157	100	17.503	95	100	

Peptide Information

Calc. Mass	Obsrv. Mass	± da	± ppm	Start Seq.	End Seq.	Sequence	Ion Score	C. I. %	Modification	Rank	Result Type
1066.5164	1066.5685	0.0521	49	269	276	YEDEINKR					Mascot
1074.579	1074.5909	0.0119	11	181	189	EQIKSLNDK					Mascot
1082.5994	1082.5487	-0.0507	-47	190	198	FASFIDKVR					Mascot
1139.5579	1139.6287	0.0708	62	267	275	TKYEDEINK					Mascot
1141.5194	1141.6199	0.1005	88	463	471	DYQELMNTK					Mascot
1232.5907	1232.6776	0.0869	71	615	625	FVSTTYSRGTN					Mascot
1265.6372	1265.688	0.0508	40	277	287	TNAENEFVTIK					Mascot
1344.7006	1344.7557	0.0551	41	443	454	LNEIEDALTQAK					Mascot
1393.7322	1393.7693	0.0371	27	277	288	TNAENEFVTIKK					Mascot
1421.7383	1421.7313	-0.007	-5	276	287	RTNAENEFVTIK					Mascot
1456.718	1456.7714	0.0534	37	21	34	NFSSGSAGLVSFQR					Mascot
1475.7489	1475.8389	0.09	61	211	222	WELLQQVDTSTR	96	100			Mascot
1475.7853	1475.8389	0.0536	36	199	210	FLEQQNQVLQTK					Mascot
1487.7999	1487.8263	0.0264	18	395	406	MEISELNRVIQR					Mascot
1791.829	1791.8446	0.0156	9	417	431	QISQMQQNISDAEQR			Oxidation (M)[5]		Mascot
1791.829	1791.8446	0.0156	9	417	431	QISQMQQNISDAEQR			Oxidation (M)[5]		Mascot
2406.1257	2406.0745	-0.0512	-21	52	77	FSGGGFCGSSGGGFGS KSLVNLGGGR			Carbamidomethyl (C)[7]		Mascot
2705.1448	2705.3398	0.195	72	492	517	MSGECTPNVSVSVSTSH TSMSGTSSR			Carbamidomethyl (C)[5], Oxidation (M)[1,20]		Mascot
2705.1448	2705.3398	0.195	72	492	517	MSGECTPNVSVSVSTSH TSMSGTSSR			Carbamidomethyl (C)[5], Oxidation (M)[1,20]		Mascot

5	Keratin, type II cytoskeletal 1 OS=Canis familiaris GN=KRT1 PE=1 SV=1	K2C1_CANFA	63922.1	7.66	12	119	100	12.492	95	100	

Peptide Information

Calc. Mass	Obsrv. Mass	± da	± ppm	Start Seq.	End Seq.	Sequence	Ion Score	C. I. %	Modification	Rank	Result Type
802.4417	802.4954	0.0537	67	181	186	EREQIK					Mascot
999.5218	999.4979	-0.0239	-24	388	396	HGDNLKSTK					Mascot
1033.516	1033.5781	0.0621	60	485	493	TLLEGEESR					Mascot

Calc. Mass	Obsrv. Mass	± da	± ppm	Start Seq.	End Seq.	Sequence	Ion Score	C. I. %	Modification	Rank	Result Type
1066.5164	1066.5685	0.0521	49	271	278	YEDEINKR					Mascot
1141.5194	1141.6199	0.1005	88	465	473	DYQELMNTK					Mascot
1265.6372	1265.688	0.0508	40	279	289	TNAENEFVTIK					Mascot
1307.6624	1307.7587	0.0963	74	394	404	STKMEISELNR					Mascot
1323.6573	1323.7312	0.0739	56	394	404	STKMEISELNR			Oxidation (M)[4]		Mascot
1323.6573	1323.7312	0.0739	56	394	404	STKMEISELNR			Oxidation (M)[4]		Mascot
1393.7322	1393.7693	0.0371	27	279	290	TNAENEFVTIKK					Mascot
1421.7383	1421.7313	-0.007	-5	278	289	RTNAENEFVTIK					Mascot
1475.7489	1475.8389	0.09	61	213	224	WELLQQVDTSTR	96	100			Mascot
1475.7853	1475.8389	0.0536	36	201	212	FLEQQNQVLQTK					Mascot
1638.8599	1638.9562	0.0963	59	187	200	SLNNQFASFIDKVR					Mascot

6 Flagellum-specific ATP synthase OS=Buchnera aphidicola subsp. Acyrthosiphon pisum (strain APS) GN=fliI PE=3 SV=1 | FLII_BUCAI | 52029.7 | 9.05 | 15 | 69 | 92.872 | 6.132

Peptide Information

Calc. Mass	Obsrv. Mass	± da	± ppm	Start Seq.	End Seq.	Sequence	Ion Score	C. I. %	Modification	Rank	Result Type
832.4709	832.5322	0.0613	74	12	18	GILMNLR			Oxidation (M)[4]		Mascot
973.5862	973.5909	0.0047	5	202	210	SILLGMIAR					Mascot
973.5862	973.5909	0.0047	5	202	210	SILLGMIAR					Mascot
999.5655	999.4979	-0.0676	-68	388	396	VMPNIINAK					Mascot
1065.594	1065.5627	-0.0313	-29	191	201	IGIFSSSGIGK					Mascot
1106.6066	1106.5803	-0.0263	-24	407	415	LVASYQRNR					Mascot
1140.6049	1140.6317	0.0268	23	112	121	IASNSNFFIK					Mascot
1158.5977	1158.65	0.0523	45	2	11	AAIMSSYLFR					Mascot
1227.6515	1227.6412	-0.0103	-8	358	368	SVLDGHIMLSR					Mascot
1297.7297	1297.728	-0.0017	-1	279	289	HVLLIMDSLTR					Mascot
1491.8053	1491.833	0.0277	19	441	452	LEKFLQQEISEK					Mascot
1493.8938	1493.8334	-0.0604	-40	245	259	SVIIAAPADVSPLLK					Mascot
1638.936	1638.9562	0.0202	12	123	137	LPLGMELLGRVLDGR					Mascot
1674.9425	1674.8341	-0.1084	-65	211	225	YTQADIIVIALIGER					Mascot
1988.0457	1988.129	0.0833	42	2	18	AAIMSSYLFRGILMNLR			Oxidation (M)[4,14]		Mascot
3312.8083	3312.5403	-0.268	-81	45	75	LVSINGLILEVVGLNTSIG SECLIERTIDGK			Carbamidomethyl (C)[22]		Mascot
3312.8083	3312.5403	-0.268	-81	45	75	LVSINGLILEVVGLNTSIG SECLIERTIDGK			Carbamidomethyl (C)[22]		Mascot

7 Microtubule-actin cross-linking factor 1 OS=Rattus norvegicus GN=Macf1 PE=1 SV=1 | MACF1_RAT | 623207.6 | 5.28 | 56 | 66 | 85.777 | 28.932

Peptide Information

Calc. Mass	Obsrv. Mass	± da	± ppm	Start Seq.	End Seq.	Sequence	Ion Score	C. I. %	Modification	Rank	Result Type
802.4781	802.4954	0.0173	22	3578	3584	LDGIKTR					Mascot
807.4471	807.4536	0.0065	8	3814	3819	YARLER					Mascot
832.4523	832.5322	0.0799	96	1906	1912	LKDATER					Mascot
870.5229	870.585	0.0621	71	3736	3743	LMALGPIR					Mascot
948.5513	948.4907	-0.0606	-64	4748	4755	VFQKELGK					Mascot
951.4629	951.5168	0.0539	57	1454	1462	TSASTDIEK					Mascot
973.5425	973.5909	0.0484	50	804	811	NLQDSIKR					Mascot
973.5425	973.5909	0.0484	50	804	811	NLQDSIKR					Mascot
1006.4874	1006.492	0.0046	5	2423	2432	GMDASLSPTK					Mascot
1018.5601	1018.5725	0.0124	12	408	415	LIVEMLER			Oxidation (M)[5]		Mascot
1057.5636	1057.5587	-0.0049	-5	3344	3353	IAQAAELADR					Mascot
1060.5647	1060.6218	0.0571	54	1226	1234	AVAEQLHHR					Mascot
1068.5143	1068.5731	0.0588	55	521	528	LECTNLYR			Carbamidomethyl (C)[3]		Mascot
1074.6453	1074.5909	-0.0544	-51	5204	5212	ILRSTVMVR					Mascot
1092.5685	1092.5721	0.0036	3	4647	4655	QPVYDTTIR					Mascot
1106.5034	1106.5803	0.0769	69	1018	1026	IEEEVEACK			Carbamidomethyl (C)[8]		Mascot
1127.5691	1127.5874	0.0183	16	1235	1244	AAEPNLDLER					Mascot
1141.6688	1141.6199	-0.0489	-43	3322	3330	LLQRLLDDR					Mascot
1158.6154	1158.65	0.0346	30	341	350	SFPQNPVELK					Mascot
1165.6259	1165.6493	0.0234	20	676	684	MRHLQSLHK			Oxidation (M)[1]		Mascot
1182.63	1182.6475	0.0175	15	4215	4224	LNHQGELMLK					Mascot
1232.6117	1232.6776	0.0659	53	2659	2668	QQLEETSEIR					Mascot
1252.5804	1252.6274	0.047	38	5153	5163	DAYRPTTDADK					Mascot
1259.7028	1259.7482	0.0454	36	408	417	LIVEMLEREK					Mascot
1297.6205	1297.728	0.1075	83	2026	2036	FQSLSCSLAER			Carbamidomethyl (C)[6]		Mascot
1307.655	1307.7587	0.1037	79	1070	1081	QIQSSASSKTDR					Mascot
1320.7271	1320.6729	-0.0542	-41	1870	1881	GDLRFVTISGQK					Mascot
1323.7532	1323.7312	-0.022	-17	678	688	HLQSLHKFVSK					Mascot
1323.7532	1323.7312	-0.022	-17	678	688	HLQSLHKFVSK					Mascot
1332.7191	1332.6359	-0.0832	-62	1783	1793	QMLQEKLGELK			Oxidation (M)[2]		Mascot
1339.7329	1339.6949	-0.038	-28	1103	1114	HLRSDLDAVSVK					Mascot
1344.6754	1344.7557	0.0803	60	56	67	KSQDSVLDPAER					Mascot
1365.6758	1365.7278	0.052	38	1245	1255	YQEKGSQLQER					Mascot

Calc. Mass	Obsrv. Mass	± da	± ppm	Start Seq.	End Seq.	Sequence	Ion Score	C. I. %	Modification	Rank	Result Type
1376.7057	1376.7865	0.0808	59	2406	2417	EASSVLQWLESK					Mascot
1379.6948	1379.7948	0.1	72	2100	2111	SSLEATREMVTR					Mascot
1412.7202	1412.6652	-0.055	-39	869	880	STLSVKAICDYR			Carbamidomethyl (C)[9]		Mascot
1421.7019	1421.7313	0.0294	21	1908	1919	DATERYTTLHSK					Mascot
1458.8064	1458.812	0.0056	4	4565	4576	NLLVSVQSRWEK					Mascot
1487.7952	1487.8263	0.0311	21	3565	3577	QTTGEEVLLIQEK					Mascot
1493.7595	1493.8334	0.0739	49	2240	2251	DFTELQKTVQER					Mascot
1605.8306	1605.8174	-0.0132	-8	3211	3223	MFQKEQVDPLQVK			Oxidation (M)[1]		Mascot
1638.8599	1638.9562	0.0963	59	4990	5003	NIEPTHAPFIEKSR					Mascot
1641.8555	1641.9426	0.0871	53	57	71	SQDSVLDPAERAVVR					Mascot
1707.964	1707.8805	-0.0835	-49	108	123	DGHNLISLLEVLSGIK					Mascot
1708.8389	1708.8435	0.0046	3	3681	3695	EGLDKLVSDANEQYK					Mascot
1715.9326	1715.9122	-0.0204	-12	2445	2459	AFLAELEQNSPKIQK					Mascot
1716.92	1716.9579	0.0379	22	3124	3138	MLEEEGTLDLLGLKR					Mascot
1716.92	1716.9579	0.0379	22	3124	3138	MLEEEGTLDLLGLKR	5	0			Mascot
1738.8091	1738.8689	0.0598	34	5323	5341	TSLAGDTSNSSSPASTGAK					Mascot
1791.8356	1791.8446	0.009	5	4328	4345	AGNELLESSAGDDASSLR					Mascot
1791.9851	1791.8446	-0.1405	-78	1479	1494	EQVSEAIKTSQIFLAK					Mascot
1813.8942	1813.8425	-0.0517	-29	4020	4033	LDQMVFFWEDIKAR			Oxidation (M)[4]		Mascot
1926.959	1926.9812	0.0222	12	1333	1348	LDQCQKLSQQYSTTVK			Carbamidomethyl (C)[4]		Mascot
1947.9231	1948.0273	0.1042	53	588	602	FVYELLSWVEEMQMK			Oxidation (M)[12]		Mascot
1993.9825	1994.1018	0.1193	60	3431	3448	ALEEEIENHAADVQQAVK					Mascot
1993.9825	1994.1018	0.1193	60	3431	3448	ALEEEIENHAADVQQAVK					Mascot
2341.218	2341.3452	0.1272	54	2705	2725	LETVALPLQGLEDLAADRMNR			Oxidation (M)[19]		Mascot
2687.353	2687.3975	0.0445	17	4203	4224	VEVYQQQIEMEKLNHQGELMLK					Mascot
3223.6482	3223.5027	-0.1455	-45	2780	2809	NLNQHSGSYEVIVAEGESLLLSVPPGEEKK					Mascot
3312.6411	3312.5403	-0.1008	-30	270	299	LLDAEDVDVPSPDEKSVITYVSSIYDAFPK					Mascot
3312.6411	3312.5403	-0.1008	-30	270	299	LLDAEDVDVPSPDEKSVITYVSSIYDAFPK					Mascot

8	O-phosphoseryl-tRNA(Sec) selenium transferase OS=Methanocaldococcus jannaschii (strain ATCC 43067 / DSM 2661 / JAL-1 / JCM 10045 / NBRC 100440) GN=spcS PE=1 SV=1	SPCS_METJA	48995.2	9	16	64	76.396	8.686

Peptide Information

Calc. Mass	Obsrv. Mass	± da	± ppm	Start Seq.	End Seq.	Sequence	Ion Score	C. I. %	Modification	Rank	Result Type
1018.5414	1018.5725	0.0311	31	226	234	SDDIKEIAK					Mascot

Calc. Mass	Obsrv. Mass	± da	± ppm	Start Seq.	End Seq.	Sequence	Ion Score	C. I. %	Modification	Rank	Result Type
1057.614	1057.5587	-0.0553	-52	426	434	VIEKLDEVL					Mascot
1068.5685	1068.5731	0.0046	4	94	103	SGNLIDPQPK					Mascot
1127.642	1127.5874	-0.0546	-48	27	35	IIENILEQR					Mascot
1201.5817	1201.6703	0.0886	74	171	180	ATSFIGMRMR			Oxidation (M)[7,9]		Mascot
1297.6456	1297.728	0.0824	63	326	335	DYLNLMKEQK			Oxidation (M)[6]		Mascot
1300.736	1300.6179	-0.1181	-91	181	192	LVETVLDGDIVK					Mascot
1307.714	1307.7587	0.0447	34	167	178	SPIKATSFIGMR					Mascot
1323.709	1323.7312	0.0222	17	167	178	SPIKATSFIGMR			Oxidation (M)[11]		Mascot
1323.709	1323.7312	0.0222	17	167	178	SPIKATSFIGMR			Oxidation (M)[11]		Mascot
1339.6853	1339.6949	0.0096	7	266	277	YRIDAVVSSSDK					Mascot
1365.6006	1365.7278	0.1272	93	82	93	DGVFDFCHGVGR			Carbamidomethyl (C)[7]		Mascot
1379.7351	1379.7948	0.0597	43	104	116	APGASVMYKLTNK					Mascot
1493.8363	1493.8334	-0.0029	-2	278	291	NLFTPIGGGIIYTK					Mascot
1638.8275	1638.9562	0.1287	79	153	166	YNSNVVIYPYAAHK					Mascot
1674.8732	1674.8341	-0.0391	-23	356	370	VLNVENPISSCITTK			Carbamidomethyl (C)[11]		Mascot
1708.9164	1708.8435	-0.0729	-43	2	16	GLNITGLIPKHMENR			Oxidation (M)[12]		Mascot
1847.0021	1846.8806	-0.1215	-66	37	52	APENGIDEEHIKLLLR					Mascot

9	Keratin, type II cytoskeletal 2 epidermal OS=Homo sapiens GN=KRT2 PE=1 SV=2	K22E_HUMAN	65678.3	8.07	10	63	72.899	14.477	32	42.753

Peptide Information

Calc. Mass	Obsrv. Mass	± da	± ppm	Start Seq.	End Seq.	Sequence	Ion Score	C. I. %	Modification	Rank	Result Type
973.5312	973.5909	0.0597	61	394	401	IEISELNR					Mascot
973.5312	973.5909	0.0597	61	394	401	IEISELNR	32	42.753			Mascot
1066.5164	1066.5685	0.0521	49	268	275	YEDEINKR					Mascot
1068.5031	1068.5731	0.07	66	288	296	DVDNAYMIK					Mascot
1082.5994	1082.5487	-0.0507	-47	189	197	FASFIDKVR					Mascot
1107.543	1107.6064	0.0634	57	354	362	AQYEEIAQR					Mascot
1139.5402	1139.6287	0.0885	78	462	470	DYQELMNVK					Mascot
1320.5829	1320.6729	0.09	68	46	61	HGGGGGGFGGGGFGSR					Mascot
1475.7853	1475.8389	0.0536	36	198	209	FLEQQNQVLQTK					Mascot
1475.7853	1475.8389	0.0536	36	198	209	FLEQQNQVLQTK	11	0			Mascot
1490.8802	1490.8395	-0.0407	-27	402	414	VIQRLQGEIAHVK					Mascot
2384.2166	2384.1062	-0.1104	-46	462	481	DYQELMNVKLALDVEIAT YR					Mascot

10	Nuclear mitotic apparatus protein 1 OS=Homo sapiens GN=NUMA1 PE=1 SV=2	NUMA1_HUMAN	239198.6	5.63	28	62	64.274	13.129

Peptide Information

Calc. Mass	Obsrv. Mass	± da	± ppm	Start Seq.	End Seq.	Sequence	Ion Score	C. I. %	Modification	Rank	Result Type
802.4417	802.4954	0.0537	67	598	604	ERDAALK					Mascot
832.4523	832.5322	0.0799	96	644	650	EKAELSR					Mascot
847.4705	847.4894	0.0189	22	1612	1618	LQMEKAK					Mascot
973.5061	973.5909	0.0848	87	1647	1654	ELRAEAER					Mascot
973.5061	973.5909	0.0848	87	1647	1654	ELRAEAER	11	0			Mascot
1006.4873	1006.492	0.0047	5	436	443	VEMLETER					Mascot
1018.5275	1018.5725	0.045	44	1149	1157	SLEAERASR					Mascot
1127.6281	1127.5874	-0.0407	-36	1920	1928	IAELQQRNR					Mascot
1201.6423	1201.6703	0.028	23	564	573	EQQLKEVAEK					Mascot
1232.637	1232.6776	0.0406	33	898	908	LADDLSTLQEK					Mascot
1235.6743	1235.6145	-0.0598	-48	1700	1710	FQVATDALKSR					Mascot
1265.6406	1265.688	0.0474	37	1254	1264	LVMAESEKSQK			Oxidation (M)[3]		Mascot
1297.6859	1297.728	0.0421	32	741	751	AETRSLVEQHK					Mascot
1405.7686	1405.7604	-0.0082	-6	1696	1708	DLGKFQVATDALK					Mascot
1458.766	1458.812	0.046	32	1491	1503	LAEVQREAQSTAR					Mascot
1513.8473	1513.8529	0.0056	4	406	419	GEVLGDVLQLETLK					Mascot
1513.8473	1513.9755	0.1282	85	406	419	GEVLGDVLQLETLK					Mascot
1584.7938	1584.8206	0.0268	17	1428	1441	AEKASYAEQLSMLK			Oxidation (M)[12]		Mascot
1674.79	1674.8341	0.0441	26	98	111	MTMLLLYHSTMSSK			Oxidation (M)[1,3]		Mascot
1698.8341	1698.9609	0.1268	75	1668	1681	EAEQTCRHLTAQVR			Carbamidomethyl (C)[6]		Mascot
1699.8722	1699.9323	0.0601	35	420	435	QEAATLAANNTQLQAR					Mascot
1707.8054	1707.8805	0.0751	44	2006	2019	ATSCFPRPMTPRDR			Carbamidomethyl (C)[4], Oxidation (M)[9]		Mascot
1715.9287	1715.9122	-0.0165	-10	2090	2108	IATTTASAATAAAIGATPR					Mascot
1791.9092	1791.8446	-0.0646	-36	243	257	LLTEKDAQIAMMQQR			Oxidation (M)[11]		Mascot
1791.9092	1791.8446	-0.0646	-36	243	257	LLTEKDAQIAMMQQR			Oxidation (M)[11]		Mascot
1816.9916	1816.9534	-0.0382	-21	1531	1545	QKLTAQVEQLEVFQR					Mascot
1837.9211	1838.0459	0.1248	68	898	914	LADDLSTLQEKMAATSK			Oxidation (M)[12]		Mascot
2023.1294	2023.017	-0.1124	-56	614	631	AAKLEILQQQLQVANEAR					Mascot
2341.2007	2341.3452	0.1445	62	1175	1196	AQELGHSQSALASAQRE LAAFR					Mascot
2501.1758	2501.4143	0.2385	95	787	808	RELAEAMAAQHTAESEC EQLVK			Carbamidomethyl (C)[17]		Mascot
2687.3418	2687.3975	0.0557	21	420	443	QEAATLAANNTQLQARV EMLETER					Mascot

Gel Idx/Pos	112/E11	Instr./Gel Origin	BA2151/Sample Project 20140814	Process Status	Analysis Succeeded
Plate [#] Name	[1] Sample Project 20140814	Instrument Sample Name		Spectra	11

Rank	Protein Name	Accession No.	Protein MW	Protein PI	Pep. Count	Protein Score	Protein Score C. I. %	Intensity Matched	Total Ion Score	Total Ion C. I. %	Confirmed
1	17.4 kDa class I heat shock protein OS=Arabidopsis thaliana GN=HSP17.4A PE=2 SV=2	HSP17_ARATH	17428.8	5.21	6	288	100	8.076	244	100	

Peptide Information

Calc. Mass	Obsrv. Mass	± da	± ppm	Start Seq.	End Seq.	Sequence	Ion Score	C. I. %	Modification	Rank	Result Type
827.4985	827.499	0.0005	1	64	71	ADVPGLKK					Mascot
974.5417	974.5937	0.052	53	115	122	FRLPENAK					Mascot
974.5417	974.5937	0.052	53	115	122	FRLPENAK	18	0			Mascot
1057.5314	1057.5701	0.0387	37	55	63	ETPEAHVFK					Mascot
1066.5681	1066.5667	-0.0014	-1	2	11	SLVPSFFGGR					Mascot
1657.8392	1657.9315	0.0923	56	76	90	VEVEDGNILQISGER					Mascot
1657.8392	1657.9315	0.0923	56	76	90	VEVEDGNILQISGER	139	100			Mascot
2143.0876	2143.2131	0.1255	59	72	90	EEVKVEVEDGNILQISGE R					Mascot
2143.0876	2143.2131	0.1255	59	72	90	EEVKVEVEDGNILQISGE R	104	100			Mascot

Rank	Protein Name	Accession No.	Protein MW	Protein PI	Pep. Count	Protein Score	Protein Score C. I. %	Intensity Matched	Total Ion Score	Total Ion C. I. %	Confirmed
2	17.6 kDa class I heat shock protein 3 OS=Arabidopsis thaliana GN=HSP17.6C PE=2 SV=2	HS17C_ARATH	17593	5.36	5	282	100	8.37	244	100	

Peptide Information

Calc. Mass	Obsrv. Mass	± da	± ppm	Start Seq.	End Seq.	Sequence	Ion Score	C. I. %	Modification	Rank	Result Type
974.5417	974.5937	0.052	53	116	123	FRLPENAK					Mascot
974.5417	974.5937	0.052	53	116	123	FRLPENAK	18	0			Mascot
1057.5314	1057.5701	0.0387	37	56	64	ETPEAHVFK					Mascot
1193.6348	1193.6835	0.0487	41	1	11	MSLIPSIFGGR			Oxidation (M)[1]		Mascot
1657.8392	1657.9315	0.0923	56	77	91	VEVEDGNILQISGER					Mascot
1657.8392	1657.9315	0.0923	56	77	91	VEVEDGNILQISGER	139	100			Mascot
2143.0876	2143.2131	0.1255	59	73	91	EEVKVEVEDGNILQISGE R					Mascot
2143.0876	2143.2131	0.1255	59	73	91	EEVKVEVEDGNILQISGE R	104	100			Mascot

Rank	Protein Name	Accession No.	Protein MW	Protein PI	Pep. Count	Protein Score	Protein Score C. I. %	Intensity Matched	Total Ion Score	Total Ion C. I. %	Confirmed
3	Keratin, type II cytoskeletal 1 OS=Homo sapiens GN=KRT1 PE=1 SV=6	K2C1_HUMAN	66170.1	8.15	18	189	100	14.822	110	100	

Peptide Information

Calc. Mass	Obsrv. Mass	± da	± ppm	Start Seq.	End Seq.	Sequence	Ion Score	C. I. %	Modification	Rank	Result Type
802.4417	802.4793	0.0376	47	180	185	EREQIK					Mascot
973.5312	973.5822	0.051	52	396	403	IEISELNR					Mascot
1033.516	1033.5695	0.0535	52	484	492	TLLEGEESR					Mascot
1065.5211	1065.5698	0.0487	46	356	364	AQYEDIAQK					Mascot
1066.5164	1066.5667	0.0503	47	270	277	YEDEINKR					Mascot
1092.5029	1092.5798	0.0769	70	603	616	GSGGGSSGGSIGGR					Mascot
1141.5194	1141.5607	0.0413	36	464	472	DYQELMNTK					Mascot
1179.6005	1179.6644	0.0639	54	377	386	YEELQITAGR					Mascot
1277.71	1277.7808	0.0708	55	473	483	LALDLEIATYR					Mascot
1300.5297	1300.6263	0.0966	74	258	267	NMQDMVEDYR					Mascot
1302.7152	1302.7207	0.0055	4	344	355	SLDLDSIIAEVK					Mascot
1475.7489	1475.8331	0.0842	57	212	223	WELLQQVDTSTR	46	97.486			Mascot
1475.7853	1475.8331	0.0478	32	200	211	FLEQQNQVLQTK					Mascot
1657.793	1657.9315	0.1385	84	13	29	SGGGFSSGSAGIINYQR					Mascot
1657.793	1657.9315	0.1385	84	13	29	SGGGFSSGSAGIINYQR					Mascot
1716.8511	1716.9508	0.0997	58	418	432	QISNLQQSISDAEQR					Mascot
1716.8511	1716.9508	0.0997	58	418	432	QISNLQQSISDAEQR	66	99.973			Mascot
1993.9827	1994.1001	0.1174	59	625	644	SSGGSSSVKFVSTTYSG VTR					Mascot
2383.9519	2384.0981	0.1462	61	519	549	GGGGGGYGSGGSSYGS GGGSYGSGGGGGGGR					Mascot
3312.3083	3312.5334	0.2251	68	550	588	GSYGSGGSSYGSGGGS YGSGGGGGGHGSYGSG SSSGGYR					Mascot

4 Keratin, type II cytoskeletal 1 OS=Pan troglodytes GN=KRT1 PE=1 SV=1 | K2C1_PANTR | 65620.8 | 7.62 | 17 | 183 | 100 | 14.66 | 110 | 100

Peptide Information

Calc. Mass	Obsrv. Mass	± da	± ppm	Start Seq.	End Seq.	Sequence	Ion Score	C. I. %	Modification	Rank	Result Type
802.4417	802.4793	0.0376	47	175	180	EREQIK					Mascot
973.5312	973.5822	0.051	52	391	398	IEISELNR					Mascot
1033.516	1033.5695	0.0535	52	479	487	TLLEGEESR					Mascot
1065.5211	1065.5698	0.0487	46	351	359	AQYEDIAQK					Mascot
1066.5164	1066.5667	0.0503	47	265	272	YEDEINKR					Mascot
1092.5029	1092.5798	0.0769	70	596	609	GSGGGSSGGSIGGR					Mascot
1141.5194	1141.5607	0.0413	36	459	467	DYQELMNTK					Mascot
1179.6005	1179.6644	0.0639	54	372	381	YEELQITAGR					Mascot

Calc. Mass	Obsrv. Mass	± da	± ppm	Start Seq.	End Seq.	Sequence	Ion Score	C. I. %	Modification	Rank	Result Type
1277.71	1277.7808	0.0708	55	468	478	LALDLEIATYR					Mascot
1302.7152	1302.7207	0.0055	4	339	350	SLDLDSIIAEVK					Mascot
1475.7489	1475.8331	0.0842	57	207	218	WELLQQVDTSTR	46	97.486			Mascot
1475.7853	1475.8331	0.0478	32	195	206	FLEQQNQVLQTK					Mascot
1657.793	1657.9315	0.1385	84	13	29	SGGGFSSGSAGIINYQR					Mascot
1657.793	1657.9315	0.1385	84	13	29	SGGGFSSGSAGIINYQR					Mascot
1716.8511	1716.9508	0.0997	58	413	427	QISNLQQSISDAEQR					Mascot
1716.8511	1716.9508	0.0997	58	413	427	QISNLQQSISDAEQR	66	99.973			Mascot
1993.9827	1994.1001	0.1174	59	618	637	SSGGSSSVKFVSTTYSG VTR					Mascot
2383.9519	2384.0981	0.1462	61	514	544	GGGGGGYGSGGSSYGS GGGSYGSGGGGGGGR					Mascot
3312.3083	3312.5334	0.2251	68	545	583	GSYGSGGSSYGSGGGS YGSGGGGGGHGSYGSG SSSGGYR					Mascot

5	Keratin, type I cytoskeletal 10 OS=Homo sapiens GN=KRT10 PE=1 SV=6	K1C10_HUMAN	59019.8	5.13	16	130	100	10.661	69	99.987		

Peptide Information

Calc. Mass	Obsrv. Mass	± da	± ppm	Start Seq.	End Seq.	Sequence	Ion Score	C. I. %	Modification	Rank	Result Type
807.3995	807.4481	0.0486	60	229	235	LAADDFR					Mascot
847.452	847.4241	-0.0279	-33	363	369	SEITELR					Mascot
993.4999	993.5576	0.0577	58	238	245	YENEVALR					Mascot
1060.5092	1060.6028	0.0936	88	1	9	MSVRYSSSK			Oxidation (M)[1]		Mascot
1090.531	1090.5891	0.0581	53	148	156	VTMQNLNDR					Mascot
1106.5259	1106.5785	0.0526	48	148	156	VTMQNLNDR			Oxidation (M)[3]		Mascot
1109.4899	1109.561	0.0711	64	335	343	DAEAWFNEK					Mascot
1118.5087	1118.5767	0.068	61	185	194	HGNSHQGEPR					Mascot
1165.5848	1165.6494	0.0646	55	442	450	LENEIQTYR					Mascot
1201.6172	1201.6801	0.0629	52	246	256	QSVEADINGLR					Mascot
1234.6791	1234.7441	0.065	53	236	245	LKYENEVALR					Mascot
1300.5951	1300.6263	0.0312	24	286	295	NHEEEMKDLR					Mascot
1365.6393	1365.7185	0.0792	58	323	333	SQYEQLAEQNR					Mascot
1365.6393	1365.7185	0.0792	58	323	333	SQYEQLAEQNR	39	86.277			Mascot
1390.6809	1390.7574	0.0765	55	387	399	QSLEASLAETEGR					Mascot
1493.7343	1493.8207	0.0864	58	323	334	SQYEQLAEQNRK					Mascot
1493.7343	1493.8207	0.0864	58	323	334	SQYEQLAEQNRK	30	4.403			Mascot
1707.7722	1707.8722	0.1	59	41	59	GSLGGGFSSGGFSGGSF SR					Mascot

Calc. Mass	Obsrv. Mass	± da	± ppm	Start Seq.	End Seq.	Sequence	Ion Score	C. I. %	Modification	Rank	Result Type
2367.2627	2367.4055	0.1428	60	208	228	NQILNLTTDNANILLQIDN AR					Mascot

6	Keratin, type II cytoskeletal 1 OS=Rattus norvegicus GN=Krt1 PE=2 SV=1	K2C1_RAT	65190.2	8	14	81	99.59	4.499	45	96.513

Peptide Information

Calc. Mass	Obsrv. Mass	± da	± ppm	Start Seq.	End Seq.	Sequence	Ion Score	C. I. %	Modification	Rank	Result Type
827.4297	827.499	0.0693	84	190	196	FASFIDK					Mascot
845.4839	845.4955	0.0116	14	239	245	RQVDSLK					Mascot
905.4938	905.5045	0.0107	12	409	416	SEIDSVKK					Mascot
960.4785	960.5528	0.0743	77	615	622	FVSTTYSR					Mascot
1066.5164	1066.5667	0.0503	47	269	276	YEDEINKR					Mascot
1082.5994	1082.5859	-0.0135	-12	190	198	FASFIDKVR					Mascot
1139.5579	1139.6312	0.0733	64	267	275	TKYEDEINK					Mascot
1141.5194	1141.5607	0.0413	36	463	471	DYQELMNTK					Mascot
1232.5907	1232.671	0.0803	65	615	625	FVSTTYSRGTN					Mascot
1475.7489	1475.8331	0.0842	57	211	222	WELLQQVDTSTR	46	97.486			Mascot
1475.7853	1475.8331	0.0478	32	199	210	FLEQQNQVLQTK					Mascot
1612.8191	1612.8988	0.0797	49	21	35	NFSSGSAGLVSFQRR					Mascot
1791.829	1791.8383	0.0093	5	417	431	QISQMQQNISDAEQR			Oxidation (M)[5]		Mascot
2705.1448	2705.3376	0.1928	71	492	517	MSGECTPNVSVSVSTSH TSMSGTSSR			Carbamidomethyl (C)[5], Oxidation (M)[1,20]		Mascot

7	Keratin, type II cytoskeletal 6A OS=Rattus norvegicus GN=Krt6a PE=1 SV=1	K2C6A_RAT	59554.8	8.06	18	74	98.037	11.967	

Peptide Information

Calc. Mass	Obsrv. Mass	± da	± ppm	Start Seq.	End Seq.	Sequence	Ion Score	C. I. %	Modification	Rank	Result Type
819.4141	819.4181	0.004	5	426	432	AKQDMAR					Mascot
827.4297	827.499	0.0693	84	163	169	FASFIDK					Mascot
835.4091	835.4603	0.0512	61	426	432	AKQDMAR			Oxidation (M)[5]		Mascot
951.5292	951.5234	-0.0058	-6	1	8	MSTKTVIR			Oxidation (M)[1]		Mascot
974.5451	974.5937	0.0486	50	428	435	QDMARLLK					Mascot
974.5451	974.5937	0.0486	50	428	435	QDMARLLK	1	0			Mascot
1057.5637	1057.5701	0.0064	6	213	221	ELDNILGQR					Mascot
1082.5994	1082.5859	-0.0135	-12	163	171	FASFIDKVR					Mascot
1106.5146	1106.5785	0.0639	58	456	464	TLLEGEECR			Carbamidomethyl (C)[8]		Mascot
1107.5793	1107.6007	0.0214	19	328	336	AQYEEIAKR					Mascot

1139.5402	1139.6312	0.091	80	436	444	EYQDLMNVK					Mascot
1154.551	1154.6094	0.0584	51	261	270	KDVDAAYMNK					Mascot
1154.551	1154.6094	0.0584	51	261	270	KDVDAAYMNK					Mascot
1179.6005	1179.6644	0.0639	54	349	358	YEELQITAGR					Mascot
1263.6943	1263.7345	0.0402	32	445	455	LALDVEIATYR					Mascot
1300.7107	1300.6263	-0.0844	-65	414	425	GKLEGLEDALQK					Mascot
1302.7152	1302.7207	0.0055	4	316	327	SLDLDSIIAEVK					Mascot
1493.8032	1493.8207	0.0175	12	433	444	LLKEYQDLMNVK					Mascot
1493.8032	1493.8207	0.0175	12	433	444	LLKEYQDLMNVK					Mascot
1838.9283	1839.0276	0.0993	54	197	211	QGLETLFEQYINDLR					Mascot
2271.176	2271.3147	0.1387	61	390	410	QIANLQAAIAEAEQRGEM ALK			Oxidation (M)[18]		Mascot
2384.2166	2384.0981	-0.1185	-50	436	455	EYQDLMNVKLALDVEIAT YR					Mascot

8	ATP-dependent Clp protease ATP-binding subunit ClpX OS=Tropheryma whipplei (strain Twist) GN=clpX PE=3 SV=1	CLPX_TROWT	47018.4	5.49	11	68	91.026	3.425

Protein Group

ATP-dependent Clp protease ATP-binding subunit ClpX OS=Tropheryma whipplei (strain TW08/27) GN=clpX PE=3 SV=1	CLPX_TROW8	46990.4	5.4899 997711 1816

Peptide Information

Calc. Mass	Obsrv. Mass	± da	± ppm	Start Seq.	End Seq.	Sequence	Ion Score	C. I. %	Modification	Rank	Result Type
810.4104	810.4756	0.0652	80	417	423	SNYIRSA					Mascot
846.4316	846.4981	0.0665	79	199	206	ADSPSITR					Mascot
974.5265	974.5937	0.0672	69	198	206	KADSPSITR					Mascot
974.5265	974.5937	0.0672	69	198	206	KADSPSITR	22	0			Mascot
1039.5103	1039.5658	0.0555	53	414	421	CARSNYIR			Carbamidomethyl (C)[1]		Mascot
1308.6583	1308.7406	0.0823	63	91	101	DLSVAVYNHYK					Mascot
1329.7195	1329.7198	0.0003	0	119	131	SNILLIGPTGCGK			Carbamidomethyl (C)[11]		Mascot
1432.6989	1432.7712	0.0723	50	182	194	AEAGIVCIDEIDK			Carbamidomethyl (C)[7]		Mascot
1522.7795	1522.8512	0.0717	47	268	281	IGRSNMGFGSDLLR					Mascot
2367.2085	2367.4055	0.197	83	391	413	GDIESVRITAETVAGGGP HLTMR					Mascot
2720.3447	2720.4277	0.083	31	171	194	LLQDADFDITRAEAGIVCI DEIDK			Carbamidomethyl (C)[18]		Mascot
2807.4866	2807.4912	0.0046	2	63	87	SVLEGLPKPAEIYAFLDE YVIGQQK					Mascot

9	Keratin, type II cytoskeletal 2 epidermal OS=Homo sapiens GN=KRT2 PE=1 SV=2	K22E_HUMAN	65678.3	8.07	17	67	88.703	8.163

Peptide Information

Calc. Mass	Obsrv. Mass	± da	± ppm	Start Seq.	End Seq.	Sequence	Ion Score	C. I. %	Modification	Rank	Result Type
827.4297	827.499	0.0693	84	189	195	FASFIDK					Mascot
831.4935	831.4421	-0.0514	-62	62	70	SLVGLGGTK					Mascot
973.5312	973.5822	0.051	52	394	401	IEISELNR					Mascot
1037.5262	1037.5718	0.0456	44	239	247	YLDGLTAER					Mascot
1066.5164	1066.5667	0.0503	47	268	275	YEDEINKR					Mascot
1082.5994	1082.5859	-0.0135	-12	189	197	FASFIDKVR					Mascot
1107.543	1107.6007	0.0577	52	354	362	AQYEEIAQR					Mascot
1139.5402	1139.6312	0.091	80	462	470	DYQELMNVK					Mascot
1193.6273	1193.6835	0.0562	47	238	247	RYLDGLTAER					Mascot
1263.6943	1263.7345	0.0402	32	471	481	LALDVEIATYR					Mascot
1320.5829	1320.6658	0.0829	63	46	61	HGGGGGGFGGGGFGSR					Mascot
1320.5829	1320.6658	0.0829	63	46	61	HGGGGGGFGGGGFGSR					Mascot
1329.7261	1329.7198	-0.0063	-5	342	353	NLDLDSIIAEVK					Mascot
1460.7996	1460.8413	0.0417	29	303	314	VDLLNQEIEFLK					Mascot
1475.7853	1475.8331	0.0478	32	198	209	FLEQQNQVLQTK					Mascot
1475.7853	1475.8331	0.0478	32	198	209	FLEQQNQVLQTK					Mascot
1838.9144	1839.0276	0.1132	62	71	92	SISISVAGGGGGFGAAGGFGGR					Mascot
2384.2166	2384.0981	-0.1185	-50	462	481	DYQELMNVKLALDVEIATYR					Mascot
2399.0186	2399.1624	0.1438	60	93	122	GGGFGGGSSFGGGSGFSGGGFGGGGFGGGR					Mascot

10	Keratin, type I cytoskeletal 10 OS=Bos taurus GN=KRT10 PE=3 SV=1	K1C10_BOVIN	54986.3	5.05	10	66	86.417	6.585	39	86.277

Peptide Information

Calc. Mass	Obsrv. Mass	± da	± ppm	Start Seq.	End Seq.	Sequence	Ion Score	C. I. %	Modification	Rank	Result Type
807.3995	807.4481	0.0486	60	210	216	LAADDFR					Mascot
847.452	847.4241	-0.0279	-33	344	350	SEITELR					Mascot
948.4493	948.499	0.0497	52	16	27	SGGGGGGGSSLR					Mascot
1060.5092	1060.6028	0.0936	88	1	9	MSVRYSSSK			Oxidation (M)[1]		Mascot
1109.4899	1109.561	0.0711	64	316	324	DAEAWFNEK					Mascot
1165.5848	1165.6494	0.0646	55	423	431	LENEIQTYR					Mascot
1201.6172	1201.6801	0.0629	52	227	237	QSVEADINGLR					Mascot
1365.6758	1365.7185	0.0427	31	304	314	SQYEQLAEKNR					Mascot

1365.6758	1365.7185	0.0427	31	304	314	SQYEQLAEKNR	39	86.277	Mascot
1390.6809	1390.7574	0.0765	55	368	380	QSLEASLAETEGR			Mascot
1450.7245	1450.7423	0.0178	12	16	32	SGGGGGGGSSLRISSSK			Mascot

Gel Idx/Pos	113/E12	Instr./Gel Origin	BA2151/Sample Project 20140814	Process Status	Analysis Succeeded
Plate [#] Name	[1] Sample Project 20140814	Instrument Sample Name		Spectra	11

Rank	Protein Name	Accession No.	Protein MW	Protein PI	Pep. Count	Protein Score	Protein Score C. I. %	Intensity Matched	Total Ion Score	Total Ion C. I. %	Confirmed
1	Uncharacterized 6.1 kDa protein OS=Thermoproteus tenax virus 1 (strain KRA1) PE=4 SV=1	YORJ_TTV1K	6225.3	10.73	2	14	0	.54			

Peptide Information

Calc. Mass	Obsrv. Mass	± da	± ppm	Start Seq.	End Seq.	Sequence	Ion Score	C. I. %	Modification	Rank	Result Type
818.3647	818.3382	-0.0265	-32	2	7	DMHMLR			Oxidation (M)[2]		Mascot
832.4457	832.3728	-0.0729	-88	19	24	LQERMR					Mascot

Rank	Protein Name	Accession No.	Protein MW	Protein PI	Pep. Count	Protein Score	Protein Score C. I. %	Intensity Matched	Total Ion Score	Total Ion C. I. %	Confirmed
2	30S ribosomal protein S21 OS=Methylobacterium sp. (strain 4-46) GN=rpsU PE=3 SV=1	RS21_METS4	10889.2	11.73	2	14	0	.602			

Peptide Information

Calc. Mass	Obsrv. Mass	± da	± ppm	Start Seq.	End Seq.	Sequence	Ion Score	C. I. %	Modification	Rank	Result Type
1771.0701	1771.2119	0.1418	80	65	81	EGLIAAPKPKPRAVAPR					Mascot
1961.0563	1961.2361	0.1798	92	77	97	AVAPRRPAAAPAPASSP TTTA					Mascot

Rank	Protein Name	Accession No.	Protein MW	Protein PI	Pep. Count	Protein Score	Protein Score C. I. %	Intensity Matched	Total Ion Score	Total Ion C. I. %	Confirmed
3	Histone H2B.9 OS=Oryza sativa subsp. japonica GN=H2B.9 PE=3 SV=1	H2B9_ORYSJ	16246	10.05	3	14	0	1.688			

Protein Group

Histone H2B.9 OS=Oryza sativa subsp. indica GN=H2B.9 PE=3 SV=1	H2B9_ORYSI	16246	10.050 000190 7349

Peptide Information

Calc. Mass	Obsrv. Mass	± da	± ppm	Start Seq.	End Seq.	Sequence	Ion Score	C. I. %	Modification	Rank	Result Type
832.4523	832.3728	-0.0795	-96	121	127	EIQTSVR					Mascot
842.5094	842.5699	0.0605	72	5	12	AEKKPAAK					Mascot
889.4374	889.3856	-0.0518	-58	42	51	GEKGGAGEGK					Mascot

Rank	Protein Name	Accession No.	Protein MW	Protein PI	Pep. Count	Protein Score	Protein Score C. I. %	Intensity Matched	Total Ion Score	Total Ion C. I. %	Confirmed
4	50S ribosomal protein L32 OS=Pseudomonas putida (strain GB-1) GN=rpmF PE=3 SV=1	RL32_PSEPG	6751.4	9.69	2	13	0	.54			

Protein Group

50S ribosomal protein L32 OS=Pseudomonas putida (strain F1 / ATCC 700007) GN=rpmF PE=3 SV=1	RL32_PSEP1	6751.4	9.6899 995803 833

50S ribosomal protein L32 OS=Pseudomonas putida (strain KT2440) GN=rpmF PE=3 SV=3	RL32_PSEPK	6751.4	9.6899995803833

Peptide Information

Calc. Mass	Obsrv. Mass	± da	± ppm	Start Seq.	End Seq.	Sequence	Ion Score	C. I. %	Modification	Rank	Result Type
818.4189	818.3382	-0.0807	-99	1	7	MAVQQNK					Mascot
832.4047	832.3728	-0.0319	-38	53	60	VVDKGADE					Mascot

5	50S ribosomal protein L37e OS=Methanococcus aeolicus (strain Nankai-3 / ATCC BAA-1280) GN=rpl37e PE=3 SV=1	RL37_META3	7240.8	11.6	2	13	0	1.364	

Peptide Information

Calc. Mass	Obsrv. Mass	± da	± ppm	Start Seq.	End Seq.	Sequence	Ion Score	C. I. %	Modification	Rank	Result Type
842.4955	842.5699	0.0744	88	56	62	INGQRVR					Mascot
889.4738	889.3856	-0.0882	-99	2	10	SKGTPSQGK					Mascot

6	Regulatory protein RecX OS=Klebsiella pneumoniae (strain 342) GN=recX PE=3 SV=1	RECX_KLEP3	19547	8.58	3	12	0	.735

Peptide Information

Calc. Mass	Obsrv. Mass	± da	± ppm	Start Seq.	End Seq.	Sequence	Ion Score	C. I. %	Modification	Rank	Result Type
818.3792	818.3382	-0.041	-50	68	73	YLDDHR					Mascot
832.4271	832.3728	-0.0543	-65	2	8	TEPSSRR					Mascot
856.5363	856.5657	0.0294	34	14	20	LLDRAIR					Mascot

7	Myelin-associated oligodendrocyte basic protein OS=Rattus norvegicus GN=Mobp PE=1 SV=1	MOBP_RAT	19490.2	11.04	3	12	0	1.036

Peptide Information

Calc. Mass	Obsrv. Mass	± da	± ppm	Start Seq.	End Seq.	Sequence	Ion Score	C. I. %	Modification	Rank	Result Type
832.4271	832.3728	-0.0543	-65	116	122	SPSRTER					Mascot
856.4886	856.5657	0.0771	90	72	79	ATSPQKPK					Mascot
889.3873	889.3856	-0.0017	-2	49	55	SGCFYQK			Carbamidomethyl (C)[3]		Mascot

8	30S ribosomal protein S21 OS=Buchnera aphidicola subsp. Acyrthosiphon pisum (strain APS) GN=rpsU PE=3 SV=1	RS21_BUCAI	8629.8	11.15	2	12	0	1.043

Protein Group

30S ribosomal protein S21 OS=Buchnera aphidicola	RS21_BUCA5	8629.8	11.149

subsp. Acyrthosiphon pisum (strain 5A) GN=rpsU PE=3 SV=1			999618 5303
30S ribosomal protein S21 OS=Buchnera aphidicola subsp. Acyrthosiphon pisum (strain Tuc7) GN=rpsU PE=3 SV=1	RS21_BUCAT	8629.8	11.149 999618 5303
30S ribosomal protein S21 OS=Buchnera aphidicola subsp. Schizaphis graminum (strain Sg) GN=rpsU PE=3 SV=1	RS21_BUCAP	8643.8	11.149 999618 5303

Peptide Information

Calc. Mass	Obsrv. Mass	± da	± ppm	Start Seq.	End Seq.	Sequence	Ion Score	C. I. %	Modification	Rank	Result Type
842.5094	842.5699	0.0605	72	26	33	AGILAEIR					Mascot
856.5436	856.5657	0.0221	26	1	7	MPIIKVR					Mascot

9	Mitotic-spindle organizing protein 1 OS=Aspergillus clavatus (strain ATCC 1007 / CBS 513.65 / DSM 816 / NCTC 3887 / NRRL 1) GN=ACLA_026210 PE=3 SV=1	MZT1_ASPCL	7869.1	4.85	2	12	0	.731

Peptide Information

Calc. Mass	Obsrv. Mass	± da	± ppm	Start Seq.	End Seq.	Sequence	Ion Score	C. I. %	Modification	Rank	Result Type
818.3712	818.3382	-0.033	-40	1	7	MPQAEDK					Mascot
889.4738	889.3856	-0.0882	-99	59	66	KETAPTSR					Mascot

10	Uncharacterized protein YraL OS=Bacillus subtilis (strain 168) GN=yraL PE=4 SV=1	YRAL_BACSU	9958.4	9.68	2	12	0	.928

Peptide Information

Calc. Mass	Obsrv. Mass	± da	± ppm	Start Seq.	End Seq.	Sequence	Ion Score	C. I. %	Modification	Rank	Result Type
842.4982	842.5699	0.0717	85	6	13	ATAILPEK					Mascot
1653.9785	1654.1134	0.1349	82	6	20	ATAILPEKLISEIQK					Mascot

Gel Idx/Pos	114/E13	Instr./Gel Origin	BA2151/Sample Project 20140814	Process Status	Analysis Succeeded
Plate [#] Name	[1] Sample Project 20140814	Instrument Sample Name		Spectra	11

Rank	Protein Name	Accession No.	Protein MW	Protein PI	Pep. Count	Protein Score	Protein Score C. I. %	Intensity Matched	Total Ion Score	Total Ion C. I. %	Confirmed
1	Glycerol-3-phosphate dehydrogenase [NAD(+)], cytoplasmic OS=Drosophila ezoana GN=Gpdh1 PE=3 SV=4	GPDA_DROEZ	38941.1	6.33	13	68	91.43	3.85			

Peptide Information

Calc. Mass	Obsrv. Mass	± da	± ppm	Start Seq.	End Seq.	Sequence	Ion Score	C. I. %	Modification	Rank	Result Type
904.4557	904.5082	0.0525	58	338	344	DLIDCIR			Carbamidomethyl (C)[5]		Mascot
1158.583	1158.5596	-0.0234	-20	240	249	FVDVFYPGSK					Mascot
1593.7175	1593.7689	0.0514	32	37	49	VTMFVYEEMIDGK			Oxidation (M)[3,9]		Mascot
1667.8962	1668.0333	0.1371	82	50	63	KLTEIINETHENVK					Mascot
1721.8125	1721.8431	0.0306	18	37	50	VTMFVYEEMIDGKK			Oxidation (M)[3,9]		Mascot
1728.0419	1727.9558	-0.0861	-50	110	125	IKPNAIAISLIKGFDK					Mascot
1731.8848	1731.9838	0.099	57	5	21	VNVCIVGSGNWGSAIAK			Carbamidomethyl (C)[4]		Mascot
1731.8848	1731.9838	0.099	57	5	21	VNVCIVGSGNWGSAIAK			Carbamidomethyl (C)[4]		Mascot
1866.9695	1866.9907	0.0212	11	274	290	VSEAFVTSGKTIEELEK					Mascot
1944.0437	1944.093	0.0493	25	51	66	LTEIINETHENVKYLK					Mascot
1947.9845	1948.1257	0.1412	72	298	314	LQGPPTAEEVNYMLKNK			Oxidation (M)[13]		Mascot
1958.1069	1957.9847	-0.1222	-62	67	85	GHKLPTNVVAVPDLVEAA K					Mascot
2118.0762	2118.0901	0.0139	7	232	249	LGLMEMIRFVDVFYPGSK			Oxidation (M)[4]		Mascot
2272.1948	2272.2488	0.054	24	86	104	NADILIFVVPHQFIPNFCK			Carbamidomethyl (C)[18]		Mascot

Rank	Protein Name	Accession No.	Protein MW	Protein PI	Pep. Count	Protein Score	Protein Score C. I. %	Intensity Matched	Total Ion Score	Total Ion C. I. %	Confirmed
2	Glycerol-3-phosphate dehydrogenase [NAD(+)], cytoplasmic OS=Drosophila kanekoi GN=Gpdh1 PE=3 SV=3	GPDA_DROKA	40066.7	6.33	13	66	85.446	3.85			

Peptide Information

Calc. Mass	Obsrv. Mass	± da	± ppm	Start Seq.	End Seq.	Sequence	Ion Score	C. I. %	Modification	Rank	Result Type
904.4557	904.5082	0.0525	58	338	344	DLIDCIR			Carbamidomethyl (C)[5]		Mascot
1158.583	1158.5596	-0.0234	-20	240	249	FVDVFYPGSK					Mascot
1593.7175	1593.7689	0.0514	32	37	49	VTMFVYEEMIDGK			Oxidation (M)[3,9]		Mascot
1667.8962	1668.0333	0.1371	82	50	63	KLTEIINETHENVK					Mascot
1721.8125	1721.8431	0.0306	18	37	50	VTMFVYEEMIDGKK			Oxidation (M)[3,9]		Mascot
1728.0419	1727.9558	-0.0861	-50	110	125	IKPNAIAISLIKGFDK					Mascot

1731.8848	1731.9838	0.099	57	5	21	VNVCIVGSGNWGSAIAK			Carbamidomethyl (C)[4]		Mascot
1731.8848	1731.9838	0.099	57	5	21	VNVCIVGSGNWGSAIAK			Carbamidomethyl (C)[4]		Mascot
1866.9695	1866.9907	0.0212	11	274	290	VSEAFVTSGKTIEELEK					Mascot
1944.0437	1944.093	0.0493	25	51	66	LTEIINETHENVKYLK					Mascot
1947.9845	1948.1257	0.1412	72	298	314	LQGPPTAEEVNYMLKNK			Oxidation (M)[13]		Mascot
1958.1069	1957.9847	-0.1222	-62	67	85	GHKLPTNVVAVPDLVEAAK					Mascot
2118.0762	2118.0901	0.0139	7	232	249	LGLMEMIRFVDVFYPGSK			Oxidation (M)[4]		Mascot
2272.1948	2272.2488	0.054	24	86	104	NADILIFVVPHQFIPNFCK			Carbamidomethyl (C)[18]		Mascot

3	Glycerol-3-phosphate dehydrogenase [NAD(+)], cytoplasmic OS=Drosophila americana GN=Gpdh PE=3 SV=4	GPDA_DROAE	38925.1	6.33	12	60	42.06	3.756

Peptide Information

Calc. Mass	Obsrv. Mass	± da	± ppm	Start Seq.	End Seq.	Sequence	Ion Score	C. I. %	Modification	Rank	Result Type
904.4557	904.5082	0.0525	58	338	344	DLIDCIR			Carbamidomethyl (C)[5]		Mascot
1158.583	1158.5596	-0.0234	-20	240	249	FVDVFYPGSK					Mascot
1593.7175	1593.7689	0.0514	32	37	49	VTMFVYEEMIDGK			Oxidation (M)[3,9]		Mascot
1667.8962	1668.0333	0.1371	82	50	63	KLTEIINETHENVK					Mascot
1721.8125	1721.8431	0.0306	18	37	50	VTMFVYEEMIDGKK			Oxidation (M)[3,9]		Mascot
1728.0419	1727.9558	-0.0861	-50	110	125	IKPNAIAISLIKGFDK					Mascot
1731.8848	1731.9838	0.099	57	5	21	VNVCIVGSGNWGSAIAK			Carbamidomethyl (C)[4]		Mascot
1731.8848	1731.9838	0.099	57	5	21	VNVCIVGSGNWGSAIAK			Carbamidomethyl (C)[4]		Mascot
1944.0437	1944.093	0.0493	25	51	66	LTEIINETHENVKYLK					Mascot
1947.9845	1948.1257	0.1412	72	298	314	LQGPPTAEEVNYMLKNK			Oxidation (M)[13]		Mascot
1958.1069	1957.9847	-0.1222	-62	67	85	GHKLPTNVVAVPDLVEAAK					Mascot
2118.0762	2118.0901	0.0139	7	232	249	LGLMEMIRFVDVFYPGSK			Oxidation (M)[4]		Mascot
2272.1948	2272.2488	0.054	24	86	104	NADILIFVVPHQFIPNFCK			Carbamidomethyl (C)[18]		Mascot

4	Glycerol-3-phosphate dehydrogenase [NAD(+)], cytoplasmic OS=Drosophila virilis GN=Gpdh PE=1 SV=3	GPDA_DROVI	39253.2	6.33	12	60	39.329	3.756

Peptide Information

Calc. Mass	Obsrv. Mass	± da	± ppm	Start Seq.	End Seq.	Sequence	Ion Score	C. I. %	Modification	Rank	Result Type
904.4557	904.5082	0.0525	58	338	344	DLIDCIR			Carbamidomethyl (C)[5]		Mascot
1158.583	1158.5596	-0.0234	-20	240	249	FVDVFYPGSK					Mascot
1593.7175	1593.7689	0.0514	32	37	49	VTMFVYEEMIDGK			Oxidation (M)[3,9]		Mascot

1667.8962	1668.0333	0.1371	82	50	63	KLTEIINETHENVK					Mascot
1721.8125	1721.8431	0.0306	18	37	50	VTMFVYEEMIDGKK			Oxidation (M)[3,9]		Mascot
1728.0419	1727.9558	-0.0861	-50	110	125	IKPNAIAISLIKGFDK					Mascot
1731.8848	1731.9838	0.099	57	5	21	VNVCIVGSGNWGSAIAK			Carbamidomethyl (C)[4]		Mascot
1731.8848	1731.9838	0.099	57	5	21	VNVCIVGSGNWGSAIAK			Carbamidomethyl (C)[4]		Mascot
1944.0437	1944.093	0.0493	25	51	66	LTEIINETHENVKYLK					Mascot
1947.9845	1948.1257	0.1412	72	298	314	LQGPPTAEEVNYMLKNK			Oxidation (M)[13]		Mascot
1958.1069	1957.9847	-0.1222	-62	67	85	GHKLPTNVVAVPDLVEAA K					Mascot
2118.0762	2118.0901	0.0139	7	232	249	LGLMEMIRFVDVFYPGSK			Oxidation (M)[4]		Mascot
2272.1948	2272.2488	0.054	24	86	104	NADILIFVVPHQFIPNFCK			Carbamidomethyl (C)[18]		Mascot

5	Exodeoxyribonuclease V beta chain OS=Borrelia burgdorferi (strain ATCC 35210 / B31 / CIP 102532 / DSM 4680) GN=recB PE=3 SV=1	EX5B_BORBU	138085.4	9.13	23	58	3.843	24.96

Peptide Information

Calc. Mass	Obsrv. Mass	± da	± ppm	Start Seq.	End Seq.	Sequence	Ion Score	C. I. %	Modification	Rank	Result Type
904.5502	904.5082	-0.042	-46	1049	1056	GIVDLIFK					Mascot
905.5818	905.5005	-0.0813	-90	1098	1105	IYALGIKK					Mascot
906.5658	906.5256	-0.0402	-44	317	323	ILKYIEK					Mascot
926.5094	926.5532	0.0438	47	400	406	QIIYSFR					Mascot
1342.7253	1342.7705	0.0452	34	254	264	FFSTLIEKETK					Mascot
1513.8771	1513.8973	0.0202	13	276	288	IKNDLICLGINIK			Carbamidomethyl (C)[7]		Mascot
1528.837	1528.8854	0.0484	32	583	595	QSFKTLNYILSSK					Mascot
1666.8945	1667.0117	0.1172	70	278	291	NDLICLGINIKHEK			Carbamidomethyl (C)[5]		Mascot
1704.8744	1704.912	0.0376	22	1029	1041	YLFDKHFEDLHIK					Mascot
1730.9283	1730.9679	0.0396	23	8	24	IQNNTTILIEASAGTGK					Mascot
1765.9193	1765.9735	0.0542	31	1013	1026	EMEFLIKINPEFQK					Mascot
1792.9229	1792.9319	0.009	5	217	231	DEILSFYNKHIQTGK					Mascot
1822.9545	1823.0076	0.0531	29	66	81	VIENAYSNSKTNEILK					Mascot
1822.9545	1823.0076	0.0531	29	66	81	VIENAYSNSKTNEILK					Mascot
1838.9899	1839.0151	0.0252	14	685	699	TYETTLEIISKIYHK					Mascot
1845.0369	1844.9812	-0.0557	-30	1151	1166	FNDVDLDKIILELGIK					Mascot
1848.9644	1849.1047	0.1403	76	1113	1127	EYNQKFGGIIYLFTR					Mascot
1861.043	1860.9644	-0.0786	-42	630	645	NEITLINAINKITFEK					Mascot
1921.9403	1922.13	0.1897	99	883	899	KEYTSSFSSLTAQAHHK					Mascot
2073.0686	2073.1521	0.0835	40	933	951	DIGNILHAAMEEIIFSTAK					Mascot

Calc. Mass	Obsrv. Mass	± da	± ppm	Start Seq.	End Seq.	Sequence	Ion Score	C. I. %	Modification	Rank	Result Type
2112.0391	2112.0408	0.0017	1	726	743	INNINNDNESIELMTIHK					Mascot
2272.2812	2272.2488	-0.0324	-14	587	605	TLNYILSSKILNVPWNLQR					Mascot
2288.1235	2288.2026	0.0791	35	1140	1158	FENGIYFNLPKFNDVDLDK					Mascot
2327.166	2327.1606	-0.0054	-2	726	745	INNINNDNESIELMTIHKSK					Mascot

6	Glycerol-3-phosphate dehydrogenase [NAD(+)], cytoplasmic OS=Drosophila pseudoobscura pseudoobscura GN=Gpdh PE=3 SV=2	GPDA_DROPS	40049.7	6.52	12	57	0	3.593	

Peptide Information

Calc. Mass	Obsrv. Mass	± da	± ppm	Start Seq.	End Seq.	Sequence	Ion Score	C. I. %	Modification	Rank	Result Type
904.4557	904.5082	0.0525	58	338	344	DLIDCIR			Carbamidomethyl (C)[5]		Mascot
1158.583	1158.5596	-0.0234	-20	240	249	FVDVFYPGSK					Mascot
1593.7175	1593.7689	0.0514	32	37	49	VTMFVYEEMIDGK			Oxidation (M)[3,9]		Mascot
1667.8962	1668.0333	0.1371	82	50	63	KLTEIINETHENVK					Mascot
1721.8125	1721.8431	0.0306	18	37	50	VTMFVYEEMIDGKK			Oxidation (M)[3,9]		Mascot
1728.0419	1727.9558	-0.0861	-50	110	125	IKPNAIAISLIKGFDK					Mascot
1731.8848	1731.9838	0.099	57	5	21	VNVCIVGSGNWGSAIAK			Carbamidomethyl (C)[4]		Mascot
1731.8848	1731.9838	0.099	57	5	21	VNVCIVGSGNWGSAIAK			Carbamidomethyl (C)[4]		Mascot
1866.9695	1866.9907	0.0212	11	274	290	VSEAFVTSGKTIEELEK					Mascot
1944.0437	1944.093	0.0493	25	51	66	LTEIINETHENVKYLK					Mascot
1947.9845	1948.1257	0.1412	72	298	314	LQGPPTAEEVNYMLKNK			Oxidation (M)[13]		Mascot
2118.0762	2118.0901	0.0139	7	232	249	LGLMEMIRFVDVFYPGSK			Oxidation (M)[4]		Mascot
2272.1948	2272.2488	0.054	24	86	104	NADILIFVVPHQFIPNFCK			Carbamidomethyl (C)[18]		Mascot

7	ATP synthase subunit 4, mitochondrial OS=Paracoccidioides brasiliensis GN=ATP4 PE=2 SV=1	ATPF_PARBR	26606.2	9.39	10	54	0	1.911	

Peptide Information

Calc. Mass	Obsrv. Mass	± da	± ppm	Start Seq.	End Seq.	Sequence	Ion Score	C. I. %	Modification	Rank	Result Type
886.5104	886.5752	0.0648	73	139	145	QRIENVK					Mascot
913.5577	913.6075	0.0498	55	15	22	VRPVLSSR					Mascot
1059.6045	1059.6454	0.0409	39	146	155	QLSGVVDITK					Mascot
1132.5634	1132.6508	0.0874	77	113	121	EWAETQIQK					Mascot
1388.7169	1388.7994	0.0825	59	113	123	EWAETQIQKQK					Mascot
1388.7169	1388.7994	0.0825	59	113	123	EWAETQIQKQK					Mascot
1593.8456	1593.7689	-0.0767	-48	124	138	DILNGARANHTNAVK					Mascot

Calc. Mass	Obsrv. Mass	± da	± ppm	Start Seq.	End Seq.	Sequence	Ion Score	C. I. %	Modification	Rank	Result Type
1612.8905	1612.8604	-0.0301	-19	49	64	AQSIIDALPGNSLVSK					Mascot
1642.9375	1642.9989	0.0614	37	141	155	IENVKQLSGVVDITK					Mascot
1678.88	1678.9332	0.0532	32	186	199	KVLDSWVQYEGQVK					Mascot
1805.9545	1806.0344	0.0799	44	187	201	VLDSWVQYEGQVKVR					Mascot

8	Proteasome-activating nucleotidase OS=Methanococcus maripaludis (strain C6 / ATCC BAA-1332) GN=pan PE=3 SV=1	PAN_METM6	45996.3	5.34	14	52	0	5.118

Peptide Information

Calc. Mass	Obsrv. Mass	± da	± ppm	Start Seq.	End Seq.	Sequence	Ion Score	C. I. %	Modification	Rank	Result Type
820.4927	820.5025	0.0098	12	232	238	DVFKLAK					Mascot
830.4982	830.4728	-0.0254	-31	213	220	VVGSELVK					Mascot
870.5771	870.5731	-0.004	-5	34	40	VLRLELK					Mascot
876.5302	876.5251	-0.0051	-6	229	235	LVRDVFK					Mascot
926.5557	926.5532	-0.0025	-3	162	169	EVVELPLK					Mascot
1018.5414	1018.5989	0.0575	56	25	33	TQIAELESK					Mascot
1158.6517	1158.5596	-0.0921	-79	184	195	GVLLYGPPGTGK					Mascot
1565.6312	1565.736	0.1048	67	1	13	MSYPDDYSTDVEK			Oxidation (M)[1]		Mascot
1565.6312	1565.736	0.1048	67	1	13	MSYPDDYSTDVEK	4	0	Oxidation (M)[1]		Mascot
1684.9996	1684.9836	-0.016	-9	184	200	GVLLYGPPGTGKTLLAK					Mascot
1684.9996	1684.9836	-0.016	-9	184	200	GVLLYGPPGTGKTLLAK	3	0			Mascot
1777.9041	1777.9739	0.0698	39	241	256	SPCIIFIDEIDAVASK			Carbamidomethyl (C)[3]		Mascot
1792.8534	1792.9319	0.0785	44	310	324	FDRIIEISMPDEDGR					Mascot
1849.0946	1849.1047	0.0101	5	177	195	VGIVPPKGVLLYGPPGTG K					Mascot
1934.0052	1934.167	0.1618	84	241	257	SPCIIFIDEIDAVASKR			Carbamidomethyl (C)[3]		Mascot
2271.0745	2271.2261	0.1516	67	267	285	EVQRTLMQLLAEMDGFD SR			Oxidation (M)[7,13]		Mascot

9	WASH complex subunit strumpellin OS=Mus musculus GN=Kiaa0196 PE=2 SV=2	STRUM_MOUSE	134880.1	6.72	20	50	0	41.105

Peptide Information

Calc. Mass	Obsrv. Mass	± da	± ppm	Start Seq.	End Seq.	Sequence	Ion Score	C. I. %	Modification	Rank	Result Type
805.4414	805.4813	0.0399	50	921	928	SIVANSSK					Mascot
906.4713	906.5256	0.0543	60	276	282	MREIVDK			Oxidation (M)[1]		Mascot
944.5134	944.4857	-0.0277	-29	530	536	FLHQMIR					Mascot
1179.6481	1179.6738	0.0257	22	975	985	HLAAALDNLNK					Mascot
1376.8188	1376.7614	-0.0574	-42	32	43	LSEFIPAVFLLK					Mascot

1382.7638	1382.7468	-0.017	-12	700	711	VDPKQLLEDGIR					Mascot
1513.8672	1513.8973	0.0301	20	530	541	FLHQMIRTINIK					Mascot
1684.8766	1684.9836	0.107	64	971	985	FDSRHLAAALDNLNK					Mascot
1684.8766	1684.9836	0.107	64	971	985	FDSRHLAAALDNLNK					Mascot
1698.9214	1699.027	0.1056	62	1091	1104	YTEQFLALIGQFIR					Mascot
1790.8861	1790.9825	0.0964	54	1142	1156	VAEAHVPNFIFDEFR					Mascot
1804.97	1805.0304	0.0604	33	676	691	LTHAISIFTEGILMMK					Mascot
1820.965	1821.0348	0.0698	38	676	691	LTHAISIFTEGILMMK			Oxidation (M)[14]		Mascot
1836.9598	1837.0134	0.0536	29	676	691	LTHAISIFTEGILMMK			Oxidation (M)[14,15]		Mascot
1845.0415	1844.9812	-0.0603	-33	215	230	VPINETFISMVIGRLR					Mascot
1861.0365	1860.9644	-0.0721	-39	215	230	VPINETFISMVIGRLR			Oxidation (M)[10]		Mascot
1903.0392	1903.0487	0.0095	5	904	920	TVQETLKMLMSAVNPLK					Mascot
1906.0137	1906.1533	0.1396	73	911	928	MLMSAVNPLKSIVANSSK			Oxidation (M)[1]		Mascot
1906.0137	1906.1533	0.1396	73	911	928	MLMSAVNPLKSIVANSSK			Oxidation (M)[1]		Mascot
1922.0085	1922.13	0.1215	63	911	928	MLMSAVNPLKSIVANSSK			Oxidation (M)[1,3]		Mascot
1958.0302	1957.9847	-0.0455	-23	312	329	TALNNTLDLANVKEQASR					Mascot
1993.9834	1994.0939	0.1105	55	1	17	MLDFLAENNLCGQAILR			Carbamidomethyl (C)[11], Oxidation (M)[1]		Mascot
2073.2393	2073.1521	-0.0872	-42	626	643	VLQIIPESMFTSLLKIIK					Mascot
2289.3523	2289.2073	-0.145	-63	1035	1053	RLPYFPIVNFLFLIAQLPK					Mascot
2317.1646	2317.2341	0.0695	30	792	810	TKIQDWQSMYQSTHIPIP K			Oxidation (M)[9]		Mascot
2317.1646	2317.2341	0.0695	30	792	810	TKIQDWQSMYQSTHIPIP K			Oxidation (M)[9]		Mascot
2345.1812	2345.2444	0.0632	27	951	970	VGQMQILRQQIANELNSS CR			Carbamidomethyl (C)[19]		Mascot

10	Uncharacterized mitochondrial protein AtMg00850 OS=Arabidopsis thaliana GN=AtMg00850 PE=4 SV=1	M850_ARATH	12112.5	10.93	7	50	0	18.655	

Peptide Information

Calc. Mass	Obsrv. Mass	± da	± ppm	Start Seq.	End Seq.	Sequence	Ion Score	C. I. %	Modification	Rank	Result Type
1475.7676	1475.833	0.0654	44	45	56	LKNWLGEMLEAR			Oxidation (M)[8]		Mascot
1678.8218	1678.9332	0.1114	66	10	24	DYPQNVAGNMLSTLR					Mascot
1822.0182	1821.9874	-0.0308	-17	26	41	EQNQLTSVLTGIHILR					Mascot
1822.9116	1823.0076	0.096	53	10	25	DYPQNVAGNMLSTLRK			Oxidation (M)[10]		Mascot
1822.9116	1823.0076	0.096	53	9	24	KDYPQNVAGNMLSTLR			Oxidation (M)[11]		Mascot
1950.1132	1950.1167	0.0035	2	25	41	KEQNQLTSVLTGIHILR					Mascot
2090.0891	2090.0564	-0.0327	-16	76	95	DGGWPTARGLPSLLQAH GTR					Mascot
2090.0891	2090.0564	-0.0327	-16	76	95	DGGWPTARGLPSLLQAH					Mascot

GTR

Gel Idx/Pos	115/E14	Instr./Gel Origin	BA2151/Sample Project 20140814	Process Status	Analysis Succeeded
Plate [#] Name	[1] Sample Project 20140814	Instrument Sample Name		Spectra	11

Rank	Protein Name	Accession No.	Protein MW	Protein PI	Pep. Count	Protein Score	Protein Score C. I. %	Intensity Matched	Total Ion Score	Total Ion C. I. %	Confirmed
1	Alpha-amylase/trypsin inhibitor CM3 OS=Triticum aestivum PE=1 SV=1	IAAC3_WHEAT	18893.3	7.44	8	503	100	57.66	450	100	

Peptide Information

Calc. Mass	Obsrv. Mass	± da	± ppm	Start Seq.	End Seq.	Sequence	Ion Score	C. I. %	Modification	Rank	Result Type
1010.52	1010.6013	0.0813	80	37	44	TNLLPHCR			Carbamidomethyl (C)[7]		Mascot
1010.52	1010.6013	0.0813	80	37	44	TNLLPHCR	42	94.638	Carbamidomethyl (C)[7]		Mascot
1110.5038	1110.5927	0.0889	80	133	140	EMQWDFVR					Mascot
1126.4987	1126.579	0.0803	71	133	140	EMQWDFVR			Oxidation (M)[2]		Mascot
1126.4987	1126.579	0.0803	71	133	140	EMQWDFVR	18	0	Oxidation (M)[2]		Mascot
1698.9214	1699.0631	0.1417	83	101	115	YFIALPVPSQPVDPR					Mascot
1698.9214	1699.0631	0.1417	83	101	115	YFIALPVPSQPVDPR	90	100			Mascot
1727.8381	1727.9855	0.1474	85	116	132	SGNVGESGLIDLPGCPR			Carbamidomethyl (C)[15]		Mascot
1727.8381	1727.9855	0.1474	85	116	132	SGNVGESGLIDLPGCPR	97	100	Carbamidomethyl (C)[15]		Mascot
1801.8427	1801.9651	0.1224	68	45	60	DYVLQQTCGTFTPGSK			Carbamidomethyl (C)[8]		Mascot
1876.0222	1876.1882	0.166	88	141	157	LLVAPGQCNLATIHNVR			Carbamidomethyl (C)[8]		Mascot
1876.0222	1876.1882	0.166	88	141	157	LLVAPGQCNLATIHNVR	108	100	Carbamidomethyl (C)[8]		Mascot
1957.8564	1958.0225	0.1661	85	81	95	LYCCQELAEISQQCR			Carbamidomethyl (C)[3,4,14]		Mascot
1957.8564	1958.0225	0.1661	85	81	95	LYCCQELAEISQQCR	96	100	Carbamidomethyl (C)[3,4,14]		Mascot
2255.1416	2255.3093	0.1677	74	61	80	LPEWMTSASIYSPGKPYLAK			Oxidation (M)[5]		Mascot

Rank	Protein Name	Accession No.	Protein MW	Protein PI	Pep. Count	Protein Score	Protein Score C. I. %	Intensity Matched	Total Ion Score	Total Ion C. I. %	Confirmed
2	Alpha-amylase/trypsin inhibitor CMd OS=Hordeum vulgare GN=IAT3 PE=1 SV=2	IAAD_HORVU	19140.3	6.07	2	116	100	20.247	108	100	

Peptide Information

Calc. Mass	Obsrv. Mass	± da	± ppm	Start Seq.	End Seq.	Sequence	Ion Score	C. I. %	Modification	Rank	Result Type
1876.0222	1876.1882	0.166	88	144	160	LLVAPGQCNLATIHNVR			Carbamidomethyl (C)[8]		Mascot
1876.0222	1876.1882	0.166	88	144	160	LLVAPGQCNLATIHNVR	108	100	Carbamidomethyl (C)[8]		Mascot
1967.8772	1968.0417	0.1645	84	84	98	LYCCQELAEIPQQCR			Carbamidomethyl (C)[3,4,14]		Mascot

Rank	Protein Name	Accession No.	Protein MW	Protein PI	Pep. Count	Protein Score	Protein Score C. I. %	Intensity Matched	Total Ion Score	Total Ion C. I. %	Confirmed
3	Flagellar transcriptional regulator FlhD OS=Acidovorax sp. (strain JS42) GN=flhD PE=3 SV=1	FLHD_ACISJ	12061.3	5.29	4	51	0	4.169	27	0	

Peptide Information

Calc. Mass	Obsrv. Mass	± da	± ppm	Start Seq.	End Seq.	Sequence	Ion Score	C. I. %	Modification	Rank	Result Type
1010.5087	1010.6013	0.0926	92	61	68	NTLLCSFR			Carbamidomethyl (C)[5]		Mascot
1010.5087	1010.6013	0.0926	92	61	68	NTLLCSFR	27	0	Carbamidomethyl (C)[5]		Mascot
1749.9568	1749.9546	-0.0022	-1	12	26	EANLTYLMLAQTLIR					Mascot
1870.944	1871.0168	0.0728	39	87	104	AASEATNTLHANILMASR					Mascot
1981.9978	1981.9698	-0.028	-14	69	85	VDDELVWSLLTSHNTPR					Mascot

4	Chaperone protein ClpB OS=Bdellovibrio bacteriovorus (strain ATCC 15356 / DSM 50701 / NCIB 9529 / HD100) GN=clpB PE=3 SV=1	CLPB_BDEBA	95237.5	5.92	20	50	0	24.61	

Peptide Information

Calc. Mass	Obsrv. Mass	± da	± ppm	Start Seq.	End Seq.	Sequence	Ion Score	C. I. %	Modification	Rank	Result Type
873.5403	873.5563	0.016	18	832	839	EIISGKVK					Mascot
1010.5166	1010.6013	0.0847	84	353	360	YEVHHGIR					Mascot
1010.5226	1010.6013	0.0787	78	757	764	IDEIVMFK			Oxidation (M)[6]		Mascot
1044.5936	1044.5792	-0.0144	-14	471	479	LKADIEDLK					Mascot
1062.4996	1062.5945	0.0949	89	5	14	SQEAMQAAAR					Mascot
1067.6208	1067.5972	-0.0236	-22	838	847	VKAGDTIHVK					Mascot
1078.5792	1078.5892	0.01	9	749	756	FRPEFLNR					Mascot
1085.566	1085.6619	0.0959	88	561	569	LLHMEDSLK					Mascot
1094.5663	1094.588	0.0217	20	76	84	MFASPRLEK			Oxidation (M)[1]		Mascot
1098.579	1098.5846	0.0056	5	222	231	QDVPDNLIGK					Mascot
1130.6052	1130.5756	-0.0296	-26	488	498	EGDLGKAAELK					Mascot
1136.5582	1136.6466	0.0884	78	113	123	GGDSELNGLFK					Mascot
1136.5582	1136.6466	0.0884	78	113	123	GGDSELNGLFK					Mascot
1190.5946	1190.6523	0.0577	48	4	14	KSQEAMQAAAR					Mascot
1550.8281	1550.9136	0.0855	55	232	246	KLMSLDMGALIAGAK			Oxidation (M)[3,7]		Mascot
1709.9077	1709.9904	0.0827	48	233	248	LMSLDMGALIAGAKYR					Mascot
1722.8698	1723.0195	0.1497	87	619	633	ALAEFLFDDEQAVVR					Mascot
1725.9026	1726.0176	0.115	67	233	248	LMSLDMGALIAGAKYR			Oxidation (M)[2]		Mascot
1727.9803	1727.9855	0.0052	3	48	62	LNVPQAQFLAELRTK					Mascot
1727.9803	1727.9855	0.0052	3	48	62	LNVPQAQFLAELRTK					Mascot
1741.9265	1741.9941	0.0676	39	285	301	TEGAMDAGQLLKPALAR					Mascot
1968.1277	1968.0417	-0.086	-44	44	60	ILDKLNVPQAQFLAELR					Mascot
1982.1532	1981.9698	-0.1834	-93	820	837	VIQTELLNPLSKEIISGK					Mascot

5	Amino-acid acetyltransferase, mitochondrial OS=Ashbya gossypii (strain ATCC 10895 / CBS 109.51 / FGSC 9923 / NRRL Y-1056) GN=ARG2 PE=3 SV=1	NAGS_ASHGO	62093.3	9.27	12	48	0	28.5	8	0

Peptide Information

Calc. Mass	Obsrv. Mass	± da	± ppm	Start Seq.	End Seq.	Sequence	Ion Score	C. I. %	Modification	Rank	Result Type
873.4788	873.5563	0.0775	89	215	221	QQITDLR					Mascot
1136.5405	1136.6466	0.1061	93	395	403	NLDMSHYLK			Oxidation (M)[4]		Mascot
1136.5405	1136.6466	0.1061	93	395	403	NLDMSHYLK			Oxidation (M)[4]		Mascot
1154.5333	1154.5759	0.0426	37	166	174	ELVAYMPCR			Carbamidomethyl (C)[8], Oxidation (M)[6]		Mascot
1190.678	1190.6523	-0.0257	-22	330	340	ANPIFSTTVLK					Mascot
1725.8993	1726.0176	0.1183	69	62	77	TFSAGLINDFAIMLGR					Mascot
1741.8942	1741.9941	0.0999	57	62	77	TFSAGLINDFAIMLGR			Oxidation (M)[13]		Mascot
1743.9164	1743.9559	0.0395	23	341	355	QGVDIKLYDYSTLTK					Mascot
1755.9177	1756.0088	0.0911	52	178	192	FFIINRYGGIPSSER					Mascot
1759.8757	1759.9949	0.1192	68	448	462	GSLCISDVIFNLMFK			Carbamidomethyl (C)[4], Oxidation (M)[13]		Mascot
1823.9069	1823.9805	0.0736	40	109	125	HGLQSIPINEPMASGTR			Oxidation (M)[12]		Mascot
1831.0325	1831.1591	0.1266	69	330	346	ANPIFSTTVLKQGVDIK					Mascot
1876.0076	1876.1882	0.1806	96	6	21	LLTTKVGYHTPNYVNR					Mascot
1876.0076	1876.1882	0.1806	96	6	21	LLTTKVGYHTPNYVNR	8	0			Mascot
1913.9392	1914.0844	0.1452	76	527	541	EYITYVRDIEPSWSR					Mascot

6	GAS2-like protein 2 OS=Mus musculus GN=Gas2l2 PE=2 SV=1	GA2L2_MOUSE	95228.2	9.78	15	47	0	11.665		

Peptide Information

Calc. Mass	Obsrv. Mass	± da	± ppm	Start Seq.	End Seq.	Sequence	Ion Score	C. I. %	Modification	Rank	Result Type
919.5029	919.5851	0.0822	89	734	742	ASVCLGKGK			Carbamidomethyl (C)[4]		Mascot
1008.4792	1008.5524	0.0732	73	1	9	MSQHVGHGR					Mascot
1010.5853	1010.6013	0.016	16	572	580	HTSLALGRR					Mascot
1010.5853	1010.6013	0.016	16	572	580	HTSLALGRR					Mascot
1091.5845	1091.6376	0.0531	49	618	628	SGVYVPSLGGR					Mascot
1093.5525	1093.6249	0.0724	66	582	590	EQALYDNLK					Mascot
1110.6565	1110.5927	-0.0638	-57	246	254	ILRSHVMVR					Mascot
1126.6514	1126.579	-0.0724	-64	246	254	ILRSHVMVR			Oxidation (M)[7]		Mascot
1126.6514	1126.579	-0.0724	-64	246	254	ILRSHVMVR	18	0	Oxidation (M)[7]		Mascot
1142.6528	1142.5779	-0.0749	-66	433	442	LQIPEATSKR					Mascot

Calc. Mass	Obsrv. Mass	± da	± ppm	Start Seq.	End Seq.	Sequence	Ion Score	C. I. %	Modification	Rank	Result Type
1148.6534	1148.6239	-0.0295	-26	496	506	LSPARPPTPGR					Mascot
1176.5856	1176.6691	0.0835	71	690	700	ASLNAEDTTVR					Mascot
1176.5856	1176.6691	0.0835	71	690	700	ASLNAEDTTVR					Mascot
1190.7144	1190.6523	-0.0621	-52	642	652	ELVQGPPPLLK					Mascot
1636.9017	1636.9785	0.0768	47	427	441	APTPQRLQIPEATSK					Mascot
1741.8539	1741.9941	0.1402	80	298	313	VQDGPSQPQPTMTISR					Mascot
1743.9501	1743.9559	0.0058	3	612	628	SQAIPRSGVYVPSLGGR					Mascot
1749.9535	1749.9546	0.0011	1	182	198	DLALPSPDPPPPIPPAR					Mascot
1913.979	1914.0844	0.1054	55	20	35	SIRPFKSSEQYLEAMK					Mascot

7 ATP-dependent RNA helicase rok1 OS=Neosartorya fischeri (strain ATCC 1020 / DSM 3700 / FGSC A1164 / NRRL 181) GN=rok1 PE=3 SV=1 | ROK1_NEOFI | 82293.2 | 9.21 | 12 | 47 | 0 | 29.306

Peptide Information

Calc. Mass	Obsrv. Mass	± da	± ppm	Start Seq.	End Seq.	Sequence	Ion Score	C. I. %	Modification	Rank	Result Type
1010.5266	1010.6013	0.0747	74	693	701	ISTKSGFDR					Mascot
1010.5266	1010.6013	0.0747	74	693	701	ISTKSGFDR					Mascot
1079.5732	1079.6042	0.031	29	476	485	LVYAATEQGK					Mascot
1094.6027	1094.588	-0.0147	-13	1	9	MDAFKLLTR					Mascot
1110.5977	1110.5927	-0.005	-5	1	9	MDAFKLLTR			Oxidation (M)[1]		Mascot
1176.5604	1176.6691	0.1087	92	15	27	GGAAPSSAQSSTR					Mascot
1176.5604	1176.6691	0.1087	92	15	27	GGAAPSSAQSSTR					Mascot
1613.0149	1612.8833	-0.1316	-82	256	269	TLSFLIPVINKIVR					Mascot
1699.0054	1699.0631	0.0577	34	505	518	LRPPFLIFTQTIPR					Mascot
1699.0054	1699.0631	0.0577	34	505	518	LRPPFLIFTQTIPR	4	0			Mascot
1726.9739	1727.0891	0.1152	67	647	661	SIQKWLLDALPDLSK					Mascot
1728.0055	1727.9855	-0.02	-12	561	575	KGEIWILVTTDLLAR					Mascot
1728.0055	1727.9855	-0.02	-12	561	575	KGEIWILVTTDLLAR	11	0			Mascot
1759.8933	1759.9949	0.1016	58	15	33	GGAAPSSAQSSTRLPST GK					Mascot
1873.9906	1874.1575	0.1669	89	391	406	NIVLDEADVLLDELFR					Mascot
1897.9753	1898.1403	0.165	87	237	255	DAESIEPDLLVVAPTGSG K					Mascot
1900.9498	1900.9973	0.0475	25	139	154	DLEEIQTVRVESEEPK					Mascot

8 CDK5 regulatory subunit-associated protein 2 OS=Pan troglodytes GN=CDK5RAP2 PE=2 SV=1 | CK5P2_PANTR | 216229.9 | 5.37 | 26 | 47 | 0 | 12.041

Peptide Information

Calc. Mass	Obsrv. Mass	± da	± ppm	Start	End	Sequence	Ion	C. I. %	Modification	Rank	Result Type

				Seq.	Seq.		Score		
1045.6074	1045.6263	0.0189	18	319	328	AIQGLTMALK			Mascot
1061.6354	1061.62	-0.0154	-15	1312	1320	LEKLFLNGK			Mascot
1066.4912	1066.5836	0.0924	87	284	291	ERNSFEER			Mascot
1079.5481	1079.6042	0.0561	52	1029	1037	TTSSVWRDK			Mascot
1094.5953	1094.588	-0.0073	-7	472	480	LHNQEQVVK			Mascot
1126.5487	1126.579	0.0303	27	421	429	DLEEAHREK			Mascot
1126.5487	1126.579	0.0303	27	421	429	DLEEAHREK			Mascot
1130.5623	1130.5756	0.0133	12	261	270	GLCAAPREEK		Carbamidomethyl (C)[3]	Mascot
1150.6215	1150.5441	-0.0774	-67	1788	1796	LTLEEAYRR			Mascot
1156.5892	1156.5697	-0.0195	-17	980	988	TCNKQLHQK		Carbamidomethyl (C)[2]	Mascot
1174.5951	1174.578	-0.0171	-15	170	180	DVTAAQAELEK			Mascot
1190.6263	1190.6523	0.026	22	1409	1418	LEESIKTNEK			Mascot
1612.8945	1612.8833	-0.0112	-7	1726	1739	HVLGLIEDYEALLK			Mascot
1681.016	1681.0524	0.0364	22	1855	1868	VIFDQLVVTHKILR			Mascot
1733.9181	1733.9797	0.0616	36	593	606	QLEQDVLSYQNLRK			Mascot
1743.9236	1743.9559	0.0323	19	130	146	AVESLAEAGGSEIQRVK			Mascot
1744.8824	1744.963	0.0806	46	404	417	ITQELSDLQQERER			Mascot
1751.8303	1751.9327	0.1024	58	1321	1335	SVGVEMNTQIELMER		Oxidation (M)[6]	Mascot
1755.9309	1756.0088	0.0779	44	989	1004	LILAEAVMEGRPTPDK		Oxidation (M)[8]	Mascot
1759.9337	1759.9949	0.0612	35	1488	1502	TVSLEHLQREYASVK			Mascot
1775.9836	1775.9758	-0.0078	-4	1182	1197	HVKILGPLAPEMIDSR			Mascot
1866.8287	1867.0121	0.1834	98	1162	1178	SGSDGEEMTFSSLHQVR			Mascot
1874.0681	1874.1575	0.0894	48	938	953	LPILIKPSRSLGNMYR		Oxidation (M)[14]	Mascot
1901.0015	1900.9973	-0.0042	-2	1779	1795	FVSSVSTAKLTLEEAYR			Mascot
1940.0599	1940.0775	0.0176	9	1723	1739	NGRHVLGLIEDYEALLK			Mascot
1972.071	1972.0355	-0.0355	-18	866	884	VGLKDASVQTVATEGDLLR			Mascot
2255.1602	2255.3093	0.1491	66	1584	1602	GQDPFRDLHSLLMEIQALR		Oxidation (M)[13]	Mascot

9	V-type proton ATPase subunit E OS=Sulfolobus islandicus (strain M.16.4 / Kamchatka #3) GN=atpE PE=3 SV=1	VATE_SULIK	22543.9	5.76	11	47	0	6.518	

Protein Group

V-type proton ATPase subunit E OS=Sulfolobus islandicus (strain L.S.2.15 / Lassen #1) GN=atpE PE=3 SV=1	VATE_SULIL	22543.9	5.76000022888184
V-type proton ATPase subunit E OS=Sulfolobus islandicus (strain M.14.25 / Kamchatka #1) GN=atpE	VATE_SULIM	22543.9	5.7600002288

	PE=3 SV=1			8184				
	V-type proton ATPase subunit E OS=Sulfolobus islandicus (strain M.16.27) GN=atpE PE=3 SV=1	VATE_SULIA	22543.9	5.7600 002288 8184				
	V-type proton ATPase subunit E OS=Sulfolobus islandicus (strain Y.G.57.14 / Yellowstone #1) GN=atpE PE=3 SV=1	VATE_SULIY	22543.9	5.7600 002288 8184				

Peptide Information

Calc. Mass	Obsrv. Mass	± da	± ppm	Start Seq.	End Seq.	Sequence	Ion Score	C. I. %	Modification	Rank	Result Type
873.5404	873.5563	0.0159	18	93	100	IGEVVKTK					Mascot
1062.5314	1062.5945	0.0631	59	55	63	TKEEIEGEK					Mascot
1076.5834	1076.5725	-0.0109	-10	16	24	EEIKTELSK					Mascot
1092.5242	1092.61	0.0858	79	113	121	EIKEMEGEK					Mascot
1108.519	1108.5718	0.0528	48	113	121	EIKEMEGEK			Oxidation (M)[5]		Mascot
1152.6082	1152.5813	-0.0269	-23	184	194	GKISDMLFGGK					Mascot
1154.5398	1154.5759	0.0361	31	1	9	MDFEQLLDK			Oxidation (M)[1]		Mascot
1158.579	1158.5685	-0.0105	-9	158	167	IYYEGSGLTR					Mascot
1158.579	1158.5685	-0.0105	-9	158	167	IYYEGSGLTR					Mascot
1174.6427	1174.578	-0.0647	-55	132	142	STVEKVVGNNK					Mascot
1748.8854	1748.9598	0.0744	43	158	172	IYYEGSGLTRDFSLK					Mascot
1801.9265	1801.9651	0.0386	21	152	167	MLGGIRIYYEGSGLTR			Oxidation (M)[1]		Mascot
1915.8412	1915.9886	0.1474	77	116	131	EMEGEKITVYCSPNDK			Carbamidomethyl (C)[11], Oxidation (M)[2]		Mascot

10	Putative ATP-dependent RNA helicase DHX30 OS=Mus musculus GN=Dhx30 PE=2 SV=1	DHX30_MOUSE	137723.1	8.97	20	47	0	20.576

Peptide Information

Calc. Mass	Obsrv. Mass	± da	± ppm	Start Seq.	End Seq.	Sequence	Ion Score	C. I. %	Modification	Rank	Result Type
856.5111	856.5765	0.0654	76	797	803	ANVIQRR					Mascot
1008.5221	1008.5524	0.0303	30	498	506	YVTEGRGAR					Mascot
1094.5953	1094.588	-0.0073	-7	526	535	VSHELGPSLR					Mascot
1110.663	1110.5927	-0.0703	-63	1073	1082	TKSGNILLHK					Mascot
1130.5715	1130.5756	0.0041	4	656	663	HQYPHRHR					Mascot
1150.5488	1150.5441	-0.0047	-4	932	941	DPFSSSLQNR					Mascot
1154.5082	1154.5759	0.0677	59	1204	1213	GPCGSFDMRK			Carbamidomethyl (C)[3]		Mascot
1158.6017	1158.5685	-0.0332	-29	1096	1104	WLTYFMAVK					Mascot
1158.6017	1158.5685	-0.0332	-29	1096	1104	WLTYFMAVK					Mascot
1174.5966	1174.578	-0.0186	-16	1096	1104	WLTYFMAVK			Oxidation (M)[6]		Mascot

1182.6001	1182.5334	-0.0667	-56	119	129	SVEVEGYGSKK		Mascot
1198.6678	1198.5872	-0.0806	-67	401	411	LQSDDILPLGK		Mascot
1686.9537	1687.0203	0.0666	39	490	503	IPQLLLERYVTEGR		Mascot
1697.9446	1698.0372	0.0926	55	537	551	NVGFQVRLESKPPAR		Mascot
1698.9612	1699.0631	0.1019	60	823	836	LEKMVPFQVPEILR		Mascot
1698.9612	1699.0631	0.1019	60	823	836	LEKMVPFQVPEILR		Mascot
1714.9561	1715.0414	0.0853	50	823	836	LEKMVPFQVPEILR	Oxidation (M)[4]	Mascot
1743.9177	1743.9559	0.0382	22	146	161	GWGLLGPRNELFDAAK		Mascot
1745.0167	1744.963	-0.0537	-31	270	286	NLLAKVIQIATSSSTAK		Mascot
1775.9512	1775.9758	0.0246	14	190	206	QLNPENIRPGGPAGLSR		Mascot
1823.9174	1823.9805	0.0631	35	982	996	ENYLEENLLYAPSLR		Mascot
1839.9501	1839.9568	0.0067	4	448	463	GPIWQEAPQLPVDPHR		Mascot
1900.9626	1900.9973	0.0347	18	689	705	GEPGGILCFLPGWQEIK	Carbamidomethyl (C)[8]	Mascot
1986.0437	1986.0487	0.005	3	604	621	LNPALRLVLMSATGDNE R	Oxidation (M)[10]	Mascot

Gel Idx/Pos	116/E15	Instr./Gel Origin	BA2151/Sample Project 20140814	Process Status	Analysis Succeeded
Plate [#] Name	[1] Sample Project 20140814	Instrument Sample Name		Spectra	11

Rank	Protein Name	Accession No.	Protein MW	Protein PI	Pep. Count	Protein Score	Protein Score C. I. %	Intensity Matched	Total Ion Score	Total Ion C. I. %	Confirmed
1	Nucleoside diphosphate kinase 1 OS=Oryza sativa subsp. indica GN=NDKR PE=1 SV=1	NDK1_ORYSI	16834.6	6.3	7	179	100	14.111	138	100	

Peptide Information

Calc. Mass	Obsrv. Mass	± da	± ppm	Start Seq.	End Seq.	Sequence	Ion Score	C. I. %	Modification	Rank	Result Type
943.5571	943.6226	0.0655	69	16	24	GLIGDIISR					Mascot
943.5571	943.6226	0.0655	69	16	24	GLIGDIISR	47	98.561			Mascot
955.5182	955.5574	0.0392	41	29	36	GFFLRGMK					Mascot
1647.8523	1647.944	0.0917	56	2	15	EQSFIMIKPDGVQR					Mascot
1708.9493	1708.913	-0.0363	-21	87	102	IIGATRPWEAAPGTIR					Mascot
1716.8744	1717.0065	0.1321	77	126	139	EIALWFPEGLAEWR					Mascot
1716.8744	1717.0065	0.1321	77	126	139	EIALWFPEGLAEWR	91	100			Mascot
1794.8877	1795.0537	0.166	92	1	15	MEQSFIMIKPDGVQR			Oxidation (M)[1]		Mascot
1810.8827	1811.0403	0.1576	87	1	15	MEQSFIMIKPDGVQR			Oxidation (M)[1,7]		Mascot
1844.9694	1845.1071	0.1377	75	125	139	KEIALWFPEGLAEWR					Mascot

Rank	Protein Name	Accession No.	Protein MW	Protein PI	Pep. Count	Protein Score	Protein Score C. I. %	Intensity Matched	Total Ion Score	Total Ion C. I. %	Confirmed
2	Nucleoside diphosphate kinase 1 OS=Mesembryanthemum crystallinum GN=NDKP1 PE=2 SV=1	NDK1_MESCR	16340.6	6.3	4	179	100	33.411	132	100	

Peptide Information

Calc. Mass	Obsrv. Mass	± da	± ppm	Start Seq.	End Seq.	Sequence	Ion Score	C. I. %	Modification	Rank	Result Type
943.5571	943.6226	0.0655	69	16	24	GLVGEIISR					Mascot
943.5571	943.6226	0.0655	69	16	24	GLVGEIISR	27	0			Mascot
1609.8909	1610.0118	0.1209	75	87	102	IIGATNPLASEPGTIR					Mascot
1609.8909	1610.0118	0.1209	75	87	102	IIGATNPLASEPGTIR	102	100			Mascot
1647.9218	1647.944	0.0222	13	34	47	ALKFINVDRPFAEK					Mascot
1737.9858	1738.1172	0.1314	76	86	102	KIIGATNPLASEPGTIR					Mascot
1737.9858	1738.1172	0.1314	76	86	102	KIIGATNPLASEPGTIR	30	23.065			Mascot

Rank	Protein Name	Accession No.	Protein MW	Protein PI	Pep. Count	Protein Score	Protein Score C. I. %	Intensity Matched	Total Ion Score	Total Ion C. I. %	Confirmed
3	Nucleoside diphosphate kinase 1 OS=Spinacia oleracea GN=NDPK1 PE=2 SV=1	NDK1_SPIOL	16279.5	6.42	3	172	100	33.299	132	100	

Peptide Information

Calc. Mass	Obsrv. Mass	± da	± ppm	Start Seq.	End Seq.	Sequence	Ion Score	C. I. %	Modification	Rank	Result Type
943.5571	943.6226	0.0655	69	16	24	GLVGEIISR					Mascot
943.5571	943.6226	0.0655	69	16	24	GLVGEIISR	27	0			Mascot
1609.8909	1610.0118	0.1209	75	87	102	LIGATNPLASEPGTIR					Mascot
1609.8909	1610.0118	0.1209	75	87	102	LIGATNPLASEPGTIR	102	100			Mascot
1737.9858	1738.1172	0.1314	76	86	102	KLIGATNPLASEPGTIR					Mascot
1737.9858	1738.1172	0.1314	76	86	102	KLIGATNPLASEPGTIR	30	23.065			Mascot

4	Nucleoside diphosphate kinase 1 OS=Oryza sativa subsp. japonica GN=NDKR PE=1 SV=1	NDK1_ORYSJ	16850.6	6.3	6	171	100	14.03	138	100

Peptide Information

Calc. Mass	Obsrv. Mass	± da	± ppm	Start Seq.	End Seq.	Sequence	Ion Score	C. I. %	Modification	Rank	Result Type
943.5571	943.6226	0.0655	69	16	24	GLIGDIISR					Mascot
943.5571	943.6226	0.0655	69	16	24	GLIGDIISR	47	98.561			Mascot
1647.8523	1647.944	0.0917	56	2	15	EQSFIMIKPDGVQR					Mascot
1708.9493	1708.913	-0.0363	-21	87	102	IIGATRPWEAAPGTIR					Mascot
1716.8744	1717.0065	0.1321	77	126	139	EIALWFPEGLAEWR					Mascot
1716.8744	1717.0065	0.1321	77	126	139	EIALWFPEGLAEWR	91	100			Mascot
1794.8877	1795.0537	0.166	92	1	15	MEQSFIMIKPDGVQR			Oxidation (M)[1]		Mascot
1810.8827	1811.0403	0.1576	87	1	15	MEQSFIMIKPDGVQR			Oxidation (M)[1,7]		Mascot
1844.9694	1845.1071	0.1377	75	125	139	KEIALWFPEGLAEWR					Mascot

5	Nucleoside diphosphate kinase 1 OS=Arabidopsis thaliana GN=NDPK1 PE=1 SV=1	NDK1_ARATH	16546.6	6.3	3	118	100	4.312	106	100

Peptide Information

Calc. Mass	Obsrv. Mass	± da	± ppm	Start Seq.	End Seq.	Sequence	Ion Score	C. I. %	Modification	Rank	Result Type
886.5469	886.597	0.0501	57	79	86	NVVLTGRK					Mascot
1370.6659	1370.7637	0.0978	71	112	124	NVIHGSDSVESAR					Mascot
1370.6659	1370.7637	0.0978	71	112	124	NVIHGSDSVESAR	106	100			Mascot
1498.7609	1498.7765	0.0156	10	112	125	NVIHGSDSVESARK					Mascot

6	Nucleoside diphosphate kinase OS=Cyanothece sp. (strain ATCC 51142) GN=ndk PE=3 SV=1	NDK_CYAA5	16533.4	5.29	2	116	100	24.08	108	100

Peptide Information

Calc. Mass	Obsrv. Mass	± da	± ppm	Start Seq.	End Seq.	Sequence	Ion Score	C. I. %	Modification	Rank	Result Type

Calc. Mass	Obsrv. Mass	± da	± ppm	Start Seq.	End Seq.	Sequence	Ion Score	C. I. %	Modification	Rank	Result Type
1609.8909	1610.0118	0.1209	75	87	102	LIGATNPLSAEPGTIR					Mascot
1609.8909	1610.0118	0.1209	75	87	102	LIGATNPLSAEPGTIR	79	99.999			Mascot
1737.9858	1738.1172	0.1314	76	86	102	KLIGATNPLSAEPGTIR					Mascot
1737.9858	1738.1172	0.1314	76	86	102	KLIGATNPLSAEPGTIR	29	5.131			Mascot

7	Nucleoside diphosphate kinase OS=Capsicum annuum PE=2 SV=1	NDK_CAPAN	16372.5	6.31	3	102	99.997	24.278	88	100	

Protein Group

Nucleoside diphosphate kinase 1 OS=Nicotiana tabacum PE=2 SV=1	NDK1_TOBAC	16285.5	6.3099 999427 7954

Peptide Information

Calc. Mass	Obsrv. Mass	± da	± ppm	Start Seq.	End Seq.	Sequence	Ion Score	C. I. %	Modification	Rank	Result Type
1482.766	1482.7775	0.0115	8	112	125	NVIHGSDAVESARK					Mascot
1609.8909	1610.0118	0.1209	75	87	102	IIGATNPLESAPGTIR					Mascot
1609.8909	1610.0118	0.1209	75	87	102	IIGATNPLESAPGTIR	66	99.981			Mascot
1737.9858	1738.1172	0.1314	76	86	102	KIIGATNPLESAPGTIR					Mascot
1737.9858	1738.1172	0.1314	76	86	102	KIIGATNPLESAPGTIR	22	0			Mascot

8	Nucleoside diphosphate kinase OS=Symbiobacterium thermophilum (strain T / IAM 14863) GN=ndk PE=3 SV=1	NDK_SYMTH	16843.6	5.76	3	76	98.642	9.55	64	99.965	

Peptide Information

Calc. Mass	Obsrv. Mass	± da	± ppm	Start Seq.	End Seq.	Sequence	Ion Score	C. I. %	Modification	Rank	Result Type
943.5571	943.6226	0.0655	69	16	24	GLIGEVISR					Mascot
943.5571	943.6226	0.0655	69	16	24	GLIGEVISR	64	99.965			Mascot
1647.8635	1647.944	0.0805	49	2	15	ERSFVMVKPDGVQR					Mascot
1794.899	1795.0537	0.1547	86	1	15	MERSFVMVKPDGVQR			Oxidation (M)[1]		Mascot
1810.8939	1811.0403	0.1464	81	1	15	MERSFVMVKPDGVQR			Oxidation (M)[1,7]		Mascot

9	Nucleoside diphosphate kinase OS=Acaryochloris marina (strain MBIC 11017) GN=ndk PE=3 SV=1	NDK_ACAM1	16368.5	5.44	3	74	97.991	16.013	36	79.388	

Peptide Information

Calc. Mass	Obsrv. Mass	± da	± ppm	Start Seq.	End Seq.	Sequence	Ion Score	C. I. %	Modification	Rank	Result Type
943.5571	943.6226	0.0655	69	16	24	GLVGEIISR					Mascot
943.5571	943.6226	0.0655	69	16	24	GLVGEIISR	27	0			Mascot

949.4738	949.5352	0.0614	65	103	111	GDFGIDIGR					Mascot
949.4738	949.5352	0.0614	65	103	111	GDFGIDIGR	36	79.388			Mascot
1746.932	1746.9801	0.0481	28	1	15	MERTFLAVKPDGVQR					Mascot

10	UPF0215 protein STK_03040 OS=Sulfolobus tokodaii (strain DSM 16993 / JCM 10545 / NBRC 100140 / 7) GN=STK_03040 PE=3 SV=2	Y304_SULTO	20334.7	5.67	10	68	91.23	7.356

Peptide Information

Calc. Mass	Obsrv. Mass	± da	± ppm	Start Seq.	End Seq.	Sequence	Ion Score	C. I. %	Modification	Rank	Result Type
973.4598	973.4818	0.022	23	104	110	HFNDERR					Mascot
1272.608	1272.593	-0.015	-12	100	109	ALDRHFNDER					Mascot
1370.8002	1370.7637	-0.0365	-27	111	122	DIIINVISNLTR					Mascot
1370.8002	1370.7637	-0.0365	-27	111	122	DIIINVISNLTR					Mascot
1570.7822	1570.9143	0.1321	84	1	14	MLVSGIDDGYFPLK			Oxidation (M)[1]		Mascot
1714.905	1714.9927	0.0877	51	2	16	LVSGIDDGYFPLKYK					Mascot
1732.948	1733.0016	0.0536	31	160	175	IAHEISSSLSTFLLSK					Mascot
1755.8734	1756.0209	0.1475	84	140	153	MAKNIIEEYQVFDR					Mascot
1759.8936	1760.0526	0.159	90	128	142	GDVYIYTNLDLKMAK			Oxidation (M)[13]		Mascot
1771.8684	1771.9921	0.1237	70	140	153	MAKNIIEEYQVFDR			Oxidation (M)[1]		Mascot
1861.043	1861.1113	0.0683	37	160	176	IAHEISSSLSTFLLSKK					Mascot
1861.9406	1862.0841	0.1435	77	1	16	MLVSGIDDGYFPLKYK			Oxidation (M)[1]		Mascot

Gel Idx/Pos	117/E16	Instr./Gel Origin	BA2151/Sample Project 20140814	Process Status	Analysis Succeeded
Plate [#] Name	[1] Sample Project 20140814	Instrument Sample Name		Spectra	11

Rank	Protein Name	Accession No.	Protein MW	Protein PI	Pep. Count	Protein Score	Protein Score C. I. %	Intensity Matched	Total Ion Score	Total Ion C. I. %	Confirmed
1	Alpha-amylase inhibitor 0.19 OS=Triticum aestivum PE=1 SV=1	IAA1_WHEAT	13898.6	6.66	5	376	100	38.784	345	100	

Peptide Information

Calc. Mass	Obsrv. Mass	± da	± ppm	Start Seq.	End Seq.	Sequence	Ion Score	C. I. %	Modification	Rank	Result Type
1162.6249	1162.7083	0.0834	72	90	100	LTAASITAVCR			Carbamidomethyl (C)[10]		Mascot
1162.6249	1162.7083	0.0834	72	90	100	LTAASITAVCR	73	99.995	Carbamidomethyl (C)[10]		Mascot
1570.8007	1570.9241	0.1234	79	26	39	LQCNGSQVPEAVLR			Carbamidomethyl (C)[3]		Mascot
1612.7463	1612.8733	0.127	79	67	82	EHGAQEGQAGTGAFPR					Mascot
1612.7463	1612.8733	0.127	79	67	82	EHGAQEGQAGTGAFPR	138	100			Mascot
1663.8361	1663.9354	0.0993	60	101	116	LPIVVDASGDGAYVCK			Carbamidomethyl (C)[15]		Mascot
1862.7731	1862.9274	0.1543	83	40	53	DCCQQLAHISEWCR			Carbamidomethyl (C)[2,3,13]		Mascot
1862.7731	1862.9274	0.1543	83	40	53	DCCQQLAHISEWCR	135	100	Carbamidomethyl (C)[2,3,13]		Mascot

Rank	Protein Name	Accession No.	Protein MW	Protein PI	Pep. Count	Protein Score	Protein Score C. I. %	Intensity Matched	Total Ion Score	Total Ion C. I. %	Confirmed
2	Alpha-amylase inhibitor 0.53 OS=Triticum aestivum PE=1 SV=1	IAA5_WHEAT	13689.5	5.23	3	87	99.899	6.83	73	99.995	

Peptide Information

Calc. Mass	Obsrv. Mass	± da	± ppm	Start Seq.	End Seq.	Sequence	Ion Score	C. I. %	Modification	Rank	Result Type
1162.6249	1162.7083	0.0834	72	90	100	LTAASITAVCR			Carbamidomethyl (C)[10]		Mascot
1162.6249	1162.7083	0.0834	72	90	100	LTAASITAVCR	73	99.995	Carbamidomethyl (C)[10]		Mascot
1570.8007	1570.9241	0.1234	79	26	39	LQCNGSQVPEAVLR			Carbamidomethyl (C)[3]		Mascot
1663.8361	1663.9354	0.0993	60	101	116	LPIVVDASGDGAYVCK			Carbamidomethyl (C)[15]		Mascot

Rank	Protein Name	Accession No.	Protein MW	Protein PI	Pep. Count	Protein Score	Protein Score C. I. %	Intensity Matched	Total Ion Score	Total Ion C. I. %	Confirmed
3	Virulence-associated V antigen OS=Yersinia pseudotuberculosis serotype I (strain IP32953) GN=lcrV PE=4 SV=1	LCRV_YERPS	37370.2	5.49	13	60	39.329	6.428			

Peptide Information

Calc. Mass	Obsrv. Mass	± da	± ppm	Start Seq.	End Seq.	Sequence	Ion Score	C. I. %	Modification	Rank	Result Type
866.4254	866.4423	0.0169	20	238	244	NFLESEK					Mascot
898.4087	898.4189	0.0102	11	312	318	YDSVMQR					Mascot
994.5203	994.5861	0.0658	66	238	245	NFLESEKK					Mascot

1158.6616	1158.674	0.0124	11	63	72	VITDDIELLK					Mascot
1514.8424	1514.8656	0.0232	15	153	165	LREELAELTAELK					Mascot
1594.8047	1594.8619	0.0572	36	117	130	AFMAVIHFSLTADR			Oxidation (M)[3]		Mascot
1594.8047	1594.8619	0.0572	36	117	130	AFMAVIHFSLTADR			Oxidation (M)[3]		Mascot
1599.7987	1599.9438	0.1451	91	87	100	GGHYDNQLQNGIKR					Mascot
1633.9564	1633.8265	-0.1299	-80	73	86	KILAYFLPEDAILK					Mascot
1650.8962	1650.8127	-0.0835	-51	298	311	FNSAIEALNRFIQK					Mascot
1727.8269	1727.9701	0.1432	83	312	326	YDSVMQRLLDDTSGK					Mascot
1743.8218	1743.9756	0.1538	88	312	326	YDSVMQRLLDDTSGK			Oxidation (M)[5]		Mascot
1860.8763	1860.9132	0.0369	20	4	18	AYEQNPQHFIEDLEK					Mascot
2359.2576	2359.3123	0.0547	23	277	297	SRPLNDLVSQKTTQLSDI TSR					Mascot
2391.2378	2391.304	0.0662	28	117	137	AFMAVIHFSLTADRIDDDI LK					Mascot
2407.2327	2407.2957	0.063	26	117	137	AFMAVIHFSLTADRIDDDI LK			Oxidation (M)[3]		Mascot

4	Ribosomal RNA small subunit methyltransferase G OS=Bacillus anthracis (strain CDC 684 / NRRL 3495) GN=rsmG PE=3 SV=1	RSMG_BACAC	27406.2	6.17	10	59	37.916	3.435	

Protein Group

Ribosomal RNA small subunit methyltransferase G OS=Bacillus anthracis (strain A0248) GN=rsmG PE=3 SV=1	RSMG_BACAA	27406.2	6.1700 000762 9395
Ribosomal RNA small subunit methyltransferase G OS=Bacillus anthracis GN=rsmG PE=3 SV=1	RSMG_BACAN	27406.2	6.1700 000762 9395
Ribosomal RNA small subunit methyltransferase G OS=Bacillus cereus (strain 03BB102) GN=rsmG PE=3 SV=1	RSMG_BACC3	27406.2	6.1700 000762 9395
Ribosomal RNA small subunit methyltransferase G OS=Bacillus cereus (strain AH820) GN=rsmG PE=3 SV=1	RSMG_BACC0	27406.2	6.1700 000762 9395
Ribosomal RNA small subunit methyltransferase G OS=Bacillus cereus (strain ATCC 10987) GN=rsmG PE=3 SV=1	RSMG_BACC1	27406.2	6.1700 000762 9395
Ribosomal RNA small subunit methyltransferase G OS=Bacillus cereus (strain Q1) GN=rsmG PE=3 SV=1	RSMG_BACCQ	27406.2	6.1700 000762 9395
Ribosomal RNA small subunit methyltransferase G OS=Bacillus thuringiensis (strain Al Hakam) GN=rsmG PE=3 SV=1	RSMG_BACAH	27406.2	6.1700 000762 9395
Ribosomal RNA small subunit methyltransferase G OS=Bacillus thuringiensis subsp. konkukian (strain 97-27) GN=rsmG PE=3 SV=1	RSMG_BACHK	27406.2	6.1700 000762 9395

Peptide Information

Calc. Mass	Obsrv. Mass	± da	± ppm	Start Seq.	End Seq.	Sequence	Ion Score	C. I. %	Modification	Rank	Result Type

842.5345	842.5685	0.034	40	211	217	NILLIEK					Mascot
1158.6842	1158.674	-0.0102	-9	96	105	VTIVDSLQKR					Mascot
1480.7577	1480.8849	0.1272	86	139	151	EAYDIVMARAVAR			Oxidation (M)[7]		Mascot
1480.7577	1480.8849	0.1272	86	139	151	EAYDIVMARAVAR			Oxidation (M)[7]		Mascot
1508.7526	1508.9005	0.1479	98	135	147	EGVREAYDIVMAR					Mascot
1511.7047	1511.8361	0.1314	87	2	13	NIEQFQSMLEEK			Oxidation (M)[8]		Mascot
1626.7502	1626.8413	0.0911	56	1	13	MNIEQFQSMLEEK					Mascot
2345.1335	2345.3555	0.222	95	21	38	QLEQFEIYFETLVEWNEK					Mascot
2345.1335	2345.3555	0.222	95	21	38	QLEQFEIYFETLVEWNEK					Mascot
2357.1475	2357.3167	0.1692	72	1	20	MNIEQFQSMLEEKGITLSSR			Oxidation (M)[1]		Mascot
2361.1931	2361.3115	0.1184	50	174	196	GAAANEEIENGKYALEVLGGDLK					Mascot
2373.1426	2373.321	0.1784	75	1	20	MNIEQFQSMLEEKGITLSSR			Oxidation (M)[1,9]		Mascot
2391.3391	2391.304	-0.0351	-15	152	173	LSVLSELCLPLVKVGGTFIAMK			Carbamidomethyl (C)[8], Oxidation (M)[21]		Mascot

5	Arginine--tRNA ligase OS=Methanosarcina barkeri (strain Fusaro / DSM 804) GN=argS PE=3 SV=1	SYR_METBF	63590	5.55	14	53	0	38.389

Peptide Information

Calc. Mass	Obsrv. Mass	± da	± ppm	Start Seq.	End Seq.	Sequence	Ion Score	C. I. %	Modification	Rank	Result Type
1229.6671	1229.7339	0.0668	54	408	419	QVAEMVGIGAVR					Mascot
1480.7577	1480.8849	0.1272	86	492	504	MASFDRVIDLAAR			Oxidation (M)[1]		Mascot
1480.7577	1480.8849	0.1272	86	492	504	MASFDRVIDLAAR			Oxidation (M)[1]		Mascot
1511.8104	1511.8361	0.0257	17	283	296	GALVVDLSDYGFKK					Mascot
1553.7708	1553.8446	0.0738	47	373	385	RGQFISADELFDR					Mascot
1570.9526	1570.9241	-0.0285	-18	231	245	AVSLAISGIKETLLR					Mascot
1571.7952	1571.9146	0.1194	76	217	230	VEAGDVKTIDSFYK					Mascot
1571.7952	1571.9146	0.1194	76	217	230	VEAGDVKTIDSFYK					Mascot
1592.9081	1592.8567	-0.0514	-32	1	14	MFLELKAQATSILK					Mascot
1669.996	1669.8923	-0.1037	-62	337	352	LISGQLRATLNSIGVK					Mascot
1727.8851	1727.9701	0.085	49	280	295	TDKGALVVDLSDYGFK					Mascot
1863.0011	1862.9274	-0.0737	-40	246	261	LNVVHDKFVSESTFLK					Mascot
1863.0011	1862.9274	-0.0737	-40	246	261	LNVVHDKFVSESTFLK					Mascot
1876.011	1876.1667	0.1557	83	408	424	QVAEMVGIGAVRYDIVR					Mascot
1878.8328	1878.9159	0.0831	44	150	165	AGYDVEVQYYVNDMGR					Mascot
1894.8276	1894.9077	0.0801	42	150	165	AGYDVEVQYYVNDMGR			Oxidation (M)[14]		Mascot
1894.8276	1894.9077	0.0801	42	150	165	AGYDVEVQYYVNDMGR			Oxidation (M)[14]		Mascot

Calc. Mass	Obsrv. Mass	± da	± ppm	Start Seq.	End Seq.	Sequence	Ion Score	C. I. %	Modification	Rank	Result Type
2409.2119	2409.2942	0.0823	34	353	373	EPEVVIFEFVSLPEGSMSTRR					Mascot
2425.2068	2425.2898	0.083	34	353	373	EPEVVIFEFVSLPEGSMSTRR			Oxidation (M)[17]		Mascot
2449.2205	2449.3162	0.0957	39	386	406	VTEAALEQVETRRPETSEEFK					Mascot

6	Mediator of RNA polymerase II transcription subunit 14 OS=Magnaporthe oryzae (strain 70-15 / ATCC MYA-4617 / FGSC 8958) GN=RGR1 PE=3 SV=1	MED14_MAGO7	125562.4	9.42	20	52	0	30.204

Peptide Information

Calc. Mass	Obsrv. Mass	± da	± ppm	Start Seq.	End Seq.	Sequence	Ion Score	C. I. %	Modification	Rank	Result Type
832.3916	832.3714	-0.0202	-24	180	185	MMHNKR			Oxidation (M)[1]		Mascot
1158.6266	1158.674	0.0474	41	630	639	THVQLQFASK					Mascot
1162.5997	1162.7083	0.1086	93	346	355	RQAMELSVGR			Oxidation (M)[4]		Mascot
1162.5997	1162.7083	0.1086	93	346	355	RQAMELSVGR			Oxidation (M)[4]		Mascot
1508.822	1508.9005	0.0785	52	815	827	LYEFLGSISRAPR					Mascot
1553.8727	1553.8446	-0.0281	-18	906	918	LPFYLPTTLAVYR					Mascot
1568.7349	1568.8777	0.1428	91	173	184	YDFVLDRMMHNK					Mascot
1587.8701	1587.865	-0.0051	-3	699	714	TSEVLPSLSGKGSLGR					Mascot
1593.8496	1593.8718	0.0222	14	365	377	LNRSLAVQYWTSR					Mascot
1609.7089	1609.8553	0.1464	91	982	995	TDAEPNKSNDEFSR					Mascot
1612.829	1612.8733	0.0443	27	84	97	SHNELEDTIRSLAK					Mascot
1612.829	1612.8733	0.0443	27	84	97	SHNELEDTIRSLAK					Mascot
1669.8698	1669.8923	0.0225	13	368	381	SLAVQYWTSRYPAK					Mascot
1698.9174	1699.0388	0.1214	71	466	479	FVNKEDHLVLELSR					Mascot
1745.8262	1745.9774	0.1512	87	425	439	DIEIPLDEADLSMER					Mascot
1771.8578	1771.9052	0.0474	27	514	529	TRPMPMAESNLNTPGR					Mascot
1771.8578	1771.9052	0.0474	27	514	529	TRPMPMAESNLNTPGR	7	0			Mascot
1787.8528	1787.9104	0.0576	32	514	529	TRPMPMAESNLNTPGR			Oxidation (M)[4]		Mascot
1803.8477	1803.9078	0.0601	33	514	529	TRPMPMAESNLNTPGR			Oxidation (M)[4,6]		Mascot
1803.8477	1803.9078	0.0601	33	514	529	TRPMPMAESNLNTPGR			Oxidation (M)[4,6]		Mascot
1876.9321	1876.9304	-0.0017	-1	1010	1028	SLSTSACGPASAGVEELLK			Carbamidomethyl (C)[7]		Mascot
1967.9392	1968.0306	0.0914	46	570	585	NILSNREQTDAMAWFR			Oxidation (M)[12]		Mascot
2302.2014	2302.2859	0.0845	37	530	549	DPVAVIGWVRNTFAMEELVR					Mascot
2318.1963	2318.2764	0.0801	35	530	549	DPVAVIGWVRNTFAMEELVR			Oxidation (M)[15]		Mascot
2357.188	2357.3167	0.1287	55	310	330	MYLELKVNEVLGAEGLAGCYK			Carbamidomethyl (C)[19]		Mascot
2373.1829	2373.321	0.1381	58	310	330	MYLELKVNEVLGAEGLAGCYK			Carbamidomethyl (C)[19], Oxidation (M)[1]		Mascot

2411.2454	2411.2932	0.0478	20	231	250	WLEDLDTLLSLRLTIDDHDK					Mascot
2433.2053	2433.3286	0.1233	51	1052	1073	QPVVQPGQQPQVQNQANGVMNR			Oxidation (M)[20]		Mascot

7	Aspartyl/glutamyl-tRNA(Asn/Gln) amidotransferase subunit C OS=Heliobacterium modesticaldum (strain ATCC 51547 / Ice1) GN=gatC PE=3 SV=1	GATC_HELMI	10806.5	4.7	7	52	0	2.315	

Peptide Information

Calc. Mass	Obsrv. Mass	± da	± ppm	Start Seq.	End Seq.	Sequence	Ion Score	C. I. %	Modification	Rank	Result Type
1593.7544	1593.8718	0.1174	74	76	89	IVANAPEEEDGFFR					Mascot
1669.8545	1669.8923	0.0378	23	27	40	YTTQLNAILDYAQR					Mascot
1680.8989	1680.8879	-0.011	-7	2	16	ALTKAEVEYVAMLAR			Oxidation (M)[12]		Mascot
1845.9429	1845.9733	0.0304	16	48	63	DVPPTAHVFPLHNVMR			Oxidation (M)[15]		Mascot
1878.8981	1878.9159	0.0178	9	74	89	ERIVANAPEEEDGFFR					Mascot
2407.2173	2407.2957	0.0784	33	6	26	AEVEYVAMLARLELSEADLER					Mascot
2425.2722	2425.2898	0.0176	7	27	47	YTTQLNAILDYAQRLQGLDTK					Mascot

8	Cyclic 2,3-diphosphoglycerate synthetase OS=Thermococcus sibiricus (strain MM 739 / DSM 12597) GN=cpgS PE=3 SV=1	CPGS_THESM	48138.8	7.03	12	51	0	40.662	

Peptide Information

Calc. Mass	Obsrv. Mass	± da	± ppm	Start Seq.	End Seq.	Sequence	Ion Score	C. I. %	Modification	Rank	Result Type
1162.7671	1162.7083	-0.0588	-51	90	100	IAAVLLKHGIK					Mascot
1162.7671	1162.7083	-0.0588	-51	90	100	IAAVLLKHGIK					Mascot
1571.8176	1571.9146	0.097	62	345	357	HLENRYDVEIVGK					Mascot
1571.8176	1571.9146	0.097	62	345	357	HLENRYDVEIVGK					Mascot
1612.8978	1612.8733	-0.0245	-15	327	341	KAILVMTAPPEGLEK			Oxidation (M)[6]		Mascot
1612.8978	1612.8733	-0.0245	-15	327	341	KAILVMTAPPEGLEK			Oxidation (M)[6]		Mascot
1628.8643	1628.8457	-0.0186	-11	420	433	EAVLEIGWELKGER					Mascot
1669.9272	1669.8923	-0.0349	-21	417	430	NLREAVLEIGWELK					Mascot
1680.8625	1680.8879	0.0254	15	21	36	KIGDVSCAVFLGGTEK			Carbamidomethyl (C)[7]		Mascot
1685.9983	1685.936	-0.0623	-37	142	156	TLKEISKPIIVTMGR					Mascot
1701.9932	1701.9323	-0.0609	-36	142	156	TLKEISKPIIVTMGR			Oxidation (M)[13]		Mascot
1766.9833	1766.9823	-0.001	-1	328	344	AILVMTAPPEGLEKAAR					Mascot
1772.0238	1771.9052	-0.1186	-67	275	291	IGLADLIVITLADMVSK					Mascot
1772.0238	1771.9052	-0.1186	-67	275	291	IGLADLIVITLADMVSK					Mascot
1788.0188	1787.9104	-0.1084	-61	275	291	IGLADLIVITLADMVSK			Oxidation (M)[14]		Mascot

1890.899	1890.944	0.045	24	207	223	RCGGGMVGFSFFDIVNK			Carbamidomethyl (C)[2]		Mascot
2345.178	2345.3555	0.1775	76	1	20	MRMVLIDGEHYPDVTAW AIK					Mascot
2345.178	2345.3555	0.1775	76	1	20	MRMVLIDGEHYPDVTAW AIK					Mascot
2357.2964	2357.3167	0.0203	9	227	249	LAEKLEGDIVILEGSGATF PAVK					Mascot
2361.1731	2361.3115	0.1384	59	1	20	MRMVLIDGEHYPDVTAW AIK			Oxidation (M)[1]		Mascot
2377.168	2377.3125	0.1445	61	1	20	MRMVLIDGEHYPDVTAW AIK			Oxidation (M)[1,3]		Mascot

9	Ribosomal RNA small subunit methyltransferase G OS=Bacillus cereus (strain AH187) GN=rsmG PE=3 SV=1	RSMG_BACC7	27402.2	6.17	9	49	0	3.248	

Peptide Information

Calc. Mass	Obsrv. Mass	± da	± ppm	Start Seq.	End Seq.	Sequence	Ion Score	C. I. %	Modification	Rank	Result Type
842.5345	842.5685	0.034	40	211	217	NILLIEK					Mascot
1158.6842	1158.674	-0.0102	-9	96	105	VTIVDSLQKR					Mascot
1480.7577	1480.8849	0.1272	86	139	151	EAYDIVMARAVAR			Oxidation (M)[7]		Mascot
1480.7577	1480.8849	0.1272	86	139	151	EAYDIVMARAVAR			Oxidation (M)[7]		Mascot
1508.7526	1508.9005	0.1479	98	135	147	EGVREAYDIVMAR					Mascot
1511.7047	1511.8361	0.1314	87	2	13	NIEQFQSMLEEK			Oxidation (M)[8]		Mascot
1626.7502	1626.8413	0.0911	56	1	13	MNIEQFQSMLEEK					Mascot
2345.1335	2345.3555	0.222	95	21	38	QLEQFEIYFETLVEWNEK					Mascot
2345.1335	2345.3555	0.222	95	21	38	QLEQFEIYFETLVEWNEK					Mascot
2361.1931	2361.3115	0.1184	50	174	196	GAAANEEIENGKYALEVL GGDLK					Mascot
2391.3391	2391.304	-0.0351	-15	152	173	LSVLSELCLPLVKVGGTFI AMK			Carbamidomethyl (C)[8], Oxidation (M)[21]		Mascot

10	Ribosomal RNA small subunit methyltransferase G OS=Bacillus cereus (strain ZK / E33L) GN=rsmG PE=3 SV=1	RSMG_BACCZ	27420.3	6.47	9	49	0	3.26	

Peptide Information

Calc. Mass	Obsrv. Mass	± da	± ppm	Start Seq.	End Seq.	Sequence	Ion Score	C. I. %	Modification	Rank	Result Type
842.5345	842.5685	0.034	40	211	217	NILLIEK					Mascot
1158.6842	1158.674	-0.0102	-9	96	105	VTIVDSLQKR					Mascot
1480.7577	1480.8849	0.1272	86	139	151	EAYDIVMARAVAR			Oxidation (M)[7]		Mascot
1480.7577	1480.8849	0.1272	86	139	151	EAYDIVMARAVAR			Oxidation (M)[7]		Mascot
1508.7526	1508.9005	0.1479	98	135	147	EGVREAYDIVMAR					Mascot
1511.7047	1511.8361	0.1314	87	2	13	NIEQFQSMLEEK			Oxidation (M)[8]		Mascot

1626.7502	1626.8413	0.0911	56	1	13	MNIEQFQSMLEEK		Mascot
2345.1335	2345.3555	0.222	95	21	38	QLEQFEIYFETLVEWNEK		Mascot
2345.1335	2345.3555	0.222	95	21	38	QLEQFEIYFETLVEWNEK		Mascot
2357.1475	2357.3167	0.1692	72	1	20	MNIEQFQSMLEEKGITLS SR	Oxidation (M)[1]	Mascot
2373.1426	2373.321	0.1784	75	1	20	MNIEQFQSMLEEKGITLS SR	Oxidation (M)[1,9]	Mascot
2391.3391	2391.304	-0.0351	-15	152	173	LSVLSELCLPLVKVGGTFI AMK	Carbamidomethyl (C)[8], Oxidation (M)[21]	Mascot

Gel Idx/Pos	118/E17	Instr./Gel Origin	BA2151/Sample Project 20140814	Process Status	Analysis Succeeded
Plate [#] Name	[1] Sample Project 20140814	Instrument Sample Name		Spectra	11

Rank	Protein Name	Accession No.	Protein MW	Protein PI	Pep. Count	Protein Score	Protein Score C. I. %	Intensity Matched	Total Ion Score	Total Ion C. I. %	Confirmed
1	16.9 kDa class I heat shock protein 1 OS=Triticum aestivum GN=hsp16.9A PE=2 SV=1	HS16A_WHEAT	16867.8	5.83	6	324	100	27.362	290	100	

Peptide Information

Calc. Mass	Obsrv. Mass	± da	± ppm	Start Seq.	End Seq.	Sequence	Ion Score	C. I. %	Modification	Rank	Result Type
975.5258	975.5861	0.0603	62	110	117	FRLPEDAK					Mascot
975.5258	975.5861	0.0603	62	110	117	FRLPEDAK	30	20.401			Mascot
1027.6146	1027.5669	-0.0477	-46	137	145	AEVKKPEVK					Mascot
1057.5314	1057.5671	0.0357	34	50	58	ETPEAHVFK					Mascot
1600.8177	1600.9133	0.0956	60	71	85	VEVEDGNVLVVSGER					Mascot
1600.8177	1600.9133	0.0956	60	71	85	VEVEDGNVLVVSGER	97	100			Mascot
1905.9666	1906.083	0.1164	61	26	45	SIVPAISGGSSETAAFAN AR					Mascot
1905.9666	1906.083	0.1164	61	26	45	SIVPAISGGSSETAAFAN AR	164	100			Mascot
2086.0664	2086.1868	0.1204	58	67	85	EEVKVEVEDGNVLVVSG ER					Mascot
2086.0664	2086.1868	0.1204	58	67	85	EEVKVEVEDGNVLVVSG ER					Mascot

Rank	Protein Name	Accession No.	Protein MW	Protein PI	Pep. Count	Protein Score	Protein Score C. I. %	Intensity Matched	Total Ion Score	Total Ion C. I. %	Confirmed
2	16.9 kDa class I heat shock protein 2 OS=Triticum aestivum GN=hsp16.9B PE=1 SV=1	HS16B_WHEAT	16856.8	6.19	5	124	100	4.484	97	100	

Peptide Information

Calc. Mass	Obsrv. Mass	± da	± ppm	Start Seq.	End Seq.	Sequence	Ion Score	C. I. %	Modification	Rank	Result Type
1027.6146	1027.5669	-0.0477	-46	137	145	AEVKKPEVK					Mascot
1057.5314	1057.5671	0.0357	34	50	58	ETPEAHVFK					Mascot
1600.8177	1600.9133	0.0956	60	71	85	VEVEDGNVLVVSGER					Mascot
1600.8177	1600.9133	0.0956	60	71	85	VEVEDGNVLVVSGER	97	100			Mascot
1875.9559	1876.0944	0.1385	74	26	45	SIVPAISGGGSETAAFAN AR					Mascot
2086.0664	2086.1868	0.1204	58	67	85	EEVKVEVEDGNVLVVSG ER					Mascot
2086.0664	2086.1868	0.1204	58	67	85	EEVKVEVEDGNVLVVSG ER					Mascot

Rank	Protein Name	Accession No.	Protein MW	Protein PI	Pep. Count	Protein Score	Protein Score C. I. %	Intensity Matched	Total Ion Score	Total Ion C. I. %	Confirmed
3	Mitogen-activated protein kinase kinase kinase 7 OS=Homo sapiens GN=MAP3K7 PE=1 SV=1	M3K7_HUMAN	67894.6	6.69	12	59	33.475	4.849			

Peptide Information

Calc. Mass	Obsrv. Mass	± da	± ppm	Start Seq.	End Seq.	Sequence	Ion Score	C. I. %	Modification	Rank	Result Type
814.5145	814.4923	-0.0222	-27	352	358	LLKNQAK					Mascot
837.4287	837.4843	0.0556	66	45	52	GAFGVVCK			Carbamidomethyl (C)[7]		Mascot
847.5036	847.4811	-0.0225	-27	73	79	AFIVELR					Mascot
900.3953	900.465	0.0697	77	529	535	MAQEYMK					Mascot
975.5985	975.5861	-0.0124	-13	72	79	KAFIVELR					Mascot
975.5985	975.5861	-0.0124	-13	72	79	KAFIVELR	22	0			Mascot
1232.5978	1232.673	0.0752	61	355	365	NQAKQQSESGR					Mascot
1365.5927	1365.7269	0.1342	98	518	528	ESMAVFEQHCK			Carbamidomethyl (C)[10]		Mascot
1475.7561	1475.8302	0.0741	50	359	372	QQSESGRLSLGASR					Mascot
1919.9127	1920.0708	0.1581	82	331	347	SDTNMEQVPATNDTIKR					Mascot
1994.0205	1994.104	0.0835	42	210	225	CDVFSWGIILWEVITR			Carbamidomethyl (C)[1]		Mascot
2059.0667	2059.2166	0.1499	73	366	386	LSLGASRGSSVESLPPTS EGK					Mascot
2440.2944	2440.2034	-0.091	-37	415	437	TASFGNILDVPEIVISGNG QPRR					Mascot

4	NADH-quinone oxidoreductase subunit I OS=Rickettsia africae (strain ESF-5) GN=nuoI PE=3 SV=1	NUOI_RICAE	19057.4	6.82	10	57	0	2.461	

Peptide Information

Calc. Mass	Obsrv. Mass	± da	± ppm	Start Seq.	End Seq.	Sequence	Ion Score	C. I. %	Modification	Rank	Result Type
804.4436	804.492	0.0484	60	1	6	MIHYLK					Mascot
815.437	815.4922	0.0552	68	137	143	LLQNGDR					Mascot
829.4607	829.4934	0.0327	39	23	28	YFFKPK					Mascot
838.4642	838.467	0.0028	3	46	52	GEHALRR					Mascot
932.4836	932.5412	0.0576	62	144	151	WEQALASK					Mascot
1126.5779	1126.5951	0.0172	15	29	37	VTINYPYEK					Mascot
1201.6688	1201.6802	0.0114	9	144	153	WEQALASKLR					Mascot
1259.5936	1259.6865	0.0929	74	89	98	TTRYDIDMTK			Oxidation (M)[8]		Mascot
1888.0402	1888.1011	0.0609	32	7	22	SFFLYEIVRGMALTLK					Mascot
1959.0562	1958.9761	-0.0801	-41	1	15	MIHYLKSFFLYEIVR					Mascot

5	26S proteasome regulatory subunit RPN9 OS=Saccharomyces cerevisiae (strain ATCC 204508 / S288c) GN=RPN9 PE=1 SV=1	RPN9_YEAST	45810.8	5.51	14	55	0	10.966	

Peptide Information

Calc. Mass	Obsrv. Mass	± da	± ppm	Start	End	Sequence	Ion	C. I. %	Modification	Rank	Result Type

				Seq.	Seq.		Score				
807.4974	807.4653	-0.0321	-40	81	87	YLLASLK					Mascot
814.5396	814.4923	-0.0473	-58	331	338	AISLGLLK					Mascot
815.4733	815.4922	0.0189	23	141	147	NDLVKAR					Mascot
828.4938	828.4824	-0.0114	-14	160	166	KDSIPLR					Mascot
842.5206	842.5685	0.0479	57	54	60	STPLRLR					Mascot
900.5513	900.465	-0.0863	-96	73	80	INQLSVVK					Mascot
960.4884	960.563	0.0746	78	148	155	DLLDDLEK					Mascot
1065.5211	1065.5854	0.0643	60	104	112	AQFQELDSK					Mascot
1193.6161	1193.6819	0.0658	55	104	113	AQFQELDSKK					Mascot
1259.6267	1259.6865	0.0598	47	370	379	LVEWNDQVEK					Mascot
1600.7742	1600.9133	0.1391	87	91	103	DFDESLKYLDDLK					Mascot
1600.7742	1600.9133	0.1391	87	91	103	DFDESLKYLDDLK					Mascot
1699.7963	1699.9316	0.1353	80	167	180	ITNSFYSTNSQYFK					Mascot
1716.8989	1716.9459	0.047	27	307	321	MLSFEDISKATHLPK					Mascot
1839.0118	1839.0294	0.0176	10	289	303	QKICLMTLIETVFVK			Carbamidomethyl (C)[4], Oxidation (M)[6]		Mascot

6	S-adenosylmethionine:tRNA ribosyltransferase-isomerase OS=Rhodobacter sphaeroides (strain KD131 / KCTC 12085) GN=queA PE=3 SV=1	QUEA_RHOSK	37620.6	6.52	12	55	0	8.302	

Peptide Information

Calc. Mass	Obsrv. Mass	± da	± ppm	Start Seq.	End Seq.	Sequence	Ion Score	C. I. %	Modification	Rank	Result Type
802.4603	802.5089	0.0486	61	94	100	AMAKPLR			Oxidation (M)[2]		Mascot
804.4686	804.492	0.0234	29	66	72	LTGTRTR					Mascot
829.489	829.4934	0.0044	5	54	60	LVLNNTR					Mascot
889.4486	889.4902	0.0416	47	71	78	TRGEAEAR					Mascot
1320.6907	1320.6748	-0.0159	-12	122	132	GETDLRLVFDR					Mascot
1365.8325	1365.7269	-0.1056	-77	54	65	LVLNNTRVIPAR					Mascot
1438.8489	1438.771	-0.0779	-54	247	261	AAGGRVIPVGTTALR					Mascot
1600.8152	1600.9133	0.0981	61	79	93	IEVTLMEPAAAGGWR					Mascot
1600.8152	1600.9133	0.0981	61	79	93	IEVTLMEPAAAGGWR					Mascot
1934.0562	1934.1	0.0438	23	304	320	STLLMLVSALMGQERIR			Oxidation (M)[5]		Mascot
2085.991	2086.1868	0.1958	94	227	246	MHAEWGEVTEAAAAEIA ATK					Mascot
2085.991	2086.1868	0.1958	94	227	246	MHAEWGEVTEAAAAEIA ATK					Mascot
2214.0972	2214.2949	0.1977	89	73	93	GEAEARIEVTLMEPAAAG					Mascot

Calc. Mass	Obsrv. Mass	± da	± ppm	Start Seq.	End Seq.	Sequence	Ion Score	C. I. %	Modification	Rank	Result Type
						GWR					
2214.0972	2214.2949	0.1977	89	73	93	GEAEARIEVTLMEPAAAG GWR					Mascot
2384.2578	2384.1011	-0.1567	-66	79	100	IEVTLMEPAAAGGWRAM AKPLR			Oxidation (M)[6]		Mascot

7	Probable cellulose synthase A catalytic subunit 8 [UDP-forming] OS=Oryza sativa subsp. japonica GN=CESA8 PE=2 SV=1	CESA8_ORYSJ	121971.4	8.03	18	54	0	4.905

Peptide Information

Calc. Mass	Obsrv. Mass	± da	± ppm	Start Seq.	End Seq.	Sequence	Ion Score	C. I. %	Modification	Rank	Result Type
832.4464	832.3781	-0.0683	-82	327	332	WFPINR					Mascot
845.4879	845.5289	0.041	48	209	215	VDGWKLK					Mascot
849.4465	849.504	0.0575	68	1062	1068	IDPFTTR					Mascot
889.4526	889.4902	0.0376	42	207	213	ERVDGWK					Mascot
906.5155	906.5209	0.0054	6	339	345	LALRYDR					Mascot
908.4333	908.5132	0.0799	88	604	610	NDRYANR					Mascot
919.4995	919.4804	-0.0191	-21	422	428	KYNIEPR					Mascot
993.5727	993.5936	0.0209	21	522	529	LVYVSREK					Mascot
1029.5323	1029.5725	0.0402	39	807	816	GSAPINLSDR					Mascot
1082.5365	1082.5869	0.0504	47	129	138	YDSGEIGLTK					Mascot
1107.5178	1107.6165	0.0987	89	598	606	FDGIDRNDR					Mascot
1140.5466	1140.6416	0.095	83	655	665	GSFLSSLCGGR			Carbamidomethyl (C)[8]		Mascot
1365.6157	1365.7269	0.1112	81	835	845	HCPIWYGYGGR			Carbamidomethyl (C)[2]		Mascot
1438.7393	1438.771	0.0317	22	711	722	SLLMSQMSLEKR			Oxidation (M)[4]		Mascot
1609.8121	1609.9492	0.1371	85	327	338	WFPINRETYLDR					Mascot
1628.8867	1628.948	0.0613	38	802	816	RPAFKGSAPINLSDR					Mascot
1628.8867	1628.948	0.0613	38	802	816	RPAFKGSAPINLSDR	17	0			Mascot
1742.8973	1742.9565	0.0592	34	607	620	YANRNTVFFDINLR					Mascot
1927.9337	1928.0139	0.0802	42	423	437	YNIEPRAPEWYFSQK					Mascot

8	Acetyl-CoA carboxylase, mitochondrial OS=Saccharomyces cerevisiae (strain JAY291) GN=HFA1 PE=3 SV=2	HFA1_YEAS2	260133.2	8.83	30	54	0	10.131

Peptide Information

Calc. Mass	Obsrv. Mass	± da	± ppm	Start Seq.	End Seq.	Sequence	Ion Score	C. I. %	Modification	Rank	Result Type
806.477	806.483	0.006	7	669	674	YLELLR					Mascot
807.4141	807.4653	0.0512	63	150	155	EMRSIR			Oxidation (M)[2]		Mascot
814.5257	814.4923	-0.0334	-41	1088	1094	ARSVLLR					Mascot

818.4254	818.4744	0.049	60	1280	1286	SENDLIK		Mascot
819.4512	819.4633	0.0121	15	1572	1577	YFPKHK		Mascot
832.4271	832.3781	-0.049	-59	747	753	DDIRGTR		Mascot
874.4741	874.5138	0.0397	45	2114	2120	QNDLTKR		Mascot
900.4825	900.465	-0.0175	-19	995	1001	KYYAVEK		Mascot
919.4883	919.4804	-0.0079	-9	1875	1882	DVDFKPAK		Mascot
932.5233	932.5412	0.0179	19	589	596	QNMVLALK	Oxidation (M)[3]	Mascot
968.516	968.5056	-0.0104	-11	1717	1725	GNSVVVEHK		Mascot
993.5615	993.5936	0.0321	32	1528	1536	SLSLPYSVK		Mascot
1027.4764	1027.5669	0.0905	88	2008	2015	DMYNEVLK	Oxidation (M)[2]	Mascot
1033.5538	1033.5801	0.0263	25	59	66	GQRPEYRK		Mascot
1104.626	1104.6376	0.0116	11	263	273	ISSTIVAQSAK		Mascot
1128.6412	1128.7089	0.0677	60	676	685	GQVPPKDFLK		Mascot
1128.6412	1128.7089	0.0677	60	676	685	GQVPPKDFLK		Mascot
1182.6226	1182.6326	0.01	8	1292	1301	LHENERGLSK		Mascot
1182.6226	1182.6326	0.01	8	1292	1301	LHENERGLSK		Mascot
1232.7435	1232.673	-0.0705	-57	318	328	AKLIGFPVMIK	Oxidation (M)[9]	Mascot
1320.7886	1320.6748	-0.1138	-86	1488	1499	LLISNKSGYVVK		Mascot
1438.671	1438.771	0.1	70	1002	1012	IFENHDIHEER		Mascot
1628.9153	1628.948	0.0327	20	138	152	ILIANNGIAAVKEMR	Oxidation (M)[14]	Mascot
1628.9153	1628.948	0.0327	20	138	152	ILIANNGIAAVKEMR	Oxidation (M)[14]	Mascot
1657.912	1657.9301	0.0181	11	2256	2270	GNIALEELTRLVDSK		Mascot
1707.8523	1707.886	0.0337	20	1186	1200	GAPDLLMSWQFSSLR		Mascot
1716.801	1716.9459	0.1449	84	2001	2015	GFSGGQRDMYNEVLK	Oxidation (M)[9]	Mascot
1928.035	1928.0139	-0.0211	-11	1697	1712	GFQYLYLAPKDMQLLK		Mascot
2086.0903	2086.1868	0.0965	46	1183	1200	IHKGAPDLLMSWQFSSLR		Mascot
2086.0903	2086.1868	0.0965	46	1183	1200	IHKGAPDLLMSWQFSSLR		Mascot
2239.218	2239.2456	0.0276	12	822	843	QPGSIIEAGDVIAKLTLDSPSK		Mascot
2807.3379	2807.5007	0.1628	58	1601	1625	EPGLNNIGMVAFEIMVQTPEYPEGR	Oxidation (M)[9]	Mascot
2811.3684	2811.4961	0.1277	45	619	643	DFESNNISTGWLDDLILKNLSSDSK		Mascot
2823.3328	2823.5073	0.1745	62	1601	1625	EPGLNNIGMVAFEIMVQTPEYPEGR	Oxidation (M)[9,15]	Mascot
2839.2913	2839.4963	0.205	72	2043	2067	GGSWVVIDPTINPEQMEMYADVESR	Oxidation (M)[16]	Mascot

9	3-phosphoshikimate 1-carboxyvinyltransferase OS=Bacillus licheniformis (strain DSM 13 / ATCC 14580) GN=aroA PE=3 SV=1	AROA_BACLD	45721.8	5.89	14	54	0	13.549

Peptide Information

Calc. Mass	Obsrv. Mass	± da	± ppm	Start Seq.	End Seq.	Sequence	Ion Score	C. I. %	Modification	Rank	Result Type
842.5094	842.5685	0.0591	70	124	130	VTEPLRK					Mascot
847.4454	847.4811	0.0357	42	131	138	MGAKIDGR					Mascot
858.5295	858.5243	-0.0052	-6	261	268	TGIIDVLK					Mascot
870.5156	870.5833	0.0677	78	123	129	RVTEPLR					Mascot
900.4282	900.465	0.0368	41	190	196	SRDHTER					Mascot
975.5357	975.5861	0.0504	52	344	352	IDTVASELK					Mascot
975.5357	975.5861	0.0504	52	344	352	IDTVASELK					Mascot
1140.5905	1140.6416	0.0511	45	197	206	MLSMFGVSLR					Mascot
1179.6844	1179.671	-0.0134	-11	367	378	IHGKTPLTGGAK					Mascot
1475.77	1475.8302	0.0602	41	340	352	ETNRIDTVASELK					Mascot
1493.8323	1493.8356	0.0033	2	6	19	ISSLKGELHIPGDK					Mascot
1600.8074	1600.9133	0.1059	66	25	39	SVMFGAMAEGKTVIK			Oxidation (M)[3,7]		Mascot
1600.8074	1600.9133	0.1059	66	25	39	SVMFGAMAEGKTVIK			Oxidation (M)[3,7]		Mascot
1641.8707	1641.9226	0.0519	32	139	154	AGGEYTPLSVRGGHLK					Mascot
1657.912	1657.9301	0.0181	11	294	309	TSSLKAAEISGDLIPR					Mascot
1707.8306	1707.886	0.0554	32	20	35	SISHRSVMFGAMAEGK					Mascot

10	Chaperone protein ClpB OS=Thermus thermophilus (strain HB27 / ATCC BAA-163 / DSM 7039) GN=clpB PE=3 SV=1	CLPB_THET2	96081.8	5.62	20	54	0	11.375	

Peptide Information

Calc. Mass	Obsrv. Mass	± da	± ppm	Start Seq.	End Seq.	Sequence	Ion Score	C. I. %	Modification	Rank	Result Type
814.4305	814.4923	0.0618	76	218	225	GDVPEGLK					Mascot
815.4733	815.4922	0.0189	23	456	461	EREILR					Mascot
837.4577	837.4843	0.0266	32	371	376	YITERR					Mascot
842.5094	842.5685	0.0591	70	67	73	ELSRLPK					Mascot
870.5043	870.5833	0.079	91	53	61	AGADPKALK					Mascot
887.4985	887.4702	-0.0283	-32	533	540	WTGIPVSK					Mascot
897.4676	897.4907	0.0231	26	552	558	LEEELHK					Mascot
900.5149	900.465	-0.0499	-55	411	417	LQLEIER					Mascot
906.5043	906.5209	0.0166	18	728	734	IRDEVFK					Mascot
1028.5623	1028.5295	-0.0328	-32	815	823	ELETPLAQK					Mascot
1033.5234	1033.5801	0.0567	55	94	102	AEALMEELK					Mascot

1057.6001	1057.5671	-0.033	-31	205	214	TAIVEGLAQR		Mascot
1154.678	1154.6058	-0.0722	-63	215	225	IVKGDVPEGLK		Mascot
1320.6464	1320.6748	0.0284	22	94	104	AEALMEELKDR	Oxidation (M)[5]	Mascot
1628.9105	1628.948	0.0375	23	434	448	AIEAEIAKLTEEIAK		Mascot
1628.9105	1628.948	0.0375	23	434	448	AIEAEIAKLTEEIAK		Mascot
1699.8796	1699.9316	0.052	31	281	297	AEGAVDAGNMLKPALAR	Oxidation (M)[10]	Mascot
1888.0651	1888.1011	0.036	19	760	774	EQIRQIVEIQLSYLR		Mascot
1942.9902	1942.983	-0.0072	-4	392	408	LRMALESAPEEIDALER		Mascot
1958.9851	1958.9761	-0.009	-5	392	408	LRMALESAPEEIDALER	Oxidation (M)[3]	Mascot
1994.1071	1994.104	-0.0031	-2	835	854	VQVDVGPAGLVFAVPAR VEA		Mascot
2341.3027	2341.3445	0.0418	18	579	601	AGLKDPNRPIGSFLFLGP TGVGK		Mascot

Gel Idx/Pos	119/E18	Instr./Gel Origin	BA2151/Sample Project 20140814	Process Status	Analysis Succeeded
Plate [#] Name	[1] Sample Project 20140814	Instrument Sample Name		Spectra	11

Rank	Protein Name	Accession No.	Protein MW	Protein PI	Pep. Count	Protein Score	Protein Score C. I. %	Intensity Matched	Total Ion Score	Total Ion C. I. %	Confirmed
1	Alpha-amylase inhibitor 0.19 OS=Triticum aestivum PE=1 SV=1	IAA1_WHEAT	13898.6	6.66	6	191	100	7.628	148	100	

Peptide Information

Calc. Mass	Obsrv. Mass	± da	± ppm	Start Seq.	End Seq.	Sequence	Ion Score	C. I. %	Modification	Rank	Result Type
1162.6249	1162.7152	0.0903	78	90	100	LTAASITAVCR			Carbamidomethyl (C)[10]		Mascot
1162.6249	1162.7152	0.0903	78	90	100	LTAASITAVCR			Carbamidomethyl (C)[10]		Mascot
1570.8007	1570.8739	0.0732	47	26	39	LQCNGSQVPEAVLR			Carbamidomethyl (C)[3]		Mascot
1570.8007	1570.8739	0.0732	47	26	39	LQCNGSQVPEAVLR	44	96.702	Carbamidomethyl (C)[3]		Mascot
1612.7463	1612.8792	0.1329	82	67	82	EHGAQEGQAGTGAFPR					Mascot
1612.7463	1612.8792	0.1329	82	67	82	EHGAQEGQAGTGAFPR	104	100			Mascot
1663.8361	1663.931	0.0949	57	101	116	LPIVVDASGDGAYVCK			Carbamidomethyl (C)[15]		Mascot
1862.7731	1862.9211	0.148	79	40	53	DCCQQLAHISEWCR			Carbamidomethyl (C)[2,3,13]		Mascot
1862.7731	1862.9211	0.148	79	40	53	DCCQQLAHISEWCR			Carbamidomethyl (C)[2,3,13]		Mascot
2807.4431	2807.5427	0.0996	35	90	116	LTAASITAVCRLPIVVDAS GDGAYVCK			Carbamidomethyl (C)[10,26]		Mascot

Rank	Protein Name	Accession No.	Protein MW	Protein PI	Pep. Count	Protein Score	Protein Score C. I. %	Intensity Matched	Total Ion Score	Total Ion C. I. %	Confirmed
2	Alpha-amylase inhibitor 0.53 OS=Triticum aestivum PE=1 SV=1	IAA5_WHEAT	13689.5	5.23	4	69	92.872	3.116	44	96.702	

Peptide Information

Calc. Mass	Obsrv. Mass	± da	± ppm	Start Seq.	End Seq.	Sequence	Ion Score	C. I. %	Modification	Rank	Result Type
1162.6249	1162.7152	0.0903	78	90	100	LTAASITAVCR			Carbamidomethyl (C)[10]		Mascot
1162.6249	1162.7152	0.0903	78	90	100	LTAASITAVCR			Carbamidomethyl (C)[10]		Mascot
1570.8007	1570.8739	0.0732	47	26	39	LQCNGSQVPEAVLR			Carbamidomethyl (C)[3]		Mascot
1570.8007	1570.8739	0.0732	47	26	39	LQCNGSQVPEAVLR	44	96.702	Carbamidomethyl (C)[3]		Mascot
1663.8361	1663.931	0.0949	57	101	116	LPIVVDASGDGAYVCK			Carbamidomethyl (C)[15]		Mascot
2807.4431	2807.5427	0.0996	35	90	116	LTAASITAVCRLPIVVDAS GDGAYVCK			Carbamidomethyl (C)[10,26]		Mascot

Rank	Protein Name	Accession No.	Protein MW	Protein PI	Pep. Count	Protein Score	Protein Score C. I. %	Intensity Matched	Total Ion Score	Total Ion C. I. %	Confirmed
3	Thioredoxin domain-containing protein 2 OS=Homo sapiens GN=TXNDC2 PE=1 SV=4	TXND2_HUMAN	60709	4.81	12	48	0	43.623			

Peptide Information

Calc. Mass	Obsrv. Mass	± da	± ppm	Start Seq.	End Seq.	Sequence	Ion Score	C. I. %	Modification	Rank	Result Type

Calc. Mass	Obsrv. Mass	± da	± ppm	Start Seq.	End Seq.	Sequence	Ion Score	C. I. %	Modification	Rank	Result Type
983.5408	983.6041	0.0633	64	392	400	SLEEAIPPK					Mascot
1570.7959	1570.8739	0.078	50	192	205	EDLPKSSEEAIQPK					Mascot
1570.8435	1570.8739	0.0304	19	272	286	ASVKPSQPKEGDISK					Mascot
1594.8799	1594.8702	-0.0097	-6	251	265	EGDIPKSSAKPIQPK					Mascot
1623.9065	1623.9751	0.0686	42	146	160	ESNIPKSSAKPIQPK					Mascot
1627.8174	1627.9435	0.1261	77	281	295	EGDISKSPEEAIQPK					Mascot
1640.8854	1640.9381	0.0527	32	227	241	TSVKPSQPKESDIPK					Mascot
1641.833	1641.9668	0.1338	81	176	190	EGDIPKAPEETIQSK					Mascot
1641.833	1641.9668	0.1338	81	116	130	EGDIPKAPEETIQSK					Mascot
1653.8694	1653.9658	0.0964	58	332	346	SLEEAIQPKEGDIPK					Mascot
1657.828	1657.9525	0.1245	75	107	121	SSEETIQPKEGDIPK					Mascot
1723.9113	1724.0227	0.1114	65	401	415	EIDIPKSPEETIQPK					Mascot
1862.9041	1862.9211	0.017	9	62	76	EKAFLPMVSHTFHMR			Oxidation (M)[7,14]		Mascot
1862.9041	1862.9211	0.017	9	62	76	EKAFLPMVSHTFHMR			Oxidation (M)[7,14]		Mascot

4	Imidazole glycerol phosphate synthase subunit HisF OS=Uncultured termite group 1 bacterium phylotype Rs-D17 GN=hisF PE=3 SV=1	HIS6_UNCTG	28115.9	7.71	9	43	0	7.147	

Peptide Information

Calc. Mass	Obsrv. Mass	± da	± ppm	Start Seq.	End Seq.	Sequence	Ion Score	C. I. %	Modification	Rank	Result Type
882.5043	882.4459	-0.0584	-66	237	243	EVKEHLK					Mascot
904.5098	904.5393	0.0295	33	100	108	VSLNSSAVK					Mascot
904.5098	904.5393	0.0295	33	100	108	VSLNSSAVK					Mascot
1039.4943	1039.5626	0.0683	66	181	189	DGYDSELLK					Mascot
1570.8622	1570.8739	0.0117	7	6	19	VIPCLDVTDGRVVK			Carbamidomethyl (C)[4]		Mascot
1570.8622	1570.8739	0.0117	7	6	19	VIPCLDVTDGRVVK			Carbamidomethyl (C)[4]		Mascot
1594.8146	1594.8702	0.0556	35	161	176	AAAFGAGEILLTSMDK					Mascot
1612.9269	1612.8792	-0.0477	-30	190	207	AISSSVVIPVIASGGAGK					Mascot
1612.9269	1612.8792	-0.0477	-30	190	207	AISSSVVIPVIASGGAGK					Mascot
1623.814	1623.9751	0.1611	99	134	147	TGEHKWNVFVHGGR					Mascot
1630.8395	1630.9902	0.1507	92	85	99	TTEDIRNLLNAGADK					Mascot
1657.9636	1657.9525	-0.0111	-7	69	84	TAEKVFIPLTVGGGIR					Mascot

5	2,3-bisphosphoglycerate-independent phosphoglycerate mutase OS=Pyrobaculum islandicum (strain DSM 4184 / JCM 9189) GN=apgM PE=3 SV=1	APGM_PYRIL	45076.6	6.19	9	43	0	2.721	

Peptide Information

Calc. Mass	Obsrv. Mass	± da	± ppm	Start Seq.	End Seq.	Sequence	Ion Score	C. I. %	Modification	Rank	Result Type
904.4921	904.5393	0.0472	52	287	294	LAVELMGR			Oxidation (M)[6]		Mascot
904.4921	904.5393	0.0472	52	287	294	LAVELMGR	12	0	Oxidation (M)[6]		Mascot
984.54	984.6108	0.0708	72	137	144	YGVEIIYK					Mascot
1025.6466	1025.5859	-0.0607	-59	247	257	AAAIAGVALIR					Mascot
1552.7689	1552.7949	0.026	17	377	389	FSELTCWRGALGR			Carbamidomethyl (C)[6]		Mascot
1611.816	1611.9656	0.1496	93	262	277	AVGMDVYTARGLGGTK			Oxidation (M)[4]		Mascot
1640.933	1640.9381	0.0051	3	171	185	VGAKLLESRPLENSK					Mascot
1663.7957	1663.931	0.1353	81	94	108	TNLATIDDNGMVLDR			Oxidation (M)[11]		Mascot
1693.8909	1693.9386	0.0477	28	137	150	YGVEIIYKSTVEHR					Mascot
1803.9019	1803.9283	0.0264	15	94	109	TNLATIDDNGMVLDRR					Mascot

6	30S ribosomal protein S7 OS=Wolinella succinogenes (strain ATCC 29543 / DSM 1740 / LMG 7466 / NCTC 11488 / FDC 602W) GN=rpsG PE=3 SV=1	RS7_WOLSU	18087.7	10.14	4	43	0	20.325	25	0	

Peptide Information

Calc. Mass	Obsrv. Mass	± da	± ppm	Start Seq.	End Seq.	Sequence	Ion Score	C. I. %	Modification	Rank	Result Type
858.4832	858.4295	-0.0537	-63	102	108	WLLEAAR					Mascot
1001.556	1001.6293	0.0733	73	68	75	VRPMVEVR			Oxidation (M)[4]		Mascot
1001.556	1001.6293	0.0733	73	68	75	VRPMVEVR	25	0	Oxidation (M)[4]		Mascot
1627.8802	1627.9435	0.0633	39	6	20	APVREVLGDPIYGNK					Mascot
1702.9236	1702.9659	0.0423	25	79	94	VGGATYQVPVEVRATR					Mascot

7	Sulfate/thiosulfate import ATP-binding protein CysA OS=Nocardia farcinica (strain IFM 10152) GN=cysA PE=3 SV=1	CYSA_NOCFA	36210.1	6.38	6	42	0	1.559	25	0	

Peptide Information

Calc. Mass	Obsrv. Mass	± da	± ppm	Start Seq.	End Seq.	Sequence	Ion Score	C. I. %	Modification	Rank	Result Type
904.5032	904.5393	0.0361	40	202	209	IAVMNKGR			Oxidation (M)[4]		Mascot
904.5032	904.5393	0.0361	40	202	209	IAVMNKGR	25	0	Oxidation (M)[4]		Mascot
1013.5738	1013.6171	0.0433	43	64	72	DVTRVAPQK					Mascot
1030.5792	1030.6249	0.0457	44	168	175	ADLRTWLR					Mascot
1552.8959	1552.7949	-0.101	-65	278	290	VVHLGFEVRVELR					Mascot
1611.8966	1611.9656	0.069	43	273	286	ATVERVVHLGFEVR					Mascot

1698.8751	1698.9547	0.0796	47	73	86	RDIGFVFQHYAAFK					Mascot

8	Transmembrane anterior posterior transformation protein 1 homolog OS=Gallus gallus GN=TAPT1 PE=2 SV=2	TAPT1_CHICK	66417	8.73	10	41	0	26.104

Peptide Information

Calc. Mass	Obsrv. Mass	± da	± ppm	Start Seq.	End Seq.	Sequence	Ion Score	C. I. %	Modification	Rank	Result Type
856.5138	856.591	0.0772	90	118	124	IPKELEK					Mascot
883.4858	883.5193	0.0335	38	148	154	VFLAMFR					Mascot
899.4808	899.4022	-0.0786	-87	148	154	VFLAMFR			Oxidation (M)[5]		Mascot
1001.5626	1001.6293	0.0667	67	565	572	KDLLEIDR					Mascot
1001.5626	1001.6293	0.0667	67	565	572	KDLLEIDR	17	0			Mascot
1129.6688	1129.6851	0.0163	14	77	86	RGLSDLSLLR					Mascot
1570.9211	1570.8739	-0.0472	-30	416	429	MGFIPLPLAVLLMR					Mascot
1570.9211	1570.8739	-0.0472	-30	416	429	MGFIPLPLAVLLMR					Mascot
1612.8403	1612.8792	0.0389	24	210	222	LYIIYNMLEVADR					Mascot
1612.8403	1612.8792	0.0389	24	210	222	LYIIYNMLEVADR					Mascot
1623.9292	1623.9751	0.0459	28	325	337	FTNYVLLLIVCLR			Carbamidomethyl (C)[11]		Mascot
1695.8119	1695.8855	0.0736	43	311	324	NNLFQMSNSDIKER					Mascot
1743.0172	1743.0001	-0.0171	-10	415	429	RMGFIPLPLAVLLMR			Oxidation (M)[2]		Mascot
1771.8969	1771.9019	0.005	3	287	301	SLLTIMMSNNFVEIK			Oxidation (M)[6,7]		Mascot

9	ATP synthase subunit delta OS=Verminephrobacter eiseniae (strain EF01-2) GN=atpH PE=3 SV=1	ATPD_VEREI	19453.4	9.09	7	40	0	2.58

Peptide Information

Calc. Mass	Obsrv. Mass	± da	± ppm	Start Seq.	End Seq.	Sequence	Ion Score	C. I. %	Modification	Rank	Result Type
875.4767	875.4564	-0.0203	-23	172	178	ARLEQMK					Mascot
1345.7209	1345.7061	-0.0148	-11	159	171	VVVGDEVLDSSVK					Mascot
1679.9368	1679.8894	-0.0474	-28	54	68	VTADQVFALFTQVLK					Mascot
1693.9596	1693.9386	-0.021	-12	143	158	LNLAVQQDPSLIGGIR					Mascot
1702.8694	1702.9659	0.0965	57	69	84	DAARAALPEMAGNFLR					Mascot
1895.0095	1894.9135	-0.096	-51	1	17	MAELATIARPYAEALFK					Mascot
2093.1389	2093.2698	0.1309	63	54	72	VTADQVFALFTQVLKDAA R					Mascot

10	DNA gyrase subunit B OS=Spiroplasma citri GN=gyrB PE=3 SV=1	GYRB_SPICI	72889.1	6.45	14	39	0	66.623

Peptide Information

Calc. Mass	Obsrv. Mass	± da	± ppm	Start Seq.	End Seq.	Sequence	Ion Score	C. I. %	Modification	Rank	Result Type
850.4669	850.4578	-0.0091	-11	634	640	YVKNLDV					Mascot
930.5618	930.5845	0.0227	24	345	352	LLNSEVKK					Mascot
957.588	957.5973	0.0093	10	445	452	FQAILPLR					Mascot
959.4905	959.4135	-0.077	-80	541	548	VENSNQIR					Mascot
1001.5625	1001.6293	0.0668	67	561	568	EELLKQNK					Mascot
1001.5625	1001.6293	0.0668	67	561	568	EELLKQNK					Mascot
1296.6947	1296.7959	0.1012	78	216	226	TVLYQFNNGIK					Mascot
1296.6947	1296.7959	0.1012	78	216	226	TVLYQFNNGIK					Mascot
1479.8318	1479.9012	0.0694	47	290	301	LAIVREINNYFK					Mascot
1623.8967	1623.9751	0.0784	48	512	523	ILLLTFFYRYMK			Oxidation (M)[11]		Mascot
1641.8265	1641.9668	0.1403	85	497	511	VIIMTDADVDGAHIR			Oxidation (M)[4]		Mascot
1641.8265	1641.9668	0.1403	85	497	511	VIIMTDADVDGAHIR			Oxidation (M)[4]		Mascot
1663.8538	1663.931	0.0772	46	361	375	GLSSYLLENPEDAKK					Mascot
1665.9283	1665.9174	-0.0109	-7	110	127	ISGGLHGVGASVVNALSK					Mascot
1665.9283	1665.9174	-0.0109	-7	110	127	ISGGLHGVGASVVNALSK					Mascot
1679.8486	1679.8894	0.0408	24	422	438	IAELYIVEGDSAGGSAK					Mascot
1799.9862	1800.0577	0.0715	40	60	75	IILNKDESITVIDNGR					Mascot
1862.947	1862.9211	-0.0259	-14	140	155	YVMEFHNGGQILTPIK			Oxidation (M)[3]		Mascot
1862.947	1862.9211	-0.0259	-14	140	155	YVMEFHNGGQILTPIK			Oxidation (M)[3]		Mascot

Gel Idx/Pos	120/E19	Instr./Gel Origin	BA2151/Sample Project 20140814	Process Status	Analysis Succeeded
Plate [#] Name	[1] Sample Project 20140814	Instrument Sample Name		Spectra	11

Rank	Protein Name	Accession No.	Protein MW	Protein PI	Pep. Count	Protein Score	Protein Score C. I. %	Intensity Matched	Total Ion Score	Total Ion C. I. %	Confirmed
1	Alpha-amylase/trypsin inhibitor CM3 OS=Triticum aestivum PE=1 SV=1	IAAC3_WHEAT	18893.3	7.44	8	583	100	57.249	531	100	

Peptide Information

Calc. Mass	Obsrv. Mass	± da	± ppm	Start Seq.	End Seq.	Sequence	Ion Score	C. I. %	Modification	Rank	Result Type
1010.52	1010.5914	0.0714	71	37	44	TNLLPHCR			Carbamidomethyl (C)[7]		Mascot
1010.52	1010.5914	0.0714	71	37	44	TNLLPHCR	42	93.98	Carbamidomethyl (C)[7]		Mascot
1110.5038	1110.5851	0.0813	73	133	140	EMQWDFVR					Mascot
1126.4987	1126.5664	0.0677	60	133	140	EMQWDFVR			Oxidation (M)[2]		Mascot
1126.4987	1126.5664	0.0677	60	133	140	EMQWDFVR	57	99.814	Oxidation (M)[2]		Mascot
1698.9214	1699.0544	0.133	78	101	115	YFIALPVPSQPVDPR					Mascot
1698.9214	1699.0544	0.133	78	101	115	YFIALPVPSQPVDPR	88	100			Mascot
1727.8381	1727.9695	0.1314	76	116	132	SGNVGESGLIDLPGCPR			Carbamidomethyl (C)[15]		Mascot
1727.8381	1727.9695	0.1314	76	116	132	SGNVGESGLIDLPGCPR	122	100	Carbamidomethyl (C)[15]		Mascot
1801.8427	1801.9408	0.0981	54	45	60	DYVLQQTCGTFTPGSK			Carbamidomethyl (C)[8]		Mascot
1876.0222	1876.1837	0.1615	86	141	157	LLVAPGQCNLATIHNVR			Carbamidomethyl (C)[8]		Mascot
1876.0222	1876.1837	0.1615	86	141	157	LLVAPGQCNLATIHNVR	98	100	Carbamidomethyl (C)[8]		Mascot
1957.8564	1958.0208	0.1644	84	81	95	LYCCQELAEISQQCR			Carbamidomethyl (C)[3,4,14]		Mascot
1957.8564	1958.0208	0.1644	84	81	95	LYCCQELAEISQQCR	124	100	Carbamidomethyl (C)[3,4,14]		Mascot
2255.1416	2255.2786	0.137	61	61	80	LPEWMTSASIYSPGKPYLAK			Oxidation (M)[5]		Mascot

Rank	Protein Name	Accession No.	Protein MW	Protein PI	Pep. Count	Protein Score	Protein Score C. I. %	Intensity Matched	Total Ion Score	Total Ion C. I. %	Confirmed
2	Alpha-amylase/trypsin inhibitor CMd OS=Hordeum vulgare GN=IAT3 PE=1 SV=2	IAAD_HORVU	19140.3	6.07	2	106	99.999	17.475	98	100	

Peptide Information

Calc. Mass	Obsrv. Mass	± da	± ppm	Start Seq.	End Seq.	Sequence	Ion Score	C. I. %	Modification	Rank	Result Type
1876.0222	1876.1837	0.1615	86	144	160	LLVAPGQCNLATIHNVR			Carbamidomethyl (C)[8]		Mascot
1876.0222	1876.1837	0.1615	86	144	160	LLVAPGQCNLATIHNVR	98	100	Carbamidomethyl (C)[8]		Mascot
1967.8772	1968.0333	0.1561	79	84	98	LYCCQELAEIPQQCR			Carbamidomethyl (C)[3,4,14]		Mascot

Rank	Protein Name	Accession No.	Protein MW	Protein PI	Pep. Count	Protein Score	Protein Score C. I. %	Intensity Matched	Total Ion Score	Total Ion C. I. %	Confirmed
3	Uncharacterized RNA methyltransferase BH0687 OS=Bacillus halodurans (strain ATCC BAA-125 / DSM 18197 / FERM 7344 / JCM 9153 / C-125) GN=BH0687 PE=3 SV=1	Y687_BACHD	51921.9	8.02	14	60	40.71	3.224			

Peptide Information

Calc. Mass	Obsrv. Mass	± da	± ppm	Start Seq.	End Seq.	Sequence	Ion Score	C. I. %	Modification	Rank	Result Type
1008.6061	1008.5428	-0.0633	-63	198	206	GTLRHVVAR					Mascot
1049.5739	1049.5916	0.0177	17	253	261	RTNVIFGDK					Mascot
1110.5314	1110.5851	0.0537	48	425	434	VLEDGGYETK					Mascot
1130.5986	1130.563	-0.0356	-31	1	10	MSKQQAPVNK					Mascot
1146.5936	1146.6166	0.023	20	1	10	MSKQQAPVNK			Oxidation (M)[1]		Mascot
1154.6641	1154.5677	-0.0964	-83	244	253	SIVQNVNPKR					Mascot
1709.9255	1709.9583	0.0328	19	207	221	YGKNTGEIMVVLITR			Oxidation (M)[9]		Mascot
1726.9011	1727.0719	0.1708	99	334	349	HVYGVEIVPEAISDAK					Mascot
1883.0022	1883.1118	0.1096	58	334	350	HVYGVEIVPEAISDAKR					Mascot
1897.9225	1898.1085	0.186	98	57	73	GYGFGRVLNMIEASPDR			Oxidation (M)[10]		Mascot
1913.9531	1914.045	0.0919	48	264	279	VLWGEEYIYDTIGDIK					Mascot
1941.0375	1941.0542	0.0167	9	115	131	ITAVTVRPTIGMNEPWR					Mascot
1974.0146	1973.9926	-0.022	-11	392	408	GCDEALLKTILNMKPDR			Carbamidomethyl (C)[2]		Mascot
1986.0079	1986.0424	0.0345	17	280	296	FAISARSFYQVNPEQTK					Mascot
2255.2329	2255.2786	0.0457	20	112	131	IGKITAVTVRPTIGMNEP WR			Oxidation (M)[15]		Mascot

4	Putative cobalt-precorrin-6A synthase [deacetylating] OS=Agrobacterium tumefaciens (strain C58 / ATCC 33970) GN=cbiD PE=3 SV=2	CBID_AGRT5	39291.4	6.03	11	56	0	3.545	

Peptide Information

Calc. Mass	Obsrv. Mass	± da	± ppm	Start Seq.	End Seq.	Sequence	Ion Score	C. I. %	Modification	Rank	Result Type
815.4985	815.4954	-0.0031	-4	250	258	VTIAGGVAK					Mascot
919.4843	919.5724	0.0881	96	2	9	ETDGKTLR					Mascot
1001.5778	1001.6198	0.042	42	326	335	AWVTAAAALK					Mascot
1066.5198	1066.5786	0.0588	55	1	9	METDGKTLR			Oxidation (M)[1]		Mascot
1156.6222	1156.5559	-0.0663	-57	241	249	YLRSHPVER					Mascot
1176.6121	1176.6429	0.0308	26	88	98	RGEPGSGITFR					Mascot
1176.6121	1176.6429	0.0308	26	88	98	RGEPGSGITFR	9	0			Mascot
1198.6249	1198.6265	0.0016	1	262	272	LAQGMLDVHSK					Mascot
1590.7979	1590.9554	0.1575	99	259	272	MTKLAQGMLDVHSK			Oxidation (M)[1,8]		Mascot
1941.1168	1941.0542	-0.0626	-32	336	353	TPAIALDILVFDRQGALK					Mascot
1967.9305	1968.0333	0.1028	52	69	87	DAGDDPDVTHGALIESTV R					Mascot

Calc. Mass	Obsrv. Mass	± da	± ppm	Start Seq.	End Seq.	Sequence	Ion Score	C. I. %	Modification	Rank	Result Type
1985.9425	1986.0424	0.0999	50	354	372	GRTTSTPSHQPAPSSFGDR					Mascot

5	Myotrophin OS=Xenopus laevis GN=mtpn PE=3 SV=1	MTPN_XENLA	12907.4	5.27	7	50	0	1.088	

Peptide Information

Calc. Mass	Obsrv. Mass	± da	± ppm	Start Seq.	End Seq.	Sequence	Ion Score	C. I. %	Modification	Rank	Result Type
981.5074	981.5356	0.0282	29	83	90	CVELFVSK			Carbamidomethyl (C)[1]		Mascot
1109.6024	1109.5797	-0.0227	-20	82	90	KCVELFVSK			Carbamidomethyl (C)[2]		Mascot
1162.5852	1162.6079	0.0227	20	20	30	EFVAGGVDVNR					Mascot
1710.8381	1710.9543	0.1162	68	67	81	HGITPLLSACYEGHR			Carbamidomethyl (C)[10]		Mascot
1736.8677	1736.9744	0.1067	61	5	19	EFMWALKNGDLDAVK					Mascot
1752.8625	1752.9395	0.077	44	5	19	EFMWALKNGDLDAVK			Oxidation (M)[3]		Mascot
1775.9036	1775.9755	0.0719	40	20	36	EFVAGGVDVNRTLEGGR					Mascot
2255.094	2255.2786	0.1846	82	98	118	GPDGLNAFESTDNQAIKDLLH					Mascot

6	tRNA pseudouridine synthase A OS=Pelobacter carbinolicus (strain DSM 2380 / Gra Bd 1) GN=truA PE=3 SV=1	TRUA_PELCD	27932.1	8.98	10	50	0	16.967	

Peptide Information

Calc. Mass	Obsrv. Mass	± da	± ppm	Start Seq.	End Seq.	Sequence	Ion Score	C. I. %	Modification	Rank	Result Type
1062.5613	1062.583	0.0217	20	133	141	TVLDLQAMR			Oxidation (M)[8]		Mascot
1136.5994	1136.6389	0.0395	35	188	197	GSGFLRNMVR					Mascot
1136.5994	1136.6389	0.0395	35	188	197	GSGFLRNMVR					Mascot
1140.6161	1140.5767	-0.0394	-35	112	120	YSIYRSPVR					Mascot
1142.6099	1142.5651	-0.0448	-39	117	126	SPVRSPLACR			Carbamidomethyl (C)[9]		Mascot
1173.666	1173.5831	-0.0829	-71	198	208	IMVGTLVEVGR					Mascot
1727.8317	1727.9695	0.1378	80	209	225	GARPPEEVGAMLAGGCR			Carbamidomethyl (C)[16]		Mascot
1727.8317	1727.9695	0.1378	80	209	225	GARPPEEVGAMLAGGCR			Carbamidomethyl (C)[16]		Mascot
1743.8265	1743.9325	0.106	61	209	225	GARPPEEVGAMLAGGCR			Carbamidomethyl (C)[16], Oxidation (M)[11]		Mascot
1809.9706	1809.9728	0.0022	1	21	37	QPNGLSVQQVVEEALAK					Mascot
1874.9695	1875.1484	0.1789	95	127	141	FSWHVRTVLDLQAMR			Oxidation (M)[14]		Mascot
1979.928	1979.9819	0.0539	27	91	108	DAALMPDDFHARYSAVGK			Oxidation (M)[5]		Mascot
1986.0089	1986.0424	0.0335	17	59	76	GMVAHFVTPQPLPMAAYR					Mascot

7	Elongation factor G OS=Carsonella ruddii (strain PV) GN=fusA PE=3 SV=1	EFG_CARRP	77441.8	9.07	15	49	0	7.453	

Peptide Information

Calc. Mass	Obsrv. Mass	± da	± ppm	Start Seq.	End Seq.	Sequence	Ion Score	C. I. %	Modification	Rank	Result Type
1010.6245	1010.5914	-0.0331	-33	517	526	IEVVGGVIPK					Mascot
1010.6245	1010.5914	-0.0331	-33	517	526	IEVVGGVIPK					Mascot
1066.6659	1066.5786	-0.0873	-82	297	305	SKFLALLFK					Mascot
1085.5626	1085.6533	0.0907	84	227	235	YINSNFSIK					Mascot
1092.63	1092.592	-0.038	-35	145	153	YLSIIENIK					Mascot
1156.5746	1156.5559	-0.0187	-16	201	209	NFDISNKYR					Mascot
1158.6552	1158.5547	-0.1005	-87	250	260	IIPIACGSSLK			Carbamidomethyl (C)[6]		Mascot
1158.6552	1158.5547	-0.1005	-87	250	260	IIPIACGSSLK			Carbamidomethyl (C)[6]		Mascot
1182.6306	1182.6119	-0.0187	-16	27	36	ILFFSGFSHK					Mascot
1590.9075	1590.9554	0.0479	30	6	20	NIRNIGIIAHVDAGK					Mascot
1711	1710.9543	-0.0457	-27	355	371	DLNIASAGDIVVLIGLK					Mascot
1714.9486	1715.0264	0.0778	45	325	339	IEPGQIIFNNSKNIK					Mascot
1775.9943	1775.9755	-0.0188	-11	511	526	DDFIFKIEVVGGVIPK					Mascot
1809.9996	1809.9728	-0.0268	-15	527	541	EYFLSIEKGILEQIK					Mascot
1912.0175	1912.0106	-0.0069	-4	227	242	YINSNFSIKDIIESIR					Mascot
1974.1093	1973.9926	-0.1167	-59	535	552	GILEQIKCGVVLGYPVTK			Carbamidomethyl (C)[8]		Mascot
2029.1403	2029.2979	0.1576	78	587	604	ANSFLLEPIMKVEIISPK					Mascot

8	ATP-dependent RNA helicase rok1 OS=Neosartorya fischeri (strain ATCC 1020 / DSM 3700 / FGSC A1164 / NRRL 181) GN=rok1 PE=3 SV=1	ROK1_NEOFI	82293.2	9.21	13	49	0	29.114

Peptide Information

Calc. Mass	Obsrv. Mass	± da	± ppm	Start Seq.	End Seq.	Sequence	Ion Score	C. I. %	Modification	Rank	Result Type
832.4457	832.3792	-0.0665	-80	316	322	GMRVVDR					Mascot
1010.5266	1010.5914	0.0648	64	693	701	ISTKSGFDR					Mascot
1010.5266	1010.5914	0.0648	64	693	701	ISTKSGFDR					Mascot
1094.6027	1094.5773	-0.0254	-23	1	9	MDAFKLLTR					Mascot
1110.5977	1110.5851	-0.0126	-11	1	9	MDAFKLLTR			Oxidation (M)[1]		Mascot
1176.5604	1176.6429	0.0825	70	15	27	GGAAPSSAQSSTR					Mascot
1176.5604	1176.6429	0.0825	70	15	27	GGAAPSSAQSSTR					Mascot
1613.0149	1612.8743	-0.1406	-87	256	269	TLSFLIPVINKIVR					Mascot
1699.0054	1699.0544	0.049	29	505	518	LRPPFLIFTQTIPR					Mascot
1699.0054	1699.0544	0.049	29	505	518	LRPPFLIFTQTIPR	3	0			Mascot

Calc. Mass	Obsrv. Mass	± da	± ppm	Start Seq.	End Seq.	Sequence	Ion Score	C. I. %	Modification	Rank	Result Type
1720.8535	1721.0106	0.1571	91	134	147	VTDMRDLEEIQTVR			Oxidation (M)[4]		Mascot
1726.9739	1727.0719	0.098	57	647	661	SIQKWLLDALPDLSK					Mascot
1728.0055	1727.9695	-0.036	-21	561	575	KGEIWILVTTDLLAR					Mascot
1728.0055	1727.9695	-0.036	-21	561	575	KGEIWILVTTDLLAR	13	0			Mascot
1809.8688	1809.9728	0.104	57	423	439	ASLWSATMGSNIEDLAK			Oxidation (M)[8]		Mascot
1873.9906	1874.1099	0.1193	64	391	406	NIVLDEADVLLDELFR					Mascot
1897.9753	1898.1085	0.1332	70	237	255	DAESIEPDLLVVAPTGSGK					Mascot
1900.9498	1900.9803	0.0305	16	139	154	DLEEIQTVRVESEEPK					Mascot

9	50S ribosomal protein L11 OS=Methanocorpusculum labreanum (strain ATCC 43576 / DSM 4855 / Z) GN=rpl11 PE=3 SV=1	RL11_METLZ	16176.5	5.22	8	49	0	1.878	

Peptide Information

Calc. Mass	Obsrv. Mass	± da	± ppm	Start Seq.	End Seq.	Sequence	Ion Score	C. I. %	Modification	Rank	Result Type
873.4676	873.5439	0.0763	87	33	40	AVVDDINK					Mascot
1001.5626	1001.6198	0.0572	57	33	41	AVVDDINKK					Mascot
1685.813	1685.9717	0.1587	94	142	157	QAIAAVNAGEWDEQLA					Mascot
1883.1035	1883.1118	0.0083	4	60	77	KNVTLTVGIPPTTALVMK					Mascot
1939.9141	1940.0787	0.1646	85	42	59	TAEFNGMSVPVTVMVDDK					Mascot
1955.9089	1956.0895	0.1806	92	42	59	TAEFNGMSVPVTVMVDDK			Oxidation (M)[7]		Mascot
1967.9778	1968.0333	0.0555	28	123	141	EVMGTCVSVGVTVEGKTAK			Carbamidomethyl (C)[6], Oxidation (M)[3]		Mascot
1971.9039	1972.0272	0.1233	63	42	59	TAEFNGMSVPVTVMVDDK			Oxidation (M)[7,14]		Mascot
1985.9927	1986.0424	0.0497	25	139	157	TAKQAIAAVNAGEWDEQLA					Mascot
1994.0665	1994.1057	0.0392	20	84	103	GSGTPNTQAVGNLPLEAVIR					Mascot

10	Proteasome-activating nucleotidase OS=Archaeoglobus fulgidus (strain ATCC 49558 / VC-16 / DSM 4304 / JCM 9628 / NBRC 100126) GN=pan PE=1 SV=1	PAN_ARCFU	44993.1	5.43	13	49	0	9.241	

Peptide Information

Calc. Mass	Obsrv. Mass	± da	± ppm	Start Seq.	End Seq.	Sequence	Ion Score	C. I. %	Modification	Rank	Result Type
1001.5989	1001.6198	0.0209	21	77	86	VVVKSSTGPK					Mascot
1012.6037	1012.5822	-0.0215	-21	385	393	KTTPIPDLK					Mascot
1049.5739	1049.5916	0.0177	17	36	43	FIESERIR					Mascot
1061.5474	1061.6239	0.0765	72	342	352	ITEGASGADIK					Mascot
1108.4978	1108.5729	0.0751	68	250	259	RTNSDTSGDR					Mascot

1158.6517	1158.5547	-0.097	-84	177	188	GVLLYGPPGTGK		Mascot
1158.6517	1158.5547	-0.097	-84	177	188	GVLLYGPPGTGK		Mascot
1535.8315	1535.9565	0.125	81	2	14	GDSEIQYLLEKLK		Mascot
1682.8669	1682.9562	0.0893	53	1	14	MGDSEIQYLLEKLK	Oxidation (M)[1]	Mascot
1714.9374	1715.0264	0.089	52	234	249	APSIIFIDELDAIAAR		Mascot
1736.8346	1736.9744	0.1398	80	264	278	TMMQLLAELDGFDPR		Mascot
1752.8296	1752.9395	0.1099	63	264	278	TMMQLLAELDGFDPR	Oxidation (M)[2]	Mascot
1867.0548	1867.0076	-0.0472	-25	290	305	IDILDPAILRPGRFDR		Mascot
1871.0386	1871.0052	-0.0334	-18	234	250	APSIIFIDELDAIAARR		Mascot
1972.075	1972.0272	-0.0478	-24	232	249	EKAPSIIFIDELDAIAAR		Mascot

Gel Idx/Pos	121/E20	Instr./Gel Origin	BA2151/Sample Project 20140814	Process Status	Analysis Succeeded
Plate [#] Name	[1] Sample Project 20140814	Instrument Sample Name		Spectra	11

Rank	Protein Name	Accession No.	Protein MW	Protein PI	Pep. Count	Protein Score	Protein Score C. I. %	Intensity Matched	Total Ion Score	Total Ion C. I. %	Confirmed
1	Alpha-amylase inhibitor 0.19 OS=Triticum aestivum PE=1 SV=1	IAA1_WHEAT	13898.6	6.66	6	283	100	27.661	243	100	

Peptide Information

Calc. Mass	Obsrv. Mass	± da	± ppm	Start Seq.	End Seq.	Sequence	Ion Score	C. I. %	Modification	Rank	Result Type
1162.6249	1162.7305	0.1056	91	90	100	LTAASITAVCR			Carbamidomethyl (C)[10]		Mascot
1570.8007	1570.9443	0.1436	91	26	39	LQCNGSQVPEAVLR			Carbamidomethyl (C)[3]		Mascot
1612.7463	1612.8998	0.1535	95	67	82	EHGAQEGQAGTGAFPR					Mascot
1612.7463	1612.8998	0.1535	95	67	82	EHGAQEGQAGTGAFPR	141	100			Mascot
1617.8993	1617.8964	-0.0029	-2	86	100	EVVKLTAASITAVCR			Carbamidomethyl (C)[14]		Mascot
1663.8361	1663.9633	0.1272	76	101	116	LPIVVDASGDGAYVCK			Carbamidomethyl (C)[15]		Mascot
1862.7731	1862.9509	0.1778	95	40	53	DCCQQLAHISEWCR			Carbamidomethyl (C)[2,3,13]		Mascot
1862.7731	1862.9509	0.1778	95	40	53	DCCQQLAHISEWCR	102	100	Carbamidomethyl (C)[2,3,13]		Mascot

Rank	Protein Name	Accession No.	Protein MW	Protein PI	Pep. Count	Protein Score	Protein Score C. I. %	Intensity Matched	Total Ion Score	Total Ion C. I. %	Confirmed
2	30S ribosomal protein S4, chloroplastic (Fragment) OS=Lygeum spartum GN=rps4 PE=3 SV=1	RR4_LYGSP	22769.9	11.16	9	54	0	3.662			

Peptide Information

Calc. Mass	Obsrv. Mass	± da	± ppm	Start Seq.	End Seq.	Sequence	Ion Score	C. I. %	Modification	Rank	Result Type
864.4686	864.3842	-0.0844	-98	42	47	EQYRIR					Mascot
1555.8928	1555.8473	-0.0455	-29	109	121	QLVNHRHILVNGR					Mascot
1570.8839	1570.9443	0.0604	38	160	172	LPKHLTIDTLEYK					Mascot
1587.8337	1587.86	0.0263	17	144	159	SKGLVQNSIASSDPGK					Mascot
1674.9465	1674.9434	-0.0031	-2	183	196	WVGLKINELLVVEY					Mascot
1710.9385	1711.0081	0.0696	41	146	162	GLVQNSIASSDPGKLPK					Mascot
1743.964	1743.9984	0.0344	20	163	177	HLTIDTLEYKGLVNK					Mascot
1820.9912	1820.9557	-0.0355	-19	98	114	LGMASTIPGARQLVNHR					Mascot
1961.0637	1961.0715	0.0078	4	91	108	LDNILFRLGMASTIPGAR			Oxidation (M)[10]		Mascot

Rank	Protein Name	Accession No.	Protein MW	Protein PI	Pep. Count	Protein Score	Protein Score C. I. %	Intensity Matched	Total Ion Score	Total Ion C. I. %	Confirmed
3	30S ribosomal protein S4, chloroplastic (Fragment) OS=Melica uniflora GN=rps4 PE=3 SV=1	RR4_MELUN	22689.8	10.97	9	53	0	3.662			

Protein Group

30S ribosomal protein S4, chloroplastic (Fragment)	RR4_MELAL	22705.8	10.970

OS=Melica altissima GN=rps4 PE=3 SV=1 000267 0288

Peptide Information

Calc. Mass	Obsrv. Mass	± da	± ppm	Start Seq.	End Seq.	Sequence	Ion Score	C. I. %	Modification	Rank	Result Type
864.4686	864.3842	-0.0844	-98	42	47	EQYRIR					Mascot
1555.8928	1555.8473	-0.0455	-29	109	121	QLVNHRHILVNGR					Mascot
1570.8839	1570.9443	0.0604	38	160	172	LPKHLTIDTLEYK					Mascot
1587.8337	1587.86	0.0263	17	144	159	SKGLVQNSIASSDPGK					Mascot
1674.9465	1674.9434	-0.0031	-2	183	196	WVGLKINELLVVEY					Mascot
1710.9385	1711.0081	0.0696	41	146	162	GLVQNSIASSDPGKLPK					Mascot
1743.964	1743.9984	0.0344	20	163	177	HLTIDTLEYKGLVNK					Mascot
1820.9912	1820.9557	-0.0355	-19	98	114	LGMASTIPGARQLVNHR					Mascot
1961.0637	1961.0715	0.0078	4	91	108	LDNILFRLGMASTIPGAR			Oxidation (M)[10]		Mascot

4	4-hydroxy-3-methylbut-2-enyl diphosphate reductase OS=Dehalococcoides sp. (strain CBDB1) GN=ispH PE=3 SV=1	ISPH_DEHSC	30656.9	6.24	10	51	0	4.287	

Peptide Information

Calc. Mass	Obsrv. Mass	± da	± ppm	Start Seq.	End Seq.	Sequence	Ion Score	C. I. %	Modification	Rank	Result Type
898.4815	898.4229	-0.0586	-65	226	232	HLLDLCK			Carbamidomethyl (C)[6]		Mascot
1045.6187	1045.6442	0.0255	24	48	57	LSGMGVRVVK					Mascot
1478.756	1478.902	0.146	99	86	98	GLEIVDTTCPFVK			Carbamidomethyl (C)[9]		Mascot
1583.8751	1583.9449	0.0698	44	174	187	NVIDQALVKDAEIR					Mascot
1634.8571	1634.8708	0.0137	8	86	99	GLEIVDTTCPFVKR			Carbamidomethyl (C)[9]		Mascot
1712.8749	1712.9332	0.0583	34	210	225	VDLMLVIGGHNSANTR			Oxidation (M)[4]		Mascot
1743.8306	1743.9984	0.1678	96	2	16	KVECASNIGFCFGVR			Carbamidomethyl (C)[4,11]		Mascot
1771.8368	1771.9348	0.098	55	3	17	VECASNIGFCFGVRR			Carbamidomethyl (C)[3,10]		Mascot
1771.8368	1771.9348	0.098	55	3	17	VECASNIGFCFGVRR			Carbamidomethyl (C)[3,10]		Mascot
1890.866	1890.9769	0.1109	59	1	16	MKVECASNIGFCFGVR			Carbamidomethyl (C)[5,12], Oxidation (M)[1]		Mascot
2449.2905	2449.3445	0.054	22	25	47	TASERGGVETLGALVHN QQVLNR					Mascot

5	Thiamine-monophosphate kinase OS=Aquifex aeolicus (strain VF5) GN=thiL PE=1 SV=1	THIL_AQUAE	34618.9	5.37	11	51	0	4.616	

Peptide Information

Calc. Mass	Obsrv. Mass	± da	± ppm	Start Seq.	End Seq.	Sequence	Ion Score	C. I. %	Modification	Rank	Result Type

Calc. Mass	Obsrv. Mass	± da	± ppm	Start Seq.	End Seq.	Sequence	Ion Score	C. I. %	Modification	Rank	Result Type
817.3549	817.416	0.0611	75	107	112	ACEFYK			Carbamidomethyl (C)[2]		Mascot
850.5005	850.4699	-0.0306	-36	185	191	HLRPTAR					Mascot
882.5196	882.4621	-0.0575	-65	100	106	FYIGVKR					Mascot
1509.7544	1509.8857	0.1313	87	147	161	LGDSVFVSGTLGDSR					Mascot
1553.7152	1553.8669	0.1517	98	23	36	VIGDDTAPVEYCSK			Carbamidomethyl (C)[12]		Mascot
1587.9357	1587.86	-0.0757	-48	3	16	LKELGEFGLIDLIK					Mascot
1640.8022	1640.9122	0.11	67	234	246	LPLSNELKMYCEK			Carbamidomethyl (C)[11], Oxidation (M)[9]		Mascot
1653.917	1653.9619	0.0449	27	64	80	AISVNVSDVIANGGLPK					Mascot
1860.8619	1860.9423	0.0804	43	107	122	ACEFYKCEVVGGNISK			Carbamidomethyl (C)[2,7]		Mascot
1879.0437	1878.9403	-0.1034	-55	126	142	IGISVFLVGETERFVGR					Mascot
1968.1277	1968.0828	-0.0449	-23	37	53	KLLLTTDVLNEGVHFLR					Mascot

6	Cytochrome c oxidase subunit 8A, mitochondrial OS=Homo sapiens GN=COX8A PE=1 SV=2	COX8A_HUMAN	7631.2	10.27	6	50	0	1.86	

Peptide Information

Calc. Mass	Obsrv. Mass	± da	± ppm	Start Seq.	End Seq.	Sequence	Ion Score	C. I. %	Modification	Rank	Result Type
817.4639	817.416	-0.0479	-59	11	18	GLTGSARR					Mascot
1011.6561	1011.6255	-0.0306	-30	2	10	SVLTPLLLR					Mascot
1142.6967	1142.6034	-0.0933	-82	1	10	MSVLTPLLLR					Mascot
1158.6915	1158.5983	-0.0932	-80	1	10	MSVLTPLLLR			Oxidation (M)[1]		Mascot
1176.6736	1176.6868	0.0132	11	24	34	AKIHSLPPEGK					Mascot
1654.0011	1653.9619	-0.0392	-24	2	17	SVLTPLLLRGLTGSAR					Mascot
1785.0415	1785.1989	0.1574	88	1	17	MSVLTPLLLRGLTGSAR					Mascot

7	DNA mismatch repair protein MutS OS=Rhodopseudomonas palustris (strain ATCC BAA-98 / CGA009) GN=mutS PE=3 SV=1	MUTS_RHOPA	97982.3	5.77	17	50	0	12.542	

Peptide Information

Calc. Mass	Obsrv. Mass	± da	± ppm	Start Seq.	End Seq.	Sequence	Ion Score	C. I. %	Modification	Rank	Result Type
882.4203	882.4621	0.0418	47	483	490	YADDTGIK					Mascot
1110.5062	1110.6162	0.11	99	226	235	DVFDSATAER					Mascot
1142.6099	1142.6034	-0.0065	-6	370	380	AAPDMARALAR					Mascot
1158.6049	1158.5983	-0.0066	-6	370	380	AAPDMARALAR			Oxidation (M)[5]		Mascot
1174.5997	1174.6041	0.0044	4	366	376	SSLRAAPDMAR					Mascot
1176.6848	1176.6868	0.002	2	619	628	HPVVEQALKR					Mascot
1480.8093	1480.9141	0.1048	71	514	526	LMAPPLNATFIHR					Mascot

Calc. Mass	Obsrv. Mass	± da	± ppm	Start Seq.	End Seq.	Sequence	Ion Score	C. I. %	Modification	Rank	Result Type
1480.8093	1480.9141	0.1048	71	514	526	LMAPPLNATFIHR					Mascot
1599.8312	1599.9589	0.1277	80	648	662	NGQIWLLTGPNMAGK					Mascot
1610.7843	1610.8864	0.1021	63	255	269	LEATAAAACVTYVDR			Carbamidomethyl (C)[9]		Mascot
1650.8091	1650.8472	0.0381	23	74	88	HLGADIPMCGVPVER			Carbamidomethyl (C)[9]		Mascot
1653.9323	1653.9619	0.0296	18	578	594	AAAHAFALLDVATALAK					Mascot
1724.8966	1725.0562	0.1596	93	796	811	GEVVFLHEVLPGSADR					Mascot
1743.8442	1743.9984	0.1542	88	103	118	VAVCEQTEDPAAARAR			Carbamidomethyl (C)[4]		Mascot
1837.9478	1837.9567	0.0089	5	474	490	LVVASMQARYADDTGIK					Mascot
1853.9426	1853.9149	-0.0277	-15	474	490	LVVASMQARYADDTGIK			Oxidation (M)[6]		Mascot
1862.939	1862.9509	0.0119	6	311	328	RGSLLDAIDCTVTAAGSR			Carbamidomethyl (C)[10]		Mascot
1862.939	1862.9509	0.0119	6	311	328	RGSLLDAIDCTVTAAGSR			Carbamidomethyl (C)[10]		Mascot
1910.8879	1910.9321	0.0442	23	450	466	DGGFVREGYEPALDETR					Mascot
2254.1536	2254.3462	0.1926	85	881	900	ALHPDEMTPREALDALYA LK					Mascot
2334.2864	2334.2954	0.009	4	514	534	LMAPPLNATFIHRQTLAG QVR					Mascot

8	Aspartyl/glutamyl-tRNA(Asn/Gln) amidotransferase subunit C OS=Heliobacterium modesticaldum (strain ATCC 51547 / Ice1) GN=gatC PE=3 SV=1	GATC_HELMI	10806.5	4.7	7	50	0	2.969	

Peptide Information

Calc. Mass	Obsrv. Mass	± da	± ppm	Start Seq.	End Seq.	Sequence	Ion Score	C. I. %	Modification	Rank	Result Type
1174.595	1174.6041	0.0091	8	17	26	LELSEADLER					Mascot
1593.7544	1593.8995	0.1451	91	76	89	IVANAPEEEDGFFR					Mascot
1669.8545	1669.9221	0.0676	40	27	40	YTTQLNAILDYAQR					Mascot
1680.8989	1680.9149	0.016	10	2	16	ALTKAEVEYVAMLAR			Oxidation (M)[12]		Mascot
1845.9429	1845.9938	0.0509	28	48	63	DVPPTAHVFPLHNVMR			Oxidation (M)[15]		Mascot
1878.8981	1878.9403	0.0422	22	74	89	ERIVANAPEEEDGFFR					Mascot
2425.2722	2425.321	0.0488	20	27	47	YTTQLNAILDYAQRLQGL DTK					Mascot

9	DNA mismatch repair protein MutS OS=Rhodopseudomonas palustris (strain BisB5) GN=mutS PE=3 SV=1	MUTS_RHOPS	99559.1	6.1	17	49	0	12.459	

Peptide Information

Calc. Mass	Obsrv. Mass	± da	± ppm	Start Seq.	End Seq.	Sequence	Ion Score	C. I. %	Modification	Rank	Result Type
882.4203	882.4621	0.0418	47	487	494	YADETGVK					Mascot
1110.5062	1110.6162	0.11	99	230	239	DVFDSATAER					Mascot
1136.5881	1136.6709	0.0828	73	149	158	ANNYLMAIAR					Mascot

Calc. Mass	Obsrv. Mass	± da	± ppm	Start Seq.	End Seq.	Sequence	Ion Score	C. I. %	Modification	Rank	Result Type
1142.6099	1142.6034	-0.0065	-6	374	384	AAPDMARALAR					Mascot
1158.6049	1158.5983	-0.0066	-6	374	384	AAPDMARALAR			Oxidation (M)[5]		Mascot
1174.5997	1174.6041	0.0044	4	370	380	SALRAAPDMAR			Oxidation (M)[9]		Mascot
1176.6848	1176.6868	0.002	2	623	632	HPVVEQALKR					Mascot
1480.8093	1480.9141	0.1048	71	518	530	LMAPPLNATFIHR					Mascot
1480.8093	1480.9141	0.1048	71	518	530	LMAPPLNATFIHR					Mascot
1550.7883	1550.9199	0.1316	85	864	877	TLADDLPLFAMTAR			Oxidation (M)[11]		Mascot
1650.8091	1650.8472	0.0381	23	78	92	HLGADIPMCGVPVER			Carbamidomethyl (C)[9]		Mascot
1653.9323	1653.9619	0.0296	18	582	598	AAAHAFALLDVATALAK					Mascot
1712.9153	1712.9332	0.0179	10	661	677	VGAGQIWLLTGPNMAGK					Mascot
1724.8966	1725.0562	0.1596	93	811	826	GEVVFLHEVLPGSADR					Mascot
1743.8442	1743.9984	0.1542	88	107	122	VAVCEQTEDPAAARAR			Carbamidomethyl (C)[4]		Mascot
1837.9478	1837.9567	0.0089	5	478	494	LVVAAMQARYADETGVK			Oxidation (M)[6]		Mascot
1862.939	1862.9509	0.0119	6	315	332	RGSLLDAIDCTVTAAGSR			Carbamidomethyl (C)[10]		Mascot
1862.939	1862.9509	0.0119	6	315	332	RGSLLDAIDCTVTAAGSR			Carbamidomethyl (C)[10]		Mascot
2334.2864	2334.2954	0.009	4	518	538	LMAPPLNATFIHRQTLAG QVR					Mascot
2385.3071	2385.3611	0.054	23	408	430	VLAQLSQLAQPPHDIAAA MAALR					Mascot

10	2,3,4,5-tetrahydropyridine-2,6-dicarboxylate N-succinyltransferase OS=Ralstonia solanacearum (strain GMI1000) GN=dapD PE=3 SV=1	DAPD_RALSO	29729.2	5.72	9	48	0	4.64	

Peptide Information

Calc. Mass	Obsrv. Mass	± da	± ppm	Start Seq.	End Seq.	Sequence	Ion Score	C. I. %	Modification	Rank	Result Type
817.4526	817.416	-0.0366	-45	257	263	KVDAQTR					Mascot
1594.7609	1594.8899	0.129	81	216	228	IYDRETGEVHYGR					Mascot
1594.7609	1594.8899	0.129	81	216	228	IYDRETGEVHYGR					Mascot
1653.8918	1653.9619	0.0701	42	30	45	AAVANVIDQLDQGALR					Mascot
1674.8123	1674.9434	0.1311	78	88	102	VPSKFASYTADDFAR					Mascot
1680.7765	1680.9149	0.1384	82	92	106	FASYTADDFARGGFR					Mascot
1830.8981	1830.9264	0.0283	15	2	16	SQQLQSLIEQAWEDR					Mascot
2377.2583	2377.3345	0.0762	32	23	45	AAPNDVRAAVANVIDQLD QGALR					Mascot
2425.1494	2425.321	0.1716	71	70	91	LEDNAPMTAGGFTHFYD KVPSK					Mascot
2449.2834	2449.3445	0.0611	25	220	243	ETGEVHYGRVPAGSVVV PGNLPSK					Mascot

Gel Idx/Pos	122/E21	Instr./Gel Origin	BA2151/Sample Project 20140814	Process Status	Analysis Succeeded
Plate [#] Name	[1] Sample Project 20140814	Instrument Sample Name		Spectra	11

Rank	Protein Name	Accession No.	Protein MW	Protein PI	Pep. Count	Protein Score	Protein Score C. I. %	Intensity Matched	Total Ion Score	Total Ion C. I. %	Confirmed
1	Ribulose bisphosphate carboxylase small chain clone 512 (Fragment) OS=Triticum aestivum PE=3 SV=1	RBS3_WHEAT	13274.5	5.84	11	309	100	36.132	212	100	

Peptide Information

Calc. Mass	Obsrv. Mass	± da	± ppm	Start Seq.	End Seq.	Sequence	Ion Score	C. I. %	Modification	Rank	Result Type
906.5043	906.5795	0.0752	83	14	20	QVDYLIR					Mascot
914.4229	914.481	0.0581	64	50	55	YWTMWK					Mascot
930.4178	930.4788	0.061	66	50	55	YWTMWK			Oxidation (M)[4]		Mascot
965.4873	965.5659	0.0786	81	85	92	IIGFDNMR					Mascot
965.4873	965.5659	0.0786	81	85	92	IIGFDNMR	25	0			Mascot
981.4822	981.5511	0.0689	70	85	92	IIGFDNMR			Oxidation (M)[7]		Mascot
981.4822	981.5511	0.0689	70	85	92	IIGFDNMR	15	0	Oxidation (M)[7]		Mascot
1012.4734	1012.5549	0.0815	80	77	84	EYPDAYVR					Mascot
1012.4734	1012.5549	0.0815	80	77	84	EYPDAYVR	59	99.858			Mascot
1140.5684	1140.6562	0.0878	77	76	84	KEYPDAYVR					Mascot
1165.571	1165.6488	0.0778	67	23	31	WVPCLEFSK			Carbamidomethyl (C)[4]		Mascot
1165.571	1165.6488	0.0778	67	23	31	WVPCLEFSK	29	0	Carbamidomethyl (C)[4]		Mascot
1365.5819	1365.696	0.1141	84	38	49	EHNASPGYYDGR	100	100			Mascot
2268.0789	2268.2593	0.1804	80	93	112	QVQCVSFIAFKPPGCEESGK			Carbamidomethyl (C)[4,15]		Mascot
2296.0837	2296.2825	0.1988	87	56	75	LPMFGCTDATQVINEVEEVK			Carbamidomethyl (C)[6], Oxidation (M)[3]		Mascot
2339.116	2339.302	0.186	80	93	113	QVQCVSFIAFKPPGCEESGKA			Carbamidomethyl (C)[4,15]		Mascot
2408.1836	2408.375	0.1914	79	56	76	LPMFGCTDATQVINEVEEVKK			Carbamidomethyl (C)[6]		Mascot
2424.1785	2424.363	0.1845	76	56	76	LPMFGCTDATQVINEVEEVKK			Carbamidomethyl (C)[6], Oxidation (M)[3]		Mascot

Rank	Protein Name	Accession No.	Protein MW	Protein PI	Pep. Count	Protein Score	Protein Score C. I. %	Intensity Matched	Total Ion Score	Total Ion C. I. %	Confirmed
2	Ribulose bisphosphate carboxylase small chain, chloroplastic OS=Hordeum vulgare PE=2 SV=1	RBS_HORVU	19692.8	8.98	12	297	100	36.261	212	100	

Peptide Information

Calc. Mass	Obsrv. Mass	± da	± ppm	Start Seq.	End Seq.	Sequence	Ion Score	C. I. %	Modification	Rank	Result Type
906.5043	906.5795	0.0752	83	75	81	QVDYLIR					Mascot
914.4229	914.481	0.0581	64	111	116	YWTMWK					Mascot

930.4178	930.4788	0.061	66	111	116	YWTMWK			Oxidation (M)[4]		Mascot
965.4873	965.5659	0.0786	81	146	153	IIGFDNMR					Mascot
965.4873	965.5659	0.0786	81	146	153	IIGFDNMR	25	0			Mascot
981.4822	981.5511	0.0689	70	146	153	IIGFDNMR			Oxidation (M)[7]		Mascot
981.4822	981.5511	0.0689	70	146	153	IIGFDNMR	15	0	Oxidation (M)[7]		Mascot
1012.4734	1012.5549	0.0815	80	138	145	EYPDAYVR					Mascot
1012.4734	1012.5549	0.0815	80	138	145	EYPDAYVR	59	99.858			Mascot
1140.5684	1140.6562	0.0878	77	137	145	KEYPDAYVR					Mascot
1165.571	1165.6488	0.0778	67	84	92	WVPCLEFSK			Carbamidomethyl (C)[4]		Mascot
1165.571	1165.6488	0.0778	67	84	92	WVPCLEFSK	29	0	Carbamidomethyl (C)[4]		Mascot
1365.5819	1365.696	0.1141	84	99	110	EHNASPGYYDGR	100	100			Mascot
1862.9681	1862.9376	-0.0305	-16	2	20	APTVMASSATSVAPFQGLK					Mascot
1922.0521	1922.1836	0.1315	68	58	74	FETLSYLPPLSTEALLK					Mascot
2050.147	2050.2974	0.1504	73	57	74	KFETLSYLPPLSTEALLK					Mascot
2296.0837	2296.2825	0.1988	87	117	136	LPMFGCTDATQVLNEVEEVK			Carbamidomethyl (C)[6], Oxidation (M)[3]		Mascot
2408.1836	2408.375	0.1914	79	117	137	LPMFGCTDATQVLNEVEEVKK			Carbamidomethyl (C)[6]		Mascot
2424.1785	2424.363	0.1845	76	117	137	LPMFGCTDATQVLNEVEEVKK			Carbamidomethyl (C)[6], Oxidation (M)[3]		Mascot

3	Ribulose bisphosphate carboxylase small chain PW9, chloroplastic OS=Triticum aestivum PE=3 SV=1	RBS2_WHEAT	19783.8	8.52	12	175	100	25.01	87	100

Peptide Information

Calc. Mass	Obsrv. Mass	± da	± ppm	Start Seq.	End Seq.	Sequence	Ion Score	C. I. %	Modification	Rank	Result Type
906.5043	906.5795	0.0752	83	76	82	QVDYLIR					Mascot
914.4229	914.481	0.0581	64	112	117	YWTMWK					Mascot
930.4178	930.4788	0.061	66	112	117	YWTMWK			Oxidation (M)[4]		Mascot
951.4717	951.5367	0.065	68	147	154	VIGFDNMR					Mascot
1012.4734	1012.5549	0.0815	80	139	146	EYPDAYVR					Mascot
1012.4734	1012.5549	0.0815	80	139	146	EYPDAYVR	59	99.858			Mascot
1140.5684	1140.6562	0.0878	77	138	146	KEYPDAYVR					Mascot
1165.571	1165.6488	0.0778	67	85	93	WVPCLEFSK			Carbamidomethyl (C)[4]		Mascot
1165.571	1165.6488	0.0778	67	85	93	WVPCLEFSK	29	0	Carbamidomethyl (C)[4]		Mascot
1381.5768	1381.6897	0.1129	82	100	111	EHNSSPGYYDGR					Mascot
1862.9681	1862.9376	-0.0305	-16	2	20	APAVMASSATTVAPFQGLK			Oxidation (M)[5]		Mascot
1922.0521	1922.1836	0.1315	68	59	75	FETLSYLPPLSTEALLK					Mascot
2050.147	2050.2974	0.1504	73	58	75	KFETLSYLPPLSTEALLK					Mascot

2296.085	2296.2825	0.1975	86	155	174	QVQCVSFIAFRPPGCEES GK			Carbamidomethyl (C)[4,15]		Mascot
2408.1836	2408.375	0.1914	79	118	138	LPMFGCTDATQVLNEVE EVKK			Carbamidomethyl (C)[6]		Mascot
2424.1785	2424.363	0.1845	76	118	138	LPMFGCTDATQVLNEVE EVKK			Carbamidomethyl (C)[6], Oxidation (M)[3]		Mascot

4	Ribulose bisphosphate carboxylase small chain PWS4.3, chloroplastic OS=Triticum aestivum PE=3 SV=2	RBS1_WHEAT	19689.9	8.99	10	149	100	24.502	87	100

Peptide Information

Calc. Mass	Obsrv. Mass	± da	± ppm	Start Seq.	End Seq.	Sequence	Ion Score	C. I. %	Modification	Rank	Result Type
906.5043	906.5795	0.0752	83	75	81	QVDYLIR					Mascot
914.4229	914.481	0.0581	64	111	116	YWTMWK					Mascot
930.4178	930.4788	0.061	66	111	116	YWTMWK			Oxidation (M)[4]		Mascot
933.5152	933.5845	0.0693	74	146	153	VIGFDNLR					Mascot
1012.4734	1012.5549	0.0815	80	138	145	EYPDAYVR					Mascot
1012.4734	1012.5549	0.0815	80	138	145	EYPDAYVR	59	99.858			Mascot
1140.5684	1140.6562	0.0878	77	137	145	KEYPDAYVR					Mascot
1165.571	1165.6488	0.0778	67	84	92	WVPCLEFSK			Carbamidomethyl (C)[4]		Mascot
1165.571	1165.6488	0.0778	67	84	92	WVPCLEFSK	29	0	Carbamidomethyl (C)[4]		Mascot
1381.5768	1381.6897	0.1129	82	99	110	EHNSSPGYYDGR					Mascot
1862.9681	1862.9376	-0.0305	-16	2	20	APAVMASSATTVAPFQG LK			Oxidation (M)[5]		Mascot
2296.085	2296.2825	0.1975	86	154	173	QVQCVSFIAFRPPGCEES GK			Carbamidomethyl (C)[4,15]		Mascot
2408.1836	2408.375	0.1914	79	117	137	LPMFGCTDATQVLNEVE EVKK			Carbamidomethyl (C)[6]		Mascot
2424.1785	2424.363	0.1845	76	117	137	LPMFGCTDATQVLNEVE EVKK			Carbamidomethyl (C)[6], Oxidation (M)[3]		Mascot

5	Alpha-amylase/trypsin inhibitor CM3 OS=Triticum aestivum PE=1 SV=1	IAAC3_WHEAT	18893.3	7.44	6	132	100	5.113	99	100

Peptide Information

Calc. Mass	Obsrv. Mass	± da	± ppm	Start Seq.	End Seq.	Sequence	Ion Score	C. I. %	Modification	Rank	Result Type
1010.52	1010.5105	-0.0095	-9	37	44	TNLLPHCR			Carbamidomethyl (C)[7]		Mascot
1110.5038	1110.6021	0.0983	89	133	140	EMQWDFVR					Mascot
1126.4987	1126.5851	0.0864	77	133	140	EMQWDFVR			Oxidation (M)[2]		Mascot
1698.9214	1699.0543	0.1329	78	101	115	YFIALPVPSQPVDPR					Mascot
1698.9214	1699.0543	0.1329	78	101	115	YFIALPVPSQPVDPR	55	99.689			Mascot
1727.8381	1727.9868	0.1487	86	116	132	SGNVGESGLIDLPGCPR			Carbamidomethyl (C)[15]		Mascot
1876.0222	1876.1796	0.1574	84	141	157	LLVAPGQCNLATIHNVR			Carbamidomethyl (C)[8]		Mascot

Calc. Mass	Obsrv. Mass	± da	± ppm	Start Seq.	End Seq.	Sequence	Ion Score	C. I. %	Modification	Rank	Result Type
1876.0222	1876.1796	0.1574	84	141	157	LLVAPGQCNLATIHNVR	44	95.459	Carbamidomethyl (C)[8]		Mascot
1957.8564	1958.025	0.1686	86	81	95	LYCCQELAEISQQCR			Carbamidomethyl (C)[3,4,14]		Mascot

6	Ribulose bisphosphate carboxylase small chain SSU40B, chloroplastic OS=Lemna gibba GN=SSU40B PE=3 SV=1	RBS4_LEMGI	20145.8	7.57	6	109	100	4.735	79	99.999

Protein Group

Ribulose bisphosphate carboxylase small chain SSU40A, chloroplastic OS=Lemna gibba GN=SSU40A PE=3 SV=1	RBS3_LEMGI	20073.8	8.2399997711816
Ribulose bisphosphate carboxylase small chain SSU5A, chloroplastic OS=Lemna gibba GN=SSU5A PE=3 SV=1	RBS5_LEMGI	20017.8	7.59999990463257
Ribulose bisphosphate carboxylase small chain SSU5B, chloroplastic OS=Lemna gibba GN=SSU5B PE=3 SV=1	RBS6_LEMGI	20086.9	7.59999990463257

Peptide Information

Calc. Mass	Obsrv. Mass	± da	± ppm	Start Seq.	End Seq.	Sequence	Ion Score	C. I. %	Modification	Rank	Result Type
914.4229	914.481	0.0581	64	122	127	YWTMWK					Mascot
930.4178	930.4788	0.061	66	122	127	YWTMWK			Oxidation (M)[4]		Mascot
962.5417	962.4765	-0.0652	-68	157	164	IIGFDNKR					Mascot
1365.5819	1365.696	0.1141	84	110	121	ENHASPGYYDGR	79	99.999			Mascot
1922.0157	1922.1836	0.1679	87	69	85	FETLSYLPPLSVEDLAK					Mascot
2050.1106	2050.2974	0.1868	91	68	85	KFETLSYLPPLSVEDLAK					Mascot
2339.1257	2339.302	0.1763	75	128	148	LPMFGCTDASQVIAEVEEAKK			Carbamidomethyl (C)[6], Oxidation (M)[3]		Mascot

7	Ribulose bisphosphate carboxylase small chain SSU26, chloroplastic OS=Lemna gibba GN=SSU26 PE=3 SV=1	RBS2_LEMGI	20029.9	8.62	6	107	99.999	4.735	79	99.999

Peptide Information

Calc. Mass	Obsrv. Mass	± da	± ppm	Start Seq.	End Seq.	Sequence	Ion Score	C. I. %	Modification	Rank	Result Type
914.4229	914.481	0.0581	64	122	127	YWTMWK					Mascot
930.4178	930.4788	0.061	66	122	127	YWTMWK			Oxidation (M)[4]		Mascot
962.5417	962.4765	-0.0652	-68	157	164	IIGFDNKR					Mascot
1365.5819	1365.696	0.1141	84	110	121	ENHASPGYYDGR	79	99.999			Mascot
1922.0157	1922.1836	0.1679	87	69	85	FETLSYLPPLSVEDLAK					Mascot
2050.1106	2050.2974	0.1868	91	68	85	KFETLSYLPPLSVEDLAK					Mascot
2339.1257	2339.302	0.1763	75	128	148	LPMFGCTDASQVIAEVEEAKK			Carbamidomethyl (C)[6], Oxidation (M)[3]		Mascot

8	Ribulose bisphosphate carboxylase small chain 6, chloroplastic OS=Mesembryanthemum crystallinum	RBS6_MESCR	20709.2	7.63	4	81	99.55	2.136	66	99.972

GN=RBCS-6 PE=3 SV=1

Peptide Information

Calc. Mass	Obsrv. Mass	± da	± ppm	Start Seq.	End Seq.	Sequence	Ion Score	C. I. %	Modification	Rank	Result Type
914.4229	914.481	0.0581	64	127	132	YWTMWK					Mascot
930.4178	930.4788	0.061	66	127	132	YWTMWK			Oxidation (M)[4]		Mascot
933.5152	933.5845	0.0693	74	162	169	IIGFDNVR					Mascot
1365.5819	1365.696	0.1141	84	115	126	EHGNTPGYYDGR	66	99.972			Mascot
1490.7446	1490.8339	0.0893	60	44	58	KSSNDLSTVPSNGGK					Mascot

9	Ribulose bisphosphate carboxylase small chain 2B, chloroplastic OS=Arabidopsis thaliana GN=RBCS-2B PE=2 SV=2	RBS2B_ARATH	20622.2	7.59	3	75	98.441	1.114	66	99.972

Protein Group

Ribulose bisphosphate carboxylase small chain 1B, chloroplastic OS=Arabidopsis thaliana GN=RBCS-1B PE=1 SV=1	RBS1B_ARATH	20558.2	7.5900 001525 8789

Peptide Information

Calc. Mass	Obsrv. Mass	± da	± ppm	Start Seq.	End Seq.	Sequence	Ion Score	C. I. %	Modification	Rank	Result Type
914.4229	914.481	0.0581	64	121	126	YWTMWK					Mascot
930.4178	930.4788	0.061	66	121	126	YWTMWK			Oxidation (M)[4]		Mascot
951.4894	951.5367	0.0473	50	29	37	SSASFPVTR					Mascot
1365.5819	1365.696	0.1141	84	109	120	EHGNTPGYYDGR	66	99.972			Mascot

10	Ribulose bisphosphate carboxylase small chain 2, chloroplastic OS=Mesembryanthemum crystallinum GN=RBCS-2 PE=2 SV=1	RBS2_MESCR	20299	7.63	3	75	98.441	2.013	66	99.972

Peptide Information

Calc. Mass	Obsrv. Mass	± da	± ppm	Start Seq.	End Seq.	Sequence	Ion Score	C. I. %	Modification	Rank	Result Type
914.4229	914.481	0.0581	64	121	126	YWTMWK					Mascot
930.4178	930.4788	0.061	66	121	126	YWTMWK			Oxidation (M)[4]		Mascot
933.5152	933.5845	0.0693	74	156	163	IIGFDNVR					Mascot
1365.5819	1365.696	0.1141	84	109	120	EHGNTPGYYDGR	66	99.972			Mascot

Gel Idx/Pos	123/E22	Instr./Gel Origin	BA2151/Sample Project 20140814	Process Status	Analysis Succeeded
Plate [#] Name	[1] Sample Project 20140814	Instrument Sample Name		Spectra	11

Rank	Protein Name	Accession No.	Protein MW	Protein PI	Pep. Count	Protein Score	Protein Score C. I. %	Intensity Matched	Total Ion Score	Total Ion C. I. %	Confirmed
1	Ribulose bisphosphate carboxylase small chain clone 512 (Fragment) OS=Triticum aestivum PE=3 SV=1	RBS3_WHEAT	13274.5	5.84	6	165	100	3.098	128	100	

Peptide Information

Calc. Mass	Obsrv. Mass	± da	± ppm	Start Seq.	End Seq.	Sequence	Ion Score	C. I. %	Modification	Rank	Result Type
906.5043	906.5706	0.0663	73	14	20	QVDYLIR					Mascot
914.4229	914.4921	0.0692	76	50	55	YWTMWK					Mascot
965.4873	965.5562	0.0689	71	85	92	IIGFDNMR					Mascot
981.4822	981.5389	0.0567	58	85	92	IIGFDNMR			Oxidation (M)[7]		Mascot
1012.4734	1012.5547	0.0813	80	77	84	EYPDAYVR					Mascot
1012.4734	1012.5547	0.0813	80	77	84	EYPDAYVR	26	0			Mascot
1165.571	1165.6528	0.0818	70	23	31	WVPCLEFSK			Carbamidomethyl (C)[4]		Mascot
1365.5819	1365.6908	0.1089	80	38	49	EHNASPGYYDGR	103	100			Mascot

Rank	Protein Name	Accession No.	Protein MW	Protein PI	Pep. Count	Protein Score	Protein Score C. I. %	Intensity Matched	Total Ion Score	Total Ion C. I. %	Confirmed
2	Ribulose bisphosphate carboxylase small chain, chloroplastic OS=Hordeum vulgare PE=2 SV=1	RBS_HORVU	19692.8	8.98	7	162	100	3.382	128	100	

Peptide Information

Calc. Mass	Obsrv. Mass	± da	± ppm	Start Seq.	End Seq.	Sequence	Ion Score	C. I. %	Modification	Rank	Result Type
906.5043	906.5706	0.0663	73	75	81	QVDYLIR					Mascot
914.4229	914.4921	0.0692	76	111	116	YWTMWK					Mascot
965.4873	965.5562	0.0689	71	146	153	IIGFDNMR					Mascot
981.4822	981.5389	0.0567	58	146	153	IIGFDNMR			Oxidation (M)[7]		Mascot
1012.4734	1012.5547	0.0813	80	138	145	EYPDAYVR					Mascot
1012.4734	1012.5547	0.0813	80	138	145	EYPDAYVR	26	0			Mascot
1165.571	1165.6528	0.0818	70	84	92	WVPCLEFSK			Carbamidomethyl (C)[4]		Mascot
1365.5819	1365.6908	0.1089	80	99	110	EHNASPGYYDGR	103	100			Mascot
1862.9681	1862.929	-0.0391	-21	2	20	APTVMASSATSVAPFQGLK					Mascot

Rank	Protein Name	Accession No.	Protein MW	Protein PI	Pep. Count	Protein Score	Protein Score C. I. %	Intensity Matched	Total Ion Score	Total Ion C. I. %	Confirmed
3	Alpha-amylase inhibitor 0.19 OS=Triticum aestivum PE=1 SV=1	IAA1_WHEAT	13898.6	6.66	5	133	100	4.086	103	100	

Peptide Information

Calc. Mass	Obsrv. Mass	± da	± ppm	Start	End	Sequence	Ion	C. I. %	Modification	Rank	Result Type

				Seq.	Seq.		Score				
1162.6249	1162.7157	0.0908	78	90	100	LTAASITAVCR			Carbamidomethyl (C)[10]		Mascot
1570.8007	1570.91	0.1093	70	26	39	LQCNGSQVPEAVLR			Carbamidomethyl (C)[3]		Mascot
1612.7463	1612.8781	0.1318	82	67	82	EHGAQEGQAGTGAFPR					Mascot
1612.7463	1612.8781	0.1318	82	67	82	EHGAQEGQAGTGAFPR	103	100			Mascot
1663.8361	1663.9304	0.0943	57	101	116	LPIVVDASGDGAYVCK			Carbamidomethyl (C)[15]		Mascot
1862.7731	1862.929	0.1559	84	40	53	DCCQQLAHISEWCR			Carbamidomethyl (C)[2,3,13]		Mascot

4	Ribulose bisphosphate carboxylase small chain SSU26, chloroplastic OS=Lemna gibba GN=SSU26 PE=3 SV=1	RBS2_LEMGI	20029.9	8.62	6	104	99.998	1.465	78	99.999	

Peptide Information

Calc. Mass	Obsrv. Mass	± da	± ppm	Start Seq.	End Seq.	Sequence	Ion Score	C. I. %	Modification	Rank	Result Type
914.4229	914.4921	0.0692	76	122	127	YWTMWK					Mascot
935.4945	935.5002	0.0057	6	31	39	SSVAFPATR					Mascot
962.5417	962.4761	-0.0656	-68	157	164	IIGFDNKR					Mascot
1365.5819	1365.6908	0.1089	80	110	121	ENHASPGYYDGR	78	99.999			Mascot
1400.7493	1400.8	0.0507	36	41	54	ANKNLSTLPSNGGK					Mascot
1546.7393	1546.8203	0.081	52	55	67	VSCMQVWPPEGLK			Carbamidomethyl (C)[3], Oxidation (M)[4]		Mascot

5	Ribulose bisphosphate carboxylase small chain SSU5B, chloroplastic OS=Lemna gibba GN=SSU5B PE=3 SV=1	RBS6_LEMGI	20086.9	7.6	5	99	99.993	1.283	78	99.999	

Protein Group

Ribulose bisphosphate carboxylase small chain SSU5A, chloroplastic OS=Lemna gibba GN=SSU5A PE=3 SV=1	RBS5_LEMGI	20017.8	7.5999999046 3257

Peptide Information

Calc. Mass	Obsrv. Mass	± da	± ppm	Start Seq.	End Seq.	Sequence	Ion Score	C. I. %	Modification	Rank	Result Type
914.4229	914.4921	0.0692	76	122	127	YWTMWK					Mascot
935.4945	935.5002	0.0057	6	31	39	SSVAFPATR					Mascot
962.5417	962.4761	-0.0656	-68	157	164	IIGFDNKR					Mascot
1365.5819	1365.6908	0.1089	80	110	121	ENHASPGYYDGR	78	99.999			Mascot
1546.7393	1546.8203	0.081	52	55	67	VSCMQVWPPEGLK			Carbamidomethyl (C)[3], Oxidation (M)[4]		Mascot

6	Ribulose bisphosphate carboxylase small chain SSU40B, chloroplastic OS=Lemna gibba GN=SSU40B PE=3 SV=1	RBS4_LEMGI	20145.8	7.57	4	94	99.976	.547	78	99.999	

Protein Group

	Ribulose bisphosphate carboxylase small chain SSU40A, chloroplastic OS=Lemna gibba GN=SSU40A PE=3 SV=1	RBS3_LEMGI	20073.8	8.2399 997711 1816

Peptide Information

Calc. Mass	Obsrv. Mass	± da	± ppm	Start Seq.	End Seq.	Sequence	Ion Score	C. I. %	Modification	Rank	Result Type
914.4229	914.4921	0.0692	76	122	127	YWTMWK					Mascot
962.5417	962.4761	-0.0656	-68	157	164	IIGFDNKR					Mascot
1365.5819	1365.6908	0.1089	80	110	121	ENHASPGYYDGR	78	99.999			Mascot
1546.7393	1546.8203	0.081	52	55	67	VSCMQVWPPEGLK			Carbamidomethyl (C)[3], Oxidation (M)[4]		Mascot

7	Ribulose bisphosphate carboxylase small chain 2, chloroplastic OS=Mesembryanthemum crystallinum GN=RBCS-2 PE=2 SV=1	RBS2_MESCR	20299	7.63	6	87	99.882	1.351	59	99.872

Peptide Information

Calc. Mass	Obsrv. Mass	± da	± ppm	Start Seq.	End Seq.	Sequence	Ion Score	C. I. %	Modification	Rank	Result Type
914.4229	914.4921	0.0692	76	121	126	YWTMWK					Mascot
933.5152	933.5565	0.0413	44	156	163	IIGFDNVR					Mascot
935.5197	935.5002	-0.0195	-21	30	38	SVSAFPVTK					Mascot
1082.563	1082.6304	0.0674	62	147	155	KAYPEAFTR					Mascot
1365.5819	1365.6908	0.1089	80	109	120	EHGNTPGYYDGR	59	99.872			Mascot
1570.8232	1570.91	0.0868	55	53	65	VQCMQVWPPLGKK			Carbamidomethyl (C)[3]		Mascot

8	Ribulose bisphosphate carboxylase small chain 6, chloroplastic OS=Mesembryanthemum crystallinum GN=RBCS-6 PE=3 SV=1	RBS6_MESCR	20709.2	7.63	5	80	99.459	.629	59	99.872

Peptide Information

Calc. Mass	Obsrv. Mass	± da	± ppm	Start Seq.	End Seq.	Sequence	Ion Score	C. I. %	Modification	Rank	Result Type
914.4229	914.4921	0.0692	76	127	132	YWTMWK					Mascot
933.5152	933.5565	0.0413	44	162	169	IIGFDNVR					Mascot
1365.5819	1365.6908	0.1089	80	115	126	EHGNTPGYYDGR	59	99.872			Mascot
1490.7446	1490.8396	0.095	64	44	58	KSSNDLSTVPSNGGK					Mascot
1570.8232	1570.91	0.0868	55	59	71	VQCMQVWPPLGKK			Carbamidomethyl (C)[3]		Mascot

9	Ribulose bisphosphate carboxylase small chain 2B, chloroplastic OS=Arabidopsis thaliana GN=RBCS-2B PE=2 SV=2	RBS2B_ARATH	20622.2	7.59	4	76	98.732	1.134	59	99.872

Peptide Information

Calc. Mass	Obsrv. Mass	± da	± ppm	Start	End	Sequence	Ion	C. I. %	Modification	Rank	Result Type

				Seq.	Seq.		Score				
914.4229	914.4921	0.0692	76	121	126	YWTMWK					Mascot
935.4945	935.5002	0.0057	6	156	163	IIGFDNTR					Mascot
1365.5819	1365.6908	0.1089	80	109	120	EHGNTPGYYDGR	59	99.872			Mascot
2703.3369	2703.5413	0.2044	76	2	28	ASSMFSSTAVVTSPAQA TMVAPFTGLK			Oxidation (M)[4]		Mascot

10	Ribulose bisphosphate carboxylase small chain 3, chloroplastic OS=Mesembryanthemum crystallinum GN=RBCS-3 PE=2 SV=1	RBS3_MESCR	20600.1	6.73	4	74	97.64	.482	59	99.872	

Protein Group

Ribulose bisphosphate carboxylase small chain 1, chloroplastic OS=Mesembryanthemum crystallinum GN=RBCS-1 PE=2 SV=2	RBS1_MESCR	20491.1	7.6300 001144 4092

Peptide Information

Calc. Mass	Obsrv. Mass	± da	± ppm	Start Seq.	End Seq.	Sequence	Ion Score	C. I. %	Modification	Rank	Result Type
914.4229	914.4921	0.0692	76	124	129	YWTMWK					Mascot
933.5152	933.5565	0.0413	44	159	166	IIGFDNVR					Mascot
1365.5819	1365.6908	0.1089	80	112	123	EHGNTPGYYDGR	59	99.872			Mascot
1570.8232	1570.91	0.0868	55	56	68	VQCMQVWPPLGKK			Carbamidomethyl (C)[3]		Mascot

Gel Idx/Pos	124/E23	Instr./Gel Origin	BA2151/Sample Project 20140814	Process Status	Analysis Succeeded
Plate [#] Name	[1] Sample Project 20140814	Instrument Sample Name		Spectra	11

Rank	Protein Name	Accession No.	Protein MW	Protein PI	Pep. Count	Protein Score	Protein Score C. I. %	Intensity Matched	Total Ion Score	Total Ion C. I. %	Confirmed
1	Solute carrier family 25 member 38 homolog OS=Sclerotinia sclerotiorum (strain ATCC 18683 / 1980 / Ss-1) GN=SS1G_14414 PE=3 SV=1	S2538_SCLS1	35603.9	10.23	11	49	0	33.677			

Peptide Information

Calc. Mass	Obsrv. Mass	± da	± ppm	Start Seq.	End Seq.	Sequence	Ion Score	C. I. %	Modification	Rank	Result Type
832.4271	832.3878	-0.0393	-47	2	10	SNGGNAGKK					Mascot
854.5458	854.5988	0.053	62	264	270	IQLQPKK					Mascot
864.4131	864.493	0.0799	92	281	288	MVGEEGVK			Oxidation (M)[1]		Mascot
979.4625	979.4661	0.0036	4	1	10	MSNGGNAGKK			Oxidation (M)[1]		Mascot
983.5996	983.5421	-0.0575	-58	262	269	TRIQLQPK					Mascot
1821.9745	1822.0182	0.0437	24	302	317	AVSSALAWTIYEELIR					Mascot
1822.8793	1823.0492	0.1699	93	152	167	YESNLYAYKSIMGAGR					Mascot
1822.9368	1823.0492	0.1124	62	281	297	MVGEEGVKSLFDGLGLR			Oxidation (M)[1]		Mascot
1839.0195	1839.0602	0.0407	22	39	54	TRLQQSNHASLLTTIR					Mascot
1906.0393	1906.2008	0.1615	85	103	121	TIGVVDAKNGPVHSSSLPK					Mascot
1906.0393	1906.2008	0.1615	85	103	121	TIGVVDAKNGPVHSSSLPK					Mascot
2018.0634	2018.1365	0.0731	36	189	205	DAPYAGLYVLFYEQLKK					Mascot

Rank	Protein Name	Accession No.	Protein MW	Protein PI	Pep. Count	Protein Score	Protein Score C. I. %	Intensity Matched	Total Ion Score	Total Ion C. I. %	Confirmed
2	3-hydroxyasparagine phosphotransferase OS=Streptomyces coelicolor (strain ATCC BAA-471 / A3(2) / M145) GN=hasP PE=4 SV=1	HASP_STRCO	33336.3	5.77	9	42	0	14.209			

Peptide Information

Calc. Mass	Obsrv. Mass	± da	± ppm	Start Seq.	End Seq.	Sequence	Ion Score	C. I. %	Modification	Rank	Result Type
930.4891	930.5039	0.0148	16	234	241	IEVEVADR					Mascot
955.5571	955.5062	-0.0509	-53	173	181	LLELSGAPR					Mascot
955.5571	955.5062	-0.0509	-53	173	181	LLELSGAPR					Mascot
967.4592	967.5065	0.0473	49	268	275	RFTDDASR					Mascot
1657.9008	1658.0123	0.1115	67	41	57	TSLVVGGELDGVEALAK					Mascot
1657.9008	1658.0123	0.1115	67	41	57	TSLVVGGELDGVEALAK					Mascot
1822.8905	1823.0492	0.1587	87	25	40	EVCPGFAPGEVVYRSR			Carbamidomethyl (C)[3]		Mascot

Calc. Mass	Obsrv. Mass	± da	± ppm	Start Seq.	End Seq.	Sequence	Ion Score	C. I. %	Modification	Rank	Result Type
1822.8905	1823.0492	0.1587	87	25	40	EVCPGFAPGEVVYRSR			Carbamidomethyl (C)[3]		Mascot
1913.0703	1913.2228	0.1525	80	41	59	TSLVVGGELDGVEALAKVR					Mascot
1928.0021	1928.1366	0.1345	70	246	261	DGMMPFLVNLTLLLYR			Oxidation (M)[3,4]		Mascot
1954.0757	1954.1312	0.0555	28	104	121	LTGEVLAPDRFPVTPVSR					Mascot
1996.0413	1996.1115	0.0702	35	134	150	LRHWRPAAAGAWAVDYR					Mascot

3	Glucose 1-dehydrogenase OS=Bacillus subtilis (strain 168) GN=gdh PE=2 SV=2	DHG_BACSU	28072.4	5.26	8	36	0	1.24		

Peptide Information

Calc. Mass	Obsrv. Mass	± da	± ppm	Start Seq.	End Seq.	Sequence	Ion Score	C. I. %	Modification	Rank	Result Type
807.4359	807.4763	0.0404	50	27	33	FGKEQAK					Mascot
886.5356	886.5894	0.0538	61	74	81	NIVQTAIK					Mascot
967.4917	967.5065	0.0148	15	1	8	MYPDLKGK			Oxidation (M)[1]		Mascot
1805.983	1806.074	0.091	50	167	182	LMTETLALEYAPKGIR					Mascot
1821.9779	1822.0182	0.0403	22	167	182	LMTETLALEYAPKGIR			Oxidation (M)[2]		Mascot
1847.0386	1846.9899	-0.0487	-26	112	129	VIGTNLTGAFLGSREAIK					Mascot
1928.0336	1928.1366	0.103	53	50	68	EEVIKAGGEAVVVQGDVTK					Mascot
1929.9764	1930.1416	0.1652	86	55	73	AGGEAVVVQGDVTKEEDVK					Mascot
2840.3196	2840.5842	0.2646	93	235	261	EASYVTGITLFADGGMTQYPSFQAGRG			Oxidation (M)[16]		Mascot

4	6-hydroxy-3-succinoylpyridine 3-monooxygenase HspA OS=Pseudomonas putida GN=nicB PE=1 SV=1	HSPA_PSEPU	35758.6	7.16	7	35	0	27.918	10	0

Peptide Information

Calc. Mass	Obsrv. Mass	± da	± ppm	Start Seq.	End Seq.	Sequence	Ion Score	C. I. %	Modification	Rank	Result Type
807.4471	807.4763	0.0292	36	263	270	GSRAAAFK					Mascot
886.5244	886.5894	0.065	73	62	69	LLESPSIK					Mascot
888.5049	888.5419	0.037	42	92	98	YHTALRK					Mascot
1819.9993	1819.993	-0.0063	-3	28	42	GTPYKWLDLLPLFEK					Mascot
1906.0254	1906.2008	0.1754	92	184	201	AHTDVRIGVVVPTSGQNR					Mascot
1906.0254	1906.2008	0.1754	92	184	201	AHTDVRIGVVVPTSGQNR	10	0			Mascot
1954.007	1954.1312	0.1242	64	297	312	VLQYIHSWIAQQEELP					Mascot
1990.0393	1990.1107	0.0714	36	99	115	LHDGRIELIEGYYAVNK					Mascot
1990.0393	1990.1107	0.0714	36	99	115	LHDGRIELIEGYYAVNK					Mascot

5	GTPase Era OS=Lactobacillus helveticus (strain DPC 4571) GN=era PE=3 SV=1	ERA_LACH4	34027.1	7.79	8	33	0	13.481

Peptide Information

Calc. Mass	Obsrv. Mass	± da	± ppm	Start Seq.	End Seq.	Sequence	Ion Score	C. I. %	Modification	Rank	Result Type
842.5458	842.5921	0.0463	55	242	250	GIIIGKGGK					Mascot
983.5044	983.5421	0.0377	38	47	55	ISGIYTSDK					Mascot
1315.7329	1315.7896	0.0567	43	33	44	VAITSNKPQTTR					Mascot
1365.7539	1365.7241	-0.0298	-22	275	284	LWVKVQHNWR					Mascot
1819.0226	1818.995	-0.0276	-15	5	21	EFKSGFVALLGRPNVGK					Mascot
1822.9772	1823.0492	0.072	39	133	147	LLLIMDSYHKLEGFK			Oxidation (M)[5]		Mascot
1822.9772	1823.0492	0.072	39	133	147	LLLIMDSYHKLEGFK			Oxidation (M)[5]		Mascot
1930.0256	1930.1416	0.116	60	56	72	MQVVFVDTPGIFKPHSK					Mascot
1960.1379	1960.2885	0.1506	77	116	132	VPVFLVINKVDQIHPNK					Mascot

6 Testis-specific gene 13 protein OS=Macaca fascicularis GN=TSGA13 PE=2 SV=1 | TSG13_MACFA | 31212.4 | 10.04 | 11 | 33 | 0 | 2.405

Peptide Information

Calc. Mass	Obsrv. Mass	± da	± ppm	Start Seq.	End Seq.	Sequence	Ion Score	C. I. %	Modification	Rank	Result Type
876.5049	876.5278	0.0229	26	73	79	FLAQNRK					Mascot
922.4628	922.5396	0.0768	83	34	41	EIYDAVGR					Mascot
934.5026	934.582	0.0794	85	25	33	EKGMVVSGK					Mascot
955.5281	955.5062	-0.0219	-23	79	86	KTTSFMLK					Mascot
955.5281	955.5062	-0.0219	-23	79	86	KTTSFMLK					Mascot
979.4803	979.4661	-0.0142	-14	16	24	TSENSSAKR					Mascot
983.6036	983.5421	-0.0615	-63	258	265	APQWIIKK					Mascot
1105.6365	1105.6703	0.0338	31	42	50	SKFVLENLR					Mascot
1182.6742	1182.6604	-0.0138	-12	255	264	NGRAPQWIIK					Mascot
1821.8575	1822.0182	0.1607	88	80	94	TTSFMLKVTEYDQDK			Oxidation (M)[5]		Mascot
1928.0852	1928.1366	0.0514	27	156	172	LKPIFPLTLSDDPTSKR					Mascot
1972.0439	1972.1152	0.0713	36	51	66	HYTVHPNLAQYYKPLK					Mascot

7 SsrA-binding protein OS=Acinetobacter baumannii (strain AB0057) GN=smpB PE=3 SV=1 | SSRP_ACIB5 | 18182.7 | 10 | 4 | 33 | 0 | 24.126 | 15 | 0

Protein Group

SsrA-binding protein OS=Acinetobacter baumannii (strain AB307-0294) GN=smpB PE=3 SV=1	SSRP_ACIB3	18182.7	10
SsrA-binding protein OS=Acinetobacter baumannii	SSRP_ACIBC	18182.7	10

(strain ACICU) GN=smpB PE=3 SV=1

SsrA-binding protein OS=Acinetobacter baumannii (strain ATCC 17978 / NCDC KC 755) GN=smpB PE=3 SV=2	SSRP_ACIBT	18182.7	10
SsrA-binding protein OS=Acinetobacter baumannii (strain AYE) GN=smpB PE=3 SV=1	SSRP_ACIBY	18182.7	10

Peptide Information

Calc. Mass	Obsrv. Mass	± da	± ppm	Start Seq.	End Seq.	Sequence	Ion Score	C. I. %	Modification	Rank	Result Type
876.4608	876.5278	0.067	76	101	108	LMGAVNQK			Oxidation (M)[2]		Mascot
889.4275	889.4092	-0.0183	-21	145	150	ERDWQR					Mascot
1333.6709	1333.7976	0.1267	95	50	60	MSLTESYVIFK			Oxidation (M)[1]		Mascot
1333.6709	1333.7976	0.1267	95	50	60	MSLTESYVIFK			Oxidation (M)[1]		Mascot
1822.952	1823.0492	0.0972	53	31	46	FEAGMSLLGWEVKSLR					Mascot
1822.952	1823.0492	0.0972	53	31	46	FEAGMSLLGWEVKSLR	15	0			Mascot
1838.9469	1839.0602	0.1133	62	31	46	FEAGMSLLGWEVKSLR			Oxidation (M)[5]		Mascot

8	Interleukin-18 OS=Gallus gallus GN=IL18 PE=2 SV=1	IL18_CHICK	23285.3	5.39	4	33	0	1.275	19	0

Peptide Information

Calc. Mass	Obsrv. Mass	± da	± ppm	Start Seq.	End Seq.	Sequence	Ion Score	C. I. %	Modification	Rank	Result Type
807.4658	807.4763	0.0105	13	112	117	MVVRFR					Mascot
917.4033	917.4254	0.0221	24	137	144	TFTSCSSK			Carbamidomethyl (C)[5]		Mascot
955.5142	955.5062	-0.008	-8	108	115	EHGKMVVR					Mascot
955.5142	955.5062	-0.008	-8	108	115	EHGKMVVR	19	0			Mascot
1349.677	1349.7954	0.1184	88	88	99	MPVAFSVQVEDK					Mascot
1365.672	1365.7241	0.0521	38	88	99	MPVAFSVQVEDK			Oxidation (M)[1]		Mascot

9	Adenine phosphoribosyltransferase OS=Shewanella sediminis (strain HAW-EB3) GN=apt PE=3 SV=1	APT_SHESH	20284.7	4.97	6	32	0	24.22

Peptide Information

Calc. Mass	Obsrv. Mass	± da	± ppm	Start Seq.	End Seq.	Sequence	Ion Score	C. I. %	Modification	Rank	Result Type
870.5519	870.6113	0.0594	68	142	149	LIRNLGGK					Mascot
1315.7402	1315.7896	0.0494	38	167	177	RLQAMDLELVK					Mascot
1790.968	1790.9614	-0.0066	-4	2	17	ISMNNDTLAVIKQSIK			Oxidation (M)[3]		Mascot
1790.968	1790.9614	-0.0066	-4	2	17	ISMNNDTLAVIKQSIK			Oxidation (M)[3]		Mascot
1906.0137	1906.2008	0.1871	98	1	17	MISMNNDTLAVIKQSIK					Mascot

Calc. Mass	Obsrv. Mass	± da	± ppm	Start Seq.	End Seq.	Sequence	Ion Score	C. I. %	Modification	Rank	Result Type
1906.0137	1906.2008	0.1871	98	1	17	MISMNNDTLAVIKQSIK					Mascot
1922.0085	1922.1959	0.1874	98	1	17	MISMNNDTLAVIKQSIK			Oxidation (M)[1]		Mascot
1928.0951	1928.1366	0.0415	22	123	141	VLVIDDLLATGGTIEATVK					Mascot
2025.9508	2026.0072	0.0564	28	168	184	LQAMDLELVKLCEFDGE			Carbamidomethyl (C)[12], Oxidation (M)[4]		Mascot

10	Phosphoglucosamine mutase OS=Corynebacterium diphtheriae (strain ATCC 700971 / NCTC 13129 / Biotype gravis) GN=glmM PE=3 SV=1	GLMM_CORDI	46814.1	5.28	7	32	0	19.24	13	0	

Peptide Information

Calc. Mass	Obsrv. Mass	± da	± ppm	Start Seq.	End Seq.	Sequence	Ion Score	C. I. %	Modification	Rank	Result Type
842.5709	842.5921	0.0212	25	18	25	LTALLALK					Mascot
853.5366	853.5552	0.0186	22	44	51	RPVAVVGR					Mascot
864.4574	864.493	0.0356	41	4	11	LFGTDGVR					Mascot
876.4495	876.5278	0.0783	89	423	430	VMVEAPSK			Oxidation (M)[2]		Mascot
1333.6781	1333.7976	0.1195	90	423	434	VMVEAPSKETAR			Oxidation (M)[2]		Mascot
1333.6781	1333.7976	0.1195	90	423	434	VMVEAPSKETAR			Oxidation (M)[2]		Mascot
1921.0609	1921.1929	0.132	69	281	298	STLVATVMSNLGLRLAMK			Oxidation (M)[8]		Mascot
1989.9845	1990.1107	0.1262	63	52	71	DPRVSGEMLAAALSAGM ASR					Mascot
1989.9845	1990.1107	0.1262	63	52	71	DPRVSGEMLAAALSAGM ASR	13	0			Mascot

Gel Idx/Pos	125/E24	Instr./Gel Origin	BA2151/Sample Project 20140814	Process Status	Analysis Succeeded
Plate [#] Name	[1] Sample Project 20140814	Instrument Sample Name		Spectra	11

Rank	Protein Name	Accession No.	Protein MW	Protein PI	Pep. Count	Protein Score	Protein Score C. I. %	Intensity Matched	Total Ion Score	Total Ion C. I. %	Confirmed
1	Keratin, type II cytoskeletal 1 OS=Homo sapiens GN=KRT1 PE=1 SV=6	K2C1_HUMAN	66170.1	8.15	13	123	100	9.116	82	100	

Peptide Information

Calc. Mass	Obsrv. Mass	± da	± ppm	Start Seq.	End Seq.	Sequence	Ion Score	C. I. %	Modification	Rank	Result Type
874.4993	874.499	-0.0003	0	66	74	SLVNLGGSK					Mascot
973.5312	973.6044	0.0732	75	396	403	IEISELNR					Mascot
1033.516	1033.5986	0.0826	80	484	492	TLLEGEESR					Mascot
1066.5164	1066.5927	0.0763	72	270	277	YEDEINKR					Mascot
1073.595	1073.6378	0.0428	40	408	416	LRSEIDNVK					Mascot
1179.6005	1179.7	0.0995	84	377	386	YEELQITAGR					Mascot
1277.71	1277.8147	0.1047	82	473	483	LALDLEIATYR					Mascot
1277.71	1277.8147	0.1047	82	473	483	LALDLEIATYR	22	0			Mascot
1383.6903	1383.7797	0.0894	65	186	197	SLNNQFASFIDK					Mascot
1475.7489	1475.8694	0.1205	82	212	223	WELLQQVDTSTR	35	72.382			Mascot
1475.7853	1475.8694	0.0841	57	200	211	FLEQQNQVLQTK					Mascot
1716.8511	1716.9916	0.1405	82	418	432	QISNLQQSISDAEQR					Mascot
1716.8511	1716.9916	0.1405	82	418	432	QISNLQQSISDAEQR	27	0			Mascot
1993.9827	1994.1547	0.172	86	625	644	SSGGSSSVKFVSTTYSG VTR					Mascot
2383.9519	2384.1689	0.217	91	519	549	GGGGGGYGSGGSSYGS GGGSYGSGGGGGGGR					Mascot

Rank	Protein Name	Accession No.	Protein MW	Protein PI	Pep. Count	Protein Score	Protein Score C. I. %	Intensity Matched	Total Ion Score	Total Ion C. I. %	Confirmed
2	Keratin, type II cytoskeletal 1 OS=Pan troglodytes GN=KRT1 PE=1 SV=1	K2C1_PANTR	65620.8	7.62	13	122	100	9.116	82	100	

Peptide Information

Calc. Mass	Obsrv. Mass	± da	± ppm	Start Seq.	End Seq.	Sequence	Ion Score	C. I. %	Modification	Rank	Result Type
874.4993	874.499	-0.0003	0	66	74	SLVNLGGSK					Mascot
973.5312	973.6044	0.0732	75	391	398	IEISELNR					Mascot
1033.516	1033.5986	0.0826	80	479	487	TLLEGEESR					Mascot
1066.5164	1066.5927	0.0763	72	265	272	YEDEINKR					Mascot
1073.595	1073.6378	0.0428	40	403	411	LRSEIDNVK					Mascot
1179.6005	1179.7	0.0995	84	372	381	YEELQITAGR					Mascot

Calc. Mass	Obsrv. Mass	± da	± ppm	Start Seq.	End Seq.	Sequence	Ion Score	C. I. %	Modification	Rank	Result Type
1277.71	1277.8147	0.1047	82	468	478	LALDLEIATYR					Mascot
1277.71	1277.8147	0.1047	82	468	478	LALDLEIATYR	22	0			Mascot
1383.6903	1383.7797	0.0894	65	181	192	SLNNQFASFIDK					Mascot
1475.7489	1475.8694	0.1205	82	207	218	WELLQQVDTSTR	35	72.382			Mascot
1475.7853	1475.8694	0.0841	57	195	206	FLEQQNQVLQTK					Mascot
1716.8511	1716.9916	0.1405	82	413	427	QISNLQQSISDAEQR					Mascot
1716.8511	1716.9916	0.1405	82	413	427	QISNLQQSISDAEQR	27	0			Mascot
1993.9827	1994.1547	0.172	86	618	637	SSGGSSSVKFVSTTYSG VTR					Mascot
2383.9519	2384.1689	0.217	91	514	544	GGGGGGYGSGGSSYGS GGGSYGSGGGGGGGR					Mascot

3	Keratin, type I cytoskeletal 10 OS=Homo sapiens GN=KRT10 PE=1 SV=6	K1C10_HUMAN	59019.8	5.13	15	72	96.888	5.687	

Peptide Information

Calc. Mass	Obsrv. Mass	± da	± ppm	Start Seq.	End Seq.	Sequence	Ion Score	C. I. %	Modification	Rank	Result Type
807.3995	807.4669	0.0674	83	229	235	LAADDFR					Mascot
809.4403	809.4898	0.0495	61	157	163	LASYLDK					Mascot
847.452	847.5265	0.0745	88	363	369	SEITELR					Mascot
1090.531	1090.6218	0.0908	83	148	156	VTMQNLNDR					Mascot
1118.5087	1118.6057	0.097	87	185	194	HGNSHQGEPR					Mascot
1165.5848	1165.6805	0.0957	82	442	450	LENEIQTYR					Mascot
1165.5848	1165.6805	0.0957	82	442	450	LENEIQTYR					Mascot
1201.6172	1201.7164	0.0992	83	246	256	QSVEADINGLR					Mascot
1234.6791	1234.7744	0.0953	77	236	245	LKYENEVALR					Mascot
1300.5951	1300.682	0.0869	67	286	295	NHEEEMKDLR					Mascot
1365.6393	1365.7504	0.1111	81	323	333	SQYEQLAEQNR					Mascot
1365.6393	1365.7504	0.1111	81	323	333	SQYEQLAEQNR	18	0			Mascot
1390.6809	1390.797	0.1161	83	387	399	QSLEASLAETEGR					Mascot
1434.7699	1434.8754	0.1055	74	440	450	IRLENEIQTYR					Mascot
1493.7343	1493.8571	0.1228	82	323	334	SQYEQLAEQNRK					Mascot
1707.7722	1707.9216	0.1494	87	41	59	GSLGGGFSSGGFSGGSF SR					Mascot
2367.2627	2367.4746	0.2119	90	208	228	NQILNLTTDNANILLQIDN AR					Mascot

4	Ribose import ATP-binding protein RbsA OS=Vibrio fischeri (strain ATCC 700601 / ES114) GN=rbsA PE=3 SV=1	RBSA_VIBF1	55553.6	5.2	16	66	87.324	5.635	

Peptide Information

Calc. Mass	Obsrv. Mass	± da	± ppm	Start Seq.	End Seq.	Sequence	Ion Score	C. I. %	Modification	Rank	Result Type
807.4359	807.4669	0.031	38	108	115	TNAFGGIK					Mascot
898.4338	898.5197	0.0859	96	473	480	ISGEFMAK			Oxidation (M)[6]		Mascot
943.5029	943.55	0.0471	50	316	323	MINPISPR			Oxidation (M)[1]		Mascot
990.5591	990.5675	0.0084	8	130	137	LNVKHHSR					Mascot
1055.6096	1055.6603	0.0507	48	416	424	VLILDEPTR					Mascot
1300.777	1300.682	-0.095	-73	404	415	VAIAKGLMTRPK			Oxidation (M)[8]		Mascot
1323.6692	1323.7491	0.0799	60	64	75	YEGKAVNFNGPK					Mascot
1475.7775	1475.8694	0.0919	62	1	13	MTQAILELNGIEK			Oxidation (M)[1]		Mascot
1475.7775	1475.8694	0.0919	62	1	13	MTQAILELNGIEK			Oxidation (M)[1]		Mascot
1493.723	1493.8571	0.1341	90	324	337	DGLANGIAYISEDR					Mascot
1507.8011	1507.8632	0.0621	41	298	311	IIYGALPMTCGAIK			Carbamidomethyl (C)[10]		Mascot
1584.7211	1584.8693	0.1482	94	473	486	ISGEFMAKDADQEK			Oxidation (M)[6]		Mascot
1839.0521	1839.0751	0.023	13	409	424	GLMTRPKVLILDEPTR					Mascot
1926.9993	1927.046	0.0467	24	386	403	TPTRDQIIGNLSGGNQQK					Mascot
1989.9586	1990.0985	0.1399	70	50	67	SLTGIYAMDAGEIRYEGK			Oxidation (M)[8]		Mascot
2384.1426	2384.1689	0.0263	11	163	183	VIIMDEPTDALTDTETESL FK			Oxidation (M)[4]		Mascot
2399.114	2399.2385	0.1245	52	443	464	AEGMSIILVSSEMPEVLG MSDR			Oxidation (M)[4,13,19]		Mascot

5 Threonine--tRNA ligase OS=Desulfotomaculum reducens (strain MI-1) GN=thrS PE=3 SV=1 | SYT_DESRM | 73548.5 | 6.04 | 19 | 64 | 78.473 | 5.877

Peptide Information

Calc. Mass	Obsrv. Mass	± da	± ppm	Start Seq.	End Seq.	Sequence	Ion Score	C. I. %	Modification	Rank	Result Type
806.4883	806.488	-0.0003	0	576	581	INYKIR					Mascot
813.4828	813.5146	0.0318	39	542	548	VLPITDR					Mascot
826.5145	826.5173	0.0028	3	294	300	TPIILNR					Mascot
906.5043	906.5355	0.0312	34	511	518	VVFGSIER					Mascot
973.5425	973.6044	0.0619	64	580	587	IREAQTQK					Mascot
1066.5792	1066.5927	0.0135	13	354	362	WGELGLVHR					Mascot
1073.5586	1073.6378	0.0792	74	567	575	VELDARNEK					Mascot
1107.5219	1107.6311	0.1092	99	274	281	NELENFWR					Mascot
1259.6565	1259.7604	0.1039	82	225	234	KAMLDEHLFR					Mascot
1303.6576	1303.7542	0.0966	74	344	353	MHSYRDLPLR			Oxidation (M)[1]		Mascot
1320.6842	1320.7004	0.0162	12	363	374	HELSGALHGLMR					Mascot
1320.6842	1320.7004	0.0162	12	363	374	HELSGALHGLMR					Mascot

Calc. Mass	Obsrv. Mass	± da	± ppm	Start Seq.	End Seq.	Sequence	Ion Score	C. I. %	Modification	Rank	Result Type
1379.7417	1379.8333	0.0916	66	13	25	EYAVGTTVLEVAK					Mascot
1383.6539	1383.7797	0.1258	91	491	502	FNLNYVGEDGQK					Mascot
1390.6743	1390.797	0.1227	88	598	609	EMEQGAVAVRER			Oxidation (M)[2]		Mascot
1487.8468	1487.8589	0.0121	8	40	52	VNGKIVDLEYPLK					Mascot
1584.7839	1584.8693	0.0854	54	197	210	LMSIAGAYWRGSEK			Oxidation (M)[2]		Mascot
1790.8054	1790.9393	0.1339	75	448	463	MDYKVNEGDGAFYGPK					Mascot
2384.2405	2384.1689	-0.0716	-30	354	374	WGELGLVHRHELSGALH GLMR			Oxidation (M)[20]		Mascot
2399.1621	2399.2385	0.0764	32	321	341	IDEADYAVKPMNCPGSIL VYK			Carbamidomethyl (C)[13], Oxidation (M)[11]		Mascot

6	Coiled-coil domain-containing protein 105 OS=Homo sapiens GN=CCDC105 PE=2 SV=3	CC105_HUMAN	57386.1	9.89	18	58	16.25	12.914

Peptide Information

Calc. Mass	Obsrv. Mass	± da	± ppm	Start Seq.	End Seq.	Sequence	Ion Score	C. I. %	Modification	Rank	Result Type
813.5053	813.5146	0.0093	11	153	158	QLLRQR					Mascot
815.4846	815.5014	0.0168	21	132	139	GARLTAAR					Mascot
844.525	844.5616	0.0366	43	302	308	RLLVESK					Mascot
857.5315	857.5762	0.0447	52	135	142	LTAARLGR					Mascot
868.5363	868.5245	-0.0118	-14	393	399	LDRPLVR					Mascot
890.4829	890.5129	0.03	34	340	347	ASETLELK					Mascot
906.4601	906.5355	0.0754	83	309	316	DTLVEMAK					Mascot
973.6305	973.6044	-0.0261	-27	109	116	LPLPLHRK					Mascot
1036.6262	1036.6017	-0.0245	-24	2	10	RVLVPPAER					Mascot
1307.7008	1307.785	0.0842	64	65	74	AAYIQPWRFR					Mascot
1308.6655	1308.761	0.0955	73	451	462	NIGHEVDGNVVR					Mascot
1320.6881	1320.7004	0.0123	9	98	108	GKMKPPAWYAR			Oxidation (M)[3]		Mascot
1320.6881	1320.7004	0.0123	9	98	108	GKMKPPAWYAR			Oxidation (M)[3]		Mascot
1329.6256	1329.7509	0.1253	94	232	241	DTLNFCFKER			Carbamidomethyl (C)[6]		Mascot
1333.7079	1333.7825	0.0746	56	348	358	ERLNMTLGLMR					Mascot
1365.6978	1365.7504	0.0526	39	348	358	ERLNMTLGLMR			Oxidation (M)[5,10]		Mascot
1365.6978	1365.7504	0.0526	39	348	358	ERLNMTLGLMR			Oxidation (M)[5,10]		Mascot
1584.8064	1584.8693	0.0629	40	16	30	VGAPAWREAAQAMAR					Mascot
1600.8013	1600.9283	0.127	79	16	30	VGAPAWREAAQAMAR			Oxidation (M)[13]		Mascot
1699.8082	1699.9738	0.1656	97	467	479	QRQPHVCYEQAQR			Carbamidomethyl (C)[7]		Mascot
1716.9253	1716.9916	0.0663	39	119	134	ALQTTEVVHAHARGAR					Mascot
1716.9253	1716.9916	0.0663	39	119	134	ALQTTEVVHAHARGAR	5	0			Mascot

1822.9698	1823.0372	0.0674	37	80	97	GGGTLEKPPPGEGVTLWK					Mascot

7	Golgin IMH1 OS=Saccharomyces cerevisiae (strain ATCC 204508 / S288c) GN=IMH1 PE=1 SV=1	IMH1_YEAST	105332.7	5.52	25	54	0	13.714	

Peptide Information

Calc. Mass	Obsrv. Mass	± da	± ppm	Start Seq.	End Seq.	Sequence	Ion Score	C. I. %	Modification	Rank	Result Type
806.4883	806.488	-0.0003	0	796	801	LLKNYR					Mascot
809.4152	809.4898	0.0746	92	145	150	KEQNYK					Mascot
830.5345	830.4927	-0.0418	-50	456	462	IELLSKK					Mascot
832.4047	832.3934	-0.0113	-14	151	157	NDIDDLK					Mascot
844.4886	844.5616	0.073	86	721	727	LREEGLK					Mascot
847.4631	847.5265	0.0634	75	449	455	NTELRSK					Mascot
857.5203	857.5762	0.0559	65	43	49	EIQAKLR					Mascot
888.4785	888.5284	0.0499	56	670	676	QKELENK					Mascot
990.5466	990.5675	0.0209	21	763	771	LSLSKADEK					Mascot
1033.5413	1033.5986	0.0573	55	203	211	EEVSISELK					Mascot
1037.515	1037.6063	0.0913	88	137	144	DKEEEFLK					Mascot
1060.5997	1060.6432	0.0435	41	802	811	SLSSQLNALK					Mascot
1066.6367	1066.5927	-0.044	-41	714	722	SEIHALKLR					Mascot
1118.5801	1118.6057	0.0256	23	175	184	NDTVSGLREK					Mascot
1179.6555	1179.7	0.0445	38	1	10	MFKQLSQIGK					Mascot
1201.6787	1201.7164	0.0377	31	426	435	TVKLELDDLR					Mascot
1232.6117	1232.6953	0.0836	68	387	396	EKNSELEEVR					Mascot
1308.6431	1308.761	0.1179	90	528	538	QFSEQKDVAEK					Mascot
1323.6512	1323.7491	0.0979	74	276	287	AISHQNVGNNNR					Mascot
1365.6896	1365.7504	0.0608	45	344	354	QKYEDIEAELK					Mascot
1365.6896	1365.7504	0.0608	45	344	354	QKYEDIEAELK					Mascot
1493.8071	1493.8571	0.05	33	799	811	NYRSLSSQLNALK					Mascot
1657.912	1657.9591	0.0471	28	4	18	QLSQIGKNLTDELAK					Mascot
1716.9265	1716.9916	0.0651	38	203	217	EEVSISELKEELAIK					Mascot
1716.9265	1716.9916	0.0651	38	203	217	EEVSISELKEELAIK					Mascot
1790.9257	1790.9393	0.0136	8	272	287	ELPKAISHQNVGNNNR					Mascot
2152.1052	2152.2019	0.0967	45	239	257	STIMEEKSSELAELNITLK			Oxidation (M)[4]		Mascot

8	Lon protease homolog, mitochondrial OS=Kluyveromyces lactis (strain ATCC 8585 / CBS 2359 / DSM 70799 / NBRC 1267 / NRRL Y-1140 / WM37) GN=PIM1 PE=3 SV=1	LONM_KLULA	124453.5	5.33	22	54	0	15.146	

Peptide Information

Calc. Mass	Obsrv. Mass	± da	± ppm	Start Seq.	End Seq.	Sequence	Ion Score	C. I. %	Modification	Rank	Result Type
815.4297	815.5014	0.0717	88	952	958	LAYSFSK					Mascot
832.4523	832.3934	-0.0589	-71	831	837	VKQSENK					Mascot
842.5458	842.5815	0.0357	42	802	809	KAALNVVK					Mascot
874.4265	874.499	0.0725	83	486	493	ELGIDDGR					Mascot
888.4785	888.5284	0.0499	56	202	209	AVVISDER					Mascot
1055.6096	1055.6603	0.0507	48	507	515	LQLPETVQK					Mascot
1090.531	1090.6218	0.0908	83	63	71	KQMNDPVSR			Oxidation (M)[3]		Mascot
1179.5861	1179.7	0.1139	97	317	326	ASLQKMENMK					Mascot
1182.5426	1182.6561	0.1135	96	1091	1100	GNTVWSEDFK					Mascot
1182.5426	1182.6561	0.1135	96	1091	1100	GNTVWSEDFK					Mascot
1232.5868	1232.6953	0.1085	88	473	481	EYYLMEQLK			Oxidation (M)[5]		Mascot
1259.6631	1259.7604	0.0973	77	74	83	LEQLESQWVK					Mascot
1277.7252	1277.8147	0.0895	70	221	231	QQPYIGAFLLK					Mascot
1277.7252	1277.8147	0.0895	70	221	231	QQPYIGAFLLK					Mascot
1320.7158	1320.7004	-0.0154	-12	749	760	IAENYLSPSAKK					Mascot
1320.7158	1320.7004	-0.0154	-12	749	760	IAENYLSPSAKK					Mascot
1333.678	1333.7825	0.1045	78	448	458	KELMNAELQNK			Oxidation (M)[4]		Mascot
1383.7056	1383.7797	0.0741	54	965	975	FPENRFFEVAK					Mascot
1407.6897	1407.8213	0.1316	93	939	951	TGQLGDVMKESSR					Mascot
1628.8466	1628.9979	0.1513	93	375	387	VFKEISQLNTMFR			Oxidation (M)[11]		Mascot
1716.885	1716.9916	0.1066	62	266	280	TGAETMTALLYPHRR					Mascot
1716.885	1716.9916	0.1066	62	266	280	TGAETMTALLYPHRR					Mascot
1790.8477	1790.9393	0.0916	51	810	824	QLSIDDKPMENEEVK			Oxidation (M)[9]		Mascot
1906.011	1906.1669	0.1559	82	539	554	NYLDWITSLPWGIISK					Mascot
1906.011	1906.1669	0.1559	82	539	554	NYLDWITSLPWGIISK					Mascot
1994.0858	1994.1547	0.0689	35	193	209	RPLFPGFYKAVVISDER					Mascot
2088.137	2088.2114	0.0744	36	1008	1027	GLDPTVAMTGELTLTGKVLR			Oxidation (M)[8]		Mascot

9	Membrane-bound lytic murein transglycosylase F OS=Shewanella denitrificans (strain OS217 / ATCC BAA-1090 / DSM 15013) GN=mltF PE=3 SV=1	MLTF_SHEDO	56094.7	6.18	12	54	0	3.747	

Peptide Information

Calc. Mass	Obsrv. Mass	± da	± ppm	Start Seq.	End Seq.	Sequence	Ion Score	C. I. %	Modification	Rank	Result Type

809.4225	809.4898	0.0673	83	113	119	MGPTLYK					Mascot
847.54	847.5265	-0.0135	-16	84	90	QLFKALK					Mascot
888.4421	888.5284	0.0863	97	465	472	VDANDNIK					Mascot
890.4651	890.5129	0.0478	54	209	216	AGMILEEK					Mascot
906.4944	906.5355	0.0411	45	255	261	YFGHVKR					Mascot
990.5731	990.5675	-0.0056	-6	120	127	VNQVLVYR					Mascot
1193.7292	1193.7139	-0.0153	-13	217	226	IEVVWLLPPK					Mascot
1365.7195	1365.7504	0.0309	23	345	356	GGAIYLKDMIER					Mascot
1365.7195	1365.7504	0.0309	23	345	356	GGAIYLKDMIER	18	0			Mascot
1487.7714	1487.8589	0.0875	59	424	436	GSQAVHYVDSIRR					Mascot
1791.8541	1791.8975	0.0434	24	352	366	DMIERLPESISDSQR			Oxidation (M)[2]		Mascot
1800.8916	1800.9937	0.1021	57	436	449	RYYDTLVWVDNQTK					Mascot
1989.9626	1990.0985	0.1359	68	66	82	FADYLGVSLEMIPFTDR			Oxidation (M)[11]		Mascot

10	Probable histone-lysine N-methyltransferase ATXR3 OS=Arabidopsis thaliana GN=ATXR3 PE=2 SV=2	ATXR3_ARATH	269117.4	6.44	23	54	0	13.45

Peptide Information

Calc. Mass	Obsrv. Mass	± da	± ppm	Start Seq.	End Seq.	Sequence	Ion Score	C. I. %	Modification	Rank	Result Type
815.441	815.5014	0.0604	74	1775	1780	YVSYRK					Mascot
819.4108	819.4697	0.0589	72	1012	1018	SHSSVFR					Mascot
823.4607	823.5065	0.0458	56	2057	2062	VRYVMR					Mascot
832.441	832.3934	-0.0476	-57	1434	1440	VSQIEEK					Mascot
833.4152	833.39	-0.0252	-30	236	242	GEFIPDR					Mascot
844.5251	844.5616	0.0365	43	887	894	ASLLSVVR					Mascot
868.5073	868.5245	0.0172	20	1089	1095	LHQLVMK					Mascot
905.4799	905.5175	0.0376	42	297	304	SGLDRTTR					Mascot
906.468	906.5355	0.0675	74	717	723	EIFVENR					Mascot
990.485	990.5675	0.0825	83	959	967	TDTSVNTPR					Mascot
1033.5347	1033.5986	0.0639	62	1033	1041	SPETIAMLR			Oxidation (M)[7]		Mascot
1036.5687	1036.6017	0.033	32	2279	2286	QPQRPWPK					Mascot
1090.6104	1090.6218	0.0114	10	1329	1338	SLKQTTDVAK					Mascot
1107.5714	1107.6311	0.0597	54	1434	1442	VSQIEEKMK			Oxidation (M)[8]		Mascot
1165.575	1165.6805	0.1055	91	1443	1452	NGYYVSHGLR					Mascot
1165.575	1165.6805	0.1055	91	1443	1452	NGYYVSHGLR	18	0			Mascot
1193.679	1193.7139	0.0349	29	1996	2004	LVRFINFER					Mascot
1300.7372	1300.682	-0.0552	-42	2136	2145	SLLWLRDEIR					Mascot

1307.693	1307.785	0.092	70	1202	1211	VFHMLRYDVK				Mascot
1320.6332	1320.7004	0.0672	51	284	293	FVNEDIYHQR				Mascot
1320.6332	1320.7004	0.0672	51	284	293	FVNEDIYHQR				Mascot
1323.6879	1323.7491	0.0612	46	1202	1211	VFHMLRYDVK			Oxidation (M)[4]	Mascot
1365.7638	1365.7504	-0.0134	-10	1941	1951	VLKDWHGLLER				Mascot
1365.7638	1365.7504	-0.0134	-10	1941	1951	VLKDWHGLLER	18	0		Mascot
1493.7635	1493.8571	0.0936	63	2021	2032	KYFSDIHLDVEK				Mascot
1699.8181	1699.9738	0.1557	92	920	935	CPSRPARPSPASSDSK			Carbamidomethyl (C)[1]	Mascot
1791.9496	1791.8975	-0.0521	-29	829	843	KIVLNDGFPLCLMQK			Carbamidomethyl (C)[11], Oxidation (M)[13]	Mascot

Gel Idx/Pos	126/F1	Instr./Gel Origin	BA2151/Sample Project 20140814	Process Status	Analysis Succeeded
Plate [#] Name	[1] Sample Project 20140814	Instrument Sample Name		Spectra	11

Rank	Protein Name	Accession No.	Protein MW	Protein PI	Pep. Count	Protein Score	Protein Score C. I. %	Intensity Matched	Total Ion Score	Total Ion C. I. %	Confirmed
1	Valine--tRNA ligase OS=Rattus norvegicus GN=Vars PE=2 SV=2	SYVC_RAT	141534.5	7.54	26	71	95.803	11.828			

Peptide Information

Calc. Mass	Obsrv. Mass	± da	± ppm	Start Seq.	End Seq.	Sequence	Ion Score	C. I. %	Modification	Rank	Result Type
806.4519	806.4321	-0.0198	-25	247	252	FQQKQK					Mascot
807.4467	807.4438	-0.0029	-4	833	839	MVMLGLK			Oxidation (M)[1]		Mascot
811.4461	811.45	0.0039	5	50	56	TPFPPPR					Mascot
854.5458	854.4635	-0.0823	-96	264	270	KPKPEKK					Mascot
878.4254	878.4912	0.0658	75	770	777	EFGVSPDK					Mascot
886.5356	886.4849	-0.0507	-57	1200	1207	ELGKLQAK					Mascot
920.4836	920.5696	0.086	93	174	181	YVLDPSAR					Mascot
920.52	920.5696	0.0496	54	244	250	LEKFQQK					Mascot
963.537	963.5128	-0.0242	-25	700	707	ILPEAHQR					Mascot
982.5006	982.5057	0.0051	5	724	730	QLWWGHR					Mascot
1001.5639	1001.5497	-0.0142	-14	182	189	RIWGNVTR					Mascot
1003.5934	1003.5793	-0.0141	-14	840	847	LTEKLPFR					Mascot
1028.5596	1028.532	-0.0276	-27	1214	1221	QAQRLQER					Mascot
1073.5447	1073.5959	0.0512	48	1209	1217	SEAQRQAQR					Mascot
1115.5844	1115.5979	0.0135	12	462	470	LHEEGVIYR					Mascot
1154.5988	1154.6056	0.0068	6	40	49	ICLQPPPSSR			Carbamidomethyl (C)[2]		Mascot
1154.5988	1154.6056	0.0068	6	40	49	ICLQPPPSSR			Carbamidomethyl (C)[2]		Mascot
1193.6525	1193.6578	0.0053	4	451	461	LSATVTEAFVR					Mascot
1240.6685	1240.6931	0.0246	20	138	147	ALNPLEEWLR					Mascot
1262.7211	1262.6655	-0.0556	-44	833	843	MVMLGLKLTEK					Mascot
1314.6294	1314.7166	0.0872	66	655	665	DNPMVVPLCNR			Carbamidomethyl (C)[9]		Mascot
1333.7474	1333.7375	-0.0099	-7	202	214	AVLGEVVLYSGAR					Mascot
1333.7474	1333.7375	-0.0099	-7	202	214	AVLGEVVLYSGAR					Mascot
1355.7053	1355.7042	-0.0011	-1	1231	1242	VPLEVQEADEVK					Mascot
1379.7278	1379.6986	-0.0292	-21	123	137	GPGQDPQAALGALGK					Mascot
1507.7612	1507.7996	0.0384	25	251	263	QKTQQQQPAHGEK					Mascot

1615.873	1615.8756	0.0026	2	508	521	EKVEFGVLVSFAYK					Mascot
1691.8083	1691.913	0.1047	62	522	537	VQGSDSDEEVVVATTR					Mascot

2 Vinculin OS=Homo sapiens GN=VCL PE=1 SV=4 VINC_HUMAN 124292 5.5 28 70 93.933 7.263

Peptide Information

Calc. Mass	Obsrv. Mass	± da	± ppm	Start Seq.	End Seq.	Sequence	Ion Score	C. I. %	Modification	Rank	Result Type
802.4417	802.4725	0.0308	38	647	655	AAAVGTANK					Mascot
807.3777	807.4438	0.0661	82	1039	1044	QCTDKR			Carbamidomethyl (C)[2]		Mascot
815.4733	815.4153	-0.058	-71	410	417	GALAEARK					Mascot
820.4233	820.4497	0.0264	32	320	326	EILGTCK			Carbamidomethyl (C)[6]		Mascot
821.3821	821.4584	0.0763	93	81	87	VENACTK			Carbamidomethyl (C)[5]		Mascot
824.4625	824.4713	0.0088	11	489	496	AAVHLEGK					Mascot
868.4271	868.5136	0.0865	100	904	910	QLHDEAR					Mascot
880.4523	880.4979	0.0456	52	1118	1125	TDAGFTLR					Mascot
934.4702	934.5311	0.0609	65	73	80	DMPPAFIK			Oxidation (M)[2]		Mascot
944.4472	944.4956	0.0484	51	816	823	SFLDSGYR					Mascot
993.4855	993.5475	0.062	62	171	178	MAKMIDER					Mascot
1001.5261	1001.5497	0.0236	24	220	228	NQGIEEALK					Mascot
1028.5847	1028.532	-0.0527	-51	984	993	GNDIIAAAKR					Mascot
1037.5295	1037.5587	0.0292	28	373	381	KLEAMTNSK			Oxidation (M)[5]		Mascot
1060.4979	1060.5878	0.0899	85	418	426	IAELCDDPK			Carbamidomethyl (C)[5]		Mascot
1078.5198	1078.5739	0.0541	50	1035	1043	EVAKQCTDK			Carbamidomethyl (C)[6]		Mascot
1090.5714	1090.5863	0.0149	14	72	80	RDMPPAFIK			Oxidation (M)[3]		Mascot
1105.5306	1105.6265	0.0959	87	342	352	GQGSSPVAMQK			Oxidation (M)[9]		Mascot
1105.61	1105.6265	0.0165	15	434	444	SLGEISALTSK					Mascot
1118.5623	1118.5729	0.0106	9	539	547	QDLLAKCDR			Carbamidomethyl (C)[7]		Mascot
1152.5574	1152.6266	0.0692	60	994	1003	MALLMAEMSR					Mascot
1308.6584	1308.7041	0.0457	35	993	1003	RMALLMAEMSR					Mascot
1314.7377	1314.7166	-0.0211	-16	465	476	QVATALQNLQTK					Mascot
1332.6689	1332.7048	0.0359	27	340	352	ARGQGSSPVAMQK			Oxidation (M)[11]		Mascot
1349.6332	1349.7333	0.1001	74	770	780	EVENSEDPKFR					Mascot
1436.7413	1436.7327	-0.0086	-6	374	386	LEAMTNSKQSIAK			Oxidation (M)[4]		Mascot
1436.7413	1436.7327	-0.0086	-6	374	386	LEAMTNSKQSIAK			Oxidation (M)[4]		Mascot
1819.9557	1819.9797	0.024	13	529	544	LANVMMGPYRQDLLAK					Mascot
2384.1267	2384.0923	-0.0344	-14	890	910	AGEVINQPMMMAARQLH DEAR			Oxidation (M)[9]		Mascot

3	Dystonin OS=Homo sapiens GN=DST PE=1 SV=4	DYST_HUMAN	865259.2	5.14	74	69	93.792	34.087	15	0		

Peptide Information

Calc. Mass	Obsrv. Mass	± da	± ppm	Start Seq.	End Seq.	Sequence	Ion Score	C. I. %	Modification	Rank	Result Type
802.4457	802.4725	0.0268	33	4639	4644	QWLVEK					Mascot
807.373	807.4438	0.0708	88	6338	6344	VTEESDK					Mascot
814.3916	814.4446	0.053	65	7202	7207	VMDFFR					Mascot
815.4006	815.4153	0.0147	18	2610	2616	DENGKPR					Mascot
820.441	820.4497	0.0087	11	4410	4416	SLEDTKK					Mascot
821.4515	821.4584	0.0069	8	5929	5934	YLQLER					Mascot
827.4006	827.478	0.0774	94	4018	4024	LDHATDR					Mascot
829.4414	829.4451	0.0037	4	7086	7092	KQPDVDK					Mascot
834.4719	834.4493	-0.0226	-27	7453	7460	LPGYLSGK					Mascot
836.4294	836.4729	0.0435	52	4240	4246	MKDLSAR			Oxidation (M)[1]		Mascot
846.493	846.5065	0.0135	16	4950	4956	LKESLEK					Mascot
847.4341	847.448	0.0139	16	471	477	DEIMALR					Mascot
854.473	854.4635	-0.0095	-11	6960	6966	TLIDQHK					Mascot
878.4651	878.4912	0.0261	30	4261	4267	MEELKTK					Mascot
886.5244	886.4849	-0.0395	-45	6412	6419	AIEIELAK					Mascot
888.4785	888.5007	0.0222	25	4130	4137	TLDDIVGR					Mascot
906.4349	906.5106	0.0757	84	1374	1381	AMVDSQQK					Mascot
911.4945	911.4946	0.0001	0	3067	3074	KHVEDVGK					Mascot
920.4869	920.5696	0.0827	90	5851	5858	LMSLGDIR			Oxidation (M)[2]		Mascot
920.52	920.5696	0.0496	54	6859	6866	AFQKELGK					Mascot
934.5026	934.5311	0.0285	30	862	870	STIANLMGK					Mascot
942.5479	942.4948	-0.0531	-56	5265	5272	LLDRAQAR					Mascot
944.5159	944.4956	-0.0203	-21	7165	7172	KLNDALDR					Mascot
963.42	963.5128	0.0928	96	5801	5808	MVAEDNER					Mascot
975.4854	975.5643	0.0789	81	4144	4151	SVNERNEK					Mascot
981.5112	981.5219	0.0107	11	1761	1769	AAHEGLIDR					Mascot
989.5738	989.5548	-0.019	-19	6868	6876	TSSVQALKR					Mascot
992.5411	992.5588	0.0177	18	4386	4393	SWIKETTK					Mascot
993.5098	993.5475	0.0377	38	1476	1484	ISSEEISTK					Mascot
995.5044	995.5937	0.0893	90	3285	3293	LLADGYSEK					Mascot
1010.5339	1010.5422	0.0083	8	5873	5880	TFTMEILR					Mascot
1012.5898	1012.5197	-0.0701	-69	1089	1096	QIRTPLER					Mascot

1028.5847	1028.532	-0.0527	-51	6478	6486	KQQLDGALR				Mascot
1042.5415	1042.5468	0.0053	5	5330	5338	EEIEPLQGK				Mascot
1057.5248	1057.5532	0.0284	27	7200	7207	SRVMDFFR				Mascot
1060.582	1060.5878	0.0058	5	1718	1726	VSLIDMVQR				Mascot
1066.6368	1066.5559	-0.0809	-76	6740	6748	IKTQLAQHK				Mascot
1073.5198	1073.5959	0.0761	71	7200	7207	SRVMDFFR			Oxidation (M)[4]	Mascot
1078.5375	1078.5739	0.0364	34	6116	6126	SGGTDKDISAK				Mascot
1084.5674	1084.6068	0.0394	36	1046	1053	QYYQELLK				Mascot
1090.6401	1090.5863	-0.0538	-49	7324	7332	ILRSTVMVR			Oxidation (M)[7]	Mascot
1105.5419	1105.6265	0.0846	77	6107	6115	QRGEEMIAR			Oxidation (M)[6]	Mascot
1105.5419	1105.6265	0.0846	77	6107	6115	QRGEEMIAR	15	0	Oxidation (M)[6]	Mascot
1107.5463	1107.6112	0.0649	59	6894	6902	VQMQELSTR			Oxidation (M)[3]	Mascot
1115.642	1115.5979	-0.0441	-40	4108	4117	KTAEVLLDAR				Mascot
1143.5641	1143.5796	0.0155	14	2813	2823	DPQGNGSDLIK				Mascot
1152.5784	1152.6266	0.0482	42	647	656	AIEEFESSLK				Mascot
1162.6831	1162.6561	-0.027	-23	4819	4828	QLEGILKSFK				Mascot
1165.6953	1165.6305	-0.0648	-56	7003	7011	HWITIIRAR				Mascot
1182.5287	1182.6128	0.0841	71	2548	2557	FDDSGSWRGR				Mascot
1182.5287	1182.6128	0.0841	71	2548	2557	FDDSGSWRGR				Mascot
1193.5765	1193.6578	0.0813	68	1699	1707	VLERQNMCK			Carbamidomethyl (C)[8], Oxidation (M)[7]	Mascot
1201.6787	1201.6705	-0.0082	-7	3766	3776	VAAVKQLEESK				Mascot
1205.6525	1205.6478	-0.0047	-4	4070	4080	HLSEPIAVDPK				Mascot
1223.634	1223.6602	0.0262	21	3452	3461	DMKLAEEFLK				Mascot
1227.6804	1227.676	-0.0044	-4	6479	6489	QQLDGALRQAK				Mascot
1243.6027	1243.714	0.1113	89	4001	4012	DGGKVDTSATHR				Mascot
1243.6893	1243.714	0.0247	20	5459	5470	IATTAEPADKVK				Mascot
1248.5599	1248.6755	0.1156	93	1936	1946	MPDLGDLEACK			Carbamidomethyl (C)[10]	Mascot
1308.6252	1308.7041	0.0789	60	4895	4905	TLTAQSHMYEK				Mascot
1314.6648	1314.7166	0.0518	39	1315	1325	KIQENQPENSK				Mascot
1331.7318	1331.7196	-0.0122	-9	1448	1458	ELQKWVSNISK				Mascot
1332.6504	1332.7048	0.0544	41	1241	1251	AISDEMFKTYK				Mascot
1333.6781	1333.7375	0.0594	45	1374	1385	AMVDSQQKSPVK			Oxidation (M)[2]	Mascot
1333.6859	1333.7375	0.0516	39	7294	7304	RFQVEQIGDNK				Mascot
1355.6987	1355.7042	0.0055	4	2697	2707	ENLLNHEMVLK			Oxidation (M)[8]	Mascot
1417.791	1417.7399	-0.0511	-36	5557	5569	HLHQAVSIGQSLK				Mascot
1435.7064	1435.7716	0.0652	45	3881	3892	QGQYLSPEEKEK				Mascot
1436.7566	1436.7327	-0.0239	-17	888	899	TSIPIKAICDYR			Carbamidomethyl (C)[9]	Mascot

1436.7566	1436.7327	-0.0239	-17	888	899	TSIPIKAICDYR			Carbamidomethyl (C)[9]		Mascot
1527.7802	1527.791	0.0108	7	1046	1057	QYYQELLKSAER					Mascot
1562.8148	1562.8428	0.028	18	40	51	TFTKWINQHLMK			Oxidation (M)[11]		Mascot
1615.8286	1615.8756	0.047	29	5859	5872	LEQDQTSAQLQVQK					Mascot
1636.9017	1636.8833	-0.0184	-11	3112	3125	IEQQLPKEQALSPR					Mascot
1657.7804	1657.9304	0.15	90	227	241	LLDPEDVDVSSPDEK					Mascot
1657.7804	1657.9304	0.15	90	227	241	LLDPEDVDVSSPDEK					Mascot
1819.9701	1819.9797	0.0096	5	157	172	LLLWTQQATEGYAGIR					Mascot
1883.1001	1882.9701	-0.13	-69	3434	3450	QLETFELGLAPIAVILR					Mascot
2227.125	2227.1355	0.0105	5	7327	7346	STVMVRVGGGWMALDE FLVK			Oxidation (M)[4,12]		Mascot

4 Pre-mRNA-splicing factor CWC21 OS=Saccharomyces cerevisiae (strain ATCC 204508 / S288c) GN=CWC21 PE=1 SV=1 | CWC21_YEAST | 15810 | 9.67 | 11 | 66 | 84.76 | 2.256

Peptide Information

Calc. Mass	Obsrv. Mass	± da	± ppm	Start Seq.	End Seq.	Sequence	Ion Score	C. I. %	Modification	Rank	Result Type
847.4454	847.448	0.0026	3	99	105	CEALRAK			Carbamidomethyl (C)[1]		Mascot
982.5026	982.5057	0.0031	3	1	9	MSYNGIGLK					Mascot
1082.5663	1082.551	-0.0153	-14	116	124	MSSLYTPRK					Mascot
1084.5607	1084.6068	0.0461	43	31	39	RPQGSQQQR					Mascot
1201.6423	1201.6705	0.0282	23	72	81	EIEVQVSELR					Mascot
1240.6617	1240.6931	0.0314	25	30	39	RRPQGSQQQR					Mascot
1331.6339	1331.7196	0.0857	64	106	115	LTNEWQEQQR					Mascot
1507.834	1507.7996	-0.0344	-23	49	62	ASHDKASRPLAVQK					Mascot
1691.7858	1691.913	0.1272	75	84	97	LEEEETLSEEQIDK					Mascot
1757.8638	1757.906	0.0422	24	13	29	GSSTSGHVQRSLASNNR					Mascot
1819.8807	1819.9797	0.099	54	84	98	LEEEETLSEEQIDKK					Mascot

5 Vinculin OS=Sus scrofa GN=VCL PE=1 SV=4 | VINC_PIG | 124437.2 | 5.62 | 27 | 64 | 79.91 | 7.323

Peptide Information

Calc. Mass	Obsrv. Mass	± da	± ppm	Start Seq.	End Seq.	Sequence	Ion Score	C. I. %	Modification	Rank	Result Type
802.4417	802.4725	0.0308	38	648	656	AAAVGTANK					Mascot
807.3777	807.4438	0.0661	82	1040	1045	QCTDKR			Carbamidomethyl (C)[2]		Mascot
815.4733	815.4153	-0.058	-71	411	418	GALAEARK					Mascot
820.4233	820.4497	0.0264	32	321	327	EILGTCK			Carbamidomethyl (C)[6]		Mascot

821.3821	821.4584	0.0763	93	81	87	VENACTK			Carbamidomethyl (C)[5]		Mascot
824.4625	824.4713	0.0088	11	490	497	AAVHLEGK					Mascot
868.4271	868.5136	0.0865	100	905	911	QLHDEAR					Mascot
880.4523	880.4979	0.0456	52	1119	1126	TDAGFTLR					Mascot
934.4702	934.5311	0.0609	65	73	80	DMPPAFIK			Oxidation (M)[2]		Mascot
944.4472	944.4956	0.0484	51	817	824	SFLDSGYR					Mascot
993.4855	993.5475	0.062	62	171	178	MAKMIDER					Mascot
1001.5261	1001.5497	0.0236	24	220	228	NQGIEEALK					Mascot
1028.5847	1028.532	-0.0527	-51	985	994	GNDIIAAAKR					Mascot
1037.5295	1037.5587	0.0292	28	374	382	KLEAMTNSK			Oxidation (M)[5]		Mascot
1060.4979	1060.5878	0.0899	85	419	427	IAELCDDPK			Carbamidomethyl (C)[5]		Mascot
1073.5409	1073.5959	0.055	51	343	353	GQGASPVAMQK					Mascot
1078.5198	1078.5739	0.0541	50	1036	1044	EVAKQCTDK			Carbamidomethyl (C)[6]		Mascot
1090.5714	1090.5863	0.0149	14	72	80	RDMPPAFIK			Oxidation (M)[3]		Mascot
1105.61	1105.6265	0.0165	15	435	445	SLGEISALTSK					Mascot
1105.61	1105.6265	0.0165	15	435	445	SLGEISALTSK					Mascot
1118.5623	1118.5729	0.0106	9	540	548	QDLLAKCDR			Carbamidomethyl (C)[7]		Mascot
1152.5574	1152.6266	0.0692	60	995	1004	MALLMAEMSR					Mascot
1308.6584	1308.7041	0.0457	35	994	1004	RMALLMAEMSR					Mascot
1314.7377	1314.7166	-0.0211	-16	466	477	QVATALQNLQTK					Mascot
1349.6332	1349.7333	0.1001	74	771	781	EVENSEDPKFR					Mascot
1436.7413	1436.7327	-0.0086	-6	375	387	LEAMTNSKQSIAK			Oxidation (M)[4]		Mascot
1436.7413	1436.7327	-0.0086	-6	375	387	LEAMTNSKQSIAK			Oxidation (M)[4]		Mascot
1819.9557	1819.9797	0.024	13	530	545	LANVMMGPYRQDLLAK					Mascot
2384.1267	2384.0923	-0.0344	-14	891	911	AGEVINQPMMMAARQLH DEAR			Oxidation (M)[9]		Mascot

6	Peptide chain release factor 1 OS=Aquifex aeolicus (strain VF5) GN=prfA PE=3 SV=1	RF1_AQUAE	41865.3	6.62	17	62	68.159	7.651

Peptide Information

Calc. Mass	Obsrv. Mass	± da	± ppm	Start Seq.	End Seq.	Sequence	Ion Score	C. I. %	Modification	Rank	Result Type
820.4159	820.4497	0.0338	41	72	78	SSDKDLR					Mascot
836.4625	836.4729	0.0104	12	13	18	LQEKYR					Mascot
847.4883	847.448	-0.0403	-48	149	156	VSILSSNK					Mascot
856.525	856.5681	0.0431	50	113	119	NVILEIR					Mascot
864.5189	864.5106	-0.0083	-10	324	330	INLTLYK					Mascot
878.4651	878.4912	0.0261	30	88	94	LTEEMKK					Mascot

888.5036	888.5007	-0.0029	-3	94	100	KLEEELK					Mascot
1003.5167	1003.5793	0.0626	62	297	305	KEQVGTGER					Mascot
1010.4941	1010.5422	0.0481	48	141	148	YAEEKGWK					Mascot
1057.5889	1057.5532	-0.0357	-34	339	347	LDEIIDALR					Mascot
1126.5925	1126.571	-0.0215	-19	1	9	MLKEAYISR			Oxidation (M)[1]		Mascot
1205.5507	1205.6478	0.0971	81	137	145	MYQKYAEEK			Oxidation (M)[1]		Mascot
1308.6808	1308.7041	0.0233	18	309	318	IRTYNFPQNR					Mascot
1314.7125	1314.7166	0.0041	3	187	198	VQRVPVTESSGR					Mascot
1435.7063	1435.7716	0.0653	45	45	55	ELQEINELYER					Mascot
1805.928	1805.9062	-0.0218	-12	42	55	ELKELQEINELYER					Mascot
1819.8741	1819.9797	0.1056	58	79	93	ELAEEEVNRLTEEMK					Mascot

7	Nitrogenase iron-molybdenum cofactor biosynthesis protein NifE OS=Methanococcus maripaludis GN=nifE PE=3 SV=1	NIFE_METMI	54272.1	5.49	16	59	36.47	10.199	

Peptide Information

Calc. Mass	Obsrv. Mass	± da	± ppm	Start Seq.	End Seq.	Sequence	Ion Score	C. I. %	Modification	Rank	Result Type
811.4349	811.45	0.0151	19	293	299	YGIPSFK					Mascot
864.4462	864.5106	0.0644	74	129	136	DIVFGGEK					Mascot
880.5138	880.4979	-0.0159	-18	431	437	EKYLSLK					Mascot
888.5149	888.5007	-0.0142	-16	327	333	RTEELIK					Mascot
911.4833	911.4946	0.0113	12	111	118	STGDKLYK					Mascot
939.5298	939.5062	-0.0236	-25	292	299	KYGIPSFK					Mascot
989.5163	989.5548	0.0385	39	194	202	SFNKTAGHK					Mascot
992.5411	992.5588	0.0177	18	129	137	DIVFGGEKK					Mascot
1073.5586	1073.5959	0.0373	35	2	10	VLNLDTENR					Mascot
1201.6536	1201.6705	0.0169	14	2	11	VLNLDTENRK					Mascot
1227.5674	1227.676	0.1086	88	281	291	SSNYLAAQMDK					Mascot
1332.6941	1332.7048	0.0107	8	1	11	MVLNLDTENRK					Mascot
1333.7362	1333.7375	0.0013	1	419	430	EYKPDILISGAK					Mascot
1333.7362	1333.7375	0.0013	1	419	430	EYKPDILISGAK					Mascot
1355.6624	1355.7042	0.0418	31	281	292	SSNYLAAQMDKK					Mascot
1657.7891	1657.9304	0.1413	85	314	327	AVAEFFGDEEMIKR			Oxidation (M)[11]		Mascot
1657.7891	1657.9304	0.1413	85	314	327	AVAEFFGDEEMIKR			Oxidation (M)[11]		Mascot
1691.8541	1691.913	0.0589	35	450	464	ITAFSGYQGFINFAR					Mascot

8	DNA-directed RNA polymerase subunit beta' OS=Herminiimonas arsenicoxydans GN=rpoC PE=3	RPOC_HERAR	156853.2	7.01	28	58	6.032	7.695	

SV=1

Peptide Information

Calc. Mass	Obsrv. Mass	± da	± ppm	Start Seq.	End Seq.	Sequence	Ion Score	C. I. %	Modification	Rank	Result Type
802.4305	802.4725	0.042	52	1126	1132	IPTESQK					Mascot
807.428	807.4438	0.0158	20	326	332	SLAEMIK			Oxidation (M)[5]		Mascot
814.4669	814.4446	-0.0223	-27	22	29	IGLASPEK					Mascot
827.5098	827.478	-0.0318	-38	1075	1081	TVRPQVK					Mascot
829.489	829.4451	-0.0439	-53	973	980	GGQIVISR					Mascot
836.4005	836.4729	0.0724	87	697	703	AMMDQLK					Mascot
846.468	846.5065	0.0385	45	704	710	VEDVTKR					Mascot
847.452	847.448	-0.004	-5	203	209	QELKDSK					Mascot
934.4992	934.5311	0.0319	34	1143	1150	VAELFEAR					Mascot
963.4564	963.5128	0.0564	59	885	892	TPLTCDTR			Carbamidomethyl (C)[5]		Mascot
975.5734	975.5643	-0.0091	-9	338	345	FRQNLLGK					Mascot
982.4952	982.5057	0.0105	11	791	799	TANSGYLTR					Mascot
992.5444	992.5588	0.0144	15	326	334	SLAEMIKGK			Oxidation (M)[5]		Mascot
995.5091	995.5937	0.0846	85	418	425	EHPVMLNR					Mascot
1028.6099	1028.532	-0.0779	-76	876	884	LGIDEVKVR					Mascot
1060.6401	1060.5878	-0.0523	-49	999	1008	VPYGATLIVK					Mascot
1078.6329	1078.5739	-0.059	-55	1	9	MKALLDLFK					Mascot
1084.6473	1084.6068	-0.0405	-37	1347	1356	GLKENVIVGR					Mascot
1090.5011	1090.5863	0.0852	78	1284	1292	SELLDENDR					Mascot
1205.681	1205.6478	-0.0332	-28	385	395	LELMGLATTIK			Oxidation (M)[4]		Mascot
1232.6304	1232.6476	0.0172	14	197	206	DAEMLRQELK					Mascot
1248.6252	1248.6755	0.0503	40	197	206	DAEMLRQELK			Oxidation (M)[4]		Mascot
1314.7893	1314.7166	-0.0727	-55	587	598	GLPFSVLNRALK					Mascot
1355.7529	1355.7042	-0.0487	-36	825	837	ALVEGGEVIEALR					Mascot
1417.7104	1417.7399	0.0295	21	191	202	AIDIDRDAEMLR					Mascot
1435.6846	1435.7716	0.087	61	1175	1186	QRLEITDMDGNK			Oxidation (M)[8]		Mascot
1507.7495	1507.7996	0.0501	33	697	709	AMMDQLKVEDVTK					Mascot
1657.7938	1657.9304	0.1366	82	893	906	FGLCALCYGRDLGR			Carbamidomethyl (C)[4,7]		Mascot
1657.7938	1657.9304	0.1366	82	893	906	FGLCALCYGRDLGR			Carbamidomethyl (C)[4,7]		Mascot
1905.9188	1906.0681	0.1493	78	1374	1389	ESWEAEERTALLQSEK					Mascot

9	DNA-directed RNA polymerase subunit omega OS=Staphylococcus epidermidis (strain ATCC 12228) GN=rpoZ PE=3 SV=1	RPOZ_STAES	7786.2	8.01	7	57	1.603	1.854	

Protein Group

DNA-directed RNA polymerase subunit omega OS=Staphylococcus epidermidis (strain ATCC 35984 / RP62A) GN=rpoZ PE=3 SV=1	RPOZ_STAEQ	7761.2	7.9800 000190 7349

Peptide Information

Calc. Mass	Obsrv. Mass	± da	± ppm	Start Seq.	End Seq.	Sequence	Ion Score	C. I. %	Modification	Rank	Result Type
888.4533	888.5007	0.0474	53	27	33	ARELDER					Mascot
1107.6521	1107.6112	-0.0409	-37	17	26	YLIATTAAKR					Mascot
1243.6932	1243.714	0.0208	17	60	70	IEPVVPKEYLG					Mascot
1243.6932	1243.714	0.0208	17	60	70	IEPVVPKEYLG					Mascot
1355.7351	1355.7042	-0.0309	-23	1	12	MLNPPLNQLTAK			Oxidation (M)[1]		Mascot
1379.7893	1379.6986	-0.0907	-66	13	25	VNSKYLIATTAAK					Mascot
1636.9381	1636.8833	-0.0548	-33	2	16	LNPPLNQLTAKVNSK					Mascot
1707.9528	1707.8776	-0.0752	-44	51	66	ALEEIADGKIEPVVPK					Mascot

10	Metalloendopeptidase G1 OS=Vaccinia virus (strain Copenhagen) GN=G1L PE=3 SV=1	G1_VACCC	68266.6	6.3	17	57	0	3.134	

Peptide Information

Calc. Mass	Obsrv. Mass	± da	± ppm	Start Seq.	End Seq.	Sequence	Ion Score	C. I. %	Modification	Rank	Result Type
806.444	806.4321	-0.0119	-15	568	574	CLVVSTK			Carbamidomethyl (C)[1]		Mascot
814.4855	814.4446	-0.0409	-50	1	7	MIVLPNK					Mascot
815.4482	815.4153	-0.0329	-40	412	418	RNAEGIR					Mascot
820.5039	820.4497	-0.0542	-66	482	488	SKILFGR					Mascot
886.5104	886.4849	-0.0255	-29	413	420	NAEGIRVK					Mascot
1082.5477	1082.551	0.0033	3	471	479	DSTLEFSKR					Mascot
1162.582	1162.6561	0.0741	64	153	161	DMLVNRMQR					Mascot
1223.6267	1223.6602	0.0335	27	575	585	GDVENFSSLKK					Mascot
1232.7184	1232.6476	-0.0708	-57	370	379	KPYRSIPLMK					Mascot
1248.7059	1248.6755	-0.0304	-24	380	389	RLTSNEIFIR					Mascot
1262.5688	1262.6655	0.0967	77	110	118	ELENEYYFR					Mascot
1308.7198	1308.7041	-0.0157	-12	334	344	DIIVIYPNFSK					Mascot
1331.6987	1331.7196	0.0209	16	142	152	IDMIDNLNIVR			Oxidation (M)[3]		Mascot
1332.7998	1332.7048	-0.095	-71	162	173	ISGSNIVIFVKR					Mascot
1615.8401	1615.8756	0.0355	22	526	539	GLIYSMEFTNLLSK					Mascot
1882.958	1882.9701	0.0121	6	568	584	CLVVSTKGDVENFSSLK			Carbamidomethyl (C)[1]		Mascot

2384.1438	2384.0923	-0.0515	-22	419	439	VKHSFSADDIQAIMESDS FLK	Oxidation (M)[14]	Mascot

Gel Idx/Pos	127/F2	Instr./Gel Origin	BA2151/Sample Project 20140814	Process Status	Analysis Succeeded
Plate [#] Name	[1] Sample Project 20140814	Instrument Sample Name		Spectra	11

Rank	Protein Name	Accession No.	Protein MW	Protein PI	Pep. Count	Protein Score	Protein Score C. I. %	Intensity Matched	Total Ion Score	Total Ion C. I. %	Confirmed
1	16.9 kDa class I heat shock protein 1 OS=Triticum aestivum GN=hsp16.9A PE=2 SV=1	HS16A_WHEAT	16867.8	5.83	6	226	100	25.752	180	100	

Peptide Information

Calc. Mass	Obsrv. Mass	± da	± ppm	Start Seq.	End Seq.	Sequence	Ion Score	C. I. %	Modification	Rank	Result Type
975.5258	975.5746	0.0488	50	110	117	FRLPEDAK					Mascot
975.5258	975.5746	0.0488	50	110	117	FRLPEDAK	24	0			Mascot
1057.5314	1057.5516	0.0202	19	50	58	ETPEAHVFK					Mascot
1057.5314	1057.5516	0.0202	19	50	58	ETPEAHVFK	14	0			Mascot
1600.8177	1600.8934	0.0757	47	71	85	VEVEDGNVLVVSGER					Mascot
1600.8177	1600.8934	0.0757	47	71	85	VEVEDGNVLVVSGER	9	0			Mascot
1905.9666	1906.0603	0.0937	49	26	45	SIVPAISGGSSETAAFAN AR					Mascot
1905.9666	1906.0603	0.0937	49	26	45	SIVPAISGGSSETAAFAN AR	142	100			Mascot
2086.0664	2086.155	0.0886	42	67	85	EEVKVEVEDGNVLVVSG ER					Mascot
2260.0347	2260.1248	0.0901	40	7	25	SNVFDPFADLWADPFDT FR					Mascot

Rank	Protein Name	Accession No.	Protein MW	Protein PI	Pep. Count	Protein Score	Protein Score C. I. %	Intensity Matched	Total Ion Score	Total Ion C. I. %	Confirmed
2	17.4 kDa class I heat shock protein OS=Arabidopsis thaliana GN=HSP17.4A PE=2 SV=2	HSP17_ARATH	17428.8	5.21	8	71	95.398	4.425	14	0	

Peptide Information

Calc. Mass	Obsrv. Mass	± da	± ppm	Start Seq.	End Seq.	Sequence	Ion Score	C. I. %	Modification	Rank	Result Type
812.4083	812.4328	0.0245	30	107	113	SSGKFMR					Mascot
822.3475	822.4102	0.0627	76	91	97	SSENEEK					Mascot
827.4985	827.4829	-0.0156	-19	64	71	ADVPGLKK					Mascot
828.4033	828.4686	0.0653	79	107	113	SSGKFMR			Oxidation (M)[6]		Mascot
974.5417	974.5853	0.0436	45	115	122	FRLPENAK					Mascot
1057.5314	1057.5516	0.0202	19	55	63	ETPEAHVFK					Mascot
1057.5314	1057.5516	0.0202	19	55	63	ETPEAHVFK	14	0			Mascot
1492.7544	1492.7943	0.0399	27	42	54	DVAAFTNAKVDWR					Mascot
1657.8392	1657.9052	0.066	40	76	90	VEVEDGNILQISGER					Mascot
1657.8392	1657.9052	0.066	40	76	90	VEVEDGNILQISGER	17	0			Mascot

	2143.0876	2143.1763	0.0887	41	72	90	EEVKVEVEDGNILQISGE R					Mascot

3	Nebulin-related-anchoring protein OS=Mus musculus GN=Nrap PE=1 SV=3	NRAP_MOUSE	196716	9.34	34	59	31.926	11.469	

Peptide Information

Calc. Mass	Obsrv. Mass	± da	± ppm	Start Seq.	End Seq.	Sequence	Ion Score	C. I. %	Modification	Rank	Result Type
800.405	800.405	0	0	1203	1209	QHPGSFK					Mascot
803.3893	803.4076	0.0183	23	993	999	EQGENVK					Mascot
808.4312	808.4181	-0.0131	-16	1035	1041	DGGYKLR					Mascot
812.3971	812.4328	0.0357	44	553	559	GKGFEMK			Oxidation (M)[6]		Mascot
821.3676	821.4275	0.0599	73	90	96	EDGEPFK					Mascot
868.5185	868.4354	-0.0831	-96	425	431	RMLHALK					Mascot
887.4944	887.4644	-0.03	-34	717	723	QRVDELK					Mascot
904.4669	904.4432	-0.0237	-26	478	484	QNIDRMK					Mascot
906.4791	906.4818	0.0027	3	1550	1556	EAFLRDR					Mascot
974.5516	974.5853	0.0337	35	949	957	AGELISEKK					Mascot
975.5218	975.5746	0.0528	54	589	598	GRALGATDSK					Mascot
975.5218	975.5746	0.0528	54	589	598	GRALGATDSK					Mascot
981.4241	981.5099	0.0858	87	1	8	MNVQACSR			Carbamidomethyl (C)[6], Oxidation (M)[1]		Mascot
1039.5419	1039.5503	0.0084	8	396	404	ISKFTSDNK					Mascot
1053.4596	1053.5323	0.0727	69	226	234	YTEDGGQQR					Mascot
1057.5386	1057.5516	0.013	12	216	225	AGGQLQNDVR					Mascot
1057.5386	1057.5516	0.013	12	216	225	AGGQLQNDVR					Mascot
1093.5636	1093.5691	0.0055	5	1233	1242	LYKAAGEDAR					Mascot
1106.4684	1106.567	0.0986	89	415	424	GHYDGVGMDR					Mascot
1107.5681	1107.571	0.0029	3	534	543	ANAELFSEVK					Mascot
1127.5957	1127.6078	0.0121	11	958	966	YRQHPDALK					Mascot
1165.5961	1165.6324	0.0363	31	1538	1547	VSRDIASDFR					Mascot
1235.6379	1235.6399	0.002	2	990	999	LYREQGENVK					Mascot
1320.6907	1320.6368	-0.0539	-41	181	192	AANQLASQVQYK					Mascot
1398.7263	1398.7932	0.0669	48	534	545	ANAELFSEVKYK					Mascot
1435.7653	1435.8223	0.057	40	1611	1622	RSQQLASDVLYR					Mascot
1438.7147	1438.7618	0.0471	33	166	178	GSFPAMITPAYQR					Mascot
1657.9021	1657.9052	0.0031	2	1709	1723	GVNPDASEILHIHKK					Mascot
1657.9021	1657.9052	0.0031	2	1709	1723	GVNPDASEILHIHKK					Mascot
1685.8857	1685.946	0.0603	36	1418	1433	GTGWLALQSPQIESAK					Mascot

Calc. Mass	Obsrv. Mass	± da	± ppm	Start Seq.	End Seq.	Sequence	Ion Score	C. I. %	Modification	Rank	Result Type
1791.9501	1791.823	-0.1271	-71	340	356	GAAHFHSLAAQDNLVLK					Mascot
1948.0511	1948.0288	-0.0223	-11	340	357	GAAHFHSLAAQDNLVLKR					Mascot
2086.054	2086.155	0.101	48	929	947	WMKGTGWVATGSLHVEQAK					Mascot
2143.0642	2143.1763	0.1121	52	671	688	AYGLQSELQYKADLAWMR					Mascot
2276.0837	2276.1562	0.0725	32	1380	1398	TQCHEFTALPEDLKMAWAK			Carbamidomethyl (C)[3]		Mascot
2292.0789	2292.1221	0.0432	19	1380	1398	TQCHEFTALPEDLKMAWAK			Carbamidomethyl (C)[3], Oxidation (M)[15]		Mascot
2721.3591	2721.4224	0.0633	23	739	763	SQGLQNAVAYKAGNEQSVHQYTISK					Mascot
2749.271	2749.46	0.189	69	1453	1475	FTTVVDSPDLIHAKESYMHCNER			Carbamidomethyl (C)[20]		Mascot

4	Iron-dependent extradiol dioxygenase OS=Rhodococcus sp. (strain RHA1) GN=hsaC PE=1 SV=1	HSAC_RHOSR	33664.7	5.41	12	57	0	19.454	

Peptide Information

Calc. Mass	Obsrv. Mass	± da	± ppm	Start Seq.	End Seq.	Sequence	Ion Score	C. I. %	Modification	Rank	Result Type
832.4597	832.3773	-0.0824	-99	26	33	VLGMVEGK					Mascot
851.3716	851.4453	0.0737	87	44	50	MDDFPAR					Mascot
975.5734	975.5746	0.0012	1	162	169	DVLGFRLR					Mascot
975.5734	975.5746	0.0012	1	162	169	DVLGFRLR					Mascot
1092.5685	1092.562	-0.0065	-6	79	89	DNLSAAGVAFK					Mascot
1106.5477	1106.567	0.0193	17	34	43	GSDPDALYLR					Mascot
1182.6266	1182.6119	-0.0147	-12	51	60	LVIFPGEHDR					Mascot
1232.5906	1232.6401	0.0495	40	273	282	QVEDESWIAR					Mascot
1438.7611	1438.7618	0.0007	0	21	33	EYGLKVLGMVEGK			Oxidation (M)[9]		Mascot
1932.8943	1933.0692	0.1749	90	5	20	SLAYMRIEATDMSAWR			Oxidation (M)[5,12]		Mascot
1932.8943	1933.0692	0.1749	90	5	20	SLAYMRIEATDMSAWR			Oxidation (M)[5,12]		Mascot
1947.8906	1948.0288	0.1382	71	283	300	ESTAVSLWGHDFSVGMQP					Mascot
1959.9771	1960.082	0.1049	54	61	78	LSVSGWETANAAELQEVR					Mascot
1959.9771	1960.082	0.1049	54	61	78	LSVSGWETANAAELQEVR					Mascot
2214.0869	2214.249	0.1621	73	240	258	MSATLGRHVNDLMLSFYMK					Mascot
2214.0869	2214.249	0.1621	73	240	258	MSATLGRHVNDLMLSFYMK					Mascot

5	Altered inheritance of mitochondria protein 9, mitochondrial OS=Zygosaccharomyces rouxii (strain ATCC 2623 / CBS 732 / NBRC 1130 / NCYC 568 / NRRL Y-229) GN=AIM9 PE=3 SV=1	AIM9_ZYGRC	70682.7	6.25	15	53	0	3.517	

Peptide Information

Calc. Mass	Obsrv. Mass	± da	± ppm	Start Seq.	End Seq.	Sequence	Ion Score	C. I. %	Modification	Rank	Result Type
805.4778	805.4047	-0.0731	-91	4	11	STTAKLGK					Mascot
888.4244	888.4561	0.0317	36	142	149	MASIHEGK			Oxidation (M)[1]		Mascot
1016.4894	1016.4987	0.0093	9	242	250	DSLPDDNLK					Mascot
1079.4867	1079.5725	0.0858	79	475	482	AQYEFMYK					Mascot
1090.5164	1090.5929	0.0765	70	279	287	DYERPGEPK					Mascot
1235.5878	1235.6399	0.0521	42	475	483	AQYEFMYKR					Mascot
1435.7468	1435.8223	0.0755	53	216	227	QPFVLEEFIEGK					Mascot
1492.7179	1492.7943	0.0764	51	486	497	NQFLWDSALNER					Mascot
1605.8306	1605.802	-0.0286	-18	182	195	LKSEVATMDFAHLK			Oxidation (M)[8]		Mascot
1685.8898	1685.946	0.0562	33	328	342	FLGPWSANEPLEVVK					Mascot
1707.8073	1707.8615	0.0542	32	37	51	ADEVFTKLSDENDPK					Mascot
1839.0123	1839.0043	-0.008	-4	168	183	IPYALENGHVIESRLK					Mascot
2086.1001	2086.155	0.0549	26	184	202	SEVATMDFAHLKLGINVP K			Oxidation (M)[6]		Mascot
2214.1804	2214.249	0.0686	31	323	342	EALSKFLGPWSANEPLE VVK					Mascot
2214.1804	2214.249	0.0686	31	323	342	EALSKFLGPWSANEPLE VVK					Mascot
2691.3962	2691.4343	0.0381	14	579	601	LISRPFAATQGWIPQDMF ENLIK			Oxidation (M)[17]		Mascot

6	Ferredoxin--NADP reductase OS=Ignicoccus hospitalis (strain KIN4/I / DSM 18386 / JCM 14125) GN=Igni_0617 PE=3 SV=1	FENR_IGNH4	33003.6	9.04	10	52	0	12.071	

Peptide Information

Calc. Mass	Obsrv. Mass	± da	± ppm	Start Seq.	End Seq.	Sequence	Ion Score	C. I. %	Modification	Rank	Result Type
828.5049	828.4686	-0.0363	-44	57	63	RAIEIAR					Mascot
831.4458	831.4038	-0.042	-51	255	261	EILVDDK					Mascot
833.4152	833.382	-0.0332	-40	85	91	AWKEGDK					Mascot
887.5019	887.4644	-0.0375	-42	194	201	MVLNSVPK					Mascot
906.4825	906.4818	-0.0007	-1	183	189	SIERMVR			Oxidation (M)[5]		Mascot
975.504	975.5746	0.0706	72	179	186	APMRSIER			Oxidation (M)[3]		Mascot
975.504	975.5746	0.0706	72	179	186	APMRSIER	16	0	Oxidation (M)[3]		Mascot
1016.5622	1016.4987	-0.0635	-62	253	261	GKEILVDDK					Mascot
1053.5615	1053.5323	-0.0292	-28	71	79	FFGVEVVEK					Mascot
1685.9221	1685.946	0.0239	14	264	280	TNVKGVFAAGDITPAPK					Mascot
1932.9413	1933.0692	0.1279	66	64	79	AFEEMQKFFGVEVVEK			Oxidation (M)[5]		Mascot

Calc. Mass	Obsrv. Mass	± da	± ppm	Start Seq.	End Seq.	Sequence	Ion Score	C. I. %	Modification	Rank	Result Type
1932.9413	1933.0692	0.1279	66	64	79	AFEEMQKFFGVEVVEK			Oxidation (M)[5]		Mascot

7	30S ribosomal protein S4 OS=Gloeobacter violaceus (strain PCC 7421) GN=rpsD PE=3 SV=1	RS4_GLOVI	23581.4	10.25	11	52	0	2.426		

Peptide Information

Calc. Mass	Obsrv. Mass	± da	± ppm	Start Seq.	End Seq.	Sequence	Ion Score	C. I. %	Modification	Rank	Result Type
821.4515	821.4275	-0.024	-29	132	138	VSIPSYR					Mascot
851.4621	851.4453	-0.0168	-20	50	56	LSEYAIR					Mascot
904.4669	904.4432	-0.0237	-26	23	30	GIRCSSPK			Carbamidomethyl (C)[4]		Mascot
981.504	981.5099	0.0059	6	65	72	WSYGVLEK					Mascot
1092.6411	1092.562	-0.0791	-72	50	58	LSEYAIRLK					Mascot
1107.6157	1107.571	-0.0447	-40	48	56	QKLSEYAIR					Mascot
1182.5758	1182.6119	0.0361	31	1	10	MARYTGPVCK			Carbamidomethyl (C)[9]		Mascot
1398.7952	1398.7932	-0.002	-1	176	188	ETLAATVKALPER					Mascot
1888.995	1889.0582	0.0632	33	139	156	VRPGDAITVMPNSAAFVK			Oxidation (M)[10]		Mascot
1947.9797	1948.0288	0.0491	25	114	130	AQARQWVNHGHFAVNG R					Mascot
2691.4287	2691.4343	0.0056	2	132	156	VSIPSYRVRPGDAITVMP NSAAFVK			Oxidation (M)[17]		Mascot

8	10 kDa chaperonin OS=Alkaliphilus oremlandii (strain OhILAs) GN=groS PE=3 SV=1	CH10_ALKOO	10081.4	5.03	7	51	0	9.091		

Peptide Information

Calc. Mass	Obsrv. Mass	± da	± ppm	Start Seq.	End Seq.	Sequence	Ion Score	C. I. %	Modification	Rank	Result Type
833.4437	833.382	-0.0617	-74	53	59	EVVMEVK					Mascot
906.4415	906.4818	0.0403	44	15	22	VEAEETTK					Mascot
912.5261	912.4716	-0.0545	-60	2	9	NIKPLGDR					Mascot
928.5462	928.4882	-0.058	-62	23	32	SGIVLPGSAK					Mascot
1232.6555	1232.6401	-0.0154	-12	53	63	EVVMEVKVGDK					Mascot
1932.9954	1933.0692	0.0738	38	69	85	YAGTEVKFDGVEYTILK					Mascot
1932.9954	1933.0692	0.0738	38	69	85	YAGTEVKFDGVEYTILK					Mascot
2839.458	2839.4666	0.0086	3	33	59	EQPQLAEVMAVGPGGVI EGKEVVMEVK			Oxidation (M)[9]		Mascot

9	17.6 kDa class I heat shock protein 3 OS=Arabidopsis thaliana GN=HSP17.6C PE=2 SV=2	HS17C_ARATH	17593	5.36	5	51	0	2.786	14	0

Peptide Information

Calc. Mass	Obsrv. Mass	± da	± ppm	Start Seq.	End Seq.	Sequence	Ion Score	C. I. %	Modification	Rank	Result Type

Calc. Mass	Obsrv. Mass	± da	± ppm	Start Seq.	End Seq.	Sequence	Ion Score	C. I. %	Modification	Rank	Result Type
974.5417	974.5853	0.0436	45	116	123	FRLPENAK					Mascot
1053.6302	1053.5323	-0.0979	-93	143	151	VPEKKPEVK					Mascot
1057.5314	1057.5516	0.0202	19	56	64	ETPEAHVFK					Mascot
1057.5314	1057.5516	0.0202	19	56	64	ETPEAHVFK	14	0			Mascot
1657.8392	1657.9052	0.066	40	77	91	VEVEDGNILQISGER					Mascot
1657.8392	1657.9052	0.066	40	77	91	VEVEDGNILQISGER	17	0			Mascot
2143.0876	2143.1763	0.0887	41	73	91	EEVKVEVEDGNILQISGE R					Mascot

10	Genome polyprotein 1 OS=Barley yellow mosaic virus (strain Japanese II-1) PE=3 SV=1	POL1_BAYMJ	272708.1	7.68	32	51	0	9.467	

Peptide Information

Calc. Mass	Obsrv. Mass	± da	± ppm	Start Seq.	End Seq.	Sequence	Ion Score	C. I. %	Modification	Rank	Result Type
803.4304	803.4076	-0.0228	-28	2309	2315	MNGGLRR					Mascot
807.3851	807.4084	0.0233	29	1139	1144	MEPKMR			Oxidation (M)[1]		Mascot
819.3954	819.4077	0.0123	15	1489	1495	KEENSGR					Mascot
829.489	829.4341	-0.0549	-66	2183	2190	LKTPSGAR					Mascot
830.4227	830.4091	-0.0136	-16	758	763	NERQQR					Mascot
832.4523	832.3773	-0.075	-90	1795	1801	VEAEKTR					Mascot
859.54	859.468	-0.072	-84	834	840	LPIFLTR					Mascot
865.489	865.4293	-0.0597	-69	1480	1486	KFTQSVR					Mascot
868.4788	868.4354	-0.0434	-50	1107	1112	WREHLK					Mascot
912.4066	912.4716	0.065	71	132	138	GWISMMR			Oxidation (M)[5,6]		Mascot
968.4618	968.478	0.0162	17	1686	1692	DLCKYNR			Carbamidomethyl (C)[3]		Mascot
981.4193	981.5099	0.0906	92	1150	1158	DMDAAVESK			Oxidation (M)[2]		Mascot
1068.5837	1068.5457	-0.038	-36	607	615	KFYVSATPR					Mascot
1092.5433	1092.562	0.0187	17	2136	2144	FELADADRR					Mascot
1093.5194	1093.5691	0.0497	45	1149	1158	KDMDAAVESK					Mascot
1141.5558	1141.5861	0.0303	27	824	833	AQADTMTIFK			Oxidation (M)[6]		Mascot
1182.5637	1182.6119	0.0482	41	2400	2410	VLDSDGHPELT					Mascot
1232.5651	1232.6401	0.075	61	985	994	QSMVTNVMYK			Oxidation (M)[3,8]		Mascot
1600.7966	1600.8934	0.0968	60	2191	2206	IPSSAADGNWSVPATK					Mascot
1600.7966	1600.8934	0.0968	60	2191	2206	IPSSAADGNWSVPATK					Mascot
1628.8503	1628.9115	0.0612	38	972	984	NYKTLIENNQHVR					Mascot
1707.8748	1707.8615	-0.0133	-8	2333	2348	LVAHWSMKHGASANAK					Mascot
1791.8621	1791.823	-0.0391	-22	2395	2410	TTNHRVLDSDGHPELT					Mascot

1887.9131	1888.0402	0.1271	67	932	948	HRASMSNVFAGIEEPAR	Oxidation (M)[5]	Mascot
1943.0961	1943.0315	-0.0646	-33	834	851	LPIFLTRDLINADGSVAK		Mascot
2086.0063	2086.155	0.1487	71	190	207	IEYGLNGTYFGEHVAMLR	Oxidation (M)[16]	Mascot
2214.1072	2214.249	0.1418	64	2373	2393	LAALGTGTYNTMLTSDTT NLR		Mascot
2214.1072	2214.249	0.1418	64	2223	2242	SVPKSVMEHNNSVALES ELK	Oxidation (M)[7]	Mascot
2260.1987	2260.1248	-0.0739	-33	299	316	HSIYFMFLTIVAILWSFR	Oxidation (M)[6]	Mascot
2691.3481	2691.4343	0.0862	32	91	114	HEMRSCVVPIVTSDASFE TVAVIK	Carbamidomethyl (C)[6], Oxidation (M)[3]	Mascot
2721.4565	2721.4224	-0.0341	-13	1894	1917	AIDLIYDEILNTTICLANGM VIKK	Carbamidomethyl (C)[15]	Mascot
2807.3801	2807.4749	0.0948	34	1113	1138	QKGSNEILTNTMPVSEG GEILAEVMK	Oxidation (M)[12,25]	Mascot
3312.741	3312.5049	-0.2361	-71	642	672	FIAAQGGGDLLDISKHDT ALVFLAGRPECIK	Carbamidomethyl (C)[29]	Mascot

Gel Idx/Pos	128/F3	Instr./Gel Origin	BA2151/Sample Project 20140814	Process Status	Analysis Succeeded
Plate [#] Name	[1] Sample Project 20140814	Instrument Sample Name		Spectra	11

Rank	Protein Name	Accession No.	Protein MW	Protein PI	Pep. Count	Protein Score	Protein Score C. I. %	Intensity Matched	Total Ion Score	Total Ion C. I. %	Confirmed
1	Alpha-amylase inhibitor 0.19 OS=Triticum aestivum PE=1 SV=1	IAA1_WHEAT	13898.6	6.66	6	350	100	35.685	310	100	

Peptide Information

Calc. Mass	Obsrv. Mass	± da	± ppm	Start Seq.	End Seq.	Sequence	Ion Score	C. I. %	Modification	Rank	Result Type
1162.6249	1162.6868	0.0619	53	90	100	LTAASITAVCR			Carbamidomethyl (C)[10]		Mascot
1162.6249	1162.6868	0.0619	53	90	100	LTAASITAVCR	36	73.943	Carbamidomethyl (C)[10]		Mascot
1570.8007	1570.892	0.0913	58	26	39	LQCNGSQVPEAVLR			Carbamidomethyl (C)[3]		Mascot
1612.7463	1612.8384	0.0921	57	67	82	EHGAQEGQAGTGAFPR					Mascot
1612.7463	1612.8384	0.0921	57	67	82	EHGAQEGQAGTGAFPR	144	100			Mascot
1617.8993	1617.8442	-0.0551	-34	86	100	EVVKLTAASITAVCR			Carbamidomethyl (C)[14]		Mascot
1663.8361	1663.8826	0.0465	28	101	116	LPIVVDASGDGAYVCK			Carbamidomethyl (C)[15]		Mascot
1862.7731	1862.8889	0.1158	62	40	53	DCCQQLAHISEWCR			Carbamidomethyl (C)[2,3,13]		Mascot
1862.7731	1862.8889	0.1158	62	40	53	DCCQQLAHISEWCR	130	100	Carbamidomethyl (C)[2,3,13]		Mascot

Rank	Protein Name	Accession No.	Protein MW	Protein PI	Pep. Count	Protein Score	Protein Score C. I. %	Intensity Matched	Total Ion Score	Total Ion C. I. %	Confirmed
2	Acetyl-CoA carboxylase 1 OS=Arabidopsis thaliana GN=ACC1 PE=1 SV=1	ACC1_ARATH	252819.5	6.09	34	66	85.107	51.995			

Peptide Information

Calc. Mass	Obsrv. Mass	± da	± ppm	Start Seq.	End Seq.	Sequence	Ion Score	C. I. %	Modification	Rank	Result Type
850.4669	850.438	-0.0289	-34	451	457	VQELSFK					Mascot
1017.5574	1017.5997	0.0423	42	996	1004	ASQLLEQTK					Mascot
1017.5574	1017.5997	0.0423	42	996	1004	ASQLLEQTK					Mascot
1052.5959	1052.4934	-0.1025	-97	1412	1420	EIHRSVGVR					Mascot
1158.6589	1158.5784	-0.0805	-69	1005	1014	LSELRSNIAR					Mascot
1162.5773	1162.6868	0.1095	94	636	646	DGGLLMQLDGK			Oxidation (M)[6]		Mascot
1162.5773	1162.6868	0.1095	94	636	646	DGGLLMQLDGK			Oxidation (M)[6]		Mascot
1170.5691	1170.6844	0.1153	98	1241	1249	HSFHWSLEK					Mascot
1229.7286	1229.7083	-0.0203	-17	493	504	ALAIANMVLGLK			Oxidation (M)[7]		Mascot
1507.7244	1507.8191	0.0947	63	2046	2057	ELLECMGRLDQK			Carbamidomethyl (C)[5], Oxidation (M)[6]		Mascot
1526.7961	1526.8381	0.042	28	1274	1286	LKGYSNIQYTPSR					Mascot
1555.8163	1555.7913	-0.025	-16	533	545	IHTGWLDSRIAMR					Mascot

1570.8146	1570.892	0.0774	49	1329	1342	QLSQTLISMAFTSK			Oxidation (M)[9]		Mascot
1571.8112	1571.8813	0.0701	45	533	545	IHTGWLDSRIAMR			Oxidation (M)[12]		Mascot
1571.8112	1571.8813	0.0701	45	533	545	IHTGWLDSRIAMR			Oxidation (M)[12]		Mascot
1587.752	1587.8105	0.0585	37	1421	1432	MHRLGVCEWEVR			Carbamidomethyl (C)[7], Oxidation (M)[1]		Mascot
1609.7679	1609.8175	0.0496	31	2164	2176	LIQDWFCNSDIAK			Carbamidomethyl (C)[7]		Mascot
1612.8442	1612.8384	-0.0058	-4	1378	1390	EQQIDDLVPFPRR					Mascot
1612.8442	1612.8384	-0.0058	-4	1378	1390	EQQIDDLVPFPRR					Mascot
1623.7618	1623.8729	0.1111	68	2102	2115	FAELHDTSMRMAAK			Oxidation (M)[9]		Mascot
1626.8633	1626.8342	-0.0291	-18	622	635	MNKSEVVAEIHTLR					Mascot
1640.9404	1640.8561	-0.0843	-51	877	891	GALERLIEPLMSLAK					Mascot
1656.9353	1656.7957	-0.1396	-84	877	891	GALERLIEPLMSLAK			Oxidation (M)[11]		Mascot
1674.8123	1674.8899	0.0776	46	55	68	SVRTWAYETFGTEK					Mascot
1679.8599	1679.8538	-0.0061	-4	2193	2206	DNVSNYELKLSELR					Mascot
1680.8414	1680.8505	0.0091	5	962	975	LMEQLVYPNPAAYR			Oxidation (M)[2]		Mascot
1701.917	1701.9255	0.0085	5	2029	2043	GNVLEPEGTIEIKFR					Mascot
1707.7821	1707.8693	0.0872	51	435	450	VTSEDPDDGFKPTSGR					Mascot
1711.8546	1711.9137	0.0591	35	1990	2003	QPVFVYIPMMGELR			Oxidation (M)[9,10]		Mascot
1743.833	1743.9104	0.0774	44	862	876	GILEAHLSSCDEKER			Carbamidomethyl (C)[10]		Mascot
1744.9303	1744.9352	0.0049	3	145	162	GIIFLGPPASSMAALGDK					Mascot
1771.8187	1771.873	0.0543	31	394	408	FYGIEHGGGYDSWRK					Mascot
1771.8187	1771.873	0.0543	31	394	408	FYGIEHGGGYDSWRK					Mascot
1787.9005	1787.8693	-0.0312	-17	712	727	MCMPLLSPASGVIHFK			Carbamidomethyl (C)[2]		Mascot
1799.825	1799.9004	0.0754	42	393	407	RFYGIEHGGGYDSWR					Mascot
1801.8069	1801.8833	0.0764	42	2179	2192	EEAWTDDQVFFTWK					Mascot
1803.8955	1803.87	-0.0255	-14	712	727	MCMPLLSPASGVIHFK			Carbamidomethyl (C)[2], Oxidation (M)[1]		Mascot
1817.8964	1817.9156	0.0192	11	1456	1470	TCTVHIYREVETPGR			Carbamidomethyl (C)[2]		Mascot
1821.0164	1820.8965	-0.1199	-66	35	51	RPIHSILIANNGMAAVK			Oxidation (M)[13]		Mascot
1830.912	1830.8683	-0.0437	-24	565	582	ASATSAAVVSDYVGYLEK					Mascot
1875.9811	1876.1326	0.1515	81	2070	2085	QSEAYANIELLQQQIK					Mascot
1907.9684	1907.88	-0.0884	-46	962	977	LMEQLVYPNPAAYRDK					Mascot

3	Phosphoribosylaminoimidazole-succinocarboxamide synthase OS=Saccharophagus degradans (strain 2-40 / ATCC 43961 / DSM 17024) GN=purC PE=3 SV=1	PUR7_SACD2	41429.2	4.99	13	66	85.107	27.003

Peptide Information

Calc. Mass	Obsrv. Mass	± da	± ppm	Start Seq.	End Seq.	Sequence	Ion Score	C. I. %	Modification	Rank	Result Type
1052.4796	1052.4934	0.0138	13	270	277	IWDGENYR					Mascot

Calc. Mass	Obsrv. Mass	± da	± ppm	Start Seq.	End Seq.	Sequence	Ion Score	C. I. %	Modification	Rank	Result Type
1507.7209	1507.8191	0.0982	65	149	161	NFCGINLEDGLQK			Carbamidomethyl (C)[3]		Mascot
1514.7274	1514.8323	0.1049	69	195	207	ADIENNFAAFQFK					Mascot
1566.8123	1566.8246	0.0123	8	180	194	GVPGVPEVDDVNITR					Mascot
1590.7733	1590.8229	0.0496	31	133	145	QYITGSMWRAYSK					Mascot
1598.7367	1598.8851	0.1484	93	256	269	LIYMDEVGTPDSSR			Oxidation (M)[4]		Mascot
1606.7683	1606.8374	0.0691	43	133	145	QYITGSMWRAYSK			Oxidation (M)[7]		Mascot
1612.9884	1612.8384	-0.15	-93	165	179	LSELLITPSTKGILK					Mascot
1612.9884	1612.8384	-0.15	-93	165	179	LSELLITPSTKGILK					Mascot
1770.881	1770.9363	0.0553	31	195	209	ADIENNFAAFQFKQK					Mascot
1775.817	1775.8689	0.0519	29	64	78	LSAFDCIWHAEGDVR			Carbamidomethyl (C)[6]		Mascot
1845.9395	1845.9252	-0.0143	-8	84	99	GAALNAISNHWFELFR					Mascot
1875.9634	1876.1326	0.1692	90	46	63	GYNVAADAPLAIMVISDR					Mascot
1878.9015	1878.8792	-0.0223	-12	149	164	NFCGINLEDGLQKDQK			Carbamidomethyl (C)[3]		Mascot
1891.0219	1890.894	-0.1279	-68	1	17	MSLAHQVLAVNNDLPIR					Mascot

4	DNA-directed RNA polymerase subunit beta OS=Cuscuta reflexa GN=rpoB PE=2 SV=2	RPOB_CUSRE	121098.4	8.67	23	63	73.516	34.752

Peptide Information

Calc. Mass	Obsrv. Mass	± da	± ppm	Start Seq.	End Seq.	Sequence	Ion Score	C. I. %	Modification	Rank	Result Type
814.4893	814.5068	0.0175	21	783	789	GRVIDVR					Mascot
850.4529	850.438	-0.0149	-18	925	931	SRIFDGR					Mascot
878.5206	878.4377	-0.0829	-94	466	473	VLHLSPGR					Mascot
882.3951	882.431	0.0359	41	891	897	YEQEASR					Mascot
1010.4901	1010.5731	0.083	82	891	898	YEQEASRK					Mascot
1055.646	1055.6432	-0.0028	-3	761	771	AILGIPVSASK					Mascot
1069.4766	1069.526	0.0494	46	542	550	ALMSSNMQR			Oxidation (M)[3,7]		Mascot
1126.5779	1126.5397	-0.0382	-34	220	228	ENAILEFYK					Mascot
1158.6154	1158.5784	-0.037	-32	551	560	QAVPLTWSEK					Mascot
1511.8104	1511.8254	0.015	10	216	228	IGSKENAILEFYK					Mascot
1612.9818	1612.8384	-0.1434	-89	178	192	ISILVLLSAMGLNLR					Mascot
1612.9818	1612.8384	-0.1434	-89	178	192	ISILVLLSAMGLNLR					Mascot
1628.9768	1628.8262	-0.1506	-92	178	192	ISILVLLSAMGLNLR			Oxidation (M)[10]		Mascot
1641.9071	1641.9178	0.0107	7	705	718	VTNEIPHLEAHLIR					Mascot
1641.9071	1641.9178	0.0107	7	705	718	VTNEIPHLEAHLIR					Mascot
1662.8922	1662.8602	-0.032	-19	393	409	VSSLGPGGLTGRTASFR					Mascot
1674.8157	1674.8899	0.0742	44	691	704	YEIQIHMTTQGPEK					Mascot

1696.948	1696.8567	-0.0913	-54	1017	1033	QEVLGTTIVGGTIPSPK					Mascot
1701.8741	1701.9255	0.0514	30	833	846	ILPRQDMPYLQDGR					Mascot
1743.8984	1743.9104	0.012	7	570	586	QAALDSGSLAIAEREGR					Mascot
1744.9592	1744.9352	-0.024	-14	316	331	SVADLLQDQFGLALVR					Mascot
1754.9647	1754.9452	-0.0195	-11	635	653	GQILAGGAATVEGELALG K					Mascot
1777.0007	1776.8727	-0.128	-72	121	135	IVINQILQSPGIYYR					Mascot
1799.9916	1799.9004	-0.0912	-51	270	284	LHLDIPHNNTFLLPR					Mascot
1802.9105	1802.9163	0.0058	3	690	704	KYEIQIHMTTQGPEK					Mascot
1892.8743	1892.8872	0.0129	7	298	313	FGMGTLDDMNHLQNKR			Oxidation (M)[3]		Mascot

5	60 kDa chaperonin 2 OS=Sorangium cellulosum (strain So ce56) GN=groL2 PE=3 SV=1	CH602_SORC5	58066.5	5.61	16	61	60.827	3.448

Peptide Information

Calc. Mass	Obsrv. Mass	± da	± ppm	Start Seq.	End Seq.	Sequence	Ion Score	C. I. %	Modification	Rank	Result Type
1011.5218	1011.5891	0.0673	67	396	404	VEDALHATR					Mascot
1052.4929	1052.4934	0.0005	0	381	390	VGAATETEMK			Oxidation (M)[9]		Mascot
1136.6422	1136.6133	-0.0289	-25	123	132	AVEAIVEHLR					Mascot
1174.65	1174.5524	-0.0976	-83	161	171	LLADAMEKVGK					Mascot
1479.8279	1479.8657	0.0378	26	123	136	AVEAIVEHLRGSAK					Mascot
1511.7522	1511.8254	0.0732	48	1	13	MAAKEIIYNESAR			Oxidation (M)[1]		Mascot
1593.8484	1593.8391	-0.0093	-6	43	58	SFGSPTVTKDGVTVAK					Mascot
1656.8275	1656.7957	-0.0318	-19	269	284	GTLHCAAVKAPGFGDR			Carbamidomethyl (C)[5]		Mascot
1669.7664	1669.9071	0.1407	84	351	364	AQIENTTSDYDREK					Mascot
1680.8738	1680.8505	-0.0233	-14	102	117	EGSKLVAAGHNPMEIK					Mascot
1696.8687	1696.8567	-0.012	-7	102	117	EGSKLVAAGHNPMEIK			Oxidation (M)[13]		Mascot
1712.8094	1712.8558	0.0464	27	66	80	FENMGAQMVREVASK			Oxidation (M)[4]		Mascot
1755.0084	1754.9452	-0.0632	-36	227	242	ISNMKDLLPVLEAIAK					Mascot
1771.0034	1770.9363	-0.0671	-38	227	242	ISNMKDLLPVLEAIAK			Oxidation (M)[4]		Mascot
1783.8168	1783.8816	0.0648	36	182	197	SADTTLDVVEGMQFDR					Mascot
1799.8116	1799.9004	0.0888	49	182	197	SADTTLDVVEGMQFDR			Oxidation (M)[12]		Mascot
1802.9131	1802.9163	0.0032	2	143	160	EIAQVGTISANGDETIGK					Mascot
1875.0256	1874.8934	-0.1322	-71	372	390	LVGGVAVVKVGAATETE MK			Oxidation (M)[18]		Mascot
1885.0138	1884.8556	-0.1582	-84	452	469	QISANAGEEGSIVVQKVR					Mascot

6	Nebulin OS=Homo sapiens GN=NEB PE=1 SV=4	NEBU_HUMAN	775406.4	9.11	69	60	48.361	71.76

Peptide Information

Calc. Mass	Obsrv. Mass	± da	± ppm	Start Seq.	End Seq.	Sequence	Ion Score	C. I. %	Modification	Rank	Result Type
814.4682	814.5068	0.0386	47	1829	1835	GKHIGFR					Mascot
832.4159	832.3668	-0.0491	-59	696	703	VAAQNSDK					Mascot
878.44	878.4377	-0.0023	-3	3685	3692	NNALTMSK					Mascot
882.4203	882.431	0.0107	12	6084	6090	YKEDAEK					Mascot
994.5568	994.5671	0.0103	10	236	244	ALSDVAYKK					Mascot
1010.5516	1010.5731	0.0215	21	5964	5972	YATKIASEK					Mascot
1011.5258	1011.5891	0.0633	63	4963	4971	LPGDTPHFK					Mascot
1017.5574	1017.5997	0.0423	42	975	983	ASDILNEKK					Mascot
1017.5574	1017.5997	0.0423	42	975	983	ASDILNEKK					Mascot
1052.5371	1052.4934	-0.0437	-42	3798	3805	IQSEREYK					Mascot
1055.6207	1055.6432	0.0225	21	1068	1077	TDAIPIRAAK					Mascot
1136.505	1136.6133	0.1083	95	3060	3068	MMWSMHVAK			Oxidation (M)[1]		Mascot
1174.5851	1174.5524	-0.0327	-28	4671	4679	ENYHQIKDK					Mascot
1229.6848	1229.7083	0.0235	19	5999	6009	AKNATQILNEK					Mascot
1473.7771	1473.9083	0.1312	89	3150	3163	GIGWVPIGSMDVVK			Oxidation (M)[10]		Mascot
1507.6768	1507.8191	0.1423	94	5986	5998	GLTEMEDTPDMLR					Mascot
1509.7518	1509.8234	0.0716	47	5393	5404	HFKYATQLMNEK					Mascot
1511.7523	1511.8254	0.0731	48	3829	3840	KCQTLVTDIDYR			Carbamidomethyl (C)[2]		Mascot
1514.7598	1514.8323	0.0725	48	3234	3245	QNKINYSESLYR					Mascot
1526.7784	1526.8381	0.0597	39	3879	3892	GIGWMPQGSPEVLR					Mascot
1537.7931	1537.7909	-0.0022	-1	1237	1250	FTSIVDSPVMVQAK			Oxidation (M)[10]		Mascot
1551.7761	1551.8519	0.0758	49	5748	5761	ATQAAKQASEVEYR					Mascot
1555.8367	1555.7913	-0.0454	-29	5190	5202	LQSQYLYVELATK					Mascot
1566.704	1566.8246	0.1206	77	6023	6036	GLNAMANETPDFMR					Mascot
1568.9006	1568.848	-0.0526	-34	6128	6141	GKITVVQDTPEILR					Mascot
1570.8839	1570.892	0.0081	5	2526	2540	GYDLPVDAIPIKAAK					Mascot
1571.7635	1571.8813	0.1178	75	4259	4272	GHYVGVPTMRDDPK					Mascot
1571.7635	1571.8813	0.1178	75	4259	4272	GHYVGVPTMRDDPK					Mascot
1583.9155	1583.8816	-0.0339	-21	5242	5255	YTPVPDTPILIRAK					Mascot
1587.7584	1587.8105	0.0521	33	4259	4272	GHYVGVPTMRDDPK			Oxidation (M)[9]		Mascot
1590.8925	1590.8229	-0.0696	-44	3572	3586	FSSPVDMLGVVLAKK					Mascot
1593.8306	1593.8391	0.0085	5	2248	2261	IHVMPDTPDILQAK			Oxidation (M)[4]		Mascot
1598.8901	1598.8851	-0.005	-3	1551	1565	GYDLRPDAIPIVAAK					Mascot
1606.8873	1606.8374	-0.0499	-31	3572	3586	FSSPVDMLGVVLAKK			Oxidation (M)[7]		Mascot

1610.9476	1610.818	-0.1296	-80	6130	6143	ITVVQDTPEILRVK		Mascot
1612.7135	1612.8384	0.1249	77	958	971	GCGWVPFGSLEMEK	Carbamidomethyl (C)[2], Oxidation (M)[12]	Mascot
1612.7135	1612.8384	0.1249	77	958	971	GCGWVPFGSLEMEK	Carbamidomethyl (C)[2], Oxidation (M)[12]	Mascot
1626.8738	1626.8342	-0.0396	-24	5470	5482	QISDILYKLEYNK		Mascot
1628.8643	1628.8262	-0.0381	-23	2178	2193	GLGWSPAGSLEVEKAK		Mascot
1634.8861	1634.8024	-0.0837	-51	5032	5045	IHTTPDTPEIRQVK		Mascot
1640.9404	1640.8561	-0.0843	-51	4297	4312	AKINIPADMVSVLAAK		Mascot
1641.8483	1641.9178	0.0695	42	407	420	FKLDTVLQNFSSDK		Mascot
1641.8483	1641.9178	0.0695	42	407	420	FKLDTVLQNFSSDK		Mascot
1656.9353	1656.7957	-0.1396	-84	4297	4312	AKINIPADMVSVLAAK	Oxidation (M)[9]	Mascot
1662.8738	1662.8602	-0.0136	-8	1516	1529	YTIDPELPQFIQAK		Mascot
1663.8901	1663.8826	-0.0075	-5	6187	6202	EAIGQGTPIPDLPEVK		Mascot
1672.8177	1672.8746	0.0569	34	4625	4639	SYDLQSQLQYTAAGK		Mascot
1685.8857	1685.8871	0.0014	1	2421	2436	GIGWSPLGSLEAEKNK		Mascot
1696.8627	1696.8567	-0.006	-4	5203	5217	ERPHHHAGNQTTALK		Mascot
1701.8918	1701.9255	0.0337	20	5658	5672	KHLAQGSYTTLPETR		Mascot
1702.8833	1702.8988	0.0155	9	1867	1882	TSFHTPVDMLSVVAAK		Mascot
1707.8622	1707.8693	0.0071	4	5698	5712	GKSNYSIMLEPPEVK	Oxidation (M)[8]	Mascot
1711.9377	1711.9137	-0.024	-14	1934	1949	GIGWLPLGSLEAEKNK		Mascot
1712.8602	1712.8558	-0.0044	-3	655	668	YQLDTQLKNFSEAR		Mascot
1713.8378	1713.8588	0.021	12	3893	3906	VKNAQNIFCDSVYR	Carbamidomethyl (C)[9]	Mascot
1714.9486	1714.8574	-0.0912	-53	5149	5162	VQELKTHLSELVYR		Mascot
1724.9946	1724.9625	-0.0321	-19	1446	1461	GIGWIPIGSLEVEKVK		Mascot
1727.8707	1727.9348	0.0641	37	3462	3476	TIHVMPDTPEIMLAK	Oxidation (M)[5,12]	Mascot
1744.9229	1744.9352	0.0123	7	5452	5466	FTSIVDTPEHLRTTK		Mascot
1745.9078	1745.9364	0.0286	16	3150	3165	GIGWVPIGSMDVVKCK	Carbamidomethyl (C)[15]	Mascot
1754.9688	1754.9452	-0.0236	-13	1690	1705	GIGWVPIESLEVEKAK		Mascot
1765.9518	1765.9591	0.0073	4	3327	3342	TRYSSPVDMLGIVLAK	Oxidation (M)[9]	Mascot
1771.8901	1771.873	-0.0171	-10	469	483	GFFPQTITQEYEAIK		Mascot
1771.8901	1771.873	-0.0171	-10	469	483	GFFPQTITQEYEAIK		Mascot
1796.9211	1796.8944	-0.0267	-15	2080	2095	SLEDDPKLVHSMQVAK		Mascot
1799.9148	1799.9004	-0.0144	-8	3044	3059	QLGHHIGARNIEDDPK		Mascot
1801.8868	1801.8833	-0.0035	-2	4433	4448	GKYTFSPDTPHISHSK		Mascot
1803.8993	1803.87	-0.0293	-16	4542	4557	ANVHIPNDMMNHVLAK		Mascot
1806.9783	1806.8693	-0.109	-60	2246	2261	TKIHVMPDTPDILQAK		Mascot
1818.0596	1817.9156	-0.144	-79	5947	5963	GRNLTGLEVTPALLHVK		Mascot
1830.9093	1830.8683	-0.041	-22	6488	6502	NDQDQETITGLRVWR		Mascot

Calc. Mass	Obsrv. Mass	± da	± ppm	Start Seq.	End Seq.	Sequence	Ion Score	C. I. %	Modification	Rank	Result Type
1860.9789	1860.8782	-0.1007	-54	3296	3311	AVHDDPKIMWSLHIAK					Mascot
1862.8854	1862.8889	0.0035	2	1530	1545	VNALNMSDAHYKADWK					Mascot
1862.8854	1862.8889	0.0035	2	1530	1545	VNALNMSDAHYKADWK					Mascot
1874.8701	1874.8934	0.0233	12	6362	6376	NQENFSSVLYKENMR			Oxidation (M)[14]		Mascot
1878.8804	1878.8792	-0.0012	-1	1530	1545	VNALNMSDAHYKADWK			Oxidation (M)[6]		Mascot
1890.8943	1890.894	-0.0003	0	716	730	CYFPQTITQEYEAIK			Carbamidomethyl (C)[1]		Mascot
1919.9242	1919.9135	-0.0107	-6	1723	1738	LKFTYAMDTMEQALNK			Oxidation (M)[7]		Mascot
1935.0256	1934.9003	-0.1253	-65	2456	2473	FTSIPDAMDIVLAKTNAK					Mascot

7 Probable peroxidase 61 OS=Arabidopsis thaliana GN=PER61 PE=3 SV=1 | PER61_ARATH | 37744.9 | 6.94 | 12 | 60 | 45.927 | 5.124

Peptide Information

Calc. Mass	Obsrv. Mass	± da	± ppm	Start Seq.	End Seq.	Sequence	Ion Score	C. I. %	Modification	Rank	Result Type
882.4502	882.431	-0.0192	-22	306	313	SFALAMSR					Mascot
1010.5451	1010.5731	0.028	28	305	313	KSFALAMSR					Mascot
1136.5154	1136.6133	0.0979	86	203	211	THCSYVVDR			Carbamidomethyl (C)[3]		Mascot
1162.6038	1162.6868	0.083	71	235	243	YLCPPRTQK			Carbamidomethyl (C)[3]		Mascot
1162.6038	1162.6868	0.083	71	235	243	YLCPPRTQK			Carbamidomethyl (C)[3]		Mascot
1674.8956	1674.8899	-0.0057	-3	314	329	MGSINVLTGTAGEIRR					Mascot
1685.9333	1685.8871	-0.0462	-27	98	113	TAPQNRGLGGFVIIDK					Mascot
1745.8673	1745.9364	0.0691	40	122	137	CPGVVSCADILNLATR			Carbamidomethyl (C)[1,7]		Mascot
1775.8534	1775.8689	0.0155	9	138	154	DAVHMAGAPSYPVFTGR					Mascot
1801.869	1801.8833	0.0143	8	203	216	THCSYVVDRLYNFK			Carbamidomethyl (C)[3]		Mascot
1803.8549	1803.87	0.0151	8	291	305	EITQEFASGFEDFRK					Mascot
1874.9351	1874.8934	-0.0417	-22	185	202	GLDVLDMTTLLGAHSMGK			Oxidation (M)[7]		Mascot
1890.9301	1890.894	-0.0361	-19	185	202	GLDVLDMTTLLGAHSMGK			Oxidation (M)[7,16]		Mascot
1919.9094	1919.9135	0.0041	2	244	261	GQTDPLVYLNPDSGSSNR					Mascot

8 ATP-dependent helicase/deoxyribonuclease subunit B OS=Streptococcus mutans serotype c (strain ATCC 700610 / UA159) GN=rexB PE=3 SV=1 | ADDB_STRMU | 123829.9 | 5.71 | 21 | 59 | 36.47 | 34.54

Peptide Information

Calc. Mass	Obsrv. Mass	± da	± ppm	Start Seq.	End Seq.	Sequence	Ion Score	C. I. %	Modification	Rank	Result Type
878.5094	878.4377	-0.0717	-82	599	605	RFDIVTK					Mascot
1514.7889	1514.8323	0.0434	29	30	42	VFYIAPNSLSFEK					Mascot

1526.8424	1526.8381	-0.0043	-3	711	724	LAKEGLTIPEVNDK					Mascot
1555.8003	1555.7913	-0.009	-6	963	975	NLSYKGLFLEDEK					Mascot
1568.8167	1568.848	0.0313	20	883	897	LSDGSLGIVDYKSSK					Mascot
1609.8196	1609.8175	-0.0021	-1	561	574	FVFALGMTQSHFPK					Mascot
1612.8152	1612.8384	0.0232	14	379	391	YNFRAEDVMNLLK					Mascot
1612.8152	1612.8384	0.0232	14	379	391	YNFRAEDVMNLLK					Mascot
1626.9247	1626.8342	-0.0905	-56	441	454	LRAEVMSPLQELIK					Mascot
1628.8101	1628.8262	0.0161	10	379	391	YNFRAEDVMNLLK			Oxidation (M)[9]		Mascot
1641.8595	1641.9178	0.0583	36	968	982	GLFLEDEKAHLANGK					Mascot
1641.8595	1641.9178	0.0583	36	968	982	GLFLEDEKAHLANGK					Mascot
1650.8698	1650.7789	-0.0909	-55	880	894	IDRLSDGSLGIVDYK					Mascot
1653.8517	1653.8241	-0.0276	-17	383	397	AEDVMNLLKSGLYGK			Oxidation (M)[5]		Mascot
1696.9156	1696.8567	-0.0589	-35	325	339	YKDILVLLGDADAYK					Mascot
1702.868	1702.8988	0.0308	18	714	728	EGLTIPEVNDKMQTK					Mascot
1714.9333	1714.8574	-0.0759	-44	680	694	VIELNRSAIDQELSK					Mascot
1724.8966	1724.9625	0.0659	38	292	305	DKEHVTIWDVINQK					Mascot
1783.9126	1783.8816	-0.031	-17	695	708	EEQTFWSVAVRYLR					Mascot
1796.9905	1796.8944	-0.0961	-53	213	228	GVDIIIGTYISQKAYR					Mascot
1799.9326	1799.9004	-0.0322	-18	30	44	VFYIAPNSLSFEKER					Mascot
1830.8705	1830.8683	-0.0022	-1	1037	1051	AITHFEADRHMPYAR			Oxidation (M)[11]		Mascot
1843.976	1843.8983	-0.0777	-42	127	143	ASNLTVLELNQLNSAEK					Mascot
1845.9705	1845.9252	-0.0453	-25	663	679	LAADPEDIGNYKALLSR					Mascot

9	Genome polyprotein OS=Parsnip yellow fleck virus (isolate P-121) PE=1 SV=1	POLG_PYFV1	339225	6.57	37	59	33.475	34.451

Peptide Information

Calc. Mass	Obsrv. Mass	± da	± ppm	Start Seq.	End Seq.	Sequence	Ion Score	C. I. %	Modification	Rank	Result Type
832.3546	832.3668	0.0122	15	2131	2136	YFMDEK					Mascot
878.4916	878.4377	-0.0539	-61	1622	1628	IMKYPAR					Mascot
1136.5001	1136.6133	0.1132	100	2972	2981	LSQDDVGCSR			Carbamidomethyl (C)[8]		Mascot
1158.6049	1158.5784	-0.0265	-23	343	352	CPNSIGKNLR			Carbamidomethyl (C)[1]		Mascot
1170.6842	1170.6844	0.0002	0	1065	1076	LAVGQAQGLVSK					Mascot
1229.6848	1229.7083	0.0235	19	353	364	IGVAQASIQNTK					Mascot
1479.8054	1479.8657	0.0603	41	1161	1175	GVLSSLVDSISGAFK					Mascot
1507.725	1507.8191	0.0941	62	1345	1355	TLCEFKDYIYR			Carbamidomethyl (C)[3]		Mascot
1509.8424	1509.8234	-0.019	-13	2989	3001	FLTLDGQLPQHIK					Mascot

1526.7672	1526.8381	0.0709	46	1527	1539	GFSPTYNQIIQMK					Mascot
1566.7871	1566.8246	0.0375	24	1961	1974	GRNFITSGDELTTR					Mascot
1570.8411	1570.892	0.0509	32	638	651	VIIAEFALHPMSSR					Mascot
1598.8333	1598.8851	0.0518	32	2489	2502	HKHGIMVGINPHSR			Oxidation (M)[6]		Mascot
1602.7733	1602.877	0.1037	65	865	876	MFTLWRGDIEYR			Oxidation (M)[1]		Mascot
1612.913	1612.8384	-0.0746	-46	350	364	NLRIGVAQASIQNTK					Mascot
1612.913	1612.8384	-0.0746	-46	350	364	NLRIGVAQASIQNTK					Mascot
1617.8669	1617.8442	-0.0227	-14	2276	2289	STIVPSLIQPYMPR			Oxidation (M)[12]		Mascot
1626.8738	1626.8342	-0.0396	-24	1233	1246	AAISIVTKYEEQFK					Mascot
1640.9078	1640.8561	-0.0517	-32	223	236	LERTQNTPITVNVR					Mascot
1653.7902	1653.8241	0.0339	20	83	96	DTEQVLSAERCAFK			Carbamidomethyl (C)[11]		Mascot
1662.7428	1662.8602	0.1174	71	2400	2412	EYLFETMESGERR			Oxidation (M)[7]		Mascot
1679.9578	1679.8538	-0.104	-62	1891	1905	ILSNNILEELPSPIK					Mascot
1696.7643	1696.8567	0.0924	54	2046	2059	FQNGADYYFAFEPK					Mascot
1698.9789	1699.0005	0.0216	13	1049	1064	ELGFKPKVVESAAVPK					Mascot
1707.8193	1707.8693	0.05	29	1540	1554	TNNCFIVPMADLANK			Carbamidomethyl (C)[4]		Mascot
1713.8959	1713.8588	-0.0371	-22	997	1010	LKVYSKPGENFEFR					Mascot
1754.8741	1754.9452	0.0711	41	674	689	GSLVLTFEINCSASTR			Carbamidomethyl (C)[11]		Mascot
1765.9847	1765.9591	-0.0256	-14	205	219	EAIAADRLQLYLYVK					Mascot
1801.8578	1801.8833	0.0255	14	2953	2966	VCETFVSKEWQNFK			Carbamidomethyl (C)[2]		Mascot
1803.964	1803.87	-0.094	-52	1028	1043	KPFQNSVPDVFITPSK					Mascot
1817.9176	1817.9156	-0.002	-1	2186	2200	YVSTYVPITCDMIKK			Carbamidomethyl (C)[10]		Mascot
1830.8691	1830.8683	-0.0008	0	2851	2866	YGNSLFNLVCDNSLSK			Carbamidomethyl (C)[10]		Mascot
1844.0177	1843.8983	-0.1194	-65	2691	2706	ADELIPSLFHAPLHKR					Mascot
1845.9415	1845.9252	-0.0163	-9	510	525	DSDGIKIPIEFMSIHK			Oxidation (M)[12]		Mascot
1860.8806	1860.8782	-0.0024	-1	1629	1643	MKYVDFLCVCVAEAR			Carbamidomethyl (C)[8,10]		Mascot
1895.017	1894.8744	-0.1426	-75	1216	1232	ALMMVAITSISALYWPK					Mascot
1895.017	1894.8744	-0.1426	-75	1216	1232	ALMMVAITSISALYWPK					Mascot
1907.8699	1907.88	0.0101	5	2972	2988	LSQDDVGCSRTTPVCGR			Carbamidomethyl (C)[8,15]		Mascot
1911.0118	1910.8466	-0.1652	-86	1216	1232	ALMMVAITSISALYWPK			Oxidation (M)[3]		Mascot
1957.8882	1957.9752	0.087	44	1942	1958	EEDFEMISLNALMGQAK			Oxidation (M)[6,13]		Mascot

10	Arginine--tRNA ligase OS=Methanosarcina barkeri (strain Fusaro / DSM 804) GN=argS PE=3 SV=1	SYR_METBF	63590	5.55	15	58	23.62	28.968	

Peptide Information

Calc. Mass	Obsrv. Mass	± da	± ppm	Start Seq.	End Seq.	Sequence	Ion Score	C. I. %	Modification	Rank	Result Type

1229.6671	1229.7083	0.0412	34	408	419	QVAEMVGIGAVR		Mascot
1480.7577	1480.8571	0.0994	67	492	504	MASFDRVIDLAAR	Oxidation (M)[1]	Mascot
1480.7577	1480.8571	0.0994	67	492	504	MASFDRVIDLAAR	Oxidation (M)[1]	Mascot
1511.8104	1511.8254	0.015	10	283	296	GALVVDLSDYGFKK		Mascot
1526.7771	1526.8381	0.061	40	92	104	YMDKTVATVLEEK		Mascot
1553.7708	1553.808	0.0372	24	373	385	RGQFISADELFDR		Mascot
1570.9526	1570.892	-0.0606	-39	231	245	AVSLAISGIKETLLR		Mascot
1571.7952	1571.8813	0.0861	55	217	230	VEAGDVKTIDSFYK		Mascot
1571.7952	1571.8813	0.0861	55	217	230	VEAGDVKTIDSFYK		Mascot
1662.8254	1662.8602	0.0348	21	209	223	EIDALMEKVEAGDVK	Oxidation (M)[6]	Mascot
1669.996	1669.9071	-0.0889	-53	337	352	LISGQLRATLNSIGVK		Mascot
1713.8483	1713.8588	0.0105	6	430	443	STVFNWKEALDFEK		Mascot
1727.8851	1727.9348	0.0497	29	280	295	TDKGALVVDLSDYGFK		Mascot
1830.9518	1830.8683	-0.0835	-46	552	569	IVLANSLDTLGIAAPESM	Oxidation (M)[18]	Mascot
1863.0011	1862.8889	-0.1122	-60	246	261	LNVVHDKFVSESTFLK		Mascot
1863.0011	1862.8889	-0.1122	-60	246	261	LNVVHDKFVSESTFLK		Mascot
1876.011	1876.1326	0.1216	65	408	424	QVAEMVGIGAVRYDIVR		Mascot
1878.8328	1878.8792	0.0464	25	150	165	AGYDVEVQYYVNDMGR		Mascot
1894.8276	1894.8744	0.0468	25	150	165	AGYDVEVQYYVNDMGR	Oxidation (M)[14]	Mascot
1894.8276	1894.8744	0.0468	25	150	165	AGYDVEVQYYVNDMGR	Oxidation (M)[14]	Mascot

Rank	Protein Name	Accession No.	Protein MW	Protein PI	Pep. Count	Protein Score	Protein Score C. I. %	Intensity Matched	Total Ion Score	Total Ion C. I. %	Confirmed
1	Alpha-amylase inhibitor 0.19 OS=Triticum aestivum PE=1 SV=1	IAA1_WHEAT	13898.6	6.66	6	317	100	36.012	278	100	

Peptide Information

Calc. Mass	Obsrv. Mass	± da	± ppm	Start Seq.	End Seq.	Sequence	Ion Score	C. I. %	Modification	Rank	Result Type
1162.6249	1162.6742	0.0493	42	90	100	LTAASITAVCR			Carbamidomethyl (C)[10]		Mascot
1570.8007	1570.8644	0.0637	41	26	39	LQCNGSQVPEAVLR			Carbamidomethyl (C)[3]		Mascot
1612.7463	1612.8197	0.0734	46	67	82	EHGAQEGQAGTGAFPR					Mascot
1612.7463	1612.8197	0.0734	46	67	82	EHGAQEGQAGTGAFPR	147	100			Mascot
1617.8993	1617.8162	-0.0831	-51	86	100	EVVKLTAASITAVCR			Carbamidomethyl (C)[14]		Mascot
1663.8361	1663.8524	0.0163	10	101	116	LPIVVDASGDGAYVCK			Carbamidomethyl (C)[15]		Mascot
1862.7731	1862.8665	0.0934	50	40	53	DCCQQLAHISEWCR			Carbamidomethyl (C)[2,3,13]		Mascot
1862.7731	1862.8665	0.0934	50	40	53	DCCQQLAHISEWCR	131	100	Carbamidomethyl (C)[2,3,13]		Mascot

Rank	Protein Name	Accession No.	Protein MW	Protein PI	Pep. Count	Protein Score	Protein Score C. I. %	Intensity Matched	Total Ion Score	Total Ion C. I. %	Confirmed
2	Alpha-amylase/trypsin inhibitor CM3 OS=Triticum aestivum PE=1 SV=1	IAAC3_WHEAT	18893.3	7.44	7	236	100	13.203	195	100	

Peptide Information

Calc. Mass	Obsrv. Mass	± da	± ppm	Start Seq.	End Seq.	Sequence	Ion Score	C. I. %	Modification	Rank	Result Type
1010.52	1010.5569	0.0369	37	37	44	TNLLPHCR			Carbamidomethyl (C)[7]		Mascot
1110.5038	1110.5485	0.0447	40	133	140	EMQWDFVR					Mascot
1126.4987	1126.5249	0.0262	23	133	140	EMQWDFVR			Oxidation (M)[2]		Mascot
1698.9214	1698.9817	0.0603	35	101	115	YFIALPVPSQPVDPR					Mascot
1698.9214	1698.9817	0.0603	35	101	115	YFIALPVPSQPVDPR	75	99.997			Mascot
1727.8381	1727.9132	0.0751	43	116	132	SGNVGESGLIDLPGCPR			Carbamidomethyl (C)[15]		Mascot
1801.8427	1801.869	0.0263	15	45	60	DYVLQQTCGTFTPGSK			Carbamidomethyl (C)[8]		Mascot
1876.0222	1876.1145	0.0923	49	141	157	LLVAPGQCNLATIHNVR			Carbamidomethyl (C)[8]		Mascot
1876.0222	1876.1145	0.0923	49	141	157	LLVAPGQCNLATIHNVR	35	69.283	Carbamidomethyl (C)[8]		Mascot
1957.8564	1957.9537	0.0973	50	81	95	LYCCQELAEISQQCR			Carbamidomethyl (C)[3,4,14]		Mascot
1957.8564	1957.9537	0.0973	50	81	95	LYCCQELAEISQQCR	85	100	Carbamidomethyl (C)[3,4,14]		Mascot

Rank	Protein Name	Accession No.	Protein MW	Protein PI	Pep. Count	Protein Score	Protein Score C. I. %	Intensity Matched	Total Ion Score	Total Ion C. I. %	Confirmed
3	Arginine--tRNA ligase OS=Methanosarcina barkeri (strain Fusaro / DSM 804) GN=argS PE=3 SV=1	SYR_METBF	63590	5.55	15	58	14.3	36.759			

Peptide Information

Calc. Mass	Obsrv. Mass	± da	± ppm	Start Seq.	End Seq.	Sequence	Ion Score	C. I. %	Modification	Rank	Result Type
1061.5051	1061.5972	0.0921	87	313	320	DLAYHEWK					Mascot
1229.6671	1229.6963	0.0292	24	408	419	QVAEMVGIGAVR					Mascot
1480.7577	1480.8339	0.0762	51	492	504	MASFDRVIDLAAR			Oxidation (M)[1]		Mascot
1511.8104	1511.803	-0.0074	-5	283	296	GALVVDLSDYGFKK					Mascot
1553.7708	1553.8466	0.0758	49	373	385	RGQFISADELFDR					Mascot
1570.9526	1570.8644	-0.0882	-56	231	245	AVSLAISGIKETLLR					Mascot
1571.7952	1571.8567	0.0615	39	217	230	VEAGDVKTIDSFYK					Mascot
1571.7952	1571.8567	0.0615	39	217	230	VEAGDVKTIDSFYK					Mascot
1669.996	1669.8574	-0.1386	-83	337	352	LISGQLRATLNSIGVK					Mascot
1713.8483	1713.83	-0.0183	-11	430	443	STVFNWKEALDFEK					Mascot
1726.8872	1726.9327	0.0455	26	321	336	AGQADRIIDIFGADHK					Mascot
1727.8851	1727.9132	0.0281	16	280	295	TDKGALVVDLSDYGFK					Mascot
1863.0011	1862.8665	-0.1346	-72	246	261	LNVVHDKFVSESTFLK					Mascot
1863.0011	1862.8665	-0.1346	-72	246	261	LNVVHDKFVSESTFLK					Mascot
1876.011	1876.1145	0.1035	55	408	424	QVAEMVGIGAVRYDIVR					Mascot
1876.011	1876.1145	0.1035	55	408	424	QVAEMVGIGAVRYDIVR					Mascot
1878.8328	1878.8632	0.0304	16	150	165	AGYDVEVQYYVNDMGR					Mascot
1894.8276	1894.8521	0.0245	13	150	165	AGYDVEVQYYVNDMGR			Oxidation (M)[14]		Mascot
1894.8276	1894.8521	0.0245	13	150	165	AGYDVEVQYYVNDMGR			Oxidation (M)[14]		Mascot
2409.2119	2409.1948	-0.0171	-7	353	373	EPEVVIFEFVSLPEGSMS TRR					Mascot

4 Glutamyl-tRNA(Gln) amidotransferase subunit B, chloroplastic/mitochondrial OS=Oryza sativa subsp. indica GN=GATB PE=2 SV=1 GATB_ORYSI 60456.1 6.03 15 57 0 13.859

Peptide Information

Calc. Mass	Obsrv. Mass	± da	± ppm	Start Seq.	End Seq.	Sequence	Ion Score	C. I. %	Modification	Rank	Result Type
1011.533	1011.5706	0.0376	37	38	47	AESARAAAHK					Mascot
1108.5746	1108.5385	-0.0361	-33	500	508	QLEQYRSGK					Mascot
1126.531	1126.5249	-0.0061	-5	280	289	NMNSFSAISR					Mascot
1142.5259	1142.5298	0.0039	3	280	289	NMNSFSAISR			Oxidation (M)[2]		Mascot
1193.6493	1193.6592	0.0099	8	1	10	MALTLLRGMR			Oxidation (M)[1,9]		Mascot
1479.7843	1479.8215	0.0372	25	150	162	GYQISQFDIPIAK					Mascot
1483.9094	1483.8014	-0.108	-73	456	469	EILIELIAKGGTVK					Mascot

1490.7883	1490.8871	0.0988	66	123	136	LGLALNCEIATTSK			Carbamidomethyl (C)[7]		Mascot
1507.7388	1507.8048	0.066	44	226	239	TGIEAAEYGAELQR					Mascot
1511.804	1511.803	-0.001	-1	511	524	LQGFFAGQVMKASK					Mascot
1611.8159	1611.7891	-0.0268	-17	276	289	VEIKNMNSFSAISR			Oxidation (M)[6]		Mascot
1624.7748	1624.8218	0.047	29	243	257	YLGVSNGNMQEGSLR					Mascot
1640.7698	1640.8411	0.0713	43	243	257	YLGVSNGNMQEGSLR			Oxidation (M)[9]		Mascot
1641.8517	1641.894	0.0423	26	211	225	AGVPLLEIVSEPDMR			Oxidation (M)[14]		Mascot
1698.9537	1698.9817	0.028	16	290	303	AIDYEISRQILLHK					Mascot
1698.9537	1698.9817	0.028	16	290	303	AIDYEISRQILLHK					Mascot
1875.9923	1876.1145	0.1222	65	226	242	TGIEAAEYGAELQRLVR					Mascot
1875.9923	1876.1145	0.1222	65	226	242	TGIEAAEYGAELQRLVR					Mascot
1908.9847	1908.8904	-0.0943	-49	123	139	LGLALNCEIATTSKFDR			Carbamidomethyl (C)[7]		Mascot

5	Protein PA-X OS=Influenza A virus (strain A/Swine/Iowa/15/1930 H1N1) GN=PA PE=3 SV=1	PAX_I30A0	29466.8	8.5	11	56	0	26.365	

Peptide Information

Calc. Mass	Obsrv. Mass	± da	± ppm	Start Seq.	End Seq.	Sequence	Ion Score	C. I. %	Modification	Rank	Result Type
1107.6521	1107.5778	-0.0743	-67	239	248	TATLRASFLK					Mascot
1193.6201	1193.6592	0.0391	33	126	134	EVHIYYLEK					Mascot
1320.5824	1320.6436	0.0612	46	228	238	TLGHMWMDSSR					Mascot
1594.7604	1594.8126	0.0522	33	7	19	QCFNPMIVELAEK			Carbamidomethyl (C)[2], Oxidation (M)[6]		Mascot
1594.7604	1594.8126	0.0522	33	7	19	QCFNPMIVELAEK			Carbamidomethyl (C)[2], Oxidation (M)[6]		Mascot
1641.8694	1641.894	0.0246	15	58	73	GESIIVESGDPNALLK					Mascot
1837.865	1837.9143	0.0493	27	180	195	QEMASRGLWDSFASPR					Mascot
1853.86	1853.8263	-0.0337	-18	180	195	QEMASRGLWDSFASPR			Oxidation (M)[3]		Mascot
1862.9	1862.8665	-0.0335	-18	228	243	TLGHMWMDSSRTATLR					Mascot
1862.9	1862.8665	-0.0335	-18	228	243	TLGHMWMDSSRTATLR					Mascot
1878.8949	1878.8632	-0.0317	-17	228	243	TLGHMWMDSSRTATLR			Oxidation (M)[5]		Mascot
1894.8899	1894.8521	-0.0378	-20	228	243	TLGHMWMDSSRTATLR			Oxidation (M)[5,7]		Mascot
1894.8899	1894.8521	-0.0378	-20	228	243	TLGHMWMDSSRTATLR			Oxidation (M)[5,7]		Mascot
1908.938	1908.8904	-0.0476	-25	7	22	QCFNPMIVELAEKAMK			Carbamidomethyl (C)[2]		Mascot
1935.0294	1934.8783	-0.1511	-78	58	75	GESIIVESGDPNALLKHR					Mascot
1957.9987	1957.9537	-0.045	-23	222	238	ISPVLKTLGHMWMDSSR					Mascot
1957.9987	1957.9537	-0.045	-23	222	238	ISPVLKTLGHMWMDSSR					Mascot
3373.5166	3373.6626	0.146	43	140	168	SEETHIHIFSFTGEEMATK ADYTLDEESR					Mascot

6	Trem-like transcript 2 protein OS=Homo sapiens	TRML2_HUMAN	35503.2	9.68	11	56	0	6.612	

GN=TREML2 PE=1 SV=2

Peptide Information

Calc. Mass	Obsrv. Mass	± da	± ppm	Start Seq.	End Seq.	Sequence	Ion Score	C. I. %	Modification	Rank	Result Type
1483.8413	1483.8014	-0.0399	-27	250	262	SLLNRLPSMPSIR					Mascot
1487.6519	1487.7988	0.1469	99	97	107	LQDSGRYWCMR			Carbamidomethyl (C)[9], Oxidation (M)[10]		Mascot
1507.717	1507.8048	0.0878	58	205	219	TMGSQTVTASPSNAR					Mascot
1626.8043	1626.8011	-0.0032	-2	31	44	LLEGETLSVQCSYK			Carbamidomethyl (C)[11]		Mascot
1663.8181	1663.8524	0.0343	21	204	219	RTMGSQTVTASPSNAR					Mascot
1743.8953	1743.8887	-0.0066	-4	307	321	DPPGRPEPYVEVYLI					Mascot
1775.9432	1775.8542	-0.089	-50	239	254	SPTTGLCLTSRSLLNR			Carbamidomethyl (C)[7]		Mascot
1801.7415	1801.869	0.1275	71	292	306	RHMASYSMCSDPSTR			Carbamidomethyl (C)[9], Oxidation (M)[3]		Mascot
1817.8777	1817.8982	0.0205	11	188	204	TGYSFTATSTTSQGPRR					Mascot
1894.8989	1894.8521	-0.0468	-25	220	238	DSSAGPESISTKSGDLST R					Mascot
1894.8989	1894.8521	-0.0468	-25	220	238	DSSAGPESISTKSGDLST R					Mascot
1908.9445	1908.8904	-0.0541	-28	232	249	SGDLSTRSPTTGLCLTSR			Carbamidomethyl (C)[14]		Mascot

7 Heat shock-related 70 kDa protein 2 (Fragments) OS=Mesocricetus auratus GN=HSPA2 PE=1 SV=1 HSP72_MESAU 21270.9 5.52 9 56 0 6.276

Peptide Information

Calc. Mass	Obsrv. Mass	± da	± ppm	Start Seq.	End Seq.	Sequence	Ion Score	C. I. %	Modification	Rank	Result Type
1017.5687	1017.5867	0.018	18	167	175	ITITNDKGR					Mascot
1229.6848	1229.6963	0.0115	9	69	80	DAGTITGLNVLR					Mascot
1480.7543	1480.8339	0.0796	54	125	136	ARFEELNADLFR					Mascot
1487.7013	1487.7988	0.0975	66	12	24	TTPSYVAFTDTER					Mascot
1657.9232	1657.8042	-0.119	-72	65	80	QATKDAGTITGLNVLR					Mascot
1663.8109	1663.8524	0.0415	25	32	46	NQVAMNPTNTIFDAK					Mascot
1787.9901	1787.8477	-0.1424	-80	81	97	IINEPTAAAIAYGLDKK					Mascot
1819.912	1819.8943	-0.0177	-10	32	47	NQVAMNPTNTIFDAKR					Mascot
1894.9658	1894.8521	-0.1137	-60	48	64	VQSAVITVPAYFNDSQR					Mascot
1894.9658	1894.8521	-0.1137	-60	48	64	VQSAVITVPAYFNDSQR					Mascot

8 Sorting nexin-5 OS=Bos taurus GN=SNX5 PE=2 SV=1 SNX5_BOVIN 47075.2 6.31 12 55 0 34.227

Peptide Information

Calc. Mass	Obsrv. Mass	± da	± ppm	Start Seq.	End Seq.	Sequence	Ion Score	C. I. %	Modification	Rank	Result Type

882.4389	882.4204	-0.0185	-21	119	125	EEFAKMK					Mascot
898.4338	898.369	-0.0648	-72	119	125	EEFAKMK			Oxidation (M)[6]		Mascot
1593.8119	1593.7898	-0.0221	-14	315	328	ALIDYENSNKALDK					Mascot
1611.7974	1611.7891	-0.0083	-5	2	15	AAVPEVLQQQEEDR					Mascot
1612.7523	1612.8197	0.0674	42	109	123	LGEGEGSMTKEEFAK					Mascot
1612.7523	1612.8197	0.0674	42	109	123	LGEGEGSMTKEEFAK					Mascot
1617.8694	1617.8162	-0.0532	-33	283	296	VSSDEDLKLTELLR					Mascot
1650.8521	1650.7456	-0.1065	-65	389	402	NNVSLLQSCIDLFK			Carbamidomethyl (C)[9]		Mascot
1698.8843	1698.9817	0.0974	57	375	388	NLIEMSELEIKHAR			Oxidation (M)[5]		Mascot
1698.8843	1698.9817	0.0974	57	375	388	NLIEMSELEIKHAR	4	0	Oxidation (M)[5]		Mascot
1710.8698	1710.8799	0.0101	6	53	67	TTLPTFQSPEFSVTR					Mascot
1875.9673	1876.1145	0.1472	78	297	311	YYMLNIEAAKDLLYR					Mascot
1875.9673	1876.1145	0.1472	78	297	311	YYMLNIEAAKDLLYR					Mascot
1878.9379	1878.8632	-0.0747	-40	389	404	NNVSLLQSCIDLFKNN			Carbamidomethyl (C)[9]		Mascot
1957.9647	1957.9537	-0.011	-6	1	17	MAAVPEVLQQQEEDRSK					Mascot
1957.9647	1957.9537	-0.011	-6	1	17	MAAVPEVLQQQEEDRSK					Mascot
2424.2559	2424.1562	-0.0997	-41	47	67	FTVHTKTTLPTFQSPEFS VTR					Mascot

9	GTPase Der OS=Buchnera aphidicola subsp. Baizongia pistaciae (strain Bp) GN=der PE=3 SV=1	DER_BUCBP	53202.1	9.95	15	54	0	13.613

Peptide Information

Calc. Mass	Obsrv. Mass	± da	± ppm	Start Seq.	End Seq.	Sequence	Ion Score	C. I. %	Modification	Rank	Result Type
882.4832	882.4204	-0.0628	-71	391	397	HQPPIYK					Mascot
1093.6252	1093.5519	-0.0733	-67	423	431	LEKLSNVYK					Mascot
1142.6317	1142.5298	-0.1019	-89	43	52	HGFIIVNNTK					Mascot
1158.6115	1158.5144	-0.0971	-84	226	236	VIVDSNPGTTR					Mascot
1513.8109	1513.7977	-0.0132	-9	53	66	IVLIDTPGINEDSK					Mascot
1571.7952	1571.8567	0.0615	39	179	191	DFDIIYSITNDKK					Mascot
1571.7952	1571.8567	0.0615	39	179	191	DFDIIYSITNDKK					Mascot
1585.9458	1585.8591	-0.0867	-55	1	15	MVITIALIGRTNVGK					Mascot
1587.8523	1587.776	-0.0763	-48	310	323	NGCSVIIILNKNDK			Carbamidomethyl (C)[3]		Mascot
1626.8706	1626.8011	-0.0695	-43	99	111	LMHKDVEIIEMLR					Mascot
1640.8099	1640.8411	0.0312	19	27	41	NDALASNHASLTRDR					Mascot
1641.9058	1641.894	-0.0118	-7	53	67	IVLIDTPGINEDSKK					Mascot
1726.9963	1726.9327	-0.0636	-37	411	425	HNPLTIIIHGNKLEK					Mascot

1805.9943	1805.8433	-0.151	-84	81	96	FSIKQADLVCLVVSAR			Carbamidomethyl (C)[10]		Mascot
1910.9791	1910.8416	-0.1375	-72	22	39	LTGNRNDALASNHASLT R					Mascot
2424.2744	2424.1562	-0.1182	-49	353	373	HNMGTSIIFKLINEAFFNSI K					Mascot

10	Dynein beta chain, ciliary OS=Tripneustes gratilla PE=1 SV=1	DYHC_TRIGR	515561.2	5.22	47	54	0	61.58

Peptide Information

Calc. Mass	Obsrv. Mass	± da	± ppm	Start Seq.	End Seq.	Sequence	Ion Score	C. I. %	Modification	Rank	Result Type
850.4451	850.4298	-0.0153	-18	1347	1353	NMLTSLR			Oxidation (M)[2]		Mascot
882.4753	882.4204	-0.0549	-62	1634	1641	LALGMYSK					Mascot
994.4952	994.5512	0.056	56	3458	3465	QKYGDDLR					Mascot
1094.5333	1094.5391	0.0058	5	4242	4250	MNMLTSEIR					Mascot
1108.4728	1108.5385	0.0657	59	559	566	EIYDEHMR			Oxidation (M)[7]		Mascot
1110.55	1110.5485	-0.0015	-1	2090	2098	DMDFEKVVK					Mascot
1126.5231	1126.5249	0.0018	2	4242	4250	MNMLTSEIR			Oxidation (M)[1,3]		Mascot
1136.6311	1136.6021	-0.029	-26	148	158	SSVYVVAGQVK					Mascot
1142.6165	1142.5298	-0.0867	-76	4414	4423	AIPVDKQDTR					Mascot
1158.6228	1158.5144	-0.1084	-94	2005	2013	FITLYTLCK			Carbamidomethyl (C)[8]		Mascot
1165.632	1165.6436	0.0116	10	1770	1779	DVVAMMVLKK			Oxidation (M)[5,6]		Mascot
1193.6525	1193.6592	0.0067	6	1571	1580	ALAEYLETKR					Mascot
1229.7075	1229.6963	-0.0112	-9	1286	1295	ALWDLIMIVR					Mascot
1320.5775	1320.6436	0.0661	50	4186	4200	DAGGGGGGGSSREEK					Mascot
1480.837	1480.8339	-0.0031	-2	2628	2641	LSPTVVSATLDLHK					Mascot
1483.7573	1483.8014	0.0441	30	978	989	VQTIMTKAQEYR			Oxidation (M)[5]		Mascot
1511.8944	1511.803	-0.0914	-60	842	854	IHSLIKENLGLFK					Mascot
1553.9414	1553.8466	-0.0948	-61	4452	4466	AAKWTLAGVALLLQV					Mascot
1555.9054	1555.8025	-0.1029	-66	3594	3606	IILKELEDNLLSR					Mascot
1568.7162	1568.8199	0.1037	66	4374	4387	EGSYVHGLFMEGAR			Oxidation (M)[10]		Mascot
1593.8536	1593.7898	-0.0638	-40	4435	4447	QRGPTFVWTFNLK					Mascot
1594.6857	1594.8126	0.1269	80	2547	2557	QHMDYKHWYDR			Oxidation (M)[3]		Mascot
1594.7101	1594.8126	0.1025	64	2907	2919	GMGLQDTRENCWK			Carbamidomethyl (C)[11]		Mascot
1598.7263	1598.8704	0.1441	90	4212	4224	LPEEFNMMEIMAK			Oxidation (M)[7]		Mascot
1599.8207	1599.8834	0.0627	39	2378	2389	VEFSKWWITEFK					Mascot
1612.9495	1612.8197	-0.1298	-80	4400	4413	LKELAPNMPVIFIK					Mascot
1612.9495	1612.8197	-0.1298	-80	4400	4413	LKELAPNMPVIFIK					Mascot
1624.7418	1624.8218	0.08	49	3428	3441	MSTENATILSNCQR			Carbamidomethyl (C)[12]		Mascot

1640.7367	1640.8411	0.1044	64	3428	3441	MSTENATILSNCQR	Carbamidomethyl (C)[12], Oxidation (M)[1]	Mascot
1669.8367	1669.8574	0.0207	12	3994	4008	ITNEPPTGMFANLHK		Mascot
1674.9037	1674.88	-0.0237	-14	3379	3391	VDLQERMWLPFLK		Mascot
1680.8494	1680.8317	-0.0177	-11	1469	1482	HIAHFLEEVSGWQK		Mascot
1698.8486	1698.9817	0.1331	78	716	729	YVANLDLTQAWYNK		Mascot
1698.8486	1698.9817	0.1331	78	716	729	YVANLDLTQAWYNK		Mascot
1710.8157	1710.8799	0.0642	38	643	656	VFENWTKGVDEVCK	Carbamidomethyl (C)[13]	Mascot
1712.8497	1712.8481	-0.0016	-1	2041	2054	RGDPQRPEDQVLMR	Oxidation (M)[13]	Mascot
1803.9501	1803.8561	-0.094	-52	1779	1793	KVDNAQAFQWLSQLR		Mascot
1803.9501	1803.8561	-0.094	-52	1779	1793	KVDNAQAFQWLSQLR		Mascot
1805.9293	1805.8433	-0.086	-48	127	141	NHEQWPVVVSQDVLR		Mascot
1817.9401	1817.8982	-0.0419	-23	522	536	MLDCYGPLLDRPVIR	Carbamidomethyl (C)[4]	Mascot
1820.0099	1819.8943	-0.1156	-64	2928	2943	QLKTVLCFSPVGTTLR	Carbamidomethyl (C)[7]	Mascot
1845.8258	1845.9197	0.0939	51	4009	4023	ALYNFNQDTLEMCAR	Carbamidomethyl (C)[13]	Mascot
1859.0209	1859.1053	0.0844	45	3237	3254	GKSLAAGGLCSWVVNIV K	Carbamidomethyl (C)[10]	Mascot
1862.9331	1862.8665	-0.0666	-36	2731	2747	AKPNIHCHFATGIGDPK	Carbamidomethyl (C)[7]	Mascot
1862.9331	1862.8665	-0.0666	-36	2731	2747	AKPNIHCHFATGIGDPK	Carbamidomethyl (C)[7]	Mascot
1875.9698	1876.1145	0.1447	77	3290	3306	IAELDANLAELTAQFEK		Mascot
1875.9698	1876.1145	0.1447	77	3290	3306	IAELDANLAELTAQFEK		Mascot
1884.8644	1884.8215	-0.0429	-23	1625	1641	QDDEGNDTKLALGMYSK		Mascot
1894.991	1894.8521	-0.1389	-73	1484	1499	LSTTDSVITIWFEVQR		Mascot
1894.991	1894.8521	-0.1389	-73	1484	1499	LSTTDSVITIWFEVQR		Mascot
1935.0433	1934.8783	-0.165	-85	2099	2115	QSTLDLKLQAEDSFVLK		Mascot
1968.1165	1967.9696	-0.1469	-75	2817	2833	LASYISSLEVFQITLRK		Mascot
2369.1401	2369.135	-0.0051	-2	567	588	VEENDGNAPLNKNMPDV AGQLK	Oxidation (M)[14]	Mascot
2399.3545	2399.1494	-0.2051	-85	2106	2126	LQAEDSFVLKVVQLEELL AVR		Mascot
2424.3684	2424.1562	-0.2122	-88	3157	3181	SFGSPPSAVLKVAAAVM VLLAPNGK		Mascot

Gel Idx/Pos	130/F5	Instr./Gel Origin	BA2151/Sample Project 20140814	Process Status	Analysis Succeeded
Plate [#] Name	[1] Sample Project 20140814	Instrument Sample Name		Spectra	11

Rank	Protein Name	Accession No.	Protein MW	Protein PI	Pep. Count	Protein Score	Protein Score C. I. %	Intensity Matched	Total Ion Score	Total Ion C. I. %	Confirmed
1	Keratin, type I cytoskeletal 10 OS=Homo sapiens GN=KRT10 PE=1 SV=6	K1C10_HUMAN	59019.8	5.13	16	70	94.467	11.381			

Peptide Information

Calc. Mass	Obsrv. Mass	± da	± ppm	Start Seq.	End Seq.	Sequence	Ion Score	C. I. %	Modification	Rank	Result Type
807.3995	807.4478	0.0483	60	229	235	LAADDFR					Mascot
809.4403	809.4584	0.0181	22	157	163	LASYLDK					Mascot
847.452	847.5054	0.0534	63	363	369	SEITELR					Mascot
993.4999	993.5597	0.0598	60	238	245	YENEVALR					Mascot
1003.553	1003.5864	0.0334	33	363	370	SEITELRR					Mascot
1090.531	1090.5977	0.0667	61	148	156	VTMQNLNDR					Mascot
1106.5259	1106.5709	0.045	41	148	156	VTMQNLNDR			Oxidation (M)[3]		Mascot
1109.4899	1109.5765	0.0866	78	335	343	DAEAWFNEK					Mascot
1118.5087	1118.5695	0.0608	54	185	194	HGNSHQGEPR					Mascot
1165.5848	1165.6541	0.0693	59	442	450	LENEIQTYR					Mascot
1165.5848	1165.6541	0.0693	59	442	450	LENEIQTYR					Mascot
1201.6172	1201.699	0.0818	68	246	256	QSVEADINGLR					Mascot
1234.6791	1234.7545	0.0754	61	236	245	LKYENEVALR					Mascot
1365.6393	1365.7216	0.0823	60	323	333	SQYEQLAEQNR					Mascot
1390.6809	1390.7526	0.0717	52	387	399	QSLEASLAETEGR					Mascot
1493.7343	1493.825	0.0907	61	323	334	SQYEQLAEQNRK					Mascot
1707.7722	1707.8678	0.0956	56	41	59	GSLGGGFSSGGFSGGSFSR					Mascot
2367.2627	2367.3945	0.1318	56	208	228	NQILNLTTDNANILLQIDNAR					Mascot

Rank	Protein Name	Accession No.	Protein MW	Protein PI	Pep. Count	Protein Score	Protein Score C. I. %	Intensity Matched	Total Ion Score	Total Ion C. I. %	Confirmed
2	Keratin, type I cytoskeletal 10 OS=Canis familiaris GN=KRT10 PE=2 SV=1	K1C10_CANFA	57847.2	5.09	12	42	0	7.795			

Peptide Information

Calc. Mass	Obsrv. Mass	± da	± ppm	Start Seq.	End Seq.	Sequence	Ion Score	C. I. %	Modification	Rank	Result Type
807.3995	807.4478	0.0483	60	219	225	LAADDFR					Mascot
809.4403	809.4584	0.0181	22	147	153	LASYLDK					Mascot
847.452	847.5054	0.0534	63	353	359	SEITELR					Mascot

Calc. Mass	Obsrv. Mass	± da	± ppm	Start Seq.	End Seq.	Sequence	Ion Score	C. I. %	Modification	Rank	Result Type
993.4999	993.5597	0.0598	60	228	235	YENEVALR					Mascot
1003.553	1003.5864	0.0334	33	353	360	SEITELRR					Mascot
1090.531	1090.5977	0.0667	61	138	146	VTMQNLNDR					Mascot
1106.5259	1106.5709	0.045	41	138	146	VTMQNLNDR			Oxidation (M)[3]		Mascot
1109.5559	1109.5765	0.0206	19	175	184	HGNSSQRAPR					Mascot
1165.5848	1165.6541	0.0693	59	432	440	LENEIQTYR					Mascot
1165.5848	1165.6541	0.0693	59	432	440	LENEIQTYR					Mascot
1201.6172	1201.699	0.0818	68	236	246	QSVEADINGLR					Mascot
1234.6791	1234.7545	0.0754	61	226	235	LKYENEVALR					Mascot
1707.7722	1707.8678	0.0956	56	35	53	GSIGGGFSSGGFSGGSF SR					Mascot
2367.2627	2367.3945	0.1318	56	198	218	NQILNLTTDNANILLQIDN AR					Mascot

3	16.9 kDa class I heat shock protein 1 OS=Triticum aestivum GN=hsp16.9A PE=2 SV=1	HS16A_WHEAT	16867.8	5.83	3	40	0	3.592	26	0	

Peptide Information

Calc. Mass	Obsrv. Mass	± da	± ppm	Start Seq.	End Seq.	Sequence	Ion Score	C. I. %	Modification	Rank	Result Type
975.5258	975.5727	0.0469	48	110	117	FRLPEDAK					Mascot
1027.6146	1027.5535	-0.0611	-59	137	145	AEVKKPEVK					Mascot
1905.9666	1906.0742	0.1076	56	26	45	SIVPAISGGSSETAAFAN AR					Mascot
1905.9666	1906.0742	0.1076	56	26	45	SIVPAISGGSSETAAFAN AR	26	0			Mascot

4	UPF0102 protein DICTH_1420 OS=Dictyoglomus thermophilum (strain ATCC 35947 / DSM 3960 / H-6-12) GN=DICTH_1420 PE=3 SV=1	Y1420_DICT6	13741.6	9.66	7	36	0	5.046	

Peptide Information

Calc. Mass	Obsrv. Mass	± da	± ppm	Start Seq.	End Seq.	Sequence	Ion Score	C. I. %	Modification	Rank	Result Type
802.4417	802.4953	0.0536	67	2	8	NNKEIGK					Mascot
847.5036	847.5054	0.0018	2	22	28	GFIILER					Mascot
1003.6047	1003.5864	-0.0183	-18	21	28	RGFIILER					Mascot
1165.6827	1165.6541	-0.0286	-25	77	86	KIAELYISTK					Mascot
1165.6827	1165.6541	-0.0286	-25	77	86	KIAELYISTK					Mascot
1182.6439	1182.6407	-0.0032	-3	95	104	FDIMSIILSK			Oxidation (M)[4]		Mascot
1390.8304	1390.7526	-0.0778	-56	78	89	IAELYISTKKPK					Mascot
1838.9535	1839.0247	0.0712	39	5	20	EIGKLGEDFTIDFLNK					Mascot
1838.9535	1839.0247	0.0712	39	5	20	EIGKLGEDFTIDFLNK					Mascot

5	Cell division topological specificity factor OS=Burkholderia cenocepacia (strain MC0-3) GN=minE PE=3 SV=1	MINE_BURCC	9415.2	5.63	5	36	0	5.911		

Protein Group

Cell division topological specificity factor OS=Burkholderia cenocepacia (strain AU 1054) GN=minE PE=3 SV=1	MINE_BURCA	9415.2	5.6300 001144 4092
Cell division topological specificity factor OS=Burkholderia cenocepacia (strain HI2424) GN=minE PE=3 SV=1	MINE_BURCH	9415.2	5.6300 001144 4092
Cell division topological specificity factor OS=Burkholderia cepacia (strain J2315 / LMG 16656) GN=minE PE=3 SV=1	MINE_BURCJ	9414.2	6.0799 999237 0605
Cell division topological specificity factor OS=Burkholderia multivorans (strain ATCC 17616 / 249) GN=minE PE=3 SV=1	MINE_BURM1	9405.2	5.7800 002098 0835
Cell division topological specificity factor OS=Burkholderia sp. (strain 383) GN=minE PE=3 SV=1	MINE_BURS3	9414.2	6.0799 999237 0605
Cell division topological specificity factor OS=Burkholderia vietnamiensis (strain G4 / LMG 22486) GN=minE PE=3 SV=1	MINE_BURVG	9405.2	5.7800 002098 0835

Peptide Information

Calc. Mass	Obsrv. Mass	± da	± ppm	Start Seq.	End Seq.	Sequence	Ion Score	C. I. %	Modification	Rank	Result Type
847.4631	847.5054	0.0423	50	14	21	SASVAKER					Mascot
1106.6456	1106.5709	-0.0747	-68	2	11	SILSFLLGEK					Mascot
1234.7406	1234.7545	0.0139	11	2	12	SILSFLLGEKK					Mascot
1365.781	1365.7216	-0.0594	-43	1	12	MSILSFLLGEKK					Mascot
1838.9746	1839.0247	0.0501	27	69	84	QDDLEVLEVKIEIPQA					Mascot
1838.9746	1839.0247	0.0501	27	69	84	QDDLEVLEVKIEIPQA					Mascot

6	Phosphocarrier protein HPr OS=Pseudomonas aeruginosa (strain ATCC 15692 / PAO1 / 1C / PRS 101 / LMG 12228) GN=ptsH PE=3 SV=1	PTHP_PSEAE	9810	5.56	3	36	0	3.192	17	0

Peptide Information

Calc. Mass	Obsrv. Mass	± da	± ppm	Start Seq.	End Seq.	Sequence	Ion Score	C. I. %	Modification	Rank	Result Type
1254.6255	1254.6699	0.0444	35	47	58	SIMAVMMLAAGK			Oxidation (M)[3,6]		Mascot
1258.7075	1258.6863	-0.0212	-17	1	11	MPALEITIINK			Oxidation (M)[1]		Mascot
1906.0942	1906.0742	-0.02	-10	1	17	MPALEITIINKLGLHAR			Oxidation (M)[1]		Mascot
1906.0942	1906.0742	-0.02	-10	1	17	MPALEITIINKLGLHAR	17	0	Oxidation (M)[1]		Mascot

7	ATP-dependent Clp protease adapter protein ClpS	CLPS_SALAR	12358.1	4.49	5	36	0	3.67		

OS=Salmonella arizonae (strain ATCC BAA-731 / CDC346-86 / RSK2980) GN=clpS PE=3 SV=1

Peptide Information

Calc. Mass	Obsrv. Mass	± da	± ppm	Start Seq.	End Seq.	Sequence	Ion Score	C. I. %	Modification	Rank	Result Type
1109.5408	1109.5765	0.0357	32	85	93	VEMVNQYAR					Mascot
1165.5922	1165.6541	0.0619	53	20	29	DALKPPSMYK			Oxidation (M)[8]		Mascot
1165.5922	1165.6541	0.0619	53	20	29	DALKPPSMYK			Oxidation (M)[8]		Mascot
1475.7676	1475.8262	0.0586	40	58	70	ATQLMLAVHYQGK			Oxidation (M)[5]		Mascot
1475.7676	1475.8262	0.0586	40	58	70	ATQLMLAVHYQGK			Oxidation (M)[5]		Mascot
1993.9138	1994.1021	0.1883	94	4	19	TNDWLDFDQLVEDDLR					Mascot
2367.2153	2367.3945	0.1792	76	30	49	VILVNDDYTPMEFVIDVLQK			Oxidation (M)[11]		Mascot

8	Riboflavin biosynthesis protein RibBA OS=Bacillus cereus (strain G9842) GN=ribBA PE=3 SV=1	RIBBA_BACC2	44384.7	5.41	9	35	0	5.684

Peptide Information

Calc. Mass	Obsrv. Mass	± da	± ppm	Start Seq.	End Seq.	Sequence	Ion Score	C. I. %	Modification	Rank	Result Type
809.4879	809.4584	-0.0295	-36	232	238	EHIALVK					Mascot
827.4733	827.458	-0.0153	-18	348	354	LLTNNPR					Mascot
1165.6398	1165.6541	0.0143	12	282	291	EGKGVLLYMR					Mascot
1165.6398	1165.6541	0.0143	12	282	291	EGKGVLLYMR					Mascot
1182.6113	1182.6407	0.0294	25	139	150	AGHTEAAVDLAK					Mascot
1184.6746	1184.6407	-0.0339	-29	292	302	QEGRGIGLLNK					Mascot
1254.7053	1254.6699	-0.0354	-28	239	250	GDISIGEPVLVR					Mascot
1493.8145	1493.825	0.0105	7	187	198	MITIEDLIAYRR					Mascot
1838.8636	1839.0247	0.1611	88	266	281	CDCGPQLHAALAQIER			Carbamidomethyl (C)[1,3]		Mascot
1838.8636	1839.0247	0.1611	88	266	281	CDCGPQLHAALAQIER			Carbamidomethyl (C)[1,3]		Mascot
1960.0862	1960.2197	0.1335	68	330	347	DYGIGAQILKDLGLQSLR					Mascot

9	Aspartate carbamoyltransferase OS=Methanoculleus marisnigri (strain ATCC 35101 / DSM 1498 / JR1) GN=pyrB PE=3 SV=1	PYRB_METMJ	33125.1	5.54	8	35	0	2.826

Peptide Information

Calc. Mass	Obsrv. Mass	± da	± ppm	Start Seq.	End Seq.	Sequence	Ion Score	C. I. %	Modification	Rank	Result Type
975.5105	975.5727	0.0622	64	80	88	GETLADTIR					Mascot
1107.6045	1107.6008	-0.0037	-3	213	221	ELDVLYVTR					Mascot

1109.5474	1109.5765	0.0291	26	13	21	SDLDYLLDR					Mascot
1258.6031	1258.6863	0.0832	66	51	61	TRMSFATAMAR			Oxidation (M)[3]		Mascot
1390.7988	1390.7526	-0.0462	-33	252	262	ERLMILHPLPR			Oxidation (M)[4]		Mascot
1707.9397	1707.8678	-0.0719	-42	284	298	NGVPIRMALLHEVMK					Mascot
1708.8323	1708.844	0.0117	7	198	212	GMEVVEHPNVEEAIR					Mascot
1993.9761	1994.1021	0.126	63	196	212	ERGMEVVEHPNVEEAIR					Mascot

10	Cell division topological specificity factor OS=Burkholderia mallei (strain NCTC 10229) GN=minE PE=3 SV=1	MINE_BURM9	9389.2	6.58	5	35	0	5.589

Protein Group

Cell division topological specificity factor OS=Burkholderia mallei (strain ATCC 23344) GN=minE PE=3 SV=1	MINE_BURMA	9389.2	6.5799 999237 0605
Cell division topological specificity factor OS=Burkholderia mallei (strain NCTC 10247) GN=minE PE=3 SV=1	MINE_BURM7	9389.2	6.5799 999237 0605
Cell division topological specificity factor OS=Burkholderia mallei (strain SAVP1) GN=minE PE=3 SV=1	MINE_BURMS	9389.2	6.5799 999237 0605
Cell division topological specificity factor OS=Burkholderia pseudomallei (strain 1106a) GN=minE PE=3 SV=1	MINE_BURP0	9389.2	6.5799 999237 0605
Cell division topological specificity factor OS=Burkholderia pseudomallei (strain 1710b) GN=minE PE=3 SV=1	MINE_BURP1	9389.2	6.5799 999237 0605
Cell division topological specificity factor OS=Burkholderia pseudomallei (strain 668) GN=minE PE=3 SV=1	MINE_BURP6	9389.2	6.5799 999237 0605
Cell division topological specificity factor OS=Burkholderia pseudomallei (strain K96243) GN=minE PE=3 SV=1	MINE_BURPS	9389.2	6.5799 999237 0605
Cell division topological specificity factor OS=Burkholderia thailandensis (strain E264 / ATCC 700388 / DSM 13276 / CIP 106301) GN=minE PE=3 SV=1	MINE_BURTA	9389.2	6.5799 999237 0605

Peptide Information

Calc. Mass	Obsrv. Mass	± da	± ppm	Start Seq.	End Seq.	Sequence	Ion Score	C. I. %	Modification	Rank	Result Type
831.4683	831.4162	-0.0521	-63	14	21	SAAVAKER					Mascot
1106.6456	1106.5709	-0.0747	-68	2	11	SILSFLLGEK					Mascot
1234.7406	1234.7545	0.0139	11	2	12	SILSFLLGEKK					Mascot
1365.781	1365.7216	-0.0594	-43	1	12	MSILSFLLGEKK					Mascot
1838.9746	1839.0247	0.0501	27	69	84	QDDLEVLEVKIEIPQA					Mascot
1838.9746	1839.0247	0.0501	27	69	84	QDDLEVLEVKIEIPQA					Mascot

Gel Idx/Pos	131/F6	Instr./Gel Origin	BA2151/Sample Project 20140814	Process Status	Analysis Succeeded
Plate [#] Name	[1] Sample Project 20140814	Instrument Sample Name		Spectra	11

Rank	Protein Name	Accession No.	Protein MW	Protein PI	Pep. Count	Protein Score	Protein Score C. I. %	Intensity Matched	Total Ion Score	Total Ion C. I. %	Confirmed
1	Keratin, type II cytoskeletal 1 OS=Homo sapiens GN=KRT1 PE=1 SV=6	K2C1_HUMAN	66170.1	8.15	18	183	100	11.582	99	100	

Peptide Information

Calc. Mass	Obsrv. Mass	± da	± ppm	Start Seq.	End Seq.	Sequence	Ion Score	C. I. %	Modification	Rank	Result Type
1006.4297	1006.488	0.0583	58	589	602	GGSGGGGGGSSGGR					Mascot
1033.516	1033.5616	0.0456	44	484	492	TLLEGEESR					Mascot
1065.5211	1065.5483	0.0272	26	356	364	AQYEDIAQK					Mascot
1066.5164	1066.559	0.0426	40	270	277	YEDEINKR					Mascot
1092.5029	1092.5649	0.062	57	603	616	GSGGGSSGGSIGGR					Mascot
1127.5402	1127.572	0.0318	28	289	298	KDVDGAYMTK					Mascot
1141.5194	1141.5803	0.0609	53	464	472	DYQELMNTK					Mascot
1265.6372	1265.6681	0.0309	24	278	288	TNAENEFVTIK					Mascot
1277.71	1277.765	0.055	43	473	483	LALDLEIATYR	3	0			Mascot
1332.5195	1332.6061	0.0866	65	258	267	NMQDMVEDYR			Oxidation (M)[2,5]		Mascot
1475.7489	1475.8168	0.0679	46	212	223	WELLQQVDTSTR	40	90.026			Mascot
1657.793	1657.8805	0.0875	53	13	29	SGGGFSSGSAGIINYQR					Mascot
1716.8511	1716.9358	0.0847	49	418	432	QISNLQQSISDAEQR	26	0			Mascot
1993.9767	1994.0825	0.1058	53	224	239	THNLEPYFESFINNLR	36	76.13			Mascot
1993.9827	1994.0825	0.0998	50	625	644	SSGGSSSVKFVSTTYSG VTR					Mascot
2286.1248	2286.0864	-0.0384	-17	367	386	AEAESLYQSKYEELQITA GR					Mascot
2383.9519	2384.0759	0.124	52	519	549	GGGGGGYGSGGSSYGS GGGSYGSGGGGGGGR					Mascot
3312.3083	3312.5071	0.1988	60	550	588	GSYGSGGSSYGSGGGS YGSGGGGGGHGSYGSG SSSGGYR					Mascot

Rank	Protein Name	Accession No.	Protein MW	Protein PI	Pep. Count	Protein Score	Protein Score C. I. %	Intensity Matched	Total Ion Score	Total Ion C. I. %	Confirmed
2	Keratin, type II cytoskeletal 1 OS=Pan troglodytes GN=KRT1 PE=1 SV=1	K2C1_PANTR	65620.8	7.62	16	170	100	10.906	99	100	

Peptide Information

Calc. Mass	Obsrv. Mass	± da	± ppm	Start Seq.	End Seq.	Sequence	Ion Score	C. I. %	Modification	Rank	Result Type
1033.516	1033.5616	0.0456	44	479	487	TLLEGEESR					Mascot
1065.5211	1065.5483	0.0272	26	351	359	AQYEDIAQK					Mascot

Calc. Mass	Obsrv. Mass	± da	± ppm	Start Seq.	End Seq.	Sequence	Ion Score	C. I. %	Modification	Rank	Result Type
1066.5164	1066.559	0.0426	40	265	272	YEDEINKR					Mascot
1092.5029	1092.5649	0.062	57	596	609	GSGGGSSGGSIGGR					Mascot
1127.5402	1127.572	0.0318	28	284	293	KDVDGAYMTK					Mascot
1141.5194	1141.5803	0.0609	53	459	467	DYQELMNTK					Mascot
1265.6372	1265.6681	0.0309	24	273	283	TNAENEFVTIK					Mascot
1277.71	1277.765	0.055	43	468	478	LALDLEIATYR	3	0			Mascot
1475.7489	1475.8168	0.0679	46	207	218	WELLQQVDTSTR	40	90.026			Mascot
1657.793	1657.8805	0.0875	53	13	29	SGGGFSSGSAGIINYQR					Mascot
1716.8511	1716.9358	0.0847	49	413	427	QISNLQQSISDAEQR	26	0			Mascot
1993.9767	1994.0825	0.1058	53	219	234	THNLEPYFESFINNLR	36	76.13			Mascot
1993.9827	1994.0825	0.0998	50	618	637	SSGGSSSVKFVSTTYSG VTR					Mascot
2286.1248	2286.0864	-0.0384	-17	362	381	AEAESLYQSKYEELQITA GR					Mascot
2383.9519	2384.0759	0.124	52	514	544	GGGGGGYGSGGSSYGS GGGSYGSGGGGGGGR					Mascot
3312.3083	3312.5071	0.1988	60	545	583	GSYGSGGSSYGSGGGS YGSGGGGGGHGSYGSG SSSGGYR					Mascot

3 7-cyano-7-deazaguanine synthase OS=Cupriavidus taiwanensis (strain R1 / LMG 19424) GN=queC PE=3 SV=1 QUEC_CUPTR 24285.1 5.11 12 78 99.103 5.437

Peptide Information

Calc. Mass	Obsrv. Mass	± da	± ppm	Start Seq.	End Seq.	Sequence	Ion Score	C. I. %	Modification	Rank	Result Type
827.5098	827.4303	-0.0795	-96	50	58	VAAALGAVR					Mascot
1006.5065	1006.488	-0.0185	-18	151	159	AGVEGDRFR					Mascot
1127.5803	1127.572	-0.0083	-7	40	49	HSSELEAAKR					Mascot
1140.6082	1140.6119	0.0037	3	160	169	VHAPIIDMTK			Oxidation (M)[8]		Mascot
1265.696	1265.6681	-0.0279	-22	59	68	HEIVDLDLRR					Mascot
1373.6519	1373.729	0.0771	56	24	35	AQGFETYALSMR					Mascot
1427.7828	1427.8231	0.0403	28	158	169	FRVHAPIIDMTK					Mascot
1475.7238	1475.8168	0.093	63	36	48	YGQRHSSELEAAK					Mascot
1708.9415	1708.8358	-0.1057	-62	160	174	VHAPIIDMTKGEIIR			Oxidation (M)[8]		Mascot
1791.8661	1791.8201	-0.046	-26	210	227	AGFEAAGVPDPTRYQAA A					Mascot
1791.8661	1791.8201	-0.046	-26	210	227	AGFEAAGVPDPTRYQAA A					Mascot
1874.0416	1874.0637	0.0221	12	5	23	AIVLLSGGLDSATVLAMA R			Oxidation (M)[17]		Mascot
2243.2542	2243.2505	-0.0037	-2	2	23	TQRAIVLLSGGLDSATVL AMAR					Mascot

4 Protein translocase subunit SecA OS=Ehrlichia SECA_EHRCR 99255 6.08 24 68 92.184 15.402

chaffeensis (strain Arkansas) GN=secA PE=3 SV=1

Peptide Information

Calc. Mass	Obsrv. Mass	± da	± ppm	Start Seq.	End Seq.	Sequence	Ion Score	C. I. %	Modification	Rank	Result Type
808.4312	808.426	-0.0052	-6	631	636	NYDIRK					Mascot
815.4443	815.4041	-0.0402	-49	1	7	MLSIAHK			Oxidation (M)[1]		Mascot
817.4413	817.4223	-0.019	-23	51	57	EELKNGK					Mascot
827.3637	827.4303	0.0666	80	96	102	GMISEMK			Oxidation (M)[2,6]		Mascot
897.4247	897.4391	0.0144	16	188	194	DNMKFSR					Mascot
913.4196	913.4401	0.0205	22	188	194	DNMKFSR			Oxidation (M)[3]		Mascot
982.5469	982.4804	-0.0665	-68	750	757	AWDHVVKK					Mascot
993.5615	993.5444	-0.0171	-17	521	528	EAIEIKYK					Mascot
1037.6215	1037.5621	-0.0594	-57	461	469	LKHSVLNAR					Mascot
1081.6041	1081.5455	-0.0586	-54	810	817	FYELIIQR					Mascot
1082.5841	1082.5272	-0.0569	-53	769	778	DHLAALDSLK					Mascot
1140.5797	1140.6119	0.0322	28	833	841	IEYNIHHSK					Mascot
1165.6497	1165.6404	-0.0093	-8	449	458	SEMLSKLLTK			Oxidation (M)[3]		Mascot
1235.7107	1235.6068	-0.1039	-84	8	18	IFGSTNSRIIK					Mascot
1259.6888	1259.6901	0.0013	1	779	789	CGINLRSIAQK			Carbamidomethyl (C)[1]		Mascot
1262.6488	1262.6583	0.0095	8	641	650	FDNVINEQRK					Mascot
1299.5919	1299.6729	0.081	62	96	107	GMISEMKTGEGK			Oxidation (M)[2,6]		Mascot
1299.5919	1299.6729	0.081	62	96	107	GMISEMKTGEGK			Oxidation (M)[2,6]		Mascot
1405.772	1405.7343	-0.0377	-27	508	520	MLAKTALANITDK			Oxidation (M)[1]		Mascot
1458.712	1458.8102	0.0982	67	758	768	VMIMSLDYLWR			Oxidation (M)[2,4]		Mascot
1487.7601	1487.8018	0.0417	28	335	347	RYSDGLHQALEAK					Mascot
1584.8857	1584.8008	-0.0849	-54	124	137	GVHIVTVNDYLAKR					Mascot
1629.9058	1629.8849	-0.0209	-13	512	526	TALANITDKEAIEIK					Mascot
1629.9058	1629.8849	-0.0209	-13	512	526	TALANITDKEAIEIK					Mascot
1908.9451	1908.9504	0.0053	3	724	739	VLEHLNETVDEHFAQK					Mascot
2384.2466	2384.0759	-0.1707	-72	82	102	HFDVQLIGGIVLHKGMISE MK			Oxidation (M)[16,20]		Mascot
3312.5874	3312.5071	-0.0803	-24	137	165	RDAEWMGELYSALGITV GCILTETNDLER			Carbamidomethyl (C)[19]		Mascot

5	Protein translocase subunit SecA OS=Ehrlichia canis (strain Jake) GN=secA PE=3 SV=1	SECA_EHRCJ	99374.3	6.27	25	67	88.439	13.952	

Peptide Information

Calc. Mass	Obsrv. Mass	± da	± ppm	Start Seq.	End Seq.	Sequence	Ion Score	C. I. %	Modification	Rank	Result Type

Calc. Mass	Obsrv. Mass	± da	± ppm	Start Seq.	End Seq.	Sequence	Ion Score	C. I. %	Modification	Rank	Result Type
808.4312	808.426	-0.0052	-6	631	636	NYDIRK					Mascot
815.4443	815.4041	-0.0402	-49	1	7	MLSIAHK			Oxidation (M)[1]		Mascot
817.4413	817.4223	-0.019	-23	51	57	EELKNGK					Mascot
827.3637	827.4303	0.0666	80	96	102	GMISEMK			Oxidation (M)[2,6]		Mascot
831.4934	831.4349	-0.0585	-70	529	535	QLSEKVK					Mascot
897.4247	897.4391	0.0144	16	188	194	DNMKFSR					Mascot
913.4196	913.4401	0.0205	22	188	194	DNMKFSR			Oxidation (M)[3]		Mascot
952.5098	952.4902	-0.0196	-21	697	704	ILSSEFTR					Mascot
993.5615	993.5444	-0.0171	-17	521	528	EAIEIKYK					Mascot
1033.5538	1033.5616	0.0078	8	651	658	VVFDQRNR					Mascot
1037.6215	1037.5621	-0.0594	-57	461	469	LKHSVLNAR					Mascot
1082.5841	1082.5272	-0.0569	-53	769	778	DHLAALDSLK					Mascot
1139.5626	1139.6016	0.039	34	192	200	FSRSEMVQR					Mascot
1194.6365	1194.6404	0.0039	3	258	268	NIFLTEAGTTK					Mascot
1259.6888	1259.6901	0.0013	1	779	789	CGINLRSIAQK			Carbamidomethyl (C)[1]		Mascot
1262.6488	1262.6583	0.0095	8	641	650	FDNVINEQRK					Mascot
1265.6525	1265.6681	0.0156	12	747	756	DQKLWDYAVK					Mascot
1299.5919	1299.6729	0.081	62	96	107	GMISEMKTGEGK			Oxidation (M)[2,6]		Mascot
1299.5919	1299.6729	0.081	62	96	107	GMISEMKTGEGK			Oxidation (M)[2,6]		Mascot
1323.574	1323.703	0.129	97	687	696	YYNLDDETYK					Mascot
1407.7512	1407.7469	-0.0043	-3	449	460	SEMLSKLLTQNK			Oxidation (M)[3]		Mascot
1458.712	1458.8102	0.0982	67	758	768	VMIMSLDYLWR			Oxidation (M)[2,4]		Mascot
1487.7601	1487.8018	0.0417	28	335	347	RYSDGLHQALEAK					Mascot
1584.8857	1584.8008	-0.0849	-54	124	137	GVHIVTVNDYLAKR					Mascot
1699.8538	1699.9058	0.052	31	659	672	ILDNDSYDISIIYR					Mascot
2239.1838	2239.1973	0.0135	6	77	95	VLNMRHFDVQLIGGMVL HK			Oxidation (M)[4,15]		Mascot
2384.1755	2384.0759	-0.0996	-42	237	255	KIDNLIYELVEEDYELEEK					Mascot

6	50S ribosomal protein L2 OS=Burkholderia xenovorans (strain LB400) GN=rplB PE=3 SV=1	RL2_BURXL	30202	11.2	12	64	79.442	9.693

Peptide Information

Calc. Mass	Obsrv. Mass	± da	± ppm	Start Seq.	End Seq.	Sequence	Ion Score	C. I. %	Modification	Rank	Result Type
951.4928	951.5015	0.0087	9	264	271	TTSMIVQR			Oxidation (M)[4]		Mascot
952.5574	952.4902	-0.0672	-71	19	26	VVNKDLHK					Mascot
1107.594	1107.5959	0.0019	2	264	272	TTSMIVQRR			Oxidation (M)[4]		Mascot

Calc. Mass	Obsrv. Mass	± da	± ppm	Start Seq.	End Seq.	Sequence	Ion Score	C. I. %	Modification	Rank	Result Type
1140.6749	1140.6119	-0.063	-55	213	221	WRGIRPTVR					Mascot
1184.5947	1184.6274	0.0327	28	244	254	DPVSPWGTPTK					Mascot
1491.7802	1491.8051	0.0249	17	88	101	SANIALVLYADGER					Mascot
1674.7654	1674.816	0.0506	30	222	238	GVAMNPVDHPHGGGEGK			Oxidation (M)[4]		Mascot
1707.8629	1707.861	-0.0019	-1	151	167	GAQMARSAGTSAMLLAR			Oxidation (M)[4]		Mascot
1707.8629	1707.861	-0.0019	-1	151	167	GAQMARSAGTSAMLLAR			Oxidation (M)[4]		Mascot
1994.0488	1994.0825	0.0337	17	157	175	SAGTSAMLLAREGIYAQVR					Mascot
1994.0488	1994.0825	0.0337	17	157	175	SAGTSAMLLAREGIYAQVR	2	0			Mascot
2115.0149	2115.1475	0.1326	63	222	243	GVAMNPVDHPHGGGEGKTAAGR					Mascot
2239.1831	2239.1973	0.0142	6	23	43	DLHKGKPFAPLLDSQSTTAGR					Mascot
2510.2676	2510.239	-0.0286	-11	134	156	NIPVGTTIHCIEMLPGKGAQMAR			Carbamidomethyl (C)[10], Oxidation (M)[13]		Mascot

7 Tektin-4 OS=Rattus norvegicus GN=Tekt4 PE=2 SV=1 TEKT4_RAT 52742.4 5.83 17 63 72.268 7.524

Peptide Information

Calc. Mass	Obsrv. Mass	± da	± ppm	Start Seq.	End Seq.	Sequence	Ion Score	C. I. %	Modification	Rank	Result Type
819.3843	819.3833	-0.001	-1	288	294	DTAEDLR					Mascot
864.4686	864.4551	-0.0135	-16	163	169	QHPDLVR					Mascot
1006.4258	1006.488	0.0622	62	308	315	CEELNDAR			Carbamidomethyl (C)[1]		Mascot
1057.6113	1057.5515	-0.0598	-57	271	279	ERLASVNLR					Mascot
1060.5569	1060.6061	0.0492	46	193	202	TMGQAVGQIR					Mascot
1107.5933	1107.5959	0.0026	2	170	178	DYVEVELLK					Mascot
1118.4758	1118.5629	0.0871	78	107	114	LEDMHCWK			Carbamidomethyl (C)[6]		Mascot
1140.6484	1140.6119	-0.0365	-32	347	356	EAPLRVAQTR					Mascot
1232.6528	1232.6542	0.0014	1	192	202	RTMGQAVGQIR			Oxidation (M)[3]		Mascot
1262.5793	1262.6583	0.079	63	308	317	CEELNDARQK			Carbamidomethyl (C)[1]		Mascot
1491.8167	1491.8051	-0.0116	-8	415	427	DIAVKTNSLFIDR					Mascot
1507.7322	1507.8065	0.0743	49	295	307	LQCDAVNLAFSNR			Carbamidomethyl (C)[3]		Mascot
1657.7354	1657.8805	0.1451	88	64	76	YHQAFADRDYSER					Mascot
1738.7531	1738.9048	0.1517	87	51	63	YLMDEWFQNSYAR			Oxidation (M)[3]		Mascot
1838.9316	1839.0095	0.0779	42	11	26	EPAPQSIDVCELPQKK			Carbamidomethyl (C)[10]		Mascot
2022.9379	2022.9906	0.0527	26	48	63	TAKYLMDEWFQNSYAR					Mascot
2286.074	2286.0864	0.0124	5	371	389	DNAQFRLMSEVEELNMSLK			Oxidation (M)[8,16]		Mascot

8 T-complex protein 1 subunit epsilon OS=Dictyostelium discoideum GN=cct5 PE=3 SV=1 TCPE_DICDI 59662.8 5.58 18 62 64.274 8.207

Peptide Information

Calc. Mass	Obsrv. Mass	± da	± ppm	Start Seq.	End Seq.	Sequence	Ion Score	C. I. %	Modification	Rank	Result Type
806.444	806.4064	-0.0376	-47	39	45	TISNIMK					Mascot
817.4414	817.4223	-0.0191	-23	207	214	LEGKEGGK					Mascot
819.428	819.3833	-0.0447	-55	389	395	MIVEEAK					Mascot
835.423	835.4144	-0.0086	-10	389	395	MIVEEAK			Oxidation (M)[1]		Mascot
847.4705	847.4191	-0.0514	-61	475	481	AMQIKEK					Mascot
863.4655	863.4727	0.0072	8	475	481	AMQIKEK			Oxidation (M)[2]		Mascot
951.5482	951.5015	-0.0467	-49	24	31	LRGIEAHR					Mascot
1016.5411	1016.5536	0.0125	12	320	328	WVGGLDLEK					Mascot
1032.4707	1032.5415	0.0708	69	280	287	YFTDMVEK					Mascot
1033.5889	1033.5616	-0.0273	-26	258	266	TKNSIEITK					Mascot
1126.5627	1126.5674	0.0047	4	146	155	TISDSIEFSK					Mascot
1201.7225	1201.6404	-0.0821	-68	522	531	MILKIDDIIK					Mascot
1259.634	1259.6901	0.0561	45	280	289	YFTDMVEKVK					Mascot
1345.7046	1345.7233	0.0187	14	177	187	FHRQMSEIAVK					Mascot
1373.6617	1373.729	0.0673	49	526	538	IDDIIKMGPGADE					Mascot
1584.9208	1584.8008	-0.12	-76	215	228	LEDTQLVKGIIIDK					Mascot
1605.8344	1605.7875	-0.0469	-29	352	366	AGLVREVGFGTTQDR					Mascot
1699.891	1699.9058	0.0148	9	244	257	ICLLTCPFEPPKPK			Carbamidomethyl (C)[2,6]		Mascot
1874.0933	1874.0637	-0.0296	-16	510	525	TQQFLLANQVVKMILK					Mascot
1890.9663	1891.046	0.0797	42	156	172	DNIEPLIKTAMTCLGSK			Carbamidomethyl (C)[13]		Mascot

9	4-hydroxythreonine-4-phosphate dehydrogenase OS=Campylobacter jejuni (strain RM1221) GN=pdxA PE=3 SV=1	PDXA_CAMJR	41526.1	9.29	14	60	48.361	12.273	

Peptide Information

Calc. Mass	Obsrv. Mass	± da	± ppm	Start Seq.	End Seq.	Sequence	Ion Score	C. I. %	Modification	Rank	Result Type
815.3934	815.4041	0.0107	13	349	355	SYFEAAK					Mascot
829.405	829.3665	-0.0385	-46	22	28	SHEELSK					Mascot
993.4887	993.5444	0.0557	56	90	97	VDENFEIK					Mascot
1037.494	1037.5621	0.0681	66	159	167	NAIMMLGCK			Carbamidomethyl (C)[8]		Mascot
1060.5535	1060.6061	0.0526	50	145	153	YKGHTDALR					Mascot
1118.5881	1118.5629	-0.0252	-23	105	114	SGLYGFLSFK					Mascot
1165.589	1165.6404	0.0514	44	158	167	KNAIMMLGCK			Carbamidomethyl (C)[9]		Mascot

Calc. Mass	Obsrv. Mass	± da	± ppm	Start Seq.	End Seq.	Sequence	Ion Score	C. I. %	Modification	Rank	Result Type
1306.6539	1306.6534	-0.0005	0	147	157	GHTDALRDFFK					Mascot
1323.8035	1323.703	-0.1005	-76	187	197	ITFKNLSIFLK					Mascot
1699.9418	1699.9058	-0.036	-21	168	182	ELFVGLFSEHIPLAK					Mascot
1707.8436	1707.861	0.0174	10	90	104	VDENFEIKAGEIDTK					Mascot
1707.8436	1707.861	0.0174	10	90	104	VDENFEIKAGEIDTK					Mascot
1994.2008	1994.0825	-0.1183	-59	3	21	KLAISIGDINGIGLEILVR					Mascot
1994.2008	1994.0825	-0.1183	-59	3	21	KLAISIGDINGIGLEILVR					Mascot
2115.0837	2115.1475	0.0638	30	292	309	NCNRLVAMYHDLALAPL K			Carbamidomethyl (C)[2], Oxidation (M)[8]		Mascot
2807.3379	2807.4719	0.134	48	208	233	MGLLGFNPHAGDYGVIG GEEEKIMEK			Oxidation (M)[1]		Mascot

10	Keratin, type II cytoskeletal 1 OS=Rattus norvegicus GN=Krt1 PE=2 SV=1	K2C1_RAT	65190.2	8	11	59	33.475	5.345	38	86.169

Peptide Information

Calc. Mass	Obsrv. Mass	± da	± ppm	Start Seq.	End Seq.	Sequence	Ion Score	C. I. %	Modification	Rank	Result Type
827.4297	827.4303	0.0006	1	190	196	FASFIDK					Mascot
1066.5164	1066.559	0.0426	40	269	276	YEDEINKR					Mascot
1082.5994	1082.5272	-0.0722	-67	190	198	FASFIDKVR					Mascot
1139.5579	1139.6016	0.0437	38	267	275	TKYEDEINK					Mascot
1140.5103	1140.6119	0.1016	89	355	363	AQYDSICQR			Carbamidomethyl (C)[7]		Mascot
1141.5194	1141.5803	0.0609	53	463	471	DYQELMNTK					Mascot
1232.5907	1232.6542	0.0635	52	615	625	FVSTTYSRGTN					Mascot
1265.6372	1265.6681	0.0309	24	277	287	TNAENEFVTIK					Mascot
1475.7489	1475.8168	0.0679	46	211	222	WELLQQVDTSTR	40	90.026			Mascot
1487.7999	1487.8018	0.0019	1	395	406	MEISELNRVIQR					Mascot
1791.829	1791.8201	-0.0089	-5	417	431	QISQMQQNISDAEQR			Oxidation (M)[5]		Mascot
1791.829	1791.8201	-0.0089	-5	417	431	QISQMQQNISDAEQR			Oxidation (M)[5]		Mascot

Gel Idx/Pos	132/F7	Instr./Gel Origin	BA2151/Sample Project 20140814	Process Status	Analysis Succeeded
Plate [#] Name	[1] Sample Project 20140814	Instrument Sample Name		Spectra	11

Rank	Protein Name	Accession No.	Protein MW	Protein PI	Pep. Count	Protein Score	Protein Score C. I. %	Intensity Matched	Total Ion Score	Total Ion C. I. %	Confirmed
1	Keratin, type I cytoskeletal 9 OS=Homo sapiens GN=KRT9 PE=1 SV=3	K1C9_HUMAN	62254.9	5.14	12	209	100	5.619	170	100	

Peptide Information

Calc. Mass	Obsrv. Mass	± da	± ppm	Start Seq.	End Seq.	Sequence	Ion Score	C. I. %	Modification	Rank	Result Type
897.4135	897.4661	0.0526	59	234	240	MTLDDFR					Mascot
913.4084	913.4512	0.0428	47	234	240	MTLDDFR			Oxidation (M)[1]		Mascot
982.4337	982.4886	0.0549	56	35	46	FSSSGGGGGGGR					Mascot
1060.5634	1060.6169	0.0535	50	225	233	TLLDIDNTR					Mascot
1066.4987	1066.5596	0.0609	57	243	250	FEMEQNLR					Mascot
1082.4935	1082.5405	0.047	43	243	250	FEMEQNLR			Oxidation (M)[3]		Mascot
1232.5979	1232.6649	0.067	54	14	29	SGGGGGGGLGSGGSIR					Mascot
1235.5288	1235.6135	0.0847	69	47	59	FSSSSGYGGGSSR					Mascot
1307.6776	1307.7468	0.0692	53	241	250	IKFEMEQNLR					Mascot
1323.6726	1323.7111	0.0385	29	241	250	IKFEMEQNLR			Oxidation (M)[5]		Mascot
1605.7432	1605.7992	0.056	35	200	212	NYSPYYNTIDDLK					Mascot
1707.7504	1707.8672	0.1168	68	47	63	FSSSSGYGGGSSRVCGR			Carbamidomethyl (C)[15]		Mascot
1791.7278	1791.8416	0.1138	64	491	513	GGSGGSYGGGGSGGGY GGGSGSR					Mascot
1837.9655	1837.9916	0.0261	14	375	390	HGVQELEIELQSQLSK					Mascot
2705.1611	2705.3333	0.1722	64	64	95	GGGGSFGYSYGGGSGG GFSASSLGGGFGGGSR					Mascot
2705.1611	2705.3333	0.1722	64	64	95	GGGGSFGYSYGGGSGG GFSASSLGGGFGGGSR	170	100			Mascot

Rank	Protein Name	Accession No.	Protein MW	Protein PI	Pep. Count	Protein Score	Protein Score C. I. %	Intensity Matched	Total Ion Score	Total Ion C. I. %	Confirmed
2	Keratin, type II cytoskeletal 1 OS=Homo sapiens GN=KRT1 PE=1 SV=6	K2C1_HUMAN	66170.1	8.15	17	172	100	9.111	100	100	

Peptide Information

Calc. Mass	Obsrv. Mass	± da	± ppm	Start Seq.	End Seq.	Sequence	Ion Score	C. I. %	Modification	Rank	Result Type
1006.4297	1006.4913	0.0616	61	589	602	GGSGGGGGGSSGGR					Mascot
1033.516	1033.564	0.048	46	484	492	TLLEGEESR					Mascot
1066.5164	1066.5596	0.0432	41	270	277	YEDEINKR					Mascot
1092.5029	1092.5701	0.0672	62	603	616	GSGGGSSGGSIGGR					Mascot

1141.5194	1141.6035	0.0841	74	464	472	DYQELMNTK					Mascot
1179.6005	1179.6605	0.06	51	377	386	YEELQITAGR					Mascot
1277.71	1277.7743	0.0643	50	473	483	LALDLEIATYR					Mascot
1475.7489	1475.824	0.0751	51	212	223	WELLQQVDTSTR	59	99.888			Mascot
1475.7853	1475.824	0.0387	26	200	211	FLEQQNQVLQTK					Mascot
1523.7887	1523.8939	0.1052	69	461	472	LLRDYQELMNTK					Mascot
1638.8599	1638.9734	0.1135	69	186	199	SLNNQFASFIDKVR					Mascot
1716.8511	1716.9432	0.0921	54	418	432	QISNLQQSISDAEQR					Mascot
1716.8511	1716.9432	0.0921	54	418	432	QISNLQQSISDAEQR	42	93.68			Mascot
1730.9548	1730.9976	0.0428	25	198	211	VRFLEQQNQVLQTK					Mascot
1844.946	1844.9774	0.0314	17	417	432	KQISNLQQSISDAEQR					Mascot
1993.9827	1994.092	0.1093	55	625	644	SSGGSSSVKFVSTTYSG VTR					Mascot
2383.9519	2384.0955	0.1436	60	519	549	GGGGGGYGSGGSSYGS GGGSYGSGGGGGGGR					Mascot
3312.3083	3312.5312	0.2229	67	550	588	GSYGSGGSSYGSGGGS YGSGGGGGGHGSYGSG SSSGGYR					Mascot

3	Keratin, type II cytoskeletal 1 OS=Pan troglodytes GN=KRT1 PE=1 SV=1	K2C1_PANTR	65620.8	7.62	16	164	100	8.945	100	100	

Peptide Information

Calc. Mass	Obsrv. Mass	± da	± ppm	Start Seq.	End Seq.	Sequence	Ion Score	C. I. %	Modification	Rank	Result Type
1033.516	1033.564	0.048	46	479	487	TLLEGEESR					Mascot
1066.5164	1066.5596	0.0432	41	265	272	YEDEINKR					Mascot
1092.5029	1092.5701	0.0672	62	596	609	GSGGGSSGGSIGGR					Mascot
1141.5194	1141.6035	0.0841	74	459	467	DYQELMNTK					Mascot
1179.6005	1179.6605	0.06	51	372	381	YEELQITAGR					Mascot
1277.71	1277.7743	0.0643	50	468	478	LALDLEIATYR					Mascot
1475.7489	1475.824	0.0751	51	207	218	WELLQQVDTSTR	59	99.888			Mascot
1475.7853	1475.824	0.0387	26	195	206	FLEQQNQVLQTK					Mascot
1523.7887	1523.8939	0.1052	69	456	467	LLRDYQELMNTK					Mascot
1638.8599	1638.9734	0.1135	69	181	194	SLNNQFASFIDKVR					Mascot
1716.8511	1716.9432	0.0921	54	413	427	QISNLQQSISDAEQR					Mascot
1716.8511	1716.9432	0.0921	54	413	427	QISNLQQSISDAEQR	42	93.68			Mascot
1730.9548	1730.9976	0.0428	25	193	206	VRFLEQQNQVLQTK					Mascot
1844.946	1844.9774	0.0314	17	412	427	KQISNLQQSISDAEQR					Mascot
1993.9827	1994.092	0.1093	55	618	637	SSGGSSSVKFVSTTYSG VTR					Mascot
2383.9519	2384.0955	0.1436	60	514	544	GGGGGGYGSGGSSYGS					Mascot

Calc. Mass	Obsrv. Mass	± da	± ppm	Start Seq.	End Seq.	Sequence	Ion Score	C. I. %	Modification	Rank	Result Type
3312.3083	3312.5312	0.2229	67	545	583	GGGSYGSGGGGGGGR GSYGSGGSSYGSGGGS YGSGGGGGGHGSYGSG SSSGGYR					Mascot

4	Keratin, type II cytoskeletal 1 OS=Rattus norvegicus GN=Krt1 PE=2 SV=1	K2C1_RAT	65190.2	8	14	94	99.979	5.751	58	99.845	

Peptide Information

Calc. Mass	Obsrv. Mass	± da	± ppm	Start Seq.	End Seq.	Sequence	Ion Score	C. I. %	Modification	Rank	Result Type
1066.5164	1066.5596	0.0432	41	269	276	YEDEINKR					Mascot
1082.5994	1082.5405	-0.0589	-54	190	198	FASFIDKVR					Mascot
1139.5579	1139.6292	0.0713	63	267	275	TKYEDEINK					Mascot
1141.5194	1141.6035	0.0841	74	463	471	DYQELMNTK					Mascot
1232.5907	1232.6649	0.0742	60	615	625	FVSTTYSRGTN					Mascot
1475.7489	1475.824	0.0751	51	211	222	WELLQQVDTSTR	59	99.888			Mascot
1475.7853	1475.824	0.0387	26	199	210	FLEQQNQVLQTK					Mascot
1487.7999	1487.8179	0.018	12	395	406	MEISELNRVIQR					Mascot
1523.7887	1523.8939	0.1052	69	460	471	LLRDYQELMNTK					Mascot
1730.9548	1730.9976	0.0428	25	197	210	VRFLEQQNQVLQTK					Mascot
1791.829	1791.8416	0.0126	7	417	431	QISQMQQNISDAEQR			Oxidation (M)[5]		Mascot
1903.9291	1904.0781	0.149	78	416	431	KQISQMQQNISDAEQR					Mascot
1943.9498	1944.0605	0.1107	57	223	238	TQNLDPFFESYISNLR					Mascot
2705.1448	2705.3333	0.1885	70	492	517	MSGECTPNVSVSVSTSH TSMSGTSSR			Carbamidomethyl (C)[5], Oxidation (M)[1,20]		Mascot
2705.1448	2705.3333	0.1885	70	492	517	MSGECTPNVSVSVSTSH TSMSGTSSR			Carbamidomethyl (C)[5], Oxidation (M)[1,20]		Mascot

5	Keratin, type II cytoskeletal 1 OS=Canis familiaris GN=KRT1 PE=1 SV=1	K2C1_CANFA	63922.1	7.66	10	75	98.25	3.195	58	99.845	

Peptide Information

Calc. Mass	Obsrv. Mass	± da	± ppm	Start Seq.	End Seq.	Sequence	Ion Score	C. I. %	Modification	Rank	Result Type
1033.516	1033.564	0.048	46	485	493	TLLEGEESR					Mascot
1066.5164	1066.5596	0.0432	41	271	278	YEDEINKR					Mascot
1141.5194	1141.6035	0.0841	74	465	473	DYQELMNTK					Mascot
1307.6624	1307.7468	0.0844	65	394	404	STKMEISELNR					Mascot
1323.6573	1323.7111	0.0538	41	394	404	STKMEISELNR			Oxidation (M)[4]		Mascot
1475.7489	1475.824	0.0751	51	213	224	WELLQQVDTSTR	59	99.888			Mascot
1475.7853	1475.824	0.0387	26	201	212	FLEQQNQVLQTK					Mascot
1523.7887	1523.8939	0.1052	69	462	473	LLRDYQELMNTK					Mascot

1600.7715	1600.8983	0.1268	79	13	29	SGGGFSSGSAGLVSFQR					Mascot
1638.8599	1638.9734	0.1135	69	187	200	SLNNQFASFIDKVR					Mascot
1730.9548	1730.9976	0.0428	25	199	212	VRFLEQQNQVLQTK					Mascot

6	Microtubule-actin cross-linking factor 1 OS=Rattus norvegicus GN=Macf1 PE=1 SV=1	MACF1_RAT	623207.6	5.28	56	74	98.037	54.901

Peptide Information

Calc. Mass	Obsrv. Mass	± da	± ppm	Start Seq.	End Seq.	Sequence	Ion Score	C. I. %	Modification	Rank	Result Type
807.4471	807.4484	0.0013	2	3814	3819	YARLER					Mascot
832.4523	832.3693	-0.083	-100	1906	1912	LKDATER					Mascot
870.5229	870.5677	0.0448	51	3736	3743	LMALGPIR					Mascot
886.5468	886.5641	0.0173	20	5403	5410	RGLTKPSK					Mascot
974.5266	974.5925	0.0659	68	3696	3704	LVSDTVGQR					Mascot
1006.4874	1006.4913	0.0039	4	2423	2432	GMDASLSPTK					Mascot
1037.5449	1037.5734	0.0285	27	2551	2558	QQVQFMLK			Oxidation (M)[6]		Mascot
1055.5819	1055.6652	0.0833	79	87	94	WVNKHLMK					Mascot
1060.5647	1060.6169	0.0522	49	1226	1234	AVAEQLHHR					Mascot
1092.5685	1092.5701	0.0016	1	4647	4655	QPVYDTTIR					Mascot
1101.615	1101.6012	-0.0138	-13	2313	2322	GVLVEEINTK					Mascot
1141.6688	1141.6035	-0.0653	-57	3322	3330	LLQRLLDDR					Mascot
1158.6154	1158.6248	0.0094	8	341	350	SFPQNPVELK					Mascot
1165.6259	1165.627	0.0011	1	676	684	MRHLQSLHK			Oxidation (M)[1]		Mascot
1165.6259	1165.627	0.0011	1	676	684	MRHLQSLHK			Oxidation (M)[1]		Mascot
1181.5343	1181.6407	0.1064	90	5077	5084	YMRWMNHK			Oxidation (M)[2]		Mascot
1197.5292	1197.5919	0.0627	52	5077	5084	YMRWMNHK			Oxidation (M)[2,5]		Mascot
1232.6117	1232.6649	0.0532	43	2659	2668	QQLEETSEIR					Mascot
1262.6158	1262.6707	0.0549	43	1329	1338	NQTKLDQCQK			Carbamidomethyl (C)[8]		Mascot
1307.655	1307.7468	0.0918	70	1070	1081	QIQSSASSKTDR					Mascot
1313.7787	1313.7223	-0.0564	-43	2809	2819	KTLQNQLVELK					Mascot
1320.7271	1320.6606	-0.0665	-50	1870	1881	GDLRFVTISGQK					Mascot
1323.7532	1323.7111	-0.0421	-32	678	688	HLQSLHKFVSK					Mascot
1331.6631	1331.728	0.0649	49	499	508	YYQLEELAFR					Mascot
1331.6631	1331.728	0.0649	49	499	508	YYQLEELAFR					Mascot
1353.5852	1353.7109	0.1257	93	813	823	YTCDHNTSLSR			Carbamidomethyl (C)[3]		Mascot
1359.7227	1359.7566	0.0339	25	1809	1819	QTQTLRDELQK					Mascot
1365.6758	1365.713	0.0372	27	1245	1255	YQEKGSQLQER					Mascot

1379.6948	1379.7778	0.083	60	2100	2111	SSLEATREMVTR				Mascot
1412.7202	1412.6616	-0.0586	-41	869	880	STLSVKAICDYR			Carbamidomethyl (C)[9]	Mascot
1417.8413	1417.7914	-0.0499	-35	4553	4564	FLSQKQDVVLIK				Mascot
1457.8475	1457.7926	-0.0549	-38	1431	1443	VKDLLGWVSTLAR				Mascot
1487.7952	1487.8179	0.0227	15	3565	3577	QTTGEEVLLIQEK				Mascot
1493.7595	1493.8148	0.0553	37	2240	2251	DFTELQKTVQER				Mascot
1523.8217	1523.8939	0.0722	47	4989	5001	KNIEPTHAPFIEK				Mascot
1565.7019	1565.7141	0.0122	8	784	796	ENAAYFQFFSDAR				Mascot
1605.8306	1605.7992	-0.0314	-20	3211	3223	MFQKEQVDPLQVK			Oxidation (M)[1]	Mascot
1638.8599	1638.9734	0.1135	69	4990	5003	NIEPTHAPFIEKSR				Mascot
1685.932	1685.9408	0.0088	5	1463	1477	AILEQQVLAEELTTK				Mascot
1685.9697	1685.9408	-0.0289	-17	136	149	LQNVQIALDFLKQR				Mascot
1707.964	1707.8672	-0.0968	-57	108	123	DGHNLISLLEVLSGIK				Mascot
1716.92	1716.9432	0.0232	14	3124	3138	MLEEEGTLDLLGLKR				Mascot
1716.92	1716.9432	0.0232	14	3124	3138	MLEEEGTLDLLGLKR	15	0		Mascot
1731.9752	1731.9601	-0.0151	-9	1390	1404	TRYTALVTLTTQHVK				Mascot
1740.8551	1740.823	-0.0321	-18	1636	1649	LYQAKEQYEGLQDR				Mascot
1765.8248	1765.9454	0.1206	68	4348	4361	LETMNQCWESVLQK			Carbamidomethyl (C)[7]	Mascot
1791.8356	1791.8416	0.006	3	4328	4345	AGNELLESSAGDDASSL R				Mascot
1804.8811	1805.0232	0.1421	79	1656	1671	ELEEAVTSALQQETEK				Mascot
1822.8462	1822.9807	0.1345	74	4960	4974	ALITEHQSFMEEMTR				Mascot
1822.8462	1822.9807	0.1345	74	4960	4974	ALITEHQSFMEEMTR				Mascot
1838.8412	1839.0139	0.1727	94	4960	4974	ALITEHQSFMEEMTR			Oxidation (M)[10]	Mascot
1844.9613	1844.9774	0.0161	9	1091	1105	IAEQEHVQEDLKHLR				Mascot
1887.9407	1888.1119	0.1712	91	1577	1593	ASQQELSALQQNQSDLK				Mascot
1904.9746	1905.097	0.1224	64	2390	2405	QESLQTVLSRMEEVQK				Mascot
1905.9926	1906.1257	0.1331	70	1142	1157	MDHVYGLSIVCLNKLK			Carbamidomethyl (C)[11], Oxidation (M)[1]	Mascot
1905.9926	1906.1257	0.1331	70	1142	1157	MDHVYGLSIVCLNKLK			Carbamidomethyl (C)[11], Oxidation (M)[1]	Mascot
1926.959	1927.0072	0.0482	25	1333	1348	LDQCQKLSQQYSTTVK			Carbamidomethyl (C)[4]	Mascot
1927.947	1928.0828	0.1358	70	4200	4214	EFKVEVYQQQIEMEK				Mascot
1943.9419	1944.0605	0.1186	61	4200	4214	EFKVEVYQQQIEMEK			Oxidation (M)[13]	Mascot
1993.9825	1994.092	0.1095	55	3431	3448	ALEEEIENHAADVQQAVK				Mascot
2227.125	2227.0925	-0.0325	-15	5207	5226	STVMVRVGGGWMALDE FLVK			Oxidation (M)[4,12]	Mascot
2426.1411	2426.2397	0.0986	41	784	803	ENAAYFQFFSDARDLESF LR				Mascot
2426.1411	2426.2397	0.0986	41	784	803	ENAAYFQFFSDARDLESF LR	2	0		Mascot
2831.5303	2831.376	-0.1543	-54	530	555	GHFTSLELVPPSTLTTTH				Mascot

3312.6411	3312.5312	-0.1099	-33	270	299	LKAEPLNK LLDAEDVDVPSPDEKSVI TYVSSIYDAFPK					Mascot

7	tRNA dimethylallyltransferase OS=Burkholderia mallei (strain NCTC 10229) GN=miaA PE=3 SV=1	MIAA_BURM9	35415.3	7.64	12	60	39.329	4.486	

Protein Group

tRNA dimethylallyltransferase OS=Burkholderia mallei (strain ATCC 23344) GN=miaA PE=3 SV=1	MIAA_BURMA	35415.3	7.6399998664856
tRNA dimethylallyltransferase OS=Burkholderia mallei (strain NCTC 10247) GN=miaA PE=3 SV=1	MIAA_BURM7	35415.3	7.6399998664856
tRNA dimethylallyltransferase OS=Burkholderia mallei (strain SAVP1) GN=miaA PE=3 SV=1	MIAA_BURMS	35415.3	7.6399998664856
tRNA dimethylallyltransferase OS=Burkholderia pseudomallei (strain 1106a) GN=miaA PE=3 SV=1	MIAA_BURP0	35416.3	6.96000003814697
tRNA dimethylallyltransferase OS=Burkholderia pseudomallei (strain 1710b) GN=miaA PE=3 SV=2	MIAA_BURP1	35416.3	6.96000003814697
tRNA dimethylallyltransferase OS=Burkholderia pseudomallei (strain K96243) GN=miaA PE=3 SV=1	MIAA_BURPS	35416.3	6.96000003814697

Peptide Information

Calc. Mass	Obsrv. Mass	± da	± ppm	Start Seq.	End Seq.	Sequence	Ion Score	C. I. %	Modification	Rank	Result Type
1055.5844	1055.6652	0.0808	77	149	159	LAGIDPATAAR					Mascot
1092.5215	1092.5701	0.0486	44	1	10	MSERNAASAR					Mascot
1193.646	1193.6736	0.0276	23	275	284	GIFATRQLCK			Carbamidomethyl (C)[9]		Mascot
1313.7173	1313.7223	0.005	4	310	321	AVDALERVLDGR					Mascot
1475.6982	1475.824	0.1258	85	297	309	IVVDCCAPDATVR			Carbamidomethyl (C)[5,6]		Mascot
1475.6982	1475.824	0.1258	85	297	309	IVVDCCAPDATVR			Carbamidomethyl (C)[5,6]		Mascot
1668.889	1668.9749	0.0859	51	99	113	GRTPLLAGGTMLYYR					Mascot
1716.9644	1716.9432	-0.0212	-12	34	48	RPIEIVSVDSALVYR					Mascot
1716.9644	1716.9432	-0.0212	-12	34	48	RPIEIVSVDSALVYR					Mascot
1765.908	1765.9454	0.0374	21	114	130	ALTQGLNDLPAADPDVR					Mascot
1844.9647	1844.9774	0.0127	7	5	23	NAASARTVACLLGPTAS GK			Carbamidomethyl (C)[10]		Mascot
1891.0548	1891.0885	0.0337	18	201	217	FVPVALEPSERAVLHAR					Mascot

1904.9106	1905.097	0.1864	98	241	256	DDLHLGLPSMRCVGYR			Carbamidomethyl (C)[12], Oxidation (M)[10]		Mascot
2227.2156	2227.0925	-0.1231	-55	171	191	ALEVYLLTGQPMSALLAA PPR			Oxidation (M)[12]		Mascot

8	DNA-directed RNA polymerase subunit beta' OS=Acidothermus cellulolyticus (strain ATCC 43068 / 11B) GN=rpoC PE=3 SV=1	RPOC_ACIC1	146372.1	6.77	24	59	30.34	17.177

Peptide Information

Calc. Mass	Obsrv. Mass	± da	± ppm	Start Seq.	End Seq.	Sequence	Ion Score	C. I. %	Modification	Rank	Result Type
832.4345	832.3693	-0.0652	-78	254	262	GGMGAQAIK					Mascot
974.5815	974.5925	0.011	11	892	900	AISMRIGVK					Mascot
975.5734	975.5868	0.0134	14	413	420	FRQNLLGK					Mascot
1101.5786	1101.6012	0.0226	21	619	628	DLGELDIQAK					Mascot
1126.5133	1126.5715	0.0582	52	967	976	LGVCAACYGR			Carbamidomethyl (C)[4,7]		Mascot
1139.5804	1139.6292	0.0488	43	863	872	TADSGYLTRR					Mascot
1193.6049	1193.6736	0.0687	58	739	748	QEILEAYEAK					Mascot
1213.6899	1213.5991	-0.0908	-75	897	908	IGVKGPDGTLTR					Mascot
1235.6631	1235.6135	-0.0496	-40	909	919	LPTVETSVYAR					Mascot
1262.6157	1262.6707	0.055	44	166	175	MNVELEERAR			Oxidation (M)[1]		Mascot
1313.6808	1313.7223	0.0415	32	1253	1263	NIRVEPTEEAR					Mascot
1331.7253	1331.728	0.0027	2	1241	1252	LIPAGTGMPRYR					Mascot
1331.7253	1331.728	0.0027	2	1241	1252	LIPAGTGMPRYR					Mascot
1379.8192	1379.7778	-0.0414	-30	1137	1147	HIEIIVRQMLK					Mascot
1473.7908	1473.7731	-0.0177	-12	687	699	KDLSTIVNDLAER					Mascot
1507.7291	1507.8145	0.0854	57	805	817	GNMMQVRQIAGMR			Oxidation (M)[3]		Mascot
1637.9109	1637.9283	0.0174	11	357	371	LLDLGAPEIIVNNEK					Mascot
1685.9255	1685.9408	0.0153	9	723	738	AGVTISISDVVMPPRK			Oxidation (M)[12]		Mascot
1685.9255	1685.9408	0.0153	9	723	738	AGVTISISDVVMPPRK			Oxidation (M)[12]		Mascot
1707.9137	1707.8672	-0.0465	-27	460	474	LVDLNHAQNIKSAER					Mascot
1716.9644	1716.9432	-0.0212	-12	422	437	VDYSGRSVIVVGPQLK					Mascot
1716.9644	1716.9432	-0.0212	-12	422	437	VDYSGRSVIVVGPQLK					Mascot
1740.954	1740.823	-0.131	-75	446	459	VMALELFKPFVMKR			Oxidation (M)[2,12]		Mascot
1765.9993	1765.9454	-0.0539	-31	1234	1250	ENVIIGKLIPAGTGMPR					Mascot
1839.0586	1839.0139	-0.0447	-24	1216	1233	VLTDAAINAKSDPLLGLK					Mascot
2384.0833	2384.0955	0.0122	5	784	804	EMEANFPKDNPVFMMVN SGAR					Mascot
2705.4832	2705.3333	-0.1499	-55	931	956	VLAAKGSDIGDLTITELLA HGVEQVR					Mascot
2705.4832	2705.3333	-0.1499	-55	931	956	VLAAKGSDIGDLTITELLA					Mascot

HGVEQVR

9	50S ribosomal protein L1 OS=Streptomyces coelicolor (strain ATCC BAA-471 / A3(2) / M145) GN=rplA PE=3 SV=2	RL1_STRCO	25747.8	9.52	11	57	0	10.301

Peptide Information

Calc. Mass	Obsrv. Mass	± da	± ppm	Start Seq.	End Seq.	Sequence	Ion Score	C. I. %	Modification	Rank	Result Type
847.4454	847.4233	-0.0221	-26	54	60	KADQMVR					Mascot
906.5043	906.5155	0.0112	12	161	167	IEFRVDK					Mascot
988.5309	988.5723	0.0414	42	142	151	TGTVTPDVAK					Mascot
1165.6477	1165.627	-0.0207	-18	168	177	HSNLHFIIGK					Mascot
1165.6477	1165.627	-0.0207	-18	168	177	HSNLHFIIGK					Mascot
1507.838	1507.8145	-0.0235	-16	165	177	VDKHSNLHFIIGK					Mascot
1628.8855	1628.9452	0.0597	37	142	157	TGTVTPDVAKAVNDIK					Mascot
1716.8585	1716.9432	0.0847	49	212	228	AALSTTMGPGIPLDSNR			Oxidation (M)[7]		Mascot
1716.8585	1716.9432	0.0847	49	212	228	AALSTTMGPGIPLDSNR			Oxidation (M)[7]		Mascot
1804.9263	1805.0232	0.0969	54	107	123	GRLDFDAVVATPDLMGK					Mascot
1844.9535	1844.9774	0.0239	13	211	228	KAALSTTMGPGIPLDSNR			Oxidation (M)[8]		Mascot
1903.9946	1904.0781	0.0835	44	109	126	LDFDAVVATPDLMGKVGR					Mascot
2384.198	2384.0955	-0.1025	-43	178	198	TSFDDTKLVENYGAALEEILR					Mascot

10	Myosin-6 OS=Mus musculus GN=Myh6 PE=1 SV=2	MYH6_MOUSE	224224.8	5.57	32	56	0	17.778

Peptide Information

Calc. Mass	Obsrv. Mass	± da	± ppm	Start Seq.	End Seq.	Sequence	Ion Score	C. I. %	Modification	Rank	Result Type
807.4108	807.4484	0.0376	47	1448	1453	QRNFDK					Mascot
832.4047	832.3693	-0.0354	-43	1320	1326	QLEEEGK					Mascot
988.5057	988.5723	0.0666	67	1319	1326	RQLEEEGK					Mascot
1060.5171	1060.6169	0.0998	94	1366	1374	ANSEVAQWR					Mascot
1158.6266	1158.6248	-0.0018	-2	551	560	AKLYDNHLGK					Mascot
1165.6827	1165.627	-0.0557	-48	1478	1487	SLSTELFKLK					Mascot
1165.6827	1165.627	-0.0557	-48	1478	1487	SLSTELFKLK					Mascot
1313.6444	1313.7223	0.0779	59	1773	1783	KEQDTSAHLER					Mascot
1323.6539	1323.7111	0.0572	43	1085	1095	KEFDISQQNSK					Mascot
1329.7122	1329.7091	-0.0031	-2	1280	1291	AKLQTENGELAR					Mascot
1331.7179	1331.728	0.0101	8	561	571	SNNFQKPRNVK					Mascot
1331.7179	1331.728	0.0101	8	561	571	SNNFQKPRNVK					Mascot

1373.7522	1373.7445	-0.0077	-6	954	965	KDIDDLELTLAK		Mascot
1412.7184	1412.6616	-0.0568	-40	828	837	NWPWMKLYFK		Mascot
1417.7546	1417.7914	0.0368	26	642	654	KGSSFQTVSALHR		Mascot
1475.774	1475.824	0.05	34	600	612	DPLNETVVGLYQK		Mascot
1475.774	1475.824	0.05	34	600	612	DPLNETVVGLYQK		Mascot
1487.7965	1487.8179	0.0214	14	1362	1374	VLSKANSEVAQWR		Mascot
1523.8217	1523.8939	0.0722	47	191	204	VIQYFASIAAIGDR		Mascot
1565.7476	1565.7141	-0.0335	-21	1595	1608	MVDSLQTSLDAETR		Mascot
1740.928	1740.823	-0.105	-60	726	741	ILNPAAIPEGQFIDSR		Mascot
1765.816	1765.9454	0.1294	73	928	942	LEDEEEMNAELTAKK	Oxidation (M)[7]	Mascot
1804.9109	1805.0232	0.1123	62	1152	1168	LEEAGGATSVQIEMNKK		Mascot
1821.9463	1821.9591	0.0128	7	681	696	KAPGVMDNPLVMHQLR	Oxidation (M)[6]	Mascot
1837.9412	1837.9916	0.0504	27	681	696	KAPGVMDNPLVMHQLR	Oxidation (M)[6,12]	Mascot
1838.9243	1839.0139	0.0896	49	1179	1195	DLEEATLQHEATAAALR		Mascot
1856.896	1857.0701	0.1741	94	2	18	TDAQMADFGAAAQYLRK		Mascot
1873.9939	1874.0587	0.0648	35	977	993	VKNLTEEMAGLDEIIAK		Mascot
1887.9672	1888.1119	0.1447	77	643	659	GSSFQTVSALHRENLNK		Mascot
1891.0172	1891.0885	0.0713	38	600	616	DPLNETVVGLYQKSSLK		Mascot
1910.9091	1910.9396	0.0305	16	1654	1670	DTQLQLDDAVHANDDLK		Mascot
1993.8954	1994.092	0.1966	99	1620	1636	MEGDLNEMEIQLSQANR	Oxidation (M)[1]	Mascot
1995.0254	1995.0848	0.0594	30	1178	1195	RDLEEATLQHEATAAALR		Mascot
2023.0203	2023.0099	-0.0104	-5	1197	1214	KHADSVAELGEQIDNLQR		Mascot
2255.1111	2255.124	0.0129	6	1224	1242	SEFKLELDDVTSNMEQIIK	Oxidation (M)[14]	Mascot
2705.2869	2705.3333	0.0464	17	1620	1643	MEGDLNEMEIQLSQANRI ASEAQK		Mascot
2705.2869	2705.3333	0.0464	17	1620	1643	MEGDLNEMEIQLSQANRI ASEAQK		Mascot

Gel Idx/Pos	133/F8	Instr./Gel Origin	BA2151/Sample Project 20140814	Process Status	Analysis Succeeded
Plate [#] Name	[1] Sample Project 20140814	Instrument Sample Name		Spectra	11

Rank	Protein Name	Accession No.	Protein MW	Protein PI	Pep. Count	Protein Score	Protein Score C. I. %	Intensity Matched	Total Ion Score	Total Ion C. I. %	Confirmed
1	Phosphoenolpyruvate-protein phosphotransferase OS=Haemophilus influenzae (strain ATCC 51907 / DSM 11121 / KW20 / Rd) GN=ptsI PE=3 SV=1	PT1_HAEIN	63765.3	4.68	14	54	0	4.762			

Peptide Information

Calc. Mass	Obsrv. Mass	± da	± ppm	Start Seq.	End Seq.	Sequence	Ion Score	C. I. %	Modification	Rank	Result Type
816.4322	816.4282	-0.004	-5	125	131	ERAGDIR					Mascot
822.3046	822.3141	0.0095	12	280	286	DCDGAER			Carbamidomethyl (C)[2]		Mascot
836.3818	836.356	-0.0258	-31	333	340	TMDIGGDK					Mascot
927.5734	927.548	-0.0254	-27	359	366	AIRIALDR					Mascot
1477.8083	1477.835	0.0267	18	1	15	MISGILASPGIAFGK			Oxidation (M)[1]		Mascot
1569.921	1569.9546	0.0336	21	196	210	SLELPAIVGTNKVTK					Mascot
2118.1702	2118.073	-0.0972	-46	386	403	LAVMFPMIISVEEIRELK					Mascot
2118.1702	2118.073	-0.0972	-46	386	403	LAVMFPMIISVEEIRELK					Mascot
2153.0684	2153.009	-0.0594	-28	107	124	ILDQQVTMLSEIDDEYLK					Mascot
2309.2397	2309.2681	0.0284	12	380	400	ASAFGKLAVMFPMIISVE EIR					Mascot
2317.1858	2317.2192	0.0334	14	419	440	LFDNNIQVGVMVETPSAA VNAK					Mascot
2317.2874	2317.2192	-0.0682	-29	258	279	DLPAITLDGHKVDVVANI GTIR					Mascot
2617.177	2617.3359	0.1589	61	487	510	QVIDASHAEGKWTGMCG ELAGDER			Carbamidomethyl (C)[16]		Mascot
3319.6401	3319.9202	0.2801	84	70	97	AAIFEGHLMILEDEELEEE ILDYLRSNK					Mascot
3347.7368	3347.97	0.2332	70	211	240	LVNTGDYLILDAINNQVYI NPTASQIDELK					Mascot

Rank	Protein Name	Accession No.	Protein MW	Protein PI	Pep. Count	Protein Score	Protein Score C. I. %	Intensity Matched	Total Ion Score	Total Ion C. I. %	Confirmed
2	Argininosuccinate synthase OS=Polaromonas naphthalenivorans (strain CJ2) GN=argG PE=3 SV=1	ASSY_POLNA	49227	5.22	12	50	0	9.536			

Peptide Information

Calc. Mass	Obsrv. Mass	± da	± ppm	Start Seq.	End Seq.	Sequence	Ion Score	C. I. %	Modification	Rank	Result Type
816.4937	816.4282	-0.0655	-80	283	289	IIEAKSR					Mascot
834.4502	834.3815	-0.0687	-82	402	408	IGQLTMR			Oxidation (M)[6]		Mascot
874.4628	874.4152	-0.0476	-54	228	234	DEVEVKR					Mascot
888.4897	888.52	0.0303	34	234	240	REEVTVR					Mascot

1926.9702	1927.0009	0.0307	16	271	287	HGLGMSDQIENRIIEAK			Oxidation (M)[5]		Mascot
1997.0426	1996.918	-0.1246	-62	330	345	LLYQGRWFDSQAIMLR					Mascot
1997.0426	1996.918	-0.1246	-62	330	345	LLYQGRWFDSQAIMLR					Mascot
2090.0991	2090.0396	-0.0595	-28	216	233	IVNPIMGVAFWKDEVEVK			Oxidation (M)[6]		Mascot
2090.0991	2090.0396	-0.0595	-28	216	233	IVNPIMGVAFWKDEVEVK			Oxidation (M)[6]		Mascot
2230.1826	2230.3457	0.1631	73	346	365	ETAQRWVASAITGEVTIE LR					Mascot
2274.0012	2274.2271	0.2259	99	185	205	MSAEKAYSTDSNMLGAT HEAK			Oxidation (M)[1,13]		Mascot
2290.9666	2290.7656	-0.201	-88	170	189	AEMSEFMTQAGFGYKMS AEK			Oxidation (M)[3,7,16]		Mascot
3347.7383	3347.97	0.2317	69	75	107	SQLAAEGLAALQAGAFHI TTAGVTYFNTTPLGR					Mascot
3664.9009	3665.1602	0.2593	71	290	321	GIYEAPGLALLFIAYERLV TGIHNEDTIEQYR					Mascot

3 Recombination protein RecR OS=Clostridium botulinum (strain Alaska E43 / Type E3) GN=recR PE=3 SV=1 | RECR_CLOBA | 22230.6 | 6 | 9 | 47 | 0 | 2.492

Peptide Information

Calc. Mass	Obsrv. Mass	± da	± ppm	Start Seq.	End Seq.	Sequence	Ion Score	C. I. %	Modification	Rank	Result Type
849.4716	849.3922	-0.0794	-93	11	17	LIEEFAK					Mascot
856.5363	856.5782	0.0419	49	125	131	IRELVAR					Mascot
899.3849	899.339	-0.0459	-51	91	97	DIMTMEK			Oxidation (M)[3,5]		Mascot
1333.678	1333.7251	0.0471	35	127	137	ELVARMNEEVK			Oxidation (M)[6]		Mascot
2024.9702	2024.9546	-0.0156	-8	81	97	STICVVEQPKDIMTMEK			Carbamidomethyl (C)[4], Oxidation (M)[13]		Mascot
2073.0613	2073.0957	0.0344	17	1	17	MEFYPVAIEKLIEEFAK			Oxidation (M)[1]		Mascot
2118.0964	2118.073	-0.0234	-11	170	190	IAAGIPVGGDLEYADEVT LSK					Mascot
2118.0964	2118.073	-0.0234	-11	170	190	IAAGIPVGGDLEYADEVT LSK					Mascot
2123.1497	2123.115	-0.0347	-16	29	46	LTLHILNLPDDEVREFAK					Mascot
2807.4045	2807.5112	0.1067	38	100	124	EFNGLYHVLHGNISPMQ GRGPQDIK					Mascot

4 Uncharacterized protein YabE OS=Bacillus subtilis (strain 168) GN=yabE PE=4 SV=1 | YABE_BACSU | 47852.3 | 8.94 | 11 | 46 | 0 | 19.471

Peptide Information

Calc. Mass	Obsrv. Mass	± da	± ppm	Start Seq.	End Seq.	Sequence	Ion Score	C. I. %	Modification	Rank	Result Type
834.3886	834.3815	-0.0071	-9	1	7	MGEREGR					Mascot
889.4924	889.5203	0.0279	31	208	214	QQKMNIK					Mascot
930.5618	930.5588	-0.003	-3	307	315	DKVIAVGTK					Mascot
930.5618	930.5588	-0.003	-3	307	315	DKVIAVGTK					Mascot

Calc. Mass	Obsrv. Mass	± da	± ppm	Start Seq.	End Seq.	Sequence	Ion Score	C. I. %	Modification	Rank	Result Type
1342.7213	1342.7738	0.0525	39	215	226	DEDKIKPALDAK					Mascot
1738.8745	1738.9376	0.0631	36	8	22	VDSLLDTLYNLSEEK					Mascot
1997.0663	1996.918	-0.1483	-74	83	100	THANTVGDLLETLDIKTR					Mascot
1997.0663	1996.918	-0.1483	-74	83	100	THANTVGDLLETLDIKTR					Mascot
2090.0586	2090.0396	-0.019	-9	173	191	DMKINIEPAFQVTVNDAGK					Mascot
2090.0586	2090.0396	-0.019	-9	173	191	DMKINIEPAFQVTVNDAGK					Mascot
2123.1052	2123.115	0.0098	5	110	128	QTKITADMDVVYEAAKPVK			Oxidation (M)[8]		Mascot
2221.1248	2221.2585	0.1337	60	391	412	VHVEGYGYAIAADTGSAIKGNK					Mascot
2289.1973	2289.1877	-0.0096	-4	137	157	TLWSTAKTVGALLDEQDVDVK					Mascot
2442.3425	2442.301	-0.0415	-17	44	67	VILVAACLLLAGSGTAYAAHELTK			Carbamidomethyl (C)[7]		Mascot

5	Cysteine--tRNA ligase OS=Bartonella tribocorum (strain CIP 105476 / IBS 506) GN=cysS PE=3 SV=1	SYC_BART1	57980.5	6.93	12	45	0	7.78

Peptide Information

Calc. Mass	Obsrv. Mass	± da	± ppm	Start Seq.	End Seq.	Sequence	Ion Score	C. I. %	Modification	Rank	Result Type
807.4215	807.3519	-0.0696	-86	1	6	MMKELR					Mascot
888.5189	888.52	0.0011	1	495	501	ITVWEIK					Mascot
1182.5725	1182.6188	0.0463	39	338	346	FSMLQTHYR					Mascot
1301.71	1301.6923	-0.0177	-14	15	25	KENFIPIDPTK					Mascot
1439.858	1439.8177	-0.0403	-28	472	484	IRNELAAEGILLK					Mascot
1908.8884	1909.0396	0.1512	79	281	296	MANFWMHNGFLQVEGK					Mascot
2090.0376	2090.0396	0.002	1	356	371	LMQSSSELYRWYELLR			Oxidation (M)[2]		Mascot
2090.0376	2090.0396	0.002	1	356	371	LMQSSSELYRWYELLR			Oxidation (M)[2]		Mascot
2221.2161	2221.2585	0.0424	19	407	427	FYKAGDALALANGMNLLGLLR					Mascot
2309.1343	2309.2681	0.1338	58	347	365	EPLNWTAQRLMQSSSELYR					Mascot
2317.1208	2317.2192	0.0984	42	59	78	HVYGSDHVLYARNITDVDDK					Mascot
2317.1208	2317.2192	0.0984	42	59	78	HVYGSDHVLYARNITDVDDK					Mascot
3664.9824	3665.1602	0.1778	49	28	58	LYVCGPTVYDYAHIGNARPLIVFDILFRLLR			Carbamidomethyl (C)[4]		Mascot
3680.7837	3681.1355	0.3518	96	103	133	TYFQFQQDTIALGCLLPTSQPRATEHLEEMR			Carbamidomethyl (C)[14]		Mascot

6	E3 ubiquitin-protein ligase UPL5 OS=Arabidopsis thaliana GN=UPL5 PE=1 SV=1	UPL5_ARATH	101156	6.25	17	45	0	8.611

Peptide Information

Calc. Mass	Obsrv. Mass	± da	± ppm	Start Seq.	End Seq.	Sequence	Ion Score	C. I. %	Modification	Rank	Result Type

Calc. Mass	Obsrv. Mass	± da	± ppm	Start Seq.	End Seq.	Sequence	Ion Score	C. I. %	Modification	Rank	Result Type
817.4665	817.3932	-0.0733	-90	634	640	ISLEDIK					Mascot
847.4308	847.3521	-0.0787	-93	584	591	FSPNPASK					Mascot
874.4451	874.4152	-0.0299	-34	285	291	KVCPDQK			Carbamidomethyl (C)[3]		Mascot
915.4062	915.3291	-0.0771	-84	645	651	IMYNSCK			Carbamidomethyl (C)[6]		Mascot
995.607	995.6909	0.0839	84	606	614	VIALALMHK					Mascot
1301.7576	1301.6923	-0.0653	-50	711	721	RFATPILEQVK					Mascot
1382.7283	1382.7384	0.0101	7	95	106	LQIFVRMMSGGK			Oxidation (M)[7]		Mascot
1388.709	1388.8042	0.0952	69	682	693	DTIELCPDGKLK			Carbamidomethyl (C)[6]		Mascot
1388.7169	1388.8042	0.0873	63	860	872	LIAQDHVSSSFGK					Mascot
1439.8192	1439.8177	-0.0015	-1	493	504	RHLAMLLFPDVK					Mascot
1997.03	1996.918	-0.112	-56	204	221	IVTFFAMIPVESDESIAK					Mascot
1997.03	1996.918	-0.112	-56	204	221	IVTFFAMIPVESDESIAK					Mascot
2271.0823	2271.1941	0.1118	49	51	70	QEIDADHMAASAQQTLISWR					Mascot
2300.1404	2300.25	0.1096	48	148	167	ENSLTYYSIEQDASLQLVAR					Mascot
2311.2729	2311.1843	-0.0886	-38	369	389	VAFPIPIVLPMQSTALEAEIR			Oxidation (M)[11]		Mascot
2345.0237	2345.2429	0.2192	93	6	28	SSADDSTNNANRSYSAVAGTDNK					Mascot
2617.3271	2617.3359	0.0088	3	557	576	EWFYLVCQEIFNPKNTLFLR			Carbamidomethyl (C)[7]		Mascot
3681.7776	3682.1257	0.3481	95	417	449	EVGNSETMSSSWSQYLSILKIINSMSNIYQGAK			Oxidation (M)[8]		Mascot
3681.7776	3682.1257	0.3481	95	417	449	EVGNSETMSSSWSQYLSILKIINSMSNIYQGAK			Oxidation (M)[8]		Mascot

7	Notchless protein homolog OS=Arabidopsis thaliana GN=NLE1 PE=2 SV=1	NLE1_ARATH	53434.1	8.63	11	45	0	7.355	

Peptide Information

Calc. Mass	Obsrv. Mass	± da	± ppm	Start Seq.	End Seq.	Sequence	Ion Score	C. I. %	Modification	Rank	Result Type
817.4526	817.3932	-0.0594	-73	459	466	VVSGGKDR					Mascot
888.5189	888.52	0.0011	1	225	231	IWDITLK					Mascot
889.4374	889.5203	0.0829	93	324	331	TKGDSPER					Mascot
1182.575	1182.6188	0.0438	37	214	224	FVTSSKDGDAR					Mascot
1958.9211	1958.993	0.0719	37	165	181	HLVSGSKSGEICCWNPK			Carbamidomethyl (C)[12,13]		Mascot
2025.0011	2024.9546	-0.0465	-23	196	212	WITGISWEPVHLSSPCR			Carbamidomethyl (C)[16]		Mascot
2153.0962	2153.009	-0.0872	-40	195	212	KWITGISWEPVHLSSPCR			Carbamidomethyl (C)[17]		Mascot
2300.335	2300.25	-0.085	-37	82	100	VLTIVYQQQAVFRIRPVNR					Mascot
2312.0466	2312.1604	0.1138	49	438	458	QDLPGHADEVFAVDWSPDGEK					Mascot

2839.3535	2839.4954	0.1419	50	438	464	QDLPGHADEVFAVDWSP DGEKVVSGGK					Mascot
3681.792	3682.1257	0.3337	91	46	76	FLDNEEMLPYSFYVSDEE LLVPVGTYLEKNK					Mascot
3681.792	3682.1257	0.3337	91	46	76	FLDNEEMLPYSFYVSDEE LLVPVGTYLEKNK					Mascot

8	Aliphatic sulfonates import ATP-binding protein SsuB 2 OS=Pseudomonas syringae pv. phaseolicola (strain 1448A / Race 6) GN=ssuB2 PE=3 SV=1	SSUB2_PSE14	29064.9	9.74	9	44	0	2.595

Peptide Information

Calc. Mass	Obsrv. Mass	± da	± ppm	Start Seq.	End Seq.	Sequence	Ion Score	C. I. %	Modification	Rank	Result Type
849.4498	849.3922	-0.0576	-68	1	7	MATLELR			Oxidation (M)[1]		Mascot
874.4993	874.4152	-0.0841	-96	255	262	DVTLSALR					Mascot
954.6207	954.6233	0.0026	3	138	146	VALARGLVR					Mascot
1359.6499	1359.7456	0.0957	70	242	254	TQAADTATQEPAR					Mascot
1439.8118	1439.8177	0.0059	4	74	86	VPVARAAVFQEPR					Mascot
1474.7384	1474.7878	0.0494	33	60	73	TLAGLDDIDSGELR					Mascot
1997.0663	1996.918	-0.1483	-74	60	78	TLAGLDDIDSGELRVPVA R					Mascot
1997.0663	1996.918	-0.1483	-74	60	78	TLAGLDDIDSGELRVPVA R					Mascot
2113.1433	2113.0164	-0.1269	-60	1	19	MATLELRNTSSVTPPVQL R					Mascot
2153.1924	2153.009	-0.1834	-85	234	254	LLGLLGVKTQAADTATQE PAR					Mascot

9	50S ribosomal protein L7/L12 OS=Synechococcus sp. (strain CC9311) GN=rplL PE=3 SV=1	RL7_SYNS3	12937.8	4.69	7	43	0	23.375

Peptide Information

Calc. Mass	Obsrv. Mass	± da	± ppm	Start Seq.	End Seq.	Sequence	Ion Score	C. I. %	Modification	Rank	Result Type
802.4305	802.3547	-0.0758	-94	117	124	AIEEVGGK					Mascot
816.4283	816.4282	-0.0001	0	93	100	AMVEAAPK					Mascot
832.4233	832.3776	-0.0457	-55	93	100	AMVEAAPK			Oxidation (M)[2]		Mascot
889.4625	889.5203	0.0578	65	109	116	DDAEALKK					Mascot
930.5255	930.5588	0.0333	36	116	124	KAIEEVGGK					Mascot
930.5255	930.5588	0.0333	36	116	124	KAIEEVGGK					Mascot
1333.7209	1333.7251	0.0042	3	2	13	SAKTDEILESLK					Mascot
1641.8007	1641.9208	0.1201	73	59	73	TEFDVVLESFDAAAK					Mascot
2317.2749	2317.2192	-0.0557	-24	5	25	TDEILESLKSLSLLEASEL VK					Mascot
2317.2749	2317.2192	-0.0557	-24	5	25	TDEILESLKSLSLLEASEL VK					Mascot

10	Elongation factor 4 OS=Clostridium novyi (strain NT) GN=lepA PE=3 SV=1	LEPA_CLONN	67805.1	5.22	13	42	0	19.408	

Peptide Information

Calc. Mass	Obsrv. Mass	± da	± ppm	Start Seq.	End Seq.	Sequence	Ion Score	C. I. %	Modification	Rank	Result Type
807.3777	807.3519	-0.0258	-32	1	6	MQNDRK			Oxidation (M)[1]		Mascot
816.4938	816.4282	-0.0656	-80	54	60	ERGITIK					Mascot
831.428	831.3934	-0.0346	-42	404	410	MEEPVVK					Mascot
834.4502	834.3815	-0.0687	-82	546	552	ETVKAMR					Mascot
847.423	847.3521	-0.0709	-84	404	410	MEEPVVK			Oxidation (M)[1]		Mascot
848.4294	848.3487	-0.0807	-95	382	388	RDGEMLK					Mascot
1641.9421	1641.9208	-0.0213	-13	170	184	TGLNIKDVLEAIVEK					Mascot
2090.0837	2090.0396	-0.0441	-21	583	601	QIGSVEVPQEAFMSILKVD					Mascot
2090.0837	2090.0396	-0.0441	-21	583	601	QIGSVEVPQEAFMSILKVD					Mascot
2113.1475	2113.0164	-0.1311	-62	523	541	EVIPRQMFEIPIQAAVGAK			Oxidation (M)[7]		Mascot
2230.1472	2230.3457	0.1985	89	345	362	CGFLGLLHMEIIQERIER			Carbamidomethyl (C)[1], Oxidation (M)[9]		Mascot
2271.1292	2271.1941	0.0649	29	324	344	LNDAALSYEPETSIALGFGFR					Mascot
2289.1755	2289.1877	0.0122	5	29	48	LLEATGTLTQREMENQVLDK					Mascot
2345.1729	2345.2429	0.07	30	489	509	LDMLLNGDVVDALSMIVPEER			Oxidation (M)[3]		Mascot
2839.4626	2839.4954	0.0328	12	445	467	VVVNYYIPLNEIIYDFFDMLKSK			Oxidation (M)[19]		Mascot

Rank	Protein Name	Accession No.	Protein MW	Protein PI	Pep. Count	Protein Score	Protein Score C. I. %	Intensity Matched	Total Ion Score	Total Ion C. I. %	Confirmed
1	Cyclin-dependent kinase 1-A OS=Xenopus laevis GN=cdk1-a PE=1 SV=1	CDK1A_XENLA	34598.3	8.3	10	57	0	6.456			

Peptide Information

Calc. Mass	Obsrv. Mass	± da	± ppm	Start Seq.	End Seq.	Sequence	Ion Score	C. I. %	Modification	Rank	Result Type
832.4523	832.3834	-0.0689	-83	246	254	GGSLSANVK					Mascot
906.4832	906.5375	0.0543	60	239	245	NSFPKWK					Mascot
1169.646	1169.7087	0.0627	54	23	33	HKATGQVVAMK					Mascot
1565.8112	1565.7477	-0.0635	-41	159	170	VYTHEVVTLWYR					Mascot
1647.8702	1647.8679	-0.0023	-1	76	88	LYLIFEFLSMDLK			Oxidation (M)[10]		Mascot
1791.9562	1791.8669	-0.0893	-50	259	274	DGLDLLAKMLIYDPAK			Oxidation (M)[9]		Mascot
1889.0967	1889.1967	0.1	53	124	139	VLHRDLKPQNLLIDSK					Mascot
1958.978	1959.0035	0.0255	13	90	106	YLDSIPSGQYIDTMLVK			Oxidation (M)[14]		Mascot
2289.1033	2289.1997	0.0964	42	219	238	ALGTPNNEVWPEVESLQDYK					Mascot
2705.3569	2705.3823	0.0254	9	216	238	IFRALGTPNNEVWPEVESLQDYK					Mascot

Rank	Protein Name	Accession No.	Protein MW	Protein PI	Pep. Count	Protein Score	Protein Score C. I. %	Intensity Matched	Total Ion Score	Total Ion C. I. %	Confirmed
2	Ribosomal RNA small subunit methyltransferase G OS=Shewanella piezotolerans (strain WP3 / JCM 13877) GN=rsmG PE=3 SV=1	RSMG_SHEPW	23626.1	5.41	7	38	0	19.881			

Peptide Information

Calc. Mass	Obsrv. Mass	± da	± ppm	Start Seq.	End Seq.	Sequence	Ion Score	C. I. %	Modification	Rank	Result Type
1179.6593	1179.6768	0.0175	15	194	202	LDEQRHLLR					Mascot
1475.804	1475.8553	0.0513	35	54	66	HIMDSLVVSPHLK					Mascot
1707.8888	1707.9053	0.0165	10	24	37	QLVDFVGMLNKWNK			Oxidation (M)[8]		Mascot
1791.9535	1791.8669	-0.0866	-48	54	69	HIMDSLVVSPHLKGSR			Oxidation (M)[3]		Mascot
1905.9851	1906.1636	0.1785	94	38	53	AFNLTSVRDPEQMLIR			Oxidation (M)[13]		Mascot
1905.9851	1906.1636	0.1785	94	38	53	AFNLTSVRDPEQMLIR			Oxidation (M)[13]		Mascot
1933.9978	1934.1898	0.192	99	108	124	QVQFELGINNISSIESR					Mascot
2384.1438	2384.1333	-0.0105	-4	2	22	LFAQLNEYMAEVGLDASEQQK					Mascot

Rank	Protein Name	Accession No.	Protein MW	Protein PI	Pep. Count	Protein Score	Protein Score C. I. %	Intensity Matched	Total Ion Score	Total Ion C. I. %	Confirmed
3	Gamma-glutamyl phosphate reductase OS=Geobacter sulfurreducens (strain ATCC 51573 / DSM 12127 /	PROA_GEOSL	45841.1	5.72	5	36	0	12.558	21	0	

PCA) GN=proA PE=3 SV=1

Peptide Information

Calc. Mass	Obsrv. Mass	± da	± ppm	Start Seq.	End Seq.	Sequence	Ion Score	C. I. %	Modification	Rank	Result Type
832.4047	832.3834	-0.0213	-26	54	61	DLEAGEAK					Mascot
1360.7617	1360.7939	0.0322	24	16	29	QAAIAMAKLSASAK					Mascot
1360.7617	1360.7939	0.0322	24	16	29	QAAIAMAKLSASAK					Mascot
1565.7919	1565.7477	-0.0442	-28	323	336	VVDDLDEAVAHINR					Mascot
1822.915	1823.0137	0.0987	54	62	77	GLSTAMLDRLMLNEAR			Oxidation (M)[6,11]		Mascot
1822.915	1823.0137	0.0987	54	62	77	GLSTAMLDRLMLNEAR	21	0	Oxidation (M)[6,11]		Mascot
1934.0641	1934.1898	0.1257	65	252	268	VQRPGVCNALETLLVHK			Carbamidomethyl (C)[7]		Mascot

4 Very-long-chain 3-oxoacyl-CoA reductase 1 OS=Arabidopsis thaliana GN=KCR1 PE=1 SV=1 | KCR1_ARATH | 36024 | 9.51 | 7 | 35 | 0 | 22.461

Peptide Information

Calc. Mass	Obsrv. Mass	± da	± ppm	Start Seq.	End Seq.	Sequence	Ion Score	C. I. %	Modification	Rank	Result Type
906.4713	906.5375	0.0662	73	309	316	GLQKDSMK					Mascot
1791.9388	1791.8669	-0.0719	-40	49	65	RYGSWAIITGPTDGIGK					Mascot
1906.0104	1906.1636	0.1532	80	226	242	KSGIDVQCQVPLYVATK			Carbamidomethyl (C)[8]		Mascot
1906.0104	1906.1636	0.1532	80	226	242	KSGIDVQCQVPLYVATK			Carbamidomethyl (C)[8]		Mascot
1930.9368	1931.1014	0.1646	85	211	225	TYVDQFTKCLHVEYK			Carbamidomethyl (C)[9]		Mascot
2461.1804	2461.2722	0.0918	37	99	120	YSQTQILTVVMDFSGDID EGVK			Oxidation (M)[11]		Mascot
2705.3643	2705.3823	0.018	7	184	210	GAIINMGSGAAALIPSYPF YSVYAGAK			Oxidation (M)[6]		Mascot
2718.3984	2718.3132	-0.0852	-31	147	169	YFHEVDEELINNLIKINVE GTTK					Mascot

5 RlpA-like protein OS=Rickettsia conorii (strain ATCC VR-613 / Malish 7) GN=rlpA PE=3 SV=1 | RLPA_RICCN | 36441.6 | 9.96 | 7 | 35 | 0 | 1.403

Peptide Information

Calc. Mass	Obsrv. Mass	± da	± ppm	Start Seq.	End Seq.	Sequence	Ion Score	C. I. %	Modification	Rank	Result Type
888.4897	888.5308	0.0411	46	212	219	VTNKANNK					Mascot
982.5866	982.5107	-0.0759	-77	1	8	MIIVKNHK					Mascot
1169.7001	1169.7087	0.0086	7	272	281	NINLKKPQSK					Mascot
1475.804	1475.8553	0.0513	35	220	232	AVILMVNDRGPFK			Oxidation (M)[5]		Mascot
1889.1041	1889.1967	0.0926	49	195	211	NLLTAAHKTLPLPCLVK			Carbamidomethyl (C)[14]		Mascot
2808.3647	2808.5261	0.1614	57	39	65	EDASTGLTYTADISKVGN					Mascot

Calc. Mass	Obsrv. Mass	± da	± ppm	Start Seq.	End Seq.	Sequence	Ion Score	C. I. %	Modification	Rank	Result Type
3313.553	3313.5818	0.0288	9	23	53	QISGEPAQR FEGNTACSTAAYTLVRED ASTGLTYTADISK			Carbamidomethyl (C)[7]		Mascot

6	SsrA-binding protein OS=Shewanella woodyi (strain ATCC 51908 / MS32) GN=smpB PE=3 SV=1	SSRP_SHEWM	18965.9	9.92	3	34	0	6.678	21	0

Protein Group

SsrA-binding protein OS=Shewanella sediminis (strain HAW-EB3) GN=smpB PE=3 SV=1	SSRP_SHESH	18951.9	9.9200 000762 9395

Peptide Information

Calc. Mass	Obsrv. Mass	± da	± ppm	Start Seq.	End Seq.	Sequence	Ion Score	C. I. %	Modification	Rank	Result Type
805.4162	805.4794	0.0632	78	11	18	NSSASIAR					Mascot
832.4199	832.3834	-0.0365	-44	149	154	EWQIEK					Mascot
1822.9191	1823.0137	0.0946	52	32	47	MEAGLSLMGWEVKSIR			Oxidation (M)[1]		Mascot
1822.9191	1823.0137	0.0946	52	32	47	MEAGLSLMGWEVKSIR	21	0	Oxidation (M)[1]		Mascot
1838.9139	1839.0477	0.1338	73	32	47	MEAGLSLMGWEVKSIR			Oxidation (M)[1,8]		Mascot

7	Glutamate--tRNA ligase OS=Bifidobacterium longum (strain NCC 2705) GN=gltX PE=3 SV=1	SYE_BIFLO	56702.9	5.04	9	34	0	7.35

Protein Group

Glutamate--tRNA ligase OS=Bifidobacterium longum (strain DJO10A) GN=gltX PE=3 SV=1	SYE_BIFLD	56702.9	5.0399 999618 5303

Peptide Information

Calc. Mass	Obsrv. Mass	± da	± ppm	Start Seq.	End Seq.	Sequence	Ion Score	C. I. %	Modification	Rank	Result Type
933.4523	933.3978	-0.0545	-58	421	429	DSAGEVLDK					Mascot
1342.6849	1342.7579	0.073	54	2	13	TDAENTKPELPK					Mascot
1360.7518	1360.7939	0.0421	31	465	477	LAFGPVRVAMSGR					Mascot
1360.7518	1360.7939	0.0421	31	465	477	LAFGPVRVAMSGR					Mascot
1565.7152	1565.7477	0.0325	21	168	180	MPDEDIAFDDLIR			Oxidation (M)[1]		Mascot
1927.963	1928.1187	0.1557	81	17	33	VRFCPSPTGTPHVGMIR			Carbamidomethyl (C)[4], Oxidation (M)[15]		Mascot
1930.9155	1931.1014	0.1859	96	270	285	DPESNLFNHRDNGFIR					Mascot
1959.0811	1959.0035	-0.0776	-40	454	471	ALVEEGGYKPRLAFGPV R					Mascot
2384.2205	2384.1333	-0.0872	-37	444	464	TDNLHETLNKALVEEGGY KPR					Mascot
3313.6118	3313.5818	-0.03	-9	286	314	EGLLNYLALLGWSIAPDR DVFSMDEMTEK					Mascot

8	3-oxoadipate CoA-transferase subunit A	PCAI_PSEPU	24275.5	5.28	3	33	0	6.439

OS=Pseudomonas putida GN=pcaI PE=1 SV=2

Peptide Information

Calc. Mass	Obsrv. Mass	± da	± ppm	Start Seq.	End Seq.	Sequence	Ion Score	C. I. %	Modification	Rank	Result Type
1182.5646	1182.6519	0.0873	74	176	186	NFGPIMAMAAK			Oxidation (M)[6,8]		Mascot
1823.0022	1823.0137	0.0115	6	93	109	AGKIELEVVPQGNLAER					Mascot
1823.0022	1823.0137	0.0115	6	93	109	AGKIELEVVPQGNLAER	20	0			Mascot
2461.2617	2461.2722	0.0105	4	138	158	EIDGRMYVLEMPLHADFA LIK					Mascot

9	30S ribosomal protein S6 OS=Lactobacillus acidophilus (strain ATCC 700396 / NCK56 / N2 / NCFM) GN=rpsF PE=3 SV=1	RS6_LACAC	11328.7	5.39	4	32	0	10.865	

Peptide Information

Calc. Mass	Obsrv. Mass	± da	± ppm	Start Seq.	End Seq.	Sequence	Ion Score	C. I. %	Modification	Rank	Result Type
1320.6465	1320.6969	0.0504	38	31	43	VIADNGGTMVESK					Mascot
1360.7617	1360.7939	0.0322	24	84	95	IDNAILRSMTVK					Mascot
1360.7617	1360.7939	0.0322	24	84	95	IDNAILRSMTVK	6	0			Mascot
1822.864	1823.0137	0.1497	82	31	47	VIADNGGTMVESKDWGK			Oxidation (M)[9]		Mascot
1822.864	1823.0137	0.1497	82	31	47	VIADNGGTMVESKDWGK			Oxidation (M)[9]		Mascot
2808.2683	2808.5261	0.2578	92	57	80	YREGTYHIMTFTADNADA VNEFSR					Mascot

10	Interferon-inducible GTPase 5 OS=Mus musculus GN=Irgc PE=2 SV=1	IIGP5_MOUSE	45458.7	9.65	8	32	0	1.979	

Peptide Information

Calc. Mass	Obsrv. Mass	± da	± ppm	Start Seq.	End Seq.	Sequence	Ion Score	C. I. %	Modification	Rank	Result Type
818.4301	818.3643	-0.0658	-80	406	412	MSPRGVR			Oxidation (M)[1]		Mascot
888.4799	888.5308	0.0509	57	296	302	SLRGYHR					Mascot
1342.7227	1342.7579	0.0352	26	394	405	TPNASASKPWRK					Mascot
1928.0521	1928.1187	0.0666	35	2	19	ATSRLPAVPEETTILMAK					Mascot
1959.0123	1959.0035	-0.0088	-4	126	141	QVDFGRYDFFLLVSPR					Mascot
1961.1178	1961.2535	0.1357	69	240	257	RHAGLLSLPDISLEALQK					Mascot
2318.1833	2318.2429	0.0596	26	43	65	ELLANSETTRLEVGVTGE SGAGK					Mascot
2384.1604	2384.1333	-0.0271	-11	222	240	YDFPMLVTTWEHDLPAH RR					Mascot

Rank	Protein Name	Accession No.	Protein MW	Protein PI	Pep. Count	Protein Score	Protein Score C. I. %	Intensity Matched	Total Ion Score	Total Ion C. I. %	Confirmed
1	GMP synthase [glutamine-hydrolyzing] subunit B OS=Methanosarcina mazei (strain ATCC BAA-159 / DSM 3647 / Goe1 / Go1 / JCM 11833 / OCM 88) GN=guaAB PE=3 SV=1	GUAAB_METMA	34267	5.27	11	55	0	8.653			

Peptide Information

Calc. Mass	Obsrv. Mass	± da	± ppm	Start Seq.	End Seq.	Sequence	Ion Score	C. I. %	Modification	Rank	Result Type
832.441	832.378	-0.063	-76	132	139	IESEGGIK					Mascot
888.4897	888.5261	0.0364	41	236	244	GTGVKGDVR					Mascot
906.5043	906.5305	0.0262	29	101	108	AIGETFIR					Mascot
1232.7072	1232.6791	-0.0281	-23	1	10	MVKPEKFIPK			Oxidation (M)[1]		Mascot
1475.8118	1475.8414	0.0296	20	241	253	GDVRAYGWIVAIR					Mascot
1731.8912	1731.9841	0.0929	54	83	98	DRFLAALAGVTDPEEK					Mascot
1948.0321	1948.1429	0.1108	57	44	60	AIGDRLQPIYIDTGLMR			Oxidation (M)[16]		Mascot
1959.027	1959.004	-0.023	-12	67	82	IRHIFSHMNLDVVYAK			Oxidation (M)[8]		Mascot
2440.2178	2440.2507	0.0329	13	259	279	DGMTAEALELPWEVLKH LESR			Oxidation (M)[3]		Mascot
2454.2083	2454.2939	0.0856	35	132	154	IESEGGIKSHHNVGGLPS VMDFK			Oxidation (M)[20]		Mascot
2564.3137	2564.3748	0.0611	24	24	48	AIIALSGGVDSSVCAELAY RAIGDR			Carbamidomethyl (C)[14]		Mascot

Rank	Protein Name	Accession No.	Protein MW	Protein PI	Pep. Count	Protein Score	Protein Score C. I. %	Intensity Matched	Total Ion Score	Total Ion C. I. %	Confirmed
2	Gamma-glutamyl phosphate reductase OS=Geobacter sulfurreducens (strain ATCC 51573 / DSM 12127 / PCA) GN=proA PE=3 SV=1	PROA_GEOSL	45841.1	5.72	10	48	0	13.835	17	0	

Peptide Information

Calc. Mass	Obsrv. Mass	± da	± ppm	Start Seq.	End Seq.	Sequence	Ion Score	C. I. %	Modification	Rank	Result Type
819.4393	819.4332	-0.0061	-7	16	23	QAAIAMAK			Oxidation (M)[6]		Mascot
832.4047	832.378	-0.0267	-32	54	61	DLEAGEAK					Mascot
934.4913	934.5605	0.0692	74	187	194	EGVLEMLK			Oxidation (M)[6]		Mascot
957.5727	957.5659	-0.0068	-7	163	170	ILGEELKR					Mascot
999.5693	999.5004	-0.0689	-69	7	15	IRNIAADAR					Mascot
1565.7919	1565.7424	-0.0495	-32	323	336	VVDDLDEAVAHINR					Mascot
1822.915	1823.0027	0.0877	48	62	77	GLSTAMLDRLMLNEAR			Oxidation (M)[6,11]		Mascot
1822.915	1823.0027	0.0877	48	62	77	GLSTAMLDRLMLNEAR	17	0	Oxidation (M)[6,11]		Mascot

Calc. Mass	Obsrv. Mass	± da	± ppm	Start Seq.	End Seq.	Sequence	Ion Score	C. I. %	Modification	Rank	Result Type
1934.0641	1934.1827	0.1186	61	252	268	VQRPGVCNALETLLVHK			Carbamidomethyl (C)[7]		Mascot
1934.0641	1934.1827	0.1186	61	252	268	VQRPGVCNALETLLVHK			Carbamidomethyl (C)[7]		Mascot
1944.0946	1944.0599	-0.0347	-18	24	42	LSASAKNELLLAMAGSLVR					Mascot
2454.0854	2454.2939	0.2085	85	225	245	HYKGVCHVFVDADADFDMAEK			Carbamidomethyl (C)[6]		Mascot

3	Ribosomal RNA small subunit methyltransferase G OS=Shewanella piezotolerans (strain WP3 / JCM 13877) GN=rsmG PE=3 SV=1	RSMG_SHEPW	23626.1	5.41	9	47	0	36.769	

Peptide Information

Calc. Mass	Obsrv. Mass	± da	± ppm	Start Seq.	End Seq.	Sequence	Ion Score	C. I. %	Modification	Rank	Result Type
864.5778	864.5156	-0.0622	-72	199	205	HLLRVVK					Mascot
1060.4768	1060.5781	0.1013	96	158	166	SDGCFYALK			Carbamidomethyl (C)[4]		Mascot
1179.6593	1179.6788	0.0195	17	194	202	LDEQRHLLR					Mascot
1475.804	1475.8414	0.0374	25	54	66	HIMDSLVVSPHLK					Mascot
1707.8888	1707.8939	0.0051	3	24	37	QLVDFVGMLNKWNK			Oxidation (M)[8]		Mascot
1791.9535	1791.864	-0.0895	-50	54	69	HIMDSLVVSPHLKGSR			Oxidation (M)[3]		Mascot
1905.9851	1906.1558	0.1707	90	38	53	AFNLTSVRDPEQMLIR			Oxidation (M)[13]		Mascot
1905.9851	1906.1558	0.1707	90	38	53	AFNLTSVRDPEQMLIR			Oxidation (M)[13]		Mascot
1933.9978	1934.1827	0.1849	96	108	124	QVQFELGINNISSIESR					Mascot
1933.9978	1934.1827	0.1849	96	108	124	QVQFELGINNISSIESR					Mascot
2384.1438	2384.1318	-0.012	-5	2	22	LFAQLNEYMAEVGLDASEQQK					Mascot

4	Uracil phosphoribosyltransferase OS=Hahella chejuensis (strain KCTC 2396) GN=upp PE=3 SV=1	UPP_HAHCH	22824.4	6.99	9	46	0	1.386	

Peptide Information

Calc. Mass	Obsrv. Mass	± da	± ppm	Start Seq.	End Seq.	Sequence	Ion Score	C. I. %	Modification	Rank	Result Type
804.4573	804.4938	0.0365	45	20	26	RAEISTK					Mascot
821.4879	821.464	-0.0239	-29	204	210	IFGTKQK					Mascot
854.5029	854.5865	0.0836	98	13	19	HKIGLMR					Mascot
886.537	886.5599	0.0229	26	8	14	HPLVRHK					Mascot
933.4523	933.3954	-0.0569	-61	104	111	NEETLEAK					Mascot
1707.845	1707.8939	0.0489	29	172	186	AHPDVHIYTAAIDER					Mascot
1721.8956	1721.8359	-0.0597	-35	30	45	ELAQEVGALLTYEASK					Mascot
2450.4165	2450.2527	-0.1638	-67	79	103	AGLGMLDGVLSLIPAAKVSVVGQVR					Mascot
3312.8157	3312.5615	-0.2542	-77	117	147	LVGELDQRLALIIDPMLAT			Oxidation (M)[16]		Mascot

GGSMISTIDLLK

5	GDSL esterase/lipase EXL6 OS=Arabidopsis thaliana GN=EXL6 PE=1 SV=1	EXL6_ARATH	38970.2	9.49	11	46	0	2.8

Peptide Information

Calc. Mass	Obsrv. Mass	± da	± ppm	Start Seq.	End Seq.	Sequence	Ion Score	C. I. %	Modification	Rank	Result Type
807.4182	807.4564	0.0382	47	193	198	MVVWTR			Oxidation (M)[1]		Mascot
916.5098	916.5445	0.0347	38	127	135	VLSAGDQVK					Mascot
922.4628	922.531	0.0682	74	203	210	DLYDLGAR					Mascot
928.4774	928.5101	0.0327	35	136	142	DFKDYLK					Mascot
1721.9836	1721.8359	-0.1477	-86	326	340	AYEVISKPIVYQIAK					Mascot
1791.9238	1791.864	-0.0598	-33	270	284	FVYVDIYGTLMDLVK			Oxidation (M)[11]		Mascot
1844.8901	1844.9637	0.0736	40	228	243	ASFGGVFGWCNFLLNR			Carbamidomethyl (C)[10]		Mascot
1928.0068	1928.0995	0.0927	48	211	227	KFAVMGVMPVGCLPIHR			Carbamidomethyl (C)[12], Oxidation (M)[5]		Mascot
1944.0017	1944.0599	0.0582	30	211	227	KFAVMGVMPVGCLPIHR			Carbamidomethyl (C)[12], Oxidation (M)[5,8]		Mascot
1963.1263	1963.1896	0.0633	32	326	343	AYEVISKPIVYQIAKGLA					Mascot
2823.3381	2823.5266	0.1885	67	228	251	ASFGGVFGWCNFLLNRIT EDFNMK			Carbamidomethyl (C)[10]		Mascot
2839.3333	2839.5134	0.1801	63	228	251	ASFGGVFGWCNFLLNRIT EDFNMK			Carbamidomethyl (C)[10], Oxidation (M)[23]		Mascot
2921.5408	2921.5774	0.0366	13	156	182	EIVSNAVFLISEGNNDLG YFVAPALLR					Mascot

6	Glutamate 5-kinase OS=Burkholderia mallei (strain NCTC 10229) GN=proB PE=3 SV=1	PROB_BURM9	39265.9	6.42	10	44	0	13.41

Protein Group

Glutamate 5-kinase OS=Burkholderia mallei (strain ATCC 23344) GN=proB PE=3 SV=1	PROB_BURMA	39265.9	6.4200 000762 9395
Glutamate 5-kinase OS=Burkholderia mallei (strain NCTC 10247) GN=proB PE=3 SV=1	PROB_BURM7	39265.9	6.4200 000762 9395
Glutamate 5-kinase OS=Burkholderia mallei (strain SAVP1) GN=proB PE=3 SV=1	PROB_BURMS	39265.9	6.4200 000762 9395
Glutamate 5-kinase OS=Burkholderia pseudomallei (strain 1710b) GN=proB PE=3 SV=1	PROB_BURP1	39265.9	6.4200 000762 9395
Glutamate 5-kinase OS=Burkholderia pseudomallei (strain 668) GN=proB PE=3 SV=1	PROB_BURP6	39265.9	6.4200 000762 9395
Glutamate 5-kinase OS=Burkholderia pseudomallei	PROB_BURPS	39265.9	6.4200

(strain K96243) GN=proB PE=3 SV=1 | 000762 9395

Peptide Information

Calc. Mass	Obsrv. Mass	± da	± ppm	Start Seq.	End Seq.	Sequence	Ion Score	C. I. %	Modification	Rank	Result Type
829.4315	829.4873	0.0558	67	95	101	FAEHGIR					Mascot
886.5104	886.5599	0.0495	56	242	248	ERDVLVR					Mascot
916.5826	916.5445	-0.0381	-42	123	130	STLLTLLR					Mascot
999.5734	999.5004	-0.073	-73	35	43	WAAQIAALR					Mascot
1543.8802	1543.9767	0.0965	63	11	25	LVVKVGSSLVTNDGR					Mascot
1707.9388	1707.8939	-0.0449	-26	281	298	GHVVIDAGAVDKLTAGG K					Mascot
1822.9771	1823.0027	0.0256	14	102	117	TAQILLTHADLADRER					Mascot
1822.9771	1823.0027	0.0256	14	102	117	TAQILLTHADLADRER	2	0			Mascot
1934.0355	1934.1827	0.1472	76	26	43	GLDHDAIGRWAAQIAALR					Mascot
1934.0355	1934.1827	0.1472	76	26	43	GLDHDAIGRWAAQIAALR					Mascot
1959.0328	1959.004	-0.0288	-15	44	63	GAGKEVVLVSSGAIAEG MQR					Mascot
2921.5918	2921.5774	-0.0144	-5	342	366	LIQRKPSGEIETVLGYML EPELIHR					Mascot

7	Methionyl-tRNA formyltransferase OS=Bacillus anthracis (strain CDC 684 / NRRL 3495) GN=fmt PE=3 SV=1	FMT_BACAC	34828.4	5.4	9	43	0	4.437

Protein Group

Methionyl-tRNA formyltransferase OS=Bacillus anthracis (strain A0248) GN=fmt PE=3 SV=1	FMT_BACAA	34828.4	5.4000 000953 6743
Methionyl-tRNA formyltransferase OS=Bacillus anthracis GN=fmt PE=1 SV=1	FMT_BACAN	34828.4	5.4000 000953 6743
Methionyl-tRNA formyltransferase OS=Bacillus cereus (strain 03BB102) GN=fmt PE=3 SV=1	FMT_BACC3	34828.4	5.4000 000953 6743
Methionyl-tRNA formyltransferase OS=Bacillus cereus (strain AH187) GN=fmt PE=3 SV=1	FMT_BACC7	34814.4	5.4000 000953 6743
Methionyl-tRNA formyltransferase OS=Bacillus cereus (strain AH820) GN=fmt PE=3 SV=1	FMT_BACC0	34828.4	5.4000 000953 6743
Methionyl-tRNA formyltransferase OS=Bacillus cereus (strain Q1) GN=fmt PE=3 SV=1	FMT_BACCQ	34814.4	5.4000 000953

			6743
Methionyl-tRNA formyltransferase OS=Bacillus cereus (strain ZK / E33L) GN=fmt PE=3 SV=1	FMT_BACCZ	34828.4	5.4000 000953 6743
Methionyl-tRNA formyltransferase OS=Bacillus thuringiensis (strain Al Hakam) GN=fmt PE=3 SV=1	FMT_BACAH	34828.4	5.4000 000953 6743

Peptide Information

Calc. Mass	Obsrv. Mass	± da	± ppm	Start Seq.	End Seq.	Sequence	Ion Score	C. I. %	Modification	Rank	Result Type
854.5345	854.5865	0.052	61	43	50	VLTPTPVK					Mascot
930.5255	930.5034	-0.0221	-24	301	309	GTKPEIGTK					Mascot
1359.6726	1359.7766	0.104	76	117	129	GGAPIHYAIMEGK			Oxidation (M)[10]		Mascot
1679.8826	1679.9193	0.0367	22	4	18	VVFMGTPDFSVPVLR			Oxidation (M)[4]		Mascot
1685.9697	1685.9565	-0.0132	-8	51	65	VEAEKHGIPVLQPLR					Mascot
1905.0668	1905.1392	0.0724	38	2	18	IKVVFMGTPDFSVPVLR					Mascot
2426.304	2426.2661	-0.0379	-16	20	41	LIEDGYDVIGVVTQPDRP VGRK					Mascot
2426.304	2426.2661	-0.0379	-16	20	41	LIEDGYDVIGVVTQPDRP VGRK					Mascot
2454.3101	2454.2939	-0.0162	-7	19	40	RLIEDGYDVIGVVTQPDR PVGR					Mascot
2564.2727	2564.3748	0.1021	40	256	281	SAEAGTIVAIEEDGFVVAT GNETGVK					Mascot

8	Uncharacterized protein slr1128 OS=Synechocystis sp. (strain PCC 6803 / Kazusa) GN=slr1128 PE=3 SV=1	Y1128_SYNY3	35761.9	5.55	11	43	0	3.233	

Peptide Information

Calc. Mass	Obsrv. Mass	± da	± ppm	Start Seq.	End Seq.	Sequence	Ion Score	C. I. %	Modification	Rank	Result Type
872.5312	872.5765	0.0453	52	154	160	VTRVELR					Mascot
887.5196	887.5483	0.0287	32	208	215	VLEAEAKK					Mascot
893.455	893.4988	0.0438	49	280	286	VMFLDPR			Oxidation (M)[2]		Mascot
922.4628	922.531	0.0682	74	27	33	NEYLVER					Mascot
1359.6791	1359.7766	0.0975	72	142	153	ELDISTDPWGVK					Mascot
1593.7611	1593.7775	0.0164	10	167	180	AVLDSMELQMTAER					Mascot
1707.8912	1707.8939	0.0027	2	118	132	SEIGKLELDQTFTAR					Mascot
1721.8561	1721.8359	-0.0202	-12	167	181	AVLDSMELQMTAERK					Mascot
1928.0634	1928.0995	0.0361	19	101	117	VENLQSAMVNLVLTQIR					Mascot
1944.0583	1944.0599	0.0016	1	101	117	VENLQSAMVNLVLTQIR			Oxidation (M)[8]		Mascot
2227.1838	2227.1238	-0.06	-27	2	21	EAFFLFFLVFFGSAIGTSV					Mascot

						K						
2227.1838	2227.1238	-0.06	-27	2	21	EAFFLFFLVFFGSAIGTSVK					Mascot
2718.3291	2718.3101	-0.019	-7	255	279	EALQFLLAQQYLNMGTTIGSSDSSK			Oxidation (M)[14]		Mascot

9 Fatty acid oxidation complex subunit alpha OS=Klebsiella pneumoniae subsp. pneumoniae (strain ATCC 700721 / MGH 78578) GN=fadJ PE=3 SV=1 | FADJ_KLEP7 | 77889.1 | 9.31 | 14 | 42 | 0 | 5.474

Peptide Information

Calc. Mass	Obsrv. Mass	± da	± ppm	Start Seq.	End Seq.	Sequence	Ion Score	C. I. %	Modification	Rank	Result Type
842.4982	842.5762	0.078	93	483	490	TPIVVADK					Mascot
855.5522	855.5712	0.019	22	41	47	GLIRQLR					Mascot
869.5203	869.5076	-0.0127	-15	337	343	IKDIQPR					Mascot
893.4475	893.4988	0.0513	57	33	40	AEFGSQVR					Mascot
1105.6001	1105.587	-0.0131	-12	239	248	KTQGNYPAVK					Mascot
1565.8278	1565.7424	-0.0854	-55	158	172	LIGVSTALDMMLTGK			Oxidation (M)[10]		Mascot
1649.901	1649.9337	0.0327	20	483	497	TPIVVADKAGFYVNR					Mascot
1707.8734	1707.8939	0.0205	12	666	681	YMDTIGAGEVAAILQR					Mascot
1845.0117	1844.9637	-0.048	-26	390	405	DVVIEAVFEDLALKQR					Mascot
1851.0474	1851.0479	0.0005	0	509	525	LLVEGEPIEVIDNALVK					Mascot
1948.0607	1948.1429	0.0822	42	155	172	LPRLIGVSTALDMMLTGK			Oxidation (M)[13,14]		Mascot
1963.0219	1963.1896	0.1677	85	682	698	LAAQFGPRFTPCDTLLR			Carbamidomethyl (C)[12]		Mascot
2450.2617	2450.2527	-0.009	-4	642	665	SARDGDIGAVFGIGFPPFLGGPFR					Mascot
2564.3291	2564.3748	0.0457	18	666	689	YMDTIGAGEVAAILQRLAAQFGPR			Oxidation (M)[2]		Mascot

10 ATP phosphoribosyltransferase regulatory subunit OS=Cyanothece sp. (strain ATCC 51142) GN=hisZ PE=3 SV=1 | HISZ_CYAA5 | 45554.1 | 7.17 | 11 | 42 | 0 | 9.207

Peptide Information

Calc. Mass	Obsrv. Mass	± da	± ppm	Start Seq.	End Seq.	Sequence	Ion Score	C. I. %	Modification	Rank	Result Type
847.4268	847.496	0.0692	82	360	366	KDDNSLR					Mascot
868.4635	868.5492	0.0857	99	276	283	AIGQTNHK					Mascot
888.5189	888.5261	0.0072	8	390	396	TIVWIEK					Mascot
889.4625	889.3951	-0.0674	-76	375	381	NEEEIKK					Mascot
906.5155	906.5305	0.015	17	354	360	YAQQLRK					Mascot
922.4628	922.531	0.0682	74	382	389	YAQENGIK					Mascot
1105.5736	1105.587	0.0134	12	344	353	TSESQIEALK					Mascot

1565.7853	1565.7424	-0.0429	-27	85	98	AAVTRMANTSYPQR		Mascot
1731.8846	1731.9841	0.0995	57	178	191	QEVRHCLATLDYVK	Carbamidomethyl (C)[6]	Mascot
1791.9573	1791.864	-0.0933	-52	276	292	AIGQTNHKLQNLGQGGR		Mascot
2384.2471	2384.1318	-0.1153	-48	284	304	LQNLGQGGRYDQLLGVY HPQK		Mascot

Gel Idx/Pos	136/F11	Instr./Gel Origin	BA2151/Sample Project 20140814	Process Status	Analysis Succeeded
Plate [#] Name	[1] Sample Project 20140814	Instrument Sample Name		Spectra	11

Rank	Protein Name	Accession No.	Protein MW	Protein PI	Pep. Count	Protein Score	Protein Score C. I. %	Intensity Matched	Total Ion Score	Total Ion C. I. %	Confirmed
1	50S ribosomal protein L14 OS=Thermus thermophilus (strain HB8 / ATCC 27634 / DSM 579) GN=rplN PE=1 SV=1	RL14_THET8	13351.3	9.81	7	48	0	18.911			

Peptide Information

Calc. Mass	Obsrv. Mass	± da	± ppm	Start Seq.	End Seq.	Sequence	Ion Score	C. I. %	Modification	Rank	Result Type
1001.5626	1001.6186	0.056	56	50	59	GAVKEGDVVK					Mascot
1170.6842	1170.7004	0.0162	14	54	64	EGDVVKAVVVR					Mascot
1241.696	1241.6997	0.0037	3	68	78	EIKRPDGSAIR					Mascot
1778.9647	1778.9586	-0.0061	-3	27	44	GSNAKYATVGDVIVASVK					Mascot
1828.9188	1828.963	0.0442	24	79	94	FDDNAAVIINNQLEPR					Mascot
1888.0538	1887.9731	-0.0807	-43	32	49	YATVGDVIVASVKEAIPR					Mascot
1888.0538	1887.9731	-0.0807	-43	32	49	YATVGDVIVASVKEAIPR					Mascot
1903.9872	1903.9358	-0.0514	-27	2	18	IQPQTYLEVADNTGARK					Mascot

Rank	Protein Name	Accession No.	Protein MW	Protein PI	Pep. Count	Protein Score	Protein Score C. I. %	Intensity Matched	Total Ion Score	Total Ion C. I. %	Confirmed
2	Foldase protein PrsA OS=Streptococcus agalactiae serotype III (strain NEM316) GN=prsA PE=3 SV=1	PRSA_STRA3	33928.7	9.53	10	42	0	5.86			

Peptide Information

Calc. Mass	Obsrv. Mass	± da	± ppm	Start Seq.	End Seq.	Sequence	Ion Score	C. I. %	Modification	Rank	Result Type
842.5709	842.5787	0.0078	9	256	262	LKEVILK					Mascot
889.4625	889.3905	-0.072	-81	156	163	LDAEDKAK					Mascot
1050.5942	1050.6381	0.0439	42	269	277	SFQNKVISK					Mascot
1170.6075	1170.7004	0.0929	79	117	126	TTMLVEYAVK			Oxidation (M)[3]		Mascot
1569.8193	1569.874	0.0547	35	117	130	TTMLVEYAVKEAAK			Oxidation (M)[3]		Mascot
1587.7538	1587.8307	0.0769	48	38	51	GDTITVSDFYDQVK					Mascot
1763.9684	1763.873	-0.0954	-54	52	67	TSKAAQQSMLTLILSR			Oxidation (M)[9]		Mascot
1869.9592	1869.9636	0.0044	2	140	155	EAYKNYTPETSVQVIK					Mascot
1869.9592	1869.9636	0.0044	2	140	155	EAYKNYTPETSVQVIK					Mascot
1885.9364	1885.9412	0.0048	3	195	212	FDSAGTSLPKEVMSAAFK					Mascot
1901.9314	1901.965	0.0336	18	195	212	FDSAGTSLPKEVMSAAFK			Oxidation (M)[13]		Mascot
1903.9285	1903.9358	0.0073	4	38	54	GDTITVSDFYDQVKTSK					Mascot

3	50S ribosomal protein L14 OS=Thermus thermophilus (strain HB27 / ATCC BAA-163 / DSM 7039) GN=rplN PE=1 SV=1	RL14_THET2	13337.3	9.81	6	40	0	18.85

Protein Group

50S ribosomal protein L14 OS=Thermus aquaticus GN=rplN PE=3 SV=1	RL14_THEAQ	13337.3	9.8100004196167
50S ribosomal protein L14 OS=Thermus thermophilus GN=rplN PE=3 SV=1	RL14_THETH	13337.3	9.8100004196167

Peptide Information

Calc. Mass	Obsrv. Mass	± da	± ppm	Start Seq.	End Seq.	Sequence	Ion Score	C. I. %	Modification	Rank	Result Type
1001.5626	1001.6186	0.056	56	50	59	GAVKEGDVVK					Mascot
1170.6842	1170.7004	0.0162	14	54	64	EGDVVKAVVVR					Mascot
1778.9647	1778.9586	-0.0061	-3	27	44	GSNAKYATVGDVIVASVK					Mascot
1828.9188	1828.963	0.0442	24	79	94	FDDNAAVIINNQLEPR					Mascot
1888.0538	1887.9731	-0.0807	-43	32	49	YATVGDVIVASVKEAIPR					Mascot
1888.0538	1887.9731	-0.0807	-43	32	49	YATVGDVIVASVKEAIPR					Mascot
1903.9872	1903.9358	-0.0514	-27	2	18	IQPQTYLEVADNTGARK					Mascot

4	Foldase protein PrsA OS=Streptococcus agalactiae serotype Ia (strain ATCC 27591 / A909 / CDC SS700) GN=prsA PE=3 SV=1	PRSA_STRA1	33912.7	9.53	9	38	0	5.795

Peptide Information

Calc. Mass	Obsrv. Mass	± da	± ppm	Start Seq.	End Seq.	Sequence	Ion Score	C. I. %	Modification	Rank	Result Type
842.5709	842.5787	0.0078	9	256	262	LKEVILK					Mascot
889.4625	889.3905	-0.072	-81	156	163	LDAEDKAK					Mascot
1170.6075	1170.7004	0.0929	79	117	126	TTMLVEYAVK			Oxidation (M)[3]		Mascot
1569.8193	1569.874	0.0547	35	117	130	TTMLVEYAVKEAAK			Oxidation (M)[3]		Mascot
1587.7538	1587.8307	0.0769	48	38	51	GDTITVSDFYDQVK					Mascot
1763.9684	1763.873	-0.0954	-54	52	67	TSKAAQQSMLTLILSR			Oxidation (M)[9]		Mascot
1869.9592	1869.9636	0.0044	2	140	155	EAYKNYTPETSVQVIK					Mascot
1869.9592	1869.9636	0.0044	2	140	155	EAYKNYTPETSVQVIK					Mascot
1885.9364	1885.9412	0.0048	3	195	212	FDSAGTSLPKEVMSAAFK					Mascot
1901.9314	1901.965	0.0336	18	195	212	FDSAGTSLPKEVMSAAFK			Oxidation (M)[13]		Mascot
1903.9285	1903.9358	0.0073	4	38	54	GDTITVSDFYDQVKTSK					Mascot

5	Telomere repeat-binding factor 2 OS=Arabidopsis thaliana GN=TRB2 PE=1 SV=1	TRB2_ARATH	33050.4	9.87	8	34	0	1.755

Peptide Information

Calc. Mass	Obsrv. Mass	± da	± ppm	Start Seq.	End Seq.	Sequence	Ion Score	C. I. %	Modification	Rank	Result Type
832.4271	832.377	-0.0501	-60	138	144	ELRGSDR					Mascot
1055.5845	1055.6667	0.0822	78	171	180	HLSSNGTLVK					Mascot
1479.8306	1479.8748	0.0442	30	30	42	TILSDTEFSLILK					Mascot
1587.9218	1587.8307	-0.0911	-57	128	140	IIFEAITNLRELR					Mascot
1641.8846	1641.9323	0.0477	29	8	22	WTPEEEAALKAGVLK					Mascot
1749.884	1749.881	-0.003	-2	272	288	AEAEAEAAQIFAKAAMK					Mascot
1859.903	1859.8984	-0.0046	-2	100	119	AKPTSPGGSGGGSPRTC ASK			Carbamidomethyl (C)[17]		Mascot
1903.9508	1903.9358	-0.015	-8	212	228	DPTKPEENGANSLTKFR					Mascot

6	Thiamine-phosphate synthase OS=Clostridium botulinum (strain Alaska E43 / Type E3) GN=thiE PE=3 SV=1	THIE_CLOBA	23279.4	5.58	7	34	0	2.086

Peptide Information

Calc. Mass	Obsrv. Mass	± da	± ppm	Start Seq.	End Seq.	Sequence	Ion Score	C. I. %	Modification	Rank	Result Type
832.4523	832.377	-0.0753	-90	194	200	DIEKATR					Mascot
1241.7576	1241.6997	-0.0579	-47	30	41	SILGGATLIQLR					Mascot
1595.7469	1595.8385	0.0916	57	16	29	DLMSTNTLEEAVEK			Oxidation (M)[3]		Mascot
1597.9094	1597.8231	-0.0863	-54	157	171	VSMPVVVIGGINKER					Mascot
1599.8378	1599.9204	0.0826	52	3	15	NKIDYSIYLVTDR					Mascot
1677.8153	1677.9301	0.1148	68	125	140	ADYIGVGAMYSTGTKK			Oxidation (M)[9]		Mascot
1912.0936	1911.9061	-0.1875	-98	152	169	EITQKVSMPVVVIGGINK					Mascot

7	2-succinylbenzoate--CoA ligase OS=Lactococcus lactis subsp. lactis (strain IL1403) GN=menE PE=3 SV=2	MENE_LACLA	50716.6	5.63	9	34	0	3.529

Peptide Information

Calc. Mass	Obsrv. Mass	± da	± ppm	Start Seq.	End Seq.	Sequence	Ion Score	C. I. %	Modification	Rank	Result Type
856.525	856.5931	0.0681	80	69	75	EVLLLNR					Mascot
1508.8207	1508.8323	0.0116	8	91	103	IDKVFTSDLLTEK					Mascot
1594.8007	1594.8427	0.042	26	32	45	MAEHLAPLIDNQSR					Mascot
1597.8618	1597.8231	-0.0387	-24	132	146	IAVIMNTSATTGKFK			Oxidation (M)[5]		Mascot
1779.1143	1778.9586	-0.1557	-88	243	258	LILLGGEFIPQPLIKK					Mascot

1852.9626	1852.8817	-0.0809	-44	424	438	YKRPQSITFMDELPK					Mascot
1854.9895	1854.8948	-0.0947	-51	147	163	SVPITWGMISNHVKASK					Mascot
1854.9895	1854.8948	-0.0947	-51	147	163	SVPITWGMISNHVKASK					Mascot
1859.9836	1859.8984	-0.0852	-46	145	160	FKSVPITWGMISNHVK			Oxidation (M)[10]		Mascot
1902.0121	1901.965	-0.0471	-25	396	412	WGQVPILFVSGNISQEK					Mascot

8	DNA-directed RNA polymerase subunit omega OS=Neisseria gonorrhoeae (strain NCCP11945) GN=rpoZ PE=3 SV=1	RPOZ_NEIG2	7552	9.19	4	33	0	4.991	

Protein Group

DNA-directed RNA polymerase subunit omega OS=Neisseria gonorrhoeae (strain ATCC 700825 / FA 1090) GN=rpoZ PE=3 SV=1	RPOZ_NEIG1	7552	9.1899995803833
DNA-directed RNA polymerase subunit omega OS=Neisseria meningitidis serogroup A / serotype 4A (strain Z2491) GN=rpoZ PE=3 SV=1	RPOZ_NEIMA	7552	9.1899995803833
DNA-directed RNA polymerase subunit omega OS=Neisseria meningitidis serogroup B (strain MC58) GN=rpoZ PE=3 SV=1	RPOZ_NEIMB	7552	9.1899995803833
DNA-directed RNA polymerase subunit omega OS=Neisseria meningitidis serogroup C / serotype 2a (strain ATCC 700532 / FAM18) GN=rpoZ PE=3 SV=1	RPOZ_NEIMF	7552	9.1899995803833

Peptide Information

Calc. Mass	Obsrv. Mass	± da	± ppm	Start Seq.	End Seq.	Sequence	Ion Score	C. I. %	Modification	Rank	Result Type
1480.8119	1480.8875	0.0756	51	53	66	EIAAGHIGTELLTR					Mascot
1569.7867	1569.874	0.0873	56	29	42	QLENGNTPLVDDVR					Mascot
1612.8918	1612.8481	-0.0437	-27	13	26	ISNHFDLTLVAARR					Mascot
1796.925	1796.9584	0.0334	19	27	42	ARQLENGNTPLVDDVR					Mascot
1796.925	1796.9584	0.0334	19	27	42	ARQLENGNTPLVDDVR					Mascot

9	Elongation factor Ts OS=Dictyoglomus thermophilum (strain ATCC 35947 / DSM 3960 / H-6-12) GN=tsf PE=3 SV=1	EFTS_DICT6	22526	5.42	7	33	0	3.062	

Peptide Information

Calc. Mass	Obsrv. Mass	± da	± ppm	Start Seq.	End Seq.	Sequence	Ion Score	C. I. %	Modification	Rank	Result Type
1050.5942	1050.6381	0.0439	42	124	131	EIYRTQLK					Mascot
1055.6321	1055.6667	0.0346	33	181	189	LGENIVVRR					Mascot
1749.9493	1749.881	-0.0683	-39	128	142	TQLKNEGKPEHVIEK					Mascot

1763.8997	1763.873	-0.0267	-15	96	111	DIALQIAGMNPQYVSK			Oxidation (M)[9]		Mascot
1828.8939	1828.963	0.0691	38	151	164	FYEEVCLLEQPFVR			Carbamidomethyl (C)[6]		Mascot
1859.9531	1859.8984	-0.0547	-29	25	41	ALEEANGDMEKAVTILR					Mascot
1870.0645	1869.9636	-0.1009	-54	172	188	DLITEAISKLGENIVVR					Mascot
1870.0645	1869.9636	-0.1009	-54	172	188	DLITEAISKLGENIVVR					Mascot

10	Type III pantothenate kinase OS=Burkholderia thailandensis (strain E264 / ATCC 700388 / DSM 13276 / CIP 106301) GN=coaX PE=3 SV=1	COAX_BURTA	27215.9	6.09	7	33	0	3.983	

Peptide Information

Calc. Mass	Obsrv. Mass	± da	± ppm	Start Seq.	End Seq.	Sequence	Ion Score	C. I. %	Modification	Rank	Result Type
889.4526	889.3905	-0.0621	-70	17	24	WALADTGR					Mascot
917.4839	917.4086	-0.0753	-82	209	215	AWRDLEK					Mascot
1241.7212	1241.6997	-0.0215	-17	223	235	LVLSGGAADAIVR					Mascot
1508.7196	1508.8323	0.1127	75	1	14	MSGVCLLIDAGNSR			Carbamidomethyl (C)[5], Oxidation (M)[1]		Mascot
1587.8643	1587.8307	-0.0336	-21	64	77	IDALIDAHWPALPR					Mascot
1749.8987	1749.881	-0.0177	-10	1	16	MSGVCLLIDAGNSRIK			Carbamidomethyl (C)[5], Oxidation (M)[1]		Mascot
1795.785	1795.9215	0.1365	76	82	98	ACAAQCGVTNGYAEPAR			Carbamidomethyl (C)[2,6]		Mascot

Gel Idx/Pos	137/F12	Instr./Gel Origin	BA2151/Sample Project 20140814	Process Status	Analysis Succeeded
Plate [#] Name	[1] Sample Project 20140814	Instrument Sample Name		Spectra	11

Rank	Protein Name	Accession No.	Protein MW	Protein PI	Pep. Count	Protein Score	Protein Score C. I. %	Intensity Matched	Total Ion Score	Total Ion C. I. %	Confirmed
1	Alpha-amylase inhibitor 0.53 OS=Triticum aestivum PE=1 SV=1	IAA5_WHEAT	13689.5	5.23	4	344	100	41.573	322	100	

Peptide Information

Calc. Mass	Obsrv. Mass	± da	± ppm	Start Seq.	End Seq.	Sequence	Ion Score	C. I. %	Modification	Rank	Result Type
1162.6249	1162.7124	0.0875	75	90	100	LTAASITAVCR			Carbamidomethyl (C)[10]		Mascot
1162.6249	1162.7124	0.0875	75	90	100	LTAASITAVCR	58	99.877	Carbamidomethyl (C)[10]		Mascot
1570.8007	1570.9186	0.1179	75	26	39	LQCNGSQVPEAVLR			Carbamidomethyl (C)[3]		Mascot
1663.8361	1663.9296	0.0935	56	101	116	LPIVVDASGDGAYVCK			Carbamidomethyl (C)[15]		Mascot
1663.8361	1663.9296	0.0935	56	101	116	LPIVVDASGDGAYVCK	104	100	Carbamidomethyl (C)[15]		Mascot
1846.8137	1846.9543	0.1406	76	67	84	EHGVSEGQAGTGAFPSC R			Carbamidomethyl (C)[17]		Mascot
1846.8137	1846.9543	0.1406	76	67	84	EHGVSEGQAGTGAFPSC R	159	100	Carbamidomethyl (C)[17]		Mascot

Rank	Protein Name	Accession No.	Protein MW	Protein PI	Pep. Count	Protein Score	Protein Score C. I. %	Intensity Matched	Total Ion Score	Total Ion C. I. %	Confirmed
2	Alpha-amylase inhibitor 0.19 OS=Triticum aestivum PE=1 SV=1	IAA1_WHEAT	13898.6	6.66	5	193	100	16.352	163	100	

Peptide Information

Calc. Mass	Obsrv. Mass	± da	± ppm	Start Seq.	End Seq.	Sequence	Ion Score	C. I. %	Modification	Rank	Result Type
1162.6249	1162.7124	0.0875	75	90	100	LTAASITAVCR			Carbamidomethyl (C)[10]		Mascot
1162.6249	1162.7124	0.0875	75	90	100	LTAASITAVCR	58	99.877	Carbamidomethyl (C)[10]		Mascot
1570.8007	1570.9186	0.1179	75	26	39	LQCNGSQVPEAVLR			Carbamidomethyl (C)[3]		Mascot
1612.7463	1612.8715	0.1252	78	67	82	EHGAQEGQAGTGAFPR					Mascot
1663.8361	1663.9296	0.0935	56	101	116	LPIVVDASGDGAYVCK			Carbamidomethyl (C)[15]		Mascot
1663.8361	1663.9296	0.0935	56	101	116	LPIVVDASGDGAYVCK	104	100	Carbamidomethyl (C)[15]		Mascot
1862.7731	1862.9368	0.1637	88	40	53	DCCQQLAHISEWCR			Carbamidomethyl (C)[2,3,13]		Mascot

Rank	Protein Name	Accession No.	Protein MW	Protein PI	Pep. Count	Protein Score	Protein Score C. I. %	Intensity Matched	Total Ion Score	Total Ion C. I. %	Confirmed
3	50S ribosomal protein L10 OS=Acidithiobacillus ferrooxidans (strain ATCC 53993) GN=rplJ PE=3 SV=1	RL10_ACIF5	18710.4	10.32	9	50	0	3.077			

Protein Group

50S ribosomal protein L10 OS=Acidithiobacillus ferrooxidans (strain ATCC 23270 / DSM 14882 / NCIB 8455) GN=rplJ PE=3 SV=1	RL10_ACIF2	18710.4	10.319 999694 8242

Peptide Information

Calc. Mass	Obsrv. Mass	± da	± ppm	Start Seq.	End Seq.	Sequence	Ion Score	C. I. %	Modification	Rank	Result Type
1023.5945	1023.5461	-0.0484	-47	48	56	QSVHVQVVK					Mascot
1055.6824	1055.6676	-0.0148	-14	105	115	LVIIGGVLSGK					Mascot
1144.6321	1144.6804	0.0483	42	9	18	EQVVATLQTR					Mascot
1475.804	1475.8405	0.0365	25	138	151	LLGTMQAPVAGFVR			Oxidation (M)[5]		Mascot
1583.8615	1583.9053	0.0438	28	63	77	ALAGTPFAVMDHLLK					Mascot
1585.8909	1585.9095	0.0186	12	5	18	LAEKEQVVATLQTR					Mascot
1592.9482	1592.8314	-0.1168	-73	48	61	QSVHVQVVKNTLLK					Mascot
1599.8563	1599.943	0.0867	54	63	77	ALAGTPFAVMDHLLK			Oxidation (M)[10]		Mascot
1749.9316	1749.8699	-0.0617	-35	32	47	GLTVAQMTVFRAEAQK					Mascot
1755.9575	1755.944	-0.0135	-8	62	77	RALAGTPFAVMDHLLK			Oxidation (M)[11]		Mascot

4	Thymidylate kinase OS=Bacillus amyloliquefaciens (strain FZB42) GN=tmk PE=3 SV=1	KTHY_BACA2	23871.5	5.69	3	49	0	3.268	40	92.046	

Peptide Information

Calc. Mass	Obsrv. Mass	± da	± ppm	Start Seq.	End Seq.	Sequence	Ion Score	C. I. %	Modification	Rank	Result Type
997.5789	997.6003	0.0214	21	82	90	VQPALKEGR					Mascot
997.5789	997.6003	0.0214	21	82	90	VQPALKEGR	40	92.046			Mascot
1049.5222	1049.6204	0.0982	94	146	154	ITSNDSREK					Mascot
1593.8496	1593.8453	-0.0043	-3	155	167	NRLDLEALHFHTK					Mascot

5	6,7-dimethyl-8-ribityllumazine synthase OS=Methylobacterium populi (strain ATCC BAA-705 / NCIMB 13946 / BJ001) GN=ribH PE=3 SV=1	RISB_METPB	17569.2	4.99	2	47	0	3.11	41	92.628	

Peptide Information

Calc. Mass	Obsrv. Mass	± da	± ppm	Start Seq.	End Seq.	Sequence	Ion Score	C. I. %	Modification	Rank	Result Type
997.5537	997.6003	0.0466	47	161	169	RAANLEPAR					Mascot
997.5537	997.6003	0.0466	47	161	169	RAANLEPAR	41	92.628			Mascot
1584.754	1584.9103	0.1563	99	30	43	YYDDIADELLAGAR					Mascot

6	6,7-dimethyl-8-ribityllumazine synthase OS=Methylobacterium extorquens (strain PA1) GN=ribH PE=3 SV=1	RISB_METEP	17922.3	4.98	2	47	0	3.11	41	92.628	

Protein Group

6,7-dimethyl-8-ribityllumazine synthase OS=Methylobacterium extorquens (strain CM4 / NCIMB 13688) GN=ribH PE=3 SV=1	RISB_METC4	17922.3	4.9800 000190 7349

Peptide Information

Calc. Mass	Obsrv. Mass	± da	± ppm	Start Seq.	End Seq.	Sequence	Ion Score	C. I. %	Modification	Rank	Result Type
997.5537	997.6003	0.0466	47	165	173	RAANLEPAR					Mascot
997.5537	997.6003	0.0466	47	165	173	RAANLEPAR	41	92.628			Mascot
1584.754	1584.9103	0.1563	99	34	47	YYDDIADELLAGAR					Mascot

7	CinA-like protein OS=Gemmatimonas aurantiaca (strain T-27 / DSM 14586 / JCM 11422 / NBRC 100505) GN=GAU_1122 PE=3 SV=1	CINAL_GEMAT	45610.6	5.39	11	45	0	3.561		

Peptide Information

Calc. Mass	Obsrv. Mass	± da	± ppm	Start Seq.	End Seq.	Sequence	Ion Score	C. I. %	Modification	Rank	Result Type
980.5887	980.5746	-0.0141	-14	174	182	VPKDGPVIR					Mascot
1169.65	1169.7032	0.0532	45	147	156	WVVMLPGVPR			Oxidation (M)[4]		Mascot
1506.6853	1506.8026	0.1173	78	40	54	ATCGDDAESIAAAVR			Carbamidomethyl (C)[3]		Mascot
1569.8392	1569.8976	0.0584	37	147	159	WVVMLPGVPREMR					Mascot
1583.8938	1583.9053	0.0115	7	160	173	GMLADTILPLLRDR					Mascot
1585.8342	1585.9095	0.0753	47	147	159	WVVMLPGVPREMR			Oxidation (M)[4]		Mascot
1599.8887	1599.943	0.0543	34	160	173	GMLADTILPLLRDR			Oxidation (M)[2]		Mascot
1707.8041	1707.9033	0.0992	58	88	102	GMVMDAEVLANLEQR			Oxidation (M)[2,4]		Mascot
1870.9982	1870.9	-0.0982	-52	188	205	TANIAESALADRLGELAR					Mascot
2331.2666	2331.3154	0.0488	21	292	314	LTAIPGSSRTVQGGVIAY ANEVK					Mascot
2331.2666	2331.3154	0.0488	21	292	314	LTAIPGSSRTVQGGVIAY ANEVK					Mascot
2377.2292	2377.2246	-0.0046	-2	315	337	TRELGVPAEMIAAHGAVS EPVAR			Oxidation (M)[10]		Mascot
2393.0459	2393.2302	0.1843	77	253	273	FIYGEGDDDLAALMLSEC AMR			Carbamidomethyl (C)[18], Oxidation (M)[14]		Mascot
2409.0408	2409.2427	0.2019	84	253	273	FIYGEGDDDLAALMLSEC AMR			Carbamidomethyl (C)[18], Oxidation (M)[14,20]		Mascot
2427.2991	2427.2258	-0.0733	-30	225	247	LTSWSLPSRDAEGALTQ AAALLR					Mascot

8	Pre-mRNA-splicing factor SLT11 OS=Kluyveromyces lactis (strain ATCC 8585 / CBS 2359 / DSM 70799 / NBRC 1267 / NRRL Y-1140 / WM37) GN=SLT11 PE=3 SV=1	SLT11_KLULA	35184	9.22	4	45	0	3.845	35	74.439	

Peptide Information

Calc. Mass	Obsrv. Mass	± da	± ppm	Start Seq.	End Seq.	Sequence	Ion Score	C. I. %	Modification	Rank	Result Type
997.5676	997.6003	0.0327	33	114	122	QVAPLNIDK					Mascot

Calc. Mass	Obsrv. Mass	± da	± ppm	Start Seq.	End Seq.	Sequence	Ion Score	C. I. %	Modification	Rank	Result Type
997.5676	997.6003	0.0327	33	114	122	QVAPLNIDK	35	74.439			Mascot
1023.4676	1023.5461	0.0785	77	27	34	YPNGRECK			Carbamidomethyl (C)[7]		Mascot
1553.8066	1553.8959	0.0893	57	216	228	VGPTVEKCYLLMK			Carbamidomethyl (C)[8], Oxidation (M)[12]		Mascot
1900.9797	1900.8883	-0.0914	-48	84	100	NSMKQLLNENADSVIPK					Mascot

9	DNA polymerase I OS=Bacillus caldotenax GN=polA PE=1 SV=1	DPO1_BACCA	99527	5.49	14	44	0	9.876	

Peptide Information

Calc. Mass	Obsrv. Mass	± da	± ppm	Start Seq.	End Seq.	Sequence	Ion Score	C. I. %	Modification	Rank	Result Type
997.5425	997.6003	0.0578	58	678	685	RDLDIHTK					Mascot
997.5425	997.6003	0.0578	58	678	685	RDLDIHTK	17	0			Mascot
1144.6508	1144.6804	0.0296	26	808	817	AMIDLNARLK					Mascot
1215.6957	1215.7262	0.0305	25	762	771	GYVTTLLHRR					Mascot
1585.8446	1585.9095	0.0649	41	603	616	VHTIFNQALTQTGR					Mascot
1594.8336	1594.8717	0.0381	24	773	785	YLPDITSRNFNVR					Mascot
1660.8397	1660.9456	0.1059	64	791	806	MAMNTPIQGSAADIIK					Mascot
1663.8286	1663.9296	0.101	61	121	135	AEQEGFEVKVISGDR					Mascot
1663.8286	1663.9296	0.101	61	121	135	AEQEGFEVKVISGDR					Mascot
1701.8589	1701.9208	0.0619	36	499	512	RLEQMGEELAEQLR					Mascot
1731.0051	1730.9758	-0.0293	-17	825	839	LLLQVHDELILEAPK					Mascot
1820.9244	1820.9926	0.0682	37	791	807	MAMNTPIQGSAADIIKK			Oxidation (M)[1,3]		Mascot
1828.8721	1828.9471	0.075	41	29	44	GIHTNAVYGFTMMLNK			Oxidation (M)[12,13]		Mascot
1828.8721	1828.9471	0.075	41	29	44	GIHTNAVYGFTMMLNK			Oxidation (M)[12,13]		Mascot
1855.0365	1854.9142	-0.1223	-66	269	284	VVALFKELGFQSFLEK					Mascot
1871.0175	1870.9	-0.1175	-63	451	466	AAAIWALERPFLDELR					Mascot
2336.188	2336.2432	0.0552	24	513	532	TVEQRIYELAGQEFNINS PK					Mascot

10	Intraflagellar transport protein 43 homolog A OS=Salmo salar GN=ift43a PE=2 SV=1	IF43A_SALSA	23125.5	4.46	8	44	0	1.825	

Peptide Information

Calc. Mass	Obsrv. Mass	± da	± ppm	Start Seq.	End Seq.	Sequence	Ion Score	C. I. %	Modification	Rank	Result Type
1023.5721	1023.5461	-0.026	-25	199	207	EEQVPLPVL					Mascot
1495.8843	1495.8715	-0.0128	-9	158	170	LLTKVLAPEQEVR					Mascot
1506.7104	1506.8026	0.0922	61	1	14	MDDNLQLGDSGVVK			Oxidation (M)[1]		Mascot
1584.809	1584.9103	0.1013	64	73	86	SGRRPAVVEDVEDR					Mascot

1599.7902	1599.943	0.1528	96	144	157	YSAFQTLDGEIDLK			Mascot
1730.8378	1730.9758	0.138	80	42	58	SSSSTSMGEVPPPKPAR		Oxidation (M)[7]	Mascot
1858.9327	1858.9229	-0.0098	-5	41	58	KSSSSTSMGEVPPPKPA R		Oxidation (M)[8]	Mascot
1870.944	1870.9	-0.044	-24	42	59	SSSSTSMGEVPPPKPAR R			Mascot

Gel Idx/Pos	138/F13	Instr./Gel Origin	BA2151/Sample Project 20140814	Process Status	Analysis Succeeded
Plate [#] Name	[1] Sample Project 20140814	Instrument Sample Name		Spectra	11

Rank	Protein Name	Accession No.	Protein MW	Protein PI	Pep. Count	Protein Score	Protein Score C. I. %	Intensity Matched	Total Ion Score	Total Ion C. I. %	Confirmed
1	Alpha-amylase/trypsin inhibitor CMb OS=Hordeum vulgare GN=IAT2 PE=1 SV=2	IAAB_HORVU	17199.2	5.77	6	302	100	37.252	268	100	

Peptide Information

Calc. Mass	Obsrv. Mass	± da	± ppm	Start Seq.	End Seq.	Sequence	Ion Score	C. I. %	Modification	Rank	Result Type
801.4076	801.4696	0.062	77	86	91	FFMGRK			Oxidation (M)[3]		Mascot
1023.4928	1023.5602	0.0674	66	108	115	EVQMDFVR					Mascot
1023.4928	1023.5602	0.0674	66	108	115	EVQMDFVR	20	0			Mascot
1039.4878	1039.5409	0.0531	51	108	115	EVQMDFVR			Oxidation (M)[4]		Mascot
1039.4878	1039.5409	0.0531	51	108	115	EVQMDFVR	36	70.678	Oxidation (M)[4]		Mascot
1168.5052	1168.5846	0.0794	68	46	54	DYVEQQACR			Carbamidomethyl (C)[8]		Mascot
1168.5052	1168.5846	0.0794	68	46	54	DYVEQQACR	74	99.996	Carbamidomethyl (C)[8]		Mascot
1285.6293	1285.7178	0.0885	69	81	90	CQALRFFMGR			Carbamidomethyl (C)[1]		Mascot
1799.8528	1799.9772	0.1244	69	92	107	SRPDQSGLMELPGCPR			Carbamidomethyl (C)[14]		Mascot
1815.8477	1815.9524	0.1047	58	92	107	SRPDQSGLMELPGCPR			Carbamidomethyl (C)[14], Oxidation (M)[9]		Mascot
1815.8477	1815.9524	0.1047	58	92	107	SRPDQSGLMELPGCPR	46	97.504	Carbamidomethyl (C)[14], Oxidation (M)[9]		Mascot
1861.8102	1861.9398	0.1296	70	66	80	QQCCGELANIPQQCR			Carbamidomethyl (C)[3,4,14]		Mascot
1861.8102	1861.9398	0.1296	70	66	80	QQCCGELANIPQQCR	112	100	Carbamidomethyl (C)[3,4,14]		Mascot

Rank	Protein Name	Accession No.	Protein MW	Protein PI	Pep. Count	Protein Score	Protein Score C. I. %	Intensity Matched	Total Ion Score	Total Ion C. I. %	Confirmed
2	Alpha-amylase/trypsin inhibitor CM16 OS=Triticum aestivum PE=1 SV=1	IAC16_WHEAT	16398.8	5.31	5	296	100	37.2	268	100	

Peptide Information

Calc. Mass	Obsrv. Mass	± da	± ppm	Start Seq.	End Seq.	Sequence	Ion Score	C. I. %	Modification	Rank	Result Type
1023.4928	1023.5602	0.0674	66	108	115	EVQMDFVR					Mascot
1023.4928	1023.5602	0.0674	66	108	115	EVQMDFVR	20	0			Mascot
1039.4878	1039.5409	0.0531	51	108	115	EVQMDFVR			Oxidation (M)[4]		Mascot
1039.4878	1039.5409	0.0531	51	108	115	EVQMDFVR	36	70.678	Oxidation (M)[4]		Mascot
1168.5052	1168.5846	0.0794	68	46	54	DYVEQQACR			Carbamidomethyl (C)[8]		Mascot
1168.5052	1168.5846	0.0794	68	46	54	DYVEQQACR	74	99.996	Carbamidomethyl (C)[8]		Mascot
1175.6307	1175.6776	0.0469	40	55	65	IETPGSPYLAK					Mascot
1799.8528	1799.9772	0.1244	69	92	107	SRPDQSGLMELPGCPR			Carbamidomethyl (C)[14]		Mascot

1815.8477	1815.9524	0.1047	58	92	107	SRPDQSGLMELPGCPR			Carbamidomethyl (C)[14], Oxidation (M)[9]		Mascot
1815.8477	1815.9524	0.1047	58	92	107	SRPDQSGLMELPGCPR	46	97.504	Carbamidomethyl (C)[14], Oxidation (M)[9]		Mascot
1861.8102	1861.9398	0.1296	70	66	80	QQCCGELANIPQQCR			Carbamidomethyl (C)[3,4,14]		Mascot
1861.8102	1861.9398	0.1296	70	66	80	QQCCGELANIPQQCR	112	100	Carbamidomethyl (C)[3,4,14]		Mascot

3	RNA-directed RNA polymerase L OS=Avian metapneumovirus (isolate Canada goose/Minnesota/15a/2001) GN=L PE=3 SV=1	L_AMPV1	214431.8	9.11	32	68	90.603	19.643

Peptide Information

Calc. Mass	Obsrv. Mass	± da	± ppm	Start Seq.	End Seq.	Sequence	Ion Score	C. I. %	Modification	Rank	Result Type
810.4832	810.4111	-0.0721	-89	784	790	DIGHKLK					Mascot
858.5295	858.4742	-0.0553	-64	1376	1383	LDLVTLGK					Mascot
956.6251	956.5419	-0.0832	-87	342	349	TIRLLVNK					Mascot
974.5153	974.5739	0.0586	60	757	765	LSQGVDEVK					Mascot
1005.5588	1005.5474	-0.0114	-11	60	67	LRNAFQTR					Mascot
1023.5656	1023.5602	-0.0054	-5	1511	1518	RVMLFDVK			Oxidation (M)[3]		Mascot
1023.5656	1023.5602	-0.0054	-5	1511	1518	RVMLFDVK			Oxidation (M)[3]		Mascot
1061.5474	1061.5189	-0.0285	-27	123	132	STSENPSVLK					Mascot
1077.5588	1077.5746	0.0158	15	1760	1767	THWDLIHR					Mascot
1077.5588	1077.5746	0.0158	15	1760	1767	THWDLIHR					Mascot
1184.598	1184.5673	-0.0307	-26	1833	1843	SIATYVMQGSK					Mascot
1190.5648	1190.559	-0.0058	-5	1079	1089	TSAINGEDIDR					Mascot
1210.65	1210.6742	0.0242	20	1237	1247	ICIGSLGISYK			Carbamidomethyl (C)[2]		Mascot
1230.7417	1230.6882	-0.0535	-43	888	898	QLSKTLAITQK					Mascot
1508.7665	1508.8304	0.0639	42	1771	1783	DALLITLCDAEFK			Carbamidomethyl (C)[8]		Mascot
1566.7767	1566.777	0.0003	0	557	570	ELSVGRMFAMQPGK			Oxidation (M)[7]		Mascot
1570.7384	1570.8727	0.1343	86	683	696	HAPPETEGVYDIDK					Mascot
1605.7466	1605.8197	0.0731	46	501	513	FTEEMFNSSEKLK			Oxidation (M)[5]		Mascot
1679.9401	1679.9097	-0.0304	-18	1233	1247	LLDKICIGSLGISYK			Carbamidomethyl (C)[6]		Mascot
1696.9017	1696.9159	0.0142	8	630	644	ASIVTDLSKFNQAFR					Mascot
1733.8251	1733.9413	0.1162	67	1650	1662	NYMVMLPWQHINR			Oxidation (M)[3,5]		Mascot
1769.9467	1769.9449	-0.0018	-1	466	481	TNLEMVLNDKAISPPK					Mascot
1785.9415	1785.963	0.0215	12	466	481	TNLEMVLNDKAISPPK			Oxidation (M)[5]		Mascot
1789.9769	1789.9257	-0.0512	-29	718	733	LWTMEAVSLLDVVSVK					Mascot
1802.9283	1802.9677	0.0394	22	539	554	QEYLGDKEHVVSLTGK					Mascot
1803.8582	1803.9585	0.1003	56	791	805	EGETYISRDLQFMSK					Mascot
1837.8485	1837.9198	0.0713	39	1742	1759	VIDGGEGLSMDTTDATQ					Mascot

						K					
1842.9596	1842.9612	0.0016	1	1360	1375	LVEKISSDQHIFSPDK					Mascot
1861.9437	1861.9398	-0.0039	-2	977	993	ATLTTLMRDPQAVGSER			Oxidation (M)[7]		Mascot
1861.9437	1861.9398	-0.0039	-2	977	993	ATLTTLMRDPQAVGSER			Oxidation (M)[7]		Mascot
1872.9962	1872.9381	-0.0581	-31	175	191	TIGSIMFIISSFGCVIK			Carbamidomethyl (C)[14]		Mascot
1883.86	1883.8893	0.0293	16	1439	1454	EWGDGFVTDHAFIDFK					Mascot
1887.9885	1887.9604	-0.0281	-15	935	951	TPDFLTEAVSHMSVLLK					Mascot
1890.0477	1889.9658	-0.0819	-43	1062	1078	VVNMISGTKSITNLLQR			Oxidation (M)[4]		Mascot
1994.0165	1994.1213	0.1048	53	1265	1282	LSVSSRPMEFPASVPAY<br>R					Mascot
2836.3799	2836.6248	0.2449	86	905	929	ETDVVNLWMNVPMQFG<br>GGDPVVLYR					Mascot

4	ATP synthase gamma chain OS=Carboxydothermus hydrogenoformans (strain Z-2901 / DSM 6008) GN=atpG PE=3 SV=1	ATPG_CARHZ	31150.6	9.52	12	66	87.613	9.67

Peptide Information

Calc. Mass	Obsrv. Mass	± da	± ppm	Start Seq.	End Seq.	Sequence	Ion Score	C. I. %	Modification	Rank	Result Type
832.4523	832.3814	-0.0709	-85	16	22	NTQQITK					Mascot
858.5155	858.4742	-0.0413	-48	261	268	ARQAAITK					Mascot
874.4563	874.3775	-0.0788	-90	2	8	PSMRDLR					Mascot
1005.4968	1005.5474	0.0506	50	1	8	MPSMRDLR					Mascot
1021.4917	1021.5346	0.0429	42	1	8	MPSMRDLR			Oxidation (M)[1]		Mascot
1023.5543	1023.5602	0.0059	6	147	155	VIAGFIMEK			Oxidation (M)[7]		Mascot
1023.5543	1023.5602	0.0059	6	147	155	VIAGFIMEK			Oxidation (M)[7]		Mascot
1055.5521	1055.5548	0.0027	3	216	223	YVEVQVYR					Mascot
1179.6368	1179.6786	0.0418	35	75	85	ATGYVLITADR					Mascot
1605.853	1605.8197	-0.0333	-21	35	48	AQEMVVASRPYAKR					Mascot
1663.949	1663.8829	-0.0661	-40	56	71	AAAATTETAHPLLIKR					Mascot
1696.9269	1696.9159	-0.011	-6	216	229	YVEVQVYRALLESK					Mascot
1844.8075	1844.9202	0.1127	61	237	253	MTAMDTATENATEMIAK			Oxidation (M)[1]		Mascot
1844.8075	1844.9202	0.1127	61	237	253	MTAMDTATENATEMIAK			Oxidation (M)[1]		Mascot
2452.2766	2452.1951	-0.0815	-33	75	97	ATGYVLITADRGLCGGFN<br>ANLIR			Carbamidomethyl (C)[14]		Mascot

5	Exodeoxyribonuclease 7 large subunit OS=Streptococcus mutans serotype c (strain ATCC 700610 / UA159) GN=xseA PE=3 SV=1	EX7L_STRMU	51172.1	9.1	16	61	58.026	15.233

Peptide Information

Calc. Mass	Obsrv. Mass	± da	± ppm	Start Seq.	End Seq.	Sequence	Ion Score	C. I. %	Modification	Rank	Result Type

Calc. Mass	Obsrv. Mass	± da	± ppm	Start Seq.	End Seq.	Sequence	Ion Score	C. I. %	Modification	Rank	Result Type
874.4628	874.3775	-0.0853	-98	288	294	QKDEQVK					Mascot
1005.584	1005.5474	-0.0366	-36	129	136	QRLSQFVK					Mascot
1168.5555	1168.5846	0.0291	25	66	75	LGFELEEGMK			Oxidation (M)[9]		Mascot
1168.5555	1168.5846	0.0291	25	66	75	LGFELEEGMK			Oxidation (M)[9]		Mascot
1175.6783	1175.6776	-0.0007	-1	265	274	ADLLAFLKER					Mascot
1210.5852	1210.6742	0.089	74	18	26	FDRDPYLER					Mascot
1285.614	1285.7178	0.1038	81	275	284	QMRSYQAVMR			Oxidation (M)[2]		Mascot
1424.7493	1424.7662	0.0169	12	409	420	DNHIIQSTQQIK					Mascot
1445.7761	1445.7721	-0.004	-3	38	48	RRPNHQYFSLK					Mascot
1508.7413	1508.8304	0.0891	59	367	379	LLLSHMSSQYDSK					Mascot
1661.8395	1661.9397	0.1002	60	39	51	RPNHQYFSLKDEK					Mascot
1707.8912	1707.9062	0.015	9	383	397	FEKAQDALLSLDTTR					Mascot
1734.8983	1734.9948	0.0965	56	1	15	MSDYLSVSSLTKYLK					Mascot
1785.9381	1785.963	0.0249	14	98	114	AEPDGIGALAVQFEQLK					Mascot
1804.9626	1804.9165	-0.0461	-26	66	81	LGFELEEGMKINVIGR					Mascot
1883.932	1883.8893	-0.0427	-23	49	64	DEKAVIQATMWSGIYR			Oxidation (M)[10]		Mascot
2384.3435	2384.1272	-0.2163	-91	249	272	AATPTAAAELATPVTKAD LLAFLK					Mascot

6	Threonine--tRNA ligase OS=Rickettsia peacockii (strain Rustic) GN=thrS PE=3 SV=1	SYT_RICPU	72779.1	5.52	17	61	57.048	10.171

Peptide Information

Calc. Mass	Obsrv. Mass	± da	± ppm	Start Seq.	End Seq.	Sequence	Ion Score	C. I. %	Modification	Rank	Result Type
810.3992	810.4111	0.0119	15	287	293	NGYTEVK					Mascot
828.5049	828.468	-0.0369	-45	602	608	NKQVAIR					Mascot
858.5043	858.4742	-0.0301	-35	512	519	AILGSLER					Mascot
908.5247	908.4642	-0.0605	-67	505	511	RPVMLHR					Mascot
1005.4636	1005.5474	0.0838	83	427	435	AGSNEVWDK					Mascot
1021.5425	1021.5346	-0.0079	-8	581	588	IREFSNQK					Mascot
1190.6416	1190.559	-0.0826	-69	2	12	INISFPDGSIK					Mascot
1210.6401	1210.6742	0.0341	28	197	206	LMKVAGAYWR			Oxidation (M)[2]		Mascot
1237.6073	1237.6249	0.0176	14	200	210	VAGAYWRGDSR					Mascot
1321.6821	1321.713	0.0309	23	1	12	MINISFPDGSIK					Mascot
1445.7133	1445.7721	0.0588	41	131	141	NEQVTRELWDR					Mascot
1770.9174	1770.9294	0.012	7	269	282	GWSIYNTIEQYIRK					Mascot
1770.9174	1770.9294	0.012	7	269	282	GWSIYNTIEQYIRK					Mascot

1781.9003	1781.9882	0.0879	49	211	225	NEMLQRIYGTAWATK					Mascot
1795.9259	1795.907	-0.0189	-11	17	33	NITAYEVANAISMSLAK					Mascot
1797.8953	1798.0042	0.1089	61	211	225	NEMLQRIYGTAWATK			Oxidation (M)[3]		Mascot
1814.0059	1813.9893	-0.0166	-9	157	173	AEIIASIPAGEPITLYR					Mascot
2373.1008	2373.2698	0.169	71	354	374	MSEFGLCHRNEASGALH GLMR			Carbamidomethyl (C)[7]		Mascot
2389.0959	2389.271	0.1751	73	354	374	MSEFGLCHRNEASGALH GLMR			Carbamidomethyl (C)[7], Oxidation (M)[1]		Mascot
2405.0908	2405.2759	0.1851	77	354	374	MSEFGLCHRNEASGALH GLMR			Carbamidomethyl (C)[7], Oxidation (M)[1,20]		Mascot
2480.2227	2480.2866	0.0639	26	109	130	DTPFTTDDLAVIEAKMQE LSQK					Mascot
2496.2175	2496.2991	0.0816	33	109	130	DTPFTTDDLAVIEAKMQE LSQK			Oxidation (M)[16]		Mascot

7	Exocyst complex protein EXO70 OS=Debaryomyces hansenii (strain ATCC 36239 / CBS 767 / JCM 1990 / NBRC 0083 / IGC 2968) GN=EXO70 PE=3 SV=2	EXO70_DEBHA	70361	9.04	12	58	8.171	13.36	28	0

Peptide Information

Calc. Mass	Obsrv. Mass	± da	± ppm	Start Seq.	End Seq.	Sequence	Ion Score	C. I. %	Modification	Rank	Result Type
828.4614	828.468	0.0066	8	597	602	YIKYNK					Mascot
911.4291	911.5076	0.0785	86	222	228	YNNEMIK					Mascot
1023.5656	1023.5602	-0.0054	-5	177	184	TIDKIFMR					Mascot
1023.5656	1023.5602	-0.0054	-5	177	184	TIDKIFMR					Mascot
1039.5605	1039.5409	-0.0196	-19	177	184	TIDKIFMR			Oxidation (M)[7]		Mascot
1039.5605	1039.5409	-0.0196	-19	177	184	TIDKIFMR	28	0	Oxidation (M)[7]		Mascot
1179.662	1179.6786	0.0166	14	368	377	LSEYNISLLK					Mascot
1230.6552	1230.6882	0.033	27	447	456	KSMQGYYLIK					Mascot
1285.7474	1285.7178	-0.0296	-23	457	467	NTILIETIVNR					Mascot
1663.9418	1663.8829	-0.0589	-35	121	134	FKGILINFENLIDK					Mascot
1804.0215	1803.9585	-0.063	-35	84	99	YEAVLNNSIELIGLKK					Mascot
1844.8813	1844.9202	0.0389	21	536	550	DLFKNFNESFEDALR					Mascot
1844.8813	1844.9202	0.0389	21	536	550	DLFKNFNESFEDALR					Mascot
1875.8508	1875.9397	0.0889	47	540	554	NFNESFEDALRNYEK					Mascot
2063.0889	2063.1348	0.0459	22	190	206	MAEIMRPHEEPTKPIKR					Mascot
2432.2668	2432.3628	0.096	39	2	23	SFNVDIDEADVAVLNQNL IKSK					Mascot

8	Threonine--tRNA ligase OS=Rickettsia conorii (strain ATCC VR-613 / Malish 7) GN=thrS PE=3 SV=1	SYT_RICCN	72820	5.42	16	58	8.171	9.761	

Peptide Information

Calc. Mass	Obsrv. Mass	± da	± ppm	Start Seq.	End Seq.	Sequence	Ion Score	C. I. %	Modification	Rank	Result Type

Calc. Mass	Obsrv. Mass	± da	± ppm	Start Seq.	End Seq.	Sequence	Ion Score	C. I. %	Modification	Rank	Result Type
810.3992	810.4111	0.0119	15	287	293	NGYTEVK					Mascot
858.5043	858.4742	-0.0301	-35	512	519	AILGSLER					Mascot
908.5247	908.4642	-0.0605	-67	505	511	RPVMLHR					Mascot
1005.4636	1005.5474	0.0838	83	427	435	AGSNEVWDK					Mascot
1021.5425	1021.5346	-0.0079	-8	581	588	IREFSNQK					Mascot
1190.6416	1190.559	-0.0826	-69	2	12	INISFPDGSIK					Mascot
1210.6401	1210.6742	0.0341	28	197	206	LMKVAGAYWR			Oxidation (M)[2]		Mascot
1237.6073	1237.6249	0.0176	14	200	210	VAGAYWRGDSR					Mascot
1321.6821	1321.713	0.0309	23	1	12	MINISFPDGSIK					Mascot
1445.7133	1445.7721	0.0588	41	131	141	NEQVTRELWDR					Mascot
1662.8585	1662.9066	0.0481	29	109	123	DKPFTTDDLAVIEAK					Mascot
1770.9174	1770.9294	0.012	7	269	282	GWSIYNTIEQYIRK					Mascot
1770.9174	1770.9294	0.012	7	269	282	GWSIYNTIEQYIRK					Mascot
1781.9003	1781.9882	0.0879	49	211	225	NEMLQRIYGTAWATK					Mascot
1795.9259	1795.907	-0.0189	-11	17	33	NITAYEVANAISMSLAK					Mascot
1797.8953	1798.0042	0.1089	61	211	225	NEMLQRIYGTAWATK			Oxidation (M)[3]		Mascot
1814.0059	1813.9893	-0.0166	-9	157	173	AEIIASIPAGEPITLYR					Mascot
2373.1008	2373.2698	0.169	71	354	374	MSEFGLCHRNEASGALH GLMR			Carbamidomethyl (C)[7]		Mascot
2389.0959	2389.271	0.1751	73	354	374	MSEFGLCHRNEASGALH GLMR			Carbamidomethyl (C)[7], Oxidation (M)[1]		Mascot
2405.0908	2405.2759	0.1851	77	354	374	MSEFGLCHRNEASGALH GLMR			Carbamidomethyl (C)[7], Oxidation (M)[1,20]		Mascot

9	Probable tRNA pseudouridine synthase B OS=Nanoarchaeum equitans (strain Kin4-M) GN=truB PE=3 SV=1	TRUB_NANEQ	39519.3	9.2	11	56	0	9.226	

Peptide Information

Calc. Mass	Obsrv. Mass	± da	± ppm	Start Seq.	End Seq.	Sequence	Ion Score	C. I. %	Modification	Rank	Result Type
810.4542	810.4111	-0.0431	-53	165	170	KVYCIK			Carbamidomethyl (C)[4]		Mascot
908.4692	908.4642	-0.005	-6	2	8	LCLSLMR			Carbamidomethyl (C)[2], Oxidation (M)[6]		Mascot
1023.5148	1023.5602	0.0454	44	1	8	MLCLSLMR			Carbamidomethyl (C)[3]		Mascot
1023.5148	1023.5602	0.0454	44	1	8	MLCLSLMR			Carbamidomethyl (C)[3]		Mascot
1039.5098	1039.5409	0.0311	30	1	8	MLCLSLMR			Carbamidomethyl (C)[3], Oxidation (M)[1]		Mascot
1039.5098	1039.5409	0.0311	30	1	8	MLCLSLMR	11	0	Carbamidomethyl (C)[3], Oxidation (M)[7]		Mascot
1055.5046	1055.5548	0.0502	48	1	8	MLCLSLMR			Carbamidomethyl (C)[3], Oxidation (M)[1,7]		Mascot
1179.6595	1179.6786	0.0191	16	333	342	VFMKPGLYPK					Mascot
1210.6361	1210.6742	0.0381	31	202	212	LGVGAHMQELR					Mascot

1508.8835	1508.8304	-0.0531	-35	249	261	VFLPVEEAVKHLK					Mascot
1803.0198	1802.9677	-0.0521	-29	139	154	VMSKFVGTIIQTPPLR			Oxidation (M)[2]		Mascot
1883.9547	1883.8893	-0.0654	-35	333	348	VFMKPGLYPKMWVSQG			Oxidation (M)[3]		Mascot
2063.0764	2063.1348	0.0584	28	304	322	GELVAIGIALMDSKEMLEK			Oxidation (M)[11]		Mascot
2405.4741	2405.2759	-0.1982	-82	96	119	VTGVLPIAIGEATKVLQTLLIAGK					Mascot
2480.3794	2480.2866	-0.0928	-37	294	317	GDLVSIHTLKGELVAIGIALMDSK					Mascot
2496.3743	2496.2991	-0.0752	-30	294	317	GDLVSIHTLKGELVAIGIALMDSK			Oxidation (M)[21]		Mascot

10	Putative sporulation transcription regulator WhiA OS=Bacillus clausii (strain KSM-K16) GN=whiA PE=3 SV=1	WHIA_BACSK	37196.7	9.57	10	55	0	13.742	22	0

Peptide Information

Calc. Mass	Obsrv. Mass	± da	± ppm	Start Seq.	End Seq.	Sequence	Ion Score	C. I. %	Modification	Rank	Result Type
911.4404	911.5076	0.0672	74	83	89	NNVYMVR			Oxidation (M)[5]		Mascot
956.5887	956.5419	-0.0468	-49	262	269	LQEVAKLR					Mascot
991.4738	991.5533	0.0795	80	216	223	DMRNSVNR					Mascot
1021.5425	1021.5346	-0.0079	-8	308	316	LRNGTFDAK					Mascot
1023.5404	1023.5602	0.0198	19	82	89	KNNVYMVR					Mascot
1023.5404	1023.5602	0.0198	19	82	89	KNNVYMVR					Mascot
1039.5354	1039.5409	0.0055	5	82	89	KNNVYMVR			Oxidation (M)[6]		Mascot
1039.5354	1039.5409	0.0055	5	82	89	KNNVYMVR	22	0	Oxidation (M)[6]		Mascot
1111.4758	1111.558	0.0822	74	279	288	ELGEMMQGGK			Oxidation (M)[5,6]		Mascot
1845.0746	1844.9202	-0.1544	-84	63	77	HLYPNIHIELLVQKK					Mascot
1845.0746	1844.9202	-0.1544	-84	63	77	HLYPNIHIELLVQKK					Mascot
1890.0114	1889.9658	-0.0456	-24	23	40	AELSALIRMNGSLSLGNK			Oxidation (M)[9]		Mascot
2432.2637	2432.3628	0.0991	41	10	30	ELTQLELVSCCARAELSALIR			Carbamidomethyl (C)[10,11]		Mascot
2448.4377	2448.3171	-0.1206	-49	57	76	IYTLIKHLYPNIHIELLVQK					Mascot

Gel Idx/Pos	139/F14	Instr./Gel Origin	BA2151/Sample Project 20140814	Process Status	Analysis Succeeded
Plate [#] Name	[1] Sample Project 20140814	Instrument Sample Name		Spectra	11

Rank	Protein Name	Accession No.	Protein MW	Protein PI	Pep. Count	Protein Score	Protein Score C. I. %	Intensity Matched	Total Ion Score	Total Ion C. I. %	Confirmed
1	Alpha-amylase/trypsin inhibitor CMb OS=Hordeum vulgare GN=IAT2 PE=1 SV=2	IAAB_HORVU	17199.2	5.77	6	185	100	17.133	133	100	

Peptide Information

Calc. Mass	Obsrv. Mass	± da	± ppm	Start Seq.	End Seq.	Sequence	Ion Score	C. I. %	Modification	Rank	Result Type
801.4076	801.452	0.0444	55	86	91	FFMGRK			Oxidation (M)[3]		Mascot
1023.4928	1023.5446	0.0518	51	108	115	EVQMDFVR					Mascot
1039.4878	1039.5559	0.0681	66	108	115	EVQMDFVR			Oxidation (M)[4]		Mascot
1039.4878	1039.5559	0.0681	66	108	115	EVQMDFVR	8	0	Oxidation (M)[4]		Mascot
1168.5052	1168.6005	0.0953	82	46	54	DYVEQQACR			Carbamidomethyl (C)[8]		Mascot
1168.5052	1168.6005	0.0953	82	46	54	DYVEQQACR	57	99.8	Carbamidomethyl (C)[8]		Mascot
1285.6293	1285.7179	0.0886	69	81	90	CQALRFFMGR			Carbamidomethyl (C)[1]		Mascot
1799.8528	1800.002	0.1492	83	92	107	SRPDQSGLMELPGCPR			Carbamidomethyl (C)[14]		Mascot
1815.8477	1815.9718	0.1241	68	92	107	SRPDQSGLMELPGCPR			Carbamidomethyl (C)[14], Oxidation (M)[9]		Mascot
1815.8477	1815.9718	0.1241	68	92	107	SRPDQSGLMELPGCPR	10	0	Carbamidomethyl (C)[14], Oxidation (M)[9]		Mascot
1861.8102	1861.9623	0.1521	82	66	80	QQCCGELANIPQQCR			Carbamidomethyl (C)[3,4,14]		Mascot
1861.8102	1861.9623	0.1521	82	66	80	QQCCGELANIPQQCR	76	99.998	Carbamidomethyl (C)[3,4,14]		Mascot

Rank	Protein Name	Accession No.	Protein MW	Protein PI	Pep. Count	Protein Score	Protein Score C. I. %	Intensity Matched	Total Ion Score	Total Ion C. I. %	Confirmed
2	Alpha-amylase/trypsin inhibitor CM16 OS=Triticum aestivum PE=1 SV=1	IAC16_WHEAT	16398.8	5.31	5	178	100	16.726	133	100	

Peptide Information

Calc. Mass	Obsrv. Mass	± da	± ppm	Start Seq.	End Seq.	Sequence	Ion Score	C. I. %	Modification	Rank	Result Type
1023.4928	1023.5446	0.0518	51	108	115	EVQMDFVR					Mascot
1039.4878	1039.5559	0.0681	66	108	115	EVQMDFVR			Oxidation (M)[4]		Mascot
1039.4878	1039.5559	0.0681	66	108	115	EVQMDFVR	8	0	Oxidation (M)[4]		Mascot
1168.5052	1168.6005	0.0953	82	46	54	DYVEQQACR			Carbamidomethyl (C)[8]		Mascot
1168.5052	1168.6005	0.0953	82	46	54	DYVEQQACR	57	99.8	Carbamidomethyl (C)[8]		Mascot
1175.6307	1175.6857	0.055	47	55	65	IETPGSPYLAK					Mascot
1799.8528	1800.002	0.1492	83	92	107	SRPDQSGLMELPGCPR			Carbamidomethyl (C)[14]		Mascot
1815.8477	1815.9718	0.1241	68	92	107	SRPDQSGLMELPGCPR			Carbamidomethyl (C)[14], Oxidation (M)[9]		Mascot
1815.8477	1815.9718	0.1241	68	92	107	SRPDQSGLMELPGCPR	10	0	Carbamidomethyl (C)[14], Oxidation (M)[9]		Mascot

1861.8102	1861.9623	0.1521	82	66	80	QQCCGELANIPQQCR			Carbamidomethyl (C)[3,4,14]		Mascot
1861.8102	1861.9623	0.1521	82	66	80	QQCCGELANIPQQCR	76	99.998	Carbamidomethyl (C)[3,4,14]		Mascot

3	UDP-N-acetylenolpyruvoylglucosamine reductase OS=Prochlorococcus marinus (strain NATL2A) GN=murB PE=3 SV=1	MURB_PROMT	32301	9.38	12	61	57.048	10.496

Peptide Information

Calc. Mass	Obsrv. Mass	± da	± ppm	Start Seq.	End Seq.	Sequence	Ion Score	C. I. %	Modification	Rank	Result Type
808.4199	808.39	-0.0299	-37	239	246	FGGAEISK					Mascot
831.4934	831.4185	-0.0749	-90	2	8	NSIKLEK					Mascot
836.4117	836.3975	-0.0142	-17	68	74	GLSLCMR			Carbamidomethyl (C)[5]		Mascot
884.4261	884.4411	0.015	17	159	165	DLNFGYR					Mascot
1037.5415	1037.5526	0.0111	11	38	45	YVINWTNK					Mascot
1165.6365	1165.6744	0.0379	33	38	46	YVINWTNKK					Mascot
1168.611	1168.6005	-0.0105	-9	236	246	GFRFGGAEISK					Mascot
1168.611	1168.6005	-0.0105	-9	236	246	GFRFGGAEISK					Mascot
1184.7249	1184.6152	-0.1097	-93	225	235	AAKLIEELGLK					Mascot
1612.8469	1612.8801	0.0332	21	274	287	VFDSYGILLETEVK					Mascot
1770.9419	1770.9562	0.0143	8	48	64	IPCSVIGAGSNLLINDK			Carbamidomethyl (C)[3]		Mascot
1770.9419	1770.9562	0.0143	8	48	64	IPCSVIGAGSNLLINDK			Carbamidomethyl (C)[3]		Mascot
1883.9432	1883.9255	-0.0177	-9	203	218	LKTQPYQAQTCGSVFR			Carbamidomethyl (C)[11]		Mascot
1927.0317	1926.9982	-0.0335	-17	84	101	NNGIIEVLSGEMLPTLAR					Mascot

4	Flagellum-specific ATP synthase OS=Treponema pallidum (strain Nichols) GN=fliI PE=3 SV=1	FLII_TREPA	48050.3	6.23	14	58	12.304	11.396

Peptide Information

Calc. Mass	Obsrv. Mass	± da	± ppm	Start Seq.	End Seq.	Sequence	Ion Score	C. I. %	Modification	Rank	Result Type
806.4189	806.3947	-0.0242	-30	143	149	QMVTGVR			Oxidation (M)[2]		Mascot
842.5206	842.5838	0.0632	75	369	375	EAVRIVR					Mascot
1001.5527	1001.5838	0.0311	31	21	29	YVGHVTAVR					Mascot
1011.4962	1011.5402	0.044	43	174	182	STLMGMIAR			Oxidation (M)[4,6]		Mascot
1021.5677	1021.5485	-0.0192	-19	163	173	LGIFSGSGVGK					Mascot
1165.6688	1165.6744	0.0056	5	328	338	GIVDGHIVLSR					Mascot
1175.6201	1175.6857	0.0656	56	404	413	AIAMRAELER			Oxidation (M)[4]		Mascot
1232.6998	1232.6945	-0.0053	-4	105	116	VLNAFGKAIDGK					Mascot
1475.7272	1475.8579	0.1307	89	125	137	SEVLRASSNPMER					Mascot

1770.0021	1770.0162	0.0141	8	21	36	YVGHVTAVRGLLIESR					Mascot
1772.8524	1772.9491	0.0967	55	200	215	EVMDFVAHDLGPEGLK			Oxidation (M)[3]		Mascot
1819.9952	1819.9672	-0.028	-15	281	296	GYTPGVFETLPKLLER					Mascot
1861.908	1861.9623	0.0543	29	234	250	GAYTATAIAEYFRDQGK					Mascot
1861.908	1861.9623	0.0543	29	234	250	GAYTATAIAEYFRDQGK					Mascot
2066.9924	2067.1704	0.178	86	390	408	VGAYQQGSDAELDRAIAMR			Oxidation (M)[18]		Mascot

5	50S ribosomal protein L1 OS=Streptomyces aureofaciens GN=rplA PE=3 SV=1	RL1_STRAU	25989.9	9.52	12	58	12.304	3.765

Peptide Information

Calc. Mass	Obsrv. Mass	± da	± ppm	Start Seq.	End Seq.	Sequence	Ion Score	C. I. %	Modification	Rank	Result Type
806.4519	806.3947	-0.0572	-71	159	165	GGKIEFR					Mascot
811.3832	811.4166	0.0334	41	179	185	VSFSDEK					Mascot
845.4475	845.4466	-0.0009	-1	9	16	AADAKVDR					Mascot
847.4454	847.4274	-0.018	-21	53	59	KADQMVR					Mascot
906.5043	906.4583	-0.046	-51	162	168	IEFRVDK					Mascot
1165.6477	1165.6744	0.0267	23	169	178	HSNLHFIIGK					Mascot
1679.8884	1679.9415	0.0531	32	143	158	TGTVTMDVAKAVTEIK			Oxidation (M)[6]		Mascot
1787.8811	1787.9642	0.0831	46	31	46	ETSATKFDSTVEVAFR					Mascot
1791.8979	1791.9139	0.016	9	136	152	GLMPNPKTGTVTMDVAK			Oxidation (M)[3,13]		Mascot
1796.9072	1796.9917	0.0845	47	54	70	ADQMVRGTVNLPHGTGK			Oxidation (M)[4]		Mascot
1859.9644	1859.9629	-0.0015	-1	212	229	KAALSTTMGPGIQLDSNR					Mascot
2063.0227	2063.1641	0.1414	69	106	124	GNRLNEFDAVVATPDLMGK			Oxidation (M)[17]		Mascot

6	NAD-reducing hydrogenase HoxS subunit gamma OS=Cupriavidus necator (strain ATCC 17699 / H16 / DSM 428 / Stanier 337) GN=hoxU PE=1 SV=2	HOXU_CUPNH	26840.5	6.82	10	58	12.304	8.581

Peptide Information

Calc. Mass	Obsrv. Mass	± da	± ppm	Start Seq.	End Seq.	Sequence	Ion Score	C. I. %	Modification	Rank	Result Type
884.4472	884.4411	-0.0061	-7	130	137	VVDHASEK					Mascot
906.4527	906.4583	0.0056	6	11	18	TLTTEEGR					Mascot
974.5516	974.564	0.0124	13	2	10	SIQITIDGK					Mascot
1106.5082	1106.589	0.0808	73	39	47	DKPCLGTCR			Carbamidomethyl (C)[4,8]		Mascot
1679.8026	1679.9415	0.1389	83	39	52	DKPCLGTCRVCSVK			Carbamidomethyl (C)[4,8,11]		Mascot
1804.9156	1804.9391	0.0235	13	48	64	VCSVKVNGNVAAACTVR			Carbamidomethyl (C)[2,14]		Mascot
1843.9219	1843.9946	0.0727	39	68	83	GLNVEVNDPELVDMRK			Oxidation (M)[14]		Mascot

1861.9866	1861.9623	-0.0243	-13	2	18	SIQITIDGKTLTTEEGR					Mascot
1861.9866	1861.9623	-0.0243	-13	2	18	SIQITIDGKTLTTEEGR					Mascot
1883.9419	1883.9255	-0.0164	-9	176	192	IEIDAELANAMPPEQVK			Oxidation (M)[11]		Mascot
1993.0271	1993.0671	0.04	20	1	18	MSIQITIDGKTLTTEEGR					Mascot

7 Protein GrpE OS=Actinobacillus succinogenes (strain ATCC 55618 / 130Z) GN=grpE PE=3 SV=1 | GRPE_ACTSZ | 22204.2 | 4.52 | 10 | 58 | 3.843 | 5.013

Peptide Information

Calc. Mass	Obsrv. Mass	± da	± ppm	Start Seq.	End Seq.	Sequence	Ion Score	C. I. %	Modification	Rank	Result Type
818.389	818.3757	-0.0133	-16	80	86	AEQDVEK					Mascot
822.4468	822.3979	-0.0489	-59	182	188	GYLLNSR					Mascot
834.3774	834.3803	0.0029	3	71	77	AEVDNMR					Mascot
844.4523	844.3815	-0.0708	-84	62	68	EQDALLR					Mascot
872.52	872.4678	-0.0522	-60	134	141	ELLATVAR					Mascot
974.4901	974.564	0.0739	76	79	86	RAEQDVEK					Mascot
1077.5106	1077.5876	0.077	71	69	77	ARAEVDNMR			Oxidation (M)[8]		Mascot
1077.5106	1077.5876	0.077	71	69	77	ARAEVDNMR			Oxidation (M)[8]		Mascot
1571.8275	1571.9174	0.0899	57	48	61	VQELEEQLADAAKK					Mascot
1571.8275	1571.9174	0.0899	57	48	61	VQELEEQLADAAKK					Mascot
1993.0721	1993.0671	-0.005	-3	182	199	GYLLNSRVIRPAMVMVAA			Oxidation (M)[13,15]		Mascot
2045.1642	2045.1516	-0.0126	-6	123	141	GLFDGVELTLKELLATVAR					Mascot

8 PEP2-like protein NECHADRAFT_97050 OS=Nectria haematococca (strain 77-13-4 / ATCC MYA-4622 / FGSC 9596 / MPVI) GN=NECHADRAFT_97050 PE=3 SV=2 | PEP2L_NECH7 | 27069.5 | 6.76 | 11 | 56 | 0 | 6.815

Peptide Information

Calc. Mass	Obsrv. Mass	± da	± ppm	Start Seq.	End Seq.	Sequence	Ion Score	C. I. %	Modification	Rank	Result Type
831.4294	831.4185	-0.0109	-13	85	91	GAFIMHR					Mascot
847.4243	847.4274	0.0031	4	85	91	GAFIMHR			Oxidation (M)[5]		Mascot
869.4661	869.5018	0.0357	41	158	165	MIPANPAR					Mascot
870.5771	870.5858	0.0087	10	25	31	LVLERLK					Mascot
1024.4597	1024.5574	0.0977	95	133	139	FCFFFEK			Carbamidomethyl (C)[2]		Mascot
1037.5562	1037.5526	-0.0036	-3	196	204	VLLDMPGHR					Mascot
1050.5731	1050.62	0.0469	45	174	182	LKGYPPGYR					Mascot
1365.6403	1365.7491	0.1088	80	80	91	AGMDKGAFIMHR			Oxidation (M)[3,10]		Mascot

1571.8064	1571.9174	0.111	71	221	233	QAKQWLEGEQIEI					Mascot
1571.8064	1571.9174	0.111	71	221	233	QAKQWLEGEQIEI					Mascot
1770.8732	1770.9562	0.083	47	69	84	VPYQDFITASKAGMDK					Mascot
1770.8732	1770.9562	0.083	47	69	84	VPYQDFITASKAGMDK					Mascot
1791.9501	1791.9139	-0.0362	-20	206	220	HVGTPNLEKHDLLYR					Mascot
1812.9061	1813.017	0.1109	61	158	173	MIPANPARFPAVDEER					Mascot

9	Nebulin-related-anchoring protein OS=Homo sapiens GN=NRAP PE=2 SV=2	NRAP_HUMAN	197919.7	9.24	38	56	0	21.484

Peptide Information

Calc. Mass	Obsrv. Mass	± da	± ppm	Start Seq.	End Seq.	Sequence	Ion Score	C. I. %	Modification	Rank	Result Type
803.353	803.4114	0.0584	73	1567	1573	SVDDDPR					Mascot
806.4519	806.3947	-0.0572	-71	1560	1566	GLQIGYR					Mascot
808.3618	808.39	0.0282	35	594	601	AMGTADSR					Mascot
812.3971	812.3626	-0.0345	-42	556	562	GKGFEMK			Oxidation (M)[6]		Mascot
817.405	817.4033	-0.0017	-2	996	1002	EQGENIK					Mascot
818.4366	818.3757	-0.0609	-74	1657	1663	SDLNLTR					Mascot
820.4675	820.3961	-0.0714	-87	795	801	AKGFELR					Mascot
824.4148	824.4098	-0.005	-6	619	625	KGFEESK					Mascot
831.457	831.4185	-0.0385	-46	709	716	AGQLVSEK					Mascot
832.4312	832.3828	-0.0484	-58	1105	1111	KGFEHSK					Mascot
834.4178	834.3803	-0.0375	-45	685	691	ADLAWMK					Mascot
837.4101	837.4123	0.0022	3	1544	1550	EIASDFR					Mascot
860.4043	860.4125	0.0082	10	2	8	NVQPCSR			Carbamidomethyl (C)[5]		Mascot
869.5203	869.5018	-0.0185	-21	1319	1326	LIGPQSVR					Mascot
872.4771	872.4678	-0.0093	-11	428	434	RTLHAMK			Oxidation (M)[6]		Mascot
906.4791	906.4583	-0.0208	-23	1553	1559	EAFLRDR					Mascot
914.4617	914.4559	-0.0058	-6	1065	1071	YKEAFEK					Mascot
974.5516	974.564	0.0124	13	952	960	AGELISEKK					Mascot
1021.4982	1021.5485	0.0503	49	1429	1437	SPQMESAKK			Oxidation (M)[4]		Mascot
1023.5074	1023.5446	0.0372	36	1072	1080	MKGQMLGSR			Oxidation (M)[1]		Mascot
1024.5533	1024.5574	0.0041	4	1403	1411	AHALQSELR					Mascot
1039.5419	1039.5559	0.014	13	399	407	ISKFTSDNK					Mascot
1039.5419	1039.5559	0.014	13	399	407	ISKFTSDNK					Mascot
1054.5602	1054.5491	-0.0111	-11	1412	1420	YKSDLIGMK					Mascot
1077.5323	1077.5876	0.0553	51	788	796	SSWENQKAK					Mascot

Calc. Mass	Obsrv. Mass	± da	± ppm	Start Seq.	End Seq.	Sequence	Ion Score	C. I. %	Modification	Rank	Result Type
1077.58	1077.5876	0.0076	7	1558	1566	DRGLQIGYR					Mascot
1196.559	1196.637	0.078	65	1327	1335	DDPRIQHCR			Carbamidomethyl (C)[8]		Mascot
1320.5862	1320.694	0.1078	82	410	419	ENYQNHMRGR			Oxidation (M)[7]		Mascot
1445.786	1445.7733	-0.0127	-9	1531	1543	LDAIPFQTARASR					Mascot
1508.7638	1508.863	0.0992	66	1337	1348	MGQLQSELQYRR					Mascot
1570.8258	1570.9159	0.0901	57	1178	1192	GVACVIPGTLEIEGR			Carbamidomethyl (C)[4]		Mascot
1779.9463	1780.0184	0.0721	41	237	253	GKGSFPAMITPAYQIAK					Mascot
1791.9059	1791.9139	0.008	4	935	951	GMGWVATGSLNVEQAK K			Oxidation (M)[2]		Mascot
1796.9	1796.9917	0.0917	51	307	323	GKGSFPAMITPAYQNAK			Oxidation (M)[8]		Mascot
1804.9592	1804.9391	-0.0201	-11	1003	1018	HHYTPTADLPEVLLAK					Mascot
1819.9086	1819.9672	0.0586	32	1576	1590	HFLNVGRLQSDNEYK					Mascot
1843.8345	1843.9946	0.1601	87	151	165	KSLGEEYTEDYEQPR					Mascot
1861.9259	1861.9623	0.0364	20	271	288	EMRGMAGPAIGAEGILTR			Oxidation (M)[2,5]		Mascot
1861.9259	1861.9623	0.0364	20	271	288	EMRGMAGPAIGAEGILTR	1	0	Oxidation (M)[2,5]		Mascot
1870.0433	1869.9614	-0.0819	-44	519	534	NKLNYTLPQDVPQLVK					Mascot

10	DNA topoisomerase 1 OS=Campylobacter jejuni subsp. jejuni serotype O:2 (strain NCTC 11168) GN=topA PE=3 SV=1	TOP1_CAMJE	79896.5	9.06	20	55	0	11.95

Peptide Information

Calc. Mass	Obsrv. Mass	± da	± ppm	Start Seq.	End Seq.	Sequence	Ion Score	C. I. %	Modification	Rank	Result Type
803.4774	803.4114	-0.066	-82	268	274	LGFNPKK					Mascot
804.4461	804.4014	-0.0447	-56	553	559	IEEGKTK					Mascot
822.4212	822.3979	-0.0233	-28	275	281	TMMIAQK					Mascot
844.4159	844.3815	-0.0344	-41	102	108	DENTLPR					Mascot
845.4727	845.4466	-0.0261	-31	117	124	SAIENALK					Mascot
849.4828	849.4266	-0.0562	-66	16	23	TIGNFLGK					Mascot
853.4302	853.4349	0.0047	6	486	492	DYVTIDK					Mascot
872.52	872.4678	-0.0522	-60	175	181	IIVDREK					Mascot
974.5669	974.564	-0.0029	-3	425	432	DKILPNFK					Mascot
975.5833	975.5757	-0.0076	-8	560	568	IASQKTVTK					Mascot
1024.4946	1024.5574	0.0628	61	511	519	NFSDIVDSK					Mascot
1168.5132	1168.6005	0.0873	75	544	551	EFYYPFMR			Oxidation (M)[7]		Mascot
1168.5132	1168.6005	0.0873	75	544	551	EFYYPFMR			Oxidation (M)[7]		Mascot
1570.8588	1570.9159	0.0571	36	87	101	EGEAIAYHIAKAIGK					Mascot
1641.8207	1641.9644	0.1437	88	585	598	GRFGEFVACLNFPK			Carbamidomethyl (C)[9]		Mascot

1707.8292	1707.9233	0.0941	55	669	682	CEECGETLVIKELK	Carbamidomethyl (C)[1,4]	Mascot
1716.8237	1716.985	0.1613	94	587	600	FGEFVACLNFPKCK	Carbamidomethyl (C)[7,13]	Mascot
1754.9324	1754.9247	-0.0077	-4	16	31	TIGNFLGKDYEVIASK		Mascot
1813.0219	1813.017	-0.0049	-3	109	124	IVFHEITKSAIENALK		Mascot
2044.9862	2045.1516	0.1654	81	40	56	SSFGIKIEDDEFIPEYR		Mascot
2062.9897	2063.1641	0.1744	85	565	583	TVTKLGESCPDCGGELAI R	Carbamidomethyl (C)[9,12]	Mascot
