# Supplementary material for: Proteomic analysis of middle and late stages of bread wheat (Triticum aestivum L.) grain development
Source: Front Plant Sci. 2015 Sep 15;6:735. doi: 10.3389/fpls.2015.00735 (PMC4569854; doi:10.3389/fpls.2015.00735)
Supplement: Supplementary file 6 [file DataSheet5.PDF]

Analysis Information

Report Type	Protein-Peptide Summary by Spot	Analysis Type	Combined (MS+MS/MS)
Sample Set Name	Sample set_20140814	Database	SwissProt
Analysis Name	BSA0928	Creation Date	09/28/2014 13:23:38
Reported By	09/30/2014 14:59:33 - admin	Last Modified	09/28/2014 13:42:02

MS Acq. : Proc. Methods	(Unspecified) : (Unspecified)
Interpretation Method	(Unspecified)

**Gel Idx/Pos**	151/G2	**Instr./Gel Origin**	BA2151/Sample Project 20140814	**Process Status**	Analysis Succeeded
**Plate [#] Name**	[1] Sample Project 20140814	**Instrument Sample Name**		**Spectra**	11

Rank	Protein Name	Accession No.	Protein MW	Protein PI	Pep. Count	Protein Score	Protein Score C. I. %	Intensity Matched	Total Ion Score	Total Ion C. I. %	Confirmed
1	Alpha-amylase inhibitor 0.53 OS=Triticum aestivum PE=1 SV=1	IAA5_WHEAT	13689.5	5.23	5	299	100	21.61	268	100	

Peptide Information

Calc. Mass	Obsrv. Mass	± da	± ppm	Start Seq.	End Seq.	Sequence	Ion Score	C. I. %	Modification	Rank	Result Type
1162.6249	1162.6165	-0.0084	-7	90	100	LTAASITAVCR			Carbamidomethyl (C)[10]		Mascot
1162.6249	1162.6165	-0.0084	-7	90	100	LTAASITAVCR	49	98.995	Carbamidomethyl (C)[10]		Mascot
1554.6637	1554.7523	0.0886	57	54	66	CGALYSMLDSMYK			Carbamidomethyl (C)[1], Oxidation (M)[7]		Mascot
1570.8007	1570.7888	-0.0119	-8	26	39	LQCNGSQVPEAVLR			Carbamidomethyl (C)[3]		Mascot
1663.8361	1663.7917	-0.0444	-27	101	116	LPIVVDASGDGAYVCK			Carbamidomethyl (C)[15]		Mascot
1663.8361	1663.7917	-0.0444	-27	101	116	LPIVVDASGDGAYVCK	86	100	Carbamidomethyl (C)[15]		Mascot
1846.8137	1846.8	-0.0137	-7	67	84	EHGVSEGQAGTGAFPSCR			Carbamidomethyl (C)[17]		Mascot
1846.8137	1846.8	-0.0137	-7	67	84	EHGVSEGQAGTGAFPSCR	134	100	Carbamidomethyl (C)[17]		Mascot

Rank	Protein Name	Accession No.	Protein MW	Protein PI	Pep. Count	Protein Score	Protein Score C. I. %	Intensity Matched	Total Ion Score	Total Ion C. I. %	Confirmed
2	Alpha-amylase inhibitor 0.19 OS=Triticum aestivum PE=1 SV=1	IAA1_WHEAT	13898.6	6.66	6	174	100	5.722	135	100	

Peptide Information

Calc. Mass	Obsrv. Mass	± da	± ppm	Start Seq.	End Seq.	Sequence	Ion Score	C. I. %	Modification	Rank	Result Type
1162.6249	1162.6165	-0.0084	-7	90	100	LTAASITAVCR			Carbamidomethyl (C)[10]		Mascot
1162.6249	1162.6165	-0.0084	-7	90	100	LTAASITAVCR	49	98.995	Carbamidomethyl (C)[10]		Mascot

Calc. Mass	Obsrv. Mass	± da	± ppm	Start Seq.	End Seq.	Sequence	Ion Score	C. I. %	Modification	Rank	Result Type
1554.6637	1554.7523	0.0886	57	54	66	CGALYSMLDSMYK			Carbamidomethyl (C)[1], Oxidation (M)[7]		Mascot
1570.8007	1570.7888	-0.0119	-8	26	39	LQCNGSQVPEAVLR			Carbamidomethyl (C)[3]		Mascot
1612.7463	1612.728	-0.0183	-11	67	82	EHGAQEGQAGTGAFPR					Mascot
1663.8361	1663.7917	-0.0444	-27	101	116	LPIVVDASGDGAYVCK			Carbamidomethyl (C)[15]		Mascot
1663.8361	1663.7917	-0.0444	-27	101	116	LPIVVDASGDGAYVCK	86	100	Carbamidomethyl (C)[15]		Mascot
1862.7731	1862.7512	-0.0219	-12	40	53	DCCQQLAHISEWCR			Carbamidomethyl (C)[2,3,13]		Mascot

3	Alpha-amylase/trypsin inhibitor CM3 OS=Triticum aestivum PE=1 SV=1	IAAC3_WHEAT	18893.3	7.44	6	51	0	2.691	18	0

Peptide Information

Calc. Mass	Obsrv. Mass	± da	± ppm	Start Seq.	End Seq.	Sequence	Ion Score	C. I. %	Modification	Rank	Result Type
1010.52	1010.481	-0.039	-39	37	44	TNLLPHCR			Carbamidomethyl (C)[7]		Mascot
1126.4987	1126.4783	-0.0204	-18	133	140	EMQWDFVR			Oxidation (M)[2]		Mascot
1698.9214	1698.8981	-0.0233	-14	101	115	YFIALPVPSQPVDPR					Mascot
1698.9214	1698.8981	-0.0233	-14	101	115	YFIALPVPSQPVDPR	18	0			Mascot
1727.8381	1727.8289	-0.0092	-5	116	132	SGNVGESGLIDLPGCPR			Carbamidomethyl (C)[15]		Mascot
1876.0222	1876.0044	-0.0178	-9	141	157	LLVAPGQCNLATIHNVR			Carbamidomethyl (C)[8]		Mascot
1957.8564	1957.8517	-0.0047	-2	81	95	LYCCQELAEISQQCR			Carbamidomethyl (C)[3,4,14]		Mascot

4	Tubulin polymerization-promoting protein family member 3 OS=Bos taurus GN=TPPP3 PE=1 SV=1	TPPP3_BOVIN	19101.8	9.18	9	50	0	2.986

Peptide Information

Calc. Mass	Obsrv. Mass	± da	± ppm	Start Seq.	End Seq.	Sequence	Ion Score	C. I. %	Modification	Rank	Result Type
823.3801	823.4341	0.054	66	38	43	LCKDCK			Carbamidomethyl (C)[2,5]		Mascot
839.3893	839.4026	0.0133	16	166	173	NAGTYDAK					Mascot
870.493	870.5163	0.0233	27	78	85	ALEELAPK					Mascot
1550.752	1550.7289	-0.0231	-15	93	106	EEAFDAICQLVAGK			Carbamidomethyl (C)[8]		Mascot
1599.7319	1599.8081	0.0762	48	1	15	MAASTDVAGLEESFR			Oxidation (M)[1]		Mascot
1727.8269	1727.8289	0.002	1	1	16	MAASTDVAGLEESFRK			Oxidation (M)[1]		Mascot
1765.8789	1765.7094	-0.1695	-96	91	106	SKEEAFDAICQLVAGK			Carbamidomethyl (C)[10]		Mascot
1795.8545	1795.7262	-0.1283	-71	17	33	FAIHGDPKASGHEMNGK					Mascot
1821.9594	1821.795	-0.1644	-90	44	61	VADGKAVTGTDVDIVFSK					Mascot

5	U1-cyrtautoxin-As1d OS=Apomastus schlingeri PE=1 SV=1	TXP9_APOSC	8817	6.93	6	49	0	1.099

Peptide Information

Calc. Mass	Obsrv. Mass	± da	± ppm	Start Seq.	End Seq.	Sequence	Ion Score	C. I. %	Modification	Rank	Result Type
882.3808	882.4042	0.0234	27	34	40	SECCKAK			Carbamidomethyl (C)[3,4]		Mascot
948.4567	948.4624	0.0057	6	25	33	TPGALCSSR			Carbamidomethyl (C)[6]		Mascot
1612.6876	1612.728	0.0404	25	25	38	TPGALCSSRSECCK			Carbamidomethyl (C)[6,12,13]		Mascot
1641.7286	1641.8136	0.085	52	39	53	AKHSDSVTYSSGCSR			Carbamidomethyl (C)[13]		Mascot
1779.8232	1779.731	-0.0922	-52	54	67	QWSDQQGLFINQCR			Carbamidomethyl (C)[13]		Mascot
1931.9215	1932.0769	0.1554	80	17	33	LPNGQWCKTPGALCSSR			Carbamidomethyl (C)[7,14]		Mascot

6	Succinyl-diaminopimelate desuccinylase OS=Campylobacter curvus (strain 525.92) GN=dapE PE=3 SV=1	DAPE_CAMC5	40220.7	5.67	11	47	0	4.7	

Peptide Information

Calc. Mass	Obsrv. Mass	± da	± ppm	Start Seq.	End Seq.	Sequence	Ion Score	C. I. %	Modification	Rank	Result Type
839.437	839.4026	-0.0344	-41	338	344	IHAVDER					Mascot
841.4202	841.4214	0.0012	1	85	91	DGFIYAR					Mascot
1480.7643	1480.7665	0.0022	1	278	290	QSSKPFLTDKDSK					Mascot
1487.6948	1487.7277	0.0329	22	85	97	DGFIYARGAQDMK			Oxidation (M)[12]		Mascot
1553.901	1553.7733	-0.1277	-82	298	312	AVQKISGVAPQLNTK					Mascot
1570.786	1570.7888	0.0028	2	313	327	GGTSDARYLAEFGVK					Mascot
1598.8828	1598.8342	-0.0486	-30	353	365	LYLVFKELIENFA					Mascot
1795.7957	1795.7262	-0.0695	-39	206	222	IAGHDMDAGSEFFSPSK					Mascot
1843.8248	1843.7267	-0.0981	-53	92	108	GAQDMKSGVAAFVCACR			Carbamidomethyl (C)[14,16], Oxidation (M)[5]		Mascot
1931.9902	1932.0769	0.0867	45	26	41	FIAEFLGDFEARFIEK					Mascot
1968.0298	1967.8601	-0.1697	-86	328	344	VVEFGVINDRIHAVDER					Mascot

7	10 kDa chaperonin OS=Sphingomonas wittichii (strain RW1 / DSM 6014 / JCM 10273) GN=groS PE=3 SV=1	CH10_SPHWW	10121.4	5.16	7	46	0	3.229	

Peptide Information

Calc. Mass	Obsrv. Mass	± da	± ppm	Start Seq.	End Seq.	Sequence	Ion Score	C. I. %	Modification	Rank	Result Type
806.4043	806.4021	-0.0022	-3	70	76	WSGTEVK					Mascot
860.4472	860.4194	-0.0278	-32	14	20	RVEAEEK					Mascot
902.4829	902.409	-0.0739	-82	87	95	ESDILGIVG					Mascot
948.5513	948.4624	-0.0889	-94	61	69	AGDKILFGK					Mascot
1158.5837	1158.4768	-0.1069	-92	1	9	MSFRPLHDR					Mascot
1174.5786	1174.4778	-0.1008	-86	1	9	MSFRPLHDR			Oxidation (M)[1]		Mascot

Calc. Mass	Obsrv. Mass	± da	± ppm	Start Seq.	End Seq.	Sequence	Ion Score	C. I. %	Modification	Rank	Result Type
1570.8071	1570.7888	-0.0183	-12	33	48	EKPQEGEVVAVGGGSK					Mascot
1641.9006	1641.8136	-0.087	-53	1	13	MSFRPLHDRVLVR			Oxidation (M)[1]		Mascot

8	Photoreceptor-specific nuclear receptor OS=Mus musculus GN=Nr2e3 PE=1 SV=1	NR2E3_MOUSE	43947	7.92	11	43	0	5.759

Peptide Information

Calc. Mass	Obsrv. Mass	± da	± ppm	Start Seq.	End Seq.	Sequence	Ion Score	C. I. %	Modification	Rank	Result Type
1593.8153	1593.7281	-0.0872	-55	2	18	SSTVAASTMPVSVAASK					Mascot
1685.767	1685.8029	0.0359	21	76	90	CQVGAGMCPVDKAHR			Carbamidomethyl (C)[1,8]		Mascot
1721.9103	1721.9182	0.0079	5	2	19	SSTVAASTMPVSVAASK K					Mascot
1724.8558	1724.7135	-0.1423	-82	1	18	MSSTVAASTMPVSVAAS K					Mascot
1737.8993	1737.7867	-0.1126	-65	295	309	FRALAVDPTEFACLK			Carbamidomethyl (C)[13]		Mascot
1749.7643	1749.722	-0.0423	-24	101	115	CLQAGMNQDAVQNER			Carbamidomethyl (C)[1], Oxidation (M)[6]		Mascot
1780.7571	1780.7659	0.0088	5	51	65	HYGIYACNGCSGFFK			Carbamidomethyl (C)[7,10]		Mascot
1828.8647	1828.7939	-0.0708	-39	25	41	WGLGEDPTGVGPSLQC R			Carbamidomethyl (C)[16]		Mascot
1828.8647	1828.7939	-0.0708	-39	25	41	WGLGEDPTGVGPSLQC R			Carbamidomethyl (C)[16]		Mascot
1844.8267	1844.7267	-0.1	-54	119	135	SMAQVHLDAMETGSDPR					Mascot
1852.9507	1852.7719	-0.1788	-96	1	19	MSSTVAASTMPVSVAAS KK					Mascot
1860.8215	1860.7366	-0.0849	-46	119	135	SMAQVHLDAMETGSDPR			Oxidation (M)[2]		Mascot
1861.8644	1861.7672	-0.0972	-52	100	115	KCLQAGMNQDAVQNER			Carbamidomethyl (C)[2]		Mascot

9	50S ribosomal protein L6 OS=Frankia sp. (strain EAN1pec) GN=rplF PE=3 SV=1	RL6_FRASN	19344.6	10.27	7	42	0	3.625

Peptide Information

Calc. Mass	Obsrv. Mass	± da	± ppm	Start Seq.	End Seq.	Sequence	Ion Score	C. I. %	Modification	Rank	Result Type
1162.6328	1162.6165	-0.0163	-14	161	170	GVRYQGEVVR					Mascot
1162.6328	1162.6165	-0.0163	-14	161	170	GVRYQGEVVR					Mascot
1593.8595	1593.7281	-0.1314	-82	31	45	GSLSHTVAEPIEVVR					Mascot
1641.8517	1641.8136	-0.0381	-23	70	85	TLVSNMVVGVTAGYSK			Oxidation (M)[6]		Mascot
1810.9154	1810.7822	-0.1332	-74	46	60	EDGQLIVNRPNDERR					Mascot
1815.9276	1815.7642	-0.1634	-90	100	116	GSDLEFALGYSHPVPVK					Mascot
1876.0287	1876.0044	-0.0243	-13	28	45	GPKGSLSHTVAEPIEVVR					Mascot
2390.2861	2390.1233	-0.1628	-68	63	85	ALHGLTRTLVSNMVVGV TAGYSK			Oxidation (M)[13]		Mascot

10	Zinc finger protein 252 OS=Canis familiaris	ZN252_CANFA	102064.4	8.97	16	42	0	7.176

GN=ZNF252 PE=2 SV=1

Peptide Information

Calc. Mass	Obsrv. Mass	± da	± ppm	Start Seq.	End Seq.	Sequence	Ion Score	C. I. %	Modification	Rank	Result Type
805.3937	805.3331	-0.0606	-75	149	155	SAEEELK					Mascot
823.4785	823.4341	-0.0444	-54	497	503	AQVIQHK					Mascot
839.4482	839.4026	-0.0456	-54	279	285	ALSQHQR					Mascot
1158.4619	1158.4768	0.0149	13	129	137	ESEFEEMNK			Oxidation (M)[7]		Mascot
1182.5936	1182.5453	-0.0483	-41	107	116	QEISKAMGFR			Oxidation (M)[7]		Mascot
1550.83	1550.7289	-0.1011	-65	716	728	AFNVRSSLVQHHR					Mascot
1554.6741	1554.7523	0.0782	50	88	100	SHESESFQSLMEK			Oxidation (M)[11]		Mascot
1570.7053	1570.7888	0.0835	53	125	137	NVSKESEFEEMNK					Mascot
1724.8337	1724.7135	-0.1202	-70	183	198	SSSLEVDYTVDASPVR					Mascot
1751.7112	1751.7216	0.0104	6	673	687	THNGEKPYECGDCGK			Carbamidomethyl (C)[10,13]		Mascot
1780.7629	1780.7659	0.003	2	421	435	IHSGEKPYDCTECGK			Carbamidomethyl (C)[10,13]		Mascot
1795.7374	1795.7262	-0.0112	-6	645	659	SHTGEKPYECNECGK			Carbamidomethyl (C)[10,13]		Mascot
1821.7894	1821.795	0.0056	3	561	575	IHTGEKPYECNECGK			Carbamidomethyl (C)[10,13]		Mascot
1823.7622	1823.8145	0.0523	29	813	827	VHSGERPYMCNECGK			Carbamidomethyl (C)[10,13]		Mascot
1828.9148	1828.7939	-0.1209	-66	248	263	QSSVLSENQRVNNPEK					Mascot
1828.9148	1828.7939	-0.1209	-66	248	263	QSSVLSENQRVNNPEK					Mascot
1958.9403	1958.8407	-0.0996	-51	741	756	CGKAFSQHSQFIQHQR			Carbamidomethyl (C)[1]		Mascot

Gel Idx/Pos	152/G3	Instr./Gel Origin	BA2151/Sample Project 20140814	Process Status	Analysis Succeeded
Plate [#] Name	[1] Sample Project 20140814	Instrument Sample Name		Spectra	11

Rank	Protein Name	Accession No.	Protein MW	Protein PI	Pep. Count	Protein Score	Protein Score C. I. %	Intensity Matched	Total Ion Score	Total Ion C. I. %	Confirmed
1	Alpha-amylase inhibitor 0.53 OS=Triticum aestivum PE=1 SV=1	IAA5_WHEAT	13689.5	5.23	6	420	100	36.586	378	100	

Peptide Information

Calc. Mass	Obsrv. Mass	± da	± ppm	Start Seq.	End Seq.	Sequence	Ion Score	C. I. %	Modification	Rank	Result Type
1162.6249	1162.6138	-0.0111	-10	90	100	LTAASITAVCR			Carbamidomethyl (C)[10]		Mascot
1162.6249	1162.6138	-0.0111	-10	90	100	LTAASITAVCR	70	99.992	Carbamidomethyl (C)[10]		Mascot
1554.6637	1554.6335	-0.0302	-19	54	66	CGALYSMLDSMYK			Carbamidomethyl (C)[1], Oxidation (M)[7]		Mascot
1570.8007	1570.7839	-0.0168	-11	26	39	LQCNGSQVPEAVLR			Carbamidomethyl (C)[3]		Mascot
1663.8361	1663.7701	-0.066	-40	101	116	LPIVVDASGDGAYVCK			Carbamidomethyl (C)[15]		Mascot
1663.8361	1663.7701	-0.066	-40	101	116	LPIVVDASGDGAYVCK	141	100	Carbamidomethyl (C)[15]		Mascot
1846.8137	1846.7911	-0.0226	-12	67	84	EHGVSEGGQAGTGAFPSC R			Carbamidomethyl (C)[17]		Mascot
1846.8137	1846.7911	-0.0226	-12	67	84	EHGVSEGGQAGTGAFPSC R	167	100	Carbamidomethyl (C)[17]		Mascot
2807.4431	2807.3105	-0.1326	-47	90	116	LTAASITAVCRLPIVVDAS GDGAYVCK			Carbamidomethyl (C)[10,26]		Mascot

Rank	Protein Name	Accession No.	Protein MW	Protein PI	Pep. Count	Protein Score	Protein Score C. I. %	Intensity Matched	Total Ion Score	Total Ion C. I. %	Confirmed
2	Alpha-amylase inhibitor 0.19 OS=Triticum aestivum PE=1 SV=1	IAA1_WHEAT	13898.6	6.66	6	253	100	12.85	211	100	

Peptide Information

Calc. Mass	Obsrv. Mass	± da	± ppm	Start Seq.	End Seq.	Sequence	Ion Score	C. I. %	Modification	Rank	Result Type
1162.6249	1162.6138	-0.0111	-10	90	100	LTAASITAVCR			Carbamidomethyl (C)[10]		Mascot
1162.6249	1162.6138	-0.0111	-10	90	100	LTAASITAVCR	70	99.992	Carbamidomethyl (C)[10]		Mascot
1554.6637	1554.6335	-0.0302	-19	54	66	CGALYSMLDSMYK			Carbamidomethyl (C)[1], Oxidation (M)[7]		Mascot
1570.8007	1570.7839	-0.0168	-11	26	39	LQCNGSQVPEAVLR			Carbamidomethyl (C)[3]		Mascot
1663.8361	1663.7701	-0.066	-40	101	116	LPIVVDASGDGAYVCK			Carbamidomethyl (C)[15]		Mascot
1663.8361	1663.7701	-0.066	-40	101	116	LPIVVDASGDGAYVCK	141	100	Carbamidomethyl (C)[15]		Mascot
1862.7731	1862.7668	-0.0063	-3	40	53	DCCQQLAHISEWCR			Carbamidomethyl (C)[2,3,13]		Mascot
2807.4431	2807.3105	-0.1326	-47	90	116	LTAASITAVCRLPIVVDAS GDGAYVCK			Carbamidomethyl (C)[10,26]		Mascot

Rank	Protein Name	Accession No.	Protein MW	Protein PI	Pep. Count	Protein Score	Protein Score C. I. %	Intensity Matched	Total Ion Score	Total Ion C. I. %	Confirmed
3	H-2 class I histocompatibility antigen, K-W28 alpha chain OS=Mus musculus GN=H2-K1 PE=1 SV=2	HA1W_MOUSE	41476.8	6.64	11	48	0	3.138			

Peptide Information

Calc. Mass	Obsrv. Mass	± da	± ppm	Start Seq.	End Seq.	Sequence	Ion Score	C. I. %	Modification	Rank	Result Type
1055.6249	1055.5773	-0.0476	-45	265	274	WASVVVPLGK					Mascot
1105.5459	1105.5618	0.0159	14	359	368	VMVHDPHSLA					Mascot
1232.4672	1232.578	0.1108	90	119	129	MYGCDVGSDGR			Carbamidomethyl (C)[4], Oxidation (M)[1]		Mascot
1553.7417	1553.5964	-0.1453	-94	179	191	AYLEGACVEWLSR			Carbamidomethyl (C)[7]		Mascot
1595.7085	1595.7083	-0.0002	0	57	69	FDSDAENPRYEPR					Mascot
1598.7415	1598.8308	0.0893	56	119	132	MYGCDVGSDGRLLR			Carbamidomethyl (C)[4]		Mascot
1598.7415	1598.8308	0.0893	56	119	132	MYGCDVGSDGRLLR			Carbamidomethyl (C)[4]		Mascot
1657.873	1657.7758	-0.0972	-59	153	167	TWTAADMAALITKHK					Mascot
1685.9235	1685.782	-0.1415	-84	28	42	YFHTAVSRPGLGKPR					Mascot
1908.8698	1908.791	-0.0788	-41	70	83	ARWMEQVEPEYWER					Mascot
1931.9796	1932.0626	0.083	43	179	194	AYLEGACVEWLSRHLK			Carbamidomethyl (C)[7]		Mascot
2336.0388	2336.0134	-0.0254	-11	133	152	GYEQVAYDGCDYIALNE DLK			Carbamidomethyl (C)[10]		Mascot

4	Translation initiation factor IF-1 1 OS=Novosphingobium aromaticivorans (strain DSM 12444) GN=infA1 PE=3 SV=2	IF11_NOVAD	9191.8	9.3	7	46	0	1.756	

Peptide Information

Calc. Mass	Obsrv. Mass	± da	± ppm	Start Seq.	End Seq.	Sequence	Ion Score	C. I. %	Modification	Rank	Result Type
1118.5874	1118.504	-0.0834	-75	2	10	AKEELLEMR					Mascot
1140.5983	1140.5547	-0.0436	-38	67	75	ITYRFMPGR					Mascot
1533.7656	1533.6395	-0.1261	-82	26	39	LENDHEILGHTAGK					Mascot
1788.9352	1788.8099	-0.1253	-70	24	39	VRLENDHEILGHTAGK					Mascot
1820.9072	1820.8322	-0.075	-41	26	41	LENDHEILGHTAGKMR					Mascot
2272.28	2272.1055	-0.1745	-77	45	64	IRVLVGDEVLVELTPYDLT K					Mascot
2379.2046	2379.0547	-0.1499	-63	4	23	EELLEMRGTVVELLPNA MFR			Oxidation (M)[6,18]		Mascot

5	Pentatricopeptide repeat-containing protein At2g45350, chloroplastic OS=Arabidopsis thaliana GN=CRR4 PE=3 SV=2	PP202_ARATH	70052.3	6.78	16	46	0	3.526	

Peptide Information

Calc. Mass	Obsrv. Mass	± da	± ppm	Start Seq.	End Seq.	Sequence	Ion Score	C. I. %	Modification	Rank	Result Type
1037.5562	1037.5144	-0.0418	-40	130	138	ACSRLGFVK			Carbamidomethyl (C)[2]		Mascot
1106.5775	1106.5116	-0.0659	-60	273	281	GLFDVMPRR			Oxidation (M)[6]		Mascot
1144.5569	1144.5826	0.0257	22	304	312	TLFDQMPHR					Mascot

Calc. Mass	Obsrv. Mass	± da	± ppm	Start Seq.	End Seq.	Sequence	Ion Score	C. I. %	Modification	Rank	Result Type
1201.5784	1201.6221	0.0437	36	524	533	TFLTACSHHK			Carbamidomethyl (C)[6]		Mascot
1215.6667	1215.6044	-0.0623	-51	139	149	GGMQIHGFLKK					Mascot
1241.5869	1241.6071	0.0202	16	24	34	TSDDVNQIHGR					Mascot
1506.7621	1506.6346	-0.1275	-85	268	280	IEDAKGLFDVMPR			Oxidation (M)[11]		Mascot
1585.7567	1585.7523	-0.0044	-3	282	295	DVVTWATMIDGYAK			Oxidation (M)[8]		Mascot
1593.7102	1593.6982	-0.012	-8	186	199	DSVSYNSMIDGYVK			Oxidation (M)[8]		Mascot
1595.85	1595.7083	-0.1417	-89	24	37	TSDDVNQIHGRLIK					Mascot
1599.7189	1599.806	0.0871	54	169	181	CGCLGLSRQMFDR			Carbamidomethyl (C)[1,3]		Mascot
1749.8113	1749.7186	-0.0927	-53	185	199	RDSVSYNSMIDGYVK			Oxidation (M)[9]		Mascot
1789.8248	1789.7885	-0.0363	-20	313	328	DVVAYNSMMAGYVQNK					Mascot
2272.0845	2272.1055	0.021	9	200	218	CGLIVSARELFDLMPMEM K			Carbamidomethyl (C)[1], Oxidation (M)[14,16]		Mascot
2393.1953	2393.0225	-0.1728	-72	487	507	LQHYGCMVDILSRSGSIE LAK			Carbamidomethyl (C)[6], Oxidation (M)[7]		Mascot
2434.1741	2434.0493	-0.1248	-51	186	207	DSVSYNSMIDGYVKCGLI VSAR			Carbamidomethyl (C)[15]		Mascot

6	3-deoxy-manno-octulosonate cytidylyltransferase OS=Bdellovibrio bacteriovorus (strain ATCC 15356 / DSM 50701 / NCIB 9529 / HD100) GN=kdsB PE=3 SV=1	KDSB_BDEBA	27132.1	6	9	46	0	7.919	

Peptide Information

Calc. Mass	Obsrv. Mass	± da	± ppm	Start Seq.	End Seq.	Sequence	Ion Score	C. I. %	Modification	Rank	Result Type
1105.5922	1105.5618	-0.0304	-27	238	247	LEKLLSSQGM					Mascot
1599.8101	1599.806	-0.0041	-3	177	189	HIGMYAYSRNFLK					Mascot
1660.9534	1660.8115	-0.1419	-85	11	25	FGSTRFPGKPLVNLK					Mascot
1685.8705	1685.782	-0.0885	-52	222	237	VKEASVGVDTPEDLAR					Mascot
1828.9287	1828.7888	-0.1399	-76	224	240	EASVGVDTPEDLARLEK					Mascot
1828.9287	1828.7888	-0.1399	-76	224	240	EASVGVDTPEDLARLEK					Mascot
1839.0222	1838.8777	-0.1445	-79	40	56	SKLLSEVIVATDHEGIK					Mascot
2329.1855	2329.0867	-0.0988	-42	190	209	QFCEAPPALIEKAESLEQ LR			Carbamidomethyl (C)[3]		Mascot
2379.0237	2379.0547	0.031	13	156	176	YPMPYSRMSAQEAGSM DGCLK			Carbamidomethyl (C)[19]		Mascot
2395.0186	2395.0366	0.018	8	156	176	YPMPYSRMSAQEAGSM DGCLK			Carbamidomethyl (C)[19], Oxidation (M)[3]		Mascot
2395.0186	2395.0366	0.018	8	156	176	YPMPYSRMSAQEAGSM DGCLK			Carbamidomethyl (C)[19], Oxidation (M)[3]		Mascot
2411.0134	2411.0488	0.0354	15	156	176	YPMPYSRMSAQEAGSM DGCLK			Carbamidomethyl (C)[19], Oxidation (M)[3,8]		Mascot
2419.1301	2419.062	-0.0681	-28	120	141	MDMATLAHPISAEELQS MNSVK			Oxidation (M)[1]		Mascot
2427.0083	2427.0215	0.0132	5	156	176	YPMPYSRMSAQEAGSM DGCLK			Carbamidomethyl (C)[19], Oxidation (M)[3,8,16]		Mascot

Calc. Mass	Obsrv. Mass	± da	± ppm	Start Seq.	End Seq.	Sequence	Ion Score	C. I. %	Modification	Rank	Result Type
2435.1252	2435.0625	-0.0627	-26	120	141	MDMATLAHPISAEELQS MNSVK			Oxidation (M)[1,3]		Mascot
2451.1201	2451.0442	-0.0759	-31	120	141	MDMATLAHPISAEELQS MNSVK			Oxidation (M)[1,3,18]		Mascot

7	Keratin, type I cytoskeletal 39 OS=Homo sapiens GN=KRT39 PE=1 SV=2	K1C39_HUMAN	57155.1	5.19	12	45	0	2.951	

Peptide Information

Calc. Mass	Obsrv. Mass	± da	± ppm	Start Seq.	End Seq.	Sequence	Ion Score	C. I. %	Modification	Rank	Result Type
1105.6285	1105.5618	-0.0667	-60	461	469	ICTITKEIK			Carbamidomethyl (C)[2]		Mascot
1201.6423	1201.6221	-0.0202	-17	214	224	ADLEAQVQSLK					Mascot
1448.705	1448.8206	0.1156	80	116	127	MLERENAELESK					Mascot
1480.7729	1480.7648	-0.0081	-5	68	79	KPIYLMNNFNAR					Mascot
1491.7689	1491.6917	-0.0772	-52	375	386	QNQEYEILLDVK					Mascot
1551.6956	1551.7278	0.0322	21	334	346	DSQECILTETEAR			Carbamidomethyl (C)[5]		Mascot
1584.7145	1584.7305	0.016	10	270	281	CQYEPIMETNRK			Carbamidomethyl (C)[1], Oxidation (M)[7]		Mascot
1838.8372	1838.8777	0.0405	22	332	346	MRDSQECILTETEAR			Carbamidomethyl (C)[7]		Mascot
1926.9814	1926.8472	-0.1342	-70	318	333	SVNTLEVELQAQHRMR			Oxidation (M)[15]		Mascot
2271.9387	2272.1055	0.1668	73	2	21	DTKGCTTTNSPSTPCQN CSR			Carbamidomethyl (C)[5,15,18]		Mascot
2329.1704	2329.0867	-0.0837	-36	249	269	LDIEVTAAPSADLNQVLQ EMR			Oxidation (M)[20]		Mascot
2418.9741	2419.062	0.0879	36	1	21	MDTKGCTTTNSPSTPCQ NCSR			Carbamidomethyl (C)[6,16,19], Oxidation (M)[1]		Mascot

8	Peptidyl-prolyl cis-trans isomerase-like 2 OS=Rhizopus delemar (strain RA 99-880 / ATCC MYA-4621 / FGSC 9543 / NRRL 43880) GN=cyp14 PE=3 SV=1	PPIL2_RHIO9	61309.1	8.98	14	44	0	14.96	

Peptide Information

Calc. Mass	Obsrv. Mass	± da	± ppm	Start Seq.	End Seq.	Sequence	Ion Score	C. I. %	Modification	Rank	Result Type
1065.5211	1065.5105	-0.0106	-10	76	85	YGTNPVTGEK					Mascot
1105.5499	1105.5618	0.0119	11	477	486	MGWFGPSVPK					Mascot
1162.5449	1162.6138	0.0689	59	171	179	DMSKFDYLK			Oxidation (M)[2]		Mascot
1162.5449	1162.6138	0.0689	59	171	179	DMSKFDYLK			Oxidation (M)[2]		Mascot
1193.6161	1193.5995	-0.0166	-14	75	85	KYGTNPVTGEK					Mascot
1215.6368	1215.6044	-0.0324	-27	227	236	EEIPTSFHKK					Mascot
1232.6093	1232.578	-0.0313	-25	309	318	TCHNFIELAK			Carbamidomethyl (C)[2]		Mascot
1491.7301	1491.6917	-0.0384	-26	474	486	EEKMGWFGPSVPK					Mascot
1507.725	1507.7111	-0.0139	-9	474	486	EEKMGWFGPSVPK			Oxidation (M)[4]		Mascot
1570.837	1570.7839	-0.0531	-34	190	204	KPINNINVAGMGNTK					Mascot

Calc. Mass	Obsrv. Mass	± da	± ppm	Start Seq.	End Seq.	Sequence	Ion Score	C. I. %	Modification	Rank	Result Type
1585.8519	1585.7523	-0.0996	-63	306	318	KPKTCHNFIELAK			Carbamidomethyl (C)[5]		Mascot
1657.8334	1657.7758	-0.0576	-35	143	155	HWKDLLTDEPFTR					Mascot
1828.8242	1828.7888	-0.0354	-19	454	468	LTHEANAERENEEMR					Mascot
1828.8242	1828.7888	-0.0354	-19	454	468	LTHEANAERENEEMR					Mascot
1844.8192	1844.7358	-0.0834	-45	454	468	LTHEANAERENEEMR			Oxidation (M)[14]		Mascot
1927.0172	1926.8472	-0.17	-88	124	140	TTGNVFAYDTLEKLNIK					Mascot
2605.2644	2605.1865	-0.0779	-30	102	123	DEYFCPVTYKVFSDHTTI AAIK			Carbamidomethyl (C)[5]		Mascot
2807.3789	2807.3105	-0.0684	-24	380	405	DTNGSQFFITYAAAPHLD GLHTVFGK					Mascot

9	Enolase OS=Alkaliphilus metalliredigens (strain QYMF) GN=eno PE=3 SV=1	ENO_ALKMQ	46386.9	4.64	11	44	0	4.927

Peptide Information

Calc. Mass	Obsrv. Mass	± da	± ppm	Start Seq.	End Seq.	Sequence	Ion Score	C. I. %	Modification	Rank	Result Type
806.4519	806.4119	-0.04	-50	403	408	YNQLLR					Mascot
1037.5626	1037.5144	-0.0482	-46	2	10	TIISDVYAR					Mascot
1168.6031	1168.4957	-0.1074	-92	1	10	MTIISDVYAR					Mascot
1232.6191	1232.578	-0.0411	-33	93	103	IMLDLDGTPNK			Oxidation (M)[2]		Mascot
1431.7512	1431.8011	0.0499	35	93	105	IMLDLDGTPNKAK			Oxidation (M)[2]		Mascot
1660.7775	1660.8115	0.034	20	266	279	TAEEMVDFYEALVK			Oxidation (M)[5]		Mascot
1788.8724	1788.8099	-0.0625	-35	266	280	TAEEMVDFYEALVKK			Oxidation (M)[5]		Mascot
1828.8488	1828.7888	-0.06	-33	239	254	LALDVAATEFYDEDEK					Mascot
1828.8488	1828.7888	-0.06	-33	239	254	LALDVAATEFYDEDEK					Mascot
1932.0073	1932.0626	0.0553	29	17	34	GNPTIEVEVYLESGVLGR					Mascot
2360.2456	2360.031	-0.2146	-91	305	325	LGDNIQIVGDDLFVTNTE RLK					Mascot
2451.1902	2451.0442	-0.146	-60	409	429	IEDMLGFTGQYIGNEVFY NIK					Mascot
2467.1851	2467.0513	-0.1338	-54	409	429	IEDMLGFTGQYIGNEVFY NIK			Oxidation (M)[4]		Mascot

10	H-2 class I histocompatibility antigen, K-Q alpha chain (Fragment) OS=Mus musculus GN=H2-K1 PE=2 SV=1	HA1Q_MOUSE	37174.5	5.96	10	44	0	2.849

Peptide Information

Calc. Mass	Obsrv. Mass	± da	± ppm	Start Seq.	End Seq.	Sequence	Ion Score	C. I. %	Modification	Rank	Result Type
1055.6249	1055.5773	-0.0476	-45	225	234	WASVVVPLGK					Mascot
1105.5459	1105.5618	0.0159	14	319	328	VMVHDPHSLA					Mascot
1232.4672	1232.578	0.1108	90	79	89	MYGCDVGSDGR			Carbamidomethyl (C)[4], Oxidation (M)[1]		Mascot
1553.7417	1553.5964	-0.1453	-94	139	151	AYLEGACVEWLSR			Carbamidomethyl (C)[7]		Mascot

1595.7085	1595.7083	-0.0002	0	17	29	FDSDAENPRYEPR		Mascot
1598.7415	1598.8308	0.0893	56	79	92	MYGCDVGSDGRLLR	Carbamidomethyl (C)[4]	Mascot
1598.7415	1598.8308	0.0893	56	79	92	MYGCDVGSDGRLLR	Carbamidomethyl (C)[4]	Mascot
1657.873	1657.7758	-0.0972	-59	113	127	TWTAADMAALITKHK		Mascot
1908.8698	1908.791	-0.0788	-41	30	43	ARWMEQVEPEYWER		Mascot
1931.9796	1932.0626	0.083	43	139	154	AYLEGACVEWLSRHLK	Carbamidomethyl (C)[7]	Mascot
2336.0388	2336.0134	-0.0254	-11	93	112	GYEQVAYDGCDYIALNE DLK	Carbamidomethyl (C)[10]	Mascot

Gel Idx/Pos	153/G4	Instr./Gel Origin	BA2151/Sample Project 20140814	Process Status	Analysis Succeeded
Plate [#] Name	[1] Sample Project 20140814	Instrument Sample Name		Spectra	11

Rank	Protein Name	Accession No.	Protein MW	Protein PI	Pep. Count	Protein Score	Protein Score C. I. %	Intensity Matched	Total Ion Score	Total Ion C. I. %	Confirmed
1	Alpha-amylase/trypsin inhibitor CMb OS=Hordeum vulgare GN=IAT2 PE=1 SV=2	IAAB_HORVU	17199.2	5.77	6	95	99.981	13.807	60	99.935	

Peptide Information

Calc. Mass	Obsrv. Mass	± da	± ppm	Start Seq.	End Seq.	Sequence	Ion Score	C. I. %	Modification	Rank	Result Type
801.4076	801.3926	-0.015	-19	86	91	FFMGRK			Oxidation (M)[3]		Mascot
1023.4928	1023.462	-0.0308	-30	108	115	EVQMDFVR					Mascot
1039.4878	1039.4647	-0.0231	-22	108	115	EVQMDFVR			Oxidation (M)[4]		Mascot
1168.5052	1168.486	-0.0192	-16	46	54	DYVEQQACR			Carbamidomethyl (C)[8]		Mascot
1168.5052	1168.486	-0.0192	-16	46	54	DYVEQQACR	19	0	Carbamidomethyl (C)[8]		Mascot
1799.8528	1799.8123	-0.0405	-23	92	107	SRPDQSGLMELPGCPR			Carbamidomethyl (C)[14]		Mascot
1799.8528	1799.8123	-0.0405	-23	92	107	SRPDQSGLMELPGCPR	41	94.962	Carbamidomethyl (C)[14]		Mascot
1815.8477	1815.7909	-0.0568	-31	92	107	SRPDQSGLMELPGCPR			Carbamidomethyl (C)[14], Oxidation (M)[9]		Mascot
1815.8477	1815.7909	-0.0568	-31	92	107	SRPDQSGLMELPGCPR	28	0	Carbamidomethyl (C)[14], Oxidation (M)[9]		Mascot
1861.8102	1861.7733	-0.0369	-20	66	80	QQCCGELANIPQQCR			Carbamidomethyl (C)[3,4,14]		Mascot
1927.9478	1927.7932	-0.1546	-80	91	107	KSRPDQSGLMELPGCPR			Carbamidomethyl (C)[15]		Mascot

Rank	Protein Name	Accession No.	Protein MW	Protein PI	Pep. Count	Protein Score	Protein Score C. I. %	Intensity Matched	Total Ion Score	Total Ion C. I. %	Confirmed
2	Alpha-amylase/trypsin inhibitor CM16 OS=Triticum aestivum PE=1 SV=1	IAC16_WHEAT	16398.8	5.31	4	80	99.393	13.591	60	99.935	

Peptide Information

Calc. Mass	Obsrv. Mass	± da	± ppm	Start Seq.	End Seq.	Sequence	Ion Score	C. I. %	Modification	Rank	Result Type
1023.4928	1023.462	-0.0308	-30	108	115	EVQMDFVR					Mascot
1039.4878	1039.4647	-0.0231	-22	108	115	EVQMDFVR			Oxidation (M)[4]		Mascot
1168.5052	1168.486	-0.0192	-16	46	54	DYVEQQACR			Carbamidomethyl (C)[8]		Mascot
1168.5052	1168.486	-0.0192	-16	46	54	DYVEQQACR	19	0	Carbamidomethyl (C)[8]		Mascot
1799.8528	1799.8123	-0.0405	-23	92	107	SRPDQSGLMELPGCPR			Carbamidomethyl (C)[14]		Mascot
1799.8528	1799.8123	-0.0405	-23	92	107	SRPDQSGLMELPGCPR	41	94.962	Carbamidomethyl (C)[14]		Mascot
1815.8477	1815.7909	-0.0568	-31	92	107	SRPDQSGLMELPGCPR			Carbamidomethyl (C)[14], Oxidation (M)[9]		Mascot
1815.8477	1815.7909	-0.0568	-31	92	107	SRPDQSGLMELPGCPR	28	0	Carbamidomethyl (C)[14], Oxidation (M)[9]		Mascot
1861.8102	1861.7733	-0.0369	-20	66	80	QQCCGELANIPQQCR			Carbamidomethyl (C)[3,4,14]		Mascot

Rank	Protein Name	Accession No.	Protein MW	Protein PI	Pep. Count	Protein Score	Protein Score C. I. %	Intensity Matched	Total Ion Score	Total Ion C. I. %	Confirmed
3	Uncharacterized ribulose bisphosphate carboxylase-like	RBLL_SYMSP	57729.2	6.3	12	49	0	3.526			

protein (Fragment) OS=Symbiodinium sp. GN=rbcG PE=3 SV=1

Peptide Information

Calc. Mass	Obsrv. Mass	± da	± ppm	Start Seq.	End Seq.	Sequence	Ion Score	C. I. %	Modification	Rank	Result Type
986.4955	986.469	-0.0265	-27	270	276	QFVHYHR					Mascot
1227.6077	1227.5857	-0.022	-18	511	522	SIHPASSSNTAR					Mascot
1345.6893	1345.6583	-0.031	-23	147	161	GASNGGLAAGAMKPK			Oxidation (M)[12]		Mascot
1781.9069	1781.8518	-0.0551	-31	79	94	IAYPTVLFDSNLTDGR					Mascot
1798.9844	1798.8446	-0.1398	-78	143	161	ILDKGASNGGLAAGAMKPK					Mascot
1822.7661	1822.7942	0.0281	15	220	235	HSGGNYTDEPKEMDAR			Oxidation (M)[13]		Mascot
1839.8906	1839.8215	-0.0691	-38	492	510	MASQSTAGSAVNPYTGGLK					Mascot
1855.8855	1855.7639	-0.1216	-66	492	510	MASQSTAGSAVNPYTGGLK			Oxidation (M)[1]		Mascot
1944.9232	1944.8005	-0.1227	-63	389	406	DGPASGAIACRQVEEAWK			Carbamidomethyl (C)[10]		Mascot
2157.0532	2156.9851	-0.0681	-32	99	118	SFLTLAIGNTQGMEDVEFGK					Mascot
2173.0483	2172.9834	-0.0649	-30	99	118	SFLTLAIGNTQGMEDVEFGK			Oxidation (M)[13]		Mascot
2262.9907	2263	0.0093	4	34	54	MGYDYLATAAHFAAESCTGAR			Carbamidomethyl (C)[17]		Mascot
2278.9856	2279.0071	0.0215	9	34	54	MGYDYLATAAHFAAESCTGAR			Carbamidomethyl (C)[17], Oxidation (M)[1]		Mascot
2807.3022	2807.2622	-0.04	-14	340	363	LCNQEWEGMRQNAPIISGSMNALR			Carbamidomethyl (C)[2], Oxidation (M)[9,20]		Mascot
3029.5037	3029.4021	-0.1016	-34	290	317	GYSAFVHTKISQVISTTSIHADTMSFGK			Oxidation (M)[24]		Mascot

4	Zinc finger protein 77 OS=Homo sapiens GN=ZNF77 PE=2 SV=2	ZNF77_HUMAN	64546	9.04	13	47	0	10.74

Peptide Information

Calc. Mass	Obsrv. Mass	± da	± ppm	Start Seq.	End Seq.	Sequence	Ion Score	C. I. %	Modification	Rank	Result Type
809.3748	809.3411	-0.0337	-42	51	58	TSGSSSQR					Mascot
1023.4598	1023.462	0.0022	2	31	38	DVMLETCR			Carbamidomethyl (C)[7]		Mascot
1039.4547	1039.4647	0.01	10	31	38	DVMLETCR			Carbamidomethyl (C)[7], Oxidation (M)[3]		Mascot
1345.5988	1345.6583	0.0595	44	317	327	THTGEKPCQCK			Carbamidomethyl (C)[8,10]		Mascot
1788.7977	1788.7603	-0.0374	-21	74	88	FTGSDSWSIFGENWR					Mascot
1814.8239	1814.7915	-0.0324	-18	341	355	EHGRTHSGEKPYECK			Carbamidomethyl (C)[14]		Mascot
1815.8265	1815.7909	-0.0356	-20	429	443	THTGEKPFECKHCGK			Carbamidomethyl (C)[10,13]		Mascot
1815.8265	1815.7909	-0.0356	-20	429	443	THTGEKPFECKHCGK			Carbamidomethyl (C)[10,13]		Mascot
1822.8397	1822.7942	-0.0455	-25	373	387	MHTGEKPYVCKQCGK			Carbamidomethyl (C)[10,13]		Mascot

1827.8048	1827.7761	-0.0287	-16	317	331	THTGEKPCQCKHCGK			Carbamidomethyl (C)[8,10,13]		Mascot
1831.8214	1831.7776	-0.0438	-24	289	303	THTGEKPYECKHCGK			Carbamidomethyl (C)[10,13]		Mascot
1842.8361	1842.7883	-0.0478	-26	134	149	SYPTEAKPSECTKCGK			Carbamidomethyl (C)[11,14]		Mascot
1842.8361	1842.7883	-0.0478	-26	134	149	SYPTEAKPSECTKCGK			Carbamidomethyl (C)[11,14]		Mascot
1870.8865	1870.8108	-0.0757	-40	285	299	EHVRTHTGEKPYECK			Carbamidomethyl (C)[14]		Mascot
1876.8616	1876.7734	-0.0882	-47	369	383	AHMRMHTGEKPYVCK			Carbamidomethyl (C)[14], Oxidation (M)[3,5]		Mascot
1961.969	1961.8221	-0.1469	-75	89	104	FDNTGDQHQIPQRHLR					Mascot
1961.969	1961.8221	-0.1469	-75	89	104	FDNTGDQHQIPQRHLR					Mascot

5	Cysteine-rich receptor-like protein kinase 37 OS=Arabidopsis thaliana GN=CRK37 PE=3 SV=1	CRK37_ARATH	74191.3	7.15	13	46	0	2.076	

Peptide Information

Calc. Mass	Obsrv. Mass	± da	± ppm	Start Seq.	End Seq.	Sequence	Ion Score	C. I. %	Modification	Rank	Result Type
820.4312	820.3605	-0.0707	-86	255	261	AFDNVVR					Mascot
1039.4514	1039.4647	0.0133	13	221	228	ECVNDFQK			Carbamidomethyl (C)[2]		Mascot
1768.9229	1768.7838	-0.1391	-79	262	278	VPAPPPQASSTIIDYGR					Mascot
1771.9259	1771.7704	-0.1555	-88	470	485	DLKASNILLDAEMNPK					Mascot
1831.8678	1831.7776	-0.0902	-49	87	101	QACKTCLEHVIEDTK			Carbamidomethyl (C)[3,6]		Mascot
1873.8572	1873.8174	-0.0398	-21	486	501	VADFGMARLFDMDETR					Mascot
1875.9231	1875.7806	-0.1425	-76	528	544	SDVYSFGVMLLEMISGK					Mascot
1889.8521	1889.812	-0.0401	-21	486	501	VADFGMARLFDMDETR			Oxidation (M)[6]		Mascot
2262.1248	2261.9968	-0.128	-57	171	191	TLEAASTAENSSVLKYYS ATR					Mascot
2263.0652	2263	-0.0652	-29	549	566	LEKEEEEEEEELPAFVWK					Mascot
2276.0725	2276.0083	-0.0642	-28	313	332	QSHTIINDVFDSNNGQSM LR					Mascot
2279.116	2279.0071	-0.1089	-48	473	493	ASNILLDAEMNPKVADFG MAR			Oxidation (M)[10]		Mascot
2807.353	2807.2622	-0.0908	-32	313	336	QSHTIINDVFDSNNGQSM LRFDLR					Mascot
3029.4165	3029.4021	-0.0144	-5	192	217	TEFTQISDVYALMQCVPD LSPGNCKR			Carbamidomethyl (C)[15,24]		Mascot

6	CWF19-like protein 2 OS=Xenopus tropicalis GN=cwf19l2 PE=2 SV=1	C19L2_XENTR	108315.2	8.78	17	46	0	15.403	

Peptide Information

Calc. Mass	Obsrv. Mass	± da	± ppm	Start Seq.	End Seq.	Sequence	Ion Score	C. I. %	Modification	Rank	Result Type
1039.4561	1039.4647	0.0086	8	690	697	MQAMHEHR					Mascot
1099.5994	1099.5369	-0.0625	-57	134	143	AEVKAEVPEK					Mascot

Calc. Mass	Obsrv. Mass	± da	± ppm	Start Seq.	End Seq.	Sequence	Ion Score	C. I. %	Modification	Rank	Result Type
1196.5763	1196.5199	-0.0564	-47	698	707	SLTATMEKCR			Carbamidomethyl (C)[9]		Mascot
1237.6396	1237.6034	-0.0362	-29	494	503	ERPQSPRPDR					Mascot
1556.6985	1556.6936	-0.0049	-3	706	717	CRFCFDNAELPK			Carbamidomethyl (C)[1,4]		Mascot
1771.9014	1771.7704	-0.131	-74	193	208	ELNPYWKDGGTGLPPK					Mascot
1788.8107	1788.7603	-0.0504	-28	265	281	LEAAEQAASQNSNDGQR					Mascot
1797.968	1797.8259	-0.1421	-79	879	893	EIIGGMLDLEPRIWR					Mascot
1815.8481	1815.7909	-0.0572	-32	297	311	YPRSSQHTDDNSRPR					Mascot
1815.8481	1815.7909	-0.0572	-32	297	311	YPRSSQHTDDNSRPR	3	0			Mascot
1870.7913	1870.8108	0.0195	10	621	635	YFHDDDNQSLQDMVK			Oxidation (M)[13]		Mascot
1876.8052	1876.7734	-0.0318	-17	1	17	MAADGGMFESSRSIEEK			Oxidation (M)[1,7]		Mascot
1916.021	1915.8358	-0.1852	-97	494	509	ERPQSPRPDRSPQPIR					Mascot
1916.8776	1916.7744	-0.1032	-54	690	705	MQAMHEHRSLTATMEK			Oxidation (M)[1]		Mascot
1916.9056	1916.7744	-0.1312	-68	264	281	KLEAAEQAASQNSNDGQR					Mascot
1932.8724	1932.7712	-0.1012	-52	690	705	MQAMHEHRSLTATMEK			Oxidation (M)[1,4]		Mascot
1948.8674	1948.7908	-0.0766	-39	690	705	MQAMHEHRSLTATMEK			Oxidation (M)[1,4,14]		Mascot
2134.0928	2133.9463	-0.1465	-69	357	375	SVSFKPKNPFLKPSDDDGR					Mascot
2173.1475	2172.9834	-0.1641	-76	708	726	FCFDNAELPKHLIVAIGTK			Carbamidomethyl (C)[2]		Mascot
2279.0835	2279.0071	-0.0764	-34	774	792	MFESKGLDCVFLESNIYAR			Carbamidomethyl (C)[9]		Mascot

7	Phosphopantetheine adenylyltransferase OS=Thermosipho melanesiensis (strain BI429 / DSM 12029) GN=coaD PE=3 SV=1	COAD_THEM4	18742.8	9.21	8	44	0	20.172	

Peptide Information

Calc. Mass	Obsrv. Mass	± da	± ppm	Start Seq.	End Seq.	Sequence	Ion Score	C. I. %	Modification	Rank	Result Type
1556.803	1556.6936	-0.1094	-70	26	38	IFSEVYVVVMENK					Mascot
1572.7979	1572.6685	-0.1294	-82	26	38	IFSEVYVVVMENK			Oxidation (M)[10]		Mascot
1799.8013	1799.8123	0.011	6	106	120	EMCPNVDTVFLMTDK			Carbamidomethyl (C)[3]		Mascot
1799.8013	1799.8123	0.011	6	106	120	EMCPNVDTVFLMTDK			Carbamidomethyl (C)[3]		Mascot
1815.7963	1815.7909	-0.0054	-3	106	120	EMCPNVDTVFLMTDK			Carbamidomethyl (C)[3], Oxidation (M)[2]		Mascot
1815.7963	1815.7909	-0.0054	-3	106	120	EMCPNVDTVFLMTDK			Carbamidomethyl (C)[3], Oxidation (M)[2]		Mascot
1831.7911	1831.7776	-0.0135	-7	106	120	EMCPNVDTVFLMTDK			Carbamidomethyl (C)[3], Oxidation (M)[2,12]		Mascot
1872.9777	1872.8197	-0.158	-84	23	38	ATKIFSEVYVVVMENK			Oxidation (M)[13]		Mascot
1876.8456	1876.7734	-0.0722	-38	90	105	AVTDFEYELQMAMANK			Oxidation (M)[11]		Mascot
1927.8962	1927.7932	-0.103	-53	106	121	EMCPNVDTVFLMTDKK			Carbamidomethyl (C)[3]		Mascot
2203.0522	2202.99	-0.0622	-28	87	105	GLRAVTDFEYELQMAMANK			Oxidation (M)[14]		Mascot
2248.2012	2247.9958	-0.2054	-91	2	21	KAIYPGSFDPITYGHLDIIK					Mascot

2248.2012	2247.9958	-0.2054	-91	2	21	KAIYPGSFDPITYGHLDIIK					Mascot
2276.2075	2276.0083	-0.1992	-88	3	22	AIYPGSFDPITYGHLDIIKR					Mascot

8 Ornithine aminotransferase OS=Geobacillus sp. (strain WCH70) GN=rocD PE=3 SV=1 | OAT_GEOSW | 44411.7 | 5.93 | 10 | 42 | 0 | 8.105

Peptide Information

Calc. Mass	Obsrv. Mass	± da	± ppm	Start Seq.	End Seq.	Sequence	Ion Score	C. I. %	Modification	Rank	Result Type
1345.7726	1345.6583	-0.1143	-85	170	181	IIPYGDVEALKK					Mascot
1798.9116	1798.8446	-0.067	-37	358	372	QEGLLCKETHDTVIR			Carbamidomethyl (C)[6]		Mascot
1842.8262	1842.7883	-0.0379	-21	42	57	YMDMLSAYSAVNQGHR					Mascot
1842.8262	1842.7883	-0.0379	-21	42	57	YMDMLSAYSAVNQGHR					Mascot
1890.9556	1890.8241	-0.1315	-70	218	234	ENNVLYIADEIQSGLGR					Mascot
1955.9089	1955.771	-0.1379	-71	98	115	DMVLPMNTGAEAVETAF K			Oxidation (M)[2,6]		Mascot
2157.134	2156.9851	-0.1489	-69	10	28	LTEQYGANNYHPLPVVLT K					Mascot
2203.9861	2203.9839	-0.0022	-1	238	255	MFACDWENVVPDMYILG K			Carbamidomethyl (C)[4], Oxidation (M)[1]		Mascot
2231.2144	2231.0403	-0.1741	-78	160	180	GFGPMLPGIKIIPYGDVEA LK			Oxidation (M)[5]		Mascot
2276.146	2276.0083	-0.1377	-60	72	90	ITLTSRAFHNDQLGPWYE K					Mascot
2290.176	2290.0178	-0.1582	-69	336	355	GRGLFIGVELHTSARPYC EK			Carbamidomethyl (C)[18]		Mascot

9 UPF0538 protein C2orf76 homolog OS=Xenopus laevis PE=2 SV=1 | CB076_XENLA | 14658.5 | 7.06 | 7 | 42 | 0 | 2.338

Peptide Information

Calc. Mass	Obsrv. Mass	± da	± ppm	Start Seq.	End Seq.	Sequence	Ion Score	C. I. %	Modification	Rank	Result Type
801.4254	801.3926	-0.0328	-41	120	126	ANPVSKW					Mascot
986.553	986.469	-0.084	-85	66	74	VIHQPHGAK					Mascot
1077.5721	1077.4797	-0.0924	-86	2	11	MSQGVTVTVR					Mascot
1167.7208	1167.6178	-0.103	-88	88	97	LILAPDRTLR					Mascot
1785.9396	1785.7627	-0.1769	-99	20	34	NFRPVVYHGVNLDQK					Mascot
1831.8433	1831.7776	-0.0657	-36	98	113	DAGVAHETELAFFCHK			Carbamidomethyl (C)[14]		Mascot
1845.9164	1845.7786	-0.1378	-75	35	49	VQEFIMQINDDIPQR					Mascot
1861.9114	1861.7733	-0.1381	-74	35	49	VQEFIMQINDDIPQR			Oxidation (M)[6]		Mascot

10 Elongation factor 2 OS=Ashbya gossypii (strain ATCC 10895 / CBS 109.51 / FGSC 9923 / NRRL Y-1056) GN=EFT1 PE=3 SV=1 | EF2_ASHGO | 93943.5 | 6.07 | 16 | 42 | 0 | 5.998

Peptide Information

Calc. Mass	Obsrv. Mass	± da	± ppm	Start Seq.	End Seq.	Sequence	Ion Score	C. I. %	Modification	Rank	Result Type
804.3846	804.3373	-0.0473	-59	61	66	KDEQER					Mascot
809.4008	809.3411	-0.0597	-74	434	440	AVLMMGR			Oxidation (M)[4,5]		Mascot
1039.5493	1039.4647	-0.0846	-81	376	384	ADLMLYVSK					Mascot
1077.5179	1077.4797	-0.0382	-35	701	710	GAGQIMPTMR			Oxidation (M)[6]		Mascot
1771.7957	1771.7704	-0.0253	-14	618	632	VMADEYGWDVTDARK			Oxidation (M)[2]		Mascot
1773.8087	1773.7592	-0.0495	-28	240	253	MMERLWGDSYFNPK					Mascot
1785.8875	1785.7627	-0.1248	-70	1	15	MVAFTVDQIRSLMDK			Oxidation (M)[1,13]		Mascot
1788.8208	1788.7603	-0.0605	-34	72	87	STAISLFSEMSEEDVK			Oxidation (M)[10]		Mascot
1797.9126	1797.8259	-0.0867	-48	376	391	ADLMLYVSKMVPTSDK					Mascot
1799.8964	1799.8123	-0.0841	-47	770	785	AYLPVNESFGFTGELR					Mascot
1799.8964	1799.8123	-0.0841	-47	770	785	AYLPVNESFGFTGELR					Mascot
1829.9023	1829.7933	-0.109	-60	376	391	ADLMLYVSKMVPTSDK			Oxidation (M)[4,10]		Mascot
1870.8389	1870.8108	-0.0281	-15	616	631	ARVMADEYGWDVTDAR			Oxidation (M)[4]		Mascot
1889.9451	1889.812	-0.1331	-70	565	582	ETVEGESSQVALSKSPN K					Mascot
1927.9253	1927.7932	-0.1321	-69	466	482	TGTLTTFESAHNMKVMK			Oxidation (M)[13,16]		Mascot
1962.9344	1962.8198	-0.1146	-58	826	841	HGLKENVPGWQEYYDK					Mascot
2173.175	2172.9834	-0.1916	-88	586	605	IYLKAQPIDEEVSLAIEGG K					Mascot
3039.6157	3039.4082	-0.2075	-68	326	352	KFLPAADALLEMIIMHLPS PVTAQNYR					Mascot

Gel Idx/Pos	154/G5	Instr./Gel Origin	BA2151/Sample Project 20140814	Process Status	Analysis Succeeded
Plate [#] Name	[1] Sample Project 20140814	Instrument Sample Name		Spectra	11

Rank	Protein Name	Accession No.	Protein MW	Protein PI	Pep. Count	Protein Score	Protein Score C. I. %	Intensity Matched	Total Ion Score	Total Ion C. I. %	Confirmed
1	Alpha-amylase/trypsin inhibitor CMb OS=Hordeum vulgare GN=IAT2 PE=1 SV=2	IAAB_HORVU	17199.2	5.77	5	111	100	20.323	86	100	

Peptide Information

Calc. Mass	Obsrv. Mass	± da	± ppm	Start Seq.	End Seq.	Sequence	Ion Score	C. I. %	Modification	Rank	Result Type
801.4076	801.3785	-0.0291	-36	86	91	FFMGRK			Oxidation (M)[3]		Mascot
1039.4878	1039.4535	-0.0343	-33	108	115	EVQMDFVR			Oxidation (M)[4]		Mascot
1168.5052	1168.489	-0.0162	-14	46	54	DYVEQQACR			Carbamidomethyl (C)[8]		Mascot
1168.5052	1168.489	-0.0162	-14	46	54	DYVEQQACR	25	0	Carbamidomethyl (C)[8]		Mascot
1799.8528	1799.8337	-0.0191	-11	92	107	SRPDQSGLMELPGCPR			Carbamidomethyl (C)[14]		Mascot
1815.8477	1815.7843	-0.0634	-35	92	107	SRPDQSGLMELPGCPR			Carbamidomethyl (C)[14], Oxidation (M)[9]		Mascot
1815.8477	1815.7843	-0.0634	-35	92	107	SRPDQSGLMELPGCPR	61	99.931	Carbamidomethyl (C)[14], Oxidation (M)[9]		Mascot
1861.8102	1861.7701	-0.0401	-22	66	80	QQCCGELANIPQQCR			Carbamidomethyl (C)[3,4,14]		Mascot

Rank	Protein Name	Accession No.	Protein MW	Protein PI	Pep. Count	Protein Score	Protein Score C. I. %	Intensity Matched	Total Ion Score	Total Ion C. I. %	Confirmed
2	Alpha-amylase/trypsin inhibitor CM16 OS=Triticum aestivum PE=1 SV=1	IAC16_WHEAT	16398.8	5.31	4	105	99.998	20.146	86	100	

Peptide Information

Calc. Mass	Obsrv. Mass	± da	± ppm	Start Seq.	End Seq.	Sequence	Ion Score	C. I. %	Modification	Rank	Result Type
1039.4878	1039.4535	-0.0343	-33	108	115	EVQMDFVR			Oxidation (M)[4]		Mascot
1168.5052	1168.489	-0.0162	-14	46	54	DYVEQQACR			Carbamidomethyl (C)[8]		Mascot
1168.5052	1168.489	-0.0162	-14	46	54	DYVEQQACR	25	0	Carbamidomethyl (C)[8]		Mascot
1799.8528	1799.8337	-0.0191	-11	92	107	SRPDQSGLMELPGCPR			Carbamidomethyl (C)[14]		Mascot
1815.8477	1815.7843	-0.0634	-35	92	107	SRPDQSGLMELPGCPR			Carbamidomethyl (C)[14], Oxidation (M)[9]		Mascot
1815.8477	1815.7843	-0.0634	-35	92	107	SRPDQSGLMELPGCPR	61	99.931	Carbamidomethyl (C)[14], Oxidation (M)[9]		Mascot
1861.8102	1861.7701	-0.0401	-22	66	80	QQCCGELANIPQQCR			Carbamidomethyl (C)[3,4,14]		Mascot

Rank	Protein Name	Accession No.	Protein MW	Protein PI	Pep. Count	Protein Score	Protein Score C. I. %	Intensity Matched	Total Ion Score	Total Ion C. I. %	Confirmed
3	Ketol-acid reductoisomerase OS=Desulfovibrio desulfuricans (strain G20) GN=ilvC PE=3 SV=1	ILVC_DESDG	36391.4	5.7	10	45	0	6.748			

Peptide Information

Calc. Mass	Obsrv. Mass	± da	± ppm	Start Seq.	End Seq.	Sequence	Ion Score	C. I. %	Modification	Rank	Result Type
908.4368	908.384	-0.0528	-58	322	328	SMMPWLK			Oxidation (M)[2]		Mascot

Calc. Mass	Obsrv. Mass	± da	± ppm	Start Seq.	End Seq.	Sequence	Ion Score	C. I. %	Modification	Rank	Result Type
1150.5813	1150.4761	-0.1052	-91	121	130	DVDVFMIAPK			Oxidation (M)[6]		Mascot
1182.5936	1182.5389	-0.0547	-46	164	175	ALAYAMGVGGTR			Oxidation (M)[6]		Mascot
1193.6169	1193.5889	-0.028	-23	320	328	LRSMMPWLK			Oxidation (M)[4,5]		Mascot
1196.6171	1196.52	-0.0971	-81	296	305	ANYPTFTARR					Mascot
1768.9116	1768.78	-0.1316	-74	3	17	VYYEQDANLEVLKGK					Mascot
1770.793	1770.7762	-0.0168	-9	248	263	HSISDTAEYGDYVTGR					Mascot
1842.9307	1842.7971	-0.1336	-72	1	15	MKVYYEQDANLEVLK					Mascot
1842.9307	1842.7971	-0.1336	-72	1	15	MKVYYEQDANLEVLK					Mascot
1926.894	1926.8394	-0.0546	-28	248	264	HSISDTAEYGDYVTGRR					Mascot
2157.1121	2156.989	-0.1231	-57	164	184	ALAYAMGVGGTRSGVIE TTFR					Mascot
2173.1072	2172.9656	-0.1416	-65	164	184	ALAYAMGVGGTRSGVIE TTFR			Oxidation (M)[6]		Mascot

4	Tetrahydroxynaphthalene reductase OS=Magnaporthe oryzae (strain 70-15 / ATCC MYA-4617 / FGSC 8958) GN=MGG_02252 PE=1 SV=2	T4HR_MAGO7	30433.4	6.24	10	45	0	18.569	

Peptide Information

Calc. Mass	Obsrv. Mass	± da	± ppm	Start Seq.	End Seq.	Sequence	Ion Score	C. I. %	Modification	Rank	Result Type
848.4261	848.3469	-0.0792	-93	175	182	HAVYSGSK					Mascot
1054.4366	1054.4471	0.0105	10	191	199	CMAIDMADK			Carbamidomethyl (C)[1]		Mascot
1107.4954	1107.5171	0.0217	20	125	133	DVTPEEFDR					Mascot
1167.7096	1167.6013	-0.1083	-93	201	212	ITVNVVAPGGIK					Mascot
1168.4874	1168.489	0.0016	1	213	221	TDMYHAVCR			Carbamidomethyl (C)[8], Oxidation (M)[3]		Mascot
1168.4874	1168.489	0.0016	1	213	221	TDMYHAVCR			Carbamidomethyl (C)[8], Oxidation (M)[3]		Mascot
1182.5316	1182.5389	0.0073	6	191	200	CMAIDMADKK			Carbamidomethyl (C)[1]		Mascot
1231.7079	1231.59	-0.1179	-96	159	170	LILMGSITGQAK					Mascot
1231.7079	1231.59	-0.1179	-96	159	170	LILMGSITGQAK					Mascot
1799.9175	1799.8337	-0.0838	-47	13	30	YDAIPGPLGPQSASLEGK					Mascot
1915.8711	1915.83	-0.0411	-21	183	199	GAIETFARCMAIDMADK			Carbamidomethyl (C)[9], Oxidation (M)[10]		Mascot
2263.1904	2263.019	-0.1714	-76	104	124	IFGKLDIVCSNSGVVSFG HVK			Carbamidomethyl (C)[9]		Mascot

5	DNA-directed RNA polymerase subunit beta' OS=Mesorhizobium sp. (strain BNC1) GN=rpoC PE=3 SV=1	RPOC_MESSB	155903	6.65	23	44	0	12.785	

Peptide Information

Calc. Mass	Obsrv. Mass	± da	± ppm	Start Seq.	End Seq.	Sequence	Ion Score	C. I. %	Modification	Rank	Result Type
823.3688	823.3573	-0.0115	-14	693	699	VADEMMK					Mascot

Calc. Mass	Obsrv. Mass	± da	± ppm	Start Seq.	End Seq.	Sequence	Ion Score	C. I. %	Modification	Rank	Result Type
908.436	908.384	-0.052	-57	998	1005	IFVDDGDK					Mascot
913.4448	913.3907	-0.0541	-59	604	610	MIDTVYR			Oxidation (M)[1]		Mascot
982.4952	982.4262	-0.069	-70	786	794	TANSGYLTR					Mascot
1039.4878	1039.4535	-0.0343	-33	616	623	ETVIFCDR			Carbamidomethyl (C)[6]		Mascot
1068.5685	1068.4811	-0.0874	-82	391	400	GYSSTVKQAK					Mascot
1105.575	1105.4919	-0.0831	-75	988	997	ASHKVTYGSR					Mascot
1151.5791	1151.4907	-0.0884	-77	205	215	SELASTTSDLK					Mascot
1193.6313	1193.5889	-0.0424	-36	679	688	YNKVVDAWAK					Mascot
1231.6277	1231.59	-0.0377	-31	1368	1377	SRDDLILDER					Mascot
1231.6277	1231.59	-0.0377	-31	1368	1377	SRDDLILDER					Mascot
1234.6072	1234.6041	-0.0031	-3	624	634	IMALGFSHACK			Carbamidomethyl (C)[10]		Mascot
1265.6121	1265.582	-0.0301	-24	703	713	AIEFDDSGRQK					Mascot
1320.6365	1320.5688	-0.0677	-51	682	692	VVDAWAKCSER			Carbamidomethyl (C)[8]		Mascot
1507.7145	1507.7124	-0.0021	-1	604	615	MIDTVYRHCGQK			Carbamidomethyl (C)[9]		Mascot
1649.7523	1649.8076	0.0553	34	611	623	HCGQKETVIFCDR			Carbamidomethyl (C)[2,11]		Mascot
1734.8593	1734.7842	-0.0751	-43	300	314	MLQEAVDALFDNGRR					Mascot
1751.9287	1751.8254	-0.1033	-59	778	794	GLADTALKTANSGYLTR					Mascot
1751.9287	1751.8254	-0.1033	-59	778	794	GLADTALKTANSGYLTR					Mascot
1827.8331	1827.7661	-0.067	-37	761	776	EGLTVMEYFNSTHGAR			Oxidation (M)[6]		Mascot
1906.007	1905.9261	-0.0809	-42	1201	1218	GDFILDGNPAPHDILAIK					Mascot
1916.8412	1916.78	-0.0612	-32	712	727	QKQMNSIYMMSHSGAR			Oxidation (M)[4,9,10]		Mascot
1916.8412	1916.78	-0.0612	-32	712	727	QKQMNSIYMMSHSGAR			Oxidation (M)[4,9,10]		Mascot
1955.928	1955.7363	-0.1917	-98	761	777	EGLTVMEYFNSTHGARK			Oxidation (M)[6]		Mascot
2098.1138	2097.9436	-0.1702	-81	859	877	LIDERDVEAIEAAGIQTVR					Mascot
2839.4482	2839.2646	-0.1836	-65	575	598	MIIGELLPKNHNVPFDICN QELTK			Carbamidomethyl (C)[18], Oxidation (M)[1]		Mascot

6	Paramyosin OS=Caenorhabditis elegans GN=unc-15 PE=1 SV=1	MYSP_CAEEL	102002.2	5.28	16	43	0	6.776

Peptide Information

Calc. Mass	Obsrv. Mass	± da	± ppm	Start Seq.	End Seq.	Sequence	Ion Score	C. I. %	Modification	Rank	Result Type
866.44	866.355	-0.085	-98	551	557	MKSEISR			Oxidation (M)[1]		Mascot
1150.5409	1150.4761	-0.0648	-56	523	531	EEEMEALRK			Oxidation (M)[4]		Mascot
1193.6637	1193.5889	-0.0748	-63	239	248	VQLDNLQHVK					Mascot
1231.6165	1231.59	-0.0265	-22	60	69	LLQEDLESER					Mascot
1231.6165	1231.59	-0.0265	-22	60	69	LLQEDLESER					Mascot
1234.6062	1234.6041	-0.0021	-2	195	205	FEAQANELANK					Mascot

Calc. Mass	Obsrv. Mass	± da	± ppm	Start Seq.	End Seq.	Sequence	Ion Score	C. I. %	Modification	Rank	Result Type
1751.8521	1751.8254	-0.0267	-15	340	353	QAEYEEQIEIMLQK					Mascot
1751.8521	1751.8254	-0.0267	-15	340	353	QAEYEEQIEIMLQK					Mascot
1771.8457	1771.7725	-0.0732	-41	288	303	TALDEESIARSDAEHK					Mascot
1862.923	1862.7776	-0.1454	-78	674	690	LETELSTAQADLDEVTK					Mascot
1890.9299	1890.8411	-0.0888	-47	1	15	MERLESLEPLELMER			Oxidation (M)[1]		Mascot
1905.9447	1905.9261	-0.0186	-10	824	839	RQLAESESVTMQNLQR			Oxidation (M)[11]		Mascot
1908.9087	1908.7847	-0.124	-65	318	333	FDAEVALHHEEVEDLR					Mascot
1932.9662	1932.7833	-0.1829	-95	195	211	FEAQANELANKVEDLNK					Mascot
2098.0564	2097.9436	-0.1128	-54	298	315	SDAEHKLNLANTEITQWK					Mascot
2263.1135	2263.019	-0.0945	-42	709	727	AVEQLHEEQEHSMKIDALR					Mascot
2276.0613	2275.9841	-0.0772	-34	139	156	HQDSCLDYQDQIEQLQKK			Carbamidomethyl (C)[5]		Mascot
2839.3931	2839.2646	-0.1285	-45	30	57	SPSQAAFGAPFGSMSVADLGSLTRLEDK					Mascot

7	Protein OS-9 homolog OS=Candida glabrata (strain ATCC 2001 / CBS 138 / JCM 3761 / NBRC 0622 / NRRL Y-65) GN=YOS9 PE=3 SV=1	OS9_CANGA	78267.3	4.84	12	43	0	7.024

Peptide Information

Calc. Mass	Obsrv. Mass	± da	± ppm	Start Seq.	End Seq.	Sequence	Ion Score	C. I. %	Modification	Rank	Result Type
1054.481	1054.4471	-0.0339	-32	64	71	CFLPNMTR			Carbamidomethyl (C)[1], Oxidation (M)[6]		Mascot
1077.4695	1077.4811	0.0116	11	680	688	DEEVVESDR					Mascot
1077.4695	1077.4811	0.0116	11	680	688	DEEVVESDR	8	0			Mascot
1190.576	1190.4581	-0.1179	-99	581	592	ALGSNIDNSSGR					Mascot
1734.7413	1734.7842	0.0429	25	538	554	GDTDTPPQSSQSSANDK					Mascot
1799.856	1799.8337	-0.0223	-12	72	86	LHEQNVQSVNDPEYK					Mascot
1825.8572	1825.7783	-0.0789	-43	235	250	NAVPIICHMSNDVPDK			Carbamidomethyl (C)[7], Oxidation (M)[9]		Mascot
1825.8572	1825.7783	-0.0789	-43	235	250	NAVPIICHMSNDVPDK			Carbamidomethyl (C)[7], Oxidation (M)[9]		Mascot
1841.9678	1841.8524	-0.1154	-63	89	105	LIDTLDMGAHIIDASLK			Oxidation (M)[7]		Mascot
1888.907	1888.7942	-0.1128	-60	558	575	SGMGQKEPGVDEIGSELR					Mascot
1890.8922	1890.8411	-0.0511	-27	370	384	SINFTGPNNFQWYFR					Mascot
1932.8669	1932.7833	-0.0836	-43	680	696	DEEVVESDRNGVIDDEL					Mascot
1934.9706	1934.7971	-0.1735	-90	46	63	NKTALESGELFDLGPDTK					Mascot
2098.1213	2097.9436	-0.1777	-85	87	105	QKLIDTLDMGAHIIDASLK			Oxidation (M)[9]		Mascot

8	Peptide chain release factor 1 OS=Burkholderia xenovorans (strain LB400) GN=prfA PE=3 SV=1	RF1_BURXL	40442.5	5.51	11	41	0	9.924

Peptide Information

Calc. Mass	Obsrv. Mass	± da	± ppm	Start Seq.	End Seq.	Sequence	Ion Score	C. I. %	Modification	Rank	Result Type
808.4059	808.3455	-0.0604	-75	138	143	YAERNR					Mascot
909.4676	909.3979	-0.0697	-77	166	174	IAGEAAYSK					Mascot
913.423	913.3907	-0.0323	-35	1	7	MKTSMQR			Oxidation (M)[1,5]		Mascot
982.4741	982.4262	-0.0479	-49	309	316	TYNFPQGR					Mascot
1039.528	1039.4535	-0.0745	-72	229	239	ASGAGGQHINK					Mascot
1649.813	1649.8076	-0.0054	-3	117	133	AGTGGDESALFAGDLLR					Mascot
1838.9066	1838.8573	-0.0493	-27	246	261	VTHLPTGIVVECQDDR			Carbamidomethyl (C)[12]		Mascot
1905.9606	1905.9261	-0.0345	-18	41	56	EHAELGPVVEHYALWR					Mascot
1933.8307	1933.8021	-0.0286	-15	144	160	WQVEMMSASESDLGGYK			Oxidation (M)[5]		Mascot
1933.8307	1933.8021	-0.0286	-15	144	160	WQVEMMSASESDLGGYK			Oxidation (M)[5]		Mascot
1949.8256	1949.7786	-0.047	-24	144	160	WQVEMMSASESDLGGYK			Oxidation (M)[5,6]		Mascot
2187.9797	2187.9866	0.0069	3	142	160	NRWQVEMMSASESDLGGYK					Mascot
2276.1934	2275.9841	-0.2093	-92	38	56	LTREHAELGPVVEHYALWR					Mascot

9	Phosphopantetheine adenylyltransferase OS=Mycobacterium bovis (strain BCG / Pasteur 1173P2) GN=coaD PE=3 SV=1	COAD_MYCBP	17731	5.53	7	41	0	2.469	

Protein Group

Phosphopantetheine adenylyltransferase OS=Mycobacterium bovis (strain ATCC BAA-935 / AF2122/97) GN=coaD PE=3 SV=1	COAD_MYCBO	17731	5.5300 002098 0835
Phosphopantetheine adenylyltransferase OS=Mycobacterium bovis (strain BCG / Tokyo 172 / ATCC 35737 / TMC 1019) GN=coaD PE=3 SV=1	COAD_MYCBT	17731	5.5300 002098 0835
Phosphopantetheine adenylyltransferase OS=Mycobacterium tuberculosis (strain ATCC 25177 / H37Ra) GN=coaD PE=3 SV=1	COAD_MYCTA	17731	5.5300 002098 0835
Phosphopantetheine adenylyltransferase OS=Mycobacterium tuberculosis GN=coaD PE=1 SV=1	COAD_MYCTU	17731	5.5300 002098 0835

Peptide Information

Calc. Mass	Obsrv. Mass	± da	± ppm	Start Seq.	End Seq.	Sequence	Ion Score	C. I. %	Modification	Rank	Result Type
982.4696	982.4262	-0.0434	-44	79	87	SCGMTAIVK			Carbamidomethyl (C)[2], Oxidation (M)[4]		Mascot
1099.4725	1099.5061	0.0336	31	42	50	TGMFDLDER			Oxidation (M)[3]		Mascot
1167.6117	1167.6013	-0.0104	-9	56	65	ESTTHLPNLR					Mascot
1308.6763	1308.6238	-0.0525	-40	79	90	SCGMTAIVKGLR			Carbamidomethyl (C)[2], Oxidation (M)[4]		Mascot
1657.7925	1657.7815	-0.011	-7	42	55	TGMFDLDERIAMVK			Oxidation (M)[3,12]		Mascot

1905.8357	1905.9261	0.0904	47	91	106	TGTDFEYELQMAQMNK					Mascot
2248.0374	2248.0044	-0.033	-15	88	106	GLRTGTDFEYELQMAQMNK			Oxidation (M)[14]		Mascot
2264.0322	2263.9922	-0.04	-18	88	106	GLRTGTDFEYELQMAQMNK			Oxidation (M)[14,17]		Mascot

10	Acireductone dioxygenase OS=Synechococcus sp. (strain WH8102) GN=mtnD PE=3 SV=1	MTND_SYNPX	20843.1	4.83	7	41	0	2.844	

Peptide Information

Calc. Mass	Obsrv. Mass	± da	± ppm	Start Seq.	End Seq.	Sequence	Ion Score	C. I. %	Modification	Rank	Result Type
982.441	982.4262	-0.0148	-15	85	92	MTPDHPER					Mascot
1118.5874	1118.4944	-0.093	-83	127	135	EVLVTLCER			Carbamidomethyl (C)[7]		Mascot
1771.8644	1771.7725	-0.0919	-52	23	38	LVCNDPASIQAELADR			Carbamidomethyl (C)[3]		Mascot
1837.8149	1837.767	-0.0479	-26	147	161	HWFDMGPTPSFCALR			Carbamidomethyl (C)[12], Oxidation (M)[5]		Mascot
1905.9706	1905.9261	-0.0445	-23	4	22	LSIFPDHGDGGDGALPLPK					Mascot
2248.0305	2248.0044	-0.0261	-12	162	181	FFNNSEGWVATFTGDSIAER					Mascot
2280.1443	2279.9905	-0.1538	-67	1	22	MSRLSIFPDHGDGGDGALPLPK					Mascot
2280.1443	2279.9905	-0.1538	-67	1	22	MSRLSIFPDHGDGGDGALPLPK					Mascot

Gel Idx/Pos	155/G6	Instr./Gel Origin	BA2151/Sample Project 20140814	Process Status	Analysis Succeeded
Plate [#] Name	[1] Sample Project 20140814	Instrument Sample Name		Spectra	11

Rank	Protein Name	Accession No.	Protein MW	Protein PI	Pep. Count	Protein Score	Protein Score C. I. %	Intensity Matched	Total Ion Score	Total Ion C. I. %	Confirmed
1	30S ribosomal protein S15, chloroplastic OS=Vitis vinifera GN=rps15 PE=3 SV=1	RR15_VITVI	10495	11.42	3	21	0	.663			

Peptide Information

Calc. Mass	Obsrv. Mass	± da	± ppm	Start Seq.	End Seq.	Sequence	Ion Score	C. I. %	Modification	Rank	Result Type
1107.5906	1107.5387	-0.0519	-47	44	52	DYLSQRGLR					Mascot
1618.8911	1619.0524	0.1613	100	4	17	NLFISVISQKEANR					Mascot
1959.9559	1960.0977	0.1418	72	14	30	EANRGSVEFQVFSFTNK					Mascot

Rank	Protein Name	Accession No.	Protein MW	Protein PI	Pep. Count	Protein Score	Protein Score C. I. %	Intensity Matched	Total Ion Score	Total Ion C. I. %	Confirmed
2	Putative cytochrome c oxidase subunit II PS17 (Fragments) OS=Pinus strobus PE=1 SV=1	PS17_PINST	1707	9.63	2	21	0	1.204			

Peptide Information

Calc. Mass	Obsrv. Mass	± da	± ppm	Start Seq.	End Seq.	Sequence	Ion Score	C. I. %	Modification	Rank	Result Type
856.5251	856.5172	-0.0079	-9	1	8	SPTVIALR					Mascot
870.5043	870.5399	0.0356	41	9	16	VVEALSPR					Mascot

Rank	Protein Name	Accession No.	Protein MW	Protein PI	Pep. Count	Protein Score	Protein Score C. I. %	Intensity Matched	Total Ion Score	Total Ion C. I. %	Confirmed
3	Acetyl-coenzyme A carboxylase carboxyl transferase subunit alpha OS=Bartonella tribocorum (strain CIP 105476 / IBS 506) GN=accA PE=3 SV=1	ACCA_BART1	35051.3	8.49	5	20	0	1.388			

Peptide Information

Calc. Mass	Obsrv. Mass	± da	± ppm	Start Seq.	End Seq.	Sequence	Ion Score	C. I. %	Modification	Rank	Result Type
1308.6907	1308.64	-0.0507	-39	43	53	RSQTALNDIYK					Mascot
1728.9603	1729.1053	0.145	84	173	189	GQAEAIAQSTAATLRLK					Mascot
1789.0331	1789.0601	0.027	15	258	274	LKIIDGIISEPLGGAHR					Mascot
1838.8953	1838.8875	-0.0078	-4	24	39	IFQEKGSLDMSDEIAR					Mascot
1959.9519	1960.0977	0.1458	74	107	124	GEAVAYIGQEKGHDTQTR					Mascot

Rank	Protein Name	Accession No.	Protein MW	Protein PI	Pep. Count	Protein Score	Protein Score C. I. %	Intensity Matched	Total Ion Score	Total Ion C. I. %	Confirmed
4	UPF0145 protein BC_1816 OS=Bacillus cereus (strain ATCC 14579 / DSM 31) GN=BC_1816 PE=3 SV=1	Y1816_BACCR	11025.7	4.73	3	20	0	.612			

Peptide Information

Calc. Mass	Obsrv. Mass	± da	± ppm	Start Seq.	End Seq.	Sequence	Ion Score	C. I. %	Modification	Rank	Result Type

807.4359	807.3845	-0.0514	-64	34	40	DLFASVR						Mascot
1308.6543	1308.64	-0.0143	-11	41	53	DVVGGRAGAYESK						Mascot
1960.0498	1960.0977	0.0479	24	71	88	QKGANAIIGIDVDYEVVR						Mascot

5 Nucleoid-associated protein VP2178 OS=Vibrio parahaemolyticus serotype O3:K6 (strain RIMD 2210633) GN=VP2178 PE=3 SV=1 | Y2178_VIBPA | 12014.8 | 4.88 | 3 | 20 | 0 | .438

Peptide Information

Calc. Mass	Obsrv. Mass	± da	± ppm	Start Seq.	End Seq.	Sequence	Ion Score	C. I. %	Modification	Rank	Result Type
807.3851	807.3845	-0.0006	-1	5	12	GGMGNLMK					Mascot
1838.8306	1838.8875	0.0569	31	5	20	GGMGNLMKQAQQMQER			Oxidation (M)[3,7]		Mascot
1927.8785	1927.8556	-0.0229	-12	92	109	MASVTGGMQLPPGMKMPF			Oxidation (M)[1,8,14]		Mascot

6 Desiccation stress protein DSP-22, chloroplastic OS=Craterostigma plantagineum GN=DSP-22 PE=2 SV=1 | DS22_CRAPL | 22091.2 | 8.61 | 4 | 19 | 0 | .982

Peptide Information

Calc. Mass	Obsrv. Mass	± da	± ppm	Start Seq.	End Seq.	Sequence	Ion Score	C. I. %	Modification	Rank	Result Type
917.4323	917.3455	-0.0868	-95	59	65	EEQQQQK					Mascot
1308.6729	1308.64	-0.0329	-25	34	43	QSYELPLMRR			Oxidation (M)[8]		Mascot
1788.9604	1789.0601	0.0997	56	177	192	FAMIGLVALAFTEYVK			Oxidation (M)[3]		Mascot
1838.754	1838.8875	0.1335	73	1	16	MASSTCYATIPAMSCR			Carbamidomethyl (C)[6,15], Oxidation (M)[1,13]		Mascot

7 Parvalbumin alpha OS=Homo sapiens GN=PVALB PE=1 SV=2 | PRVA_HUMAN | 12051 | 4.98 | 3 | 19 | 0 | .5

Peptide Information

Calc. Mass	Obsrv. Mass	± da	± ppm	Start Seq.	End Seq.	Sequence	Ion Score	C. I. %	Modification	Rank	Result Type
1194.5857	1194.6129	0.0272	23	82	92	ETKMLMAAGDK					Mascot
1308.5923	1308.64	0.0477	36	85	97	MLMAAGDKDGDGK					Mascot
1838.8655	1838.8875	0.022	12	93	110	DGDGKIGVDEFSTLVAES					Mascot

8 Bifunctional chitinase/lysozyme (Fragments) OS=Carica papaya PE=1 SV=1 | CHLY_CARPA | 2875.5 | 9.98 | 2 | 19 | 0 | .633

Peptide Information

Calc. Mass	Obsrv. Mass	± da	± ppm	Start Seq.	End Seq.	Sequence	Ion Score	C. I. %	Modification	Rank	Result Type

Calc. Mass	Obsrv. Mass	± da	± ppm	Start Seq.	End Seq.	Sequence	Ion Score	C. I. %	Modification	Rank	Result Type
999.4637	999.4119	-0.0518	-52	9	16	SMFDQMLK					Mascot
1308.6188	1308.64	0.0212	16	9	18	SMFDQMLKHR			Oxidation (M)[2]		Mascot

9	Putative defensin-like protein 237 OS=Arabidopsis thaliana GN=SCRL21 PE=3 SV=2	DF237_ARATH	11641.6	9.2	3	19	0	.519	

Peptide Information

Calc. Mass	Obsrv. Mass	± da	± ppm	Start Seq.	End Seq.	Sequence	Ion Score	C. I. %	Modification	Rank	Result Type
807.3777	807.3845	0.0068	8	40	46	CGTDRAK			Carbamidomethyl (C)[1]		Mascot
1107.4484	1107.5387	0.0903	82	71	79	CDDDRQGNK			Carbamidomethyl (C)[1]		Mascot
1838.812	1838.8875	0.0755	41	61	75	QVLNTISSCRCDDDR			Carbamidomethyl (C)[9,11]		Mascot

10	10 kDa chaperonin OS=Prochlorococcus marinus (strain SARG / CCMP1375 / SS120) GN=groS PE=3 SV=1	CH10_PROMA	10824.7	4.92	3	19	0	1.918

Protein Group

10 kDa chaperonin OS=Prochlorococcus marinus (strain MIT 9211) GN=groS PE=3 SV=1	CH10_PROM4	10824.7	4.9499998092 6514
10 kDa chaperonin OS=Prochlorococcus marinus (strain MIT 9313) GN=groS PE=3 SV=1	CH10_PROMM	10882.8	4.9200000762 9395
10 kDa chaperonin OS=Synechococcus sp. (strain WH7803) GN=groS PE=3 SV=1	CH10_SYNPW	10767.7	4.7800002098 0835
10 kDa chaperonin OS=Synechococcus sp. (strain WH8102) GN=groS PE=3 SV=1	CH10_SYNPX	10834.8	4.9200000762 9395

Peptide Information

Calc. Mass	Obsrv. Mass	± da	± ppm	Start Seq.	End Seq.	Sequence	Ion Score	C. I. %	Modification	Rank	Result Type
807.373	807.3845	0.0115	14	22	28	VSESEEK					Mascot
999.5106	999.4119	-0.0987	-99	63	72	QAPEVGVGDK					Mascot
1599.9065	1600.0537	0.1472	92	2	17	AAVSLSVSTVKPLGDR					Mascot
1599.9065	1600.0537	0.1472	92	2	17	AAVSLSVSTVKPLGDR					Mascot

Gel Idx/Pos	156/G7	Instr./Gel Origin	BA2151/Sample Project 20140814	Process Status	Analysis Succeeded
Plate [#] Name	[1] Sample Project 20140814	Instrument Sample Name		Spectra	11

Rank	Protein Name	Accession No.	Protein MW	Protein PI	Pep. Count	Protein Score	Protein Score C. I. %	Intensity Matched	Total Ion Score	Total Ion C. I. %	Confirmed
1	Keratin, type I cytoskeletal 10 OS=Homo sapiens GN=KRT10 PE=1 SV=6	K1C10_HUMAN	59019.8	5.13	18	187	100	4.102	104	100	

Peptide Information

Calc. Mass	Obsrv. Mass	± da	± ppm	Start Seq.	End Seq.	Sequence	Ion Score	C. I. %	Modification	Rank	Result Type
807.3995	807.3857	-0.0138	-17	229	235	LAADDFR					Mascot
847.452	847.4329	-0.0191	-23	363	369	SEITELR					Mascot
1003.553	1003.5164	-0.0366	-36	363	370	SEITELRR					Mascot
1106.5259	1106.5032	-0.0227	-21	148	156	VTMQNLNDR			Oxidation (M)[3]		Mascot
1118.5087	1118.4897	-0.019	-17	185	194	HGNSHQGEPR					Mascot
1201.6172	1201.5826	-0.0346	-29	246	256	QSVEADINGLR					Mascot
1262.5972	1262.5918	-0.0054	-4	451	464	SLLEGEGSSGGGGR					Mascot
1316.59	1316.5868	-0.0032	-2	286	295	NHEEEMKDLR			Oxidation (M)[6]		Mascot
1357.7183	1357.6669	-0.0514	-38	246	257	QSVEADINGLRR					Mascot
1365.6393	1365.6191	-0.0202	-15	323	333	SQYEQLAEQNR	32	35.056			Mascot
1381.6482	1381.6163	-0.0319	-23	166	177	ALEESNYELEGK					Mascot
1390.6809	1390.6545	-0.0264	-19	387	399	QSLEASLAETEGR					Mascot
1434.7699	1434.7395	-0.0304	-21	440	450	IRLENEIQTYR	4	0			Mascot
1493.7343	1493.7051	-0.0292	-20	323	334	SQYEQLAEQNRK	49	98.656			Mascot
1707.7722	1707.7456	-0.0266	-16	41	59	GSLGGGFSSGGFSGGSFSR	23	0			Mascot
1797.0116	1796.92	-0.0916	-51	371	386	NVQALEIELQSQLALK					Mascot
2904.3826	2904.3218	-0.0608	-21	296	322	NVSTGDVNVEMNAAPGVDLTQLLNNMR			Oxidation (M)[11,26]		Mascot
3052.6272	3052.573	-0.0542	-18	202	228	TIDDLKNQILNLTTDNANILLQIDNAR					Mascot

Rank	Protein Name	Accession No.	Protein MW	Protein PI	Pep. Count	Protein Score	Protein Score C. I. %	Intensity Matched	Total Ion Score	Total Ion C. I. %	Confirmed
2	Polyadenylate-binding protein 2-B OS=Xenopus laevis GN=pabpn1-b PE=2 SV=1	PAB2B_XENLA	32306.9	5.56	13	69	92.536	6.479			

Peptide Information

Calc. Mass	Obsrv. Mass	± da	± ppm	Start Seq.	End Seq.	Sequence	Ion Score	C. I. %	Modification	Rank	Result Type
848.4546	848.4283	-0.0263	-31	191	197	VTILCDK			Carbamidomethyl (C)[5]		Mascot
870.4904	870.5199	0.0295	34	59	67	VGGRTAPGR					Mascot

Calc. Mass	Obsrv. Mass	± da	± ppm	Start Seq.	End Seq.	Sequence	Ion Score	C. I. %	Modification	Rank	Result Type
1091.5878	1091.5054	-0.0824	-75	1	11	MAAVSSVASLR					Mascot
1107.5826	1107.5212	-0.0614	-55	1	11	MAAVSSVASLR			Oxidation (M)[1]		Mascot
1229.6736	1229.5732	-0.1004	-82	126	135	LKELQNEVEK					Mascot
1265.5679	1265.6066	0.0387	31	116	125	VREMEEEAEK			Oxidation (M)[4]		Mascot
1373.7245	1373.6379	-0.0866	-63	238	249	RTNRPGISTTDR					Mascot
1456.7445	1456.698	-0.0465	-32	269	280	FYSGYTPRPRGR					Mascot
1515.7988	1515.7372	-0.0616	-41	191	203	VTILCDKFTGHPK			Carbamidomethyl (C)[5]		Mascot
1515.7988	1515.7372	-0.0616	-41	191	203	VTILCDKFTGHPK			Carbamidomethyl (C)[5]		Mascot
1543.8326	1543.7606	-0.072	-47	41	54	GTLDLDLELLTQGR					Mascot
1674.8671	1674.7028	-0.1643	-98	239	253	TNRPGISTTDRGFPR					Mascot
1699.9337	1699.7977	-0.136	-80	41	55	GTLDLDLELLTQGRR					Mascot
1829.7356	1829.8708	0.1352	74	21	40	GGAGPSGGGQDPGEDD PMGR			Oxidation (M)[18]		Mascot
3052.3855	3052.573	0.1875	61	163	190	SIYVGNVDYGATAEELEA HFHGCGSVNR			Carbamidomethyl (C)[23]		Mascot

3	Myosin heavy chain, muscle OS=Drosophila melanogaster GN=Mhc PE=1 SV=4	MYSA_DROME	225411	5.91	36	63	70.285	11.621	

Peptide Information

Calc. Mass	Obsrv. Mass	± da	± ppm	Start Seq.	End Seq.	Sequence	Ion Score	C. I. %	Modification	Rank	Result Type
802.4417	802.4217	-0.02	-25	1043	1049	VRGDVEK					Mascot
807.4359	807.3857	-0.0502	-62	1645	1650	YQQQLK					Mascot
826.4166	826.4093	-0.0073	-9	1214	1220	AKAEHDR					Mascot
829.4638	829.3951	-0.0687	-83	1134	1140	QRADLAR					Mascot
848.4182	848.4283	0.0101	12	1863	1869	MQDLVDK					Mascot
856.4676	856.5104	0.0428	50	249	256	IHFGPTGK					Mascot
896.4108	896.4196	0.0088	10	1374	1381	YESDGVAR					Mascot
1026.4739	1026.4803	0.0064	6	542	550	ATDQTFSEK					Mascot
1033.579	1033.4937	-0.0853	-83	933	940	NQLFQQKK					Mascot
1058.5953	1058.5005	-0.0948	-90	1354	1362	ADLQRQLSK					Mascot
1068.5585	1068.4973	-0.0612	-57	131	138	RYPVYTNR					Mascot
1091.5942	1091.5054	-0.0888	-81	1083	1092	DKELSSITAK					Mascot
1104.528	1104.5208	-0.0072	-7	1317	1325	LADEESRER					Mascot
1106.559	1106.5032	-0.0558	-50	405	414	VGNEFVTQGR					Mascot
1163.5474	1163.4836	-0.0638	-55	1232	1241	TACDQLGRDK			Carbamidomethyl (C)[3]		Mascot
1182.6338	1182.5424	-0.0914	-77	1925	1937	AGSVGRGASPAPR					Mascot
1262.6055	1262.5918	-0.0137	-11	351	362	ITAAVMHMGGMK			Oxidation (M)[6]		Mascot
1286.7175	1286.6093	-0.1082	-84	270	280	ARVISQQSLER					Mascot

Calc. Mass	Obsrv. Mass	± da	± ppm	Start Seq.	End Seq.	Sequence	Ion Score	C. I. %	Modification	Rank	Result Type
1308.6794	1308.619	-0.0604	-46	26	36	IDQSKPYDSKK					Mascot
1316.6191	1316.5868	-0.0323	-25	386	396	LFGCDTAELYK			Carbamidomethyl (C)[4]		Mascot
1321.6781	1321.6213	-0.0568	-43	654	664	EQLNSLMTTLR			Oxidation (M)[7]		Mascot
1321.7362	1321.6213	-0.1149	-87	45	56	EGYLLGEIKATK					Mascot
1336.6855	1336.6111	-0.0744	-56	25	35	RIDQSKPYDSK					Mascot
1373.6655	1373.6379	-0.0276	-20	1677	1688	ANALQNELEESR					Mascot
1390.6849	1390.6545	-0.0304	-22	599	610	DPLNDTVVDQFK					Mascot
1479.6996	1479.7128	0.0132	9	1592	1605	ALDSMQASLEAEAK			Oxidation (M)[5]		Mascot
1487.7085	1487.7126	0.0041	3	1820	1831	ELENELDGEQRR					Mascot
1529.7666	1529.7037	-0.0629	-41	1676	1688	RANALQNELEESR					Mascot
1542.8235	1542.7236	-0.0999	-65	907	919	LTAQKNDLENQLR					Mascot
1738.8242	1738.8005	-0.0237	-14	1262	1276	LDETNRTLNDFDASK					Mascot
1801.9054	1801.845	-0.0604	-34	762	776	VFFRAGVLGQMEEFR			Oxidation (M)[11]		Mascot
1801.9054	1801.845	-0.0604	-34	762	776	VFFRAGVLGQMEEFR			Oxidation (M)[11]		Mascot
1829.8876	1829.8708	-0.0168	-9	1114	1128	IEELEEEVEAERQAR					Mascot
1890.9556	1890.8982	-0.0574	-30	542	558	ATDQTFSEKLTNTHLGK					Mascot
1908.913	1908.7762	-0.1368	-72	346	362	EDVYRITAAVMHMGGMK					Mascot
1929.9877	1929.907	-0.0807	-42	1418	1433	QRLSTEVEDLQLEVDR					Mascot
1941.9877	1941.8958	-0.0919	-47	1503	1520	NLADEVKDLLDQIGEGGR					Mascot

4	Kelch-like protein 3 OS=Danio rerio GN=klhl3 PE=3 SV=1	KLHL3_DANRE	67907.6	6.08	17	62	68.884	5.86	

Peptide Information

Calc. Mass	Obsrv. Mass	± da	± ppm	Start Seq.	End Seq.	Sequence	Ion Score	C. I. %	Modification	Rank	Result Type
800.4083	800.374	-0.0343	-43	249	254	LMEHVR			Oxidation (M)[2]		Mascot
838.4529	838.3813	-0.0716	-85	101	107	ANHVEIR					Mascot
982.4913	982.4394	-0.0519	-53	281	288	DFLIEAMK			Oxidation (M)[7]		Mascot
992.4531	992.464	0.0109	11	24	32	TLDSQDDAK					Mascot
1003.5419	1003.5164	-0.0255	-25	446	456	SSVGVGVVDGK					Mascot
1037.5931	1037.499	-0.0941	-91	2	9	VLWGFFLR					Mascot
1184.6285	1184.5457	-0.0828	-70	1	9	MVLWGFFLR			Oxidation (M)[1]		Mascot
1193.6388	1193.5961	-0.0427	-36	227	236	VFEAMVAWIK					Mascot
1229.453	1229.5732	0.1202	98	533	541	QVCDMNMCR			Carbamidomethyl (C)[3,8], Oxidation (M)[5]		Mascot
1320.5348	1320.5608	0.026	20	484	493	WCYVSDMSTR			Carbamidomethyl (C)[2], Oxidation (M)[7]		Mascot
1390.7227	1390.6545	-0.0682	-49	341	351	WYQVADLPSRR					Mascot
1456.8127	1456.698	-0.1147	-79	316	329	VMMVVGGQAPKAIR					Mascot

1460.641	1460.7269	0.0859	59	484	494	WCYVSDMSTRR			Carbamidomethyl (C)[2]		Mascot
1471.8031	1471.6912	-0.1119	-76	1	11	MVLWGFFLRFR					Mascot
1479.6533	1479.7128	0.0595	40	386	397	DQWSSIPSMQER			Oxidation (M)[9]		Mascot
1487.7981	1487.7126	-0.0855	-57	1	11	MVLWGFFLRFR			Oxidation (M)[1]		Mascot
1543.7509	1543.7606	0.0097	6	12	23	FFLTCGRNCIQK			Carbamidomethyl (C)[5,9]		Mascot
1738.8152	1738.8005	-0.0147	-8	432	445	ANEWMFVAPMNTRR			Oxidation (M)[5]		Mascot

5	Keratin, type I cytoskeletal 10 OS=Bos taurus GN=KRT10 PE=3 SV=1	K1C10_BOVIN	54986.3	5.05	10	61	58.026	2.582	32	35.056	

Peptide Information

Calc. Mass	Obsrv. Mass	± da	± ppm	Start Seq.	End Seq.	Sequence	Ion Score	C. I. %	Modification	Rank	Result Type
807.3995	807.3857	-0.0138	-17	210	216	LAADDFR					Mascot
847.452	847.4329	-0.0191	-23	344	350	SEITELR					Mascot
1003.553	1003.5164	-0.0366	-36	344	351	SEITELRR					Mascot
1104.5466	1104.5208	-0.0258	-23	129	137	ITMQNLNDR					Mascot
1201.6172	1201.5826	-0.0346	-29	227	237	QSVEADINGLR					Mascot
1265.5909	1265.6066	0.0157	12	315	324	RDAEAWFNEK					Mascot
1357.7183	1357.6669	-0.0514	-38	227	238	QSVEADINGLRR					Mascot
1365.6758	1365.6191	-0.0567	-42	304	314	SQYEQLAEKNR	32	35.056			Mascot
1390.6809	1390.6545	-0.0264	-19	368	380	QSLEASLAETEGR					Mascot
1434.7699	1434.7395	-0.0304	-21	421	431	IRLENEIQTYR	4	0			Mascot

6	Retinoic acid receptor RXR-beta-A OS=Danio rerio GN=rxrba PE=2 SV=1	RXRBA_DANRE	52362.1	7.53	18	60	48.361	8.013			

Peptide Information

Calc. Mass	Obsrv. Mass	± da	± ppm	Start Seq.	End Seq.	Sequence	Ion Score	C. I. %	Modification	Rank	Result Type
810.3671	810.3807	0.0136	17	185	191	CLAMGMK			Carbamidomethyl (C)[1]		Mascot
826.3619	826.4093	0.0474	57	185	191	CLAMGMK			Carbamidomethyl (C)[1], Oxidation (M)[4]		Mascot
848.4294	848.4283	-0.0011	-1	374	380	TELGCLR			Carbamidomethyl (C)[5]		Mascot
863.4006	863.429	0.0284	33	417	423	YPDQQGR					Mascot
874.4265	874.4199	-0.0066	-8	193	199	EVVQDER					Mascot
914.4036	914.3859	-0.0177	-19	156	162	DLSYTCR			Carbamidomethyl (C)[6]		Mascot
982.4631	982.4394	-0.0237	-24	185	192	CLAMGMKR			Carbamidomethyl (C)[1], Oxidation (M)[4]		Mascot
1106.5146	1106.5032	-0.0114	-10	163	171	DNKDCLVDK			Carbamidomethyl (C)[5]		Mascot
1229.584	1229.5732	-0.0108	-9	182	191	YQKCLAMGMK			Carbamidomethyl (C)[4]		Mascot

Calc. Mass	Obsrv. Mass	± da	± ppm	Start Seq.	End Seq.	Sequence	Ion Score	C. I. %	Modification	Rank	Result Type
1286.6951	1286.6093	-0.0858	-67	391	402	GLSSPSEVELLR					Mascot
1323.6144	1323.6398	0.0254	19	124	135	LCAICGDRSSGK			Carbamidomethyl (C)[2,5]		Mascot
1390.7842	1390.6545	-0.1297	-93	270	280	QLFTLVEWAKR					Mascot
1460.709	1460.7269	0.0179	12	403	414	EKVYASLEAYCK			Carbamidomethyl (C)[11]		Mascot
1487.7312	1487.7126	-0.0186	-13	405	416	VYASLEAYCKQR			Carbamidomethyl (C)[9]		Mascot
1515.7186	1515.7372	0.0186	12	344	357	ESAHNAEVGAIFDR					Mascot
1515.7186	1515.7372	0.0186	12	344	357	ESAHNAEVGAIFDR					Mascot
1543.8326	1543.7606	-0.072	-47	391	404	GLSSPSEVELLREK					Mascot
1628.7078	1628.8151	0.1073	66	368	380	DMQMDKTELGCLR			Carbamidomethyl (C)[11], Oxidation (M)[2,4]		Mascot
1738.9349	1738.8005	-0.1344	-77	436	449	SIGLKCLEHLFFFK			Carbamidomethyl (C)[6]		Mascot
1838.799	1838.8856	0.0866	47	136	150	HYGVYSCEGCKGFFK			Carbamidomethyl (C)[7,10]		Mascot

7	Oxidoreductase HTATIP2 OS=Pongo pygmaeus GN=HTATIP2 PE=3 SV=1	HTAI2_PONPY	27402.1	8.57	12	59	25.358	3.517

Peptide Information

Calc. Mass	Obsrv. Mass	± da	± ppm	Start Seq.	End Seq.	Sequence	Ion Score	C. I. %	Modification	Rank	Result Type
835.4421	835.4028	-0.0393	-47	10	15	LREDFR					Mascot
848.4359	848.4283	-0.0076	-9	2	9	AETEALSK					Mascot
1033.579	1033.4937	-0.0853	-83	97	106	VKAGAEGFVR					Mascot
1091.5514	1091.5054	-0.046	-42	115	125	SAELAKAGGCK			Carbamidomethyl (C)[10]		Mascot
1201.6293	1201.5826	-0.0467	-39	209	218	AMLNNMVRPR					Mascot
1263.625	1263.624	-0.001	-1	219	228	DKQMELLENK			Oxidation (M)[4]		Mascot
1316.6958	1316.5868	-0.109	-83	126	137	HFNLLSSKGADK					Mascot
1320.6431	1320.5608	-0.0823	-62	64	74	NVNQEVVDFEK					Mascot
1460.7461	1460.7269	-0.0192	-13	209	220	AMLNNMVRPRDK			Oxidation (M)[2]		Mascot
1471.838	1471.6912	-0.1468	-100	229	242	AIHDLGKVHGSLKP					Mascot
1738.9156	1738.8005	-0.1151	-66	221	235	QMELLENKAIHDLGK					Mascot
2717.2446	2717.0457	-0.1989	-73	75	98	LDDYASAFQGHDVGFCC LGTTRVK			Carbamidomethyl (C)[16,17]		Mascot

8	Zinc finger protein 484 OS=Homo sapiens GN=ZNF484 PE=1 SV=1	ZN484_HUMAN	100951.9	8.68	19	58	16.25	7.346

Peptide Information

Calc. Mass	Obsrv. Mass	± da	± ppm	Start Seq.	End Seq.	Sequence	Ion Score	C. I. %	Modification	Rank	Result Type
891.5159	891.4424	-0.0735	-82	794	800	HQKIHTK					Mascot
896.4724	896.4196	-0.0528	-59	4	11	SLESVSFK					Mascot
992.4279	992.464	0.0361	36	195	203	NNATENSDK					Mascot

1026.5116	1026.4803	-0.0313	-30	593	600	SHFITHER					Mascot
1201.5089	1201.5826	0.0737	61	666	675	CSDCGKAFTR			Carbamidomethyl (C)[1,4]		Mascot
1229.5262	1229.5732	0.047	38	174	183	RPHNCNSCGK			Carbamidomethyl (C)[5,8]		Mascot
1265.6168	1265.6066	-0.0102	-8	541	550	IHQKCHTGER			Carbamidomethyl (C)[5]		Mascot
1302.6219	1302.6377	0.0158	12	128	138	CGENQNKPLSR			Carbamidomethyl (C)[1]		Mascot
1323.6726	1323.6398	-0.0328	-25	801	811	QKPYKCSDLGK			Carbamidomethyl (C)[6]		Mascot
1332.614	1332.5303	-0.0837	-63	300	310	VYAGICTEYEK			Carbamidomethyl (C)[6]		Mascot
1357.6212	1357.6669	0.0457	34	173	183	KRPHNCNSCGK			Carbamidomethyl (C)[6,9]		Mascot
1365.7168	1365.6191	-0.0977	-72	565	575	STLSMHQRIHR					Mascot
1373.6696	1373.6379	-0.0317	-23	20	30	DEWQQLDLAQK					Mascot
1381.7118	1381.6163	-0.0955	-69	565	575	STLSMHQRIHR			Oxidation (M)[5]		Mascot
1390.6638	1390.6545	-0.0093	-7	319	329	QKTPYEGNYYK					Mascot
1427.6294	1427.7582	0.1288	90	576	587	GEKPYVCTECGK			Carbamidomethyl (C)[7,10]		Mascot
1507.7316	1507.7136	-0.018	-12	109	120	DDPYSILEELWK					Mascot
1805.8309	1805.8442	0.0133	7	713	727	IHTGEKPYICNECGK			Carbamidomethyl (C)[10,13]		Mascot
1815.9124	1815.8121	-0.1003	-55	4	19	SLESVSFKDVTVDFSR					Mascot
1833.8735	1833.8386	-0.0349	-19	573	587	IHRGEKPYVCTECGK			Carbamidomethyl (C)[10,13]		Mascot
1833.8735	1833.8386	-0.0349	-19	573	587	IHRGEKPYVCTECGK			Carbamidomethyl (C)[10,13]		Mascot

9	Membrane-bound lytic murein transglycosylase F OS=Shewanella sp. (strain MR-7) GN=mltF PE=3 SV=1	MLTF_SHESR	54852	5.9	15	57	0	3.186	

Peptide Information

Calc. Mass	Obsrv. Mass	± da	± ppm	Start Seq.	End Seq.	Sequence	Ion Score	C. I. %	Modification	Rank	Result Type
802.4417	802.4217	-0.02	-25	328	334	EIGITNR					Mascot
874.488	874.4199	-0.0681	-78	207	214	SGLVLEEK					Mascot
886.4529	886.4082	-0.0447	-50	415	421	TRYGYAR					Mascot
896.4294	896.4196	-0.0098	-11	74	80	MVPYTNR			Oxidation (M)[1]		Mascot
922.488	922.4426	-0.0454	-49	81	88	AELYDALK					Mascot
1229.6848	1229.5732	-0.1116	-91	328	338	EIGITNRLDAK					Mascot
1332.6543	1332.5303	-0.124	-93	422	433	GSEAVHYVDSIR					Mascot
1336.6791	1336.6111	-0.068	-51	343	354	GGAAYLRDMINR					Mascot
1460.7301	1460.7269	-0.0032	-2	126	138	EGMPTPKDITDLK			Oxidation (M)[3]		Mascot
1674.8268	1674.7028	-0.124	-74	384	397	KLAESMELNPNAWR			Oxidation (M)[6]		Mascot
1799.8997	1799.8578	-0.0419	-23	74	88	MVPYTNRAELYDALK			Oxidation (M)[1]		Mascot
1805.9071	1805.8442	-0.0629	-35	311	327	SPTGVRGMMMLTQPTAK					Mascot
1829.9426	1829.8708	-0.0718	-39	89	105	KNEIDIIAAGMTETPAR					Mascot

1942.0182	1941.8958	-0.1224	-63	242	258	LAGTLDHLNEKYFGHVK					Mascot
1966.9829	1966.9036	-0.0793	-40	184	200	EIDYTIADSSSVQINRR					Mascot

10	Uncharacterized protein C11orf35 OS=Homo sapiens GN=C11orf35 PE=2 SV=2	CK035_HUMAN	71133.5	9.12	16	57	0	5.096

Peptide Information

Calc. Mass	Obsrv. Mass	± da	± ppm	Start Seq.	End Seq.	Sequence	Ion Score	C. I. %	Modification	Rank	Result Type
802.369	802.4217	0.0527	66	105	111	SSHSQEK					Mascot
810.458	810.3807	-0.0773	-95	584	589	LQKEHR					Mascot
812.3896	812.3741	-0.0155	-19	136	141	EHLEER					Mascot
826.4529	826.4093	-0.0436	-53	469	475	RETPAPR					Mascot
834.3839	834.3375	-0.0464	-56	323	329	DSEDLQK					Mascot
926.469	926.4061	-0.0629	-68	458	465	GEVLSEHR					Mascot
1037.5527	1037.499	-0.0537	-52	398	405	GFPERLYR					Mascot
1091.5779	1091.5054	-0.0725	-66	170	178	SSWVGRMLR					Mascot
1107.5729	1107.5212	-0.0517	-47	170	178	SSWVGRMLR			Oxidation (M)[7]		Mascot
1308.6431	1308.619	-0.0241	-18	476	487	VFADGTDLSIDR					Mascot
1543.7097	1543.7606	0.0509	33	224	236	YPNLFTNMEPSSK			Oxidation (M)[8]		Mascot
1674.8367	1674.7028	-0.1339	-80	148	161	TLQEMEAELQNLQK					Mascot
1699.8109	1699.7977	-0.0132	-8	223	236	RYPNLFTNMEPSSK			Oxidation (M)[9]		Mascot
1799.8633	1799.8578	-0.0055	-3	224	238	YPNLFTNMEPSSKQK			Oxidation (M)[8]		Mascot
1961.9424	1961.9165	-0.0259	-13	305	322	DHRASSEQALVQAGSYSR					Mascot
1961.9424	1961.9165	-0.0259	-13	305	322	DHRASSEQALVQAGSYSR					Mascot
1966.9618	1966.9036	-0.0582	-30	239	256	QPRPWPQLDTGSPESSGK					Mascot
2369.1216	2368.9724	-0.1492	-63	308	329	ASSEQALVQAGSYSRDSEDLQK					Mascot

Gel Idx/Pos	157/G8	Instr./Gel Origin	BA2151/Sample Project 20140814	Process Status	Analysis Succeeded
Plate [#] Name	[1] Sample Project 20140814	Instrument Sample Name		Spectra	11

Rank	Protein Name	Accession No.	Protein MW	Protein PI	Pep. Count	Protein Score	Protein Score C. I. %	Intensity Matched	Total Ion Score	Total Ion C. I. %	Confirmed
1	Tryptophan synthase beta chain (Fragment) OS=Buchnera aphidicola subsp. Rhopalosiphum maidis GN=trpB PE=3 SV=1	TRPB_BUCRM	24872.7	8.59	10	54	0	5.038			

Peptide Information

Calc. Mass	Obsrv. Mass	± da	± ppm	Start Seq.	End Seq.	Sequence	Ion Score	C. I. %	Modification	Rank	Result Type
847.4421	847.3937	-0.0484	-57	26	32	QSPNVFR					Mascot
932.4546	932.4264	-0.0282	-30	159	166	TGIYFGMK			Oxidation (M)[7]		Mascot
948.4203	948.4483	0.028	30	52	59	DACNEALR			Carbamidomethyl (C)[3]		Mascot
1122.5725	1122.4967	-0.0758	-68	26	34	QSPNVFRMK			Oxidation (M)[8]		Mascot
1349.728	1349.6318	-0.0962	-71	33	44	MKLMGAEVISVR			Oxidation (M)[1]		Mascot
1491.7108	1491.6871	-0.0237	-16	167	179	SSLMQNQEGQIEK					Mascot
1507.7057	1507.7032	-0.0025	-2	167	179	SSLMQNQEGQIEK			Oxidation (M)[4]		Mascot
1543.8088	1543.7375	-0.0713	-46	144	158	GIETGKHGAPLNHGR					Mascot
1871.9333	1871.8287	-0.1046	-56	150	166	HGAPLNHGRTGIYFGMK			Oxidation (M)[16]		Mascot
1926.84	1926.8087	-0.0313	-16	52	67	DACNEALRDWSGNYQK			Carbamidomethyl (C)[3]		Mascot
2239.1855	2239.0542	-0.1313	-59	205	224	AQYVSITDIEALEAFQILSK					Mascot

Rank	Protein Name	Accession No.	Protein MW	Protein PI	Pep. Count	Protein Score	Protein Score C. I. %	Intensity Matched	Total Ion Score	Total Ion C. I. %	Confirmed
2	Ribosome-recycling factor OS=Thermosynechococcus elongatus (strain BP-1) GN=frr PE=3 SV=1	RRF_THEEB	20146.7	6.77	7	49	0	4.469			

Peptide Information

Calc. Mass	Obsrv. Mass	± da	± ppm	Start Seq.	End Seq.	Sequence	Ion Score	C. I. %	Modification	Rank	Result Type
831.4207	831.4418	0.0211	25	3	9	LADVEER					Mascot
835.423	835.3668	-0.0562	-67	176	182	EKDIMTV					Mascot
848.4724	848.3998	-0.0726	-86	70	77	STLASIEK					Mascot
859.5247	859.4466	-0.0781	-91	109	116	ELVKTAAK					Mascot
1543.7462	1543.7375	-0.0087	-6	37	49	VMVDYYGTETPLR					Mascot
1817.8889	1817.822	-0.0669	-37	13	28	SVEATQHDFNSIRTGR					Mascot
1817.8889	1817.822	-0.0669	-37	13	28	SVEATQHDFNSIRTGR	13	0			Mascot
2368.1965	2368.2007	0.0042	2	29	49	ANAALLDRVMVDYYGTETPLR					Mascot

Rank	Protein Name	Accession No.	Protein MW	Protein PI	Pep. Count	Protein Score	Protein Score C. I. %	Intensity Matched	Total Ion Score	Total Ion C. I. %	Confirmed
3	50S ribosomal protein L9 OS=Caulobacter crescentus	RL9_CAUCR	20896.7	5.16	7	44	0	10.014			

(strain ATCC 19089 / CB15) GN=rplI PE=3 SV=1

Protein Group

50S ribosomal protein L9 OS=Caulobacter crescentus (strain NA1000 / CB15N) GN=rplI PE=3 SV=1	RL9_CAUCN	20896.7	5.1599 998474 1211

Peptide Information

Calc. Mass	Obsrv. Mass	± da	± ppm	Start Seq.	End Seq.	Sequence	Ion Score	C. I. %	Modification	Rank	Result Type
831.4319	831.4418	0.0099	12	104	111	AEGGKVDR					Mascot
834.3588	834.4421	0.0833	100	147	153	SQDEAER					Mascot
870.5771	870.5206	-0.0565	-65	2	8	KVILLER					Mascot
1321.7012	1321.6199	-0.0813	-62	23	33	DGFARNYLLPR					Mascot
1321.7012	1321.6199	-0.0813	-62	23	33	DGFARNYLLPR					Mascot
1420.7543	1420.6823	-0.072	-51	39	51	ATKANLATFEAQR					Mascot
1479.7188	1479.7188	0	0	82	96	QAGESGQLYGSVAGR					Mascot
1515.8563	1515.7192	-0.1371	-90	109	121	VDRSMVVLDKPIK			Oxidation (M)[5]		Mascot
1515.8563	1515.7192	-0.1371	-90	109	121	VDRSMVVLDKPIK	13	0	Oxidation (M)[5]		Mascot

4	Uncharacterized protein ORF23 OS=Ostreid herpesvirus 1 (isolate France) GN=ORF23 PE=4 SV=1	Y023_OSHVF	145788.5	4.85	18	44	0	7.408	

Peptide Information

Calc. Mass	Obsrv. Mass	± da	± ppm	Start Seq.	End Seq.	Sequence	Ion Score	C. I. %	Modification	Rank	Result Type
810.3879	810.3676	-0.0203	-25	321	326	YEELEK					Mascot
858.5294	858.4929	-0.0365	-43	611	617	DLEKLIK					Mascot
859.4744	859.4466	-0.0278	-32	1221	1227	QQQATRK					Mascot
908.4254	908.3735	-0.0519	-57	702	708	RMTDTER					Mascot
955.3859	955.4586	0.0727	76	802	810	GMMAGVDDK			Oxidation (M)[2,3]		Mascot
993.4921	993.4468	-0.0453	-46	283	291	MLGEESIAK			Oxidation (M)[1]		Mascot
1232.6416	1232.5519	-0.0897	-73	382	392	TAANMINKQNK					Mascot
1323.6427	1323.6218	-0.0209	-16	28	38	TFQNDIELDTK					Mascot
1338.6649	1338.6079	-0.057	-43	618	628	LVNDHDQEIQK					Mascot
1349.6617	1349.6318	-0.0299	-22	1247	1257	TLEQQMVDELK			Oxidation (M)[6]		Mascot
1420.7352	1420.6823	-0.0529	-37	283	295	MLGEESIAKDSLK					Mascot
1442.5774	1442.6907	0.1133	79	948	961	GDDGDVAMMTSDTK					Mascot
1838.8743	1838.8655	-0.0088	-5	703	717	MTDTERGLFTYFTTR					Mascot
1929.9805	1929.9089	-0.0716	-37	1228	1244	TTTTVSIEAEDFIAYLR					Mascot
1944.9985	1944.8307	-0.1678	-86	643	661	AVTDGTRTENINDVGGV					Mascot

						VK						
1945.9866	1945.8882	-0.0984	-51	321	336	YEELEKIQADTHTQIK						Mascot
2258.2466	2258.1106	-0.136	-60	731	749	VYEVLLEKIHSQTMLDLVK						Mascot
2840.4395	2840.269	-0.1705	-60	297	320	YYKIVTDDLVYGGLNDYINVVFEK						Mascot

5 Probable WRKY transcription factor 32 OS=Arabidopsis thaliana GN=WRKY32 PE=2 SV=1 | WRK32_ARATH | 52132.8 | 5.75 | 11 | 44 | 0 | 6.909

Peptide Information

Calc. Mass	Obsrv. Mass	± da	± ppm	Start Seq.	End Seq.	Sequence	Ion Score	C. I. %	Modification	Rank	Result Type
801.4213	801.4565	0.0352	44	57	63	DQVQGVR					Mascot
810.3529	810.3676	0.0147	18	169	174	DGYNWR					Mascot
822.4105	822.408	-0.0025	-3	26	32	DGLSQFR					Mascot
835.4155	835.3668	-0.0487	-58	433	439	ESEKQSK					Mascot
850.4781	850.3942	-0.0839	-99	176	182	YGQKQVK					Mascot
1253.5203	1253.6033	0.083	66	1	11	MEEDTGIDEAK			Oxidation (M)[1]		Mascot
1320.5382	1320.5499	0.0117	9	193	202	CTYTECCAKK			Carbamidomethyl (C)[1,6,7]		Mascot
1323.6176	1323.6218	0.0042	3	26	36	DGLSQFRDEEK					Mascot
1792.8436	1792.9717	0.1281	71	319	335	FVVHAAGDVGICGDGYR			Carbamidomethyl (C)[12]		Mascot
1792.8436	1792.9717	0.1281	71	319	335	FVVHAAGDVGICGDGYR			Carbamidomethyl (C)[12]		Mascot
1930.0645	1929.9089	-0.1556	-81	366	382	HIETAVENTKAVIITYK					Mascot
1958.8899	1958.8148	-0.0751	-38	1	17	MEEDTGIDEAKTYTVEK					Mascot

6 Pyridoxal biosynthesis lyase PdxS OS=Anoxybacillus flavithermus (strain DSM 21510 / WK1) GN=pdxS PE=3 SV=1 | PDXS_ANOFW | 31708.3 | 5.54 | 10 | 44 | 0 | 4.937

Peptide Information

Calc. Mass	Obsrv. Mass	± da	± ppm	Start Seq.	End Seq.	Sequence	Ion Score	C. I. %	Modification	Rank	Result Type
810.3484	810.3676	0.0192	24	12	18	GMAEMQK			Oxidation (M)[2]		Mascot
822.3563	822.408	0.0517	63	289	294	MQERGW			Oxidation (M)[1]		Mascot
826.3433	826.397	0.0537	65	12	18	GMAEMQK			Oxidation (M)[2,5]		Mascot
864.4244	864.4283	0.0039	5	1	8	MAVTGTER					Mascot
1107.5828	1107.5027	-0.0801	-72	1	10	MAVTGTERVK			Oxidation (M)[1]		Mascot
1107.5828	1107.5027	-0.0801	-72	1	10	MAVTGTERVK			Oxidation (M)[1]		Mascot
1442.8253	1442.6907	-0.1346	-93	188	200	NLGAPFEVLLEIK					Mascot
1738.8654	1738.7531	-0.1123	-65	150	165	GEPGTGNIVEAVRHMR			Oxidation (M)[15]		Mascot
1871.9644	1871.8287	-0.1357	-72	277	292	GIDISSLLPEQRMQER					Mascot

1942.0426	1941.8744	-0.1682	-87	270	288	GLGGAMKGIDISSLLPEQR					Mascot
1944.9882	1944.8307	-0.1575	-81	54	72	AAGGVARMADPTVIEEVMK					Mascot
2222.0615	2222.094	0.0325	15	12	32	GMAEMQKGGVIMDVVNAEQAK			Oxidation (M)[2]		Mascot

7	Kelch-like protein 38 OS=Rattus norvegicus GN=Klhl38 PE=2 SV=1	KLH38_RAT	66655.2	7.53	13	44	0	8.534

Peptide Information

Calc. Mass	Obsrv. Mass	± da	± ppm	Start Seq.	End Seq.	Sequence	Ion Score	C. I. %	Modification	Rank	Result Type
854.4843	854.4434	-0.0409	-48	324	331	ASAVTLHR					Mascot
864.3767	864.4283	0.0516	60	189	195	LCGEEEK			Carbamidomethyl (C)[2]		Mascot
895.376	895.4143	0.0383	43	503	509	CADMKDR			Carbamidomethyl (C)[1]		Mascot
1182.5507	1182.5209	-0.0298	-25	511	521	MHHGATVMGNK					Mascot
1182.5507	1182.5209	-0.0298	-25	511	521	MHHGATVMGNK					Mascot
1193.6388	1193.5756	-0.0632	-53	196	205	VFEALMAWVK					Mascot
1194.575	1194.5687	-0.0063	-5	13	22	DQNFSSDLLR					Mascot
1323.6726	1323.6218	-0.0508	-38	214	223	YMQELLQQVR			Oxidation (M)[2]		Mascot
1338.6519	1338.6079	-0.044	-33	510	521	RMHHGATVMGNK					Mascot
1871.8514	1871.8287	-0.0227	-12	174	188	ELCVMELRDYLGDDK			Carbamidomethyl (C)[3], Oxidation (M)[5]		Mascot
1930.0004	1929.9089	-0.0915	-47	196	211	VFEALMAWVKHDLQAR			Oxidation (M)[6]		Mascot
1944.9531	1944.8307	-0.1224	-63	511	528	MHHGATVMGNKLYVTGGR			Oxidation (M)[1]		Mascot
2258.0759	2258.1106	0.0347	15	382	402	NFIFSIGGTGEGQELLGSMER			Oxidation (M)[19]		Mascot
2840.3633	2840.269	-0.0943	-33	377	402	STTHRNFIFSIGGTGEGQELLGSMER			Oxidation (M)[24]		Mascot

8	Serine--tRNA ligase OS=Listeria welshimeri serovar 6b (strain ATCC 35897 / DSM 20650 / SLCC5334) GN=serS PE=3 SV=1	SYS_LISW6	49323.1	5.17	13	43	0	9.898

Peptide Information

Calc. Mass	Obsrv. Mass	± da	± ppm	Start Seq.	End Seq.	Sequence	Ion Score	C. I. %	Modification	Rank	Result Type
801.4002	801.4565	0.0563	70	278	283	QHQFNK					Mascot
831.4763	831.4418	-0.0345	-41	163	168	FVFYKK					Mascot
856.5615	856.4998	-0.0617	-72	2	8	LDVKLLR					Mascot
859.4883	859.4466	-0.0417	-49	85	91	TLDIELR					Mascot
864.4574	864.4283	-0.0291	-34	31	37	FGELDKR					Mascot
883.4196	883.3487	-0.0709	-80	132	138	TFDFEPK					Mascot
1122.5038	1122.4967	-0.0071	-6	254	262	YTAFSACFR			Carbamidomethyl (C)[7]		Mascot

1194.6188	1194.5687	-0.0501	-42	412	422	VLQGYMGGIEK					Mascot
1321.6052	1321.6199	0.0147	11	67	77	QDADAKIEEMR			Oxidation (M)[10]		Mascot
1321.6052	1321.6199	0.0147	11	67	77	QDADAKIEEMR			Oxidation (M)[10]		Mascot
1805.8381	1805.8282	-0.0099	-5	335	348	YDLEVWIPSYDSYR					Mascot
1817.8235	1817.822	-0.0015	-1	349	363	EISSCSNFESFQARR			Carbamidomethyl (C)[5]		Mascot
1817.8235	1817.822	-0.0015	-1	349	363	EISSCSNFESFQARR			Carbamidomethyl (C)[5]		Mascot
1837.8286	1837.8566	0.028	15	254	270	YTAFSACFRSEAGSAGR			Carbamidomethyl (C)[7]		Mascot
1933.9331	1933.8823	-0.0508	-26	334	348	KYDLEVWIPSYDSYR					Mascot

9	UPF0145 protein Patl_2194 OS=Pseudoalteromonas atlantica (strain T6c / ATCC BAA-1087) GN=Patl_2194 PE=3 SV=1	Y2194_PSEA6	11503.7	4.62	6	43	0	4.101		

Peptide Information

Calc. Mass	Obsrv. Mass	± da	± ppm	Start Seq.	End Seq.	Sequence	Ion Score	C. I. %	Modification	Rank	Result Type
817.405	817.3376	-0.0674	-82	64	70	ELEAEAR					Mascot
1193.6083	1193.5756	-0.0327	-27	2	12	IMTTTPSIEGK			Oxidation (M)[2]		Mascot
1308.6538	1308.5863	-0.0675	-52	1	12	MIMTTTPSIEGK					Mascot
1321.7032	1321.6199	-0.0833	-63	2	13	IMTTTPSIEGKK			Oxidation (M)[2]		Mascot
1321.7032	1321.6199	-0.0833	-63	2	13	IMTTTPSIEGKK			Oxidation (M)[2]		Mascot
1871.8549	1871.8287	-0.0262	-14	90	108	GGSMLMVSISGTAVSCSDL			Carbamidomethyl (C)[16]		Mascot
1926.9304	1926.8087	-0.1217	-63	47	63	SGAYEDELTNARQIGFR					Mascot

10	Chaperone protein HscA homolog OS=Bordetella petrii (strain ATCC BAA-461 / DSM 12804 / CCUG 43448) GN=hscA PE=3 SV=1	HSCA_BORPD	65692.2	4.76	4	42	0	4.6	34	57.182

Peptide Information

Calc. Mass	Obsrv. Mass	± da	± ppm	Start Seq.	End Seq.	Sequence	Ion Score	C. I. %	Modification	Rank	Result Type
859.4268	859.4466	0.0198	23	534	540	EQQVEAR					Mascot
1253.6484	1253.6033	-0.0451	-36	249	261	ASLGDAPLAPADR					Mascot
1515.7285	1515.7192	-0.0093	-6	548	562	AALAADGDLLDADER					Mascot
1515.7285	1515.7192	-0.0093	-6	548	562	AALAADGDLLDADER	34	57.182			Mascot
1871.9645	1871.8287	-0.1358	-73	323	342	DAGLAVGDINGVVMVGGATR					Mascot

Gel Idx/Pos	158/G9	Instr./Gel Origin	BA2151/Sample Project 20140814	Process Status	Analysis Succeeded
Plate [#] Name	[1] Sample Project 20140814	Instrument Sample Name		Spectra	11

Rank	Protein Name	Accession No.	Protein MW	Protein PI	Pep. Count	Protein Score	Protein Score C. I. %	Intensity Matched	Total Ion Score	Total Ion C. I. %	Confirmed
1	Keratin, type II cytoskeletal 1 OS=Homo sapiens GN=KRT1 PE=1 SV=6	K2C1_HUMAN	66170.1	8.15	11	71	95.502	2.714	43	94.452	

Peptide Information

Calc. Mass	Obsrv. Mass	± da	± ppm	Start Seq.	End Seq.	Sequence	Ion Score	C. I. %	Modification	Rank	Result Type
832.4886	832.4798	-0.0088	-11	75	82	SISISVAR					Mascot
1033.516	1033.4933	-0.0227	-22	484	492	TLLEGEESR					Mascot
1157.5143	1157.5315	0.0172	15	464	472	DYQELMNTK			Oxidation (M)[6]		Mascot
1277.71	1277.683	-0.027	-21	473	483	LALDLEIATYR	14	0			Mascot
1302.7012	1302.6357	-0.0655	-50	393	403	NSKIEISELNR					Mascot
1332.5195	1332.4734	-0.0461	-35	258	267	NMQDMVEDYR			Oxidation (M)[2,5]		Mascot
1340.6692	1340.6299	-0.0393	-29	365	376	SKAEAESLYQSK					Mascot
1393.7322	1393.6746	-0.0576	-41	278	289	TNAENEFVTIKK					Mascot
1475.7489	1475.7231	-0.0258	-17	212	223	WELLQQVDTSTR	32	32.676			Mascot
1657.793	1657.7297	-0.0633	-38	13	29	SGGGFSSGSAGIINYQR					Mascot
3312.3083	3312.2607	-0.0476	-14	550	588	GSYGSGGSSYGSGGGS YGSGGGGGGHGSYGSG SSSGGYR					Mascot

Rank	Protein Name	Accession No.	Protein MW	Protein PI	Pep. Count	Protein Score	Protein Score C. I. %	Intensity Matched	Total Ion Score	Total Ion C. I. %	Confirmed
2	Keratin, type II cytoskeletal 1 OS=Pan troglodytes GN=KRT1 PE=1 SV=1	K2C1_PANTR	65620.8	7.62	10	70	94.338	2.47	45	95.999	

Peptide Information

Calc. Mass	Obsrv. Mass	± da	± ppm	Start Seq.	End Seq.	Sequence	Ion Score	C. I. %	Modification	Rank	Result Type
832.4886	832.4798	-0.0088	-11	75	82	SISISVAR					Mascot
1033.516	1033.4933	-0.0227	-22	479	487	TLLEGEESR					Mascot
1157.5143	1157.5315	0.0172	15	459	467	DYQELMNTK			Oxidation (M)[6]		Mascot
1277.71	1277.683	-0.027	-21	468	478	LALDLEIATYR	14	0			Mascot
1302.7012	1302.6357	-0.0655	-50	388	398	NSKIEISELNR					Mascot
1340.6692	1340.6299	-0.0393	-29	360	371	SKAEAESLYQSK					Mascot
1393.7322	1393.6746	-0.0576	-41	273	284	TNAENEFVTIKK					Mascot
1475.7489	1475.7231	-0.0258	-17	207	218	WELLQQVDTSTR	32	32.676			Mascot
1657.793	1657.7297	-0.0633	-38	13	29	SGGGFSSGSAGIINYQR					Mascot

Calc. Mass	Obsrv. Mass	± da	± ppm	Start Seq.	End Seq.	Sequence	Ion Score	C. I. %	Modification	Rank	Result Type
3312.3083	3312.2607	-0.0476	-14	545	583	GSYGSGGSSYGSGGGS YGSGGGGGGHGSYGSG SSSGGYR					Mascot

3	Tuftelin-interacting protein 11 OS=Xenopus tropicalis GN=tfip11 PE=2 SV=2	TFP11_XENTR	95634	5.96	20	64	80.367	5.931	14	0

Peptide Information

Calc. Mass	Obsrv. Mass	± da	± ppm	Start Seq.	End Seq.	Sequence	Ion Score	C. I. %	Modification	Rank	Result Type
800.405	800.368	-0.037	-46	427	432	DWNPLR					Mascot
807.4029	807.3817	-0.0212	-26	264	270	VIDMTGR			Oxidation (M)[4]		Mascot
810.3926	810.384	-0.0086	-11	156	162	MGYVQGR					Mascot
894.3879	894.4447	0.0568	64	400	405	YYEEYK					Mascot
938.44	938.4265	-0.0135	-14	2	9	SMSHLYGK			Oxidation (M)[2]		Mascot
964.4669	964.4389	-0.028	-29	217	223	EMSQWRK					Mascot
1165.6147	1165.5651	-0.0496	-43	156	166	MGYVQGRGLGK					Mascot
1259.6519	1259.6483	-0.0036	-3	234	243	YSYKTVEELK					Mascot
1262.6562	1262.5579	-0.0983	-78	750	762	GIGAAAAVPMNFK			Oxidation (M)[10]		Mascot
1263.5886	1263.642	0.0534	42	365	374	VLETVEECER			Carbamidomethyl (C)[8]		Mascot
1308.7094	1308.6294	-0.08	-61	152	162	LLQKMGYVQGR			Oxidation (M)[5]		Mascot
1332.5525	1332.4734	-0.0791	-59	731	740	DFQYEAMQER			Oxidation (M)[7]		Mascot
1358.674	1358.6504	-0.0236	-17	274	284	VYYSYSQLAHK					Mascot
1475.7887	1475.7231	-0.0656	-44	258	270	EISQVKVIDMTGR	14	0			Mascot
1491.7836	1491.7136	-0.07	-47	258	270	EISQVKVIDMTGR			Oxidation (M)[10]		Mascot
1627.7826	1627.6421	-0.1405	-86	586	599	EVFTPGSWEAFMVK					Mascot
1926.9916	1926.8328	-0.1588	-82	406	422	MSEKADLSVAIVYPLMK			Oxidation (M)[1,16]		Mascot
1931.0419	1930.8529	-0.189	-98	750	768	GIGAAAAVPMNFKDLIQS K					Mascot
2010.9226	2011.0842	0.1616	80	427	443	DWNPLRDPTYGTDVMSK			Oxidation (M)[15]		Mascot
2217.0857	2217.1399	0.0542	24	330	347	QLQYEQDMVVNLTHELE K					Mascot
2263.1716	2263.1274	-0.0442	-20	70	90	DYSAPVNFISAGIRKPAA EEK					Mascot

4	Keratin, type I cytoskeletal 10 OS=Homo sapiens GN=KRT10 PE=1 SV=6	K1C10_HUMAN	59019.8	5.13	11	58	6.032	2.513	29	0

Peptide Information

Calc. Mass	Obsrv. Mass	± da	± ppm	Start Seq.	End Seq.	Sequence	Ion Score	C. I. %	Modification	Rank	Result Type
807.3995	807.3817	-0.0178	-22	229	235	LAADDFR					Mascot
847.452	847.4286	-0.0234	-28	363	369	SEITELR					Mascot

Calc. Mass	Obsrv. Mass	± da	± ppm	Start Seq.	End Seq.	Sequence	Ion Score	C. I. %	Modification	Rank	Result Type
993.4999	993.4618	-0.0381	-38	238	245	YENEVALR					Mascot
1060.5092	1060.5392	0.03	28	1	9	MSVRYSSSK			Oxidation (M)[1]		Mascot
1106.5259	1106.4882	-0.0377	-34	148	156	VTMQNLNDR			Oxidation (M)[3]		Mascot
1118.5087	1118.495	-0.0137	-12	185	194	HGNSHQGEPR					Mascot
1165.5848	1165.5651	-0.0197	-17	442	450	LENEIQTYR	29	0			Mascot
1201.6172	1201.5695	-0.0477	-40	246	256	QSVEADINGLR					Mascot
1262.5972	1262.5579	-0.0393	-31	451	464	SLLEGEGSSGGGGR					Mascot
1493.7343	1493.7026	-0.0317	-21	323	334	SQYEQLAEQNRK					Mascot
1707.7722	1707.7434	-0.0288	-17	41	59	GSLGGGFSSGGFSGGSF SR					Mascot

5	Late embryogenesis abundant protein Lea14-A OS=Gossypium hirsutum GN=LEA14-A PE=2 SV=1	LEA14_GOSHI	16526.6	4.84	3	56	0	1.283	45	96.579	

Peptide Information

Calc. Mass	Obsrv. Mass	± da	± ppm	Start Seq.	End Seq.	Sequence	Ion Score	C. I. %	Modification	Rank	Result Type
864.4495	864.4537	0.0042	5	1	7	MSQLLEK			Oxidation (M)[1]		Mascot
905.4979	905.4672	-0.0307	-34	144	151	LPTLSDIF					Mascot
905.4979	905.4672	-0.0307	-34	144	151	LPTLSDIF	45	96.579			Mascot
1287.6791	1287.6256	-0.0535	-42	21	32	KPEASVSDVDLK					Mascot

6	Ribosomal RNA small subunit methyltransferase G OS=Bordetella pertussis (strain Tohama I / ATCC BAA-589 / NCTC 13251) GN=rsmG PE=3 SV=1	RSMG_BORPE	25261.2	8.78	10	56	0	3.943	

Protein Group

Ribosomal RNA small subunit methyltransferase G OS=Bordetella parapertussis (strain 12822 / ATCC BAA-587 / NCTC 13253) GN=rsmG PE=3 SV=1	RSMG_BORPA	25261.2	8.7799997329 7119

Peptide Information

Calc. Mass	Obsrv. Mass	± da	± ppm	Start Seq.	End Seq.	Sequence	Ion Score	C. I. %	Modification	Rank	Result Type
856.4635	856.5093	0.0458	53	77	86	GLPAAGSGAR					Mascot
878.4375	878.4086	-0.0289	-33	219	224	CLIWMR			Carbamidomethyl (C)[1]		Mascot
894.4324	894.4447	0.0123	14	219	224	CLIWMR			Carbamidomethyl (C)[1], Oxidation (M)[5]		Mascot
905.4761	905.4672	-0.0089	-10	176	184	EGGTLVAMK					Mascot
905.4761	905.4672	-0.0089	-10	176	184	EGGTLVAMK					Mascot
1106.5874	1106.4882	-0.0992	-90	176	186	EGGTLVAMKGK			Oxidation (M)[8]		Mascot
1349.7358	1349.6364	-0.0994	-74	34	43	LLRYIEQMQR					Mascot
1423.6899	1423.6981	0.0082	6	37	46	YIEQMQRWNR					Mascot
1657.8003	1657.7297	-0.0706	-43	108	121	AHWDVTCVDAVEKK			Carbamidomethyl (C)[7]		Mascot

Calc. Mass	Obsrv. Mass	± da	± ppm	Start Seq.	End Seq.	Sequence	Ion Score	C. I. %	Modification	Rank	Result Type
1874.0205	1873.9125	-0.108	-58	89	107	LFDVGSGGGLPGVVLAIM R			Oxidation (M)[18]		Mascot
2054.0447	2054.0684	0.0237	12	15	33	LAQACDALRLPADAGQQ QK			Carbamidomethyl (C)[5]		Mascot
2263.1611	2263.1274	-0.0337	-15	2	23	SAVPDIPGGPAQRLAQA CDALR			Carbamidomethyl (C)[18]		Mascot

7	Chorismate synthase OS=Clostridium sticklandii (strain ATCC 12662 / DSM 519 / JCM 1433 / NCIB 10654) GN=aroC PE=3 SV=1	AROC_CLOSD	40211.3	5.72	12	55	0	4.3		

Peptide Information

Calc. Mass	Obsrv. Mass	± da	± ppm	Start Seq.	End Seq.	Sequence	Ion Score	C. I. %	Modification	Rank	Result Type
838.4338	838.3748	-0.059	-70	1	8	MSTTLGTK					Mascot
993.4935	993.4618	-0.0317	-32	332	339	HDPCIVPR			Carbamidomethyl (C)[4]		Mascot
1302.661	1302.6357	-0.0253	-19	182	192	QMELPVISEEK					Mascot
1308.5452	1308.6294	0.0842	64	86	96	NSNQHSSDYEK					Mascot
1327.6272	1327.6471	0.0199	15	35	45	LDMDYIEAVMK					Mascot
1332.5778	1332.4734	-0.1044	-78	249	260	GIEFGDGFEMSK			Oxidation (M)[10]		Mascot
1358.7501	1358.6504	-0.0997	-73	237	248	LSHMLFSIPAVK			Oxidation (M)[4]		Mascot
1393.6199	1393.6746	0.0547	39	48	59	MPGQNDMSTPRK			Oxidation (M)[1,7]		Mascot
1493.7781	1493.7026	-0.0755	-51	193	204	EIFMRNLIQDAK			Oxidation (M)[4]		Mascot
2010.9875	2011.0842	0.0967	48	182	197	QMELPVISEEKEIFMR			Oxidation (M)[2,15]		Mascot
2054.0852	2054.0684	-0.0168	-8	281	300	SNHNGGILGGITNAMPIIF K					Mascot
2218.0591	2218.1646	0.1055	48	261	280	ARASMVNDQMSIIDGEVT HK			Oxidation (M)[5]		Mascot
2234.054	2234.1016	0.0476	21	261	280	ARASMVNDQMSIIDGEVT HK			Oxidation (M)[5,10]		Mascot

8	Tuftelin-interacting protein 11 OS=Xenopus laevis GN=tfip11 PE=2 SV=1	TFP11_XENLA	95526.8	5.86	18	54	0	6.316	14	0

Peptide Information

Calc. Mass	Obsrv. Mass	± da	± ppm	Start Seq.	End Seq.	Sequence	Ion Score	C. I. %	Modification	Rank	Result Type
800.405	800.368	-0.037	-46	427	432	DWNPLR					Mascot
807.4029	807.3817	-0.0212	-26	264	270	VIDMTGR			Oxidation (M)[4]		Mascot
827.3538	827.4014	0.0476	58	156	162	MGYMPGR			Oxidation (M)[1]		Mascot
894.3879	894.4447	0.0568	64	400	405	YYEEYK					Mascot
938.44	938.4265	-0.0135	-14	2	9	SMSHLYGK			Oxidation (M)[2]		Mascot
964.4669	964.4389	-0.028	-29	217	223	EMSQWRK					Mascot
1182.5758	1182.5366	-0.0392	-33	156	166	MGYMPGRGLGK			Oxidation (M)[1]		Mascot
1182.5758	1182.5366	-0.0392	-33	156	166	MGYMPGRGLGK			Oxidation (M)[1]		Mascot

Calc. Mass	Obsrv. Mass	± da	± ppm	Start Seq.	End Seq.	Sequence	Ion Score	C. I. %	Modification	Rank	Result Type
1259.6519	1259.6483	-0.0036	-3	234	243	YSYKTVEELK					Mascot
1262.6562	1262.5579	-0.0983	-78	749	761	GIGAAAAVPMNFK			Oxidation (M)[10]		Mascot
1263.5886	1263.642	0.0534	42	365	374	VLETVEECER			Carbamidomethyl (C)[8]		Mascot
1332.5525	1332.4734	-0.0791	-59	730	739	DFQYEAMQER			Oxidation (M)[7]		Mascot
1349.6736	1349.6364	-0.0372	-28	274	284	VYYSYSQLAQK					Mascot
1475.7887	1475.7231	-0.0656	-44	258	270	EISQVKVIDMTGR	14	0			Mascot
1491.7836	1491.7136	-0.07	-47	258	270	EISQVKVIDMTGR			Oxidation (M)[10]		Mascot
1627.7826	1627.6421	-0.1405	-86	585	598	EVFTPGSWEAFMVK					Mascot
1926.9916	1926.8328	-0.1588	-82	406	422	MSEKADLSVAIVYPLMK			Oxidation (M)[1,16]		Mascot
1931.0419	1930.8529	-0.189	-98	749	767	GIGAAAAVPMNFKDLIQSK					Mascot
2217.0857	2217.1399	0.0542	24	330	347	QLQYEQDMVVNLTHELEK					Mascot
2263.1716	2263.1274	-0.0442	-20	70	90	DYSAPVNFISAGIRKPAAEEK					Mascot

9	Uridylate kinase OS=Pseudoalteromonas atlantica (strain T6c / ATCC BAA-1087) GN=pyrH PE=3 SV=1	PYRH_PSEA6	26842.8	5.78	11	53	0	3.163			

Peptide Information

Calc. Mass	Obsrv. Mass	± da	± ppm	Start Seq.	End Seq.	Sequence	Ion Score	C. I. %	Modification	Rank	Result Type
864.4686	864.4537	-0.0149	-17	210	216	DHNIPIR					Mascot
919.4883	919.3972	-0.0911	-99	178	185	NPDAVLYK					Mascot
1060.5535	1060.5392	-0.0143	-13	2	10	STTPKHAYR					Mascot
1103.5626	1103.5062	-0.0564	-51	63	73	GEGLAKAGMNR					Mascot
1157.6412	1157.5315	-0.1097	-95	153	163	GIEIEADAVLK					Mascot
1165.5372	1165.5651	0.0279	24	167	177	VDGVYDSDPAK					Mascot
1232.6667	1232.5679	-0.0988	-80	33	42	VLERMAQEIK			Oxidation (M)[5]		Mascot
1308.6188	1308.6294	0.0106	8	217	227	VFNMNTPGCLR			Carbamidomethyl (C)[9]		Mascot
1423.7679	1423.6981	-0.0698	-49	187	198	LSYSEVLDKELK					Mascot
2217.1836	2217.1399	-0.0437	-20	12	32	ILLKLSGEALMGEEGFGIDPK					Mascot
2263.1274	2263.1274	0	0	16	36	LSGEALMGEEGFGIDPKVLER			Oxidation (M)[7]		Mascot

10	Tuftelin-interacting protein 11 OS=Sus scrofa GN=TFIP11 PE=2 SV=1	TFP11_PIG	96838.5	5.49	17	53	0	6.877	12	0		

Peptide Information

Calc. Mass	Obsrv. Mass	± da	± ppm	Start Seq.	End Seq.	Sequence	Ion Score	C. I. %	Modification	Rank	Result Type
807.4029	807.3817	-0.0212	-26	268	274	VIDMTGR			Oxidation (M)[4]		Mascot
948.4203	948.3887	-0.0316	-33	752	759	EAENMAQR					Mascot

964.4152	964.4389	0.0237	25	752	759	EAENMAQR			Oxidation (M)[5]	Mascot
993.5251	993.4618	-0.0633	-64	402	409	IFETLQDK				Mascot
1259.6519	1259.6483	-0.0036	-3	238	247	YSYKTVEELK				Mascot
1277.7035	1277.683	-0.0205	-16	156	166	LLQKMGYVPGR			Oxidation (M)[5]	Mascot
1302.7416	1302.6357	-0.1059	-81	562	572	LEPLYSPIRSK				Mascot
1323.5812	1323.6279	0.0467	35	58	69	DSDEERPSFGGK				Mascot
1323.5812	1323.6279	0.0467	35	58	69	DSDEERPSFGGK				Mascot
1327.7581	1327.6471	-0.111	-84	256	267	LSAPQKEISQVK				Mascot
1332.5525	1332.4734	-0.0791	-59	741	750	DFQYEAMQER			Oxidation (M)[7]	Mascot
1340.6521	1340.6299	-0.0222	-17	433	442	DYFKEWDPLK				Mascot
1393.7145	1393.6746	-0.0399	-29	760	773	GIGVAASSVPMNFK			Oxidation (M)[11]	Mascot
1475.7887	1475.7231	-0.0656	-44	262	274	EISQVKVIDMTGR	14	0		Mascot
1479.6823	1479.7106	0.0283	19	58	70	DSDEERPSFGGKR				Mascot
1491.7817	1491.7136	-0.0681	-46	479	489	LIWEVWMPFVR			Oxidation (M)[7]	Mascot
1507.7322	1507.7065	-0.0257	-17	713	727	AVSSNVGAYMQPGAR				Mascot
1627.7323	1627.6421	-0.0902	-55	138	150	SFMDFGSWERHTK				Mascot
1629.7618	1629.6421	-0.1197	-73	596	609	DVFTPGSWEAFMVK			Oxidation (M)[12]	Mascot

Gel Idx/Pos	159/G10	Instr./Gel Origin	BA2151/Sample Project 20140814	Process Status	Analysis Succeeded
Plate [#] Name	[1] Sample Project 20140814	Instrument Sample Name		Spectra	11

Rank	Protein Name	Accession No.	Protein MW	Protein PI	Pep. Count	Protein Score	Protein Score C. I. %	Intensity Matched	Total Ion Score	Total Ion C. I. %	Confirmed
1	Keratin, type II cytoskeletal 1 OS=Homo sapiens GN=KRT1 PE=1 SV=6	K2C1_HUMAN	66170.1	8.15	22	372	100	7.39	272	100	

Peptide Information

Calc. Mass	Obsrv. Mass	± da	± ppm	Start Seq.	End Seq.	Sequence	Ion Score	C. I. %	Modification	Rank	Result Type
802.4417	802.4233	-0.0184	-23	180	185	EREQIK					Mascot
832.4886	832.4731	-0.0155	-19	75	82	SISISVAR					Mascot
973.5312	973.508	-0.0232	-24	396	403	IEISELNR	37	75.744			Mascot
999.4452	999.424	-0.0212	-21	290	298	DVDGAYMTK					Mascot
1006.4297	1006.4092	-0.0205	-20	589	602	GGSGGGGGGSSGGR					Mascot
1033.516	1033.4932	-0.0228	-22	484	492	TLLEGEESR					Mascot
1065.5211	1065.4773	-0.0438	-41	356	364	AQYEDIAQK					Mascot
1066.5164	1066.4847	-0.0317	-30	270	277	YEDEINKR					Mascot
1073.595	1073.5188	-0.0762	-71	408	416	LRSEIDNVK					Mascot
1127.5402	1127.5016	-0.0386	-34	289	298	KDVDGAYMTK					Mascot
1141.5194	1141.5021	-0.0173	-15	464	472	DYQELMNTK					Mascot
1265.6372	1265.6135	-0.0237	-19	278	288	TNAENEFVTIK					Mascot
1277.71	1277.6824	-0.0276	-22	473	483	LALDLEIATYR	72	99.993			Mascot
1302.7012	1302.6577	-0.0435	-33	393	403	NSKIEISELNR					Mascot
1316.5247	1316.5229	-0.0018	-1	258	267	NMQDMVEDYR			Oxidation (M)[2]		Mascot
1332.5195	1332.5005	-0.019	-14	258	267	NMQDMVEDYR			Oxidation (M)[2,5]		Mascot
1357.6958	1357.6211	-0.0747	-55	444	455	LNDLEDALQQAK					Mascot
1383.6903	1383.6462	-0.0441	-32	186	197	SLNNQFASFIDK	30	0			Mascot
1475.7489	1475.7178	-0.0311	-21	212	223	WELLQQVDTSTR	79	99.999			Mascot
1657.793	1657.7561	-0.0369	-22	13	29	SGGGFSSGSAGIINYQR					Mascot
1716.8511	1716.8074	-0.0437	-25	418	432	QISNLQQSISDAEQR	57	99.757			Mascot
2286.1248	2285.938	-0.1868	-82	367	386	AEAESLYQSKYEELQITAGR					Mascot
3312.3083	3312.2476	-0.0607	-18	550	588	GSYGSGGSSYGSGGGSYGSGGGGGGHGSYGSGSSSGGYR					Mascot

Rank	Protein Name	Accession No.	Protein MW	Protein PI	Pep. Count	Protein Score	Protein Score C. I. %	Intensity Matched	Total Ion Score	Total Ion C. I. %	Confirmed
2	Keratin, type II cytoskeletal 1 OS=Pan troglodytes GN=KRT1 PE=1 SV=1	K2C1_PANTR	65620.8	7.62	21	365	100	7.139	273	100	

Peptide Information

Calc. Mass	Obsrv. Mass	± da	± ppm	Start Seq.	End Seq.	Sequence	Ion Score	C. I. %	Modification	Rank	Result Type
802.4417	802.4233	-0.0184	-23	175	180	EREQIK					Mascot
832.4886	832.4731	-0.0155	-19	75	82	SISISVAR					Mascot
973.5312	973.508	-0.0232	-24	391	398	IEISELNR	37	75.744			Mascot
999.4452	999.424	-0.0212	-21	285	293	DVDGAYMTK					Mascot
1033.516	1033.4932	-0.0228	-22	479	487	TLLEGEESR					Mascot
1065.5211	1065.4773	-0.0438	-41	351	359	AQYEDIAQK					Mascot
1066.5164	1066.4847	-0.0317	-30	265	272	YEDEINKR					Mascot
1073.595	1073.5188	-0.0762	-71	403	411	LRSEIDNVK					Mascot
1127.5402	1127.5016	-0.0386	-34	284	293	KDVDGAYMTK					Mascot
1141.5194	1141.5021	-0.0173	-15	459	467	DYQELMNTK					Mascot
1265.6372	1265.6135	-0.0237	-19	273	283	TNAENEFVTIK					Mascot
1277.71	1277.6824	-0.0276	-22	468	478	LALDLEIATYR	72	99.993			Mascot
1302.7012	1302.6577	-0.0435	-33	388	398	NSKIEISELNR					Mascot
1357.6958	1357.6211	-0.0747	-55	439	450	LNDLEDALQQAK					Mascot
1383.6903	1383.6462	-0.0441	-32	181	192	SLNNQFASFIDK	30	0			Mascot
1475.7489	1475.7178	-0.0311	-21	207	218	WELLQQVDTSTR	79	99.999			Mascot
1657.793	1657.7561	-0.0369	-22	13	29	SGGGFSSGSAGIINYQR					Mascot
1716.8511	1716.8074	-0.0437	-25	413	427	QISNLQQSISDAEQR	57	99.757			Mascot
1987.8372	1987.9645	0.1273	64	247	262	LDSELNNMQDMVEDYR			Oxidation (M)[8]		Mascot
2003.8322	2003.9404	0.1082	54	247	262	LDSELNNMQDMVEDYR			Oxidation (M)[8,11]		Mascot
2286.1248	2285.938	-0.1868	-82	362	381	AEAESLYQSKYEELQITA GR					Mascot
3312.3083	3312.2476	-0.0607	-18	545	583	GSYGSGGSSYGSGGGS YGSGGGGGHGSYGSG SSSGGYR					Mascot

3	Keratin, type II cytoskeletal 1 OS=Canis familiaris GN=KRT1 PE=1 SV=1	K2C1_CANFA	63922.1	7.66	11	129	100	4.645	108	100	

Peptide Information

Calc. Mass	Obsrv. Mass	± da	± ppm	Start Seq.	End Seq.	Sequence	Ion Score	C. I. %	Modification	Rank	Result Type
802.4417	802.4233	-0.0184	-23	181	186	EREQIK					Mascot
905.4938	905.4398	-0.054	-60	411	418	SEIDSVKK					Mascot
999.5218	999.424	-0.0978	-98	388	396	HGDNLKSTK					Mascot
1003.5353	1003.5281	-0.0072	-7	457	464	EDMARLLR					Mascot
1033.516	1033.4932	-0.0228	-22	485	493	TLLEGEESR					Mascot

1066.5164	1066.4847	-0.0317	-30	271	278	YEDEINKR					Mascot
1141.5194	1141.5021	-0.0173	-15	465	473	DYQELMNTK					Mascot
1265.6372	1265.6135	-0.0237	-19	279	289	TNAENEFVTIK					Mascot
1323.6573	1323.6371	-0.0202	-15	394	404	STKMEISELNR			Oxidation (M)[4]		Mascot
1323.6573	1323.6371	-0.0202	-15	394	404	STKMEISELNR	7	0	Oxidation (M)[4]		Mascot
1383.6903	1383.6462	-0.0441	-32	187	198	SLNNQFASFIDK	30	0			Mascot
1475.7489	1475.7178	-0.0311	-21	213	224	WELLQQVDTSTR	79	99.999			Mascot

4	Keratin, type II cytoskeletal 4 OS=Homo sapiens GN=KRT4 PE=1 SV=4	K2C4_HUMAN	57649	6.25	12	111	100	3.349	72	99.993		

Peptide Information

Calc. Mass	Obsrv. Mass	± da	± ppm	Start Seq.	End Seq.	Sequence	Ion Score	C. I. %	Modification	Rank	Result Type
832.4821	832.4731	-0.009	-11	375	380	MIQRLR			Oxidation (M)[1]		Mascot
1003.5054	1003.5281	0.0227	23	212	220	QLDTLGNDK					Mascot
1033.5459	1033.4932	-0.0527	-51	427	434	EELARMLR			Oxidation (M)[6]		Mascot
1057.532	1057.4642	-0.0678	-64	5	13	QQCVRGGPR			Carbamidomethyl (C)[3]		Mascot
1107.543	1107.5167	-0.0263	-24	327	335	AQYEEIAQR					Mascot
1126.5449	1126.4749	-0.07	-62	435	443	EYQELMSVK					Mascot
1277.71	1277.6824	-0.0276	-22	444	454	LALDIEIATYR	72	99.993			Mascot
1309.653	1309.6316	-0.0214	-16	57	69	GNKSISMSVAGSR			Oxidation (M)[7]		Mascot
1357.7322	1357.6211	-0.1111	-82	315	326	NLDLDSIIAEVR					Mascot
1475.7635	1475.7178	-0.0457	-31	367	378	SEIAELNRMIQR			Oxidation (M)[9]		Mascot
1563.7697	1563.7153	-0.0544	-35	10	26	GGPRGFSCGSAIVGGGK			Carbamidomethyl (C)[8]		Mascot
3052.4351	3052.5518	0.1167	38	288	314	VLYDAELSQMQTHVSDT SVVLSMDNNR					Mascot

5	Keratin, type II cytoskeletal 73 OS=Mus musculus GN=Krt73 PE=1 SV=1	K2C73_MOUSE	59501.9	8.36	11	109	100	3.837	87	100		

Peptide Information

Calc. Mass	Obsrv. Mass	± da	± ppm	Start Seq.	End Seq.	Sequence	Ion Score	C. I. %	Modification	Rank	Result Type
973.5312	973.508	-0.0232	-24	393	400	AKLDELER					Mascot
999.4451	999.424	-0.0211	-21	241	249	DVDAAYMSK					Mascot
1033.5459	1033.4932	-0.0527	-51	407	414	EELARMLR			Oxidation (M)[6]		Mascot
1060.5997	1060.5413	-0.0584	-55	359	367	LRSEIESVK					Mascot
1127.5402	1127.5016	-0.0386	-34	240	249	KDVDAAYMSK					Mascot
1194.6226	1194.5734	-0.0492	-41	33	45	AAGKGLSGGFSSR					Mascot
1265.6736	1265.6135	-0.0601	-47	307	317	AQYEDIALKSK					Mascot

Calc. Mass	Obsrv. Mass	± da	± ppm	Start Seq.	End Seq.	Sequence	Ion Score	C. I. %	Modification	Rank	Result Type
1277.71	1277.6824	-0.0276	-22	424	434	LALDIEIATYR	72	99.993			Mascot
1357.7322	1357.6211	-0.1111	-82	295	306	NLDLDSIIAEVR					Mascot
1475.7853	1475.7178	-0.0675	-46	151	162	FLEQQNQVLQTK	17	0			Mascot
1674.8268	1674.7006	-0.1262	-75	163	175	WELLQQLDLSNCR			Carbamidomethyl (C)[12]		Mascot

6	Keratin, type II cytoskeletal 73 OS=Rattus norvegicus GN=Krt73 PE=1 SV=1	K2C73_RAT	60976.6	8.17	10	108	100	3.769	89	100	

Peptide Information

Calc. Mass	Obsrv. Mass	± da	± ppm	Start Seq.	End Seq.	Sequence	Ion Score	C. I. %	Modification	Rank	Result Type
999.4451	999.424	-0.0211	-21	241	249	DVDAAYMSK					Mascot
1033.5459	1033.4932	-0.0527	-51	407	414	EELARMLR			Oxidation (M)[6]		Mascot
1060.5997	1060.5413	-0.0584	-55	359	367	LRSEIESVK					Mascot
1127.5402	1127.5016	-0.0386	-34	240	249	KDVDAAYMSK					Mascot
1265.6736	1265.6135	-0.0601	-47	307	317	AQYEDIALKSK					Mascot
1277.71	1277.6824	-0.0276	-22	424	434	LALDIEIATYR	72	99.993			Mascot
1357.7322	1357.6211	-0.1111	-82	295	306	NLDLDSIIAEVR					Mascot
1475.7853	1475.7178	-0.0675	-46	151	162	FLEQQNQVLQTK	17	0			Mascot
1674.8268	1674.7006	-0.1262	-75	163	175	WELLQQLDLSNCR			Carbamidomethyl (C)[12]		Mascot
1976.9574	1976.9419	-0.0155	-8	55	74	SISFNVASGSGRTGGYG FGR					Mascot

7	Keratin, type II cytoskeletal 8 OS=Homo sapiens GN=KRT8 PE=1 SV=7	K2C8_HUMAN	53671.1	5.52	13	104	99.998	8.321	72	99.993	

Peptide Information

Calc. Mass	Obsrv. Mass	± da	± ppm	Start Seq.	End Seq.	Sequence	Ion Score	C. I. %	Modification	Rank	Result Type
1003.5418	1003.5281	-0.0137	-14	89	96	TQEKEQIK					Mascot
1033.5208	1033.4932	-0.0276	-27	365	372	QDMARQLR			Oxidation (M)[3]		Mascot
1060.5634	1060.5413	-0.0221	-21	393	401	KLLEGEESR					Mascot
1066.5164	1066.4847	-0.0317	-30	179	186	YEDEINKR					Mascot
1081.5637	1081.4749	-0.0888	-82	9	18	SYKVSTSGPR					Mascot
1201.6536	1201.5873	-0.0663	-55	149	158	RQLETLGQEK					Mascot
1277.71	1277.6824	-0.0276	-22	382	392	LALDIEIATYR	72	99.993			Mascot
1320.6716	1320.5592	-0.1124	-85	253	264	SLDMDSIIAEVK					Mascot
1412.6838	1412.5449	-0.1389	-98	274	285	SRAEAESMYQIK					Mascot
1473.7795	1473.6688	-0.1107	-75	470	483	DGKLVSESSDVLPK					Mascot
1475.6981	1475.7178	0.0197	13	402	414	LESGMQNMSIHTK					Mascot

Calc. Mass	Obsrv. Mass	± da	± ppm	Start Seq.	End Seq.	Sequence	Ion Score	C. I. %	Modification	Rank	Result Type
1491.6931	1491.7074	0.0143	10	402	414	LESGMQNMSIHTK			Oxidation (M)[5]		Mascot
1507.688	1507.7042	0.0162	11	402	414	LESGMQNMSIHTK			Oxidation (M)[5,8]		Mascot
1507.688	1507.7042	0.0162	11	402	414	LESGMQNMSIHTK			Oxidation (M)[5,8]		Mascot
1628.8602	1628.8247	-0.0355	-22	348	362	DANAKLSELEAALQR					Mascot
2003.9062	2003.9404	0.0342	17	134	149	SNMDNMFESYINNLRR					Mascot

8	Keratin, type II cytoskeletal 7 OS=Rattus norvegicus GN=Krt7 PE=2 SV=1	K2C7_RAT	50678	5.67	7	100	99.994	2.256	72	99.993	

Protein Group

Keratin, type II cytoskeletal 7 OS=Mus musculus GN=Krt7 PE=1 SV=1	K2C7_MOUSE	50678	5.6700000762 9395

Peptide Information

Calc. Mass	Obsrv. Mass	± da	± ppm	Start Seq.	End Seq.	Sequence	Ion Score	C. I. %	Modification	Rank	Result Type
973.5425	973.508	-0.0345	-35	125	133	SAKSSQLPR	12	0			Mascot
1060.5634	1060.5413	-0.0221	-21	388	396	KLLEGEESR					Mascot
1194.575	1194.5734	-0.0016	-1	172	180	NKYEEEINR					Mascot
1262.66	1262.5753	-0.0847	-67	9	20	STAYPGRGAQVR					Mascot
1277.71	1277.6824	-0.0276	-22	377	387	LALDIEIATYR	72	99.993			Mascot
1357.707	1357.6211	-0.0859	-63	144	155	QQLETLQLDGGR					Mascot
1390.7689	1390.644	-0.1249	-90	181	192	RTAAENEFVLLK					Mascot

9	Keratin, type II cytoskeletal 1 OS=Rattus norvegicus GN=Krt1 PE=2 SV=1	K2C1_RAT	65190.2	8	11	100	99.994	3.584	78	99.998	

Peptide Information

Calc. Mass	Obsrv. Mass	± da	± ppm	Start Seq.	End Seq.	Sequence	Ion Score	C. I. %	Modification	Rank	Result Type
905.4938	905.4398	-0.054	-60	409	416	SEIDSVKK					Mascot
1066.5164	1066.4847	-0.0317	-30	269	276	YEDEINKR					Mascot
1074.579	1074.4915	-0.0875	-81	181	189	EQIKSLNDK					Mascot
1139.5579	1139.5244	-0.0335	-29	267	275	TKYEDEINK					Mascot
1140.5103	1140.5468	0.0365	32	355	363	AQYDSICQR			Carbamidomethyl (C)[7]		Mascot
1141.5194	1141.5021	-0.0173	-15	463	471	DYQELMNTK					Mascot
1232.5907	1232.5718	-0.0189	-15	615	625	FVSTTYSRGTN					Mascot
1265.6372	1265.6135	-0.0237	-19	277	287	TNAENEFVTIK					Mascot
1475.7489	1475.7178	-0.0311	-21	211	222	WELLQQVDTSTR	79	99.999			Mascot
1487.7999	1487.7064	-0.0935	-63	395	406	MEISELNRVIQR					Mascot
2689.1499	2689.3337	0.1838	68	492	517	MSGECTPNVSVSVSTSH			Carbamidomethyl (C)[5], Oxidation (M)[1]		Mascot

TSMSGTSSR

10	Keratin, type I cytoskeletal 10 OS=Homo sapiens GN=KRT10 PE=1 SV=6	K1C10_HUMAN	59019.8	5.13	19	99	99.993	5.909	13	0

Peptide Information

Calc. Mass	Obsrv. Mass	± da	± ppm	Start Seq.	End Seq.	Sequence	Ion Score	C. I. %	Modification	Rank	Result Type
807.3995	807.3778	-0.0217	-27	229	235	LAADDFR					Mascot
809.4403	809.4012	-0.0391	-48	157	163	LASYLDK					Mascot
847.452	847.439	-0.013	-15	363	369	SEITELR					Mascot
913.4738	913.3878	-0.086	-94	2	9	SVRYSSSK					Mascot
993.4999	993.476	-0.0239	-24	238	245	YENEVALR					Mascot
1003.553	1003.5281	-0.0249	-25	363	370	SEITELRR					Mascot
1060.5092	1060.5413	0.0321	30	1	9	MSVRYSSSK			Oxidation (M)[1]		Mascot
1106.5259	1106.496	-0.0299	-27	148	156	VTMQNLNDR			Oxidation (M)[3]		Mascot
1118.5087	1118.4799	-0.0288	-26	185	194	HGNSHQGEPR					Mascot
1158.6041	1158.5542	-0.0499	-43	199	207	YYKTIDDLK					Mascot
1201.6172	1201.5873	-0.0299	-25	246	256	QSVEADINGLR					Mascot
1262.5972	1262.5753	-0.0219	-17	451	464	SLLEGEGSSGGGGR					Mascot
1316.59	1316.5229	-0.0671	-51	286	295	NHEEEMKDLR			Oxidation (M)[6]		Mascot
1357.7183	1357.6211	-0.0972	-72	246	257	QSVEADINGLRR					Mascot
1390.6809	1390.644	-0.0369	-27	387	399	QSLEASLAETEGR					Mascot
1707.7722	1707.7317	-0.0405	-24	41	59	GSLGGGFSSGGFSGGSF SR	2	0			Mascot
1797.0116	1796.9375	-0.0741	-41	371	386	NVQALEIELQSQLALK					Mascot
2367.2627	2367.2104	-0.0523	-22	208	228	NQILNLTTDNANILLQIDN AR	13	0			Mascot
3052.6272	3052.5518	-0.0754	-25	202	228	TIDDLKNQILNLTTDNANI LLQIDNAR					Mascot

Gel Idx/Pos	160/G11	Instr./Gel Origin	BA2151/Sample Project 20140814	Process Status	Analysis Succeeded
Plate [#] Name	[1] Sample Project 20140814	Instrument Sample Name		Spectra	11

Rank	Protein Name	Accession No.	Protein MW	Protein PI	Pep. Count	Protein Score	Protein Score C. I. %	Intensity Matched	Total Ion Score	Total Ion C. I. %	Confirmed
1	Translationally-controlled tumor protein homolog OS=Triticum aestivum GN=TCTP PE=2 SV=1	TCTP_WHEAT	18851.3	4.55	11	459	100	78.453	383	100	

Peptide Information

Calc. Mass	Obsrv. Mass	± da	± ppm	Start Seq.	End Seq.	Sequence	Ion Score	C. I. %	Modification	Rank	Result Type
890.4553	890.4107	-0.0446	-50	86	92	QFISHMK					Mascot
896.4546	896.4383	-0.0163	-18	1	7	MLVYQDK					Mascot
906.4502	906.4373	-0.0129	-14	86	92	QFISHMK			Oxidation (M)[6]		Mascot
912.4495	912.4365	-0.013	-14	1	7	MLVYQDK			Oxidation (M)[1]		Mascot
912.4495	912.4365	-0.013	-14	1	7	MLVYQDK	25	0	Oxidation (M)[1]		Mascot
1046.5564	1046.5015	-0.0549	-52	86	93	QFISHMKR					Mascot
1062.5514	1062.5211	-0.0303	-29	86	93	QFISHMKR			Oxidation (M)[6]		Mascot
1063.5784	1063.5646	-0.0138	-13	67	75	VVDIVDTFR					Mascot
1063.5784	1063.5646	-0.0138	-13	67	75	VVDIVDTFR	72	99.992			Mascot
1075.5419	1075.5221	-0.0198	-18	76	84	LQEQPAFDK					Mascot
1150.5627	1150.5693	0.0066	6	102	111	LEGDDLDVFK					Mascot
1203.6368	1203.6132	-0.0236	-20	76	85	LQEQPAFDKK					Mascot
1203.6368	1203.6132	-0.0236	-20	76	85	LQEQPAFDKK	48	98.045			Mascot
1278.6577	1278.6486	-0.0091	-7	102	112	LEGDDLDVFKK					Mascot
1598.7697	1598.7454	-0.0243	-15	8	21	LSGDELLSDSFPYR					Mascot
1598.7697	1598.7454	-0.0243	-15	8	21	LSGDELLSDSFPYR	139	100			Mascot
1736.8643	1736.8264	-0.0379	-22	149	164	EGAADPTFLYFAHGLK					Mascot
1736.8643	1736.8264	-0.0379	-22	149	164	EGAADPTFLYFAHGLK	99	100			Mascot
2781.323	2781.2561	-0.0669	-24	125	148	LKDLQFFVGESMHDDGG VVFAYYK			Oxidation (M)[12]		Mascot

Rank	Protein Name	Accession No.	Protein MW	Protein PI	Pep. Count	Protein Score	Protein Score C. I. %	Intensity Matched	Total Ion Score	Total Ion C. I. %	Confirmed
2	Translationally-controlled tumor protein homolog OS=Hordeum vulgare GN=TCTP PE=2 SV=2	TCTP_HORVU	18929.4	4.53	8	429	100	77.799	383	100	

Peptide Information

Calc. Mass	Obsrv. Mass	± da	± ppm	Start Seq.	End Seq.	Sequence	Ion Score	C. I. %	Modification	Rank	Result Type
896.4546	896.4383	-0.0163	-18	1	7	MLVYQDK					Mascot
912.4495	912.4365	-0.013	-14	1	7	MLVYQDK			Oxidation (M)[1]		Mascot

912.4495	912.4365	-0.013	-14	1	7	MLVYQDK	25	0	Oxidation (M)[1]		Mascot
1063.5784	1063.5646	-0.0138	-13	67	75	VVDIVDTFR					Mascot
1063.5784	1063.5646	-0.0138	-13	67	75	VVDIVDTFR	72	99.992			Mascot
1075.5419	1075.5221	-0.0198	-18	76	84	LQEQPAFDK					Mascot
1150.5626	1150.5693	0.0067	6	102	111	LEGEELDAFK					Mascot
1203.6368	1203.6132	-0.0236	-20	76	85	LQEQPAFDKK					Mascot
1203.6368	1203.6132	-0.0236	-20	76	85	LQEQPAFDKK	48	98.045			Mascot
1278.6576	1278.6486	-0.009	-7	102	112	LEGEELDAFKK					Mascot
1598.7697	1598.7454	-0.0243	-15	8	21	LSGDELLSDSFPYR					Mascot
1598.7697	1598.7454	-0.0243	-15	8	21	LSGDELLSDSFPYR	139	100			Mascot
1736.8643	1736.8264	-0.0379	-22	149	164	EGAADPTFLYFAHGLK					Mascot
1736.8643	1736.8264	-0.0379	-22	149	164	EGAADPTFLYFAHGLK	99	100			Mascot

3	Translationally-controlled tumor protein homolog OS=Glycine max GN=TCTP PE=2 SV=1	TCTP_SOYBN	19097.5	4.57	4	135	100	26.93	119	100	

Peptide Information

Calc. Mass	Obsrv. Mass	± da	± ppm	Start Seq.	End Seq.	Sequence	Ion Score	C. I. %	Modification	Rank	Result Type
1063.5784	1063.5646	-0.0138	-13	67	75	VVDIVDTFR					Mascot
1063.5784	1063.5646	-0.0138	-13	67	75	VVDIVDTFR	72	99.992			Mascot
1075.5419	1075.5221	-0.0198	-18	76	84	LQEQPAFDK					Mascot
1203.6368	1203.6132	-0.0236	-20	76	85	LQEQPAFDKK					Mascot
1203.6368	1203.6132	-0.0236	-20	76	85	LQEQPAFDKK	48	98.045			Mascot
1348.7107	1348.644	-0.0667	-49	102	112	LDAEQQELFKK					Mascot

4	Translationally-controlled tumor protein homolog OS=Solanum tuberosum GN=TCTP PE=2 SV=2	TCTP_SOLTU	18891.5	4.58	4	135	100	26.892	119	100	

Peptide Information

Calc. Mass	Obsrv. Mass	± da	± ppm	Start Seq.	End Seq.	Sequence	Ion Score	C. I. %	Modification	Rank	Result Type
890.4941	890.4107	-0.0834	-94	112	119	KNIESATK					Mascot
1063.5784	1063.5646	-0.0138	-13	67	75	VVDIVDTFR					Mascot
1063.5784	1063.5646	-0.0138	-13	67	75	VVDIVDTFR	72	99.992			Mascot
1075.5419	1075.5221	-0.0198	-18	76	84	LQEQPAFDK					Mascot
1203.6368	1203.6132	-0.0236	-20	76	85	LQEQPAFDKK					Mascot
1203.6368	1203.6132	-0.0236	-20	76	85	LQEQPAFDKK	48	98.045			Mascot

5	Translationally-controlled tumor protein homolog OS=Hevea brasiliensis GN=TCTP PE=2 SV=1	TCTP_HEVBR	19083.6	4.5	3	130	100	26.809	119	100	

Protein Group

Translationally-controlled tumor protein homolog OS=Elaeis guineensis var. tenera GN=TCTP PE=1 SV=1	TCTP_ELAGV	19209.6	4.6199998855 5908
Translationally-controlled tumor protein homolog OS=Solanum lycopersicum GN=TCTP PE=2 SV=1	TCTP_SOLLC	19010.6	4.5500001907 3486

Peptide Information

Calc. Mass	Obsrv. Mass	± da	± ppm	Start Seq.	End Seq.	Sequence	Ion Score	C. I. %	Modification	Rank	Result Type
1063.5784	1063.5646	-0.0138	-13	67	75	VVDIVDTFR					Mascot
1063.5784	1063.5646	-0.0138	-13	67	75	VVDIVDTFR	72	99.992			Mascot
1075.5419	1075.5221	-0.0198	-18	76	84	LQEQPAFDK					Mascot
1203.6368	1203.6132	-0.0236	-20	76	85	LQEQPAFDKK					Mascot
1203.6368	1203.6132	-0.0236	-20	76	85	LQEQPAFDKK	48	98.045			Mascot

6	Translationally-controlled tumor protein homolog OS=Cucumis melo GN=TCTP PE=2 SV=1	TCTP_CUCME	18803.5	4.51	3	114	100	26.809	103	100	

Peptide Information

Calc. Mass	Obsrv. Mass	± da	± ppm	Start Seq.	End Seq.	Sequence	Ion Score	C. I. %	Modification	Rank	Result Type
1063.5784	1063.5646	-0.0138	-13	67	75	VVDIVDTFR					Mascot
1063.5784	1063.5646	-0.0138	-13	67	75	VVDIVDTFR	72	99.992			Mascot
1075.5089	1075.5221	0.0132	12	76	84	LQEQPSMDK					Mascot
1203.6039	1203.6132	0.0093	8	76	85	LQEQPSMDKK					Mascot
1203.6039	1203.6132	0.0093	8	76	85	LQEQPSMDKK	32	21.997			Mascot

7	Translationally-controlled tumor protein homolog OS=Nicotiana tabacum GN=TCTP PE=2 SV=1	TCTP_TOBAC	18729.2	4.36	2	84	99.79	26.813	72	99.992	

Peptide Information

Calc. Mass	Obsrv. Mass	± da	± ppm	Start Seq.	End Seq.	Sequence	Ion Score	C. I. %	Modification	Rank	Result Type
1063.5784	1063.5646	-0.0138	-13	67	75	VVDIVDTFR					Mascot
1063.5784	1063.5646	-0.0138	-13	67	75	VVDIVDTFR	72	99.992			Mascot
1718.8749	1718.8237	-0.0512	-30	149	164	DGATDPTFLYLAHGLK					Mascot
1718.8749	1718.8237	-0.0512	-30	149	164	DGATDPTFLYLAHGLK	5	0			Mascot

8	Translationally-controlled tumor protein homolog OS=Pseudotsuga menziesii GN=TCTP PE=2 SV=1	TCTP_PSEMZ	18882.6	4.68	3	83	99.747	25.859	72	99.992	

Peptide Information

Calc. Mass	Obsrv. Mass	± da	± ppm	Start Seq.	End Seq.	Sequence	Ion Score	C. I. %	Modification	Rank	Result Type
1063.5784	1063.5646	-0.0138	-13	66	74	VVDIVDTFR					Mascot
1063.5784	1063.5646	-0.0138	-13	66	74	VVDIVDTFR	72	99.992			Mascot
1101.5575	1101.5083	-0.0492	-45	75	83	LQEQPPFDK					Mascot
2087.0332	2087.0073	-0.0259	-12	148	166	DGATDPTFLYFADGLKEV K					Mascot

9	Translationally-controlled tumor protein homolog OS=Zea mays GN=TCTP PE=2 SV=1	TCTP_MAIZE	18735.4	4.52	3	82	99.667	25.862	72	99.992

Peptide Information

Calc. Mass	Obsrv. Mass	± da	± ppm	Start Seq.	End Seq.	Sequence	Ion Score	C. I. %	Modification	Rank	Result Type
1063.5784	1063.5646	-0.0138	-13	66	74	VVDIVDTFR					Mascot
1063.5784	1063.5646	-0.0138	-13	66	74	VVDIVDTFR	72	99.992			Mascot
1101.5575	1101.5083	-0.0492	-45	75	83	LQEQPPFDK					Mascot
1316.6191	1316.6167	-0.0024	-2	126	136	DLQFFVGESMK			Oxidation (M)[10]		Mascot

10	Translationally-controlled tumor protein homolog 2 (Fragments) OS=Pseudotsuga menziesii PE=1 SV=1	TCTP2_PSEMZ	1889	5.91	1	80	99.447	25.69	72	99.992

Peptide Information

Calc. Mass	Obsrv. Mass	± da	± ppm	Start Seq.	End Seq.	Sequence	Ion Score	C. I. %	Modification	Rank	Result Type
1063.5784	1063.5646	-0.0138	-13	1	9	VVDIVDTFR					Mascot
1063.5784	1063.5646	-0.0138	-13	1	9	VVDIVDTFR	72	99.992			Mascot

Gel Idx/Pos	161/G12	Instr./Gel Origin	BA2151/Sample Project 20140814	Process Status	Analysis Succeeded
Plate [#] Name	[1] Sample Project 20140814	Instrument Sample Name		Spectra	11

Rank	Protein Name	Accession No.	Protein MW	Protein PI	Pep. Count	Protein Score	Protein Score C. I. %	Intensity Matched	Total Ion Score	Total Ion C. I. %	Confirmed
1	2-Cys peroxiredoxin BAS1, chloroplastic (Fragment) OS=Triticum aestivum GN=TSA PE=1 SV=2	BAS1_WHEAT	23426.2	5.71	6	208	100	50.838	146	100	

Protein Group

2-Cys peroxiredoxin BAS1, chloroplastic (Fragment) OS=Hordeum vulgare GN=BAS1 PE=2 SV=1	BAS1_HORVU	23398.1	5.4800 000190 7349

Peptide Information

Calc. Mass	Obsrv. Mass	± da	± ppm	Start Seq.	End Seq.	Sequence	Ion Score	C. I. %	Modification	Rank	Result Type
805.4818	805.4572	-0.0246	-31	141	147	GLFIIDK					Mascot
819.4207	819.4034	-0.0173	-21	164	170	SVDETLR					Mascot
1021.5565	1021.5298	-0.0267	-26	114	122	YPLVSDVTK					Mascot
1360.7107	1360.6843	-0.0264	-19	11	23	AAAEYDLPLVGNK					Mascot
1485.8424	1485.8036	-0.0388	-26	127	140	SFGVLIPDQGIALR					Mascot
1485.8424	1485.8036	-0.0388	-26	127	140	SFGVLIPDQGIALR	109	100			Mascot
1707.9137	1707.8635	-0.0502	-29	148	163	EGVIQHSTINNLGIGR					Mascot
1707.9137	1707.8635	-0.0502	-29	148	163	EGVIQHSTINNLGIGR	37	81.26			Mascot

Rank	Protein Name	Accession No.	Protein MW	Protein PI	Pep. Count	Protein Score	Protein Score C. I. %	Intensity Matched	Total Ion Score	Total Ion C. I. %	Confirmed
2	2-Cys peroxiredoxin BAS1-like, chloroplastic OS=Arabidopsis thaliana GN=At5g06290 PE=2 SV=3	BAS1B_ARATH	29932.3	5.55	3	168	100	44.981	146	100	

Peptide Information

Calc. Mass	Obsrv. Mass	± da	± ppm	Start Seq.	End Seq.	Sequence	Ion Score	C. I. %	Modification	Rank	Result Type
805.4818	805.4572	-0.0246	-31	203	209	GLFIIDK					Mascot
1485.8424	1485.8036	-0.0388	-26	189	202	SFGVLIPDQGIALR					Mascot
1485.8424	1485.8036	-0.0388	-26	189	202	SFGVLIPDQGIALR	109	100			Mascot
1707.9137	1707.8635	-0.0502	-29	210	225	EGVIQHSTINNLGIGR					Mascot
1707.9137	1707.8635	-0.0502	-29	210	225	EGVIQHSTINNLGIGR	37	81.26			Mascot

Rank	Protein Name	Accession No.	Protein MW	Protein PI	Pep. Count	Protein Score	Protein Score C. I. %	Intensity Matched	Total Ion Score	Total Ion C. I. %	Confirmed
3	2-Cys peroxiredoxin BAS1, chloroplastic OS=Oryza sativa subsp. japonica GN=BAS1 PE=1 SV=1	BAS1_ORYSJ	28307.5	5.67	3	131	100	33.691	109	100	

Peptide Information

Calc. Mass	Obsrv. Mass	± da	± ppm	Start Seq.	End Seq.	Sequence	Ion Score	C. I. %	Modification	Rank	Result Type

Calc. Mass	Obsrv. Mass	± da	± ppm	Start Seq.	End Seq.	Sequence	Ion Score	C. I. %	Modification	Rank	Result Type
805.4818	805.4572	-0.0246	-31	191	197	GLFIIDK					Mascot
1021.5385	1021.5298	-0.0087	-9	44	53	LSASSSRSAR					Mascot
1485.8424	1485.8036	-0.0388	-26	177	190	SFGVLIPDQGIALR					Mascot
1485.8424	1485.8036	-0.0388	-26	177	190	SFGVLIPDQGIALR	109	100			Mascot

4	2-Cys peroxiredoxin BAS1, chloroplastic OS=Arabidopsis thaliana GN=BAS1 PE=1 SV=2	BAS1A_ARATH	29188	6.92	2	50	0	14.257	37	81.26		

Protein Group

2-Cys peroxiredoxin BAS1, chloroplastic OS=Spinacia oleracea GN=BAS1 PE=2 SV=1	BAS1_SPIOL	29048.9	7.6999 998092 6514

Peptide Information

Calc. Mass	Obsrv. Mass	± da	± ppm	Start Seq.	End Seq.	Sequence	Ion Score	C. I. %	Modification	Rank	Result Type
805.4818	805.4572	-0.0246	-31	196	202	GLFIIDK					Mascot
1707.9137	1707.8635	-0.0502	-29	203	218	EGVIQHSTINNLGIGR					Mascot
1707.9137	1707.8635	-0.0502	-29	203	218	EGVIQHSTINNLGIGR	37	81.26			Mascot

5	Spermatogenesis-defective protein 39 OS=Caenorhabditis elegans GN=spe-39 PE=1 SV=1	SPE39_CAEEL	59640.9	9.55	2	46	0	14.098	37	81.26

Peptide Information

Calc. Mass	Obsrv. Mass	± da	± ppm	Start Seq.	End Seq.	Sequence	Ion Score	C. I. %	Modification	Rank	Result Type
1509.7076	1509.751	0.0434	29	290	302	TMLHGSFSIPEMK			Oxidation (M)[2,12]		Mascot
1707.9541	1707.8635	-0.0906	-53	429	443	WGVSINNHNLLTIVK					Mascot
1707.9541	1707.8635	-0.0906	-53	429	443	WGVSINNHNLLTIVK	37	81.26			Mascot

6	1-deoxy-D-xylulose-5-phosphate synthase OS=Methylobacillus flagellatus (strain KT / ATCC 51484 / DSM 6875) GN=dxs PE=3 SV=1	DXS_METFK	66684.7	5.67	2	36	0	31.16	28	0

Peptide Information

Calc. Mass	Obsrv. Mass	± da	± ppm	Start Seq.	End Seq.	Sequence	Ion Score	C. I. %	Modification	Rank	Result Type
1410.8468	1410.9866	0.1398	99	209	221	VLGVVPPVREFAK					Mascot
1485.8094	1485.8036	-0.0058	-4	481	493	DMQLLPIGKGELR			Oxidation (M)[2]		Mascot
1485.8094	1485.8036	-0.0058	-4	481	493	DMQLLPIGKGELR	28	0	Oxidation (M)[2]		Mascot

7	Glutamine--tRNA ligase OS=Bradyrhizobium japonicum (strain USDA 110) GN=glnS PE=3 SV=1	SYQ_BRAJA	63838.1	6.03	2	36	0	12.853	27	0

Peptide Information

Calc. Mass	Obsrv. Mass	± da	± ppm	Start Seq.	End Seq.	Sequence	Ion Score	C. I. %	Modification	Rank	Result Type
1690.8582	1690.8481	-0.0101	-6	1	15	MTEPVAAEVGRDFIR					Mascot
1707.7504	1707.8635	0.1131	66	439	454	CTYDPATRGGNAPDGR			Carbamidomethyl (C)[1]		Mascot
1707.7504	1707.8635	0.1131	66	439	454	CTYDPATRGGNAPDGR	27	0	Carbamidomethyl (C)[1]		Mascot

8	NADH pyrophosphatase OS=Pasteurella multocida (strain Pm70) GN=nudC PE=3 SV=1	NUDC_PASMU	30204	5.24	2	35	0	42.976	22	0

Peptide Information

Calc. Mass	Obsrv. Mass	± da	± ppm	Start Seq.	End Seq.	Sequence	Ion Score	C. I. %	Modification	Rank	Result Type
1485.8397	1485.8036	-0.0361	-24	141	153	GAQILLANHQRHK					Mascot
1485.8397	1485.8036	-0.0361	-24	141	153	GAQILLANHQRHK					Mascot
1707.9541	1707.8635	-0.0906	-53	71	85	SLLALPEAHFNLLNR					Mascot
1707.9541	1707.8635	-0.0906	-53	71	85	SLLALPEAHFNLLNR	22	0			Mascot

9	Nitrogenase iron protein (Fragment) OS=Enterobacter agglomerans GN=nifH PE=3 SV=1	NIFH_ENTAG	3884	9.86	3	35	0	13.416

Peptide Information

Calc. Mass	Obsrv. Mass	± da	± ppm	Start Seq.	End Seq.	Sequence	Ion Score	C. I. %	Modification	Rank	Result Type
1251.6515	1251.6959	0.0444	35	5	16	QCAIYGKGGIGK			Carbamidomethyl (C)[2]		Mascot
1360.6171	1360.6843	0.0672	49	1	11	MAMRQCAIYGK			Carbamidomethyl (C)[6], Oxidation (M)[1,3]		Mascot
1707.8582	1707.8635	0.0053	3	17	32	STTTQNLVAALAEMNK			Oxidation (M)[14]		Mascot
1707.8582	1707.8635	0.0053	3	17	32	STTTQNLVAALAEMNK			Oxidation (M)[14]		Mascot

10	B3 domain-containing protein At2g31720 OS=Arabidopsis thaliana GN=ARF70 PE=2 SV=1	Y2172_ARATH	36163.6	6.92	5	34	0	48.191

Peptide Information

Calc. Mass	Obsrv. Mass	± da	± ppm	Start Seq.	End Seq.	Sequence	Ion Score	C. I. %	Modification	Rank	Result Type
805.4427	805.4572	0.0145	18	186	191	IDRHHK					Mascot
1360.693	1360.6843	-0.0087	-6	128	138	TPTEWLIDVMR					Mascot
1485.7544	1485.8036	0.0492	33	152	164	VLPNSDVDELQTR					Mascot
1485.7544	1485.8036	0.0492	33	152	164	VLPNSDVDELQTR					Mascot
1707.8469	1707.8635	0.0166	10	298	312	MSSLVSDTELDLELR					Mascot
1707.8469	1707.8635	0.0166	10	298	312	MSSLVSDTELDLELR					Mascot
2033.9186	2033.932	0.0134	7	28	46	TVSAFAYEEIGETSTEGDK					Mascot

Gel Idx/Pos	162/G13	Instr./Gel Origin	BA2151/Sample Project 20140814	Process Status	Analysis Succeeded
Plate [#] Name	[1] Sample Project 20140814	Instrument Sample Name		Spectra	11

Rank	Protein Name	Accession No.	Protein MW	Protein PI	Pep. Count	Protein Score	Protein Score C. I. %	Intensity Matched	Total Ion Score	Total Ion C. I. %	Confirmed
1	Serpin-Z2B OS=Triticum aestivum PE=1 SV=1	SPZ2B_WHEAT	43011.4	5.18	12	631	100	44.5	571	100	

Peptide Information

Calc. Mass	Obsrv. Mass	± da	± ppm	Start Seq.	End Seq.	Sequence	Ion Score	C. I. %	Modification	Rank	Result Type
925.5214	925.5009	-0.0205	-22	11	18	LSIAHQTR					Mascot
925.5214	925.5009	-0.0205	-22	11	18	LSIAHQTR	47	97.854			Mascot
947.5156	947.4725	-0.0431	-45	2	10	ATTLATDVR					Mascot
1137.6667	1137.6322	-0.0345	-30	172	181	LVLGNALYFK					Mascot
1137.6667	1137.6322	-0.0345	-30	172	181	LVLGNALYFK	56	99.715			Mascot
1192.5382	1192.5111	-0.0271	-23	182	191	GAWTDQFDPR					Mascot
1192.5382	1192.5111	-0.0271	-23	182	191	GAWTDQFDPR	71	99.992			Mascot
1223.5903	1223.5408	-0.0495	-40	127	137	AEAQSVDFQTK					Mascot
1372.7068	1372.6772	-0.0296	-22	159	171	DILPAGSIDNTTR					Mascot
1372.7068	1372.6772	-0.0296	-22	159	171	DILPAGSIDNTTR	91	100			Mascot
1446.7965	1446.7487	-0.0478	-33	11	22	LSIAHQTRFAFR					Mascot
1514.7485	1514.7039	-0.0446	-29	125	137	YKAEAQSVDFQTK					Mascot
1514.7485	1514.7039	-0.0446	-29	125	137	YKAEAQSVDFQTK	106	100			Mascot
1531.7751	1531.7251	-0.05	-33	138	151	AAEVTAQVNSWVEK					Mascot
2085.155	2085.105	-0.05	-24	152	171	VTTGLIKDILPAGSIDNTT R					Mascot
2085.155	2085.105	-0.05	-24	152	171	VTTGLIKDILPAGSIDNTT R	200	100			Mascot
2838.4858	2838.4041	-0.0817	-29	99	124	VAFANGVFVDASLQLKPS FQELAVCK			Carbamidomethyl (C)[25]		Mascot
3751.9614	3751.9146	-0.0468	-12	23	61	LASAISSNPESTVNNAAF SPVSLHVALSLITAGAGG ATR					Mascot

Rank	Protein Name	Accession No.	Protein MW	Protein PI	Pep. Count	Protein Score	Protein Score C. I. %	Intensity Matched	Total Ion Score	Total Ion C. I. %	Confirmed
2	Serpin-Z2A OS=Triticum aestivum PE=1 SV=1	SPZ2A_WHEAT	43341.5	5.46	7	228	100	18.112	209	100	

Peptide Information

Calc. Mass	Obsrv. Mass	± da	± ppm	Start Seq.	End Seq.	Sequence	Ion Score	C. I. %	Modification	Rank	Result Type
925.5214	925.5009	-0.0205	-22	11	18	LSIAHQTR					Mascot
925.5214	925.5009	-0.0205	-22	11	18	LSIAHQTR	47	97.854			Mascot
947.5156	947.4725	-0.0431	-45	2	10	ATTLATDVR					Mascot

Calc. Mass	Obsrv. Mass	± da	± ppm	Start Seq.	End Seq.	Sequence	Ion Score	C. I. %	Modification	Rank	Result Type
1137.6667	1137.6322	-0.0345	-30	172	181	LVLGNALYFK					Mascot
1137.6667	1137.6322	-0.0345	-30	172	181	LVLGNALYFK	56	99.715			Mascot
1182.5175	1182.5314	0.0139	12	182	191	GAWTDQFDSR					Mascot
1223.5903	1223.5408	-0.0495	-40	127	137	AEAQSVDFQTK					Mascot
1514.7485	1514.7039	-0.0446	-29	125	137	YKAEAQSVDFQTK					Mascot
1514.7485	1514.7039	-0.0446	-29	125	137	YKAEAQSVDFQTK	106	100			Mascot
1531.7751	1531.7251	-0.05	-33	138	151	AAEVTAQVNSWVEK					Mascot

3	Serpin-ZX OS=Hordeum vulgare GN=PAZX PE=1 SV=1	SPZX_HORVU	42920.3	6.77	6	117	100	12.358	102	100	

Peptide Information

Calc. Mass	Obsrv. Mass	± da	± ppm	Start Seq.	End Seq.	Sequence	Ion Score	C. I. %	Modification	Rank	Result Type
868.5138	868.5022	-0.0116	-13	357	364	SLPVEPVK					Mascot
925.5214	925.5009	-0.0205	-22	8	15	LSIAHQTR					Mascot
925.5214	925.5009	-0.0205	-22	8	15	LSIAHQTR	47	97.854			Mascot
1137.6667	1137.6322	-0.0345	-30	171	180	LVLGNALYFK					Mascot
1137.6667	1137.6322	-0.0345	-30	171	180	LVLGNALYFK	56	99.715			Mascot
1345.6958	1345.619	-0.0768	-57	158	170	EILPAGSVDSTTR					Mascot
1513.7645	1513.7068	-0.0577	-38	137	150	APEVAGQVNSWVEK					Mascot
1558.7869	1558.6805	-0.1064	-68	270	282	HMPMQKVPVGQFK			Oxidation (M)[2,4]		Mascot

4	Serpin-Z7 OS=Hordeum vulgare GN=PAZ7 PE=1 SV=2	BSZ7_HORVU	42851.2	5.45	4	110	100	11.913	102	100	

Peptide Information

Calc. Mass	Obsrv. Mass	± da	± ppm	Start Seq.	End Seq.	Sequence	Ion Score	C. I. %	Modification	Rank	Result Type
925.5214	925.5009	-0.0205	-22	11	18	LSIAHQTR					Mascot
925.5214	925.5009	-0.0205	-22	11	18	LSIAHQTR	47	97.854			Mascot
1137.6667	1137.6322	-0.0345	-30	175	184	LVLGNALYFK					Mascot
1137.6667	1137.6322	-0.0345	-30	175	184	LVLGNALYFK	56	99.715			Mascot
1320.6431	1320.5927	-0.0504	-38	264	274	LSTEPDFLENR					Mascot
1496.7969	1496.707	-0.0899	-60	230	242	VLKLPYQHGGDNR					Mascot

5	Serpin-ZXA OS=Oryza sativa subsp. japonica GN=Os03g0610650 PE=1 SV=1	SPZXA_ORYSJ	42113.9	5.75	7	75	98.125	3.833	56	99.715	

Peptide Information

Calc. Mass	Obsrv. Mass	± da	± ppm	Start Seq.	End Seq.	Sequence	Ion Score	C. I. %	Modification	Rank	Result Type

Calc. Mass	Obsrv. Mass	± da	± ppm	Start Seq.	End Seq.	Sequence	Ion Score	C. I. %	Modification	Rank	Result Type
893.4727	893.4134	-0.0593	-66	116	124	TFGDVAVGK					Mascot
906.5043	906.4158	-0.0885	-98	276	283	QVTVGQFK					Mascot
1137.6667	1137.6322	-0.0345	-30	172	181	LVLGNALYFK					Mascot
1137.6667	1137.6322	-0.0345	-30	172	181	LVLGNALYFK	56	99.715			Mascot
1182.5824	1182.5314	-0.051	-43	205	215	SVQAPFMSTSK					Mascot
1262.6012	1262.5374	-0.0638	-51	127	137	AETHSVDFQTK					Mascot
1345.7474	1345.619	-0.1284	-95	227	238	VLKLPYQQGGDK					Mascot
1553.7595	1553.7238	-0.0357	-23	125	137	YKAETHSVDFQTK					Mascot

6	2-Cys peroxiredoxin BAS1, chloroplastic (Fragment) OS=Triticum aestivum GN=TSA PE=1 SV=2	BAS1_WHEAT	23426.2	5.71	6	69	93.193	2.473	41	91.878	

Protein Group

2-Cys peroxiredoxin BAS1, chloroplastic (Fragment) OS=Hordeum vulgare GN=BAS1 PE=2 SV=1	BAS1_HORVU	23398.1	5.4800 000190 7349

Peptide Information

Calc. Mass	Obsrv. Mass	± da	± ppm	Start Seq.	End Seq.	Sequence	Ion Score	C. I. %	Modification	Rank	Result Type
818.4077	818.3915	-0.0162	-20	195	201	SMKPDPK			Oxidation (M)[2]		Mascot
819.4207	819.399	-0.0217	-26	164	170	SVDETLR					Mascot
923.5197	923.4603	-0.0594	-64	42	49	LSDYIGKK					Mascot
1485.8424	1485.8077	-0.0347	-23	127	140	SFGVLIPDQGIALR					Mascot
1485.8424	1485.8077	-0.0347	-23	127	140	SFGVLIPDQGIALR	41	91.878			Mascot
1707.9137	1707.8701	-0.0436	-26	148	163	EGVIQHSTINNLGIGR					Mascot
2494.3777	2494.3174	-0.0603	-24	141	163	GLFIIDKEGVIQHSTINNL GIGR					Mascot

7	Serpin-ZXB OS=Oryza sativa subsp. japonica GN=Os03g0610800 PE=2 SV=1	SPZXB_ORYSJ	43315.8	8.84	5	67	89.211	3.67	56	99.715	

Peptide Information

Calc. Mass	Obsrv. Mass	± da	± ppm	Start Seq.	End Seq.	Sequence	Ion Score	C. I. %	Modification	Rank	Result Type
1137.6667	1137.6322	-0.0345	-30	183	192	LVLGNALYFK					Mascot
1137.6667	1137.6322	-0.0345	-30	183	192	LVLGNALYFK	56	99.715			Mascot
1232.594	1232.5385	-0.0555	-45	323	334	ADLTGMVGSPER					Mascot
1248.5889	1248.4979	-0.091	-73	323	334	ADLTGMVGSPER			Oxidation (M)[6]		Mascot
1262.6012	1262.5374	-0.0638	-51	138	148	AETHSVDFQTK					Mascot
1531.75	1531.7251	-0.0249	-16	149	162	AAEVASQVNSWVDR					Mascot
1553.7595	1553.7238	-0.0357	-23	136	148	YKAETHSVDFQTK					Mascot

8	Sulfate adenylyltransferase OS=Archaeoglobus fulgidus (strain ATCC 49558 / VC-16 / DSM 4304 / JCM 9628 / NBRC 100126) GN=sat PE=3 SV=2	SAT_ARCFU	53260.9	6.59	8	66	87.324	21.67	47	97.854

Peptide Information

Calc. Mass	Obsrv. Mass	± da	± ppm	Start Seq.	End Seq.	Sequence	Ion Score	C. I. %	Modification	Rank	Result Type
908.4665	908.4548	-0.0117	-13	195	201	FWFPPSK					Mascot
925.5214	925.5009	-0.0205	-22	214	221	TVIAHQTR					Mascot
925.5214	925.5009	-0.0205	-22	214	221	TVIAHQTR	47	97.854			Mascot
1159.5929	1159.6261	0.0332	29	399	409	GMVAEGVFPPR					Mascot
1175.5878	1175.5488	-0.039	-33	399	409	GMVAEGVFPPR			Oxidation (M)[2]		Mascot
1224.5983	1224.4994	-0.0989	-81	195	203	FWFPPSKCR			Carbamidomethyl (C)[8]		Mascot
1224.5983	1224.4994	-0.0989	-81	195	203	FWFPPSKCR			Carbamidomethyl (C)[8]		Mascot
1372.777	1372.6772	-0.0998	-73	1	13	MPLIKTPPPHGGK					Mascot
1372.777	1372.6772	-0.0998	-73	1	13	MPLIKTPPPHGGK					Mascot
1468.6713	1468.6671	-0.0042	-3	284	294	HMVTFTLWDMR			Oxidation (M)[2,10]		Mascot
1507.5989	1507.7175	0.1186	79	309	320	QNMGCTHHMFGR			Carbamidomethyl (C)[5], Oxidation (M)[3,9]		Mascot
1707.8378	1707.8701	0.0323	19	156	169	EPFDDKHPGYVIYK					Mascot

9	Serpin-Z1B OS=Triticum aestivum PE=1 SV=1	SPZ1B_WHEAT	43119.9	5.44	5	58	10.261	10.261	47	97.854

Peptide Information

Calc. Mass	Obsrv. Mass	± da	± ppm	Start Seq.	End Seq.	Sequence	Ion Score	C. I. %	Modification	Rank	Result Type
925.5214	925.5009	-0.0205	-22	11	18	LSIAHQTR					Mascot
925.5214	925.5009	-0.0205	-22	11	18	LSIAHQTR	47	97.854			Mascot
947.5156	947.4725	-0.0431	-45	2	10	ATTLATDVR					Mascot
1176.5896	1176.5562	-0.0334	-28	262	271	LSAEPDFLER					Mascot
1345.6958	1345.619	-0.0768	-57	159	171	NILPSGSVDNTTK					Mascot
1585.8295	1585.7068	-0.1227	-77	288	301	FKISFGMEASDLLK					Mascot

10	Alanine--tRNA ligase OS=Burkholderia vietnamiensis (strain G4 / LMG 22486) GN=alaS PE=3 SV=1	SYA_BURVG	95655.9	5.69	12	56	0	21.07	23	0

Peptide Information

Calc. Mass	Obsrv. Mass	± da	± ppm	Start Seq.	End Seq.	Sequence	Ion Score	C. I. %	Modification	Rank	Result Type
818.4553	818.3915	-0.0638	-78	1	7	MKAAEIR					Mascot
856.4523	856.5015	0.0492	57	142	149	EVGVPAER					Mascot
906.5043	906.4158	-0.0885	-98	655	662	VLDLGFSR					Mascot

908.389	908.4548	0.0658	72	218	225	DAQGNMTR			Oxidation (M)[6]	Mascot
989.5486	989.5022	-0.0464	-47	60	68	ATTAQRSVR				Mascot
989.5486	989.5022	-0.0464	-47	60	68	ATTAQRSVR	11	0		Mascot
1175.5739	1175.5488	-0.0251	-21	563	572	NHSATHLMHK				Mascot
1192.5627	1192.5111	-0.0516	-43	229	239	QSVDTGMGLER				Mascot
1192.5627	1192.5111	-0.0516	-43	229	239	QSVDTGMGLER	23	0		Mascot
1208.5576	1208.504	-0.0536	-44	229	239	QSVDTGMGLER			Oxidation (M)[7]	Mascot
1208.5576	1208.504	-0.0536	-44	229	239	QSVDTGMGLER			Oxidation (M)[7]	Mascot
1230.626	1230.5133	-0.1127	-92	218	228	DAQGNMTRLPK				Mascot
1320.7059	1320.5927	-0.1132	-86	13	23	FFESKGHTIVR				Mascot
1531.791	1531.7251	-0.0659	-43	563	575	NHSATHLMHKALR			Oxidation (M)[8]	Mascot
1535.8317	1535.7102	-0.1215	-79	469	483	VVALYVEGSSVGEVK				Mascot
1926.832	1926.8248	-0.0072	-4	414	430	ERGMTVDEPAFDDAMAR			Oxidation (M)[4]	Mascot

Gel Idx/Pos	163/G14	Instr./Gel Origin	BA2151/Sample Project 20140814	Process Status	Analysis Succeeded
Plate [#] Name	[1] Sample Project 20140814	Instrument Sample Name		Spectra	11

Rank	Protein Name	Accession No.	Protein MW	Protein PI	Pep. Count	Protein Score	Protein Score C. I. %	Intensity Matched	Total Ion Score	Total Ion C. I. %	Confirmed
1	2-Cys peroxiredoxin BAS1, chloroplastic (Fragment) OS=Triticum aestivum GN=TSA PE=1 SV=2	BAS1_WHEAT	23426.2	5.71	11	278	100	68.743	186	100	

Protein Group

2-Cys peroxiredoxin BAS1, chloroplastic (Fragment) OS=Hordeum vulgare GN=BAS1 PE=2 SV=1	BAS1_HORVU	23398.1	5.4800 000190 7349

Peptide Information

Calc. Mass	Obsrv. Mass	± da	± ppm	Start Seq.	End Seq.	Sequence	Ion Score	C. I. %	Modification	Rank	Result Type
805.4818	805.4611	-0.0207	-26	141	147	GLFIIDK					Mascot
818.4077	818.3916	-0.0161	-20	195	201	SMKPDPK			Oxidation (M)[2]		Mascot
819.4207	819.4026	-0.0181	-22	164	170	SVDETLR					Mascot
1021.5565	1021.5464	-0.0101	-10	114	122	YPLVSDVTK					Mascot
1360.7107	1360.6747	-0.036	-26	11	23	AAAEYDLPLVGNK					Mascot
1485.8424	1485.8185	-0.0239	-16	127	140	SFGVLIPDQGIALR					Mascot
1485.8424	1485.8185	-0.0239	-16	127	140	SFGVLIPDQGIALR	134	100			Mascot
1707.9137	1707.8865	-0.0272	-16	148	163	EGVIQHSTINNLGIGR					Mascot
1707.9137	1707.8865	-0.0272	-16	148	163	EGVIQHSTINNLGIGR	17	0			Mascot
1748.943	1748.8667	-0.0763	-44	106	122	SGGLGDLKYPLVSDVTK					Mascot
2010.9808	2010.9224	-0.0584	-29	24	41	APDFAAEAVFDQEFINVK					Mascot
2010.9808	2010.9224	-0.0584	-29	24	41	APDFAAEAVFDQEFINVK					Mascot
2494.3777	2494.3333	-0.0444	-18	141	163	GLFIIDKEGVIQHSTINNL GIGR					Mascot
2494.3777	2494.3333	-0.0444	-18	141	163	GLFIIDKEGVIQHSTINNL GIGR	34	57.899			Mascot
2700.3992	2700.3516	-0.0476	-18	81	104	INTEILGVSVDSVFSHLA WVQTER					Mascot
2700.3992	2700.3516	-0.0476	-18	81	104	INTEILGVSVDSVFSHLA WVQTER					Mascot

Rank	Protein Name	Accession No.	Protein MW	Protein PI	Pep. Count	Protein Score	Protein Score C. I. %	Intensity Matched	Total Ion Score	Total Ion C. I. %	Confirmed
2	2-Cys peroxiredoxin BAS1-like, chloroplastic OS=Arabidopsis thaliana GN=At5g06290 PE=2 SV=3	BAS1B_ARATH	29932.3	5.55	8	237	100	61.384	186	100	

Peptide Information

Calc. Mass	Obsrv. Mass	± da	± ppm	Start Seq.	End Seq.	Sequence	Ion Score	C. I. %	Modification	Rank	Result Type
805.4818	805.4611	-0.0207	-26	203	209	GLFIIDK					Mascot

Calc. Mass	Obsrv. Mass	± da	± ppm	Start Seq.	End Seq.	Sequence	Ion Score	C. I. %	Modification	Rank	Result Type
818.4077	818.3916	-0.0161	-20	258	264	SMKPDPK			Oxidation (M)[2]		Mascot
1485.8424	1485.8185	-0.0239	-16	189	202	SFGVLIPDQGIALR					Mascot
1485.8424	1485.8185	-0.0239	-16	189	202	SFGVLIPDQGIALR	134	100			Mascot
1707.9137	1707.8865	-0.0272	-16	210	225	EGVIQHSTINNLGIGR					Mascot
1707.9137	1707.8865	-0.0272	-16	210	225	EGVIQHSTINNLGIGR	17	0			Mascot
1748.9066	1748.8667	-0.0399	-23	168	184	SGGLGDLNYPLVSDITK					Mascot
1935.8998	1935.8794	-0.0204	-11	1	19	MSMASIASSSSTTLLSSS R			Oxidation (M)[1,3]		Mascot
2494.3777	2494.3333	-0.0444	-18	203	225	GLFIIDKEGVIQHSTINNL GIGR					Mascot
2494.3777	2494.3333	-0.0444	-18	203	225	GLFIIDKEGVIQHSTINNL GIGR	34	57.899			Mascot
2857.3826	2857.3193	-0.0633	-22	233	257	TLQALQYVQENPDEVCP AGWKPGEK			Carbamidomethyl (C)[16]		Mascot

3	2-Cys peroxiredoxin BAS1, chloroplastic OS=Oryza sativa subsp. japonica GN=BAS1 PE=1 SV=1	BAS1_ORYSJ	28307.5	5.67	7	175	100	53.175	134	100	

Peptide Information

Calc. Mass	Obsrv. Mass	± da	± ppm	Start Seq.	End Seq.	Sequence	Ion Score	C. I. %	Modification	Rank	Result Type
805.4818	805.4611	-0.0207	-26	191	197	GLFIIDK					Mascot
818.4077	818.3916	-0.0161	-20	246	252	SMKPDPK			Oxidation (M)[2]		Mascot
1021.5385	1021.5464	0.0079	8	44	53	LSASSSRSAR					Mascot
1485.8424	1485.8185	-0.0239	-16	177	190	SFGVLIPDQGIALR					Mascot
1485.8424	1485.8185	-0.0239	-16	177	190	SFGVLIPDQGIALR	134	100			Mascot
1539.8715	1539.7283	-0.1432	-93	36	50	APAARPLRLSASSSR					Mascot
2054.9707	2054.9089	-0.0618	-30	74	91	APDFDAEAVFDQEFINVK					Mascot
2700.3992	2700.3516	-0.0476	-18	131	154	LNTEILGVSIDSVFSHLAW VQTDR					Mascot
2700.3992	2700.3516	-0.0476	-18	131	154	LNTEILGVSIDSVFSHLAW VQTDR					Mascot

4	2-Cys peroxiredoxin BAS1, chloroplastic OS=Arabidopsis thaliana GN=BAS1 PE=1 SV=2	BAS1A_ARATH	29188	6.92	7	92	99.968	13.2	51	99.181	

Peptide Information

Calc. Mass	Obsrv. Mass	± da	± ppm	Start Seq.	End Seq.	Sequence	Ion Score	C. I. %	Modification	Rank	Result Type
805.4818	805.4611	-0.0207	-26	196	202	GLFIIDK					Mascot
818.4077	818.3916	-0.0161	-20	251	257	SMKPDPK			Oxidation (M)[2]		Mascot
1550.8021	1550.8054	0.0033	2	2	17	ASVASSTTLISSPSSR					Mascot
1694.8821	1694.8005	-0.0816	-48	35	51	TLSSPSASASLRSGFAR					Mascot
1707.9137	1707.8865	-0.0272	-16	203	218	EGVIQHSTINNLGIGR					Mascot
1707.9137	1707.8865	-0.0272	-16	203	218	EGVIQHSTINNLGIGR	17	0			Mascot

1748.9066	1748.8667	-0.0399	-23	161	177	SGGLGDLNYPLISDVTK					Mascot
2494.3777	2494.3333	-0.0444	-18	196	218	GLFIIDKEGVIQHSTINNL GIGR					Mascot
2494.3777	2494.3333	-0.0444	-18	196	218	GLFIIDKEGVIQHSTINNL GIGR	34	57.899			Mascot

5 2-Cys peroxiredoxin BAS1, chloroplastic OS=Spinacia oleracea GN=BAS1 PE=2 SV=1 | BAS1_SPIOL | 29048.9 | 7.7 | 6 | 84 | 99.78 | 12.962 | 51 | 99.181

Peptide Information

Calc. Mass	Obsrv. Mass	± da	± ppm	Start Seq.	End Seq.	Sequence	Ion Score	C. I. %	Modification	Rank	Result Type
805.4818	805.4611	-0.0207	-26	196	202	GLFIIDK					Mascot
818.4077	818.3916	-0.0161	-20	250	256	SMKPDPK			Oxidation (M)[2]		Mascot
1694.8821	1694.8005	-0.0816	-48	35	51	TLSSPSASASLRSGFAR					Mascot
1707.9137	1707.8865	-0.0272	-16	203	218	EGVIQHSTINNLGIGR					Mascot
1707.9137	1707.8865	-0.0272	-16	203	218	EGVIQHSTINNLGIGR	17	0			Mascot
1748.9066	1748.8667	-0.0399	-23	161	177	SGGLGDLNYPLISDVTK					Mascot
2494.3777	2494.3333	-0.0444	-18	196	218	GLFIIDKEGVIQHSTINNL GIGR					Mascot
2494.3777	2494.3333	-0.0444	-18	196	218	GLFIIDKEGVIQHSTINNL GIGR	34	57.899			Mascot

6 Putative late blight resistance protein homolog R1B-11 OS=Solanum demissum GN=R1B-11 PE=5 SV=1 | R1B11_SOLDE | 145639 | 6.05 | 18 | 53 | 0 | 9.031

Peptide Information

Calc. Mass	Obsrv. Mass	± da	± ppm	Start Seq.	End Seq.	Sequence	Ion Score	C. I. %	Modification	Rank	Result Type
805.493	805.4611	-0.0319	-40	128	133	EFKLLR					Mascot
819.4433	819.4026	-0.0407	-50	1	6	MIEIWK					Mascot
841.409	841.392	-0.017	-20	249	255	AEYSFPK					Mascot
858.468	858.4847	0.0167	19	387	394	SIEPGISR					Mascot
870.5771	870.5106	-0.0665	-76	1241	1248	LVVGKLNK					Mascot
1043.5408	1043.5166	-0.0242	-23	544	552	YSDSLAFLK					Mascot
1377.7347	1377.7139	-0.0208	-15	736	748	IFGPSHPMLGPPK					Mascot
1467.7084	1467.8075	0.0991	68	1	11	MIEIWKSQCTR			Carbamidomethyl (C)[9], Oxidation (M)[1]		Mascot
1471.7687	1471.8124	0.0437	30	749	761	SKLPTHQMLSTGR			Oxidation (M)[8]		Mascot
1529.8298	1529.7916	-0.0382	-25	131	142	LLRFFVCFVSNK			Carbamidomethyl (C)[7]		Mascot
1547.7588	1547.7235	-0.0353	-23	1066	1078	VEDYLSETVDHLK					Mascot
1824.9451	1824.8235	-0.1216	-67	256	272	TSLAVNKVDDVNTHSPK					Mascot
1839.0051	1838.8975	-0.1076	-59	1029	1043	LIYLYIPNFSPENKK					Mascot
2048.9592	2048.9133	-0.0459	-22	483	500	EDTVLDDMNQALGFDLP					Mascot

Calc. Mass	Obsrv. Mass	± da	± ppm	Start Seq.	End Seq.	Sequence	Ion Score	C. I. %	Modification	Rank	Result Type
						R					
2110.0396	2109.8547	-0.1849	-88	507	523	AMVYLVMQKAFHCNLPR			Carbamidomethyl (C)[13], Oxidation (M)[2,7]		Mascot
2700.3879	2700.3516	-0.0363	-13	1066	1087	VEDYLSETVDHLKHLEVL ELYR					Mascot
2700.3879	2700.3516	-0.0363	-13	1066	1087	VEDYLSETVDHLKHLEVL ELYR					Mascot
2724.2798	2724.3223	0.0425	16	595	616	AYEVEYVVDACINKETPH WCLK			Carbamidomethyl (C)[11,20]		Mascot
2733.4241	2733.3354	-0.0887	-32	832	855	SLEDIAEGYLENLIGRNLV MVTQR					Mascot

7	50S ribosomal protein L9 OS=Wigglesworthia glossinidia brevipalpis GN=rplI PE=3 SV=1	RL9_WIGBR	17491	10.16	8	53	0	3.005

Peptide Information

Calc. Mass	Obsrv. Mass	± da	± ppm	Start Seq.	End Seq.	Sequence	Ion Score	C. I. %	Modification	Rank	Result Type
858.5481	858.4847	-0.0634	-74	1	7	MQIILLK					Mascot
1282.6711	1282.6028	-0.0683	-53	74	83	SLKEIEIYCK			Carbamidomethyl (C)[9]		Mascot
1398.774	1398.7511	-0.0229	-16	90	102	LFGSIGANHIIEK					Mascot
1467.7004	1467.8075	0.1071	73	136	147	SNMCTEICVILK			Carbamidomethyl (C)[4,8]		Mascot
1527.7689	1527.832	0.0631	41	53	64	ILEEQSKYENFK					Mascot
1639.953	1639.8872	-0.0658	-40	90	104	LFGSIGANHIIEKIK					Mascot
1694.8638	1694.8005	-0.0633	-37	134	147	VKSNMCTEICVILK			Carbamidomethyl (C)[6,10]		Mascot
1824.8652	1824.8235	-0.0417	-23	136	150	SNMCTEICVILKNNI			Carbamidomethyl (C)[4,8], Oxidation (M)[3]		Mascot

8	ATP synthase subunit alpha OS=Clostridium thermocellum (strain ATCC 27405 / DSM 1237) GN=atpA PE=3 SV=1	ATPA_CLOTH	56012.9	5.36	11	49	0	4.365

Peptide Information

Calc. Mass	Obsrv. Mass	± da	± ppm	Start Seq.	End Seq.	Sequence	Ion Score	C. I. %	Modification	Rank	Result Type
805.3937	805.4611	0.0674	84	119	125	GEIETEK					Mascot
815.4621	815.4952	0.0331	41	165	171	ELIIGDR					Mascot
819.4756	819.4026	-0.073	-89	273	279	AMSLLLR			Oxidation (M)[2]		Mascot
828.4825	828.4495	-0.033	-40	94	101	TVEVPVGK					Mascot
1382.8049	1382.6809	-0.124	-90	273	284	AMSLLLRRPPGR			Oxidation (M)[2]		Mascot
1471.8479	1471.8124	-0.0355	-24	176	189	TAIAVDTILNQKGK					Mascot
1511.6895	1511.8101	0.1206	80	478	491	ATGDLSSETENMLK			Oxidation (M)[12]		Mascot
1539.769	1539.7283	-0.0407	-26	392	405	ELAVFAQFGSDLDK					Mascot
1553.7384	1553.7893	0.0509	33	285	297	EAYPGDVFYLHSR					Mascot
1639.7844	1639.8872	0.1028	63	478	492	ATGDLSSETENMLKK			Oxidation (M)[12]		Mascot
2039.0188	2038.892	-0.1268	-62	427	444	ATMPVEDQVIVLYMATNK			Oxidation (M)[3]		Mascot

2055.0137	2054.9089	-0.1048	-51	427	444	ATMPVEDQVIVLYMATNK			Oxidation (M)[3,14]		Mascot

9	Xylose isomerase OS=Rhizobium etli (strain CFN 42 / ATCC 51251) GN=xylA PE=3 SV=1	XYLA_RHIEC	49170.4	5.48	10	49	0	57.619

Protein Group

Xylose isomerase OS=Rhizobium etli (strain CIAT 652) GN=xylA PE=3 SV=1	XYLA_RHIE6	49153.4	5.5100 002288 8184

Peptide Information

Calc. Mass	Obsrv. Mass	± da	± ppm	Start Seq.	End Seq.	Sequence	Ion Score	C. I. %	Modification	Rank	Result Type
800.3719	800.301	-0.0709	-89	39	44	MEDHLR					Mascot
1395.7002	1395.6685	-0.0317	-23	191	202	EGYETLLNTDLK					Mascot
1471.7614	1471.8124	0.051	35	1	13	MSTGFFGDIQKIK					Mascot
1485.802	1485.8185	0.0165	11	422	434	SGKQELLENVVNR					Mascot
1485.802	1485.8185	0.0165	11	422	434	SGKQELLENVVNR					Mascot
1547.8441	1547.7235	-0.1206	-78	136	148	LLWGTANLFSNRR					Mascot
1550.8789	1550.8054	-0.0735	-47	226	239	GTILIEPKPQEPTK					Mascot
1551.8013	1551.7709	-0.0304	-20	191	203	EGYETLLNTDLKR					Mascot
1707.8701	1707.8865	0.0164	10	12	26	IKYEGPDSTNPLAFR					Mascot
1707.8701	1707.8865	0.0164	10	12	26	IKYEGPDSTNPLAFR					Mascot
1936.0538	1935.8794	-0.1744	-90	204	219	ELDQLGRFLNLVVEYK					Mascot
2049.0876	2048.9133	-0.1743	-85	129	147	QAATGTKLLWGTANLFSNR					Mascot

10	DNA repair protein RecO OS=Rhizobium meliloti (strain 1021) GN=recO PE=3 SV=2	RECO_RHIME	26837.9	6.36	8	49	0	4.511

Peptide Information

Calc. Mass	Obsrv. Mass	± da	± ppm	Start Seq.	End Seq.	Sequence	Ion Score	C. I. %	Modification	Rank	Result Type
805.4413	805.4611	0.0198	25	239	247	ALKSASSAA					Mascot
819.4254	819.4026	-0.0228	-28	167	173	SGRAICR			Carbamidomethyl (C)[6]		Mascot
1021.5564	1021.5464	-0.01	-10	158	166	SELVYVSPK					Mascot
1527.7662	1527.832	0.0658	43	216	229	HVYEPRGVDAASAR					Mascot
1547.7887	1547.7235	-0.0652	-42	1	13	MQWSDEAIILGIR			Oxidation (M)[1]		Mascot
1694.853	1694.8005	-0.0525	-31	151	166	CGATGARSELVYVSPK			Carbamidomethyl (C)[1]		Mascot
1927.9695	1927.8445	-0.125	-65	43	59	TMQPVLQPGNSVEVSWR					Mascot
1935.9454	1935.8794	-0.066	-34	14	31	RHGESSVIAEVMTPGHGR			Oxidation (M)[12]		Mascot

Gel Idx/Pos	164/G15	Instr./Gel Origin	BA2151/Sample Project 20140814	Process Status	Analysis Succeeded
Plate [#] Name	[1] Sample Project 20140814	Instrument Sample Name		Spectra	11

Rank	Protein Name	Accession No.	Protein MW	Protein PI	Pep. Count	Protein Score	Protein Score C. I. %	Intensity Matched	Total Ion Score	Total Ion C. I. %	Confirmed
1	Serpin-Z1A OS=Triticum aestivum GN=WZCI PE=1 SV=1	SPZ1A_WHEAT	43262.2	5.6	7	534	100	51.094	508	100	

Peptide Information

Calc. Mass	Obsrv. Mass	± da	± ppm	Start Seq.	End Seq.	Sequence	Ion Score	C. I. %	Modification	Rank	Result Type
1176.5896	1176.5861	-0.0035	-3	261	270	LSAEPDFLER					Mascot
1176.5896	1176.5861	-0.0035	-3	261	270	LSAEPDFLER	94	100			Mascot
1292.7097	1292.6851	-0.0246	-19	289	300	ISFGIEASDLLK					Mascot
1292.7097	1292.6851	-0.0246	-19	289	300	ISFGIEASDLLK	83	100			Mascot
1567.873	1567.8324	-0.0406	-26	287	300	FKISFGIEASDLLK					Mascot
1679.8864	1679.8699	-0.0165	-10	261	274	LSAEPDFLERHIPR					Mascot
2113.0999	2113.052	-0.0479	-23	379	398	EDISGVVLFMGHVVNPLLSS					Mascot
2113.0999	2113.052	-0.0479	-23	379	398	EDISGVVLFMGHVVNPLLSS	63	99.953			Mascot
2129.0947	2129.0298	-0.0649	-30	379	398	EDISGVVLFMGHVVNPLLSS			Oxidation (M)[10]		Mascot
2129.0947	2129.0298	-0.0649	-30	379	398	EDISGVVLFMGHVVNPLLSS	73	99.995	Oxidation (M)[10]		Mascot
2720.3525	2720.3323	-0.0202	-7	328	353	VSSVFHQAFVEVNEQGTEAAASTAIK					Mascot
2720.3525	2720.3323	-0.0202	-7	328	353	VSSVFHQAFVEVNEQGTEAAASTAIK	259	100			Mascot
2944.5642	2944.7695	0.2053	70	101	126	FANGVFVDASLLLKPSFQEIAVCKYK			Carbamidomethyl (C)[23]		Mascot

Rank	Protein Name	Accession No.	Protein MW	Protein PI	Pep. Count	Protein Score	Protein Score C. I. %	Intensity Matched	Total Ion Score	Total Ion C. I. %	Confirmed
2	Serpin-Z1B OS=Triticum aestivum PE=1 SV=1	SPZ1B_WHEAT	43119.9	5.44	6	506	100	46.058	486	100	

Peptide Information

Calc. Mass	Obsrv. Mass	± da	± ppm	Start Seq.	End Seq.	Sequence	Ion Score	C. I. %	Modification	Rank	Result Type
1176.5896	1176.5861	-0.0035	-3	262	271	LSAEPDFLER					Mascot
1176.5896	1176.5861	-0.0035	-3	262	271	LSAEPDFLER	94	100			Mascot
1679.8864	1679.8699	-0.0165	-10	262	275	LSAEPDFLERHIPR					Mascot
2113.0999	2113.052	-0.0479	-23	380	399	EDISGVVLFMGHVVNPLLSS					Mascot
2113.0999	2113.052	-0.0479	-23	380	399	EDISGVVLFMGHVVNPLLSS	63	99.953			Mascot
2129.0947	2129.0298	-0.0649	-30	380	399	EDISGVVLFMGHVVNPLLSS			Oxidation (M)[10]		Mascot
2129.0947	2129.0298	-0.0649	-30	380	399	EDISGVVLFMGHVVNPLL	73	99.995	Oxidation (M)[10]		Mascot

Calc. Mass	Obsrv. Mass	± da	± ppm	Start Seq.	End Seq.	Sequence	Ion Score	C. I. %	Modification	Rank	Result Type
						SS					
2267.1628	2267.1016	-0.0612	-27	241	261	QFSMYILLPEAPGGLSSL AEK			Oxidation (M)[4]		Mascot
2720.3525	2720.3323	-0.0202	-7	329	354	VSSVFHQAFVEVNEQGT EAAASTAIK					Mascot
2720.3525	2720.3323	-0.0202	-7	329	354	VSSVFHQAFVEVNEQGT EAAASTAIK	259	100			Mascot
3039.3896	3039.376	-0.0136	-4	302	328	CLGLQLPFSDEADFSEM VDSPMPQGLR			Carbamidomethyl (C)[1]		Mascot
3055.3845	3055.3569	-0.0276	-9	302	328	CLGLQLPFSDEADFSEM VDSPMPQGLR			Carbamidomethyl (C)[1], Oxidation (M)[17]		Mascot
3071.3796	3071.3579	-0.0217	-7	302	328	CLGLQLPFSDEADFSEM VDSPMPQGLR			Carbamidomethyl (C)[1], Oxidation (M)[17,22]		Mascot
3071.3796	3071.3579	-0.0217	-7	302	328	CLGLQLPFSDEADFSEM VDSPMPQGLR	60	99.91	Carbamidomethyl (C)[1], Oxidation (M)[17,22]		Mascot

3	Serpin-Z1C OS=Triticum aestivum PE=1 SV=1	SPZ1C_WHEAT	42969	5.62	5	442	100	44.052	426	100	

Peptide Information

Calc. Mass	Obsrv. Mass	± da	± ppm	Start Seq.	End Seq.	Sequence	Ion Score	C. I. %	Modification	Rank	Result Type
1176.5896	1176.5861	-0.0035	-3	261	270	LSAEPDFLER					Mascot
1176.5896	1176.5861	-0.0035	-3	261	270	LSAEPDFLER	94	100			Mascot
1679.8864	1679.8699	-0.0165	-10	261	274	LSAEPDFLERHIPR					Mascot
2113.0999	2113.052	-0.0479	-23	379	398	EDISGVVLFMGHVVNPLL SS					Mascot
2113.0999	2113.052	-0.0479	-23	379	398	EDISGVVLFMGHVVNPLL SS	63	99.953			Mascot
2129.0947	2129.0298	-0.0649	-30	379	398	EDISGVVLFMGHVVNPLL SS			Oxidation (M)[10]		Mascot
2129.0947	2129.0298	-0.0649	-30	379	398	EDISGVVLFMGHVVNPLL SS	73	99.995	Oxidation (M)[10]		Mascot
2720.3525	2720.3323	-0.0202	-7	328	353	VSSVFHQAFVEVNEQGT EAAASTAIK					Mascot
2720.3525	2720.3323	-0.0202	-7	328	353	VSSVFHQAFVEVNEQGT EAAASTAIK	259	100			Mascot
3037.3853	3037.4165	0.0312	10	301	327	CLGLQLPFSNEADFSEM VDSPMAHGLR			Carbamidomethyl (C)[1], Oxidation (M)[17]		Mascot

4	Serpin-Z2A OS=Triticum aestivum PE=1 SV=1	SPZ2A_WHEAT	43341.5	5.46	3	88	99.916	7.327	83	100	

Peptide Information

Calc. Mass	Obsrv. Mass	± da	± ppm	Start Seq.	End Seq.	Sequence	Ion Score	C. I. %	Modification	Rank	Result Type
1182.5175	1182.5504	0.0329	28	182	191	GAWTDQFDSR					Mascot
1292.7097	1292.6851	-0.0246	-19	289	300	ISFGIEASDLLK					Mascot
1292.7097	1292.6851	-0.0246	-19	289	300	ISFGIEASDLLK	83	100			Mascot
1567.873	1567.8324	-0.0406	-26	287	300	FKISFGIEASDLLK					Mascot

5	14-3-3-like protein A OS=Hordeum vulgare PE=2 SV=1	1433A_HORVU	29447.8	4.83	12	66	85.777	1.994			

Peptide Information

Calc. Mass	Obsrv. Mass	± da	± ppm	Start Seq.	End Seq.	Sequence	Ion Score	C. I. %	Modification	Rank	Result Type
819.4458	819.4134	-0.0324	-40	95	101	IETELSK					Mascot
922.4199	922.4544	0.0345	37	130	136	MKGDYHR			Oxidation (M)[1]		Mascot
1051.5419	1051.516	-0.0259	-25	80	89	GNEAYVASIK					Mascot
1198.5334	1198.5574	0.024	20	251	262	EAASKPEGEGHS					Mascot
1336.7107	1336.6428	-0.0679	-51	148	159	KEAAENTLVAYK					Mascot
1616.849	1616.8638	0.0148	9	34	48	VAKTADVGELTVEER					Mascot
1632.7648	1632.84	0.0752	46	24	36	YEEMVEFMEKVAK					Mascot
1699.7955	1699.7889	-0.0066	-4	2	16	STAEATREENVYMAK					Mascot
1846.8309	1846.7903	-0.0406	-22	1	16	MSTAEATREENVYMAK			Oxidation (M)[1]		Mascot
2131.9675	2132.0564	0.0889	42	17	33	LAEQAERYEEMVEFMEK					Mascot
2289.249	2289.0933	-0.1557	-68	109	129	LLDSHLVPSATAAESKVF YLK					Mascot
3008.5688	3008.7617	0.1929	64	149	176	EAAENTLVAYKSAQDIAL ADLPTTHPIR					Mascot

6	tRNA dimethylallyltransferase OS=Shewanella piezotolerans (strain WP3 / JCM 13877) GN=miaA PE=3 SV=1	MIAA_SHEPW	34667.1	6.1	11	64	78.473	43.731

Peptide Information

Calc. Mass	Obsrv. Mass	± da	± ppm	Start Seq.	End Seq.	Sequence	Ion Score	C. I. %	Modification	Rank	Result Type
845.4727	845.428	-0.0447	-53	89	95	IEDIISR					Mascot
1174.65	1174.5868	-0.0632	-54	9	20	VITLMGPTASGK					Mascot
1176.6835	1176.5861	-0.0974	-83	174	184	ISGKSLTELTK					Mascot
1176.6835	1176.5861	-0.0974	-83	174	184	ISGKSLTELTK					Mascot
1292.6919	1292.6851	-0.0068	-5	217	228	AMLGLGFVEEVK					Mascot
1292.6919	1292.6851	-0.0068	-5	217	228	AMLGLGFVEEVK	4	0			Mascot
1341.6467	1341.7599	0.1132	84	234	244	DDLHLELPSMR			Oxidation (M)[10]		Mascot
1341.6467	1341.7599	0.1132	84	234	244	DDLHLELPSMR			Oxidation (M)[10]		Mascot
1385.7999	1385.7474	-0.0525	-38	84	95	DALLKIEDIISR					Mascot
1473.748	1473.679	-0.069	-47	98	110	TPLLVGGTMMYFK			Oxidation (M)[9]		Mascot
1567.8553	1567.8324	-0.0229	-15	215	228	FKAMLGLGFVEEVK					Mascot
2113.2302	2113.052	-0.1782	-84	9	29	VITLMGPTASGKTALAIEL VK					Mascot
2113.2302	2113.052	-0.1782	-84	9	29	VITLMGPTASGKTALAIEL VK	1	0			Mascot
2129.2251	2129.0298	-0.1953	-92	9	29	VITLMGPTASGKTALAIEL VK			Oxidation (M)[5]		Mascot
2129.2251	2129.0298	-0.1953	-92	9	29	VITLMGPTASGKTALAIEL VK	5	0	Oxidation (M)[5]		Mascot

2167.0818	2167.0034	-0.0784	-36	187	205	SEAFPYEAIQFAIAPNDR K					Mascot
2942.3447	2942.4395	0.0948	32	250	273	QCWQHLDGEYDYETMV EKAIVATR			Carbamidomethyl (C)[2]		Mascot

7 Protein HIRA OS=Zea mays PE=1 SV=1 HIRA_MAIZE 106526.1 7.81 19 63 70.961 12.436

Peptide Information

Calc. Mass	Obsrv. Mass	± da	± ppm	Start Seq.	End Seq.	Sequence	Ion Score	C. I. %	Modification	Rank	Result Type
821.4338	821.426	-0.0078	-9	190	195	TVMIWR			Oxidation (M)[3]		Mascot
855.4683	855.4509	-0.0174	-20	573	579	LSIEHTR					Mascot
885.4213	885.4675	0.0462	52	269	275	FNNSTFR					Mascot
908.4948	908.4161	-0.0787	-87	241	248	HSAPVLER					Mascot
958.484	958.52	0.036	38	885	894	VGSASPTDPK					Mascot
1063.5531	1063.5066	-0.0465	-44	640	648	GTKTLWSDR					Mascot
1130.5988	1130.5557	-0.0431	-38	756	766	CGSPLVALASR			Carbamidomethyl (C)[1]		Mascot
1232.594	1232.6211	0.0271	22	913	923	EDILPSMASNR					Mascot
1292.6515	1292.6851	0.0336	26	618	631	GAGDMIGVGALSTK			Oxidation (M)[5]		Mascot
1292.6515	1292.6851	0.0336	26	618	631	GAGDMIGVGALSTK			Oxidation (M)[5]		Mascot
1575.7485	1575.8232	0.0747	47	64	76	DHFGSVNCVRWAK			Carbamidomethyl (C)[8]		Mascot
1711.847	1711.8552	0.0082	5	563	579	ASAGAGNDGRLSIEHTR					Mascot
1846.9269	1846.7903	-0.1366	-74	190	204	TVMIWRTSDWSLAHK			Oxidation (M)[3]		Mascot
2113.0781	2113.052	-0.0261	-12	580	599	SMAPSSLTPCSALSIHVIN K			Carbamidomethyl (C)[10]		Mascot
2113.0781	2113.052	-0.0261	-12	580	599	SMAPSSLTPCSALSIHVIN K			Carbamidomethyl (C)[10]		Mascot
2129.073	2129.0298	-0.0432	-20	580	599	SMAPSSLTPCSALSIHVIN K			Carbamidomethyl (C)[10], Oxidation (M)[2]		Mascot
2129.073	2129.0298	-0.0432	-20	580	599	SMAPSSLTPCSALSIHVIN K			Carbamidomethyl (C)[10], Oxidation (M)[2]		Mascot
2131.9131	2132.0564	0.1433	67	524	543	MNGTKPSYGSNSNSNNC GVK			Carbamidomethyl (C)[17], Oxidation (M)[1]		Mascot
2150.9626	2151.033	0.0704	33	96	116	KAGSGTSEFGSGEPPDA ENWK					Mascot
2702.3745	2702.4214	0.0469	17	388	413	YGDVGGRQSNLAESPAQ LLLEQASAK					Mascot
2742.3616	2742.3228	-0.0388	-14	721	747	ACILHDSLASLVASPDES SAKDAGTVK			Carbamidomethyl (C)[2]		Mascot
3006.6523	3006.4106	-0.2417	-80	2	27	ILEKPSWIRHEGLQIFSIDI QTGGLR					Mascot
3037.532	3037.4165	-0.1155	-38	249	275	GEWAATFDFLGHNAPIVV VKFNNSTFR					Mascot

8 DNA repair protein RecO OS=Rhizobium loti (strain MAFF303099) GN=recO PE=3 SV=1 RECO_RHILO 27787.4 7.18 11 59 33.475 2.023

Peptide Information

Calc. Mass	Obsrv. Mass	± da	± ppm	Start Seq.	End Seq.	Sequence	Ion Score	C. I. %	Modification	Rank	Result Type

800.405	800.4015	-0.0035	-4	217	222	HVYEPR					Mascot
1029.5112	1029.5459	0.0347	34	174	182	EAGAPWRDK					Mascot
1161.5535	1161.5653	0.0118	10	198	208	ADPAAVEDAFR					Mascot
1175.6605	1175.5708	-0.0897	-76	183	192	MLALPAFLQR			Oxidation (M)[1]		Mascot
1320.6907	1320.6599	-0.0308	-23	158	169	QDLAYVSPKSGR					Mascot
1575.7948	1575.8232	0.0284	18	1	13	MEWRDEGIILGTR					Mascot
1864.9447	1864.8802	-0.0645	-35	15	31	HGETSAILEVMTRAHGR					Mascot
2063.0413	2063.0562	0.0149	7	81	99	LMDSAVAVYGLQTMAAH LR			Oxidation (M)[2]		Mascot
2132.0078	2132.0564	0.0486	23	62	80	LDEHLGTFQAEAIEMNAA R			Oxidation (M)[15]		Mascot
2359.146	2359.1489	0.0029	1	60	80	ARLDEHLGTFQAEAIEMN AAR			Oxidation (M)[17]		Mascot
3008.4783	3008.7617	0.2834	94	105	133	DAHGGLYEALSVMIVHLD DADAAGELVAR					Mascot

9	Glycine--tRNA ligase OS=Borrelia burgdorferi (strain ZS7) GN=glyQS PE=3 SV=1	SYG_BORBZ	52716.3	6.62	14	58	3.843	3.425	

Protein Group

Glycine--tRNA ligase OS=Borrelia burgdorferi (strain ATCC 35210 / B31 / CIP 102532 / DSM 4680) GN=glyQS PE=3 SV=1	SYG_BORBU	52689.3	6.6199 998855 5908

Peptide Information

Calc. Mass	Obsrv. Mass	± da	± ppm	Start Seq.	End Seq.	Sequence	Ion Score	C. I. %	Modification	Rank	Result Type
856.4999	856.5028	0.0029	3	227	233	ISPDRLR					Mascot
908.4724	908.4161	-0.0563	-62	98	105	ADFIDLSK					Mascot
1029.6093	1029.5459	-0.0634	-62	165	174	IPFGIAQVGK					Mascot
1146.5538	1146.541	-0.0128	-11	270	279	GNYDLTQHAK					Mascot
1158.5864	1158.5844	-0.002	-2	218	226	MNFFIETLK			Oxidation (M)[1]		Mascot
1175.6453	1175.5708	-0.0745	-63	4	13	MEDIISLAKR					Mascot
1274.7137	1274.6344	-0.0793	-62	2	12	VRMEDIISLAK					Mascot
1290.7086	1290.6093	-0.0993	-77	2	12	VRMEDIISLAK			Oxidation (M)[3]		Mascot
1341.6547	1341.7599	0.1052	78	236	247	AHDSTQLAHYAK					Mascot
1341.6547	1341.7599	0.1052	78	236	247	AHDSTQLAHYAK					Mascot
1616.818	1616.8638	0.0458	28	234	247	FKAHDSTQLAHYAK					Mascot
1711.7051	1711.8552	0.1501	88	75	90	ASGHVDGFSDSMVDCK			Carbamidomethyl (C)[15]		Mascot
2103.8975	2103.9978	0.1003	48	189	204	TCEFEQMEMQFFVHPK			Carbamidomethyl (C)[2], Oxidation (M)[7]		Mascot
2250.2056	2250.0781	-0.1275	-57	423	440	IFINDLYSYIKTEILNYK					Mascot
2990.4934	2990.4666	-0.0268	-9	15	42	GFVFQSSEVYGGLSGAW DYGPLGVELKK					Mascot

3087.4575	3087.3564	-0.1011	-33	388	413	RQDEIGTPYCVTIDYNTIE DETVTVR			Carbamidomethyl (C)[10]		Mascot

10	Kelch-like protein 38 OS=Homo sapiens GN=KLHL38 PE=1 SV=3	KLH38_HUMAN	66524.5	8.19	15	56	0	14.028	

Peptide Information

Calc. Mass	Obsrv. Mass	± da	± ppm	Start Seq.	End Seq.	Sequence	Ion Score	C. I. %	Modification	Rank	Result Type
811.3879	811.4218	0.0339	42	44	49	EIPCHR			Carbamidomethyl (C)[4]		Mascot
908.4692	908.4161	-0.0531	-58	462	468	METRMIK					Mascot
958.4702	958.52	0.0498	52	214	220	YMQELFK					Mascot
1021.423	1021.4904	0.0674	66	61	68	AMFCSSFR			Carbamidomethyl (C)[4], Oxidation (M)[2]		Mascot
1130.5663	1130.5557	-0.0106	-9	213	220	RYMQELFK			Oxidation (M)[3]		Mascot
1146.5902	1146.541	-0.0492	-43	308	317	QTGQWQSLAK					Mascot
1182.5507	1182.5504	-0.0003	0	512	522	MHHGATVMGNK					Mascot
1198.5674	1198.5574	-0.01	-8	457	465	NSWFKMETR					Mascot
1204.5593	1204.6133	0.054	45	13	22	DHDFSSDLLR					Mascot
1204.5593	1204.6133	0.054	45	13	22	DHDFSSDLLR	4	0			Mascot
1214.5405	1214.5251	-0.0154	-13	512	522	MHHGATVMGNK			Oxidation (M)[1,8]		Mascot
1292.6766	1292.6851	0.0085	7	142	152	LSEILSCETLK			Carbamidomethyl (C)[7]		Mascot
1292.6766	1292.6851	0.0085	7	142	152	LSEILSCETLK	8	0	Carbamidomethyl (C)[7]		Mascot
1341.6984	1341.7599	0.0615	46	214	223	YMQELFKQVR					Mascot
1341.6984	1341.7599	0.0615	46	214	223	YMQELFKQVR					Mascot
1354.6467	1354.696	0.0493	36	511	522	RMHHGATVMGNK			Oxidation (M)[2]		Mascot
1506.8315	1506.7181	-0.1134	-75	347	359	SLVSHNVYIFSLK					Mascot
2151.0652	2151.033	-0.0322	-15	430	447	DQRLYLFGGEDIMQNPV R					Mascot
2167.0601	2167.0034	-0.0567	-26	430	447	DQRLYLFGGEDIMQNPV R			Oxidation (M)[13]		Mascot
2272.0737	2272.0854	0.0117	5	383	403	NFIFSIGGIGEGQELMGS MER					Mascot

Gel Idx/Pos	165/G16	Instr./Gel Origin	BA2151/Sample Project 20140814	Process Status	Analysis Succeeded
Plate [#] Name	[1] Sample Project 20140814	Instrument Sample Name		Spectra	11

Rank	Protein Name	Accession No.	Protein MW	Protein PI	Pep. Count	Protein Score	Protein Score C. I. %	Intensity Matched	Total Ion Score	Total Ion C. I. %	Confirmed
1	14-3-3-like protein A OS=Hordeum vulgare PE=2 SV=1	1433A_HORVU	29447.8	4.83	21	677	100	67.885	515	100	

Peptide Information

Calc. Mass	Obsrv. Mass	± da	± ppm	Start Seq.	End Seq.	Sequence	Ion Score	C. I. %	Modification	Rank	Result Type
816.421	816.4076	-0.0134	-16	17	23	LAEQAER					Mascot
907.5247	907.4792	-0.0455	-50	49	56	NLLSVAYK					Mascot
917.5302	917.5087	-0.0215	-23	68	75	IISSIEQK					Mascot
922.4199	922.4067	-0.0132	-14	130	136	MKGDYHR			Oxidation (M)[1]		Mascot
1051.5419	1051.522	-0.0199	-19	80	89	GNEAYVASIK					Mascot
1076.5946	1076.5731	-0.0215	-20	93	101	TRIETELSK					Mascot
1189.6609	1189.6497	-0.0112	-9	222	231	DSTLIMQLLR					Mascot
1189.6609	1189.6497	-0.0112	-9	222	231	DSTLIMQLLR	87	100			Mascot
1205.6559	1205.6342	-0.0217	-18	222	231	DSTLIMQLLR			Oxidation (M)[6]		Mascot
1205.6559	1205.6342	-0.0217	-18	222	231	DSTLIMQLLR	35	70.927	Oxidation (M)[6]		Mascot
1208.6157	1208.611	-0.0047	-4	149	159	EAAENTLVAYK					Mascot
1318.6486	1318.6364	-0.0122	-9	37	48	TADVGELTVEER					Mascot
1318.6486	1318.6364	-0.0122	-9	37	48	TADVGELTVEER	118	100			Mascot
1334.5643	1334.542	-0.0223	-17	24	33	YEEMVEFMEK					Mascot
1336.7107	1336.6345	-0.0762	-57	148	159	KEAAENTLVAYK					Mascot
1350.5592	1350.5243	-0.0349	-26	24	33	YEEMVEFMEK			Oxidation (M)[4]		Mascot
1366.5542	1366.511	-0.0432	-32	24	33	YEEMVEFMEK			Oxidation (M)[4,8]		Mascot
1418.7485	1418.7345	-0.014	-10	68	79	IISSIEQKEESR					Mascot
1517.8799	1517.8373	-0.0426	-28	49	62	NLLSVAYKNVIGAR					Mascot
1552.7601	1552.745	-0.0151	-10	76	89	EESRGNEAYVASIK					Mascot
1818.9708	1818.9503	-0.0205	-11	160	176	SAQDIALADLPTTHPIR					Mascot
1818.9708	1818.9503	-0.0205	-11	160	176	SAQDIALADLPTTHPIR	140	100			Mascot
1846.8309	1846.968	0.1371	74	1	16	MSTAEATREENVYMAK			Oxidation (M)[1]		Mascot
2106.9463	2106.8904	-0.0559	-27	232	250	DNLTLWTSDNAEEGGDEIK					Mascot
2114.9763	2114.9133	-0.063	-30	203	221	QAFDEAIAELDSLGEESYK					Mascot
2131.9675	2131.9492	-0.0183	-9	17	33	LAEQAERYEEMVEFMEK					Mascot
2131.9675	2131.9492	-0.0183	-9	17	33	LAEQAERYEEMVEFMEK	34	60.962			Mascot

2147.9624	2147.9199	-0.0425	-20	17	33	LAEQAERYEEMVEFMEK			Oxidation (M)[11]		Mascot
2147.9624	2147.9199	-0.0425	-20	17	33	LAEQAERYEEMVEFMEK	19	0	Oxidation (M)[15]		Mascot
2163.9573	2163.8999	-0.0574	-27	17	33	LAEQAERYEEMVEFMEK			Oxidation (M)[11,15]		Mascot
2163.9573	2163.8999	-0.0574	-27	17	33	LAEQAERYEEMVEFMEK	44	96.194	Oxidation (M)[11,15]		Mascot
2331.2019	2331.1804	-0.0215	-9	177	196	LGLALNFSVFYYEILNSPD R					Mascot
2331.2019	2331.1804	-0.0215	-9	177	196	LGLALNFSVFYYEILNSPD R	125	100			Mascot
3285.6196	3285.6016	-0.018	-5	203	231	QAFDEAIAELDSLGEESY KDSTLIMQLLR					Mascot
3301.6145	3301.5984	-0.0161	-5	203	231	QAFDEAIAELDSLGEESY KDSTLIMQLLR			Oxidation (M)[25]		Mascot

2	14-3-3-like protein GF14-F OS=Oryza sativa subsp. japonica GN=GF14F PE=1 SV=2	14336_ORYSJ	29273.7	4.81	17	514	100	56.838	396	100	

Peptide Information

Calc. Mass	Obsrv. Mass	± da	± ppm	Start Seq.	End Seq.	Sequence	Ion Score	C. I. %	Modification	Rank	Result Type
816.421	816.4076	-0.0134	-16	17	23	LAEQAER					Mascot
907.5247	907.4792	-0.0455	-50	49	56	NLLSVAYK					Mascot
917.5302	917.5087	-0.0215	-23	68	75	IISSIEQK					Mascot
922.4199	922.4067	-0.0132	-14	130	136	MKGDYHR			Oxidation (M)[1]		Mascot
1051.5419	1051.522	-0.0199	-19	80	89	GNEAYVASIK					Mascot
1189.6609	1189.6497	-0.0112	-9	222	231	DSTLIMQLLR					Mascot
1189.6609	1189.6497	-0.0112	-9	222	231	DSTLIMQLLR	87	100			Mascot
1205.6559	1205.6342	-0.0217	-18	222	231	DSTLIMQLLR			Oxidation (M)[6]		Mascot
1205.6559	1205.6342	-0.0217	-18	222	231	DSTLIMQLLR	35	70.927	Oxidation (M)[6]		Mascot
1208.6157	1208.611	-0.0047	-4	149	159	EAAENTLVAYK					Mascot
1334.5643	1334.542	-0.0223	-17	24	33	YEEMVEFMEK					Mascot
1336.7107	1336.6345	-0.0762	-57	148	159	KEAAENTLVAYK					Mascot
1350.5592	1350.5243	-0.0349	-26	24	33	YEEMVEFMEK			Oxidation (M)[4]		Mascot
1366.5542	1366.511	-0.0432	-32	24	33	YEEMVEFMEK			Oxidation (M)[4,8]		Mascot
1418.7485	1418.7345	-0.014	-10	68	79	IISSIEQKEESR					Mascot
1517.8799	1517.8373	-0.0426	-28	49	62	NLLSVAYKNVIGAR					Mascot
1552.7601	1552.745	-0.0151	-10	76	89	EESRGNEAYVASIK					Mascot
1818.9708	1818.9503	-0.0205	-11	160	176	SAQDIALADLPTTHPIR					Mascot
1818.9708	1818.9503	-0.0205	-11	160	176	SAQDIALADLPTTHPIR	140	100			Mascot
2114.9766	2114.9133	-0.0633	-30	203	221	QAFDDAIAELDTLGEESY K					Mascot
2131.9675	2131.9492	-0.0183	-9	17	33	LAEQAERYEEMVEFMEK					Mascot
2131.9675	2131.9492	-0.0183	-9	17	33	LAEQAERYEEMVEFMEK	34	60.962			Mascot

Calc. Mass	Obsrv. Mass	± da	± ppm	Start Seq.	End Seq.	Sequence	Ion Score	C. I. %	Modification	Rank	Result Type
2147.9624	2147.9199	-0.0425	-20	17	33	LAEQAERYEEMVEFMEK			Oxidation (M)[11]		Mascot
2147.9624	2147.9199	-0.0425	-20	17	33	LAEQAERYEEMVEFMEK	19	0	Oxidation (M)[15]		Mascot
2163.9573	2163.8999	-0.0574	-27	17	33	LAEQAERYEEMVEFMEK			Oxidation (M)[11,15]		Mascot
2163.9573	2163.8999	-0.0574	-27	17	33	LAEQAERYEEMVEFMEK	44	96.194	Oxidation (M)[11,15]		Mascot
2331.2019	2331.1804	-0.0215	-9	177	196	LGLALNFSVFYYEILNSPDR					Mascot
2331.2019	2331.1804	-0.0215	-9	177	196	LGLALNFSVFYYEILNSPDR	125	100			Mascot
3285.6196	3285.6016	-0.018	-5	203	231	QAFDDAIAELDTLGEESYKDSTLIMQLLR					Mascot
3301.6145	3301.5984	-0.0161	-5	203	231	QAFDDAIAELDTLGEESYKDSTLIMQLLR			Oxidation (M)[25]		Mascot

3 14-3-3-like protein OS=Solanum tuberosum PE=2 SV=1 14331_SOLTU 29490.7 4.71 13 328 100 31.593 256 100

Peptide Information

Calc. Mass	Obsrv. Mass	± da	± ppm	Start Seq.	End Seq.	Sequence	Ion Score	C. I. %	Modification	Rank	Result Type
816.421	816.4076	-0.0134	-16	14	20	LAEQAER					Mascot
907.5247	907.4792	-0.0455	-50	45	52	NLLSVAYK					Mascot
917.5302	917.5087	-0.0215	-23	64	71	IISSIEQK					Mascot
922.4199	922.4067	-0.0132	-14	126	132	MKGDYHR			Oxidation (M)[1]		Mascot
1189.6609	1189.6497	-0.0112	-9	219	228	DSTLIMQLLR					Mascot
1189.6609	1189.6497	-0.0112	-9	219	228	DSTLIMQLLR	87	100			Mascot
1205.6559	1205.6342	-0.0217	-18	219	228	DSTLIMQLLR			Oxidation (M)[6]		Mascot
1205.6559	1205.6342	-0.0217	-18	219	228	DSTLIMQLLR	35	70.927	Oxidation (M)[6]		Mascot
1334.5643	1334.542	-0.0223	-17	21	30	YEEMVEFMEK					Mascot
1350.5592	1350.5243	-0.0349	-26	21	30	YEEMVEFMEK			Oxidation (M)[4]		Mascot
1366.5542	1366.511	-0.0432	-32	21	30	YEEMVEFMEK			Oxidation (M)[4,8]		Mascot
1418.7485	1418.7345	-0.014	-10	64	75	IISSIEQKEESR					Mascot
1517.8799	1517.8373	-0.0426	-28	45	58	NLLSVAYKNVIGAR					Mascot
1533.7292	1533.774	0.0448	29	76	88	GNEDHVSSIKEYR					Mascot
2114.9766	2114.9133	-0.0633	-30	200	218	QAFDDAIAELDTLGEESYK					Mascot
2131.9675	2131.9492	-0.0183	-9	14	30	LAEQAERYEEMVEFMEK					Mascot
2131.9675	2131.9492	-0.0183	-9	14	30	LAEQAERYEEMVEFMEK	34	60.962			Mascot
2147.9624	2147.9199	-0.0425	-20	14	30	LAEQAERYEEMVEFMEK			Oxidation (M)[11]		Mascot
2147.9624	2147.9199	-0.0425	-20	14	30	LAEQAERYEEMVEFMEK	19	0	Oxidation (M)[15]		Mascot
2163.9573	2163.8999	-0.0574	-27	14	30	LAEQAERYEEMVEFMEK			Oxidation (M)[11,15]		Mascot
2163.9573	2163.8999	-0.0574	-27	14	30	LAEQAERYEEMVEFMEK	44	96.194	Oxidation (M)[11,15]		Mascot
2331.2019	2331.1804	-0.0215	-9	174	193	LGLALNFSVFYYEILNSPDR					Mascot

2331.2019	2331.1804	-0.0215	-9	174	193	LGLALNFSVFYYEILNSPD R	125	100			Mascot
3285.6196	3285.6016	-0.018	-5	200	228	QAFDDAIAELDTLGEESY KDSTLIMQLLR					Mascot
3301.6145	3301.5984	-0.0161	-5	200	228	QAFDDAIAELDTLGEESY KDSTLIMQLLR			Oxidation (M)[25]		Mascot

4	14-3-3-like protein C OS=Nicotiana tabacum PE=1 SV=1	1433C_TOBAC	29459.7	4.78	13	325	100	30.142	256	100	

Peptide Information

Calc. Mass	Obsrv. Mass	± da	± ppm	Start Seq.	End Seq.	Sequence	Ion Score	C. I. %	Modification	Rank	Result Type
816.421	816.4076	-0.0134	-16	17	23	LAEQAER					Mascot
907.5247	907.4792	-0.0455	-50	49	56	NLLSVAYK					Mascot
917.5302	917.5087	-0.0215	-23	68	75	IISSIEQK					Mascot
922.4199	922.4067	-0.0132	-14	130	136	MKGDYHR			Oxidation (M)[1]		Mascot
1142.5325	1142.6431	0.1106	97	251	260	EDPKPDEAKN					Mascot
1189.6609	1189.6497	-0.0112	-9	222	231	DSTLIMQLLR					Mascot
1189.6609	1189.6497	-0.0112	-9	222	231	DSTLIMQLLR	87	100			Mascot
1205.6559	1205.6342	-0.0217	-18	222	231	DSTLIMQLLR			Oxidation (M)[6]		Mascot
1205.6559	1205.6342	-0.0217	-18	222	231	DSTLIMQLLR	35	70.927	Oxidation (M)[6]		Mascot
1311.6791	1311.6558	-0.0233	-18	148	159	KEAAESTLTAYK					Mascot
1334.5643	1334.542	-0.0223	-17	24	33	YEEMVEFMEK					Mascot
1350.5592	1350.5243	-0.0349	-26	24	33	YEEMVEFMEK			Oxidation (M)[4]		Mascot
1366.5542	1366.511	-0.0432	-32	24	33	YEEMVEFMEK			Oxidation (M)[4,8]		Mascot
1418.7485	1418.7345	-0.014	-10	68	79	IISSIEQKEESR					Mascot
1517.8799	1517.8373	-0.0426	-28	49	62	NLLSVAYKNVIGAR					Mascot
1631.8673	1631.7383	-0.129	-79	95	108	IENELSKICDGILK			Carbamidomethyl (C)[9]		Mascot
2131.9675	2131.9492	-0.0183	-9	17	33	LAEQAERYEEMVEFMEK					Mascot
2131.9675	2131.9492	-0.0183	-9	17	33	LAEQAERYEEMVEFMEK	34	60.962			Mascot
2147.9624	2147.9199	-0.0425	-20	17	33	LAEQAERYEEMVEFMEK			Oxidation (M)[11]		Mascot
2147.9624	2147.9199	-0.0425	-20	17	33	LAEQAERYEEMVEFMEK	19	0	Oxidation (M)[15]		Mascot
2163.9573	2163.8999	-0.0574	-27	17	33	LAEQAERYEEMVEFMEK			Oxidation (M)[11,15]		Mascot
2163.9573	2163.8999	-0.0574	-27	17	33	LAEQAERYEEMVEFMEK	44	96.194	Oxidation (M)[11,15]		Mascot
2331.2019	2331.1804	-0.0215	-9	177	196	LGLALNFSVFYYEILNSPD R					Mascot
2331.2019	2331.1804	-0.0215	-9	177	196	LGLALNFSVFYYEILNSPD R	125	100			Mascot

5	14-3-3-like protein OS=Helianthus annuus PE=2 SV=1	1433_HELAN	29043.5	4.65	12	325	100	31.417	256	100	

Peptide Information

Calc. Mass	Obsrv. Mass	± da	± ppm	Start	End	Sequence	Ion	C. I. %	Modification	Rank	Result Type

				Seq.	Seq.		Score				
816.421	816.4076	-0.0134	-16	17	23	LAEQAER					Mascot
907.5247	907.4792	-0.0455	-50	50	57	NLLSVAYK					Mascot
917.5302	917.5087	-0.0215	-23	69	76	IISSIEQK					Mascot
1189.6609	1189.6497	-0.0112	-9	223	232	DSTLIMQLLR					Mascot
1189.6609	1189.6497	-0.0112	-9	223	232	DSTLIMQLLR	87	100			Mascot
1205.6559	1205.6342	-0.0217	-18	223	232	DSTLIMQLLR			Oxidation (M)[6]		Mascot
1205.6559	1205.6342	-0.0217	-18	223	232	DSTLIMQLLR	35	70.927	Oxidation (M)[6]		Mascot
1334.5643	1334.542	-0.0223	-17	24	33	YEEMVEFMEK					Mascot
1350.5592	1350.5243	-0.0349	-26	24	33	YEEMVEFMEK			Oxidation (M)[4]		Mascot
1366.5542	1366.511	-0.0432	-32	24	33	YEEMVEFMEK			Oxidation (M)[4,8]		Mascot
1418.7485	1418.7345	-0.014	-10	69	80	IISSIEQKEESR					Mascot
1517.8799	1517.8373	-0.0426	-28	50	63	NLLSVAYKNVIGAR					Mascot
1533.8119	1533.774	-0.0379	-25	110	125	VLDSKLIGSASGGDSK					Mascot
2114.9766	2114.9133	-0.0633	-30	204	222	QAFDEAIAELDTLGEDSYK					Mascot
2131.9675	2131.9492	-0.0183	-9	17	33	LAEQAERYEEMVEFMEK					Mascot
2131.9675	2131.9492	-0.0183	-9	17	33	LAEQAERYEEMVEFMEK	34	60.962			Mascot
2147.9624	2147.9199	-0.0425	-20	17	33	LAEQAERYEEMVEFMEK			Oxidation (M)[11]		Mascot
2147.9624	2147.9199	-0.0425	-20	17	33	LAEQAERYEEMVEFMEK	19	0	Oxidation (M)[15]		Mascot
2163.9573	2163.8999	-0.0574	-27	17	33	LAEQAERYEEMVEFMEK			Oxidation (M)[11,15]		Mascot
2163.9573	2163.8999	-0.0574	-27	17	33	LAEQAERYEEMVEFMEK	44	96.194	Oxidation (M)[11,15]		Mascot
2331.2019	2331.1804	-0.0215	-9	178	197	LGLALNFSVFYYEILNSPDR					Mascot
2331.2019	2331.1804	-0.0215	-9	178	197	LGLALNFSVFYYEILNSPDR	125	100			Mascot
3285.6196	3285.6016	-0.018	-5	204	232	QAFDEAIAELDTLGEDSYKDSTLIMQLLR					Mascot
3301.6145	3301.5984	-0.0161	-5	204	232	QAFDEAIAELDTLGEDSYKDSTLIMQLLR			Oxidation (M)[25]		Mascot

6	14-3-3-like protein A OS=Vicia faba PE=2 SV=1	1433A_VICFA	29515.7	4.71	13	324	100	54.297	256	100

Peptide Information

Calc. Mass	Obsrv. Mass	± da	± ppm	Start Seq.	End Seq.	Sequence	Ion Score	C. I. %	Modification	Rank	Result Type
816.421	816.4076	-0.0134	-16	17	23	LAEQAER					Mascot
844.4345	844.448	0.0135	16	1	8	MATAPTPR					Mascot
907.5247	907.4792	-0.0455	-50	49	56	NLLSVAYK					Mascot
917.5302	917.5087	-0.0215	-23	68	75	IISSIEQK					Mascot
922.4199	922.4067	-0.0132	-14	130	136	MKGDYHR			Oxidation (M)[1]		Mascot

1189.6609	1189.6497	-0.0112	-9	222	231	DSTLIMQLLR					Mascot
1189.6609	1189.6497	-0.0112	-9	222	231	DSTLIMQLLR	87	100			Mascot
1205.6559	1205.6342	-0.0217	-18	222	231	DSTLIMQLLR			Oxidation (M)[6]		Mascot
1205.6559	1205.6342	-0.0217	-18	222	231	DSTLIMQLLR	35	70.927	Oxidation (M)[6]		Mascot
1334.5643	1334.542	-0.0223	-17	24	33	YEEMVEFMEK					Mascot
1350.5592	1350.5243	-0.0349	-26	24	33	YEEMVEFMEK			Oxidation (M)[4]		Mascot
1366.5542	1366.511	-0.0432	-32	24	33	YEEMVEFMEK			Oxidation (M)[4,8]		Mascot
1418.7485	1418.7345	-0.014	-10	68	79	IISSIEQKEESR					Mascot
1517.8799	1517.8373	-0.0426	-28	49	62	NLLSVAYKNVIGAR					Mascot
1559.7561	1559.8628	0.1067	68	80	92	GNDEHVSVIRDYR					Mascot
1818.963	1818.9503	-0.0127	-7	93	108	SKIETELSNICNGILK			Carbamidomethyl (C)[11]		Mascot
1818.963	1818.9503	-0.0127	-7	93	108	SKIETELSNICNGILK			Carbamidomethyl (C)[11]		Mascot
2131.9675	2131.9492	-0.0183	-9	17	33	LAEQAERYEEMVEFMEK					Mascot
2131.9675	2131.9492	-0.0183	-9	17	33	LAEQAERYEEMVEFMEK	34	60.962			Mascot
2147.9624	2147.9199	-0.0425	-20	17	33	LAEQAERYEEMVEFMEK			Oxidation (M)[11]		Mascot
2147.9624	2147.9199	-0.0425	-20	17	33	LAEQAERYEEMVEFMEK	19	0	Oxidation (M)[15]		Mascot
2163.9573	2163.8999	-0.0574	-27	17	33	LAEQAERYEEMVEFMEK			Oxidation (M)[11,15]		Mascot
2163.9573	2163.8999	-0.0574	-27	17	33	LAEQAERYEEMVEFMEK	44	96.194	Oxidation (M)[11,15]		Mascot
2331.2019	2331.1804	-0.0215	-9	177	196	LGLALNFSVFYYEILNSPD R					Mascot
2331.2019	2331.1804	-0.0215	-9	177	196	LGLALNFSVFYYEILNSPD R	125	100			Mascot

7	14-3-3-like protein GF14 chi OS=Arabidopsis thaliana GN=GRF1 PE=1 SV=3	14331_ARATH	30027	4.68	13	324	100	30.537	256	100

Peptide Information

Calc. Mass	Obsrv. Mass	± da	± ppm	Start Seq.	End Seq.	Sequence	Ion Score	C. I. %	Modification	Rank	Result Type
816.421	816.4076	-0.0134	-16	19	25	LAEQAER					Mascot
907.5247	907.4792	-0.0455	-50	51	58	NLLSVAYK					Mascot
917.5302	917.5087	-0.0215	-23	70	77	IISSIEQK					Mascot
922.4199	922.4067	-0.0132	-14	132	138	MKGDYHR			Oxidation (M)[1]		Mascot
1189.6609	1189.6497	-0.0112	-9	224	233	DSTLIMQLLR					Mascot
1189.6609	1189.6497	-0.0112	-9	224	233	DSTLIMQLLR	87	100			Mascot
1205.6559	1205.6342	-0.0217	-18	224	233	DSTLIMQLLR			Oxidation (M)[6]		Mascot
1205.6559	1205.6342	-0.0217	-18	224	233	DSTLIMQLLR	35	70.927	Oxidation (M)[6]		Mascot
1334.5643	1334.542	-0.0223	-17	26	35	YEEMVEFMEK					Mascot
1350.5592	1350.5243	-0.0349	-26	26	35	YEEMVEFMEK			Oxidation (M)[4]		Mascot
1366.5542	1366.511	-0.0432	-32	26	35	YEEMVEFMEK			Oxidation (M)[4,8]		Mascot

Calc. Mass	Obsrv. Mass	± da	± ppm	Start Seq.	End Seq.	Sequence	Ion Score	C. I. %	Modification	Rank	Result Type
1418.7485	1418.7345	-0.014	-10	70	81	IISSIEQKEESR					Mascot
1517.8799	1517.8373	-0.0426	-28	51	64	NLLSVAYKNVIGAR					Mascot
1559.7561	1559.8628	0.1067	68	82	94	GNDDHVSLIRDYR					Mascot
1799.8633	1799.8657	0.0024	1	11	25	DEFVYMAKLAEQAER					Mascot
1800.8585	1800.9512	0.0927	51	2	18	ATPGASSARDEFVYMAK					Mascot
1816.8534	1816.8915	0.0381	21	2	18	ATPGASSARDEFVYMAK			Oxidation (M)[15]		Mascot
2131.9675	2131.9492	-0.0183	-9	19	35	LAEQAERYEEMVEFMEK					Mascot
2131.9675	2131.9492	-0.0183	-9	19	35	LAEQAERYEEMVEFMEK	34	60.962			Mascot
2147.9624	2147.9199	-0.0425	-20	19	35	LAEQAERYEEMVEFMEK			Oxidation (M)[11]		Mascot
2147.9624	2147.9199	-0.0425	-20	19	35	LAEQAERYEEMVEFMEK	19	0	Oxidation (M)[15]		Mascot
2163.9573	2163.8999	-0.0574	-27	19	35	LAEQAERYEEMVEFMEK			Oxidation (M)[11,15]		Mascot
2163.9573	2163.8999	-0.0574	-27	19	35	LAEQAERYEEMVEFMEK	44	96.194	Oxidation (M)[11,15]		Mascot
2331.2019	2331.1804	-0.0215	-9	179	198	LGLALNFSVFYYEILNSPDR					Mascot
2331.2019	2331.1804	-0.0215	-9	179	198	LGLALNFSVFYYEILNSPDR	125	100			Mascot

8	14-3-3-like protein GF14 psi OS=Arabidopsis thaliana GN=GRF3 PE=1 SV=2	14333_ARATH	28702.4	4.75	12	317	100	30.522	256	100	

Peptide Information

Calc. Mass	Obsrv. Mass	± da	± ppm	Start Seq.	End Seq.	Sequence	Ion Score	C. I. %	Modification	Rank	Result Type
805.4301	805.4165	-0.0136	-17	91	97	IESELSK					Mascot
816.421	816.4076	-0.0134	-16	13	19	LAEQAER					Mascot
907.5247	907.4792	-0.0455	-50	45	52	NLLSVAYK					Mascot
917.5302	917.5087	-0.0215	-23	64	71	IISSIEQK					Mascot
922.4199	922.4067	-0.0132	-14	126	132	MKGDYHR			Oxidation (M)[1]		Mascot
1189.6609	1189.6497	-0.0112	-9	218	227	DSTLIMQLLR					Mascot
1189.6609	1189.6497	-0.0112	-9	218	227	DSTLIMQLLR	87	100			Mascot
1205.6559	1205.6342	-0.0217	-18	218	227	DSTLIMQLLR			Oxidation (M)[6]		Mascot
1205.6559	1205.6342	-0.0217	-18	218	227	DSTLIMQLLR	35	70.927	Oxidation (M)[6]		Mascot
1334.5643	1334.542	-0.0223	-17	20	29	YEEMVEFMEK					Mascot
1350.5592	1350.5243	-0.0349	-26	20	29	YEEMVEFMEK			Oxidation (M)[4]		Mascot
1366.5542	1366.511	-0.0432	-32	20	29	YEEMVEFMEK			Oxidation (M)[4,8]		Mascot
1517.8799	1517.8373	-0.0426	-28	45	58	NLLSVAYKNVIGAR					Mascot
2114.9766	2114.9133	-0.0633	-30	199	217	QAFDDAIAELDTLGEESYK					Mascot
2131.9675	2131.9492	-0.0183	-9	13	29	LAEQAERYEEMVEFMEK					Mascot
2131.9675	2131.9492	-0.0183	-9	13	29	LAEQAERYEEMVEFMEK	34	60.962			Mascot

Calc. Mass	Obsrv. Mass	± da	± ppm	Start Seq.	End Seq.	Sequence	Ion Score	C. I. %	Modification	Rank	Result Type
2147.9624	2147.9199	-0.0425	-20	13	29	LAEQAERYEEMVEFMEK			Oxidation (M)[11]		Mascot
2147.9624	2147.9199	-0.0425	-20	13	29	LAEQAERYEEMVEFMEK	19	0	Oxidation (M)[15]		Mascot
2163.9573	2163.8999	-0.0574	-27	13	29	LAEQAERYEEMVEFMEK			Oxidation (M)[11,15]		Mascot
2163.9573	2163.8999	-0.0574	-27	13	29	LAEQAERYEEMVEFMEK	44	96.194	Oxidation (M)[11,15]		Mascot
2331.2019	2331.1804	-0.0215	-9	173	192	LGLALNFSVFYYEILNSPD R					Mascot
2331.2019	2331.1804	-0.0215	-9	173	192	LGLALNFSVFYYEILNSPD R	125	100			Mascot
3285.6196	3285.6016	-0.018	-5	199	227	QAFDDAIAELDTLGEESY KDSTLIMQLLR					Mascot
3301.6145	3301.5984	-0.0161	-5	199	227	QAFDDAIAELDTLGEESY KDSTLIMQLLR			Oxidation (M)[25]		Mascot

9	14-3-3-like protein OS=Mesembryanthemum crystallinum GN=GBF PE=2 SV=1	1433_MESCR	30007.9	4.79	12	316	100	30.326	256	100		

Peptide Information

Calc. Mass	Obsrv. Mass	± da	± ppm	Start Seq.	End Seq.	Sequence	Ion Score	C. I. %	Modification	Rank	Result Type
816.421	816.4076	-0.0134	-16	16	22	LAEQAER					Mascot
907.5247	907.4792	-0.0455	-50	48	55	NLLSVAYK					Mascot
917.5302	917.5087	-0.0215	-23	67	74	IISSIEQK					Mascot
922.4199	922.4067	-0.0132	-14	129	135	MKGDYHR			Oxidation (M)[1]		Mascot
1187.5651	1187.6262	0.0611	51	255	264	RESGEEKPQQ					Mascot
1189.6609	1189.6497	-0.0112	-9	221	230	DSTLIMQLLR					Mascot
1189.6609	1189.6497	-0.0112	-9	221	230	DSTLIMQLLR	87	100			Mascot
1205.6559	1205.6342	-0.0217	-18	221	230	DSTLIMQLLR			Oxidation (M)[6]		Mascot
1205.6559	1205.6342	-0.0217	-18	221	230	DSTLIMQLLR	35	70.927	Oxidation (M)[6]		Mascot
1334.5643	1334.542	-0.0223	-17	23	32	YEEMVEFMEK					Mascot
1350.5592	1350.5243	-0.0349	-26	23	32	YEEMVEFMEK			Oxidation (M)[4]		Mascot
1366.5542	1366.511	-0.0432	-32	23	32	YEEMVEFMEK			Oxidation (M)[4,8]		Mascot
1418.7485	1418.7345	-0.014	-10	67	78	IISSIEQKEESR					Mascot
1517.8799	1517.8373	-0.0426	-28	48	61	NLLSVAYKNVIGAR					Mascot
2106.9463	2106.8904	-0.0559	-27	231	249	DNLTLWTSDNAEEGGDE IK					Mascot
2131.9675	2131.9492	-0.0183	-9	16	32	LAEQAERYEEMVEFMEK					Mascot
2131.9675	2131.9492	-0.0183	-9	16	32	LAEQAERYEEMVEFMEK	34	60.962			Mascot
2147.9624	2147.9199	-0.0425	-20	16	32	LAEQAERYEEMVEFMEK			Oxidation (M)[11]		Mascot
2147.9624	2147.9199	-0.0425	-20	16	32	LAEQAERYEEMVEFMEK	19	0	Oxidation (M)[15]		Mascot
2163.9573	2163.8999	-0.0574	-27	16	32	LAEQAERYEEMVEFMEK			Oxidation (M)[11,15]		Mascot
2163.9573	2163.8999	-0.0574	-27	16	32	LAEQAERYEEMVEFMEK	44	96.194	Oxidation (M)[11,15]		Mascot
2331.2019	2331.1804	-0.0215	-9	176	195	LGLALNFSVFYYEILNSPD R					Mascot

2331.2019	2331.1804	-0.0215	-9	176	195	LGLALNFSVFYYEILNSPD R	125	100			Mascot

10 14-3-3-like protein OS=Oenothera elata subsp. hookeri PE=2 SV=2 | 1433_OENEH | 29408.5 | 4.61 | 11 | 313 | 100 | 29.917 | 256 | 100

Peptide Information

Calc. Mass	Obsrv. Mass	± da	± ppm	Start Seq.	End Seq.	Sequence	Ion Score	C. I. %	Modification	Rank	Result Type
816.421	816.4076	-0.0134	-16	17	23	LAEQAER					Mascot
907.5247	907.4792	-0.0455	-50	49	56	NLLSVAYK					Mascot
917.5302	917.5087	-0.0215	-23	68	75	IISSIEQK					Mascot
922.4199	922.4067	-0.0132	-14	130	136	MKGDYHR			Oxidation (M)[1]		Mascot
1189.6609	1189.6497	-0.0112	-9	222	231	DSTLIMQLLR					Mascot
1189.6609	1189.6497	-0.0112	-9	222	231	DSTLIMQLLR	87	100			Mascot
1205.6559	1205.6342	-0.0217	-18	222	231	DSTLIMQLLR			Oxidation (M)[6]		Mascot
1205.6559	1205.6342	-0.0217	-18	222	231	DSTLIMQLLR	35	70.927	Oxidation (M)[6]		Mascot
1334.5643	1334.542	-0.0223	-17	24	33	YEEMVEFMEK					Mascot
1350.5592	1350.5243	-0.0349	-26	24	33	YEEMVEFMEK			Oxidation (M)[4]		Mascot
1366.5542	1366.511	-0.0432	-32	24	33	YEEMVEFMEK			Oxidation (M)[4,8]		Mascot
1418.7485	1418.7345	-0.014	-10	68	79	IISSIEQKEESR					Mascot
1517.8799	1517.8373	-0.0426	-28	49	62	NLLSVAYKNVIGAR					Mascot
2131.9675	2131.9492	-0.0183	-9	17	33	LAEQAERYEEMVEFMEK					Mascot
2131.9675	2131.9492	-0.0183	-9	17	33	LAEQAERYEEMVEFMEK	34	60.962			Mascot
2147.9624	2147.9199	-0.0425	-20	17	33	LAEQAERYEEMVEFMEK			Oxidation (M)[11]		Mascot
2147.9624	2147.9199	-0.0425	-20	17	33	LAEQAERYEEMVEFMEK	19	0	Oxidation (M)[15]		Mascot
2152.9341	2152.9175	-0.0166	-8	232	250	DNLTLWTSDMQDDGGD EIK					Mascot
2163.9573	2163.8999	-0.0574	-27	17	33	LAEQAERYEEMVEFMEK			Oxidation (M)[11,15]		Mascot
2163.9573	2163.8999	-0.0574	-27	17	33	LAEQAERYEEMVEFMEK	44	96.194	Oxidation (M)[11,15]		Mascot
2331.2019	2331.1804	-0.0215	-9	177	196	LGLALNFSVFYYEILNSPD R					Mascot
2331.2019	2331.1804	-0.0215	-9	177	196	LGLALNFSVFYYEILNSPD R	125	100			Mascot

Gel Idx/Pos	166/G17	Instr./Gel Origin	BA2151/Sample Project 20140814	Process Status	Analysis Succeeded
Plate [#] Name	[1] Sample Project 20140814	Instrument Sample Name		Spectra	11

Rank	Protein Name	Accession No.	Protein MW	Protein PI	Pep. Count	Protein Score	Protein Score C. I. %	Intensity Matched	Total Ion Score	Total Ion C. I. %	Confirmed
1	14-3-3-like protein GF14-6 OS=Zea mays GN=GRF1 PE=1 SV=1	14331_MAIZE	29758	4.76	17	451	100	41.96	350	100	

Peptide Information

Calc. Mass	Obsrv. Mass	± da	± ppm	Start Seq.	End Seq.	Sequence	Ion Score	C. I. %	Modification	Rank	Result Type
816.421	816.4134	-0.0076	-9	17	23	LAEQAER					Mascot
818.444	818.4239	-0.0201	-25	102	108	ICDGILK			Carbamidomethyl (C)[2]		Mascot
907.5247	907.4822	-0.0425	-47	49	56	NLLSVAYK					Mascot
917.5302	917.5153	-0.0149	-16	68	75	IISSIEQK					Mascot
932.4294	932.4268	-0.0026	-3	130	136	MKGDYYR					Mascot
948.4244	948.4129	-0.0115	-12	130	136	MKGDYYR			Oxidation (M)[1]		Mascot
999.4451	999.4496	0.0045	5	9	16	EENVYMAK			Oxidation (M)[6]		Mascot
1144.6321	1144.6272	-0.0049	-4	80	89	GNEDRVTLIK					Mascot
1189.6609	1189.6603	-0.0006	-1	222	231	DSTLIMQLLR					Mascot
1189.6609	1189.6603	-0.0006	-1	222	231	DSTLIMQLLR	22	0			Mascot
1205.6559	1205.6425	-0.0134	-11	222	231	DSTLIMQLLR			Oxidation (M)[6]		Mascot
1205.6559	1205.6425	-0.0134	-11	222	231	DSTLIMQLLR	23	0	Oxidation (M)[6]		Mascot
1212.5565	1212.6031	0.0466	38	149	159	DAAENTMVAYK					Mascot
1228.5514	1228.5819	0.0305	25	149	159	DAAENTMVAYK			Oxidation (M)[7]		Mascot
1366.5542	1366.5227	-0.0315	-23	24	33	YEEMVEFMEK			Oxidation (M)[4,8]		Mascot
1388.738	1388.7262	-0.0118	-8	68	79	IISSIEQKEEGR					Mascot
1388.738	1388.7262	-0.0118	-8	68	79	IISSIEQKEEGR	54	99.592			Mascot
1406.6646	1406.6599	-0.0047	-3	37	48	TVDSEELTVEER					Mascot
1406.6646	1406.6599	-0.0047	-3	37	48	TVDSEELTVEER	109	100			Mascot
1708.9116	1708.85	-0.0616	-36	109	124	LLETHLVPSSTAPESK					Mascot
1786.9811	1786.9723	-0.0088	-5	160	176	AAQDIALAELAPTHPIR					Mascot
1786.9811	1786.9723	-0.0088	-5	160	176	AAQDIALAELAPTHPIR	156	100			Mascot
2147.9624	2147.9368	-0.0256	-12	17	33	LAEQAERYEEMVEFMEK			Oxidation (M)[11]		Mascot
2163.9573	2163.9104	-0.0469	-22	17	33	LAEQAERYEEMVEFMEK			Oxidation (M)[11,15]		Mascot
2163.9573	2163.9104	-0.0469	-22	17	33	LAEQAERYEEMVEFMEK	8	0	Oxidation (M)[11,15]		Mascot
2174.9976	2174.946	-0.0516	-24	203	221	QAFDEAISELDTLSEESYK					Mascot

2331.2019	2331.2019	0	0	177	196	LGLALNFSVFYYEILNSPDR					Mascot

2	14-3-3-like protein GF14-12 OS=Zea mays GN=GRF2 PE=2 SV=2	14332_MAIZE	29731.9	4.75	15	409	100	37.347	328	100		

Peptide Information

Calc. Mass	Obsrv. Mass	± da	± ppm	Start Seq.	End Seq.	Sequence	Ion Score	C. I. %	Modification	Rank	Result Type
816.421	816.4134	-0.0076	-9	17	23	LAEQAER					Mascot
818.444	818.4239	-0.0201	-25	102	108	ICDGILK			Carbamidomethyl (C)[2]		Mascot
907.5247	907.4822	-0.0425	-47	49	56	NLLSVAYK					Mascot
917.5302	917.5153	-0.0149	-16	68	75	IISSIEQK					Mascot
932.4294	932.4268	-0.0026	-3	130	136	MKGDYYR					Mascot
948.4244	948.4129	-0.0115	-12	130	136	MKGDYYR			Oxidation (M)[1]		Mascot
999.4451	999.4496	0.0045	5	9	16	EENVYMAK			Oxidation (M)[6]		Mascot
1144.6321	1144.6272	-0.0049	-4	80	89	GNEDRVTLIK					Mascot
1212.5565	1212.6031	0.0466	38	149	159	DAAENTMVAYK					Mascot
1228.5514	1228.5819	0.0305	25	149	159	DAAENTMVAYK			Oxidation (M)[7]		Mascot
1366.5542	1366.5227	-0.0315	-23	24	33	YEEMVEFMEK			Oxidation (M)[4,8]		Mascot
1388.738	1388.7262	-0.0118	-8	68	79	IISSIEQKEEGR					Mascot
1388.738	1388.7262	-0.0118	-8	68	79	IISSIEQKEEGR	54	99.592			Mascot
1406.6646	1406.6599	-0.0047	-3	37	48	TVDSEELTVEER					Mascot
1406.6646	1406.6599	-0.0047	-3	37	48	TVDSEELTVEER	109	100			Mascot
1786.9811	1786.9723	-0.0088	-5	160	176	AAQDIALAELAPTHPIR					Mascot
1786.9811	1786.9723	-0.0088	-5	160	176	AAQDIALAELAPTHPIR	156	100			Mascot
2147.9624	2147.9368	-0.0256	-12	17	33	LAEQAERYEEMVEFMEK			Oxidation (M)[11]		Mascot
2163.9573	2163.9104	-0.0469	-22	17	33	LAEQAERYEEMVEFMEK			Oxidation (M)[11,15]		Mascot
2163.9573	2163.9104	-0.0469	-22	17	33	LAEQAERYEEMVEFMEK	8	0	Oxidation (M)[11,15]		Mascot
2174.9976	2174.946	-0.0516	-24	203	221	QAFDEAISELDTLSEESYK					Mascot
2331.2019	2331.2019	0	0	177	196	LGLALNFSVFYYEILNSPDR					Mascot

3	14-3-3-like protein GF14-B OS=Oryza sativa subsp. japonica GN=GF14B PE=1 SV=2	14332_ORYSJ	29959.1	4.76	15	287	100	17.853	209	100		

Peptide Information

Calc. Mass	Obsrv. Mass	± da	± ppm	Start Seq.	End Seq.	Sequence	Ion Score	C. I. %	Modification	Rank	Result Type
816.421	816.4134	-0.0076	-9	18	24	LAEQAER					Mascot
818.444	818.4239	-0.0201	-25	103	109	ICDGILK			Carbamidomethyl (C)[2]		Mascot

907.5247	907.4822	-0.0425	-47	50	57	NLLSVAYK					Mascot
917.5302	917.5153	-0.0149	-16	69	76	IISSIEQK					Mascot
932.4294	932.4268	-0.0026	-3	131	137	MKGDYYR					Mascot
948.4244	948.4129	-0.0115	-12	131	137	MKGDYYR			Oxidation (M)[1]		Mascot
999.4451	999.4496	0.0045	5	10	17	EENVYMAK			Oxidation (M)[6]		Mascot
1144.6321	1144.6272	-0.0049	-4	81	90	GNEDRVTLIK					Mascot
1189.6609	1189.6603	-0.0006	-1	223	232	DSTLIMQLLR					Mascot
1189.6609	1189.6603	-0.0006	-1	223	232	DSTLIMQLLR	22	0			Mascot
1205.6559	1205.6425	-0.0134	-11	223	232	DSTLIMQLLR			Oxidation (M)[6]		Mascot
1205.6559	1205.6425	-0.0134	-11	223	232	DSTLIMQLLR	23	0	Oxidation (M)[6]		Mascot
1212.5565	1212.6031	0.0466	38	150	160	DAAENTMVAYK					Mascot
1228.5514	1228.5819	0.0305	25	150	160	DAAENTMVAYK			Oxidation (M)[7]		Mascot
1366.5542	1366.5227	-0.0315	-23	25	34	YEEMVEFMEK			Oxidation (M)[4,8]		Mascot
1406.6646	1406.6599	-0.0047	-3	38	49	TVDSEELTVEER					Mascot
1406.6646	1406.6599	-0.0047	-3	38	49	TVDSEELTVEER	109	100			Mascot
1418.7485	1418.7394	-0.0091	-6	69	80	IISSIEQKEESR					Mascot
1418.7485	1418.7394	-0.0091	-6	69	80	IISSIEQKEESR	69	99.988			Mascot
2147.9624	2147.9368	-0.0256	-12	18	34	LAEQAERYEEMVEFMEK			Oxidation (M)[11]		Mascot
2163.9573	2163.9104	-0.0469	-22	18	34	LAEQAERYEEMVEFMEK			Oxidation (M)[11,15]		Mascot
2163.9573	2163.9104	-0.0469	-22	18	34	LAEQAERYEEMVEFMEK	8	0	Oxidation (M)[11,15]		Mascot
2174.9976	2174.946	-0.0516	-24	204	222	QAFDEAISELDTLSEESY K					Mascot
2331.2019	2331.2019	0	0	178	197	LGLALNFSVFYYEILNSPD R					Mascot

4	14-3-3-like protein GF14-E OS=Oryza sativa subsp. japonica GN=GF14E PE=2 SV=1	14335_ORYSJ	29844.9	4.71	14	280	100	17.585	208	100		

Peptide Information

Calc. Mass	Obsrv. Mass	± da	± ppm	Start Seq.	End Seq.	Sequence	Ion Score	C. I. %	Modification	Rank	Result Type
816.421	816.4134	-0.0076	-9	18	24	LAEQAER					Mascot
818.444	818.4239	-0.0201	-25	103	109	ICDGILK			Carbamidomethyl (C)[2]		Mascot
907.5247	907.4822	-0.0425	-47	50	57	NLLSVAYK					Mascot
917.5302	917.5153	-0.0149	-16	69	76	IISSIEQK					Mascot
932.4294	932.4268	-0.0026	-3	131	137	MKGDYYR					Mascot
948.4244	948.4129	-0.0115	-12	131	137	MKGDYYR			Oxidation (M)[1]		Mascot
999.4451	999.4496	0.0045	5	10	17	EENVYMAK			Oxidation (M)[6]		Mascot
1189.6609	1189.6603	-0.0006	-1	223	232	DSTLIMQLLR					Mascot
1189.6609	1189.6603	-0.0006	-1	223	232	DSTLIMQLLR	22	0			Mascot

1205.6559	1205.6425	-0.0134	-11	223	232	DSTLIMQLLR			Oxidation (M)[6]		Mascot
1205.6559	1205.6425	-0.0134	-11	223	232	DSTLIMQLLR	23	0	Oxidation (M)[6]		Mascot
1212.5565	1212.6031	0.0466	38	150	160	DAAENTMVAYK					Mascot
1228.5514	1228.5819	0.0305	25	150	160	DAAENTMVAYK			Oxidation (M)[7]		Mascot
1366.5542	1366.5227	-0.0315	-23	25	34	YEEMVEFMEK			Oxidation (M)[4,8]		Mascot
1406.6646	1406.6599	-0.0047	-3	38	49	TVDSEELTVEER					Mascot
1406.6646	1406.6599	-0.0047	-3	38	49	TVDSEELTVEER	109	100			Mascot
1418.7485	1418.7394	-0.0091	-6	69	80	IISSIEQKEESR					Mascot
1418.7485	1418.7394	-0.0091	-6	69	80	IISSIEQKEESR	69	99.988			Mascot
2147.9624	2147.9368	-0.0256	-12	18	34	LAEQAERYEEMVEFMEK			Oxidation (M)[11]		Mascot
2163.9573	2163.9104	-0.0469	-22	18	34	LAEQAERYEEMVEFMEK			Oxidation (M)[11,15]		Mascot
2163.9573	2163.9104	-0.0469	-22	18	34	LAEQAERYEEMVEFMEK	8	0	Oxidation (M)[11,15]		Mascot
2174.9976	2174.946	-0.0516	-24	204	222	QAFDEAISELDTLSEESYK					Mascot
2331.2019	2331.2019	0	0	178	197	LGLALNFSVFYYEILNSPDR					Mascot

5	14-3-3-like protein B OS=Hordeum vulgare PE=2 SV=1	1433B_HORVU	29786.9	4.67	14	276	100	17.653	209	100	

Peptide Information

Calc. Mass	Obsrv. Mass	± da	± ppm	Start Seq.	End Seq.	Sequence	Ion Score	C. I. %	Modification	Rank	Result Type
816.421	816.4134	-0.0076	-9	18	24	LAEQAER					Mascot
818.444	818.4239	-0.0201	-25	103	109	ICDGILK			Carbamidomethyl (C)[2]		Mascot
907.5247	907.4822	-0.0425	-47	50	57	NLLSVAYK					Mascot
917.5302	917.5153	-0.0149	-16	69	76	IISSIEQK					Mascot
932.4294	932.4268	-0.0026	-3	131	137	MKGDYYR					Mascot
948.4244	948.4129	-0.0115	-12	131	137	MKGDYYR			Oxidation (M)[1]		Mascot
999.4451	999.4496	0.0045	5	10	17	EENVYMAK			Oxidation (M)[6]		Mascot
1144.6321	1144.6272	-0.0049	-4	81	90	GNEDRVTLIK					Mascot
1189.6609	1189.6603	-0.0006	-1	223	232	DSTLIMQLLR					Mascot
1189.6609	1189.6603	-0.0006	-1	223	232	DSTLIMQLLR	22	0			Mascot
1205.6559	1205.6425	-0.0134	-11	223	232	DSTLIMQLLR			Oxidation (M)[6]		Mascot
1205.6559	1205.6425	-0.0134	-11	223	232	DSTLIMQLLR	23	0	Oxidation (M)[6]		Mascot
1212.5565	1212.6031	0.0466	38	150	160	DAAENTMVAYK					Mascot
1228.5514	1228.5819	0.0305	25	150	160	DAAENTMVAYK			Oxidation (M)[7]		Mascot
1366.5542	1366.5227	-0.0315	-23	25	34	YEEMVEFMEK			Oxidation (M)[4,8]		Mascot
1406.6646	1406.6599	-0.0047	-3	38	49	TVDSEELTVEER					Mascot
1406.6646	1406.6599	-0.0047	-3	38	49	TVDSEELTVEER	109	100			Mascot

1418.7485	1418.7394	-0.0091	-6	69	80	IISSIEQKEESR					Mascot
1418.7485	1418.7394	-0.0091	-6	69	80	IISSIEQKEESR	69	99.988			Mascot
2147.9624	2147.9368	-0.0256	-12	18	34	LAEQAERYEEMVEFMEK			Oxidation (M)[11]		Mascot
2163.9573	2163.9104	-0.0469	-22	18	34	LAEQAERYEEMVEFMEK			Oxidation (M)[11,15]		Mascot
2163.9573	2163.9104	-0.0469	-22	18	34	LAEQAERYEEMVEFMEK	8	0	Oxidation (M)[11,15]		Mascot
2331.2019	2331.2019	0	0	178	197	LGLALNFSVFYYEILNSPDR					Mascot

6	14-3-3-like protein GF14 nu OS=Arabidopsis thaliana GN=GRF7 PE=1 SV=1	14337_ARATH	29920	4.74	11	244	100	16.431	192	100	

Peptide Information

Calc. Mass	Obsrv. Mass	± da	± ppm	Start Seq.	End Seq.	Sequence	Ion Score	C. I. %	Modification	Rank	Result Type
816.421	816.4134	-0.0076	-9	14	20	LAEQAER					Mascot
907.5247	907.4822	-0.0425	-47	46	53	NLLSVAYK					Mascot
917.5302	917.5153	-0.0149	-16	65	72	IISSIEQK					Mascot
1189.6609	1189.6603	-0.0006	-1	219	228	DSTLIMQLLR					Mascot
1189.6609	1189.6603	-0.0006	-1	219	228	DSTLIMQLLR	22	0			Mascot
1205.6559	1205.6425	-0.0134	-11	219	228	DSTLIMQLLR			Oxidation (M)[6]		Mascot
1205.6559	1205.6425	-0.0134	-11	219	228	DSTLIMQLLR	23	0	Oxidation (M)[6]		Mascot
1366.5542	1366.5227	-0.0315	-23	21	30	YEEMVEFMEK			Oxidation (M)[4,8]		Mascot
1382.691	1382.6038	-0.0872	-63	2	13	SSSREENVYLAK					Mascot
1406.6646	1406.6599	-0.0047	-3	34	45	TVDTDELTVEER					Mascot
1406.6646	1406.6599	-0.0047	-3	34	45	TVDTDELTVEER	91	100			Mascot
1418.7485	1418.7394	-0.0091	-6	65	76	IISSIEQKEESR					Mascot
1418.7485	1418.7394	-0.0091	-6	65	76	IISSIEQKEESR	69	99.988			Mascot
2147.9624	2147.9368	-0.0256	-12	14	30	LAEQAERYEEMVEFMEK			Oxidation (M)[11]		Mascot
2163.9573	2163.9104	-0.0469	-22	14	30	LAEQAERYEEMVEFMEK			Oxidation (M)[11,15]		Mascot
2163.9573	2163.9104	-0.0469	-22	14	30	LAEQAERYEEMVEFMEK	8	0	Oxidation (M)[11,15]		Mascot
2331.2019	2331.2019	0	0	174	193	LGLALNFSVFYYEILNSPDR					Mascot
2634.2166	2634.3469	0.1303	49	229	252	DNLTLWNSDINDEAGGDEIKEASK					Mascot

7	14-3-3-like protein GF14-D OS=Oryza sativa subsp. japonica GN=GF14D PE=2 SV=1	14334_ORYSJ	29357.6	4.83	10	226	100	11.033	183	100	

Peptide Information

Calc. Mass	Obsrv. Mass	± da	± ppm	Start Seq.	End Seq.	Sequence	Ion Score	C. I. %	Modification	Rank	Result Type
816.421	816.4134	-0.0076	-9	17	23	LAEQAER					Mascot

Calc. Mass	Obsrv. Mass	± da	± ppm	Start Seq.	End Seq.	Sequence	Ion Score	C. I. %	Modification	Rank	Result Type
907.5247	907.4822	-0.0425	-47	53	60	NLLSVAYK					Mascot
917.5302	917.5153	-0.0149	-16	72	79	IISSIEQK					Mascot
1025.5123	1025.5107	-0.0016	-2	84	93	GNDAHAATIR					Mascot
1189.6609	1189.6603	-0.0006	-1	226	235	DSTLIMQLLR					Mascot
1189.6609	1189.6603	-0.0006	-1	226	235	DSTLIMQLLR	22	0			Mascot
1205.6559	1205.6425	-0.0134	-11	226	235	DSTLIMQLLR			Oxidation (M)[6]		Mascot
1205.6559	1205.6425	-0.0134	-11	226	235	DSTLIMQLLR	23	0	Oxidation (M)[6]		Mascot
1388.738	1388.7262	-0.0118	-8	72	83	IISSIEQKEEGR					Mascot
1388.738	1388.7262	-0.0118	-8	72	83	IISSIEQKEEGR	54	99.592			Mascot
1394.5603	1394.526	-0.0343	-25	24	33	YEEMVEYMER			Oxidation (M)[4]		Mascot
1772.9653	1772.9572	-0.0081	-5	164	180	AAQDIALADLAPTHPIR					Mascot
1772.9653	1772.9572	-0.0081	-5	164	180	AAQDIALADLAPTHPIR	106	100			Mascot
2191.9634	2191.9226	-0.0408	-19	17	33	LAEQAERYEEMVEYMER			Oxidation (M)[11]		Mascot
2331.2019	2331.2019	0	0	181	200	LGLALNFSVFYYEILNSPDR					Mascot

8	14-3-3-like protein D OS=Nicotiana tabacum PE=2 SV=1	1433D_TOBAC	28410.3	4.76	5	196	100	30.301	179	100		

Peptide Information

Calc. Mass	Obsrv. Mass	± da	± ppm	Start Seq.	End Seq.	Sequence	Ion Score	C. I. %	Modification	Rank	Result Type
816.421	816.4134	-0.0076	-9	18	24	LAEQAER					Mascot
907.5247	907.4822	-0.0425	-47	50	57	NLLSVAYK					Mascot
932.4294	932.4268	-0.0026	-3	131	137	MKGDYYR					Mascot
948.4244	948.4129	-0.0115	-12	131	137	MKGDYYR			Oxidation (M)[1]		Mascot
1189.6609	1189.6603	-0.0006	-1	223	232	DSTLIMQLLR					Mascot
1189.6609	1189.6603	-0.0006	-1	223	232	DSTLIMQLLR	22	0			Mascot
1205.6559	1205.6425	-0.0134	-11	223	232	DSTLIMQLLR			Oxidation (M)[6]		Mascot
1205.6559	1205.6425	-0.0134	-11	223	232	DSTLIMQLLR	23	0	Oxidation (M)[6]		Mascot
1786.9811	1786.9723	-0.0088	-5	161	177	AAQDIALAELAPTHPIR					Mascot
1786.9811	1786.9723	-0.0088	-5	161	177	AAQDIALAELAPTHPIR	156	100			Mascot

9	14-3-3-like protein GF14-C OS=Oryza sativa subsp. japonica GN=GF14C PE=1 SV=1	14333_ORYSJ	28979.5	4.78	10	189	100	12.262	141	100		

Peptide Information

Calc. Mass	Obsrv. Mass	± da	± ppm	Start Seq.	End Seq.	Sequence	Ion Score	C. I. %	Modification	Rank	Result Type
816.421	816.4134	-0.0076	-9	12	18	LAEQAER					Mascot
818.444	818.4239	-0.0201	-25	97	103	ICDGILK			Carbamidomethyl (C)[2]		Mascot

907.5247	907.4822	-0.0425	-47	44	51	NLLSVAYK					Mascot
999.4451	999.4496	0.0045	5	4	11	EENVYMAK			Oxidation (M)[6]		Mascot
1139.6056	1139.6025	-0.0031	-3	75	84	GNEEHVTLIK					Mascot
1189.6609	1189.6603	-0.0006	-1	217	226	DSTLIMQLLR					Mascot
1189.6609	1189.6603	-0.0006	-1	217	226	DSTLIMQLLR	22	0			Mascot
1205.6559	1205.6425	-0.0134	-11	217	226	DSTLIMQLLR			Oxidation (M)[6]		Mascot
1205.6559	1205.6425	-0.0134	-11	217	226	DSTLIMQLLR	23	0	Oxidation (M)[6]		Mascot
1366.5542	1366.5227	-0.0315	-23	19	28	YEEMVEYMEK			Oxidation (M)[4]		Mascot
1382.5491	1382.6038	0.0547	40	19	28	YEEMVEYMEK			Oxidation (M)[4,8]		Mascot
1418.7009	1418.7394	0.0385	27	32	43	TVDVEELTVEER					Mascot
1418.7009	1418.7394	0.0385	27	32	43	TVDVEELTVEER	11	0			Mascot
1772.9653	1772.9572	-0.0081	-5	155	171	AAQDIALADLAPTHPIR					Mascot
1772.9653	1772.9572	-0.0081	-5	155	171	AAQDIALADLAPTHPIR	106	100			Mascot
2147.9624	2147.9368	-0.0256	-12	12	28	LAEQAERYEEMVEYMEK					Mascot
2163.9573	2163.9104	-0.0469	-22	12	28	LAEQAERYEEMVEYMEK			Oxidation (M)[11]		Mascot
2163.9573	2163.9104	-0.0469	-22	12	28	LAEQAERYEEMVEYMEK	12	0	Oxidation (M)[15]		Mascot
2179.9521	2179.9221	-0.03	-14	12	28	LAEQAERYEEMVEYMEK			Oxidation (M)[11,15]		Mascot

10	14-3-3-like protein A OS=Hordeum vulgare PE=2 SV=1	1433A_HORVU	29447.8	4.83	14	171	100	12.698	100	100	

Peptide Information

Calc. Mass	Obsrv. Mass	± da	± ppm	Start Seq.	End Seq.	Sequence	Ion Score	C. I. %	Modification	Rank	Result Type
816.421	816.4134	-0.0076	-9	17	23	LAEQAER					Mascot
818.444	818.4239	-0.0201	-25	102	108	ICDGILK			Carbamidomethyl (C)[2]		Mascot
882.3986	882.3768	-0.0218	-25	1	8	MSTAEATR			Oxidation (M)[1]		Mascot
907.5247	907.4822	-0.0425	-47	49	56	NLLSVAYK					Mascot
917.5302	917.5153	-0.0149	-16	68	75	IISSIEQK					Mascot
999.4451	999.4496	0.0045	5	9	16	EENVYMAK			Oxidation (M)[6]		Mascot
1051.5419	1051.5254	-0.0165	-16	80	89	GNEAYVASIK					Mascot
1189.6609	1189.6603	-0.0006	-1	222	231	DSTLIMQLLR					Mascot
1189.6609	1189.6603	-0.0006	-1	222	231	DSTLIMQLLR	22	0			Mascot
1205.6559	1205.6425	-0.0134	-11	222	231	DSTLIMQLLR			Oxidation (M)[6]		Mascot
1205.6559	1205.6425	-0.0134	-11	222	231	DSTLIMQLLR	23	0	Oxidation (M)[6]		Mascot
1318.6486	1318.6445	-0.0041	-3	37	48	TADVGELTVEER					Mascot
1366.5542	1366.5227	-0.0315	-23	24	33	YEEMVEFMEK			Oxidation (M)[4,8]		Mascot
1418.7485	1418.7394	-0.0091	-6	68	79	IISSIEQKEESR					Mascot

1418.7485	1418.7394	-0.0091	-6	68	79	IISSIEQKEESR	69	99.988		Mascot
1818.9708	1818.9535	-0.0173	-10	160	176	SAQDIALADLPTTHPIR				Mascot
2147.9624	2147.9368	-0.0256	-12	17	33	LAEQAERYEEMVEFMEK			Oxidation (M)[11]	Mascot
2163.9573	2163.9104	-0.0469	-22	17	33	LAEQAERYEEMVEFMEK			Oxidation (M)[11,15]	Mascot
2163.9573	2163.9104	-0.0469	-22	17	33	LAEQAERYEEMVEFMEK	8	0	Oxidation (M)[11,15]	Mascot
2331.2019	2331.2019	0	0	177	196	LGLALNFSVFYYEILNSPD R				Mascot

Gel Idx/Pos	167/G18	Instr./Gel Origin	BA2151/Sample Project 20140814	Process Status	Analysis Succeeded
Plate [#] Name	[1] Sample Project 20140814	Instrument Sample Name		Spectra	11

Rank	Protein Name	Accession No.	Protein MW	Protein PI	Pep. Count	Protein Score	Protein Score C. I. %	Intensity Matched	Total Ion Score	Total Ion C. I. %	Confirmed
1	14-3-3-like protein A OS=Hordeum vulgare PE=2 SV=1	1433A_HORVU	29447.8	4.83	18	334	100	41.338	206	100	

Peptide Information

Calc. Mass	Obsrv. Mass	± da	± ppm	Start Seq.	End Seq.	Sequence	Ion Score	C. I. %	Modification	Rank	Result Type
816.421	816.4305	0.0095	12	17	23	LAEQAER					Mascot
819.4458	819.4396	-0.0062	-8	95	101	IETELSK					Mascot
906.425	906.4412	0.0162	18	130	136	MKGDYHR					Mascot
907.5247	907.4802	-0.0445	-49	49	56	NLLSVAYK					Mascot
917.5302	917.5102	-0.02	-22	68	75	IISSIEQK					Mascot
922.4199	922.43	0.0101	11	130	136	MKGDYHR			Oxidation (M)[1]		Mascot
922.4199	922.43	0.0101	11	130	136	MKGDYHR			Oxidation (M)[1]		Mascot
928.5324	928.4614	-0.071	-76	125	131	VFYLKMK					Mascot
1051.5419	1051.551	0.0091	9	80	89	GNEAYVASIK					Mascot
1051.5419	1051.551	0.0091	9	80	89	GNEAYVASIK					Mascot
1076.5946	1076.6132	0.0186	17	93	101	TRIETELSK					Mascot
1076.5946	1076.6132	0.0186	17	93	101	TRIETELSK					Mascot
1189.6609	1189.6735	0.0126	11	222	231	DSTLIMQLLR					Mascot
1205.6559	1205.6677	0.0118	10	222	231	DSTLIMQLLR			Oxidation (M)[6]		Mascot
1205.6559	1205.6677	0.0118	10	222	231	DSTLIMQLLR	13	0	Oxidation (M)[6]		Mascot
1208.6157	1208.6475	0.0318	26	149	159	EAAENTLVAYK					Mascot
1318.6486	1318.6693	0.0207	16	37	48	TADVGELTVEER					Mascot
1318.6486	1318.6693	0.0207	16	37	48	TADVGELTVEER	89	100			Mascot
1336.7107	1336.722	0.0113	8	148	159	KEAAENTLVAYK					Mascot
1366.5542	1366.5656	0.0114	8	24	33	YEEMVEFMEK			Oxidation (M)[4,8]		Mascot
1418.7485	1418.7694	0.0209	15	68	79	IISSIEQKEESR					Mascot
1418.7485	1418.7694	0.0209	15	68	79	IISSIEQKEESR	28	0			Mascot
1552.7601	1552.7839	0.0238	15	76	89	EESRGNEAYVASIK					Mascot
1664.7546	1664.8337	0.0791	48	24	36	YEEMVEFMEKVAK			Oxidation (M)[4,8]		Mascot
1818.9708	1818.9923	0.0215	12	160	176	SAQDIALADLPTTHPIR					Mascot
1818.9708	1818.9923	0.0215	12	160	176	SAQDIALADLPTTHPIR	90	100			Mascot
2163.9573	2163.9624	0.0051	2	17	33	LAEQAERYEEMVEFMEK			Oxidation (M)[11,15]		Mascot

2	14-3-3-like protein GF14-F OS=Oryza sativa subsp. japonica GN=GF14F PE=1 SV=2	14336_ORYSJ	29273.7	4.81	16	226	100	32.281	117	100

Peptide Information

Calc. Mass	Obsrv. Mass	± da	± ppm	Start Seq.	End Seq.	Sequence	Ion Score	C. I. %	Modification	Rank	Result Type
816.421	816.4305	0.0095	12	17	23	LAEQAER					Mascot
819.4458	819.4396	-0.0062	-8	95	101	IETELSK					Mascot
906.425	906.4412	0.0162	18	130	136	MKGDYHR					Mascot
907.5247	907.4802	-0.0445	-49	49	56	NLLSVAYK					Mascot
917.5302	917.5102	-0.02	-22	68	75	IISSIEQK					Mascot
922.4199	922.43	0.0101	11	130	136	MKGDYHR			Oxidation (M)[1]		Mascot
922.4199	922.43	0.0101	11	130	136	MKGDYHR			Oxidation (M)[1]		Mascot
928.5324	928.4614	-0.071	-76	125	131	VFYLKMK					Mascot
1051.5419	1051.551	0.0091	9	80	89	GNEAYVASIK					Mascot
1051.5419	1051.551	0.0091	9	80	89	GNEAYVASIK					Mascot
1189.6609	1189.6735	0.0126	11	222	231	DSTLIMQLLR					Mascot
1205.6559	1205.6677	0.0118	10	222	231	DSTLIMQLLR			Oxidation (M)[6]		Mascot
1205.6559	1205.6677	0.0118	10	222	231	DSTLIMQLLR	13	0	Oxidation (M)[6]		Mascot
1208.6157	1208.6475	0.0318	26	149	159	EAAENTLVAYK					Mascot
1336.7107	1336.722	0.0113	8	148	159	KEAAENTLVAYK					Mascot
1366.5542	1366.5656	0.0114	8	24	33	YEEMVEFMEK			Oxidation (M)[4,8]		Mascot
1418.7485	1418.7694	0.0209	15	68	79	IISSIEQKEESR					Mascot
1418.7485	1418.7694	0.0209	15	68	79	IISSIEQKEESR	28	0			Mascot
1552.7601	1552.7839	0.0238	15	76	89	EESRGNEAYVASIK					Mascot
1664.7546	1664.8337	0.0791	48	24	36	YEEMVEFMEKVAK			Oxidation (M)[4,8]		Mascot
1818.9708	1818.9923	0.0215	12	160	176	SAQDIALADLPTTHPIR					Mascot
1818.9708	1818.9923	0.0215	12	160	176	SAQDIALADLPTTHPIR	90	100			Mascot
2163.9573	2163.9624	0.0051	2	17	33	LAEQAERYEEMVEFMEK			Oxidation (M)[11,15]		Mascot

3	14-3-3-like protein B OS=Hordeum vulgare PE=2 SV=1	1433B_HORVU	29786.9	4.67	15	118	100	13.429	28	0

Peptide Information

Calc. Mass	Obsrv. Mass	± da	± ppm	Start Seq.	End Seq.	Sequence	Ion Score	C. I. %	Modification	Rank	Result Type
816.421	816.4305	0.0095	12	18	24	LAEQAER					Mascot
907.5247	907.4802	-0.0445	-49	50	57	NLLSVAYK					Mascot
917.5302	917.5102	-0.02	-22	69	76	IISSIEQK					Mascot
928.5324	928.4614	-0.071	-76	126	132	VFYLKMK					Mascot

948.4244	948.4443	0.0199	21	131	137	MKGDYYR			Oxidation (M)[1]		Mascot
1091.4712	1091.5502	0.079	72	77	85	EESRGNEDR					Mascot
1189.6609	1189.6735	0.0126	11	223	232	DSTLIMQLLR					Mascot
1205.6559	1205.6677	0.0118	10	223	232	DSTLIMQLLR			Oxidation (M)[6]		Mascot
1205.6559	1205.6677	0.0118	10	223	232	DSTLIMQLLR	13	0	Oxidation (M)[6]		Mascot
1212.5565	1212.6271	0.0706	58	150	160	DAAENTMVAYK					Mascot
1228.5514	1228.6252	0.0738	60	150	160	DAAENTMVAYK			Oxidation (M)[7]		Mascot
1340.6515	1340.6578	0.0063	5	149	160	KDAAENTMVAYK					Mascot
1356.6464	1356.6136	-0.0328	-24	149	160	KDAAENTMVAYK			Oxidation (M)[8]		Mascot
1366.5542	1366.5656	0.0114	8	25	34	YEEMVEFMEK			Oxidation (M)[4,8]		Mascot
1406.6646	1406.6847	0.0201	14	38	49	TVDSEELTVEER					Mascot
1418.7485	1418.7694	0.0209	15	69	80	IISSIEQKEESR					Mascot
1418.7485	1418.7694	0.0209	15	69	80	IISSIEQKEESR	28	0			Mascot
1664.7546	1664.8337	0.0791	48	25	37	YEEMVEFMEKVAK			Oxidation (M)[4,8]		Mascot
1827.0123	1827.0353	0.023	13	161	177	AAQEIALAELPPTHPIR					Mascot
2163.9573	2163.9624	0.0051	2	18	34	LAEQAERYEEMVEFMEK			Oxidation (M)[11,15]		Mascot

4	14-3-3-like protein GF14-E OS=Oryza sativa subsp. japonica GN=GF14E PE=2 SV=1	14335_ORYSJ	29844.9	4.71	15	115	100	13.428	28	0

Peptide Information

Calc. Mass	Obsrv. Mass	± da	± ppm	Start Seq.	End Seq.	Sequence	Ion Score	C. I. %	Modification	Rank	Result Type
816.421	816.4305	0.0095	12	18	24	LAEQAER					Mascot
819.4458	819.4396	-0.0062	-8	96	102	IETELSK					Mascot
907.5247	907.4802	-0.0445	-49	50	57	NLLSVAYK					Mascot
917.5302	917.5102	-0.02	-22	69	76	IISSIEQK					Mascot
928.5324	928.4614	-0.071	-76	126	132	VFYLKMK					Mascot
948.4244	948.4443	0.0199	21	131	137	MKGDYYR			Oxidation (M)[1]		Mascot
1091.4712	1091.5502	0.079	72	77	85	EESRGNEDR					Mascot
1189.6609	1189.6735	0.0126	11	223	232	DSTLIMQLLR					Mascot
1205.6559	1205.6677	0.0118	10	223	232	DSTLIMQLLR			Oxidation (M)[6]		Mascot
1205.6559	1205.6677	0.0118	10	223	232	DSTLIMQLLR	13	0	Oxidation (M)[6]		Mascot
1212.5565	1212.6271	0.0706	58	150	160	DAAENTMVAYK					Mascot
1228.5514	1228.6252	0.0738	60	150	160	DAAENTMVAYK			Oxidation (M)[7]		Mascot
1340.6515	1340.6578	0.0063	5	149	160	KDAAENTMVAYK					Mascot
1356.6464	1356.6136	-0.0328	-24	149	160	KDAAENTMVAYK			Oxidation (M)[8]		Mascot
1366.5542	1366.5656	0.0114	8	25	34	YEEMVEFMEK			Oxidation (M)[4,8]		Mascot

Calc. Mass	Obsrv. Mass	± da	± ppm	Start Seq.	End Seq.	Sequence	Ion Score	C. I. %	Modification	Rank	Result Type
1406.6646	1406.6847	0.0201	14	38	49	TVDSEELTVEER					Mascot
1418.7485	1418.7694	0.0209	15	69	80	IISSIEQKEESR					Mascot
1418.7485	1418.7694	0.0209	15	69	80	IISSIEQKEESR	28	0			Mascot
1664.7546	1664.8337	0.0791	48	25	37	YEEMVEFMEKVAK			Oxidation (M)[4,8]		Mascot
2163.9573	2163.9624	0.0051	2	18	34	LAEQAERYEEMVEFMEK			Oxidation (M)[11,15]		Mascot

5	14-3-3-like protein GF14-B OS=Oryza sativa subsp. japonica GN=GF14B PE=1 SV=2	14332_ORYSJ	29959.1	4.76	14	108	100	13.229	28	0

Peptide Information

Calc. Mass	Obsrv. Mass	± da	± ppm	Start Seq.	End Seq.	Sequence	Ion Score	C. I. %	Modification	Rank	Result Type
816.421	816.4305	0.0095	12	18	24	LAEQAER					Mascot
907.5247	907.4802	-0.0445	-49	50	57	NLLSVAYK					Mascot
917.5302	917.5102	-0.02	-22	69	76	IISSIEQK					Mascot
928.5324	928.4614	-0.071	-76	126	132	VFYLKMK					Mascot
948.4244	948.4443	0.0199	21	131	137	MKGDYYR			Oxidation (M)[1]		Mascot
1091.4712	1091.5502	0.079	72	77	85	EESRGNEDR					Mascot
1189.6609	1189.6735	0.0126	11	223	232	DSTLIMQLLR					Mascot
1205.6559	1205.6677	0.0118	10	223	232	DSTLIMQLLR			Oxidation (M)[6]		Mascot
1205.6559	1205.6677	0.0118	10	223	232	DSTLIMQLLR	13	0	Oxidation (M)[6]		Mascot
1212.5565	1212.6271	0.0706	58	150	160	DAAENTMVAYK					Mascot
1228.5514	1228.6252	0.0738	60	150	160	DAAENTMVAYK			Oxidation (M)[7]		Mascot
1340.6515	1340.6578	0.0063	5	149	160	KDAAENTMVAYK					Mascot
1356.6464	1356.6136	-0.0328	-24	149	160	KDAAENTMVAYK			Oxidation (M)[8]		Mascot
1366.5542	1366.5656	0.0114	8	25	34	YEEMVEFMEK			Oxidation (M)[4,8]		Mascot
1406.6646	1406.6847	0.0201	14	38	49	TVDSEELTVEER					Mascot
1418.7485	1418.7694	0.0209	15	69	80	IISSIEQKEESR					Mascot
1418.7485	1418.7694	0.0209	15	69	80	IISSIEQKEESR	28	0			Mascot
1664.7546	1664.8337	0.0791	48	25	37	YEEMVEFMEKVAK			Oxidation (M)[4,8]		Mascot
2163.9573	2163.9624	0.0051	2	18	34	LAEQAERYEEMVEFMEK			Oxidation (M)[11,15]		Mascot

6	14-3-3-like protein OS=Lilium longiflorum PE=2 SV=1	1433_LILLO	29348.8	4.79	13	105	99.998	16.846	28	0

Peptide Information

Calc. Mass	Obsrv. Mass	± da	± ppm	Start Seq.	End Seq.	Sequence	Ion Score	C. I. %	Modification	Rank	Result Type
816.421	816.4305	0.0095	12	17	23	LAEQAER					Mascot
874.4087	874.4389	0.0302	35	1	8	MSPAEPSR					Mascot

Calc. Mass	Obsrv. Mass	± da	± ppm	Start Seq.	End Seq.	Sequence	Ion Score	C. I. %	Modification	Rank	Result Type
906.425	906.4412	0.0162	18	130	136	MKGDYHR					Mascot
907.5247	907.4802	-0.0445	-49	49	56	NLLSVAYK					Mascot
917.5302	917.5102	-0.02	-22	68	75	IISSIEQK					Mascot
922.4199	922.43	0.0101	11	130	136	MKGDYHR			Oxidation (M)[1]		Mascot
922.4199	922.43	0.0101	11	130	136	MKGDYHR			Oxidation (M)[1]		Mascot
928.5324	928.4614	-0.071	-76	125	131	VFYLKMK					Mascot
1189.6609	1189.6735	0.0126	11	222	231	DSTLIMQLLR					Mascot
1205.6559	1205.6677	0.0118	10	222	231	DSTLIMQLLR			Oxidation (M)[6]		Mascot
1205.6559	1205.6677	0.0118	10	222	231	DSTLIMQLLR	13	0	Oxidation (M)[6]		Mascot
1323.7155	1323.6761	-0.0394	-30	148	159	KEAAESTLLAYK					Mascot
1366.5542	1366.5656	0.0114	8	24	33	YEEMVEFMEK			Oxidation (M)[4,8]		Mascot
1418.7485	1418.7694	0.0209	15	68	79	IISSIEQKEESR					Mascot
1418.7485	1418.7694	0.0209	15	68	79	IISSIEQKEESR	28	0			Mascot
1660.771	1660.8722	0.1012	61	24	36	YEEMVEFMEKVAR					Mascot
1676.7659	1676.8392	0.0733	44	24	36	YEEMVEFMEKVAR			Oxidation (M)[4]		Mascot
1838.8412	1838.9473	0.1061	58	1	16	MSPAEPSREENVYMAK					Mascot
2163.9573	2163.9624	0.0051	2	17	33	LAEQAERYEEMVEFMEK			Oxidation (M)[11,15]		Mascot

7	14-3-3-like protein 2 (Fragments) OS=Pseudotsuga menziesii PE=1 SV=1	14332_PSEMZ	7715.8	4.28	7	104	99.998	11.103	28	0

Peptide Information

Calc. Mass	Obsrv. Mass	± da	± ppm	Start Seq.	End Seq.	Sequence	Ion Score	C. I. %	Modification	Rank	Result Type
816.421	816.4305	0.0095	12	1	7	LAEQAER					Mascot
907.5247	907.4802	-0.0445	-49	18	25	NLLSVAYK					Mascot
917.5302	917.5102	-0.02	-22	26	33	IISSIEQK					Mascot
1189.6609	1189.6735	0.0126	11	57	66	DSTLIMQLLR					Mascot
1205.6559	1205.6677	0.0118	10	57	66	DSTLIMQLLR			Oxidation (M)[6]		Mascot
1205.6559	1205.6677	0.0118	10	57	66	DSTLIMQLLR	13	0	Oxidation (M)[6]		Mascot
1366.5542	1366.5656	0.0114	8	8	17	YEEMVEYMEK			Oxidation (M)[4]		Mascot
1418.7485	1418.7694	0.0209	15	26	37	IISSIEQKEESR					Mascot
1418.7485	1418.7694	0.0209	15	26	37	IISSIEQKEESR	28	0			Mascot
2163.9573	2163.9624	0.0051	2	1	17	LAEQAERYEEMVEYMEK			Oxidation (M)[11]		Mascot

8	14-3-3-like protein OS=Helianthus annuus PE=2 SV=1	1433_HELAN	29043.5	4.65	12	100	99.994	12.583	28	0

Peptide Information

Calc. Mass	Obsrv. Mass	± da	± ppm	Start Seq.	End Seq.	Sequence	Ion Score	C. I. %	Modification	Rank	Result Type

Calc. Mass	Obsrv. Mass	± da	± ppm	Start Seq.	End Seq.	Sequence	Ion Score	C. I. %	Modification	Rank	Result Type
806.3825	806.4345	0.052	64	1	8	MAAASSPR			Oxidation (M)[1]		Mascot
816.421	816.4305	0.0095	12	17	23	LAEQAER					Mascot
907.5247	907.4802	-0.0445	-49	50	57	NLLSVAYK					Mascot
917.5302	917.5102	-0.02	-22	69	76	IISSIEQK					Mascot
928.5324	928.4614	-0.071	-76	126	132	VFYLKMK					Mascot
948.4244	948.4443	0.0199	21	131	137	MKGDYYR			Oxidation (M)[1]		Mascot
1189.6609	1189.6735	0.0126	11	223	232	DSTLIMQLLR					Mascot
1205.6559	1205.6677	0.0118	10	223	232	DSTLIMQLLR			Oxidation (M)[6]		Mascot
1205.6559	1205.6677	0.0118	10	223	232	DSTLIMQLLR	13	0	Oxidation (M)[6]		Mascot
1308.7158	1308.6849	-0.0309	-24	149	160	KLAAENTLSAYK					Mascot
1366.5542	1366.5656	0.0114	8	24	33	YEEMVEFMEK			Oxidation (M)[4,8]		Mascot
1418.7485	1418.7694	0.0209	15	69	80	IISSIEQKEESR					Mascot
1418.7485	1418.7694	0.0209	15	69	80	IISSIEQKEESR	28	0			Mascot
1533.8119	1533.8105	-0.0014	-1	110	125	VLDSKLIGSASGGDSK					Mascot
2163.9573	2163.9624	0.0051	2	17	33	LAEQAERYEEMVEFMEK			Oxidation (M)[11,15]		Mascot

9	14-3-3-like protein GF14 chi OS=Arabidopsis thaliana GN=GRF1 PE=1 SV=3	14331_ARATH	30027	4.68	12	95	99.983	16.498	28	0	

Peptide Information

Calc. Mass	Obsrv. Mass	± da	± ppm	Start Seq.	End Seq.	Sequence	Ion Score	C. I. %	Modification	Rank	Result Type
816.421	816.4305	0.0095	12	19	25	LAEQAER					Mascot
906.425	906.4412	0.0162	18	132	138	MKGDYHR					Mascot
907.5247	907.4802	-0.0445	-49	51	58	NLLSVAYK					Mascot
917.5302	917.5102	-0.02	-22	70	77	IISSIEQK					Mascot
922.4199	922.43	0.0101	11	132	138	MKGDYHR			Oxidation (M)[1]		Mascot
922.4199	922.43	0.0101	11	132	138	MKGDYHR			Oxidation (M)[1]		Mascot
928.5324	928.4614	-0.071	-76	127	133	VFYLKMK					Mascot
948.4567	948.4443	-0.0124	-13	1	10	MATPGASSAR					Mascot
1189.6609	1189.6735	0.0126	11	224	233	DSTLIMQLLR					Mascot
1205.6559	1205.6677	0.0118	10	224	233	DSTLIMQLLR			Oxidation (M)[6]		Mascot
1205.6559	1205.6677	0.0118	10	224	233	DSTLIMQLLR	13	0	Oxidation (M)[6]		Mascot
1219.5953	1219.6635	0.0682	56	151	161	DAAEHTLTAYK					Mascot
1366.5542	1366.5656	0.0114	8	26	35	YEEMVEFMEK			Oxidation (M)[4,8]		Mascot
1418.7485	1418.7694	0.0209	15	70	81	IISSIEQKEESR					Mascot
1418.7485	1418.7694	0.0209	15	70	81	IISSIEQKEESR	28	0			Mascot

1664.7546	1664.8337	0.0791	48	26	38	YEEMVEFMEKVAK			Oxidation (M)[4,8]		Mascot
2163.9573	2163.9624	0.0051	2	19	35	LAEQAERYEEMVEFMEK			Oxidation (M)[11,15]		Mascot

10	14-3-3-like protein OS=Mesembryanthemum crystallinum GN=GBF PE=2 SV=1	1433_MESCR	30007.9	4.79	12	94	99.976	16.129	28	0	

Peptide Information

Calc. Mass	Obsrv. Mass	± da	± ppm	Start Seq.	End Seq.	Sequence	Ion Score	C. I. %	Modification	Rank	Result Type
816.421	816.4305	0.0095	12	16	22	LAEQAER					Mascot
819.4458	819.4396	-0.0062	-8	94	100	IETELSK					Mascot
906.425	906.4412	0.0162	18	129	135	MKGDYHR					Mascot
907.5247	907.4802	-0.0445	-49	48	55	NLLSVAYK					Mascot
917.5302	917.5102	-0.02	-22	67	74	IISSIEQK					Mascot
922.4199	922.43	0.0101	11	129	135	MKGDYHR			Oxidation (M)[1]		Mascot
922.4199	922.43	0.0101	11	129	135	MKGDYHR			Oxidation (M)[1]		Mascot
928.5324	928.4614	-0.071	-76	124	130	VFYLKMK					Mascot
1031.464	1031.5134	0.0494	48	256	264	ESGEEKPQQ					Mascot
1189.6609	1189.6735	0.0126	11	221	230	DSTLIMQLLR					Mascot
1205.6559	1205.6677	0.0118	10	221	230	DSTLIMQLLR			Oxidation (M)[6]		Mascot
1205.6559	1205.6677	0.0118	10	221	230	DSTLIMQLLR	13	0	Oxidation (M)[6]		Mascot
1366.5542	1366.5656	0.0114	8	23	32	YEEMVEFMEK			Oxidation (M)[4,8]		Mascot
1418.7485	1418.7694	0.0209	15	67	78	IISSIEQKEESR					Mascot
1418.7485	1418.7694	0.0209	15	67	78	IISSIEQKEESR	28	0			Mascot
1664.7546	1664.8337	0.0791	48	23	35	YEEMVEFMEKVAK			Oxidation (M)[4,8]		Mascot
2163.9573	2163.9624	0.0051	2	16	32	LAEQAERYEEMVEFMEK			Oxidation (M)[11,15]		Mascot

Gel Idx/Pos	168/G19	Instr./Gel Origin	BA2151/Sample Project 20140814	Process Status	Analysis Succeeded
Plate [#] Name	[1] Sample Project 20140814	Instrument Sample Name		Spectra	11

Rank	Protein Name	Accession No.	Protein MW	Protein PI	Pep. Count	Protein Score	Protein Score C. I. %	Intensity Matched	Total Ion Score	Total Ion C. I. %	Confirmed
1	14-3-3-like protein GF14-6 OS=Zea mays GN=GRF1 PE=1 SV=1	14331_MAIZE	29758	4.76	16	483	100	58.969	374	100	

Peptide Information

Calc. Mass	Obsrv. Mass	± da	± ppm	Start Seq.	End Seq.	Sequence	Ion Score	C. I. %	Modification	Rank	Result Type
816.421	816.4236	0.0026	3	17	23	LAEQAER					Mascot
907.5247	907.5185	-0.0062	-7	49	56	NLLSVAYK					Mascot
917.5302	917.5294	-0.0008	-1	68	75	IISSIEQK					Mascot
917.5302	917.5294	-0.0008	-1	68	75	IISSIEQK	43	94.356			Mascot
932.4294	932.4377	0.0083	9	130	136	MKGDYYR					Mascot
948.4244	948.4241	-0.0003	0	130	136	MKGDYYR			Oxidation (M)[1]		Mascot
948.4244	948.4241	-0.0003	0	130	136	MKGDYYR	15	0	Oxidation (M)[1]		Mascot
999.4451	999.4538	0.0087	9	9	16	EENVYMAK			Oxidation (M)[6]		Mascot
1189.6609	1189.6698	0.0089	7	222	231	DSTLIMQLLR					Mascot
1189.6609	1189.6698	0.0089	7	222	231	DSTLIMQLLR	80	99.999			Mascot
1205.6559	1205.6586	0.0027	2	222	231	DSTLIMQLLR			Oxidation (M)[6]		Mascot
1205.6559	1205.6586	0.0027	2	222	231	DSTLIMQLLR	37	77.059	Oxidation (M)[6]		Mascot
1212.5565	1212.5978	0.0413	34	149	159	DAAENTMVAYK					Mascot
1228.5514	1228.6074	0.056	46	149	159	DAAENTMVAYK			Oxidation (M)[7]		Mascot
1366.5542	1366.5485	-0.0057	-4	24	33	YEEMVEFMEK			Oxidation (M)[4,8]		Mascot
1366.5542	1366.5485	-0.0057	-4	24	33	YEEMVEFMEK	1	0	Oxidation (M)[4,8]		Mascot
1388.738	1388.7393	0.0013	1	68	79	IISSIEQKEEGR					Mascot
1406.6646	1406.6774	0.0128	9	37	48	TVDSEELTVEER					Mascot
1406.6646	1406.6774	0.0128	9	37	48	TVDSEELTVEER	94	100			Mascot
1708.9116	1708.8749	-0.0367	-21	109	124	LLETHLVPSSTAPESK					Mascot
1786.9811	1786.9965	0.0154	9	160	176	AAQDIALAELAPTHPIR					Mascot
1786.9811	1786.9965	0.0154	9	160	176	AAQDIALAELAPTHPIR	156	100			Mascot
1828.8568	1829.0045	0.1477	81	1	16	MASAELSREENVYMAK					Mascot
2163.9573	2163.936	-0.0213	-10	17	33	LAEQAERYEEMVEFMEK			Oxidation (M)[11,15]		Mascot
2174.9976	2174.9839	-0.0137	-6	203	221	QAFDEAISELDTLSEESY K					Mascot
2331.2019	2331.2153	0.0134	6	177	196	LGLALNFSVFYYEILNSPD R					Mascot

2	14-3-3-like protein GF14-12 OS=Zea mays GN=GRF2 PE=2 SV=2	14332_MAIZE	29731.9	4.75	14	384	100	48.539	293	100	

Peptide Information

Calc. Mass	Obsrv. Mass	± da	± ppm	Start Seq.	End Seq.	Sequence	Ion Score	C. I. %	Modification	Rank	Result Type
816.421	816.4236	0.0026	3	17	23	LAEQAER					Mascot
907.5247	907.5185	-0.0062	-7	49	56	NLLSVAYK					Mascot
917.5302	917.5294	-0.0008	-1	68	75	IISSIEQK					Mascot
917.5302	917.5294	-0.0008	-1	68	75	IISSIEQK	43	94.356			Mascot
932.4294	932.4377	0.0083	9	130	136	MKGDYYR					Mascot
948.4244	948.4241	-0.0003	0	130	136	MKGDYYR			Oxidation (M)[1]		Mascot
948.4244	948.4241	-0.0003	0	130	136	MKGDYYR	15	0	Oxidation (M)[1]		Mascot
999.4451	999.4538	0.0087	9	9	16	EENVYMAK			Oxidation (M)[6]		Mascot
1212.5565	1212.5978	0.0413	34	149	159	DAAENTMVAYK					Mascot
1228.5514	1228.6074	0.056	46	149	159	DAAENTMVAYK			Oxidation (M)[7]		Mascot
1366.5542	1366.5485	-0.0057	-4	24	33	YEEMVEFMEK			Oxidation (M)[4,8]		Mascot
1366.5542	1366.5485	-0.0057	-4	24	33	YEEMVEFMEK	1	0	Oxidation (M)[4,8]		Mascot
1388.738	1388.7393	0.0013	1	68	79	IISSIEQKEEGR					Mascot
1406.6646	1406.6774	0.0128	9	37	48	TVDSEELTVEER					Mascot
1406.6646	1406.6774	0.0128	9	37	48	TVDSEELTVEER	94	100			Mascot
1786.9811	1786.9965	0.0154	9	160	176	AAQDIALAELAPTHPIR					Mascot
1786.9811	1786.9965	0.0154	9	160	176	AAQDIALAELAPTHPIR	156	100			Mascot
1828.8568	1829.0045	0.1477	81	1	16	MASAELSREENVYMAK					Mascot
2163.9573	2163.936	-0.0213	-10	17	33	LAEQAERYEEMVEFMEK			Oxidation (M)[11,15]		Mascot
2174.9976	2174.9839	-0.0137	-6	203	221	QAFDEAISELDTLSEESYK					Mascot
2331.2019	2331.2153	0.0134	6	177	196	LGLALNFSVFYYEILNSPDR					Mascot

3	14-3-3-like protein GF14-E OS=Oryza sativa subsp. japonica GN=GF14E PE=2 SV=1	14335_ORYSJ	29844.9	4.71	14	305	100	18.856	217	100	

Peptide Information

Calc. Mass	Obsrv. Mass	± da	± ppm	Start Seq.	End Seq.	Sequence	Ion Score	C. I. %	Modification	Rank	Result Type
816.421	816.4236	0.0026	3	18	24	LAEQAER					Mascot
907.5247	907.5185	-0.0062	-7	50	57	NLLSVAYK					Mascot
917.5302	917.5294	-0.0008	-1	69	76	IISSIEQK					Mascot
917.5302	917.5294	-0.0008	-1	69	76	IISSIEQK	43	94.356			Mascot

932.4294	932.4377	0.0083	9	131	137	MKGDYYR					Mascot
948.4244	948.4241	-0.0003	0	131	137	MKGDYYR			Oxidation (M)[1]		Mascot
948.4244	948.4241	-0.0003	0	131	137	MKGDYYR	15	0	Oxidation (M)[1]		Mascot
999.4451	999.4538	0.0087	9	10	17	EENVYMAK			Oxidation (M)[6]		Mascot
1091.4712	1091.5469	0.0757	69	77	85	EESRGNEDR					Mascot
1189.6609	1189.6698	0.0089	7	223	232	DSTLIMQLLR					Mascot
1189.6609	1189.6698	0.0089	7	223	232	DSTLIMQLLR	80	99.999			Mascot
1205.6559	1205.6586	0.0027	2	223	232	DSTLIMQLLR			Oxidation (M)[6]		Mascot
1205.6559	1205.6586	0.0027	2	223	232	DSTLIMQLLR	37	77.059	Oxidation (M)[6]		Mascot
1212.5565	1212.5978	0.0413	34	150	160	DAAENTMVAYK					Mascot
1228.5514	1228.6074	0.056	46	150	160	DAAENTMVAYK			Oxidation (M)[7]		Mascot
1366.5542	1366.5485	-0.0057	-4	25	34	YEEMVEFMEK			Oxidation (M)[4,8]		Mascot
1366.5542	1366.5485	-0.0057	-4	25	34	YEEMVEFMEK	1	0	Oxidation (M)[4,8]		Mascot
1406.6646	1406.6774	0.0128	9	38	49	TVDSEELTVEER					Mascot
1406.6646	1406.6774	0.0128	9	38	49	TVDSEELTVEER	94	100			Mascot
1418.7485	1418.7595	0.011	8	69	80	IISSIEQKEESR					Mascot
2163.9573	2163.936	-0.0213	-10	18	34	LAEQAERYEEMVEFMEK			Oxidation (M)[11,15]		Mascot
2174.9976	2174.9839	-0.0137	-6	204	222	QAFDEAISELDTLSEESYK					Mascot
2331.2019	2331.2153	0.0134	6	178	197	LGLALNFSVFYYEILNSPDR					Mascot

4	14-3-3-like protein GF14-B OS=Oryza sativa subsp. japonica GN=GF14B PE=1 SV=2	14332_ORYSJ	29959.1	4.76	14	303	100	18.856	217	100

Peptide Information

Calc. Mass	Obsrv. Mass	± da	± ppm	Start Seq.	End Seq.	Sequence	Ion Score	C. I. %	Modification	Rank	Result Type
816.421	816.4236	0.0026	3	18	24	LAEQAER					Mascot
907.5247	907.5185	-0.0062	-7	50	57	NLLSVAYK					Mascot
917.5302	917.5294	-0.0008	-1	69	76	IISSIEQK					Mascot
917.5302	917.5294	-0.0008	-1	69	76	IISSIEQK	43	94.356			Mascot
932.4294	932.4377	0.0083	9	131	137	MKGDYYR					Mascot
948.4244	948.4241	-0.0003	0	131	137	MKGDYYR			Oxidation (M)[1]		Mascot
948.4244	948.4241	-0.0003	0	131	137	MKGDYYR	15	0	Oxidation (M)[1]		Mascot
999.4451	999.4538	0.0087	9	10	17	EENVYMAK			Oxidation (M)[6]		Mascot
1091.4712	1091.5469	0.0757	69	77	85	EESRGNEDR					Mascot
1189.6609	1189.6698	0.0089	7	223	232	DSTLIMQLLR					Mascot
1189.6609	1189.6698	0.0089	7	223	232	DSTLIMQLLR	80	99.999			Mascot
1205.6559	1205.6586	0.0027	2	223	232	DSTLIMQLLR			Oxidation (M)[6]		Mascot

Calc. Mass	Obsrv. Mass	± da	± ppm	Start Seq.	End Seq.	Sequence	Ion Score	C. I. %	Modification	Rank	Result Type
1205.6559	1205.6586	0.0027	2	223	232	DSTLIMQLLR	37	77.059	Oxidation (M)[6]		Mascot
1212.5565	1212.5978	0.0413	34	150	160	DAAENTMVAYK					Mascot
1228.5514	1228.6074	0.056	46	150	160	DAAENTMVAYK			Oxidation (M)[7]		Mascot
1366.5542	1366.5485	-0.0057	-4	25	34	YEEMVEFMEK			Oxidation (M)[4,8]		Mascot
1366.5542	1366.5485	-0.0057	-4	25	34	YEEMVEFMEK	1	0	Oxidation (M)[4,8]		Mascot
1406.6646	1406.6774	0.0128	9	38	49	TVDSEELTVEER					Mascot
1406.6646	1406.6774	0.0128	9	38	49	TVDSEELTVEER	94	100			Mascot
1418.7485	1418.7595	0.011	8	69	80	IISSIEQKEESR					Mascot
2163.9573	2163.936	-0.0213	-10	18	34	LAEQAERYEEMVEFMEK			Oxidation (M)[11,15]		Mascot
2174.9976	2174.9839	-0.0137	-6	204	222	QAFDEAISELDTLSEESY K					Mascot
2331.2019	2331.2153	0.0134	6	178	197	LGLALNFSVFYYEILNSPD R					Mascot

5	14-3-3-like protein B OS=Hordeum vulgare PE=2 SV=1	1433B_HORVU	29786.9	4.67	13	293	100	18.674	217	100	

Peptide Information

Calc. Mass	Obsrv. Mass	± da	± ppm	Start Seq.	End Seq.	Sequence	Ion Score	C. I. %	Modification	Rank	Result Type
816.421	816.4236	0.0026	3	18	24	LAEQAER					Mascot
907.5247	907.5185	-0.0062	-7	50	57	NLLSVAYK					Mascot
917.5302	917.5294	-0.0008	-1	69	76	IISSIEQK					Mascot
917.5302	917.5294	-0.0008	-1	69	76	IISSIEQK	43	94.356			Mascot
932.4294	932.4377	0.0083	9	131	137	MKGDYYR					Mascot
948.4244	948.4241	-0.0003	0	131	137	MKGDYYR			Oxidation (M)[1]		Mascot
948.4244	948.4241	-0.0003	0	131	137	MKGDYYR	15	0	Oxidation (M)[1]		Mascot
999.4451	999.4538	0.0087	9	10	17	EENVYMAK			Oxidation (M)[6]		Mascot
1091.4712	1091.5469	0.0757	69	77	85	EESRGNEDR					Mascot
1189.6609	1189.6698	0.0089	7	223	232	DSTLIMQLLR					Mascot
1189.6609	1189.6698	0.0089	7	223	232	DSTLIMQLLR	80	99.999			Mascot
1205.6559	1205.6586	0.0027	2	223	232	DSTLIMQLLR			Oxidation (M)[6]		Mascot
1205.6559	1205.6586	0.0027	2	223	232	DSTLIMQLLR	37	77.059	Oxidation (M)[6]		Mascot
1212.5565	1212.5978	0.0413	34	150	160	DAAENTMVAYK					Mascot
1228.5514	1228.6074	0.056	46	150	160	DAAENTMVAYK			Oxidation (M)[7]		Mascot
1366.5542	1366.5485	-0.0057	-4	25	34	YEEMVEFMEK			Oxidation (M)[4,8]		Mascot
1366.5542	1366.5485	-0.0057	-4	25	34	YEEMVEFMEK	1	0	Oxidation (M)[4,8]		Mascot
1406.6646	1406.6774	0.0128	9	38	49	TVDSEELTVEER					Mascot
1406.6646	1406.6774	0.0128	9	38	49	TVDSEELTVEER	94	100			Mascot
1418.7485	1418.7595	0.011	8	69	80	IISSIEQKEESR					Mascot

2163.9573	2163.936	-0.0213	-10	18	34	LAEQAERYEEMVEFMEK			Oxidation (M)[11,15]		Mascot
2331.2019	2331.2153	0.0134	6	178	197	LGLALNFSVFYYEILNSPD R					Mascot

6	14-3-3-like protein D OS=Nicotiana tabacum PE=2 SV=1	1433D_TOBAC	28410.3	4.76	5	269	100	53.221	237	100	

Peptide Information

Calc. Mass	Obsrv. Mass	± da	± ppm	Start Seq.	End Seq.	Sequence	Ion Score	C. I. %	Modification	Rank	Result Type
816.421	816.4236	0.0026	3	18	24	LAEQAER					Mascot
907.5247	907.5185	-0.0062	-7	50	57	NLLSVAYK					Mascot
932.4294	932.4377	0.0083	9	131	137	MKGDYYR					Mascot
948.4244	948.4241	-0.0003	0	131	137	MKGDYYR			Oxidation (M)[1]		Mascot
948.4244	948.4241	-0.0003	0	131	137	MKGDYYR	15	0	Oxidation (M)[1]		Mascot
1189.6609	1189.6698	0.0089	7	223	232	DSTLIMQLLR					Mascot
1189.6609	1189.6698	0.0089	7	223	232	DSTLIMQLLR	80	99.999			Mascot
1205.6559	1205.6586	0.0027	2	223	232	DSTLIMQLLR			Oxidation (M)[6]		Mascot
1205.6559	1205.6586	0.0027	2	223	232	DSTLIMQLLR	37	77.059	Oxidation (M)[6]		Mascot
1786.9811	1786.9965	0.0154	9	161	177	AAQDIALAELAPTHPIR					Mascot
1786.9811	1786.9965	0.0154	9	161	177	AAQDIALAELAPTHPIR	156	100			Mascot

7	14-3-3-like protein GF14 nu OS=Arabidopsis thaliana GN=GRF7 PE=1 SV=1	14337_ARATH	29920	4.74	10	239	100	17.462	198	100	

Peptide Information

Calc. Mass	Obsrv. Mass	± da	± ppm	Start Seq.	End Seq.	Sequence	Ion Score	C. I. %	Modification	Rank	Result Type
816.421	816.4236	0.0026	3	14	20	LAEQAER					Mascot
907.5247	907.5185	-0.0062	-7	46	53	NLLSVAYK					Mascot
917.5302	917.5294	-0.0008	-1	65	72	IISSIEQK					Mascot
917.5302	917.5294	-0.0008	-1	65	72	IISSIEQK	43	94.356			Mascot
1189.6609	1189.6698	0.0089	7	219	228	DSTLIMQLLR					Mascot
1189.6609	1189.6698	0.0089	7	219	228	DSTLIMQLLR	80	99.999			Mascot
1205.6559	1205.6586	0.0027	2	219	228	DSTLIMQLLR			Oxidation (M)[6]		Mascot
1205.6559	1205.6586	0.0027	2	219	228	DSTLIMQLLR	37	77.059	Oxidation (M)[6]		Mascot
1366.5542	1366.5485	-0.0057	-4	21	30	YEEMVEFMEK			Oxidation (M)[4,8]		Mascot
1366.5542	1366.5485	-0.0057	-4	21	30	YEEMVEFMEK	1	0	Oxidation (M)[4,8]		Mascot
1382.691	1382.5944	-0.0966	-70	2	13	SSSREENVYLAK					Mascot
1406.6646	1406.6774	0.0128	9	34	45	TVDTDELTVEER					Mascot
1406.6646	1406.6774	0.0128	9	34	45	TVDTDELTVEER	75	99.996			Mascot

Calc. Mass	Obsrv. Mass	± da	± ppm	Start Seq.	End Seq.	Sequence	Ion Score	C. I. %	Modification	Rank	Result Type
1418.7485	1418.7595	0.011	8	65	76	IISSIEQKEESR					Mascot
2163.9573	2163.936	-0.0213	-10	14	30	LAEQAERYEEMVEFMEK			Oxidation (M)[11,15]		Mascot
2331.2019	2331.2153	0.0134	6	174	193	LGLALNFSVFYYEILNSPD R					Mascot

8	14-3-3-like protein GF14-D OS=Oryza sativa subsp. japonica GN=GF14D PE=2 SV=1	14334_ORYSJ	29357.6	4.83	10	222	100	14.822	179	100

Peptide Information

Calc. Mass	Obsrv. Mass	± da	± ppm	Start Seq.	End Seq.	Sequence	Ion Score	C. I. %	Modification	Rank	Result Type
816.421	816.4236	0.0026	3	17	23	LAEQAER					Mascot
907.5247	907.5185	-0.0062	-7	53	60	NLLSVAYK					Mascot
917.5302	917.5294	-0.0008	-1	72	79	IISSIEQK					Mascot
917.5302	917.5294	-0.0008	-1	72	79	IISSIEQK	43	94.356			Mascot
1025.5123	1025.5297	0.0174	17	84	93	GNDAHAATIR					Mascot
1112.5219	1112.5162	-0.0057	-5	255	265	EAAAPKEPGDQ					Mascot
1189.6609	1189.6698	0.0089	7	226	235	DSTLIMQLLR					Mascot
1189.6609	1189.6698	0.0089	7	226	235	DSTLIMQLLR	80	99.999			Mascot
1205.6559	1205.6586	0.0027	2	226	235	DSTLIMQLLR			Oxidation (M)[6]		Mascot
1205.6559	1205.6586	0.0027	2	226	235	DSTLIMQLLR	37	77.059	Oxidation (M)[6]		Mascot
1378.5653	1378.6094	0.0441	32	24	33	YEEMVEYMER					Mascot
1388.738	1388.7393	0.0013	1	72	83	IISSIEQKEEGR					Mascot
1394.5603	1394.5698	0.0095	7	24	33	YEEMVEYMER			Oxidation (M)[4]		Mascot
1772.9653	1772.9747	0.0094	5	164	180	AAQDIALADLAPTHPIR					Mascot
1772.9653	1772.9747	0.0094	5	164	180	AAQDIALADLAPTHPIR	56	99.735			Mascot
2331.2019	2331.2153	0.0134	6	181	200	LGLALNFSVFYYEILNSPD R					Mascot

9	14-3-3-like protein RA215 OS=Solanum tuberosum PE=2 SV=1	14332_SOLTU	28691.3	4.78	13	203	100	14.339	123	100

Peptide Information

Calc. Mass	Obsrv. Mass	± da	± ppm	Start Seq.	End Seq.	Sequence	Ion Score	C. I. %	Modification	Rank	Result Type
907.5247	907.5185	-0.0062	-7	45	52	NLLSVAYK					Mascot
917.5302	917.5294	-0.0008	-1	64	71	IISSIEQK					Mascot
917.5302	917.5294	-0.0008	-1	64	71	IISSIEQK	43	94.356			Mascot
932.4294	932.4377	0.0083	9	126	132	MKGDYYR					Mascot
948.4244	948.4241	-0.0003	0	126	132	MKGDYYR			Oxidation (M)[1]		Mascot
948.4244	948.4241	-0.0003	0	126	132	MKGDYYR	15	0	Oxidation (M)[1]		Mascot

1189.6609	1189.6698	0.0089	7	218	227	DSTLIMQLLR					Mascot
1189.6609	1189.6698	0.0089	7	218	227	DSTLIMQLLR	80	99.999			Mascot
1197.6222	1197.6633	0.0411	34	76	86	GNEDHVASIKK					Mascot
1205.6559	1205.6586	0.0027	2	218	227	DSTLIMQLLR			Oxidation (M)[6]		Mascot
1205.6559	1205.6586	0.0027	2	218	227	DSTLIMQLLR	37	77.059	Oxidation (M)[6]		Mascot
1324.6743	1324.6505	-0.0238	-18	144	155	KEAAENTLSAYK					Mascot
1366.5542	1366.5485	-0.0057	-4	21	30	YEEMVEFMEK			Oxidation (M)[4,8]		Mascot
1366.5542	1366.5485	-0.0057	-4	21	30	YEEMVEFMEK	1	0	Oxidation (M)[4,8]		Mascot
1382.637	1382.5944	-0.0426	-31	6	17	EENVYMANVAAR			Oxidation (M)[6]		Mascot
1418.7485	1418.7595	0.011	8	64	75	IISSIEQKEESR					Mascot
1738.8177	1738.8654	0.0477	27	6	20	EENVYMANVAARAER			Oxidation (M)[6]		Mascot
1818.9266	1818.9784	0.0518	28	89	104	SQIENELTSICNGILK			Carbamidomethyl (C)[11]		Mascot
1940.8953	1941.0648	0.1695	87	1	17	MASPREENVYMANVAAR			Oxidation (M)[1,11]		Mascot
2331.2019	2331.2153	0.0134	6	173	192	LGLALNFSVFYYEILNSPDR					Mascot

10	14-3-3-like protein 2 (Fragments) OS=Pseudotsuga menziesii PE=1 SV=1	14332_PSEMZ	7715.8	4.28	7	195	100	14.611	123	100	

Peptide Information

Calc. Mass	Obsrv. Mass	± da	± ppm	Start Seq.	End Seq.	Sequence	Ion Score	C. I. %	Modification	Rank	Result Type
816.421	816.4236	0.0026	3	1	7	LAEQAER					Mascot
907.5247	907.5185	-0.0062	-7	18	25	NLLSVAYK					Mascot
917.5302	917.5294	-0.0008	-1	26	33	IISSIEQK					Mascot
917.5302	917.5294	-0.0008	-1	26	33	IISSIEQK	43	94.356			Mascot
1189.6609	1189.6698	0.0089	7	57	66	DSTLIMQLLR					Mascot
1189.6609	1189.6698	0.0089	7	57	66	DSTLIMQLLR	80	99.999			Mascot
1205.6559	1205.6586	0.0027	2	57	66	DSTLIMQLLR			Oxidation (M)[6]		Mascot
1205.6559	1205.6586	0.0027	2	57	66	DSTLIMQLLR	37	77.059	Oxidation (M)[6]		Mascot
1366.5542	1366.5485	-0.0057	-4	8	17	YEEMVEYMEK			Oxidation (M)[4]		Mascot
1366.5542	1366.5485	-0.0057	-4	8	17	YEEMVEYMEK	8	0	Oxidation (M)[4]		Mascot
1382.5491	1382.5944	0.0453	33	8	17	YEEMVEYMEK			Oxidation (M)[4,8]		Mascot
1418.7485	1418.7595	0.011	8	26	37	IISSIEQKEESR					Mascot
2163.9573	2163.936	-0.0213	-10	1	17	LAEQAERYEEMVEYMEK			Oxidation (M)[11]		Mascot

Gel Idx/Pos	169/G20	Instr./Gel Origin	BA2151/Sample Project 20140814	Process Status	Analysis Succeeded
Plate [#] Name	[1] Sample Project 20140814	Instrument Sample Name		Spectra	11

Rank	Protein Name	Accession No.	Protein MW	Protein PI	Pep. Count	Protein Score	Protein Score C. I. %	Intensity Matched	Total Ion Score	Total Ion C. I. %	Confirmed
1	14-3-3-like protein GF14-6 OS=Zea mays GN=GRF1 PE=1 SV=1	14331_MAIZE	29758	4.76	17	418	100	63.572	304	100	

Peptide Information

Calc. Mass	Obsrv. Mass	± da	± ppm	Start Seq.	End Seq.	Sequence	Ion Score	C. I. %	Modification	Rank	Result Type
816.421	816.4315	0.0105	13	17	23	LAEQAER					Mascot
818.444	818.4407	-0.0033	-4	102	108	ICDGILK			Carbamidomethyl (C)[2]		Mascot
907.5247	907.5327	0.008	9	49	56	NLLSVAYK					Mascot
917.5302	917.538	0.0078	9	68	75	IISSIEQK					Mascot
917.5302	917.538	0.0078	9	68	75	IISSIEQK	25	0			Mascot
932.4294	932.4701	0.0407	44	130	136	MKGDYYR					Mascot
948.4244	948.4344	0.01	11	130	136	MKGDYYR			Oxidation (M)[1]		Mascot
948.4244	948.4344	0.01	11	130	136	MKGDYYR	15	0	Oxidation (M)[1]		Mascot
999.4451	999.4573	0.0122	12	9	16	EENVYMAK			Oxidation (M)[6]		Mascot
1144.6321	1144.6542	0.0221	19	80	89	GNEDRVTLIK					Mascot
1189.6609	1189.678	0.0171	14	222	231	DSTLIMQLLR					Mascot
1205.6559	1205.6694	0.0135	11	222	231	DSTLIMQLLR			Oxidation (M)[6]		Mascot
1205.6559	1205.6694	0.0135	11	222	231	DSTLIMQLLR	18	0	Oxidation (M)[6]		Mascot
1212.5565	1212.6193	0.0628	52	149	159	DAAENTMVAYK					Mascot
1228.5514	1228.6051	0.0537	44	149	159	DAAENTMVAYK			Oxidation (M)[7]		Mascot
1366.5542	1366.5502	-0.004	-3	24	33	YEEMVEFMEK			Oxidation (M)[4,8]		Mascot
1388.738	1388.7384	0.0004	0	68	79	IISSIEQKEEGR					Mascot
1406.6646	1406.6904	0.0258	18	37	48	TVDSEELTVEER					Mascot
1406.6646	1406.6904	0.0258	18	37	48	TVDSEELTVEER	101	100			Mascot
1708.9116	1708.9102	-0.0014	-1	109	124	LLETHLVPSSTAPESK					Mascot
1786.9811	1787.0148	0.0337	19	160	176	AAQDIALAELAPTHPIR					Mascot
1786.9811	1787.0148	0.0337	19	160	176	AAQDIALAELAPTHPIR	146	100			Mascot
2163.9573	2163.9636	0.0063	3	17	33	LAEQAERYEEMVEFMEK			Oxidation (M)[11,15]		Mascot
2163.9573	2163.9636	0.0063	3	17	33	LAEQAERYEEMVEFMEK	13	0	Oxidation (M)[11,15]		Mascot
2174.9976	2175.0115	0.0139	6	203	221	QAFDEAISELDTLSEESY K					Mascot
2331.2019	2331.2488	0.0469	20	177	196	LGLALNFSVFYYEILNSPD R					Mascot

2	14-3-3-like protein GF14-12 OS=Zea mays GN=GRF2 PE=2 SV=2	14332_MAIZE	29731.9	4.75	15	382	100	57.527	286	100

Peptide Information

Calc. Mass	Obsrv. Mass	± da	± ppm	Start Seq.	End Seq.	Sequence	Ion Score	C. I. %	Modification	Rank	Result Type
816.421	816.4315	0.0105	13	17	23	LAEQAER					Mascot
818.444	818.4407	-0.0033	-4	102	108	ICDGILK			Carbamidomethyl (C)[2]		Mascot
907.5247	907.5327	0.008	9	49	56	NLLSVAYK					Mascot
917.5302	917.538	0.0078	9	68	75	IISSIEQK					Mascot
917.5302	917.538	0.0078	9	68	75	IISSIEQK	25	0			Mascot
932.4294	932.4701	0.0407	44	130	136	MKGDYYR					Mascot
948.4244	948.4344	0.01	11	130	136	MKGDYYR			Oxidation (M)[1]		Mascot
948.4244	948.4344	0.01	11	130	136	MKGDYYR	15	0	Oxidation (M)[1]		Mascot
999.4451	999.4573	0.0122	12	9	16	EENVYMAK			Oxidation (M)[6]		Mascot
1144.6321	1144.6542	0.0221	19	80	89	GNEDRVTLIK					Mascot
1212.5565	1212.6193	0.0628	52	149	159	DAAENTMVAYK					Mascot
1228.5514	1228.6051	0.0537	44	149	159	DAAENTMVAYK			Oxidation (M)[7]		Mascot
1366.5542	1366.5502	-0.004	-3	24	33	YEEMVEFMEK			Oxidation (M)[4,8]		Mascot
1388.738	1388.7384	0.0004	0	68	79	IISSIEQKKEEGR					Mascot
1406.6646	1406.6904	0.0258	18	37	48	TVDSEELTVEER					Mascot
1406.6646	1406.6904	0.0258	18	37	48	TVDSEELTVEER	101	100			Mascot
1786.9811	1787.0148	0.0337	19	160	176	AAQDIALAELAPTHPIR					Mascot
1786.9811	1787.0148	0.0337	19	160	176	AAQDIALAELAPTHPIR	146	100			Mascot
2163.9573	2163.9636	0.0063	3	17	33	LAEQAERYEEMVEFMEK			Oxidation (M)[11,15]		Mascot
2163.9573	2163.9636	0.0063	3	17	33	LAEQAERYEEMVEFMEK	13	0	Oxidation (M)[11,15]		Mascot
2174.9976	2175.0115	0.0139	6	203	221	QAFDEAISELDTLSEESYK					Mascot
2331.2019	2331.2488	0.0469	20	177	196	LGLALNFSVFYYEILNSPDR					Mascot

3	14-3-3-like protein GF14-E OS=Oryza sativa subsp. japonica GN=GF14E PE=2 SV=1	14335_ORYSJ	29844.9	4.71	17	300	100	22.977	190	100

Peptide Information

Calc. Mass	Obsrv. Mass	± da	± ppm	Start Seq.	End Seq.	Sequence	Ion Score	C. I. %	Modification	Rank	Result Type
816.421	816.4315	0.0105	13	18	24	LAEQAER					Mascot
818.444	818.4407	-0.0033	-4	103	109	ICDGILK			Carbamidomethyl (C)[2]		Mascot
819.4458	819.4365	-0.0093	-11	96	102	IETELSK					Mascot

Calc. Mass	Obsrv. Mass	± da	± ppm	Start Seq.	End Seq.	Sequence	Ion Score	C. I. %	Modification	Rank	Result Type
907.5247	907.5327	0.008	9	50	57	NLLSVAYK					Mascot
917.5302	917.538	0.0078	9	69	76	IISSIEQK					Mascot
917.5302	917.538	0.0078	9	69	76	IISSIEQK	25	0			Mascot
932.4294	932.4701	0.0407	44	131	137	MKGDYYR					Mascot
948.4244	948.4344	0.01	11	131	137	MKGDYYR			Oxidation (M)[1]		Mascot
948.4244	948.4344	0.01	11	131	137	MKGDYYR	15	0	Oxidation (M)[1]		Mascot
999.4451	999.4573	0.0122	12	10	17	EENVYMAK			Oxidation (M)[6]		Mascot
1004.5622	1004.5597	-0.0025	-2	94	102	GKIETELSK					Mascot
1091.4712	1091.5339	0.0627	57	77	85	EESRGNEDR					Mascot
1189.6609	1189.678	0.0171	14	223	232	DSTLIMQLLR					Mascot
1205.6559	1205.6694	0.0135	11	223	232	DSTLIMQLLR			Oxidation (M)[6]		Mascot
1205.6559	1205.6694	0.0135	11	223	232	DSTLIMQLLR	18	0	Oxidation (M)[6]		Mascot
1212.5565	1212.6193	0.0628	52	150	160	DAAENTMVAYK					Mascot
1228.5514	1228.6051	0.0537	44	150	160	DAAENTMVAYK			Oxidation (M)[7]		Mascot
1366.5542	1366.5502	-0.004	-3	25	34	YEEMVEFMEK			Oxidation (M)[4,8]		Mascot
1406.6646	1406.6904	0.0258	18	38	49	TVDSEELTVEER					Mascot
1406.6646	1406.6904	0.0258	18	38	49	TVDSEELTVEER	101	100			Mascot
1418.7485	1418.777	0.0285	20	69	80	IISSIEQKEESR					Mascot
1418.7485	1418.777	0.0285	20	69	80	IISSIEQKEESR	33	45.081			Mascot
2163.9573	2163.9636	0.0063	3	18	34	LAEQAERYEEMVEFMEK			Oxidation (M)[11,15]		Mascot
2163.9573	2163.9636	0.0063	3	18	34	LAEQAERYEEMVEFMEK	13	0	Oxidation (M)[11,15]		Mascot
2174.9976	2175.0115	0.0139	6	204	222	QAFDEAISELDTLSEESYK					Mascot
2331.2595	2331.2488	-0.0107	-5	110	130	LLDSHLVPSSTAPESKVFYLK					Mascot

4	14-3-3-like protein GF14-B OS=Oryza sativa subsp. japonica GN=GF14B PE=1 SV=2	14332_ORYSJ	29959.1	4.76	16	292	100	23.018	191	100

Peptide Information

Calc. Mass	Obsrv. Mass	± da	± ppm	Start Seq.	End Seq.	Sequence	Ion Score	C. I. %	Modification	Rank	Result Type
816.421	816.4315	0.0105	13	18	24	LAEQAER					Mascot
818.444	818.4407	-0.0033	-4	103	109	ICDGILK			Carbamidomethyl (C)[2]		Mascot
907.5247	907.5327	0.008	9	50	57	NLLSVAYK					Mascot
917.5302	917.538	0.0078	9	69	76	IISSIEQK					Mascot
917.5302	917.538	0.0078	9	69	76	IISSIEQK	25	0			Mascot
932.4294	932.4701	0.0407	44	131	137	MKGDYYR					Mascot
948.4244	948.4344	0.01	11	131	137	MKGDYYR			Oxidation (M)[1]		Mascot
948.4244	948.4344	0.01	11	131	137	MKGDYYR	15	0	Oxidation (M)[1]		Mascot

Calc. Mass	Obsrv. Mass	± da	± ppm	Start Seq.	End Seq.	Sequence	Ion Score	C. I. %	Modification	Rank	Result Type
999.4451	999.4573	0.0122	12	10	17	EENVYMAK			Oxidation (M)[6]		Mascot
1091.4712	1091.5339	0.0627	57	77	85	EESRGNEDR					Mascot
1144.6321	1144.6542	0.0221	19	81	90	GNEDRVTLIK					Mascot
1189.6609	1189.678	0.0171	14	223	232	DSTLIMQLLR					Mascot
1205.6559	1205.6694	0.0135	11	223	232	DSTLIMQLLR			Oxidation (M)[6]		Mascot
1205.6559	1205.6694	0.0135	11	223	232	DSTLIMQLLR	18	0	Oxidation (M)[6]		Mascot
1212.5565	1212.6193	0.0628	52	150	160	DAAENTMVAYK					Mascot
1228.5514	1228.6051	0.0537	44	150	160	DAAENTMVAYK			Oxidation (M)[7]		Mascot
1366.5542	1366.5502	-0.004	-3	25	34	YEEMVEFMEK			Oxidation (M)[4,8]		Mascot
1406.6646	1406.6904	0.0258	18	38	49	TVDSEELTVEER					Mascot
1406.6646	1406.6904	0.0258	18	38	49	TVDSEELTVEER	101	100			Mascot
1418.7485	1418.777	0.0285	20	69	80	IISSIEQKEESR					Mascot
1418.7485	1418.777	0.0285	20	69	80	IISSIEQKEESR	33	45.081			Mascot
2163.9573	2163.9636	0.0063	3	18	34	LAEQAERYEEMVEFMEK			Oxidation (M)[11,15]		Mascot
2163.9573	2163.9636	0.0063	3	18	34	LAEQAERYEEMVEFMEK	13	0	Oxidation (M)[11,15]		Mascot
2174.9976	2175.0115	0.0139	6	204	222	QAFDEAISELDTLSEESY K					Mascot
2331.2019	2331.2488	0.0469	20	178	197	LGLALNFSVFYYEILNSPD R					Mascot

5	14-3-3-like protein B OS=Hordeum vulgare PE=2 SV=1	1433B_HORVU	29786.9	4.67	16	291	100	23.077	191	100		

Peptide Information

Calc. Mass	Obsrv. Mass	± da	± ppm	Start Seq.	End Seq.	Sequence	Ion Score	C. I. %	Modification	Rank	Result Type
816.421	816.4315	0.0105	13	18	24	LAEQAER					Mascot
818.444	818.4407	-0.0033	-4	103	109	ICDGILK			Carbamidomethyl (C)[2]		Mascot
907.5247	907.5327	0.008	9	50	57	NLLSVAYK					Mascot
917.5302	917.538	0.0078	9	69	76	IISSIEQK					Mascot
917.5302	917.538	0.0078	9	69	76	IISSIEQK	25	0			Mascot
932.4294	932.4701	0.0407	44	131	137	MKGDYYR					Mascot
948.4244	948.4344	0.01	11	131	137	MKGDYYR			Oxidation (M)[1]		Mascot
948.4244	948.4344	0.01	11	131	137	MKGDYYR	15	0	Oxidation (M)[1]		Mascot
999.4451	999.4573	0.0122	12	10	17	EENVYMAK			Oxidation (M)[6]		Mascot
1091.4712	1091.5339	0.0627	57	77	85	EESRGNEDR					Mascot
1144.6321	1144.6542	0.0221	19	81	90	GNEDRVTLIK					Mascot
1189.6609	1189.678	0.0171	14	223	232	DSTLIMQLLR					Mascot
1205.6559	1205.6694	0.0135	11	223	232	DSTLIMQLLR			Oxidation (M)[6]		Mascot
1205.6559	1205.6694	0.0135	11	223	232	DSTLIMQLLR	18	0	Oxidation (M)[6]		Mascot

1212.5565	1212.6193	0.0628	52	150	160	DAAENTMVAYK					Mascot
1228.5514	1228.6051	0.0537	44	150	160	DAAENTMVAYK			Oxidation (M)[7]		Mascot
1366.5542	1366.5502	-0.004	-3	25	34	YEEMVEFMEK			Oxidation (M)[4,8]		Mascot
1406.6646	1406.6904	0.0258	18	38	49	TVDSEELTVEER					Mascot
1406.6646	1406.6904	0.0258	18	38	49	TVDSEELTVEER	101	100			Mascot
1418.7485	1418.777	0.0285	20	69	80	IISSIEQKEESR					Mascot
1418.7485	1418.777	0.0285	20	69	80	IISSIEQKEESR	33	45.081			Mascot
1827.0123	1827.0242	0.0119	7	161	177	AAQEIALAELPPTHPIR					Mascot
2163.9573	2163.9636	0.0063	3	18	34	LAEQAERYEEMVEFMEK			Oxidation (M)[11,15]		Mascot
2163.9573	2163.9636	0.0063	3	18	34	LAEQAERYEEMVEFMEK	13	0	Oxidation (M)[11,15]		Mascot
2331.2595	2331.2488	-0.0107	-5	110	130	LLDSHLVPSSTAPESKVF YLK					Mascot

6	14-3-3-like protein GF14 nu OS=Arabidopsis thaliana GN=GRF7 PE=1 SV=1	14337_ARATH	29920	4.74	12	231	100	20.66	174	100	

Peptide Information

Calc. Mass	Obsrv. Mass	± da	± ppm	Start Seq.	End Seq.	Sequence	Ion Score	C. I. %	Modification	Rank	Result Type
816.421	816.4315	0.0105	13	14	20	LAEQAER					Mascot
819.4458	819.4365	-0.0093	-11	92	98	IETELSK					Mascot
907.5247	907.5327	0.008	9	46	53	NLLSVAYK					Mascot
917.5302	917.538	0.0078	9	65	72	IISSIEQK					Mascot
917.5302	917.538	0.0078	9	65	72	IISSIEQK	25	0			Mascot
1004.5622	1004.5597	-0.0025	-2	90	98	GKIETELSK					Mascot
1189.6609	1189.678	0.0171	14	219	228	DSTLIMQLLR					Mascot
1205.6559	1205.6694	0.0135	11	219	228	DSTLIMQLLR			Oxidation (M)[6]		Mascot
1205.6559	1205.6694	0.0135	11	219	228	DSTLIMQLLR	18	0	Oxidation (M)[6]		Mascot
1366.5542	1366.5502	-0.004	-3	21	30	YEEMVEFMEK			Oxidation (M)[4,8]		Mascot
1406.6646	1406.6904	0.0258	18	34	45	TVDTDELTVEER					Mascot
1406.6646	1406.6904	0.0258	18	34	45	TVDTDELTVEER	84	100			Mascot
1418.7485	1418.777	0.0285	20	65	76	IISSIEQKEESR					Mascot
1418.7485	1418.777	0.0285	20	65	76	IISSIEQKEESR	33	45.081			Mascot
2163.9573	2163.9636	0.0063	3	14	30	LAEQAERYEEMVEFMEK			Oxidation (M)[11,15]		Mascot
2163.9573	2163.9636	0.0063	3	14	30	LAEQAERYEEMVEFMEK	13	0	Oxidation (M)[11,15]		Mascot
2331.2019	2331.2488	0.0469	20	174	193	LGLALNFSVFYYEILNSPD R					Mascot
2775.303	2775.3718	0.0688	25	194	218	ACSLAKQAFDEAISELDT LGEESYK			Carbamidomethyl (C)[2]		Mascot

7	14-3-3-like protein D OS=Nicotiana tabacum PE=2 SV=1	1433D_TOBAC	28410.3	4.76	5	195	100	51.118	164	100	

Peptide Information

Calc. Mass	Obsrv. Mass	± da	± ppm	Start Seq.	End Seq.	Sequence	Ion Score	C. I. %	Modification	Rank	Result Type
816.421	816.4315	0.0105	13	18	24	LAEQAER					Mascot
907.5247	907.5327	0.008	9	50	57	NLLSVAYK					Mascot
932.4294	932.4701	0.0407	44	131	137	MKGDYYR					Mascot
948.4244	948.4344	0.01	11	131	137	MKGDYYR			Oxidation (M)[1]		Mascot
948.4244	948.4344	0.01	11	131	137	MKGDYYR	15	0	Oxidation (M)[1]		Mascot
1189.6609	1189.678	0.0171	14	223	232	DSTLIMQLLR					Mascot
1205.6559	1205.6694	0.0135	11	223	232	DSTLIMQLLR			Oxidation (M)[6]		Mascot
1205.6559	1205.6694	0.0135	11	223	232	DSTLIMQLLR	18	0	Oxidation (M)[6]		Mascot
1786.9811	1787.0148	0.0337	19	161	177	AAQDIALAELAPTHPIR					Mascot
1786.9811	1787.0148	0.0337	19	161	177	AAQDIALAELAPTHPIR	146	100			Mascot

8	14-3-3-like protein OS=Lilium longiflorum PE=2 SV=1	1433_LILLO	29348.8	4.79	13	156	100	13.894	90	100	

Peptide Information

Calc. Mass	Obsrv. Mass	± da	± ppm	Start Seq.	End Seq.	Sequence	Ion Score	C. I. %	Modification	Rank	Result Type
816.421	816.4315	0.0105	13	17	23	LAEQAER					Mascot
818.444	818.4407	-0.0033	-4	102	108	ICDGILK			Carbamidomethyl (C)[2]		Mascot
907.5247	907.5327	0.008	9	49	56	NLLSVAYK					Mascot
917.5302	917.538	0.0078	9	68	75	IISSIEQK					Mascot
917.5302	917.538	0.0078	9	68	75	IISSIEQK	25	0			Mascot
999.4451	999.4573	0.0122	12	9	16	EENVYMAK			Oxidation (M)[6]		Mascot
1189.6609	1189.678	0.0171	14	222	231	DSTLIMQLLR					Mascot
1205.6559	1205.6694	0.0135	11	222	231	DSTLIMQLLR			Oxidation (M)[6]		Mascot
1205.6559	1205.6694	0.0135	11	222	231	DSTLIMQLLR	18	0	Oxidation (M)[6]		Mascot
1323.7155	1323.6851	-0.0304	-23	148	159	KEAAESTLLAYK					Mascot
1366.5542	1366.5502	-0.004	-3	24	33	YEEMVEFMEK			Oxidation (M)[4,8]		Mascot
1418.7485	1418.777	0.0285	20	68	79	IISSIEQKEESR					Mascot
1418.7485	1418.777	0.0285	20	68	79	IISSIEQKEESR	33	45.081			Mascot
1707.8007	1707.8818	0.0811	47	2	16	SPAEPSREENVYMAK					Mascot
1802.976	1803.0045	0.0285	16	160	176	SAQDIALAELAPTHPIR					Mascot
2163.9573	2163.9636	0.0063	3	17	33	LAEQAERYEEMVEFMEK			Oxidation (M)[11,15]		Mascot
2163.9573	2163.9636	0.0063	3	17	33	LAEQAERYEEMVEFMEK	13	0	Oxidation (M)[11,15]		Mascot
2331.2595	2331.2488	-0.0107	-5	109	129	LLDSHLVPSSTAPESKVF YLK					Mascot

9	14-3-3-like protein A OS=Hordeum vulgare PE=2 SV=1	1433A_HORVU	29447.8	4.83	13	154	100	13.784	90	100	

Peptide Information

Calc. Mass	Obsrv. Mass	± da	± ppm	Start Seq.	End Seq.	Sequence	Ion Score	C. I. %	Modification	Rank	Result Type
816.421	816.4315	0.0105	13	17	23	LAEQAER					Mascot
818.444	818.4407	-0.0033	-4	102	108	ICDGILK			Carbamidomethyl (C)[2]		Mascot
819.4458	819.4365	-0.0093	-11	95	101	IETELSK					Mascot
907.5247	907.5327	0.008	9	49	56	NLLSVAYK					Mascot
917.5302	917.538	0.0078	9	68	75	IISSIEQK					Mascot
917.5302	917.538	0.0078	9	68	75	IISSIEQK	25	0			Mascot
999.4451	999.4573	0.0122	12	9	16	EENVYMAK			Oxidation (M)[6]		Mascot
1189.6609	1189.678	0.0171	14	222	231	DSTLIMQLLR					Mascot
1205.6559	1205.6694	0.0135	11	222	231	DSTLIMQLLR			Oxidation (M)[6]		Mascot
1205.6559	1205.6694	0.0135	11	222	231	DSTLIMQLLR	18	0	Oxidation (M)[6]		Mascot
1366.5542	1366.5502	-0.004	-3	24	33	YEEMVEFMEK			Oxidation (M)[4,8]		Mascot
1418.7485	1418.777	0.0285	20	68	79	IISSIEQKEESR					Mascot
1418.7485	1418.777	0.0285	20	68	79	IISSIEQKEESR	33	45.081			Mascot
1818.9708	1818.988	0.0172	9	160	176	SAQDIALADLPTTHPIR					Mascot
1846.8309	1846.9272	0.0963	52	1	16	MSTAEATREENVYMAK			Oxidation (M)[1]		Mascot
2163.9573	2163.9636	0.0063	3	17	33	LAEQAERYEEMVEFMEK			Oxidation (M)[11,15]		Mascot
2163.9573	2163.9636	0.0063	3	17	33	LAEQAERYEEMVEFMEK	13	0	Oxidation (M)[11,15]		Mascot
2331.2019	2331.2488	0.0469	20	177	196	LGLALNFSVFYYEILNSPDR					Mascot

10	14-3-3-like protein 2 (Fragments) OS=Pseudotsuga menziesii PE=1 SV=1	14332_PSEMZ	7715.8	4.28	7	149	100	13.246	86	100	

Peptide Information

Calc. Mass	Obsrv. Mass	± da	± ppm	Start Seq.	End Seq.	Sequence	Ion Score	C. I. %	Modification	Rank	Result Type
816.421	816.4315	0.0105	13	1	7	LAEQAER					Mascot
907.5247	907.5327	0.008	9	18	25	NLLSVAYK					Mascot
917.5302	917.538	0.0078	9	26	33	IISSIEQK					Mascot
917.5302	917.538	0.0078	9	26	33	IISSIEQK	25	0			Mascot
1189.6609	1189.678	0.0171	14	57	66	DSTLIMQLLR					Mascot
1205.6559	1205.6694	0.0135	11	57	66	DSTLIMQLLR			Oxidation (M)[6]		Mascot
1205.6559	1205.6694	0.0135	11	57	66	DSTLIMQLLR	18	0	Oxidation (M)[6]		Mascot
1366.5542	1366.5502	-0.004	-3	8	17	YEEMVEYMEK			Oxidation (M)[4]		Mascot

1418.7485	1418.777	0.0285	20	26	37	IISSIEQKEESR				Mascot
1418.7485	1418.777	0.0285	20	26	37	IISSIEQKEESR	33	56.944		Mascot
2163.9573	2163.9636	0.0063	3	1	17	LAEQAERYEEMVEYMEK			Oxidation (M)[11]	Mascot
2163.9573	2163.9636	0.0063	3	1	17	LAEQAERYEEMVEYMEK	10	0	Oxidation (M)[15]	Mascot
2179.9521	2179.9736	0.0215	10	1	17	LAEQAERYEEMVEYMEK			Oxidation (M)[11,15]	Mascot

Gel Idx/Pos	170/G21	Instr./Gel Origin	BA2151/Sample Project 20140814	Process Status	Analysis Succeeded
Plate [#] Name	[1] Sample Project 20140814	Instrument Sample Name		Spectra	11

Rank	Protein Name	Accession No.	Protein MW	Protein PI	Pep. Count	Protein Score	Protein Score C. I. %	Intensity Matched	Total Ion Score	Total Ion C. I. %	Confirmed
1	14-3-3-like protein B OS=Hordeum vulgare PE=2 SV=1	1433B_HORVU	29786.9	4.67	14	87	99.887	11.057			

Peptide Information

Calc. Mass	Obsrv. Mass	± da	± ppm	Start Seq.	End Seq.	Sequence	Ion Score	C. I. %	Modification	Rank	Result Type
816.421	816.4322	0.0112	14	18	24	LAEQAER					Mascot
818.444	818.4481	0.0041	5	103	109	ICDGILK			Carbamidomethyl (C)[2]		Mascot
907.5247	907.5406	0.0159	18	50	57	NLLSVAYK					Mascot
917.5302	917.5402	0.01	11	69	76	IISSIEQK					Mascot
932.4294	932.4441	0.0147	16	131	137	MKGDYYR					Mascot
948.4244	948.4377	0.0133	14	131	137	MKGDYYR			Oxidation (M)[1]		Mascot
999.4451	999.462	0.0169	17	10	17	EENVYMAK			Oxidation (M)[6]		Mascot
1016.5986	1016.6001	0.0015	1	94	102	GKIEVELTK					Mascot
1189.6609	1189.6863	0.0254	21	223	232	DSTLIMQLLR					Mascot
1189.6609	1189.6863	0.0254	21	223	232	DSTLIMQLLR					Mascot
1205.6559	1205.6742	0.0183	15	223	232	DSTLIMQLLR			Oxidation (M)[6]		Mascot
1366.5542	1366.5648	0.0106	8	25	34	YEEMVEFMEK			Oxidation (M)[4,8]		Mascot
1406.6646	1406.691	0.0264	19	38	49	TVDSEELTVEER					Mascot
1406.6646	1406.691	0.0264	19	38	49	TVDSEELTVEER					Mascot
1418.7485	1418.7678	0.0193	14	69	80	IISSIEQKEESR					Mascot
1827.0123	1827.0411	0.0288	16	161	177	AAQEIALAELPPTHPIR					Mascot
2131.9675	2132.0005	0.033	15	18	34	LAEQAERYEEMVEFMEK					Mascot
2163.9573	2163.9697	0.0124	6	18	34	LAEQAERYEEMVEFMEK			Oxidation (M)[11,15]		Mascot
2331.2019	2331.2139	0.012	5	178	197	LGLALNFSVFYYEILNSPDR					Mascot

Rank	Protein Name	Accession No.	Protein MW	Protein PI	Pep. Count	Protein Score	Protein Score C. I. %	Intensity Matched	Total Ion Score	Total Ion C. I. %	Confirmed
2	14-3-3-like protein 2 (Fragments) OS=Pseudotsuga menziesii PE=1 SV=1	14332_PSEMZ	7715.8	4.28	7	72	96.959	7.445			

Peptide Information

Calc. Mass	Obsrv. Mass	± da	± ppm	Start Seq.	End Seq.	Sequence	Ion Score	C. I. %	Modification	Rank	Result Type
816.421	816.4322	0.0112	14	1	7	LAEQAER					Mascot
907.5247	907.5406	0.0159	18	18	25	NLLSVAYK					Mascot

917.5302	917.5402	0.01	11	26	33	IISSIEQK						Mascot
1189.6609	1189.6863	0.0254	21	57	66	DSTLIMQLLR						Mascot
1189.6609	1189.6863	0.0254	21	57	66	DSTLIMQLLR						Mascot
1205.6559	1205.6742	0.0183	15	57	66	DSTLIMQLLR			Oxidation (M)[6]			Mascot
1366.5542	1366.5648	0.0106	8	8	17	YEEMVEYMEK			Oxidation (M)[4]			Mascot
1418.7485	1418.7678	0.0193	14	26	37	IISSIEQKEESR						Mascot
2163.9573	2163.9697	0.0124	6	1	17	LAEQAERYEEMVEYMEK			Oxidation (M)[11]			Mascot

3 14-3-3-like protein GF14-E OS=Oryza sativa subsp. japonica GN=GF14E PE=2 SV=1 | 14335_ORYSJ | 29844.9 | 4.71 | 12 | 68 | 91.026 | 10.534

Peptide Information

Calc. Mass	Obsrv. Mass	± da	± ppm	Start Seq.	End Seq.	Sequence	Ion Score	C. I. %	Modification	Rank	Result Type
816.421	816.4322	0.0112	14	18	24	LAEQAER					Mascot
818.444	818.4481	0.0041	5	103	109	ICDGILK			Carbamidomethyl (C)[2]		Mascot
907.5247	907.5406	0.0159	18	50	57	NLLSVAYK					Mascot
917.5302	917.5402	0.01	11	69	76	IISSIEQK					Mascot
932.4294	932.4441	0.0147	16	131	137	MKGDYYR					Mascot
948.4244	948.4377	0.0133	14	131	137	MKGDYYR			Oxidation (M)[1]		Mascot
999.4451	999.462	0.0169	17	10	17	EENVYMAK			Oxidation (M)[6]		Mascot
1189.6609	1189.6863	0.0254	21	223	232	DSTLIMQLLR					Mascot
1189.6609	1189.6863	0.0254	21	223	232	DSTLIMQLLR					Mascot
1205.6559	1205.6742	0.0183	15	223	232	DSTLIMQLLR			Oxidation (M)[6]		Mascot
1366.5542	1366.5648	0.0106	8	25	34	YEEMVEFMEK			Oxidation (M)[4,8]		Mascot
1406.6646	1406.691	0.0264	19	38	49	TVDSEELTVEER					Mascot
1406.6646	1406.691	0.0264	19	38	49	TVDSEELTVEER					Mascot
1418.7485	1418.7678	0.0193	14	69	80	IISSIEQKEESR					Mascot
2131.9675	2132.0005	0.033	15	18	34	LAEQAERYEEMVEFMEK					Mascot
2163.9573	2163.9697	0.0124	6	18	34	LAEQAERYEEMVEFMEK			Oxidation (M)[11,15]		Mascot
2331.2019	2331.2139	0.012	5	178	197	LGLALNFSVFYYEILNSPDR					Mascot

4 14-3-3-like protein GF14-B OS=Oryza sativa subsp. japonica GN=GF14B PE=1 SV=2 | 14332_ORYSJ | 29959.1 | 4.76 | 12 | 67 | 89.457 | 10.534

Peptide Information

Calc. Mass	Obsrv. Mass	± da	± ppm	Start Seq.	End Seq.	Sequence	Ion Score	C. I. %	Modification	Rank	Result Type
816.421	816.4322	0.0112	14	18	24	LAEQAER					Mascot
818.444	818.4481	0.0041	5	103	109	ICDGILK			Carbamidomethyl (C)[2]		Mascot

907.5247	907.5406	0.0159	18	50	57	NLLSVAYK					Mascot
917.5302	917.5402	0.01	11	69	76	IISSIEQK					Mascot
932.4294	932.4441	0.0147	16	131	137	MKGDYYR					Mascot
948.4244	948.4377	0.0133	14	131	137	MKGDYYR			Oxidation (M)[1]		Mascot
999.4451	999.462	0.0169	17	10	17	EENVYMAK			Oxidation (M)[6]		Mascot
1189.6609	1189.6863	0.0254	21	223	232	DSTLIMQLLR					Mascot
1189.6609	1189.6863	0.0254	21	223	232	DSTLIMQLLR					Mascot
1205.6559	1205.6742	0.0183	15	223	232	DSTLIMQLLR			Oxidation (M)[6]		Mascot
1366.5542	1366.5648	0.0106	8	25	34	YEEMVEFMEK			Oxidation (M)[4,8]		Mascot
1406.6646	1406.691	0.0264	19	38	49	TVDSEELTVEER					Mascot
1406.6646	1406.691	0.0264	19	38	49	TVDSEELTVEER					Mascot
1418.7485	1418.7678	0.0193	14	69	80	IISSIEQKEESR					Mascot
2131.9675	2132.0005	0.033	15	18	34	LAEQAERYEEMVEFMEK					Mascot
2163.9573	2163.9697	0.0124	6	18	34	LAEQAERYEEMVEFMEK			Oxidation (M)[11,15]		Mascot
2331.2019	2331.2139	0.012	5	178	197	LGLALNFSVFYYEILNSPDR					Mascot

5	14-3-3-like protein OS=Lilium longiflorum PE=2 SV=1	1433_LILLO	29348.8	4.79	11	62	61.719	9.253

Peptide Information

Calc. Mass	Obsrv. Mass	± da	± ppm	Start Seq.	End Seq.	Sequence	Ion Score	C. I. %	Modification	Rank	Result Type
816.421	816.4322	0.0112	14	17	23	LAEQAER					Mascot
818.444	818.4481	0.0041	5	102	108	ICDGILK			Carbamidomethyl (C)[2]		Mascot
907.5247	907.5406	0.0159	18	49	56	NLLSVAYK					Mascot
917.5302	917.5402	0.01	11	68	75	IISSIEQK					Mascot
999.4451	999.462	0.0169	17	9	16	EENVYMAK			Oxidation (M)[6]		Mascot
1189.6609	1189.6863	0.0254	21	222	231	DSTLIMQLLR					Mascot
1189.6609	1189.6863	0.0254	21	222	231	DSTLIMQLLR					Mascot
1205.6559	1205.6742	0.0183	15	222	231	DSTLIMQLLR			Oxidation (M)[6]		Mascot
1366.5542	1366.5648	0.0106	8	24	33	YEEMVEFMEK			Oxidation (M)[4,8]		Mascot
1418.7485	1418.7678	0.0193	14	68	79	IISSIEQKEESR					Mascot
1420.6803	1420.7113	0.031	22	37	48	TVDTEELTVEER					Mascot
1420.6803	1420.7113	0.031	22	37	48	TVDTEELTVEER					Mascot
2131.9675	2132.0005	0.033	15	17	33	LAEQAERYEEMVEFMEK					Mascot
2163.9573	2163.9697	0.0124	6	17	33	LAEQAERYEEMVEFMEK			Oxidation (M)[11,15]		Mascot
2331.2019	2331.2139	0.012	5	177	196	LGLALNFSVFYYEILNSPDR					Mascot

6	14-3-3-like protein GF14-6 OS=Zea mays GN=GRF1	14331_MAIZE	29758	4.76	11	58	20.02	10.149

PE=1 SV=1

Peptide Information

Calc. Mass	Obsrv. Mass	± da	± ppm	Start Seq.	End Seq.	Sequence	Ion Score	C. I. %	Modification	Rank	Result Type
816.421	816.4322	0.0112	14	17	23	LAEQAER					Mascot
818.444	818.4481	0.0041	5	102	108	ICDGILK			Carbamidomethyl (C)[2]		Mascot
907.5247	907.5406	0.0159	18	49	56	NLLSVAYK					Mascot
917.5302	917.5402	0.01	11	68	75	IISSIEQK					Mascot
932.4294	932.4441	0.0147	16	130	136	MKGDYYR					Mascot
948.4244	948.4377	0.0133	14	130	136	MKGDYYR			Oxidation (M)[1]		Mascot
999.4451	999.462	0.0169	17	9	16	EENVYMAK			Oxidation (M)[6]		Mascot
1189.6609	1189.6863	0.0254	21	222	231	DSTLIMQLLR					Mascot
1189.6609	1189.6863	0.0254	21	222	231	DSTLIMQLLR					Mascot
1205.6559	1205.6742	0.0183	15	222	231	DSTLIMQLLR			Oxidation (M)[6]		Mascot
1366.5542	1366.5648	0.0106	8	24	33	YEEMVEFMEK			Oxidation (M)[4,8]		Mascot
1406.6646	1406.691	0.0264	19	37	48	TVDSEELTVEER					Mascot
1406.6646	1406.691	0.0264	19	37	48	TVDSEELTVEER					Mascot
2131.9675	2132.0005	0.033	15	17	33	LAEQAERYEEMVEFMEK					Mascot
2163.9573	2163.9697	0.0124	6	17	33	LAEQAERYEEMVEFMEK			Oxidation (M)[11,15]		Mascot
2331.2019	2331.2139	0.012	5	177	196	LGLALNFSVFYYEILNSPDR					Mascot

7	14-3-3-like protein GF14 nu OS=Arabidopsis thaliana GN=GRF7 PE=1 SV=1	14337_ARATH	29920	4.74	10	57	0	9.375	

Peptide Information

Calc. Mass	Obsrv. Mass	± da	± ppm	Start Seq.	End Seq.	Sequence	Ion Score	C. I. %	Modification	Rank	Result Type
816.421	816.4322	0.0112	14	14	20	LAEQAER					Mascot
907.5247	907.5406	0.0159	18	46	53	NLLSVAYK					Mascot
917.5302	917.5402	0.01	11	65	72	IISSIEQK					Mascot
1189.6609	1189.6863	0.0254	21	219	228	DSTLIMQLLR					Mascot
1189.6609	1189.6863	0.0254	21	219	228	DSTLIMQLLR					Mascot
1205.6559	1205.6742	0.0183	15	219	228	DSTLIMQLLR			Oxidation (M)[6]		Mascot
1366.5542	1366.5648	0.0106	8	21	30	YEEMVEFMEK			Oxidation (M)[4,8]		Mascot
1406.6646	1406.691	0.0264	19	34	45	TVDTDELTVEER					Mascot
1406.6646	1406.691	0.0264	19	34	45	TVDTDELTVEER					Mascot
1418.7485	1418.7678	0.0193	14	65	76	IISSIEQKEESR					Mascot

1788.9603	1789.1007	0.1404	78	157	173	SAQDIALADLAPTHPIR						Mascot
2131.9675	2132.0005	0.033	15	14	30	LAEQAERYEEMVEFMEK						Mascot
2163.9573	2163.9697	0.0124	6	14	30	LAEQAERYEEMVEFMEK			Oxidation (M)[11,15]			Mascot
2331.2019	2331.2139	0.012	5	174	193	LGLALNFSVFYYEILNSPDR						Mascot

8	14-3-3 protein 6 OS=Solanum lycopersicum GN=TFT6 PE=2 SV=2	14336_SOLLC	29063.4	4.7	10	56	0	7.995	

Protein Group

14-3-3-like protein 16R OS=Solanum tuberosum PE=2 SV=1	14335_SOLTU	29032.4	4.7399 997711 1816

Peptide Information

Calc. Mass	Obsrv. Mass	± da	± ppm	Start Seq.	End Seq.	Sequence	Ion Score	C. I. %	Modification	Rank	Result Type
816.421	816.4322	0.0112	14	14	20	LAEQAER					Mascot
907.5247	907.5406	0.0159	18	47	54	NLLSVAYK					Mascot
917.5302	917.5402	0.01	11	66	73	IISSIEQK					Mascot
999.4451	999.462	0.0169	17	6	13	EENVYMAK			Oxidation (M)[6]		Mascot
1189.6609	1189.6863	0.0254	21	220	229	DSTLIMQLLR					Mascot
1189.6609	1189.6863	0.0254	21	220	229	DSTLIMQLLR					Mascot
1205.6559	1205.6742	0.0183	15	220	229	DSTLIMQLLR			Oxidation (M)[6]		Mascot
1324.6743	1324.6525	-0.0218	-16	146	157	KEAAENTLSAYK					Mascot
1366.5542	1366.5648	0.0106	8	21	30	YEEMVEFMEK			Oxidation (M)[4,8]		Mascot
1418.7485	1418.7678	0.0193	14	66	77	IISSIEQKEESR					Mascot
2131.9675	2132.0005	0.033	15	14	30	LAEQAERYEEMVEFMEK					Mascot
2163.9573	2163.9697	0.0124	6	14	30	LAEQAERYEEMVEFMEK			Oxidation (M)[11,15]		Mascot
2331.2019	2331.2139	0.012	5	175	194	LGLALNFSVFYYEILNSPDR					Mascot

9	14-3-3-like protein GF14-F OS=Oryza sativa subsp. japonica GN=GF14F PE=1 SV=2	14336_ORYSJ	29273.7	4.81	10	52	0	8.216	

Peptide Information

Calc. Mass	Obsrv. Mass	± da	± ppm	Start Seq.	End Seq.	Sequence	Ion Score	C. I. %	Modification	Rank	Result Type
816.421	816.4322	0.0112	14	17	23	LAEQAER					Mascot
818.444	818.4481	0.0041	5	102	108	ICDGILK			Carbamidomethyl (C)[2]		Mascot
907.5247	907.5406	0.0159	18	49	56	NLLSVAYK					Mascot
917.5302	917.5402	0.01	11	68	75	IISSIEQK					Mascot
999.4451	999.462	0.0169	17	9	16	EENVYMAK			Oxidation (M)[6]		Mascot
1189.6609	1189.6863	0.0254	21	222	231	DSTLIMQLLR					Mascot

1189.6609	1189.6863	0.0254	21	222	231	DSTLIMQLLR					Mascot
1205.6559	1205.6742	0.0183	15	222	231	DSTLIMQLLR			Oxidation (M)[6]		Mascot
1366.5542	1366.5648	0.0106	8	24	33	YEEMVEFMEK			Oxidation (M)[4,8]		Mascot
1418.7485	1418.7678	0.0193	14	68	79	IISSIEQKEESR					Mascot
2131.9675	2132.0005	0.033	15	17	33	LAEQAERYEEMVEFMEK					Mascot
2163.9573	2163.9697	0.0124	6	17	33	LAEQAERYEEMVEFMEK			Oxidation (M)[11,15]		Mascot
2331.2019	2331.2139	0.012	5	177	196	LGLALNFSVFYYEILNSPDR					Mascot

10	14-3-3-like protein A OS=Hordeum vulgare PE=2 SV=1	1433A_HORVU	29447.8	4.83	10	52	0	8.216

Peptide Information

Calc. Mass	Obsrv. Mass	± da	± ppm	Start Seq.	End Seq.	Sequence	Ion Score	C. I. %	Modification	Rank	Result Type
816.421	816.4322	0.0112	14	17	23	LAEQAER					Mascot
818.444	818.4481	0.0041	5	102	108	ICDGILK			Carbamidomethyl (C)[2]		Mascot
907.5247	907.5406	0.0159	18	49	56	NLLSVAYK					Mascot
917.5302	917.5402	0.01	11	68	75	IISSIEQK					Mascot
999.4451	999.462	0.0169	17	9	16	EENVYMAK			Oxidation (M)[6]		Mascot
1189.6609	1189.6863	0.0254	21	222	231	DSTLIMQLLR					Mascot
1189.6609	1189.6863	0.0254	21	222	231	DSTLIMQLLR					Mascot
1205.6559	1205.6742	0.0183	15	222	231	DSTLIMQLLR			Oxidation (M)[6]		Mascot
1366.5542	1366.5648	0.0106	8	24	33	YEEMVEFMEK			Oxidation (M)[4,8]		Mascot
1418.7485	1418.7678	0.0193	14	68	79	IISSIEQKEESR					Mascot
2131.9675	2132.0005	0.033	15	17	33	LAEQAERYEEMVEFMEK					Mascot
2163.9573	2163.9697	0.0124	6	17	33	LAEQAERYEEMVEFMEK			Oxidation (M)[11,15]		Mascot
2331.2019	2331.2139	0.012	5	177	196	LGLALNFSVFYYEILNSPDR					Mascot

Gel Idx/Pos	171/G22	Instr./Gel Origin	BA2151/Sample Project 20140814	Process Status	Analysis Succeeded
Plate [#] Name	[1] Sample Project 20140814	Instrument Sample Name		Spectra	11

Rank	Protein Name	Accession No.	Protein MW	Protein PI	Pep. Count	Protein Score	Protein Score C. I. %	Intensity Matched	Total Ion Score	Total Ion C. I. %	Confirmed
1	14-3-3-like protein B OS=Hordeum vulgare PE=2 SV=1	1433B_HORVU	29786.9	4.67	22	416	100	72.463	248	100	

Peptide Information

Calc. Mass	Obsrv. Mass	± da	± ppm	Start Seq.	End Seq.	Sequence	Ion Score	C. I. %	Modification	Rank	Result Type
816.421	816.4395	0.0185	23	18	24	LAEQAER					Mascot
831.4822	831.5016	0.0194	23	96	102	IEVELTK					Mascot
907.5247	907.5403	0.0156	17	50	57	NLLSVAYK					Mascot
917.5302	917.5472	0.017	19	69	76	IISSIEQK					Mascot
932.4294	932.4494	0.02	21	131	137	MKGDYYR					Mascot
948.4244	948.4431	0.0187	20	131	137	MKGDYYR			Oxidation (M)[1]		Mascot
948.4244	948.4431	0.0187	20	131	137	MKGDYYR	15	0	Oxidation (M)[1]		Mascot
999.4451	999.4665	0.0214	21	10	17	EENVYMAK			Oxidation (M)[6]		Mascot
1016.5986	1016.6107	0.0121	12	94	102	GKIEVELTK					Mascot
1091.4712	1091.5564	0.0852	78	77	85	EESRGNEDR					Mascot
1189.6609	1189.6909	0.03	25	223	232	DSTLIMQLLR					Mascot
1205.6559	1205.6827	0.0268	22	223	232	DSTLIMQLLR			Oxidation (M)[6]		Mascot
1205.6559	1205.6827	0.0268	22	223	232	DSTLIMQLLR	32	32.079	Oxidation (M)[6]		Mascot
1212.5565	1212.6049	0.0484	40	150	160	DAAENTMVAYK					Mascot
1228.5514	1228.5693	0.0179	15	150	160	DAAENTMVAYK			Oxidation (M)[7]		Mascot
1300.6532	1300.6512	-0.002	-2	138	148	YLAEFKSGTER					Mascot
1356.6464	1356.6509	0.0045	3	149	160	KDAAENTMVAYK			Oxidation (M)[8]		Mascot
1366.5542	1366.5667	0.0125	9	25	34	YEEMVEFMEK			Oxidation (M)[4,8]		Mascot
1366.5542	1366.5667	0.0125	9	25	34	YEEMVEFMEK			Oxidation (M)[4,8]		Mascot
1406.6646	1406.7013	0.0367	26	38	49	TVDSEELTVEER					Mascot
1406.6646	1406.7013	0.0367	26	38	49	TVDSEELTVEER	102	100			Mascot
1418.7485	1418.7878	0.0393	28	69	80	IISSIEQKEESR					Mascot
1517.8799	1517.9158	0.0359	24	50	63	NLLSVAYKNVIGAR					Mascot
1680.8804	1680.9084	0.028	17	110	125	LLDSHLVPSSTAPESK					Mascot
1827.0123	1827.0615	0.0492	27	161	177	AAQEIALAELPPTHPIR					Mascot
1827.0123	1827.0615	0.0492	27	161	177	AAQEIALAELPPTHPIR	114	100			Mascot
2160.9819	2160.9971	0.0152	7	204	222	QAFDEAISELDSLSEESY K					Mascot

2163.9573	2163.9719	0.0146	7	18	34	LAEQAERYEEMVEFMEK			Oxidation (M)[11,15]		Mascot
2331.2595	2331.269	0.0095	4	110	130	LLDSHLVPSSTAPESKVF YLK					Mascot
2595.1768	2595.2207	0.0439	17	233	255	DNLTLWTSDISEDAAEEM KDAPK			Oxidation (M)[18]		Mascot

2	14-3-3-like protein GF14-E OS=Oryza sativa subsp. japonica GN=GF14E PE=2 SV=1	14335_ORYSJ	29844.9	4.71	17	247	100	28.234	132	100

Peptide Information

Calc. Mass	Obsrv. Mass	± da	± ppm	Start Seq.	End Seq.	Sequence	Ion Score	C. I. %	Modification	Rank	Result Type
816.421	816.4395	0.0185	23	18	24	LAEQAER					Mascot
907.5247	907.5403	0.0156	17	50	57	NLLSVAYK					Mascot
917.5302	917.5472	0.017	19	69	76	IISSIEQK					Mascot
932.4294	932.4494	0.02	21	131	137	MKGDYYR					Mascot
948.4244	948.4431	0.0187	20	131	137	MKGDYYR			Oxidation (M)[1]		Mascot
948.4244	948.4431	0.0187	20	131	137	MKGDYYR	15	0	Oxidation (M)[1]		Mascot
999.4451	999.4665	0.0214	21	10	17	EENVYMAK			Oxidation (M)[6]		Mascot
1091.4712	1091.5564	0.0852	78	77	85	EESRGNEDR					Mascot
1189.6609	1189.6909	0.03	25	223	232	DSTLIMQLLR					Mascot
1205.6559	1205.6827	0.0268	22	223	232	DSTLIMQLLR			Oxidation (M)[6]		Mascot
1205.6559	1205.6827	0.0268	22	223	232	DSTLIMQLLR	32	32.079	Oxidation (M)[6]		Mascot
1212.5565	1212.6049	0.0484	40	150	160	DAAENTMVAYK					Mascot
1228.5514	1228.5693	0.0179	15	150	160	DAAENTMVAYK			Oxidation (M)[7]		Mascot
1356.6464	1356.6509	0.0045	3	149	160	KDAAENTMVAYK			Oxidation (M)[8]		Mascot
1366.5542	1366.5667	0.0125	9	25	34	YEEMVEFMEK			Oxidation (M)[4,8]		Mascot
1366.5542	1366.5667	0.0125	9	25	34	YEEMVEFMEK			Oxidation (M)[4,8]		Mascot
1406.6646	1406.7013	0.0367	26	38	49	TVDSEELTVEER					Mascot
1406.6646	1406.7013	0.0367	26	38	49	TVDSEELTVEER	102	100			Mascot
1418.7485	1418.7878	0.0393	28	69	80	IISSIEQKEESR					Mascot
1517.8799	1517.9158	0.0359	24	50	63	NLLSVAYKNVIGAR					Mascot
1680.8804	1680.9084	0.028	17	110	125	LLDSHLVPSSTAPESK					Mascot
1867.8855	1867.9791	0.0936	50	2	17	SQPAELSREENVYMAK			Oxidation (M)[14]		Mascot
2163.9573	2163.9719	0.0146	7	18	34	LAEQAERYEEMVEFMEK			Oxidation (M)[11,15]		Mascot
2331.2595	2331.269	0.0095	4	110	130	LLDSHLVPSSTAPESKVF YLK					Mascot

3	14-3-3-like protein GF14-B OS=Oryza sativa subsp. japonica GN=GF14B PE=1 SV=2	14332_ORYSJ	29959.1	4.76	15	226	100	27.713	133	100

Peptide Information

Calc. Mass	Obsrv. Mass	± da	± ppm	Start Seq.	End Seq.	Sequence	Ion Score	C. I. %	Modification	Rank	Result Type
816.421	816.4395	0.0185	23	18	24	LAEQAER					Mascot
907.5247	907.5403	0.0156	17	50	57	NLLSVAYK					Mascot
917.5302	917.5472	0.017	19	69	76	IISSIEQK					Mascot
932.4294	932.4494	0.02	21	131	137	MKGDYYR					Mascot
948.4244	948.4431	0.0187	20	131	137	MKGDYYR			Oxidation (M)[1]		Mascot
948.4244	948.4431	0.0187	20	131	137	MKGDYYR	15	0	Oxidation (M)[1]		Mascot
999.4451	999.4665	0.0214	21	10	17	EENVYMAK			Oxidation (M)[6]		Mascot
1091.4712	1091.5564	0.0852	78	77	85	EESRGNEDR					Mascot
1189.6609	1189.6909	0.03	25	223	232	DSTLIMQLLR					Mascot
1205.6559	1205.6827	0.0268	22	223	232	DSTLIMQLLR			Oxidation (M)[6]		Mascot
1205.6559	1205.6827	0.0268	22	223	232	DSTLIMQLLR	32	32.079	Oxidation (M)[6]		Mascot
1212.5565	1212.6049	0.0484	40	150	160	DAAENTMVAYK					Mascot
1228.5514	1228.5693	0.0179	15	150	160	DAAENTMVAYK			Oxidation (M)[7]		Mascot
1356.6464	1356.6509	0.0045	3	149	160	KDAAENTMVAYK			Oxidation (M)[8]		Mascot
1366.5542	1366.5667	0.0125	9	25	34	YEEMVEFMEK			Oxidation (M)[4,8]		Mascot
1366.5542	1366.5667	0.0125	9	25	34	YEEMVEFMEK			Oxidation (M)[4,8]		Mascot
1406.6646	1406.7013	0.0367	26	38	49	TVDSEELTVEER					Mascot
1406.6646	1406.7013	0.0367	26	38	49	TVDSEELTVEER	102	100			Mascot
1418.7485	1418.7878	0.0393	28	69	80	IISSIEQKEESR					Mascot
1517.8799	1517.9158	0.0359	24	50	63	NLLSVAYKNVIGAR					Mascot
2163.9573	2163.9719	0.0146	7	18	34	LAEQAERYEEMVEFMEK			Oxidation (M)[11,15]		Mascot
2331.2019	2331.269	0.0671	29	178	197	LGLALNFSVFYYEILNSPD R					Mascot

4	14-3-3-like protein GF14-6 OS=Zea mays GN=GRF1 PE=1 SV=1	14331_MAIZE	29758	4.76	13	211	100	27.23	133	100	

Peptide Information

Calc. Mass	Obsrv. Mass	± da	± ppm	Start Seq.	End Seq.	Sequence	Ion Score	C. I. %	Modification	Rank	Result Type
816.421	816.4395	0.0185	23	17	23	LAEQAER					Mascot
907.5247	907.5403	0.0156	17	49	56	NLLSVAYK					Mascot
917.5302	917.5472	0.017	19	68	75	IISSIEQK					Mascot
932.4294	932.4494	0.02	21	130	136	MKGDYYR					Mascot
948.4244	948.4431	0.0187	20	130	136	MKGDYYR			Oxidation (M)[1]		Mascot
948.4244	948.4431	0.0187	20	130	136	MKGDYYR	15	0	Oxidation (M)[1]		Mascot
999.4451	999.4665	0.0214	21	9	16	EENVYMAK			Oxidation (M)[6]		Mascot

1189.6609	1189.6909	0.03	25	222	231	DSTLIMQLLR					Mascot
1205.6559	1205.6827	0.0268	22	222	231	DSTLIMQLLR			Oxidation (M)[6]		Mascot
1205.6559	1205.6827	0.0268	22	222	231	DSTLIMQLLR	32	32.079	Oxidation (M)[6]		Mascot
1212.5565	1212.6049	0.0484	40	149	159	DAAENTMVAYK					Mascot
1228.5514	1228.5693	0.0179	15	149	159	DAAENTMVAYK			Oxidation (M)[7]		Mascot
1356.6464	1356.6509	0.0045	3	148	159	KDAAENTMVAYK			Oxidation (M)[8]		Mascot
1366.5542	1366.5667	0.0125	9	24	33	YEEMVEFMEK			Oxidation (M)[4,8]		Mascot
1366.5542	1366.5667	0.0125	9	24	33	YEEMVEFMEK			Oxidation (M)[4,8]		Mascot
1406.6646	1406.7013	0.0367	26	37	48	TVDSEELTVEER					Mascot
1406.6646	1406.7013	0.0367	26	37	48	TVDSEELTVEER	102	100			Mascot
1517.8799	1517.9158	0.0359	24	49	62	NLLSVAYKNVIGAR					Mascot
2163.9573	2163.9719	0.0146	7	17	33	LAEQAERYEEMVEFMEK			Oxidation (M)[11,15]		Mascot
2331.2019	2331.269	0.0671	29	177	196	LGLALNFSVFYYEILNSPD R					Mascot

5	14-3-3-like protein GF14-12 OS=Zea mays GN=GRF2 PE=2 SV=2	14332_MAIZE	29731.9	4.75	12	172	100	14.974	102	100	

Peptide Information

Calc. Mass	Obsrv. Mass	± da	± ppm	Start Seq.	End Seq.	Sequence	Ion Score	C. I. %	Modification	Rank	Result Type
816.421	816.4395	0.0185	23	17	23	LAEQAER					Mascot
907.5247	907.5403	0.0156	17	49	56	NLLSVAYK					Mascot
917.5302	917.5472	0.017	19	68	75	IISSIEQK					Mascot
932.4294	932.4494	0.02	21	130	136	MKGDYYR					Mascot
948.4244	948.4431	0.0187	20	130	136	MKGDYYR			Oxidation (M)[1]		Mascot
948.4244	948.4431	0.0187	20	130	136	MKGDYYR	15	0	Oxidation (M)[1]		Mascot
999.4451	999.4665	0.0214	21	9	16	EENVYMAK			Oxidation (M)[6]		Mascot
1212.5565	1212.6049	0.0484	40	149	159	DAAENTMVAYK					Mascot
1228.5514	1228.5693	0.0179	15	149	159	DAAENTMVAYK			Oxidation (M)[7]		Mascot
1356.6464	1356.6509	0.0045	3	148	159	KDAAENTMVAYK			Oxidation (M)[8]		Mascot
1366.5542	1366.5667	0.0125	9	24	33	YEEMVEFMEK			Oxidation (M)[4,8]		Mascot
1366.5542	1366.5667	0.0125	9	24	33	YEEMVEFMEK			Oxidation (M)[4,8]		Mascot
1406.6646	1406.7013	0.0367	26	37	48	TVDSEELTVEER					Mascot
1406.6646	1406.7013	0.0367	26	37	48	TVDSEELTVEER	102	100			Mascot
1517.8799	1517.9158	0.0359	24	49	62	NLLSVAYKNVIGAR					Mascot
2163.9573	2163.9719	0.0146	7	17	33	LAEQAERYEEMVEFMEK			Oxidation (M)[11,15]		Mascot
2331.2019	2331.269	0.0671	29	177	196	LGLALNFSVFYYEILNSPD R					Mascot

6	14-3-3-like protein GF14 nu OS=Arabidopsis thaliana GN=GRF7 PE=1 SV=1	14337_ARATH	29920	4.74	11	164	100	25.023	116	100

Peptide Information

Calc. Mass	Obsrv. Mass	± da	± ppm	Start Seq.	End Seq.	Sequence	Ion Score	C. I. %	Modification	Rank	Result Type
816.421	816.4395	0.0185	23	14	20	LAEQAER					Mascot
907.5247	907.5403	0.0156	17	46	53	NLLSVAYK					Mascot
917.5302	917.5472	0.017	19	65	72	IISSIEQK					Mascot
1189.6609	1189.6909	0.03	25	219	228	DSTLIMQLLR					Mascot
1205.6559	1205.6827	0.0268	22	219	228	DSTLIMQLLR			Oxidation (M)[6]		Mascot
1205.6559	1205.6827	0.0268	22	219	228	DSTLIMQLLR	32	32.079	Oxidation (M)[6]		Mascot
1366.5542	1366.5667	0.0125	9	21	30	YEEMVEFMEK			Oxidation (M)[4,8]		Mascot
1366.5542	1366.5667	0.0125	9	21	30	YEEMVEFMEK			Oxidation (M)[4,8]		Mascot
1382.691	1382.5564	-0.1346	-97	2	13	SSSREENVYLAK					Mascot
1406.6646	1406.7013	0.0367	26	34	45	TVDTDELTVEER					Mascot
1406.6646	1406.7013	0.0367	26	34	45	TVDTDELTVEER	84	100			Mascot
1418.7485	1418.7878	0.0393	28	65	76	IISSIEQKEESR					Mascot
1517.8799	1517.9158	0.0359	24	46	59	NLLSVAYKNVIGAR					Mascot
2163.9573	2163.9719	0.0146	7	14	30	LAEQAERYEEMVEFMEK			Oxidation (M)[11,15]		Mascot
2331.2019	2331.269	0.0671	29	174	193	LGLALNFSVFYYEILNSPD R					Mascot

7	14-3-3-like protein RA215 OS=Solanum tuberosum PE=2 SV=1	14332_SOLTU	28691.3	4.78	13	109	100	18.933	32	32.079

Peptide Information

Calc. Mass	Obsrv. Mass	± da	± ppm	Start Seq.	End Seq.	Sequence	Ion Score	C. I. %	Modification	Rank	Result Type
889.3897	889.3541	-0.0356	-40	247	254	EPSKADNE					Mascot
907.5247	907.5403	0.0156	17	45	52	NLLSVAYK					Mascot
917.5302	917.5472	0.017	19	64	71	IISSIEQK					Mascot
932.4294	932.4494	0.02	21	126	132	MKGDYYR					Mascot
948.4244	948.4431	0.0187	20	126	132	MKGDYYR			Oxidation (M)[1]		Mascot
948.4244	948.4431	0.0187	20	126	132	MKGDYYR	15	0	Oxidation (M)[1]		Mascot
1189.6609	1189.6909	0.03	25	218	227	DSTLIMQLLR					Mascot
1197.6222	1197.6924	0.0702	59	76	86	GNEDHVASIKK					Mascot
1205.6559	1205.6827	0.0268	22	218	227	DSTLIMQLLR			Oxidation (M)[6]		Mascot
1205.6559	1205.6827	0.0268	22	218	227	DSTLIMQLLR	32	32.079	Oxidation (M)[6]		Mascot
1324.6743	1324.6709	-0.0034	-3	144	155	KEAAENTLSAYK					Mascot

Calc. Mass	Obsrv. Mass	± da	± ppm	Start Seq.	End Seq.	Sequence	Ion Score	C. I. %	Modification	Rank	Result Type
1366.5542	1366.5667	0.0125	9	21	30	YEEMVEFMEK			Oxidation (M)[4,8]		Mascot
1366.642	1366.5667	-0.0753	-55	6	17	EENVYMANVAAR					Mascot
1382.637	1382.5564	-0.0806	-58	6	17	EENVYMANVAAR			Oxidation (M)[6]		Mascot
1418.7485	1418.7878	0.0393	28	64	75	IISSIEQKEESR					Mascot
1517.8799	1517.9158	0.0359	24	45	58	NLLSVAYKNVIGAR					Mascot
1818.9266	1819.0199	0.0933	51	89	104	SQIENELTSICNGILK			Carbamidomethyl (C)[11]		Mascot
2331.2019	2331.269	0.0671	29	173	192	LGLALNFSVFYYEILNSPDR					Mascot

8	14-3-3-like protein 2 (Fragments) OS=Pseudotsuga menziesii PE=1 SV=1	14332_PSEMZ	7715.8	4.28	7	109	100	21.128	46	97.271

Peptide Information

Calc. Mass	Obsrv. Mass	± da	± ppm	Start Seq.	End Seq.	Sequence	Ion Score	C. I. %	Modification	Rank	Result Type
816.421	816.4395	0.0185	23	1	7	LAEQAER					Mascot
907.5247	907.5403	0.0156	17	18	25	NLLSVAYK					Mascot
917.5302	917.5472	0.017	19	26	33	IISSIEQK					Mascot
1189.6609	1189.6909	0.03	25	57	66	DSTLIMQLLR					Mascot
1205.6559	1205.6827	0.0268	22	57	66	DSTLIMQLLR			Oxidation (M)[6]		Mascot
1205.6559	1205.6827	0.0268	22	57	66	DSTLIMQLLR	32	32.079	Oxidation (M)[6]		Mascot
1366.5542	1366.5667	0.0125	9	8	17	YEEMVEYMEK			Oxidation (M)[4]		Mascot
1366.5542	1366.5667	0.0125	9	8	17	YEEMVEYMEK	14	0	Oxidation (M)[4]		Mascot
1382.5491	1382.5564	0.0073	5	8	17	YEEMVEYMEK			Oxidation (M)[4,8]		Mascot
1418.7485	1418.7878	0.0393	28	26	37	IISSIEQKEESR					Mascot
2163.9573	2163.9719	0.0146	7	1	17	LAEQAERYEEMVEYMEK			Oxidation (M)[11]		Mascot

9	14-3-3-like protein OS=Helianthus annuus PE=2 SV=1	1433_HELAN	29043.5	4.65	12	108	100	23.354	32	32.079

Peptide Information

Calc. Mass	Obsrv. Mass	± da	± ppm	Start Seq.	End Seq.	Sequence	Ion Score	C. I. %	Modification	Rank	Result Type
806.3825	806.3615	-0.021	-26	1	8	MAAASSPR			Oxidation (M)[1]		Mascot
816.421	816.4395	0.0185	23	17	23	LAEQAER					Mascot
907.5247	907.5403	0.0156	17	50	57	NLLSVAYK					Mascot
917.5302	917.5472	0.017	19	69	76	IISSIEQK					Mascot
932.4294	932.4494	0.02	21	131	137	MKGDYYR					Mascot
948.4244	948.4431	0.0187	20	131	137	MKGDYYR			Oxidation (M)[1]		Mascot
948.4244	948.4431	0.0187	20	131	137	MKGDYYR	15	0	Oxidation (M)[1]		Mascot
1189.6609	1189.6909	0.03	25	223	232	DSTLIMQLLR					Mascot

1205.6559	1205.6827	0.0268	22	223	232	DSTLIMQLLR			Oxidation (M)[6]		Mascot
1205.6559	1205.6827	0.0268	22	223	232	DSTLIMQLLR	32	32.079	Oxidation (M)[6]		Mascot
1328.6481	1328.6814	0.0333	25	138	148	YLAEFKTGDER					Mascot
1366.5542	1366.5667	0.0125	9	24	33	YEEMVEFMEK			Oxidation (M)[4,8]		Mascot
1366.5542	1366.5667	0.0125	9	24	33	YEEMVEFMEK			Oxidation (M)[4,8]		Mascot
1418.7485	1418.7878	0.0393	28	69	80	IISSIEQKEESR					Mascot
1517.8799	1517.9158	0.0359	24	50	63	NLLSVAYKNVIGAR					Mascot
2163.9573	2163.9719	0.0146	7	17	33	LAEQAERYEEMVEFMEK			Oxidation (M)[11,15]		Mascot
2331.2019	2331.269	0.0671	29	178	197	LGLALNFSVFYYEILNSPD R					Mascot

10	14-3-3-like protein A OS=Hordeum vulgare PE=2 SV=1	1433A_HORVU	29447.8	4.83	14	106	99.999	22.217	32	32.079	

Peptide Information

Calc. Mass	Obsrv. Mass	± da	± ppm	Start Seq.	End Seq.	Sequence	Ion Score	C. I. %	Modification	Rank	Result Type
816.421	816.4395	0.0185	23	17	23	LAEQAER					Mascot
907.5247	907.5403	0.0156	17	49	56	NLLSVAYK					Mascot
917.5302	917.5472	0.017	19	68	75	IISSIEQK					Mascot
999.4451	999.4665	0.0214	21	9	16	EENVYMAK			Oxidation (M)[6]		Mascot
1051.5419	1051.5702	0.0283	27	80	89	GNEAYVASIK					Mascot
1189.6609	1189.6909	0.03	25	222	231	DSTLIMQLLR					Mascot
1205.6559	1205.6827	0.0268	22	222	231	DSTLIMQLLR			Oxidation (M)[6]		Mascot
1205.6559	1205.6827	0.0268	22	222	231	DSTLIMQLLR	32	32.079	Oxidation (M)[6]		Mascot
1318.6486	1318.6783	0.0297	23	37	48	TADVGELTVEER					Mascot
1366.5542	1366.5667	0.0125	9	24	33	YEEMVEFMEK			Oxidation (M)[4,8]		Mascot
1366.5542	1366.5667	0.0125	9	24	33	YEEMVEFMEK			Oxidation (M)[4,8]		Mascot
1418.7485	1418.7878	0.0393	28	68	79	IISSIEQKEESR					Mascot
1517.8799	1517.9158	0.0359	24	49	62	NLLSVAYKNVIGAR					Mascot
1818.9708	1819.0199	0.0491	27	160	176	SAQDIALADLPTTHPIR					Mascot
1846.8309	1847.0034	0.1725	93	1	16	MSTAEATREENVYMAK			Oxidation (M)[1]		Mascot
2163.9573	2163.9719	0.0146	7	17	33	LAEQAERYEEMVEFMEK			Oxidation (M)[11,15]		Mascot
2331.2019	2331.269	0.0671	29	177	196	LGLALNFSVFYYEILNSPD R					Mascot

Gel Idx/Pos	172/G23	Instr./Gel Origin	BA2151/Sample Project 20140814	Process Status	Analysis Succeeded
Plate [#] Name	[1] Sample Project 20140814	Instrument Sample Name		Spectra	11

Rank	Protein Name	Accession No.	Protein MW	Protein PI	Pep. Count	Protein Score	Protein Score C. I. %	Intensity Matched	Total Ion Score	Total Ion C. I. %	Confirmed
1	14-3-3-like protein B OS=Hordeum vulgare PE=2 SV=1	1433B_HORVU	29786.9	4.67	12	206	100	7.351	153	100	

Peptide Information

Calc. Mass	Obsrv. Mass	± da	± ppm	Start Seq.	End Seq.	Sequence	Ion Score	C. I. %	Modification	Rank	Result Type
816.421	816.4482	0.0272	33	18	24	LAEQAER					Mascot
818.444	818.468	0.024	29	103	109	ICDGILK			Carbamidomethyl (C)[2]		Mascot
917.5302	917.5295	-0.0007	-1	69	76	IISSIEQK					Mascot
948.4244	948.4635	0.0391	41	131	137	MKGDYYR			Oxidation (M)[1]		Mascot
1018.4985	1018.5401	0.0416	41	1	9	MAQPAELSR			Oxidation (M)[1]		Mascot
1189.6609	1189.7001	0.0392	33	223	232	DSTLIMQLLR					Mascot
1205.6559	1205.6904	0.0345	29	223	232	DSTLIMQLLR			Oxidation (M)[6]		Mascot
1212.5565	1212.6239	0.0674	56	150	160	DAAENTMVAYK					Mascot
1212.5565	1212.6239	0.0674	56	150	160	DAAENTMVAYK					Mascot
1228.5514	1228.6569	0.1055	86	150	160	DAAENTMVAYK			Oxidation (M)[7]		Mascot
1366.5542	1366.6586	0.1044	76	25	34	YEEMVEFMEK			Oxidation (M)[4,8]		Mascot
1406.6646	1406.7145	0.0499	35	38	49	TVDSEELTVEER					Mascot
1406.6646	1406.7145	0.0499	35	38	49	TVDSEELTVEER	49	98.587			Mascot
1418.7485	1418.7648	0.0163	11	69	80	IISSIEQKEESR					Mascot
1827.0123	1827.0712	0.0589	32	161	177	AAQEIALAELPPTHPIR					Mascot
1827.0123	1827.0712	0.0589	32	161	177	AAQEIALAELPPTHPIR	104	100			Mascot
2183.9648	2184.0884	0.1236	57	233	251	DNLTLWTSDISEDAAEEMK			Oxidation (M)[18]		Mascot

Rank	Protein Name	Accession No.	Protein MW	Protein PI	Pep. Count	Protein Score	Protein Score C. I. %	Intensity Matched	Total Ion Score	Total Ion C. I. %	Confirmed
2	14-3-3-like protein GF14-E OS=Oryza sativa subsp. japonica GN=GF14E PE=2 SV=1	14335_ORYSJ	29844.9	4.71	12	96	99.985	4.62	49	98.587	

Peptide Information

Calc. Mass	Obsrv. Mass	± da	± ppm	Start Seq.	End Seq.	Sequence	Ion Score	C. I. %	Modification	Rank	Result Type
816.421	816.4482	0.0272	33	18	24	LAEQAER					Mascot
818.444	818.468	0.024	29	103	109	ICDGILK			Carbamidomethyl (C)[2]		Mascot
819.4458	819.4619	0.0161	20	96	102	IETELSK					Mascot
887.4581	887.4862	0.0281	32	2	9	SQPAELSR					Mascot

Calc. Mass	Obsrv. Mass	± da	± ppm	Start Seq.	End Seq.	Sequence	Ion Score	C. I. %	Modification	Rank	Result Type
917.5302	917.5295	-0.0007	-1	69	76	IISSIEQK					Mascot
948.4244	948.4635	0.0391	41	131	137	MKGDYYR			Oxidation (M)[1]		Mascot
1018.4985	1018.5401	0.0416	41	1	9	MSQPAELSR					Mascot
1189.6609	1189.7001	0.0392	33	223	232	DSTLIMQLLR					Mascot
1205.6559	1205.6904	0.0345	29	223	232	DSTLIMQLLR			Oxidation (M)[6]		Mascot
1212.5565	1212.6239	0.0674	56	150	160	DAAENTMVAYK					Mascot
1212.5565	1212.6239	0.0674	56	150	160	DAAENTMVAYK					Mascot
1228.5514	1228.6569	0.1055	86	150	160	DAAENTMVAYK			Oxidation (M)[7]		Mascot
1366.5542	1366.6586	0.1044	76	25	34	YEEMVEFMEK			Oxidation (M)[4,8]		Mascot
1406.6646	1406.7145	0.0499	35	38	49	TVDSEELTVEER					Mascot
1406.6646	1406.7145	0.0499	35	38	49	TVDSEELTVEER	49	98.587			Mascot
1418.7485	1418.7648	0.0163	11	69	80	IISSIEQKEESR					Mascot

3 Kinesin-like protein NACK2 OS=Nicotiana tabacum GN=NACK2 PE=1 SV=1 NACK2_TOBAC 108131.8 8.46 26 95 99.984 8.834

Peptide Information

Calc. Mass	Obsrv. Mass	± da	± ppm	Start Seq.	End Seq.	Sequence	Ion Score	C. I. %	Modification	Rank	Result Type
802.4781	802.4758	-0.0023	-3	27	33	TTPSKIR					Mascot
812.4009	812.4586	0.0577	71	278	284	EGSHINR					Mascot
818.4254	818.468	0.0426	52	907	914	DSAEVVAK					Mascot
822.4104	822.4509	0.0405	49	96	102	VYEQGAR					Mascot
823.456	823.4778	0.0218	26	722	728	AYVTELK					Mascot
828.505	828.467	-0.038	-46	14	20	IVRTPSR					Mascot
829.4315	829.4429	0.0114	14	304	310	SGHIPYR					Mascot
864.4356	864.499	0.0634	73	296	303	LSCSGGKR			Carbamidomethyl (C)[3]		Mascot
870.5519	870.5682	0.0163	19	888	894	RLQLVNK					Mascot
913.4634	913.5018	0.0384	42	705	711	MQTMFKK					Mascot
977.5778	977.5485	-0.0293	-30	731	738	VAKLQYQK					Mascot
993.4595	993.5327	0.0732	74	266	275	ASQTSADGTR					Mascot
1201.6536	1201.6555	0.0019	2	379	388	EVSRLEAELR					Mascot
1201.6536	1201.6555	0.0019	2	379	388	EVSRLEAELR					Mascot
1211.6168	1211.6366	0.0198	16	877	886	WDIPPDGKQR					Mascot
1212.7198	1212.6239	-0.0959	-79	2	13	VIGTPVTTPLSK					Mascot
1212.7198	1212.6239	-0.0959	-79	2	13	VIGTPVTTPLSK					Mascot
1301.6696	1301.6438	-0.0258	-20	424	434	DLAQSQLELER					Mascot
1301.6696	1301.6438	-0.0258	-20	424	434	DLAQSQLELER	11	0			Mascot

Calc. Mass	Obsrv. Mass	± da	± ppm	Start Seq.	End Seq.	Sequence	Ion Score	C. I. %	Modification	Rank	Result Type
1324.5872	1324.6782	0.091	69	412	421	MEEEMNELKR			Oxidation (M)[1]		Mascot
1324.5872	1324.6782	0.091	69	412	421	MEEEMNELKR			Oxidation (M)[1]		Mascot
1333.6594	1333.6329	-0.0265	-20	578	589	SQESNIASLEQK					Mascot
1472.7314	1472.7424	0.011	7	895	906	LWSDPLNMQNVR					Mascot
1619.8309	1619.8855	0.0546	34	356	370	EVTTTAQVNMVVAEK					Mascot
1635.8701	1635.8854	0.0153	9	132	146	GITESAVNDIYGRIK					Mascot
1909.0614	1908.8799	-0.1815	-95	278	294	EGSHINRSLLTVTNVIR					Mascot
2182.9856	2183.0886	0.103	47	596	614	SIDELVMHLPSCHESADS R			Carbamidomethyl (C)[12]		Mascot
2182.9856	2183.0886	0.103	47	596	614	SIDELVMHLPSCHESADS R			Carbamidomethyl (C)[12]		Mascot
2184.1594	2184.0884	-0.071	-33	889	906	LQLVNKLWSDPLNMQNV R			Oxidation (M)[14]		Mascot
2208.9543	2209.1475	0.1932	87	77	95	HTGPYSFDYVFDPTCSTS K			Carbamidomethyl (C)[15]		Mascot
2680.3538	2680.4312	0.0774	29	250	275	SFLATLNLVDLAGSERAS QTSADGTR					Mascot

4 14-3-3-like protein GF14-6 OS=Zea mays GN=GRF1 PE=1 SV=1 14331_MAIZE 29758 4.76 11 91 99.956 4.589 49 98.587

Peptide Information

Calc. Mass	Obsrv. Mass	± da	± ppm	Start Seq.	End Seq.	Sequence	Ion Score	C. I. %	Modification	Rank	Result Type
816.421	816.4482	0.0272	33	17	23	LAEQAER					Mascot
818.444	818.468	0.024	29	102	108	ICDGILK			Carbamidomethyl (C)[2]		Mascot
864.4243	864.499	0.0747	86	1	8	MASAELSR					Mascot
917.5302	917.5295	-0.0007	-1	68	75	IISSIEQK					Mascot
948.4244	948.4635	0.0391	41	130	136	MKGDYYR			Oxidation (M)[1]		Mascot
1018.5779	1018.5401	-0.0378	-37	93	101	GKIETELTK					Mascot
1189.6609	1189.7001	0.0392	33	222	231	DSTLIMQLLR					Mascot
1205.6559	1205.6904	0.0345	29	222	231	DSTLIMQLLR			Oxidation (M)[6]		Mascot
1212.5565	1212.6239	0.0674	56	149	159	DAAENTMVAYK					Mascot
1212.5565	1212.6239	0.0674	56	149	159	DAAENTMVAYK					Mascot
1228.5514	1228.6569	0.1055	86	149	159	DAAENTMVAYK			Oxidation (M)[7]		Mascot
1366.5542	1366.6586	0.1044	76	24	33	YEEMVEFMEK			Oxidation (M)[4,8]		Mascot
1406.6646	1406.7145	0.0499	35	37	48	TVDSEELTVEER					Mascot
1406.6646	1406.7145	0.0499	35	37	48	TVDSEELTVEER	49	98.587			Mascot
1786.9811	1787.038	0.0569	32	160	176	AAQDIALAELAPTHPIR					Mascot

5 14-3-3-like protein GF14-12 OS=Zea mays GN=GRF2 PE=2 SV=2 14332_MAIZE 29731.9 4.75 10 85 99.821 3.423 49 98.587

Peptide Information

Calc. Mass	Obsrv. Mass	± da	± ppm	Start Seq.	End Seq.	Sequence	Ion Score	C. I. %	Modification	Rank	Result Type
816.421	816.4482	0.0272	33	17	23	LAEQAER					Mascot
818.444	818.468	0.024	29	102	108	ICDGILK			Carbamidomethyl (C)[2]		Mascot
864.4243	864.499	0.0747	86	1	8	MASAELSR					Mascot
917.5302	917.5295	-0.0007	-1	68	75	IISSIEQK					Mascot
948.4244	948.4635	0.0391	41	130	136	MKGDYYR			Oxidation (M)[1]		Mascot
1018.5779	1018.5401	-0.0378	-37	93	101	GKIETELTK					Mascot
1212.5565	1212.6239	0.0674	56	149	159	DAAENTMVAYK					Mascot
1212.5565	1212.6239	0.0674	56	149	159	DAAENTMVAYK					Mascot
1228.5514	1228.6569	0.1055	86	149	159	DAAENTMVAYK			Oxidation (M)[7]		Mascot
1366.5542	1366.6586	0.1044	76	24	33	YEEMVEFMEK			Oxidation (M)[4,8]		Mascot
1406.6646	1406.7145	0.0499	35	37	48	TVDSEELTVEER					Mascot
1406.6646	1406.7145	0.0499	35	37	48	TVDSEELTVEER	49	98.587			Mascot
1786.9811	1787.038	0.0569	32	160	176	AAQDIALAELAPTHPIR					Mascot

6 Nesprin-1 OS=Mus musculus GN=Syne1 PE=1 SV=2 SYNE1_MOUSE 1016650.4 5.43 84 84 99.808 25.293

Peptide Information

Calc. Mass	Obsrv. Mass	± da	± ppm	Start Seq.	End Seq.	Sequence	Ion Score	C. I. %	Modification	Rank	Result Type
800.4122	800.3878	-0.0244	-30	6	12	ASSRSHR					Mascot
802.4053	802.4758	0.0705	88	5318	5323	QELQER					Mascot
803.4872	803.4896	0.0024	3	868	874	SLTLIEK					Mascot
804.4977	804.4658	-0.0319	-40	974	980	VLNAFLK					Mascot
806.3712	806.448	0.0768	95	5610	5615	EMEELR					Mascot
807.3995	807.4329	0.0334	41	1555	1560	FEENLR					Mascot
812.4009	812.4586	0.0577	71	921	927	HVEANSR					Mascot
814.4682	814.4694	0.0012	1	4073	4078	QVKHFR					Mascot
815.437	815.4548	0.0178	22	4079	4085	ALQEQAR					Mascot
816.4574	816.4482	-0.0092	-11	596	602	NLSVEVR					Mascot
818.4254	818.468	0.0426	52	5232	5239	LPGSSTEK					Mascot
819.4611	819.4619	0.0008	1	3201	3207	LYDLPAK					Mascot
820.4233	820.4488	0.0255	31	195	201	QMGIEVK			Oxidation (M)[2]		Mascot
822.4576	822.4509	-0.0067	-8	5813	5819	MLTMKAK					Mascot
829.5254	829.4429	-0.0825	-99	1522	1528	AKLTQIR					Mascot
835.438	835.4996	0.0616	74	2	9	ATSRASSR					Mascot
837.4828	837.4694	-0.0134	-16	3852	3858	AQLSKYK					Mascot

838.4525	838.4747	0.0222	26	5813	5819	MLTMKAK	Oxidation (M)[1]	Mascot
839.4886	839.4791	-0.0095	-11	1004	1009	LHKQWK		Mascot
847.4155	847.4778	0.0623	74	2288	2294	EITEEAR		Mascot
849.4577	849.4724	0.0147	17	8650	8656	EVSHHIK		Mascot
850.4451	850.4597	0.0146	17	5073	5079	MASLEKR	Oxidation (M)[1]	Mascot
858.4355	858.4931	0.0576	67	8792	8799	YTNGPPPL		Mascot
859.4785	859.4933	0.0148	17	1655	1660	WQRLEK		Mascot
864.4573	864.499	0.0417	48	7416	7422	LNELGYR		Mascot
866.4586	866.4695	0.0109	13	6063	6069	MSTIRMK		Mascot
872.4584	872.4678	0.0094	11	6090	6096	QEQALQR		Mascot
874.4628	874.4664	0.0036	4	7058	7065	NSVENALK		Mascot
881.3999	881.4678	0.0679	77	8114	8120	EEFETAR		Mascot
887.4581	887.4862	0.0281	32	7719	7726	ELENAVGR		Mascot
906.5295	906.4913	-0.0382	-42	5122	5129	LSEFAVLK		Mascot
913.5353	913.5018	-0.0335	-37	2898	2906	VESLAPAVK		Mascot
917.5302	917.5295	-0.0007	-1	3480	3487	EAVTKLEK		Mascot
937.4989	937.5152	0.0163	17	565	572	QTADIYVK		Mascot
977.4622	977.5485	0.0863	88	1069	1076	GPHHLCEK	Carbamidomethyl (C)[6]	Mascot
1018.5316	1018.5401	0.0085	8	7112	7119	QTWISLDR		Mascot
1022.5516	1022.5552	0.0036	4	5966	5973	TLYEVLER		Mascot
1030.4987	1030.5656	0.0669	65	635	643	MLSQSEHAK		Mascot
1057.5889	1057.5544	-0.0345	-33	6624	6632	EEIQQLLGK		Mascot
1059.6595	1059.6129	-0.0466	-44	4396	4404	QVLLKSLMK		Mascot
1064.5769	1064.6239	0.047	44	3312	3320	SVLDSRMLK	Oxidation (M)[7]	Mascot
1072.6361	1072.5725	-0.0636	-59	2256	2264	INNLKELTK		Mascot
1107.5826	1107.5754	-0.0072	-7	4493	4502	TCKTAQASLK	Carbamidomethyl (C)[2]	Mascot
1111.563	1111.6084	0.0454	41	2139	2148	ELDSFTSKGK		Mascot
1182.6001	1182.6014	0.0013	1	2137	2146	QKELDSFTSK		Mascot
1205.6862	1205.6904	0.0042	3	2006	2015	VPTRQALQHR		Mascot
1228.6394	1228.6569	0.0175	14	503	511	MEFLELKYR		Mascot
1229.6372	1229.678	0.0408	33	21	30	LQDEQEIVQK		Mascot
1245.6798	1245.6827	0.0029	2	5324	5334	EAVETRINSVK		Mascot
1267.6351	1267.752	0.1169	92	195	205	QMGIEVKDFGK	Oxidation (M)[2]	Mascot
1301.7172	1301.6438	-0.0734	-56	6875	6886	VDTAALRAELSR		Mascot
1301.7498	1301.6438	-0.106	-81	2662	2672	LSQIQDILLMK		Mascot
1320.6616	1320.6375	-0.0241	-18	8423	8433	WELLQAQAMSK	Oxidation (M)[9]	Mascot

1324.6281	1324.6782	0.0501	38	1353	1363	YLFQTGSSHER		Mascot
1324.6281	1324.6782	0.0501	38	1353	1363	YLFQTGSSHER		Mascot
1333.6958	1333.6329	-0.0629	-47	1315	1326	LESTLTGLEQSR		Mascot
1347.6461	1347.7228	0.0767	57	5109	5120	EGVIELMNDAEK		Mascot
1369.7046	1369.7151	0.0105	8	7869	7879	WQHLLDLMAAR	Oxidation (M)[8]	Mascot
1406.7638	1406.7145	-0.0493	-35	5966	5976	TLYEVLERQQK		Mascot
1406.7638	1406.7145	-0.0493	-35	5966	5976	TLYEVLERQQK		Mascot
1418.7638	1418.7648	0.001	1	5330	5341	INSVKSWVQETK		Mascot
1420.7036	1420.7468	0.0432	30	5684	5695	MQAVQLCQSALR	Carbamidomethyl (C)[7], Oxidation (M)[1]	Mascot
1467.7162	1467.8503	0.1341	91	5182	5192	SMTTVWQRWTR	Oxidation (M)[2]	Mascot
1472.6873	1472.7424	0.0551	37	5423	5434	AMSQEFSCKIQK	Carbamidomethyl (C)[8], Oxidation (M)[2]	Mascot
1475.7411	1475.7928	0.0517	35	5109	5121	EGVIELMNDAEKK		Mascot
1558.9203	1558.7932	-0.1271	-82	3684	3696	YQALLLQVLEQIK		Mascot
1619.7871	1619.8855	0.0984	61	2429	2443	ESSNLTGDSQILEAR		Mascot
1635.7432	1635.8854	0.1422	87	2741	2753	SQLEQWMESVDQR		Mascot
1657.8644	1657.8036	-0.0608	-37	6597	6611	ALQDLVDLLDTGQEK		Mascot
1826.9355	1827.0712	0.1357	74	2575	2591	LSQRGQLLSEESHSAGK		Mascot
1826.9355	1827.0712	0.1357	74	2575	2591	LSQRGQLLSEESHSAGK		Mascot
1838.0382	1838.0308	-0.0074	-4	2596	2611	STQLLTSYQSLLRVTK		Mascot
1839.0626	1838.9919	-0.0707	-38	6498	6513	VLFTSLADSKYIILQK		Mascot
1908.9154	1908.8799	-0.0355	-19	2094	2110	STVCNVLEDASNVVVMR	Carbamidomethyl (C)[4], Oxidation (M)[16]	Mascot
1926.9994	1926.931	-0.0684	-35	1227	1242	LLSNFGECVQYKEIVK	Carbamidomethyl (C)[8]	Mascot
1981.9358	1981.9778	0.042	21	3966	3982	AMMEEIAGFEDRLDNLK		Mascot
2015.9049	2015.9983	0.0934	46	5415	5431	VQEIEEGKAMSQEFSCK	Carbamidomethyl (C)[16], Oxidation (M)[10]	Mascot
2084.0505	2084.1589	0.1084	52	8180	8197	SEPLDAAVIEEELDELRR		Mascot
2101.0166	2101.1707	0.1541	73	4780	4796	EHAQMCRQLESQLEVVK	Carbamidomethyl (C)[6], Oxidation (M)[5]	Mascot
2101.0166	2101.1707	0.1541	73	4780	4796	EHAQMCRQLESQLEVVK	Carbamidomethyl (C)[6], Oxidation (M)[5]	Mascot
2149.1387	2149.0837	-0.055	-26	834	852	INEILSVLEQEAQSSTLFK		Mascot
2165.0007	2165.0256	0.0249	12	3642	3659	WHEDLSAHRDEVEEVGT R		Mascot
2166.1726	2166.0991	-0.0735	-34	3119	3137	LNTMLFKGELLSSLLTEE K		Mascot
2183.0298	2183.0886	0.0588	27	7699	7716	LSQPLPDHHEELHAEQM R	Oxidation (M)[17]	Mascot
2183.0298	2183.0886	0.0588	27	7699	7716	LSQPLPDHHEELHAEQM R	Oxidation (M)[17]	Mascot
2184.0376	2184.0884	0.0508	23	1107	1125	ELKASIDNTYTMLVDDPD K	Oxidation (M)[12]	Mascot
2209.0125	2209.1475	0.135	61	7140	7158	AACDEINGHLMEARYSLS R	Carbamidomethyl (C)[3], Oxidation (M)[11]	Mascot
2399.166	2399.1946	0.0286	12	8318	8339	EFYLRGAVGLSGDPSSL ESQMR		Mascot

2680.3247	2680.4312	0.1065	40	7097	7119	EEVSGSVMSTLQELRQT WISLDR			Oxidation (M)[8]		Mascot
2807.4146	2807.4307	0.0161	6	4449	4472	ALSEKTQFLMAVFQATS QIQQHER			Oxidation (M)[10]		Mascot

7	14-3-3-like protein GF14-B OS=Oryza sativa subsp. japonica GN=GF14B PE=1 SV=2	14332_ORYSJ	29959.1	4.76	10	83	99.716	4.233	49	98.587

Peptide Information

Calc. Mass	Obsrv. Mass	± da	± ppm	Start Seq.	End Seq.	Sequence	Ion Score	C. I. %	Modification	Rank	Result Type
816.421	816.4482	0.0272	33	18	24	LAEQAER					Mascot
818.444	818.468	0.024	29	103	109	ICDGILK			Carbamidomethyl (C)[2]		Mascot
917.5302	917.5295	-0.0007	-1	69	76	IISSIEQK					Mascot
948.4244	948.4635	0.0391	41	131	137	MKGDYYR			Oxidation (M)[1]		Mascot
1018.5779	1018.5401	-0.0378	-37	94	102	GKIETELTK					Mascot
1189.6609	1189.7001	0.0392	33	223	232	DSTLIMQLLR					Mascot
1205.6559	1205.6904	0.0345	29	223	232	DSTLIMQLLR			Oxidation (M)[6]		Mascot
1212.5565	1212.6239	0.0674	56	150	160	DAAENTMVAYK					Mascot
1212.5565	1212.6239	0.0674	56	150	160	DAAENTMVAYK					Mascot
1228.5514	1228.6569	0.1055	86	150	160	DAAENTMVAYK			Oxidation (M)[7]		Mascot
1366.5542	1366.6586	0.1044	76	25	34	YEEMVEFMEK			Oxidation (M)[4,8]		Mascot
1406.6646	1406.7145	0.0499	35	38	49	TVDSEELTVEER					Mascot
1406.6646	1406.7145	0.0499	35	38	49	TVDSEELTVEER	49	98.587			Mascot
1418.7485	1418.7648	0.0163	11	69	80	IISSIEQKEESR					Mascot

8	14-3-3-like protein GF14 nu OS=Arabidopsis thaliana GN=GRF7 PE=1 SV=1	14337_ARATH	29920	4.74	8	71	96.083	3.359	49	98.587

Peptide Information

Calc. Mass	Obsrv. Mass	± da	± ppm	Start Seq.	End Seq.	Sequence	Ion Score	C. I. %	Modification	Rank	Result Type
816.421	816.4482	0.0272	33	14	20	LAEQAER					Mascot
819.4458	819.4619	0.0161	20	92	98	IETELSK					Mascot
906.425	906.4913	0.0663	73	127	133	MKGDYHR					Mascot
917.5302	917.5295	-0.0007	-1	65	72	IISSIEQK					Mascot
1189.6609	1189.7001	0.0392	33	219	228	DSTLIMQLLR					Mascot
1205.6559	1205.6904	0.0345	29	219	228	DSTLIMQLLR			Oxidation (M)[6]		Mascot
1366.5542	1366.6586	0.1044	76	21	30	YEEMVEFMEK			Oxidation (M)[4,8]		Mascot
1406.6646	1406.7145	0.0499	35	34	45	TVDTDELTVEER					Mascot
1406.6646	1406.7145	0.0499	35	34	45	TVDTDELTVEER	49	98.587			Mascot

1418.7485	1418.7648	0.0163	11	65	76	IISSIEQKEESR						Mascot

9	Structural maintenance of chromosomes protein 6 OS=Takifugu rubripes GN=smc6 PE=2 SV=1	SMC6_TAKRU	125928	7.18	30	66	86.417	10.671	

Peptide Information

Calc. Mass	Obsrv. Mass	± da	± ppm	Start Seq.	End Seq.	Sequence	Ion Score	C. I. %	Modification	Rank	Result Type
804.425	804.4658	0.0408	51	318	323	VDEWKK					Mascot
811.4421	811.4589	0.0168	21	577	583	VHDVSVR					Mascot
815.5098	815.4548	-0.055	-67	881	887	VKIATQR					Mascot
816.4461	816.4482	0.0021	3	458	464	ELEGLQK					Mascot
823.4308	823.4778	0.047	57	256	261	RDYLEK					Mascot
834.3774	834.3536	-0.0238	-29	984	990	ADLNDMR					Mascot
838.3988	838.4747	0.0759	91	816	821	CKHHEK			Carbamidomethyl (C)[1]		Mascot
839.437	839.4791	0.0421	50	447	454	HAIEGQGK					Mascot
850.3975	850.4597	0.0622	73	809	815	TDQEVMK					Mascot
859.4883	859.4933	0.005	6	324	331	KVEVAEGK					Mascot
866.473	866.4695	-0.0035	-4	156	162	EGIRTYK					Mascot
881.4662	881.4678	0.0016	2	941	947	FLSARCK			Carbamidomethyl (C)[6]		Mascot
913.5577	913.5018	-0.0559	-61	565	572	RPAIITSR					Mascot
917.4938	917.5295	0.0357	39	847	854	ELQESIAK					Mascot
937.5023	937.5152	0.0129	14	718	725	TTEVKTMK					Mascot
948.4819	948.4635	-0.0184	-19	220	227	ATQLEQMK					Mascot
1037.4972	1037.5547	0.0575	55	296	303	EFEPMKEK					Mascot
1057.5637	1057.5544	-0.0093	-9	165	174	SQSGHIISTK					Mascot
1072.6249	1072.5725	-0.0524	-49	726	734	LQLELTDLK					Mascot
1111.5201	1111.6084	0.0883	79	309	317	CATNKFNEK			Carbamidomethyl (C)[1]		Mascot
1189.6212	1189.7001	0.0789	66	228	237	DDFVHIKSTK					Mascot
1201.6172	1201.6555	0.0383	32	701	710	NISENQDLLR					Mascot
1201.6172	1201.6555	0.0383	32	701	710	NISENQDLLR	6	0			Mascot
1232.6481	1232.6174	-0.0307	-25	836	846	TLENNVASKEK					Mascot
1406.7209	1406.7145	-0.0064	-5	916	926	NLNNFIKCLDR			Carbamidomethyl (C)[8]		Mascot
1406.7209	1406.7145	-0.0064	-5	916	926	NLNNFIKCLDR			Carbamidomethyl (C)[8]		Mascot
1472.7704	1472.7424	-0.028	-19	458	470	ELEGLQKSIDANR					Mascot
1501.7542	1501.8018	0.0476	32	216	227	FFMKATQLEQMK					Mascot
1826.9105	1827.0712	0.1607	88	487	503	FGDQMPALLAAIDEAHK					Mascot
1826.9105	1827.0712	0.1607	88	487	503	FGDQMPALLAAIDEAHK					Mascot

1870.9467	1871.0712	0.1245	67	267	282	SLASVNEMYTKLEELK			Oxidation (M)[8]		Mascot
2209.1863	2209.1475	-0.0388	-18	115	135	GFVKEGESFAVVSITLNNI GK					Mascot
2807.3418	2807.4307	0.0889	32	958	983	GFTGNMTFDHKNETLSIS VQPGQGNK					Mascot

10 RNA-binding protein AU-1 OS=Pyrobaculum neutrophilum (strain DSM 2338 / JCM 9278 / V24Sta) GN=aubA PE=3 SV=1 | AUBA_PYRNV | 48937.1 | 9.09 | 16 | 66 | 85.107 | 3.922

Peptide Information

Calc. Mass	Obsrv. Mass	± da	± ppm	Start Seq.	End Seq.	Sequence	Ion Score	C. I. %	Modification	Rank	Result Type
811.4421	811.4589	0.0168	21	423	430	RPEAPGGK					Mascot
812.4988	812.4586	-0.0402	-49	251	258	AAVVPTVR					Mascot
818.4553	818.468	0.0127	16	309	315	MRGEVVK					Mascot
850.4781	850.4597	-0.0184	-22	183	190	AYASIGLR					Mascot
937.5353	937.5152	-0.0201	-21	8	16	GIYATALTK					Mascot
1032.4932	1032.5603	0.0671	65	413	420	YAHMLPER			Oxidation (M)[4]		Mascot
1037.4899	1037.5547	0.0648	62	197	205	YAGEEELAR					Mascot
1064.5809	1064.6239	0.043	40	232	240	CLAVVLFDK			Carbamidomethyl (C)[1]		Mascot
1165.6688	1165.6324	-0.0364	-31	217	228	LSQGGPPGALLR					Mascot
1182.673	1182.6014	-0.0716	-61	138	149	GVAVPEIVVDGK					Mascot
1228.6433	1228.6569	0.0136	11	85	96	ASAGLHDVFVGR					Mascot
1301.6783	1301.6438	-0.0345	-27	413	422	YAHMLPERLR			Oxidation (M)[4]		Mascot
1301.6783	1301.6438	-0.0345	-27	413	422	YAHMLPERLR			Oxidation (M)[4]		Mascot
1420.743	1420.7468	0.0038	3	206	216	EAEELYRELLR					Mascot
1838.0436	1838.0308	-0.0128	-7	293	308	GRVAIYHVKPWGEVVK					Mascot
2121.1228	2121.1135	-0.0093	-4	385	403	VVYIDLLVDKAYGPDGVE R					Mascot
2475.2449	2475.2102	-0.0347	-14	271	292	CLDLLDHVGADVYERAA AFLAR			Carbamidomethyl (C)[1]		Mascot

Gel Idx/Pos	173/G24	Instr./Gel Origin	BA2151/Sample Project 20140814	Process Status	Analysis Succeeded
Plate [#] Name	[1] Sample Project 20140814	Instrument Sample Name		Spectra	11

Rank	Protein Name	Accession No.	Protein MW	Protein PI	Pep. Count	Protein Score	Protein Score C. I. %	Intensity Matched	Total Ion Score	Total Ion C. I. %	Confirmed
1	Actin OS=Nicotiana tabacum PE=3 SV=1	ACT1_TOBAC	41940	5.46	14	158	100	7.987	88	100	

Peptide Information

Calc. Mass	Obsrv. Mass	± da	± ppm	Start Seq.	End Seq.	Sequence	Ion Score	C. I. %	Modification	Rank	Result Type
976.4483	976.4879	0.0396	41	21	30	AGFAGDDAPR					Mascot
1132.527	1132.574	0.047	42	199	208	GYSFTTTAER					Mascot
1182.5273	1182.6241	0.0968	82	53	63	DAYVGDEAQSK					Mascot
1198.7056	1198.7493	0.0437	36	31	41	AVFPSIVGRPR					Mascot
1459.6813	1459.8174	0.1361	93	362	374	AEYDESGPSIVHR					Mascot
1459.6813	1459.8174	0.1361	93	362	374	AEYDESGPSIVHR					Mascot
1493.7703	1493.8156	0.0453	30	315	328	MSKEITALAPSSMK					Mascot
1509.7651	1509.8357	0.0706	47	315	328	MSKEITALAPSSMK			Oxidation (M)[1]		Mascot
1515.7491	1515.8109	0.0618	41	87	97	IWHHTFYNELR					Mascot
1525.76	1525.7906	0.0306	20	315	328	MSKEITALAPSSMK			Oxidation (M)[1,13]		Mascot
1600.8363	1600.8148	-0.0215	-13	186	198	DLTDHLMKILTER			Oxidation (M)[7]		Mascot
1620.8414	1620.8264	-0.015	-9	299	314	IVLSGGSTMFPGIADR					Mascot
1747.8861	1747.9551	0.069	39	241	256	SYELPDGQVITIGAER					Mascot
1747.8861	1747.9551	0.069	39	241	256	SYELPDGQVITIGAER	88	100			Mascot
1919.8943	1920.0375	0.1432	75	218	233	LSYIALDFEQEMETSK			Oxidation (M)[12]		Mascot
1948.8746	1948.9633	0.0887	46	71	86	YPIEHGIVSNWDDMEK			Oxidation (M)[14]		Mascot
1954.0645	1954.1154	0.0509	26	98	115	VAPEEHPVLLTEAPLNPK					Mascot
3151.6423	3151.7866	0.1443	46	150	179	TTGIVLDSGDGVSHTVPI YEGYALPHAILR					Mascot

Rank	Protein Name	Accession No.	Protein MW	Protein PI	Pep. Count	Protein Score	Protein Score C. I. %	Intensity Matched	Total Ion Score	Total Ion C. I. %	Confirmed
2	Actin-58 OS=Solanum tuberosum GN=AC58 PE=3 SV=1	ACT3_SOLTU	41987.2	5.46	13	150	100	7.226	88	100	

Peptide Information

Calc. Mass	Obsrv. Mass	± da	± ppm	Start Seq.	End Seq.	Sequence	Ion Score	C. I. %	Modification	Rank	Result Type
976.4483	976.4879	0.0396	41	21	30	AGFAGDDAPR					Mascot
1132.527	1132.574	0.047	42	199	208	GYSFTTTAER					Mascot
1182.5273	1182.6241	0.0968	82	53	63	DAYVGDEAQSK					Mascot

1459.6813	1459.8174	0.1361	93	362	374	AEYDESGPSIVHR					Mascot
1459.6813	1459.8174	0.1361	93	362	374	AEYDESGPSIVHR					Mascot
1493.7703	1493.8156	0.0453	30	315	328	MSKELTALAPSSMK					Mascot
1509.7651	1509.8357	0.0706	47	315	328	MSKELTALAPSSMK			Oxidation (M)[1]		Mascot
1515.7491	1515.8109	0.0618	41	87	97	IWHHTFYNELR					Mascot
1525.76	1525.7906	0.0306	20	315	328	MSKELTALAPSSMK			Oxidation (M)[1,13]		Mascot
1546.7761	1546.7697	-0.0064	-4	21	35	AGFAGDDAPRAVFPR					Mascot
1600.8363	1600.8148	-0.0215	-13	186	198	DLTDHLMKILTER			Oxidation (M)[7]		Mascot
1747.8861	1747.9551	0.069	39	241	256	SYELPDGQVITIGAER					Mascot
1747.8861	1747.9551	0.069	39	241	256	SYELPDGQVITIGAER	88	100			Mascot
1822.9891	1822.9315	-0.0576	-32	36	52	IVGRPRHTGVMVGMGQK					Mascot
1948.8746	1948.9633	0.0887	46	71	86	YPIEHGIVSNWDDMEK			Oxidation (M)[14]		Mascot
1954.0645	1954.1154	0.0509	26	98	115	VAPEEHPVLLTEAPLNPK					Mascot
3151.6423	3151.7866	0.1443	46	150	179	TTGIVLDSGDGVSHTVPI YEGYALPHAILR					Mascot

3	Actin-97 OS=Solanum tuberosum GN=AC97 PE=1 SV=1	ACT11_SOLTU	41844	5.31	12	143	100	6.991	88	100	

Peptide Information

Calc. Mass	Obsrv. Mass	± da	± ppm	Start Seq.	End Seq.	Sequence	Ion Score	C. I. %	Modification	Rank	Result Type
976.4483	976.4879	0.0396	41	21	30	AGFAGDDAPR					Mascot
1118.5114	1118.5582	0.0468	42	199	208	GYSFTTSAER					Mascot
1182.5273	1182.6241	0.0968	82	53	63	DAYVGDEAQSK					Mascot
1198.7056	1198.7493	0.0437	36	31	41	AVFPSIVGRPR					Mascot
1459.6813	1459.8174	0.1361	93	362	374	AEYDESGPSIVHR					Mascot
1459.6813	1459.8174	0.1361	93	362	374	AEYDESGPSIVHR					Mascot
1493.7703	1493.8156	0.0453	30	315	328	MSKEITALAPSSMK					Mascot
1509.7651	1509.8357	0.0706	47	315	328	MSKEITALAPSSMK			Oxidation (M)[1]		Mascot
1515.7491	1515.8109	0.0618	41	87	97	IWHHTFYNELR					Mascot
1525.76	1525.7906	0.0306	20	315	328	MSKEITALAPSSMK			Oxidation (M)[1,13]		Mascot
1547.8098	1547.7852	-0.0246	-16	180	193	LDLAGRDLTDSLMK					Mascot
1747.8861	1747.9551	0.069	39	241	256	SYELPDGQVITIGAER					Mascot
1747.8861	1747.9551	0.069	39	241	256	SYELPDGQVITIGAER	88	100			Mascot
1948.8746	1948.9633	0.0887	46	71	86	YPIEHGIVSNWDDMEK			Oxidation (M)[14]		Mascot
1954.0645	1954.1154	0.0509	26	98	115	VAPEEHPVLLTEAPLNPK					Mascot
3151.6423	3151.7866	0.1443	46	150	179	TTGIVLDSGDGVSHTVPI YEGYALPHAILR					Mascot

4	Actin-66 (Fragment) OS=Solanum tuberosum PE=3	ACT5_SOLTU	37285.9	5.54	11	143	100	5.547	88	100	

SV=1

Peptide Information

Calc. Mass	Obsrv. Mass	± da	± ppm	Start Seq.	End Seq.	Sequence	Ion Score	C. I. %	Modification	Rank	Result Type
976.4483	976.4879	0.0396	41	1	10	AGFAGDDAPR					Mascot
1132.527	1132.574	0.047	42	179	188	GYSFTTTAER					Mascot
1182.5273	1182.6241	0.0968	82	33	43	DAYVGDEAQSK					Mascot
1198.7056	1198.7493	0.0437	36	11	21	AVFPSIVGRPR					Mascot
1493.7703	1493.8156	0.0453	30	295	308	MSKEITALAPSSMK					Mascot
1509.7651	1509.8357	0.0706	47	295	308	MSKEITALAPSSMK			Oxidation (M)[1]		Mascot
1515.7491	1515.8109	0.0618	41	67	77	IWHHTFYNELR					Mascot
1525.76	1525.7906	0.0306	20	295	308	MSKEITALAPSSMK			Oxidation (M)[1,13]		Mascot
1600.8363	1600.8148	-0.0215	-13	166	178	DLTDHLMKILTER			Oxidation (M)[7]		Mascot
1747.8861	1747.9551	0.069	39	221	236	SYELPDGQVITIGAER					Mascot
1747.8861	1747.9551	0.069	39	221	236	SYELPDGQVITIGAER	88	100			Mascot
1948.8746	1948.9633	0.0887	46	51	66	YPIEHGIVSNWDDMEK			Oxidation (M)[14]		Mascot
1954.0645	1954.1154	0.0509	26	78	95	VAPEEHPVLLTEAPLNPK					Mascot
3151.6423	3151.7866	0.1443	46	130	159	TTGIVLDSGDGVSHTVPI YEGYALPHAILR					Mascot

5	Actin-1 OS=Oryza sativa subsp. japonica GN=ACT1 PE=2 SV=1	ACT1_ORYSJ	42014	5.3	12	142	100	7.059	88	100	

Protein Group

Actin-1 OS=Oryza sativa subsp. indica GN=ACT1 PE=1 SV=1	ACT1_ORYSI	42014	5.3000 001907 3486

Peptide Information

Calc. Mass	Obsrv. Mass	± da	± ppm	Start Seq.	End Seq.	Sequence	Ion Score	C. I. %	Modification	Rank	Result Type
976.4483	976.4879	0.0396	41	21	30	AGFAGDDAPR					Mascot
1132.527	1132.574	0.047	42	199	208	GYSFTTTAER					Mascot
1182.5273	1182.6241	0.0968	82	53	63	DAYVGDEAQSK					Mascot
1198.7056	1198.7493	0.0437	36	31	41	AVFPSIVGRPR					Mascot
1459.6813	1459.8174	0.1361	93	362	374	AEYDESGPSIVHR					Mascot
1459.6813	1459.8174	0.1361	93	362	374	AEYDESGPSIVHR					Mascot
1493.7703	1493.8156	0.0453	30	315	328	MSKEITALAPSSMK					Mascot
1509.7651	1509.8357	0.0706	47	315	328	MSKEITALAPSSMK			Oxidation (M)[1]		Mascot
1515.7491	1515.8109	0.0618	41	87	97	IWHHTFYNELR					Mascot

1525.76	1525.7906	0.0306	20	315	328	MSKEITALAPSSMK			Oxidation (M)[1,13]		Mascot
1623.8411	1623.933	0.0919	57	180	193	LDLAGRDLTDYLMK					Mascot
1747.8861	1747.9551	0.069	39	241	256	SYELPDGQVITIGAER					Mascot
1747.8861	1747.9551	0.069	39	241	256	SYELPDGQVITIGAER	88	100			Mascot
1948.8746	1948.9633	0.0887	46	71	86	YPIEHGIVSNWDDMEK			Oxidation (M)[14]		Mascot
1954.0645	1954.1154	0.0509	26	98	115	VAPEEHPVLLTEAPLNPK					Mascot
3151.6423	3151.7866	0.1443	46	150	179	TTGIVLDSGDGVSHTVPI YEGYALPHAILR					Mascot

6	Actin-100 (Fragment) OS=Solanum tuberosum GN=AC100 PE=3 SV=1	ACT12_SOLTU	39756	5.56	11	140	100	5.032	88	100

Peptide Information

Calc. Mass	Obsrv. Mass	± da	± ppm	Start Seq.	End Seq.	Sequence	Ion Score	C. I. %	Modification	Rank	Result Type
976.4483	976.4879	0.0396	41	1	10	AGFAGDDAPR					Mascot
1118.5114	1118.5582	0.0468	42	179	188	GYSFTTSAER					Mascot
1182.5273	1182.6241	0.0968	82	33	43	DAYVGDEAQSK					Mascot
1198.7056	1198.7493	0.0437	36	11	21	AVFPSIVGRPR					Mascot
1493.7703	1493.8156	0.0453	30	295	308	MSKEITALAPSSMK					Mascot
1509.7651	1509.8357	0.0706	47	295	308	MSKEITALAPSSMK			Oxidation (M)[1]		Mascot
1515.7491	1515.8109	0.0618	41	67	77	IWHHTFYNELR					Mascot
1525.76	1525.7906	0.0306	20	295	308	MSKEITALAPSSMK			Oxidation (M)[1,13]		Mascot
1620.8085	1620.8264	0.0179	11	160	173	LDLAGRDLTDCLMK			Carbamidomethyl (C)[11]		Mascot
1623.8081	1623.933	0.1249	77	166	178	DLTDCLMKILTER			Carbamidomethyl (C)[5], Oxidation (M)[7]		Mascot
1747.8861	1747.9551	0.069	39	221	236	SYELPDGQVITIGAER					Mascot
1747.8861	1747.9551	0.069	39	221	236	SYELPDGQVITIGAER	88	100			Mascot
1948.8746	1948.9633	0.0887	46	51	66	YPIEHGIVSNWDDMEK			Oxidation (M)[14]		Mascot
3151.6423	3151.7866	0.1443	46	130	159	TTGIVLDSGDGVSHTVPI YEGYALPHAILR					Mascot

7	Actin OS=Gossypium hirsutum PE=3 SV=1	ACT_GOSHI	41867	5.31	11	136	100	7.44	88	100

Peptide Information

Calc. Mass	Obsrv. Mass	± da	± ppm	Start Seq.	End Seq.	Sequence	Ion Score	C. I. %	Modification	Rank	Result Type
976.4483	976.4879	0.0396	41	21	30	AGFAGDDAPR					Mascot
1132.527	1132.574	0.047	42	199	208	GYSFTTTAER					Mascot
1182.5273	1182.6241	0.0968	82	53	63	DAYVGDEAQSK					Mascot
1198.7056	1198.7493	0.0437	36	31	41	AVFPSIVGRPR					Mascot
1459.6813	1459.8174	0.1361	93	362	374	AEYDESGPSIVHR					Mascot

1459.6813	1459.8174	0.1361	93	362	374	AEYDESGPSIVHR					Mascot
1493.7703	1493.8156	0.0453	30	315	328	MSKEITALAPSSMK					Mascot
1509.7651	1509.8357	0.0706	47	315	328	MSKEITALAPSSMK			Oxidation (M)[1]		Mascot
1515.7491	1515.8109	0.0618	41	87	97	IWHHTFYNELR					Mascot
1525.76	1525.7906	0.0306	20	315	328	MSKEITALAPSSMK			Oxidation (M)[1,13]		Mascot
1531.8148	1531.8087	-0.0061	-4	180	193	LDLAGRDLTDALMK					Mascot
1547.8098	1547.7852	-0.0246	-16	180	193	LDLAGRDLTDALMK			Oxidation (M)[13]		Mascot
1747.8861	1747.9551	0.069	39	241	256	SYELPDGQVITIGAER					Mascot
1747.8861	1747.9551	0.069	39	241	256	SYELPDGQVITIGAER	88	100			Mascot
1954.0645	1954.1154	0.0509	26	98	115	VAPEEHPVLLTEAPLNPK					Mascot
3151.6423	3151.7866	0.1443	46	150	179	TTGIVLDSGDGVSHTVPI YEGYALPHAILR					Mascot

8	Actin-101 OS=Solanum tuberosum GN=AC101 PE=3 SV=1	ACT13_SOLTU	41902	5.23	11	135	100	5.032	88	100	

Peptide Information

Calc. Mass	Obsrv. Mass	± da	± ppm	Start Seq.	End Seq.	Sequence	Ion Score	C. I. %	Modification	Rank	Result Type
976.4483	976.4879	0.0396	41	21	30	AGFAGDDAPR					Mascot
1118.5114	1118.5582	0.0468	42	199	208	GYSFTTSAER					Mascot
1182.5273	1182.6241	0.0968	82	53	63	DAYVGDEAQSK					Mascot
1198.7056	1198.7493	0.0437	36	31	41	AVFPSIVGRPR					Mascot
1493.7703	1493.8156	0.0453	30	315	328	MSKEITALAPSSMK					Mascot
1509.7651	1509.8357	0.0706	47	315	328	MSKEITALAPSSMK			Oxidation (M)[1]		Mascot
1515.7491	1515.8109	0.0618	41	87	97	IWHHTFYNELR					Mascot
1525.76	1525.7906	0.0306	20	315	328	MSKEITALAPSSMK			Oxidation (M)[1,13]		Mascot
1620.8085	1620.8264	0.0179	11	180	193	LDLAGRDLTDCLMK			Carbamidomethyl (C)[11]		Mascot
1623.8081	1623.933	0.1249	77	186	198	DLTDCLMKILTER			Carbamidomethyl (C)[5], Oxidation (M)[7]		Mascot
1747.8861	1747.9551	0.069	39	241	256	SYELPDGQVITIGAER					Mascot
1747.8861	1747.9551	0.069	39	241	256	SYELPDGQVITIGAER	88	100			Mascot
1948.8746	1948.9633	0.0887	46	71	86	YPIEHGIVSNWDDMEK			Oxidation (M)[14]		Mascot
3151.6423	3151.7866	0.1443	46	150	179	TTGIVLDSGDGVSHTVPI YEGYALPHAILR					Mascot

9	Actin-82 (Fragment) OS=Solanum tuberosum PE=3 SV=1	ACT9_SOLTU	37322.8	5.54	10	132	100	5.378	88	100	

Peptide Information

Calc. Mass	Obsrv. Mass	± da	± ppm	Start Seq.	End Seq.	Sequence	Ion Score	C. I. %	Modification	Rank	Result Type

976.4483	976.4879	0.0396	41	1	10	AGFAGDDAPR					Mascot
1132.527	1132.574	0.047	42	179	188	GYSFTTTAER					Mascot
1182.5273	1182.6241	0.0968	82	33	43	DAYVGDEAQSK					Mascot
1198.7056	1198.7493	0.0437	36	11	21	AVFPSIVGRPR					Mascot
1493.7703	1493.8156	0.0453	30	295	308	MSKEITALAPSSMK					Mascot
1509.7651	1509.8357	0.0706	47	295	308	MSKEITALAPSSMK			Oxidation (M)[1]		Mascot
1515.7491	1515.8109	0.0618	41	67	77	IWHHTFYNELR					Mascot
1525.76	1525.7906	0.0306	20	295	308	MSKEITALAPSSMK			Oxidation (M)[1,13]		Mascot
1600.8363	1600.8148	-0.0215	-13	166	178	DLTDHLMKILTER			Oxidation (M)[7]		Mascot
1747.8861	1747.9551	0.069	39	221	236	SYELPDGQVITIGAER					Mascot
1747.8861	1747.9551	0.069	39	221	236	SYELPDGQVITIGAER	88	100			Mascot
1948.8746	1948.9633	0.0887	46	51	66	YPIEHGIVSNWDDMEK			Oxidation (M)[14]		Mascot
1954.0645	1954.1154	0.0509	26	78	95	VAPEEHPVLLTEAPLNPK					Mascot

10	Actin-1 OS=Sorghum bicolor GN=AC1 PE=2 SV=1	ACT1_SORBI	42121	5.44	11	129	100	6.443	83	100

Peptide Information

Calc. Mass	Obsrv. Mass	± da	± ppm	Start Seq.	End Seq.	Sequence	Ion Score	C. I. %	Modification	Rank	Result Type
976.4483	976.4879	0.0396	41	21	30	AGFAGDDAPR					Mascot
1132.527	1132.574	0.047	42	199	208	GYSFTTTAER					Mascot
1182.5273	1182.6241	0.0968	82	53	63	DAYVGDEAQSK					Mascot
1198.7056	1198.7493	0.0437	36	31	41	AVFPSIVGRPR					Mascot
1459.6813	1459.8174	0.1361	93	362	374	AEYDESGPSIVHR					Mascot
1459.6813	1459.8174	0.1361	93	362	374	AEYDESGPSIVHR					Mascot
1515.7491	1515.8109	0.0618	41	87	97	IWHHTFYNELR					Mascot
1623.8411	1623.933	0.0919	57	180	193	LDLAGRDLTDYLMK					Mascot
1747.8861	1747.9551	0.069	39	241	256	SYELPDGQVITIAADR					Mascot
1747.8861	1747.9551	0.069	39	241	256	SYELPDGQVITIAADR	83	100			Mascot
1948.8746	1948.9633	0.0887	46	71	86	YPIEHGIVSNWDDMEK			Oxidation (M)[14]		Mascot
1954.0645	1954.1154	0.0509	26	98	115	VAPEEHPVLLTEAPLNPK					Mascot
3151.6423	3151.7866	0.1443	46	150	179	TTGIVLDSGDGVSHTVPI YEGYALPHAILR					Mascot

Gel Idx/Pos	174/H1	Instr./Gel Origin	BA2151/Sample Project 20140814	Process Status	Analysis Succeeded
Plate [#] Name	[1] Sample Project 20140814	Instrument Sample Name		Spectra	11

Rank	Protein Name	Accession No.	Protein MW	Protein PI	Pep. Count	Protein Score	Protein Score C. I. %	Intensity Matched	Total Ion Score	Total Ion C. I. %	Confirmed
1	Spermidine synthase 1 OS=Oryza sativa subsp. japonica GN=SPDSYN1 PE=2 SV=1	SPD1_ORYSJ	35522.7	5.23	7	428	100	17.641	402	100	

Peptide Information

Calc. Mass	Obsrv. Mass	± da	± ppm	Start Seq.	End Seq.	Sequence	Ion Score	C. I. %	Modification	Rank	Result Type
976.488	976.5054	0.0174	18	1	9	MEAEAAAKR					Mascot
1211.7107	1211.7185	0.0078	6	111	123	VLVIGGGDGGVLR					Mascot
1211.7107	1211.7185	0.0078	6	111	123	VLVIGGGDGGVLR	126	100			Mascot
1339.8057	1339.8129	0.0072	5	110	123	KVLVIGGGDGGVLR					Mascot
1339.8057	1339.8129	0.0072	5	110	123	KVLVIGGGDGGVLR	94	100			Mascot
1355.7682	1355.7615	-0.0067	-5	163	175	VSLHIGDGVAFLK					Mascot
1355.7682	1355.7615	-0.0067	-5	163	175	VSLHIGDGVAFLK	90	100			Mascot
1440.8422	1440.8477	0.0055	4	76	88	VLVLDGVIQVTER					Mascot
1440.8422	1440.8477	0.0055	4	76	88	VLVLDGVIQVTER	92	100			Mascot
1672.7847	1672.7766	-0.0081	-5	128	141	HSSVEQIDICEIDK			Carbamidomethyl (C)[10]		Mascot
2447.1792	2447.1609	-0.0183	-7	128	148	HSSVEQIDICEIDKMVVD VSK			Carbamidomethyl (C)[10], Oxidation (M)[15]		Mascot

Rank	Protein Name	Accession No.	Protein MW	Protein PI	Pep. Count	Protein Score	Protein Score C. I. %	Intensity Matched	Total Ion Score	Total Ion C. I. %	Confirmed
2	Spermidine synthase 1 OS=Arabidopsis thaliana GN=SPDSYN1 PE=1 SV=1	SPD1_ARATH	36986.2	4.72	5	236	100	12.284	219	100	

Peptide Information

Calc. Mass	Obsrv. Mass	± da	± ppm	Start Seq.	End Seq.	Sequence	Ion Score	C. I. %	Modification	Rank	Result Type
1211.7107	1211.7185	0.0078	6	124	136	VLVIGGGDGGVLR					Mascot
1211.7107	1211.7185	0.0078	6	124	136	VLVIGGGDGGVLR	126	100			Mascot
1323.6539	1323.6692	0.0153	12	291	302	HPLNPIDESSSK					Mascot
1339.8057	1339.8129	0.0072	5	123	136	KVLVIGGGDGGVLR					Mascot
1339.8057	1339.8129	0.0072	5	123	136	KVLVIGGGDGGVLR	94	100			Mascot
2176.0405	2176.1184	0.0779	36	189	210	NAAEGSYDAVIVDSSDPI GPAK					Mascot
2447.1616	2447.1609	-0.0007	0	141	161	HASIEQIDMCEIDKMVVD VSK			Carbamidomethyl (C)[10]		Mascot

Rank	Protein Name	Accession No.	Protein MW	Protein PI	Pep. Count	Protein Score	Protein Score C. I. %	Intensity Matched	Total Ion Score	Total Ion C. I. %	Confirmed
3	Spermidine synthase OS=Solanum lycopersicum GN=SPDSYN PE=2 SV=1	SPDE_SOLLC	38110.9	4.81	5	234	100	12.834	219	100	

Peptide Information

Calc. Mass	Obsrv. Mass	± da	± ppm	Start Seq.	End Seq.	Sequence	Ion Score	C. I. %	Modification	Rank	Result Type
1211.7107	1211.7185	0.0078	6	131	143	VLVIGGGDGGVLR					Mascot
1211.7107	1211.7185	0.0078	6	131	143	VLVIGGGDGGVLR	126	100			Mascot
1339.8057	1339.8129	0.0072	5	130	143	KVLVIGGGDGGVLR					Mascot
1339.8057	1339.8129	0.0072	5	130	143	KVLVIGGGDGGVLR	94	100			Mascot
1672.7847	1672.7766	-0.0081	-5	148	161	HSSVEQIDICEIDK			Carbamidomethyl (C)[10]		Mascot
1782.8401	1782.8182	-0.0219	-12	2	17	ADECAAFMKGTELPVK			Carbamidomethyl (C)[4], Oxidation (M)[8]		Mascot
1835.8811	1835.8411	-0.04	-22	80	95	SDYQNVLVFQSSTYGK					Mascot

4	Spermidine synthase 2 OS=Arabidopsis thaliana GN=SPDSYN2 PE=1 SV=2	SPD2_ARATH	37629.6	4.72	5	234	100	13.218	219	100	

Peptide Information

Calc. Mass	Obsrv. Mass	± da	± ppm	Start Seq.	End Seq.	Sequence	Ion Score	C. I. %	Modification	Rank	Result Type
1211.7107	1211.7185	0.0078	6	128	140	VLVIGGGDGGVLR					Mascot
1211.7107	1211.7185	0.0078	6	128	140	VLVIGGGDGGVLR	126	100			Mascot
1339.8057	1339.8129	0.0072	5	127	140	KVLVIGGGDGGVLR					Mascot
1339.8057	1339.8129	0.0072	5	127	140	KVLVIGGGDGGVLR	94	100			Mascot
1654.786	1654.7916	0.0056	3	166	179	QYFPNVAVGYEDPR					Mascot
1672.7847	1672.7766	-0.0081	-5	145	158	HSSVEQIDICEIDK			Carbamidomethyl (C)[10]		Mascot
1870.9982	1871.0101	0.0119	6	2	18	SSTQEASVTDLPVKRPR					Mascot

5	Spermidine synthase OS=Saccharomyces cerevisiae (strain ATCC 204508 / S288c) GN=SPE3 PE=1 SV=1	SPEE_YEAST	33530.9	5.33	4	231	100	12.751	219	100	

Peptide Information

Calc. Mass	Obsrv. Mass	± da	± ppm	Start Seq.	End Seq.	Sequence	Ion Score	C. I. %	Modification	Rank	Result Type
1211.7107	1211.7185	0.0078	6	92	104	VLVIGGGDGGVLR					Mascot
1211.7107	1211.7185	0.0078	6	92	104	VLVIGGGDGGVLR	126	100			Mascot
1339.8057	1339.8129	0.0072	5	91	104	KVLVIGGGDGGVLR					Mascot
1339.8057	1339.8129	0.0072	5	91	104	KVLVIGGGDGGVLR	94	100			Mascot
1517.8224	1517.8235	0.0011	1	144	156	VKTHIGDGFQFLR					Mascot
2064.0142	2064.1116	0.0974	47	17	34	EISDTMWPGQAMTLKVEK					Mascot

6	Spermidine synthase 2 OS=Hyoscyamus niger PE=2 SV=1	SPD2_HYONI	34351.1	4.99	4	230	100	12.639	219	100	

Protein Group

Spermidine synthase 1 OS=Datura stramonium PE=2 SV=1	SPD1_DATST	34410.3	5.2300 000190 7349

Peptide Information

Calc. Mass	Obsrv. Mass	± da	± ppm	Start Seq.	End Seq.	Sequence	Ion Score	C. I. %	Modification	Rank	Result Type
1155.6521	1155.592	-0.0601	-52	150	160	LHVGDGVAFLK					Mascot
1211.7107	1211.7185	0.0078	6	96	108	VLVIGGGDGGVLR					Mascot
1211.7107	1211.7185	0.0078	6	96	108	VLVIGGGDGGVLR	126	100			Mascot
1339.8057	1339.8129	0.0072	5	95	108	KVLVIGGGDGGVLR					Mascot
1339.8057	1339.8129	0.0072	5	95	108	KVLVIGGGDGGVLR	94	100			Mascot
1672.7847	1672.7766	-0.0081	-5	113	126	HSSVEQIDICEIDK			Carbamidomethyl (C)[10]		Mascot

7	Spermidine synthase OS=Coffea arabica PE=2 SV=1	SPDE_COFAR	34877.6	5.05	3	228	100	11.837	219	100	

Peptide Information

Calc. Mass	Obsrv. Mass	± da	± ppm	Start Seq.	End Seq.	Sequence	Ion Score	C. I. %	Modification	Rank	Result Type
1211.7107	1211.7185	0.0078	6	104	116	VLVIGGGDGGVLR					Mascot
1211.7107	1211.7185	0.0078	6	104	116	VLVIGGGDGGVLR	126	100			Mascot
1339.8057	1339.8129	0.0072	5	103	116	KVLVIGGGDGGVLR					Mascot
1339.8057	1339.8129	0.0072	5	103	116	KVLVIGGGDGGVLR	94	100			Mascot
2382.1799	2382.1924	0.0125	5	135	155	MVVDVSKQFFPDVAVGF EDPR					Mascot

8	Spermidine synthase OS=Neurospora crassa (strain ATCC 24698 / 74-OR23-1A / CBS 708.71 / DSM 1257 / FGSC 987) GN=spe-3 PE=3 SV=1	SPEE_NEUCR	33395.8	5.54	3	227	100	15.327	219	100	

Peptide Information

Calc. Mass	Obsrv. Mass	± da	± ppm	Start Seq.	End Seq.	Sequence	Ion Score	C. I. %	Modification	Rank	Result Type
1211.7107	1211.7185	0.0078	6	90	102	VLVIGGGDGGVLR					Mascot
1211.7107	1211.7185	0.0078	6	90	102	VLVIGGGDGGVLR	126	100			Mascot
1339.8057	1339.8129	0.0072	5	89	102	KVLVIGGGDGGVLR					Mascot
1339.8057	1339.8129	0.0072	5	89	102	KVLVIGGGDGGVLR	94	100			Mascot
1656.8129	1656.8247	0.0118	7	2	15	SEIAHPTIQDGWFR					Mascot
1656.8129	1656.8247	0.0118	7	2	15	SEIAHPTIQDGWFR					Mascot

9	Spermidine synthase 1 OS=Hyoscyamus niger PE=2 SV=1	SPD1_HYONI	35037.5	4.96	3	227	100	12.519	219	100	

Protein Group

Spermidine synthase 2 OS=Datura stramonium PE=2 SV=1	SPD2_DATST	35337.5	4.9499 998092 6514
Spermidine synthase OS=Nicotiana sylvestris PE=2 SV=1	SPDE_NICSY	34889.4	5.2100 000381 4697

Peptide Information

Calc. Mass	Obsrv. Mass	± da	± ppm	Start Seq.	End Seq.	Sequence	Ion Score	C. I. %	Modification	Rank	Result Type
1211.7107	1211.7185	0.0078	6	104	116	VLVIGGGDGGVLR					Mascot
1211.7107	1211.7185	0.0078	6	104	116	VLVIGGGDGGVLR	126	100			Mascot
1339.8057	1339.8129	0.0072	5	103	116	KVLVIGGGDGGVLR					Mascot
1339.8057	1339.8129	0.0072	5	103	116	KVLVIGGGDGGVLR	94	100			Mascot
1672.7847	1672.7766	-0.0081	-5	121	134	HSSVEQIDICEIDK			Carbamidomethyl (C)[10]		Mascot

10	Spermidine synthase 1 OS=Pisum sativum GN=SPDSYN1 PE=1 SV=1	SPD1_PEA	37076.5	4.98	3	226	100	12.519	219	100	

Peptide Information

Calc. Mass	Obsrv. Mass	± da	± ppm	Start Seq.	End Seq.	Sequence	Ion Score	C. I. %	Modification	Rank	Result Type
1211.7107	1211.7185	0.0078	6	123	135	VLVIGGGDGGVLR					Mascot
1211.7107	1211.7185	0.0078	6	123	135	VLVIGGGDGGVLR	126	100			Mascot
1339.8057	1339.8129	0.0072	5	122	135	KVLVIGGGDGGVLR					Mascot
1339.8057	1339.8129	0.0072	5	122	135	KVLVIGGGDGGVLR	94	100			Mascot
1672.821	1672.7766	-0.0444	-27	140	153	HSSVEKIDICEIDK			Carbamidomethyl (C)[10]		Mascot

Gel Idx/Pos	175/H2	Instr./Gel Origin	BA2151/Sample Project 20140814	Process Status	Analysis Succeeded
Plate [#] Name	[1] Sample Project 20140814	Instrument Sample Name		Spectra	11

Rank	Protein Name	Accession No.	Protein MW	Protein PI	Pep. Count	Protein Score	Protein Score C. I. %	Intensity Matched	Total Ion Score	Total Ion C. I. %	Confirmed
1	Oxygen-evolving enhancer protein 1, chloroplastic OS=Nicotiana tabacum GN=PSBO PE=2 SV=1	PSBO_TOBAC	35377.1	5.89	14	394	100	26.49	314	100	

Peptide Information

Calc. Mass	Obsrv. Mass	± da	± ppm	Start Seq.	End Seq.	Sequence	Ion Score	C. I. %	Modification	Rank	Result Type
850.4305	850.4356	0.0051	6	238	245	GSSFLDPK					Mascot
988.521	988.5354	0.0144	15	37	46	AFGVEPAAAR					Mascot
1080.5573	1080.563	0.0057	5	92	100	LTFDEIQSK					Mascot
1236.6583	1236.6716	0.0133	11	91	100	RLTFDEIQSK					Mascot
1236.6583	1236.6716	0.0133	11	91	100	RLTFDEIQSK	34	63.061			Mascot
1328.6555	1328.6542	-0.0013	-1	136	146	FCLEPTSFTVK			Carbamidomethyl (C)[2]		Mascot
1444.7867	1444.7297	-0.057	-39	24	36	NNLQLRSAQSVSK					Mascot
1456.7505	1456.749	-0.0015	-1	135	146	KFCLEPTSFTVK			Carbamidomethyl (C)[3]		Mascot
1544.7704	1544.7468	-0.0236	-15	147	160	AESVNKNAPPDFQK					Mascot
1562.7559	1562.7727	0.0168	11	248	264	GGSTGYDNAVALPAGGR					Mascot
1562.7559	1562.7727	0.0168	11	248	264	GGSTGYDNAVALPAGGR	145	100			Mascot
1664.8346	1664.8987	0.0641	39	1	16	MAASLQAAATLMQPTK			Oxidation (M)[1,12]		Mascot
1760.8813	1760.9	0.0187	11	192	208	DGIDYAAVTVQLPGGER					Mascot
1760.8813	1760.9	0.0187	11	192	208	DGIDYAAVTVQLPGGER	136	100			Mascot
1775.8784	1775.8879	0.0095	5	246	264	GRGGSTGYDNAVALPAG GR					Mascot
2152.1907	2152.1355	-0.0552	-26	2	23	AASLQAAATLMQPTKVG VAPAR					Mascot
2294.1299	2294.1667	0.0368	16	188	208	FEEKDGIDYAAVTVQLPG GER					Mascot

Rank	Protein Name	Accession No.	Protein MW	Protein PI	Pep. Count	Protein Score	Protein Score C. I. %	Intensity Matched	Total Ion Score	Total Ion C. I. %	Confirmed
2	Oxygen-evolving enhancer protein 1, chloroplastic OS=Solanum tuberosum GN=PSBO PE=2 SV=1	PSBO_SOLTU	35595.1	5.84	11	368	100	25.684	315	100	

Peptide Information

Calc. Mass	Obsrv. Mass	± da	± ppm	Start Seq.	End Seq.	Sequence	Ion Score	C. I. %	Modification	Rank	Result Type
850.4305	850.4356	0.0051	6	238	245	GSSFLDPK					Mascot
1080.5573	1080.563	0.0057	5	92	100	LTFDEIQSK					Mascot
1135.5742	1135.5557	-0.0185	-16	153	162	NSAPDFQKTK					Mascot

1236.6583	1236.6716	0.0133	11	91	100	RLTFDEIQSK					Mascot
1236.6583	1236.6716	0.0133	11	91	100	RLTFDEIQSK	34	63.061			Mascot
1328.6555	1328.6542	-0.0013	-1	136	146	FCLEPTSFTVK			Carbamidomethyl (C)[2]		Mascot
1456.7505	1456.749	-0.0015	-1	135	146	KFCLEPTSFTVK			Carbamidomethyl (C)[3]		Mascot
1562.7559	1562.7727	0.0168	11	248	264	GGSTGYDNAVALPAGGR					Mascot
1562.7559	1562.7727	0.0168	11	248	264	GGSTGYDNAVALPAGGR	145	100			Mascot
1664.8346	1664.8987	0.0641	39	1	16	MAASLQAAATLMQPTK			Oxidation (M)[1,12]		Mascot
1760.8813	1760.9	0.0187	11	192	208	DGIDYAAVTVQLPGGER					Mascot
1760.8813	1760.9	0.0187	11	192	208	DGIDYAAVTVQLPGGER	136	100			Mascot
1775.8784	1775.8879	0.0095	5	246	264	GRGGSTGYDNAVALPAG GR					Mascot
2294.1299	2294.1667	0.0368	16	188	208	FEEKDGIDYAAVTVQLPG GER					Mascot

3	Oxygen-evolving enhancer protein 1, chloroplastic OS=Solanum lycopersicum GN=PSBO PE=2 SV=2	PSBO_SOLLC	35154	5.91	11	334	100	16.9	279	100	

Peptide Information

Calc. Mass	Obsrv. Mass	± da	± ppm	Start Seq.	End Seq.	Sequence	Ion Score	C. I. %	Modification	Rank	Result Type
850.4305	850.4356	0.0051	6	235	242	GSSFLDPK					Mascot
1135.5742	1135.5557	-0.0185	-16	150	159	NSAPDFQKTK					Mascot
1328.6555	1328.6542	-0.0013	-1	133	143	FCLEPTSFTVK			Carbamidomethyl (C)[2]		Mascot
1444.7867	1444.7297	-0.057	-39	21	33	NNLQLRSAQSVSK					Mascot
1456.7505	1456.749	-0.0015	-1	132	143	KFCLEPTSFTVK			Carbamidomethyl (C)[3]		Mascot
1562.7559	1562.7727	0.0168	11	245	261	GGSTGYDNAVALPAGGR					Mascot
1562.7559	1562.7727	0.0168	11	245	261	GGSTGYDNAVALPAGGR	145	100			Mascot
1664.8346	1664.8987	0.0641	39	1	16	MAASLQAAATLMQPTK			Oxidation (M)[1,12]		Mascot
1760.8813	1760.9	0.0187	11	189	205	DGIDYAAVTVQLPGGER					Mascot
1760.8813	1760.9	0.0187	11	189	205	DGIDYAAVTVQLPGGER	136	100			Mascot
1775.8784	1775.8879	0.0095	5	243	261	GRGGSTGYDNAVALPAG GR					Mascot
2181.0969	2180.9963	-0.1006	-46	34	53	AFGVEQGSGRLTCSLQT EIK			Carbamidomethyl (C)[13]		Mascot
2294.1299	2294.1667	0.0368	16	185	205	FEEKDGIDYAAVTVQLPG GER					Mascot

4	Oxygen-evolving enhancer protein 1, chloroplastic OS=Helianthus annuus GN=PSBO PE=1 SV=1	PSBO_HELAN	34487.4	5.4	10	329	100	34.072	281	100	

Peptide Information

Calc. Mass	Obsrv. Mass	± da	± ppm	Start Seq.	End Seq.	Sequence	Ion Score	C. I. %	Modification	Rank	Result Type
850.4305	850.4356	0.0051	6	230	237	GSSFLDPK					Mascot

Calc. Mass	Obsrv. Mass	± da	± ppm	Start Seq.	End Seq.	Sequence	Ion Score	C. I. %	Modification	Rank	Result Type
996.4996	996.4797	-0.0199	-20	29	38	AFGIESTGSK					Mascot
1328.6555	1328.6542	-0.0013	-1	128	138	FCLEPTSFTVK			Carbamidomethyl (C)[2]		Mascot
1416.7217	1416.6793	-0.0424	-30	257	268	GDEEELLKENIK					Mascot
1562.7559	1562.7727	0.0168	11	240	256	GGSTGYDNAVALPAGGR					Mascot
1562.7559	1562.7727	0.0168	11	240	256	GGSTGYDNAVALPAGGR	145	100			Mascot
1760.8813	1760.9	0.0187	11	184	200	DGIDYAAVTVQLPGGER					Mascot
1760.8813	1760.9	0.0187	11	184	200	DGIDYAAVTVQLPGGER	136	100			Mascot
1775.8784	1775.8879	0.0095	5	238	256	GRGGSTGYDNAVALPAG GR					Mascot
1786.8463	1786.9854	0.1391	78	1	17	MAASLQAAATFMTPTSR			Oxidation (M)[1,12]		Mascot
2280.1494	2280.2263	0.0769	34	159	179	LTYTLDEIEGPLEVSSDG TIK					Mascot
2280.1494	2280.2263	0.0769	34	159	179	LTYTLDEIEGPLEVSSDG TIK					Mascot
2294.1299	2294.1667	0.0368	16	180	200	FEEKDGIDYAAVTVQLPG GER					Mascot

5	Oxygen-evolving enhancer protein 1, chloroplastic (Fragments) OS=Populus euphratica GN=PSBO PE=1 SV=1	PSBO_POPEU	10664.5	5.36	4	308	100	14.714	281	100

Peptide Information

Calc. Mass	Obsrv. Mass	± da	± ppm	Start Seq.	End Seq.	Sequence	Ion Score	C. I. %	Modification	Rank	Result Type
1544.7704	1544.7468	-0.0236	-15	11	24	AEGINKNSPPDFQK					Mascot
1562.7559	1562.7727	0.0168	11	56	72	GGSTGYDNAVALPAGGR					Mascot
1562.7559	1562.7727	0.0168	11	56	72	GGSTGYDNAVALPAGGR	145	100			Mascot
1760.8813	1760.9	0.0187	11	31	47	DGIDYAAVTVQLPGGER					Mascot
1760.8813	1760.9	0.0187	11	31	47	DGIDYAAVTVQLPGGER	136	100			Mascot
2262.1548	2262.2466	0.0918	41	27	47	LMTRDGIDYAAVTVQLPG GER					Mascot
2278.1497	2278.2119	0.0622	27	27	47	LMTRDGIDYAAVTVQLPG GER			Oxidation (M)[2]		Mascot

6	Putative oxygen-evolving enhancer protein 1 (Fragments) OS=Pinus strobus GN=PSBO PE=1 SV=1	PSBO_PINST	13643.7	4.46	4	303	100	14.7	281	100

Peptide Information

Calc. Mass	Obsrv. Mass	± da	± ppm	Start Seq.	End Seq.	Sequence	Ion Score	C. I. %	Modification	Rank	Result Type
850.3975	850.4356	0.0381	45	35	42	GSSMLDPK			Oxidation (M)[4]		Mascot
1562.7559	1562.7727	0.0168	11	18	34	GGSTGYDNAVALPAGGR					Mascot
1562.7559	1562.7727	0.0168	11	18	34	GGSTGYDNAVALPAGGR	145	100			Mascot
1760.8813	1760.9	0.0187	11	1	17	DGIDYAAVTVQLPGGER					Mascot
1760.8813	1760.9	0.0187	11	1	17	DGIDYAAVTVQLPGGER	136	100			Mascot

1767.8185	1767.9192	0.1007	57	57	71	SYHDTNAENEFVTIK					Mascot

7	Oxygen-evolving enhancer protein 1, chloroplastic OS=Triticum aestivum GN=PSBO PE=1 SV=1	PSBO_WHEAT	34946.8	8.73	8	301	100	20.132	273	100

Peptide Information

Calc. Mass	Obsrv. Mass	± da	± ppm	Start Seq.	End Seq.	Sequence	Ion Score	C. I. %	Modification	Rank	Result Type
850.4305	850.4356	0.0051	6	231	238	GSSFLDPK					Mascot
1080.5573	1080.563	0.0057	5	86	94	LTFDEIQSK					Mascot
1236.6583	1236.6716	0.0133	11	85	94	RLTFDEIQSK					Mascot
1236.6583	1236.6716	0.0133	11	85	94	RLTFDEIQSK	34	63.061			Mascot
1328.6555	1328.6542	-0.0013	-1	130	140	FCLEPTSFTVK			Carbamidomethyl (C)[2]		Mascot
1456.7505	1456.749	-0.0015	-1	129	140	KFCLEPTSFTVK			Carbamidomethyl (C)[3]		Mascot
1760.8813	1760.9	0.0187	11	186	202	DGIDYAAVTVQLPGGER					Mascot
1760.8813	1760.9	0.0187	11	186	202	DGIDYAAVTVQLPGGER	136	100			Mascot
2168.9917	2168.9822	-0.0095	-4	101	121	GTGTANQCPTIDGGVDS FPFK			Carbamidomethyl (C)[8]		Mascot
2168.9917	2168.9822	-0.0095	-4	101	121	GTGTANQCPTIDGGVDS FPFK	103	100	Carbamidomethyl (C)[8]		Mascot
2294.1299	2294.1667	0.0368	16	182	202	FEEKDGIDYAAVTVQLPG GER					Mascot

8	Oxygen-evolving enhancer protein 1, chloroplastic OS=Spinacia oleracea GN=PSBO PE=1 SV=1	PSBO_SPIOL	35377	5.58	5	297	100	14.514	281	100

Peptide Information

Calc. Mass	Obsrv. Mass	± da	± ppm	Start Seq.	End Seq.	Sequence	Ion Score	C. I. %	Modification	Rank	Result Type
850.4305	850.4356	0.0051	6	236	243	GSSFLDPK					Mascot
1562.7559	1562.7727	0.0168	11	246	262	GGSTGYDNAVALPAGGR					Mascot
1562.7559	1562.7727	0.0168	11	246	262	GGSTGYDNAVALPAGGR	145	100			Mascot
1760.8813	1760.9	0.0187	11	190	206	DGIDYAAVTVQLPGGER					Mascot
1760.8813	1760.9	0.0187	11	190	206	DGIDYAAVTVQLPGGER	136	100			Mascot
1775.8784	1775.8879	0.0095	5	244	262	GRGGSTGYDNAVALPAG GR					Mascot
2294.1299	2294.1667	0.0368	16	186	206	FEEKDGIDYAAVTVQLPG GER					Mascot

9	Oxygen-evolving enhancer protein 1, chloroplastic OS=Fritillaria agrestis GN=PSBO PE=2 SV=1	PSBO_FRIAG	35075.9	6.26	12	289	100	28.937	229	100

Peptide Information

Calc. Mass	Obsrv. Mass	± da	± ppm	Start Seq.	End Seq.	Sequence	Ion Score	C. I. %	Modification	Rank	Result Type

810.4719	810.4111	-0.0608	-75	125	131	TGKYTLK					Mascot
850.4305	850.4356	0.0051	6	235	242	GSSFLDPK					Mascot
930.468	930.4703	0.0023	2	150	157	NAPPEFQK					Mascot
950.571	950.5679	-0.0031	-3	206	213	VPFLFTVK					Mascot
950.571	950.5679	-0.0031	-3	206	213	VPFLFTVK	59	99.87			Mascot
1080.5573	1080.563	0.0057	5	89	97	LTFDEIQSK					Mascot
1159.6106	1159.5874	-0.0232	-20	150	159	NAPPEFQKTK					Mascot
1236.6583	1236.6716	0.0133	11	88	97	RLTFDEIQSK					Mascot
1236.6583	1236.6716	0.0133	11	88	97	RLTFDEIQSK	34	63.061			Mascot
1264.6379	1264.6732	0.0353	28	270	281	ENIKDVSSSTGK					Mascot
1562.8214	1562.7727	-0.0487	-31	317	329	DVKIQGIWYAQLE					Mascot
1562.8214	1562.7727	-0.0487	-31	317	329	DVKIQGIWYAQLE					Mascot
1760.8813	1760.9	0.0187	11	189	205	DGIDYAAVTVQLPGGER					Mascot
1760.8813	1760.9	0.0187	11	189	205	DGIDYAAVTVQLPGGER	136	100			Mascot
2294.1438	2294.1667	0.0229	10	164	184	LTYTLDEIEGPFEVAPDG TVK					Mascot
2302.0881	2302.1626	0.0745	32	33	53	AFGFDNSTAGRLTCSINS DLR			Carbamidomethyl (C)[14]		Mascot

10	Oxygen-evolving enhancer protein 1-2, chloroplastic OS=Arabidopsis thaliana GN=PSBO2 PE=1 SV=1	PSBO2_ARATH	35225.8	5.92	7	230	100	12.139	204	100

Peptide Information

Calc. Mass	Obsrv. Mass	± da	± ppm	Start Seq.	End Seq.	Sequence	Ion Score	C. I. %	Modification	Rank	Result Type
850.4305	850.4356	0.0051	6	237	244	GSSFLDPK					Mascot
950.571	950.5679	-0.0031	-3	208	215	VPFLFTVK					Mascot
950.571	950.5679	-0.0031	-3	208	215	VPFLFTVK	59	99.87			Mascot
1562.7559	1562.7727	0.0168	11	247	263	GGSTGYDNAVALPAGGR					Mascot
1562.7559	1562.7727	0.0168	11	247	263	GGSTGYDNAVALPAGGR	145	100			Mascot
1664.8677	1664.8987	0.031	19	1	16	MATSLQAAATFLQPAK			Oxidation (M)[1]		Mascot
1673.9069	1673.8508	-0.0561	-34	272	287	ENVKNTAASVGEITLK					Mascot
1775.8784	1775.8879	0.0095	5	245	263	GRGGSTGYDNAVALPAG GR					Mascot
2294.1299	2294.1667	0.0368	16	187	207	FKEEDGIDYAAVTVQLPG GER					Mascot

Gel Idx/Pos	176/H3	Instr./Gel Origin	BA2151/Sample Project 20140814	Process Status	Analysis Succeeded
Plate [#] Name	[1] Sample Project 20140814	Instrument Sample Name		Spectra	11

Rank	Protein Name	Accession No.	Protein MW	Protein PI	Pep. Count	Protein Score	Protein Score C. I. %	Intensity Matched	Total Ion Score	Total Ion C. I. %	Confirmed
1	Glucose-1-phosphate adenylyltransferase small subunit, chloroplastic/amyloplastic OS=Triticum aestivum GN=AGP-S PE=2 SV=1	GLGS_WHEAT	52399.6	5.54	20	465	100	40.291	348	100	

Peptide Information

Calc. Mass	Obsrv. Mass	± da	± ppm	Start Seq.	End Seq.	Sequence	Ion Score	C. I. %	Modification	Rank	Result Type
854.444	854.4503	0.0063	7	200	207	ATAFGLMK			Oxidation (M)[7]		Mascot
972.4673	972.473	0.0057	6	448	455	ETDGYFIK					Mascot
1009.5975	1009.6042	0.0067	7	260	267	HVMLQLLR					Mascot
1017.5952	1017.603	0.0078	8	366	374	IHHSVVGLR					Mascot
1017.5952	1017.603	0.0078	8	366	374	IHHSVVGLR	69	99.986			Mascot
1025.5925	1025.5934	0.0009	1	260	267	HVMLQLLR			Oxidation (M)[3]		Mascot
1025.5925	1025.5934	0.0009	1	260	267	HVMLQLLR			Oxidation (M)[3]		Mascot
1032.5472	1032.5532	0.006	6	330	338	SAPIYTQPR					Mascot
1032.5472	1032.5532	0.006	6	330	338	SAPIYTQPR	43	94.114			Mascot
1074.6194	1074.6204	0.001	1	214	222	IIEFAEKPK					Mascot
1256.5801	1256.6061	0.026	21	18	27	REQCNIDGHK			Carbamidomethyl (C)[4]		Mascot
1384.6896	1384.7012	0.0116	8	319	329	KPIPDFSFYDR					Mascot
1384.6896	1384.7012	0.0116	8	319	329	KPIPDFSFYDR	103	100			Mascot
1390.8165	1390.6974	-0.1191	-86	407	420	GGIPIGIGKNSHIK					Mascot
1489.67	1489.7454	0.0754	51	19	31	EQCNIDGHKSSSK			Carbamidomethyl (C)[3]		Mascot
1522.7761	1522.7578	-0.0183	-12	106	119	HLSRAYGSNIGGYK					Mascot
1553.7628	1553.8525	0.0897	58	200	213	ATAFGLMKIDEEGR			Oxidation (M)[7]		Mascot
1621.7925	1621.7865	-0.006	-4	228	242	AMMVDTTILGLDDAR					Mascot
1637.7874	1637.7786	-0.0088	-5	228	242	AMMVDTTILGLDDAR			Oxidation (M)[2]		Mascot
1653.7822	1653.774	-0.0082	-5	228	242	AMMVDTTILGLDDAR			Oxidation (M)[2,3]		Mascot
1657.8796	1657.8049	-0.0747	-45	2	17	DVPLASKTFPSPSPSK					Mascot
1786.9368	1786.9772	0.0404	23	76	91	LIDIPVSNCLNSNISK			Carbamidomethyl (C)[9]		Mascot
1889.9525	1889.9342	-0.0183	-10	345	362	VLDADVTDSVIGEGCVIK			Carbamidomethyl (C)[15]		Mascot
1970.9965	1971.0115	0.015	8	430	447	IGDNVMIINVDNVQEAAR					Mascot
1970.9965	1971.0115	0.015	8	430	447	IGDNVMIINVDNVQEAAR	133	100			Mascot
1986.9913	1986.9746	-0.0167	-8	430	447	IGDNVMIINVDNVQEAAR			Oxidation (M)[6]		Mascot

1986.9913	1986.9746	-0.0167	-8	430	447	IGDNVMIINVDNVQEAAR	96	100	Oxidation (M)[6]		Mascot
2318.2537	2318.1223	-0.1314	-57	76	95	LIDIPVSNCLNSNISKIYVR			Carbamidomethyl (C)[9]		Mascot
2368.0874	2368.1069	0.0195	8	268	290	EQFPGANDFGSEVIPGAT STGMR					Mascot
2398.219	2398.1899	-0.0291	-12	319	338	KPIPDFSFYDRSAPIYTQP R					Mascot

2	Glucose-1-phosphate adenylyltransferase small subunit, chloroplastic/amyloplastic OS=Hordeum vulgare PE=2 SV=1	GLGS_HORVU	56412.8	6.11	17	431	100	39.071	348	100		

Peptide Information

Calc. Mass	Obsrv. Mass	± da	± ppm	Start Seq.	End Seq.	Sequence	Ion Score	C. I. %	Modification	Rank	Result Type
854.444	854.4503	0.0063	7	240	247	ATAFGLMK			Oxidation (M)[7]		Mascot
972.4673	972.473	0.0057	6	488	495	ETDGYFIK					Mascot
1009.5975	1009.6042	0.0067	7	300	307	HVMLQLLR					Mascot
1017.5952	1017.603	0.0078	8	406	414	IHHSVVGLR					Mascot
1017.5952	1017.603	0.0078	8	406	414	IHHSVVGLR	69	99.986			Mascot
1025.5925	1025.5934	0.0009	1	300	307	HVMLQLLR			Oxidation (M)[3]		Mascot
1025.5925	1025.5934	0.0009	1	300	307	HVMLQLLR			Oxidation (M)[3]		Mascot
1032.5472	1032.5532	0.006	6	370	378	SAPIYTQPR					Mascot
1032.5472	1032.5532	0.006	6	370	378	SAPIYTQPR	43	94.114			Mascot
1074.6194	1074.6204	0.001	1	254	262	IIEFAEKPK					Mascot
1256.7109	1256.6061	-0.1048	-83	104	115	AKPAVPLGANYR					Mascot
1384.6896	1384.7012	0.0116	8	359	369	KPIPDFSFYDR					Mascot
1384.6896	1384.7012	0.0116	8	359	369	KPIPDFSFYDR	103	100			Mascot
1390.8165	1390.6974	-0.1191	-86	447	460	GGIPIGIGKNSHIK					Mascot
1522.7761	1522.7578	-0.0183	-12	146	159	HLSRAYGSNIGGYK					Mascot
1553.7628	1553.8525	0.0897	58	240	253	ATAFGLMKIDEEGR			Oxidation (M)[7]		Mascot
1786.9368	1786.9772	0.0404	23	116	131	LIDIPVSNCLNSNISK			Carbamidomethyl (C)[9]		Mascot
1889.9525	1889.9342	-0.0183	-10	385	402	VLDADVTDSVIGEGCVIK			Carbamidomethyl (C)[15]		Mascot
1894.0038	1894.0343	0.0305	16	1	18	MAMAAAASPSKILIPPHR			Oxidation (M)[1,3]		Mascot
1970.9965	1971.0115	0.015	8	470	487	IGDNVMIINVDNVQEAAR					Mascot
1970.9965	1971.0115	0.015	8	470	487	IGDNVMIINVDNVQEAAR	133	100			Mascot
1986.9913	1986.9746	-0.0167	-8	470	487	IGDNVMIINVDNVQEAAR			Oxidation (M)[6]		Mascot
1986.9913	1986.9746	-0.0167	-8	470	487	IGDNVMIINVDNVQEAAR	96	100	Oxidation (M)[6]		Mascot
2368.0874	2368.1069	0.0195	8	308	330	EQFPGANDFGSEVIPGAT STGMR					Mascot
2398.219	2398.1899	-0.0291	-12	359	378	KPIPDFSFYDRSAPIYTQP R					Mascot

3	Glucose-1-phosphate adenylyltransferase small subunit, chloroplastic OS=Arabidopsis thaliana GN=APS1 PE=2 SV=2	GLGS_ARATH	56957.2	6.13	11	146	100	13.489	112	100	

Peptide Information

Calc. Mass	Obsrv. Mass	± da	± ppm	Start Seq.	End Seq.	Sequence	Ion Score	C. I. %	Modification	Rank	Result Type
854.444	854.4503	0.0063	7	247	254	ATAFGLMK			Oxidation (M)[7]		Mascot
950.5087	950.5363	0.0276	29	50	57	STVSRLCK			Carbamidomethyl (C)[7]		Mascot
972.4673	972.473	0.0057	6	495	502	ETDGYFIK					Mascot
1017.5952	1017.603	0.0078	8	413	421	IHHSVVGLR					Mascot
1017.5952	1017.603	0.0078	8	413	421	IHHSVVGLR	69	99.986			Mascot
1032.5472	1032.5532	0.006	6	377	385	SAPIYTQPR					Mascot
1032.5472	1032.5532	0.006	6	377	385	SAPIYTQPR	43	94.114			Mascot
1074.6194	1074.6204	0.001	1	261	269	IIEFAEKPK					Mascot
1256.7109	1256.6061	-0.1048	-83	111	122	AKPAVPLGANYR					Mascot
1553.7628	1553.8525	0.0897	58	247	260	ATAFGLMKIDEEGR			Oxidation (M)[7]		Mascot
1731.8445	1731.8688	0.0243	14	292	306	EMPFIASMGIYVVSR			Oxidation (M)[2,8]		Mascot
1786.9368	1786.9772	0.0404	23	123	138	LIDIPVSNCLNSNISK			Carbamidomethyl (C)[9]		Mascot
1971.0142	1971.0115	-0.0027	-1	12	31	VPPASTSNSTGKATEAVPTR					Mascot
1971.0142	1971.0115	-0.0027	-1	12	31	VPPASTSNSTGKATEAVPTR					Mascot

4	Glucose-1-phosphate adenylyltransferase small subunit, chloroplastic OS=Solanum lycopersicum PE=2 SV=1	GLGS_SOLLC	57733.5	6.49	11	143	100	11.572	112	100	

Peptide Information

Calc. Mass	Obsrv. Mass	± da	± ppm	Start Seq.	End Seq.	Sequence	Ion Score	C. I. %	Modification	Rank	Result Type
854.444	854.4503	0.0063	7	248	255	ATAFGLMK			Oxidation (M)[7]		Mascot
918.5077	918.4451	-0.0626	-68	46	53	LMPVSSLR			Oxidation (M)[2]		Mascot
972.4673	972.473	0.0057	6	496	503	ETDGYFIK					Mascot
1017.5952	1017.603	0.0078	8	414	422	IHHSVVGLR					Mascot
1017.5952	1017.603	0.0078	8	414	422	IHHSVVGLR	69	99.986			Mascot
1032.5472	1032.5532	0.006	6	378	386	SAPIYTQPR					Mascot
1032.5472	1032.5532	0.006	6	378	386	SAPIYTQPR	43	94.114			Mascot
1232.6667	1232.6057	-0.061	-49	308	317	DVMLNLLRDK			Oxidation (M)[3]		Mascot
1256.7109	1256.6061	-0.1048	-83	112	123	AKPAVPLGANYR					Mascot
1505.8145	1505.7706	-0.0439	-29	455	468	GSVPIGIGKNCLYK			Carbamidomethyl (C)[11]		Mascot

1553.7628	1553.8525	0.0897	58	248	261	ATAFGLMKIDEEGR			Oxidation (M)[7]		Mascot
1786.9368	1786.9772	0.0404	23	124	139	LIDIPVSNCLNSNISK			Carbamidomethyl (C)[9]		Mascot
2366.1445	2366.0886	-0.0559	-24	316	338	DKFPGANDFGSEVIPGAT SLGMR					Mascot
2382.1394	2382.1074	-0.032	-13	316	338	DKFPGANDFGSEVIPGAT SLGMR			Oxidation (M)[22]		Mascot

5	Glucose-1-phosphate adenylyltransferase small subunit, chloroplastic/amyloplastic OS=Solanum tuberosum PE=1 SV=2	GLGS_SOLTU	57603.4	6.73	10	138	100	11.368	112	100	

Peptide Information

Calc. Mass	Obsrv. Mass	± da	± ppm	Start Seq.	End Seq.	Sequence	Ion Score	C. I. %	Modification	Rank	Result Type
854.444	854.4503	0.0063	7	248	255	ATAFGLMK			Oxidation (M)[7]		Mascot
918.5077	918.4451	-0.0626	-68	46	53	LMPVSSLR			Oxidation (M)[2]		Mascot
972.4673	972.473	0.0057	6	496	503	ETDGYFIK					Mascot
1017.5952	1017.603	0.0078	8	414	422	IHHSVVGLR					Mascot
1017.5952	1017.603	0.0078	8	414	422	IHHSVVGLR	69	99.986			Mascot
1032.5472	1032.5532	0.006	6	378	386	SAPIYTQPR					Mascot
1032.5472	1032.5532	0.006	6	378	386	SAPIYTQPR	43	94.114			Mascot
1232.6667	1232.6057	-0.061	-49	308	317	DVMLNLLRDK			Oxidation (M)[3]		Mascot
1256.7109	1256.6061	-0.1048	-83	112	123	AKPAVPLGANYR					Mascot
1553.7628	1553.8525	0.0897	58	248	261	ATAFGLMKIDEEGR			Oxidation (M)[7]		Mascot
1786.9368	1786.9772	0.0404	23	124	139	LIDIPVSNCLNSNISK			Carbamidomethyl (C)[9]		Mascot
2366.1445	2366.0886	-0.0559	-24	316	338	DKFPGANDFGSEVIPGAT SLGMR					Mascot
2382.1394	2382.1074	-0.032	-13	316	338	DKFPGANDFGSEVIPGAT SLGMR			Oxidation (M)[22]		Mascot

6	Glucose-1-phosphate adenylyltransferase small subunit, chloroplastic/amyloplastic OS=Oryza sativa subsp. japonica GN=AGPS PE=2 SV=4	GLGS_ORYSJ	56467.8	6.58	9	135	100	11.353	112	100	

Peptide Information

Calc. Mass	Obsrv. Mass	± da	± ppm	Start Seq.	End Seq.	Sequence	Ion Score	C. I. %	Modification	Rank	Result Type
854.444	854.4503	0.0063	7	241	248	ATAFGLMK			Oxidation (M)[7]		Mascot
972.4673	972.473	0.0057	6	489	496	ETDGYFIK					Mascot
1017.5952	1017.603	0.0078	8	407	415	IHHSVVGLR					Mascot
1017.5952	1017.603	0.0078	8	407	415	IHHSVVGLR	69	99.986			Mascot
1032.5472	1032.5532	0.006	6	371	379	SAPIYTQPR					Mascot
1032.5472	1032.5532	0.006	6	371	379	SAPIYTQPR	43	94.114			Mascot
1060.6038	1060.5721	-0.0317	-30	255	263	IVEFAEKPK					Mascot

Calc. Mass	Obsrv. Mass	± da	± ppm	Start Seq.	End Seq.	Sequence	Ion Score	C. I. %	Modification	Rank	Result Type
1256.7109	1256.6061	-0.1048	-83	105	116	AKPAVPLGANYR					Mascot
1553.7628	1553.8525	0.0897	58	241	254	ATAFGLMKIDEEGR			Oxidation (M)[7]		Mascot
1786.9368	1786.9772	0.0404	23	117	132	LIDIPVSNCLNSNISK			Carbamidomethyl (C)[9]		Mascot
1889.9525	1889.9342	-0.0183	-10	386	403	VLDADVTDSVIGEGCVIK			Carbamidomethyl (C)[15]		Mascot

7 Glucose-1-phosphate adenylyltransferase small subunit 2, chloroplastic OS=Vicia faba GN=AGPP PE=2 SV=1 | GLGS2_VICFA | 56309.7 | 6.19 | 7 | 85 | 99.829 | 5.23 | 69 | 99.986

Peptide Information

Calc. Mass	Obsrv. Mass	± da	± ppm	Start Seq.	End Seq.	Sequence	Ion Score	C. I. %	Modification	Rank	Result Type
854.444	854.4503	0.0063	7	239	246	ATAFGLMK			Oxidation (M)[7]		Mascot
1017.5952	1017.603	0.0078	8	405	413	IHHSVVGLR					Mascot
1017.5952	1017.603	0.0078	8	405	413	IHHSVVGLR	69	99.986			Mascot
1256.7109	1256.6061	-0.1048	-83	103	114	AKPAVPLGANYR					Mascot
1302.7166	1302.6393	-0.0773	-59	51	62	TSGRNPFIVSPK					Mascot
1553.7628	1553.8525	0.0897	58	239	252	ATAFGLMKIDEEGR			Oxidation (M)[7]		Mascot
1786.9368	1786.9772	0.0404	23	115	130	LIDIPVSNCLNSNISK			Carbamidomethyl (C)[9]		Mascot
2400.1982	2400.1528	-0.0454	-19	358	377	KPVPDFSFYDRSSPIYTQPR					Mascot

8 Glucose-1-phosphate adenylyltransferase small subunit (Fragment) OS=Zea mays GN=GLG1 PE=2 SV=1 | GLGS_MAIZE | 13409 | 5.56 | 2 | 76 | 98.703 | 4.307 | 69 | 99.986

Peptide Information

Calc. Mass	Obsrv. Mass	± da	± ppm	Start Seq.	End Seq.	Sequence	Ion Score	C. I. %	Modification	Rank	Result Type
972.4673	972.473	0.0057	6	100	107	ETDGYFIK					Mascot
1017.5952	1017.603	0.0078	8	17	25	IHHSVVGLR					Mascot
1017.5952	1017.603	0.0078	8	17	25	IHHSVVGLR	69	99.986			Mascot

9 Keratin, type II cytoskeletal 1 OS=Pan troglodytes GN=KRT1 PE=1 SV=1 | K2C1_PANTR | 65620.8 | 7.62 | 11 | 69 | 92.872 | 2.71 | 36 | 72.72

Peptide Information

Calc. Mass	Obsrv. Mass	± da	± ppm	Start Seq.	End Seq.	Sequence	Ion Score	C. I. %	Modification	Rank	Result Type
945.4999	945.5195	0.0196	21	428	436	GENALKDAK					Mascot
999.4452	999.4621	0.0169	17	285	293	DVDGAYMTK					Mascot
1065.5211	1065.5074	-0.0137	-13	351	359	AQYEDIAQK					Mascot
1127.5402	1127.5374	-0.0028	-2	284	293	KDVDGAYMTK					Mascot
1265.6372	1265.6404	0.0032	3	273	283	TNAENEFVTIK					Mascot

Calc. Mass	Obsrv. Mass	± da	± ppm	Start Seq.	End Seq.	Sequence	Ion Score	C. I. %	Modification	Rank	Result Type
1302.7012	1302.6393	-0.0619	-48	388	398	NSKIEISELNR					Mascot
1475.7489	1475.7617	0.0128	9	207	218	WELLQQVDTSTR	38	80.328			Mascot
1653.8153	1653.774	-0.0413	-25	285	299	DVDGAYMTKVDLQAK					Mascot
1657.793	1657.8049	0.0119	7	13	29	SGGGFSSGSAGIINYQR					Mascot
1669.8102	1669.7965	-0.0137	-8	285	299	DVDGAYMTKVDLQAK			Oxidation (M)[7]		Mascot
2400.2114	2400.1528	-0.0586	-24	459	478	DYQELMNTKLALDLEIATYR					Mascot
3312.3083	3312.3525	0.0442	13	545	583	GSYGSGGSSYGSGGGSYGSGGGGGGHGSYGSGSSSGGYR					Mascot

10	Keratin, type II cytoskeletal 1 OS=Homo sapiens GN=KRT1 PE=1 SV=6	K2C1_HUMAN	66170.1	8.15	11	67	90.16	2.71	36	72.72

Peptide Information

Calc. Mass	Obsrv. Mass	± da	± ppm	Start Seq.	End Seq.	Sequence	Ion Score	C. I. %	Modification	Rank	Result Type
945.4999	945.5195	0.0196	21	433	441	GENALKDAK					Mascot
999.4452	999.4621	0.0169	17	290	298	DVDGAYMTK					Mascot
1065.5211	1065.5074	-0.0137	-13	356	364	AQYEDIAQK					Mascot
1127.5402	1127.5374	-0.0028	-2	289	298	KDVDGAYMTK					Mascot
1265.6372	1265.6404	0.0032	3	278	288	TNAENEFVTIK					Mascot
1302.7012	1302.6393	-0.0619	-48	393	403	NSKIEISELNR					Mascot
1475.7489	1475.7617	0.0128	9	212	223	WELLQQVDTSTR	38	80.328			Mascot
1653.8153	1653.774	-0.0413	-25	290	304	DVDGAYMTKVDLQAK					Mascot
1657.793	1657.8049	0.0119	7	13	29	SGGGFSSGSAGIINYQR					Mascot
1669.8102	1669.7965	-0.0137	-8	290	304	DVDGAYMTKVDLQAK			Oxidation (M)[7]		Mascot
2400.2114	2400.1528	-0.0586	-24	464	483	DYQELMNTKLALDLEIATYR					Mascot
3312.3083	3312.3525	0.0442	13	550	588	GSYGSGGSSYGSGGGSYGSGGGGGGHGSYGSGSSSGGYR					Mascot

Gel Idx/Pos	177/H4	Instr./Gel Origin	BA2151/Sample Project 20140814	Process Status	Analysis Succeeded
Plate [#] Name	[1] Sample Project 20140814	Instrument Sample Name		Spectra	11

Rank	Protein Name	Accession No.	Protein MW	Protein PI	Pep. Count	Protein Score	Protein Score C. I. %	Intensity Matched	Total Ion Score	Total Ion C. I. %	Confirmed
1	Oxygen-evolving enhancer protein 1, chloroplastic OS=Nicotiana tabacum GN=PSBO PE=2 SV=1	PSBO_TOBAC	35377.1	5.89	12	330	100	28.877	267	100	

Peptide Information

Calc. Mass	Obsrv. Mass	± da	± ppm	Start Seq.	End Seq.	Sequence	Ion Score	C. I. %	Modification	Rank	Result Type
988.521	988.5256	0.0046	5	37	46	AFGVEPAAAR					Mascot
1080.5573	1080.5521	-0.0052	-5	92	100	LTFDEIQSK					Mascot
1236.6583	1236.6652	0.0069	6	91	100	RLTFDEIQSK					Mascot
1236.6583	1236.6652	0.0069	6	91	100	RLTFDEIQSK	21	0			Mascot
1328.6555	1328.6475	-0.008	-6	136	146	FCLEPTSFTVK			Carbamidomethyl (C)[2]		Mascot
1456.7505	1456.7262	-0.0243	-17	135	146	KFCLEPTSFTVK			Carbamidomethyl (C)[3]		Mascot
1544.7704	1544.7515	-0.0189	-12	147	160	AESVNKNAPPDFQK					Mascot
1562.7559	1562.765	0.0091	6	248	264	GGSTGYDNAVALPAGGR					Mascot
1562.7559	1562.765	0.0091	6	248	264	GGSTGYDNAVALPAGGR	107	100			Mascot
1664.8346	1664.8744	0.0398	24	1	16	MAASLQAAATLMQPTK			Oxidation (M)[1,12]		Mascot
1747.9259	1747.8792	-0.0467	-27	47	61	LTCSLQTELKDLAQK			Carbamidomethyl (C)[3]		Mascot
1760.8813	1760.8878	0.0065	4	192	208	DGIDYAAVTVQLPGGER					Mascot
1760.8813	1760.8878	0.0065	4	192	208	DGIDYAAVTVQLPGGER	140	100			Mascot
2152.1907	2152.1318	-0.0589	-27	2	23	AASLQAAATLMQPTKVG VAPAR					Mascot
2294.1299	2294.1372	0.0073	3	188	208	FEEKDGIDYAAVTVQLPG GER					Mascot

Rank	Protein Name	Accession No.	Protein MW	Protein PI	Pep. Count	Protein Score	Protein Score C. I. %	Intensity Matched	Total Ion Score	Total Ion C. I. %	Confirmed
2	Oxygen-evolving enhancer protein 1, chloroplastic OS=Solanum tuberosum GN=PSBO PE=2 SV=1	PSBO_SOLTU	35595.1	5.84	9	307	100	28.2	269	100	

Peptide Information

Calc. Mass	Obsrv. Mass	± da	± ppm	Start Seq.	End Seq.	Sequence	Ion Score	C. I. %	Modification	Rank	Result Type
1080.5573	1080.5521	-0.0052	-5	92	100	LTFDEIQSK					Mascot
1236.6583	1236.6652	0.0069	6	91	100	RLTFDEIQSK					Mascot
1236.6583	1236.6652	0.0069	6	91	100	RLTFDEIQSK	21	0			Mascot
1328.6555	1328.6475	-0.008	-6	136	146	FCLEPTSFTVK			Carbamidomethyl (C)[2]		Mascot
1456.7505	1456.7262	-0.0243	-17	135	146	KFCLEPTSFTVK			Carbamidomethyl (C)[3]		Mascot
1562.7559	1562.765	0.0091	6	248	264	GGSTGYDNAVALPAGGR					Mascot

1562.7559	1562.765	0.0091	6	248	264	GGSTGYDNAVALPAGGR	107	100			Mascot
1649.8534	1649.8151	-0.0383	-23	320	333	DVKIQGIWYAQLES					Mascot
1664.8346	1664.8744	0.0398	24	1	16	MAASLQAAATLMQPTK			Oxidation (M)[1,12]		Mascot
1760.8813	1760.8878	0.0065	4	192	208	DGIDYAAVTVQLPGGER					Mascot
1760.8813	1760.8878	0.0065	4	192	208	DGIDYAAVTVQLPGGER	140	100			Mascot
2294.1299	2294.1372	0.0073	3	188	208	FEEKDGIDYAAVTVQLPG GER					Mascot

3	Putative oxygen-evolving enhancer protein 1 (Fragments) OS=Pinus strobus GN=PSBO PE=1 SV=1	PSBO_PINST	13643.7	4.46	5	280	100	21.347	248	100

Peptide Information

Calc. Mass	Obsrv. Mass	± da	± ppm	Start Seq.	End Seq.	Sequence	Ion Score	C. I. %	Modification	Rank	Result Type
1562.7559	1562.765	0.0091	6	18	34	GGSTGYDNAVALPAGGR					Mascot
1562.7559	1562.765	0.0091	6	18	34	GGSTGYDNAVALPAGGR	107	100			Mascot
1760.8813	1760.8878	0.0065	4	1	17	DGIDYAAVTVQLPGGER					Mascot
1760.8813	1760.8878	0.0065	4	1	17	DGIDYAAVTVQLPGGER	140	100			Mascot
1767.8185	1767.9056	0.0871	49	57	71	SYHDTNAENEFVTIK					Mascot
1895.9135	1896.0393	0.1258	66	57	72	SYHDTNAENEFVTIKK					Mascot
2398.1628	2398.2019	0.0391	16	35	56	GSSMLDPKELGQMNIVF EGVSK			Oxidation (M)[4,13]		Mascot

4	Oxygen-evolving enhancer protein 1, chloroplastic OS=Helianthus annuus GN=PSBO PE=1 SV=1	PSBO_HELAN	34487.4	5.4	7	277	100	37.344	248	100

Peptide Information

Calc. Mass	Obsrv. Mass	± da	± ppm	Start Seq.	End Seq.	Sequence	Ion Score	C. I. %	Modification	Rank	Result Type
1328.6555	1328.6475	-0.008	-6	128	138	FCLEPTSFTVK			Carbamidomethyl (C)[2]		Mascot
1562.7559	1562.765	0.0091	6	240	256	GGSTGYDNAVALPAGGR					Mascot
1562.7559	1562.765	0.0091	6	240	256	GGSTGYDNAVALPAGGR	107	100			Mascot
1760.8813	1760.8878	0.0065	4	184	200	DGIDYAAVTVQLPGGER					Mascot
1760.8813	1760.8878	0.0065	4	184	200	DGIDYAAVTVQLPGGER	140	100			Mascot
1786.8463	1786.9529	0.1066	60	1	17	MAASLQAAATFMTPTSR			Oxidation (M)[1,12]		Mascot
2268.166	2268.1057	-0.0603	-27	209	229	ELVATGKPESFGGNFLVP SYR					Mascot
2280.1494	2280.2117	0.0623	27	159	179	LTYTLDEIEGPLEVSSDG TIK					Mascot
2280.1494	2280.2117	0.0623	27	159	179	LTYTLDEIEGPLEVSSDG TIK					Mascot
2294.1299	2294.1372	0.0073	3	180	200	FEEKDGIDYAAVTVQLPG GER					Mascot

5	Oxygen-evolving enhancer protein 1, chloroplastic	PSBO_POPEU	10664.5	5.36	4	275	100	21.334	248	100

(Fragments) OS=Populus euphratica GN=PSBO PE=1 SV=1

Peptide Information

Calc. Mass	Obsrv. Mass	± da	± ppm	Start Seq.	End Seq.	Sequence	Ion Score	C. I. %	Modification	Rank	Result Type
1544.7704	1544.7515	-0.0189	-12	11	24	AEGINKNSPPDFQK					Mascot
1562.7559	1562.765	0.0091	6	56	72	GGSTGYDNAVALPAGGR					Mascot
1562.7559	1562.765	0.0091	6	56	72	GGSTGYDNAVALPAGGR	107	100			Mascot
1760.8813	1760.8878	0.0065	4	31	47	DGIDYAAVTVQLPGGER					Mascot
1760.8813	1760.8878	0.0065	4	31	47	DGIDYAAVTVQLPGGER	140	100			Mascot
2262.1548	2262.2004	0.0456	20	27	47	LMTRDGIDYAAVTVQLPGGER					Mascot
2278.1497	2278.1426	-0.0071	-3	27	47	LMTRDGIDYAAVTVQLPGGER			Oxidation (M)[2]		Mascot

6	Oxygen-evolving enhancer protein 1, chloroplastic OS=Solanum lycopersicum GN=PSBO PE=2 SV=2	PSBO_SOLLC	35154	5.91	6	267	100	21.968	246	100	

Peptide Information

Calc. Mass	Obsrv. Mass	± da	± ppm	Start Seq.	End Seq.	Sequence	Ion Score	C. I. %	Modification	Rank	Result Type
1328.6555	1328.6475	-0.008	-6	133	143	FCLEPTSFTVK			Carbamidomethyl (C)[2]		Mascot
1456.7505	1456.7262	-0.0243	-17	132	143	KFCLEPTSFTVK			Carbamidomethyl (C)[3]		Mascot
1562.7559	1562.765	0.0091	6	245	261	GGSTGYDNAVALPAGGR					Mascot
1562.7559	1562.765	0.0091	6	245	261	GGSTGYDNAVALPAGGR	107	100			Mascot
1664.8346	1664.8744	0.0398	24	1	16	MAASLQAAATLMQPTK			Oxidation (M)[1,12]		Mascot
1760.8813	1760.8878	0.0065	4	189	205	DGIDYAAVTVQLPGGER					Mascot
1760.8813	1760.8878	0.0065	4	189	205	DGIDYAAVTVQLPGGER	140	100			Mascot
2294.1299	2294.1372	0.0073	3	185	205	FEEKDGIDYAAVTVQLPGGER					Mascot

7	Oxygen-evolving enhancer protein 1, chloroplastic OS=Fritillaria agrestis GN=PSBO PE=2 SV=1	PSBO_FRIAG	35075.9	6.26	9	259	100	29.578	220	100	

Peptide Information

Calc. Mass	Obsrv. Mass	± da	± ppm	Start Seq.	End Seq.	Sequence	Ion Score	C. I. %	Modification	Rank	Result Type
930.468	930.4618	-0.0062	-7	150	157	NAPPEFQK					Mascot
950.571	950.5623	-0.0087	-9	206	213	VPFLFTVK					Mascot
950.571	950.5623	-0.0087	-9	206	213	VPFLFTVK	58	99.856			Mascot
1080.5573	1080.5521	-0.0052	-5	89	97	LTFDEIQSK					Mascot
1236.6583	1236.6652	0.0069	6	88	97	RLTFDEIQSK					Mascot

1236.6583	1236.6652	0.0069	6	88	97	RLTFDEIQSK	21	0			Mascot
1562.8214	1562.765	-0.0564	-36	317	329	DVKIQGIWYAQLE					Mascot
1562.8214	1562.765	-0.0564	-36	317	329	DVKIQGIWYAQLE					Mascot
1760.8813	1760.8878	0.0065	4	189	205	DGIDYAAVTVQLPGGER					Mascot
1760.8813	1760.8878	0.0065	4	189	205	DGIDYAAVTVQLPGGER	140	100			Mascot
1895.8995	1896.0393	0.1398	74	26	43	SNSHLSKAFGFDNSTAG R					Mascot
2294.1438	2294.1372	-0.0066	-3	164	184	LTYTLDEIEGPFEVAPDG TVK					Mascot
2302.0881	2302.1621	0.074	32	33	53	AFGFDNSTAGRLTCSINS DLR			Carbamidomethyl (C)[14]		Mascot

8	Oxygen-evolving enhancer protein 1, chloroplastic OS=Spinacia oleracea GN=PSBO PE=1 SV=1	PSBO_SPIOL	35377	5.58	3	257	100	21.283	248	100

Peptide Information

Calc. Mass	Obsrv. Mass	± da	± ppm	Start Seq.	End Seq.	Sequence	Ion Score	C. I. %	Modification	Rank	Result Type
1562.7559	1562.765	0.0091	6	246	262	GGSTGYDNAVALPAGGR					Mascot
1562.7559	1562.765	0.0091	6	246	262	GGSTGYDNAVALPAGGR	107	100			Mascot
1760.8813	1760.8878	0.0065	4	190	206	DGIDYAAVTVQLPGGER					Mascot
1760.8813	1760.8878	0.0065	4	190	206	DGIDYAAVTVQLPGGER	140	100			Mascot
2294.1299	2294.1372	0.0073	3	186	206	FEEKDGIDYAAVTVQLPG GER					Mascot

9	Oxygen-evolving enhancer protein 1, chloroplastic OS=Pisum sativum GN=PSBO PE=1 SV=1	PSBO_PEA	35099.9	6.25	9	203	100	24.972	162	100

Peptide Information

Calc. Mass	Obsrv. Mass	± da	± ppm	Start Seq.	End Seq.	Sequence	Ion Score	C. I. %	Modification	Rank	Result Type
1035.5066	1035.5609	0.0543	52	269	278	ENNKSAASSK					Mascot
1080.5573	1080.5521	-0.0052	-5	88	96	LTFDEIQSK					Mascot
1236.6583	1236.6652	0.0069	6	87	96	RLTFDEIQSK					Mascot
1236.6583	1236.6652	0.0069	6	87	96	RLTFDEIQSK	21	0			Mascot
1664.8346	1664.8744	0.0398	24	1	16	MAASLQAAATLMQPTK			Oxidation (M)[1,12]		Mascot
1760.8813	1760.8878	0.0065	4	188	204	DGIDYAAVTVQLPGGER					Mascot
1760.8813	1760.8878	0.0065	4	188	204	DGIDYAAVTVQLPGGER	140	100			Mascot
1786.9844	1786.9529	-0.0315	-18	2	18	AASLQAAATLMQPTKLR			Oxidation (M)[11]		Mascot
1895.9784	1896.0393	0.0609	32	132	148	LCLEPTSFTVKSEGVTK			Carbamidomethyl (C)[2]		Mascot
2294.1299	2294.1372	0.0073	3	184	204	FEEKDGIDYAAVTVQLPG GER					Mascot
2434.1482	2434.1592	0.011	5	244	268	GASTGYDNAVALPAGGR GDEEELGK					Mascot

2434.1482	2434.1592	0.011	5	244	268	GASTGYDNAVALPAGGR GDEEELGK						Mascot

10	Oxygen-evolving enhancer protein 1, chloroplastic OS=Triticum aestivum GN=PSBO PE=1 SV=1	PSBO_WHEAT	34946.8	8.73	9	199	100	24.731	162	100	

Peptide Information

Calc. Mass	Obsrv. Mass	± da	± ppm	Start Seq.	End Seq.	Sequence	Ion Score	C. I. %	Modification	Rank	Result Type
1080.5573	1080.5521	-0.0052	-5	86	94	LTFDEIQSK					Mascot
1236.6583	1236.6652	0.0069	6	85	94	RLTFDEIQSK					Mascot
1236.6583	1236.6652	0.0069	6	85	94	RLTFDEIQSK	21	0			Mascot
1328.6555	1328.6475	-0.008	-6	130	140	FCLEPTSFTVK			Carbamidomethyl (C)[2]		Mascot
1360.6591	1360.7015	0.0424	31	258	269	GDEEELAKENVK					Mascot
1456.7505	1456.7262	-0.0243	-17	129	140	KFCLEPTSFTVK			Carbamidomethyl (C)[3]		Mascot
1760.8813	1760.8878	0.0065	4	186	202	DGIDYAAVTVQLPGGER					Mascot
1760.8813	1760.8878	0.0065	4	186	202	DGIDYAAVTVQLPGGER	140	100			Mascot
2168.9917	2168.9763	-0.0154	-7	101	121	GTGTANQCPTIDGGVDS FPFK			Carbamidomethyl (C)[8]		Mascot
2290.2224	2290.0703	-0.1521	-66	62	85	MAGFALATSALLVSGATA EGAPKR					Mascot
2294.1299	2294.1372	0.0073	3	182	202	FEEKDGIDYAAVTVQLPG GER					Mascot

Gel Idx/Pos	178/H5	Instr./Gel Origin	BA2151/Sample Project 20140814	Process Status	Analysis Succeeded
Plate [#] Name	[1] Sample Project 20140814	Instrument Sample Name		Spectra	11

Rank	Protein Name	Accession No.	Protein MW	Protein PI	Pep. Count	Protein Score	Protein Score C. I. %	Intensity Matched	Total Ion Score	Total Ion C. I. %	Confirmed
1	L-ascorbate peroxidase 2, cytosolic OS=Oryza sativa subsp. japonica GN=APX2 PE=1 SV=1	APX2_ORYSJ	27214.6	5.21	11	399	100	34.091	331	100	

Peptide Information

Calc. Mass	Obsrv. Mass	± da	± ppm	Start Seq.	End Seq.	Sequence	Ion Score	C. I. %	Modification	Rank	Result Type
911.4291	911.4346	0.0055	6	54	62	TGGPFGTMK			Oxidation (M)[8]		Mascot
974.491	974.4979	0.0069	7	32	39	NCAPLMLR			Carbamidomethyl (C)[2]		Mascot
990.4859	990.4873	0.0014	1	32	39	NCAPLMLR			Carbamidomethyl (C)[2], Oxidation (M)[6]		Mascot
1249.6172	1249.6227	0.0055	4	121	131	QDKPEPPPEGR					Mascot
1249.6172	1249.6227	0.0055	4	121	131	QDKPEPPPEGR	70	99.991			Mascot
1309.6495	1309.6583	0.0088	7	132	143	LPDATQGSDHLR					Mascot
1309.6495	1309.6583	0.0088	7	132	143	LPDATQGSDHLR	112	100			Mascot
1533.7445	1533.7635	0.019	12	40	53	LAWHSAGTFDVSSR					Mascot
1557.8458	1557.7363	-0.1095	-70	211	224	ALMADPAFRPLVEK					Mascot
1585.8552	1585.8997	0.0445	28	26	39	GLIAEKNCAPLMLR			Carbamidomethyl (C)[8]		Mascot
1585.8552	1585.8997	0.0445	28	26	39	GLIAEKNCAPLMLR			Carbamidomethyl (C)[8]		Mascot
1601.8502	1601.8862	0.036	22	26	39	GLIAEKNCAPLMLR			Carbamidomethyl (C)[8], Oxidation (M)[12]		Mascot
1798.9222	1798.8157	-0.1065	-59	5	21	SYPTVSDEYLAAVGKAK					Mascot
2046.908	2046.8977	-0.0103	-5	225	242	YAADEDAFFADYAEAHLK					Mascot
2046.908	2046.8977	-0.0103	-5	225	242	YAADEDAFFADYAEAHLK	149	100			Mascot
2601.2727	2601.3193	0.0466	18	132	155	LPDATQGSDHLRQVFSA QMGLSDK					Mascot
2712.3159	2712.3928	0.0769	28	54	80	TGGPFGTMKNPGEQSHA ANAGLDIAVR			Oxidation (M)[8]		Mascot

Rank	Protein Name	Accession No.	Protein MW	Protein PI	Pep. Count	Protein Score	Protein Score C. I. %	Intensity Matched	Total Ion Score	Total Ion C. I. %	Confirmed
2	L-ascorbate peroxidase, cytosolic OS=Pisum sativum GN=APX1 PE=1 SV=2	APX1_PEA	27233	5.52	3	125	100	9.528	112	100	

Peptide Information

Calc. Mass	Obsrv. Mass	± da	± ppm	Start Seq.	End Seq.	Sequence	Ion Score	C. I. %	Modification	Rank	Result Type
1309.6859	1309.6583	-0.0276	-21	131	142	LPDATKGSDHLR					Mascot
1309.6859	1309.6583	-0.0276	-21	131	142	LPDATKGSDHLR	112	100			Mascot
2074.9392	2074.9314	-0.0078	-4	224	241	YAADEDVFFADYAEAHLK					Mascot
3689.864	3689.927	0.063	17	86	119	EQFPIVSYADFYQLAGVV					Mascot

AVEITGGPEVPFHPGR

3	L-ascorbate peroxidase 1, cytosolic OS=Oryza sativa subsp. japonica GN=APX1 PE=1 SV=1	APX1_ORYSJ	27252.8	5.42	3	120	100	15.383	112	100	

Protein Group

L-ascorbate peroxidase 1, cytosolic OS=Oryza sativa subsp. indica GN=APX1 PE=2 SV=1	APX1_ORYSI	27256.7	5.3099 999427 7954

Peptide Information

Calc. Mass	Obsrv. Mass	± da	± ppm	Start Seq.	End Seq.	Sequence	Ion Score	C. I. %	Modification	Rank	Result Type
911.4291	911.4346	0.0055	6	53	61	TGGPFGTMK			Oxidation (M)[8]		Mascot
1309.6859	1309.6583	-0.0276	-21	131	142	LPDATKGSDHLR					Mascot
1309.6859	1309.6583	-0.0276	-21	131	142	LPDATKGSDHLR	112	100			Mascot
1817.9392	1817.9055	-0.0337	-19	120	136	EDKPAPPPEGRLPDATK					Mascot
1817.9392	1817.9055	-0.0337	-19	120	136	EDKPAPPPEGRLPDATK					Mascot

4	Protein ORF1940 OS=Acidianus two-tailed virus PE=4 SV=1	Y1940_ATV	212473.6	5.24	34	72	96.172	15.095			

Peptide Information

Calc. Mass	Obsrv. Mass	± da	± ppm	Start Seq.	End Seq.	Sequence	Ion Score	C. I. %	Modification	Rank	Result Type
899.5309	899.4767	-0.0542	-60	859	866	LTVKPGER					Mascot
908.4724	908.3921	-0.0803	-88	648	655	ISPTSFEK					Mascot
960.4996	960.5494	0.0498	52	893	901	AETNLEGVK					Mascot
974.5153	974.4979	-0.0174	-18	1619	1626	VIDEIETR					Mascot
1041.4922	1041.5337	0.0415	40	496	504	IGDLMSDFK			Oxidation (M)[5]		Mascot
1042.6144	1042.6051	-0.0093	-9	943	951	IDKLPDTLK					Mascot
1052.635	1052.5769	-0.0581	-55	1780	1788	IEEVKPLPK					Mascot
1056.5796	1056.5825	0.0029	3	217	226	NIAQAANNLK					Mascot
1081.571	1081.5358	-0.0352	-33	1071	1079	ASMFKQIEK					Mascot
1113.6514	1113.6182	-0.0332	-30	548	557	IEVKAPDITK					Mascot
1209.6184	1209.6808	0.0624	52	976	985	GLPKMYEDLK			Oxidation (M)[5]		Mascot
1231.6528	1231.6073	-0.0455	-37	1617	1626	EKVIDEIETR					Mascot
1248.7198	1248.613	-0.1068	-86	1276	1286	LVDDIKASIFK					Mascot
1260.6835	1260.6693	-0.0142	-11	988	998	VIDPVDTFLNK					Mascot
1423.6926	1423.7698	0.0772	54	722	733	KYFDAIMTGYAK			Oxidation (M)[7]		Mascot
1451.72	1451.7046	-0.0154	-11	611	623	IPYDLQGKSMGDK					Mascot
1456.7318	1456.7734	0.0416	29	833	844	TEFDYLVTNLNK					Mascot

1503.765	1503.7513	-0.0137	-9	1672	1685	KDNNATEVLTDVGK					Mascot
1507.8003	1507.7562	-0.0441	-29	734	747	GDFSDVKVAELLSK					Mascot
1535.7272	1535.7472	0.02	13	342	355	QFVAEAQANMNANK					Mascot
1535.7272	1535.7472	0.02	13	342	355	QFVAEAQANMNANK					Mascot
1557.8007	1557.7363	-0.0644	-41	1632	1645	VDEIEGIVNDVIDK					Mascot
1601.8857	1601.8862	0.0005	0	741	755	VAELLSKVNSSDLAR					Mascot
1613.9473	1613.8496	-0.0977	-61	706	721	GVAITTDKATTIVPVK					Mascot
1669.8756	1669.7628	-0.1128	-68	505	520	EKISIGLHGISDDASK					Mascot
1686.8909	1686.7753	-0.1156	-69	893	908	AETNLEGVKAVDALEK					Mascot
1697.8414	1697.8232	-0.0182	-11	1649	1663	LYDSAVKDGMLNDLK			Oxidation (M)[10]		Mascot
1739.0426	1738.9407	-0.1019	-59	569	584	NVNVKLVKPLSTDLAK					Mascot
1743.8656	1743.9214	0.0558	32	1358	1372	TDLMQTYMDVKATVK					Mascot
1761.9204	1761.8989	-0.0215	-12	1060	1075	GLPDNLVNDIKASMFK					Mascot
1777.9153	1777.9075	-0.0078	-4	1060	1075	GLPDNLVNDIKASMFK			Oxidation (M)[14]		Mascot
1790.9316	1790.9156	-0.016	-9	748	763	VNSSDLARLMTDDIIK					Mascot
1815.8867	1815.9233	0.0366	20	1354	1368	IVDKTDLMQTYMDVK			Oxidation (M)[8]		Mascot
1831.8817	1831.8876	0.0059	3	1354	1368	IVDKTDLMQTYMDVK			Oxidation (M)[8,12]		Mascot
1856.9712	1856.8738	-0.0974	-52	884	901	SLPGGSEIRAETNLEGVK					Mascot
2029.056	2028.9647	-0.0913	-45	1373	1392	GPGTTQNTTTPSVAQNIT LK					Mascot
2047.0264	2046.8977	-0.1287	-63	962	979	MENSSLEEEVDLIKGLPK			Oxidation (M)[1]		Mascot
2047.0264	2046.8977	-0.1287	-63	962	979	MENSSLEEEVDLIKGLPK			Oxidation (M)[1]		Mascot

5	DNA-directed RNA polymerase subunit beta OS=Pelobacter carbinolicus (strain DSM 2380 / Gra Bd 1) GN=rpoB PE=3 SV=1	RPOB_PELCD	152682	5.31	23	69	93.792	14.823	15	0

Peptide Information

Calc. Mass	Obsrv. Mass	± da	± ppm	Start Seq.	End Seq.	Sequence	Ion Score	C. I. %	Modification	Rank	Result Type
859.4632	859.4189	-0.0443	-52	114	121	ESGVQAIR					Mascot
1006.4912	1006.4913	0.0001	0	1319	1327	SDDVTGRTR					Mascot
1033.5459	1033.5662	0.0203	20	1087	1096	LSVGDKMAGR					Mascot
1041.6051	1041.5337	-0.0714	-69	537	547	LSALGPGGLTR					Mascot
1054.5602	1054.5565	-0.0037	-4	1328	1336	MYEAIVKGK			Oxidation (M)[1]		Mascot
1081.5175	1081.5358	0.0183	17	556	564	DVHPTHYGR					Mascot
1232.594	1232.5992	0.0052	4	702	713	ADAPLVGTGMER			Oxidation (M)[10]		Mascot
1248.6696	1248.613	-0.0566	-45	2	12	AYSVANNQLLR					Mascot
1249.7375	1249.6227	-0.1148	-92	376	386	RLRPGDPPTIK					Mascot

1249.7375	1249.6227	-0.1148	-92	376	386	RLRPGDPPTIK	15	0			Mascot
1263.7419	1263.6342	-0.1077	-85	927	939	LPPGVEGVVIGAR					Mascot
1291.6641	1291.6434	-0.0207	-16	462	472	AVGELLENQYR					Mascot
1451.7499	1451.7046	-0.0453	-31	93	105	GMTFAAPVKVCVR			Carbamidomethyl (C)[11], Oxidation (M)[2]		Mascot
1507.8049	1507.7562	-0.0487	-32	1	13	MAYSVANNQLLRK					Mascot
1580.7737	1580.7927	0.019	12	363	375	MATPEDARIEIYR			Oxidation (M)[1]		Mascot
1738.9269	1738.9407	0.0138	8	1134	1149	MNVGQILETHLGLAAR			Oxidation (M)[1]		Mascot
1816.0076	1815.9233	-0.0843	-46	462	477	AVGELLENQYRVGLVR					Mascot
1816.952	1816.9022	-0.0498	-27	686	701	ALMGSNMQRQAVPLLR			Oxidation (M)[3,7]		Mascot
1835.1001	1834.9248	-0.1753	-96	880	897	IGAEVKPGDILVGKITPK	1	0			Mascot
2069.0762	2068.8875	-0.1887	-91	852	870	LGKEEITNDIPNLGEDALK					Mascot
2144.1243	2144.0728	-0.0515	-24	1249	1266	VTVGIMYMLKLHHLVDDK			Oxidation (M)[6,8]		Mascot
2568.2463	2568.3245	0.0782	30	743	765	IDEGEVDEDGTGVDIYNLI KFAR					Mascot
2631.3203	2631.2825	-0.0378	-14	387	409	SANALFESLFFNPERYDL SVVGR					Mascot
2633.3557	2633.3027	-0.053	-20	739	762	IVVKIDEGEVDEDGTGVDI YNLIK					Mascot
2633.3557	2633.3027	-0.053	-20	739	762	IVVKIDEGEVDEDGTGVDI YNLIK					Mascot

6 Putative ciliary rootlet coiled-coil protein-like 3 protein OS=Homo sapiens PE=5 SV=2 CROL3_HUMAN 250066.2 5.64 32 60 47.158 11.637

Peptide Information

Calc. Mass	Obsrv. Mass	± da	± ppm	Start Seq.	End Seq.	Sequence	Ion Score	C. I. %	Modification	Rank	Result Type
814.4781	814.4724	-0.0057	-7	1663	1669	EVLGLQR					Mascot
859.438	859.4189	-0.0191	-22	1526	1532	EAQEARR					Mascot
899.4152	899.4767	0.0615	68	1724	1730	LDQACHR			Carbamidomethyl (C)[5]		Mascot
900.501	900.4582	-0.0428	-48	857	863	LQQERAR					Mascot
932.4618	932.3951	-0.0667	-72	881	887	LEVQQCR			Carbamidomethyl (C)[6]		Mascot
974.5265	974.4979	-0.0286	-29	1319	1327	SAAEREALK					Mascot
997.5173	997.4758	-0.0415	-42	2225	2233	HVGNISTNR					Mascot
1006.4734	1006.4913	0.0179	18	437	444	DELASRCR			Carbamidomethyl (C)[7]		Mascot
1033.5525	1033.5662	0.0137	13	1415	1423	SLLSEELSR					Mascot
1042.535	1042.6051	0.0701	67	498	505	EQLEHMKK					Mascot
1056.5731	1056.5825	0.0094	9	1979	1986	QVLCRPQR			Carbamidomethyl (C)[4]		Mascot
1113.6375	1113.6182	-0.0193	-17	524	532	LQGELELRR					Mascot
1143.6171	1143.6047	-0.0124	-11	53	62	VAGGPFRFHR					Mascot
1209.6335	1209.6808	0.0473	39	2144	2153	LQDLTAQHQR					Mascot

1231.6603	1231.6073	-0.053	-43	898	908	AALEMVVEELK					Mascot
1260.6907	1260.6693	-0.0214	-17	1415	1425	SLLSEELSRAR					Mascot
1331.7101	1331.647	-0.0631	-47	486	497	STGLCQVNALLR			Carbamidomethyl (C)[5]		Mascot
1507.7289	1507.7562	0.0273	18	46	59	DFSPSDRVAGGPFR					Mascot
1519.7281	1519.7373	0.0092	6	180	193	TEACVGARDTGINR			Carbamidomethyl (C)[4]		Mascot
1669.8868	1669.7628	-0.124	-74	1107	1121	QEQLEGQAALLGREK					Mascot
1686.877	1686.7753	-0.1017	-60	603	618	VATERGLADLQADTAR					Mascot
1743.9113	1743.9214	0.0101	6	801	815	KPWWASVGKACLGQR			Carbamidomethyl (C)[11]		Mascot
1832.9348	1832.8988	-0.036	-20	832	848	SSQALVASLQEQLSESR					Mascot
1833.9429	1833.8986	-0.0443	-24	281	296	LGFFGADLQSLRPPCR			Carbamidomethyl (C)[15]		Mascot
1839.9196	1839.9088	-0.0108	-6	463	478	RSELEHSVDLEEALGR					Mascot
1845.9236	1845.9285	0.0049	3	506	523	ANDALGRELAGMTGSVQR					Mascot
1850.9681	1850.8995	-0.0686	-37	816	831	GPQLTPPEQELCLQLK			Carbamidomethyl (C)[12]		Mascot
1861.9186	1861.9364	0.0178	10	506	523	ANDALGRELAGMTGSVQR			Oxidation (M)[12]		Mascot
1888.8958	1888.8297	-0.0661	-35	2078	2093	QDQGEPEVKMEQETLK					Mascot
2069.0234	2068.8875	-0.1359	-66	542	561	GGLDAQPLHAMGTTGKDFPR					Mascot
2085.0183	2084.863	-0.1553	-74	542	561	GGLDAQPLHAMGTTGKDFPR			Oxidation (M)[11]		Mascot
2552.1968	2552.3953	0.1985	78	1021	1044	SEGVEQRDSLAAMAALMEGLAQDK			Oxidation (M)[13,17]		Mascot
2599.4243	2599.322	-0.1023	-39	195	217	DRVLALLEFLVLSHFAIGDSDLR					Mascot
2646.2854	2646.3123	0.0269	10	1431	1454	VQQEAQSQQEQAQATISATTEELK					Mascot

7	Replicase large subunit OS=Tobacco mosaic virus (strain vulgare) PE=1 SV=2	RDRP_TMV	185060.6	6.57	27	58	23.62	29.742

Peptide Information

Calc. Mass	Obsrv. Mass	± da	± ppm	Start Seq.	End Seq.	Sequence	Ion Score	C. I. %	Modification	Rank	Result Type
926.4512	926.4913	0.0401	43	895	901	STRCQFK			Carbamidomethyl (C)[4]		Mascot
948.4342	948.3906	-0.0436	-46	638	645	EVEEPSMK					Mascot
974.52	974.4979	-0.0221	-23	860	867	QAAEMIRR					Mascot
990.5149	990.4873	-0.0276	-28	860	867	QAAEMIRR			Oxidation (M)[5]		Mascot
997.4329	997.4758	0.0429	43	1177	1184	DCILDMSK			Carbamidomethyl (C)[2], Oxidation (M)[6]		Mascot
1052.5623	1052.5769	0.0146	14	1017	1025	ILTFTQSDK					Mascot
1081.4766	1081.5358	0.0592	55	345	353	TLAMCNSER			Carbamidomethyl (C)[5]		Mascot
1113.547	1113.6182	0.0712	64	134	142	DIMRHEGQK					Mascot
1209.5715	1209.6808	0.1093	90	344	353	KTLAMCNSER			Carbamidomethyl (C)[6]		Mascot
1225.5664	1225.6646	0.0982	80	344	353	KTLAMCNSER			Carbamidomethyl (C)[6], Oxidation (M)[5]		Mascot

Calc. Mass	Obsrv. Mass	± da	± ppm	Start Seq.	End Seq.	Sequence	Ion Score	C. I. %	Modification	Rank	Result Type
1232.6232	1232.5992	-0.024	-19	1101	1110	LSSYLLDMYK					Mascot
1248.618	1248.613	-0.005	-4	1101	1110	LSSYLLDMYK			Oxidation (M)[8]		Mascot
1260.5645	1260.6693	0.1048	83	1548	1556	DWEHLEEFR					Mascot
1309.7661	1309.6583	-0.1078	-82	1191	1201	DQIKPLIPMVR					Mascot
1309.7661	1309.6583	-0.1078	-82	1191	1201	DQIKPLIPMVR					Mascot
1331.6987	1331.647	-0.0517	-39	537	548	ASVDMPALDIRK			Oxidation (M)[5]		Mascot
1451.7278	1451.7046	-0.0232	-16	158	169	TVPNFQKEAFDR					Mascot
1585.9094	1585.8997	-0.0097	-6	1209	1222	QTGLLENLVAMIKR					Mascot
1585.9094	1585.8997	-0.0097	-6	1209	1222	QTGLLENLVAMIKR					Mascot
1601.9044	1601.8862	-0.0182	-11	1209	1222	QTGLLENLVAMIKR			Oxidation (M)[11]		Mascot
1743.8582	1743.9214	0.0632	36	793	808	RVAVSSESVVYSDMAK			Oxidation (M)[14]		Mascot
1748.7983	1748.923	0.1247	71	880	894	DNVKTVDSFMMNFGK			Oxidation (M)[10]		Mascot
1816.9124	1816.9022	-0.0102	-6	965	979	TTLRCPADVTHYLNR			Carbamidomethyl (C)[5]		Mascot
1847.8462	1847.951	0.1048	57	119	133	GRAYVHCCMPNLDVR			Carbamidomethyl (C)[7,8]		Mascot
1856.9423	1856.8738	-0.0685	-37	794	810	VAVSSESVVYSDMAKLR			Oxidation (M)[13]		Mascot
1863.0885	1862.944	-0.1445	-78	1185	1201	SVAAPKDQIKPLIPMVR					Mascot
1970.908	1970.9799	0.0719	36	1512	1526	QYGYFCGRYVIHHDR			Carbamidomethyl (C)[6]		Mascot
2074.8813	2074.9314	0.0501	24	1150	1167	CLPGNSTMMNNFDAVTM R			Carbamidomethyl (C)[1], Oxidation (M)[8]		Mascot
2085.0474	2084.863	-0.1844	-88	354	370	ILLEDSSSVNYWFPKMR					Mascot
2567.2124	2567.3604	0.148	58	1127	1149	GSNLFVAAPKTGDISDM QFYYDK					Mascot
2615.3674	2615.3994	0.032	12	1222	1245	RNFNAPELSGIIDIENTAS LVVDK					Mascot
2646.429	2646.3123	-0.1167	-44	197	220	VYAIALHSIYDIPADEFGA ALLRK					Mascot
2993.3213	2993.4258	0.1045	35	568	593	ESDKFDVDVFSQMCQSL EVDPMTAAK			Carbamidomethyl (C)[14], Oxidation (M)[13]		Mascot

8	DNA topoisomerase 2 OS=Bombyx mori GN=TOP2 PE=2 SV=1	TOP2_BOMMO	176872.2	8.9	25	58	10.261	20.342

Peptide Information

Calc. Mass	Obsrv. Mass	± da	± ppm	Start Seq.	End Seq.	Sequence	Ion Score	C. I. %	Modification	Rank	Result Type
814.5032	814.4724	-0.0308	-38	345	351	NVLEVLK					Mascot
899.5197	899.4767	-0.043	-48	887	894	LLDGEKPK					Mascot
960.476	960.5494	0.0734	76	895	901	VMHPWYK					Mascot
1033.4796	1033.5662	0.0866	84	449	458	LEDANDAGTK					Mascot
1052.483	1052.5769	0.0939	89	200	208	QTWGSNMTK					Mascot
1081.5525	1081.5358	-0.0167	-15	529	537	KYNSVDDLK					Mascot
1225.6423	1225.6646	0.0223	18	983	993	LSEIEAEGIHK					Mascot

Calc. Mass	Obsrv. Mass	± da	± ppm	Start Seq.	End Seq.	Sequence	Ion Score	C. I. %	Modification	Rank	Result Type
1232.5946	1232.5992	0.0046	4	1087	1097	GYAPDPIADWK					Mascot
1351.6965	1351.6731	-0.0234	-17	251	263	RAYDVAASSQGVK					Mascot
1456.7101	1456.7734	0.0633	43	388	400	ENMTLQAKSFGSK			Oxidation (M)[3]		Mascot
1557.7479	1557.7363	-0.0116	-7	671	682	EWLTNHMDEVKR					Mascot
1580.7374	1580.7927	0.0553	35	200	213	QTWGSNMTKASEPK			Oxidation (M)[7]		Mascot
1607.7911	1607.8595	0.0684	43	1040	1053	KDYLEGQLQAEADK					Mascot
1761.913	1761.8989	-0.0141	-8	92	106	IYDEILVNAADNKQR					Mascot
1816.8501	1816.9022	0.0521	29	648	663	YSGPTDDHHIELAFSK					Mascot
1839.8661	1839.9088	0.0427	23	609	622	ETENHHTYNIKYYK					Mascot
1845.9415	1845.9285	-0.013	-7	177	192	LCNIFSTKFTVETASK			Carbamidomethyl (C)[2]		Mascot
1856.9728	1856.8738	-0.099	-53	887	901	LLDGEKPKVMHPWYK			Oxidation (M)[10]		Mascot
1862.9365	1862.944	0.0075	4	1145	1159	KFNYLLGMSMWMLTK					Mascot
1891.9535	1891.9397	-0.0138	-7	1190	1205	EDLDAFLIKLDEVEDK					Mascot
1894.9264	1894.9009	-0.0255	-13	1145	1159	KFNYLLGMSMWMLTK			Oxidation (M)[8,10]		Mascot
1971.0005	1970.9799	-0.0206	-10	807	823	DSASPRYIFTLMSPLTR			Oxidation (M)[12]		Mascot
2046.9695	2046.8977	-0.0718	-35	592	607	DKEISFYSLPEFEEWK					Mascot
2046.9695	2046.8977	-0.0718	-35	592	607	DKEISFYSLPEFEEWK					Mascot
2551.2473	2551.406	0.1587	62	367	387	THMWLFVNCLIVNPTFDSQTK			Carbamidomethyl (C)[9]		Mascot
2567.2422	2567.3604	0.1182	46	367	387	THMWLFVNCLIVNPTFDSQTK			Carbamidomethyl (C)[9], Oxidation (M)[3]		Mascot
2601.217	2601.3193	0.1023	39	1329	1353	KMTFSGSSSGEMSASDAEVEVLVPR					Mascot
2616.0908	2616.2896	0.1988	76	997	1017	LQTTISMTCMNAFDYNCCLEK			Carbamidomethyl (C)[9,17,18], Oxidation (M)[7]		Mascot
2616.0908	2616.2896	0.1988	76	997	1017	LQTTISMTCMNAFDYNCCLEK			Carbamidomethyl (C)[9,17,18], Oxidation (M)[7]		Mascot
2632.0857	2632.2976	0.2119	81	997	1017	LQTTISMTCMNAFDYNCCLEK			Carbamidomethyl (C)[9,17,18], Oxidation (M)[7,10]		Mascot
2633.207	2633.3027	0.0957	36	1329	1353	KMTFSGSSSGEMSASDAEVEVLVPR			Oxidation (M)[2,12]		Mascot
2633.207	2633.3027	0.0957	36	1329	1353	KMTFSGSSSGEMSASDAEVEVLVPR			Oxidation (M)[2,12]		Mascot

9	Replicase large subunit OS=Tobacco mosaic virus (strain Korean NC 82) PE=3 SV=2	RDRP_TMVKR	185097.7	6.62	27	58	10.261	29.926

Peptide Information

Calc. Mass	Obsrv. Mass	± da	± ppm	Start Seq.	End Seq.	Sequence	Ion Score	C. I. %	Modification	Rank	Result Type
926.4512	926.4913	0.0401	43	895	901	STRCQFK			Carbamidomethyl (C)[4]		Mascot
948.4342	948.3906	-0.0436	-46	638	645	EVEEPSMK					Mascot
974.52	974.4979	-0.0221	-23	860	867	QAAEMIRR					Mascot
990.5149	990.4873	-0.0276	-28	860	867	QAAEMIRR			Oxidation (M)[5]		Mascot

997.4329	997.4758	0.0429	43	1177	1184	DCILDMSK			Carbamidomethyl (C)[2], Oxidation (M)[6]		Mascot
1033.5677	1033.5662	-0.0015	-1	1127	1136	GSNLFVATPK					Mascot
1052.5623	1052.5769	0.0146	14	1017	1025	ILTFTQSDK					Mascot
1081.4766	1081.5358	0.0592	55	345	353	TLAMCNSER			Carbamidomethyl (C)[5]		Mascot
1113.547	1113.6182	0.0712	64	134	142	DIMRHEGQK					Mascot
1209.5715	1209.6808	0.1093	90	344	353	KTLAMCNSER			Carbamidomethyl (C)[6]		Mascot
1225.5664	1225.6646	0.0982	80	344	353	KTLAMCNSER			Carbamidomethyl (C)[6], Oxidation (M)[5]		Mascot
1232.6232	1232.5992	-0.024	-19	1101	1110	LSSYLLDMYK					Mascot
1248.618	1248.613	-0.005	-4	1101	1110	LSSYLLDMYK			Oxidation (M)[8]		Mascot
1260.5645	1260.6693	0.1048	83	1548	1556	DWEHLEEFR					Mascot
1309.7661	1309.6583	-0.1078	-82	1191	1201	DQIKPLIPMVR					Mascot
1309.7661	1309.6583	-0.1078	-82	1191	1201	DQIKPLIPMVR					Mascot
1331.6987	1331.647	-0.0517	-39	537	548	ASVDMPALDIRK			Oxidation (M)[5]		Mascot
1451.7278	1451.7046	-0.0232	-16	158	169	TVPNFQKEAFDR					Mascot
1585.9094	1585.8997	-0.0097	-6	1209	1222	QTGLLENLVAMIKR					Mascot
1585.9094	1585.8997	-0.0097	-6	1209	1222	QTGLLENLVAMIKR					Mascot
1601.9044	1601.8862	-0.0182	-11	1209	1222	QTGLLENLVAMIKR			Oxidation (M)[11]		Mascot
1743.8582	1743.9214	0.0632	36	793	808	RVAVSSESVVYSDMAK			Oxidation (M)[14]		Mascot
1748.7983	1748.923	0.1247	71	880	894	DNVKTVDSFMMNFGK			Oxidation (M)[10]		Mascot
1816.9124	1816.9022	-0.0102	-6	965	979	TTLRCPADVTHYLNR			Carbamidomethyl (C)[5]		Mascot
1847.8462	1847.951	0.1048	57	119	133	GRAYVHCCMPNLDVR			Carbamidomethyl (C)[7,8]		Mascot
1856.9423	1856.8738	-0.0685	-37	794	810	VAVSSESVVYSDMAKLR			Oxidation (M)[13]		Mascot
1863.0885	1862.944	-0.1445	-78	1185	1201	SVAAPKDQIKPLIPMVR					Mascot
1970.908	1970.9799	0.0719	36	1512	1526	QYGYFCGRYVIHHDR			Carbamidomethyl (C)[6]		Mascot
2074.8813	2074.9314	0.0501	24	1150	1167	CLPGNSTMMNNFDAVTMR			Carbamidomethyl (C)[1], Oxidation (M)[8]		Mascot
2085.0474	2084.863	-0.1844	-88	354	370	ILLEDSSSVNYWFPKMR					Mascot
2615.3674	2615.3994	0.032	12	1222	1245	RNFNAPELSGIIDIENTASLVVDK					Mascot
2646.429	2646.3123	-0.1167	-44	197	220	VYAIALHSIYDIPADEFGAALLRK					Mascot
2993.3213	2993.4258	0.1045	35	568	593	ESDKFDVDVFSQMCQSLEVDPMTAAK			Carbamidomethyl (C)[14], Oxidation (M)[13]		Mascot

10	Nuclear distribution protein PAC1 OS=Ustilago maydis (strain 521 / FGSC 9021) GN=PAC1 PE=3 SV=1	LIS1_USTMA	50399.3	6.93	14	56	0	2.461

Peptide Information

Calc. Mass	Obsrv. Mass	± da	± ppm	Start Seq.	End Seq.	Sequence	Ion Score	C. I. %	Modification	Rank	Result Type
911.3961	911.4346	0.0385	42	78	84	IMEMESR			Oxidation (M)[2]		Mascot

974.5054	974.4979	-0.0075	-8	384	391	LWDLQSGR		Mascot
992.5775	992.4864	-0.0911	-92	58	66	AKYAGLLEK		Mascot
1143.6005	1143.6047	0.0042	4	310	320	QLASLDPNASK		Mascot
1351.6886	1351.6731	-0.0155	-11	372	383	SLLSVSDDKTMR		Mascot
1423.6635	1423.7698	0.1063	75	263	274	WLVSCSTDQTAR	Carbamidomethyl (C)[5]	Mascot
1507.7792	1507.7562	-0.023	-15	21	32	DELHKSILDYFK		Mascot
1533.7479	1533.7635	0.0156	10	33	45	TNNLHESFATLMR		Mascot
1557.7656	1557.7363	-0.0293	-19	46	59	EANQEGFVPDPRAK		Mascot
1743.8145	1743.9214	0.1069	61	2	18	SGFNGTSTSSNSILSER		Mascot
1777.908	1777.9075	-0.0005	0	413	429	IEAPIPPAQDGEEAGRK		Mascot
1795.865	1795.9327	0.0677	38	148	161	LWDWETGDFERTLK		Mascot
1798.8389	1798.8157	-0.0232	-13	321	338	DASASMAGQFVATGSRD K		Mascot
1834.9546	1834.9248	-0.0298	-16	363	380	GLAFSPNGKSLLSVSDDK		Mascot

Gel Idx/Pos	179/H6	Instr./Gel Origin	BA2151/Sample Project 20140814	Process Status	Analysis Succeeded
Plate [#] Name	[1] Sample Project 20140814	Instrument Sample Name		Spectra	11

Rank	Protein Name	Accession No.	Protein MW	Protein PI	Pep. Count	Protein Score	Protein Score C. I. %	Intensity Matched	Total Ion Score	Total Ion C. I. %	Confirmed
1	L-ascorbate peroxidase 2, cytosolic OS=Oryza sativa subsp. japonica GN=APX2 PE=1 SV=1	APX2_ORYSJ	27214.6	5.21	9	224	100	42.341	177	100	

Peptide Information

Calc. Mass	Obsrv. Mass	± da	± ppm	Start Seq.	End Seq.	Sequence	Ion Score	C. I. %	Modification	Rank	Result Type
899.5672	899.4792	-0.088	-98	24	31	LRGLIAEK					Mascot
911.4291	911.4317	0.0026	3	54	62	TGGPFGTMK			Oxidation (M)[8]		Mascot
974.491	974.4974	0.0064	7	32	39	NCAPLMLR			Carbamidomethyl (C)[2]		Mascot
990.4859	990.4855	-0.0004	0	32	39	NCAPLMLR			Carbamidomethyl (C)[2], Oxidation (M)[6]		Mascot
1249.6172	1249.6228	0.0056	4	121	131	QDKPEPPPEGR					Mascot
1249.6172	1249.6228	0.0056	4	121	131	QDKPEPPPEGR	67	99.981			Mascot
1309.6495	1309.656	0.0065	5	132	143	LPDATQGSDHLR					Mascot
1309.6495	1309.656	0.0065	5	132	143	LPDATQGSDHLR	110	100			Mascot
1557.8458	1557.7429	-0.1029	-66	211	224	ALMADPAFRPLVEK					Mascot
1585.8552	1585.8948	0.0396	25	26	39	GLIAEKNCAPLMLR			Carbamidomethyl (C)[8]		Mascot
1585.8552	1585.8948	0.0396	25	26	39	GLIAEKNCAPLMLR			Carbamidomethyl (C)[8]		Mascot
1601.8502	1601.8773	0.0271	17	26	39	GLIAEKNCAPLMLR			Carbamidomethyl (C)[8], Oxidation (M)[12]		Mascot
2046.908	2046.9011	-0.0069	-3	225	242	YAADEDAFFADYAEAHLK					Mascot
2617.2676	2617.2988	0.0312	12	132	155	LPDATQGSDHLRQVFSA QMGLSDK			Oxidation (M)[19]		Mascot

Rank	Protein Name	Accession No.	Protein MW	Protein PI	Pep. Count	Protein Score	Protein Score C. I. %	Intensity Matched	Total Ion Score	Total Ion C. I. %	Confirmed
2	L-ascorbate peroxidase 1, cytosolic OS=Oryza sativa subsp. japonica GN=APX1 PE=1 SV=1	APX1_ORYSJ	27252.8	5.42	5	127	100	22.385	110	100	

Protein Group

L-ascorbate peroxidase 1, cytosolic OS=Oryza sativa subsp. indica GN=APX1 PE=2 SV=1	APX1_ORYSI	27256.7	5.3099 999427 7954

Peptide Information

Calc. Mass	Obsrv. Mass	± da	± ppm	Start Seq.	End Seq.	Sequence	Ion Score	C. I. %	Modification	Rank	Result Type
911.4291	911.4317	0.0026	3	53	61	TGGPFGTMK			Oxidation (M)[8]		Mascot
1192.5957	1192.5811	-0.0146	-12	120	130	EDKPAPPPEGR					Mascot
1309.6859	1309.656	-0.0299	-23	131	142	LPDATKGSDHLR					Mascot
1309.6859	1309.656	-0.0299	-23	131	142	LPDATKGSDHLR	110	100			Mascot

1596.7217	1596.7545	0.0328	21	224	236	YAADEKAFFEDYK					Mascot
1817.9392	1817.907	-0.0322	-18	120	136	EDKPAPPPEGRLPDATK					Mascot
1817.9392	1817.907	-0.0322	-18	120	136	EDKPAPPPEGRLPDATK					Mascot

3	L-ascorbate peroxidase, cytosolic OS=Pisum sativum GN=APX1 PE=1 SV=2	APX1_PEA	27233	5.52	2	117	100	15.541	110	100		

Peptide Information

Calc. Mass	Obsrv. Mass	± da	± ppm	Start Seq.	End Seq.	Sequence	Ion Score	C. I. %	Modification	Rank	Result Type
1309.6859	1309.656	-0.0299	-23	131	142	LPDATKGSDHLR					Mascot
1309.6859	1309.656	-0.0299	-23	131	142	LPDATKGSDHLR	110	100			Mascot
2550.2729	2550.3416	0.0687	27	148	172	AMGLSDQDIVALSGGHTIGAAHKER			Oxidation (M)[2]		Mascot

4	T-complex protein 1 subunit theta OS=Pongo abelii GN=CCT8 PE=2 SV=3	TCPQ_PONAB	60152.7	5.42	15	58	20.02	8.006		

Protein Group

T-complex protein 1 subunit theta OS=Homo sapiens GN=CCT8 PE=1 SV=4	TCPQ_HUMAN	60152.7	5.4200000762 9395

Peptide Information

Calc. Mass	Obsrv. Mass	± da	± ppm	Start Seq.	End Seq.	Sequence	Ion Score	C. I. %	Modification	Rank	Result Type
1052.483	1052.4873	0.0043	4	45	54	TAYGPNGMNK					Mascot
1121.595	1121.5509	-0.0441	-39	75	84	ELEVQHPAAK					Mascot
1192.6056	1192.5811	-0.0245	-21	225	235	KETEGDVTSVK					Mascot
1232.6093	1232.5988	-0.0105	-9	297	307	VADMALHYANK					Mascot
1232.6093	1232.5988	-0.0105	-9	297	307	VADMALHYANK					Mascot
1249.5729	1249.6228	0.0499	40	271	281	GEENLMDAQVK			Oxidation (M)[6]		Mascot
1249.5729	1249.6228	0.0499	40	271	281	GEENLMDAQVK			Oxidation (M)[6]		Mascot
1366.7511	1366.6698	-0.0813	-59	308	318	YNIMLVRLNSK			Oxidation (M)[4]		Mascot
1567.8439	1567.8712	0.0273	17	365	378	HEKEDGAISTIVLR					Mascot
1607.9091	1607.8722	-0.0369	-23	2	16	ALHVPKAPGFAQMLK					Mascot
1623.9039	1623.834	-0.0699	-43	2	16	ALHVPKAPGFAQMLK			Oxidation (M)[13]		Mascot
1739.8932	1739.8785	-0.0147	-8	138	152	KAHEILPNLVCCSAK			Carbamidomethyl (C)[11,12]		Mascot
1790.0323	1789.922	-0.1103	-62	505	520	YWAIKLATNAAVTVLR					Mascot
1793.9314	1793.8667	-0.0647	-36	156	171	DIDEVSSLLRTSIMSK					Mascot
1815.9309	1815.9576	0.0267	15	166	181	TSIMSKQYGNEVFLAK					Mascot
1831.9259	1831.8951	-0.0308	-17	166	181	TSIMSKQYGNEVFLAK			Oxidation (M)[4]		Mascot

Calc. Mass	Obsrv. Mass	± da	± ppm	Start Seq.	End Seq.	Sequence	Ion Score	C. I. %	Modification	Rank	Result Type
1834.9731	1834.9218	-0.0513	-28	207	224	ILGSGISSSSVLHGMVFK			Oxidation (M)[15]		Mascot
1847.8555	1847.9479	0.0924	50	239	254	IAVYSCPFDGMITETK			Carbamidomethyl (C)[6], Oxidation (M)[11]		Mascot
1891.047	1890.9143	-0.1327	-70	521	539	VDQIIMAKPAGGPKPPSG K					Mascot

5	1-phosphatidylinositol 4,5-bisphosphate phosphodiesterase beta-1 OS=Rattus norvegicus GN=Plcb1 PE=1 SV=1	PLCB1_RAT	139112.5	5.86	22	58	18.157	9.866

Peptide Information

Calc. Mass	Obsrv. Mass	± da	± ppm	Start Seq.	End Seq.	Sequence	Ion Score	C. I. %	Modification	Rank	Result Type
899.4945	899.4792	-0.0153	-17	161	168	LQVTPEGR					Mascot
960.5109	960.5406	0.0297	31	1113	1120	LEEAQSKR					Mascot
974.4465	974.4974	0.0509	52	552	559	FESFETSK					Mascot
1052.5194	1052.4873	-0.0321	-30	816	823	YVNLMEQR					Mascot
1271.5872	1271.5897	0.0025	2	287	297	KGQMSVDGFMR			Oxidation (M)[4]		Mascot
1291.5875	1291.6532	0.0657	51	564	574	SFEMSSFVETK					Mascot
1507.7275	1507.7518	0.0243	16	298	311	YLSGEENGVVSPEK					Mascot
1519.7421	1519.757	0.0149	10	185	198	VETALEACSLPSSR			Carbamidomethyl (C)[8]		Mascot
1525.8373	1525.757	-0.0803	-53	1101	1112	SYIQEVVQYIKR					Mascot
1549.7203	1549.6447	-0.0756	-49	562	574	NKSFEMSSFVETK			Oxidation (M)[6]		Mascot
1553.7152	1553.7679	0.0527	34	1142	1153	LQMELEQEYQDK					Mascot
1567.8843	1567.8712	-0.0131	-8	255	267	DPRLNEILYPPLK					Mascot
1601.8745	1601.8773	0.0028	2	60	73	ETELLDLSLVKDAR					Mascot
1789.8749	1789.922	0.0471	26	1042	1056	LTDVAEECQNNQLKK			Carbamidomethyl (C)[8]		Mascot
1795.8134	1795.9415	0.1281	71	199	213	NDSIPQEDFTPDVYR					Mascot
1816.0038	1815.9576	-0.0462	-25	1156	1170	RLPLEILEFVQEAMK					Mascot
1831.9987	1831.8951	-0.1036	-57	1156	1170	RLPLEILEFVQEAMK			Oxidation (M)[14]		Mascot
1862.0243	1861.9078	-0.1165	-63	86	101	LRELLDVGNIGHLEQR					Mascot
2068.9971	2068.9031	-0.094	-45	444	461	YPLESGVPLPSPMDLMY K			Oxidation (M)[13,16]		Mascot
2259.1655	2259.0864	-0.0791	-35	661	681	HFDPFTEGIVDGIVANTLS VK					Mascot
2550.353	2550.3416	-0.0114	-4	1	24	MAGAQPGVHALQLKPVC VSDSLKK			Carbamidomethyl (C)[17], Oxidation (M)[1]		Mascot
2616.207	2616.2866	0.0796	30	288	311	GQMSVDGFMRYLSGEE NGVVSPEK					Mascot
2616.207	2616.2866	0.0796	30	288	311	GQMSVDGFMRYLSGEE NGVVSPEK					Mascot
2632.2019	2632.2778	0.0759	29	288	311	GQMSVDGFMRYLSGEE NGVVSPEK			Oxidation (M)[3]		Mascot
2633.3127	2633.3018	-0.0109	-4	1133	1153	QQILDEKPKLQMELEQEY QDK					Mascot
2633.3127	2633.3018	-0.0109	-4	1133	1153	QQILDEKPKLQMELEQEY					Mascot

2649.3076	2649.3081	0.0005	0	1133	1153	QDK QQILDEKPKLQMELEQEY QDK			Oxidation (M)[12]		Mascot

6 Elongation factor Ts OS=Saccharopolyspora erythraea (strain NRRL 23338) GN=tsf PE=3 SV=1 | EFTS_SACEN | 29015.1 | 5.07 | 11 | 56 | 0 | 6.162

Peptide Information

Calc. Mass	Obsrv. Mass	± da	± ppm	Start Seq.	End Seq.	Sequence	Ion Score	C. I. %	Modification	Rank	Result Type
859.4785	859.42	-0.0585	-68	144	150	VATYLHR					Mascot
1192.5845	1192.5811	-0.0034	-3	82	91	NDEFIELANK					Mascot
1323.6427	1323.6517	0.009	7	25	36	KALEATEGDFDK					Mascot
1484.734	1484.8405	0.1065	72	191	203	DEVPADIVADERR					Mascot
1519.8115	1519.757	-0.0545	-36	251	265	ALLDEAGVTVTGFAR					Mascot
1557.7755	1557.7429	-0.0326	-21	233	246	DNVLLDQPSVQDSK					Mascot
1577.8435	1577.8168	-0.0267	-17	137	150	VATFDGKVATYLHR					Mascot
1685.8705	1685.7423	-0.1282	-76	233	247	DNVLLDQPSVQDSKK					Mascot
1686.9272	1686.7644	-0.1628	-97	116	131	TVGEVVQELSAKIGEK					Mascot
1848.0226	1847.9479	-0.0747	-40	248	265	TVKALLDEAGVTVTGFAR					Mascot
2633.3306	2633.3018	-0.0288	-11	56	81	ATAEGLVAADNGVLVELN SETDFVAK					Mascot
2633.3306	2633.3018	-0.0288	-11	56	81	ATAEGLVAADNGVLVELN SETDFVAK					Mascot

7 DNA-directed RNA polymerase subunit beta OS=Pelobacter carbinolicus (strain DSM 2380 / Gra Bd 1) GN=rpoB PE=3 SV=1 | RPOB_PELCD | 152682 | 5.31 | 19 | 56 | 0 | 14.037 | 15 | 0

Peptide Information

Calc. Mass	Obsrv. Mass	± da	± ppm	Start Seq.	End Seq.	Sequence	Ion Score	C. I. %	Modification	Rank	Result Type
859.4632	859.42	-0.0432	-50	114	121	ESGVQAIR					Mascot
909.4676	909.4584	-0.0092	-10	1241	1248	TGEAFKEK					Mascot
1006.4912	1006.4825	-0.0087	-9	1319	1327	SDDVTGRTR					Mascot
1033.5459	1033.5713	0.0254	25	1087	1096	LSVGDKMAGR					Mascot
1081.5175	1081.5422	0.0247	23	556	564	DVHPTHYGR					Mascot
1232.594	1232.5988	0.0048	4	702	713	ADAPLVGTGMER			Oxidation (M)[10]		Mascot
1232.594	1232.5988	0.0048	4	702	713	ADAPLVGTGMER			Oxidation (M)[10]		Mascot
1249.7375	1249.6228	-0.1147	-92	376	386	RLRPGDPPTIK					Mascot
1249.7375	1249.6228	-0.1147	-92	376	386	RLRPGDPPTIK	15	0			Mascot
1265.6962	1265.64	-0.0562	-44	714	726	IVAHDSGAAVVAR					Mascot
1291.6641	1291.6532	-0.0109	-8	462	472	AVGELLENQYR					Mascot

Calc. Mass	Obsrv. Mass	± da	± ppm	Start Seq.	End Seq.	Sequence	Ion Score	C. I. %	Modification	Rank	Result Type
1366.6063	1366.6698	0.0635	46	187	197	GSWLDFDFDHK					Mascot
1507.8049	1507.7518	-0.0531	-35	1	13	MAYSVANNQLLRK					Mascot
1580.7737	1580.7908	0.0171	11	363	375	MATPEDARIEIYR			Oxidation (M)[1]		Mascot
1816.0076	1815.9576	-0.05	-28	462	477	AVGELLENQYRVGLVR					Mascot
1835.1001	1834.9218	-0.1783	-97	880	897	IGAEVKPGDILVGKITPK	1	0			Mascot
2069.0762	2068.9031	-0.1731	-84	852	870	LGKEEITNDIPNLGEDALK					Mascot
2568.2463	2568.3601	0.1138	44	743	765	IDEGEVDEDGTGVDIYNLI KFAR					Mascot
2617.3469	2617.2988	-0.0481	-18	976	1001	ESTHGKLESLLVGQTSAS ALFDAAGK					Mascot
2631.3203	2631.2839	-0.0364	-14	387	409	SANALFESLFFNPERYDL SVVGR					Mascot
2633.3557	2633.3018	-0.0539	-20	739	762	IVVKIDEGEVDEDGTGVDI YNLIK					Mascot
2633.3557	2633.3018	-0.0539	-20	739	762	IVVKIDEGEVDEDGTGVDI YNLIK					Mascot

8	Flagellar biosynthesis protein FlhA OS=Helicobacter pylori (strain ATCC 700392 / 26695) GN=flhA PE=1 SV=2	FLHA_HELPY	80858.1	6.14	16	55	0	9.896	

Peptide Information

Calc. Mass	Obsrv. Mass	± da	± ppm	Start Seq.	End Seq.	Sequence	Ion Score	C. I. %	Modification	Rank	Result Type
960.536	960.5406	0.0046	5	336	344	EGKDGLLTK					Mascot
1192.6144	1192.5811	-0.0333	-28	157	167	ARFALDAMPGK			Oxidation (M)[8]		Mascot
1247.6552	1247.6232	-0.032	-26	667	677	LIEVVSEEAMK					Mascot
1249.7375	1249.6228	-0.1147	-92	366	376	IKPHTPTTRAK					Mascot
1249.7375	1249.6228	-0.1147	-92	366	376	IKPHTPTTRAK					Mascot
1291.6423	1291.6532	0.0109	8	696	706	KALSNQMEQAR			Oxidation (M)[7]		Mascot
1308.6682	1308.6586	-0.0096	-7	554	564	DYPTIVEESKK					Mascot
1567.8115	1567.8712	0.0597	38	444	456	DNLQLPPTHYEIK					Mascot
1607.7775	1607.8722	0.0947	59	491	504	EPAFGMDALWIETK					Mascot
1623.7723	1623.834	0.0617	38	491	504	EPAFGMDALWIETK			Oxidation (M)[6]		Mascot
1789.9008	1789.922	0.0212	12	633	647	FLTFSTDSEQFLLNK					Mascot
1795.9524	1795.9415	-0.0109	-6	429	443	IASDYGFLMPQIRIR			Oxidation (M)[9]		Mascot
1831.8168	1831.8951	0.0783	43	190	207	AALSQEADFYGAMDGAS K					Mascot
1846.0909	1845.9078	-0.1831	-99	565	581	IPTGAIRSVLQALLHEK					Mascot
1847.8116	1847.9479	0.1363	74	190	207	AALSQEADFYGAMDGAS K			Oxidation (M)[13]		Mascot
1849.9153	1849.9266	0.0113	6	491	506	EPAFGMDALWIETKNK					Mascot
2047.0012	2046.9011	-0.1001	-49	168	186	QMAIDADLNSGLIDDKEA K					Mascot

2568.3022	2568.3601	0.0579	23	459	482	GIVIGEGMVMPDKFLAMNTGFVNK					Mascot
2584.2974	2584.2869	-0.0105	-4	459	482	GIVIGEGMVMPDKFLAMNTGFVNK			Oxidation (M)[8]		Mascot
2600.2922	2600.3152	0.023	9	459	482	GIVIGEGMVMPDKFLAMNTGFVNK			Oxidation (M)[8,10]		Mascot
2616.2871	2616.2866	-0.0005	0	459	482	GIVIGEGMVMPDKFLAMNTGFVNK			Oxidation (M)[8,10,17]		Mascot
2616.2871	2616.2866	-0.0005	0	459	482	GIVIGEGMVMPDKFLAMNTGFVNK			Oxidation (M)[8,10,17]		Mascot
2649.2898	2649.3081	0.0183	7	159	183	FALDAMPGKQMAIDADLNSGLIDDK					Mascot

9	30S ribosomal protein S18 OS=Flavobacterium psychrophilum (strain JIP02/86 / ATCC 49511) GN=rpsR PE=3 SV=1	RS18_FLAPJ	11382.2	10.12	8	54	0	2.21	

Peptide Information

Calc. Mass	Obsrv. Mass	± da	± ppm	Start Seq.	End Seq.	Sequence	Ion Score	C. I. %	Modification	Rank	Result Type
1006.5164	1006.4825	-0.0339	-34	2	11	STLQQSASGK					Mascot
1265.6519	1265.64	-0.0119	-9	1	12	MSTLQQSASGKK					Mascot
1291.6893	1291.6532	-0.0361	-28	18	28	YLTPLNIDTNK					Mascot
1503.7729	1503.7535	-0.0194	-13	42	53	YIDYKDADFLLK					Mascot
1517.8185	1517.77	-0.0485	-32	86	98	HLAFMPYVADLLK					Mascot
1623.8378	1623.834	-0.0038	-2	47	60	DADFLLKFVNEQGK					Mascot
1744.9568	1744.9086	-0.0482	-28	84	98	ARHLAFMPYVADLLK					Mascot
1847.9498	1847.9479	-0.0019	-1	13	28	DGDIRYLTPLNIDTNK					Mascot

10	Vinculin OS=Drosophila melanogaster GN=Vinc PE=1 SV=1	VINC_DROME	107034.4	6.27	19	54	0	16.688	

Peptide Information

Calc. Mass	Obsrv. Mass	± da	± ppm	Start Seq.	End Seq.	Sequence	Ion Score	C. I. %	Modification	Rank	Result Type
859.4495	859.42	-0.0295	-34	1	7	MPVFHTK					Mascot
974.5629	974.4974	-0.0655	-67	267	275	KLSNAISNK					Mascot
1056.6161	1056.5752	-0.0409	-39	710	718	LLGDVREVR					Mascot
1081.5459	1081.5422	-0.0037	-3	567	575	IRMNYPGSK			Oxidation (M)[3]		Mascot
1113.6262	1113.6061	-0.0201	-18	410	419	AINLIVEEGR					Mascot
1231.6641	1231.6105	-0.0536	-44	929	940	ETVRAAEGASIK					Mascot
1249.6093	1249.6228	0.0135	11	236	245	MSDELQEIIR			Oxidation (M)[1]		Mascot
1249.6093	1249.6228	0.0135	11	236	245	MSDELQEIIR			Oxidation (M)[1]		Mascot
1265.7213	1265.64	-0.0813	-64	364	375	SLVHQAVLGVDK					Mascot
1306.6234	1306.6155	-0.0079	-6	56	67	VGRDTINSSDDK					Mascot

1366.7186	1366.6698	-0.0488	-36	376	388	AGVQQTAHTIQGR		Mascot
1601.8064	1601.8773	0.0709	44	631	644	QPQKMVDNTSNIAR		Mascot
1632.8486	1632.7925	-0.0561	-34	635	648	MVDNTSNIARLINR	Oxidation (M)[1]	Mascot
1636.7086	1636.8055	0.0969	59	655	668	QEADNSEDPVFTER		Mascot
1817.9868	1817.907	-0.0798	-44	178	192	EKELTHQVHSEILVR		Mascot
1817.9868	1817.907	-0.0798	-44	178	192	EKELTHQVHSEILVR		Mascot
1839.9998	1839.9187	-0.0811	-44	359	375	MGELKSLVHQAVLGVDK	Oxidation (M)[1]	Mascot
2552.3542	2552.3501	-0.0041	-2	679	704	SLPAMVGDAKLVATNIAD PAAAAAWK		Mascot
2552.3682	2552.5674	0.1992	78	140	161	VLDYLAVAEVINTMEQLV QFLK	Oxidation (M)[14]	Mascot
2568.3491	2568.3601	0.011	4	679	704	SLPAMVGDAKLVATNIAD PAAAAAWK	Oxidation (M)[5]	Mascot
2569.3994	2569.375	-0.0244	-9	199	220	TLAPILICSMKVYIHIVEQQ GR	Carbamidomethyl (C)[8]	Mascot
2569.3994	2569.375	-0.0244	-9	199	220	TLAPILICSMKVYIHIVEQQ GR	Carbamidomethyl (C)[8]	Mascot
2585.3943	2585.3171	-0.0772	-30	199	220	TLAPILICSMKVYIHIVEQQ GR	Carbamidomethyl (C)[8], Oxidation (M)[10]	Mascot
2649.3088	2649.3081	-0.0007	0	268	290	LSNAISNKMEQANEWLS NPYALR		Mascot

Gel Idx/Pos	180/H7	Instr./Gel Origin	BA2151/Sample Project 20140814	Process Status	Analysis Succeeded
Plate [#] Name	[1] Sample Project 20140814	Instrument Sample Name		Spectra	11

Rank	Protein Name	Accession No.	Protein MW	Protein PI	Pep. Count	Protein Score	Protein Score C. I. %	Intensity Matched	Total Ion Score	Total Ion C. I. %	Confirmed
1	1,2-dihydroxy-3-keto-5-methylthiopentene dioxygenase 2 OS=Oryza sativa subsp. japonica GN=ARD2 PE=2 SV=1	MTND2_ORYSJ	23654.5	5.08	10	294	100	25.074	243	100	

Protein Group

1,2-dihydroxy-3-keto-5-methylthiopentene dioxygenase 2 OS=Oryza sativa subsp. indica GN=ARD2 PE=2 SV=2	MTND2_ORYSI	23654.5	5.0799 999237 0605

Peptide Information

Calc. Mass	Obsrv. Mass	± da	± ppm	Start Seq.	End Seq.	Sequence	Ion Score	C. I. %	Modification	Rank	Result Type
834.4355	834.4135	-0.022	-26	85	91	LPNYEAK					Mascot
1013.5639	1013.5618	-0.0021	-2	27	34	LPHHREPK					Mascot
1074.4963	1074.5231	0.0268	25	120	127	DQNDQWIR					Mascot
1215.5892	1215.5856	-0.0036	-3	146	155	FTLDSDNYIK					Mascot
1417.7079	1417.6674	-0.0405	-29	133	145	GGMIVLPAGMYHR			Oxidation (M)[3]		Mascot
1433.7029	1433.6815	-0.0214	-15	133	145	GGMIVLPAGMYHR			Oxidation (M)[3,10]		Mascot
1433.7029	1433.6815	-0.0214	-15	133	145	GGMIVLPAGMYHR			Oxidation (M)[3,10]		Mascot
1465.6417	1465.6401	-0.0016	-1	108	119	YCLEGSGYFDVR			Carbamidomethyl (C)[2]		Mascot
1465.6417	1465.6401	-0.0016	-1	108	119	YCLEGSGYFDVR	103	100	Carbamidomethyl (C)[2]		Mascot
1561.7979	1561.755	-0.0429	-27	132	145	KGGMIVLPAGMYHR			Oxidation (M)[4,11]		Mascot
1573.7679	1573.7462	-0.0217	-14	146	158	FTLDSDNYIKAMR					Mascot
1589.7628	1589.777	0.0142	9	146	158	FTLDSDNYIKAMR			Oxidation (M)[12]		Mascot
1815.8297	1815.8312	0.0015	1	94	107	NFFEEHLHTDEEIR					Mascot
1815.8297	1815.8312	0.0015	1	94	107	NFFEEHLHTDEEIR	140	100			Mascot
2057.0088	2057.0054	-0.0034	-2	92	107	LKNFFEEHLHTDEEIR					Mascot

Rank	Protein Name	Accession No.	Protein MW	Protein PI	Pep. Count	Protein Score	Protein Score C. I. %	Intensity Matched	Total Ion Score	Total Ion C. I. %	Confirmed
2	1,2-dihydroxy-3-keto-5-methylthiopentene dioxygenase 1 OS=Sorghum bicolor GN=Sb01g021500 PE=3 SV=2	MTND1_SORBI	21606.7	5.21	8	282	100	23.655	243	100	

Peptide Information

Calc. Mass	Obsrv. Mass	± da	± ppm	Start Seq.	End Seq.	Sequence	Ion Score	C. I. %	Modification	Rank	Result Type
1075.4803	1075.5006	0.0203	19	103	110	DENDQWIR					Mascot
1417.7079	1417.6674	-0.0405	-29	116	128	GGMIVLPAGMYHR			Oxidation (M)[3]		Mascot

1433.7029	1433.6815	-0.0214	-15	116	128	GGMIVLPAGMYHR			Oxidation (M)[3,10]		Mascot
1433.7029	1433.6815	-0.0214	-15	116	128	GGMIVLPAGMYHR			Oxidation (M)[3,10]		Mascot
1465.6417	1465.6401	-0.0016	-1	91	102	YCLEGSGYFDVR			Carbamidomethyl (C)[2]		Mascot
1465.6417	1465.6401	-0.0016	-1	91	102	YCLEGSGYFDVR	103	100	Carbamidomethyl (C)[2]		Mascot
1561.7979	1561.755	-0.0429	-27	115	128	KGGMIVLPAGMYHR			Oxidation (M)[4,11]		Mascot
1600.7238	1600.7271	0.0033	2	35	47	LNPDNWENDENLK					Mascot
1815.8297	1815.8312	0.0015	1	77	90	NFFEEHLHTDEEIR					Mascot
1815.8297	1815.8312	0.0015	1	77	90	NFFEEHLHTDEEIR	140	100			Mascot
1959.0546	1958.8679	-0.1867	-95	164	180	EYLDKLLKPEGQAVEAR					Mascot
2057.0088	2057.0054	-0.0034	-2	75	90	IKNFFEEHLHTDEEIR					Mascot

3	1,2-dihydroxy-3-keto-5-methylthiopentene dioxygenase 1 OS=Oryza sativa subsp. japonica GN=ARD1 PE=2 SV=1	MTND1_ORYSJ	23854.6	5.07	6	224	100	17.096	201	100	

Protein Group

1,2-dihydroxy-3-keto-5-methylthiopentene dioxygenase 1 OS=Oryza sativa subsp. indica GN=ARD1 PE=1 SV=2	MTND1_ORYSI	23854.6	5.0700 001716 6138

Peptide Information

Calc. Mass	Obsrv. Mass	± da	± ppm	Start Seq.	End Seq.	Sequence	Ion Score	C. I. %	Modification	Rank	Result Type
1013.5639	1013.5618	-0.0021	-2	27	34	LPHHREPK					Mascot
1074.4963	1074.5231	0.0268	25	120	127	DQNDQWIR					Mascot
1417.7079	1417.6674	-0.0405	-29	133	145	GGMIVLPAGMYHR			Oxidation (M)[3]		Mascot
1433.7029	1433.6815	-0.0214	-15	133	145	GGMIVLPAGMYHR			Oxidation (M)[3,10]		Mascot
1433.7029	1433.6815	-0.0214	-15	133	145	GGMIVLPAGMYHR			Oxidation (M)[3,10]		Mascot
1465.6417	1465.6401	-0.0016	-1	108	119	YCLEGSGYFDVR			Carbamidomethyl (C)[2]		Mascot
1465.6417	1465.6401	-0.0016	-1	108	119	YCLEGSGYFDVR	103	100	Carbamidomethyl (C)[2]		Mascot
1561.7979	1561.755	-0.0429	-27	132	145	KGGMIVLPAGMYHR			Oxidation (M)[4,11]		Mascot
1704.7245	1704.6937	-0.0308	-18	71	84	GYSYVDICDVCPEK			Carbamidomethyl (C)[8,11]		Mascot
1704.7245	1704.6937	-0.0308	-18	71	84	GYSYVDICDVCPEK	98	100	Carbamidomethyl (C)[8,11]		Mascot

4	1,2-dihydroxy-3-keto-5-methylthiopentene dioxygenase 2 OS=Vitis vinifera GN=VIT_05s0020g04080 PE=2 SV=1	MTND2_VITVI	23586.4	4.9	5	159	100	11.548	140	100	

Peptide Information

Calc. Mass	Obsrv. Mass	± da	± ppm	Start Seq.	End Seq.	Sequence	Ion Score	C. I. %	Modification	Rank	Result Type
1212.5896	1212.6326	0.043	35	179	188	QEYLEAFGQK					Mascot
1212.5896	1212.6326	0.043	35	179	188	QEYLEAFGQK					Mascot

1554.6807	1554.8038	0.1231	79	51	63	LDADNYETDEELK					Mascot
1815.8297	1815.8312	0.0015	1	93	106	NFFEEHLHTDEEIR					Mascot
1815.8297	1815.8312	0.0015	1	93	106	NFFEEHLHTDEEIR	140	100			Mascot
2057.0088	2057.0054	-0.0034	-2	91	106	IKNFFEEHLHTDEEIR					Mascot
2515.0315	2515.2703	0.2388	95	6	25	DDREEVLQAWYMDDSD EDQR					Mascot

5	1,2-dihydroxy-3-keto-5-methylthiopentene dioxygenase homolog 2 OS=Sorghum bicolor GN=Sb01g046360 PE=3 SV=1	MTND2_SORBI	21646.7	5.33	8	134	100	17.14	103	100

Peptide Information

Calc. Mass	Obsrv. Mass	± da	± ppm	Start Seq.	End Seq.	Sequence	Ion Score	C. I. %	Modification	Rank	Result Type
834.4355	834.4135	-0.022	-26	68	74	LPNYEAK					Mascot
1013.5639	1013.5618	-0.0021	-2	10	17	LPHHREPK					Mascot
1075.4803	1075.5006	0.0203	19	103	110	DENDQWIR					Mascot
1215.662	1215.5856	-0.0764	-63	15	24	EPKEFIPLDK					Mascot
1417.7079	1417.6674	-0.0405	-29	116	128	GGMIVLPAGMYHR			Oxidation (M)[3]		Mascot
1433.7029	1433.6815	-0.0214	-15	116	128	GGMIVLPAGMYHR			Oxidation (M)[3,10]		Mascot
1433.7029	1433.6815	-0.0214	-15	116	128	GGMIVLPAGMYHR			Oxidation (M)[3,10]		Mascot
1465.6417	1465.6401	-0.0016	-1	91	102	YCLEGSGYFDVR			Carbamidomethyl (C)[2]		Mascot
1465.6417	1465.6401	-0.0016	-1	91	102	YCLEGSGYFDVR	103	100	Carbamidomethyl (C)[2]		Mascot
1561.7979	1561.755	-0.0429	-27	115	128	KGGMIVLPAGMYHR			Oxidation (M)[4,11]		Mascot
1589.7441	1589.777	0.0329	21	35	47	LNADDWENDEKLK					Mascot

6	1,2-dihydroxy-3-keto-5-methylthiopentene dioxygenase 4 OS=Oryza sativa subsp. japonica GN=ARD4 PE=2 SV=1	MTND4_ORYSJ	22062.9	5.07	5	121	100	17.27	103	100

Peptide Information

Calc. Mass	Obsrv. Mass	± da	± ppm	Start Seq.	End Seq.	Sequence	Ion Score	C. I. %	Modification	Rank	Result Type
959.4581	959.4835	0.0254	26	175	184	GDTGFALAAH					Mascot
1212.626	1212.6326	0.0066	5	133	142	FIVDSNNYIK					Mascot
1212.626	1212.6326	0.0066	5	133	142	FIVDSNNYIK					Mascot
1320.6642	1320.635	-0.0292	-22	44	54	SESEEELTKIR					Mascot
1465.6417	1465.6401	-0.0016	-1	95	106	YCLEGSGYFDVR			Carbamidomethyl (C)[2]		Mascot
1465.6417	1465.6401	-0.0016	-1	95	106	YCLEGSGYFDVR	103	100	Carbamidomethyl (C)[2]		Mascot
1600.8152	1600.7271	-0.0881	-55	119	132	EGDMIILPAGIYHR			Oxidation (M)[4]		Mascot

7	1,2-dihydroxy-3-keto-5-methylthiopentene dioxygenase	MTND1_VITVI	22751.2	5.36	2	110	100	13.383	103	100

1 OS=Vitis vinifera GN=VIT_05s0020g04070 PE=3 SV=1

Peptide Information

Calc. Mass	Obsrv. Mass	± da	± ppm	Start Seq.	End Seq.	Sequence	Ion Score	C. I. %	Modification	Rank	Result Type
1465.6417	1465.6401	-0.0016	-1	99	110	YCLEGSGYFDVR			Carbamidomethyl (C)[2]		Mascot
1465.6417	1465.6401	-0.0016	-1	99	110	YCLEGSGYFDVR	103	100	Carbamidomethyl (C)[2]		Mascot
1573.8043	1573.7462	-0.0581	-37	137	149	FTLDTGNYVKLMR			Oxidation (M)[12]		Mascot

8 60 kDa chaperonin OS=Thermoanaerobacter brockii GN=groL PE=1 SV=2 CH60_THEBR 58080.7 5.05 15 66 87.029 17.306

Peptide Information

Calc. Mass	Obsrv. Mass	± da	± ppm	Start Seq.	End Seq.	Sequence	Ion Score	C. I. %	Modification	Rank	Result Type
800.4512	800.4377	-0.0135	-17	491	498	AGIVDPTK					Mascot
931.5095	931.4581	-0.0514	-55	394	402	IEDALAATK					Mascot
931.5142	931.4581	-0.0561	-60	312	319	LDMLGRAR	17	0			Mascot
982.5026	982.4711	-0.0315	-32	267	275	GTFTCVAVK			Carbamidomethyl (C)[5]		Mascot
1074.5725	1074.5231	-0.0494	-46	309	317	DVRLDMLGR					Mascot
1224.6696	1224.599	-0.0706	-58	392	402	HRIEDALAATK					Mascot
1455.5371	1455.6479	0.1108	76	528	541	NTGMPNPGAGMDMM			Oxidation (M)[4,11]		Mascot
1465.7534	1465.6401	-0.1133	-77	427	440	VVDSLEGDFKTGAK					Mascot
1465.7534	1465.6401	-0.1133	-77	427	440	VVDSLEGDFKTGAK					Mascot
1473.693	1473.701	0.008	5	196	208	GYISPYMVTDAEK					Mascot
1487.527	1487.6613	0.1343	90	528	541	NTGMPNPGAGMDMM			Oxidation (M)[4,11,13,14]		Mascot
1600.8541	1600.7271	-0.127	-79	452	467	QIATNAGVDGSVIVEK					Mascot
1699.9187	1699.8047	-0.114	-67	209	223	MEAVLEEPVILITDK					Mascot
1798.991	1798.8671	-0.1239	-69	370	388	LAGGVAVIQAGAATETEL K					Mascot
1826.9131	1826.922	0.0089	5	141	158	ESIAHVASISAADEEIGK					Mascot
1843.8783	1843.8586	-0.0197	-11	483	498	EEFTDMFKAGIVDPTK			Oxidation (M)[6]		Mascot
2547.3083	2547.2698	-0.0385	-15	80	104	TNDIAGDGTTTATLLAQA MVREGLK					Mascot

9 Serrate RNA effector molecule homolog OS=Danio rerio GN=srrt PE=2 SV=1 SRRT_DANRE 103836.9 5.37 25 65 84.042 25.793

Peptide Information

Calc. Mass	Obsrv. Mass	± da	± ppm	Start Seq.	End Seq.	Sequence	Ion Score	C. I. %	Modification	Rank	Result Type

931.5029	931.4581	-0.0448	-48	466	473	AEIVALCR			Carbamidomethyl (C)[7]		Mascot
931.5029	931.4581	-0.0448	-48	466	473	AEIVALCR			Carbamidomethyl (C)[7]		Mascot
942.4614	942.4789	0.0175	19	474	480	RYPGFMR			Oxidation (M)[6]		Mascot
986.436	986.4793	0.0433	44	75	82	HDMSPQQK			Oxidation (M)[3]		Mascot
1088.5922	1088.5109	-0.0813	-75	744	752	WLCPLSGKK			Carbamidomethyl (C)[3]		Mascot
1088.5922	1088.5109	-0.0813	-75	744	752	WLCPLSGKK			Carbamidomethyl (C)[3]		Mascot
1107.4735	1107.5133	0.0398	36	261	270	MEGGAENDLR			Oxidation (M)[1]		Mascot
1130.5259	1130.5486	0.0227	20	520	529	DCELAPGVNR			Carbamidomethyl (C)[2]		Mascot
1142.5371	1142.4917	-0.0454	-40	75	83	HDMSPQQKR			Oxidation (M)[3]		Mascot
1146.5637	1146.5089	-0.0548	-48	285	296	LSSGGTPSEPSK					Mascot
1159.5491	1159.6044	0.0553	48	203	212	SKYHPDEAGR					Mascot
1177.61	1177.5857	-0.0243	-21	344	354	EAPAEPIPEPK					Mascot
1224.6444	1224.599	-0.0454	-37	214	224	KAEAHSALQNR					Mascot
1232.6382	1232.5762	-0.062	-50	876	885	GDPRNIIEYR					Mascot
1265.7073	1265.6313	-0.076	-60	535	545	VRNVNGITQHK					Mascot
1373.7034	1373.6299	-0.0735	-54	860	872	GQGGYPGKPRNSR					Mascot
1374.5227	1374.6404	0.1177	86	1	11	MGDSDDEYDRR			Oxidation (M)[1]		Mascot
1374.7377	1374.6404	-0.0973	-71	685	695	ITLREVSDWQK					Mascot
1387.6462	1387.7767	0.1305	94	35	45	DDWNDRRPSAR					Mascot
1408.6678	1408.6185	-0.0493	-35	186	196	QQMQDFFLAHK			Oxidation (M)[3]		Mascot
1448.7744	1448.6487	-0.1257	-87	731	743	FVVANTQELGKDK					Mascot
1465.6587	1465.6401	-0.0186	-13	709	721	ENLSEDEAAKMGR			Oxidation (M)[11]		Mascot
1465.6587	1465.6401	-0.0186	-13	709	721	ENLSEDEAAKMGR			Oxidation (M)[11]		Mascot
1487.686	1487.6613	-0.0247	-17	271	282	ILEQPSEEEEER					Mascot
1554.7482	1554.8038	0.0556	36	70	82	FSPPRHDMSPQQK					Mascot
1573.772	1573.7462	-0.0258	-16	777	789	EVVFFNNFLMDAK					Mascot
1589.7668	1589.777	0.0102	6	777	789	EVVFFNNFLMDAK			Oxidation (M)[10]		Mascot
2056.967	2057.0054	0.0384	19	298	315	DEPKPADTENKPSEEKD K					Mascot
2520.0266	2520.1194	0.0928	37	399	420	EPEEVEEKEEEEEEEGE AADGK					Mascot

10	Tripartite motif-containing protein 29 OS=Homo sapiens GN=TRIM29 PE=1 SV=2	TRI29_HUMAN	66477.6	6.73	17	64	80.367	17.931

Peptide Information

Calc. Mass	Obsrv. Mass	± da	± ppm	Start Seq.	End Seq.	Sequence	Ion Score	C. I. %	Modification	Rank	Result Type
915.4166	915.476	0.0594	65	579	588	GNGIGSNEAP					Mascot
931.4488	931.4581	0.0093	10	423	429	HVEKMCK			Carbamidomethyl (C)[6]		Mascot

931.4488	931.4581	0.0093	10	423	429	HVEKMCK			Carbamidomethyl (C)[6]	Mascot
953.505	953.4387	-0.0663	-70	129	136	KSIFSESR				Mascot
982.5204	982.4711	-0.0493	-50	493	500	FTKETTQK				Mascot
1120.611	1120.5024	-0.1086	-97	210	218	DHQLLEPIR				Mascot
1120.611	1120.5024	-0.1086	-97	210	218	DHQLLEPIR	5	0		Mascot
1130.5953	1130.5486	-0.0467	-41	555	564	AQPQTWKSGK				Mascot
1158.5977	1158.5803	-0.0174	-15	470	478	RYSMYLTPK				Mascot
1182.4706	1182.5538	0.0832	70	440	449	NHMENGGDHR			Oxidation (M)[3]	Mascot
1387.7039	1387.7767	0.0728	52	471	482	YSMYLTPKGGVR			Oxidation (M)[3]	Mascot
1448.7241	1448.6487	-0.0754	-52	479	492	GGVRTSYQPSSPGR				Mascot
1455.7227	1455.6479	-0.0748	-51	483	495	TSYQPSSPGRFTK				Mascot
1473.8173	1473.701	-0.1163	-79	308	319	AILEQNFRDLVR				Mascot
1600.8475	1600.7271	-0.1204	-75	137	150	KPTVSIMEPGETRR				Mascot
1604.726	1604.8096	0.0836	52	2	17	EAADASRSNGSSPEAR				Mascot
1657.8181	1657.8011	-0.017	-10	496	509	ETTQKNFNNLYGTK				Mascot
1699.861	1699.8047	-0.0563	-33	330	344	AALEQREQDAVDQVK				Mascot
2251.1387	2251.2656	0.1269	56	130	149	SIFSESRKPTVSIMEPGE<br>TR				Mascot

Gel Idx/Pos	181/H8	Instr./Gel Origin	BA2151/Sample Project 20140814	Process Status	Analysis Succeeded
Plate [#] Name	[1] Sample Project 20140814	Instrument Sample Name		Spectra	11

Rank	Protein Name	Accession No.	Protein MW	Protein PI	Pep. Count	Protein Score	Protein Score C. I. %	Intensity Matched	Total Ion Score	Total Ion C. I. %	Confirmed
1	UPF0403 protein BLi02323/BL05224 OS=Bacillus licheniformis (strain DSM 13 / ATCC 14580) GN=BLi02323 PE=3 SV=1	Y2323_BACLD	16128.7	4.64	10	74	97.693	4.947			

Peptide Information

Calc. Mass	Obsrv. Mass	± da	± ppm	Start Seq.	End Seq.	Sequence	Ion Score	C. I. %	Modification	Rank	Result Type
806.4189	806.3979	-0.021	-26	88	94	EATAKMR					Mascot
856.5073	856.5198	0.0125	15	11	17	QLVLPMR					Mascot
897.4056	897.4244	0.0188	21	128	134	EMTEIMK			Oxidation (M)[2]		Mascot
937.4625	937.3888	-0.0737	-79	135	142	NITDAFEK					Mascot
1165.483	1165.5382	0.0552	47	2	10	STAYEEYMR			Oxidation (M)[8]		Mascot
1181.679	1181.5842	-0.0948	-80	112	121	GKEVVHFIPR					Mascot
1491.7075	1491.7024	-0.0051	-3	74	87	GPDETVTVFAGQDR					Mascot
1799.8553	1799.8931	0.0378	21	128	142	EMTEIMKNITDAFEK					Mascot
2002.9725	2003.0315	0.059	29	2	17	STAYEEYMRQLVLPMR			Oxidation (M)[8]		Mascot
2018.9674	2019.0319	0.0645	32	2	17	STAYEEYMRQLVLPMR			Oxidation (M)[8,15]		Mascot
2166.0029	2166.1641	0.1612	74	1	17	MSTAYEEYMRQLVLPMR			Oxidation (M)[1,9,16]		Mascot

Rank	Protein Name	Accession No.	Protein MW	Protein PI	Pep. Count	Protein Score	Protein Score C. I. %	Intensity Matched	Total Ion Score	Total Ion C. I. %	Confirmed
2	Archaeal glutamate synthase [NADPH] OS=Methanocaldococcus jannaschii (strain ATCC 43067 / DSM 2661 / JAL-1 / JCM 10045 / NBRC 100440) GN=MJ1351 PE=3 SV=1	AGLUS_METJA	56331.1	8.56	16	62	62.59	9.467			

Peptide Information

Calc. Mass	Obsrv. Mass	± da	± ppm	Start Seq.	End Seq.	Sequence	Ion Score	C. I. %	Modification	Rank	Result Type
826.3763	826.3533	-0.023	-28	395	401	CSADVFK			Carbamidomethyl (C)[1]		Mascot
828.421	828.3628	-0.0582	-70	12	18	VEVDPNR					Mascot
852.4573	852.399	-0.0583	-68	41	47	IISYSNR					Mascot
856.4523	856.5198	0.0675	79	448	455	LDPEVGAR					Mascot
875.437	875.3522	-0.0848	-97	67	73	ENAISWR					Mascot
884.4625	884.4624	-0.0001	0	463	469	AWTHEIK					Mascot
921.4103	921.4919	0.0816	89	54	60	CVVMCPR			Carbamidomethyl (C)[1,5]		Mascot
921.4103	921.4919	0.0816	89	54	60	CVVMCPR			Carbamidomethyl (C)[1,5]		Mascot

Calc. Mass	Obsrv. Mass	± da	± ppm	Start Seq.	End Seq.	Sequence	Ion Score	C. I. %	Modification	Rank	Result Type
937.4053	937.3888	-0.0165	-18	54	60	CVVMCPR			Carbamidomethyl (C)[1,5], Oxidation (M)[4]		Mascot
1060.5898	1060.5209	-0.0689	-65	348	358	GGTGAAPKVFR					Mascot
1119.5793	1119.516	-0.0633	-57	10	18	YKVEVDPNR					Mascot
1309.6495	1309.6454	-0.0041	-3	37	47	EGDRIISYSNR					Mascot
1321.6603	1321.6331	-0.0272	-21	92	104	TGCILLSGMGNAK			Carbamidomethyl (C)[3]		Mascot
1542.8486	1542.7349	-0.1137	-74	470	484	ELLGAAGINSIESLR					Mascot
1556.672	1556.7242	0.0522	34	202	216	ECGTFMGTGEGGLPK			Carbamidomethyl (C)[2], Oxidation (M)[6]		Mascot
1597.916	1597.7969	-0.1191	-75	492	506	GVGLNEKELEVLGIK					Mascot
1834.948	1834.8755	-0.0725	-40	359	375	DHVGIPIEMAIAAVDQR					Mascot
1838.8776	1838.8862	0.0086	5	199	216	AVKECGTFMGTGEGGLP K			Carbamidomethyl (C)[5]		Mascot

3	Non-neuronal cytoplasmic intermediate filament protein B OS=Helix pomatia PE=1 SV=1	IFEB_HELPO	51311.1	5.21	15	60	47.158	8.216

Peptide Information

Calc. Mass	Obsrv. Mass	± da	± ppm	Start Seq.	End Seq.	Sequence	Ion Score	C. I. %	Modification	Rank	Result Type
856.4999	856.5198	0.0199	23	14	20	QSKIQPR					Mascot
921.4788	921.4919	0.0131	14	349	356	FASLQAER					Mascot
921.4788	921.4919	0.0131	14	349	356	FASLQAER					Mascot
932.4683	932.3976	-0.0707	-76	415	422	LLEGEESR					Mascot
1021.5312	1021.4678	-0.0634	-62	339	348	NAAYAELAAK					Mascot
1060.5634	1060.5209	-0.0425	-40	414	422	KLLEGEESR					Mascot
1068.4844	1068.4905	0.0061	6	283	291	DIQSAYDEK					Mascot
1119.6256	1119.516	-0.1096	-98	130	139	KVIDELSSTK					Mascot
1184.543	1184.5591	0.0161	14	4	13	ISTTYEEEGR					Mascot
1391.6583	1391.6547	-0.0036	-3	193	204	IRCGSLEDENAK			Carbamidomethyl (C)[3]		Mascot
1527.7285	1527.7109	-0.0176	-12	4	16	ISTTYEEEGRQSK					Mascot
1527.7285	1527.7109	-0.0176	-12	4	16	ISTTYEEEGRQSK					Mascot
1542.7655	1542.7349	-0.0306	-20	403	414	ITMELEIACYRK			Carbamidomethyl (C)[9], Oxidation (M)[3]		Mascot
1556.7374	1556.7242	-0.0132	-8	317	329	DGMQLQHVQEEVK			Oxidation (M)[3]		Mascot
1823.8956	1823.8336	-0.062	-34	74	88	EMQNLNERLAGYIEK			Oxidation (M)[2]		Mascot
1842.9919	1842.8755	-0.1164	-63	140	157	GVSEAKVAGLQDEIASLR					Mascot
3166.584	3166.5615	-0.0225	-7	249	274	DLLDQLELLKPEPMQIKG MDYAEFWK			Oxidation (M)[14]		Mascot

4	Phorbol ester/diacylglycerol-binding protein unc-13 OS=Caenorhabditis elegans GN=unc-13 PE=1 SV=4	UNC13_CAEEL	246902.4	6.43	31	60	43.378	16.089

Peptide Information

Calc. Mass	Obsrv. Mass	± da	± ppm	Start	End	Sequence	Ion	C. I. %	Modification	Rank	Result Type

				Seq.	Seq.		Score		
805.3951	805.4141	0.019	24	434	440	TGANTWR			Mascot
806.4189	806.3979	-0.021	-26	2131	2136	LKTECR		Carbamidomethyl (C)[5]	Mascot
831.3454	831.2968	-0.0486	-58	2066	2071	DYCFAR		Carbamidomethyl (C)[3]	Mascot
837.4465	837.4219	-0.0246	-29	1424	1430	IDLSAYR			Mascot
848.3897	848.3289	-0.0608	-72	1913	1920	DSFHASGK			Mascot
852.4574	852.399	-0.0584	-69	1953	1959	TFITSQR			Mascot
903.4756	903.3887	-0.0869	-96	876	882	SCPILWK		Carbamidomethyl (C)[2]	Mascot
909.4901	909.4176	-0.0725	-80	469	476	GSRVSFTR			Mascot
925.4924	925.4077	-0.0847	-92	886	893	TPHPMKSK			Mascot
976.4846	976.4183	-0.0663	-68	1322	1329	VRGEDSWK			Mascot
1013.4357	1013.4067	-0.029	-29	338	346	YDTPMSSGR			Mascot
1068.5507	1068.4905	-0.0602	-56	883	891	TEKTPHPMK			Mascot
1119.619	1119.516	-0.103	-92	1834	1842	ITIVNMEKR		Oxidation (M)[6]	Mascot
1182.5134	1182.5597	0.0463	39	727	736	ESYEDRNGGR			Mascot
1182.5134	1182.5597	0.0463	39	727	736	ESYEDRNGGR			Mascot
1184.6093	1184.5591	-0.0502	-42	1556	1565	MALEEHAQKK			Mascot
1217.5005	1217.5461	0.0456	37	2066	2074	DYCFARDDR		Carbamidomethyl (C)[3]	Mascot
1321.6133	1321.6331	0.0198	15	1569	1578	TPEYMNLYFK		Oxidation (M)[5]	Mascot
1354.6671	1354.6846	0.0175	13	1815	1825	YADQCEKTVLK		Carbamidomethyl (C)[5]	Mascot
1374.6285	1374.6411	0.0126	9	1632	1643	ADNFPQTSEHTK			Mascot
1449.5964	1449.723	0.1266	87	1208	1218	FHFECHNSTDR		Carbamidomethyl (C)[5]	Mascot
1491.7074	1491.7024	-0.005	-3	307	321	EFGESAVPPAASSSR			Mascot
1492.6882	1492.7777	0.0895	60	918	930	MKPPAARCESDSK		Carbamidomethyl (C)[8], Oxidation (M)[1]	Mascot
1527.8352	1527.7109	-0.1243	-81	91	103	LIGVHYMPLSEIR			Mascot
1527.8352	1527.7109	-0.1243	-81	91	103	LIGVHYMPLSEIR			Mascot
1542.7581	1542.7349	-0.0232	-15	1552	1564	NDLKMALEEHAQK		Oxidation (M)[5]	Mascot
1594.9275	1594.9169	-0.0106	-7	1461	1475	VLELASPPRASTVVR			Mascot
1700.7837	1700.8585	0.0748	44	1255	1268	TLSGEMDVWYNLEK		Oxidation (M)[6]	Mascot
1812.7307	1812.8462	0.1155	64	233	248	EGAASYEDEEDAYHAR			Mascot
1840.8898	1840.8756	-0.0142	-8	1255	1269	TLSGEMDVWYNLEKR			Mascot
2019.0514	2019.0319	-0.0195	-10	1112	1127	MKIQEQNKPEVFQMIR			Mascot
2239.197	2239.1106	-0.0864	-39	30	49	LQGAVDEFNSYVTVKLQ TVK			Mascot
3166.4873	3166.5615	0.0742	23	1293	1318	LAPYHVQYTCLHEHLFAA HCVDEEVK		Carbamidomethyl (C)[10,20]	Mascot

5	DNA polymerase III subunit alpha OS=Yersinia pestis GN=dnaE PE=3 SV=2	DPO3A_YERPE	131256.6	5.73	25	59	37.916	11.522

Peptide Information

Calc. Mass	Obsrv. Mass	± da	± ppm	Start Seq.	End Seq.	Sequence	Ion Score	C. I. %	Modification	Rank	Result Type
810.3848	810.3364	-0.0484	-60	728	734	MKSMGEK					Mascot
813.3481	813.2859	-0.0622	-76	724	729	DMEKMK			Oxidation (M)[2,5]		Mascot
820.3618	820.4221	0.0603	74	764	770	CEDGALR			Carbamidomethyl (C)[1]		Mascot
826.3797	826.3533	-0.0264	-32	728	734	MKSMGEK			Oxidation (M)[1]		Mascot
838.3294	838.3524	0.023	27	936	942	ACACNSR			Carbamidomethyl (C)[2,4]		Mascot
903.4781	903.3887	-0.0894	-99	494	501	EQLASLDK					Mascot
909.4611	909.4176	-0.0435	-48	551	557	CIAFDKR			Carbamidomethyl (C)[1]		Mascot
941.4475	941.3932	-0.0543	-58	811	818	NGAYQFNK					Mascot
958.5316	958.4549	-0.0767	-80	1123	1131	SKPSDLVGR					Mascot
958.5316	958.4549	-0.0767	-80	1123	1131	SKPSDLVGR					Mascot
1034.5854	1034.4829	-0.1025	-99	234	241	IDQPHRLR					Mascot
1119.5826	1119.516	-0.0666	-59	997	1006	SAEVNVLMTR					Mascot
1165.5453	1165.5382	-0.0071	-6	85	93	CMESLVRDR			Carbamidomethyl (C)[1]		Mascot
1181.5402	1181.5842	0.044	37	85	93	CMESLVRDR			Carbamidomethyl (C)[1], Oxidation (M)[2]		Mascot
1184.5769	1184.5591	-0.0178	-15	506	515	YPEAFKAACK			Carbamidomethyl (C)[9]		Mascot
1193.6129	1193.5845	-0.0284	-24	512	521	AACKLQSLMR			Carbamidomethyl (C)[3], Oxidation (M)[9]		Mascot
1217.6201	1217.5461	-0.074	-61	502	511	YATKYPEAFK					Mascot
1255.6464	1255.5903	-0.0561	-45	223	233	DIAHMVTNNIK					Mascot
1309.6127	1309.6454	0.0327	25	109	119	DVCELMTLANK			Carbamidomethyl (C)[3], Oxidation (M)[6]		Mascot
1366.7213	1366.6445	-0.0768	-56	494	505	EQLASLDKYATK					Mascot
1374.5995	1374.6411	0.0416	30	1087	1097	DEIGQFTDFMR			Oxidation (M)[10]		Mascot
1449.7189	1449.723	0.0041	3	109	120	DVCELMTLANKR			Carbamidomethyl (C)[3]		Mascot
1518.8639	1518.7317	-0.1322	-87	158	172	DFAKIIGTLVTAGGR					Mascot
1584.7428	1584.7383	-0.0045	-3	922	934	FESLEQFEEAVEK					Mascot
1594.8258	1594.9169	0.0911	57	704	718	LLAGFTMAEADGVRK			Oxidation (M)[7]		Mascot
1901.9823	1901.9127	-0.0696	-37	301	318	MADDEPATLMKLAVAGL R					Mascot
1959.9236	1959.9055	-0.0181	-9	803	818	EIWDAFEKNGAYQFNK					Mascot
2167.1143	2167.1941	0.0798	37	861	880	DALTYGIHVLPPDVNVSS NR					Mascot

6	Proteasome subunit alpha type-3 OS=Oryza sativa subsp. japonica GN=PAG1 PE=2 SV=1	PSA3_ORYSJ	27505.9	5.75	10	56	0	3.968	

Peptide Information

Calc. Mass	Obsrv. Mass	± da	± ppm	Start Seq.	End Seq.	Sequence	Ion Score	C. I. %	Modification	Rank	Result Type

Calc. Mass	Obsrv. Mass	± da	± ppm	Start Seq.	End Seq.	Sequence	Ion Score	C. I. %	Modification	Rank	Result Type
884.3995	884.4624	0.0629	71	94	101	SEAASYEK					Mascot
937.423	937.3888	-0.0342	-36	58	65	MMLEGSNR					Mascot
1119.619	1119.516	-0.103	-92	179	187	LKLSELTCR			Carbamidomethyl (C)[8]		Mascot
1217.6559	1217.5461	-0.1098	-90	42	52	CKDGIVLGVEK			Carbamidomethyl (C)[1]		Mascot
1349.589	1349.6361	0.0471	35	237	249	VAAQAALEEMDAD			Oxidation (M)[10]		Mascot
1447.7574	1447.6946	-0.0628	-43	30	43	AVDNSGTVVGIKCK			Carbamidomethyl (C)[13]		Mascot
1497.74	1497.7069	-0.0331	-22	53	65	LVTSKMMLEGSNR			Oxidation (M)[6,7]		Mascot
1799.8521	1799.8931	0.041	23	144	159	DGPQLYMIEPSGVSYK			Oxidation (M)[7]		Mascot
1959.9294	1959.9055	-0.0239	-12	2	20	SSIGTGYDLSVTTFSPDG R					Mascot
2225.1814	2225.0032	-0.1782	-80	21	41	VFQVEYATKAVDNSGTV VGIK					Mascot

7	Nebulin-related-anchoring protein OS=Mus musculus GN=Nrap PE=1 SV=3	NRAP_MOUSE	196716	9.34	34	55	0	18.283

Peptide Information

Calc. Mass	Obsrv. Mass	± da	± ppm	Start Seq.	End Seq.	Sequence	Ion Score	C. I. %	Modification	Rank	Result Type
800.405	800.3356	-0.0694	-87	1203	1209	QHPGSFK					Mascot
803.3893	803.3351	-0.0542	-67	993	999	EQGENVK					Mascot
806.4519	806.3979	-0.054	-67	1557	1563	GLQIGYR					Mascot
820.4675	820.4221	-0.0454	-55	792	798	AKGFELR					Mascot
821.3676	821.3494	-0.0182	-22	90	96	EDGEPFK					Mascot
824.4148	824.3596	-0.0552	-67	616	622	KGFEESK					Mascot
834.3886	834.3089	-0.0797	-96	2	8	NVQACSR			Carbamidomethyl (C)[5]		Mascot
852.421	852.399	-0.022	-26	859	865	KGFEDTR					Mascot
884.5134	884.4624	-0.051	-58	425	431	RMLHALK			Oxidation (M)[2]		Mascot
909.4321	909.4176	-0.0145	-16	925	931	ADMKWMK					Mascot
925.427	925.4077	-0.0193	-21	925	931	ADMKWMK			Oxidation (M)[3]		Mascot
941.4219	941.3932	-0.0287	-30	925	931	ADMKWMK			Oxidation (M)[3,6]		Mascot
1021.4982	1021.4678	-0.0304	-30	79	89	SVAAMGGIDGK			Oxidation (M)[5]		Mascot
1062.4885	1062.5435	0.055	52	972	980	DTPEMVQAR			Oxidation (M)[5]		Mascot
1119.5695	1119.516	-0.0535	-48	1201	1209	YRQHPGSFK					Mascot
1165.5961	1165.5382	-0.0579	-50	1538	1547	VSRDIASDFR					Mascot
1216.6222	1216.5658	-0.0564	-46	1269	1277	YKEAWHNLR					Mascot
1217.6484	1217.5461	-0.1023	-84	1257	1268	AKTNAANLSEAK					Mascot
1278.6801	1278.6652	-0.0149	-12	706	716	AGQLISEKNYR					Mascot
1309.6747	1309.6454	-0.0293	-22	1259	1270	TNAANLSEAKYK					Mascot

1316.6958	1316.5839	-0.1119	-85	1643	1653	AHQLQSDVKYK					Mascot
1349.696	1349.6361	-0.0599	-44	1400	1410	AHALQSEFRYK					Mascot
1374.676	1374.6411	-0.0349	-25	1368	1379	KAQALASDHDYR					Mascot
1419.6587	1419.7095	0.0508	36	262	271	YHQQYHREMK					Mascot
1484.7856	1484.6619	-0.1237	-83	981	992	ISYTQAVDRLYR					Mascot
1497.7114	1497.7069	-0.0045	-3	1334	1345	MGQLQSENQYRK			Oxidation (M)[1]		Mascot
1542.8163	1542.7349	-0.0814	-53	1453	1466	FTTVVDSPDLIHAK					Mascot
1553.6901	1553.7379	0.0478	31	724	737	FTSVADSSQMEHAK			Oxidation (M)[10]		Mascot
1582.8258	1582.7416	-0.0842	-53	1175	1189	GVPCVVPGTLEIEGR			Carbamidomethyl (C)[4]		Mascot
1582.8258	1582.7416	-0.0842	-53	1175	1189	GVPCVVPGTLEIEGR			Carbamidomethyl (C)[4]		Mascot
1594.8298	1594.9169	0.0871	55	237	251	GSFPAMITPAYQIAK					Mascot
1640.8391	1640.869	0.0299	18	932	947	GTGWVATGSLHVEQAK					Mascot
1813.0219	1812.8462	-0.1757	-97	518	533	LNYTLPQDAPQLLKAK					Mascot
1823.848	1823.8336	-0.0144	-8	79	96	SVAAMGGIDGKEDGEPFK			Oxidation (M)[5]		Mascot
1840.8494	1840.8756	0.0262	14	144	159	MVEARQSLGEGYTEDR					Mascot
1844.984	1844.9204	-0.0636	-34	1109	1124	AHFHLPLDMVTLVHAK			Oxidation (M)[9]		Mascot
1901.9175	1901.9127	-0.0048	-3	1160	1174	LQSENLYRSDLNFMR			Oxidation (M)[14]		Mascot

8	Ribulose bisphosphate carboxylase small chain, chloroplastic OS=Trifolium repens GN=RBCS PE=1 SV=1	RBS_TRIRP	20253.3	8.86	9	54	0	7.63	

Peptide Information

Calc. Mass	Obsrv. Mass	± da	± ppm	Start Seq.	End Seq.	Sequence	Ion Score	C. I. %	Modification	Rank	Result Type
893.4727	893.4232	-0.0495	-55	30	38	SSAGFPVTK					Mascot
921.504	921.4919	-0.0121	-13	84	90	EVEYLLR					Mascot
921.504	921.4919	-0.0121	-13	84	90	EVEYLLR					Mascot
958.424	958.4549	0.0309	32	121	126	YWTMWR			Oxidation (M)[4]		Mascot
958.424	958.4549	0.0309	32	121	126	YWTMWR			Oxidation (M)[4]		Mascot
1021.5676	1021.4678	-0.0998	-98	30	39	SSAGFPVTKK					Mascot
1447.7937	1447.6946	-0.0991	-68	1	14	MALISSAAVTTINR					Mascot
1492.7141	1492.7777	0.0636	43	92	103	GWVPCVEFELEK			Carbamidomethyl (C)[5]		Mascot
1527.853	1527.7109	-0.1421	-93	15	29	APVQANLATPFTGLK					Mascot
1527.853	1527.7109	-0.1421	-93	15	29	APVQANLATPFTGLK					Mascot
1542.792	1542.7349	-0.0571	-37	53	65	VNCMQVWPPVGKK			Carbamidomethyl (C)[3]		Mascot
2002.9155	2003.0315	0.116	58	104	120	GFVHRQYNSSPGYYDGR					Mascot

9	Valine--tRNA ligase OS=Parabacteroides distasonis	SYV_PARD8	100178.8	5.37	19	53	0	11.51	

(strain ATCC 8503 / DSM 20701 / NCTC 11152)
GN=valS PE=3 SV=1

Peptide Information

Calc. Mass	Obsrv. Mass	± da	± ppm	Start Seq.	End Seq.	Sequence	Ion Score	C. I. %	Modification	Rank	Result Type
806.4307	806.3979	-0.0328	-41	586	591	IWNAFR					Mascot
828.4362	828.3628	-0.0734	-89	385	390	FKNTYR					Mascot
852.4097	852.399	-0.0107	-13	333	339	VEAYENK					Mascot
897.504	897.4244	-0.0796	-89	346	353	TNVPIEPK					Mascot
957.4611	957.3837	-0.0774	-81	391	397	HWMENIK					Mascot
958.4186	958.4549	0.0363	38	141	148	TAFTMDEK			Oxidation (M)[5]		Mascot
958.4186	958.4549	0.0363	38	141	148	TAFTMDEK			Oxidation (M)[5]		Mascot
1068.5547	1068.4905	-0.0642	-60	354	361	LSMQWFLK			Oxidation (M)[3]		Mascot
1165.5017	1165.5382	0.0365	31	503	511	MIMAGYEYR			Oxidation (M)[1,3]		Mascot
1201.6147	1201.6393	0.0246	20	169	178	GVRMVNWDPK					Mascot
1211.5791	1211.5447	-0.0344	-28	622	632	TIAEVDDSFSK					Mascot
1217.6096	1217.5461	-0.0635	-52	169	178	GVRMVNWDPK			Oxidation (M)[4]		Mascot
1284.6947	1284.5963	-0.0984	-77	518	528	SVYFTGIVRDK					Mascot
1374.7377	1374.6411	-0.0966	-70	833	845	LGNENFVSKAPAK					Mascot
1461.7229	1461.7311	0.0082	6	633	644	YRLSEAMMAVYK					Mascot
1491.7162	1491.7024	-0.0138	-9	387	397	NTYRHWMENIK					Mascot
1527.7438	1527.7109	-0.0329	-22	333	345	VEAYENKVGFSER					Mascot
1527.7438	1527.7109	-0.0329	-22	333	345	VEAYENKVGFSER					Mascot
1650.7574	1650.7777	0.0203	12	436	449	EKCGNPNLTMSDLR			Carbamidomethyl (C)[3], Oxidation (M)[10]		Mascot
1799.8547	1799.8931	0.0384	21	398	410	DWCISRQLWWGHR			Carbamidomethyl (C)[3]		Mascot
1842.879	1842.8755	-0.0035	-2	617	632	MQLDKTIAEVDDSFSK			Oxidation (M)[1]		Mascot
2002.8634	2003.0315	0.1681	84	132	148	LGASCDWDRTAFTMDEK			Carbamidomethyl (C)[5]		Mascot
2018.8583	2019.0319	0.1736	86	132	148	LGASCDWDRTAFTMDEK			Carbamidomethyl (C)[5], Oxidation (M)[14]		Mascot

10	70 kDa neurofilament protein OS=Doryteuthis pealeii PE=2 SV=1	NF70_DORPE	71129.5	5.31	21	52	0	13.874	

Peptide Information

Calc. Mass	Obsrv. Mass	± da	± ppm	Start Seq.	End Seq.	Sequence	Ion Score	C. I. %	Modification	Rank	Result Type
805.4301	805.4141	-0.016	-20	219	225	SIESLEK					Mascot
806.3825	806.3979	0.0154	19	96	101	EKQDMR					Mascot
810.4104	810.3364	-0.074	-91	39	45	TSGYINR					Mascot

824.4122	824.3596	-0.0526	-64	569	575	NHQAQAR		Mascot
837.4498	837.4219	-0.0279	-33	1	7	MSVTQKK	Oxidation (M)[1]	Mascot
884.4512	884.4624	0.0112	13	107	113	FANYIEK		Mascot
893.4145	893.4232	0.0087	10	343	349	QNMEVTR	Oxidation (M)[3]	Mascot
906.46	906.3746	-0.0854	-94	412	419	GEMESILK		Mascot
912.3437	912.3717	0.028	31	237	243	MNDEMEK	Oxidation (M)[1]	Mascot
912.3437	912.3717	0.028	31	237	243	MNDEMEK	Oxidation (M)[1]	Mascot
932.4683	932.3976	-0.0707	-76	441	448	LLEGEESR		Mascot
976.4734	976.4183	-0.0551	-56	530	537	EENLTGWK		Mascot
1060.5634	1060.5209	-0.0425	-40	440	448	KLLEGEESR		Mascot
1062.5677	1062.5435	-0.0242	-23	219	227	SIESLEKEK		Mascot
1119.5575	1119.516	-0.0415	-37	343	351	QNMEVTRNK		Mascot
1201.5485	1201.6393	0.0908	76	606	615	ANYTQNTVYQ		Mascot
1216.6783	1216.5658	-0.1125	-92	124	134	LAGELEELKSK		Mascot
1527.6996	1527.7109	0.0113	7	144	155	EMYETELEEARK		Mascot
1527.6996	1527.7109	0.0113	7	144	155	EMYETELEEARK		Mascot
1569.6962	1569.7494	0.0532	34	393	404	DRQNELESCQYK	Carbamidomethyl (C)[9]	Mascot
1650.7826	1650.7777	-0.0049	-3	230	243	QSNILAKMNDEMEK		Mascot
1799.7688	1799.8931	0.1243	69	309	322	DIQQEYDAKCDQMR	Carbamidomethyl (C)[10]	Mascot
1844.9177	1844.9204	0.0027	1	323	337	GDIEAYYNLKVQEFR		Mascot

Gel Idx/Pos	182/H9	Instr./Gel Origin	BA2151/Sample Project 20140814	Process Status	Analysis Succeeded
Plate [#] Name	[1] Sample Project 20140814	Instrument Sample Name		Spectra	11

Rank	Protein Name	Accession No.	Protein MW	Protein PI	Pep. Count	Protein Score	Protein Score C. I. %	Intensity Matched	Total Ion Score	Total Ion C. I. %	Confirmed
1	Protein IN2-1 homolog B OS=Oryza sativa subsp. japonica GN=GSTZ5 PE=2 SV=1	IN21B_ORYSJ	27458.1	5.35	9	115	100	4.485	69	99.988	

Protein Group

Protein Name	Accession No.	Protein MW	Protein PI
Protein IN2-1 homolog B OS=Oryza sativa subsp. indica GN=GSTZ5 PE=1 SV=1	IN21B_ORYSI	27458.1	5.3499 999046 3257

Peptide Information

Calc. Mass	Obsrv. Mass	± da	± ppm	Start Seq.	End Seq.	Sequence	Ion Score	C. I. %	Modification	Rank	Result Type
812.4737	812.415	-0.0587	-72	206	212	GRPNLQK					Mascot
925.4989	925.4219	-0.077	-83	138	146	ASYSSIVAK					Mascot
962.4941	962.4642	-0.0299	-31	220	227	IHAYTETK					Mascot
965.505	965.4653	-0.0397	-41	53	60	NYKGLQDK					Mascot
1086.5983	1086.59	-0.0083	-8	191	199	FQIFFSGIK					Mascot
1540.7366	1540.7316	-0.005	-3	36	47	LYVAYHCPYAQR			Carbamidomethyl (C)[7]		Mascot
1540.7366	1540.7316	-0.005	-3	36	47	LYVAYHCPYAQR	69	99.988	Carbamidomethyl (C)[7]		Mascot
1630.8951	1630.8925	-0.0026	-2	63	76	IVAIDLADRPAWYK					Mascot
2075.0325	2075.0427	0.0102	5	147	166	GDVCDEAVAALDKIEAAL SK			Carbamidomethyl (C)[4]		Mascot
2138.0752	2138.0918	0.0166	8	36	52	LYVAYHCPYAQRAWIAR			Carbamidomethyl (C)[7]		Mascot

Rank	Protein Name	Accession No.	Protein MW	Protein PI	Pep. Count	Protein Score	Protein Score C. I. %	Intensity Matched	Total Ion Score	Total Ion C. I. %	Confirmed
2	Argininosuccinate lyase OS=Geobacter sulfurreducens (strain ATCC 51573 / DSM 12127 / PCA) GN=argH PE=3 SV=1	ARLY_GEOSL	51286.9	5.26	18	74	97.896	12.174			

Peptide Information

Calc. Mass	Obsrv. Mass	± da	± ppm	Start Seq.	End Seq.	Sequence	Ion Score	C. I. %	Modification	Rank	Result Type
816.4573	816.3838	-0.0735	-90	449	455	AEIERAK					Mascot
823.3436	823.3916	0.048	58	185	190	MEDCLR			Carbamidomethyl (C)[4]		Mascot
844.4886	844.4527	-0.0359	-43	447	453	VKAEIER					Mascot
851.4443	851.4188	-0.0255	-30	343	349	IFADMVR					Mascot
979.4448	979.4631	0.0183	19	185	191	MEDCLRR			Carbamidomethyl (C)[4]		Mascot
979.4448	979.4631	0.0183	19	185	191	MEDCLRR			Carbamidomethyl (C)[4]		Mascot
995.4397	995.4653	0.0256	26	185	191	MEDCLRR			Carbamidomethyl (C)[4], Oxidation (M)[1]		Mascot

Calc. Mass	Obsrv. Mass	± da	± ppm	Start Seq.	End Seq.	Sequence	Ion Score	C. I. %	Modification	Rank	Result Type
1038.5579	1038.5021	-0.0558	-54	287	295	NPDVPELVR					Mascot
1201.6899	1201.6466	-0.0433	-36	437	448	VATGGTALERVK					Mascot
1267.6287	1267.6044	-0.0243	-19	343	352	IFADMVREMR					Mascot
1299.6184	1299.5959	-0.0225	-17	343	352	IFADMVREMR			Oxidation (M)[5,9]		Mascot
1468.7903	1468.7197	-0.0706	-48	301	313	VYGNLMALLTVMK			Oxidation (M)[6]		Mascot
1570.7795	1570.7246	-0.0549	-35	379	392	QGMPFRDAHEVVGK					Mascot
1651.7996	1651.838	0.0384	23	314	327	ALPLAYNKDMQEDK			Oxidation (M)[10]		Mascot
1662.8949	1662.882	-0.0129	-8	328	342	EPLFDTIDTVKGSLK					Mascot
1675.8757	1675.8369	-0.0388	-23	46	60	MLGKQGILPMAEVEK			Oxidation (M)[1,10]		Mascot
1798.9554	1798.8801	-0.0753	-42	298	313	TGRVYGNLMALLTVMK			Oxidation (M)[9,15]		Mascot
1896.832	1896.9326	0.1006	53	403	418	DLPDLTMDEWQGFSDK					Mascot
1926.9919	1926.8624	-0.1295	-67	360	378	AAAAKGFSTATDVADYLV R					Mascot
2175.0461	2175.0713	0.0252	12	268	286	FVELTDSFCTGSSIMPQK K			Carbamidomethyl (C)[9]		Mascot
2188.0237	2188.0457	0.022	10	235	253	DFALEFMGASSILMMHLS R			Oxidation (M)[7,14]		Mascot

3	Protein translocase subunit SecA OS=Enterobacter sp. (strain 638) GN=secA PE=3 SV=1	SECA_ENT38	102307.7	5.29	22	69	92.706	34.294

Peptide Information

Calc. Mass	Obsrv. Mass	± da	± ppm	Start Seq.	End Seq.	Sequence	Ion Score	C. I. %	Modification	Rank	Result Type
815.4522	815.4328	-0.0194	-24	316	322	AHALFTR					Mascot
816.3781	816.3838	0.0057	7	895	901	SCHGRLS			Carbamidomethyl (C)[2]		Mascot
851.4509	851.4188	-0.0321	-38	323	329	DVDYIVK					Mascot
871.5108	871.4397	-0.0711	-82	626	633	AIANAQRK					Mascot
1018.5414	1018.4628	-0.0786	-77	38	46	LSDDELKAK					Mascot
1086.6014	1086.59	-0.0114	-10	269	277	ARQVNLTER					Mascot
1180.5337	1180.597	0.0633	54	751	760	EEVVGTEMMR					Mascot
1180.5337	1180.597	0.0633	54	751	760	EEVVGTEMMR					Mascot
1267.5769	1267.6044	0.0275	22	98	108	CIAEMRTGEGK			Carbamidomethyl (C)[1], Oxidation (M)[5]		Mascot
1340.6594	1340.6887	0.0293	22	349	360	WSDGLHQAVEAK					Mascot
1476.6458	1476.7216	0.0758	51	833	844	MPEEVEAMEQQR					Mascot
1492.6406	1492.7394	0.0988	66	833	844	MPEEVEAMEQQR			Oxidation (M)[1]		Mascot
1508.6356	1508.7002	0.0646	43	833	844	MPEEVEAMEQQR			Oxidation (M)[1,8]		Mascot
1570.8337	1570.7246	-0.1091	-69	125	138	GVHVVTVNDYLAQR					Mascot
1593.7499	1593.7705	0.0206	13	24	37	AVTVINAMEPEMEK			Oxidation (M)[8,12]		Mascot
1669.7812	1669.7584	-0.0228	-14	421	434	KDMPDLVYMTEAEK					Mascot
1727.8898	1727.8372	-0.0526	-30	83	97	HFDVQLLGGMVLNER					Mascot

1743.8848	1743.8324	-0.0524	-30	83	97	HFDVQLLGGMVLNER			Oxidation (M)[10]		Mascot
1743.8848	1743.8324	-0.0524	-30	83	97	HFDVQLLGGMVLNER	3	0	Oxidation (M)[10]		Mascot
1851.8793	1851.8801	0.0008	0	221	237	TPLIISGPAEDSSDMYR					Mascot
1869.0441	1868.8864	-0.1577	-84	444	461	ERTAAGQPVLVGTISIEK					Mascot
1926.7988	1926.8624	0.0636	33	252	268	EDSDTFQGEGHFSVDEK					Mascot
1947.9998	1947.9958	-0.004	-2	761	776	HFEKGVMLQTLDSLWK			Oxidation (M)[7]		Mascot
1958.9423	1958.8541	-0.0882	-45	829	844	VQVRMPEEVEAMEQQR					Mascot
1979.9742	1979.9742	0	0	221	238	TPLIISGPAEDSSDMYRK					Mascot
2132.1233	2132.0559	-0.0674	-32	57	76	GETVESLIPEAFAVVREA SK					Mascot
2133.9692	2134.092	0.1228	58	578	596	QGDAGSSRFYLSMEDAL MR					Mascot
2133.9692	2134.092	0.1228	58	578	596	QGDAGSSRFYLSMEDAL MR					Mascot
2149.9641	2150.0847	0.1206	56	578	596	QGDAGSSRFYLSMEDAL MR			Oxidation (M)[13]		Mascot
2165.959	2166.0786	0.1196	55	578	596	QGDAGSSRFYLSMEDAL MR			Oxidation (M)[13,18]		Mascot
2165.959	2166.0786	0.1196	55	578	596	QGDAGSSRFYLSMEDAL MR			Oxidation (M)[13,18]		Mascot

4	Dynein gamma chain, flagellar outer arm OS=Chlamydomonas reinhardtii GN=ODA2 PE=1 SV=1	DYHG_CHLRE	515882.8	6.19	55	67	88.17	51.811

Peptide Information

Calc. Mass	Obsrv. Mass	± da	± ppm	Start Seq.	End Seq.	Sequence	Ion Score	C. I. %	Modification	Rank	Result Type
806.4519	806.3834	-0.0685	-85	3836	3841	LNKFER					Mascot
815.4985	815.4328	-0.0657	-81	851	857	EVSLLVR					Mascot
823.4057	823.3916	-0.0141	-17	3847	3852	TFREDR					Mascot
853.3938	853.4214	0.0276	32	4102	4108	ITDDFDK					Mascot
870.3848	870.4536	0.0688	79	2976	2982	FTMACPK			Carbamidomethyl (C)[5], Oxidation (M)[3]		Mascot
871.4349	871.4397	0.0048	6	985	990	YDFLWK					Mascot
891.4968	891.4105	-0.0863	-97	373	380	VTLIGNMK			Oxidation (M)[7]		Mascot
925.456	925.4219	-0.0341	-37	670	676	EAKYMQR					Mascot
965.5051	965.4653	-0.0398	-41	973	980	TQVGEYIR					Mascot
979.5063	979.4631	-0.0432	-44	1692	1699	TIMNKNMK					Mascot
979.5063	979.4631	-0.0432	-44	1692	1699	TIMNKNMK					Mascot
995.4792	995.4653	-0.0139	-14	2442	2450	FNAETTANK					Mascot
996.5876	996.4883	-0.0993	-100	2840	2848	GLYKIAGFK					Mascot
996.5876	996.4883	-0.0993	-100	2840	2848	GLYKIAGFK					Mascot
1001.4985	1001.4518	-0.0467	-47	1335	1341	HWKEVMR			Oxidation (M)[6]		Mascot
1034.4941	1034.4584	-0.0357	-35	3005	3011	EYFEKYR					Mascot

1038.5466	1038.5021	-0.0445	-43	1542	1550	SLSAYLEQK		Mascot
1068.533	1068.5437	0.0107	10	3839	3846	FERMCVVK	Carbamidomethyl (C)[5]	Mascot
1086.5691	1086.59	0.0209	19	1056	1064	TQFAQNLHK		Mascot
1106.5378	1106.5179	-0.0199	-18	1986	1994	QAHYDFGLR		Mascot
1180.5515	1180.597	0.0455	39	1134	1143	EETTMVSDLR		Mascot
1180.6409	1180.597	-0.0439	-37	2123	2131	HVIWRMNPK		Mascot
1290.5709	1290.6243	0.0534	41	4133	4143	DTRSSDGFSYR		Mascot
1388.5822	1388.5784	-0.0038	-3	1602	1612	MTEMFSQQNEK	Oxidation (M)[1]	Mascot
1427.7543	1427.656	-0.0983	-69	697	707	FYHNQLTHLVR		Mascot
1476.6754	1476.7216	0.0462	31	797	808	SFTYEGFVEQNR		Mascot
1492.7642	1492.7394	-0.0248	-17	1957	1970	LAAAGYQENDILSK		Mascot
1507.7872	1507.7366	-0.0506	-34	1937	1949	ALFRGVTMMVPNR	Oxidation (M)[8]	Mascot
1524.7615	1524.6851	-0.0764	-50	3173	3185	MLPVTKAEYTDEK		Mascot
1540.7563	1540.7316	-0.0247	-16	3173	3185	MLPVTKAEYTDEK	Oxidation (M)[1]	Mascot
1540.8441	1540.7316	-0.1125	-73	813	825	QAELLAIRNEEVR		Mascot
1593.7876	1593.7705	-0.0171	-11	2128	2141	MNPKAITAPQMFGR	Oxidation (M)[1,11]	Mascot
1630.8945	1630.8925	-0.002	-1	1701	1714	VDALLRDMVNITVR	Oxidation (M)[8]	Mascot
1651.7666	1651.838	0.0714	43	681	694	CSSPSQMVLLQEEK	Carbamidomethyl (C)[1], Oxidation (M)[7]	Mascot
1662.8256	1662.882	0.0564	34	4208	4221	EDVVDKICEDLLSK	Carbamidomethyl (C)[8]	Mascot
1675.9014	1675.8369	-0.0645	-38	3853	3868	TLIAAADYIAEALGQR		Mascot
1699.7626	1699.8292	0.0666	39	1425	1439	LEESQMTLGSMATNR	Oxidation (M)[6,11]	Mascot
1727.8204	1727.8372	0.0168	10	1723	1736	TNLETCITVHMHQK	Carbamidomethyl (C)[6], Oxidation (M)[11]	Mascot
1743.8231	1743.8324	0.0093	5	3655	3668	INEACEEYRPVAHR	Carbamidomethyl (C)[5]	Mascot
1743.9309	1743.8324	-0.0985	-56	1087	1101	IEDLEDVRNVMAVLK		Mascot
1798.8971	1798.8801	-0.017	-9	1899	1914	KSFQFTDGTTVSLDPR		Mascot
1835.9718	1835.8971	-0.0747	-41	2194	2210	VLTLANGDRILMSAAMK	Oxidation (M)[12,16]	Mascot
1851.8145	1851.8801	0.0656	35	226	240	DGEERGPDTELEYWR		Mascot
1869.0262	1868.8864	-0.1398	-75	588	604	IEAPMQLAQNKNLLAAK	Oxidation (M)[5]	Mascot
1896.9637	1896.9326	-0.0311	-16	2532	2549	FITDVRYVAAMNTPGGG K		Mascot
1897.9452	1897.9169	-0.0283	-15	2616	2630	MLPTPAKFHYLFNMR	Oxidation (M)[1,14]	Mascot
1926.9299	1926.8624	-0.0675	-35	681	696	CSSPSQMVLLQEEKFK	Carbamidomethyl (C)[1], Oxidation (M)[7]	Mascot
1947.9229	1947.9958	0.0729	37	2211	2228	AMFEPENLNNASPATVS R		Mascot
1958.8735	1958.8541	-0.0194	-10	4009	4024	ASYQWVNQDMLDMVSR	Oxidation (M)[10]	Mascot
2075.1133	2075.0427	-0.0706	-34	3758	3777	ATDLDVFLKGGAALDINS VR		Mascot
2132.0264	2132.0559	0.0295	14	3298	3316	VQAKLDEMQAQFDAAMA HK		Mascot

2146.9753	2147.0427	0.0674	31	862	879	NMYNAIMQCTLNSLQAMK			Carbamidomethyl (C)[9], Oxidation (M)[2]		Mascot
2149.9617	2150.0847	0.123	57	935	952	QLPAWGMDNVATYHEMMR					Mascot
2153.0618	2153.0476	-0.0142	-7	2513	2531	QLLEQGGMYSLEKPIGDMK			Oxidation (M)[8]		Mascot
2164.0161	2164.0813	0.0652	30	3298	3316	VQAKLDEMQAQFDAAMAHK			Oxidation (M)[8,16]		Mascot
2166.0681	2166.0786	0.0105	5	677	694	FNIRCSSPSQMVLLQEEK			Carbamidomethyl (C)[5]		Mascot
2166.0681	2166.0786	0.0105	5	677	694	FNIRCSSPSQMVLLQEEK	3	0	Carbamidomethyl (C)[5]		Mascot
2205.1594	2205.0461	-0.1133	-51	2770	2787	VMKLELVLFTDCVTHLMR			Carbamidomethyl (C)[12]		Mascot
2221.1543	2221.0999	-0.0544	-24	2770	2787	VMKLELVLFTDCVTHLMR			Carbamidomethyl (C)[12], Oxidation (M)[2]		Mascot
2338.1306	2338.1433	0.0127	5	1107	1126	ESEIDNLIGPIEEMYGLLMR			Oxidation (M)[14]		Mascot
2356.1172	2356.1624	0.0452	19	4005	4024	AGLRASYQWVNQDMLDMVSR			Oxidation (M)[14]		Mascot
2356.1172	2356.1624	0.0452	19	4005	4024	AGLRASYQWVNQDMLDMVSR			Oxidation (M)[14]		Mascot

5	Replicase large subunit OS=Tobacco mild green mosaic virus PE=3 SV=2	RDRP_TMGMV	184566.8	6.85	31	59	25.358	15.893	

Peptide Information

Calc. Mass	Obsrv. Mass	± da	± ppm	Start Seq.	End Seq.	Sequence	Ion Score	C. I. %	Modification	Rank	Result Type
806.3825	806.3834	0.0009	1	875	881	ATMDNVR					Mascot
816.4574	816.3838	-0.0736	-90	390	396	SEVIVNR					Mascot
828.3743	828.4153	0.041	49	577	582	FKDMCK			Carbamidomethyl (C)[5]		Mascot
838.424	838.3746	-0.0494	-59	473	478	FRCLDK			Carbamidomethyl (C)[3]		Mascot
844.3691	844.4527	0.0836	99	577	582	FKDMCK			Carbamidomethyl (C)[5], Oxidation (M)[4]		Mascot
871.3978	871.4397	0.0419	48	1156	1162	YDAVTMR			Oxidation (M)[6]		Mascot
916.5145	916.502	-0.0125	-14	858	865	QAAAMIRR					Mascot
925.489	925.4219	-0.0671	-73	1514	1520	YIIHHDK					Mascot
994.4874	994.4592	-0.0282	-28	989	998	SVSSEMIGGK					Mascot
1162.6077	1162.5836	-0.0241	-21	188	197	IHPPENSGRR					Mascot
1180.6208	1180.597	-0.0238	-20	538	547	SEELPHLDIK					Mascot
1180.6208	1180.597	-0.0238	-20	538	547	SEELPHLDIK					Mascot
1290.6471	1290.6243	-0.0228	-18	950	959	LQVDEVEMRR			Oxidation (M)[8]		Mascot
1299.6614	1299.5959	-0.0655	-50	689	700	QMASVVYTGSLK			Oxidation (M)[2]		Mascot
1427.593	1427.656	0.063	44	170	181	YAEAPNEVCCSK			Carbamidomethyl (C)[9,10]		Mascot
1454.7792	1454.7075	-0.0717	-49	491	502	FFGNVFPTIKER					Mascot
1468.6849	1468.7197	0.0348	24	813	825	TMRDGEPHEPTAK					Mascot
1470.6708	1470.7018	0.031	21	725	737	SLKDEVGYDSDSR					Mascot
1570.7723	1570.7246	-0.0477	-30	358	370	DTASVNFWFPKMK					Mascot

1651.8612	1651.838	-0.0232	-14	371	385	DMVIVPLFEGSITSK			Oxidation (M)[2]		Mascot
1652.8022	1652.8206	0.0184	11	1172	1185	DCVLDFSKSIPMPK			Carbamidomethyl (C)[2], Oxidation (M)[12]		Mascot
1675.8109	1675.8369	0.026	16	946	958	HFEKLQVDEVEMR			Oxidation (M)[12]		Mascot
1692.801	1692.8411	0.0401	24	138	151	HEGQKDSIEMYLSR					Mascot
1699.8625	1699.8292	-0.0333	-20	297	309	EFLVTRVNTWFCK			Carbamidomethyl (C)[12]		Mascot
1757.8739	1757.8328	-0.0411	-23	791	806	RVAVSSDTMVYSDIAK			Oxidation (M)[9]		Mascot
1798.9276	1798.8801	-0.0475	-26	354	368	AIFRDTASVNFWFPK					Mascot
1813.8398	1813.8994	0.0596	33	182	196	TFQDCRIHPPENSGR			Carbamidomethyl (C)[5]		Mascot
1897.0079	1896.9326	-0.0753	-40	1403	1418	LGLNEFLAEVWKQGHR					Mascot
1897.9874	1897.9169	-0.0705	-37	689	705	QMASVVYTGSLKVQQMK					Mascot
1926.9916	1926.8624	-0.1292	-67	369	385	MKDMVIVPLFEGSITSK			Oxidation (M)[1,4]		Mascot
2009.0923	2008.9662	-0.1261	-63	1180	1196	SIPMPKEVKPCLEPVLR			Carbamidomethyl (C)[11], Oxidation (M)[4]		Mascot
2215.1792	2215.0254	-0.1538	-69	845	864	FDLDEDLILVPGKQAAAMIR					Mascot
2356.0737	2356.1624	0.0887	38	1482	1501	GLDFPDIQSCANLMWNFEAK			Carbamidomethyl (C)[10]		Mascot
2356.0737	2356.1624	0.0887	38	1482	1501	GLDFPDIQSCANLMWNFEAK			Carbamidomethyl (C)[10]		Mascot

6	DNA-directed RNA polymerase subunit beta' OS=Lysinibacillus sphaericus (strain C3-41) GN=rpoC PE=3 SV=2	RPOC_LYSSC	137891.9	6.93	26	57	0	22.035

Peptide Information

Calc. Mass	Obsrv. Mass	± da	± ppm	Start Seq.	End Seq.	Sequence	Ion Score	C. I. %	Modification	Rank	Result Type
800.4512	800.3898	-0.0614	-77	12	19	IGLASPDK					Mascot
809.4008	809.4021	0.0013	2	668	673	MLDRMK			Oxidation (M)[1]		Mascot
815.4443	815.4328	-0.0115	-14	315	321	SLSHMLK					Mascot
825.3957	825.3926	-0.0031	-4	668	673	MLDRMK			Oxidation (M)[1,5]		Mascot
853.46	853.4214	-0.0386	-45	672	678	MKNLGFK			Oxidation (M)[1]		Mascot
916.5462	916.502	-0.0442	-48	814	822	KGLADTALK					Mascot
962.4941	962.4642	-0.0299	-31	660	667	FHITETSK					Mascot
978.5255	978.4762	-0.0493	-50	210	217	LEVVESFR					Mascot
996.4931	996.4883	-0.0048	-5	70	77	YKGVVCDR			Carbamidomethyl (C)[6]		Mascot
996.4931	996.4883	-0.0048	-5	70	77	YKGVVCDR			Carbamidomethyl (C)[6]		Mascot
1038.6055	1038.5021	-0.1034	-100	378	387	GLAHNIKSAK					Mascot
1299.6427	1299.5959	-0.0468	-36	182	192	IDLEDETHSLK					Mascot
1427.743	1427.656	-0.087	-61	634	645	HFESVPVVNPFR					Mascot
1454.84	1454.7075	-0.1325	-91	471	483	LLMLAAQNILNPK			Oxidation (M)[3]		Mascot
1458.6353	1458.7119	0.0766	53	752	764	TNPIFMMSDSGAR			Oxidation (M)[6,7]		Mascot

Calc. Mass	Obsrv. Mass	± da	± ppm	Start Seq.	End Seq.	Sequence	Ion Score	C. I. %	Modification	Rank	Result Type
1470.7336	1470.7018	-0.0318	-22	249	260	FATSDLNDLYRR					Mascot
1521.8523	1521.8235	-0.0288	-19	1066	1078	VKDVSTVQEYLLK					Mascot
1652.9343	1652.8206	-0.1137	-69	407	420	EHPVLLNRAPTLHR					Mascot
1692.9829	1692.8411	-0.1418	-84	778	793	GLMANPAGRIIELPIK					Mascot
1699.865	1699.8292	-0.0358	-21	393	406	LNNDVWDVLEDVIR					Mascot
1725.9746	1725.8477	-0.1269	-74	1050	1065	GQVLTEGSIDPKQLLK					Mascot
1798.9069	1798.8801	-0.0268	-15	182	196	IDLEDETHSLKEELK					Mascot
1870.0546	1869.8793	-0.1753	-94	421	437	LGIQAFEPTLVEGRAIR					Mascot
1870.0546	1869.8793	-0.1753	-94	421	437	LGIQAFEPTLVEGRAIR					Mascot
2074.9927	2075.0427	0.05	24	1155	1173	ASLETESFLSAASFQETTR					Mascot
2147.0901	2147.0427	-0.0474	-22	852	871	GLTIGALMEGTELIEALDER			Oxidation (M)[8]		Mascot
2175.0347	2175.0713	0.0366	17	833	851	LVDVAQDVIVREDDCGTDR			Carbamidomethyl (C)[15]		Mascot
2205.2139	2205.0461	-0.1678	-76	415	434	APTLHRLGIQAFEPTLVEGR					Mascot
3580.7952	3580.6394	-0.1558	-44	1107	1140	VRVIEAGDTDLLPGSLLDIHQFAEANADAVMDGK					Mascot

7	DNA-directed RNA polymerase subunit beta' OS=Burkholderia sp. (strain 383) GN=rpoC PE=3 SV=1	RPOC_BURS3	156906.1	6.47	23	57	0	40.44

Peptide Information

Calc. Mass	Obsrv. Mass	± da	± ppm	Start Seq.	End Seq.	Sequence	Ion Score	C. I. %	Modification	Rank	Result Type
800.4512	800.3898	-0.0614	-77	22	29	IGLASPDK					Mascot
809.4152	809.4021	-0.0131	-16	666	671	VKEYDR					Mascot
816.4574	816.3838	-0.0736	-90	1348	1354	DDLRGLK					Mascot
828.4574	828.4153	-0.0421	-51	1140	1147	DITGGLPR					Mascot
916.5462	916.502	-0.0442	-48	786	794	KGLADTALK					Mascot
962.5339	962.4642	-0.0697	-72	326	334	SLADMIKGK					Mascot
978.5288	978.4762	-0.0526	-54	326	334	SLADMIKGK			Oxidation (M)[5]		Mascot
995.5091	995.4653	-0.0438	-44	418	425	EHPVMLNR					Mascot
1180.6064	1180.597	-0.0094	-8	124	133	LGMVLDMTLR			Oxidation (M)[3,7]		Mascot
1180.6064	1180.597	-0.0094	-8	124	133	LGMVLDMTLR			Oxidation (M)[3,7]		Mascot
1470.7336	1470.7018	-0.0318	-22	260	271	FATSDLNDLYRR					Mascot
1662.8553	1662.882	0.0267	16	82	96	GVICEKCGVEVTLAK			Carbamidomethyl (C)[4,7]		Mascot
1669.8367	1669.7584	-0.0783	-47	621	635	ATVVFADQLMQSGFR					Mascot
1686.8857	1686.8055	-0.0802	-48	418	431	EHPVMLNRAPTLHR			Oxidation (M)[5]		Mascot
1699.8182	1699.8292	0.011	6	61	74	IFGPIKDYECLCGK			Carbamidomethyl (C)[10,12]		Mascot
1742.8816	1742.8527	-0.0289	-17	640	655	AGISICVDDMLVPPQK			Carbamidomethyl (C)[6]		Mascot

1743.9388	1743.8324	-0.1064	-61	1140	1155	DITGGLPRVAELFEAR				Mascot
1743.9388	1743.8324	-0.1064	-61	1140	1155	DITGGLPRVAELFEAR				Mascot
1813.7579	1813.8994	0.1415	78	1289	1303	SDMLDENDRMIAEDK			Oxidation (M)[3,10]	Mascot
2147.1165	2147.0427	-0.0738	-34	750	769	GLMAKPDGSIIETPITANF R			Oxidation (M)[3]	Mascot
2148.9624	2149.0576	0.0952	44	889	906	TPLTCETRYGLCAACYG R			Carbamidomethyl (C)[5,12,15]	Mascot
2149.9819	2150.0847	0.1028	48	1280	1297	FIPGEQVERSDMLDEND R				Mascot
2166.1514	2166.0786	-0.0728	-34	1118	1137	DGQQVQVGEVLARIPTE AQK				Mascot
2166.1514	2166.0786	-0.0728	-34	1118	1137	DGQQVQVGEVLARIPTE AQK				Mascot
2192.97	2193.0747	0.1047	48	718	736	ETRQESFNSIYMMADSG AR				Mascot
2192.97	2193.0747	0.1047	48	718	736	ETRQESFNSIYMMADSG AR				Mascot
2215.1604	2215.0254	-0.135	-61	10	29	QVQQEEVFDAIKIGLASP DK				Mascot
3336.8752	3336.6396	-0.2356	-71	230	259	SGIKPEWMILEVLPVLPP ELRPLVPLDGGR			Oxidation (M)[8]	Mascot

8	DNA-directed RNA polymerase subunit beta' OS=Xanthobacter autotrophicus (strain ATCC BAA-1158 / Py2) GN=rpoC PE=3 SV=1	RPOC_XANP2	155570.3	8.48	29	56	0	27.354	

Peptide Information

Calc. Mass	Obsrv. Mass	± da	± ppm	Start Seq.	End Seq.	Sequence	Ion Score	C. I. %	Modification	Rank	Result Type
828.4574	828.4153	-0.0421	-51	1126	1133	DITGGLPR					Mascot
838.3876	838.3746	-0.013	-16	55	61	DGLFCAR			Carbamidomethyl (C)[5]		Mascot
844.4774	844.4527	-0.0247	-29	23	30	ITIASPDK					Mascot
853.3794	853.4214	0.042	49	693	699	VAEEMMK			Oxidation (M)[5]		Mascot
870.4931	870.4536	-0.0395	-45	1334	1341	VDPLEGLK					Mascot
916.5462	916.502	-0.0442	-48	778	786	KGLADTALK					Mascot
931.4917	931.4193	-0.0724	-78	642	649	DDMVVPKK					Mascot
962.5339	962.4642	-0.0697	-72	328	336	SLADMLKGK					Mascot
965.472	965.4653	-0.0067	-7	881	888	SVLTCETR			Carbamidomethyl (C)[5]		Mascot
978.5288	978.4762	-0.0526	-54	328	336	SLADMLKGK			Oxidation (M)[5]		Mascot
995.5091	995.4653	-0.0438	-44	420	427	EHPVMLNR					Mascot
1018.535	1018.4628	-0.0722	-71	1117	1125	VPMESAKTR					Mascot
1034.5299	1034.4584	-0.0715	-69	1117	1125	VPMESAKTR			Oxidation (M)[3]		Mascot
1201.6357	1201.6466	0.0109	9	1349	1360	LIPAGTGAQMAR			Oxidation (M)[10]		Mascot
1299.7056	1299.5959	-0.1097	-84	584	594	HPKLSYDVVNK					Mascot
1333.6781	1333.7086	0.0305	23	193	204	GMDLDKIAADLR			Oxidation (M)[2]		Mascot

1427.6802	1427.656	-0.0242	-17	554	566	YNGVDAEGKPYSK					Mascot
1454.7308	1454.7075	-0.0233	-16	599	610	KEISNMIDAVYR			Oxidation (M)[6]		Mascot
1458.7799	1458.7119	-0.068	-47	797	809	LVDVAQDSIITER					Mascot
1470.7336	1470.7018	-0.0318	-22	262	273	FATSDLNDLYRR					Mascot
1630.8662	1630.8925	0.0263	16	374	386	MALELFKPFIYSR			Oxidation (M)[1]		Mascot
1646.8279	1646.8737	0.0458	28	960	974	NVARNSEGDLVVMAR			Oxidation (M)[13]		Mascot
1686.8857	1686.8055	-0.0802	-48	420	433	EHPVMLNRAPTLHR			Oxidation (M)[5]		Mascot
1699.8182	1699.8292	0.011	6	62	75	IFGPIKDYECLCGK			Carbamidomethyl (C)[10,12]		Mascot
1742.9662	1742.8527	-0.1135	-65	373	386	KMALELFKPFIYSR					Mascot
1743.9388	1743.8324	-0.1064	-61	1126	1141	DITGGLPRVAELFEAR					Mascot
1743.9388	1743.8324	-0.1064	-61	1126	1141	DITGGLPRVAELFEAR					Mascot
1757.8639	1757.8328	-0.0311	-18	858	871	GTMIEEWHVERINK			Oxidation (M)[3]		Mascot
1851.884	1851.8801	-0.0039	-2	713	728	EKQINSIYMMSHSGAR					Mascot
1870.0658	1869.8793	-0.1865	-100	1236	1251	LQGVHINDKHIEVIVR					Mascot
1870.0658	1869.8793	-0.1865	-100	1236	1251	LQGVHINDKHIEVIVR					Mascot
2031.1597	2030.9945	-0.1652	-81	1285	1304	AAEEAKKPATGHPVLLGI TK					Mascot
2147.1416	2147.0427	-0.0989	-46	742	761	GLMAKPSGEIIESPIISNF K			Oxidation (M)[3]		Mascot

9 DNA-directed RNA polymerase subunit beta' OS=Burkholderia phymatum (strain DSM 17167 / STM815) GN=rpoC PE=3 SV=1 | RPOC_BURP8 | 157122 | 6.4 | 23 | 55 | 0 | 29.264

Peptide Information

Calc. Mass	Obsrv. Mass	± da	± ppm	Start Seq.	End Seq.	Sequence	Ion Score	C. I. %	Modification	Rank	Result Type
800.4512	800.3898	-0.0614	-77	22	29	IGLASPDK					Mascot
809.4152	809.4021	-0.0131	-16	666	671	VKEYDR					Mascot
816.4574	816.3838	-0.0736	-90	1348	1354	DDLRGLK					Mascot
828.4574	828.4153	-0.0421	-51	1140	1147	DITGGLPR					Mascot
916.5462	916.502	-0.0442	-48	786	794	KGLADTALK					Mascot
962.5339	962.4642	-0.0697	-72	326	334	SLADMIKGK					Mascot
978.5288	978.4762	-0.0526	-54	326	334	SLADMIKGK			Oxidation (M)[5]		Mascot
995.5091	995.4653	-0.0438	-44	418	425	EHPVMLNR					Mascot
1180.6064	1180.597	-0.0094	-8	124	133	LGMVLDMTLR			Oxidation (M)[3,7]		Mascot
1180.6064	1180.597	-0.0094	-8	124	133	LGMVLDMTLR			Oxidation (M)[3,7]		Mascot
1340.674	1340.6887	0.0147	11	959	970	SNGTVRFTATMR					Mascot
1356.6689	1356.5829	-0.086	-63	959	970	SNGTVRFTATMR			Oxidation (M)[11]		Mascot
1470.7336	1470.7018	-0.0318	-22	260	271	FATSDLNDLYRR					Mascot

1662.8553	1662.882	0.0267	16	82	96	GVICEKCGVEVTLAK			Carbamidomethyl (C)[4,7]		Mascot
1686.8857	1686.8055	-0.0802	-48	418	431	EHPVMLNRAPTLHR			Oxidation (M)[5]		Mascot
1699.8182	1699.8292	0.011	6	61	74	IFGPIKDYECLCGK			Carbamidomethyl (C)[10,12]		Mascot
1742.8816	1742.8527	-0.0289	-17	640	655	AGISICVDDMLVPPQK			Carbamidomethyl (C)[6]		Mascot
1743.9388	1743.8324	-0.1064	-61	1140	1155	DITGGLPRVAELFEAR					Mascot
1743.9388	1743.8324	-0.1064	-61	1140	1155	DITGGLPRVAELFEAR					Mascot
1757.8528	1757.8328	-0.02	-11	621	635	ETVIFADQLMQSGFR			Oxidation (M)[10]		Mascot
1798.7218	1798.8801	0.1583	88	1289	1303	SDMLDENDRMNAEDK			Oxidation (M)[3]		Mascot
2147.1165	2147.0427	-0.0738	-34	750	769	GLMAKPDGSIIETPITANF R			Oxidation (M)[3]		Mascot
2149.9819	2150.0847	0.1028	48	1280	1297	FIPGEQVERSDMLDEND R					Mascot
2153.0945	2153.0476	-0.0469	-22	977	996	GEQIVISRSGEALITDDHG R					Mascot
2166.1514	2166.0786	-0.0728	-34	1118	1137	DGQQVQVGEVLARIPTE AQK					Mascot
2166.1514	2166.0786	-0.0728	-34	1118	1137	DGQQVQVGEVLARIPTE AQK					Mascot
2215.0701	2215.0254	-0.0447	-20	552	571	ITEMVHNEDKSEGAPAFV PK			Oxidation (M)[4]		Mascot
3336.8752	3336.6396	-0.2356	-71	230	259	SGIKPEWMILEVLPVLPP ELRPLVPLDGGR			Oxidation (M)[8]		Mascot

10	Transcription elongation factor SPT5 OS=Gallus gallus GN=SUPT5H PE=2 SV=1	SPT5H_CHICK	120306.2	5	20	55	0	21.014

Peptide Information

Calc. Mass	Obsrv. Mass	± da	± ppm	Start Seq.	End Seq.	Sequence	Ion Score	C. I. %	Modification	Rank	Result Type
851.4178	851.4188	0.001	1	256	262	EMTDVLK			Oxidation (M)[2]		Mascot
891.4352	891.4105	-0.0247	-28	181	187	CKIGEER			Carbamidomethyl (C)[1]		Mascot
965.5414	965.4653	-0.0761	-79	211	219	SVVAPEHVK					Mascot
979.4818	979.4631	-0.0187	-19	617	624	GFAFLHCK			Carbamidomethyl (C)[7]		Mascot
979.4818	979.4631	-0.0187	-19	617	624	GFAFLHCK			Carbamidomethyl (C)[7]		Mascot
1106.5841	1106.5179	-0.0662	-60	482	491	FEGDTGLIVR					Mascot
1162.6038	1162.5836	-0.0202	-17	304	312	MIPRIDFDR					Mascot
1290.5559	1290.6243	0.0684	53	131	140	EEELGEYYMK					Mascot
1340.7242	1340.6887	-0.0355	-26	445	455	ITIMPKHEDLK			Oxidation (M)[4]		Mascot
1476.7516	1476.7216	-0.03	-20	456	467	DMLEFPAQELRK					Mascot
1492.7465	1492.7394	-0.0071	-5	456	467	DMLEFPAQELRK			Oxidation (M)[2]		Mascot
1508.8472	1508.7002	-0.147	-97	704	717	ISQGPYKGYIGVVK					Mascot
1521.7769	1521.8235	0.0466	31	599	612	VIDGPHSGREGEIR					Mascot
1646.7592	1646.8737	0.1145	70	776	790	TPMYGSQTPLHDGSR					Mascot
1652.7776	1652.8206	0.043	26	791	805	TPHYGSQTPLHDGSR					Mascot

1662.7542	1662.882	0.1278	77	776	790	TPMYGSQTPLHDGSR	Oxidation (M)[3]	Mascot
1684.8654	1684.8153	-0.0501	-30	580	594	FAVALDSEQNNIHVK		Mascot
1725.8265	1725.8477	0.0212	12	650	665	DVTNFTVGSFAPMSPR		Mascot
1727.8785	1727.8372	-0.0413	-24	550	563	LERETFQVLNMYGK		Mascot
1743.8734	1743.8324	-0.041	-24	550	563	LERETFQVLNMYGK	Oxidation (M)[11]	Mascot
1743.8734	1743.8324	-0.041	-24	550	563	LERETFQVLNMYGK	Oxidation (M)[11]	Mascot
1757.928	1757.8328	-0.0952	-54	1042	1058	EATGILLSIDGEDGIVR		Mascot
1896.9709	1896.9326	-0.0383	-20	742	759	LTTVGSRRPGGMTSTYG R		Mascot
2164.0996	2164.0813	-0.0183	-8	492	509	VEENFVILFSDLTMHELK		Mascot
2188.0134	2188.0457	0.0323	15	666	688	ISSPMHPSGAGQRGGFG GGGMSR		Mascot

Rank	Protein Name	Accession No.	Protein MW	Protein PI	Pep. Count	Protein Score	Protein Score C. I. %	Intensity Matched	Total Ion Score	Total Ion C. I. %	Confirmed
1	LL-diaminopimelate aminotransferase OS=Leptospira interrogans serogroup Icterohaemorrhagiae serovar Lai (strain 56601) GN=dapL PE=3 SV=1	DAPAT_LEPIN	45047.8	6.41	13	60	50.685	6.388			

Protein Group

LL-diaminopimelate aminotransferase OS=Leptospira interrogans serogroup Icterohaemorrhagiae serovar copenhageni (strain Fiocruz L1-130) GN=dapL PE=1 SV=1	DAPAT_LEPIC	45075.8	6.4099 998474 1211

Peptide Information

Calc. Mass	Obsrv. Mass	± da	± ppm	Start Seq.	End Seq.	Sequence	Ion Score	C. I. %	Modification	Rank	Result Type
822.4468	822.4144	-0.0324	-39	250	257	TAGFTGLR					Mascot
863.4621	863.4581	-0.004	-5	239	245	EVAIEFR					Mascot
866.4406	866.4357	-0.0049	-6	200	206	AWVEYAK					Mascot
1167.5099	1167.5276	0.0177	15	301	311	GAEACYSPQGK			Carbamidomethyl (C)[5]		Mascot
1280.6998	1280.6161	-0.0837	-65	13	23	AGYLFPEISKR					Mascot
1316.6555	1316.6222	-0.0333	-25	154	164	YSNLIYMPATK			Oxidation (M)[7]		Mascot
1323.6943	1323.6246	-0.0697	-53	196	206	ESLKAWVEYAK					Mascot
1490.7234	1490.6664	-0.057	-38	273	285	SGEEVSLNSLWNR					Mascot
1490.7234	1490.6664	-0.057	-38	273	285	SGEEVSLNSLWNR					Mascot
1609.8334	1609.7908	-0.0426	-26	287	300	HTTKFNGVSYVTQK					Mascot
1959.0004	1958.8525	-0.1479	-75	313	329	EIQTSIAYYMANASKIR					Mascot
2166.0901	2166.0857	-0.0044	-2	125	144	IAVADPVYPVYVDTNVMAGR			Oxidation (M)[17]		Mascot
2166.0901	2166.0857	-0.0044	-2	125	144	IAVADPVYPVYVDTNVMAGR			Oxidation (M)[17]		Mascot
2199.075	2199.0483	-0.0267	-12	145	164	TGEIGPDGRYSNLIYMPATK			Oxidation (M)[16]		Mascot
2440.2217	2440.0295	-0.1922	-79	154	174	YSNLIYMPATKENGFQPEIPK					Mascot

Rank	Protein Name	Accession No.	Protein MW	Protein PI	Pep. Count	Protein Score	Protein Score C. I. %	Intensity Matched	Total Ion Score	Total Ion C. I. %	Confirmed
2	Protein PA-X OS=Influenza A virus (strain A/USA:Phila/1935 H1N1) GN=PA PE=3 SV=1	PAX_I35A3	29774.1	8.43	12	59	37.916	5.187			

Peptide Information

Calc. Mass	Obsrv. Mass	± da	± ppm	Start Seq.	End Seq.	Sequence	Ion Score	C. I. %	Modification	Rank	Result Type
812.3607	812.3954	0.0347	43	1	6	MEDFVR			Oxidation (M)[1]		Mascot

Calc. Mass	Obsrv. Mass	± da	± ppm	Start Seq.	End Seq.	Sequence	Ion Score	C. I. %	Modification	Rank	Result Type
853.3937	853.4183	0.0246	29	23	29	EYGEDLK					Mascot
863.4621	863.4581	-0.004	-5	76	82	FEIIEGR					Mascot
950.5128	950.4682	-0.0446	-47	244	251	ASFLKCPK			Carbamidomethyl (C)[6]		Mascot
1229.5719	1229.624	0.0521	42	20	29	TMKEYGEDLK			Oxidation (M)[2]		Mascot
1349.7212	1349.6477	-0.0735	-54	125	134	REVHIYYLEK					Mascot
1367.7101	1367.6545	-0.0556	-41	175	185	LFTIRQEMASR			Oxidation (M)[8]		Mascot
1506.7952	1506.6841	-0.1111	-74	126	137	EVHIYYLEKANK					Mascot
1594.7604	1594.9049	0.1445	91	7	19	QCFNPMIVELAEK			Carbamidomethyl (C)[2], Oxidation (M)[6]		Mascot
1970.9385	1970.9543	0.0158	8	7	22	QCFNPMIVELAEKTMK			Carbamidomethyl (C)[2], Oxidation (M)[6,15]		Mascot
2004.9882	2005.0004	0.0122	6	222	238	TSPALKILEPMWMDSNR			Oxidation (M)[11]		Mascot
2193.0645	2193.0718	0.0073	3	140	158	SEKTHIHIFSFTGEEMATK					Mascot
2193.0645	2193.0718	0.0073	3	140	158	SEKTHIHIFSFTGEEMATK					Mascot

3	Protein PA-X OS=Influenza A virus (strain A/Russia:St.Petersburg/8/2006 H1N1) GN=PA PE=3 SV=1	PAX_I06A0	29803.1	8.38	12	59	37.916	5.197	

Protein Group

Protein PA-X OS=Influenza A virus (strain A/Puerto Rico/8/1934 H1N1) GN=PA PE=3 SV=1	PAX_I34A1	29803.1	8.3800 001144 4092
Protein PA-X OS=Influenza A virus (strain A/X-31 H3N2) GN=PA PE=3 SV=1	PAX_I000X	29776.1	8.3800 001144 4092

Peptide Information

Calc. Mass	Obsrv. Mass	± da	± ppm	Start Seq.	End Seq.	Sequence	Ion Score	C. I. %	Modification	Rank	Result Type
812.3607	812.3954	0.0347	43	1	6	MEDFVR			Oxidation (M)[1]		Mascot
853.3937	853.4183	0.0246	29	23	29	EYGEDLK					Mascot
863.4621	863.4581	-0.004	-5	76	82	FEIIEGR					Mascot
1229.5719	1229.624	0.0521	42	20	29	TMKEYGEDLK			Oxidation (M)[2]		Mascot
1349.7212	1349.6477	-0.0735	-54	125	134	REVHIYYLEK					Mascot
1367.7101	1367.6545	-0.0556	-41	175	185	LFTIRQEMASR			Oxidation (M)[8]		Mascot
1506.7952	1506.6841	-0.1111	-74	126	137	EVHIYYLEKANK					Mascot
1549.7645	1549.762	-0.0025	-2	186	198	GLWDSFVSPREEK					Mascot
1594.7604	1594.9049	0.1445	91	7	19	QCFNPMIVELAEK			Carbamidomethyl (C)[2], Oxidation (M)[6]		Mascot
1970.9385	1970.9543	0.0158	8	7	22	QCFNPMIVELAEKTMK			Carbamidomethyl (C)[2], Oxidation (M)[6,15]		Mascot
2004.9882	2005.0004	0.0122	6	222	238	TSPALKILEPMWMDSNR			Oxidation (M)[11]		Mascot
2193.0645	2193.0718	0.0073	3	140	158	SEKTHIHIFSFTGEEMATK					Mascot

Calc. Mass	Obsrv. Mass	± da	± ppm	Start Seq.	End Seq.	Sequence	Ion Score	C. I. %	Modification	Rank	Result Type
2193.0645	2193.0718	0.0073	3	140	158	SEKTHIHIFSFTGEEMATK					Mascot

4	Protein PA-X OS=Influenza A virus (strain A/Henry/1936 H1N1) GN=PA PE=3 SV=1	PAX_I36A0	29807.1	8.63	12	57	0	4.905

Peptide Information

Calc. Mass	Obsrv. Mass	± da	± ppm	Start Seq.	End Seq.	Sequence	Ion Score	C. I. %	Modification	Rank	Result Type
812.3607	812.3954	0.0347	43	1	6	MEDFVR			Oxidation (M)[1]		Mascot
852.4097	852.4203	0.0106	12	23	29	EYGENLK					Mascot
863.4621	863.4581	-0.004	-5	76	82	FEIIEGR					Mascot
950.5128	950.4682	-0.0446	-47	244	251	ASFLKCPK			Carbamidomethyl (C)[6]		Mascot
1349.7212	1349.6477	-0.0735	-54	125	134	REVHIYYLEK					Mascot
1367.7101	1367.6545	-0.0556	-41	175	185	LFTIRQEMASR			Oxidation (M)[8]		Mascot
1506.7952	1506.6841	-0.1111	-74	126	137	EVHIYYLEKANK					Mascot
1549.7645	1549.762	-0.0025	-2	186	198	GLWDSFVSPREEK					Mascot
1594.7604	1594.9049	0.1445	91	7	19	QCFNPMIVELAEK			Carbamidomethyl (C)[2], Oxidation (M)[6]		Mascot
1970.9385	1970.9543	0.0158	8	7	22	QCFNPMIVELAEKTMK			Carbamidomethyl (C)[2], Oxidation (M)[6,15]		Mascot
2004.9882	2005.0004	0.0122	6	222	238	TSPALKILEPMWMDSNR			Oxidation (M)[11]		Mascot
2193.0645	2193.0718	0.0073	3	140	158	SEKTHIHIFSFTGEEMATK					Mascot
2193.0645	2193.0718	0.0073	3	140	158	SEKTHIHIFSFTGEEMATK					Mascot

5	Uncharacterized protein S3 OS=Diadromus pulchellus idnoreovirus 1 GN=S2 PE=4 SV=1	S3_DPIRV	138984.2	7.85	23	55	0	8.813

Peptide Information

Calc. Mass	Obsrv. Mass	± da	± ppm	Start Seq.	End Seq.	Sequence	Ion Score	C. I. %	Modification	Rank	Result Type
800.4485	800.4299	-0.0186	-23	690	695	NQRNLR					Mascot
804.4825	804.4244	-0.0581	-72	655	662	TLSGSVLK					Mascot
841.4414	841.4304	-0.011	-13	371	377	YTTNKSK					Mascot
849.5192	849.4452	-0.074	-87	191	197	KVNFITK					Mascot
858.5043	858.4796	-0.0247	-29	874	880	RLDVVEK					Mascot
870.5156	870.5117	-0.0039	-4	403	411	GNGGILAIR					Mascot
919.4479	919.434	-0.0139	-15	449	456	TQSNNDIK					Mascot
950.5128	950.4682	-0.0446	-47	292	299	QFIGMQVK					Mascot
968.5272	968.4766	-0.0506	-52	176	183	HNLREATK					Mascot
1021.4619	1021.4922	0.0303	30	1157	1164	EEMINVDR			Oxidation (M)[3]		Mascot
1075.5718	1075.5291	-0.0427	-40	589	597	GNFPMIIQR					Mascot
1105.6113	1105.5255	-0.0858	-78	782	790	QGQKNIYVR					Mascot

Calc. Mass	Obsrv. Mass	± da	± ppm	Start Seq.	End Seq.	Sequence	Ion Score	C. I. %	Modification	Rank	Result Type
1180.4946	1180.5895	0.0949	80	551	559	RDNNMCTNR			Carbamidomethyl (C)[6]		Mascot
1211.6743	1211.566	-0.1083	-89	475	485	VSEGVIHTTLR					Mascot
1303.6602	1303.6537	-0.0065	-5	1182	1194	TSVGINSNNVNGK					Mascot
1320.7158	1320.6069	-0.1089	-82	300	311	VIEGAYVINSQK					Mascot
1518.7581	1518.7103	-0.0478	-31	1157	1168	EEMINVDRDIIR			Oxidation (M)[3]		Mascot
1527.7142	1527.7136	-0.0006	0	444	456	MTMTKTQSNNDIK			Oxidation (M)[1]		Mascot
1527.7142	1527.7136	-0.0006	0	444	456	MTMTKTQSNNDIK			Oxidation (M)[1]		Mascot
1539.806	1539.8059	-0.0001	0	1108	1121	GKMSINAIVHGEQR					Mascot
1680.7435	1680.8041	0.0606	36	791	806	DGTSSGGYVANWHAMK					Mascot
1739.9175	1739.8219	-0.0956	-55	566	581	FTTTSRSSPSVTLDIK					Mascot
2104.155	2103.9575	-0.1975	-94	16	33	QFIFGILNDNVREINIAK					Mascot
2839.4434	2839.3105	-0.1329	-47	1028	1054	ANNTLTGTTDIEIVNDVIN GQSAHITK					Mascot

6 UPF0403 protein YphP OS=Bacillus subtilis (strain 168) GN=yphP PE=1 SV=1 | YPHP_BACSU | 16035.7 | 4.81 | 8 | 55 | 0 | 5.601

Peptide Information

Calc. Mass	Obsrv. Mass	± da	± ppm	Start Seq.	End Seq.	Sequence	Ion Score	C. I. %	Modification	Rank	Result Type
806.4189	806.4187	-0.0002	0	88	94	EATAKMR					Mascot
822.4138	822.4144	0.0006	1	88	94	EATAKMR			Oxidation (M)[6]		Mascot
858.4866	858.4796	-0.007	-8	11	17	QLVVPMR			Oxidation (M)[6]		Mascot
996.5625	996.4843	-0.0782	-78	114	121	EVVHFIPR					Mascot
996.5625	996.4843	-0.0782	-78	114	121	EVVHFIPR					Mascot
1211.4707	1211.566	0.0953	79	2	10	SMAYEEYMR			Oxidation (M)[2,8]		Mascot
1492.7279	1492.7629	0.035	23	74	87	TPDNTVTVFAGQDK					Mascot
1597.6985	1597.8176	0.1191	75	122	134	HEIEGHDMEEIMK					Mascot
2034.9446	2034.9753	0.0307	15	2	17	SMAYEEYMRQLVVPMR			Oxidation (M)[2,8]		Mascot
2165.9851	2166.0857	0.1006	46	1	17	MSMAYEEYMRQLVVPM R			Oxidation (M)[1,3]		Mascot
2165.9851	2166.0857	0.1006	46	1	17	MSMAYEEYMRQLVVPM R	3	0	Oxidation (M)[1,3]		Mascot

7 Methyl-CpG-binding protein 2 OS=Macaca fascicularis GN=MECP2 PE=2 SV=1 | MECP2_MACFA | 52565.7 | 9.95 | 16 | 54 | 0 | 7.451

Protein Group

Methyl-CpG-binding protein 2 OS=Homo sapiens GN=MECP2 PE=1 SV=1 | MECP2_HUMAN | 52579.7 | 9.9499 998092 6514

Peptide Information

Calc. Mass	Obsrv. Mass	± da	± ppm	Start Seq.	End Seq.	Sequence	Ion Score	C. I. %	Modification	Rank	Result Type
805.3985	805.4028	0.0043	5	338	344	TCKSPGR			Carbamidomethyl (C)[2]		Mascot
847.4156	847.4567	0.0411	49	355	363	SSSASSPPK					Mascot
870.4792	870.5117	0.0325	37	178	186	SPKAPGTGR					Mascot
875.5349	875.4537	-0.0812	-93	23	29	DKPLKFK					Mascot
889.5101	889.4669	-0.0432	-49	287	294	AVKESSIR					Mascot
904.4985	904.4572	-0.0413	-46	310	317	ETVSIEVK					Mascot
979.5063	979.4592	-0.0471	-48	1	9	MVAGMLGLR			Oxidation (M)[1,5]		Mascot
1161.6475	1161.532	-0.1155	-99	308	317	TRETVSIEVK					Mascot
1167.6368	1167.5276	-0.1092	-94	257	267	AEADPQAIPKK					Mascot
1349.6915	1349.6477	-0.0438	-32	1	12	MVAGMLGLREEK			Oxidation (M)[1]		Mascot
1486.7795	1486.6873	-0.0922	-62	459	471	KDIVSSSMPRPNR					Mascot
1490.6726	1490.6664	-0.0062	-4	418	431	MPRGGSLESDGCPK			Carbamidomethyl (C)[12]		Mascot
1490.6726	1490.6664	-0.0062	-4	418	431	MPRGGSLESDGCPK			Carbamidomethyl (C)[12]		Mascot
1506.6676	1506.6841	0.0165	11	418	431	MPRGGSLESDGCPK			Carbamidomethyl (C)[12], Oxidation (M)[1]		Mascot
1518.7283	1518.7103	-0.018	-12	10	22	EEKSEDQDLQGLK					Mascot
1549.7891	1549.762	-0.0271	-17	234	249	AEGGGATTSTQVMVIK					Mascot
2440.1377	2440.0295	-0.1082	-44	145	167	VGDTSLDPNDFDFTVTG RGSPSR					Mascot
2596.2747	2596.1548	-0.1199	-46	224	249	MPFQTSPGGKAEGGGAT TSTQVMVIK			Oxidation (M)[1]		Mascot

8 Protein PA-X OS=Influenza A virus (strain A/Duck/England/1/1956 H11N6) GN=PA PE=3 SV=1 PAX_I56A2 29660 8.14 12 54 0 5.455

Peptide Information

Calc. Mass	Obsrv. Mass	± da	± ppm	Start Seq.	End Seq.	Sequence	Ion Score	C. I. %	Modification	Rank	Result Type
812.3607	812.3954	0.0347	43	1	6	MEDFVR			Oxidation (M)[1]		Mascot
837.3624	837.402	0.0396	47	23	29	EYGEDPK					Mascot
863.4621	863.4581	-0.004	-5	76	82	FEIIEGR					Mascot
950.5128	950.4682	-0.0446	-47	244	251	ASFLKCPK			Carbamidomethyl (C)[6]		Mascot
1161.5205	1161.532	0.0115	10	207	217	SQEPCAGSPTK			Carbamidomethyl (C)[5]		Mascot
1167.535	1167.5276	-0.0074	-6	20	29	AMKEYGEDPK					Mascot
1349.7212	1349.6477	-0.0735	-54	125	134	REVHIYYLEK					Mascot
1367.7101	1367.6545	-0.0556	-41	175	185	LFTIRQEMASR			Oxidation (M)[8]		Mascot
1506.7952	1506.6841	-0.1111	-74	126	137	EVHIYYLEKANK					Mascot
1518.77	1518.7103	-0.0597	-39	186	198	GLWDSFVNPREAK					Mascot
1594.7604	1594.9049	0.1445	91	7	19	QCFNPMIVELAEK			Carbamidomethyl (C)[2], Oxidation (M)[6]		Mascot

2193.0645	2193.0718	0.0073	3	140	158	SEKTHIHIFSFTGEEMATK						Mascot
2193.0645	2193.0718	0.0073	3	140	158	SEKTHIHIFSFTGEEMATK						Mascot

9	CTP synthase OS=Actinobacillus pleuropneumoniae serotype 5b (strain L20) GN=pyrG PE=3 SV=1	PYRG_ACTP2	60431.2	5.7	14	54	0	7.001

Peptide Information

Calc. Mass	Obsrv. Mass	± da	± ppm	Start Seq.	End Seq.	Sequence	Ion Score	C. I. %	Modification	Rank	Result Type
804.421	804.4244	0.0034	4	488	495	VSGLSADR					Mascot
838.4053	838.4058	0.0005	1	87	94	ANNFTSGK					Mascot
875.5019	875.4537	-0.0482	-55	33	40	GLNVTIMK					Mascot
919.4268	919.434	0.0072	8	538	545	AYQDSHKA					Mascot
1167.4948	1167.5276	0.0328	28	430	440	SDESDLGGTMR					Mascot
1184.5729	1184.5519	-0.021	-18	84	94	MSKANNFTSGK					Mascot
1211.5791	1211.566	-0.0131	-11	327	336	YIDSQDIETK					Mascot
1265.6372	1265.6163	-0.0209	-17	456	466	EVYGAETIVER					Mascot
1297.5842	1297.6198	0.0356	27	253	263	SQGLDTFVCDR			Carbamidomethyl (C)[9]		Mascot
1492.7754	1492.7629	-0.0125	-8	454	466	AREVYGAETIVER					Mascot
1698.881	1698.8364	-0.0446	-26	87	101	ANNFTSGKIYSEVLR					Mascot
2166.1665	2166.0857	-0.0808	-37	469	487	HRYEVNNTLLPQIEAAGL K					Mascot
2166.1665	2166.0857	-0.0808	-37	469	487	HRYEVNNTLLPQIEAAGL K					Mascot
2199.2529	2199.0483	-0.2046	-93	19	40	GIAAASLASILEARGLNVT IMK					Mascot
2596.2964	2596.1548	-0.1416	-55	283	306	QANPTGEVTIGMVGKYV ELPDAYK			Oxidation (M)[12]		Mascot

10	2-dehydro-3-deoxyphosphooctonate aldolase OS=Leptospira interrogans serogroup Icterohaemorrhagiae serovar Lai (strain 56601) GN=kdsA PE=3 SV=2	KDSA_LEPIN	31968.4	6.96	11	54	0	5.804

Protein Group

2-dehydro-3-deoxyphosphooctonate aldolase OS=Leptospira interrogans serogroup Icterohaemorrhagiae serovar copenhageni (strain Fiocruz L1-130) GN=kdsA PE=3 SV=2	KDSA_LEPIC	31968.4	6.9600 000381 4697

Peptide Information

Calc. Mass	Obsrv. Mass	± da	± ppm	Start Seq.	End Seq.	Sequence	Ion Score	C. I. %	Modification	Rank	Result Type
812.3897	812.3954	0.0057	7	68	74	SSVNSYR					Mascot
841.4778	841.4304	-0.0474	-56	75	83	GPGLAEGIK					Mascot
849.4135	849.4452	0.0317	37	268	274	EMIGLDR			Oxidation (M)[2]		Mascot

858.5407	858.4796	-0.0611	-71	278	284	KEILISR		Mascot
866.4036	866.4357	0.0321	37	2	8	KDNTCTK	Carbamidomethyl (C)[5]	Mascot
893.3781	893.4225	0.0444	50	159	166	MNESGNNK		Mascot
909.373	909.4121	0.0391	43	159	166	MNESGNNK	Oxidation (M)[1]	Mascot
950.5054	950.4682	-0.0372	-39	9	16	RDFLNGTK		Mascot
1021.5676	1021.4922	-0.0754	-74	84	91	NLEYIKNK		Mascot
1075.5531	1075.5291	-0.024	-22	144	153	GQFLAPADTR		Mascot
1537.7059	1537.8041	0.0982	64	41	53	VCAEMIEVCGELK	Carbamidomethyl (C)[2,9]	Mascot
1537.7059	1537.8041	0.0982	64	41	53	VCAEMIEVCGELK	Carbamidomethyl (C)[2,9]	Mascot
1553.7008	1553.7684	0.0676	44	41	53	VCAEMIEVCGELK	Carbamidomethyl (C)[2,9], Oxidation (M)[5]	Mascot
1553.7008	1553.7684	0.0676	44	41	53	VCAEMIEVCGELK	Carbamidomethyl (C)[2,9], Oxidation (M)[5]	Mascot
2166.0239	2166.0857	0.0618	29	36	53	DLLDRVCAEMIEVCGELK	Carbamidomethyl (C)[7,14], Oxidation (M)[10]	Mascot
2166.0239	2166.0857	0.0618	29	36	53	DLLDRVCAEMIEVCGELK	Carbamidomethyl (C)[7,14], Oxidation (M)[10]	Mascot

Gel Idx/Pos	184/H11	Instr./Gel Origin	BA2151/Sample Project 20140814	Process Status	Analysis Succeeded
Plate [#] Name	[1] Sample Project 20140814	Instrument Sample Name		Spectra	11

Rank	Protein Name	Accession No.	Protein MW	Protein PI	Pep. Count	Protein Score	Protein Score C. I. %	Intensity Matched	Total Ion Score	Total Ion C. I. %	Confirmed
1	Sec-independent protein translocase protein TatB OS=Rhizobium meliloti (strain 1021) GN=tatB PE=3 SV=1	TATB_RHIME	22740	9.95	12	68	90.603	4.375			

Peptide Information

Calc. Mass	Obsrv. Mass	± da	± ppm	Start Seq.	End Seq.	Sequence	Ion Score	C. I. %	Modification	Rank	Result Type
816.4032	816.393	-0.0102	-12	80	86	DAMNPLR					Mascot
832.3981	832.321	-0.0771	-93	80	86	DAMNPLR			Oxidation (M)[3]		Mascot
897.4611	897.4288	-0.0323	-36	31	38	AFGRMTSK					Mascot
925.4737	925.4243	-0.0494	-53	193	202	APGEAPAANK					Mascot
932.4319	932.4513	0.0194	21	56	63	EADLDDVR					Mascot
955.4414	955.4329	-0.0085	-9	41	48	GMASDFRR			Oxidation (M)[2]		Mascot
989.5374	989.4658	-0.0716	-72	134	144	VAAAAVSSASR					Mascot
1060.527	1060.5587	0.0317	30	56	64	EADLDDVRK					Mascot
1140.6008	1140.5684	-0.0324	-28	193	204	APGEAPAANKSK					Mascot
1372.743	1372.6571	-0.0859	-63	87	98	QLGNEIKSDLQK					Mascot
1535.7595	1535.705	-0.0545	-35	134	148	VAAAAVSSASRQMDR			Oxidation (M)[13]		Mascot
1557.8231	1557.7355	-0.0876	-56	65	79	TISDAQSLNPTAALR					Mascot
1791.8508	1791.7368	-0.114	-64	49	63	QFDEALREADLDDVR					Mascot

Rank	Protein Name	Accession No.	Protein MW	Protein PI	Pep. Count	Protein Score	Protein Score C. I. %	Intensity Matched	Total Ion Score	Total Ion C. I. %	Confirmed
2	Phenylalanine--tRNA ligase alpha subunit OS=Shewanella sediminis (strain HAW-EB3) GN=pheS PE=3 SV=1	SYFA_SHESH	37277.9	5.41	12	63	73.516	15.738			

Peptide Information

Calc. Mass	Obsrv. Mass	± da	± ppm	Start Seq.	End Seq.	Sequence	Ion Score	C. I. %	Modification	Rank	Result Type
955.4587	955.4329	-0.0258	-27	36	43	GKITDMMK			Oxidation (M)[6,7]		Mascot
989.5374	989.4658	-0.0716	-72	168	176	TQTSGVQIR					Mascot
1060.6262	1060.5587	-0.0675	-64	187	195	IISPGRVYR					Mascot
1178.6277	1178.5804	-0.0473	-40	101	111	IDNGGLHPVTR					Mascot
1201.5448	1201.6628	0.118	98	38	47	ITDMMKMMGK			Oxidation (M)[4]		Mascot
1201.5448	1201.6628	0.118	98	38	47	ITDMMKMMGK			Oxidation (M)[4]		Mascot
1217.5397	1217.6083	0.0686	56	38	47	ITDMMKMMGK			Oxidation (M)[4,5]		Mascot

1320.6042	1320.5967	-0.0075	-6	290	301	YSGFAFGMGVER					Mascot
1473.7625	1473.7142	-0.0483	-33	116	128	IETFFGELGFSVK					Mascot
1837.9952	1837.9287	-0.0665	-36	177	192	TMENEKPPLRIISPGR					Mascot
1844.8926	1844.9404	0.0478	26	307	321	YGVNDLRSFFENDLR					Mascot
1869.8953	1869.8965	0.0012	1	153	167	ADHDTFYFNPKVMLR			Oxidation (M)[13]		Mascot
1869.8953	1869.8965	0.0012	1	153	167	ADHDTFYFNPKVMLR			Oxidation (M)[13]		Mascot
2258.1763	2258.2048	0.0285	13	2	22	SQLTEIVEQALAAIEGTED LK					Mascot
2955.552	2955.4148	-0.1372	-46	2	28	SQLTEIVEQALAAIEGTED LKALDELR					Mascot
2955.552	2955.4148	-0.1372	-46	2	28	SQLTEIVEQALAAIEGTED LKALDELR					Mascot

3	Vimentin OS=Mus musculus GN=Vim PE=1 SV=3	VIME_MOUSE	53712.1	5.06	17	63	71.622	5.799

Peptide Information

Calc. Mass	Obsrv. Mass	± da	± ppm	Start Seq.	End Seq.	Sequence	Ion Score	C. I. %	Modification	Rank	Result Type
905.4323	905.4269	-0.0054	-6	335	342	GTNESLER					Mascot
906.468	906.4423	-0.0257	-28	123	129	FLEQQNK					Mascot
925.4009	925.4243	0.0234	25	314	320	QESNEYR					Mascot
932.4683	932.4513	-0.017	-18	403	410	LLEGEESR					Mascot
1060.5634	1060.5587	-0.0047	-4	402	410	KLLEGEESR					Mascot
1081.5021	1081.5175	0.0154	14	314	321	QESNEYRR					Mascot
1118.551	1118.5259	-0.0251	-22	365	373	LQDEIQNMK					Mascot
1125.6051	1125.5221	-0.083	-74	114	122	FANYIDKVR					Mascot
1270.562	1270.5626	0.0006	0	146	155	LGDLYEEEMR			Oxidation (M)[9]		Mascot
1270.562	1270.5626	0.0006	0	146	155	LGDLYEEEMR			Oxidation (M)[9]		Mascot
1308.6543	1308.6567	0.0024	2	293	304	SKFADLSEAANR					Mascot
1309.606	1309.6417	0.0357	27	283	292	NLQEAEEWYK					Mascot
1320.6324	1320.5967	-0.0357	-27	335	345	GTNESLERQMR					Mascot
1490.752	1490.6857	-0.0663	-44	322	334	QVQSLTCEVDALK			Carbamidomethyl (C)[7]		Mascot
1527.6969	1527.722	0.0251	16	14	28	MFGGSGTSSRPSSNR					Mascot
1539.9105	1539.7926	-0.1179	-77	130	143	ILLAELEQLKGQGK					Mascot
1699.793	1699.8229	0.0299	18	13	28	RMFGGSGTSSRPSSNR			Oxidation (M)[2]		Mascot
1838.9607	1838.9227	-0.038	-21	425	440	ETNLESLPLVDTHSKR					Mascot

4	Vimentin OS=Rattus norvegicus GN=Vim PE=1 SV=2	VIME_RAT	53757.1	5.06	17	62	63.442	5.799

Peptide Information

Calc. Mass	Obsrv. Mass	± da	± ppm	Start Seq.	End Seq.	Sequence	Ion Score	C. I. %	Modification	Rank	Result Type

Calc. Mass	Obsrv. Mass	± da	± ppm	Start Seq.	End Seq.	Sequence	Ion Score	C. I. %	Modification	Rank	Result Type
905.4323	905.4269	-0.0054	-6	335	342	GTNESLER					Mascot
906.468	906.4423	-0.0257	-28	123	129	FLEQQNK					Mascot
925.4009	925.4243	0.0234	25	314	320	QESNEYR					Mascot
932.4683	932.4513	-0.017	-18	403	410	LLEGEESR					Mascot
1060.5634	1060.5587	-0.0047	-4	402	410	KLLEGEESR					Mascot
1081.5021	1081.5175	0.0154	14	314	321	QESNEYRR					Mascot
1118.551	1118.5259	-0.0251	-22	365	373	LQDEIQNMK					Mascot
1125.6051	1125.5221	-0.083	-74	114	122	FANYIDKVR					Mascot
1270.562	1270.5626	0.0006	0	146	155	LGDLYEEEMR			Oxidation (M)[9]		Mascot
1270.562	1270.5626	0.0006	0	146	155	LGDLYEEEMR			Oxidation (M)[9]		Mascot
1308.6543	1308.6567	0.0024	2	293	304	SKFADLSEAANR					Mascot
1309.606	1309.6417	0.0357	27	283	292	NLQEAEEWYK					Mascot
1320.6324	1320.5967	-0.0357	-27	335	345	GTNESLERQMR					Mascot
1490.752	1490.6857	-0.0663	-44	322	334	QVQSLTCEVDALK			Carbamidomethyl (C)[7]		Mascot
1527.6969	1527.722	0.0251	16	14	28	MFGGSGTSSRPSSNR					Mascot
1539.9105	1539.7926	-0.1179	-77	130	143	ILLAELEQLKGQGK					Mascot
1699.793	1699.8229	0.0299	18	13	28	RMFGGSGTSSRPSSNR			Oxidation (M)[2]		Mascot
1838.9607	1838.9227	-0.038	-21	425	440	ETNLESLPLVDTHSKR					Mascot

5 Vimentin OS=Sus scrofa GN=VIM PE=1 SV=2 VIME_PIG 53692.1 5.06 17 60 50.685 5.654

Peptide Information

Calc. Mass	Obsrv. Mass	± da	± ppm	Start Seq.	End Seq.	Sequence	Ion Score	C. I. %	Modification	Rank	Result Type
886.4265	886.4622	0.0357	40	5	12	TVSSSSYR					Mascot
905.4323	905.4269	-0.0054	-6	335	342	GTNESLER					Mascot
906.468	906.4423	-0.0257	-28	123	129	FLEQQNK					Mascot
925.4009	925.4243	0.0234	25	314	320	QESNEYR					Mascot
932.4683	932.4513	-0.017	-18	403	410	LLEGEESR					Mascot
1060.5634	1060.5587	-0.0047	-4	402	410	KLLEGEESR					Mascot
1081.5021	1081.5175	0.0154	14	314	321	QESNEYRR					Mascot
1118.551	1118.5259	-0.0251	-22	365	373	LQDEIQNMK					Mascot
1125.6051	1125.5221	-0.083	-74	114	122	FANYIDKVR					Mascot
1270.562	1270.5626	0.0006	0	146	155	LGDLYEEEMR			Oxidation (M)[9]		Mascot
1270.562	1270.5626	0.0006	0	146	155	LGDLYEEEMR			Oxidation (M)[9]		Mascot
1308.6543	1308.6567	0.0024	2	293	304	SKFADLSEAANR					Mascot
1309.606	1309.6417	0.0357	27	283	292	NLQEAEEWYK					Mascot

1320.6324	1320.5967	-0.0357	-27	335	345	GTNESLERQMR					Mascot
1490.752	1490.6857	-0.0663	-44	322	334	QVQSLTCEVDALK			Carbamidomethyl (C)[7]		Mascot
1527.8279	1527.722	-0.1059	-69	379	390	HLREYQDLLNVK					Mascot
1539.9105	1539.7926	-0.1179	-77	130	143	ILLAELEQLKGQGK					Mascot
1838.9607	1838.9227	-0.038	-21	425	440	ETNLESLPLVDTHSKR					Mascot

6	Serine/threonine-protein phosphatase 2B catalytic subunit A1 OS=Saccharomyces cerevisiae (strain ATCC 204508 / S288c) GN=CNA1 PE=1 SV=2	PP2B1_YEAST	63532.1	6.25	18	60	48.361	5.142	

Peptide Information

Calc. Mass	Obsrv. Mass	± da	± ppm	Start Seq.	End Seq.	Sequence	Ion Score	C. I. %	Modification	Rank	Result Type
813.3923	813.3849	-0.0074	-9	326	331	MYKNNK			Oxidation (M)[1]		Mascot
852.4097	852.3857	-0.024	-28	31	37	YELENGK					Mascot
853.46	853.4216	-0.0384	-45	300	306	ASCKFLK			Carbamidomethyl (C)[3]		Mascot
870.5771	870.5472	-0.0299	-34	460	467	NKILAIAK					Mascot
906.4639	906.4423	-0.0216	-24	2	9	SKDLNSSR					Mascot
932.5159	932.4513	-0.0646	-69	4	11	DLNSSRIK					Mascot
958.5139	958.4507	-0.0632	-66	487	495	TMNAGVLPR					Mascot
963.4265	963.4689	0.0424	44	435	443	ETGTPSDEK					Mascot
1037.5044	1037.5251	0.0207	20	1	9	MSKDLNSSR					Mascot
1064.4871	1064.4967	0.0096	9	291	299	GCSFAFTFK			Carbamidomethyl (C)[2]		Mascot
1141.6113	1141.5553	-0.056	-49	67	76	TGLPNHSFLR					Mascot
1167.5463	1167.5627	0.0164	14	360	368	YEENVMNIR					Mascot
1192.6606	1192.5551	-0.1055	-88	93	103	ILNMSTVALSK			Oxidation (M)[4]		Mascot
1265.5896	1265.6313	0.0417	33	56	66	IPSDEEVFDSK					Mascot
1309.6635	1309.6417	-0.0218	-17	27	37	DLTKYELENGK					Mascot
1539.7009	1539.7926	0.0917	60	316	328	AHEAQDAGYRMYK					Mascot
1665.8629	1665.8556	-0.0073	-4	355	368	AAVLKYEENVMNIR			Oxidation (M)[11]		Mascot
1911.9423	1912.0369	0.0946	49	184	198	HLTSYFTFKNEMLHK			Oxidation (M)[12]		Mascot

7	Mannose-6-phosphate isomerase OS=Rhizobium meliloti (strain 1021) GN=pmi PE=3 SV=1	MANA_RHIME	43803.8	5.17	10	57	0	2.527	14	0

Peptide Information

Calc. Mass	Obsrv. Mass	± da	± ppm	Start Seq.	End Seq.	Sequence	Ion Score	C. I. %	Modification	Rank	Result Type
820.3254	820.3516	0.0262	32	276	282	DGTCPDR			Carbamidomethyl (C)[4]		Mascot
891.3744	891.4144	0.04	45	70	76	GWDGDWR					Mascot

Calc. Mass	Obsrv. Mass	± da	± ppm	Start Seq.	End Seq.	Sequence	Ion Score	C. I. %	Modification	Rank	Result Type
1064.5371	1064.4967	-0.0404	-38	315	324	FAALTEGAER					Mascot
1298.6423	1298.6138	-0.0285	-22	59	69	QVYCFAAAGRR			Carbamidomethyl (C)[4]		Mascot
1475.7965	1475.7523	-0.0442	-30	311	324	AAIRFAALTEGAER					Mascot
1479.6863	1479.7594	0.0731	49	155	167	HPLAGFEEDDPPR					Mascot
1911.9381	1912.0369	0.0988	52	194	210	VAWANLADEIAHLAMDR			Oxidation (M)[15]		Mascot
2214.9973	2215.1887	0.1914	86	28	48	GFDGEGGGFVETIDMKG EPTR			Oxidation (M)[15]		Mascot
2955.3765	2955.4148	0.0383	13	168	193	LPLGSNPHMHLFEACLAS EEVEGFDR			Carbamidomethyl (C)[15]		Mascot
2955.478	2955.4148	-0.0632	-21	194	220	VAWANLADEIAHLAMDR FIDAESGALR	16	0			Mascot

8	DNA-directed RNA polymerase subunit beta' OS=Rickettsia massiliae (strain Mtu5) GN=rpoC PE=3 SV=1	RPOC_RICM5	154389.9	8.03	26	57	0	7.538

Peptide Information

Calc. Mass	Obsrv. Mass	± da	± ppm	Start Seq.	End Seq.	Sequence	Ion Score	C. I. %	Modification	Rank	Result Type
807.4359	807.3985	-0.0374	-46	728	734	GSFQQIK					Mascot
845.4727	845.3984	-0.0743	-88	778	785	GQIDTALK					Mascot
852.4322	852.3857	-0.0465	-55	348	354	RVDYSGR					Mascot
886.4992	886.4622	-0.037	-42	1135	1142	VAELVEAR					Mascot
955.4843	955.4329	-0.0514	-54	786	794	TASSGYLTR					Mascot
977.4833	977.4943	0.011	11	81	88	NRGITCEK			Carbamidomethyl (C)[6]		Mascot
979.5458	979.4709	-0.0749	-76	193	200	ELDFSKLK					Mascot
1006.4986	1006.4384	-0.0602	-60	89	97	CGVEVTVSR			Carbamidomethyl (C)[1]		Mascot
1033.5347	1033.5221	-0.0126	-12	1035	1043	DMVEGITIR					Mascot
1217.6447	1217.6083	-0.0364	-30	999	1009	LLVDDGDMVIK					Mascot
1223.6235	1223.6138	-0.0097	-8	735	745	QLGGMRGLMTK			Oxidation (M)[5,9]		Mascot
1232.6006	1232.5959	-0.0047	-4	1044	1055	DVTDEATGIPSK					Mascot
1298.645	1298.6138	-0.0312	-24	1350	1361	LVPAGTGYFMDK					Mascot
1372.5654	1372.6571	0.0917	67	689	699	CTDRVANDMMK			Carbamidomethyl (C)[1], Oxidation (M)[9,10]		Mascot
1473.7584	1473.7142	-0.0442	-30	408	419	SEVWDVLEEVIR					Mascot
1475.77	1475.7523	-0.0177	-12	1121	1134	ESTTTKDITGGLPR					Mascot
1479.7777	1479.7594	-0.0183	-12	55	67	DGLFCARIFGPVK			Carbamidomethyl (C)[5]		Mascot
1491.8782	1491.733	-0.1452	-97	387	400	LELYGIATTIKAAK					Mascot
1507.8777	1507.7452	-0.1325	-88	1246	1257	HIEVIIRQMLQK					Mascot
1539.8629	1539.7926	-0.0703	-46	820	834	SVIEGGEVIVPLAEK					Mascot
1585.7866	1585.8348	0.0482	30	1350	1363	LVPAGTGYFMDKMR					Mascot
1659.9065	1659.8273	-0.0792	-48	746	760	SNGQIIQTPIISNFK					Mascot

Calc. Mass	Obsrv. Mass	± da	± ppm	Start Seq.	End Seq.	Sequence	Ion Score	C. I. %	Modification	Rank	Result Type
1665.855	1665.8556	0.0006	0	125	138	ISTLLDMTMRDIEK					Mascot
1911.9546	1912.0369	0.0823	43	201	216	QELYEELQTTSSEVKK					Mascot
2215.1362	2215.1887	0.0525	24	715	734	INAIYMMAISGARGSFQQIK			Oxidation (M)[6]		Mascot
2633.4185	2633.2781	-0.1404	-53	1093	1117	YYLPVGAVLSVEDGVQISVGDIIAR					Mascot

9	Pyruvate kinase PKM OS=Pongo abelii GN=PKM PE=2 SV=3	KPYM_PONAB	58494.1	7.98	17	55	0	6.52

Peptide Information

Calc. Mass	Obsrv. Mass	± da	± ppm	Start Seq.	End Seq.	Sequence	Ion Score	C. I. %	Modification	Rank	Result Type
884.4771	884.4672	-0.0099	-11	377	383	MQHLIAR			Oxidation (M)[1]		Mascot
905.5091	905.4269	-0.0822	-91	394	400	LFEELVR					Mascot
912.4534	912.4173	-0.0361	-40	248	255	ASDVHEVR					Mascot
979.5029	979.4709	-0.032	-33	490	498	VNFAMNVGK					Mascot
1033.6041	1033.5221	-0.082	-79	393	400	KLFEELVR					Mascot
1037.4801	1037.5251	0.045	43	312	319	MMIGRCNR			Carbamidomethyl (C)[6]		Mascot
1118.6052	1118.5259	-0.0793	-71	126	136	GSGTAEVELKK					Mascot
1141.61	1141.5553	-0.0547	-48	295	305	GDLGIEIPAEK					Mascot
1193.6447	1193.6221	-0.0226	-19	57	66	SVETLKEMIK			Oxidation (M)[8]		Mascot
1232.6667	1232.5959	-0.0708	-57	163	173	NICKVVEVGSK			Carbamidomethyl (C)[3]		Mascot
1309.7119	1309.6417	-0.0702	-54	306	316	VFLAQKMMIGR			Oxidation (M)[7]		Mascot
1473.8094	1473.7142	-0.0952	-65	423	436	CLAAALIVLTESGR			Carbamidomethyl (C)[1]		Mascot
1507.8704	1507.7452	-0.1252	-83	448	461	APIIAVTRNPQTAR					Mascot
1665.8153	1665.8556	0.0403	24	280	294	FDEILEASDGIMVAR					Mascot
1837.9113	1837.9287	0.0174	9	279	294	RFDEILEASDGIMVAR			Oxidation (M)[13]		Mascot
1908.9592	1908.8296	-0.1296	-68	320	336	AGKPVICATQMLESMIK			Carbamidomethyl (C)[7], Oxidation (M)[11,15]		Mascot
1926.9425	1926.8954	-0.0471	-24	377	392	MQHLIAREAEAAMFHR			Oxidation (M)[1]		Mascot
1942.9375	1942.8795	-0.058	-30	377	392	MQHLIAREAEAAMFHR			Oxidation (M)[1,13]		Mascot

10	Acyl-CoA dehydrogenase family member 11 OS=Mus musculus GN=Acad11 PE=1 SV=2	ACD11_MOUSE	88051.9	8.67	18	54	0	5.811

Peptide Information

Calc. Mass	Obsrv. Mass	± da	± ppm	Start Seq.	End Seq.	Sequence	Ion Score	C. I. %	Modification	Rank	Result Type
807.4359	807.3985	-0.0374	-46	82	87	IDREFK					Mascot
834.3951	834.3265	-0.0686	-82	555	562	TESPSASR					Mascot
849.4465	849.3749	-0.0716	-84	177	183	QVSTWTK					Mascot

853.392	853.4216	0.0296	35	632	637	IHHCMR	Carbamidomethyl (C)[4]	Mascot
893.4475	893.4317	-0.0158	-18	619	626	GFEISQGR		Mascot
955.4996	955.4329	-0.0667	-70	391	398	QHVFPAEK		Mascot
958.5428	958.4507	-0.0921	-96	377	384	RGQEVLTR		Mascot
979.4843	979.4709	-0.0134	-14	525	534	DGGGYIVNGK		Mascot
1006.4622	1006.4384	-0.0238	-24	766	773	MELQDQAR	Oxidation (M)[1]	Mascot
1064.5227	1064.4967	-0.026	-24	645	652	ILQIMCDR	Carbamidomethyl (C)[6], Oxidation (M)[5]	Mascot
1107.5793	1107.5435	-0.0358	-32	525	535	DGGGYIVNGKK		Mascot
1217.6062	1217.6083	0.0021	2	535	545	KWWSSGAGNPK		Mascot
1298.7063	1298.6138	-0.0925	-71	357	368	TLRTTPPQADAK		Mascot
1527.7737	1527.722	-0.0517	-34	223	234	LDNIVFHPKECR	Carbamidomethyl (C)[11]	Mascot
1535.8064	1535.705	-0.1014	-66	751	765	LADGPDEVHLSAIAK		Mascot
1556.8755	1556.7396	-0.1359	-87	548	562	IAIVLGRTESPSASR		Mascot
1556.8755	1556.7396	-0.1359	-87	548	562	IAIVLGRTESPSASR		Mascot
1837.9708	1837.9287	-0.0421	-23	663	677	LYEHEVVAHWIAKSR		Mascot
2258.1104	2258.2048	0.0944	42	296	314	GIDPNLPNWNFFMALSFF K		Mascot

Gel Idx/Pos	185/H12	Instr./Gel Origin	BA2151/Sample Project 20140814	Process Status	Analysis Succeeded
Plate [#] Name	[1] Sample Project 20140814	Instrument Sample Name		Spectra	11

Rank	Protein Name	Accession No.	Protein MW	Protein PI	Pep. Count	Protein Score	Protein Score C. I. %	Intensity Matched	Total Ion Score	Total Ion C. I. %	Confirmed
1	Glycerophosphodiester phosphodiesterase domain-containing protein 1 OS=Homo sapiens GN=GDPD1 PE=1 SV=2	GDPD1_HUMAN	36429.1	8.66	14	70	94.206	12.392			

Peptide Information

Calc. Mass	Obsrv. Mass	± da	± ppm	Start Seq.	End Seq.	Sequence	Ion Score	C. I. %	Modification	Rank	Result Type
802.5032	802.5666	0.0634	79	162	168	KVSELVK					Mascot
830.5094	830.4372	-0.0722	-87	163	169	VSELVKR					Mascot
852.3339	852.3701	0.0362	42	123	129	ACQCEGK			Carbamidomethyl (C)[2,4]		Mascot
864.4574	864.3787	-0.0787	-91	116	122	LDVSFQR					Mascot
950.4366	950.4304	-0.0062	-7	307	314	DFLHNFSA					Mascot
1155.6633	1155.5795	-0.0838	-73	26	34	YPTLLHQRK					Mascot
1219.6218	1219.5808	-0.041	-34	305	314	LRDFLHNFSA					Mascot
1219.6218	1219.5808	-0.041	-34	305	314	LRDFLHNFSA					Mascot
1511.7336	1511.7952	0.0616	41	80	92	DEQVVVSHDENLK					Mascot
1661.7874	1661.7654	-0.022	-13	66	79	IGTDMLELDCHITK			Carbamidomethyl (C)[10], Oxidation (M)[5]		Mascot
1879.051	1878.8839	-0.1671	-89	247	261	SQKFLIWLSDLLLMR			Oxidation (M)[14]		Mascot
1901.9392	1901.8466	-0.0926	-49	173	188	EHLTVWGNANYEIVEK					Mascot
1944.9232	1944.8665	-0.0567	-29	47	65	GGAGENLENTMAAFQHAVK					Mascot
1944.9232	1944.8665	-0.0567	-29	47	65	GGAGENLENTMAAFQHAVK					Mascot
1960.9182	1960.8771	-0.0411	-21	47	65	GGAGENLENTMAAFQHAVK			Oxidation (M)[11]		Mascot
1983.0005	1982.9539	-0.0466	-23	189	204	CYKENSDIPILFSLQR			Carbamidomethyl (C)[1]		Mascot
2011.0171	2011.0291	0.012	6	272	287	GIQVYIWVLNEEQEYK					Mascot

Rank	Protein Name	Accession No.	Protein MW	Protein PI	Pep. Count	Protein Score	Protein Score C. I. %	Intensity Matched	Total Ion Score	Total Ion C. I. %	Confirmed
2	Multifunctional CCA protein OS=Cupriavidus taiwanensis (strain R1 / LMG 19424) GN=cca PE=3 SV=1	CCA_CUPTR	46135.7	6.3	15	64	78.473	5.497			

Peptide Information

Calc. Mass	Obsrv. Mass	± da	± ppm	Start Seq.	End Seq.	Sequence	Ion Score	C. I. %	Modification	Rank	Result Type
810.4178	810.3467	-0.0711	-88	197	202	MFEVLR			Oxidation (M)[1]		Mascot
824.4485	824.3699	-0.0786	-95	395	401	DRVHAAR					Mascot

1032.5255	1032.4746	-0.0509	-49	188	196	GLMEARPSR			Oxidation (M)[3]		Mascot
1127.5997	1127.5596	-0.0401	-36	45	53	DFPVFLHPR					Mascot
1145.6096	1145.5809	-0.0287	-25	326	334	LLERCDALR			Carbamidomethyl (C)[5]		Mascot
1201.6172	1201.5857	-0.0315	-26	372	384	EAAASVDAGAIAR					Mascot
1203.6705	1203.5867	-0.0838	-70	274	283	HIGHELRSVR					Mascot
1203.6705	1203.5867	-0.0838	-70	274	283	HIGHELRSVR	4	0			Mascot
1342.6387	1342.6772	0.0385	29	129	140	HVSDAFAEDPVR					Mascot
1507.7322	1507.7184	-0.0138	-9	339	351	FAQALQACEADKR			Carbamidomethyl (C)[8]		Mascot
1551.7771	1551.7361	-0.041	-26	197	209	MFEVLRECGALAR			Carbamidomethyl (C)[8]		Mascot
1705.9749	1705.813	-0.1619	-95	210	223	LLPELERLWGVPQR					Mascot
1853.8785	1853.8596	-0.0189	-10	151	165	FHDFNVAAETMRLMR			Oxidation (M)[11]		Mascot
1901.9617	1901.8466	-0.1151	-61	355	371	GFEHSDYPQAARLLAAR					Mascot
1927.0145	1926.8427	-0.1718	-89	264	280	GTTPEDVLPRHIGHELR					Mascot
2002.0096	2001.9182	-0.0914	-46	163	180	LMREMVAAGEVDALVPE R			Oxidation (M)[2]		Mascot

3	50S ribosomal protein L5 OS=Clostridium botulinum (strain Loch Maree / Type A3) GN=rplE PE=3 SV=1	RL5_CLOBM	20538.9	9.3	13	56	0	15.358

Peptide Information

Calc. Mass	Obsrv. Mass	± da	± ppm	Start Seq.	End Seq.	Sequence	Ion Score	C. I. %	Modification	Rank	Result Type
830.444	830.4372	-0.0068	-8	25	31	NIMEVPK					Mascot
1032.533	1032.4746	-0.0584	-57	1	8	MMPRLQEK					Mascot
1145.4932	1145.5809	0.0877	77	95	103	QNMYEFADK					Mascot
1195.6392	1195.6018	-0.0374	-31	152	162	GMDIIFVTTAK					Mascot
1217.6129	1217.5725	-0.0404	-33	80	89	IRENMPLGCK			Carbamidomethyl (C)[9]		Mascot
1231.6277	1231.5842	-0.0435	-35	163	172	TDEEARELLR					Mascot
1273.5881	1273.5939	0.0058	5	94	103	KQNMYEFADK					Mascot
1325.6923	1325.6569	-0.0354	-27	21	31	FGYKNIMEVPK					Mascot
1397.7173	1397.6453	-0.072	-52	122	134	SFDGRGNYAIGIK					Mascot
1433.724	1433.678	-0.046	-32	82	93	ENMPLGCKVTLR			Carbamidomethyl (C)[7], Oxidation (M)[3]		Mascot
1433.724	1433.678	-0.046	-32	82	93	ENMPLGCKVTLR			Carbamidomethyl (C)[7], Oxidation (M)[3]		Mascot
1449.8036	1449.6652	-0.1384	-95	169	180	ELLRFLGMPFAR					Mascot
1464.8243	1464.7979	-0.0264	-18	150	162	IRGMDIIFVTTAK					Mascot
1465.7985	1465.8096	0.0111	8	169	180	ELLRFLGMPFAR			Oxidation (M)[8]		Mascot
1465.7985	1465.8096	0.0111	8	169	180	ELLRFLGMPFAR			Oxidation (M)[8]		Mascot
1507.7679	1507.7184	-0.0495	-33	135	146	EQLIFPEIEFDK					Mascot

4	Huntingtin-interacting protein 1 OS=Mus musculus	HIP1_MOUSE	116099.6	5.31	20	55	0	23.806

GN=Hip1 PE=1 SV=2

Peptide Information

Calc. Mass	Obsrv. Mass	± da	± ppm	Start Seq.	End Seq.	Sequence	Ion Score	C. I. %	Modification	Rank	Result Type
813.4213	813.3412	-0.0801	-98	532	538	HELATSR					Mascot
824.3607	824.3699	0.0092	11	150	155	MEYHTK			Oxidation (M)[1]		Mascot
1145.5685	1145.5809	0.0124	11	768	777	QEELGDLVDK					Mascot
1187.6129	1187.5911	-0.0218	-18	262	270	FMEQFTKLK			Oxidation (M)[2]		Mascot
1187.6129	1187.5911	-0.0218	-18	262	270	FMEQFTKLK			Oxidation (M)[2]		Mascot
1191.5575	1191.5863	0.0288	24	150	158	MEYHTKNPR			Oxidation (M)[1]		Mascot
1194.5862	1194.5979	0.0117	10	461	470	AQANEQRYSK					Mascot
1195.5889	1195.6018	0.0129	11	62	71	TCILGTHHEK			Carbamidomethyl (C)[2]		Mascot
1201.567	1201.5857	0.0187	16	260	268	DRFMEQFTK					Mascot
1203.59	1203.5867	-0.0033	-3	22	33	RGVGAGMEAAER					Mascot
1203.59	1203.5867	-0.0033	-3	22	33	RGVGAGMEAAER					Mascot
1217.562	1217.5725	0.0105	9	260	268	DRFMEQFTK			Oxidation (M)[4]		Mascot
1219.5848	1219.5808	-0.004	-3	22	33	RGVGAGMEAAER			Oxidation (M)[7]		Mascot
1219.5848	1219.5808	-0.004	-3	22	33	RGVGAGMEAAER			Oxidation (M)[7]		Mascot
1273.6747	1273.5939	-0.0808	-63	835	845	DLQKEIVESGR					Mascot
1359.7478	1359.6335	-0.1143	-84	901	914	EIAASTAQLVAASK					Mascot
1415.7601	1415.6722	-0.0879	-62	920	932	GSLNLTQLQQASR					Mascot
1444.7278	1444.6819	-0.0459	-32	520	531	TQEQQDVLENLK					Mascot
1449.6825	1449.6652	-0.0173	-12	597	609	LAGAQESMCQQVK			Carbamidomethyl (C)[9]		Mascot
1465.6774	1465.8096	0.1322	90	597	609	LAGAQESMCQQVK			Carbamidomethyl (C)[9], Oxidation (M)[8]		Mascot
1465.7203	1465.8096	0.0893	61	653	665	VSSVSSCLEQLEK			Carbamidomethyl (C)[7]		Mascot
1513.6952	1513.8158	0.1206	80	705	718	APPEPADSLTEACR			Carbamidomethyl (C)[13]		Mascot
1557.802	1557.8049	0.0029	2	475	487	YSELVQNHADLLR					Mascot
1853.9426	1853.8596	-0.083	-45	380	395	LYREISGLTGQLDNMK			Oxidation (M)[15]		Mascot
2018.8318	2018.906	0.0742	37	330	346	DDLMDMDASQQTLFDNK			Oxidation (M)[4,6]		Mascot
2322.1858	2322.1433	-0.0425	-18	778	799	EMAATSAAIEAATTRIEEI LSK			Oxidation (M)[2]		Mascot

5	ATP synthase subunit beta OS=Salinispora arenicola (strain CNS-205) GN=atpD PE=3 SV=1	ATPB_SALAI	52710	4.85	13	55	0	24.721

Peptide Information

Calc. Mass	Obsrv. Mass	± da	± ppm	Start Seq.	End Seq.	Sequence	Ion Score	C. I. %	Modification	Rank	Result Type

864.4131	864.3787	-0.0344	-40	478	485	AKELMAEG			Oxidation (M)[5]		Mascot
1203.6766	1203.5867	-0.0899	-75	177	186	TVLIQEMITR					Mascot
1203.6766	1203.5867	-0.0899	-75	177	186	TVLIQEMITR					Mascot
1219.6715	1219.5808	-0.0907	-74	177	186	TVLIQEMITR			Oxidation (M)[7]		Mascot
1219.6715	1219.5808	-0.0907	-74	177	186	TVLIQEMITR			Oxidation (M)[7]		Mascot
1322.6257	1322.5936	-0.0321	-24	1	14	MTVSATADGPAGTK			Oxidation (M)[1]		Mascot
1397.6809	1397.6453	-0.0356	-25	190	203	NFGGTSVFAGVGER					Mascot
1432.7432	1432.6614	-0.0818	-57	351	364	GIYPAVDPLASSSR					Mascot
1465.7645	1465.8096	0.0451	31	269	282	FTQAGSEVSTLLGR					Mascot
1465.7645	1465.8096	0.0451	31	269	282	FTQAGSEVSTLLGR					Mascot
1487.7563	1487.7373	-0.019	-13	240	252	VALSALTMAEYFR			Oxidation (M)[8]		Mascot
1529.8833	1529.7975	-0.0858	-56	177	189	TVLIQEMITRVAR					Mascot
1529.8833	1529.7975	-0.0858	-56	177	189	TVLIQEMITRVAR					Mascot
1545.8782	1545.7981	-0.0801	-52	177	189	TVLIQEMITRVAR			Oxidation (M)[7]		Mascot
1634.7843	1634.7689	-0.0154	-9	223	237	TALVYGQMDEPPGTR					Mascot
1661.8453	1661.7654	-0.0799	-48	2	19	TVSATADGPAGTKTATG R					Mascot
1756.9414	1756.8777	-0.0637	-36	238	252	LRVALSALTMAEYFR			Oxidation (M)[10]		Mascot
1977.0804	1976.8921	-0.1883	-95	253	268	DVQKQEVLLFIDNIFR					Mascot
2322.074	2322.1433	0.0693	30	283	303	MPSAVGYQPTLADEMGE LQER					Mascot

6	Hemoglobin subunit beta OS=Pagrus major GN=hbb PE=2 SV=3	HBB_PAGMA	16680.6	7.83	8	54	0	12.673	

Peptide Information

Calc. Mass	Obsrv. Mass	± da	± ppm	Start Seq.	End Seq.	Sequence	Ion Score	C. I. %	Modification	Rank	Result Type
864.4244	864.3787	-0.0457	-53	67	74	TVMGGLDR			Oxidation (M)[3]		Mascot
1322.6926	1322.5936	-0.099	-75	32	41	LMIVYPWTQR			Oxidation (M)[2]		Mascot
1433.7173	1433.678	-0.0393	-27	123	134	VFNANVQEAWQK					Mascot
1433.7173	1433.678	-0.0393	-27	123	134	VFNANVQEAWQK					Mascot
1513.754	1513.8158	0.0618	41	61	74	VAEHGRTVMGGLDR			Oxidation (M)[9]		Mascot
1551.7472	1551.7361	-0.0111	-7	1	13	MVEWTDAERSAIK			Oxidation (M)[1]		Mascot
1828.9705	1828.9332	-0.0373	-20	119	134	LGPKVFNANVQEAWQK					Mascot
1828.9705	1828.9332	-0.0373	-20	119	134	LGPKVFNANVQEAWQK					Mascot
1844.9535	1844.9418	-0.0117	-6	67	83	TVMGGLDRAVQNLDDIK					Mascot
1905.0337	1904.8768	-0.1569	-82	106	122	LLAECFSICVGIKLGPK			Carbamidomethyl (C)[5,9]		Mascot

7	Photosystem I reaction center subunit II OS=Cyanidium caldarium GN=psaD PE=3 SV=1	PSAD_CYACA	15960.4	9.38	9	52	0	3.739	

Peptide Information

Calc. Mass	Obsrv. Mass	± da	± ppm	Start Seq.	End Seq.	Sequence	Ion Score	C. I. %	Modification	Rank	Result Type
830.473	830.4372	-0.0358	-43	2	8	QQSLNLK					Mascot
893.488	893.4068	-0.0812	-91	134	140	FTHKTIF					Mascot
909.5556	909.5027	-0.0529	-58	79	85	FKILNFK					Mascot
1273.6569	1273.5939	-0.063	-49	66	76	EQCLALGNQLK			Carbamidomethyl (C)[3]		Mascot
1397.7537	1397.6453	-0.1084	-78	126	137	NLNPAEVKFTHK					Mascot
1514.8359	1514.8027	-0.0332	-22	66	78	EQCLALGNQLKIK			Carbamidomethyl (C)[3]		Mascot
1661.897	1661.7654	-0.1316	-79	119	133	IDHNIGKNLNPAEVK					Mascot
1743.8436	1743.834	-0.0096	-6	24	38	SSDIEEKYAITWSSK					Mascot
1764.8837	1764.9802	0.0965	55	39	54	NEDIFEIPTGGVAKMK			Oxidation (M)[15]		Mascot

8	Exocyst complex component 8 OS=Rattus norvegicus GN=Exoc8 PE=1 SV=1	EXOC8_RAT	81676.5	5.3	17	52	0	19.019	

Peptide Information

Calc. Mass	Obsrv. Mass	± da	± ppm	Start Seq.	End Seq.	Sequence	Ion Score	C. I. %	Modification	Rank	Result Type
810.341	810.3467	0.0057	7	1	8	MSDSGASR					Mascot
864.4574	864.3787	-0.0787	-91	65	71	QFIETAR					Mascot
909.5264	909.5027	-0.0237	-26	427	435	AAAVHTAIR					Mascot
1032.5209	1032.4746	-0.0463	-45	707	716	VNPESTTSVV					Mascot
1194.5671	1194.5979	0.0308	26	504	514	ESLSTAAECVK			Carbamidomethyl (C)[9]		Mascot
1209.6949	1209.579	-0.1159	-96	551	560	EIIIEATKHR					Mascot
1217.6637	1217.5725	-0.0912	-75	683	693	RFEEGVGKPAK					Mascot
1244.5867	1244.6057	0.019	15	140	151	EGPGTGEEGKQR					Mascot
1249.6284	1249.5618	-0.0666	-53	11	21	RQLESGGFEAR					Mascot
1260.5538	1260.651	0.0972	77	559	567	HRNSEEMWR			Oxidation (M)[7]		Mascot
1322.5756	1322.5936	0.018	14	486	497	SAMGMFVDAFSK			Oxidation (M)[3,5]		Mascot
1431.7478	1431.7122	-0.0356	-25	263	273	EWLEVLEETKR					Mascot
1461.7804	1461.7361	-0.0443	-30	569	581	MNLMTPEALGKLK			Oxidation (M)[1]		Mascot
1465.791	1465.8096	0.0186	13	124	139	GAAQAGFLPGPAGVPR					Mascot
1465.791	1465.8096	0.0186	13	124	139	GAAQAGFLPGPAGVPR					Mascot
1513.7542	1513.8158	0.0616	41	240	251	DMFKLLMFPESR					Mascot
1529.7491	1529.7975	0.0484	32	240	251	DMFKLLMFPESR			Oxidation (M)[2]		Mascot
1529.7491	1529.7975	0.0484	32	240	251	DMFKLLMFPESR			Oxidation (M)[2]		Mascot
1545.744	1545.7981	0.0541	35	240	251	DMFKLLMFPESR			Oxidation (M)[2,7]		Mascot

Calc. Mass	Obsrv. Mass	± da	± ppm	Start Seq.	End Seq.	Sequence	Ion Score	C. I. %	Modification	Rank	Result Type
2002.0968	2001.9182	-0.1786	-89	380	396	QLTEVLVFELSPDRSLR					Mascot
2010.9301	2011.0291	0.099	49	486	503	SAMGMFVDAFSKQVFDSK			Oxidation (M)[3]		Mascot

9	Sickle tail protein homolog OS=Homo sapiens GN=KIAA1217 PE=1 SV=2	SKT_HUMAN	214609.9	6.59	31	52	0	30.969	

Peptide Information

Calc. Mass	Obsrv. Mass	± da	± ppm	Start Seq.	End Seq.	Sequence	Ion Score	C. I. %	Modification	Rank	Result Type
824.3389	824.3699	0.031	38	276	282	TMNGDMR					Mascot
830.4843	830.4372	-0.0471	-57	651	657	RSVAELR					Mascot
852.4461	852.3701	-0.076	-89	584	591	GPITSYSK					Mascot
864.388	864.3787	-0.0093	-11	21	27	QMQEQGK			Oxidation (M)[2]		Mascot
876.4318	876.3638	-0.068	-78	1748	1755	MPVPMSAK			Oxidation (M)[1]		Mascot
1032.5181	1032.4746	-0.0435	-42	45	53	ERLSNGNSR					Mascot
1182.5857	1182.5507	-0.035	-30	809	818	SMTDVLTMLR			Oxidation (M)[2]		Mascot
1194.6188	1194.5979	-0.0209	-17	1133	1142	IMAELQAFQK			Oxidation (M)[2]		Mascot
1203.6005	1203.5867	-0.0138	-11	712	720	QKYLHEEEK					Mascot
1203.6005	1203.5867	-0.0138	-11	712	720	QKYLHEEEK					Mascot
1209.5529	1209.579	0.0261	22	1687	1697	ALVDTSCSSNR			Carbamidomethyl (C)[7]		Mascot
1241.6671	1241.5906	-0.0765	-62	337	348	SMVVPGNATIPR					Mascot
1244.613	1244.6057	-0.0073	-6	976	985	RQNLDHYNGK					Mascot
1259.6664	1259.5978	-0.0686	-54	990	1000	LLEEAQANIMK					Mascot
1322.6919	1322.5936	-0.0983	-74	809	819	SMTDVLTMLRR					Mascot
1325.7101	1325.6569	-0.0532	-40	544	555	QVFAYSTATIPK					Mascot
1342.6923	1342.6772	-0.0151	-11	1232	1242	TSEYKTEIIMK					Mascot
1432.7656	1432.6614	-0.1042	-73	470	482	TPPASPHRVSDLR					Mascot
1433.7173	1433.678	-0.0393	-27	1179	1189	NLEFFHEDVRK					Mascot
1433.7173	1433.678	-0.0393	-27	1178	1188	KNLEFFHEDVR					Mascot
1464.6826	1464.7979	0.1153	79	606	620	NHTDSAGTPHVSGGK					Mascot
1529.8687	1529.7975	-0.0712	-47	513	525	KEPGTLVYIEKPR					Mascot
1529.8687	1529.7975	-0.0712	-47	513	525	KEPGTLVYIEKPR					Mascot
1551.7412	1551.7361	-0.0051	-3	1873	1886	GHHLSFSPQSQNGR					Mascot
1705.8942	1705.813	-0.0812	-48	1001	1016	SIPNLEMPPATGPLPR			Oxidation (M)[7]		Mascot
1743.7888	1743.834	0.0452	26	74	88	SSKEILGMQTSEMDR			Oxidation (M)[8,13]		Mascot
1853.9314	1853.8596	-0.0718	-39	226	241	MLESPSVAIYIKDESR			Oxidation (M)[1]		Mascot
1870.9546	1870.8864	-0.0682	-36	181	196	ERSLGVLYLQYGDETK					Mascot
1944.8644	1944.8665	0.0021	1	1189	1204	KSDVEYENGPQMEFQK			Oxidation (M)[12]		Mascot

1944.8644	1944.8665	0.0021	1	1189	1204	KSDVEYENGPQMEFQK			Oxidation (M)[12]		Mascot
1958.9414	1958.875	-0.0664	-34	1077	1095	ENITAKASSEDAGPSPQTR					Mascot
1983.0546	1982.9539	-0.1007	-51	183	199	SLGVLYLQYGDETKQLR					Mascot
2002.054	2001.9182	-0.1358	-68	330	348	IPYGGTRSMVVPGNATIPR			Oxidation (M)[9]		Mascot
2011.0198	2011.0291	0.0093	5	1237	1253	TEIIMKENSISNMSLLR			Oxidation (M)[5,13]		Mascot

10	Rho GTPase-activating protein 21 OS=Homo sapiens GN=ARHGAP21 PE=1 SV=1	RHG21_HUMAN	218566.7	7.85	28	51	0	14.889

Peptide Information

Calc. Mass	Obsrv. Mass	± da	± ppm	Start Seq.	End Seq.	Sequence	Ion Score	C. I. %	Modification	Rank	Result Type
820.3981	820.3426	-0.0555	-68	1513	1519	SPTLSCR			Carbamidomethyl (C)[6]		Mascot
824.338	824.3699	0.0319	39	913	920	SSEDSGSR					Mascot
858.4177	858.4106	-0.0071	-8	439	445	RSTSHDR					Mascot
893.4475	893.4068	-0.0407	-46	1392	1400	SKGSWGSGK					Mascot
950.4863	950.4304	-0.0559	-59	462	469	LEDSVLMK			Oxidation (M)[7]		Mascot
1032.5685	1032.4746	-0.0939	-91	1741	1750	STGSLLTPTR					Mascot
1145.6677	1145.5809	-0.0868	-76	894	903	LSFKHVSSLK					Mascot
1155.6746	1155.5795	-0.0951	-82	949	958	RVGGSIRPWK					Mascot
1195.5814	1195.6018	0.0204	17	1931	1942	NASSAANAQPHK					Mascot
1201.5848	1201.5857	0.0009	1	390	398	TYKEYIDNR					Mascot
1217.6848	1217.5725	-0.1123	-92	1739	1750	GKSTGSLLTPTR					Mascot
1219.6166	1219.5808	-0.0358	-29	6	17	TGLSEGDGDKLK					Mascot
1219.6166	1219.5808	-0.0358	-29	6	17	TGLSEGDGDKLK					Mascot
1273.6635	1273.5939	-0.0696	-55	1603	1614	LSPEVQSVAESK					Mascot
1322.6699	1322.5936	-0.0763	-58	1089	1100	SEPKTQSPHSPK					Mascot
1373.5751	1373.6248	0.0497	36	1444	1454	ENVEQCHNDTK			Carbamidomethyl (C)[6]		Mascot
1375.6337	1375.6134	-0.0203	-15	668	680	TDSAPDQQVETGK					Mascot
1464.7767	1464.7979	0.0212	14	1725	1737	EAPSLTKVFDVMK					Mascot
1491.8529	1491.7179	-0.135	-90	1406	1419	ELLVSSIFAAASRK					Mascot
1511.7775	1511.7952	0.0177	12	245	258	MEIQVPPSPTDVAK					Mascot
1514.7963	1514.8027	0.0064	4	627	640	APSTHVTKPSFSQK					Mascot
1557.7795	1557.8049	0.0254	16	170	183	DVTALAYSQDAYLK					Mascot
1812.8287	1812.901	0.0723	40	1427	1442	AQPSSSEDELDNVFFK					Mascot
1829.0103	1828.9332	-0.0771	-42	1292	1307	MEPRNLAIVFGPTLVR			Oxidation (M)[1]		Mascot
1829.0103	1828.9332	-0.0771	-42	1292	1307	MEPRNLAIVFGPTLVR			Oxidation (M)[1]		Mascot

1844.7789	1844.9418	0.1629	88	1010	1024	LTTSDCECLFQAEDR	Carbamidomethyl (C)[6,8]	Mascot
1901.979	1901.8466	-0.1324	-70	242	258	AYRMEIQVPPSPTDVAK		Mascot
1905.0527	1904.8768	-0.1759	-92	950	965	VGGSIRPWKQMYVVLR	Oxidation (M)[11]	Mascot
1926.9264	1926.8427	-0.0837	-43	1820	1836	VHEQSGERESELSAVNR		Mascot
2001.926	2001.9182	-0.0078	-4	1495	1512	ESTPSEEPSPPHNSKHN K		Mascot

Gel Idx/Pos	186/H13	Instr./Gel Origin	BA2151/Sample Project 20140814	Process Status	Analysis Succeeded
Plate [#] Name	[1] Sample Project 20140814	Instrument Sample Name		Spectra	11

Rank	Protein Name	Accession No.	Protein MW	Protein PI	Pep. Count	Protein Score	Protein Score C. I. %	Intensity Matched	Total Ion Score	Total Ion C. I. %	Confirmed
1	1-(5-phosphoribosyl)-5-[(5-phosphoribosylamino)methylideneamino] imidazole-4-carboxamide isomerase OS=Thermotoga neapolitana (strain ATCC 49049 / DSM 4359 / NS-E) GN=hisA PE=3 SV=1	HIS4_THENN	27992	5.71	13	69	93.348	13.087			

Peptide Information

Calc. Mass	Obsrv. Mass	± da	± ppm	Start Seq.	End Seq.	Sequence	Ion Score	C. I. %	Modification	Rank	Result Type
809.4338	809.3976	-0.0362	-45	91	97	LLGMGFR			Oxidation (M)[4]		Mascot
817.489	817.471	-0.018	-22	98	104	RQIVSSK					Mascot
830.4982	830.4315	-0.0667	-80	183	190	IALETGVK					Mascot
1175.6671	1175.5537	-0.1134	-96	105	114	VLEDPSFLKK					Mascot
1182.6113	1182.5563	-0.055	-47	202	211	SLEEALEVHR					Mascot
1187.642	1187.5876	-0.0544	-46	143	152	DIDPVFLVNR					Mascot
1187.642	1187.5876	-0.0544	-46	143	152	DIDPVFLVNR					Mascot
1447.8341	1447.6998	-0.1343	-93	1	12	MLVIPAIDLYRK			Oxidation (M)[1]		Mascot
1593.8669	1593.7478	-0.1191	-75	225	238	AFLEGTLTVEVMKR					Mascot
1812.9855	1812.9301	-0.0554	-31	39	54	LVEEGFSLIHVVDLSR					Mascot
1871.0597	1870.8809	-0.1788	-96	183	201	IALETGVKVIAAGGISSER					Mascot
1945.0026	1944.8658	-0.1368	-70	174	190	EHDFSLTERIALETGVK					Mascot
1945.0026	1944.8658	-0.1368	-70	174	190	EHDFSLTERIALETGVK					Mascot
1967.0009	1966.8827	-0.1182	-60	23	38	ENTIFYEKDPIELVEK					Mascot
2019.1307	2018.9503	-0.1804	-89	219	237	GVIVGRAFLEGTLTVEVMK					Mascot

Rank	Protein Name	Accession No.	Protein MW	Protein PI	Pep. Count	Protein Score	Protein Score C. I. %	Intensity Matched	Total Ion Score	Total Ion C. I. %	Confirmed
2	ATP-dependent dethiobiotin synthetase BioD OS=Vibrio fischeri (strain MJ11) GN=bioD PE=3 SV=1	BIOD_VIBFM	24507.6	5.08	9	67	89.931	10.46	18	0	

Protein Group

Protein Name	Accession No.	Protein MW	Protein PI
ATP-dependent dethiobiotin synthetase BioD OS=Vibrio fischeri (strain ATCC 700601 / ES114) GN=bioD PE=3 SV=1	BIOD_VIBF1	24507.6	5.0799999237 0605

Peptide Information

Calc. Mass	Obsrv. Mass	± da	± ppm	Start Seq.	End Seq.	Sequence	Ion Score	C. I. %	Modification	Rank	Result Type
871.4996	871.425	-0.0746	-86	61	68	AVANVELR					Mascot

1175.58	1175.5537	-0.0263	-22	191	200	MLEDKMPAPK			Oxidation (M)[1]		Mascot
1191.5748	1191.5807	0.0059	5	191	200	MLEDKMPAPK			Oxidation (M)[1,6]		Mascot
1233.6548	1233.5664	-0.0884	-72	201	211	LGEIPYMPSVK					Mascot
1314.7562	1314.6304	-0.1258	-96	22	33	AILDALNMKGLR					Mascot
1405.7509	1405.6421	-0.1088	-77	201	212	LGEIPYMPSVKR			Oxidation (M)[7]		Mascot
1405.7509	1405.6421	-0.1088	-77	201	212	LGEIPYMPSVKR	18	0	Oxidation (M)[7]		Mascot
1447.7428	1447.6998	-0.043	-30	178	190	VNPGTENYAEIIK					Mascot
1517.7417	1517.7479	0.0062	4	214	226	NMGKYIHLEALDN					Mascot
1533.7366	1533.7327	-0.0039	-3	214	226	NMGKYIHLEALDN			Oxidation (M)[2]		Mascot
1700.8201	1700.894	0.0739	43	1	16	MIDAFFIAGTDTDVGK					Mascot
2018.9852	2018.9503	-0.0349	-17	123	139	VPVSKDDCLSTWVQQEK			Carbamidomethyl (C)[8]		Mascot

3	Cysteine--tRNA ligase OS=Mycobacterium sp. (strain KMS) GN=cysS PE=3 SV=1	SYC_MYCSK	53037.4	5.35	14	61	59.915	13.602	

Protein Group

Cysteine--tRNA ligase OS=Mycobacterium sp. (strain JLS) GN=cysS PE=3 SV=1	SYC_MYCSJ	53051.4	5.3499 999046 3257
Cysteine--tRNA ligase OS=Mycobacterium sp. (strain MCS) GN=cysS PE=3 SV=1	SYC_MYCSS	53037.4	5.3499 999046 3257

Peptide Information

Calc. Mass	Obsrv. Mass	± da	± ppm	Start Seq.	End Seq.	Sequence	Ion Score	C. I. %	Modification	Rank	Result Type
809.4192	809.3976	-0.0216	-27	192	197	DFTLWK					Mascot
1126.5562	1126.5515	-0.0047	-4	19	28	LYDTMTGAVR					Mascot
1203.6117	1203.5789	-0.0328	-27	443	452	KDWATADQIR					Mascot
1387.7428	1387.6543	-0.0885	-64	81	92	NVTDIDDKILNK					Mascot
1427.7238	1427.6283	-0.0955	-67	5	18	ADADRPATSPTGLR					Mascot
1512.817	1512.7388	-0.0782	-52	339	352	TRVGTVVPGDWTPK					Mascot
1543.8326	1543.7567	-0.0759	-49	416	430	DETSAALAAIDVLVR					Mascot
1577.712	1577.7471	0.0351	22	398	411	AMMGILGCDPLDER			Carbamidomethyl (C)[8]		Mascot
1577.712	1577.7471	0.0351	22	398	411	AMMGILGCDPLDER			Carbamidomethyl (C)[8]		Mascot
1593.707	1593.7478	0.0408	26	398	411	AMMGILGCDPLDER			Carbamidomethyl (C)[8], Oxidation (M)[2]		Mascot
1618.7126	1618.7729	0.0603	37	380	394	ALDSGDHETAMTQAR			Oxidation (M)[11]		Mascot
1668.8149	1668.7778	-0.0371	-22	311	325	SMLEFSETALQDAVK					Mascot
1813.0365	1812.9301	-0.1064	-59	278	294	MSKSLGNVLSIPAVLQR					Mascot
1829.0314	1828.9254	-0.106	-58	278	294	MSKSLGNVLSIPAVLQR			Oxidation (M)[1]		Mascot

	1829.0314	1828.9254	-0.106	-58	278	294	MSKSLGNVLSIPAVLQR			Oxidation (M)[1]		Mascot
	1853.9579	1853.8694	-0.0885	-48	19	34	LYDTMTGAVRDFVPLR					Mascot
	1949.9242	1949.9172	-0.007	-4	395	411	SIRAMMGILGCDPLDER			Carbamidomethyl (C)[11], Oxidation (M)[5]		Mascot
	1958.9349	1958.8408	-0.0941	-48	380	397	ALDSGDHETAMTQARSIR					Mascot

4	Cytochrome c oxidase subunit 4 isoform 1, mitochondrial (Fragment) OS=Pongo pygmaeus GN=COX4I1 PE=3 SV=1	COX41_PONPY	16816.6	9.44	11	60	51.807	8.19	

Peptide Information

Calc. Mass	Obsrv. Mass	± da	± ppm	Start Seq.	End Seq.	Sequence	Ion Score	C. I. %	Modification	Rank	Result Type
809.4008	809.3976	-0.0032	-4	119	124	RMLDMK			Oxidation (M)[2]		Mascot
1241.6637	1241.5496	-0.1141	-92	18	28	DHPLPEVAHVK					Mascot
1297.5848	1297.6224	0.0376	29	135	143	WDYEKNEWK					Mascot
1387.7006	1387.6543	-0.0463	-33	99	110	HVYGPLPQSFDK					Mascot
1388.6329	1388.6151	-0.0178	-13	5	16	SEDFSLSAYVDR					Mascot
1388.6329	1388.6151	-0.0178	-13	5	16	SEDFSLSAYVDR					Mascot
1397.7649	1397.6353	-0.1296	-93	17	28	RDHPLPEVAHVK					Mascot
1497.689	1497.7743	0.0853	57	41	53	EKASWSSLSMDEK					Mascot
1513.684	1513.7732	0.0892	59	41	53	EKASWSSLSMDEK			Oxidation (M)[10]		Mascot
1513.684	1513.7732	0.0892	59	41	53	EKASWSSLSMDEK			Oxidation (M)[10]		Mascot
1543.8016	1543.7567	-0.0449	-29	98	110	RHVYGPLPQSFDK					Mascot
1660.8761	1660.7781	-0.098	-59	120	134	MLDMKVNPIQGLASK			Oxidation (M)[1]		Mascot
1700.7333	1700.894	0.1607	94	63	76	ESFAEMNRGSNEWK			Oxidation (M)[6]		Mascot
1900.9109	1900.8373	-0.0736	-39	43	58	ASWSSLSMDEKVELYR					Mascot

5	Ribonuclease HII OS=Bacillus clausii (strain KSM-K16) GN=rnhB PE=3 SV=1	RNH2_BACSK	29024	8.79	12	57	0	22.894	

Peptide Information

Calc. Mass	Obsrv. Mass	± da	± ppm	Start Seq.	End Seq.	Sequence	Ion Score	C. I. %	Modification	Rank	Result Type
809.4039	809.3976	-0.0063	-8	59	64	YEQELK					Mascot
830.473	830.4315	-0.0415	-50	153	160	AINGLSQK					Mascot
870.5229	870.535	0.0121	14	1	8	MVPIAAIR					Mascot
1107.4888	1107.5214	0.0326	29	234	242	TYGMTPEHR			Oxidation (M)[4]		Mascot
1126.6038	1126.5515	-0.0523	-46	46	54	QQTLIQMHK					Mascot
1219.5889	1219.578	-0.0109	-9	234	243	TYGMTPEHRK					Mascot
1219.5889	1219.578	-0.0109	-9	234	243	TYGMTPEHRK					Mascot

Calc. Mass	Obsrv. Mass	± da	± ppm	Start Seq.	End Seq.	Sequence	Ion Score	C. I. %	Modification	Rank	Result Type
1235.5837	1235.5642	-0.0195	-16	234	243	TYGMTPEHRK			Oxidation (M)[4]		Mascot
1261.6932	1261.6027	-0.0905	-72	149	160	AMTKAINGLSQK					Mascot
1517.8534	1517.7479	-0.1055	-70	183	198	GDAKSLSIAASSVLAK					Mascot
1700.816	1700.894	0.078	46	11	25	LMDGSISHNELDELK					Mascot
1828.9109	1828.9254	0.0145	8	11	26	LMDGSISHNELDELKK					Mascot
1828.9109	1828.9254	0.0145	8	10	25	KLMDGSISHNELDELK					Mascot
1844.9059	1844.9261	0.0202	11	11	26	LMDGSISHNELDELKK			Oxidation (M)[2]		Mascot
2002.0175	2001.8895	-0.128	-64	39	54	YQAQQEKQQTLIQMHK					Mascot

6	Sepiapterin reductase OS=Xenopus tropicalis GN=spr PE=2 SV=1	SPRE_XENTR	28568.8	7.67	10	57	0	12.547	

Peptide Information

Calc. Mass	Obsrv. Mass	± da	± ppm	Start Seq.	End Seq.	Sequence	Ion Score	C. I. %	Modification	Rank	Result Type
809.4338	809.3976	-0.0362	-45	222	227	FLMDRK					Mascot
817.4301	817.471	0.0409	50	184	190	VLAEEEK					Mascot
1175.6453	1175.5537	-0.0916	-78	230	240	GKMVDIQVSAK					Mascot
1187.6267	1187.5876	-0.0391	-33	184	193	VLAEEEKDVR					Mascot
1187.6267	1187.5876	-0.0391	-33	184	193	VLAEEEKDVR	14	0			Mascot
1191.6403	1191.5807	-0.0596	-50	230	240	GKMVDIQVSAK			Oxidation (M)[3]		Mascot
1297.6344	1297.6224	-0.012	-9	242	252	MLDLLEADAYK			Oxidation (M)[1]		Mascot
1497.8108	1497.7743	-0.0365	-24	20	32	GFGRTLAHLLCPR			Carbamidomethyl (C)[11]		Mascot
1513.8584	1513.7732	-0.0852	-56	96	110	LLIINNAGSIGDVSK					Mascot
1513.8584	1513.7732	-0.0852	-56	96	110	LLIINNAGSIGDVSK					Mascot
1850.9463	1850.8871	-0.0592	-32	1	19	MAATGALGSVLCVLTGA SR			Carbamidomethyl (C)[12], Oxidation (M)[1]		Mascot
1974.0039	1973.8999	-0.104	-53	65	83	VRWEAADLGTSEGVSAA VR					Mascot
1983.9957	1983.9065	-0.0892	-45	194	211	VLNYAPGPLDTDMHVVA R			Oxidation (M)[13]		Mascot

7	Chorismate synthase OS=Caldicellulosiruptor bescii (strain ATCC BAA-1888 / DSM 6725 / Z-1320) GN=aroC PE=3 SV=1	AROC_CALBD	42903	6.77	13	56	0	8.101	

Peptide Information

Calc. Mass	Obsrv. Mass	± da	± ppm	Start Seq.	End Seq.	Sequence	Ion Score	C. I. %	Modification	Rank	Result Type
805.4236	805.4	-0.0236	-29	48	53	RMEIEK					Mascot
817.4301	817.471	0.0409	50	200	206	EEIDIAK					Mascot
882.4059	882.4167	0.0108	12	193	199	EAEMKMK			Oxidation (M)[4]		Mascot
1487.858	1487.7291	-0.1289	-87	350	363	AAIAYVLANALIER					Mascot

Calc. Mass	Obsrv. Mass	± da	± ppm	Start Seq.	End Seq.	Sequence	Ion Score	C. I. %	Modification	Rank	Result Type
1497.7618	1497.7743	0.0125	8	364	377	LGGDSAKTMLETFK					Mascot
1513.7567	1513.7732	0.0165	11	364	377	LGGDSAKTMLETFK			Oxidation (M)[9]		Mascot
1513.7567	1513.7732	0.0165	11	364	377	LGGDSAKTMLETFK			Oxidation (M)[9]		Mascot
1517.6248	1517.7479	0.1231	81	86	98	SFMDATQCDVDTK			Carbamidomethyl (C)[8]		Mascot
1533.6196	1533.7327	0.1131	74	86	98	SFMDATQCDVDTK			Carbamidomethyl (C)[8], Oxidation (M)[3]		Mascot
1545.7942	1545.7767	-0.0175	-11	207	221	QIGDSVGGIAEVICK			Carbamidomethyl (C)[14]		Mascot
1764.8949	1764.9271	0.0322	18	64	79	NSFTTGAPITLMIENR					Mascot
1764.8949	1764.9271	0.0322	18	64	79	NSFTTGAPITLMIENR					Mascot
1780.8899	1780.9359	0.046	26	64	79	NSFTTGAPITLMIENR			Oxidation (M)[12]		Mascot
1788.924	1788.8773	-0.0467	-26	320	335	SVDIRTFQPAEAAVER					Mascot
1844.8337	1844.9261	0.0924	50	266	280	FGSEVHDEIYYDDKK					Mascot
1871.0055	1870.8809	-0.1246	-67	236	252	KLDAQIAHSVMSIQSVK			Oxidation (M)[11]		Mascot
1973.9709	1973.8999	-0.071	-36	286	305	TNNAGGIEGGISNGMDIV VR					Mascot
1989.9658	1989.915	-0.0508	-26	286	305	TNNAGGIEGGISNGMDIV VR			Oxidation (M)[15]		Mascot
2644.2461	2644.2983	0.0522	20	64	85	NSFTTGAPITLMIENRDYE NWR			Oxidation (M)[12]		Mascot

8	Aspartate--tRNA ligase OS=Halothermothrix orenii (strain H 168 / OCM 544 / DSM 9562) GN=aspS PE=3 SV=1	SYD_HALOH	66830.8	5.66	15	54	0	22.614

Peptide Information

Calc. Mass	Obsrv. Mass	± da	± ppm	Start Seq.	End Seq.	Sequence	Ion Score	C. I. %	Modification	Rank	Result Type
1175.6605	1175.5537	-0.1068	-91	25	34	KVTVMGWVQK					Mascot
1191.6555	1191.5807	-0.0748	-63	25	34	KVTVMGWVQK			Oxidation (M)[5]		Mascot
1203.6667	1203.5789	-0.0878	-73	26	35	VTVMGWVQKR					Mascot
1219.6205	1219.578	-0.0425	-35	421	431	EEGLIPDGVYK					Mascot
1219.6616	1219.578	-0.0836	-69	26	35	VTVMGWVQKR	6	0	Oxidation (M)[4]		Mascot
1241.6637	1241.5496	-0.1141	-92	37	47	DHGGVIFIDLR					Mascot
1397.7649	1397.6353	-0.1296	-93	36	47	RDHGGVIFIDLR					Mascot
1447.725	1447.6998	-0.0252	-17	278	289	IPVMPYQEAIDR			Oxidation (M)[4]		Mascot
1487.7588	1487.7291	-0.0297	-20	566	580	ASSPLTEAPSTVAEK					Mascot
1507.7938	1507.7123	-0.0815	-54	253	265	EDVFSLVNGLMKR					Mascot
1512.7919	1512.7388	-0.0531	-35	37	49	DHGGVIFIDLRDR					Mascot
1559.8428	1559.7472	-0.0956	-61	418	431	IAREEGLIPDGVYK					Mascot
1796.8813	1796.9534	0.0721	40	479	495	AYDFVLNGEELGGGSIR					Mascot
1844.9601	1844.9261	-0.034	-18	563	580	TQKASSPLTEAPSTVAEK					Mascot
1878.963	1878.8932	-0.0698	-37	500	515	DLQMKVFNALNISEEK					Mascot

Calc. Mass	Obsrv. Mass	± da	± ppm	Start Seq.	End Seq.	Sequence	Ion Score	C. I. %	Modification	Rank	Result Type
1958.9679	1958.8408	-0.1271	-65	48	64	DRSGIVQVVFNPDNNER					Mascot
2723.2698	2723.271	0.0012	0	432	453	FVWVVDFPLMEYDEDEG RYVAK			Oxidation (M)[10]		Mascot

9	Naringenin,2-oxoglutarate 3-dioxygenase OS=Callistephus chinensis GN=FHT PE=2 SV=1	FL3H_CALCH	40526.5	5.59	12	54	0	6.527	

Peptide Information

Calc. Mass	Obsrv. Mass	± da	± ppm	Start Seq.	End Seq.	Sequence	Ion Score	C. I. %	Modification	Rank	Result Type
830.4804	830.4315	-0.0489	-59	1	8	MAAPISLK					Mascot
907.4739	907.5023	0.0284	31	161	168	VLMGLACK			Carbamidomethyl (C)[7], Oxidation (M)[3]		Mascot
1241.6559	1241.5496	-0.1063	-86	203	213	CPQPDLTLGLK			Carbamidomethyl (C)[1]		Mascot
1261.6212	1261.6027	-0.0185	-15	89	98	DFFHLPTQEK					Mascot
1276.693	1276.5894	-0.1036	-81	325	335	MSTDLELARLK					Mascot
1397.757	1397.6353	-0.1217	-87	203	214	CPQPDLTLGLKR			Carbamidomethyl (C)[1]		Mascot
1447.7712	1447.6998	-0.0714	-49	169	181	LLEVLSEAMGLEK			Oxidation (M)[9]		Mascot
1748.814	1748.9541	0.1401	80	140	152	DYSRWPDKPNEWR					Mascot
1814.9027	1814.9299	0.0272	15	153	168	AVTEEYSKVLMGLACK			Carbamidomethyl (C)[15], Oxidation (M)[11]		Mascot
1926.8356	1926.8472	0.0116	6	309	323	SIMEEPMTFMEMYKK			Oxidation (M)[3,7]		Mascot
1942.8306	1942.8535	0.0229	12	309	323	SIMEEPMTFMEMYKK			Oxidation (M)[3,7,10]		Mascot
1944.9194	1944.8658	-0.0536	-28	187	202	ACVDMDQKVVVNYYPK			Carbamidomethyl (C)[2], Oxidation (M)[5]		Mascot
1944.9194	1944.8658	-0.0536	-28	187	202	ACVDMDQKVVVNYYPK			Carbamidomethyl (C)[2], Oxidation (M)[5]		Mascot
1958.8254	1958.8408	0.0154	8	309	323	SIMEEPMTFMEMYKK			Oxidation (M)[3,7,10,12]		Mascot
1974.0828	1973.8999	-0.1829	-93	169	186	LLEVLSEAMGLEKEALTK					Mascot
1990.0776	1989.915	-0.1626	-82	169	186	LLEVLSEAMGLEKEALTK			Oxidation (M)[9]		Mascot

10	Beta-1,3-galactosyltransferase 4 OS=Mus musculus GN=B3galt4 PE=2 SV=1	B3GT4_MOUSE	41665.8	9.23	12	54	0	8.232	

Peptide Information

Calc. Mass	Obsrv. Mass	± da	± ppm	Start Seq.	End Seq.	Sequence	Ion Score	C. I. %	Modification	Rank	Result Type
1195.6365	1195.6024	-0.0341	-29	293	303	RGGLAPTHCVK			Carbamidomethyl (C)[9]		Mascot
1198.6652	1198.5801	-0.0851	-71	227	236	VRPTRTPESR					Mascot
1201.5961	1201.5734	-0.0227	-19	187	197	GGPSEQWQKGK					Mascot
1213.6324	1213.6056	-0.0268	-22	304	314	LAGATHYPLDR					Mascot
1314.6697	1314.6304	-0.0393	-30	158	167	YCPMARYILK			Carbamidomethyl (C)[2]		Mascot
1433.6519	1433.6738	0.0219	15	327	337	VDPWQMQEAWK			Oxidation (M)[6]		Mascot
1512.7401	1512.7388	-0.0013	-1	117	130	QQLADLSSESAAHR					Mascot

1513.7291	1513.7732	0.0441	29	315	326	CCYGKFLLTSHK	Carbamidomethyl (C)[1,2]	Mascot
1513.7291	1513.7732	0.0441	29	315	326	CCYGKFLLTSHK	Carbamidomethyl (C)[1,2]	Mascot
1668.8412	1668.7778	-0.0634	-38	116	130	RQQLADLSSESAAHR		Mascot
1700.9694	1700.894	-0.0754	-44	143	157	NLTLKTLSGLNWVNK		Mascot
1765.0021	1764.9271	-0.075	-42	212	226	GQAVPLLYLGRVHWR		Mascot
1765.0021	1764.9271	-0.075	-42	212	226	GQAVPLLYLGRVHWR		Mascot
1853.9368	1853.8694	-0.0674	-36	347	362	TAPFCSWLQGFLGTLR	Carbamidomethyl (C)[5]	Mascot

Gel Idx/Pos	187/H14	Instr./Gel Origin	BA2151/Sample Project 20140814	Process Status	Analysis Succeeded
Plate [#] Name	[1] Sample Project 20140814	Instrument Sample Name		Spectra	11

Rank	Protein Name	Accession No.	Protein MW	Protein PI	Pep. Count	Protein Score	Protein Score C. I. %	Intensity Matched	Total Ion Score	Total Ion C. I. %	Confirmed
1	Triosephosphate isomerase (Fragment) OS=Anopheles merus GN=Tpi PE=3 SV=1	TPIS_ANOME	22206.4	5.33	5	109	100	15.878	87	100	

Peptide Information

Calc. Mass	Obsrv. Mass	± da	± ppm	Start Seq.	End Seq.	Sequence	Ion Score	C. I. %	Modification	Rank	Result Type
1388.7202	1388.7308	0.0106	8	104	115	VIACIGETLQER			Carbamidomethyl (C)[4]		Mascot
1388.7202	1388.7308	0.0106	8	104	115	VIACIGETLQER	87	100	Carbamidomethyl (C)[4]		Mascot
1591.8326	1591.8215	-0.0111	-7	80	93	RAIFGETDELIAEK					Mascot
1619.8826	1619.8365	-0.0461	-28	50	65	VAKGAFTGEISPAMLK					Mascot
1619.8826	1619.8365	-0.0461	-28	50	65	VAKGAFTGEISPAMLK					Mascot
1635.8774	1635.8441	-0.0333	-20	50	65	VAKGAFTGEISPAMLK			Oxidation (M)[14]		Mascot
1749.9242	1749.8872	-0.037	-21	156	171	TATPEQAQEVHAALRK					Mascot
1763.8997	1763.9388	0.0391	22	34	49	SLLPETIGVAAQNCYK			Carbamidomethyl (C)[14]		Mascot

Rank	Protein Name	Accession No.	Protein MW	Protein PI	Pep. Count	Protein Score	Protein Score C. I. %	Intensity Matched	Total Ion Score	Total Ion C. I. %	Confirmed
2	Triosephosphate isomerase (Fragment) OS=Culex pipiens GN=Tpi PE=3 SV=1	TPIS_CULPI	22281.5	5.7	3	98	99.991	15.285	87	100	

Peptide Information

Calc. Mass	Obsrv. Mass	± da	± ppm	Start Seq.	End Seq.	Sequence	Ion Score	C. I. %	Modification	Rank	Result Type
1388.7202	1388.7308	0.0106	8	104	115	VIACIGETLQER			Carbamidomethyl (C)[4]		Mascot
1388.7202	1388.7308	0.0106	8	104	115	VIACIGETLQER	87	100	Carbamidomethyl (C)[4]		Mascot
1619.8826	1619.8365	-0.0461	-28	50	65	VAKGAFTGEISPAMLK					Mascot
1619.8826	1619.8365	-0.0461	-28	50	65	VAKGAFTGEISPAMLK					Mascot
1635.8774	1635.8441	-0.0333	-20	50	65	VAKGAFTGEISPAMLK			Oxidation (M)[14]		Mascot
1749.9242	1749.8872	-0.037	-21	156	171	TATPEQAQEVHAALRK					Mascot

Rank	Protein Name	Accession No.	Protein MW	Protein PI	Pep. Count	Protein Score	Protein Score C. I. %	Intensity Matched	Total Ion Score	Total Ion C. I. %	Confirmed
3	Triosephosphate isomerase OS=Culex tarsalis GN=Tpi PE=1 SV=2	TPIS_CULTA	26828.7	5.98	3	96	99.988	18.624	87	100	

Peptide Information

Calc. Mass	Obsrv. Mass	± da	± ppm	Start Seq.	End Seq.	Sequence	Ion Score	C. I. %	Modification	Rank	Result Type
1388.7202	1388.7308	0.0106	8	122	133	VIACIGETLQER			Carbamidomethyl (C)[4]		Mascot
1388.7202	1388.7308	0.0106	8	122	133	VIACIGETLQER	87	100	Carbamidomethyl (C)[4]		Mascot

1607.8136	1607.8387	0.0251	16	174	188	TASPEQAQEVHAALR						Mascot
1607.8136	1607.8387	0.0251	16	174	188	TASPEQAQEVHAALR						Mascot
1735.9086	1735.9298	0.0212	12	174	189	TASPEQAQEVHAALRK						Mascot

4	Triosephosphate isomerase (Fragment) OS=Aedes togoi GN=Tpi PE=3 SV=1	TPIS_AEDTO	22253.4	5.3	2	94	99.977	15.142	87	100

Peptide Information

Calc. Mass	Obsrv. Mass	± da	± ppm	Start Seq.	End Seq.	Sequence	Ion Score	C. I. %	Modification	Rank	Result Type
1388.7202	1388.7308	0.0106	8	104	115	VIACIGETLQER			Carbamidomethyl (C)[4]		Mascot
1388.7202	1388.7308	0.0106	8	104	115	VIACIGETLQER	87	100	Carbamidomethyl (C)[4]		Mascot
1619.8826	1619.8365	-0.0461	-28	50	65	VAKGAFTGEISPAMLK					Mascot
1619.8826	1619.8365	-0.0461	-28	50	65	VAKGAFTGEISPAMLK					Mascot
1635.8774	1635.8441	-0.0333	-20	50	65	VAKGAFTGEISPAMLK			Oxidation (M)[14]		Mascot

5	Pyrroline-5-carboxylate reductase 2 OS=Bacillus subtilis (strain 168) GN=proI PE=3 SV=1	P5CR2_BACSU	30491	6.62	10	72	96.888	22.994	23	0

Peptide Information

Calc. Mass	Obsrv. Mass	± da	± ppm	Start Seq.	End Seq.	Sequence	Ion Score	C. I. %	Modification	Rank	Result Type
1297.6456	1297.7203	0.0747	58	181	191	YIEAMEKAAQK			Oxidation (M)[5]		Mascot
1388.7017	1388.7308	0.0291	21	227	240	EITSPGGTTEAGLR					Mascot
1388.7017	1388.7308	0.0291	21	227	240	EITSPGGTTEAGLR	23	0			Mascot
1410.6794	1410.6998	0.0204	14	47	57	ETYSVRPCRDK			Carbamidomethyl (C)[8]		Mascot
1577.817	1577.8138	-0.0032	-2	75	88	DAAESIDSIRPYIK					Mascot
1577.817	1577.8138	-0.0032	-2	75	88	DAAESIDSIRPYIK					Mascot
1611.9363	1611.8346	-0.1017	-63	112	126	LAVIRVMPNTSAAIR					Mascot
1619.8387	1619.8365	-0.0022	-1	262	276	SAEIKEQFAGAALER					Mascot
1619.8387	1619.8365	-0.0022	-1	262	276	SAEIKEQFAGAALER					Mascot
1627.9313	1627.8253	-0.106	-65	112	126	LAVIRVMPNTSAAIR			Oxidation (M)[7]		Mascot
1629.8694	1629.8319	-0.0375	-23	148	162	ALLETIGDATLVEER					Mascot
1689.8153	1689.8467	0.0314	19	247	260	FEEAIIHCIEETAK			Carbamidomethyl (C)[8]		Mascot
1763.9473	1763.9388	-0.0085	-5	42	55	LIELKETYSVRPCR			Carbamidomethyl (C)[13]		Mascot
1997.0186	1996.9937	-0.0249	-12	128	147	SATGFSVSTEASKNDIIAA K					Mascot

6	Nesprin-2 OS=Homo sapiens GN=SYNE2 PE=1 SV=3	SYNE2_HUMAN	801816.8	5.26	69	64	77.972	45.527	

Peptide Information

Calc. Mass	Obsrv. Mass	± da	± ppm	Start	End	Sequence	Ion	C. I. %	Modification	Rank	Result Type

				Seq.	Seq.		Score		
807.3739	807.4046	0.0307	38	1082	1088	AMEPTMK			Mascot
832.3215	832.3271	0.0056	7	5106	5111	MEMDYK		Oxidation (M)[1]	Mascot
871.4052	871.4444	0.0392	45	1383	1389	MLDMSFK			Mascot
891.4166	891.4346	0.018	20	2429	2435	NTEDERK			Mascot
954.5302	954.4868	-0.0434	-45	5154	5161	VHGMLNRK			Mascot
1003.5418	1003.5474	0.0056	6	2570	2578	EKDSLGNLK			Mascot
1033.5533	1033.5706	0.0173	17	2993	3000	CCILQVLK		Carbamidomethyl (C)[1,2]	Mascot
1037.5375	1037.5319	-0.0056	-5	4637	4644	SYQNEIKR			Mascot
1098.511	1098.5739	0.0629	57	6217	6225	QMVHEGNQR			Mascot
1106.551	1106.5262	-0.0248	-22	2330	2338	DDIKSLQCK		Carbamidomethyl (C)[8]	Mascot
1114.5059	1114.5746	0.0687	62	6217	6225	QMVHEGNQR		Oxidation (M)[2]	Mascot
1118.4637	1118.5159	0.0522	47	319	327	DSENDTYFK			Mascot
1182.6477	1182.572	-0.0757	-64	4032	4041	LPQLQGEIER			Mascot
1193.5765	1193.6252	0.0487	41	1927	1936	MESICQARAK		Carbamidomethyl (C)[5]	Mascot
1297.7185	1297.7203	0.0018	1	5878	5888	HVLVEDVMVLK		Oxidation (M)[8]	Mascot
1308.6041	1308.6594	0.0553	42	3357	3365	YLENYKCYR		Carbamidomethyl (C)[7]	Mascot
1320.6174	1320.5928	-0.0246	-19	5793	5803	DMEPQLAEMIK		Oxidation (M)[2]	Mascot
1353.7737	1353.6589	-0.1148	-85	2085	2096	EIILLKPEGDAR			Mascot
1357.7012	1357.7043	0.0031	2	6003	6013	WQHLFDVIGSR			Mascot
1370.7712	1370.7581	-0.0131	-10	3136	3147	VLQNMVLELSPK			Mascot
1371.7307	1371.7534	0.0227	17	4397	4407	DFIKFIEFNAK			Mascot
1374.6028	1374.7111	0.1083	79	1383	1393	MLDMSFKDAER		Oxidation (M)[1,4]	Mascot
1388.7322	1388.7308	-0.0014	-1	6131	6140	IEETWRLWQK			Mascot
1388.7817	1388.7308	-0.0509	-37	2152	2162	EDLRLMLIELK		Oxidation (M)[6]	Mascot
1410.6583	1410.6998	0.0415	29	1304	1314	YKTQFEGMNHR			Mascot
1416.6425	1416.7568	0.1143	81	1669	1679	MELHQLTEEDR		Oxidation (M)[1]	Mascot
1434.7952	1434.7882	-0.007	-5	1324	1336	ALEDFLASLRTAK			Mascot
1485.7948	1485.7375	-0.0573	-39	742	754	LIGQVEIWEAEAK			Mascot
1487.7013	1487.7531	0.0518	35	1582	1593	EEFNEHLEVVDK			Mascot
1491.7955	1491.7511	-0.0444	-30	5916	5927	LNTWVVFNEKNK			Mascot
1559.9012	1559.7791	-0.1221	-78	2988	3000	LTAIKCCILQVLK		Carbamidomethyl (C)[6,7]	Mascot
1590.8594	1590.8282	-0.0312	-20	665	677	TQLEMNLPLMIKK		Oxidation (M)[5,10]	Mascot
1591.7931	1591.8215	0.0284	18	3429	3441	LRCTENDGICLLK		Carbamidomethyl (C)[3,10]	Mascot
1603.7573	1603.8354	0.0781	49	3722	3733	KMWDELDLWHSK		Oxidation (M)[2]	Mascot
1607.8387	1607.8387	0	0	5442	5455	TKEAFLQNSSVLDR			Mascot

1607.8387	1607.8387	0	0	5442	5455	TKEAFLQNSSVLDR				Mascot
1611.9039	1611.8346	-0.0693	-43	3956	3969	LPQTGMKPLPVFQR				Mascot
1615.865	1615.8154	-0.0496	-31	1156	1169	LTDLQVIKNETDAR				Mascot
1617.8079	1617.8246	0.0167	10	4972	4985	EQSLNVSQDLDTIR				Mascot
1627.8989	1627.8253	-0.0736	-45	3956	3969	LPQTGMKPLPVFQR			Oxidation (M)[6]	Mascot
1633.7415	1633.8154	0.0739	45	2524	2537	DFQEYLAAVESSMK			Oxidation (M)[13]	Mascot
1648.9269	1648.8564	-0.0705	-43	3942	3955	KTIAEIVSYQVELR				Mascot
1689.8555	1689.8467	-0.0088	-5	4945	4958	HLLSYNRDSDQLTK				Mascot
1691.8176	1691.851	0.0334	20	4959	4971	WLESSQHTLNYWK				Mascot
1704.9353	1704.8618	-0.0735	-43	1428	1441	LLEACIFKNNELLK			Carbamidomethyl (C)[5]	Mascot
1710.7471	1710.8197	0.0726	42	559	571	QYMMVKSDVCMYR			Carbamidomethyl (C)[10]	Mascot
1726.7419	1726.8116	0.0697	40	559	571	QYMMVKSDVCMYR			Carbamidomethyl (C)[10], Oxidation (M)[3]	Mascot
1731.8986	1731.8478	-0.0508	-29	5037	5050	WTMLITQLPDIQEK			Oxidation (M)[3]	Mascot
1733.817	1733.9089	0.0919	53	6141	6153	FLDDYSRFEDWLK				Mascot
1739.8125	1739.9189	0.1064	61	2351	2364	QEMECCLNSILKSK			Carbamidomethyl (C)[5,6]	Mascot
1743.8184	1743.864	0.0456	26	3349	3362	AQETEAERYLENYK				Mascot
1747.8973	1747.9216	0.0243	14	6285	6299	QLNGFQQEITLNTNK				Mascot
1761.8688	1761.9474	0.0786	45	3065	3080	MTEVVLKAPDSSPESR			Oxidation (M)[1]	Mascot
1763.9426	1763.9388	-0.0038	-2	2544	2559	ESLKVGPLDSVTYLDK				Mascot
1767.921	1767.9196	-0.0014	-1	6535	6548	WEMIQAQELHNKLK				Mascot
1838.0205	1837.929	-0.0915	-50	5873	5888	ADLTRHVLVEDVMVLK				Mascot
1838.7605	1838.9265	0.166	90	850	863	MEESQKELESYMMR			Oxidation (M)[1,12,13]	Mascot
1881.8549	1882.0166	0.1617	86	5135	5149	YERTEFAEHLGEMNR				Mascot
1896.8929	1896.9996	0.1067	56	3888	3904	MADDVVAIESEVKSMEK			Oxidation (M)[1]	Mascot
1908.9662	1908.8147	-0.1515	-79	5020	5036	ETDTATLRASLAQFEQK				Mascot
1913.8699	1914.0105	0.1406	73	5804	5818	QFQSTVETWDQCEKK			Carbamidomethyl (C)[12]	Mascot
1926.8988	1926.8969	-0.0019	-1	6217	6231	QMVHEGNQRWDNLQR			Oxidation (M)[2]	Mascot
1956.0471	1956.0685	0.0214	11	6717	6733	QPQVDMLQEISNSLLIK				Mascot
1967.9379	1967.988	0.0501	25	3232	3247	EFEDLQMQLNTSIDLR			Oxidation (M)[7]	Mascot
1971.0505	1971.0166	-0.0339	-17	1436	1452	NNELLKNIQDVQSQISK				Mascot
1972.042	1972.0454	0.0034	2	6717	6733	QPQVDMLQEISNSLLIK			Oxidation (M)[6]	Mascot
1972.957	1973.0276	0.0706	36	4083	4101	LPAVTSEEGGVAERDASER				Mascot
1972.957	1973.0276	0.0706	36	4083	4101	LPAVTSEEGGVAERDASER	1	0		Mascot
1977.0739	1977.0271	-0.0468	-24	584	599	VLACWATYVENLRLLR			Carbamidomethyl (C)[4]	Mascot
1987.1335	1987.0134	-0.1201	-60	64	81	GHVLLDLLEVLSGQQLPR				Mascot
1989.011	1989.0219	0.0109	5	4381	4396	DFQQQQVLELKPMEQK				Mascot

Calc. Mass	Obsrv. Mass	± da	± ppm	Start Seq.	End Seq.	Sequence	Ion Score	C. I. %	Modification	Rank	Result Type
1989.011	1989.0219	0.0109	5	4381	4396	DFQQQQVLELKPMEQK					Mascot
1997.0814	1996.9937	-0.0877	-44	5179	5194	IQILNNWLEAQEERLK					Mascot
2001.0573	2001.002	-0.0553	-28	3136	3152	VLQNMVLELSPKELDEK			Oxidation (M)[5]		Mascot
2005.006	2005.015	0.009	4	4381	4396	DFQQQQVLELKPMEQK			Oxidation (M)[13]		Mascot
2005.006	2005.015	0.009	4	4381	4396	DFQQQQVLELKPMEQK			Oxidation (M)[13]		Mascot
2011.0342	2011.0284	-0.0058	-3	5162	5178	IQHLEQLLESITESENK					Mascot
2831.2869	2831.2188	-0.0681	-24	1727	1751	MEHVQKCLTGESNCHAL SGSTAELR			Carbamidomethyl (C)[7,14], Oxidation (M)[1]		Mascot

7	Phosphate import ATP-binding protein PstB OS=Pseudomonas putida GN=pstB PE=3 SV=1	PSTB_PSEPU	31143.1	6.15	12	64	76.933	3.759

Peptide Information

Calc. Mass	Obsrv. Mass	± da	± ppm	Start Seq.	End Seq.	Sequence	Ion Score	C. I. %	Modification	Rank	Result Type
1082.5114	1082.5771	0.0657	61	267	275	QTEDYITGR					Mascot
1107.627	1107.5504	-0.0766	-69	70	78	STLLRTFNR					Mascot
1193.5984	1193.6252	0.0268	22	58	69	VTAFIGPSGCGK			Carbamidomethyl (C)[10]		Mascot
1410.661	1410.6998	0.0388	28	238	249	VSDYTAFMYLGK			Oxidation (M)[8]		Mascot
1660.8993	1660.8295	-0.0698	-42	112	125	RVGMVFQKPNPFPK			Oxidation (M)[4]		Mascot
1662.8633	1662.8446	-0.0187	-11	44	57	QALFDVSMNIPKQR			Oxidation (M)[8]		Mascot
1760.9113	1760.9535	0.0422	24	223	237	YTIVIVTHNMQQAAR			Oxidation (M)[10]		Mascot
1763.9473	1763.9388	-0.0085	-5	58	74	VTAFIGPSGCGKSTLLR			Carbamidomethyl (C)[10]		Mascot
1767.88	1767.9196	0.0396	22	250	265	LVEFGDTDTLFTNPAK					Mascot
1960.0433	1960.0547	0.0114	6	221	237	SKYTIVIVTHNMQQAAR					Mascot
1980.0913	1980.0134	-0.0779	-39	126	142	TIYENVVYGLRIQGINK					Mascot
2010.941	2011.0284	0.0874	43	2	19	QQDSHTHGIDMSALGRN K			Oxidation (M)[11]		Mascot

8	Nebulin OS=Homo sapiens GN=NEB PE=1 SV=4	NEBU_HUMAN	775406.4	9.11	72	62	69.592	50.589

Peptide Information

Calc. Mass	Obsrv. Mass	± da	± ppm	Start Seq.	End Seq.	Sequence	Ion Score	C. I. %	Modification	Rank	Result Type
807.4359	807.4046	-0.0313	-39	4956	4962	TLPTGYR					Mascot
871.4996	871.4444	-0.0552	-63	3421	3427	QRPETLK					Mascot
891.4207	891.4346	0.0139	16	2482	2488	EAWDKDK					Mascot
905.4938	905.4636	-0.0302	-33	5291	5298	KVTDDISK					Mascot
1003.5782	1003.5474	-0.0308	-31	3746	3755	ADAISIKSAK					Mascot
1037.468	1037.5319	0.0639	62	302	310	MNADNISTR			Oxidation (M)[1]		Mascot
1098.6129	1098.5739	-0.039	-35	3303	3311	IMWSLHIAK					Mascot

1107.5542	1107.5504	-0.0038	-3	4040	4047	LQNEREYR		Mascot
1114.6078	1114.5746	-0.0332	-30	3303	3311	IMWSLHIAK	Oxidation (M)[2]	Mascot
1118.5486	1118.5159	-0.0327	-29	3546	3554	MMWSIHVAK	Oxidation (M)[1]	Mascot
1193.6776	1193.6252	-0.0524	-44	3486	3495	LYKLALEESK		Mascot
1289.6406	1289.6503	0.0097	8	855	866	MIGALSINDDPK	Oxidation (M)[1]	Mascot
1308.7019	1308.6594	-0.0425	-32	6580	6590	HAKTTELPQQR		Mascot
1316.5753	1316.5984	0.0231	18	457	466	NYKAEYEEDR		Mascot
1323.7155	1323.6578	-0.0577	-44	5608	5618	NTLIESDLKYK		Mascot
1340.6879	1340.6927	0.0048	4	6254	6265	GIPTPITPEMER		Mascot
1353.6467	1353.6589	0.0122	9	1395	1406	MAQDVATNVNYK		Mascot
1357.7474	1357.7043	-0.0431	-32	2039	2050	GYDLRPDAIPIK		Mascot
1370.7063	1370.7581	0.0518	38	5415	5425	DKYHLVVDEPR		Mascot
1371.6573	1371.7534	0.0961	70	834	845	ANTKNTSDVMYK		Mascot
1388.7104	1388.7308	0.0204	15	4257	4268	QKGHYVGVPTMR	Oxidation (M)[11]	Mascot
1388.7104	1388.7308	0.0204	15	4257	4268	QKGHYVGVPTMR	Oxidation (M)[11]	Mascot
1402.6056	1402.733	0.1274	91	6464	6474	NMQDFSDINYR		Mascot
1410.6648	1410.6998	0.035	25	3450	3460	RLYTEAWDNDK		Mascot
1416.7482	1416.7568	0.0086	6	4485	4496	HADLVNSELKYK		Mascot
1434.7257	1434.7882	0.0625	44	5819	5831	VMKDANNLASEVK	Oxidation (M)[2]	Mascot
1485.8424	1485.7375	-0.1049	-71	2038	2050	KGYDLRPDAIPIK		Mascot
1491.8165	1491.7511	-0.0654	-44	230	243	VAQAQKALSDVAYK		Mascot
1507.6768	1507.7521	0.0753	50	5986	5998	GLTEMEDTPDMLR		Mascot
1530.7006	1530.795	0.0944	62	6463	6474	KNMQDFSDINYR		Mascot
1577.8356	1577.8138	-0.0218	-14	2248	2261	IHVMPDTPDILQAK		Mascot
1577.8356	1577.8138	-0.0218	-14	2248	2261	IHVMPDTPDILQAK		Mascot
1590.8925	1590.8282	-0.0643	-40	3572	3586	FSSPVDMLGVVLAKK		Mascot
1591.7786	1591.8215	0.0429	27	6447	6460	GCFTPVVTDPITER	Carbamidomethyl (C)[2]	Mascot
1603.8149	1603.8354	0.0205	13	6347	6360	ATPTPFTPEMERVK		Mascot
1605.8451	1605.8469	0.0018	1	2331	2344	LVLSMNVAKMQSER		Mascot
1607.7015	1607.8387	0.1372	85	2813	2825	DDPKMMWSMHVAK	Oxidation (M)[5,6]	Mascot
1607.835	1607.8387	0.0037	2	3671	3684	FTCITDTPEIVLAK	Carbamidomethyl (C)[3]	Mascot
1615.7897	1615.8154	0.0257	16	4558	4569	RCQYILSDLEYR	Carbamidomethyl (C)[2]	Mascot
1619.8098	1619.8365	0.0267	16	6347	6360	ATPTPFTPEMERVK	Oxidation (M)[10]	Mascot
1619.8098	1619.8365	0.0267	16	6347	6360	ATPTPFTPEMERVK	Oxidation (M)[10]	Mascot
1623.6964	1623.832	0.1356	84	2813	2825	DDPKMMWSMHVAK	Oxidation (M)[5,6,9]	Mascot
1627.9054	1627.8253	-0.0801	-49	5240	5253	DKYTPVPDTPILIR		Mascot
1629.8199	1629.8319	0.012	7	2087	2100	LVHSMQVAKMQSDR		Mascot

1634.8861	1634.8401	-0.046	-28	5032	5045	IHTTPDTPEIRQVK		Mascot
1635.8854	1635.8441	-0.0413	-25	4938	4951	ALHAYKLQSSNLYK		Mascot
1648.8541	1648.8564	0.0023	1	4149	4162	TKIETLNFTPVDDR		Mascot
1660.8265	1660.8295	0.003	2	537	550	CHIPPDTPAFIQHK	Carbamidomethyl (C)[1]	Mascot
1662.8738	1662.8446	-0.0292	-18	1516	1529	YTIDPELPQFIQAK		Mascot
1676.8677	1676.8687	0.001	1	1625	1639	YHTPLDMVSVTAAKK	Oxidation (M)[7]	Mascot
1687.826	1687.866	0.04	24	5301	5313	YKENYMSQLGIWR		Mascot
1689.8563	1689.8467	-0.0096	-6	1843	1856	LVHFMQVAKMQSDR		Mascot
1691.8673	1691.851	-0.0163	-10	5698	5712	GKSNYSIMLEPPEVK		Mascot
1704.9531	1704.8618	-0.0913	-54	4892	4907	LVTDTPVYVQAVKSGK		Mascot
1705.8513	1705.9072	0.0559	33	1843	1856	LVHFMQVAKMQSDR	Oxidation (M)[5]	Mascot
1705.8513	1705.9072	0.0559	33	1843	1856	LVHFMQVAKMQSDR	Oxidation (M)[5]	Mascot
1710.9789	1710.8197	-0.1592	-93	1202	1217	GIGWIPIGSLDVEKVK		Mascot
1726.866	1726.8116	-0.0544	-32	4273	4286	LVWFEHAGQIQNER		Mascot
1731.8549	1731.8478	-0.0071	-4	4680	4693	YTTVLETVDYDRTR		Mascot
1733.8462	1733.9089	0.0627	36	3546	3559	MMWSIHVAKIQSDR	Oxidation (M)[1,2]	Mascot
1735.8457	1735.9298	0.0841	48	6536	6552	EQSRSASALSISGGEEK		Mascot
1747.9048	1747.9216	0.0168	10	2907	2922	GIGWVSIGSLDVEKCK	Carbamidomethyl (C)[15]	Mascot
1749.9568	1749.8872	-0.0696	-40	3327	3342	TRYSSPVDMLGIVLAK		Mascot
1751.8269	1751.9213	0.0944	54	2664	2679	GIGWMTSGSLEDEKNK		Mascot
1761.9204	1761.9474	0.027	15	1623	1638	TKYHTPLDMVSVTAAK		Mascot
1767.9712	1767.9196	-0.0516	-29	1307	1324	GNNVLGDAIPITAAKASR		Mascot
1808.9463	1808.9431	-0.0032	-2	891	906	TIYTAPLDMLQVTQAK	Oxidation (M)[9]	Mascot
1837.8901	1837.929	0.0389	21	1135	1150	SKYNTPHDMFNVVAAK	Oxidation (M)[9]	Mascot
1867.9406	1867.91	-0.0306	-16	3218	3233	TQVHIMPDTPEIMLAR	Oxidation (M)[6]	Mascot
1882.0004	1882.0166	0.0162	9	5487	5502	GYTTIHDTPMLLHVRK		Mascot
1885.9541	1886.0022	0.0481	26	2994	3009	INYSETLYKLANEEAK		Mascot
1955.0485	1955.0406	-0.0079	-4	6185	6202	YKEAIGQGTPIPDLPEVK		Mascot
1960.0004	1960.0547	0.0543	28	4542	4558	ANVHIPNDMMNHVLAKR		Mascot
1963.8711	1963.9949	0.1238	63	3053	3068	NIEDDPKMMWSMHVAK	Oxidation (M)[8,9]	Mascot
1979.866	1980.0134	0.1474	74	3053	3068	NIEDDPKMMWSMHVAK	Oxidation (M)[8,9,12]	Mascot
1986.0266	1986.0027	-0.0239	-12	4608	4624	GIGCYVWDTPQILHAKK	Carbamidomethyl (C)[4]	Mascot
2011.0715	2011.0284	-0.0431	-21	608	625	MIGVLSINDDPKMLHSLK		Mascot
2353.1143	2353.1724	0.0581	25	2869	2887	NYLHQWTCLPDQSDVIH AR	Carbamidomethyl (C)[8]	Mascot
2369.25	2369.0232	-0.2268	-96	3629	3649	SDLEWLRGIGWVPIGSVE VEK		Mascot
2450.3079	2450.2573	-0.0506	-21	1021	1041	GEEIIHKYNLPPDLPQFIQ		Mascot

						AK					
2831.3591	2831.2188	-0.1403	-50	5911	5935	GKPMLDFETPTYITAKES QQMQSGK			Oxidation (M)[4]		Mascot

9	ATP-dependent RNA helicase Dhx29 OS=Mus musculus GN=Dhx29 PE=2 SV=1	DHX29_MOUSE	155019.5	8.13	26	61	60.827	31.256

Peptide Information

Calc. Mass	Obsrv. Mass	± da	± ppm	Start Seq.	End Seq.	Sequence	Ion Score	C. I. %	Modification	Rank	Result Type
891.4352	891.4346	-0.0006	-1	480	486	METNKPR			Oxidation (M)[1]		Mascot
905.4363	905.4636	0.0273	30	392	399	SPNPSFEK					Mascot
986.549	986.4833	-0.0657	-67	972	980	ASALQRQGR					Mascot
1114.5198	1114.5746	0.0548	49	1055	1064	IGACEPNEPK			Carbamidomethyl (C)[4]		Mascot
1308.7345	1308.6594	-0.0751	-57	674	684	LLYCTTGVLLR			Carbamidomethyl (C)[4]		Mascot
1323.6461	1323.6578	0.0117	9	218	228	KEETTVEVNMK			Oxidation (M)[10]		Mascot
1357.7111	1357.7043	-0.0068	-5	388	399	NLPKSPNPSFEK					Mascot
1388.7686	1388.7308	-0.0378	-27	376	386	SPKQFLIDWVR					Mascot
1388.7686	1388.7308	-0.0378	-27	376	386	SPKQFLIDWVR					Mascot
1402.7219	1402.733	0.0111	8	10	24	APGAAAMRAAVSASR			Oxidation (M)[7]		Mascot
1410.6968	1410.6998	0.003	2	1227	1238	IMCTKSVDVTEK			Carbamidomethyl (C)[3]		Mascot
1487.8805	1487.7531	-0.1274	-86	40	54	KPVARPAPAVPTGAR					Mascot
1530.8527	1530.795	-0.0577	-38	1200	1213	GPQTLSFQDIALLK					Mascot
1577.8184	1577.8138	-0.0046	-3	256	268	FDPNQRYLNLAAR					Mascot
1577.8184	1577.8138	-0.0046	-3	256	268	FDPNQRYLNLAAR					Mascot
1590.7395	1590.8282	0.0887	56	249	261	GLEEEEKFDPNQR					Mascot
1619.7561	1619.8365	0.0804	50	358	370	EPHDVRNFDYTAR					Mascot
1619.7561	1619.8365	0.0804	50	358	370	EPHDVRNFDYTAR					Mascot
1629.7472	1629.8319	0.0847	52	655	667	NSLCGYQIRMESR			Carbamidomethyl (C)[4], Oxidation (M)[10]		Mascot
1674.8561	1674.8303	-0.0258	-15	122	135	LQDLYMALQAFSFK					Mascot
1726.7402	1726.8116	0.0714	41	234	248	YAEQQDEEEKGEGSK					Mascot
1735.9418	1735.9298	-0.012	-7	1306	1320	LLSVDGWIYFQAPVK					Mascot
1747.951	1747.9216	-0.0294	-17	1168	1182	TSLLTLEDVKQELMK					Mascot
1760.8735	1760.9535	0.08	45	725	740	SDLHLILMSATVDSDK			Oxidation (M)[8]		Mascot
1763.9459	1763.9388	-0.0071	-4	1168	1182	TSLLTLEDVKQELMK			Oxidation (M)[14]		Mascot
1896.9922	1896.9996	0.0074	4	1214	1231	AVLAAGLYDSVGKIMCTK			Carbamidomethyl (C)[16]		Mascot
1954.9956	1955.0406	0.045	23	741	756	FSTYFTHCPILRISGR			Carbamidomethyl (C)[8]		Mascot
1973.1682	1973.0276	-0.1406	-71	707	723	SVQSDFLLVILKEILQK					Mascot
1973.1682	1973.0276	-0.1406	-71	707	723	SVQSDFLLVILKEILQK					Mascot

2001.13	2001.002	-0.128	-64	1345	1361	MSLENDKILQIITELIK					Mascot
2021.0854	2020.9664	-0.119	-59	1304	1320	ERLLSVDGWIYFQAPVK					Mascot

10	CinA-like protein OS=Leptospira borgpetersenii serovar Hardjo-bovis (strain L550) GN=LBL_1257 PE=3 SV=1	CINAL_LEPBL	46328.6	5.67	7	60	51.807	13.217	42	92.679

Peptide Information

Calc. Mass	Obsrv. Mass	± da	± ppm	Start Seq.	End Seq.	Sequence	Ion Score	C. I. %	Modification	Rank	Result Type
871.4308	871.4444	0.0136	16	188	194	ELYSGFR					Mascot
1098.6194	1098.5739	-0.0455	-41	174	182	EELAPWILK					Mascot
1308.6365	1308.6594	0.0229	17	117	127	NFQEAMQTAIR					Mascot
1370.7791	1370.7581	-0.021	-15	214	227	AIADGKAIWGVAAK					Mascot
1388.7744	1388.7308	-0.0436	-31	6	19	VIVVSTGSELTAGR					Mascot
1388.7744	1388.7308	-0.0436	-31	6	19	VIVVSTGSELTAGR	42	92.679			Mascot
1591.8361	1591.8215	-0.0146	-9	275	290	ITIGTAESCTGGLIAK			Carbamidomethyl (C)[9]		Mascot
2023.043	2023.0468	0.0038	2	117	133	NFQEAMQTAIRQVFVPK			Oxidation (M)[6]		Mascot

Gel Idx/Pos	188/H15	Instr./Gel Origin	BA2151/Sample Project 20140814	Process Status	Analysis Succeeded
Plate [#] Name	[1] Sample Project 20140814	Instrument Sample Name		Spectra	11

Rank	Protein Name	Accession No.	Protein MW	Protein PI	Pep. Count	Protein Score	Protein Score C. I. %	Intensity Matched	Total Ion Score	Total Ion C. I. %	Confirmed
1	GTP-binding protein Rheb homolog 1 OS=Caenorhabditis briggsae GN=rheb-1 PE=3 SV=1	RHEB1_CAEBR	23704.8	6.22	11	69	93.499	2.231			

Peptide Information

Calc. Mass	Obsrv. Mass	± da	± ppm	Start Seq.	End Seq.	Sequence	Ion Score	C. I. %	Modification	Rank	Result Type
849.3883	849.4256	0.0373	44	128	134	CDLGTQR			Carbamidomethyl (C)[1]		Mascot
916.4734	916.4604	-0.013	-14	135	142	VVQQEEGK					Mascot
1231.7046	1231.608	-0.0966	-78	163	172	VNEVFELLLR					Mascot
1301.7035	1301.6265	-0.077	-59	15	26	KVAVMGYPHVGK			Oxidation (M)[5]		Mascot
1335.7015	1335.6426	-0.0589	-44	152	162	FVEITARETNR					Mascot
1348.609	1348.6592	0.0502	37	99	109	SFDICSSIYEK			Carbamidomethyl (C)[5]		Mascot
1357.7322	1357.7228	-0.0094	-7	135	146	VVQQEEGKELAK					Mascot
1364.6329	1364.6674	0.0345	25	42	52	YETTIEDQHTK					Mascot
1898.0997	1897.9833	-0.1164	-61	110	127	IISVYGDPSIPIIIVGNK					Mascot
2357.1521	2357.2146	0.0625	27	60	79	DFNLRVTDTAGQQEYTV FPR					Mascot
2501.1401	2501.2498	0.1097	44	33	52	FTQNMFPDRYETTIEDQH TK					Mascot

Rank	Protein Name	Accession No.	Protein MW	Protein PI	Pep. Count	Protein Score	Protein Score C. I. %	Intensity Matched	Total Ion Score	Total Ion C. I. %	Confirmed
2	Glycine--tRNA ligase beta subunit OS=Actinobacillus pleuropneumoniae serotype 5b (strain L20) GN=glyS PE=3 SV=1	SYGB_ACTP2	76503	5.13	17	62	65.882	8.328			

Peptide Information

Calc. Mass	Obsrv. Mass	± da	± ppm	Start Seq.	End Seq.	Sequence	Ion Score	C. I. %	Modification	Rank	Result Type
807.4359	807.3835	-0.0524	-65	507	514	SAALFGNK					Mascot
816.4322	816.4616	0.0294	36	340	345	QRLEDR					Mascot
923.4226	923.3699	-0.0527	-57	147	153	MMRWGDK					Mascot
942.5255	942.4316	-0.0939	-100	120	128	AVIEGQPTK					Mascot
1089.58	1089.5977	0.0177	16	473	482	GSADPFALRR					Mascot
1231.61	1231.608	-0.002	-2	655	665	VMVNAEDANLR					Mascot
1233.6508	1233.6416	-0.0092	-7	446	457	SLVACSVALADK			Carbamidomethyl (C)[5]		Mascot
1233.6508	1233.6416	-0.0092	-7	446	457	SLVACSVALADK			Carbamidomethyl (C)[5]		Mascot
1247.6049	1247.6178	0.0129	10	655	665	VMVNAEDANLR			Oxidation (M)[2]		Mascot

Calc. Mass	Obsrv. Mass	± da	± ppm	Start Seq.	End Seq.	Sequence	Ion Score	C. I. %	Modification	Rank	Result Type
1259.6478	1259.6282	-0.0196	-16	593	603	VEGEISIEIDR					Mascot
1265.6307	1265.6317	0.001	1	213	223	GMVVADFNERK					Mascot
1265.6307	1265.6317	0.001	1	213	223	GMVVADFNERK					Mascot
1373.6736	1373.6976	0.024	17	283	293	GDQKYFPIYDK					Mascot
1768.9803	1768.8287	-0.1516	-86	368	385	IEALAGEIAAQIGADKAK					Mascot
1898.0342	1897.9833	-0.0509	-27	365	383	TARIEALAGEIAAQIGADK					Mascot
1997.0413	1997.0109	-0.0304	-15	269	286	FLAVPAEALVYTMKGDQ K			Oxidation (M)[13]		Mascot
2012.1652	2012.0647	-0.1005	-50	346	362	LPRLETVLFQQQLGTLR					Mascot
2035.0052	2034.9897	-0.0155	-8	1	18	MTTQNFLAEIGTEELPPK			Oxidation (M)[1]		Mascot
2341.113	2341.2322	0.1192	51	646	665	ETVDNFFEKVMVNAEDA NLR					Mascot
2357.1079	2357.2146	0.1067	45	646	665	ETVDNFFEKVMVNAEDA NLR			Oxidation (M)[11]		Mascot
2453.1831	2453.1562	-0.0269	-11	23	44	LATAFAENVENELNQADL SFEK					Mascot

3	Vacuolar protein sorting-associated protein 54 OS=Rattus norvegicus GN=Vps54 PE=2 SV=1	VPS54_RAT	109913.5	6.04	18	59	37.916	21.828

Peptide Information

Calc. Mass	Obsrv. Mass	± da	± ppm	Start Seq.	End Seq.	Sequence	Ion Score	C. I. %	Modification	Rank	Result Type
816.4461	816.4616	0.0155	19	22	28	KEVDPTK					Mascot
888.5037	888.4516	-0.0521	-59	575	582	LTDLELGK					Mascot
967.5393	967.4514	-0.0879	-91	602	609	AVKFLMSR			Oxidation (M)[6]		Mascot
1219.6907	1219.6005	-0.0902	-74	135	144	TLLHIHDKSR					Mascot
1233.6124	1233.6416	0.0292	24	659	667	FVNRFHEER					Mascot
1233.6124	1233.6416	0.0292	24	659	667	FVNRFHEER					Mascot
1265.681	1265.6317	-0.0493	-39	849	859	LIAIMDSLFDK					Mascot
1265.681	1265.6317	-0.0493	-39	849	859	LIAIMDSLFDK	9	0			Mascot
1301.7423	1301.6265	-0.1158	-89	669	679	TKLSLLLDNER					Mascot
1304.7533	1304.6792	-0.0741	-57	789	800	TITTKNLALSSR					Mascot
1304.7533	1304.6792	-0.0741	-57	789	800	TITTKNLALSSR					Mascot
1329.6871	1329.6992	0.0121	9	885	895	MHEAIFDLLPK			Oxidation (M)[1]		Mascot
1373.8152	1373.6976	-0.1176	-86	370	381	LVSLVFGLLEQR					Mascot
1501.9102	1501.7865	-0.1237	-82	370	382	LVSLVFGLLEQRK					Mascot
1782.9419	1782.9763	0.0344	19	326	340	HLGSQLCELEKLIDK			Carbamidomethyl (C)[7]		Mascot
1827.8616	1827.7991	-0.0625	-34	629	643	LMETYIVDTEQICGR			Carbamidomethyl (C)[13]		Mascot
2034.9194	2034.9897	0.0703	35	864	880	CEVEAPAPSPCFRNICK			Carbamidomethyl (C)[1,11,16]		Mascot
2166.0461	2166.1382	0.0921	43	2	22	ASSHSSSPVPQGSSSDV FFKK					Mascot

2248.1616	2248.1287	-0.0329	-15	423	440	LADQMRMLNFPQWIDLLK			Oxidation (M)[5]		Mascot
2313.0815	2313.186	0.1045	45	1	22	MASSHSSSPVPQGSSSDVFFKK			Oxidation (M)[1]		Mascot
2313.0815	2313.186	0.1045	45	1	22	MASSHSSSPVPQGSSSDVFFKK			Oxidation (M)[1]		Mascot
2335.2856	2335.158	-0.1276	-55	801	819	CLQLIVHYIPVIRAHFEAR			Carbamidomethyl (C)[1]		Mascot

4	Sterol 3-beta-glucosyltransferase OS=Neosartorya fumigata (strain ATCC MYA-4609 / Af293 / CBS 101355 / FGSC A1100) GN=atg26 PE=3 SV=2	ATG26_ASPFU	158277.9	6.46	22	59	31.926	5.646	20	0

Peptide Information

Calc. Mass	Obsrv. Mass	± da	± ppm	Start Seq.	End Seq.	Sequence	Ion Score	C. I. %	Modification	Rank	Result Type
807.3591	807.3835	0.0244	30	611	618	TDGNTSGR					Mascot
856.4635	856.386	-0.0775	-90	145	151	RTISDHK					Mascot
888.54	888.4516	-0.0884	-99	1170	1177	TVIESVLK					Mascot
948.4493	948.4637	0.0144	15	583	590	RNSDETAR					Mascot
1001.5778	1001.5844	0.0066	7	374	381	EWVKALQK					Mascot
1108.5455	1108.6488	0.1033	93	1	9	MRPFLDDAK			Oxidation (M)[1]		Mascot
1203.5675	1203.5669	-0.0006	0	1380	1390	AISDADMLPDR					Mascot
1203.5675	1203.5669	-0.0006	0	1380	1390	AISDADMLPDR	20	0			Mascot
1219.5624	1219.6005	0.0381	31	1380	1390	AISDADMLPDR			Oxidation (M)[7]		Mascot
1249.6365	1249.6362	-0.0003	0	302	310	YSRYWFSLK					Mascot
1259.6888	1259.6282	-0.0606	-48	1291	1301	MIIRAQDLGAR			Oxidation (M)[1]		Mascot
1264.6393	1264.6171	-0.0222	-18	482	492	NRWSLTSGTSR					Mascot
1287.7572	1287.6305	-0.1267	-98	794	804	FGYHGLVIIIR					Mascot
1302.7263	1302.6642	-0.0621	-48	1079	1090	NELGLKATTLDK					Mascot
1303.7369	1303.6538	-0.0831	-64	283	294	STVAIKSGYLHK					Mascot
1319.7253	1319.6538	-0.0715	-54	763	773	LCFRSLIPGTR			Carbamidomethyl (C)[2]		Mascot
1320.6139	1320.673	0.0591	45	584	595	NSDETARSPSTR					Mascot
1329.7737	1329.6992	-0.0745	-56	1170	1181	TVIESVLKADVR					Mascot
1342.7074	1342.6519	-0.0555	-41	223	235	GPRDASVSPLSTR					Mascot
1348.6964	1348.6592	-0.0372	-28	1261	1272	VEDLGVGICMKK			Carbamidomethyl (C)[9]		Mascot
1364.6913	1364.6674	-0.0239	-18	1261	1272	VEDLGVGICMKK			Carbamidomethyl (C)[9], Oxidation (M)[10]		Mascot
1926.9226	1926.8667	-0.0559	-29	195	211	MVEAQAQFDLKASSTER			Oxidation (M)[1]		Mascot
2335.0562	2335.158	0.1018	44	1049	1067	MGGAYNYITYVMFDNVFWK			Oxidation (M)[1]		Mascot
2355.1221	2355.1719	0.0498	21	58	78	EGHLQYMQQSIFGMIAAVGSR			Oxidation (M)[7,14]		Mascot
2389.2834	2389.1343	-0.1491	-62	450	470	ILINDQSLKISSQHLSPQPDR					Mascot

5	Threonine--tRNA ligase OS=Marinomonas sp. (strain MWYL1) GN=thrS PE=3 SV=1	SYT_MARMS	74244.2	5.43	18	57	0	18.033

Peptide Information

Calc. Mass	Obsrv. Mass	± da	± ppm	Start Seq.	End Seq.	Sequence	Ion Score	C. I. %	Modification	Rank	Result Type
807.4108	807.3835	-0.0273	-34	562	568	LNSNGFR					Mascot
839.4145	839.3613	-0.0532	-63	130	136	TDYDVVK					Mascot
888.4608	888.4516	-0.0092	-10	138	145	MTPIAEAR					Mascot
979.5571	979.4996	-0.0575	-59	228	235	ELKGYLTR					Mascot
1120.6072	1120.5626	-0.0446	-40	590	599	VPYMIVVGDK					Mascot
1182.6478	1182.5615	-0.0863	-73	2	12	PVITLPDGSQR					Mascot
1302.7067	1302.6642	-0.0425	-33	73	84	HSFAHLVGHAVK					Mascot
1308.6794	1308.6771	-0.0023	-2	231	241	GYLTRIEEAEK					Mascot
1326.6953	1326.6379	-0.0574	-43	302	312	VLWEKSGHWGK					Mascot
1329.6831	1329.6992	0.0161	12	1	12	MPVITLPDGSQR			Oxidation (M)[1]		Mascot
1336.6301	1336.6696	0.0395	30	157	168	VALIDDMDESTK					Mascot
1336.6301	1336.6696	0.0395	30	157	168	VALIDDMDESTK					Mascot
1722.7839	1722.8527	0.0688	40	350	363	DLPFRMAEFGSCHR			Carbamidomethyl (C)[12]		Mascot
1822.9956	1822.9637	-0.0319	-17	505	520	QTPVMLHRAIVGSLER			Oxidation (M)[5]		Mascot
1994.8081	1994.9933	0.1852	93	169	183	EVGLYYHEEYMDMCR			Carbamidomethyl (C)[14]		Mascot
1996.9294	1997.0109	0.0815	41	201	217	LAGAYWRGDSNNEMLQ R			Oxidation (M)[14]		Mascot
2296.1221	2296.2537	0.1316	57	251	269	LDLFHVQEEAPGMVFWH PK			Oxidation (M)[13]		Mascot
2357.0696	2357.2146	0.145	62	355	375	MAEFGSCHRNEPSGALH GIMR			Carbamidomethyl (C)[7]		Mascot
2373.0645	2373.1953	0.1308	55	355	375	MAEFGSCHRNEPSGALH GIMR			Carbamidomethyl (C)[7], Oxidation (M)[1]		Mascot
2389.0596	2389.1343	0.0747	31	355	375	MAEFGSCHRNEPSGALH GIMR			Carbamidomethyl (C)[7], Oxidation (M)[1,20]		Mascot
2437.1753	2437.1423	-0.033	-14	472	492	DAIGRVWQCGTIQVDFS MPTR			Carbamidomethyl (C)[9]		Mascot
2437.1753	2437.1423	-0.033	-14	472	492	DAIGRVWQCGTIQVDFS MPTR			Carbamidomethyl (C)[9]		Mascot
2453.1702	2453.1562	-0.014	-6	472	492	DAIGRVWQCGTIQVDFS MPTR			Carbamidomethyl (C)[9], Oxidation (M)[18]		Mascot

6	Protein Brevis radix-like 1 OS=Oryza sativa subsp. japonica GN=BRXL1 PE=2 SV=1	BRXL1_ORYSJ	44403.3	5.5	13	56	0	2.648

Peptide Information

Calc. Mass	Obsrv. Mass	± da	± ppm	Start Seq.	End Seq.	Sequence	Ion Score	C. I. %	Modification	Rank	Result Type
807.373	807.3835	0.0105	13	221	227	SEDESLK					Mascot

856.41	856.386	-0.024	-28	99	104	FHYAYR					Mascot
979.5683	979.4996	-0.0687	-70	10	19	QLAGGAPPLR					Mascot
1182.5936	1182.5615	-0.0321	-27	55	65	DMALKASGAYR					Mascot
1231.4929	1231.608	0.1151	93	306	316	TETSSMDASMR			Oxidation (M)[6]		Mascot
1259.6281	1259.6282	0.0001	0	383	391	LWWEENRAR					Mascot
1302.745	1302.6642	-0.0808	-62	48	59	ALTAQIKDMALK					Mascot
1318.7399	1318.66	-0.0799	-61	48	59	ALTAQIKDMALK			Oxidation (M)[9]		Mascot
1319.6855	1319.6538	-0.0317	-24	182	191	EIFNKWQAQR					Mascot
1335.6135	1335.6426	0.0291	22	317	328	SSSSPEEVDRSR					Mascot
1371.7267	1371.7052	-0.0215	-16	209	220	FNQQTPLPTTPK					Mascot
1768.7899	1768.8287	0.0388	22	84	98	RHHPYHAYADSGSDR					Mascot
1794.8347	1794.7769	-0.0578	-32	187	199	WQAQRWWAENYEK					Mascot
1958.9158	1958.8638	-0.052	-27	298	316	LSSISGAKTETSSMDASM R					Mascot

7	Uncharacterized protein UL27 OS=Human cytomegalovirus (strain Merlin) GN=UL27 PE=3 SV=1	UL27_HCMVM	70126	9.33	15	56	0	7.917

Peptide Information

Calc. Mass	Obsrv. Mass	± da	± ppm	Start Seq.	End Seq.	Sequence	Ion Score	C. I. %	Modification	Rank	Result Type
807.4471	807.3835	-0.0636	-79	450	455	ELSRFR					Mascot
867.4683	867.4164	-0.0519	-60	317	323	LEHAELR					Mascot
934.4893	934.4159	-0.0734	-79	153	160	WLNAGAFR					Mascot
1152.5505	1152.5472	-0.0033	-3	532	541	HGDAPQDRTR					Mascot
1231.7058	1231.608	-0.0978	-79	329	339	HLGAFHLPAIR					Mascot
1265.696	1265.6317	-0.0643	-51	317	326	LEHAELRLER					Mascot
1265.696	1265.6317	-0.0643	-51	317	326	LEHAELRLER					Mascot
1287.7202	1287.6305	-0.0897	-70	418	428	MLSGDVQRLIR					Mascot
1303.7151	1303.6538	-0.0613	-47	418	428	MLSGDVQRLIR			Oxidation (M)[1]		Mascot
1320.7383	1320.673	-0.0653	-49	513	524	APPTPEAVRVQR					Mascot
1501.8499	1501.7865	-0.0634	-42	327	339	NRHLGAFHLPAIR					Mascot
1768.9526	1768.8287	-0.1239	-70	384	399	RGYAMYLASNAVLALR					Mascot
1827.9382	1827.7991	-0.1391	-76	72	89	AGLLCRTPEDLAAAGGQ K			Carbamidomethyl (C)[5]		Mascot
1968.016	1967.9647	-0.0513	-26	257	274	AAVFFHATFMARAEAALK			Oxidation (M)[10]		Mascot
1995.1208	1994.9933	-0.1275	-64	385	402	GYAMYLASNAVLALRIIR					Mascot
2341.1494	2341.2322	0.0828	35	1	21	MNPVDQPPPPPLTQQPE EQAK					Mascot
2357.1443	2357.2146	0.0703	30	1	21	MNPVDQPPPPPLTQQPE EQAK			Oxidation (M)[1]		Mascot

2373.1365	2373.1953	0.0588	25	542	562	LPQFSSALSDAELSNHAN RCR			Carbamidomethyl (C)[20]		Mascot

8	Nuclear receptor corepressor 2 OS=Mus musculus GN=Ncor2 PE=1 SV=3	NCOR2_MOUSE	270325.9	7	29	54	0	9.853	15	0		

Peptide Information

Calc. Mass	Obsrv. Mass	± da	± ppm	Start Seq.	End Seq.	Sequence	Ion Score	C. I. %	Modification	Rank	Result Type
807.4471	807.3835	-0.0636	-79	1119	1125	HPGVLER					Mascot
979.5207	979.4996	-0.0211	-22	1587	1595	GVTGVDLYR					Mascot
1253.5354	1253.5465	0.0111	9	611	620	WTEEEMETAK					Mascot
1264.6757	1264.6171	-0.0586	-46	1390	1400	EAGRSIHEIPR					Mascot
1265.6121	1265.6317	0.0196	15	1265	1277	SSSGPPHETAAPK					Mascot
1265.6121	1265.6317	0.0196	15	1265	1277	SSSGPPHETAAPK	15	0			Mascot
1290.5743	1290.6478	0.0735	57	2203	2214	DGEQGEPRMGSK					Mascot
1317.6547	1317.6649	0.0102	8	2	13	SGSTQPVAQTWR					Mascot
1318.7842	1318.66	-0.1242	-94	1495	1507	GAPVVVPELGKPR					Mascot
1319.6888	1319.6538	-0.035	-27	629	640	NWSAIARMVGSK					Mascot
1331.8046	1331.6841	-0.1205	-90	1406	1417	TPELPLAPRPLK					Mascot
1335.6838	1335.6426	-0.0412	-31	629	640	NWSAIARMVGSK			Oxidation (M)[8]		Mascot
1346.5828	1346.6832	0.1004	75	1279	1289	TYDMMEGRVGR			Oxidation (M)[4,5]		Mascot
1348.6855	1348.6592	-0.0263	-20	1688	1700	ESSLALNYAAGPR					Mascot
1349.7059	1349.644	-0.0619	-46	225	235	SLVQIIYDENR					Mascot
1351.7076	1351.6572	-0.0504	-37	79	89	SQELHLRPESR					Mascot
1358.6547	1358.6569	0.0022	2	1925	1937	EPASSPSKSSEPR					Mascot
1378.7438	1378.699	-0.0448	-32	1394	1404	SIHEIPREELR					Mascot
1381.6304	1381.6381	0.0077	6	611	621	WTEEEMETAKK					Mascot
1407.6322	1407.702	0.0698	50	427	437	QVTNMWSEQER					Mascot
1501.7322	1501.7865	0.0543	36	331	341	EYYEKQFPEIR					Mascot
1541.6797	1541.8086	0.1289	84	409	421	FINMNGLMDDPMK			Oxidation (M)[4]		Mascot
1722.8042	1722.8527	0.0485	28	1261	1277	EDGRSSSGPPHETAAPK					Mascot
1782.8588	1782.9763	0.1175	66	407	421	IKFINMNGLMDDPMK			Oxidation (M)[6]		Mascot
1823.0215	1822.9637	-0.0578	-32	1055	1070	WSSGLPFPIPPREVIK					Mascot
1926.8763	1926.8667	-0.0096	-5	427	441	QVTNMWSEQERDTFR					Mascot
2035.0582	2034.9897	-0.0685	-34	444	460	FMQHPKNFGLIASFLER					Mascot
2335.1929	2335.158	-0.0349	-15	145	166	LEPVSPPSPPHADPELEL APSR					Mascot
2404.2878	2404.197	-0.0908	-38	1119	1140	HPGVLERQLGAISQQGM SVQLR					Mascot
2437.3159	2437.1423	-0.1736	-71	1482	1507	SGTSSGAGGSITRGAPV					Mascot

						VVPELGKPR						
2437.3159	2437.1423	-0.1736	-71	1482	1507	SGTSSGAGGSITRGAPV VVPELGKPR						Mascot
2501.3042	2501.2498	-0.0544	-22	1126	1148	QLGAISQQGMSVQLRVP HSEHAK						Mascot

9	Elongation factor Ts OS=Thiobacillus denitrificans (strain ATCC 25259) GN=tsf PE=3 SV=1	EFTS_THIDA	31132.1	5.82	12	54	0	4.263

Peptide Information

Calc. Mass	Obsrv. Mass	± da	± ppm	Start Seq.	End Seq.	Sequence	Ion Score	C. I. %	Modification	Rank	Result Type
979.4996	979.4996	0	0	152	160	FASYIHGGK					Mascot
1120.5303	1120.5626	0.0323	29	26	36	ALTEAGGDMQK					Mascot
1233.7202	1233.6416	-0.0786	-64	223	233	IAEGTVQKFLK					Mascot
1233.7202	1233.6416	-0.0786	-64	223	233	IAEGTVQKFLK					Mascot
1249.6423	1249.6362	-0.0061	-5	277	289	VSDFAAEVAAAAK					Mascot
1264.6202	1264.6171	-0.0031	-2	25	36	KALTEAGGDMQK			Oxidation (M)[10]		Mascot
1302.6901	1302.6642	-0.0259	-20	245	255	DDKQTVEQVLK					Mascot
1348.7107	1348.6592	-0.0515	-38	277	290	VSDFAAEVAAAAKV					Mascot
1349.7094	1349.644	-0.0654	-48	2	13	AEITASMVKDLR			Oxidation (M)[7]		Mascot
1770.8037	1770.8367	0.033	19	72	87	SAAMVEVNCETDFVAK			Carbamidomethyl (C)[9]		Mascot
1786.7987	1786.8215	0.0228	13	72	87	SAAMVEVNCETDFVAK			Carbamidomethyl (C)[9], Oxidation (M)[4]		Mascot
1898.0342	1897.9833	-0.0509	-27	52	71	SAGRVAAEGVVTIAISPD GK					Mascot
2029.0634	2029.0111	-0.0523	-26	212	230	EAGKPEAMLEKIAEGTVQ K					Mascot
2271.0532	2271.2261	0.1729	76	256	275	SKNSQCHGFTMYVVGEG IEK			Carbamidomethyl (C)[6]		Mascot

10	Vacuolar protein sorting-associated protein 21 OS=Saccharomyces cerevisiae (strain ATCC 204508 / S288c) GN=VPS21 PE=1 SV=1	VPS21_YEAST	23180.8	5.25	9	53	0	3.579

Peptide Information

Calc. Mass	Obsrv. Mass	± da	± ppm	Start Seq.	End Seq.	Sequence	Ion Score	C. I. %	Modification	Rank	Result Type
849.4676	849.4256	-0.042	-49	2	9	NTSVTSIK					Mascot
1291.6311	1291.6179	-0.0132	-10	122	132	IDMLQEGGERK			Oxidation (M)[3]		Mascot
1303.7006	1303.6538	-0.0468	-36	37	48	EPTIGAAFLTQR					Mascot
1335.5771	1335.6426	0.0655	49	175	186	TAEEQNSASNER					Mascot
1351.6277	1351.6572	0.0295	22	58	68	FEIWDTAGQER					Mascot
1786.8929	1786.8215	-0.0714	-40	171	186	IPLKTAEEQNSASNER					Mascot
1826.728	1826.769	0.041	22	193	210	VDLNAANDGTSANSACS C			Carbamidomethyl (C)[16,18]		Mascot

2325.2561	2325.1631	-0.093	-40	37	57	EPTIGAAFLTQRVTINEHT VK	Mascot
2373.1833	2373.1953	0.012	5	49	68	VTINEHTVKFEIWDTAGQ ER	Mascot

Rank	Protein Name	Accession No.	Protein MW	Protein PI	Pep. Count	Protein Score	Protein Score C. I. %	Intensity Matched	Total Ion Score	Total Ion C. I. %	Confirmed
1	Chaperone protein HtpG OS=Nitrosococcus oceani (strain ATCC 19707 / NCIMB 11848) GN=htpG PE=3 SV=1	HTPG_NITOC	72322.1	5.13	18	55	0	6.789			

Peptide Information

Calc. Mass	Obsrv. Mass	± da	± ppm	Start Seq.	End Seq.	Sequence	Ion Score	C. I. %	Modification	Rank	Result Type
854.4077	854.351	-0.0567	-66	383	389	EFGQVMK			Oxidation (M)[6]		Mascot
886.4741	886.459	-0.0151	-17	343	349	EILQNNR					Mascot
896.506	896.4395	-0.0665	-74	532	538	EVRVTHR					Mascot
1033.5103	1033.5269	0.0166	16	290	297	APFDLWER					Mascot
1060.5707	1060.5751	0.0044	4	625	634	LNSLLLAMAD					Mascot
1118.6125	1118.5343	-0.0782	-70	362	371	ILGLLEDMAK			Oxidation (M)[8]		Mascot
1182.6127	1182.5759	-0.0368	-31	146	156	RAGFGPEHGVR					Mascot
1201.694	1201.5886	-0.1054	-88	615	624	LEEPALFVKR					Mascot
1203.5852	1203.5775	-0.0077	-6	37	48	ELISNGADAADK					Mascot
1232.6667	1232.6047	-0.062	-50	624	634	RLNSLLLAMAD			Oxidation (M)[9]		Mascot
1265.6372	1265.6492	0.012	9	107	118	AFLESLSGDQAK					Mascot
1304.6382	1304.6917	0.0535	41	290	299	APFDLWERDR					Mascot
1323.7856	1323.6796	-0.106	-80	173	184	ATRGTVVTLHLR					Mascot
1334.6733	1334.65	-0.0233	-17	78	90	TLTIADNGIGMGR			Oxidation (M)[11]		Mascot
1472.7704	1472.7251	-0.0453	-31	37	50	ELISNGADAADKLR					Mascot
1490.7744	1490.7582	-0.0162	-11	77	90	RTLTIADNGIGMGR			Oxidation (M)[12]		Mascot
1693.8043	1693.7076	-0.0967	-57	377	389	YQSFWKEFGQVMK			Oxidation (M)[12]		Mascot
1838.8337	1838.9313	0.0976	53	383	399	EFGQVMKEGVGEDSGNR					Mascot

Rank	Protein Name	Accession No.	Protein MW	Protein PI	Pep. Count	Protein Score	Protein Score C. I. %	Intensity Matched	Total Ion Score	Total Ion C. I. %	Confirmed
2	Protein translocase subunit SecA OS=Maricaulis maris (strain MCS10) GN=secA PE=3 SV=1	SECA_MARMM	107824.7	5.18	20	55	0	9.194			

Peptide Information

Calc. Mass	Obsrv. Mass	± da	± ppm	Start Seq.	End Seq.	Sequence	Ion Score	C. I. %	Modification	Rank	Result Type
847.4594	847.4501	-0.0093	-11	489	495	TLPEMLK			Oxidation (M)[5]		Mascot
856.5251	856.5345	0.0094	11	220	227	TPLIISGR					Mascot

Calc. Mass	Obsrv. Mass	± da	± ppm	Start Seq.	End Seq.	Sequence	Ion Score	C. I. %	Modification	Rank	Result Type
870.5308	870.553	0.0222	26	302	308	AHKIFVR					Mascot
896.3567	896.4395	0.0828	92	138	144	DSEWMGR			Oxidation (M)[5]		Mascot
1060.527	1060.5751	0.0481	45	561	569	TPTQTEIDR					Mascot
1068.5758	1068.526	-0.0498	-47	454	462	LMSDKALFK			Oxidation (M)[2]		Mascot
1201.6106	1201.5886	-0.022	-18	787	797	AANAGPELMRR			Oxidation (M)[9]		Mascot
1325.6188	1325.6858	0.067	51	850	860	MMDNLRGEVTK			Oxidation (M)[1,2]		Mascot
1472.7566	1472.7251	-0.0315	-21	464	476	MLGHLYPKDEAAK					Mascot
1592.8279	1592.7451	-0.0828	-52	718	732	QSTAEDLVSAHIPPK					Mascot
1592.8279	1592.7451	-0.0828	-52	718	732	QSTAEDLVSAHIPPK					Mascot
1693.8214	1693.7076	-0.1138	-67	421	434	YAAIIDEIVDCQQR			Carbamidomethyl (C)[11]		Mascot
1818.9419	1818.8596	-0.0823	-45	59	75	LDALMPEAFAAVREAAK			Oxidation (M)[5]		Mascot
1886.8888	1886.7604	-0.1284	-68	841	855	TEAFNLFERMMDNLR					Mascot
1942.9514	1942.8821	-0.0693	-36	646	662	TLGMKEGEAIQHPWMSK					Mascot
1958.9463	1958.8876	-0.0587	-30	646	662	TLGMKEGEAIQHPWMSK			Oxidation (M)[4]		Mascot
1997.045	1997.0073	-0.0377	-19	870	888	APAPPKEPEGLTATHIDPR					Mascot
2012.1023	2012.0151	-0.0872	-43	435	453	GQPVLVGTASIEKSEIIDR					Mascot
2020.9944	2021.0497	0.0553	27	316	333	DNAVVLIDEFTGRMMPGR					Mascot
2094.0034	2093.9812	-0.0222	-11	892	911	NEMADPGDGPVAPRVAPTQR			Oxidation (M)[3]		Mascot
2094.0034	2093.9812	-0.0222	-11	892	911	NEMADPGDGPVAPRVAPTQR			Oxidation (M)[3]		Mascot
2313.1431	2313.1821	0.039	17	695	714	AVFEQRIDFMVSDDVADVIK			Oxidation (M)[10]		Mascot
2815.4304	2815.1584	-0.272	-97	77	102	ALGLRPYDVQLMGGMVLHEGSIAEMK					Mascot

3	Elongation factor G OS=Clostridium phytofermentans (strain ATCC 700394 / DSM 18823 / ISDg) GN=fusA PE=3 SV=1	EFG_CLOPH	78595.4	5.08	18	54	0	6.391

Peptide Information

Calc. Mass	Obsrv. Mass	± da	± ppm	Start Seq.	End Seq.	Sequence	Ion Score	C. I. %	Modification	Rank	Result Type
806.4042	806.431	0.0268	33	5	10	EYPLER					Mascot
816.4461	816.4059	-0.0402	-49	204	210	GEQIEIK					Mascot
824.4335	824.4014	-0.0321	-39	596	603	IAGSMAFK					Mascot
847.3866	847.4501	0.0635	75	211	217	EIPDDMK					Mascot
849.3672	849.432	0.0648	76	515	521	GQYGHCK			Carbamidomethyl (C)[6]		Mascot
910.3901	910.4533	0.0632	69	218	224	DQAEEYR					Mascot
993.5074	993.4911	-0.0163	-16	327	335	IMADPFVGK			Oxidation (M)[2]		Mascot
1090.564	1090.593	0.029	27	135	143	QADKYNVPR					Mascot

1233.6514	1233.6423	-0.0091	-7	31	40	ILYYTGVNYK					Mascot
1237.5994	1237.6304	0.031	25	1	10	MAGREYPLER			Oxidation (M)[1]		Mascot
1265.6559	1265.6492	-0.0067	-5	111	122	VLDSAVGVFCAK			Carbamidomethyl (C)[10]		Mascot
1334.6019	1334.65	0.0481	36	510	521	QSGGRGQYGHCK			Carbamidomethyl (C)[11]		Mascot
1406.6919	1406.6732	-0.0187	-13	596	608	IAGSMAFKDAMHK					Mascot
1627.8666	1627.8448	-0.0218	-13	327	340	IMADPFVGKLAFFR			Oxidation (M)[2]		Mascot
1786.8719	1786.8252	-0.0467	-26	123	138	GGVEPQSETVWRQADK					Mascot
1927.0066	1926.8881	-0.1185	-61	13	30	NIGIMAHIDAGKTTLSER					Mascot
1943.0015	1942.8821	-0.1194	-61	13	30	NIGIMAHIDAGKTTLSER			Oxidation (M)[5]		Mascot
2182.0273	2182.1174	0.0901	41	675	693	TQGRGAYSMFFSTYEPV PK			Oxidation (M)[9]		Mascot
2322.1111	2322.1763	0.0652	28	679	698	GAYSMFFSTYEPVPKNV QEK					Mascot

4	Uncharacterized protein B0304.2 OS=Caenorhabditis elegans GN=B0304.2 PE=4 SV=1	YT22_CAEEL	24329.3	10.85	12	54	0	5.405	

Peptide Information

Calc. Mass	Obsrv. Mass	± da	± ppm	Start Seq.	End Seq.	Sequence	Ion Score	C. I. %	Modification	Rank	Result Type
816.421	816.4059	-0.0151	-18	188	194	QSPLESR					Mascot
993.4921	993.4911	-0.001	-1	56	64	SSMVLSPEK			Oxidation (M)[3]		Mascot
1090.5925	1090.593	0.0005	0	174	183	KMATPSQSLK					Mascot
1106.5874	1106.5249	-0.0625	-56	174	183	KMATPSQSLK			Oxidation (M)[2]		Mascot
1118.5986	1118.5343	-0.0643	-57	175	184	MATPSQSLKR					Mascot
1133.5984	1133.6221	0.0237	21	56	65	SSMVLSPEKR					Mascot
1182.5862	1182.5759	-0.0103	-9	148	157	RTENGSIYSR					Mascot
1201.6172	1201.5886	-0.0286	-24	185	194	QEKQSPLESR					Mascot
1231.7046	1231.6066	-0.098	-80	160	172	LGATFSPLVAGAK					Mascot
1232.623	1232.6047	-0.0183	-15	68	78	LNTSNDSNLVR					Mascot
1319.6451	1319.6652	0.0201	15	149	159	TENGSIYSRHR					Mascot
1336.708	1336.6875	-0.0205	-15	188	199	QSPLESRHGGLR					Mascot
1336.708	1336.6875	-0.0205	-15	188	199	QSPLESRHGGLR					Mascot
1501.8081	1501.8094	0.0013	1	66	78	LRLNTSNDSNLVR					Mascot

5	Adenylate kinase OS=Lodderomyces elongisporus (strain ATCC 11503 / CBS 2605 / JCM 1781 / NBRC 1676 / NRRL YB-4239) GN=ADK1 PE=3 SV=1	KAD2_LODEL	28423.9	8.22	12	54	0	7.038	

Peptide Information

Calc. Mass	Obsrv. Mass	± da	± ppm	Start Seq.	End Seq.	Sequence	Ion Score	C. I. %	Modification	Rank	Result Type

Calc. Mass	Obsrv. Mass	± da	± ppm	Start Seq.	End Seq.	Sequence	Ion Score	C. I. %	Modification	Rank	Result Type
849.4312	849.432	0.0008	1	51	58	GTQSTDLK					Mascot
886.5356	886.459	-0.0766	-86	17	23	IQQLEKK					Mascot
993.5033	993.4911	-0.0122	-12	134	141	LDSMLKDR			Oxidation (M)[4]		Mascot
1090.5375	1090.593	0.0555	51	199	208	SDDNEAALKK					Mascot
1127.6306	1127.5261	-0.1045	-93	143	152	TPLENAIELK					Mascot
1203.6521	1203.5775	-0.0746	-62	174	183	SYHKIFSPPK					Mascot
1284.6794	1284.5867	-0.0927	-72	2	12	SVEELKDTVHK					Mascot
1415.72	1415.656	-0.064	-45	1	12	MSVEELKDTVHK					Mascot
1472.8108	1472.7251	-0.0857	-58	23	36	KVGVLPSSQPDFAK					Mascot
1794.8085	1794.8135	0.005	3	59	73	DKFCACHLATGDMLR			Carbamidomethyl (C)[4,6]		Mascot
2013.0917	2013.0465	-0.0452	-22	227	244	TGIWQGIDASQKPAKVW K					Mascot
2013.0917	2013.0465	-0.0452	-22	227	244	TGIWQGIDASQKPAKVW K					Mascot
2110.0967	2109.9753	-0.1214	-58	209	225	RLVTYHAQTEPIVEYYK					Mascot
2110.0967	2109.9753	-0.1214	-58	209	225	RLVTYHAQTEPIVEYYK					Mascot

6	RAF proto-oncogene serine/threonine-protein kinase OS=Pongo abelii GN=RAF1 PE=2 SV=1	RAF1_PONAB	73803.2	9.33	16	53	0	10.187	

Protein Group

RAF proto-oncogene serine/threonine-protein kinase OS=Homo sapiens GN=RAF1 PE=1 SV=1	RAF1_HUMAN	73803.2	9.3299 999237 0605

Peptide Information

Calc. Mass	Obsrv. Mass	± da	± ppm	Start Seq.	End Seq.	Sequence	Ion Score	C. I. %	Modification	Rank	Result Type
847.4341	847.4501	0.016	19	276	282	MIEDAIR					Mascot
864.4355	864.4284	-0.0071	-8	217	223	MRESVSR					Mascot
928.5574	928.4812	-0.0762	-82	392	399	NEVAVLRK					Mascot
960.5295	960.4807	-0.0488	-51	583	590	RLVADCVK			Carbamidomethyl (C)[6]		Mascot
1078.5714	1078.5558	-0.0156	-14	555	563	DQIIFMVGR					Mascot
1201.6172	1201.5886	-0.0286	-24	317	327	ERAPVSGTQEK					Mascot
1233.5958	1233.6423	0.0465	38	42	53	ASDDGKLTDPSK					Mascot
1265.7365	1265.6492	-0.0873	-69	368	378	WHGDVAVKILK					Mascot
1336.6136	1336.6875	0.0739	55	90	100	GLQPECCAVFR			Carbamidomethyl (C)[6,7]		Mascot
1336.6136	1336.6875	0.0739	55	90	100	GLQPECCAVFR			Carbamidomethyl (C)[6,7]		Mascot
1381.7297	1381.6605	-0.0692	-50	440	450	FQMFQLIDIAR					Mascot
1942.7993	1942.8821	0.0828	43	165	179	CQTCGYKFHEHCSTK			Carbamidomethyl (C)[1,4,12]		Mascot
1997.0161	1997.0073	-0.0088	-4	555	572	DQIIFMVGRGYASPDLSK					Mascot

2012.0131	2012.0151	0.002	1	451	467	QTAQGMDYLHAKNIIHR			Oxidation (M)[6]		Mascot
2013.011	2013.0465	0.0355	18	555	572	DQIIFMVGRGYASPDLSK			Oxidation (M)[6]		Mascot
2013.011	2013.0465	0.0355	18	555	572	DQIIFMVGRGYASPDLSK	3	0	Oxidation (M)[6]		Mascot
2094.0371	2093.9812	-0.0559	-27	90	106	GLQPECCAVFRLLHEHK			Carbamidomethyl (C)[6,7]		Mascot
2094.0371	2093.9812	-0.0559	-27	90	106	GLQPECCAVFRLLHEHK			Carbamidomethyl (C)[6,7]		Mascot
2181.9646	2182.1174	0.1528	70	337	354	DSSYYWEIEASEVMLSTR			Oxidation (M)[14]		Mascot
2313.1616	2313.1821	0.0205	9	255	275	QRSTSTPNVHMVSTTLPVDSR					Mascot

7	Uncharacterized protein YqbO OS=Bacillus subtilis (strain 168) GN=yqbO PE=1 SV=2	YQBO_BACSU	171075.8	9.74	25	52	0	11.643	

Peptide Information

Calc. Mass	Obsrv. Mass	± da	± ppm	Start Seq.	End Seq.	Sequence	Ion Score	C. I. %	Modification	Rank	Result Type
807.4141	807.4138	-0.0003	0	44	49	TERVMR			Oxidation (M)[5]		Mascot
847.5036	847.4501	-0.0535	-63	962	968	SIWKSVK					Mascot
960.536	960.4807	-0.0553	-58	1315	1323	GLDSVDVKK					Mascot
1033.5273	1033.5269	-0.0004	0	243	252	NLSQQTGASK					Mascot
1078.535	1078.5558	0.0208	19	1201	1209	MPFQNTVNK					Mascot
1090.6367	1090.593	-0.0437	-40	1488	1497	GRYLLAQAAK					Mascot
1133.6348	1133.6221	-0.0127	-11	1071	1081	GKMLLSQAASK					Mascot
1145.6022	1145.5854	-0.0168	-15	682	692	SGRTVGTNPTR					Mascot
1182.575	1182.5759	0.0009	1	1290	1299	EELDIHSPSR					Mascot
1193.6525	1193.6261	-0.0264	-22	814	823	KLNSTLFDQK					Mascot
1203.6077	1203.5775	-0.0302	-25	685	695	TVGTNPTRDSR					Mascot
1232.6667	1232.6047	-0.062	-50	328	339	LTGGQIANAMIK			Oxidation (M)[10]		Mascot
1284.6583	1284.5867	-0.0716	-56	293	303	QSGDLIAYVYR					Mascot
1304.6627	1304.6917	0.029	22	62	72	ARLLEMGLDDR			Oxidation (M)[6]		Mascot
1379.6591	1379.6825	0.0234	17	918	929	WDSASSVWQSVK					Mascot
1490.6362	1490.7582	0.122	82	461	473	DNNPMTPVNDAMR			Oxidation (M)[5]		Mascot
1818.8175	1818.8596	0.0421	23	198	214	STMSQDAYVSATSNVNK			Oxidation (M)[3]		Mascot
1838.9066	1838.9313	0.0247	13	1328	1347	QAGSLAAAYSGMGAVSGNVK					Mascot
1956.963	1956.9739	0.0109	6	1290	1306	EELDIHSPSRVMMSLGR					Mascot
2011.9542	2012.0151	0.0609	30	1157	1174	NVGQPMNNLISYSPNYGK			Oxidation (M)[6]		Mascot
2094.0039	2093.9812	-0.0227	-11	873	890	SWAQNKWNSASSVWESVK					Mascot
2094.0039	2093.9812	-0.0227	-11	873	890	SWAQNKWNSASSVWESVK					Mascot
2313.0122	2313.1821	0.1699	73	461	480	DNNPMTPVNDAMRDFES			Oxidation (M)[5,12]		Mascot

						ISK					
2501.2268	2501.2637	0.0369	15	1175	1198	QVVNGYAKGQNSTSTGT DGFLQTK					Mascot
2501.2268	2501.2637	0.0369	15	1175	1198	QVVNGYAKGQNSTSTGT DGFLQTK					Mascot
2815.3105	2815.1584	-0.1521	-54	1538	1561	EVIIQFNGDQHFHNDQD MNSLVAK			Oxidation (M)[18]		Mascot
3263.6716	3263.3474	-0.3242	-99	417	447	AAIATELIGTQYEDLKQPI LDMAEGIGTSAK			Oxidation (M)[22]		Mascot

8	Alanine--tRNA ligase OS=Burkholderia sp. (strain 383) GN=alaS PE=3 SV=2	SYA_BURS3	95745.8	5.68	18	52	0	10.292

Peptide Information

Calc. Mass	Obsrv. Mass	± da	± ppm	Start Seq.	End Seq.	Sequence	Ion Score	C. I. %	Modification	Rank	Result Type
816.4573	816.4059	-0.0514	-63	3	9	AAEIREK					Mascot
864.4825	864.4284	-0.0541	-63	518	525	FAVADTLK					Mascot
886.4629	886.459	-0.0039	-4	142	149	EVGVPTER					Mascot
906.5043	906.4753	-0.029	-32	655	662	VLDLGFSR					Mascot
928.5574	928.4812	-0.0762	-82	150	157	IIRIGDNK					Mascot
967.5068	967.4578	-0.049	-51	550	557	AEIDAHRR					Mascot
993.4999	993.4911	-0.0088	-9	705	712	YVQELDAR					Mascot
1108.611	1108.6591	0.0481	43	576	585	EVLGAHVQQK					Mascot
1140.6736	1140.5684	-0.1052	-92	681	692	IVVEGGVAAGIR					Mascot
1233.6838	1233.6423	-0.0415	-34	385	396	GGKVLDGELAFK					Mascot
1320.7059	1320.6492	-0.0567	-43	13	23	FFESKGHTIVR					Mascot
1522.7649	1522.6498	-0.1151	-76	47	59	DVFLGTDPRPYSR					Mascot
1592.853	1592.7451	-0.1079	-68	469	483	IVALYVDGSSVNEVK					Mascot
1592.853	1592.7451	-0.1079	-68	469	483	IVALYVDGSSVNEVK					Mascot
1794.7753	1794.8135	0.0382	21	596	610	FDFAHNAPMTDDEIR			Oxidation (M)[9]		Mascot
1926.832	1926.8881	0.0561	29	414	430	ERGMTVDEPAFDDAMAR			Oxidation (M)[4]		Mascot
1942.827	1942.8821	0.0551	28	414	430	ERGMTVDEPAFDDAMAR			Oxidation (M)[4,15]		Mascot
1950.8763	1950.9423	0.066	34	596	611	FDFAHNAPMTDDEIRR			Oxidation (M)[9]		Mascot
1966.9441	1966.9479	0.0038	2	663	680	ELCGGTHVSRSGDIGFFK			Carbamidomethyl (C)[3]		Mascot
2012.9821	2013.0465	0.0644	32	630	648	VMPYDEAVKGGAMALFG EK					Mascot
2012.9821	2013.0465	0.0644	32	630	648	VMPYDEAVKGGAMALFG EK					Mascot

9	Porphobilinogen deaminase 2 OS=Streptomyces coelicolor (strain ATCC BAA-471 / A3(2) / M145) GN=hemC2 PE=3 SV=1	HEM32_STRCO	33677.6	6.42	11	52	0	5.402

Peptide Information

Calc. Mass	Obsrv. Mass	± da	± ppm	Start	End	Sequence	Ion	C. I. %	Modification	Rank	Result Type

Calc. Mass	Obsrv. Mass	± da	± ppm	Start Seq.	End Seq.	Sequence	Ion Score	C. I. %	Modification	Rank	Result Type
867.457	867.4467	-0.0103	-12	306	313	EIIDGIAH					Mascot
1133.6426	1133.6221	-0.0205	-18	28	38	AELAALHPGVR					Mascot
1203.6337	1203.5775	-0.0562	-47	1	10	MRMSAPELIR					Mascot
1231.6318	1231.6066	-0.0252	-20	51	61	WLGDLSQVEGK					Mascot
1232.594	1232.6047	0.0107	9	15	25	DSPMALAQVER			Oxidation (M)[4]		Mascot
1301.7061	1301.6255	-0.0806	-62	39	50	TEVVPVRTTGDK					Mascot
1308.692	1308.6833	-0.0087	-7	275	285	VRLNAHEWAGR					Mascot
1371.7777	1371.7228	-0.0549	-40	3	14	MSAPELIRIVSR					Mascot
1487.7635	1487.7646	0.0011	1	15	27	DSPMALAQVERVR			Oxidation (M)[4]		Mascot
1997.0009	1997.0073	0.0064	3	67	85	EVDAALLSGEADLAVHCV K			Carbamidomethyl (C)[17]		Mascot
2501.2705	2501.2637	-0.0068	-3	62	85	GAFTKEVDAALLSGEADL AVHCVK			Carbamidomethyl (C)[22]		Mascot
2501.2705	2501.2637	-0.0068	-3	62	85	GAFTKEVDAALLSGEADL AVHCVK			Carbamidomethyl (C)[22]		Mascot

10 GTP-binding protein Rheb homolog 1 OS=Caenorhabditis briggsae GN=rheb-1 PE=3 SV=1 | RHEB1_CAEBR | 23704.8 | 6.22 | 9 | 52 | 0 | 3.98

Peptide Information

Calc. Mass	Obsrv. Mass	± da	± ppm	Start Seq.	End Seq.	Sequence	Ion Score	C. I. %	Modification	Rank	Result Type
847.4268	847.4501	0.0233	27	2	9	SSTATQPR					Mascot
849.3883	849.432	0.0437	51	128	134	CDLGTQR			Carbamidomethyl (C)[1]		Mascot
1231.7046	1231.6066	-0.098	-80	163	172	VNEVFELLLR					Mascot
1301.7035	1301.6255	-0.078	-60	15	26	KVAVMGYPHVGK			Oxidation (M)[5]		Mascot
1323.7128	1323.6796	-0.0332	-25	179	190	GNLRGPPQQQTK					Mascot
1348.609	1348.7158	0.1068	79	99	109	SFDICSSIYEK			Carbamidomethyl (C)[5]		Mascot
1627.7856	1627.8448	0.0592	36	1	14	MSSTATQPRYSLNR			Oxidation (M)[1]		Mascot
1827.9899	1827.8334	-0.1565	-86	16	32	VAVMGYPHVGKSSIVQR					Mascot
2501.1401	2501.2637	0.1236	49	33	52	FTQNMFPDRYETTIEDQH TK					Mascot
2501.1401	2501.2637	0.1236	49	33	52	FTQNMFPDRYETTIEDQH TK					Mascot

Gel Idx/Pos	190/H17	Instr./Gel Origin	BA2151/Sample Project 20140814	Process Status	Analysis Succeeded
Plate [#] Name	[1] Sample Project 20140814	Instrument Sample Name		Spectra	11

Rank	Protein Name	Accession No.	Protein MW	Protein PI	Pep. Count	Protein Score	Protein Score C. I. %	Intensity Matched	Total Ion Score	Total Ion C. I. %	Confirmed
1	Serpin-Z2B OS=Triticum aestivum PE=1 SV=1	SPZ2B_WHEAT	43011.4	5.18	5	105	99.998	8.133	93	100	

Peptide Information

Calc. Mass	Obsrv. Mass	± da	± ppm	Start Seq.	End Seq.	Sequence	Ion Score	C. I. %	Modification	Rank	Result Type
925.5214	925.5336	0.0122	13	11	18	LSIAHQTR					Mascot
925.5214	925.5336	0.0122	13	11	18	LSIAHQTR					Mascot
1005.5363	1005.5119	-0.0244	-24	230	238	LPYKQGGDK					Mascot
1223.5903	1223.6508	0.0605	49	127	137	AEAQSVDFQTK					Mascot
1258.7253	1258.7123	-0.013	-10	289	300	ISLGIEASDLLK					Mascot
1665.8595	1665.8937	0.0342	21	261	274	LSAEPEFLEQHIPR					Mascot
1665.8595	1665.8937	0.0342	21	261	274	LSAEPEFLEQHIPR	93	100			Mascot

Rank	Protein Name	Accession No.	Protein MW	Protein PI	Pep. Count	Protein Score	Protein Score C. I. %	Intensity Matched	Total Ion Score	Total Ion C. I. %	Confirmed
2	Keratin, type II cytoskeletal 75 OS=Mus musculus GN=Krt75 PE=1 SV=1	K2C75_MOUSE	59932	8.46	19	79	99.303	5.555			

Peptide Information

Calc. Mass	Obsrv. Mass	± da	± ppm	Start Seq.	End Seq.	Sequence	Ion Score	C. I. %	Modification	Rank	Result Type
816.4872	816.4369	-0.0503	-62	374	379	MIQRLR					Mascot
835.4091	835.4909	0.0818	98	424	430	AKQDMAR			Oxidation (M)[5]		Mascot
852.4574	852.4548	-0.0026	-3	32	39	FSSVSVAR					Mascot
888.4785	888.4644	-0.0141	-16	529	538	GPVGSGSSIK					Mascot
929.5163	929.4803	-0.036	-39	220	227	GRLDAELR					Mascot
972.5472	972.4764	-0.0708	-73	403	411	GELALKDAR					Mascot
1005.4669	1005.5119	0.045	45	455	462	LLEGEECR			Carbamidomethyl (C)[7]		Mascot
1066.5164	1066.5413	0.0249	23	240	247	YEDEINKR					Mascot
1136.6171	1136.5609	-0.0562	-49	61	70	SLYNLGGTRR					Mascot
1189.6133	1189.6564	0.0431	36	414	423	LMELEDALQK					Mascot
1205.6083	1205.6394	0.0311	26	414	423	LMELEDALQK			Oxidation (M)[2]		Mascot
1263.6943	1263.6604	-0.0339	-27	443	453	LALDVEIATYR					Mascot
1302.7152	1302.686	-0.0292	-22	314	325	SLDLDSIIAEVK					Mascot
1304.6958	1304.691	-0.0048	-4	247	258	RAAAENEFVGLK					Mascot
1323.6587	1323.6802	0.0215	16	70	82	RVSIGGCAGSGFR			Carbamidomethyl (C)[7]		Mascot

Calc. Mass	Obsrv. Mass	± da	± ppm	Start Seq.	End Seq.	Sequence	Ion Score	C. I. %	Modification	Rank	Result Type
1349.6478	1349.7034	0.0556	41	363	373	NTKQEISEMNR					Mascot
1455.6863	1455.7954	0.1091	75	335	346	SRAEAESWYQTK					Mascot
1493.7417	1493.7631	0.0214	14	228	239	NMQEVVEDFKVR					Mascot
1693.781	1693.9113	0.1303	77	388	402	QCSSLQTAISDAEQR			Carbamidomethyl (C)[2]		Mascot
1693.781	1693.9113	0.1303	77	388	402	QCSSLQTAISDAEQR			Carbamidomethyl (C)[2]		Mascot
1739.8923	1739.9204	0.0281	16	529	546	GPVGSGSSIKFVSSTSSR					Mascot

3	Keratin, type II cytoskeletal 1 OS=Canis familiaris GN=KRT1 PE=1 SV=1	K2C1_CANFA	63922.1	7.66	16	68	92.184	4.982	

Peptide Information

Calc. Mass	Obsrv. Mass	± da	± ppm	Start Seq.	End Seq.	Sequence	Ion Score	C. I. %	Modification	Rank	Result Type
802.4417	802.4518	0.0101	13	181	186	EREQIK					Mascot
836.3931	836.4257	0.0326	39	455	461	AKEDMAR			Oxidation (M)[5]		Mascot
874.4993	874.4697	-0.0296	-34	68	76	SLVNLGGSK					Mascot
905.4938	905.5015	0.0077	9	411	418	SEIDSVKK					Mascot
1003.5353	1003.5573	0.022	22	457	464	EDMARLLR					Mascot
1026.4562	1026.5063	0.0501	49	291	299	DVDAAFMNK			Oxidation (M)[7]		Mascot
1033.516	1033.5393	0.0233	23	485	493	TLLEGEESR					Mascot
1066.5164	1066.5413	0.0249	23	271	278	YEDEINKR					Mascot
1117.5961	1117.6404	0.0443	40	77	89	SISISVAGGGGGR					Mascot
1318.7101	1318.6818	-0.0283	-21	345	356	SLDLDSIISEVK					Mascot
1323.6573	1323.6802	0.0229	17	394	404	STKMEISELNR			Oxidation (M)[4]		Mascot
1393.7322	1393.6858	-0.0464	-33	279	290	TNAENEFVTIKK					Mascot
1475.7489	1475.7739	0.025	17	213	224	WELLQQVDTSTR	17	0			Mascot
1664.8313	1664.8496	0.0183	11	291	305	DVDAAFMNKVDLQAK					Mascot
1673.8453	1673.7948	-0.0505	-30	419	433	QISALQQSISDAEQR					Mascot
1828.7893	1828.8724	0.0831	45	46	67	FSGGRCGGGGGGGAGG GGFGSR			Carbamidomethyl (C)[6]		Mascot
1828.7893	1828.8724	0.0831	45	46	67	FSGGRCGGGGGGGAGG GGFGSR			Carbamidomethyl (C)[6]		Mascot

4	Retinoic acid receptor RXR-alpha OS=Xenopus laevis GN=rxra PE=1 SV=1	RXRA_XENLA	54290	8.11	16	68	91.026	5.317	

Peptide Information

Calc. Mass	Obsrv. Mass	± da	± ppm	Start Seq.	End Seq.	Sequence	Ion Score	C. I. %	Modification	Rank	Result Type
810.3671	810.4369	0.0698	86	221	227	CLAMGMK			Carbamidomethyl (C)[1]		Mascot
836.3931	836.4257	0.0326	39	18	24	TCADTLR			Carbamidomethyl (C)[2]		Mascot

846.4104	846.4658	0.0554	65	434	440	YPEQPGR				Mascot
848.4294	848.4423	0.0129	15	391	397	TELGCLR		Carbamidomethyl (C)[5]		Mascot
888.54	888.4644	-0.0756	-85	375	382	VLTELVSK				Mascot
982.4631	982.4998	0.0367	37	221	228	CLAMGMKR		Carbamidomethyl (C)[1], Oxidation (M)[4]		Mascot
1016.5119	1016.5201	0.0082	8	228	235	REAVQEER				Mascot
1070.4427	1070.5375	0.0948	89	383	390	MRDMQMDK		Oxidation (M)[1]		Mascot
1103.6096	1103.594	-0.0156	-14	398	407	AIVLFNPDSK				Mascot
1144.5706	1144.5996	0.029	25	229	237	EAVQEERQR				Mascot
1205.519	1205.6394	0.1204	100	213	220	CQYCRYQK		Carbamidomethyl (C)[1,4]		Mascot
1245.5789	1245.6302	0.0513	41	218	227	YQKCLAMGMK		Carbamidomethyl (C)[4], Oxidation (M)[7]		Mascot
1687.8585	1687.8617	0.0032	2	2	17	SSAAMDTKHFLPLGGR				Mascot
1703.8534	1703.8182	-0.0352	-21	2	17	SSAAMDTKHFLPLGGR		Oxidation (M)[5]		Mascot
1738.9349	1738.8406	-0.0943	-54	453	466	SIGLKCLEHLFFFK		Carbamidomethyl (C)[6]		Mascot
1818.8989	1818.9581	0.0592	33	1	17	MSSAAMDTKHFLPLGGR				Mascot
1838.799	1838.9561	0.1571	85	172	186	HYGVYSCEGCKGFFK		Carbamidomethyl (C)[7,10]		Mascot

5 FYVE and coiled-coil domain-containing protein 1 OS=Homo sapiens GN=FYCO1 PE=1 SV=3 | FYCO1_HUMAN | 168590 | 4.86 | 30 | 68 | 90.817 | 9.208

Peptide Information

Calc. Mass	Obsrv. Mass	± da	± ppm	Start Seq.	End Seq.	Sequence	Ion Score	C. I. %	Modification	Rank	Result Type
806.3825	806.4419	0.0594	74	253	258	MQQLDR			Oxidation (M)[1]		Mascot
808.3683	808.439	0.0707	87	379	386	ADTASDTK					Mascot
816.4614	816.4369	-0.0245	-30	139	145	SPFLQPK					Mascot
829.5142	829.4441	-0.0701	-85	825	831	TLVQQLK					Mascot
844.525	844.5013	-0.0237	-28	61	68	ATLLGNKK					Mascot
872.52	872.4808	-0.0392	-45	537	543	KQLIQDK					Mascot
888.4533	888.4644	0.0111	12	943	949	EREGLER					Mascot
914.5054	914.4769	-0.0285	-31	624	631	ELQNVVGR					Mascot
916.4482	916.4991	0.0509	56	1030	1037	GQLEEQGR					Mascot
929.505	929.4803	-0.0247	-27	1430	1437	ENIQGQLK					Mascot
974.5265	974.515	-0.0115	-12	415	422	TKVEEVNR					Mascot
1003.5207	1003.5573	0.0366	36	1147	1154	DKDALWQK					Mascot
1015.5418	1015.5372	-0.0046	-5	502	509	ELLEQEVR					Mascot
1021.4908	1021.5103	0.0195	19	379	388	ADTASDTKGR					Mascot
1065.5211	1065.5699	0.0488	46	1155	1163	SDALEFQQK					Mascot
1088.5443	1088.587	0.0427	39	281	290	GRTAAEDNVR					Mascot

1126.5463	1126.5544	0.0081	7	1185	1192	EFSWMVRR			Oxidation (M)[5]		Mascot
1136.5438	1136.5609	0.0171	15	689	698	EAMKAQMAEK					Mascot
1184.6746	1184.5907	-0.0839	-71	1430	1439	ENIQGQLKVR					Mascot
1223.7107	1223.6508	-0.0599	-49	822	831	EHKTLVQQLK					Mascot
1258.6387	1258.7123	0.0736	58	1080	1090	EGAALREDLER					Mascot
1304.6376	1304.691	0.0534	41	1109	1118	LCQEVTNRER			Carbamidomethyl (C)[2]		Mascot
1347.6461	1347.7191	0.073	54	451	462	EMAPLQEELSGK			Oxidation (M)[2]		Mascot
1412.6951	1412.6868	-0.0083	-6	544	555	DHLSQQVGMLER					Mascot
1687.8936	1687.8617	-0.0319	-19	448	462	LVKEMAPLQEELSGK			Oxidation (M)[5]		Mascot
1739.7556	1739.9204	0.1648	95	68	80	KDYWDYFCACLAK			Carbamidomethyl (C)[8,10]		Mascot
1828.9261	1828.8724	-0.0537	-29	1005	1019	FQLSAEIMDYQSRLK					Mascot
1828.9473	1828.8724	-0.0749	-41	340	355	LESMLQPLAQELEATR					Mascot
1838.8241	1838.9561	0.132	72	69	82	DYWDYFCACLAKVK			Carbamidomethyl (C)[7,9]		Mascot
1926.9644	1926.903	-0.0614	-32	362	378	NQHLASFPGWLAMAQQ K					Mascot

6	Golgin subfamily B member 1 OS=Homo sapiens GN=GOLGB1 PE=1 SV=2	GOGB1_HUMAN	377215.2	4.96	53	67	89.931	15.915

Peptide Information

Calc. Mass	Obsrv. Mass	± da	± ppm	Start Seq.	End Seq.	Sequence	Ion Score	C. I. %	Modification	Rank	Result Type
800.4737	800.4326	-0.0411	-51	1479	1484	QQIQRK					Mascot
802.4669	802.4518	-0.0151	-19	2204	2210	VIDEAKK					Mascot
803.4006	803.4647	0.0641	80	987	992	ENEQRK					Mascot
812.3858	812.4508	0.065	80	110	115	YIEEMK					Mascot
824.4512	824.4363	-0.0149	-18	2760	2766	KYDASLK					Mascot
826.4893	826.465	-0.0243	-29	854	860	VRHISSK					Mascot
829.4526	829.4441	-0.0085	-10	2773	2779	EQGLLNR					Mascot
831.457	831.4246	-0.0324	-39	1494	1500	EALKENK					Mascot
846.4679	846.4658	-0.0021	-2	2103	2109	KELQSNK					Mascot
848.4108	848.4423	0.0315	37	308	314	NTVETER					Mascot
872.4948	872.4808	-0.014	-16	2510	2516	EEIRGLR					Mascot
874.5356	874.4697	-0.0659	-75	102	109	AKLTSLNK					Mascot
878.4214	878.4695	0.0481	55	663	670	STKQDGDK					Mascot
884.5312	884.4904	-0.0408	-46	1235	1241	QLQIQVR					Mascot
888.4672	888.4644	-0.0028	-3	1849	1856	IAGLEEEK					Mascot
916.5098	916.4991	-0.0107	-12	3155	3161	KLLEEER					Mascot
943.5571	943.4969	-0.0602	-64	2806	2813	LNQQLLSK					Mascot

972.5472	972.4764	-0.0708	-73	933	940	EQLNLLSR		Mascot
989.5336	989.5588	0.0252	25	878	885	MDQLLLEK		Mascot
989.5336	989.5588	0.0252	25	878	885	MDQLLLEK		Mascot
1003.5054	1003.5573	0.0519	52	1770	1778	QANLEATEK		Mascot
1005.5285	1005.5119	-0.0166	-17	878	885	MDQLLLEK	Oxidation (M)[1]	Mascot
1009.4731	1009.5217	0.0486	48	2739	2747	SMSSLQNSR		Mascot
1013.5084	1013.5285	0.0201	20	2373	2380	LHEEINMK		Mascot
1015.553	1015.5372	-0.0158	-16	3148	3155	QEVNELRK		Mascot
1016.5054	1016.5201	0.0147	14	3228	3235	SLCHSRTR	Carbamidomethyl (C)[3]	Mascot
1021.4731	1021.5103	0.0372	36	2882	2890	AMSSLQNDR		Mascot
1026.4739	1026.5063	0.0324	32	1042	1049	ENKEYSEK		Mascot
1060.5569	1060.5763	0.0194	18	2023	2030	QLQKDCIR	Carbamidomethyl (C)[6]	Mascot
1065.5211	1065.5699	0.0488	46	2589	2597	SFNALQEEK		Mascot
1066.4912	1066.5413	0.0501	47	1207	1214	QQKDDYNR		Mascot
1088.5834	1088.587	0.0036	3	1574	1583	LALEGLTEDK		Mascot
1103.5844	1103.594	0.0096	9	363	371	YSALEQKHK		Mascot
1117.6285	1117.6404	0.0119	11	878	886	MDQLLLEKK		Mascot
1136.5616	1136.5609	-0.0007	-1	1549	1557	DKLITEMDR	Oxidation (M)[7]	Mascot
1144.6208	1144.5996	-0.0212	-19	1849	1858	IAGLEEEKQK		Mascot
1145.6161	1145.5726	-0.0435	-38	1501	1510	SLQEELSLAR		Mascot
1149.5681	1149.6006	0.0325	28	2881	2890	KAMSSLQNDR		Mascot
1205.6195	1205.6394	0.0199	17	2228	2237	LKEDNCSVLK	Carbamidomethyl (C)[6]	Mascot
1223.6743	1223.6508	-0.0235	-19	2644	2654	LSALFSSSQKR		Mascot
1245.5966	1245.6302	0.0336	27	2242	2251	QMSIHMEELK		Mascot
1308.5961	1308.699	0.1029	79	2882	2892	AMSSLQNDRDR	Oxidation (M)[2]	Mascot
1340.7421	1340.6893	-0.0528	-39	1389	1399	ELQPKLDELQK		Mascot
1347.6135	1347.7191	0.1056	78	2578	2588	ESEEANEDLRR		Mascot
1349.6696	1349.7034	0.0338	25	2476	2486	IVGDYQQLEER		Mascot
1400.7057	1400.6951	-0.0106	-8	975	985	EELQHEFDLLK		Mascot
1412.7169	1412.6868	-0.0301	-21	2257	2267	LEHDKQIWESK		Mascot
1455.824	1455.7954	-0.0286	-20	556	567	HKELSVLLLEMK	Oxidation (M)[11]	Mascot
1475.7336	1475.7739	0.0403	27	1519	1532	SLADVESQVSAQNK		Mascot
1493.7781	1493.7631	-0.015	-10	2928	2939	TKAFQIMQEELR		Mascot
1576.8217	1576.8002	-0.0215	-14	717	729	EISNLNQLIEEFK		Mascot
1693.9232	1693.9113	-0.0119	-7	1374	1388	LESSQLQIAGLEHLR		Mascot
1693.9232	1693.9113	-0.0119	-7	1374	1388	LESSQLQIAGLEHLR		Mascot
1828.9036	1828.8724	-0.0312	-17	72	86	DVQLQQKDEALQEER		Mascot

1828.9036	1828.8724	-0.0312	-17	72	86	DVQLQQKDEALQEER					Mascot
1859.887	1859.8619	-0.0251	-13	1026	1041	EIPLSETERGEVEEDK					Mascot
1927.0244	1926.903	-0.1214	-63	2548	2563	DSQQKQLLEVQLQQNK					Mascot

7	Cysteine--tRNA ligase OS=Thermosipho melanesiensis (strain BI429 / DSM 12029) GN=cysS PE=3 SV=1	SYC_THEM4	53906.7	6.28	18	67	89.697	5.491

Peptide Information

Calc. Mass	Obsrv. Mass	± da	± ppm	Start Seq.	End Seq.	Sequence	Ion Score	C. I. %	Modification	Rank	Result Type
802.457	802.4518	-0.0052	-6	390	395	YHLITR					Mascot
803.4509	803.4647	0.0138	17	170	176	VEVSELK					Mascot
812.4161	812.4508	0.0347	43	108	114	AANFHPR					Mascot
824.3784	824.4363	0.0579	70	434	440	DYNVADK					Mascot
856.5363	856.5348	-0.0015	-2	441	447	IRDALLR					Mascot
874.4628	874.4697	0.0069	8	383	389	EEEALKR					Mascot
886.4305	886.4823	0.0518	58	149	155	EFKDYGK					Mascot
916.4985	916.4991	0.0006	1	121	128	EIVEAVEK					Mascot
943.5207	943.4969	-0.0238	-25	161	169	LEDLVAGAR					Mascot
1103.5885	1103.594	0.0055	5	178	186	NPLDFALWK					Mascot
1136.5253	1136.5609	0.0356	31	373	382	DLNKAMDEGK			Oxidation (M)[6]		Mascot
1223.6783	1223.6508	-0.0275	-22	396	406	VFGSVLGIFER					Mascot
1318.7213	1318.6818	-0.0395	-30	448	460	AGVKILDTSEGTK					Mascot
1347.7267	1347.7191	-0.0076	-6	241	253	AQSEALTGKPFAK					Mascot
1349.6254	1349.7034	0.078	58	377	388	AMDEGKEEEALK					Mascot
1455.7512	1455.7954	0.0442	30	1	13	MAIYITNTESGKK					Mascot
1475.7642	1475.7739	0.0097	7	75	87	IINEANAWGVNFK					Mascot
1693.8254	1693.9113	0.0859	51	301	314	TPIEFSHEIMFNTK					Mascot
1693.8254	1693.9113	0.0859	51	301	314	TPIEFSHEIMFNTK					Mascot

8	Fibronectin type III domain-containing protein 1 OS=Homo sapiens GN=FNDC1 PE=1 SV=4	FNDC1_HUMAN	205889.3	9.35	31	67	89.211	11.601

Peptide Information

Calc. Mass	Obsrv. Mass	± da	± ppm	Start Seq.	End Seq.	Sequence	Ion Score	C. I. %	Modification	Rank	Result Type
802.4417	802.4518	0.0101	13	303	309	EKGELAR					Mascot
808.4675	808.439	-0.0285	-35	653	660	AVGSLHPK					Mascot
826.4893	826.465	-0.0243	-29	565	571	TLRPPSR					Mascot

829.5029	829.4441	-0.0588	-71	504	510	DLLLDLK					Mascot
831.3955	831.4246	0.0291	35	967	974	AQPGSTDR					Mascot
834.4064	834.3451	-0.0613	-73	166	172	VRSSDDR					Mascot
840.4872	840.4402	-0.047	-56	588	594	MPALPRR					Mascot
856.4821	856.5348	0.0527	62	588	594	MPALPRR			Oxidation (M)[1]		Mascot
882.5156	882.46	-0.0556	-63	1150	1158	VPSRAAPGK					Mascot
911.5243	911.4898	-0.0345	-38	586	593	ARMPALPR					Mascot
925.5829	925.5336	-0.0493	-53	45	52	HVKLLSTK					Mascot
925.5829	925.5336	-0.0493	-53	45	52	HVKLLSTK					Mascot
929.4556	929.4803	0.0247	27	1300	1306	QRMMHAR					Mascot
974.5166	974.515	-0.0016	-2	1314	1321	QPARPSYR					Mascot
982.5905	982.4998	-0.0907	-92	564	571	RTLRPPSR					Mascot
989.501	989.5588	0.0578	58	337	345	ISQGERDGK					Mascot
989.501	989.5588	0.0578	58	337	345	ISQGERDGK					Mascot
1009.5135	1009.5217	0.0082	8	205	212	MNYVPLTR			Oxidation (M)[1]		Mascot
1016.5193	1016.5201	0.0008	1	1	10	MAPEAGATLR					Mascot
1070.5338	1070.5375	0.0037	3	1023	1032	DAGRSPSQPR					Mascot
1088.5695	1088.587	0.0175	16	800	809	QAEATAQTLR					Mascot
1117.575	1117.6404	0.0654	59	1651	1660	SDLPPQHAPR					Mascot
1144.6069	1144.5996	-0.0073	-6	675	684	QSPSSVLRDR					Mascot
1155.5793	1155.6353	0.056	48	193	203	GFLLGYGESGR					Mascot
1193.6062	1193.6523	0.0461	39	310	318	WDYKQIANR					Mascot
1245.6699	1245.6302	-0.0397	-32	1650	1660	KSDLPPQHAPR					Mascot
1263.6879	1263.6604	-0.0275	-22	1795	1805	FVGVVLCNSLR			Carbamidomethyl (C)[7]		Mascot
1318.6499	1318.6818	0.0319	24	1322	1333	QGYNGRPNVEGK					Mascot
1323.7128	1323.6802	-0.0326	-25	1334	1346	VLPGSNGKPNGQR					Mascot
1340.7103	1340.6893	-0.021	-16	1	13	MAPEAGATLRAPR					Mascot
1393.6893	1393.6858	-0.0035	-3	205	215	MNYVPLTRDER					Mascot
1673.8354	1673.7948	-0.0406	-24	484	500	APASSQHPSVPASPQGR					Mascot
1703.7845	1703.8182	0.0337	20	1110	1126	HQQVESPTGAGAGGDHR					Mascot
2334.1306	2334.1328	0.0022	1	529	549	KAEELDLQSTEITGEEELGSR					Mascot

9	Phosphoglucan phosphatase LSF1, chloroplastic OS=Arabidopsis thaliana GN=LSF1 PE=1 SV=1	LSF1_ARATH	66269.8	9.17	18	67	89.211	6.512	

Peptide Information

Calc. Mass	Obsrv. Mass	± da	± ppm	Start Seq.	End Seq.	Sequence	Ion Score	C. I. %	Modification	Rank	Result Type

808.4199	808.439	0.0191	24	89	96	FALSADGK					Mascot
810.3992	810.4369	0.0377	47	136	142	DFGDTKK					Mascot
836.4625	836.4257	-0.0368	-44	38	45	GIAYLGSR					Mascot
897.4577	897.4619	0.0042	5	46	52	EKFGFNR					Mascot
916.521	916.4991	-0.0219	-24	190	197	NLLSSNLR					Mascot
989.5699	989.5588	-0.0111	-11	113	121	IIMVGDTLK					Mascot
989.5699	989.5588	-0.0111	-11	113	121	IIMVGDTLK					Mascot
1005.5649	1005.5119	-0.053	-53	113	121	IIMVGDTLK			Oxidation (M)[3]		Mascot
1036.5211	1036.5175	-0.0036	-3	508	515	YIINGDWR					Mascot
1066.5164	1066.5413	0.0249	23	359	367	DADSFDLRK					Mascot
1117.665	1117.6404	-0.0246	-22	113	122	IIMVGDTLKK					Mascot
1136.6058	1136.5609	-0.0449	-40	562	570	VLTESERFR					Mascot
1193.6426	1193.6523	0.0097	8	180	189	GRVSFVTWNK					Mascot
1232.7031	1232.6149	-0.0882	-72	111	121	ARIIMVGDTLK			Oxidation (M)[5]		Mascot
1263.6427	1263.6604	0.0177	14	123	135	ASDSSGGTLVEIK					Mascot
1302.6147	1302.686	0.0713	55	386	396	VFVTCTTGFDR			Carbamidomethyl (C)[5]		Mascot
1347.6475	1347.7191	0.0716	53	280	291	GELSYNHALGMR					Mascot
1605.714	1605.7922	0.0782	49	198	214	ASSQGSGNSGYAAFSSK					Mascot
1828.8429	1828.8724	0.0295	16	16	32	SCPSIMIGSSFRSGNGR			Carbamidomethyl (C)[2], Oxidation (M)[6]		Mascot
1828.8429	1828.8724	0.0295	16	16	32	SCPSIMIGSSFRSGNGR			Carbamidomethyl (C)[2], Oxidation (M)[6]		Mascot
1926.9292	1926.903	-0.0262	-14	123	141	ASDSSGGTLVEIKDFGDT K					Mascot

10 Adenosine deaminase domain-containing protein 1 OS=Mus musculus GN=Adad1 PE=1 SV=1 ADAD1_MOUSE 68367.1 9.01 17 67 88.96 6.108

Peptide Information

Calc. Mass	Obsrv. Mass	± da	± ppm	Start Seq.	End Seq.	Sequence	Ion Score	C. I. %	Modification	Rank	Result Type
808.3981	808.439	0.0409	51	543	550	SGLSMASR					Mascot
824.3931	824.4363	0.0432	52	543	550	SGLSMASR			Oxidation (M)[5]		Mascot
836.364	836.4257	0.0617	74	338	344	NPAMMEK			Oxidation (M)[4]		Mascot
848.436	848.4423	0.0063	7	477	484	VDDALTSK					Mascot
922.4523	922.4858	0.0335	36	1	9	MATAGGSRR			Oxidation (M)[1]		Mascot
974.5015	974.515	0.0135	14	127	133	EFIMKYK			Oxidation (M)[4]		Mascot
1005.5801	1005.5119	-0.0682	-68	124	131	LPKEFIMK					Mascot
1013.5738	1013.5285	-0.0453	-45	468	476	GLEIAINQR					Mascot
1021.575	1021.5103	-0.0647	-63	124	131	LPKEFIMK			Oxidation (M)[7]		Mascot
1033.6041	1033.5393	-0.0648	-63	560	568	FNLLAKEAK					Mascot

1036.5496	1036.5175	-0.0321	-31	55	63	VPSFAQMLK	Oxidation (M)[7]	Mascot
1065.5543	1065.5699	0.0156	15	551	559	LCKAAMLSR	Carbamidomethyl (C)[2], Oxidation (M)[6]	Mascot
1117.6154	1117.6404	0.025	22	322	329	SLLRYFYR		Mascot
1308.7522	1308.699	-0.0532	-41	110	122	GLSSISNPVLPPK		Mascot
1318.7227	1318.6818	-0.0409	-31	309	320	VLHDTHAVVTAR		Mascot
1340.6475	1340.6893	0.0418	31	422	433	VNSMSSSDKLTR	Oxidation (M)[4]	Mascot
1693.8829	1693.9113	0.0284	17	345	359	SIFCTEPASNLLTLK	Carbamidomethyl (C)[4]	Mascot
1693.8829	1693.9113	0.0284	17	345	359	SIFCTEPASNLLTLK	Carbamidomethyl (C)[4]	Mascot
1828.9336	1828.8724	-0.0612	-33	330	344	QLLLFYSKNPAMMEK	Oxidation (M)[12]	Mascot
1828.9336	1828.8724	-0.0612	-33	330	344	QLLLFYSKNPAMMEK	Oxidation (M)[12]	Mascot
2444.3145	2444.1562	-0.1583	-65	17	42	LGLPLAAHLPASLGGEGA KDSVGGEK		Mascot

Gel Idx/Pos	191/H18	Instr./Gel Origin	BA2151/Sample Project 20140814	Process Status	Analysis Succeeded
Plate [#] Name	[1] Sample Project 20140814	Instrument Sample Name		Spectra	11

Rank	Protein Name	Accession No.	Protein MW	Protein PI	Pep. Count	Protein Score	Protein Score C. I. %	Intensity Matched	Total Ion Score	Total Ion C. I. %	Confirmed
1	1-(5-phosphoribosyl)-5-[(5-phosphoribosylamino)methylideneamino] imidazole-4-carboxamide isomerase OS=Corynebacterium glutamicum (strain ATCC 13032 / DSM 20300 / JCM 1318 / LMG 3730 / NCIMB 10025) GN=hisA PE=3 SV=2	HIS4_CORGL	26649.6	4.49	11	64	77.459	3.261			

Peptide Information

Calc. Mass	Obsrv. Mass	± da	± ppm	Start Seq.	End Seq.	Sequence	Ion Score	C. I. %	Modification	Rank	Result Type
856.5251	856.5355	0.0104	12	124	131	IAVDIAVR					Mascot
904.4159	904.4666	0.0507	56	132	138	LEDGEWR					Mascot
906.4098	906.4791	0.0693	76	158	165	LDSQGCAR			Carbamidomethyl (C)[6]		Mascot
1047.4954	1047.5476	0.0522	50	19	28	LDQGEAGTEK					Mascot
1161.5647	1161.6694	0.1047	90	132	140	LEDGEWRTR					Mascot
1320.7046	1320.697	-0.0076	-6	233	244	FTLEEALAAVEK					Mascot
1333.7474	1333.7	-0.0474	-36	120	131	YGEKIAVDIAVR					Mascot
1741.9232	1741.9788	0.0556	32	124	138	IAVDIAVRLEDGEWR					Mascot
2146.0312	2146.0002	-0.031	-14	139	157	TRGNGWVSDGGDLWEVLER					Mascot
2178.1077	2177.9924	-0.1153	-53	214	232	YQDEGIDSVIIGKALYEHK					Mascot
2194.0874	2193.9834	-0.104	-47	19	39	LDQGEAGTEKSYGTPLESALK					Mascot

Rank	Protein Name	Accession No.	Protein MW	Protein PI	Pep. Count	Protein Score	Protein Score C. I. %	Intensity Matched	Total Ion Score	Total Ion C. I. %	Confirmed
2	Cyclic 2,3-diphosphoglycerate synthetase OS=Methanothermobacter thermautotrophicus (strain ATCC 29096 / DSM 1053 / JCM 10044 / NBRC 100330 / Delta H) GN=cpgS PE=1 SV=1	CPGS_METTH	51686.3	4.83	14	63	74.708	4.941			

Peptide Information

Calc. Mass	Obsrv. Mass	± da	± ppm	Start Seq.	End Seq.	Sequence	Ion Score	C. I. %	Modification	Rank	Result Type
813.5192	813.4472	-0.072	-89	78	84	LIGELIR					Mascot
818.4553	818.433	-0.0223	-27	220	226	ILTVGCR			Carbamidomethyl (C)[6]		Mascot
884.4413	884.4766	0.0353	40	288	294	NFFGPFR					Mascot
909.4788	909.446	-0.0328	-36	152	160	TAVSAYAAR					Mascot
1047.5946	1047.5476	-0.047	-45	454	462	AIADFNLRK					Mascot
1048.6038	1048.5896	-0.0142	-14	39	48	ALIFIGGTEK					Mascot

1333.7474	1333.7	-0.0474	-36	108	120	IASVVLGEGAVYR					Mascot
1391.655	1391.7126	0.0576	41	177	189	GGPEEPEIVHGDR					Mascot
1741.8184	1741.9788	0.1604	92	228	243	CGGGMVGDVFITNMKR			Carbamidomethyl (C)[1]		Mascot
1757.8132	1757.9246	0.1114	63	228	243	CGGGMVGDVFITNMKR			Carbamidomethyl (C)[1], Oxidation (M)[5]		Mascot
1958.9688	1959.0446	0.0758	39	161	176	LIHERQYNPCVVAMGR			Carbamidomethyl (C)[10], Oxidation (M)[14]		Mascot
2146.085	2146.0002	-0.0848	-40	418	436	DALEAGLEVIYCDNIPVV R			Carbamidomethyl (C)[12]		Mascot
2581.24	2581.2151	-0.0249	-10	204	226	GVHAASDHWEDALMSRI LTVGCR			Carbamidomethyl (C)[22]		Mascot
2597.2349	2597.2502	0.0153	6	204	226	GVHAASDHWEDALMSRI LTVGCR			Carbamidomethyl (C)[22], Oxidation (M)[14]		Mascot
2687.396	2687.3103	-0.0857	-32	24	48	AAVETLDSMEHIDVKALIF IGGTEK					Mascot
2742.3123	2742.27	-0.0423	-15	1	23	MFMKATETMICLVDGEH YLPVTR			Carbamidomethyl (C)[11]		Mascot

3	Cobyric acid synthase OS=Clostridium difficile (strain 630) GN=cobQ PE=3 SV=1	COBQ_CLOD6	56657.4	5.93	16	61	57.048	9.637	

Peptide Information

Calc. Mass	Obsrv. Mass	± da	± ppm	Start Seq.	End Seq.	Sequence	Ion Score	C. I. %	Modification	Rank	Result Type
807.3665	807.4217	0.0552	68	51	57	EGLEMGR			Oxidation (M)[5]		Mascot
813.4941	813.4472	-0.0469	-58	454	460	AILNNIR					Mascot
861.4676	861.4595	-0.0081	-9	310	317	SGLESQIK					Mascot
870.5771	870.5201	-0.057	-65	190	197	VKGVIINK					Mascot
883.452	883.463	0.011	12	492	498	EHLDIEK					Mascot
928.5714	928.4925	-0.0789	-85	109	116	LELVDVLK					Mascot
1320.6729	1320.697	0.0241	18	396	406	GYEIHMGITKR			Oxidation (M)[6]		Mascot
1346.7461	1346.7493	0.0032	2	4	16	KIMLQGTASNVGK					Mascot
1353.6831	1353.746	0.0629	46	39	50	SQNMALNSFITK					Mascot
1353.6831	1353.746	0.0629	46	39	50	SQNMALNSFITK					Mascot
1375.7402	1375.6714	-0.0688	-50	394	405	VKGYEIHMGITK					Mascot
1391.7352	1391.7126	-0.0226	-16	394	405	VKGYEIHMGITK			Oxidation (M)[8]		Mascot
1556.7964	1556.7747	-0.0217	-14	325	338	LIFGICGGYQMLGK			Carbamidomethyl (C)[6]		Mascot
1634.8789	1634.7892	-0.0897	-55	104	116	YHDYKLELVDVLK					Mascot
1634.8789	1634.7892	-0.0897	-55	104	116	YHDYKLELVDVLK					Mascot
2158.0266	2158.0427	0.0161	7	39	57	SQNMALNSFITKEGLEM GR			Oxidation (M)[4,17]		Mascot
2174.1785	2174.0112	-0.1673	-77	5	25	IMLQGTASNVGKSVLVAG LCR			Carbamidomethyl (C)[20]		Mascot
2194.0808	2193.9834	-0.0974	-44	126	145	YDIVVMEGAGSNAEINLK DR					Mascot
2701.3059	2701.3364	0.0305	11	368	390	EKTTTQVEATIVECISEYM NNLK			Carbamidomethyl (C)[14]		Mascot

	2717.3008	2717.3303	0.0295	11	368	390	EKTTTQVEATIVECISEYM NNLK			Carbamidomethyl (C)[14], Oxidation (M)[19]		Mascot

4	Probable ATP-dependent helicase DinG OS=Salmonella choleraesuis (strain SC-B67) GN=dinG PE=3 SV=1	DING_SALCH	81455.5	6.92	17	60	39.329	15.693	

Peptide Information

Calc. Mass	Obsrv. Mass	± da	± ppm	Start Seq.	End Seq.	Sequence	Ion Score	C. I. %	Modification	Rank	Result Type
810.4104	810.4244	0.014	17	464	470	SLNSFSR					Mascot
818.4804	818.433	-0.0474	-58	1	8	MALTAALK					Mascot
832.4159	832.3384	-0.0775	-93	41	48	TLAGEEGR					Mascot
856.5073	856.5355	0.0282	33	500	506	LVIPQMR					Mascot
861.4676	861.4595	-0.0081	-9	527	533	EQLESKK					Mascot
884.4584	884.4766	0.0182	21	374	381	TGSHDIVR					Mascot
921.4709	921.538	0.0671	73	471	478	LQEMSGLK			Oxidation (M)[4]		Mascot
963.551	963.4902	-0.0608	-63	274	281	LQLDLFSK					Mascot
1333.6781	1333.7	0.0219	16	405	418	LASMAQSSGAPVSK					Mascot
1570.9064	1570.8223	-0.0841	-54	559	571	LLLLVQGDQPRYR					Mascot
1618.9315	1618.791	-0.1405	-87	2	16	ALTAALKAQIAAWYK					Mascot
1965.9926	1965.9501	-0.0425	-22	385	401	VILQMNWALGMFEAQSK					Mascot
1965.9926	1965.9501	-0.0425	-22	385	401	VILQMNWALGMFEAQSK					Mascot
2380.2295	2380.1516	-0.0779	-33	636	656	YPFEVQSLPSASFNLIQQ VGR					Mascot
2380.2295	2380.1516	-0.0779	-33	636	656	YPFEVQSLPSASFNLIQQ VGR					Mascot
2565.2781	2565.2175	-0.0606	-24	426	446	EGQLHVWFHCVGIRVSD QLER			Carbamidomethyl (C)[10]		Mascot
2581.4026	2581.2151	-0.1875	-73	613	635	IAFPPIDSPVVITEGEWLK SLNR					Mascot
2687.3386	2687.3103	-0.0283	-11	259	281	DALEMSAEITASWYRLQL DLFSK					Mascot
2931.6304	2931.4702	-0.1602	-55	586	612	SVLVGLQSFAEGLDLKGE LLTQVHIHK					Mascot

5	Cytochrome c OS=Nigella damascena PE=1 SV=1	CYC_NIGDA	12158.1	9.79	8	58	23.62	3.016	

Peptide Information

Calc. Mass	Obsrv. Mass	± da	± ppm	Start Seq.	End Seq.	Sequence	Ion Score	C. I. %	Modification	Rank	Result Type
806.4771	806.4398	-0.0373	-46	81	87	KYIPGTK					Mascot
807.4433	807.4217	-0.0216	-27	88	94	MVFPGLK			Oxidation (M)[1]		Mascot
875.4257	875.4569	0.0312	36	64	70	AVNWEEK					Mascot
906.5294	906.4791	-0.0503	-55	100	107	ABLLAYLK					Mascot

1429.7985	1429.7078	-0.0907	-63	88	99	MVFPGLKKPZZR						Mascot
1536.6925	1536.6725	-0.02	-13	1	16	ASFBZAPAGBSASGEK						Mascot
1536.6925	1536.6725	-0.02	-13	1	16	ASFBZAPAGBSASGEK						Mascot
1568.6693	1568.8074	0.1381	88	22	35	CAZCHTVBZGAGHK			Carbamidomethyl (C)[1,4]			Mascot
1926.908	1926.9143	0.0063	3	1	19	ASFBZAPAGBSASGEKIFK						Mascot

6 Acetylglutamate kinase OS=Agrobacterium radiobacter (strain K84 / ATCC BAA-868) GN=argB PE=3 SV=1 | ARGB_AGRRK | 31012.4 | 5.31 | 11 | 58 | 12.304 | 3.43

Peptide Information

Calc. Mass	Obsrv. Mass	± da	± ppm	Start Seq.	End Seq.	Sequence	Ion Score	C. I. %	Modification	Rank	Result Type
878.4553	878.4639	0.0086	10	16	22	ALPYMQR					Mascot
1010.4611	1010.4754	0.0143	14	128	136	DGNMVFAEK					Mascot
1026.4562	1026.4967	0.0405	39	128	136	DGNMVFAEK			Oxidation (M)[4]		Mascot
1048.5344	1048.5896	0.0552	53	254	262	VETCIDAIK			Carbamidomethyl (C)[4]		Mascot
1320.6001	1320.697	0.0969	73	32	44	YGGHAMGNAELGK			Oxidation (M)[6]		Mascot
1346.6522	1346.7493	0.0971	72	128	139	DGNMVFAEKAHK					Mascot
1429.8302	1429.7078	-0.1224	-86	212	224	LLFLTDVPGVLDK					Mascot
1474.7748	1474.7936	0.0188	13	2	15	SEAQSELQASLLAK					Mascot
1618.8721	1618.791	-0.0811	-50	94	108	TVEIVEMVLAGSINK			Oxidation (M)[7]		Mascot
1860.9637	1860.8376	-0.1261	-68	27	44	TIVVKYGGHAMGNAELGK			Oxidation (M)[11]		Mascot
2174.1738	2174.0112	-0.1626	-75	89	108	VTDQKTVEIVEMVLAGSINK					Mascot
2184.2058	2184.0283	-0.1775	-81	254	274	VETCIDAIKAGVQGVVILNGK			Carbamidomethyl (C)[4]		Mascot

7 Probable ATP-dependent helicase DinG OS=Salmonella paratyphi A (strain ATCC 9150 / SARB42) GN=dinG PE=3 SV=1 | DING_SALPA | 81548.5 | 6.92 | 17 | 57 | 0 | 15.733

Peptide Information

Calc. Mass	Obsrv. Mass	± da	± ppm	Start Seq.	End Seq.	Sequence	Ion Score	C. I. %	Modification	Rank	Result Type
810.4104	810.4244	0.014	17	464	470	SLNSFSR					Mascot
818.4804	818.433	-0.0474	-58	1	8	MALTAALK					Mascot
832.4159	832.3384	-0.0775	-93	41	48	TLAGEEGR					Mascot
856.5073	856.5355	0.0282	33	500	506	LVIPQMR					Mascot
860.4472	860.4551	0.0079	9	440	446	VSEQLER					Mascot
861.4676	861.4595	-0.0081	-9	527	533	EQLESKK					Mascot
921.4709	921.538	0.0671	73	471	478	LQEMSGLK			Oxidation (M)[4]		Mascot

963.551	963.4902	-0.0608	-63	274	281	LQLDLFSK					Mascot
1333.6781	1333.7	0.0219	16	405	418	LASMAQSSGAPVSK					Mascot
1536.7992	1536.6725	-0.1267	-82	392	404	ALGMFEAQSKLWR					Mascot
1536.7992	1536.6725	-0.1267	-82	392	404	ALGMFEAQSKLWR					Mascot
1570.9064	1570.8223	-0.0841	-54	559	571	LLLLVQGDQPRYR					Mascot
1618.9315	1618.791	-0.1405	-87	2	16	ALTAALKAQIAAWYK					Mascot
1966.0944	1965.9501	-0.1443	-73	447	463	LLWCSVPHIIVTSATLR			Carbamidomethyl (C)[4]		Mascot
1966.0944	1965.9501	-0.1443	-73	447	463	LLWCSVPHIIVTSATLR	1	0	Carbamidomethyl (C)[4]		Mascot
2380.2295	2380.1516	-0.0779	-33	636	656	YPFEVQSLPSASFNLIQQ VGR					Mascot
2380.2295	2380.1516	-0.0779	-33	636	656	YPFEVQSLPSASFNLIQQ VGR					Mascot
2581.4026	2581.2151	-0.1875	-73	613	635	IAFPPIDSPVVITEGEWLK SLNR					Mascot
2687.3386	2687.3103	-0.0283	-11	259	281	DALEMSAEITASWYRLQL DLFSK					Mascot
2931.6304	2931.4702	-0.1602	-55	586	612	SVLVGLQSFAEGLDLKGE LLTQVHIHK					Mascot

8	Probable ATP-dependent helicase DinG OS=Salmonella typhimurium (strain LT2 / SGSC1412 / ATCC 700720) GN=dinG PE=3 SV=1	DING_SALTY	81498.5	7.11	17	57	0	13.621

Peptide Information

Calc. Mass	Obsrv. Mass	± da	± ppm	Start Seq.	End Seq.	Sequence	Ion Score	C. I. %	Modification	Rank	Result Type
810.4104	810.4244	0.014	17	464	470	SLNSFSR					Mascot
818.4804	818.433	-0.0474	-58	1	8	MALTAALK					Mascot
832.4159	832.3384	-0.0775	-93	41	48	TLAGEEGR					Mascot
856.5073	856.5355	0.0282	33	500	506	LVIPQMR					Mascot
861.4676	861.4595	-0.0081	-9	527	533	EQLESKK					Mascot
884.4584	884.4766	0.0182	21	374	381	TGSHDIVR					Mascot
921.4709	921.538	0.0671	73	471	478	LQEMSGLK			Oxidation (M)[4]		Mascot
963.551	963.4902	-0.0608	-63	274	281	LQLDLFSK					Mascot
1333.6781	1333.7	0.0219	16	405	418	LASMAQSSGAPVSK					Mascot
1536.7992	1536.6725	-0.1267	-82	392	404	ALGMFEAQSKLWR					Mascot
1536.7992	1536.6725	-0.1267	-82	392	404	ALGMFEAQSKLWR					Mascot
1570.9064	1570.8223	-0.0841	-54	559	571	LLLLVQGDQPRYR					Mascot
1618.9315	1618.791	-0.1405	-87	2	16	ALTAALKAQIAAWYK					Mascot
2380.2295	2380.1516	-0.0779	-33	636	656	YPFEVQSLPSASFNLIQQ VGR					Mascot
2380.2295	2380.1516	-0.0779	-33	636	656	YPFEVQSLPSASFNLIQQ VGR					Mascot
2565.2781	2565.2175	-0.0606	-24	426	446	EGQLHVWFHCVGIRVSD QLER			Carbamidomethyl (C)[10]		Mascot

Calc. Mass	Obsrv. Mass	± da	± ppm	Start Seq.	End Seq.	Sequence	Ion Score	C. I. %	Modification	Rank	Result Type
2581.4026	2581.2151	-0.1875	-73	613	635	IAFPPIDSPVVITEGEWLK SLNR					Mascot
2687.3386	2687.3103	-0.0283	-11	259	281	DALEMSAEITASWYRLQL DLFSK					Mascot
2931.6304	2931.4702	-0.1602	-55	586	612	SVLVGLQSFAEGLDLKGE LLTQVHIHK					Mascot

9	40S ribosomal protein S3a OS=Biphyllus lunatus PE=2 SV=1	RS3A_BIPLU	30046.9	9.66	12	55	0	4.052	

Peptide Information

Calc. Mass	Obsrv. Mass	± da	± ppm	Start Seq.	End Seq.	Sequence	Ion Score	C. I. %	Modification	Rank	Result Type
834.4203	834.343	-0.0773	-93	178	185	DISGSDLK					Mascot
856.5138	856.5355	0.0217	25	191	198	LLPDSIAK					Mascot
861.4862	861.4595	-0.0267	-31	171	177	MVEIITR					Mascot
1010.5741	1010.4754	-0.0987	-98	57	65	IASEGLKHR					Mascot
1068.5685	1068.5385	-0.03	-28	231	240	LLELHGDSGK					Mascot
1346.6951	1346.7493	0.0542	40	120	131	WQSLIEASADVK					Mascot
1381.6715	1381.76	0.0885	64	35	46	APCMFATRQVGK			Carbamidomethyl (C)[3], Oxidation (M)[4]		Mascot
1391.6849	1391.7126	0.0277	20	155	165	KTCYAQHTQVR			Carbamidomethyl (C)[3]		Mascot
1474.79	1474.7936	0.0036	2	119	131	KWQSLIEASADVK					Mascot
1656.8115	1656.8723	0.0608	37	253	267	VDKPEGYEPPVQEAV					Mascot
2266.1714	2266.1055	-0.0659	-29	120	139	WQSLIEASADVKTTDGYL LR					Mascot
2266.1714	2266.1055	-0.0659	-29	120	139	WQSLIEASADVKTTDGYL LR					Mascot
2687.3457	2687.3103	-0.0354	-13	89	112	LIAEDVQGRNVLTNFHG MDLTTDK					Mascot

10	Holliday junction resolvase RecU OS=Streptococcus equi subsp. zooepidemicus (strain H70) GN=recU PE=3 SV=1	RECU_STRS7	23407.2	9.57	11	55	0	3.983	

Peptide Information

Calc. Mass	Obsrv. Mass	± da	± ppm	Start Seq.	End Seq.	Sequence	Ion Score	C. I. %	Modification	Rank	Result Type
805.4315	805.4856	0.0541	67	65	70	VDYPRR					Mascot
821.4515	821.4166	-0.0349	-42	173	179	KNGFEVK					Mascot
849.4213	849.4715	0.0502	59	24	30	QVDFANR					Mascot
869.4767	869.4692	-0.0075	-9	75	81	IVEAYFK					Mascot
1010.4975	1010.4754	-0.0221	-22	165	172	SMPLDYIR			Oxidation (M)[2]		Mascot
1068.6088	1068.5385	-0.0703	-66	73	81	AKIVEAYFK					Mascot
1333.6271	1333.7	0.0729	55	82	93	QASTTDYSGIYK					Mascot
1742.1051	1741.9788	-0.1263	-72	49	64	GIAVIHKKPTPIQIVK					Mascot

2184.0859	2184.0283	-0.0576	-26	75	93	IVEAYFKQASTTDYSGIYK	Mascot
2219.1382	2219.2107	0.0725	33	144	161	ETYYLPAKALIDFYQIDR	Mascot
2597.3286	2597.2502	-0.0784	-30	180	202	LGAFPQVPYLDIIEQKFLG GDYN	Mascot

Gel Idx/Pos	192/H19	Instr./Gel Origin	BA2151/Sample Project 20140814	Process Status	Analysis Succeeded
Plate [#] Name	[1] Sample Project 20140814	Instrument Sample Name		Spectra	11

Rank	Protein Name	Accession No.	Protein MW	Protein PI	Pep. Count	Protein Score	Protein Score C. I. %	Intensity Matched	Total Ion Score	Total Ion C. I. %	Confirmed
1	Triosephosphate isomerase, cytosolic OS=Hordeum vulgare PE=1 SV=3	TPIS_HORVU	26948	5.39	9	442	100	51.484	394	100	

Peptide Information

Calc. Mass	Obsrv. Mass	± da	± ppm	Start Seq.	End Seq.	Sequence	Ion Score	C. I. %	Modification	Rank	Result Type
954.4832	954.5059	0.0227	24	5	12	FFVGGNWK					Mascot
954.4832	954.5059	0.0227	24	5	12	FFVGGNWK	63	99.953			Mascot
1033.6041	1033.6255	0.0214	21	114	123	VAYALAQGLK					Mascot
1289.6332	1289.6741	0.0409	32	195	206	TNVSPEVAESTR					Mascot
1289.6332	1289.6741	0.0409	32	195	206	TNVSPEVAESTR	53	99.525			Mascot
1312.6566	1312.6898	0.0332	25	207	219	IIYGGSVTGASCK			Carbamidomethyl (C)[12]		Mascot
1374.7046	1374.7457	0.0411	30	124	135	VIACVGETLEQR			Carbamidomethyl (C)[4]		Mascot
1374.7046	1374.7457	0.0411	30	124	135	VIACVGETLEQR	94	100	Carbamidomethyl (C)[4]		Mascot
1604.8503	1604.8995	0.0492	31	176	190	VATPAQAQEVHANLR					Mascot
1604.8503	1604.8995	0.0492	31	176	190	VATPAQAQEVHANLR	88	100			Mascot
1811.9585	1812.0146	0.0561	31	56	70	LRPEIQVAAQNCWVK			Carbamidomethyl (C)[12]		Mascot
1811.9585	1812.0146	0.0561	31	56	70	LRPEIQVAAQNCWVK	95	100	Carbamidomethyl (C)[12]		Mascot
2011.0906	2011.0872	-0.0034	-2	54	70	AKLRPEIQVAAQNCWVK			Carbamidomethyl (C)[14]		Mascot
3053.5625	3053.6904	0.1279	42	72	100	GGAFTGEVSAEMLANLG VPWVILGHSERR					Mascot
3069.5576	3069.6748	0.1172	38	72	100	GGAFTGEVSAEMLANLG VPWVILGHSERR			Oxidation (M)[12]		Mascot

Rank	Protein Name	Accession No.	Protein MW	Protein PI	Pep. Count	Protein Score	Protein Score C. I. %	Intensity Matched	Total Ion Score	Total Ion C. I. %	Confirmed
2	Triosephosphate isomerase, cytosolic OS=Secale cereale PE=2 SV=3	TPIS_SECCE	27138.1	5.24	7	337	100	34.433	306	100	

Peptide Information

Calc. Mass	Obsrv. Mass	± da	± ppm	Start Seq.	End Seq.	Sequence	Ion Score	C. I. %	Modification	Rank	Result Type
954.4832	954.5059	0.0227	24	5	12	FFVGGNWK					Mascot
954.4832	954.5059	0.0227	24	5	12	FFVGGNWK	63	99.953			Mascot
1033.6041	1033.6255	0.0214	21	114	123	VAYALAQGLK					Mascot
1289.6332	1289.6741	0.0409	32	195	206	TNVSPEVAESTR					Mascot
1289.6332	1289.6741	0.0409	32	195	206	TNVSPEVAESTR	53	99.525			Mascot
1312.6566	1312.6898	0.0332	25	207	219	IIYGGSVTGASCK			Carbamidomethyl (C)[12]		Mascot

1374.7046	1374.7457	0.0411	30	124	135	VIACVGETLEQR			Carbamidomethyl (C)[4]		Mascot
1374.7046	1374.7457	0.0411	30	124	135	VIACVGETLEQR	94	100	Carbamidomethyl (C)[4]		Mascot
1811.9585	1812.0146	0.0561	31	56	70	LRPEIQVAAQNCWVK			Carbamidomethyl (C)[12]		Mascot
1811.9585	1812.0146	0.0561	31	56	70	LRPEIQVAAQNCWVK	95	100	Carbamidomethyl (C)[12]		Mascot
2346.2493	2346.2766	0.0273	12	155	175	IKDWTNVVVAYEPVWAIG TGK					Mascot

3	Triosephosphate isomerase, cytosolic OS=Zea mays PE=3 SV=3	TPIS_MAIZE	27236.1	5.52	4	175	100	12.357	158	100	

Peptide Information

Calc. Mass	Obsrv. Mass	± da	± ppm	Start Seq.	End Seq.	Sequence	Ion Score	C. I. %	Modification	Rank	Result Type
954.4832	954.5059	0.0227	24	5	12	FFVGGNWK					Mascot
954.4832	954.5059	0.0227	24	5	12	FFVGGNWK	63	99.953			Mascot
1374.7046	1374.7457	0.0411	30	124	135	VIACVGETLEQR			Carbamidomethyl (C)[4]		Mascot
1374.7046	1374.7457	0.0411	30	124	135	VIACVGETLEQR	94	100	Carbamidomethyl (C)[4]		Mascot
2925.4929	2925.5884	0.0955	33	72	99	GGAFTGEVSAEMLVNLG VPWVILGHSER					Mascot
2941.4878	2941.5771	0.0893	30	72	99	GGAFTGEVSAEMLVNLG VPWVILGHSER			Oxidation (M)[12]		Mascot
3053.5876	3053.6904	0.1028	34	71	99	KGGAFTGEVSAEMLVNL GVPWVILGHSER					Mascot
3069.5828	3069.6748	0.092	30	71	99	KGGAFTGEVSAEMLVNL GVPWVILGHSER			Oxidation (M)[13]		Mascot

4	Triosephosphate isomerase, cytosolic OS=Oryza sativa subsp. japonica GN=TPI PE=1 SV=3	TPIS_ORYSJ	27274.1	5.38	3	166	100	11.559	158	100	

Peptide Information

Calc. Mass	Obsrv. Mass	± da	± ppm	Start Seq.	End Seq.	Sequence	Ion Score	C. I. %	Modification	Rank	Result Type
954.4832	954.5059	0.0227	24	5	12	FFVGGNWK					Mascot
954.4832	954.5059	0.0227	24	5	12	FFVGGNWK	63	99.953			Mascot
1374.7046	1374.7457	0.0411	30	124	135	VIACVGETLEQR			Carbamidomethyl (C)[4]		Mascot
1374.7046	1374.7457	0.0411	30	124	135	VIACVGETLEQR	94	100	Carbamidomethyl (C)[4]		Mascot
2346.2493	2346.2766	0.0273	12	155	175	IKDWTNVVVAYEPVWAIG TGK					Mascot

5	Triosephosphate isomerase, cytosolic OS=Coptis japonica PE=2 SV=1	TPIS_COPJA	27244.2	5.54	3	165	100	11.528	158	100	

Peptide Information

Calc. Mass	Obsrv. Mass	± da	± ppm	Start Seq.	End Seq.	Sequence	Ion Score	C. I. %	Modification	Rank	Result Type
954.4832	954.5059	0.0227	24	5	12	FFVGGNWK					Mascot

Calc. Mass	Obsrv. Mass	± da	± ppm	Start Seq.	End Seq.	Sequence	Ion Score	C. I. %	Modification	Rank	Result Type
954.4832	954.5059	0.0227	24	5	12	FFVGGNWK	63	99.953			Mascot
1374.7046	1374.7457	0.0411	30	124	135	VIACVGETLEQR			Carbamidomethyl (C)[4]		Mascot
1374.7046	1374.7457	0.0411	30	124	135	VIACVGETLEQR	94	100	Carbamidomethyl (C)[4]		Mascot
1809.9606	1809.9985	0.0379	21	176	191	VASPAQAQEVHFELRK					Mascot

6	Triosephosphate isomerase, cytosolic (Fragment) OS=Lactuca sativa PE=2 SV=1	TPIS_LACSA	20755.6	5.28	2	103	99.997	9.654	94	100

Peptide Information

Calc. Mass	Obsrv. Mass	± da	± ppm	Start Seq.	End Seq.	Sequence	Ion Score	C. I. %	Modification	Rank	Result Type
1374.7046	1374.7457	0.0411	30	65	76	VIACVGETLEQR			Carbamidomethyl (C)[4]		Mascot
1374.7046	1374.7457	0.0411	30	65	76	VIACVGETLEQR	94	100	Carbamidomethyl (C)[4]		Mascot
3053.5625	3053.6904	0.1279	42	13	41	GGAFTGEVSAEMLANLG VPWVILGHSERR					Mascot
3069.5576	3069.6748	0.1172	38	13	41	GGAFTGEVSAEMLANLG VPWVILGHSERR			Oxidation (M)[12]		Mascot

7	Triosephosphate isomerase, chloroplastic OS=Spinacia oleracea GN=TPIP1 PE=1 SV=1	TPIC_SPIOL	34786.7	6.45	6	85	99.817	26.573	63	99.953

Peptide Information

Calc. Mass	Obsrv. Mass	± da	± ppm	Start Seq.	End Seq.	Sequence	Ion Score	C. I. %	Modification	Rank	Result Type
954.4832	954.5059	0.0227	24	73	80	FFVGGNWK					Mascot
954.4832	954.5059	0.0227	24	73	80	FFVGGNWK	63	99.953			Mascot
1289.6454	1289.6741	0.0287	22	60	72	CPRGVVAMAGSGK			Carbamidomethyl (C)[1]		Mascot
1289.6454	1289.6741	0.0287	22	60	72	CPRGVVAMAGSGK			Carbamidomethyl (C)[1]		Mascot
1811.9261	1812.0146	0.0885	49	63	80	GVVAMAGSGKFFVGGN WK					Mascot
1811.9261	1812.0146	0.0885	49	63	80	GVVAMAGSGKFFVGGN WK					Mascot
1825.9126	1826.0011	0.0885	48	152	166	DLGCQWVILGHSERR			Carbamidomethyl (C)[4]		Mascot
1827.9211	1828.0045	0.0834	46	63	80	GVVAMAGSGKFFVGGN WK			Oxidation (M)[5]		Mascot
2006.0125	2006.0277	0.0152	8	120	137	SSLTGRVEISAQNCWIGK			Carbamidomethyl (C)[14]		Mascot
2346.2122	2346.2766	0.0644	27	180	201	AAYALNQGVGVIACIGEL LEER			Carbamidomethyl (C)[14]		Mascot

8	Triosephosphate isomerase OS=Gallus gallus GN=TPI1 PE=1 SV=2	TPIS_CHICK	26831.9	6.71	5	84	99.769	3.864	63	99.953

Peptide Information

Calc. Mass	Obsrv. Mass	± da	± ppm	Start Seq.	End Seq.	Sequence	Ion Score	C. I. %	Modification	Rank	Result Type
954.4832	954.5059	0.0227	24	6	13	FFVGGNWK					Mascot

Calc. Mass	Obsrv. Mass	± da	± ppm	Start Seq.	End Seq.	Sequence	Ion Score	C. I. %	Modification	Rank	Result Type
954.4832	954.5059	0.0227	24	6	13	FFVGGNWK	63	99.953			Mascot
1325.6519	1325.712	0.0601	45	206	218	IIYGGSVTGGNCK			Carbamidomethyl (C)[12]		Mascot
1614.8235	1614.8829	0.0594	37	99	112	RHVFGESDELIGQK					Mascot
1741.8981	1741.9128	0.0147	8	190	205	GWLKSHVSDAVAQSTR					Mascot
3069.5891	3069.6748	0.0857	28	219	247	ELASQHDVDGFLVGGAS LKPEFVDIINAK					Mascot

9	Triosephosphate isomerase B OS=Danio rerio GN=tpi1b PE=2 SV=1	TPISB_DANRE	27095.9	6.45	5	81	99.56	2.729	63	99.953

Peptide Information

Calc. Mass	Obsrv. Mass	± da	± ppm	Start Seq.	End Seq.	Sequence	Ion Score	C. I. %	Modification	Rank	Result Type
954.4832	954.5059	0.0227	24	6	13	FFVGGNWK					Mascot
954.4832	954.5059	0.0227	24	6	13	FFVGGNWK	63	99.953			Mascot
1312.6566	1312.6898	0.0332	25	206	218	IIYGGSVTGGTCK			Carbamidomethyl (C)[12]		Mascot
1614.8235	1614.8829	0.0594	37	99	112	RHVFGESDELIGQK					Mascot
1620.7687	1620.8802	0.1115	69	55	68	LDPNIDVAAQNCYK			Carbamidomethyl (C)[12]		Mascot
1801.9556	1801.9786	0.023	13	190	205	QWLKTNVSEAVANSVR					Mascot

10	Triosephosphate isomerase OS=Schistosoma mansoni GN=TPI PE=1 SV=1	TPIS_SCHMA	28446.6	7.64	5	80	99.447	2.953	63	99.953

Peptide Information

Calc. Mass	Obsrv. Mass	± da	± ppm	Start Seq.	End Seq.	Sequence	Ion Score	C. I. %	Modification	Rank	Result Type
954.4832	954.5059	0.0227	24	7	14	FFVGGNWK					Mascot
954.4832	954.5059	0.0227	24	7	14	FFVGGNWK	63	99.953			Mascot
976.5098	976.4907	-0.0191	-20	155	162	IKSADEWK					Mascot
1505.7384	1505.8064	0.068	45	195	207	WFKTNAPNGVDEK					Mascot
1620.8228	1620.8802	0.0574	35	100	113	RNIFGESDELIAEK					Mascot
1865.998	1865.9346	-0.0634	-34	179	194	VATPQQAQEVHNFLRK					Mascot

Gel Idx/Pos	193/H20	Instr./Gel Origin	BA2151/Sample Project 20140814	Process Status	Analysis Succeeded
Plate [#] Name	[1] Sample Project 20140814	Instrument Sample Name		Spectra	11

Rank	Protein Name	Accession No.	Protein MW	Protein PI	Pep. Count	Protein Score	Protein Score C. I. %	Intensity Matched	Total Ion Score	Total Ion C. I. %	Confirmed
1	Keratin, type II cytoskeletal 6B OS=Homo sapiens GN=KRT6B PE=1 SV=5	K2C6B_HUMAN	60315.4	8.09	13	78	99.038	2.786	47	98.177	

Peptide Information

Calc. Mass	Obsrv. Mass	± da	± ppm	Start Seq.	End Seq.	Sequence	Ion Score	C. I. %	Modification	Rank	Result Type
906.468	906.4875	0.0195	22	183	189	FLEQQNK					Mascot
972.5109	972.5209	0.01	10	379	386	QEIAEINR					Mascot
1043.548	1043.5565	0.0085	8	224	232	QLDNIVGER					Mascot
1066.5164	1066.5424	0.026	24	253	260	YEDEINKR					Mascot
1107.543	1107.5734	0.0304	27	339	347	AQYEEIAQR					Mascot
1220.5828	1220.6515	0.0687	56	241	250	NMQDLVEDLK			Oxidation (M)[2]		Mascot
1263.6943	1263.7169	0.0226	18	456	466	LALDVEIATYR					Mascot
1357.7322	1357.7451	0.0129	10	425	436	NKLEGLEDALQK					Mascot
1407.7114	1407.7445	0.0331	24	288	299	ADTLTDEINFLR					Mascot
1455.6863	1455.7355	0.0492	34	348	359	SRAEAESWYQTK					Mascot
1507.8188	1507.7864	-0.0324	-21	444	455	LLKEYQELMNVK					Mascot
1890.9708	1891.0214	0.0506	27	208	222	QNLEPLFEQYINNLR					Mascot
1890.9708	1891.0214	0.0506	27	208	222	QNLEPLFEQYINNLR	47	98.177			Mascot
2304.1072	2304.2551	0.1479	64	401	421	QCANLQAAIADAEQRGE MALK			Carbamidomethyl (C)[2], Oxidation (M)[18]		Mascot

Rank	Protein Name	Accession No.	Protein MW	Protein PI	Pep. Count	Protein Score	Protein Score C. I. %	Intensity Matched	Total Ion Score	Total Ion C. I. %	Confirmed
2	Tuftelin-interacting protein 11 OS=Oryctolagus cuniculus GN=TFIP11 PE=2 SV=1	TFP11_RABIT	96644.6	5.63	19	76	98.61	9.701	15	0	

Peptide Information

Calc. Mass	Obsrv. Mass	± da	± ppm	Start Seq.	End Seq.	Sequence	Ion Score	C. I. %	Modification	Rank	Result Type
807.4029	807.4201	0.0172	21	268	274	VIDMTGR			Oxidation (M)[4]		Mascot
948.4203	948.4719	0.0516	54	753	760	EAENMAQR					Mascot
1022.5087	1022.5522	0.0435	43	1	8	MSLSHLYR			Oxidation (M)[1]		Mascot
1141.6113	1141.5421	-0.0692	-61	491	499	NIVTQWQPR					Mascot
1253.6736	1253.5927	-0.0809	-65	107	117	QEELPKDLGPK					Mascot
1259.6519	1259.6898	0.0379	30	238	247	YSYKTVEELK					Mascot
1374.6688	1374.729	0.0602	44	278	288	VYYSYSQISHK					Mascot

Calc. Mass	Obsrv. Mass	± da	± ppm	Start Seq.	End Seq.	Sequence	Ion Score	C. I. %	Modification	Rank	Result Type
1389.687	1389.7164	0.0294	21	2	13	SLSHLYRDGEGR					Mascot
1444.6526	1444.7816	0.129	89	741	751	KDFQYEAMQER					Mascot
1475.7887	1475.7908	0.0021	1	262	274	EISQVKVIDMTGR	15	0			Mascot
1491.7836	1491.7732	-0.0104	-7	262	274	EISQVKVIDMTGR			Oxidation (M)[10]		Mascot
1507.7322	1507.7864	0.0542	36	714	728	AVSSNVGAYMQPGAR					Mascot
1741.8868	1741.9064	0.0196	11	574	589	LSSALQKWHPSDSSAK					Mascot
1756.9745	1756.939	-0.0355	-20	800	813	QLYTFGRIVIYIDR					Mascot
1844.863	1844.978	0.115	62	387	402	LQPACSNPLTLDECAR			Carbamidomethyl (C)[5,14]		Mascot
1891.0184	1891.0214	0.003	2	70	86	RPRDYSAPVNFISAGLK					Mascot
1891.0184	1891.0214	0.003	2	70	86	RPRDYSAPVNFISAGLK					Mascot
1953.0184	1953.0179	-0.0005	0	417	433	MSDRVDLAVAIVYPLMK			Oxidation (M)[1,16]		Mascot
2018.8673	2018.9867	0.1194	59	88	106	GAAEEAELDDSEDEEKP GK					Mascot
2181.105	2181.1062	0.0012	1	597	615	DVFTPGSWEAFMVKNIV PK			Oxidation (M)[12]		Mascot
3671.928	3671.8105	-0.1175	-32	309	340	EAKAPGFALPELEHNLQL LIELTEQEIIQNDR					Mascot
3671.928	3671.8105	-0.1175	-32	309	340	EAKAPGFALPELEHNLQL LIELTEQEIIQNDR					Mascot

3	Keratin, type I cytoskeletal 16 OS=Homo sapiens GN=KRT16 PE=1 SV=4	K1C16_HUMAN	51578.3	4.99	16	73	97.352	3.027	

Peptide Information

Calc. Mass	Obsrv. Mass	± da	± ppm	Start Seq.	End Seq.	Sequence	Ion Score	C. I. %	Modification	Rank	Result Type
807.3995	807.4201	0.0206	26	197	203	LAADDFR					Mascot
1036.5422	1036.5469	0.0047	5	197	205	LAADDFRTK					Mascot
1106.5624	1106.5507	-0.0117	-11	31	41	ISSVLAGGSCR			Carbamidomethyl (C)[10]		Mascot
1122.579	1122.6133	0.0343	31	410	418	LEQEIATYR					Mascot
1141.483	1141.5421	0.0591	52	291	299	DQYEQMAEK					Mascot
1201.6172	1201.6433	0.0261	22	214	224	QTVEADVNGLR					Mascot
1220.6005	1220.6515	0.051	42	355	365	ASLENSLEETK					Mascot
1259.6743	1259.6898	0.0155	12	204	213	TKYEHELALR					Mascot
1338.6649	1338.707	0.0421	31	42	55	APSTYGGGLSVSSR					Mascot
1357.7183	1357.7451	0.0268	20	214	225	QTVEADVNGLRR					Mascot
1379.7278	1379.7577	0.0299	22	408	418	TRLEQEIATYR					Mascot
1757.8857	1757.8977	0.012	7	156	169	QRPSEIKDYSPYFK					Mascot
2064.1448	2064.2024	0.0576	28	178	196	IIAATIENAQPILQIDNAR					Mascot
2167.0952	2167.0752	-0.02	-9	235	252	TDLEMQIEGLKEELAYLR			Oxidation (M)[5]		Mascot
2278.0188	2278.2344	0.2156	95	7	30	QFTSSSSMKGSCGIGGGI			Carbamidomethyl (C)[12], Oxidation (M)[8]		Mascot

						GGGSSR				
2350.0906	2350.2412	0.1506	64	420	441	LLEGEDAHLSSQQASGQ SYSSR				Mascot

4	Keratin, type II cytoskeletal 6A OS=Homo sapiens GN=KRT6A PE=1 SV=3	K2C6A_HUMAN	60293.4	8.09	11	70	94.071	2.578	47	98.177

Peptide Information

Calc. Mass	Obsrv. Mass	± da	± ppm	Start Seq.	End Seq.	Sequence	Ion Score	C. I. %	Modification	Rank	Result Type
906.468	906.4875	0.0195	22	183	189	FLEQQNK					Mascot
972.5109	972.5209	0.01	10	379	386	QEIAEINR					Mascot
1066.5164	1066.5424	0.026	24	253	260	YEDEINKR					Mascot
1107.543	1107.5734	0.0304	27	339	347	AQYEEIAQR					Mascot
1263.6943	1263.7169	0.0226	18	456	466	LALDVEIATYR					Mascot
1357.7322	1357.7451	0.0129	10	425	436	NKLEGLEDALQK					Mascot
1407.7114	1407.7445	0.0331	24	288	299	ADTLTDEINFLR					Mascot
1455.6863	1455.7355	0.0492	34	348	359	SRAEAESWYQTK					Mascot
1507.8188	1507.7864	-0.0324	-21	444	455	LLKEYQELMNVK					Mascot
1890.9708	1891.0214	0.0506	27	208	222	QNLEPLFEQYINNLR					Mascot
1890.9708	1891.0214	0.0506	27	208	222	QNLEPLFEQYINNLR	47	98.177			Mascot
2304.1072	2304.2551	0.1479	64	401	421	QCANLQAAIADAEQRGE MALK			Carbamidomethyl (C)[2], Oxidation (M)[18]		Mascot

5	Keratin, type II cytoskeletal 6C OS=Homo sapiens GN=KRT6C PE=1 SV=3	K2C6C_HUMAN	60273.4	8.09	11	66	87.029	2.446	47	98.177

Peptide Information

Calc. Mass	Obsrv. Mass	± da	± ppm	Start Seq.	End Seq.	Sequence	Ion Score	C. I. %	Modification	Rank	Result Type
906.468	906.4875	0.0195	22	183	189	FLEQQNK					Mascot
972.5109	972.5209	0.01	10	379	386	QEIAEINR					Mascot
1066.5164	1066.5424	0.026	24	253	260	YEDEINKR					Mascot
1107.543	1107.5734	0.0304	27	339	347	AQYEEIAQR					Mascot
1220.5828	1220.6515	0.0687	56	241	250	NMQDLVEDLK			Oxidation (M)[2]		Mascot
1263.6943	1263.7169	0.0226	18	456	466	LALDVEIATYR					Mascot
1357.7322	1357.7451	0.0129	10	425	436	NKLEGLEDALQK					Mascot
1407.7114	1407.7445	0.0331	24	288	299	ADTLTDEINFLR					Mascot
1455.6863	1455.7355	0.0492	34	348	359	SRAEAESWYQTK					Mascot
1507.8188	1507.7864	-0.0324	-21	444	455	LLKEYQELMNVK					Mascot
1890.9708	1891.0214	0.0506	27	208	222	QNLEPLFEQYINNLR					Mascot
1890.9708	1891.0214	0.0506	27	208	222	QNLEPLFEQYINNLR	47	98.177			Mascot

6	Keratin, type II cytoskeletal 1 OS=Canis familiaris GN=KRT1 PE=1 SV=1	K2C1_CANFA	63922.1	7.66	10	66	85.777	1.786	46	98.047

Peptide Information

Calc. Mass	Obsrv. Mass	± da	± ppm	Start Seq.	End Seq.	Sequence	Ion Score	C. I. %	Modification	Rank	Result Type
839.4105	839.4333	0.0228	27	600	608	TSSGSSSVK					Mascot
1033.516	1033.5406	0.0246	24	485	493	TLLEGEESR					Mascot
1066.5164	1066.5424	0.026	24	271	278	YEDEINKR					Mascot
1141.5194	1141.5421	0.0227	20	465	473	DYQELMNTK					Mascot
1323.6573	1323.6747	0.0174	13	394	404	STKMEISELNR			Oxidation (M)[4]		Mascot
1338.6899	1338.707	0.0171	13	366	377	SKAEAEALYQTK					Mascot
1475.7489	1475.7908	0.0419	28	213	224	WELLQQVDTSTR	48	98.591			Mascot
1756.8726	1756.939	0.0664	38	13	30	SGGGFSSGSAGLVSFQR R					Mascot
1982.9607	1983.0228	0.0621	31	225	240	THSLEPYFENYISNLR					Mascot
1982.9607	1983.0228	0.0621	31	225	240	THSLEPYFENYISNLR	0	0			Mascot
2286.1685	2286.0391	-0.1294	-57	419	439	QISALQQSISDAEQRGEN ALK					Mascot

7	Keratin, type II cytoskeletal 1 OS=Pan troglodytes GN=KRT1 PE=1 SV=1	K2C1_PANTR	65620.8	7.62	9	64	80.367	.98	46	98.047

Peptide Information

Calc. Mass	Obsrv. Mass	± da	± ppm	Start Seq.	End Seq.	Sequence	Ion Score	C. I. %	Modification	Rank	Result Type
804.4097	804.3704	-0.0393	-49	405	411	SEIDNVK					Mascot
1033.516	1033.5406	0.0246	24	479	487	TLLEGEESR					Mascot
1066.5164	1066.5424	0.026	24	265	272	YEDEINKR					Mascot
1141.5194	1141.5421	0.0227	20	459	467	DYQELMNTK					Mascot
1357.6958	1357.7451	0.0493	36	439	450	LNDLEDALQQAK					Mascot
1475.7489	1475.7908	0.0419	28	207	218	WELLQQVDTSTR	48	98.591			Mascot
1657.793	1657.8511	0.0581	35	13	29	SGGGFSSGSAGIINYQR					Mascot
1844.946	1844.978	0.032	17	412	427	KQISNLQQSISDAEQR					Mascot
2286.1248	2286.0391	-0.0857	-37	362	381	AEAESLYQSKYEELQITA GR					Mascot

8	Keratin, type II cytoskeletal 1 OS=Homo sapiens GN=KRT1 PE=1 SV=6	K2C1_HUMAN	66170.1	8.15	9	64	79.442	.98	46	98.047

Peptide Information

Calc. Mass	Obsrv. Mass	± da	± ppm	Start Seq.	End Seq.	Sequence	Ion Score	C. I. %	Modification	Rank	Result Type

804.4097	804.3704	-0.0393	-49	410	416	SEIDNVK					Mascot
1033.516	1033.5406	0.0246	24	484	492	TLLEGEESR					Mascot
1066.5164	1066.5424	0.026	24	270	277	YEDEINKR					Mascot
1141.5194	1141.5421	0.0227	20	464	472	DYQELMNTK					Mascot
1357.6958	1357.7451	0.0493	36	444	455	LNDLEDALQQAK					Mascot
1475.7489	1475.7908	0.0419	28	212	223	WELLQQVDTSTR	48	98.591			Mascot
1657.793	1657.8511	0.0581	35	13	29	SGGGFSSGSAGIINYQR					Mascot
1844.946	1844.978	0.032	17	417	432	KQISNLQQSISDAEQR					Mascot
2286.1248	2286.0391	-0.0857	-37	367	386	AEAESLYQSKYEELQITA GR					Mascot

9	Keratin, type II cytoskeletal 5 OS=Homo sapiens GN=KRT5 PE=1 SV=3	K2C5_HUMAN	62568.1	7.59	8	60	50.685	1.612	47	98.177	

Peptide Information

Calc. Mass	Obsrv. Mass	± da	± ppm	Start Seq.	End Seq.	Sequence	Ion Score	C. I. %	Modification	Rank	Result Type
810.4105	810.4314	0.0209	26	4	10	QSSVSFR					Mascot
906.468	906.4875	0.0195	22	188	194	FLEQQNK					Mascot
972.5472	972.5209	-0.0263	-27	421	429	GELALKDAR					Mascot
1066.5164	1066.5424	0.026	24	258	265	YEDEINKR					Mascot
1263.6943	1263.7169	0.0226	18	461	471	LALDVEIATYR					Mascot
1336.6604	1336.7051	0.0447	33	344	354	AQYEEIANRSR					Mascot
1374.6431	1374.729	0.0859	62	381	391	NTKHEISEMNR			Oxidation (M)[9]		Mascot
1890.9708	1891.0214	0.0506	27	213	227	QNLEPLFEQYINNLR					Mascot
1890.9708	1891.0214	0.0506	27	213	227	QNLEPLFEQYINNLR	47	98.177			Mascot

10	Keratin, type II cytoskeletal 5 OS=Pan troglodytes GN=KRT5 PE=2 SV=1	K2C5_PANTR	62728.1	6.89	8	60	49.536	1.612	47	98.177	

Peptide Information

Calc. Mass	Obsrv. Mass	± da	± ppm	Start Seq.	End Seq.	Sequence	Ion Score	C. I. %	Modification	Rank	Result Type
810.4105	810.4314	0.0209	26	4	10	QSSVSFR					Mascot
906.468	906.4875	0.0195	22	190	196	FLEQQNK					Mascot
972.5472	972.5209	-0.0263	-27	423	431	GELALKDAR					Mascot
1066.5164	1066.5424	0.026	24	260	267	YEDEINKR					Mascot
1263.6943	1263.7169	0.0226	18	463	473	LALDVEIATYR					Mascot
1336.6604	1336.7051	0.0447	33	346	356	AQYEEIANRSR					Mascot
1374.6431	1374.729	0.0859	62	383	393	NTKHEISEMNR			Oxidation (M)[9]		Mascot

1890.9708	1891.0214	0.0506	27	215	229	QNLEPLFEQYINNLR			Mascot
1890.9708	1891.0214	0.0506	27	215	229	QNLEPLFEQYINNLR	47	98.389	Mascot

Gel Idx/Pos	194/H21	Instr./Gel Origin	BA2151/Sample Project 20140814	Process Status	Analysis Succeeded
Plate [#] Name	[1] Sample Project 20140814	Instrument Sample Name		Spectra	11

Rank	Protein Name	Accession No.	Protein MW	Protein PI	Pep. Count	Protein Score	Protein Score C. I. %	Intensity Matched	Total Ion Score	Total Ion C. I. %	Confirmed
1	Triosephosphate isomerase, cytosolic OS=Hordeum vulgare PE=1 SV=3	TPIS_HORVU	26948	5.39	8	99	99.993	4.893	60	99.931	

Peptide Information

Calc. Mass	Obsrv. Mass	± da	± ppm	Start Seq.	End Seq.	Sequence	Ion Score	C. I. %	Modification	Rank	Result Type
954.4832	954.5046	0.0214	22	5	12	FFVGGNWK					Mascot
1033.6041	1033.6332	0.0291	28	114	123	VAYALAQGLK					Mascot
1289.6332	1289.6774	0.0442	34	195	206	TNVSPEVAESTR					Mascot
1374.7046	1374.7511	0.0465	34	124	135	VIACVGETLEQR			Carbamidomethyl (C)[4]		Mascot
1604.8503	1604.9038	0.0535	33	176	190	VATPAQAQEVHANLR					Mascot
1604.8503	1604.9038	0.0535	33	176	190	VATPAQAQEVHANLR	60	99.931			Mascot
1811.9585	1812.0214	0.0629	35	56	70	LRPEIQVAAQNCWVK			Carbamidomethyl (C)[12]		Mascot
2011.0906	2011.0569	-0.0337	-17	54	70	AKLRPEIQVAAQNCWVK			Carbamidomethyl (C)[14]		Mascot
2835.3862	2835.3923	0.0061	2	124	149	VIACVGETLEQREAGSTM EVVAEQTK			Carbamidomethyl (C)[4]		Mascot

Rank	Protein Name	Accession No.	Protein MW	Protein PI	Pep. Count	Protein Score	Protein Score C. I. %	Intensity Matched	Total Ion Score	Total Ion C. I. %	Confirmed
2	Imidazole glycerol phosphate synthase subunit HisF OS=Bacillus cereus (strain ATCC 14579 / DSM 31) GN=hisF PE=3 SV=1	HIS6_BACCR	27159.2	5.63	10	59	33.475	3.953			

Peptide Information

Calc. Mass	Obsrv. Mass	± da	± ppm	Start Seq.	End Seq.	Sequence	Ion Score	C. I. %	Modification	Rank	Result Type
929.505	929.5223	0.0173	19	245	252	DANIEVRL					Mascot
1085.6062	1085.6288	0.0226	21	243	251	LRDANIEVR					Mascot
1351.6199	1351.7256	0.1057	78	148	159	VDTGMDAIEWAK			Oxidation (M)[5]		Mascot
1358.7386	1358.7045	-0.0341	-25	95	108	AGADKVSINSAAVR					Mascot
1491.7261	1491.7885	0.0624	42	148	160	VDTGMDAIEWAKR					Mascot
1691.8521	1691.8612	0.0091	5	164	180	LGAGEILLTSMDADGTK					Mascot
1845.0845	1844.9824	-0.1021	-55	69	87	TAAKVFIPLTVGGGISSVK					Mascot
1845.0845	1844.9824	-0.1021	-55	69	87	TAAKVFIPLTVGGGISSVK					Mascot
2381.1343	2381.2573	0.123	52	139	159	WNVYVNGGRVDTGMDAI EWAK					Mascot
2442.2446	2442.228	-0.0166	-7	112	133	LIEEGAEHFGSQCIVVAID ARK			Carbamidomethyl (C)[13]		Mascot
3351.7253	3351.7539	0.0286	9	187	217	LTEEISKSVSIPVIASGGC			Carbamidomethyl (C)[19]		Mascot

GHTDHIIEVFQK

3	Mitochondrial presequence protease OS=Emericella nidulans (strain FGSC A4 / ATCC 38163 / CBS 112.46 / NRRL 194 / M139) GN=cym1 PE=3 SV=1	CYM1_EMENI	117252.6	5.89	19	54	0	4.766

Peptide Information

Calc. Mass	Obsrv. Mass	± da	± ppm	Start Seq.	End Seq.	Sequence	Ion Score	C. I. %	Modification	Rank	Result Type
986.5629	986.507	-0.0559	-57	423	430	VLQETLQR					Mascot
1022.5516	1022.5771	0.0255	25	842	850	FDLSTPSKK					Mascot
1033.571	1033.6332	0.0622	60	1013	1021	SVCLLGEKK			Carbamidomethyl (C)[3]		Mascot
1035.5217	1035.5721	0.0504	49	270	278	KNYNPSNAK					Mascot
1084.6725	1084.6752	0.0027	2	783	791	LAELIEKLR					Mascot
1109.6062	1109.6063	0.0001	0	588	596	EHKPVRESK					Mascot
1308.614	1308.709	0.095	73	648	657	TMEQWEDLIK			Oxidation (M)[2]		Mascot
1351.6025	1351.7256	0.1231	91	183	192	EEDFRQEGWR					Mascot
1491.6863	1491.7885	0.1022	69	1022	1033	DWAESEGWEVRK					Mascot
1523.8693	1523.8282	-0.0411	-27	437	449	VQGFLHQLELALR					Mascot
1688.7909	1688.8789	0.088	52	1033	1049	KLSMNPNGSNIPSGDAA			Oxidation (M)[4]		Mascot
1688.7909	1688.8789	0.088	52	1033	1049	KLSMNPNGSNIPSGDAA			Oxidation (M)[4]		Mascot
1819.9298	1819.9508	0.021	12	997	1012	DVNEVAQTFLVDGTRR					Mascot
1819.9298	1819.9508	0.021	12	997	1012	DVNEVAQTFLVDGTRR					Mascot
1938.8862	1938.9795	0.0933	48	660	678	TGGVSTSNFHTTSPTEM GK					Mascot
1965.9388	1966.057	0.1182	60	973	987	YFLNGITHEMDQRWR					Mascot
1981.9338	1982.0155	0.0817	41	973	987	YFLNGITHEMDQRWR			Oxidation (M)[10]		Mascot
2167.0925	2167.1758	0.0833	38	567	585	AQHADLSCLPSLRVEDIS R			Carbamidomethyl (C)[8]		Mascot
2196.0603	2196.1731	0.1128	51	658	678	LKTGGVSTSNFHTTSPTE MGK			Oxidation (M)[19]		Mascot
2335.1099	2335.2791	0.1692	72	503	522	YLINDNCLTFTMVGTPSF NK			Carbamidomethyl (C)[7]		Mascot
2351.1047	2351.2314	0.1267	54	503	522	YLINDNCLTFTMVGTPSF NK			Carbamidomethyl (C)[7], Oxidation (M)[12]		Mascot
3425.658	3425.8298	0.1718	50	493	522	GGYLEALMQKYLINDNCL TFTMVGTPSFNK			Carbamidomethyl (C)[17]		Mascot
3693.8184	3693.8044	-0.014	-4	597	629	VEGTDIVWREAPTNGLTY FQAVNAFADLPDDLR					Mascot

4	Elongation factor G OS=Lactobacillus helveticus (strain DPC 4571) GN=fusA PE=3 SV=1	EFG_LACH4	76773.8	4.94	16	54	0	4.006

Peptide Information

Calc. Mass	Obsrv. Mass	± da	± ppm	Start Seq.	End Seq.	Sequence	Ion Score	C. I. %	Modification	Rank	Result Type

Calc. Mass	Obsrv. Mass	± da	± ppm	Start Seq.	End Seq.	Sequence	Ion Score	C. I. %	Modification	Rank	Result Type
1085.5521	1085.6288	0.0767	71	357	365	LLQMHANSR			Oxidation (M)[4]		Mascot
1107.5317	1107.5854	0.0537	48	432	440	LTEEDPTFR					Mascot
1109.6024	1109.6063	0.0039	4	133	141	IVFVNKMDK			Oxidation (M)[7]		Mascot
1358.7678	1358.7045	-0.0633	-47	553	565	NGVLAGYPLIDVK					Mascot
1691.8309	1691.8612	0.0303	18	538	552	EFIPSVDQGLQEAMK					Mascot
1720.9381	1720.9299	-0.0082	-5	124	138	QAETYGVPRIVFVNK					Mascot
1727.8098	1727.8839	0.0741	43	668	682	GTFTMVFDHYSPTPK					Mascot
1743.8048	1743.8992	0.0944	54	668	682	GTFTMVFDHYSPTPK			Oxidation (M)[5]		Mascot
1803.8682	1803.9613	0.0931	52	215	231	GELIEAVADVDDGIMEK					Mascot
1819.863	1819.9508	0.0878	48	215	231	GELIEAVADVDDGIMEK			Oxidation (M)[15]		Mascot
1819.863	1819.9508	0.0878	48	215	231	GELIEAVADVDDGIMEK			Oxidation (M)[15]		Mascot
1835.9287	1835.9595	0.0308	17	470	485	EFNVDAKIGEPQVAYR					Mascot
1959.9692	1960.0253	0.0561	29	214	231	RGELIEAVADVDDGIMEK					Mascot
2132.0618	2132.1311	0.0693	33	332	351	VYTGSLESGSYVLNASKNSR					Mascot
2145.105	2145.0393	-0.0657	-31	643	661	VINSFVPLSEMFGYATTLR					Mascot
2322.1758	2322.2332	0.0574	25	609	629	VQVTTPEEYLGDVMGSITARR					Mascot
2502.2625	2502.3115	0.049	20	76	96	DYRINIIDTPGHVDFTIEVER					Mascot
2531.0813	2531.3113	0.23	91	39	59	IHKIGETHEGDSQMDWMDEEK			Oxidation (M)[14]		Mascot
3654.9114	3654.8074	-0.104	-28	384	417	NTTTGDSLTDPDHPLILESLKVPDPVIQVSVEPK					Mascot

5	Aspartate carbamoyltransferase OS=Novosphingobium aromaticivorans (strain DSM 12444) GN=pyrB PE=3 SV=1	PYRB_NOVAD	37283.1	6.64	11	52	0	25.277	

Peptide Information

Calc. Mass	Obsrv. Mass	± da	± ppm	Start Seq.	End Seq.	Sequence	Ion Score	C. I. %	Modification	Rank	Result Type
1004.5379	1004.6089	0.071	71	236	244	GANIVMMLR					Mascot
1084.5997	1084.6752	0.0755	70	169	179	LPAGSDLNGLK					Mascot
1085.5739	1085.6288	0.0549	51	263	271	HLYGLTPER					Mascot
1231.5702	1231.6735	0.1033	84	330	341	RTPGWEAEGASA					Mascot
1270.6572	1270.7268	0.0696	55	180	190	VTICGDILHSR			Carbamidomethyl (C)[4]		Mascot
1727.9109	1727.8839	-0.027	-16	86	102	LGADVVNMHAATSSVKK					Mascot
1743.9059	1743.8992	-0.0067	-4	86	102	LGADVVNMHAATSSVKK			Oxidation (M)[8]		Mascot
1755.9171	1755.9794	0.0623	35	85	101	RLGADVVNMHAATSSVK					Mascot
1982.9688	1983.0311	0.0623	31	272	289	LARAEADAFVMHPGPMNR					Mascot
1982.9688	1983.0311	0.0623	31	272	289	LARAEADAFVMHPGPMNR					Mascot

2196.1057	2196.1731	0.0674	31	2	22	QSQNTPAAGRYPAGGLA FPHR						Mascot
2375.2422	2375.2063	-0.0359	-15	103	124	GETLIDTAMTLNAMRADA IVIR						Mascot
3425.6511	3425.8298	0.1787	52	138	168	VDCPVLNAGDGQHEHPT QALLDALTMRHAMK			Carbamidomethyl (C)[3]			Mascot

6	Ethylene receptor 1 OS=Solanum lycopersicum GN=ETR1 PE=1 SV=1	ETR1_SOLLC	84717.8	7.99	16	52	0	25.552

Peptide Information

Calc. Mass	Obsrv. Mass	± da	± ppm	Start Seq.	End Seq.	Sequence	Ion Score	C. I. %	Modification	Rank	Result Type
946.5641	946.5666	0.0025	3	394	401	LMVETILK					Mascot
1109.5554	1109.6063	0.0509	46	1	9	MGSLLRMNR			Oxidation (M)[1,7]		Mascot
1221.6475	1221.6392	-0.0083	-7	491	502	EGNVSISAFVAK					Mascot
1471.7686	1471.7883	0.0197	13	163	174	MLTHEIRSTLDR					Mascot
1604.9081	1604.9038	-0.0043	-3	723	737	VGMDGVILKPVSVYK					Mascot
1604.9081	1604.9038	-0.0043	-3	723	737	VGMDGVILKPVSVYK					Mascot
1619.8977	1619.8414	-0.0563	-35	700	714	HGRPLIVALTGNTDR					Mascot
1719.8054	1719.8934	0.088	51	357	370	ARNDFLAVMNHEMR			Oxidation (M)[9]		Mascot
1754.8928	1754.9846	0.0918	52	286	300	RYALMVLMLPSDSAR			Oxidation (M)[5,8]		Mascot
1827.9745	1828.0031	0.0286	16	333	348	ARDLLMEQNVALDLAR					Mascot
1844.0125	1844.0031	-0.0094	-5	402	418	SSNLLATLINDVLDLSR					Mascot
2145.1155	2145.0393	-0.0762	-36	641	660	MVTKGLLTHLGCDVTTV GSR			Carbamidomethyl (C)[12]		Mascot
2225.1587	2225.1672	0.0085	4	453	472	LFVTLSLSSDLPEYVIGDE K					Mascot
2351.2395	2351.2314	-0.0081	-3	266	285	VPLLHLSNFQINDWPELS TK					Mascot
2353.2537	2353.2297	-0.024	-10	452	472	KLFVTLSLSSDLPEYVIGD EK					Mascot
2353.2537	2353.2297	-0.024	-10	452	472	KLFVTLSLSSDLPEYVIGD EK					Mascot
2381.26	2381.2573	-0.0027	-1	453	473	LFVTLSLSSDLPEYVIGDE KR					Mascot
2507.3406	2507.3152	-0.0254	-10	266	286	VPLLHLSNFQINDWPELS TKR					Mascot

7	Ribosomal RNA small subunit methyltransferase A OS=Zymomonas mobilis subsp. mobilis (strain ATCC 31821 / ZM4 / CP4) GN=rsmA PE=3 SV=1	RSMA_ZYMMO	30800.4	9.38	10	52	0	10.342

Peptide Information

Calc. Mass	Obsrv. Mass	± da	± ppm	Start Seq.	End Seq.	Sequence	Ion Score	C. I. %	Modification	Rank	Result Type
875.5131	875.5337	0.0206	24	232	238	MLRQSLK					Mascot
1084.5997	1084.6752	0.0755	70	2	10	LDQTLEPLR					Mascot

1231.6351	1231.6735	0.0384	31	1	10	MLDQTLEPLR			Oxidation (M)[1]		Mascot
1270.6903	1270.7268	0.0365	29	178	188	SEAKLSFPVHR					Mascot
1828.0327	1828.0031	-0.0296	-16	49	66	TVYEVGPGPGGLTRALLK					Mascot
1938.9572	1938.9795	0.0223	12	138	152	TWQPWWSSLTLMFQK					Mascot
2005.1304	2004.9884	-0.142	-71	189	207	SAFVPPPKVMSAVVHLTPK					Mascot
2225.1826	2225.1672	-0.0154	-7	257	275	RPETVSVAEFIAIGRHWEK					Mascot
2502.2327	2502.3115	0.0788	31	235	256	QSLKNIEHMMEALELAGIDATR			Oxidation (M)[9,10]		Mascot
3671.8521	3671.8333	-0.0188	-5	239	271	NIEHMMEALELAGIDATRRPETVSVAEFIAIGR			Oxidation (M)[5,6]		Mascot
3671.8521	3671.8333	-0.0188	-5	239	271	NIEHMMEALELAGIDATRRPETVSVAEFIAIGR			Oxidation (M)[5,6]		Mascot

8	Large structural protein OS=Australian bat lyssavirus (isolate Human/AUS/1998) GN=L PE=3 SV=1	L_ABLVH	244403.7	8.54	27	52	0	9.944	

Peptide Information

Calc. Mass	Obsrv. Mass	± da	± ppm	Start Seq.	End Seq.	Sequence	Ion Score	C. I. %	Modification	Rank	Result Type
875.4733	875.5337	0.0604	69	1103	1109	EISWGRK					Mascot
929.5163	929.5223	0.006	6	482	489	SHSLTRTK					Mascot
986.4941	986.507	0.0129	13	1779	1786	YFQSVQSK					Mascot
1004.5622	1004.6089	0.0467	46	201	209	DLSSVDIKK					Mascot
1283.6478	1283.7423	0.0945	74	1881	1891	DVEYLTSSTLR					Mascot
1289.6995	1289.6774	-0.0221	-17	1564	1574	ADLRQLSSLMR					Mascot
1471.723	1471.7883	0.0653	44	370	380	HWGHPYIDYRK					Mascot
1604.8313	1604.9038	0.0725	45	1951	1964	MVDDLELQRGTLSK					Mascot
1604.8313	1604.9038	0.0725	45	1951	1964	MVDDLELQRGTLSK					Mascot
1720.8977	1720.9299	0.0322	19	596	610	LIDRVTGQGLQDYSR					Mascot
1743.8813	1743.8992	0.0179	10	1773	1786	NLSTWRYFQSVQSK					Mascot
1755.8999	1755.9794	0.0795	45	549	562	IEGRFFALMSWNLR			Oxidation (M)[9]		Mascot
1819.9225	1819.9508	0.0283	16	1758	1772	VVDFESIWEKPSDLR					Mascot
1820.0211	1819.9508	-0.0703	-39	1421	1437	GLARGVLIGSSICFLTR			Carbamidomethyl (C)[13]		Mascot
1835.9585	1835.9595	0.001	1	1501	1514	SVLCYLQHVLRYER			Carbamidomethyl (C)[4]		Mascot
1843.9596	1844.0031	0.0435	24	1497	1511	EGNRSVLCYLQHVLR			Carbamidomethyl (C)[8]		Mascot
1938.959	1938.9795	0.0205	11	394	410	VIDGTYQECLASDLAKR			Carbamidomethyl (C)[9]		Mascot
1959.9998	1960.0253	0.0255	13	1358	1374	LPEVYLKPGHFDSLCGK			Carbamidomethyl (C)[15]		Mascot
1979.106	1979.0253	-0.0807	-41	525	543	SIDLGGLPDDDLIIGLKPK					Mascot
2025.0903	2025.0753	-0.015	-7	628	645	LESTKDVFSVLDQVFGLK					Mascot
2351.2983	2351.2314	-0.0669	-28	1665	1685	LQNPLISGLRVVQWATG					Mascot

Calc. Mass	Obsrv. Mass	± da	± ppm	Start Seq.	End Seq.	Sequence	Ion Score	C. I. %	Modification	Rank	Result Type
						AHYK					
2375.2871	2375.2063	-0.0808	-34	428	447	LLAQDHPLTPYIRTQTWPPK					Mascot
2394.2009	2394.3521	0.1512	63	1575	1596	QVLGGHGEDSLDSGEDIQRLLR					Mascot
2492.3694	2492.4226	0.0532	21	1537	1556	MTYLTLITYQSHILLQRIER					Mascot
2499.2766	2499.2859	0.0093	4	1853	1874	ATGYITQLTSSFSSELYLRFSK					Mascot
3470.7017	3470.7981	0.0964	28	166	193	YLANIYASYLFFHVIILYMNALDWDEEK			Oxidation (M)[19]		Mascot
3509.7178	3509.7595	0.0417	12	1779	1808	YFQSVQSKLNMSYDLIICDAEVTDIISVNK			Carbamidomethyl (C)[18], Oxidation (M)[11]		Mascot
3509.7178	3509.7595	0.0417	12	1779	1808	YFQSVQSKLNMSYDLIICDAEVTDIISVNK			Carbamidomethyl (C)[18], Oxidation (M)[11]		Mascot
3693.989	3693.8044	-0.1846	-50	1019	1051	SIEPLFPRFLSELFSSSFLGIPESIIGLIQNSR					Mascot

9	Uncharacterized protein C17C9.14 OS=Schizosaccharomyces pombe (strain 972 / ATCC 24843) GN=SPAC17C9.14 PE=4 SV=1	YDFE_SCHPO	26231.7	4.3	9	51	0	5.259	

Peptide Information

Calc. Mass	Obsrv. Mass	± da	± ppm	Start Seq.	End Seq.	Sequence	Ion Score	C. I. %	Modification	Rank	Result Type
1004.5662	1004.6089	0.0427	43	147	154	EILYEPLK					Mascot
1231.5549	1231.6735	0.1186	96	2	12	SNPTIEGDENR					Mascot
1471.7136	1471.7883	0.0747	51	50	62	NIDEHKQTGNTSK					Mascot
1627.7421	1627.8811	0.139	85	185	197	CIQIFESPEYDAR			Carbamidomethyl (C)[1]		Mascot
1751.7938	1751.8947	0.1009	58	69	83	NDDNLNSLIQEMMSK					Mascot
1755.837	1755.9794	0.1424	81	185	198	CIQIFESPEYDARK			Carbamidomethyl (C)[1]		Mascot
1996.9797	1997.0426	0.0629	31	182	197	IQKCIQIFESPEYDAR			Carbamidomethyl (C)[4]		Mascot
2024.9628	2025.0753	0.1125	56	67	83	IKNDDNLNSLIQEMMSK			Oxidation (M)[14,15]		Mascot
3509.6443	3509.7595	0.1152	33	14	44	ENTKSNTKPNFDDLDDLDDILDDLDVPSAFK					Mascot
3509.6443	3509.7595	0.1152	33	14	44	ENTKSNTKPNFDDLDDLDDILDDLDVPSAFK					Mascot

10	N-acetyl-gamma-glutamyl-phosphate reductase OS=Pseudomonas aeruginosa (strain UCBPP-PA14) GN=argC PE=3 SV=1	ARGC_PSEAB	36978.1	6.1	10	51	0	2.054	

Peptide Information

Calc. Mass	Obsrv. Mass	± da	± ppm	Start Seq.	End Seq.	Sequence	Ion Score	C. I. %	Modification	Rank	Result Type
946.5065	946.5666	0.0601	63	179	189	SGVSGAGRGAK					Mascot
1033.5902	1033.6332	0.043	42	20	28	LLAQHPQAR					Mascot
1035.547	1035.5721	0.0251	24	95	103	VIDLSADFR					Mascot
1374.7588	1374.7511	-0.0077	-6	29	41	VEVITSRSEAGVK					Mascot

1590.885	1590.8871	0.0021	1	4	19	VGIVGGTGYTGVELLR		Mascot
1965.8755	1966.057	0.1815	92	190	207	VGSLFCEAGESMMAYAV K	Carbamidomethyl (C)[6], Oxidation (M)[12]	Mascot
1979.0995	1979.0253	-0.0742	-37	1	19	MIKVGIVGGTGYTGVELL R	Oxidation (M)[1]	Mascot
1981.8705	1982.0155	0.145	73	190	207	VGSLFCEAGESMMAYAV K	Carbamidomethyl (C)[6], Oxidation (M)[12,13]	Mascot
2835.3521	2835.3923	0.0402	14	42	65	VADMYPNLRGHYDDLQF SVPDVQR		Mascot
3258.6875	3258.8091	0.1216	37	211	240	HLPEISQGLCRASGGDV GLTFVPHLTPMIR	Carbamidomethyl (C)[10]	Mascot
3693.8083	3693.8044	-0.0039	-1	104	136	LADAEEWARWYGQPHG APALLDEAVYGLPEVNR		Mascot

Gel Idx/Pos	195/H22	Instr./Gel Origin	BA2151/Sample Project 20140814	Process Status	Analysis Succeeded
Plate [#] Name	[1] Sample Project 20140814	Instrument Sample Name		Spectra	11

Rank	Protein Name	Accession No.	Protein MW	Protein PI	Pep. Count	Protein Score	Protein Score C. I. %	Intensity Matched	Total Ion Score	Total Ion C. I. %	Confirmed
1	Nesprin-1 OS=Mus musculus GN=Syne1 PE=1 SV=2	SYNE1_MOUSE	1016650.4	5.43	78	66	86.727	67.643			

Peptide Information

Calc. Mass	Obsrv. Mass	± da	± ppm	Start Seq.	End Seq.	Sequence	Ion Score	C. I. %	Modification	Rank	Result Type
807.3995	807.4291	0.0296	37	1555	1560	FEENLR					Mascot
849.4577	849.457	-0.0007	-1	8650	8656	EVSHHIK					Mascot
856.4271	856.3976	-0.0295	-34	8731	8737	SDPRPER					Mascot
866.4586	866.447	-0.0116	-13	6063	6069	MSTIRMK					Mascot
906.5295	906.4809	-0.0486	-54	5122	5129	LSEFAVLK					Mascot
909.475	909.5072	0.0322	35	503	509	MEFLELK					Mascot
929.5302	929.5046	-0.0256	-28	3002	3009	EILDALQK					Mascot
951.5145	951.5133	-0.0012	-1	1719	1727	EALFSVASK					Mascot
967.4955	967.4861	-0.0094	-10	7050	7057	EQHLLGDR					Mascot
969.4822	969.5003	0.0181	19	356	362	VQYEMKR			Oxidation (M)[5]		Mascot
987.537	987.5645	0.0275	28	7005	7012	SWQLLQGR					Mascot
1005.4734	1005.5252	0.0518	52	7276	7284	DIADDEVTK					Mascot
1006.5139	1006.5233	0.0094	9	3935	3942	WLLQMSGR			Oxidation (M)[5]		Mascot
1034.6245	1034.5588	-0.0657	-64	5121	5129	KLSEFAVLK					Mascot
1057.5889	1057.5444	-0.0445	-42	6624	6632	EEIQQLLGK					Mascot
1078.5891	1078.5786	-0.0105	-10	3910	3918	ERVFSLEAK					Mascot
1078.5891	1078.5786	-0.0105	-10	3910	3918	ERVFSLEAK					Mascot
1088.5946	1088.5938	-0.0008	-1	2111	2120	ATIKDQGDLK					Mascot
1094.5663	1094.5826	0.0163	15	6958	6966	GFKIDLNCK			Carbamidomethyl (C)[8]		Mascot
1096.5687	1096.6034	0.0347	32	348	355	YQAFKHFR					Mascot
1097.595	1097.6506	0.0556	51	5448	5457	AKTDDLVHAK					Mascot
1102.5925	1102.6025	0.01	9	7336	7345	LGALEQALCK			Carbamidomethyl (C)[9]		Mascot
1106.5874	1106.6005	0.0131	12	2830	2838	MRTVEDLVK			Oxidation (M)[1]		Mascot
1112.7037	1112.6166	-0.0871	-78	3633	3641	EIQLLQLKK					Mascot
1114.658	1114.6252	-0.0328	-29	8602	8610	QELLKSQLR					Mascot
1120.6361	1120.6154	-0.0207	-18	7193	7202	FGSITNQLLK					Mascot
1130.5325	1130.6245	0.092	81	5130	5139	TSSIHEAEEK					Mascot

1130.5325	1130.6245	0.092	81	5130	5139	TSSIHEAEEK		Mascot
1136.606	1136.6084	0.0024	2	2295	2304	GTLKDFTAQR		Mascot
1144.6208	1144.5885	-0.0323	-28	1968	1978	QSEADALVALK		Mascot
1145.714	1145.615	-0.099	-86	5438	5447	DLTTILTKLK		Mascot
1145.714	1145.615	-0.099	-86	5438	5447	DLTTILTKLK		Mascot
1169.5222	1169.564	0.0418	36	3389	3397	WTSYQDDVR		Mascot
1173.5933	1173.6523	0.059	50	3754	3763	DMEKGHSLLK	Oxidation (M)[2]	Mascot
1182.6001	1182.5983	-0.0018	-2	2137	2146	QKELDSFTSK		Mascot
1196.5946	1196.6097	0.0151	13	2490	2499	ANEEFQAFLK		Mascot
1202.6449	1202.7219	0.077	64	1078	1087	LQLIEELCGK	Carbamidomethyl (C)[8]	Mascot
1202.6449	1202.7219	0.077	64	1078	1087	LQLIEELCGK	Carbamidomethyl (C)[8]	Mascot
1208.6746	1208.6094	-0.0652	-54	7048	7057	LKEQHLLGDR		Mascot
1228.6394	1228.5991	-0.0403	-33	503	511	MEFLELKYR		Mascot
1233.5352	1233.6422	0.107	87	1096	1106	DTCGACHTALK	Carbamidomethyl (C)[3,6]	Mascot
1238.6119	1238.636	0.0241	19	7396	7405	MEELKGQMLK	Oxidation (M)[1,8]	Mascot
1278.6338	1278.6633	0.0295	23	3641	3650	KWHEDLSAHR		Mascot
1304.6667	1304.7118	0.0451	35	8423	8433	WELLQAQAMSK		Mascot
1315.6965	1315.662	-0.0345	-26	2883	2894	ELIDSREIGAGR		Mascot
1320.6696	1320.6687	-0.0009	-1	3605	3614	FQQADEWLKR		Mascot
1331.743	1331.6748	-0.0682	-51	8350	8360	FQIQQTANILR		Mascot
1347.6461	1347.6643	0.0182	14	5109	5120	EGVIELMNDAEK		Mascot
1405.6992	1405.7078	0.0086	6	1643	1654	EMQTALEDLLAR	Oxidation (M)[2]	Mascot
1406.7638	1406.6857	-0.0781	-56	5966	5976	TLYEVLERQQK		Mascot
1422.6859	1422.6721	-0.0138	-10	6936	6947	DEEDIRNAIGYK		Mascot
1438.7173	1438.6798	-0.0375	-26	1203	1215	RGDELAELSSSFK		Mascot
1467.7162	1467.7849	0.0687	47	5182	5192	SMTTVWQRWTR	Oxidation (M)[2]	Mascot
1490.7744	1490.8031	0.0287	19	8607	8619	SQLRVASLQDMSR		Mascot
1519.7024	1519.8446	0.1422	94	2057	2069	EINGLEATWDDTR		Mascot
1559.8387	1559.8219	-0.0168	-11	1386	1399	RTESIATQAENLVK		Mascot
1572.8479	1572.8794	0.0315	20	3878	3891	SEALLELVQDQSLK		Mascot
1574.7843	1574.9065	0.1222	78	5107	5120	AREGVIELMNDAEK		Mascot
1582.8336	1582.8146	-0.019	-12	7126	7139	IQLTSALGQWSNHK		Mascot
1590.7792	1590.8862	0.107	67	5107	5120	AREGVIELMNDAEK	Oxidation (M)[9]	Mascot
1602.9102	1602.8824	-0.0278	-17	730	743	KLSEPLEVSFINVK		Mascot
1606.7894	1606.9036	0.1142	71	2401	2414	IQDSLEKHFSGSMK		Mascot
1613.8104	1613.8188	0.0084	5	2524	2535	KLNLWIHEMEER	Oxidation (M)[9]	Mascot
1629.9323	1629.884	-0.0483	-30	2754	2767	LEHPLQLQPGLKEK		Mascot

1635.7432	1635.8901	0.1469	90	2741	2753	SQLEQWMESVDQR					Mascot
1768.9164	1768.9301	0.0137	8	900	914	QIADVHHAFQSMIKK			Oxidation (M)[12]		Mascot
1809.884	1809.9303	0.0463	26	3600	3613	LMEQKFQQADEWLK			Oxidation (M)[2]		Mascot
1822.9407	1823.0471	0.1064	58	7050	7065	EQHLLGDRNSVENALK					Mascot
1827.8556	1827.9144	0.0588	32	8083	8097	QMVHGGNQRWDDLQK			Oxidation (M)[2]		Mascot
1827.8556	1827.9144	0.0588	32	8083	8097	QMVHGGNQRWDDLQK			Oxidation (M)[2]		Mascot
1833.8105	1833.9481	0.1376	75	5458	5473	AEHKMLGEELDGCNSK			Carbamidomethyl (C)[13], Oxidation (M)[5]		Mascot
1836.9675	1837.0265	0.059	32	5502	5516	LTELHQQTIRQAENR					Mascot
1851.8979	1851.8506	-0.0473	-26	44	58	RKPPMVVDDLFEDMK			Oxidation (M)[5,14]		Mascot
1866.9597	1866.9868	0.0271	15	7346	7362	QQAVLQAGVVDYETFAK					Mascot
1875.9381	1875.9786	0.0405	22	1643	1657	EMQTALEDLLARWQR			Oxidation (M)[2]		Mascot
1892.9205	1893.01	0.0895	47	2094	2110	STVCNVLEDASNVVVMR			Carbamidomethyl (C)[4]		Mascot
1902.8512	1903.0134	0.1622	85	4231	4245	EQDLQRTSSYHDHMR					Mascot
1914.9879	1915.0133	0.0254	13	4104	4119	NTAKDIQQTEQLIEQR					Mascot
1926.9994	1926.9629	-0.0365	-19	1227	1242	LLSNFGECVQYKEIVK			Carbamidomethyl (C)[8]		Mascot
1927.9495	1928.0159	0.0664	34	8180	8196	SEPLDAAVIEEELDELR					Mascot
1927.9495	1928.0159	0.0664	34	8180	8196	SEPLDAAVIEEELDELR					Mascot
1943.9994	1943.9769	-0.0225	-12	3002	3018	EILDALQKAEPMTEDLK					Mascot
1950.9702	1951.0414	0.0712	36	5600	5615	LSQQVTQFGREMEELR					Mascot
1952.8842	1953.0007	0.1165	60	6662	6678	ETQFHTDCMAQASAVLK			Carbamidomethyl (C)[8], Oxidation (M)[9]		Mascot
1966.9651	1966.9906	0.0255	13	5600	5615	LSQQVTQFGREMEELR			Oxidation (M)[12]		Mascot
2109.0244	2109.1589	0.1345	64	45	62	KPPMVVDDLFEDMKDGI K			Oxidation (M)[4,13]		Mascot
2109.0244	2109.1589	0.1345	64	45	62	KPPMVVDDLFEDMKDGI K			Oxidation (M)[4,13]		Mascot

2	ATP-dependent RNA helicase dbp7 OS=Sclerotinia sclerotiorum (strain ATCC 18683 / 1980 / Ss-1) GN=dbp7 PE=3 SV=1	DBP7_SCLS1	96922.2	9.33	17	64	77.972	7.345

Peptide Information

Calc. Mass	Obsrv. Mass	± da	± ppm	Start Seq.	End Seq.	Sequence	Ion Score	C. I. %	Modification	Rank	Result Type
866.4982	866.447	-0.0512	-59	579	586	VDPPTLPK					Mascot
951.4781	951.5133	0.0352	37	172	179	AISFQEEK					Mascot
971.6499	971.6036	-0.0463	-48	526	534	LVTLTALLK					Mascot
971.6499	971.6036	-0.0463	-48	526	534	LVTLTALLK	21	0			Mascot
1096.6473	1096.6034	-0.0439	-40	385	395	GVNILVATPGR					Mascot
1098.5361	1098.6393	0.1032	94	188	197	LPHGSIDGMR			Oxidation (M)[9]		Mascot
1102.5527	1102.6025	0.0498	45	413	421	WLVLDEGDR					Mascot

Calc. Mass	Obsrv. Mass	± da	± ppm	Start Seq.	End Seq.	Sequence	Ion Score	C. I. %	Modification	Rank	Result Type
1173.5382	1173.6523	0.1141	97	828	837	EEEKAPEGER					Mascot
1196.6746	1196.6097	-0.0649	-54	160	171	LPPGSINSGARK					Mascot
1224.7423	1224.7079	-0.0344	-28	384	395	KGVNILVATPGR					Mascot
1380.6292	1380.7648	0.1356	98	722	733	GFGGTGREWEER					Mascot
1582.7563	1582.8146	0.0583	37	442	456	SVASGSSEMMSLPKR			Oxidation (M)[9]		Mascot
1783.8864	1783.8997	0.0133	7	458	472	VTILCSATMKMNVQR			Carbamidomethyl (C)[5], Oxidation (M)[9,11]		Mascot
1809.9495	1809.9303	-0.0192	-11	116	131	KTTFQEETRPAFVAGK					Mascot
1836.9855	1837.0265	0.041	22	214	230	KPGQVVSSLFTFNPTSK					Mascot
1875.9447	1875.9786	0.0339	18	497	514	ANGIEADDKAFSAPTQLK					Mascot
1927.0219	1926.9629	-0.059	-31	180	197	KPAYISGKLPHGSIDGMR					Mascot
2109.0837	2109.1589	0.0752	36	709	728	ALTHHTAEDLIQKGFGGTGR					Mascot
2109.0837	2109.1589	0.0752	36	709	728	ALTHHTAEDLIQKGFGGTGR					Mascot

3 Muscle M-line assembly protein unc-89 OS=Caenorhabditis elegans GN=unc-89 PE=1 SV=3 | UNC89_CAEEL | 899002.9 | 5.42 | 67 | 62 | 66.659 | 51.963

Peptide Information

Calc. Mass	Obsrv. Mass	± da	± ppm	Start Seq.	End Seq.	Sequence	Ion Score	C. I. %	Modification	Rank	Result Type
807.3553	807.4291	0.0738	91	1783	1789	MADDEVK					Mascot
866.477	866.447	-0.03	-35	4520	4525	EIKWYK					Mascot
867.5298	867.4686	-0.0612	-71	3017	3024	VIGEPKPK					Mascot
870.5043	870.5741	0.0698	80	4438	4445	EIPNAKAK					Mascot
906.4462	906.4809	0.0347	38	5114	5120	DDRITMR					Mascot
930.5255	930.4752	-0.0503	-54	4041	4049	VSGTKPDVK					Mascot
944.5047	944.5848	0.0801	85	1409	1417	EKSPASPTK					Mascot
971.5156	971.6036	0.088	91	4337	4345	NGKEIPDAK					Mascot
971.5156	971.6036	0.088	91	4337	4345	NGKEIPDAK					Mascot
987.4966	987.5645	0.0679	69	1180	1188	VRDGQEGAR					Mascot
1005.5284	1005.5252	-0.0032	-3	3204	3211	EEIKMEVK					Mascot
1006.5679	1006.5233	-0.0446	-44	4927	4934	KYELAINR					Mascot
1023.5105	1023.4998	-0.0107	-10	696	704	LQYDGQTAK					Mascot
1088.5946	1088.5938	-0.0008	-1	2697	2705	KLEESQNIK					Mascot
1092.5466	1092.5817	0.0351	32	3856	3864	EVEMSARVR			Oxidation (M)[4]		Mascot
1094.5477	1094.5826	0.0349	32	4413	4422	QGETATFNVK					Mascot
1098.6517	1098.6393	-0.0124	-11	4626	4636	ELPGAAAKTIK					Mascot
1102.5739	1102.6025	0.0286	26	6885	6894	TEPLSADTLR					Mascot

1112.6786	1112.6166	-0.062	-56	2926	2934	EIELIARIR		Mascot
1114.6102	1114.6252	0.015	13	1730	1739	AEEKPKSPTK		Mascot
1120.5481	1120.6154	0.0673	60	1249	1258	QDLSSSEVQK		Mascot
1126.6466	1126.6205	-0.0261	-23	254	262	LLEEPEIKR		Mascot
1128.626	1128.6136	-0.0124	-11	1535	1544	VDEKPKSPTK		Mascot
1130.6205	1130.6245	0.004	4	3815	3824	GAPEFVELLR		Mascot
1130.6205	1130.6245	0.004	4	3815	3824	GAPEFVELLR		Mascot
1144.5957	1144.5885	-0.0072	-6	5107	5116	DGVVLQKDDR		Mascot
1145.511	1145.615	0.104	91	431	439	DFETSEYVR		Mascot
1145.562	1145.615	0.053	46	2622	2632	IHGVTADMTGK	Oxidation (M)[8]	Mascot
1151.5361	1151.5935	0.0574	50	3853	3862	EGKEVEMSAR	Oxidation (M)[7]	Mascot
1152.5684	1152.606	0.0376	33	4244	4252	WYKNGDEIK		Mascot
1165.6147	1165.6284	0.0137	12	6723	6731	ETCVRVFVR	Carbamidomethyl (C)[3]	Mascot
1169.6525	1169.564	-0.0885	-76	1583	1593	SPEKPASPTKK		Mascot
1173.6627	1173.6523	-0.0104	-9	5924	5934	APGFTTPLTIR		Mascot
1182.5637	1182.5983	0.0346	29	163	172	SEEEFVSSLR		Mascot
1187.5837	1187.6726	0.0889	75	8024	8033	MRTDEALSHK		Mascot
1202.6263	1202.7219	0.0956	79	1814	1824	TPEKSAAEELK		Mascot
1202.6429	1202.7219	0.079	66	5279	5287	FNVPLWDRR		Mascot
1208.5867	1208.6094	0.0227	19	38	48	SESLTSRTDGR		Mascot
1228.5481	1228.5991	0.051	42	216	226	GLNYYSDDPGK		Mascot
1233.6686	1233.6422	-0.0264	-21	1922	1933	EITTAQGVTVSK		Mascot
1278.6399	1278.6633	0.0234	18	7441	7452	EELFGMGAPTVK		Mascot
1304.6844	1304.7118	0.0274	21	1354	1364	RVSFAEEELPK		Mascot
1320.6067	1320.6687	0.062	47	2500	2511	DGVEITSDGHYK		Mascot
1336.6162	1336.7045	0.0883	66	2247	2259	AKNAAGECETSAK	Carbamidomethyl (C)[8]	Mascot
1380.7522	1380.7648	0.0126	9	6306	6316	YPQVTYVIEIR		Mascot
1412.7897	1412.7742	-0.0155	-11	5618	5630	VTGLPNPSVKWSK		Mascot
1422.7435	1422.6721	-0.0714	-50	3167	3181	AIATNSIGTATSTSK		Mascot
1438.7788	1438.6798	-0.099	-69	181	193	VLDDPEVPEAVKK		Mascot
1497.7809	1497.8668	0.0859	57	4803	4814	IQGEPEDVRWLR		Mascot
1497.7809	1497.8668	0.0859	57	4803	4814	IQGEPEDVRWLR		Mascot
1559.8118	1559.8219	0.0101	6	5275	5286	YPPRFNVPLWDR		Mascot
1572.842	1572.8794	0.0374	24	3603	3615	VEGKPEPEVKWFK		Mascot
1582.8071	1582.8146	0.0075	5	3337	3351	VEGSGSYSITIKDAR		Mascot
1607.8639	1607.9105	0.0466	29	7196	7210	ETIPKPTPSPTSPQK		Mascot
1635.9218	1635.8901	-0.0317	-19	6955	6968	KPPPTVEYVPQPRK		Mascot

1783.8531	1783.8997	0.0466	26	3978	3992	LTVTNAKLDDMDEYR		Mascot
1809.8865	1809.9303	0.0438	24	4846	4862	DAGEYTVEVINESGKAK		Mascot
1822.9004	1823.0471	0.1467	80	2635	2651	CVAYNKAGEVSTEGPLK	Carbamidomethyl (C)[1]	Mascot
1833.9957	1833.9481	-0.0476	-26	4830	4845	IDDTTYRLIIPSADLK		Mascot
1836.9259	1837.0265	0.1006	55	2973	2988	DTCEATLTVIESLEKK	Carbamidomethyl (C)[3]	Mascot
1843.9987	1843.8727	-0.126	-68	6385	6400	VIPLLDPYAEKALDMR		Mascot
1875.9309	1875.9786	0.0477	25	6322	6336	QWSLLEYNIPEPVCK	Carbamidomethyl (C)[14]	Mascot
1902.963	1903.0134	0.0504	26	1996	2012	GDTVQMDVIALHSPLYK	Oxidation (M)[6]	Mascot
1914.9742	1915.0133	0.0391	20	7541	7558	KNQPAIFECAVSASPAPK	Carbamidomethyl (C)[9]	Mascot
1926.887	1926.9629	0.0759	39	3112	3128	GSPQPHVDFYSFSETTK		Mascot
1943.9669	1943.9769	0.01	5	2031	2048	NEENKSSLIIPNAQDSGK		Mascot
1951.1111	1951.0414	-0.0697	-36	4226	4243	GTPLVLEVEIEGKPKDVK		Mascot
1966.9399	1966.9906	0.0507	26	7801	7818	DAVDSTTEGHAHCAVKIR	Carbamidomethyl (C)[13]	Mascot
2109.1128	2109.1589	0.0461	22	3613	3630	WFKDGVPIAIDNQHVIEK		Mascot
2109.1128	2109.1589	0.0461	22	3613	3630	WFKDGVPIAIDNQHVIEK		Mascot
3419.7654	3419.8794	0.114	33	3679	3708	QTAEEVKPLFIEPLKETFA VEGDTVVLECK	Carbamidomethyl (C)[29]	Mascot

4	30S ribosomal protein S4, chloroplastic OS=Stigeoclonium helveticum GN=rps4 PE=3 SV=1	RR4_STIHE	340918.4	10.21	46	60	51.807	30.456

Peptide Information

Calc. Mass	Obsrv. Mass	± da	± ppm	Start Seq.	End Seq.	Sequence	Ion Score	C. I. %	Modification	Rank	Result Type
848.4737	848.496	0.0223	26	2	8	SRYLGPR					Mascot
855.4974	855.5323	0.0349	41	454	460	FYATILK					Mascot
870.6022	870.5741	-0.0281	-32	1329	1336	TLLVLIAK					Mascot
930.493	930.4752	-0.0178	-19	436	442	EKYVYTK					Mascot
951.5145	951.5133	-0.0012	-1	1643	1650	IDNVYLSK					Mascot
987.5655	987.5645	-0.001	-1	924	931	QMIQLNIK					Mascot
1005.5397	1005.5252	-0.0145	-14	2603	2610	LQLNDKMK			Oxidation (M)[7]		Mascot
1034.6245	1034.5588	-0.0657	-64	1764	1772	TFVISILNK					Mascot
1078.4874	1078.5786	0.0912	85	1683	1691	SSMYQTFAK			Oxidation (M)[3]		Mascot
1078.4874	1078.5786	0.0912	85	1683	1691	SSMYQTFAK			Oxidation (M)[3]		Mascot
1088.6133	1088.5938	-0.0195	-18	197	206	GVTMPTKNLK					Mascot
1092.5718	1092.5817	0.0099	9	1534	1542	NMKSLLENK			Oxidation (M)[2]		Mascot
1098.663	1098.6393	-0.0237	-22	2193	2201	INVQIQKQK					Mascot
1102.6255	1102.6025	-0.023	-21	1900	1909	LKSLGPWSSK					Mascot
1114.683	1114.6252	-0.0578	-52	2018	2027	LNVSLLKAEK					Mascot

1126.6255	1126.6205	-0.005	-4	1713	1721	SVYLKQFNK		Mascot
1130.635	1130.6245	-0.0105	-9	2134	2142	CQNNLIKLK	Carbamidomethyl (C)[1]	Mascot
1130.635	1130.6245	-0.0105	-9	2134	2142	CQNNLIKLK	Carbamidomethyl (C)[1]	Mascot
1136.6674	1136.6084	-0.059	-52	595	604	NIFSKLSSLK		Mascot
1165.6252	1165.6284	0.0032	3	2345	2354	AFEFNPSLLK		Mascot
1208.6932	1208.6094	-0.0838	-69	120	130	LHMAPTIKAAR		Mascot
1224.6881	1224.7079	0.0198	16	120	130	LHMAPTIKAAR	Oxidation (M)[3]	Mascot
1233.6871	1233.6422	-0.0449	-36	346	357	SISALILMNGAK	Oxidation (M)[8]	Mascot
1304.6991	1304.7118	0.0127	10	2666	2676	LTVMLNNSLQR	Oxidation (M)[4]	Mascot
1320.6583	1320.6687	0.0104	8	2845	2855	FGDHLQTFIDK		Mascot
1336.6815	1336.7045	0.023	17	1491	1502	SSISERTLNSSR		Mascot
1405.8049	1405.7078	-0.0971	-69	1643	1654	IDNVYLSKVLNK		Mascot
1406.7526	1406.6857	-0.0669	-48	2327	2338	LSYLPKDLDSQK		Mascot
1438.7246	1438.6798	-0.0448	-31	1664	1675	LDLLMVNYTNDK		Mascot
1497.8887	1497.8668	-0.0219	-15	736	748	IQNDISLLLLLDK		Mascot
1497.8887	1497.8668	-0.0219	-15	736	748	IQNDISLLLLLDK		Mascot
1519.8816	1519.8446	-0.037	-24	235	248	IGAVLNVTINHRGR		Mascot
1559.8138	1559.8219	0.0081	5	2226	2238	LIYSSILMDVFSR	Oxidation (M)[8]	Mascot
1572.7839	1572.8794	0.0955	61	1950	1962	QISMFQTLSFAQR	Oxidation (M)[4]	Mascot
1574.84	1574.9065	0.0665	42	1725	1736	FVIYLYNLNMLR	Oxidation (M)[10]	Mascot
1578.885	1578.8715	-0.0135	-9	408	421	QKLLNVSSFQSSLK		Mascot
1590.9102	1590.8862	-0.024	-15	1128	1141	AEFTQTVLITILNK		Mascot
1607.8387	1607.9105	0.0718	45	846	859	LVNLNSNQSNTLYK		Mascot
1613.905	1613.8188	-0.0862	-53	2109	2121	FQKSQFLTNILFK		Mascot
1629.8231	1629.884	0.0609	37	2046	2059	TTGVDLTNYKQFSR		Mascot
1635.845	1635.8901	0.0451	28	2056	2069	QFSRIVTNNSNIDK		Mascot
1783.9888	1783.8997	-0.0891	-50	113	127	LDNIVFRLHMAPTIK	Oxidation (M)[10]	Mascot
1822.9546	1823.0471	0.0925	51	816	832	ISQQSFLGSAASEKLEK		Mascot
1836.9888	1837.0265	0.0377	21	1664	1678	LDLLMVNYTNDKLLR	Oxidation (M)[5]	Mascot
1851.9884	1851.8506	-0.1378	-74	2614	2628	MYKTNLLQELVNLEK	Oxidation (M)[1]	Mascot
1876.1127	1875.9786	-0.1341	-71	230	246	IQSLKIGAVLNVTINHR		Mascot
1892.9712	1893.01	0.0388	20	2632	2648	SPNAQSFEKSEILSISR		Mascot
1953.0328	1953.0007	-0.0321	-16	872	888	AVFLVDYSENQISLLNK		Mascot
1967.0485	1966.9906	-0.0579	-29	1083	1099	NLEKITETFGSISTLWK		Mascot
2109.1338	2109.1589	0.0251	12	658	675	TNLNFKIDNILSISQNFK		Mascot
2109.1338	2109.1589	0.0251	12	658	675	TNLNFKIDNILSISQNFK		Mascot

5	10 kDa chaperonin OS=Legionella pneumophila (strain Corby) GN=groS PE=3 SV=1	CH10_LEGPC	10468.6	5.85	9	59	34.99	17.356

Protein Group

10 kDa chaperonin OS=Legionella pneumophila (strain Lens) GN=groS PE=3 SV=1	CH10_LEGPL	10468.6	5.8499 999046 3257
10 kDa chaperonin OS=Legionella pneumophila (strain Paris) GN=groS PE=3 SV=1	CH10_LEGPA	10468.6	5.8499 999046 3257
10 kDa chaperonin OS=Legionella pneumophila GN=groS PE=1 SV=1	CH10_LEGPN	10468.6	5.8499 999046 3257
10 kDa chaperonin OS=Legionella pneumophila subsp. pneumophila (strain Philadelphia 1 / ATCC 33152 / DSM 7513) GN=groS PE=3 SV=1	CH10_LEGPH	10468.6	5.8499 999046 3257

Peptide Information

Calc. Mass	Obsrv. Mass	± da	± ppm	Start Seq.	End Seq.	Sequence	Ion Score	C. I. %	Modification	Rank	Result Type
849.3883	849.457	0.0687	81	14	19	RMEEER					Mascot
906.5268	906.4809	-0.0459	-51	3	9	IRPLHDR					Mascot
1034.6218	1034.5588	-0.063	-61	2	9	KIRPLHDR					Mascot
1145.6348	1145.615	-0.0198	-17	77	86	VDGKELVVMR					Mascot
1145.6348	1145.615	-0.0198	-17	77	86	VDGKELVVMR					Mascot
1165.6622	1165.6284	-0.0338	-29	1	9	MKIRPLHDR					Mascot
1182.6001	1182.5983	-0.0018	-2	70	80	YSGTEVKVDGK					Mascot
1796.9866	1796.9415	-0.0451	-25	38	55	GEIIAVGAGKVLENGDVR					Mascot
1843.9583	1843.8727	-0.0856	-46	20	37	TTAGGIVIPDSATEKPMR					Mascot
1875.9554	1875.9786	0.0232	12	81	96	ELVVMREDDIMGVIEK					Mascot

6	Arginine--tRNA ligase OS=Ignicoccus hospitalis (strain KIN4/I / DSM 18386 / JCM 14125) GN=argS PE=3 SV=1	SYR_IGNH4	71180.4	5.66	19	58	12.304	11.892

Peptide Information

Calc. Mass	Obsrv. Mass	± da	± ppm	Start Seq.	End Seq.	Sequence	Ion Score	C. I. %	Modification	Rank	Result Type
867.541	867.4686	-0.0724	-83	395	401	LPQIQLR					Mascot
951.5509	951.5133	-0.0376	-40	369	376	DIAYTIKK					Mascot
1112.6422	1112.6166	-0.0256	-23	385	394	VINVIAAEQR					Mascot
1114.5376	1114.6252	0.0876	79	359	368	SDGTTLYTTR					Mascot

1128.6372	1128.6136	-0.0236	-21	70	80	GEIEGRLAGVK					Mascot
1151.5878	1151.5935	0.0057	5	140	149	NMFLGDSLVR					Mascot
1167.5828	1167.5771	-0.0057	-5	140	149	NMFLGDSLVR			Oxidation (M)[2]		Mascot
1187.6241	1187.6726	0.0485	41	233	241	WPEIVREMK					Mascot
1193.5685	1193.6403	0.0718	60	259	268	KYEEGDPEVK					Mascot
1202.7507	1202.7219	-0.0288	-24	170	180	QVAVLVYGLLK					Mascot
1202.7507	1202.7219	-0.0288	-24	170	180	QVAVLVYGLLK					Mascot
1331.6326	1331.6748	0.0422	32	23	33	GLEVEEEELER					Mascot
1347.6638	1347.6643	0.0005	0	460	471	SEAEDVDEVVKK					Mascot
1412.7719	1412.7742	0.0023	2	610	622	GLWLLGVEAPARM					Mascot
1521.8458	1521.8353	-0.0105	-7	140	152	NMFLGDSLVRILK			Oxidation (M)[2]		Mascot
1559.8138	1559.8219	0.0081	5	1	13	MYVLSETKSNFLK					Mascot
1590.8196	1590.8862	0.0666	42	540	553	DMILSIAEFPEVAR					Mascot
1606.8146	1606.9036	0.089	55	540	553	DMILSIAEFPEVAR			Oxidation (M)[2]		Mascot
1768.7946	1768.9301	0.1355	77	244	258	FDDEDPEAKVSELMK			Oxidation (M)[14]		Mascot
1833.9164	1833.9481	0.0317	17	436	450	GRMITLDWLLDEAER			Oxidation (M)[3]		Mascot
1902.9291	1903.0134	0.0843	44	454	470	ELVEGRSEAEDVDEVVK					Mascot
2108.0559	2108.1692	0.1133	54	181	198	LGRLEPPEGEKPDHWYG K					Mascot

7	Serine/threonine-protein kinase GIN4 OS=Saccharomyces cerevisiae (strain ATCC 204508 / S288c) GN=GIN4 PE=1 SV=1	GIN4_YEAST	130007	9.22	26	57	1.603	14.452

Peptide Information

Calc. Mass	Obsrv. Mass	± da	± ppm	Start Seq.	End Seq.	Sequence	Ion Score	C. I. %	Modification	Rank	Result Type
866.4003	866.447	0.0467	54	899	906	FNGSNEAK					Mascot
906.4791	906.4809	0.0018	2	1012	1018	DHLKEHK					Mascot
930.468	930.4752	0.0072	8	13	20	DNTIGPWK					Mascot
942.5255	942.5574	0.0319	34	269	276	ILTVDPER					Mascot
944.5272	944.5848	0.0576	61	394	401	VSTTPQRR					Mascot
987.572	987.5645	-0.0075	-8	595	602	LLEKEDLK					Mascot
1023.5656	1023.4998	-0.0658	-64	1106	1114	GKMSVVQFK					Mascot
1078.5739	1078.5786	0.0047	4	405	414	ESLISVTSSR					Mascot
1078.5748	1078.5786	0.0038	4	501	509	MSLIPSMKR			Oxidation (M)[1]		Mascot
1094.5697	1094.5826	0.0129	12	501	509	MSLIPSMKR			Oxidation (M)[1,7]		Mascot
1097.6313	1097.6506	0.0193	18	2	12	AINGNSIPAIK					Mascot
1098.6266	1098.6393	0.0127	12	269	277	ILTVDPERR					Mascot

Calc. Mass	Obsrv. Mass	± da	± ppm	Start Seq.	End Seq.	Sequence	Ion Score	C. I. %	Modification	Rank	Result Type
1128.5969	1128.6136	0.0167	15	811	820	LSVLSMYSTK					Mascot
1144.5919	1144.5885	-0.0034	-3	811	820	LSVLSMYSTK			Oxidation (M)[6]		Mascot
1151.6056	1151.5935	-0.0121	-11	1085	1094	LSSDNIFSLR					Mascot
1152.6008	1152.606	0.0052	5	963	971	EEKKPEQHK					Mascot
1228.6719	1228.5991	-0.0728	-59	1	12	MAINGNSIPAIK					Mascot
1278.6941	1278.6633	-0.0308	-24	821	831	ESFTNLVDILK					Mascot
1304.707	1304.7118	0.0048	4	664	674	NFSLQTRPVSR					Mascot
1320.6616	1320.6687	0.0071	5	732	742	MEYPSIIAPQR			Oxidation (M)[1]		Mascot
1347.7631	1347.6643	-0.0988	-73	1048	1059	ISAKQLFNGLEK					Mascot
1497.7948	1497.8668	0.072	48	716	728	FLGSSFNIDKELK					Mascot
1497.7948	1497.8668	0.072	48	716	728	FLGSSFNIDKELK					Mascot
1568.8333	1568.8164	-0.0169	-11	126	138	GPLPEHEAIRFFR					Mascot
1582.7609	1582.8146	0.0537	34	893	906	LNFADRFNGSNEAK					Mascot
1833.993	1833.9481	-0.0449	-24	648	662	NINHLEVDIDNILRR					Mascot
1866.8539	1866.9868	0.1329	71	245	260	VQKGEFEMPSDDEISR					Mascot
1902.9226	1903.0134	0.0908	48	631	647	LDGNIRSISAPMENEEK					Mascot
1953.0803	1953.0007	-0.0796	-41	156	171	DLKPENLLLDHKYNIK					Mascot

8 Nebulin-related-anchoring protein OS=Homo sapiens GN=NRAP PE=2 SV=2 NRAP_HUMAN 197919.7 9.24 35 57 0 41.865

Peptide Information

Calc. Mass	Obsrv. Mass	± da	± ppm	Start Seq.	End Seq.	Sequence	Ion Score	C. I. %	Modification	Rank	Result Type
856.4821	856.3976	-0.0845	-99	428	434	RTLHAMK					Mascot
867.4207	867.4686	0.0479	55	514	520	ADYEKNK					Mascot
906.4791	906.4809	0.0018	2	1553	1559	EAFLRDR					Mascot
930.4792	930.4752	-0.004	-4	1310	1316	HDFVKER					Mascot
969.486	969.5003	0.0143	15	196	203	GHDERISR					Mascot
971.538	971.6036	0.0656	68	1680	1688	RAAELANAR					Mascot
971.538	971.6036	0.0656	68	1680	1688	RAAELANAR					Mascot
1005.5033	1005.5252	0.0219	22	1429	1437	SPQMESAKK					Mascot
1023.5074	1023.4998	-0.0076	-7	1072	1080	MKGQMLGSR			Oxidation (M)[1]		Mascot
1078.6144	1078.5786	-0.0358	-33	802	811	LDSLTFLAAK					Mascot
1078.6144	1078.5786	-0.0358	-33	802	811	LDSLTFLAAK					Mascot
1088.5735	1088.5938	0.0203	19	767	775	DEPLFLQAR					Mascot
1094.6093	1094.5826	-0.0267	-24	367	375	LVSEVEYKK					Mascot
1128.5684	1128.6136	0.0452	40	1544	1552	EIASDFRYK					Mascot

1130.5913	1130.6245	0.0332	29	254	263	RANELASDVR					Mascot
1130.5913	1130.6245	0.0332	29	254	263	RANELASDVR					Mascot
1136.631	1136.6084	-0.0226	-20	537	546	TNAKLFSEVK					Mascot
1151.5803	1151.5935	0.0132	11	1541	1550	ASREIASDFR					Mascot
1152.6484	1152.606	-0.0424	-37	1402	1411	KAHALQSELR					Mascot
1196.559	1196.6097	0.0507	42	1327	1335	DDPRIQHCR			Carbamidomethyl (C)[8]		Mascot
1202.6165	1202.7219	0.1054	88	886	896	AQHLATDVGYK					Mascot
1202.6165	1202.7219	0.1054	88	886	896	AQHLATDVGYK					Mascot
1238.6012	1238.636	0.0348	28	1129	1139	AQTLASNQDYK					Mascot
1304.5913	1304.7118	0.1205	92	410	419	ENYQNHMRGR					Mascot
1315.7117	1315.662	-0.0497	-38	1403	1413	AHALQSELRYK					Mascot
1320.5862	1320.6687	0.0825	62	410	419	ENYQNHMRGR			Oxidation (M)[7]		Mascot
1380.7118	1380.7648	0.053	38	643	654	AQTLASDLDYRK					Mascot
1406.7386	1406.6857	-0.0529	-38	184	195	ANQLASQVEYKR					Mascot
1438.7147	1438.6798	-0.0349	-24	168	180	GSFPAMITPAYQR					Mascot
1490.7196	1490.8031	0.0835	56	656	667	LHEYTVLPEDMK			Oxidation (M)[11]		Mascot
1590.7871	1590.8862	0.0991	62	1560	1573	GLQIGYRSVDDDPR					Mascot
1602.8196	1602.8824	0.0628	39	655	667	KLHEYTVLPEDMK					Mascot
1629.9146	1629.884	-0.0306	-19	1414	1428	SDLIGMKGIGWLALR					Mascot
1796.9	1796.9415	0.0415	23	307	323	GKGSFPAMITPAYQNAK			Oxidation (M)[8]		Mascot
1809.7743	1809.9303	0.156	86	838	853	DVQGDSQMSHSLQMSK			Oxidation (M)[8,14]		Mascot
1827.9746	1827.9144	-0.0602	-33	594	610	AMGTADSRLLHSLQIAK			Oxidation (M)[2]		Mascot
1827.9746	1827.9144	-0.0602	-33	594	610	AMGTADSRLLHSLQIAK			Oxidation (M)[2]		Mascot
1833.9606	1833.9481	-0.0125	-7	343	359	GAAHYHSLPAQDNLVLK					Mascot
1843.8345	1843.8727	0.0382	21	151	165	KSLGEEYTEDYEQPR					Mascot
1927.9661	1928.0159	0.0498	26	1524	1540	AGSYDFRLDAIPFQTAR					Mascot
1927.9661	1928.0159	0.0498	26	1524	1540	AGSYDFRLDAIPFQTAR					Mascot
2109.0911	2109.1589	0.0678	32	341	359	MKGAAHYHSLPAQDNLVLK			Oxidation (M)[1]		Mascot
2109.0911	2109.1589	0.0678	32	341	359	MKGAAHYHSLPAQDNLVLK			Oxidation (M)[1]		Mascot

9	30S ribosomal protein S8 OS=Gluconacetobacter diazotrophicus (strain ATCC 49037 / DSM 5601 / PAI5) GN=rpsH PE=3 SV=1	RS8_GLUDA	14700	10.13	9	56	0	20.646

Peptide Information

Calc. Mass	Obsrv. Mass	± da	± ppm	Start Seq.	End Seq.	Sequence	Ion Score	C. I. %	Modification	Rank	Result Type
951.453	951.5133	0.0603	63	48	55	GYAQEDLR					Mascot

1126.6943	1126.6205	-0.0738	-66	57	66	GVAQLRIELK						Mascot
1145.5732	1145.615	0.0418	36	120	130	AANVGGEVLCR			Carbamidomethyl (C)[10]			Mascot
1145.5732	1145.615	0.0418	36	120	130	AANVGGEVLCR			Carbamidomethyl (C)[10]			Mascot
1238.6423	1238.636	-0.0063	-5	20	31	ARHAACVAPASK			Carbamidomethyl (C)[6]			Mascot
1304.6515	1304.7118	0.0603	46	2	13	SLSDPLGDMLTR						Mascot
1320.6465	1320.6687	0.0222	17	2	13	SLSDPLGDMLTR			Oxidation (M)[9]			Mascot
1467.6819	1467.7849	0.103	70	1	13	MSLSDPLGDMLTR			Oxidation (M)[1,10]			Mascot
1568.8431	1568.8164	-0.0267	-17	67	79	YLDGEPVIKEIHR						Mascot
1736.8669	1736.9037	0.0368	21	1	15	MSLSDPLGDMLTRIR			Oxidation (M)[1,10]			Mascot
1928.0964	1928.0159	-0.0805	-42	93	110	ELPRVYAGLGVSILSTPR						Mascot
1928.0964	1928.0159	-0.0805	-42	93	110	ELPRVYAGLGVSILSTPR						Mascot

10	Serine hydroxymethyltransferase OS=Roseiflexus sp. (strain RS-1) GN=glyA PE=3 SV=1	GLYA_ROSS1	47553.3	6.37	13	56	0	8.76

Peptide Information

Calc. Mass	Obsrv. Mass	± da	± ppm	Start Seq.	End Seq.	Sequence	Ion Score	C. I. %	Modification	Rank	Result Type
849.4135	849.457	0.0435	51	17	23	IIDGEMR			Oxidation (M)[6]		Mascot
929.5414	929.5046	-0.0368	-40	378	386	LGTPALTTR					Mascot
1005.5146	1005.5252	0.0106	11	17	24	IIDGEMRR			Oxidation (M)[6]		Mascot
1078.502	1078.5786	0.0766	71	418	426	EEVMALCAR			Carbamidomethyl (C)[7]		Mascot
1078.502	1078.5786	0.0766	71	418	426	EEVMALCAR	1	0	Carbamidomethyl (C)[7]		Mascot
1094.4969	1094.5826	0.0857	78	418	426	EEVMALCAR			Carbamidomethyl (C)[7], Oxidation (M)[4]		Mascot
1151.5878	1151.5935	0.0057	5	1	9	MSTLQTLWR			Oxidation (M)[1]		Mascot
1196.5902	1196.6097	0.0195	16	259	268	TLMMSELLDK			Oxidation (M)[3]		Mascot
1315.6965	1315.662	-0.0345	-26	341	353	AVSGKAAQEALDR					Mascot
1572.834	1572.8794	0.0454	29	346	360	AAQEALDRAAITTNK					Mascot
1613.8759	1613.8188	-0.0571	-35	219	232	YAHVVTSTTHKTLR					Mascot
1796.9501	1796.9415	-0.0086	-5	361	377	NAVPNDDKSPLITSGIR					Mascot
1834.0078	1833.9481	-0.0597	-33	269	286	MVIPGVQGGPLMHVIAAK			Oxidation (M)[1]		Mascot
1836.92	1837.0265	0.1065	58	236	252	GGIILMGEDFENPFGLK					Mascot
1927.9185	1928.0159	0.0974	51	287	303	AVGFGENLQPEFETYAR					Mascot
1927.9185	1928.0159	0.0974	51	287	303	AVGFGENLQPEFETYAR					Mascot

Rank	Protein Name	Accession No.	Protein MW	Protein PI	Pep. Count	Protein Score	Protein Score C. I. %	Intensity Matched	Total Ion Score	Total Ion C. I. %	Confirmed
1	Dephospho-CoA kinase OS=Rhodopseudomonas palustris (strain ATCC BAA-98 / CGA009) GN=coaE PE=3 SV=1	COAE_RHOPA	21540.4	5.98	11	74	97.797	9.264			

Peptide Information

Calc. Mass	Obsrv. Mass	± da	± ppm	Start Seq.	End Seq.	Sequence	Ion Score	C. I. %	Modification	Rank	Result Type
818.3712	818.4409	0.0697	85	157	163	QMPDAEK					Mascot
967.5029	967.5002	-0.0027	-3	69	77	VVHDPAAMK					Mascot
967.5029	967.5002	-0.0027	-3	69	77	VVHDPAAMK					Mascot
1228.6719	1228.6025	-0.0694	-56	186	196	EILEAAAKMPR					Mascot
1245.7236	1245.7362	0.0126	10	2	14	LVLGLTGSIGMGK					Mascot
1303.6675	1303.7183	0.0508	39	139	149	ILARDNMTPEK			Oxidation (M)[7]		Mascot
1320.757	1320.7295	-0.0275	-21	78	88	RLEGIVHPMLR					Mascot
1336.7518	1336.7333	-0.0185	-14	78	88	RLEGIVHPMLR			Oxidation (M)[9]		Mascot
1336.7518	1336.7333	-0.0185	-14	78	88	RLEGIVHPMLR			Oxidation (M)[9]		Mascot
1376.764	1376.7657	0.0017	1	1	14	MLVLGLTGSIGMGK					Mascot
1733.983	1734.0138	0.0308	18	2	19	LVLGLTGSIGMGKSTTAK					Mascot
1754.976	1754.92	-0.056	-32	121	136	RVDAVVVVTTSPEVQR					Mascot
1902.9597	1903.0477	0.088	46	20	37	LFAEAGVPVYDADATVHK					Mascot
1902.9597	1903.0477	0.088	46	20	37	LFAEAGVPVYDADATVHK					Mascot
2248.1497	2248.2319	0.0822	37	38	59	IYENEAVPAIEAAFPGTTLGGK					Mascot

Rank	Protein Name	Accession No.	Protein MW	Protein PI	Pep. Count	Protein Score	Protein Score C. I. %	Intensity Matched	Total Ion Score	Total Ion C. I. %	Confirmed
2	Exocyst complex component 5 OS=Caenorhabditis elegans GN=sec-10 PE=3 SV=1	EXOC5_CAEEL	76500.9	5.82	19	69	93.647	6.149			

Peptide Information

Calc. Mass	Obsrv. Mass	± da	± ppm	Start Seq.	End Seq.	Sequence	Ion Score	C. I. %	Modification	Rank	Result Type
805.4315	805.4517	0.0202	25	136	142	FANVQAR					Mascot
817.4315	817.4504	0.0189	23	476	481	WQQSLR					Mascot
849.4828	849.4609	-0.0219	-26	653	659	SIKNFQI					Mascot
866.473	866.4438	-0.0292	-34	649	655	TDFRSIK					Mascot
906.4349	906.4817	0.0468	52	27	33	QMNEEKK					Mascot

1120.6361	1120.6395	0.0034	3	494	503	QLNSIVGYVK						Mascot
1145.5732	1145.6306	0.0574	50	525	534	VSPQCQLASR			Carbamidomethyl (C)[5]			Mascot
1179.6844	1179.6649	-0.0195	-17	336	346	KQIGGGLHELK						Mascot
1200.6835	1200.6013	-0.0822	-68	465	475	ETIVEPSVAKK						Mascot
1253.6484	1253.6095	-0.0389	-31	42	52	LHEKNGEAIDK						Mascot
1259.6968	1259.6926	-0.0042	-3	136	146	FANVQARIGQR						Mascot
1291.7555	1291.6893	-0.0662	-51	234	244	LVLNIFTGRMK						Mascot
1349.7094	1349.7072	-0.0022	-2	482	493	SLESKMSLGLER						Mascot
1358.6733	1358.731	0.0577	42	55	66	QLDHTMQAVSTK						Mascot
1376.6403	1376.7657	0.1254	91	304	314	YIATYCSEELK			Carbamidomethyl (C)[6]			Mascot
1381.7396	1381.7048	-0.0348	-25	168	179	MAEVAKILSEFK			Oxidation (M)[1]			Mascot
1903.0647	1903.0477	-0.017	-9	391	406	EELPRHVENIVDVLLK						Mascot
1903.0647	1903.0477	-0.017	-9	391	406	EELPRHVENIVDVLLK						Mascot
2248.1609	2248.2319	0.071	32	284	303	LHISPDSAFLSTLTDSIFQ R						Mascot
3402.6887	3402.8711	0.1824	54	353	383	LMNVETYGGETFVAEDV AISILQETKNAFGR						Mascot

3 ATP-dependent RNA helicase MSS116, mitochondrial OS=Lodderomyces elongisporus (strain ATCC 11503 / CBS 2605 / JCM 1781 / NBRC 1676 / NRRL YB-4239) GN=MSS116 PE=3 SV=1 | MS116_LODEL | 77277.4 | 9.06 | 19 | 66 | 87.895 | 26.655

Peptide Information

Calc. Mass	Obsrv. Mass	± da	± ppm	Start Seq.	End Seq.	Sequence	Ion Score	C. I. %	Modification	Rank	Result Type
832.4384	832.3593	-0.0791	-95	435	441	SASQRQR					Mascot
835.4785	835.4278	-0.0507	-61	442	448	ALSNFKR					Mascot
849.4135	849.4609	0.0474	56	371	377	CKDAIDK			Carbamidomethyl (C)[1]		Mascot
963.537	963.5076	-0.0294	-31	440	447	QRALSNFK					Mascot
1179.6368	1179.6649	0.0281	24	324	334	SLLFSATVDAR					Mascot
1228.6896	1228.6025	-0.0871	-71	133	143	NTGLIDDVILR					Mascot
1253.6848	1253.6095	-0.0753	-60	256	267	NPSEIVIATPGR					Mascot
1303.6349	1303.7183	0.0834	64	102	112	NINTNTNKNDR					Mascot
1320.6642	1320.7295	0.0653	49	81	92	GSQVTEQTELTK					Mascot
1329.7347	1329.762	0.0273	21	238	249	KPHISYVIGGMK					Mascot
1381.6859	1381.7048	0.0189	14	608	618	DVVREFFEQGR					Mascot
1733.9181	1734.0138	0.0957	55	569	583	LRADDLVAENVSLYR					Mascot
1903.0219	1903.0477	0.0258	14	232	249	GISGSRKPHISYVIGGMK			Oxidation (M)[17]		Mascot
1903.0219	1903.0477	0.0258	14	232	249	GISGSRKPHISYVIGGMK			Oxidation (M)[17]		Mascot

Calc. Mass	Obsrv. Mass	± da	± ppm	Start Seq.	End Seq.	Sequence	Ion Score	C. I. %	Modification	Rank	Result Type
1951.0608	1951.0365	-0.0243	-12	256	273	NPSEIVIATPGRLEADLR					Mascot
1952.031	1952.0386	0.0076	4	497	513	EGKAVLFITQPEMAYVR					Mascot
1994.0593	1994.0789	0.0196	10	268	285	LEADLRSPLFASAFTDIK					Mascot
2013.0818	2013.1245	0.0427	21	21	37	IHFPVISRGFHNSFINK					Mascot
2013.0818	2013.1245	0.0427	21	21	37	IHFPVISRGFHNSFINK					Mascot
2035.0343	2035.0688	0.0345	17	316	334	SDDAEPLKSLLFSATVDAR					Mascot
2226.1555	2226.271	0.1155	52	468	487	GVTHVVQLFPSSEIADYVHK					Mascot
2226.1555	2226.271	0.1155	52	468	487	GVTHVVQLFPSSEIADYVHK					Mascot

4 Glutamate--tRNA ligase 2 OS=Sulfurimonas denitrificans (strain ATCC 33889 / DSM 1251) GN=gltX2 PE=3 SV=1 | SYE2_SULDN | 50168 | 5.91 | 15 | 59 | 31.926 | 16.002

Peptide Information

Calc. Mass	Obsrv. Mass	± da	± ppm	Start Seq.	End Seq.	Sequence	Ion Score	C. I. %	Modification	Rank	Result Type
805.405	805.4517	0.0467	58	106	112	EEAKNSK					Mascot
839.437	839.4061	-0.0309	-37	110	116	NSKTAYR					Mascot
935.5197	935.5031	-0.0166	-18	425	432	NYLGEIVK					Mascot
939.5258	939.5032	-0.0226	-24	151	158	DHIKGEIK					Mascot
1291.6827	1291.6893	0.0066	5	291	301	SPARFDINMLK					Mascot
1302.7198	1302.7197	-0.0001	0	80	90	AMALQLLQDKR			Oxidation (M)[2]		Mascot
1349.7423	1349.7072	-0.0351	-26	386	397	NYIIQESGLKGK					Mascot
1358.725	1358.731	0.006	4	295	305	FDINMLKHVNK					Mascot
1467.7479	1467.7992	0.0513	35	319	332	YVGFADAQIGEVAK					Mascot
1467.7479	1467.7992	0.0513	35	319	332	YVGFADAQIGEVAK					Mascot
1706.9185	1706.9257	0.0072	4	197	211	GEDHVSNTPKQILIR					Mascot
1794.9272	1794.8594	-0.0678	-38	356	370	NIPDEFKEYAQTIVK					Mascot
1859.9572	1859.9945	0.0373	20	405	421	ILLMGSEHGPDIATVYK			Oxidation (M)[4]		Mascot
1953.0439	1953.0508	0.0069	4	219	235	NIEYAHLPIILNDDGKK					Mascot
2013.0157	2013.1245	0.1088	54	1	18	MLRFAPSPTGDMHIGNLR					Mascot
2013.0157	2013.1245	0.1088	54	1	18	MLRFAPSPTGDMHIGNLR	1	0			Mascot
2297.1699	2297.2886	0.1187	52	272	290	EIFTIQEAIEWFSLENISK					Mascot

5 Thiazole synthase OS=Moorella thermoacetica (strain ATCC 39073) GN=thiG PE=3 SV=1 | THIG_MOOTA | 27186.2 | 5.27 | 11 | 58 | 3.843 | 5.036

Peptide Information

Calc. Mass	Obsrv. Mass	± da	± ppm	Start Seq.	End Seq.	Sequence	Ion Score	C. I. %	Modification	Rank	Result Type

805.4349	805.4517	0.0168	21	165	170	TREMIR					Mascot
1120.5555	1120.6395	0.084	75	1	10	MADELIIGDK			Oxidation (M)[1]		Mascot
1179.619	1179.6649	0.0459	39	231	241	MAYLAGLAPTR			Oxidation (M)[1]		Mascot
1302.6736	1302.7197	0.0461	35	86	97	IARAAGCGNWVK			Carbamidomethyl (C)[7]		Mascot
1501.822	1501.8647	0.0427	28	2	15	ADELIIGDKALTSR					Mascot
1706.8353	1706.9257	0.0904	53	60	75	FIPAGCVLMPNTSGAR			Carbamidomethyl (C)[6], Oxidation (M)[9]		Mascot
1794.8625	1794.8594	-0.0031	-2	214	230	DPVAMARAFSMAVEAGR			Oxidation (M)[5]		Mascot
1902.9491	1903.0477	0.0986	52	89	105	AAGCGNWVKIEVVTDQR			Carbamidomethyl (C)[4]		Mascot
1902.9491	1903.0477	0.0986	52	89	105	AAGCGNWVKIEVVTDQR			Carbamidomethyl (C)[4]		Mascot
1953.0586	1953.0508	-0.0078	-4	142	161	LKEAGAAAIMPLGAPIGS NR			Oxidation (M)[10]		Mascot
1994.0852	1994.0789	-0.0063	-3	144	164	EAGAAAIMPLGAPIGSNR GLK					Mascot
3218.6992	3218.6941	-0.0051	-2	16	46	LFIGTGKFASHEIMGQAV SQSGAQVVTVALR			Oxidation (M)[14]		Mascot

6 Methionyl-tRNA formyltransferase, mitochondrial OS=Homo sapiens GN=MTFMT PE=1 SV=2 | FMT_HUMAN | 44203.4 | 9.7 | 12 | 57 | 0 | 25.105

Peptide Information

Calc. Mass	Obsrv. Mass	± da	± ppm	Start Seq.	End Seq.	Sequence	Ion Score	C. I. %	Modification	Rank	Result Type
812.4413	812.4322	-0.0091	-11	44	49	EKPPWR					Mascot
888.5189	888.4931	-0.0258	-29	182	189	FDVGPILK					Mascot
1107.6382	1107.6079	-0.0303	-27	61	70	EALRALHAAR					Mascot
1303.7216	1303.7183	-0.0033	-3	199	210	STAKELEAVLSR					Mascot
1320.6252	1320.7295	0.1043	79	232	243	QQPMEGATYAPK					Mascot
1336.6202	1336.7333	0.1131	85	232	243	QQPMEGATYAPK			Oxidation (M)[4]		Mascot
1336.6202	1336.7333	0.1131	85	232	243	QQPMEGATYAPK			Oxidation (M)[4]		Mascot
1376.7355	1376.7657	0.0302	22	326	337	DGWIGVRSVMLK			Oxidation (M)[10]		Mascot
1381.7686	1381.7048	-0.0638	-46	190	202	QETVPVPPKSTAK					Mascot
1795.0034	1794.8594	-0.144	-80	81	97	LEVVTMPSPSPKGLPVK			Oxidation (M)[6]		Mascot
2013.1201	2013.1245	0.0044	2	267	284	AIGNIIPLQTLWMANTIK			Oxidation (M)[13]		Mascot
2013.1201	2013.1245	0.0044	2	267	284	AIGNIIPLQTLWMANTIK			Oxidation (M)[13]		Mascot
2035.0793	2035.0688	-0.0105	-5	316	332	QSQILLVYCKDGWIGVR			Carbamidomethyl (C)[9]		Mascot
2226.2275	2226.271	0.0435	20	211	231	LGANMLISVLKNLPESLS NGR					Mascot
2226.2275	2226.271	0.0435	20	211	231	LGANMLISVLKNLPESLS NGR					Mascot
3402.6277	3402.8711	0.2434	72	98	128	QYAVQSQLPVYEWPDVG SGEYDVGVVASFGR					Mascot

7 Chromosome partition protein MukE OS=Haemophilus | MUKE_HAES1 | 28006.4 | 5.04 | 10 | 55 | 0 | 8.067

somnus (strain 129Pt) GN=mukE PE=3 SV=1

Peptide Information

Calc. Mass	Obsrv. Mass	± da	± ppm	Start Seq.	End Seq.	Sequence	Ion Score	C. I. %	Modification	Rank	Result Type
906.5043	906.4817	-0.0226	-25	60	66	YNVELIR					Mascot
1200.5856	1200.6013	0.0157	13	193	203	SGDDPLEAQLR					Mascot
1259.6842	1259.6926	0.0084	7	2	12	TDNIQDLLSIK					Mascot
1304.713	1304.7412	0.0282	22	85	96	SVLSELEMLVGK					Mascot
1304.713	1304.7412	0.0282	22	85	96	SVLSELEMLVGK					Mascot
1320.708	1320.7295	0.0215	16	85	96	SVLSELEMLVGK			Oxidation (M)[8]		Mascot
1329.7122	1329.762	0.0498	37	135	147	AINLRAGGSDLDK					Mascot
1406.7196	1406.7023	-0.0173	-12	1	12	MTDNIQDLLSIK			Oxidation (M)[1]		Mascot
1490.6969	1490.8264	0.1295	87	207	220	EGEAATAQSLQEEK					Mascot
1582.8547	1582.8483	-0.0064	-4	193	206	SGDDPLEAQLRLIR					Mascot
1859.9247	1859.9945	0.0698	38	187	203	FGAEVRSGDDPLEAQLR					Mascot
1902.9403	1903.0477	0.1074	56	207	224	EGEAATAQSLQEEKNGL K					Mascot
1902.9403	1903.0477	0.1074	56	207	224	EGEAATAQSLQEEKNGL K					Mascot

8	ATP synthase gamma chain OS=Streptococcus mutans serotype c (strain ATCC 700610 / UA159) GN=atpG PE=3 SV=2	ATPG_STRMU	32385.6	5.38	11	53	0	6.023

Peptide Information

Calc. Mass	Obsrv. Mass	± da	± ppm	Start Seq.	End Seq.	Sequence	Ion Score	C. I. %	Modification	Rank	Result Type
817.4666	817.4504	-0.0162	-20	261	267	VIEDLTK					Mascot
820.4788	820.467	-0.0118	-14	268	273	LYNRVR					Mascot
834.4567	834.4919	0.0352	42	2	9	TGSLSEIK					Mascot
963.5469	963.5076	-0.0393	-41	12	20	ITSTQKTGK					Mascot
971.4833	971.5494	0.0661	68	42	49	DFQIYASK					Mascot
1253.6592	1253.6095	-0.0497	-40	21	32	ITSAMKMVSSAK					Mascot
1308.7535	1308.7341	-0.0194	-15	131	141	ARHIPVAFELR					Mascot
1927.9971	1928.0287	0.0316	16	274	292	QAAITQEITEIVAGANALD					Mascot
1950.8903	1951.0365	0.1462	75	113	130	GDDYTIISIGGMGSDFFR					Mascot
1966.8853	1967.0161	0.1308	67	113	130	GDDYTIISIGGMGSDFFR			Oxidation (M)[12]		Mascot
1966.8853	1967.0161	0.1308	67	113	130	GDDYTIISIGGMGSDFFR			Oxidation (M)[12]		Mascot
1994.0991	1994.0789	-0.0202	-10	89	107	GLVGAYNSTILKAVMDTI K					Mascot

2034.8856	2035.0688	0.1832	90	241	260	TAEHAAGMTAMQTATDN ADK					Mascot

9	Uncharacterized protein YtoI OS=Bacillus subtilis (strain 168) GN=ytoI PE=4 SV=1	YTOI_BACSU	49006.8	6.55	14	53	0	5.366

Peptide Information

Calc. Mass	Obsrv. Mass	± da	± ppm	Start Seq.	End Seq.	Sequence	Ion Score	C. I. %	Modification	Rank	Result Type
805.46	805.4517	-0.0083	-10	293	299	LIGMISR			Oxidation (M)[4]		Mascot
817.4414	817.4504	0.009	11	319	325	LDDIVSR					Mascot
963.5509	963.5076	-0.0433	-45	178	185	AIYDQLIK					Mascot
1078.5238	1078.5891	0.0653	61	225	233	FPVVDDQMK					Mascot
1120.603	1120.6395	0.0365	33	249	258	NASIEKVMTK					Mascot
1253.6051	1253.6095	0.0044	4	429	438	AMLMVQLMER			Oxidation (M)[2,4]		Mascot
1302.745	1302.7197	-0.0253	-19	255	266	VMTKNPVTVIGK			Oxidation (M)[2]		Mascot
1308.6473	1308.7341	0.0868	66	429	439	AMLMVQLMERS					Mascot
1582.8687	1582.8483	-0.0204	-13	187	200	EIVLVEDILTPADR					Mascot
1903.0284	1903.0477	0.0193	10	72	89	LTYAEVVNVIDGQVLGGR					Mascot
1903.0284	1903.0477	0.0193	10	72	89	LTYAEVVNVIDGQVLGGR					Mascot
1928.031	1928.0287	-0.0023	-1	225	241	FPVVDDQMKIHGILTSK					Mascot
1953.0778	1953.0508	-0.027	-14	112	129	YTAAGNLLIVGNRINAHR					Mascot
1955.9751	1956.0511	0.076	39	95	111	TLNKFVIGAMELDAMMR			Oxidation (M)[10]		Mascot
2297.25	2297.2886	0.0386	17	5	24	HEQILTYIDSLPVGEKISV R					Mascot

10	Isoleucine--tRNA ligase OS=Methanocorpusculum labreanum (strain ATCC 43576 / DSM 4855 / Z) GN=ileS PE=3 SV=1	SYI_METLZ	123085.9	5.17	21	52	0	10.802

Peptide Information

Calc. Mass	Obsrv. Mass	± da	± ppm	Start Seq.	End Seq.	Sequence	Ion Score	C. I. %	Modification	Rank	Result Type
817.4413	817.4504	0.0091	11	103	109	NKADIEK					Mascot
820.4345	820.467	0.0325	40	981	986	IQEMRK			Oxidation (M)[4]		Mascot
888.4859	888.4931	0.0072	8	1007	1014	VMPLVDSK					Mascot
943.5458	943.5114	-0.0344	-36	260	267	ELAEQILK					Mascot
967.4666	967.5002	0.0336	35	611	618	QFGVDVMR			Oxidation (M)[7]		Mascot
967.4666	967.5002	0.0336	35	611	618	QFGVDVMR			Oxidation (M)[7]		Mascot
1097.5586	1097.6644	0.1058	96	1015	1023	HDVIETEVR					Mascot
1120.5316	1120.6395	0.1079	96	450	457	DWCISRQR			Carbamidomethyl (C)[3]		Mascot
1228.6896	1228.6025	-0.0871	-71	996	1006	IKAEVVIDDAR					Mascot

1231.7046	1231.6536	-0.051	-41	867	877	TVTVVKGVWDK		Mascot
1253.5719	1253.6095	0.0376	30	739	748	MWLEEESVSK	Oxidation (M)[1]	Mascot
1259.6155	1259.6926	0.0771	61	271	280	YQDYSIIETK		Mascot
1291.7256	1291.6893	-0.0363	-28	260	270	ELAEQILKYGK		Mascot
1320.7158	1320.7295	0.0137	10	902	913	AFIEEANGTKLK		Mascot
1329.666	1329.762	0.096	72	644	653	MFNVLWNVYK	Oxidation (M)[1]	Mascot
1490.7308	1490.8264	0.0956	64	580	593	SVLMHGFALDAEGK	Oxidation (M)[4]	Mascot
1949.979	1950.0818	0.1028	53	576	593	APYKSVLMHGFALDAEG K	Oxidation (M)[8]	Mascot
1967.0267	1967.0161	-0.0106	-5	1007	1023	VMPLVDSKHDVIETEVR		Mascot
1967.0267	1967.0161	-0.0106	-5	1007	1023	VMPLVDSKHDVIETEVR		Mascot
2035.0244	2035.0688	0.0444	22	474	490	YHVVGRYEELEQLSGQK		Mascot
2248.1802	2248.2319	0.0517	23	560	579	GWFYSQLALSTIAFGKAP YK		Mascot
2421.0598	2421.2732	0.2134	88	783	803	CEGDLPSVHMVDWFSG NDALR	Carbamidomethyl (C)[1], Oxidation (M)[10]	Mascot
3402.6497	3402.8711	0.2214	65	308	338	VVIADYVAMENTGMVHIA PGHGWDDYLVGLK	Oxidation (M)[9,14]	Mascot

Gel Idx/Pos	197/H24	Instr./Gel Origin	BA2151/Sample Project 20140814	Process Status	Analysis Succeeded
Plate [#] Name	[1] Sample Project 20140814	Instrument Sample Name		Spectra	11

Rank	Protein Name	Accession No.	Protein MW	Protein PI	Pep. Count	Protein Score	Protein Score C. I. %	Intensity Matched	Total Ion Score	Total Ion C. I. %	Confirmed
1	Carbonic anhydrase, chloroplastic OS=Hordeum vulgare PE=2 SV=1	CAHC_HORVU	35736.1	8.93	8	83	99.735	5.659	57	99.841	

Peptide Information

Calc. Mass	Obsrv. Mass	± da	± ppm	Start Seq.	End Seq.	Sequence	Ion Score	C. I. %	Modification	Rank	Result Type
856.4563	856.4152	-0.0411	-48	132	138	TGFEKFK					Mascot
1120.6038	1120.6555	0.0517	46	145	153	KPDFFEPLK					Mascot
1187.6685	1187.6923	0.0238	20	13	22	SPVFVFAHKR					Mascot
1219.5234	1219.6055	0.0821	67	160	169	YMVFACADSR			Carbamidomethyl (C)[6]		Mascot
1235.5184	1235.5841	0.0657	53	160	169	YMVFACADSR			Carbamidomethyl (C)[6], Oxidation (M)[2]		Mascot
1278.6478	1278.718	0.0702	55	307	318	LVGGHYDFVSGK					Mascot
1794.7719	1794.8651	0.0932	52	239	253	DGADDSFHFVEDWVR					Mascot
1794.7719	1794.8651	0.0932	52	239	253	DGADDSFHFVEDWVR	41	93.846			Mascot
1855.9476	1856.0358	0.0882	48	139	153	TEVYDKKPDFFEPLK					Mascot
1946.0052	1946.1089	0.1037	53	170	187	VCPSVTLGLEPGEAFTIR			Carbamidomethyl (C)[2]		Mascot
1946.0052	1946.1089	0.1037	53	170	187	VCPSVTLGLEPGEAFTIR	16	0	Carbamidomethyl (C)[2]		Mascot

Rank	Protein Name	Accession No.	Protein MW	Protein PI	Pep. Count	Protein Score	Protein Score C. I. %	Intensity Matched	Total Ion Score	Total Ion C. I. %	Confirmed
2	Nesprin-1 OS=Mus musculus GN=Syne1 PE=1 SV=2	SYNE1_MOUSE	1016650.4	5.43	68	64	77.459	43.048			

Peptide Information

Calc. Mass	Obsrv. Mass	± da	± ppm	Start Seq.	End Seq.	Sequence	Ion Score	C. I. %	Modification	Rank	Result Type
849.4577	849.4711	0.0134	16	8650	8656	EVSHHIK					Mascot
856.4271	856.4152	-0.0119	-14	8731	8737	SDPRPER					Mascot
866.4586	866.452	-0.0066	-8	6063	6069	MSTIRMK					Mascot
871.5472	871.5052	-0.042	-48	1332	1338	RIQVSLR					Mascot
889.4924	889.4626	-0.0298	-34	8437	8443	MKQNLQK					Mascot
905.4873	905.4216	-0.0657	-73	8437	8443	MKQNLQK			Oxidation (M)[1]		Mascot
906.5295	906.4916	-0.0379	-42	5122	5129	LSEFAVLK					Mascot
938.5458	938.516	-0.0298	-32	5736	5743	HSLIFPPK					Mascot
1057.5889	1057.4905	-0.0984	-93	6624	6632	EEIQQLLGK					Mascot
1078.5891	1078.5875	-0.0016	-1	3910	3918	ERVFSLEAK					Mascot
1097.595	1097.6626	0.0676	62	5448	5457	AKTDDLVHAK					Mascot

1120.6361	1120.6555	0.0194	17	7193	7202	FGSITNQLLK		Mascot
1142.6528	1142.6293	-0.0235	-21	5315	5323	LVKQELQER		Mascot
1144.6208	1144.6077	-0.0131	-11	1968	1978	QSEADALVALK		Mascot
1145.714	1145.6301	-0.0839	-73	5438	5447	DLTTILTKLK		Mascot
1179.6191	1179.6605	0.0414	35	4175	4183	DFIKQLQCK	Carbamidomethyl (C)[8]	Mascot
1182.6001	1182.6144	0.0143	12	2137	2146	QKELDSFTSK		Mascot
1196.5946	1196.6228	0.0282	24	2490	2499	ANEEFQAFLK		Mascot
1208.6746	1208.6232	-0.0514	-43	7048	7057	LKEQHLLGDR		Mascot
1208.6746	1208.6232	-0.0514	-43	7048	7057	LKEQHLLGDR		Mascot
1228.6394	1228.5996	-0.0398	-32	503	511	MEFLELKYR		Mascot
1278.6338	1278.718	0.0842	66	3641	3650	KWHEDLSAHR		Mascot
1281.6508	1281.6295	-0.0213	-17	2817	2827	TQFQDIMTVAK		Mascot
1297.6458	1297.6373	-0.0085	-7	2817	2827	TQFQDIMTVAK	Oxidation (M)[7]	Mascot
1406.7638	1406.7052	-0.0586	-42	5966	5976	TLYEVLERQQK		Mascot
1490.8578	1490.8168	-0.041	-28	7295	7307	GLETVKDSLFILR		Mascot
1493.7855	1493.8135	0.028	19	756	768	VPVMDAQYKMIAK		Mascot
1631.821	1631.8483	0.0273	17	3772	3785	AMKFLAEHEAEALR	Oxidation (M)[2]	Mascot
1647.845	1647.8419	-0.0031	-2	8216	8229	LPVRLPDDHDLSDR		Mascot
1678.8258	1678.8385	0.0127	8	489	502	FHFVSSTSELHLMK	Oxidation (M)[13]	Mascot
1706.8014	1706.8285	0.0271	16	8323	8339	GAVGLSGDPSSLESQMR	Oxidation (M)[16]	Mascot
1794.877	1794.8651	-0.0119	-7	7439	7453	HWSLTSSQTTERFSK		Mascot
1794.877	1794.8651	-0.0119	-7	7439	7453	HWSLTSSQTTERFSK		Mascot
1810.9294	1810.8536	-0.0758	-42	2444	2460	LHNLQGVLDSLSDGQSK		Mascot
1811.8302	1811.973	0.1428	79	7792	7805	NKELCEWLTQMESK	Carbamidomethyl (C)[5], Oxidation (M)[11]	Mascot
1811.8606	1811.973	0.1124	62	8083	8097	QMVHGGNQRWDDLQK		Mascot
1822.9407	1822.8593	-0.0814	-45	7050	7065	EQHLLGDRNSVENALK		Mascot
1826.9355	1826.8625	-0.073	-40	2575	2591	LSQRGQLLSEESHSAGK		Mascot
1851.9408	1852.0175	0.0767	41	4279	4294	LALALQEEMYAIDDLK	Oxidation (M)[9]	Mascot
1859.0022	1859.0078	0.0056	3	6335	6351	LLSGVLPFRGEAQTQDK		Mascot
1859.9432	1860.0336	0.0904	49	1643	1657	EMQTALEDLLARWQR		Mascot
1861.0178	1861.0289	0.0111	6	7186	7202	QEGGLQKFGSITNQLLK		Mascot
1874.8953	1875.0205	0.1252	67	337	352	AQMTESSLQDKYQAFK		Mascot
1875.9381	1876.0226	0.0845	45	1643	1657	EMQTALEDLLARWQR	Oxidation (M)[2]	Mascot
1875.9381	1876.0226	0.0845	45	1643	1657	EMQTALEDLLARWQR	Oxidation (M)[2]	Mascot
1892.9205	1893.0314	0.1109	59	2094	2110	STVCNVLEDASNVVVMR	Carbamidomethyl (C)[4]	Mascot
1902.0114	1902.057	0.0456	24	7885	7901	ETLVAVQQLDKNMGSLR		Mascot

1909.0542	1909.0375	-0.0167	-9	7616	7632	VFLRQQGSYILTVEAGK				Mascot
1918.0062	1918.0648	0.0586	31	7885	7901	ETLVAVQQLDKNMGSLR			Oxidation (M)[13]	Mascot
1922.9818	1923.0242	0.0424	22	5407	5422	IHDQIQEKVQEIEEGK				Mascot
1924.8958	1925.0469	0.1511	78	2167	2182	TDMESTLDKWLDVSER				Mascot
1926.9994	1926.9918	-0.0076	-4	1227	1242	LLSNFGECVQYKEIVK			Carbamidomethyl (C)[8]	Mascot
1928.9681	1929.0305	0.0624	32	6468	6483	LSLLDSVVDQRCHQMK			Carbamidomethyl (C)[12]	Mascot
1931.9896	1932.0483	0.0587	30	7285	7300	WIQDCNDLLKGLETVK			Carbamidomethyl (C)[5]	Mascot
1943.9994	1944.0503	0.0509	26	3002	3018	EILDALQKAEPMTEDLK				Mascot
1949.9716	1950.0786	0.107	55	7776	7791	QQVSERLNEWAVFSEK				Mascot
1950.9702	1951.0499	0.0797	41	5600	5615	LSQQVTQFGREMEELR				Mascot
1951.943	1952.0452	0.1022	52	3088	3104	CFDLPQNLSEVSSSLQK			Carbamidomethyl (C)[1]	Mascot
1955.1324	1955.0426	-0.0898	-46	4643	4660	LSALPQQFNVIVALAKDK				Mascot
1966.9651	1967.0344	0.0693	35	5600	5615	LSQQVTQFGREMEELR	4	0	Oxidation (M)[12]	Mascot
1967.0306	1967.0344	0.0038	2	4474	4489	IVFREYICLLPDDVSK			Carbamidomethyl (C)[8]	Mascot
1972.9467	1973.0416	0.0949	48	6566	6581	LQDMYDELLMTVSSRR			Oxidation (M)[4]	Mascot
1983.0038	1983.0267	0.0229	12	3038	3054	VSGLIKEYNCLCLQASK			Carbamidomethyl (C)[10,12]	Mascot
1983.0038	1983.0267	0.0229	12	3038	3054	VSGLIKEYNCLCLQASK			Carbamidomethyl (C)[10,12]	Mascot
1988.9996	1989.0048	0.0052	3	2425	2443	AAARESSNLTGDSQILEAR				Mascot
1990.0096	1990.0015	-0.0081	-4	5294	5310	AITALQDQCLNMQEKVK			Carbamidomethyl (C)[9]	Mascot
1996.9281	1997.0676	0.1395	70	1031	1047	FSAAVEECRAELEQETK			Carbamidomethyl (C)[8]	Mascot
2322.1792	2322.283	0.1038	45	2094	2114	STVCNVLEDASNVVVMRATIK			Carbamidomethyl (C)[4], Oxidation (M)[16]	Mascot
2588.2231	2588.324	0.1009	39	2616	2637	SCQLALKEHEALEEATQSMWAR			Carbamidomethyl (C)[2]	Mascot
2635.2854	2635.3235	0.0381	14	5517	5538	LSKLNQALSHMEEYNEMLETVR				Mascot
2651.2803	2651.3323	0.052	20	5517	5538	LSKLNQALSHMEEYNEMLETVR			Oxidation (M)[11]	Mascot
3217.5869	3217.7222	0.1353	42	5281	5308	AFPGTEEEVPILRAITALQDQCLNMQEK			Carbamidomethyl (C)[22], Oxidation (M)[25]	Mascot
3217.5869	3217.7222	0.1353	42	5281	5308	AFPGTEEEVPILRAITALQDQCLNMQEK			Carbamidomethyl (C)[22], Oxidation (M)[25]	Mascot
3234.5559	3234.707	0.1511	47	7902	7928	TWLAHMESELAKPIVYDSCNSEEIQRK			Carbamidomethyl (C)[19]	Mascot
3239.4475	3239.7078	0.2603	80	3389	3415	WTSYQDDVRQFSSWMDSVEVSLTESEK				Mascot
3243.6165	3243.696	0.0795	25	7727	7754	WTDDLTELMLVRDALAVYLSAEDISMLK			Oxidation (M)[9,26]	Mascot
3255.4424	3255.6965	0.2541	78	3389	3415	WTSYQDDVRQFSSWMDSVEVSLTESEK			Oxidation (M)[15]	Mascot
3268.5422	3268.7007	0.1585	48	7794	7820	ELCEWLTQMESKVSQNGDILIEEMIEK			Carbamidomethyl (C)[3], Oxidation (M)[9]	Mascot
3272.6025	3272.6904	0.0879	27	6553	6580	LQVEQSAVQELSKLQDMYDELLMTVSSR			Oxidation (M)[17,23]	Mascot
3403.5281	3403.8267	0.2986	88	3283	3311	FSQGVKELQDWMSDAVHMLDSYCLPTSDK			Carbamidomethyl (C)[23], Oxidation (M)[12]	Mascot

3	tRNA-specific 2-thiouridylase MnmA OS=Aromatoleum aromaticum (strain EbN1) GN=mnmA PE=3 SV=1	MNMA_AROAE	42033.9	5.49	12	58	21.84	43.581

Peptide Information

Calc. Mass	Obsrv. Mass	± da	± ppm	Start Seq.	End Seq.	Sequence	Ion Score	C. I. %	Modification	Rank	Result Type
867.4723	867.4846	0.0123	14	86	92	VFADFLR					Mascot
1138.6005	1138.6073	0.0068	6	84	92	DRVFADFLR					Mascot
1208.6633	1208.6232	-0.0401	-33	188	199	IAAEAGLHVAEK					Mascot
1208.6633	1208.6232	-0.0401	-33	188	199	IAAEAGLHVAEK					Mascot
1278.757	1278.718	-0.039	-30	173	183	TLFPLGALYKR					Mascot
1860.0127	1860.0336	0.0209	11	281	297	ANVLYAVQGHEHPALLK					Mascot
1901.9651	1902.057	0.0919	48	220	235	YLPMRPGEIRNLDDGR					Mascot
1917.96	1918.0648	0.1048	55	220	235	YLPMRPGEIRNLDDGR			Oxidation (M)[4]		Mascot
1926.9722	1926.9918	0.0196	10	312	326	DPHTHWVYTAKPRYR					Mascot
1943.9471	1944.0503	0.1032	53	127	143	IATGHYAQVREWANDGR					Mascot
1967.0281	1967.0344	0.0063	3	236	252	VIGEHQGLMYHTIGQRK					Mascot
1967.0281	1967.0344	0.0063	3	236	252	VIGEHQGLMYHTIGQRK					Mascot
1983.0229	1983.0267	0.0038	2	236	252	VIGEHQGLMYHTIGQRK			Oxidation (M)[9]		Mascot
1983.0229	1983.0267	0.0038	2	236	252	VIGEHQGLMYHTIGQRK			Oxidation (M)[9]		Mascot
1989.1532	1989.0048	-0.1484	-75	165	182	LNQAQLAKTLFPLGALYK					Mascot
3215.6692	3215.7126	0.0434	13	12	42	VVVGMSGGVDSSVTALL LKQQGFDVTGLFMK			Oxidation (M)[5,30]		Mascot
3268.6042	3268.7007	0.0965	30	57	85	QDLVDVASVCDVIGIDLE VVNFSAEYKDR			Carbamidomethyl (C)[10]		Mascot

4	Immunoglobulin-like and fibronectin type III domain-containing protein 1 OS=Mus musculus GN=Igfn1 PE=1 SV=3	IGFN1_MOUSE	306492.7	6.15	30	55	0	39.451

Peptide Information

Calc. Mass	Obsrv. Mass	± da	± ppm	Start Seq.	End Seq.	Sequence	Ion Score	C. I. %	Modification	Rank	Result Type
849.4424	849.4711	0.0287	34	877	884	SSLSGDRK					Mascot
867.4543	867.4846	0.0303	35	1024	1030	VDARNHR					Mascot
938.4764	938.516	0.0396	42	300	306	ELMDFRK					Mascot
1142.5801	1142.6293	0.0492	43	290	298	QKEPQEDLR					Mascot
1144.6083	1144.6077	-0.0006	-1	2004	2013	QGPHRHLGSR					Mascot
1187.6016	1187.6923	0.0907	76	652	662	VTLPGDSQSQR					Mascot
1196.551	1196.6228	0.0718	60	1	9	MNEMRTQVR			Oxidation (M)[1,4]		Mascot
1228.6719	1228.5996	-0.0723	-59	154	165	EAGTMAAKPPKK					Mascot

1235.5917	1235.5841	-0.0076	-6	2684	2693	SWHEVADHVR					Mascot
1278.655	1278.718	0.063	49	1850	1862	AGFTGSSSVPGRR					Mascot
1406.7023	1406.7052	0.0029	2	1298	1310	EGDGERISFLGAR					Mascot
1493.723	1493.8135	0.0905	61	1365	1378	TAYGEESKGLGPER					Mascot
1557.8054	1557.953	0.1476	95	470	482	VVVPLAETRCEER			Carbamidomethyl (C)[10]		Mascot
1631.8024	1631.8483	0.0459	28	2743	2756	SPTYQDPDLSQKPR					Mascot
1706.7803	1706.8285	0.0482	28	1274	1290	EGDFGNGTGVPLSMGPR			Oxidation (M)[14]		Mascot
1794.8473	1794.8651	0.0178	10	1006	1023	EGSVGGSQVAALMMSSQR					Mascot
1794.8473	1794.8651	0.0178	10	1006	1023	EGSVGGSQVAALMMSSQR					Mascot
1810.8423	1810.8536	0.0113	6	1006	1023	EGSVGGSQVAALMMSSQR			Oxidation (M)[13]		Mascot
1826.8372	1826.8625	0.0253	14	1006	1023	EGSVGGSQVAALMMSSQR			Oxidation (M)[13,14]		Mascot
1858.9125	1859.0078	0.0953	51	341	355	ICMQYGIVDFRGMLR			Carbamidomethyl (C)[2]		Mascot
1874.9075	1875.0205	0.113	60	341	355	ICMQYGIVDFRGMLR			Carbamidomethyl (C)[2], Oxidation (M)[3]		Mascot
1875.9171	1876.0226	0.1055	56	2401	2415	YTVVGLRQGCQYEFR			Carbamidomethyl (C)[10]		Mascot
1875.9171	1876.0226	0.1055	56	2401	2415	YTVVGLRQGCQYEFR	9	0	Carbamidomethyl (C)[10]		Mascot
1891.9807	1892.0197	0.039	21	1526	1545	MHLADGSGRLGGPGSLAAPK					Mascot
1909.9767	1910.0164	0.0397	21	1304	1321	ISFLGARTSTVGTGNWDK					Mascot
1954.9366	1955.0426	0.106	54	1973	1991	RATPQGAGGLEPWAGSEDR					Mascot
1956.0006	1956.0698	0.0692	35	927	946	SGLQEIQGRNGQGSAGALGR					Mascot
1982.8583	1983.0267	0.1684	85	776	792	KEGTEMWGDCLDVTEGR			Carbamidomethyl (C)[10]		Mascot
1982.8583	1983.0267	0.1684	85	776	792	KEGTEMWGDCLDVTEGR			Carbamidomethyl (C)[10]		Mascot
1989.931	1990.0015	0.0705	35	2774	2790	MTCAVQGSPQPHVTWFK			Carbamidomethyl (C)[3], Oxidation (M)[1]		Mascot
2252.0759	2252.2139	0.138	61	1006	1027	EGSVGGSQVAALMMSSQRVDAR			Oxidation (M)[13]		Mascot
2322.2349	2322.283	0.0481	21	1031	1053	LVTSPGLGVQGSGRTLGHMEGLR					Mascot
2635.4204	2635.3235	-0.0969	-37	2566	2589	VPLSFEAAPMPEVTWLKDGLPLPK					Mascot
2651.4153	2651.3323	-0.083	-31	2566	2589	VPLSFEAAPMPEVTWLKDGLPLPK			Oxidation (M)[10]		Mascot
3233.4592	3233.7139	0.2547	79	1130	1161	APCGAPAETESGKWGTYSHDSGVPGGLWSGSK			Carbamidomethyl (C)[3]		Mascot
3247.707	3247.7231	0.0161	5	2093	2122	LTAQDGVIFEQDGLTHRLILTHVEGTQAGK					Mascot
3403.6475	3403.8267	0.1792	53	2123	2153	YTFVAGCQHSEASLTVQDPPTIAPDVTETLR			Carbamidomethyl (C)[7]		Mascot

5	Riboflavin biosynthesis protein RibF OS=Pseudomonas fluorescens GN=ribF PE=3 SV=1	RIBF_PSEFL	34105	8.45	10	51	0	2.168

Peptide Information

Calc. Mass	Obsrv. Mass	± da	± ppm	Start	End	Sequence	Ion	C. I. %	Modification	Rank	Result Type

				Seq.	Seq.		Score				
865.5002	865.4634	-0.0368	-43	30	37	GHQAILAR					Mascot
1078.6481	1078.5875	-0.0606	-56	189	198	IAGRVLHGQK					Mascot
1120.5933	1120.6555	0.0622	56	88	96	VLCLAFNQR			Carbamidomethyl (C)[3]		Mascot
1131.5389	1131.6425	0.1036	92	76	87	QAATAGGEGVDR					Mascot
1228.6658	1228.5996	-0.0662	-54	6	15	GLHNLRPEHR					Mascot
1856.0184	1856.0358	0.0174	9	1	15	MQLVRGLHNLRPEHR					Mascot
1932.0549	1932.0483	-0.0066	-3	284	302	FASLEALKTAINADVAAAR					Mascot
1997.0525	1997.0676	0.0151	8	42	59	AVELGVPSCVVIFEPQPR			Carbamidomethyl (C)[9]		Mascot
3233.5862	3233.7139	0.1277	39	132	161	VGDFDFLQHAGVNQGFTVEAAQTVELDGLR					Mascot
3243.6509	3243.696	0.0451	14	42	70	AVELGVPSCVVIFEPQPREFFTPETAPAR			Carbamidomethyl (C)[9]		Mascot

6	NAD(P)H-quinone oxidoreductase subunit H 1 OS=Gloeobacter violaceus (strain PCC 7421) GN=ndhH1 PE=3 SV=1	NDHH1_GLOVI	45101	6.12	13	49	0	27.462	

Peptide Information

Calc. Mass	Obsrv. Mass	± da	± ppm	Start Seq.	End Seq.	Sequence	Ion Score	C. I. %	Modification	Rank	Result Type
867.4062	867.4846	0.0784	90	1	7	MSMLETR					Mascot
883.4012	883.4744	0.0732	83	1	7	MSMLETR			Oxidation (M)[1]		Mascot
1092.5466	1092.6106	0.064	59	2	10	SMLETRAER					Mascot
1135.5776	1135.4916	-0.086	-76	265	273	IQEMRESVK			Oxidation (M)[4]		Mascot
1144.5681	1144.6077	0.0396	35	321	329	MPRGEHYVR					Mascot
1145.6062	1145.6301	0.0239	21	226	235	ASGVNWDLRK					Mascot
1380.6729	1380.7871	0.1142	83	175	185	ARDFCNYLPPK			Carbamidomethyl (C)[5]		Mascot
1678.7451	1678.8385	0.0934	56	139	152	EMIYDLFEAATGMR			Oxidation (M)[2,13]		Mascot
1892.9865	1893.0314	0.0449	24	186	200	IDEYERLITNNPIFR					Mascot
1923.0698	1923.0242	-0.0456	-24	54	69	IAESRTIIQYLPYVTR					Mascot
1924.944	1925.0469	0.1029	53	335	351	GELGVYLIGDDSTFPWR					Mascot
1926.979	1926.9918	0.0128	7	11	27	MVINLGPHHPSMHGVLR			Oxidation (M)[1,12]		Mascot
1931.899	1932.0483	0.1493	77	137	152	EREMIYDLFEAATGMR					Mascot
3255.6812	3255.6965	0.0153	5	111	138	IASHLLWLGPFMADIGATSPFFYIFRER					Mascot

7	DNA-directed RNA polymerase subunit alpha OS=Streptococcus suis (strain 05ZYH33) GN=rpoA PE=3 SV=1	RPOA_STRSY	34320.9	4.78	10	49	0	5.311	

Protein Group

DNA-directed RNA polymerase subunit alpha OS=Streptococcus suis (strain 98HAH33) GN=rpoA PE=3 SV=1	RPOA_STRS2	34320.9	4.7800002098 0835

Peptide Information

Calc. Mass	Obsrv. Mass	± da	± ppm	Start Seq.	End Seq.	Sequence	Ion Score	C. I. %	Modification	Rank	Result Type
865.3794	865.4634	0.084	97	279	285	TEPEMMK					Mascot
899.556	899.6173	0.0613	68	301	309	LADLGLGLK					Mascot
1120.5634	1120.6555	0.0921	82	145	154	GYVPAEDNKK					Mascot
1138.5852	1138.6073	0.0221	19	29	39	GYGTTLGNSLR					Mascot
1493.7748	1493.8135	0.0387	26	17	28	DYGRFVIEPLER					Mascot
1860.959	1861.0289	0.0699	38	155	172	DDAPVGTLAVDSIYTPVK					Mascot
1946.0304	1946.1089	0.0785	40	214	230	ILMEHLGLFTDLTEVAK			Oxidation (M)[3]		Mascot
1946.0304	1946.1089	0.0785	40	214	230	ILMEHLGLFTDLTEVAK			Oxidation (M)[3]		Mascot
1952.0045	1952.0452	0.0407	21	1	16	MIEFEKPTITKIDENK			Oxidation (M)[1]		Mascot
1989.054	1989.0048	-0.0492	-25	155	173	DDAPVGTLAVDSIYTPVK K					Mascot
3230.6541	3230.7385	0.0844	26	183	213	VGSNDGFDKLTLEINTNG TIIPEDALGLSAR					Mascot

8	Protein thf1 OS=Prochlorococcus marinus (strain SARG / CCMP1375 / SS120) GN=thf1 PE=3 SV=1	THF1_PROMA	24684.9	6.04	9	49	0	30.662	

Peptide Information

Calc. Mass	Obsrv. Mass	± da	± ppm	Start Seq.	End Seq.	Sequence	Ion Score	C. I. %	Modification	Rank	Result Type
849.4498	849.4711	0.0213	25	188	194	NSMEKIK					Mascot
865.4447	865.4634	0.0187	22	188	194	NSMEKIK			Oxidation (M)[3]		Mascot
867.4207	867.4846	0.0639	74	93	99	EYSNNIK					Mascot
1179.6368	1179.6605	0.0237	20	90	99	AIKEYSNNIK					Mascot
1196.5464	1196.6228	0.0764	64	1	11	MGEQTTISDSK					Mascot
1647.8337	1647.8419	0.0082	5	2	16	GEQTTISDSKGLFHK					Mascot
1678.8138	1678.8385	0.0247	15	76	89	ILESLCNSCNIDIK			Carbamidomethyl (C)[6,9]		Mascot
1794.8691	1794.8651	-0.004	-2	1	16	MGEQTTISDSKGLFHK			Oxidation (M)[1]		Mascot
1794.8691	1794.8651	-0.004	-2	1	16	MGEQTTISDSKGLFHK			Oxidation (M)[1]		Mascot
1962.0636	1962.0658	0.0022	1	12	27	GLFHKEFPYVIPPVYR					Mascot
3234.6357	3234.707	0.0713	22	48	75	IDTIFSYGLIISFERFTVGY EPDSHISK					Mascot

9	Ribosomal RNA large subunit methyltransferase M OS=Shewanella sp. (strain W3-18-1) GN=rlmM PE=3 SV=1	RLMM_SHESW	41381.8	6.42	11	49	0	18.714	

Peptide Information

Calc. Mass	Obsrv. Mass	± da	± ppm	Start Seq.	End Seq.	Sequence	Ion Score	C. I. %	Modification	Rank	Result Type
905.4761	905.4216	-0.0545	-60	247	254	LMETGQVK					Mascot
1097.5925	1097.6626	0.0701	64	2	9	KNLFLFCR			Carbamidomethyl (C)[7]		Mascot
1228.6329	1228.5996	-0.0333	-27	1	9	MKNLFLFCR			Carbamidomethyl (C)[8]		Mascot
1812.0226	1811.973	-0.0496	-27	86	103	ISPIVAALSDVSKAGEVR					Mascot
1812.0226	1811.973	-0.0496	-27	86	103	ISPIVAALSDVSKAGEVR					Mascot
1909.9299	1910.0164	0.0865	45	269	283	NIYWLVCDMVEKPAR			Carbamidomethyl (C)[7], Oxidation (M)[9]		Mascot
1949.9274	1950.0786	0.1512	78	69	85	QMFAASDLLVDLPENDR			Oxidation (M)[2]		Mascot
1950.9821	1951.0499	0.0678	35	346	361	DEVTVHLWLRPNTAWN					Mascot
1966.9175	1967.0344	0.1169	59	104	120	VETPDTNEAKELSAFCR			Carbamidomethyl (C)[16]		Mascot
1966.9175	1967.0344	0.1169	59	104	120	VETPDTNEAKELSAFCR			Carbamidomethyl (C)[16]		Mascot
2252.0588	2252.2139	0.1551	69	210	230	SGMNAVDLGACPGGWT YQLVR			Carbamidomethyl (C)[11]		Mascot
2635.1616	2635.3235	0.1619	61	35	57	ANNNDAYVVYQCFEDDA ADTLVK			Carbamidomethyl (C)[12]		Mascot
3230.6614	3230.7385	0.0771	24	69	98	QMFAASDLLVDLPENDRI SPIVAALSDVSK			Oxidation (M)[2]		Mascot

10	Glutamine amidotransferase subunit PdxT OS=Staphylococcus haemolyticus (strain JCSC1435) GN=pdxT PE=3 SV=1	PDXT_STAHJ	20449.8	5.07	8	49	0	2.086	

Peptide Information

Calc. Mass	Obsrv. Mass	± da	± ppm	Start Seq.	End Seq.	Sequence	Ion Score	C. I. %	Modification	Rank	Result Type
1142.6027	1142.6293	0.0266	23	53	61	RLMDLYGFK					Mascot
1380.6981	1380.7871	0.089	64	174	184	VTQYFIDHMVK					Mascot
1406.7386	1406.7052	-0.0334	-24	95	106	LDITVERNSFGR					Mascot
1631.9705	1631.8483	-0.1222	-75	3	17	IGVLALQGAVREHIR					Mascot
1811.86	1811.973	0.113	62	159	173	YLGVSFHPELTDDYR					Mascot
1811.86	1811.973	0.113	62	159	173	YLGVSFHPELTDDYR					Mascot
1925.0352	1925.0469	0.0117	6	14	30	EHIRHIELSGHEGIAVK					Mascot
1954.9506	1955.0426	0.092	47	102	118	NSFGRQVDSFEAELDIK					Mascot
2588.4294	2588.324	-0.1054	-41	131	155	APHIANVEEGVEILSTVG DKIVAVK					Mascot

Gel Idx/Pos	201/I1	Instr./Gel Origin	BA2151/Sample Project 20140814	Process Status	Analysis Succeeded
Plate [#] Name	[1] Sample Project 20140814	Instrument Sample Name		Spectra	11

Rank	Protein Name	Accession No.	Protein MW	Protein PI	Pep. Count	Protein Score	Protein Score C. I. %	Intensity Matched	Total Ion Score	Total Ion C. I. %	Confirmed
1	Glucose 1-dehydrogenase OS=Sulfolobus solfataricus GN=gdh PE=1 SV=1	GLCDH_SULSF	41264.6	6.68	11	56	0	6.021			

Protein Group

Glucose 1-dehydrogenase 1 OS=Sulfolobus solfataricus (strain ATCC 35092 / DSM 1617 / JCM 11322 / P2) GN=gdh1 PE=3 SV=1	GLCD1_SULSO	41264.6	6.6799 998283 3862

Peptide Information

Calc. Mass	Obsrv. Mass	± da	± ppm	Start Seq.	End Seq.	Sequence	Ion Score	C. I. %	Modification	Rank	Result Type
905.4575	905.3804	-0.0771	-85	338	345	TVSINDEK					Mascot
1138.484	1138.4963	0.0123	11	126	133	EWWYDDPK					Mascot
1182.6477	1182.5458	-0.1019	-86	291	300	TLQEIVHTNK					Mascot
1187.6519	1187.6215	-0.0304	-26	158	167	SIEEILEVQK					Mascot
1507.8037	1507.6649	-0.1388	-92	333	345	MLITKTVSINDEK			Oxidation (M)[1]		Mascot
1875.783	1875.8912	0.1082	58	120	133	MDGFMREWWYDDPK					Mascot
1875.783	1875.8912	0.1082	58	120	133	MDGFMREWWYDDPK					Mascot
1891.7778	1891.8856	0.1078	57	120	133	MDGFMREWWYDDPK			Oxidation (M)[1]		Mascot
1909.9437	1909.8794	-0.0643	-34	33	49	TIYNGICGTDREIVNGK			Carbamidomethyl (C)[7]		Mascot
2993.6292	2993.3516	-0.2776	-93	141	167	SIEDIGILAQPLADIEKSIE EILEVQK					Mascot
3221.6841	3221.5283	-0.1558	-48	271	300	NGVLGLFGFSTSGSVPL DYKTLQEIVHTNK					Mascot
3221.7908	3221.5283	-0.2625	-81	184	212	VLVVGTGPIGVLFTLLFRT YGLEVWMANR					Mascot
3262.4172	3262.5371	0.1199	37	98	125	NCLVGRPDFCETGEFGE AGIHKMDGFMR			Carbamidomethyl (C)[2,10], Oxidation (M)[23,27]		Mascot

Rank	Protein Name	Accession No.	Protein MW	Protein PI	Pep. Count	Protein Score	Protein Score C. I. %	Intensity Matched	Total Ion Score	Total Ion C. I. %	Confirmed
2	Aspartate--tRNA ligase OS=Lactobacillus johnsonii (strain CNCM I-12250 / La1 / NCC 533) GN=aspS PE=3 SV=2	SYD_LACJO	70114.5	5.08	15	50	0	7.267			

Peptide Information

Calc. Mass	Obsrv. Mass	± da	± ppm	Start Seq.	End Seq.	Sequence	Ion Score	C. I. %	Modification	Rank	Result Type
849.4465	849.4175	-0.029	-34	178	184	DYLVPSR					Mascot
866.4254	866.3941	-0.0313	-36	370	376	FLTDENK					Mascot
905.3846	905.3804	-0.0042	-5	601	608	EAQEDADK					Mascot

938.4941	938.453	-0.0411	-44	461	468	LLDTDPHK						Mascot
1335.701	1335.6204	-0.0806	-60	258	268	IMKDVMNIDLK			Oxidation (M)[2]			Mascot
1637.6562	1637.774	0.1178	72	601	614	EAQEDADKNSTWDE						Mascot
1811.9175	1811.9285	0.011	6	305	322	DSDFKVFSGAIADGGVVK						Mascot
1811.9175	1811.9285	0.011	6	305	322	DSDFKVFSGAIADGGVVK						Mascot
1942.9869	1942.8536	-0.1333	-69	108	124	VPPFEIKDDINAAEQTR						Mascot
1946.9917	1946.9407	-0.051	-26	90	107	TGEVEVDATEIDVLNKSK						Mascot
1956.0266	1955.9575	-0.0691	-35	185	201	IYPGSFYALPQSPQLFK						Mascot
1964.9324	1964.9341	0.0017	1	444	460	WIAAHHPFTMPDDEGIK						Mascot
1980.9274	1980.9288	0.0014	1	444	460	WIAAHHPFTMPDDEGIK			Oxidation (M)[10]			Mascot
1983.0083	1982.9014	-0.1069	-54	15	30	YEGGQEVTLYGWVQRVR						Mascot
1983.0083	1982.9014	-0.1069	-54	15	30	YEGGQEVTLYGWVQRVR						Mascot
2252.0183	2252.0735	0.0552	25	469	489	AHARSYDIVMNGDEMGG GSIR			Oxidation (M)[10]			Mascot
3277.5093	3277.5459	0.0366	11	229	256	QPEFTQIDMETSFLDEQG VQDYTEGLLK			Oxidation (M)[9]			Mascot
3389.6094	3389.6235	0.0141	4	229	257	QPEFTQIDMETSFLDEQG VQDYTEGLLKK						Mascot

3	Putative competence-damage inducible protein OS=Pelotomaculum thermopropionicum (strain DSM 13744 / JCM 10971 / SI) GN=cinA PE=3 SV=1	CINA_PELTS	44741.3	5.86	11	49	0	7.579

Peptide Information

Calc. Mass	Obsrv. Mass	± da	± ppm	Start Seq.	End Seq.	Sequence	Ion Score	C. I. %	Modification	Rank	Result Type
849.4716	849.4175	-0.0541	-64	408	414	YLLTKPD					Mascot
920.4393	920.4496	0.0103	11	234	241	MVDELADK					Mascot
1298.641	1298.5768	-0.0642	-49	300	311	GGVVAYSNEMKK			Oxidation (M)[10]		Mascot
1335.6825	1335.6204	-0.0621	-46	185	196	LTGITESMVQEK					Mascot
1818.8903	1818.828	-0.0623	-34	225	241	AGNSELAQKMVDELADK					Mascot
1860.996	1860.9155	-0.0805	-43	270	287	RLSLSVAESCTGGLIAAR			Carbamidomethyl (C)[10]		Mascot
1947.0294	1946.9407	-0.0887	-46	312	329	EILGVPQDILDQHGAVSR					Mascot
2651.3499	2651.147	-0.2029	-77	242	264	IRPRLEEYIFGCGEETLE GVVGK			Carbamidomethyl (C)[12]		Mascot
2651.3499	2651.147	-0.2029	-77	242	264	IRPRLEEYIFGCGEETLE GVVGK			Carbamidomethyl (C)[12]		Mascot
2993.6057	2993.3516	-0.2541	-85	145	171	IILILPGPPHELTAMFENS ALPFLSEK			Oxidation (M)[15]		Mascot
3233.7642	3233.5259	-0.2383	-74	145	174	IILILPGPPHELTAMFENS ALPFLSEKAGK					Mascot
3249.7593	3249.5183	-0.241	-74	145	174	IILILPGPPHELTAMFENS ALPFLSEKAGK			Oxidation (M)[15]		Mascot
3249.7593	3249.5183	-0.241	-74	145	174	IILILPGPPHELTAMFENS ALPFLSEKAGK			Oxidation (M)[15]		Mascot
3277.7542	3277.5459	-0.2083	-64	142	171	DGKIILILPGPPHELTAMF					Mascot

ENSALPFLSEK

4	Beta-glucosidase A OS=Clostridium thermocellum (strain ATCC 27405 / DSM 1237) GN=bglA PE=3 SV=1	BGLA_CLOTH	51507.1	5.69	11	49	0	4.042

Peptide Information

Calc. Mass	Obsrv. Mass	± da	± ppm	Start Seq.	End Seq.	Sequence	Ion Score	C. I. %	Modification	Rank	Result Type
920.4836	920.4496	-0.034	-37	441	448	EVIKNNGF					Mascot
1298.6641	1298.5768	-0.0873	-67	75	84	SYRFSISWPR					Mascot
1490.7737	1490.7335	-0.0402	-27	270	282	KGIELSFPEDDLK					Mascot
1678.8282	1678.7141	-0.1141	-68	233	248	AEDIEAAELSFSLAGR					Mascot
1857.8429	1857.8888	0.0459	25	138	152	DTTDYFTEYSEVIFK					Mascot
1927.892	1927.906	0.014	7	303	320	YDPSSESGFSPANSILEK					Mascot
1933.0865	1932.8988	-0.1877	-97	189	206	TSLEVSHNLLLSHGKAVK					Mascot
2593.2603	2593.1467	-0.1136	-44	210	232	EMNIDAQIGIALNLSYHYP ASEK			Oxidation (M)[2]		Mascot
2993.519	2993.3516	-0.1674	-56	207	232	LFREMNIDAQIGIALNLSY HYPASEK					Mascot
3249.4761	3249.5183	0.0422	13	9	37	DFIWGSATAAYQIEGAYN EDGKGESIWDR					Mascot
3249.4761	3249.5183	0.0422	13	9	37	DFIWGSATAAYQIEGAYN EDGKGESIWDR					Mascot
3277.7654	3277.5459	-0.2195	-67	104	131	LTNLLLENGIMPAITLYHW DLPQKLQDK					Mascot

5	Ribose import ATP-binding protein RbsA 1 OS=Pasteurella multocida (strain Pm70) GN=rbsA1 PE=3 SV=1	RBSA1_PASMU	54929.6	5.7	12	49	0	2.2

Peptide Information

Calc. Mass	Obsrv. Mass	± da	± ppm	Start Seq.	End Seq.	Sequence	Ion Score	C. I. %	Modification	Rank	Result Type
1228.6355	1228.5298	-0.1057	-86	120	129	MHQEADKLLK			Oxidation (M)[1]		Mascot
1490.7196	1490.7335	0.0139	9	351	363	ENMSLTALAYFSK			Oxidation (M)[3]		Mascot
1678.8582	1678.7141	-0.1441	-86	1	14	MQHETLLHIQGIDK			Oxidation (M)[1]		Mascot
1919.0089	1918.9167	-0.0922	-48	280	298	GEILGVSGLMGAGRTEL MK					Mascot
1928.0376	1927.906	-0.1316	-68	51	68	VLTGIYSKDAGTIEYLGK					Mascot
1950.9988	1950.929	-0.0698	-36	280	298	GEILGVSGLMGAGRTEL MK			Oxidation (M)[10,18]		Mascot
1997.0273	1996.9279	-0.0994	-50	15	33	SFPGVKALSNACLSVYA GR			Carbamidomethyl (C)[12]		Mascot
2120.0474	2120.0249	-0.0225	-11	226	243	AVNVLDEDQLIEMMVGR R			Oxidation (M)[13,14]		Mascot
2323.3167	2323.1211	-0.1956	-84	405	425	VAIAKGLMTRPNVLILDEP TR			Oxidation (M)[8]		Mascot
2544.2004	2544.1472	-0.0532	-21	21	45	ALSNACLSVYAGRAMAL MGENGAGK			Carbamidomethyl (C)[6], Oxidation (M)[15,18]		Mascot

Calc. Mass	Obsrv. Mass	± da	± ppm	Start Seq.	End Seq.	Sequence	Ion Score	C. I. %	Modification	Rank	Result Type
2608.3367	2608.1658	-0.1709	-66	256	279	GELLLEVENLSGSGVHD VSFTLHR					Mascot
2839.3489	2839.3015	-0.0474	-17	218	242	DGEFIGEKAVNVLDEDQL IEMMVGR			Oxidation (M)[21,22]		Mascot
3262.6023	3262.5371	-0.0652	-20	311	338	VRLAQQEIHNTCPQDGL NHGIVYISEDR			Carbamidomethyl (C)[12]		Mascot

6	Uncharacterized 18.9 kDa protein in mobE 3'region OS=Thiobacillus ferrooxidans PE=4 SV=1	YME1_THIFE	19219.3	4.88	7	49	0	5.069	

Peptide Information

Calc. Mass	Obsrv. Mass	± da	± ppm	Start Seq.	End Seq.	Sequence	Ion Score	C. I. %	Modification	Rank	Result Type
1875.9554	1875.8912	-0.0642	-34	2	19	GVAMIDALNIATMPIADK			Oxidation (M)[4,13]		Mascot
1875.9554	1875.8912	-0.0642	-34	2	19	GVAMIDALNIATMPIADK			Oxidation (M)[4,13]		Mascot
1949.9386	1949.9401	0.0015	1	115	131	LQEGQQAYALLAAEECR			Carbamidomethyl (C)[16]		Mascot
2252.189	2252.0735	-0.1155	-51	2	22	GVAMIDALNIATMPIADKV HR			Oxidation (M)[4]		Mascot
2544.3027	2544.1472	-0.1555	-61	20	41	VHRHMLASYYALQLDAL QAEAK			Oxidation (M)[5]		Mascot
3247.5591	3247.5312	-0.0279	-9	43	71	LGYFFAQADTAATMPPVL ADCLAWAVEYR			Carbamidomethyl (C)[21]		Mascot
3262.6406	3262.5371	-0.1035	-32	136	165	ASLPPEPSPLSEVEVSSF VDEFFTKLDAPK					Mascot
3403.6602	3403.6716	0.0114	3	43	72	LGYFFAQADTAATMPPVL ADCLAWAVEYRR			Carbamidomethyl (C)[21]		Mascot
3419.6553	3419.696	0.0407	12	43	72	LGYFFAQADTAATMPPVL ADCLAWAVEYRR			Carbamidomethyl (C)[21], Oxidation (M)[14]		Mascot

7	Tyrosine-protein kinase isoform SRK1 OS=Spongilla lacustris GN=SRK1 PE=2 SV=1	SRK1_SPOLA	58227	5.38	12	47	0	3.445	

Peptide Information

Calc. Mass	Obsrv. Mass	± da	± ppm	Start Seq.	End Seq.	Sequence	Ion Score	C. I. %	Modification	Rank	Result Type
821.4264	821.3973	-0.0291	-35	448	453	IQQNYR					Mascot
1074.453	1074.4609	0.0079	7	454	462	MPCPANCPK			Carbamidomethyl (C)[3,7]		Mascot
1325.5944	1325.671	0.0766	58	401	411	WTAPEAAMYNR			Oxidation (M)[8]		Mascot
1520.6774	1520.7272	0.0498	33	463	473	QFHDIMLDCWR			Carbamidomethyl (C)[9]		Mascot
1637.788	1637.774	-0.014	-9	434	447	FPYPGMTNPEVLEK			Oxidation (M)[6]		Mascot
1811.9539	1811.9285	-0.0254	-14	317	332	YGSLLEYLRGEDGVLK					Mascot
1811.9539	1811.9285	-0.0254	-14	317	332	YGSLLEYLRGEDGVLK					Mascot
1901.9564	1901.9995	0.0431	23	16	34	ATAGSTVDSHELSQSVK GK					Mascot
1996.9095	1996.9279	0.0184	9	150	167	DSETTPGDFSLSVKDQD R					Mascot
2004.955	2004.8679	-0.0871	-43	98	115	DTAGKEGYIPSNYVAEYK					Mascot
2593.3701	2593.1467	-0.2234	-86	412	433	FTIKSDVWSFGVVLYEIIT YGR					Mascot

Calc. Mass	Obsrv. Mass	± da	± ppm	Start Seq.	End Seq.	Sequence	Ion Score	C. I. %	Modification	Rank	Result Type
2608.1023	2608.1658	0.0635	24	454	473	MPCPANCPKQFHDIMLDCWR			Carbamidomethyl (C)[3,7,18], Oxidation (M)[1,15]		Mascot
3217.5835	3217.5295	-0.054	-17	135	163	MLNQSFNQVGSFLIRDSETTPGDFSLSVK					Mascot
3233.5784	3233.5259	-0.0525	-16	135	163	MLNQSFNQVGSFLIRDSETTPGDFSLSVK			Oxidation (M)[1]		Mascot

8	DNA mismatch repair protein MutS OS=Burkholderia phytofirmans (strain DSM 17436 / PsJN) GN=mutS PE=3 SV=1	MUTS_BURPP	97688.2	5.47	14	47	0	18.965	

Peptide Information

Calc. Mass	Obsrv. Mass	± da	± ppm	Start Seq.	End Seq.	Sequence	Ion Score	C. I. %	Modification	Rank	Result Type
920.4584	920.4496	-0.0088	-10	504	510	VPDDYRR					Mascot
1461.8339	1461.7557	-0.0782	-53	318	328	LLRHWLHHPPR					Mascot
1664.7883	1664.7518	-0.0365	-22	133	146	SDVYLLAMCVAHNR			Carbamidomethyl (C)[9], Oxidation (M)[8]		Mascot
1851.9819	1851.8625	-0.1194	-64	627	643	LLLITGPNMGGKSTFMR			Oxidation (M)[9]		Mascot
1891.9786	1891.8856	-0.093	-49	321	336	HWLHHPPRESAVAQAR					Mascot
1897.9291	1897.8732	-0.0559	-29	272	287	VEYESEYIGLDPATRR					Mascot
1909.8961	1909.8794	-0.0167	-9	457	472	DISENCGQFLIDLETR			Carbamidomethyl (C)[6]		Mascot
1922.0164	1921.9042	-0.1122	-58	848	864	AEPAVPPAMQELVERLR			Oxidation (M)[9]		Mascot
1950.9954	1950.929	-0.0664	-34	845	862	DAKAEPAVPPAMQELVER					Mascot
1966.9902	1966.9025	-0.0877	-45	845	862	DAKAEPAVPPAMQELVER			Oxidation (M)[12]		Mascot
1966.9902	1966.9025	-0.0877	-45	845	862	DAKAEPAVPPAMQELVER	5	0	Oxidation (M)[12]		Mascot
2544.3643	2544.1472	-0.2171	-85	639	661	STFMRQTALIALLAYVGSYVPAR			Oxidation (M)[4]		Mascot
2591.2163	2591.2151	-0.0012	0	1	23	MGIQTAAANDVAQHTPMMQQYLR			Oxidation (M)[1]		Mascot
2667.3196	2667.1499	-0.1697	-64	604	626	HPVVEAQVEQFIANDCSLTPERK			Carbamidomethyl (C)[16]		Mascot
3247.6992	3247.5312	-0.168	-52	397	427	TQVAAVAPNADSLARIDASLEPPQACVELLK			Carbamidomethyl (C)[26]		Mascot
3389.854	3389.6235	-0.2305	-68	148	181	GVATSVGLAWLNLASGALRLAEVAPDQVAAALER					Mascot

9	Calmodulin OS=Physarum polycephalum PE=2 SV=3	CALM_PHYPO	16725.8	4.08	8	47	0	1.056	

Peptide Information

Calc. Mass	Obsrv. Mass	± da	± ppm	Start Seq.	End Seq.	Sequence	Ion Score	C. I. %	Modification	Rank	Result Type
821.4185	821.3973	-0.0212	-26	32	38	ELGTVMR			Oxidation (M)[6]		Mascot
920.4319	920.4496	0.0177	19	23	31	DGDGNITTK					Mascot
1044.5143	1044.4125	-0.1018	-97	108	116	HVMTNLGEK			Oxidation (M)[3]		Mascot
1325.5526	1325.671	0.1184	89	77	87	MADTDTEEEIR			Oxidation (M)[1]		Mascot

1335.6097	1335.6204	0.0107	8	117	127	LSDEEVDEMIR				Mascot
1857.8866	1857.8888	0.0022	1	15	31	EAFSLFDKDGDGNITTK				Mascot
2593.2378	2593.1467	-0.0911	-35	1	22	MVDSLTEEQIAEFKEAFS LFDK			Oxidation (M)[1]	Mascot
3216.4163	3216.5098	0.0935	29	117	144	LSDEEVDEMIREADVDG DGQVNYDEFVK				Mascot

10	Ribosomal RNA large subunit methyltransferase H OS=Campylobacter lari (strain RM2100 / D67 / ATCC BAA-1060) GN=rlmH PE=3 SV=1	RLMH_CAMLR	17753.9	8.81	8	47	0	3.864

Peptide Information

Calc. Mass	Obsrv. Mass	± da	± ppm	Start Seq.	End Seq.	Sequence	Ion Score	C. I. %	Modification	Rank	Result Type
905.4363	905.3804	-0.0559	-62	19	25	IDEHYTK					Mascot
1057.5201	1057.4225	-0.0976	-92	79	87	EFTSVEFAK					Mascot
1182.6188	1182.5458	-0.073	-62	131	139	TMLLEQIYR			Oxidation (M)[2]		Mascot
1187.6816	1187.6215	-0.0601	-51	1	10	MQINLLSIQK					Mascot
1298.674	1298.5768	-0.0972	-75	57	67	SYTNALNPYKK					Mascot
1678.7352	1678.7141	-0.0211	-13	30	42	FCSFNEICVFNNK			Carbamidomethyl (C)[2,8]		Mascot
2005.0349	2004.8679	-0.167	-83	2	18	QINLLSIQKNNNDEFSK					Mascot
3249.5925	3249.5183	-0.0742	-23	93	121	NEITFFIGGAYGFEQEFIA QMHTSIALSK					Mascot
3249.5925	3249.5183	-0.0742	-23	93	121	NEITFFIGGAYGFEQEFIA QMHTSIALSK					Mascot

Gel Idx/Pos	202/I2	Instr./Gel Origin	BA2151/Sample Project 20140814	Process Status	Analysis Succeeded
Plate [#] Name	[1] Sample Project 20140814	Instrument Sample Name		Spectra	11

Rank	Protein Name	Accession No.	Protein MW	Protein PI	Pep. Count	Protein Score	Protein Score C. I. %	Intensity Matched	Total Ion Score	Total Ion C. I. %	Confirmed
1	Muscle M-line assembly protein unc-89 OS=Caenorhabditis elegans GN=unc-89 PE=1 SV=3	UNC89_CAEEL	899002.9	5.42	69	82	99.643	68.198			

Peptide Information

Calc. Mass	Obsrv. Mass	± da	± ppm	Start Seq.	End Seq.	Sequence	Ion Score	C. I. %	Modification	Rank	Result Type
867.5298	867.4488	-0.081	-93	3017	3024	VIGEPKPK					Mascot
887.5197	887.4387	-0.081	-91	4023	4030	ELSAIQVK					Mascot
906.4462	906.4601	0.0139	15	5114	5120	DDRITMR					Mascot
1058.5114	1058.5356	0.0242	23	5631	5640	DGGPLIEDSR					Mascot
1060.5786	1060.5676	-0.011	-10	2317	2325	VKYVHETGK					Mascot
1076.5518	1076.5511	-0.0007	-1	3856	3864	EVEMSARVR					Mascot
1085.5837	1085.5514	-0.0323	-30	1801	1810	VEEKPASPTK					Mascot
1088.5443	1088.5459	0.0016	1	1182	1191	DGQEGARVTR					Mascot
1099.5854	1099.572	-0.0134	-12	5419	5428	DGLPLRADSR					Mascot
1104.5685	1104.5519	-0.0166	-15	5807	5816	DVFDANKAPK					Mascot
1107.6005	1107.5505	-0.05	-45	3129	3138	VETKITSSSR					Mascot
1118.5576	1118.5621	0.0045	4	2512	2521	IVEEEDGSLK					Mascot
1120.5481	1120.5863	0.0382	34	1249	1258	QDLSSSEVQK					Mascot
1129.567	1129.5697	0.0027	2	2622	2632	IHGVTADMTGK					Mascot
1131.5641	1131.6042	0.0401	35	4253	4263	DGKVEDLGNGK					Mascot
1131.5641	1131.6042	0.0401	35	4253	4263	DGKVEDLGNGK					Mascot
1135.5413	1135.5942	0.0529	47	3853	3862	EGKEVEMSAR					Mascot
1143.6368	1143.5775	-0.0593	-52	2923	2932	DGKEIELIAR					Mascot
1145.562	1145.5773	0.0153	13	2622	2632	IHGVTADMTGK			Oxidation (M)[8]		Mascot
1151.5361	1151.5947	0.0586	51	3853	3862	EGKEVEMSAR			Oxidation (M)[7]		Mascot
1159.6007	1159.5918	-0.0089	-8	6898	6905	YQHKWLER					Mascot
1161.5874	1161.5846	-0.0028	-2	6144	6154	GGMPFPPGFVR					Mascot
1163.5837	1163.5946	0.0109	9	826	836	LGSASCDGRLK			Carbamidomethyl (C)[6]		Mascot
1163.5837	1163.5946	0.0109	9	826	836	LGSASCDGRLK			Carbamidomethyl (C)[6]		Mascot
1177.5824	1177.5775	-0.0049	-4	6144	6154	GGMPFPPGFVR			Oxidation (M)[3]		Mascot
1187.5837	1187.5979	0.0142	12	8024	8033	MRTDEALSHK					Mascot

1190.59	1190.5598	-0.0302	-25	1640	1650	SPEKSATEDVK		Mascot
1192.6243	1192.5831	-0.0412	-35	3007	3016	TSEKVVLECK	Carbamidomethyl (C)[9]	Mascot
1195.5854	1195.5868	0.0014	1	5851	5860	DGERVFPYGR		Mascot
1195.6205	1195.5868	-0.0337	-28	4160	4169	IVQVTESEYK		Mascot
1199.54	1199.5828	0.0428	36	920	930	AQNEHGTAESR		Mascot
1211.5944	1211.5697	-0.0247	-20	5482	5491	KEGEEPFFTK		Mascot
1227.5422	1227.5518	0.0096	8	5888	5898	CVAENTYGSAR	Carbamidomethyl (C)[1]	Mascot
1236.5718	1236.571	-0.0008	-1	289	297	MVQYQNYFK	Oxidation (M)[1]	Mascot
1267.5913	1267.6483	0.057	45	4744	4754	NAVRDDADTYK		Mascot
1299.7419	1299.649	-0.0929	-71	644	655	EAAPPAFVTKLR		Mascot
1306.642	1306.6305	-0.0115	-9	5971	5982	IKMEAAADGTQR	Oxidation (M)[3]	Mascot
1320.6067	1320.6163	0.0096	7	2500	2511	DGVEITSDGHYK		Mascot
1336.6162	1336.6594	0.0432	32	2247	2259	AKNAAGECETSAK	Carbamidomethyl (C)[8]	Mascot
1343.7529	1343.7368	-0.0161	-12	6883	6894	LKTEPLSADTLR		Mascot
1365.7373	1365.7122	-0.0251	-18	1874	1886	SKSPEAEKPPAPK		Mascot
1371.705	1371.7085	0.0035	3	5571	5583	TQATAHVQMALGK	Oxidation (M)[9]	Mascot
1380.7522	1380.7369	-0.0153	-11	6306	6316	YPQVTYVIEIR		Mascot
1380.7522	1380.7369	-0.0153	-11	6306	6316	YPQVTYVIEIR		Mascot
1401.7042	1401.7177	0.0135	10	7312	7323	QEHSMEVLIATK	Oxidation (M)[5]	Mascot
1408.6638	1408.7352	0.0714	51	3358	3370	YACRATNPAGEAK	Carbamidomethyl (C)[3]	Mascot
1410.6934	1410.7175	0.0241	17	5236	5247	EEDFGTLKCIAK	Carbamidomethyl (C)[9]	Mascot
1412.7897	1412.7311	-0.0586	-41	5618	5630	VTGLPNPSVKWSK		Mascot
1412.8512	1412.7311	-0.1201	-85	1042	1053	KPPKFVEILVDK		Mascot
1418.691	1418.6959	0.0049	3	5003	5015	NGQELKPDADGFK		Mascot
1422.7435	1422.7004	-0.0431	-30	3167	3181	AIATNSIGTATSTSK		Mascot
1424.6805	1424.7264	0.0459	32	7521	7533	NDDGTFAPIFTAR		Mascot
1425.7584	1425.7273	-0.0311	-22	1846	1857	EKSPEKPEEKPK		Mascot
1430.8076	1430.6981	-0.1095	-77	2880	2892	TPTPVMAPKFITK		Mascot
1443.7689	1443.6909	-0.078	-54	7983	7994	EEEIKENVINVK		Mascot
1447.7209	1447.7092	-0.0117	-8	5707	5719	MGDVRTTEGQPLK	Oxidation (M)[1]	Mascot
1498.7748	1498.8478	0.073	49	1693	1706	SPQTVEEKPASPTK		Mascot
1503.7551	1503.6906	-0.0645	-43	5656	5668	IKNATVHDEGTYR		Mascot
1505.7992	1505.6841	-0.1151	-76	3825	3837	SCTVTEKQQAILK	Carbamidomethyl (C)[2]	Mascot
1607.8639	1607.827	-0.0369	-23	7196	7210	ETIPKPTPSPTSPQK		Mascot
1818.9081	1818.8566	-0.0515	-28	4469	4487	VVVSNDAGDADSSAALT VK		Mascot
1836.9259	1836.932	0.0061	3	2973	2988	DTCEATLTVIESLEKK	Carbamidomethyl (C)[3]	Mascot
1966.9399	1966.952	0.0121	6	7801	7818	DAVDSTTEGHAHCAVKIR	Carbamidomethyl (C)[13]	Mascot

2138.1201	2138.0757	-0.0444	-21	7486	7505	SHEQSTALLQKAPSATAIER					Mascot
2234.0354	2234.1318	0.0964	43	7765	7784	TANEDMKSNLQLQTDDPTGR					Mascot
2234.0354	2234.1318	0.0964	43	7765	7784	TANEDMKSNLQLQTDDPTGR					Mascot
2235.2019	2235.1545	-0.0474	-21	839	858	VPPAPPTFNKPLEDKTVQEK					Mascot
2250.0964	2250.0808	-0.0156	-7	6250	6268	FNGEIDVTPYLTEEYGFKK					Mascot
2252.1167	2252.1233	0.0066	3	3133	3151	ITSSSRIAIEHDQTNTHWR					Mascot
2252.2021	2252.1233	-0.0788	-35	79	98	EDVEAIPLEQGQIVEVLDKK					Mascot
2274.0649	2274.0916	0.0267	12	6401	6419	YSEQYACAPWFSPGVVEKR			Carbamidomethyl (C)[7]		Mascot
2280.1846	2280.1387	-0.0459	-20	2288	2307	FSAIVTGKPMPNVTWYLNNK					Mascot
2285.1296	2285.1548	0.0252	11	951	970	DIEDQTVKTGEFAVFETTVR					Mascot
2714.4043	2714.3528	-0.0515	-19	7109	7133	TQHPVATPILASPGGDQQQQKIPMR			Oxidation (M)[24]		Mascot
2715.3157	2715.3311	0.0154	6	3435	3459	QTAVGSFSLTINDARQGDVGIYSCR			Carbamidomethyl (C)[24]		Mascot
2731.3186	2731.304	-0.0146	-5	1899	1921	TDLAHFEVVVEHATECKWFLDGK			Carbamidomethyl (C)[16]		Mascot

2	2,3,4,5-tetrahydropyridine-2,6-dicarboxylate N-succinyltransferase OS=Shewanella pealeana (strain ATCC 700345 / ANG-SQ1) GN=dapD PE=3 SV=1	DAPD_SHEPA	29792.5	5.92	7	75	98.25	7.335	46	97.319	

Peptide Information

Calc. Mass	Obsrv. Mass	± da	± ppm	Start Seq.	End Seq.	Sequence	Ion Score	C. I. %	Modification	Rank	Result Type
906.4679	906.4601	-0.0078	-9	8	15	IEAAFEAR					Mascot
913.5465	913.5012	-0.0453	-50	264	271	VGINELLR					Mascot
1147.5776	1147.5962	0.0186	16	34	44	AIAMLDTGEAR					Mascot
1147.5776	1147.5962	0.0186	16	34	44	AIAMLDTGEAR	46	97.319			Mascot
1163.5725	1163.5946	0.0221	19	34	44	AIAMLDTGEAR			Oxidation (M)[4]		Mascot
1163.5725	1163.5946	0.0221	19	34	44	AIAMLDTGEAR			Oxidation (M)[4]		Mascot
1190.6277	1190.5598	-0.0679	-57	6	15	QRIEAAFEAR					Mascot
1392.7006	1392.6808	-0.0198	-14	69	81	IFDNGVIEGAETK					Mascot
1394.8003	1394.7161	-0.0842	-60	227	241	VPAGSVVVAGNLPSK					Mascot
2754.3867	2754.3176	-0.0691	-25	188	213	SEIVEGVIVEEGSVISMGVYIGQSTR			Oxidation (M)[17]		Mascot

3	Protein translocase subunit SecA OS=Hydrogenobaculum sp. (strain Y04AAS1) GN=secA PE=3 SV=1	SECA_HYDS0	108738	5.31	24	70	94.593	13.161			

Peptide Information

Calc. Mass	Obsrv. Mass	± da	± ppm	Start	End	Sequence	Ion	C. I. %	Modification	Rank	Result Type

				Seq.	Seq.		Score		
832.4047	832.3247	-0.08	-96	799	805	EQEIGEK			Mascot
906.4713	906.4601	-0.0112	-12	806	812	TMREIEK			Mascot
907.4342	907.4711	0.0369	41	365	371	DVHYMVK		Oxidation (M)[5]	Mascot
913.5254	913.5012	-0.0242	-26	190	196	DIRVWPK			Mascot
1078.578	1078.5381	-0.0399	-37	719	727	SILFQDDIK			Mascot
1079.5402	1079.552	0.0118	11	108	117	IAEMKTGEGK		Oxidation (M)[4]	Mascot
1085.6313	1085.5514	-0.0799	-74	605	615	AGGLLVIGTER			Mascot
1120.5746	1120.5863	0.0117	10	688	696	VELQNFQSR			Mascot
1129.5345	1129.5697	0.0352	31	629	639	AGRQGDPGESR			Mascot
1131.5892	1131.6042	0.015	13	28	37	INALEESLDK			Mascot
1131.5892	1131.6042	0.015	13	28	37	INALEESLDK			Mascot
1135.5742	1135.5942	0.02	18	311	319	DFIVDEKNR			Mascot
1147.6357	1147.5962	-0.0395	-34	464	473	DLPDAIFKTK			Mascot
1147.6357	1147.5962	-0.0395	-34	464	473	DLPDAIFKTK			Mascot
1159.6582	1159.5918	-0.0664	-57	345	354	HIDLVHAINK			Mascot
1161.6045	1161.5846	-0.0199	-17	540	551	GAVTIATNMAGR			Mascot
1175.6089	1175.588	-0.0209	-18	183	192	AMEAIEKDIR			Mascot
1177.5994	1177.5775	-0.0219	-19	540	551	GAVTIATNMAGR		Oxidation (M)[9]	Mascot
1191.6038	1191.5857	-0.0181	-15	183	192	AMEAIEKDIR		Oxidation (M)[2]	Mascot
1236.5889	1236.571	-0.0179	-14	799	808	EQEIGEKTMR		Oxidation (M)[9]	Mascot
1283.6704	1283.6488	-0.0216	-17	858	867	LFEELMLNFK			Mascot
1299.6654	1299.649	-0.0164	-13	858	867	LFEELMLNFK		Oxidation (M)[6]	Mascot
1392.7039	1392.6808	-0.0231	-17	902	914	SMAISEALENIAK		Oxidation (M)[2]	Mascot
1401.7373	1401.7177	-0.0196	-14	214	225	TSYFTKAISVER			Mascot
1452.7482	1452.7034	-0.0448	-31	841	852	SYAQRDPLVEFK			Mascot
1503.8199	1503.6906	-0.1293	-86	868	881	ISAIQSIMNAQISK			Mascot
1818.9055	1818.8566	-0.0489	-27	197	213	GMVGDAIDYSKIDVHAK			Mascot
2264.1553	2264.1145	-0.0408	-18	660	679	LMEFMKIPEGEPIESSIVS K			Mascot
2280.1501	2280.1387	-0.0114	-5	660	679	LMEFMKIPEGEPIESSIVS K		Oxidation (M)[2]	Mascot
2715.4248	2715.3311	-0.0937	-35	540	566	GAVTIATNMAGRGTDILL GGNPEFLAR			Mascot
2731.4197	2731.304	-0.1157	-42	540	566	GAVTIATNMAGRGTDILL GGNPEFLAR		Oxidation (M)[9]	Mascot

4	Protein PA-X OS=Influenza A virus (strain A/Equine/London/1416/1973 H7N7) GN=PA PE=3 SV=1	PAX_I73A4	29653	8.44	12	67	90.16	7.27

Peptide Information

Calc. Mass	Obsrv. Mass	± da	± ppm	Start Seq.	End Seq.	Sequence	Ion Score	C. I. %	Modification	Rank	Result Type
1076.6212	1076.5511	-0.0701	-65	117	125	FVEIGVTRR					Mascot
1163.5845	1163.5946	0.0101	9	186	195	GLWDSFVSPR					Mascot
1163.5845	1163.5946	0.0101	9	186	195	GLWDSFVSPR	12	0			Mascot
1167.535	1167.5881	0.0531	45	20	29	AMKEYGEDPK					Mascot
1183.53	1183.5747	0.0447	38	20	29	AMKEYGEDPK			Oxidation (M)[2]		Mascot
1193.6201	1193.5875	-0.0326	-27	126	134	EVHIYYLEK					Mascot
1349.7212	1349.6985	-0.0227	-17	125	134	REVHIYYLEK					Mascot
1351.7151	1351.6703	-0.0448	-33	175	185	LFTIRQEMASR					Mascot
1422.6747	1422.7004	0.0257	18	23	34	EYGEDPKIETNK					Mascot
1425.6605	1425.7273	0.0668	47	159	170	ADYTLDEESRAR					Mascot
1864.8899	1864.9832	0.0933	50	143	158	THIHIFSFTGEEMATK			Oxidation (M)[13]		Mascot
1967.0128	1966.952	-0.0608	-31	204	221	DLKSQGQCAGLPITVSHR			Carbamidomethyl (C)[8]		Mascot
2020.983	2021.0447	0.0617	31	222	238	TSPALKILEPMWMDSNR			Oxidation (M)[11,13]		Mascot
2040.959	2041.0183	0.0593	29	83	99	DRTMAWTVVNSICNTTR			Carbamidomethyl (C)[13], Oxidation (M)[4]		Mascot

5 Uncharacterized protein KIAA1211 homolog OS=Danio rerio GN=si:dkeyp-117h8.2 PE=4 SV=3 K1211_DANRE 123825.7 6.92 21 66 85.446 5.368

Peptide Information

Calc. Mass	Obsrv. Mass	± da	± ppm	Start Seq.	End Seq.	Sequence	Ion Score	C. I. %	Modification	Rank	Result Type
887.4832	887.4387	-0.0445	-50	329	335	IQELQEK					Mascot
906.4163	906.4601	0.0438	48	2	9	SQENVSDK					Mascot
1078.4834	1078.5381	0.0547	51	273	280	QEEEERMK					Mascot
1107.4801	1107.5505	0.0704	64	972	982	ESAGSSPTEDK					Mascot
1118.5477	1118.5621	0.0144	13	163	172	EDYGIIHGSK					Mascot
1120.5706	1120.5863	0.0157	14	838	847	RTSGDTISQR					Mascot
1147.6582	1147.5962	-0.062	-54	790	799	FSRSTLPLAR					Mascot
1147.6582	1147.5962	-0.062	-54	790	799	FSRSTLPLAR	10	0			Mascot
1161.5858	1161.5846	-0.0012	-1	2	11	SQENVSDKVR					Mascot
1336.6379	1336.6594	0.0215	16	603	614	NSEIFGQEEGVK					Mascot
1378.6082	1378.7106	0.1024	74	970	982	DRESAGSSPTEDK					Mascot
1422.7085	1422.7004	-0.0081	-6	67	78	EKSVSHDTVQHR					Mascot
1424.6904	1424.7264	0.036	25	615	627	TESAFTETSPVQK					Mascot
1430.691	1430.6981	0.0071	5	699	712	AQGGDEQPSPFGIK					Mascot

1467.7625	1467.7151	-0.0474	-32	581	594	TMSASAKFSITPAR						Mascot
1469.8363	1469.7185	-0.1178	-80	929	941	ETGPLWITLALQK						Mascot
1864.9624	1864.9832	0.0208	11	993	1008	HQTADENKRPDTLLAR						Mascot
1928.0488	1927.9574	-0.0914	-47	925	941	SQDKETGPLWITLALQK						Mascot
2137.948	2138.0757	0.1277	60	672	691	SQRSESGSPIQAESEDS DTK						Mascot
2250.1499	2250.0808	-0.0691	-31	615	635	TESAFTETSPVQKELPSL GTK						Mascot
2285.1926	2285.1548	-0.0378	-17	1046	1066	FPPGDTTQVSTEPAWLA LAKR						Mascot
2288.1406	2288.0762	-0.0644	-28	764	784	SSPISPQQENIEFQTTVA PSK						Mascot

6 Uncharacterized protein K11H3.2 OS=Caenorhabditis elegans GN=K11H3.2 PE=4 SV=1 | YM42_CAEEL | 25989 | 5.24 | 11 | 65 | 82.901 | 2.885

Peptide Information

Calc. Mass	Obsrv. Mass	± da	± ppm	Start Seq.	End Seq.	Sequence	Ion Score	C. I. %	Modification	Rank	Result Type
1104.5797	1104.5519	-0.0278	-25	157	166	FASSTQHAKK					Mascot
1107.5867	1107.5505	-0.0362	-33	40	48	DPKLCAIYK			Carbamidomethyl (C)[5]		Mascot
1151.5944	1151.5947	0.0003	0	102	112	AELASPTSFTK					Mascot
1159.6357	1159.5918	-0.0439	-38	73	82	VFGPIIEEQK					Mascot
1378.7325	1378.7106	-0.0219	-16	100	112	ARAELASPTSFTK					Mascot
1401.7737	1401.7177	-0.056	-40	73	84	VFGPIIEEQKNK					Mascot
1440.6788	1440.6863	0.0075	5	181	192	SNSMYLEDVLNR					Mascot
1927.8695	1927.9574	0.0879	46	85	99	EDEIIEFEEFEEIEK					Mascot
2021.0591	2021.0447	-0.0144	-7	66	82	TVEEFQKVFGPIIEEQK					Mascot
2234.2651	2234.1318	-0.1333	-60	43	61	LCAIYKMILQNSALLLIEK			Carbamidomethyl (C)[2]		Mascot
2234.2651	2234.1318	-0.1333	-60	43	61	LCAIYKMILQNSALLLIEK			Carbamidomethyl (C)[2]		Mascot
2250.26	2250.0808	-0.1792	-80	43	61	LCAIYKMILQNSALLLIEK			Carbamidomethyl (C)[2], Oxidation (M)[7]		Mascot
2714.2224	2714.3528	0.1304	48	9	31	SNLNSEMNDLVQQAEEW AQSQHR					Mascot

7 Zinc finger protein 860 OS=Homo sapiens GN=ZNF860 PE=2 SV=3 | ZN860_HUMAN | 75591.1 | 9.46 | 16 | 64 | 76.396 | 7.318

Peptide Information

Calc. Mass	Obsrv. Mass	± da	± ppm	Start Seq.	End Seq.	Sequence	Ion Score	C. I. %	Modification	Rank	Result Type
1060.5422	1060.5676	0.0254	24	447	455	THTGEKPYK					Mascot
1099.5742	1099.572	-0.0022	-2	59	68	NLHSVDISSK					Mascot
1104.6022	1104.5519	-0.0503	-46	612	620	HQRIHTGQK					Mascot
1143.6157	1143.5775	-0.0382	-33	502	511	AIHTGEKPYK					Mascot

1147.6218	1147.5962	-0.0256	-22	518	527	VFNQQATLAR					Mascot
1147.6218	1147.5962	-0.0256	-22	518	527	VFNQQATLAR	24	0			Mascot
1175.5803	1175.588	0.0077	7	419	427	IHNEERSYK					Mascot
1183.5702	1183.5747	0.0045	4	134	143	LTGSTDRYDR					Mascot
1195.6252	1195.5868	-0.0384	-32	238	247	AFNCSSLLRK			Carbamidomethyl (C)[4]		Mascot
1195.6252	1195.5868	-0.0384	-32	238	247	AFNCSSLLRK			Carbamidomethyl (C)[4]		Mascot
1211.6393	1211.5697	-0.0696	-57	602	611	AFTSHSHRIR					Mascot
1392.7053	1392.6808	-0.0245	-18	40	50	CLDPTQRALYR			Carbamidomethyl (C)[1]		Mascot
1422.7965	1422.7004	-0.0961	-68	490	501	TFRHNSALVIHK					Mascot
1430.6039	1430.6981	0.0942	66	540	550	CEECDTVFSRK			Carbamidomethyl (C)[1,4]		Mascot
1447.8057	1447.7092	-0.0965	-67	248	259	HQIIYLGGKQYK					Mascot
1893.847	1893.9363	0.0893	47	307	321	LHTGEKPYKCEECDK			Carbamidomethyl (C)[10,13]		Mascot
2040.9833	2041.0183	0.035	17	73	91	KFSSTAQGNTEVDTGTL ER					Mascot
2286.0251	2286.0735	0.0484	21	288	306	CNECGKVFNQQSNLASH HR			Carbamidomethyl (C)[1,4]		Mascot

8	(Dimethylallyl)adenosine tRNA methylthiotransferase MiaB OS=Haemophilus ducreyi (strain 35000HP / ATCC 700724) GN=miaB PE=3 SV=1	MIAB_HAEDU	54003.4	5.25	13	63	72.268	9.346

Peptide Information

Calc. Mass	Obsrv. Mass	± da	± ppm	Start Seq.	End Seq.	Sequence	Ion Score	C. I. %	Modification	Rank	Result Type
832.4047	832.3247	-0.08	-96	463	470	EDDLGVGK					Mascot
1078.5562	1078.5381	-0.0181	-17	394	402	KDIMELTGR			Oxidation (M)[4]		Mascot
1120.6262	1120.5863	-0.0399	-36	58	66	VFSQLGRWK					Mascot
1129.6034	1129.5697	-0.0337	-30	110	118	LPEMINQIR			Oxidation (M)[4]		Mascot
1131.5906	1131.6042	0.0136	12	278	286	RNHTALEYK					Mascot
1131.6368	1131.6042	-0.0326	-29	449	459	IVESASSVIAR	23	0			Mascot
1136.6283	1136.5918	-0.0365	-32	359	367	DRLAHLQQR					Mascot
1167.6005	1167.5881	-0.0124	-11	426	435	ITDVYSNSLR					Mascot
1247.595	1247.6451	0.0501	40	368	377	INHQAMQFSR			Oxidation (M)[6]		Mascot
1392.7006	1392.6808	-0.0198	-14	463	475	EDDLGVGKYAVNL					Mascot
1428.7958	1428.7225	-0.0733	-51	279	290	NHTALEYKAIIR					Mascot
1503.7584	1503.6906	-0.0678	-45	436	448	GEVIRTEDQMGLR					Mascot
2285.0325	2285.1548	0.1223	54	2	20	AKLHITTWGCQMNEYDS SK			Carbamidomethyl (C)[10], Oxidation (M)[12]		Mascot

9	Plectin OS=Homo sapiens GN=PLEC PE=1 SV=3	PLEC_HUMAN	533461.8	5.74	60	61	60.827	40.509

Peptide Information

Calc. Mass	Obsrv. Mass	± da	± ppm	Start Seq.	End Seq.	Sequence	Ion Score	C. I. %	Modification	Rank	Result Type
887.4581	887.4387	-0.0194	-22	2266	2274	AQAQAAAEK					Mascot
906.4536	906.4601	0.0065	7	2	9	VAGMLMPR			Oxidation (M)[4,6]		Mascot
907.4666	907.4711	0.0045	5	785	792	NTNMTAKK					Mascot
1058.5425	1058.5356	-0.0069	-7	935	943	HPAHPMRGR					Mascot
1060.5157	1060.5676	0.0519	49	3638	3647	VLADPSDDTK					Mascot
1078.5238	1078.5381	0.0143	13	765	772	ELMWLNEK			Oxidation (M)[3]		Mascot
1085.4607	1085.5514	0.0907	84	2576	2584	QQSDHDAER					Mascot
1088.547	1088.5459	-0.0011	-1	1825	1833	ELAEQELEK					Mascot
1107.5729	1107.5505	-0.0224	-20	3107	3115	GRLCFEGLR			Carbamidomethyl (C)[4]		Mascot
1118.5735	1118.5621	-0.0114	-10	2526	2535	VAEMSRAQAR					Mascot
1120.611	1120.5863	-0.0247	-22	2310	2318	FAEQTLRQK					Mascot
1129.5848	1129.5697	-0.0151	-13	2340	2348	NLLDEELQR					Mascot
1131.5388	1131.6042	0.0654	58	2225	2234	RAAEEAEEAR	3	0			Mascot
1131.6256	1131.6042	-0.0214	-19	1987	1996	TEAEIALKEK					Mascot
1143.6005	1143.5775	-0.023	-20	1783	1792	LQAEEVAQQK					Mascot
1145.5658	1145.5773	0.0115	10	1638	1646	RQVQDESQR					Mascot
1147.5089	1147.5962	0.0873	76	4288	4296	EMSVYEAYR					Mascot
1147.5089	1147.5962	0.0873	76	4288	4296	EMSVYEAYR					Mascot
1151.5579	1151.5947	0.0368	32	4339	4348	QYDIDDAIAK					Mascot
1159.6066	1159.5918	-0.0148	-13	3146	3155	DVAEVDTVRR					Mascot
1161.6045	1161.5846	-0.0199	-17	2685	2694	LVASMEEARR					Mascot
1163.5725	1163.5946	0.0221	19	578	587	DLDKADSMIR	12	0			Mascot
1163.6056	1163.5946	-0.011	-9	3463	3472	AQFEQLKDGK					Mascot
1175.5361	1175.588	0.0519	44	3667	3676	CVEDPETGLR			Carbamidomethyl (C)[1]		Mascot
1177.5994	1177.5775	-0.0219	-19	2685	2694	LVASMEEARR			Oxidation (M)[5]		Mascot
1183.6106	1183.5747	-0.0359	-30	3221	3231	AGLVGPEFHEK					Mascot
1187.6266	1187.5979	-0.0287	-24	2587	2596	EAIAELEREK					Mascot
1190.678	1190.5598	-0.1182	-99	588	597	LLFNDVQTLK					Mascot
1191.6117	1191.5857	-0.026	-22	1933	1942	FRELAEEAAR					Mascot
1192.6433	1192.5831	-0.0602	-50	3437	3446	QERLSFSGLR					Mascot
1193.5765	1193.5875	0.011	9	1119	1127	CQRCISELK			Carbamidomethyl (C)[1,4]		Mascot
1195.6833	1195.5868	-0.0965	-81	1408	1417	FAKQYINAIK					Mascot
1195.6833	1195.5868	-0.0965	-81	1408	1417	FAKQYINAIK					Mascot
1199.6742	1199.5828	-0.0914	-76	2585	2594	LREAIAELER					Mascot

Calc. Mass	Obsrv. Mass	± da	± ppm	Start Seq.	End Seq.	Sequence	Ion Score	C. I. %	Modification	Rank	Result Type
1207.5889	1207.5801	-0.0088	-7	3739	3749	AQLMADFQAGR					Mascot
1209.6334	1209.5621	-0.0713	-59	2016	2025	RLEEQAAQHK					Mascot
1227.6692	1227.5518	-0.1174	-96	2172	2182	QLQLAQEAAQK					Mascot
1236.6293	1236.571	-0.0583	-47	4130	4140	MGIVGPEFKDK			Oxidation (M)[1]		Mascot
1283.7188	1283.6488	-0.07	-55	102	112	RPVAMVMPARR					Mascot
1284.7019	1284.6481	-0.0538	-42	811	821	ELQNAGDRLLR					Mascot
1299.6328	1299.649	0.0162	12	3771	3781	QQGLASYDYVR					Mascot
1306.642	1306.6305	-0.0115	-9	2451	2461	EKMQAVQEATR			Oxidation (M)[3]		Mascot
1320.6907	1320.6163	-0.0744	-56	3783	3793	RLTAEDLFEAR					Mascot
1343.8218	1343.7368	-0.085	-63	3755	3765	MIIIIIEIIEK			Oxidation (M)[1]		Mascot
1365.6646	1365.7122	0.0476	35	3358	3368	QEELYSELQAR					Mascot
1380.7166	1380.7369	0.0203	15	3286	3297	SHRVPLDVACAR			Carbamidomethyl (C)[10]		Mascot
1380.7166	1380.7369	0.0203	15	3286	3297	SHRVPLDVACAR			Carbamidomethyl (C)[10]		Mascot
1384.7291	1384.7305	0.0014	1	1604	1616	QRGGAEGELQALR					Mascot
1401.7333	1401.7177	-0.0156	-11	2127	2139	VQKSLAAEEEAAR					Mascot
1410.7336	1410.7175	-0.0161	-11	2809	2821	LAQGHTTVDELAR					Mascot
1412.7856	1412.7311	-0.0545	-39	1781	1792	LRLQAEEVAQQK					Mascot
1412.7856	1412.7311	-0.0545	-39	1781	1792	LRLQAEEVAQQK					Mascot
1418.7244	1418.6959	-0.0285	-20	2	13	VAGMLMPRDQLR			Oxidation (M)[4,6]		Mascot
1422.7335	1422.7004	-0.0331	-23	2733	2743	EQLQLLEEQHR					Mascot
1428.7078	1428.7225	0.0147	10	2707	2717	QEELQQLEQQR					Mascot
1447.6958	1447.7092	0.0134	9	2510	2521	QRQLEMSAEAER					Mascot
1469.8184	1469.7185	-0.0999	-68	1697	1709	ARQVQVALETAQR					Mascot
1503.7584	1503.6906	-0.0678	-45	1128	1139	DIRLQLEACETR			Carbamidomethyl (C)[9]		Mascot
1592.8027	1592.7312	-0.0715	-45	3356	3368	ARQEELYSELQAR					Mascot
1607.7772	1607.827	0.0498	31	714	728	SDEGQLSPATRGAYR					Mascot
1893.876	1893.9363	0.0603	32	2488	2503	EQMAQQLAEETQGFQR					Mascot
1966.856	1966.952	0.096	49	1076	1093	DSQDAGGFGPEDRLMAER			Oxidation (M)[15]		Mascot
2273.9617	2274.0916	0.1299	57	773	791	EEEEVGFDWSDRNTNMTAK			Oxidation (M)[16]		Mascot
2714.261	2714.3528	0.0918	34	63	83	ETFAWCHFYWYLTNEGIAHLR			Carbamidomethyl (C)[6]		Mascot
2731.3025	2731.304	0.0015	1	538	561	LQMEAGLCEEQLNQADALLQSDVR			Carbamidomethyl (C)[8]		Mascot

10	Titin OS=Mus musculus GN=Ttn PE=1 SV=1	TITIN_MOUSE	3933362.2	5.91	115	61	59.915	82.734

Peptide Information

Calc. Mass	Obsrv. Mass	± da	± ppm	Start Seq.	End Seq.	Sequence	Ion Score	C. I. %	Modification	Rank	Result Type

832.3254	832.3247	-0.0007	-1	33559	33565	HGEDSCK			Carbamidomethyl (C)[6]	Mascot
867.4094	867.4488	0.0394	45	9947	9953	EEAVYEK				Mascot
887.4304	887.4387	0.0083	9	24572	24577	WTRCHK			Carbamidomethyl (C)[4]	Mascot
906.468	906.4601	-0.0079	-9	22375	22382	NTATVSWK				Mascot
907.4883	907.4711	-0.0172	-19	11277	11284	GKAVFEEK				Mascot
913.4924	913.5012	0.0088	10	11988	11995	TRPMAPPK			Oxidation (M)[4]	Mascot
1058.5365	1058.5356	-0.0009	-1	6254	6263	DDVELVSGPK				Mascot
1060.5997	1060.5676	-0.0321	-30	16254	16264	LGSATASINVK				Mascot
1076.5371	1076.5511	0.014	13	21317	21325	ETQAVNWTK				Mascot
1078.531	1078.5381	0.0071	7	28738	28748	NAMGSASATIR				Mascot
1079.5944	1079.552	-0.0424	-39	15534	15543	KSTVTITDSK				Mascot
1085.5878	1085.5514	-0.0364	-34	18241	18249	ITGYIVEYK				Mascot
1088.5483	1088.5459	-0.0024	-2	14659	14667	FEKDGSIHR				Mascot
1099.5782	1099.572	-0.0062	-6	20619	20627	IDGYIISYR				Mascot
1104.4956	1104.5519	0.0563	51	2513	2522	THASDEGPYK				Mascot
1107.5503	1107.5505	0.0002	0	22103	22111	DLPSKSWMK			Oxidation (M)[8]	Mascot
1118.5801	1118.5621	-0.018	-16	3010	3019	NGVEIKSTDR				Mascot
1120.4993	1120.5863	0.087	78	1723	1731	FGPAHFECR			Carbamidomethyl (C)[8]	Mascot
1129.6034	1129.5697	-0.0337	-30	21427	21435	CNLPEKLQK			Carbamidomethyl (C)[1]	Mascot
1131.5753	1131.6042	0.0289	26	20029	20038	DATDLTRSPR				Mascot
1131.5753	1131.6042	0.0289	26	20029	20038	DATDLTRSPR	14	0		Mascot
1135.5743	1135.5942	0.0199	18	19438	19449	GTWGVVSAGSSK				Mascot
1136.598	1136.5918	-0.0062	-5	34832	34841	TLSTQMNITK				Mascot
1143.5826	1143.5775	-0.0051	-4	10513	10521	HMKITQEEK				Mascot
1145.5797	1145.5773	-0.0024	-2	27615	27624	DGVEIEERAK				Mascot
1147.5994	1147.5962	-0.0032	-3	20533	20542	TWSTVTPEVK				Mascot
1147.5994	1147.5962	-0.0032	-3	20533	20542	TWSTVTPEVK	4	0		Mascot
1151.5944	1151.5947	0.0003	0	3281	3290	EEQLLSTGFK				Mascot
1159.5776	1159.5918	0.0142	12	10513	10521	HMKITQEEK			Oxidation (M)[2]	Mascot
1161.6263	1161.5846	-0.0417	-36	4286	4296	GNLVPSDGKFK				Mascot
1163.5426	1163.5946	0.052	45	8230	8239	DEVEIQESSK				Mascot
1163.5426	1163.5946	0.052	45	8230	8239	DEVEIQESSK				Mascot
1167.6039	1167.5881	-0.0158	-14	30492	30501	TTLTVKDSMR			Oxidation (M)[9]	Mascot
1175.6307	1175.588	-0.0427	-36	9717	9726	IEAEPIQFTK				Mascot
1177.6324	1177.5775	-0.0549	-47	14119	14128	YEIVADGRVR				Mascot
1183.5776	1183.5747	-0.0029	-2	32730	32739	YQSNATLVCK			Carbamidomethyl (C)[9]	Mascot
1185.5858	1185.5563	-0.0295	-25	34358	34368	SITVHEGESAR				Mascot

1187.6783	1187.5979	-0.0804	-68	9132	9141	ERLIPPSFTK		Mascot
1190.6085	1190.5598	-0.0487	-41	29306	29315	TCEIEIGQLK	Carbamidomethyl (C)[2]	Mascot
1191.6005	1191.5857	-0.0148	-12	31959	31968	WTKEGQDISK		Mascot
1192.6572	1192.5831	-0.0741	-62	17096	17105	TKSTITLDWK		Mascot
1193.5869	1193.5875	0.0006	1	34046	34056	AQRESSSSVSR		Mascot
1195.6934	1195.5868	-0.1066	-89	19294	19304	LVPPSVELDVK		Mascot
1195.6934	1195.5868	-0.1066	-89	19294	19304	LVPPSVELDVK		Mascot
1199.5804	1199.5828	0.0024	2	29513	29521	DSEYQFRVR		Mascot
1207.5702	1207.5801	0.0099	8	30409	30419	AKNDAGYSEPR		Mascot
1211.6089	1211.5697	-0.0392	-32	5236	5246	MSFAESTAVLR		Mascot
1227.6005	1227.5518	-0.0487	-40	31685	31694	LTEGNEYVFR		Mascot
1236.6041	1236.571	-0.0331	-27	33794	33803	IRDQYEMPGK		Mascot
1247.6591	1247.6451	-0.014	-11	20091	20101	NLKITDVSSDR		Mascot
1267.6715	1267.6483	-0.0232	-18	17697	17708	VMDAPGPPKDLK		Mascot
1283.642	1283.6488	0.0068	5	23166	23176	WAKPEYTGGFK		Mascot
1284.6505	1284.6481	-0.0024	-2	27116	27126	ITGYVVEMQTK	Oxidation (M)[8]	Mascot
1299.6216	1299.649	0.0274	21	30397	30406	LVENTEYEFR		Mascot
1306.7267	1306.6305	-0.0962	-74	17815	17825	NTVHLSWKPPK		Mascot
1320.6115	1320.6163	0.0048	4	28112	28122	AWVSVTNNCNR	Carbamidomethyl (C)[9]	Mascot
1336.7809	1336.6594	-0.1215	-91	30154	30165	QTHIVRAGVSIR		Mascot
1343.7213	1343.7368	0.0155	12	20662	20674	VAARNAVGVSMPR	Oxidation (M)[11]	Mascot
1349.761	1349.6985	-0.0625	-46	21496	21507	AGVTMRLYVPVK	Oxidation (M)[5]	Mascot
1351.6423	1351.6703	0.028	21	23880	23891	GSDRWATCATVK	Carbamidomethyl (C)[8]	Mascot
1365.6566	1365.7122	0.0556	41	2146	2158	DVTAEDSASIMVK		Mascot
1366.6962	1366.6991	0.0029	2	21632	21643	TSFRVSNLEEGK		Mascot
1371.7128	1371.7085	-0.0043	-3	2661	2672	DGKHLALSNNFR		Mascot
1378.7074	1378.7106	0.0032	2	29595	29606	VNTSPISGREYR		Mascot
1380.6827	1380.7369	0.0542	39	8213	8224	YECKIGGSPEIK	Carbamidomethyl (C)[3]	Mascot
1380.7958	1380.7369	-0.0589	-43	2790	2802	LGASARLHVETVK		Mascot
1384.7505	1384.7305	-0.02	-14	21721	21732	VMKSLSLQYSTK		Mascot
1392.6729	1392.6808	0.0079	6	9482	9492	YARMYGITDFR		Mascot
1394.7162	1394.7161	-0.0001	0	3756	3768	QEITAFAEGTISK		Mascot
1401.7373	1401.7177	-0.0196	-14	15887	15898	NTADLKWTVPEK		Mascot
1406.6985	1406.6692	-0.0293	-21	2385	2396	VSLENVEGVWMK	Oxidation (M)[11]	Mascot
1408.7107	1408.7352	0.0245	17	20281	20293	DPIEPPGPPTNFK		Mascot
1410.7046	1410.7175	0.0129	9	16313	16324	RTYIPVMSGENK	Oxidation (M)[7]	Mascot

1412.7632	1412.7311	-0.0321	-23	16505	16516	DENVIVPEEIKK		Mascot
1412.8148	1412.7311	-0.0837	-59	10305	10317	EEVPPPKVPVPPK		Mascot
1418.7135	1418.6959	-0.0176	-12	8136	8147	DNREIRPGGNYK		Mascot
1422.7006	1422.7004	-0.0002	0	20094	20105	ITDVSSDRCTIR	Carbamidomethyl (C)[9]	Mascot
1424.7533	1424.7264	-0.0269	-19	1585	1597	ATGNPNPDIVWLK		Mascot
1425.7406	1425.7273	-0.0133	-9	21553	21564	YILTLENSCGKK	Carbamidomethyl (C)[9]	Mascot
1428.8097	1428.7225	-0.0872	-61	6773	6785	ALLTVQEPPSFVK		Mascot
1430.775	1430.6981	-0.0769	-54	23088	23099	ERNGILWQTVSK		Mascot
1432.7035	1432.696	-0.0075	-5	1415	1427	SPIRMSPAMSPAR	Oxidation (M)[5,9]	Mascot
1440.6676	1440.6863	0.0187	13	33262	33273	EISLEAMDFVDR	Oxidation (M)[7]	Mascot
1443.6719	1443.6909	0.019	13	9199	9211	RASMADAGLYTCK	Carbamidomethyl (C)[12]	Mascot
1447.7177	1447.7092	-0.0085	-6	3188	3199	DGIEINFQVQER		Mascot
1452.7264	1452.7034	-0.023	-16	15022	15033	TSIRWDTAMTVR	Oxidation (M)[9]	Mascot
1467.6962	1467.7151	0.0189	13	31989	32001	NDSGTYDLVLENK		Mascot
1469.7999	1469.7185	-0.0814	-55	28520	28532	VTTISAGLIYEFR		Mascot
1490.7446	1490.7556	0.011	7	3977	3990	STGQDVALRTEEGK		Mascot
1498.7319	1498.8478	0.1159	77	18678	18690	YCVVVENSTGSRK	Carbamidomethyl (C)[2]	Mascot
1503.7325	1503.6906	-0.0419	-28	22560	22572	EVAPDFELDAELR		Mascot
1505.7021	1505.6841	-0.018	-12	1419	1432	MSPAMSPARMSPAR	Oxidation (M)[1]	Mascot
1592.7512	1592.7312	-0.02	-13	34004	34016	LSEYKSELDYMSK		Mascot
1607.8025	1607.827	0.0245	15	20724	20738	GDDEVVTSSHLAIHK		Mascot
1818.8909	1818.8566	-0.0343	-19	24189	24203	FKTTGLEEGIEYEFR		Mascot
1827.9236	1827.8459	-0.0777	-43	30502	30518	GDSGRYFLTLENTAGVK		Mascot
1836.9233	1836.932	0.0087	5	5355	5371	VTNEVGSSTCSARVTLR	Carbamidomethyl (C)[10]	Mascot
1864.9222	1864.9832	0.061	33	20821	20837	CDVSRGDWVTALASVTK	Carbamidomethyl (C)[1]	Mascot
1893.999	1893.9363	-0.0627	-33	33262	33277	EISLEAMDFVDRLLVK	Oxidation (M)[7]	Mascot
1927.9681	1927.9574	-0.0107	-6	22750	22767	ASEAPLPPDSLNIMDITK	Oxidation (M)[14]	Mascot
1955.8916	1955.9537	0.0621	32	26308	26324	DQMLVQWHEPVNDGGS K	Oxidation (M)[3]	Mascot
1966.9506	1966.952	0.0014	1	22457	22475	DVAYPPGPPSNAHVTDT TK		Mascot
2021.1431	2021.0447	-0.0984	-49	25603	25621	NPFVLPGPPKSLEVTNIA K		Mascot
2041.0964	2041.0183	-0.0781	-38	16348	16366	IGGGEYIELKNPVIAQDPK		Mascot
2137.9932	2138.0757	0.0825	39	25863	25881	GINFTQLSIDNCDRNDAG K	Carbamidomethyl (C)[12]	Mascot
2234.1345	2234.1318	-0.0027	-1	14306	14324	RILIIQNAQLEDAGSYNCR	Carbamidomethyl (C)[18]	Mascot
2234.1345	2234.1318	-0.0027	-1	14306	14324	RILIIQNAQLEDAGSYNCR	Carbamidomethyl (C)[18]	Mascot
2235.0784	2235.1545	0.0761	34	14130	14148	LIIHGCTPEDIKTYTCDAK	Carbamidomethyl (C)[6,16]	Mascot
2250.1184	2250.0808	-0.0376	-17	25858	25876	VQIEKGINFTQLSIDNCDR	Carbamidomethyl (C)[17]	Mascot

2252.104	2252.1233	0.0193	9	34027	34044	QRQVTEITEIEEEYEISR				Mascot
2252.104	2252.1233	0.0193	9	34027	34044	QRQVTEITEIEEEYEISR	1	0		Mascot
2264.1843	2264.1145	-0.0698	-31	24500	24520	IMAVNKYGVGEPLESESLIAK			Oxidation (M)[2]	Mascot
2268.2852	2268.1216	-0.1636	-72	21565	21585	EYTIVVKVLDTPGPPVNVTVK				Mascot
2274.0771	2274.0916	0.0145	6	3460	3479	EIASLLSAEEDFQTYSSDLR				Mascot
2280.1885	2280.1387	-0.0498	-22	31662	31681	DAHRPGWLPVSESVTRPTFK				Mascot
2285.1443	2285.1548	0.0105	5	18286	18307	AVNVAGVGEPGEVTDVIEMKDR				Mascot
2286.2163	2286.0735	-0.1428	-62	20594	20613	LEVTEMTKNSATLAWLPPLR			Oxidation (M)[6]	Mascot
2288.1592	2288.0762	-0.083	-36	18775	18795	IHAENLYGISDPLVSDSMKAK				Mascot
2715.3196	2715.3311	0.0115	4	25525	25548	CTLAWSPPLQDGGSDISHYVVEKR			Carbamidomethyl (C)[1]	Mascot
2731.2993	2731.304	0.0047	2	26008	26032	VTLTDVSQTSASLMWEKPEHDGGSR				Mascot
2732.4868	2732.3357	-0.1511	-55	9526	9548	EFEELVAFIQQRLTQTEPVTLIK				Mascot
2754.3152	2754.3176	0.0024	1	16025	16048	TSGPDCNFRVTDVIEGTEVQFQVR			Carbamidomethyl (C)[6]	Mascot

Gel Idx/Pos	203/I3	Instr./Gel Origin	BA2151/Sample Project 20140814	Process Status	Analysis Succeeded
Plate [#] Name	[1] Sample Project 20140814	Instrument Sample Name		Spectra	11

Rank	Protein Name	Accession No.	Protein MW	Protein PI	Pep. Count	Protein Score	Protein Score C. I. %	Intensity Matched	Total Ion Score	Total Ion C. I. %	Confirmed
1	Histidine--tRNA ligase OS=Yersinia pestis bv. Antiqua (strain Antiqua) GN=hisS PE=3 SV=1	SYH_YERPA	47460.3	5.24	13	65	81.251	3.224			

Protein Group

Protein Name	Accession No.	Protein MW	Protein PI
Histidine--tRNA ligase OS=Yersinia pestis (strain Pestoides F) GN=hisS PE=3 SV=1	SYH_YERPP	47460.3	5.2399997711816
Histidine--tRNA ligase OS=Yersinia pestis GN=hisS PE=3 SV=1	SYH_YERPE	47460.3	5.2399997711816
Histidine--tRNA ligase OS=Yersinia pestis bv. Antiqua (strain Angola) GN=hisS PE=3 SV=1	SYH_YERPG	47460.3	5.2399997711816
Histidine--tRNA ligase OS=Yersinia pestis bv. Antiqua (strain Nepal516) GN=hisS PE=3 SV=1	SYH_YERPN	47460.3	5.2399997711816
Histidine--tRNA ligase OS=Yersinia pseudotuberculosis serotype I (strain IP32953) GN=hisS PE=3 SV=1	SYH_YERPS	47460.3	5.2399997711816
Histidine--tRNA ligase OS=Yersinia pseudotuberculosis serotype IB (strain PB1/+) GN=hisS PE=3 SV=1	SYH_YERPB	47460.3	5.2399997711816
Histidine--tRNA ligase OS=Yersinia pseudotuberculosis serotype O:1b (strain IP 31758) GN=hisS PE=3 SV=1	SYH_YERP3	47450.2	5.2399997711816
Histidine--tRNA ligase OS=Yersinia pseudotuberculosis serotype O:3 (strain YPIII) GN=hisS PE=3 SV=1	SYH_YERPY	47460.3	5.2399997711816

Peptide Information

Calc. Mass	Obsrv. Mass	± da	± ppm	Start Seq.	End Seq.	Sequence	Ion Score	C. I. %	Modification	Rank	Result Type
865.4526	865.4037	-0.0489	-57	372	378	QFTRADK					Mascot
1060.5931	1060.5466	-0.0465	-44	1	9	MAKNIQAIR			Oxidation (M)[1]		Mascot
1182.6129	1182.547	-0.0659	-56	105	113	LWYIGPMFR					Mascot
1281.662	1281.5493	-0.1127	-88	360	371	IMTNYGGGNVKK					Mascot

1315.7217	1315.6066	-0.1151	-87	54	65	RAIGEVTDVVEK						Mascot
1422.741	1422.6154	-0.1256	-88	200	211	MYSNPLRVLDSK						Mascot
1438.736	1438.6079	-0.1281	-89	200	211	MYSNPLRVLDSK			Oxidation (M)[1]			Mascot
1517.8435	1517.7177	-0.1258	-83	247	259	ASIPYTVNERLVR						Mascot
1891.9946	1891.9083	-0.0863	-46	353	370	DALPTLKIMTNYGGGNVK						Mascot
1956.9734	1956.9514	-0.022	-11	401	418	DLRNGEQETLAQADVAA R						Mascot
2561.2817	2561.1772	-0.1045	-41	235	256	QHFAGLCELLDKASIPYT VNER			Carbamidomethyl (C)[7]			Mascot
3239.7212	3239.5442	-0.177	-55	122	150	YRQFHQLGAEVFGLPGP DIDAELILLTAR						Mascot
3420.6755	3420.7087	0.0332	10	267	298	TVFEWVTHSLGAQGTVC AGGRYDGLVEQLGGR			Carbamidomethyl (C)[17]			Mascot
3448.8164	3448.7388	-0.0776	-23	124	153	QFHQLGAEVFGLPGPDID AELILLTARWWR						Mascot

2	Probable acetoacetate decarboxylase OS=Clostridium botulinum (strain Eklund 17B / Type B) GN=adc PE=3 SV=1	ADC_CLOBB	27953.3	6.31	11	57	1.603	9.593

Peptide Information

Calc. Mass	Obsrv. Mass	± da	± ppm	Start Seq.	End Seq.	Sequence	Ion Score	C. I. %	Modification	Rank	Result Type
889.4738	889.4243	-0.0495	-56	38	45	TDADALRK					Mascot
930.489	930.4361	-0.0529	-57	152	159	QLDLNEAK					Mascot
969.5074	969.4487	-0.0587	-61	141	149	VAIATMGYK			Oxidation (M)[6]		Mascot
1046.528	1046.5054	-0.0226	-22	22	29	GPYRFHNR					Mascot
1145.595	1145.5648	-0.0302	-26	171	180	IIPNYDGTPR					Mascot
1145.595	1145.5648	-0.0302	-26	171	180	IIPNYDGTPR					Mascot
1195.6429	1195.5608	-0.0821	-69	150	159	HKQLDLNEAK					Mascot
1412.7897	1412.7141	-0.0756	-54	9	21	QITTPLTAPAFPR					Mascot
1886.0283	1885.9266	-0.1017	-54	9	25	QITTPLTAPAFPRGPYR					Mascot
1943.0597	1942.8854	-0.1743	-90	4	21	SEVSKQITTPLTAPAFPR					Mascot
1956.0049	1955.9403	-0.0646	-33	204	220	LQLFDHAMAPFNDLPVK					Mascot
3389.71	3389.6492	-0.0608	-18	191	220	DITVHEAWTGPARLQLFD HAMAPFNDLPVK					Mascot
3405.7048	3405.6458	-0.059	-17	191	220	DITVHEAWTGPARLQLFD HAMAPFNDLPVK			Oxidation (M)[21]		Mascot

3	Uncharacterized protein YAE1 OS=Lodderomyces elongisporus (strain ATCC 11503 / CBS 2605 / JCM 1781 / NBRC 1676 / NRRL YB-4239) GN=YAE1 PE=3 SV=1	YAE1_LODEL	20886.8	4.78	9	56	0	7.786

Peptide Information

Calc. Mass	Obsrv. Mass	± da	± ppm	Start Seq.	End Seq.	Sequence	Ion Score	C. I. %	Modification	Rank	Result Type

Calc. Mass	Obsrv. Mass	± da	± ppm	Start Seq.	End Seq.	Sequence	Ion Score	C. I. %	Modification	Rank	Result Type
1078.5891	1078.5316	-0.0575	-53	80	89	KQGYVDGLAK					Mascot
1078.5891	1078.5316	-0.0575	-53	80	89	KQGYVDGLAK					Mascot
1088.5443	1088.5442	-0.0001	0	55	64	DGVGSRQQNK					Mascot
1195.5446	1195.5608	0.0162	14	146	155	ECMNALNISK			Carbamidomethyl (C)[2], Oxidation (M)[3]		Mascot
1517.7853	1517.7177	-0.0676	-45	65	77	QTQSLAMNEIARR					Mascot
1732.9269	1732.8812	-0.0457	-26	156	169	VLDLKYFDDHLNLK					Mascot
1875.9342	1875.8877	-0.0465	-25	61	76	QQNKQTQSLAMNEIAR			Oxidation (M)[11]		Mascot
1875.9342	1875.8877	-0.0465	-25	61	76	QQNKQTQSLAMNEIAR			Oxidation (M)[11]		Mascot
1885.9807	1885.9266	-0.0541	-29	98	115	GFDFAYSIGADLGIKVGR					Mascot
1956.9774	1956.9514	-0.026	-13	81	97	QGYVDGLAKHQEENLQK					Mascot
2252.0647	2252.093	0.0283	13	134	155	SGGSGNDLGSLKECMNA LNISK			Carbamidomethyl (C)[14]		Mascot

4	Putative HTH-type transcriptional regulator YhjB OS=Escherichia coli (strain K12) GN=yhjB PE=4 SV=1	YHJB_ECOLI	22703.7	6.32	9	56	0	2.919	

Peptide Information

Calc. Mass	Obsrv. Mass	± da	± ppm	Start Seq.	End Seq.	Sequence	Ion Score	C. I. %	Modification	Rank	Result Type
889.5101	889.4243	-0.0858	-96	138	145	DLKSLSAR					Mascot
1046.5881	1046.5054	-0.0827	-79	68	76	TVVQFPEVK					Mascot
1060.5609	1060.5466	-0.0143	-13	9	17	QSIFIHGMK					Mascot
1144.6222	1144.5391	-0.0831	-73	175	183	AHLESLYRR					Mascot
1182.5493	1182.547	-0.0023	-2	190	200	TQAAMMLNISS			Oxidation (M)[5]		Mascot
1676.8636	1676.7921	-0.0715	-43	146	160	QREILTMLAAGESNK			Oxidation (M)[7]		Mascot
1926.9919	1926.8879	-0.104	-54	24	41	IPGVSIQGASQADELWQK					Mascot
1950.0089	1949.941	-0.0679	-35	2	17	QIVMFDRQSIFIHGMK					Mascot
1966.0038	1965.9446	-0.0592	-30	2	17	QIVMFDRQSIFIHGMK			Oxidation (M)[4]		Mascot
3373.5901	3373.6201	0.03	9	104	134	DSTVETFALAVNSAAMG MMFLPGDWRTTPEK					Mascot
3389.5852	3389.6492	0.064	19	104	134	DSTVETFALAVNSAAMG MMFLPGDWRTTPEK			Oxidation (M)[16]		Mascot
3405.5801	3405.6458	0.0657	19	104	134	DSTVETFALAVNSAAMG MMFLPGDWRTTPEK			Oxidation (M)[16,18]		Mascot

5	FACT complex subunit Ssrp1 OS=Drosophila melanogaster GN=Ssrp PE=1 SV=2	SSRP1_DROME	81710.4	5.45	17	54	0	4.918	

Peptide Information

Calc. Mass	Obsrv. Mass	± da	± ppm	Start Seq.	End Seq.	Sequence	Ion Score	C. I. %	Modification	Rank	Result Type
866.3461	866.4011	0.055	63	613	618	YHDEMR			Oxidation (M)[5]		Mascot

Calc. Mass	Obsrv. Mass	± da	± ppm	Start Seq.	End Seq.	Sequence	Ion Score	C. I. %	Modification	Rank	Result Type
930.5077	930.4361	-0.0716	-77	234	241	IPMDSVLR					Mascot
1144.578	1144.5391	-0.0389	-34	423	432	LHVSNMGKDK			Oxidation (M)[6]		Mascot
1298.6376	1298.599	-0.0386	-30	371	381	FEEISSVNFAR					Mascot
1331.6954	1331.6165	-0.0789	-59	397	408	NGTVHIFSSIEK					Mascot
1380.7556	1380.7098	-0.0458	-33	23	33	LKMTEQNIIFK			Oxidation (M)[3]		Mascot
1490.7196	1490.7427	0.0231	15	114	126	FMGSVLSFDKESK			Oxidation (M)[2]		Mascot
1507.8115	1507.6714	-0.1401	-93	189	203	ASVISASGESIAIFR					Mascot
1550.8287	1550.7809	-0.0478	-31	252	264	QMFFVLSLDPPIK			Oxidation (M)[2]		Mascot
1647.8265	1647.7654	-0.0611	-37	409	421	EEYAKLFDYITQK					Mascot
1678.8721	1678.7565	-0.1156	-69	302	316	LEKEISGPVYEVMGK					Mascot
1796.9915	1796.84	-0.1515	-84	234	248	IPMDSVLRLFMLPHK					Mascot
1818.8071	1818.8202	0.0131	7	91	105	AAYSQEMVEKEMCVK			Carbamidomethyl (C)[13], Oxidation (M)[7]		Mascot
1828.9812	1828.8572	-0.124	-68	234	248	IPMDSVLRLFMLPHK			Oxidation (M)[3,11]		Mascot
1901.979	1901.934	-0.045	-24	127	143	TIFEVPLSHVSQCVTGK			Carbamidomethyl (C)[13]		Mascot
1908.9889	1908.8411	-0.1478	-77	249	264	DSRQMFFVLSLDPPIK			Oxidation (M)[5]		Mascot
1920.783	1920.9264	0.1434	75	659	675	SKEYISDDDSTSSDDEK					Mascot
1951.976	1951.9329	-0.0431	-22	397	413	NGTVHIFSSIEKEEYAK					Mascot
1951.976	1951.9329	-0.0431	-22	397	413	NGTVHIFSSIEKEEYAK					Mascot

6 Threonine--tRNA ligase OS=Neisseria meningitidis serogroup C (strain 053442) GN=thrS PE=3 SV=1 SYT_NEIM0 73046.9 5.86 15 53 0 10.879

Peptide Information

Calc. Mass	Obsrv. Mass	± da	± ppm	Start Seq.	End Seq.	Sequence	Ion Score	C. I. %	Modification	Rank	Result Type
848.4182	848.4304	0.0122	14	294	300	TPQIMDK			Oxidation (M)[5]		Mascot
865.4413	865.4037	-0.0376	-43	283	290	ELNAAGYK					Mascot
1037.5197	1037.463	-0.0567	-55	346	353	SYRDLPMR					Mascot
1145.6348	1145.5648	-0.07	-61	137	146	IMTPRAEAIK			Oxidation (M)[2]		Mascot
1145.6348	1145.5648	-0.07	-61	137	146	IMTPRAEAIK	5	0	Oxidation (M)[2]		Mascot
1163.5845	1163.5836	-0.0009	-1	409	418	QFGFHDVSVK					Mascot
1173.5093	1173.5994	0.0901	77	314	323	DNMFVTSSEK			Oxidation (M)[3]		Mascot
1281.6368	1281.5493	-0.0875	-68	363	374	NEPSGALHGLMR					Mascot
1298.6376	1298.599	-0.0386	-30	147	156	IFQERGEEYK					Mascot
1315.6688	1315.6066	-0.0622	-47	73	84	HSCAHLVGHAVK			Carbamidomethyl (C)[3]		Mascot
1331.6989	1331.6165	-0.0824	-62	1	12	MLNITLPDGSVR			Oxidation (M)[1]		Mascot
1380.639	1380.7098	0.0708	51	492	503	LGAEYVTENNDR					Mascot
1980.9344	1980.9137	-0.0207	-10	200	216	LAGAYWRGDSNNEMLQ R					Mascot

3238.4248	3238.552	0.1272	39	157	182	LRLIDDMPEVEAMGMYH HQEYVDMCR			Carbamidomethyl (C)[25]		Mascot
3239.7634	3239.5442	-0.2192	-68	2	33	LNITLPDGSVRQYESPVT VAQIAASIGAGLAK					Mascot
3254.646	3254.533	-0.113	-35	44	72	LVDACDPITEDSSVQIITP KDQEGIEIIR			Carbamidomethyl (C)[5]		Mascot
3254.646	3254.533	-0.113	-35	44	72	LVDACDPITEDSSVQIITP KDQEGIEIIR			Carbamidomethyl (C)[5]		Mascot

7	Keratin, type I cytoskeletal 13 OS=Homo sapiens GN=KRT13 PE=1 SV=4	K1C13_HUMAN	49899.5	4.91	13	52	0	18.92

Peptide Information

Calc. Mass	Obsrv. Mass	± da	± ppm	Start Seq.	End Seq.	Sequence	Ion Score	C. I. %	Modification	Rank	Result Type
823.4559	823.4077	-0.0482	-59	115	121	LASYLEK					Mascot
867.3989	867.432	0.0331	38	28	35	GVSTCSTR			Carbamidomethyl (C)[5]		Mascot
1060.4728	1060.5466	0.0738	70	241	248	KNHEEEMK			Oxidation (M)[7]		Mascot
1078.6255	1078.5316	-0.0939	-87	115	123	LASYLEKVR					Mascot
1078.6255	1078.5316	-0.0939	-87	115	123	LASYLEKVR					Mascot
1144.6321	1144.5391	-0.093	-81	166	175	ILTATIENNR					Mascot
1173.6838	1173.5994	-0.0844	-72	213	222	RVLDELTLSK					Mascot
1412.6223	1412.7141	0.0918	65	279	289	EQYEAMAERNR			Oxidation (M)[6]		Mascot
1550.771	1550.7809	0.0099	6	138	150	DWHLKQSPASPER					Mascot
1676.8167	1676.7921	-0.0246	-15	151	163	DYSPYYKTIEELR					Mascot
1857.8469	1857.9412	0.0943	51	273	287	VLAEMREQYEAMAER			Oxidation (M)[5,12]		Mascot
1908.9847	1908.8411	-0.1436	-75	106	121	ITMQNLNDRLASYLEK					Mascot
2252.1438	2252.093	-0.0508	-23	306	325	EVSTNTAMIQTSKTEITEL R					Mascot
2635.2668	2635.1633	-0.1035	-39	249	272	EFSNQVVGQVNVEMDAT PGIDLTR			Oxidation (M)[14]		Mascot

8	Phosphomethylpyrimidine synthase OS=Bordetella pertussis (strain Tohama I / ATCC BAA-589 / NCTC 13251) GN=thiC PE=3 SV=1	THIC_BORPE	71322.9	5.79	15	51	0	14.526

Peptide Information

Calc. Mass	Obsrv. Mass	± da	± ppm	Start Seq.	End Seq.	Sequence	Ion Score	C. I. %	Modification	Rank	Result Type
1060.5422	1060.5466	0.0044	4	164	171	EHYLETLR					Mascot
1138.5375	1138.5322	-0.0053	-5	602	612	DYAAAQGVSEK					Mascot
1145.6348	1145.5648	-0.07	-61	112	121	LTDPKLTAMR					Mascot
1145.6348	1145.5648	-0.07	-61	112	121	LTDPKLTAMR					Mascot
1163.5361	1163.5836	0.0475	41	613	622	DALQQGMQEK			Oxidation (M)[7]		Mascot
1406.7499	1406.6199	-0.13	-92	532	545	GHPGAAIRDNALSK					Mascot

1406.7499	1406.6199	-0.13	-92	532	545	GHPGAAIRDNALSK					Mascot
1490.7281	1490.7427	0.0146	10	133	145	AGANVTQMHYARR			Oxidation (M)[8]		Mascot
1631.8938	1631.7623	-0.1315	-81	145	158	RGIVTPEMEFIALR					Mascot
1647.8887	1647.7654	-0.1233	-75	145	158	RGIVTPEMEFIALR			Oxidation (M)[8]		Mascot
1836.916	1836.912	-0.004	-2	613	628	DALQQGMQEKAVEFVK			Oxidation (M)[7]		Mascot
1875.8959	1875.8877	-0.0082	-4	365	378	WCLAHHKESFLYER			Carbamidomethyl (C)[2]		Mascot
1875.8959	1875.8877	-0.0082	-4	365	378	WCLAHHKESFLYER			Carbamidomethyl (C)[2]		Mascot
1921.0034	1920.9264	-0.077	-40	341	357	LPFIPMTADRMTGIVSR			Oxidation (M)[6]		Mascot
1955.7843	1955.9403	0.156	80	580	595	VAHFCSMCGPHFCSMK			Carbamidomethyl (C)[5,8,13]		Mascot
2252.1921	2252.093	-0.0991	-44	2	24	NANPTFLAATAEVDAAAV APLPK					Mascot
2592.4045	2592.1531	-0.2514	-97	184	207	LLRQHPGQSFGAALPSAI TPEFVR					Mascot
2684.166	2684.1558	-0.0102	-4	580	601	VAHFCSMCGPHFCSMKI TQDVR			Carbamidomethyl (C)[5,8,13], Oxidation (M)[7]		Mascot
3420.7114	3420.7087	-0.0027	-1	418	447	TLGELTQVAWKHDVQVM IEGPGHVPMQMIK			Oxidation (M)[17,26,28]		Mascot

9	Autonomous glycyl radical cofactor OS=Shigella flexneri serotype 5b (strain 8401) GN=grcA PE=3 SV=1	GRCA_SHIF8	14332.4	4.95	7	51	0	1.71

Peptide Information

Calc. Mass	Obsrv. Mass	± da	± ppm	Start Seq.	End Seq.	Sequence	Ion Score	C. I. %	Modification	Rank	Result Type
865.4414	865.4037	-0.0377	-44	49	55	LGDIEYR					Mascot
1060.5634	1060.5466	-0.0168	-16	80	88	RETLEDAVK					Mascot
1337.6583	1337.6547	-0.0036	-3	36	48	AGYAEDEVVAVSK					Mascot
1590.8823	1590.7378	-0.1445	-91	67	80	VEGGQHLNVNVLRR					Mascot
1908.9736	1908.8411	-0.1325	-69	31	48	CIVAKAGYAEDEVVAVSK			Carbamidomethyl (C)[1]		Mascot
1951.9985	1951.9329	-0.0656	-34	100	116	VSGYAVRFNSLTPEQQR					Mascot
1951.9985	1951.9329	-0.0656	-34	100	116	VSGYAVRFNSLTPEQQR					Mascot
3218.6328	3218.5381	-0.0947	-29	2	30	ITGIQITKAANDDLLNSFW LLDSEQGEAR					Mascot

10	Mediator of RNA polymerase II transcription subunit 16 OS=Emericella nidulans (strain FGSC A4 / ATCC 38163 / CBS 112.46 / NRRL 194 / M139) GN=sin4 PE=3 SV=2	MED16_EMENI	103397	6.5	14	50	0	14.424

Peptide Information

Calc. Mass	Obsrv. Mass	± da	± ppm	Start Seq.	End Seq.	Sequence	Ion Score	C. I. %	Modification	Rank	Result Type
823.442	823.4077	-0.0343	-42	835	840	SELYRR					Mascot
987.5469	987.5199	-0.027	-27	841	848	ERDVDIIK					Mascot
1088.5555	1088.5442	-0.0113	-10	875	884	NGNGSQLSRR					Mascot

1406.6515	1406.6199	-0.0316	-22	805	816	QTVPAMRGEMDR			Oxidation (M)[6]	Mascot
1406.6515	1406.6199	-0.0316	-22	805	816	QTVPAMRGEMDR	16	0	Oxidation (M)[6]	Mascot
1421.7604	1421.6545	-0.1059	-74	199	211	GALLCITRSGVMK			Carbamidomethyl (C)[5], Oxidation (M)[12]	Mascot
1422.6465	1422.6154	-0.0311	-22	805	816	QTVPAMRGEMDR			Oxidation (M)[6,10]	Mascot
1732.9011	1732.8812	-0.0199	-11	856	874	SGKGSGLGSVEAVMGLGAR				Mascot
1846.0433	1845.8855	-0.1578	-85	126	142	VSIYSIPIALNSVNVTR				Mascot
1851.9236	1851.8595	-0.0641	-35	551	566	QLSPDAQTAFLNEVYR				Mascot
1857.9739	1857.9412	-0.0327	-18	534	550	GCGGEVNTDDILLILIR			Carbamidomethyl (C)[2]	Mascot
1875.9706	1875.8877	-0.0829	-44	58	74	LGCIASISQDSTRVNVR			Carbamidomethyl (C)[3]	Mascot
1875.9706	1875.8877	-0.0829	-44	58	74	LGCIASISQDSTRVNVR			Carbamidomethyl (C)[3]	Mascot
1902.8729	1902.957	0.0841	44	766	783	HAYTGAGFGDNERPGPEK				Mascot
1902.8729	1902.957	0.0841	44	766	783	HAYTGAGFGDNERPGPEK				Mascot
1983.0481	1982.8906	-0.1575	-79	294	310	LVHMKVEVPYNIPSSNR				Mascot
2561.3147	2561.1772	-0.1375	-54	261	281	ISLYRVSIQWTPSQWDPTQQK				Mascot
2651.2712	2651.1372	-0.134	-51	817	839	LAIFMGDYGWLGFGNDARSELYR				Mascot
2667.2661	2667.1472	-0.1189	-45	817	839	LAIFMGDYGWLGFGNDARSELYR			Oxidation (M)[5]	Mascot

Gel Idx/Pos	204/I4	Instr./Gel Origin	BA2151/Sample Project 20140814	Process Status	Analysis Succeeded
Plate [#] Name	[1] Sample Project 20140814	Instrument Sample Name		Spectra	11

Rank	Protein Name	Accession No.	Protein MW	Protein PI	Pep. Count	Protein Score	Protein Score C. I. %	Intensity Matched	Total Ion Score	Total Ion C. I. %	Confirmed
1	L-ascorbate peroxidase 1, cytosolic OS=Arabidopsis thaliana GN=APX1 PE=1 SV=2	APX1_ARATH	27828.9	5.72	2	64	77.459	3.557	58	99.774	

Peptide Information

Calc. Mass	Obsrv. Mass	± da	± ppm	Start Seq.	End Seq.	Sequence	Ion Score	C. I. %	Modification	Rank	Result Type
976.4703	976.47	-0.0003	0	31	38	NCAPIMVR			Carbamidomethyl (C)[2], Oxidation (M)[6]		Mascot
1249.6172	1249.6107	-0.0065	-5	120	130	EDKPQPPPEGR					Mascot
1249.6172	1249.6107	-0.0065	-5	120	130	EDKPQPPPEGR	58	99.774			Mascot

Rank	Protein Name	Accession No.	Protein MW	Protein PI	Pep. Count	Protein Score	Protein Score C. I. %	Intensity Matched	Total Ion Score	Total Ion C. I. %	Confirmed
2	Proteasome subunit alpha type-4 OS=Caenorhabditis elegans GN=pas-3 PE=3 SV=2	PSA4_CAEEL	28492.5	6.62	4	63	72.268	8.444	49	98.327	

Peptide Information

Calc. Mass	Obsrv. Mass	± da	± ppm	Start Seq.	End Seq.	Sequence	Ion Score	C. I. %	Modification	Rank	Result Type
918.5077	918.4532	-0.0545	-59	208	215	VEMAVLTR					Mascot
1007.5156	1007.5112	-0.0044	-4	9	17	TTIFSPEGR					Mascot
1007.5156	1007.5112	-0.0044	-4	9	17	TTIFSPEGR	49	98.327			Mascot
1034.5	1034.4529	-0.0471	-46	241	249	EKEAETAEK					Mascot
1050.5691	1050.4817	-0.0874	-83	119	127	QRYTQIGGK					Mascot

Rank	Protein Name	Accession No.	Protein MW	Protein PI	Pep. Count	Protein Score	Protein Score C. I. %	Intensity Matched	Total Ion Score	Total Ion C. I. %	Confirmed
3	Proteasome subunit alpha type-4-1 OS=Oryza sativa subsp. japonica GN=PAC1 PE=2 SV=1	PSA4A_ORYSJ	27197.7	6.44	3	61	52.904	8.36	49	98.327	

Protein Group

Proteasome subunit alpha type-4-1 OS=Oryza sativa subsp. indica GN=OsI_021120 PE=1 SV=2	PSA4A_ORYSI	27197.7	6.4400 000572 2046
Proteasome subunit alpha type-4-2 OS=Oryza sativa subsp. indica GN=OsI_021067 PE=1 SV=1	PSA4B_ORYSI	27179.7	6.4400 000572 2046
Proteasome subunit alpha type-4-2 OS=Oryza sativa subsp. japonica GN=Os06g0167600 PE=2 SV=1	PSA4B_ORYSJ	27179.7	6.4400 000572 2046

Peptide Information

Calc. Mass	Obsrv. Mass	± da	± ppm	Start	End	Sequence	Ion	C. I. %	Modification	Rank	Result Type

Calc. Mass	Obsrv. Mass	± da	± ppm	Start Seq.	End Seq.	Sequence	Ion Score	C. I. %	Modification	Rank	Result Type
943.5458	943.478	-0.0678	-72	186	194	EEAVALALK					Mascot
1007.5156	1007.5112	-0.0044	-4	9	17	TTIFSPEGR					Mascot
1007.5156	1007.5112	-0.0044	-4	9	17	TTIFSPEGR	49	98.327			Mascot
2542.2751	2542.2664	-0.0087	-3	68	91	IDSHLACAVAGIMSDANIL LNTAR			Carbamidomethyl (C)[7], Oxidation (M)[13]		Mascot

4	Keratin, type I cytoskeletal 23 OS=Homo sapiens GN=KRT23 PE=1 SV=2	K1C23_HUMAN	48272.3	6.09	13	59	37.916	16.909

Peptide Information

Calc. Mass	Obsrv. Mass	± da	± ppm	Start Seq.	End Seq.	Sequence	Ion Score	C. I. %	Modification	Rank	Result Type
929.5051	929.4233	-0.0818	-88	64	73	SSPLLGGNGK					Mascot
1035.5503	1035.4946	-0.0557	-54	388	397	SSMKVSATPK					Mascot
1050.5439	1050.4817	-0.0622	-59	31	42	APTVHGGAGGAR					Mascot
1051.5453	1051.4642	-0.0811	-77	388	397	SSMKVSATPK			Oxidation (M)[3]		Mascot
1052.5082	1052.4778	-0.0304	-29	152	160	MAVDDFNLK					Mascot
1052.5082	1052.4778	-0.0304	-29	152	160	MAVDDFNLK					Mascot
1067.4575	1067.4625	0.005	5	209	216	HHEQEMEK					Mascot
1068.5031	1068.4725	-0.0306	-29	152	160	MAVDDFNLK			Oxidation (M)[1]		Mascot
1068.5031	1068.4725	-0.0306	-29	152	160	MAVDDFNLK			Oxidation (M)[1]		Mascot
1078.4946	1078.5327	0.0381	35	74	82	ATMQNLNDR			Oxidation (M)[3]		Mascot
1090.5375	1090.4667	-0.0708	-65	374	383	LLEGESEGTR					Mascot
1242.6688	1242.6216	-0.0472	-38	229	239	VDTGPREDLIK					Mascot
1537.7983	1537.7174	-0.0809	-53	27	42	SFPRAPTVHGGAGGAR					Mascot
1885.9542	1885.8928	-0.0614	-33	293	308	TFQALEIDLQTQYSTK					Mascot
1956.041	1955.9791	-0.0619	-32	31	50	APTVHGGAGGARISLSFT TR					Mascot
2086.9539	2086.9973	0.0434	21	152	168	MAVDDFNLKYENEHSFK					Mascot
2542.1719	2542.2664	0.0945	37	2	26	NSGHSFSQTPSASFHGA GGGWGRPR					Mascot

5	Proteasome subunit alpha type-4-like OS=Drosophila melanogaster GN=Prosalpha3T PE=2 SV=1	PSA4L_DROME	28550.2	8.42	3	59	34.99	8.697	49	98.327

Peptide Information

Calc. Mass	Obsrv. Mass	± da	± ppm	Start Seq.	End Seq.	Sequence	Ion Score	C. I. %	Modification	Rank	Result Type
1007.5156	1007.5112	-0.0044	-4	9	17	TTIFSPEGR					Mascot
1007.5156	1007.5112	-0.0044	-4	9	17	TTIFSPEGR	49	98.327			Mascot
1029.4935	1029.4753	-0.0182	-18	1	8	MARFFDSR					Mascot

Calc. Mass	Obsrv. Mass	± da	± ppm	Start Seq.	End Seq.	Sequence	Ion Score	C. I. %	Modification	Rank	Result Type
2071.072	2071.0107	-0.0613	-30	221	237	YGNTTVFHILEKNEIHR					Mascot

6	Proteasome subunit alpha type-3 OS=Saccharomyces cerevisiae (strain ATCC 204508 / S288c) GN=PRE9 PE=1 SV=1	PSA3_YEAST	28696.6	5.06	3	59	34.99	7.862	49	98.327	

Peptide Information

Calc. Mass	Obsrv. Mass	± da	± ppm	Start Seq.	End Seq.	Sequence	Ion Score	C. I. %	Modification	Rank	Result Type
1007.5156	1007.5112	-0.0044	-4	10	18	TTIFSPEGR					Mascot
1007.5156	1007.5112	-0.0044	-4	10	18	TTIFSPEGR	49	98.327			Mascot
1080.4956	1080.5028	0.0072	7	219	228	GANDGEVYQK					Mascot
1956.0172	1955.9791	-0.0381	-19	52	68	VTSTLLEQDTSTEKLYK					Mascot

7	Proteasome subunit alpha type-4 OS=Petunia hybrida GN=PAC1 PE=2 SV=1	PSA4_PETHY	27385.6	5.6	3	59	31.926	7.83	49	98.327	

Peptide Information

Calc. Mass	Obsrv. Mass	± da	± ppm	Start Seq.	End Seq.	Sequence	Ion Score	C. I. %	Modification	Rank	Result Type
915.5146	915.4647	-0.0499	-55	41	49	DGVVLVGEK					Mascot
1007.5156	1007.5112	-0.0044	-4	9	17	TTIFSPEGR					Mascot
1007.5156	1007.5112	-0.0044	-4	9	17	TTIFSPEGR	49	98.327			Mascot
1642.8204	1642.9016	0.0812	49	195	209	VLSKTMDSTSLTSEK			Oxidation (M)[6]		Mascot

8	Proteasome subunit alpha type-4 OS=Dictyostelium discoideum GN=psmA4 PE=2 SV=1	PSA4_DICDI	28197.1	5.65	3	58	18.157	7.798	49	98.327	

Peptide Information

Calc. Mass	Obsrv. Mass	± da	± ppm	Start Seq.	End Seq.	Sequence	Ion Score	C. I. %	Modification	Rank	Result Type
915.5145	915.4647	-0.0498	-54	41	49	DGIVLAAEK					Mascot
1007.5156	1007.5112	-0.0044	-4	9	17	TTIFSPEGR					Mascot
1007.5156	1007.5112	-0.0044	-4	9	17	TTIFSPEGR	49	98.327			Mascot
1034.5194	1034.4529	-0.0665	-64	212	219	LEFSYFTK					Mascot

9	Proteasome subunit alpha type-4-A OS=Arabidopsis thaliana GN=PAC1 PE=1 SV=1	PSA4A_ARATH	27571.9	6.6	2	57	0	7.18	49	98.327	

Peptide Information

Calc. Mass	Obsrv. Mass	± da	± ppm	Start Seq.	End Seq.	Sequence	Ion Score	C. I. %	Modification	Rank	Result Type
1007.5156	1007.5112	-0.0044	-4	9	17	TTIFSPEGR					Mascot

1007.5156	1007.5112	-0.0044	-4	9	17	TTIFSPEGR	49	98.327			Mascot
2559.2747	2559.2598	-0.0149	-6	199	221	TMDSTSLTSEKLELAEVY LTPSK			Oxidation (M)[2]		Mascot

10	Putative proteasome subunit alpha type-4-B OS=Arabidopsis thaliana GN=PAC2 PE=5 SV=1	PSA4B_ARATH	22830.6	6.96	2	56	0	7.279	49	98.327	

Peptide Information

Calc. Mass	Obsrv. Mass	± da	± ppm	Start Seq.	End Seq.	Sequence	Ion Score	C. I. %	Modification	Rank	Result Type
915.5146	915.4647	-0.0499	-55	41	49	DGVVLVGEK					Mascot
1007.5156	1007.5112	-0.0044	-4	9	17	TTIFSPEGR					Mascot
1007.5156	1007.5112	-0.0044	-4	9	17	TTIFSPEGR	49	98.327			Mascot

Gel Idx/Pos	205/I5	Instr./Gel Origin	BA2151/Sample Project 20140814	Process Status	Analysis Succeeded
Plate [#] Name	[1] Sample Project 20140814	Instrument Sample Name		Spectra	11

Rank	Protein Name	Accession No.	Protein MW	Protein PI	Pep. Count	Protein Score	Protein Score C. I. %	Intensity Matched	Total Ion Score	Total Ion C. I. %	Confirmed
1	Nucleoid-associated protein Tola_2216 OS=Tolumonas auensis (strain DSM 9187 / TA4) GN=Tola_2216 PE=3 SV=1	Y2216_TOLAT	12171.9	5.15	9	55	0	9.774			

Peptide Information

Calc. Mass	Obsrv. Mass	± da	± ppm	Start Seq.	End Seq.	Sequence	Ion Score	C. I. %	Modification	Rank	Result Type
839.3749	839.3144	-0.0605	-72	5	12	GGMGNLMK			Oxidation (M)[3,7]		Mascot
1097.6062	1097.6028	-0.0034	-3	44	53	ITITGSHNVR					Mascot
1139.5701	1139.5272	-0.0429	-38	2	12	FGKGGMGNLMK					Mascot
1807.9082	1807.8781	-0.0301	-17	91	107	AKMAEITGGMQLPPGFK			Oxidation (M)[3,10]		Mascot
1857.8075	1857.9448	0.1373	74	5	20	GGMGNLMKQAQMMQER			Oxidation (M)[3,7,12]		Mascot
1951.9479	1951.9303	-0.0176	-9	93	110	MAEITGGMQLPPGFKMPF					Mascot
3233.5518	3233.4875	-0.0643	-20	55	83	VTIDPSLLQDDDQEMLEDLIAAAFNDAVR			Oxidation (M)[15]		Mascot
3373.658	3373.6094	-0.0486	-14	55	84	VTIDPSLLQDDDQEMLEDLIAAAFNDAVRR					Mascot
3373.658	3373.6094	-0.0486	-14	54	83	RVTIDPSLLQDDDQEMLEDLIAAAFNDAVR					Mascot
3389.6531	3389.6125	-0.0406	-12	55	84	VTIDPSLLQDDDQEMLEDLIAAAFNDAVRR			Oxidation (M)[15]		Mascot

Rank	Protein Name	Accession No.	Protein MW	Protein PI	Pep. Count	Protein Score	Protein Score C. I. %	Intensity Matched	Total Ion Score	Total Ion C. I. %	Confirmed
2	Lipid-A-disaccharide synthase OS=Sodalis glossinidius (strain morsitans) GN=lpxB PE=3 SV=1	LPXB_SODGM	42579.5	8.8	10	50	0	21.967			

Peptide Information

Calc. Mass	Obsrv. Mass	± da	± ppm	Start Seq.	End Seq.	Sequence	Ion Score	C. I. %	Modification	Rank	Result Type
1147.6656	1147.6825	0.0169	15	294	303	MKPLTFALAR					Mascot
1812.0061	1811.936	-0.0701	-39	351	365	AALLAMFRQLHQQIR			Oxidation (M)[6]		Mascot
1857.976	1857.9448	-0.0312	-17	116	130	TIHYVSPSVWAWRQK					Mascot
1893.9487	1893.9132	-0.0355	-19	201	217	QSEVAMLSADFLRAAER					Mascot
1967.0854	1966.9166	-0.1688	-86	182	200	QALGIAAEARCLALLPGSR			Carbamidomethyl (C)[11]		Mascot
1967.0854	1966.9166	-0.1688	-86	182	200	QALGIAAEARCLALLPGSR			Carbamidomethyl (C)[11]		Mascot
2126.1072	2126.2385	0.1313	62	286	303	CPMVVGYRMKPLTFALAR			Carbamidomethyl (C)[1], Oxidation (M)[3]		Mascot
3164.3799	3164.499	0.1191	38	151	177	AFYDCYNVPCQFIGHTLADAMSLDPDK			Carbamidomethyl (C)[5,10], Oxidation (M)[21]		Mascot

Calc. Mass	Obsrv. Mass	± da	± ppm	Start Seq.	End Seq.	Sequence	Ion Score	C. I. %	Modification	Rank	Result Type
3233.6326	3233.4875	-0.1451	-45	255	285	LLDNQARQAMIAADAALL ASGTASLECMLAK			Carbamidomethyl (C)[27], Oxidation (M)[10]		Mascot
3249.6277	3249.5203	-0.1074	-33	255	285	LLDNQARQAMIAADAALL ASGTASLECMLAK			Carbamidomethyl (C)[27], Oxidation (M)[10,28]		Mascot
3401.6394	3401.6414	0.002	1	262	293	QAMIAADAALLASGTASL ECMLAKCPMVVGYR			Carbamidomethyl (C)[20,25], Oxidation (M)[3,21]		Mascot
3417.6343	3417.6863	0.052	15	262	293	QAMIAADAALLASGTASL ECMLAKCPMVVGYR			Carbamidomethyl (C)[20,25], Oxidation (M)[3,21,27]		Mascot
3445.5784	3445.6343	0.0559	16	45	73	MQAEGMEAWYDMEELA VMGIVEVVERLPR			Oxidation (M)[1,6,12,18]		Mascot

3	Nucleoporin NUP2 OS=Saccharomyces cerevisiae (strain ATCC 204508 / S288c) GN=NUP2 PE=1 SV=2	NUP2_YEAST	77890.7	6.81	14	48	0	27.526	

Peptide Information

Calc. Mass	Obsrv. Mass	± da	± ppm	Start Seq.	End Seq.	Sequence	Ion Score	C. I. %	Modification	Rank	Result Type
1106.5735	1106.542	-0.0315	-28	30	39	VASSAVMNRR			Oxidation (M)[7]		Mascot
1145.5797	1145.5819	0.0022	2	229	238	LNPSTDKNEK					Mascot
1228.5627	1228.5308	-0.0319	-26	625	635	SYDSRGVGEMK					Mascot
1228.5627	1228.5308	-0.0319	-26	625	635	SYDSRGVGEMK					Mascot
1244.5576	1244.4998	-0.0578	-46	625	635	SYDSRGVGEMK			Oxidation (M)[10]		Mascot
1885.9291	1885.9272	-0.0019	-1	432	449	KDSKPAFSFGISNGSESK					Mascot
1901.9087	1901.8928	-0.0159	-8	450	467	DSDKPSLPSAVDGENDK K					Mascot
1902.7723	1902.928	0.1557	82	13	29	ETYDSNESDDDVTPSTK					Mascot
1902.8352	1902.928	0.0928	49	342	359	SSDSNDSNPSFSFSIPSK					Mascot
1951.8767	1951.9303	0.0536	27	201	217	DESDSENDIEIKGPEFK					Mascot
1955.0233	1954.9728	-0.0505	-26	467	484	KEATKPAFSFGINTNTTK					Mascot
1966.9506	1966.9166	-0.034	-17	360	378	NTPDASKPSFSFGVPNS SK					Mascot
1966.9506	1966.9166	-0.034	-17	360	378	NTPDASKPSFSFGVPNS SK					Mascot
1997.9814	1998.1549	0.1735	87	490	508	APTFTFGSSALADNKEDV K					Mascot
2014.9717	2014.9128	-0.0589	-29	552	569	FSLPFEQKGSQTTTNDS K					Mascot
2254.1714	2254.303	0.1316	58	670	691	YEPLAPGNDNLIKAPTVA ADGK					Mascot
3233.5193	3233.4875	-0.0318	-10	397	426	EASQEDDNNNVEKPSSK PAFNLISNAGTEK					Mascot

4	Uncharacterized protein R159 OS=Acanthamoeba polyphaga mimivirus GN=MIMI_R159 PE=4 SV=1	YR159_MIMIV	15771	8.45	7	46	0	8.936	

Peptide Information

Calc. Mass	Obsrv. Mass	± da	± ppm	Start Seq.	End Seq.	Sequence	Ion Score	C. I. %	Modification	Rank	Result Type
1089.6051	1089.561	-0.0441	-40	91	100	FPIGGRDSIK					Mascot

1518.77	1518.7906	0.0206	14	101	112	TFRESVISWEHK					Mascot
1534.7595	1534.7936	0.0341	22	2	17	SASIGDLIGDSIGDSK					Mascot
1550.6904	1550.7864	0.096	62	38	50	QVKDTEAHYCDGK			Carbamidomethyl (C)[10]		Mascot
1665.8	1665.8008	0.0008	0	1	17	MSASIGDLIGDSIGDSK					Mascot
1809.8899	1810.0663	0.1764	97	1	18	MSASIGDLIGDSIGDSKK			Oxidation (M)[1]		Mascot
1903.0437	1902.928	-0.1157	-61	51	66	RFDVVISDVLIGWVER					Mascot
1903.0437	1902.928	-0.1157	-61	51	66	RFDVVISDVLIGWVER					Mascot

5 Alpha-(1,3)-fucosyltransferase B OS=Drosophila melanogaster GN=FucTB PE=1 SV=2 | FUCTB_DROME | 52338 | 8.73 | 11 | 46 | 0 | 7.495

Peptide Information

Calc. Mass	Obsrv. Mass	± da	± ppm	Start Seq.	End Seq.	Sequence	Ion Score	C. I. %	Modification	Rank	Result Type
1097.599	1097.6028	0.0038	3	79	88	GVLFYGSNIK					Mascot
1154.5762	1154.536	-0.0402	-35	187	195	EDYVKELMK					Mascot
1199.6379	1199.5419	-0.096	-80	208	217	NRDLPESLQK					Mascot
1208.5399	1208.5667	0.0268	22	398	408	AAMEVGQCQAK			Carbamidomethyl (C)[8], Oxidation (M)[3]		Mascot
1313.558	1313.5408	-0.0172	-13	49	58	DMSWNYDVQR					Mascot
1490.7097	1490.7404	0.0307	21	372	383	HYNCPLEPVYAK			Carbamidomethyl (C)[4]		Mascot
1811.8977	1811.936	0.0383	21	120	133	EFLRHFHFTSTFSR					Mascot
1918.9514	1918.9171	-0.0343	-18	192	207	ELMKHLPIDSYGSCLR			Carbamidomethyl (C)[14]		Mascot
1934.9463	1934.9248	-0.0215	-11	192	207	ELMKHLPIDSYGSCLR			Carbamidomethyl (C)[14], Oxidation (M)[3]		Mascot
3218.686	3218.5151	-0.1709	-53	254	280	FWRPLIMGVIPIYFGSPTI KDWEPNNK					Mascot
3401.8218	3401.6414	-0.1804	-53	7	36	YGIALVALLMVGATVLFF WSENIINYENIK					Mascot
3417.8167	3417.6863	-0.1304	-38	7	36	YGIALVALLMVGATVLFF WSENIINYENIK			Oxidation (M)[10]		Mascot
3433.6885	3433.6763	-0.0122	-4	124	153	HFHFTSTFSRYSNLPLTT MYLPSGEALTSK					Mascot

6 Methoxy mycolic acid synthase MmaA3 OS=Mycobacterium tuberculosis (strain ATCC 25177 / H37Ra) GN=mmaA3 PE=1 SV=1 | MMAA3_MYCTA | 33526.6 | 5.27 | 9 | 45 | 0 | 6.751

Protein Group

Methoxy mycolic acid synthase MmaA3 OS=Mycobacterium bovis (strain ATCC BAA-935 / AF2122/97) GN=cmaB PE=1 SV=1	MMAA3_MYCBO	33526.6	5.2699 999809 2651
Methoxy mycolic acid synthase MmaA3 OS=Mycobacterium tuberculosis GN=mmaA3 PE=1 SV=1	MMAA3_MYCTU	33526.6	5.2699 999809 2651

Peptide Information

Calc. Mass	Obsrv. Mass	± da	± ppm	Start Seq.	End Seq.	Sequence	Ion Score	C. I. %	Modification	Rank	Result Type
812.3971	812.4363	0.0392	48	271	277	YLTGCAK			Carbamidomethyl (C)[5]		Mascot
834.3668	834.3173	-0.0495	-59	153	158	YDDFFK					Mascot
1106.5585	1106.542	-0.0165	-15	187	196	GIPLTMEMAK			Oxidation (M)[6]		Mascot
1196.5042	1196.5425	0.0383	32	38	46	TYSCAYFER			Carbamidomethyl (C)[4]		Mascot
1196.5042	1196.5425	0.0383	32	38	46	TYSCAYFER			Carbamidomethyl (C)[4]		Mascot
1637.9163	1637.806	-0.1103	-67	197	210	FIRFIVTDIFPGGR					Mascot
1901.9868	1901.8928	-0.094	-49	224	240	AGFTITDIQSLQPHFAR					Mascot
1934.9252	1934.9248	-0.0004	0	278	293	AFRMGYIDCNQFTLAK			Carbamidomethyl (C)[9]		Mascot
3378.7593	3378.627	-0.1323	-39	224	253	AGFTITDIQSLQPHFARTL DLWAEALQAHK					Mascot
3389.7166	3389.6125	-0.1041	-31	124	152	VLLSDWANFSEPVDRIVT IEAIEHFGFER					Mascot

7 Elongation factor Tu OS=Rickettsia rhipicephali GN=tuf PE=3 SV=1 EFTU_RICRH 42992.1 5.28 9 44 0 6.703

Peptide Information

Calc. Mass	Obsrv. Mass	± da	± ppm	Start Seq.	End Seq.	Sequence	Ion Score	C. I. %	Modification	Rank	Result Type
1809.9719	1810.0663	0.0944	52	9	25	TKPHVNIGTIGHVDHGK					Mascot
1811.0022	1810.8914	-0.1108	-61	264	280	KLLDEGQAGDNVGILLR					Mascot
1919.0525	1918.9171	-0.1354	-71	156	172	ELLSKYGFPGDEIPIIK					Mascot
1935.0045	1934.9248	-0.0797	-41	208	224	INPFLMPIEDVFSISGR					Mascot
1935.8931	1935.925	0.0319	16	39	57	TGGAQATAYDQIDAAPE EK					Mascot
2221.0369	2221.0715	0.0346	16	39	59	TGGAQATAYDQIDAAPE EKER					Mascot
3218.6792	3218.5151	-0.1641	-51	26	57	TSLTAAITIVLAKTGGAQA TAYDQIDAAPEEK					Mascot
3396.7366	3396.6084	-0.1282	-38	349	378	QMVMPGDNATFTVELIKP IAMQEGLKFSIR			Oxidation (M)[2,4]		Mascot
3401.7517	3401.6414	-0.1103	-32	344	374	LPADKQMVMPGDNATFT VELIKPIAMQEGLK			Oxidation (M)[7]		Mascot
3417.7466	3417.6863	-0.0603	-18	344	374	LPADKQMVMPGDNATFT VELIKPIAMQEGLK			Oxidation (M)[7,9]		Mascot
3433.7417	3433.6763	-0.0654	-19	344	374	LPADKQMVMPGDNATFT VELIKPIAMQEGLK			Oxidation (M)[7,9,26]		Mascot

8 Ethylene-responsive transcription factor ERF095 OS=Arabidopsis thaliana GN=ERF095 PE=1 SV=1 ERF95_ARATH 15755.4 5.24 7 43 0 4.941

Peptide Information

Calc. Mass	Obsrv. Mass	± da	± ppm	Start Seq.	End Seq.	Sequence	Ion Score	C. I. %	Modification	Rank	Result Type
1228.5514	1228.5308	-0.0206	-17	4	13	IESYNTNEMK					Mascot

1228.5514	1228.5308	-0.0206	-17	4	13	IESYNTNEMK					Mascot
1244.5464	1244.4998	-0.0466	-37	4	13	IESYNTNEMK			Oxidation (M)[9]		Mascot
1550.7598	1550.7864	0.0266	17	39	52	VWLGTFNTAEDAAR					Mascot
1637.7815	1637.806	0.0245	15	66	78	AILNFPHEYQMMK			Oxidation (M)[11]		Mascot
1891.8165	1891.9205	0.104	55	79	97	DGPNGSHENAVASSSSG YR					Mascot
1891.8165	1891.9205	0.104	55	79	97	DGPNGSHENAVASSSSG YR					Mascot
1971.9784	1971.9203	-0.0581	-29	35	52	HGARVWLGTFNTAEDAA R					Mascot
1994.9575	1994.9521	-0.0054	-3	63	78	GQRAILNFPHEYQMMK			Oxidation (M)[14,15]		Mascot
3417.5605	3417.6863	0.1258	37	98	128	GGGGGDDGREVIEFEYL DDSLLEELLDYGER					Mascot

9	Holliday junction ATP-dependent DNA helicase RuvA OS=Synechococcus sp. (strain RCC307) GN=ruvA PE=3 SV=1	RUVA_SYNR3	23148.8	5.45	7	43	0	21.763

Peptide Information

Calc. Mass	Obsrv. Mass	± da	± ppm	Start Seq.	End Seq.	Sequence	Ion Score	C. I. %	Modification	Rank	Result Type
1224.6484	1224.5448	-0.1036	-85	2	12	IGWLHGTIGDR					Mascot
1298.6423	1298.5831	-0.0592	-46	198	208	HCLAWLSRQAG			Carbamidomethyl (C)[2]		Mascot
1875.9672	1875.913	-0.0542	-29	41	57	GTALESPTTVHTHLQQR					Mascot
1875.9672	1875.913	-0.0542	-29	41	57	GTALESPTTVHTHLQQR					Mascot
1994.9944	1994.9521	-0.0423	-21	2	18	IGWLHGTIGDRWQEGNR					Mascot
2126.0349	2126.2385	0.2036	96	1	18	MIGWLHGTIGDRWQEGN R					Mascot
3419.9182	3419.6731	-0.2451	-72	77	111	LLISVNGVGPQVALGLIS GLGAVSLLQAMAAEDVK			Oxidation (M)[29]		Mascot
3419.9182	3419.6731	-0.2451	-72	77	111	LLISVNGVGPQVALGLIS GLGAVSLLQAMAAEDVK			Oxidation (M)[29]		Mascot
3448.6929	3448.6882	-0.0047	-1	13	40	WQEGNRCWLLLICGPVG YELQVSESLWR			Carbamidomethyl (C)[7,13]		Mascot
3448.6929	3448.6882	-0.0047	-1	13	40	WQEGNRCWLLLICGPVG YELQVSESLWR			Carbamidomethyl (C)[7,13]		Mascot

10	Chaperone protein ClpB OS=Escherichia coli O6:H1 (strain CFT073 / ATCC 700928 / UPEC) GN=clpB PE=3 SV=1	CLPB_ECOL6	95697	5.37	14	43	0	19.46

Protein Group

Chaperone protein ClpB OS=Escherichia coli (strain K12) GN=clpB PE=1 SV=1	CLPB_ECOLI	95697	5.3699 998855 5908
Chaperone protein ClpB OS=Escherichia coli O157:H7 GN=clpB PE=3 SV=1	CLPB_ECO57	95697	5.3699 998855 5908

Chaperone protein ClpB OS=Shigella flexneri GN=clpB PE=3 SV=2	CLPB_SHIFL	95672	5.3699998555908

Peptide Information

Calc. Mass	Obsrv. Mass	± da	± ppm	Start Seq.	End Seq.	Sequence	Ion Score	C. I. %	Modification	Rank	Result Type
1145.4854	1145.5819	0.0965	84	632	640	IDMSEFMEK			Oxidation (M)[3]		Mascot
1187.6267	1187.5797	-0.047	-40	512	522	QLEAATQLEGK					Mascot
1210.5562	1210.552	-0.0042	-3	729	738	FGELDYAHMK					Mascot
1226.551	1226.5571	0.0061	5	729	738	FGELDYAHMK			Oxidation (M)[9]		Mascot
1733.8309	1733.9216	0.0907	52	289	305	ADGAMDAGNMLKPALAR			Oxidation (M)[5,10]		Mascot
1811.9723	1811.936	-0.0363	-20	570	586	VIGQNEAVDAVSNAIRR					Mascot
1891.9912	1891.9205	-0.0707	-37	802	818	LLSENGYDPVYGARPLK					Mascot
1891.9912	1891.9205	-0.0707	-37	802	818	LLSENGYDPVYGARPLK					Mascot
1901.8948	1901.8928	-0.002	-1	145	162	GGESVNDQGAEDQRQALK					Mascot
1922.0131	1921.9318	-0.0813	-42	682	698	AHPDVFNILLQVLDDGR					Mascot
1955.0444	1954.9728	-0.0716	-37	385	402	QLPDKAIDLIDEAASSIR					Mascot
1995.0143	1994.9521	-0.0622	-31	71	89	LPQVEGTGGDVQPSQDLVR					Mascot
2126.1506	2126.2385	0.0879	41	739	756	ELVLGVVSHNFRPEFINR					Mascot
2875.2292	2875.3521	0.1229	43	617	640	ALANFMFDSDEAMVRIDMSEFMEK			Oxidation (M)[6,13,18]		Mascot
3419.614	3419.6731	0.0591	17	289	320	ADGAMDAGNMLKPALARGELHCVGATTLDEYR			Carbamidomethyl (C)[22], Oxidation (M)[5]		Mascot
3419.614	3419.6731	0.0591	17	289	320	ADGAMDAGNMLKPALARGELHCVGATTLDEYR			Carbamidomethyl (C)[22], Oxidation (M)[5]		Mascot
3463.8584	3463.6655	-0.1929	-56	669	698	RPYSVILLDEVEKAHPDVFNILLQVLDDGR					Mascot

Gel Idx/Pos	206/I6	Instr./Gel Origin	BA2151/Sample Project 20140814	Process Status	Analysis Succeeded
Plate [#] Name	[1] Sample Project 20140814	Instrument Sample Name		Spectra	11

Rank	Protein Name	Accession No.	Protein MW	Protein PI	Pep. Count	Protein Score	Protein Score C. I. %	Intensity Matched	Total Ion Score	Total Ion C. I. %	Confirmed
1	Elongation factor G OS=Arthrobacter aurescens (strain TC1) GN=fusA PE=3 SV=1	EFG_ARTAT	77515.2	4.97	16	60	39.329	8.86			

Peptide Information

Calc. Mass	Obsrv. Mass	± da	± ppm	Start Seq.	End Seq.	Sequence	Ion Score	C. I. %	Modification	Rank	Result Type
1170.5646	1170.5618	-0.0028	-2	133	141	ICFVNKMDK			Carbamidomethyl (C)[2], Oxidation (M)[7]		Mascot
1263.625	1263.6754	0.0504	40	1	11	MAQDVLTDLNK			Oxidation (M)[1]		Mascot
1430.775	1430.7245	-0.0505	-35	479	491	VEANVGKPQVAYR					Mascot
1507.6992	1507.7377	0.0385	26	638	651	RGQMQSMEDAAGVK					Mascot
1518.7944	1518.798	0.0036	2	1	13	MAQDVLTDLNKVR			Oxidation (M)[1]		Mascot
1523.6941	1523.78	0.0859	56	638	651	RGQMQSMEDAAGVK			Oxidation (M)[4]		Mascot
1839.0045	1838.8999	-0.1046	-57	606	621	KANPVLLEPLMDVEVR			Oxidation (M)[11]		Mascot
1858.8931	1858.8917	-0.0014	-1	112	127	EGVEPQSETVWRQADK					Mascot
1892.9158	1892.9152	-0.0006	0	220	236	AQLVETVAEASEELMEK			Oxidation (M)[15]		Mascot
1902.0443	1901.9048	-0.1395	-73	479	495	VEANVGKPQVAYRETIK					Mascot
1927.9946	1927.9271	-0.0675	-35	371	389	EMPVEGATAGHIYAAIGL K					Mascot
1927.9946	1927.9271	-0.0675	-35	371	389	EMPVEGATAGHIYAAIGL K					Mascot
1935.923	1935.9364	0.0134	7	622	638	TPEEYMGDVIGDLNARR					Mascot
1943.9895	1943.8976	-0.0919	-47	371	389	EMPVEGATAGHIYAAIGL K			Oxidation (M)[2]		Mascot
1951.9178	1951.9337	0.0159	8	622	638	TPEEYMGDVIGDLNARR			Oxidation (M)[6]		Mascot
1951.9178	1951.9337	0.0159	8	622	638	TPEEYMGDVIGDLNARR			Oxidation (M)[6]		Mascot
1979.9242	1979.9556	0.0314	16	255	271	MTINSELYPVFCGSAFK			Carbamidomethyl (C)[12], Oxidation (M)[1]		Mascot
2238.0596	2238.1487	0.0891	40	194	214	GDVTMGASYEVQEIPAD LQAK			Oxidation (M)[5]		Mascot
2588.2991	2588.1743	-0.1248	-48	100	123	VLDGAVAVFDGKEGVEP QSETVWR					Mascot
2652.374	2652.1565	-0.2175	-82	77	99	NQINIIDTPGHVDFTVEVE RSLR					Mascot
3223.5002	3223.5273	0.0271	8	32	59	ILFYTGVNHKIGETHDGA STTDWMEQEK			Oxidation (M)[24]		Mascot

Rank	Protein Name	Accession No.	Protein MW	Protein PI	Pep. Count	Protein Score	Protein Score C. I. %	Intensity Matched	Total Ion Score	Total Ion C. I. %	Confirmed
2	Polyphosphate kinase OS=Rhodopseudomonas palustris (strain HaA2) GN=ppk PE=3 SV=1	PPK_RHOP2	82483.2	7.06	16	54	0	13.484			

Peptide Information

Calc. Mass	Obsrv. Mass	± da	± ppm	Start	End	Sequence	Ion	C. I. %	Modification	Rank	Result Type

Calc. Mass	Obsrv. Mass	± da	± ppm	Start Seq.	End Seq.	Sequence	Ion Score	C. I. %	Modification	Rank	Result Type
987.5656	987.5455	-0.0201	-20	521	529	MAVSPLTLR					Mascot
1158.6226	1158.5764	-0.0462	-40	81	90	AQVREGITER					Mascot
1187.6168	1187.6139	-0.0029	-2	111	120	LASDQQAIWR					Mascot
1406.6812	1406.6254	-0.0558	-40	424	434	FDEEANIRWAR					Mascot
1517.8323	1517.7848	-0.0475	-31	381	393	DPDVVAIKQTLYR					Mascot
1811.0386	1810.8898	-0.1488	-82	121	137	DLRGILTEAGIVLLDGR					Mascot
1819.8457	1819.8225	-0.0232	-13	671	686	DTEQSWQLLPDGSSTR					Mascot
1844.9977	1844.9073	-0.0904	-49	42	57	RVLEEAVNPSHPVLER					Mascot
1853.0181	1852.8778	-0.1403	-76	538	554	GETAFARHGKPAAIWLK					Mascot
1860.0378	1859.9141	-0.1237	-67	439	455	AGVQVVYGFLELKTHAK					Mascot
1860.9379	1860.9288	-0.0091	-5	485	500	IYTDLSYFTSDPIIGR					Mascot
1902.9419	1902.9312	-0.0107	-6	60	75	FLSISANNLDEFFMVR					Mascot
1902.9419	1902.9312	-0.0107	-6	60	75	FLSISANNLDEFFMVR					Mascot
1918.9368	1918.9395	0.0027	1	60	75	FLSISANNLDEFFMVR			Oxidation (M)[14]		Mascot
1925.9208	1925.8792	-0.0416	-22	627	643	AAVYISSADMMPRNLDR			Oxidation (M)[10]		Mascot
1936.0538	1935.9364	-0.1174	-61	435	451	DLERAGVQVVYGFLELK					Mascot
1944.0662	1943.8976	-0.1686	-87	43	59	VLEEAVNPSHPVLERVR					Mascot
3155.7432	3155.47	-0.2732	-87	644	670	RVEILCPLLNPTVHQQVL EQIMVANLK			Carbamidomethyl (C)[6]		Mascot

3	ATP synthase subunit b OS=Francisella philomiragia subsp. philomiragia (strain ATCC 25017) GN=atpF PE=3 SV=1	ATPF_FRAP2	17403.3	6.78	9	53	0	2.359

Peptide Information

Calc. Mass	Obsrv. Mass	± da	± ppm	Start Seq.	End Seq.	Sequence	Ion Score	C. I. %	Modification	Rank	Result Type
991.5822	991.5373	-0.0449	-45	148	155	ILKDFVEK					Mascot
1144.6321	1144.5614	-0.0707	-62	60	69	QSAEILREAK					Mascot
1158.6365	1158.5764	-0.0601	-52	92	102	EEAIAAADKIK					Mascot
1266.5704	1266.5482	-0.0222	-18	103	113	SMAMAEIEQEK					Mascot
1507.7495	1507.7377	-0.0118	-8	101	113	IKSMAMAEIEQEK					Mascot
1523.7444	1523.78	0.0356	23	101	113	IKSMAMAEIEQEK			Oxidation (M)[4]		Mascot
1600.8442	1600.7502	-0.094	-59	72	85	ATEIVENAYVRAHK					Mascot
1812.9524	1812.9358	-0.0166	-9	118	134	EELKHEVVSLAMAAASK					Mascot
2652.3486	2652.1565	-0.1921	-72	1	23	MDINITLIGQMITFAIFVGF TMK			Oxidation (M)[1,11,22]		Mascot
3384.8252	3384.6343	-0.1909	-56	2	30	DINITLIGQMITFAIFVGFT MKFVWPPLR			Oxidation (M)[10]		Mascot

4	Putative membrane protein insertion efficiency factor OS=Shewanella sp. (strain MR-4) GN=Shewmr4_3941 PE=3 SV=1	YIDD_SHESM	9710	9.72	6	51	0	19.355	

Protein Group

Putative membrane protein insertion efficiency factor OS=Shewanella sp. (strain ANA-3) GN=Shewana3_0007 PE=3 SV=1	YIDD_SHESA	9710	9.7200 002670 2881
Putative membrane protein insertion efficiency factor OS=Shewanella sp. (strain MR-7) GN=Shewmr7_4033 PE=3 SV=1	YIDD_SHESR	9710	9.7200 002670 2881

Peptide Information

Calc. Mass	Obsrv. Mass	± da	± ppm	Start Seq.	End Seq.	Sequence	Ion Score	C. I. %	Modification	Rank	Result Type
1037.535	1037.5201	-0.0149	-14	53	60	GCWFALKR			Carbamidomethyl (C)[2]		Mascot
1860.9967	1860.9288	-0.0679	-36	2	17	AQTQSPLQWLATTFIR					Mascot
1949.0426	1948.9332	-0.1094	-56	61	78	ILKCHPLHPGGSDPVPPK			Carbamidomethyl (C)[4]		Mascot
1966.9263	1966.9214	-0.0049	-2	31	46	CRFNPTCSHYAIEAIK			Carbamidomethyl (C)[1,7]		Mascot
1966.9263	1966.9214	-0.0049	-2	31	46	CRFNPTCSHYAIEAIK			Carbamidomethyl (C)[1,7]		Mascot
1979.9506	1979.9556	0.005	3	64	81	CHPLHPGGSDPVPPKND R			Carbamidomethyl (C)[1]		Mascot
1992.0372	1991.897	-0.1402	-70	1	17	MAQTQSPLQWLATTFIR					Mascot

5	ATP synthase gamma chain OS=Protochlamydia amoebophila (strain UWE25) GN=atpG PE=3 SV=1	ATPG_PARUW	32872.4	9.48	10	51	0	3.218	

Peptide Information

Calc. Mass	Obsrv. Mass	± da	± ppm	Start Seq.	End Seq.	Sequence	Ion Score	C. I. %	Modification	Rank	Result Type
991.5353	991.5373	0.002	2	1	8	MASLREIR			Oxidation (M)[1]		Mascot
1187.6783	1187.6139	-0.0644	-54	186	195	FLNIQQISPK					Mascot
1590.8738	1590.8092	-0.0646	-41	105	118	TYAQEQVELILVGK					Mascot
1811.9619	1811.9358	-0.0261	-14	23	38	AMEMVAASRLHQVQIK					Mascot
1811.9619	1811.9358	-0.0261	-14	23	38	AMEMVAASRLHQVQIK	5	0			Mascot
1919.0709	1918.9395	-0.1314	-68	56	71	LSQITQDIKHPLLEQR					Mascot
1935.96	1935.9364	-0.0236	-12	133	148	FEFGEWGEKLTSHQIK					Mascot
1950.9609	1950.9211	-0.0398	-20	127	141	RPWNIRFEFGEWGEK					Mascot
1992.0317	1991.897	-0.1347	-68	267	285	QAGITKEMLEITSGAEGL K			Oxidation (M)[8]		Mascot
2652.3701	2652.1565	-0.2136	-81	76	101	VGVVIVAADMGLSGPYN KDIFSAANK			Oxidation (M)[10]		Mascot
3124.5159	3124.4956	-0.0203	-6	199	224	SPVDYIFEPNPKQIYGAIL FNYCMTK			Carbamidomethyl (C)[23], Oxidation (M)[24]		Mascot

6 Spectrin alpha chain OS=Drosophila melanogaster GN=alpha-Spec PE=1 SV=2 SPTCA_DROME 279101.4 5.08 32 51 0 46.285

Peptide Information

Calc. Mass	Obsrv. Mass	± da	± ppm	Start Seq.	End Seq.	Sequence	Ion Score	C. I. %	Modification	Rank	Result Type
804.3522	804.2994	-0.0528	-66	512	517	HEDFEK					Mascot
987.5469	987.5455	-0.0014	-1	1743	1751	DLTGVQNLK					Mascot
991.5029	991.5373	0.0344	35	2387	2394	MKPFSEPR					Mascot
1106.5212	1106.5375	0.0163	15	924	933	DEDSSEALLK					Mascot
1144.5593	1144.5614	0.0021	2	1201	1211	ANALNNDDLGK					Mascot
1145.6161	1145.5636	-0.0525	-46	712	721	DLTSVQNLQK					Mascot
1145.6161	1145.5636	-0.0525	-46	712	721	DLTSVQNLQK					Mascot
1187.6168	1187.6139	-0.0029	-2	2282	2291	SGKLNHQEFK					Mascot
1208.5212	1208.5538	0.0326	27	1569	1579	QCSGSEDAVQK			Carbamidomethyl (C)[2]		Mascot
1266.5961	1266.5482	-0.0479	-38	411	421	GEIDAREDSFK					Mascot
1347.6427	1347.6012	-0.0415	-31	266	277	DADETVAWIAEK					Mascot
1406.7209	1406.6254	-0.0955	-68	2025	2035	DRLLAMQEQFR					Mascot
1422.7158	1422.6089	-0.1069	-75	2025	2035	DRLLAMQEQFR			Oxidation (M)[6]		Mascot
1617.8418	1617.7544	-0.0874	-54	2387	2400	MKPFSEPRSGQPIK			Oxidation (M)[1]		Mascot
1631.8136	1631.8994	0.0858	53	1648	1661	HQLVEADIVAHEDR					Mascot
1700.9065	1700.816	-0.0905	-53	8	21	EVKILETVEDIQER					Mascot
1838.9283	1838.8999	-0.0284	-15	2395	2410	SGQPIKDALDYIDFTR					Mascot
1853.0392	1852.8778	-0.1614	-87	780	794	KQHLLDSLQVQQLFR					Mascot
1860.9563	1860.9288	-0.0275	-15	1048	1063	QNQINSQYDNLLALAR					Mascot
1867.8868	1867.932	0.0452	24	614	628	MQKHQNFEHELNANK					Mascot
1891.9081	1891.9485	0.0404	21	464	477	ILYEQCMDLQLFYR			Carbamidomethyl (C)[6]		Mascot
1892.9283	1892.9152	-0.0131	-7	762	779	EGNLSARYAALAAPMGE R			Oxidation (M)[15]		Mascot
1900.9626	1900.8934	-0.0692	-36	1917	1932	KGALLDNSAYLQFMWK			Oxidation (M)[14]		Mascot
1901.9868	1901.9048	-0.082	-43	1580	1594	RLTQIADQWEYLTHK					Mascot
1902.9708	1902.9312	-0.0396	-21	994	1009	GDVLTLLNSNNKDWWK					Mascot
1902.9708	1902.9312	-0.0396	-21	994	1009	GDVLTLLNSNNKDWWK					Mascot
1927.9735	1927.9271	-0.0464	-24	369	384	FLADFRDLVSWINGMK			Oxidation (M)[15]		Mascot
1927.9735	1927.9271	-0.0464	-24	369	384	FLADFRDLVSWINGMK			Oxidation (M)[15]		Mascot
1935.942	1935.9364	-0.0056	-3	1537	1553	HQKHQAFEAELAANADR					Mascot
1949.0273	1948.9332	-0.0941	-48	1386	1400	RLQLEQNLDLQLYMR			Oxidation (M)[14]		Mascot
1950.959	1950.9211	-0.0379	-19	1264	1279	EINEMWDQIITKSTAR			Oxidation (M)[5]		Mascot

1957.8774	1957.9929	0.1155	59	2341	2356	ETENVQSYEEIENAFR					Mascot
1966.9209	1966.9214	0.0005	0	2174	2191	TSMMEGSGSLEQQLEAL R					Mascot
1966.9209	1966.9214	0.0005	0	2174	2191	TSMMEGSGSLEQQLEAL R					Mascot
1979.9161	1979.9556	0.0395	20	1872	1888	DRCSLICDQGSELVEAK			Carbamidomethyl (C)[3,7]		Mascot
1982.9158	1982.9019	-0.0139	-7	2174	2191	TSMMEGSGSLEQQLEAL R			Oxidation (M)[3]		Mascot
1982.9158	1982.9019	-0.0139	-7	2174	2191	TSMMEGSGSLEQQLEAL R			Oxidation (M)[3]		Mascot
1991.9491	1991.897	-0.0521	-26	1247	1263	LMQSHPDTAEQTYAKQK			Oxidation (M)[2]		Mascot
3377.6604	3377.6091	-0.0513	-15	1298	1328	DLLAWINSMMSLVTSDEL ANDVTGAEALIER					Mascot
3393.6553	3393.6404	-0.0149	-4	1298	1328	DLLAWINSMMSLVTSDEL ANDVTGAEALIER			Oxidation (M)[9]		Mascot

7	Coproporphyrinogen-III oxidase, aerobic OS=Prochlorococcus marinus (strain SARG / CCMP1375 / SS120) GN=hemF PE=3 SV=1	HEM6_PROMA	39830.7	6.65	10	50	0	3.699	

Peptide Information

Calc. Mass	Obsrv. Mass	± da	± ppm	Start Seq.	End Seq.	Sequence	Ion Score	C. I. %	Modification	Rank	Result Type
991.4526	991.5373	0.0847	85	336	343	CRPHHAVD			Carbamidomethyl (C)[1]		Mascot
1308.6835	1308.6357	-0.0478	-37	220	230	LSWEDLFSLAK					Mascot
1518.7522	1518.798	0.0458	30	93	105	WFATGTSMVLHPR			Oxidation (M)[8]		Mascot
1824.9326	1824.9395	0.0069	4	90	105	GHKWFATGTSMVLHPR					Mascot
1949.0314	1948.9332	-0.0982	-50	231	247	ACGNAFLPSYIPIIEKR			Carbamidomethyl (C)[2]		Mascot
1950.9597	1950.9211	-0.0386	-20	183	200	GVGGIFFDYQDGSGKLY K					Mascot
1992.0033	1991.897	-0.1063	-53	201	219	GQNPNGLSSIASKELGEY K					Mascot
2238.1628	2238.1487	-0.0141	-6	288	306	TESILMSLPPLARWEYGF K					Mascot
2588.3865	2588.1743	-0.2122	-82	277	300	GTIFGLQTNGRTESILMS LPPLAR			Oxidation (M)[17]		Mascot
3408.6482	3408.6533	0.0051	1	307	335	APPESRESLLTDVFTKPQ EWFTDESLEEK					Mascot

8	21S rRNA pseudouridine(2819) synthase OS=Saccharomyces cerevisiae (strain ATCC 204508 / S288c) GN=PUS5 PE=1 SV=1	PUS5_YEAST	29625.4	9.41	9	50	0	6.543	

Peptide Information

Calc. Mass	Obsrv. Mass	± da	± ppm	Start Seq.	End Seq.	Sequence	Ion Score	C. I. %	Modification	Rank	Result Type
1106.5702	1106.5375	-0.0327	-30	97	106	GGNNGYKLQR					Mascot
1145.5474	1145.5636	0.0162	14	246	254	EVLLENWDQ					Mascot
1145.5474	1145.5636	0.0162	14	246	254	EVLLENWDQ					Mascot

1208.6635	1208.5538	-0.1097	-91	107	117	KYVAIVESSGR					Mascot
1266.6477	1266.5482	-0.0995	-79	118	127	FNKPNNYEIK					Mascot
1430.7131	1430.7245	0.0114	8	70	82	LDHCVTGGMLIAK			Carbamidomethyl (C)[4], Oxidation (M)[9]		Mascot
1844.9786	1844.9073	-0.0713	-39	148	163	EVDENCIVLQLVTGKK			Carbamidomethyl (C)[6]		Mascot
1992.0471	1991.897	-0.1501	-75	146	162	FKEVDENCIVLQLVTGK			Carbamidomethyl (C)[8]		Mascot
3405.7776	3405.6074	-0.1702	-50	5	33	KQIPIIFENTHYFIVNKPPG IPSQPPDCR			Carbamidomethyl (C)[28]		Mascot
3452.7996	3452.657	-0.1426	-41	184	214	HGSTVNFPELFNDQIALH SACIITKIGLQTK			Carbamidomethyl (C)[21]		Mascot

9 Zinc finger C2H2 protein ECU08_0560 OS=Encephalitozoon cuniculi (strain GB-M1) GN=ECU08_0560 PE=4 SV=2 | Z856_ENCCU | 34331.8 | 8.65 | 10 | 50 | 0 | 12.652

Peptide Information

Calc. Mass	Obsrv. Mass	± da	± ppm	Start Seq.	End Seq.	Sequence	Ion Score	C. I. %	Modification	Rank	Result Type
991.5319	991.5373	0.0054	5	2	10	KQDAGLGFR					Mascot
1078.5276	1078.5322	0.0046	4	126	135	DAGPELSHPR					Mascot
1138.5674	1138.5516	-0.0158	-14	1	10	MKQDAGLGFR			Oxidation (M)[1]		Mascot
1491.705	1491.7422	0.0372	25	136	147	FSIGEYKFGCQR			Carbamidomethyl (C)[10]		Mascot
1824.9954	1824.9395	-0.0559	-31	20	37	VLPGADVSEVLLPDTATK					Mascot
1902.9344	1902.9312	-0.0032	-2	126	142	DAGPELSHPRFSIGEYK					Mascot
1902.9344	1902.9312	-0.0032	-2	126	142	DAGPELSHPRFSIGEYK					Mascot
1919.0134	1918.9395	-0.0739	-39	3	19	QDAGLGFRLWVESVVSR					Mascot
1982.7864	1982.9019	0.1155	58	60	75	CFGVCADSSQICEYMR			Carbamidomethyl (C)[1,5,12]		Mascot
1982.7864	1982.9019	0.1155	58	60	75	CFGVCADSSQICEYMR			Carbamidomethyl (C)[1,5,12]		Mascot
2385.1001	2384.958	-0.1421	-60	263	282	SDNLSQHYKVHSTTNEM HTR					Mascot
3452.6357	3452.657	0.0213	6	47	75	VLFNLSDLVDILKCFGVC ADSSQICEYMR			Carbamidomethyl (C)[14,18,25]		Mascot

10 Serine/threonine-protein kinase PAK 7 OS=Rattus norvegicus GN=Pak7 PE=1 SV=1 | PAK7_RAT | 81354.2 | 8.35 | 14 | 49 | 0 | 13.235

Peptide Information

Calc. Mass	Obsrv. Mass	± da	± ppm	Start Seq.	End Seq.	Sequence	Ion Score	C. I. %	Modification	Rank	Result Type
1060.5027	1060.5651	0.0624	59	168	176	QNGHAMKMK			Oxidation (M)[6]		Mascot
1170.6477	1170.5618	-0.0859	-73	353	363	GPTKLPQSQSK					Mascot
1507.8129	1507.7377	-0.0752	-50	554	566	ALSYLHNQGVIHR					Mascot
1518.8461	1518.798	-0.0481	-32	487	498	RELLFNEVVIMR					Mascot
1534.7782	1534.7842	0.006	4	454	468	IGEGSTGIVCIATEK			Carbamidomethyl (C)[10]		Mascot

1811.8997	1811.7454	-0.1543	-85	583	598	LSDFGFCAQVSKEVPK	Carbamidomethyl (C)[7]	Mascot
1811.8997	1811.9358	0.0361	20	583	598	LSDFGFCAQVSKEVPK	Carbamidomethyl (C)[7]	Mascot
1811.8997	1811.9358	0.0361	20	583	598	LSDFGFCAQVSKEVPK	Carbamidomethyl (C)[7]	Mascot
1852.8899	1852.8778	-0.0121	-7	336	352	AQMVFSPPLSGSDTYPR		Mascot
1857.9827	1857.9287	-0.054	-29	701	716	LAGPPSCIVPLMRQYR	Carbamidomethyl (C)[7]	Mascot
1902.924	1902.9312	0.0072	4	270	285	SSYLHQTSPQPAMRQR	Oxidation (M)[13]	Mascot
1902.924	1902.9312	0.0072	4	270	285	SSYLHQTSPQPAMRQR	Oxidation (M)[13]	Mascot
1950.0154	1949.9384	-0.077	-39	600	616	KSLVGTPYWMAPEVISR	Oxidation (M)[10]	Mascot
1958.0012	1957.9929	-0.0083	-4	454	472	IGEGSTGIVCIATEKHTGK	Carbamidomethyl (C)[10]	Mascot
2652.28	2652.1565	-0.1235	-47	9	31	IEISGPSNFEHRVHTGFD PQEQK		Mascot
3223.6714	3223.5273	-0.1441	-45	539	566	MNEEQIATVCLSVLKALS YLHNQGVIHR	Carbamidomethyl (C)[10]	Mascot
3402.4768	3402.6047	0.1279	38	116	145	IQGHSEENGFITFSQYSS ESDTTTDYTTEK		Mascot

Gel Idx/Pos	207/I7	Instr./Gel Origin	BA2151/Sample Project 20140814	Process Status	Analysis Succeeded
Plate [#] Name	[1] Sample Project 20140814	Instrument Sample Name		Spectra	11

Rank	Protein Name	Accession No.	Protein MW	Protein PI	Pep. Count	Protein Score	Protein Score C. I. %	Intensity Matched	Total Ion Score	Total Ion C. I. %	Confirmed
1	Mitotic chromosome and X-chromosome-associated protein mix-1 OS=Caenorhabditis elegans GN=mix-1 PE=1 SV=2	MIX1_CAEEL	141166.8	8.31	26	64	75.846	11.037			

Peptide Information

Calc. Mass	Obsrv. Mass	± da	± ppm	Start Seq.	End Seq.	Sequence	Ion Score	C. I. %	Modification	Rank	Result Type
812.397	812.3837	-0.0133	-16	174	179	MYDQKK					Mascot
847.4706	847.4771	0.0065	8	306	312	QSIMLQK					Mascot
872.52	872.4965	-0.0235	-27	1041	1047	KVDELIR					Mascot
1094.6066	1094.5299	-0.0767	-70	422	430	GERLHNQIK					Mascot
1110.55	1110.5111	-0.0389	-35	1023	1031	ITEDFNMLK					Mascot
1116.5869	1116.5048	-0.0821	-74	895	903	NNQRQISTR					Mascot
1117.567	1117.5453	-0.0217	-19	581	590	TMIPVSENAR					Mascot
1238.6449	1238.5703	-0.0746	-60	1023	1032	ITEDFNMLKK					Mascot
1312.6315	1312.7092	0.0777	59	61	72	SMHELISHGGTK			Oxidation (M)[2]		Mascot
1347.7267	1347.5969	-0.1298	-96	268	278	FNQLDLDLKNK					Mascot
1402.6996	1402.7367	0.0371	26	1186	1198	LNGGDIAVLCQDK			Carbamidomethyl (C)[10]		Mascot
1422.7377	1422.6168	-0.1209	-85	5	16	SIHLDGFKSYQK					Mascot
1441.7621	1441.6571	-0.105	-73	1	12	MHIKSIHLDGFK			Oxidation (M)[1]		Mascot
1462.7133	1462.6199	-0.0934	-64	931	943	ELTSLQQSEASNR					Mascot
1511.7635	1511.7238	-0.0397	-26	59	72	AKSMHELISHGGTK			Oxidation (M)[4]		Mascot
1549.7639	1549.7284	-0.0355	-23	716	728	ELQLTEASSQKCR			Carbamidomethyl (C)[12]		Mascot
1562.7526	1562.7413	-0.0113	-7	727	739	CRDLNNQLATAMR			Carbamidomethyl (C)[1]		Mascot
1590.8082	1590.7792	-0.029	-18	931	944	ELTSLQQSEASNRK					Mascot
1613.7742	1613.7007	-0.0735	-46	1162	1175	QGMFSNADVLFQTR					Mascot
1651.7388	1651.7041	-0.0347	-21	953	965	FEWLSDEEAHFNK					Mascot
1818.9708	1818.8329	-0.1379	-76	746	761	TNINNSEFGIVVRDLK					Mascot
1865.9803	1865.9438	-0.0365	-20	134	150	GVGLNVNNPHFLIMQGR					Mascot
1867.9032	1867.917	0.0138	7	361	376	DIQSQSDDEALVTKYR					Mascot
1972.0248	1971.9398	-0.085	-43	1087	1105	VSFGGVVKDSLHELSGGQR					Mascot
2355.1252	2355.1641	0.0389	17	17	38	HTDILDFSPTFNAITGYNGSGK					Mascot

3187.5571	3187.5171	-0.04	-13	151	178	ITTVLNMKPEEILGMVEE AAGTKMYDQK			Oxidation (M)[7,15,24]		Mascot

2	Trigger factor OS=Lactobacillus delbrueckii subsp. bulgaricus (strain ATCC BAA-365) GN=tig PE=3 SV=1	TIG_LACDB	48600.4	4.61	12	54	0	3.967

Protein Group

Trigger factor OS=Lactobacillus delbrueckii subsp. bulgaricus (strain ATCC 11842 / DSM 20081) GN=tig PE=3 SV=1	TIG_LACDA	48600.4	4.6100 001335 144

Peptide Information

Calc. Mass	Obsrv. Mass	± da	± ppm	Start Seq.	End Seq.	Sequence	Ion Score	C. I. %	Modification	Rank	Result Type
922.4451	922.4555	0.0104	11	101	107	NKAWEMK			Oxidation (M)[6]		Mascot
1046.5226	1046.4946	-0.028	-27	347	356	SQLSANAAER					Mascot
1060.5634	1060.5416	-0.0218	-21	137	145	DIDAELTKR					Mascot
1139.5514	1139.5443	-0.0071	-6	1	10	MSANWKTTGK			Oxidation (M)[1]		Mascot
1273.6859	1273.5621	-0.1238	-97	347	358	SQLSANAAERVK					Mascot
1315.5471	1315.6038	0.0567	43	385	395	DLADNYNMSEK			Oxidation (M)[8]		Mascot
1345.6893	1345.6501	-0.0392	-29	145	155	RQEQNAEMVLK					Mascot
1422.7476	1422.6168	-0.1308	-92	133	144	VYQKDIDAELTK					Mascot
1477.6904	1477.7775	0.0871	59	256	268	DVDDSVETLDELK					Mascot
1490.7883	1490.7308	-0.0575	-39	415	428	AMDLITDSAKQVAK					Mascot
1490.7883	1490.7308	-0.0575	-39	415	428	AMDLITDSAKQVAK	10	0			Mascot
1562.7479	1562.7413	-0.0066	-4	146	158	QEQNAEMVLKNDK			Oxidation (M)[7]		Mascot
1955.0631	1954.9359	-0.1272	-65	82	100	ETGIKAVGQPQIVPVSMG K			Oxidation (M)[17]		Mascot

3	Ribonuclease 3 OS=Lactobacillus salivarius (strain UCC118) GN=rnc PE=3 SV=1	RNC_LACS1	27167.6	5.93	12	53	0	8.242

Peptide Information

Calc. Mass	Obsrv. Mass	± da	± ppm	Start Seq.	End Seq.	Sequence	Ion Score	C. I. %	Modification	Rank	Result Type
816.4872	816.4512	-0.036	-44	80	86	LRAAMVR					Mascot
1060.5707	1060.5416	-0.0291	-27	2	10	IEGLQEMLK					Mascot
1092.6049	1092.5381	-0.0668	-61	139	147	DEVVRFISK					Mascot
1182.5936	1182.549	-0.0446	-38	82	92	AAMVRADSFSK					Mascot
1347.7123	1347.5969	-0.1154	-86	1	11	MIEGLQEMLKR					Mascot
1422.741	1422.6168	-0.1242	-87	217	229	AAEQMAAYQALKK					Mascot
1438.7358	1438.6074	-0.1284	-89	217	229	AAEQMAAYQALKK			Oxidation (M)[5]		Mascot
1438.7358	1438.6074	-0.1284	-89	216	228	KAAEQMAAYQALK			Oxidation (M)[6]		Mascot

1635.7295	1635.7319	0.0024	1	153	165	LELGWFDHMMDNK					Mascot
1651.7244	1651.7041	-0.0203	-12	153	165	LELGWFDHMMDNK			Oxidation (M)[9]		Mascot
1675.7957	1675.705	-0.0907	-54	166	179	TELQEVLQQNGECK			Carbamidomethyl (C)[13]		Mascot
1896.9425	1896.9192	-0.0233	-12	93	107	FAIECHFNEYIRLGK			Carbamidomethyl (C)[5]		Mascot
2220.0981	2220.1172	0.0191	9	148	165	VIFPKLELGWFDHMMDN K					Mascot
2234.0334	2234.1152	0.0818	37	87	104	ADSFSKFAIECHFNEYIR			Carbamidomethyl (C)[11]		Mascot
2236.093	2236.1377	0.0447	20	148	165	VIFPKLELGWFDHMMDN K			Oxidation (M)[14]		Mascot
2236.093	2236.1377	0.0447	20	148	165	VIFPKLELGWFDHMMDN K			Oxidation (M)[14]		Mascot

4	Transcription elongation factor GreA OS=Listeria welshimeri serovar 6b (strain ATCC 35897 / DSM 20650 / SLCC5334) GN=greA PE=3 SV=1	GREA_LISW6	17514.9	4.62	8	51	0	4.297

Peptide Information

Calc. Mass	Obsrv. Mass	± da	± ppm	Start Seq.	End Seq.	Sequence	Ion Score	C. I. %	Modification	Rank	Result Type
872.52	872.4965	-0.0235	-27	31	37	EVVERIK					Mascot
1021.5387	1021.5018	-0.0369	-36	6	14	VFPMTLEGK					Mascot
1106.5874	1106.5508	-0.0366	-33	65	73	ITTIENMIR			Oxidation (M)[7]		Mascot
1450.7611	1450.6208	-0.1403	-97	2	14	ATEKVFPMTLEGK					Mascot
1549.8698	1549.7284	-0.1414	-91	122	136	ISNDSPIAKGLLGHK					Mascot
1613.7914	1613.7007	-0.0907	-56	1	14	MATEKVFPMTLEGK			Oxidation (M)[1,9]		Mascot
2188.0803	2188.1265	0.0462	21	137	156	EGEEVTIQTPAGDMNVKI EK					Mascot
2204.0752	2204.1172	0.042	19	137	156	EGEEVTIQTPAGDMNVKI EK			Oxidation (M)[14]		Mascot
2680.1646	2680.1682	0.0036	1	41	64	SFGDLSENSEYDSAKDE QAFVEGR					Mascot

5	Cx9C motif-containing protein 4 OS=Homo sapiens GN=CMC4 PE=1 SV=1	CMC4_HUMAN	8140.8	8.5	7	50	0	1.66

Peptide Information

Calc. Mass	Obsrv. Mass	± da	± ppm	Start Seq.	End Seq.	Sequence	Ion Score	C. I. %	Modification	Rank	Result Type
1054.4808	1054.4314	-0.0494	-47	37	44	KCCAQYPK			Carbamidomethyl (C)[2,3]		Mascot
1116.583	1116.5048	-0.0782	-70	28	36	CQAVIQELR			Carbamidomethyl (C)[1]		Mascot
1139.5085	1139.5443	0.0358	31	38	46	CCAQYPKGR			Carbamidomethyl (C)[1,2]		Mascot
1147.5234	1147.5221	-0.0013	-1	1	9	MPQKDPCQK			Carbamidomethyl (C)[7], Oxidation (M)[1]		Mascot
1330.5767	1330.6598	0.0831	62	17	27	CLQANSYMESK			Carbamidomethyl (C)[1]		Mascot
2012.923	2012.9529	0.0299	15	47	63	SVVCSGFEKEEEENLTR			Carbamidomethyl (C)[4]		Mascot
2187.9832	2188.1265	0.1433	65	10	27	QACEIQKCLQANSYMES			Carbamidomethyl (C)[3,8]		Mascot

2203.978	2204.1172	0.1392	63	10	27	K QACEIQKCLQANSYMES K			Carbamidomethyl (C)[3,8], Oxidation (M)[15]		Mascot

6	Alpha-(1,3)-fucosyltransferase OS=Gorilla gorilla gorilla GN=FUT6 PE=3 SV=1	FUT6_GORGO	42173.9	8.97	11	50	0	3.601	

Peptide Information

Calc. Mass	Obsrv. Mass	± da	± ppm	Start Seq.	End Seq.	Sequence	Ion Score	C. I. %	Modification	Rank	Result Type
1060.5535	1060.5416	-0.0119	-11	218	226	VDVYGRSHK					Mascot
1250.6528	1250.567	-0.0858	-69	208	217	YYQSLQAHLK					Mascot
1420.689	1420.6033	-0.0857	-60	111	122	EVMYNPSAQLPR			Oxidation (M)[3]		Mascot
1450.7029	1450.6208	-0.0821	-57	227	239	SLPQGTMMETLSR					Mascot
1511.7424	1511.7238	-0.0186	-12	2	14	DPLGPAKPQWSCR			Carbamidomethyl (C)[12]		Mascot
1562.7559	1562.7413	-0.0146	-9	36	49	VSRDDPTVYPNGSR					Mascot
1632.897	1632.8257	-0.0713	-44	97	110	KVYPQADAVIVHHR					Mascot
1818.8837	1818.8329	-0.0508	-28	224	239	SHKSLPQGTMMETLSR			Oxidation (M)[10]		Mascot
1926.7991	1926.8663	0.0672	35	80	96	CSEMLPGTADCNITADR			Carbamidomethyl (C)[1,11], Oxidation (M)[4]		Mascot
1940.0024	1939.937	-0.0654	-34	208	223	YYQSLQAHLKVDVYGR					Mascot
2250.0422	2250.0649	0.0227	10	39	59	DDPTVYPNGSRFPDSTG TPAR					Mascot

7	Inner centromere protein-related protein pic1 OS=Schizosaccharomyces pombe (strain 972 / ATCC 24843) GN=pic1 PE=1 SV=2	PIC1_SCHPO	114463	9.54	21	50	0	7.069	

Peptide Information

Calc. Mass	Obsrv. Mass	± da	± ppm	Start Seq.	End Seq.	Sequence	Ion Score	C. I. %	Modification	Rank	Result Type
812.4083	812.3837	-0.0246	-30	874	881	ASMHAPAK					Mascot
816.4322	816.4512	0.019	23	724	729	RQEDLR					Mascot
1060.5746	1060.5416	-0.033	-31	445	453	IQEVSSNKR					Mascot
1094.4783	1094.5299	0.0516	47	884	893	NSSMQEPSSK					Mascot
1110.4731	1110.5111	0.038	34	884	893	NSSMQEPSSK			Oxidation (M)[4]		Mascot
1117.5605	1117.5453	-0.0152	-14	80	88	CALPTPRMR			Carbamidomethyl (C)[1], Oxidation (M)[8]		Mascot
1159.6066	1159.5585	-0.0481	-41	732	742	KPLTDNGATSR					Mascot
1238.5801	1238.5703	-0.0098	-8	2	12	GSNGSELWFNK					Mascot
1312.712	1312.7092	-0.0028	-2	69	79	YSTPRLSPVHR					Mascot
1317.7063	1317.6372	-0.0691	-52	178	187	RFTEWNVPLR					Mascot
1385.6155	1385.6241	0.0086	6	1	12	MGSNGSELWFNK			Oxidation (M)[1]		Mascot
1409.6907	1409.6029	-0.0878	-62	13	24	ELEYSQQLTNGK					Mascot

Calc. Mass	Obsrv. Mass	± da	± ppm	Start Seq.	End Seq.	Sequence	Ion Score	C. I. %	Modification	Rank	Result Type
1420.6598	1420.6033	-0.0565	-40	611	624	AKNGAANASNMESR					Mascot
1477.7897	1477.7775	-0.0122	-8	49	60	LQLESDIYELVR					Mascot
1497.7697	1497.7302	-0.0395	-26	939	951	VNLPSWAESPELR					Mascot
1549.7605	1549.7284	-0.0321	-21	642	654	ELSNNEFPSRQTK					Mascot
1632.8262	1632.8257	-0.0005	0	884	898	NSSMQEPSSKSPLLK					Mascot
1845.8826	1845.8875	0.0049	3	326	343	SSGVAFSSETVTSSSKER					Mascot
1905.9222	1905.8704	-0.0518	-27	780	797	MEPDSVTSVTQPSVGSL R			Oxidation (M)[1]		Mascot
1971.9698	1971.9398	-0.03	-15	402	418	SQEFDFFEAKIPDSIAK					Mascot
2012.9794	2012.9529	-0.0265	-13	839	855	TMHHNVNPFTKQNGIMK			Oxidation (M)[2]		Mascot
2018.0223	2017.8745	-0.1478	-73	779	797	KMEPDSVTSVTQPSVGS LR					Mascot

8 30S ribosomal protein S17 OS=Lactobacillus sakei subsp. sakei (strain 23K) GN=rpsQ PE=3 SV=1 RS17_LACSS 10533.5 9.73 7 49 0 26.858 9 0

Peptide Information

Calc. Mass	Obsrv. Mass	± da	± ppm	Start Seq.	End Seq.	Sequence	Ion Score	C. I. %	Modification	Rank	Result Type
987.5006	987.5192	0.0186	19	34	41	THPEYGKR					Mascot
1145.5116	1145.4414	-0.0702	-61	1	9	MSEESRNHR					Mascot
1145.5116	1145.4414	-0.0702	-61	1	9	MSEESRNHR	9	0			Mascot
1317.7307	1317.6372	-0.0935	-71	65	75	VMETRPLSRTK					Mascot
1330.5546	1330.6598	0.1052	79	48	58	YYAQDDNNEAK					Mascot
1590.7754	1590.7792	0.0038	2	21	33	MDKTITVMVETYK			Oxidation (M)[1,8]		Mascot
1955.9094	1955.9551	0.0457	23	48	64	YYAQDDNNEAKVGDVVR					Mascot
1955.9094	1955.9551	0.0457	23	48	64	YYAQDDNNEAKVGDVVR					Mascot
2012.9998	2012.9529	-0.0469	-23	24	40	TITVMVETYKTHPEYGK			Oxidation (M)[5]		Mascot

9 Beta-1,3-N-acetylglucosaminyltransferase radical fringe OS=Notophthalmus viridescens GN=RFNG PE=2 SV=1 RFNG_NOTVI 45703 8.21 11 49 0 2.142

Peptide Information

Calc. Mass	Obsrv. Mass	± da	± ppm	Start Seq.	End Seq.	Sequence	Ion Score	C. I. %	Modification	Rank	Result Type
812.4526	812.3837	-0.0689	-85	32	37	TPWRPR					Mascot
1092.5896	1092.5381	-0.0515	-47	113	123	SVLASSINSSK					Mascot
1317.5819	1317.6372	0.0553	42	101	112	HEGPTDNPGEHK					Mascot
1434.7336	1434.6283	-0.1053	-73	346	358	QVTLSYGGPDNKR					Mascot
1632.7574	1632.8257	0.0683	42	198	210	MAVEYDKFIESER			Oxidation (M)[1]		Mascot
1803.9236	1804.0383	0.1147	64	359	375	NVVSVGGIFSLENDPTR					Mascot

1867.98	1867.917	-0.063	-34	124	139	DALEFEDLFIAVKTTR					Mascot
1976.0409	1975.9426	-0.0983	-50	304	321	LPDDCTIGYIIEGLLGVK			Carbamidomethyl (C)[5]		Mascot
2012.9872	2012.9529	-0.0343	-17	322	337	MHHTPLFHSHLENLQR			Oxidation (M)[1]		Mascot
2355.1274	2355.1641	0.0367	16	376	393	FRTVHCLLYPDTHWCPP R			Carbamidomethyl (C)[6,15]		Mascot
3187.6284	3187.5171	-0.1113	-35	229	256	HLLASFSHSQDVYLGRP SLDHPIEAIER					Mascot

10 Myosin-7B OS=Homo sapiens GN=MYH7B PE=2 SV=3 MYH7B_HUMAN 222391.7 5.77 29 48 0 23.564

Peptide Information

Calc. Mass	Obsrv. Mass	± da	± ppm	Start Seq.	End Seq.	Sequence	Ion Score	C. I. %	Modification	Rank	Result Type
816.421	816.4512	0.0302	37	1473	1479	ELEAAQR					Mascot
847.4706	847.4771	0.0065	8	1652	1658	LMQAQLK			Oxidation (M)[2]		Mascot
922.4662	922.4555	-0.0107	-12	1418	1425	CSSLEKAK			Carbamidomethyl (C)[1]		Mascot
1046.5226	1046.4946	-0.028	-27	1642	1651	QATEAQAATR					Mascot
1078.4834	1078.5276	0.0442	41	1917	1926	ADMAETQANK					Mascot
1078.4834	1078.5276	0.0442	41	1917	1926	ADMAETQANK	3	0			Mascot
1094.4783	1094.5299	0.0516	47	1917	1926	ADMAETQANK			Oxidation (M)[3]		Mascot
1116.5756	1116.5048	-0.0708	-63	1697	1706	AALEQGERSR					Mascot
1145.5546	1145.4414	-0.1132	-99	1157	1168	LEEAGGASAGQR					Mascot
1145.5546	1145.4414	-0.1132	-99	1157	1168	LEEAGGASAGQR					Mascot
1147.579	1147.5221	-0.0569	-50	711	719	ICRQGFPNR			Carbamidomethyl (C)[2]		Mascot
1315.7191	1315.6038	-0.1153	-88	355	366	IVGALLHFGNMK			Oxidation (M)[11]		Mascot
1345.7257	1345.6501	-0.0756	-56	660	670	ENLNKLMTNLR					Mascot
1347.6686	1347.5969	-0.0717	-53	1917	1928	ADMAETQANKLR					Mascot
1402.7061	1402.7367	0.0306	22	1828	1839	ELEAELDAEQKK					Mascot
1409.665	1409.6029	-0.0621	-44	1765	1777	AITDAAMMAEELK			Oxidation (M)[7]		Mascot
1437.7472	1437.6572	-0.09	-63	261	273	LASADIDSYLLEK					Mascot
1441.7546	1441.6571	-0.0975	-68	563	574	SPNFQQPRPDKK					Mascot
1450.7173	1450.6208	-0.0965	-67	1854	1865	VKELAYQAEEDR					Mascot
1497.7307	1497.7302	-0.0005	0	827	838	AFNAVKNWSWMK			Oxidation (M)[11]		Mascot
1507.7057	1507.7239	0.0182	12	169	183	DNQSMLITGESGAGK					Mascot
1590.8826	1590.7792	-0.1034	-65	355	368	IVGALLHFGNMKFK			Oxidation (M)[11]		Mascot
1632.8262	1632.8257	-0.0005	0	1101	1114	VEDEQLLGAQMQKK			Oxidation (M)[11]		Mascot
1732.8977	1732.8788	-0.0189	-11	1493	1507	HGHEEALEALETLKR					Mascot
1796.8046	1796.8546	0.05	28	1352	1366	EQHEEEAEAQAELQR					Mascot
1796.8046	1796.8546	0.05	28	1352	1366	EQHEEEAEAQAELQR					Mascot

1845.9817	1845.8875	-0.0942	-51	1491	1506	LRHGHEEALEALETLK		Mascot
1909.9702	1909.9011	-0.0691	-36	671	686	ATQPHFVRCIVPNENK	Carbamidomethyl (C)[9]	Mascot
1975.9819	1975.9426	-0.0393	-20	1511	1528	NLQEEISDLTDQVSLSGK		Mascot
2017.9801	2017.8745	-0.1056	-52	16	32	QGYQEMTKVHTIPWDGK		Mascot
2188.0364	2188.1265	0.0901	41	928	946	ELSERLEDEEEVNADLAA R		Mascot
2236.0952	2236.1377	0.0425	19	1665	1683	DEEQRLAAELHEQAQAL ER		Mascot
2236.0952	2236.1377	0.0425	19	1665	1683	DEEQRLAAELHEQAQAL ER		Mascot
2250.1108	2250.0649	-0.0459	-20	1004	1023	KALQEAHQQALGDLQAE EDR		Mascot

Gel Idx/Pos	208/I8	Instr./Gel Origin	BA2151/Sample Project 20140814	Process Status	Analysis Succeeded
Plate [#] Name	[1] Sample Project 20140814	Instrument Sample Name		Spectra	11

Rank	Protein Name	Accession No.	Protein MW	Protein PI	Pep. Count	Protein Score	Protein Score C. I. %	Intensity Matched	Total Ion Score	Total Ion C. I. %	Confirmed
1	Internal virion protein D OS=Enterobacteria phage T7 GN=16 PE=3 SV=1	VIVD_BPT7	143748.2	6.56	31	73	97.412	6.773			

Peptide Information

Calc. Mass	Obsrv. Mass	± da	± ppm	Start Seq.	End Seq.	Sequence	Ion Score	C. I. %	Modification	Rank	Result Type
837.4464	837.4545	0.0081	10	1168	1173	KEYLER					Mascot
847.4957	847.4274	-0.0683	-81	625	631	IVMDIIK			Oxidation (M)[3]		Mascot
951.4352	951.4813	0.0461	48	1061	1068	QAFSMDPR					Mascot
987.6019	987.5245	-0.0774	-78	625	632	IVMDIIKR					Mascot
1021.5247	1021.5082	-0.0165	-16	799	806	IMPAYDRR					Mascot
1048.6038	1048.6067	0.0029	3	511	520	LAGFTEIGLK					Mascot
1128.6194	1128.5417	-0.0777	-69	1041	1049	SLIKEHMVR			Oxidation (M)[7]		Mascot
1167.5277	1167.5487	0.021	18	126	136	GDFASISEEGR					Mascot
1173.5317	1173.5951	0.0634	54	1045	1054	EHMVRGEDGK			Oxidation (M)[3]		Mascot
1206.5902	1206.606	0.0158	13	663	672	GTYVPHVYDR					Mascot
1230.6113	1230.5765	-0.0348	-28	596	604	EETRYTIYR					Mascot
1346.8042	1346.6976	-0.1066	-79	508	520	GIKLAGFTEIGLK					Mascot
1364.6805	1364.6722	-0.0083	-6	19	31	AADANGVSYDLLR					Mascot
1404.6826	1404.6066	-0.076	-54	436	448	ETARNANSADLSR					Mascot
1418.7421	1418.625	-0.1171	-83	806	819	RVNGDIAIMGSTGK					Mascot
1428.7516	1428.6201	-0.1315	-92	841	853	KTGEVHALMDTVK					Mascot
1434.7369	1434.6251	-0.1118	-78	806	819	RVNGDIAIMGSTGK			Oxidation (M)[9]		Mascot
1444.7465	1444.7384	-0.0081	-6	841	853	KTGEVHALMDTVK			Oxidation (M)[9]		Mascot
1463.7198	1463.7432	0.0234	16	639	651	ELMENPAIFGNTK					Mascot
1490.7883	1490.7308	-0.0575	-39	1002	1016	QGMLGDVISATLTGK					Mascot
1534.8224	1534.7111	-0.1113	-73	988	1001	LLNGTTNYLLDAAR					Mascot
1613.8026	1613.7079	-0.0947	-59	48	62	TGPLGMMQFTKATAK			Oxidation (M)[6,7]		Mascot
1635.8159	1635.7402	-0.0757	-46	638	651	RELMENPAIFGNTK			Oxidation (M)[4]		Mascot
1818.9419	1818.8286	-0.1133	-62	148	165	SPMAGQQLETFGGITPKG K					Mascot
1838.9283	1838.8961	-0.0322	-18	252	267	LDNGFDVFKDTITPTR					Mascot
1856.9899	1856.9032	-0.0867	-47	842	858	TGEVHALMDTVKILTGR			Oxidation (M)[8]		Mascot

Calc. Mass	Obsrv. Mass	± da	± ppm	Start Seq.	End Seq.	Sequence	Ion Score	C. I. %	Modification	Rank	Result Type
1864.9797	1864.9446	-0.0351	-19	1002	1019	QGMLGDVISATLTGKTTR			Oxidation (M)[3]		Mascot
1926.0656	1925.9548	-0.1108	-58	955	973	LREATDTGPAVANIVGTLK					Mascot
1937.9868	1937.9778	-0.009	-5	268	282	WNSHIWTPEELEKIR					Mascot
1953.9302	1953.9396	0.0094	5	97	114	FDGDELKAALAYNQGEGR					Mascot
2010.9741	2010.9703	-0.0038	-2	551	568	FGATASDIHERLHGTDQR					Mascot
2323.1533	2323.1287	-0.0246	-11	883	904	NAYMGAQNITEIAGMIVTGNVR					Mascot
3369.5659	3369.6255	0.0596	18	196	224	GIEQEATAKPFAKDFWETHGETLDEYNSR					Mascot

2	Glutamate synthase 1 [NADH], chloroplastic OS=Oryza sativa subsp. japonica GN=Os01g0681900 PE=2 SV=1	GLT1_ORYSJ	238797.4	6.69	32	72	96.172	38.172

Peptide Information

Calc. Mass	Obsrv. Mass	± da	± ppm	Start Seq.	End Seq.	Sequence	Ion Score	C. I. %	Modification	Rank	Result Type
837.4465	837.4545	0.008	10	188	194	SKAEFQK					Mascot
987.4894	987.5245	0.0351	36	948	955	AAYKEYSR					Mascot
1048.5343	1048.6067	0.0724	69	1781	1789	SIECAIIDK			Carbamidomethyl (C)[4]		Mascot
1060.5634	1060.5507	-0.0127	-12	706	714	KGLEETLDR					Mascot
1076.635	1076.55	-0.085	-79	694	702	VLDITYPKK					Mascot
1230.6338	1230.5765	-0.0573	-47	26	36	RSHSSVAAPYR					Mascot
1238.5988	1238.5806	-0.0182	-15	1828	1837	MGHFVTVFER			Oxidation (M)[1]		Mascot
1273.6271	1273.5808	-0.0463	-36	537	547	DIVESVPETER					Mascot
1390.5807	1390.6548	0.0741	53	828	838	YFYASNYGMMK			Oxidation (M)[9]		Mascot
1406.5756	1406.6235	0.0479	34	828	838	YFYASNYGMMK			Oxidation (M)[9,10]		Mascot
1406.5756	1406.6235	0.0479	34	828	838	YFYASNYGMMK			Oxidation (M)[9,10]		Mascot
1410.6942	1410.6219	-0.0723	-51	1841	1853	IGGLMMYGVPNMK					Mascot
1428.7628	1428.6201	-0.1427	-100	1389	1401	AVGTMLSHEVTKR					Mascot
1444.7577	1444.7384	-0.0193	-13	1389	1401	AVGTMLSHEVTKR			Oxidation (M)[5]		Mascot
1497.8346	1497.7245	-0.1101	-74	839	852	VLAKMGISTLASYK			Oxidation (M)[5]		Mascot
1517.774	1517.7039	-0.0701	-46	114	126	RATVNDALEMLER					Mascot
1534.6705	1534.7111	0.0406	26	827	838	KYFYASNYGMMK			Oxidation (M)[10,11]		Mascot
1768.8544	1768.8678	0.0134	8	1838	1853	ADRIGGLMMYGVPNMK			Oxidation (M)[8]		Mascot
1784.8492	1784.9188	0.0696	39	1838	1853	ADRIGGLMMYGVPNMK			Oxidation (M)[8,9]		Mascot
1785.8702	1785.9167	0.0465	26	828	842	YFYASNYGMMKVLAK					Mascot
1818.879	1818.8286	-0.0504	-28	1308	1323	SDMLEVDPEVVKSNEK					Mascot
1856.9105	1856.9032	-0.0073	-4	115	130	ATVNDALEMLERMAHR					Mascot
1859.8593	1859.8853	0.026	14	98	114	DSCGVGFVAELSGDYKR			Carbamidomethyl (C)[3]		Mascot

1859.8593	1859.8853	0.026	14	98	114	DSCGVGFVAELSGDYKR			Carbamidomethyl (C)[3]		Mascot
1876.9434	1876.9094	-0.034	-18	1952	1970	VVVIGGGDTGTDCIGTSIR			Carbamidomethyl (C)[13]		Mascot
1876.9434	1876.9094	-0.034	-18	1952	1970	VVVIGGGDTGTDCIGTSIR			Carbamidomethyl (C)[13]		Mascot
1898.9872	1898.926	-0.0612	-32	138	155	NTGDGAGILVALPHNFFR					Mascot
1956.0721	1955.9619	-0.1102	-56	667	684	LALEGPLVSIDEMEAIKK					Mascot
1956.0721	1955.9619	-0.1102	-56	667	684	LALEGPLVSIDEMEAIKK					Mascot
1968.0042	1967.9226	-0.0816	-41	1291	1307	EIMSQLGFRTITEMVGR					Mascot
1972.0671	1971.9507	-0.1164	-59	667	684	LALEGPLVSIDEMEAIKK			Oxidation (M)[13]		Mascot
2013.0436	2012.9756	-0.068	-34	577	594	AFGYTVEALEMLLLPMAK			Oxidation (M)[11]		Mascot
2264.115	2264.1284	0.0134	6	1300	1319	TITEMVGRSDMLEVDPEVVK			Oxidation (M)[5]		Mascot
2323.1375	2323.1287	-0.0088	-4	972	992	FKDTADMISVDEVEPASEIVK					Mascot
2356.241	2356.1257	-0.1153	-49	138	159	NTGDGAGILVALPHNFFREVTK					Mascot
2933.3552	2933.4104	0.0552	19	398	422	SLPEAVMMMIPEAWQNDVNMEPEKK			Oxidation (M)[7]		Mascot
3079.5332	3079.48	-0.0532	-17	156	183	EVTKDAGFELPQPGEYAVGMVFLPIDEK					Mascot
3079.5332	3079.48	-0.0532	-17	156	183	EVTKDAGFELPQPGEYAVGMVFLPIDEK					Mascot
3095.5283	3095.4785	-0.0498	-16	156	183	EVTKDAGFELPQPGEYAVGMVFLPIDEK			Oxidation (M)[20]		Mascot
3109.5623	3109.4917	-0.0706	-23	1863	1890	RVNLMAEEGITFVVNANVGSDPLYSIER			Oxidation (M)[5]		Mascot
3457.7073	3457.7588	0.0515	15	1271	1299	FAGEPEHVINFFFMLAEELREIMSQLGFR					Mascot
3469.8359	3469.71	-0.1259	-36	1320	1350	SNEKLENIDLSLILKPAAEIRPGAAQYCVEK			Carbamidomethyl (C)[28]		Mascot

3	Probable pyruvate kinase, cytosolic isozyme OS=Arabidopsis thaliana GN=At4g26390 PE=3 SV=1	KPYC_ARATH	54968.1	8.14	16	64	79.442	5.546

Peptide Information

Calc. Mass	Obsrv. Mass	± da	± ppm	Start Seq.	End Seq.	Sequence	Ion Score	C. I. %	Modification	Rank	Result Type
918.4059	918.4587	0.0528	57	110	116	TICMSYK			Carbamidomethyl (C)[3], Oxidation (M)[4]		Mascot
978.4924	978.5208	0.0284	29	139	146	VLSCDKEK			Carbamidomethyl (C)[4]		Mascot
1046.501	1046.5059	0.0049	5	110	117	TICMSYKK			Carbamidomethyl (C)[3], Oxidation (M)[4]		Mascot
1048.5642	1048.6067	0.0425	41	28	37	LLMAGMSVAR					Mascot
1088.5881	1088.5469	-0.0412	-38	2	10	AMEQRPKTK					Mascot
1202.6892	1202.585	-0.1042	-87	438	449	GLVPVLYAGSAR					Mascot
1331.597	1331.6117	0.0147	11	106	116	GDEKTICMSYK			Carbamidomethyl (C)[7]		Mascot
1347.5919	1347.6084	0.0165	12	106	116	GDEKTICMSYK			Carbamidomethyl (C)[7], Oxidation (M)[8]		Mascot
1444.8523	1444.7384	-0.1139	-79	79	91	TGFLKDGKPIQLK					Mascot
1818.9639	1818.8286	-0.1353	-74	21	37	SVPMVEKLLMAGMSVAR					Mascot

1859.8732	1859.8853	0.0121	7	338	353	ICVEAESTLDYGDIFK			Carbamidomethyl (C)[2]		Mascot
1859.9718	1859.8853	-0.0865	-47	11	27	IVCTLGPASRSVPMVEK			Carbamidomethyl (C)[3], Oxidation (M)[14]		Mascot
1972.1127	1971.9507	-0.162	-82	432	449	HSLIYRGLVPVLYAGSAR					Mascot
2246.0979	2246.1194	0.0215	10	355	375	IMLHAAVPMSPMESLASSAVR			Oxidation (M)[2,9,12]		Mascot
2357.0581	2357.1438	0.0857	36	38	56	FNFSHGSYEYHQETLDNLR					Mascot
2933.478	2933.4104	-0.0676	-23	118	144	LAQDVNPGMVILCADGTISLKVLSCDK			Carbamidomethyl (C)[13,25], Oxidation (M)[9]		Mascot
3499.7449	3499.6301	-0.1148	-33	221	251	TILLMSKVENQEGVANFDDILINSDAFMIAR			Oxidation (M)[5,28]		Mascot

4	Putative pentatricopeptide repeat-containing protein At5g08490 OS=Arabidopsis thaliana GN=PCMP-E32 PE=3 SV=1	PP370_ARATH	95908.7	6.98	18	64	76.933	25.997

Peptide Information

Calc. Mass	Obsrv. Mass	± da	± ppm	Start Seq.	End Seq.	Sequence	Ion Score	C. I. %	Modification	Rank	Result Type
918.468	918.4587	-0.0093	-10	453	460	EVHGYSVK					Mascot
978.5077	978.5208	0.0131	13	143	150	SMHSYIIK					Mascot
1046.52	1046.5059	-0.0141	-13	589	596	QCHGYIIR			Carbamidomethyl (C)[2]		Mascot
1129.6113	1129.5535	-0.0578	-51	248	256	QIHSYVVQR					Mascot
1145.6313	1145.5669	-0.0644	-56	451	460	VKEVHGYSVK					Mascot
1145.6313	1145.5669	-0.0644	-56	451	460	VKEVHGYSVK					Mascot
1230.5065	1230.5765	0.07	57	72	80	MDDCQKMFR			Carbamidomethyl (C)[4]		Mascot
1436.703	1436.6234	-0.0796	-55	620	631	HAYSVFQSDARR					Mascot
1448.7856	1448.7418	-0.0438	-30	276	288	VGRIEEAASLFTR					Mascot
1497.7618	1497.7245	-0.0373	-25	156	169	DTLVGNALVSMYAK			Oxidation (M)[11]		Mascot
1530.723	1530.7861	0.0631	41	817	829	NVFVSGDCSHPRR			Carbamidomethyl (C)[8]		Mascot
1785.9092	1785.9167	0.0075	4	830	844	DSIFDLVNALYLQMK			Oxidation (M)[14]		Mascot
1898.863	1898.926	0.063	33	379	395	FGDTSAAYWAFSLMSTK			Oxidation (M)[14]		Mascot
1926.0154	1925.9548	-0.0606	-31	829	844	RDSIFDLVNALYLQMK					Mascot
2356.1907	2356.1257	-0.065	-28	257	275	SWLQTHVFVCNSLVSFYLR			Carbamidomethyl (C)[10]		Mascot
2357.2097	2357.1438	-0.0659	-28	830	849	DSIFDLVNALYLQMKEPVVF			Oxidation (M)[14]		Mascot
3079.501	3079.48	-0.021	-7	81	107	QMDSLDPVVWNIVLTGLSVSCGRETMR			Carbamidomethyl (C)[21], Oxidation (M)[2]		Mascot
3079.501	3079.48	-0.021	-7	81	107	QMDSLDPVVWNIVLTGLSVSCGRETMR			Carbamidomethyl (C)[21], Oxidation (M)[2]		Mascot
3095.4958	3095.4785	-0.0173	-6	81	107	QMDSLDPVVWNIVLTGLSVSCGRETMR			Carbamidomethyl (C)[21], Oxidation (M)[2,26]		Mascot
3457.7024	3457.7588	0.0564	16	156	187	DTLVGNALVSMYAKFGFIFPDAYTAFDGIADK					Mascot
3469.6047	3469.71	0.1053	30	188	217	DVVSWNAIIAGFSENNM			Carbamidomethyl (C)[26], Oxidation		Mascot

			MADAFRSFCLMLK					(M)[17,18]	
5	Cellulose synthase operon protein C OS=Escherichia coli (strain K12) GN=bcsC PE=1 SV=3	BCSC_ECOLI	127759.6	5.88	22	64	76.933	8.945	

Peptide Information

Calc. Mass	Obsrv. Mass	± da	± ppm	Start Seq.	End Seq.	Sequence	Ion Score	C. I. %	Modification	Rank	Result Type
847.4156	847.4274	0.0118	14	451	457	SIDDIER					Mascot
1127.4867	1127.5385	0.0518	46	983	990	WMTGYYYK			Oxidation (M)[2]		Mascot
1129.6477	1129.5535	-0.0942	-83	342	350	WNSLLKVNR					Mascot
1144.6222	1144.5374	-0.0848	-74	264	273	QLADPAFRAR					Mascot
1187.5917	1187.606	0.0143	12	974	982	NVEDNWRVR					Mascot
1207.5988	1207.6091	0.0103	9	103	113	TTMLLSTPDGR			Oxidation (M)[3]		Mascot
1301.6597	1301.5677	-0.092	-71	501	511	LSQDLWQAGQR					Mascot
1406.6659	1406.6235	-0.0424	-30	861	874	SQSDSGASVAVGWR					Mascot
1406.6659	1406.6235	-0.0424	-30	861	874	SQSDSGASVAVGWR					Mascot
1436.6686	1436.6234	-0.0452	-31	585	597	ESGKEAEAEAMLR			Oxidation (M)[11]		Mascot
1448.6873	1448.7418	0.0545	38	703	715	SQPPSMESAMVLR			Oxidation (M)[6]		Mascot
1463.7411	1463.7432	0.0021	1	227	240	DMPVSDASVSALKK			Oxidation (M)[2]		Mascot
1490.8003	1490.7308	-0.0695	-47	488	500	LALDPGSVWITYR					Mascot
1635.8701	1635.7402	-0.1299	-79	54	68	LELIDPNNPDVVAAR					Mascot
1719.8523	1719.8309	-0.0214	-12	999	1012	VTIGLNNMIWHYDK			Oxidation (M)[8]		Mascot
1761.8225	1761.8845	0.062	35	806	821	AHTTMLQVDAPYSDGR					Mascot
1768.8389	1768.8678	0.0289	16	720	735	FEAQAGDPTQALETYK					Mascot
1838.8701	1838.8961	0.026	14	198	214	DEGFAVLEQMAKSNAGR			Oxidation (M)[10]		Mascot
1859.9585	1859.8853	-0.0732	-39	998	1012	RVTIGLNNMIWHYDK					Mascot
1859.9585	1859.8853	-0.0732	-39	998	1012	RVTIGLNNMIWHYDK					Mascot
1939.0397	1938.9674	-0.0723	-37	54	70	LELIDPNNPDVVAARFR					Mascot
2264.1235	2264.1284	0.0049	2	138	158	LFNGAPPEGDIAVEYWST VAK					Mascot
3109.4609	3109.4917	0.0308	10	520	546	NLAQQKSNDPEQVYAYG LYLSGHDQDR					Mascot
3469.6521	3469.71	0.0579	17	1013	1042	DLSGYSLGQGGYYSPQE YLSFAIPVMWRER					Mascot

6	DNA mismatch repair protein MSH3 OS=Ashbya gossypii (strain ATCC 10895 / CBS 109.51 / FGSC 9923 / NRRL Y-1056) GN=MSH3 PE=3 SV=1	MSH3_ASHGO	117750.6	7.59	23	61	56.048	14.222

Peptide Information

Calc. Mass	Obsrv. Mass	± da	± ppm	Start Seq.	End Seq.	Sequence	Ion Score	C. I. %	Modification	Rank	Result Type

Calc. Mass	Obsrv. Mass	± da	± ppm	Start Seq.	End Seq.	Sequence	Ion Score	C. I. %	Modification	Rank	Result Type
832.3795	832.3132	-0.0663	-80	504	510	DGQDLER					Mascot
918.5367	918.4587	-0.078	-85	673	681	VASTKAVSR					Mascot
997.5353	997.5127	-0.0226	-23	402	409	NESLLFFK					Mascot
1078.6003	1078.5328	-0.0675	-63	455	463	TNYGLRNLK					Mascot
1078.6003	1078.5328	-0.0675	-63	455	463	TNYGLRNLK					Mascot
1127.5878	1127.5385	-0.0493	-44	149	157	DLKLQHMDK					Mascot
1128.5281	1128.5417	0.0136	12	243	253	NSGTSSSVFSR					Mascot
1167.6368	1167.5487	-0.0881	-75	716	725	ITGEYTSLRK					Mascot
1173.6011	1173.5951	-0.006	-5	955	965	AHNSYGLNVAK					Mascot
1187.5593	1187.606	0.0467	39	81	89	GFEDFRFNR					Mascot
1231.6538	1231.5796	-0.0742	-60	791	802	IMIITGPNMGGK					Mascot
1301.6372	1301.5677	-0.0695	-53	262	273	ATYGINETFGTK					Mascot
1346.7162	1346.6976	-0.0186	-14	229	241	VGVVEQTETSAVK					Mascot
1364.7686	1364.6722	-0.0964	-71	2	12	LQQPTISRFFK					Mascot
1448.7678	1448.7418	-0.026	-18	51	64	KPEAAPMHVGLGNK					Mascot
1461.7445	1461.7588	0.0143	10	97	108	EQEVGFAERLQR					Mascot
1477.7897	1477.7103	-0.0794	-54	140	151	LTELDQQFKDLK					Mascot
1490.7672	1490.7308	-0.0364	-24	277	290	VLGDSASVWGLVCK			Carbamidomethyl (C)[13]		Mascot
1511.804	1511.7034	-0.1006	-67	1	12	MLQQPTISRFFK			Oxidation (M)[1]		Mascot
1549.7428	1549.7175	-0.0253	-16	777	789	YMPNDVNLNREGK					Mascot
1856.9866	1856.9032	-0.0834	-45	185	201	LVPGKLTVHETDPADHK					Mascot
1939.0059	1938.9674	-0.0385	-20	699	714	ELLQDLALQEYESFIK					Mascot
2263.9619	2264.1284	0.1665	74	114	133	EGGCVEEDETELDNEAP RSK			Carbamidomethyl (C)[4]		Mascot
3469.7249	3469.71	-0.0149	-4	921	949	SPILGNYHMSYIEEKRPG ENWPSVVFLYK			Oxidation (M)[9]		Mascot

7	Cell division protein FtsQ OS=Bradyrhizobium japonicum (strain USDA 110) GN=ftsQ PE=3 SV=1	FTSQ_BRAJA	37709.5	9.93	11	59	25.358	2.657	

Peptide Information

Calc. Mass	Obsrv. Mass	± da	± ppm	Start Seq.	End Seq.	Sequence	Ion Score	C. I. %	Modification	Rank	Result Type
918.4713	918.4587	-0.0126	-14	302	309	DIVAVDMR					Mascot
1444.7002	1444.7384	0.0382	26	1	13	MDGAGSLTRSFFR					Mascot
1549.822	1549.7175	-0.1045	-67	152	165	SSLLFLDADAVRDK					Mascot
1613.8315	1613.7079	-0.1236	-77	277	291	LPEQDVGNALAMLSR					Mascot
1706.9146	1706.7568	-0.1578	-92	180	193	LYPGQLMIELTERK			Oxidation (M)[7]		Mascot
1784.9866	1784.9188	-0.0678	-38	135	151	KQLTQDEILAIGGVSGR					Mascot

1839.0487	1838.8961	-0.1526	-83	23	39	AAAIGAVVLLREWVQDK				Mascot
1970.0376	1969.9535	-0.0841	-43	277	294	LPEQDVGNALAMLSRLD K				Mascot
1978.0287	1977.9333	-0.0954	-48	116	134	NAMANSAGFRITSVVING R				Mascot
2933.5156	2933.4104	-0.1052	-36	194	220	AFALWQEAGRLSVIADD GAVLEPYVSR				Mascot
3631.9341	3631.7476	-0.1865	-51	81	115	VGISMTVLLLIGSCGFGIV KGGHLQDFVTAISDAR			Carbamidomethyl (C)[14]	Mascot

8	Benign gonial cell neoplasm protein OS=Drosophila melanogaster GN=bgcn PE=1 SV=2	BGCN_DROME	140785.4	6.71	21	58	8.171	28.915	

Peptide Information

Calc. Mass	Obsrv. Mass	± da	± ppm	Start Seq.	End Seq.	Sequence	Ion Score	C. I. %	Modification	Rank	Result Type
837.3957	837.4545	0.0588	70	198	203	IMCIER			Carbamidomethyl (C)[3], Oxidation (M)[2]		Mascot
1046.6027	1046.5059	-0.0968	-92	1188	1196	LMSALIDKR					Mascot
1060.6302	1060.5507	-0.0795	-75	1129	1136	HIFVILYR					Mascot
1301.6783	1301.5677	-0.1106	-85	916	925	TPWIVCNRQK			Carbamidomethyl (C)[6]		Mascot
1346.6084	1346.6976	0.0892	66	33	44	TSEHEAFANNAR					Mascot
1347.7419	1347.6084	-0.1335	-99	603	613	FEWVAKDALLR					Mascot
1404.6974	1404.6066	-0.0908	-65	710	721	LMDIPVSCQLGR			Carbamidomethyl (C)[8], Oxidation (M)[2]		Mascot
1497.752	1497.7245	-0.0275	-18	576	587	VPFKYQIDTACR			Carbamidomethyl (C)[11]		Mascot
1534.79	1534.7111	-0.0789	-51	349	361	GPDIYKEIPEAFR					Mascot
1559.6665	1559.7373	0.0708	45	22	32	RCCQQFPCTFR			Carbamidomethyl (C)[2,3,8]		Mascot
1864.9415	1864.9446	0.0031	2	1148	1162	AKFVNGVFMLQEYFR			Oxidation (M)[9]		Mascot
1876.8746	1876.9094	0.0348	19	694	709	IDVLDDAEDVTWLGCR			Carbamidomethyl (C)[15]		Mascot
1876.8746	1876.9094	0.0348	19	694	709	IDVLDDAEDVTWLGCR			Carbamidomethyl (C)[15]		Mascot
1908.9154	1908.8176	-0.0978	-51	948	963	IRLSPICDSEMSLTDR			Carbamidomethyl (C)[7], Oxidation (M)[11]		Mascot
1940.0521	1939.9827	-0.0694	-36	77	94	TLVLSSGATLNMFTLLSR			Oxidation (M)[12]		Mascot
1954.0288	1953.9396	-0.0892	-46	45	62	SLGLRSQVVHVNGNSCV K			Carbamidomethyl (C)[16]		Mascot
1955.9268	1955.9619	0.0351	18	1012	1028	DCEPVSQYTVLTDTLSK			Carbamidomethyl (C)[2]		Mascot
1955.9268	1955.9619	0.0351	18	1012	1028	DCEPVSQYTVLTDTLSK			Carbamidomethyl (C)[2]		Mascot
2010.9817	2010.9703	-0.0114	-6	964	979	NVNVFIDEWIWMVMSK					Mascot
2264.1909	2264.1284	-0.0625	-28	1197	1214	VGNLIMELFAFRHHWIHK			Oxidation (M)[6]		Mascot
2323.2373	2323.1287	-0.1086	-47	287	307	MALSSHQNLRVVLLSQM GNPK					Mascot
3457.7427	3457.7588	0.0161	5	136	165	FWTEAQLNFLTAFLGHSL SDEILQSLYASR					Mascot
3631.6941	3631.7476	0.0535	15	950	979	LSPICDSEMSLTDRNVNV FIDEWIWMVMSK			Carbamidomethyl (C)[5], Oxidation (M)[9]		Mascot

9	Chromodomain-helicase-DNA-binding protein 4 OS=Homo sapiens GN=CHD4 PE=1 SV=2	CHD4_HUMAN	219406.7	5.62	24	58	6.032	22.042

Peptide Information

Calc. Mass	Obsrv. Mass	± da	± ppm	Start Seq.	End Seq.	Sequence	Ion Score	C. I. %	Modification	Rank	Result Type
951.508	951.4813	-0.0267	-28	1423	1430	AFLNAIMR			Oxidation (M)[7]		Mascot
1078.4283	1078.5328	0.1045	97	1229	1239	DEATDGGGDNK					Mascot
1078.4283	1078.5328	0.1045	97	1229	1239	DEATDGGGDNK					Mascot
1206.5195	1206.606	0.0865	72	1627	1636	TEEPMETEPK			Oxidation (M)[5]		Mascot
1238.6207	1238.5806	-0.0401	-32	201	210	MMMVLGAKWR			Oxidation (M)[1]		Mascot
1346.7209	1346.6976	-0.0233	-17	1885	1895	LQMSERNILSR					Mascot
1390.7188	1390.6548	-0.064	-46	1455	1465	EFKAYVSLFMR					Mascot
1406.7137	1406.6235	-0.0902	-64	1455	1465	EFKAYVSLFMR			Oxidation (M)[10]		Mascot
1406.7137	1406.6235	-0.0902	-64	1455	1465	EFKAYVSLFMR	2	0	Oxidation (M)[10]		Mascot
1410.7517	1410.6219	-0.1298	-92	196	208	IAVSKMMMVLGAK			Oxidation (M)[6,7]		Mascot
1463.761	1463.7432	-0.0178	-12	945	956	KLHDMLGPHMLR			Oxidation (M)[5]		Mascot
1497.7115	1497.7245	0.013	9	1093	1105	YERIDGGITGNMR			Oxidation (M)[12]		Mascot
1511.8363	1511.7034	-0.1329	-88	1878	1890	IPPVAVRLQMSER			Oxidation (M)[10]		Mascot
1761.8549	1761.8845	0.0296	17	1096	1111	IDGGITGNMRQEAIDR			Oxidation (M)[9]		Mascot
1785.9481	1785.9167	-0.0314	-18	1648	1663	SAIDLTPIVVEDKEEK					Mascot
1856.9113	1856.9032	-0.0081	-4	1112	1127	FNAPGAQQFCFLLSTR			Carbamidomethyl (C)[10]		Mascot
1876.848	1876.9094	0.0614	33	1627	1643	TEEPMETEPKGAADVEK			Oxidation (M)[5]		Mascot
1876.848	1876.9094	0.0614	33	1627	1643	TEEPMETEPKGAADVEK			Oxidation (M)[5]		Mascot
1898.9661	1898.926	-0.0401	-21	1733	1748	HDYWLLAGIINHGYAR					Mascot
1925.9215	1925.9548	0.0333	17	1431	1446	YGMPPQDAFTTQWLVR			Oxidation (M)[3]		Mascot
1977.8958	1977.9333	0.0375	19	1289	1304	EEEMGEEEEVEREIIK					Mascot
1982.792	1982.9535	0.1615	81	577	594	KNDMDEPPSGDFGGDE EK			Oxidation (M)[4]		Mascot
2246.1819	2246.1194	-0.0625	-28	1192	1212	MMLTHLVVRPGLGSKTG SMSK			Oxidation (M)[1]		Mascot
3079.4348	3079.48	0.0452	15	76	102	MLLCRQLGDSSGEGPEF VEEEEEVALR			Carbamidomethyl (C)[4]		Mascot
3079.4348	3079.48	0.0452	15	76	102	MLLCRQLGDSSGEGPEF VEEEEEVALR			Carbamidomethyl (C)[4]		Mascot
3095.4297	3095.4785	0.0488	16	76	102	MLLCRQLGDSSGEGPEF VEEEEEVALR			Carbamidomethyl (C)[4], Oxidation (M)[1]		Mascot
3241.4268	3241.5405	0.1137	35	147	173	EPKSSAQLLEDWGMEDI DHVFSEEDYR			Oxidation (M)[14]		Mascot
3241.4268	3241.5405	0.1137	35	147	173	EPKSSAQLLEDWGMEDI DHVFSEEDYR			Oxidation (M)[14]		Mascot
3457.427	3457.7588	0.3318	96	307	338	RSSSEDDDLDVESDFDD ASINSYSVSDGSTSR					Mascot
3469.7612	3469.71	-0.0512	-15	510	541	WGQPPSPTPVPRPPDAD PNTPSPKPLEGRPER					Mascot
3631.491	3631.7476	0.2566	71	308	341	SSSEDDDLDVESDFDDA					Mascot

SINSYSVSDGSTSRSSR

10	Nuclear rim protein 1 OS=Ashbya gossypii (strain ATCC 10895 / CBS 109.51 / FGSC 9923 / NRRL Y-1056) GN=NUR1 PE=3 SV=2	NUR1_ASHGO	67763.9	9.15	15	58	3.843	30.227

Peptide Information

Calc. Mass	Obsrv. Mass	± da	± ppm	Start Seq.	End Seq.	Sequence	Ion Score	C. I. %	Modification	Rank	Result Type
837.4213	837.4545	0.0332	40	379	384	EKYNQR					Mascot
951.4094	951.4813	0.0719	76	137	143	EYDYFSK					Mascot
997.4407	997.5127	0.072	72	320	328	GMAAEYNNK					Mascot
1076.635	1076.55	-0.085	-79	311	319	IKDELAIFK					Mascot
1145.6062	1145.5669	-0.0393	-34	580	589	GSIESRPPFR					Mascot
1145.6062	1145.5669	-0.0393	-34	580	589	GSIESRPPFR					Mascot
1406.7209	1406.6235	-0.0974	-69	545	556	NLHTHTTVVCPK			Carbamidomethyl (C)[10]		Mascot
1406.7209	1406.6235	-0.0974	-69	545	556	NLHTHTTVVCPK			Carbamidomethyl (C)[10]		Mascot
1410.7297	1410.6219	-0.1078	-76	11	22	LEDLVLTSMSFR					Mascot
1428.6866	1428.6201	-0.0665	-47	120	131	INRTTDGFDFSR					Mascot
1534.8159	1534.7111	-0.1048	-68	544	556	KNLHTHTTVVCPK			Carbamidomethyl (C)[11]		Mascot
1549.6931	1549.7175	0.0244	16	144	156	YQHGASWSSANWR					Mascot
1768.8469	1768.8678	0.0209	12	421	436	KINGAAMSVNPMSFNR			Oxidation (M)[7,12]		Mascot
1784.8926	1784.9188	0.0262	15	387	402	AFEDIPTGSHSQNIIR					Mascot
1899.066	1898.926	-0.14	-74	169	184	CLYILLLVSNSVLTYK			Carbamidomethyl (C)[1]		Mascot
1925.9576	1925.9548	-0.0028	-1	522	538	EDNHLAFGVNSPNIRSR					Mascot
3241.478	3241.5405	0.0625	19	474	504	AIPTDHSTGGNISGGNYVEYTSNDVHGEPPR					Mascot
3241.478	3241.5405	0.0625	19	474	504	AIPTDHSTGGNISGGNYVEYTSNDVHGEPPR					Mascot

Gel Idx/Pos	209/I9	Instr./Gel Origin	BA2151/Sample Project 20140814	Process Status	Analysis Succeeded
Plate [#] Name	[1] Sample Project 20140814	Instrument Sample Name		Spectra	11

Rank	Protein Name	Accession No.	Protein MW	Protein PI	Pep. Count	Protein Score	Protein Score C. I. %	Intensity Matched	Total Ion Score	Total Ion C. I. %	Confirmed
1	Vitellogenin-3 OS=Solenopsis invicta PE=2 SV=1	VIT3_SOLIN	202010.1	8.44	30	67	89.211	25.72			

Peptide Information

Calc. Mass	Obsrv. Mass	± da	± ppm	Start Seq.	End Seq.	Sequence	Ion Score	C. I. %	Modification	Rank	Result Type
921.4887	921.4862	-0.0025	-3	1071	1078	IESDTTKK					Mascot
987.5258	987.5131	-0.0127	-13	1146	1153	LDWKPNSK					Mascot
1046.5365	1046.4967	-0.0398	-38	519	528	GEEAAELLSK					Mascot
1060.4471	1060.5336	0.0865	82	239	246	MTKENMMK			Oxidation (M)[1,6,7]		Mascot
1078.5891	1078.5236	-0.0655	-61	47	55	QQYTGIQLK					Mascot
1078.5891	1078.5236	-0.0655	-61	47	55	QQYTGIQLK					Mascot
1106.5259	1106.5458	0.0199	18	1723	1731	NNESMDLKR					Mascot
1187.5613	1187.5753	0.014	12	267	276	SFTIQSTEMK			Oxidation (M)[9]		Mascot
1250.5868	1250.563	-0.0238	-19	1290	1299	QCMEEIQKGK			Carbamidomethyl (C)[2]		Mascot
1269.5933	1269.5499	-0.0434	-34	229	238	QLYHYGMDVK			Oxidation (M)[7]		Mascot
1312.6896	1312.6602	-0.0294	-22	905	915	LKTTAFNNFEK					Mascot
1330.6824	1330.6354	-0.047	-35	126	136	NVPTWEVNMLK					Mascot
1340.7434	1340.6639	-0.0795	-59	115	125	HGVIRDLLFDR					Mascot
1345.6206	1345.6273	0.0067	5	953	963	SGVYETLFCNR			Carbamidomethyl (C)[9]		Mascot
1389.7472	1389.6681	-0.0791	-57	1079	1091	ESVLEDIVTGISK					Mascot
1406.6758	1406.6101	-0.0657	-47	350	361	NSEQENSLETKK					Mascot
1406.6758	1406.6101	-0.0657	-47	350	361	NSEQENSLETKK					Mascot
1420.7444	1420.6115	-0.1329	-94	1536	1547	HVRLYANGATYR					Mascot
1422.7476	1422.608	-0.1396	-98	1065	1077	GPFSIKIESDTTK					Mascot
1422.7476	1422.608	-0.1396	-98	1065	1077	GPFSIKIESDTTK					Mascot
1434.7046	1434.649	-0.0556	-39	1749	1761	IQNFQIPVSCSAA			Carbamidomethyl (C)[10]		Mascot
1545.8119	1545.6985	-0.1134	-73	514	528	SGQVKGEEAAELLSK					Mascot
1613.7815	1613.6996	-0.0819	-51	229	241	QLYHYGMDVKMTK					Mascot
1618.8873	1618.7343	-0.153	-95	101	114	DIPMSGKPFEIKLK			Oxidation (M)[4]		Mascot
1705.8949	1705.821	-0.0739	-43	795	808	YVYVGLDSIFNFLR					Mascot
1845.9349	1845.8745	-0.0604	-33	652	666	YQRMLMVAALYDLSR			Oxidation (M)[4]		Mascot
1864.9122	1864.9387	0.0265	14	1139	1153	EMQHHSKLDWKPNSK					Mascot

Calc. Mass	Obsrv. Mass	± da	± ppm	Start Seq.	End Seq.	Sequence	Ion Score	C. I. %	Modification	Rank	Result Type
1867.9657	1867.9077	-0.058	-31	655	670	MLMVAALYDLSRDIPK			Oxidation (M)[1,3]		Mascot
1898.9185	1898.9174	-0.0011	-1	573	588	NNYVVPSYYPVHSFGR					Mascot
1904.9388	1904.8774	-0.0614	-32	466	480	KQYTELEQYVSQFNK					Mascot
1977.9409	1977.9203	-0.0206	-10	1115	1131	CEIQIDMTFDAVTIHGK			Carbamidomethyl (C)[1]		Mascot
2037.0975	2036.9698	-0.1277	-63	1479	1497	VGNDLKVAINGQNVEIPE K					Mascot
2839.3621	2839.2961	-0.066	-23	864	887	NNLEGHVLFNSVYGSMV YPYDKHR					Mascot

2	Short neurotoxin 1 OS=Enhydrina schistosa PE=1 SV=1	NXS1_ENHSC	7206.1	8.42	7	65	81.678	1.61

Peptide Information

Calc. Mass	Obsrv. Mass	± da	± ppm	Start Seq.	End Seq.	Sequence	Ion Score	C. I. %	Modification	Rank	Result Type
1300.6501	1300.6139	-0.0362	-28	38	49	GCGCPQVKPGIK			Carbamidomethyl (C)[2,4]		Mascot
1337.5574	1337.6232	0.0658	49	2	12	TCCNQQSSQPK			Carbamidomethyl (C)[2,3]		Mascot
1421.5671	1421.6682	0.1011	71	13	24	TTTNCAESSCYK			Carbamidomethyl (C)[5,10]		Mascot
1450.5144	1450.6421	0.1277	88	50	60	LECCHTNECNN			Carbamidomethyl (C)[3,4,9]		Mascot
1468.5978	1468.6727	0.0749	51	1	12	MTCCNQQSSQPK			Carbamidomethyl (C)[3,4]		Mascot
1549.6621	1549.7122	0.0501	32	13	25	TTTNCAESSCYKK			Carbamidomethyl (C)[5,10]		Mascot
1698.8778	1698.7634	-0.1144	-67	35	49	IERGCGCPQVKPGIK			Carbamidomethyl (C)[5,7]		Mascot

3	Short neurotoxin 1 OS=Hydrophis lapemoides PE=1 SV=1	NXS1_HYDLA	7285.2	8.65	7	61	60.827	1.61

Peptide Information

Calc. Mass	Obsrv. Mass	± da	± ppm	Start Seq.	End Seq.	Sequence	Ion Score	C. I. %	Modification	Rank	Result Type
1300.6501	1300.6139	-0.0362	-28	38	49	GCGCPQVKPGIK			Carbamidomethyl (C)[2,4]		Mascot
1337.5574	1337.6232	0.0658	49	2	12	TCCNQQSSQPK			Carbamidomethyl (C)[2,3]		Mascot
1421.5671	1421.6682	0.1011	71	13	24	TTTNCAESSCYK			Carbamidomethyl (C)[5,10]		Mascot
1450.5144	1450.6421	0.1277	88	50	60	LECCHTNECNN			Carbamidomethyl (C)[3,4,9]		Mascot
1468.5978	1468.6727	0.0749	51	1	12	MTCCNQQSSQPK			Carbamidomethyl (C)[3,4]		Mascot
1549.6621	1549.7122	0.0501	32	13	25	TTTNCAESSCYKK			Carbamidomethyl (C)[5,10]		Mascot
1698.8778	1698.7634	-0.1144	-67	35	49	IERGCGCPQVKPGIK			Carbamidomethyl (C)[5,7]		Mascot

4	Nebulin-related-anchoring protein OS=Mus musculus GN=Nrap PE=1 SV=3	NRAP_MOUSE	196716	9.34	31	60	49.536	53.958

Peptide Information

Calc. Mass	Obsrv. Mass	± da	± ppm	Start Seq.	End Seq.	Sequence	Ion Score	C. I. %	Modification	Rank	Result Type

1021.4982	1021.4931	-0.0051	-5	79	89	SVAAMGGIDGK	Oxidation (M)[5]	Mascot
1046.4935	1046.4967	0.0032	3	972	980	DTPEMVQAR		Mascot
1078.4656	1078.5236	0.058	54	1502	1510	MNAMHLSDK	Oxidation (M)[1,4]	Mascot
1078.6144	1078.5236	-0.0908	-84	799	808	LDSLAFLTAK		Mascot
1094.6093	1094.5156	-0.0937	-86	364	372	LVSEVEYKK		Mascot
1106.4684	1106.5458	0.0774	70	415	424	GHYDGVGMDR		Mascot
1117.5961	1117.5424	-0.0537	-48	252	261	RATELASDVR		Mascot
1117.5961	1117.5424	-0.0537	-48	252	261	RATELASDVR		Mascot
1230.63	1230.5531	-0.0769	-62	97	106	SVLHWDMKSK		Mascot
1238.576	1238.5681	-0.0079	-6	226	236	YTEDGGQQRGK		Mascot
1238.576	1238.5681	-0.0079	-6	226	236	YTEDGGQQRGK		Mascot
1252.6532	1252.5513	-0.1019	-81	1435	1445	AGDLISETKYR		Mascot
1273.614	1273.5624	-0.0516	-41	1500	1510	ARMNAMHLSDK		Mascot
1319.6484	1319.59	-0.0584	-44	107	119	AGAGAASRLMNER	Oxidation (M)[10]	Mascot
1320.6907	1320.6083	-0.0824	-62	181	192	AANQLASQVQYK		Mascot
1389.6897	1389.6681	-0.0216	-16	1052	1063	ASSEVISDYKYK		Mascot
1438.7147	1438.5996	-0.1151	-80	166	178	GSFPAMITPAYQR		Mascot
1438.7147	1438.5996	-0.1151	-80	166	178	GSFPAMITPAYQR		Mascot
1484.7856	1484.817	0.0314	21	981	992	ISYTQAVDRLYR		Mascot
1497.7114	1497.7047	-0.0067	-4	1334	1345	MGQLQSENQYRK	Oxidation (M)[1]	Mascot
1619.767	1619.7397	-0.0273	-17	623	635	THFNLPMDMVNLR	Oxidation (M)[7,9]	Mascot
1628.6316	1628.7892	0.1576	97	291	303	CGQVYSEECDEPR	Carbamidomethyl (C)[1,9]	Mascot
1705.873	1705.821	-0.052	-30	1243	1256	HQYTMTLGLPEFIR		Mascot
1779.9463	1779.8074	-0.1389	-78	235	251	GKGSFPAMITPAYQIAK		Mascot
1796.9	1796.8391	-0.0609	-34	304	320	GKGSFPAMITPAYQNAK	Oxidation (M)[8]	Mascot
1796.9	1796.8391	-0.0609	-34	304	320	GKGSFPAMITPAYQNAK	Oxidation (M)[8]	Mascot
1856.8444	1856.8613	0.0169	9	144	159	MVEARQSLGEGYTEDR	Oxidation (M)[1]	Mascot
1864.8528	1864.9387	0.0859	46	272	290	GMASPVGAEGGMTKDS VDR		Mascot
1896.8427	1896.9003	0.0576	30	272	290	GMASPVGAEGGMTKDS VDR	Oxidation (M)[2,12]	Mascot
1905.0051	1904.8774	-0.1277	-67	1243	1258	HQYTMTLGLPEFIRAK		Mascot
1954.9658	1954.9196	-0.0462	-24	1295	1311	ASGDIASDFLYRHEFVK		Mascot
1955.9579	1955.949	-0.0089	-5	623	638	THFNLPMDMVNLRHAK	Oxidation (M)[7,9]	Mascot
1955.9579	1955.949	-0.0089	-5	623	638	THFNLPMDMVNLRHAK	Oxidation (M)[7,9]	Mascot
1978.0314	1977.9203	-0.1111	-56	599	615	LLHSLQVAKMSSEVEYK	Oxidation (M)[10]	Mascot
2036.9607	2036.9698	0.0091	4	379	395	GHSINYCETPQFRNVSK	Carbamidomethyl (C)[7]	Mascot
2143.0642	2142.9604	-0.1038	-48	671	688	AYGLQSELQYKADLAWM R		Mascot

2236.0696	2236.1265	0.0569	25	830	850	LIGAKSAQGDSQMSHSL QMSK			Oxidation (M)[13,19]		Mascot
2236.0696	2236.1265	0.0569	25	830	850	LIGAKSAQGDSQMSHSL QMSK			Oxidation (M)[13,19]		Mascot

5	Bifunctional DNA-directed RNA polymerase subunit beta-beta' OS=Helicobacter pylori (strain J99) GN=rpoBC PE=3 SV=1	RPOBC_HELPJ	324458.9	6.61	41	58	14.3	55.818

Peptide Information

Calc. Mass	Obsrv. Mass	± da	± ppm	Start Seq.	End Seq.	Sequence	Ion Score	C. I. %	Modification	Rank	Result Type
856.5111	856.4855	-0.0256	-30	2338	2344	NIIANRR					Mascot
1021.4917	1021.4931	0.0014	1	693	701	ALMGTNMQR					Mascot
1036.5422	1036.4789	-0.0633	-61	2794	2801	LFKEENTR					Mascot
1078.4623	1078.5236	0.0613	57	966	973	LDMEHFDR			Oxidation (M)[3]		Mascot
1078.5813	1078.5236	-0.0577	-53	2841	2850	VLTEASIAMK			Oxidation (M)[9]		Mascot
1094.5953	1094.5156	-0.0797	-73	2155	2164	TANAGYLTRK					Mascot
1110.5249	1110.5044	-0.0205	-18	2263	2272	CYGLNLGEGK			Carbamidomethyl (C)[1]		Mascot
1117.575	1117.5424	-0.0326	-29	725	734	DSWGAIKANR					Mascot
1117.575	1117.5424	-0.0326	-29	725	734	DSWGAIKANR					Mascot
1139.5436	1139.5282	-0.0154	-14	2072	2081	EMMSAIAKDK			Oxidation (M)[2]		Mascot
1205.595	1205.5809	-0.0141	-12	1409	1418	IHSWSYGEVK					Mascot
1242.5891	1242.558	-0.0311	-25	2069	2079	MSKEMMSAIAK			Oxidation (M)[1]		Mascot
1249.5956	1249.564	-0.0316	-25	1257	1266	VNVGYMYMIK			Oxidation (M)[6,8]		Mascot
1300.5839	1300.6139	0.03	23	1242	1252	MDLYDGRTGEK			Oxidation (M)[1]		Mascot
1312.6532	1312.6602	0.007	5	1649	1659	FAVSDVNELYR					Mascot
1319.6743	1319.59	-0.0843	-64	2325	2334	FYNLRTYTNK					Mascot
1320.6827	1320.6083	-0.0744	-56	1321	1331	EMLTIKSDDIR					Mascot
1323.625	1323.6306	0.0056	4	252	262	MEFDLKDPQGK			Oxidation (M)[1]		Mascot
1340.7606	1340.6639	-0.0967	-72	1671	1682	LMELGAPEIIVR					Mascot
1404.7377	1404.6033	-0.1344	-96	2097	2109	GSAAQIRQLSAMR			Oxidation (M)[12]		Mascot
1406.7063	1406.6101	-0.0962	-68	2467	2477	YYVLEANHLER					Mascot
1406.7063	1406.6101	-0.0962	-68	2467	2477	YYVLEANHLER					Mascot
1454.6798	1454.5496	-0.1302	-90	835	846	DDVFTSTHIYEK					Mascot
1468.7543	1468.6727	-0.0816	-56	1649	1660	FAVSDVNELYRR					Mascot
1484.6873	1484.817	0.1297	87	1236	1248	IAMDGKMDLYDGR					Mascot
1490.7771	1490.7065	-0.0706	-47	2011	2024	AGISISMEDIITPK			Oxidation (M)[7]		Mascot
1507.7904	1507.6989	-0.0915	-61	239	251	YLIPFASLDANQR					Mascot
1511.7411	1511.6993	-0.0418	-28	1151	1163	EFGKQIASMLEDK			Oxidation (M)[9]		Mascot

Calc. Mass	Obsrv. Mass	± da	± ppm	Start Seq.	End Seq.	Sequence	Ion Score	C. I. %	Modification	Rank	Result Type
1698.8657	1698.7634	-0.1023	-60	1007	1021	EGDQIPKEEIASINR					Mascot
1705.9227	1705.821	-0.1017	-60	1871	1886	VLMLSSMNILLPASGK			Oxidation (M)[3,7]		Mascot
1717.9041	1717.8705	-0.0336	-20	2011	2026	AGISISMEDIITPKDK					Mascot
1732.9303	1732.8687	-0.0616	-36	1211	1226	GVKMAIPVFEGISQEK					Mascot
1796.9066	1796.8391	-0.0675	-38	832	846	ITKDDVFTSTHIYEK					Mascot
1796.9066	1796.8391	-0.0675	-38	832	846	ITKDDVFTSTHIYEK					Mascot
1812.9126	1812.8297	-0.0829	-46	754	768	EEAYIDAYSLQKNLR					Mascot
1867.8743	1867.9077	0.0334	18	1528	1544	EPGEAAYDNEGTKLVMK			Oxidation (M)[16]		Mascot
1884.0437	1883.8615	-0.1822	-97	2390	2408	SDIVKPSELAGVGGKIEG K					Mascot
1896.955	1896.9003	-0.0547	-29	2822	2840	AAIGSDSIISAASFQETTK					Mascot
1904.9812	1904.8774	-0.1038	-54	2614	2632	TSIAISDGSSVEQAEVLAK					Mascot
1926.9808	1926.8483	-0.1325	-69	852	868	ELKHGVEEFTADIPDVK					Mascot
1938.0253	1937.9448	-0.0805	-42	2007	2024	YATKAGISISMEDIITPK					Mascot
1954.0201	1953.9296	-0.0905	-46	2007	2024	YATKAGISISMEDIITPK			Oxidation (M)[11]		Mascot
1956.0293	1955.949	-0.0803	-41	1742	1759	SVIVVGPNLKMDECGLPK			Carbamidomethyl (C)[14]		Mascot
1956.0293	1955.949	-0.0803	-41	1742	1759	SVIVVGPNLKMDECGLPK			Carbamidomethyl (C)[14]		Mascot
1972.0243	1971.9243	-0.1	-51	1742	1759	SVIVVGPNLKMDECGLPK			Carbamidomethyl (C)[14], Oxidation (M)[11]		Mascot
2037.1161	2036.9698	-0.1463	-72	467	485	AVGELLANELHSGLVKM QK					Mascot
2236.1343	2236.1265	-0.0078	-3	2365	2384	IETVYEEVVVSVKNGDQE AK					Mascot
2236.1343	2236.1265	-0.0078	-3	2365	2384	IETVYEEVVVSVKNGDQE AK					Mascot

6	Enhancer of yellow 2 transcription factor OS=Drosophila erecta GN=e(y)2 PE=3 SV=1	ENY2_DROER	11650.9	4.7	8	56	0	14.898

Peptide Information

Calc. Mass	Obsrv. Mass	± da	± ppm	Start Seq.	End Seq.	Sequence	Ion Score	C. I. %	Modification	Rank	Result Type
856.5138	856.4855	-0.0283	-33	69	76	TLVPDAIK					Mascot
921.4247	921.4862	0.0615	67	28	34	LTECGWR			Carbamidomethyl (C)[4]		Mascot
1078.5132	1078.5236	0.0104	10	35	42	DEVRLMCR			Carbamidomethyl (C)[7]		Mascot
1078.5132	1078.5236	0.0104	10	35	42	DEVRLMCR			Carbamidomethyl (C)[7]		Mascot
1094.5082	1094.5156	0.0074	7	35	42	DEVRLMCR			Carbamidomethyl (C)[7], Oxidation (M)[6]		Mascot
1420.6638	1420.6115	-0.0523	-37	28	38	LTECGWRDEVR			Carbamidomethyl (C)[4]		Mascot
1812.8796	1812.8297	-0.0499	-28	1	17	MSVSAAVDQYTVLTGDR					Mascot
1896.9662	1896.9003	-0.0659	-35	2	19	SVSAAVDQYTVLTGDRS K					Mascot
1978.0128	1977.9203	-0.0925	-47	49	66	GTNNSFTVEQLITEVTPK					Mascot
2218.9834	2219.0229	0.0395	18	83	101	IRTILTENEEEADEPEDES					Mascot

7	Dystonin OS=Homo sapiens GN=DST PE=1 SV=4	DYST_HUMAN	865259.2	5.14	63	56	0	37.725

Peptide Information

Calc. Mass	Obsrv. Mass	± da	± ppm	Start Seq.	End Seq.	Sequence	Ion Score	C. I. %	Modification	Rank	Result Type
921.4346	921.4862	0.0516	56	6090	6097	NVSVDMEK					Mascot
987.5356	987.5131	-0.0225	-23	6650	6657	EQIIELDK					Mascot
1036.5092	1036.4789	-0.0303	-29	7380	7387	TNMELREK			Oxidation (M)[3]		Mascot
1046.5411	1046.4967	-0.0444	-42	5261	5268	QCNKLLDR			Carbamidomethyl (C)[2]		Mascot
1060.5634	1060.5336	-0.0298	-28	5203	5211	DAETLQKQK					Mascot
1078.4656	1078.5236	0.058	54	2652	2660	MHSCSSLEK			Carbamidomethyl (C)[4]		Mascot
1078.5375	1078.5236	-0.0139	-13	6116	6126	SGGTDKDISAK					Mascot
1094.4606	1094.5156	0.055	50	2652	2660	MHSCSSLEK			Carbamidomethyl (C)[4], Oxidation (M)[1]		Mascot
1113.5106	1113.45	-0.0606	-54	289	297	HHVTTMSER			Oxidation (M)[6]		Mascot
1117.5273	1117.5424	0.0151	14	1097	1105	DDLHESVFR					Mascot
1117.5273	1117.5424	0.0151	14	1097	1105	DDLHESVFR					Mascot
1139.5765	1139.5282	-0.0483	-42	1239	1248	AKAISDEMFK					Mascot
1141.5889	1141.5007	-0.0882	-77	5064	5072	FKEFQEVSK					Mascot
1147.5413	1147.5582	0.0169	15	4974	4982	CQNNLEEIK			Carbamidomethyl (C)[1]		Mascot
1182.5287	1182.5426	0.0139	12	2548	2557	FDDSGSWRGR					Mascot
1193.5765	1193.5795	0.003	3	1699	1707	VLERQNMCK			Carbamidomethyl (C)[8], Oxidation (M)[7]		Mascot
1205.6525	1205.5809	-0.0716	-59	4070	4080	HLSEPIAVDPK					Mascot
1230.6549	1230.5531	-0.1018	-83	687	696	LLNTSRNQER					Mascot
1242.5485	1242.558	0.0095	8	2315	2325	YISNTSGEDEK					Mascot
1249.5961	1249.564	-0.0321	-26	1957	1966	FQPSTVHDYR					Mascot
1252.6644	1252.5513	-0.1131	-90	3005	3015	EHLQLGVNNTK					Mascot
1269.6508	1269.5499	-0.1009	-79	5100	5109	YLTMLQTQQK			Oxidation (M)[4]		Mascot
1273.5089	1273.5624	0.0535	42	173	181	CENFTTCWR			Carbamidomethyl (C)[1,7]		Mascot
1300.6631	1300.6139	-0.0492	-38	318	328	ETEIPPKETEK					Mascot
1312.6995	1312.6602	-0.0393	-30	7060	7070	EVIPQEIEEVK					Mascot
1319.5936	1319.59	-0.0036	-3	1364	1373	DYELQTMTYR					Mascot
1323.7267	1323.6306	-0.0961	-73	5809	5819	YRLVSDTITQK					Mascot
1330.7002	1330.6354	-0.0648	-49	708	718	ATNELIWLNEK					Mascot
1331.6372	1331.5981	-0.0391	-29	5752	5763	GEEASQAQMRPK					Mascot
1340.7256	1340.6639	-0.0617	-46	44	53	WINQHLMKVR			Oxidation (M)[7]		Mascot
1347.6322	1347.5935	-0.0387	-29	5752	5763	GEEASQAQMRPK			Oxidation (M)[9]		Mascot
1347.6652	1347.5935	-0.0717	-53	4242	4253	DLSARFSEASHK					Mascot

1389.7195	1389.6681	-0.0514	-37	6960	6970	TLIDQHKEFMK		Mascot
1404.7118	1404.6033	-0.1085	-77	3966	3977	QKSFSEDVISHK		Mascot
1406.6581	1406.6101	-0.048	-34	5221	5233	LEALMASNDNANK	Oxidation (M)[5]	Mascot
1406.7274	1406.6101	-0.1173	-83	4560	4571	SFEAELKQNVNK		Mascot
1410.686	1410.604	-0.082	-58	2098	2110	NTVINSEFSQSGK		Mascot
1422.6859	1422.608	-0.0779	-55	3706	3717	NTENFLKENGEK		Mascot
1422.7332	1422.608	-0.1252	-88	1336	1347	MLVSEIEMKQSK		Mascot
1438.728	1438.5996	-0.1284	-89	1336	1347	MLVSEIEMKQSK	Oxidation (M)[1]	Mascot
1438.728	1438.5996	-0.1284	-89	1336	1347	MLVSEIEMKQSK	Oxidation (M)[1]	Mascot
1490.6838	1490.7065	0.0227	15	2652	2663	MHSCSSLEKQQR	Carbamidomethyl (C)[4]	Mascot
1511.6794	1511.6993	0.0199	13	6315	6326	SEAYQQQIEMER		Mascot
1530.8123	1530.7283	-0.084	-55	5123	5136	RLAQDLVVEASDSK		Mascot
1545.7279	1545.6985	-0.0294	-19	6726	6739	SLDSELEIANDPDK		Mascot
1562.8148	1562.7217	-0.0931	-60	40	51	TFTKWINQHLMK	Oxidation (M)[11]	Mascot
1573.6522	1573.7382	0.086	55	173	184	CENFTTCWRDGK	Carbamidomethyl (C)[1,7]	Mascot
1613.7112	1613.6996	-0.0116	-7	2057	2070	EKDECTATPSSFNK	Carbamidomethyl (C)[5]	Mascot
1619.8058	1619.7397	-0.0661	-41	1794	1807	MTVEEAVREGVIDR	Oxidation (M)[1]	Mascot
1628.7446	1628.7892	0.0446	27	908	921	DDECVLANNSHRAK	Carbamidomethyl (C)[4]	Mascot
1678.8582	1678.7368	-0.1214	-72	6903	6916	WETVCALSISKQTR	Carbamidomethyl (C)[5]	Mascot
1698.7462	1698.7634	0.0172	10	4518	4531	AQEESSAMMQWLQK	Oxidation (M)[8,9]	Mascot
1717.8538	1717.8705	0.0167	10	5752	5766	GEEASQAQMRPKELK	Oxidation (M)[9]	Mascot
1779.8364	1779.8074	-0.029	-16	5221	5236	LEALMASNDNANKTCK	Carbamidomethyl (C)[15]	Mascot
1818.9531	1818.8351	-0.118	-65	5985	5999	ELIAEHKPHIDKMNK	Oxidation (M)[13]	Mascot
1838.8412	1838.8784	0.0372	20	7071	7085	ALIAEHQTFMEEMTR	Oxidation (M)[10,13]	Mascot
1845.8978	1845.8745	-0.0233	-13	1097	1111	DDLHESVFRITEQEK		Mascot
1857.0441	1856.8613	-0.1828	-98	5706	5722	LSTDVAKTLEQALQLAR		Mascot
1896.9194	1896.9003	-0.0191	-10	1389	1404	MQSSADLIIQEFMDLR		Mascot
1904.8984	1904.8774	-0.021	-11	5083	5099	EQLDIHDSLGSQAYSNK		Mascot
1910.9415	1910.8895	-0.052	-27	1190	1205	LYETKLCEEEAVIADK	Carbamidomethyl (C)[7]	Mascot
1926.9708	1926.8483	-0.1225	-64	349	364	LLQGYHPNDIEKEWGK		Mascot
1937.9862	1937.9448	-0.0414	-21	999	1015	TMLPGEHQQVLSNLQSR		Mascot
1953.9811	1953.9296	-0.0515	-26	999	1015	TMLPGEHQQVLSNLQSR	Oxidation (M)[2]	Mascot
1954.9691	1954.9196	-0.0495	-25	7231	7247	FPTSRLEMSAVADIFDR		Mascot
2037.0447	2036.9698	-0.0749	-37	7388	7406	FILADGASQGMAAFRPR GR	Oxidation (M)[11]	Mascot
2219.1047	2219.0229	-0.0818	-37	1708	1726	DLIDPCTSEKVSLIDMVQ R	Carbamidomethyl (C)[6]	Mascot
3382.7417	3382.7234	-0.0183	-5	6000	6030	TGPQLLELSPGEGFSIQE KYVAADTLYSQIK		Mascot

3399.7141	3399.7427	0.0286	8	5724	5751	LHSTHEELCTWLDKVEV ELLSYETQVLK			Carbamidomethyl (C)[9]		Mascot

8 Tyrosine--tRNA ligase OS=Nitrosospira multiformis (strain ATCC 25196 / NCIMB 11849) GN=tyrS PE=3 SV=1 | SYY_NITMU | 45510.6 | 6.78 | 13 | 55 | 0 | 4.03

Peptide Information

Calc. Mass	Obsrv. Mass	± da	± ppm	Start Seq.	End Seq.	Sequence	Ion Score	C. I. %	Modification	Rank	Result Type
1110.5249	1110.5044	-0.0205	-18	147	155	MLERDDFGK					Mascot
1187.5553	1187.5753	0.02	17	274	283	QWQQDVGNGR					Mascot
1252.6355	1252.5513	-0.0842	-67	394	403	FARVTICEEK			Carbamidomethyl (C)[7]		Mascot
1396.6599	1396.5894	-0.0705	-50	249	259	LMSISDELMWR			Oxidation (M)[2]		Mascot
1418.7308	1418.6205	-0.1103	-78	359	371	MIEQGGVKLNGEK			Oxidation (M)[1]		Mascot
1422.6495	1422.608	-0.0415	-29	304	316	QDAEAALADFESR					Mascot
1422.6495	1422.608	-0.0415	-29	304	316	QDAEAALADFESR	3	0			Mascot
1434.7444	1434.649	-0.0954	-66	379	391	LSCGQMVIVQVGK			Carbamidomethyl (C)[3], Oxidation (M)[6]		Mascot
1468.8053	1468.6727	-0.1326	-90	138	150	LAATHTVARMLER					Mascot
1484.8003	1484.817	0.0167	11	138	150	LAATHTVARMLER			Oxidation (M)[10]		Mascot
1530.8447	1530.7283	-0.1164	-76	1	13	MTIPISEQIEIIK			Oxidation (M)[1]		Mascot
1818.8903	1818.8351	-0.0552	-30	14	28	RGCSELLLEDELEQK			Carbamidomethyl (C)[3]		Mascot
1838.9617	1838.8784	-0.0833	-45	376	391	TFRLSCGQMVIVQVGK			Carbamidomethyl (C)[6], Oxidation (M)[9]		Mascot
1911.0043	1910.8895	-0.1148	-60	87	103	NTTRPPLSREQVAENAK					Mascot
1978.0062	1977.9203	-0.0859	-43	183	200	ADIELGGTDQKFNLLMGR					Mascot

9 Aspartyl/glutamyl-tRNA(Asn/Gln) amidotransferase subunit B OS=Clostridium botulinum (strain 657 / Type Ba4) GN=gatB PE=3 SV=1 | GATB_CLOB6 | 54317.7 | 5.55 | 16 | 54 | 0 | 8.771

Peptide Information

Calc. Mass	Obsrv. Mass	± da	± ppm	Start Seq.	End Seq.	Sequence	Ion Score	C. I. %	Modification	Rank	Result Type
1036.4979	1036.4789	-0.019	-18	468	476	LVSEEANKM			Oxidation (M)[9]		Mascot
1139.5765	1139.5282	-0.0483	-42	390	399	VLDDMFKSGK					Mascot
1230.6437	1230.5531	-0.0906	-74	239	248	GEILEQETRR					Mascot
1300.726	1300.6139	-0.1121	-86	365	375	FNPEQLAQLIK					Mascot
1320.6365	1320.6083	-0.0282	-21	340	351	AASNWLMGDISR					Mascot
1331.6089	1331.5981	-0.0108	-8	79	88	KNYFYPDCPK			Carbamidomethyl (C)[8]		Mascot
1337.6617	1337.6232	-0.0385	-29	173	184	SILSSIEVSDCK			Carbamidomethyl (C)[11]		Mascot
1406.7427	1406.6101	-0.1326	-94	301	312	AERFVNEFGIPK					Mascot

Calc. Mass	Obsrv. Mass	± da	± ppm	Start Seq.	End Seq.	Sequence	Ion Score	C. I. %	Modification	Rank	Result Type
1406.7427	1406.6101	-0.1326	-94	301	312	AERFVNEFGIPK					Mascot
1420.689	1420.6115	-0.0775	-55	249	260	WDEANSVTVLMR					Mascot
1613.7952	1613.6996	-0.0956	-59	117	130	IGIERIHMEEDAGK			Oxidation (M)[8]		Mascot
1618.7526	1618.7343	-0.0183	-11	313	326	YDAMVLTLTMDMAK			Oxidation (M)[4]		Mascot
1635.7795	1635.7322	-0.0473	-29	89	101	NYQITQDEVPICR			Carbamidomethyl (C)[12]		Mascot
1651.8109	1651.7448	-0.0661	-40	249	262	WDEANSVTVLMRSK			Oxidation (M)[11]		Mascot
1838.9607	1838.8784	-0.0823	-45	232	247	HVEAVTKGEILEQETR					Mascot
1845.9341	1845.8745	-0.0596	-32	426	441	IIENNPQSIEDFKNGK					Mascot
1868.0752	1867.9077	-0.1675	-90	365	380	FNPEQLAQLIKLINAR					Mascot

10	UDP-N-acetylhexosamine pyrophosphorylase-like protein 1 OS=Xenopus tropicalis GN=uap1l1 PE=2 SV=1	UAP1L_XENTR	57718.8	5.39	15	54	0	30.949

Peptide Information

Calc. Mass	Obsrv. Mass	± da	± ppm	Start Seq.	End Seq.	Sequence	Ion Score	C. I. %	Modification	Rank	Result Type
941.5414	941.5354	-0.006	-6	349	357	AVIGSLEPR					Mascot
1094.5994	1094.5156	-0.0838	-77	400	408	NFVAFEVLR					Mascot
1110.5249	1110.5044	-0.0205	-18	198	206	SDVVMFEQR					Mascot
1127.5375	1127.4762	-0.0613	-54	51	58	ELREHCQR			Carbamidomethyl (C)[6]		Mascot
1208.5502	1208.5681	0.0179	15	4	14	SESAESAESRR					Mascot
1273.6132	1273.5624	-0.0508	-40	66	76	ESSAPQRLDDR					Mascot
1323.5771	1323.6306	0.0535	40	2	13	DRSESAESAESR					Mascot
1454.6177	1454.5496	-0.0681	-47	1	13	MDRSESAESAESR					Mascot
1530.7013	1530.7283	0.027	18	186	197	FFEDHAYFGLER					Mascot
1549.7792	1549.7122	-0.067	-43	223	237	AKLAMAPDGNGGLYR			Oxidation (M)[5]		Mascot
1618.7854	1618.7343	-0.0511	-32	238	251	ALSDNRILEDMEGR					Mascot
1635.824	1635.7322	-0.0918	-56	387	399	MEKFVFDVFQFAK					Mascot
1651.819	1651.7448	-0.0742	-45	387	399	MEKFVFDVFQFAK			Oxidation (M)[1]		Mascot
1845.9779	1845.8745	-0.1034	-56	207	224	MLPAVGFDGAAILEDKAK					Mascot
1867.9987	1867.9077	-0.091	-49	125	142	LGVTYPKGMYSVGLPSAK					Mascot
1883.9935	1883.8615	-0.132	-70	125	142	LGVTYPKGMYSVGLPSAK			Oxidation (M)[9]		Mascot
1956.0009	1955.949	-0.0519	-27	73	89	LDDRMQPVPPEFLGSVR					Mascot
1956.0009	1955.949	-0.0519	-27	73	89	LDDRMQPVPPEFLGSVR					Mascot
1971.9957	1971.9243	-0.0714	-36	73	89	LDDRMQPVPPEFLGSVR			Oxidation (M)[5]		Mascot

Gel Idx/Pos	210/I10	Instr./Gel Origin	BA2151/Sample Project 20140814	Process Status	Analysis Succeeded
Plate [#] Name	[1] Sample Project 20140814	Instrument Sample Name		Spectra	11

Rank	Protein Name	Accession No.	Protein MW	Protein PI	Pep. Count	Protein Score	Protein Score C. I. %	Intensity Matched	Total Ion Score	Total Ion C. I. %	Confirmed
1	1-Cys peroxiredoxin PER1 OS=Triticum aestivum GN=PER1 PE=2 SV=1	REHY_WHEAT	24178.2	6.08	13	195	100	20.187	112	100	

Peptide Information

Calc. Mass	Obsrv. Mass	± da	± ppm	Start Seq.	End Seq.	Sequence	Ion Score	C. I. %	Modification	Rank	Result Type
878.4036	878.4022	-0.0014	-2	151	157	NMDEVVR			Oxidation (M)[2]		Mascot
972.4745	972.4695	-0.005	-5	119	127	DAEGQLPSR					Mascot
979.5571	979.5389	-0.0182	-19	128	136	TLHIVGPDK					Mascot
988.5673	988.5319	-0.0354	-36	158	167	AVDSLLTAAK					Mascot
1107.5681	1107.5558	-0.0123	-11	84	93	DIEAYKPGSK					Mascot
1253.7212	1253.6019	-0.1193	-95	158	169	AVDSLLTAAKHK					Mascot
1277.6195	1277.6052	-0.0143	-11	94	104	VTYPIMADPDR					Mascot
1293.6144	1293.5979	-0.0165	-13	94	104	VTYPIMADPDR	24	0	Oxidation (M)[6]		Mascot
1300.6355	1300.6293	-0.0062	-5	140	150	LSFLYPSCTGR			Carbamidomethyl (C)[8]		Mascot
1300.6355	1300.6293	-0.0062	-5	140	150	LSFLYPSCTGR	88	100	Carbamidomethyl (C)[8]		Mascot
1471.7209	1471.7075	-0.0134	-9	67	79	LLGISCDDVQSHK			Carbamidomethyl (C)[6]		Mascot
1583.7411	1583.6924	-0.0487	-31	196	209	MFPQGFETADLPSK			Oxidation (M)[1]		Mascot
1711.8361	1711.7836	-0.0525	-31	196	210	MFPQGFETADLPSKK			Oxidation (M)[1]		Mascot
2064.0608	2064.0044	-0.0564	-27	2	21	PGLTIGDTVPNLELDSTHGK					Mascot
2144.0264	2143.9248	-0.1016	-47	140	157	LSFLYPSCTGRNMDEVVR			Carbamidomethyl (C)[8]		Mascot

Rank	Protein Name	Accession No.	Protein MW	Protein PI	Pep. Count	Protein Score	Protein Score C. I. %	Intensity Matched	Total Ion Score	Total Ion C. I. %	Confirmed
2	1-Cys peroxiredoxin PER1 OS=Hordeum vulgare GN=PER1 PE=2 SV=1	REHY_HORVU	24176.3	6.31	12	186	100	20.021	112	100	

Peptide Information

Calc. Mass	Obsrv. Mass	± da	± ppm	Start Seq.	End Seq.	Sequence	Ion Score	C. I. %	Modification	Rank	Result Type
878.4036	878.4022	-0.0014	-2	151	157	NMDEVVR			Oxidation (M)[2]		Mascot
979.5571	979.5389	-0.0182	-19	128	136	TLHIVGPDK					Mascot
988.5673	988.5319	-0.0354	-36	158	167	AVDSLLTAAK					Mascot
1107.5681	1107.5558	-0.0123	-11	84	93	DIEAYKPGSK					Mascot
1253.7212	1253.6019	-0.1193	-95	158	169	AVDSLLTAAKHK					Mascot
1277.6195	1277.6052	-0.0143	-11	94	104	VTYPIMADPDR					Mascot

1293.6144	1293.5979	-0.0165	-13	94	104	VTYPIMADPDR	24	0	Oxidation (M)[6]		Mascot
1300.6355	1300.6293	-0.0062	-5	140	150	LSFLYPSCTGR			Carbamidomethyl (C)[8]		Mascot
1300.6355	1300.6293	-0.0062	-5	140	150	LSFLYPSCTGR	88	100	Carbamidomethyl (C)[8]		Mascot
1471.7209	1471.7075	-0.0134	-9	67	79	LLGISCDDVQSHK			Carbamidomethyl (C)[6]		Mascot
1583.7411	1583.6924	-0.0487	-31	196	209	MFPQGFETADLPSK			Oxidation (M)[1]		Mascot
1711.8361	1711.7836	-0.0525	-31	196	210	MFPQGFETADLPSKK			Oxidation (M)[1]		Mascot
2064.0608	2064.0044	-0.0564	-27	2	21	PGLTIGDTVPNLELDSTHGK					Mascot
2144.0264	2143.9248	-0.1016	-47	140	157	LSFLYPSCTGRNMDEVVR			Carbamidomethyl (C)[8]		Mascot

3 Probable 1-Cys peroxiredoxin (Fragment) OS=Bromus secalinus PE=2 SV=1 REHY_BROSE 22593.6 8.24 10 164 100 18.883 112 100

Peptide Information

Calc. Mass	Obsrv. Mass	± da	± ppm	Start Seq.	End Seq.	Sequence	Ion Score	C. I. %	Modification	Rank	Result Type
878.4036	878.4022	-0.0014	-2	135	141	NMDEVVR			Oxidation (M)[2]		Mascot
972.4745	972.4695	-0.005	-5	103	111	DAEGQLPSR					Mascot
979.5571	979.5389	-0.0182	-19	112	120	TLHIVGPDK					Mascot
988.5673	988.5319	-0.0354	-36	142	151	AVDSLLTAAK					Mascot
1107.5681	1107.5558	-0.0123	-11	68	77	DIEAYKPGSK					Mascot
1253.7212	1253.6019	-0.1193	-95	142	153	AVDSLLTAAKHK					Mascot
1277.6195	1277.6052	-0.0143	-11	78	88	VTYPIMADPDR					Mascot
1293.6144	1293.5979	-0.0165	-13	78	88	VTYPIMADPDR	24	0	Oxidation (M)[6]		Mascot
1300.6355	1300.6293	-0.0062	-5	124	134	LSFLYPSCTGR			Carbamidomethyl (C)[8]		Mascot
1300.6355	1300.6293	-0.0062	-5	124	134	LSFLYPSCTGR	88	100	Carbamidomethyl (C)[8]		Mascot
1471.7209	1471.7075	-0.0134	-9	51	63	LLGISCDDVQSHK			Carbamidomethyl (C)[6]		Mascot
2144.0264	2143.9248	-0.1016	-47	124	141	LSFLYPSCTGRNMDEVVR			Carbamidomethyl (C)[8]		Mascot

4 Lamin-A OS=Gallus gallus GN=LMNA PE=2 SV=1 LMNA_CHICK 73348.5 6.5 20 58 18.157 6.878

Peptide Information

Calc. Mass	Obsrv. Mass	± da	± ppm	Start Seq.	End Seq.	Sequence	Ion Score	C. I. %	Modification	Rank	Result Type
873.4424	873.5043	0.0619	71	156	162	NLENEVR					Mascot
917.4938	917.4501	-0.0437	-48	171	179	LEGALSEAK					Mascot
958.4952	958.4544	-0.0408	-43	388	396	LSPSPSSQR					Mascot
978.4785	978.5108	0.0323	33	1	8	MSTPSQRR			Oxidation (M)[1]		Mascot
1048.5092	1048.5846	0.0754	72	181	188	QLQDEMLR			Oxidation (M)[6]		Mascot

Calc. Mass	Obsrv. Mass	± da	± ppm	Start Seq.	End Seq.	Sequence	Ion Score	C. I. %	Modification	Rank	Result Type
1076.6462	1076.5466	-0.0996	-93	41	49	LAVYIDKVR					Mascot
1085.6677	1085.5709	-0.0968	-89	101	109	LQLELSKVR					Mascot
1094.5841	1094.5343	-0.0498	-45	265	274	TYGAKLENAK					Mascot
1117.5233	1117.5487	0.0254	23	416	425	LEDGEGREGR					Mascot
1145.5909	1145.5634	-0.0275	-24	270	279	LENAKQSAER					Mascot
1145.5909	1145.5634	-0.0275	-24	270	279	LENAKQSAER					Mascot
1243.6641	1243.6019	-0.0622	-50	378	387	LLEGEEERLR					Mascot
1314.676	1314.618	-0.058	-44	400	413	SSGLQHSGAGSAKK					Mascot
1324.6566	1324.5721	-0.0845	-64	366	376	LALDMEINAYR			Oxidation (M)[5]		Mascot
1406.6404	1406.6166	-0.0238	-17	588	600	TVLCGTCGQPADK			Carbamidomethyl (C)[4,7]		Mascot
1438.7397	1438.62	-0.1197	-83	221	232	HETRLVEIDNGR					Mascot
1488.7078	1488.7438	0.036	24	459	471	SNEDQALGNWQVK					Mascot
1520.7373	1520.7249	-0.0124	-8	529	542	TALINSNGEEVAMR			Oxidation (M)[13]		Mascot
1535.7925	1535.8141	0.0216	14	620	635	GYRSSGGGIGEGLLGR					Mascot
1648.8323	1648.7747	-0.0576	-35	529	543	TALINSNGEEVAMRK			Oxidation (M)[13]		Mascot
1979.9352	1979.9379	0.0027	1	280	297	NSSMAGAAHEELQQTHIR					Mascot

5	DNA-directed RNA polymerase subunit beta OS=Anaplasma phagocytophilum GN=rpoB PE=3 SV=1	RPOB_ANAPH	156473.6	5.36	25	57	0	14.04

Peptide Information

Calc. Mass	Obsrv. Mass	± da	± ppm	Start Seq.	End Seq.	Sequence	Ion Score	C. I. %	Modification	Rank	Result Type
837.4213	837.456	0.0347	41	1255	1261	SGQKFDR					Mascot
856.441	856.5192	0.0782	91	872	879	DTPLGPEK					Mascot
978.5288	978.5108	-0.018	-18	1044	1052	ISAKVADMK			Oxidation (M)[8]		Mascot
979.5571	979.5389	-0.0182	-19	258	265	VLYVRSDK					Mascot
988.5033	988.5319	0.0286	29	669	677	HGGNFVMVK					Mascot
1007.4761	1007.476	-0.0001	0	710	718	ALMGSNMQR					Mascot
1094.559	1094.5343	-0.0247	-23	980	988	QSEVNSFRK					Mascot
1106.6357	1106.5503	-0.0854	-77	269	277	VPFVVDRFK					Mascot
1182.6517	1182.551	-0.1007	-85	222	230	DIIYFRIDK					Mascot
1229.7365	1229.6184	-0.1181	-96	758	767	VDRVLYPVIR					Mascot
1243.6138	1243.6019	-0.0119	-10	1333	1343	SDDTSPGRRPR					Mascot
1259.642	1259.5912	-0.0508	-40	615	624	YGFIESPYRK					Mascot
1314.749	1314.618	-0.131	-100	1262	1272	KVTVGYIYMLK					Mascot
1339.6536	1339.6194	-0.0342	-26	1095	1106	HNLQPGDKMAGR			Oxidation (M)[9]		Mascot
1372.743	1372.7045	-0.0385	-28	968	979	GVEENDRSLLIK					Mascot

1444.8192	1444.7382	-0.081	-56	1372	1383	ELRSLCLDVVLK			Carbamidomethyl (C)[6]		Mascot
1453.8022	1453.7374	-0.0648	-45	1273	1284	LHHLVDDKIHAR					Mascot
1471.7727	1471.7075	-0.0652	-44	1216	1229	RGVPMAAPVFEGPK			Oxidation (M)[5]		Mascot
1490.8512	1490.736	-0.1152	-77	105	117	GITFSVPMRITLR					Mascot
1490.8512	1490.736	-0.1152	-77	105	117	GITFSVPMRITLR					Mascot
1520.8431	1520.7249	-0.1182	-78	975	987	SLLIKQSEVNSFR					Mascot
1648.8462	1648.7747	-0.0715	-43	1195	1209	LKEDVASMSDEALLK					Mascot
1680.8599	1680.7344	-0.1255	-75	789	802	SNHNTCINQRPIVK			Carbamidomethyl (C)[6]		Mascot
1956.0801	1955.9583	-0.1218	-62	1030	1047	SLEALWEIGLSNPKISAK					Mascot
1956.0801	1955.9583	-0.1218	-62	1030	1047	SLEALWEIGLSNPKISAK					Mascot
1979.9532	1979.9379	-0.0153	-8	856	871	DVFTSIHIEEFECVVR			Carbamidomethyl (C)[13]		Mascot
2064.0879	2064.0044	-0.0835	-40	786	802	VQRSNHNTCINQRPIVK			Carbamidomethyl (C)[9]		Mascot

6	Myosin heavy chain kinase A OS=Dictyostelium discoideum GN=mhkA PE=1 SV=2	MHCKA_DICDI	130021.2	8.01	25	56	0	12.969

Peptide Information

Calc. Mass	Obsrv. Mass	± da	± ppm	Start Seq.	End Seq.	Sequence	Ion Score	C. I. %	Modification	Rank	Result Type
848.3995	848.4667	0.0672	79	384	390	EEIDNTK					Mascot
873.504	873.5043	0.0003	0	374	380	VEEKLQK					Mascot
917.4574	917.4501	-0.0073	-8	240	247	IEGEVNEK					Mascot
958.5316	958.4544	-0.0772	-81	809	818	LGGVLSGNNK					Mascot
986.5781	986.5275	-0.0506	-51	677	684	FNQKKPPK					Mascot
988.5574	988.5319	-0.0255	-26	584	592	KPFAEGALR					Mascot
1007.4714	1007.476	0.0046	5	365	372	NEIMEEVK			Oxidation (M)[4]		Mascot
1048.5092	1048.5846	0.0754	72	629	637	NNEAMTQLK					Mascot
1064.5042	1064.5398	0.0356	33	629	637	NNEAMTQLK			Oxidation (M)[5]		Mascot
1073.5586	1073.5094	-0.0492	-46	239	247	RIEGEVNEK					Mascot
1088.631	1088.5505	-0.0805	-74	229	238	STGNIDIKIK					Mascot
1117.5848	1117.5487	-0.0361	-32	382	390	IREEIDNTK					Mascot
1124.6534	1124.5916	-0.0618	-55	936	945	VHIIRSGNTK					Mascot
1182.5137	1182.551	0.0373	32	966	974	YLFSCSYDK			Carbamidomethyl (C)[5]		Mascot
1199.6379	1199.623	-0.0149	-12	142	151	VEQQQQQLAK					Mascot
1229.7001	1229.6184	-0.0817	-66	785	795	AGINKFITTHK					Mascot
1259.6453	1259.5912	-0.0541	-43	52	62	KFGCSSFLVSK			Carbamidomethyl (C)[4]		Mascot
1372.7695	1372.7045	-0.065	-47	581	592	VERKPFAEGALR					Mascot
1453.7468	1453.7374	-0.0094	-6	858	869	DLKCVSTIQSFR			Carbamidomethyl (C)[4]		Mascot

1471.8229	1471.7075	-0.1154	-78	616	628	LFPPIEMISPISK					Mascot
1488.8091	1488.7438	-0.0653	-44	435	447	SDSLLLLMQNNLK					Mascot
1490.7343	1490.736	0.0017	1	796	808	CNAVCALLDLDVK			Carbamidomethyl (C)[1,5]		Mascot
1490.7343	1490.736	0.0017	1	796	808	CNAVCALLDLDVK			Carbamidomethyl (C)[1,5]		Mascot
1711.8361	1711.7836	-0.0525	-31	1059	1072	IWDLSNFSCIDTLK			Carbamidomethyl (C)[9]		Mascot
1818.963	1818.834	-0.129	-71	198	212	QELQQELQSLSIKMK			Oxidation (M)[14]		Mascot
2144.0515	2143.9248	-0.1267	-59	1121	1139	CLMVFNNQIISAAFDGSIK			Carbamidomethyl (C)[1], Oxidation (M)[3]		Mascot
2501.3142	2501.2224	-0.0918	-37	616	637	LFPPIEMISPISKNNEAMTQLK					Mascot

7	Annexin A2 OS=Gallus gallus GN=ANXA2 PE=1 SV=2	ANXA2_CHICK	38901	6.92	16	55	0	7.528

Peptide Information

Calc. Mass	Obsrv. Mass	± da	± ppm	Start Seq.	End Seq.	Sequence	Ion Score	C. I. %	Modification	Rank	Result Type
808.4675	808.3878	-0.0797	-99	221	227	SVPHLQK					Mascot
873.559	873.5043	-0.0547	-63	169	176	KLMVALAK					Mascot
894.4059	894.4257	0.0198	22	296	302	CEVDMLK			Carbamidomethyl (C)[1]		Mascot
941.4761	941.5412	0.0651	69	274	281	LYDSMKGK					Mascot
957.4709	957.4382	-0.0327	-34	274	281	LYDSMKGK			Oxidation (M)[5]		Mascot
972.5359	972.4695	-0.0664	-68	38	47	DAAALEAAIK					Mascot
988.492	988.5319	0.0399	40	146	152	VYREMYK					Mascot
1050.5579	1050.562	0.0041	4	197	205	ELYDAGVKR					Mascot
1078.535	1078.5311	-0.0039	-4	213	220	WINIMTER			Oxidation (M)[5]		Mascot
1078.535	1078.5311	-0.0039	-4	213	220	WINIMTER			Oxidation (M)[5]		Mascot
1225.5696	1225.5769	0.0073	6	158	168	DIISDTSGDFR					Mascot
1230.6073	1230.5964	-0.0109	-9	136	145	TNQELNEINR					Mascot
1238.59	1238.5782	-0.0118	-10	105	115	TPSQYDASELK					Mascot
1339.7482	1339.6194	-0.1288	-96	221	231	SVPHLQKVFER					Mascot
1353.6646	1353.7111	0.0465	34	158	169	DIISDTSGDFRK					Mascot
1432.6665	1432.6996	0.0331	23	234	245	SYSPYDMLESIK					Mascot
1648.8402	1648.7747	-0.0655	-40	136	148	TNQELNEINRVYR					Mascot
1680.7646	1680.7344	-0.0302	-18	325	339	GDYQRALLNLCGGED			Carbamidomethyl (C)[11]		Mascot

8	Kinesin-like protein NACK1 OS=Nicotiana tabacum GN=NACK1 PE=1 SV=1	NACK1_TOBAC	109277.9	6.7	22	54	0	23.183

Peptide Information

Calc. Mass	Obsrv. Mass	± da	± ppm	Start Seq.	End Seq.	Sequence	Ion Score	C. I. %	Modification	Rank	Result Type

878.4479	878.4022	-0.0457	-52	688	694	EGTPYRR					Mascot
957.504	957.4382	-0.0658	-69	882	889	WEVPLEGK					Mascot
1096.4987	1096.5376	0.0389	35	467	475	NTMGRQSMR			Oxidation (M)[3]		Mascot
1124.5946	1124.5916	-0.003	-3	720	728	SYVTELKER					Mascot
1145.6096	1145.5634	-0.0462	-40	530	538	LQAEIREMR					Mascot
1145.6096	1145.5634	-0.0462	-40	530	538	LQAEIREMR					Mascot
1199.6089	1199.623	0.0141	12	124	133	TYTMRGITEK					Mascot
1226.631	1226.6396	0.0086	7	204	213	HLISICEAQR			Carbamidomethyl (C)[6]		Mascot
1229.6737	1229.6184	-0.0553	-45	546	557	EVEVGSVVAVNK					Mascot
1230.6324	1230.5964	-0.036	-29	518	529	LGNQDAAETIAK					Mascot
1259.6451	1259.5912	-0.0539	-43	264	275	ASQTNADGARLR					Mascot
1300.6168	1300.6293	0.0125	10	385	395	TPDPANEKDWK					Mascot
1300.6168	1300.6293	0.0125	10	385	395	TPDPANEKDWK					Mascot
1314.7377	1314.618	-0.1197	-91	623	634	LLPLTSSNSINR					Mascot
1316.7169	1316.5878	-0.1291	-98	558	569	SVSANLKEEIAR					Mascot
1339.5947	1339.6194	0.0247	18	706	716	MFQEAAEENVR			Oxidation (M)[1]		Mascot
1406.7274	1406.6166	-0.1108	-79	154	165	ISGLEIYNENVR					Mascot
1520.7335	1520.7249	-0.0086	-6	396	407	IQQMEMEIEELK					Mascot
1535.8765	1535.8141	-0.0624	-41	310	323	LTRILQHSLGGNAR					Mascot
1557.798	1557.8629	0.0649	42	409	421	QRDLAQSQVDELR					Mascot
1647.8007	1647.7832	-0.0175	-11	352	366	EVTNNAQVNMVVSDK					Mascot
1663.7957	1663.7274	-0.0683	-41	352	366	EVTNNAQVNMVVSDK			Oxidation (M)[10]		Mascot
1706.8279	1706.7325	-0.0954	-56	706	719	MFQEAAEENVRNIR					Mascot
1839.9011	1839.876	-0.0251	-14	83	99	VFGPDSITEAVYEEGVK					Mascot
1982.9713	1982.9559	-0.0154	-8	512	529	EVACHRLGNQDAAETIAK			Carbamidomethyl (C)[4]		Mascot

9	ORC1-type DNA replication protein OS=Cenarchaeum symbiosum (strain A) GN=cdc6 PE=3 SV=1	CDC6_CENSY	45688.3	8.84	14	54	0	4.595

Peptide Information

Calc. Mass	Obsrv. Mass	± da	± ppm	Start Seq.	End Seq.	Sequence	Ion Score	C. I. %	Modification	Rank	Result Type
808.4563	808.3878	-0.0685	-85	104	110	LVYTNAK					Mascot
856.5363	856.5192	-0.0171	-20	271	277	RAIDLLR					Mascot
873.4465	873.5043	0.0578	66	314	320	SYPLHEK					Mascot
878.4036	878.4022	-0.0014	-2	278	285	VAGEMAER			Oxidation (M)[5]		Mascot
986.5992	986.5275	-0.0717	-73	147	155	LIKAIDTGR					Mascot
1007.552	1007.476	-0.076	-75	178	185	DVLYQLTR					Mascot

1182.5862	1182.551	-0.0352	-30	286	297	AAADGVTEGHVR					Mascot
1316.6804	1316.5878	-0.0926	-70	303	313	IEENKENTALR					Mascot
1477.7758	1477.8043	0.0285	19	178	189	DVLYQLTRANER					Mascot
1535.7336	1535.8141	0.0805	52	329	342	SKGSSTGEIYSTYR					Mascot
1557.802	1557.8629	0.0609	39	308	320	ENTALRSYPLHEK					Mascot
1663.8901	1663.7274	-0.1627	-98	192	207	EGSLTIVGISNDLAFK					Mascot
1680.8665	1680.7344	-0.1321	-79	286	302	AAADGVTEGHVRDAALK					Mascot
2323.1169	2323.1233	0.0064	3	241	264	AGEAFVPGAVSSSALNL CAAMAGR			Carbamidomethyl (C)[18], Oxidation (M)[21]		Mascot

10	RNA-directed RNA polymerase L OS=Lettuce necrotic yellows virus (isolate 318) GN=L PE=3 SV=1	L_LNYV3	238482.8	8.15	31	52	0	35.47

Peptide Information

Calc. Mass	Obsrv. Mass	± da	± ppm	Start Seq.	End Seq.	Sequence	Ion Score	C. I. %	Modification	Rank	Result Type
866.473	866.3929	-0.0801	-92	1214	1220	FRDTSLK					Mascot
972.5221	972.4695	-0.0526	-54	643	651	GGQEGLRQK					Mascot
975.5179	975.5283	0.0104	11	750	758	GVPLSMDLK			Oxidation (M)[6]		Mascot
979.4778	979.5389	0.0611	62	113	119	ESVRMWR			Oxidation (M)[5]		Mascot
1048.5391	1048.5846	0.0455	43	860	867	RLMMNVPR			Oxidation (M)[3,4]		Mascot
1060.5885	1060.5504	-0.0381	-36	428	437	SVSPTLSELK					Mascot
1073.5837	1073.5094	-0.0743	-69	377	386	GNGELETLIK					Mascot
1078.502	1078.5311	0.0291	27	1676	1684	NIEGIMNCK			Carbamidomethyl (C)[8]		Mascot
1078.502	1078.5311	0.0291	27	1676	1684	NIEGIMNCK			Carbamidomethyl (C)[8]		Mascot
1094.4969	1094.5343	0.0374	34	1676	1684	NIEGIMNCK			Carbamidomethyl (C)[8], Oxidation (M)[6]		Mascot
1096.611	1096.5376	-0.0734	-67	840	849	GAHQVKTDIK					Mascot
1107.5503	1107.5558	0.0055	5	1133	1142	NPPYLGSMTK					Mascot
1117.6113	1117.5487	-0.0626	-56	1853	1862	SSGKLLGYHR					Mascot
1145.5369	1145.5634	0.0265	23	491	499	EREMNPTPR			Oxidation (M)[4]		Mascot
1145.5369	1145.5634	0.0265	23	491	499	EREMNPTPR			Oxidation (M)[4]		Mascot
1187.6995	1187.5979	-0.1016	-86	1307	1317	SSKLSQLIPSK					Mascot
1187.6995	1187.5979	-0.1016	-86	1307	1317	SSKLSQLIPSK					Mascot
1225.6609	1225.5769	-0.084	-69	1330	1340	ISMTLEHVPAK					Mascot
1226.5801	1226.6396	0.0595	49	1235	1244	YHVSTDTFTR					Mascot
1229.5541	1229.6184	0.0643	52	565	574	TVCMSLDFEK			Carbamidomethyl (C)[3]		Mascot
1230.5209	1230.5964	0.0755	61	1408	1416	DFYDQLCNR			Carbamidomethyl (C)[7]		Mascot
1300.7372	1300.6293	-0.1079	-83	1863	1873	SVIRQIWDGVK					Mascot
1300.7372	1300.6293	-0.1079	-83	1863	1873	SVIRQIWDGVK					Mascot

1309.6747	1309.6012	-0.0735	-56	1685	1698	TAFVTGNGLGGTSK		Mascot
1316.7184	1316.5878	-0.1306	-99	915	924	WLKPIFMPER		Mascot
1507.7097	1507.6736	-0.0361	-24	1	12	MDLWNEETTPKK	Oxidation (M)[1]	Mascot
1535.7523	1535.8141	0.0618	40	1609	1621	ITEWAPLTSTTCR	Carbamidomethyl (C)[12]	Mascot
1591.8003	1591.8486	0.0483	30	1169	1182	VIGWFVPEESETAK		Mascot
1591.8003	1591.8486	0.0483	30	1169	1182	VIGWFVPEESETAK		Mascot
1620.7898	1620.7551	-0.0347	-21	846	859	TDIKGEMVDETNLR		Mascot
1647.7156	1647.7832	0.0676	41	120	131	CEFPHDDWVRMR	Carbamidomethyl (C)[1]	Mascot
1648.7822	1648.7747	-0.0075	-5	1788	1801	QTMIATVTMAHDWK	Oxidation (M)[3]	Mascot
1663.7104	1663.7274	0.017	10	120	131	CEFPHDDWVRMR	Carbamidomethyl (C)[1], Oxidation (M)[11]	Mascot
1678.8615	1678.7002	-0.1613	-96	132	146	GMQNLLIVMNAISSR	Oxidation (M)[2,9]	Mascot
1680.7759	1680.7344	-0.0415	-25	1201	1215	GMTEGTSGSEIHRFR	Oxidation (M)[2]	Mascot
1706.887	1706.7325	-0.1545	-91	915	927	WLKPIFMPERSMR	Oxidation (M)[7]	Mascot
1982.931	1982.9559	0.0249	13	1425	1441	CMSLSDWKTPTTEAISR	Carbamidomethyl (C)[1]	Mascot
2323.2554	2323.1233	-0.1321	-57	2012	2030	STLIHVYDYIPMLTLIMKR	Oxidation (M)[12]	Mascot

Gel Idx/Pos	211/I11	Instr./Gel Origin	BA2151/Sample Project 20140814	Process Status	Analysis Succeeded
Plate [#] Name	[1] Sample Project 20140814	Instrument Sample Name		Spectra	11

Rank	Protein Name	Accession No.	Protein MW	Protein PI	Pep. Count	Protein Score	Protein Score C. I. %	Intensity Matched	Total Ion Score	Total Ion C. I. %	Confirmed
1	1-Cys peroxiredoxin PER1 OS=Triticum aestivum GN=PER1 PE=2 SV=1	REHY_WHEAT	24178.2	6.08	10	320	100	23.538	261	100	

Peptide Information

Calc. Mass	Obsrv. Mass	± da	± ppm	Start Seq.	End Seq.	Sequence	Ion Score	C. I. %	Modification	Rank	Result Type
862.4087	862.4234	0.0147	17	151	157	NMDEVVR					Mascot
878.4036	878.4014	-0.0022	-3	151	157	NMDEVVR			Oxidation (M)[2]		Mascot
972.4745	972.4718	-0.0027	-3	119	127	DAEGQLPSR					Mascot
1277.6195	1277.613	-0.0065	-5	94	104	VTYPIMADPDR					Mascot
1293.6144	1293.5973	-0.0171	-13	94	104	VTYPIMADPDR	15	0	Oxidation (M)[6]		Mascot
1300.6355	1300.6281	-0.0074	-6	140	150	LSFLYPSCTGR			Carbamidomethyl (C)[8]		Mascot
1300.6355	1300.6281	-0.0074	-6	140	150	LSFLYPSCTGR	77	99.998	Carbamidomethyl (C)[8]		Mascot
1471.7209	1471.7009	-0.02	-14	67	79	LLGISCDDVQSHK			Carbamidomethyl (C)[6]		Mascot
1583.7411	1583.7004	-0.0407	-26	196	209	MFPQGFETADLPSK			Oxidation (M)[1]		Mascot
1711.8361	1711.7919	-0.0442	-26	196	210	MFPQGFETADLPSKK			Oxidation (M)[1]		Mascot
2064.0608	2064.0266	-0.0342	-17	2	21	PGLTIGDTVPNLELDSTHGK					Mascot
2064.0608	2064.0266	-0.0342	-17	2	21	PGLTIGDTVPNLELDSTHGK	80	99.999			Mascot
2144.0264	2143.95	-0.0764	-36	140	157	LSFLYPSCTGRNMDEVVR			Carbamidomethyl (C)[8]		Mascot
2158.0081	2157.9651	-0.043	-20	109	127	QLNMVDPDEKDAEGQLPSR			Oxidation (M)[4]		Mascot
2158.0081	2157.9651	-0.043	-20	109	127	QLNMVDPDEKDAEGQLPSR	89	100	Oxidation (M)[4]		Mascot

Rank	Protein Name	Accession No.	Protein MW	Protein PI	Pep. Count	Protein Score	Protein Score C. I. %	Intensity Matched	Total Ion Score	Total Ion C. I. %	Confirmed
2	1-Cys peroxiredoxin PER1 OS=Hordeum vulgare GN=PER1 PE=2 SV=1	REHY_HORVU	24176.3	6.31	9	222	100	22.644	172	100	

Peptide Information

Calc. Mass	Obsrv. Mass	± da	± ppm	Start Seq.	End Seq.	Sequence	Ion Score	C. I. %	Modification	Rank	Result Type
862.4087	862.4234	0.0147	17	151	157	NMDEVVR					Mascot
878.4036	878.4014	-0.0022	-3	151	157	NMDEVVR			Oxidation (M)[2]		Mascot
1277.6195	1277.613	-0.0065	-5	94	104	VTYPIMADPDR					Mascot
1293.6144	1293.5973	-0.0171	-13	94	104	VTYPIMADPDR	15	0	Oxidation (M)[6]		Mascot
1300.6355	1300.6281	-0.0074	-6	140	150	LSFLYPSCTGR			Carbamidomethyl (C)[8]		Mascot

Calc. Mass	Obsrv. Mass	± da	± ppm	Start Seq.	End Seq.	Sequence	Ion Score	C. I. %	Modification	Rank	Result Type
1300.6355	1300.6281	-0.0074	-6	140	150	LSFLYPSCTGR	77	99.998	Carbamidomethyl (C)[8]		Mascot
1471.7209	1471.7009	-0.02	-14	67	79	LLGISCDDVQSHK			Carbamidomethyl (C)[6]		Mascot
1583.7411	1583.7004	-0.0407	-26	196	209	MFPQGFETADLPSK			Oxidation (M)[1]		Mascot
1711.8361	1711.7919	-0.0442	-26	196	210	MFPQGFETADLPSKK			Oxidation (M)[1]		Mascot
2064.0608	2064.0266	-0.0342	-17	2	21	PGLTIGDTVPNLELDSTHGK					Mascot
2064.0608	2064.0266	-0.0342	-17	2	21	PGLTIGDTVPNLELDSTHGK	80	99.999			Mascot
2141.0293	2140.9465	-0.0828	-39	109	127	QLNMVDPDEKDAQGQLPSR					Mascot
2141.0293	2140.9465	-0.0828	-39	109	127	QLNMVDPDEKDAQGQLPSR					Mascot
2144.0264	2143.95	-0.0764	-36	140	157	LSFLYPSCTGRNMDEVVR			Carbamidomethyl (C)[8]		Mascot

3	Probable 1-Cys peroxiredoxin (Fragment) OS=Bromus secalinus PE=2 SV=1	REHY_BROSE	22593.6	8.24	7	213	100	20.811	181	100

Peptide Information

Calc. Mass	Obsrv. Mass	± da	± ppm	Start Seq.	End Seq.	Sequence	Ion Score	C. I. %	Modification	Rank	Result Type
862.4087	862.4234	0.0147	17	135	141	NMDEVVR					Mascot
878.4036	878.4014	-0.0022	-3	135	141	NMDEVVR			Oxidation (M)[2]		Mascot
972.4745	972.4718	-0.0027	-3	103	111	DAEGQLPSR					Mascot
1277.6195	1277.613	-0.0065	-5	78	88	VTYPIMADPDR					Mascot
1293.6144	1293.5973	-0.0171	-13	78	88	VTYPIMADPDR	15	0	Oxidation (M)[6]		Mascot
1300.6355	1300.6281	-0.0074	-6	124	134	LSFLYPSCTGR			Carbamidomethyl (C)[8]		Mascot
1300.6355	1300.6281	-0.0074	-6	124	134	LSFLYPSCTGR	77	99.998	Carbamidomethyl (C)[8]		Mascot
1471.7209	1471.7009	-0.02	-14	51	63	LLGISCDDVQSHK			Carbamidomethyl (C)[6]		Mascot
2144.0264	2143.95	-0.0764	-36	124	141	LSFLYPSCTGRNMDEVVR			Carbamidomethyl (C)[8]		Mascot
2158.0081	2157.9651	-0.043	-20	93	111	QLNMVDPDEKDAEGQLPSR			Oxidation (M)[4]		Mascot
2158.0081	2157.9651	-0.043	-20	93	111	QLNMVDPDEKDAEGQLPSR	89	100	Oxidation (M)[4]		Mascot

4	1-Cys peroxiredoxin A OS=Oryza sativa subsp. indica GN=OsI_27030 PE=2 SV=1	REHYA_ORYSI	24228.3	5.97	5	101	99.996	4.901	80	99.999

Peptide Information

Calc. Mass	Obsrv. Mass	± da	± ppm	Start Seq.	End Seq.	Sequence	Ion Score	C. I. %	Modification	Rank	Result Type
862.4087	862.4234	0.0147	17	152	158	NMDEVVR					Mascot
878.4036	878.4014	-0.0022	-3	152	158	NMDEVVR			Oxidation (M)[2]		Mascot
987.5469	987.5217	-0.0252	-26	159	168	AVDALQTAAK					Mascot
1471.7209	1471.7009	-0.02	-14	67	79	LLGISCDDVQSHK			Carbamidomethyl (C)[6]		Mascot

Calc. Mass	Obsrv. Mass	± da	± ppm	Start Seq.	End Seq.	Sequence	Ion Score	C. I. %	Modification	Rank	Result Type
2064.0608	2064.0266	-0.0342	-17	2	21	PGLTIGDTVPNLELDSTHGK					Mascot
2064.0608	2064.0266	-0.0342	-17	2	21	PGLTIGDTVPNLELDSTHGK	80	99.999			Mascot
2158.042	2157.9651	-0.0769	-36	141	158	LSFLYPSCVGRNMDEVVR			Carbamidomethyl (C)[8], Oxidation (M)[13]		Mascot
2158.042	2157.9651	-0.0769	-36	141	158	LSFLYPSCVGRNMDEVVR			Carbamidomethyl (C)[8], Oxidation (M)[13]		Mascot

5 1-Cys peroxiredoxin PER1 OS=Zea mays GN=PER1 PE=2 SV=1 REHY_MAIZE 25060.6 6.31 4 95 99.983 2.959 80 99.999

Peptide Information

Calc. Mass	Obsrv. Mass	± da	± ppm	Start Seq.	End Seq.	Sequence	Ion Score	C. I. %	Modification	Rank	Result Type
1225.6576	1225.6198	-0.0378	-31	149	159	LSFLYPATTGR					Mascot
1583.7411	1583.7004	-0.0407	-26	207	220	MFPQGFETADLPSK			Oxidation (M)[1]		Mascot
1711.8361	1711.7919	-0.0442	-26	207	221	MFPQGFETADLPSKK			Oxidation (M)[1]		Mascot
2064.0608	2064.0266	-0.0342	-17	2	21	PGLTIGDTVPNLELDSTHGK					Mascot
2064.0608	2064.0266	-0.0342	-17	2	21	PGLTIGDTVPNLELDSTHGK	80	99.999			Mascot

6 1-Cys peroxiredoxin A OS=Oryza sativa subsp. japonica GN=Os07g0638300 PE=2 SV=1 REHYA_ORYSJ 24198.3 5.97 4 94 99.98 2.959 80 99.999

Peptide Information

Calc. Mass	Obsrv. Mass	± da	± ppm	Start Seq.	End Seq.	Sequence	Ion Score	C. I. %	Modification	Rank	Result Type
862.4087	862.4234	0.0147	17	152	158	NMDEVVR					Mascot
878.4036	878.4014	-0.0022	-3	152	158	NMDEVVR			Oxidation (M)[2]		Mascot
987.5469	987.5217	-0.0252	-26	159	168	AVDALQTAAK					Mascot
1471.7209	1471.7009	-0.02	-14	67	79	LLGISCDDVQSHK			Carbamidomethyl (C)[6]		Mascot
2064.0608	2064.0266	-0.0342	-17	2	21	PGLTIGDTVPNLELDSTHGK					Mascot
2064.0608	2064.0266	-0.0342	-17	2	21	PGLTIGDTVPNLELDSTHGK	80	99.999			Mascot

7 Microtubule-associated protein 6 OS=Mus musculus GN=Map6 PE=1 SV=2 MAP6_MOUSE 96675.8 9.51 22 76 98.578 8.008

Peptide Information

Calc. Mass	Obsrv. Mass	± da	± ppm	Start Seq.	End Seq.	Sequence	Ion Score	C. I. %	Modification	Rank	Result Type
895.5261	895.4439	-0.0822	-92	160	166	AWPLPRR					Mascot
950.473	950.4767	0.0037	4	15	21	FWNQLDK					Mascot
1004.4982	1004.5391	0.0409	41	331	339	AGPAWMVTR			Oxidation (M)[6]		Mascot
1076.5079	1076.5358	0.0279	26	319	328	ASGADQRDTR					Mascot

1096.6361	1096.5288	-0.1073	-98	641	651	DQGAVLLGPVK					Mascot
1106.6205	1106.5609	-0.0596	-54	608	618	DQGPVVPGLPK					Mascot
1173.6879	1173.5924	-0.0955	-81	22	32	ADIAVPLVFTK					Mascot
1208.598	1208.5988	0.0008	1	819	829	DQGPMLPEPPK					Mascot
1226.6964	1226.6398	-0.0566	-46	409	419	AVADALNRQIR					Mascot
1243.6429	1243.6035	-0.0394	-32	707	717	DQSFPTPAPRK					Mascot
1269.6434	1269.5785	-0.0649	-51	212	226	DPEGAGGAGVLAAGK					Mascot
1346.6434	1346.6281	-0.0153	-11	541	553	TTEGPSATKPDDK					Mascot
1372.7253	1372.6962	-0.0291	-21	739	750	KDQHVSIMASLK			Oxidation (M)[8]		Mascot
1438.7173	1438.6106	-0.1067	-74	773	785	DTGTDTTAPRYLK					Mascot
1441.8373	1441.697	-0.1403	-97	503	515	VEKPSVQSSKPKK					Mascot
1448.738	1448.7361	-0.0019	-1	718	730	DPGPVIPEPEKDR					Mascot
1711.861	1711.7919	-0.0691	-40	417	431	QIREEVASTVSSSYR					Mascot
1818.8716	1818.8328	-0.0388	-21	541	557	TTEGPSATKPDDKEQSK					Mascot
1926.9563	1926.866	-0.0903	-47	195	211	RPQSQERGPMQLSADA R					Mascot
2071.1797	2071.0967	-0.083	-40	676	695	DSVPLAPAKAQSPLLPEP LK					Mascot
2141.0833	2140.9465	-0.1368	-64	762	782	NQGLAGPELVKDTGTDT TAPR					Mascot
2141.0833	2140.9465	-0.1368	-64	762	782	NQGLAGPELVKDTGTDT TAPR					Mascot
2610.2253	2610.2419	0.0166	6	459	482	MVHETSYSAQFKGEANK PSAADNK					Mascot

8	Adenylate kinase OS=Magnaporthe oryzae (strain 70-15 / ATCC MYA-4617 / FGSC 8958) GN=ADK1 PE=3 SV=1	KAD2_MAGO7	29920.5	8.85	13	68	91.816	10.538

Peptide Information

Calc. Mass	Obsrv. Mass	± da	± ppm	Start Seq.	End Seq.	Sequence	Ion Score	C. I. %	Modification	Rank	Result Type
948.4268	948.5135	0.0867	91	202	210	SDDNADALK					Mascot
1076.5219	1076.5358	0.0139	13	202	211	SDDNADALKK					Mascot
1078.5681	1078.5282	-0.0399	-37	120	129	GGFILDGFPR					Mascot
1078.5681	1078.5282	-0.0399	-37	120	129	GGFILDGFPR					Mascot
1145.6161	1145.5577	-0.0584	-51	11	21	LGSVLDSLDAR					Mascot
1145.6161	1145.5577	-0.0584	-51	11	21	LGSVLDSLDAR					Mascot
1178.5293	1178.5653	0.036	31	1	10	MGAMEDQLQR					Mascot
1319.6743	1319.6462	-0.0281	-21	177	187	SYHTTFNPPKK					Mascot
1320.7158	1320.6849	-0.0309	-23	248	259	SLLNIFDGDAKK					Mascot
1372.7795	1372.6962	-0.0833	-61	11	23	LGSVLDSLDARVK					Mascot

1530.8235	1530.777	-0.0465	-30	260	276	ASSAGGGILDKLAASGR				Mascot
1583.6764	1583.7004	0.024	15	64	76	FSCCHLATGDMLR			Carbamidomethyl (C)[3,4], Oxidation (M)[11]	Mascot
1628.8425	1628.7738	-0.0687	-42	130	144	TVPQAQSLDAMLQAR				Mascot
1644.8374	1644.8552	0.0178	11	130	144	TVPQAQSLDAMLQAR			Oxidation (M)[11]	Mascot
2064.0715	2064.0266	-0.0449	-22	91	109	KIMDQGGLVSDDIVIGMIK			Oxidation (M)[3,17]	Mascot
2064.0715	2064.0266	-0.0449	-22	91	109	KIMDQGGLVSDDIVIGMIK			Oxidation (M)[3,17]	Mascot
2141.1389	2140.9465	-0.1924	-90	229	247	KTGIWKPIDASQEPGTVWK				Mascot
2141.1389	2140.9465	-0.1924	-90	229	247	KTGIWKPIDASQEPGTVWK				Mascot

9 E3 ubiquitin-protein ligase RNF213 OS=Mus musculus GN=Rnf213 PE=2 SV=1 | RN213_MOUSE | 591425.6 | 6.33 | 52 | 66 | 85.446 | 39.152

Peptide Information

Calc. Mass	Obsrv. Mass	± da	± ppm	Start Seq.	End Seq.	Sequence	Ion Score	C. I. %	Modification	Rank	Result Type
807.422	807.4065	-0.0155	-19	4059	4064	HREHTK					Mascot
878.4479	878.4014	-0.0465	-53	4742	4748	QFQNVSR					Mascot
895.452	895.4439	-0.0081	-9	1174	1181	LPNSSSYK					Mascot
941.5891	941.5426	-0.0465	-49	4257	4264	KVIAQQVR					Mascot
967.4843	967.4795	-0.0048	-5	2197	2205	SAFTGDTLR					Mascot
972.4567	972.4718	0.0151	16	4267	4274	DHVSLMDR					Mascot
978.5037	978.5118	0.0081	8	777	784	ISNQNMKK			Oxidation (M)[6]		Mascot
987.5693	987.5217	-0.0476	-48	4901	4908	IQRQISSR					Mascot
1021.5537	1021.5037	-0.05	-49	3772	3779	TRLQNFSR					Mascot
1037.531	1037.5084	-0.0226	-22	5011	5018	AFSRCQLR			Carbamidomethyl (C)[5]		Mascot
1154.6052	1154.7151	0.1099	95	3228	3239	LSGSSLGSFTAK					Mascot
1173.5648	1173.5924	0.0276	24	3992	4001	FSPTVSQDHR					Mascot
1187.6783	1187.5625	-0.1158	-98	913	922	RLVEIGFPEK					Mascot
1187.6783	1187.5625	-0.1158	-98	913	922	RLVEIGFPEK					Mascot
1205.6195	1205.61	-0.0095	-8	4287	4297	DATAKAVLECK			Carbamidomethyl (C)[10]		Mascot
1208.6304	1208.5988	-0.0316	-26	3405	3415	RSTIMASDVTK					Mascot
1209.5603	1209.6177	0.0574	47	5001	5010	MCDQTAQVLK			Carbamidomethyl (C)[2], Oxidation (M)[1]		Mascot
1209.5603	1209.6177	0.0574	47	5001	5010	MCDQTAQVLK			Carbamidomethyl (C)[2], Oxidation (M)[1]		Mascot
1230.6008	1230.6077	0.0069	6	1841	1851	RCLTSGSQGHK			Carbamidomethyl (C)[2]		Mascot
1258.5957	1258.6422	0.0465	37	4265	4274	NRDHVSLMDR			Oxidation (M)[8]		Mascot
1300.6128	1300.6281	0.0153	12	1561	1571	KDHNSNTEVEK					Mascot
1300.6128	1300.6281	0.0153	12	1561	1571	KDHNSNTEVEK					Mascot
1319.6987	1319.6462	-0.0525	-40	4572	4582	DLEQLTKMLGR			Oxidation (M)[8]		Mascot

1347.6461	1347.5977	-0.0484	-36	2097	2107	EVIDMELTPER		Oxidation (M)[5]	Mascot
1385.7206	1385.6602	-0.0604	-44	890	901	SRMLSISSAYVR		Oxidation (M)[3]	Mascot
1390.642	1390.6829	0.0409	29	1183	1193	ETHYCLSPDIR		Carbamidomethyl (C)[5]	Mascot
1399.5803	1399.6564	0.0761	54	1285	1295	FMCTMNPQDQK		Carbamidomethyl (C)[3]	Mascot
1435.8348	1435.705	-0.1298	-90	2078	2089	APLFKFLDLFPK			Mascot
1441.7695	1441.697	-0.0725	-50	2209	2220	NFVVTFMILMAR			Mascot
1448.738	1448.7361	-0.0019	-1	3332	3344	VLVIQADFDDGTR			Mascot
1453.8525	1453.7524	-0.1001	-69	3118	3129	QFPVPLINRLEK			Mascot
1477.776	1477.7992	0.0232	16	2915	2926	DYYSLIKMVFAK			Mascot
1506.7911	1506.7382	-0.0529	-35	1460	1472	ALDNDQHLPDKLK			Mascot
1529.7992	1529.7621	-0.0371	-24	3214	3227	SILLDCATPDAVVR		Carbamidomethyl (C)[6]	Mascot
1562.8762	1562.7469	-0.1293	-83	2932	2945	RGLSPQDITHAVLR			Mascot
1591.7642	1591.8313	0.0671	42	2372	2386	CGIPVIIMGETGCGK		Carbamidomethyl (C)[1,13]	Mascot
1620.7612	1620.7449	-0.0163	-10	3552	3566	EAANQDALQEAGTFR			Mascot
1628.8854	1628.7738	-0.1116	-69	3505	3518	VTILLDLLNEDNTR			Mascot
1662.9174	1662.9125	-0.0049	-3	2992	3006	GLDGAESRYLLVLTR			Mascot
1711.801	1711.7919	-0.0091	-5	3381	3396	MGSGTSYVGFHGGLWR			Mascot
1796.8154	1796.8629	0.0475	26	1697	1712	CTVQDLVQATSACESK		Carbamidomethyl (C)[1,13]	Mascot
1818.9968	1818.8328	-0.164	-90	1679	1694	KPRPSEAALMMLSFIK			Mascot
1864.9078	1864.946	0.0382	20	2372	2388	CGIPVIIMGETGCGKTR		Carbamidomethyl (C)[1,13], Oxidation (M)[8]	Mascot
1926.9275	1926.866	-0.0615	-32	3727	3741	EELMFLQMALWSCLR		Carbamidomethyl (C)[13]	Mascot
1952.884	1952.9392	0.0552	28	1183	1198	ETHYCLSPDIREMASK		Carbamidomethyl (C)[5], Oxidation (M)[13]	Mascot
2064.073	2064.0266	-0.0464	-22	1766	1784	CLAHLATMGGTPVERPL PK		Carbamidomethyl (C)[1], Oxidation (M)[8]	Mascot
2064.073	2064.0266	-0.0464	-22	1766	1784	CLAHLATMGGTPVERPL PK		Carbamidomethyl (C)[1], Oxidation (M)[8]	Mascot
2071.1094	2071.0967	-0.0127	-6	1582	1598	LVHVFIKLHCAGNMLFR		Carbamidomethyl (C)[10], Oxidation (M)[14]	Mascot
2085.8838	2086.0115	0.1277	61	3445	3461	ELAEEQMEVEDSEEMKK		Oxidation (M)[7,15]	Mascot
2091.9917	2092.0098	0.0181	9	4267	4283	DHVSLMDRYLVHGNEYK		Oxidation (M)[6]	Mascot
2094.0359	2094.0215	-0.0144	-7	3631	3648	SNSEMSFILVQSHMNLLK		Oxidation (M)[5]	Mascot
2121.1538	2121.0376	-0.1162	-55	5015	5031	CQLRHIIALWQFLSAHK		Carbamidomethyl (C)[1]	Mascot
2135.1504	2135.0557	-0.0947	-44	664	681	WLGILPLLHTCMQKSPPK		Carbamidomethyl (C)[11], Oxidation (M)[12]	Mascot
2141.0818	2140.9465	-0.1353	-63	661	677	CDRWLGILPLLHTCMQK		Carbamidomethyl (C)[1,14]	Mascot
2141.0818	2140.9465	-0.1353	-63	661	677	CDRWLGILPLLHTCMQK		Carbamidomethyl (C)[1,14]	Mascot
2143.9358	2143.95	0.0142	7	1285	1301	FMCTMNPQDQKGWISER		Carbamidomethyl (C)[3], Oxidation (M)[2]	Mascot
2264.2068	2264.1147	-0.0921	-41	1934	1954	LLSAATVFRDGLCVGIVT SER		Carbamidomethyl (C)[13]	Mascot

10	Nuclear receptor coactivator 7 OS=Gallus gallus GN=NCOA7 PE=2 SV=1	NCOA7_CHICK	102796.7	5.55	21	63	71.622	8.566

Peptide Information

Calc. Mass	Obsrv. Mass	± da	± ppm	Start Seq.	End Seq.	Sequence	Ion Score	C. I. %	Modification	Rank	Result Type
815.492	815.4208	-0.0712	-87	677	683	VGKPMRK					Mascot
870.468	870.5426	0.0746	86	197	204	LPDADLAR					Mascot
1004.5622	1004.5391	-0.0231	-23	521	529	VETALDTKK					Mascot
1106.5737	1106.5609	-0.0128	-12	668	676	TPPMFLCIK			Carbamidomethyl (C)[7]		Mascot
1187.6631	1187.5625	-0.1006	-85	712	721	EIITVEEAKR					Mascot
1187.6631	1187.5625	-0.1006	-85	712	721	EIITVEEAKR					Mascot
1205.5719	1205.61	0.0381	32	494	503	ADLDLELCEK			Carbamidomethyl (C)[8]		Mascot
1247.5176	1247.6077	0.0901	72	98	107	YYSTDDNQNK					Mascot
1258.6208	1258.6422	0.0214	17	436	446	VPSPMDRGLDR			Oxidation (M)[5]		Mascot
1277.677	1277.613	-0.064	-50	567	578	AIGIDLTLSCSK			Carbamidomethyl (C)[10]		Mascot
1346.5819	1346.6281	0.0462	34	544	556	DGSGSQSSEDLHK					Mascot
1385.7345	1385.6602	-0.0743	-54	651	662	IQVPIEDMLPSK			Oxidation (M)[8]		Mascot
1441.6233	1441.697	0.0737	51	589	600	SMELDSCCVGRK			Carbamidomethyl (C)[7,8]		Mascot
1529.8687	1529.7621	-0.1066	-70	142	154	FNITPNKLVELNK					Mascot
1562.775	1562.7469	-0.0281	-18	866	878	FGLWLDADLYHGR					Mascot
1583.77	1583.7004	-0.0696	-44	398	411	FAADPHVKEPSEEK					Mascot
1915.8992	1915.9664	0.0672	35	544	561	DGSGSQSSEDLHKAEIQ K					Mascot
1953.0441	1952.9392	-0.1049	-54	771	787	LAYSTLEHGTSLKTLYR					Mascot
2071.0613	2071.0967	0.0354	17	743	759	HHSALLENMHIEQLARR			Oxidation (M)[9]		Mascot
2094.021	2094.0215	0.0005	0	341	360	QQNGDSPNVPGAKQINP SDK					Mascot
2124.0608	2124.0225	-0.0383	-18	380	397	SKSLEFYNNITATEHLEK					Mascot
2141.0884	2140.9465	-0.1419	-66	241	260	GVVGGVMIVTPNNIMFDP HK			Oxidation (M)[7]		Mascot
2141.0884	2140.9465	-0.1419	-66	241	260	GVVGGVMIVTPNNIMFDP HK			Oxidation (M)[7]		Mascot

Gel Idx/Pos	212/I12	Instr./Gel Origin	BA2151/Sample Project 20140814	Process Status	Analysis Succeeded
Plate [#] Name	[1] Sample Project 20140814	Instrument Sample Name		Spectra	11

Rank	Protein Name	Accession No.	Protein MW	Protein PI	Pep. Count	Protein Score	Protein Score C. I. %	Intensity Matched	Total Ion Score	Total Ion C. I. %	Confirmed
1	L-ascorbate peroxidase 1, cytosolic OS=Arabidopsis thaliana GN=APX1 PE=1 SV=2	APX1_ARATH	27828.9	5.72	4	83	99.703	2.489	70	99.989	

Peptide Information

Calc. Mass	Obsrv. Mass	± da	± ppm	Start Seq.	End Seq.	Sequence	Ion Score	C. I. %	Modification	Rank	Result Type
1249.6172	1249.6124	-0.0048	-4	120	130	EDKPQPPPEGR					Mascot
1249.6172	1249.6124	-0.0048	-4	120	130	EDKPQPPPEGR	70	99.989			Mascot
1444.6954	1444.73	0.0346	24	2	13	TKNYPTVSEDYK					Mascot
1635.7333	1635.7223	-0.011	-7	39	52	LAWHSAGTFDCQSR			Carbamidomethyl (C)[11]		Mascot
1734.9069	1734.8104	-0.0965	-56	155	170	DIVALSGAHTLGRCHK			Carbamidomethyl (C)[14]		Mascot

Rank	Protein Name	Accession No.	Protein MW	Protein PI	Pep. Count	Protein Score	Protein Score C. I. %	Intensity Matched	Total Ion Score	Total Ion C. I. %	Confirmed
2	Gamma-glutamyl phosphate reductase OS=Brachyspira hyodysenteriae (strain ATCC 49526 / WA1) GN=proA PE=3 SV=1	PROA_BRAHW	49175.5	5.84	15	63	70.285	18.47			

Peptide Information

Calc. Mass	Obsrv. Mass	± da	± ppm	Start Seq.	End Seq.	Sequence	Ion Score	C. I. %	Modification	Rank	Result Type
875.4468	875.4254	-0.0214	-24	36	42	IEENKDK					Mascot
911.4767	911.4224	-0.0543	-60	66	72	SMFNRLK			Oxidation (M)[2]		Mascot
924.4785	924.4166	-0.0619	-67	9	16	NAKEATYK					Mascot
992.4279	992.4663	0.0384	39	149	158	GGSEGENTNK					Mascot
998.5087	998.4706	-0.0381	-38	63	70	ISKSMFNR			Oxidation (M)[5]		Mascot
1034.5518	1034.4625	-0.0893	-86	268	276	NIASEYLPK					Mascot
1078.5891	1078.5057	-0.0834	-77	205	214	GGNSLVQYIK					Mascot
1320.6715	1320.6066	-0.0649	-49	186	196	EDIKELLSMDK					Mascot
1490.7156	1490.7324	0.0168	11	283	295	ENEIKMNADAEVK					Mascot
1537.8625	1537.7144	-0.1481	-96	395	408	GPVGLEGLTIYKYK					Mascot
1587.7399	1587.6609	-0.079	-50	336	349	YGSHHTDSIISENK					Mascot
1627.9153	1627.9331	0.0178	11	98	111	VLLETELDDNLLLK					Mascot
1627.9153	1627.9331	0.0178	11	98	111	VLLETELDDNLLLK					Mascot
1812.9895	1812.9407	-0.0488	-27	268	282	NIASEYLPKLYNLFK					Mascot
1819.0034	1818.8752	-0.1282	-70	190	204	ELLSMDKYIDLIIPR					Mascot

1982.8735	1982.9631	0.0896	45	355	371	FMTFVDSANVYCNASTR			Carbamidomethyl (C)[12]		Mascot

3	Myosin-7 OS=Homo sapiens GN=MYH7 PE=1 SV=5	MYH7_HUMAN	223757	5.63	29	62	61.719	26.233

Peptide Information

Calc. Mass	Obsrv. Mass	± da	± ppm	Start Seq.	End Seq.	Sequence	Ion Score	C. I. %	Modification	Rank	Result Type
924.5037	924.4166	-0.0871	-94	1476	1483	SLSTELFK					Mascot
1024.4806	1024.4702	-0.0104	-10	238	246	NDNSSRFGK					Mascot
1048.5131	1048.4572	-0.0559	-53	235	243	TVRNDNSSR					Mascot
1060.5171	1060.5332	0.0161	15	1364	1372	ANSEVAQWR					Mascot
1066.5714	1066.4674	-0.104	-98	794	802	GVLARMEYK					Mascot
1084.5067	1084.4668	-0.0399	-37	435	442	MFNWMVTR					Mascot
1374.6028	1374.6842	0.0814	59	848	858	EMASMKEEFTR			Oxidation (M)[2]		Mascot
1444.6849	1444.73	0.0451	31	1772	1783	EQDTSAHLERMK					Mascot
1507.8115	1507.7217	-0.0898	-60	1306	1317	LTYTQQLEDLKR					Mascot
1525.8109	1525.7373	-0.0736	-48	59	72	VTAETEYGKTVTVK					Mascot
1525.8109	1525.7373	-0.0736	-48	59	72	VTAETEYGKTVTVK					Mascot
1537.76	1537.7144	-0.0456	-30	1758	1771	AITDAAMMAEELKK			Oxidation (M)[7]		Mascot
1547.6344	1547.7114	0.077	50	370	383	EEQAEPDGTEEADK					Mascot
1610.8182	1610.8978	0.0796	49	352	365	LTGAIMHFGNMKFK			Oxidation (M)[6]		Mascot
1626.8131	1626.8962	0.0831	51	352	365	LTGAIMHFGNMKFK			Oxidation (M)[6,11]		Mascot
1643.8962	1643.8926	-0.0036	-2	1290	1303	QLDEKEALISQLTR					Mascot
1646.8306	1646.8035	-0.0271	-16	977	991	NLTEEMAGLDEIIAK					Mascot
1703.8058	1703.8724	0.0666	39	73	86	EDQVMQQNPPKFDK					Mascot
1734.8091	1734.8104	0.0013	1	430	442	AVYERMFNWMVTR			Oxidation (M)[6,10]		Mascot
1740.928	1740.833	-0.095	-55	724	739	ILNPAAIPEGQFIDSR					Mascot
1831.7941	1831.9717	0.1776	97	368	383	QREEQAEPDGTEEADK					Mascot
1831.7941	1831.9717	0.1776	97	368	383	QREEQAEPDGTEEADK					Mascot
1859.887	1859.9668	0.0798	43	1027	1042	LEQQVDDLEGSLEQEK					Mascot
1958.9286	1958.8513	-0.0773	-39	347	363	NSMYKLTGAIMHFGNMK			Oxidation (M)[3]		Mascot
1987.9818	1988.0076	0.0258	13	1027	1043	LEQQVDDLEGSLEQEKK					Mascot
1989.0573	1989.0907	0.0334	17	977	994	NLTEEMAGLDEIIAKLTK					Mascot
1990.0942	1990.0204	-0.0738	-37	809	825	DSLLVIQWNIRAFMGVK					Mascot
1995.0254	1994.9786	-0.0468	-23	1176	1193	RDLEEATLQHEATAAALR					Mascot
2005.0522	2004.9878	-0.0644	-32	977	994	NLTEEMAGLDEIIAKLTK			Oxidation (M)[6]		Mascot
2006.0892	2005.9946	-0.0946	-47	809	825	DSLLVIQWNIRAFMGVK			Oxidation (M)[14]		Mascot
2054.0625	2053.9731	-0.0894	-44	1589	1606	NHLRVVDSLQTSLDAETR					Mascot

2054.0625	2053.9731	-0.0894	-44	1589	1606	NHLRVVDSLQTSLDAETR					Mascot
2087.0503	2087.0125	-0.0378	-18	1025	1042	VKLEQQVDDLEGSLEQE K					Mascot
2716.2488	2716.3118	0.063	23	1618	1641	MEGDLNEMEIQLSHANR MAAEAQK					Mascot
2738.3933	2738.302	-0.0913	-33	84	106	FDKIEDMAMLTFLHEPAV LYNLK					Mascot

4 Ribosomal RNA large subunit methyltransferase K/L OS=Pseudoalteromonas haloplanktis (strain TAC 125) GN=rlmL PE=3 SV=1 | RLMKL_PSEHT | 80136.8 | 8.89 | 19 | 61 | 59.915 | 19.278

Peptide Information

Calc. Mass	Obsrv. Mass	± da	± ppm	Start Seq.	End Seq.	Sequence	Ion Score	C. I. %	Modification	Rank	Result Type
916.4985	916.4686	-0.0299	-33	276	283	AELDDIIK					Mascot
924.4785	924.4166	-0.0619	-67	284	291	FKQSDATK					Mascot
990.554	990.4738	-0.0802	-81	56	63	VLMLIEEK			Oxidation (M)[3]		Mascot
1006.5051	1006.4717	-0.0334	-33	379	387	QVSLTTDDK					Mascot
1010.4611	1010.3917	-0.0694	-69	596	603	FEQADCLK			Carbamidomethyl (C)[6]		Mascot
1078.5262	1078.5057	-0.0205	-19	452	460	EIDEKTSEK					Mascot
1108.5634	1108.5121	-0.0513	-46	535	543	YIQENAKDK					Mascot
1490.8214	1490.7324	-0.089	-60	648	661	ILSPSGTLIFSNNK					Mascot
1507.6886	1507.7217	0.0331	22	146	158	YGVSMYIDYSGPR					Mascot
1508.7526	1508.7277	-0.0249	-17	500	512	QNRTMVVEEFGAK					Mascot
1523.6836	1523.7227	0.0391	26	146	158	YGVSMYIDYSGPR			Oxidation (M)[5]		Mascot
1547.8502	1547.7114	-0.1388	-90	663	677	GFVMDEVGLIGLGLK					Mascot
1587.7584	1587.6609	-0.0975	-61	388	401	KALNFEGSMSFANR			Oxidation (M)[9]		Mascot
1610.8999	1610.8978	-0.0021	-1	240	253	ELKEELLGNIIDPK					Mascot
1618.9163	1618.8385	-0.0778	-48	647	661	KILSPSGTLIFSNNK					Mascot
1627.7711	1627.9331	0.162	100	64	77	EGVDDKNSLYNFAR					Mascot
1627.7711	1627.9331	0.162	100	64	77	EGVDDKNSLYNFAR					Mascot
1646.9225	1646.8035	-0.119	-72	648	662	ILSPSGTLIFSNNKR					Mascot
1703.9513	1703.8724	-0.0789	-46	662	677	RGFVMDEVGLIGLGLK					Mascot
1837.9478	1838.0114	0.0636	35	36	51	FEADSLLAQKVCLSTR			Carbamidomethyl (C)[12]		Mascot
1845.9229	1845.9391	0.0162	9	363	378	NGPLDVELNLYQLDDK					Mascot

5 L-ascorbate peroxidase 1, cytosolic OS=Oryza sativa subsp. japonica GN=APX1 PE=1 SV=1 | APX1_ORYSJ | 27252.8 | 5.42 | 4 | 61 | 58.981 | 7.443 | 50 | 98.965

Protein Group

L-ascorbate peroxidase 1, cytosolic OS=Oryza sativa subsp. indica GN=APX1 PE=2 SV=1 | APX1_ORYSI | 27256.7 | 5.3099999427

Peptide Information

Calc. Mass	Obsrv. Mass	± da	± ppm	Start Seq.	End Seq.	Sequence	Ion Score	C. I. %	Modification	Rank	Result Type
911.4291	911.4224	-0.0067	-7	53	61	TGGPFGTMK			Oxidation (M)[8]		Mascot
919.4196	919.4149	-0.0047	-5	230	236	AFFEDYK					Mascot
1036.4847	1036.4802	-0.0045	-4	173	181	SGFEGPWTR					Mascot
1036.4847	1036.4802	-0.0045	-4	173	181	SGFEGPWTR	50	98.965			Mascot
1505.7384	1505.7155	-0.0229	-15	39	52	LAWHSAGTFDVSSK					Mascot

6	DNA mismatch repair protein MutS OS=Escherichia fergusonii (strain ATCC 35469 / DSM 13698 / CDC 0568-73) GN=mutS PE=3 SV=1	MUTS_ESCF3	95586.3	5.36	12	58	20.02	28.835	31	22.03	

Protein Group

DNA mismatch repair protein MutS OS=Escherichia coli O17:K52:H18 (strain UMN026 / ExPEC) GN=mutS PE=3 SV=1	MUTS_ECOLU	95586.3	5.3600001335144

Peptide Information

Calc. Mass	Obsrv. Mass	± da	± ppm	Start Seq.	End Seq.	Sequence	Ion Score	C. I. %	Modification	Rank	Result Type
1048.5092	1048.4572	-0.052	-50	164	172	ETMAAELQR					Mascot
1064.5042	1064.4897	-0.0145	-14	164	172	ETMAAELQR			Oxidation (M)[3]		Mascot
1078.568	1078.5057	-0.0623	-58	841	848	QALEWIYR					Mascot
1204.6433	1204.5304	-0.1129	-94	211	220	QQLNLQFGTR					Mascot
1231.6538	1231.6027	-0.0511	-41	609	620	MLIITGPNMGGK					Mascot
1247.6487	1247.6113	-0.0374	-30	609	620	MLIITGPNMGGK			Oxidation (M)[1]		Mascot
1505.7377	1505.7155	-0.0222	-15	263	275	EQDSIIMDAATRR					Mascot
1525.8057	1525.7373	-0.0684	-45	368	379	MRHAFQQLPELR					Mascot
1525.8057	1525.7373	-0.0684	-45	368	379	MRHAFQQLPELR	31	22.03			Mascot
1541.8005	1541.7266	-0.0739	-48	368	379	MRHAFQQLPELR			Oxidation (M)[1]		Mascot
1610.8497	1610.8978	0.0481	30	380	394	AQLENVDSAPVQALR					Mascot
1627.9319	1627.9331	0.0012	1	20	32	LKAQHPEILLFYR					Mascot
1627.9319	1627.9331	0.0012	1	20	32	LKAQHPEILLFYR					Mascot
1646.8286	1646.8035	-0.0251	-15	142	156	GFGYATLDISSGRFR					Mascot
1885.9333	1885.87	-0.0633	-34	609	625	MLIITGPNMGGKSTYMR			Oxidation (M)[1]		Mascot
2054.0991	2053.9731	-0.126	-61	626	644	QTALIALMAYIGSYVPAQK			Oxidation (M)[8]		Mascot
2054.0991	2053.9731	-0.126	-61	626	644	QTALIALMAYIGSYVPAQK			Oxidation (M)[8]		Mascot

2738.52	2738.302	-0.218	-80	320	343	VLLERQQTIGALQDFTAE LQPVLR						Mascot

7	DNA mismatch repair protein MutS OS=Escherichia coli O139:H28 (strain E24377A / ETEC) GN=mutS PE=3 SV=2	MUTS_ECO24	95887.5	5.36	12	58	10.261	29.423	31	22.03	

Peptide Information

Calc. Mass	Obsrv. Mass	± da	± ppm	Start Seq.	End Seq.	Sequence	Ion Score	C. I. %	Modification	Rank	Result Type
1048.5092	1048.4572	-0.052	-50	166	174	ETMAAELQR					Mascot
1064.5042	1064.4897	-0.0145	-14	166	174	ETMAAELQR			Oxidation (M)[3]		Mascot
1078.568	1078.5057	-0.0623	-58	843	850	QALEWIYR					Mascot
1204.6433	1204.5304	-0.1129	-94	213	222	QQLNLQFGTR					Mascot
1231.6538	1231.6027	-0.0511	-41	611	622	MLIITGPNMGGK					Mascot
1247.6487	1247.6113	-0.0374	-30	611	622	MLIITGPNMGGK			Oxidation (M)[1]		Mascot
1505.7377	1505.7155	-0.0222	-15	265	277	EQDSIIMDAATRR					Mascot
1525.8057	1525.7373	-0.0684	-45	370	381	MRHAFQQLPELR					Mascot
1525.8057	1525.7373	-0.0684	-45	370	381	MRHAFQQLPELR	31	22.03			Mascot
1541.8005	1541.7266	-0.0739	-48	370	381	MRHAFQQLPELR			Oxidation (M)[1]		Mascot
1627.9319	1627.9331	0.0012	1	22	34	LKAQHPEILLFYR					Mascot
1627.9319	1627.9331	0.0012	1	22	34	LKAQHPEILLFYR					Mascot
1646.8286	1646.8035	-0.0251	-15	144	158	GFGYATLDISSGRFR					Mascot
1712.7771	1712.7953	0.0182	11	8	21	DFSSHTPMMQQYLK					Mascot
1885.9333	1885.87	-0.0633	-34	611	627	MLIITGPNMGGKSTYMR			Oxidation (M)[1]		Mascot
2054.0991	2053.9731	-0.126	-61	628	646	QTALIALMAYIGSYVPAQ K			Oxidation (M)[8]		Mascot
2054.0991	2053.9731	-0.126	-61	628	646	QTALIALMAYIGSYVPAQ K			Oxidation (M)[8]		Mascot
2738.52	2738.302	-0.218	-80	322	345	VLLERQQTIGALQDFTAE LQPVLR					Mascot

8	Myosin-7 OS=Canis familiaris GN=MYH7 PE=1 SV=3	MYH7_CANFA	223635.9	5.58	28	55	0	15.75	

Peptide Information

Calc. Mass	Obsrv. Mass	± da	± ppm	Start Seq.	End Seq.	Sequence	Ion Score	C. I. %	Modification	Rank	Result Type
892.4556	892.4305	-0.0251	-28	919	925	VKEMTER					Mascot
924.5037	924.4166	-0.0871	-94	1476	1483	SLSTELFK					Mascot
990.5115	990.4738	-0.0377	-38	560	567	SNNFQKPR					Mascot
1024.4806	1024.4702	-0.0104	-10	238	246	NDNSSRFGK					Mascot
1048.5131	1048.4572	-0.0559	-53	235	243	TVRNDNSSR					Mascot
1060.5171	1060.5332	0.0161	15	1364	1372	ANSEVAQWR					Mascot

1084.5067	1084.4668	-0.0399	-37	435	442	MFNWMVTR					Mascot
1374.6028	1374.6842	0.0814	59	848	858	EMATMKEEFAR			Oxidation (M)[2,5]		Mascot
1444.6849	1444.73	0.0451	31	1772	1783	EQDTSAHLERMK					Mascot
1507.8115	1507.7217	-0.0898	-60	1306	1317	LTYTQQLEDLKR					Mascot
1537.76	1537.7144	-0.0456	-30	1758	1771	AITDAAMMAEELKK			Oxidation (M)[7]		Mascot
1547.6344	1547.7114	0.077	50	370	383	EEQAEPDGTEEADK					Mascot
1610.8182	1610.8978	0.0796	49	352	365	LTGAIMHFGNMKFK			Oxidation (M)[6]		Mascot
1618.8547	1618.8385	-0.0162	-10	414	429	GQNVQQVAYATGALAK					Mascot
1626.8131	1626.8962	0.0831	51	352	365	LTGAIMHFGNMKFK			Oxidation (M)[6,11]		Mascot
1643.8962	1643.8926	-0.0036	-2	1290	1303	QLDEKEALISQLTR					Mascot
1646.8306	1646.8035	-0.0271	-16	977	991	NLTEEMAGLDEIIAK					Mascot
1703.8058	1703.8724	0.0666	39	73	86	EDQVMQQNPPKFDK					Mascot
1740.928	1740.833	-0.095	-55	724	739	ILNPAAIPEGQFIDSR					Mascot
1831.7941	1831.9717	0.1776	97	368	383	QREEQAEPDGTEEADK					Mascot
1831.7941	1831.9717	0.1776	97	368	383	QREEQAEPDGTEEADK					Mascot
1859.887	1859.9668	0.0798	43	1027	1042	LEQQVDDLEGSLEQEK					Mascot
1958.9286	1958.8513	-0.0773	-39	347	363	NSMYKLTGAIMHFGNMK			Oxidation (M)[3]		Mascot
1987.9818	1988.0076	0.0258	13	1027	1043	LEQQVDDLEGSLEQEKK					Mascot
1989.0573	1989.0907	0.0334	17	977	994	NLTEEMAGLDEIIAKLTK					Mascot
1995.0254	1994.9786	-0.0468	-23	1176	1193	RDLEEATLQHEATAAALR					Mascot
2005.0522	2004.9878	-0.0644	-32	977	994	NLTEEMAGLDEIIAKLTK			Oxidation (M)[6]		Mascot
2054.0625	2053.9731	-0.0894	-44	1589	1606	NHLRVVDSLQTSLDAETR					Mascot
2054.0625	2053.9731	-0.0894	-44	1589	1606	NHLRVVDSLQTSLDAETR					Mascot
2087.0503	2087.0125	-0.0378	-18	1025	1042	VKLEQQVDDLEGSLEQE K					Mascot
2716.2488	2716.3118	0.063	23	1618	1641	MEGDLNEMEIQLSHANR MAAEAQK					Mascot
2738.3933	2738.302	-0.0913	-33	84	106	FDKIEDMAMLTFLHEPAV LYNLK					Mascot

9	Transaldolase OS=Photorhabdus luminescens subsp. laumondii (strain TT01) GN=tal PE=3 SV=1	TAL_PHOLL	35516.4	5.19	11	55	0	2.394	

Peptide Information

Calc. Mass	Obsrv. Mass	± da	± ppm	Start Seq.	End Seq.	Sequence	Ion Score	C. I. %	Modification	Rank	Result Type
1061.5911	1061.585	-0.0061	-6	309	317	LEKMISDLL					Mascot
1064.5769	1064.4897	-0.0872	-82	1	9	MTDKLTSLR					Mascot
1132.5481	1132.4781	-0.07	-62	67	76	EQQIVDASDK					Mascot
1374.6859	1374.6842	-0.0017	-1	251	262	ELSEAQGEVERK					Mascot
1498.9104	1498.8119	-0.0985	-66	129	141	ILIKLASTWQGIR					Mascot

1505.7881	1505.7155	-0.0726	-48	11	25	LTTVVADTGDIAAMK					Mascot
1583.7272	1583.8131	0.0859	54	215	228	QHGYDTVVMGASFR			Oxidation (M)[9]		Mascot
1649.8779	1649.8871	0.0092	6	10	25	KLTTVVADTGDIAAMK			Oxidation (M)[15]		Mascot
1740.84	1740.833	-0.007	-4	93	107	ISTEVDARLSYDSER					Mascot
1859.9246	1859.9668	0.0422	23	51	66	LIDEAIAWAREQSDSR					Mascot
2716.394	2716.3118	-0.0822	-30	26	49	LYQPQDATTNPSLILNAA QIPEYR					Mascot

10	DNA mismatch repair protein MutS OS=Escherichia coli O45:K1 (strain S88 / ExPEC) GN=mutS PE=3 SV=1	MUTS_ECO45	95629.3	5.39	11	54	0	28.604	31	22.03

Protein Group

DNA mismatch repair protein MutS OS=Escherichia coli (strain UTI89 / UPEC) GN=mutS PE=3 SV=1	MUTS_ECOUT	95629.3	5.3899998664856
DNA mismatch repair protein MutS OS=Escherichia coli O127:H6 (strain E2348/69 / EPEC) GN=mutS PE=3 SV=1	MUTS_ECO27	95659.3	5.3899998664856
DNA mismatch repair protein MutS OS=Escherichia coli O1:K1 / APEC GN=mutS PE=3 SV=1	MUTS_ECOK1	95629.3	5.3899998664856
DNA mismatch repair protein MutS OS=Escherichia coli O6:H1 (strain CFT073 / ATCC 700928 / UPEC) GN=mutS PE=3 SV=1	MUTS_ECOL6	95615.3	5.3899998664856
DNA mismatch repair protein MutS OS=Escherichia coli O6:K15:H31 (strain 536 / UPEC) GN=mutS PE=3 SV=1	MUTS_ECOL5	95599.3	5.3899998664856

Peptide Information

Calc. Mass	Obsrv. Mass	± da	± ppm	Start Seq.	End Seq.	Sequence	Ion Score	C. I. %	Modification	Rank	Result Type
1048.5092	1048.4572	-0.052	-50	164	172	ETMAAELQR					Mascot
1064.5042	1064.4897	-0.0145	-14	164	172	ETMAAELQR			Oxidation (M)[3]		Mascot
1078.568	1078.5057	-0.0623	-58	841	848	QALEWIYR					Mascot
1204.6433	1204.5304	-0.1129	-94	211	220	QQLNLQFGTR					Mascot
1231.6538	1231.6027	-0.0511	-41	609	620	MLIITGPNMGGK					Mascot
1247.6487	1247.6113	-0.0374	-30	609	620	MLIITGPNMGGK			Oxidation (M)[1]		Mascot
1525.8057	1525.7373	-0.0684	-45	368	379	MRHAFQQLPELR					Mascot
1525.8057	1525.7373	-0.0684	-45	368	379	MRHAFQQLPELR	31	22.03			Mascot
1541.8005	1541.7266	-0.0739	-48	368	379	MRHAFQQLPELR			Oxidation (M)[1]		Mascot
1610.8497	1610.8978	0.0481	30	380	394	AQLENVDSAPVQALR					Mascot

1627.9319	1627.9331	0.0012	1	20	32	LKAQHPEILLFYR		Mascot
1627.9319	1627.9331	0.0012	1	20	32	LKAQHPEILLFYR		Mascot
1646.8286	1646.8035	-0.0251	-15	142	156	GFGYATLDISSGRFR		Mascot
1885.9333	1885.87	-0.0633	-34	609	625	MLIITGPNMGGKSTYMR	Oxidation (M)[1]	Mascot
2054.0991	2053.9731	-0.126	-61	626	644	QTALIALMAYIGSYVPAQ K	Oxidation (M)[8]	Mascot
2054.0991	2053.9731	-0.126	-61	626	644	QTALIALMAYIGSYVPAQ K	Oxidation (M)[8]	Mascot
2738.52	2738.302	-0.218	-80	320	343	VLLERQQTIGALQDFTAE LQPVLR		Mascot

Gel Idx/Pos	213/I13	Instr./Gel Origin	BA2151/Sample Project 20140814	Process Status	Analysis Succeeded
Plate [#] Name	[1] Sample Project 20140814	Instrument Sample Name		Spectra	11

Rank	Protein Name	Accession No.	Protein MW	Protein PI	Pep. Count	Protein Score	Protein Score C. I. %	Intensity Matched	Total Ion Score	Total Ion C. I. %	Confirmed
1	Proteasome subunit alpha type-6 OS=Oryza sativa subsp. japonica GN=PAA1 PE=2 SV=1	PSA6_ORYSJ	27670.2	6.19	10	168	100	31.336	124	100	

Peptide Information

Calc. Mass	Obsrv. Mass	± da	± ppm	Start Seq.	End Seq.	Sequence	Ion Score	C. I. %	Modification	Rank	Result Type
807.4108	807.4277	0.0169	21	223	228	DDRVFR					Mascot
836.3896	836.4016	0.012	14	96	102	NEAAEFR					Mascot
946.5316	946.533	0.0014	1	34	43	SAGVTSIGVR					Mascot
946.5316	946.533	0.0014	1	34	43	SAGVTSIGVR					Mascot
1072.5997	1072.6053	0.0056	5	212	221	ATEIEVGVVR					Mascot
1072.5997	1072.6053	0.0056	5	212	221	ATEIEVGVVR	64	99.951			Mascot
1156.611	1156.6171	0.0061	5	12	21	HITIFSPEGR					Mascot
1156.611	1156.6171	0.0061	5	12	21	HITIFSPEGR	60	99.894			Mascot
1160.5986	1160.5919	-0.0067	-6	22	30	LYQVEYAFK					Mascot
1220.6157	1220.6034	-0.0123	-10	172	181	EQEAINFLEK					Mascot
1272.5579	1272.5577	-0.0002	0	154	164	CDPAGHFFGHK			Carbamidomethyl (C)[1]		Mascot
1407.6978	1407.6737	-0.0241	-17	105	116	WGYEMPVDVLAK					Mascot
1926.9767	1926.8766	-0.1001	-52	229	245	ALTTEEIDQHLTAISER					Mascot

Rank	Protein Name	Accession No.	Protein MW	Protein PI	Pep. Count	Protein Score	Protein Score C. I. %	Intensity Matched	Total Ion Score	Total Ion C. I. %	Confirmed
2	Proteasome subunit alpha type-6-B OS=Arabidopsis thaliana GN=PAA2 PE=1 SV=1	PSA6B_ARATH	27446.9	5.75	7	146	100	25.217	123	100	

Peptide Information

Calc. Mass	Obsrv. Mass	± da	± ppm	Start Seq.	End Seq.	Sequence	Ion Score	C. I. %	Modification	Rank	Result Type
836.3896	836.4016	0.012	14	96	102	NEAAEFR					Mascot
1025.476	1025.4946	0.0186	18	2	11	SRGSGAGYDR					Mascot
1072.5997	1072.6053	0.0056	5	212	221	ATEIEVGVVR					Mascot
1072.5997	1072.6053	0.0056	5	212	221	ATEIEVGVVR	64	99.951			Mascot
1156.611	1156.6171	0.0061	5	12	21	HITIFSPEGR					Mascot
1156.611	1156.6171	0.0061	5	12	21	HITIFSPEGR	60	99.894			Mascot
1172.5114	1172.613	0.1016	87	1	11	MSRGSGAGYDR			Oxidation (M)[1]		Mascot
1206.6001	1206.6058	0.0057	5	172	181	EQEAVNFLEK					Mascot

1220.6304	1220.6034	-0.027	-22	44	54	GKDSVCVVTQK			Carbamidomethyl (C)[6]		Mascot

3	Proteasome subunit alpha type-6-A OS=Arabidopsis thaliana GN=PAA1 PE=1 SV=2	PSA6A_ARATH	27390.8	5.6	6	141	100	19.999	123	100		

Peptide Information

Calc. Mass	Obsrv. Mass	± da	± ppm	Start Seq.	End Seq.	Sequence	Ion Score	C. I. %	Modification	Rank	Result Type
1025.476	1025.4946	0.0186	18	2	11	SRGSGAGYDR					Mascot
1072.5997	1072.6053	0.0056	5	212	221	ATEIEVGVVR					Mascot
1072.5997	1072.6053	0.0056	5	212	221	ATEIEVGVVR	64	99.951			Mascot
1156.611	1156.6171	0.0061	5	12	21	HITIFSPEGR					Mascot
1156.611	1156.6171	0.0061	5	12	21	HITIFSPEGR	60	99.894			Mascot
1172.5114	1172.613	0.1016	87	1	11	MSRGSGAGYDR			Oxidation (M)[1]		Mascot
1206.6001	1206.6058	0.0057	5	172	181	EQEAVNFLEK					Mascot
1220.6304	1220.6034	-0.027	-22	44	54	GKDSVCVVTQK			Carbamidomethyl (C)[6]		Mascot

4	Proteasome subunit alpha type-6 OS=Glycine max GN=PAA1 PE=1 SV=2	PSA6_SOYBN	27488.9	5.83	4	136	100	23.606	124	100		

Peptide Information

Calc. Mass	Obsrv. Mass	± da	± ppm	Start Seq.	End Seq.	Sequence	Ion Score	C. I. %	Modification	Rank	Result Type
836.3896	836.4016	0.012	14	96	102	NEAAEFR					Mascot
1072.5997	1072.6053	0.0056	5	212	221	ATEIEVGVVR					Mascot
1072.5997	1072.6053	0.0056	5	212	221	ATEIEVGVVR	64	99.951			Mascot
1156.611	1156.6171	0.0061	5	12	21	HITIFSPEGR					Mascot
1156.611	1156.6171	0.0061	5	12	21	HITIFSPEGR	60	99.894			Mascot
1206.6001	1206.6058	0.0057	5	172	181	DQEAINFLEK					Mascot

5	Proteasome subunit alpha type-6 OS=Nicotiana tabacum GN=PAA1 PE=1 SV=1	PSA6_TOBAC	27400	5.92	6	80	99.507	19.666	60	99.894		

Peptide Information

Calc. Mass	Obsrv. Mass	± da	± ppm	Start Seq.	End Seq.	Sequence	Ion Score	C. I. %	Modification	Rank	Result Type
836.3896	836.4016	0.012	14	96	102	NEAAEFR					Mascot
1156.611	1156.6171	0.0061	5	12	21	HITIFSPEGR					Mascot
1156.611	1156.6171	0.0061	5	12	21	HITIFSPEGR	60	99.894			Mascot
1220.6157	1220.6034	-0.0123	-10	172	181	EQEAINFLEK					Mascot
1272.5579	1272.5577	-0.0002	0	154	164	CDPAGHFFGHK			Carbamidomethyl (C)[1]		Mascot

Calc. Mass	Obsrv. Mass	± da	± ppm	Start Seq.	End Seq.	Sequence	Ion Score	C. I. %	Modification	Rank	Result Type
1942.9381	1942.9749	0.0368	19	148	164	GPQLFKCDPAGHFFGHK			Carbamidomethyl (C)[7]		Mascot
1955.9921	1955.9672	-0.0249	-13	229	245	VLTTEEIDEHLTAISER					Mascot

6	Proteasome subunit alpha type-1 OS=Saccharomyces cerevisiae (strain ATCC 204508 / S288c) GN=SCL1 PE=1 SV=1	PSA1_YEAST	28154.2	5.89	6	78	99.218	15.139	60	99.894	

Peptide Information

Calc. Mass	Obsrv. Mass	± da	± ppm	Start Seq.	End Seq.	Sequence	Ion Score	C. I. %	Modification	Rank	Result Type
858.4567	858.444	-0.0127	-15	245	252	LVAIAEQD					Mascot
1049.5297	1049.5005	-0.0292	-28	49	57	DCTVVISQK			Carbamidomethyl (C)[2]		Mascot
1156.611	1156.6171	0.0061	5	15	24	HITIFSPEGR					Mascot
1156.611	1156.6171	0.0061	5	15	24	HITIFSPEGR	60	99.894			Mascot
1160.5986	1160.5919	-0.0067	-6	25	33	LYQVEYAFK					Mascot
1455.7114	1455.6522	-0.0592	-41	233	244	FFTLSAENIEER					Mascot
1738.7815	1738.8336	0.0521	30	106	119	YKYGYDMPCDVLAK			Carbamidomethyl (C)[9], Oxidation (M)[7]		Mascot

7	Proteasome subunit alpha type-6 OS=Caenorhabditis elegans GN=pas-1 PE=1 SV=1	PSA6_CAEEL	27259.9	8.22	3	69	93.034	12.031	60	99.894	

Peptide Information

Calc. Mass	Obsrv. Mass	± da	± ppm	Start Seq.	End Seq.	Sequence	Ion Score	C. I. %	Modification	Rank	Result Type
1156.611	1156.6171	0.0061	5	12	21	HITIFSPEGR					Mascot
1156.611	1156.6171	0.0061	5	12	21	HITIFSPEGR	60	99.894			Mascot
1198.6903	1198.6031	-0.0872	-73	44	55	GADAAVIAVQKR					Mascot
1272.6041	1272.5577	-0.0464	-36	154	164	VDPAGYYRGMK			Oxidation (M)[10]		Mascot

8	Cell division topological specificity factor OS=Rhizobium etli (strain CFN 42 / ATCC 51251) GN=minE PE=3 SV=1	MINE_RHIEC	9639.2	5.52	8	69	93.034	5.193	

Peptide Information

Calc. Mass	Obsrv. Mass	± da	± ppm	Start Seq.	End Seq.	Sequence	Ion Score	C. I. %	Modification	Rank	Result Type
805.4679	805.4406	-0.0273	-34	6	11	LFNKQR					Mascot
870.4904	870.5242	0.0338	39	10	17	QRTAPAAR					Mascot
985.5928	985.5165	-0.0763	-77	41	49	EEILAVIAK					Mascot
1078.6368	1078.535	-0.1018	-94	20	28	LQVLLAHER					Mascot
1078.6368	1078.535	-0.1018	-94	20	28	LQVLLAHER					Mascot
1182.6453	1182.578	-0.0673	-57	1	9	MNIFRLFNK					Mascot

1198.6401	1198.6031	-0.037	-31	1	9	MNIFRLFNK			Oxidation (M)[1]		Mascot
1204.6532	1204.6029	-0.0503	-42	29	40	SSAGSDLVSLLR					Mascot
1959.0546	1958.915	-0.1396	-71	41	57	EEILAVIAKHVELDHDK					Mascot
2264.2722	2264.1421	-0.1301	-57	20	40	LQVLLAHERSSAGSDLVS LLR					Mascot

9	Proteasome subunit alpha type-6 OS=Homo sapiens GN=PSMA6 PE=1 SV=1	PSA6_HUMAN	27838	6.34	3	68	92.184	13.983	60	99.894

Protein Group

Proteasome subunit alpha type-6 OS=Bos taurus GN=PSMA6 PE=1 SV=1	PSA6_BOVIN	27838	6.3400 001525 8789
Proteasome subunit alpha type-6 OS=Mus musculus GN=Psma6 PE=1 SV=1	PSA6_MOUSE	27811	6.3400 001525 8789
Proteasome subunit alpha type-6 OS=Rattus norvegicus GN=Psma6 PE=1 SV=1	PSA6_RAT	27838	6.3400 001525 8789

Peptide Information

Calc. Mass	Obsrv. Mass	± da	± ppm	Start Seq.	End Seq.	Sequence	Ion Score	C. I. %	Modification	Rank	Result Type
1156.611	1156.6171	0.0061	5	12	21	HITIFSPEGR					Mascot
1156.611	1156.6171	0.0061	5	12	21	HITIFSPEGR	60	99.894			Mascot
1160.5986	1160.5919	-0.0067	-6	22	30	LYQVEYAFK					Mascot
1172.5735	1172.613	0.0395	34	96	104	YEAANWKYK					Mascot

10	Proteasome subunit alpha type-6 OS=Drosophila melanogaster GN=Prosalpha1 PE=2 SV=2	PSA6_DROME	27486.9	7.55	2	66	86.101	13.578	60	99.894

Peptide Information

Calc. Mass	Obsrv. Mass	± da	± ppm	Start Seq.	End Seq.	Sequence	Ion Score	C. I. %	Modification	Rank	Result Type
1156.611	1156.6171	0.0061	5	12	21	HITIFSPEGR					Mascot
1156.611	1156.6171	0.0061	5	12	21	HITIFSPEGR	60	99.894			Mascot
1160.5986	1160.5919	-0.0067	-6	22	30	LYQVEYAFK					Mascot

Gel Idx/Pos	214/I14	Instr./Gel Origin	BA2151/Sample Project 20140814	Process Status	Analysis Succeeded
Plate [#] Name	[1] Sample Project 20140814	Instrument Sample Name		Spectra	11

Rank	Protein Name	Accession No.	Protein MW	Protein PI	Pep. Count	Protein Score	Protein Score C. I. %	Intensity Matched	Total Ion Score	Total Ion C. I. %	Confirmed
1	Fructokinase-2 OS=Oryza sativa subsp. japonica GN=FRK2 PE=1 SV=1	SCRK2_ORYSJ	35893.1	5.02	13	235	100	29.924	165	100	

Protein Group

Protein Name	Accession No.	Protein MW	Protein PI
Fructokinase-2 OS=Oryza sativa subsp. indica GN=FRK2 PE=1 SV=2	SCRK2_ORYSI	35893.1	5.0199 999809 2651

Peptide Information

Calc. Mass	Obsrv. Mass	± da	± ppm	Start Seq.	End Seq.	Sequence	Ion Score	C. I. %	Modification	Rank	Result Type
842.4301	842.4733	0.0432	51	160	167	SAHVAAMR					Mascot
858.425	858.4254	0.0004	0	160	167	SAHVAAMR			Oxidation (M)[7]		Mascot
892.4022	892.4059	0.0037	4	117	122	EFMFYR					Mascot
908.3971	908.395	-0.0021	-2	117	122	EFMFYR			Oxidation (M)[3]		Mascot
908.3971	908.395	-0.0021	-2	117	122	EFMFYR	25	0	Oxidation (M)[3]		Mascot
963.5873	963.5838	-0.0035	-4	103	111	TALAFVTLK					Mascot
987.5721	987.5706	-0.0015	-2	238	246	LLIVTDGEK					Mascot
1233.5382	1233.6298	0.0916	74	287	296	DDSIFHNEEK					Mascot
1360.7253	1360.687	-0.0383	-28	238	249	LLIVTDGEKGCR			Carbamidomethyl (C)[11]		Mascot
1451.6948	1451.6985	0.0037	3	171	183	SAGILCSYDPNVR			Carbamidomethyl (C)[6]		Mascot
1451.6948	1451.6985	0.0037	3	171	183	SAGILCSYDPNVR	57	99.778	Carbamidomethyl (C)[6]		Mascot
1735.8361	1735.871	0.0349	20	72	86	FGDDEFGHMLVDILK					Mascot
1792.9052	1792.9064	0.0012	1	145	159	IFHYGSISLITEPCR			Carbamidomethyl (C)[14]		Mascot
1792.9052	1792.9064	0.0012	1	145	159	IFHYGSISLITEPCR	85	100	Carbamidomethyl (C)[14]		Mascot
1816.8395	1816.8103	-0.0292	-16	87	102	KNGVNAEGCLFDEHAR			Carbamidomethyl (C)[9]		Mascot
1816.8395	1816.8103	-0.0292	-16	87	102	KNGVNAEGCLFDEHAR			Carbamidomethyl (C)[9]		Mascot
1863.931	1863.9353	0.0043	2	72	87	FGDDEFGHMLVDILKK					Mascot
1952.8721	1952.9612	0.0891	46	209	226	VSDDEVAFLTQGDANDE K					Mascot
2128.0955	2128.0962	0.0007	0	123	141	NPSADMLLTEAELNLDLI R					Mascot
2144.0903	2144.0696	-0.0207	-10	123	141	NPSADMLLTEAELNLDLI R			Oxidation (M)[6]		Mascot

Rank	Protein Name	Accession No.	Protein MW	Protein PI	Pep. Count	Protein Score	Protein Score C. I. %	Intensity Matched	Total Ion Score	Total Ion C. I. %	Confirmed
2	Voltage-dependent L-type calcium channel subunit beta-2 OS=Oryctolagus cuniculus GN=CACNB2 PE=1 SV=1	CACB2_RABIT	71242.6	8.62	16	60	43.378	25.479			

Peptide Information

Calc. Mass	Obsrv. Mass	± da	± ppm	Start Seq.	End Seq.	Sequence	Ion Score	C. I. %	Modification	Rank	Result Type
858.468	858.4254	-0.0426	-50	303	310	SVLNNPSK					Mascot
873.4537	873.4371	-0.0166	-19	476	483	TDRSAPAR					Mascot
908.436	908.395	-0.041	-45	536	543	DSAYVEPK					Mascot
908.436	908.395	-0.041	-45	536	543	DSAYVEPK					Mascot
920.4908	920.4221	-0.0687	-75	515	523	SGTSRGLSR					Mascot
1009.5537	1009.5046	-0.0491	-49	156	163	LQHEQRAK					Mascot
1201.5228	1201.5342	0.0114	9	265	274	GYEVTDMMQK					Mascot
1207.5643	1207.6512	0.0869	72	126	134	FNNDWWIGR					Mascot
1225.6357	1225.6718	0.0361	29	390	400	HLNVQMVAADK					Mascot
1225.6357	1225.6718	0.0361	29	390	400	HLNVQMVAADK					Mascot
1233.5127	1233.6298	0.1171	95	265	274	GYEVTDMMQK			Oxidation (M)[7,8]		Mascot
1241.6307	1241.6672	0.0365	29	390	400	HLNVQMVAADK			Oxidation (M)[6]		Mascot
1241.6307	1241.6672	0.0365	29	390	400	HLNVQMVAADK			Oxidation (M)[6]		Mascot
1464.7019	1464.7437	0.0418	29	124	134	EKFNNDWWIGR					Mascot
1700.9078	1700.8988	-0.009	-5	435	450	ATHPPSSNLPNPLLSR					Mascot
1702.015	1701.8953	-0.1197	-70	359	374	TSLAPIVVYVKISSPK					Mascot
1773.8324	1773.8701	0.0377	21	484	499	SASQAEEEPCLEPAKK			Carbamidomethyl (C)[10]		Mascot
1780.9188	1780.8276	-0.0912	-51	217	233	SPKPSANSVTSPHSKEK					Mascot
1805.8988	1805.8282	-0.0706	-39	317	332	SNTRSSLAEVQSEIER					Mascot
2067.9766	2068.0317	0.0551	27	265	281	GYEVTDMMQKALFDFLK			Oxidation (M)[7,8]		Mascot
2067.9766	2068.0317	0.0551	27	265	281	GYEVTDMMQKALFDFLK			Oxidation (M)[7,8]		Mascot
2127.9976	2128.0962	0.0986	46	479	498	SAPARSASQAEEEPCLE PAK			Carbamidomethyl (C)[15]		Mascot

3	Uroporphyrinogen decarboxylase OS=Bacillus anthracis GN=hemE PE=3 SV=1	DCUP_BACAN	39283.2	5.76	12	58	8.171	1.63

Peptide Information

Calc. Mass	Obsrv. Mass	± da	± ppm	Start Seq.	End Seq.	Sequence	Ion Score	C. I. %	Modification	Rank	Result Type
963.5298	963.5838	0.054	56	175	182	AWFALIDK					Mascot
965.5302	965.4636	-0.0666	-69	4	11	TINETFLK					Mascot
1182.6439	1182.6022	-0.0417	-35	183	192	LADMVITYLK			Oxidation (M)[4]		Mascot
1191.5865	1191.6652	0.0787	66	28	37	QAGRSQPEYR					Mascot
1237.6899	1237.6586	-0.0313	-25	92	103	SGIGPVIDNPIR					Mascot

1394.7427	1394.6755	-0.0672	-48	333	343	RLTTFIHEYSK				Mascot
1729.8115	1729.8168	0.0053	3	18	31	TDYVPAWYMRQAGR			Oxidation (M)[9]	Mascot
1735.9377	1735.871	-0.0667	-38	334	348	LTTFIHEYSKGQLAK				Mascot
1916.9615	1917.0919	0.1304	68	167	182	AFMYAEPKAWFALIDK			Oxidation (M)[3]	Mascot
1920.0623	1919.9467	-0.1156	-60	183	200	LADMVITYLKAQINAGAK				Mascot
2040.0186	2039.9331	-0.0855	-42	201	218	AVQIFDSWVGTVNVADY R				Mascot
2127.156	2127.0386	-0.1174	-55	175	192	AWFALIDKLADMVITYLK			Oxidation (M)[12]	Mascot

4	Chromosome partition protein Smc OS=Fusobacterium nucleatum subsp. nucleatum (strain ATCC 25586 / CIP 101130 / JCM 8532 / LMG 13131) GN=smc PE=3 SV=2	SMC_FUSNN	136558.7	5.19	24	58	3.843	15.104	

Peptide Information

Calc. Mass	Obsrv. Mass	± da	± ppm	Start Seq.	End Seq.	Sequence	Ion Score	C. I. %	Modification	Rank	Result Type
860.4472	860.4041	-0.0431	-50	999	1005	ERDDVVK					Mascot
873.5152	873.4371	-0.0781	-89	262	268	LNKTLER					Mascot
924.3914	924.3965	0.0051	6	1039	1045	MCDETIR			Carbamidomethyl (C)[2]		Mascot
981.5074	981.4624	-0.045	-46	561	568	CIAFLTEK			Carbamidomethyl (C)[1]		Mascot
1105.6001	1105.593	-0.0071	-6	378	386	FDLIENRAK					Mascot
1175.6519	1175.6442	-0.0077	-7	678	687	TLEEKVTDLK					Mascot
1182.5273	1182.6022	0.0749	63	411	420	SSQDEVENFK					Mascot
1231.6641	1231.6525	-0.0116	-9	305	315	AVTSERLDNVK					Mascot
1233.709	1233.6298	-0.0792	-64	921	930	FEKIVEVIEK					Mascot
1281.7202	1281.6918	-0.0284	-22	2	12	YLKAVEINGFK					Mascot
1327.6199	1327.7024	0.0825	62	1148	1159	ESDSIFGVTMNK					Mascot
1407.759	1407.6786	-0.0804	-57	136	148	TAYSVIGQGKVER					Mascot
1481.6472	1481.7051	0.0579	39	1039	1050	MCDETIRNTEGR			Carbamidomethyl (C)[2]		Mascot
1481.6472	1481.7051	0.0579	39	1039	1050	MCDETIRNTEGR			Carbamidomethyl (C)[2]		Mascot
1679.9214	1679.8285	-0.0929	-55	223	237	SSLAKGIYITELEQK					Mascot
1723.8319	1723.8422	0.0103	6	352	366	NIVEMAAANKEFENK			Oxidation (M)[5]		Mascot
1789.9695	1789.808	-0.1615	-90	814	828	ILFLNNQSTIEQLEK					Mascot
1806.9385	1806.8787	-0.0598	-33	1129	1143	DFTDKSQFILITHNK					Mascot
1816.8456	1816.8103	-0.0353	-19	1144	1159	ETMKESDSIFGVTMNK					Mascot
1816.8456	1816.8103	-0.0353	-19	1144	1159	ETMKESDSIFGVTMNK					Mascot
1820.9541	1820.8286	-0.1255	-69	972	987	INNFGDVNLLAINEFK					Mascot
1865.8988	1865.8585	-0.0403	-22	878	892	IYNSENRDIETLNER					Mascot
1895.0121	1895.0588	0.0467	25	538	555	AVEAAIPGNLQDIIVEDK					Mascot

1937.9338	1937.9644	0.0306	16	698	713	EDLSIKLENYENEVDK					Mascot
1954.979	1954.9463	-0.0327	-17	1148	1165	ESDSIFGVTMNKEIGISK					Mascot
2068.1899	2068.0317	-0.1582	-76	942	959	LNELVEITAQVIEVEKLK					Mascot
2068.1899	2068.0317	-0.1582	-76	942	959	LNELVEITAQVIEVEKLK					Mascot

5	Probable succinate dehydrogenase [ubiquinone] flavoprotein subunit, mitochondrial OS=Schizosaccharomyces pombe (strain 972 / ATCC 24843) GN=sdh1 PE=2 SV=1	DHSA_SCHPO	71049	8.57	16	55	0	3.602

Peptide Information

Calc. Mass	Obsrv. Mass	± da	± ppm	Start Seq.	End Seq.	Sequence	Ion Score	C. I. %	Modification	Rank	Result Type
812.4447	812.4322	-0.0125	-15	460	466	ACALHIK			Carbamidomethyl (C)[2]		Mascot
844.4635	844.4085	-0.055	-65	542	548	DIGIRDR					Mascot
924.4355	924.3965	-0.039	-42	316	322	GERFMER					Mascot
983.5408	983.4583	-0.0825	-84	19	27	LFSTSSTLK					Mascot
1156.5634	1156.5503	-0.0131	-11	165	175	AFGGQSLEYGK					Mascot
1182.5605	1182.6022	0.0417	35	507	515	LDMQKTMQR			Oxidation (M)[3,7]		Mascot
1213.6034	1213.6744	0.071	59	319	328	FMERYAPTAK					Mascot
1394.6594	1394.6755	0.0161	12	176	188	GGQAYRCAAVADR			Carbamidomethyl (C)[7]		Mascot
1729.9014	1729.8168	-0.0846	-49	564	579	NLLTCAVQTANAALNR			Carbamidomethyl (C)[5]		Mascot
1747.8572	1747.8489	-0.0083	-5	624	638	TTMDENEVKPVPPFK			Oxidation (M)[3]		Mascot
1771.8684	1771.8171	-0.0513	-29	69	85	ATFGLAEAGFNTACITK			Carbamidomethyl (C)[14]		Mascot
1788.9742	1789.0099	0.0357	20	373	390	LPGISETAAIFAGVDVTK					Mascot
1814.933	1814.8474	-0.0856	-47	226	241	GVIAMNLEDGSIHRFR					Mascot
1836.8221	1836.8644	0.0423	23	122	137	GSDWLGDQDAIHYMTK					Mascot
2144.1135	2144.0696	-0.0439	-20	48	68	YPVIDHTYDAIVVGAGGA GLR					Mascot
2341.1719	2341.05	-0.1219	-52	423	445	DKIVPGLYAAGEAACVSV HGGNR			Carbamidomethyl (C)[15]		Mascot

6	Fructokinase-2 OS=Zea mays GN=FRK2 PE=1 SV=1	SCRK2_MAIZE	35858.1	5.34	8	54	0	8.083	25	0

Peptide Information

Calc. Mass	Obsrv. Mass	± da	± ppm	Start Seq.	End Seq.	Sequence	Ion Score	C. I. %	Modification	Rank	Result Type
874.4022	874.4091	0.0069	8	160	167	SAHMAAMR					Mascot
890.3971	890.3925	-0.0046	-5	160	167	SAHMAAMR			Oxidation (M)[4]		Mascot
892.4022	892.4059	0.0037	4	117	122	EFMFYR					Mascot
908.3971	908.395	-0.0021	-2	117	122	EFMFYR			Oxidation (M)[3]		Mascot
908.3971	908.395	-0.0021	-2	117	122	EFMFYR	25	0	Oxidation (M)[3]		Mascot

963.5873	963.5838	-0.0035	-4	103	111	TALAFVTLK						Mascot
1233.5382	1233.6298	0.0916	74	287	296	DDSIFHNEEK						Mascot
1327.7845	1327.7024	-0.0821	-62	100	111	HARTALAFVTLK						Mascot
1332.6941	1332.6498	-0.0443	-33	238	249	LLVVTDGDKGCR			Carbamidomethyl (C)[11]			Mascot
1750.011	1749.8501	-0.1609	-92	316	333	GAIPALPTVATAQDLIAK						Mascot
1954.9757	1954.9463	-0.0294	-15	203	219	EADFIKVSDDEVAFLTR						Mascot

7	3-oxosteroid 1-dehydrogenase OS=Comamonas testosteroni PE=1 SV=1	3O1D_COMTE	62975.1	9.96	16	54	0	27.532

Peptide Information

Calc. Mass	Obsrv. Mass	± da	± ppm	Start Seq.	End Seq.	Sequence	Ion Score	C. I. %	Modification	Rank	Result Type
981.5363	981.4624	-0.0739	-75	474	482	NPNLGPIEK					Mascot
1209.6051	1209.6798	0.0747	62	412	420	SWLNNVYWK					Mascot
1209.6051	1209.6798	0.0747	62	412	420	SWLNNVYWK					Mascot
1231.6907	1231.6525	-0.0382	-31	263	274	RGVLLGAGGFER					Mascot
1263.6879	1263.6395	-0.0484	-38	104	114	QIGIPYRAMAK			Oxidation (M)[9]		Mascot
1327.7441	1327.7024	-0.0417	-31	245	256	VTGVIVERNGQR					Mascot
1337.7001	1337.6886	-0.0115	-9	411	420	KSWLNNVYWK					Mascot
1679.8613	1679.8285	-0.0328	-20	305	321	AGQAWAHTGADGLVLG R					Mascot
1723.9524	1723.8422	-0.1102	-64	358	374	VRPVSGIPAAMLAENAK					Mascot
1749.8701	1749.8501	-0.02	-11	264	279	GVLLGAGGFERNQEMR			Oxidation (M)[15]		Mascot
1771.9847	1771.8171	-0.1676	-95	206	223	RMTGGQALVAGLLTAAN K					Mascot
1792.8759	1792.9064	0.0305	17	275	288	NQEMRDQYLNKPTR					Mascot
1792.8759	1792.9064	0.0305	17	275	288	NQEMRDQYLNKPTR					Mascot
1803.921	1803.8069	-0.1141	-63	474	489	NPNLGPIEKGPFYAMR					Mascot
1808.8708	1808.8906	0.0198	11	275	288	NQEMRDQYLNKPTR			Oxidation (M)[4]		Mascot
1814.8352	1814.8474	0.0122	7	289	304	LVDGNPCGRQYGDAHR			Carbamidomethyl (C)[7]		Mascot
1838.9258	1838.8496	-0.0762	-41	483	498	GPFYAMRLWPGEIGTK			Oxidation (M)[6]		Mascot
1952.9569	1952.9612	0.0043	2	163	179	MSISAFEAHSMLSRELK			Oxidation (M)[1]		Mascot
2068.0315	2068.0317	0.0002	0	391	410	AQNPMGPLMPGSAVPDS KVR			Oxidation (M)[5]		Mascot
2068.0315	2068.0317	0.0002	0	391	410	AQNPMGPLMPGSAVPDS KVR			Oxidation (M)[5]		Mascot

8	Glycine--tRNA ligase OS=Chlamydia pneumoniae GN=glyQS PE=3 SV=2	SYG_CHLPN	114417.1	5.48	19	54	0	20.143

Peptide Information

Calc. Mass	Obsrv. Mass	± da	± ppm	Start	End	Sequence	Ion	C. I. %	Modification	Rank	Result Type

Calc. Mass	Obsrv. Mass	± da	± ppm	Start Seq.	End Seq.	Sequence	Ion Score	C. I. %	Modification	Rank	Result Type
842.4268	842.4733	0.0465	55	69	75	YGVHPNR					Mascot
874.4669	874.4091	-0.0578	-66	47	54	ALGPEPYK					Mascot
892.4523	892.4059	-0.0464	-52	55	62	AAYVEPSR					Mascot
983.552	983.4583	-0.0937	-95	371	379	NVAPEVVQK					Mascot
1225.7151	1225.6718	-0.0433	-35	812	822	LPIDLASLLDR					Mascot
1225.7151	1225.6718	-0.0433	-35	812	822	LPIDLASLLDR					Mascot
1231.614	1231.6525	0.0385	31	855	864	TFMGSLEFRK			Oxidation (M)[3]		Mascot
1237.6212	1237.6586	0.0374	30	275	284	LVADSYVEWR					Mascot
1464.7805	1464.7437	-0.0368	-25	551	564	AHSSDTISAIPLPR					Mascot
1473.8159	1473.6735	-0.1424	-97	864	877	KDEIAAVLIDSATK					Mascot
1735.9126	1735.871	-0.0416	-24	271	284	QLTRLVADSYVEWR					Mascot
1747.9014	1747.8489	-0.0525	-30	47	62	ALGPEPYKAAYVEPSR					Mascot
1774.995	1774.8218	-0.1732	-98	653	667	QDLQTPLTTFIEKLK					Mascot
1781.0055	1780.8276	-0.1779	-100	878	893	NPIEILDTAEALQLLK					Mascot
1792.88	1792.9064	0.0264	15	468	482	MVWDNSGVEYARPIR					Mascot
1792.88	1792.9064	0.0264	15	468	482	MVWDNSGVEYARPIR					Mascot
1806.9484	1806.8787	-0.0697	-39	285	301	ASLNYPLLSLSSTSEPK					Mascot
1808.8749	1808.8906	0.0157	9	468	482	MVWDNSGVEYARPIR			Oxidation (M)[1]		Mascot
1823.9327	1823.8079	-0.1248	-68	933	948	ESNFKQVLDAFPGFPK					Mascot
1941.0297	1940.8657	-0.164	-84	1	17	MSEHPLTLQSMIATILR					Mascot
1976.9965	1977.0205	0.024	12	668	684	SVTYFEALGSLYDKVER					Mascot
1976.9965	1977.0205	0.024	12	668	684	SVTYFEALGSLYDKVER					Mascot
2197.2373	2197.1462	-0.0911	-41	754	773	HITMGQKLSTIGTLLSLLDR					Mascot

9 Centromere-associated protein E OS=Mus musculus GN=Cenpe PE=1 SV=1 | CENPE_MOUSE | 287830.4 | 5.25 | 41 | 54 | 0 | 42.076

Peptide Information

Calc. Mass	Obsrv. Mass	± da	± ppm	Start Seq.	End Seq.	Sequence	Ion Score	C. I. %	Modification	Rank	Result Type
804.425	804.4033	-0.0217	-27	181	187	WLATGEK					Mascot
842.4553	842.4733	0.018	21	1773	1779	IAHMNLK			Oxidation (M)[4]		Mascot
844.4886	844.4085	-0.0801	-95	351	357	EIADLRK					Mascot
873.5152	873.4371	-0.0781	-89	721	727	RITDLQK					Mascot
920.4683	920.4221	-0.0462	-50	1405	1412	ETELSSVR					Mascot
965.4357	965.4636	0.0279	29	2467	2474	DVSQCKTQ			Carbamidomethyl (C)[5]		Mascot

987.5734	987.5706	-0.0028	-3	2426	2434	SLPAPHPIR		Mascot
1030.451	1030.4441	-0.0069	-7	428	435	MKDSNYEK	Oxidation (M)[1]	Mascot
1105.567	1105.593	0.026	24	1895	1903	DNLREAMLK	Oxidation (M)[7]	Mascot
1156.6433	1156.5503	-0.093	-80	2334	2342	LKQQNEQLR		Mascot
1223.5977	1223.6556	0.0579	47	2031	2040	EMAFIQEVEK		Mascot
1225.6536	1225.6718	0.0182	15	1675	1684	EKEHQLSQVK		Mascot
1225.6536	1225.6718	0.0182	15	1675	1684	EKEHQLSQVK		Mascot
1231.6277	1231.6525	0.0248	20	936	945	QLQESLQTER		Mascot
1241.6307	1241.6672	0.0365	29	975	984	QHQETINMLK		Mascot
1241.6307	1241.6672	0.0365	29	975	984	QHQETINMLK		Mascot
1263.6263	1263.6395	0.0132	10	190	199	HYGITKMNQR	Oxidation (M)[7]	Mascot
1305.705	1305.7113	0.0063	5	692	702	ELQLAFQEISK		Mascot
1327.6852	1327.7024	0.0172	13	767	777	SEELHIITTER		Mascot
1332.6577	1332.6498	-0.0079	-6	1564	1574	VEMENVNLTQR		Mascot
1337.6188	1337.6886	0.0698	52	1	12	MAEEASVAVCVR	Carbamidomethyl (C)[10], Oxidation (M)[1]	Mascot
1360.7682	1360.687	-0.0812	-60	1729	1740	ITLVTKENDSLK		Mascot
1364.6726	1364.6731	0.0005	0	1689	1699	ETVDQMEQLKK	Oxidation (M)[6]	Mascot
1481.7628	1481.7051	-0.0577	-39	450	462	TSVTSLRENSLMK	Oxidation (M)[12]	Mascot
1481.7628	1481.7051	-0.0577	-39	450	462	TSVTSLRENSLMK	Oxidation (M)[12]	Mascot
1532.756	1532.7548	-0.0012	-1	975	986	QHQETINMLKMK	Oxidation (M)[8,11]	Mascot
1700.8926	1700.8988	0.0062	4	788	802	DSRIQGLLEEIGNTR		Mascot
1701.7935	1701.8953	0.1018	60	550	563	DQEMQLMHEVSNLK		Mascot
1723.8353	1723.8422	0.0069	4	1330	1343	KLEESQMEISCLTR	Carbamidomethyl (C)[11]	Mascot
1735.8345	1735.871	0.0365	21	894	908	EELESRDSSLQSVEK		Mascot
1747.8241	1747.8489	0.0248	14	2066	2079	LSQEMDLHIEEMLK	Oxidation (M)[5,12]	Mascot
1749.7959	1749.8501	0.0542	31	1070	1083	ENIEMSIENQEELR	Oxidation (M)[5]	Mascot
1774.8566	1774.8218	-0.0348	-20	2203	2219	DSERPQAASGAEQLTSK		Mascot
1788.8722	1789.0099	0.1377	77	1756	1770	SLQQTEARDLENQEK		Mascot
1792.8534	1792.9064	0.053	30	1590	1603	DELWSMEERLTVER		Mascot
1792.8534	1792.9064	0.053	30	1590	1603	DELWSMEERLTVER		Mascot
1803.8187	1803.8069	-0.0118	-7	93	108	THTMMGSEDCLGVIPR	Carbamidomethyl (C)[10]	Mascot
1805.8916	1805.8282	-0.0634	-35	331	346	NTPYVNEVSNDEALLK		Mascot
1808.8484	1808.8906	0.0422	23	1590	1603	DELWSMEERLTVER	Oxidation (M)[6]	Mascot
1820.8451	1820.8286	-0.0165	-9	46	60	SFQFDRVFDSNETTK		Mascot
1863.9481	1863.9353	-0.0128	-7	625	641	QAMRTLSDLDTVALDAK	Oxidation (M)[3]	Mascot
1954.8699	1954.9463	0.0764	39	1644	1660	MVSDYTDEISHTQGDLK	Oxidation (M)[1]	Mascot
1976.9052	1977.0205	0.1153	58	1780	1795	EHQETIDRLMETMSEK		Mascot

1976.9052	1977.0205	0.1153	58	1780	1795	EHQETIDRLMETMSEK				Mascot
2039.9446	2039.9331	-0.0115	-6	1796	1812	TEEISNMKMELENVNMK				Mascot
2144.1062	2144.0696	-0.0366	-17	1	19	MAEEASVAVCVRVRPLN SR			Carbamidomethyl (C)[10]	Mascot
2440.2214	2440.208	-0.0134	-5	1621	1640	EEELRVAHVHLEEHQETI NK				Mascot

10 Microtubule-actin cross-linking factor 1 OS=Rattus norvegicus GN=Macf1 PE=1 SV=1 MACF1_RAT 623207.6 5.28 54 54 0 41.389

Peptide Information

Calc. Mass	Obsrv. Mass	± da	± ppm	Start Seq.	End Seq.	Sequence	Ion Score	C. I. %	Modification	Rank	Result Type
804.421	804.4033	-0.0177	-22	4587	4593	SLDDARK					Mascot
842.4519	842.4733	0.0214	25	4465	4471	WHVVSSK					Mascot
844.4523	844.4085	-0.0438	-52	3315	3321	AQIQEQK					Mascot
858.4791	858.4254	-0.0537	-63	2093	2099	QDIARQK					Mascot
860.4221	860.4041	-0.018	-21	72	78	VADERDR					Mascot
890.4578	890.3925	-0.0653	-73	4034	4040	SEEREIK					Mascot
904.521	904.5242	0.0032	4	4798	4805	LSVSKQSR					Mascot
908.4393	908.395	-0.0443	-49	3978	3985	SATMELEK					Mascot
908.4393	908.395	-0.0443	-49	3978	3985	SATMELEK					Mascot
921.4346	921.426	-0.0086	-9	2885	2892	AMLNEAEK			Oxidation (M)[2]		Mascot
924.4342	924.3965	-0.0377	-41	3978	3985	SATMELEK			Oxidation (M)[4]		Mascot
963.5118	963.5838	0.072	75	1495	1502	HGHKLSER					Mascot
965.505	965.4636	-0.0414	-43	638	645	EARLYEGK					Mascot
1156.5746	1156.5503	-0.0243	-21	2820	2828	SHWEDLNKK					Mascot
1182.6188	1182.6022	-0.0166	-14	661	669	LETQYCKLK			Carbamidomethyl (C)[6]		Mascot
1191.6005	1191.6652	0.0647	54	5181	5190	FQVEQIGENK					Mascot
1209.6548	1209.6798	0.025	21	3958	3968	MPPLIPAEVDK					Mascot
1209.6548	1209.6798	0.025	21	3958	3968	MPPLIPAEVDK					Mascot
1225.6497	1225.6718	0.0221	18	3958	3968	MPPLIPAEVDK			Oxidation (M)[1]		Mascot
1225.7263	1225.6718	-0.0545	-44	486	495	QLQVDLQILR					Mascot
1231.6528	1231.6525	-0.0003	0	4662	4672	EKTLLADDAQK					Mascot
1263.5999	1263.6395	0.0396	31	3332	3343	ATVDMLQAEGGR			Oxidation (M)[5]		Mascot
1281.6144	1281.6918	0.0774	60	4193	4202	DQLNEMKEFK					Mascot
1305.6798	1305.7113	0.0315	24	1359	1370	AFVESQQKSPGK					Mascot
1327.7006	1327.7024	0.0018	1	1198	1208	TTLQHWLSDVK					Mascot
1332.7191	1332.6498	-0.0693	-52	1783	1793	QMLQEKLGELK			Oxidation (M)[2]		Mascot

1360.7219	1360.687	-0.0349	-26	2602	2612	WVELTDKLNSR		Mascot
1394.6659	1394.6755	0.0096	7	1641	1651	EQYEGLQDRTR		Mascot
1407.7261	1407.6786	-0.0475	-34	4440	4453	LMLLSRGDSGSGSK		Mascot
1451.657	1451.6985	0.0415	29	824	835	LEDLLQDSMDEK	Oxidation (M)[9]	Mascot
1451.6583	1451.6985	0.0402	28	2483	2493	MQEDLNSRWEK	Oxidation (M)[1]	Mascot
1464.6636	1464.7437	0.0801	55	3112	3123	LDIEDSEAECRK	Carbamidomethyl (C)[10]	Mascot
1481.6802	1481.7051	0.0249	17	812	823	KYTCDHNTSLSR	Carbamidomethyl (C)[4]	Mascot
1481.6802	1481.7051	0.0249	17	812	823	KYTCDHNTSLSR	Carbamidomethyl (C)[4]	Mascot
1589.8356	1589.7793	-0.0563	-35	3211	3223	MFQKEQVDPLQVK		Mascot
1700.8966	1700.8988	0.0022	1	966	979	DIDAVQTWNLEKLR		Mascot
1701.9495	1701.8953	-0.0542	-32	836	851	EQLIQSKSSVASLVGR		Mascot
1725.9547	1725.8115	-0.1432	-83	5038	5050	WQQVWLLALERQR		Mascot
1765.8248	1765.8038	-0.021	-12	4348	4361	LETMNQCWESVLQK	Carbamidomethyl (C)[7]	Mascot
1773.7378	1773.8701	0.1323	75	1	15	MSSSDEETLSERSCR	Carbamidomethyl (C)[14]	Mascot
1774.8719	1774.8218	-0.0501	-28	1858	1873	QGSFSEDVISHKGDLR		Mascot
1776.9127	1776.8344	-0.0783	-44	1979	1993	QLQEELAEHQVPVEK		Mascot
1789.7328	1789.808	0.0752	42	1	15	MSSSDEETLSERSCR	Carbamidomethyl (C)[14], Oxidation (M)[1]	Mascot
1791.8356	1791.8419	0.0063	4	4328	4345	AGNELLESSAGDDASSL R		Mascot
1813.8942	1813.8052	-0.089	-49	4020	4033	LDQMVFFWEDIKAR	Oxidation (M)[4]	Mascot
1820.896	1820.8286	-0.0674	-37	4783	4797	GQLQELSTRWDTVCK	Carbamidomethyl (C)[14]	Mascot
1838.8412	1838.8496	0.0084	5	4960	4974	ALITEHQSFMEEMTR	Oxidation (M)[10]	Mascot
1849.8497	1849.895	0.0453	24	4127	4141	KSIDEMNNAWENLNR	Oxidation (M)[6]	Mascot
1865.9603	1865.8585	-0.1018	-55	738	754	SLQDTAELLSLENHPAK		Mascot
1894.9757	1895.0588	0.0831	44	2623	2638	STQYQDLLQDLSEKVK		Mascot
1917.011	1917.0919	0.0809	42	3942	3957	IEPMLETLENLSSRLR	Oxidation (M)[4]	Mascot
1919.9036	1919.9467	0.0431	22	392	407	LPQGYHPNHVEEEWGK		Mascot
1937.9889	1937.9644	-0.0245	-13	3978	3994	SATMELEKLQPSFEALK	Oxidation (M)[4]	Mascot
1954.9944	1954.9463	-0.0481	-25	5117	5133	FPTTKLEMTAVADIFDR		Mascot
1976.9845	1977.0205	0.036	18	2323	2341	GTALESLIMDITAPDSQA K	Oxidation (M)[9]	Mascot
1976.9845	1977.0205	0.036	18	2323	2341	GTALESLIMDITAPDSQA K	Oxidation (M)[9]	Mascot
2040.0702	2039.9331	-0.1371	-67	2843	2858	AQKYQWHVEDLVPWIK		Mascot
2068.0168	2068.0317	0.0149	7	755	772	QTVEAYSAAVQSQLQW MK		Mascot
2068.0168	2068.0317	0.0149	7	755	772	QTVEAYSAAVQSQLQW MK		Mascot
2080.1396	2080.093	-0.0466	-22	2704	2722	RLETVALPLQGLEDLAAD R		Mascot
2090.0837	2090.0027	-0.081	-39	4108	4126	ILGADLIFACGETEKPEVK	Carbamidomethyl (C)[10]	Mascot

2341.0911	2341.05	-0.0411	-18	2107	2127	EMVTRFMETADGNSAAV LQDR	Mascot

Gel Idx/Pos	215/I15	Instr./Gel Origin	BA2151/Sample Project 20140814	Process Status	Analysis Succeeded
Plate [#] Name	[1] Sample Project 20140814	Instrument Sample Name		Spectra	11

Rank	Protein Name	Accession No.	Protein MW	Protein PI	Pep. Count	Protein Score	Protein Score C. I. %	Intensity Matched	Total Ion Score	Total Ion C. I. %	Confirmed
1	Glutenin, high molecular weight subunit DX5 OS=Triticum aestivum GN=GLU-1D-1D PE=3 SV=5	GLT5_WHEAT	90523.6	6.43	4	110	100	4.685	110	100	

Protein Group

Glutenin, high molecular weight subunit PW212 OS=Triticum aestivum PE=3 SV=1	GLT4_WHEAT	89348.1	5.8200 001716 6138

Peptide Information

Calc. Mass	Obsrv. Mass	± da	± ppm	Start Seq.	End Seq.	Sequence	Ion Score	C. I. %	Modification	Rank	Result Type
1044.532	1044.5123	-0.0197	-19	34	41	ELQELQER					Mascot
1392.6359	1392.6281	-0.0078	-6	45	55	ACQQVMDQQLR			Carbamidomethyl (C)[2], Oxidation (M)[6]		Mascot
1473.7301	1473.7666	0.0365	25	825	837	AQQLAAQLPAMCR			Carbamidomethyl (C)[12], Oxidation (M)[11]		Mascot
1990.9618	1990.96	-0.0018	-1	83	100	GGSFYPGETTPPQQLQQ R					Mascot
1990.9618	1990.96	-0.0018	-1	83	100	GGSFYPGETTPPQQLQQ R	110	100			Mascot

Rank	Protein Name	Accession No.	Protein MW	Protein PI	Pep. Count	Protein Score	Protein Score C. I. %	Intensity Matched	Total Ion Score	Total Ion C. I. %	Confirmed
2	Microtubule-actin cross-linking factor 1, isoforms 1/2/3/5 OS=Homo sapiens GN=MACF1 PE=1 SV=4	MACF1_HUMAN	843033	5.28	65	63	74.708	42.753			

Peptide Information

Calc. Mass	Obsrv. Mass	± da	± ppm	Start Seq.	End Seq.	Sequence	Ion Score	C. I. %	Modification	Rank	Result Type
820.4597	820.4283	-0.0314	-38	7369	7375	IPTMSKK			Oxidation (M)[4]		Mascot
890.4618	890.4591	-0.0027	-3	4669	4675	WVELTDK					Mascot
892.4193	892.4098	-0.0095	-11	928	935	DAIEMASR					Mascot
908.4142	908.3867	-0.0275	-30	928	935	DAIEMASR			Oxidation (M)[5]		Mascot
972.5472	972.4761	-0.0711	-73	5205	5212	RELEALNK					Mascot
991.4989	991.5569	0.058	58	5213	5220	QCGKLTER			Carbamidomethyl (C)[2]		Mascot
993.5363	993.5036	-0.0327	-33	3004	3011	LYQETAIR					Mascot
1044.5936	1044.5123	-0.0813	-78	1133	1141	SELNLLVEK					Mascot
1046.5564	1046.4756	-0.0808	-77	1027	1034	ARFQHLMK			Oxidation (M)[7]		Mascot
1057.5248	1057.4238	-0.101	-96	7044	7051	SRVMDFFR					Mascot
1064.4895	1064.5681	0.0786	74	2001	2009	DEYQTSPPK					Mascot
1082.5187	1082.554	0.0353	33	3949	3957	VLDMENSFK					Mascot

1096.6514	1096.5692	-0.0822	-75	5628	5636	ALLELVPWR		Mascot
1156.5858	1156.5378	-0.048	-42	6487	6496	VFANEVNAHR		Mascot
1182.5316	1182.562	0.0304	26	3805	3813	LDKQCEMMK	Carbamidomethyl (C)[5]	Mascot
1209.6548	1209.6578	0.003	2	5916	5926	MPPLIPAEVDK		Mascot
1214.5214	1214.6326	0.1112	92	3805	3813	LDKQCEMMK	Carbamidomethyl (C)[5], Oxidation (M)[7,8]	Mascot
1247.6049	1247.6075	0.0026	2	5399	5410	ATVDMLQAEGGR		Mascot
1252.5804	1252.5829	0.0025	2	7111	7121	DAYRPTTDADK		Mascot
1262.6158	1262.6775	0.0617	49	1329	1338	NQTKLDQCQK	Carbamidomethyl (C)[8]	Mascot
1262.6158	1262.6775	0.0617	49	1329	1338	NQTKLDQCQK	Carbamidomethyl (C)[8]	Mascot
1280.6416	1280.6505	0.0089	7	4233	4242	LQQFMENKSR		Mascot
1281.6144	1281.6591	0.0447	35	6151	6160	DQLNEMKEFK		Mascot
1284.5382	1284.6371	0.0989	77	5112	5122	VNSGCVMMENK	Carbamidomethyl (C)[5], Oxidation (M)[7]	Mascot
1300.5331	1300.6146	0.0815	63	5112	5122	VNSGCVMMENK	Carbamidomethyl (C)[5], Oxidation (M)[7,8]	Mascot
1303.6239	1303.6859	0.062	48	1349	1358	DYELQLMTYK		Mascot
1303.7151	1303.6859	-0.0292	-22	1737	1749	QLAGGMVSLKSGR		Mascot
1310.725	1310.5969	-0.1281	-98	6173	6183	LNHQGELMLKK		Mascot
1310.725	1310.5969	-0.1281	-98	6173	6183	LNHQGELMLKK		Mascot
1326.7198	1326.6074	-0.1124	-85	6173	6183	LNHQGELMLKK	Oxidation (M)[8]	Mascot
1328.7057	1328.6915	-0.0142	-11	3961	3972	EPSEIGNLVKDK		Mascot
1328.7057	1328.6915	-0.0142	-11	3961	3972	EPSEIGNLVKDK		Mascot
1344.6754	1344.686	0.0106	8	56	67	KSQDSVLDPAER		Mascot
1346.6951	1346.7379	0.0428	32	4512	4523	AFLAELEQNSPK		Mascot
1359.6863	1359.6877	0.0014	1	5955	5967	GEELIGRSQGADK		Mascot
1360.5984	1360.7285	0.1301	96	980	991	SSAPGECHQIMK	Carbamidomethyl (C)[7], Oxidation (M)[11]	Mascot
1360.7219	1360.7285	0.0066	5	4669	4679	WVELTDKLNSR		Mascot
1403.7278	1403.7317	0.0039	3	5828	5839	ESIAEHKPHIDK		Mascot
1415.7278	1415.725	-0.0028	-2	97	107	KHINDLYEDLR		Mascot
1424.7744	1424.7144	-0.06	-42	5543	5555	YADITVTSSKALR		Mascot
1428.7693	1428.7058	-0.0635	-44	7010	7021	LNDALDRLEELK		Mascot
1436.7162	1436.6782	-0.038	-26	5007	5018	TGSLEEMTQRLR	Oxidation (M)[7]	Mascot
1451.657	1451.6829	0.0259	18	824	835	LEDLLQDSMDEK	Oxidation (M)[9]	Mascot
1456.6261	1456.7053	0.0792	54	3679	3690	DIEGFMEENQTK	Oxidation (M)[6]	Mascot
1456.827	1456.7053	-0.1217	-84	3501	3513	AWVGNKNLILNSK		Mascot
1473.7584	1473.7666	0.0082	6	966	977	DLDLVQTWNLEK		Mascot
1480.7941	1480.7096	-0.0845	-57	5336	5348	IAQLQEALLHCGK	Carbamidomethyl (C)[11]	Mascot
1528.7966	1528.7076	-0.089	-58	2018	2031	QKTPEGLQESANVK		Mascot
1551.8126	1551.7551	-0.0575	-37	6905	6917	DQEPIPQNIDRVK		Mascot

Calc. Mass	Obsrv. Mass	± da	± ppm	Start Seq.	End Seq.	Sequence	Ion Score	C. I. %	Modification	Rank	Result Type
1569.8846	1569.799	-0.0856	-55	4936	4949	VTLDPVQLESSLLR					Mascot
1588.8541	1588.8538	-0.0003	0	2391	2405	DAVTVGLLDKETATR					Mascot
1588.8541	1588.8538	-0.0003	0	2391	2405	DAVTVGLLDKETATR					Mascot
1596.7687	1596.7831	0.0144	9	5865	5877	AENMYAQIKEEVR			Oxidation (M)[4]		Mascot
1610.8319	1610.8207	-0.0112	-7	2962	2975	LPSEQVLQQPMNAR					Mascot
1615.8148	1615.7594	-0.0554	-34	4219	4232	LLPQAEMFEHLSGK			Oxidation (M)[7]		Mascot
1621.8077	1621.8403	0.0326	20	5278	5290	MFQKEQVDPLQMK					Mascot
1645.8289	1645.8457	0.0168	10	6136	6150	LCTMPPVGTDLNTVK			Carbamidomethyl (C)[2]		Mascot
1792.8059	1792.8774	0.0715	40	3330	3346	ESPGSEQTPFMTAPEGK					Mascot
1798.8939	1798.7986	-0.0953	-53	1797	1811	HNLIDQDMACAILIR			Carbamidomethyl (C)[10], Oxidation (M)[8]		Mascot
1838.8412	1838.8809	0.0397	22	6918	6932	ALIAEHQTFMEEMTR			Oxidation (M)[10,13]		Mascot
1885.7911	1885.9521	0.161	85	2797	2812	DMLIACNQTAEMSCNK			Carbamidomethyl (C)[6,14]		Mascot
1933.9688	1933.9921	0.0233	12	6370	6385	EQLDTHMELYSQLKAK					Mascot
1947.9231	1947.915	-0.0081	-4	588	602	FVYELLSWVEEMQMK			Oxidation (M)[12]		Mascot
1958.9893	1958.8542	-0.1351	-69	3941	3957	FVTISGQKVLDMENSFK			Oxidation (M)[12]		Mascot
2031.9474	2031.921	-0.0264	-13	1373	1389	RMLSSSDAITQEFMDLR			Oxidation (M)[2,14]		Mascot
2084.0117	2083.9597	-0.052	-25	755	772	QTVEAYSAAVQSQLQWMK			Oxidation (M)[17]		Mascot
2091.9805	2092.043	0.0625	30	496	511	DENYYQLEELAFRVMR			Oxidation (M)[15]		Mascot
2092.0266	2092.043	0.0164	8	4342	4360	WLKETEGSIPPTETSMSAK					Mascot
2153.0332	2153.1294	0.0962	45	2880	2898	GKSLGQVSLTHPYSECDFK			Carbamidomethyl (C)[16]		Mascot
2182.1616	2182.0283	-0.1333	-61	2725	2744	IIASHQVLNGGIVDIFSDQR					Mascot
2201.01	2201.1213	0.1113	51	4431	4448	DLTEIQCDMSDVNLKYEK			Carbamidomethyl (C)[7]		Mascot
2217.0051	2217.1045	0.0994	45	4431	4448	DLTEIQCDMSDVNLKYEK			Carbamidomethyl (C)[7], Oxidation (M)[9]		Mascot
2322.3181	2322.1709	-0.1472	-63	5617	5636	LVLDTVNEVSRALLELVPWR					Mascot
2450.2866	2450.2693	-0.0173	-7	341	360	TFPQNPVELKALYNQYIHFK					Mascot

3	Sister chromatid cohesion protein PDS5 homolog A OS=Mus musculus GN=Pds5a PE=3 SV=3	PDS5A_MOUSE	151771.5	8.06	26	59	25.358	9.065

Peptide Information

Calc. Mass	Obsrv. Mass	± da	± ppm	Start Seq.	End Seq.	Sequence	Ion Score	C. I. %	Modification	Rank	Result Type
820.4159	820.4283	0.0124	15	812	819	STGEKNGK					Mascot
892.4193	892.4098	-0.0095	-11	805	811	DLLMNDR			Oxidation (M)[4]		Mascot
894.4904	894.45	-0.0404	-45	729	736	RGTPHQAK					Mascot
939.4564	939.4343	-0.0221	-24	879	886	ISKSDMSR			Oxidation (M)[6]		Mascot
1096.5898	1096.5692	-0.0206	-19	331	339	FNDIHVPVR					Mascot

1280.7573	1280.6505	-0.1068	-83	377	388	HDVIVTIITAAK					Mascot
1300.6361	1300.6146	-0.0215	-17	507	516	CQNMLRSHVR			Carbamidomethyl (C)[1]		Mascot
1303.6715	1303.6859	0.0144	11	601	611	QPTNPFLEMVK					Mascot
1303.6715	1303.6859	0.0144	11	601	611	QPTNPFLEMVK					Mascot
1332.639	1332.6976	0.0586	44	1297	1310	AAGSQESLEAGNAK					Mascot
1342.7074	1342.6617	-0.0457	-34	1283	1294	NDDLNKPVSKGR					Mascot
1344.6866	1344.686	-0.0006	0	292	303	SNDGEERLAVVR					Mascot
1346.7539	1346.7379	-0.016	-12	513	523	SHVRELLDLHK					Mascot
1382.717	1382.6981	-0.0189	-14	416	427	EAMMGLAQLYKK					Mascot
1428.7482	1428.7058	-0.0424	-30	544	556	NLPDPGKAQDFVK					Mascot
1451.7198	1451.6829	-0.0369	-25	980	991	EYIKQNPMATEK					Mascot
1524.774	1524.7439	-0.0301	-20	737	749	QAVHCIHAIFSNK			Carbamidomethyl (C)[5]		Mascot
1621.822	1621.8403	0.0183	11	449	461	LLHIYYQNSIDDK					Mascot
1635.8081	1635.8278	0.0197	12	570	583	SQLELLISPTCSCK			Carbamidomethyl (C)[11,13]		Mascot
1699.8257	1699.8195	-0.0062	-4	1028	1040	ECLWFMLEVLMTK			Carbamidomethyl (C)[2]		Mascot
1858.0182	1857.8992	-0.119	-64	390	405	DLALVNDQLLGFVRER					Mascot
1904.9933	1904.9753	-0.018	-9	568	583	LRSQLELLISPTCSCK			Carbamidomethyl (C)[13,15]		Mascot
1958.9971	1958.8542	-0.1429	-73	467	482	IFAQYLVPHNLETEER					Mascot
2031.9941	2031.921	-0.0731	-36	1274	1292	SESQGNATKNDDLNKPVSK					Mascot
2201.04	2201.1213	0.0813	37	524	543	QPTSEANCSAMFGKLMTIAK			Carbamidomethyl (C)[8], Oxidation (M)[11]		Mascot
2213.0842	2213.0586	-0.0256	-12	340	358	LESVKFASHCLMNHPDLAK			Carbamidomethyl (C)[10], Oxidation (M)[12]		Mascot
2217.0349	2217.1045	0.0696	31	524	543	QPTSEANCSAMFGKLMTIAK			Carbamidomethyl (C)[8], Oxidation (M)[11,16]		Mascot
2450.1501	2450.2693	0.1192	49	1158	1182	SAGTETGSNINANSELSPSAGSRSR					Mascot

4	Outer dense fiber protein 2 OS=Mus musculus GN=Odf2 PE=1 SV=1	ODFP2_MOUSE	96337.1	7.52	25	57	0	7.96	

Peptide Information

Calc. Mass	Obsrv. Mass	± da	± ppm	Start Seq.	End Seq.	Sequence	Ion Score	C. I. %	Modification	Rank	Result Type
892.4119	892.4098	-0.0021	-2	2	11	SASSSGGSPR					Mascot
972.5585	972.4761	-0.0824	-85	756	763	TELGQLRR					Mascot
993.5363	993.5036	-0.0327	-33	448	455	YNQVVKDK					Mascot
1026.5289	1026.5	-0.0289	-28	191	198	DFTMLQKK			Oxidation (M)[4]		Mascot
1044.4996	1044.5123	0.0127	12	497	505	QTAEYSAFK					Mascot
1096.527	1096.5692	0.0422	38	260	268	LSTFEETNR					Mascot
1131.5641	1131.6167	0.0526	46	714	723	QLESAIEDAR					Mascot

Calc. Mass	Obsrv. Mass	± da	± ppm	Start Seq.	End Seq.	Sequence	Ion Score	C. I. %	Modification	Rank	Result Type
1156.6184	1156.5378	-0.0806	-70	63	72	TKVPWMPPGK			Oxidation (M)[6]		Mascot
1182.5974	1182.562	-0.0354	-30	369	378	NLERSGNQHK					Mascot
1203.6766	1203.6522	-0.0244	-20	224	234	QVMALKDTIGK					Mascot
1246.5409	1246.6187	0.0778	62	538	547	NYEGMIDNYK					Mascot
1252.6243	1252.5829	-0.0414	-33	511	521	LKASFAPMEDK			Oxidation (M)[8]		Mascot
1276.6454	1276.5808	-0.0646	-51	379	389	AEVEAIMEQLK			Oxidation (M)[7]		Mascot
1284.614	1284.6371	0.0231	18	188	197	EEKDFTMLQK			Oxidation (M)[7]		Mascot
1328.678	1328.6915	0.0135	10	65	76	VPWMPPGKSSAR			Oxidation (M)[4]		Mascot
1328.678	1328.6915	0.0135	10	65	76	VPWMPPGKSSAR	6	0	Oxidation (M)[4]		Mascot
1332.7305	1332.6976	-0.0329	-25	286	296	LMEQQGTLLKR			Oxidation (M)[2]		Mascot
1344.7118	1344.686	-0.0258	-19	661	671	QLSQKVDELER					Mascot
1388.738	1388.6987	-0.0393	-28	725	736	QVEQTKEQALSK					Mascot
1456.677	1456.7053	0.0283	19	334	346	TASELSKSMESMR					Mascot
1456.677	1456.7053	0.0283	19	334	346	TASELSKSMESMR					Mascot
1569.8054	1569.799	-0.0064	-4	31	45	TPCGAPSVTVTKSHK			Carbamidomethyl (C)[3]		Mascot
1699.7527	1699.8195	0.0668	39	1	17	MSASSSGGSPRFPSCGK			Carbamidomethyl (C)[15]		Mascot
1792.8746	1792.8774	0.0028	2	281	295	EDSERLMEQQGTLLK			Oxidation (M)[7]		Mascot
1811.8627	1811.8757	0.013	7	237	251	TEKQMTCTDINTLTR			Carbamidomethyl (C)[7]		Mascot
1904.9131	1904.9753	0.0622	33	707	723	ECGSLARQLESAIEDAR			Carbamidomethyl (C)[2]		Mascot
1933.9648	1933.9921	0.0273	14	469	484	VTDLVNQQQSLEEKMR			Oxidation (M)[15]		Mascot

5	Threonine--tRNA ligase OS=Parabacteroides distasonis (strain ATCC 8503 / DSM 20701 / NCTC 11152) GN=thrS PE=3 SV=1	SYT_PARD8	75326.1	6	18	56	0	3.903

Peptide Information

Calc. Mass	Obsrv. Mass	± da	± ppm	Start Seq.	End Seq.	Sequence	Ion Score	C. I. %	Modification	Rank	Result Type
892.3683	892.4098	0.0415	47	62	68	WEDEEGK					Mascot
894.4138	894.45	0.0362	40	520	527	APFGSMER					Mascot
991.5571	991.5569	-0.0002	0	350	357	SYKDLPLR					Mascot
1178.649	1178.6841	0.0351	30	597	606	IPYMLIVGEK			Oxidation (M)[4]		Mascot
1246.6313	1246.6187	-0.0126	-10	59	68	LLKWEDEEGK					Mascot
1247.6227	1247.6075	-0.0152	-12	607	617	EAENSEVSVRK					Mascot
1280.6667	1280.6505	-0.0162	-13	473	483	LDFMVKDAIGR			Oxidation (M)[4]		Mascot
1284.7311	1284.6371	-0.094	-73	528	539	FVAVLIEHTAGK					Mascot
1344.6471	1344.686	0.0389	29	461	472	IEYGEAAFYGPK					Mascot
1436.7567	1436.6782	-0.0785	-55	1	12	MIKITFPDNSVR			Oxidation (M)[1]		Mascot

Calc. Mass	Obsrv. Mass	± da	± ppm	Start Seq.	End Seq.	Sequence	Ion Score	C. I. %	Modification	Rank	Result Type
1451.7159	1451.6829	-0.033	-23	17	30	GTTAMQIAESISSR					Mascot
1524.8533	1524.7439	-0.1094	-72	183	196	GPHLPNTSYLKAVK					Mascot
1551.6707	1551.7551	0.0844	54	249	261	EMDLFMFSDTVGK			Oxidation (M)[2,6]		Mascot
1567.7924	1567.7251	-0.0673	-43	227	239	MLDEYLTLLEEAK					Mascot
1627.8398	1627.8575	0.0177	11	568	581	ELGNQEIRAIVDDR					Mascot
1699.8109	1699.8195	0.0086	5	413	426	TMNFENFEAQISLR					Mascot
1811.9612	1811.8757	-0.0855	-47	398	412	QEFLNVMDIISIVFK			Oxidation (M)[7]		Mascot
1958.9276	1958.8542	-0.0734	-37	413	428	TMNFENFEAQISLRDK			Oxidation (M)[2]		Mascot

6	Apolipoprotein A-I OS=Sus scrofa GN=APOA1 PE=1 SV=4	APOA1_PIG	30306.7	5.48	12	56	0	3.393	

Peptide Information

Calc. Mass	Obsrv. Mass	± da	± ppm	Start Seq.	End Seq.	Sequence	Ion Score	C. I. %	Modification	Rank	Result Type
991.5029	991.5569	0.054	54	142	150	MAPLGAEFR					Mascot
1064.6323	1064.5681	-0.0642	-60	175	183	LRAHVEALR					Mascot
1209.5746	1209.6578	0.0832	69	206	217	EGGGSLAEYQAK					Mascot
1214.674	1214.6326	-0.0414	-34	218	228	AQEQLKALGEK					Mascot
1241.6412	1241.6448	0.0036	3	36	46	DFATVYVDAIK					Mascot
1247.6565	1247.6075	-0.049	-39	140	150	QKMAPLGAEFR					Mascot
1300.6281	1300.6146	-0.0135	-10	184	194	QHVAPYSDDLR					Mascot
1344.7733	1344.686	-0.0873	-65	249	261	VSILAAIDEASKK					Mascot
1404.7052	1404.7207	0.0155	11	142	154	MAPLGAEFREGAR					Mascot
1596.7904	1596.7831	-0.0073	-5	69	82	LLDNWDSLGSTFTK					Mascot
1906.9504	1907.0546	0.1042	55	206	223	EGGGSLAEYQAKAQEQL K					Mascot
2217.2966	2217.1045	-0.1921	-87	229	248	AKPALEDLRQGLLPVLEN LK					Mascot

7	Clustered mitochondria protein homolog OS=Brugia malayi GN=Bm1_28595 PE=3 SV=2	CLU_BRUMA	164876.5	6.07	22	56	0	12.374	10	0	

Peptide Information

Calc. Mass	Obsrv. Mass	± da	± ppm	Start Seq.	End Seq.	Sequence	Ion Score	C. I. %	Modification	Rank	Result Type
820.4233	820.4283	0.005	6	1295	1301	TALQMEK					Mascot
823.3978	823.3947	-0.0031	-4	1177	1183	ATLMSER			Oxidation (M)[4]		Mascot
890.528	890.4591	-0.0689	-77	125	131	FMVLPRK					Mascot
972.5724	972.4761	-0.0963	-99	1411	1419	AVEDLAVKK					Mascot
1082.4758	1082.554	0.0782	72	356	363	YHITCCTK			Carbamidomethyl (C)[5,6]		Mascot

Calc. Mass	Obsrv. Mass	± da	± ppm	Start Seq.	End Seq.	Sequence	Ion Score	C. I. %	Modification	Rank	Result Type
1276.6753	1276.5808	-0.0945	-74	934	943	VICITELLCR			Carbamidomethyl (C)[3,9]		Mascot
1295.6412	1295.7664	0.1252	97	1291	1301	GDFRTALQMEK					Mascot
1295.6412	1295.7664	0.1252	97	1291	1301	GDFRTALQMEK					Mascot
1300.7008	1300.6146	-0.0862	-66	1327	1337	HLTQQAVTFQK					Mascot
1382.6951	1382.6981	0.003	2	717	727	QELIDAFVEYR					Mascot
1436.7314	1436.6782	-0.0532	-37	833	845	AVGSIRMDAFDVR					Mascot
1456.802	1456.7053	-0.0967	-66	1327	1338	HLTQQAVTFQKR					Mascot
1456.802	1456.7053	-0.0967	-66	1327	1338	HLTQQAVTFQKR	10	0			Mascot
1473.7697	1473.7666	-0.0031	-2	1106	1119	AVDAHSLFLSGQTK					Mascot
1588.9357	1588.8538	-0.0819	-52	1062	1074	RFVQIVGIQMLLR			Oxidation (M)[10]		Mascot
1588.9357	1588.8538	-0.0819	-52	1062	1074	RFVQIVGIQMLLR			Oxidation (M)[10]		Mascot
1603.8247	1603.8112	-0.0135	-8	789	802	IDEMPLLETEAAKK			Oxidation (M)[4]		Mascot
1635.8379	1635.8278	-0.0101	-6	934	946	VICITELLCRCAK			Carbamidomethyl (C)[3,9,11]		Mascot
1857.9739	1857.8992	-0.0747	-40	893	909	TIMLLDGASLIDSLHSR			Oxidation (M)[3]		Mascot
1885.9655	1885.9521	-0.0134	-7	1160	1176	LSYILGDPSEALSQQHK					Mascot
1933.8323	1933.9921	0.1598	83	2	16	CVCACARVCVYVCTR			Carbamidomethyl (C)[1,3,5,9,13]		Mascot
2031.8978	2031.921	0.0232	11	1012	1027	ENDDWAQMSSHKLWER					Mascot
2120.0442	2120.0559	0.0117	6	1320	1337	ESSDCLKHLTQQAVTFQK			Carbamidomethyl (C)[5]		Mascot
2153.2078	2153.1294	-0.0784	-36	385	403	TVHHSLLDLLSSISISFKR					Mascot
2230.1523	2230.1558	0.0035	2	782	801	EITADIKIDEMPLLETEAAK					Mascot

8	30S ribosomal protein S13 OS=Streptococcus equi subsp. zooepidemicus (strain MGCS10565) GN=rpsM PE=3 SV=1	RS13_STREM	13415.5	10.52	5	56	0	5.5	25	0	

Protein Group

30S ribosomal protein S13 OS=Streptococcus equi subsp. equi (strain 4047) GN=rpsM PE=3 SV=1	RS13_STRE4	13415.5	10.5200004577637
30S ribosomal protein S13 OS=Streptococcus equi subsp. zooepidemicus (strain H70) GN=rpsM PE=3 SV=1	RS13_STRS7	13415.5	10.5200004577637
30S ribosomal protein S13 OS=Streptococcus mutans serotype c (strain ATCC 700610 / UA159) GN=rpsM PE=3 SV=1	RS13_STRMU	13397.5	10.5200004577637

Peptide Information

Calc. Mass	Obsrv. Mass	± da	± ppm	Start Seq.	End Seq.	Sequence	Ion Score	C. I. %	Modification	Rank	Result Type
1214.674	1214.6326	-0.0414	-34	33	44	ILAAAGVSEDIR					Mascot

1262.5859	1262.6775	0.0916	73	47	57	DLTSDQEDAIR				Mascot
1262.5859	1262.6775	0.0916	73	47	57	DLTSDQEDAIR	9	0		Mascot
1310.6886	1310.5969	-0.0917	-70	80	90	LMEIGSYRGIR			Oxidation (M)[2]	Mascot
1310.6886	1310.5969	-0.0917	-70	80	90	LMEIGSYRGIR	25	0	Oxidation (M)[2]	Mascot
1342.7689	1342.6617	-0.1072	-80	32	44	KILAAAGVSEDIR				Mascot
1415.7312	1415.725	-0.0062	-4	1	13	MARIAGVDIPNDK			Oxidation (M)[1]	Mascot

9	30S ribosomal protein S13 OS=Streptococcus pneumoniae (strain 70585) GN=rpsM PE=3 SV=1	RS13_STRP7	13413.5	10.63	5	56	0	5.5	25	0

Protein Group

30S ribosomal protein S13 OS=Streptococcus pneumoniae (strain ATCC 700669 / Spain 23F-1) GN=rpsM PE=3 SV=1	RS13_STRPJ	13413.5	10.6300001144409
30S ribosomal protein S13 OS=Streptococcus pneumoniae (strain ATCC BAA-255 / R6) GN=rpsM PE=3 SV=1	RS13_STRR6	13413.5	10.6300001144409
30S ribosomal protein S13 OS=Streptococcus pneumoniae (strain CGSP14) GN=rpsM PE=3 SV=1	RS13_STRPS	13413.5	10.6300001144409
30S ribosomal protein S13 OS=Streptococcus pneumoniae (strain Hungary19A-6) GN=rpsM PE=3 SV=1	RS13_STRPI	13413.5	10.6300001144409
30S ribosomal protein S13 OS=Streptococcus pneumoniae (strain JJA) GN=rpsM PE=3 SV=1	RS13_STRZJ	13413.5	10.6300001144409
30S ribosomal protein S13 OS=Streptococcus pneumoniae (strain P1031) GN=rpsM PE=3 SV=1	RS13_STRZP	13413.5	10.6300001144409
30S ribosomal protein S13 OS=Streptococcus pneumoniae serotype 2 (strain D39 / NCTC 7466) GN=rpsM PE=3 SV=1	RS13_STRP2	13413.5	10.6300001144409
30S ribosomal protein S13 OS=Streptococcus pneumoniae serotype 4 (strain ATCC BAA-334 / TIGR4) GN=rpsM PE=3 SV=1	RS13_STRPN	13413.5	10.6300001144409
30S ribosomal protein S13 OS=Streptococcus suis (strain 05ZYH33) GN=rpsM PE=3 SV=1	RS13_STRSY	13385.5	10.5200004577637
30S ribosomal protein S13 OS=Streptococcus suis (strain 98HAH33) GN=rpsM PE=3 SV=1	RS13_STRS2	13385.5	10.5200004577637
30S ribosomal protein S13 OS=Streptococcus uberis	RS13_STRU0	13417.5	10.520

(strain ATCC BAA-854 / 0140J) GN=rpsM PE=3 SV=1		000457<br>7637								

Peptide Information

Calc. Mass	Obsrv. Mass	± da	± ppm	Start Seq.	End Seq.	Sequence	Ion Score	C. I. %	Modification	Rank	Result Type
1214.674	1214.6326	-0.0414	-34	33	44	ILAAAGISEDVR					Mascot
1262.5859	1262.6775	0.0916	73	47	57	DLTSDQEDAIR					Mascot
1262.5859	1262.6775	0.0916	73	47	57	DLTSDQEDAIR	9	0			Mascot
1310.6886	1310.5969	-0.0917	-70	80	90	LMEIGSYRGIR			Oxidation (M)[2]		Mascot
1310.6886	1310.5969	-0.0917	-70	80	90	LMEIGSYRGIR	25	0	Oxidation (M)[2]		Mascot
1342.7689	1342.6617	-0.1072	-80	32	44	KILAAAGISEDVR					Mascot
1415.7312	1415.725	-0.0062	-4	1	13	MARIAGVDIPNDK			Oxidation (M)[1]		Mascot

10	Mitochondrial presequence protease OS=Schizosaccharomyces pombe (strain 972 / ATCC 24843) GN=cym1 PE=3 SV=1	CYM1_SCHPO	99307.8	6.78	18	55	0	17.964	

Peptide Information

Calc. Mass	Obsrv. Mass	± da	± ppm	Start Seq.	End Seq.	Sequence	Ion Score	C. I. %	Modification	Rank	Result Type
894.4753	894.45	-0.0253	-28	560	567	FLDIAGMK					Mascot
1241.6888	1241.6448	-0.044	-35	2	12	NYAKLSIAFSK					Mascot
1246.6499	1246.6187	-0.0312	-25	784	794	SRGVNQQTQTK					Mascot
1281.6726	1281.6591	-0.0135	-11	148	158	DVYLDATLFPK					Mascot
1344.7634	1344.686	-0.0774	-58	277	287	RELNLPNTFLK					Mascot
1360.7583	1360.7285	-0.0298	-22	43	53	KVPELQLNYTR					Mascot
1360.7583	1360.7285	-0.0298	-22	43	53	KVPELQLNYTR					Mascot
1388.7242	1388.6987	-0.0255	-18	1	12	MNYAKLSIAFSK			Oxidation (M)[1]		Mascot
1473.8424	1473.7666	-0.0758	-51	44	55	VPELQLNYTRLK					Mascot
1480.7192	1480.7096	-0.0096	-6	240	251	FHRSHYHPSNAK					Mascot
1508.7758	1508.741	-0.0348	-23	26	37	WLHVGDKVHDFR					Mascot
1524.8455	1524.7439	-0.1016	-67	410	422	LEAILHQMEISLK					Mascot
1610.8247	1610.8207	-0.004	-2	181	194	SPIIFNGVVYNEMK					Mascot
1615.8149	1615.7594	-0.0555	-34	295	309	VVEYGPLDPVMAPGR			Oxidation (M)[11]		Mascot
1657.8407	1657.7843	-0.0564	-34	484	497	FVFTMLPSSTFPQR					Mascot
1664.9006	1664.9608	0.0602	36	145	158	NLRDVYLDATLFPK					Mascot
1904.9713	1904.9753	0.004	2	687	705	TSVSGITDGIAEKGHSFA K					Mascot
2153.0833	2153.1294	0.0461	21	623	643	RYTGGISISPSAVTNNSD VSK					Mascot

2576.3242	2576.1985	-0.1257	-49	310	332	QVKTSISFLANDTSNVYE TFALK	Mascot

Rank	Protein Name	Accession No.	Protein MW	Protein PI	Pep. Count	Protein Score	Protein Score C. I. %	Intensity Matched	Total Ion Score	Total Ion C. I. %	Confirmed
1	Sedoheptulose-1,7-bisphosphatase, chloroplastic OS=Triticum aestivum PE=2 SV=1	S17P_WHEAT	42547.2	6.04	15	126	100	16.784	52	99.349	

Peptide Information

Calc. Mass	Obsrv. Mass	± da	± ppm	Start Seq.	End Seq.	Sequence	Ion Score	C. I. %	Modification	Rank	Result Type
853.4414	853.4326	-0.0088	-10	360	367	TQVAYGSK					Mascot
921.4611	921.455	-0.0061	-7	259	266	MFSPGNLR					Mascot
930.5255	930.532	0.0065	7	352	359	VISVLDER					Mascot
937.456	937.4582	0.0022	2	259	266	MFSPGNLR			Oxidation (M)[1]		Mascot
1185.5059	1185.5493	0.0434	37	267	276	ATFDNPDYDK					Mascot
1188.5532	1188.5646	0.0114	10	373	382	FEETLYGSSR					Mascot
1225.5737	1225.5691	-0.0046	-4	95	104	LLICMGEAMR			Carbamidomethyl (C)[4], Oxidation (M)[5,9]		Mascot
1235.6631	1235.6394	-0.0237	-19	307	318	GIFTNVTSPTAK					Mascot
1585.797	1585.8354	0.0384	24	245	258	WQHVKDTTSIGEGK					Mascot
1588.9349	1588.8877	-0.0472	-30	323	336	LLFEVAPLGFLIEK					Mascot
1608.8091	1608.8002	-0.0089	-6	138	150	LLFEALEYSHVCK			Carbamidomethyl (C)[12]		Mascot
1649.8568	1649.8385	-0.0183	-11	290	304	YTGGMVPDVNQIIVK			Oxidation (M)[5]		Mascot
1649.8568	1649.8385	-0.0183	-11	290	304	YTGGMVPDVNQIIVK			Oxidation (M)[5]		Mascot
1730.8054	1730.8131	0.0077	4	230	244	DCPGTHEFLLLDEGK			Carbamidomethyl (C)[2]		Mascot
1906.9944	1907.0732	0.0788	41	290	306	YTGGMVPDVNQIIVKEK			Oxidation (M)[5]		Mascot
1933.9801	1934.0062	0.0261	13	202	221	LTGVTGGDQVAAAMGIYGPR					Mascot
1949.975	1949.9709	-0.0041	-2	202	221	LTGVTGGDQVAAAMGIYGPR			Oxidation (M)[14]		Mascot
1949.975	1949.9709	-0.0041	-2	202	221	LTGVTGGDQVAAAMGIYGPR	52	99.349	Oxidation (M)[14]		Mascot
2064.9915	2064.97	-0.0215	-10	267	283	ATFDNPDYDKLVNYYVK					Mascot

Rank	Protein Name	Accession No.	Protein MW	Protein PI	Pep. Count	Protein Score	Protein Score C. I. %	Intensity Matched	Total Ion Score	Total Ion C. I. %	Confirmed
2	Glutamate-ammonia-ligase adenylyltransferase OS=Bordetella parapertussis (strain 12822 / ATCC BAA-587 / NCTC 13253) GN=glnE PE=3 SV=1	GLNE_BORPA	104532	5.8	23	75	98.329	21.635			

Peptide Information

Calc. Mass	Obsrv. Mass	± da	± ppm	Start Seq.	End Seq.	Sequence	Ion Score	C. I. %	Modification	Rank	Result Type
871.493	871.4277	-0.0653	-75	333	339	MPALQRR					Mascot

930.5077	930.532	0.0243	26	795	802	ADILVMPR			Oxidation (M)[6]		Mascot
985.5359	985.5052	-0.0307	-31	330	338	GGRMPALQR					Mascot
993.5952	993.5057	-0.0895	-90	896	903	AQHQLRLK					Mascot
1081.491	1081.4752	-0.0158	-15	779	789	YAAGDTEAGAR					Mascot
1199.6929	1199.5977	-0.0952	-79	793	802	IRADILVMPR			Oxidation (M)[8]		Mascot
1245.6223	1245.6464	0.0241	19	826	835	SELFDVKHDR					Mascot
1245.7678	1245.6464	-0.1214	-97	74	83	ERVFLTLIVR					Mascot
1268.707	1268.5988	-0.1082	-85	484	494	TETLLGSHRVR					Mascot
1278.6338	1278.6135	-0.0203	-16	171	180	RISNHEFYGR					Mascot
1280.6422	1280.6726	0.0304	24	231	239	EWERYAWLK					Mascot
1322.6304	1322.6655	0.0351	27	617	626	QMNLMRDVQR			Oxidation (M)[2,5]		Mascot
1335.6903	1335.6327	-0.0576	-43	709	720	EDAAELYAKLGR					Mascot
1596.889	1596.7979	-0.0911	-57	795	808	ADILVMPRDVQALR					Mascot
1632.7469	1632.8032	0.0563	34	721	734	RMTSWLSTMTSSGR			Oxidation (M)[2,9]		Mascot
1719.8297	1719.843	0.0133	8	380	394	EDEQTHLLPADPAQR					Mascot
1781.8197	1781.9213	0.1016	57	185	200	MMPALSEVDADGQVFR			Oxidation (M)[1]		Mascot
1858.0546	1857.9182	-0.1364	-73	317	332	EIEFIVQLSQLIRGGR					Mascot
1858.0546	1857.9182	-0.1364	-73	317	332	EIEFIVQLSQLIRGGR					Mascot
1885.9377	1885.9645	0.0268	14	1	18	MSSAPPFAAALAWSGHL R			Oxidation (M)[1]		Mascot
1885.9377	1885.9645	0.0268	14	1	18	MSSAPPFAAALAWSGHL R			Oxidation (M)[1]		Mascot
1934.097	1934.0062	-0.0908	-47	672	690	RPGAEPHLAVIAYGKLGG K					Mascot
1937.9208	1938.037	0.1162	60	184	200	RMMPALSEVDADGQVFR			Oxidation (M)[2]		Mascot
2820.4429	2820.3298	-0.1131	-40	205	230	LRPDGDAGPLAWSLDAL EHYLIGQGR					Mascot
2911.4092	2911.3374	-0.0718	-25	833	857	HDRGGMVDVEFVTQYLV LCHAATHR			Carbamidomethyl (C)[19]		Mascot
2911.4092	2911.3374	-0.0718	-25	833	857	HDRGGMVDVEFVTQYLV LCHAATHR			Carbamidomethyl (C)[19]		Mascot

3	Glutamate-ammonia-ligase adenylyltransferase OS=Bordetella bronchiseptica (strain ATCC BAA-588 / NCTC 13252 / RB50) GN=glnE PE=3 SV=1	GLNE_BORBR	104526	5.75	22	68	92.362	24.043

Peptide Information

Calc. Mass	Obsrv. Mass	± da	± ppm	Start Seq.	End Seq.	Sequence	Ion Score	C. I. %	Modification	Rank	Result Type
871.493	871.4277	-0.0653	-75	333	339	MPALQRR					Mascot
930.5077	930.532	0.0243	26	795	802	ADILVMPR			Oxidation (M)[6]		Mascot
985.5359	985.5052	-0.0307	-31	330	338	GGRMPALQR					Mascot
993.5952	993.5057	-0.0895	-90	896	903	AQHQLRLK					Mascot

1081.491	1081.4752	-0.0158	-15	779	789	YAAGDTEAGAR					Mascot
1199.6929	1199.5977	-0.0952	-79	793	802	IRADILVMPR			Oxidation (M)[8]		Mascot
1245.6223	1245.6464	0.0241	19	826	835	SELFDVKHDR					Mascot
1245.7678	1245.6464	-0.1214	-97	74	83	ERVFLTLIVR					Mascot
1268.707	1268.5988	-0.1082	-85	484	494	TETLLGSHRVR					Mascot
1278.6338	1278.6135	-0.0203	-16	171	180	RISNHEFYGR					Mascot
1280.6422	1280.6726	0.0304	24	231	239	EWERYAWLK					Mascot
1322.6304	1322.6655	0.0351	27	617	626	QMNLMRDVQR			Oxidation (M)[2,5]		Mascot
1335.6903	1335.6327	-0.0576	-43	709	720	EDAAELYAKLGR					Mascot
1596.889	1596.7979	-0.0911	-57	795	808	ADILVMPRDVQALR					Mascot
1632.7469	1632.8032	0.0563	34	721	734	RMTSWLSTMTSSGR			Oxidation (M)[2,9]		Mascot
1719.8297	1719.843	0.0133	8	380	394	EDEQTHLLPADPAQR					Mascot
1858.0546	1857.9182	-0.1364	-73	317	332	EIEFIVQLSQLIRGGR					Mascot
1858.0546	1857.9182	-0.1364	-73	317	332	EIEFIVQLSQLIRGGR					Mascot
1885.9377	1885.9645	0.0268	14	1	18	MSSAPPFAAALAWSGHL R			Oxidation (M)[1]		Mascot
1885.9377	1885.9645	0.0268	14	1	18	MSSAPPFAAALAWSGHL R			Oxidation (M)[1]		Mascot
1934.097	1934.0062	-0.0908	-47	672	690	RPGAEPHLAVIAYGKLGG K					Mascot
1949.9573	1949.9709	0.0136	7	184	200	RMMPVLSEVDADGQVFR					Mascot
1949.9573	1949.9709	0.0136	7	184	200	RMMPVLSEVDADGQVFR					Mascot
1965.9521	1965.9929	0.0408	21	184	200	RMMPVLSEVDADGQVFR			Oxidation (M)[2]		Mascot
2820.4429	2820.3298	-0.1131	-40	205	230	LRPDGDAGPLAWSLDAL EHYLIGQGR					Mascot
2911.4092	2911.3374	-0.0718	-25	833	857	HDRGGMVDVEFVTQYLV LCHAATHR			Carbamidomethyl (C)[19]		Mascot
2911.4092	2911.3374	-0.0718	-25	833	857	HDRGGMVDVEFVTQYLV LCHAATHR			Carbamidomethyl (C)[19]		Mascot

4	Pyruvate dehydrogenase E1 component subunit beta-1, mitochondrial OS=Oryza sativa subsp. japonica GN=Os08g0536000 PE=2 SV=1	ODPB1_ORYSJ	40204.4	5.25	8	67	90.16	7.363	41	92.332

Peptide Information

Calc. Mass	Obsrv. Mass	± da	± ppm	Start Seq.	End Seq.	Sequence	Ion Score	C. I. %	Modification	Rank	Result Type
856.4999	856.5052	0.0053	6	278	284	SIRPLDR					Mascot
873.5403	873.4637	-0.0766	-88	77	84	ISKGLLDK					Mascot
959.5632	959.5163	-0.0469	-49	285	293	ATINASVRK					Mascot
1200.6583	1200.6696	0.0113	9	267	277	EGISAEVINLR					Mascot
1256.6667	1256.6428	-0.0239	-19	348	358	MAVPQVEDIVR					Mascot
1632.8956	1632.8032	-0.0924	-57	182	196	VLTPYSAEDARGLLK					Mascot

1687.8473	1687.8282	-0.0191	-11	332	347	IAGADVPMPYAANLER					Mascot
1703.8422	1703.8239	-0.0183	-11	332	347	IAGADVPMPYAANLER			Oxidation (M)[8]		Mascot
1795.916	1795.9297	0.0137	8	138	153	SNYMSAGQINVPIVFR					Mascot
1811.9109	1811.9072	-0.0037	-2	138	153	SNYMSAGQINVPIVFR			Oxidation (M)[4]		Mascot
1811.9109	1811.9072	-0.0037	-2	138	153	SNYMSAGQINVPIVFR	41	92.332	Oxidation (M)[4]		Mascot

5 Alanine--tRNA ligase OS=Sinorhizobium medicae (strain WSM419) GN=alaS PE=3 SV=1 SYA_SINMW 95836.7 5.37 19 66 86.417 6.722

Peptide Information

Calc. Mass	Obsrv. Mass	± da	± ppm	Start Seq.	End Seq.	Sequence	Ion Score	C. I. %	Modification	Rank	Result Type
921.4458	921.455	0.0092	10	1	8	MSGVNEIR			Oxidation (M)[1]		Mascot
1199.6201	1199.5977	-0.0224	-19	655	666	VVSMGQGLHGSK					Mascot
1228.7008	1228.6168	-0.084	-68	143	153	IAGLSDDRIIR					Mascot
1259.6842	1259.5955	-0.0887	-70	252	264	ALIAASEEATGVK					Mascot
1549.8221	1549.722	-0.1001	-65	808	824	TLGSGVVAFVGVSGDGK					Mascot
1569.8245	1569.8461	0.0216	14	562	575	ANHSATHLLHEALR					Mascot
1608.9432	1608.8002	-0.143	-89	726	741	ALAAALKVQPSDVLGR					Mascot
1677.9171	1677.8925	-0.0246	-15	807	824	KTLGSGVVAFVGVSGDG K					Mascot
1687.8147	1687.8282	0.0135	8	70	85	AGGKHNDLDNVGYTAR					Mascot
1839.0096	1838.9103	-0.0993	-54	560	575	LRANHSATHLLHEALR					Mascot
1907.0345	1907.0732	0.0387	20	576	593	EVLGTHVSQKGSLVAPE R					Mascot
1966.015	1965.9929	-0.0221	-11	309	325	AMRHAQLLGAQDPLMW K					Mascot
1980.9307	1980.9951	0.0644	33	1	16	MSGVNEIRSMFLDYFR			Oxidation (M)[1]		Mascot
1996.9255	1996.9857	0.0602	30	1	16	MSGVNEIRSMFLDYFR			Oxidation (M)[1,10]		Mascot
2007.0222	2006.991	-0.0312	-16	125	141	LLVTVYHTDDEAFGLWK					Mascot
2048.1062	2047.9648	-0.1414	-69	106	123	AIELAWNLITKEYGLDAK					Mascot
2065.0171	2064.97	-0.0471	-23	526	543	RGEGLFVHYCVVEEGSV K			Carbamidomethyl (C)[10]		Mascot
2082.0615	2081.937	-0.1245	-60	48	66	NVFTGLEQRPYSTAATA QK					Mascot
2163.1233	2163.0159	-0.1074	-50	124	141	RLLVTVYHTDDEAFGLW K					Mascot
2703.2642	2703.2292	-0.035	-13	630	654	LMSVDDAIAEGAMALFGE KYGDEVR			Oxidation (M)[2]		Mascot

6 Dystonin OS=Homo sapiens GN=DST PE=1 SV=4 DYST_HUMAN 865259.2 5.14 65 64 78.963 50.893

Peptide Information

Calc. Mass	Obsrv. Mass	± da	± ppm	Start Seq.	End Seq.	Sequence	Ion Score	C. I. %	Modification	Rank	Result Type

801.4114	801.4723	0.0609	76	7352	7358	VHHHGSK		Mascot
871.4996	871.4277	-0.0719	-83	6460	6466	LEVLNQR		Mascot
873.4828	873.4637	-0.0191	-22	5482	5488	WEALLNK		Mascot
921.4346	921.455	0.0204	22	6090	6097	NVSVDMEK		Mascot
930.5142	930.532	0.0178	19	5883	5890	DIIDDLVK		Mascot
937.4295	937.4582	0.0287	31	6090	6097	NVSVDMEK	Oxidation (M)[6]	Mascot
985.5537	985.5052	-0.0485	-49	7487	7495	KTPSRPGSR		Mascot
993.5098	993.5057	-0.0041	-4	1476	1484	ISSEEISTK		Mascot
1008.4745	1008.4807	0.0062	6	3075	3082	NDFLQSER		Mascot
1026.5289	1026.4851	-0.0438	-43	5873	5880	TFTMEILR	Oxidation (M)[4]	Mascot
1081.571	1081.4752	-0.0958	-89	3247	3255	ITGVFELMR	Oxidation (M)[8]	Mascot
1178.6892	1178.6509	-0.0383	-32	6327	6336	LNHQAELLLK		Mascot
1182.5287	1182.5994	0.0707	60	2548	2557	FDDSGSWRGR		Mascot
1188.5977	1188.5646	-0.0331	-28	6550	6559	GQQMLARCPK	Carbamidomethyl (C)[8]	Mascot
1199.6743	1199.5977	-0.0766	-64	4567	4576	QNVNKVQELK		Mascot
1200.6835	1200.6696	-0.0139	-12	4906	4916	TIAEGENLLLK		Mascot
1207.6205	1207.6014	-0.0191	-16	4268	4277	VELFENLSEK		Mascot
1233.6322	1233.6409	0.0087	7	6773	6783	EKTSLADDNLK		Mascot
1235.6412	1235.6394	-0.0018	-1	2264	2274	TMLSGQRIGEK	Oxidation (M)[2]	Mascot
1245.6321	1245.6464	0.0143	11	747	756	ELDQKEENIK		Mascot
1245.6321	1245.6464	0.0143	11	747	756	ELDQKEENIK		Mascot
1252.6644	1252.7351	0.0707	56	3005	3015	EHLQLGVNNTK		Mascot
1252.6644	1252.7351	0.0707	56	3005	3015	EHLQLGVNNTK		Mascot
1256.6191	1256.6428	0.0237	19	6540	6549	EETYKSLMQK		Mascot
1259.6412	1259.5955	-0.0457	-36	4200	4210	NIMLEQDIAGR		Mascot
1273.6172	1273.6152	-0.002	-2	55	64	HVNDLYEDLR		Mascot
1276.6016	1276.5928	-0.0088	-7	3713	3723	ENGEKLSQEDK		Mascot
1280.6481	1280.6726	0.0245	19	3682	3691	YQSKQEELQK		Mascot
1300.6631	1300.7095	0.0464	36	318	328	ETEIPPKETEK		Mascot
1310.6774	1310.6073	-0.0701	-53	6345	6355	HTVQDPLMELK		Mascot
1326.6722	1326.6162	-0.056	-42	6345	6355	HTVQDPLMELK	Oxidation (M)[8]	Mascot
1335.5886	1335.6327	0.0441	33	1364	1373	DYELQTMTYR	Oxidation (M)[7]	Mascot
1360.6281	1360.743	0.1149	84	1252	1261	ERDLDFDWHK		Mascot
1404.7118	1404.6904	-0.0214	-15	3966	3977	QKSFSEDVISHK		Mascot
1569.7755	1569.8461	0.0706	45	257	272	VPEGGEGIGANDVEVK		Mascot
1585.8254	1585.8354	0.01	6	3915	3927	MKLTHSLQEELEK		Mascot

1603.8109	1603.8038	-0.0071	-4	1794	1807	MTVEEAVREGVIDR		Mascot
1645.8115	1645.8647	0.0532	32	1761	1774	AAHEGLIDRETMFR		Mascot
1649.8179	1649.8385	0.0206	12	6163	6175	FWCDHMSLIVTIK	Carbamidomethyl (C)[3]	Mascot
1649.8997	1649.8385	-0.0612	-37	5738	5751	VEVELLSYETQVLK		Mascot
1671.7386	1671.7992	0.0606	36	5895	5908	IMTACSEEEKQSMK	Carbamidomethyl (C)[5]	Mascot
1677.8932	1677.8925	-0.0007	0	1271	1283	WQNVHVQIDNRLR		Mascot
1687.7335	1687.8282	0.0947	56	5895	5908	IMTACSEEEKQSMK	Carbamidomethyl (C)[5], Oxidation (M)[2]	Mascot
1703.7285	1703.8239	0.0954	56	5895	5908	IMTACSEEEKQSMK	Carbamidomethyl (C)[5], Oxidation (M)[2,13]	Mascot
1719.8549	1719.843	-0.0119	-7	6239	6253	KSIDELNSAWDSLNK		Mascot
1730.8861	1730.8131	-0.073	-42	975	988	SVVSWHYLINEIDR		Mascot
1772.8562	1772.9188	0.0626	35	3068	3082	HVEDVGKNDFLQSER		Mascot
1796.0276	1795.9297	-0.0979	-55	5664	5679	KQNVDQALLNGLELLK		Mascot
1804.8923	1804.9218	0.0295	16	1535	1550	QLQESQTSGDVKVEEK		Mascot
1812.059	1811.9072	-0.1518	-84	1173	1189	TVNLVLKNTQAAEALVK		Mascot
1812.059	1811.9072	-0.1518	-84	1173	1189	TVNLVLKNTQAAEALVK		Mascot
1838.8412	1838.9103	0.0691	38	7071	7085	ALIAEHQTFMEEMTR	Oxidation (M)[10,13]	Mascot
1855.9661	1855.9205	-0.0456	-25	5339	5355	QQDVNWLGQGLIQSAAK		Mascot
1857.8899	1857.9182	0.0283	15	4223	4239	KFMETTDPSTASSLQAK	Oxidation (M)[3]	Mascot
1858.0334	1857.9182	-0.1152	-62	90	104	FHRLQNVQIALDYLK		Mascot
1879.8954	1879.8922	-0.0032	-2	591	606	SSLLDQNLTEEEINMK	Oxidation (M)[15]	Mascot
1886.0416	1885.9645	-0.0771	-41	4792	4808	QIKVAQALCEDLSALVK	Carbamidomethyl (C)[9]	Mascot
1886.0416	1885.9645	-0.0771	-41	4792	4808	QIKVAQALCEDLSALVK	Carbamidomethyl (C)[9]	Mascot
1901.8732	1901.9668	0.0936	49	471	486	DEIMALRNECSSVYSK	Carbamidomethyl (C)[10]	Mascot
1906.9613	1907.0732	0.1119	59	5237	5253	MMLATEETSPDLVGIKR	Oxidation (M)[1]	Mascot
1914.9015	1914.9475	0.046	24	6312	6326	QFKSEAYQQQIEMER		Mascot
1926.9708	1926.9021	-0.0687	-36	349	364	LLQGYHPNDIEKEWGK		Mascot
1932.9597	1932.9972	0.0375	19	3240	3255	QDQHSQKITGVFELMR	Oxidation (M)[15]	Mascot
1937.9862	1938.037	0.0508	26	999	1015	TMLPGEHQQVLSNLQSR		Mascot
1942.9539	1943.0226	0.0687	35	2453	2471	ENENSMVPQGAPVGSLS VK		Mascot
1958.9489	1958.8848	-0.0641	-33	2453	2471	ENENSMVPQGAPVGSLS VK	Oxidation (M)[6]	Mascot
1990.9287	1990.9954	0.0667	34	2298	2314	SLNMISSNPQVQYHNDK	Oxidation (M)[4]	Mascot
2007.0468	2006.991	-0.0558	-28	6090	6106	NVSVDMEKLQPLYETLK		Mascot
2023.0416	2022.9879	-0.0537	-27	6090	6106	NVSVDMEKLQPLYETLK	Oxidation (M)[6]	Mascot
2048.0369	2047.9648	-0.0721	-35	4485	4502	KDMTDISHGYEDLGLLLK		Mascot
2064.9622	2064.97	0.0078	4	625	642	TEWGSDLPSVESHLENH K		Mascot
2082.0906	2081.937	-0.1536	-74	308	324	ALYNQYLQFKETEIPPK		Mascot

Calc. Mass	Obsrv. Mass	± da	± ppm	Start Seq.	End Seq.	Sequence	Ion Score	C. I. %	Modification	Rank	Result Type
2163.064	2163.0159	-0.0481	-22	4486	4504	DMTDISHGYEDLGLLLKD K					Mascot
2820.3132	2820.3298	0.0166	6	6716	6739	LMEWLEESEKSLDSELEI ANDPDK					Mascot
2939.4568	2939.3745	-0.0823	-28	6833	6858	VEPQLAEDQPVHGDIDLV MNLIDNHK					Mascot

7	Argininosuccinate lyase OS=Methanoculleus marisnigri (strain ATCC 35101 / DSM 1498 / JR1) GN=argH PE=3 SV=1	ARLY_METMJ	53587.4	5.47	15	61	58.026	11.536

Peptide Information

Calc. Mass	Obsrv. Mass	± da	± ppm	Start Seq.	End Seq.	Sequence	Ion Score	C. I. %	Modification	Rank	Result Type
856.457	856.5052	0.0482	56	105	111	LHMGRSR					Mascot
921.457	921.455	-0.002	-2	1	7	MRSDQIR			Oxidation (M)[1]		Mascot
937.456	937.4582	0.0022	2	314	321	GLPMSYNR					Mascot
959.5017	959.5163	0.0146	15	3	10	SDQIRQGR					Mascot
1188.6041	1188.5646	-0.0395	-33	286	295	KNPDVAEIMR			Oxidation (M)[9]		Mascot
1259.6412	1259.5955	-0.0457	-36	287	297	NPDVAEIMRAK			Oxidation (M)[8]		Mascot
1346.6846	1346.7938	0.1092	81	112	122	NDEVATCIRIR			Carbamidomethyl (C)[7]		Mascot
1346.6964	1346.7938	0.0974	72	379	389	EYGLPFRTAHR					Mascot
1424.7493	1424.714	-0.0353	-25	397	410	HGSLDLATLEAAAR					Mascot
1639.8837	1639.8578	-0.0259	-16	35	49	ADLLVDMAHLLGLSR			Oxidation (M)[7]		Mascot
1763.9175	1763.8907	-0.0268	-15	411	426	EAADFSVVELGLTREK					Mascot
1906.9982	1907.0732	0.075	39	441	459	NIVGGPAPEAVAAQISEQ R					Mascot
1991.0458	1990.9954	-0.0504	-25	322	338	DLQELTPHLWRGVEAAR					Mascot
2007.0182	2006.991	-0.0272	-14	192	211	VDVSPLGSAAFASTGFPL DR					Mascot
2086.9785	2086.9919	0.0134	6	235	253	DFAIEVLADASICMTSTSR			Carbamidomethyl (C)[13]		Mascot

8	Gamma-glutamyl phosphate reductase OS=Anoxybacillus flavithermus (strain DSM 21510 / WK1) GN=proA PE=3 SV=1	PROA_ANOFW	45811.9	5.36	13	61	55.024	12.978

Peptide Information

Calc. Mass	Obsrv. Mass	± da	± ppm	Start Seq.	End Seq.	Sequence	Ion Score	C. I. %	Modification	Rank	Result Type
985.5425	985.5052	-0.0373	-38	75	83	VQQIADGVR					Mascot
1026.4674	1026.4851	0.0177	17	184	191	ETAQQMFR			Oxidation (M)[6]		Mascot
1200.6219	1200.6696	0.0477	40	57	67	ENGLSPSLLDR					Mascot
1207.6794	1207.6014	-0.078	-65	403	413	TLVYGSGQVRK					Mascot
1216.6606	1216.5867	-0.0739	-61	238	248	EMAIDIVINAK					Mascot
1245.6984	1245.6464	-0.052	-42	391	402	GPMGLRAITTTK					Mascot

1245.6984	1245.6464	-0.052	-42	391	402	GPMGLRAITTTK						Mascot
1747.9259	1747.9344	0.0085	5	11	26	LKQAASQLAMLSTEEK						Mascot
1747.9259	1747.9344	0.0085	5	11	26	LKQAASQLAMLSTEEK						Mascot
1763.9208	1763.8907	-0.0301	-17	11	26	LKQAASQLAMLSTEEK			Oxidation (M)[10]			Mascot
1811.9723	1811.9072	-0.0651	-36	138	155	TGNAVLLRGSSSAIHSNK						Mascot
1811.9723	1811.9072	-0.0651	-36	138	155	TGNAVLLRGSSSAIHSNK						Mascot
1873.9324	1873.9147	-0.0177	-9	51	67	DMEIGKENGLSPSLLDR						Mascot
1915.0131	1914.9475	-0.0656	-34	167	183	QSKIPTDAIQLLEDTSR						Mascot
1927.0323	1926.9021	-0.1302	-68	266	280	QWPYIKELLETLHEK						Mascot
1950.059	1949.9709	-0.0881	-45	249	265	LQRPSVCNAVETVIVHK			Carbamidomethyl (C)[7]			Mascot
1950.059	1949.9709	-0.0881	-45	249	265	LQRPSVCNAVETVIVHK			Carbamidomethyl (C)[7]			Mascot
2047.9608	2047.9648	0.004	2	368	386	FTDGEQFGYGAEIGISTQK						Mascot

9	Uncharacterized protein M4 OS=Microplitis demolitor bracovirus (isolate Webb) GN=M4 PE=4 SV=1	YM4_MDBVW	13590.9	9.08	9	60	42.06	7.083

Peptide Information

Calc. Mass	Obsrv. Mass	± da	± ppm	Start Seq.	End Seq.	Sequence	Ion Score	C. I. %	Modification		Rank	Result Type
853.4488	853.4326	-0.0162	-19	68	74	IGIMEYK						Mascot
982.4662	982.4322	-0.034	-35	75	82	TGKCPYEK			Carbamidomethyl (C)[4]			Mascot
1252.6719	1252.7351	0.0632	50	65	74	QVRIGIMEYK			Oxidation (M)[7]			Mascot
1252.6719	1252.7351	0.0632	50	65	74	QVRIGIMEYK			Oxidation (M)[7]			Mascot
1268.6232	1268.5988	-0.0244	-19	16	25	FLELPFMDEK						Mascot
1310.6813	1310.6073	-0.074	-56	78	87	CPYEKILYPK			Carbamidomethyl (C)[1]			Mascot
1632.7357	1632.8032	0.0675	41	2	15	SQSSSDNKCFMVVK			Carbamidomethyl (C)[9], Oxidation (M)[11]			Mascot
1747.7812	1747.9344	0.1532	88	1	15	MSQSSSDNKCFMVVK			Carbamidomethyl (C)[10]			Mascot
1747.7812	1747.9344	0.1532	88	1	15	MSQSSSDNKCFMVVK			Carbamidomethyl (C)[10]			Mascot
1763.7761	1763.8907	0.1146	65	1	15	MSQSSSDNKCFMVVK			Carbamidomethyl (C)[10], Oxidation (M)[1]			Mascot
2064.9844	2064.97	-0.0144	-7	10	25	CFMVVKFLELPFMDEK			Carbamidomethyl (C)[1], Oxidation (M)[3,13]			Mascot
2703.3044	2703.2292	-0.0752	-28	40	62	TQEYVMVAFPLQNELSPSLFDMK			Oxidation (M)[6]			Mascot

10	Glutamate-ammonia-ligase adenylyltransferase OS=Bordetella pertussis (strain Tohama I / ATCC BAA-589 / NCTC 13251) GN=glnE PE=3 SV=2	GLNE_BORPE	104532	5.85	21	60	40.71	21.379

Peptide Information

Calc. Mass	Obsrv. Mass	± da	± ppm	Start Seq.	End Seq.	Sequence	Ion Score	C. I. %	Modification		Rank	Result Type

871.493	871.4277	-0.0653	-75	333	339	MPALQRR		Mascot
930.5077	930.532	0.0243	26	795	802	ADILVMPR	Oxidation (M)[6]	Mascot
985.5359	985.5052	-0.0307	-31	330	338	GGRMPALQR		Mascot
993.5952	993.5057	-0.0895	-90	896	903	AQHQLRLK		Mascot
1081.491	1081.4752	-0.0158	-15	779	789	YAAGDTEAGAR		Mascot
1199.6929	1199.5977	-0.0952	-79	793	802	IRADILVMPR	Oxidation (M)[8]	Mascot
1245.6223	1245.6464	0.0241	19	826	835	SELFDVKHDR		Mascot
1245.7678	1245.6464	-0.1214	-97	74	83	ERVFLTLIVR		Mascot
1268.707	1268.5988	-0.1082	-85	484	494	TETLLGSHRVR		Mascot
1278.6338	1278.6135	-0.0203	-16	171	180	RISNHEFYGR		Mascot
1280.6422	1280.6726	0.0304	24	231	239	EWERYAWLK		Mascot
1322.6304	1322.6655	0.0351	27	617	626	QMNLMRDVQR	Oxidation (M)[2,5]	Mascot
1335.6903	1335.6327	-0.0576	-43	709	720	EDAAELYAKLGR		Mascot
1596.889	1596.7979	-0.0911	-57	795	808	ADILVMPRDVQALR		Mascot
1632.7469	1632.8032	0.0563	34	721	734	RMTSWLSTMTSSGR	Oxidation (M)[2,9]	Mascot
1719.8297	1719.843	0.0133	8	380	394	EDEQTHLLPADPAQR		Mascot
1858.0546	1857.9182	-0.1364	-73	317	332	EIEFIVQLSQLIRGGR		Mascot
1858.0546	1857.9182	-0.1364	-73	317	332	EIEFIVQLSQLIRGGR		Mascot
1885.9377	1885.9645	0.0268	14	1	18	MSSAPPFAAALAWSGHL R	Oxidation (M)[1]	Mascot
1885.9377	1885.9645	0.0268	14	1	18	MSSAPPFAAALAWSGHL R	Oxidation (M)[1]	Mascot
1934.097	1934.0062	-0.0908	-47	672	690	RPGAEPHLAVIAYGKLGG K		Mascot
1949.9573	1949.9709	0.0136	7	184	200	RMMPVLSEVDADGQVFR		Mascot
1949.9573	1949.9709	0.0136	7	184	200	RMMPVLSEVDADGQVFR		Mascot
1965.9521	1965.9929	0.0408	21	184	200	RMMPVLSEVDADGQVFR	Oxidation (M)[2]	Mascot
2820.4429	2820.3298	-0.1131	-40	205	230	LRPDGDAGPLAWSLDAL EHYLIGQGR		Mascot

Gel Idx/Pos	217/I17	Instr./Gel Origin	BA2151/Sample Project 20140814	Process Status	Analysis Succeeded
Plate [#] Name	[1] Sample Project 20140814	Instrument Sample Name		Spectra	11

Rank	Protein Name	Accession No.	Protein MW	Protein PI	Pep. Count	Protein Score	Protein Score C. I. %	Intensity Matched	Total Ion Score	Total Ion C. I. %	Confirmed
1	Microtubule-actin cross-linking factor 1, isoforms 1/2/3/5 OS=Homo sapiens GN=MACF1 PE=1 SV=4	MACF1_HUMAN	843033	5.28	66	94	99.979	33.411			

Peptide Information

Calc. Mass	Obsrv. Mass	± da	± ppm	Start Seq.	End Seq.	Sequence	Ion Score	C. I. %	Modification	Rank	Result Type
807.4471	807.4316	-0.0155	-19	5772	5777	YARLER					Mascot
853.489	853.4373	-0.0517	-61	3701	3707	EKLHQAK					Mascot
871.4996	871.4281	-0.0715	-82	3323	3329	TPQEKLR					Mascot
937.5214	937.4711	-0.0503	-54	2010	2017	VVEIGHQR					Mascot
1105.567	1105.656	0.089	81	4952	4960	AMLNEVEKR			Oxidation (M)[2]		Mascot
1182.6188	1182.5792	-0.0396	-33	661	669	LETQYCKLK			Carbamidomethyl (C)[6]		Mascot
1186.6427	1186.5852	-0.0575	-48	5225	5234	QEQLELTLGR					Mascot
1199.6896	1199.5938	-0.0958	-80	5716	5725	AFSIDIIRHK					Mascot
1213.6536	1213.661	0.0074	6	7376	7387	TTTASPRTPGPK					Mascot
1245.6686	1245.6591	-0.0095	-8	6412	6422	TEQSVALLEQK					Mascot
1262.6158	1262.6621	0.0463	37	1329	1338	NQTKLDQCQK			Carbamidomethyl (C)[8]		Mascot
1273.7184	1273.6179	-0.1005	-79	408	417	LIIEMLEREK					Mascot
1303.7151	1303.7041	-0.011	-8	1737	1749	QLAGGMVSLKSGR					Mascot
1310.725	1310.6139	-0.1111	-85	6173	6183	LNHQGELMLKK					Mascot
1328.7057	1328.6826	-0.0231	-17	3961	3972	EPSEIGNLVKDK					Mascot
1346.6951	1346.8013	0.1062	79	4512	4523	AFLAELEQNSPK					Mascot
1360.7219	1360.8065	0.0846	62	4669	4679	WVELTDKLNSR					Mascot
1384.605	1384.6951	0.0901	65	5736	5747	SEIFGTCGEEQK			Carbamidomethyl (C)[7]		Mascot
1386.569	1386.7031	0.1341	97	1	12	MSSSDEETLSER			Oxidation (M)[1]		Mascot
1424.7744	1424.7365	-0.0379	-27	5543	5555	YADITVTSSKALR					Mascot
1515.7948	1515.7552	-0.0396	-26	1226	1238	VVAEQMSRLTPER					Mascot
1517.7893	1517.7593	-0.03	-20	1607	1618	NLINPQMYQQLR					Mascot
1588.808	1588.8275	0.0195	12	6009	6021	FWYDMAALLTTIK			Oxidation (M)[5]		Mascot
1649.8745	1649.8367	-0.0378	-23	1211	1225	NSVFSVLDEEIAKAK					Mascot
1707.964	1707.8503	-0.1137	-67	108	123	DGHNLISLLEVLSGIK					Mascot
1731.0276	1730.8745	-0.1531	-88	854	868	TIVQLKPRSPDHVLK					Mascot

1735.8142	1735.8698	0.0556	32	6306	6319	LEAMNQCWESVLQK	Carbamidomethyl (C)[7]	Mascot
1758.9232	1758.8623	-0.0609	-35	2688	2705	VLEAQANTGGIIDTATGK		Mascot
1770.8326	1770.9122	0.0796	45	928	942	DAIEMASRVEQSYQK	Oxidation (M)[5]	Mascot
1772.9655	1772.938	-0.0275	-16	1433	1448	ELLGWVSTLARNTQGK		Mascot
1772.9655	1772.938	-0.0275	-16	1433	1448	ELLGWVSTLARNTQGK		Mascot
1788.8473	1788.9294	0.0821	46	719	732	RNYFSELTMELEEK		Mascot
1795.9337	1795.9189	-0.0148	-8	2543	2557	VENLNIHQIFNPETK		Mascot
1800.9563	1800.9573	0.001	1	2113	2128	QLIGTQREDQTAVSVR		Mascot
1804.8422	1804.9277	0.0855	47	719	732	RNYFSELTMELEEK	Oxidation (M)[9]	Mascot
1804.8811	1804.9277	0.0466	26	3723	3738	ELEEAVTSALQQETEK		Mascot
1811.075	1810.9216	-0.1534	-85	6516	6531	QDVVLIKNLLVSVQSR		Mascot
1813.8942	1813.912	0.0178	10	5978	5991	LDQMVFFWEDIKAR	Oxidation (M)[4]	Mascot
1830.0219	1829.9071	-0.1148	-63	1463	1478	AILEQQVLSEELTTKK		Mascot
1838.8412	1838.9241	0.0829	45	6918	6932	ALIAEHQTFMEEMTR	Oxidation (M)[10,13]	Mascot
1843.8564	1843.9121	0.0557	30	1374	1389	MLSSSDAITQEFMDLR		Mascot
1863.9025	1863.9171	0.0146	8	3887	3900	FLQDHKEFESWLER		Mascot
1869.9739	1869.9657	-0.0082	-4	5190	5205	HMLEEEGTLDLLGLKR	Oxidation (M)[2]	Mascot
1872.0338	1871.9022	-0.1316	-70	5801	5817	ALIAQLPSPAIDHEQLR		Mascot
1879.9232	1879.9	-0.0232	-12	556	571	ATHSSSTSWFRKPMTR		Mascot
1885.7911	1885.9497	0.1586	84	2797	2812	DMLIACNQTAEMSCNK	Carbamidomethyl (C)[6,14]	Mascot
1885.7911	1885.9497	0.1586	84	2797	2812	DMLIACNQTAEMSCNK	Carbamidomethyl (C)[6,14]	Mascot
1890.0219	1889.9321	-0.0898	-48	5936	5952	SATVELEKLQPSFEALK		Mascot
1895.9181	1895.8781	-0.04	-21	556	571	ATHSSSTSWFRKPMTR	Oxidation (M)[14]	Mascot
1901.786	1901.9435	0.1575	83	2797	2812	DMLIACNQTAEMSCNK	Carbamidomethyl (C)[6,14], Oxidation (M)[2]	Mascot
1917.011	1916.9454	-0.0656	-34	5900	5915	IEPMLETLENLSSRLR	Oxidation (M)[4]	Mascot
1933.9688	1933.9926	0.0238	12	6370	6385	EQLDTHMELYSQLKAK		Mascot
1940.9858	1941.0311	0.0453	23	4457	4472	QESLQAILNRMEEVHK	Oxidation (M)[11]	Mascot
1942.9944	1943.0565	0.0621	32	3941	3957	FVTISGQKVLDMENSFK		Mascot
1942.9944	1943.0565	0.0621	32	3941	3957	FVTISGQKVLDMENSFK		Mascot
1949.9637	1949.9559	-0.0078	-4	6370	6385	EQLDTHMELYSQLKAK	Oxidation (M)[7]	Mascot
1958.9893	1958.962	-0.0273	-14	3941	3957	FVTISGQKVLDMENSFK	Oxidation (M)[12]	Mascot
2023.068	2022.9916	-0.0764	-38	3636	3654	ALAGHQGRTTQQDLSAL QK		Mascot
2084.0117	2083.9565	-0.0552	-26	755	772	QTVEAYSAAVQSQLQW MK	Oxidation (M)[17]	Mascot
2178.0747	2178.1689	0.0942	43	4371	4389	SLLDDWASKGTLVEEINC K	Carbamidomethyl (C)[18]	Mascot
2226.1587	2226.1304	-0.0283	-13	332	350	QHTILMSDKTFPQNPVEL K		Mascot
2293.2551	2293.1672	-0.0879	-38	1392	1411	YTALVTLTTQHVKYISDAL		Mascot

						R					
2294.1047	2294.1538	0.0491	21	5673	5692	SQQYEQAADAELAWVAE TKR					Mascot
2304.0964	2304.0203	-0.0761	-33	641	660	LYEGKMSQNFHTSYAET LGK					Mascot
2315.0166	2315.1794	0.1628	70	5135	5155	EMFSQLADLDDELDGMG AIGR			Oxidation (M)[2,16]		Mascot
2703.3481	2703.2261	-0.122	-45	6161	6182	VEVYQQQIEMEKLNHQG ELMLK			Oxidation (M)[10]		Mascot
2725.4395	2725.2405	-0.199	-73	943	964	VMALWHQLHVNTKSLIS WNYLR			Oxidation (M)[2]		Mascot
2867.3848	2867.3521	-0.0327	-11	2605	2630	KEGLLTNEAVLSPGMMH GIVDPENCR			Carbamidomethyl (C)[25]		Mascot
2910.6035	2910.3157	-0.2878	-99	2425	2452	VSVTLASTLGLVDVADQP ELINLEKASK					Mascot
2911.3843	2911.344	-0.0403	-14	6562	6586	KLIDWLEDAESHLDSELEI SNDPDK					Mascot
2911.4294	2911.344	-0.0854	-29	392	415	LPQGYHPNDVEEEWGKL IIEMLER			Oxidation (M)[21]		Mascot
2933.3372	2933.3538	0.0166	6	3330	3357	ESPGSEQTPFMTAPEGK GNGGVNPEPFR			Oxidation (M)[11]		Mascot
2968.5054	2968.3787	-0.1267	-43	6823	6849	RVDVNSAVAMGEVILAVC HPDCITTIK			Carbamidomethyl (C)[18,22]		Mascot
3024.4685	3024.4753	0.0068	2	6563	6588	LIDWLEDAESHLDSELEIS NDPDKIK					Mascot

2	DNA-directed RNA polymerase subunit beta OS=Francisella tularensis subsp. novicida (strain U112) GN=rpoB PE=3 SV=1	RPOB_FRATN	151804.2	5.42	24	69	93.499	9.262	

Protein Group

DNA-directed RNA polymerase subunit beta OS=Francisella tularensis subsp. tularensis (strain FSC 198) GN=rpoB PE=3 SV=1	RPOB_FRAT1	151790.2	5.4200 000762 9395
DNA-directed RNA polymerase subunit beta OS=Francisella tularensis subsp. tularensis (strain SCHU S4 / Schu 4) GN=rpoB PE=3 SV=1	RPOB_FRATT	151790.2	5.4200 000762 9395
DNA-directed RNA polymerase subunit beta OS=Francisella tularensis subsp. tularensis (strain WY96-3418) GN=rpoB PE=3 SV=1	RPOB_FRATW	151790.2	5.4200 000762 9395

Peptide Information

Calc. Mass	Obsrv. Mass	± da	± ppm	Start Seq.	End Seq.	Sequence	Ion Score	C. I. %	Modification	Rank	Result Type
937.5578	937.4711	-0.0867	-92	149	156	VVVSQLHR					Mascot
1027.5895	1027.6213	0.0318	31	533	543	ISALGPGGLSR					Mascot
1105.6252	1105.656	0.0308	28	1037	1046	IAIDAKFEAK					Mascot
1328.7209	1328.6826	-0.0383	-29	1190	1203	GGVPIATPVFDGAK					Mascot
1515.8378	1515.7552	-0.0826	-54	975	988	SSIEQEVVGAKIQK					Mascot
1571.7853	1571.7805	-0.0048	-3	584	596	VNDYGFLEAPYRK					Mascot
1603.869	1603.795	-0.074	-46	254	267	FDIVDAQDNVIVKK					Mascot

1721.8817	1721.8636	-0.0181	-11	906	921	AIFNEKASNVADSSLR					Mascot
1721.8817	1721.8636	-0.0181	-11	906	921	AIFNEKASNVADSSLR	12	0			Mascot
1730.9469	1730.8745	-0.0724	-42	698	713	SEKPLVGTGMEKIVAR			Oxidation (M)[10]		Mascot
1733.8892	1733.8594	-0.0298	-17	1325	1339	LTMNVDVPESFNVLR					Mascot
1735.9993	1735.8698	-0.1295	-75	995	1010	GAILTKEFLATLPFSK					Mascot
1772.951	1772.938	-0.013	-7	682	697	VLMGANMQRQAVPTLK			Oxidation (M)[3]		Mascot
1772.951	1772.938	-0.013	-7	682	697	VLMGANMQRQAVPTLK			Oxidation (M)[3]		Mascot
1786.9963	1786.8899	-0.1064	-60	149	164	VVVSQLHRSPGVFFSK					Mascot
1788.9459	1788.9294	-0.0165	-9	682	697	VLMGANMQRQAVPTLK			Oxidation (M)[3,7]		Mascot
1801.0066	1800.9573	-0.0493	-27	1049	1065	SITQSNELSPGVLKTVK					Mascot
1839.0773	1838.9241	-0.1532	-83	490	506	LMPKDIVNSKPITAAIK					Mascot
1843.98	1843.9121	-0.0679	-37	744	759	SQTSNLVDIYSLTKFK					Mascot
1901.8811	1901.9435	0.0624	33	626	640	LDENNHFVEDLIQCR			Carbamidomethyl (C)[14]		Mascot
1913.9539	1913.8792	-0.0747	-39	458	473	SVGEMVENQFRIGLYR			Oxidation (M)[5]		Mascot
1942.9757	1943.0565	0.0808	42	355	370	YDLTRNTDEALVEIYK					Mascot
1943.0121	1943.0565	0.0444	23	1190	1208	GGVPIATPVFDGAKEEDI K					Mascot
2022.9801	2022.9916	0.0115	6	922	940	MPSGTSGTVINVQVFEN DK					Mascot
2083.9753	2083.9565	-0.0188	-9	827	843	DDKYTSIHIEEFTCVAR			Carbamidomethyl (C)[14]		Mascot
2213.2112	2213.0493	-0.1619	-73	1121	1141	MNIGQILEAHLGLASYGL GKK					Mascot
2293.1355	2293.1672	0.0317	14	1236	1254	HVTVGYMYMLKLDHLVD DK			Oxidation (M)[7]		Mascot

3 Myosin-D OS=Toxoplasma gondii PE=2 SV=1 MYOD_TOXGO 91803.6 8.6 18 64 76.933 47.967

Peptide Information

Calc. Mass	Obsrv. Mass	± da	± ppm	Start Seq.	End Seq.	Sequence	Ion Score	C. I. %	Modification	Rank	Result Type
1027.5782	1027.6213	0.0431	42	448	456	KLNADIEPK					Mascot
1105.6252	1105.656	0.0308	28	622	631	GLFEGVKVEK					Mascot
1186.6368	1186.5852	-0.0516	-43	161	170	LPPHVFYASR					Mascot
1245.6191	1245.6591	0.04	32	171	180	RAMTNMHQLK			Oxidation (M)[3]		Mascot
1703.8785	1703.8556	-0.0229	-13	421	435	WTVPDSEMLRASLAK					Mascot
1770.932	1770.9122	-0.0198	-11	249	264	FMLLDVAREGGIQHGK					Mascot
1772.8755	1772.938	0.0625	35	281	293	DERNYHIFYQFLK					Mascot
1772.8755	1772.938	0.0625	35	281	293	DERNYHIFYQFLK					Mascot
1786.9269	1786.8899	-0.037	-21	249	264	FMLLDVAREGGIQHGK			Oxidation (M)[2]		Mascot
1857.9449	1857.9213	-0.0236	-13	201	217	MLMKYLATSAGGNLDLK			Oxidation (M)[1,3]		Mascot
1857.9449	1857.9213	-0.0236	-13	201	217	MLMKYLATSAGGNLDLK			Oxidation (M)[1,3]		Mascot

1865.0266	1865.0181	-0.0085	-5	518	533	IEYTTNEKLIETLLGK					Mascot
1873.9623	1873.9156	-0.0467	-25	1	17	MAAKPEQDCKTAAALIR			Carbamidomethyl (C)[9]		Mascot
1873.9623	1873.9156	-0.0467	-25	1	17	MAAKPEQDCKTAAALIR			Carbamidomethyl (C)[9]		Mascot
1889.9572	1889.9321	-0.0251	-13	1	17	MAAKPEQDCKTAAALIR			Carbamidomethyl (C)[9], Oxidation (M)[1]		Mascot
1914.0654	1913.8792	-0.1862	-97	11	30	TAAALIRAGSLIEGVESAG K					Mascot
1926.9708	1926.8949	-0.0759	-39	154	170	DAPDVDKLPPHVFYASR					Mascot
1959.0369	1958.962	-0.0749	-38	218	236	IQTAIMAANPVLEAFGNA K					Mascot
1974.0219	1974.0164	-0.0055	-3	495	510	NFTDIVFEKELQLYSK					Mascot
2023.1198	2022.9916	-0.1282	-63	431	447	ASLAKGMFEQLFLWIIR					Mascot
2178.0232	2178.1689	0.1457	67	534	554	GTSVLAALEDQCISPSGT DEK			Carbamidomethyl (C)[12]		Mascot
2315.2539	2315.1794	-0.0745	-32	218	239	IQTAIMAANPVLEAFGNA KTVR					Mascot
2332.2371	2332.0894	-0.1477	-63	771	791	EALSAWEPLVGVFEGMT VLKR					Mascot

4	Protein translocase subunit SecA, chloroplastic OS=Pisum sativum GN=secA PE=2 SV=1	SECA_PEA	114632.4	6.4	20	61	60.827	47.473

Peptide Information

Calc. Mass	Obsrv. Mass	± da	± ppm	Start Seq.	End Seq.	Sequence	Ion Score	C. I. %	Modification	Rank	Result Type
807.4835	807.4316	-0.0519	-64	872	877	SYLRLR					Mascot
937.4407	937.4711	0.0304	32	979	986	MENQKSGK			Oxidation (M)[1]		Mascot
1245.6984	1245.6591	-0.0393	-32	480	490	AVVVEISRMNK					Mascot
1328.6879	1328.6826	-0.0053	-4	887	898	DIVEQQAPGLMK					Mascot
1384.7981	1384.6951	-0.103	-74	452	463	VTIVPTNKPMIR			Oxidation (M)[10]		Mascot
1424.7017	1424.7365	0.0348	24	465	477	DESDVVFRATTGK					Mascot
1500.7839	1500.7501	-0.0338	-23	886	898	RDIVEQQAPGLMK			Oxidation (M)[12]		Mascot
1517.8839	1517.7593	-0.1246	-82	903	914	FLILSNIDRLWK					Mascot
1603.8473	1603.795	-0.0523	-33	216	229	VGLIQQNMTSEQKK					Mascot
1758.9749	1758.8623	-0.1126	-64	81	96	KQYAAIVNTINGLEPK					Mascot
1812.0266	1811.9083	-0.1183	-65	583	599	LVAEGEFVSVKKPPPSK					Mascot
1813.9113	1813.912	0.0007	0	887	902	DIVEQQAPGLMKEAER					Mascot
1829.9062	1829.9071	0.0009	0	887	902	DIVEQQAPGLMKEAER			Oxidation (M)[11]		Mascot
1843.9081	1843.9121	0.004	2	943	957	LEGYNLFLEMMAQIR			Oxidation (M)[10]		Mascot
1858.0546	1857.9213	-0.1333	-72	53	71	TRQSGPVASLGGLLGGIF K					Mascot
1858.0546	1857.9213	-0.1333	-72	53	71	TRQSGPVASLGGLLGGIF K					Mascot
1864.9586	1865.0181	0.0595	32	375	390	EVLIVDEFTGRVMQGR			Oxidation (M)[13]		Mascot

1873.9991	1873.9156	-0.0835	-45	684	701	VVAAGGLHVVGTERHES R					Mascot
1873.9991	1873.9156	-0.0835	-45	684	701	VVAAGGLHVVGTERHES R					Mascot
2084.0515	2083.9565	-0.095	-46	211	228	FLGMKVGLIQQNMTSEQ K			Oxidation (M)[4,13]		Mascot
2315.2136	2315.1794	-0.0342	-15	532	554	EAEIVAQSGRLGAVTIAT NMAGR					Mascot
2820.4495	2820.3445	-0.105	-37	542	569	LGAVTIATNMAGRGTDIIL GGNAEFMAR					Mascot
2894.5745	2894.3984	-0.1761	-61	140	165	VLGLRPFDVQLIGGMVLH KGEIAEMR			Oxidation (M)[15]		Mascot
2910.5693	2910.3157	-0.2536	-87	140	165	VLGLRPFDVQLIGGMVLH KGEIAEMR			Oxidation (M)[15,25]		Mascot

5	DNA-directed RNA polymerase subunit beta OS=Francisella tularensis subsp. mediasiatica (strain FSC147) GN=rpoB PE=3 SV=1	RPOB_FRATM	151744.2	5.4	22	60	49.536	8.218

Peptide Information

Calc. Mass	Obsrv. Mass	± da	± ppm	Start Seq.	End Seq.	Sequence	Ion Score	C. I. %	Modification	Rank	Result Type
937.5578	937.4711	-0.0867	-92	149	156	VVVSQLHR					Mascot
1027.5895	1027.6213	0.0318	31	533	543	ISALGPGGLSR					Mascot
1105.6252	1105.656	0.0308	28	1037	1046	IAIDAKFEAK					Mascot
1328.7209	1328.6826	-0.0383	-29	1190	1203	GGVPIATPVFDGAK					Mascot
1515.8378	1515.7552	-0.0826	-54	975	988	SSIEQEVVGAKIQK					Mascot
1571.7853	1571.7805	-0.0048	-3	584	596	VNDYGFLEAPYRK					Mascot
1721.8817	1721.8636	-0.0181	-11	906	921	AIFNEKASNVADSSLR					Mascot
1721.8817	1721.8636	-0.0181	-11	906	921	AIFNEKASNVADSSLR	12	0			Mascot
1730.9469	1730.8745	-0.0724	-42	698	713	SEKPLVGTGMEKIVAR			Oxidation (M)[10]		Mascot
1733.8892	1733.8594	-0.0298	-17	1325	1339	LTMNVDVPESFNVLR					Mascot
1735.9993	1735.8698	-0.1295	-75	995	1010	GAILTKEFLATLPFSK					Mascot
1772.951	1772.938	-0.013	-7	682	697	VLMGANMQRQAVPTLK			Oxidation (M)[3]		Mascot
1772.951	1772.938	-0.013	-7	682	697	VLMGANMQRQAVPTLK			Oxidation (M)[3]		Mascot
1786.9963	1786.8899	-0.1064	-60	149	164	VVVSQLHRSPGVFFSK					Mascot
1788.9459	1788.9294	-0.0165	-9	682	697	VLMGANMQRQAVPTLK			Oxidation (M)[3,7]		Mascot
1801.0066	1800.9573	-0.0493	-27	1049	1065	SITQSNELSPGVLKTVK					Mascot
1839.0773	1838.9241	-0.1532	-83	490	506	LMPKDIVNSKPITAAIK					Mascot
1843.98	1843.9121	-0.0679	-37	744	759	SQTSNLVDIYSLTKFK					Mascot
1913.9539	1913.8792	-0.0747	-39	458	473	SVGEMVENQFRIGLYR			Oxidation (M)[5]		Mascot
1942.9757	1943.0565	0.0808	42	355	370	YDLTRNTDEALVEIYK					Mascot
1943.0121	1943.0565	0.0444	23	1190	1208	GGVPIATPVFDGAKEEDI K					Mascot
2022.9801	2022.9916	0.0115	6	922	940	MPSGTSGTVINVQVFEN					Mascot

Calc. Mass	Obsrv. Mass	± da	± ppm	Start Seq.	End Seq.	Sequence	Ion Score	C. I. %	Modification	Rank	Result Type
						DK					
2083.9753	2083.9565	-0.0188	-9	827	843	DDKYTSIHIEEFTCVAR			Carbamidomethyl (C)[14]		Mascot
2213.2112	2213.0493	-0.1619	-73	1121	1141	MNIGQILEAHLGLASYGL GKK					Mascot
2293.1355	2293.1672	0.0317	14	1236	1254	HVTVGYMYMLKLDHLVD DK			Oxidation (M)[7]		Mascot

6	50S ribosomal protein L11 OS=Buchnera aphidicola subsp. Baizongia pistaciae (strain Bp) GN=rplK PE=3 SV=1	RL11_BUCBP	15478.4	9.91	8	59	31.926	1.741

Peptide Information

Calc. Mass	Obsrv. Mass	± da	± ppm	Start Seq.	End Seq.	Sequence	Ion Score	C. I. %	Modification	Rank	Result Type
1182.6552	1182.5792	-0.076	-64	1	10	MAKTIQAYIK			Oxidation (M)[1]		Mascot
1500.8129	1500.7501	-0.0628	-42	83	97	AAGITKGSSKPNQNK					Mascot
1735.9993	1735.8698	-0.1295	-75	66	81	SFSFITKTPPASVLLK					Mascot
1872.0437	1871.9022	-0.1415	-76	128	146	SIIGTANSIGLIIAEGTNK					Mascot
1900.0936	1899.9515	-0.1421	-75	98	114	VGTITMQQILDIAQIKK					Mascot
2003.092	2002.9561	-0.1359	-68	11	31	LQVSAGTANPSPPIGPAL GQK					Mascot
2712.4712	2712.2407	-0.2305	-85	89	113	GSSKPNQNKVGTITMQQI LDIAQIK					Mascot
2820.5618	2820.3445	-0.2173	-77	4	31	TIQAYIKLQVSAGTANPS PPIGPALGQK					Mascot

7	50S ribosomal protein L5 OS=Desulfococcus oleovorans (strain DSM 6200 / Hxd3) GN=rplE PE=3 SV=1	RL5_DESOH	20518.9	9.43	11	57	1.603	2.931

Peptide Information

Calc. Mass	Obsrv. Mass	± da	± ppm	Start Seq.	End Seq.	Sequence	Ion Score	C. I. %	Modification	Rank	Result Type
937.4634	937.4711	0.0077	8	172	179	LMGMPFKN					Mascot
1199.6816	1199.5938	-0.0878	-73	24	33	NVMQIPKLEK					Mascot
1386.626	1386.7031	0.0771	56	93	102	HERMYDFFNK					Mascot
1571.8826	1571.7805	-0.1021	-65	34	48	IVLNMGLGEAISNIK					Mascot
1649.8285	1649.8367	0.0082	5	79	92	LRQGMPVGCMVTLR			Carbamidomethyl (C)[9], Oxidation (M)[5,10]		Mascot
1770.8561	1770.9122	0.0561	32	81	95	QGMPVGCMVTLRHER			Carbamidomethyl (C)[7]		Mascot
1786.851	1786.8899	0.0389	22	81	95	QGMPVGCMVTLRHER			Carbamidomethyl (C)[7], Oxidation (M)[3]		Mascot
1795.9412	1795.9189	-0.0223	-12	16	30	LQETFGYKNVMQIPK					Mascot
1811.936	1811.9083	-0.0277	-15	16	30	LQETFGYKNVMQIPK			Oxidation (M)[11]		Mascot
1839.8984	1839.9252	0.0268	15	116	133	GISGQAFDGQGNYTLGV R					Mascot
1840.9779	1840.9182	-0.0597	-32	96	110	MYDFFNKLVNIALPR					Mascot
1856.9728	1856.9169	-0.0559	-30	96	110	MYDFFNKLVNIALPR			Oxidation (M)[1]		Mascot

1879.9688	1879.9	-0.0688	-37	134	148	EQIIFPEIEYDKIDK					Mascot
1958.0991	1957.9976	-0.1015	-52	31	48	LEKIVLNMGLGEAISNIK			Oxidation (M)[8]		Mascot

8	Translation initiation factor IF-2 OS=Rickettsia conorii (strain ATCC VR-613 / Malish 7) GN=infB PE=3 SV=1	IF2_RICCN	91073.8	6.52	17	57	0	29.655

Peptide Information

Calc. Mass	Obsrv. Mass	± da	± ppm	Start Seq.	End Seq.	Sequence	Ion Score	C. I. %	Modification	Rank	Result Type
871.4883	871.4281	-0.0602	-69	239	246	LVSQAPEK					Mascot
1199.5361	1199.5938	0.0577	48	734	743	EQYIGSAEMR			Oxidation (M)[9]		Mascot
1213.6423	1213.661	0.0187	15	441	450	IDKPDIDIER					Mascot
1273.6747	1273.6179	-0.0568	-45	692	704	AGANALTAAEKEK					Mascot
1310.6475	1310.6139	-0.0336	-26	174	184	YGIESEKELDK					Mascot
1549.8584	1549.7339	-0.1245	-80	534	548	NSDIIIAGTAYGKVK					Mascot
1571.77	1571.7805	0.0105	7	24	38	SFDSLTGAQSFVNAK					Mascot
1603.7922	1603.795	0.0028	2	129	142	QQNTEENKVEVSAK					Mascot
1703.9011	1703.8556	-0.0455	-27	329	344	TRAPVVTVMGHVDHGK					Mascot
1786.8971	1786.8899	-0.0072	-4	24	40	SFDSLTGAQSFVNAKSK					Mascot
1857.8284	1857.9213	0.0929	50	205	221	KADLFNMLSDDESGSGR			Oxidation (M)[7]		Mascot
1857.8284	1857.9213	0.0929	50	205	221	KADLFNMLSDDESGSGR	2	0	Oxidation (M)[7]		Mascot
1937.9062	1938.075	0.1688	87	380	396	AITFIDTPGHEAFSEMR			Oxidation (M)[16]		Mascot
1943.0444	1943.0565	0.0121	6	87	104	AAEQSQLNDPLKISTLSK					Mascot
1943.0444	1943.0565	0.0121	6	87	104	AAEQSQLNDPLKISTLSK					Mascot
2028.0219	2028.0004	-0.0215	-11	734	750	EQYIGSAEMRQIFNITK					Mascot
2304.238	2304.0203	-0.2177	-94	331	352	APVVTVMGHVDHGKTSL LDSLK					Mascot
2939.541	2939.3716	-0.1694	-58	709	733	YYSIIYHLIDDIKAIMSGML DPIVR					Mascot
2968.5701	2968.3787	-0.1914	-64	453	479	NELYVHEIIGEEAGGDIMII PISALKK			Oxidation (M)[18]		Mascot

9	Chromosome partition protein MukB OS=Klebsiella pneumoniae subsp. pneumoniae (strain ATCC 700721 / MGH 78578) GN=mukB PE=3 SV=1	MUKB_KLEP7	170021.5	5.27	24	57	0	21.108

Peptide Information

Calc. Mass	Obsrv. Mass	± da	± ppm	Start Seq.	End Seq.	Sequence	Ion Score	C. I. %	Modification	Rank	Result Type
807.4471	807.4316	-0.0155	-19	288	293	ALEYRR					Mascot
937.5465	937.4711	-0.0754	-80	1443	1450	GTTYKLVR					Mascot
1199.6492	1199.5938	-0.0554	-46	500	509	ELLRDGVNQR					Mascot

1245.6182	1245.6591	0.0409	33	991	1000	QRLEQAESER					Mascot
1262.6376	1262.6621	0.0245	19	219	229	DYLLPENSGVR					Mascot
1295.6412	1295.7493	0.1081	83	230	240	KAFQDMEAALR			Oxidation (M)[6]		Mascot
1360.7067	1360.8065	0.0998	73	1222	1232	LTEELTNREQK					Mascot
1515.8391	1515.7552	-0.0839	-55	955	967	QQAFALAEVVQRR					Mascot
1517.8071	1517.7593	-0.0478	-31	413	425	AIQYNQALQALER					Mascot
1588.7926	1588.8275	0.0349	22	566	580	IASLADSVSNAQEQR					Mascot
1721.8462	1721.8636	0.0174	10	1108	1122	EQVVTAKAGWCAVMR			Carbamidomethyl (C)[11], Oxidation (M)[14]		Mascot
1721.8462	1721.8636	0.0174	10	1108	1122	EQVVTAKAGWCAVMR			Carbamidomethyl (C)[11], Oxidation (M)[14]		Mascot
1747.8909	1747.9017	0.0108	6	1315	1328	LYQRLNPQIDMGQR			Oxidation (M)[11]		Mascot
1843.8743	1843.9121	0.0378	21	1137	1152	ELAYLSADELRSMSDK			Oxidation (M)[13]		Mascot
1856.939	1856.9169	-0.0221	-12	795	810	ESLSERFATLSFDVQK					Mascot
1863.9196	1863.9171	-0.0025	-1	397	412	SQLADYQQALDVQQTR					Mascot
1869.88	1869.9657	0.0857	46	261	276	HLISEATNYVAADYMR			Oxidation (M)[15]		Mascot
1871.9094	1871.9022	-0.0072	-4	895	910	VDEIQERLDEAQEAAR					Mascot
1896.0146	1895.8781	-0.1365	-72	1426	1442	LEMQLIIAAPENISPEK					Mascot
1973.9974	1974.0164	0.019	10	83	99	AGVCYSVLDVINSRHQR			Carbamidomethyl (C)[4]		Mascot
1990.9274	1991.0117	0.0843	42	1205	1221	TDDPVEAIEQMEIELSR			Oxidation (M)[11]		Mascot
2293.1572	2293.1672	0.01	4	902	921	LDEAQEAARFIQQYGNQ LAK					Mascot
2315.1924	2315.1794	-0.013	-6	1005	1025	DAMRAHAAQLSQYNQVL ASLK					Mascot
2703.3406	2703.2261	-0.1145	-42	1319	1341	LNPQIDMGQRTPQTIGEE LLDYR			Oxidation (M)[7]		Mascot
2911.4143	2911.344	-0.0703	-24	1329	1352	TPQTIGEELLDYRNYLEM EVEVNR					Mascot
2911.4143	2911.344	-0.0703	-24	1329	1352	TPQTIGEELLDYRNYLEM EVEVNR					Mascot

10	Nodulation protein D OS=Azorhizobium caulinodans (strain ATCC 43989 / DSM 5975 / ORS 571) GN=nodD PE=4 SV=2	NODD_AZOC5	35756.3	6.67	12	54	0	28.303

Peptide Information

Calc. Mass	Obsrv. Mass	± da	± ppm	Start Seq.	End Seq.	Sequence	Ion Score	C. I. %	Modification	Rank	Result Type
1384.8159	1384.6951	-0.1208	-87	114	127	GVISAASTSLPLLR					Mascot
1386.8315	1386.7031	-0.1284	-93	75	86	DILSRIQSTIIK					Mascot
1588.8112	1588.8275	0.0163	10	29	43	SINLSQPAMSAAVQR			Oxidation (M)[9]		Mascot
1603.6887	1603.795	0.1063	66	170	182	MVCVGCARNHEDR			Carbamidomethyl (C)[3,6]		Mascot
1703.9302	1703.8556	-0.0746	-44	242	255	IAIMPYRLVEHFSK					Mascot
1773.0284	1772.938	-0.0904	-51	256	270	FMPLKVFALPFPLPR					Mascot
1773.0284	1772.938	-0.0904	-51	256	270	FMPLKVFALPFPLPR					Mascot

1789.0233	1788.9294	-0.0939	-52	256	270	FMPLKVFALPFPLPR	Oxidation (M)[2]	Mascot
1856.9655	1856.9169	-0.0486	-26	44	58	LRIYFNDDLFTINGR		Mascot
1857.9963	1857.9213	-0.075	-40	29	45	SINLSQPAMSAAVQRLR	Oxidation (M)[9]	Mascot
1857.9963	1857.9213	-0.075	-40	29	45	SINLSQPAMSAAVQRLR	Oxidation (M)[9]	Mascot
1872.924	1872.901	-0.023	-12	46	60	IYFNDDLFTINGRER		Mascot
1991.0378	1991.0117	-0.0261	-13	183	199	NISSIQEYLSMRHVVAK	Oxidation (M)[11]	Mascot
2213.0049	2213.0493	0.0444	20	203	220	GMRPSLEEWFMAENGM RR	Oxidation (M)[2]	Mascot
2867.5876	2867.3521	-0.2355	-82	101	127	IISSDYSTSIFIRGVISAAS TSLPLLR		Mascot

Gel Idx/Pos	218/I18	Instr./Gel Origin	BA2151/Sample Project 20140814	Process Status	Analysis Succeeded
Plate [#] Name	[1] Sample Project 20140814	Instrument Sample Name		Spectra	11

Rank	Protein Name	Accession No.	Protein MW	Protein PI	Pep. Count	Protein Score	Protein Score C. I. %	Intensity Matched	Total Ion Score	Total Ion C. I. %	Confirmed
1	40S ribosomal protein SA OS=Brassica napus GN=LRP PE=2 SV=1	RSSA_BRANA	32237.9	5.14	8	140	100	26.995	105	100	

Peptide Information

Calc. Mass	Obsrv. Mass	± da	± ppm	Start Seq.	End Seq.	Sequence	Ion Score	C. I. %	Modification	Rank	Result Type
809.4008	809.4124	0.0116	14	182	187	MVLQMR			Oxidation (M)[1,5]		Mascot
838.4417	838.4663	0.0246	29	130	136	TDHQPIK					Mascot
870.5156	870.5576	0.042	48	188	195	GTIRPAQK					Mascot
940.5826	940.6027	0.0201	21	122	129	LLILTDPR					Mascot
940.5826	940.6027	0.0201	21	122	129	LLILTDPR	61	99.932			Mascot
1339.6754	1339.7114	0.036	27	91	103	FAQYTGANAIAGR					Mascot
2065.9397	2065.9922	0.0525	25	104	121	HTPGTFTNQMQTSFSEPR					Mascot
2065.9397	2065.9922	0.0525	25	104	121	HTPGTFTNQMQTSFSEPR					Mascot
2081.9346	2081.9744	0.0398	19	104	121	HTPGTFTNQMQTSFSEPR			Oxidation (M)[10]		Mascot
2081.9346	2081.9744	0.0398	19	104	121	HTPGTFTNQMQTSFSEPR	44	97.014	Oxidation (M)[10]		Mascot
2193.0315	2193.041	0.0095	4	137	156	EGALGNIPIIAFCDTDSPMR			Carbamidomethyl (C)[13], Oxidation (M)[19]		Mascot
2466.3464	2466.4006	0.0542	22	65	86	VIVAIENPQDIIVQSARPYGQR					Mascot

Rank	Protein Name	Accession No.	Protein MW	Protein PI	Pep. Count	Protein Score	Protein Score C. I. %	Intensity Matched	Total Ion Score	Total Ion C. I. %	Confirmed
2	40S ribosomal protein Sa-1 OS=Arabidopsis thaliana GN=RPSaA PE=1 SV=3	RSSA1_ARATH	32498.9	5.05	7	134	100	26.851	105	100	

Peptide Information

Calc. Mass	Obsrv. Mass	± da	± ppm	Start Seq.	End Seq.	Sequence	Ion Score	C. I. %	Modification	Rank	Result Type
809.4008	809.4124	0.0116	14	185	190	MVLQMR			Oxidation (M)[1,5]		Mascot
838.4417	838.4663	0.0246	29	133	139	TDHQPIK					Mascot
940.5826	940.6027	0.0201	21	125	132	LLILTDPR					Mascot
940.5826	940.6027	0.0201	21	125	132	LLILTDPR	61	99.932			Mascot
1339.6754	1339.7114	0.036	27	94	106	FAQYTGANAIAGR					Mascot
2065.9397	2065.9922	0.0525	25	107	124	HTPGTFTNQMQTSFSEPR					Mascot
2065.9397	2065.9922	0.0525	25	107	124	HTPGTFTNQMQTSFSEPR					Mascot

Calc. Mass	Obsrv. Mass	± da	± ppm	Start Seq.	End Seq.	Sequence	Ion Score	C. I. %	Modification	Rank	Result Type
2081.9346	2081.9744	0.0398	19	107	124	HTPGTFTNQMQTSFSEPR			Oxidation (M)[10]		Mascot
2081.9346	2081.9744	0.0398	19	107	124	HTPGTFTNQMQTSFSEPR	44	97.014	Oxidation (M)[10]		Mascot
2193.0315	2193.041	0.0095	4	140	159	EGALGNIPIIAFCDTDSPMR			Carbamidomethyl (C)[13], Oxidation (M)[19]		Mascot
2466.3464	2466.4006	0.0542	22	68	89	VIVAIENPQDIIVQSARPYGQR					Mascot

3	40S ribosomal protein Sa-2 OS=Arabidopsis thaliana GN=RPSaB PE=2 SV=2	RSSA2_ARATH	30805.3	5.04	5	122	100	26.463	105	100	

Peptide Information

Calc. Mass	Obsrv. Mass	± da	± ppm	Start Seq.	End Seq.	Sequence	Ion Score	C. I. %	Modification	Rank	Result Type
809.4008	809.4124	0.0116	14	186	191	MVLQMR			Oxidation (M)[1,5]		Mascot
838.4417	838.4663	0.0246	29	134	140	TDHQPIK					Mascot
940.5826	940.6027	0.0201	21	126	133	LLILTDPR					Mascot
940.5826	940.6027	0.0201	21	126	133	LLILTDPR	61	99.932			Mascot
2065.9397	2065.9922	0.0525	25	108	125	HTPGTFTNQMQTSFSEPR					Mascot
2065.9397	2065.9922	0.0525	25	108	125	HTPGTFTNQMQTSFSEPR					Mascot
2081.9346	2081.9744	0.0398	19	108	125	HTPGTFTNQMQTSFSEPR			Oxidation (M)[10]		Mascot
2081.9346	2081.9744	0.0398	19	108	125	HTPGTFTNQMQTSFSEPR	44	97.014	Oxidation (M)[10]		Mascot
2466.3828	2466.4006	0.0178	7	69	90	VIVAIENPKDIIVQSARPYGQR					Mascot

4	40S ribosomal protein SA OS=Glycine max PE=1 SV=1	RSSA_SOYBN	34056.1	5.1	7	116	100	23.407	90	100	

Peptide Information

Calc. Mass	Obsrv. Mass	± da	± ppm	Start Seq.	End Seq.	Sequence	Ion Score	C. I. %	Modification	Rank	Result Type
809.4008	809.4124	0.0116	14	188	193	MVLQMR			Oxidation (M)[1,5]		Mascot
838.4417	838.4663	0.0246	29	136	142	TDHQPIK					Mascot
940.5826	940.6027	0.0201	21	128	135	LLILTDPR					Mascot
940.5826	940.6027	0.0201	21	128	135	LLILTDPR	61	99.932			Mascot
1099.4296	1099.4526	0.023	21	36	43	NCDFQMER			Carbamidomethyl (C)[2]		Mascot
1099.4296	1099.4526	0.023	21	36	43	NCDFQMER	29	3.821	Carbamidomethyl (C)[2]		Mascot
1115.4244	1115.4442	0.0198	18	36	43	NCDFQMER			Carbamidomethyl (C)[2], Oxidation (M)[6]		Mascot
1115.4244	1115.4442	0.0198	18	36	43	NCDFQMER	12	0	Carbamidomethyl (C)[2], Oxidation (M)[6]		Mascot
1203.6368	1203.655	0.0182	15	163	173	YVDIGIPANNK					Mascot
2195.0107	2195.0593	0.0486	22	143	162	EAALGNIPTIAFCDTDSPMR			Carbamidomethyl (C)[13], Oxidation (M)[19]		Mascot
2466.3464	2466.4006	0.0542	22	71	92	VIVAIENPQDIIVQSARPYGQR					Mascot

5	40S ribosomal protein SA OS=Daucus carota GN=179B PE=2 SV=1	RSSA_DAUCA	32660.2	4.88	7	115	100	23.375	90	100	

Peptide Information

Calc. Mass	Obsrv. Mass	± da	± ppm	Start Seq.	End Seq.	Sequence	Ion Score	C. I. %	Modification	Rank	Result Type
809.4008	809.4124	0.0116	14	181	186	MVLQMR			Oxidation (M)[1,5]		Mascot
825.4577	825.4378	-0.0199	-24	187	194	GVISQGHK					Mascot
838.4417	838.4663	0.0246	29	129	135	TDHQPIK					Mascot
940.5826	940.6027	0.0201	21	121	128	LLILTDPR					Mascot
940.5826	940.6027	0.0201	21	121	128	LLILTDPR	61	99.932			Mascot
1099.4296	1099.4526	0.023	21	29	36	NCDFQMER			Carbamidomethyl (C)[2]		Mascot
1099.4296	1099.4526	0.023	21	29	36	NCDFQMER	29	3.821	Carbamidomethyl (C)[2]		Mascot
1115.4244	1115.4442	0.0198	18	29	36	NCDFQMER			Carbamidomethyl (C)[2], Oxidation (M)[6]		Mascot
1115.4244	1115.4442	0.0198	18	29	36	NCDFQMER	12	0	Carbamidomethyl (C)[2], Oxidation (M)[6]		Mascot
1203.6368	1203.655	0.0182	15	156	166	YVDIGIPANNK					Mascot
2195.0107	2195.0593	0.0486	22	136	155	EAALGNIPTIAFCDTDSPMR			Carbamidomethyl (C)[13], Oxidation (M)[19]		Mascot

6	40S ribosomal protein SA OS=Vitis vinifera GN=GSVIVT00034021001 PE=2 SV=1	RSSA_VITVI	34058.2	5.43	6	81	99.59	19.018	61	99.932	

Peptide Information

Calc. Mass	Obsrv. Mass	± da	± ppm	Start Seq.	End Seq.	Sequence	Ion Score	C. I. %	Modification	Rank	Result Type
809.4008	809.4124	0.0116	14	181	186	MVLQMR			Oxidation (M)[1,5]		Mascot
838.4417	838.4663	0.0246	29	129	135	TDHQPIK					Mascot
940.5826	940.6027	0.0201	21	121	128	LLILTDPR					Mascot
940.5826	940.6027	0.0201	21	121	128	LLILTDPR	61	99.932			Mascot
1203.6515	1203.655	0.0035	3	1	11	MATPTRALSQK					Mascot
2195.0107	2195.0593	0.0486	22	136	155	EAALGNIPTIAFCDTDSPMR			Carbamidomethyl (C)[13], Oxidation (M)[19]		Mascot
2466.3828	2466.4006	0.0178	7	64	85	VIVAIENPKDIIVQSARPYGQR					Mascot

7	40S ribosomal protein SA OS=Cicer arietinum GN=RAP40 PE=2 SV=1	RSSA_CICAR	33264.8	5.1	3	67	88.703	17.821	61	99.932	

Peptide Information

Calc. Mass	Obsrv. Mass	± da	± ppm	Start Seq.	End Seq.	Sequence	Ion Score	C. I. %	Modification	Rank	Result Type
809.4008	809.4124	0.0116	14	184	189	MVLQMR			Oxidation (M)[1,5]		Mascot
838.4417	838.4663	0.0246	29	132	138	TDHQPIK					Mascot

940.5826	940.6027	0.0201	21	124	131	LLILTDPR					Mascot
940.5826	940.6027	0.0201	21	124	131	LLILTDPR	61	99.932			Mascot

8	1-(5-phosphoribosyl)-5-[(5-phosphoribosylamino)methylideneamino] imidazole-4-carboxamide isomerase OS=Brachyspira hyodysenteriae (strain ATCC 49526 / WA1) GN=hisA PE=3 SV=1	HIS4_BRAHW	26903	5.19	12	65	81.678	4.894	

Peptide Information

Calc. Mass	Obsrv. Mass	± da	± ppm	Start Seq.	End Seq.	Sequence	Ion Score	C. I. %	Modification	Rank	Result Type
807.4611	807.4128	-0.0483	-60	92	98	YLDIGVK					Mascot
847.452	847.4722	0.0202	24	85	91	NEDTIKK					Mascot
1051.5055	1051.449	-0.0565	-54	17	25	LVEGNYDNK					Mascot
1126.5852	1126.6113	0.0261	23	122	131	EHIAVSIDSR					Mascot
1145.6348	1145.655	0.0202	18	210	220	DMKINGAIVGK					Mascot
1192.6647	1192.6821	0.0174	15	1	10	MYILPAIDLK			Oxidation (M)[1]		Mascot
1397.7133	1397.7533	0.04	29	122	133	EHIAVSIDSRDR					Mascot
1417.7434	1417.7758	0.0324	23	120	131	YKEHIAVSIDSR					Mascot
1705.8505	1705.9476	0.0971	57	11	25	NGEVVRLVEGNYDNK					Mascot
1800.8936	1800.9945	0.1009	56	155	170	LDSMGIDTIIYTDISK			Oxidation (M)[4]		Mascot
2066.0474	2065.9922	-0.0552	-27	189	207	ISCNIIASGGVTFEDEIIK			Carbamidomethyl (C)[3]		Mascot
2066.0474	2065.9922	-0.0552	-27	189	207	ISCNIIASGGVTFEDEIIK			Carbamidomethyl (C)[3]		Mascot
2195.1013	2195.0593	-0.042	-19	100	119	TILGTAAVENIDFTNNMINK			Oxidation (M)[17]		Mascot

9	Ubiquinone biosynthesis protein coq4, mitochondrial OS=Schizosaccharomyces pombe (strain 972 / ATCC 24843) GN=coq4 PE=3 SV=1	COQ4_SCHPO	31194.5	9.56	13	64	79.91	21.375	

Peptide Information

Calc. Mass	Obsrv. Mass	± da	± ppm	Start Seq.	End Seq.	Sequence	Ion Score	C. I. %	Modification	Rank	Result Type
809.4767	809.4124	-0.0643	-79	265	272	IEAAPPLV					Mascot
940.5462	940.6027	0.0565	60	128	136	TLPPNTLGK					Mascot
940.5462	940.6027	0.0565	60	128	136	TLPPNTLGK					Mascot
962.5087	962.5842	0.0755	78	112	119	DKPRMTSK					Mascot
977.4469	977.4871	0.0402	41	217	224	LNCEQASR			Carbamidomethyl (C)[3]		Mascot
978.5037	978.5623	0.0586	60	112	119	DKPRMTSK			Oxidation (M)[5]		Mascot
1051.5459	1051.449	-0.0969	-92	137	144	IYVDWIDK					Mascot
1078.6619	1078.5864	-0.0755	-70	263	272	IRIEAAPPLV					Mascot

1203.5852	1203.655	0.0698	58	251	260	ELTNDIEDVR					Mascot
1335.7705	1335.6775	-0.093	-70	59	71	IFLVAGSAIMGLK			Oxidation (M)[10]		Mascot
1406.7825	1406.7751	-0.0074	-5	116	127	MTSKSLNLPFLR					Mascot
1487.8693	1487.7911	-0.0782	-53	23	35	KFLKPSVASNQLR					Mascot
1846.0408	1845.8821	-0.1587	-86	59	75	IFLVAGSAIMGLKAPWR			Oxidation (M)[10]		Mascot
2055.9441	2056.0388	0.0947	46	153	169	SPTRFVDDPEEAYVMQR			Oxidation (M)[15]		Mascot
2104.0281	2103.9651	-0.063	-30	76	95	GGDMISVLGDASGQPFF LHR					Mascot
2120.0229	2120.0159	-0.007	-3	76	95	GGDMISVLGDASGQPFF LHR			Oxidation (M)[4]		Mascot

10 J domain-containing protein required for chloroplast accumulation response 1 OS=Arabidopsis thaliana GN=JAC1 PE=1 SV=1 | JAC1_ARATH | 71615.1 | 6.88 | 16 | 63 | 74.708 | 23.325

Peptide Information

Calc. Mass	Obsrv. Mass	± da	± ppm	Start Seq.	End Seq.	Sequence	Ion Score	C. I. %	Modification	Rank	Result Type
905.4687	905.4918	0.0231	26	47	54	VTSNEVTR					Mascot
1003.5782	1003.5734	-0.0048	-5	261	270	SGLSGLKDVK					Mascot
1099.5419	1099.4526	-0.0893	-81	292	301	SEQAFFGVSK					Mascot
1099.5419	1099.4526	-0.0893	-81	292	301	SEQAFFGVSK	15	0			Mascot
1145.5685	1145.655	0.0865	76	383	393	SSLDSSPIPDK					Mascot
1156.6473	1156.6448	-0.0025	-2	214	224	GVPVVIWGSSR					Mascot
1170.575	1170.6051	0.0301	26	448	458	TDIIHDGSNAK					Mascot
1193.6372	1193.6666	0.0294	25	171	182	TSSKADVVSTAK					Mascot
1214.7216	1214.6683	-0.0533	-44	272	282	TSLKRPGVQTK					Mascot
1299.6903	1299.6346	-0.0557	-43	459	469	ETVNIPDQQKK					Mascot
1313.6155	1313.7035	0.088	67	622	633	GASANQKYMAEK			Oxidation (M)[9]		Mascot
1313.6155	1313.7035	0.088	67	622	633	GASANQKYMAEK			Oxidation (M)[9]		Mascot
1390.6809	1390.7084	0.0275	20	254	267	NEEGDGKSGLSGLK					Mascot
1406.6467	1406.7751	0.1284	91	543	554	MEEANKDAEEIK					Mascot
1465.7493	1465.7946	0.0453	31	161	174	ETVSSSPLSRTSSK					Mascot
1465.7493	1465.7946	0.0453	31	161	174	ETVSSSPLSRTSSK					Mascot
1723.8497	1723.9309	0.0812	47	394	411	SSFASSSAAPEVGKDGV K					Mascot
1789.963	1789.9362	-0.0268	-15	214	230	GVPVVIWGSSRLSSMSK					Mascot
2147.0042	2147.0608	0.0566	26	23	43	SPGGDDVDIDFGDVFGG PPKR					Mascot

Gel Idx/Pos	219/I19	Instr./Gel Origin	BA2151/Sample Project 20140814	Process Status	Analysis Succeeded
Plate [#] Name	[1] Sample Project 20140814	Instrument Sample Name		Spectra	11

Rank	Protein Name	Accession No.	Protein MW	Protein PI	Pep. Count	Protein Score	Protein Score C. I. %	Intensity Matched	Total Ion Score	Total Ion C. I. %	Confirmed
1	Glucose-1-phosphate adenylyltransferase large subunit 1, chloroplastic/amyloplastic OS=Hordeum vulgare PE=2 SV=2	GLGL1_HORVU	58580.1	6.17	13	71	95.502	12.368	28	0	

Peptide Information

Calc. Mass	Obsrv. Mass	± da	± ppm	Start Seq.	End Seq.	Sequence	Ion Score	C. I. %	Modification	Rank	Result Type
866.4618	866.447	-0.0148	-17	252	259	ASEYGLVK					Mascot
952.4887	952.5057	0.017	18	381	388	TPFFTSPR					Mascot
952.4887	952.5057	0.017	18	381	388	TPFFTSPR	25	0			Mascot
1023.5945	1023.6124	0.0179	17	416	424	IEHSIIGVR					Mascot
1266.7278	1266.6205	-0.1073	-85	416	426	IEHSIIGVRSR					Mascot
1302.6624	1302.6932	0.0308	24	402	412	EAIISHGCFLR			Carbamidomethyl (C)[8]		Mascot
1302.6624	1302.6932	0.0308	24	402	412	EAIISHGCFLR			Carbamidomethyl (C)[8]		Mascot
1306.6243	1306.662	0.0377	29	469	479	ISNCIIDMNAR			Carbamidomethyl (C)[4]		Mascot
1322.6191	1322.6451	0.026	20	469	479	ISNCIIDMNAR			Carbamidomethyl (C)[4], Oxidation (M)[8]		Mascot
1543.8413	1543.8767	0.0354	23	400	412	IKEAIISHGCFLR			Carbamidomethyl (C)[10]		Mascot
1646.8174	1646.8459	0.0285	17	322	335	YAELHDFGSEILPR					Mascot
1646.8174	1646.8459	0.0285	17	322	335	YAELHDFGSEILPR	3	0			Mascot
1756.9592	1756.9988	0.0396	23	209	223	SIEHILILSGDQLYR					Mascot
1838.8776	1838.976	0.0984	54	124	139	LIDIPMSNCFNSGINK			Carbamidomethyl (C)[9], Oxidation (M)[6]		Mascot
1910.8879	1910.9208	0.0329	17	490	505	EGVQEADRPEEGYYIR					Mascot
2055.9399	2055.9924	0.0525	26	233	251	HVDDNADITLSCAPVGES R			Carbamidomethyl (C)[12]		Mascot
2763.3162	2763.3708	0.0546	20	336	358	ALHDHNVQAYVFTDYWE DIGTIR					Mascot

Rank	Protein Name	Accession No.	Protein MW	Protein PI	Pep. Count	Protein Score	Protein Score C. I. %	Intensity Matched	Total Ion Score	Total Ion C. I. %	Confirmed
2	Glucose-1-phosphate adenylyltransferase large subunit, chloroplastic/amyloplastic OS=Triticum aestivum GN=AGP-L PE=2 SV=2	GLGL2_WHEAT	58399.1	6.12	13	70	94.593	12.368	28	0	

Peptide Information

Calc. Mass	Obsrv. Mass	± da	± ppm	Start Seq.	End Seq.	Sequence	Ion Score	C. I. %	Modification	Rank	Result Type
866.4618	866.447	-0.0148	-17	251	258	ASEYGLVK					Mascot
952.4887	952.5057	0.017	18	380	387	TPFFTSPR					Mascot

Calc. Mass	Obsrv. Mass	± da	± ppm	Start Seq.	End Seq.	Sequence	Ion Score	C. I. %	Modification	Rank	Result Type
952.4887	952.5057	0.017	18	380	387	TPFFTSPR	25	0			Mascot
1023.5945	1023.6124	0.0179	17	415	423	IEHSIIGVR					Mascot
1266.7278	1266.6205	-0.1073	-85	415	425	IEHSIIGVRSR					Mascot
1302.6624	1302.6932	0.0308	24	401	411	EAIISHGCFLR			Carbamidomethyl (C)[8]		Mascot
1302.6624	1302.6932	0.0308	24	401	411	EAIISHGCFLR			Carbamidomethyl (C)[8]		Mascot
1306.6243	1306.662	0.0377	29	468	478	ISNCIIDMNAR			Carbamidomethyl (C)[4]		Mascot
1322.6191	1322.6451	0.026	20	468	478	ISNCIIDMNAR			Carbamidomethyl (C)[4], Oxidation (M)[8]		Mascot
1543.8413	1543.8767	0.0354	23	399	411	IKEAIISHGCFLR			Carbamidomethyl (C)[10]		Mascot
1646.8174	1646.8459	0.0285	17	321	334	YAELHDFGSEILPR					Mascot
1646.8174	1646.8459	0.0285	17	321	334	YAELHDFGSEILPR	3	0			Mascot
1756.9592	1756.9988	0.0396	23	208	222	SIEHILILSGDQLYR					Mascot
1838.8776	1838.976	0.0984	54	123	138	LIDIPMSNCFNSGINK			Carbamidomethyl (C)[9], Oxidation (M)[6]		Mascot
1910.8879	1910.9208	0.0329	17	489	504	EGVQEADRPEEGYYIR					Mascot
2055.9399	2055.9924	0.0525	26	232	250	HVDDNADITLSCAPVGES R			Carbamidomethyl (C)[12]		Mascot
2763.3162	2763.3708	0.0546	20	335	357	ALHDHNVQAYVFTDYWE DIGTIR					Mascot

3	Glucose-1-phosphate adenylyltransferase large subunit, chloroplastic/amyloplastic (Fragment) OS=Triticum aestivum GN=AGA.7 PE=2 SV=1	GLGL3_WHEAT	56037.9	6.61	11	64	75.846	9.844	28	0	

Peptide Information

Calc. Mass	Obsrv. Mass	± da	± ppm	Start Seq.	End Seq.	Sequence	Ion Score	C. I. %	Modification	Rank	Result Type
866.4618	866.447	-0.0148	-17	232	239	ASEYGLVK					Mascot
908.5424	908.486	-0.0564	-62	10	17	APLRAPQR					Mascot
952.4887	952.5057	0.017	18	361	368	TPFFTSPR					Mascot
952.4887	952.5057	0.017	18	361	368	TPFFTSPR	25	0			Mascot
1306.6243	1306.662	0.0377	29	446	456	ISNCIIDMNAR			Carbamidomethyl (C)[4]		Mascot
1322.6191	1322.6451	0.026	20	446	456	ISNCIIDMNAR			Carbamidomethyl (C)[4], Oxidation (M)[8]		Mascot
1646.8174	1646.8459	0.0285	17	302	315	YAELHDFGSEILPR					Mascot
1646.8174	1646.8459	0.0285	17	302	315	YAELHDFGSEILPR	3	0			Mascot
1745.8826	1745.9778	0.0952	55	382	395	EAIILHGCFLRECK			Carbamidomethyl (C)[8,13]		Mascot
1756.9592	1756.9988	0.0396	23	189	203	SIEHILILSGDQLYR					Mascot
1838.8776	1838.976	0.0984	54	104	119	LIDIPMSNCFNSGINK			Carbamidomethyl (C)[9], Oxidation (M)[6]		Mascot
1910.8879	1910.9208	0.0329	17	467	482	EGVQEADRPEEGYYIR					Mascot
2055.9399	2055.9924	0.0525	26	213	231	HVDDNADITLSCAPVGES R			Carbamidomethyl (C)[12]		Mascot
2763.3162	2763.3708	0.0546	20	316	338	ALHDHNVQAYVFTDYWE DIGTIR					Mascot

4	Probable transcriptional regulatory protein NarL	NARL_MYCTU	23016.2	6.11	10	58	20.02	5.613	

OS=Mycobacterium tuberculosis GN=narL PE=1 SV=1

Peptide Information

Calc. Mass	Obsrv. Mass	± da	± ppm	Start Seq.	End Seq.	Sequence	Ion Score	C. I. %	Modification	Rank	Result Type
989.5197	989.5475	0.0278	28	121	129	AVLDCAKGR			Carbamidomethyl (C)[5]		Mascot
1173.6376	1173.6871	0.0495	42	188	196	THVQRLYEK					Mascot
1189.6106	1189.5946	-0.016	-13	203	213	AAAVAEAMRQR			Oxidation (M)[8]		Mascot
1201.5994	1201.6438	0.0444	37	1	10	MSNPQPEKVR			Oxidation (M)[1]		Mascot
1253.6638	1253.6312	-0.0326	-26	11	21	VVVGDDHPLFR					Mascot
1335.7855	1335.6698	-0.1157	-87	146	158	QRAAPVAPVLSAR					Mascot
1389.6614	1389.6842	0.0228	16	64	77	MPGMDGAQVAAAVR			Oxidation (M)[1]		Mascot
1508.8333	1508.7112	-0.1221	-81	9	21	VRVVVGDDHPLFR					Mascot
1552.8694	1552.7592	-0.1102	-71	130	145	DVVAPSLVGGLAGEIR					Mascot
1793.9658	1793.9658	0	0	11	26	VVVGDDHPLFREGVVR					Mascot

5	Conjugal transfer protein TraA OS=Rhizobium radiobacter GN=traA PE=3 SV=1	TRAA_RHIRD	123705	9.48	23	55	0	19.502	

Peptide Information

Calc. Mass	Obsrv. Mass	± da	± ppm	Start Seq.	End Seq.	Sequence	Ion Score	C. I. %	Modification	Rank	Result Type
809.3577	809.4084	0.0507	63	196	201	DGWFER					Mascot
858.5156	858.4623	-0.0533	-62	806	812	LVDLSRR					Mascot
866.4222	866.447	0.0248	29	604	610	MLNVMAR			Oxidation (M)[1,5]		Mascot
925.4594	925.5168	0.0574	62	411	418	TTMMKAAR			Oxidation (M)[3]		Mascot
936.4785	936.4868	0.0083	9	945	951	DLEQYLR					Mascot
1016.5445	1016.5753	0.0308	30	833	842	ETAPMVAGIK					Mascot
1180.603	1180.6284	0.0254	22	886	896	AMNFAALLADK			Oxidation (M)[2]		Mascot
1193.5909	1193.6833	0.0924	77	550	560	GNVENALSAYR					Mascot
1236.6188	1236.6171	-0.0017	-1	601	610	DVRMLNVMAR			Oxidation (M)[4,8]		Mascot
1322.7175	1322.6451	-0.0724	-55	14	26	GSGRSVVLSAAYR					Mascot
1389.7307	1389.6842	-0.0465	-33	1	13	MAIAHFSASIVSR					Mascot
1390.7438	1390.7068	-0.037	-27	366	378	GVSPTALDATFRR					Mascot
1461.7631	1461.8458	0.0827	57	18	30	SVVLSAAYRHCAK			Carbamidomethyl (C)[11]		Mascot
1524.8533	1524.7169	-0.1364	-89	813	827	LAAFAAHLGITQSPK					Mascot
1646.9225	1646.8459	-0.0766	-47	223	238	QGINLEPTIHLGVGAK					Mascot
1646.9225	1646.8459	-0.0766	-47	223	238	QGINLEPTIHLGVGAK					Mascot
1746.9069	1746.9836	0.0767	44	1	17	MAIAHFSASIVSRGSGR					Mascot

1793.8606	1793.9658	0.1052	59	686	700	FYNNLDHGYATTIHK					Mascot
1803.9196	1804.033	0.1134	63	954	968	ETVTQRLQTDEQSLR					Mascot
1817.998	1817.8987	-0.0993	-55	1068	1083	TAQQLVAHERTVHSLK					Mascot
1817.998	1817.8987	-0.0993	-55	1068	1083	TAQQLVAHERTVHSLK					Mascot
1827.9388	1827.9662	0.0274	15	419	436	EAWELAGYHVVGGALAG K					Mascot
1842.0344	1841.9247	-0.1097	-60	202	218	LNHHLALGGIDLKIDGR					Mascot
1849.9291	1849.8888	-0.0403	-22	1010	1025	ETKLEIDGFNQAVTER					Mascot
1849.9291	1849.8888	-0.0403	-22	1010	1025	ETKLEIDGFNQAVTER					Mascot
2550.3206	2550.2988	-0.0218	-9	653	677	NGMIGHVVVAAANRIVAT VGEGDQR			Oxidation (M)[3]		Mascot

6	Glutamine--tRNA ligase OS=Neisseria meningitidis serogroup B (strain MC58) GN=glnS PE=3 SV=1	SYQ_NEIMB	65065.5	5.74	15	48	0	16.863	

Peptide Information

Calc. Mass	Obsrv. Mass	± da	± ppm	Start Seq.	End Seq.	Sequence	Ion Score	C. I. %	Modification	Rank	Result Type
821.4628	821.4135	-0.0493	-60	473	478	VRLYDR					Mascot
908.4328	908.486	0.0532	59	294	301	MPTISGMR			Oxidation (M)[1]		Mascot
950.4214	950.4604	0.039	41	164	172	NGEFPDGSK					Mascot
1180.6824	1180.6284	-0.054	-46	265	274	LELLYTITSK					Mascot
1221.5715	1221.6427	0.0712	58	178	188	IDMASGNINMR					Mascot
1233.6208	1233.6455	0.0247	20	15	25	TIIEEDLESGK					Mascot
1237.5664	1237.6108	0.0444	36	178	188	IDMASGNINMR			Oxidation (M)[3]		Mascot
1253.5613	1253.6312	0.0699	56	178	188	IDMASGNINMR			Oxidation (M)[3,10]		Mascot
1302.7053	1302.6932	-0.0121	-9	552	562	TVTLKDSWQPK					Mascot
1302.7166	1302.6932	-0.0234	-18	479	489	LFTVERPDAVR					Mascot
1322.6587	1322.6451	-0.0136	-10	151	161	SVEENLDLFTR					Mascot
1390.7689	1390.7068	-0.0621	-45	545	556	DTPVFNRTVTLK					Mascot
1524.6649	1524.7169	0.052	34	370	382	AAFHPNHEEMGER					Mascot
1540.6598	1540.7168	0.057	37	370	382	AAFHPNHEEMGER			Oxidation (M)[10]		Mascot
1646.865	1646.8459	-0.0191	-12	458	472	GVIHWVSAEHAAEIK					Mascot
1646.865	1646.8459	-0.0191	-12	458	472	GVIHWVSAEHAAEIK					Mascot
1803.8794	1804.033	0.1536	85	424	439	CDEVVKDEAGNVVELK			Carbamidomethyl (C)[1]		Mascot
1827.845	1827.9662	0.1212	66	85	99	EDVEWLGFHWAGEPR					Mascot
1849.9919	1849.8888	-0.1031	-56	475	489	LYDRLFTVERPDAVR					Mascot
1849.9919	1849.8888	-0.1031	-56	475	489	LYDRLFTVERPDAVR					Mascot

7	Glutamine synthetase cytosolic isozyme 1-3 OS=Arabidopsis thaliana GN=GLN1-3 PE=1 SV=1	GLN13_ARATH	38798.3	5.72	7	47	0	9.983	22	0	

Peptide Information

Calc. Mass	Obsrv. Mass	± da	± ppm	Start Seq.	End Seq.	Sequence	Ion Score	C. I. %	Modification	Rank	Result Type
870.5771	870.5433	-0.0338	-39	269	276	AIGKLQLK					Mascot
1345.6132	1345.646	0.0328	24	279	290	EHIAAYGEGNER					Mascot
1345.6132	1345.646	0.0328	24	279	290	EHIAAYGEGNER					Mascot
1501.7142	1501.728	0.0138	9	279	291	EHIAAYGEGNERR					Mascot
1501.7142	1501.728	0.0138	9	279	291	EHIAAYGEGNERR					Mascot
1610.767	1610.807	0.04	25	277	290	HKEHIAAYGEGNER					Mascot
1793.9255	1793.9658	0.0403	22	19	34	IIAEYIWIGGSGMDIR					Mascot
1809.9204	1809.9457	0.0253	14	19	34	IIAEYIWIGGSGMDIR			Oxidation (M)[13]		Mascot
1817.8566	1817.8987	0.0421	23	296	311	HETADINTFSWGVANR					Mascot
1817.8566	1817.8987	0.0421	23	296	311	HETADINTFSWGVANR	22	0			Mascot
2288.1167	2288.1177	0.001	0	296	316	HETADINTFSWGVANRG ASVR					Mascot

8	Cryptochrome DASH OS=Idiomarina loihiensis (strain ATCC BAA-735 / DSM 15497 / L2-TR) GN=cry PE=3 SV=1	CRYD_IDILO	52299.8	5.86	12	46	0	6.09

Peptide Information

Calc. Mass	Obsrv. Mass	± da	± ppm	Start Seq.	End Seq.	Sequence	Ion Score	C. I. %	Modification	Rank	Result Type
889.405	889.3402	-0.0648	-73	318	324	TSFYSER					Mascot
925.4778	925.5168	0.039	42	192	199	NISYPGFK					Mascot
1026.5619	1026.5419	-0.02	-19	305	314	LFAFSGLSGK					Mascot
1182.663	1182.5835	-0.0795	-67	304	314	RLFAFSGLSGK					Mascot
1193.5773	1193.6833	0.106	89	56	66	YGHYGIPAMGK					Mascot
1201.5848	1201.6438	0.059	49	315	324	SPKTSFYSER					Mascot
1266.6194	1266.6205	0.0011	1	292	300	EYFFWYALK					Mascot
1399.7039	1399.7975	0.0936	67	5	15	QQLVFIMADYR			Oxidation (M)[7]		Mascot
1502.7322	1502.7791	0.0469	31	56	68	YGHYGIPAMGKHR			Oxidation (M)[9]		Mascot
1508.8584	1508.7112	-0.1472	-98	69	80	FTFLQQSLKQLR					Mascot
1610.8901	1610.807	-0.0831	-52	177	191	NFSVQPVNALPSLPK					Mascot
2292.271	2292.1802	-0.0908	-40	171	191	VEPLSRNFSVQPVNALPS LPK					Mascot

9	Unconventional myosin-IXa OS=Mus musculus GN=Myo9a PE=1 SV=2	MYO9A_MOUSE	294274.6	9.1	29	45	0	23.444

Peptide Information

Calc. Mass	Obsrv. Mass	± da	± ppm	Start Seq.	End Seq.	Sequence	Ion Score	C. I. %	Modification	Rank	Result Type
839.3893	839.4678	0.0785	94	98	104	LSGEDYR					Mascot
858.5043	858.4623	-0.042	-49	755	761	SLEILQR					Mascot
872.4836	872.5273	0.0437	50	1629	1635	ELPKTER					Mascot
893.488	893.4727	-0.0153	-17	1954	1960	VWVNTFK					Mascot
908.52	908.486	-0.034	-37	318	324	YLLEKSR					Mascot
936.4277	936.4868	0.0591	63	1518	1524	EMMEQIR					Mascot
949.4883	949.5086	0.0203	21	1939	1945	TMRLEQR			Oxidation (M)[2]		Mascot
952.4226	952.5057	0.0831	87	1518	1524	EMMEQIR			Oxidation (M)[2]		Mascot
952.4226	952.5057	0.0831	87	1518	1524	EMMEQIR			Oxidation (M)[2]		Mascot
982.4913	982.4487	-0.0426	-43	1869	1876	SMDEFLLK					Mascot
1236.6583	1236.6171	-0.0412	-33	2171	2181	GVYSVIDQLSR					Mascot
1302.5995	1302.6932	0.0937	72	1219	1229	ISRESSMDFSK			Oxidation (M)[7]		Mascot
1302.5995	1302.6932	0.0937	72	1219	1229	ISRESSMDFSK			Oxidation (M)[7]		Mascot
1389.7518	1389.6842	-0.0676	-49	488	500	LAEAVTVRNSMAK					Mascot
1399.842	1399.7975	-0.0445	-32	924	935	LPLRFSDALVLR					Mascot
1420.6849	1420.7067	0.0218	15	1293	1305	VRSLGGMSPSEER			Oxidation (M)[7]		Mascot
1476.7953	1476.7665	-0.0288	-20	402	414	LQLAMEMVGFLPK					Mascot
1492.7754	1492.7664	-0.009	-6	987	999	KININSDNYQVGK					Mascot
1501.7241	1501.728	0.0039	3	1387	1401	GASSDSEHLKNGTAK					Mascot
1501.7241	1501.728	0.0039	3	1387	1401	GASSDSEHLKNGTAK					Mascot
1502.7809	1502.7791	-0.0018	-1	39	52	NSTAAEVIDSLINR					Mascot
1508.7852	1508.7112	-0.074	-49	402	414	LQLAMEMVGFLPK			Oxidation (M)[5,7]		Mascot
1571.8639	1571.7972	-0.0667	-42	2337	2349	VLTEQIENLQKEK					Mascot
1610.7843	1610.807	0.0227	14	1206	1218	AIEECKYVIESNR			Carbamidomethyl (C)[5]		Mascot
1646.8174	1646.8459	0.0285	17	357	369	QPEEYHFLNQITK					Mascot
1646.8174	1646.8459	0.0285	17	357	369	QPEEYHFLNQITK					Mascot
1745.8124	1745.9778	0.1654	95	2456	2473	AASACEAQGTEGPLGQA K			Carbamidomethyl (C)[5]		Mascot
1746.9393	1746.9836	0.0443	25	2206	2221	MSANALAIVFAPCILR			Carbamidomethyl (C)[13]		Mascot
1821.9495	1821.8834	-0.0661	-36	1545	1560	TEASVLAPSFYQPRQK					Mascot
1831.8545	1831.9281	0.0736	40	740	754	SMDSFSFLQHPVHQR			Oxidation (M)[2]		Mascot
1833.8324	1833.9004	0.068	37	1905	1921	QNIFSSYSSALAMDDGK					Mascot
1838.9607	1838.976	0.0153	8	514	529	INHALLNSKDLEQDTK					Mascot
1849.8273	1849.8888	0.0615	33	1905	1921	QNIFSSYSSALAMDDGK			Oxidation (M)[13]		Mascot
1849.8273	1849.8888	0.0615	33	1905	1921	QNIFSSYSSALAMDDGK			Oxidation (M)[13]		Mascot

2056.0454	2055.9924	-0.053	-26	2238	2254	TTTCVELIVVEQMNKYK			Carbamidomethyl (C)[4]		Mascot
2607.4082	2607.3159	-0.0923	-35	501	522	SLYSALFDWIVFRINHALLNSK					Mascot
2763.3472	2763.3708	0.0236	9	2113	2137	QGLDTDAESVNLDDYNIHVIASVFK					Mascot

10	50S ribosomal protein L10 OS=Helicobacter pylori (strain Shi470) GN=rplJ PE=3 SV=1	RL10_HELPS	18696.9	8.99	8	44	0	5.878	

Peptide Information

Calc. Mass	Obsrv. Mass	± da	± ppm	Start Seq.	End Seq.	Sequence	Ion Score	C. I. %	Modification	Rank	Result Type
989.5197	989.5475	0.0278	28	45	53	NKACAQGIK			Carbamidomethyl (C)[4]		Mascot
1276.6685	1276.6715	0.003	2	94	103	LVFDFQKDHK					Mascot
1389.7526	1389.6842	-0.0684	-49	104	115	DHFVLKAGLFDK					Mascot
1646.9113	1646.8459	-0.0654	-40	11	25	VELVANLKTQFADAK					Mascot
1646.9113	1646.8459	-0.0654	-40	11	25	VELVANLKTQFADAK					Mascot
1756.8939	1756.9988	0.1049	60	19	33	TQFADAKALLICDYK			Carbamidomethyl (C)[12]		Mascot
1793.9756	1793.9658	-0.0098	-5	116	132	ESVSVAHVEAVSKLPNK					Mascot
1803.9132	1804.033	0.1198	66	133	148	EELMGMLLSVWTAPAR					Mascot
2288.1777	2288.1177	-0.06	-26	129	148	LPNKEELMGMLLSVWTAPAR			Oxidation (M)[8,10]		Mascot

Gel Idx/Pos	220/I20	Instr./Gel Origin	BA2151/Sample Project 20140814	Process Status	Analysis Succeeded
Plate [#] Name	[1] Sample Project 20140814	Instrument Sample Name		Spectra	11

Rank	Protein Name	Accession No.	Protein MW	Protein PI	Pep. Count	Protein Score	Protein Score C. I. %	Intensity Matched	Total Ion Score	Total Ion C. I. %	Confirmed
1	Glutamine synthetase cytosolic isozyme OS=Daucus carota GN=GLN1 PE=2 SV=1	GLNA1_DAUCA	38658.3	5.69	4	140	100	12.464	129	100	

Peptide Information

Calc. Mass	Obsrv. Mass	± da	± ppm	Start Seq.	End Seq.	Sequence	Ion Score	C. I. %	Modification	Rank	Result Type
1467.8054	1467.8813	0.0759	52	38	51	TLSGPVDDPTKLPK					Mascot
1467.8054	1467.8813	0.0759	52	38	51	TLSGPVDDPTKLPK					Mascot
1552.7251	1552.7704	0.0453	29	276	289	HAQHIAAYGEGNER					Mascot
1552.7251	1552.7704	0.0453	29	276	289	HAQHIAAYGEGNER	129	100			Mascot
1708.8263	1708.8668	0.0405	24	276	290	HAQHIAAYGEGNERR					Mascot
2600.2595	2600.325	0.0655	25	84	106	GNHILVMCDTYTPAGEPI PTNKR			Carbamidomethyl (C)[8], Oxidation (M)[7]		Mascot

Rank	Protein Name	Accession No.	Protein MW	Protein PI	Pep. Count	Protein Score	Protein Score C. I. %	Intensity Matched	Total Ion Score	Total Ion C. I. %	Confirmed
2	Nesprin-1 OS=Homo sapiens GN=SYNE1 PE=1 SV=4	SYNE1_HUMAN	1017127.1	5.37	77	86	99.864	39.108			

Peptide Information

Calc. Mass	Obsrv. Mass	± da	± ppm	Start Seq.	End Seq.	Sequence	Ion Score	C. I. %	Modification	Rank	Result Type
832.4159	832.3414	-0.0745	-89	5715	5721	LQEEASR					Mascot
837.4141	837.4224	0.0083	10	8011	8016	FEDWLK					Mascot
857.5454	857.4862	-0.0592	-69	5317	5323	LVKQELK					Mascot
866.4586	866.468	0.0094	11	6054	6060	MSTIRMK					Mascot
925.489	925.5417	0.0527	57	8041	8047	KFEAFQR					Mascot
927.446	927.5176	0.0716	77	5802	5808	CKMLTMK			Carbamidomethyl (C)[1], Oxidation (M)[3]		Mascot
950.44	950.4577	0.0177	19	5337	5343	CWVQETK			Carbamidomethyl (C)[1]		Mascot
952.4887	952.5087	0.02	21	187	194	WVQYTAGK					Mascot
960.536	960.5875	0.0515	54	5417	5424	IKEVEQSK					Mascot
960.536	960.5875	0.0515	54	5417	5424	IKEVEQSK					Mascot
980.5047	980.5263	0.0216	22	844	852	EAQSSALFK					Mascot
989.603	989.5602	-0.0428	-43	6332	6340	LLSGVPLYK					Mascot
1012.5244	1012.5301	0.0057	6	6902	6909	LHQLQMDK					Mascot
1132.5997	1132.6221	0.0224	20	2159	2168	IHSSDFSLVK					Mascot
1149.5754	1149.6318	0.0564	49	5304	5312	CLNMQEKVK			Carbamidomethyl (C)[1]		Mascot

1173.5933	1173.6902	0.0969	83	3756	3765	DMEKGHSLLK	Oxidation (M)[2]	Mascot
1182.5242	1182.5966	0.0724	61	6289	6298	SSAEDQMRMK		Mascot
1192.5997	1192.5765	-0.0232	-19	3607	3615	FQQVDEWLK		Mascot
1201.7039	1201.6725	-0.0314	-26	1216	1226	ALVTLLSEVEK		Mascot
1224.6008	1224.5675	-0.0333	-27	7036	7044	TWFETQEKR		Mascot
1233.6322	1233.6418	0.0096	8	2399	2409	TQASLQESLEK		Mascot
1302.5922	1302.6931	0.1009	77	8722	8735	GGSDSSLSEPGPGR		Mascot
1337.6696	1337.7076	0.038	28	5790	5801	GYQEQIASLNSK		Mascot
1345.707	1345.6591	-0.0479	-36	2885	2896	ELIDSREIGASR		Mascot
1358.6006	1358.7225	0.1219	90	4015	4026	DSYSAICSTAQR	Carbamidomethyl (C)[7]	Mascot
1389.7122	1389.7111	-0.0011	-1	2369	2380	KYHSAELESLGR		Mascot
1390.6995	1390.7296	0.0301	22	5624	5635	LQNLQDAAKDMK	Oxidation (M)[11]	Mascot
1415.5745	1415.7003	0.1258	89	2866	2878	WSDMSGDSSATQK	Oxidation (M)[4]	Mascot
1433.7635	1433.7799	0.0164	11	5943	5954	SWETLKNVISEK		Mascot
1457.821	1457.7734	-0.0476	-33	5159	5171	IVALEEKASQLEK		Mascot
1467.8054	1467.8813	0.0759	52	3032	3045	DLSTYSGKVSGLIK		Mascot
1467.8054	1467.8813	0.0759	52	3032	3045	DLSTYSGKVSGLIK		Mascot
1473.806	1473.7697	-0.0363	-25	6483	6494	LQQILNFQNDLK		Mascot
1492.7716	1492.8175	0.0459	31	731	743	LSEPLEVSFMNVK		Mascot
1494.7812	1494.7866	0.0054	4	1737	1747	LHLEQLDERWR		Mascot
1501.754	1501.7388	-0.0152	-10	3057	3068	GCQNKEQILQQR	Carbamidomethyl (C)[2]	Mascot
1511.8805	1511.8696	-0.0109	-7	363	374	KQIEHLIQPLHR		Mascot
1540.7676	1540.7919	0.0243	16	2819	2831	TQFNDIMTVAKEK	Oxidation (M)[7]	Mascot
1552.79	1552.7704	-0.0196	-13	5179	5191	ATLSRSMTTVWQR	Oxidation (M)[7]	Mascot
1552.833	1552.7704	-0.0626	-40	3861	3874	SLQQTVLSHEPSVK		Mascot
1575.7908	1575.7838	-0.007	-4	5085	5098	MSRDSGAQVDLLQR		Mascot
1577.7727	1577.7385	-0.0342	-22	5111	5123	EEVIELMNDTEKK		Mascot
1580.8292	1580.7953	-0.0339	-21	4854	4866	ARHQIQSWQGELK		Mascot
1598.8385	1598.8107	-0.0278	-17	4266	4280	SDAESTAVHLEALKK		Mascot
1608.7401	1608.8066	0.0665	41	6438	6451	NLFSQAFPENGDNR		Mascot
1623.7982	1623.8337	0.0355	22	3368	3381	DMWASLLSAGIRCK	Carbamidomethyl (C)[13], Oxidation (M)[2]	Mascot
1663.8802	1663.9525	0.0723	43	6939	6952	NSIGYKAIHEYLQK		Mascot
1666.7894	1666.9081	0.1187	71	6297	6309	MKWESLHQEFSTK	Oxidation (M)[1]	Mascot
1675.869	1675.9905	0.1215	72	7298	7311	DSLFFLHELGEQLK		Mascot
1679.802	1679.9083	0.1063	63	45	58	KPPMVVDDLFEDMK	Oxidation (M)[4]	Mascot
1683.929	1683.8945	-0.0345	-20	364	377	QIEHLIQPLHRDGK		Mascot
1730.9258	1730.9242	-0.0016	-1	900	914	QIADIHVAFQSMVKK	Oxidation (M)[12]	Mascot

1739.8633	1739.9308	0.0675	39	7200	7214	ECHPPVTETLTNTLK			Carbamidomethyl (C)[2]		Mascot
1739.8633	1739.9308	0.0675	39	7200	7214	ECHPPVTETLTNTLK			Carbamidomethyl (C)[2]		Mascot
1742.9899	1742.943	-0.0469	-27	6615	6629	IIVSSKEEIQQLLDK					Mascot
1756.9229	1757.0048	0.0819	47	4122	4136	LVQAQNLTQGWEEIK					Mascot
1789.9192	1790.0315	0.1123	63	446	460	LEQHKDLLQNTDAHK					Mascot
1789.9192	1790.0315	0.1123	63	446	460	LEQHKDLLQNTDAHK					Mascot
1810.0334	1809.9603	-0.0731	-40	86	102	IHAVANIGTALKFLEGR					Mascot
1817.8678	1817.9065	0.0387	21	3577	3590	QDWQAYQHRLSETR					Mascot
1817.8678	1817.9065	0.0387	21	3577	3590	QDWQAYQHRLSETR					Mascot
1821.9204	1821.9871	0.0667	37	3602	3615	LMEQKFQQVDEWLK					Mascot
1826.0018	1825.9365	-0.0653	-36	7077	7092	EVEKIEQNGLALIQNK					Mascot
1827.9017	1827.9741	0.0724	40	6182	6197	ALHDKQLNMQGTAQEK			Oxidation (M)[9]		Mascot
1830.0007	1829.9457	-0.055	-30	6332	6348	LLSGVPLYKGDVPTQDK					Mascot
1849.8815	1849.8967	0.0152	8	4770	4785	VSLLEDTTSAYQEHEK					Mascot
1861.9502	1861.9452	-0.005	-3	4106	4121	TTAKDIQQTEQTIEQK					Mascot
1873.9655	1874.0134	0.0479	26	2463	2479	LDAVTQEGQTLYAHLSK					Mascot
1882.0215	1881.9762	-0.0453	-24	8594	8608	QLMQIKHELLESQLR			Oxidation (M)[3]		Mascot
1891.9178	1891.9536	0.0358	19	5602	5617	MSQQVAELGRETEELR			Oxidation (M)[1]		Mascot
1898.8689	1899.0466	0.1777	94	2169	2184	TDMESTVDKWLDVSEK			Oxidation (M)[3]		Mascot
1898.9495	1899.0466	0.0971	51	7343	7359	QQTSLQAGVLDYETFAK					Mascot
1908.9563	1908.9158	-0.0405	-21	1350	1365	ETVVRYLFQTGSSHER					Mascot
1959.0468	1958.8795	-0.1673	-85	7803	7819	VSQNGDILIEEMIEKLK					Mascot
1983.9594	1983.9059	-0.0535	-27	7403	7419	FSSMAPDLDRLNELGYR					Mascot
1984.0797	1983.9059	-0.1738	-88	897	913	LQRQIADIHVAFQSMVK					Mascot
2000.0746	2000.0529	-0.0217	-11	897	913	LQRQIADIHVAFQSMVK			Oxidation (M)[15]		Mascot
2045.1138	2045.1345	0.0207	10	3252	3269	HRVSQLSSQYLALSNLTK					Mascot
2055.9507	2056.0344	0.0837	41	6383	6400	LYDGVSATSTWLDDVEE R					Mascot
2516.3064	2516.3865	0.0801	32	744	764	LLIQDLEDIEQRVPVMDA QYK					Mascot
2573.3167	2573.2998	-0.0169	-7	2263	2284	ELTKNLETPPDLQFIEADL MQK					Mascot
2648.3601	2648.3406	-0.0195	-7	1783	1805	LFLSELQTTSEISIMDHQV ALTR			Oxidation (M)[15]		Mascot
2972.4934	2972.4504	-0.043	-14	2743	2767	NQLEQWMESVDQKIEHP LQPQPGLK					Mascot

3	Glutamate 5-kinase OS=Dictyoglomus turgidum (strain Z-1310 / DSM 6724) GN=proB PE=3 SV=1	PROB_DICTD	41407.2	6.43	14	72	96.509	15.401

Peptide Information

Calc. Mass	Obsrv. Mass	± da	± ppm	Start	End	Sequence	Ion	C. I. %	Modification	Rank	Result Type

				Seq.	Seq.		Score				
837.4101	837.4224	0.0123	15	302	308	VEGEFTR					Mascot
1311.6726	1311.7821	0.1095	83	207	219	GTGGMFTKVQAAK			Oxidation (M)[5]		Mascot
1337.6882	1337.7076	0.0194	15	201	214	IAGPGKGTGGMFTK			Oxidation (M)[11]		Mascot
1358.7638	1358.7225	-0.0413	-30	44	57	ELVLVSSGAIASGR					Mascot
1399.7977	1399.8088	0.0111	8	220	232	IVTEAGIPMILAR			Oxidation (M)[9]		Mascot
1511.8792	1511.8696	-0.0096	-6	233	245	ADVENILERIVLK					Mascot
1552.8806	1552.7704	-0.1102	-71	68	82	DLPAKQALSAVGQVR					Mascot
1552.8806	1552.7704	-0.1102	-71	68	82	DLPAKQALSAVGQVR					Mascot
1580.9006	1580.7953	-0.1053	-67	96	109	QPIAQILLTAEDLR					Mascot
1608.8479	1608.8066	-0.0413	-26	325	338	GITNYDSLELDKIK					Mascot
1646.8295	1646.8601	0.0306	19	264	276	KHWMLFMAKPEGR			Oxidation (M)[4]		Mascot
1646.8295	1646.8601	0.0306	19	264	276	KHWMLFMAKPEGR			Oxidation (M)[4]		Mascot
1666.8588	1666.9081	0.0493	30	249	262	VGTFFYPSEKHLNK					Mascot
1683.8451	1683.8945	0.0494	29	83	95	LMQYYSQLFSIFK			Oxidation (M)[2]		Mascot
1827.9375	1827.9741	0.0366	20	114	128	YINISQTFETLLEEK					Mascot
1984.0386	1983.9059	-0.1327	-67	113	128	RYINISQTFETLLEEK					Mascot
1984.0386	1983.9059	-0.1327	-67	113	128	RYINISQTFETLLEEK					Mascot

4	Serum albumin OS=Homo sapiens GN=ALB PE=1 SV=2	ALBU_HUMAN	71317.2	5.92	14	68	90.384	7.651	27	0	

Peptide Information

Calc. Mass	Obsrv. Mass	± da	± ppm	Start Seq.	End Seq.	Sequence	Ion Score	C. I. %	Modification	Rank	Result Type
927.4934	927.5176	0.0242	26	162	168	YLYEIAR					Mascot
960.5625	960.5875	0.025	26	427	434	FQNALLVR					Mascot
960.5625	960.5875	0.025	26	427	434	FQNALLVR	19	0			Mascot
1149.615	1149.6318	0.0168	15	66	75	LVNEVTEFAK					Mascot
1311.7419	1311.7821	0.0402	31	362	372	HPDYSVVLLLR					Mascot
1358.6298	1358.7225	0.0927	68	570	581	AVMDDFAAFVEK			Oxidation (M)[3]		Mascot
1467.8431	1467.8813	0.0382	26	361	372	RHPDYSVVLLLR					Mascot
1467.8431	1467.8813	0.0382	26	361	372	RHPDYSVVLLLR	8	0			Mascot
1511.8429	1511.8696	0.0267	18	439	452	VPQVSTPTLVEVSR					Mascot
1623.7876	1623.8337	0.0461	28	348	360	DVFLGMFLYEYAR					Mascot
1639.7826	1639.8265	0.0439	27	348	360	DVFLGMFLYEYAR			Oxidation (M)[6]		Mascot
1639.9379	1639.9789	0.041	25	438	452	KVPQVSTPTLVEVSR					Mascot
1742.8942	1742.943	0.0488	28	170	183	HPYFYAPELLFFAK					Mascot

1898.9952	1899.0466	0.0514	27	170	184	HPYFYAPELLFFAKR					Mascot
1898.9952	1899.0466	0.0514	27	169	183	RHPYFYAPELLFFAK					Mascot
1910.9318	1910.9399	0.0081	4	509	524	RPCFSALEVDETYVPK			Carbamidomethyl (C)[3]		Mascot
2045.0955	2045.1345	0.039	19	397	413	VFDEFKPLVEEPQNLIK					Mascot

5 60S ribosomal protein L5 OS=Caenorhabditis briggsae GN=rpl-5 PE=3 SV=2 RL5_CAEBR 33445.7 9.76 12 67 88.439 4.064

Peptide Information

Calc. Mass	Obsrv. Mass	± da	± ppm	Start Seq.	End Seq.	Sequence	Ion Score	C. I. %	Modification	Rank	Result Type
927.5621	927.5176	-0.0445	-48	143	151	AVLDIGLAR					Mascot
952.4846	952.5087	0.0241	25	268	274	RLTYDER					Mascot
1192.6031	1192.5765	-0.0266	-22	202	211	HVADYMSLLK			Oxidation (M)[6]		Mascot
1345.7474	1345.6591	-0.0883	-66	109	120	HLKAIGLDSTYK					Mascot
1370.8154	1370.7211	-0.0943	-69	139	151	APFKAVLDIGLAR					Mascot
1440.8057	1440.8202	0.0145	10	283	294	ALLLQLKEQQQE					Mascot
1494.7296	1494.7866	0.057	38	164	178	GVADGGINVPHSESR					Mascot
1809.9857	1809.9603	-0.0254	-14	90	107	AGLTNYAAAYATGLLLAR					Mascot
1825.9443	1825.9365	-0.0078	-4	69	85	IEGDVVVASAYAHELPR					Mascot
1885.9113	1885.9873	0.076	40	225	241	FLSNGMNADNLVATYQK					Mascot
2271.2495	2271.1506	-0.0989	-44	86	107	YGLKAGLTNYAAAYATGLLLAR					Mascot
2287.2083	2287.1633	-0.045	-20	69	89	IEGDVVVASAYAHELPRYGLK					Mascot

6 Protein translocase subunit SecA OS=Chlamydophila abortus (strain S26/3) GN=secA PE=3 SV=1 SECA_CHLAB 111440.4 5.71 20 62 61.719 8.319

Peptide Information

Calc. Mass	Obsrv. Mass	± da	± ppm	Start Seq.	End Seq.	Sequence	Ion Score	C. I. %	Modification	Rank	Result Type
927.5985	927.5176	-0.0809	-87	916	923	DIRIAIVK					Mascot
1173.6587	1173.6902	0.0315	27	886	896	SEVGLRTVGQK					Mascot
1182.6378	1182.5966	-0.0412	-35	745	753	QRQTIYAFR					Mascot
1224.677	1224.5675	-0.1095	-89	876	885	IHLVDMDLLR					Mascot
1345.6893	1345.6591	-0.0302	-22	852	863	VDANGVCKDVIR			Carbamidomethyl (C)[7]		Mascot
1389.7162	1389.7111	-0.0051	-4	245	255	HNPVYFELKDK					Mascot
1390.7213	1390.7296	0.0083	6	825	836	VADDLIEVFQNK					Mascot
1610.7731	1610.8025	0.0294	18	837	851	FASMVQEITEAAGEK					Mascot
1675.9088	1675.9905	0.0817	49	276	289	RGLELFLDMDILPK			Oxidation (M)[9]		Mascot

1683.8622	1683.8945	0.0323	19	506	521	LAGMTGTAITESKEFK					Mascot
1745.9004	1745.9574	0.057	33	435	448	AHLLMERDVDYIVR			Oxidation (M)[5]		Mascot
1790.943	1791.0042	0.0612	34	616	633	LGAVTVATNMAGRGTDIK			Oxidation (M)[10]		Mascot
1810.0255	1809.9603	-0.0652	-36	768	784	EAISHVALMIASLIVSR					Mascot
1826.0205	1825.9365	-0.084	-46	768	784	EAISHVALMIASLIVSR			Oxidation (M)[9]		Mascot
1882.0215	1881.9762	-0.0453	-24	876	891	IHLVDMDLLRSEVGLR			Oxidation (M)[6]		Mascot
1927.1012	1926.9119	-0.1893	-98	161	178	WLGLTTGVLISGSPLEKR					Mascot
2000.0093	2000.0529	0.0436	22	860	875	DVIRSVMIMHIDEQWK					Mascot
2045.0524	2045.1345	0.0821	40	449	466	NDQIVIIDEHTGRPQPGR					Mascot
2186.1016	2186.1926	0.091	42	215	233	GFYFAIIDEVDSILIDEAR					Mascot
2454.1641	2454.2661	0.102	42	837	859	FASMVQEITEAAGEKVDA NGVCK			Carbamidomethyl (C)[22]		Mascot
2664.3887	2664.3223	-0.0664	-25	602	628	NHAQEAEIIAAAGKLGAV TVATNMAGR					Mascot

7	Genome polyprotein OS=Parsnip yellow fleck virus (isolate P-121) PE=1 SV=1	POLG_PYFV1	339225	6.57	38	60	51.807	16.264

Peptide Information

Calc. Mass	Obsrv. Mass	± da	± ppm	Start Seq.	End Seq.	Sequence	Ion Score	C. I. %	Modification	Rank	Result Type
832.3546	832.3414	-0.0132	-16	2131	2136	YFMDEK					Mascot
856.5502	856.5103	-0.0399	-47	692	699	LIVSVTPK					Mascot
857.4951	857.4862	-0.0089	-10	1875	1881	INGIRER					Mascot
866.4149	866.468	0.0531	61	1450	1457	CKGASSTR			Carbamidomethyl (C)[1]		Mascot
925.5214	925.5417	0.0203	22	129	136	TLEAHRAK					Mascot
950.4941	950.4577	-0.0364	-38	2670	2678	ASEAKPYGK					Mascot
952.5726	952.5087	-0.0639	-67	2938	2944	IHIKQWK					Mascot
1012.5058	1012.5301	0.0243	24	1442	1449	ELLDETHR					Mascot
1192.6321	1192.5765	-0.0556	-47	2668	2678	NKASEAKPYGK					Mascot
1233.6368	1233.6418	0.005	4	2555	2564	DNLTRMLTNR					Mascot
1343.6637	1343.7076	0.0439	33	1576	1587	HGLADPGAFMRR			Oxidation (M)[10]		Mascot
1345.6028	1345.6591	0.0563	42	569	578	NVKADYMMWR			Oxidation (M)[7,8]		Mascot
1415.6229	1415.7003	0.0774	55	160	169	ECIMYLCDRR			Carbamidomethyl (C)[2,7]		Mascot
1433.6478	1433.7799	0.1321	92	1276	1288	ACATNFEDLAHGK			Carbamidomethyl (C)[2]		Mascot
1440.749	1440.8202	0.0712	49	2931	2941	TFTMFMRIHIK			Oxidation (M)[4]		Mascot
1457.7158	1457.7734	0.0576	40	2742	2752	EYYEELKDQIK					Mascot
1473.7333	1473.7697	0.0364	25	2801	2813	NHFSALTGQEEIK					Mascot
1489.7567	1489.7677	0.011	7	1747	1759	ATKQEMPDDILTK					Mascot
1492.7729	1492.8175	0.0446	30	497	509	VFHSMPGRTVFAK			Oxidation (M)[5]		Mascot

1501.8262	1501.7388	-0.0874	-58	1202	1215	LVENSVPDALGIFK					Mascot
1580.8431	1580.7953	-0.0478	-30	1800	1811	NLIYKQEQEFLR					Mascot
1598.8333	1598.8107	-0.0226	-14	2489	2502	HKHGIMVGINPHSR			Oxidation (M)[6]		Mascot
1608.8269	1608.8066	-0.0203	-13	2110	2124	VPFGGEFISEQTVAK					Mascot
1617.8669	1617.87	0.0031	2	2276	2289	STIVPSLIQPYMPR			Oxidation (M)[12]		Mascot
1646.7479	1646.8601	0.1122	68	2400	2412	EYLFETMESGERR					Mascot
1646.7479	1646.8601	0.1122	68	2400	2412	EYLFETMESGERR					Mascot
1675.8915	1675.9905	0.099	59	2320	2332	YEEQARPIKISWR					Mascot
1679.9578	1679.9083	-0.0495	-29	1891	1905	ILSNNILEELPSPIK					Mascot
1708.9303	1708.8668	-0.0635	-37	1335	1350	TVGGISIGVKTLCEFK			Carbamidomethyl (C)[13]		Mascot
1710.7705	1710.8741	0.1036	61	1086	1099	FDEEEETLNSELQK					Mascot
1817.9176	1817.9065	-0.0111	-6	2186	2200	YVSTYVPITCDMIKK			Carbamidomethyl (C)[10]		Mascot
1817.9176	1817.9065	-0.0111	-6	2186	2200	YVSTYVPITCDMIKK			Carbamidomethyl (C)[10]		Mascot
1826.0497	1825.9365	-0.1132	-62	1906	1922	MLLVLATSVGSLYLAFK					Mascot
1829.9467	1829.9457	-0.001	-1	510	525	DSDGIKIPIEFMSIHK					Mascot
1833.9126	1833.941	0.0284	15	2186	2200	YVSTYVPITCDMIKK			Carbamidomethyl (C)[10], Oxidation (M)[12]		Mascot
1842.0446	1841.9354	-0.1092	-59	1906	1922	MLLVLATSVGSLYLAFK			Oxidation (M)[1]		Mascot
1855.9371	1855.9418	0.0047	3	2623	2638	YVRACVYGDDNIVAIK			Carbamidomethyl (C)[5]		Mascot
1881.8865	1881.9762	0.0897	48	1380	1394	EWFEEVESVTLQETR					Mascot
1898.8615	1899.0466	0.1851	97	324	342	AIDAGTSYEDSDVVGSNA K					Mascot
1898.8615	1899.0466	0.1851	97	324	342	AIDAGTSYEDSDVVGSNA K					Mascot
1911.0118	1910.9399	-0.0719	-38	1216	1232	ALMMVAITSISALYWPK			Oxidation (M)[3]		Mascot
1927.0068	1926.9119	-0.0949	-49	1216	1232	ALMMVAITSISALYWPK			Oxidation (M)[3,4]		Mascot
1984.011	1983.9059	-0.1051	-53	2945	2960	HVLQAYFRVCETFVSK			Carbamidomethyl (C)[10]		Mascot
1984.011	1983.9059	-0.1051	-53	2945	2960	HVLQAYFRVCETFVSK			Carbamidomethyl (C)[10]		Mascot
2020.9907	2020.9868	-0.0039	-2	526	544	AVSAHDSNSHNALSRVE AR					Mascot

8	6-phosphofructokinase 1 OS=Clostridium perfringens (strain 13 / Type A) GN=pfkA1 PE=3 SV=1	K6PF1_CLOPE	34158.8	5.34	12	60	45.927	2.463

Peptide Information

Calc. Mass	Obsrv. Mass	± da	± ppm	Start Seq.	End Seq.	Sequence	Ion Score	C. I. %	Modification	Rank	Result Type
989.5448	989.5602	0.0154	16	163	171	VSIVEVMGR					Mascot
1201.592	1201.6725	0.0805	67	153	162	LRDTSTSHER					Mascot
1233.6322	1233.6418	0.0096	8	233	243	EIESVTGIETR					Mascot
1337.6519	1337.7076	0.0557	42	73	82	CLEFKQEEVR			Carbamidomethyl (C)[1]		Mascot

Calc. Mass	Obsrv. Mass	± da	± ppm	Start Seq.	End Seq.	Sequence	Ion Score	C. I. %	Modification	Rank	Result Type
1358.691	1358.7225	0.0315	23	253	266	GGSPTADDIVLASR					Mascot
1360.7505	1360.7415	-0.009	-7	267	279	MGAKAVEVLLEGK			Oxidation (M)[1]		Mascot
1577.804	1577.7385	-0.0655	-42	26	39	MALHHGLEVMGVQR					Mascot
1683.8523	1683.8945	0.0422	25	40	54	GYAGLINGELFKMDR					Mascot
1710.7891	1710.8741	0.085	50	291	304	IFDMDIDEALDIER			Oxidation (M)[4]		Mascot
1745.8851	1745.9574	0.0723	41	253	270	GGSPTADDIVLASRMGA K					Mascot
1841.9902	1841.9354	-0.0548	-30	3	21	KIAVLTSGGDAPGMNAAL R					Mascot
2021.0532	2020.9868	-0.0664	-33	22	39	AVTRMALHHGLEVMGVQ R			Oxidation (M)[5]		Mascot

9 Probable dual-specificity RNA methyltransferase RlmN OS=Rhodopirellula baltica (strain SH1) GN=rlmN PE=3 SV=1 RLMN_RHOBA 41016.3 8.84 13 60 44.667 4.048

Peptide Information

Calc. Mass	Obsrv. Mass	± da	± ppm	Start Seq.	End Seq.	Sequence	Ion Score	C. I. %	Modification	Rank	Result Type
837.4002	837.4224	0.0222	27	269	275	YFHASGR					Mascot
857.5203	857.4862	-0.0341	-40	296	302	QLSQILR					Mascot
1303.606	1303.71	0.104	80	350	361	GDEINAACGQLR			Carbamidomethyl (C)[8]		Mascot
1389.7485	1389.7111	-0.0374	-27	334	346	AILESAGVNVNFR					Mascot
1473.7729	1473.7697	-0.0032	-2	151	163	NLTGGEILEQMLR					Mascot
1489.7679	1489.7677	-0.0002	0	151	163	NLTGGEILEQMLR			Oxidation (M)[11]		Mascot
1580.937	1580.7953	-0.1417	-90	211	225	RITISTVGLPPAIDK					Mascot
1608.8856	1608.8066	-0.079	-49	25	37	KNHLLNWSLDQLK					Mascot
1708.8574	1708.8668	0.0094	6	62	76	ATSFEEMTDLPAKLR					Mascot
1730.8973	1730.9242	0.0269	16	38	51	DWLQEQGQKPFRAK					Mascot
1855.9331	1855.9418	0.0087	5	107	123	LADGGEVECVLLRDGPR			Carbamidomethyl (C)[9]		Mascot
1912.0685	1911.9385	-0.13	-68	103	119	ILVRLADGGEVECVLLR			Carbamidomethyl (C)[13]		Mascot
2021.0348	2020.9868	-0.048	-24	305	322	SVMMNVIPYNPVAGLPY R					Mascot
2186.0586	2186.1926	0.134	61	56	74	WLFSGRATSFEEMTDLP AK					Mascot

10 Replication factor C small subunit OS=Pyrococcus horikoshii (strain ATCC 700860 / DSM 12428 / JCM 9974 / NBRC 100139 / OT-3) GN=rfcS PE=3 SV=1 RFCS_PYRHO 98645.3 7.94 22 60 42.06 8.315

Peptide Information

Calc. Mass	Obsrv. Mass	± da	± ppm	Start Seq.	End Seq.	Sequence	Ion Score	C. I. %	Modification	Rank	Result Type
837.4828	837.4224	-0.0604	-72	240	246	VYVNSKK					Mascot
856.4635	856.5103	0.0468	55	761	767	ARPEDIR					Mascot

980.5154	980.5263	0.0109	11	768	775	EMMLLALK	Oxidation (M)[2,3]	Mascot
1173.5898	1173.6902	0.1004	86	480	489	KYSSYAEIGR		Mascot
1201.5961	1201.6725	0.0764	64	481	490	YSSYAEIGRR		Mascot
1233.5239	1233.6418	0.1179	96	660	669	TMEMFSSNVR	Oxidation (M)[2,4]	Mascot
1311.7631	1311.7821	0.019	14	735	747	AINILQAAAALDK		Mascot
1389.625	1389.7111	0.0861	62	659	669	RTMEMFSSNVR	Oxidation (M)[3,5]	Mascot
1399.7581	1399.8088	0.0507	36	155	166	WVKAEELKPGDK		Mascot
1433.6512	1433.7799	0.1287	90	1	11	MHNMEEVREVK	Oxidation (M)[1,4]	Mascot
1467.8641	1467.8813	0.0172	12	734	747	RAINILQAAAALDK		Mascot
1467.8641	1467.8813	0.0172	12	734	747	RAINILQAAAALDK		Mascot
1473.7737	1473.7697	-0.004	-3	109	119	LREFEVQYVYK		Mascot
1535.854	1535.8058	-0.0482	-31	26	38	LDEIVGQEHIVKR		Mascot
1598.8207	1598.8107	-0.01	-6	462	475	VLGIDVEHTIEAMR	Oxidation (M)[13]	Mascot
1665.8782	1665.9008	0.0226	14	45	61	TGSMPHLLFAGPPGVGK		Mascot
1805.9028	1806.0167	0.1139	63	230	245	EITHENGTSKVYVNSK		Mascot
1817.9612	1817.9065	-0.0547	-30	761	775	ARPEDIREMMLLALK	Oxidation (M)[9,10]	Mascot
1817.9612	1817.9065	-0.0547	-30	761	775	ARPEDIREMMLLALK	Oxidation (M)[9,10]	Mascot
1828.0228	1827.9741	-0.0487	-27	12	25	VLEKPWVEKYRPQR		Mascot
1829.9756	1829.9457	-0.0299	-16	410	425	ESLSRGILIDWNEVAK		Mascot
1911.0369	1910.9399	-0.097	-51	459	475	IAKVLGIDVEHTIEAMR	Oxidation (M)[16]	Mascot
2056.1008	2056.0344	-0.0664	-32	462	479	VLGIDVEHTIEAMRVFAR		Mascot
2287.2041	2287.1633	-0.0408	-18	640	659	IIFLDEADALTQDAQQALR R		Mascot

Gel Idx/Pos	221/I21	Instr./Gel Origin	BA2151/Sample Project 20140814	Process Status	Analysis Succeeded
Plate [#] Name	[1] Sample Project 20140814	Instrument Sample Name		Spectra	11

Rank	Protein Name	Accession No.	Protein MW	Protein PI	Pep. Count	Protein Score	Protein Score C. I. %	Intensity Matched	Total Ion Score	Total Ion C. I. %	Confirmed
1	Probable fructose-bisphosphate aldolase 2, chloroplastic OS=Arabidopsis thaliana GN=FBA2 PE=1 SV=2	ALFC2_ARATH	43132.1	6.78	7	160	100	4.929	141	100	

Peptide Information

Calc. Mass	Obsrv. Mass	± da	± ppm	Start Seq.	End Seq.	Sequence	Ion Score	C. I. %	Modification	Rank	Result Type
873.4577	873.501	0.0433	50	203	210	EAAWGLAR					Mascot
901.5101	901.4962	-0.0139	-15	369	377	ANSLAQLGK					Mascot
947.4979	947.5263	0.0284	30	340	347	ALQNTCLK			Carbamidomethyl (C)[6]		Mascot
1153.5736	1153.6294	0.0558	48	47	57	AASSYADELVK					Mascot
1194.55	1194.5503	0.0003	0	389	398	EGMFVKGYTY					Mascot
1431.7074	1431.7681	0.0607	42	83	95	LDSIGLENTEANR					Mascot
1431.7074	1431.7681	0.0607	42	83	95	LDSIGLENTEANR	94	100			Mascot
1587.8085	1587.8683	0.0598	38	82	95	RLDSIGLENTEANR					Mascot
1587.8085	1587.8683	0.0598	38	82	95	RLDSIGLENTEANR	48	98.176			Mascot

Rank	Protein Name	Accession No.	Protein MW	Protein PI	Pep. Count	Protein Score	Protein Score C. I. %	Intensity Matched	Total Ion Score	Total Ion C. I. %	Confirmed
2	Fructose-bisphosphate aldolase 2, chloroplastic OS=Pisum sativum PE=1 SV=2	ALFC2_PEA	38031.5	5.48	6	157	100	4.61	141	100	

Peptide Information

Calc. Mass	Obsrv. Mass	± da	± ppm	Start Seq.	End Seq.	Sequence	Ion Score	C. I. %	Modification	Rank	Result Type
822.4355	822.4597	0.0242	29	191	197	TFEVAQK					Mascot
873.4577	873.501	0.0433	50	155	162	EAAWGLAR					Mascot
1197.527	1197.6063	0.0793	66	330	340	YIGDGESEEAK					Mascot
1328.7322	1328.7007	-0.0315	-24	238	248	VAEYTLNLLHR					Mascot
1431.7074	1431.7681	0.0607	42	35	47	LDSIGLENTEANR					Mascot
1431.7074	1431.7681	0.0607	42	35	47	LDSIGLENTEANR	94	100			Mascot
1587.8085	1587.8683	0.0598	38	34	47	RLDSIGLENTEANR					Mascot
1587.8085	1587.8683	0.0598	38	34	47	RLDSIGLENTEANR	48	98.176			Mascot

Rank	Protein Name	Accession No.	Protein MW	Protein PI	Pep. Count	Protein Score	Protein Score C. I. %	Intensity Matched	Total Ion Score	Total Ion C. I. %	Confirmed
3	Fructose-bisphosphate aldolase, chloroplastic OS=Oryza sativa subsp. japonica GN=Os11g0171300 PE=1 SV=2	ALFC_ORYSJ	42207.6	6.38	13	134	100	24.762	75	99.996	

Peptide Information

Calc. Mass	Obsrv. Mass	± da	± ppm	Start Seq.	End Seq.	Sequence	Ion Score	C. I. %	Modification	Rank	Result Type
822.4355	822.4597	0.0242	29	229	235	TFEVAQK					Mascot
873.4577	873.501	0.0433	50	193	200	EAAWGLAR					Mascot
901.5101	901.4962	-0.0139	-15	359	367	ANSLAQLGK					Mascot
947.4979	947.5263	0.0284	30	330	337	ALQNTCLK			Carbamidomethyl (C)[6]		Mascot
1141.5007	1141.5876	0.0869	76	368	378	YTSDGEAAEAK					Mascot
1156.5382	1156.5863	0.0481	42	163	172	EAAYYQQGAR					Mascot
1156.5382	1156.5863	0.0481	42	163	172	EAAYYQQGAR	75	99.996			Mascot
1169.7001	1169.6417	-0.0584	-50	348	358	AAQDALLLRAK					Mascot
1387.7175	1387.7709	0.0534	38	73	85	LASIGLENTEANR					Mascot
1510.8475	1510.8711	0.0236	16	178	192	TVVSIPNGPSELAVK					Mascot
1543.8187	1543.9049	0.0862	56	72	85	RLASIGLENTEANR					Mascot
1569.8782	1569.8785	0.0003	0	23	37	QAAAPKPVTVSMVVR			Oxidation (M)[12]		Mascot
1666.015	1665.9258	-0.0892	-54	122	136	IVDILTEQKIVPGIK					Mascot
2341.2446	2341.2424	-0.0022	-1	16	37	SEWGATRQAAAPKPVTV SMVVR					Mascot

4 Glycine--tRNA ligase beta subunit OS=Hyphomonas neptunium (strain ATCC 15444) GN=glyS PE=3 SV=1 SYGB_HYPNA 75534.1 5.6 20 77 98.921 17.905

Peptide Information

Calc. Mass	Obsrv. Mass	± da	± ppm	Start Seq.	End Seq.	Sequence	Ion Score	C. I. %	Modification	Rank	Result Type
871.5247	871.4879	-0.0368	-42	217	225	AAILGGIEK					Mascot
905.4839	905.488	0.0041	5	42	50	TAFAVSGPR					Mascot
905.4839	905.488	0.0041	5	42	50	TAFAVSGPR	7	0			Mascot
947.468	947.5263	0.0583	62	351	359	EELGSLGDK					Mascot
984.5948	984.6262	0.0314	32	360	368	VERVAALAR					Mascot
984.5948	984.6262	0.0314	32	360	368	VERVAALAR					Mascot
1027.6259	1027.5435	-0.0824	-80	216	225	RAAILGGIEK					Mascot
1074.55	1074.5715	0.0215	20	334	343	ATPLEVMGEK					Mascot
1143.6368	1143.6388	0.002	2	52	62	LTAVVEGLDAR					Mascot
1143.6368	1143.6388	0.002	2	52	62	LTAVVEGLDAR					Mascot
1155.6844	1155.5908	-0.0936	-81	485	494	INLENGVRLK					Mascot
1172.6521	1172.6132	-0.0389	-33	21	32	AEADLLAALTGK					Mascot
1194.6266	1194.5503	-0.0763	-64	280	290	YFAATHAKTGK					Mascot
1200.6484	1200.5704	-0.078	-65	693	703	GALHKVADFSR					Mascot

1299.7379	1299.7012	-0.0367	-28	51	62	RLTAVVEGLDAR					Mascot
1328.7322	1328.7007	-0.0315	-24	75	87	VGAPEQAIAGFLR					Mascot
1357.6423	1357.7185	0.0762	56	188	199	GPYTVTGWADYK					Mascot
1409.7496	1409.8662	0.1166	83	200	212	SQLEGQGHVVLSR					Mascot
1413.8312	1413.7448	-0.0864	-61	21	34	AEADLLAALTGKLK					Mascot
1431.6903	1431.7681	0.0778	54	106	118	GDFYVAYSTVPGR					Mascot
1431.6903	1431.7681	0.0778	54	106	118	GDFYVAYSTVPGR					Mascot
1507.8003	1507.7993	-0.001	-1	346	359	TIAFKEELGSLGDK					Mascot
1630.8833	1630.9734	0.0901	55	17	32	MQAKAEADLLAALTGK					Mascot
1660.8806	1660.8351	-0.0455	-27	35	50	EAGLAWKTAFAVSGPR					Mascot

5	DNA ligase OS=Desulforudis audaxviator (strain MP104C) GN=ligA PE=3 SV=1	DNLJ_DESAP	74761.6	6.53	18	74	97.64	5.664

Peptide Information

Calc. Mass	Obsrv. Mass	± da	± ppm	Start Seq.	End Seq.	Sequence	Ion Score	C. I. %	Modification	Rank	Result Type
877.4427	877.4959	0.0532	61	269	274	HLEHWR					Mascot
1155.6732	1155.5908	-0.0824	-71	487	497	IGPKSADNLLK					Mascot
1299.6692	1299.7012	0.032	25	190	201	EDAGLPLFANPR					Mascot
1342.7074	1342.7056	-0.0018	-1	612	624	AEAAARIEELGGR					Mascot
1347.7267	1347.804	0.0773	57	633	645	TDYVVLGKNPGGK					Mascot
1387.7468	1387.7709	0.0241	17	652	663	LGVNIIEEPEFK					Mascot
1413.6831	1413.7448	0.0617	44	173	184	GEVYMPKEAFAR			Oxidation (M)[5]		Mascot
1431.7843	1431.7681	-0.0162	-11	466	478	LVADPADLFTLTR					Mascot
1431.7843	1431.7681	-0.0162	-11	466	478	LVADPADLFTLTR					Mascot
1459.8744	1459.7859	-0.0885	-61	505	517	NSLARLIFSLGIR					Mascot
1543.8479	1543.9049	0.057	37	652	664	LGVNIIEEPEFKR					Mascot
1587.8854	1587.8683	-0.0171	-11	465	478	RLVADPADLFTLTR					Mascot
1587.8854	1587.8683	-0.0171	-11	465	478	RLVADPADLFTLTR	5	0			Mascot
1600.7867	1600.9432	0.1565	98	239	252	HSEALAWLGEQGFR					Mascot
1665.8218	1665.9258	0.104	62	102	116	TGLADEDVDYVVELK					Mascot
1827.9559	1828.0005	0.0446	24	138	154	GDGVTGEEITPNLRTIR					Mascot
1927.0033	1926.952	-0.0513	-27	253	268	VNPHTRIFSTLEEVER					Mascot
1953.0513	1952.9324	-0.1189	-61	348	365	LAGTTVSRASLHNEDLIR					Mascot
1984.0321	1983.9323	-0.0998	-50	162	179	QPGAPNVIEVRGEVYMP K					Mascot
2186.1646	2186.2415	0.0769	35	220	238	KLDIWLYGIGYTDGIVFG R					Mascot

6	Gamma-glutamyl phosphate reductase OS=Clostridium cellulolyticum (strain ATCC 35319 / DSM 5812 / JCM 6584 / H10) GN=proA PE=3 SV=1	PROA_CLOCE	47951.6	5.15	10	66	87.613	14.472

Peptide Information

Calc. Mass	Obsrv. Mass	± da	± ppm	Start Seq.	End Seq.	Sequence	Ion Score	C. I. %	Modification	Rank	Result Type
905.4761	905.488	0.0119	13	17	25	MAALSGEVK					Mascot
905.4761	905.488	0.0119	13	17	25	MAALSGEVK	27	0			Mascot
984.5724	984.6262	0.0538	55	57	65	ENLASPLLK					Mascot
984.5724	984.6262	0.0538	55	57	65	ENLASPLLK	12	0			Mascot
1171.6793	1171.6323	-0.047	-40	32	42	IADALLANSKR					Mascot
1172.7037	1172.6132	-0.0905	-77	263	273	AVAKEFLPGLK					Mascot
1187.634	1187.7223	0.0883	74	83	93	SLMALEEPIGK					Mascot
1328.7419	1328.7007	-0.0412	-31	54	65	SEKENLASPLLK					Mascot
1587.8271	1587.8683	0.0412	26	2	16	DIRQLCGNAGVASVK			Carbamidomethyl (C)[6]		Mascot
1587.8271	1587.8683	0.0412	26	2	16	DIRQLCGNAGVASVK			Carbamidomethyl (C)[6]		Mascot
1828.0248	1828.0005	-0.0243	-13	83	99	SLMALEEPIGKTLLANK					Mascot
1952.9746	1952.9324	-0.0422	-22	246	262	TQYVAVCNATETLLVDR			Carbamidomethyl (C)[7]		Mascot
1974.9954	1975.1183	0.1229	62	172	188	GWIGLLESRDDVNEMLK					Mascot

7	50S ribosomal protein L6 OS=Neisseria meningitidis serogroup B (strain MC58) GN=rplF PE=1 SV=1	RL6_NEIMB	18892	9.63	10	65	82.095	2.729

Peptide Information

Calc. Mass	Obsrv. Mass	± da	± ppm	Start Seq.	End Seq.	Sequence	Ion Score	C. I. %	Modification	Rank	Result Type
877.4811	877.4959	0.0148	17	70	77	ALVSNMVK			Oxidation (M)[6]		Mascot
889.4778	889.4929	0.0151	17	153	160	APEPYKGK					Mascot
947.556	947.5263	-0.0297	-31	19	27	FGAEALVIK					Mascot
1169.6637	1169.6417	-0.022	-19	139	149	QVVGQVAAEIR					Mascot
1335.7454	1335.7206	-0.0248	-19	161	172	GVRYVGEVVVMK					Mascot
1507.8843	1507.7993	-0.085	-56	4	18	VAKNPVTVPAGVEVK					Mascot
1543.8704	1543.9049	0.0345	22	139	152	QVVGQVAAEIRAFR					Mascot
1864.9368	1865.0157	0.0789	42	60	77	QANAMSGTARALVSNMVK			Oxidation (M)[5]		Mascot
2084.019	2084.1321	0.1131	54	50	69	LTFVANNSSKQANAMSGTAR			Oxidation (M)[15]		Mascot
2138.2219	2138.1433	-0.0786	-37	7	27	NPVTVPAGVEVKFGAEALVIK					Mascot

8	Plastin-1 OS=Mus musculus GN=Pls1 PE=2 SV=1	PLSI_MOUSE	70762.5	5.23	15	64	79.91	7.184

Peptide Information

Calc. Mass	Obsrv. Mass	± da	± ppm	Start Seq.	End Seq.	Sequence	Ion Score	C. I. %	Modification	Rank	Result Type
856.441	856.5128	0.0718	84	537	544	STSISSFK					Mascot
984.6088	984.6262	0.0174	18	247	255	NEALIALLK					Mascot
984.6088	984.6262	0.0174	18	247	255	NEALIALLK	10	0			Mascot
1074.583	1074.5715	-0.0115	-11	42	51	EASLPLPGYK					Mascot
1155.5311	1155.5908	0.0597	52	1	10	MENSTTTISR			Oxidation (M)[1]		Mascot
1178.5245	1178.5887	0.0642	54	256	265	DGEDLEELMK					Mascot
1194.5195	1194.5503	0.0308	26	256	265	DGEDLEELMK			Oxidation (M)[9]		Mascot
1285.7111	1285.7229	0.0118	9	59	70	ILVVADNNKDGK					Mascot
1387.6605	1387.7709	0.1104	80	613	624	MVMTVFACLMGK			Carbamidomethyl (C)[8]		Mascot
1532.7261	1532.8721	0.146	95	168	180	MINLSEPDTIDER					Mascot
1583.9003	1583.8232	-0.0771	-49	54	67	EIVEKILVVADNNK					Mascot
1742.9337	1743.0765	0.1428	82	300	314	AYFHLLNQIAPKGDR					Mascot
1786.011	1785.9917	-0.0193	-11	518	532	VTDDIIIKWVNQTLK					Mascot
1827.91	1828.0005	0.0905	50	613	628	MVMTVFACLMGKGLNR			Carbamidomethyl (C)[8]		Mascot
1974.9801	1975.1183	0.1382	70	168	184	MINLSEPDTIDERAINK			Oxidation (M)[1]		Mascot
2185.259	2185.2559	-0.0031	-1	236	255	VGLFADIEISRNEALIALLK					Mascot
3046.603	3046.7024	0.0994	33	475	502	FSLVGIAGQDLNEGNATL TLALVWQLMR			Oxidation (M)[27]		Mascot

9	Chaperone protein ClpB OS=Clostridium perfringens (strain 13 / Type A) GN=clpB PE=3 SV=1	CLPB_CLOPE	97935.6	5.18	19	63	73.516	10.022	

Peptide Information

Calc. Mass	Obsrv. Mass	± da	± ppm	Start Seq.	End Seq.	Sequence	Ion Score	C. I. %	Modification	Rank	Result Type
889.5505	889.4929	-0.0576	-65	782	788	IIDIFLR					Mascot
901.485	901.4962	0.0112	12	789	795	DVENRLR					Mascot
984.62	984.6262	0.0062	6	89	96	RINEVLIK					Mascot
984.62	984.6262	0.0062	6	89	96	RINEVLIK	10	0			Mascot
1182.6842	1182.6044	-0.0798	-67	228	238	IVRGDVPEGLK					Mascot
1187.6281	1187.7223	0.0942	79	381	389	LSHRYIQDR					Mascot
1387.725	1387.7709	0.0459	33	395	407	AIDLIDEAGAMIR					Mascot
1487.8502	1487.8141	-0.0361	-24	241	255	VIISLDMGALIAGAK			Oxidation (M)[7]		Mascot
1507.8367	1507.7993	-0.0374	-25	828	840	YISNVLETEIAKK					Mascot
1566.8051	1566.8159	0.0108	7	676	688	NPYSVILFDEIEK					Mascot
1600.8429	1600.9432	0.1003	63	408	421	SEIDSLPTELDIIR					Mascot

1614.885	1614.9985	0.1135	70	550	563	WTGIPVTKLVESER					Mascot
1617.9364	1617.8929	-0.0435	-27	603	618	KPIGSFIFLGPTGVGK					Mascot
1791.0197	1791.001	-0.0187	-10	241	257	VIISLDMGALIAGAKYR					Mascot
1864.8593	1865.0157	0.1564	84	520	535	EQEASMEKDNENALLK			Oxidation (M)[6]		Mascot
1948.0573	1948.0806	0.0233	12	765	781	VDDIIMFRPLSSEGIKK					Mascot
2185.175	2185.2559	0.0809	37	338	356	FQPVIVEEPTVEETVSILR					Mascot
2341.2761	2341.2424	-0.0337	-14	337	356	RFQPVIVEEPTVEETVSIL R					Mascot
2430.1394	2430.2747	0.1353	56	311	330	GELHCIGATTFDEYRQYI EK			Carbamidomethyl (C)[5]		Mascot
2448.1057	2448.249	0.1433	59	628	647	NLFDSEDNIVRIDMSEYM EK					Mascot
2448.1057	2448.249	0.1433	59	628	647	NLFDSEDNIVRIDMSEYM EK					Mascot
2464.1006	2464.2488	0.1482	60	628	647	NLFDSEDNIVRIDMSEYM EK			Oxidation (M)[14]		Mascot

10 Spastin OS=Drosophila virilis GN=spas PE=3 SV=1 SPAST_DROVI 83580.5 9.67 19 63 70.285 3.985

Peptide Information

Calc. Mass	Obsrv. Mass	± da	± ppm	Start Seq.	End Seq.	Sequence	Ion Score	C. I. %	Modification	Rank	Result Type
856.4999	856.5128	0.0129	15	372	380	AVPAASGRK					Mascot
1027.5796	1027.5435	-0.0361	-35	142	149	VIYRSPHR					Mascot
1135.6106	1135.6478	0.0372	33	567	576	YVGDGEKLVR					Mascot
1169.6565	1169.6417	-0.0148	-13	227	236	AFEYISKALK					Mascot
1172.5403	1172.6132	0.0729	62	603	612	SSNEHEASRR					Mascot
1178.6528	1178.5887	-0.0641	-54	353	363	ITLRSSGYGPK					Mascot
1182.6841	1182.6044	-0.0797	-67	346	356	ASAEPPKITLR					Mascot
1194.5308	1194.5503	0.0195	16	42	52	SCSEALIDDGK			Carbamidomethyl (C)[2]		Mascot
1197.5422	1197.6063	0.0641	54	760	769	WSQDYGDITI					Mascot
1200.6696	1200.5704	-0.0992	-83	397	407	SQTLPRNLGSK					Mascot
1418.6945	1418.7769	0.0824	58	151	162	DCNIEIVVQNSK			Carbamidomethyl (C)[2]		Mascot
1431.7551	1431.7681	0.013	9	364	379	TGGATTSKAVPAASGR					Mascot
1431.7551	1431.7681	0.013	9	364	379	TGGATTSKAVPAASGR					Mascot
1459.7766	1459.7859	0.0093	6	123	133	SLIYQLFCIFR			Carbamidomethyl (C)[8]		Mascot
1511.7588	1511.8571	0.0983	65	693	707	ITDGYSGSDLTALAK					Mascot
1569.8595	1569.8785	0.019	12	678	692	QGSPLDTEALARLAK					Mascot
1583.7218	1583.8232	0.1014	64	42	56	SCSEALIDDGKSSSK			Carbamidomethyl (C)[2]		Mascot
1665.9758	1665.9258	-0.05	-30	381	396	LTIGNKRPGNLAVANK					Mascot
2138.2068	2138.1433	-0.0635	-30	471	490	GVEQKLVQLILDEIVEGG AK					Mascot

3075.6003	3075.7417	0.1414	46	163	189	EQQAIICPLEGSGVNIEQ AQILPQRQR	Carbamidomethyl (C)[7]	Mascot

Gel Idx/Pos	222/I22	Instr./Gel Origin	BA2151/Sample Project 20140814	Process Status	Analysis Succeeded
Plate [#] Name	[1] Sample Project 20140814	Instrument Sample Name		Spectra	11

Rank	Protein Name	Accession No.	Protein MW	Protein PI	Pep. Count	Protein Score	Protein Score C. I. %	Intensity Matched	Total Ion Score	Total Ion C. I. %	Confirmed
1	Fructose-bisphosphate aldolase, chloroplastic OS=Oryza sativa subsp. japonica GN=Os11g0171300 PE=1 SV=2	ALFC_ORYSJ	42207.6	6.38	10	223	100	31.517	185	100	

Peptide Information

Calc. Mass	Obsrv. Mass	± da	± ppm	Start Seq.	End Seq.	Sequence	Ion Score	C. I. %	Modification	Rank	Result Type
822.4355	822.4487	0.0132	16	229	235	TFEVAQK					Mascot
873.4577	873.4947	0.037	42	193	200	EAAWGLAR					Mascot
901.5101	901.495	-0.0151	-17	359	367	ANSLAQLGK					Mascot
947.4979	947.5132	0.0153	16	330	337	ALQNTCLK			Carbamidomethyl (C)[6]		Mascot
1156.5382	1156.5806	0.0424	37	163	172	EAAYYQQGAR					Mascot
1156.5382	1156.5806	0.0424	37	163	172	EAAYYQQGAR	74	99.996			Mascot
1387.7175	1387.769	0.0515	37	73	85	LASIGLENTEANR					Mascot
1387.7175	1387.769	0.0515	37	73	85	LASIGLENTEANR	112	100			Mascot
1510.8475	1510.8488	0.0013	1	178	192	TVVSIPNGPSELAVK					Mascot
1543.8187	1543.8806	0.0619	40	72	85	RLASIGLENTEANR					Mascot
1622.8021	1622.8186	0.0165	10	269	282	DRATPEQVSDYTLK					Mascot
2067.0981	2067.1934	0.0953	46	338	356	TWGGQPENVKAAQDALLR					Mascot

Rank	Protein Name	Accession No.	Protein MW	Protein PI	Pep. Count	Protein Score	Protein Score C. I. %	Intensity Matched	Total Ion Score	Total Ion C. I. %	Confirmed
2	Probable fructose-bisphosphate aldolase 1, chloroplastic OS=Arabidopsis thaliana GN=FBA1 PE=1 SV=2	ALFC1_ARATH	43075	6.18	7	130	100	4.547	112	100	

Peptide Information

Calc. Mass	Obsrv. Mass	± da	± ppm	Start Seq.	End Seq.	Sequence	Ion Score	C. I. %	Modification	Rank	Result Type
873.4577	873.4947	0.037	42	204	211	EAAWGLAR					Mascot
901.5101	901.495	-0.0151	-17	370	378	ANSLAQLGK					Mascot
947.4979	947.5132	0.0153	16	341	348	ALQNTCLK			Carbamidomethyl (C)[6]		Mascot
1194.55	1194.5437	-0.0063	-5	390	399	EGMFVKGYTY					Mascot
1387.7175	1387.769	0.0515	37	84	96	LASIGLENTEANR					Mascot
1387.7175	1387.769	0.0515	37	84	96	LASIGLENTEANR	112	100			Mascot
1522.7968	1522.9061	0.1093	72	1	15	MASSTATMLKASPVK					Mascot
1543.8187	1543.8806	0.0619	40	83	96	RLASIGLENTEANR					Mascot

3	Fructose-bisphosphate aldolase, chloroplastic OS=Spinacia oleracea PE=1 SV=3	ALFC_SPIOL	42726.8	6.85	6	126	100	30.253	112	100		

Peptide Information

Calc. Mass	Obsrv. Mass	± da	± ppm	Start Seq.	End Seq.	Sequence	Ion Score	C. I. %	Modification	Rank	Result Type
873.4577	873.4947	0.037	42	201	208	EAAWGLAR					Mascot
947.4979	947.5132	0.0153	16	338	345	ALQNTCLK			Carbamidomethyl (C)[6]		Mascot
1156.4753	1156.5806	0.1053	91	373	382	YTGEGESEER					Mascot
1156.4753	1156.5806	0.1053	91	373	382	YTGEGESEER					Mascot
1387.7175	1387.769	0.0515	37	81	93	LASIGLENTEANR					Mascot
1387.7175	1387.769	0.0515	37	81	93	LASIGLENTEANR	112	100			Mascot
1543.8187	1543.8806	0.0619	40	80	93	RLASIGLENTEANR					Mascot
1622.7626	1622.8186	0.056	35	66	80	GILAMDESNATCGKR			Carbamidomethyl (C)[12]		Mascot

4	Enolase OS=Mesembryanthemum crystallinum GN=PGH1 PE=2 SV=1	ENO_MESCR	48660.6	5.62	7	96	99.987	5.04	78	99.998

Peptide Information

Calc. Mass	Obsrv. Mass	± da	± ppm	Start Seq.	End Seq.	Sequence	Ion Score	C. I. %	Modification	Rank	Result Type
932.4571	932.4723	0.0152	16	422	429	IEEELGDK					Mascot
1008.5221	1008.473	-0.0491	-49	9	16	ARQIYDSR					Mascot
1138.6102	1138.5708	-0.0394	-35	234	244	TAIEKAGYTGK					Mascot
1138.6102	1138.5708	-0.0394	-35	234	244	TAIEKAGYTGK					Mascot
1215.6052	1215.6906	0.0854	70	371	381	KAGWGVMASHR			Oxidation (M)[7]		Mascot
1510.8477	1510.8488	0.0011	1	322	335	VQIVGDDLLVTNPK					Mascot
1790.9283	1790.99	0.0617	34	35	52	AAVPSGASTGVYEALELR					Mascot
1790.9283	1790.99	0.0617	34	35	52	AAVPSGASTGVYEALELR	78	99.998			Mascot
2106.0576	2106.1365	0.0789	37	239	258	AGYTGKVVIGMDVAASE FYK					Mascot

5	Enolase OS=Alnus glutinosa GN=PGH1 PE=2 SV=1	ENO_ALNGL	47795.5	5.41	6	92	99.969	28.216	78	99.998

Peptide Information

Calc. Mass	Obsrv. Mass	± da	± ppm	Start Seq.	End Seq.	Sequence	Ion Score	C. I. %	Modification	Rank	Result Type
1156.5793	1156.5806	0.0013	1	368	377	RAGWGVMAHR			Oxidation (M)[7]		Mascot
1156.6685	1156.5806	-0.0879	-76	62	72	GVLKAVDNVNK					Mascot
1308.6794	1308.6915	0.0121	9	53	65	DGGSDYLGKGVLK					Mascot

1510.8477	1510.8488	0.0011	1	319	332	VQIVGDDLLVTNPK					Mascot
1790.9283	1790.99	0.0617	34	35	52	AAVPSGASTGVYEALELR					Mascot
1790.9283	1790.99	0.0617	34	35	52	AAVPSGASTGVYEALELR	78	99.998			Mascot
1884.9598	1885.0559	0.0961	51	169	185	LAMQEFMILPVGASSFK			Oxidation (M)[3]		Mascot

6	Enolase OS=Oryza sativa subsp. japonica GN=ENO1 PE=1 SV=2	ENO_ORYSJ	48284.5	5.41	5	90	99.945	5.813	78	99.998

Peptide Information

Calc. Mass	Obsrv. Mass	± da	± ppm	Start Seq.	End Seq.	Sequence	Ion Score	C. I. %	Modification	Rank	Result Type
1138.6102	1138.5708	-0.0394	-35	235	245	TAIEKAGYTGK					Mascot
1138.6102	1138.5708	-0.0394	-35	235	245	TAIEKAGYTGK					Mascot
1143.5681	1143.6222	0.0541	47	262	270	DKTYDLNFK					Mascot
1790.9283	1790.99	0.0617	34	36	53	AAVPSGASTGVYEALELR					Mascot
1790.9283	1790.99	0.0617	34	36	53	AAVPSGASTGVYEALELR	78	99.998			Mascot
1983.8536	1983.9185	0.0649	33	18	35	GNPTVEVDVCCSDGTFAR			Carbamidomethyl (C)[10,11]		Mascot
2067.2358	2067.1934	-0.0424	-21	112	132	LGANAILAVSLAICKAGAIIK			Carbamidomethyl (C)[14]		Mascot

7	Enolase (Fragments) OS=Populus euphratica PE=1 SV=1	ENO_POPEU	2749.4	4.79	1	87	99.897	1.169	78	99.998

Peptide Information

Calc. Mass	Obsrv. Mass	± da	± ppm	Start Seq.	End Seq.	Sequence	Ion Score	C. I. %	Modification	Rank	Result Type
1790.9283	1790.99	0.0617	34	1	18	AAVPSGASTGVYEALELR					Mascot
1790.9283	1790.99	0.0617	34	1	18	AAVPSGASTGVYEALELR	78	99.998			Mascot

8	Enolase 2 OS=Zea mays GN=ENO2 PE=2 SV=1	ENO2_MAIZE	48417.6	5.7	4	85	99.833	5.316	78	99.998

Peptide Information

Calc. Mass	Obsrv. Mass	± da	± ppm	Start Seq.	End Seq.	Sequence	Ion Score	C. I. %	Modification	Rank	Result Type
1138.6102	1138.5708	-0.0394	-35	235	245	TAIEKAGYTGK					Mascot
1138.6102	1138.5708	-0.0394	-35	235	245	TAIEKAGYTGK					Mascot
1143.5317	1143.6222	0.0905	79	262	270	DQTYDLNFK					Mascot
1175.6453	1175.6224	-0.0229	-19	345	354	EKSCNALLLK			Carbamidomethyl (C)[4]		Mascot
1790.9283	1790.99	0.0617	34	36	53	AAVPSGASTGVYEALELR					Mascot
1790.9283	1790.99	0.0617	34	36	53	AAVPSGASTGVYEALELR	78	99.998			Mascot

9	Translation initiation factor IF-2 OS=Streptococcus equi subsp. zooepidemicus (strain MGCS10565) GN=infB	IF2_STREM	103963.1	9.23	23	84	99.764	8.772		

PE=3 SV=1

Peptide Information

Calc. Mass	Obsrv. Mass	± da	± ppm	Start Seq.	End Seq.	Sequence	Ion Score	C. I. %	Modification	Rank	Result Type
873.5192	873.4947	-0.0245	-28	826	832	LHSIIYK					Mascot
877.4196	877.4873	0.0677	77	667	674	AMTNDLGR					Mascot
885.4901	885.4633	-0.0268	-30	292	298	KQNRPDK					Mascot
889.4373	889.4843	0.047	53	234	240	EQEEAKR					Mascot
901.5101	901.495	-0.0151	-17	67	74	QVAEQKAK					Mascot
904.4523	904.4595	0.0072	8	906	912	HYKDDVK					Mascot
932.5411	932.4723	-0.0688	-74	464	471	TTLLDTLR					Mascot
1200.6332	1200.5619	-0.0713	-59	241	250	LEQQARQEAK					Mascot
1246.6288	1246.6606	0.0318	26	315	324	KSWNSQNQVR					Mascot
1335.64	1335.6957	0.0557	42	218	228	AEQNAEYARQR					Mascot
1347.7632	1347.8099	0.0467	35	889	900	VIRDSVVIFDGK					Mascot
1387.7037	1387.769	0.0653	47	134	145	QLGRSQGQQTER					Mascot
1387.7037	1387.769	0.0653	47	134	145	QLGRSQGQQTER					Mascot
1592.8829	1592.9685	0.0856	54	865	880	VSKVGTIGGFMVVNGK					Mascot
1592.8829	1592.9685	0.0856	54	865	880	VSKVGTIGGFMVVNGK					Mascot
1764.8738	1765.0166	0.1428	81	499	514	ITFLDTPGHAAFTSMR					Mascot
1892.9688	1892.8943	-0.0745	-39	498	514	KITFLDTPGHAAFTSMR					Mascot
1958.9131	1958.944	0.0309	16	434	449	FFTDDSYLNPENIVER					Mascot
2047.078	2047.1085	0.0305	15	826	842	LHSIIYKVIEEVEEAMK			Oxidation (M)[16]		Mascot
2050.9751	2051.1526	0.1775	87	913	931	EIGNAQEGGLMIEGFNDI K			Oxidation (M)[11]		Mascot
2057.0146	2057.168	0.1534	75	26	45	SLGLDVKSHASSVEEAD ANK					Mascot
2339.3545	2339.2502	-0.1043	-45	747	769	TVNVIIKADVQGSVEALA ASLLK					Mascot
2369.238	2369.2905	0.0525	22	265	288	EASAKATEPIASMAAAPV AKPVDK			Oxidation (M)[13]		Mascot
2428.2468	2428.281	0.0342	14	475	498	VATGEAGGITQHIGAYQI EEAGKK					Mascot
2434.3049	2434.2593	-0.0456	-19	78	101	ATTPQPAAATQEASQPV AVKPKSR					Mascot

10 Thymidylate kinase OS=Tropheryma whipplei (strain Twist) GN=tmk PE=3 SV=1 KTHY_TROWT 22480.7 6.43 11 74 97.896 24.331

Protein Group

Thymidylate kinase OS=Tropheryma whipplei (strain TW08/27) GN=tmk PE=3 SV=1 KTHY_TROW8 22480.7 6.4299 998283 3862

Peptide Information

Calc. Mass	Obsrv. Mass	± da	± ppm	Start Seq.	End Seq.	Sequence	Ion Score	C. I. %	Modification	Rank	Result Type
905.5567	905.4818	-0.0749	-83	154	160	VRNIYLK					Mascot
905.5567	905.4818	-0.0749	-83	154	160	VRNIYLK					Mascot
1081.5525	1081.5139	-0.0386	-36	171	180	VIDASQSFSK					Mascot
1138.5739	1138.5708	-0.0031	-3	56	65	TEALLYSADR					Mascot
1138.5739	1138.5708	-0.0031	-3	56	65	TEALLYSADR					Mascot
1369.7587	1369.7133	-0.0454	-33	143	153	IERKPIDFHSK					Mascot
1387.6562	1387.769	0.1128	81	1	13	MFVTFEGIDGSGK					Mascot
1387.6562	1387.769	0.1128	81	1	13	MFVTFEGIDGSGK					Mascot
1522.786	1522.9061	0.1201	79	156	168	NIYLKLSADNTDR					Mascot
1592.8643	1592.9685	0.1042	65	14	29	TAQLTAAGEFLKASGK					Mascot
1592.8643	1592.9685	0.1042	65	14	29	TAQLTAAGEFLKASGK					Mascot
1622.9224	1622.8186	-0.1038	-64	66	81	AENIAKNVIPALNAGK					Mascot
1706.9912	1706.9454	-0.0458	-27	72	87	NVIPALNAGKVVLQDR					Mascot
1764.9126	1765.0166	0.104	59	56	71	TEALLYSADRAENIAK					Mascot
2371.2292	2371.2515	0.0223	9	82	101	VVLQDRYLDSFLAYQLAD NK					Mascot

Gel Idx/Pos	223/I23	Instr./Gel Origin	BA2151/Sample Project 20140814	Process Status	Analysis Succeeded
Plate [#] Name	[1] Sample Project 20140814	Instrument Sample Name		Spectra	11

Rank	Protein Name	Accession No.	Protein MW	Protein PI	Pep. Count	Protein Score	Protein Score C. I. %	Intensity Matched	Total Ion Score	Total Ion C. I. %	Confirmed
1	Actin-97 OS=Solanum tuberosum GN=AC97 PE=1 SV=1	ACT11_SOLTU	41844	5.31	15	509	100	60.746	424	100	

Peptide Information

Calc. Mass	Obsrv. Mass	± da	± ppm	Start Seq.	End Seq.	Sequence	Ion Score	C. I. %	Modification	Rank	Result Type
895.5359	895.569	0.0331	37	331	338	VVAPPERK					Mascot
976.4483	976.486	0.0377	39	21	30	AGFAGDDAPR					Mascot
1118.5114	1118.5577	0.0463	41	199	208	GYSFTTSAER					Mascot
1118.5114	1118.5577	0.0463	41	199	208	GYSFTTSAER	22	0			Mascot
1182.5273	1182.587	0.0597	50	53	63	DAYVGDEAQSK					Mascot
1198.7056	1198.7498	0.0442	37	31	41	AVFPSIVGRPR					Mascot
1198.7056	1198.7498	0.0442	37	31	41	AVFPSIVGRPR	43	94.913			Mascot
1459.6813	1459.7402	0.0589	40	362	374	AEYDESGPSIVHR					Mascot
1515.7491	1515.8101	0.061	40	87	97	IWHHTFYNELR					Mascot
1515.7491	1515.8101	0.061	40	87	97	IWHHTFYNELR	75	99.997			Mascot
1547.8098	1547.8048	-0.005	-3	180	193	LDLAGRDLTDSLMK					Mascot
1547.8098	1547.8048	-0.005	-3	180	193	LDLAGRDLTDSLMK					Mascot
1563.8047	1563.7969	-0.0078	-5	180	193	LDLAGRDLTDSLMK			Oxidation (M)[13]		Mascot
1587.7762	1587.8502	0.074	47	362	375	AEYDESGPSIVHRK					Mascot
1747.8861	1747.9542	0.0681	39	241	256	SYELPDGQVITIGAER					Mascot
1747.8861	1747.9542	0.0681	39	241	256	SYELPDGQVITIGAER	135	100			Mascot
1954.0645	1954.1345	0.07	36	98	115	VAPEEHPVLLTEAPLNPK					Mascot
1954.0645	1954.1345	0.07	36	98	115	VAPEEHPVLLTEAPLNPK	97	100			Mascot
2149.9563	2149.8726	-0.0837	-39	1	20	MADGEDIQPLVCDNGTG MVK			Carbamidomethyl (C)[12]		Mascot
2197.0959	2197.1804	0.0845	38	294	314	DLYGNIVLSGGTTMFPGI ADR					Mascot
2213.0908	2213.1753	0.0845	38	294	314	DLYGNIVLSGGTTMFPGI ADR			Oxidation (M)[14]		Mascot
2325.1909	2325.2661	0.0752	32	293	314	KDLYGNIVLSGGTTMFPG IADR					Mascot
2341.1858	2341.2668	0.081	35	293	314	KDLYGNIVLSGGTTMFPG IADR			Oxidation (M)[15]		Mascot
3151.6423	3151.7881	0.1458	46	150	179	TTGIVLDSGDGVSHTVPI YEGYALPHAILR					Mascot
3151.6423	3151.7881	0.1458	46	150	179	TTGIVLDSGDGVSHTVPI YEGYALPHAILR	52	99.253			Mascot

2	Actin OS=Gossypium hirsutum PE=3 SV=1	ACT_GOSHI	41867	5.31	16	496	100	62.152	402	100	

Peptide Information

Calc. Mass	Obsrv. Mass	± da	± ppm	Start Seq.	End Seq.	Sequence	Ion Score	C. I. %	Modification	Rank	Result Type
895.5359	895.569	0.0331	37	331	338	VVAPPERK					Mascot
976.4483	976.486	0.0377	39	21	30	AGFAGDDAPR					Mascot
1132.527	1132.5756	0.0486	43	199	208	GYSFTTTAER					Mascot
1176.55	1176.6389	0.0889	76	42	52	HTGVMVGMGQK			Oxidation (M)[5,8]		Mascot
1182.5273	1182.587	0.0597	50	53	63	DAYVGDEAQSK					Mascot
1198.7056	1198.7498	0.0442	37	31	41	AVFPSIVGRPR					Mascot
1198.7056	1198.7498	0.0442	37	31	41	AVFPSIVGRPR	43	94.913			Mascot
1459.6813	1459.7402	0.0589	40	362	374	AEYDESGPSIVHR					Mascot
1515.7491	1515.8101	0.061	40	87	97	IWHHTFYNELR					Mascot
1515.7491	1515.8101	0.061	40	87	97	IWHHTFYNELR	75	99.997			Mascot
1531.8148	1531.7997	-0.0151	-10	180	193	LDLAGRDLTDALMK					Mascot
1547.8098	1547.8048	-0.005	-3	180	193	LDLAGRDLTDALMK			Oxidation (M)[13]		Mascot
1547.8098	1547.8048	-0.005	-3	180	193	LDLAGRDLTDALMK			Oxidation (M)[13]		Mascot
1587.7762	1587.8502	0.074	47	362	375	AEYDESGPSIVHRK					Mascot
1747.8861	1747.9542	0.0681	39	241	256	SYELPDGQVITIGAER					Mascot
1747.8861	1747.9542	0.0681	39	241	256	SYELPDGQVITIGAER	135	100			Mascot
1954.0645	1954.1345	0.07	36	98	115	VAPEEHPVLLTEAPLNPK					Mascot
1954.0645	1954.1345	0.07	36	98	115	VAPEEHPVLLTEAPLNPK	97	100			Mascot
2149.9563	2149.8726	-0.0837	-39	1	20	MADGEDIQPLVCDNGTG MVK			Carbamidomethyl (C)[12]		Mascot
2197.0959	2197.1804	0.0845	38	294	314	DLYGNIVLSGGTTMFPGI ADR					Mascot
2213.0908	2213.1753	0.0845	38	294	314	DLYGNIVLSGGTTMFPGI ADR			Oxidation (M)[14]		Mascot
2325.1909	2325.2661	0.0752	32	293	314	KDLYGNIVLSGGTTMFPG IADR					Mascot
2341.1858	2341.2668	0.081	35	293	314	KDLYGNIVLSGGTTMFPG IADR			Oxidation (M)[15]		Mascot
3151.6423	3151.7881	0.1458	46	150	179	TTGIVLDSGDGVSHTVPI YEGYALPHAILR					Mascot
3151.6423	3151.7881	0.1458	46	150	179	TTGIVLDSGDGVSHTVPI YEGYALPHAILR	52	99.253			Mascot

3	Actin-1 OS=Oryza sativa subsp. japonica GN=ACT1 PE=2 SV=1	ACT1_ORYSJ	42014	5.3	15	484	100	55.06	402	100

Protein Group

Actin-1 OS=Oryza sativa subsp. indica GN=ACT1 PE=1 SV=1	ACT1_ORYSI	42014	5.3000 001907 3486

Peptide Information

Calc. Mass	Obsrv. Mass	± da	± ppm	Start Seq.	End Seq.	Sequence	Ion Score	C. I. %	Modification	Rank	Result Type
890.4764	890.4863	0.0099	11	209	215	EIVRDMK					Mascot
895.5359	895.569	0.0331	37	331	338	VVAPPERK					Mascot
976.4483	976.486	0.0377	39	21	30	AGFAGDDAPR					Mascot
1132.527	1132.5756	0.0486	43	199	208	GYSFTTTAER					Mascot
1176.55	1176.6389	0.0889	76	42	52	HTGVMVGMGQK			Oxidation (M)[5,8]		Mascot
1182.5273	1182.587	0.0597	50	53	63	DAYVGDEAQSK					Mascot
1198.7056	1198.7498	0.0442	37	31	41	AVFPSIVGRPR					Mascot
1198.7056	1198.7498	0.0442	37	31	41	AVFPSIVGRPR	43	94.913			Mascot
1459.6813	1459.7402	0.0589	40	362	374	AEYDESGPSIVHR					Mascot
1515.7491	1515.8101	0.061	40	87	97	IWHHTFYNELR					Mascot
1515.7491	1515.8101	0.061	40	87	97	IWHHTFYNELR	75	99.997			Mascot
1587.7762	1587.8502	0.074	47	362	375	AEYDESGPSIVHRK					Mascot
1747.8861	1747.9542	0.0681	39	241	256	SYELPDGQVITIGAER					Mascot
1747.8861	1747.9542	0.0681	39	241	256	SYELPDGQVITIGAER	135	100			Mascot
1954.0645	1954.1345	0.07	36	98	115	VAPEEHPVLLTEAPLNPK					Mascot
1954.0645	1954.1345	0.07	36	98	115	VAPEEHPVLLTEAPLNPK	97	100			Mascot
2197.0959	2197.1804	0.0845	38	294	314	DLYGNIVLSGGTTMFPGI ADR					Mascot
2213.0908	2213.1753	0.0845	38	294	314	DLYGNIVLSGGTTMFPGI ADR			Oxidation (M)[14]		Mascot
2325.1909	2325.2661	0.0752	32	293	314	KDLYGNIVLSGGTTMFPG IADR					Mascot
2341.1858	2341.2668	0.081	35	293	314	KDLYGNIVLSGGTTMFPG IADR			Oxidation (M)[15]		Mascot
3151.6423	3151.7881	0.1458	46	150	179	TTGIVLDSGDGVSHTVPI YEGYALPHAILR					Mascot
3151.6423	3151.7881	0.1458	46	150	179	TTGIVLDSGDGVSHTVPI YEGYALPHAILR	52	99.253			Mascot

4	Actin OS=Nicotiana tabacum PE=3 SV=1	ACT1_TOBAC	41940	5.46	15	483	100	54.825	402	100	

Peptide Information

Calc. Mass	Obsrv. Mass	± da	± ppm	Start Seq.	End Seq.	Sequence	Ion Score	C. I. %	Modification	Rank	Result Type
895.5359	895.569	0.0331	37	331	338	VVAPPERK					Mascot
976.4483	976.486	0.0377	39	21	30	AGFAGDDAPR					Mascot
1132.527	1132.5756	0.0486	43	199	208	GYSFTTTAER					Mascot
1176.55	1176.6389	0.0889	76	42	52	HTGVMVGMGQK			Oxidation (M)[5,8]		Mascot
1182.5273	1182.587	0.0597	50	53	63	DAYVGDEAQSK					Mascot

1198.7056	1198.7498	0.0442	37	31	41	AVFPSIVGRPR					Mascot
1198.7056	1198.7498	0.0442	37	31	41	AVFPSIVGRPR	43	94.913			Mascot
1459.6813	1459.7402	0.0589	40	362	374	AEYDESGPSIVHR					Mascot
1515.7491	1515.8101	0.061	40	87	97	IWHHTFYNELR					Mascot
1515.7491	1515.8101	0.061	40	87	97	IWHHTFYNELR	75	99.997			Mascot
1587.7762	1587.8502	0.074	47	362	375	AEYDESGPSIVHRK					Mascot
1747.8861	1747.9542	0.0681	39	241	256	SYELPDGQVITIGAER					Mascot
1747.8861	1747.9542	0.0681	39	241	256	SYELPDGQVITIGAER	135	100			Mascot
1954.0645	1954.1345	0.07	36	98	115	VAPEEHPVLLTEAPLNPK					Mascot
1954.0645	1954.1345	0.07	36	98	115	VAPEEHPVLLTEAPLNPK	97	100			Mascot
1983.0039	1983.1628	0.1589	80	299	317	IVLSGGSTMFPGIADRMS K			Oxidation (M)[9]		Mascot
2149.9563	2149.8726	-0.0837	-39	1	20	MADGEDIQPLVCDNGTG MVK			Carbamidomethyl (C)[12]		Mascot
2197.1323	2197.1804	0.0481	22	294	314	DLYGKIVLSGGSTMFPGI ADR					Mascot
2213.1272	2213.1753	0.0481	22	294	314	DLYGKIVLSGGSTMFPGI ADR			Oxidation (M)[14]		Mascot
3151.6423	3151.7881	0.1458	46	150	179	TTGIVLDSGDGVSHTVPI YEGYALPHAILR					Mascot
3151.6423	3151.7881	0.1458	46	150	179	TTGIVLDSGDGVSHTVPI YEGYALPHAILR	52	99.253			Mascot

5	Actin-1 OS=Sorghum bicolor GN=AC1 PE=2 SV=1	ACT1_SORBI	42121	5.44	17	479	100	55.413	380	100

Peptide Information

Calc. Mass	Obsrv. Mass	± da	± ppm	Start Seq.	End Seq.	Sequence	Ion Score	C. I. %	Modification	Rank	Result Type
890.4764	890.4863	0.0099	11	209	215	EIVRDMK					Mascot
895.5359	895.569	0.0331	37	331	338	VVAPPERK					Mascot
976.4483	976.486	0.0377	39	21	30	AGFAGDDAPR					Mascot
1132.527	1132.5756	0.0486	43	199	208	GYSFTTTAER					Mascot
1176.55	1176.6389	0.0889	76	42	52	HTGVMVGMGQK			Oxidation (M)[5,8]		Mascot
1182.5273	1182.587	0.0597	50	53	63	DAYVGDEAQSK					Mascot
1198.7056	1198.7498	0.0442	37	31	41	AVFPSIVGRPR					Mascot
1198.7056	1198.7498	0.0442	37	31	41	AVFPSIVGRPR	43	94.913			Mascot
1459.6813	1459.7402	0.0589	40	362	374	AEYDESGPSIVHR					Mascot
1515.7491	1515.8101	0.061	40	87	97	IWHHTFYNELR					Mascot
1515.7491	1515.8101	0.061	40	87	97	IWHHTFYNELR	75	99.997			Mascot
1570.7828	1570.7961	0.0133	8	317	330	QGNHCLAPSSMKIK			Carbamidomethyl (C)[5]		Mascot
1587.7762	1587.8502	0.074	47	362	375	AEYDESGPSIVHRK					Mascot
1616.7455	1616.8395	0.094	58	315	328	MRQGNHCLAPSSMK			Carbamidomethyl (C)[7]		Mascot

1747.8861	1747.9542	0.0681	39	241	256	SYELPDGQVITIAADR					Mascot
1747.8861	1747.9542	0.0681	39	241	256	SYELPDGQVITIAADR	112	100			Mascot
1954.0645	1954.1345	0.07	36	98	115	VAPEEHPVLLTEAPLNPK					Mascot
1954.0645	1954.1345	0.07	36	98	115	VAPEEHPVLLTEAPLNPK	97	100			Mascot
2197.0959	2197.1804	0.0845	38	294	314	DLYGNIVLSGGTTMFPGI ADR					Mascot
2213.0908	2213.1753	0.0845	38	294	314	DLYGNIVLSGGTTMFPGI ADR			Oxidation (M)[14]		Mascot
2325.1909	2325.2661	0.0752	32	293	314	KDLYGNIVLSGGTTMFPG IADR					Mascot
2341.1858	2341.2668	0.081	35	293	314	KDLYGNIVLSGGTTMFPG IADR			Oxidation (M)[15]		Mascot
3151.6423	3151.7881	0.1458	46	150	179	TTGIVLDSGDGVSHTVPI YEGYALPHAILR					Mascot
3151.6423	3151.7881	0.1458	46	150	179	TTGIVLDSGDGVSHTVPI YEGYALPHAILR	52	99.253			Mascot

6	Actin-66 (Fragment) OS=Solanum tuberosum PE=3 SV=1	ACT5_SOLTU	37285.9	5.54	12	469	100	52.83	402	100		

Peptide Information

Calc. Mass	Obsrv. Mass	± da	± ppm	Start Seq.	End Seq.	Sequence	Ion Score	C. I. %	Modification	Rank	Result Type
895.5359	895.569	0.0331	37	311	318	VVAPPERK					Mascot
976.4483	976.486	0.0377	39	1	10	AGFAGDDAPR					Mascot
1132.527	1132.5756	0.0486	43	179	188	GYSFTTTAER					Mascot
1176.55	1176.6389	0.0889	76	22	32	HTGVMVGMGQK			Oxidation (M)[5,8]		Mascot
1182.5273	1182.587	0.0597	50	33	43	DAYVGDEAQSK					Mascot
1198.7056	1198.7498	0.0442	37	11	21	AVFPSIVGRPR					Mascot
1198.7056	1198.7498	0.0442	37	11	21	AVFPSIVGRPR	43	94.913			Mascot
1515.7491	1515.8101	0.061	40	67	77	IWHHTFYNELR					Mascot
1515.7491	1515.8101	0.061	40	67	77	IWHHTFYNELR	75	99.997			Mascot
1747.8861	1747.9542	0.0681	39	221	236	SYELPDGQVITIGAER					Mascot
1747.8861	1747.9542	0.0681	39	221	236	SYELPDGQVITIGAER	135	100			Mascot
1954.0645	1954.1345	0.07	36	78	95	VAPEEHPVLLTEAPLNPK					Mascot
1954.0645	1954.1345	0.07	36	78	95	VAPEEHPVLLTEAPLNPK	97	100			Mascot
2197.0959	2197.1804	0.0845	38	274	294	DLYGNIVLSGGTTMFPGI ADR					Mascot
2213.0908	2213.1753	0.0845	38	274	294	DLYGNIVLSGGTTMFPGI ADR			Oxidation (M)[14]		Mascot
2325.1909	2325.2661	0.0752	32	273	294	KDLYGNIVLSGGTTMFPG IADR					Mascot
2341.1858	2341.2668	0.081	35	273	294	KDLYGNIVLSGGTTMFPG IADR			Oxidation (M)[15]		Mascot
3151.6423	3151.7881	0.1458	46	130	159	TTGIVLDSGDGVSHTVPI YEGYALPHAILR					Mascot
3151.6423	3151.7881	0.1458	46	130	159	TTGIVLDSGDGVSHTVPI	52	99.253			Mascot

YEGYALPHAILR

7	Actin-104 (Fragment) OS=Nicotiana tabacum PE=3 SV=1	ACT7_TOBAC	37177.9	5.57	10	452	100	49.333	402	100	

Peptide Information

Calc. Mass	Obsrv. Mass	± da	± ppm	Start Seq.	End Seq.	Sequence	Ion Score	C. I. %	Modification	Rank	Result Type
895.5359	895.569	0.0331	37	311	318	VVAPPERK					Mascot
976.4483	976.486	0.0377	39	1	10	AGFAGDDAPR					Mascot
1182.5273	1182.587	0.0597	50	33	43	DAYVGDEAQSK					Mascot
1198.7056	1198.7498	0.0442	37	11	21	AVFPSIVGRPR					Mascot
1198.7056	1198.7498	0.0442	37	11	21	AVFPSIVGRPR	43	94.913			Mascot
1515.7491	1515.8101	0.061	40	67	77	IWHHTFYNELR					Mascot
1515.7491	1515.8101	0.061	40	67	77	IWHHTFYNELR	75	99.997			Mascot
1747.8861	1747.9542	0.0681	39	221	236	SYELPDGQVITIGAER					Mascot
1747.8861	1747.9542	0.0681	39	221	236	SYELPDGQVITIGAER	135	100			Mascot
1774.8793	1774.958	0.0787	44	174	188	ILTERGYMFTTSAER					Mascot
1954.0645	1954.1345	0.07	36	78	95	VAPEEHPVLLTEAPLNPK					Mascot
1954.0645	1954.1345	0.07	36	78	95	VAPEEHPVLLTEAPLNPK	97	100			Mascot
2183.0801	2183.1589	0.0788	36	274	294	DLYGNIVLSGGSTMFPGI ADR					Mascot
2199.0752	2199.1707	0.0955	43	274	294	DLYGNIVLSGGSTMFPGI ADR			Oxidation (M)[14]		Mascot
3151.6423	3151.7881	0.1458	46	130	159	TTGIVLDSGDGVSHTVPI YEGYALPHAILR					Mascot
3151.6423	3151.7881	0.1458	46	130	159	TTGIVLDSGDGVSHTVPI YEGYALPHAILR	52	99.253			Mascot

8	Actin-1 OS=Glycine max GN=SAC1 PE=3 SV=2	ACT1_SOYBN	41671.7	5.36	7	427	100	47.131	402	100	

Peptide Information

Calc. Mass	Obsrv. Mass	± da	± ppm	Start Seq.	End Seq.	Sequence	Ion Score	C. I. %	Modification	Rank	Result Type
1149.582	1149.6763	0.0943	82	318	328	EISALAPSSMK			Oxidation (M)[10]		Mascot
1198.7056	1198.7498	0.0442	37	31	41	AVFPSIVGRPR					Mascot
1198.7056	1198.7498	0.0442	37	31	41	AVFPSIVGRPR	43	94.913			Mascot
1515.7491	1515.8101	0.061	40	87	97	IWHHTFYNELR					Mascot
1515.7491	1515.8101	0.061	40	87	97	IWHHTFYNELR	75	99.997			Mascot
1747.8861	1747.9542	0.0681	39	241	256	SYELPDGQVITIGAER					Mascot
1747.8861	1747.9542	0.0681	39	241	256	SYELPDGQVITIGAER	135	100			Mascot
1954.0645	1954.1345	0.07	36	98	115	VAPEEHPVLLTEAPLNPK					Mascot
1954.0645	1954.1345	0.07	36	98	115	VAPEEHPVLLTEAPLNPK	97	100			Mascot

Calc. Mass	Obsrv. Mass	± da	± ppm	Start Seq.	End Seq.	Sequence	Ion Score	C. I. %	Modification	Rank	Result Type
1976.0448	1976.1086	0.0638	32	53	70	DAYVGDEAQVQRGILTLK					Mascot
3151.6423	3151.7881	0.1458	46	150	179	TTGIVLDSGDGVSHTVPI YEGYALPHAILR					Mascot
3151.6423	3151.7881	0.1458	46	150	179	TTGIVLDSGDGVSHTVPI YEGYALPHAILR	52	99.253			Mascot

9	Actin-58 OS=Solanum tuberosum GN=AC58 PE=3 SV=1	ACT3_SOLTU	41987.2	5.46	13	424	100	49.842	359	100	

Peptide Information

Calc. Mass	Obsrv. Mass	± da	± ppm	Start Seq.	End Seq.	Sequence	Ion Score	C. I. %	Modification	Rank	Result Type
895.5359	895.569	0.0331	37	331	338	VVAPPERK					Mascot
976.4483	976.486	0.0377	39	21	30	AGFAGDDAPR					Mascot
1132.527	1132.5756	0.0486	43	199	208	GYSFTTTAER					Mascot
1176.55	1176.6389	0.0889	76	42	52	HTGVMVGMGQK			Oxidation (M)[5,8]		Mascot
1182.5273	1182.587	0.0597	50	53	63	DAYVGDEAQSK					Mascot
1459.6813	1459.7402	0.0589	40	362	374	AEYDESGPSIVHR					Mascot
1515.7491	1515.8101	0.061	40	87	97	IWHHTFYNELR					Mascot
1515.7491	1515.8101	0.061	40	87	97	IWHHTFYNELR	75	99.997			Mascot
1587.7762	1587.8502	0.074	47	362	375	AEYDESGPSIVHRK					Mascot
1747.8861	1747.9542	0.0681	39	241	256	SYELPDGQVITIGAER					Mascot
1747.8861	1747.9542	0.0681	39	241	256	SYELPDGQVITIGAER	135	100			Mascot
1954.0645	1954.1345	0.07	36	98	115	VAPEEHPVLLTEAPLNPK					Mascot
1954.0645	1954.1345	0.07	36	98	115	VAPEEHPVLLTEAPLNPK	97	100			Mascot
2197.0959	2197.1804	0.0845	38	294	314	DLYGNIVLSGGTTMFPGI ADR					Mascot
2213.0908	2213.1753	0.0845	38	294	314	DLYGNIVLSGGTTMFPGI ADR			Oxidation (M)[14]		Mascot
2325.1909	2325.2661	0.0752	32	293	314	KDLYGNIVLSGGTTMFPG IADR					Mascot
2341.1858	2341.2668	0.081	35	293	314	KDLYGNIVLSGGTTMFPG IADR			Oxidation (M)[15]		Mascot
3151.6423	3151.7881	0.1458	46	150	179	TTGIVLDSGDGVSHTVPI YEGYALPHAILR					Mascot
3151.6423	3151.7881	0.1458	46	150	179	TTGIVLDSGDGVSHTVPI YEGYALPHAILR	52	99.253			Mascot

10	Actin-100 (Fragment) OS=Solanum tuberosum GN=AC100 PE=3 SV=1	ACT12_SOLTU	39756	5.56	15	414	100	52.078	327	100	

Peptide Information

Calc. Mass	Obsrv. Mass	± da	± ppm	Start Seq.	End Seq.	Sequence	Ion Score	C. I. %	Modification	Rank	Result Type
890.4764	890.4863	0.0099	11	189	195	EIVRDMK					Mascot
895.5359	895.569	0.0331	37	311	318	VVAPPERK					Mascot

976.4483	976.486	0.0377	39	1	10	AGFAGDDAPR				Mascot
1118.5114	1118.5577	0.0463	41	179	188	GYSFTTSAER				Mascot
1118.5114	1118.5577	0.0463	41	179	188	GYSFTTSAER	22	0		Mascot
1176.55	1176.6389	0.0889	76	22	32	HTGVMVGMGQK			Oxidation (M)[5,8]	Mascot
1182.5273	1182.587	0.0597	50	33	43	DAYVGDEAQSK				Mascot
1198.7056	1198.7498	0.0442	37	11	21	AVFPSIVGRPR				Mascot
1198.7056	1198.7498	0.0442	37	11	21	AVFPSIVGRPR	43	94.913		Mascot
1445.6655	1445.7344	0.0689	48	342	354	GEYDESGPSIVHR				Mascot
1515.7491	1515.8101	0.061	40	67	77	IWHHTFYNELR				Mascot
1515.7491	1515.8101	0.061	40	67	77	IWHHTFYNELR	75	99.997		Mascot
1573.7605	1573.8466	0.0861	55	342	355	GEYDESGPSIVHRK				Mascot
1747.8861	1747.9542	0.0681	39	221	236	SYELPDGQVITIGAER				Mascot
1747.8861	1747.9542	0.0681	39	221	236	SYELPDGQVITIGAER	135	100		Mascot
1855.9324	1855.9874	0.055	30	198	213	LAYVALDYEQELETAK				Mascot
2197.0959	2197.1804	0.0845	38	274	294	DLYGNIVLSGGTTMFPGI<br>ADR				Mascot
2213.0908	2213.1753	0.0845	38	274	294	DLYGNIVLSGGTTMFPGI<br>ADR			Oxidation (M)[14]	Mascot
2325.1909	2325.2661	0.0752	32	273	294	KDLYGNIVLSGGTTMFPG<br>IADR				Mascot
2341.1858	2341.2668	0.081	35	273	294	KDLYGNIVLSGGTTMFPG<br>IADR			Oxidation (M)[15]	Mascot
3151.6423	3151.7881	0.1458	46	130	159	TTGIVLDSGDGVSHTVPI<br>YEGYALPHAILR				Mascot
3151.6423	3151.7881	0.1458	46	130	159	TTGIVLDSGDGVSHTVPI<br>YEGYALPHAILR	52	99.253		Mascot
