# Supplementary material for: Proteomic analysis of middle and late stages of bread wheat (Triticum aestivum L.) grain development
Source: Front Plant Sci. 2015 Sep 15;6:735. doi: 10.3389/fpls.2015.00735 (PMC4569854; doi:10.3389/fpls.2015.00735)
Supplement: Supplementary file 7 [file DataSheet6.PDF]

Analysis Information

Report Type	Protein-Peptide Summary by Spot	Analysis Type	Combined (MS+MS/MS)
Sample Set Name	Sample set_20140814	Database	SwissProt
Analysis Name	BSA0929	Creation Date	09/30/2014 08:38:36
Reported By	09/30/2014 14:55:28 - admin	Last Modified	09/30/2014 09:08:18

MS Acq. : Proc. Methods	(Unspecified) : (Unspecified)
Interpretation Method	(Unspecified)

**Gel Idx/Pos**	240/J16	**Instr./Gel Origin**	BA2151/Sample Project 20140814	**Process Status**	Analysis Succeeded
**Plate [#] Name**	[1] Sample Project 20140814	**Instrument Sample Name**		**Spectra**	11

Rank	Protein Name	Accession No.	Protein MW	Protein PI	Pep. Count	Protein Score	Protein Score C. I. %	Intensity Matched	Total Ion Score	Total Ion C. I. %	Confirmed
1	Actin-51 (Fragment) OS=Solanum lycopersicum PE=3 SV=1	ACT2_SOLLC	37264.8	5.28	10	156	100	9.155	109	100	

Peptide Information

Calc. Mass	Obsrv. Mass	± da	± ppm	Start Seq.	End Seq.	Sequence	Ion Score	C. I. %	Modification	Rank	Result Type
800.5352	800.4624	-0.0728	-91	44	50	RGILTLK					Mascot
976.4483	976.4434	-0.0049	-5	1	10	AGFAGDDAPR					Mascot
1176.55	1176.594	0.044	37	22	32	HTGVMVGMGQK			Oxidation (M)[5,8]		Mascot
1198.7056	1198.6981	-0.0075	-6	11	21	AVFPSIVGRPR					Mascot
1515.7491	1515.7528	0.0037	2	67	77	IWHHTFYNELR					Mascot
1883.9385	1883.9622	0.0237	13	198	213	LAYVALDYEQEIETAR					Mascot
1883.9385	1883.9622	0.0237	13	198	213	LAYVALDYEQEIETAR	109	100			Mascot
1954.0645	1954.0579	-0.0066	-3	78	95	VAPEEHPVLLTEAPLNPK					Mascot
2141.0762	2140.9949	-0.0813	-38	196	213	EKLAYVALDYEQEIETAR					Mascot
2199.0752	2199.0608	-0.0144	-7	274	294	DLYGNIVLSGGSTMFPGI ADR			Oxidation (M)[14]		Mascot
3151.6423	3151.7039	0.0616	20	130	159	TTGIVLDSGDGVSHTVPI YEGYALPHAILR					Mascot

Rank	Protein Name	Accession No.	Protein MW	Protein PI	Pep. Count	Protein Score	Protein Score C. I. %	Intensity Matched	Total Ion Score	Total Ion C. I. %	Confirmed
2	Actin-54 (Fragment) OS=Nicotiana tabacum PE=3 SV=1	ACT3_TOBAC	37521.1	5.66	10	153	100	11.713	109	100	

Peptide Information

Calc. Mass	Obsrv. Mass	± da	± ppm	Start	End	Sequence	Ion	C. I. %	Modification	Rank	Result Type

				Seq.	Seq.		Score				
800.5352	800.4624	-0.0728	-91	44	50	RGILTLK					Mascot
976.4483	976.4434	-0.0049	-5	1	10	AGFAGDDAPR					Mascot
1176.55	1176.594	0.044	37	22	32	HTGVMVGMGQK			Oxidation (M)[5,8]		Mascot
1198.7056	1198.6981	-0.0075	-6	11	21	AVFPSIVGRPR					Mascot
1515.7491	1515.7528	0.0037	2	67	77	IWHHTFYNELR					Mascot
1547.8098	1547.7478	-0.062	-40	162	175	LDLAGRDLTDSLMK					Mascot
1547.8098	1547.7478	-0.062	-40	162	175	LDLAGRDLTDSLMK					Mascot
1883.9385	1883.9622	0.0237	13	200	215	LAYVALDYEQELETAR					Mascot
1883.9385	1883.9622	0.0237	13	200	215	LAYVALDYEQELETAR	109	100			Mascot
1954.0645	1954.0579	-0.0066	-3	78	95	VAPEEHPVLLTEAPLNPK					Mascot
2141.0762	2140.9949	-0.0813	-38	198	215	EKLAYVALDYEQELETAR					Mascot
2199.0752	2199.0608	-0.0144	-7	276	296	DLYGNIVLSGGSTMFPGI ADR			Oxidation (M)[14]		Mascot

3	Monodehydroascorbate reductase OS=Solanum lycopersicum GN=AFRR PE=1 SV=1	MDAR_SOLLC	47120.4	5.77	9	125	100	17.303	91	100	

Peptide Information

Calc. Mass	Obsrv. Mass	± da	± ppm	Start Seq.	End Seq.	Sequence	Ion Score	C. I. %	Modification	Rank	Result Type
978.4752	978.4617	-0.0135	-14	312	319	VEHVDHSR					Mascot
1430.7638	1430.762	-0.0018	-1	40	52	EAVAPYERPALSK					Mascot
1439.7642	1439.7705	0.0063	4	8	22	YVIVGGGVSAGYAAR					Mascot
1439.7642	1439.7705	0.0063	4	8	22	YVIVGGGVSAGYAAR	19	0			Mascot
1471.7112	1471.7186	0.0074	5	63	76	LPGFHVCVGSGGER			Carbamidomethyl (C)[7]		Mascot
1471.7112	1471.7186	0.0074	5	63	76	LPGFHVCVGSGGER	72	99.993	Carbamidomethyl (C)[7]		Mascot
1507.873	1507.7401	-0.1329	-88	115	128	YQTLVIATGTTVLK					Mascot
1984.0135	1983.9939	-0.0196	-10	225	243	GTVAVGFDTHPNGEVKE VK					Mascot
1984.0135	1983.9939	-0.0196	-10	225	243	GTVAVGFDTHPNGEVKE VK					Mascot
2100.0972	2100.1575	0.0603	29	413	432	VQPPATLDQLAQEGISFA SK					Mascot
2213.1812	2213.0962	-0.085	-38	413	433	VQPPATLDQLAQEGISFA SKI					Mascot
2685.3594	2685.5132	0.1538	57	287	310	TSVPDVYAVGDVATFPLK MYNEIR					Mascot

4	Monodehydroascorbate reductase, seedling isozyme OS=Cucumis sativus PE=2 SV=1	MDARS_CUCSA	47500.7	5.29	4	79	99.271	6.275	72	99.993	

Peptide Information

Calc. Mass	Obsrv. Mass	± da	± ppm	Start Seq.	End Seq.	Sequence	Ion Score	C. I. %	Modification	Rank	Result Type

Calc. Mass	Obsrv. Mass	± da	± ppm	Start Seq.	End Seq.	Sequence	Ion Score	C. I. %	Modification	Rank	Result Type
934.5032	934.4917	-0.0115	-12	77	83	LLPDWYK					Mascot
978.4752	978.4617	-0.0135	-14	312	319	VEHVDHSR					Mascot
1430.7638	1430.762	-0.0018	-1	40	52	EAVAPYERPALSK					Mascot
1471.7112	1471.7186	0.0074	5	63	76	LPGFHVCVGSGGER			Carbamidomethyl (C)[7]		Mascot
1471.7112	1471.7186	0.0074	5	63	76	LPGFHVCVGSGGER	72	99.993	Carbamidomethyl (C)[7]		Mascot

5 Actin-71 OS=Solanum tuberosum GN=AC71 PE=3 SV=2 | ACT6_SOLTU | 41983.1 | 5.38 | 14 | 74 | 97.64 | 12.039

Peptide Information

Calc. Mass	Obsrv. Mass	± da	± ppm	Start Seq.	End Seq.	Sequence	Ion Score	C. I. %	Modification	Rank	Result Type
800.5352	800.4624	-0.0728	-91	64	70	RGILTLK					Mascot
976.4483	976.4434	-0.0049	-5	21	30	AGFAGDDAPR					Mascot
1198.7056	1198.6981	-0.0075	-6	31	41	AVFPSIVGRPR					Mascot
1380.6754	1380.7014	0.026	19	53	64	DAYVVDEAQSKR					Mascot
1459.6813	1459.7009	0.0196	13	362	374	AEYDESGPSIVHR					Mascot
1515.7491	1515.7528	0.0037	2	87	97	IWHHTFYNELR					Mascot
1536.776	1536.7622	-0.0138	-9	315	328	MSKEIQALAPSSMK			Oxidation (M)[1]		Mascot
1601.817	1601.7889	-0.0281	-18	199	212	GYSFTTTAEKEIVR					Mascot
1623.8411	1623.776	-0.0651	-40	180	193	LDLAGRDLTEYMVK					Mascot
1639.8361	1639.9377	0.1016	62	180	193	LDLAGRDLTEYMVK			Oxidation (M)[12]		Mascot
1883.9637	1883.9622	-0.0015	-1	218	233	LAYLALDFEQELETTK					Mascot
1883.9637	1883.9622	-0.0015	-1	218	233	LAYLALDFEQELETTK					Mascot
1954.0645	1954.0579	-0.0066	-3	98	115	VAPEEHPVLLTEAPLNPK					Mascot
2141.1013	2140.9949	-0.1064	-50	216	233	EKLAYLALDFEQELETTK					Mascot
3151.6423	3151.7039	0.0616	20	150	179	TTGIVLDSGDGVSHTVPI YEGYALPHAILR					Mascot
3181.4236	3181.7122	0.2886	91	1	30	MADVEDIQPLVCDNGTG MVKAGFAGDDAPR			Carbamidomethyl (C)[12], Oxidation (M)[1,18]		Mascot
3181.4236	3181.7122	0.2886	91	1	30	MADVEDIQPLVCDNGTG MVKAGFAGDDAPR			Carbamidomethyl (C)[12], Oxidation (M)[1,18]		Mascot

6 Actin OS=Gossypium hirsutum PE=3 SV=1 | ACT_GOSHI | 41867 | 5.31 | 13 | 63 | 72.268 | 8.588

Peptide Information

Calc. Mass	Obsrv. Mass	± da	± ppm	Start Seq.	End Seq.	Sequence	Ion Score	C. I. %	Modification	Rank	Result Type
800.5352	800.4624	-0.0728	-91	64	70	RGILTLK					Mascot
976.4483	976.4434	-0.0049	-5	21	30	AGFAGDDAPR					Mascot
1132.527	1132.5295	0.0025	2	199	208	GYSFTTTAER					Mascot

1176.55	1176.594	0.044	37	42	52	HTGVMVGMGQK			Oxidation (M)[5,8]		Mascot
1198.7056	1198.6981	-0.0075	-6	31	41	AVFPSIVGRPR					Mascot
1459.6813	1459.7009	0.0196	13	362	374	AEYDESGPSIVHR					Mascot
1515.7491	1515.7528	0.0037	2	87	97	IWHHTFYNELR					Mascot
1531.8148	1531.7411	-0.0737	-48	180	193	LDLAGRDLTDALMK					Mascot
1547.8098	1547.7478	-0.062	-40	180	193	LDLAGRDLTDALMK			Oxidation (M)[13]		Mascot
1547.8098	1547.7478	-0.062	-40	180	193	LDLAGRDLTDALMK			Oxidation (M)[13]		Mascot
1747.8861	1747.8982	0.0121	7	241	256	SYELPDGQVITIGAER					Mascot
1954.0645	1954.0579	-0.0066	-3	98	115	VAPEEHPVLLTEAPLNPK					Mascot
1975.8855	1976.0479	0.1624	82	71	86	YPIEHGIVNNWDDMEK			Oxidation (M)[14]		Mascot
2197.0959	2197.0081	-0.0878	-40	294	314	DLYGNIVLSGGTTMFPGI ADR					Mascot
2213.0908	2213.0962	0.0054	2	294	314	DLYGNIVLSGGTTMFPGI ADR			Oxidation (M)[14]		Mascot
3151.6423	3151.7039	0.0616	20	150	179	TTGIVLDSGDGVSHTVPI YEGYALPHAILR					Mascot

7	Proline--tRNA ligase OS=Prochlorococcus marinus (strain NATL1A) GN=proS PE=3 SV=1	SYP_PROM1	68227.2	6.32	16	63	70.961	12.236	

Peptide Information

Calc. Mass	Obsrv. Mass	± da	± ppm	Start Seq.	End Seq.	Sequence	Ion Score	C. I. %	Modification	Rank	Result Type
809.3788	809.3802	0.0014	2	232	238	YGANQEK					Mascot
1098.5579	1098.516	-0.0419	-38	182	190	NAYQNIFTK					Mascot
1405.7369	1405.7806	0.0437	31	144	154	DEIRPRFGLMR			Oxidation (M)[10]		Mascot
1412.7897	1412.7198	-0.0699	-49	431	443	GIEIGHIFQLGTK					Mascot
1430.7791	1430.762	-0.0171	-12	542	553	FKDADLIGIPWR					Mascot
1439.7601	1439.7705	0.0104	7	559	571	EASSGLVELHNRK					Mascot
1439.7601	1439.7705	0.0104	7	559	571	EASSGLVELHNRK					Mascot
1641.871	1641.8778	0.0068	4	35	48	ITGGIYAYMPLLWK			Oxidation (M)[9]		Mascot
1738.8065	1738.8892	0.0827	48	508	521	NNDQKCLAEDIYQK			Carbamidomethyl (C)[6]		Mascot
1746.0061	1745.9579	-0.0482	-28	130	143	QLPINIFQIQTKFR					Mascot
1747.832	1747.8982	0.0662	38	88	102	SYTQGEGIMFSLKDR			Oxidation (M)[9]		Mascot
1763.9109	1763.891	-0.0199	-11	513	526	CLAEDIYQKLIQNR			Carbamidomethyl (C)[1]		Mascot
1791.96	1791.9362	-0.0238	-13	13	28	DVPSEADIISHQLLVR					Mascot
1797.972	1797.8264	-0.1456	-81	34	48	RITGGIYAYMPLLWK			Oxidation (M)[10]		Mascot
1963.9154	1964.0367	0.1213	62	457	473	GIEDHLWMGCYGIGISR			Carbamidomethyl (C)[10]		Mascot
1979.9103	1980.0222	0.1119	57	457	473	GIEDHLWMGCYGIGISR			Carbamidomethyl (C)[10], Oxidation (M)[8]		Mascot
1982.1184	1981.9966	-0.1218	-61	35	51	ITGGIYAYMPLLWKVLK			Oxidation (M)[9]		Mascot

2100.0906	2100.1575	0.0669	32	426	443	LEECRGIEIGHIFQLGTK			Carbamidomethyl (C)[4]		Mascot

8	CoB--CoM heterodisulfide reductase iron-sulfur subunit A OS=Methanothermobacter marburgensis (strain DSM 2133 / 14651 / NBRC 100331 / OCM 82 / Marburg) GN=hdrA PE=1 SV=3	HDRA_METTM	73731.3	4.87	16	62	67.418	3.766

Peptide Information

Calc. Mass	Obsrv. Mass	± da	± ppm	Start Seq.	End Seq.	Sequence	Ion Score	C. I. %	Modification	Rank	Result Type
999.572	999.5921	0.0201	20	139	147	LLEPLEASK					Mascot
1002.5764	1002.5576	-0.0188	-19	128	136	DLVRMAVAK					Mascot
1057.6001	1057.5852	-0.0149	-14	65	73	DIKELGINR					Mascot
1412.7129	1412.7198	0.0069	5	553	567	DIPDAVAQASGAAAR					Mascot
1600.7312	1600.7819	0.0507	32	52	64	YYCSDPGQLEIQK			Carbamidomethyl (C)[3]		Mascot
1640.8901	1640.8713	-0.0188	-11	68	82	ELGINRVVVAACSPR			Carbamidomethyl (C)[12]		Mascot
1838.9695	1838.9847	0.0152	8	74	89	VVVAACSPRLHEPTFR			Carbamidomethyl (C)[6]		Mascot
1943.9742	1943.9467	-0.0275	-14	642	659	TEQIMAQIEAALNEPASK					Mascot
1981.8345	1981.9966	0.1621	82	109	125	EHDSWVHMDNPEGATE K					Mascot
2005.9246	2005.9744	0.0498	25	424	439	DKMPDTEVTLYYMDIR			Oxidation (M)[3]		Mascot
2046.0325	2046.082	0.0495	24	513	531	QTIGLSKSADGFLMEAHP K			Oxidation (M)[14]		Mascot
2170.1868	2170.0986	-0.0882	-41	532	552	LRPVDTLTDGVYLAGVA QGPK					Mascot
2187.1843	2187.0613	-0.123	-56	154	176	ALVIGGGVAGIQAALDLA DMGFK					Mascot
2196.9614	2197.0081	0.0467	21	109	127	EHDSWVHMDNPEGATE KAK			Oxidation (M)[8]		Mascot
2313.1445	2313.1672	0.0227	10	90	108	RCVEEAGLNQFLFEFANI R			Carbamidomethyl (C)[2]		Mascot
2442.1536	2442.3828	0.2292	94	190	210	MGQLDKTFPTLDCSMCIL APK			Carbamidomethyl (C)[13,16], Oxidation (M)[1]		Mascot

9	Flagellum-specific ATP synthase OS=Salmonella typhimurium (strain LT2 / SGSC1412 / ATCC 700720) GN=fliI PE=1 SV=1	FLII_SALTY	49291	6.05	13	62	61.719	15.633

Peptide Information

Calc. Mass	Obsrv. Mass	± da	± ppm	Start Seq.	End Seq.	Sequence	Ion Score	C. I. %	Modification	Rank	Result Type
978.5367	978.4617	-0.075	-77	393	400	QLLSSFQR					Mascot
1380.7053	1380.7014	-0.0039	-3	175	188	GQRMGLFAGSGVGK			Oxidation (M)[4]		Mascot
1429.729	1429.6914	-0.0376	-26	189	200	SVLLGMMARYTR			Oxidation (M)[6,7]		Mascot
1453.8486	1453.7487	-0.0999	-69	201	214	ADVIVVGLIGERGR					Mascot
1507.8843	1507.7401	-0.1442	-96	232	246	SVVIAAPADVSPLLR					Mascot

1775.9221	1775.9221	0	0	375	389	AMTALITEQHYARVR			Oxidation (M)[2]		Mascot
1865.9314	1865.9912	0.0598	32	403	420	DLVSVGAYAKGSDPMLD K					Mascot
1881.9263	1882.0273	0.101	54	403	420	DLVSVGAYAKGSDPMLD K			Oxidation (M)[15]		Mascot
1884.0299	1883.9622	-0.0677	-36	77	93	LFLMPLEEVEGILPGAR					Mascot
1884.0299	1883.9622	-0.0677	-36	77	93	LFLMPLEEVEGILPGAR					Mascot
1982.0385	1981.9966	-0.0419	-21	178	197	MGLFAGSGVGKSVLLGM MAR					Mascot
2046.0688	2046.082	0.0132	6	277	295	YAMAQREIALAIGEPPAT K			Oxidation (M)[3]		Mascot
2159.1682	2158.9907	-0.1775	-82	8	26	WLTALDNFEAKMALLPAV R					Mascot
2159.1682	2158.9907	-0.1775	-82	8	26	WLTALDNFEAKMALLPAV R					Mascot
2185.1836	2185.071	-0.1126	-52	98	119	NGHGDGLQSGKQLPLGP ALLGR					Mascot
3151.6667	3151.7039	0.0372	12	34	63	ATGLVLEATGLQLPLGAT CIIERQDGPETK			Carbamidomethyl (C)[19]		Mascot

10	Actin-1 OS=Oryza sativa subsp. japonica GN=ACT1 PE=2 SV=1	ACT1_ORYSJ	42014	5.3	13	62	61.719	5.55

Protein Group

Actin-1 OS=Oryza sativa subsp. indica GN=ACT1 PE=1 SV=1	ACT1_ORYSI	42014	5.3000 001907 3486

Peptide Information

Calc. Mass	Obsrv. Mass	± da	± ppm	Start Seq.	End Seq.	Sequence	Ion Score	C. I. %	Modification	Rank	Result Type
800.5352	800.4624	-0.0728	-91	64	70	RGILTLK					Mascot
976.4483	976.4434	-0.0049	-5	21	30	AGFAGDDAPR					Mascot
1132.527	1132.5295	0.0025	2	199	208	GYSFTTTAER					Mascot
1176.55	1176.594	0.044	37	42	52	HTGVMVGMGQK			Oxidation (M)[5,8]		Mascot
1198.7056	1198.6981	-0.0075	-6	31	41	AVFPSIVGRPR					Mascot
1459.6813	1459.7009	0.0196	13	362	374	AEYDESGPSIVHR					Mascot
1515.7491	1515.7528	0.0037	2	87	97	IWHHTFYNELR					Mascot
1623.8411	1623.776	-0.0651	-40	180	193	LDLAGRDLTDYLMK					Mascot
1639.8361	1639.9377	0.1016	62	180	193	LDLAGRDLTDYLMK			Oxidation (M)[13]		Mascot
1747.8861	1747.8982	0.0121	7	241	256	SYELPDGQVITIGAER					Mascot
1905.8787	1905.9282	0.0495	26	218	233	LSYIALDYDQEMETAK			Oxidation (M)[12]		Mascot
1954.0645	1954.0579	-0.0066	-3	98	115	VAPEEHPVLLTEAPLNPK					Mascot
2197.0959	2197.0081	-0.0878	-40	294	314	DLYGNIVLSGGTTMFPGI ADR					Mascot
2213.0908	2213.0962	0.0054	2	294	314	DLYGNIVLSGGTTMFPGI ADR			Oxidation (M)[14]		Mascot
3151.6423	3151.7039	0.0616	20	150	179	TTGIVLDSGDGVSHTVPI YEGYALPHAILR					Mascot

Gel Idx/Pos	241/J17	Instr./Gel Origin	BA2151/Sample Project 20140814	Process Status	Analysis Succeeded
Plate [#] Name	[1] Sample Project 20140814	Instrument Sample Name		Spectra	11

Rank	Protein Name	Accession No.	Protein MW	Protein PI	Pep. Count	Protein Score	Protein Score C. I. %	Intensity Matched	Total Ion Score	Total Ion C. I. %	Confirmed
1	Photosystem I assembly protein Ycf4 OS=Huperzia lucidula GN=ycf4 PE=3 SV=1	YCF4_HUPLU	21491.1	9.02	7	37	0	1.796			

Peptide Information

Calc. Mass	Obsrv. Mass	± da	± ppm	Start Seq.	End Seq.	Sequence	Ion Score	C. I. %	Modification	Rank	Result Type
870.5229	870.5418	0.0189	22	141	147	VLHMKVK			Oxidation (M)[4]		Mascot
945.5363	945.5093	-0.027	-29	157	164	ISENLTLR					Mascot
962.4479	962.3818	-0.0661	-69	107	114	WGFPGENR					Mascot
1016.487	1016.5295	0.0425	42	176	184	FLHVSMEGP					Mascot
1118.5491	1118.5861	0.037	33	107	115	WGFPGENRR					Mascot
1265.5732	1265.6566	0.0834	66	1	9	MNWQSEWLR			Oxidation (M)[1]		Mascot
1627.8262	1627.8523	0.0261	16	170	184	AAELARFLHVSMEGP					Mascot

Rank	Protein Name	Accession No.	Protein MW	Protein PI	Pep. Count	Protein Score	Protein Score C. I. %	Intensity Matched	Total Ion Score	Total Ion C. I. %	Confirmed
2	50S ribosomal protein L6 OS=Streptomyces avermitilis (strain ATCC 31267 / DSM 46492 / JCM 5070 / NCIMB 12804 / NRRL 8165 / MA-4680) GN=rplF PE=3 SV=1	RL6_STRAW	19205.5	9.92	6	31	0	1.049			

Peptide Information

Calc. Mass	Obsrv. Mass	± da	± ppm	Start Seq.	End Seq.	Sequence	Ion Score	C. I. %	Modification	Rank	Result Type
856.5251	856.5344	0.0093	11	23	30	TVQVKGPK					Mascot
1016.5887	1016.5295	-0.0592	-58	152	159	LRKPDPYK					Mascot
1149.6263	1149.5764	-0.0499	-43	162	171	GVKYEGEVIR					Mascot
1507.8479	1507.7552	-0.0927	-61	31	45	GSLSHTIAAPIEIAK					Mascot
1547.8064	1547.699	-0.1074	-69	126	139	VEAPTRFSVEGIDK					Mascot
1839.0045	1838.9272	-0.0773	-42	71	87	TLVANMITGVTQGYVKK			Oxidation (M)[6]		Mascot

Rank	Protein Name	Accession No.	Protein MW	Protein PI	Pep. Count	Protein Score	Protein Score C. I. %	Intensity Matched	Total Ion Score	Total Ion C. I. %	Confirmed
3	Mitochondrial uncoupling protein 2 OS=Cyprinus carpio GN=ucp2 PE=2 SV=1	UCP2_CYPCA	33909.2	9.62	8	29	0	2.741			

Peptide Information

Calc. Mass	Obsrv. Mass	± da	± ppm	Start Seq.	End Seq.	Sequence	Ion Score	C. I. %	Modification	Rank	Result Type
838.4022	838.4088	0.0066	8	297	303	RAMMAAR			Oxidation (M)[3,4]		Mascot
925.456	925.5131	0.0571	62	90	97	QMSFASVR					Mascot

	1050.5579	1050.5026	-0.0553	-53	166	174	TIAKEEGFR					Mascot
	1118.6317	1118.5861	-0.0456	-41	49	59	IPVNTGHGPVK					Mascot
	1263.7056	1263.7179	0.0123	10	78	89	SLYSGLVAGLQR					Mascot
	1479.7454	1479.7269	-0.0185	-13	269	280	AFYKGFMPSFLR			Oxidation (M)[7]		Mascot
	1665.8344	1665.8687	0.0343	21	106	120	QFYTKGSEHVGIGSR					Mascot
	2186.1387	2186.0127	-0.126	-58	78	97	SLYSGLVAGLQRQMSFA SVR			Oxidation (M)[14]		Mascot

4	Peptide chain release factor 1 OS=Syntrophomonas wolfei subsp. wolfei (strain DSM 2245B / Goettingen) GN=prfA PE=3 SV=1	RF1_SYNWW	40319.6	5.19	8	28	0	2.574	

Peptide Information

Calc. Mass	Obsrv. Mass	± da	± ppm	Start Seq.	End Seq.	Sequence	Ion Score	C. I. %	Modification	Rank	Result Type
831.4108	831.3587	-0.0521	-63	176	182	FESGVHR					Mascot
832.3907	832.3174	-0.0733	-88	294	301	NQVGSGDR					Mascot
847.4706	847.4102	-0.0604	-71	109	115	SVIMEIR					Mascot
945.4999	945.5093	0.0094	10	89	96	ASLEQELR					Mascot
1479.7261	1479.7269	0.0008	1	37	49	AHSALSDIVTMYR			Oxidation (M)[11]		Mascot
1547.6947	1547.699	0.0043	3	133	144	MYSRYAEEQGWK					Mascot
1570.6577	1570.7826	0.1249	80	63	74	EMLVEEEDEEFR			Oxidation (M)[2]		Mascot
1883.932	1883.9219	-0.0101	-5	37	52	AHSALSDIVTMYREYK					Mascot

5	Diaminopimelate epimerase OS=Rhodopseudomonas palustris (strain TIE-1) GN=dapF PE=1 SV=1	DAPF_RHOPT	32168.9	5.09	7	28	0	2.078

Peptide Information

Calc. Mass	Obsrv. Mass	± da	± ppm	Start Seq.	End Seq.	Sequence	Ion Score	C. I. %	Modification	Rank	Result Type
801.4036	801.3715	-0.0321	-40	207	212	QHITMR			Oxidation (M)[5]		Mascot
989.5414	989.5205	-0.0209	-21	56	64	LPGTEAFVR					Mascot
1118.5953	1118.5861	-0.0092	-8	213	222	TWERGAGLTK					Mascot
1265.6671	1265.6566	-0.0105	-8	1	11	MSALDNRLFAK					Mascot
1373.6743	1373.7026	0.0283	21	207	216	QHITMRTWER			Oxidation (M)[5]		Mascot
1665.8748	1665.8687	-0.0061	-4	182	195	FGPLLENHPIFPER					Mascot
2203.1177	2203.1479	0.0302	14	241	259	TDRTVEMTLPGGQLTIEW R					Mascot

6	Diaminopimelate epimerase OS=Rhodopseudomonas palustris (strain ATCC BAA-98 / CGA009) GN=dapF PE=1 SV=1	DAPF_RHOPA	32149.9	5.18	7	28	0	2.078

Peptide Information

Calc. Mass	Obsrv. Mass	± da	± ppm	Start Seq.	End Seq.	Sequence	Ion Score	C. I. %	Modification	Rank	Result Type
801.4036	801.3715	-0.0321	-40	207	212	QHITMR			Oxidation (M)[5]		Mascot
989.4985	989.5205	0.022	22	102	109	AGLLNCWR			Carbamidomethyl (C)[6]		Mascot
1118.5953	1118.5861	-0.0092	-8	213	222	TWERGAGLTK					Mascot
1265.6671	1265.6566	-0.0105	-8	1	11	MSALDNRLFAK					Mascot
1373.6743	1373.7026	0.0283	21	207	216	QHITMRTWER			Oxidation (M)[5]		Mascot
1665.8748	1665.8687	-0.0061	-4	182	195	FGPLLENHPIFPER					Mascot
2203.1177	2203.1479	0.0302	14	241	259	TDRTVEMTLPGGQLTIEW R					Mascot

7 Potassium channel toxin alpha-KTx 2.3 OS=Centruroides limpidus limpidus PE=1 SV=1 KAX23_CENLL 4536.2 9.3 3 28 0 .675

Peptide Information

Calc. Mass	Obsrv. Mass	± da	± ppm	Start Seq.	End Seq.	Sequence	Ion Score	C. I. %	Modification	Rank	Result Type
801.4002	801.3715	-0.0287	-36	21	28	FGQHAGGK					Mascot
1373.6743	1373.7026	0.0283	21	21	33	FGQHAGGKCINGK			Carbamidomethyl (C)[9]		Mascot
2203.1145	2203.1479	0.0334	15	1	18	ITINVKCTSPQQCLRPCK			Carbamidomethyl (C)[7,13,17]		Mascot

8 Transcription elongation factor A protein-like 7 OS=Homo sapiens GN=TCEAL7 PE=2 SV=2 TCAL7_HUMAN 12487.2 8.46 6 26 0 2.072

Peptide Information

Calc. Mass	Obsrv. Mass	± da	± ppm	Start Seq.	End Seq.	Sequence	Ion Score	C. I. %	Modification	Rank	Result Type
801.4101	801.3715	-0.0386	-48	7	13	ENEGKPK					Mascot
807.3851	807.3203	-0.0648	-80	1	6	MQKPCK			Carbamidomethyl (C)[5], Oxidation (M)[1]		Mascot
1263.6189	1263.7179	0.099	78	31	40	QQTEGNFRQR					Mascot
1372.689	1372.7269	0.0379	28	7	18	ENEGKPKCSVPK			Carbamidomethyl (C)[8]		Mascot
1439.6914	1439.7367	0.0453	31	20	30	EEKRPYGEFER					Mascot
1665.7207	1665.8687	0.148	89	65	77	EGDEMERCLEEIR			Carbamidomethyl (C)[8]		Mascot

9 SPBc2 prophage-derived uncharacterized protein YorZ OS=Bacillus subtilis (strain 168) GN=yorZ PE=4 SV=1 YORZ_BACSU 8390.1 8.48 4 26 0 1.756

Peptide Information

Calc. Mass	Obsrv. Mass	± da	± ppm	Start Seq.	End Seq.	Sequence	Ion Score	C. I. %	Modification	Rank	Result Type
1050.4884	1050.5026	0.0142	14	46	54	NECSNLSVK			Carbamidomethyl (C)[3]		Mascot
1265.7576	1265.6566	-0.101	-80	55	65	KTLEHLGLNIK					Mascot

1736.8094	1736.7794	-0.03	-17	2	15	NHICDICKEYISGK	Carbamidomethyl (C)[4,7]		Mascot
1883.8448	1883.9219	0.0771	41	1	15	MNHICDICKEYISGK	Carbamidomethyl (C)[5,8], Oxidation (M)[1]		Mascot

10	Adenylate kinase OS=Thermosipho africanus (strain TCF52B) GN=adk PE=3 SV=1	KAD_THEAB	24650.7	7.55	7	26	0	1.96	

Peptide Information

Calc. Mass	Obsrv. Mass	± da	± ppm	Start Seq.	End Seq.	Sequence	Ion Score	C. I. %	Modification	Rank	Result Type
838.3546	838.4088	0.0542	65	129	135	ICSNCGK			Carbamidomethyl (C)[2,5]		Mascot
847.4341	847.4102	-0.0239	-28	95	101	ALDEIMR					Mascot
856.525	856.5344	0.0094	11	207	213	EVLNIIR					Mascot
1118.6052	1118.5861	-0.0191	-17	37	47	EAVASKSELGK					Mascot
1570.9203	1570.7826	-0.1377	-88	136	149	IYNLITLPPKVDGK					Mascot
1838.9292	1838.9272	-0.002	-1	1	18	MNMVFLGPPGAGKGTYAK					Mascot
2159.1101	2158.9312	-0.1789	-83	19	36	RLIEMLNIPHISTGDMFR			Oxidation (M)[5]		Mascot

Gel Idx/Pos	242/J18	Instr./Gel Origin	BA2151/Sample Project 20140814	Process Status	Analysis Succeeded
Plate [#] Name	[1] Sample Project 20140814	Instrument Sample Name		Spectra	11

Rank	Protein Name	Accession No.	Protein MW	Protein PI	Pep. Count	Protein Score	Protein Score C. I. %	Intensity Matched	Total Ion Score	Total Ion C. I. %	Confirmed
1	Serpin-Z2B OS=Triticum aestivum PE=1 SV=1	SPZ2B_WHEAT	43011.4	5.18	15	577	100	41.952	494	100	

Peptide Information

Calc. Mass	Obsrv. Mass	± da	± ppm	Start Seq.	End Seq.	Sequence	Ion Score	C. I. %	Modification	Rank	Result Type
860.5604	860.4865	-0.0739	-86	227	233	VLKLPYK					Mascot
925.5214	925.5234	0.002	2	11	18	LSIAHQTR					Mascot
925.5214	925.5234	0.002	2	11	18	LSIAHQTR	60	99.89			Mascot
947.5156	947.5029	-0.0127	-13	2	10	ATTLATDVR					Mascot
1078.5562	1078.6249	0.0687	64	1	10	MATTLATDVR					Mascot
1137.6667	1137.6506	-0.0161	-14	172	181	LVLGNALYFK					Mascot
1192.5382	1192.5416	0.0034	3	182	191	GAWTDQFDPR					Mascot
1192.5382	1192.5416	0.0034	3	182	191	GAWTDQFDPR	44	96.081			Mascot
1223.5903	1223.5654	-0.0249	-20	127	137	AEAQSVDFQTK					Mascot
1258.7253	1258.7069	-0.0184	-15	289	300	ISLGIEASDLLK					Mascot
1372.7068	1372.7174	0.0106	8	159	171	DILPAGSIDNTTR					Mascot
1372.7068	1372.7174	0.0106	8	159	171	DILPAGSIDNTTR	99	100			Mascot
1514.7485	1514.7397	-0.0088	-6	125	137	YKAEAQSVDFQTK					Mascot
1665.8595	1665.8872	0.0277	17	261	274	LSAEPEFLEQHIPR					Mascot
1665.8595	1665.8872	0.0277	17	261	274	LSAEPEFLEQHIPR	118	100			Mascot
1922.9706	1922.9829	0.0123	6	335	353	AFVEVNETGTEAAATTIAK					Mascot
2083.1072	2083.1067	-0.0005	0	379	398	EDTSGVVLFIGHVVNPLLSS					Mascot
2838.4858	2838.5356	0.0498	18	99	124	VAFANGVFVDASLQLKPSFQELAVCK			Carbamidomethyl (C)[25]		Mascot
3751.9614	3752.0615	0.1001	27	23	61	LASAISSNPESTVNNAAFSPVSLHVALSLITAGAGGATR					Mascot
3751.9614	3752.0615	0.1001	27	23	61	LASAISSNPESTVNNAAFSPVSLHVALSLITAGAGGATR	174	100			Mascot

Rank	Protein Name	Accession No.	Protein MW	Protein PI	Pep. Count	Protein Score	Protein Score C. I. %	Intensity Matched	Total Ion Score	Total Ion C. I. %	Confirmed
2	Serpin-Z1B OS=Triticum aestivum PE=1 SV=1	SPZ1B_WHEAT	43119.9	5.44	14	408	100	16.349	336	100	

Peptide Information

Calc. Mass	Obsrv. Mass	± da	± ppm	Start Seq.	End Seq.	Sequence	Ion Score	C. I. %	Modification	Rank	Result Type

Calc. Mass	Obsrv. Mass	± da	± ppm	Start Seq.	End Seq.	Sequence	Ion Score	C. I. %	Modification	Rank	Result Type
806.4744	806.4302	-0.0442	-55	272	277	HIPRQR					Mascot
860.5604	860.4865	-0.0739	-86	228	234	VLKLPYK					Mascot
925.5214	925.5234	0.002	2	11	18	LSIAHQTR					Mascot
925.5214	925.5234	0.002	2	11	18	LSIAHQTR	60	99.89			Mascot
947.5156	947.5029	-0.0127	-13	2	10	ATTLATDVR					Mascot
1078.5562	1078.6249	0.0687	64	1	10	MATTLATDVR					Mascot
1151.6824	1151.661	-0.0214	-19	172	181	LVLANALYFK					Mascot
1176.5896	1176.5983	0.0087	7	262	271	LSAEPDFLER					Mascot
1176.5896	1176.5983	0.0087	7	262	271	LSAEPDFLER	82	100			Mascot
1345.6958	1345.7329	0.0371	28	159	171	NILPSGSVDNTTK					Mascot
1544.7592	1544.7599	0.0007	0	125	137	YKAETQSVDFQTK					Mascot
1561.7856	1561.7687	-0.0169	-11	138	151	AAEVTTQVNSWVEK					Mascot
1585.8295	1585.7592	-0.0703	-44	288	301	FKISFGMEASDLLK					Mascot
2685.3955	2685.448	0.0525	20	33	61	SAASNAAFSPVSLHSALS LLAAGAGSATR					Mascot
2685.3955	2685.448	0.0525	20	33	61	SAASNAAFSPVSLHSALS LLAAGAGSATR	195	100			Mascot
2720.3525	2720.3938	0.0413	15	329	354	VSSVFHQAFVEVNEQGT EAAASTAIK					Mascot
3071.3796	3071.4233	0.0437	14	302	328	CLGLQLPFSDEADFSEM VDSPMPQGLR			Carbamidomethyl (C)[1], Oxidation (M)[17,22]		Mascot

3	Serpin-Z1A OS=Triticum aestivum GN=WZCI PE=1 SV=1	SPZ1A_WHEAT	43262.2	5.6	11	185	100	11.72	141	100	

Peptide Information

Calc. Mass	Obsrv. Mass	± da	± ppm	Start Seq.	End Seq.	Sequence	Ion Score	C. I. %	Modification	Rank	Result Type
806.4744	806.4302	-0.0442	-55	271	276	HIPRQR					Mascot
860.5604	860.4865	-0.0739	-86	227	233	VLKLPYK					Mascot
925.5214	925.5234	0.002	2	11	18	LSIAHQTR					Mascot
925.5214	925.5234	0.002	2	11	18	LSIAHQTR	60	99.89			Mascot
947.5156	947.5029	-0.0127	-13	2	10	ATTLATDVR					Mascot
1078.5562	1078.6249	0.0687	64	1	10	MATTLATDVR					Mascot
1151.6824	1151.661	-0.0214	-19	172	181	LVLANALYFK					Mascot
1176.5896	1176.5983	0.0087	7	261	270	LSAEPDFLER					Mascot
1176.5896	1176.5983	0.0087	7	261	270	LSAEPDFLER	82	100			Mascot
1292.7097	1292.6886	-0.0211	-16	289	300	ISFGIEASDLLK					Mascot
1544.7592	1544.7599	0.0007	0	125	137	YKAETQSVDFQTK					Mascot
1561.7856	1561.7687	-0.0169	-11	138	151	AAEVTTQVNSWVEK					Mascot
2720.3525	2720.3938	0.0413	15	328	353	VSSVFHQAFVEVNEQGT EAAASTAIK					Mascot

4	Serpin-Z1C OS=Triticum aestivum PE=1 SV=1	SPZ1C_WHEAT	42969	5.62	8	166	100	11.179	141	100	

Peptide Information

Calc. Mass	Obsrv. Mass	± da	± ppm	Start Seq.	End Seq.	Sequence	Ion Score	C. I. %	Modification	Rank	Result Type
806.4744	806.4302	-0.0442	-55	271	276	HIPRQR					Mascot
860.5604	860.4865	-0.0739	-86	227	233	VLKLPYK					Mascot
925.5214	925.5234	0.002	2	11	18	LSIAHQTR					Mascot
925.5214	925.5234	0.002	2	11	18	LSIAHQTR	60	99.89			Mascot
947.5156	947.5029	-0.0127	-13	2	10	ATTLATDVR					Mascot
1078.5562	1078.6249	0.0687	64	1	10	MATTLATDVR					Mascot
1151.6824	1151.661	-0.0214	-19	172	181	LVLANALYFK					Mascot
1176.5896	1176.5983	0.0087	7	261	270	LSAEPDFLER					Mascot
1176.5896	1176.5983	0.0087	7	261	270	LSAEPDFLER	82	100			Mascot
2720.3525	2720.3938	0.0413	15	328	353	VSSVFHQAFVEVNEQGT EAAASTAIK					Mascot

5	Serpin-Z2A OS=Triticum aestivum PE=1 SV=1	SPZ2A_WHEAT	43341.5	5.46	9	89	99.935	7.267	60	99.89	

Peptide Information

Calc. Mass	Obsrv. Mass	± da	± ppm	Start Seq.	End Seq.	Sequence	Ion Score	C. I. %	Modification	Rank	Result Type
860.5604	860.4865	-0.0739	-86	227	233	VLKLPYK					Mascot
925.5214	925.5234	0.002	2	11	18	LSIAHQTR					Mascot
925.5214	925.5234	0.002	2	11	18	LSIAHQTR	60	99.89			Mascot
947.5156	947.5029	-0.0127	-13	2	10	ATTLATDVR					Mascot
1078.5562	1078.6249	0.0687	64	1	10	MATTLATDVR					Mascot
1137.6667	1137.6506	-0.0161	-14	172	181	LVLGNALYFK					Mascot
1223.5903	1223.5654	-0.0249	-20	127	137	AEAQSVDFQTK					Mascot
1292.7097	1292.6886	-0.0211	-16	289	300	ISFGIEASDLLK					Mascot
1514.7485	1514.7397	-0.0088	-6	125	137	YKAEAQSVDFQTK					Mascot
1922.9706	1922.9829	0.0123	6	335	353	TFVEVNETGTEAAAATIA K					Mascot

6	Serpin-Z4 OS=Hordeum vulgare GN=PAZ1 PE=1 SV=2	SPZ4_HORVU	43363.4	5.72	6	72	96.888	6.222	60	99.89	

Peptide Information

Calc. Mass	Obsrv. Mass	± da	± ppm	Start Seq.	End Seq.	Sequence	Ion Score	C. I. %	Modification	Rank	Result Type
840.3734	840.4317	0.0583	69	190	196	FDESNTK					Mascot
907.54	907.4628	-0.0772	-85	284	290	FQLPKFK					Mascot

Calc. Mass	Obsrv. Mass	± da	± ppm	Start Seq.	End Seq.	Sequence	Ion Score	C. I. %	Modification	Rank	Result Type
925.5214	925.5234	0.002	2	11	18	LSIAHQTR					Mascot
925.5214	925.5234	0.002	2	11	18	LSIAHQTR	60	99.89			Mascot
947.5156	947.5029	-0.0127	-13	2	10	ATTLATDVR					Mascot
1078.5562	1078.6249	0.0687	64	1	10	MATTLATDVR					Mascot
1151.6824	1151.661	-0.0214	-19	174	183	LILGNALYFK					Mascot

7	Serpin-ZX OS=Hordeum vulgare GN=PAZX PE=1 SV=1	SPZX_HORVU	42920.3	6.77	4	69	93.193	6.16	60	99.89

Peptide Information

Calc. Mass	Obsrv. Mass	± da	± ppm	Start Seq.	End Seq.	Sequence	Ion Score	C. I. %	Modification	Rank	Result Type
925.5214	925.5234	0.002	2	8	15	LSIAHQTR					Mascot
925.5214	925.5234	0.002	2	8	15	LSIAHQTR	60	99.89			Mascot
1137.6667	1137.6506	-0.0161	-14	171	180	LVLGNALYFK					Mascot
1345.7474	1345.7329	-0.0145	-11	226	237	VLKLPYQQGGDK					Mascot
2734.332	2734.4077	0.0757	28	126	150	GETQSVDFQTKAPEVAG QVNSWVEK					Mascot

8	Homeobox protein Hox-D11 OS=Notophthalmus viridescens GN=HOXD11 PE=2 SV=1	HXD11_NOTVI	32047.8	9.45	13	67	89.931	6.683		

Peptide Information

Calc. Mass	Obsrv. Mass	± da	± ppm	Start Seq.	End Seq.	Sequence	Ion Score	C. I. %	Modification	Rank	Result Type
866.4267	866.4454	0.0187	22	64	69	EYGWRR					Mascot
925.5101	925.5234	0.0133	14	170	177	ITHSPDKK					Mascot
925.5101	925.5234	0.0133	14	170	177	ITHSPDKK					Mascot
947.4541	947.5029	0.0488	52	196	204	SNSSATPQR					Mascot
1132.5858	1132.5553	-0.0305	-27	91	99	DFIQPSNRR					Mascot
1146.5273	1146.5642	0.0369	32	184	195	ADSPSGEVAADK					Mascot
1190.5873	1190.5728	-0.0145	-12	196	206	SNSSATPQRSR					Mascot
1218.6742	1218.6333	-0.0409	-34	248	256	QVKIWFQNR					Mascot
1320.6624	1320.632	-0.0304	-23	223	232	EFFFNVYINK					Mascot
1577.7999	1577.7871	-0.0128	-8	223	234	EFFFNVYINKEK					Mascot
1703.7911	1703.826	0.0349	20	76	90	GSYPSYYPSEEVVAR					Mascot
1747.7916	1747.907	0.1154	66	178	195	MGAEGRADSPSGEVAAD K					Mascot
2074.9636	2075.0747	0.1111	54	184	204	ADSPSGEVAADKSNSSA TPQR					Mascot
3071.5005	3071.4233	-0.0772	-25	100	128	SDVLFKADPLCAHHGTP SAASNLYSTVGR			Carbamidomethyl (C)[11]		Mascot

9	Serpin-Z7 OS=Hordeum vulgare GN=PAZ7 PE=1 SV=2	BSZ7_HORVU	42851.2	5.45	4	67	89.697	6.156	60	99.89

Peptide Information

Calc. Mass	Obsrv. Mass	± da	± ppm	Start Seq.	End Seq.	Sequence	Ion Score	C. I. %	Modification	Rank	Result Type
925.5214	925.5234	0.002	2	11	18	LSIAHQTR					Mascot
925.5214	925.5234	0.002	2	11	18	LSIAHQTR	60	99.89			Mascot
1137.6667	1137.6506	-0.0161	-14	175	184	LVLGNALYFK					Mascot
1320.6431	1320.632	-0.0111	-8	264	274	LSTEPDFLENR					Mascot
1722.9941	1722.9036	-0.0905	-53	175	189	LVLGNALYFKGLWTK					Mascot

10	Sulfate adenylyltransferase OS=Archaeoglobus fulgidus (strain ATCC 49558 / VC-16 / DSM 4304 / JCM 9628 / NBRC 100126) GN=sat PE=3 SV=2	SAT_ARCFU	53260.9	6.59	10	66	85.446	23.602	41	92.886	

Peptide Information

Calc. Mass	Obsrv. Mass	± da	± ppm	Start Seq.	End Seq.	Sequence	Ion Score	C. I. %	Modification	Rank	Result Type
907.5247	907.4628	-0.0619	-68	436	442	YLELKNK					Mascot
908.4665	908.4939	0.0274	30	195	201	FWFPPSK					Mascot
925.5214	925.5234	0.002	2	214	221	TVIAHQTR					Mascot
925.5214	925.5234	0.002	2	214	221	TVIAHQTR	41	92.886			Mascot
1175.5878	1175.5912	0.0034	3	399	409	GMVAEGVFPPR			Oxidation (M)[2]		Mascot
1224.5983	1224.5366	-0.0617	-50	195	203	FWFPPSKCR			Carbamidomethyl (C)[8]		Mascot
1224.5983	1224.5366	-0.0617	-50	195	203	FWFPPSKCR			Carbamidomethyl (C)[8]		Mascot
1372.777	1372.7174	-0.0596	-43	1	13	MPLIKTPPPHGGK					Mascot
1372.777	1372.7174	-0.0596	-43	1	13	MPLIKTPPPHGGK					Mascot
1491.6039	1491.6368	0.0329	22	309	320	QNMGCTHHMFGR			Carbamidomethyl (C)[5], Oxidation (M)[3]		Mascot
1507.5989	1507.6311	0.0322	21	309	320	QNMGCTHHMFGR			Carbamidomethyl (C)[5], Oxidation (M)[3,9]		Mascot
1609.8109	1609.8544	0.0435	27	120	133	GQPFATLDIEEVYK					Mascot
1671.8431	1671.8842	0.0411	25	423	434	WWKVYNYPFVNR					Mascot
1707.8378	1707.876	0.0382	22	156	169	EPFDDKHPGYVIYK					Mascot

Gel Idx/Pos	243/J19	Instr./Gel Origin	BA2151/Sample Project 20140814	Process Status	Analysis Succeeded
Plate [#] Name	[1] Sample Project 20140814	Instrument Sample Name		Spectra	11

Rank	Protein Name	Accession No.	Protein MW	Protein PI	Pep. Count	Protein Score	Protein Score C. I. %	Intensity Matched	Total Ion Score	Total Ion C. I. %	Confirmed
1	Serpin-Z2B OS=Triticum aestivum PE=1 SV=1	SPZ2B_WHEAT	43011.4	5.18	7	107	99.999	4.227	80	99.999	

Peptide Information

Calc. Mass	Obsrv. Mass	± da	± ppm	Start Seq.	End Seq.	Sequence	Ion Score	C. I. %	Modification	Rank	Result Type
925.5214	925.5213	-0.0001	0	11	18	LSIAHQTR					Mascot
925.5214	925.5213	-0.0001	0	11	18	LSIAHQTR	6	0			Mascot
947.5156	947.4882	-0.0274	-29	2	10	ATTLATDVR					Mascot
1137.6667	1137.6163	-0.0504	-44	172	181	LVLGNALYFK					Mascot
1192.5382	1192.6343	0.0961	81	182	191	GAWTDQFDPR					Mascot
1372.7068	1372.7135	0.0067	5	159	171	DILPAGSIDNTTR					Mascot
1372.7068	1372.7135	0.0067	5	159	171	DILPAGSIDNTTR	22	0			Mascot
1514.7485	1514.751	0.0025	2	125	137	YKAEAQSVDFQTK					Mascot
1665.8595	1665.8821	0.0226	14	261	274	LSAEPEFLEQHIPR					Mascot
1665.8595	1665.8821	0.0226	14	261	274	LSAEPEFLEQHIPR	58	99.818			Mascot

Rank	Protein Name	Accession No.	Protein MW	Protein PI	Pep. Count	Protein Score	Protein Score C. I. %	Intensity Matched	Total Ion Score	Total Ion C. I. %	Confirmed
2	Sec-independent protein translocase protein TatB OS=Rhizobium meliloti (strain 1021) GN=tatB PE=3 SV=1	TATB_RHIME	22740	9.95	13	71	96.083	5.53			

Peptide Information

Calc. Mass	Obsrv. Mass	± da	± ppm	Start Seq.	End Seq.	Sequence	Ion Score	C. I. %	Modification	Rank	Result Type
801.4465	801.3968	-0.0497	-62	87	93	QLGNEIK					Mascot
816.4032	816.3992	-0.004	-5	80	86	DAMNPLR					Mascot
817.4778	817.4156	-0.0622	-76	170	177	KPGTSVTK					Mascot
832.3981	832.3301	-0.068	-82	80	86	DAMNPLR			Oxidation (M)[3]		Mascot
841.46	841.4263	-0.0337	-40	24	30	DLPPMLR					Mascot
872.4836	872.4377	-0.0459	-53	179	187	AAGETAPKK					Mascot
878.4366	878.4318	-0.0048	-5	49	55	QFDEALR					Mascot
925.4737	925.5213	0.0476	51	193	202	APGEAPAANK					Mascot
925.4737	925.5213	0.0476	51	193	202	APGEAPAANK					Mascot
932.4319	932.4841	0.0522	56	56	63	EADLDDVR					Mascot
989.5374	989.5185	-0.0189	-19	134	144	VAAAAVSSASR					Mascot

Calc. Mass	Obsrv. Mass	± da	± ppm	Start Seq.	End Seq.	Sequence	Ion Score	C. I. %	Modification	Rank	Result Type
989.5374	989.5185	-0.0189	-19	134	144	VAAAAVSSASR					Mascot
1060.527	1060.5253	-0.0017	-2	56	64	EADLDDVRK					Mascot
1140.6008	1140.5964	-0.0044	-4	193	204	APGEAPAANKSK					Mascot
1146.5573	1146.5868	0.0295	26	145	154	QMDRAADVPK			Oxidation (M)[2]		Mascot
1372.743	1372.7135	-0.0295	-21	87	98	QLGNEIKSDLQK					Mascot
1372.743	1372.7135	-0.0295	-21	87	98	QLGNEIKSDLQK					Mascot

3	RNA-binding protein AU-1 OS=Pyrobaculum arsenaticum (strain DSM 13514 / JCM 11321) GN=aubA PE=3 SV=1	AUBA_PYRAR	48880.9	8.95	17	70	94.206	9.008		

Peptide Information

Calc. Mass	Obsrv. Mass	± da	± ppm	Start Seq.	End Seq.	Sequence	Ion Score	C. I. %	Modification	Rank	Result Type
800.4512	800.4043	-0.0469	-59	25	31	VVQPTEK					Mascot
830.4478	830.403	-0.0448	-54	245	251	ARLDEAR					Mascot
849.4386	849.4177	-0.0209	-25	214	220	EMLEISK					Mascot
856.4999	856.5173	0.0174	20	421	427	LRDPLSR					Mascot
870.4792	870.533	0.0538	62	221	229	GGSPGQVLR					Mascot
925.5465	925.5213	-0.0252	-27	423	430	DPLSRLPK					Mascot
925.5465	925.5213	-0.0252	-27	423	430	DPLSRLPK					Mascot
947.5673	947.4882	-0.0791	-83	89	96	LKEVFVGR					Mascot
949.5142	949.4977	-0.0165	-17	17	24	LALDWGFK					Mascot
1140.6736	1140.5964	-0.0772	-68	25	34	VVQPTEKIAR					Mascot
1176.6848	1176.6007	-0.0841	-71	182	191	LRQYASIGLR					Mascot
1182.663	1182.6465	-0.0165	-14	184	193	QYASIGLRFK					Mascot
1190.7031	1190.606	-0.0971	-82	386	395	VIYIDLLVDK					Mascot
1244.6971	1244.7128	0.0157	13	260	271	GHHALRAQGLGK					Mascot
1244.6971	1244.7128	0.0157	13	260	271	GHHALRAQGLGK					Mascot
1326.6624	1326.6665	0.0041	3	413	422	YAEMFPQRLR			Oxidation (M)[4]		Mascot
1487.7166	1487.7985	0.0819	55	356	368	GYVVHTYYTAEGK					Mascot
1733.8606	1733.8721	0.0115	7	369	384	AVGTYVNANTVPEWGR					Mascot
1885.9291	1885.969	0.0399	21	97	115	AVGEATVEGPGGEVFDVPR					Mascot

4	Dual serine/threonine and tyrosine protein kinase OS=Strongylocentrotus purpuratus GN=DSTYK PE=2 SV=1	DUSTY_STRPU	109565.7	6.79	22	68	92.002	6.088	25	0

Peptide Information

Calc. Mass	Obsrv. Mass	± da	± ppm	Start Seq.	End Seq.	Sequence	Ion Score	C. I. %	Modification	Rank	Result Type

Calc. Mass	Obsrv. Mass	± da	± ppm	Start Seq.	End Seq.	Sequence	Ion Score	C. I. %	Modification	Rank	Result Type
807.4471	807.3883	-0.0588	-73	402	407	RIEYAR					Mascot
811.4308	811.405	-0.0258	-32	15	21	DLAHEVK					Mascot
826.4529	826.3912	-0.0617	-75	624	630	HSGRLEK					Mascot
827.4006	827.4014	0.0008	1	716	722	SIAEHDR					Mascot
841.5002	841.4263	-0.0739	-88	881	887	KGVRPER					Mascot
856.441	856.5173	0.0763	89	695	702	SVVPPDDK					Mascot
872.4948	872.4377	-0.0571	-65	667	673	LEREIGR					Mascot
921.4458	921.4808	0.035	38	631	637	TEEQRMK					Mascot
932.4869	932.4841	-0.0028	-3	484	491	LNACVVEK			Carbamidomethyl (C)[4]		Mascot
989.5626	989.5185	-0.0441	-45	649	657	LALESTSLR					Mascot
989.5626	989.5185	-0.0441	-45	649	657	LALESTSLR					Mascot
999.6197	999.5292	-0.0905	-91	492	500	LIGSVELLR					Mascot
999.6197	999.5292	-0.0905	-91	492	500	LIGSVELLR	25	0			Mascot
1032.532	1032.5232	-0.0088	-9	628	635	LEKTEEQR					Mascot
1051.5756	1051.576	0.0004	0	293	301	HNGLDARLR					Mascot
1137.6528	1137.6163	-0.0365	-32	22	31	HFGGLTKHLK					Mascot
1140.5579	1140.5964	0.0385	34	597	605	SICSQFRSR			Carbamidomethyl (C)[3]		Mascot
1194.6742	1194.6158	-0.0584	-49	781	790	YLHSLGLVHR					Mascot
1212.6517	1212.5985	-0.0532	-44	751	760	MQRDLHTAIK					Mascot
1232.6271	1232.6104	-0.0167	-14	569	578	TPTRVDTEWK					Mascot
1263.7307	1263.7242	-0.0065	-5	921	931	LQDIYTKALAK					Mascot
1278.7166	1278.6514	-0.0652	-51	769	780	LHVALDVAEGVR					Mascot
1372.7179	1372.7135	-0.0044	-3	419	429	DLTNQKQEQLR					Mascot
1372.7179	1372.7135	-0.0044	-3	419	429	DLTNQKQEQLR	3	0			Mascot
1851.9634	1851.9326	-0.0308	-17	501	516	ESFLGTLQRCLASLEK			Carbamidomethyl (C)[10]		Mascot

5	Rhamnogalacturonan endolyase YesW OS=Bacillus subtilis (strain 168) GN=yesW PE=1 SV=1	YESW_BACSU	67658.5	6	13	65	84.405	3.442	25	0

Peptide Information

Calc. Mass	Obsrv. Mass	± da	± ppm	Start Seq.	End Seq.	Sequence	Ion Score	C. I. %	Modification	Rank	Result Type
817.4202	817.4156	-0.0046	-6	550	555	EEVVWR					Mascot
840.4097	840.4257	0.016	19	90	96	TTNYVDK					Mascot
878.4214	878.4318	0.0104	12	556	563	TEDSSALR					Mascot
949.4989	949.4977	-0.0012	-1	344	351	LWTLDSSK					Mascot
999.6098	999.5292	-0.0806	-81	200	207	LWRINLGK					Mascot

Calc. Mass	Obsrv. Mass	± da	± ppm	Start Seq.	End Seq.	Sequence	Ion Score	C. I. %	Modification	Rank	Result Type
999.6098	999.5292	-0.0806	-81	200	207	LWRINLGK					Mascot
1096.5059	1096.5728	0.0669	61	512	520	WDYQNGVSK					Mascot
1137.5688	1137.6163	0.0475	42	54	63	TDGGIFVSWR					Mascot
1140.6372	1140.5964	-0.0408	-36	79	89	DGQKLNAAPVK					Mascot
1221.6144	1221.6074	-0.007	-6	229	240	AEVAMKTADGTK					Mascot
1237.6094	1237.6204	0.011	9	229	240	AEVAMKTADGTK			Oxidation (M)[5]		Mascot
1372.6638	1372.7135	0.0497	36	449	461	DVGRGMAADIDPR					Mascot
1372.6638	1372.7135	0.0497	36	449	461	DVGRGMAADIDPR	25	0			Mascot
1423.7039	1423.7733	0.0694	49	575	585	LYTLMHDPVYR			Oxidation (M)[5]		Mascot
1987.8855	1987.9363	0.0508	26	211	228	AGAHYTQFMVYDLDGDGK					Mascot
2407.2405	2407.3208	0.0803	33	121	143	ASVWAQPYHSVPLDKPAGGTTPK					Mascot

6	Urocanate hydratase OS=Vibrio vulnificus (strain YJ016) GN=hutU PE=3 SV=1	HUTU_VIBVY	61961.8	5.43	16	64	76.933	9.545

Peptide Information

Calc. Mass	Obsrv. Mass	± da	± ppm	Start Seq.	End Seq.	Sequence	Ion Score	C. I. %	Modification	Rank	Result Type
810.4355	810.3917	-0.0438	-54	214	220	TGYVDKK					Mascot
890.4764	890.4382	-0.0382	-43	303	310	QSMAVQVK					Mascot
1003.5054	1003.4987	-0.0067	-7	291	299	QQDESAVVK					Mascot
1016.4755	1016.5453	0.0698	69	2	10	TQSQGQDPR					Mascot
1029.584	1029.5114	-0.0726	-71	403	411	IQFQGLPAR					Mascot
1051.6007	1051.576	-0.0247	-23	19	28	APHGTTLRAK					Mascot
1060.5059	1060.5253	0.0194	18	535	544	HADAGYDIAK					Mascot
1146.6089	1146.5868	-0.0221	-19	412	420	ICWVGLKDR			Carbamidomethyl (C)[2]		Mascot
1176.6405	1176.6007	-0.0398	-34	300	310	AAKQSMAVQVK			Oxidation (M)[6]		Mascot
1222.7015	1222.6179	-0.0836	-68	16	26	TIRAPHGTTLR					Mascot
1244.7097	1244.7128	0.0031	2	221	232	ATTLDEALAIVK					Mascot
1244.7097	1244.7128	0.0031	2	221	232	ATTLDEALAIVK					Mascot
1372.8046	1372.7135	-0.0911	-66	220	232	KATTLDEALAIVK					Mascot
1372.8046	1372.7135	-0.0911	-66	220	232	KATTLDEALAIVK	0	0			Mascot
1437.7155	1437.7842	0.0687	48	421	432	ERLGQAFNEMVK			Oxidation (M)[10]		Mascot
1665.8854	1665.8821	-0.0033	-2	520	534	IARVLHNDPATGVMR			Oxidation (M)[14]		Mascot
1665.8854	1665.8821	-0.0033	-2	520	534	IARVLHNDPATGVMR			Oxidation (M)[14]		Mascot
1693.8578	1693.8749	0.0171	10	423	437	LGQAFNEMVKNGELK			Oxidation (M)[8]		Mascot
1886.047	1885.969	-0.078	-41	403	418	IQFQGLPARICWVGLK			Carbamidomethyl (C)[11]		Mascot

7	DNA polymerase catalytic subunit OS=Human	DPOL_HHV1K	137631.4	7.05	24	63	71.622	6.99

herpesvirus 1 (strain KOS) GN=UL30 PE=1 SV=1

Peptide Information

Calc. Mass	Obsrv. Mass	± da	± ppm	Start Seq.	End Seq.	Sequence	Ion Score	C. I. %	Modification	Rank	Result Type
800.4161	800.4043	-0.0118	-15	164	170	AAQFHAR					Mascot
801.4036	801.3968	-0.0068	-8	909	915	MASHISR					Mascot
803.4872	803.4246	-0.0626	-78	1183	1189	ITESLLK					Mascot
807.3818	807.3883	0.0065	8	780	785	DWLAMR			Oxidation (M)[5]		Mascot
817.3985	817.4156	0.0171	21	909	915	MASHISR			Oxidation (M)[1]		Mascot
849.4498	849.4177	-0.0321	-38	843	849	EMLLATR			Oxidation (M)[2]		Mascot
850.4053	850.4424	0.0371	44	234	241	ESPGASFR					Mascot
876.4574	876.3801	-0.0773	-88	485	492	VPLDGYGR					Mascot
878.4189	878.4318	0.0129	15	874	881	APGPYSMR					Mascot
886.4741	886.4831	0.009	10	1020	1026	RITDPER					Mascot
932.4683	932.4841	0.0158	17	1082	1089	EVEETVAR					Mascot
936.4832	936.4963	0.0131	14	493	500	MNGRGVFR					Mascot
989.5163	989.5185	0.0022	2	643	652	FRGAGGEAPK					Mascot
989.5163	989.5185	0.0022	2	643	652	FRGAGGEAPK					Mascot
1016.4941	1016.5453	0.0512	50	220	227	APRDLCER			Carbamidomethyl (C)[6]		Mascot
1068.4957	1068.522	0.0263	25	293	302	YEGGVDATTR					Mascot
1116.6677	1116.5566	-0.1111	-99	762	770	LFFVKAHVR					Mascot
1117.5637	1117.5873	0.0236	21	2	14	FSGGGGPLSPGGK					Mascot
1118.5854	1118.5946	0.0092	8	117	126	VGSGGFWPRR					Mascot
1137.5946	1137.6163	0.0217	19	31	41	GAGRGPPPCLR			Carbamidomethyl (C)[9]		Mascot
1190.5988	1190.606	0.0072	6	1226	1235	MLHRAFDTLA			Oxidation (M)[1]		Mascot
1205.6195	1205.6381	0.0186	15	896	908	GLTAAGLTAMGDK					Mascot
1221.6144	1221.6074	-0.007	-6	896	908	GLTAAGLTAMGDK			Oxidation (M)[10]		Mascot
1263.7208	1263.7242	0.0034	3	1048	1057	RLAHLTVYYK					Mascot
1372.6088	1372.7135	0.1047	76	675	687	EEGGGEREPEGAR					Mascot
1372.6088	1372.7135	0.1047	76	675	687	EEGGGEREPEGAR					Mascot
1373.692	1373.7117	0.0197	14	688	700	ETAGRHVGYQGAK					Mascot
1988.0052	1987.9363	-0.0689	-35	896	915	GLTAAGLTAMGDKMASH ISR					Mascot

8	Chaperone protein ClpB OS=Wolinella succinogenes (strain ATCC 29543 / DSM 1740 / LMG 7466 / NCTC 11488 / FDC 602W) GN=clpB PE=3 SV=1	CLPB_WOLSU	96049.1	5.7	24	63	70.961	9.718

Peptide Information

Calc. Mass	Obsrv. Mass	± da	± ppm	Start Seq.	End Seq.	Sequence	Ion Score	C. I. %	Modification	Rank	Result Type
801.4036	801.3968	-0.0068	-8	7	12	MTHQLR			Oxidation (M)[1]		Mascot
829.4638	829.3928	-0.071	-86	325	331	DAALQRR					Mascot
831.4934	831.4928	-0.0006	-1	81	87	ENIKISK					Mascot
849.4386	849.4177	-0.0209	-25	426	432	EALLMEK			Oxidation (M)[5]		Mascot
860.4723	860.4653	-0.007	-8	59	66	SALELEAK					Mascot
866.4254	866.4255	0.0001	0	498	505	AAEIDYGK					Mascot
870.4679	870.533	0.0651	75	506	512	IPELENR					Mascot
904.5106	904.5279	0.0173	19	190	196	MMQILIR					Mascot
904.5106	904.5279	0.0173	19	190	196	MMQILIR					Mascot
936.5005	936.4963	-0.0042	-4	190	196	MMQILIR			Oxidation (M)[1,2]		Mascot
947.552	947.4882	-0.0638	-67	615	623	TQSAKTLAK					Mascot
960.536	960.4867	-0.0493	-51	72	80	LPQSSSLTK					Mascot
999.5986	999.5292	-0.0694	-69	546	553	WTQIPVKK					Mascot
999.5986	999.5292	-0.0694	-69	546	553	WTQIPVKK					Mascot
1032.6057	1032.5232	-0.0825	-80	190	197	MMQILIRK					Mascot
1060.5171	1060.5253	0.0082	8	515	522	ELNTQWNR					Mascot
1137.5205	1137.6163	0.0958	84	728	736	IMENSDKER			Oxidation (M)[2]		Mascot
1161.4802	1161.5367	0.0565	49	635	643	IDMSEYMEK			Oxidation (M)[3]		Mascot
1171.6681	1171.6182	-0.0499	-43	562	571	VLGVEEELRK					Mascot
1192.6208	1192.6343	0.0135	11	457	466	TLLAQFENEK					Mascot
1240.6896	1240.7271	0.0375	30	305	316	GELHTIGATTLK					Mascot
1324.7583	1324.6562	-0.1021	-77	172	183	ALENKLDPVIGR					Mascot
1514.8723	1514.751	-0.1213	-80	236	250	RVVALDMSALIAGAK					Mascot
1665.8298	1665.8821	0.0523	31	184	196	DEEISRMMQILIR			Oxidation (M)[7,8]		Mascot
1665.8298	1665.8821	0.0523	31	184	196	DEEISRMMQILIR			Oxidation (M)[7,8]		Mascot
1693.9305	1693.8749	-0.0556	-33	237	252	VVALDMSALIAGAKYR			Oxidation (M)[6]		Mascot
1746.8326	1746.9242	0.0916	52	426	440	EALLMEKESTHSEAR			Oxidation (M)[5]		Mascot
1746.8326	1746.9242	0.0916	52	426	440	EALLMEKESTHSEAR			Oxidation (M)[5]		Mascot
1988.0586	1987.9363	-0.1223	-62	384	401	YLPDKAIDLIDEAAAELK					Mascot

9	Cysteine--tRNA ligase OS=Neisseria meningitidis serogroup A / serotype 4A (strain Z2491) GN=cysS PE=3 SV=1	SYC_NEIMA	79436.7	5.6	16	60	50.685	5.25	

Peptide Information

Calc. Mass	Obsrv. Mass	± da	± ppm	Start Seq.	End Seq.	Sequence	Ion Score	C. I. %	Modification	Rank	Result Type

823.423	823.4235	0.0005	1	1	7	MTTITEK					Mascot
847.4268	847.3969	-0.0299	-35	215	221	LSSEQQR					Mascot
904.4734	904.5279	0.0545	60	41	48	IDTNEKGK					Mascot
904.4734	904.5279	0.0545	60	41	48	IDTNEKGK	11	0			Mascot
949.5101	949.4977	-0.0124	-13	395	402	VEVDGFKR					Mascot
960.536	960.4867	-0.0493	-51	111	120	DSLAGLVSAK					Mascot
999.551	999.5292	-0.0218	-22	171	178	FKLSEFTK					Mascot
999.551	999.5292	-0.0218	-22	171	178	FKLSEFTK					Mascot
1013.5302	1013.4688	-0.0614	-61	239	247	EPFAPIDPK					Mascot
1132.6038	1132.6008	-0.003	-3	403	411	DPLDFVLWK					Mascot
1137.6375	1137.6163	-0.0212	-19	676	684	IRDLLNEHK					Mascot
1146.5651	1146.5868	0.0217	19	669	677	NWAESDRIR					Mascot
1190.5835	1190.606	0.0225	19	613	623	TNDAQLAGCLK			Carbamidomethyl (C)[9]		Mascot
1205.6215	1205.6381	0.0166	14	495	503	YWLHNGFIR					Mascot
1212.6116	1212.5985	-0.0131	-11	269	278	VMVVFDMIAR			Oxidation (M)[2,7]		Mascot
1222.6844	1222.6179	-0.0665	-54	533	541	FFILRAHYR					Mascot
1733.8759	1733.8721	-0.0038	-2	495	508	YWLHNGFIRVDGEK					Mascot
1885.9291	1885.969	0.0399	21	374	390	EFSAYGQLSGKSLDDLR					Mascot

10	Microtubule-actin cross-linking factor 1 OS=Mus musculus GN=Macf1 PE=1 SV=2	MACF1_MOUSE	836892.8	5.3	68	60	48.361	26.376

Peptide Information

Calc. Mass	Obsrv. Mass	± da	± ppm	Start Seq.	End Seq.	Sequence	Ion Score	C. I. %	Modification	Rank	Result Type
800.3719	800.4043	0.0324	40	5570	5575	EVMEHR					Mascot
801.4577	801.3968	-0.0609	-76	6945	6951	INQLSAR					Mascot
803.4257	803.4246	-0.0011	-1	6683	6689	ELIEGSR					Mascot
807.4108	807.3883	-0.0225	-28	4585	4590	EFEARR					Mascot
811.4018	811.405	0.0032	4	3770	3775	QYELMK					Mascot
816.421	816.3992	-0.0218	-27	6966	6972	LNDALDR					Mascot
817.4162	817.4156	-0.0006	-1	1249	1255	GSQLQER					Mascot
827.3967	827.4014	0.0047	6	3770	3775	QYELMK			Oxidation (M)[5]		Mascot
829.4665	829.3928	-0.0737	-89	6151	6157	EPLTELK					Mascot
830.3865	830.403	0.0165	20	7006	7011	VMDFFR			Oxidation (M)[2]		Mascot
831.457	831.4928	0.0358	43	2999	3006	GKEAGIEK					Mascot
834.3839	834.3426	-0.0413	-49	1035	1041	SLENEDK					Mascot

849.4247	849.4177	-0.007	-8	3496	3502	QSMAERK				Mascot
850.4669	850.4424	-0.0245	-29	2665	2672	VDFASALK				Mascot
860.4221	860.4653	0.0432	50	72	78	VADERDR				Mascot
870.5229	870.533	0.0101	12	5653	5660	LMALGPIR				Mascot
876.4672	876.3801	-0.0871	-99	5892	5899	SATVELEK				Mascot
878.44	878.4318	-0.0082	-9	2993	3000	MIGNDKGK			Oxidation (M)[1]	Mascot
886.5179	886.4831	-0.0348	-39	5653	5660	LMALGPIR			Oxidation (M)[2]	Mascot
890.4578	890.4382	-0.0196	-22	5948	5954	SEEREIK				Mascot
904.521	904.5279	0.0069	8	6712	6719	LSVSKQSR				Mascot
904.521	904.5279	0.0069	8	6712	6719	LSVSKQSR	15	0		Mascot
921.4346	921.4808	0.0462	50	4911	4918	AMLNEAEK			Oxidation (M)[2]	Mascot
932.4796	932.4841	0.0045	5	7016	7023	DQDGKITR				Mascot
936.4897	936.4963	0.0066	7	3318	3325	ATQNVFTR				Mascot
949.4771	949.4977	0.0206	22	2155	2162	SKCQTPTK			Carbamidomethyl (C)[3]	Mascot
963.5469	963.5167	-0.0302	-31	3195	3203	SKVTTTQAK				Mascot
999.5721	999.5292	-0.0429	-43	2553	2561	LDLVSPDLK				Mascot
999.5721	999.5292	-0.0429	-43	2553	2561	LDLVSPDLK				Mascot
1003.5604	1003.4987	-0.0617	-61	1737	1746	QLAGGMVSLK				Mascot
1013.4833	1013.4688	-0.0145	-14	670	677	ETSSFRMR				Mascot
1016.5007	1016.5453	0.0446	44	4092	4099	EVEQNLER				Mascot
1017.5298	1017.5435	0.0137	13	228	235	MFNALIHR			Oxidation (M)[1]	Mascot
1017.5298	1017.5435	0.0137	13	228	235	MFNALIHR			Oxidation (M)[1]	Mascot
1021.5499	1021.5261	-0.0238	-23	4577	4584	QQVQFMLK				Mascot
1023.5622	1023.519	-0.0432	-42	83	90	TFTKWVNK				Mascot
1029.4783	1029.5114	0.0331	32	670	677	ETSSFRMR			Oxidation (M)[7]	Mascot
1032.5725	1032.5232	-0.0493	-48	5900	5908	LQPSFEALK				Mascot
1040.45	1040.5435	0.0935	90	2875	2882	EETRSCMK			Carbamidomethyl (C)[6]	Mascot
1060.582	1060.5253	-0.0567	-53	1713	1721	VGLLDLMQR			Oxidation (M)[7]	Mascot
1068.5143	1068.522	0.0077	7	521	528	LECTNLYR			Carbamidomethyl (C)[3]	Mascot
1077.5358	1077.5514	0.0156	14	4911	4919	AMLNEAEKR			Oxidation (M)[2]	Mascot
1100.6211	1100.536	-0.0851	-77	4411	4418	LWEVLRER				Mascot
1116.5896	1116.5566	-0.033	-30	5998	6007	QQVEAAETIK				Mascot
1161.611	1161.5367	-0.0743	-64	3700	3710	SKAATELAENK				Mascot
1176.6041	1176.6007	-0.0034	-3	2821	2830	KISVEMEGQR				Mascot
1182.63	1182.6465	0.0165	14	6129	6138	LNHQGELMLK				Mascot
1192.5991	1192.6343	0.0352	30	2821	2830	KISVEMEGQR			Oxidation (M)[6]	Mascot
1194.6113	1194.6158	0.0045	4	4419	4428	QESLQTVFSR				Mascot

1197.5416	1197.5466	0.005	4	4966	4975	TSSLEEMTQR			Oxidation (M)[7]	Mascot
1199.5837	1199.6254	0.0417	35	3326	3334	RLCLEHDEK			Carbamidomethyl (C)[3]	Mascot
1212.6947	1212.5985	-0.0962	-79	2466	2476	APLTVLQSIDR				Mascot
1224.5929	1224.5382	-0.0547	-45	3014	3024	IEGFPSQMTSK				Mascot
1224.5929	1224.5382	-0.0547	-45	3014	3024	IEGFPSQMTSK	1	0		Mascot
1232.6117	1232.6104	-0.0013	-1	4685	4694	QQLEETSEIR				Mascot
1244.6705	1244.7128	0.0423	34	7188	7199	SKPSSRAASPTR				Mascot
1244.6705	1244.7128	0.0423	34	7188	7199	SKPSSRAASPTR				Mascot
1263.5999	1263.7242	0.1243	98	5358	5369	ATVDMLQAEGGR			Oxidation (M)[5]	Mascot
1278.6624	1278.6514	-0.011	-9	3942	3951	YTTLHSKCIR			Carbamidomethyl (C)[8]	Mascot
1313.6556	1313.6639	0.0083	6	3596	3608	ALERHQGGSSSGK				Mascot
1320.7271	1320.6121	-0.115	-87	3899	3910	GDLRFVTISGQK				Mascot
1324.6492	1324.6562	0.007	5	6861	6871	DQEPIPQNIDR				Mascot
1326.7198	1326.6665	-0.0533	-40	6129	6139	LNHQGELMLKK			Oxidation (M)[8]	Mascot
1372.6348	1372.7135	0.0787	57	980	991	SLAPGECHQVMK			Carbamidomethyl (C)[7], Oxidation (M)[11]	Mascot
1372.6566	1372.7135	0.0569	41	6904	6915	SVEPTHAPFMEK				Mascot
1373.7383	1373.7117	-0.0266	-19	3832	3843	SEAELKQTQALR				Mascot
1423.7209	1423.7733	0.0524	37	6354	6367	LMLLSRGDSGSGSK			Oxidation (M)[2]	Mascot
1437.806	1437.7842	-0.0218	-15	862	874	NPDHVLKSTLSVK				Mascot
1487.7952	1487.7985	0.0033	2	5482	5494	QTTGEEVLLIQEK				Mascot
1526.6687	1526.7214	0.0527	35	2950	2962	REMGGEQSVQMSR			Oxidation (M)[3,11]	Mascot
1563.7247	1563.7585	0.0338	22	720	732	TYFSELTMELEGK			Oxidation (M)[8]	Mascot
1665.8442	1665.8821	0.0379	23	3823	3837	EQYAASLARSEAELK				Mascot
1665.8442	1665.8821	0.0379	23	3823	3837	EQYAASLARSEAELK				Mascot
1693.785	1693.8749	0.0899	53	4793	4805	LQCQLQENEEFQK			Carbamidomethyl (C)[3]	Mascot
1733.8679	1733.8721	0.0042	2	499	511	YYQLEELAFRVMR			Oxidation (M)[12]	Mascot
1851.9447	1851.9326	-0.0121	-7	738	754	SLQDTAEVLSLENHPAK				Mascot
1865.9603	1865.9263	-0.034	-18	2647	2664	DEAGNNVLTPPEAIQLGK				Mascot
1987.9219	1987.9363	0.0144	7	4872	4887	YQGHVEDLVPWIDECK			Carbamidomethyl (C)[15]	Mascot

Gel Idx/Pos	244/J20	Instr./Gel Origin	BA2151/Sample Project 20140814	Process Status	Analysis Succeeded
Plate [#] Name	[1] Sample Project 20140814	Instrument Sample Name		Spectra	11

Rank	Protein Name	Accession No.	Protein MW	Protein PI	Pep. Count	Protein Score	Protein Score C. I. %	Intensity Matched	Total Ion Score	Total Ion C. I. %	Confirmed
1	Collagen alpha-1(IV) chain OS=Drosophila melanogaster GN=Cg25C PE=2 SV=3	CO4A1_DROME	175390.8	8.81	30	72	96.427	14.589			

Peptide Information

Calc. Mass	Obsrv. Mass	± da	± ppm	Start Seq.	End Seq.	Sequence	Ion Score	C. I. %	Modification	Rank	Result Type
807.4359	807.3951	-0.0408	-51	398	406	GTPGPPGPK					Mascot
815.4257	815.3588	-0.0669	-82	197	204	GEKGEPAK					Mascot
819.4471	819.3804	-0.0667	-81	1435	1442	GFTGPKGR					Mascot
823.4057	823.3993	-0.0064	-8	734	741	GDKGSFGR					Mascot
837.4327	837.3818	-0.0509	-61	1	6	MLPFWK			Oxidation (M)[1]		Mascot
873.4061	873.39	-0.0161	-18	520	529	GNAGAPGDSK					Mascot
912.5261	912.4709	-0.0552	-60	304	312	GEPGLVGRK					Mascot
947.4727	947.4869	0.0142	15	84	91	CIAEKGNR			Carbamidomethyl (C)[1]		Mascot
1015.4955	1015.484	-0.0115	-11	212	220	GEKGEPGWR					Mascot
1117.6113	1117.6324	0.0211	19	968	979	GFAGVTGAPGKR					Mascot
1117.6113	1117.6324	0.0211	19	398	409	GTPGPPGPKGPR					Mascot
1149.5569	1149.5952	0.0383	33	558	569	GDMGIKGDVGGK			Oxidation (M)[3]		Mascot
1182.6226	1182.6322	0.0096	8	1064	1075	GQKGEPGPSGLR					Mascot
1193.6022	1193.611	0.0088	7	1468	1479	GEPGQPGRNGPK					Mascot
1205.5909	1205.6748	0.0839	70	517	529	GFKGNAGAPGDSK					Mascot
1205.6525	1205.6748	0.0223	18	1528	1539	GYEGAIGLIGQK					Mascot
1227.5865	1227.6271	0.0406	33	722	733	GEPGFHGRDGAK					Mascot
1251.644	1251.663	0.019	15	816	829	GNQGAVGVPGNPGK					Mascot
1255.6027	1255.6177	0.015	12	693	706	GLSGAPGNDGTPGR					Mascot
1319.6591	1319.6497	-0.0094	-7	485	498	GDAGLPGYGIQGSK					Mascot
1320.7271	1320.5986	-0.1285	-97	1213	1225	GQPGLPATVPDIR					Mascot
1326.7125	1326.6709	-0.0416	-31	1247	1261	GLTGPAGVAGAKGDR					Mascot
1412.684	1412.7172	0.0332	24	941	955	GEPGSPGLVGMPGNK			Oxidation (M)[11]		Mascot
1416.7482	1416.7977	0.0495	35	499	513	GDAGIPGYPGLKGSK					Mascot
1433.7537	1433.719	-0.0347	-24	657	671	GYPGAPGAKGVQGFK					Mascot
1463.6874	1463.7944	0.107	73	609	623	GHDGINGQTGPPGEK					Mascot

Calc. Mass	Obsrv. Mass	± da	± ppm	Start Seq.	End Seq.	Sequence	Ion Score	C. I. %	Modification	Rank	Result Type
1463.6874	1463.7944	0.107	73	609	623	GHDGINGQTGPPGEK					Mascot
1665.892	1665.8916	-0.0004	0	893	910	GDAGLPGVSGRPGIVGE K					Mascot
1676.7797	1676.9122	0.1325	79	742	757	SGEKGEPGSCALDEIK			Carbamidomethyl (C)[10]		Mascot
1692.8777	1692.8248	-0.0529	-31	816	833	GNQGAVGVPGNPGKDG LR					Mascot
1999.9105	1999.9865	0.076	38	1414	1434	GDTGYPGLNGNDGPVG APGER					Mascot

2	Phenylalanine--tRNA ligase alpha subunit OS=Verminephrobacter eiseniae (strain EF01-2) GN=pheS PE=3 SV=1	SYFA_VEREI	38954.6	6.22	14	70	95.181	9.258	

Peptide Information

Calc. Mass	Obsrv. Mass	± da	± ppm	Start Seq.	End Seq.	Sequence	Ion Score	C. I. %	Modification	Rank	Result Type
809.4152	809.3757	-0.0395	-49	330	336	YGVSDLR					Mascot
818.4631	818.3856	-0.0775	-95	182	188	HAVQHVK					Mascot
963.537	963.4664	-0.0706	-73	29	37	AQFLGKSGR					Mascot
982.4993	982.4701	-0.0292	-30	337	344	LFFDGDIR					Mascot
999.5081	999.4568	-0.0513	-51	243	250	VIFTGFCR			Carbamidomethyl (C)[7]		Mascot
1002.5843	1002.5499	-0.0344	-34	205	213	VIAPGRAYR					Mascot
1106.5333	1106.5371	0.0038	3	44	53	GMAQLCVAEK			Carbamidomethyl (C)[6]		Mascot
1122.5282	1122.5175	-0.0107	-10	44	53	GMAQLCVAEK			Carbamidomethyl (C)[6], Oxidation (M)[2]		Mascot
1182.6603	1182.6322	-0.0281	-24	182	191	HAVQHVKAHR					Mascot
1232.6117	1232.6223	0.0106	9	2	12	NELDSLVESAR					Mascot
1346.6562	1346.6886	0.0324	24	313	324	YIGFAFGMGLDR					Mascot
1379.6471	1379.7412	0.0941	68	1	12	MNELDSLVESAR			Oxidation (M)[1]		Mascot
1463.7965	1463.7944	-0.0021	-1	102	115	GQGGLHPVSLTLER					Mascot
1463.7965	1463.7944	-0.0021	-1	102	115	GQGGLHPVSLTLER					Mascot
1516.7714	1516.7666	-0.0048	-3	2	14	NELDSLVESARQR					Mascot
1516.7714	1516.7666	-0.0048	-3	2	14	NELDSLVESARQR					Mascot
1885.9814	1885.9563	-0.0251	-13	173	188	THTSPMQIRHAVQHVK			Oxidation (M)[6]		Mascot
1885.9814	1885.9563	-0.0251	-13	173	188	THTSPMQIRHAVQHVK			Oxidation (M)[6]		Mascot

3	DNA recombination protein RmuC OS=Escherichia coli (strain K12) GN=rmuC PE=1 SV=1	RMUC_ECOLI	54787.8	5.27	17	64	79.442	7.062	

Protein Group

DNA recombination protein RmuC OS=Escherichia coli O157:H7 GN=rmuC PE=3 SV=1	RMUC_ECO57	54787.8	5.2699 999809 2651
DNA recombination protein RmuC OS=Shigella flexneri GN=rmuC PE=3 SV=1	RMUC_SHIFL	54787.8	5.2699 999809 2651

Peptide Information

Calc. Mass	Obsrv. Mass	± da	± ppm	Start Seq.	End Seq.	Sequence	Ion Score	C. I. %	Modification	Rank	Result Type
816.421	816.4474	0.0264	32	32	38	AEQLAER					Mascot
819.3744	819.3804	0.006	7	361	366	YEHQSR					Mascot
824.4512	824.4043	-0.0469	-57	375	381	ASKLYDK					Mascot
841.4236	841.4668	0.0432	51	378	383	LYDKMR			Oxidation (M)[5]		Mascot
864.421	864.4019	-0.0191	-22	147	153	EQLDGFR					Mascot
902.4479	902.4413	-0.0066	-7	122	128	IFEHSNR					Mascot
982.5026	982.4701	-0.0325	-33	259	266	MTLVAYER					Mascot
1006.5428	1006.4957	-0.0471	-47	168	175	HTLTHEIR					Mascot
1058.5491	1058.5045	-0.0446	-42	122	129	IFEHSNRR					Mascot
1064.5483	1064.5563	0.008	8	154	162	RQVQDSFGK					Mascot
1193.5718	1193.611	0.0392	33	39	49	EEMVAELSAAK			Oxidation (M)[3]		Mascot
1224.579	1224.554	-0.025	-20	399	408	AQDNYRQAMK					Mascot
1227.7056	1227.6271	-0.0785	-64	136	146	QSLNSLLSPLR					Mascot
1320.6543	1320.5986	-0.0557	-42	111	121	LSEQFENLANR					Mascot
1346.637	1346.6886	0.0516	38	60	70	AECELLNNEVR			Carbamidomethyl (C)[3]		Mascot
1480.8304	1480.7672	-0.0632	-43	239	251	MQPDVIVRLPQGK					Mascot
1990.975	1990.9856	0.0106	5	32	49	AEQLAEREEMVAELSAAK			Oxidation (M)[10]		Mascot

4	Proteasome subunit beta type-7-B OS=Arabidopsis thaliana GN=PBB2 PE=1 SV=2	PSB7B_ARATH	29940.2	6.71	12	62	64.274	6.487	

Peptide Information

Calc. Mass	Obsrv. Mass	± da	± ppm	Start Seq.	End Seq.	Sequence	Ion Score	C. I. %	Modification	Rank	Result Type
830.4407	830.3871	-0.0536	-65	241	247	GYSFTKK					Mascot
841.5142	841.4668	-0.0474	-56	255	261	ITPLLER					Mascot
849.4135	849.3926	-0.0209	-25	22	28	NDMLTQK					Mascot
902.4843	902.4413	-0.043	-48	220	226	GHKEYLR					Mascot
982.47	982.4701	0.0001	0	107	114	YQTGRDSR					Mascot
1016.5371	1016.5512	0.0141	14	49	58	DGVILGADTR					Mascot
1016.5371	1016.5512	0.0141	14	49	58	DGVILGADTR					Mascot
1117.5848	1117.6324	0.0476	43	183	192	EGLTRDEGIK					Mascot
1117.5848	1117.6324	0.0476	43	183	192	EGLTRDEGIK					Mascot
1149.6879	1149.5952	-0.0927	-81	38	48	TGTTIVGLIFK					Mascot
1204.5879	1204.6403	0.0524	44	1	11	MSQSSVDIPPK			Oxidation (M)[1]		Mascot

1315.7944	1315.7001	-0.0943	-72	112	123	DSRVVTALTLLK					Mascot
1367.6842	1367.8096	0.1254	92	235	246	TYVSSKGYSFTK					Mascot
1367.6842	1367.8096	0.1254	92	235	246	TYVSSKGYSFTK					Mascot
1625.9836	1625.8792	-0.1044	-64	248	261	TEVLLTKITPLLER					Mascot

5	Kinesin-1 heavy chain OS=Mus musculus GN=Kif5b PE=1 SV=3	KINH_MOUSE	110225	6.06	27	61	58.981	14.878

Peptide Information

Calc. Mass	Obsrv. Mass	± da	± ppm	Start Seq.	End Seq.	Sequence	Ion Score	C. I. %	Modification	Rank	Result Type
816.4686	816.4474	-0.0212	-26	867	873	LRATAER					Mascot
819.3665	819.3804	0.0139	17	92	98	THTMEGK			Oxidation (M)[4]		Mascot
834.3839	834.3262	-0.0577	-69	506	512	SQEVEDK					Mascot
847.4016	847.4008	-0.0008	-1	886	892	ENASRDR					Mascot
887.4039	887.4554	0.0515	58	542	548	EMTNHQK					Mascot
904.4556	904.4779	0.0223	25	740	746	MVLEQER					Mascot
937.4374	937.4332	-0.0042	-4	895	901	YQQEVDR					Mascot
947.468	947.4869	0.0189	20	721	728	DEVEAKEK					Mascot
960.4931	960.4643	-0.0288	-30	628	635	ELAACQLR			Carbamidomethyl (C)[5]		Mascot
1006.4873	1006.4957	0.0084	8	420	427	KCEEELAK			Carbamidomethyl (C)[2]		Mascot
1015.4989	1015.484	-0.0149	-15	542	549	EMTNHQKK					Mascot
1016.5444	1016.5512	0.0068	7	858	865	CELPKLEK			Carbamidomethyl (C)[1]		Mascot
1016.5444	1016.5512	0.0068	7	858	865	CELPKLEK			Carbamidomethyl (C)[1]		Mascot
1064.5479	1064.5563	0.0084	8	551	560	AAEMMASLLK					Mascot
1096.5377	1096.5713	0.0336	31	551	560	AAEMMASLLK			Oxidation (M)[4,5]		Mascot
1232.6052	1232.6223	0.0171	14	285	295	ILQDSLGGNCR			Carbamidomethyl (C)[10]		Mascot
1244.594	1244.6871	0.0931	75	676	685	AQEKVHEMEK			Oxidation (M)[8]		Mascot
1251.7168	1251.663	-0.0538	-43	792	801	ELQTLHNLRK					Mascot
1315.6853	1315.7001	0.0148	11	729	739	LITDLQDQNQK					Mascot
1326.7124	1326.6709	-0.0415	-31	710	720	ETHQKQISSLR					Mascot
1346.7162	1346.6886	-0.0276	-20	527	539	SATLASIDAELQK					Mascot
1374.7224	1374.6686	-0.0538	-39	715	726	QISSLRDEVEAK					Mascot
1480.7166	1480.7672	0.0506	34	515	526	EYELLSDELNQK					Mascot
1497.6863	1497.8151	0.1288	86	191	203	HVAVTNMNEHSSR			Oxidation (M)[7]		Mascot
1497.6863	1497.8151	0.1288	86	191	203	HVAVTNMNEHSSR			Oxidation (M)[7]		Mascot
1665.7562	1665.8916	0.1354	81	813	829	KSAEVDSDDTGGSAAQK					Mascot
1692.9167	1692.8248	-0.0919	-54	643	656	IKSLTEYLQNVEQK					Mascot

1838.8674	1838.9359	0.0685	37	188	203	SNRHVAVTNMNEHSSR					Mascot
1885.9628	1885.9563	-0.0065	-3	942	961	GGGSFVQNNQPVGLRG GGGK					Mascot
1885.9628	1885.9563	-0.0065	-3	942	961	GGGSFVQNNQPVGLRG GGGK					Mascot
1990.8811	1990.9856	0.1045	52	51	67	VFQSSTSQEQVYNDCAK			Carbamidomethyl (C)[15]		Mascot

6 Kinesin-1 heavy chain OS=Rattus norvegicus GN=Kif5b PE=1 SV=1 KINH_RAT 110204 6.06 27 61 58.981 14.878

Peptide Information

Calc. Mass	Obsrv. Mass	± da	± ppm	Start Seq.	End Seq.	Sequence	Ion Score	C. I. %	Modification	Rank	Result Type
816.4686	816.4474	-0.0212	-26	867	873	LRATAER					Mascot
819.3665	819.3804	0.0139	17	92	98	THTMEGK			Oxidation (M)[4]		Mascot
834.3839	834.3262	-0.0577	-69	506	512	SQEVEDK					Mascot
847.4016	847.4008	-0.0008	-1	886	892	ENASRDR					Mascot
887.4039	887.4554	0.0515	58	542	548	EMTNHQK					Mascot
904.4556	904.4779	0.0223	25	740	746	MVLEQER					Mascot
937.4374	937.4332	-0.0042	-4	895	901	YQQEVDR					Mascot
947.468	947.4869	0.0189	20	721	728	DEVEAKEK					Mascot
960.4931	960.4643	-0.0288	-30	628	635	ELAACQLR			Carbamidomethyl (C)[5]		Mascot
1006.4873	1006.4957	0.0084	8	420	427	KCEEEIAK			Carbamidomethyl (C)[2]		Mascot
1015.4989	1015.484	-0.0149	-15	542	549	EMTNHQKK					Mascot
1016.5444	1016.5512	0.0068	7	858	865	CELPKLEK			Carbamidomethyl (C)[1]		Mascot
1016.5444	1016.5512	0.0068	7	858	865	CELPKLEK			Carbamidomethyl (C)[1]		Mascot
1064.5479	1064.5563	0.0084	8	551	560	AAEMMASLLK					Mascot
1096.5377	1096.5713	0.0336	31	551	560	AAEMMASLLK			Oxidation (M)[4,5]		Mascot
1232.6052	1232.6223	0.0171	14	285	295	ILQDSLGGNCR			Carbamidomethyl (C)[10]		Mascot
1244.594	1244.6871	0.0931	75	676	685	AQEKVHEMEK			Oxidation (M)[8]		Mascot
1251.7168	1251.663	-0.0538	-43	792	801	ELQTLHNLRK					Mascot
1315.6853	1315.7001	0.0148	11	729	739	LITDLQDQNQK					Mascot
1326.7124	1326.6709	-0.0415	-31	710	720	ETHQKQISSLR					Mascot
1346.7162	1346.6886	-0.0276	-20	527	539	SATLASIDAELQK					Mascot
1374.7224	1374.6686	-0.0538	-39	715	726	QISSLRDEVEAK					Mascot
1480.7166	1480.7672	0.0506	34	515	526	EYELLSDELNQK					Mascot
1497.6863	1497.8151	0.1288	86	191	203	HVAVTNMNEHSSR			Oxidation (M)[7]		Mascot
1497.6863	1497.8151	0.1288	86	191	203	HVAVTNMNEHSSR			Oxidation (M)[7]		Mascot
1665.7562	1665.8916	0.1354	81	813	829	KSAEVDSDDTGGSAAQK					Mascot

1692.9167	1692.8248	-0.0919	-54	643	656	IKSLTEYLQNVEQK					Mascot
1838.8674	1838.9359	0.0685	37	188	203	SNRHVAVTNMNEHSSR					Mascot
1885.9628	1885.9563	-0.0065	-3	942	961	GGGSFVQNNQPVGLRG GGGK					Mascot
1885.9628	1885.9563	-0.0065	-3	942	961	GGGSFVQNNQPVGLRG GGGK					Mascot
1990.8811	1990.9856	0.1045	52	51	67	VFQSSTSQEQVYNDCAK			Carbamidomethyl (C)[15]		Mascot

7 Pyruvate dehydrogenase E1 component subunit alpha, somatic form, mitochondrial (Fragment) OS=Sus scrofa GN=PDHA1 PE=1 SV=1 | ODPA_PIG | 43834.8 | 8.17 | 15 | 60 | 51.807 | 7.575

Peptide Information

Calc. Mass	Obsrv. Mass	± da	± ppm	Start Seq.	End Seq.	Sequence	Ion Score	C. I. %	Modification	Rank	Result Type
816.4362	816.4474	0.0112	14	378	384	GANQWIK					Mascot
874.4781	874.4691	-0.009	-10	245	252	GDFIPGLR					Mascot
902.4578	902.4413	-0.0165	-18	304	310	EEIQEVR					Mascot
937.4706	937.4332	-0.0374	-40	66	72	MMQTVRR			Oxidation (M)[1]		Mascot
999.4564	999.4568	0.0004	0	226	234	YGMGTSVER					Mascot
1001.5626	1001.5284	-0.0342	-34	132	140	EILAELTGR					Mascot
1015.4513	1015.484	0.0327	32	226	234	YGMGTSVER			Oxidation (M)[3]		Mascot
1117.5848	1117.6324	0.0476	43	304	312	EEIQEVRSK					Mascot
1117.5848	1117.6324	0.0476	43	304	312	EEIQEVRSK					Mascot
1182.5394	1182.6322	0.0928	78	147	157	GKGGSMHMYAK			Oxidation (M)[6]		Mascot
1193.5653	1193.611	0.0457	38	253	262	VDGMDILCVR			Carbamidomethyl (C)[8], Oxidation (M)[4]		Mascot
1263.5973	1263.7039	0.1066	84	63	71	YYRMMQTVR			Oxidation (M)[4]		Mascot
1315.6212	1315.7001	0.0789	60	263	273	EATRFAAAYCR			Carbamidomethyl (C)[10]		Mascot
1320.6981	1320.5986	-0.0995	-75	277	287	GPILMELQTYR					Mascot
1433.7305	1433.719	-0.0115	-8	323	335	MVNSNLASVEELK					Mascot
1838.097	1837.9297	-0.1673	-91	10	27	VLSGVAQKPASRVLVAS R					Mascot
1865.8348	1865.8721	0.0373	20	288	303	YHGHSMSDPGVSYRTR			Oxidation (M)[6]		Mascot

8 Gamma-glutamyl phosphate reductase OS=Clostridium beijerinckii (strain ATCC 51743 / NCIMB 8052) GN=proA PE=3 SV=1 | PROA_CLOB8 | 45059.7 | 5.81 | 14 | 59 | 37.916 | 6.317

Peptide Information

Calc. Mass	Obsrv. Mass	± da	± ppm	Start Seq.	End Seq.	Sequence	Ion Score	C. I. %	Modification	Rank	Result Type
849.475	849.3926	-0.0824	-97	1	7	MSELIIK			Oxidation (M)[1]		Mascot
887.5308	887.4554	-0.0754	-85	160	167	ALTKGIER					Mascot

935.4866	935.4622	-0.0244	-26	75	83	IESMAAGLK			Oxidation (M)[4]		Mascot
982.4332	982.4701	0.0369	38	184	191	EVATEMMR			Oxidation (M)[6]		Mascot
1015.3859	1015.484	0.0981	97	234	241	DCDFEMAK			Carbamidomethyl (C)[2]		Mascot
1016.5734	1016.5512	-0.0222	-22	40	49	AKGDIISANK					Mascot
1016.5734	1016.5512	-0.0222	-22	40	49	AKGDIISANK					Mascot
1095.6093	1095.5426	-0.0667	-61	389	398	LHARGPMGLK			Oxidation (M)[7]		Mascot
1204.6168	1204.6403	0.0235	20	173	183	ASVQLVEDTSR					Mascot
1347.7301	1347.7183	-0.0118	-9	1	12	MSELIIKGQNAK			Oxidation (M)[1]		Mascot
1374.7661	1374.6686	-0.0975	-71	75	87	IESMAAGLKDVIK					Mascot
1507.7383	1507.7513	0.013	9	27	39	DDALMIMAEELIK			Oxidation (M)[5]		Mascot
1706.8704	1706.7729	-0.0975	-57	27	41	DDALMIMAEELIKAK			Oxidation (M)[5]		Mascot
1990.9393	1990.9856	0.0463	23	370	388	FTDGSEFGFGAEIGISTQ K					Mascot
2000.0593	1999.9865	-0.0728	-36	249	266	ASRPSVCNAAEKLLINEK			Carbamidomethyl (C)[7]		Mascot

9 Peptide deformylase OS=Prochlorococcus marinus (strain NATL1A) GN=def PE=3 SV=1 DEF_PROM1 22587.7 7.66 11 59 25.358 8.003

Peptide Information

Calc. Mass	Obsrv. Mass	± da	± ppm	Start Seq.	End Seq.	Sequence	Ion Score	C. I. %	Modification	Rank	Result Type
816.4574	816.4474	-0.01	-12	51	57	VDDAIRK					Mascot
832.3981	832.3275	-0.0706	-85	143	149	DEMGRPK					Mascot
847.452	847.4008	-0.0512	-60	195	202	SDVIKATN					Mascot
960.4931	960.4643	-0.0288	-30	143	150	DEMGRPKK					Mascot
1006.4952	1006.4957	0.0005	0	186	193	QLNENNFK					Mascot
1023.5291	1023.5413	0.0122	12	1	10	MAGSFAQLAK					Mascot
1106.5446	1106.5371	-0.0075	-7	150	159	KMNADGLMAR					Mascot
1122.5394	1122.5175	-0.0219	-20	150	159	KMNADGLMAR			Oxidation (M)[2]		Mascot
1204.5588	1204.6403	0.0815	68	61	70	DMLITMYSSK			Oxidation (M)[2]		Mascot
1251.7419	1251.663	-0.0789	-63	71	83	GIGLAAPQVGIQK					Mascot
1367.6736	1367.8096	0.136	99	139	149	LSYRDEMGRPK			Oxidation (M)[7]		Mascot
1367.6736	1367.8096	0.136	99	139	149	LSYRDEMGRPK			Oxidation (M)[7]		Mascot
1516.775	1516.7666	-0.0084	-6	58	70	LAKDMLITMYSSK			Oxidation (M)[5]		Mascot
1516.775	1516.7666	-0.0084	-6	58	70	LAKDMLITMYSSK			Oxidation (M)[5]		Mascot

10 Filament-like plant protein 7 OS=Arabidopsis thaliana GN=FPP7 PE=3 SV=2 FPP7_ARATH 101987.5 5.76 23 58 21.84 8.764

Peptide Information

Calc. Mass	Obsrv. Mass	± da	± ppm	Start	End	Sequence	Ion	C. I. %	Modification	Rank	Result Type

				Seq.	Seq.		Score		
813.3961	813.4012	0.0051	6	85	90	HRSEER			Mascot
815.408	815.3588	-0.0492	-60	116	122	MHDALTK			Mascot
873.4424	873.39	-0.0524	-60	344	351	GTNIEPSR			Mascot
937.4659	937.4332	-0.0327	-35	802	809	SSLLDQMK		Oxidation (M)[7]	Mascot
1002.5942	1002.5499	-0.0443	-44	753	761	QLKALTNSK			Mascot
1003.5684	1003.5351	-0.0333	-33	870	878	TGGVKSLWR			Mascot
1023.5945	1023.5413	-0.0532	-52	29	36	IELEHRVK			Mascot
1049.511	1049.5192	0.0082	8	711	720	TASASENELK			Mascot
1067.5421	1067.5404	-0.0017	-2	2	9	DHKAWPWK			Mascot
1088.5695	1088.5596	-0.0099	-9	163	171	NKTVEDLNR			Mascot
1096.4695	1096.5713	0.1018	93	648	655	QFEWDESR			Mascot
1181.5289	1181.6011	0.0722	61	263	272	MSNEVEMLGR		Oxidation (M)[1]	Mascot
1197.5239	1197.624	0.1001	84	263	272	MSNEVEMLGR		Oxidation (M)[1,7]	Mascot
1224.5645	1224.554	-0.0105	-9	647	655	KQFEWDESR			Mascot
1263.6614	1263.7039	0.0425	34	394	405	KEMGTSLVGTPK		Oxidation (M)[3]	Mascot
1346.6798	1346.6886	0.0088	7	16	28	TVVESNGEVVADK			Mascot
1416.6714	1416.7977	0.1263	89	87	99	SEERSSHTDAGLK			Mascot
1497.7002	1497.8151	0.1149	77	275	288	VNGSPHSPMIDSEK			Mascot
1497.7002	1497.8151	0.1149	77	275	288	VNGSPHSPMIDSEK			Mascot
1665.8186	1665.8916	0.073	44	395	410	EMGTSLVGTPKAAEMK		Oxidation (M)[2]	Mascot
1706.8378	1706.7729	-0.0649	-38	725	739	QNMRTELEIAAASEK		Oxidation (M)[3]	Mascot
1837.8848	1837.9297	0.0449	24	12	28	SMEKTVVESNGEVVADK		Oxidation (M)[2]	Mascot
1838.9355	1838.9359	0.0004	0	501	515	HITQRNTDEVLEDIR			Mascot
1990.9829	1990.9856	0.0027	1	327	343	TASRLLEFESHLEESSR			Mascot
2000.0304	1999.9865	-0.0439	-22	254	272	LPGPAALSKMSNEVEML GR			Mascot

Gel Idx/Pos	245/J21	Instr./Gel Origin	BA2151/Sample Project 20140814	Process Status	Analysis Succeeded
Plate [#] Name	[1] Sample Project 20140814	Instrument Sample Name		Spectra	11

Rank	Protein Name	Accession No.	Protein MW	Protein PI	Pep. Count	Protein Score	Protein Score C. I. %	Intensity Matched	Total Ion Score	Total Ion C. I. %	Confirmed
1	Alanine--tRNA ligase OS=Flavobacterium psychrophilum (strain JIP02/86 / ATCC 49511) GN=alaS PE=3 SV=1	SYA_FLAPJ	99726.2	5.74	23	70	95.068	11.824			

Peptide Information

Calc. Mass	Obsrv. Mass	± da	± ppm	Start Seq.	End Seq.	Sequence	Ion Score	C. I. %	Modification	Rank	Result Type
817.405	817.3949	-0.0101	-12	149	155	GLIDEDR					Mascot
819.4505	819.3803	-0.0702	-86	66	72	CLRVSGK			Carbamidomethyl (C)[1]		Mascot
831.4934	831.4316	-0.0618	-74	393	400	VVAETKGK					Mascot
832.4159	832.3358	-0.0801	-96	619	625	VSDDQLR					Mascot
840.4097	840.4349	0.0252	30	273	279	YTSNEVK					Mascot
841.5142	841.4519	-0.0623	-74	738	744	KPQDILK					Mascot
864.4608	864.4142	-0.0466	-54	1	7	MTSKEIR					Mascot
871.5247	871.4689	-0.0558	-64	759	765	QIEQLLK					Mascot
923.4655	923.4854	0.0199	22	656	664	GAMALFGEK					Mascot
1015.553	1015.5254	-0.0276	-27	57	65	SPRIADTQK					Mascot
1016.5622	1016.5561	-0.0061	-6	710	719	IEAITGDAVK					Mascot
1016.5622	1016.5561	-0.0061	-6	710	719	IEAITGDAVK					Mascot
1104.583	1104.6295	0.0465	42	60	68	IADTQKCLR			Carbamidomethyl (C)[7]		Mascot
1133.595	1133.6034	0.0084	7	601	610	GSLVNPDYLR					Mascot
1172.6635	1172.6251	-0.0384	-33	709	719	RIEAITGDAVK					Mascot
1173.6111	1173.6165	0.0054	5	186	196	TPEEKAQVSGK					Mascot
1205.6008	1205.6827	0.0819	68	745	755	SVTSLQDDNVK					Mascot
1317.6838	1317.6106	-0.0732	-56	8	17	QQYLKFFESK					Mascot
1326.7528	1326.6749	-0.0779	-59	543	553	KENNLILHFAK					Mascot
1516.7802	1516.7748	-0.0054	-4	578	590	NHSATHLMHLALR			Oxidation (M)[8]		Mascot
1657.8068	1657.8276	0.0208	13	720	733	AFYTNQENTLSEIK					Mascot
1827.8661	1827.9742	0.1081	59	611	625	FDFSHFSKVSDDQLR					Mascot
1991.0239	1991.006	-0.0179	-9	573	590	ASTSKNHSATHLMHLALR			Oxidation (M)[13]		Mascot
2695.325	2695.2893	-0.0357	-13	493	517	DGILYQIVLDNTPFYPEG GGQVGDK					Mascot

Rank	Protein Name	Accession No.	Protein MW	Protein PI	Pep. Count	Protein Score	Protein Score C. I. %	Intensity Matched	Total Ion Score	Total Ion C. I. %	Confirmed
2	Protein-L-isoaspartate O-methyltransferase OS=Burkholderia phymatum (strain DSM 17167 /	PIMT_BURP8	35158	10.53	11	47	0	5.527			

STM815) GN=pcm PE=3 SV=1

Peptide Information

Calc. Mass	Obsrv. Mass	± da	± ppm	Start Seq.	End Seq.	Sequence	Ion Score	C. I. %	Modification	Rank	Result Type
808.3981	808.4072	0.0091	11	1	7	MTGERAK			Oxidation (M)[1]		Mascot
815.4158	815.4651	0.0493	60	304	310	TGPAQWR					Mascot
817.3951	817.3949	-0.0002	0	243	249	LHYGDGR					Mascot
819.4141	819.3803	-0.0338	-41	122	127	ERMVER					Mascot
881.4363	881.4303	-0.006	-7	215	221	DVYSIER					Mascot
903.4894	903.4348	-0.0546	-60	69	77	NASGIVNTK					Mascot
1068.6637	1068.558	-0.1057	-99	229	237	AKTNLRPLR					Mascot
1118.5697	1118.5272	-0.0425	-38	138	147	VLDAMAMVPR			Oxidation (M)[5]		Mascot
1205.7294	1205.6827	-0.0467	-39	317	327	VFFVPLKSGVI					Mascot
1233.7355	1233.6294	-0.1061	-86	314	323	LDRVFFVPLK					Mascot
1470.7812	1470.7897	0.0085	6	89	101	NQLPRPATAAFER					Mascot

3	Serine/threonine-protein kinase tousled-like 1 OS=Homo sapiens GN=TLK1 PE=1 SV=2	TLK1_HUMAN	87216.2	8.88	17	46	0	9.014	

Peptide Information

Calc. Mass	Obsrv. Mass	± da	± ppm	Start Seq.	End Seq.	Sequence	Ion Score	C. I. %	Modification	Rank	Result Type
810.429	810.4418	0.0128	16	714	719	CLAYRK			Carbamidomethyl (C)[1]		Mascot
811.4533	811.4084	-0.0449	-55	138	145	SIGGRGHK					Mascot
819.373	819.3803	0.0073	9	108	114	ESETPEK					Mascot
820.3948	820.3737	-0.0211	-26	492	497	SWRDEK					Mascot
821.4111	821.4008	-0.0103	-13	115	121	KQSESSR					Mascot
838.4352	838.4112	-0.024	-29	713	718	RCLAYR			Carbamidomethyl (C)[2]		Mascot
869.4219	869.3931	-0.0288	-33	270	276	CISMSKK			Carbamidomethyl (C)[1], Oxidation (M)[4]		Mascot
921.457	921.4561	-0.0009	-1	293	299	SMQDRLR			Oxidation (M)[2]		Mascot
947.468	947.4752	0.0072	8	108	115	ESETPEKK					Mascot
963.4927	963.529	0.0363	38	555	562	LMSEKEAR					Mascot
963.4927	963.529	0.0363	38	555	562	LMSEKEAR					Mascot
1016.5193	1016.5561	0.0368	36	552	559	QHKLMSEK			Oxidation (M)[5]		Mascot
1016.5193	1016.5561	0.0368	36	552	559	QHKLMSEK			Oxidation (M)[5]		Mascot
1022.6105	1022.5748	-0.0357	-35	429	436	NLHIRELK					Mascot
1096.549	1096.5804	0.0314	29	267	275	LNKCISMSK			Carbamidomethyl (C)[4], Oxidation (M)[7]		Mascot
1106.6317	1106.5505	-0.0812	-73	514	522	ELDHPRIVK					Mascot

Calc. Mass	Obsrv. Mass	± da	± ppm	Start Seq.	End Seq.	Sequence	Ion Score	C. I. %	Modification	Rank	Result Type
1319.6187	1319.6517	0.033	25	126	137	KAENQNESSQGK					Mascot
1625.77	1625.8925	0.1225	75	46	59	EGAMDELHSLDPRR					Mascot
1908.9127	1908.9491	0.0364	19	465	480	GGFSEVYKAFDLYEQR					Mascot

4	39S ribosomal protein L19, mitochondrial OS=Pongo abelii GN=MRPL19 PE=2 SV=1	RM19_PONAB	33841.6	9.39	11	42	0	6.296	

Peptide Information

Calc. Mass	Obsrv. Mass	± da	± ppm	Start Seq.	End Seq.	Sequence	Ion Score	C. I. %	Modification	Rank	Result Type
807.4029	807.3878	-0.0151	-19	97	102	KDMLER			Oxidation (M)[3]		Mascot
819.4141	819.3803	-0.0338	-41	98	103	DMLERR					Mascot
838.424	838.4112	-0.0128	-15	92	97	FQMERK					Mascot
947.5673	947.4752	-0.0921	-97	144	152	GLGATFILR					Mascot
1173.6561	1173.6165	-0.0396	-34	224	232	MKPKPWSKR			Oxidation (M)[1]		Mascot
1244.7184	1244.6997	-0.0187	-15	222	231	VKMKPKPWSK			Oxidation (M)[3]		Mascot
1263.7056	1263.7278	0.0222	18	182	191	RLDDSLLYLR					Mascot
1263.7056	1263.7278	0.0222	18	182	191	RLDDSLLYLR					Mascot
1326.7562	1326.6749	-0.0813	-61	24	35	TLLPKPASIACR			Carbamidomethyl (C)[11]		Mascot
1657.7139	1657.8276	0.1137	69	260	271	WSQPWLEFDMMR			Oxidation (M)[10,11]		Mascot
1771.0265	1771.1014	0.0749	42	104	118	KVLHIPEFYVGSILR					Mascot
2695.2556	2695.2893	0.0337	13	192	214	DALPEYSTFDVNMKPEV QEPNQK			Oxidation (M)[13]		Mascot

5	Betaine aldehyde dehydrogenase OS=Pseudomonas fluorescens (strain Pf-5 / ATCC BAA-477) GN=betB PE=3 SV=1	BETB_PSEF5	53351	4.97	12	42	0	7.111	

Peptide Information

Calc. Mass	Obsrv. Mass	± da	± ppm	Start Seq.	End Seq.	Sequence	Ion Score	C. I. %	Modification	Rank	Result Type
814.4893	814.448	-0.0413	-51	306	312	IAERVAR					Mascot
856.5363	856.5277	-0.0086	-10	74	80	RAVEILR					Mascot
881.47	881.4303	-0.0397	-45	423	429	DLNRAHR					Mascot
947.4792	947.4752	-0.004	-4	41	48	ATKEDVER					Mascot
1016.5735	1016.5561	-0.0174	-17	49	58	AVVSAEKGQK					Mascot
1016.5735	1016.5561	-0.0174	-17	49	58	AVVSAEKGQK					Mascot
1022.5451	1022.5748	0.0297	29	1	8	MARFELQK					Mascot
1067.5402	1067.6306	0.0904	85	237	247	VMASASSSSLK					Mascot
1069.5161	1069.5288	0.0127	12	225	235	VSFTGGTDTGK					Mascot
1105.6	1105.6094	0.0094	9	300	309	AAFEAKIAER					Mascot

	1324.6644	1324.6554	-0.009	-7	211	221	EVGTWLTEHPR					Mascot
	1335.6838	1335.6156	-0.0682	-51	451	463	MPVGGYKQSGVGR					Mascot
	1335.6838	1335.6156	-0.0682	-51	451	463	MPVGGYKQSGVGR					Mascot
	1991.0193	1991.006	-0.0133	-7	408	426	ANDTDFGLAAGLVTKDLNR					Mascot

6	Tyrosine-protein kinase Yes OS=Xenopus laevis GN=yes1 PE=2 SV=3	YES_XENLA	60775.6	7.04	13	41	0	3.583	

Peptide Information

Calc. Mass	Obsrv. Mass	± da	± ppm	Start Seq.	End Seq.	Sequence	Ion Score	C. I. %	Modification	Rank	Result Type
819.3632	819.3803	0.0171	21	194	199	DWDEVR					Mascot
1096.5634	1096.5804	0.017	16	100	108	TTEDLSFRK					Mascot
1180.535	1180.6313	0.0963	82	482	490	GYRMPCPQR			Carbamidomethyl (C)[6], Oxidation (M)[4]		Mascot
1233.6157	1233.6294	0.0137	11	384	392	MNYIHRDLR			Oxidation (M)[1]		Mascot
1245.6521	1245.6625	0.0104	8	463	473	GRVPYPGMVNR					Mascot
1367.7166	1367.8048	0.0882	64	174	185	GTFLVRESETTK					Mascot
1507.6383	1507.7622	0.1239	82	233	244	HYSEHADGLCYR			Carbamidomethyl (C)[10]		Mascot
1770.9531	1771.1014	0.1483	84	390	405	DLRAANILVGDNLVCK			Carbamidomethyl (C)[15]		Mascot
1827.9998	1827.9742	-0.0256	-14	245	261	LTTVCPSVKPQTQGLAK			Carbamidomethyl (C)[5]		Mascot
1830.87	1830.9702	0.1002	55	491	504	CPESLHELMKLCWK			Carbamidomethyl (C)[1,12]		Mascot
1991.0961	1991.006	-0.0901	-45	447	464	SDVWSFGILLTELVAKGR					Mascot
2695.4236	2695.2893	-0.1343	-50	245	268	LTTVCPSVKPQTQGLAKDAWEIPR			Carbamidomethyl (C)[5]		Mascot
2723.418	2723.3367	-0.0813	-30	296	319	VAIKTLKPGTMMPEAFLQEAQIMK			Oxidation (M)[11,12,23]		Mascot

7	Chaperone protein ClpB OS=Vibrio parahaemolyticus serotype O3:K6 (strain RIMD 2210633) GN=clpB PE=3 SV=1	CLPB_VIBPA	95984.1	5.31	17	41	0	10.087	

Peptide Information

Calc. Mass	Obsrv. Mass	± da	± ppm	Start Seq.	End Seq.	Sequence	Ion Score	C. I. %	Modification	Rank	Result Type
832.4159	832.3358	-0.0801	-96	477	483	SELEQAR					Mascot
903.4781	903.4348	-0.0433	-48	128	135	EVGLTEQK					Mascot
931.3648	931.3156	-0.0492	-53	484	490	MDMEFAR			Oxidation (M)[1,3]		Mascot
931.3648	931.3156	-0.0492	-53	484	490	MDMEFAR			Oxidation (M)[1,3]		Mascot
1016.5734	1016.5561	-0.0173	-17	773	781	SIASIQLER					Mascot
1016.5734	1016.5561	-0.0173	-17	773	781	SIASIQLER					Mascot
1114.683	1114.6848	0.0018	2	259	268	LKSVLNELAK					Mascot

Calc. Mass	Obsrv. Mass	± da	± ppm	Start Seq.	End Seq.	Sequence	Ion Score	C. I. %	Modification	Rank	Result Type
1317.6897	1317.6106	-0.0791	-60	531	542	VTDNEIAEVLSK					Mascot
1324.7219	1324.6554	-0.0665	-50	820	831	AIQQNVENPLAK					Mascot
1335.6903	1335.6156	-0.0747	-56	321	331	QYIEKDAALER					Mascot
1335.6903	1335.6156	-0.0747	-56	321	331	QYIEKDAALER					Mascot
1373.7271	1373.7078	-0.0193	-14	390	402	AIDLIDEAASSIR					Mascot
1507.7751	1507.7622	-0.0129	-9	164	176	FTIDLTERAEQGK					Mascot
1516.8516	1516.7748	-0.0768	-51	236	250	RVLSLDMGALVAGAK			Oxidation (M)[7]		Mascot
1625.77	1625.8925	0.1225	75	492	505	AGDLNRMSELQYGR			Oxidation (M)[7]		Mascot
1770.9596	1771.1014	0.1418	80	403	417	LQIDSKPESLDKLER					Mascot
1827.8356	1827.9742	0.1386	76	424	439	IEQQALSNEHDEASEK					Mascot
1838.9719	1838.947	-0.0249	-14	467	483	AALSGTQHIKSELEQAR					Mascot
1990.9937	1991.006	0.0123	6	512	528	QLDLATQAEMQEMTLLR					Mascot
2723.3743	2723.3367	-0.0376	-14	71	96	LPKVSGIGGDVQLSSSM GTLFNLCDK			Carbamidomethyl (C)[24]		Mascot

8 Phosphatidylserine decarboxylase proenzyme OS=Acinetobacter baumannii (strain SDF) GN=psd PE=3 SV=1 | PSD_ACIBS | 31320.5 | 8.79 | 9 | 40 | 0 | 4.894

Peptide Information

Calc. Mass	Obsrv. Mass	± da	± ppm	Start Seq.	End Seq.	Sequence	Ion Score	C. I. %	Modification	Rank	Result Type
838.4781	838.4112	-0.0669	-80	2	8	SFTSRLK					Mascot
856.5039	856.5277	0.0238	28	41	48	AAVIHAFK					Mascot
881.4363	881.4303	-0.006	-7	115	122	GQSFSVEK					Mascot
947.4832	947.4752	-0.008	-8	259	265	DKIEWEK					Mascot
1324.6321	1324.6554	0.0233	18	66	75	YKSFNDFFTR					Mascot
1367.6989	1367.8048	0.1059	77	267	278	FKAESVVVMGER			Oxidation (M)[9]		Mascot
1516.8481	1516.7748	-0.0733	-48	228	239	IELQHHELKLEK					Mascot
1770.9973	1771.1014	0.1041	59	222	236	VKPSGRIELQHHELK					Mascot
2695.4148	2695.2893	-0.1255	-47	83	109	LVDENPDSIVSPADGAIS QIGKITAGK					Mascot

9 DNA ligase OS=Thermoanaerobacter tengcongensis (strain DSM 15242 / JCM 11007 / NBRC 100824 / MB4) GN=ligA PE=3 SV=1 | DNLJ_THETN | 75201.6 | 5.71 | 18 | 40 | 0 | 10.166

Peptide Information

Calc. Mass	Obsrv. Mass	± da	± ppm	Start Seq.	End Seq.	Sequence	Ion Score	C. I. %	Modification	Rank	Result Type
807.4069	807.3878	-0.0191	-24	396	402	AFVMPDK					Mascot
815.4733	815.4651	-0.0082	-10	9	14	RIEELR					Mascot

838.3797	838.4112	0.0315	38	528	534	SMDNIMK					Mascot
864.4574	864.4142	-0.0432	-50	226	232	IEGRDFK					Mascot
871.5359	871.4689	-0.067	-77	611	618	LIVERGGK					Mascot
887.4217	887.4655	0.0438	49	56	63	TPDSPSQR					Mascot
963.4377	963.529	0.0913	95	388	395	EERTGDEK					Mascot
963.4377	963.529	0.0913	95	388	395	EERTGDEK					Mascot
1015.5894	1015.5254	-0.064	-63	292	300	REILGQTAK					Mascot
1016.5119	1016.5561	0.0442	43	180	187	LNEERAER					Mascot
1016.5119	1016.5561	0.0442	43	180	187	LNEERAER					Mascot
1069.5017	1069.5288	0.0271	25	528	536	SMDNIMKAK			Oxidation (M)[2,6]		Mascot
1118.5874	1118.5272	-0.0602	-54	570	579	LKEAGVNMEK					Mascot
1326.7124	1326.6749	-0.0375	-28	197	209	NAAAGSVRQLDPK					Mascot
1335.6791	1335.6156	-0.0635	-48	627	639	TDYLIVGADPGSK					Mascot
1335.6791	1335.6156	-0.0635	-48	627	639	TDYLIVGADPGSK					Mascot
1470.745	1470.7897	0.0447	30	304	315	WAIAFKYPAEMK			Oxidation (M)[11]		Mascot
1509.8312	1509.8456	0.0144	10	460	472	GLIQNVADLYYLK					Mascot
1516.7472	1516.7748	0.0276	18	421	433	CTGLNCPAQIARR			Carbamidomethyl (C)[1,6]		Mascot
1830.8804	1830.9702	0.0898	49	550	564	MARSIVTFFSEEQNR			Oxidation (M)[1]		Mascot
1990.979	1991.006	0.027	14	650	666	IINEEQFEAMLKGDIQP			Oxidation (M)[10]		Mascot

10	Riboflavin biosynthesis protein RibBA OS=Mycobacterium ulcerans (strain Agy99) GN=ribBA PE=3 SV=1	RIBBA_MYCUA	46419.6	5.5	11	40	0	5.221

Protein Group

Riboflavin biosynthesis protein RibBA OS=Mycobacterium marinum (strain ATCC BAA-535 / M) GN=ribBA PE=3 SV=1	RIBBA_MYCMM	46353.5	5.3499 999046 3257

Peptide Information

Calc. Mass	Obsrv. Mass	± da	± ppm	Start Seq.	End Seq.	Sequence	Ion Score	C. I. %	Modification	Rank	Result Type
811.4494	811.4084	-0.041	-51	393	398	YLMTKR					Mascot
812.4625	812.4225	-0.04	-49	332	339	LGLPADAR					Mascot
815.4734	815.4651	-0.0083	-10	133	140	AKDGGVLR					Mascot
870.5043	870.5417	0.0374	43	358	365	LLTNNPAK					Mascot
1106.5623	1106.5505	-0.0118	-11	1	9	MTRLDSVER					Mascot
1133.5546	1133.6034	0.0488	43	94	105	NGVGTGISASDR					Mascot
1233.666	1233.6294	-0.0366	-30	41	51	ATPELVAFMVR					Mascot
1244.678	1244.6997	0.0217	17	355	365	SMRLLTNNPAK					Mascot
1335.6427	1335.6156	-0.0271	-20	29	40	ENEGDLIFAAEK					Mascot

1335.6427	1335.6156	-0.0271	-20	29	40	ENEGDLIFAAEK	Mascot
1373.7107	1373.7078	-0.0029	-2	294	305	GVVLYMRGHEGR	Mascot
1461.777	1461.869	0.092	63	69	81	LGLLPMYAVNQDK	Mascot

Gel Idx/Pos	246/J22	Instr./Gel Origin	BA2151/Sample Project 20140814	Process Status	Analysis Succeeded
Plate [#] Name	[1] Sample Project 20140814	Instrument Sample Name		Spectra	11

Rank	Protein Name	Accession No.	Protein MW	Protein PI	Pep. Count	Protein Score	Protein Score C. I. %	Intensity Matched	Total Ion Score	Total Ion C. I. %	Confirmed
1	ATP synthase subunit beta, mitochondrial OS=Oryza sativa subsp. japonica GN=ATPB PE=1 SV=2	ATPBM_ORYSJ	59011.8	5.95	20	710	100	37.226	596	100	

Peptide Information

Calc. Mass	Obsrv. Mass	± da	± ppm	Start Seq.	End Seq.	Sequence	Ion Score	C. I. %	Modification	Rank	Result Type
866.4003	866.4259	0.0256	30	263	269	EGNDLYR					Mascot
1173.6627	1173.6957	0.033	28	210	219	VVDLLAPYQR					Mascot
1173.6627	1173.6957	0.033	28	210	219	VVDLLAPYQR	47	98.09			Mascot
1278.6359	1278.6624	0.0265	21	131	142	TIAMDGTEGLVR			Oxidation (M)[4]		Mascot
1390.6863	1390.7303	0.044	32	247	260	AHGGFSVFAGVGER					Mascot
1390.6863	1390.7303	0.044	32	247	260	AHGGFSVFAGVGER	109	100			Mascot
1399.7693	1399.8136	0.0443	32	305	317	VGLTGLTVAEHFR					Mascot
1399.7693	1399.8136	0.0443	32	305	317	VGLTGLTVAEHFR	45	96.866			Mascot
1409.8112	1409.854	0.0428	30	146	159	VLNTGSPITVPVGR					Mascot
1418.756	1418.7501	-0.0059	-4	270	282	EMIESGVIKLGDK					Mascot
1473.8346	1473.8148	-0.0198	-13	234	246	TVLIMELINNVAK			Oxidation (M)[5]		Mascot
1492.7755	1492.8224	0.0469	31	334	347	FTQANSEVSALLGR					Mascot
1492.7755	1492.8224	0.0469	31	334	347	FTQANSEVSALLGR	71	99.993			Mascot
1520.7969	1520.829	0.0321	21	177	189	GDITTNHFLPIHR					Mascot
1678.7676	1678.7964	0.0288	17	288	302	CALVYGQMNEPPGAR			Carbamidomethyl (C)[1], Oxidation (M)[8]		Mascot
1697.8871	1697.9668	0.0797	47	28	42	GPLHRPSPSGYLFNR					Mascot
1852.9109	1853.0294	0.1185	64	263	278	EGNDLYREMIESGVIK					Mascot
1864.944	1865.0048	0.0608	33	318	333	DAEGQDVLLFIDNIFR					Mascot
1864.944	1865.0048	0.0608	33	318	333	DAEGQDVLLFIDNIFR	117	100			Mascot
1868.9059	1869.0281	0.1222	65	263	278	EGNDLYREMIESGVIK			Oxidation (M)[9]		Mascot
2061.0498	2061.1155	0.0657	32	411	429	QISELGIYPAVDPLDSTSR					Mascot
2172.1548	2172.2068	0.052	24	190	209	EAPAFVEQATEQQILVTG IK					Mascot
2186.1453	2186.2266	0.0813	37	348	368	IPSAVGYQPTLATDLGGL QER					Mascot
2186.1453	2186.2266	0.0813	37	348	368	IPSAVGYQPTLATDLGGL QER	206	100			Mascot
2688.376	2688.4717	0.0957	36	456	479	NLQDIIAILGMDELSEDDK LTVAR			Oxidation (M)[11]		Mascot
3714.8862	3715.0525	0.1663	45	375	410	GSITSVQAIYVPADDLTD					Mascot

3842.9812	3843.1782	0.197	51	374	410	PAPATTFAHLDATTVLSR KGSITSVQAIYVPADDLT DPAPATTFAHLDATTVLS R					Mascot

2	ATP synthase subunit beta, mitochondrial OS=Nicotiana plumbaginifolia GN=ATPB PE=1 SV=1	ATPBM_NICPL	59933.3	5.95	20	709	100	37.401	596	100

Peptide Information

Calc. Mass	Obsrv. Mass	± da	± ppm	Start Seq.	End Seq.	Sequence	Ion Score	C. I. %	Modification	Rank	Result Type
803.437	803.4128	-0.0242	-30	37	44	ASSRASPK					Mascot
821.4224	821.3947	-0.0277	-34	33	40	SASRASSR					Mascot
866.4003	866.4259	0.0256	30	271	277	EGNDLYR					Mascot
1173.6627	1173.6957	0.033	28	218	227	VVDLLAPYQR					Mascot
1173.6627	1173.6957	0.033	28	218	227	VVDLLAPYQR	47	98.09			Mascot
1278.6359	1278.6624	0.0265	21	139	150	TIAMDGTEGLVR			Oxidation (M)[4]		Mascot
1390.6863	1390.7303	0.044	32	255	268	AHGGFSVFAGVGER					Mascot
1390.6863	1390.7303	0.044	32	255	268	AHGGFSVFAGVGER	109	100			Mascot
1399.7693	1399.8136	0.0443	32	313	325	VGLTGLTVAEHFR					Mascot
1399.7693	1399.8136	0.0443	32	313	325	VGLTGLTVAEHFR	45	96.866			Mascot
1409.8112	1409.854	0.0428	30	154	167	VLNTGSPITVPVGR					Mascot
1473.8346	1473.8148	-0.0198	-13	242	254	TVLIMELINNVAK			Oxidation (M)[5]		Mascot
1492.7755	1492.8224	0.0469	31	342	355	FTQANSEVSALLGR					Mascot
1492.7755	1492.8224	0.0469	31	342	355	FTQANSEVSALLGR	71	99.993			Mascot
1678.7676	1678.7964	0.0288	17	296	310	CALVYGQMNEPPGAR			Carbamidomethyl (C)[1], Oxidation (M)[8]		Mascot
1852.9109	1853.0294	0.1185	64	271	286	EGNDLYREMIESGVIK					Mascot
1853.8964	1854.0549	0.1585	85	438	453	MLSPHILGEDHYNTAR					Mascot
1864.944	1865.0048	0.0608	33	326	341	DAEGQDVLLFIDNIFR					Mascot
1864.944	1865.0048	0.0608	33	326	341	DAEGQDVLLFIDNIFR	117	100			Mascot
1868.9059	1869.0281	0.1222	65	271	286	EGNDLYREMIESGVIK			Oxidation (M)[9]		Mascot
2061.0498	2061.1155	0.0657	32	419	437	QISELGIYPAVDPLDSTSR					Mascot
2172.1548	2172.2068	0.052	24	198	217	EAPAFVEQATEQQILVTG IK					Mascot
2186.1453	2186.2266	0.0813	37	356	376	IPSAVGYQPTLATDLGGL QER					Mascot
2186.1453	2186.2266	0.0813	37	356	376	IPSAVGYQPTLATDLGGL QER	206	100			Mascot
2591.3499	2591.3718	0.0219	8	79	103	ITDEFTGAGSIGKVCQVIG AVVDVR			Carbamidomethyl (C)[15]		Mascot
3714.8862	3715.0525	0.1663	45	383	418	GSITSVQAIYVPADDLTD PAPATTFAHLDATTVLSR					Mascot
3842.9812	3843.1782	0.197	51	382	418	KGSITSVQAIYVPADDLT DPAPATTFAHLDATTVLS R					Mascot

3	ATP synthase subunit beta, mitochondrial OS=Hevea brasiliensis GN=ATPB PE=2 SV=1	ATPBM_HEVBR	60335.4	5.95	19	700	100	37.047	596	100

Peptide Information

Calc. Mass	Obsrv. Mass	± da	± ppm	Start Seq.	End Seq.	Sequence	Ion Score	C. I. %	Modification	Rank	Result Type
866.4003	866.4259	0.0256	30	273	279	EGNDLYR					Mascot
1173.6627	1173.6957	0.033	28	220	229	VVDLLAPYQR					Mascot
1173.6627	1173.6957	0.033	28	220	229	VVDLLAPYQR	47	98.09			Mascot
1278.6359	1278.6624	0.0265	21	141	152	TIAMDGTEGLVR			Oxidation (M)[4]		Mascot
1390.6863	1390.7303	0.044	32	257	270	AHGGFSVFAGVGER					Mascot
1390.6863	1390.7303	0.044	32	257	270	AHGGFSVFAGVGER	109	100			Mascot
1399.7693	1399.8136	0.0443	32	315	327	VGLTGLTVAEHFR					Mascot
1399.7693	1399.8136	0.0443	32	315	327	VGLTGLTVAEHFR	45	96.866			Mascot
1409.8112	1409.854	0.0428	30	156	169	VLNTGSPITVPVGR					Mascot
1418.756	1418.7501	-0.0059	-4	280	292	EMIESGVIKLGDK					Mascot
1473.8346	1473.8148	-0.0198	-13	244	256	TVLIMELINNVAK			Oxidation (M)[5]		Mascot
1492.7755	1492.8224	0.0469	31	344	357	FTQANSEVSALLGR					Mascot
1492.7755	1492.8224	0.0469	31	344	357	FTQANSEVSALLGR	71	99.993			Mascot
1520.8333	1520.829	-0.0043	-3	187	199	GDIKTSHFLPIHR					Mascot
1678.7676	1678.7964	0.0288	17	298	312	CALVYGQMNEPPGAR			Carbamidomethyl (C)[1], Oxidation (M)[8]		Mascot
1852.9109	1853.0294	0.1185	64	273	288	EGNDLYREMIESGVIK					Mascot
1864.944	1865.0048	0.0608	33	328	343	DAEGQDVLLFIDNIFR					Mascot
1864.944	1865.0048	0.0608	33	328	343	DAEGQDVLLFIDNIFR	117	100			Mascot
1868.9059	1869.0281	0.1222	65	273	288	EGNDLYREMIESGVIK			Oxidation (M)[9]		Mascot
2061.0498	2061.1155	0.0657	32	421	439	QISELGIYPAVDPLDSTSR					Mascot
2186.1453	2186.2266	0.0813	37	358	378	IPSAVGYQPTLATDLGGL QER					Mascot
2186.1453	2186.2266	0.0813	37	358	378	IPSAVGYQPTLATDLGGL QER	206	100			Mascot
2208.0173	2208.1892	0.1719	78	293	312	QADSKCALVYGQMNEPP GAR			Carbamidomethyl (C)[6], Oxidation (M)[13]		Mascot
2688.376	2688.4717	0.0957	36	466	489	NLQDIIAILGMDELSEDDK LTVAR			Oxidation (M)[11]		Mascot
3714.8862	3715.0525	0.1663	45	385	420	GSITSVQAIYVPADDLTD PAPATTFAHLDATTVLSR					Mascot
3842.9812	3843.1782	0.197	51	384	420	KGSITSVQAIYVPADDLT DPAPATTFAHLDATTVLS R					Mascot

4	ATP synthase subunit beta, mitochondrial OS=Zea mays GN=ATPB PE=1 SV=1	ATPBM_MAIZE	59181	6.01	17	684	100	36.856	594	100

Peptide Information

Calc. Mass	Obsrv. Mass	± da	± ppm	Start Seq.	End Seq.	Sequence	Ion Score	C. I. %	Modification	Rank	Result Type
866.4003	866.4259	0.0256	30	264	270	EGNDLYR					Mascot
1173.6627	1173.6957	0.033	28	211	220	VVDLLAPYQR					Mascot
1173.6627	1173.6957	0.033	28	211	220	VVDLLAPYQR	47	98.09			Mascot
1278.6359	1278.6624	0.0265	21	132	143	TIAMDGTEGLVR			Oxidation (M)[4]		Mascot
1390.6863	1390.7303	0.044	32	248	261	AHGGFSVFAGVGER					Mascot
1390.6863	1390.7303	0.044	32	248	261	AHGGFSVFAGVGER	109	100			Mascot
1399.7693	1399.8136	0.0443	32	306	318	VGLTGLTVAEHFR					Mascot
1399.7693	1399.8136	0.0443	32	306	318	VGLTGLTVAEHFR	45	96.866			Mascot
1409.8112	1409.854	0.0428	30	147	160	VLNTGSPITVPVGR					Mascot
1473.8346	1473.8148	-0.0198	-13	235	247	TVLIMELINNVAK			Oxidation (M)[5]		Mascot
1492.7755	1492.8224	0.0469	31	335	348	FTQANSEVSALLGR					Mascot
1492.7755	1492.8224	0.0469	31	335	348	FTQANSEVSALLGR	71	99.993			Mascot
1678.7676	1678.7964	0.0288	17	289	303	CALVYGQMNEPPGAR			Carbamidomethyl (C)[1], Oxidation (M)[8]		Mascot
1852.9109	1853.0294	0.1185	64	264	279	EGNDLYREMIESGVIK					Mascot
1864.944	1865.0048	0.0608	33	319	334	DAEGQDVLLFIDNIFR					Mascot
1864.944	1865.0048	0.0608	33	319	334	DAEGQDVLLFIDNIFR	117	100			Mascot
1868.9059	1869.0281	0.1222	65	264	279	EGNDLYREMIESGVIK			Oxidation (M)[9]		Mascot
2061.0498	2061.1155	0.0657	32	412	430	QISELGIYPAVDPLDSTSR					Mascot
2172.1548	2172.2068	0.052	24	191	210	EAPAFVEQATEQQILVTG IK					Mascot
2186.1453	2186.2266	0.0813	37	349	369	IPSAVGYQPTLATDLGGL QER					Mascot
2186.1453	2186.2266	0.0813	37	349	369	IPSAVGYQPTLATDLGGL QER	206	100			Mascot
2688.376	2688.4717	0.0957	36	457	480	NLQDIIAILGMDELSEDDK LTVAR			Oxidation (M)[11]		Mascot
3714.8862	3715.0525	0.1663	45	376	411	GSITSVQAIYVPADDLTD PAPATTFAHLDATTVLSR					Mascot
3842.9812	3843.1782	0.197	51	375	411	KGSITSVQAIYVPADDLT DPAPATTFAHLDATTVLS R					Mascot

5	ATP synthase subunit beta-3, mitochondrial OS=Arabidopsis thaliana GN=At5g08680 PE=2 SV=1	ATPBO_ARATH	59993.2	6.06	17	558	100	32.112	471	100	

Peptide Information

Calc. Mass	Obsrv. Mass	± da	± ppm	Start Seq.	End Seq.	Sequence	Ion Score	C. I. %	Modification	Rank	Result Type
803.357	803.4128	0.0558	69	77	83	TYDYGGK					Mascot
866.4003	866.4259	0.0256	30	270	276	EGNDLYR					Mascot
931.452	931.5323	0.0803	86	76	83	KTYDYGGK					Mascot

1173.6627	1173.6957	0.033	28	217	226	VVDLLAPYQR					Mascot
1173.6627	1173.6957	0.033	28	217	226	VVDLLAPYQR	47	98.09			Mascot
1278.6359	1278.6624	0.0265	21	138	149	TIAMDGTEGLVR			Oxidation (M)[4]		Mascot
1390.6863	1390.7303	0.044	32	254	267	AHGGFSVFAGVGER					Mascot
1390.6863	1390.7303	0.044	32	254	267	AHGGFSVFAGVGER	109	100			Mascot
1473.8346	1473.8148	-0.0198	-13	241	253	TVLIMELINNVAK			Oxidation (M)[5]		Mascot
1492.7755	1492.8224	0.0469	31	341	354	FTQANSEVSALLGR					Mascot
1492.7755	1492.8224	0.0469	31	341	354	FTQANSEVSALLGR	71	99.993			Mascot
1678.7676	1678.7964	0.0288	17	295	309	CALVYGQMNEPPGAR			Carbamidomethyl (C)[1], Oxidation (M)[8]		Mascot
1852.9109	1853.0294	0.1185	64	270	285	EGNDLYREMIESGVIK					Mascot
1864.944	1865.0048	0.0608	33	325	340	DAEGQDVLLFIDNIFR					Mascot
1864.944	1865.0048	0.0608	33	325	340	DAEGQDVLLFIDNIFR	117	100			Mascot
1868.9059	1869.0281	0.1222	65	270	285	EGNDLYREMIESGVIK			Oxidation (M)[9]		Mascot
2061.0498	2061.1155	0.0657	32	418	436	QISELGIYPAVDPLDSTSR					Mascot
2186.1453	2186.2266	0.0813	37	355	375	IPSAVGYQPTLASDLGAL QER					Mascot
2186.1453	2186.2266	0.0813	37	355	375	IPSAVGYQPTLASDLGAL QER	126	100			Mascot
2212.1768	2212.2603	0.0835	38	29	50	NPRLLSPGPAHGAAPCG TLLGR			Carbamidomethyl (C)[16]		Mascot
2212.1768	2212.2603	0.0835	38	29	50	NPRLLSPGPAHGAAPCG TLLGR			Carbamidomethyl (C)[16]		Mascot
2688.376	2688.4717	0.0957	36	463	486	NLQDIIAILGMDELSEDDK LTVAR			Oxidation (M)[11]		Mascot
3714.8862	3715.0525	0.1663	45	382	417	GSITSVQAIYVPADDLTD PAPATTFAHLDATTVLSR					Mascot
3842.9812	3843.1782	0.197	51	381	417	KGSITSVQAIYVPADDLT DPAPATTFAHLDATTVLS R					Mascot

6	ATP synthase subunit beta-2, mitochondrial OS=Arabidopsis thaliana GN=At5g08690 PE=1 SV=1	ATPBN_ARATH	59847.2	6.18	16	549	100	29.236	471	100		

Protein Group

ATP synthase subunit beta-1, mitochondrial OS=Arabidopsis thaliana GN=At5g08670 PE=1 SV=1	ATPBM_ARATH	59805.1	6.1799 998283 3862

Peptide Information

Calc. Mass	Obsrv. Mass	± da	± ppm	Start Seq.	End Seq.	Sequence	Ion Score	C. I. %	Modification	Rank	Result Type
803.357	803.4128	0.0558	69	74	80	TYDYGGK					Mascot
866.4003	866.4259	0.0256	30	267	273	EGNDLYR					Mascot
931.452	931.5323	0.0803	86	73	80	KTYDYGGK					Mascot
1173.6627	1173.6957	0.033	28	214	223	VVDLLAPYQR					Mascot
1173.6627	1173.6957	0.033	28	214	223	VVDLLAPYQR	47	98.09			Mascot

1278.6359	1278.6624	0.0265	21	135	146	TIAMDGTEGLVR			Oxidation (M)[4]		Mascot
1390.6863	1390.7303	0.044	32	251	264	AHGGFSVFAGVGER					Mascot
1390.6863	1390.7303	0.044	32	251	264	AHGGFSVFAGVGER	109	100			Mascot
1473.8346	1473.8148	-0.0198	-13	238	250	TVLIMELINNVAK			Oxidation (M)[5]		Mascot
1492.7755	1492.8224	0.0469	31	338	351	FTQANSEVSALLGR					Mascot
1492.7755	1492.8224	0.0469	31	338	351	FTQANSEVSALLGR	71	99.993			Mascot
1678.7676	1678.7964	0.0288	17	292	306	CALVYGQMNEPPGAR			Carbamidomethyl (C)[1], Oxidation (M)[8]		Mascot
1852.9109	1853.0294	0.1185	64	267	282	EGNDLYREMIESGVIK					Mascot
1864.944	1865.0048	0.0608	33	322	337	DAEGQDVLLFIDNIFR					Mascot
1864.944	1865.0048	0.0608	33	322	337	DAEGQDVLLFIDNIFR	117	100			Mascot
1868.9059	1869.0281	0.1222	65	267	282	EGNDLYREMIESGVIK			Oxidation (M)[9]		Mascot
2061.0498	2061.1155	0.0657	32	415	433	QISELGIYPAVDPLDSTSR					Mascot
2186.1453	2186.2266	0.0813	37	352	372	IPSAVGYQPTLASDLGAL QER					Mascot
2186.1453	2186.2266	0.0813	37	352	372	IPSAVGYQPTLASDLGAL QER	126	100			Mascot
2688.376	2688.4717	0.0957	36	460	483	NLQDIIAILGMDELSEDDK LTVAR			Oxidation (M)[11]		Mascot
3714.8862	3715.0525	0.1663	45	379	414	GSITSVQAIYVPADDLTD PAPATTFAHLDATTVLSR					Mascot
3842.9812	3843.1782	0.197	51	378	414	KGSITSVQAIYVPADDLT DPAPATTFAHLDATTVLS R					Mascot

7	ATP synthase subunit beta, mitochondrial OS=Chlamydomonas reinhardtii GN=ATP2 PE=1 SV=1	ATPBM_CHLRE	61954	4.99	10	462	100	26.393	433	100	

Peptide Information

Calc. Mass	Obsrv. Mass	± da	± ppm	Start Seq.	End Seq.	Sequence	Ion Score	C. I. %	Modification	Rank	Result Type
866.4003	866.4259	0.0256	30	219	225	EGNDLYR					Mascot
1173.6627	1173.6957	0.033	28	166	175	VVDLLAPYQR					Mascot
1173.6627	1173.6957	0.033	28	166	175	VVDLLAPYQR	47	98.09			Mascot
1390.6863	1390.7303	0.044	32	203	216	AHGGFSVFAGVGER					Mascot
1390.6863	1390.7303	0.044	32	203	216	AHGGFSVFAGVGER	109	100			Mascot
1418.756	1418.7501	-0.0059	-4	226	238	EMIESGVIKLGDK					Mascot
1473.8346	1473.8148	-0.0198	-13	190	202	TVLIMELINNVAK			Oxidation (M)[5]		Mascot
1492.7755	1492.8224	0.0469	31	290	303	FTQANSEVSALLGR					Mascot
1492.7755	1492.8224	0.0469	31	290	303	FTQANSEVSALLGR	71	99.993			Mascot
1697.864	1697.9668	0.1028	61	72	86	LVLEVAQHMGDNTVR			Oxidation (M)[9]		Mascot
1852.9109	1853.0294	0.1185	64	219	234	EGNDLYREMIESGVIK					Mascot
1864.8956	1865.0048	0.1092	59	552	568	AEAISSENMVLNEKGEK			Oxidation (M)[9]		Mascot
1864.8956	1865.0048	0.1092	59	552	568	AEAISSENMVLNEKGEK			Oxidation (M)[9]		Mascot

Calc. Mass	Obsrv. Mass	± da	± ppm	Start Seq.	End Seq.	Sequence	Ion Score	C. I. %	Modification	Rank	Result Type
1868.9059	1869.0281	0.1222	65	219	234	EGNDLYREMIESGVIK			Oxidation (M)[9]		Mascot
2186.1453	2186.2266	0.0813	37	304	324	IPSAVGYQPTLATDLGGL QER					Mascot
2186.1453	2186.2266	0.0813	37	304	324	IPSAVGYQPTLATDLGGL QER	206	100			Mascot

8	ATP synthase subunit beta, mitochondrial OS=Daucus carota GN=ATPB PE=3 SV=1	ATPBM_DAUCA	59326.8	5.62	14	391	100	26.37	322	100	

Peptide Information

Calc. Mass	Obsrv. Mass	± da	± ppm	Start Seq.	End Seq.	Sequence	Ion Score	C. I. %	Modification	Rank	Result Type
1278.6359	1278.6624	0.0265	21	130	141	TIAMDGTEGLVR			Oxidation (M)[4]		Mascot
1342.6962	1342.6866	-0.0096	-7	53	67	EPAASKPAGTAGTGK					Mascot
1390.7478	1390.7303	-0.0175	-13	219	232	GGKIGLFGGDWVGK					Mascot
1390.7478	1390.7303	-0.0175	-13	219	232	GGKIGLFGGDWVGK					Mascot
1399.7693	1399.8136	0.0443	32	301	313	VGLTGLTVAEHFR					Mascot
1399.7693	1399.8136	0.0443	32	301	313	VGLTGLTVAEHFR	45	96.866			Mascot
1473.8346	1473.8148	-0.0198	-13	233	245	TVLIMELINNVAK			Oxidation (M)[5]		Mascot
1492.7755	1492.8224	0.0469	31	330	343	FTQANSEVSALLGR					Mascot
1492.7755	1492.8224	0.0469	31	330	343	FTQANSEVSALLGR	71	99.993			Mascot
1678.7676	1678.7964	0.0288	17	284	298	CALVYGQMNEPPGSR			Carbamidomethyl (C)[1]		Mascot
2061.0498	2061.1155	0.0657	32	407	425	QISELGIYPAVDPLDSTSR					Mascot
2087.0615	2087.1243	0.0628	30	53	74	EPAASKPAGTAGTGKGTI TDEK					Mascot
2116.1033	2116.1692	0.0659	31	45	67	YATAAPAKEPAASKPAGT AGTGK					Mascot
2186.1453	2186.2266	0.0813	37	344	364	IPSAVGYQPTLATDLGGL QER					Mascot
2186.1453	2186.2266	0.0813	37	344	364	IPSAVGYQPTLATDLGGL QER	206	100			Mascot
2688.376	2688.4717	0.0957	36	451	474	NLQDIIAILGMDELSEDDK LTVAR			Oxidation (M)[11]		Mascot
3714.8862	3715.0525	0.1663	45	371	406	GSITSVQAIYVPADDLTD PAPATTFAHLDATTVLSR					Mascot
3842.9812	3843.1782	0.197	51	370	406	KGSITSVQAIYVPADDLT DPAPATTFAHLDATTVLS R					Mascot

9	ATP synthase subunit beta, mitochondrial (Fragments) OS=Vitis sp. PE=1 SV=1	ATPBM_VITSX	6430.4	6.73	4	356	100	19.164	322	100	

Peptide Information

Calc. Mass	Obsrv. Mass	± da	± ppm	Start Seq.	End Seq.	Sequence	Ion Score	C. I. %	Modification	Rank	Result Type
1399.7693	1399.8136	0.0443	32	15	27	VGLTGLTVAEHFR					Mascot
1399.7693	1399.8136	0.0443	32	15	27	VGLTGLTVAEHFR	45	96.866			Mascot

1409.8112	1409.854	0.0428	30	1	14	VLNTGSPITVPVGR					Mascot
1492.7755	1492.8224	0.0469	31	28	41	FTQANSEVSALLGR					Mascot
1492.7755	1492.8224	0.0469	31	28	41	FTQANSEVSALLGR	71	99.993			Mascot
2186.1453	2186.2266	0.0813	37	42	62	IPSAVGYQPTLATDLGGL QER					Mascot
2186.1453	2186.2266	0.0813	37	42	62	IPSAVGYQPTLATDLGGL QER	206	100			Mascot

10	ATP synthase subunit beta OS=Clostridium cellulolyticum (strain ATCC 35319 / DSM 5812 / JCM 6584 / H10) GN=atpD PE=3 SV=1	ATPB_CLOCE	50526	4.85	7	188	100	10.106	168	100	

Peptide Information

Calc. Mass	Obsrv. Mass	± da	± ppm	Start Seq.	End Seq.	Sequence	Ion Score	C. I. %	Modification	Rank	Result Type
1230.6729	1230.6603	-0.0126	-10	90	100	IFNVLGEPVDK					Mascot
1473.7407	1473.8148	0.0741	50	222	234	VGLTGLTMAEYFR			Oxidation (M)[8]		Mascot
1520.822	1520.829	0.007	5	101	114	AGPVEPTAYLPIHR					Mascot
1606.8475	1606.869	0.0215	13	20	33	FENGILPDIYNAIK					Mascot
1892.8928	1892.9484	0.0556	29	205	221	TAMVFGQMNEPPGARM R					Mascot
2044.1987	2044.0914	-0.1073	-52	148	167	IGLFGGAGVGKTVLIMELI R					Mascot
2186.1453	2186.2266	0.0813	37	265	285	IPSAVGYQPTLATDVGAL QER					Mascot
2186.1453	2186.2266	0.0813	37	265	285	IPSAVGYQPTLATDVGAL QER	168	100			Mascot

Gel Idx/Pos	247/J23	Instr./Gel Origin	BA2151/Sample Project 20140814	Process Status	Analysis Succeeded
Plate [#] Name	[1] Sample Project 20140814	Instrument Sample Name		Spectra	11

Rank	Protein Name	Accession No.	Protein MW	Protein PI	Pep. Count	Protein Score	Protein Score C. I. %	Intensity Matched	Total Ion Score	Total Ion C. I. %	Confirmed
1	UTP--glucose-1-phosphate uridylyltransferase OS=Hordeum vulgare PE=2 SV=1	UGPA_HORVU	51783.2	5.2	18	554	100	51.614	459	100	

Peptide Information

Calc. Mass	Obsrv. Mass	± da	± ppm	Start Seq.	End Seq.	Sequence	Ion Score	C. I. %	Modification	Rank	Result Type
839.5098	839.5275	0.0177	21	350	357	AIGINVPR					Mascot
918.5519	918.5631	0.0112	12	406	413	KVANFLAR					Mascot
949.5465	949.5652	0.0187	20	31	39	AGFISLVSR					Mascot
949.5465	949.5652	0.0187	20	31	39	AGFISLVSR	62	99.936			Mascot
1014.5942	1014.6111	0.0169	17	310	318	RLVDAEALK					Mascot
1052.5371	1052.5609	0.0238	23	261	270	GGTLISYEGR					Mascot
1052.5371	1052.5609	0.0238	23	261	270	GGTLISYEGR	64	99.961			Mascot
1300.7358	1300.7156	-0.0202	-16	416	427	SIPSIVELDSLK					Mascot
1312.7583	1312.7961	0.0378	29	333	345	VLQLETAAGAAIR					Mascot
1312.7583	1312.7961	0.0378	29	333	345	VLQLETAAGAAIR	110	100			Mascot
1350.7264	1350.6886	-0.0378	-28	428	441	VSGDVSFGSGVVLK					Mascot
1358.7566	1358.7612	0.0046	3	172	183	IVTEDFLPLPSK					Mascot
1390.7842	1390.6754	-0.1088	-78	346	357	FFEKAIGINVPR					Mascot
1390.7842	1390.6754	-0.1088	-78	346	357	FFEKAIGINVPR					Mascot
1428.7805	1428.7368	-0.0437	-31	2	16	AAAAVAADSKIDGLR					Mascot
1539.7325	1539.7522	0.0197	13	40	52	YLSGEAEQIEWSK					Mascot
1641.8846	1641.9037	0.0191	12	391	405	VKPSNPSIELGPEFK					Mascot
1940.0812	1940.1102	0.029	15	327	345	EVDGVKVLQLETAAGAAIR					Mascot
1967.0121	1967.0576	0.0455	23	218	235	EYVFVANSDNLGAIVDIK					Mascot
2143.9832	2144.0635	0.0803	37	189	208	DGWYPPGHGDVFPSLNNSGK					Mascot
2198.0261	2198.1274	0.1013	46	154	171	YSNSNIEIHTFNQSQYPR					Mascot
2198.0261	2198.1274	0.1013	46	154	171	YSNSNIEIHTFNQSQYPR	119	100			Mascot
2454.3127	2454.4099	0.0972	40	365	386	ATSDLLLVQSDLYTLVDGYVIR					Mascot
2454.3127	2454.4099	0.0972	40	365	386	ATSDLLLVQSDLYTLVDGYVIR	104	100			Mascot

Rank	Protein Name	Accession No.	Protein MW	Protein PI	Pep. Count	Protein Score	Protein Score C. I. %	Intensity Matched	Total Ion Score	Total Ion C. I. %	Confirmed
2	UTP--glucose-1-phosphate uridylyltransferase OS=Astragalus penduliflorus GN=UGP PE=1 SV=1	UGPA_ASTPN	51632.3	5.92	11	213	100	11.242	174	100	

Peptide Information

Calc. Mass	Obsrv. Mass	± da	± ppm	Start Seq.	End Seq.	Sequence	Ion Score	C. I. %	Modification	Rank	Result Type
839.5098	839.5275	0.0177	21	348	355	AIGINVPR					Mascot
1014.5942	1014.6111	0.0169	17	308	316	RLVEADALK					Mascot
1052.5371	1052.5609	0.0238	23	259	268	GGTLISYEGR					Mascot
1052.5371	1052.5609	0.0238	23	259	268	GGTLISYEGR	64	99.961			Mascot
1300.7358	1300.7156	-0.0202	-16	414	425	SIPSIVELDSLK					Mascot
1312.7583	1312.7961	0.0378	29	331	343	VLQLETAAGAAIR					Mascot
1312.7583	1312.7961	0.0378	29	331	343	VLQLETAAGAAIR	110	100			Mascot
1342.7617	1342.8049	0.0432	32	170	181	LVVDDFLPLPSK					Mascot
1342.7617	1342.8049	0.0432	32	170	181	LVVDDFLPLPSK					Mascot
1492.7788	1492.8119	0.0331	22	1	14	MATATATDRLSNLK					Mascot
1898.9607	1898.968	0.0073	4	387	403	ARTNPENPSIELGPEFK					Mascot
1940.0812	1940.1102	0.029	15	325	343	EVDGVKVLQLETAAGAAIR					Mascot
1967.0121	1967.0576	0.0455	23	216	233	EYVFVANSDNLGAIVDLK					Mascot
2145.1802	2145.0654	-0.1148	-54	108	126	DGLTFLDLIVIQIENLNSK					Mascot

3	Probable UTP--glucose-1-phosphate uridylyltransferase 2 OS=Arabidopsis thaliana GN=At3g03250 PE=1 SV=1	UGPA2_ARATH	51877.2	5.8	6	205	100	6.318	191	100	

Peptide Information

Calc. Mass	Obsrv. Mass	± da	± ppm	Start Seq.	End Seq.	Sequence	Ion Score	C. I. %	Modification	Rank	Result Type
965.5414	965.559	0.0176	18	27	35	SGFISLVSR					Mascot
1300.7358	1300.7156	-0.0202	-16	412	423	SIPSIVELDSLK					Mascot
1312.7583	1312.7961	0.0378	29	329	341	VLQLETAAGAAIR					Mascot
1312.7583	1312.7961	0.0378	29	329	341	VLQLETAAGAAIR	110	100			Mascot
1940.0812	1940.1102	0.029	15	323	341	EVDGVKVLQLETAAGAAIR					Mascot
2108.1023	2108.1689	0.0666	32	267	284	VQLLEIAQVPDEHVNEFK					Mascot
2108.1023	2108.1689	0.0666	32	267	284	VQLLEIAQVPDEHVNEFK	81	100			Mascot
2159.0015	2159.1079	0.1064	49	185	204	EGWYPPGHGDVFPALMNSGK					Mascot

4	UTP--glucose-1-phosphate uridylyltransferase 1 OS=Arabidopsis thaliana GN=At5g17310 PE=2 SV=1	UGPA1_ARATH	52058.2	5.73	4	199	100	5.473	191	100	

Peptide Information

Calc. Mass	Obsrv. Mass	± da	± ppm	Start Seq.	End Seq.	Sequence	Ion Score	C. I. %	Modification	Rank	Result Type

Calc. Mass	Obsrv. Mass	± da	± ppm	Start Seq.	End Seq.	Sequence	Ion Score	C. I. %	Modification	Rank	Result Type
1300.7358	1300.7156	-0.0202	-16	413	424	SIPSIVELDSLK					Mascot
1312.7583	1312.7961	0.0378	29	330	342	VLQLETAAGAAIR					Mascot
1312.7583	1312.7961	0.0378	29	330	342	VLQLETAAGAAIR	110	100			Mascot
1940.0812	1940.1102	0.029	15	324	342	EVDGVKVLQLETAAGAAI R					Mascot
2108.1023	2108.1689	0.0666	32	268	285	VQLLEIAQVPDEHVNEFK					Mascot
2108.1023	2108.1689	0.0666	32	268	285	VQLLEIAQVPDEHVNEFK	81	100			Mascot

5	UTP--glucose-1-phosphate uridylyltransferase OS=Pyrus pyrifolia PE=2 SV=1	UGPA_PYRPY	51984.4	5.99	8	197	100	8.616	174	100	

Peptide Information

Calc. Mass	Obsrv. Mass	± da	± ppm	Start Seq.	End Seq.	Sequence	Ion Score	C. I. %	Modification	Rank	Result Type
1014.5942	1014.6111	0.0169	17	308	316	RLVEADALK					Mascot
1052.5371	1052.5609	0.0238	23	259	268	GGTLISYEGR					Mascot
1052.5371	1052.5609	0.0238	23	259	268	GGTLISYEGR	64	99.961			Mascot
1312.7583	1312.7961	0.0378	29	331	343	VLQLETAAGAAIR					Mascot
1312.7583	1312.7961	0.0378	29	331	343	VLQLETAAGAAIR	110	100			Mascot
1648.7603	1648.8499	0.0896	54	37	50	YVSGEEAQHVEWSK					Mascot
1801.9695	1801.9606	-0.0089	-5	170	186	LVVEDFSPLPSKGQTGK					Mascot
1940.0812	1940.1102	0.029	15	325	343	EVDGVKVLQLETAAGAAI R					Mascot
1967.0121	1967.0576	0.0455	23	216	233	EYVFIANSDNLGAVVDLK					Mascot
2197.0786	2197.1072	0.0286	13	152	169	YSKSNVQIHTFNQSQYP R					Mascot

6	UTP--glucose-1-phosphate uridylyltransferase OS=Musa acuminata GN=UGPA PE=2 SV=1	UGPA_MUSAC	51501	5.48	8	192	100	10.154	174	100	

Peptide Information

Calc. Mass	Obsrv. Mass	± da	± ppm	Start Seq.	End Seq.	Sequence	Ion Score	C. I. %	Modification	Rank	Result Type
847.4705	847.3915	-0.079	-93	1	8	MADAKIAK					Mascot
965.5414	965.559	0.0176	18	25	33	SGFISLVSR					Mascot
1014.5942	1014.6111	0.0169	17	304	312	RLVEADALK					Mascot
1052.5371	1052.5609	0.0238	23	255	264	GGTLISYEGR					Mascot
1052.5371	1052.5609	0.0238	23	255	264	GGTLISYEGR	64	99.961			Mascot
1300.7358	1300.7156	-0.0202	-16	410	421	SIPSIVELDSLK					Mascot
1312.7583	1312.7961	0.0378	29	327	339	VLQLETAAGAAIR					Mascot
1312.7583	1312.7961	0.0378	29	327	339	VLQLETAAGAAIR	110	100			Mascot

	1539.7325	1539.7522	0.0197	13	34	46	YLSGEAEQIEWSK								Mascot
	1940.0812	1940.1102	0.029	15	321	339	EVDGVKVLQLETAAGAAI R								Mascot

7	UTP--glucose-1-phosphate uridylyltransferase OS=Solanum tuberosum PE=1 SV=3	UGPA_SOLTU	52069.4	5.7	6	95	99.984	2.676	81	100	

Peptide Information

Calc. Mass	Obsrv. Mass	± da	± ppm	Start Seq.	End Seq.	Sequence	Ion Score	C. I. %	Modification	Rank	Result Type
1014.5942	1014.6111	0.0169	17	313	321	RLVEADALK					Mascot
1300.7358	1300.7156	-0.0202	-16	420	431	SIPSIIDLDSLK					Mascot
1967.0121	1967.0576	0.0455	23	221	238	EYVFVANSDNLGAIVDLK					Mascot
2108.1023	2108.1689	0.0666	32	274	291	VQLLEIAQVPDEHVNEFK					Mascot
2108.1023	2108.1689	0.0666	32	274	291	VQLLEIAQVPDEHVNEFK	81	100			Mascot
2152.1284	2152.127	-0.0014	-1	219	238	GKEYVFVANSDNLGAIVD LK					Mascot
2168.0156	2168.1306	0.115	53	157	174	YANSNIDIHTFNQSQYPR					Mascot

8	T-complex protein 1 subunit gamma OS=Schizosaccharomyces pombe (strain 972 / ATCC 24843) GN=cct3 PE=1 SV=1	TCPG_SCHPO	59127.7	6.88	16	70	93.933	8.795			

Peptide Information

Calc. Mass	Obsrv. Mass	± da	± ppm	Start Seq.	End Seq.	Sequence	Ion Score	C. I. %	Modification	Rank	Result Type
1064.5735	1064.5604	-0.0131	-12	67	76	EIEVAHPAAK					Mascot
1218.6549	1218.6691	0.0142	12	177	188	AVRTVASTSNGR					Mascot
1342.7665	1342.8049	0.0384	29	115	125	IHPVVMIRSFK			Oxidation (M)[6]		Mascot
1342.7665	1342.8049	0.0384	29	115	125	IHPVVMIRSFK			Oxidation (M)[6]		Mascot
1390.74	1390.6754	-0.0646	-46	236	246	IVLLDCPLEYR			Carbamidomethyl (C)[6]		Mascot
1390.74	1390.6754	-0.0646	-46	236	246	IVLLDCPLEYR			Carbamidomethyl (C)[6]		Mascot
1444.6777	1444.7742	0.0965	67	340	352	DVGTGCGLFYIDK			Carbamidomethyl (C)[6]		Mascot
1447.7209	1447.8389	0.118	82	405	419	LSPGGGATEMAVSVR			Oxidation (M)[10]		Mascot
1701.8153	1701.9419	0.1266	74	338	352	EKDVGTGCGLFYIDK			Carbamidomethyl (C)[8]		Mascot
1801.9589	1801.9606	0.0017	1	20	36	AQMSNIQAAKAVADVIR			Oxidation (M)[3]		Mascot
2073.0393	2073.1272	0.0879	42	380	397	DIINEVERNLQDAMAVAR			Oxidation (M)[14]		Mascot
2144.0488	2144.0635	0.0147	7	1	19	MQSPVFVMNTNGNRQV GHK					Mascot
2164.1504	2164.1487	-0.0017	-1	275	292	RMCDYIIAVKPDLVITEK			Carbamidomethyl (C)[3]		Mascot
2176.0386	2176.0674	0.0288	13	1	19	MQSPVFVMNTNGNRQV GHK			Oxidation (M)[1,8]		Mascot
2180.1455	2180.1731	0.0276	13	275	292	RMCDYIIAVKPDLVITEK			Carbamidomethyl (C)[3], Oxidation (M)[2]		Mascot

Calc. Mass	Obsrv. Mass	± da	± ppm	Start Seq.	End Seq.	Sequence	Ion Score	C. I. %	Modification	Rank	Result Type
2187.1802	2187.2407	0.0605	28	505	524	TAIESACLLLRVDDIVSGVR			Carbamidomethyl (C)[7]		Mascot
2317.1758	2317.1914	0.0156	7	398	419	NVFFHPKLSPGGGATEMAVSVR			Oxidation (M)[17]		Mascot
2454.314	2454.4099	0.0959	39	426	447	SIEGVAQWPYRAVADAIEIIPR					Mascot
2454.314	2454.4099	0.0959	39	426	447	SIEGVAQWPYRAVADAIEIIPR					Mascot
2457.2483	2457.2017	-0.0466	-19	484	504	VVDMHEYGVWEPEAVKLQSIK					Mascot
3047.4878	3047.675	0.1872	61	126	152	QALEDALSIIDEITLPVNVDDNAEMFR			Oxidation (M)[25]		Mascot

9	tRNA(Ile2) 2-agmatinylcytidine synthetase TiaS OS=Thermoplasma volcanium (strain ATCC 51530 / DSM 4299 / JCM 9571 / NBRC 15438 / GSS1) GN=tiaS PE=3 SV=1	TIAS_THEVO	49927.3	8.07	10	59	30.34	5.634	29	0	

Peptide Information

Calc. Mass	Obsrv. Mass	± da	± ppm	Start Seq.	End Seq.	Sequence	Ion Score	C. I. %	Modification	Rank	Result Type
918.5043	918.5631	0.0588	64	408	415	YDVPVVAR					Mascot
949.5135	949.5652	0.0517	54	351	358	MQIISVSR			Oxidation (M)[1]		Mascot
949.5135	949.5652	0.0517	54	351	358	MQIISVSR	29	0	Oxidation (M)[1]		Mascot
1074.6055	1074.5496	-0.0559	-52	408	416	YDVPVVARR					Mascot
1371.6427	1371.7751	0.1324	97	181	191	YPHPEEIETEK					Mascot
1456.7908	1456.767	-0.0238	-16	157	171	GIIGSGAAISWPATR					Mascot
1513.885	1513.8071	-0.0779	-51	27	39	VNLDVIGYPRLVR					Mascot
1529.8323	1529.8113	-0.021	-14	402	415	EISPGKYDVPVVAR					Mascot
2209.1611	2209.1162	-0.0449	-20	195	214	LSILADTFRGTFNNVDIANK					Mascot
2220.1448	2220.0837	-0.0611	-28	204	223	GTFNNVDIANKYPAIFPNPK					Mascot
2454.2075	2454.4099	0.2024	82	172	191	TTYEILAYKYPHPEEIETEK					Mascot
2454.2075	2454.4099	0.2024	82	172	191	TTYEILAYKYPHPEEIETEK					Mascot

10	Chaperone protein HscA homolog OS=Haemophilus influenzae (strain ATCC 51907 / DSM 11121 / KW20 / Rd) GN=hscA PE=3 SV=1	HSCA_HAEIN	66989.9	4.89	15	58	12.304	5.576	

Peptide Information

Calc. Mass	Obsrv. Mass	± da	± ppm	Start Seq.	End Seq.	Sequence	Ion Score	C. I. %	Modification	Rank	Result Type
949.5577	949.5652	0.0075	8	449	456	SLGRFTLR					Mascot
949.5577	949.5652	0.0075	8	449	456	SLGRFTLR					Mascot
1350.8217	1350.6886	-0.1331	-99	407	418	IIPRNTTIPVAR					Mascot
1358.7023	1358.7612	0.0589	43	574	586	QGSDRDAIAQGIK					Mascot

1371.7114	1371.7751	0.0637	46	607	619	ALTGKNLSDIENP		Mascot
1480.6663	1480.7419	0.0756	51	514	526	SSFDNAQEDLQAR		Mascot
1492.8046	1492.8119	0.0073	5	297	308	EQFNELIYPLVK		Mascot
1641.8014	1641.9037	0.1023	62	427	441	DGQTAMTVHVLQGER		Mascot
1648.9058	1648.8499	-0.0559	-34	297	309	EQFNELIYPLVKR		Mascot
1939.9681	1940.1102	0.1421	73	470	487	VTYQVDADGLLSVTAMEK		Mascot
2055.0552	2055.0527	-0.0025	-1	1	19	MALLQIAEPGQAAAPHQHR	Oxidation (M)[1]	Mascot
2090.0876	2090.0583	-0.0293	-14	579	598	DAIAQGIKALDTATQEFAAR		Mascot
2108.0547	2108.1689	0.1142	54	345	363	EQVGEFFGKTPLTSIDPDK		Mascot
2108.0547	2108.1689	0.1142	54	345	363	EQVGEFFGKTPLTSIDPDK		Mascot
2207.1343	2207.1404	0.0061	3	145	165	LGGELSGVVITVPAYFDDAQR		Mascot
2256.1428	2256.1047	-0.0381	-17	470	490	VTYQVDADGLLSVTAMEKSTK		Mascot
2264.1995	2264.1724	-0.0271	-12	125	144	SPIEVSSDILSRLNHIAEQR		Mascot

Gel Idx/Pos	248/J24	Instr./Gel Origin	BA2151/Sample Project 20140814	Process Status	Analysis Succeeded
Plate [#] Name	[1] Sample Project 20140814	Instrument Sample Name		Spectra	11

Rank	Protein Name	Accession No.	Protein MW	Protein PI	Pep. Count	Protein Score	Protein Score C. I. %	Intensity Matched	Total Ion Score	Total Ion C. I. %	Confirmed
1	ATP synthase subunit beta, mitochondrial OS=Nicotiana plumbaginifolia GN=ATPB PE=1 SV=1	ATPBM_NICPL	59933.3	5.95	19	1,100	100	58.549	997	100	

Peptide Information

Calc. Mass	Obsrv. Mass	± da	± ppm	Start Seq.	End Seq.	Sequence	Ion Score	C. I. %	Modification	Rank	Result Type
866.4003	866.4203	0.02	23	271	277	EGNDLYR					Mascot
1173.6627	1173.6893	0.0266	23	218	227	VVDLLAPYQR					Mascot
1173.6627	1173.6893	0.0266	23	218	227	VVDLLAPYQR	72	99.994			Mascot
1278.6359	1278.6506	0.0147	11	139	150	TIAMDGTEGLVR			Oxidation (M)[4]		Mascot
1355.8118	1355.782	-0.0298	-22	6	17	LLASLLRQSAQR					Mascot
1390.6863	1390.7252	0.0389	28	255	268	AHGGFSVFAGVGER					Mascot
1390.6863	1390.7252	0.0389	28	255	268	AHGGFSVFAGVGER	130	100			Mascot
1399.7693	1399.8077	0.0384	27	313	325	VGLTGLTVAEHFR					Mascot
1399.7693	1399.8077	0.0384	27	313	325	VGLTGLTVAEHFR	37	79.704			Mascot
1409.8112	1409.847	0.0358	25	154	167	VLNTGSPITVPVGR					Mascot
1473.8346	1473.8335	-0.0011	-1	242	254	TVLIMELINNVAK			Oxidation (M)[5]		Mascot
1492.7755	1492.8168	0.0413	28	342	355	FTQANSEVSALLGR					Mascot
1492.7755	1492.8168	0.0413	28	342	355	FTQANSEVSALLGR	123	100			Mascot
1678.7676	1678.7976	0.03	18	296	310	CALVYGQMNEPPGAR			Carbamidomethyl (C)[1], Oxidation (M)[8]		Mascot
1864.944	1865.0068	0.0628	34	326	341	DAEGQDVLLFIDNIFR					Mascot
1864.944	1865.0068	0.0628	34	326	341	DAEGQDVLLFIDNIFR	142	100			Mascot
1869.8912	1870.0319	0.1407	75	438	453	MLSPHILGEDHYNTAR			Oxidation (M)[1]		Mascot
2061.0498	2061.1125	0.0627	30	419	437	QISELGIYPAVDPLDSTSR					Mascot
2061.0498	2061.1125	0.0627	30	419	437	QISELGIYPAVDPLDSTSR	93	100			Mascot
2172.1548	2172.2222	0.0674	31	198	217	EAPAFVEQATEQQILVTG IK					Mascot
2186.1453	2186.2305	0.0852	39	356	376	IPSAVGYQPTLATDLGGL QER					Mascot
2186.1453	2186.2305	0.0852	39	356	376	IPSAVGYQPTLATDLGGL QER	207	100			Mascot
2591.3499	2591.4268	0.0769	30	79	103	ITDEFTGAGSIGKVCQVIG AVVDVR			Carbamidomethyl (C)[15]		Mascot
2591.3499	2591.4268	0.0769	30	79	103	ITDEFTGAGSIGKVCQVIG AVVDVR	194	100	Carbamidomethyl (C)[15]		Mascot
2722.3274	2722.426	0.0986	36	464	487	NLQDIIAILGMDELSEDDK MTVAR			Oxidation (M)[11,20]		Mascot

3714.8862	3715.0779	0.1917	52	383	418	GSITSVQAIYVPADDLTDPAPATTFAHLDATTVLSR					Mascot
3842.9812	3843.1807	0.1995	52	382	418	KGSITSVQAIYVPADDLTDPAPATTFAHLDATTVLSR					Mascot

2	ATP synthase subunit beta, mitochondrial OS=Oryza sativa subsp. japonica GN=ATPB PE=1 SV=2	ATPBM_ORYSJ	59011.8	5.95	19	909	100	48.96	803	100

Peptide Information

Calc. Mass	Obsrv. Mass	± da	± ppm	Start Seq.	End Seq.	Sequence	Ion Score	C. I. %	Modification	Rank	Result Type
866.4003	866.4203	0.02	23	263	269	EGNDLYR					Mascot
1173.6627	1173.6893	0.0266	23	210	219	VVDLLAPYQR					Mascot
1173.6627	1173.6893	0.0266	23	210	219	VVDLLAPYQR	72	99.994			Mascot
1278.6359	1278.6506	0.0147	11	131	142	TIAMDGTEGLVR			Oxidation (M)[4]		Mascot
1390.6863	1390.7252	0.0389	28	247	260	AHGGFSVFAGVGER					Mascot
1390.6863	1390.7252	0.0389	28	247	260	AHGGFSVFAGVGER	130	100			Mascot
1399.7693	1399.8077	0.0384	27	305	317	VGLTGLTVAEHFR					Mascot
1399.7693	1399.8077	0.0384	27	305	317	VGLTGLTVAEHFR	37	79.704			Mascot
1409.8112	1409.847	0.0358	25	146	159	VLNTGSPITVPVGR					Mascot
1418.756	1418.7535	-0.0025	-2	270	282	EMIESGVIKLGDK					Mascot
1473.8346	1473.8335	-0.0011	-1	234	246	TVLIMELINNVAK			Oxidation (M)[5]		Mascot
1492.7755	1492.8168	0.0413	28	334	347	FTQANSEVSALLGR					Mascot
1492.7755	1492.8168	0.0413	28	334	347	FTQANSEVSALLGR	123	100			Mascot
1520.7969	1520.8304	0.0335	22	177	189	GDITTNHFLPIHR					Mascot
1678.7676	1678.7976	0.03	18	288	302	CALVYGQMNEPPGAR			Carbamidomethyl (C)[1], Oxidation (M)[8]		Mascot
1697.8871	1697.9658	0.0787	46	28	42	GPLHRPSPSGYLFNR					Mascot
1864.944	1865.0068	0.0628	34	318	333	DAEGQDVLLFIDNIFR					Mascot
1864.944	1865.0068	0.0628	34	318	333	DAEGQDVLLFIDNIFR	142	100			Mascot
2061.0498	2061.1125	0.0627	30	411	429	QISELGIYPAVDPLDSTSR					Mascot
2061.0498	2061.1125	0.0627	30	411	429	QISELGIYPAVDPLDSTSR	93	100			Mascot
2172.1548	2172.2222	0.0674	31	190	209	EAPAFVEQATEQQILVTGIK					Mascot
2186.1453	2186.2305	0.0852	39	348	368	IPSAVGYQPTLATDLGGLQER					Mascot
2186.1453	2186.2305	0.0852	39	348	368	IPSAVGYQPTLATDLGGLQER	207	100			Mascot
2658.3899	2658.4358	0.0459	17	28	53	GPLHRPSPSGYLFNRAAAYATAAAAK					Mascot
3714.8862	3715.0779	0.1917	52	375	410	GSITSVQAIYVPADDLTDPAPATTFAHLDATTVLSR					Mascot
3842.9812	3843.1807	0.1995	52	374	410	KGSITSVQAIYVPADDLTDPAPATTFAHLDATTVLSR					Mascot

3	ATP synthase subunit beta, mitochondrial OS=Hevea brasiliensis GN=ATPB PE=2 SV=1	ATPBM_HEVBR	60335.4	5.95	17	889	100	48.577	803	100	

Peptide Information

Calc. Mass	Obsrv. Mass	± da	± ppm	Start Seq.	End Seq.	Sequence	Ion Score	C. I. %	Modification	Rank	Result Type
866.4003	866.4203	0.02	23	273	279	EGNDLYR					Mascot
1173.6627	1173.6893	0.0266	23	220	229	VVDLLAPYQR					Mascot
1173.6627	1173.6893	0.0266	23	220	229	VVDLLAPYQR	72	99.994			Mascot
1278.6359	1278.6506	0.0147	11	141	152	TIAMDGTEGLVR			Oxidation (M)[4]		Mascot
1390.6863	1390.7252	0.0389	28	257	270	AHGGFSVFAGVGER					Mascot
1390.6863	1390.7252	0.0389	28	257	270	AHGGFSVFAGVGER	130	100			Mascot
1399.7693	1399.8077	0.0384	27	315	327	VGLTGLTVAEHFR					Mascot
1399.7693	1399.8077	0.0384	27	315	327	VGLTGLTVAEHFR	37	79.704			Mascot
1409.8112	1409.847	0.0358	25	156	169	VLNTGSPITVPVGR					Mascot
1418.756	1418.7535	-0.0025	-2	280	292	EMIESGVIKLGDK					Mascot
1473.8346	1473.8335	-0.0011	-1	244	256	TVLIMELINNVAK			Oxidation (M)[5]		Mascot
1492.7755	1492.8168	0.0413	28	344	357	FTQANSEVSALLGR					Mascot
1492.7755	1492.8168	0.0413	28	344	357	FTQANSEVSALLGR	123	100			Mascot
1520.8333	1520.8304	-0.0029	-2	187	199	GDIKTSHFLPIHR					Mascot
1678.7676	1678.7976	0.03	18	298	312	CALVYGQMNEPPGAR			Carbamidomethyl (C)[1], Oxidation (M)[8]		Mascot
1864.944	1865.0068	0.0628	34	328	343	DAEGQDVLLFIDNIFR					Mascot
1864.944	1865.0068	0.0628	34	328	343	DAEGQDVLLFIDNIFR	142	100			Mascot
2061.0498	2061.1125	0.0627	30	421	439	QISELGIYPAVDPLDSTSR					Mascot
2061.0498	2061.1125	0.0627	30	421	439	QISELGIYPAVDPLDSTSR	93	100			Mascot
2186.1453	2186.2305	0.0852	39	358	378	IPSAVGYQPTLATDLGGL QER					Mascot
2186.1453	2186.2305	0.0852	39	358	378	IPSAVGYQPTLATDLGGL QER	207	100			Mascot
2208.0173	2208.1965	0.1792	81	293	312	QADSKCALVYGQMNEPP GAR			Carbamidomethyl (C)[6], Oxidation (M)[13]		Mascot
3714.8862	3715.0779	0.1917	52	385	420	GSITSVQAIYVPADDLTD PAPATTFAHLDATTVLSR					Mascot
3842.9812	3843.1807	0.1995	52	384	420	KGSITSVQAIYVPADDLT DPAPATTFAHLDATTVLS R					Mascot

4	ATP synthase subunit beta, mitochondrial OS=Zea mays GN=ATPB PE=1 SV=1	ATPBM_MAIZE	59181	6.01	16	883	100	48.585	802	100	

Peptide Information

Calc. Mass	Obsrv. Mass	± da	± ppm	Start Seq.	End Seq.	Sequence	Ion Score	C. I. %	Modification	Rank	Result Type

Calc. Mass	Obsrv. Mass	± da	± ppm	Start Seq.	End Seq.	Sequence	Ion Score	C. I. %	Modification	Rank	Result Type
866.4003	866.4203	0.02	23	264	270	EGNDLYR					Mascot
1173.6627	1173.6893	0.0266	23	211	220	VVDLLAPYQR					Mascot
1173.6627	1173.6893	0.0266	23	211	220	VVDLLAPYQR	72	99.994			Mascot
1278.6359	1278.6506	0.0147	11	132	143	TIAMDGTEGLVR			Oxidation (M)[4]		Mascot
1390.6863	1390.7252	0.0389	28	248	261	AHGGFSVFAGVGER					Mascot
1390.6863	1390.7252	0.0389	28	248	261	AHGGFSVFAGVGER	130	100			Mascot
1399.7693	1399.8077	0.0384	27	306	318	VGLTGLTVAEHFR					Mascot
1399.7693	1399.8077	0.0384	27	306	318	VGLTGLTVAEHFR	37	79.704			Mascot
1409.8112	1409.847	0.0358	25	147	160	VLNTGSPITVPVGR					Mascot
1473.8346	1473.8335	-0.0011	-1	235	247	TVLIMELINNVAK			Oxidation (M)[5]		Mascot
1492.7755	1492.8168	0.0413	28	335	348	FTQANSEVSALLGR					Mascot
1492.7755	1492.8168	0.0413	28	335	348	FTQANSEVSALLGR	123	100			Mascot
1678.7676	1678.7976	0.03	18	289	303	CALVYGQMNEPPGAR			Carbamidomethyl (C)[1], Oxidation (M)[8]		Mascot
1864.944	1865.0068	0.0628	34	319	334	DAEGQDVLLFIDNIFR					Mascot
1864.944	1865.0068	0.0628	34	319	334	DAEGQDVLLFIDNIFR	142	100			Mascot
2061.0498	2061.1125	0.0627	30	412	430	QISELGIYPAVDPLDSTSR					Mascot
2061.0498	2061.1125	0.0627	30	412	430	QISELGIYPAVDPLDSTSR	93	100			Mascot
2172.1548	2172.2222	0.0674	31	191	210	EAPAFVEQATEQQILVTG IK					Mascot
2186.1453	2186.2305	0.0852	39	349	369	IPSAVGYQPTLATDLGGL QER					Mascot
2186.1453	2186.2305	0.0852	39	349	369	IPSAVGYQPTLATDLGGL QER	207	100			Mascot
2575.3186	2575.4717	0.1531	59	72	96	ITDEFTGAGAIGQVCQVI GAVVDVR			Carbamidomethyl (C)[15]		Mascot
3714.8862	3715.0779	0.1917	52	376	411	GSITSVQAIYVPADDLTD PAPATTFAHLDATTVLSR					Mascot
3842.9812	3843.1807	0.1995	52	375	411	KGSITSVQAIYVPADDLT DPAPATTFAHLDATTVLS R					Mascot

5	ATP synthase subunit beta-3, mitochondrial OS=Arabidopsis thaliana GN=At5g08680 PE=2 SV=1	ATPBO_ARATH	59993.2	6.06	13	740	100	48.549	683	100	

Peptide Information

Calc. Mass	Obsrv. Mass	± da	± ppm	Start Seq.	End Seq.	Sequence	Ion Score	C. I. %	Modification	Rank	Result Type
866.4003	866.4203	0.02	23	270	276	EGNDLYR					Mascot
1173.6627	1173.6893	0.0266	23	217	226	VVDLLAPYQR					Mascot
1173.6627	1173.6893	0.0266	23	217	226	VVDLLAPYQR	72	99.994			Mascot
1278.6359	1278.6506	0.0147	11	138	149	TIAMDGTEGLVR			Oxidation (M)[4]		Mascot
1390.6863	1390.7252	0.0389	28	254	267	AHGGFSVFAGVGER					Mascot
1390.6863	1390.7252	0.0389	28	254	267	AHGGFSVFAGVGER	130	100			Mascot

Calc. Mass	Obsrv. Mass	± da	± ppm	Start Seq.	End Seq.	Sequence	Ion Score	C. I. %	Modification	Rank	Result Type
1473.8346	1473.8335	-0.0011	-1	241	253	TVLIMELINNVAK			Oxidation (M)[5]		Mascot
1492.7755	1492.8168	0.0413	28	341	354	FTQANSEVSALLGR					Mascot
1492.7755	1492.8168	0.0413	28	341	354	FTQANSEVSALLGR	123	100			Mascot
1678.7676	1678.7976	0.03	18	295	309	CALVYGQMNEPPGAR			Carbamidomethyl (C)[1], Oxidation (M)[8]		Mascot
1864.944	1865.0068	0.0628	34	325	340	DAEGQDVLLFIDNIFR					Mascot
1864.944	1865.0068	0.0628	34	325	340	DAEGQDVLLFIDNIFR	142	100			Mascot
2061.0498	2061.1125	0.0627	30	418	436	QISELGIYPAVDPLDSTSR					Mascot
2061.0498	2061.1125	0.0627	30	418	436	QISELGIYPAVDPLDSTSR	93	100			Mascot
2186.1453	2186.2305	0.0852	39	355	375	IPSAVGYQPTLASDLGAL QER					Mascot
2186.1453	2186.2305	0.0852	39	355	375	IPSAVGYQPTLASDLGAL QER	123	100			Mascot
2212.1768	2212.2642	0.0874	40	29	50	NPRLLSPGPAHGAAPCG TLLGR			Carbamidomethyl (C)[16]		Mascot
2212.1768	2212.2642	0.0874	40	29	50	NPRLLSPGPAHGAAPCG TLLGR			Carbamidomethyl (C)[16]		Mascot
3714.8862	3715.0779	0.1917	52	382	417	GSITSVQAIYVPADDLTD PAPATTFAHLDATTVLSR					Mascot
3842.9812	3843.1807	0.1995	52	381	417	KGSITSVQAIYVPADDLT DPAPATTFAHLDATTVLS R					Mascot

6	ATP synthase subunit beta-2, mitochondrial OS=Arabidopsis thaliana GN=At5g08690 PE=1 SV=1	ATPBN_ARATH	59847.2	6.18	13	738	100	41.001	683	100		

Protein Group

ATP synthase subunit beta-1, mitochondrial OS=Arabidopsis thaliana GN=At5g08670 PE=1 SV=1	ATPBM_ARATH	59805.1	6.1799 998283 3862

Peptide Information

Calc. Mass	Obsrv. Mass	± da	± ppm	Start Seq.	End Seq.	Sequence	Ion Score	C. I. %	Modification	Rank	Result Type
866.4003	866.4203	0.02	23	267	273	EGNDLYR					Mascot
1173.6627	1173.6893	0.0266	23	214	223	VVDLLAPYQR					Mascot
1173.6627	1173.6893	0.0266	23	214	223	VVDLLAPYQR	72	99.994			Mascot
1244.6205	1244.6693	0.0488	39	37	47	HAAPCSYLLGR			Carbamidomethyl (C)[5]		Mascot
1278.6359	1278.6506	0.0147	11	135	146	TIAMDGTEGLVR			Oxidation (M)[4]		Mascot
1390.6863	1390.7252	0.0389	28	251	264	AHGGFSVFAGVGER					Mascot
1390.6863	1390.7252	0.0389	28	251	264	AHGGFSVFAGVGER	130	100			Mascot
1473.8346	1473.8335	-0.0011	-1	238	250	TVLIMELINNVAK			Oxidation (M)[5]		Mascot
1492.7755	1492.8168	0.0413	28	338	351	FTQANSEVSALLGR					Mascot
1492.7755	1492.8168	0.0413	28	338	351	FTQANSEVSALLGR	123	100			Mascot
1678.7676	1678.7976	0.03	18	292	306	CALVYGQMNEPPGAR			Carbamidomethyl (C)[1], Oxidation (M)[8]		Mascot
1864.944	1865.0068	0.0628	34	322	337	DAEGQDVLLFIDNIFR					Mascot
1864.944	1865.0068	0.0628	34	322	337	DAEGQDVLLFIDNIFR	142	100			Mascot

Calc. Mass	Obsrv. Mass	± da	± ppm	Start Seq.	End Seq.	Sequence	Ion Score	C. I. %	Modification	Rank	Result Type
2061.0498	2061.1125	0.0627	30	415	433	QISELGIYPAVDPLDSTSR					Mascot
2061.0498	2061.1125	0.0627	30	415	433	QISELGIYPAVDPLDSTSR	93	100			Mascot
2186.1453	2186.2305	0.0852	39	352	372	IPSAVGYQPTLASDLGALQER					Mascot
2186.1453	2186.2305	0.0852	39	352	372	IPSAVGYQPTLASDLGALQER	123	100			Mascot
3714.8862	3715.0779	0.1917	52	379	414	GSITSVQAIYVPADDLTDPAPATTFAHLDATTVLSR					Mascot
3842.9812	3843.1807	0.1995	52	378	414	KGSITSVQAIYVPADDLTDPAPATTFAHLDATTVLSR					Mascot

7	ATP synthase subunit beta, mitochondrial OS=Chlamydomonas reinhardtii GN=ATP2 PE=1 SV=1	ATPBM_CHLRE	61954	4.99	12	572	100	36.922	532	100

Peptide Information

Calc. Mass	Obsrv. Mass	± da	± ppm	Start Seq.	End Seq.	Sequence	Ion Score	C. I. %	Modification	Rank	Result Type
866.4003	866.4203	0.02	23	219	225	EGNDLYR					Mascot
1173.6627	1173.6893	0.0266	23	166	175	VVDLLAPYQR					Mascot
1173.6627	1173.6893	0.0266	23	166	175	VVDLLAPYQR	72	99.994			Mascot
1390.6863	1390.7252	0.0389	28	203	216	AHGGFSVFAGVGER					Mascot
1390.6863	1390.7252	0.0389	28	203	216	AHGGFSVFAGVGER	130	100			Mascot
1418.756	1418.7535	-0.0025	-2	226	238	EMIESGVIKLGDK					Mascot
1473.8346	1473.8335	-0.0011	-1	190	202	TVLIMELINNVAK			Oxidation (M)[5]		Mascot
1492.7755	1492.8168	0.0413	28	290	303	FTQANSEVSALLGR					Mascot
1492.7755	1492.8168	0.0413	28	290	303	FTQANSEVSALLGR	123	100			Mascot
1550.7367	1550.837	0.1003	65	552	565	AEAISSENMVLNEK			Oxidation (M)[9]		Mascot
1697.864	1697.9658	0.1018	60	72	86	LVLEVAQHMGDNTVR			Oxidation (M)[9]		Mascot
1825.9377	1825.9404	0.0027	1	386	401	MLNPNIIGAEHYNIAR					Mascot
1864.8956	1865.0068	0.1112	60	552	568	AEAISSENMVLNEKGEK			Oxidation (M)[9]		Mascot
1864.8956	1865.0068	0.1112	60	552	568	AEAISSENMVLNEKGEK			Oxidation (M)[9]		Mascot
2186.1453	2186.2305	0.0852	39	304	324	IPSAVGYQPTLATDLGGLQER					Mascot
2186.1453	2186.2305	0.0852	39	304	324	IPSAVGYQPTLATDLGGLQER	207	100			Mascot
2458.1306	2458.1516	0.021	9	478	498	YDDLPEMAFYMVGGIHEVVEK			Oxidation (M)[7]		Mascot

8	ATP synthase subunit beta, mitochondrial OS=Daucus carota GN=ATPB PE=3 SV=1	ATPBM_DAUCA	59326.8	5.62	14	529	100	33.102	460	100

Peptide Information

Calc. Mass	Obsrv. Mass	± da	± ppm	Start Seq.	End Seq.	Sequence	Ion Score	C. I. %	Modification	Rank	Result Type

Calc. Mass	Obsrv. Mass	± da	± ppm	Start Seq.	End Seq.	Sequence	Ion Score	C. I. %	Modification	Rank	Result Type
1278.6359	1278.6506	0.0147	11	130	141	TIAMDGTEGLVR			Oxidation (M)[4]		Mascot
1390.7478	1390.7252	-0.0226	-16	219	232	GGKIGLFGGDWVGK					Mascot
1390.7478	1390.7252	-0.0226	-16	219	232	GGKIGLFGGDWVGK					Mascot
1399.7693	1399.8077	0.0384	27	301	313	VGLTGLTVAEHFR					Mascot
1399.7693	1399.8077	0.0384	27	301	313	VGLTGLTVAEHFR	37	79.704			Mascot
1473.8346	1473.8335	-0.0011	-1	233	245	TVLIMELINNVAK			Oxidation (M)[5]		Mascot
1492.7755	1492.8168	0.0413	28	330	343	FTQANSEVSALLGR					Mascot
1492.7755	1492.8168	0.0413	28	330	343	FTQANSEVSALLGR	123	100			Mascot
1596.8956	1596.9532	0.0576	36	115	129	LVLEVAPSLGENTVR					Mascot
1678.7676	1678.7976	0.03	18	284	298	CALVYGQMNEPPGSR			Carbamidomethyl (C)[1]		Mascot
1694.7626	1694.8331	0.0705	42	284	298	CALVYGQMNEPPGSR			Carbamidomethyl (C)[1], Oxidation (M)[8]		Mascot
2061.0498	2061.1125	0.0627	30	407	425	QISELGIYPAVDPLDSTSR					Mascot
2061.0498	2061.1125	0.0627	30	407	425	QISELGIYPAVDPLDSTSR	93	100			Mascot
2087.0615	2087.124	0.0625	30	53	74	EPAASKPAGTAGTGKGTI TDEK					Mascot
2186.1453	2186.2305	0.0852	39	344	364	IPSAVGYQPTLATDLGGL QER					Mascot
2186.1453	2186.2305	0.0852	39	344	364	IPSAVGYQPTLATDLGGL QER	207	100			Mascot
2578.3704	2578.4807	0.1103	43	481	503	FLSQPFHVAEIFTGAPGK YVELK					Mascot
2635.2239	2635.4866	0.2627	100	275	298	LGDQQAESKCALVYGQM NEPPGSR			Carbamidomethyl (C)[10]		Mascot
3714.8862	3715.0779	0.1917	52	371	406	GSITSVQAIYVPADDLTD PAPATTFAHLDATTVLSR					Mascot
3842.9812	3843.1807	0.1995	52	370	406	KGSITSVQAIYVPADDLT DPAPATTFAHLDATTVLS R					Mascot

9	ATP synthase subunit beta, mitochondrial (Fragments) OS=Vitis sp. PE=1 SV=1	ATPBM_VITSX	6430.4	6.73	4	399	100	22.656	366	100

Peptide Information

Calc. Mass	Obsrv. Mass	± da	± ppm	Start Seq.	End Seq.	Sequence	Ion Score	C. I. %	Modification	Rank	Result Type
1399.7693	1399.8077	0.0384	27	15	27	VGLTGLTVAEHFR					Mascot
1399.7693	1399.8077	0.0384	27	15	27	VGLTGLTVAEHFR	37	79.704			Mascot
1409.8112	1409.847	0.0358	25	1	14	VLNTGSPITVPVGR					Mascot
1492.7755	1492.8168	0.0413	28	28	41	FTQANSEVSALLGR					Mascot
1492.7755	1492.8168	0.0413	28	28	41	FTQANSEVSALLGR	123	100			Mascot
2186.1453	2186.2305	0.0852	39	42	62	IPSAVGYQPTLATDLGGL QER					Mascot
2186.1453	2186.2305	0.0852	39	42	62	IPSAVGYQPTLATDLGGL QER	207	100			Mascot

10	ATP synthase subunit beta OS=Clostridium cellulolyticum (strain ATCC 35319 / DSM 5812 / JCM	ATPB_CLOCE	50526	4.85	6	183	100	13.058	167	100

6584 / H10) GN=atpD PE=3 SV=1

Peptide Information

Calc. Mass	Obsrv. Mass	± da	± ppm	Start Seq.	End Seq.	Sequence	Ion Score	C. I. %	Modification	Rank	Result Type
1473.7407	1473.8335	0.0928	63	222	234	VGLTGLTMAEYFR			Oxidation (M)[8]		Mascot
1520.822	1520.8304	0.0084	6	101	114	AGPVEPTAYLPIHR					Mascot
1892.8928	1893.0204	0.1276	67	205	221	TAMVFGQMNEPPGARMR					Mascot
2044.1987	2044.0897	-0.109	-53	148	167	IGLFGGAGVGKTVLIMELIR					Mascot
2186.1453	2186.2305	0.0852	39	265	285	IPSAVGYQPTLATDVGALQER					Mascot
2186.1453	2186.2305	0.0852	39	265	285	IPSAVGYQPTLATDVGALQER	167	100			Mascot
2600.364	2600.4565	0.0925	36	328	351	DIVAMGIYPAVDPLESTSRILDPK					Mascot

Gel Idx/Pos	249/K1	Instr./Gel Origin	BA2151/Sample Project 20140814	Process Status	Analysis Succeeded
Plate [#] Name	[1] Sample Project 20140814	Instrument Sample Name		Spectra	11

Rank	Protein Name	Accession No.	Protein MW	Protein PI	Pep. Count	Protein Score	Protein Score C. I. %	Intensity Matched	Total Ion Score	Total Ion C. I. %	Confirmed
1	UTP--glucose-1-phosphate uridylyltransferase OS=Hordeum vulgare PE=2 SV=1	UGPA_HORVU	51783.2	5.2	24	503	100	51.584	352	100	

Peptide Information

Calc. Mass	Obsrv. Mass	± da	± ppm	Start Seq.	End Seq.	Sequence	Ion Score	C. I. %	Modification	Rank	Result Type
839.5098	839.5059	-0.0039	-5	350	357	AIGINVPR					Mascot
858.493	858.4778	-0.0152	-18	311	318	LVDAEALK					Mascot
872.3996	872.3157	-0.0839	-96	466	473	DINGPEDI					Mascot
918.5519	918.5441	-0.0078	-8	406	413	KVANFLAR					Mascot
918.5519	918.5441	-0.0078	-8	406	413	KVANFLAR					Mascot
949.5465	949.542	-0.0045	-5	31	39	AGFISLVSR					Mascot
949.5465	949.542	-0.0045	-5	31	39	AGFISLVSR	36	68.137			Mascot
957.5073	957.4853	-0.022	-23	319	326	MEIIPNPK			Oxidation (M)[1]		Mascot
1014.5942	1014.5879	-0.0063	-6	310	318	RLVDAEALK					Mascot
1018.5051	1018.5077	0.0026	3	22	30	LGEISENEK					Mascot
1052.5371	1052.5386	0.0015	1	261	270	GGTLISYEGR					Mascot
1052.5371	1052.5386	0.0015	1	261	270	GGTLISYEGR	75	99.996			Mascot
1057.6001	1057.504	-0.0961	-91	12	21	IDGLRDAVAK					Mascot
1111.745	1111.6816	-0.0634	-57	79	88	ALLDKLVVLK					Mascot
1297.6998	1297.6887	-0.0111	-9	454	465	LEIPDGAVLENK					Mascot
1300.7358	1300.7202	-0.0156	-12	416	427	SIPSIVELDSLK					Mascot
1312.7583	1312.7721	0.0138	11	333	345	VLQLETAAGAAIR					Mascot
1312.7583	1312.7721	0.0138	11	333	345	VLQLETAAGAAIR	101	100			Mascot
1350.7264	1350.7135	-0.0129	-10	428	441	VSGDVSFGSGVVLK					Mascot
1358.7566	1358.7418	-0.0148	-11	172	183	IVTEDFLPLPSK					Mascot
1358.7566	1358.7418	-0.0148	-11	172	183	IVTEDFLPLPSK	24	0			Mascot
1390.7842	1390.6921	-0.0921	-66	346	357	FFEKAIGINVPR					Mascot
1390.7842	1390.6921	-0.0921	-66	346	357	FFEKAIGINVPR					Mascot
1641.8846	1641.8799	-0.0047	-3	391	405	VKPSNPSIELGPEFK					Mascot
1679.8964	1679.8489	-0.0475	-28	255	270	TLADVKGGTLISYEGR					Mascot
1769.9796	1769.975	-0.0046	-3	391	406	VKPSNPSIELGPEFKK					Mascot

Calc. Mass	Obsrv. Mass	± da	± ppm	Start Seq.	End Seq.	Sequence	Ion Score	C. I. %	Modification	Rank	Result Type
1967.0121	1967.0215	0.0094	5	218	235	EYVFVANSDNLGAIVDIK					Mascot
2143.9832	2144.0171	0.0339	16	189	208	DGWYPPGHGDVFPSLN NSGK					Mascot
2198.0261	2198.0654	0.0393	18	154	171	YSNSNIEIHTFNQSQYPR					Mascot
2198.0261	2198.0654	0.0393	18	154	171	YSNSNIEIHTFNQSQYPR	117	100			Mascot
2454.3127	2454.3491	0.0364	15	365	386	ATSDLLLVQSDLYTLVDG YVIR					Mascot

2	Probable UTP--glucose-1-phosphate uridylyltransferase 2 OS=Arabidopsis thaliana GN=At3g03250 PE=1 SV=1	UGPA2_ARATH	51877.2	5.8	6	278	100	11.96	265	100	

Peptide Information

Calc. Mass	Obsrv. Mass	± da	± ppm	Start Seq.	End Seq.	Sequence	Ion Score	C. I. %	Modification	Rank	Result Type
858.493	858.4778	-0.0152	-18	307	314	LVEADALK					Mascot
957.5073	957.4853	-0.022	-23	315	322	MEIIPNPK			Oxidation (M)[1]		Mascot
965.5414	965.5365	-0.0049	-5	27	35	SGFISLVSR					Mascot
965.5414	965.5365	-0.0049	-5	27	35	SGFISLVSR	31	4.876			Mascot
1300.7358	1300.7202	-0.0156	-12	412	423	SIPSIVELDSLK					Mascot
1312.7583	1312.7721	0.0138	11	329	341	VLQLETAAGAAIR					Mascot
1312.7583	1312.7721	0.0138	11	329	341	VLQLETAAGAAIR	101	100			Mascot
2108.1023	2108.1201	0.0178	8	267	284	VQLLEIAQVPDEHVNEFK					Mascot
2108.1023	2108.1201	0.0178	8	267	284	VQLLEIAQVPDEHVNEFK	133	100			Mascot

3	UTP--glucose-1-phosphate uridylyltransferase 1 OS=Arabidopsis thaliana GN=At5g17310 PE=2 SV=1	UGPA1_ARATH	52058.2	5.73	9	257	100	11.046	234	100	

Peptide Information

Calc. Mass	Obsrv. Mass	± da	± ppm	Start Seq.	End Seq.	Sequence	Ion Score	C. I. %	Modification	Rank	Result Type
822.4025	822.4134	0.0109	13	1	8	MAATATEK					Mascot
858.493	858.4778	-0.0152	-18	308	315	LVEADALK					Mascot
872.3996	872.3157	-0.0839	-96	463	470	DINGPEDL					Mascot
957.5073	957.4853	-0.022	-23	316	323	MEIIPNPK			Oxidation (M)[1]		Mascot
978.5255	978.4984	-0.0271	-28	206	214	LDAFLSQGK					Mascot
1300.7358	1300.7202	-0.0156	-12	413	424	SIPSIVELDSLK					Mascot
1312.7583	1312.7721	0.0138	11	330	342	VLQLETAAGAAIR					Mascot
1312.7583	1312.7721	0.0138	11	330	342	VLQLETAAGAAIR	101	100			Mascot
1525.6687	1525.7748	0.1061	70	14	27	SAVDGLTEMSENEK			Oxidation (M)[9]		Mascot
2108.1023	2108.1201	0.0178	8	268	285	VQLLEIAQVPDEHVNEFK					Mascot
2108.1023	2108.1201	0.0178	8	268	285	VQLLEIAQVPDEHVNEFK	133	100			Mascot

4	UTP--glucose-1-phosphate uridylyltransferase OS=Musa acuminata GN=UGPA PE=2 SV=1	UGPA_MUSAC	51501	5.48	10	232	100	22.39	206	100	

Peptide Information

Calc. Mass	Obsrv. Mass	± da	± ppm	Start Seq.	End Seq.	Sequence	Ion Score	C. I. %	Modification	Rank	Result Type
856.441	856.5117	0.0707	83	460	467	VINGPEDI					Mascot
858.493	858.4778	-0.0152	-18	305	312	LVEADALK					Mascot
957.5073	957.4853	-0.022	-23	313	320	MEIIPNPK			Oxidation (M)[1]		Mascot
965.5414	965.5365	-0.0049	-5	25	33	SGFISLVSR					Mascot
965.5414	965.5365	-0.0049	-5	25	33	SGFISLVSR	31	4.876			Mascot
1014.5942	1014.5879	-0.0063	-6	304	312	RLVEADALK					Mascot
1052.5371	1052.5386	0.0015	1	255	264	GGTLISYEGR					Mascot
1052.5371	1052.5386	0.0015	1	255	264	GGTLISYEGR	75	99.996			Mascot
1300.7358	1300.7202	-0.0156	-12	410	421	SIPSIVELDSLK					Mascot
1312.7583	1312.7721	0.0138	11	327	339	VLQLETAAGAAIR					Mascot
1312.7583	1312.7721	0.0138	11	327	339	VLQLETAAGAAIR	101	100			Mascot
1491.7843	1491.7806	-0.0037	-2	422	435	VSGDVWFGEGVVLK					Mascot
1679.8964	1679.8489	-0.0475	-28	249	264	TLADVKGGTLISYEGR					Mascot

5	UTP--glucose-1-phosphate uridylyltransferase OS=Astragalus penduliflorus GN=UGP PE=1 SV=1	UGPA_ASTPN	51632.3	5.92	13	226	100	29.126	175	100	

Peptide Information

Calc. Mass	Obsrv. Mass	± da	± ppm	Start Seq.	End Seq.	Sequence	Ion Score	C. I. %	Modification	Rank	Result Type
839.5098	839.5059	-0.0039	-5	348	355	AIGINVPR					Mascot
858.493	858.4778	-0.0152	-18	309	316	LVEADALK					Mascot
885.4676	885.4975	0.0299	34	464	471	EINGPKDL					Mascot
957.5073	957.4853	-0.022	-23	317	324	MEIIPNPK			Oxidation (M)[1]		Mascot
1014.5942	1014.5879	-0.0063	-6	308	316	RLVEADALK					Mascot
1052.5371	1052.5386	0.0015	1	259	268	GGTLISYEGR					Mascot
1052.5371	1052.5386	0.0015	1	259	268	GGTLISYEGR	75	99.996			Mascot
1300.7358	1300.7202	-0.0156	-12	414	425	SIPSIVELDSLK					Mascot
1312.7583	1312.7721	0.0138	11	331	343	VLQLETAAGAAIR					Mascot
1312.7583	1312.7721	0.0138	11	331	343	VLQLETAAGAAIR	101	100			Mascot
1342.7617	1342.7803	0.0186	14	170	181	LVVDDFLPLPSK					Mascot
1342.7617	1342.7803	0.0186	14	170	181	LVVDDFLPLPSK					Mascot
1492.7788	1492.7826	0.0038	3	1	14	MATATATDRLSNLK					Mascot

1555.8843	1555.7319	-0.1524	-98	170	183	LVVDDFLPLPSKGR					Mascot
1679.8964	1679.8489	-0.0475	-28	253	268	TLADVKGGTLISYEGR					Mascot
1967.0121	1967.0215	0.0094	5	216	233	EYVFVANSDNLGAIVDLK					Mascot

6	UTP--glucose-1-phosphate uridylyltransferase OS=Pyrus pyrifolia PE=2 SV=1	UGPA_PYRPY	51984.4	5.99	10	208	100	20.628	175	100	

Peptide Information

Calc. Mass	Obsrv. Mass	± da	± ppm	Start Seq.	End Seq.	Sequence	Ion Score	C. I. %	Modification	Rank	Result Type
858.493	858.4778	-0.0152	-18	309	316	LVEADALK					Mascot
872.3996	872.3157	-0.0839	-96	464	471	DINGPEDL					Mascot
957.5073	957.4853	-0.022	-23	317	324	MEIIPNPK			Oxidation (M)[1]		Mascot
1014.5942	1014.5879	-0.0063	-6	308	316	RLVEADALK					Mascot
1052.5371	1052.5386	0.0015	1	259	268	GGTLISYEGR					Mascot
1052.5371	1052.5386	0.0015	1	259	268	GGTLISYEGR	75	99.996			Mascot
1312.7583	1312.7721	0.0138	11	331	343	VLQLETAAGAAIR					Mascot
1312.7583	1312.7721	0.0138	11	331	343	VLQLETAAGAAIR	101	100			Mascot
1433.7788	1433.7853	0.0065	5	426	439	VSGDVWFGAGVVLK					Mascot
1679.8964	1679.8489	-0.0475	-28	253	268	TLADVKGGTLISYEGR					Mascot
1967.0121	1967.0215	0.0094	5	216	233	EYVFIANSDNLGAVVDLK					Mascot
2197.0786	2197.1367	0.0581	26	152	169	YSKSNVQIHTFNQSQYPR					Mascot

7	UTP--glucose-1-phosphate uridylyltransferase OS=Solanum tuberosum PE=1 SV=3	UGPA_SOLTU	52069.4	5.7	7	150	100	2.767	133	100	

Peptide Information

Calc. Mass	Obsrv. Mass	± da	± ppm	Start Seq.	End Seq.	Sequence	Ion Score	C. I. %	Modification	Rank	Result Type
858.493	858.4778	-0.0152	-18	314	321	LVEADALK					Mascot
872.3996	872.3157	-0.0839	-96	470	477	DINGPEDI					Mascot
957.5073	957.4853	-0.022	-23	322	329	MEIIPNPK			Oxidation (M)[1]		Mascot
1014.5942	1014.5879	-0.0063	-6	313	321	RLVEADALK					Mascot
1300.7358	1300.7202	-0.0156	-12	420	431	SIPSIIDLDSLK					Mascot
1967.0121	1967.0215	0.0094	5	221	238	EYVFVANSDNLGAIVDLK					Mascot
2108.1023	2108.1201	0.0178	8	274	291	VQLLEIAQVPDEHVNEFK					Mascot
2108.1023	2108.1201	0.0178	8	274	291	VQLLEIAQVPDEHVNEFK	133	100			Mascot

8	DNA ligase OS=Hamiltonella defensa subsp. Acyrthosiphon pisum (strain 5AT) GN=ligA PE=3 SV=1	DNLJ_HAMD5	78498.5	8.63	18	67	88.96	13.561			

Peptide Information

Calc. Mass	Obsrv. Mass	± da	± ppm	Start Seq.	End Seq.	Sequence	Ion Score	C. I. %	Modification	Rank	Result Type
858.5043	858.4778	-0.0265	-31	397	404	IGDTVVVR					Mascot
866.4474	866.4043	-0.0431	-50	1	7	MKSMLEK					Mascot
885.5152	885.4975	-0.0177	-20	360	368	TGALTPVAR					Mascot
927.5621	927.5142	-0.0479	-52	177	184	TIQSIPLR					Mascot
938.5054	938.4716	-0.0338	-36	529	536	SQKTTFAR					Mascot
965.5778	965.5365	-0.0413	-43	484	491	IIHQLVDK					Mascot
965.5778	965.5365	-0.0413	-43	484	491	IIHQLVDK	8	0			Mascot
1014.6055	1014.5879	-0.0176	-17	397	405	IGDTVVVRR					Mascot
1140.6624	1140.5513	-0.1111	-97	574	584	KVPDVGEVVAK					Mascot
1297.7952	1297.6887	-0.1065	-82	393	404	LGLRIGDTVVVR					Mascot
1312.6757	1312.7721	0.0964	73	216	226	ANHEKIFSNPR					Mascot
1312.6757	1312.7721	0.0964	73	216	226	ANHEKIFSNPR					Mascot
1371.625	1371.731	0.106	77	198	209	GEVYMPQAGFEK			Oxidation (M)[5]		Mascot
1380.7845	1380.7379	-0.0466	-34	638	650	DDLKAHLISLGAK					Mascot
1433.7748	1433.7853	0.0105	7	347	359	TTQVLGVEFQVGR					Mascot
1491.7764	1491.7806	0.0042	3	682	693	IMNEPELIEFLK			Oxidation (M)[2]		Mascot
1553.901	1553.7727	-0.1283	-83	642	657	AHLISLGAKVSGSVSK					Mascot
1663.9102	1663.8755	-0.0347	-21	269	282	LMQLKAWGLPVHDR					Mascot
1675.865	1675.9578	0.0928	55	659	674	TDFLIAGENPGSKAQK					Mascot
1679.905	1679.8489	-0.0561	-33	269	282	LMQLKAWGLPVHDR			Oxidation (M)[2]		Mascot
2178.9648	2179.0078	0.043	20	44	61	EWESQYPELMNPDSPTQ K					Mascot

9	DNA-directed RNA polymerase subunit beta' OS=Thermodesulfovibrio yellowstonii (strain ATCC 51303 / DSM 11347 / YP87) GN=rpoC PE=3 SV=1	RPOC_THEYD	154176.6	8.37	30	63	74.119	8.835	

Peptide Information

Calc. Mass	Obsrv. Mass	± da	± ppm	Start Seq.	End Seq.	Sequence	Ion Score	C. I. %	Modification	Rank	Result Type
818.4189	818.4153	-0.0036	-4	1329	1335	ENVIMGR					Mascot
856.525	856.5117	-0.0133	-16	1106	1112	IPRETIK					Mascot
885.5013	885.4975	-0.0038	-4	273	279	VINRNNR					Mascot
916.5462	916.4633	-0.0829	-90	770	778	KGLADTALK					Mascot
927.4934	927.5142	0.0208	22	984	991	YNLVYGAK					Mascot
943.5571	943.5573	0.0002	0	1321	1328	IDELRGLK					Mascot

Calc. Mass	Obsrv. Mass	± da	± ppm	Start Seq.	End Seq.	Sequence	Ion Score	C. I. %	Modification	Rank	Result Type
947.556	947.4996	-0.0564	-60	1167	1174	EYLIPKGK					Mascot
949.5353	949.542	0.0067	7	604	611	DTVLFLNK					Mascot
949.5353	949.542	0.0067	7	604	611	DTVLFLNK					Mascot
964.5244	964.451	-0.0734	-76	873	880	SVLTCRTK			Carbamidomethyl (C)[5]		Mascot
978.5288	978.4984	-0.0304	-31	327	335	SLSDMIKGK					Mascot
1057.6001	1057.504	-0.0961	-91	1113	1122	TKDITGGLPR					Mascot
1080.6049	1080.5559	-0.049	-45	390	399	GYATTIKQAK					Mascot
1102.5488	1102.6478	0.099	90	996	1005	EGQIVESGQR					Mascot
1146.6477	1146.5894	-0.0583	-51	206	216	IEAATSTGIKR					Mascot
1205.6426	1205.6532	0.0106	9	949	957	FKNIHYVER					Mascot
1300.6168	1300.7202	0.1034	80	261	271	FASSDLNDLYR					Mascot
1371.8247	1371.731	-0.0937	-68	1081	1093	YLLPAGAILVVDK					Mascot
1380.825	1380.7379	-0.0871	-63	984	995	YNLVYGAKIIVK					Mascot
1396.726	1396.7217	-0.0043	-3	592	602	LIEYIHYNFGK					Mascot
1399.7549	1399.7716	0.0167	12	1232	1242	HIEVIVRQMMK			Oxidation (M)[9]		Mascot
1405.6958	1405.7507	0.0549	39	694	705	GKEFTPEELAER					Mascot
1513.8472	1513.7909	-0.0563	-37	193	205	IDLDIVAQELKEK					Mascot
1555.8802	1555.7319	-0.1483	-95	992	1005	IIVKEGQIVESGQR					Mascot
1663.739	1663.8755	0.1365	82	881	894	FGVCSKCYGMDLAR			Carbamidomethyl (C)[4,7]		Mascot
1679.7339	1679.8489	0.115	68	881	894	FGVCSKCYGMDLAR			Carbamidomethyl (C)[4,7], Oxidation (M)[10]		Mascot
1691.8456	1691.8555	0.0099	6	83	97	GVICDKCGVEVIQSK			Carbamidomethyl (C)[4,7]		Mascot
1769.8817	1769.975	0.0933	53	655	669	QYAEGLITQGERYNK					Mascot
1967.0055	1967.0215	0.016	8	1	16	MTEDIYSLFQKPKNPR					Mascot
2130.178	2130.1218	-0.0562	-26	565	582	ILFREIVPEGVPFQMINK					Mascot
2179.1621	2179.0078	-0.1543	-71	49	67	TFKPEPEGLFCAKIFGPIK			Carbamidomethyl (C)[11]		Mascot
2186.1565	2186.1707	0.0142	6	372	389	SMALELFKPFVFNKLEEK			Oxidation (M)[2]		Mascot

10	Acetyl-CoA hydrolase OS=Neurospora crassa (strain ATCC 24698 / 74-OR23-1A / CBS 708.71 / DSM 1257 / FGSC 987) GN=acu-8 PE=3 SV=2	ACH1_NEUCR	58425.6	6.29	15	61	58.981	13.653

Peptide Information

Calc. Mass	Obsrv. Mass	± da	± ppm	Start Seq.	End Seq.	Sequence	Ion Score	C. I. %	Modification	Rank	Result Type
814.4529	814.4097	-0.0432	-53	458	464	GLSPRER					Mascot
892.5112	892.4319	-0.0793	-89	97	104	RAPHQVGK					Mascot
1034.5187	1034.5275	0.0088	9	513	522	ALVEEGSMAK					Mascot
1052.5194	1052.5386	0.0192	18	488	495	AEFECLRK			Carbamidomethyl (C)[5]		Mascot
1052.5194	1052.5386	0.0192	18	488	495	AEFECLRK			Carbamidomethyl (C)[5]		Mascot

1205.646	1205.6532	0.0072	6	205	213	KPYLIQQCR	Carbamidomethyl (C)[8]	Mascot
1218.6412	1218.6379	-0.0033	-3	88	97	WAALDMIARR	Oxidation (M)[6]	Mascot
1300.6855	1300.7202	0.0347	27	353	364	SQSVSNAPEIIR		Mascot
1350.6835	1350.7135	0.03	22	393	405	MLNGLGGSADFLR		Mascot
1409.7173	1409.8132	0.0959	68	109	120	GINEGRINFFDK		Mascot
1513.7858	1513.7909	0.0051	3	214	227	DRIGTTSVPVDPEK		Mascot
1555.6852	1555.7319	0.0467	30	338	348	FYKNWDNYYDK		Mascot
1679.8534	1679.8489	-0.0045	-3	393	408	MLNGLGGSADFLRNSK		Mascot
1833.9276	1833.9091	-0.0185	-10	406	421	NSKYSIMHTPSTRPSK		Mascot
2204.0845	2204.0605	-0.024	-11	121	138	HLSMFPVDLVYGYYTKD R		Mascot
2220.0793	2220.0249	-0.0544	-25	121	138	HLSMFPVDLVYGYYTKD R	Oxidation (M)[4]	Mascot
2454.2188	2454.3491	0.1303	53	299	319	NLNVWTEVIQDTFLDLFD SGK		Mascot

Rank	Protein Name	Accession No.	Protein MW	Protein PI	Pep. Count	Protein Score	Protein Score C. I. %	Intensity Matched	Total Ion Score	Total Ion C. I. %	Confirmed
1	ATP synthase subunit beta, chloroplastic OS=Triticum aestivum GN=atpB PE=3 SV=1	ATPB_WHEAT	53880.9	5.06	25	1,160	100	53.676	977	100	

Peptide Information

Calc. Mass	Obsrv. Mass	± da	± ppm	Start Seq.	End Seq.	Sequence	Ion Score	C. I. %	Modification	Rank	Result Type
873.5152	873.5121	-0.0031	-4	391	397	VKETLQR					Mascot
1007.5771	1007.562	-0.0151	-15	146	154	LSIFETGIK					Mascot
1201.7052	1201.7001	-0.0051	-4	155	164	VVDLLAPYRR					Mascot
1254.5818	1254.5714	-0.0104	-8	76	87	AVAMSATDGLMR			Oxidation (M)[4,11]		Mascot
1328.6707	1328.6815	0.0108	8	192	205	AHGGVSVFGGVGER					Mascot
1328.6707	1328.6815	0.0108	8	192	205	AHGGVSVFGGVGER	116	100			Mascot
1416.6866	1416.6992	0.0126	9	379	390	IVGNEHYETAQR					Mascot
1416.6866	1416.6992	0.0126	9	379	390	IVGNEHYETAQR	91	100			Mascot
1433.7748	1433.7893	0.0145	10	278	291	FVQAGSEVSALLGR					Mascot
1433.7748	1433.7893	0.0145	10	278	291	FVQAGSEVSALLGR	113	100			Mascot
1471.7614	1471.7592	-0.0022	-1	249	261	VGLTALTMAEYFR					Mascot
1487.7563	1487.7515	-0.0048	-3	249	261	VGLTALTMAEYFR			Oxidation (M)[8]		Mascot
1487.7563	1487.7515	-0.0048	-3	249	261	VGLTALTMAEYFR	30	0	Oxidation (M)[8]		Mascot
1492.8159	1492.816	0.0001	0	266	277	QDVLLFIDNIFR					Mascot
1518.6564	1518.7524	0.096	63	206	217	TREGNDLYMEMK			Oxidation (M)[9,11]		Mascot
1535.858	1535.8685	0.0105	7	40	52	LPYIYNALVVQSR					Mascot
1535.858	1535.8685	0.0105	7	40	52	LPYIYNALVVQSR	102	100			Mascot
1601.8104	1601.818	0.0076	5	232	246	VALVYGQMNEPPGAR					Mascot
1617.8054	1617.8005	-0.0049	-3	232	246	VALVYGQMNEPPGAR			Oxidation (M)[8]		Mascot
1617.8054	1617.8005	-0.0049	-3	232	246	VALVYGQMNEPPGAR	49	98.694	Oxidation (M)[8]		Mascot
1790.8928	1790.9406	0.0478	27	247	261	MRVGLTALTMAEYFR			Oxidation (M)[1,10]		Mascot
1809.0157	1808.9768	-0.0389	-22	23	39	IDQIIGPVLDVTFPPGK					Mascot
1885.955	1885.9711	0.0161	9	58	73	QINVTCEVQQLLGNNR			Carbamidomethyl (C)[6]		Mascot
1949.0491	1949.0708	0.0217	11	262	277	DVNKQDVLLFIDNIFR					Mascot
1949.0491	1949.0708	0.0217	11	262	277	DVNKQDVLLFIDNIFR	118	100			Mascot
2030.9989	2031.0533	0.0544	27	3	22	TNPTTSPPGASTIEEKSTGR					Mascot

2061.0322	2061.0659	0.0337	16	360	378	GIYPAVDPLDSTSTMLQPR					Mascot
2077.0271	2077.0442	0.0171	8	360	378	GIYPAVDPLDSTSTMLQPR			Oxidation (M)[15]		Mascot
2097.1008	2097.1152	0.0144	7	88	109	GMEVIDTGAPLSVPVGGATLGR					Mascot
2113.0959	2113.1067	0.0108	5	88	109	GMEVIDTGAPLSVPVGGATLGR			Oxidation (M)[2]		Mascot
2314.0691	2314.0925	0.0234	10	292	312	MPSAVGYQPTLSTEMGSLQER			Oxidation (M)[1,15]		Mascot
2460.1785	2460.217	0.0385	16	53	73	DTDDKQINVTCEVQQLLGNNR			Carbamidomethyl (C)[11]		Mascot
2460.1785	2460.217	0.0385	16	53	73	DTDDKQINVTCEVQQLLGNNR	190	100	Carbamidomethyl (C)[11]		Mascot
2694.3887	2694.4514	0.0627	23	110	134	IFNVLGEPVDNLGPVDSSATFPIHR					Mascot
2694.3887	2694.4514	0.0627	23	110	134	IFNVLGEPVDNLGPVDSSATFPIHR	170	100			Mascot
3325.856	3325.9202	0.0642	19	23	52	IDQIIGPVLDVTFPPGKLPYIYNALVVQSR					Mascot
3714.8862	3714.9929	0.1067	29	319	354	GSITSIQAVYVPADDLTDPAPATTFAHLDATTVLSR					Mascot
3842.9812	3843.0984	0.1172	30	318	354	KGSITSIQAVYVPADDLTDPAPATTFAHLDATTVLSR					Mascot

2	ATP synthase subunit beta, chloroplastic OS=Aegilops crassa GN=atpB PE=3 SV=1	ATPB_AEGCR	53865	5.17	23	945	100	50.025	787	100	

Protein Group

ATP synthase subunit beta, chloroplastic OS=Aegilops columnaris GN=atpB PE=3 SV=1	ATPB_AEGCO	53865	5.17000007629395
ATP synthase subunit beta, chloroplastic OS=Agrostis stolonifera GN=atpB PE=3 SV=1	ATPB_AGRST	53839.9	5.15999984741211
ATP synthase subunit beta, chloroplastic OS=Hordeum vulgare GN=atpB PE=3 SV=2	ATPB_HORVU	53898	5.17000007629395

Peptide Information

Calc. Mass	Obsrv. Mass	± da	± ppm	Start Seq.	End Seq.	Sequence	Ion Score	C. I. %	Modification	Rank	Result Type
873.5152	873.5121	-0.0031	-4	391	397	VKETLQR					Mascot
1007.5771	1007.562	-0.0151	-15	146	154	LSIFETGIK					Mascot
1201.7052	1201.7001	-0.0051	-4	155	164	VVDLLAPYRR					Mascot
1254.5818	1254.5714	-0.0104	-8	76	87	AVAMSATDGLMR			Oxidation (M)[4,11]		Mascot
1328.6707	1328.6815	0.0108	8	192	205	AHGGVSVFGGVGER					Mascot
1328.6707	1328.6815	0.0108	8	192	205	AHGGVSVFGGVGER	116	100			Mascot
1416.6866	1416.6992	0.0126	9	379	390	IVGNEHYETAQR					Mascot
1416.6866	1416.6992	0.0126	9	379	390	IVGNEHYETAQR	91	100			Mascot
1433.7748	1433.7893	0.0145	10	278	291	FVQAGSEVSALLGR					Mascot

1433.7748	1433.7893	0.0145	10	278	291	FVQAGSEVSALLGR	113	100			Mascot
1471.7614	1471.7592	-0.0022	-1	249	261	VGLTALTMAEYFR					Mascot
1487.7563	1487.7515	-0.0048	-3	249	261	VGLTALTMAEYFR			Oxidation (M)[8]		Mascot
1487.7563	1487.7515	-0.0048	-3	249	261	VGLTALTMAEYFR	30	0	Oxidation (M)[8]		Mascot
1492.8159	1492.816	0.0001	0	266	277	QDVLLFIDNIFR					Mascot
1518.6564	1518.7524	0.096	63	206	217	TREGNDLYMEMK			Oxidation (M)[9,11]		Mascot
1535.858	1535.8685	0.0105	7	40	52	LPYIYNALVVQSR					Mascot
1535.858	1535.8685	0.0105	7	40	52	LPYIYNALVVQSR	102	100			Mascot
1601.8104	1601.818	0.0076	5	232	246	VALVYGQMNEPPGAR					Mascot
1617.8054	1617.8005	-0.0049	-3	232	246	VALVYGQMNEPPGAR			Oxidation (M)[8]		Mascot
1617.8054	1617.8005	-0.0049	-3	232	246	VALVYGQMNEPPGAR	49	98.694	Oxidation (M)[8]		Mascot
1790.8928	1790.9406	0.0478	27	247	261	MRVGLTALTMAEYFR			Oxidation (M)[1,10]		Mascot
1809.0157	1808.9768	-0.0389	-22	23	39	IDQIIGPVLDVTFPPGK					Mascot
1885.955	1885.9711	0.0161	9	58	73	QINVTCEVQQLLGNNR			Carbamidomethyl (C)[6]		Mascot
1949.0491	1949.0708	0.0217	11	262	277	DVNKQDVLLFIDNIFR					Mascot
1949.0491	1949.0708	0.0217	11	262	277	DVNKQDVLLFIDNIFR	118	100			Mascot
2061.0322	2061.0659	0.0337	16	360	378	GIYPAVDPLDSTSTMLQP R					Mascot
2077.0271	2077.0442	0.0171	8	360	378	GIYPAVDPLDSTSTMLQP R			Oxidation (M)[15]		Mascot
2097.1008	2097.1152	0.0144	7	88	109	GMEVIDTGAPLSVPVGG ATLGR					Mascot
2113.0959	2113.1067	0.0108	5	88	109	GMEVIDTGAPLSVPVGG ATLGR			Oxidation (M)[2]		Mascot
2314.0691	2314.0925	0.0234	10	292	312	MPSAVGYQPTLSTEMGS LQER			Oxidation (M)[1,15]		Mascot
2694.3887	2694.4514	0.0627	23	110	134	IFNVLGEPVDNLGPVDSS ATFPIHR					Mascot
2694.3887	2694.4514	0.0627	23	110	134	IFNVLGEPVDNLGPVDSS ATFPIHR	170	100			Mascot
3325.856	3325.9202	0.0642	19	23	52	IDQIIGPVLDVTFPPGKLP YIYNALVVQSR					Mascot
3714.8862	3714.9929	0.1067	29	319	354	GSITSIQAVYVPADDLTD PAPATTFAHLDATTVLSR					Mascot
3842.9812	3843.0984	0.1172	30	318	354	KGSITSIQAVYVPADDLT DPAPATTFAHLDATTVLS R					Mascot

3	ATP synthase subunit beta, chloroplastic OS=Lolium perenne GN=atpB PE=3 SV=1	ATPB_LOLPR	53840	5.23	22	831	100	45.21	686	100	

Peptide Information

Calc. Mass	Obsrv. Mass	± da	± ppm	Start Seq.	End Seq.	Sequence	Ion Score	C. I. %	Modification	Rank	Result Type
873.5152	873.5121	-0.0031	-4	391	397	VKETLQR					Mascot
1007.5771	1007.562	-0.0151	-15	146	154	LSIFETGIK					Mascot
1201.7052	1201.7001	-0.0051	-4	155	164	VVDLLAPYRR					Mascot

1254.5818	1254.5714	-0.0104	-8	76	87	AVAMSATDGLMR			Oxidation (M)[4,11]	Mascot
1328.6707	1328.6815	0.0108	8	192	205	AHGGVSVFGGVGER				Mascot
1328.6707	1328.6815	0.0108	8	192	205	AHGGVSVFGGVGER	116	100		Mascot
1416.6866	1416.6992	0.0126	9	379	390	IVGNEHYETAQR				Mascot
1416.6866	1416.6992	0.0126	9	379	390	IVGNEHYETAQR	91	100		Mascot
1433.7748	1433.7893	0.0145	10	278	291	FVQAGSEVSALLGR				Mascot
1433.7748	1433.7893	0.0145	10	278	291	FVQAGSEVSALLGR	113	100		Mascot
1471.7614	1471.7592	-0.0022	-1	249	261	VGLTALTMAEYFR				Mascot
1487.7563	1487.7515	-0.0048	-3	249	261	VGLTALTMAEYFR			Oxidation (M)[8]	Mascot
1487.7563	1487.7515	-0.0048	-3	249	261	VGLTALTMAEYFR	30	0	Oxidation (M)[8]	Mascot
1492.8159	1492.816	0.0001	0	266	277	QDVLLFIDNIFR				Mascot
1518.6564	1518.7524	0.096	63	206	217	TREGNDLYMEMK			Oxidation (M)[9,11]	Mascot
1601.8104	1601.818	0.0076	5	232	246	VALVYGQMNEPPGAR				Mascot
1617.8054	1617.8005	-0.0049	-3	232	246	VALVYGQMNEPPGAR			Oxidation (M)[8]	Mascot
1617.8054	1617.8005	-0.0049	-3	232	246	VALVYGQMNEPPGAR	49	98.694	Oxidation (M)[8]	Mascot
1790.8928	1790.9406	0.0478	27	247	261	MRVGLTALTMAEYFR			Oxidation (M)[1,10]	Mascot
1809.0157	1808.9768	-0.0389	-22	23	39	IDQIIGPVLDVTFPPGK				Mascot
1885.955	1885.9711	0.0161	9	58	73	QINVTCEVQQLLGNNR			Carbamidomethyl (C)[6]	Mascot
1949.0491	1949.0708	0.0217	11	262	277	DVNKQDVLLFIDNIFR				Mascot
1949.0491	1949.0708	0.0217	11	262	277	DVNKQDVLLFIDNIFR	118	100		Mascot
2061.0322	2061.0659	0.0337	16	360	378	GIYPAVDPLDSTSTMLQPR				Mascot
2077.0271	2077.0442	0.0171	8	360	378	GIYPAVDPLDSTSTMLQPR			Oxidation (M)[15]	Mascot
2097.1008	2097.1152	0.0144	7	88	109	GMEVIDTGAPLSVPVGGATLGR				Mascot
2113.0959	2113.1067	0.0108	5	88	109	GMEVIDTGAPLSVPVGGATLGR			Oxidation (M)[2]	Mascot
2314.0691	2314.0925	0.0234	10	292	312	MPSAVGYQPTLSTEMGSLQER			Oxidation (M)[1,15]	Mascot
2330.218	2330.0852	-0.1328	-57	427	447	IERFLSQPFFVAEVFTGSAGK				Mascot
2694.3887	2694.4514	0.0627	23	110	134	IFNVLGEPVDNLGPVDSSATFPIHR				Mascot
2694.3887	2694.4514	0.0627	23	110	134	IFNVLGEPVDNLGPVDSSATFPIHR	170	100		Mascot
3714.8862	3714.9929	0.1067	29	319	354	GSITSIQAVYVPADDLTDPAPATTFAHLDATTVLSR				Mascot
3842.9812	3843.0984	0.1172	30	318	354	KGSITSIQAVYVPADDLTDPAPATTFAHLDATTVLSR				Mascot

4	ATP synthase subunit beta, chloroplastic OS=Oryza sativa GN=atpB PE=2 SV=1	ATPB_ORYSA	53978.1	5.38	20	741	100	40.939	617	100	

Protein Group

ATP synthase subunit beta, chloroplastic OS=Oryza nivara GN=atpB PE=3 SV=1	ATPB_ORYNI	53978.1	5.3800 001144 4092
ATP synthase subunit beta, chloroplastic OS=Oryza sativa subsp. indica GN=atpB PE=2 SV=1	ATPB_ORYSI	53978.1	5.3800 001144 4092
ATP synthase subunit beta, chloroplastic OS=Oryza sativa subsp. japonica GN=atpB PE=1 SV=2	ATPB_ORYSJ	53978.1	5.3800 001144 4092

Peptide Information

Calc. Mass	Obsrv. Mass	± da	± ppm	Start Seq.	End Seq.	Sequence	Ion Score	C. I. %	Modification	Rank	Result Type
1007.5771	1007.562	-0.0151	-15	146	154	LSIFETGIK					Mascot
1201.7052	1201.7001	-0.0051	-4	155	164	VVDLLAPYRR					Mascot
1254.5818	1254.5714	-0.0104	-8	76	87	AVAMSATDGLMR			Oxidation (M)[4,11]		Mascot
1328.6707	1328.6815	0.0108	8	192	205	AHGGVSVFGGVGER					Mascot
1328.6707	1328.6815	0.0108	8	192	205	AHGGVSVFGGVGER	116	100			Mascot
1416.6866	1416.6992	0.0126	9	379	390	IVGNEHYETAQR					Mascot
1416.6866	1416.6992	0.0126	9	379	390	IVGNEHYETAQR	91	100			Mascot
1433.7748	1433.7893	0.0145	10	278	291	FVQAGSEVSALLGR					Mascot
1433.7748	1433.7893	0.0145	10	278	291	FVQAGSEVSALLGR	113	100			Mascot
1471.7614	1471.7592	-0.0022	-1	249	261	VGLTALTMAEYFR					Mascot
1487.7563	1487.7515	-0.0048	-3	249	261	VGLTALTMAEYFR			Oxidation (M)[8]		Mascot
1487.7563	1487.7515	-0.0048	-3	249	261	VGLTALTMAEYFR	30	0	Oxidation (M)[8]		Mascot
1492.8159	1492.816	0.0001	0	266	277	QDVLLFIDNIFR					Mascot
1518.6564	1518.7524	0.096	63	206	217	TREGNDLYMEMK			Oxidation (M)[9,11]		Mascot
1535.8944	1535.8685	-0.0259	-17	40	52	LPYIYNALVVKSR					Mascot
1535.8944	1535.8685	-0.0259	-17	40	52	LPYIYNALVVKSR	102	100			Mascot
1601.8104	1601.818	0.0076	5	232	246	VALVYGQMNEPPGAR					Mascot
1617.8054	1617.8005	-0.0049	-3	232	246	VALVYGQMNEPPGAR			Oxidation (M)[8]		Mascot
1617.8054	1617.8005	-0.0049	-3	232	246	VALVYGQMNEPPGAR	49	98.694	Oxidation (M)[8]		Mascot
1790.8928	1790.9406	0.0478	27	247	261	MRVGLTALTMAEYFR			Oxidation (M)[1,10]		Mascot
1809.0157	1808.9768	-0.0389	-22	23	39	IDQIIGPVLDVTFPPGK					Mascot
1885.955	1885.9711	0.0161	9	58	73	QINVTCEVQQLLGNNR			Carbamidomethyl (C)[6]		Mascot
1949.0491	1949.0708	0.0217	11	262	277	DVNKQDVLLFIDNIFR					Mascot
1949.0491	1949.0708	0.0217	11	262	277	DVNKQDVLLFIDNIFR	118	100			Mascot
2061.0322	2061.0659	0.0337	16	360	378	GIYPAVDPLDSTSTMLQPR					Mascot
2077.0271	2077.0442	0.0171	8	360	378	GIYPAVDPLDSTSTMLQPR			Oxidation (M)[15]		Mascot
2097.1008	2097.1152	0.0144	7	88	109	GMEVIDTGAPLSVPVGGATLGR					Mascot

2113.0959	2113.1067	0.0108	5	88	109	GMEVIDTGAPLSVPVGG ATLGR			Oxidation (M)[2]		Mascot
2314.0691	2314.0925	0.0234	10	292	312	MPSAVGYQPTLSTEMGS LQER			Oxidation (M)[1,15]		Mascot
3714.8862	3714.9929	0.1067	29	319	354	GSITSIQAVYVPADDLTD PAPATTFAHLDATTVLSR					Mascot
3842.9812	3843.0984	0.1172	30	318	354	KGSITSIQAVYVPADDLT DPAPATTFAHLDATTVLS R					Mascot

5	ATP synthase subunit beta, chloroplastic OS=Saccharum officinarum GN=atpB PE=3 SV=1	ATPB_SACOF	53987.2	5.31	19	628	100	33.723	516	100	

Protein Group

ATP synthase subunit beta, chloroplastic OS=Saccharum hybrid GN=atpB PE=3 SV=1	ATPB_SACHY	53987.2	5.3099 999427 7954
ATP synthase subunit beta, chloroplastic OS=Sorghum bicolor GN=atpB PE=3 SV=1	ATPB_SORBI	54036.2	5.3099 999427 7954

Peptide Information

Calc. Mass	Obsrv. Mass	± da	± ppm	Start Seq.	End Seq.	Sequence	Ion Score	C. I. %	Modification	Rank	Result Type
873.5152	873.5121	-0.0031	-4	391	397	VKETLQR					Mascot
1007.5771	1007.562	-0.0151	-15	146	154	LSIFETGIK					Mascot
1201.7052	1201.7001	-0.0051	-4	155	164	VVDLLAPYRR					Mascot
1254.5818	1254.5714	-0.0104	-8	76	87	AVAMSATDGLMR			Oxidation (M)[4,11]		Mascot
1328.6707	1328.6815	0.0108	8	192	205	AHGGVSVFGGVGER					Mascot
1328.6707	1328.6815	0.0108	8	192	205	AHGGVSVFGGVGER	116	100			Mascot
1416.6866	1416.6992	0.0126	9	379	390	IVGNEHYETAQR					Mascot
1416.6866	1416.6992	0.0126	9	379	390	IVGNEHYETAQR	91	100			Mascot
1433.7748	1433.7893	0.0145	10	278	291	FVQAGSEVSALLGR					Mascot
1433.7748	1433.7893	0.0145	10	278	291	FVQAGSEVSALLGR	113	100			Mascot
1471.7614	1471.7592	-0.0022	-1	249	261	VGLTALTMAEYFR					Mascot
1487.7563	1487.7515	-0.0048	-3	249	261	VGLTALTMAEYFR			Oxidation (M)[8]		Mascot
1487.7563	1487.7515	-0.0048	-3	249	261	VGLTALTMAEYFR	30	0	Oxidation (M)[8]		Mascot
1492.8159	1492.816	0.0001	0	266	277	QDVLLFIDNIFR					Mascot
1518.6564	1518.7524	0.096	63	206	217	TREGNDLYMEMK			Oxidation (M)[9,11]		Mascot
1601.8104	1601.818	0.0076	5	232	246	VALVYGQMNEPPGAR					Mascot
1617.8054	1617.8005	-0.0049	-3	232	246	VALVYGQMNEPPGAR			Oxidation (M)[8]		Mascot
1617.8054	1617.8005	-0.0049	-3	232	246	VALVYGQMNEPPGAR	49	98.694	Oxidation (M)[8]		Mascot
1790.8928	1790.9406	0.0478	27	247	261	MRVGLTALTMAEYFR			Oxidation (M)[1,10]		Mascot
1885.955	1885.9711	0.0161	9	58	73	QINVTCEVQQLLGNNR			Carbamidomethyl (C)[6]		Mascot
1949.0491	1949.0708	0.0217	11	262	277	DVNKQDVLLFIDNIFR					Mascot

1949.0491	1949.0708	0.0217	11	262	277	DVNKQDVLLFIDNIFR	118	100			Mascot
2061.0322	2061.0659	0.0337	16	360	378	GIYPAVDPLDSTSTMLQP R					Mascot
2077.0271	2077.0442	0.0171	8	360	378	GIYPAVDPLDSTSTMLQP R			Oxidation (M)[15]		Mascot
2314.0691	2314.0925	0.0234	10	292	312	MPSAVGYQPTLSTEMGS LQER			Oxidation (M)[1,15]		Mascot
2722.4199	2722.4614	0.0415	15	110	134	IFNVLGEPIDNLGPVDTSA TFPIHR					Mascot
3714.8862	3714.9929	0.1067	29	319	354	GSITSIQAVYVPADDLTD PAPATTFAHLDATTVLSR					Mascot
3842.9812	3843.0984	0.1172	30	318	354	KGSITSIQAVYVPADDLT DPAPATTFAHLDATTVLS R					Mascot

6	ATP synthase subunit beta, chloroplastic OS=Zea mays GN=atpB PE=3 SV=1	ATPB_MAIZE	54064.2	5.31	18	620	100	33.626	516	100	

Peptide Information

Calc. Mass	Obsrv. Mass	± da	± ppm	Start Seq.	End Seq.	Sequence	Ion Score	C. I. %	Modification	Rank	Result Type
873.5152	873.5121	-0.0031	-4	391	397	VKETLQR					Mascot
1007.5771	1007.562	-0.0151	-15	146	154	LSIFETGIK					Mascot
1201.7052	1201.7001	-0.0051	-4	155	164	VVDLLAPYRR					Mascot
1328.6707	1328.6815	0.0108	8	192	205	AHGGVSVFGGVGER					Mascot
1328.6707	1328.6815	0.0108	8	192	205	AHGGVSVFGGVGER	116	100			Mascot
1416.6866	1416.6992	0.0126	9	379	390	IVGNEHYETAQR					Mascot
1416.6866	1416.6992	0.0126	9	379	390	IVGNEHYETAQR	91	100			Mascot
1433.7748	1433.7893	0.0145	10	278	291	FVQAGSEVSALLGR					Mascot
1433.7748	1433.7893	0.0145	10	278	291	FVQAGSEVSALLGR	113	100			Mascot
1471.7614	1471.7592	-0.0022	-1	249	261	VGLTALTMAEYFR					Mascot
1487.7563	1487.7515	-0.0048	-3	249	261	VGLTALTMAEYFR			Oxidation (M)[8]		Mascot
1487.7563	1487.7515	-0.0048	-3	249	261	VGLTALTMAEYFR	30	0	Oxidation (M)[8]		Mascot
1492.8159	1492.816	0.0001	0	266	277	QDVLLFIDNIFR					Mascot
1518.6564	1518.7524	0.096	63	206	217	TREGNDLYMEMK			Oxidation (M)[9,11]		Mascot
1601.8104	1601.818	0.0076	5	232	246	VALVYGQMNEPPGAR					Mascot
1617.8054	1617.8005	-0.0049	-3	232	246	VALVYGQMNEPPGAR			Oxidation (M)[8]		Mascot
1617.8054	1617.8005	-0.0049	-3	232	246	VALVYGQMNEPPGAR	49	98.694	Oxidation (M)[8]		Mascot
1790.8928	1790.9406	0.0478	27	247	261	MRVGLTALTMAEYFR			Oxidation (M)[1,10]		Mascot
1885.955	1885.9711	0.0161	9	58	73	QINVTCEVQQLLGNNR			Carbamidomethyl (C)[6]		Mascot
1949.0491	1949.0708	0.0217	11	262	277	DVNKQDVLLFIDNIFR					Mascot
1949.0491	1949.0708	0.0217	11	262	277	DVNKQDVLLFIDNIFR	118	100			Mascot
2061.0322	2061.0659	0.0337	16	360	378	GIYPAVDPLDSTSTMLQP					Mascot

Calc. Mass	Obsrv. Mass	± da	± ppm	Start Seq.	End Seq.	Sequence	Ion Score	C. I. %	Modification	Rank	Result Type
						R					
2077.0271	2077.0442	0.0171	8	360	378	GIYPAVDPLDSTSTMLQPR			Oxidation (M)[15]		Mascot
2314.0691	2314.0925	0.0234	10	292	312	MPSAVGYQPTLSTEMGSLQER			Oxidation (M)[1,15]		Mascot
2722.4199	2722.4614	0.0415	15	110	134	IFNVLGEPIDNLGPVDTSATFPIHR					Mascot
3714.8862	3714.9929	0.1067	29	319	354	GSITSIQAVYVPADDLTDPAPATTFAHLDATTVLSR					Mascot
3842.9812	3843.0984	0.1172	30	318	354	KGSITSIQAVYVPADDLTDPAPATTFAHLDATTVLSR					Mascot

7	ATP synthase subunit beta, chloroplastic OS=Pelargonium hortorum GN=atpB PE=3 SV=1	ATPB_PELHO	53991.3	6.64	17	520	100	30.295	425	100	

Peptide Information

Calc. Mass	Obsrv. Mass	± da	± ppm	Start Seq.	End Seq.	Sequence	Ion Score	C. I. %	Modification	Rank	Result Type
1007.5771	1007.562	-0.0151	-15	146	154	LSIFETGIK					Mascot
1201.7052	1201.7001	-0.0051	-4	155	164	VVDLLAPYRR					Mascot
1254.5818	1254.5714	-0.0104	-8	76	87	AVAMSATDGLMR			Oxidation (M)[4,11]		Mascot
1328.6707	1328.6815	0.0108	8	192	205	AHGGVSVFGGVGER					Mascot
1328.6707	1328.6815	0.0108	8	192	205	AHGGVSVFGGVGER	116	100			Mascot
1433.7748	1433.7893	0.0145	10	278	291	FVQAGSEVSALLGR					Mascot
1433.7748	1433.7893	0.0145	10	278	291	FVQAGSEVSALLGR	113	100			Mascot
1471.7614	1471.7592	-0.0022	-1	249	261	VGLTALTMAEYFR					Mascot
1487.7563	1487.7515	-0.0048	-3	249	261	VGLTALTMAEYFR			Oxidation (M)[8]		Mascot
1487.7563	1487.7515	-0.0048	-3	249	261	VGLTALTMAEYFR	30	0	Oxidation (M)[8]		Mascot
1492.8159	1492.816	0.0001	0	266	277	QDVLLFIDNIFR					Mascot
1518.6564	1518.7524	0.096	63	206	217	TREGNDLYMEMK			Oxidation (M)[9,11]		Mascot
1601.8104	1601.818	0.0076	5	232	246	VALVYGQMNEPPGAR					Mascot
1617.8054	1617.8005	-0.0049	-3	232	246	VALVYGQMNEPPGAR			Oxidation (M)[8]		Mascot
1617.8054	1617.8005	-0.0049	-3	232	246	VALVYGQMNEPPGAR	49	98.694	Oxidation (M)[8]		Mascot
1790.8928	1790.9406	0.0478	27	247	261	MRVGLTALTMAEYFR			Oxidation (M)[1,10]		Mascot
1949.0491	1949.0708	0.0217	11	262	277	DVNKQDVLLFIDNIFR					Mascot
1949.0491	1949.0708	0.0217	11	262	277	DVNKQDVLLFIDNIFR	118	100			Mascot
1963.1852	1963.0774	-0.1078	-55	20	37	LGRIVQIIGPVLDVAFPR					Mascot
2061.0322	2061.0659	0.0337	16	360	378	GIYPAVDPLDSTSTMLQPR					Mascot
2077.0271	2077.0442	0.0171	8	360	378	GIYPAVDPLDSTSTMLQPR			Oxidation (M)[15]		Mascot
2097.1008	2097.1152	0.0144	7	88	109	GMEVIDTGAPLSVPVGGATLGR					Mascot
2113.0959	2113.1067	0.0108	5	88	109	GMEVIDTGAPLSVPVGGATLGR			Oxidation (M)[2]		Mascot

2314.0691	2314.0925	0.0234	10	292	312	MPSAVGYQPTLSTEMGSLQER			Oxidation (M)[1,15]		Mascot
3714.8862	3714.9929	0.1067	29	319	354	GSITSIQAVYVPADDLTDPAPATTFAHLDATTVLSR					Mascot
3842.9812	3843.0984	0.1172	30	318	354	KGSITSIQAVYVPADDLTDPAPATTFAHLDATTVLSR					Mascot

8	ATP synthase subunit beta, chloroplastic OS=Raphanus sativus GN=atpB PE=2 SV=1	ATPB_RAPSA	53799.1	5.68	14	496	100	27.382	426	100	

Peptide Information

Calc. Mass	Obsrv. Mass	± da	± ppm	Start Seq.	End Seq.	Sequence	Ion Score	C. I. %	Modification	Rank	Result Type
1007.5771	1007.562	-0.0151	-15	146	154	LSIFETGIK					Mascot
1095.543	1095.6058	0.0628	57	206	214	TREGNDLYK					Mascot
1201.7052	1201.7001	-0.0051	-4	155	164	VVDLLAPYRR					Mascot
1328.6707	1328.6815	0.0108	8	192	205	AHGGVSVFGGVGER					Mascot
1328.6707	1328.6815	0.0108	8	192	205	AHGGVSVFGGVGER	116	100			Mascot
1433.7748	1433.7893	0.0145	10	278	291	FVQAGSEVSALLGR					Mascot
1433.7748	1433.7893	0.0145	10	278	291	FVQAGSEVSALLGR	113	100			Mascot
1471.7614	1471.7592	-0.0022	-1	249	261	VGLTALTMAEYFR					Mascot
1487.7563	1487.7515	-0.0048	-3	249	261	VGLTALTMAEYFR			Oxidation (M)[8]		Mascot
1487.7563	1487.7515	-0.0048	-3	249	261	VGLTALTMAEYFR	30	0	Oxidation (M)[8]		Mascot
1492.8159	1492.816	0.0001	0	266	277	QDVLLFIDNIFR					Mascot
1601.8104	1601.818	0.0076	5	232	246	VALVYGQMNEPPGAR					Mascot
1617.8054	1617.8005	-0.0049	-3	232	246	VALVYGQMNEPPGAR			Oxidation (M)[8]		Mascot
1617.8054	1617.8005	-0.0049	-3	232	246	VALVYGQMNEPPGAR	49	98.694	Oxidation (M)[8]		Mascot
1790.8928	1790.9406	0.0478	27	247	261	MRVGLTALTMAEYFR			Oxidation (M)[1,10]		Mascot
1949.0491	1949.0708	0.0217	11	262	277	DVNKQDVLLFIDNIFR					Mascot
1949.0491	1949.0708	0.0217	11	262	277	DVNKQDVLLFIDNIFR	118	100			Mascot
2061.0322	2061.0659	0.0337	16	360	378	GIYPAVDPLDSTSTMLQPR					Mascot
2077.0271	2077.0442	0.0171	8	360	378	GIYPAVDPLDSTSTMLQPR			Oxidation (M)[15]		Mascot
2314.1643	2314.0925	-0.0718	-31	87	109	RGMDVVDMGNPLSVPVGGATLGR			Oxidation (M)[3]		Mascot
2330.1592	2330.0852	-0.074	-32	87	109	RGMDVVDMGNPLSVPVGGATLGR			Oxidation (M)[3,8]		Mascot
3714.8862	3714.9929	0.1067	29	319	354	GSITSIQAVYVPADDLTDPAPATTFAHLDATTVLSR					Mascot
3842.9812	3843.0984	0.1172	30	318	354	KGSITSIQAVYVPADDLTDPAPATTFAHLDATTVLSR					Mascot

9	ATP synthase subunit beta, chloroplastic OS=Lobularia maritima GN=atpB PE=3 SV=1	ATPB_LOBMA	53968.2	5.69	14	494	100	27.303	425	100	

Peptide Information

Calc. Mass	Obsrv. Mass	± da	± ppm	Start Seq.	End Seq.	Sequence	Ion Score	C. I. %	Modification	Rank	Result Type
1007.5771	1007.562	-0.0151	-15	146	154	LSIFETGIK					Mascot
1201.7052	1201.7001	-0.0051	-4	155	164	VVDLLAPYRR					Mascot
1328.6707	1328.6815	0.0108	8	192	205	AHGGVSVFGGVGER					Mascot
1328.6707	1328.6815	0.0108	8	192	205	AHGGVSVFGGVGER	116	100			Mascot
1433.7748	1433.7893	0.0145	10	278	291	FVQAGSEVSALLGR					Mascot
1433.7748	1433.7893	0.0145	10	278	291	FVQAGSEVSALLGR	113	100			Mascot
1471.7614	1471.7592	-0.0022	-1	249	261	VGLTALTMAEYFR					Mascot
1487.7563	1487.7515	-0.0048	-3	249	261	VGLTALTMAEYFR			Oxidation (M)[8]		Mascot
1487.7563	1487.7515	-0.0048	-3	249	261	VGLTALTMAEYFR	30	0	Oxidation (M)[8]		Mascot
1492.8159	1492.816	0.0001	0	266	277	QDVLLFIDNIFR					Mascot
1518.6564	1518.7524	0.096	63	206	217	TREGNDLYMEMK			Oxidation (M)[9,11]		Mascot
1601.8104	1601.818	0.0076	5	232	246	VALVYGQMNEPPGAR					Mascot
1617.8054	1617.8005	-0.0049	-3	232	246	VALVYGQMNEPPGAR			Oxidation (M)[8]		Mascot
1617.8054	1617.8005	-0.0049	-3	232	246	VALVYGQMNEPPGAR	49	98.694	Oxidation (M)[8]		Mascot
1790.8928	1790.9406	0.0478	27	247	261	MRVGLTALTMAEYFR			Oxidation (M)[1,10]		Mascot
1949.0491	1949.0708	0.0217	11	262	277	DVNKQDVLLFIDNIFR					Mascot
1949.0491	1949.0708	0.0217	11	262	277	DVNKQDVLLFIDNIFR	118	100			Mascot
2061.0322	2061.0659	0.0337	16	360	378	GIYPAVDPLDSTSTMLQPR					Mascot
2077.0271	2077.0442	0.0171	8	360	378	GIYPAVDPLDSTSTMLQPR			Oxidation (M)[15]		Mascot
2314.0691	2314.0925	0.0234	10	292	312	MPSAVGYQPTLSTEMGSLQER			Oxidation (M)[1,15]		Mascot
3714.8862	3714.9929	0.1067	29	319	354	GSITSIQAVYVPADDLTDPAPATTFAHLDATTVLSR					Mascot
3842.9812	3843.0984	0.1172	30	318	354	KGSITSIQAVYVPADDLTDPAPATTFAHLDATTVLSR					Mascot

10	ATP synthase subunit beta, chloroplastic OS=Marchantia polymorpha GN=atpB PE=3 SV=1	ATPB_MARPO	53203.5	5.07	13	478	100	25.189	426	100	

Peptide Information

Calc. Mass	Obsrv. Mass	± da	± ppm	Start Seq.	End Seq.	Sequence	Ion Score	C. I. %	Modification	Rank	Result Type
1007.5771	1007.562	-0.0151	-15	144	152	LSIFETGIK					Mascot
1162.6104	1162.6659	0.0555	48	133	143	AAPAFTQLDTK					Mascot
1201.7052	1201.7001	-0.0051	-4	153	162	VVDLLAPYRR					Mascot
1240.5483	1240.6332	0.0849	68	74	85	AVAMSATDGMMR					Mascot

1328.6707	1328.6815	0.0108	8	190	203	AHGGVSVFGGVGER				Mascot
1328.6707	1328.6815	0.0108	8	190	203	AHGGVSVFGGVGER	116	100		Mascot
1433.7748	1433.7893	0.0145	10	276	289	FVQAGSEVSALLGR				Mascot
1433.7748	1433.7893	0.0145	10	276	289	FVQAGSEVSALLGR	113	100		Mascot
1471.7614	1471.7592	-0.0022	-1	247	259	VGLTALTMAEYFR				Mascot
1487.7563	1487.7515	-0.0048	-3	247	259	VGLTALTMAEYFR			Oxidation (M)[8]	Mascot
1487.7563	1487.7515	-0.0048	-3	247	259	VGLTALTMAEYFR	30	0	Oxidation (M)[8]	Mascot
1492.8159	1492.816	0.0001	0	264	275	QDVLLFIDNIFR				Mascot
1518.6564	1518.7524	0.096	63	204	215	TREGNDLYMEMK			Oxidation (M)[9,11]	Mascot
1601.8104	1601.818	0.0076	5	230	244	VALVYGQMNEPPGAR				Mascot
1617.8054	1617.8005	-0.0049	-3	230	244	VALVYGQMNEPPGAR			Oxidation (M)[8]	Mascot
1617.8054	1617.8005	-0.0049	-3	230	244	VALVYGQMNEPPGAR	49	98.695	Oxidation (M)[8]	Mascot
1790.918	1790.9406	0.0226	13	1	16	MKTNFLAFGMSTLVAK			Oxidation (M)[1,10]	Mascot
1949.0491	1949.0708	0.0217	11	260	275	DVNKQDVLLFIDNIFR				Mascot
1949.0491	1949.0708	0.0217	11	260	275	DVNKQDVLLFIDNIFR	118	100		Mascot
2198.1851	2198.0789	-0.1062	-48	86	107	GMKVIDTGAPLTVPVGEA TLGR			Oxidation (M)[2]	Mascot

Gel Idx/Pos	251/K3	Instr./Gel Origin	BA2151/Sample Project 20140814	Process Status	Analysis Succeeded
Plate [#] Name	[1] Sample Project 20140814	Instrument Sample Name		Spectra	11

Rank	Protein Name	Accession No.	Protein MW	Protein PI	Pep. Count	Protein Score	Protein Score C. I. %	Intensity Matched	Total Ion Score	Total Ion C. I. %	Confirmed
1	Tubulin alpha-1 chain OS=Eleusine indica GN=TUBA1 PE=1 SV=1	TBA1_ELEIN	50383.6	4.89	16	470	100	22.599	386	100	

Peptide Information

Calc. Mass	Obsrv. Mass	± da	± ppm	Start Seq.	End Seq.	Sequence	Ion Score	C. I. %	Modification	Rank	Result Type
1007.454	1007.4469	-0.0071	-7	97	105	EDAANNFAR					Mascot
1132.5668	1132.5575	-0.0093	-8	113	121	EIVDLCLDR			Carbamidomethyl (C)[6]		Mascot
1299.4989	1299.472	-0.0269	-21	312	320	YMACCLMYR			Carbamidomethyl (C)[4,5], Oxidation (M)[2,7]		Mascot
1396.693	1396.7186	0.0256	18	391	401	IDHKFDLMYAK			Oxidation (M)[8]		Mascot
1473.8635	1473.854	-0.0095	-6	230	243	LVSQVISSLTASLR					Mascot
1473.8635	1473.854	-0.0095	-6	230	243	LVSQVISSLTASLR	102	100			Mascot
1589.6844	1589.6995	0.0151	9	309	320	HGKYMACCLMYR			Carbamidomethyl (C)[7,8]		Mascot
1691.8711	1691.8533	-0.0178	-11	216	229	SLDIERPTYTNLNR					Mascot
1691.8711	1691.8533	-0.0178	-11	216	229	SLDIERPTYTNLNR	67	99.979			Mascot
1701.9059	1701.8979	-0.008	-5	65	79	AVFVDLEPTVIDEVR					Mascot
1701.9059	1701.8979	-0.008	-5	65	79	AVFVDLEPTVIDEVR	71	99.992			Mascot
1808.9252	1808.8848	-0.0404	-22	265	280	IHFMLSSYAPVISAEK			Oxidation (M)[4]		Mascot
1847.9723	1847.8995	-0.0728	-39	215	229	RSLDIERPTYTNLNR					Mascot
1885.9147	1885.9192	0.0045	2	374	390	AVCMISNSTSVVEVFSR			Carbamidomethyl (C)[3]		Mascot
1901.9097	1901.9373	0.0276	15	374	390	AVCMISNSTSVVEVFSR			Carbamidomethyl (C)[3], Oxidation (M)[4]		Mascot
2346.0132	2346.0142	0.001	0	403	422	AFVHWYVGEGMEEGEF SEAR			Oxidation (M)[11]		Mascot
2382.8528	2383.0151	0.1623	68	431	451	DYEEVGAEFDEGEEGDE GDEY					Mascot
2385.1946	2385.2019	0.0073	3	85	105	QLFHPEQLISGKEDAANN FAR					Mascot
2395.1746	2395.2053	0.0307	13	374	394	AVCMISNSTSVVEVFSRI DHK			Carbamidomethyl (C)[3], Oxidation (M)[4]		Mascot
2408.1882	2408.2029	0.0147	6	244	264	FDGALNVDVNEFQTNLV PYPR					Mascot
2408.1882	2408.2029	0.0147	6	244	264	FDGALNVDVNEFQTNLV PYPR	145	100			Mascot

Rank	Protein Name	Accession No.	Protein MW	Protein PI	Pep. Count	Protein Score	Protein Score C. I. %	Intensity Matched	Total Ion Score	Total Ion C. I. %	Confirmed
2	Tubulin alpha-3 chain OS=Hordeum vulgare GN=TUBA3 PE=1 SV=1	TBA3_HORVU	50381.6	4.89	16	470	100	22.494	386	100	

Protein Group

Tubulin alpha chain OS=Triticum aestivum GN=TUBA	TBA_WHEAT	50395.6	4.8899

PE=2 SV=1			998664 856
Tubulin alpha-2 chain OS=Hordeum vulgare GN=TUBA2 PE=2 SV=1	TBA2_HORVU	50353.6	4.8800 001144 4092

Peptide Information

Calc. Mass	Obsrv. Mass	± da	± ppm	Start Seq.	End Seq.	Sequence	Ion Score	C. I. %	Modification	Rank	Result Type
1007.454	1007.4469	-0.0071	-7	97	105	EDAANNFAR					Mascot
1132.5668	1132.5575	-0.0093	-8	113	121	EIVDLCLDR			Carbamidomethyl (C)[6]		Mascot
1299.4989	1299.472	-0.0269	-21	312	320	YMACCLMYR			Carbamidomethyl (C)[4,5], Oxidation (M)[2,7]		Mascot
1396.693	1396.7186	0.0256	18	391	401	IDHKFDLMYAK			Oxidation (M)[8]		Mascot
1473.8635	1473.854	-0.0095	-6	230	243	LVSQVISSLTASLR					Mascot
1473.8635	1473.854	-0.0095	-6	230	243	LVSQVISSLTASLR	102	100			Mascot
1589.6844	1589.6995	0.0151	9	309	320	HGKYMACCLMYR			Carbamidomethyl (C)[7,8]		Mascot
1691.8711	1691.8533	-0.0178	-11	216	229	SLDIERPTYTNLNR					Mascot
1691.8711	1691.8533	-0.0178	-11	216	229	SLDIERPTYTNLNR	67	99.979			Mascot
1701.9059	1701.8979	-0.008	-5	65	79	AVFVDLEPTVIDEVR					Mascot
1701.9059	1701.8979	-0.008	-5	65	79	AVFVDLEPTVIDEVR	71	99.992			Mascot
1808.9252	1808.8848	-0.0404	-22	265	280	IHFMLSSYAPVISAEK			Oxidation (M)[4]		Mascot
1847.9723	1847.8995	-0.0728	-39	215	229	RSLDIERPTYTNLNR					Mascot
1885.9147	1885.9192	0.0045	2	374	390	AVCMISNSTSVVEVFSR			Carbamidomethyl (C)[3]		Mascot
1901.9097	1901.9373	0.0276	15	374	390	AVCMISNSTSVVEVFSR			Carbamidomethyl (C)[3], Oxidation (M)[4]		Mascot
1977.8826	1977.8553	-0.0273	-14	41	60	TVGGGDDAFNTFFSETG AGK					Mascot
2346.0132	2346.0142	0.001	0	403	422	AFVHWYVGEGMEEGEF SEAR			Oxidation (M)[11]		Mascot
2385.1946	2385.2019	0.0073	3	85	105	QLFHPEQLISGKEDAANN FAR					Mascot
2395.1746	2395.2053	0.0307	13	374	394	AVCMISNSTSVVEVFSRI DHK			Carbamidomethyl (C)[3], Oxidation (M)[4]		Mascot
2408.1882	2408.2029	0.0147	6	244	264	FDGALNVDVNEFQTNLV PYPR					Mascot
2408.1882	2408.2029	0.0147	6	244	264	FDGALNVDVNEFQTNLV PYPR	145	100			Mascot

3	Tubulin alpha-2 chain OS=Zea mays GN=TUBA2 PE=3 SV=1	TBA2_MAIZE	50383.6	4.89	15	461	100	22.316	386	100	

Protein Group

Tubulin alpha-1 chain OS=Zea mays GN=TUBA1 PE=3 SV=1	TBA1_MAIZE	50383.6	4.8899 998664 856

Peptide Information

Calc. Mass	Obsrv. Mass	± da	± ppm	Start Seq.	End Seq.	Sequence	Ion Score	C. I. %	Modification	Rank	Result Type

Calc. Mass	Obsrv. Mass	± da	± ppm	Start Seq.	End Seq.	Sequence	Ion Score	C. I. %	Modification	Rank	Result Type
1007.454	1007.4469	-0.0071	-7	97	105	EDAANNFAR					Mascot
1132.5668	1132.5575	-0.0093	-8	113	121	EIVDLCLDR			Carbamidomethyl (C)[6]		Mascot
1299.4989	1299.472	-0.0269	-21	312	320	YMACCLMYR			Carbamidomethyl (C)[4,5], Oxidation (M)[2,7]		Mascot
1396.693	1396.7186	0.0256	18	391	401	IDHKFDLMYAK			Oxidation (M)[8]		Mascot
1473.8635	1473.854	-0.0095	-6	230	243	LVSQVISSLTASLR					Mascot
1473.8635	1473.854	-0.0095	-6	230	243	LVSQVISSLTASLR	102	100			Mascot
1589.6844	1589.6995	0.0151	9	309	320	HGKYMACCLMYR			Carbamidomethyl (C)[7,8]		Mascot
1691.8711	1691.8533	-0.0178	-11	216	229	SLDIERPTYTNLNR					Mascot
1691.8711	1691.8533	-0.0178	-11	216	229	SLDIERPTYTNLNR	67	99.979			Mascot
1701.9059	1701.8979	-0.008	-5	65	79	AVFVDLEPTVIDEVR					Mascot
1701.9059	1701.8979	-0.008	-5	65	79	AVFVDLEPTVIDEVR	71	99.992			Mascot
1808.9252	1808.8848	-0.0404	-22	265	280	IHFMLSSYAPVISAEK			Oxidation (M)[4]		Mascot
1847.9723	1847.8995	-0.0728	-39	215	229	RSLDIERPTYTNLNR					Mascot
1885.9147	1885.9192	0.0045	2	374	390	AVCMISNSTSVVEVFSR			Carbamidomethyl (C)[3]		Mascot
1901.9097	1901.9373	0.0276	15	374	390	AVCMISNSTSVVEVFSR			Carbamidomethyl (C)[3], Oxidation (M)[4]		Mascot
2346.0132	2346.0142	0.001	0	403	422	AFVHWYVGEGMEEGEF SEAR			Oxidation (M)[11]		Mascot
2385.1946	2385.2019	0.0073	3	85	105	QLFHPEQLISGKEDAANN FAR					Mascot
2395.1746	2395.2053	0.0307	13	374	394	AVCMISNSTSVVEVFSRI DHK			Carbamidomethyl (C)[3], Oxidation (M)[4]		Mascot
2408.1882	2408.2029	0.0147	6	244	264	FDGALNVDVNEFQTNLV PYPR					Mascot
2408.1882	2408.2029	0.0147	6	244	264	FDGALNVDVNEFQTNLV PYPR	145	100			Mascot

4	Tubulin alpha-2 chain OS=Oryza sativa subsp. japonica GN=TUBA PE=2 SV=1	TBA2_ORYSJ	50389.6	4.81	15	460	100	22.273	386	100

Peptide Information

Calc. Mass	Obsrv. Mass	± da	± ppm	Start Seq.	End Seq.	Sequence	Ion Score	C. I. %	Modification	Rank	Result Type
1007.454	1007.4469	-0.0071	-7	97	105	EDAANNFAR					Mascot
1132.5668	1132.5575	-0.0093	-8	113	121	EIVDLCLDR			Carbamidomethyl (C)[6]		Mascot
1299.4989	1299.472	-0.0269	-21	312	320	YMACCLMYR			Carbamidomethyl (C)[4,5], Oxidation (M)[2,7]		Mascot
1396.7584	1396.7186	-0.0398	-28	85	96	QLFHPEQLISGK					Mascot
1473.8635	1473.854	-0.0095	-6	230	243	LVSQVISSLTASLR					Mascot
1473.8635	1473.854	-0.0095	-6	230	243	LVSQVISSLTASLR	102	100			Mascot
1589.6844	1589.6995	0.0151	9	309	320	HGKYMACCLMYR			Carbamidomethyl (C)[7,8]		Mascot
1691.8711	1691.8533	-0.0178	-11	216	229	SLDIERPTYTNLNR					Mascot
1691.8711	1691.8533	-0.0178	-11	216	229	SLDIERPTYTNLNR	67	99.979			Mascot

1701.9059	1701.8979	-0.008	-5	65	79	AVFVDLEPTVIDEVR					Mascot
1701.9059	1701.8979	-0.008	-5	65	79	AVFVDLEPTVIDEVR	71	99.992			Mascot
1808.9252	1808.8848	-0.0404	-22	265	280	IHFMLSSYAPVISAEK			Oxidation (M)[4]		Mascot
1847.9723	1847.8995	-0.0728	-39	215	229	RSLDIERPTYTNLNR					Mascot
1885.9147	1885.9192	0.0045	2	374	390	AVCMISNSTSVVEVFSR			Carbamidomethyl (C)[3]		Mascot
1901.9097	1901.9373	0.0276	15	374	390	AVCMISNSTSVVEVFSR			Carbamidomethyl (C)[3], Oxidation (M)[4]		Mascot
1977.8826	1977.8553	-0.0273	-14	41	60	TVGGGDDAFNTFFSETGAGK					Mascot
2346.0132	2346.0142	0.001	0	403	422	AFVHWYVGEGMEEGEFSEAR			Oxidation (M)[11]		Mascot
2385.1946	2385.2019	0.0073	3	85	105	QLFHPEQLISGKEDAANNFAR					Mascot
2408.1882	2408.2029	0.0147	6	244	264	FDGALNVDVNEFQTNLVPYPR					Mascot
2408.1882	2408.2029	0.0147	6	244	264	FDGALNVDVNEFQTNLVPYPR	145	100			Mascot

5	Tubulin alpha-4 chain OS=Gossypium hirsutum PE=2 SV=1	TBA4_GOSHI	50223.6	4.93	16	322	100	17.459	240	100

Peptide Information

Calc. Mass	Obsrv. Mass	± da	± ppm	Start Seq.	End Seq.	Sequence	Ion Score	C. I. %	Modification	Rank	Result Type
1007.454	1007.4469	-0.0071	-7	97	105	EDAANNFAR					Mascot
1132.5668	1132.5575	-0.0093	-8	113	121	EIVDLCLDR			Carbamidomethyl (C)[6]		Mascot
1299.4989	1299.472	-0.0269	-21	312	320	YMACCLMYR			Carbamidomethyl (C)[4,5], Oxidation (M)[2,7]		Mascot
1396.7584	1396.7186	-0.0398	-28	85	96	QLFHPEQLISGK					Mascot
1426.7035	1426.723	0.0195	14	391	401	IDHKFDLMYTK			Oxidation (M)[8]		Mascot
1473.8635	1473.854	-0.0095	-6	230	243	LVSQVISSLTASLR					Mascot
1473.8635	1473.854	-0.0095	-6	230	243	LVSQVISSLTASLR	102	100			Mascot
1589.6844	1589.6995	0.0151	9	309	320	HGKYMACCLMYR			Carbamidomethyl (C)[7,8]		Mascot
1691.8711	1691.8533	-0.0178	-11	216	229	SLDIERPTYTNLNR					Mascot
1691.8711	1691.8533	-0.0178	-11	216	229	SLDIERPTYTNLNR	67	99.979			Mascot
1701.9059	1701.8979	-0.008	-5	65	79	AVFVDLEPTVIDEVR					Mascot
1701.9059	1701.8979	-0.008	-5	65	79	AVFVDLEPTVIDEVR	71	99.992			Mascot
1808.9252	1808.8848	-0.0404	-22	265	280	IHFMLSSYAPVISAEK			Oxidation (M)[4]		Mascot
1847.9723	1847.8995	-0.0728	-39	215	229	RSLDIERPTYTNLNR					Mascot
1885.9478	1885.9192	-0.0286	-15	353	370	CGINYQPPTVVPGGDLAK			Carbamidomethyl (C)[1]		Mascot
1977.8826	1977.8553	-0.0273	-14	41	60	TVGGGDDAFNTFFSETGAGK					Mascot
2346.0132	2346.0142	0.001	0	403	422	AFVHWYVGEGMEEGEFSEAR			Oxidation (M)[11]		Mascot
2385.1946	2385.2019	0.0073	3	85	105	QLFHPEQLISGKEDAANNFAR					Mascot

2395.1929	2395.2053	0.0124	5	244	264	FDGALNVDVTEFQTNLVPYPR					Mascot

6	Tubulin alpha-1 chain OS=Pisum sativum GN=TUBA1 PE=1 SV=1	TBA1_PEA	50305.6	4.92	15	314	100	17.222	240	100		

Protein Group

Tubulin alpha chain OS=Prunus dulcis GN=TUBA PE=2 SV=1	TBA_PRUDU	50179.5	4.9200000762 9395

Peptide Information

Calc. Mass	Obsrv. Mass	± da	± ppm	Start Seq.	End Seq.	Sequence	Ion Score	C. I. %	Modification	Rank	Result Type
1007.454	1007.4469	-0.0071	-7	97	105	EDAANNFAR					Mascot
1132.5668	1132.5575	-0.0093	-8	113	121	EIVDLCLDR			Carbamidomethyl (C)[6]		Mascot
1299.4989	1299.472	-0.0269	-21	312	320	YMACCLMYR			Carbamidomethyl (C)[4,5], Oxidation (M)[2,7]		Mascot
1396.693	1396.7186	0.0256	18	391	401	IDHKFDLMYAK			Oxidation (M)[8]		Mascot
1473.8635	1473.854	-0.0095	-6	230	243	LVSQVISSLTASLR					Mascot
1473.8635	1473.854	-0.0095	-6	230	243	LVSQVISSLTASLR	102	100			Mascot
1589.6844	1589.6995	0.0151	9	309	320	HGKYMACCLMYR			Carbamidomethyl (C)[7,8]		Mascot
1691.8711	1691.8533	-0.0178	-11	216	229	SLDIERPTYTNLNR					Mascot
1691.8711	1691.8533	-0.0178	-11	216	229	SLDIERPTYTNLNR	67	99.979			Mascot
1701.9059	1701.8979	-0.008	-5	65	79	AVFVDLEPTVIDEVR					Mascot
1701.9059	1701.8979	-0.008	-5	65	79	AVFVDLEPTVIDEVR	71	99.992			Mascot
1808.9252	1808.8848	-0.0404	-22	265	280	IHFMLSSYAPVISAEK			Oxidation (M)[4]		Mascot
1847.9723	1847.8995	-0.0728	-39	215	229	RSLDIERPTYTNLNR					Mascot
1885.9478	1885.9192	-0.0286	-15	353	370	CGINYQPPTVVPGGDLAK			Carbamidomethyl (C)[1]		Mascot
1977.8826	1977.8553	-0.0273	-14	41	60	TVGGGDDAFNTFFSETGAGK					Mascot
2346.0132	2346.0142	0.001	0	403	422	AFVHWYVGEGMEEGEFSEAR			Oxidation (M)[11]		Mascot
2385.1946	2385.2019	0.0073	3	85	105	QLFHPEQLISGKEDAANNFAR					Mascot
2395.1929	2395.2053	0.0124	5	244	264	FDGALNVDVTEFQTNLVPYPR					Mascot

7	Tubulin alpha-2 chain OS=Gossypium hirsutum PE=2 SV=1	TBA2_GOSHI	50193.6	4.93	14	306	100	17.091	240	100		

Peptide Information

Calc. Mass	Obsrv. Mass	± da	± ppm	Start Seq.	End Seq.	Sequence	Ion Score	C. I. %	Modification	Rank	Result Type
1007.454	1007.4469	-0.0071	-7	97	105	EDAANNFAR					Mascot
1132.5668	1132.5575	-0.0093	-8	113	121	EIVDLCLDR			Carbamidomethyl (C)[6]		Mascot
1299.4989	1299.472	-0.0269	-21	312	320	YMACCLMYR			Carbamidomethyl (C)[4,5], Oxidation (M)[2,7]		Mascot

1396.693	1396.7186	0.0256	18	391	401	IDHKFDLMYAK			Oxidation (M)[8]		Mascot
1473.8635	1473.854	-0.0095	-6	230	243	LVSQVISSLTASLR					Mascot
1473.8635	1473.854	-0.0095	-6	230	243	LVSQVISSLTASLR	102	100			Mascot
1589.6844	1589.6995	0.0151	9	309	320	HGKYMACCLMYR			Carbamidomethyl (C)[7,8]		Mascot
1691.8711	1691.8533	-0.0178	-11	216	229	SLDIERPTYTNLNR					Mascot
1691.8711	1691.8533	-0.0178	-11	216	229	SLDIERPTYTNLNR	67	99.979			Mascot
1701.9059	1701.8979	-0.008	-5	65	79	AVFVDLEPTVIDEVR					Mascot
1701.9059	1701.8979	-0.008	-5	65	79	AVFVDLEPTVIDEVR	71	99.992			Mascot
1808.9252	1808.8848	-0.0404	-22	265	280	IHFMLSSYAPVISAEK			Oxidation (M)[4]		Mascot
1847.9723	1847.8995	-0.0728	-39	215	229	RSLDIERPTYTNLNR					Mascot
1885.9478	1885.9192	-0.0286	-15	353	370	CGINYQPPTVVPGGDLA K			Carbamidomethyl (C)[1]		Mascot
1977.8826	1977.8553	-0.0273	-14	41	60	TVGGGDDAFNTFFSETG AGK					Mascot
2385.1946	2385.2019	0.0073	3	85	105	QLFHPEQLISGKEDAANN FAR					Mascot
2395.1929	2395.2053	0.0124	5	244	264	FDGALNVDVTEFQTNLVP YPR					Mascot

8	Protein disulfide-isomerase OS=Triticum aestivum GN=PDI PE=2 SV=1	PDI_WHEAT	56726	4.99	20	296	100	16.044	191	100		

Peptide Information

Calc. Mass	Obsrv. Mass	± da	± ppm	Start Seq.	End Seq.	Sequence	Ion Score	C. I. %	Modification	Rank	Result Type
1023.4993	1023.4863	-0.013	-13	236	244	DFDVSALEK					Mascot
1150.599	1150.5656	-0.0334	-29	137	146	EAEGIVEYLK					Mascot
1181.6201	1181.5886	-0.0315	-27	111	120	YEVQGFPTLK					Mascot
1182.6589	1182.6346	-0.0243	-21	212	222	GDAAVERPLVR					Mascot
1182.6589	1182.6346	-0.0243	-21	212	222	GDAAVERPLVR	46	97.093			Mascot
1210.6216	1210.5975	-0.0241	-20	260	269	NPDNHPYLLK					Mascot
1218.5961	1218.5593	-0.0368	-30	489	501	ETAGQAAAAATEK					Mascot
1278.694	1278.6486	-0.0454	-36	137	147	EAEGIVEYLKK					Mascot
1308.5955	1308.6018	0.0063	5	158	169	APEDATYLEDGK					Mascot
1358.674	1358.6945	0.0205	15	361	371	DYFDGKLTPFR					Mascot
1407.6427	1407.6097	-0.033	-23	294	306	SAYYGAVEEFSGK					Mascot
1423.7064	1423.6896	-0.0168	-12	373	385	SEPIPEANNEPVK					Mascot
1536.8308	1536.7975	-0.0333	-22	223	235	LFKPFDELVVDSK					Mascot
1647.8951	1647.7646	-0.1305	-79	73	87	SLAPEYEKAAQLLSK					Mascot
1647.8951	1647.7646	-0.1305	-79	73	87	SLAPEYEKAAQLLSK					Mascot
1671.8436	1671.7244	-0.1192	-71	329	343	EDQAPLILIQDSDSK					Mascot

Calc. Mass	Obsrv. Mass	± da	± ppm	Start Seq.	End Seq.	Sequence	Ion Score	C. I. %	Modification	Rank	Result Type
1674.9901	1674.916	-0.0741	-44	81	96	AAQLLSKHDPAIVLAK					Mascot
1799.9386	1799.9027	-0.0359	-20	329	344	EDQAPLILIQDSDSKK					Mascot
1835.9037	1835.9165	0.0128	7	278	293	AMLFLNFSTGPFESFK					Mascot
1865.8678	1865.8667	-0.0011	-1	196	211	SDYDFGHTVHANHLPR					Mascot
1865.8678	1865.8667	-0.0011	-1	196	211	SDYDFGHTVHANHLPR	105	100			Mascot
1899.9963	1899.9719	-0.0244	-13	254	269	VVTFDKNPDNHPYLLK					Mascot
1899.9963	1899.9719	-0.0244	-13	254	269	VVTFDKNPDNHPYLLK	41	91.577			Mascot
2135.053	2135.0745	0.0215	10	194	211	LRSDYDFGHTVHANHLPR					Mascot

9	Tubulin alpha-3 chain OS=Zea mays GN=TUBA3 PE=2 SV=1	TBA3_MAIZE	50213.7	5.09	10	284	100	11.007	248	100

Peptide Information

Calc. Mass	Obsrv. Mass	± da	± ppm	Start Seq.	End Seq.	Sequence	Ion Score	C. I. %	Modification	Rank	Result Type
1007.454	1007.4469	-0.0071	-7	97	105	EDAANNFAR					Mascot
1132.5668	1132.5575	-0.0093	-8	113	121	EIVDLCLDR			Carbamidomethyl (C)[6]		Mascot
1299.4989	1299.472	-0.0269	-21	312	320	YMACCLMYR			Carbamidomethyl (C)[4,5], Oxidation (M)[2,7]		Mascot
1396.7584	1396.7186	-0.0398	-28	85	96	QLFHPEQLISGK					Mascot
1473.8635	1473.854	-0.0095	-6	230	243	LVSQVISSLTASLR					Mascot
1473.8635	1473.854	-0.0095	-6	230	243	LVSQVISSLTASLR	102	100			Mascot
1589.6844	1589.6995	0.0151	9	309	320	HGKYMACCLMYR			Carbamidomethyl (C)[7,8]		Mascot
1885.9147	1885.9192	0.0045	2	374	390	AVCMISNSTSVVEVFSR			Carbamidomethyl (C)[3]		Mascot
1901.9097	1901.9373	0.0276	15	374	390	AVCMISNSTSVVEVFSR			Carbamidomethyl (C)[3], Oxidation (M)[4]		Mascot
2346.0132	2346.0142	0.001	0	403	422	AFVHWYVGEGMEEGEFSEAR			Oxidation (M)[11]		Mascot
2385.1946	2385.2019	0.0073	3	85	105	QLFHPEQLISGKEDAANNFAR					Mascot
2408.1882	2408.2029	0.0147	6	244	264	FDGALNVDVNEFQTNLVPYPR					Mascot
2408.1882	2408.2029	0.0147	6	244	264	FDGALNVDVNEFQTNLVPYPR	145	100			Mascot

10	Tubulin alpha-1 chain OS=Anemia phyllitidis GN=TUBA1 PE=2 SV=1	TBA1_ANEPH	50381.7	5.02	15	243	100	10.632	169	100

Peptide Information

Calc. Mass	Obsrv. Mass	± da	± ppm	Start Seq.	End Seq.	Sequence	Ion Score	C. I. %	Modification	Rank	Result Type
1007.454	1007.4469	-0.0071	-7	97	105	EDAANNFAR					Mascot
1132.5668	1132.5575	-0.0093	-8	113	121	EIVDLCLDR			Carbamidomethyl (C)[6]		Mascot
1299.4989	1299.472	-0.0269	-21	312	320	YMACCLMYR			Carbamidomethyl (C)[4,5], Oxidation (M)[2,7]		Mascot
1396.693	1396.7186	0.0256	18	391	401	IDHKFDLMYAK			Oxidation (M)[8]		Mascot

1473.8635	1473.854	-0.0095	-6	230	243	LVSQVISSLTASLR				Mascot
1473.8635	1473.854	-0.0095	-6	230	243	LVSQVISSLTASLR	102	100		Mascot
1589.6844	1589.6995	0.0151	9	309	320	HGKYMACCLMYR			Carbamidomethyl (C)[7,8]	Mascot
1691.8711	1691.8533	-0.0178	-11	216	229	SLDIERPTYTNLNR				Mascot
1691.8711	1691.8533	-0.0178	-11	216	229	SLDIERPTYTNLNR	67	99.977		Mascot
1808.9252	1808.8848	-0.0404	-22	265	280	IHFMLSSYAPVISAEK			Oxidation (M)[4]	Mascot
1847.9723	1847.8995	-0.0728	-39	215	229	RSLDIERPTYTNLNR				Mascot
1885.9478	1885.9192	-0.0286	-15	353	370	CGINYQPPTVVPGGDLA K			Carbamidomethyl (C)[1]	Mascot
1922.9866	1922.9163	-0.0703	-37	3	20	ECISIHIGQAGIQVGNAR			Carbamidomethyl (C)[2]	Mascot
1977.8826	1977.8553	-0.0273	-14	41	60	TVGGGDDAFNTFFSETG AGK				Mascot
2346.0132	2346.0142	0.001	0	403	422	AFVHWYVGEGMEEGEF SEAR			Oxidation (M)[11]	Mascot
2385.1946	2385.2019	0.0073	3	85	105	QLFHPEQLISGKEDAANN FAR				Mascot
2395.1929	2395.2053	0.0124	5	244	264	FDGALNVDVTEFQTNLVP YPR				Mascot

Gel Idx/Pos	252/K4	Instr./Gel Origin	BA2151/Sample Project 20140814	Process Status	Analysis Succeeded
Plate [#] Name	[1] Sample Project 20140814	Instrument Sample Name		Spectra	11

Rank	Protein Name	Accession No.	Protein MW	Protein PI	Pep. Count	Protein Score	Protein Score C. I. %	Intensity Matched	Total Ion Score	Total Ion C. I. %	Confirmed
1	Beta-amylase OS=Hordeum vulgare subsp. spontaneum GN=BMY1 PE=1 SV=1	AMYB_HORVS	59886.4	5.66	11	127	100	15.78	93	100	

Peptide Information

Calc. Mass	Obsrv. Mass	± da	± ppm	Start Seq.	End Seq.	Sequence	Ion Score	C. I. %	Modification	Rank	Result Type
802.4305	802.4324	0.0019	2	275	281	ILDEANK					Mascot
1016.5564	1016.5534	-0.003	-3	411	418	LFGFTYLR					Mascot
1016.5564	1016.5534	-0.003	-3	411	418	LFGFTYLR	48	98.142			Mascot
1253.6121	1253.5869	-0.0252	-20	248	258	DNGTYLTEKGR					Mascot
1285.6212	1285.6085	-0.0127	-10	61	71	GPKAYDWSAYK					Mascot
1326.6688	1326.6642	-0.0046	-3	384	394	YDPTAYNTILR					Mascot
1326.6688	1326.6642	-0.0046	-3	384	394	YDPTAYNTILR	45	96.319			Mascot
1370.6774	1370.6747	-0.0027	-2	446	457	DPYVDPMAPLPR					Mascot
1701.7247	1701.8429	0.1182	69	147	160	SAVQMYADYMTSFR			Oxidation (M)[5,10]		Mascot
1728.9221	1728.8381	-0.084	-49	257	270	GRFFLAWYSNNLIK					Mascot
1752.8916	1752.866	-0.0256	-15	419	433	LSNQLVEGQNYVNFK					Mascot
1827.9884	1827.9443	-0.0441	-24	458	474	SGPEISIEMILQAAKPK			Oxidation (M)[9]		Mascot
1842.0425	1841.9316	-0.1109	-60	288	302	VQLAIKISGIHWWYK					Mascot

Rank	Protein Name	Accession No.	Protein MW	Protein PI	Pep. Count	Protein Score	Protein Score C. I. %	Intensity Matched	Total Ion Score	Total Ion C. I. %	Confirmed
2	Beta-amylase OS=Hordeum vulgare GN=BMY1 PE=1 SV=1	AMYB_HORVU	59894.5	5.58	10	122	100	15.445	93	100	

Peptide Information

Calc. Mass	Obsrv. Mass	± da	± ppm	Start Seq.	End Seq.	Sequence	Ion Score	C. I. %	Modification	Rank	Result Type
802.4305	802.4324	0.0019	2	275	281	ILDEANK					Mascot
1016.5564	1016.5534	-0.003	-3	411	418	LFGFTYLR					Mascot
1016.5564	1016.5534	-0.003	-3	411	418	LFGFTYLR	48	98.142			Mascot
1285.6212	1285.6085	-0.0127	-10	61	71	GPKAYDWSAYK					Mascot
1326.6688	1326.6642	-0.0046	-3	384	394	YDPTAYNTILR					Mascot
1326.6688	1326.6642	-0.0046	-3	384	394	YDPTAYNTILR	45	96.319			Mascot
1370.6774	1370.6747	-0.0027	-2	446	457	DPYVDPMAPLPR					Mascot
1701.7247	1701.8429	0.1182	69	147	160	SAVQMYADYMTSFR			Oxidation (M)[5,10]		Mascot

Calc. Mass	Obsrv. Mass	± da	± ppm	Start Seq.	End Seq.	Sequence	Ion Score	C. I. %	Modification	Rank	Result Type
1728.9221	1728.8381	-0.084	-49	257	270	GRFFLAWYSNNLIK					Mascot
1752.8916	1752.866	-0.0256	-15	419	433	LSNQLVEGQNYVNFK					Mascot
1827.952	1827.9443	-0.0077	-4	458	474	SGPEISIEMILQAAQPK			Oxidation (M)[9]		Mascot
1842.0425	1841.9316	-0.1109	-60	288	302	VQLAIKISGIHWWYK					Mascot

3	Beta-amylase (Fragment) OS=Secale cereale GN=BMY1 PE=2 SV=1	AMYB_SECCE	24561.9	5.08	3	102	99.997	13.78	93	100	

Peptide Information

Calc. Mass	Obsrv. Mass	± da	± ppm	Start Seq.	End Seq.	Sequence	Ion Score	C. I. %	Modification	Rank	Result Type
1016.5564	1016.5534	-0.003	-3	107	114	LFGFTYLR					Mascot
1016.5564	1016.5534	-0.003	-3	107	114	LFGFTYLR	48	98.142			Mascot
1326.6688	1326.6642	-0.0046	-3	80	90	YDPTAYNTILR					Mascot
1326.6688	1326.6642	-0.0046	-3	80	90	YDPTAYNTILR	45	96.319			Mascot
1589.6948	1589.6962	0.0014	1	29	41	HHASLNFTCAEMR			Carbamidomethyl (C)[9], Oxidation (M)[12]		Mascot

4	Adenomatous polyposis coli protein OS=Mus musculus GN=Apc PE=1 SV=1	APC_MOUSE	313179.4	7.44	42	85	99.833	14.296			

Peptide Information

Calc. Mass	Obsrv. Mass	± da	± ppm	Start Seq.	End Seq.	Sequence	Ion Score	C. I. %	Modification	Rank	Result Type
815.4257	815.4643	0.0386	47	2603	2609	ENQVPTK					Mascot
820.441	820.3983	-0.0427	-52	2055	2061	LKSESEK					Mascot
846.5043	846.4782	-0.0261	-31	1591	1598	KLAQTASK					Mascot
847.4519	847.4324	-0.0195	-23	2180	2186	KIESENK					Mascot
982.5203	982.4401	-0.0802	-82	1052	1059	HVIEDEIK					Mascot
1032.4745	1032.5344	0.0599	58	454	461	LSFDEEHR					Mascot
1037.5925	1037.5137	-0.0788	-76	2229	2237	TMIHIPGLR					Mascot
1044.5433	1044.5336	-0.0097	-9	1685	1693	RDTIPTEGR					Mascot
1060.5157	1060.5542	0.0385	36	1551	1559	EVEKPDSEK					Mascot
1107.5649	1107.5437	-0.0212	-19	715	725	MIAMGSAAALR			Oxidation (M)[1]		Mascot
1205.6273	1205.6659	0.0386	32	2835	2845	RHSGSYLVTSV					Mascot
1232.6594	1232.5991	-0.0603	-49	364	375	DSVLLGNSRGSK					Mascot
1235.6227	1235.6245	0.0018	1	1335	1346	LQASGLSSESTR					Mascot
1297.6158	1297.6343	0.0185	14	127	137	ESTGYLEELEK					Mascot
1320.5961	1320.5922	-0.0039	-3	2400	2412	GLNQMSNGNGSNK					Mascot
1326.6438	1326.6642	0.0204	15	1627	1638	HVSFTPGDDVPR					Mascot
1326.6438	1326.6642	0.0204	15	1627	1638	HVSFTPGDDVPR					Mascot

1379.6947	1379.7136	0.0189	14	2203	2214	IRSNSEISSQMK					Mascot
1384.6825	1384.6818	-0.0007	-1	2591	2602	HVSSMPAPRQMK			Oxidation (M)[5]		Mascot
1417.6377	1417.7201	0.0824	58	2358	2371	SSGSGKMSYTSPGR			Oxidation (M)[7]		Mascot
1474.6479	1474.6841	0.0362	25	1386	1398	CTSVSSLDSFESR			Carbamidomethyl (C)[1]		Mascot
1507.7672	1507.7383	-0.0289	-19	37	49	LETEASNMKEVLK			Oxidation (M)[8]		Mascot
1507.7672	1507.7383	-0.0289	-19	37	49	LETEASNMKEVLK			Oxidation (M)[8]		Mascot
1541.8646	1541.7408	-0.1238	-80	2273	2287	GTKPAGKSELSPITR					Mascot
1547.7847	1547.7762	-0.0085	-5	12	24	QVEALKMENSNLR			Oxidation (M)[7]		Mascot
1607.8428	1607.7291	-0.1137	-71	71	84	LKEFNLDSNFPGVK					Mascot
1620.8856	1620.8102	-0.0754	-47	1047	1059	WARPKHVIEDEIK					Mascot
1635.849	1635.786	-0.063	-39	73	86	EFNLDSNFPGVKLR					Mascot
1644.7104	1644.8256	0.1152	70	938	951	SENSNRTCSMPYAK			Carbamidomethyl (C)[8]		Mascot
1698.8599	1698.8132	-0.0467	-27	154	166	DWYYAQLQNLTKR					Mascot
1701.8767	1701.8429	-0.0338	-20	2707	2723	QSVGSGSPVQTVGLETR					Mascot
1716.8665	1716.7914	-0.0751	-44	381	396	ASAALHNIIHSQPDDK					Mascot
1729.9443	1729.8627	-0.0816	-47	622	638	SQTNTLAIIESGGGILR					Mascot
1732.7806	1732.8326	0.052	30	888	902	VMEEVSAIHTSQDDR			Oxidation (M)[2]		Mascot
1740.7238	1740.7856	0.0618	36	196	210	AAMEEQLGTCQDMEK			Carbamidomethyl (C)[10]		Mascot
1801.8789	1801.9139	0.035	19	498	514	YAGMALTNLTFGDVANK			Oxidation (M)[4]		Mascot
1837.8577	1837.9485	0.0908	49	922	937	RSSASHTHSNTYNFTK					Mascot
1838.9027	1838.9227	0.02	11	310	325	VEMVYSLLSMLGTHDK			Oxidation (M)[3]		Mascot
1890.9192	1890.9586	0.0394	21	855	873	GIGLSAYHPTTENAGTSS K					Mascot
1908.996	1908.8231	-0.1729	-91	737	754	DANIMSPGSSLPSLHVRK					Mascot
2087.1035	2087.0625	-0.041	-20	715	734	MIAMGSAAALRNLMANR PAK					Mascot
2119.0933	2119.135	0.0417	20	715	734	MIAMGSAAALRNLMANR PAK			Oxidation (M)[1,4]		Mascot
2369.05	2369.03	-0.02	-8	923	943	SSASHTHSNTYNFTKSE NSNR					Mascot
2620.3135	2620.2808	-0.0327	-12	50	72	QLQGSIEDETMTSGQIDL LERLK			Oxidation (M)[11]		Mascot
2717.0337	2717.1108	0.0771	28	413	433	AYCETCWEWQEAHEQG MDQDK			Carbamidomethyl (C)[3,6], Oxidation (M)[17]		Mascot

5	Adenomatous polyposis coli protein OS=Rattus norvegicus GN=Apc PE=1 SV=1	APC_RAT	312623.2	7.05	41	72	96.816	15.053

Peptide Information

Calc. Mass	Obsrv. Mass	± da	± ppm	Start Seq.	End Seq.	Sequence	Ion Score	C. I. %	Modification	Rank	Result Type
815.4257	815.4643	0.0386	47	2603	2609	ENQVPTK					Mascot
846.468	846.4782	0.0102	12	1761	1767	NQIDTKK					Mascot

847.4519	847.4324	-0.0195	-23	2180	2186	KIESENK		Mascot
962.5167	962.4447	-0.072	-75	2591	2599	HVNSVPGPR		Mascot
982.5203	982.4401	-0.0802	-82	1052	1059	HVIEDEIK		Mascot
1032.4745	1032.5344	0.0599	58	454	461	LSFDEEHR		Mascot
1044.5433	1044.5336	-0.0097	-9	1685	1693	RDTIPTEGR		Mascot
1060.5157	1060.5542	0.0385	36	1551	1559	EVEKPDSEK		Mascot
1081.5161	1081.5354	0.0193	18	1675	1684	ASVQSGEFEK		Mascot
1107.5649	1107.5437	-0.0212	-19	715	725	MIAMGSAAALR	Oxidation (M)[1]	Mascot
1205.6273	1205.6659	0.0386	32	2832	2842	RHSGSYLVTSV		Mascot
1232.6594	1232.5991	-0.0603	-49	364	375	DSVLLGNSRGSK		Mascot
1237.6172	1237.5525	-0.0647	-52	1675	1685	ASVQSGEFEKR		Mascot
1285.6383	1285.6085	-0.0298	-23	2260	2272	SPSEGPVATTSPR		Mascot
1297.6158	1297.6343	0.0185	14	127	137	ESTGYLEELEK		Mascot
1326.6438	1326.6642	0.0204	15	1627	1638	HVSFTPGDDVPR		Mascot
1326.6438	1326.6642	0.0204	15	1627	1638	HVSFTPGDDVPR		Mascot
1379.6947	1379.7136	0.0189	14	2203	2214	IRSNSEISSQMK		Mascot
1417.6377	1417.7201	0.0824	58	2358	2371	SSGSGKMSYTSPGR	Oxidation (M)[7]	Mascot
1450.6769	1450.7041	0.0272	19	2819	2832	TDSTESSGAQSPKR		Mascot
1454.771	1454.7417	-0.0293	-20	1334	1347	LQASGLASESARHK		Mascot
1454.771	1454.7417	-0.0293	-20	1334	1347	LQASGLASESARHK		Mascot
1474.6479	1474.6841	0.0362	25	1384	1396	CTSVSSLDSFESR	Carbamidomethyl (C)[1]	Mascot
1507.6669	1507.7383	0.0714	47	105	118	SGECSPVPMGSFPR	Carbamidomethyl (C)[4]	Mascot
1507.6669	1507.7383	0.0714	47	105	118	SGECSPVPMGSFPR	Carbamidomethyl (C)[4]	Mascot
1541.8031	1541.7408	-0.0623	-40	2327	2341	NSISPGRNGISTPNK		Mascot
1547.7847	1547.7762	-0.0085	-5	12	24	QVEALKMENSNLR	Oxidation (M)[7]	Mascot
1607.8428	1607.7291	-0.1137	-71	71	84	LKEFNLDSNFPGVK		Mascot
1620.8856	1620.8102	-0.0754	-47	1047	1059	WARPKHVIEDEIK		Mascot
1635.7623	1635.786	0.0237	14	923	937	SSASHTHPNTHNFAK		Mascot
1674.7501	1674.7349	-0.0152	-9	1892	1906	VTCHTEPSSSQQSAR	Carbamidomethyl (C)[3]	Mascot
1675.7882	1675.7504	-0.0378	-23	1686	1700	DTIPTEGRSTDEAQR		Mascot
1698.8599	1698.8132	-0.0467	-27	154	166	DWYYAQLQNLTKR		Mascot
1716.8665	1716.7914	-0.0751	-44	381	396	ASAALHNIIHSQPDDK		Mascot
1729.9443	1729.8627	-0.0816	-47	622	638	SQTNTLAIIESGGGILR		Mascot
1732.8573	1732.8326	-0.0247	-14	2424	2439	SSGSESDRSERPALVR		Mascot
1740.7238	1740.7856	0.0618	36	196	210	AAMEEQLGTCQDMEK	Carbamidomethyl (C)[10]	Mascot
1801.8789	1801.9139	0.035	19	498	514	YAGMALTNLTFGDVANK	Oxidation (M)[4]	Mascot

Calc. Mass	Obsrv. Mass	± da	± ppm	Start Seq.	End Seq.	Sequence	Ion Score	C. I. %	Modification	Rank	Result Type
1838.9027	1838.9227	0.02	11	310	325	VEMVYSLLSMLGTHDK			Oxidation (M)[3]		Mascot
1841.8368	1841.9316	0.0948	51	1744	1760	IMDQVQQASMTSSGTNK			Oxidation (M)[2]		Mascot
1908.996	1908.8231	-0.1729	-91	737	754	DANIMSPGSSLPSLHVRK					Mascot
2087.1035	2087.0625	-0.041	-20	715	734	MIAMGSAAALRNLMANR PAK					Mascot
2119.0933	2119.135	0.0417	20	715	734	MIAMGSAAALRNLMANR PAK			Oxidation (M)[1,4]		Mascot
2620.3135	2620.2808	-0.0327	-12	50	72	QLQGSIEDETMTSGQIDL LERLK			Oxidation (M)[11]		Mascot
2717.0337	2717.1108	0.0771	28	413	433	AYCETCWEWQEAHEQG MDQDK			Carbamidomethyl (C)[3,6], Oxidation (M)[17]		Mascot

6	DNA-directed RNA polymerase subunit beta' OS=Pseudomonas putida (strain W619) GN=rpoC PE=3 SV=1	RPOC_PSEPW	155340.6	6.82	27	70	94.716	18.937

Peptide Information

Calc. Mass	Obsrv. Mass	± da	± ppm	Start Seq.	End Seq.	Sequence	Ion Score	C. I. %	Modification	Rank	Result Type
802.4305	802.4324	0.0019	2	1124	1130	IPQETSK					Mascot
962.5339	962.4447	-0.0892	-93	326	334	SLADMIKGK					Mascot
982.4952	982.4401	-0.0551	-56	790	798	TANSGYLTR					Mascot
1016.5272	1016.5534	0.0262	26	934	943	TFHIGGAASR					Mascot
1016.5272	1016.5534	0.0262	26	934	943	TFHIGGAASR					Mascot
1032.5573	1032.5344	-0.0229	-22	653	662	IIGSATDEVK					Mascot
1044.5432	1044.5336	-0.0096	-9	516	525	EAINAKGEGR					Mascot
1201.6722	1201.6431	-0.0291	-24	88	98	CGVEVALAKVR			Carbamidomethyl (C)[1]		Mascot
1232.7031	1232.5991	-0.104	-84	385	395	LEMRGLATTIK					Mascot
1259.6954	1259.6554	-0.04	-32	651	662	ARIIGSATDEVK					Mascot
1335.5708	1335.677	0.1062	80	67	76	DYECLCGKYK			Carbamidomethyl (C)[4,6]		Mascot
1425.7994	1425.6925	-0.1069	-75	1252	1262	HIETILRQMLR			Oxidation (M)[9]		Mascot
1525.7744	1525.7334	-0.041	-27	1263	1276	KVEISESGDSSFIK					Mascot
1541.8468	1541.7408	-0.106	-69	1073	1086	EIRPAIKMVDANGK					Mascot
1544.7737	1544.7347	-0.039	-25	298	311	MLQEAVDALLDNGR					Mascot
1603.8326	1603.7706	-0.062	-39	682	695	VIDLWSKANDEVSK					Mascot
1635.8119	1635.786	-0.0259	-16	944	959	TSAADSVQVKNGGMVR			Oxidation (M)[14]		Mascot
1644.8704	1644.8256	-0.0448	-27	187	200	ELLHAIDLEHEIGR					Mascot
1652.9343	1652.8069	-0.1274	-77	418	431	EHPVLLNRAPTLHR					Mascot
1667.9003	1667.8256	-0.0747	-45	404	417	ELPEVWDVLAEVIR					Mascot
1668.7833	1668.7889	0.0056	3	884	897	SPINCETRYGICAK			Carbamidomethyl (C)[5,12]		Mascot
1675.887	1675.7504	-0.1366	-82	124	137	IGLLMDMTLRDIER					Mascot
1684.9496	1684.7834	-0.1662	-99	371	384	KMALELFKPFIFGK			Oxidation (M)[2]		Mascot

1691.882	1691.8262	-0.0558	-33	124	137	IGLLMDMTLRDIER			Oxidation (M)[5]		Mascot
1700.8749	1700.8146	-0.0603	-35	298	312	MLQEAVDALLDNGRR					Mascot
1716.8698	1716.7914	-0.0784	-46	297	311	RMLQEAVDALLDNGR			Oxidation (M)[2]		Mascot
1729.9232	1729.8627	-0.0605	-35	1133	1148	DITGGLPRVADLFEAR					Mascot
2183.1528	2183.0332	-0.1196	-55	400	417	MVERELPEVWDVLAEVIR					Mascot
2717.3743	2717.1108	-0.2635	-97	134	156	DIERVLYFESYVVIDPGM TTLEK					Mascot

7	Beta-amylase OS=Triticum aestivum GN=BMY1 PE=2 SV=1	AMYB_WHEAT	56860.2	5.24	9	69	93.647	12.111	48	98.142	

Peptide Information

Calc. Mass	Obsrv. Mass	± da	± ppm	Start Seq.	End Seq.	Sequence	Ion Score	C. I. %	Modification	Rank	Result Type
802.4305	802.4324	0.0019	2	275	281	ILDEANK					Mascot
1016.5564	1016.5534	-0.003	-3	411	418	LFGFTYLR					Mascot
1016.5564	1016.5534	-0.003	-3	411	418	LFGFTYLR	48	98.142			Mascot
1285.6212	1285.6085	-0.0127	-10	61	71	GPKAYDWSAYK					Mascot
1297.6787	1297.6343	-0.0444	-34	210	221	YLEADFKAAAAK					Mascot
1425.6903	1425.6925	0.0022	2	371	383	EGLHVACENALGR			Carbamidomethyl (C)[7]		Mascot
1487.7893	1487.7458	-0.0435	-29	470	482	AAQPKLEPFPFDK					Mascot
1607.6512	1607.7291	0.0779	48	333	345	HHASMNFTCAEMR			Carbamidomethyl (C)[9], Oxidation (M)[5]		Mascot
1623.6461	1623.769	0.1229	76	333	345	HHASMNFTCAEMR			Carbamidomethyl (C)[9], Oxidation (M)[5,12]		Mascot
1691.8275	1691.8262	-0.0013	-1	243	256	TQFFKDNGTYLTEK					Mascot
2087.0557	2087.0625	0.0068	3	129	146	NIEYLTLGVDDQPLFHGR					Mascot

8	Pyruvate kinase PKM OS=Pongo abelii GN=PKM PE=2 SV=3	KPYM_PONAB	58494.1	7.98	18	68	91.026	7.51			

Peptide Information

Calc. Mass	Obsrv. Mass	± da	± ppm	Start Seq.	End Seq.	Sequence	Ion Score	C. I. %	Modification	Rank	Result Type
815.5349	815.4643	-0.0706	-87	264	270	NIKIISK					Mascot
1033.6041	1033.515	-0.0891	-86	393	400	KLFEELVR					Mascot
1037.4801	1037.5137	0.0336	32	312	319	MMIGRCNR			Carbamidomethyl (C)[6]		Mascot
1118.6052	1118.5227	-0.0825	-74	126	136	GSGTAEVELKK					Mascot
1141.61	1141.5322	-0.0778	-68	295	305	GDLGIEIPAEK					Mascot
1193.6447	1193.614	-0.0307	-26	57	66	SVETLKEMIK			Oxidation (M)[8]		Mascot
1197.6475	1197.6552	0.0077	6	33	43	LDIDSPPITAR					Mascot
1205.5732	1205.6659	0.0927	77	384	393	EAEAAMFHRK			Oxidation (M)[6]		Mascot

1232.6667	1232.5991	-0.0676	-55	163	173	NICKVVEVGSK			Carbamidomethyl (C)[3]		Mascot
1235.6235	1235.6245	0.001	1	63	73	EMIKSGMNVAR					Mascot
1251.6184	1251.642	0.0236	19	63	73	EMIKSGMNVAR			Oxidation (M)[2]		Mascot
1454.7611	1454.7417	-0.0194	-13	456	467	NPQTARQAHLYR					Mascot
1454.7611	1454.7417	-0.0194	-13	456	467	NPQTARQAHLYR					Mascot
1473.8094	1473.7853	-0.0241	-16	423	436	CLAAALIVLTESGR			Carbamidomethyl (C)[1]		Mascot
1507.8704	1507.7383	-0.1321	-88	448	461	APIIAVTRNPQTAR					Mascot
1507.8704	1507.7383	-0.1321	-88	448	461	APIIAVTRNPQTAR					Mascot
1667.8673	1667.8256	-0.0417	-25	137	151	GATLKITLDNAYMEK					Mascot
1701.9258	1701.8429	-0.0829	-49	462	475	QAHLYRGIFPVLCK			Carbamidomethyl (C)[13]		Mascot
1828.0215	1827.9443	-0.0772	-42	295	311	GDLGIEIPAEKVFLAQK					Mascot
1837.9113	1837.9485	0.0372	20	279	294	RFDEILEASDGIMVAR			Oxidation (M)[13]		Mascot
1908.9592	1908.8231	-0.1361	-71	320	336	AGKPVICATQMLESMIK			Carbamidomethyl (C)[7], Oxidation (M)[11,15]		Mascot

9	ATP synthase gamma chain OS=Streptococcus mutans serotype c (strain ATCC 700610 / UA159) GN=atpG PE=3 SV=2	ATPG_STRMU	32385.6	5.38	13	66	86.101	4.604	

Peptide Information

Calc. Mass	Obsrv. Mass	± da	± ppm	Start Seq.	End Seq.	Sequence	Ion Score	C. I. %	Modification	Rank	Result Type
820.4788	820.3983	-0.0805	-98	268	273	LYNRVR					Mascot
963.5469	963.5081	-0.0388	-40	12	20	ITSTQKTGK					Mascot
1081.6154	1081.5354	-0.08	-74	133	141	HIPVAFELR					Mascot
1197.6548	1197.6552	0.0004	0	155	164	IISKSVEMYK					Mascot
1235.6995	1235.6245	-0.075	-61	89	100	GLVGAYNSTILK					Mascot
1240.6685	1240.6044	-0.0641	-52	42	51	DFQIYASKIR					Mascot
1240.6685	1240.6044	-0.0641	-52	42	51	DFQIYASKIR					Mascot
1253.6592	1253.5869	-0.0723	-58	21	32	ITSAMKMVSSAK					Mascot
1285.649	1285.6085	-0.0405	-32	21	32	ITSAMKMVSSAK			Oxidation (M)[5,7]		Mascot
1308.7535	1308.6633	-0.0902	-69	131	141	ARHIPVAFELR					Mascot
1399.7726	1399.6593	-0.1133	-81	65	77	GSSNPMLISRPIK					Mascot
1417.7145	1417.7201	0.0056	4	101	112	AVMDTIKDYHPK					Mascot
1543.8625	1543.7452	-0.1173	-76	65	78	GSSNPMLISRPIKK			Oxidation (M)[6]		Mascot
1680.9392	1680.8505	-0.0887	-53	50	63	IRQITTDLLHSDLR					Mascot
2183.1667	2183.0332	-0.1335	-61	272	292	VRQAAITQEITEIVAGANALD					Mascot

10	DNA-directed RNA polymerase subunit beta' OS=Pseudomonas syringae pv. tomato (strain DC3000) GN=rpoC PE=3 SV=1	RPOC_PSESM	155321.4	6.78	26	65	84.405	22.557	

Peptide Information

Calc. Mass	Obsrv. Mass	± da	± ppm	Start Seq.	End Seq.	Sequence	Ion Score	C. I. %	Modification	Rank	Result Type
802.4305	802.4324	0.0019	2	1124	1130	IPQETSK					Mascot
962.5339	962.4447	-0.0892	-93	326	334	SLADMIKGK					Mascot
982.4952	982.4401	-0.0551	-56	790	798	TANSGYLTR					Mascot
1016.5272	1016.5534	0.0262	26	934	943	TFHIGGAASR					Mascot
1016.5272	1016.5534	0.0262	26	934	943	TFHIGGAASR					Mascot
1032.5255	1032.5344	0.0089	9	1373	1380	REMDKPTR					Mascot
1044.5432	1044.5336	-0.0096	-9	516	525	EAINAKGEGR					Mascot
1048.5204	1048.5677	0.0473	45	1373	1380	REMDKPTR			Oxidation (M)[3]		Mascot
1182.5605	1182.6229	0.0624	53	696	705	AMMSNLSKER			Oxidation (M)[2]		Mascot
1201.6722	1201.6431	-0.0291	-24	88	98	CGVEVALAKVR			Carbamidomethyl (C)[1]		Mascot
1232.7031	1232.5991	-0.104	-84	385	395	LEMRGLATTIK					Mascot
1335.5708	1335.677	0.1062	80	67	76	DYECLCGKYK			Carbamidomethyl (C)[4,6]		Mascot
1425.7994	1425.6925	-0.1069	-75	1252	1262	HIETILRQMLR			Oxidation (M)[9]		Mascot
1544.7737	1544.7347	-0.039	-25	298	311	MLQEAVDALLDNGR					Mascot
1589.8242	1589.6962	-0.128	-81	944	959	TSAADSVQVKNGGTVR					Mascot
1603.8326	1603.7706	-0.062	-39	682	695	VIDLWSKANDEVSK					Mascot
1644.8704	1644.8256	-0.0448	-27	187	200	ELLHAIDLEHEIGR					Mascot
1652.9343	1652.8069	-0.1274	-77	418	431	EHPVLLNRAPTLHR					Mascot
1667.9003	1667.8256	-0.0747	-45	404	417	ELPEVWDVLAEVIR					Mascot
1668.9546	1668.7889	-0.1657	-99	371	384	KMALELFKPFIFGK					Mascot
1675.887	1675.7504	-0.1366	-82	124	137	IGLLMDMTLRDIER					Mascot
1684.9496	1684.7834	-0.1662	-99	371	384	KMALELFKPFIFGK			Oxidation (M)[2]		Mascot
1691.882	1691.8262	-0.0558	-33	124	137	IGLLMDMTLRDIER			Oxidation (M)[5]		Mascot
1700.8749	1700.8146	-0.0603	-35	298	312	MLQEAVDALLDNGRR					Mascot
1716.8698	1716.7914	-0.0784	-46	297	311	RMLQEAVDALLDNGR			Oxidation (M)[2]		Mascot
1729.9232	1729.8627	-0.0605	-35	1133	1148	DITGGLPRVADLFEAR					Mascot
2000.0223	1999.9746	-0.0477	-24	1175	1192	LVITPNDGSDPYEELIPK					Mascot
2000.0223	1999.9746	-0.0477	-24	1175	1192	LVITPNDGSDPYEELIPK					Mascot
2183.1528	2183.0332	-0.1196	-55	400	417	MVERELPEVWDVLAEVIR					Mascot
2369.1401	2369.03	-0.1101	-46	1277	1297	GDQMELTHVLVENERLG ADDK					Mascot
2717.3743	2717.1108	-0.2635	-97	134	156	DIERVLYFESYVVIDPGM TTLEK					Mascot

Gel Idx/Pos	253/K5	Instr./Gel Origin	BA2151/Sample Project 20140814	Process Status	Analysis Succeeded
Plate [#] Name	[1] Sample Project 20140814	Instrument Sample Name		Spectra	11

Rank	Protein Name	Accession No.	Protein MW	Protein PI	Pep. Count	Protein Score	Protein Score C. I. %	Intensity Matched	Total Ion Score	Total Ion C. I. %	Confirmed
1	Tubulin alpha-2 chain OS=Oryza sativa subsp. japonica GN=TUBA PE=2 SV=1	TBA2_ORYSJ	50389.6	4.81	10	350	100	15.351	313	100	

Peptide Information

Calc. Mass	Obsrv. Mass	± da	± ppm	Start Seq.	End Seq.	Sequence	Ion Score	C. I. %	Modification	Rank	Result Type
1007.454	1007.4495	-0.0045	-4	97	105	EDAANNFAR					Mascot
1132.5668	1132.5371	-0.0297	-26	113	121	EIVDLCLDR			Carbamidomethyl (C)[6]		Mascot
1396.7584	1396.6779	-0.0805	-58	85	96	QLFHPEQLISGK					Mascot
1473.8635	1473.8313	-0.0322	-22	230	243	LVSQVISSLTASLR					Mascot
1473.8635	1473.8313	-0.0322	-22	230	243	LVSQVISSLTASLR	71	99.992			Mascot
1589.6844	1589.6282	-0.0562	-35	309	320	HGKYMACCLMYR			Carbamidomethyl (C)[7,8]		Mascot
1691.8711	1691.8196	-0.0515	-30	216	229	SLDIERPTYTNLNR					Mascot
1691.8711	1691.8196	-0.0515	-30	216	229	SLDIERPTYTNLNR	76	99.997			Mascot
1701.9059	1701.8625	-0.0434	-26	65	79	AVFVDLEPTVIDEVR					Mascot
1701.9059	1701.8625	-0.0434	-26	65	79	AVFVDLEPTVIDEVR	76	99.997			Mascot
1808.9252	1808.8646	-0.0606	-34	265	280	IHFMLSSYAPVISAEK			Oxidation (M)[4]		Mascot
2385.1946	2385.1514	-0.0432	-18	85	105	QLFHPEQLISGKEDAANN FAR					Mascot
2408.1882	2408.1445	-0.0437	-18	244	264	FDGALNVDVNEFQTNLV PYPR					Mascot
2408.1882	2408.1445	-0.0437	-18	244	264	FDGALNVDVNEFQTNLV PYPR	90	100			Mascot

Rank	Protein Name	Accession No.	Protein MW	Protein PI	Pep. Count	Protein Score	Protein Score C. I. %	Intensity Matched	Total Ion Score	Total Ion C. I. %	Confirmed
2	Tubulin alpha-1 chain OS=Zea mays GN=TUBA1 PE=3 SV=1	TBA1_MAIZE	50383.6	4.89	10	350	100	15.351	313	100	

Protein Group

Tubulin alpha chain OS=Triticum aestivum GN=TUBA PE=2 SV=1	TBA_WHEAT	50395.6	4.8899998664856
Tubulin alpha-1 chain OS=Eleusine indica GN=TUBA1 PE=1 SV=1	TBA1_ELEIN	50383.6	4.8899998664856
Tubulin alpha-2 chain OS=Hordeum vulgare GN=TUBA2 PE=2 SV=1	TBA2_HORVU	50353.6	4.88000011444092
Tubulin alpha-2 chain OS=Zea mays GN=TUBA2 PE=3 SV=1	TBA2_MAIZE	50383.6	4.8899998664856
Tubulin alpha-3 chain OS=Hordeum vulgare	TBA3_HORVU	50381.6	4.8899

GN=TUBA3 PE=1 SV=1 998664856

Peptide Information

Calc. Mass	Obsrv. Mass	± da	± ppm	Start Seq.	End Seq.	Sequence	Ion Score	C. I. %	Modification	Rank	Result Type
1007.454	1007.4495	-0.0045	-4	97	105	EDAANNFAR					Mascot
1132.5668	1132.5371	-0.0297	-26	113	121	EIVDLCLDR			Carbamidomethyl (C)[6]		Mascot
1396.693	1396.6779	-0.0151	-11	391	401	IDHKFDLMYAK			Oxidation (M)[8]		Mascot
1473.8635	1473.8313	-0.0322	-22	230	243	LVSQVISSLTASLR					Mascot
1473.8635	1473.8313	-0.0322	-22	230	243	LVSQVISSLTASLR	71	99.992			Mascot
1589.6844	1589.6282	-0.0562	-35	309	320	HGKYMACCLMYR			Carbamidomethyl (C)[7,8]		Mascot
1691.8711	1691.8196	-0.0515	-30	216	229	SLDIERPTYTNLNR					Mascot
1691.8711	1691.8196	-0.0515	-30	216	229	SLDIERPTYTNLNR	76	99.997			Mascot
1701.9059	1701.8625	-0.0434	-26	65	79	AVFVDLEPTVIDEVR					Mascot
1701.9059	1701.8625	-0.0434	-26	65	79	AVFVDLEPTVIDEVR	76	99.997			Mascot
1808.9252	1808.8646	-0.0606	-34	265	280	IHFMLSSYAPVISAEK			Oxidation (M)[4]		Mascot
2385.1946	2385.1514	-0.0432	-18	85	105	QLFHPEQLISGKEDAANNFAR					Mascot
2408.1882	2408.1445	-0.0437	-18	244	264	FDGALNVDVNEFQTNLVPYPR					Mascot
2408.1882	2408.1445	-0.0437	-18	244	264	FDGALNVDVNEFQTNLVPYPR	90	100			Mascot

3	Protein disulfide-isomerase OS=Triticum aestivum GN=PDI PE=2 SV=1	PDI_WHEAT	56726	4.99	18	291	100	30.747	203	100	

Peptide Information

Calc. Mass	Obsrv. Mass	± da	± ppm	Start Seq.	End Seq.	Sequence	Ion Score	C. I. %	Modification	Rank	Result Type
1023.4993	1023.471	-0.0283	-28	236	244	DFDVSALEK					Mascot
1104.5797	1104.5514	-0.0283	-26	128	136	NIQEYKGPR					Mascot
1150.599	1150.5203	-0.0787	-68	137	146	EAEGIVEYLK					Mascot
1181.6201	1181.5607	-0.0594	-50	111	120	YEVQGFPTLK					Mascot
1182.6589	1182.6149	-0.044	-37	212	222	GDAAVERPLVR					Mascot
1182.6589	1182.6149	-0.044	-37	212	222	GDAAVERPLVR	31	24.121			Mascot
1210.6216	1210.5952	-0.0264	-22	260	269	NPDNHPYLLK					Mascot
1278.694	1278.6205	-0.0735	-57	137	147	EAEGIVEYLKK					Mascot
1407.6427	1407.5763	-0.0664	-47	294	306	SAYYGAVEEFSGK					Mascot
1454.7373	1454.7169	-0.0204	-14	502	515	AAEPAATEPLKDEL					Mascot
1536.8308	1536.7449	-0.0859	-56	223	235	LFKPFDELVVDSK					Mascot
1647.8951	1647.7367	-0.1584	-96	73	87	SLAPEYEKAAQLLSK					Mascot

1647.8951	1647.7367	-0.1584	-96	73	87	SLAPEYEKAAQLLSK					Mascot
1654.8688	1654.7891	-0.0797	-48	245	259	FIDASSTPKVVTFDK					Mascot
1671.8436	1671.6882	-0.1554	-93	329	343	EDQAPLILIQDSDSK					Mascot
1674.9901	1674.8242	-0.1659	-99	81	96	AAQLLSKHDPAIVLAK					Mascot
1799.9386	1799.8619	-0.0767	-43	329	344	EDQAPLILIQDSDSKK					Mascot
1865.8678	1865.8357	-0.0321	-17	196	211	SDYDFGHTVHANHLPR					Mascot
1865.8678	1865.8357	-0.0321	-17	196	211	SDYDFGHTVHANHLPR	115	100			Mascot
1899.9963	1899.9279	-0.0684	-36	254	269	VVTFDKNPDNHPYLLK					Mascot
1899.9963	1899.9279	-0.0684	-36	254	269	VVTFDKNPDNHPYLLK	57	99.799			Mascot
2135.053	2135.0029	-0.0501	-23	194	211	LRSDYDFGHTVHANHLP R					Mascot

4	Tubulin alpha-4 chain OS=Gossypium hirsutum PE=2 SV=1	TBA4_GOSHI	50223.6	4.93	10	258	100	13.7	223	100	

Peptide Information

Calc. Mass	Obsrv. Mass	± da	± ppm	Start Seq.	End Seq.	Sequence	Ion Score	C. I. %	Modification	Rank	Result Type
1007.454	1007.4495	-0.0045	-4	97	105	EDAANNFAR					Mascot
1132.5668	1132.5371	-0.0297	-26	113	121	EIVDLCLDR			Carbamidomethyl (C)[6]		Mascot
1396.7584	1396.6779	-0.0805	-58	85	96	QLFHPEQLISGK					Mascot
1426.7035	1426.7113	0.0078	5	391	401	IDHKFDLMYTK			Oxidation (M)[8]		Mascot
1473.8635	1473.8313	-0.0322	-22	230	243	LVSQVISSLTASLR					Mascot
1473.8635	1473.8313	-0.0322	-22	230	243	LVSQVISSLTASLR	71	99.992			Mascot
1589.6844	1589.6282	-0.0562	-35	309	320	HGKYMACCLMYR			Carbamidomethyl (C)[7,8]		Mascot
1691.8711	1691.8196	-0.0515	-30	216	229	SLDIERPTYTNLNR					Mascot
1691.8711	1691.8196	-0.0515	-30	216	229	SLDIERPTYTNLNR	76	99.997			Mascot
1701.9059	1701.8625	-0.0434	-26	65	79	AVFVDLEPTVIDEVR					Mascot
1701.9059	1701.8625	-0.0434	-26	65	79	AVFVDLEPTVIDEVR	76	99.997			Mascot
1808.9252	1808.8646	-0.0606	-34	265	280	IHFMLSSYAPVISAEK			Oxidation (M)[4]		Mascot
2385.1946	2385.1514	-0.0432	-18	85	105	QLFHPEQLISGKEDAANN FAR					Mascot

5	Tubulin alpha chain OS=Prunus dulcis GN=TUBA PE=2 SV=1	TBA_PRUDU	50179.5	4.92	9	253	100	13.189	223	100	

Protein Group

Tubulin alpha-2 chain OS=Gossypium hirsutum PE=2 SV=1	TBA2_GOSHI	50193.6	4.9299 998283 3862

Peptide Information

Calc. Mass	Obsrv. Mass	± da	± ppm	Start Seq.	End Seq.	Sequence	Ion Score	C. I. %	Modification	Rank	Result Type

1007.454	1007.4495	-0.0045	-4	97	105	EDAANNFAR					Mascot
1132.5668	1132.5371	-0.0297	-26	113	121	EIVDLCLDR			Carbamidomethyl (C)[6]		Mascot
1396.693	1396.6779	-0.0151	-11	391	401	IDHKFDLMYAK			Oxidation (M)[8]		Mascot
1473.8635	1473.8313	-0.0322	-22	230	243	LVSQVISSLTASLR					Mascot
1473.8635	1473.8313	-0.0322	-22	230	243	LVSQVISSLTASLR	71	99.992			Mascot
1589.6844	1589.6282	-0.0562	-35	309	320	HGKYMACCLMYR			Carbamidomethyl (C)[7,8]		Mascot
1691.8711	1691.8196	-0.0515	-30	216	229	SLDIERPTYTNLNR					Mascot
1691.8711	1691.8196	-0.0515	-30	216	229	SLDIERPTYTNLNR	76	99.997			Mascot
1701.9059	1701.8625	-0.0434	-26	65	79	AVFVDLEPTVIDEVR					Mascot
1701.9059	1701.8625	-0.0434	-26	65	79	AVFVDLEPTVIDEVR	76	99.997			Mascot
1808.9252	1808.8646	-0.0606	-34	265	280	IHFMLSSYAPVISAEK			Oxidation (M)[4]		Mascot
2385.1946	2385.1514	-0.0432	-18	85	105	QLFHPEQLISGKEDAANNFAR					Mascot

6	Tubulin alpha-1 chain OS=Pisum sativum GN=TUBA1 PE=1 SV=1	TBA1_PEA	50305.6	4.92	9	252	100	13.189	223	100	

Peptide Information

Calc. Mass	Obsrv. Mass	± da	± ppm	Start Seq.	End Seq.	Sequence	Ion Score	C. I. %	Modification	Rank	Result Type
1007.454	1007.4495	-0.0045	-4	97	105	EDAANNFAR					Mascot
1132.5668	1132.5371	-0.0297	-26	113	121	EIVDLCLDR			Carbamidomethyl (C)[6]		Mascot
1396.693	1396.6779	-0.0151	-11	391	401	IDHKFDLMYAK			Oxidation (M)[8]		Mascot
1473.8635	1473.8313	-0.0322	-22	230	243	LVSQVISSLTASLR					Mascot
1473.8635	1473.8313	-0.0322	-22	230	243	LVSQVISSLTASLR	71	99.992			Mascot
1589.6844	1589.6282	-0.0562	-35	309	320	HGKYMACCLMYR			Carbamidomethyl (C)[7,8]		Mascot
1691.8711	1691.8196	-0.0515	-30	216	229	SLDIERPTYTNLNR					Mascot
1691.8711	1691.8196	-0.0515	-30	216	229	SLDIERPTYTNLNR	76	99.997			Mascot
1701.9059	1701.8625	-0.0434	-26	65	79	AVFVDLEPTVIDEVR					Mascot
1701.9059	1701.8625	-0.0434	-26	65	79	AVFVDLEPTVIDEVR	76	99.997			Mascot
1808.9252	1808.8646	-0.0606	-34	265	280	IHFMLSSYAPVISAEK			Oxidation (M)[4]		Mascot
2385.1946	2385.1514	-0.0432	-18	85	105	QLFHPEQLISGKEDAANNFAR					Mascot

7	Tubulin alpha-3 chain OS=Zea mays GN=TUBA3 PE=2 SV=1	TBA3_MAIZE	50213.7	5.09	7	181	100	7.974	161	100	

Peptide Information

Calc. Mass	Obsrv. Mass	± da	± ppm	Start Seq.	End Seq.	Sequence	Ion Score	C. I. %	Modification	Rank	Result Type
1007.454	1007.4495	-0.0045	-4	97	105	EDAANNFAR					Mascot

1132.5668	1132.5371	-0.0297	-26	113	121	EIVDLCLDR			Carbamidomethyl (C)[6]		Mascot
1396.7584	1396.6779	-0.0805	-58	85	96	QLFHPEQLISGK					Mascot
1473.8635	1473.8313	-0.0322	-22	230	243	LVSQVISSLTASLR					Mascot
1473.8635	1473.8313	-0.0322	-22	230	243	LVSQVISSLTASLR	71	99.992			Mascot
1589.6844	1589.6282	-0.0562	-35	309	320	HGKYMACCLMYR			Carbamidomethyl (C)[7,8]		Mascot
2385.1946	2385.1514	-0.0432	-18	85	105	QLFHPEQLISGKEDAANN FAR					Mascot
2408.1882	2408.1445	-0.0437	-18	244	264	FDGALNVDVNEFQTNLV PYPR					Mascot
2408.1882	2408.1445	-0.0437	-18	244	264	FDGALNVDVNEFQTNLV PYPR	90	100			Mascot

8	Tubulin alpha chain OS=Daucus carota GN=TBA PE=2 SV=1	TBA_DAUCA	50293.6	4.89	8	171	100	8.976	147	100	

Peptide Information

Calc. Mass	Obsrv. Mass	± da	± ppm	Start Seq.	End Seq.	Sequence	Ion Score	C. I. %	Modification	Rank	Result Type
1007.454	1007.4495	-0.0045	-4	97	105	EDAANNFAR					Mascot
1132.5668	1132.5371	-0.0297	-26	113	121	EIVDLCLDR			Carbamidomethyl (C)[6]		Mascot
1396.7584	1396.6779	-0.0805	-58	85	96	QLFHPEQLISGK					Mascot
1473.8635	1473.8313	-0.0322	-22	230	243	LVSQVISSLTASLR					Mascot
1473.8635	1473.8313	-0.0322	-22	230	243	LVSQVISSLTASLR	71	99.992			Mascot
1589.6844	1589.6282	-0.0562	-35	309	320	HGKYMACCLMYR			Carbamidomethyl (C)[7,8]		Mascot
1691.8711	1691.8196	-0.0515	-30	216	229	SLDIERPTYTNLNR					Mascot
1691.8711	1691.8196	-0.0515	-30	216	229	SLDIERPTYTNLNR	76	99.997			Mascot
1808.9252	1808.8646	-0.0606	-34	265	280	IHFMLSSYAPVISAEK			Oxidation (M)[4]		Mascot
2385.1946	2385.1514	-0.0432	-18	85	105	QLFHPEQLISGKEDAANN FAR					Mascot

9	Tubulin alpha-1 chain OS=Gossypium hirsutum PE=2 SV=1	TBA1_GOSHI	50390.6	4.93	8	171	100	8.976	147	100	

Protein Group

Tubulin alpha-1 chain OS=Anemia phyllitidis GN=TUBA1 PE=2 SV=1	TBA1_ANEPH	50381.7	5.0199 999809 2651

Peptide Information

Calc. Mass	Obsrv. Mass	± da	± ppm	Start Seq.	End Seq.	Sequence	Ion Score	C. I. %	Modification	Rank	Result Type
1007.454	1007.4495	-0.0045	-4	97	105	EDAANNFAR					Mascot
1132.5668	1132.5371	-0.0297	-26	113	121	EIVDLCLDR			Carbamidomethyl (C)[6]		Mascot
1396.693	1396.6779	-0.0151	-11	391	401	IDHKFDLMYAK			Oxidation (M)[8]		Mascot
1473.8635	1473.8313	-0.0322	-22	230	243	LVSQVISSLTASLR					Mascot

1473.8635	1473.8313	-0.0322	-22	230	243	LVSQVISSLTASLR	71	99.992			Mascot
1589.6844	1589.6282	-0.0562	-35	309	320	HGKYMACCLMYR			Carbamidomethyl (C)[7,8]		Mascot
1691.8711	1691.8196	-0.0515	-30	216	229	SLDIERPTYTNLNR					Mascot
1691.8711	1691.8196	-0.0515	-30	216	229	SLDIERPTYTNLNR	76	99.997			Mascot
1808.9252	1808.8646	-0.0606	-34	265	280	IHFMLSSYAPVISAEK			Oxidation (M)[4]		Mascot
2385.1946	2385.1514	-0.0432	-18	85	105	QLFHPEQLISGKEDAANN FAR					Mascot

10	Tubulin alpha-4 chain OS=Arabidopsis thaliana GN=TUBA4 PE=2 SV=2	TBA4_ARATH	50193.6	4.93	8	171	100	10.306	147	100

Protein Group

Tubulin alpha-2 chain OS=Arabidopsis thaliana GN=TUBA2 PE=2 SV=2	TBA2_ARATH	50193.6	4.9299 998283 3862
Tubulin alpha-6 chain OS=Arabidopsis thaliana GN=TUBA6 PE=2 SV=1	TBA6_ARATH	50190.6	4.9299 998283 3862

Peptide Information

Calc. Mass	Obsrv. Mass	± da	± ppm	Start Seq.	End Seq.	Sequence	Ion Score	C. I. %	Modification	Rank	Result Type
1007.454	1007.4495	-0.0045	-4	97	105	EDAANNFAR					Mascot
1132.5668	1132.5371	-0.0297	-26	113	121	EIVDLCLDR			Carbamidomethyl (C)[6]		Mascot
1396.693	1396.6779	-0.0151	-11	391	401	IDHKFDLMYAK			Oxidation (M)[8]		Mascot
1473.8635	1473.8313	-0.0322	-22	230	243	LVSQVISSLTASLR					Mascot
1473.8635	1473.8313	-0.0322	-22	230	243	LVSQVISSLTASLR	71	99.992			Mascot
1589.6844	1589.6282	-0.0562	-35	309	320	HGKYMACCLMYR			Carbamidomethyl (C)[7,8]		Mascot
1701.9059	1701.8625	-0.0434	-26	65	79	AVFVDLEPTVIDEVR					Mascot
1701.9059	1701.8625	-0.0434	-26	65	79	AVFVDLEPTVIDEVR	76	99.997			Mascot
1808.9252	1808.8646	-0.0606	-34	265	280	IHFMLSSYAPVISAEK			Oxidation (M)[4]		Mascot
2385.1946	2385.1514	-0.0432	-18	85	105	QLFHPEQLISGKEDAANN FAR					Mascot

Gel Idx/Pos	254/K6	Instr./Gel Origin	BA2151/Sample Project 20140814	Process Status	Analysis Succeeded
Plate [#] Name	[1] Sample Project 20140814	Instrument Sample Name		Spectra	11

Rank	Protein Name	Accession No.	Protein MW	Protein PI	Pep. Count	Protein Score	Protein Score C. I. %	Intensity Matched	Total Ion Score	Total Ion C. I. %	Confirmed
1	Beta-amylase OS=Hordeum vulgare subsp. spontaneum GN=BMY1 PE=1 SV=1	AMYB_HORVS	59886.4	5.66	11	285	100	23.892	249	100	

Peptide Information

Calc. Mass	Obsrv. Mass	± da	± ppm	Start Seq.	End Seq.	Sequence	Ion Score	C. I. %	Modification	Rank	Result Type
854.4301	854.4536	0.0235	28	439	445	MHANLPR			Oxidation (M)[1]		Mascot
1016.5564	1016.5394	-0.017	-17	411	418	LFGFTYLR					Mascot
1016.5564	1016.5394	-0.017	-17	411	418	LFGFTYLR	56	99.749			Mascot
1253.6121	1253.5747	-0.0374	-30	248	258	DNGTYLTEKGR					Mascot
1285.6212	1285.5829	-0.0383	-30	61	71	GPKAYDWSAYK					Mascot
1315.5769	1315.5422	-0.0347	-26	335	345	ASINFTCAEMR			Carbamidomethyl (C)[7], Oxidation (M)[10]		Mascot
1326.6688	1326.6444	-0.0244	-18	384	394	YDPTAYNTILR					Mascot
1326.6688	1326.6444	-0.0244	-18	384	394	YDPTAYNTILR	82	100			Mascot
1685.7299	1685.7437	0.0138	8	147	160	SAVQMYADYMTSFR			Oxidation (M)[5]		Mascot
1701.7247	1701.7063	-0.0184	-11	147	160	SAVQMYADYMTSFR			Oxidation (M)[5,10]		Mascot
1701.7247	1701.7063	-0.0184	-11	147	160	SAVQMYADYMTSFR			Oxidation (M)[5,10]		Mascot
1752.8916	1752.8329	-0.0587	-33	419	433	LSNQLVEGQNYVNFK					Mascot
1842.0425	1841.8951	-0.1474	-80	288	302	VQLAIKISGIHWWYK					Mascot
2013.9778	2013.9647	-0.0131	-7	303	320	VPSHAAELTAGYYNLHD R					Mascot
2013.9778	2013.9647	-0.0131	-7	303	320	VPSHAAELTAGYYNLHD R	112	100			Mascot
2733.2786	2733.2993	0.0207	8	346	370	DSEQSSQAMSAPEELVQ QVLSAGWR					Mascot
2749.2734	2749.2756	0.0022	1	346	370	DSEQSSQAMSAPEELVQ QVLSAGWR			Oxidation (M)[9]		Mascot

Rank	Protein Name	Accession No.	Protein MW	Protein PI	Pep. Count	Protein Score	Protein Score C. I. %	Intensity Matched	Total Ion Score	Total Ion C. I. %	Confirmed
2	Beta-amylase OS=Hordeum vulgare GN=BMY1 PE=1 SV=1	AMYB_HORVU	59894.5	5.58	10	277	100	23.329	249	100	

Peptide Information

Calc. Mass	Obsrv. Mass	± da	± ppm	Start Seq.	End Seq.	Sequence	Ion Score	C. I. %	Modification	Rank	Result Type
854.4301	854.4536	0.0235	28	439	445	MHANLPR			Oxidation (M)[1]		Mascot
1016.5564	1016.5394	-0.017	-17	411	418	LFGFTYLR					Mascot
1016.5564	1016.5394	-0.017	-17	411	418	LFGFTYLR	56	99.749			Mascot

Calc. Mass	Obsrv. Mass	± da	± ppm	Start Seq.	End Seq.	Sequence	Ion Score	C. I. %	Modification	Rank	Result Type
1285.6212	1285.5829	-0.0383	-30	61	71	GPKAYDWSAYK					Mascot
1315.5769	1315.5422	-0.0347	-26	335	345	ASINFTCAEMR			Carbamidomethyl (C)[7], Oxidation (M)[10]		Mascot
1326.6688	1326.6444	-0.0244	-18	384	394	YDPTAYNTILR					Mascot
1326.6688	1326.6444	-0.0244	-18	384	394	YDPTAYNTILR	82	100			Mascot
1685.7299	1685.7437	0.0138	8	147	160	SAVQMYADYMTSFR			Oxidation (M)[5]		Mascot
1701.7247	1701.7063	-0.0184	-11	147	160	SAVQMYADYMTSFR			Oxidation (M)[5,10]		Mascot
1701.7247	1701.7063	-0.0184	-11	147	160	SAVQMYADYMTSFR			Oxidation (M)[5,10]		Mascot
1705.8181	1705.7739	-0.0442	-26	243	256	TQFFRDNGTYLSEK					Mascot
1752.8916	1752.8329	-0.0587	-33	419	433	LSNQLVEGQNYVNFK					Mascot
1842.0425	1841.8951	-0.1474	-80	288	302	VQLAIKISGIHWWYK					Mascot
2013.9778	2013.9647	-0.0131	-7	303	320	VPSHAAELTAGYYNLHD R					Mascot
2013.9778	2013.9647	-0.0131	-7	303	320	VPSHAAELTAGYYNLHD R	112	100			Mascot

3	Beta-amylase OS=Triticum aestivum GN=BMY1 PE=2 SV=1	AMYB_WHEAT	56860.2	5.24	5	217	100	19.612	206	100

Peptide Information

Calc. Mass	Obsrv. Mass	± da	± ppm	Start Seq.	End Seq.	Sequence	Ion Score	C. I. %	Modification	Rank	Result Type
1016.5564	1016.5394	-0.017	-17	411	418	LFGFTYLR					Mascot
1016.5564	1016.5394	-0.017	-17	411	418	LFGFTYLR	56	99.749			Mascot
1285.6212	1285.5829	-0.0383	-30	61	71	GPKAYDWSAYK					Mascot
1685.7299	1685.7437	0.0138	8	147	160	TAVQMYADYMASFR			Oxidation (M)[5,10]		Mascot
2087.0557	2087.0425	-0.0132	-6	129	146	NIEYLTLGVDDQPLFHGR					Mascot
2087.0557	2087.0425	-0.0132	-6	129	146	NIEYLTLGVDDQPLFHGR	150	100			Mascot
2773.3274	2773.3901	0.0627	23	346	370	DSEQSEEAKSAPEELVQ QVLSAGWR					Mascot

4	Beta-amylase (Fragment) OS=Secale cereale GN=BMY1 PE=2 SV=1	AMYB_SECCE	24561.9	5.08	3	147	100	13.298	137	100

Peptide Information

Calc. Mass	Obsrv. Mass	± da	± ppm	Start Seq.	End Seq.	Sequence	Ion Score	C. I. %	Modification	Rank	Result Type
1016.5564	1016.5394	-0.017	-17	107	114	LFGFTYLR					Mascot
1016.5564	1016.5394	-0.017	-17	107	114	LFGFTYLR	56	99.749			Mascot
1326.6688	1326.6444	-0.0244	-18	80	90	YDPTAYNTILR					Mascot
1326.6688	1326.6444	-0.0244	-18	80	90	YDPTAYNTILR	82	100			Mascot
2764.3723	2764.3406	-0.0317	-11	67	90	EGLNIACENALPRYDPTA YNTILR			Carbamidomethyl (C)[7]		Mascot

5	Protein bicaudal D homolog 2 OS=Mus musculus	BICD2_MOUSE	93562	5.34	18	59	25.358	25.322		

GN=Bicd2 PE=1 SV=1

Peptide Information

Calc. Mass	Obsrv. Mass	± da	± ppm	Start Seq.	End Seq.	Sequence	Ion Score	C. I. %	Modification	Rank	Result Type
930.4752	930.4887	0.0135	15	566	574	AGRTSPEGR					Mascot
1016.5734	1016.5394	-0.034	-33	392	400	LTENLSALR					Mascot
1016.5734	1016.5394	-0.034	-33	392	400	LTENLSALR	2	0			Mascot
1326.6578	1326.6444	-0.0134	-10	716	726	AMVTETMMKLR			Oxidation (M)[2]		Mascot
1326.6578	1326.6444	-0.0134	-10	716	726	AMVTETMMKLR	3	0	Oxidation (M)[2]		Mascot
1387.7039	1387.6582	-0.0457	-33	13	23	LVMEAQPEWLR			Oxidation (M)[3]		Mascot
1622.7517	1622.782	0.0303	19	460	473	STHEAREAQHAEEK					Mascot
1646.861	1646.7638	-0.0972	-59	493	506	ASHQDRELLAHLEK					Mascot
1651.8762	1651.7631	-0.1131	-68	792	804	LELLELDHEQTRR					Mascot
1698.881	1698.8062	-0.0748	-44	200	213	QNQVEFEGLKHEIK					Mascot
1700.7255	1700.7594	0.0339	20	750	762	CDEYITQLDEMQR			Carbamidomethyl (C)[1]		Mascot
1716.7205	1716.743	0.0225	13	750	762	CDEYITQLDEMQR			Carbamidomethyl (C)[1], Oxidation (M)[11]		Mascot
1716.7205	1716.743	0.0225	13	750	762	CDEYITQLDEMQR			Carbamidomethyl (C)[1], Oxidation (M)[11]		Mascot
1752.7489	1752.8329	0.084	48	711	724	YENEKAMVTETMMK			Oxidation (M)[7,12,13]		Mascot
1773.8589	1773.7906	-0.0683	-39	734	749	EDAATFSSLRAMFATR					Mascot
1790.9535	1790.8346	-0.1189	-66	39	54	EKIQAAEYGLAVLEEK					Mascot
1801.9152	1801.886	-0.0292	-16	127	141	QLRNVLTNTQSENER					Mascot
2012.1135	2011.9863	-0.1272	-63	362	379	VGLLATLQDTQKQLEQA R					Mascot
2733.4304	2733.2993	-0.1311	-48	177	199	LLQDYSELEEENISLQKQ VSVLR					Mascot
2751.2751	2751.3047	0.0296	11	1	23	MSAPSEEEEYARLVMEA QPEWLR					Mascot
2767.2703	2767.2705	0.0002	0	1	23	MSAPSEEEEYARLVMEA QPEWLR			Oxidation (M)[1]		Mascot
2773.4983	2773.3901	-0.1082	-39	324	349	KDGLAPPSPSLVSDLLSE LHISEIQK					Mascot
2810.2209	2810.3384	0.1175	42	417	440	DRDSHEDGDYYEVDING PEILACK			Carbamidomethyl (C)[23]		Mascot

6	Tail tape measure protein gp18 OS=Bacillus phage SPP1 PE=4 SV=1	GP18_BPSPP	110791.4	10.45	20	57	0	23.576	

Peptide Information

Calc. Mass	Obsrv. Mass	± da	± ppm	Start Seq.	End Seq.	Sequence	Ion Score	C. I. %	Modification	Rank	Result Type
930.5479	930.4887	-0.0592	-64	27	34	IVRTTANR					Mascot
1016.4539	1016.5394	0.0855	84	554	562	MAASYAQMK			Oxidation (M)[1]		Mascot

1016.4539	1016.5394	0.0855	84	554	562	MAASYAQMK			Oxidation (M)[1]		Mascot
1221.6157	1221.5663	-0.0494	-40	46	55	FENAMNRLAR					Mascot
1237.6106	1237.5747	-0.0359	-29	46	55	FENAMNRLAR			Oxidation (M)[5]		Mascot
1399.6345	1399.6332	-0.0013	-1	233	244	SMQNAFKAPDMK			Oxidation (M)[2,11]		Mascot
1490.7454	1490.6362	-0.1092	-73	583	597	NMAIMAAQSAANAVK					Mascot
1547.8462	1547.7418	-0.1044	-67	479	494	TSAAGIKLAAAIADMK			Oxidation (M)[15]		Mascot
1571.7523	1571.7524	0.0001	0	415	428	MVNSFAQWTAATTK			Oxidation (M)[1]		Mascot
1572.8778	1572.7811	-0.0967	-61	158	171	IMEQIKNLQASLGK					Mascot
1622.8207	1622.782	-0.0387	-24	285	299	NMTKSIEGATAAWVK			Oxidation (M)[2]		Mascot
1670.8605	1670.7533	-0.1072	-64	495	509	LFTKTNALMAAQMAK			Oxidation (M)[9,13]		Mascot
1705.9153	1705.7739	-0.1414	-83	946	963	GITGTEGMVTKAAASLAK					Mascot
1729.953	1729.8262	-0.1268	-73	211	225	SVLNQLQPMFRGLAR					Mascot
1801.9629	1801.886	-0.0769	-43	930	945	LMRDEIGYHIGTGLVK					Mascot
1817.9579	1817.8453	-0.1126	-62	930	945	LMRDEIGYHIGTGLVK			Oxidation (M)[2]		Mascot
1842.0405	1841.8951	-0.1454	-79	397	414	TLINLAVAMAPVGSEVLK			Oxidation (M)[9]		Mascot
1991.0341	1990.9355	-0.0986	-50	554	571	MAASYAQMKITSFITALK			Oxidation (M)[1]		Mascot
2087.0437	2087.0425	-0.0012	-1	841	860	SAASSTFNALKSSVTNIM NK			Oxidation (M)[18]		Mascot
2087.0437	2087.0425	-0.0012	-1	841	860	SAASSTFNALKSSVTNIM NK			Oxidation (M)[18]		Mascot
2135.1133	2135.0303	-0.083	-39	289	309	SIEGATAAWVKWSASLG SSVK					Mascot
2183.2112	2183.0105	-0.2007	-92	449	469	ALTPVIISFQTLFGGFSSI GK					Mascot
2183.2112	2183.0105	-0.2007	-92	449	469	ALTPVIISFQTLFGGFSSI GK					Mascot
2794.5359	2794.3499	-0.186	-67	528	553	LQMSIMAAYLKQLVVAAA QQTAFAVK					Mascot
2810.5308	2810.3384	-0.1924	-68	528	553	LQMSIMAAYLKQLVVAAA QQTAFAVK			Oxidation (M)[3]		Mascot
2842.4888	2842.3542	-0.1346	-47	245	270	NFINYLNTEAPGAFVSFG KISGNIIR					Mascot

7	Replicase large subunit OS=Odontoglossum ringspot virus (isolate Singapore 1) PE=3 SV=2	RDRP_ORSVS	185074.6	6.85	25	54	0	25.499

Peptide Information

Calc. Mass	Obsrv. Mass	± da	± ppm	Start Seq.	End Seq.	Sequence	Ion Score	C. I. %	Modification	Rank	Result Type
1138.6144	1138.5857	-0.0287	-25	1521	1530	GAIVYYDPLK					Mascot
1160.5769	1160.609	0.0321	28	1123	1132	GENLFVPCPK			Carbamidomethyl (C)[8]		Mascot
1237.6787	1237.5747	-0.104	-84	1005	1015	GKIITFTQSDK					Mascot
1326.6545	1326.6444	-0.0101	-8	624	633	DLDMRFCLLK			Carbamidomethyl (C)[7], Oxidation (M)[4]		Mascot
1326.6545	1326.6444	-0.0101	-8	624	633	DLDMRFCLLK			Carbamidomethyl (C)[7], Oxidation (M)[4]		Mascot
1399.7468	1399.6332	-0.1136	-81	555	566	YYNALSELSVLK					Mascot

1571.7258	1571.7524	0.0266	17	787	801	VAVSSDSPIYSDMGK			Oxidation (M)[13]		Mascot
1620.8115	1620.7842	-0.0273	-17	721	735	VLKDVYGADPESAEK					Mascot
1636.8483	1636.7894	-0.0589	-36	312	324	VDTYFLFRGVYTR					Mascot
1643.6611	1643.7832	0.1221	74	170	182	YMNDPDAVCCDKR			Carbamidomethyl (C)[9,10]		Mascot
1645.8293	1645.7837	-0.0456	-28	138	151	HINQQDTVSTYLAR					Mascot
1680.8513	1680.7998	-0.0515	-31	370	385	DMVIVPLFDGSVTSGK			Oxidation (M)[2]		Mascot
1700.8524	1700.7594	-0.093	-55	578	592	NLCEEKDIAPDVVAK			Carbamidomethyl (C)[3]		Mascot
1701.8303	1701.7063	-0.124	-73	31	44	VYDNAVEELNHRSR					Mascot
1701.8303	1701.7063	-0.124	-73	31	44	VYDNAVEELNHRSR					Mascot
1705.9272	1705.7739	-0.1533	-90	1325	1339	QINAIFGPLFSELTR					Mascot
1711.832	1711.9052	0.0732	43	786	801	RVAVSSDSPIYSDMGK					Mascot
1727.8269	1727.7769	-0.05	-29	786	801	RVAVSSDSPIYSDMGK			Oxidation (M)[14]		Mascot
1774.8582	1774.8265	-0.0317	-18	1424	1437	DYTAGIKTCLWYQR			Carbamidomethyl (C)[9]		Mascot
1801.9443	1801.886	-0.0583	-32	429	444	VIINGVTARSEWDVDK					Mascot
1842.0154	1841.8951	-0.1203	-65	802	817	LQTLLSCLKDGEPVLR			Carbamidomethyl (C)[7]		Mascot
2012.0237	2011.9863	-0.0374	-19	396	411	DFVYTVLNHIRTYQDK					Mascot
2014.0254	2013.9647	-0.0607	-30	1181	1197	SVSVPRQQQEFFTPAHR					Mascot
2014.0254	2013.9647	-0.0607	-30	1181	1197	SVSVPRQQQEFFTPAHR					Mascot
2087.063	2087.0425	-0.0205	-10	134	151	DVARHINQQDTVSTYLAR					Mascot
2087.063	2087.0425	-0.0205	-10	134	151	DVARHINQQDTVSTYLAR					Mascot
2198.886	2199.0103	0.1243	57	325	342	GEDSEQFYTAMDEAWEYK					Mascot
2751.3149	2751.3047	-0.0102	-4	697	720	VQQMKNYMDYLSASISATVSNLCK			Carbamidomethyl (C)[23]		Mascot
2753.3755	2753.3123	-0.0632	-23	917	940	EAMVFGDAEQIPFINRVANFPYPK					Mascot
2767.3101	2767.2705	-0.0396	-14	697	720	VQQMKNYMDYLSASISATVSNLCK			Carbamidomethyl (C)[23], Oxidation (M)[4]		Mascot
2842.3142	2842.3542	0.04	14	1242	1266	FWDAYIIDELSGGNVTPMTSDAFHR					Mascot

8	DNA-directed RNA polymerase subunit beta' OS=Buchnera aphidicola subsp. Schizaphis graminum (strain Sg) GN=rpoC PE=3 SV=2	RPOC_BUCAP	158380.6	9.03	23	52	0	24.653

Peptide Information

Calc. Mass	Obsrv. Mass	± da	± ppm	Start Seq.	End Seq.	Sequence	Ion Score	C. I. %	Modification	Rank	Result Type
929.5051	929.47	-0.0351	-38	1105	1112	AIVQLDDR					Mascot
1016.5272	1016.5394	0.0122	12	934	943	TFHIGGAASR					Mascot
1016.5272	1016.5394	0.0122	12	934	943	TFHIGGAASR					Mascot
1138.5963	1138.5857	-0.0106	-9	790	799	TANSGYLTRR					Mascot

1251.7307	1251.6128	-0.1179	-94	539	549	LEIAELHSLVK					Mascot
1572.8495	1572.7811	-0.0684	-43	372	384	MALELFKPFIYGK			Oxidation (M)[1]		Mascot
1574.7843	1574.755	-0.0293	-19	298	311	MLQEAIDALLDNGR			Oxidation (M)[1]		Mascot
1600.7346	1600.7799	0.0453	28	696	709	AMMENLSTESVFNK					Mascot
1615.8724	1615.7502	-0.1222	-76	635	650	SGASVGIDDMVIPVKK					Mascot
1615.8724	1615.7502	-0.1222	-76	635	650	SGASVGIDDMVIPVKK					Mascot
1652.9343	1652.7822	-0.1521	-92	418	431	EHPVLLNRAPTLHR					Mascot
1652.9343	1652.7822	-0.1521	-92	418	431	EHPVLLNRAPTLHR					Mascot
1685.8026	1685.7437	-0.0589	-35	61	74	IFGPVKDYECLCGK			Carbamidomethyl (C)[10,12]		Mascot
1711.8975	1711.9052	0.0077	4	1124	1140	VPQESGGTKDITGGLPR					Mascot
1714.8905	1714.8019	-0.0886	-52	298	312	MLQEAIDALLDNGRR					Mascot
1729.9232	1729.8262	-0.097	-56	1133	1148	DITGGLPRVADLFEAR					Mascot
1744.8245	1744.77	-0.0545	-31	696	710	AMMENLSTESVFNKK			Oxidation (M)[2]		Mascot
1774.8251	1774.8265	0.0014	1	716	731	QISFNSIFMMADSGAR					Mascot
1790.8201	1790.8346	0.0145	8	716	731	QISFNSIFMMADSGAR			Oxidation (M)[9]		Mascot
1800.8036	1800.8634	0.0598	33	1269	1284	SGNSEFLDGEQVEFSR					Mascot
1976.0197	1975.9631	-0.0566	-29	1207	1224	GDVISDGPESPHDILRLR					Mascot
1991.0042	1990.9355	-0.0687	-35	1175	1192	LIITPVDGSDAYEEMIPK					Mascot
2041.9825	2041.9915	0.009	4	1269	1286	SGNSEFLDGEQVEFSRIK					Mascot
2070.1458	2070.0942	-0.0516	-25	372	388	MALELFKPFIYGKLEVR			Oxidation (M)[1]		Mascot
2199.1438	2199.0103	-0.1335	-61	912	933	GEAIGVIAAQSIGEPGTQL TMR					Mascot
2751.366	2751.3047	-0.0613	-22	1080	1104	IVDRDGNDVLISGTEMPA QYFLPGK			Oxidation (M)[16]		Mascot
2764.375	2764.3406	-0.0344	-12	134	156	DIERVLYFESYVVIETGMT NLEK			Oxidation (M)[18]		Mascot

9	Protein bicaudal D homolog 2 OS=Homo sapiens GN=BICD2 PE=1 SV=1	BICD2_HUMAN	93704.1	5.35	17	52	0	20.776

Peptide Information

Calc. Mass	Obsrv. Mass	± da	± ppm	Start Seq.	End Seq.	Sequence	Ion Score	C. I. %	Modification	Rank	Result Type
1016.5734	1016.5394	-0.034	-33	390	398	LTENLSALR					Mascot
1016.5734	1016.5394	-0.034	-33	390	398	LTENLSALR	2	0			Mascot
1215.6077	1215.7251	0.1174	97	567	578	TSPGGRTSPEAR					Mascot
1326.6578	1326.6444	-0.0134	-10	720	730	AMVTETMMKLR			Oxidation (M)[2]		Mascot
1326.6578	1326.6444	-0.0134	-10	720	730	AMVTETMMKLR	3	0	Oxidation (M)[2]		Mascot
1352.6298	1352.7415	0.1117	83	350	359	QQLMQMEREK			Oxidation (M)[4,6]		Mascot
1387.7039	1387.6582	-0.0457	-33	13	23	LVMEAQPEWLR			Oxidation (M)[3]		Mascot
1622.7517	1622.782	0.0303	19	458	471	STHEAREAQHAEEK					Mascot

1651.8762	1651.7631	-0.1131	-68	796	808	LELLELDHEQTRR					Mascot
1698.881	1698.8062	-0.0748	-44	200	213	QNQVEFEGLKHEIK					Mascot
1700.7255	1700.7594	0.0339	20	754	766	CDEYITQLDEMQR			Carbamidomethyl (C)[1]		Mascot
1716.7205	1716.743	0.0225	13	754	766	CDEYITQLDEMQR			Carbamidomethyl (C)[1], Oxidation (M)[11]		Mascot
1716.7205	1716.743	0.0225	13	754	766	CDEYITQLDEMQR			Carbamidomethyl (C)[1], Oxidation (M)[11]		Mascot
1752.7489	1752.8329	0.084	48	715	728	YENEKAMVTETMMK			Oxidation (M)[7,12,13]		Mascot
1773.8589	1773.7906	-0.0683	-39	738	753	EDAATFSSLRAMFATR					Mascot
1790.9535	1790.8346	-0.1189	-66	39	54	EKIQAAEYGLAVLEEK					Mascot
1801.9152	1801.886	-0.0292	-16	127	141	QLRNVLTNTQSENER					Mascot
2733.4304	2733.2993	-0.1311	-48	177	199	LLQDYSELEEENISLQKQ VSVLR					Mascot
2751.2751	2751.3047	0.0296	11	1	23	MSAPSEEEEYARLVMEA QPEWLR					Mascot
2764.4978	2764.3406	-0.1572	-57	322	347	KEGLAPPSPSLVSDLLSE LNISEIQK					Mascot
2767.2703	2767.2705	0.0002	0	1	23	MSAPSEEEEYARLVMEA QPEWLR			Oxidation (M)[1]		Mascot
2810.2209	2810.3384	0.1175	42	415	438	DRDSHEDGDYYEVDING PEILACK			Carbamidomethyl (C)[23]		Mascot

10	Unconventional myosin-X OS=Homo sapiens GN=MYO10 PE=1 SV=3	MYO10_HUMAN	239193.7	5.85	27	52	0	13.303

Peptide Information

Calc. Mass	Obsrv. Mass	± da	± ppm	Start Seq.	End Seq.	Sequence	Ion Score	C. I. %	Modification	Rank	Result Type
947.5309	947.4756	-0.0553	-58	539	547	VAVNNFGVK					Mascot
1048.5356	1048.5602	0.0246	23	1186	1194	GGLMNSWKR					Mascot
1221.5787	1221.5663	-0.0124	-10	36	45	TDYGQVFTYK					Mascot
1264.5951	1264.5359	-0.0592	-47	595	605	NNQDTLKCGSK			Carbamidomethyl (C)[8]		Mascot
1382.6191	1382.7256	0.1065	77	1931	1942	FQGMNQEQAMAK					Mascot
1572.7805	1572.7811	0.0006	0	1460	1472	ETGYWNVTVYGRK					Mascot
1645.7778	1645.7837	0.0059	4	1246	1258	LMYFENDSEEKLK					Mascot
1652.8313	1652.7822	-0.0491	-30	1604	1616	DELYCQLIKQTNK			Carbamidomethyl (C)[5]		Mascot
1652.8313	1652.7822	-0.0491	-30	1604	1616	DELYCQLIKQTNK			Carbamidomethyl (C)[5]		Mascot
1674.9174	1674.7816	-0.1358	-81	233	247	GNIQGGRIVDYLLEK					Mascot
1677.8517	1677.7592	-0.0925	-55	196	211	AILESSPIMEAFGNAK					Mascot
1680.9167	1680.7998	-0.1169	-70	1500	1514	APIDTPTQQLIQDIK					Mascot
1684.7788	1684.7556	-0.0232	-14	1740	1753	NMFALFEYNGHVDK					Mascot
1685.7913	1685.7437	-0.0476	-28	148	163	HDNQCILISGESGAGK			Carbamidomethyl (C)[5]		Mascot
1700.7738	1700.7594	-0.0144	-8	1740	1753	NMFALFEYNGHVDK			Oxidation (M)[2]		Mascot
1773.8953	1773.7906	-0.1047	-59	644	658	MPDQFDQAVVLNQLR					Mascot

1774.8436	1774.8265	-0.0171	-10	581	594	FDFIYDLFEHVSSR		Mascot
1790.9324	1790.8346	-0.0978	-55	1768	1783	FEKLAATSEVGDLPWK		Mascot
1801.7302	1801.886	0.1558	86	1698	1713	QEMTSTVYCHGGGSCK	Carbamidomethyl (C)[9,15]	Mascot
1817.7252	1817.8453	0.1201	66	1698	1713	QEMTSTVYCHGGGSCK	Carbamidomethyl (C)[9,15], Oxidation (M)[3]	Mascot
1841.8923	1841.8951	0.0028	2	147	163	RHDNQCILISGESGAGK	Carbamidomethyl (C)[6]	Mascot
2011.9828	2011.9863	0.0035	2	308	324	EVITAMDVMQFSKEEVR		Mascot
2027.9777	2028.019	0.0413	20	308	324	EVITAMDVMQFSKEEVR	Oxidation (M)[6]	Mascot
2042.0764	2041.9915	-0.0849	-42	358	376	SAELLGLDPTQLTDALTQ R		Mascot
2070.0042	2070.0942	0.09	43	1784	1799	FYFKLYCFLDTDNVPK	Carbamidomethyl (C)[7]	Mascot
2118.0181	2118.0449	0.0268	13	1931	1948	FQGMNQEQAMAKYMALI K	Oxidation (M)[4]	Mascot
2765.5137	2765.2793	-0.2344	-85	1535	1558	YTHHPLHSPLLPLPYGDI NLNLLK		Mascot
2767.4875	2767.2705	-0.217	-78	499	523	KLGLLALINEESHFPQAT DSTLLEK		Mascot
2773.3945	2773.3901	-0.0044	-2	636	658	CIKPNMQKMPDQFDQAV VLNQLR	Carbamidomethyl (C)[1]	Mascot
2797.3098	2797.3223	0.0125	4	937	959	AAQEFLESLNFDEIDECV RNIER	Carbamidomethyl (C)[17]	Mascot
2810.3965	2810.3384	-0.0581	-21	644	667	MPDQFDQAVVLNQLRYS GMLETVR		Mascot
2842.3862	2842.3542	-0.032	-11	644	667	MPDQFDQAVVLNQLRYS GMLETVR	Oxidation (M)[1,19]	Mascot

Gel Idx/Pos	255/K7	Instr./Gel Origin	BA2151/Sample Project 20140814	Process Status	Analysis Succeeded
Plate [#] Name	[1] Sample Project 20140814	Instrument Sample Name		Spectra	11

Rank	Protein Name	Accession No.	Protein MW	Protein PI	Pep. Count	Protein Score	Protein Score C. I. %	Intensity Matched	Total Ion Score	Total Ion C. I. %	Confirmed
1	Eukaryotic initiation factor 4A OS=Zea mays PE=2 SV=1	IF4A_MAIZE	46848.9	5.38	23	1,120	100	53.869	972	100	

Peptide Information

Calc. Mass	Obsrv. Mass	± da	± ppm	Start Seq.	End Seq.	Sequence	Ion Score	C. I. %	Modification	Rank	Result Type
907.4818	907.4484	-0.0334	-37	231	237	FMNKPVR			Oxidation (M)[2]		Mascot
935.5197	935.483	-0.0367	-39	127	135	ALGDYLGVK					Mascot
952.5033	952.4671	-0.0362	-38	166	172	VFDMLRR			Oxidation (M)[4]		Mascot
976.5574	976.5386	-0.0188	-19	374	382	GVAINFVTR					Mascot
1035.5768	1035.5374	-0.0394	-38	230	237	KFMNKPVR			Oxidation (M)[3]		Mascot
1070.5953	1070.5756	-0.0197	-18	173	181	QSLRPDNIK					Mascot
1104.6525	1104.6372	-0.0153	-14	373	382	KGVAINFVTR					Mascot
1104.6525	1104.6372	-0.0153	-14	373	382	KGVAINFVTR	75	99.997			Mascot
1114.6831	1114.6646	-0.0185	-17	329	338	VLITTDLLAR					Mascot
1114.6831	1114.6646	-0.0185	-17	329	338	VLITTDLLAR	74	99.996			Mascot
1142.5736	1142.5586	-0.015	-13	136	146	VHACVGGTSVR			Carbamidomethyl (C)[4]		Mascot
1142.5736	1142.5586	-0.015	-13	136	146	VHACVGGTSVR	71	99.993	Carbamidomethyl (C)[4]		Mascot
1173.6475	1173.6292	-0.0183	-16	242	251	RDELTLEGIK					Mascot
1226.6964	1226.6692	-0.0272	-22	172	181	RQSLRPDNIK					Mascot
1401.7333	1401.6835	-0.0498	-36	74	87	GLDVIQQAQSGTGK					Mascot
1461.8538	1461.8395	-0.0143	-10	151	165	ILASGVHVVVGTPGR					Mascot
1461.8538	1461.8395	-0.0143	-10	151	165	ILASGVHVVVGTPGR	102	100			Mascot
1571.708	1571.6785	-0.0295	-19	182	194	MFVLDEADEMLSR			Oxidation (M)[1]		Mascot
1579.8367	1579.7844	-0.0523	-33	198	210	DQIYDIFQLLPSK					Mascot
1587.703	1587.6549	-0.0481	-30	182	194	MFVLDEADEMLSR			Oxidation (M)[1,10]		Mascot
1587.703	1587.6549	-0.0481	-30	182	194	MFVLDEADEMLSR	26	0	Oxidation (M)[1,10]		Mascot
1800.7566	1800.7279	-0.0287	-16	300	315	DHTVSATHGDMDQNTR			Oxidation (M)[11]		Mascot
1827.9388	1827.933	-0.0058	-3	51	66	GIYAYGFEKPSAIQQR					Mascot
1827.9388	1827.933	-0.0058	-3	51	66	GIYAYGFEKPSAIQQR	138	100			Mascot
1912.0215	1911.9354	-0.0861	-45	195	210	GFKDQIYDIFQLLPSK					Mascot
2059.0754	2059.0776	0.0022	1	127	146	ALGDYLGVKVHACVGGT SVR			Carbamidomethyl (C)[13]		Mascot

2075.0842	2075.0544	-0.0298	-14	211	229	IQVGVFSATMPPEALEIT R			Oxidation (M)[10]		Mascot
2075.0842	2075.0544	-0.0298	-14	211	229	IQVGVFSATMPPEALEIT R	65	99.973	Oxidation (M)[10]		Mascot
2685.3804	2685.3704	-0.01	-4	264	286	LDTLCDLYETLAITQSVIF VNTR			Carbamidomethyl (C)[5]		Mascot
2911.4949	2911.4919	-0.003	-1	339	363	GIDVQQVSLVINYDLPTQ PENYLHR					Mascot
2911.4949	2911.4919	-0.003	-1	339	363	GIDVQQVSLVINYDLPTQ PENYLHR	183	100			Mascot
3124.5806	3124.575	-0.0056	-2	88	115	TATFCSGILQQLDYGLVE CQALVLAPTR			Carbamidomethyl (C)[5,19]		Mascot
3124.5806	3124.575	-0.0056	-2	88	115	TATFCSGILQQLDYGLVE CQALVLAPTR	237	100	Carbamidomethyl (C)[5,19]		Mascot

2	Eukaryotic initiation factor 4A-1 OS=Oryza sativa subsp. japonica GN=Os06g0701100 PE=2 SV=2	IF4A1_ORYSJ	47343.1	5.37	21	1,070	100	52.832	951	100	

Peptide Information

Calc. Mass	Obsrv. Mass	± da	± ppm	Start Seq.	End Seq.	Sequence	Ion Score	C. I. %	Modification	Rank	Result Type
907.4818	907.4484	-0.0334	-37	235	241	FMNKPVR			Oxidation (M)[2]		Mascot
935.5197	935.483	-0.0367	-39	131	139	ALGDYLGVK					Mascot
952.5033	952.4671	-0.0362	-38	170	176	VFDMLRR			Oxidation (M)[4]		Mascot
976.5574	976.5386	-0.0188	-19	378	386	GVAINFVTR					Mascot
1035.5768	1035.5374	-0.0394	-38	234	241	KFMNKPVR			Oxidation (M)[3]		Mascot
1104.6525	1104.6372	-0.0153	-14	377	386	KGVAINFVTR					Mascot
1104.6525	1104.6372	-0.0153	-14	377	386	KGVAINFVTR	75	99.997			Mascot
1114.6831	1114.6646	-0.0185	-17	333	342	VLITTDLLAR					Mascot
1114.6831	1114.6646	-0.0185	-17	333	342	VLITTDLLAR	74	99.996			Mascot
1142.5736	1142.5586	-0.015	-13	140	150	VHACVGGTSVR			Carbamidomethyl (C)[4]		Mascot
1142.5736	1142.5586	-0.015	-13	140	150	VHACVGGTSVR	71	99.993	Carbamidomethyl (C)[4]		Mascot
1173.6475	1173.6292	-0.0183	-16	246	255	RDELTLEGIK					Mascot
1401.7333	1401.6835	-0.0498	-36	78	91	GLDVIQQAQSGTGK					Mascot
1461.8538	1461.8395	-0.0143	-10	155	169	ILASGVHVVVGTPGR					Mascot
1461.8538	1461.8395	-0.0143	-10	155	169	ILASGVHVVVGTPGR	102	100			Mascot
1571.708	1571.6785	-0.0295	-19	186	198	MFVLDEADEMLSR			Oxidation (M)[1]		Mascot
1579.8367	1579.7844	-0.0523	-33	202	214	DQIYDIFQLLPSK					Mascot
1587.703	1587.6549	-0.0481	-30	186	198	MFVLDEADEMLSR			Oxidation (M)[1,10]		Mascot
1587.703	1587.6549	-0.0481	-30	186	198	MFVLDEADEMLSR	26	0	Oxidation (M)[1,10]		Mascot
1800.7566	1800.7279	-0.0287	-16	304	319	DHTVSATHGDMDQNTR			Oxidation (M)[11]		Mascot
1827.9388	1827.933	-0.0058	-3	55	70	GIYAYGFEKPSAIQQR					Mascot
1827.9388	1827.933	-0.0058	-3	55	70	GIYAYGFEKPSAIQQR	138	100			Mascot
1912.0215	1911.9354	-0.0861	-45	199	214	GFKDQIYDIFQLLPSK					Mascot

2059.0754	2059.0776	0.0022	1	131	150	ALGDYLGVKVHACVGGT SVR			Carbamidomethyl (C)[13]		Mascot
2075.0842	2075.0544	-0.0298	-14	215	233	IQVGVFSATMPPEALEIT R			Oxidation (M)[10]		Mascot
2075.0842	2075.0544	-0.0298	-14	215	233	IQVGVFSATMPPEALEIT R	65	99.973	Oxidation (M)[10]		Mascot
2685.3804	2685.3704	-0.01	-4	268	290	LDTLCDLYETLAITQSVIF VNTR			Carbamidomethyl (C)[5]		Mascot
2911.4949	2911.4919	-0.003	-1	343	367	GIDVQQVSLVINYDLPTQ PENYLHR					Mascot
2911.4949	2911.4919	-0.003	-1	343	367	GIDVQQVSLVINYDLPTQ PENYLHR	183	100			Mascot
3124.5806	3124.575	-0.0056	-2	92	119	TATFCSGILQQLDYAVVE CQALVLAPTR			Carbamidomethyl (C)[5,19]		Mascot
3124.5806	3124.575	-0.0056	-2	92	119	TATFCSGILQQLDYAVVE CQALVLAPTR	216	100	Carbamidomethyl (C)[5,19]		Mascot

3	Eukaryotic initiation factor 4A OS=Triticum aestivum PE=2 SV=1	IF4A_WHEAT	47183.1	5.31	20	898	100	50.013	790	100	

Peptide Information

Calc. Mass	Obsrv. Mass	± da	± ppm	Start Seq.	End Seq.	Sequence	Ion Score	C. I. %	Modification	Rank	Result Type
907.4818	907.4484	-0.0334	-37	235	241	FMNKPVR			Oxidation (M)[2]		Mascot
910.4702	910.4323	-0.0379	-42	391	397	MLFDIQK			Oxidation (M)[1]		Mascot
935.5197	935.483	-0.0367	-39	131	139	ALGDYLGVK					Mascot
976.5574	976.5386	-0.0188	-19	378	386	GVAINFVTR					Mascot
1035.5768	1035.5374	-0.0394	-38	234	241	KFMNKPVR			Oxidation (M)[3]		Mascot
1070.5953	1070.5756	-0.0197	-18	177	185	QSLRPDNIK					Mascot
1104.6525	1104.6372	-0.0153	-14	377	386	KGVAINFVTR					Mascot
1104.6525	1104.6372	-0.0153	-14	377	386	KGVAINFVTR	75	99.997			Mascot
1114.6831	1114.6646	-0.0185	-17	333	342	VLITTDLLAR					Mascot
1114.6831	1114.6646	-0.0185	-17	333	342	VLITTDLLAR	74	99.996			Mascot
1142.5736	1142.5586	-0.015	-13	140	150	VHACVGGTSVR			Carbamidomethyl (C)[4]		Mascot
1142.5736	1142.5586	-0.015	-13	140	150	VHACVGGTSVR	71	99.993	Carbamidomethyl (C)[4]		Mascot
1173.6475	1173.6292	-0.0183	-16	246	255	RDELTLEGIK					Mascot
1226.6964	1226.6692	-0.0272	-22	176	185	RQSLRPDNIK					Mascot
1401.7333	1401.6835	-0.0498	-36	78	91	GLDVIQQAQSGTGK					Mascot
1461.8538	1461.8395	-0.0143	-10	155	169	ILASGVHVVVGTPGR					Mascot
1461.8538	1461.8395	-0.0143	-10	155	169	ILASGVHVVVGTPGR	102	100			Mascot
1571.708	1571.6785	-0.0295	-19	186	198	MFVLDEADEMLSR			Oxidation (M)[1]		Mascot
1587.703	1587.6549	-0.0481	-30	186	198	MFVLDEADEMLSR			Oxidation (M)[1,10]		Mascot
1587.703	1587.6549	-0.0481	-30	186	198	MFVLDEADEMLSR	26	0	Oxidation (M)[1,10]		Mascot
1800.7566	1800.7279	-0.0287	-16	304	319	DHTVSATHGDMDQNTR			Oxidation (M)[11]		Mascot

1827.9388	1827.933	-0.0058	-3	55	70	GIYAYGFEKPSAIQQR					Mascot
1827.9388	1827.933	-0.0058	-3	55	70	GIYAYGFEKPSAIQQR	138	100			Mascot
2059.0754	2059.0776	0.0022	1	131	150	ALGDYLGVKVHACVGGT SVR			Carbamidomethyl (C)[13]		Mascot
2075.0842	2075.0544	-0.0298	-14	215	233	IQVGVFSATMPPEALEIT R			Oxidation (M)[10]		Mascot
2075.0842	2075.0544	-0.0298	-14	215	233	IQVGVFSATMPPEALEIT R	65	99.973	Oxidation (M)[10]		Mascot
2685.3804	2685.3704	-0.01	-4	268	290	LDTLCDLYETLAITQSVIF VNTR			Carbamidomethyl (C)[5]		Mascot
3124.5806	3124.575	-0.0056	-2	92	119	TATFCSGILQQLDYGLVE CQALVLAPTR			Carbamidomethyl (C)[5,19]		Mascot
3124.5806	3124.575	-0.0056	-2	92	119	TATFCSGILQQLDYGLVE CQALVLAPTR	237	100	Carbamidomethyl (C)[5,19]		Mascot

4 Eukaryotic initiation factor 4A-3 OS=Oryza sativa subsp. japonica GN=Os02g0146600 PE=2 SV=1 IF4A3_ORYSJ 47393.1 5.43 18 826 100 50.441 735 100

Peptide Information

Calc. Mass	Obsrv. Mass	± da	± ppm	Start Seq.	End Seq.	Sequence	Ion Score	C. I. %	Modification	Rank	Result Type
907.4818	907.4484	-0.0334	-37	235	241	FMNKPVR			Oxidation (M)[2]		Mascot
935.5197	935.483	-0.0367	-39	131	139	ALGDYLGVK					Mascot
952.5033	952.4671	-0.0362	-38	170	176	VFDMLRR			Oxidation (M)[4]		Mascot
976.5574	976.5386	-0.0188	-19	378	386	GVAINFVTR					Mascot
1035.5768	1035.5374	-0.0394	-38	234	241	KFMNKPVR			Oxidation (M)[3]		Mascot
1104.6525	1104.6372	-0.0153	-14	377	386	KGVAINFVTR					Mascot
1104.6525	1104.6372	-0.0153	-14	377	386	KGVAINFVTR	75	99.997			Mascot
1114.6831	1114.6646	-0.0185	-17	333	342	VLITTDLLAR					Mascot
1114.6831	1114.6646	-0.0185	-17	333	342	VLITTDLLAR	74	99.996			Mascot
1142.5736	1142.5586	-0.015	-13	140	150	VHACVGGTSVR			Carbamidomethyl (C)[4]		Mascot
1142.5736	1142.5586	-0.015	-13	140	150	VHACVGGTSVR	71	99.993	Carbamidomethyl (C)[4]		Mascot
1173.6475	1173.6292	-0.0183	-16	246	255	RDELTLEGIK					Mascot
1401.7333	1401.6835	-0.0498	-36	78	91	GLDVIQQAQSGTGK					Mascot
1461.8538	1461.8395	-0.0143	-10	155	169	ILASGVHVVVGTPGR					Mascot
1461.8538	1461.8395	-0.0143	-10	155	169	ILASGVHVVVGTPGR	102	100			Mascot
1571.708	1571.6785	-0.0295	-19	186	198	MFVLDEADEMLSR			Oxidation (M)[1]		Mascot
1587.703	1587.6549	-0.0481	-30	186	198	MFVLDEADEMLSR			Oxidation (M)[1,10]		Mascot
1587.703	1587.6549	-0.0481	-30	186	198	MFVLDEADEMLSR	26	0	Oxidation (M)[1,10]		Mascot
1800.7566	1800.7279	-0.0287	-16	304	319	DHTVSATHGDMDQNTR			Oxidation (M)[11]		Mascot
1827.9388	1827.933	-0.0058	-3	55	70	GIYAYGFEKPSAIQQR					Mascot
1827.9388	1827.933	-0.0058	-3	55	70	GIYAYGFEKPSAIQQR	138	100			Mascot
2059.0754	2059.0776	0.0022	1	131	150	ALGDYLGVKVHACVGGT			Carbamidomethyl (C)[13]		Mascot

						SVR					
2075.0842	2075.0544	-0.0298	-14	215	233	IQVGVFSATMPPEALEIT R			Oxidation (M)[10]		Mascot
2075.0842	2075.0544	-0.0298	-14	215	233	IQVGVFSATMPPEALEIT R	65	99.973	Oxidation (M)[10]		Mascot
2685.3804	2685.3704	-0.01	-4	268	290	LDTLCDLYETLAITQSVIF VNTR			Carbamidomethyl (C)[5]		Mascot
2911.4949	2911.4919	-0.003	-1	343	367	GIDVQQVSLVINYDLPTQ PENYLHR					Mascot
2911.4949	2911.4919	-0.003	-1	343	367	GIDVQQVSLVINYDLPTQ PENYLHR	183	100			Mascot

5	Eukaryotic initiation factor 4A-11 OS=Nicotiana tabacum PE=1 SV=1	IF411_TOBAC	47157.1	5.38	18	647	100	44.197	557	100	

Peptide Information

Calc. Mass	Obsrv. Mass	± da	± ppm	Start Seq.	End Seq.	Sequence	Ion Score	C. I. %	Modification	Rank	Result Type
907.4818	907.4484	-0.0334	-37	234	240	FMNKPVR			Oxidation (M)[2]		Mascot
910.4702	910.4323	-0.0379	-42	390	396	MLFDIQK			Oxidation (M)[1]		Mascot
935.5197	935.483	-0.0367	-39	130	138	ALGDYLGVK					Mascot
952.5033	952.4671	-0.0362	-38	169	175	VFDMLRR			Oxidation (M)[4]		Mascot
1035.5768	1035.5374	-0.0394	-38	233	240	KFMNKPVR			Oxidation (M)[3]		Mascot
1070.5953	1070.5756	-0.0197	-18	176	184	QSLRPDNIK					Mascot
1114.6831	1114.6646	-0.0185	-17	332	341	VLITTDLLAR					Mascot
1114.6831	1114.6646	-0.0185	-17	332	341	VLITTDLLAR	74	99.996			Mascot
1142.5736	1142.5586	-0.015	-13	139	149	VHACVGGTSVR			Carbamidomethyl (C)[4]		Mascot
1142.5736	1142.5586	-0.015	-13	139	149	VHACVGGTSVR	71	99.993	Carbamidomethyl (C)[4]		Mascot
1173.6475	1173.6292	-0.0183	-16	245	254	RDELTLEGIK					Mascot
1226.6964	1226.6692	-0.0272	-22	175	184	RQSLRPDNIK					Mascot
1401.7333	1401.6835	-0.0498	-36	77	90	GLDVIQQAQSGTGK					Mascot
1571.708	1571.6785	-0.0295	-19	185	197	MFVLDEADEMLSR			Oxidation (M)[1]		Mascot
1587.703	1587.6549	-0.0481	-30	185	197	MFVLDEADEMLSR			Oxidation (M)[1,10]		Mascot
1587.703	1587.6549	-0.0481	-30	185	197	MFVLDEADEMLSR	26	0	Oxidation (M)[1,10]		Mascot
1800.7566	1800.7279	-0.0287	-16	303	318	DHTVSATHGDMDQNTR			Oxidation (M)[11]		Mascot
1827.9388	1827.933	-0.0058	-3	54	69	GIYAYGFEKPSAIQQR					Mascot
1827.9388	1827.933	-0.0058	-3	54	69	GIYAYGFEKPSAIQQR	138	100			Mascot
2011.0383	2011.105	0.0667	33	246	262	DELTLEGIKQFYVNVDK					Mascot
2011.0383	2011.105	0.0667	33	246	262	DELTLEGIKQFYVNVDK					Mascot
2059.0754	2059.0776	0.0022	1	130	149	ALGDYLGVKVHACVGGT SVR			Carbamidomethyl (C)[13]		Mascot
2075.0842	2075.0544	-0.0298	-14	214	232	IQVGVFSATMPPEALEIT R			Oxidation (M)[10]		Mascot
2075.0842	2075.0544	-0.0298	-14	214	232	IQVGVFSATMPPEALEIT R	65	99.973	Oxidation (M)[10]		Mascot

2911.4949	2911.4919	-0.003	-1	342	366	GIDVQQVSLVINYDLPTQPENYLHR					Mascot
2911.4949	2911.4919	-0.003	-1	342	366	GIDVQQVSLVINYDLPTQPENYLHR	183	100			Mascot

6	Eukaryotic initiation factor 4A-2 OS=Nicotiana plumbaginifolia PE=1 SV=1	IF4A2_NICPL	47084	5.38	17	640	100	43.259	557	100	

Peptide Information

Calc. Mass	Obsrv. Mass	± da	± ppm	Start Seq.	End Seq.	Sequence	Ion Score	C. I. %	Modification	Rank	Result Type
907.4818	907.4484	-0.0334	-37	234	240	FMNKPVR			Oxidation (M)[2]		Mascot
910.4702	910.4323	-0.0379	-42	390	396	MLFDIQK			Oxidation (M)[1]		Mascot
935.5197	935.483	-0.0367	-39	130	138	ALGDYLGVK					Mascot
952.5033	952.4671	-0.0362	-38	169	175	VFDMLRR			Oxidation (M)[4]		Mascot
1016.6099	1016.5222	-0.0877	-86	376	385	KGVAINSVTK					Mascot
1035.5768	1035.5374	-0.0394	-38	233	240	KFMNKPVR			Oxidation (M)[3]		Mascot
1114.6831	1114.6646	-0.0185	-17	332	341	VLITTDLLAR					Mascot
1114.6831	1114.6646	-0.0185	-17	332	341	VLITTDLLAR	74	99.996			Mascot
1142.5736	1142.5586	-0.015	-13	139	149	VHACVGGTSVR			Carbamidomethyl (C)[4]		Mascot
1142.5736	1142.5586	-0.015	-13	139	149	VHACVGGTSVR	71	99.993	Carbamidomethyl (C)[4]		Mascot
1173.6475	1173.6292	-0.0183	-16	245	254	RDELTLEGIK					Mascot
1401.7333	1401.6835	-0.0498	-36	77	90	GLDVIQQAQSGTGK					Mascot
1571.708	1571.6785	-0.0295	-19	185	197	MFVLDEADEMLSR			Oxidation (M)[1]		Mascot
1587.703	1587.6549	-0.0481	-30	185	197	MFVLDEADEMLSR			Oxidation (M)[1,10]		Mascot
1587.703	1587.6549	-0.0481	-30	185	197	MFVLDEADEMLSR	26	0	Oxidation (M)[1,10]		Mascot
1800.7566	1800.7279	-0.0287	-16	303	318	DHTVSATHGDMDQNTR			Oxidation (M)[11]		Mascot
1827.9388	1827.933	-0.0058	-3	54	69	GIYAYGFEKPSAIQQR					Mascot
1827.9388	1827.933	-0.0058	-3	54	69	GIYAYGFEKPSAIQQR	138	100			Mascot
2011.0383	2011.105	0.0667	33	246	262	DELTLEGIKQFYVNVDK					Mascot
2011.0383	2011.105	0.0667	33	246	262	DELTLEGIKQFYVNVDK					Mascot
2059.0754	2059.0776	0.0022	1	130	149	ALGDYLGVKVHACVGGTSVR			Carbamidomethyl (C)[13]		Mascot
2075.0842	2075.0544	-0.0298	-14	214	232	IQVGVFSATMPPEALEITR			Oxidation (M)[10]		Mascot
2075.0842	2075.0544	-0.0298	-14	214	232	IQVGVFSATMPPEALEITR	65	99.973	Oxidation (M)[10]		Mascot
2911.4949	2911.4919	-0.003	-1	342	366	GIDVQQVSLVINYDLPTQPENYLHR					Mascot
2911.4949	2911.4919	-0.003	-1	342	366	GIDVQQVSLVINYDLPTQPENYLHR	183	100			Mascot

7	Eukaryotic initiation factor 4A-9 OS=Nicotiana tabacum PE=2 SV=1	IF4A9_TOBAC	47081	5.54	16	633	100	43.033	557	100	

Peptide Information

Calc. Mass	Obsrv. Mass	± da	± ppm	Start Seq.	End Seq.	Sequence	Ion Score	C. I. %	Modification	Rank	Result Type
907.4818	907.4484	-0.0334	-37	234	240	FMNKPVR			Oxidation (M)[2]		Mascot
935.5197	935.483	-0.0367	-39	130	138	ALGDYLGVK					Mascot
952.5033	952.4671	-0.0362	-38	169	175	VFDMLRR			Oxidation (M)[4]		Mascot
1035.5768	1035.5374	-0.0394	-38	233	240	KFMNKPVR			Oxidation (M)[3]		Mascot
1114.6831	1114.6646	-0.0185	-17	332	341	VLITTDLLAR					Mascot
1114.6831	1114.6646	-0.0185	-17	332	341	VLITTDLLAR	74	99.996			Mascot
1142.5736	1142.5586	-0.015	-13	139	149	VHACVGGTSVR			Carbamidomethyl (C)[4]		Mascot
1142.5736	1142.5586	-0.015	-13	139	149	VHACVGGTSVR	71	99.993	Carbamidomethyl (C)[4]		Mascot
1173.6475	1173.6292	-0.0183	-16	245	254	RDELTLEGIK					Mascot
1401.7333	1401.6835	-0.0498	-36	77	90	GLDVIQQAQSGTGK					Mascot
1477.8486	1477.7294	-0.1192	-81	154	168	ILSSGVHVVVGTPGR					Mascot
1571.708	1571.6785	-0.0295	-19	185	197	MFVLDEADEMLSR			Oxidation (M)[1]		Mascot
1587.703	1587.6549	-0.0481	-30	185	197	MFVLDEADEMLSR			Oxidation (M)[1,10]		Mascot
1587.703	1587.6549	-0.0481	-30	185	197	MFVLDEADEMLSR	26	0	Oxidation (M)[1,10]		Mascot
1800.7566	1800.7279	-0.0287	-16	303	318	DHTVSATHGDMDQNTR			Oxidation (M)[11]		Mascot
1827.9388	1827.933	-0.0058	-3	54	69	GIYAYGFEKPSAIQQR					Mascot
1827.9388	1827.933	-0.0058	-3	54	69	GIYAYGFEKPSAIQQR	138	100			Mascot
2011.0383	2011.105	0.0667	33	246	262	DELTLEGIKQFYVNVDK					Mascot
2011.0383	2011.105	0.0667	33	246	262	DELTLEGIKQFYVNVDK					Mascot
2059.0754	2059.0776	0.0022	1	130	149	ALGDYLGVKVHACVGGT SVR			Carbamidomethyl (C)[13]		Mascot
2075.0842	2075.0544	-0.0298	-14	214	232	IQVGVFSATMPPEALEIT R			Oxidation (M)[10]		Mascot
2075.0842	2075.0544	-0.0298	-14	214	232	IQVGVFSATMPPEALEIT R	65	99.973	Oxidation (M)[10]		Mascot
2911.4949	2911.4919	-0.003	-1	342	366	GIDVQQVSLVINYDLPTQ PENYLHR					Mascot
2911.4949	2911.4919	-0.003	-1	342	366	GIDVQQVSLVINYDLPTQ PENYLHR	183	100			Mascot

8	Eukaryotic initiation factor 4A-7 OS=Nicotiana tabacum PE=2 SV=1	IF4A7_TOBAC	47152.1	5.37	13	612	100	39.162	557	100	

Protein Group

Eukaryotic initiation factor 4A-10 OS=Nicotiana tabacum PE=1 SV=1	IF410_TOBAC	47098.1	5.3699 998855 5908

Peptide Information

Calc. Mass	Obsrv. Mass	± da	± ppm	Start Seq.	End Seq.	Sequence	Ion Score	C. I. %	Modification	Rank	Result Type

907.4818	907.4484	-0.0334	-37	234	240	FMNKPVR			Oxidation (M)[2]		Mascot
935.5197	935.483	-0.0367	-39	130	138	ALGDYLGVK					Mascot
952.5033	952.4671	-0.0362	-38	169	175	VFDMLRR			Oxidation (M)[4]		Mascot
1035.5768	1035.5374	-0.0394	-38	233	240	KFMNKPVR			Oxidation (M)[3]		Mascot
1114.6831	1114.6646	-0.0185	-17	332	341	VLITTDLLAR					Mascot
1114.6831	1114.6646	-0.0185	-17	332	341	VLITTDLLAR	74	99.996			Mascot
1142.5736	1142.5586	-0.015	-13	139	149	VHACVGGTSVR			Carbamidomethyl (C)[4]		Mascot
1142.5736	1142.5586	-0.015	-13	139	149	VHACVGGTSVR	71	99.993	Carbamidomethyl (C)[4]		Mascot
1401.7333	1401.6835	-0.0498	-36	77	90	GLDVIQQAQSGTGK					Mascot
1571.708	1571.6785	-0.0295	-19	185	197	MFVLDEADEMLSR			Oxidation (M)[1]		Mascot
1587.703	1587.6549	-0.0481	-30	185	197	MFVLDEADEMLSR			Oxidation (M)[1,10]		Mascot
1587.703	1587.6549	-0.0481	-30	185	197	MFVLDEADEMLSR	26	0	Oxidation (M)[1,10]		Mascot
1800.7566	1800.7279	-0.0287	-16	303	318	DHTVSATHGDMDQNTR			Oxidation (M)[11]		Mascot
1827.9388	1827.933	-0.0058	-3	54	69	GIYAYGFEKPSAIQQR					Mascot
1827.9388	1827.933	-0.0058	-3	54	69	GIYAYGFEKPSAIQQR	138	100			Mascot
2059.0754	2059.0776	0.0022	1	130	149	ALGDYLGVKVHACVGGT SVR			Carbamidomethyl (C)[13]		Mascot
2075.0842	2075.0544	-0.0298	-14	214	232	IQVGVFSATMPPEALEIT R			Oxidation (M)[10]		Mascot
2075.0842	2075.0544	-0.0298	-14	214	232	IQVGVFSATMPPEALEIT R	65	99.973	Oxidation (M)[10]		Mascot
2911.4949	2911.4919	-0.003	-1	342	366	GIDVQQVSLVINYDLPTQ PENYLHR					Mascot
2911.4949	2911.4919	-0.003	-1	342	366	GIDVQQVSLVINYDLPTQ PENYLHR	183	100			Mascot

9	Eukaryotic initiation factor 4A-14 OS=Nicotiana tabacum PE=2 SV=1	IF414_TOBAC	47131.1	5.37	13	612	100	39.022	557	100	

Peptide Information

Calc. Mass	Obsrv. Mass	± da	± ppm	Start Seq.	End Seq.	Sequence	Ion Score	C. I. %	Modification	Rank	Result Type
864.476	864.4236	-0.0524	-61	234	240	FMSKPVR					Mascot
910.4702	910.4323	-0.0379	-42	390	396	MLFDIQK			Oxidation (M)[1]		Mascot
935.5197	935.483	-0.0367	-39	130	138	ALGDYLGVK					Mascot
952.5033	952.4671	-0.0362	-38	169	175	VFDMLRR			Oxidation (M)[4]		Mascot
1114.6831	1114.6646	-0.0185	-17	332	341	VLITTDLLAR					Mascot
1114.6831	1114.6646	-0.0185	-17	332	341	VLITTDLLAR	74	99.996			Mascot
1142.5736	1142.5586	-0.015	-13	139	149	VHACVGGTSVR			Carbamidomethyl (C)[4]		Mascot
1142.5736	1142.5586	-0.015	-13	139	149	VHACVGGTSVR	71	99.993	Carbamidomethyl (C)[4]		Mascot
1401.7333	1401.6835	-0.0498	-36	77	90	GLDVIQQAQSGTGK					Mascot
1571.708	1571.6785	-0.0295	-19	185	197	MFVLDEADEMLSR			Oxidation (M)[1]		Mascot

1587.703	1587.6549	-0.0481	-30	185	197	MFVLDEADEMLSR			Oxidation (M)[1,10]		Mascot
1587.703	1587.6549	-0.0481	-30	185	197	MFVLDEADEMLSR	26	0	Oxidation (M)[1,10]		Mascot
1800.7566	1800.7279	-0.0287	-16	303	318	DHTVSATHGDMDQNTR			Oxidation (M)[11]		Mascot
1827.9388	1827.933	-0.0058	-3	54	69	GIYAYGFEKPSAIQQR					Mascot
1827.9388	1827.933	-0.0058	-3	54	69	GIYAYGFEKPSAIQQR	138	100			Mascot
2059.0754	2059.0776	0.0022	1	130	149	ALGDYLGVKVHACVGGT SVR			Carbamidomethyl (C)[13]		Mascot
2075.0842	2075.0544	-0.0298	-14	214	232	IQVGVFSATMPPEALEIT R			Oxidation (M)[10]		Mascot
2075.0842	2075.0544	-0.0298	-14	214	232	IQVGVFSATMPPEALEIT R	65	99.973	Oxidation (M)[10]		Mascot
2911.4949	2911.4919	-0.003	-1	342	366	GIDVQQVSLVINYDLPTQ PENYLHR					Mascot
2911.4949	2911.4919	-0.003	-1	342	366	GIDVQQVSLVINYDLPTQ PENYLHR	183	100			Mascot

10	Eukaryotic initiation factor 4A-8 OS=Nicotiana tabacum PE=2 SV=1	IF4A8_TOBAC	47143.1	5.57	16	563	100	38.553	492	100		

Peptide Information

Calc. Mass	Obsrv. Mass	± da	± ppm	Start Seq.	End Seq.	Sequence	Ion Score	C. I. %	Modification	Rank	Result Type
907.4818	907.4484	-0.0334	-37	234	240	FMNKPVR			Oxidation (M)[2]		Mascot
910.4702	910.4323	-0.0379	-42	390	396	MLFDIQK			Oxidation (M)[1]		Mascot
935.5197	935.483	-0.0367	-39	130	138	ALGDYLGVK					Mascot
952.5033	952.4671	-0.0362	-38	169	175	VFDMLRR			Oxidation (M)[4]		Mascot
1035.5768	1035.5374	-0.0394	-38	233	240	KFMNKPVR			Oxidation (M)[3]		Mascot
1114.6831	1114.6646	-0.0185	-17	332	341	VLITTDLLAR					Mascot
1114.6831	1114.6646	-0.0185	-17	332	341	VLITTDLLAR	74	99.996			Mascot
1142.5736	1142.5586	-0.015	-13	139	149	VHACVGGTSVR			Carbamidomethyl (C)[4]		Mascot
1142.5736	1142.5586	-0.015	-13	139	149	VHACVGGTSVR	71	99.993	Carbamidomethyl (C)[4]		Mascot
1173.6475	1173.6292	-0.0183	-16	245	254	RDELTLEGIK					Mascot
1401.7333	1401.6835	-0.0498	-36	77	90	GLDVIQQAQSGTGK					Mascot
1459.8744	1459.7487	-0.1257	-86	154	168	ILAAGVHVIVGTPGR					Mascot
1571.708	1571.6785	-0.0295	-19	185	197	MFVLDEADEMLSR			Oxidation (M)[1]		Mascot
1587.703	1587.6549	-0.0481	-30	185	197	MFVLDEADEMLSR			Oxidation (M)[1,10]		Mascot
1587.703	1587.6549	-0.0481	-30	185	197	MFVLDEADEMLSR	26	0	Oxidation (M)[1,10]		Mascot
1800.7566	1800.7279	-0.0287	-16	303	318	DHTVSATHGDMDQNTR			Oxidation (M)[11]		Mascot
1827.9388	1827.933	-0.0058	-3	54	69	GIYAYGFEKPSAIQQR					Mascot
1827.9388	1827.933	-0.0058	-3	54	69	GIYAYGFEKPSAIQQR	138	100			Mascot
2011.0383	2011.105	0.0667	33	246	262	DELTLEGIKQFYVNVDK					Mascot
2011.0383	2011.105	0.0667	33	246	262	DELTLEGIKQFYVNVDK					Mascot

2059.0754	2059.0776	0.0022	1	130	149	ALGDYLGVKVHACVGGT SVR			Carbamidomethyl (C)[13]	Mascot
2911.4949	2911.4919	-0.003	-1	342	366	GIDVQQVSLVINYDLPTQ PENYLHR				Mascot
2911.4949	2911.4919	-0.003	-1	342	366	GIDVQQVSLVINYDLPTQ PENYLHR	183	100		Mascot

Gel Idx/Pos	256/K8	Instr./Gel Origin	BA2151/Sample Project 20140814	Process Status	Analysis Succeeded
Plate [#] Name	[1] Sample Project 20140814	Instrument Sample Name		Spectra	11

Rank	Protein Name	Accession No.	Protein MW	Protein PI	Pep. Count	Protein Score	Protein Score C. I. %	Intensity Matched	Total Ion Score	Total Ion C. I. %	Confirmed
1	Eukaryotic initiation factor 4A OS=Zea mays PE=2 SV=1	IF4A_MAIZE	46848.9	5.38	21	864	100	48.263	739	100	

Peptide Information

Calc. Mass	Obsrv. Mass	± da	± ppm	Start Seq.	End Seq.	Sequence	Ion Score	C. I. %	Modification	Rank	Result Type
907.4818	907.4412	-0.0406	-45	231	237	FMNKPVR			Oxidation (M)[2]		Mascot
935.5197	935.4751	-0.0446	-48	127	135	ALGDYLGVK					Mascot
952.5033	952.4612	-0.0421	-44	166	172	VFDMLRR			Oxidation (M)[4]		Mascot
976.5574	976.5323	-0.0251	-26	374	382	GVAINFVTR					Mascot
1035.5768	1035.5188	-0.058	-56	230	237	KFMNKPVR			Oxidation (M)[3]		Mascot
1070.5953	1070.5665	-0.0288	-27	173	181	QSLRPDNIK					Mascot
1104.6525	1104.6274	-0.0251	-23	373	382	KGVAINFVTR					Mascot
1104.6525	1104.6274	-0.0251	-23	373	382	KGVAINFVTR	63	99.946			Mascot
1114.6831	1114.6573	-0.0258	-23	329	338	VLITTDLLAR					Mascot
1114.6831	1114.6573	-0.0258	-23	329	338	VLITTDLLAR	77	99.998			Mascot
1142.5736	1142.5491	-0.0245	-21	136	146	VHACVGGTSVR			Carbamidomethyl (C)[4]		Mascot
1142.5736	1142.5491	-0.0245	-21	136	146	VHACVGGTSVR	70	99.989	Carbamidomethyl (C)[4]		Mascot
1173.6475	1173.6195	-0.028	-24	242	251	RDELTLEGIK					Mascot
1226.6964	1226.662	-0.0344	-28	172	181	RQSLRPDNIK					Mascot
1401.7333	1401.6686	-0.0647	-46	74	87	GLDVIQQAQSGTGK					Mascot
1461.8538	1461.8269	-0.0269	-18	151	165	ILASGVHVVVGTPGR					Mascot
1461.8538	1461.8269	-0.0269	-18	151	165	ILASGVHVVVGTPGR	108	100			Mascot
1555.7131	1555.6875	-0.0256	-16	182	194	MFVLDEADEMLSR					Mascot
1571.708	1571.6927	-0.0153	-10	182	194	MFVLDEADEMLSR			Oxidation (M)[1]		Mascot
1587.703	1587.6492	-0.0538	-34	182	194	MFVLDEADEMLSR			Oxidation (M)[1,10]		Mascot
1827.9388	1827.9164	-0.0224	-12	51	66	GIYAYGFEKPSAIQQR					Mascot
1827.9388	1827.9164	-0.0224	-12	51	66	GIYAYGFEKPSAIQQR	141	100			Mascot
1903.8928	1903.9167	0.0239	13	182	197	MFVLDEADEMLSRGFK			Oxidation (M)[1]		Mascot
1919.8878	1919.9137	0.0259	13	182	197	MFVLDEADEMLSRGFK			Oxidation (M)[1,10]		Mascot
2059.0754	2059.063	-0.0124	-6	127	146	ALGDYLGVKVHACVGGT SVR			Carbamidomethyl (C)[13]		Mascot
2059.0894	2059.063	-0.0264	-13	211	229	IQVGVFSATMPPEALEIT R	84	100			Mascot

2075.0842	2075.033	-0.0512	-25	211	229	IQVGVFSATMPPEALEIT R			Oxidation (M)[10]		Mascot
2075.0842	2075.033	-0.0512	-25	211	229	IQVGVFSATMPPEALEIT R	66	99.971	Oxidation (M)[10]		Mascot
2685.3804	2685.3508	-0.0296	-11	264	286	LDTLCDLYETLAITQSVIF VNTR			Carbamidomethyl (C)[5]		Mascot
2911.4949	2911.4683	-0.0266	-9	339	363	GIDVQQVSLVINYDLPTQ PENYLHR					Mascot
2911.4949	2911.4683	-0.0266	-9	339	363	GIDVQQVSLVINYDLPTQ PENYLHR	197	100			Mascot
3124.5806	3124.5498	-0.0308	-10	88	115	TATFCSGILQQLDYGLVE CQALVLAPTR			Carbamidomethyl (C)[5,19]		Mascot

2	Eukaryotic initiation factor 4A-1 OS=Oryza sativa subsp. japonica GN=Os06g0701100 PE=2 SV=2	IF4A1_ORYSJ	47343.1	5.37	20	851	100	47.437	739	100

Peptide Information

Calc. Mass	Obsrv. Mass	± da	± ppm	Start Seq.	End Seq.	Sequence	Ion Score	C. I. %	Modification	Rank	Result Type
907.4818	907.4412	-0.0406	-45	235	241	FMNKPVR			Oxidation (M)[2]		Mascot
935.5197	935.4751	-0.0446	-48	131	139	ALGDYLGVK					Mascot
952.5033	952.4612	-0.0421	-44	170	176	VFDMLRR			Oxidation (M)[4]		Mascot
976.5574	976.5323	-0.0251	-26	378	386	GVAINFVTR					Mascot
1035.5768	1035.5188	-0.058	-56	234	241	KFMNKPVR			Oxidation (M)[3]		Mascot
1104.6525	1104.6274	-0.0251	-23	377	386	KGVAINFVTR					Mascot
1104.6525	1104.6274	-0.0251	-23	377	386	KGVAINFVTR	63	99.946			Mascot
1114.6831	1114.6573	-0.0258	-23	333	342	VLITTDLLAR					Mascot
1114.6831	1114.6573	-0.0258	-23	333	342	VLITTDLLAR	77	99.998			Mascot
1142.5736	1142.5491	-0.0245	-21	140	150	VHACVGGTSVR			Carbamidomethyl (C)[4]		Mascot
1142.5736	1142.5491	-0.0245	-21	140	150	VHACVGGTSVR	70	99.989	Carbamidomethyl (C)[4]		Mascot
1173.6475	1173.6195	-0.028	-24	246	255	RDELTLEGIK					Mascot
1401.7333	1401.6686	-0.0647	-46	78	91	GLDVIQQAQSGTGK					Mascot
1461.8538	1461.8269	-0.0269	-18	155	169	ILASGVHVVVGTPGR					Mascot
1461.8538	1461.8269	-0.0269	-18	155	169	ILASGVHVVVGTPGR	108	100			Mascot
1555.7131	1555.6875	-0.0256	-16	186	198	MFVLDEADEMLSR					Mascot
1571.708	1571.6927	-0.0153	-10	186	198	MFVLDEADEMLSR			Oxidation (M)[1]		Mascot
1587.703	1587.6492	-0.0538	-34	186	198	MFVLDEADEMLSR			Oxidation (M)[1,10]		Mascot
1598.7849	1598.726	-0.0589	-37	256	267	QFYVNVEKEEWK					Mascot
1827.9388	1827.9164	-0.0224	-12	55	70	GIYAYGFEKPSAIQQR					Mascot
1827.9388	1827.9164	-0.0224	-12	55	70	GIYAYGFEKPSAIQQR	141	100			Mascot
1903.8928	1903.9167	0.0239	13	186	201	MFVLDEADEMLSRGFK			Oxidation (M)[1]		Mascot
1919.8878	1919.9137	0.0259	13	186	201	MFVLDEADEMLSRGFK			Oxidation (M)[1,10]		Mascot
2059.0754	2059.063	-0.0124	-6	131	150	ALGDYLGVKVHACVGGT			Carbamidomethyl (C)[13]		Mascot

Calc. Mass	Obsrv. Mass	± da	± ppm	Start Seq.	End Seq.	Sequence	Ion Score	C. I. %	Modification	Rank	Result Type
						SVR					
2059.0894	2059.063	-0.0264	-13	215	233	IQVGVFSATMPPEALEIT R	84	100			Mascot
2075.0842	2075.033	-0.0512	-25	215	233	IQVGVFSATMPPEALEIT R			Oxidation (M)[10]		Mascot
2075.0842	2075.033	-0.0512	-25	215	233	IQVGVFSATMPPEALEIT R	66	99.971	Oxidation (M)[10]		Mascot
2685.3804	2685.3508	-0.0296	-11	268	290	LDTLCDLYETLAITQSVIF VNTR			Carbamidomethyl (C)[5]		Mascot
2911.4949	2911.4683	-0.0266	-9	343	367	GIDVQQVSLVINYDLPTQ PENYLHR					Mascot
2911.4949	2911.4683	-0.0266	-9	343	367	GIDVQQVSLVINYDLPTQ PENYLHR	197	100			Mascot
3124.5806	3124.5498	-0.0308	-10	92	119	TATFCSGILQQLDYAVVE CQALVLAPTR			Carbamidomethyl (C)[5,19]		Mascot

3 Eukaryotic initiation factor 4A-3 OS=Oryza sativa subsp. japonica GN=Os02g0146600 PE=2 SV=1 IF4A3_ORYSJ 47393.1 5.43 18 831 100 45.889 739 100

Peptide Information

Calc. Mass	Obsrv. Mass	± da	± ppm	Start Seq.	End Seq.	Sequence	Ion Score	C. I. %	Modification	Rank	Result Type
907.4818	907.4412	-0.0406	-45	235	241	FMNKPVR			Oxidation (M)[2]		Mascot
935.5197	935.4751	-0.0446	-48	131	139	ALGDYLGVK					Mascot
952.5033	952.4612	-0.0421	-44	170	176	VFDMLRR			Oxidation (M)[4]		Mascot
976.5574	976.5323	-0.0251	-26	378	386	GVAINFVTR					Mascot
1035.5768	1035.5188	-0.058	-56	234	241	KFMNKPVR			Oxidation (M)[3]		Mascot
1104.6525	1104.6274	-0.0251	-23	377	386	KGVAINFVTR					Mascot
1104.6525	1104.6274	-0.0251	-23	377	386	KGVAINFVTR	63	99.946			Mascot
1114.6831	1114.6573	-0.0258	-23	333	342	VLITTDLLAR					Mascot
1114.6831	1114.6573	-0.0258	-23	333	342	VLITTDLLAR	77	99.998			Mascot
1142.5736	1142.5491	-0.0245	-21	140	150	VHACVGGTSVR			Carbamidomethyl (C)[4]		Mascot
1142.5736	1142.5491	-0.0245	-21	140	150	VHACVGGTSVR	70	99.989	Carbamidomethyl (C)[4]		Mascot
1173.6475	1173.6195	-0.028	-24	246	255	RDELTLEGIK					Mascot
1401.7333	1401.6686	-0.0647	-46	78	91	GLDVIQQAQSGTGK					Mascot
1461.8538	1461.8269	-0.0269	-18	155	169	ILASGVHVVVGTPGR					Mascot
1461.8538	1461.8269	-0.0269	-18	155	169	ILASGVHVVVGTPGR	108	100			Mascot
1555.7131	1555.6875	-0.0256	-16	186	198	MFVLDEADEMLSR					Mascot
1571.708	1571.6927	-0.0153	-10	186	198	MFVLDEADEMLSR			Oxidation (M)[1]		Mascot
1587.703	1587.6492	-0.0538	-34	186	198	MFVLDEADEMLSR			Oxidation (M)[1,10]		Mascot
1827.9388	1827.9164	-0.0224	-12	55	70	GIYAYGFEKPSAIQQR					Mascot
1827.9388	1827.9164	-0.0224	-12	55	70	GIYAYGFEKPSAIQQR	141	100			Mascot
1903.8928	1903.9167	0.0239	13	186	201	MFVLDEADEMLSRGFK			Oxidation (M)[1]		Mascot
1919.8878	1919.9137	0.0259	13	186	201	MFVLDEADEMLSRGFK			Oxidation (M)[1,10]		Mascot

2059.0754	2059.063	-0.0124	-6	131	150	ALGDYLGVKVHACVGGTSVR			Carbamidomethyl (C)[13]		Mascot
2059.0894	2059.063	-0.0264	-13	215	233	IQVGVFSATMPPEALEITR	84	100			Mascot
2075.0842	2075.033	-0.0512	-25	215	233	IQVGVFSATMPPEALEITR			Oxidation (M)[10]		Mascot
2075.0842	2075.033	-0.0512	-25	215	233	IQVGVFSATMPPEALEITR	66	99.971	Oxidation (M)[10]		Mascot
2685.3804	2685.3508	-0.0296	-11	268	290	LDTLCDLYETLAITQSVIFVNTR			Carbamidomethyl (C)[5]		Mascot
2911.4949	2911.4683	-0.0266	-9	343	367	GIDVQQVSLVINYDLPTQPENYLHR					Mascot
2911.4949	2911.4683	-0.0266	-9	343	367	GIDVQQVSLVINYDLPTQPENYLHR	197	100			Mascot

4	Eukaryotic initiation factor 4A OS=Triticum aestivum PE=2 SV=1	IF4A_WHEAT	47183.1	5.31	23	678	100	45.591	542	100		

Peptide Information

Calc. Mass	Obsrv. Mass	± da	± ppm	Start Seq.	End Seq.	Sequence	Ion Score	C. I. %	Modification	Rank	Result Type
907.4818	907.4412	-0.0406	-45	235	241	FMNKPVR			Oxidation (M)[2]		Mascot
910.4702	910.4266	-0.0436	-48	391	397	MLFDIQK			Oxidation (M)[1]		Mascot
935.5197	935.4751	-0.0446	-48	131	139	ALGDYLGVK					Mascot
976.5574	976.5323	-0.0251	-26	378	386	GVAINFVTR					Mascot
1035.5768	1035.5188	-0.058	-56	234	241	KFMNKPVR			Oxidation (M)[3]		Mascot
1070.5953	1070.5665	-0.0288	-27	177	185	QSLRPDNIK					Mascot
1104.6525	1104.6274	-0.0251	-23	377	386	KGVAINFVTR					Mascot
1104.6525	1104.6274	-0.0251	-23	377	386	KGVAINFVTR	63	99.946			Mascot
1114.6831	1114.6573	-0.0258	-23	333	342	VLITTDLLAR					Mascot
1114.6831	1114.6573	-0.0258	-23	333	342	VLITTDLLAR	77	99.998			Mascot
1142.5736	1142.5491	-0.0245	-21	140	150	VHACVGGTSVR			Carbamidomethyl (C)[4]		Mascot
1142.5736	1142.5491	-0.0245	-21	140	150	VHACVGGTSVR	70	99.989	Carbamidomethyl (C)[4]		Mascot
1173.6475	1173.6195	-0.028	-24	246	255	RDELTLEGIK					Mascot
1226.6964	1226.662	-0.0344	-28	176	185	RQSLRPDNIK					Mascot
1401.7333	1401.6686	-0.0647	-46	78	91	GLDVIQQAQSGTGK					Mascot
1461.8538	1461.8269	-0.0269	-18	155	169	ILASGVHVVVGTPGR					Mascot
1461.8538	1461.8269	-0.0269	-18	155	169	ILASGVHVVVGTPGR	108	100			Mascot
1549.8262	1549.7515	-0.0747	-48	202	214	DQIYDIFQLLPGK					Mascot
1555.7131	1555.6875	-0.0256	-16	186	198	MFVLDEADEMLSR					Mascot
1571.708	1571.6927	-0.0153	-10	186	198	MFVLDEADEMLSR			Oxidation (M)[1]		Mascot
1587.703	1587.6492	-0.0538	-34	186	198	MFVLDEADEMLSR			Oxidation (M)[1,10]		Mascot
1598.7849	1598.726	-0.0589	-37	256	267	QFYVNVEKEEWK					Mascot
1827.9388	1827.9164	-0.0224	-12	55	70	GIYAYGFEKPSAIQQR					Mascot

1827.9388	1827.9164	-0.0224	-12	55	70	GIYAYGFEKPSAIQQR	141	100			Mascot
1882.011	1881.8552	-0.1558	-83	199	214	GFKDQIYDIFQLLPGK					Mascot
1903.8928	1903.9167	0.0239	13	186	201	MFVLDEADEMLSRGFK			Oxidation (M)[1]		Mascot
1919.8878	1919.9137	0.0259	13	186	201	MFVLDEADEMLSRGFK			Oxidation (M)[1,10]		Mascot
2059.0754	2059.063	-0.0124	-6	131	150	ALGDYLGVKVHACVGGT SVR			Carbamidomethyl (C)[13]		Mascot
2059.0894	2059.063	-0.0264	-13	215	233	IQVGVFSATMPPEALEIT R	84	100			Mascot
2075.0842	2075.033	-0.0512	-25	215	233	IQVGVFSATMPPEALEIT R			Oxidation (M)[10]		Mascot
2075.0842	2075.033	-0.0512	-25	215	233	IQVGVFSATMPPEALEIT R	66	99.971	Oxidation (M)[10]		Mascot
2685.3804	2685.3508	-0.0296	-11	268	290	LDTLCDLYETLAITQSVIF VNTR			Carbamidomethyl (C)[5]		Mascot
3124.5806	3124.5498	-0.0308	-10	92	119	TATFCSGILQQLDYGLVE CQALVLAPTR			Carbamidomethyl (C)[5,19]		Mascot

5	Eukaryotic initiation factor 4A-2 OS=Nicotiana plumbaginifolia PE=1 SV=1	IF4A2_NICPL	47084	5.38	18	664	100	41.247	568	100	

Peptide Information

Calc. Mass	Obsrv. Mass	± da	± ppm	Start Seq.	End Seq.	Sequence	Ion Score	C. I. %	Modification	Rank	Result Type
907.4818	907.4412	-0.0406	-45	234	240	FMNKPVR			Oxidation (M)[2]		Mascot
910.4702	910.4266	-0.0436	-48	390	396	MLFDIQK			Oxidation (M)[1]		Mascot
935.5197	935.4751	-0.0446	-48	130	138	ALGDYLGVK					Mascot
952.5033	952.4612	-0.0421	-44	169	175	VFDMLRR			Oxidation (M)[4]		Mascot
1016.6099	1016.5276	-0.0823	-81	376	385	KGVAINSVTK					Mascot
1016.6099	1016.5276	-0.0823	-81	376	385	KGVAINSVTK					Mascot
1035.5768	1035.5188	-0.058	-56	233	240	KFMNKPVR			Oxidation (M)[3]		Mascot
1114.6831	1114.6573	-0.0258	-23	332	341	VLITTDLLAR					Mascot
1114.6831	1114.6573	-0.0258	-23	332	341	VLITTDLLAR	77	99.998			Mascot
1142.5736	1142.5491	-0.0245	-21	139	149	VHACVGGTSVR			Carbamidomethyl (C)[4]		Mascot
1142.5736	1142.5491	-0.0245	-21	139	149	VHACVGGTSVR	70	99.989	Carbamidomethyl (C)[4]		Mascot
1173.6475	1173.6195	-0.028	-24	245	254	RDELTLEGIK					Mascot
1401.7333	1401.6686	-0.0647	-46	77	90	GLDVIQQAQSGTGK					Mascot
1555.7131	1555.6875	-0.0256	-16	185	197	MFVLDEADEMLSR					Mascot
1571.708	1571.6927	-0.0153	-10	185	197	MFVLDEADEMLSR			Oxidation (M)[1]		Mascot
1587.703	1587.6492	-0.0538	-34	185	197	MFVLDEADEMLSR			Oxidation (M)[1,10]		Mascot
1827.9388	1827.9164	-0.0224	-12	54	69	GIYAYGFEKPSAIQQR					Mascot
1827.9388	1827.9164	-0.0224	-12	54	69	GIYAYGFEKPSAIQQR	141	100			Mascot
1881.8726	1881.8552	-0.0174	-9	2	19	AGSAPEGSQFDARQFDA K					Mascot

1903.8928	1903.9167	0.0239	13	185	200	MFVLDEADEMLSRGFK			Oxidation (M)[1]		Mascot
1919.8878	1919.9137	0.0259	13	185	200	MFVLDEADEMLSRGFK			Oxidation (M)[1,10]		Mascot
2011.0383	2011.1105	0.0722	36	246	262	DELTLEGIKQFYVNVDK					Mascot
2011.0383	2011.1105	0.0722	36	246	262	DELTLEGIKQFYVNVDK					Mascot
2059.0754	2059.063	-0.0124	-6	130	149	ALGDYLGVKVHACVGGT SVR			Carbamidomethyl (C)[13]		Mascot
2059.0894	2059.063	-0.0264	-13	214	232	IQVGVFSATMPPEALEIT R	84	100			Mascot
2075.0842	2075.033	-0.0512	-25	214	232	IQVGVFSATMPPEALEIT R			Oxidation (M)[10]		Mascot
2075.0842	2075.033	-0.0512	-25	214	232	IQVGVFSATMPPEALEIT R	66	99.971	Oxidation (M)[10]		Mascot
2911.4949	2911.4683	-0.0266	-9	342	366	GIDVQQVSLVINYDLPTQ PENYLHR					Mascot
2911.4949	2911.4683	-0.0266	-9	342	366	GIDVQQVSLVINYDLPTQ PENYLHR	197	100			Mascot

6	Eukaryotic initiation factor 4A-11 OS=Nicotiana tabacum PE=1 SV=1	IF411_TOBAC	47157.1	5.38	18	659	100	40.535	568	100

Peptide Information

Calc. Mass	Obsrv. Mass	± da	± ppm	Start Seq.	End Seq.	Sequence	Ion Score	C. I. %	Modification	Rank	Result Type
907.4818	907.4412	-0.0406	-45	234	240	FMNKPVR			Oxidation (M)[2]		Mascot
910.4702	910.4266	-0.0436	-48	390	396	MLFDIQK			Oxidation (M)[1]		Mascot
935.5197	935.4751	-0.0446	-48	130	138	ALGDYLGVK					Mascot
952.5033	952.4612	-0.0421	-44	169	175	VFDMLRR			Oxidation (M)[4]		Mascot
1035.5768	1035.5188	-0.058	-56	233	240	KFMNKPVR			Oxidation (M)[3]		Mascot
1070.5953	1070.5665	-0.0288	-27	176	184	QSLRPDNIK					Mascot
1114.6831	1114.6573	-0.0258	-23	332	341	VLITTDLLAR					Mascot
1114.6831	1114.6573	-0.0258	-23	332	341	VLITTDLLAR	77	99.998			Mascot
1142.5736	1142.5491	-0.0245	-21	139	149	VHACVGGTSVR			Carbamidomethyl (C)[4]		Mascot
1142.5736	1142.5491	-0.0245	-21	139	149	VHACVGGTSVR	70	99.989	Carbamidomethyl (C)[4]		Mascot
1173.6475	1173.6195	-0.028	-24	245	254	RDELTLEGIK					Mascot
1226.6964	1226.662	-0.0344	-28	175	184	RQSLRPDNIK					Mascot
1401.7333	1401.6686	-0.0647	-46	77	90	GLDVIQQAQSGTGK					Mascot
1555.7131	1555.6875	-0.0256	-16	185	197	MFVLDEADEMLSR					Mascot
1571.708	1571.6927	-0.0153	-10	185	197	MFVLDEADEMLSR			Oxidation (M)[1]		Mascot
1587.703	1587.6492	-0.0538	-34	185	197	MFVLDEADEMLSR			Oxidation (M)[1,10]		Mascot
1827.9388	1827.9164	-0.0224	-12	54	69	GIYAYGFEKPSAIQQR					Mascot
1827.9388	1827.9164	-0.0224	-12	54	69	GIYAYGFEKPSAIQQR	141	100			Mascot
1903.8928	1903.9167	0.0239	13	185	200	MFVLDEADEMLSRGFK			Oxidation (M)[1]		Mascot
1919.8878	1919.9137	0.0259	13	185	200	MFVLDEADEMLSRGFK			Oxidation (M)[1,10]		Mascot

2011.0383	2011.1105	0.0722	36	246	262	DELTLEGIKQFYVNVDK					Mascot
2011.0383	2011.1105	0.0722	36	246	262	DELTLEGIKQFYVNVDK					Mascot
2059.0754	2059.063	-0.0124	-6	130	149	ALGDYLGVKVHACVGGT SVR			Carbamidomethyl (C)[13]		Mascot
2059.0894	2059.063	-0.0264	-13	214	232	IQVGVFSATMPPEALEIT R	84	100			Mascot
2075.0842	2075.033	-0.0512	-25	214	232	IQVGVFSATMPPEALEIT R			Oxidation (M)[10]		Mascot
2075.0842	2075.033	-0.0512	-25	214	232	IQVGVFSATMPPEALEIT R	66	99.971	Oxidation (M)[10]		Mascot
2911.4949	2911.4683	-0.0266	-9	342	366	GIDVQQVSLVINYDLPTQ PENYLHR					Mascot
2911.4949	2911.4683	-0.0266	-9	342	366	GIDVQQVSLVINYDLPTQ PENYLHR	197	100			Mascot

7	Eukaryotic initiation factor 4A-9 OS=Nicotiana tabacum PE=2 SV=1	IF4A9_TOBAC	47081	5.54	16	644	100	39.308	568	100

Peptide Information

Calc. Mass	Obsrv. Mass	± da	± ppm	Start Seq.	End Seq.	Sequence	Ion Score	C. I. %	Modification	Rank	Result Type
907.4818	907.4412	-0.0406	-45	234	240	FMNKPVR			Oxidation (M)[2]		Mascot
935.5197	935.4751	-0.0446	-48	130	138	ALGDYLGVK					Mascot
952.5033	952.4612	-0.0421	-44	169	175	VFDMLRR			Oxidation (M)[4]		Mascot
1035.5768	1035.5188	-0.058	-56	233	240	KFMNKPVR			Oxidation (M)[3]		Mascot
1114.6831	1114.6573	-0.0258	-23	332	341	VLITTDLLAR					Mascot
1114.6831	1114.6573	-0.0258	-23	332	341	VLITTDLLAR	77	99.998			Mascot
1142.5736	1142.5491	-0.0245	-21	139	149	VHACVGGTSVR			Carbamidomethyl (C)[4]		Mascot
1142.5736	1142.5491	-0.0245	-21	139	149	VHACVGGTSVR	70	99.989	Carbamidomethyl (C)[4]		Mascot
1173.6475	1173.6195	-0.028	-24	245	254	RDELTLEGIK					Mascot
1401.7333	1401.6686	-0.0647	-46	77	90	GLDVIQQAQSGTGK					Mascot
1477.8486	1477.7881	-0.0605	-41	154	168	ILSSGVHVVVGTPGR					Mascot
1555.7131	1555.6875	-0.0256	-16	185	197	MFVLDEADEMLSR					Mascot
1571.708	1571.6927	-0.0153	-10	185	197	MFVLDEADEMLSR			Oxidation (M)[1]		Mascot
1587.703	1587.6492	-0.0538	-34	185	197	MFVLDEADEMLSR			Oxidation (M)[1,10]		Mascot
1827.9388	1827.9164	-0.0224	-12	54	69	GIYAYGFEKPSAIQQR					Mascot
1827.9388	1827.9164	-0.0224	-12	54	69	GIYAYGFEKPSAIQQR	141	100			Mascot
1903.8928	1903.9167	0.0239	13	185	200	MFVLDEADEMLSRGFK			Oxidation (M)[1]		Mascot
1919.8878	1919.9137	0.0259	13	185	200	MFVLDEADEMLSRGFK			Oxidation (M)[1,10]		Mascot
2011.0383	2011.1105	0.0722	36	246	262	DELTLEGIKQFYVNVDK					Mascot
2011.0383	2011.1105	0.0722	36	246	262	DELTLEGIKQFYVNVDK					Mascot
2059.0754	2059.063	-0.0124	-6	130	149	ALGDYLGVKVHACVGGT SVR			Carbamidomethyl (C)[13]		Mascot
2059.0894	2059.063	-0.0264	-13	214	232	IQVGVFSATMPPEALEIT	84	100			Mascot

						R					
2075.0842	2075.033	-0.0512	-25	214	232	IQVGVFSATMPPEALEIT R			Oxidation (M)[10]		Mascot
2075.0842	2075.033	-0.0512	-25	214	232	IQVGVFSATMPPEALEIT R	66	99.971	Oxidation (M)[10]		Mascot
2911.4949	2911.4683	-0.0266	-9	342	366	GIDVQQVSLVINYDLPTQ PENYLHR					Mascot
2911.4949	2911.4683	-0.0266	-9	342	366	GIDVQQVSLVINYDLPTQ PENYLHR	197	100			Mascot

8	Eukaryotic initiation factor 4A-7 OS=Nicotiana tabacum PE=2 SV=1	IF4A7_TOBAC	47152.1	5.37	13	623	100	36.704	568	100	

Protein Group

Eukaryotic initiation factor 4A-10 OS=Nicotiana tabacum PE=1 SV=1	IF410_TOBAC	47098.1	5.3699 998855 5908

Peptide Information

Calc. Mass	Obsrv. Mass	± da	± ppm	Start Seq.	End Seq.	Sequence	Ion Score	C. I. %	Modification	Rank	Result Type
907.4818	907.4412	-0.0406	-45	234	240	FMNKPVR			Oxidation (M)[2]		Mascot
935.5197	935.4751	-0.0446	-48	130	138	ALGDYLGVK					Mascot
952.5033	952.4612	-0.0421	-44	169	175	VFDMLRR			Oxidation (M)[4]		Mascot
1035.5768	1035.5188	-0.058	-56	233	240	KFMNKPVR			Oxidation (M)[3]		Mascot
1114.6831	1114.6573	-0.0258	-23	332	341	VLITTDLLAR					Mascot
1114.6831	1114.6573	-0.0258	-23	332	341	VLITTDLLAR	77	99.998			Mascot
1142.5736	1142.5491	-0.0245	-21	139	149	VHACVGGTSVR			Carbamidomethyl (C)[4]		Mascot
1142.5736	1142.5491	-0.0245	-21	139	149	VHACVGGTSVR	70	99.989	Carbamidomethyl (C)[4]		Mascot
1401.7333	1401.6686	-0.0647	-46	77	90	GLDVIQQAQSGTGK					Mascot
1555.7131	1555.6875	-0.0256	-16	185	197	MFVLDEADEMLSR					Mascot
1571.708	1571.6927	-0.0153	-10	185	197	MFVLDEADEMLSR			Oxidation (M)[1]		Mascot
1587.703	1587.6492	-0.0538	-34	185	197	MFVLDEADEMLSR			Oxidation (M)[1,10]		Mascot
1827.9388	1827.9164	-0.0224	-12	54	69	GIYAYGFEKPSAIQQR					Mascot
1827.9388	1827.9164	-0.0224	-12	54	69	GIYAYGFEKPSAIQQR	141	100			Mascot
1903.8928	1903.9167	0.0239	13	185	200	MFVLDEADEMLSRGFK			Oxidation (M)[1]		Mascot
1919.8878	1919.9137	0.0259	13	185	200	MFVLDEADEMLSRGFK			Oxidation (M)[1,10]		Mascot
2059.0754	2059.063	-0.0124	-6	130	149	ALGDYLGVKVHACVGGT SVR			Carbamidomethyl (C)[13]		Mascot
2059.0894	2059.063	-0.0264	-13	214	232	IQVGVFSATMPPEALEIT R	84	100			Mascot
2075.0842	2075.033	-0.0512	-25	214	232	IQVGVFSATMPPEALEIT R			Oxidation (M)[10]		Mascot
2075.0842	2075.033	-0.0512	-25	214	232	IQVGVFSATMPPEALEIT R	66	99.971	Oxidation (M)[10]		Mascot
2911.4949	2911.4683	-0.0266	-9	342	366	GIDVQQVSLVINYDLPTQ PENYLHR					Mascot
2911.4949	2911.4683	-0.0266	-9	342	366	GIDVQQVSLVINYDLPTQ	197	100			Mascot

PENYLHR

9	Eukaryotic initiation factor 4A-14 OS=Nicotiana tabacum PE=2 SV=1	IF414_TOBAC	47131.1	5.37	12	618	100	36.586	568	100

Peptide Information

Calc. Mass	Obsrv. Mass	± da	± ppm	Start Seq.	End Seq.	Sequence	Ion Score	C. I. %	Modification	Rank	Result Type
910.4702	910.4266	-0.0436	-48	390	396	MLFDIQK			Oxidation (M)[1]		Mascot
935.5197	935.4751	-0.0446	-48	130	138	ALGDYLGVK					Mascot
952.5033	952.4612	-0.0421	-44	169	175	VFDMLRR			Oxidation (M)[4]		Mascot
1114.6831	1114.6573	-0.0258	-23	332	341	VLITTDLLAR					Mascot
1114.6831	1114.6573	-0.0258	-23	332	341	VLITTDLLAR	77	99.998			Mascot
1142.5736	1142.5491	-0.0245	-21	139	149	VHACVGGTSVR			Carbamidomethyl (C)[4]		Mascot
1142.5736	1142.5491	-0.0245	-21	139	149	VHACVGGTSVR	70	99.989	Carbamidomethyl (C)[4]		Mascot
1401.7333	1401.6686	-0.0647	-46	77	90	GLDVIQQAQSGTGK					Mascot
1555.7131	1555.6875	-0.0256	-16	185	197	MFVLDEADEMLSR					Mascot
1571.708	1571.6927	-0.0153	-10	185	197	MFVLDEADEMLSR			Oxidation (M)[1]		Mascot
1587.703	1587.6492	-0.0538	-34	185	197	MFVLDEADEMLSR			Oxidation (M)[1,10]		Mascot
1827.9388	1827.9164	-0.0224	-12	54	69	GIYAYGFEKPSAIQQR					Mascot
1827.9388	1827.9164	-0.0224	-12	54	69	GIYAYGFEKPSAIQQR	141	100			Mascot
1903.8928	1903.9167	0.0239	13	185	200	MFVLDEADEMLSRGFK			Oxidation (M)[1]		Mascot
1919.8878	1919.9137	0.0259	13	185	200	MFVLDEADEMLSRGFK			Oxidation (M)[1,10]		Mascot
2059.0754	2059.063	-0.0124	-6	130	149	ALGDYLGVKVHACVGGT SVR			Carbamidomethyl (C)[13]		Mascot
2059.0894	2059.063	-0.0264	-13	214	232	IQVGVFSATMPPEALEIT R	84	100			Mascot
2075.0842	2075.033	-0.0512	-25	214	232	IQVGVFSATMPPEALEIT R			Oxidation (M)[10]		Mascot
2075.0842	2075.033	-0.0512	-25	214	232	IQVGVFSATMPPEALEIT R	66	99.971	Oxidation (M)[10]		Mascot
2911.4949	2911.4683	-0.0266	-9	342	366	GIDVQQVSLVINYDLPTQ PENYLHR					Mascot
2911.4949	2911.4683	-0.0266	-9	342	366	GIDVQQVSLVINYDLPTQ PENYLHR	197	100			Mascot

10	Eukaryotic initiation factor 4A-8 OS=Nicotiana tabacum PE=2 SV=1	IF4A8_TOBAC	47143.1	5.57	15	548	100	36.499	485	100

Peptide Information

Calc. Mass	Obsrv. Mass	± da	± ppm	Start Seq.	End Seq.	Sequence	Ion Score	C. I. %	Modification	Rank	Result Type
907.4818	907.4412	-0.0406	-45	234	240	FMNKPVR			Oxidation (M)[2]		Mascot
910.4702	910.4266	-0.0436	-48	390	396	MLFDIQK			Oxidation (M)[1]		Mascot
935.5197	935.4751	-0.0446	-48	130	138	ALGDYLGVK					Mascot

952.5033	952.4612	-0.0421	-44	169	175	VFDMLRR			Oxidation (M)[4]	Mascot
1035.5768	1035.5188	-0.058	-56	233	240	KFMNKPVR			Oxidation (M)[3]	Mascot
1114.6831	1114.6573	-0.0258	-23	332	341	VLITTDLLAR				Mascot
1114.6831	1114.6573	-0.0258	-23	332	341	VLITTDLLAR	77	99.998		Mascot
1142.5736	1142.5491	-0.0245	-21	139	149	VHACVGGTSVR			Carbamidomethyl (C)[4]	Mascot
1142.5736	1142.5491	-0.0245	-21	139	149	VHACVGGTSVR	70	99.99	Carbamidomethyl (C)[4]	Mascot
1173.6475	1173.6195	-0.028	-24	245	254	RDELTLEGIK				Mascot
1401.7333	1401.6686	-0.0647	-46	77	90	GLDVIQQAQSGTGK				Mascot
1555.7131	1555.6875	-0.0256	-16	185	197	MFVLDEADEMLSR				Mascot
1571.708	1571.6927	-0.0153	-10	185	197	MFVLDEADEMLSR			Oxidation (M)[1]	Mascot
1587.703	1587.6492	-0.0538	-34	185	197	MFVLDEADEMLSR			Oxidation (M)[1,10]	Mascot
1827.9388	1827.9164	-0.0224	-12	54	69	GIYAYGFEKPSAIQQR				Mascot
1827.9388	1827.9164	-0.0224	-12	54	69	GIYAYGFEKPSAIQQR	141	100		Mascot
1903.8928	1903.9167	0.0239	13	185	200	MFVLDEADEMLSRGFK			Oxidation (M)[1]	Mascot
1919.8878	1919.9137	0.0259	13	185	200	MFVLDEADEMLSRGFK			Oxidation (M)[1,10]	Mascot
2011.0383	2011.1105	0.0722	36	246	262	DELTLEGIKQFYVNVDK				Mascot
2011.0383	2011.1105	0.0722	36	246	262	DELTLEGIKQFYVNVDK				Mascot
2059.0754	2059.063	-0.0124	-6	130	149	ALGDYLGVKVHACVGGT SVR			Carbamidomethyl (C)[13]	Mascot
2059.0754	2059.063	-0.0124	-6	130	149	ALGDYLGVKVHACVGGT SVR			Carbamidomethyl (C)[13]	Mascot
2911.4949	2911.4683	-0.0266	-9	342	366	GIDVQQVSLVINYDLPTQ PENYLHR				Mascot
2911.4949	2911.4683	-0.0266	-9	342	366	GIDVQQVSLVINYDLPTQ PENYLHR	197	100		Mascot

Gel Idx/Pos	257/K9	Instr./Gel Origin	BA2151/Sample Project 20140814	Process Status	Analysis Succeeded
Plate [#] Name	[1] Sample Project 20140814	Instrument Sample Name		Spectra	11

Rank	Protein Name	Accession No.	Protein MW	Protein PI	Pep. Count	Protein Score	Protein Score C. I. %	Intensity Matched	Total Ion Score	Total Ion C. I. %	Confirmed
1	Beta-amylase OS=Hordeum vulgare subsp. spontaneum GN=BMY1 PE=1 SV=1	AMYB_HORVS	59886.4	5.66	9	279	100	27.322	254	100	

Peptide Information

Calc. Mass	Obsrv. Mass	± da	± ppm	Start Seq.	End Seq.	Sequence	Ion Score	C. I. %	Modification	Rank	Result Type
1016.5564	1016.526	-0.0304	-30	411	418	LFGFTYLR					Mascot
1016.5564	1016.526	-0.0304	-30	411	418	LFGFTYLR	60	99.902			Mascot
1253.6121	1253.5538	-0.0583	-47	248	258	DNGTYLTEKGR					Mascot
1326.6688	1326.6288	-0.04	-30	384	394	YDPTAYNTILR					Mascot
1326.6688	1326.6288	-0.04	-30	384	394	YDPTAYNTILR	79	99.999			Mascot
1442.7057	1442.6742	-0.0315	-22	371	383	EGLNVACENALPR			Carbamidomethyl (C)[7]		Mascot
1442.7057	1442.6742	-0.0315	-22	371	383	EGLNVACENALPR	115	100	Carbamidomethyl (C)[7]		Mascot
1669.7349	1669.7363	0.0014	1	147	160	SAVQMYADYMTSFR					Mascot
1752.8916	1752.8192	-0.0724	-41	419	433	LSNQLVEGQNYVNFK					Mascot
1827.9884	1827.9044	-0.084	-46	458	474	SGPEISIEMILQAAKPK			Oxidation (M)[9]		Mascot
1827.9884	1827.9044	-0.084	-46	458	474	SGPEISIEMILQAAKPK			Oxidation (M)[9]		Mascot
1842.0425	1841.882	-0.1605	-87	288	302	VQLAIKISGIHWWYK					Mascot
2013.9778	2013.9648	-0.013	-6	303	320	VPSHAAELTAGYYNLHDR					Mascot

Rank	Protein Name	Accession No.	Protein MW	Protein PI	Pep. Count	Protein Score	Protein Score C. I. %	Intensity Matched	Total Ion Score	Total Ion C. I. %	Confirmed
2	Beta-amylase OS=Hordeum vulgare GN=BMY1 PE=1 SV=1	AMYB_HORVU	59894.5	5.58	8	275	100	26.501	254	100	

Peptide Information

Calc. Mass	Obsrv. Mass	± da	± ppm	Start Seq.	End Seq.	Sequence	Ion Score	C. I. %	Modification	Rank	Result Type
1016.5564	1016.526	-0.0304	-30	411	418	LFGFTYLR					Mascot
1016.5564	1016.526	-0.0304	-30	411	418	LFGFTYLR	60	99.902			Mascot
1326.6688	1326.6288	-0.04	-30	384	394	YDPTAYNTILR					Mascot
1326.6688	1326.6288	-0.04	-30	384	394	YDPTAYNTILR	79	99.999			Mascot
1442.7057	1442.6742	-0.0315	-22	371	383	EGLNVACENALPR			Carbamidomethyl (C)[7]		Mascot
1442.7057	1442.6742	-0.0315	-22	371	383	EGLNVACENALPR	115	100	Carbamidomethyl (C)[7]		Mascot
1669.7349	1669.7363	0.0014	1	147	160	SAVQMYADYMTSFR					Mascot
1752.8916	1752.8192	-0.0724	-41	419	433	LSNQLVEGQNYVNFK					Mascot

Calc. Mass	Obsrv. Mass	± da	± ppm	Start Seq.	End Seq.	Sequence	Ion Score	C. I. %	Modification	Rank	Result Type
1827.952	1827.9044	-0.0476	-26	458	474	SGPEISIEMILQAAQPK			Oxidation (M)[9]		Mascot
1827.952	1827.9044	-0.0476	-26	458	474	SGPEISIEMILQAAQPK			Oxidation (M)[9]		Mascot
1842.0425	1841.882	-0.1605	-87	288	302	VQLAIKISGIHWWYK					Mascot
2013.9778	2013.9648	-0.013	-6	303	320	VPSHAAELTAGYYNLHDR					Mascot

3	Eukaryotic initiation factor 4A OS=Triticum aestivum PE=2 SV=1	IF4A_WHEAT	47183.1	5.31	15	171	100	14.134	106	100	

Peptide Information

Calc. Mass	Obsrv. Mass	± da	± ppm	Start Seq.	End Seq.	Sequence	Ion Score	C. I. %	Modification	Rank	Result Type
976.5574	976.53	-0.0274	-28	378	386	GVAINFVTR					Mascot
1070.5953	1070.5638	-0.0315	-29	177	185	QSLRPDNIK					Mascot
1104.6525	1104.6251	-0.0274	-25	377	386	KGVAINFVTR					Mascot
1114.6831	1114.6538	-0.0293	-26	333	342	VLITTDLLAR					Mascot
1142.5736	1142.5458	-0.0278	-24	140	150	VHACVGGTSVR			Carbamidomethyl (C)[4]		Mascot
1173.6475	1173.6141	-0.0334	-28	246	255	RDELTLEGIK					Mascot
1461.8538	1461.8214	-0.0324	-22	155	169	ILASGVHVVVGTPGR					Mascot
1571.708	1571.6755	-0.0325	-21	186	198	MFVLDEADEMLSR			Oxidation (M)[1]		Mascot
1587.703	1587.6487	-0.0543	-34	186	198	MFVLDEADEMLSR			Oxidation (M)[1,10]		Mascot
1800.7566	1800.8596	0.103	57	304	319	DHTVSATHGDMDQNTR			Oxidation (M)[11]		Mascot
1827.9388	1827.9044	-0.0344	-19	55	70	GIYAYGFEKPSAIQQR					Mascot
1827.9388	1827.9044	-0.0344	-19	55	70	GIYAYGFEKPSAIQQR	106	100			Mascot
1882.011	1881.8931	-0.1179	-63	199	214	GFKDQIYDIFQLLPGK					Mascot
2013.8792	2013.9648	0.0856	43	302	319	GRDHTVSATHGDMDQNTR			Oxidation (M)[13]		Mascot
2075.0842	2075.022	-0.0622	-30	215	233	IQVGVFSATMPPEALEITR			Oxidation (M)[10]		Mascot
2685.3804	2685.3381	-0.0423	-16	268	290	LDTLCDLYETLAITQSVIFVNTR			Carbamidomethyl (C)[5]		Mascot
3124.5806	3124.5454	-0.0352	-11	92	119	TATFCSGILQQLDYGLVECQALVLAPTR			Carbamidomethyl (C)[5,19]		Mascot

4	Eukaryotic initiation factor 4A-1 OS=Oryza sativa subsp. japonica GN=Os06g0701100 PE=2 SV=2	IF4A1_ORYSJ	47343.1	5.37	14	167	100	15.159	106	100	

Peptide Information

Calc. Mass	Obsrv. Mass	± da	± ppm	Start Seq.	End Seq.	Sequence	Ion Score	C. I. %	Modification	Rank	Result Type
976.5574	976.53	-0.0274	-28	378	386	GVAINFVTR					Mascot
1104.6525	1104.6251	-0.0274	-25	377	386	KGVAINFVTR					Mascot
1114.6831	1114.6538	-0.0293	-26	333	342	VLITTDLLAR					Mascot

Calc. Mass	Obsrv. Mass	± da	± ppm	Start Seq.	End Seq.	Sequence	Ion Score	C. I. %	Modification	Rank	Result Type
1142.5736	1142.5458	-0.0278	-24	140	150	VHACVGGTSVR			Carbamidomethyl (C)[4]		Mascot
1173.6475	1173.6141	-0.0334	-28	246	255	RDELTLEGIK					Mascot
1461.8538	1461.8214	-0.0324	-22	155	169	ILASGVHVVVGTPGR					Mascot
1571.708	1571.6755	-0.0325	-21	186	198	MFVLDEADEMLSR			Oxidation (M)[1]		Mascot
1587.703	1587.6487	-0.0543	-34	186	198	MFVLDEADEMLSR			Oxidation (M)[1,10]		Mascot
1800.7566	1800.8596	0.103	57	304	319	DHTVSATHGDMDQNTR			Oxidation (M)[11]		Mascot
1827.9388	1827.9044	-0.0344	-19	55	70	GIYAYGFEKPSAIQQR					Mascot
1827.9388	1827.9044	-0.0344	-19	55	70	GIYAYGFEKPSAIQQR	106	100			Mascot
2013.8792	2013.9648	0.0856	43	302	319	GRDHTVSATHGDMDQNTR			Oxidation (M)[13]		Mascot
2075.0842	2075.022	-0.0622	-30	215	233	IQVGVFSATMPPEALEITR			Oxidation (M)[10]		Mascot
2685.3804	2685.3381	-0.0423	-16	268	290	LDTLCDLYETLAITQSVIFVNTR			Carbamidomethyl (C)[5]		Mascot
2911.4949	2911.4631	-0.0318	-11	343	367	GIDVQQVSLVINYDLPTQPENYLHR					Mascot
3124.5806	3124.5454	-0.0352	-11	92	119	TATFCSGILQQLDYAVVECQALVLAPTR			Carbamidomethyl (C)[5,19]		Mascot

5	Eukaryotic initiation factor 4A OS=Zea mays PE=2 SV=1	IF4A_MAIZE	46848.9	5.38	14	166	100	15.214	106	100	

Peptide Information

Calc. Mass	Obsrv. Mass	± da	± ppm	Start Seq.	End Seq.	Sequence	Ion Score	C. I. %	Modification	Rank	Result Type
976.5574	976.53	-0.0274	-28	374	382	GVAINFVTR					Mascot
1070.5953	1070.5638	-0.0315	-29	173	181	QSLRPDNIK					Mascot
1104.6525	1104.6251	-0.0274	-25	373	382	KGVAINFVTR					Mascot
1114.6831	1114.6538	-0.0293	-26	329	338	VLITTDLLAR					Mascot
1142.5736	1142.5458	-0.0278	-24	136	146	VHACVGGTSVR			Carbamidomethyl (C)[4]		Mascot
1173.6475	1173.6141	-0.0334	-28	242	251	RDELTLEGIK					Mascot
1461.8538	1461.8214	-0.0324	-22	151	165	ILASGVHVVVGTPGR					Mascot
1571.708	1571.6755	-0.0325	-21	182	194	MFVLDEADEMLSR			Oxidation (M)[1]		Mascot
1587.703	1587.6487	-0.0543	-34	182	194	MFVLDEADEMLSR			Oxidation (M)[1,10]		Mascot
1800.7566	1800.8596	0.103	57	300	315	DHTVSATHGDMDQNTR			Oxidation (M)[11]		Mascot
1827.9388	1827.9044	-0.0344	-19	51	66	GIYAYGFEKPSAIQQR					Mascot
1827.9388	1827.9044	-0.0344	-19	51	66	GIYAYGFEKPSAIQQR	106	100			Mascot
2075.0842	2075.022	-0.0622	-30	211	229	IQVGVFSATMPPEALEITR			Oxidation (M)[10]		Mascot
2685.3804	2685.3381	-0.0423	-16	264	286	LDTLCDLYETLAITQSVIFVNTR			Carbamidomethyl (C)[5]		Mascot
2911.4949	2911.4631	-0.0318	-11	339	363	GIDVQQVSLVINYDLPTQPENYLHR					Mascot
3124.5806	3124.5454	-0.0352	-11	88	115	TATFCSGILQQLDYGLVECQALVLAPTR			Carbamidomethyl (C)[5,19]		Mascot

6	Beta-amylase (Fragment) OS=Secale cereale GN=BMY1 PE=2 SV=1	AMYB_SECCE	24561.9	5.08	5	158	100	16.982	139	100	

Peptide Information

Calc. Mass	Obsrv. Mass	± da	± ppm	Start Seq.	End Seq.	Sequence	Ion Score	C. I. %	Modification	Rank	Result Type
1005.5112	1005.4744	-0.0368	-37	17	24	DDYRPIAR					Mascot
1016.5564	1016.526	-0.0304	-30	107	114	LFGFTYLR					Mascot
1016.5564	1016.526	-0.0304	-30	107	114	LFGFTYLR	60	99.902			Mascot
1326.6688	1326.6288	-0.04	-30	80	90	YDPTAYNTILR					Mascot
1326.6688	1326.6288	-0.04	-30	80	90	YDPTAYNTILR	79	99.999			Mascot
1589.6948	1589.6394	-0.0554	-35	29	41	HHASLNFTCAEMR			Carbamidomethyl (C)[9], Oxidation (M)[12]		Mascot
2074.9731	2075.022	0.0489	24	25	41	MLTRHHASLNFTCAEMR			Carbamidomethyl (C)[13]		Mascot
2090.968	2091.0295	0.0615	29	25	41	MLTRHHASLNFTCAEMR			Carbamidomethyl (C)[13], Oxidation (M)[1]		Mascot

7	Eukaryotic initiation factor 4A-3 OS=Oryza sativa subsp. japonica GN=Os02g0146600 PE=2 SV=1	IF4A3_ORYSJ	47393.1	5.43	12	149	100	14.552	106	100	

Peptide Information

Calc. Mass	Obsrv. Mass	± da	± ppm	Start Seq.	End Seq.	Sequence	Ion Score	C. I. %	Modification	Rank	Result Type
976.5574	976.53	-0.0274	-28	378	386	GVAINFVTR					Mascot
1104.6525	1104.6251	-0.0274	-25	377	386	KGVAINFVTR					Mascot
1114.6831	1114.6538	-0.0293	-26	333	342	VLITTDLLAR					Mascot
1142.5736	1142.5458	-0.0278	-24	140	150	VHACVGGTSVR			Carbamidomethyl (C)[4]		Mascot
1173.6475	1173.6141	-0.0334	-28	246	255	RDELTLEGIK					Mascot
1461.8538	1461.8214	-0.0324	-22	155	169	ILASGVHVVVGTPGR					Mascot
1571.708	1571.6755	-0.0325	-21	186	198	MFVLDEADEMLSR			Oxidation (M)[1]		Mascot
1587.703	1587.6487	-0.0543	-34	186	198	MFVLDEADEMLSR			Oxidation (M)[1,10]		Mascot
1800.7566	1800.8596	0.103	57	304	319	DHTVSATHGDMDQNTR			Oxidation (M)[11]		Mascot
1827.9388	1827.9044	-0.0344	-19	55	70	GIYAYGFEKPSAIQQR					Mascot
1827.9388	1827.9044	-0.0344	-19	55	70	GIYAYGFEKPSAIQQR	106	100			Mascot
2075.0842	2075.022	-0.0622	-30	215	233	IQVGVFSATMPPEALEIT R			Oxidation (M)[10]		Mascot
2685.3804	2685.3381	-0.0423	-16	268	290	LDTLCDLYETLAITQSVIF VNTR			Carbamidomethyl (C)[5]		Mascot
2911.4949	2911.4631	-0.0318	-11	343	367	GIDVQQVSLVINYDLPTQ PENYLHR					Mascot

8	Eukaryotic initiation factor 4A-2 OS=Nicotiana plumbaginifolia PE=1 SV=1	IF4A2_NICPL	47084	5.38	11	148	100	24.212	106	100	

Peptide Information

Calc. Mass	Obsrv. Mass	± da	± ppm	Start Seq.	End Seq.	Sequence	Ion Score	C. I. %	Modification	Rank	Result Type
1016.6099	1016.526	-0.0839	-83	376	385	KGVAINSVTK					Mascot
1016.6099	1016.526	-0.0839	-83	376	385	KGVAINSVTK					Mascot
1114.6831	1114.6538	-0.0293	-26	332	341	VLITTDLLAR					Mascot
1142.5736	1142.5458	-0.0278	-24	139	149	VHACVGGTSVR			Carbamidomethyl (C)[4]		Mascot
1173.6475	1173.6141	-0.0334	-28	245	254	RDELTLEGIK					Mascot
1571.708	1571.6755	-0.0325	-21	185	197	MFVLDEADEMLSR			Oxidation (M)[1]		Mascot
1587.703	1587.6487	-0.0543	-34	185	197	MFVLDEADEMLSR			Oxidation (M)[1,10]		Mascot
1800.7566	1800.8596	0.103	57	303	318	DHTVSATHGDMDQNTR			Oxidation (M)[11]		Mascot
1827.9388	1827.9044	-0.0344	-19	54	69	GIYAYGFEKPSAIQQR					Mascot
1827.9388	1827.9044	-0.0344	-19	54	69	GIYAYGFEKPSAIQQR	106	100			Mascot
1881.8726	1881.8931	0.0205	11	2	19	AGSAPEGSQFDARQFDA K					Mascot
2011.0383	2011.0721	0.0338	17	246	262	DELTLEGIKQFYVNVDK					Mascot
2075.0842	2075.022	-0.0622	-30	214	232	IQVGVFSATMPPEALEIT R			Oxidation (M)[10]		Mascot
2911.4949	2911.4631	-0.0318	-11	342	366	GIDVQQVSLVINYDLPTQ PENYLHR					Mascot

9	Eukaryotic initiation factor 4A-11 OS=Nicotiana tabacum PE=1 SV=1	IF411_TOBAC	47157.1	5.38	11	145	100	13.552	106	100	

Peptide Information

Calc. Mass	Obsrv. Mass	± da	± ppm	Start Seq.	End Seq.	Sequence	Ion Score	C. I. %	Modification	Rank	Result Type
1070.5953	1070.5638	-0.0315	-29	176	184	QSLRPDNIK					Mascot
1114.6831	1114.6538	-0.0293	-26	332	341	VLITTDLLAR					Mascot
1142.5736	1142.5458	-0.0278	-24	139	149	VHACVGGTSVR			Carbamidomethyl (C)[4]		Mascot
1173.6475	1173.6141	-0.0334	-28	245	254	RDELTLEGIK					Mascot
1571.708	1571.6755	-0.0325	-21	185	197	MFVLDEADEMLSR			Oxidation (M)[1]		Mascot
1587.703	1587.6487	-0.0543	-34	185	197	MFVLDEADEMLSR			Oxidation (M)[1,10]		Mascot
1800.7566	1800.8596	0.103	57	303	318	DHTVSATHGDMDQNTR			Oxidation (M)[11]		Mascot
1827.9388	1827.9044	-0.0344	-19	54	69	GIYAYGFEKPSAIQQR					Mascot
1827.9388	1827.9044	-0.0344	-19	54	69	GIYAYGFEKPSAIQQR	106	100			Mascot
2011.0383	2011.0721	0.0338	17	246	262	DELTLEGIKQFYVNVDK					Mascot
2013.8792	2013.9648	0.0856	43	301	318	GRDHTVSATHGDMDQNT R			Oxidation (M)[13]		Mascot
2075.0842	2075.022	-0.0622	-30	214	232	IQVGVFSATMPPEALEIT R			Oxidation (M)[10]		Mascot
2911.4949	2911.4631	-0.0318	-11	342	366	GIDVQQVSLVINYDLPTQ PENYLHR					Mascot

10	Eukaryotic initiation factor 4A-2 OS=Arabidopsis thaliana GN=TIF4A-2 PE=2 SV=1	IF4A2_ARATH	47075.1	5.45	9	136	100	11.899	106	100	

Peptide Information

Calc. Mass	Obsrv. Mass	± da	± ppm	Start Seq.	End Seq.	Sequence	Ion Score	C. I. %	Modification	Rank	Result Type
1114.6831	1114.6538	-0.0293	-26	331	340	VLITTDLLAR					Mascot
1142.5736	1142.5458	-0.0278	-24	138	148	VHACVGGTSVR			Carbamidomethyl (C)[4]		Mascot
1173.6475	1173.6141	-0.0334	-28	244	253	RDELTLEGIK					Mascot
1571.708	1571.6755	-0.0325	-21	184	196	MFVLDEADEMLSR			Oxidation (M)[1]		Mascot
1587.703	1587.6487	-0.0543	-34	184	196	MFVLDEADEMLSR			Oxidation (M)[1,10]		Mascot
1800.7566	1800.8596	0.103	57	302	317	DHTVSATHGDMDQNTR			Oxidation (M)[11]		Mascot
1827.9388	1827.9044	-0.0344	-19	53	68	GIYAYGFEKPSAIQQR					Mascot
1827.9388	1827.9044	-0.0344	-19	53	68	GIYAYGFEKPSAIQQR	106	100			Mascot
2010.9265	2011.0721	0.1456	72	2	19	AGSAPEGTQFDTRQFDQ R					Mascot
2075.0842	2075.022	-0.0622	-30	213	231	IQVGVFSATMPPEALEIT R			Oxidation (M)[10]		Mascot
2685.2681	2685.3381	0.07	26	175	196	QSLRPDCIKMFVLDEADE MLSR			Carbamidomethyl (C)[7], Oxidation (M)[10,19]		Mascot

Gel Idx/Pos	258/K10	Instr./Gel Origin	BA2151/Sample Project 20140814	Process Status	Analysis Succeeded
Plate [#] Name	[1] Sample Project 20140814	Instrument Sample Name		Spectra	11

Rank	Protein Name	Accession No.	Protein MW	Protein PI	Pep. Count	Protein Score	Protein Score C. I. %	Intensity Matched	Total Ion Score	Total Ion C. I. %	Confirmed
1	Enolase OS=Oryza sativa subsp. japonica GN=ENO1 PE=1 SV=2	ENO_ORYSJ	48284.5	5.41	19	462	100	51.591	352	100	

Peptide Information

Calc. Mass	Obsrv. Mass	± da	± ppm	Start Seq.	End Seq.	Sequence	Ion Score	C. I. %	Modification	Rank	Result Type
806.4519	806.4372	-0.0147	-18	418	423	YNQLLR					Mascot
918.5077	918.4749	-0.0328	-36	347	354	SCNALLLK			Carbamidomethyl (C)[2]		Mascot
978.5043	978.4792	-0.0251	-26	439	446	FRAPVEPY					Mascot
978.5043	978.4792	-0.0251	-26	439	446	FRAPVEPY	39	89.632			Mascot
1015.6259	1015.5479	-0.078	-77	2	11	AATIVSVKAR					Mascot
1117.5208	1117.585	0.0642	57	374	383	AGWGVMTSHR			Oxidation (M)[6]		Mascot
1146.6664	1146.5768	-0.0896	-78	1	11	MAATIVSVKAR					Mascot
1189.6034	1189.614	0.0106	9	190	199	MGVEVYHNLK					Mascot
1205.5984	1205.5643	-0.0341	-28	190	199	MGVEVYHNLK			Oxidation (M)[1]		Mascot
1551.8643	1551.8042	-0.0601	-39	134	147	IPLYQHIANLAGNK					Mascot
1573.8433	1573.7914	-0.0519	-33	355	369	VNQIGSVTESIEAVK					Mascot
1770.0306	1769.8848	-0.1458	-82	110	126	QKLGANAILAVSLAICK			Carbamidomethyl (C)[16]		Mascot
1790.9283	1790.9041	-0.0242	-14	36	53	AAVPSGASTGVYEALELR					Mascot
1790.9283	1790.9041	-0.0242	-14	36	53	AAVPSGASTGVYEALELR	155	100			Mascot
1886.9391	1886.8562	-0.0829	-44	169	185	LAMQEFMILPTGAASFK			Oxidation (M)[3,7]		Mascot
1901.8512	1901.8148	-0.0364	-19	264	279	TYDLNFKEENNDGSQK					Mascot
1983.8536	1983.8239	-0.0297	-15	18	35	GNPTVEVDVCCSDGTFA R			Carbamidomethyl (C)[10,11]		Mascot
1983.8536	1983.8239	-0.0297	-15	18	35	GNPTVEVDVCCSDGTFA R	157	100	Carbamidomethyl (C)[10,11]		Mascot
2016.9584	2016.8817	-0.0767	-38	246	263	VVIGMDVAASEFYNDKD K			Oxidation (M)[5]		Mascot
2132.1611	2132.0872	-0.0739	-35	148	168	QLVLPVPAFNVINGGSHA GNK					Mascot
2324.0425	2323.9866	-0.0559	-24	206	227	YGQDATNVGDEGGFAPN IQENK					Mascot
2346.1543	2345.9802	-0.1741	-74	169	189	LAMQEFMILPTGAASFKE AMK			Oxidation (M)[3,7]		Mascot
2452.1375	2452.0972	-0.0403	-16	205	227	KYGQDATNVGDEGGFAP NIQENK					Mascot

Rank	Protein Name	Accession No.	Protein MW	Protein PI	Pep. Count	Protein Score	Protein Score C. I. %	Intensity Matched	Total Ion Score	Total Ion C. I. %	Confirmed
2	Enolase 2 OS=Zea mays GN=ENO2 PE=2 SV=1	ENO2_MAIZE	48417.6	5.7	17	289	100	41.503	195	100	

Peptide Information

Calc. Mass	Obsrv. Mass	± da	± ppm	Start Seq.	End Seq.	Sequence	Ion Score	C. I. %	Modification	Rank	Result Type
806.4519	806.4372	-0.0147	-18	418	423	YNQLLR					Mascot
918.5077	918.4749	-0.0328	-36	347	354	SCNALLLK			Carbamidomethyl (C)[2]		Mascot
959.4469	959.5381	0.0912	95	54	62	DGGSYYLGK					Mascot
978.5043	978.4792	-0.0251	-26	439	446	FRAPVEPY					Mascot
978.5043	978.4792	-0.0251	-26	439	446	FRAPVEPY	39	89.632			Mascot
1117.5208	1117.585	0.0642	57	374	383	AGWGVMTSHR			Oxidation (M)[6]		Mascot
1551.8643	1551.8042	-0.0601	-39	134	147	IPLYQHIANLAGNK					Mascot
1573.8433	1573.7914	-0.0519	-33	355	369	VNQIGSVTESIEAVK					Mascot
1746.8256	1746.8699	0.0443	25	246	261	VVIGMDVAASEFYSDK			Oxidation (M)[5]		Mascot
1790.9283	1790.9041	-0.0242	-14	36	53	AAVPSGASTGVYEALELR					Mascot
1790.9283	1790.9041	-0.0242	-14	36	53	AAVPSGASTGVYEALELR	155	100			Mascot
1886.9391	1886.8562	-0.0829	-44	169	185	LAMQEFMILPTGAASFK			Oxidation (M)[3,7]		Mascot
2132.1611	2132.0872	-0.0739	-35	148	168	QLVLPVPAFNVINGGSHA GNK					Mascot
2251.0547	2251.0205	-0.0342	-15	83	102	DPTAQTEIDNFMVQQLD GTK					Mascot
2252.1294	2252.0425	-0.0869	-39	384	405	SGETEDTFIADLAVGLST GQIK					Mascot
2267.0496	2266.9934	-0.0562	-25	83	102	DPTAQTEIDNFMVQQLD GTK			Oxidation (M)[12]		Mascot
2324.0425	2323.9866	-0.0559	-24	206	227	YGQDATNVGDEGGFAPN IQENK					Mascot
2346.1543	2345.9802	-0.1741	-74	169	189	LAMQEFMILPTGAASFKE AMK			Oxidation (M)[3,7]		Mascot
2452.1375	2452.0972	-0.0403	-16	205	227	KYGQDATNVGDEGGFAP NIQENK					Mascot
2573.2764	2573.2383	-0.0381	-15	316	338	MTEEIGEQVQIVGDDLLV TNPTR			Oxidation (M)[1]		Mascot

3	Enolase (Fragments) OS=Populus euphratica PE=1 SV=1	ENO_POPEU	2749.4	4.79	2	213	100	33.262	195	100

Peptide Information

Calc. Mass	Obsrv. Mass	± da	± ppm	Start Seq.	End Seq.	Sequence	Ion Score	C. I. %	Modification	Rank	Result Type
978.5043	978.4792	-0.0251	-26	19	26	FRAPVEPY					Mascot
978.5043	978.4792	-0.0251	-26	19	26	FRAPVEPY	39	89.632			Mascot
1790.9283	1790.9041	-0.0242	-14	1	18	AAVPSGASTGVYEALELR					Mascot
1790.9283	1790.9041	-0.0242	-14	1	18	AAVPSGASTGVYEALELR	155	100			Mascot

4	Enolase OS=Mesembryanthemum crystallinum GN=PGH1 PE=2 SV=1	ENO_MESCR	48660.6	5.62	12	203	100	39.666	155	100

Peptide Information

Calc. Mass	Obsrv. Mass	± da	± ppm	Start Seq.	End Seq.	Sequence	Ion Score	C. I. %	Modification	Rank	Result Type
806.4519	806.4372	-0.0147	-18	416	421	YNQLLR					Mascot
932.4571	932.4705	0.0134	14	422	429	IEEELGDK					Mascot
978.5475	978.4792	-0.0683	-70	1	8	MVTIKCVK			Carbamidomethyl (C)[6]		Mascot
978.5475	978.4792	-0.0683	-70	1	8	MVTIKCVK			Carbamidomethyl (C)[6]		Mascot
1573.8433	1573.7914	-0.0519	-33	353	367	VNQIGSVTESIEAVK					Mascot
1746.8253	1746.8699	0.0446	26	269	284	EENNDGSQRISGEALK					Mascot
1790.9283	1790.9041	-0.0242	-14	35	52	AAVPSGASTGVYEALELR					Mascot
1790.9283	1790.9041	-0.0242	-14	35	52	AAVPSGASTGVYEALELR	155	100			Mascot
1886.9391	1886.8562	-0.0829	-44	168	184	LAMQEFMILPTGASSFK			Oxidation (M)[3]		Mascot
1916.931	1916.8655	-0.0655	-34	245	261	VVIGMDVAASEFYKEDK			Oxidation (M)[5]		Mascot
1943.9094	1944.0092	0.0998	51	17	34	GNPTVEADIHLDDGTYAR					Mascot
2324.0425	2323.9866	-0.0559	-24	205	226	YGQDATNVGDEGGFAPN IQENK					Mascot
2346.1543	2345.9802	-0.1741	-74	168	188	LAMQEFMILPTGASSFKE AMK			Oxidation (M)[3]		Mascot
2452.1375	2452.0972	-0.0403	-16	204	226	KYGQDATNVGDEGGFAP NIQENK					Mascot

5	Glucose-1-phosphate adenylyltransferase small subunit, chloroplastic/amyloplastic OS=Hordeum vulgare PE=2 SV=1	GLGS_HORVU	56412.8	6.11	11	196	100	2.44	158	100	

Peptide Information

Calc. Mass	Obsrv. Mass	± da	± ppm	Start Seq.	End Seq.	Sequence	Ion Score	C. I. %	Modification	Rank	Result Type
1017.5952	1017.5392	-0.056	-55	406	414	IHHSVVGLR					Mascot
1025.5925	1025.5387	-0.0538	-52	300	307	HVMLQLLR			Oxidation (M)[3]		Mascot
1032.5472	1032.5209	-0.0263	-25	370	378	SAPIYTQPR					Mascot
1256.7109	1256.597	-0.1139	-91	104	115	AKPAVPLGANYR					Mascot
1384.6896	1384.6577	-0.0319	-23	359	369	KPIPDFSFYDR					Mascot
1384.6896	1384.6577	-0.0319	-23	359	369	KPIPDFSFYDR	75	99.997			Mascot
1625.8646	1625.8336	-0.031	-19	132	145	IYVLTQFNSASLNR	83	100			Mascot
1746.9684	1746.8699	-0.0985	-56	2	18	AMAAAASPSKILIPPHR			Oxidation (M)[2]		Mascot
1769.9684	1769.8848	-0.0836	-47	488	503	ETDGYFIKSGIVTVIK					Mascot
1862.0139	1861.8931	-0.1208	-65	1	18	MAMAAAASPSKILIPPHR					Mascot
1916.9861	1916.8655	-0.1206	-63	283	299	AKEMPYIASMGIYVISK			Oxidation (M)[4]		Mascot
3061.4287	3061.4038	-0.0249	-8	160	187	NEGFVEVLAAQQSPDNP DWFQGTADAVR					Mascot

6	Enolase OS=Alnus glutinosa GN=PGH1 PE=2 SV=1	ENO_ALNGL	47795.5	5.41	7	176	100	23.01	155	100

Peptide Information

Calc. Mass	Obsrv. Mass	± da	± ppm	Start Seq.	End Seq.	Sequence	Ion Score	C. I. %	Modification	Rank	Result Type
806.4519	806.4372	-0.0147	-18	412	417	YNQLLR					Mascot
1000.4781	1000.4497	-0.0284	-28	369	377	AGWGVMAHR			Oxidation (M)[6]		Mascot
1573.8433	1573.7914	-0.0519	-33	350	364	VNQIGSVTESIEAVK					Mascot
1790.9283	1790.9041	-0.0242	-14	35	52	AAVPSGASTGVYEALELR					Mascot
1790.9283	1790.9041	-0.0242	-14	35	52	AAVPSGASTGVYEALELR	155	100			Mascot
2252.1294	2252.0425	-0.0869	-39	378	399	SGETEDTFIADLSVGLAT GQIK					Mascot
2324.0425	2323.9866	-0.0559	-24	206	227	YGQDATNVGDEGGFAPN IQENK					Mascot
2452.1375	2452.0972	-0.0403	-16	205	227	KYGQDATNVGDEGGFAP NIQENK					Mascot

7	Enolase 1 OS=Zea mays GN=ENO1 PE=1 SV=1	ENO1_MAIZE	48261.5	5.2	14	131	100	40.563	70	99.992

Peptide Information

Calc. Mass	Obsrv. Mass	± da	± ppm	Start Seq.	End Seq.	Sequence	Ion Score	C. I. %	Modification	Rank	Result Type
806.4519	806.4372	-0.0147	-18	418	423	YNQLLR					Mascot
932.5233	932.4705	-0.0528	-57	347	354	TCNALLLK			Carbamidomethyl (C)[2]		Mascot
978.5043	978.4792	-0.0251	-26	439	446	FRAPVEPY					Mascot
978.5043	978.4792	-0.0251	-26	439	446	FRAPVEPY	39	89.632			Mascot
1006.4072	1006.4995	0.0923	92	271	279	EENNDGSNK					Mascot
1189.6034	1189.614	0.0106	9	190	199	MGVEVYHNLK					Mascot
1205.5984	1205.5643	-0.0341	-28	190	199	MGVEVYHNLK			Oxidation (M)[1]		Mascot
1238.6627	1238.552	-0.1107	-89	280	290	ISGDSLKDLYK					Mascot
1551.8643	1551.8042	-0.0601	-39	134	147	IPLYQHIANLAGNK					Mascot
1790.9283	1790.9041	-0.0242	-14	36	53	GAVPSGASTGIYEALELR					Mascot
1790.9283	1790.9041	-0.0242	-14	36	53	GAVPSGASTGIYEALELR	31	28.268			Mascot
1838.9283	1838.8815	-0.0468	-25	424	440	IEEELGDAAVYAGAKFR					Mascot
1886.9391	1886.8562	-0.0829	-44	169	185	LAMQEFMILPTGASSFK			Oxidation (M)[3]		Mascot
1938.1019	1937.9597	-0.1422	-73	324	341	VQIVGDDLLVTNPTRVAK					Mascot
2324.0425	2323.9866	-0.0559	-24	206	227	YGQDATNVGDEGGFAPN IQENK					Mascot
2346.1543	2345.9802	-0.1741	-74	169	189	LAMQEFMILPTGASSFKE AMK			Oxidation (M)[3]		Mascot
2452.1375	2452.0972	-0.0403	-16	205	227	KYGQDATNVGDEGGFAP NIQENK					Mascot

8	Glucose-1-phosphate adenylyltransferase small	GLGS_SOLLC	57733.5	6.49	12	124	100	1.826	83	100

subunit, chloroplastic OS=Solanum lycopersicum PE=2
SV=1

Peptide Information

Calc. Mass	Obsrv. Mass	± da	± ppm	Start Seq.	End Seq.	Sequence	Ion Score	C. I. %	Modification	Rank	Result Type
918.5077	918.4749	-0.0328	-36	46	53	LMPVSSLR			Oxidation (M)[2]		Mascot
989.5448	989.5029	-0.0419	-42	308	315	DVMLNLLR			Oxidation (M)[3]		Mascot
1017.5952	1017.5392	-0.056	-55	414	422	IHHSVVGLR					Mascot
1032.5472	1032.5209	-0.0263	-25	378	386	SAPIYTQPR					Mascot
1189.6311	1189.614	-0.0171	-14	279	289	VDTTILGLDDK					Mascot
1256.7109	1256.597	-0.1139	-91	112	123	AKPAVPLGANYR					Mascot
1625.8646	1625.8336	-0.031	-19	140	153	IYVLTQFNSASLNR	83	100			Mascot
1769.9684	1769.8848	-0.0836	-47	496	511	ETDGYFIKSGIVTVIK					Mascot
1890.9114	1890.9189	0.0075	4	231	247	ETDADITVAALPMDEKR			Oxidation (M)[13]		Mascot
1916.9861	1916.8655	-0.1206	-63	291	307	AKEMPFIASMGIYVISK			Oxidation (M)[4,10]		Mascot
1937.9196	1937.9597	0.0401	21	393	410	MLDADVTDSVIGEGCVIK			Carbamidomethyl (C)[15], Oxidation (M)[1]		Mascot
2324.093	2323.9866	-0.1064	-46	393	413	MLDADVTDSVIGEGCVIK NCK			Carbamidomethyl (C)[15,20]		Mascot

9	Glucose-1-phosphate adenylyltransferase small subunit, chloroplastic/amyloplastic OS=Solanum tuberosum PE=1 SV=2	GLGS_SOLTU	57603.4	6.73	12	123	100	1.826	83	100	

Peptide Information

Calc. Mass	Obsrv. Mass	± da	± ppm	Start Seq.	End Seq.	Sequence	Ion Score	C. I. %	Modification	Rank	Result Type
918.5077	918.4749	-0.0328	-36	46	53	LMPVSSLR			Oxidation (M)[2]		Mascot
989.5448	989.5029	-0.0419	-42	308	315	DVMLNLLR			Oxidation (M)[3]		Mascot
1017.5952	1017.5392	-0.056	-55	414	422	IHHSVVGLR					Mascot
1032.5472	1032.5209	-0.0263	-25	378	386	SAPIYTQPR					Mascot
1189.6311	1189.614	-0.0171	-14	279	289	VDTTILGLDDK					Mascot
1256.7109	1256.597	-0.1139	-91	112	123	AKPAVPLGANYR					Mascot
1625.8646	1625.8336	-0.031	-19	140	153	IYVLTQFNSASLNR	83	100			Mascot
1769.9684	1769.8848	-0.0836	-47	496	511	ETDGYFIKSGIVTVIK					Mascot
1890.9114	1890.9189	0.0075	4	231	247	ETDADITVAALPMDEKR			Oxidation (M)[13]		Mascot
1916.9861	1916.8655	-0.1206	-63	291	307	AKEMPFIASMGIYVISK			Oxidation (M)[4,10]		Mascot
1937.9196	1937.9597	0.0401	21	393	410	MLDADVTDSVIGEGCVIK			Carbamidomethyl (C)[15], Oxidation (M)[1]		Mascot
2324.093	2323.9866	-0.1064	-46	393	413	MLDADVTDSVIGEGCVIK NCK			Carbamidomethyl (C)[15,20]		Mascot

10	Glucose-1-phosphate adenylyltransferase small	GLGS_BRANA	57294.3	5.87	10	114	100	17.252	83	100	

subunit, chloroplastic OS=Brassica napus GN=AGPS1
PE=2 SV=1

Peptide Information

Calc. Mass	Obsrv. Mass	± da	± ppm	Start Seq.	End Seq.	Sequence	Ion Score	C. I. %	Modification	Rank	Result Type
978.5288	978.4792	-0.0496	-51	2	11	ATMAAIGSLK			Oxidation (M)[3]		Mascot
978.5288	978.4792	-0.0496	-51	2	11	ATMAAIGSLK			Oxidation (M)[3]		Mascot
989.5448	989.5029	-0.0419	-42	307	314	NVMLDLLR			Oxidation (M)[3]		Mascot
1032.5472	1032.5209	-0.0263	-25	377	385	SAPIYTQPR					Mascot
1256.7109	1256.597	-0.1139	-91	111	122	AKPAVPLGANYR					Mascot
1625.8646	1625.8336	-0.031	-19	139	152	IYVLTQFNSASLNR	83	100			Mascot
1769.9684	1769.8848	-0.0836	-47	495	510	ETDGYFIKSGIVTVIK					Mascot
1886.9755	1886.8562	-0.1193	-63	290	306	AKEMPFIASMGIYVVSK			Oxidation (M)[4]		Mascot
1890.9114	1890.9189	0.0075	4	230	246	ETDADITVAALPMDEKR			Oxidation (M)[13]		Mascot
1937.9196	1937.9597	0.0401	21	392	409	MLDADVTDSVIGEGCVIK			Carbamidomethyl (C)[15], Oxidation (M)[1]		Mascot
2324.093	2323.9866	-0.1064	-46	392	412	MLDADVTDSVIGEGCVIK NCK			Carbamidomethyl (C)[15,20]		Mascot

Gel Idx/Pos	259/K11	Instr./Gel Origin	BA2151/Sample Project 20140814	Process Status	Analysis Succeeded
Plate [#] Name	[1] Sample Project 20140814	Instrument Sample Name		Spectra	11

Rank	Protein Name	Accession No.	Protein MW	Protein PI	Pep. Count	Protein Score	Protein Score C. I. %	Intensity Matched	Total Ion Score	Total Ion C. I. %	Confirmed
1	Serpin-Z1B OS=Triticum aestivum PE=1 SV=1	SPZ1B_WHEAT	43119.9	5.44	11	440	100	63.593	385	100	

Peptide Information

Calc. Mass	Obsrv. Mass	± da	± ppm	Start Seq.	End Seq.	Sequence	Ion Score	C. I. %	Modification	Rank	Result Type
925.5214	925.5015	-0.0199	-22	11	18	LSIAHQTR					Mascot
925.5214	925.5015	-0.0199	-22	11	18	LSIAHQTR	31	20.665			Mascot
1151.6824	1151.6327	-0.0497	-43	172	181	LVLANALYFK					Mascot
1176.5896	1176.572	-0.0176	-15	262	271	LSAEPDFLER					Mascot
1176.5896	1176.572	-0.0176	-15	262	271	LSAEPDFLER	76	99.998			Mascot
1345.6958	1345.7191	0.0233	17	159	171	NILPSGSVDNTTK					Mascot
1475.6438	1475.641	-0.0028	-2	182	194	GAWTDQFDSYGTK					Mascot
2062.0564	2061.9919	-0.0645	-31	138	156	AAEVTTQVNSWVEKVTS GR					Mascot
2113.0999	2113.0837	-0.0162	-8	380	399	EDISGVVLFMGHVVNPLL SS					Mascot
2129.0947	2128.9883	-0.1064	-50	380	399	EDISGVVLFMGHVVNPLL SS			Oxidation (M)[10]		Mascot
2685.3955	2685.3792	-0.0163	-6	33	61	SAASNAAFSPVSLHSALS LLAAGAGSATR					Mascot
2685.3955	2685.3792	-0.0163	-6	33	61	SAASNAAFSPVSLHSALS LLAAGAGSATR	240	100			Mascot
2720.3525	2720.3101	-0.0424	-16	329	354	VSSVFHQAFVEVNEQGT EAAASTAIK					Mascot
2720.3525	2720.3101	-0.0424	-16	329	354	VSSVFHQAFVEVNEQGT EAAASTAIK	39	89.59			Mascot
2941.5215	2941.5994	0.0779	26	355	379	MVPQQARPPSVMDFIAD HPFLFLLR			Oxidation (M)[1]		Mascot
2957.5166	2957.4834	-0.0332	-11	355	379	MVPQQARPPSVMDFIAD HPFLFLLR			Oxidation (M)[1,12]		Mascot
3055.3845	3055.3315	-0.053	-17	302	328	CLGLQLPFSDEADFSEM VDSPMPQGLR			Carbamidomethyl (C)[1], Oxidation (M)[17]		Mascot
3071.3796	3071.325	-0.0546	-18	302	328	CLGLQLPFSDEADFSEM VDSPMPQGLR			Carbamidomethyl (C)[1], Oxidation (M)[17,22]		Mascot

Rank	Protein Name	Accession No.	Protein MW	Protein PI	Pep. Count	Protein Score	Protein Score C. I. %	Intensity Matched	Total Ion Score	Total Ion C. I. %	Confirmed
2	Serpin-Z1A OS=Triticum aestivum GN=WZCI PE=1 SV=1	SPZ1A_WHEAT	43262.2	5.6	9	181	100	17.136	145	100	

Peptide Information

Calc. Mass	Obsrv. Mass	± da	± ppm	Start Seq.	End Seq.	Sequence	Ion Score	C. I. %	Modification	Rank	Result Type
925.5214	925.5015	-0.0199	-22	11	18	LSIAHQTR					Mascot

925.5214	925.5015	-0.0199	-22	11	18	LSIAHQTR	31	20.665			Mascot
1151.6824	1151.6327	-0.0497	-43	172	181	LVLANALYFK					Mascot
1176.5896	1176.572	-0.0176	-15	261	270	LSAEPDFLER					Mascot
1176.5896	1176.572	-0.0176	-15	261	270	LSAEPDFLER	76	99.998			Mascot
1292.7097	1292.6455	-0.0642	-50	289	300	ISFGIEASDLLK					Mascot
1611.8953	1611.766	-0.1293	-80	157	171	IKDILPPGSIDNTTK					Mascot
2062.0564	2061.9919	-0.0645	-31	138	156	AAEVTTQVNSWVEKVTS GR					Mascot
2113.0999	2113.0837	-0.0162	-8	379	398	EDISGVVLFMGHVVNPLL SS					Mascot
2129.0947	2128.9883	-0.1064	-50	379	398	EDISGVVLFMGHVVNPLL SS			Oxidation (M)[10]		Mascot
2720.3525	2720.3101	-0.0424	-16	328	353	VSSVFHQAFVEVNEQGT EAAASTAIK					Mascot
2720.3525	2720.3101	-0.0424	-16	328	353	VSSVFHQAFVEVNEQGT EAAASTAIK	39	89.59			Mascot
2943.5374	2943.7	0.1626	55	354	378	MVLQQARPPSVMDFIAD HPFLFLVR			Oxidation (M)[1]		Mascot
2943.5374	2943.7	0.1626	55	354	378	MVLQQARPPSVMDFIAD HPFLFLVR			Oxidation (M)[1]		Mascot

3	Serpin-Z1C OS=Triticum aestivum PE=1 SV=1	SPZ1C_WHEAT	42969	5.62	7	170	100	15.837	145	100	

Peptide Information

Calc. Mass	Obsrv. Mass	± da	± ppm	Start Seq.	End Seq.	Sequence	Ion Score	C. I. %	Modification	Rank	Result Type
925.5214	925.5015	-0.0199	-22	11	18	LSIAHQTR					Mascot
925.5214	925.5015	-0.0199	-22	11	18	LSIAHQTR	31	20.665			Mascot
1151.6824	1151.6327	-0.0497	-43	172	181	LVLANALYFK					Mascot
1176.5896	1176.572	-0.0176	-15	261	270	LSAEPDFLER					Mascot
1176.5896	1176.572	-0.0176	-15	261	270	LSAEPDFLER	76	99.998			Mascot
1239.5852	1239.6814	0.0962	78	127	137	ADTQSVDFQTK					Mascot
2113.0999	2113.0837	-0.0162	-8	379	398	EDISGVVLFMGHVVNPLL SS					Mascot
2129.0947	2128.9883	-0.1064	-50	379	398	EDISGVVLFMGHVVNPLL SS			Oxidation (M)[10]		Mascot
2720.3525	2720.3101	-0.0424	-16	328	353	VSSVFHQAFVEVNEQGT EAAASTAIK					Mascot
2720.3525	2720.3101	-0.0424	-16	328	353	VSSVFHQAFVEVNEQGT EAAASTAIK	39	89.59			Mascot
2725.4631	2725.325	-0.1381	-51	33	61	SAASNAVFSPVSLHVALS LLAAGAGSATR					Mascot

4	3-dehydroquinate synthase OS=Pyrobaculum calidifontis (strain JCM 11548 / VA1) GN=aroB PE=3 SV=1	AROB_PYRCJ	37597	6.07	12	56	0	2.965	

Peptide Information

Calc. Mass	Obsrv. Mass	± da	± ppm	Start Seq.	End Seq.	Sequence	Ion Score	C. I. %	Modification	Rank	Result Type

Calc. Mass	Obsrv. Mass	± da	± ppm	Start Seq.	End Seq.	Sequence	Ion Score	C. I. %	Modification	Rank	Result Type
804.4151	804.4279	0.0128	16	3	7	RFYYR					Mascot
836.4876	836.4518	-0.0358	-43	296	302	SLVKFDK					Mascot
837.4465	837.4443	-0.0022	-3	57	63	SFDSLLR					Mascot
887.4944	887.5195	0.0251	28	49	56	LRGGEEVK					Mascot
1198.5773	1198.5554	-0.0219	-18	256	265	MAEELGYLTR			Oxidation (M)[1]		Mascot
1239.6804	1239.6814	0.001	1	8	19	HSRGVTEVAVGK					Mascot
1292.7209	1292.6455	-0.0754	-58	194	204	AVEEVVYRSLK					Mascot
1306.675	1306.6001	-0.0749	-57	191	201	GERAVEEVVYR					Mascot
1396.6855	1396.761	0.0755	54	64	75	VYQFLAEQGADR					Mascot
1615.8663	1615.7261	-0.1402	-87	222	236	QVLNVGHTVGHAVER					Mascot
2456.2336	2456.2378	0.0042	2	256	277	MAEELGYLTRGTSEEVA TLLTR			Oxidation (M)[1]		Mascot
2742.3523	2742.2854	-0.0669	-24	168	190	YGVALDADFYQWLAENV DEVLRR					Mascot

5	Rab11 family-interacting protein 1 OS=Mus musculus GN=Rab11fip1 PE=1 SV=2	RFIP1_MOUSE	70697.3	9.47	15	54	0	12.26	

Peptide Information

Calc. Mass	Obsrv. Mass	± da	± ppm	Start Seq.	End Seq.	Sequence	Ion Score	C. I. %	Modification	Rank	Result Type
836.524	836.4518	-0.0722	-86	219	225	IKTLFSK					Mascot
874.4815	874.4594	-0.0221	-25	634	641	VPAQMGKK			Oxidation (M)[5]		Mascot
1175.6532	1175.5756	-0.0776	-66	105	114	FLGRAEVDLR					Mascot
1176.6736	1176.572	-0.1016	-86	297	307	KEGLSFLGGLR					Mascot
1176.6736	1176.572	-0.1016	-86	297	307	KEGLSFLGGLR					Mascot
1198.562	1198.5554	-0.0066	-6	382	392	LSDSSTKDSMK					Mascot
1217.6195	1217.5925	-0.027	-22	624	633	VMEETPNILR			Oxidation (M)[2]		Mascot
1345.6859	1345.7191	0.0332	25	354	365	HLFSSTENLAAR					Mascot
1475.7775	1475.641	-0.1365	-92	231	244	TPLSQSMSVLPTSK					Mascot
1491.7689	1491.6566	-0.1123	-75	612	623	ELEDYIDNLLVR					Mascot
1815.8971	1815.8557	-0.0414	-23	444	461	DAAAKGSESEPLPTVSEK					Mascot
2065.0649	2065.0388	-0.0261	-13	10	28	GPGTMWSPTHVQVTVLQ AR					Mascot
2151.1082	2151.0164	-0.0918	-43	607	623	EFQVRELEDYIDNLLVR					Mascot
2941.4773	2941.5994	0.1221	42	1	28	MSLAASAGRGPGTMWS PTHVQVTVLQAR			Oxidation (M)[1,14]		Mascot
2957.3384	2957.4834	0.145	49	185	213	DSASDTASAIVPSVTPSV DSDDESFSKDK					Mascot
3023.4309	3023.375	-0.0559	-18	393	420	SMSLPSYRPLTSGDNRE SMSPANVEAAR					Mascot
3055.4207	3055.3315	-0.0892	-29	393	420	SMSLPSYRPLTSGDNRE			Oxidation (M)[2,19]		Mascot

SMSPANVEAAR

6	Elongation factor Ts OS=Rhizobium meliloti (strain 1021) GN=tsf PE=1 SV=1	EFTS_RHIME	32138.6	5.07	11	54	0	2.393	

Peptide Information

Calc. Mass	Obsrv. Mass	± da	± ppm	Start Seq.	End Seq.	Sequence	Ion Score	C. I. %	Modification	Rank	Result Type
836.4546	836.4518	-0.0028	-3	2	9	TVTAAMVK			Oxidation (M)[6]		Mascot
1098.4741	1098.4778	0.0037	3	15	24	TGAGMMDCKK			Carbamidomethyl (C)[8]		Mascot
1173.5746	1173.6484	0.0738	63	202	212	SSEIDPAVAER					Mascot
1239.7056	1239.6814	-0.0242	-20	271	283	SVGAPIEVAGIAR					Mascot
1593.853	1593.7354	-0.1176	-74	187	201	QVAMHVAATNPLAVR			Oxidation (M)[4]		Mascot
1615.865	1615.7261	-0.1389	-86	94	111	GVANVALGTDGSVAAVS K					Mascot
1648.8541	1648.7787	-0.0754	-46	70	84	AVVVEINSETDFVAR					Mascot
2075.0227	2074.9597	-0.063	-30	25	43	ALAETNGDMEAAIDWLR AK					Mascot
2655.3738	2655.3911	0.0173	7	140	165	RSALLEVEDGVVATYVH NAAGEGIGK					Mascot
2655.3738	2655.5725	0.1987	75	140	165	RSALLEVEDGVVATYVH NAAGEGIGK					Mascot
2748.4097	2748.2622	-0.1475	-54	187	212	QVAMHVAATNPLAVRSS EIDPAVAER			Oxidation (M)[4]		Mascot
2941.5017	2941.5994	0.0977	33	241	266	KFFEEVALLSQAFVMNPD QTVEAAIK			Oxidation (M)[15]		Mascot

7	Phosphomethylpyrimidine synthase OS=Rhodopirellula baltica (strain SH1) GN=thiC PE=3 SV=1	THIC_RHOBA	49140.4	5.34	13	53	0	7.032	

Peptide Information

Calc. Mass	Obsrv. Mass	± da	± ppm	Start Seq.	End Seq.	Sequence	Ion Score	C. I. %	Modification	Rank	Result Type
901.485	901.4322	-0.0528	-59	104	110	DIDNIRR					Mascot
917.412	917.4333	0.0213	23	191	197	WMMAHNK					Mascot
1175.5652	1175.5756	0.0104	9	361	371	GAQDRDDALSK					Mascot
1204.6355	1204.6011	-0.0344	-29	422	431	ITEDIRQMAK					Mascot
1204.6355	1204.6011	-0.0344	-29	422	431	ITEDIRQMAK					Mascot
1239.7056	1239.6814	-0.0242	-20	22	31	REDLPVELIR					Mascot
1281.5173	1281.5765	0.0592	46	406	416	SAHFCSMCGPK			Carbamidomethyl (C)[5,8]		Mascot
1415.6658	1415.6708	0.005	4	417	427	YCSMKITEDIR			Carbamidomethyl (C)[2]		Mascot
1507.7032	1507.5845	-0.1187	-79	154	166	QGVDYMTIHCGVK			Carbamidomethyl (C)[10]		Mascot
1593.7941	1593.7354	-0.0587	-37	9	22	AGEITPEMEYVAKR					Mascot
1965.9045	1966.0315	0.127	65	198	213	QNPLLEAFDDLCDIMR			Carbamidomethyl (C)[12], Oxidation (M)[15]		Mascot
2223.1326	2223.1047	-0.0279	-13	2	21	TQLLSARAGEITPEMEYV			Oxidation (M)[15]		Mascot

						AK					
2720.376	2720.3101	-0.0659	-24	154	176	QGVDYMTIHCGVKLEHL HLTVNR			Carbamidomethyl (C)[10]		Mascot
2720.376	2720.3101	-0.0659	-24	154	176	QGVDYMTIHCGVKLEHL HLTVNR			Carbamidomethyl (C)[10]		Mascot
2742.3516	2742.2854	-0.0662	-24	85	109	LHTAVHFGADTVMDLST GKDIDNIR			Oxidation (M)[13]		Mascot

8	Methionine--tRNA ligase OS=Chlorobium tepidum (strain ATCC 49652 / DSM 12025 / TLS) GN=metG PE=3 SV=1	SYM_CHLTE	79959.4	5.66	16	52	0	4.427	

Peptide Information

Calc. Mass	Obsrv. Mass	± da	± ppm	Start Seq.	End Seq.	Sequence	Ion Score	C. I. %	Modification	Rank	Result Type
804.4977	804.4279	-0.0698	-87	295	301	IINFIGK					Mascot
814.4053	814.3985	-0.0068	-8	481	488	VDPEAAGR					Mascot
819.4359	819.3906	-0.0453	-55	363	369	FPADTLR					Mascot
901.4811	901.4322	-0.0489	-54	613	620	VVACEPVK			Carbamidomethyl (C)[4]		Mascot
974.469	974.511	0.042	43	278	285	QGDAELWR					Mascot
989.4972	989.5076	0.0104	11	565	572	DIEPEMKK					Mascot
989.4972	989.5076	0.0104	11	565	572	DIEPEMKK					Mascot
1173.6011	1173.6484	0.0473	40	224	233	TWLNQGLADR					Mascot
1198.5596	1198.5554	-0.0042	-4	142	151	YITGTCPVCK			Carbamidomethyl (C)[6,9]		Mascot
1345.6093	1345.7191	0.1098	82	370	380	YSIAMNYPENK			Oxidation (M)[5]		Mascot
1648.8654	1648.7787	-0.0867	-53	393	407	TNGELADTLGNFIKR					Mascot
2184.0681	2184.0813	0.0132	6	588	605	QPVPMTFKPEITFDDFQK			Oxidation (M)[5]		Mascot
2527.2034	2527.2747	0.0713	28	443	464	LEAAYDGFHFREATAQT MEIAR					Mascot
2641.304	2641.3579	0.0539	20	42	66	LCGHDVIHIGGSDEHGVP ITITADK			Carbamidomethyl (C)[2]		Mascot
2702.3647	2702.3669	0.0022	1	515	537	IWKMLGFEGTIDELVEPG NPVWR			Oxidation (M)[4]		Mascot
2725.2192	2725.325	0.1058	39	152	176	TPGANGDQCEQCGTHLS PTELIDPK			Carbamidomethyl (C)[9,12]		Mascot
2991.3755	2991.3618	-0.0137	-5	381	406	DTDFSWSDFQNRTNGEL ADTLGNFIK					Mascot

9	Elongation factor Ts OS=Sinorhizobium medicae (strain WSM419) GN=tsf PE=1 SV=1	EFTS_SINMW	32096.6	5.07	11	52	0	2.055	

Peptide Information

Calc. Mass	Obsrv. Mass	± da	± ppm	Start Seq.	End Seq.	Sequence	Ion Score	C. I. %	Modification	Rank	Result Type
836.4546	836.4518	-0.0028	-3	2	9	TVTAAMVK			Oxidation (M)[6]		Mascot
1098.4741	1098.4778	0.0037	3	15	24	TGAGMMDCKK			Carbamidomethyl (C)[8]		Mascot
1173.5746	1173.6484	0.0738	63	202	212	SSEIDPAVAER					Mascot

1239.7056	1239.6814	-0.0242	-20	271	283	SVGAPIEVAGIAR						Mascot
1593.853	1593.7354	-0.1176	-74	187	201	QVAMHVAATNPLAVR			Oxidation (M)[4]			Mascot
1615.865	1615.7261	-0.1389	-86	94	111	GVANVALGTDGSVAAVS K						Mascot
1648.8541	1648.7787	-0.0754	-46	70	84	AVVVEINSETDFVAR						Mascot
2075.0227	2074.9597	-0.063	-30	25	43	ALAETNGDMEAAIDWLR AK						Mascot
2689.325	2689.0911	-0.2339	-87	140	165	RSAMLEVEDGVVATYVH NAAGEGIGK			Oxidation (M)[4]			Mascot
2748.4097	2748.2622	-0.1475	-54	187	212	QVAMHVAATNPLAVRSS EIDPAVAER			Oxidation (M)[4]			Mascot
2941.5017	2941.5994	0.0977	33	241	266	KFFEEVALLSQAFVMNPD QTVEAAIK			Oxidation (M)[15]			Mascot

10	Recombination protein RecR OS=Flavobacterium johnsoniae (strain ATCC 17061 / DSM 2064 / UW101) GN=recR PE=3 SV=1	RECR_FLAJ1	23233	5.99	8	50	0	1.175

Peptide Information

Calc. Mass	Obsrv. Mass	± da	± ppm	Start Seq.	End Seq.	Sequence	Ion Score	C. I. %	Modification	Rank	Result Type
1225.7052	1225.6278	-0.0774	-63	192	201	SILHRVPFEK					Mascot
1384.6414	1384.6703	0.0289	21	91	102	DVMAIENTGQYK			Oxidation (M)[3]		Mascot
1623.8159	1623.8595	0.0436	27	39	52	EQTGFLSQALLNMR			Oxidation (M)[13]		Mascot
2641.3105	2641.3579	0.0474	18	137	159	VVEIIFALSSTMEGDTTNF YIYK					Mascot
2672.2703	2672.3945	0.1242	46	69	90	VCEICANSVRNHQTICVV EDIR			Carbamidomethyl (C)[2,5,16]		Mascot
2713.1587	2713.3625	0.2038	75	57	78	FCENCHNISDTKVCEICA NSVR			Carbamidomethyl (C)[2,5,14,17]		Mascot
2833.3608	2833.314	-0.0468	-17	79	102	NHQTICVVEDIRDVMAIE NTGQYK			Carbamidomethyl (C)[6]		Mascot
2991.6514	2991.3618	-0.2896	-97	103	131	GIYHVLGGKISPIEGVGPS QLNISSLVEK					Mascot
2991.6514	2991.7878	0.1364	46	103	131	GIYHVLGGKISPIEGVGPS QLNISSLVEK					Mascot

Gel Idx/Pos	260/K12	Instr./Gel Origin	BA2151/Sample Project 20140814	Process Status	Analysis Succeeded
Plate [#] Name	[1] Sample Project 20140814	Instrument Sample Name		Spectra	11

Rank	Protein Name	Accession No.	Protein MW	Protein PI	Pep. Count	Protein Score	Protein Score C. I. %	Intensity Matched	Total Ion Score	Total Ion C. I. %	Confirmed
1	Xin actin-binding repeat-containing protein 2 OS=Homo sapiens GN=XIRP2 PE=1 SV=2	XIRP2_HUMAN	383887.9	5.99	49	76	98.61	33.205			

Peptide Information

Calc. Mass	Obsrv. Mass	± da	± ppm	Start Seq.	End Seq.	Sequence	Ion Score	C. I. %	Modification	Rank	Result Type
800.4512	800.3928	-0.0584	-73	1839	1845	TEVNLPK					Mascot
846.493	846.4209	-0.0721	-85	545	551	SELKEIK					Mascot
847.4706	847.4354	-0.0352	-42	1691	1698	GNMLATLK					Mascot
856.3907	856.4612	0.0705	82	2	9	SPESGHSR					Mascot
860.4472	860.3868	-0.0604	-70	2743	2749	QEITQNK					Mascot
862.4338	862.4528	0.019	22	1723	1729	AIECLEK			Carbamidomethyl (C)[4]		Mascot
863.4655	863.4437	-0.0218	-25	1691	1698	GNMLATLK			Oxidation (M)[3]		Mascot
870.4427	870.4844	0.0417	48	3130	3136	LSEHTQR					Mascot
874.4628	874.4027	-0.0601	-69	2617	2623	NQEDKLK					Mascot
896.4836	896.3991	-0.0845	-94	755	761	EYTRTVK					Mascot
1030.5527	1030.4806	-0.0721	-70	2964	2972	NELSQSPKK					Mascot
1071.4841	1071.4359	-0.0482	-45	1114	1122	EESDYISTK					Mascot
1151.5579	1151.5886	0.0307	27	1291	1300	GEGLEYENIK					Mascot
1176.6008	1176.5571	-0.0437	-37	2052	2061	VSEKSHNTFK					Mascot
1180.4788	1180.5294	0.0506	43	1078	1087	ESQEGDECVK			Carbamidomethyl (C)[8]		Mascot
1225.5947	1225.5708	-0.0239	-20	1379	1388	ETLEDLYSQK					Mascot
1275.6791	1275.5959	-0.0832	-65	721	731	LQKITASEEEK					Mascot
1280.6118	1280.6528	0.041	32	1933	1944	DDVFNSIQSAGK					Mascot
1305.7427	1305.6398	-0.1029	-79	326	335	LYKHIHPELR					Mascot
1306.7075	1306.6619	-0.0456	-35	2365	2375	FKTPLMIAEEK					Mascot
1306.7075	1306.6619	-0.0456	-35	2365	2375	FKTPLMIAEEK					Mascot
1322.7025	1322.6145	-0.088	-67	2365	2375	FKTPLMIAEEK			Oxidation (M)[6]		Mascot
1336.6162	1336.6178	0.0016	1	3259	3271	MKTSSSHSSEAGK					Mascot
1347.6903	1347.5894	-0.1009	-75	2788	2799	QFEAEPNKSGLK					Mascot
1373.6981	1373.6519	-0.0462	-34	1337	1348	MTKEEIPPSDVK					Mascot
1377.7009	1377.588	-0.1129	-82	1153	1164	EGSYHEVTTVKK					Mascot

1389.693	1389.6786	-0.0144	-10	1337	1348	MTKEEIPPSDVK			Oxidation (M)[1]		Mascot
1433.7483	1433.7328	-0.0155	-11	966	978	DDSETAVKLQTVK					Mascot
1459.8003	1459.6652	-0.1351	-93	1123	1135	KTITEEVIQGDVK					Mascot
1471.7388	1471.7726	0.0338	23	1585	1597	QLHTESNETLTAK					Mascot
1471.7388	1471.7726	0.0338	23	1585	1597	QLHTESNETLTAK					Mascot
1499.7061	1499.7408	0.0347	23	1956	1967	NDHQKMEGFHIK			Oxidation (M)[6]		Mascot
1541.7555	1541.719	-0.0365	-24	2398	2410	TQSQNQHITEVEK					Mascot
1570.8007	1570.7356	-0.0651	-41	571	584	DGSGQMLEIKTVHR					Mascot
1573.822	1573.7517	-0.0703	-45	2710	2722	KLPQPYNSLQEEK					Mascot
1580.6969	1580.6161	-0.0808	-51	124	137	SSQEMARNEQEGSK					Mascot
1584.8452	1584.844	-0.0012	-1	1540	1553	RTIHNLLSSTSNNK					Mascot
1593.7942	1593.7253	-0.0689	-43	1323	1336	TFDSIMEAHKGITK			Oxidation (M)[6]		Mascot
1599.8126	1599.73	-0.0826	-52	2095	2108	SLNPINFNPENNVK					Mascot
1622.7883	1622.8184	0.0301	19	597	609	WMFETQPLDTINK					Mascot
1623.7717	1623.8596	0.0879	54	2290	2303	KVMVMTSSEHTETK			Oxidation (M)[3]		Mascot
1623.7717	1623.8596	0.0879	54	2290	2303	KVMVMTSSEHTETK			Oxidation (M)[3]		Mascot
1642.8435	1642.7435	-0.1	-61	2743	2756	QEITQNKSFFSSVK					Mascot
1654.7716	1654.8354	0.0638	39	481	493	WMFETRPLDSMNK					Mascot
1665.8442	1665.8066	-0.0376	-23	1933	1948	DDVFNSIQSAGKTVGK					Mascot
1670.8418	1670.8124	-0.0294	-18	2075	2089	SQDFLMKTNTSTGLK					Mascot
1673.8606	1673.7976	-0.063	-38	1463	1476	TQLLNRSTEFHAEK					Mascot
1688.8337	1688.7306	-0.1031	-61	1599	1614	QEGEKEIIGGDVEGTK					Mascot
1688.8337	1688.7306	-0.1031	-61	1599	1614	QEGEKEIIGGDVEGTK					Mascot
1746.8868	1746.8579	-0.0289	-17	2864	2880	TGKPGNKPTSLDETSSK					Mascot
1773.9495	1773.8767	-0.0728	-41	2941	2956	TRPPSPTFITIESTAR					Mascot
1873.9152	1873.8832	-0.032	-17	3179	3194	VYAKGETNHNIQQESR					Mascot
1936.0651	1935.8962	-0.1689	-87	1797	1814	NKNSLLQPKPGPFEPAA K					Mascot
1936.0651	1935.8962	-0.1689	-87	1797	1814	NKNSLLQPKPGPFEPAA K					Mascot
1981.9761	1981.9203	-0.0558	-28	1829	1845	SCHGNLVEERTEVNLPK			Carbamidomethyl (C)[2]		Mascot
2139.9541	2140.0449	0.0908	42	3199	3217	EEFGLTSLGNTSFTDFSC K			Carbamidomethyl (C)[18]		Mascot
2250.0642	2250.2	0.1358	60	2090	2108	MAMERSLNPINFNPENN VK			Oxidation (M)[1,3]		Mascot

2	Coiled-coil domain-containing protein 168 OS=Homo sapiens GN=CCDC168 PE=2 SV=2	CC168_HUMAN	279602.5	9.35	41	62	65.087	22.829

Peptide Information

Calc. Mass	Obsrv. Mass	± da	± ppm	Start Seq.	End Seq.	Sequence	Ion Score	C. I. %	Modification	Rank	Result Type

812.4988	812.4341	-0.0647	-80	263	269	EGRILPK		Mascot
856.4747	856.4612	-0.0135	-16	1009	1015	NQPSRVR		Mascot
862.4702	862.4528	-0.0174	-20	1928	1934	TLEIQMK		Mascot
874.4628	874.4027	-0.0601	-69	430	436	IQEDKNK		Mascot
913.4308	913.4075	-0.0233	-26	879	885	IHCQEAR	Carbamidomethyl (C)[3]	Mascot
928.4119	928.4622	0.0503	54	815	822	ATHADEER		Mascot
960.5109	960.4437	-0.0672	-70	1361	1368	TANLEKER		Mascot
960.5109	960.4437	-0.0672	-70	1361	1368	TANLEKER		Mascot
979.4666	979.4426	-0.024	-25	244	251	ANQQMPYK		Mascot
989.5261	989.4911	-0.035	-35	1411	1419	KELEASNAK		Mascot
1156.6321	1156.5347	-0.0974	-84	1785	1794	NKLTSHLESK		Mascot
1164.6121	1164.5533	-0.0588	-50	733	741	QHIPQKEER		Mascot
1176.5466	1176.5571	0.0105	9	172	180	EPQWGMKER	Oxidation (M)[6]	Mascot
1215.5964	1215.655	0.0586	48	82	91	EEEGEPRITR		Mascot
1215.5964	1215.655	0.0586	48	82	91	EEEGEPRITR		Mascot
1216.6005	1216.6477	0.0472	39	877	885	FRIHCQEAR	Carbamidomethyl (C)[5]	Mascot
1247.5975	1247.5989	0.0014	1	844	855	TADAEARSGDVR		Mascot
1248.5815	1248.5784	-0.0031	-2	1156	1166	EDGQSNDRISK		Mascot
1280.6707	1280.6528	-0.0179	-14	770	781	TELHVNIGGQGR		Mascot
1292.6171	1292.6412	0.0241	19	2102	2110	QYHVWFQER		Mascot
1305.6355	1305.6398	0.0043	3	519	530	VADMTSVLDPNK	Oxidation (M)[4]	Mascot
1336.6968	1336.6178	-0.079	-59	1615	1625	ERIVPEHDVSR		Mascot
1362.745	1362.6494	-0.0956	-70	1167	1178	MFSPKVLAPQTK	Oxidation (M)[1]	Mascot
1405.7587	1405.7205	-0.0382	-27	1042	1053	SGKRPEWLFTGK		Mascot
1405.7587	1405.7205	-0.0382	-27	1042	1053	SGKRPEWLFTGK		Mascot
1499.7561	1499.7408	-0.0153	-10	226	239	SRLANSNEGISSHK		Mascot
1516.7603	1516.7627	0.0024	2	1016	1028	SEEDLNQLVLNSR		Mascot
1533.722	1533.6355	-0.0865	-56	1029	1041	DEDIYFTGFGTIR		Mascot
1537.7163	1537.7323	0.016	10	1323	1335	KLEMDNDSTVNQK	Oxidation (M)[4]	Mascot
1580.6898	1580.6161	-0.0737	-47	1665	1677	DQSEPVWDMTTQK	Oxidation (M)[9]	Mascot
1593.8054	1593.7253	-0.0801	-50	1346	1358	ALHLQEEKTEMHK		Mascot
1606.8105	1606.8546	0.0441	27	1720	1733	AISESQVKNMIQDK	Oxidation (M)[10]	Mascot
1623.845	1623.8596	0.0146	9	374	389	GPVQPTAQGEEKGGLR		Mascot
1623.845	1623.8596	0.0146	9	374	389	GPVQPTAQGEEKGGLR		Mascot
1665.8014	1665.8066	0.0052	3	1939	1952	IVRESYAMTSAHER	Oxidation (M)[8]	Mascot
1670.8021	1670.8124	0.0103	6	359	373	AYTESLHGYTLSNSK		Mascot

1673.8275	1673.7976	-0.0299	-18	1386	1399	AEESQMKTQVITHR			Oxidation (M)[6]		Mascot
1738.9884	1738.8992	-0.0892	-51	1795	1809	ALEIQLNLIPEMARK					Mascot
1746.8289	1746.8579	0.029	17	1645	1659	ILTPTECPSMLEDPK			Carbamidomethyl (C)[7], Oxidation (M)[10]		Mascot
1765.865	1765.8307	-0.0343	-19	783	797	EHEGQDKPPGMIQRK			Oxidation (M)[11]		Mascot
1780.9528	1780.8284	-0.1244	-70	1953	1968	KKPLSNCIHPGFTGPK			Carbamidomethyl (C)[7]		Mascot
1864.8495	1864.9403	0.0908	49	1663	1677	QRDQSEPVWDMTTQK			Oxidation (M)[11]		Mascot
1868.8636	1869.0179	0.1543	83	314	329	SASFPPPPFYLNCDTR			Carbamidomethyl (C)[13]		Mascot
1952.9834	1952.915	-0.0684	-35	132	149	VQTVSMHGLMHPNGAVF K					Mascot
1981.9866	1981.9203	-0.0663	-33	340	357	TQFSFPPLKIQDSSDSGK					Mascot

3 Pentatricopeptide repeat-containing protein At1g28690, mitochondrial OS=Arabidopsis thaliana GN=PCMP-E34 PE=2 SV=2 PPR62_ARATH 58727.5 8.75 17 61 57.048 10.443

Peptide Information

Calc. Mass	Obsrv. Mass	± da	± ppm	Start Seq.	End Seq.	Sequence	Ion Score	C. I. %	Modification	Rank	Result Type
847.4631	847.4354	-0.0277	-33	188	195	SGKLESAR					Mascot
862.3835	862.4528	0.0693	80	321	328	CGGINDAR			Carbamidomethyl (C)[1]		Mascot
874.4815	874.4027	-0.0788	-90	149	156	GSTMILPR					Mascot
1018.4847	1018.4858	0.0011	1	321	329	CGGINDARR			Carbamidomethyl (C)[1]		Mascot
1030.5527	1030.4806	-0.0721	-70	26	35	QNVSSLSPAK					Mascot
1111.6154	1111.5736	-0.0418	-38	157	165	SLCRLVHAR			Carbamidomethyl (C)[3]		Mascot
1151.5328	1151.5886	0.0558	48	511	520	TIGRSWTSED					Mascot
1180.578	1180.5294	-0.0486	-41	329	337	RVFDQMQEK					Mascot
1215.5748	1215.655	0.0802	66	310	320	MGSSLLDMYAK					Mascot
1215.5748	1215.655	0.0802	66	310	320	MGSSLLDMYAK					Mascot
1247.5647	1247.5989	0.0342	27	310	320	MGSSLLDMYAK			Oxidation (M)[1,8]		Mascot
1275.5675	1275.5959	0.0284	22	393	402	GYEIFESMQR			Oxidation (M)[8]		Mascot
1338.6801	1338.6328	-0.0473	-35	423	434	AGDLNKAFEFAR					Mascot
1389.7267	1389.6786	-0.0481	-35	144	156	ASNSRGSTMILPR					Mascot
1405.7217	1405.7205	-0.0012	-1	144	156	ASNSRGSTMILPR			Oxidation (M)[9]		Mascot
1405.7217	1405.7205	-0.0012	-1	144	156	ASNSRGSTMILPR			Oxidation (M)[9]		Mascot
1417.6562	1417.6949	0.0387	27	256	266	RSVDMYISMQR			Oxidation (M)[5,9]		Mascot
1427.72	1427.6968	-0.0232	-16	191	202	LESARTVFETMK			Oxidation (M)[11]		Mascot
1516.8159	1516.7627	-0.0532	-35	13	25	ILPSNHYSTFPLK					Mascot
1599.7836	1599.73	-0.0536	-34	236	249	DIVVYNAMVEGFSR					Mascot
1765.9517	1765.8307	-0.121	-69	118	132	ELLLLVQRMSYSGEK					Mascot

4	Elongation factor Ts OS=Campylobacter concisus (strain 13826) GN=tsf PE=3 SV=2	EFTS_CAMC1	39456.3	5.34	14	60	43.378	5.477

Peptide Information

Calc. Mass	Obsrv. Mass	± da	± ppm	Start Seq.	End Seq.	Sequence	Ion Score	C. I. %	Modification	Rank	Result Type
862.3981	862.4528	0.0547	63	122	127	FEEYFK					Mascot
925.5829	925.4909	-0.092	-99	275	282	IIPGKIER					Mascot
1016.5523	1016.5134	-0.0389	-38	87	95	NPQFQALAK					Mascot
1216.6783	1216.6477	-0.0306	-25	307	316	KTIEQVIEEK					Mascot
1305.6322	1305.6398	0.0076	6	342	354	VDDFAAEVAAQIG					Mascot
1341.6434	1341.6488	0.0054	4	283	293	FYADNTVLDQR					Mascot
1433.7272	1433.7328	0.0056	4	341	354	KVDDFAAEVAAQIG					Mascot
1477.7567	1477.7043	-0.0524	-35	55	68	LASEGLVSVEVCSK			Carbamidomethyl (C)[12]		Mascot
1570.8323	1570.7356	-0.0967	-62	257	270	AIEEELKAEGKPEK					Mascot
1670.8822	1670.8124	-0.0698	-42	294	307	LTLLGQFYVMDDKK					Mascot
1765.8823	1765.8307	-0.0516	-29	55	70	LASEGLVSVEVCSKCK			Carbamidomethyl (C)[12,15]		Mascot
1773.9871	1773.8767	-0.1104	-62	229	243	RLGKPLHHIPEYASR					Mascot
1780.9076	1780.8284	-0.0792	-44	71	86	KATISEINSETDFVAR					Mascot
1952.9391	1952.915	-0.0241	-12	186	202	NLCMHAAAMKPSVISYK			Carbamidomethyl (C)[3], Oxidation (M)[4,9]		Mascot

5	Nuclear hormone receptor E75 OS=Metapenaeus ensis GN=E75 PE=2 SV=1	E75_METEN	69185.5	9.09	16	60	42.06	14.638

Peptide Information

Calc. Mass	Obsrv. Mass	± da	± ppm	Start Seq.	End Seq.	Sequence	Ion Score	C. I. %	Modification	Rank	Result Type
846.4792	846.4209	-0.0583	-69	555	562	SSVRNVGK					Mascot
893.4332	893.4258	-0.0074	-8	93	100	CIAVGMSR			Carbamidomethyl (C)[1]		Mascot
896.4294	896.3991	-0.0303	-34	247	254	LAGMFDAR			Oxidation (M)[4]		Mascot
963.4741	963.4682	-0.0059	-6	512	519	SSIDEKER					Mascot
1018.5098	1018.4858	-0.024	-24	72	79	NQQCSILR			Carbamidomethyl (C)[4]		Mascot
1176.5677	1176.5571	-0.0106	-9	501	511	LSSPSVCSSPR			Carbamidomethyl (C)[7]		Mascot
1341.7386	1341.6488	-0.0898	-67	563	574	RSLTPHSPPPPR					Mascot
1405.7118	1405.7205	0.0087	6	536	546	RPSSTRICSWR			Carbamidomethyl (C)[8]		Mascot
1405.7118	1405.7205	0.0087	6	536	546	RPSSTRICSWR	4	0	Carbamidomethyl (C)[8]		Mascot
1524.6498	1524.6855	0.0357	23	279	290	FLMDSMFDFAER			Oxidation (M)[3]		Mascot
1537.6249	1537.7323	0.1074	70	483	497	SDSPDDSGIESGTDR					Mascot
1573.8519	1573.7517	-0.1002	-64	542	554	ICSWRKPTTSPIK			Carbamidomethyl (C)[2]		Mascot

1665.7198	1665.8066	0.0868	52	482	497	KSDSPDDSGIESGTDR					Mascot
1701.882	1701.7955	-0.0865	-51	564	578	SLTPHSPPPPRSWSR					Mascot
1935.8654	1935.8962	0.0308	16	373	389	YKMTEHTAAGAPWDDSR					Mascot
1935.8654	1935.8962	0.0308	16	373	389	YKMTEHTAAGAPWDDSR					Mascot
1951.8604	1951.8915	0.0311	16	373	389	YKMTEHTAAGAPWDDSR			Oxidation (M)[3]		Mascot
1953.8422	1953.9014	0.0592	30	457	475	HDHSEGASSGDEATESP LK					Mascot
1954.0645	1953.9014	-0.1631	-83	220	236	LPGFQQLPQEDQVTLLK					Mascot

6	Dystonin OS=Mus musculus GN=Dst PE=1 SV=1	DYST_MOUSE	838424.5	5.19	67	59	31.926	39.855	

Peptide Information

Calc. Mass	Obsrv. Mass	± da	± ppm	Start Seq.	End Seq.	Sequence	Ion Score	C. I. %	Modification	Rank	Result Type
812.4512	812.4341	-0.0171	-21	1545	1551	VPEKPDK					Mascot
826.3069	826.3394	0.0325	39	1347	1352	MDECQK			Carbamidomethyl (C)[4], Oxidation (M)[1]		Mascot
840.4073	840.4304	0.0231	27	1949	1954	WLSFCK			Carbamidomethyl (C)[5]		Mascot
846.4316	846.4209	-0.0107	-13	3944	3950	LDQVTDR					Mascot
847.4341	847.4354	0.0013	2	471	477	DEIMALR					Mascot
857.4588	857.3925	-0.0663	-77	7307	7314	TPSRPGSR					Mascot
860.4584	860.3868	-0.0716	-83	6620	6626	SVERQNK					Mascot
862.4265	862.4528	0.0263	30	1219	1225	SEVDEKR					Mascot
863.4291	863.4437	0.0146	17	471	477	DEIMALR			Oxidation (M)[4]		Mascot
874.4781	874.4027	-0.0754	-86	5408	5414	WEALLSR					Mascot
893.4839	893.4258	-0.0581	-65	1419	1426	FAGDSLKR					Mascot
896.3414	896.3991	0.0577	64	2526	2532	EECDTSR			Carbamidomethyl (C)[3]		Mascot
925.4526	925.4909	0.0383	41	3436	3442	HEDFLHK					Mascot
960.5109	960.4437	-0.0672	-70	1725	1732	SILQENTR					Mascot
960.5109	960.4437	-0.0672	-70	1725	1732	SILQENTR	4	0			Mascot
979.5095	979.4426	-0.0669	-68	5829	5836	EGIYIQEK					Mascot
989.5738	989.4911	-0.0827	-84	6686	6694	TSSVQALKR					Mascot
1018.5275	1018.4858	-0.0417	-41	409	417	NALQSDSKR					Mascot
1111.5742	1111.5736	-0.0006	-1	5463	5471	LEEQISQHK					Mascot
1139.5765	1139.5908	0.0143	13	1238	1247	AKAISDEMFK					Mascot
1151.6532	1151.5886	-0.0646	-56	5026	5035	HLSVLQAQQK					Mascot
1156.5885	1156.5347	-0.0538	-47	3206	3215	LLFDGYATEK					Mascot
1164.6041	1164.5533	-0.0508	-44	487	496	GRMLTTEQTK					Mascot
1176.5645	1176.5571	-0.0074	-6	1216	1224	QWRSEVDEK					Mascot

1180.5991	1180.5294	-0.0697	-59	487	496	GRMLTTEQTK	Oxidation (M)[3]	Mascot
1204.6355	1204.6057	-0.0298	-25	383	392	LDMLQQIATR	Oxidation (M)[3]	Mascot
1215.658	1215.655	-0.003	-2	5872	5881	IDQILESLER		Mascot
1215.7129	1215.655	-0.0579	-48	365	374	LIIAMLEREK		Mascot
1216.6783	1216.6477	-0.0306	-25	4832	4842	TISEGENLLLK		Mascot
1232.623	1232.6259	0.0029	2	4999	5008	DTQRQLQDTK		Mascot
1247.6089	1247.5989	-0.01	-8	4654	4663	CDWIDQAIVK	Carbamidomethyl (C)[1]	Mascot
1255.7157	1255.6053	-0.1104	-88	182	192	DGKLFNAIIHK		Mascot
1275.7096	1275.5959	-0.1137	-89	337	346	LLEIWIEFGR		Mascot
1292.6515	1292.6412	-0.0103	-8	1205	1215	NNIENLMSTLK	Oxidation (M)[7]	Mascot
1305.6467	1305.6398	-0.0069	-5	5180	5190	DLEALSKQCNK	Carbamidomethyl (C)[9]	Mascot
1306.646	1306.6619	0.0159	12	1240	1250	AISDEMFKTHK		Mascot
1306.646	1306.6619	0.0159	12	1240	1250	AISDEMFKTHK		Mascot
1322.6006	1322.6145	0.0139	11	393	403	VQRDSVSCEDK	Carbamidomethyl (C)[8]	Mascot
1336.6301	1336.6178	-0.0123	-9	843	853	LEDLVQESMEK	Oxidation (M)[9]	Mascot
1338.6648	1338.6328	-0.032	-24	4168	4179	DLSARFSEASQK		Mascot
1347.6937	1347.5894	-0.1043	-77	1373	1384	AMVESQQKSPVK	Oxidation (M)[2]	Mascot
1362.6497	1362.6494	-0.0003	0	5197	5208	TREEQVDGATEK		Mascot
1373.692	1373.6519	-0.0401	-29	4643	4653	WDNLTGQLRDR		Mascot
1417.7217	1417.6949	-0.0268	-19	3658	3669	CAQLNLKAEQSR	Carbamidomethyl (C)[1]	Mascot
1433.6842	1433.7328	0.0486	34	4532	4542	WQELNQLTMDR		Mascot
1443.6659	1443.6593	-0.0066	-5	7165	7176	NDPCRVHHHGSK	Carbamidomethyl (C)[4]	Mascot
1453.8009	1453.6626	-0.1383	-95	5886	5898	LRQPPSISAEVEK		Mascot
1459.6642	1459.6652	0.001	1	6996	7006	EFANFDFDIWR		Mascot
1461.6097	1461.6963	0.0866	59	2905	2917	ETGPCTSLGHCDK	Carbamidomethyl (C)[5,11]	Mascot
1477.6904	1477.7043	0.0139	9	1427	1438	LEEEEKSLDEEK		Mascot
1487.8064	1487.7279	-0.0785	-53	5036	5048	SLQTLKQQVDEAK		Mascot
1499.6285	1499.7408	0.1123	75	719	730	EESEVAYDWSER		Mascot
1507.6516	1507.6327	-0.0189	-13	3177	3188	VLTQMDCDPEQR	Carbamidomethyl (C)[7], Oxidation (M)[5]	Mascot
1516.8887	1516.7627	-0.126	-83	337	348	LLEIWIEFGRIK		Mascot
1537.7244	1537.7323	0.0079	5	2413	2425	TPPPDDIFYDVMK		Mascot
1541.8071	1541.719	-0.0881	-57	4888	4899	EQVETLRPWIDR		Mascot
1573.6522	1573.7517	0.0995	63	173	184	CENFTTCWRDGK	Carbamidomethyl (C)[1,7]	Mascot
1606.8799	1606.8546	-0.0253	-16	7035	7048	ITRQEFIDGILSSK		Mascot
1624.8905	1624.8463	-0.0442	-27	6468	6481	EQIIELDKTGTHLK		Mascot
1746.8956	1746.8579	-0.0377	-22	1205	1218	NNIENLMSTLKQWR		Mascot
1765.9	1765.8307	-0.0693	-39	2878	2894	ADLTSAVTASEMKSQVK		Mascot

1780.8317	1780.8284	-0.0033	-2	5147	5162	LEALMASNDSANRTCK			Carbamidomethyl (C)[15]		Mascot
1864.9474	1864.9403	-0.0071	-4	1388	1403	IQSSADLVIQEFMDLR					Mascot
1868.8517	1869.0179	0.1662	89	6889	6903	TLIAEHQTFMEEMTR			Oxidation (M)[10,13]		Mascot
1874.0131	1873.8832	-0.1299	-69	6918	6934	RATDPPSLQSHIPVLDK					Mascot
1952.9131	1952.915	0.0019	1	6705	6720	DDSSWVRVQMQELSTR			Oxidation (M)[10]		Mascot
1953.9811	1953.9014	-0.0797	-41	998	1014	TMLPGEHQQVLSNLQSR			Oxidation (M)[2]		Mascot
1954.0201	1953.9014	-0.1187	-61	660	676	ISEIQMTAPLKLSYTDK			Oxidation (M)[6]		Mascot
1982.0593	1981.9203	-0.139	-70	1484	1500	KEQFSEALQTTQIFLAK					Mascot
2140.0334	2140.0449	0.0115	5	5160	5178	TCKMMLATEETSPDLIGV K			Carbamidomethyl (C)[2], Oxidation (M)[4]		Mascot
2685.2122	2685.3525	0.1403	52	3739	3764	LGAGEEDEVNGNLLETD AEGHSEATK					Mascot

7	Apolipoprotein A-I OS=Gallus gallus GN=APOA1 PE=1 SV=2	APOA1_CHICK	30661.1	5.58	13	58	12.304	7.376

Peptide Information

Calc. Mass	Obsrv. Mass	± da	± ppm	Start Seq.	End Seq.	Sequence	Ion Score	C. I. %	Modification	Rank	Result Type
989.515	989.4911	-0.0239	-24	112	119	DLEEVKEK					Mascot
1016.4393	1016.5134	0.0741	73	85	92	EDMAPYYK					Mascot
1305.6797	1305.6398	-0.0399	-31	184	194	KNLAPYSDELR					Mascot
1322.6587	1322.6145	-0.0442	-33	51	63	DAIAQFESSAVGK					Mascot
1362.7046	1362.6494	-0.0552	-41	219	229	VMEQLSNLREK			Oxidation (M)[2]		Mascot
1373.7271	1373.6519	-0.0752	-55	69	82	LADNLDTLSAAAAK					Mascot
1377.7195	1377.588	-0.1315	-95	228	238	EKMTPLVQEFR					Mascot
1405.7257	1405.7205	-0.0052	-4	230	240	MTPLVQEFRER					Mascot
1405.7257	1405.7205	-0.0052	-4	230	240	MTPLVQEFRER					Mascot
1433.7383	1433.7328	-0.0055	-4	185	196	NLAPYSDELRQK					Mascot
1537.7394	1537.7323	-0.0071	-5	131	141	WTEELEQYRQR					Mascot
1654.8357	1654.8354	-0.0003	0	36	50	DMVDVYLETVKASGK					Mascot
1665.8442	1665.8066	-0.0376	-23	47	63	ASGKDAIAQFESSAVGK					Mascot
1670.8306	1670.8124	-0.0182	-11	36	50	DMVDVYLETVKASGK			Oxidation (M)[2]		Mascot
1755.8086	1755.9146	0.106	60	20	33	SFWQHDEPQTPLDR					Mascot

8	Microtubule-actin cross-linking factor 1 OS=Mus musculus GN=Macf1 PE=1 SV=2	MACF1_MOUSE	836892.8	5.3	65	58	10.261	28.289

Peptide Information

Calc. Mass	Obsrv. Mass	± da	± ppm	Start Seq.	End Seq.	Sequence	Ion Score	C. I. %	Modification	Rank	Result Type

800.3719	800.3928	0.0209	26	5570	5575	EVMEHR		Mascot
846.468	846.4209	-0.0471	-56	4523	4530	ATEVTVAR		Mascot
857.4475	857.3925	-0.055	-64	6889	6895	KQPDVDR		Mascot
860.4221	860.3868	-0.0353	-41	72	78	VADERDR		Mascot
862.4451	862.4528	0.0077	9	2993	3000	MIGNDKGK		Mascot
870.5229	870.4844	-0.0385	-44	5653	5660	LMALGPIR		Mascot
893.4033	893.4258	0.0225	25	6107	6113	DQLNEMK	Oxidation (M)[6]	Mascot
928.4669	928.4622	-0.0047	-5	5569	5575	KEVMEHR		Mascot
933.4887	933.4069	-0.0818	-88	1908	1916	ATESGILDK		Mascot
941.4397	941.5041	0.0644	68	661	667	LETQYCK	Carbamidomethyl (C)[6]	Mascot
941.5414	941.5041	-0.0373	-40	2359	2367	AISGILDPR		Mascot
963.453	963.4682	0.0152	16	2628	2635	IDTESGWR		Mascot
979.4764	979.4426	-0.0338	-35	1106	1114	SDLDAISMK		Mascot
988.517	988.4675	-0.0495	-50	2806	2815	AGIRGSNGEK		Mascot
1002.5214	1002.4888	-0.0326	-33	1505	1512	EQISEQLR		Mascot
1011.5192	1011.4835	-0.0357	-35	1027	1034	AHFQHLMK		Mascot
1016.5007	1016.5134	0.0127	12	4092	4099	EVEQNLER		Mascot
1018.5601	1018.4858	-0.0743	-73	408	415	LIVEMLER	Oxidation (M)[5]	Mascot
1079.515	1079.5215	0.0065	6	3100	3109	GDMAAQITTR	Oxidation (M)[3]	Mascot
1111.4871	1111.5736	0.0865	78	3493	3501	DMKQSMAER	Oxidation (M)[2]	Mascot
1151.6207	1151.5886	-0.0321	-28	956	964	SLISWNYLR		Mascot
1176.6041	1176.5571	-0.047	-40	2821	2830	KISVEMEGQR		Mascot
1215.6732	1215.655	-0.0182	-15	689	699	ATAELIWLNGK		Mascot
1215.6732	1215.655	-0.0182	-15	689	699	ATAELIWLNGK		Mascot
1216.5892	1216.6477	0.0585	48	5082	5091	LEGIGQFHCR	Carbamidomethyl (C)[9]	Mascot
1225.6497	1225.5708	-0.0789	-64	5872	5882	MPPLIPAEVDK	Oxidation (M)[1]	Mascot
1232.6117	1232.6259	0.0142	12	4685	4694	QQLEETSEIR		Mascot
1247.6049	1247.5989	-0.006	-5	5358	5369	ATVDMLQAEGGR		Mascot
1255.5658	1255.6053	0.0395	31	5071	5081	VSSSCLTMENK	Carbamidomethyl (C)[5]	Mascot
1275.5997	1275.5959	-0.0038	-3	436	446	IQNGALNCEEK	Carbamidomethyl (C)[8]	Mascot
1280.6416	1280.6528	0.0112	9	4195	4204	LQQFMENKSR		Mascot
1305.6646	1305.6398	-0.0248	-19	4462	4473	TETVKAQAESNK		Mascot
1322.6774	1322.6145	-0.0629	-48	6706	6716	WDTVCKLSVSK	Carbamidomethyl (C)[5]	Mascot
1336.6315	1336.6178	-0.0137	-10	6598	6608	WDTVCGKSVER	Carbamidomethyl (C)[5]	Mascot
1338.5778	1338.6328	0.055	41	2951	2962	EMGGEQSVQMSR		Mascot
1347.7015	1347.5894	-0.1121	-83	7098	7108	RFQVEQIGENK		Mascot
1362.6318	1362.6494	0.0176	13	4129	4140	SSLEATHDMVTR	Oxidation (M)[9]	Mascot

1373.7383	1373.6519	-0.0864	-63	3832	3843	SEAELKQTQALR		Mascot
1377.6758	1377.588	-0.0878	-64	1233	1244	HPASEPNLDLER		Mascot
1417.7434	1417.6949	-0.0485	-34	4164	4174	FQQLQLQQQEK		Mascot
1459.7322	1459.6652	-0.067	-46	4777	4789	QSQQAKNCPISAK	Carbamidomethyl (C)[8]	Mascot
1461.7333	1461.6963	-0.037	-25	4771	4782	TWLDEKQSQQAK		Mascot
1471.7588	1471.7726	0.0138	9	5082	5093	LEGIGQFHCRVR	Carbamidomethyl (C)[9]	Mascot
1471.7588	1471.7726	0.0138	9	5082	5093	LEGIGQFHCRVR	Carbamidomethyl (C)[9]	Mascot
1477.7645	1477.7043	-0.0602	-41	6342	6353	EEIYNQLLDKGR		Mascot
1487.7952	1487.7279	-0.0673	-45	5482	5494	QTTGEEVLLIQEK		Mascot
1516.7465	1516.7627	0.0162	11	6903	6915	KSVEPTHAPFMEK	Oxidation (M)[11]	Mascot
1537.772	1537.7323	-0.0397	-26	7137	7150	VGGGWMALDEFLVK	Oxidation (M)[6]	Mascot
1563.7247	1563.7241	-0.0006	0	720	732	TYFSELTMELEGK	Oxidation (M)[8]	Mascot
1570.7611	1570.7356	-0.0255	-16	5934	5945	LDQMVFFWEDIK		Mascot
1573.7434	1573.7517	0.0083	5	6982	6993	EFANFDFDVWRK		Mascot
1606.8523	1606.8546	0.0023	1	943	955	VMALWHQLHINTK	Oxidation (M)[2]	Mascot
1642.6974	1642.7435	0.0461	28	2	15	SSSDEETLSERSCR	Carbamidomethyl (C)[13]	Mascot
1654.7135	1654.8354	0.1219	74	2157	2169	CQTPTKCSFTCHK	Carbamidomethyl (C)[1,7,11]	Mascot
1665.8442	1665.8066	-0.0376	-23	3823	3837	EQYAASLARSEAELK		Mascot
1669.8367	1669.7726	-0.0641	-38	4577	4589	QQVQFMLKEFEAR	Oxidation (M)[6]	Mascot
1688.8538	1688.7306	-0.1232	-73	3141	3154	LHQEIPMDPTRSHK		Mascot
1688.8538	1688.7306	-0.1232	-73	3141	3154	LHQEIPMDPTRSHK		Mascot
1701.9495	1701.7955	-0.154	-90	836	851	EQLIQSKSSVASLVGR		Mascot
1704.8486	1704.7524	-0.0962	-56	3141	3154	LHQEIPMDPTRSHK	Oxidation (M)[7]	Mascot
1738.8091	1738.8992	0.0901	52	7247	7265	TSLAGDTSNSSSPASTG AK		Mascot
1765.8248	1765.8307	0.0059	3	6262	6275	LETMNQCWESVLQK	Carbamidomethyl (C)[7]	Mascot
1773.7378	1773.8767	0.1389	78	1	15	MSSSDEETLSERSCR	Carbamidomethyl (C)[14]	Mascot
1868.9099	1869.0179	0.108	58	3733	3748	LAFQEWSSSLELAMEK		Mascot
1935.889	1935.8962	0.0072	4	1444	1462	NTQGTTTSSHTSASADIE K		Mascot
1935.889	1935.8962	0.0072	4	1444	1462	NTQGTTTSSHTSASADIE K		Mascot
1951.9178	1951.8915	-0.0263	-13	5133	5148	IQALRFDIEDSEAECR	Carbamidomethyl (C)[15]	Mascot
1952.9495	1952.915	-0.0345	-18	1115	1132	CNVFLQQSPSGSSATTL R	Carbamidomethyl (C)[1]	Mascot
2140.1431	2140.0449	-0.0982	-46	1703	1721	NLIDPNTAEKVGLLDLMQ R		Mascot
2685.3037	2685.3525	0.0488	18	3100	3123	GDMAAQITTRQETTDVQ DLYITSK		Mascot

9	Alternative oxidase, mitochondrial OS=Emericella nidulans (strain FGSC A4 / ATCC 38163 / CBS 112.46 /	AOX_EMENI	40994.1	9.5	15	56	0	8.702

NRRL 194 / M139) GN=alxA PE=3 SV=2

Peptide Information

Calc. Mass	Obsrv. Mass	± da	± ppm	Start Seq.	End Seq.	Sequence	Ion Score	C. I. %	Modification	Rank	Result Type
826.4166	826.3394	-0.0772	-93	300	306	ADEAKHR					Mascot
988.5422	988.4675	-0.0747	-76	257	265	AIKDLESGR					Mascot
1002.5804	1002.4888	-0.0916	-91	198	205	MHLLTFLK					Mascot
1018.5754	1018.4858	-0.0896	-88	198	205	MHLLTFLK			Oxidation (M)[1]		Mascot
1079.515	1079.5215	0.0065	6	2	11	NSMSTTGPIR			Oxidation (M)[3]		Mascot
1156.4915	1156.5347	0.0432	37	206	214	MAEPGWFMR			Oxidation (M)[1,8]		Mascot
1180.5568	1180.5294	-0.0274	-23	281	289	YWKMPEGNR					Mascot
1389.691	1389.6786	-0.0124	-9	106	117	NWSDWVALGSVR					Mascot
1405.7686	1405.7205	-0.0481	-34	293	304	DLLLYVRADEAK					Mascot
1405.7686	1405.7205	-0.0481	-34	293	304	DLLLYVRADEAK					Mascot
1417.7659	1417.6949	-0.071	-50	305	316	HREVNHTLGNLK					Mascot
1584.8282	1584.844	0.0158	10	18	29	HYLQFTVRTYTR					Mascot
1642.8945	1642.7435	-0.151	-92	2	17	NSMSTTGPIRVAAIPK					Mascot
1773.9351	1773.8767	-0.0584	-33	1	17	MNSMSTTGPIRVAAIPK					Mascot
1951.9032	1951.8915	-0.0117	-6	182	197	DNGWIETLLEEAYNER					Mascot
1982.0239	1981.9203	-0.1036	-52	153	171	FVFLESVAGVPGMVGGM LR			Oxidation (M)[13]		Mascot
2140.0542	2140.0449	-0.0093	-4	198	214	MHLLTFLKMAEPGWFMR			Oxidation (M)[1,9]		Mascot

10	Butyrate kinase OS=Clostridium beijerinckii (strain ATCC 51743 / NCIMB 8052) GN=buk PE=3 SV=1	BUK_CLOB8	38523.9	5.14	12	55	0	6.629

Peptide Information

Calc. Mass	Obsrv. Mass	± da	± ppm	Start Seq.	End Seq.	Sequence	Ion Score	C. I. %	Modification	Rank	Result Type
857.4628	857.3925	-0.0703	-82	154	160	FHALNQK					Mascot
870.4315	870.4844	0.0529	61	33	39	HTNEEIK					Mascot
893.4727	893.4258	-0.0469	-52	61	67	EKNFDIK					Mascot
928.408	928.4622	0.0542	58	270	276	ECESIYK			Carbamidomethyl (C)[2]		Mascot
988.4945	988.4675	-0.027	-27	345	353	VLDGEEQAK					Mascot
1232.6555	1232.6259	-0.0296	-24	285	296	AIGEMSVVLEGK					Mascot
1248.6504	1248.5784	-0.072	-58	285	296	AIGEMSVVLEGK			Oxidation (M)[5]		Mascot
1433.8112	1433.7328	-0.0784	-55	63	75	NFDIKTLSAIVGR					Mascot
1624.7643	1624.8463	0.082	50	41	52	YDTIYDQFEFRK					Mascot

1738.9124	1738.8992	-0.0132	-8	242	258	AVGKGGFVGYLNTNDVK		Mascot
1755.7717	1755.9146	0.1429	81	227	241	MCFSGKYSEAEVYGK	Carbamidomethyl (C)[2]	Mascot
1765.8976	1765.8307	-0.0669	-38	216	232	AGSVPIGDLVKMCFSGK	Carbamidomethyl (C)[13]	Mascot
1864.915	1864.9403	0.0253	14	270	284	ECESIYKAFVYQISK	Carbamidomethyl (C)[2]	Mascot

Gel Idx/Pos	261/K13	Instr./Gel Origin	BA2151/Sample Project 20140814	Process Status	Analysis Succeeded
Plate [#] Name	[1] Sample Project 20140814	Instrument Sample Name		Spectra	11

Rank	Protein Name	Accession No.	Protein MW	Protein PI	Pep. Count	Protein Score	Protein Score C. I. %	Intensity Matched	Total Ion Score	Total Ion C. I. %	Confirmed
1	Malate dehydrogenase, cytoplasmic OS=Zea mays PE=1 SV=2	MDHC_MAIZE	35909.3	5.77	9	263	100	20.705	222	100	

Peptide Information

Calc. Mass	Obsrv. Mass	± da	± ppm	Start Seq.	End Seq.	Sequence	Ion Score	C. I. %	Modification	Rank	Result Type
863.4404	863.4255	-0.0149	-17	152	158	NVTCLTR			Carbamidomethyl (C)[4]		Mascot
873.4788	873.4628	-0.016	-18	164	171	ALGQISER					Mascot
1373.7424	1373.7098	-0.0326	-24	299	310	IVQGLPIDEFSR					Mascot
1508.7928	1508.6887	-0.1041	-69	159	171	LDHNRALGQISER					Mascot
1649.9949	1649.85	-0.1449	-88	127	142	VLVVANPANTNALILK					Mascot
2000.1361	2000.0997	-0.0364	-18	8	27	VLVTGAAGQIGYALVPMI AR					Mascot
2000.1361	2000.0997	-0.0364	-18	8	27	VLVTGAAGQIGYALVPMI AR	44	97.163			Mascot
2016.1311	2016.0746	-0.0565	-28	8	27	VLVTGAAGQIGYALVPMI AR			Oxidation (M)[17]		Mascot
2016.1311	2016.0746	-0.0565	-28	8	27	VLVTGAAGQIGYALVPMI AR	96	100	Oxidation (M)[17]		Mascot
2466.2161	2466.2424	0.0263	11	181	202	NVIIWGNHSSSQYPDVN HATVK					Mascot
2605.3113	2605.2822	-0.0291	-11	68	93	GVVATTDVVEACTGVNV AVMVGGFPR			Carbamidomethyl (C)[12]		Mascot
2605.3113	2605.2822	-0.0291	-11	68	93	GVVATTDVVEACTGVNV AVMVGGFPR	126	100	Carbamidomethyl (C)[12]		Mascot
2621.3064	2621.2654	-0.041	-16	68	93	GVVATTDVVEACTGVNV AVMVGGFPR			Carbamidomethyl (C)[12], Oxidation (M)[20]		Mascot
2621.3064	2621.2654	-0.041	-16	68	93	GVVATTDVVEACTGVNV AVMVGGFPR	87	100	Carbamidomethyl (C)[12], Oxidation (M)[20]		Mascot
2648.5022	2648.2666	-0.2356	-89	127	151	VLVVANPANTNALILKEFA PSIPEK					Mascot

Rank	Protein Name	Accession No.	Protein MW	Protein PI	Pep. Count	Protein Score	Protein Score C. I. %	Intensity Matched	Total Ion Score	Total Ion C. I. %	Confirmed
2	Malate dehydrogenase, cytoplasmic 1 OS=Arabidopsis thaliana GN=At1g04410 PE=1 SV=2	MDHC1_ARATH	35890.4	6.11	10	143	100	15.357	96	100	

Peptide Information

Calc. Mass	Obsrv. Mass	± da	± ppm	Start Seq.	End Seq.	Sequence	Ion Score	C. I. %	Modification	Rank	Result Type
863.4404	863.4255	-0.0149	-17	152	158	NISCLTR			Carbamidomethyl (C)[4]		Mascot
873.4788	873.4628	-0.016	-18	164	171	ALGQISER					Mascot
1360.7545	1360.6942	-0.0603	-44	56	67	MELIDAAFPLLK					Mascot
1376.7494	1376.6934	-0.056	-41	56	67	MELIDAAFPLLK			Oxidation (M)[1]		Mascot

Calc. Mass	Obsrv. Mass	± da	± ppm	Start Seq.	End Seq.	Sequence	Ion Score	C. I. %	Modification	Rank	Result Type
1508.7928	1508.6887	-0.1041	-69	159	171	LDHNRALGQISER					Mascot
1645.7963	1645.7635	-0.0328	-20	241	256	LSSALSAASSACDHIR			Carbamidomethyl (C)[12]		Mascot
1649.9949	1649.85	-0.1449	-88	127	142	VLVVANPANTNALILK					Mascot
1773.8912	1773.8575	-0.0337	-19	240	256	KLSSALSAASSACDHIR			Carbamidomethyl (C)[13]		Mascot
2000.1361	2000.0997	-0.0364	-18	8	27	VLVTGAAGQIGYALVPMIAR					Mascot
2000.1361	2000.0997	-0.0364	-18	8	27	VLVTGAAGQIGYALVPMIAR	44	97.163			Mascot
2016.1311	2016.0746	-0.0565	-28	8	27	VLVTGAAGQIGYALVPMIAR			Oxidation (M)[17]		Mascot
2016.1311	2016.0746	-0.0565	-28	8	27	VLVTGAAGQIGYALVPMIAR	96	100	Oxidation (M)[17]		Mascot
2563.2644	2563.2583	-0.0061	-2	68	93	GVVATTDAVEGCTGVNVAVMVGGFPR			Carbamidomethyl (C)[12]		Mascot
2648.5022	2648.2666	-0.2356	-89	127	151	VLVVANPANTNALILKEFAPSIPEK					Mascot

3	Malate dehydrogenase, cytoplasmic OS=Beta vulgaris GN=NR1 PE=1 SV=1	MDHC_BETVU	35810.4	5.89	8	131	100	13.825	96	100	

Peptide Information

Calc. Mass	Obsrv. Mass	± da	± ppm	Start Seq.	End Seq.	Sequence	Ion Score	C. I. %	Modification	Rank	Result Type
873.4788	873.4628	-0.016	-18	164	171	ALGQISER					Mascot
1508.7928	1508.6887	-0.1041	-69	159	171	LDHNRALGQISER					Mascot
1645.7963	1645.7635	-0.0328	-20	241	256	LSSALSAASSACDHIR			Carbamidomethyl (C)[12]		Mascot
1649.9949	1649.85	-0.1449	-88	127	142	VLVVANPANTNALILK					Mascot
1773.8912	1773.8575	-0.0337	-19	240	256	KLSSALSAASSACDHIR			Carbamidomethyl (C)[13]		Mascot
2000.1361	2000.0997	-0.0364	-18	8	27	VLVTGAAGQIGYALVPMIAR					Mascot
2000.1361	2000.0997	-0.0364	-18	8	27	VLVTGAAGQIGYALVPMIAR	44	97.163			Mascot
2016.1311	2016.0746	-0.0565	-28	8	27	VLVTGAAGQIGYALVPMIAR			Oxidation (M)[17]		Mascot
2016.1311	2016.0746	-0.0565	-28	8	27	VLVTGAAGQIGYALVPMIAR	96	100	Oxidation (M)[17]		Mascot
2604.3274	2604.2747	-0.0527	-20	68	93	GVVATTDVAEACKGVNVAVMVGGFPR			Carbamidomethyl (C)[12]		Mascot
2620.3223	2620.2605	-0.0618	-24	68	93	GVVATTDVAEACKGVNVAVMVGGFPR			Carbamidomethyl (C)[12], Oxidation (M)[20]		Mascot
2648.3674	2648.2666	-0.1008	-38	56	80	MELVDAAFPLLKGVVATTDVAEACK			Carbamidomethyl (C)[24]		Mascot

4	Malate dehydrogenase, cytoplasmic OS=Mesembryanthemum crystallinum GN=MDH PE=2 SV=1	MDHC_MESCR	35817.4	6	7	123	100	13.451	96	100	

Peptide Information

Calc. Mass	Obsrv. Mass	± da	± ppm	Start Seq.	End Seq.	Sequence	Ion Score	C. I. %	Modification	Rank	Result Type

863.4404	863.4255	-0.0149	-17	152	158	NISCLTR			Carbamidomethyl (C)[4]		Mascot
873.4788	873.4628	-0.016	-18	164	171	ALGQISER					Mascot
1508.7928	1508.6887	-0.1041	-69	159	171	LDHNRALGQISER					Mascot
1649.9949	1649.85	-0.1449	-88	127	142	VLVVANPANTNALILK					Mascot
2000.1361	2000.0997	-0.0364	-18	8	27	VLVTGAAGQIGYALVPMIAR					Mascot
2000.1361	2000.0997	-0.0364	-18	8	27	VLVTGAAGQIGYALVPMIAR	44	97.163			Mascot
2016.1311	2016.0746	-0.0565	-28	8	27	VLVTGAAGQIGYALVPMIAR			Oxidation (M)[17]		Mascot
2016.1311	2016.0746	-0.0565	-28	8	27	VLVTGAAGQIGYALVPMIAR	96	100	Oxidation (M)[17]		Mascot
2620.3362	2620.2605	-0.0757	-29	56	80	MELVDAAFPLLKGVVATTDAAEACK			Carbamidomethyl (C)[24]		Mascot
2648.5022	2648.2666	-0.2356	-89	127	151	VLVVANPANTNALILKEFAPSIPEK					Mascot

5	Malate dehydrogenase OS=Nicotiana tabacum GN=MD1 PE=1 SV=1	MDH_TOBAC	35727.3	5.91	7	120	100	14.656	96	100	

Peptide Information

Calc. Mass	Obsrv. Mass	± da	± ppm	Start Seq.	End Seq.	Sequence	Ion Score	C. I. %	Modification	Rank	Result Type
863.4404	863.4255	-0.0149	-17	152	158	NISCLTR			Carbamidomethyl (C)[4]		Mascot
873.4788	873.4628	-0.016	-18	164	171	ALGQISER					Mascot
1508.7928	1508.6887	-0.1041	-69	159	171	LDHNRALGQISER					Mascot
1645.7963	1645.7635	-0.0328	-20	241	256	LSSALSAASSACDHIR			Carbamidomethyl (C)[12]		Mascot
1649.9949	1649.85	-0.1449	-88	127	142	VLVVANPANTNALILK					Mascot
1773.8912	1773.8575	-0.0337	-19	240	256	KLSSALSAASSACDHIR			Carbamidomethyl (C)[13]		Mascot
2000.1361	2000.0997	-0.0364	-18	8	27	VLVTGAAGQIGYALVPMIAR					Mascot
2000.1361	2000.0997	-0.0364	-18	8	27	VLVTGAAGQIGYALVPMIAR	44	97.163			Mascot
2016.1311	2016.0746	-0.0565	-28	8	27	VLVTGAAGQIGYALVPMIAR			Oxidation (M)[17]		Mascot
2016.1311	2016.0746	-0.0565	-28	8	27	VLVTGAAGQIGYALVPMIAR	96	100	Oxidation (M)[17]		Mascot

6	Malate dehydrogenase, cytoplasmic 2 OS=Arabidopsis thaliana GN=At5g43330 PE=2 SV=1	MDHC2_ARATH	35994.5	6.33	6	118	100	12.558	96	100	

Peptide Information

Calc. Mass	Obsrv. Mass	± da	± ppm	Start Seq.	End Seq.	Sequence	Ion Score	C. I. %	Modification	Rank	Result Type
1645.7963	1645.7635	-0.0328	-20	241	256	LSSALSAASSACDHIR			Carbamidomethyl (C)[12]		Mascot
1649.9949	1649.85	-0.1449	-88	127	142	VLVVANPANTNALILK					Mascot
1773.8912	1773.8575	-0.0337	-19	240	256	KLSSALSAASSACDHIR			Carbamidomethyl (C)[13]		Mascot
2000.1361	2000.0997	-0.0364	-18	8	27	VLVTGAAGQIGYALVPMI					Mascot

Calc. Mass	Obsrv. Mass	± da	± ppm	Start Seq.	End Seq.	Sequence	Ion Score	C. I. %	Modification	Rank	Result Type
						AR					
2000.1361	2000.0997	-0.0364	-18	8	27	VLVTGAAGQIGYALVPMI AR	44	97.163			Mascot
2016.1311	2016.0746	-0.0565	-28	8	27	VLVTGAAGQIGYALVPMI AR			Oxidation (M)[17]		Mascot
2016.1311	2016.0746	-0.0565	-28	8	27	VLVTGAAGQIGYALVPMI AR	96	100	Oxidation (M)[17]		Mascot
2405.2097	2405.1243	-0.0854	-36	212	231	ELVKNDEWLNGEFISTVQ QR					Mascot
2648.5022	2648.2666	-0.2356	-89	127	151	VLVVANPANTNALILKEFA PSIPEK					Mascot

7	Malate dehydrogenase, cytoplasmic OS=Medicago sativa GN=CMDH PE=1 SV=1	MDHC_MEDSA	35866.4	6.39	6	116	100	13.518	96	100	

Peptide Information

Calc. Mass	Obsrv. Mass	± da	± ppm	Start Seq.	End Seq.	Sequence	Ion Score	C. I. %	Modification	Rank	Result Type
863.4404	863.4255	-0.0149	-17	152	158	NISCLTR			Carbamidomethyl (C)[4]		Mascot
873.4788	873.4628	-0.016	-18	164	171	ALGQISER					Mascot
1508.7928	1508.6887	-0.1041	-69	159	171	LDHNRALGQISER					Mascot
1649.9949	1649.85	-0.1449	-88	127	142	VLVVANPANTNALILK					Mascot
2000.1361	2000.0997	-0.0364	-18	8	27	VLVTGAAGQIGYALVPMI AR					Mascot
2000.1361	2000.0997	-0.0364	-18	8	27	VLVTGAAGQIGYALVPMI AR	44	97.163			Mascot
2016.1311	2016.0746	-0.0565	-28	8	27	VLVTGAAGQIGYALVPMI AR			Oxidation (M)[17]		Mascot
2016.1311	2016.0746	-0.0565	-28	8	27	VLVTGAAGQIGYALVPMI AR	96	100	Oxidation (M)[17]		Mascot
2402.3337	2402.1431	-0.1906	-79	120	142	HAAANCKVLVVANPANT NALILK			Carbamidomethyl (C)[6]		Mascot

8	Malate dehydrogenase, cytoplasmic OS=Oryza sativa subsp. japonica GN=Os10g0478200 PE=1 SV=3	MDHC_ORYSJ	35888.3	5.75	4	108	100	11.106	96	100	

Peptide Information

Calc. Mass	Obsrv. Mass	± da	± ppm	Start Seq.	End Seq.	Sequence	Ion Score	C. I. %	Modification	Rank	Result Type
970.5316	970.508	-0.0236	-24	203	211	TPSGEKPVR					Mascot
1649.9949	1649.85	-0.1449	-88	127	142	VLVVANPANTNALILK					Mascot
2000.1361	2000.0997	-0.0364	-18	8	27	VLVTGAAGQIGYALVPMI AR					Mascot
2000.1361	2000.0997	-0.0364	-18	8	27	VLVTGAAGQIGYALVPMI AR	44	97.163			Mascot
2016.1311	2016.0746	-0.0565	-28	8	27	VLVTGAAGQIGYALVPMI AR			Oxidation (M)[17]		Mascot
2016.1311	2016.0746	-0.0565	-28	8	27	VLVTGAAGQIGYALVPMI AR	96	100	Oxidation (M)[17]		Mascot
2648.5022	2648.2666	-0.2356	-89	127	151	VLVVANPANTNALILKEFA PSIPEK					Mascot

9	Putative acetyl-CoA C-acetyltransferase VraB	VRAB_STAEQ	42061.6	8.68	11	59	28.718	8.329	

OS=Staphylococcus epidermidis (strain ATCC 35984 / RP62A) GN=vraB PE=3 SV=1

Peptide Information

Calc. Mass	Obsrv. Mass	± da	± ppm	Start Seq.	End Seq.	Sequence	Ion Score	C. I. %	Modification	Rank	Result Type
1908.8835	1908.8774	-0.0061	-3	168	183	NEQDDFAYRSHQLASK					Mascot
1999.9753	2000.0997	0.1244	62	99	118	MVQSGAGTIYIAGGVEST SR			Oxidation (M)[1]		Mascot
1999.9753	2000.0997	0.1244	62	99	118	MVQSGAGTIYIAGGVEST SR	0	0	Oxidation (M)[1]		Mascot
2006.9852	2006.9445	-0.0407	-20	201	217	GECFNQDESIKPQLTLK			Carbamidomethyl (C)[3]		Mascot
2372.2603	2372.1453	-0.115	-48	44	66	VMSLLDDVILGNTVGNG GNLARK			Oxidation (M)[2]		Mascot
2372.2603	2372.1453	-0.115	-48	44	66	VMSLLDDVILGNTVGNG GNLARK			Oxidation (M)[2]		Mascot
2388.2737	2388.1274	-0.1463	-61	218	239	TLGRLKPLLNEGTVTVGN SCMK			Carbamidomethyl (C)[20]		Mascot
2400.1877	2400.1523	-0.0354	-15	327	350	LNCWGGAIATGHPYGAS GAALVTR			Carbamidomethyl (C)[3]		Mascot
2403.2351	2403.1375	-0.0976	-41	356	378	HQFRTVATMGIGGGIGNA ALFER					Mascot
2404.2688	2404.1299	-0.1389	-58	218	239	TLGRLKPLLNEGTVTVGN SCMK			Carbamidomethyl (C)[20], Oxidation (M)[21]		Mascot
2419.23	2419.1465	-0.0835	-35	356	378	HQFRTVATMGIGGGIGNA ALFER			Oxidation (M)[9]		Mascot
2434.2395	2434.116	-0.1235	-51	201	221	GECFNQDESIKPQLTLKT LGR			Carbamidomethyl (C)[3]		Mascot
2466.2446	2466.2424	-0.0022	-1	99	122	MVQSGAGTIYIAGGVEST SRAPWK					Mascot
2470.2507	2470.113	-0.1377	-56	177	198	SHQLASKNMNNGNISQEI LPFK					Mascot
2588.2681	2588.2944	0.0263	10	125	145	RPQSVYESEFPQFFERA PFAR					Mascot

10	BTB/POZ domain-containing adapter for CUL3-mediated RhoA degradation protein 2 OS=Rattus norvegicus GN=Tnfaip1 PE=1 SV=1	BACD2_RAT	36536.4	8	11	56	0	18.371	

Peptide Information

Calc. Mass	Obsrv. Mass	± da	± ppm	Start Seq.	End Seq.	Sequence	Ion Score	C. I. %	Modification	Rank	Result Type
962.553	962.5208	-0.0322	-33	189	196	LSLRFNGR					Mascot
1134.6266	1134.5875	-0.0391	-34	17	28	ISGFKGGGLGNK					Mascot
1148.5555	1148.5892	0.0337	29	307	316	QLGHQSAHRD					Mascot
1425.6614	1425.7064	0.045	32	49	60	HDTMLKAMFSGR			Oxidation (M)[4,8]		Mascot
1981.8734	1982.0679	0.1945	98	275	291	SQASPSEDEDTFELRDR					Mascot
2061.8794	2061.9592	0.0798	39	202	218	DVIGDEICCWSFYGQGR			Carbamidomethyl (C)[8,9]		Mascot
2346.2202	2346.123	-0.0972	-41	79	98	HFGTILNYLRDDTVTLPQ SR					Mascot

2390.2576	2390.1448	-0.1128	-47	22	44	GGGLGNKYVQLNVGGSL HYTTVR		Mascot
2390.2576	2390.1448	-0.1128	-47	22	44	GGGLGNKYVQLNVGGSL HYTTVR		Mascot
2402.1511	2402.1431	-0.008	-3	169	188	YSYTSNSDDHLLKNIELF DK		Mascot
2405.1765	2405.1243	-0.0522	-22	129	148	DSYQPVCNIPIITSLREED R	Carbamidomethyl (C)[7]	Mascot
2418.1816	2418.1523	-0.0293	-12	89	109	DDTVTLPQSRQEIQELMA EAK	Oxidation (M)[17]	Mascot

Gel Idx/Pos	262/K14	Instr./Gel Origin	BA2151/Sample Project 20140814	Process Status	Analysis Succeeded
Plate [#] Name	[1] Sample Project 20140814	Instrument Sample Name		Spectra	11

Rank	Protein Name	Accession No.	Protein MW	Protein PI	Pep. Count	Protein Score	Protein Score C. I. %	Intensity Matched	Total Ion Score	Total Ion C. I. %	Confirmed
1	Alpha-1,4-glucan-protein synthase [UDP-forming] OS=Pisum sativum GN=UPTG PE=1 SV=1	UPTG_PEA	42059	5.73	10	215	100	16.914	175	100	

Peptide Information

Calc. Mass	Obsrv. Mass	± da	± ppm	Start Seq.	End Seq.	Sequence	Ion Score	C. I. %	Modification	Rank	Result Type
839.441	839.4164	-0.0246	-29	152	158	GYPFSLR					Mascot
989.5414	989.4857	-0.0557	-56	269	277	ASNPFVNLK					Mascot
1201.6365	1201.5807	-0.0558	-46	259	268	TGLPYIWHSK					Mascot
1283.7206	1283.6833	-0.0373	-29	13	23	DELDIVIPTIR					Mascot
1401.625	1401.5948	-0.0302	-22	76	87	ASCISFKDSACR			Carbamidomethyl (C)[3,11]		Mascot
1501.6958	1501.6659	-0.0299	-20	54	65	VPEGFDYELYNR					Mascot
1501.6958	1501.6659	-0.0299	-20	54	65	VPEGFDYELYNR	99	100			Mascot
1701.8555	1701.6964	-0.1591	-93	111	125	DPTGHEINALEQHIK					Mascot
1714.7863	1714.8383	0.052	30	202	216	GSLFPMCGMNLAFNR			Carbamidomethyl (C)[7]		Mascot
1730.8088	1730.8306	0.0218	13	301	314	DCTSVQKCYIELSK			Carbamidomethyl (C)[2,8]		Mascot
2292.1335	2292.1003	-0.0332	-14	126	144	NLLSPSTPFFFNTLYDPY R					Mascot
2292.1335	2292.1003	-0.0332	-14	126	144	NLLSPSTPFFFNTLYDPY R	76	99.998			Mascot

Rank	Protein Name	Accession No.	Protein MW	Protein PI	Pep. Count	Protein Score	Protein Score C. I. %	Intensity Matched	Total Ion Score	Total Ion C. I. %	Confirmed
2	Alpha-1,4-glucan-protein synthase [UDP-forming] OS=Zea mays GN=UPTG PE=1 SV=2	UPTG_MAIZE	41690.8	5.75	10	213	100	15.261	173	100	

Peptide Information

Calc. Mass	Obsrv. Mass	± da	± ppm	Start Seq.	End Seq.	Sequence	Ion Score	C. I. %	Modification	Rank	Result Type
839.441	839.4164	-0.0246	-29	159	165	GYPFSLR					Mascot
989.5414	989.4857	-0.0557	-56	276	284	ASNPFVNLK					Mascot
1180.6321	1180.5765	-0.0556	-47	123	132	DINALEQHIK					Mascot
1201.6365	1201.5807	-0.0558	-46	266	275	TGLPYIWHSK					Mascot
1223.5878	1223.5668	-0.021	-17	31	39	NLDFLEMWR					Mascot
1239.5826	1239.5414	-0.0412	-33	31	39	NLDFLEMWR			Oxidation (M)[7]		Mascot
1283.7206	1283.6833	-0.0373	-29	20	30	DELDIVIPTIR					Mascot
1401.625	1401.5948	-0.0302	-22	83	94	ASCISFKDSACR			Carbamidomethyl (C)[3,11]		Mascot
1501.6958	1501.6659	-0.0299	-20	61	72	VPEGFDYELYNR					Mascot

Calc. Mass	Obsrv. Mass	± da	± ppm	Start Seq.	End Seq.	Sequence	Ion Score	C. I. %	Modification	Rank	Result Type
1501.6958	1501.6659	-0.0299	-20	61	72	VPEGFDYELYNR	99	100			Mascot
1745.7809	1745.7543	-0.0266	-15	209	223	GTLFPMCGMNLAFDR			Carbamidomethyl (C)[7], Oxidation (M)[6]		Mascot
1761.7758	1761.7183	-0.0575	-33	209	223	GTLFPMCGMNLAFDR			Carbamidomethyl (C)[7], Oxidation (M)[6,9]		Mascot
2292.1335	2292.1003	-0.0332	-14	133	151	NLLSPSTPFFFNTLYDPYR					Mascot
2292.1335	2292.1003	-0.0332	-14	133	151	NLLSPSTPFFFNTLYDPYR	76	99.998			Mascot

3	UDP-arabinopyranose mutase 1 OS=Oryza sativa subsp. japonica GN=UAM1 PE=1 SV=1	RGP1_ORYSJ	41834.8	5.82	10	213	100	15.253	175	100

Peptide Information

Calc. Mass	Obsrv. Mass	± da	± ppm	Start Seq.	End Seq.	Sequence	Ion Score	C. I. %	Modification	Rank	Result Type
839.441	839.4164	-0.0246	-29	159	165	GYPFSLR					Mascot
989.5414	989.4857	-0.0557	-56	276	284	ASNPFVNLK					Mascot
1180.6321	1180.5765	-0.0556	-47	123	132	DINALEQHIK					Mascot
1201.6365	1201.5807	-0.0558	-46	266	275	TGLPYIWHSK					Mascot
1283.7206	1283.6833	-0.0373	-29	20	30	DELDIVIPTIR					Mascot
1401.625	1401.5948	-0.0302	-22	83	94	ASCISFKDSACR			Carbamidomethyl (C)[3,11]		Mascot
1501.6958	1501.6659	-0.0299	-20	61	72	VPEGFDYELYNR					Mascot
1501.6958	1501.6659	-0.0299	-20	61	72	VPEGFDYELYNR	99	100			Mascot
1592.7302	1592.6691	-0.0611	-38	105	117	YVFTIDDDCFVAK			Carbamidomethyl (C)[9]		Mascot
1745.7809	1745.7543	-0.0266	-15	209	223	GTLFPMCGMNLAFDR			Carbamidomethyl (C)[7], Oxidation (M)[6]		Mascot
1761.7758	1761.7183	-0.0575	-33	209	223	GTLFPMCGMNLAFDR			Carbamidomethyl (C)[7], Oxidation (M)[6,9]		Mascot
2292.1335	2292.1003	-0.0332	-14	133	151	NLLSPSTPFFFNTLYDPYR					Mascot
2292.1335	2292.1003	-0.0332	-14	133	151	NLLSPSTPFFFNTLYDPYR	76	99.998			Mascot

4	Beta-amylase OS=Hordeum vulgare GN=BMY1 PE=1 SV=1	AMYB_HORVU	59894.5	5.58	10	142	100	8.324	118	100

Peptide Information

Calc. Mass	Obsrv. Mass	± da	± ppm	Start Seq.	End Seq.	Sequence	Ion Score	C. I. %	Modification	Rank	Result Type
854.4301	854.4266	-0.0035	-4	439	445	MHANLPR			Oxidation (M)[1]		Mascot
993.4999	993.4631	-0.0368	-37	27	34	FEKGDELR					Mascot
1239.5964	1239.5414	-0.055	-44	248	258	DNGTYLSEKGR					Mascot
1285.6212	1285.5837	-0.0375	-29	61	71	GPKAYDWSAYK					Mascot
1299.582	1299.552	-0.03	-23	335	345	ASINFTCAEMR			Carbamidomethyl (C)[7]		Mascot
1315.5769	1315.5256	-0.0513	-39	335	345	ASINFTCAEMR			Carbamidomethyl (C)[7], Oxidation (M)[10]		Mascot
1515.7994	1515.6556	-0.1438	-95	259	270	FFLAWYSNNLIK					Mascot

1592.7421	1592.6691	-0.073	-46	333	345	HRASINFTCAEMR			Carbamidomethyl (C)[9]		Mascot
1669.7349	1669.7563	0.0214	13	147	160	SAVQMYADYMTSFR					Mascot
1701.7247	1701.6964	-0.0283	-17	147	160	SAVQMYADYMTSFR			Oxidation (M)[5,10]		Mascot
1728.9221	1728.8491	-0.073	-42	257	270	GRFFLAWYSNNLIK					Mascot
2013.9778	2013.9503	-0.0275	-14	303	320	VPSHAAELTAGYYNLHDR					Mascot
2013.9778	2013.9503	-0.0275	-14	303	320	VPSHAAELTAGYYNLHDR	118	100			Mascot

5	Beta-amylase OS=Hordeum vulgare subsp. spontaneum GN=BMY1 PE=1 SV=1	AMYB_HORVS	59886.4	5.66	10	141	100	8.572	118	100

Peptide Information

Calc. Mass	Obsrv. Mass	± da	± ppm	Start Seq.	End Seq.	Sequence	Ion Score	C. I. %	Modification	Rank	Result Type
854.4301	854.4266	-0.0035	-4	439	445	MHANLPR			Oxidation (M)[1]		Mascot
993.4999	993.4631	-0.0368	-37	27	34	FEKGDELR					Mascot
1253.6121	1253.554	-0.0581	-46	248	258	DNGTYLTEKGR					Mascot
1285.6212	1285.5837	-0.0375	-29	61	71	GPKAYDWSAYK					Mascot
1299.582	1299.552	-0.03	-23	335	345	ASINFTCAEMR			Carbamidomethyl (C)[7]		Mascot
1315.5769	1315.5256	-0.0513	-39	335	345	ASINFTCAEMR			Carbamidomethyl (C)[7], Oxidation (M)[10]		Mascot
1515.7994	1515.6556	-0.1438	-95	259	270	FFLAWYSNNLIK					Mascot
1592.7421	1592.6691	-0.073	-46	333	345	HRASINFTCAEMR			Carbamidomethyl (C)[9]		Mascot
1669.7349	1669.7563	0.0214	13	147	160	SAVQMYADYMTSFR					Mascot
1701.7247	1701.6964	-0.0283	-17	147	160	SAVQMYADYMTSFR			Oxidation (M)[5,10]		Mascot
1728.9221	1728.8491	-0.073	-42	257	270	GRFFLAWYSNNLIK					Mascot
2013.9778	2013.9503	-0.0275	-14	303	320	VPSHAAELTAGYYNLHDR					Mascot
2013.9778	2013.9503	-0.0275	-14	303	320	VPSHAAELTAGYYNLHDR	118	100			Mascot

6	Alpha-1,4-glucan-protein synthase [UDP-forming] 1 OS=Solanum tuberosum GN=UPTG1 PE=1 SV=2	UPTG1_SOLTU	42462.1	6.19	10	138	100	11.83	99	100

Peptide Information

Calc. Mass	Obsrv. Mass	± da	± ppm	Start Seq.	End Seq.	Sequence	Ion Score	C. I. %	Modification	Rank	Result Type
989.5414	989.4857	-0.0557	-56	266	274	ASNPFVNLK					Mascot
1180.6321	1180.5765	-0.0556	-47	113	122	DINALEQHIK					Mascot
1201.6365	1201.5807	-0.0558	-46	256	265	TGLPYIWHSK					Mascot
1283.7206	1283.6833	-0.0373	-29	10	20	DELDIVIPTIR					Mascot
1401.625	1401.5948	-0.0302	-22	73	84	ASCISFKDSACR			Carbamidomethyl (C)[3,11]		Mascot
1501.6958	1501.6659	-0.0299	-20	51	62	VPEGFDYELYNR					Mascot

Calc. Mass	Obsrv. Mass	± da	± ppm	Start Seq.	End Seq.	Sequence	Ion Score	C. I. %	Modification	Rank	Result Type
1501.6958	1501.6659	-0.0299	-20	51	62	VPEGFDYELYNR	99	100			Mascot
1507.7938	1507.6997	-0.0941	-62	186	198	NTRYVDAVMTIPK					Mascot
1633.7428	1633.7732	0.0304	19	142	155	DGADFVRGYPFSMR			Oxidation (M)[13]		Mascot
1745.7809	1745.7543	-0.0266	-15	199	213	GTLFPMCGMNLAFDR			Carbamidomethyl (C)[7], Oxidation (M)[6]		Mascot
1758.8037	1758.8601	0.0564	32	298	311	ECTTVQQCYLELSK			Carbamidomethyl (C)[2,8]		Mascot
1761.7758	1761.7183	-0.0575	-33	199	213	GTLFPMCGMNLAFDR			Carbamidomethyl (C)[7], Oxidation (M)[6,9]		Mascot

7	UDP-arabinopyranose mutase 3 OS=Oryza sativa subsp. japonica GN=UAM3 PE=1 SV=1	RGP3_ORYSJ	41651.9	6.01	10	138	100	12.777	99	100	

Peptide Information

Calc. Mass	Obsrv. Mass	± da	± ppm	Start Seq.	End Seq.	Sequence	Ion Score	C. I. %	Modification	Rank	Result Type
839.441	839.4164	-0.0246	-29	157	163	GYPFSLR					Mascot
989.5414	989.4857	-0.0557	-56	274	282	ASNPFVNLK					Mascot
1180.6321	1180.5765	-0.0556	-47	121	130	DINALEQHIK					Mascot
1201.6365	1201.5807	-0.0558	-46	264	273	TGLPYIWHSK					Mascot
1283.7206	1283.6833	-0.0373	-29	18	28	DELDIVIPTIR					Mascot
1401.625	1401.5948	-0.0302	-22	81	92	ASCISFKDSACR			Carbamidomethyl (C)[3,11]		Mascot
1501.6958	1501.6659	-0.0299	-20	59	70	VPEGFDYELYNR					Mascot
1501.6958	1501.6659	-0.0299	-20	59	70	VPEGFDYELYNR	99	100			Mascot
1667.8309	1667.8126	-0.0183	-11	306	319	EADTVQKCYLELAK			Carbamidomethyl (C)[8]		Mascot
1745.7809	1745.7543	-0.0266	-15	207	221	GTLFPMCGMNLAFDR			Carbamidomethyl (C)[7], Oxidation (M)[6]		Mascot
1761.7758	1761.7183	-0.0575	-33	207	221	GTLFPMCGMNLAFDR			Carbamidomethyl (C)[7], Oxidation (M)[6,9]		Mascot
2114.9778	2114.9851	0.0073	3	59	75	VPEGFDYELYNRDDINR					Mascot

8	Beta-amylase OS=Triticum aestivum GN=BMY1 PE=2 SV=1	AMYB_WHEAT	56860.2	5.24	6	132	100	7.41	121	100	

Peptide Information

Calc. Mass	Obsrv. Mass	± da	± ppm	Start Seq.	End Seq.	Sequence	Ion Score	C. I. %	Modification	Rank	Result Type
993.4999	993.4631	-0.0368	-37	27	34	FEKGDEIR					Mascot
1239.6328	1239.5414	-0.0914	-74	271	281	HGDKILDEANK					Mascot
1285.6212	1285.5837	-0.0375	-29	61	71	GPKAYDWSAYK					Mascot
1623.6461	1623.8064	0.1603	99	333	345	HHASMNFTCAEMR			Carbamidomethyl (C)[9], Oxidation (M)[5,12]		Mascot
1669.7349	1669.7563	0.0214	13	147	160	TAVQMYADYMASFR			Oxidation (M)[5]		Mascot
2087.0557	2087.0286	-0.0271	-13	129	146	NIEYLTLGVDDQPLFHGR					Mascot
2087.0557	2087.0286	-0.0271	-13	129	146	NIEYLTLGVDDQPLFHGR	121	100			Mascot

9	Alpha-1,4-glucan-protein synthase [UDP-forming] (Fragments) OS=Phoenix dactylifera PE=1 SV=1	UPTG_PHODC	6899.6	7.82	4	129	100	10.43	99	100

Peptide Information

Calc. Mass	Obsrv. Mass	± da	± ppm	Start Seq.	End Seq.	Sequence	Ion Score	C. I. %	Modification	Rank	Result Type
989.5414	989.4857	-0.0557	-56	52	60	ASNPFVNLK					Mascot
1201.6365	1201.5807	-0.0558	-46	42	51	TGLPYIWHSK					Mascot
1501.6958	1501.6659	-0.0299	-20	4	15	VPEGFDYELYNR					Mascot
1501.6958	1501.6659	-0.0299	-20	4	15	VPEGFDYELYNR	99	100			Mascot
1730.9436	1730.8306	-0.113	-65	16	30	NDINRYVDAVLTIPK					Mascot

10	Abscisic-aldehyde oxidase OS=Arabidopsis thaliana GN=AAO3 PE=1 SV=1	ALDO3_ARATH	148375	6.23	24	64	80.814	6.581	

Peptide Information

Calc. Mass	Obsrv. Mass	± da	± ppm	Start Seq.	End Seq.	Sequence	Ion Score	C. I. %	Modification	Rank	Result Type
993.4669	993.4631	-0.0038	-4	527	535	ICSLDSGNK			Carbamidomethyl (C)[2]		Mascot
1207.6569	1207.5562	-0.1007	-83	465	475	AIEVETFLTGK					Mascot
1209.5828	1209.558	-0.0248	-21	904	913	TNCLSRTAMR			Carbamidomethyl (C)[3]		Mascot
1233.5892	1233.5753	-0.0139	-11	1059	1068	CEGNEKLLDR			Carbamidomethyl (C)[1]		Mascot
1287.6296	1287.5431	-0.0865	-67	316	326	MATHMEKIGNR					Mascot
1531.7765	1531.6755	-0.101	-66	1241	1253	HFNVEIVNTGHHK					Mascot
1542.7035	1542.7496	0.0461	30	1109	1120	LKPIMDQMMMEK			Oxidation (M)[5,8,9]		Mascot
1568.853	1568.7668	-0.0862	-55	689	702	NLEQPILTVEDAVK					Mascot
1571.7523	1571.7612	0.0089	6	1	13	MDLEFAVNGERFK			Oxidation (M)[1]		Mascot
1592.8142	1592.6691	-0.1451	-91	187	199	EVMFKNLPPYNPK			Oxidation (M)[3]		Mascot
1618.833	1618.7552	-0.0778	-48	330	345	NSGSIGGNLVMAQSRK					Mascot
1629.8741	1629.7705	-0.1036	-64	658	672	CAGQRIALVVADTQK			Carbamidomethyl (C)[1]		Mascot
1633.8585	1633.7732	-0.0853	-52	643	657	TLFGPGPLFADELTR					Mascot
1667.9619	1667.8126	-0.1493	-90	476	490	LLSYSVLYEAVGLLK					Mascot
1669.8176	1669.7563	-0.0613	-37	269	282	YIDISNIPEMSMIK			Oxidation (M)[10]		Mascot
1684.9017	1684.7444	-0.1573	-93	491	505	GIIVPGKDTLHSEYR					Mascot
1686.7694	1686.7264	-0.043	-25	450	464	CFLAFGSYGGDHSIR			Carbamidomethyl (C)[1]		Mascot
1744.8939	1744.7666	-0.1273	-73	1314	1328	SLCGLYSVEKYLQGK			Carbamidomethyl (C)[3]		Mascot
1761.884	1761.7183	-0.1657	-94	673	688	HADMAAKLAVVEYDTK					Mascot
1849.9589	1849.8597	-0.0992	-54	1261	1278	ASGEPPLLLAASVHCATR			Carbamidomethyl (C)[15]		Mascot
2072.019	2072.0242	0.0052	3	266	282	FDRYIDISNIPEMSMIK					Mascot

2269.2017	2269.1716	-0.0301	-13	192	210	NLPPYNPKDHLVTFPEFL K		Mascot
2277.2131	2277.0881	-0.125	-55	1261	1282	ASGEPPLLLAASVHCATR SAIR	Carbamidomethyl (C)[15]	Mascot
3047.5032	3047.5537	0.0505	17	1286	1313	KHSLSSNFIDGSDSEFEL PVPATMPVVK	Oxidation (M)[24]	Mascot

Gel Idx/Pos	263/K15	Instr./Gel Origin	BA2151/Sample Project 20140814	Process Status	Analysis Succeeded
Plate [#] Name	[1] Sample Project 20140814	Instrument Sample Name		Spectra	11

Rank	Protein Name	Accession No.	Protein MW	Protein PI	Pep. Count	Protein Score	Protein Score C. I. %	Intensity Matched	Total Ion Score	Total Ion C. I. %	Confirmed
1	3-isopropylmalate dehydratase large subunit OS=Shewanella sp. (strain W3-18-1) GN=leuC PE=3 SV=1	LEUC_SHESW	50782.5	5.8	11	69	92.706	8.121			

Protein Group

Protein Name	Accession No.	Protein MW	Protein PI
3-isopropylmalate dehydratase large subunit OS=Shewanella putrefaciens (strain CN-32 / ATCC BAA-453) GN=leuC PE=3 SV=1	LEUC_SHEPC	50816.5	5.8000 001907 3486

Peptide Information

Calc. Mass	Obsrv. Mass	± da	± ppm	Start Seq.	End Seq.	Sequence	Ion Score	C. I. %	Modification	Rank	Result Type
970.4952	970.4947	-0.0005	-1	180	189	GHVSDGVTAK					Mascot
1028.6099	1028.5447	-0.0652	-63	2	11	TTVPKNAVAK					Mascot
1028.6099	1028.5447	-0.0652	-63	2	11	TTVPKNAVAK	25	0			Mascot
1175.6453	1175.6349	-0.0104	-9	1	11	MTTVPKNAVAK			Oxidation (M)[1]		Mascot
1291.6311	1291.6804	0.0493	38	89	99	TQVETLAQNCK			Carbamidomethyl (C)[10]		Mascot
1291.6311	1291.6804	0.0493	38	89	99	TQVETLAQNCK			Carbamidomethyl (C)[10]		Mascot
1467.7915	1467.7562	-0.0353	-24	176	189	IEVRGHVSDGVTAK					Mascot
1487.6361	1487.7085	0.0724	49	225	237	MTVCNMAIEMGAK			Carbamidomethyl (C)[4], Oxidation (M)[1,6]		Mascot
1671.6594	1671.7748	0.1154	69	412	425	LPGCSMCLAMNDDR			Carbamidomethyl (C)[4,7], Oxidation (M)[6,10]		Mascot
1736.8279	1736.8242	-0.0037	-2	260	274	GEDWAEAVAYWQAIK					Mascot
2280.1296	2279.9924	-0.1372	-60	392	411	AQAEAEGLDKIFIEAGFE WR					Mascot
2672.3157	2672.1975	-0.1182	-44	325	349	ASMEKALEYIGLSAGTPM TDISINK			Oxidation (M)[3,18]		Mascot
2764.2263	2764.1853	-0.041	-15	199	224	IGMDGGTGYVVEFCGEAI EALSMEGR			Carbamidomethyl (C)[14], Oxidation (M)[3]		Mascot
2780.2212	2780.1611	-0.0601	-22	199	224	IGMDGGTGYVVEFCGEAI EALSMEGR			Carbamidomethyl (C)[14], Oxidation (M)[3,23]		Mascot
2780.2212	2780.1611	-0.0601	-22	199	224	IGMDGGTGYVVEFCGEAI EALSMEGR			Carbamidomethyl (C)[14], Oxidation (M)[3,23]		Mascot

Rank	Protein Name	Accession No.	Protein MW	Protein PI	Pep. Count	Protein Score	Protein Score C. I. %	Intensity Matched	Total Ion Score	Total Ion C. I. %	Confirmed
2	Protein translocase subunit SecA 1 OS=Corynebacterium diphtheriae (strain ATCC 700971 / NCTC 13129 / Biotype gravis) GN=secA1 PE=3 SV=1	SECA1_CORDI	96090.5	4.91	19	67	89.931	6.644			

Peptide Information

Calc. Mass	Obsrv. Mass	± da	± ppm	Start Seq.	End Seq.	Sequence	Ion Score	C. I. %	Modification	Rank	Result Type
877.489	877.4645	-0.0245	-28	296	302	AKELFNR					Mascot

Calc. Mass	Obsrv. Mass	± da	± ppm	Start Seq.	End Seq.	Sequence	Ion Score	C. I. %	Modification	Rank	Result Type
1010.5629	1010.5342	-0.0287	-28	736	744	KAVLEDAHK					Mascot
1160.5331	1160.5334	0.0003	0	247	255	DIHYEVDNR					Mascot
1160.5331	1160.5334	0.0003	0	247	255	DIHYEVDNR					Mascot
1327.5947	1327.6813	0.0866	65	643	652	YDEVMNEQRK			Oxidation (M)[5]		Mascot
1340.6191	1340.7201	0.101	75	780	789	EHLYEMDYLK					Mascot
1501.7792	1501.6655	-0.1137	-76	474	489	AGLPGAVTVATNMAGR			Oxidation (M)[13]		Mascot
1515.79	1515.7006	-0.0894	-59	660	673	EILESADIAADIQK					Mascot
1561.7502	1561.7097	-0.0405	-26	581	592	FYLSMRDDLMVR			Oxidation (M)[5]		Mascot
1632.9181	1632.7684	-0.1497	-92	387	400	LDVIPIPTNRPNQR					Mascot
1671.8912	1671.7748	-0.1164	-70	659	673	REILESADIAADIQK					Mascot
1773.8878	1773.8527	-0.0351	-20	545	560	EAGGLYVLGTERHESR					Mascot
1784.8232	1784.7963	-0.0269	-15	624	638	SAQTSVENQNFEMRK			Oxidation (M)[13]		Mascot
1975.0244	1974.9548	-0.0696	-35	310	327	NGEVLIVDDFTGRVLDGR					Mascot
2019.9142	2019.8905	-0.0237	-12	835	853	QFAVANEQPAETEEGTV EA					Mascot
2049.0876	2049.0049	-0.0827	-40	456	473	HSVLNAKFHEQEAQIVAK					Mascot
2108.9917	2108.918	-0.0737	-35	745	763	QYAELEENVTAIGGEAQ MR					Mascot
2621.2625	2621.2588	-0.0037	-1	745	767	QYAELEENVTAIGGEAQ MRNIER					Mascot
2717.4297	2717.5981	0.1684	62	219	243	TPLIISGPVDGSSQWYSV FAQIVPR					Mascot
2764.3103	2764.1853	-0.125	-45	593	616	FVGQTMENMMNRLNVP DDVPIEAK			Oxidation (M)[6]		Mascot
2780.3052	2780.1611	-0.1441	-52	593	616	FVGQTMENMMNRLNVP DDVPIEAK			Oxidation (M)[6,9]		Mascot
2780.3052	2780.1611	-0.1441	-52	593	616	FVGQTMENMMNRLNVP DDVPIEAK			Oxidation (M)[6,9]		Mascot

3	Chaperone protein DnaK OS=Chlamydia muridarum (strain MoPn / Nigg) GN=dnaK PE=3 SV=2	DNAK_CHLMU	70642.5	4.96	16	63	74.708	3.137

Peptide Information

Calc. Mass	Obsrv. Mass	± da	± ppm	Start Seq.	End Seq.	Sequence	Ion Score	C. I. %	Modification	Rank	Result Type
804.4461	804.4453	-0.0008	-1	499	506	IEASSGLK					Mascot
863.4039	863.4173	0.0134	16	253	259	DNMALQR			Oxidation (M)[3]		Mascot
871.5359	871.504	-0.0319	-37	163	170	IAGLDVKR					Mascot
919.4479	919.4357	-0.0122	-13	585	593	AASDELSAR					Mascot
1017.5098	1017.5208	0.011	11	562	569	EIEEQIEK					Mascot
1175.5725	1175.6349	0.0624	53	507	515	EEEIQQMIR					Mascot
1487.8064	1487.7085	-0.0979	-66	62	75	QAVTNPEKTLASTK					Mascot
1695.8007	1695.868	0.0673	40	529	543	EASDIKNEADGMIFR					Mascot

1703.8599	1703.7708	-0.0891	-52	96	111	VAPNSKGDAVFEVENK					Mascot
1717.8538	1717.8146	-0.0392	-23	245	259	QEGIDLSKDNMALQR					Mascot
1753.9583	1753.8357	-0.1226	-70	555	569	IPADLVKEIEEQIEK					Mascot
2016.1376	2016.0641	-0.0735	-36	41	60	TTPSIVAFKGSETLVGIPA K					Mascot
2621.291	2621.2588	-0.0322	-12	7	31	SNKIIGIDLGTTNSCVSVM EGGQPK			Carbamidomethyl (C)[15], Oxidation (M)[19]		Mascot
2672.2581	2672.1975	-0.0606	-23	597	623	IGEAMQAQSASAANAQG GPNINSEDLK					Mascot
2694.3696	2694.3499	-0.0197	-7	102	125	GDAVFEVENKLYTPEEIG AQILMK					Mascot
2717.4468	2717.5981	0.1513	56	358	385	EPNKGVNPDEVVAIGAAI QGGVLGGEVK					Mascot

4	Putative ciliary rootlet coiled-coil protein-like 3 protein OS=Homo sapiens PE=5 SV=2	CROL3_HUMAN	250066.2	5.64	32	63	71.622	33.212

Peptide Information

Calc. Mass	Obsrv. Mass	± da	± ppm	Start Seq.	End Seq.	Sequence	Ion Score	C. I. %	Modification	Rank	Result Type
804.3846	804.4453	0.0607	75	1021	1027	SEGVEQR					Mascot
819.3843	819.3857	0.0014	2	1164	1170	DALSEER					Mascot
863.404	863.4173	0.0133	15	180	187	TEACVGAR			Carbamidomethyl (C)[4]		Mascot
878.4288	878.4692	0.0404	46	2087	2093	MEQETLK					Mascot
905.4985	905.4305	-0.068	-75	1755	1761	LCSTLRR			Carbamidomethyl (C)[2]		Mascot
944.4908	944.4796	-0.0112	-12	1059	1065	DQLREQR					Mascot
970.5679	970.4947	-0.0732	-75	2017	2025	LQGELAALR					Mascot
1056.5731	1056.5052	-0.0679	-64	1979	1986	QVLCRPQR			Carbamidomethyl (C)[4]		Mascot
1056.5731	1056.5052	-0.0679	-64	1979	1986	QVLCRPQR	4	0	Carbamidomethyl (C)[4]		Mascot
1156.6182	1156.5321	-0.0861	-74	572	582	QTRSGGLGQPR					Mascot
1166.6528	1166.609	-0.0438	-38	1910	1919	LELEHLASVR					Mascot
1185.6586	1185.6259	-0.0327	-28	1282	1291	EALAQLQREK					Mascot
1187.6492	1187.6514	0.0022	2	1889	1900	VEGALSSARAAR					Mascot
1187.6492	1187.6514	0.0022	2	1889	1900	VEGALSSARAAR	4	0			Mascot
1205.5944	1205.6221	0.0277	23	1553	1562	ATICRAEQEK			Carbamidomethyl (C)[4]		Mascot
1205.5944	1205.6221	0.0277	23	1553	1562	ATICRAEQEK			Carbamidomethyl (C)[4]		Mascot
1209.6335	1209.6112	-0.0223	-18	2144	2153	LQDLTAQHQR					Mascot
1215.6943	1215.636	-0.0583	-48	1324	1334	EALKGEIQSLK					Mascot
1233.6572	1233.5961	-0.0611	-50	1292	1302	ETLSLTLAEEK					Mascot
1314.6648	1314.646	-0.0188	-14	919	930	QGLEAEAAELQR					Mascot
1324.6315	1324.6145	-0.017	-13	42	52	MLTRDFSPSDR					Mascot
1330.6387	1330.6212	-0.0175	-13	2196	2205	EQLDQEVQWR					Mascot

1340.703	1340.7201	0.0171	13	2225	2236	HVGNISTNRSQK					Mascot
1384.7795	1384.65	-0.1295	-94	590	602	QAVVLGTDLAELR					Mascot
1431.6699	1431.6367	-0.0332	-23	73	85	GGPMGSHRFAGWR			Oxidation (M)[4]		Mascot
1442.7598	1442.6533	-0.1065	-74	1186	1198	LAAEEAADLRVER					Mascot
1507.7289	1507.7054	-0.0235	-16	46	59	DFSPSDRVAGGPFR					Mascot
1553.7742	1553.7584	-0.0158	-10	542	557	GGLDAQPLHAMGTTGK					Mascot
1632.7435	1632.7684	0.0249	15	260	273	VMPQAQGWESAQER			Oxidation (M)[2]		Mascot
1645.8326	1645.7552	-0.0774	-47	1005	1018	AECSNADLELLVRR			Carbamidomethyl (C)[3]		Mascot
1695.7902	1695.868	0.0778	46	172	187	SAVQMGSRTEACVGAR			Carbamidomethyl (C)[12], Oxidation (M)[5]		Mascot
1703.8824	1703.7708	-0.1116	-65	784	800	REQGGAPVSGVSFLGSR					Mascot
1717.865	1717.8146	-0.0504	-29	1746	1760	QDAEAQLGRLCSTLR			Carbamidomethyl (C)[11]		Mascot
2340.136	2340.1189	-0.0171	-7	1629	1648	SGEAHELQAQCSQEVLE LRR			Carbamidomethyl (C)[11]		Mascot
2626.4399	2626.5632	0.1233	47	274	296	GLQIKPRLGFFGADLQSL RPPCR			Carbamidomethyl (C)[22]		Mascot

5	DNA ligase OS=Burkholderia phytofirmans (strain DSM 17436 / PsJN) GN=ligA PE=3 SV=1	DNLJ_BURPP	75211	5.35	16	61	56.048	6.513

Peptide Information

Calc. Mass	Obsrv. Mass	± da	± ppm	Start Seq.	End Seq.	Sequence	Ion Score	C. I. %	Modification	Rank	Result Type
919.4553	919.4357	-0.0196	-21	634	642	EMLEAAGAK					Mascot
979.5393	979.4969	-0.0424	-43	187	194	GEVLMFKR					Mascot
1010.5894	1010.5342	-0.0552	-55	321	329	APRFALAHK					Mascot
1028.6212	1028.5447	-0.0765	-74	386	394	IGDTVIVRR					Mascot
1028.6212	1028.5447	-0.0765	-74	386	394	IGDTVIVRR	6	0			Mascot
1156.6011	1156.5321	-0.069	-60	453	461	QALWHFAQR					Mascot
1186.6427	1186.6293	-0.0134	-11	462	472	RALDIDGLGEK					Mascot
1327.7944	1327.6813	-0.1131	-85	617	629	TVVLTGTLPSLAR					Mascot
1330.7074	1330.6212	-0.0862	-65	216	228	NAAAGSLRQLDSK					Mascot
1376.6726	1376.6771	0.0045	3	630	642	EEAKEMLEAAGAK					Mascot
1553.8534	1553.7584	-0.095	-61	395	409	AGDVIPEVVSALLDR					Mascot
1645.7738	1645.7552	-0.0186	-11	667	681	AEELGVPVLDEDGMR			Oxidation (M)[14]		Mascot
1671.9218	1671.7748	-0.147	-88	324	338	FALAHKFPAQEALTK					Mascot
1773.8688	1773.8527	-0.0161	-9	667	682	AEELGVPVLDEDGMRK			Oxidation (M)[14]		Mascot
1957.99	1957.9441	-0.0459	-23	664	681	LAKAEELGVPVLDEDGM R			Oxidation (M)[17]		Mascot
2340.2083	2340.1189	-0.0894	-38	108	130	VGDALGKNASEPPVPVD YAAELK					Mascot
2672.3931	2672.1975	-0.1956	-73	115	139	NASEPPVPVDYAAELKFD GLAISLR					Mascot

6	Serine hydroxymethyltransferase, mitochondrial OS=Oryctolagus cuniculus GN=SHMT2 PE=1 SV=2	GLYM_RABIT	56323	8.78	16	61	52.904	4.265	

Peptide Information

Calc. Mass	Obsrv. Mass	± da	± ppm	Start Seq.	End Seq.	Sequence	Ion Score	C. I. %	Modification	Rank	Result Type
819.3414	819.3857	0.0443	54	410	416	NTCPGDR			Carbamidomethyl (C)[3]		Mascot
871.4996	871.504	0.0044	5	295	302	GVRTVDPK					Mascot
878.3924	878.4692	0.0768	87	239	245	EVCDEVK			Carbamidomethyl (C)[3]		Mascot
944.4908	944.4796	-0.0112	-12	390	398	GLDGARAER					Mascot
979.5458	979.4969	-0.0489	-50	349	356	EYSLQVLK					Mascot
1010.4546	1010.5342	0.0796	79	341	348	QACTPMFR			Carbamidomethyl (C)[3]		Mascot
1056.5109	1056.5052	-0.0057	-5	96	104	YSEGYPGKR					Mascot
1056.5109	1056.5052	-0.0057	-5	96	104	YSEGYPGKR					Mascot
1112.512	1112.5905	0.0785	71	435	442	QFREDDFR					Mascot
1139.6321	1139.6251	-0.007	-6	284	293	GARSGLIFYR					Mascot
1186.7042	1186.6293	-0.0749	-63	399	409	VLELVSITANK					Mascot
1209.6223	1209.6112	-0.0111	-9	270	280	HADVVTTTTHK					Mascot
1330.6896	1330.6212	-0.0684	-51	357	368	NARAMADALLER					Mascot
1671.8232	1671.7748	-0.0484	-29	410	425	NTCPGDRSAITPGGLR			Carbamidomethyl (C)[3]		Mascot
1696.0057	1695.868	-0.1377	-81	2	15	LPFSLLWAVRPLQR					Mascot
1717.9418	1717.8146	-0.1272	-74	246	262	AHLLADMAHISGLVAAK					Mascot
2016.076	2016.0641	-0.0119	-6	197	214	LNPQTGLIDYEQLALTAR					Mascot

7	Bifunctional protein aas OS=Escherichia fergusonii (strain ATCC 35469 / DSM 13698 / CDC 0568-73) GN=aas PE=3 SV=1	AAS_ESCF3	80664.7	9.25	17	60	44.667	3.968	

Peptide Information

Calc. Mass	Obsrv. Mass	± da	± ppm	Start Seq.	End Seq.	Sequence	Ion Score	C. I. %	Modification	Rank	Result Type
1134.5725	1134.5701	-0.0024	-2	560	569	GPNIMNGYLR					Mascot
1175.7147	1175.6349	-0.0798	-68	241	250	TLFVGRILEK					Mascot
1290.6953	1290.6262	-0.0691	-54	497	506	QLWQDKFGLR					Mascot
1327.6385	1327.6813	0.0428	32	281	292	IPAMMNYTAGVK			Oxidation (M)[4,5]		Mascot
1423.6965	1423.7172	0.0207	15	601	612	FDEQGFVQIQGR					Mascot
1467.7448	1467.7562	0.0114	8	280	292	RIPAMMNYTAGVK			Oxidation (M)[5]		Mascot
1535.7523	1535.8168	0.0645	42	204	216	ETLYESLLGAMHR			Oxidation (M)[11]		Mascot
1632.889	1632.7684	-0.1206	-74	556	569	LQLKGPNIMNGYLR			Oxidation (M)[9]		Mascot

1645.7859	1645.7552	-0.0307	-19	184	197	IAGEMLHQIMMEAR			Oxidation (M)[5]		Mascot
1695.9541	1695.868	-0.0861	-51	93	107	LVEQGRPVVIFPEGR					Mascot
1773.8809	1773.8527	-0.0282	-16	183	197	KIAGEMLHQIMMEAR			Oxidation (M)[6]		Mascot
2340.1919	2340.1189	-0.073	-31	184	203	IAGEMLHQIMMEARMAV RPR					Mascot
2586.2373	2586.343	0.1057	41	591	612	GWYDTGDIVRFDEQGFV QIQGR					Mascot
2626.4749	2626.5632	0.0883	34	93	116	LVEQGRPVVIFPEGRITV TGSLMK					Mascot
2694.3728	2694.3499	-0.0229	-8	616	640	FAKIAGEMVSLEMVEQLA LGVSPDK			Oxidation (M)[8,13]		Mascot
2717.3855	2717.5981	0.2126	78	693	716	QMPLLGSGKPDFVTLKS WVDEPEK			Oxidation (M)[2]		Mascot
2762.5063	2762.2422	-0.2641	-96	338	361	ADVTLTDKVWIFAHLLMP HLAQVK			Oxidation (M)[17]		Mascot

8	Alpha-actinin, sarcomeric OS=Anopheles gambiae GN=Actn PE=3 SV=2	ACTN_ANOGA	107087	5.6	22	60	40.71	48.417	

Peptide Information

Calc. Mass	Obsrv. Mass	± da	± ppm	Start Seq.	End Seq.	Sequence	Ion Score	C. I. %	Modification	Rank	Result Type
804.4362	804.4453	0.0091	11	633	638	KWSEVR					Mascot
981.4424	981.5081	0.0657	67	783	790	SSFNHFDK					Mascot
981.4424	981.5081	0.0657	67	783	790	SSFNHFDK					Mascot
1112.5847	1112.5905	0.0058	5	669	678	ANAVGPWIER					Mascot
1160.5841	1160.5334	-0.0507	-44	654	662	KQQNNEMLR					Mascot
1160.5994	1160.5334	-0.066	-57	322	330	RTMPWLNSR					Mascot
1180.5668	1180.5558	-0.011	-9	390	399	MVSDITNSWK					Mascot
1187.6379	1187.6514	0.0135	11	645	654	DQTLANELRK					Mascot
1187.6379	1187.6514	0.0135	11	645	654	DQTLANELRK					Mascot
1215.6732	1215.636	-0.0372	-31	312	321	LASDLLEWIR					Mascot
1308.6794	1308.626	-0.0534	-41	364	374	LETNFNTLQTK					Mascot
1308.6794	1308.626	-0.0534	-41	364	374	LETNFNTLQTK					Mascot
1314.6147	1314.646	0.0313	24	735	744	YTHYTMETLR					Mascot
1330.6096	1330.6212	0.0116	9	735	744	YTHYTMETLR			Oxidation (M)[6]		Mascot
1340.7434	1340.7201	-0.0233	-17	634	644	WSEVRALVPQR					Mascot
1400.738	1400.6851	-0.0529	-38	525	536	QGLDEAERILEK					Mascot
1442.7598	1442.6533	-0.1065	-74	757	768	NINEVENQILTR					Mascot
1467.6897	1467.7562	0.0665	45	442	453	GKEEMLQSQDFR					Mascot
1487.7502	1487.7085	-0.0417	-28	547	559	RAAPFNNWLDGAR					Mascot
1507.8115	1507.7054	-0.1061	-70	362	374	AKLETNFNTLQTK					Mascot
1717.8326	1717.8146	-0.018	-10	721	734	IHQAVQESMIFENR			Oxidation (M)[9]		Mascot

1753.8252	1753.8357	0.0105	6	466	480	KHEAFESDLAAHQDR						Mascot
1886.0746	1885.903	-0.1716	-91	594	610	EFNVIIGLVRDAEAIVK						Mascot
2625.28	2625.2393	-0.0407	-16	377	399	LSNRPAYMPTEGKMVSD ITNSWK						Mascot
2626.3181	2626.5632	0.2451	93	220	241	YLDIPRMLDPDDLINTPKP DER						Mascot
2689.333	2689.1606	-0.1724	-64	825	848	ILAVVDPNASGYVQFDAF LDFMTR						Mascot

9 Required for respiratory growth protein 9, mitochondrial OS=Coccidioides posadasii (strain C735) GN=RRG9 PE=3 SV=1 | RRG9_COCP7 | 30741.1 | 10.76 | 14 | 59 | 28.718 | 29.407

Peptide Information

Calc. Mass	Obsrv. Mass	± da	± ppm	Start Seq.	End Seq.	Sequence	Ion Score	C. I. %	Modification	Rank	Result Type
819.4029	819.3857	-0.0172	-21	173	179	MSPEAIR			Oxidation (M)[1]		Mascot
877.4349	877.4645	0.0296	34	118	123	KMEQWR					Mascot
944.4796	944.4796	0	0	222	230	RPSSPASDK					Mascot
1017.5475	1017.5208	-0.0267	-26	184	191	SKWRPSEK					Mascot
1056.5685	1056.5052	-0.0633	-60	143	152	LSPDAIEGVR					Mascot
1056.5685	1056.5052	-0.0633	-60	143	152	LSPDAIEGVR					Mascot
1072.5746	1072.5752	0.0006	1	221	230	KRPSSPASDK					Mascot
1134.5725	1134.5701	-0.0024	-2	119	126	MEQWRLQK			Oxidation (M)[1]		Mascot
1187.6208	1187.6514	0.0306	26	132	141	KFQEGWAPPK					Mascot
1187.6208	1187.6514	0.0306	26	132	141	KFQEGWAPPK					Mascot
1215.627	1215.636	0.009	7	133	142	FQEGWAPPKR					Mascot
1233.6488	1233.5961	-0.0527	-43	2	11	FGITSRPYHR					Mascot
1330.6863	1330.6212	-0.0651	-49	77	88	SGYSLKPAHTNR					Mascot
1400.7017	1400.6851	-0.0166	-12	101	113	DSQPDPQAKTVSK					Mascot
2109.1023	2108.918	-0.1843	-87	204	220	ETRIWDHMSELGLRPLR					Mascot
2280.1785	2279.9924	-0.1861	-82	2	20	FGITSRPYHRWSSFAQAL R					Mascot

10 Aspartate--tRNA ligase 1 OS=Syntrophus aciditrophicus (strain SB) GN=aspS1 PE=3 SV=1 | SYD1_SYNAS | 80799.4 | 5.4 | 16 | 58 | 21.84 | 8.151

Peptide Information

Calc. Mass	Obsrv. Mass	± da	± ppm	Start Seq.	End Seq.	Sequence	Ion Score	C. I. %	Modification	Rank	Result Type
813.4577	813.4188	-0.0389	-48	679	686	SVAPVANR					Mascot
919.4883	919.4357	-0.0526	-57	376	384	EIAPSFGAK					Mascot
1028.5676	1028.5447	-0.0229	-22	546	553	NKFGFFLR					Mascot

1028.5847	1028.5447	-0.04	-39	687	695	VREGLEAVR	10	0		Mascot
1055.5527	1055.5266	-0.0261	-25	177	184	HRISQCVR			Carbamidomethyl (C)[6]	Mascot
1112.5847	1112.5905	0.0058	5	634	642	IDHLSWVSR				Mascot
1209.6263	1209.6112	-0.0151	-12	707	717	NAPAVKGDYFK				Mascot
1246.6943	1246.6028	-0.0915	-73	38	48	VLLAGWVDAFR				Mascot
1327.5769	1327.6813	0.1044	79	385	395	GMTWMRAEEGK			Oxidation (M)[2,5]	Mascot
1561.8584	1561.7097	-0.1487	-95	532	545	RIFAALGLTEEDVK				Mascot
1632.9043	1632.7684	-0.1359	-83	290	304	MFAIGGIALSRPFPR				Mascot
1908.9525	1908.8682	-0.0843	-44	327	343	MADVTGVFSRTSYSIFK				Mascot
1957.9987	1957.9441	-0.0546	-28	234	249	QLLMIGGMERYFQLAR			Oxidation (M)[4,8]	Mascot
2020.0056	2019.8905	-0.1151	-57	191	209	GFVEVETPVLTMSTPEGAR				Mascot
2626.3025	2626.5632	0.2607	99	548	571	FGFFLRAFDFAAPPHGGLALGMDR			Oxidation (M)[22]	Mascot
3116.5754	3116.499	-0.0764	-25	415	443	VFQVEDGDVLIMVADPSCAIVNSALGQLR			Carbamidomethyl (C)[18]	Mascot

Gel Idx/Pos	264/K16	Instr./Gel Origin	BA2151/Sample Project 20140814	Process Status	Analysis Succeeded
Plate [#] Name	[1] Sample Project 20140814	Instrument Sample Name		Spectra	11

Rank	Protein Name	Accession No.	Protein MW	Protein PI	Pep. Count	Protein Score	Protein Score C. I. %	Intensity Matched	Total Ion Score	Total Ion C. I. %	Confirmed
1	Serpin-Z2B OS=Triticum aestivum PE=1 SV=1	SPZ2B_WHEAT	43011.4	5.18	9	178	100	20.611	146	100	

Peptide Information

Calc. Mass	Obsrv. Mass	± da	± ppm	Start Seq.	End Seq.	Sequence	Ion Score	C. I. %	Modification	Rank	Result Type
925.5214	925.4976	-0.0238	-26	11	18	LSIAHQTR					Mascot
925.5214	925.4976	-0.0238	-26	11	18	LSIAHQTR	37	81.742			Mascot
1137.6667	1137.6052	-0.0615	-54	172	181	LVLGNALYFK					Mascot
1192.5382	1192.5138	-0.0244	-20	182	191	GAWTDQFDPR					Mascot
1223.5903	1223.5265	-0.0638	-52	127	137	AEAQSVDFQTK					Mascot
1446.7965	1446.6721	-0.1244	-86	11	22	LSIAHQTRFAFR					Mascot
1514.7485	1514.6949	-0.0536	-35	125	137	YKAEAQSVDFQTK					Mascot
1665.8595	1665.8331	-0.0264	-16	261	274	LSAEPEFLEQHIPR					Mascot
1665.8595	1665.8331	-0.0264	-16	261	274	LSAEPEFLEQHIPR	109	100			Mascot
1922.9706	1922.9031	-0.0675	-35	335	353	AFVEVNETGTEAAATTIA K					Mascot
2083.1072	2083.0286	-0.0786	-38	379	398	EDTSGVVLFIGHVVNPLL SS					Mascot

Rank	Protein Name	Accession No.	Protein MW	Protein PI	Pep. Count	Protein Score	Protein Score C. I. %	Intensity Matched	Total Ion Score	Total Ion C. I. %	Confirmed
2	Alpha-1,4-glucan-protein synthase [UDP-forming] OS=Zea mays GN=UPTG PE=1 SV=2	UPTG_MAIZE	41690.8	5.75	7	63	73.516	2.244	42	92.994	

Peptide Information

Calc. Mass	Obsrv. Mass	± da	± ppm	Start Seq.	End Seq.	Sequence	Ion Score	C. I. %	Modification	Rank	Result Type
839.441	839.4224	-0.0186	-22	159	165	GYPFSLR					Mascot
989.5414	989.491	-0.0504	-51	276	284	ASNPFVNLK					Mascot
1223.5878	1223.5265	-0.0613	-50	31	39	NLDFLEMWR					Mascot
1446.5818	1446.6721	0.0903	62	244	254	YDDMWAGWCVK			Carbamidomethyl (C)[9], Oxidation (M)[4]		Mascot
1501.6958	1501.6715	-0.0243	-16	61	72	VPEGFDYELYNR					Mascot
1501.6958	1501.6715	-0.0243	-16	61	72	VPEGFDYELYNR	42	92.994			Mascot
1843.9225	1843.8583	-0.0642	-35	58	72	TIKVPEGFDYELYNR					Mascot
2292.1335	2292.0925	-0.041	-18	133	151	NLLSPSTPFFFNTLYDPY R					Mascot

Rank	Protein Name	Accession No.	Protein MW	Protein PI	Pep. Count	Protein Score	Protein Score C. I. %	Intensity Matched	Total Ion Score	Total Ion C. I. %	Confirmed
3	Alpha-1,4-glucan-protein synthase [UDP-forming] (Fragments) OS=Phoenix dactylifera PE=1 SV=1	UPTG_PHODC	6899.6	7.82	3	63	70.961	1.423	42	92.994	

Peptide Information

Calc. Mass	Obsrv. Mass	± da	± ppm	Start Seq.	End Seq.	Sequence	Ion Score	C. I. %	Modification	Rank	Result Type
989.5414	989.491	-0.0504	-51	52	60	ASNPFVNLK					Mascot
1501.6958	1501.6715	-0.0243	-16	4	15	VPEGFDYELYNR					Mascot
1501.6958	1501.6715	-0.0243	-16	4	15	VPEGFDYELYNR	42	92.994			Mascot
1843.9225	1843.8583	-0.0642	-35	1	15	TIKVPEGFDYELYNR					Mascot

4	Dual specificity protein phosphatase 5 OS=Homo sapiens GN=DUSP5 PE=1 SV=2	DUS5_HUMAN	42818.6	8.69	5	61	56.048	2.477	50	99.026

Peptide Information

Calc. Mass	Obsrv. Mass	± da	± ppm	Start Seq.	End Seq.	Sequence	Ion Score	C. I. %	Modification	Rank	Result Type
893.4033	893.4229	0.0196	22	376	384	SPVATATSC			Carbamidomethyl (C)[9]		Mascot
1022.5299	1022.4725	-0.0574	-56	1	9	MKVTSLDGR			Oxidation (M)[1]		Mascot
1022.5299	1022.4725	-0.0574	-56	1	9	MKVTSLDGR	50	99.026	Oxidation (M)[1]		Mascot
1126.6143	1126.5195	-0.0948	-84	286	294	LKEAFDYIK					Mascot
1330.6204	1330.6229	0.0025	2	270	280	SPTICMAYLMK			Carbamidomethyl (C)[5], Oxidation (M)[6]		Mascot
1346.6154	1346.6967	0.0813	60	270	280	SPTICMAYLMK			Carbamidomethyl (C)[5], Oxidation (M)[6,10]		Mascot
1417.653	1417.7142	0.0612	43	215	226	TSEACATHLHYK			Carbamidomethyl (C)[5]		Mascot

5	Alpha-1,4-glucan-protein synthase [UDP-forming] OS=Pisum sativum GN=UPTG PE=1 SV=1	UPTG_PEA	42059	5.73	6	58	18.157	2.315	42	92.994

Peptide Information

Calc. Mass	Obsrv. Mass	± da	± ppm	Start Seq.	End Seq.	Sequence	Ion Score	C. I. %	Modification	Rank	Result Type
837.3771	837.4459	0.0688	82	301	307	DCTSVQK			Carbamidomethyl (C)[2]		Mascot
839.441	839.4224	-0.0186	-22	152	158	GYPFSLR					Mascot
989.5414	989.491	-0.0504	-51	269	277	ASNPFVNLK					Mascot
1501.6958	1501.6715	-0.0243	-16	54	65	VPEGFDYELYNR					Mascot
1501.6958	1501.6715	-0.0243	-16	54	65	VPEGFDYELYNR	42	92.994			Mascot
1714.7863	1714.8389	0.0526	31	202	216	GSLFPMCGMNLAFNR			Carbamidomethyl (C)[7]		Mascot
2292.1335	2292.0925	-0.041	-18	126	144	NLLSPSTPFFFNTLYDPYR					Mascot

6	Peptide chain release factor 1 OS=Azoarcus sp. (strain BH72) GN=prfA PE=3 SV=1	RF1_AZOSB	40124.4	5.25	13	58	6.032	14.075

Peptide Information

Calc. Mass	Obsrv. Mass	± da	± ppm	Start Seq.	End Seq.	Sequence	Ion Score	C. I. %	Modification	Rank	Result Type

Calc. Mass	Obsrv. Mass	± da	± ppm	Start Seq.	End Seq.	Sequence	Ion Score	C. I. %	Modification	Rank	Result Type
833.4111	833.3911	-0.02	-24	287	294	QSAEAATR					Mascot
893.4839	893.4229	-0.061	-68	166	174	IVGAGAYSR					Mascot
989.5122	989.491	-0.0212	-21	287	295	QSAEAATRR					Mascot
1056.5908	1056.5651	-0.0257	-24	278	286	LNDAQLRAR					Mascot
1060.5494	1060.4994	-0.05	-47	285	294	ARQSAEAATR					Mascot
1224.579	1224.4994	-0.0796	-65	31	40	DMNAFRDLSR					Mascot
1224.579	1224.4994	-0.0796	-65	31	40	DMNAFRDLSR					Mascot
1240.574	1240.4933	-0.0807	-65	31	40	DMNAFRDLSR			Oxidation (M)[2]		Mascot
1544.7261	1544.7765	0.0504	33	67	79	ELLDDPEMQELGR					Mascot
1590.7772	1590.7349	-0.0423	-27	309	321	TYNFPQGRVTDHR					Mascot
1621.9385	1621.7931	-0.1454	-90	89	103	IAALDGDLQRALLPR					Mascot
1827.9858	1827.8425	-0.1433	-78	268	284	AQAMAVLAARLNDAQLR			Oxidation (M)[4]		Mascot
1852.9222	1852.7614	-0.1608	-87	246	261	ITHLPTGLVVECQDDR			Carbamidomethyl (C)[12]		Mascot
2014.02	2014.0059	-0.0141	-7	80	98	SELEAGEARIAALDGDLQ R					Mascot
2187.05	2187.1333	0.0833	38	117	137	AGTGGDEAALFAGDLLR MYSR			Oxidation (M)[18]		Mascot

7 Serpin-Z2A OS=Triticum aestivum PE=1 SV=1 SPZ2A_WHEAT 43341.5 5.46 7 58 6.032 4.536 37 81.742

Peptide Information

Calc. Mass	Obsrv. Mass	± da	± ppm	Start Seq.	End Seq.	Sequence	Ion Score	C. I. %	Modification	Rank	Result Type
925.5214	925.4976	-0.0238	-26	11	18	LSIAHQTR					Mascot
925.5214	925.4976	-0.0238	-26	11	18	LSIAHQTR	37	81.742			Mascot
1137.6667	1137.6052	-0.0615	-54	172	181	LVLGNALYFK					Mascot
1223.5903	1223.5265	-0.0638	-52	127	137	AEAQSVDFQTK					Mascot
1292.7097	1292.651	-0.0587	-45	289	300	ISFGIEASDLLK					Mascot
1510.7285	1510.7201	-0.0084	-6	182	194	GAWTDQFDSRVTK					Mascot
1514.7485	1514.6949	-0.0536	-35	125	137	YKAEAQSVDFQTK					Mascot
1922.9706	1922.9031	-0.0675	-35	335	353	TFVEVNETGTEAAAATIA K					Mascot

8 Protein lsd90 OS=Schizosaccharomyces pombe (strain 972 / ATCC 24843) GN=lsd90 PE=1 SV=1 LSD90_SCHPO 82446.2 6.71 20 57 1.603 5.192

Peptide Information

Calc. Mass	Obsrv. Mass	± da	± ppm	Start Seq.	End Seq.	Sequence	Ion Score	C. I. %	Modification	Rank	Result Type
800.3832	800.4001	0.0169	21	475	480	ISCHQR			Carbamidomethyl (C)[3]		Mascot
877.4526	877.4555	0.0029	3	83	90	TAYAAPQR					Mascot

Calc. Mass	Obsrv. Mass	± da	± ppm	Start Seq.	End Seq.	Sequence	Ion Score	C. I. %	Modification	Rank	Result Type
922.4489	922.4292	-0.0197	-21	194	200	ARQYNDR					Mascot
948.5182	948.4809	-0.0373	-39	454	461	QQLAMLTK			Oxidation (M)[5]		Mascot
1079.5117	1079.5386	0.0269	25	445	453	VNQFDVDSR					Mascot
1103.5803	1103.5602	-0.0201	-18	348	357	TASIREAEAR					Mascot
1115.6056	1115.502	-0.1036	-93	169	177	TLQDLLEQR					Mascot
1126.5198	1126.5195	-0.0003	0	487	496	CATQAYDAVK			Carbamidomethyl (C)[1]		Mascot
1196.5793	1196.5106	-0.0687	-57	513	522	ENEAKDLYSK					Mascot
1205.6008	1205.566	-0.0348	-29	201	211	STSEALELEAR					Mascot
1223.6267	1223.5265	-0.1002	-82	651	663	SATTPAYVGGATK					Mascot
1402.7074	1402.7021	-0.0053	-4	313	325	ARHEAAVADATYK					Mascot
1417.7897	1417.7142	-0.0755	-53	556	567	LTLEESKLTDLR					Mascot
1446.6754	1446.6721	-0.0033	-2	735	748	SSTSSGHGLMNNVR					Mascot
1498.6699	1498.782	0.1121	75	1	13	MVGTINESMQNMK			Oxidation (M)[1]		Mascot
1514.6648	1514.6949	0.0301	20	1	13	MVGTINESMQNMK			Oxidation (M)[1,9]		Mascot
1557.7438	1557.7008	-0.043	-28	475	486	ISCHQREEEITR			Carbamidomethyl (C)[3]		Mascot
1585.8545	1585.7167	-0.1378	-87	155	168	IVTTTNARIQPDEK					Mascot
1720.8721	1720.951	0.0789	46	2	17	VGTINESMQNMKIGAK					Mascot
1833.9089	1833.8804	-0.0285	-16	568	583	TAEPSQYVNDVEVARR					Mascot
2069.1238	2069.0168	-0.107	-52	264	283	LAQANADVDVANSKLDIALK					Mascot

9 Serpin-ZX OS=Hordeum vulgare GN=PAZX PE=1 SV=1 SPZX_HORVU 42920.3 6.77 7 57 0 4.355 37 81.742

Peptide Information

Calc. Mass	Obsrv. Mass	± da	± ppm	Start Seq.	End Seq.	Sequence	Ion Score	C. I. %	Modification	Rank	Result Type
807.4029	807.3633	-0.0396	-49	1	7	MATTDIR					Mascot
925.5214	925.4976	-0.0238	-26	8	15	LSIAHQTR					Mascot
925.5214	925.4976	-0.0238	-26	8	15	LSIAHQTR	37	81.742			Mascot
1137.6667	1137.6052	-0.0615	-54	171	180	LVLGNALYFK					Mascot
1187.6267	1187.6722	0.0455	38	60	71	DQLAATLGAAEK					Mascot
1313.6737	1313.656	-0.0177	-13	100	111	SFANVFVDSSLK					Mascot
1608.75	1608.7598	0.0098	6	334	348	SFVEVNEEGTEAAAR					Mascot
2014.1008	2014.0059	-0.0949	-47	100	117	SFANVFVDSSLKLKPSFK					Mascot

10 FYVE, RhoGEF and PH domain-containing protein 6 OS=Mus musculus GN=Fgd6 PE=1 SV=2 FGD6_MOUSE 156953.9 8.09 24 56 0 25.011

Peptide Information

Calc. Mass	Obsrv. Mass	± da	± ppm	Start Seq.	End Seq.	Sequence	Ion Score	C. I. %	Modification	Rank	Result Type
833.3999	833.3911	-0.0088	-11	140	146	ENLENSK					Mascot
839.3815	839.4224	0.0409	49	848	854	EIMSSEK			Oxidation (M)[3]		Mascot
893.3669	893.4229	0.056	63	832	839	QDEDAGMK					Mascot
965.5163	965.4881	-0.0282	-29	1252	1259	LDHQLSPR					Mascot
1060.5746	1060.4994	-0.0752	-71	274	283	RVASDGISQK					Mascot
1103.5942	1103.5602	-0.034	-31	491	500	KAASEELVEK					Mascot
1192.578	1192.5138	-0.0642	-54	913	921	MLTWTEQQR					Mascot
1208.5729	1208.507	-0.0659	-55	913	921	MLTWTEQQR			Oxidation (M)[1]		Mascot
1223.6123	1223.5265	-0.0858	-70	1099	1109	LNNMLSLAGMK			Oxidation (M)[4,10]		Mascot
1385.5751	1385.6683	0.0932	67	154	165	GSSWDSSSEKCR			Carbamidomethyl (C)[11]		Mascot
1385.5751	1385.6683	0.0932	67	154	165	GSSWDSSSEKCR			Carbamidomethyl (C)[11]		Mascot
1402.6016	1402.7021	0.1005	72	779	791	HQPCSSGTSQEGK			Carbamidomethyl (C)[4]		Mascot
1435.6659	1435.7214	0.0555	39	140	152	ENLENSKNGESSK					Mascot
1446.792	1446.6721	-0.1199	-83	1099	1111	LNNMLSLAGMKVR					Mascot
1463.7604	1463.689	-0.0714	-49	930	941	GPYLKMYSTYIK					Mascot
1629.8007	1629.6852	-0.1155	-71	218	232	IEFADVSSSLTGFEK					Mascot
1662.9187	1662.8546	-0.0641	-39	862	876	LLHIDFRGAVAHASR					Mascot
1665.8087	1665.8331	0.0244	15	966	979	EFEMSPRCANLALK			Carbamidomethyl (C)[8]		Mascot
1665.8087	1665.8331	0.0244	15	966	979	EFEMSPRCANLALK			Carbamidomethyl (C)[8]		Mascot
1687.8069	1687.7877	-0.0192	-11	668	682	AHSAENCSLESQKVK			Carbamidomethyl (C)[7]		Mascot
1703.9075	1703.7787	-0.1288	-76	1110	1123	VRKPTQEAYQNELK					Mascot
1720.8105	1720.951	0.1405	82	583	597	SLSAVDADRCNKPCK			Carbamidomethyl (C)[10,14]		Mascot
1727.7953	1727.7576	-0.0377	-22	200	214	APKKPEMNGDHSCTR			Carbamidomethyl (C)[13]		Mascot
1873.9398	1873.8807	-0.0591	-32	124	139	ETQCVEQLVLEPLGMK			Carbamidomethyl (C)[4]		Mascot
1922.8436	1922.9031	0.0595	31	1295	1311	EVSANTEDSTMSGYLYR					Mascot
2062.9536	2062.8857	-0.0679	-33	683	701	SWGQSSAVNGQRAESL DDR					Mascot
2095.1797	2095.0479	-0.1318	-63	284	302	TEVKGLGPLEIHLLPYTSK					Mascot

Gel Idx/Pos	265/K17	Instr./Gel Origin	BA2151/Sample Project 20140814	Process Status	Analysis Succeeded
Plate [#] Name	[1] Sample Project 20140814	Instrument Sample Name		Spectra	11

Rank	Protein Name	Accession No.	Protein MW	Protein PI	Pep. Count	Protein Score	Protein Score C. I. %	Intensity Matched	Total Ion Score	Total Ion C. I. %	Confirmed
1	Serpin-Z1C OS=Triticum aestivum PE=1 SV=1	SPZ1C_WHEAT	42969	5.62	10	148	100	10.664	105	100	

Peptide Information

Calc. Mass	Obsrv. Mass	± da	± ppm	Start Seq.	End Seq.	Sequence	Ion Score	C. I. %	Modification	Rank	Result Type
925.5214	925.5001	-0.0213	-23	11	18	LSIAHQTR					Mascot
925.5214	925.5001	-0.0213	-23	11	18	LSIAHQTR	35	69.28			Mascot
947.5156	947.4678	-0.0478	-50	2	10	ATTLATDVR					Mascot
1151.6824	1151.5846	-0.0978	-85	172	181	LVLANALYFK					Mascot
1176.5896	1176.5691	-0.0205	-17	261	270	LSAEPDFLER					Mascot
1176.5896	1176.5691	-0.0205	-17	261	270	LSAEPDFLER	70	99.99			Mascot
1239.5852	1239.6824	0.0972	78	127	137	ADTQSVDFQTK					Mascot
1352.6945	1352.6407	-0.0538	-40	289	300	ISFETEASDLLK					Mascot
1399.6125	1399.6244	0.0119	9	182	194	GAWTDQFDSSGTK					Mascot
2129.0947	2129.0059	-0.0888	-42	379	398	EDISGVVLFMGHVVNPLL SS			Oxidation (M)[10]		Mascot
2720.3525	2720.302	-0.0505	-19	328	353	VSSVFHQAFVEVNEQGT EAAASTAIK					Mascot
2725.4631	2725.4294	-0.0337	-12	33	61	SAASNAVFSPVSLHVALS LLAAGAGSATR					Mascot

Rank	Protein Name	Accession No.	Protein MW	Protein PI	Pep. Count	Protein Score	Protein Score C. I. %	Intensity Matched	Total Ion Score	Total Ion C. I. %	Confirmed
2	Serpin-Z1B OS=Triticum aestivum PE=1 SV=1	SPZ1B_WHEAT	43119.9	5.44	7	128	100	9.708	105	100	

Peptide Information

Calc. Mass	Obsrv. Mass	± da	± ppm	Start Seq.	End Seq.	Sequence	Ion Score	C. I. %	Modification	Rank	Result Type
925.5214	925.5001	-0.0213	-23	11	18	LSIAHQTR					Mascot
925.5214	925.5001	-0.0213	-23	11	18	LSIAHQTR	35	69.28			Mascot
947.5156	947.4678	-0.0478	-50	2	10	ATTLATDVR					Mascot
1151.6824	1151.5846	-0.0978	-85	172	181	LVLANALYFK					Mascot
1176.5896	1176.5691	-0.0205	-17	262	271	LSAEPDFLER					Mascot
1176.5896	1176.5691	-0.0205	-17	262	271	LSAEPDFLER	70	99.99			Mascot
1585.8295	1585.7083	-0.1212	-76	288	301	FKISFGMEASDLLK					Mascot
1601.8243	1601.7025	-0.1218	-76	288	301	FKISFGMEASDLLK			Oxidation (M)[7]		Mascot
2129.0947	2129.0059	-0.0888	-42	380	399	EDISGVVLFMGHVVNPLL SS			Oxidation (M)[10]		Mascot

2720.3525	2720.302	-0.0505	-19	329	354	VSSVFHQAFVEVNEQGT EAAASTAIK					Mascot

3	Serpin-Z1A OS=Triticum aestivum GN=WZCI PE=1 SV=1	SPZ1A_WHEAT	43262.2	5.6	6	123	100	9.412	105	100

Peptide Information

Calc. Mass	Obsrv. Mass	± da	± ppm	Start Seq.	End Seq.	Sequence	Ion Score	C. I. %	Modification	Rank	Result Type
925.5214	925.5001	-0.0213	-23	11	18	LSIAHQTR					Mascot
925.5214	925.5001	-0.0213	-23	11	18	LSIAHQTR	35	69.28			Mascot
947.5156	947.4678	-0.0478	-50	2	10	ATTLATDVR					Mascot
1151.6824	1151.5846	-0.0978	-85	172	181	LVLANALYFK					Mascot
1176.5896	1176.5691	-0.0205	-17	261	270	LSAEPDFLER					Mascot
1176.5896	1176.5691	-0.0205	-17	261	270	LSAEPDFLER	70	99.99			Mascot
2129.0947	2129.0059	-0.0888	-42	379	398	EDISGVVLFMGHVVNPLL SS			Oxidation (M)[10]		Mascot
2720.3525	2720.302	-0.0505	-19	328	353	VSSVFHQAFVEVNEQGT EAAASTAIK					Mascot

4	tRNA pseudouridine synthase B OS=Listeria innocua serovar 6a (strain CLIP 11262) GN=truB PE=3 SV=1	TRUB_LISIN	34115.9	5.82	13	69	93.034	4.81		

Peptide Information

Calc. Mass	Obsrv. Mass	± da	± ppm	Start Seq.	End Seq.	Sequence	Ion Score	C. I. %	Modification	Rank	Result Type
800.4512	800.3823	-0.0689	-86	298	304	VIELQQA					Mascot
814.4093	814.3973	-0.012	-15	127	132	LYEYAR					Mascot
842.4519	842.4773	0.0254	30	200	206	TRSGFFK					Mascot
947.556	947.4678	-0.0882	-93	271	278	VALIFQEK					Mascot
1087.5969	1087.528	-0.0689	-63	1	9	MNGIIPLWK			Oxidation (M)[1]		Mascot
1225.7052	1225.6694	-0.0358	-29	2	11	NGIIPLWKER					Mascot
1563.7571	1563.7043	-0.0528	-34	207	219	KEDCLTLAEIDEK			Carbamidomethyl (C)[4]		Mascot
1908.9927	1908.9419	-0.0508	-27	127	142	LYEYARAGIEVERPSR					Mascot
1908.9927	1908.9419	-0.0508	-27	127	142	LYEYARAGIEVERPSR					Mascot
2219.1489	2219.0793	-0.0696	-31	31	51	VGHTGTLDPEVEGVLPIC IGR			Carbamidomethyl (C)[18]		Mascot
2225.2693	2225.0818	-0.1875	-84	271	289	VALIFQEKLTAIYKPHPEK					Mascot
2253.2603	2253.0725	-0.1878	-83	251	270	VLNGVLLPKSLFQTVENE PR					Mascot
2329.2407	2329.134	-0.1067	-46	179	199	TLAVMIGELLGYPAHMSK LER					Mascot
2392.2065	2392.1055	-0.101	-42	152	173	LDGVSPLTESNPTFKLEIS CGK			Carbamidomethyl (C)[20]		Mascot

5	Conjugal transfer protein TraA OS=Agrobacterium tumefaciens (strain C58 / ATCC 33970) GN=traA PE=3	TRAA_AGRT5	123629	9.33	28	65	83.29	15.063		

SV=2

Peptide Information

Calc. Mass	Obsrv. Mass	± da	± ppm	Start Seq.	End Seq.	Sequence	Ion Score	C. I. %	Modification	Rank	Result Type
804.3958	804.364	-0.0318	-40	627	633	TADGERR					Mascot
807.4029	807.3866	-0.0163	-20	348	354	LEATMAR			Oxidation (M)[5]		Mascot
809.3577	809.3904	0.0327	40	196	201	DGWFER					Mascot
814.4669	814.3973	-0.0696	-85	856	863	LGADPTLK					Mascot
834.4324	834.3884	-0.044	-53	604	610	MLNVMAR					Mascot
857.4727	857.4324	-0.0403	-47	820	827	LGLDQSPK					Mascot
870.5519	870.4689	-0.083	-95	984	990	VVLERVR					Mascot
925.4594	925.5001	0.0407	44	411	418	TTMMKAAR			Oxidation (M)[3]		Mascot
925.4594	925.5001	0.0407	44	411	418	TTMMKAAR			Oxidation (M)[3]		Mascot
949.5214	949.4415	-0.0799	-84	336	343	VPARYSTR					Mascot
993.5146	993.4438	-0.0708	-71	952	959	MRETATLR			Oxidation (M)[1]		Mascot
1058.559	1058.5388	-0.0202	-19	256	263	IELNEERR					Mascot
1060.5746	1060.5413	-0.0333	-31	701	710	SQGATVDRVK					Mascot
1091.5667	1091.4911	-0.0756	-69	720	728	HLTYVAMTR					Mascot
1176.5677	1176.5691	0.0014	1	1047	1056	LSDGMRPEQK			Oxidation (M)[5]		Mascot
1176.5677	1176.5691	0.0014	1	1047	1056	LSDGMRPEQK			Oxidation (M)[5]		Mascot
1198.6677	1198.5815	-0.0862	-72	906	917	LEAEPASIGALK					Mascot
1204.6289	1204.5895	-0.0394	-33	601	610	DVRMLNVMAR					Mascot
1204.6289	1204.5895	-0.0394	-33	601	610	DVRMLNVMAR					Mascot
1239.615	1239.6824	0.0674	54	945	953	DLEQYLRMR			Oxidation (M)[8]		Mascot
1399.7515	1399.6244	-0.1271	-91	1057	1067	EQLKQAWPIMR					Mascot
1507.7751	1507.7097	-0.0654	-43	323	335	LQRDTIEFATGEK					Mascot
1516.7023	1516.8213	0.119	78	470	483	TVFVMDEAGMVASK			Oxidation (M)[5,10]		Mascot
1657.8591	1657.7853	-0.0738	-45	776	789	FAENRGLHIMQVAR			Oxidation (M)[10]		Mascot
1733.8817	1733.8257	-0.056	-32	444	458	EAGIESRTLSSWELR					Mascot
1891.0437	1890.9435	-0.1002	-53	45	61	KQGLVHQEFILPADAPK					Mascot
1906.1121	1905.925	-0.1871	-98	971	988	VSIDIPALSPAARVVLER					Mascot
2075.1609	2075.0117	-0.1492	-72	223	242	QGIDLEPTIHLGVGAKAIS R					Mascot
2129.1753	2129.0059	-0.1694	-80	634	652	FHAGDQIVFLKNETLLGV K					Mascot
2185.0515	2185.1646	0.1131	52	464	483	DVLDNKTVFVMDEAGMV ASK			Oxidation (M)[11]		Mascot
2742.5012	2742.3042	-0.197	-72	300	322	YVDDPAVFQQLMLRIILN PEVLR					Mascot

2758.4961	2758.2742	-0.2219	-80	300	322	YVDDPAVFQQLMLRIILN PEVLR			Oxidation (M)[12]		Mascot

6	Ribosomal RNA large subunit methyltransferase K/L OS=Thiomicrospira crunogena (strain XCL-2) GN=rlmL PE=3 SV=1	RLMKL_THICR	84291.8	6.11	18	60	45.927	7.539

Peptide Information

Calc. Mass	Obsrv. Mass	± da	± ppm	Start Seq.	End Seq.	Sequence	Ion Score	C. I. %	Modification	Rank	Result Type
814.4781	814.3973	-0.0808	-99	11	17	GLNELLR					Mascot
832.4536	832.4158	-0.0378	-45	139	145	VHGHLNR					Mascot
875.4879	875.4155	-0.0724	-83	418	424	MVSNRIR					Mascot
904.4622	904.4564	-0.0058	-6	320	327	LGEEETVK					Mascot
963.5621	963.4927	-0.0694	-72	685	692	LIFSTNLR					Mascot
981.4822	981.4249	-0.0573	-58	714	721	TMPKDYAR					Mascot
981.4822	981.4249	-0.0573	-58	714	721	TMPKDYAR					Mascot
984.4785	984.4881	0.0096	10	114	121	DGIVDYFR					Mascot
1048.5092	1048.476	-0.0332	-32	658	666	MDGTLDIQR					Mascot
1087.5929	1087.528	-0.0649	-60	176	185	ENVAAAILMR					Mascot
1204.6104	1204.5895	-0.0209	-17	657	666	RMDGTLDIQR					Mascot
1204.6104	1204.5895	-0.0209	-17	657	666	RMDGTLDIQR					Mascot
1225.6576	1225.6694	0.0118	10	112	121	IKDGIVDYFR					Mascot
1263.7056	1263.6934	-0.0122	-10	682	692	DGKLIFSTNLR					Mascot
1399.6556	1399.6244	-0.0312	-22	588	600	AGCKSSLSLDMSK			Carbamidomethyl (C)[3], Oxidation (M)[11]		Mascot
1890.9817	1890.9435	-0.0382	-20	346	362	AAILTCHTELGMFLGIK			Carbamidomethyl (C)[6], Oxidation (M)[12]		Mascot
1908.9412	1908.9419	0.0007	0	592	607	SSLSLDMSKTYLYWAK			Oxidation (M)[7]		Mascot
1908.9412	1908.9419	0.0007	0	592	607	SSLSLDMSKTYLYWAK			Oxidation (M)[7]		Mascot
2047.0283	2046.9821	-0.0462	-23	381	396	FEIEEEWFRQPALQPK					Mascot
2231.0703	2231.1436	0.0733	33	301	319	KWGDWSPGLIVCNPPYG ER			Carbamidomethyl (C)[12]		Mascot
2231.0703	2231.1436	0.0733	33	301	319	KWGDWSPGLIVCNPPYG ER			Carbamidomethyl (C)[12]		Mascot
2758.4497	2758.2742	-0.1755	-64	139	162	VHGHLNRNQLTLSLDLS GYSLHQR					Mascot

7	T-complex protein 1 subunit theta OS=Gallus gallus GN=CCT8 PE=1 SV=3	TCPQ_CHICK	60017.4	5.35	15	59	31.926	4.17

Peptide Information

Calc. Mass	Obsrv. Mass	± da	± ppm	Start Seq.	End Seq.	Sequence	Ion Score	C. I. %	Modification	Rank	Result Type
818.4366	818.3833	-0.0533	-65	38	44	ELAQTTR					Mascot

885.5152	885.4501	-0.0651	-74	327	335	TVGATALPR					Mascot
962.5128	962.4853	-0.0275	-29	8	16	APGFAQMLK					Mascot
999.5291	999.467	-0.0621	-62	55	62	MVINHLEK			Oxidation (M)[1]		Mascot
1068.4779	1068.5057	0.0278	26	45	54	TAYGPNGMNK			Oxidation (M)[8]		Mascot
1150.5891	1150.4816	-0.1075	-93	441	450	FAEAFEAIPR					Mascot
1158.5902	1158.5581	-0.0321	-28	467	476	LYAVHQEGNK					Mascot
1198.5409	1198.5815	0.0406	34	261	270	NAEELMNFSK			Oxidation (M)[6]		Mascot
1232.6093	1232.5883	-0.021	-17	297	307	VADMALHYANK					Mascot
1323.6328	1323.6239	-0.0089	-7	21	31	HYSGLEEAVYR					Mascot
1381.5901	1381.6832	0.0931	67	379	390	GSTDNLMDDIER			Oxidation (M)[7]		Mascot
1623.9039	1623.8994	-0.0045	-3	2	16	ALHVPKAPGFAQMLK			Oxidation (M)[13]		Mascot
1623.9039	1623.8994	-0.0045	-3	2	16	ALHVPKAPGFAQMLK			Oxidation (M)[13]		Mascot
1891.047	1890.9435	-0.1035	-55	521	539	VDQIIMAKPAGGPKPPSG K					Mascot
2186.1201	2186.1833	0.0632	29	491	509	DMLEAGILDTYLGKYWGI K					Mascot
2186.1201	2186.1833	0.0632	29	491	509	DMLEAGILDTYLGKYWGI K					Mascot
2260.1941	2260.1365	-0.0576	-25	204	225	VCKIVGAGVSASSVLHG MVFNK			Carbamidomethyl (C)[2]		Mascot

8	Protein RhsC OS=Escherichia coli (strain K12) GN=rhsC PE=3 SV=4	RHSC_ECOLI	158762.3	6.23	24	59	30.34	10.618

Peptide Information

Calc. Mass	Obsrv. Mass	± da	± ppm	Start Seq.	End Seq.	Sequence	Ion Score	C. I. %	Modification	Rank	Result Type
800.4011	800.3823	-0.0188	-23	1391	1397	FVFTGVM					Mascot
816.3961	816.3769	-0.0192	-24	1391	1397	FVFTGVM			Oxidation (M)[7]		Mascot
840.4686	840.4073	-0.0613	-73	942	948	HGRLTEK					Mascot
842.4413	842.4773	0.036	43	344	350	GRMVAHR			Oxidation (M)[3]		Mascot
862.4377	862.4264	-0.0113	-13	1111	1117	VSEESRR					Mascot
875.3781	875.4155	0.0374	43	335	341	SFTYDDK					Mascot
925.456	925.5001	0.0441	48	529	535	KTMTWSR			Oxidation (M)[3]		Mascot
925.456	925.5001	0.0441	48	529	535	KTMTWSR			Oxidation (M)[3]		Mascot
981.4636	981.4249	-0.0387	-39	566	573	EEGLSQYR					Mascot
981.4636	981.4249	-0.0387	-39	566	573	EEGLSQYR					Mascot
989.5414	989.5014	-0.04	-40	136	143	SESLWLVR					Mascot
993.4999	993.4438	-0.0561	-56	320	328	GELAAVYDR					Mascot
999.42	999.467	0.047	47	634	642	SMEYDAAGR					Mascot
1029.4708	1029.5009	0.0301	29	959	966	TDDERTHR					Mascot

1058.5702	1058.5388	-0.0314	-30	431	440	LRAQTDAAGR						Mascot
1204.5957	1204.5895	-0.0062	-5	899	909	IPYTTDPAGNR						Mascot
1204.5957	1204.5895	-0.0062	-5	899	909	IPYTTDPAGNR						Mascot
1232.6497	1232.5883	-0.0614	-50	1388	1397	FIRFVFTGVM			Oxidation (M)[10]			Mascot
1263.658	1263.6934	0.0354	28	807	817	LGDTPLVEYTR						Mascot
1352.7434	1352.6407	-0.1027	-76	677	687	YHHDLTGKLIR						Mascot
1381.6859	1381.6832	-0.0027	-2	738	749	VTVHYGYDSKGR						Mascot
1585.8656	1585.7083	-0.1573	-99	247	260	LVLTTQAQRAEEAR						Mascot
1657.6871	1657.7853	0.0982	59	1287	1301	GDYTCGGQDQRGESK			Carbamidomethyl (C)[5]			Mascot
1733.9585	1733.8257	-0.1328	-77	1044	1058	IQTIYQPGSFTPLIR						Mascot
2225.0371	2225.0818	0.0447	20	1191	1208	LPGQQYDEESGLYYNRH R						Mascot
2392.2356	2392.1055	-0.1301	-54	441	462	TTEYSPDVVTGLITRITTP DGR						Mascot
2725.3145	2725.4294	0.1149	42	1	27	MSGKPAARQGDMTQYG GSIVQGSAGVR			Oxidation (M)[1]			Mascot
2742.2686	2742.3042	0.0356	13	1118	1141	WLASCGLTVAQMQSQM DPVYTPAR			Carbamidomethyl (C)[5], Oxidation (M)[12,16]			Mascot

9 Acyl carrier protein OS=Akkermansia muciniphila (strain ATCC BAA-835) GN=acpP PE=3 SV=1 | ACP_AKKM8 | 8815.3 | 3.86 | 7 | 58 | 23.62 | 6.438

Peptide Information

Calc. Mass	Obsrv. Mass	± da	± ppm	Start Seq.	End Seq.	Sequence	Ion Score	C. I. %	Modification	Rank	Result Type
921.416	921.4632	0.0472	51	2	9	SDNSIEEK					Mascot
1068.4514	1068.5057	0.0543	51	1	9	MSDNSIEEK			Oxidation (M)[1]		Mascot
1176.5856	1176.5691	-0.0165	-14	2	11	SDNSIEEKVR					Mascot
1176.5856	1176.5691	-0.0165	-14	2	11	SDNSIEEKVR					Mascot
1323.621	1323.6239	0.0029	2	1	11	MSDNSIEEKVR			Oxidation (M)[1]		Mascot
1434.7839	1434.6682	-0.1157	-81	65	77	LQSVADVVAYIEK					Mascot
1657.912	1657.7853	-0.1267	-76	10	24	VRSIIVDQLGVESDK					Mascot
1718.9324	1718.7958	-0.1366	-79	65	80	LQSVADVVAYIEKVQG					Mascot

10 Alanine--tRNA ligase OS=Streptomyces coelicolor (strain ATCC BAA-471 / A3(2) / M145) GN=alaS PE=3 SV=1 | SYA_STRCO | 96069.2 | 5.19 | 20 | 57 | 0 | 12.039

Peptide Information

Calc. Mass	Obsrv. Mass	± da	± ppm	Start Seq.	End Seq.	Sequence	Ion Score	C. I. %	Modification	Rank	Result Type
804.4396	804.364	-0.0756	-94	428	433	LMKEQR					Mascot
814.4781	814.3973	-0.0808	-99	625	631	INEVLAR					Mascot

820.4345	820.3947	-0.0398	-49	428	433	LMKEQR	Oxidation (M)[2]	Mascot
842.5458	842.4773	-0.0685	-81	798	804	KLVLDVR		Mascot
851.3927	851.4117	0.019	22	1	7	MESAEIR	Oxidation (M)[1]	Mascot
857.4298	857.4324	0.0026	3	280	286	VVTDHMR		Mascot
860.4584	860.4457	-0.0127	-15	2	8	ESAEIRR		Mascot
875.4217	875.4155	-0.0062	-7	557	564	AQASIDDR		Mascot
962.5703	962.4853	-0.085	-88	741	749	VSAMLGKLK	Oxidation (M)[4]	Mascot
963.453	963.4927	0.0397	41	115	123	GGYGLDPER		Mascot
1048.4979	1048.476	-0.0219	-21	637	646	ADVMGIDEAK		Mascot
1058.5841	1058.5388	-0.0453	-43	521	530	VDTGAVIEVR		Mascot
1087.5742	1087.528	-0.0462	-42	227	236	NIDTGLGLER		Mascot
1176.5929	1176.5691	-0.0238	-20	637	647	ADVMGIDEAKK		Mascot
1176.5929	1176.5691	-0.0238	-20	637	647	ADVMGIDEAKK		Mascot
1204.6572	1204.5895	-0.0677	-56	356	366	VALAEENAFLK		Mascot
1204.6572	1204.5895	-0.0677	-56	356	366	VALAEENAFLK		Mascot
1507.8479	1507.7097	-0.1382	-92	719	731	EHTVVAQLQELIK		Mascot
1516.7504	1516.8213	0.0709	47	445	459	TGHAGAGAYREIADK		Mascot
1623.8741	1623.8994	0.0253	16	704	718	IEALVGVDAYSFLAR		Mascot
1623.8741	1623.8994	0.0253	16	704	718	IEALVGVDAYSFLAR		Mascot
1718.8379	1718.7958	-0.0421	-24	287	303	TSVMLIGDGVTPGNEGR	Oxidation (M)[4]	Mascot
1905.9204	1905.925	0.0046	2	195	208	YVEIWNLVFMQYER	Oxidation (M)[10]	Mascot
2392.1887	2392.1055	-0.0832	-35	330	350	DLIDVVIGMMGQQYPELV TDR		Mascot

Gel Idx/Pos	266/K18	Instr./Gel Origin	BA2151/Sample Project 20140814	Process Status	Analysis Succeeded
Plate [#] Name	[1] Sample Project 20140814	Instrument Sample Name		Spectra	11

Rank	Protein Name	Accession No.	Protein MW	Protein PI	Pep. Count	Protein Score	Protein Score C. I. %	Intensity Matched	Total Ion Score	Total Ion C. I. %	Confirmed
1	Phosphoglycerate kinase, cytosolic OS=Triticum aestivum PE=2 SV=1	PGKY_WHEAT	42152.7	5.64	19	651	100	57.398	530	100	

Peptide Information

Calc. Mass	Obsrv. Mass	± da	± ppm	Start Seq.	End Seq.	Sequence	Ion Score	C. I. %	Modification	Rank	Result Type
1030.6409	1030.6362	-0.0047	-5	75	83	FSLKPLVAR					Mascot
1030.6409	1030.6362	-0.0047	-5	75	83	FSLKPLVAR	46	97.437			Mascot
1089.5786	1089.5455	-0.0331	-30	6	16	SVGTLGEADLK					Mascot
1096.5521	1096.5519	-0.0002	0	307	316	TFAEALDTTK					Mascot
1245.6798	1245.6925	0.0127	10	5	16	RSVGTLGEADLK					Mascot
1274.6951	1274.6445	-0.0506	-40	6	18	SVGTLGEADLKGK					Mascot
1298.6587	1298.6355	-0.0232	-18	24	35	ADLNVPLDDAQK					Mascot
1375.8196	1375.7859	-0.0337	-24	44	55	ASIPTIKYLLEK					Mascot
1388.7421	1388.708	-0.0341	-25	179	191	ELDYLVGAVANPK					Mascot
1443.8782	1443.7881	-0.0901	-62	203	216	VSSKIGVIESLLAK					Mascot
1493.8297	1493.8295	-0.0002	0	166	178	FLRPSVAGFLMQK					Mascot
1509.8247	1509.8046	-0.0201	-13	166	178	FLRPSVAGFLMQK			Oxidation (M)[11]		Mascot
1509.8247	1509.8046	-0.0201	-13	166	178	FLRPSVAGFLMQK	25	0	Oxidation (M)[11]		Mascot
1573.8433	1573.7906	-0.0527	-33	350	366	GVTTIIGGGDSVAAVEK					Mascot
1720.9956	1721.0099	0.0143	8	106	122	LAAALPDGGVLLLENVR					Mascot
1720.9956	1721.0099	0.0143	8	106	122	LAAALPDGGVLLLENVR	152	100			Mascot
1769.8568	1769.8281	-0.0287	-16	317	331	TVIWNGPMGVFEFEK			Oxidation (M)[8]		Mascot
1919.9611	1919.9753	0.0142	7	138	155	LASVADLYVNDAFGTAH R					Mascot
1919.9611	1919.9753	0.0142	7	138	155	LASVADLYVNDAFGTAH R	151	100			Mascot
2048.0559	2048.0669	0.011	5	137	155	KLASVADLYVNDAFGTAH R					Mascot
2048.0559	2048.0669	0.011	5	137	155	KLASVADLYVNDAFGTAH R	155	100			Mascot
2089.1274	2089.0889	-0.0385	-18	241	259	SLVEEDKLELATSLIETAK					Mascot
2159.2224	2159.1467	-0.0757	-35	106	125	LAAALPDGGVLLLENVRF YK					Mascot
2446.2092	2446.1875	-0.0217	-9	84	105	LSELLGLEVVMAPDCIGE EVEK			Carbamidomethyl (C)[15], Oxidation (M)[11]		Mascot
2878.4866	2878.4949	0.0083	3	373	401	MSHISTGGGASLELLEGK PLPGVLALDEA			Oxidation (M)[1]		Mascot

2	Phosphoglycerate kinase OS=Trichodesmium erythraeum (strain IMS101) GN=pgk PE=3 SV=1	PGK_TRIEI	42536.1	5.01	7	179	100	4.837	155	100	

Peptide Information

Calc. Mass	Obsrv. Mass	± da	± ppm	Start Seq.	End Seq.	Sequence	Ion Score	C. I. %	Modification	Rank	Result Type
1360.7318	1360.791	0.0592	44	234	246	GLSVGKSLVEEDK					Mascot
1573.8507	1573.7906	-0.0601	-38	261	275	GVTMLLPTDVVVADK			Oxidation (M)[4]		Mascot
1702.9349	1702.9664	0.0315	18	219	233	LLLGGGMIFTFYKAR			Oxidation (M)[7]		Mascot
1737.8339	1737.8448	0.0109	6	316	330	TVIWNGPMGVFEMEK					Mascot
1769.8237	1769.8281	0.0044	2	316	330	TVIWNGPMGVFEMEK			Oxidation (M)[8,13]		Mascot
2048.0195	2048.0669	0.0474	23	136	154	QLASVADLYVNDAFGTA HR					Mascot
2048.0195	2048.0669	0.0474	23	136	154	QLASVADLYVNDAFGTA HR	155	100			Mascot
2105.0259	2105.0823	0.0564	27	23	41	ADFNVPVDNGSITDDTRI R					Mascot
2111.186	2111.105	-0.081	-38	181	201	FLQGAIDSPQKPLAAIIGG SK					Mascot

3	Phosphoglycerate kinase OS=Acaryochloris marina (strain MBIC 11017) GN=pgk PE=3 SV=1	PGK_ACAM1	42006.9	5.23	6	172	100	4.688	155	100	

Peptide Information

Calc. Mass	Obsrv. Mass	± da	± ppm	Start Seq.	End Seq.	Sequence	Ion Score	C. I. %	Modification	Rank	Result Type
1068.5936	1068.5649	-0.0287	-27	391	401	VLPGIAALDEA					Mascot
1158.6729	1158.624	-0.0489	-42	84	94	LSELLGQAVTK					Mascot
1702.9349	1702.9664	0.0315	18	220	234	LLIGGGMIFTFYKAR			Oxidation (M)[7]		Mascot
1714.9124	1714.8748	-0.0376	-22	19	34	VLVRADFNVPLDGDGK					Mascot
1801.9922	1801.8566	-0.1356	-75	217	232	VDKLLIGGGMIFTFYK					Mascot
2048.0195	2048.0669	0.0474	23	137	155	QLASVADLYVNDAFGTA HR					Mascot
2048.0195	2048.0669	0.0474	23	137	155	QLASVADLYVNDAFGTA HR	155	100			Mascot

4	UPF0051 protein ML0593 OS=Mycobacterium leprae (strain TN) GN=ML0593 PE=3 SV=2	Y593_MYCLE	96026.8	6.25	22	80	99.518	6.797			

Peptide Information

Calc. Mass	Obsrv. Mass	± da	± ppm	Start Seq.	End Seq.	Sequence	Ion Score	C. I. %	Modification	Rank	Result Type
842.4618	842.4789	0.0171	20	123	130	LGIPDAEK					Mascot
874.4629	874.4257	-0.0372	-43	45	52	GLSEDVVR					Mascot
1030.5641	1030.6362	0.0721	70	44	52	RGLSEDVVR					Mascot

Calc. Mass	Obsrv. Mass	± da	± ppm	Start Seq.	End Seq.	Sequence	Ion Score	C. I. %	Modification	Rank	Result Type
1030.5641	1030.6362	0.0721	70	44	52	RGLSEDVVR					Mascot
1175.5977	1175.6033	0.0056	5	859	869	LIELQMEGAVG			Oxidation (M)[6]		Mascot
1274.5834	1274.6445	0.0611	48	59	68	DEPEWMLQAR					Mascot
1375.7217	1375.7859	0.0642	47	177	189	QYLGTVIPAGDNK					Mascot
1388.738	1388.708	-0.03	-22	45	57	GLSEDVVRDISAK					Mascot
1491.7438	1491.8365	0.0927	62	118	130	NTYDRLGIPDAEK					Mascot
1493.7305	1493.8295	0.099	66	847	858	ELPMEYALELNR			Oxidation (M)[4]		Mascot
1531.7573	1531.7478	-0.0095	-6	59	70	DEPEWMLQARLK			Oxidation (M)[6]		Mascot
1701.8741	1701.8932	0.0191	11	246	261	VASGTKPVYEMHVAGR					Mascot
1801.8253	1801.8566	0.0313	17	28	44	YGYGWADSDVAGASAR R					Mascot
1864.9084	1865.0361	0.1277	68	699	714	VTMKYPAVWMTGEHAK			Oxidation (M)[3]		Mascot
1901.9982	1902.0122	0.014	7	363	380	QVAFSVPAGDPVHHTAIR					Mascot
1958.0818	1957.9142	-0.1676	-86	642	659	SDSLHSAVVEIIVKPHAR					Mascot
1977.0288	1976.9766	-0.0522	-26	152	169	ADLKDQGVVFLDTETGL R					Mascot
2028.9913	2029.0135	0.0222	11	403	420	AFETWLAELGFSGDEKT K					Mascot
2031.0652	2031.0743	0.0091	4	735	753	MLHLASNTSSNIVSKSVA R			Oxidation (M)[1]		Mascot
2089.0859	2089.0889	0.003	1	470	488	QLAIGCGLYPDGALVERT R			Carbamidomethyl (C)[6]		Mascot
2159.0557	2159.1467	0.091	42	83	100	WGSNLDGIDFDNIKYFVR					Mascot
2382.1824	2382.208	0.0256	11	306	326	VLPDDSGTIFFSESELDIK NR					Mascot
2891.3735	2891.4795	0.106	37	814	838	VSENQLFYLMSRGLAED EAMAMVVR			Oxidation (M)[10,20]		Mascot
2907.3687	2907.4668	0.0981	34	814	838	VSENQLFYLMSRGLAED EAMAMVVR			Oxidation (M)[10,20,22]		Mascot

5	Antagonist of mitotic exit network protein 1 OS=Candida glabrata (strain ATCC 2001 / CBS 138 / JCM 3761 / NBRC 0622 / NRRL Y-65) GN=AMN1 PE=3 SV=1	AMN1_CANGA	58732.5	9.43	16	70	94.954	7.705

Peptide Information

Calc. Mass	Obsrv. Mass	± da	± ppm	Start Seq.	End Seq.	Sequence	Ion Score	C. I. %	Modification	Rank	Result Type
874.5104	874.4257	-0.0847	-97	491	498	AQSLKTAR					Mascot
1156.5667	1156.5665	-0.0002	0	62	70	QESLYNMKK			Oxidation (M)[7]		Mascot
1416.8057	1416.6963	-0.1094	-77	367	381	NGSSITGLSVVALAK					Mascot
1474.743	1474.8257	0.0827	56	410	421	NIERLSLNNCNK			Carbamidomethyl (C)[10]		Mascot
1478.6653	1478.782	0.1167	79	130	141	TISNDLDDCRNR			Carbamidomethyl (C)[9]		Mascot
1494.7469	1494.8398	0.0929	62	204	217	ENTSTVVSCLAVSK			Carbamidomethyl (C)[9]		Mascot
1507.7937	1507.754	-0.0397	-26	315	326	LINDSYLIQICR			Carbamidomethyl (C)[11]		Mascot
1531.8148	1531.7478	-0.067	-44	2	15	VLPDSNIMKNGSIK			Oxidation (M)[8]		Mascot

1573.7527	1573.7906	0.0379	24	236	249	STSLTNFLSQCSTK			Carbamidomethyl (C)[11]		Mascot
1646.8605	1646.7855	-0.075	-46	1	15	MVLPDSNIMKNGSIK					Mascot
1662.8553	1662.8624	0.0071	4	1	15	MVLPDSNIMKNGSIK			Oxidation (M)[1]		Mascot
1701.8477	1701.8932	0.0455	27	236	250	STSLTNFLSQCSTKK			Carbamidomethyl (C)[11]		Mascot
1901.8845	1902.0122	0.1277	67	338	355	ACDNITDAGIVAVGTHCK			Carbamidomethyl (C)[2,17]		Mascot
1903.0032	1903.0122	0.009	5	17	32	LRDSQIENSPYSRPIK					Mascot
2048.1167	2048.0669	-0.0498	-24	367	386	NGSSITGLSVVALAKNTM IR			Oxidation (M)[18]		Mascot
2048.1167	2048.0669	-0.0498	-24	367	386	NGSSITGLSVVALAKNTM IR			Oxidation (M)[18]		Mascot
2159.0762	2159.1467	0.0705	33	261	279	MSDINNNNSRGLEIIIDPK			Oxidation (M)[1]		Mascot
2236.2158	2236.1289	-0.0869	-39	315	332	LINDSYLIQICRYLPNLK			Carbamidomethyl (C)[11]		Mascot

6	AP2/ERF and B3 domain-containing protein Os05g0549800 OS=Oryza sativa subsp. japonica GN=Os05g0549800 PE=2 SV=1	Y5498_ORYSJ	41583.3	9.51	14	68	91.026	1.869

Peptide Information

Calc. Mass	Obsrv. Mass	± da	± ppm	Start Seq.	End Seq.	Sequence	Ion Score	C. I. %	Modification	Rank	Result Type
947.4866	947.5505	0.0639	67	387	394	QCIELALT			Carbamidomethyl (C)[2]		Mascot
1060.582	1060.6387	0.0567	53	145	153	AEVVDMLRK					Mascot
1233.6475	1233.6084	-0.0391	-32	247	258	GVLLNFEDAAGK					Mascot
1363.7151	1363.6987	-0.0164	-12	303	313	NAQLFIDCKVR			Carbamidomethyl (C)[8]		Mascot
1375.6852	1375.7859	0.1007	73	154	164	HTYLEELTQNK					Mascot
1443.724	1443.7881	0.0641	44	80	90	WGAQIYERHQR					Mascot
1507.754	1507.754	0	0	91	104	VWLGTFTGEAEAAR					Mascot
1531.7864	1531.7478	-0.0386	-25	154	165	HTYLEELTQNKR					Mascot
1646.8901	1646.7855	-0.1046	-64	247	261	GVLLNFEDAAGKVWK					Mascot
1918.8005	1918.9712	0.1707	89	1	19	MDSTSCLLDDASSGAST GK			Carbamidomethyl (C)[6], Oxidation (M)[1]		Mascot
2028.9814	2029.0135	0.0321	16	262	277	FRYSYWNSSQSYVLTK					Mascot
2030.9005	2031.0743	0.1738	86	1	20	MDSTSCLLDDASSGAST GKK			Carbamidomethyl (C)[6]		Mascot
2300.1516	2300.1282	-0.0234	-10	117	137	DAVTNFRPLAESDPEAAV ELR					Mascot
2382.1838	2382.208	0.0242	10	91	112	VWLGTFTGEAEAARAYD VAAQR					Mascot

7	Acetate kinase OS=Clostridium botulinum (strain Loch Maree / Type A3) GN=ackA PE=3 SV=1	ACKA_CLOBM	43672.4	5.59	14	66	86.101	11.131

Peptide Information

Calc. Mass	Obsrv. Mass	± da	± ppm	Start Seq.	End Seq.	Sequence	Ion Score	C. I. %	Modification	Rank	Result Type

Calc. Mass	Obsrv. Mass	± da	± ppm	Start Seq.	End Seq.	Sequence	Ion Score	C. I. %	Modification	Rank	Result Type
817.4413	817.4283	-0.013	-16	353	359	IDAEKNK					Mascot
1158.6729	1158.624	-0.0489	-42	34	44	IGIEGSILTQK					Mascot
1189.5862	1189.6576	0.0714	60	175	184	KYGFHGTSHR					Mascot
1274.5756	1274.6445	0.0689	54	185	196	YVSSVASDMMGK					Mascot
1445.7305	1445.8466	0.1161	80	258	270	MSPDEVDAVINKK					Mascot
1445.7305	1445.8466	0.1161	80	258	270	MSPDEVDAVINKK	1	0			Mascot
1461.7255	1461.8082	0.0827	57	258	270	MSPDEVDAVINKK			Oxidation (M)[1]		Mascot
1646.7765	1646.7855	0.009	5	185	199	YVSSVASDMMGKDLK			Oxidation (M)[9]		Mascot
1662.7714	1662.8624	0.091	55	185	199	YVSSVASDMMGKDLK			Oxidation (M)[9,10]		Mascot
1665.7975	1665.8698	0.0723	43	241	254	CGDIDPAILLFMER			Carbamidomethyl (C)[1], Oxidation (M)[12]		Mascot
1699.8618	1699.8444	-0.0174	-10	203	218	VITCHLGNGASLCAVK			Carbamidomethyl (C)[4,13]		Mascot
1703.8521	1703.9561	0.104	61	255	269	ELKMSPDEVDAVINK			Oxidation (M)[4]		Mascot
1876.0249	1876.0261	0.0012	1	374	389	VKVFVIPTDEELMIAR			Oxidation (M)[13]		Mascot
1941.9409	1941.9265	-0.0144	-7	222	240	SVETSMGFTPLAGLAMGTR			Oxidation (M)[6]		Mascot
1957.9358	1957.9142	-0.0216	-11	222	240	SVETSMGFTPLAGLAMGTR			Oxidation (M)[6,16]		Mascot
1977.0361	1976.9766	-0.0595	-30	376	392	VFVIPTDEELMIARDTK					Mascot
1993.0311	1992.9285	-0.1026	-51	376	392	VFVIPTDEELMIARDTK			Oxidation (M)[11]		Mascot
2014.0209	2013.9899	-0.031	-15	203	221	VITCHLGNGASLCAVKEGK			Carbamidomethyl (C)[4,13]		Mascot
2065.0562	2065.0215	-0.0347	-17	271	291	SGVLGISGVSSDFRDIEGAAK					Mascot

8 Myosin-4 OS=Mus musculus GN=Myh4 PE=1 SV=1 MYH4_MOUSE 223632.3 5.58 34 65 82.095 14.724

Peptide Information

Calc. Mass	Obsrv. Mass	± da	± ppm	Start Seq.	End Seq.	Sequence	Ion Score	C. I. %	Modification	Rank	Result Type
993.5033	993.5469	0.0436	44	1788	1795	KNMEQTVK			Oxidation (M)[3]		Mascot
993.5033	993.5469	0.0436	44	1788	1795	KNMEQTVK			Oxidation (M)[3]		Mascot
1018.5163	1018.5896	0.0733	72	1321	1328	RQLEEESK					Mascot
1158.5977	1158.624	0.0263	23	798	806	GYLMRVEFK			Oxidation (M)[4]		Mascot
1189.6245	1189.6576	0.0331	28	771	781	AGLLGTLEEMR					Mascot
1224.6219	1224.5216	-0.1003	-82	1377	1386	TKYETDAIQR					Mascot
1245.6573	1245.6925	0.0352	28	957	967	DIDDLELTLAK					Mascot
1370.6667	1370.6874	0.0207	15	792	802	TQAVCRGYLMR			Carbamidomethyl (C)[5], Oxidation (M)[10]		Mascot
1416.7151	1416.6963	-0.0188	-13	1682	1693	ANLMQAEIEELR					Mascot
1443.8066	1443.7881	-0.0185	-13	563	575	SNNFQKPKPAKGK					Mascot
1465.7784	1465.8077	0.0293	20	261	273	LASADIETYLLEK					Mascot

1466.9053	1466.8478	-0.0575	-39	1562	1573	ILRIQLELNQVK					Mascot
1493.7166	1493.8295	0.1129	76	1364	1376	AMSKANSEVAQWR			Oxidation (M)[2]		Mascot
1498.7432	1498.8066	0.0634	42	1789	1800	NMEQTVKDLQHR					Mascot
1509.6737	1509.8046	0.1309	87	1065	1077	LAQESTMDIENDK			Oxidation (M)[7]		Mascot
1509.6737	1509.8046	0.1309	87	1065	1077	LAQESTMDIENDK			Oxidation (M)[7]		Mascot
1525.8262	1525.8105	-0.0157	-10	192	205	VIQYFATIAVTGDK					Mascot
1612.7975	1612.7618	-0.0357	-22	355	368	LTGAVMHYGNMKFK			Oxidation (M)[6]		Mascot
1646.8418	1646.7855	-0.0563	-34	1098	1111	IEDEQALGMQLQKK			Oxidation (M)[9]		Mascot
1647.7894	1647.7787	-0.0107	-6	981	995	NLTEEMAGLDENIAK					Mascot
1662.881	1662.8624	-0.0186	-11	417	432	GQTVQQVYNSVGALAK					Mascot
1676.8795	1676.9786	0.0991	59	131	144	WLPVYNPEVVAAYR					Mascot
1699.7633	1699.8444	0.0811	48	74	87	DDQVFSMNPPKYDK			Oxidation (M)[7]		Mascot
1702.8317	1702.9664	0.1347	79	1425	1438	LQNEVEDLMIDVER					Mascot
1714.751	1714.8748	0.1238	72	1230	1244	MEIDDLASNMETVSK			Oxidation (M)[1,10]		Mascot
1718.8265	1718.95	0.1235	72	1425	1438	LQNEVEDLMIDVER			Oxidation (M)[9]		Mascot
1737.8162	1737.8448	0.0286	16	433	445	SMYEKMFLWMVTR			Oxidation (M)[2]		Mascot
1746.8956	1746.957	0.0614	35	684	698	TPGAMEHELVLHQLR			Oxidation (M)[5]		Mascot
1769.806	1769.8281	0.0221	12	433	445	SMYEKMFLWMVTR			Oxidation (M)[2,6,10]		Mascot
1801.8199	1801.8566	0.0367	20	371	386	QREEQAEPDGTEVADK					Mascot
1902.9167	1903.0122	0.0955	50	282	296	AERSYHIFYQIMSNK			Oxidation (M)[12]		Mascot
1941.9375	1941.9265	-0.011	-6	2	19	SSDAEMAVFGEAAPYLRK					Mascot
1957.9324	1957.9142	-0.0182	-9	2	19	SSDAEMAVFGEAAPYLRK			Oxidation (M)[6]		Mascot
1976.8729	1976.9766	0.1037	52	1	18	MSSDAEMAVFGEAAPYLR			Oxidation (M)[1,7]		Mascot
2013.9117	2013.9899	0.0782	39	1622	1638	MEGDLNEMEIQLNHANR					Mascot
2069.0444	2069.0693	0.0249	12	1649	1666	NTQGMLKDTQLHLDDALR					Mascot
2089.0295	2089.0889	0.0594	28	1029	1046	TKLEQQVDDLEGSLEQEK					Mascot
2104.9678	2105.0823	0.1145	54	1	19	MSSDAEMAVFGEAAPYLRK			Oxidation (M)[1,7]		Mascot
2891.3574	2891.4795	0.1221	42	1734	1758	LETDISQIQGEMEDIVQEARNAEEK			Oxidation (M)[12]		Mascot

9	50S ribosomal protein L22P OS=Methanosaeta thermophila (strain DSM 6194 / PT) GN=rpl22p PE=3 SV=1	RL22_METTP	17340.3	11.19	11	63	74.119	10.712

Peptide Information

Calc. Mass	Obsrv. Mass	± da	± ppm	Start Seq.	End Seq.	Sequence	Ion Score	C. I. %	Modification	Rank	Result Type
817.4679	817.4283	-0.0396	-48	81	87	FPQKAAR					Mascot

1055.5493	1055.5972	0.0479	45	63	70	YNRNVPHR					Mascot
1106.6569	1106.6294	-0.0275	-25	110	119	LVIGYANTKK					Mascot
1320.6794	1320.6277	-0.0517	-39	92	103	VLENAIGNAEYK					Mascot
1362.7124	1362.728	0.0156	11	2	13	GRLNYSITPEGR					Mascot
1493.7529	1493.8295	0.0766	51	1	13	MGRLNYSITPEGR					Mascot
1509.7479	1509.8046	0.0567	38	1	13	MGRLNYSITPEGR			Oxidation (M)[1]		Mascot
1509.7479	1509.8046	0.0567	38	1	13	MGRLNYSITPEGR	4	0	Oxidation (M)[1]		Mascot
1531.8302	1531.7478	-0.0824	-54	14	27	FARAMGVELPISPK			Oxidation (M)[5]		Mascot
1701.9534	1701.8932	-0.0602	-35	41	55	VEAAERFLQDVIALK					Mascot
1747.9225	1747.8895	-0.033	-19	92	107	VLENAIGNAEYKGLEK					Mascot
1777.9	1777.9823	0.0823	46	138	152	NQETVSIEMILTEVR			Oxidation (M)[9]		Mascot
2159.1377	2159.1467	0.009	4	134	152	ATPKNQETVSIEMILTEVR					Mascot

10	Histidine--tRNA ligase, cytoplasmic OS=Takifugu rubripes GN=hars PE=3 SV=1	SYHC_TAKRU	58617.1	6.1	16	63	71.622	2.474

Peptide Information

Calc. Mass	Obsrv. Mass	± da	± ppm	Start Seq.	End Seq.	Sequence	Ion Score	C. I. %	Modification	Rank	Result Type
842.4771	842.4789	0.0018	2	249	255	LPWEAVK					Mascot
874.4022	874.4257	0.0235	27	2	8	LAMHCAR			Carbamidomethyl (C)[5], Oxidation (M)[3]		Mascot
1018.6003	1018.5896	-0.0107	-11	18	26	TTTRALSIR					Mascot
1370.725	1370.6874	-0.0376	-27	81	91	VFNTIVSCFKR			Carbamidomethyl (C)[8]		Mascot
1375.725	1375.7859	0.0609	44	421	433	TTEVQVMVAAAQK					Mascot
1443.7366	1443.7881	0.0515	36	112	123	YGEDSKLIYDLK					Mascot
1465.8009	1465.8077	0.0068	5	43	56	LLELKAHLGGDDGK					Mascot
1699.7449	1699.8444	0.0995	59	294	308	MCQSTQACAGLTDIK			Carbamidomethyl (C)[2,8], Oxidation (M)[1]		Mascot
1702.8469	1702.9664	0.1195	70	249	262	LPWEAVKNEMVNEK			Oxidation (M)[10]		Mascot
1703.8851	1703.9561	0.071	42	27	42	SFPGVTLAQIDEEVAK					Mascot
1714.9415	1714.8748	-0.0667	-39	309	322	LLFSYLQLFQVTDK					Mascot
1801.9305	1801.8566	-0.0739	-41	134	148	YDLTVPFARYLAMNK					Mascot
1941.902	1941.9265	0.0245	13	221	237	ILDGMFAVCGVPDNMFR			Carbamidomethyl (C)[9]		Mascot
1957.897	1957.9142	0.0172	9	221	237	ILDGMFAVCGVPDNMFR			Carbamidomethyl (C)[9], Oxidation (M)[5]		Mascot
1993.0826	1992.9285	-0.1541	-77	442	458	LITELWNAGIKAELMYK					Mascot
2300.2749	2300.1282	-0.1467	-64	27	47	SFPGVTLAQIDEEVAKLL ELK					Mascot
2878.3242	2878.4949	0.1707	59	221	245	ILDGMFAVCGVPDNMFR TICSTVDK			Carbamidomethyl (C)[9,20], Oxidation (M)[5,15]		Mascot

Gel Idx/Pos	267/K19	Instr./Gel Origin	BA2151/Sample Project 20140814	Process Status	Analysis Succeeded
Plate [#] Name	[1] Sample Project 20140814	Instrument Sample Name		Spectra	11

Rank	Protein Name	Accession No.	Protein MW	Protein PI	Pep. Count	Protein Score	Protein Score C. I. %	Intensity Matched	Total Ion Score	Total Ion C. I. %	Confirmed
1	Serpin-Z1C OS=Triticum aestivum PE=1 SV=1	SPZ1C_WHEAT	42969	5.62	14	345	100	60.817	269	100	

Peptide Information

Calc. Mass	Obsrv. Mass	± da	± ppm	Start Seq.	End Seq.	Sequence	Ion Score	C. I. %	Modification	Rank	Result Type
806.4744	806.4313	-0.0431	-53	271	276	HIPRQR					Mascot
925.5214	925.5156	-0.0058	-6	11	18	LSIAHQTR					Mascot
925.5214	925.5156	-0.0058	-6	11	18	LSIAHQTR	43	95.288			Mascot
947.5156	947.4764	-0.0392	-41	2	10	ATTLATDVR					Mascot
1078.5562	1078.6202	0.064	59	1	10	MATTLATDVR					Mascot
1151.6824	1151.6451	-0.0373	-32	172	181	LVLANALYFK					Mascot
1176.5896	1176.5881	-0.0015	-1	261	270	LSAEPDFLER					Mascot
1176.5896	1176.5881	-0.0015	-1	261	270	LSAEPDFLER	69	99.989			Mascot
1352.6945	1352.6516	-0.0429	-32	289	300	ISFETEASDLLK					Mascot
1399.6125	1399.5831	-0.0294	-21	182	194	GAWTDQFDSSGTK					Mascot
1530.7435	1530.7631	0.0196	13	125	137	YKADTQSVDFQTK					Mascot
2129.0947	2129.0332	-0.0615	-29	379	398	EDISGVVLFMGHVVNPLLSS			Oxidation (M)[10]		Mascot
2720.3525	2720.3455	-0.007	-3	328	353	VSSVFHQAFVEVNEQGTEAAASTAIK					Mascot
2725.4631	2725.4707	0.0076	3	33	61	SAASNAVFSPVSLHVALSLLAAGAGSATR					Mascot
2725.4631	2725.4707	0.0076	3	33	61	SAASNAVFSPVSLHVALSLLAAGAGSATR	120	100			Mascot
2930.542	2930.5276	-0.0144	-5	354	378	MALLQARPPSVMDFIADHPFLFLLR			Oxidation (M)[1,12]		Mascot
3053.3801	3053.3704	-0.0097	-3	301	327	CLGLQLPFSNEADFSEMVDSPMAHGLR			Carbamidomethyl (C)[1], Oxidation (M)[17,22]		Mascot
3053.3801	3053.3704	-0.0097	-3	301	327	CLGLQLPFSNEADFSEMVDSPMAHGLR	37	83.736	Carbamidomethyl (C)[1], Oxidation (M)[17,22]		Mascot

Rank	Protein Name	Accession No.	Protein MW	Protein PI	Pep. Count	Protein Score	Protein Score C. I. %	Intensity Matched	Total Ion Score	Total Ion C. I. %	Confirmed
2	Serpin-Z1B OS=Triticum aestivum PE=1 SV=1	SPZ1B_WHEAT	43119.9	5.44	10	151	100	26.805	112	100	

Peptide Information

Calc. Mass	Obsrv. Mass	± da	± ppm	Start Seq.	End Seq.	Sequence	Ion Score	C. I. %	Modification	Rank	Result Type
806.4744	806.4313	-0.0431	-53	272	277	HIPRQR					Mascot
925.5214	925.5156	-0.0058	-6	11	18	LSIAHQTR					Mascot
925.5214	925.5156	-0.0058	-6	11	18	LSIAHQTR	43	95.288			Mascot

947.5156	947.4764	-0.0392	-41	2	10	ATTLATDVR					Mascot
1078.5562	1078.6202	0.064	59	1	10	MATTLATDVR					Mascot
1151.6824	1151.6451	-0.0373	-32	172	181	LVLANALYFK					Mascot
1176.5896	1176.5881	-0.0015	-1	262	271	LSAEPDFLER					Mascot
1176.5896	1176.5881	-0.0015	-1	262	271	LSAEPDFLER	69	99.989			Mascot
1585.8295	1585.7332	-0.0963	-61	288	301	FKISFGMEASDLLK					Mascot
1601.8243	1601.7195	-0.1048	-65	288	301	FKISFGMEASDLLK			Oxidation (M)[7]		Mascot
2062.0564	2061.9585	-0.0979	-47	138	156	AAEVTTQVNSWVEKVTS GR					Mascot
2129.0947	2129.0332	-0.0615	-29	380	399	EDISGVVLFMGHVVNPLL SS			Oxidation (M)[10]		Mascot
2720.3525	2720.3455	-0.007	-3	329	354	VSSVFHQAFVEVNEQGT EAAASTAIK					Mascot

3	Serpin-Z1A OS=Triticum aestivum GN=WZCI PE=1 SV=1	SPZ1A_WHEAT	43262.2	5.6	9	145	100	26.392	112	100	

Peptide Information

Calc. Mass	Obsrv. Mass	± da	± ppm	Start Seq.	End Seq.	Sequence	Ion Score	C. I. %	Modification	Rank	Result Type
806.4744	806.4313	-0.0431	-53	271	276	HIPRQR					Mascot
925.5214	925.5156	-0.0058	-6	11	18	LSIAHQTR					Mascot
925.5214	925.5156	-0.0058	-6	11	18	LSIAHQTR	43	95.288			Mascot
947.5156	947.4764	-0.0392	-41	2	10	ATTLATDVR					Mascot
1078.5562	1078.6202	0.064	59	1	10	MATTLATDVR					Mascot
1151.6824	1151.6451	-0.0373	-32	172	181	LVLANALYFK					Mascot
1176.5896	1176.5881	-0.0015	-1	261	270	LSAEPDFLER					Mascot
1176.5896	1176.5881	-0.0015	-1	261	270	LSAEPDFLER	69	99.989			Mascot
2062.0564	2061.9585	-0.0979	-47	138	156	AAEVTTQVNSWVEKVTS GR					Mascot
2129.0947	2129.0332	-0.0615	-29	379	398	EDISGVVLFMGHVVNPLL SS			Oxidation (M)[10]		Mascot
2720.3525	2720.3455	-0.007	-3	328	353	VSSVFHQAFVEVNEQGT EAAASTAIK					Mascot

4	A-kinase anchor protein 10, mitochondrial (Fragment) OS=Sus scrofa GN=AKAP10 PE=2 SV=2	AKA10_PIG	72228.9	5.77	14	59	31.926	23.08

Peptide Information

Calc. Mass	Obsrv. Mass	± da	± ppm	Start Seq.	End Seq.	Sequence	Ion Score	C. I. %	Modification	Rank	Result Type
806.4631	806.4313	-0.0318	-39	235	242	ALHPAAAR					Mascot
869.4839	869.4506	-0.0333	-38	159	166	AHSLNTVK					Mascot
1034.563	1034.5166	-0.0464	-45	579	587	VSDLGQFIR					Mascot

Calc. Mass	Obsrv. Mass	± da	± ppm	Start Seq.	End Seq.	Sequence	Ion Score	C. I. %	Modification	Rank	Result Type
1176.5314	1176.5881	0.0567	48	248	257	ASLEPQESCR			Carbamidomethyl (C)[9]		Mascot
1176.5314	1176.5881	0.0567	48	248	257	ASLEPQESCR	9	0	Carbamidomethyl (C)[9]		Mascot
1352.6693	1352.6516	-0.0177	-13	280	291	SIEQDAVNTFTK					Mascot
1642.837	1642.83	-0.007	-4	574	587	MTFGRVSDLGQFIR			Oxidation (M)[1]		Mascot
1717.8955	1717.8729	-0.0226	-13	2	16	ALRPDPGPAMSFFRR					Mascot
1733.8905	1733.8416	-0.0489	-28	2	16	ALRPDPGPAMSFFRR			Oxidation (M)[10]		Mascot
1844.8695	1845.0026	0.1331	72	97	112	SCLDYQAQETKSSLSK			Carbamidomethyl (C)[2]		Mascot
2012.9454	2013.0011	0.0557	28	217	234	TSNTQNHLLLSPECDGA R			Carbamidomethyl (C)[14]		Mascot
2045.0187	2045.0061	-0.0126	-6	579	596	VSDLGQFIRESEPEPDVK					Mascot
2202.9973	2203.1309	0.1336	61	410	428	KGQYDGQEAQNDAMILY DK			Oxidation (M)[14]		Mascot
2295.0415	2295.259	0.2175	95	629	649	MIVSDVMQQAQCAQPGE TSAK			Carbamidomethyl (C)[12], Oxidation (M)[1]		Mascot
2720.4141	2720.3455	-0.0686	-25	545	568	ILKNFDEAIIVDAASLDPE SLYQR					Mascot
2742.3516	2742.3484	-0.0032	-1	47	73	NHALLEAAGPSPVAISAIS ANMDSFSR			Oxidation (M)[22]		Mascot

5	Serpin-Z4 OS=Hordeum vulgare GN=PAZ1 PE=1 SV=2	SPZ4_HORVU	43363.4	5.72	6	56	0	6.717	43	95.288	

Peptide Information

Calc. Mass	Obsrv. Mass	± da	± ppm	Start Seq.	End Seq.	Sequence	Ion Score	C. I. %	Modification	Rank	Result Type
840.3734	840.427	0.0536	64	190	196	FDESNTK					Mascot
925.5214	925.5156	-0.0058	-6	11	18	LSIAHQTR					Mascot
925.5214	925.5156	-0.0058	-6	11	18	LSIAHQTR	43	95.288			Mascot
947.5156	947.4764	-0.0392	-41	2	10	ATTLATDVR					Mascot
1078.5562	1078.6202	0.064	59	1	10	MATTLATDVR					Mascot
1151.6824	1151.6451	-0.0373	-32	174	183	LILGNALYFK					Mascot
1282.6638	1282.6517	-0.0121	-9	218	228	KQYISSSDNLK					Mascot

6	Serpin-ZX OS=Hordeum vulgare GN=PAZX PE=1 SV=1	SPZX_HORVU	42920.3	6.77	5	54	0	6.393	43	95.288	

Peptide Information

Calc. Mass	Obsrv. Mass	± da	± ppm	Start Seq.	End Seq.	Sequence	Ion Score	C. I. %	Modification	Rank	Result Type
823.3978	823.4111	0.0133	16	1	7	MATTDIR			Oxidation (M)[1]		Mascot
868.5138	868.4418	-0.072	-83	357	364	SLPVEPVK					Mascot
925.5214	925.5156	-0.0058	-6	8	15	LSIAHQTR					Mascot
925.5214	925.5156	-0.0058	-6	8	15	LSIAHQTR	43	95.288			Mascot
1530.7435	1530.7631	0.0196	13	124	136	YKGETQSVDFQTK					Mascot

Calc. Mass	Obsrv. Mass	± da	± ppm	Start Seq.	End Seq.	Sequence	Ion Score	C. I. %	Modification	Rank	Result Type
2240.2285	2240.2458	0.0173	8	137	157	APEVAGQVNSWVEKITTGLIK					Mascot

7	Catalase-peroxidase OS=Gramella forsetii (strain KT0803) GN=katG PE=3 SV=1	KATG_GRAFK	83287.5	4.99	15	52	0	8.9		

Peptide Information

Calc. Mass	Obsrv. Mass	± da	± ppm	Start Seq.	End Seq.	Sequence	Ion Score	C. I. %	Modification	Rank	Result Type
842.3978	842.4411	0.0433	51	185	192	MFGFAGGR					Mascot
857.47	857.4517	-0.0183	-21	498	506	GGANGGRIR					Mascot
869.4376	869.4506	0.013	15	603	608	NYFRNR					Mascot
1188.5797	1188.6119	0.0322	27	353	363	GPGGAYQWQPK					Mascot
1204.6395	1204.6191	-0.0204	-17	669	678	NILEMGLTWK					Mascot
1204.6395	1204.6191	-0.0204	-17	669	678	NILEMGLTWK					Mascot
1263.6151	1263.7191	0.104	82	737	746	AWDKVMNLDR			Oxidation (M)[6]		Mascot
1282.646	1282.6517	0.0057	4	741	750	VMNLDRYDLK			Oxidation (M)[2]		Mascot
1601.8719	1601.7195	-0.1524	-95	380	393	KHAPFMLTTDLSLK					Mascot
1908.9563	1908.9265	-0.0298	-16	699	715	WTGSRADLIFGSNSELR					Mascot
2013.1414	2013.0011	-0.1403	-70	545	564	VSIADLIVLAGCVGVEKAAK			Carbamidomethyl (C)[12]		Mascot
2720.1992	2720.3455	0.1463	54	669	692	NILEMGLTWKSSSDSETEFDGSDR			Oxidation (M)[5]		Mascot
2748.2756	2748.4265	0.1509	55	256	280	ETFGRMAMNDYETVALIAGGHTFGK			Oxidation (M)[6,8]		Mascot
2763.3594	2763.3354	-0.024	-9	160	184	YGKNLSWADLLVLAGNCAHESMGLK			Carbamidomethyl (C)[17], Oxidation (M)[22]		Mascot
2783.3735	2783.4736	0.1001	36	704	729	ADLIFGSNSELRAIAEVYGTDDVEAK					Mascot
3036.5679	3036.364	-0.2039	-67	623	651	AQLLTLTVPQMTVLLGGMRAMGANYDGSK					Mascot
3052.5627	3052.3298	-0.2329	-76	623	651	AQLLTLTVPQMTVLLGGMRAMGANYDGSK			Oxidation (M)[11]		Mascot

8	Sulfate adenylyltransferase OS=Archaeoglobus fulgidus (strain ATCC 49558 / VC-16 / DSM 4304 / JCM 9628 / NBRC 100126) GN=sat PE=3 SV=2	SAT_ARCFU	53260.9	6.59	5	52	0	6.487	43	95.288

Peptide Information

Calc. Mass	Obsrv. Mass	± da	± ppm	Start Seq.	End Seq.	Sequence	Ion Score	C. I. %	Modification	Rank	Result Type
813.4254	813.4012	-0.0242	-30	392	398	FSGSFLR					Mascot
925.5214	925.5156	-0.0058	-6	214	221	TVIAHQTR					Mascot
925.5214	925.5156	-0.0058	-6	214	221	TVIAHQTR	43	95.288			Mascot
985.5312	985.5208	-0.0104	-11	134	142	IDPVDVATR					Mascot
1175.5878	1175.5703	-0.0175	-15	399	409	GMVAEGVFPPR			Oxidation (M)[2]		Mascot
2012.9673	2013.0011	0.0338	17	258	275	RGDYPDEAILEGHEAVNK					Mascot

9	Chaperone protein DnaK OS=Lactobacillus brevis (strain ATCC 367 / JCM 1170) GN=dnaK PE=3 SV=1	DNAK_LACBA	66367.8	4.69	14	51	0	3.69

Peptide Information

Calc. Mass	Obsrv. Mass	± da	± ppm	Start Seq.	End Seq.	Sequence	Ion Score	C. I. %	Modification	Rank	Result Type
805.3937	805.4301	0.0364	45	530	536	VSDDEIK					Mascot
818.4366	818.4216	-0.015	-18	129	136	QATKDAGK					Mascot
848.436	848.4305	-0.0055	-6	503	509	KEEVDTK					Mascot
962.4424	962.3524	-0.09	-94	494	501	ENEEADKK					Mascot
979.4302	979.4578	0.0276	28	71	79	SHMGEAGYK					Mascot
1248.5525	1248.6134	0.0609	49	548	558	AQEANNLDDMK					Mascot
1563.7795	1563.715	-0.0645	-41	454	468	NGIVNVSAKDMGTNK			Oxidation (M)[11]		Mascot
1563.7795	1563.715	-0.0645	-41	454	468	NGIVNVSAKDMGTNK			Oxidation (M)[11]		Mascot
1871.9684	1871.798	-0.1704	-91	86	101	DYTPQQVSAMILQHLK					Mascot
1887.9634	1887.7928	-0.1706	-90	86	101	DYTPQQVSAMILQHLK			Oxidation (M)[10]		Mascot
2012.8787	2013.0011	0.1224	61	476	493	SSDGLSDEEIEKMMNEA K					Mascot
2044.8685	2045.0061	0.1376	67	476	493	SSDGLSDEEIEKMMNEA K			Oxidation (M)[13,14]		Mascot
2063.0657	2062.9014	-0.1643	-80	37	56	TTPSVVAFKDGETQVGE VAK					Mascot
2151.0452	2151.0417	-0.0035	-2	504	521	EEVDTKNEVDQLLFQTD K					Mascot
2695.5242	2695.5723	0.0481	18	6	32	IIGIDLGTTNSAVAVLEGS TPKIIANK					Mascot
2703.4233	2703.4419	0.0186	7	1	27	MASNKIIGIDLGTTNSAVA VLEGSTPK			Oxidation (M)[1]		Mascot
2758.4006	2758.3181	-0.0825	-30	333	360	EPNHSINPDEAVALGAAV QGGVITGDVK					Mascot

10	ATP synthase subunit delta OS=Clostridium kluyveri (strain ATCC 8527 / DSM 555 / NCIMB 10680) GN=atpH PE=3 SV=1	ATPD_CLOK5	21194.3	5.34	9	50	0	5.337

Protein Group

ATP synthase subunit delta OS=Clostridium kluyveri (strain NBRC 12016) GN=atpH PE=3 SV=1	ATPD_CLOK1	21194.3	5.3400 001525 8789

Peptide Information

Calc. Mass	Obsrv. Mass	± da	± ppm	Start Seq.	End Seq.	Sequence	Ion Score	C. I. %	Modification	Rank	Result Type
868.4927	868.4418	-0.0509	-59	60	66	TFINVFK					Mascot
989.4396	989.5214	0.0818	83	1	7	MYEYLDR					Mascot
989.5626	989.5214	-0.0412	-42	123	130	ETLTEKLR					Mascot

1078.6003	1078.6202	0.0199	18	84	92	GRINQLYSK		Mascot
1151.583	1151.6451	0.0621	54	23	31	VEEYLEELK		Mascot
1887.9546	1887.7928	-0.1618	-86	113	128	TVIPLEDDERETLTEK		Mascot
2060.0845	2060.1772	0.0927	45	95	112	EMGKIYLENHNTVIATVK		Mascot
2748.3647	2748.4265	0.0618	22	141	165	EELDPEIIGGVYVEVNNM VIDGTVK	Oxidation (M)[18]	Mascot
2947.4968	2947.5046	0.0078	3	141	167	EELDPEIIGGVYVEVNNM VIDGTVKSK		Mascot

Gel Idx/Pos	268/K20	Instr./Gel Origin	BA2151/Sample Project 20140814	Process Status	Analysis Succeeded
Plate [#] Name	[1] Sample Project 20140814	Instrument Sample Name		Spectra	11

Rank	Protein Name	Accession No.	Protein MW	Protein PI	Pep. Count	Protein Score	Protein Score C. I. %	Intensity Matched	Total Ion Score	Total Ion C. I. %	Confirmed
1	Beta-amylase OS=Hordeum vulgare subsp. spontaneum GN=BMY1 PE=1 SV=1	AMYB_HORVS	59886.4	5.66	11	303	100	18.164	266	100	

Peptide Information

Calc. Mass	Obsrv. Mass	± da	± ppm	Start Seq.	End Seq.	Sequence	Ion Score	C. I. %	Modification	Rank	Result Type
1016.5564	1016.5454	-0.011	-11	411	418	LFGFTYLR					Mascot
1016.5564	1016.5454	-0.011	-11	411	418	LFGFTYLR	50	98.933			Mascot
1189.6154	1189.5902	-0.0252	-21	294	302	ISGIHWWYK					Mascot
1253.6121	1253.5831	-0.029	-23	248	258	DNGTYLTEKGR					Mascot
1299.582	1299.5822	0.0002	0	335	345	ASINFTCAEMR			Carbamidomethyl (C)[7]		Mascot
1315.5769	1315.552	-0.0249	-19	335	345	ASINFTCAEMR			Carbamidomethyl (C)[7], Oxidation (M)[10]		Mascot
1326.6688	1326.6537	-0.0151	-11	384	394	YDPTAYNTILR					Mascot
1326.6688	1326.6537	-0.0151	-11	384	394	YDPTAYNTILR	66	99.97			Mascot
1669.7349	1669.7777	0.0428	26	147	160	SAVQMYADYMTSFR					Mascot
1685.7299	1685.7469	0.017	10	147	160	SAVQMYADYMTSFR			Oxidation (M)[5]		Mascot
1701.7247	1701.7129	-0.0118	-7	147	160	SAVQMYADYMTSFR			Oxidation (M)[5,10]		Mascot
1738.8732	1738.8765	0.0033	2	395	410	NARPHGINQSGPPEHK					Mascot
1752.8916	1752.8417	-0.0499	-28	419	433	LSNQLVEGQNYVNFK					Mascot
1827.9884	1827.9347	-0.0537	-29	458	474	SGPEISIEMILQAAKPK			Oxidation (M)[9]		Mascot
2013.9778	2013.9774	-0.0004	0	303	320	VPSHAAELTAGYYNLHDR					Mascot
2013.9778	2013.9774	-0.0004	0	303	320	VPSHAAELTAGYYNLHDR	150	100			Mascot
2267.1489	2267.0903	-0.0586	-26	7	26	GNYVQVYVMLPLDAVSVNNR			Oxidation (M)[9]		Mascot

Rank	Protein Name	Accession No.	Protein MW	Protein PI	Pep. Count	Protein Score	Protein Score C. I. %	Intensity Matched	Total Ion Score	Total Ion C. I. %	Confirmed
2	Beta-amylase OS=Hordeum vulgare GN=BMY1 PE=1 SV=1	AMYB_HORVU	59894.5	5.58	10	298	100	17.581	266	100	

Peptide Information

Calc. Mass	Obsrv. Mass	± da	± ppm	Start Seq.	End Seq.	Sequence	Ion Score	C. I. %	Modification	Rank	Result Type
1016.5564	1016.5454	-0.011	-11	411	418	LFGFTYLR					Mascot
1016.5564	1016.5454	-0.011	-11	411	418	LFGFTYLR	50	98.933			Mascot
1189.6154	1189.5902	-0.0252	-21	294	302	ISGIHWWYK					Mascot

Calc. Mass	Obsrv. Mass	± da	± ppm	Start Seq.	End Seq.	Sequence	Ion Score	C. I. %	Modification	Rank	Result Type
1299.582	1299.5822	0.0002	0	335	345	ASINFTCAEMR			Carbamidomethyl (C)[7]		Mascot
1315.5769	1315.552	-0.0249	-19	335	345	ASINFTCAEMR			Carbamidomethyl (C)[7], Oxidation (M)[10]		Mascot
1326.6688	1326.6537	-0.0151	-11	384	394	YDPTAYNTILR					Mascot
1326.6688	1326.6537	-0.0151	-11	384	394	YDPTAYNTILR	66	99.97			Mascot
1669.7349	1669.7777	0.0428	26	147	160	SAVQMYADYMTSFR					Mascot
1685.7299	1685.7469	0.017	10	147	160	SAVQMYADYMTSFR			Oxidation (M)[5]		Mascot
1701.7247	1701.7129	-0.0118	-7	147	160	SAVQMYADYMTSFR			Oxidation (M)[5,10]		Mascot
1738.8732	1738.8765	0.0033	2	395	410	NARPHGINQSGPPEHK					Mascot
1752.8916	1752.8417	-0.0499	-28	419	433	LSNQLVEGQNYVNFK					Mascot
1827.952	1827.9347	-0.0173	-9	458	474	SGPEISIEMILQAAQPK			Oxidation (M)[9]		Mascot
2013.9778	2013.9774	-0.0004	0	303	320	VPSHAAELTAGYYNLHDR					Mascot
2013.9778	2013.9774	-0.0004	0	303	320	VPSHAAELTAGYYNLHDR	150	100			Mascot
2267.1489	2267.0903	-0.0586	-26	7	26	GNYVQVYVMLPLDAVSVNNR			Oxidation (M)[9]		Mascot

3	Beta-amylase (Fragment) OS=Secale cereale GN=BMY1 PE=2 SV=1	AMYB_SECCE	24561.9	5.08	3	125	100	10.35	116	100	

Peptide Information

Calc. Mass	Obsrv. Mass	± da	± ppm	Start Seq.	End Seq.	Sequence	Ion Score	C. I. %	Modification	Rank	Result Type
1016.5564	1016.5454	-0.011	-11	107	114	LFGFTYLR					Mascot
1016.5564	1016.5454	-0.011	-11	107	114	LFGFTYLR	50	98.933			Mascot
1326.6688	1326.6537	-0.0151	-11	80	90	YDPTAYNTILR					Mascot
1326.6688	1326.6537	-0.0151	-11	80	90	YDPTAYNTILR	66	99.97			Mascot
1573.6998	1573.7653	0.0655	42	29	41	HHASLNFTCAEMR			Carbamidomethyl (C)[9]		Mascot

4	Beta-amylase OS=Triticum aestivum GN=BMY1 PE=2 SV=1	AMYB_WHEAT	56860.2	5.24	6	64	78.473	12.558	50	98.933	

Peptide Information

Calc. Mass	Obsrv. Mass	± da	± ppm	Start Seq.	End Seq.	Sequence	Ion Score	C. I. %	Modification	Rank	Result Type
1016.5564	1016.5454	-0.011	-11	411	418	LFGFTYLR					Mascot
1016.5564	1016.5454	-0.011	-11	411	418	LFGFTYLR	50	98.933			Mascot
1225.606	1225.6733	0.0673	55	248	258	DNGTYLTEKGK					Mascot
1225.606	1225.6733	0.0673	55	248	258	DNGTYLTEKGK					Mascot
1297.6787	1297.6333	-0.0454	-35	210	221	YLEADFKAAAAK					Mascot
1669.7349	1669.7777	0.0428	26	147	160	TAVQMYADYMASFR			Oxidation (M)[5]		Mascot
1685.7299	1685.7469	0.017	10	147	160	TAVQMYADYMASFR			Oxidation (M)[5,10]		Mascot

2087.0557	2087.041	-0.0147	-7	129	146	NIEYLTLGVDDQPLFHGR					Mascot
2233.1348	2233.0786	-0.0562	-25	483	503	NTDLPVKDHTDVGDEVL VAPV					Mascot

5	Fibrinogen silencer-binding protein OS=Rattus norvegicus GN=Fsbp PE=1 SV=1	FSBP_RAT	34369.8	7.75	12	64	76.396	5.133	

Peptide Information

Calc. Mass	Obsrv. Mass	± da	± ppm	Start Seq.	End Seq.	Sequence	Ion Score	C. I. %	Modification	Rank	Result Type
1048.5092	1048.5486	0.0394	38	203	212	MTSSPSSVPR					Mascot
1111.6146	1111.5881	-0.0265	-24	251	259	KFGLYVQEK					Mascot
1139.6208	1139.5797	-0.0411	-36	252	260	FGLYVQEKR					Mascot
1204.6104	1204.6002	-0.0102	-8	203	213	MTSSPSSVPRR					Mascot
1247.6123	1247.6047	-0.0076	-6	230	239	CDPQVLQMLK			Carbamidomethyl (C)[1], Oxidation (M)[8]		Mascot
1377.7373	1377.7174	-0.0199	-14	77	87	EYAKQELLQQK					Mascot
1453.6819	1453.7418	0.0599	41	214	225	DVFHQESGEHLR					Mascot
1620.7898	1620.7993	0.0095	6	187	202	VAEGSVSPSLSSVDMR					Mascot
1636.7848	1636.8065	0.0217	13	187	202	VAEGSVSPSLSSVDMR			Oxidation (M)[15]		Mascot
1669.7915	1669.7777	-0.0138	-8	88	102	EAQSDYKSSISEPTK					Mascot
1700.9187	1700.7792	-0.1395	-82	226	239	SLLRCDPQVLQMLK			Carbamidomethyl (C)[5]		Mascot
1716.9136	1716.7582	-0.1554	-91	226	239	SLLRCDPQVLQMLK			Carbamidomethyl (C)[5], Oxidation (M)[12]		Mascot
1752.9491	1752.8417	-0.1074	-61	28	42	ILEEHTNKNSVIVEK					Mascot
1906.0289	1905.948	-0.0809	-42	103	118	KVVEMIPQISSFCLVR			Carbamidomethyl (C)[13]		Mascot

6	Replicase large subunit OS=Odontoglossum ringspot virus (isolate Singapore 1) PE=3 SV=2	RDRP_ORSVS	185074.6	6.85	27	63	72.268	17.365	

Peptide Information

Calc. Mass	Obsrv. Mass	± da	± ppm	Start Seq.	End Seq.	Sequence	Ion Score	C. I. %	Modification	Rank	Result Type
939.4683	939.513	0.0447	48	283	290	TFFPASNR					Mascot
978.5037	978.4824	-0.0213	-22	388	395	RSEVMVNK			Oxidation (M)[5]		Mascot
1188.6583	1188.6051	-0.0532	-45	1340	1349	QLLERIDSSK					Mascot
1196.6521	1196.5886	-0.0635	-53	1420	1430	TSLKDYTAGIK					Mascot
1240.6243	1240.5956	-0.0287	-23	685	696	SMSSAVYTGPLK					Mascot
1240.6243	1240.5956	-0.0287	-23	685	696	SMSSAVYTGPLK					Mascot
1326.6545	1326.6537	-0.0008	-1	624	633	DLDMRFCLLK			Carbamidomethyl (C)[7], Oxidation (M)[4]		Mascot
1326.6545	1326.6537	-0.0008	-1	624	633	DLDMRFCLLK			Carbamidomethyl (C)[7], Oxidation (M)[4]		Mascot
1399.7468	1399.609	-0.1378	-98	555	566	YYNALSELSVLK					Mascot
1431.6097	1431.5752	-0.0345	-24	567	577	ECDEFDITQFK			Carbamidomethyl (C)[2]		Mascot

Calc. Mass	Obsrv. Mass	± da	± ppm	Start Seq.	End Seq.	Sequence	Ion Score	C. I. %	Modification	Rank	Result Type
1517.7029	1517.7496	0.0467	31	291	301	FVYHKEFMCTR			Carbamidomethyl (C)[9]		Mascot
1517.7029	1517.7496	0.0467	31	291	301	FVYHKEFMCTR			Carbamidomethyl (C)[9]		Mascot
1573.8077	1573.7653	-0.0424	-27	818	832	MPKVTLVDGVPGCGK			Carbamidomethyl (C)[13], Oxidation (M)[1]		Mascot
1620.8115	1620.7993	-0.0122	-8	721	735	VLKDVYGADPESAEK					Mascot
1636.8483	1636.8065	-0.0418	-26	312	324	VDTYFLFRGVYTR					Mascot
1643.6611	1643.7935	0.1324	81	170	182	YMNDPDAVCCDKR			Carbamidomethyl (C)[9,10]		Mascot
1649.8745	1649.772	-0.1025	-62	608	623	NPTPEALSDALSPLPK					Mascot
1680.8513	1680.8047	-0.0466	-28	370	385	DMVIVPLFDGSVTSGK			Oxidation (M)[2]		Mascot
1700.8524	1700.7792	-0.0732	-43	578	592	NLCEEKDIAPDVVAK			Carbamidomethyl (C)[3]		Mascot
1701.8303	1701.7129	-0.1174	-69	31	44	VYDNAVEELNHRSR					Mascot
1774.8582	1774.8682	0.01	6	1424	1437	DYTAGIKTCLWYQR			Carbamidomethyl (C)[9]		Mascot
1827.8442	1827.9347	0.0905	50	752	767	DKCHAWGVAELNNGEK			Carbamidomethyl (C)[3]		Mascot
1905.8517	1905.948	0.0963	51	119	133	GRDYVHCCMPNLDIR			Carbamidomethyl (C)[7,8]		Mascot
2014.0254	2013.9774	-0.048	-24	1181	1197	SVSVPRQQQEFFTPAHR					Mascot
2014.0254	2013.9774	-0.048	-24	1181	1197	SVSVPRQQQEFFTPAHR					Mascot
2087.063	2087.041	-0.022	-11	134	151	DVARHINQQDTVSTYLAR					Mascot
2140.1907	2140.0823	-0.1084	-51	1198	1217	TAAERPRSAGLLENLVAMIK					Mascot
2198.886	2199.0242	0.1382	63	325	342	GEDSEQFYTAMDEAWEYK					Mascot
2233.1499	2233.0786	-0.0713	-32	1305	1324	LGLSPQDEYAALQTIVYHSK					Mascot
2254.0452	2254.1267	0.0815	36	257	274	LSFFFQNESTLNYEHSYK					Mascot
2725.3611	2725.4678	0.1067	39	1439	1464	SGDVTTFIGNTVIIAACLASMIPMDK			Carbamidomethyl (C)[17]		Mascot

7	Myosin-2 OS=Bos taurus GN=MYH2 PE=2 SV=1	MYH2_BOVIN	224092.4	5.63	36	61	59.915	22.162

Peptide Information

Calc. Mass	Obsrv. Mass	± da	± ppm	Start Seq.	End Seq.	Sequence	Ion Score	C. I. %	Modification	Rank	Result Type
930.5618	930.4912	-0.0706	-76	916	923	TKIQLEAK					Mascot
947.4792	947.4819	0.0027	3	1695	1702	ATLEQTER					Mascot
978.5731	978.4824	-0.0907	-93	274	281	SRVTFQLK					Mascot
989.4786	989.511	0.0324	33	1585	1592	DEEIDQLK					Mascot
1038.5037	1038.5005	-0.0032	-3	1173	1180	EAEFQKMR					Mascot
1048.5131	1048.5486	0.0355	34	238	246	TVRNDNSSR					Mascot
1169.6235	1169.5549	-0.0686	-59	1481	1490	SLGTELFKMK			Oxidation (M)[9]		Mascot
1188.6107	1188.6051	-0.0056	-5	1256	1265	TLEDQVNELK					Mascot
1204.6355	1204.6002	-0.0353	-29	783	792	DEKLAQLMTR					Mascot
1224.6219	1224.5577	-0.0642	-52	1378	1387	TKYETDAIQR					Mascot

1251.6692	1251.6127	-0.0565	-45	45	55	ESFVKGTIQSR					Mascot
1260.6583	1260.5579	-0.1004	-80	25	35	IEAQNKPFDAK					Mascot
1263.6732	1263.6122	-0.061	-48	1453	1462	NFDKILAEWK					Mascot
1276.5903	1276.5999	0.0096	8	1826	1836	ELEGEVESEQK					Mascot
1299.6692	1299.5822	-0.087	-67	646	657	GSSFQTVSALFR					Mascot
1326.774	1326.6537	-0.1203	-91	1865	1875	NILRLQDLVDK					Mascot
1326.774	1326.6537	-0.1203	-91	1865	1875	NILRLQDLVDK					Mascot
1373.6907	1373.6683	-0.0224	-16	1706	1717	IAEQELLDASER					Mascot
1463.7406	1463.7959	0.0553	38	831	840	HWPWMKLFFR			Oxidation (M)[5]		Mascot
1517.6602	1517.7496	0.0894	59	373	386	EEQAEPDGTEVADK					Mascot
1517.6602	1517.7496	0.0894	59	373	386	EEQAEPDGTEVADK					Mascot
1521.7651	1521.7612	-0.0039	-3	1763	1776	AITDAAMMAEELKK					Mascot
1573.8254	1573.7653	-0.0601	-38	772	785	AGLLGLLEEMRDEK					Mascot
1594.8411	1594.7784	-0.0627	-39	355	368	LTGAVMHYGNLKFK			Oxidation (M)[6]		Mascot
1594.8411	1594.7784	-0.0627	-39	355	368	LTGAVMHYGNLKFK			Oxidation (M)[6]		Mascot
1615.865	1615.7632	-0.1018	-63	1295	1308	QLDEKEALVSQLSR					Mascot
1637.7874	1637.7552	-0.0322	-20	1598	1611	VVESMQTMLDAEIR			Oxidation (M)[5]		Mascot
1643.8436	1643.7935	-0.0501	-30	433	445	AVYEKMFLWMVAR					Mascot
1652.8014	1652.7933	-0.0081	-5	1491	1504	NAYEESLDQLETLK					Mascot
1680.7997	1680.8047	0.005	3	1231	1245	MEIDDLASNVETISK			Oxidation (M)[1]		Mascot
1681.9272	1681.7629	-0.1643	-98	191	205	RVIQYFATIAVTGDK					Mascot
1681.9272	1681.7629	-0.1643	-98	191	205	RVIQYFATIAVTGDK					Mascot
1684.9229	1684.7666	-0.1563	-93	1566	1579	IQLELNQVKSEIDR					Mascot
1738.9561	1738.8765	-0.0796	-46	768	782	VFFKAGLLGLLEEMR			Oxidation (M)[14]		Mascot
1755.9236	1755.924	0.0004	0	1797	1811	DLQNRLDEAEQLALK					Mascot
1774.8606	1774.8682	0.0076	4	1463	1477	QKYEETHAELEAAQK					Mascot
2013.9117	2013.9774	0.0657	33	1623	1639	MEGDLNEMEIQLNHANR					Mascot
2013.9117	2013.9774	0.0657	33	1623	1639	MEGDLNEMEIQLNHANR					Mascot
2087.092	2087.041	-0.051	-24	750	767	LLASIDIDHTQYKFGHTK					Mascot
2140.0149	2140.0823	0.0674	31	1227	1245	SEMKMEIDDLASNVETIS K					Mascot
2267.1853	2267.0903	-0.095	-42	387	406	AAYLQSLNSADLLKALCY PR			Carbamidomethyl (C)[17]		Mascot

8	Eukaryotic translation initiation factor 4 gamma OS=Schizosaccharomyces pombe (strain 972 / ATCC 24843) GN=tif471 PE=1 SV=1	IF4G_SCHPO	154169.6	9.25	24	60	47.158	24.019

Peptide Information

Calc. Mass	Obsrv. Mass	± da	± ppm	Start	End	Sequence	Ion	C. I. %	Modification	Rank	Result Type

				Seq.	Seq.		Score				
925.5465	925.5078	-0.0387	-42	173	181	NAAIPSKPK					Mascot
930.4536	930.4912	0.0376	40	1167	1173	IMHECIK			Carbamidomethyl (C)[5]		Mascot
1016.5622	1016.5454	-0.0168	-17	987	995	LTEKPAETK					Mascot
1016.5622	1016.5454	-0.0168	-17	987	995	LTEKPAETK					Mascot
1144.6572	1144.5651	-0.0921	-80	986	995	KLTEKPAETK					Mascot
1189.5881	1189.5902	0.0021	2	1	11	MSSKPPSNTPK			Oxidation (M)[1]		Mascot
1244.5472	1244.585	0.0378	30	1233	1242	FMLMDVMDSR					Mascot
1247.6201	1247.6047	-0.0154	-12	1207	1217	GHAAMDVYVLR			Oxidation (M)[5]		Mascot
1260.5421	1260.5579	0.0158	13	1233	1242	FMLMDVMDSR			Oxidation (M)[2]		Mascot
1276.5371	1276.5999	0.0628	49	1233	1242	FMLMDVMDSR			Oxidation (M)[2,4]		Mascot
1297.6383	1297.6333	-0.005	-4	1258	1268	TIAEIHEEAER					Mascot
1299.6176	1299.5822	-0.0354	-27	139	150	DNAAPVENVNEK					Mascot
1326.6028	1326.6537	0.0509	38	1074	1084	KMMDSIDDSIR			Oxidation (M)[2]		Mascot
1326.6028	1326.6537	0.0509	38	1074	1084	KMMDSIDDSIR			Oxidation (M)[2]		Mascot
1479.6921	1479.7738	0.0817	55	757	771	STSSLSSPSHSTSSK					Mascot
1517.7161	1517.7496	0.0335	22	1231	1242	IKFMLMDVMDSR			Oxidation (M)[4,6]		Mascot
1517.7161	1517.7496	0.0335	22	1231	1242	IKFMLMDVMDSR			Oxidation (M)[4,6]		Mascot
1529.7708	1529.7906	0.0198	13	12	25	FSYARALASSQSNK					Mascot
1563.7498	1563.7256	-0.0242	-15	717	732	SSAPLASSEANVDTSK					Mascot
1615.7533	1615.7632	0.0099	6	1105	1116	YLLSRCQEDFER			Carbamidomethyl (C)[6]		Mascot
1647.8734	1647.7715	-0.1019	-62	1027	1040	ISDQILEIAMQSRK			Oxidation (M)[10]		Mascot
1652.7722	1652.7933	0.0211	13	26	41	SNSTKASENNTATAEK					Mascot
1680.844	1680.8047	-0.0393	-23	996	1010	GEDEEALLPPEVVQR					Mascot
1755.8667	1755.924	0.0573	33	1271	1286	ALAESQRPSSGRMHGR			Oxidation (M)[13]		Mascot
1797.8953	1797.9135	0.0182	10	1	16	MSSKPPSNTPKFSYAR					Mascot
1905.8549	1905.948	0.0931	49	1058	1073	ATDEPNFSNMYARFAR			Oxidation (M)[10]		Mascot
2013.917	2013.9774	0.0604	30	925	944	SAPSRGVSHHGHGGMSGSHR			Oxidation (M)[15]		Mascot
2013.917	2013.9774	0.0604	30	925	944	SAPSRGVSHHGHGGMSGSHR			Oxidation (M)[15]		Mascot
2087.0115	2087.041	0.0295	14	1127	1145	AGEAEIMSDEYYVAAAIKR					Mascot
2140.0518	2140.0823	0.0305	14	507	526	AETPTAAPPQISEEEASQRK					Mascot

9	Killer cell lectin-like receptor 2 OS=Mus musculus GN=Klra2 PE=2 SV=1	KLRA2_MOUSE	34440.9	8.43	13	60	39.329	14.494

Peptide Information

Calc. Mass	Obsrv. Mass	± da	± ppm	Start	End	Sequence	Ion	C. I. %	Modification	Rank	Result Type

				Seq.	Seq.		Score				
925.4448	925.5078	0.063	68	87	93	QEYQVMK					Mascot
963.4265	963.4879	0.0614	64	183	190	TNDEDELK					Mascot
1016.5596	1016.5454	-0.0142	-14	275	283	GRTQSALQR					Mascot
1016.5596	1016.5454	-0.0142	-14	275	283	GRTQSALQR					Mascot
1189.6147	1189.5902	-0.0245	-21	266	276	FPIPGSCAKGR			Carbamidomethyl (C)[7]		Mascot
1299.6764	1299.5822	-0.0942	-72	224	235	LDSAARNSVPNR					Mascot
1315.6753	1315.552	-0.1233	-94	112	122	ALNDSLHYLNR					Mascot
1326.6536	1326.6537	0.0001	0	2	12	SEQEVTYTTLR					Mascot
1326.6536	1326.6537	0.0001	0	2	12	SEQEVTYTTLR					Mascot
1373.6655	1373.6683	0.0028	2	212	223	EESQQIGDRPSK					Mascot
1399.7118	1399.609	-0.1028	-73	199	209	NTYWISLTHHK					Mascot
1582.72	1582.7346	0.0146	9	94	106	NDSSLMEEMLRNK			Oxidation (M)[6]		Mascot
1636.8475	1636.8065	-0.041	-25	81	93	TLNNLRQEYQVMK					Mascot
1652.8425	1652.7933	-0.0492	-30	81	93	TLNNLRQEYQVMK			Oxidation (M)[12]		Mascot
1738.8759	1738.8765	0.0006	0	2	15	SEQEVTYTTLRFHK					Mascot
2166.9067	2167.0452	0.1385	64	236	253	QKCAYLSSFSTEEDDCAR			Carbamidomethyl (C)[3,16]		Mascot

10	General transcription factor IIH subunit 1 OS=Drosophila melanogaster GN=Tfb1 PE=2 SV=1	TF2H1_DROME	66639.7	8.95	18	59	37.916	5.23

Peptide Information

Calc. Mass	Obsrv. Mass	± da	± ppm	Start Seq.	End Seq.	Sequence	Ion Score	C. I. %	Modification	Rank	Result Type
930.4462	930.4912	0.045	48	528	534	MHETLQR			Oxidation (M)[1]		Mascot
999.4828	999.5031	0.0203	20	31	38	VAWMAEHR					Mascot
1128.5031	1128.5863	0.0832	74	215	223	MSEAEFWTK					Mascot
1144.611	1144.5651	-0.0459	-40	405	413	HFELSKVER					Mascot
1189.6146	1189.5902	-0.0244	-21	528	536	MHETLQRFK					Mascot
1204.6508	1204.6002	-0.0506	-42	537	546	MAKLVPFENR					Mascot
1311.6951	1311.6132	-0.0819	-62	525	534	LQRMHETLQR					Mascot
1373.7284	1373.6683	-0.0601	-44	440	450	LVRNSESWLNR					Mascot
1495.8115	1495.7772	-0.0343	-23	256	270	AAVQQGAGDPLLDLK					Mascot
1549.6976	1549.8265	0.1289	83	359	372	SSSDQVDKDEPQSK					Mascot
1623.9065	1623.8678	-0.0387	-24	256	271	AAVQQGAGDPLLDLKK					Mascot
1680.8109	1680.8047	-0.0062	-4	2	16	TTSSEDVLLQMGEVR			Oxidation (M)[11]		Mascot
1752.8473	1752.8417	-0.0056	-3	241	255	DIFTECGKIDDQALK			Carbamidomethyl (C)[6]		Mascot

1827.8463	1827.9347	0.0884	48	1	16	MTTSSEDVLLQMGEVR	Oxidation (M)[1,12]	Mascot
1909.0112	1908.9589	-0.0523	-27	540	555	LVPFENRAMHELSPLR		Mascot
2087.0149	2087.041	0.0261	13	1	18	MTTSSEDVLLQMGEVRY K		Mascot
2267.0332	2267.0903	0.0571	25	20	38	GDGTLYVMNERVAWMA EHR	Oxidation (M)[8,15]	Mascot
2269.1687	2269.1758	0.0071	3	186	204	YNLTSDVIHCIFKTYPAVK	Carbamidomethyl (C)[10]	Mascot

Gel Idx/Pos	269/K21	Instr./Gel Origin	BA2151/Sample Project 20140814	Process Status	Analysis Succeeded
Plate [#] Name	[1] Sample Project 20140814	Instrument Sample Name		Spectra	11

Rank	Protein Name	Accession No.	Protein MW	Protein PI	Pep. Count	Protein Score	Protein Score C. I. %	Intensity Matched	Total Ion Score	Total Ion C. I. %	Confirmed
1	Xin actin-binding repeat-containing protein 1 OS=Gallus gallus GN=Xirp1 PE=2 SV=3	XIRP1_CHICK	217505.3	5.49	32	60	51.807	26.094			

Peptide Information

Calc. Mass	Obsrv. Mass	± da	± ppm	Start Seq.	End Seq.	Sequence	Ion Score	C. I. %	Modification	Rank	Result Type
804.4098	804.4032	-0.0066	-8	944	950	TVDDVQK					Mascot
846.4567	846.4564	-0.0003	0	248	254	SEIQELK					Mascot
890.4578	890.4373	-0.0205	-23	168	175	VSETDLAR					Mascot
904.4622	904.4449	-0.0173	-19	431	438	VELSAEEK					Mascot
905.4761	905.4454	-0.0307	-34	1210	1217	GMVIQETK					Mascot
944.5047	944.493	-0.0117	-12	397	405	EQVIGGDVK					Mascot
963.4815	963.4813	-0.0002	0	1391	1399	EEIMSGGLK					Mascot
970.4839	970.5104	0.0265	27	826	833	VDETELHK					Mascot
993.5033	993.4908	-0.0125	-13	837	845	DTMSKANVK					Mascot
1004.4643	1004.4809	0.0166	17	1621	1628	EQSVQEER					Mascot
1018.535	1018.5104	-0.0246	-24	1430	1439	ATMQGIAQAK					Mascot
1031.548	1031.5057	-0.0423	-41	619	627	EISQRGDVK					Mascot
1058.5491	1058.5524	0.0033	3	917	925	GDVQAARWR					Mascot
1058.5491	1058.5524	0.0033	3	917	925	GDVQAARWR					Mascot
1106.559	1106.5085	-0.0505	-46	1919	1928	GGQGKFELDR					Mascot
1145.4966	1145.6022	0.1056	92	1347	1356	MVFACESTGK			Carbamidomethyl (C)[5], Oxidation (M)[1]		Mascot
1187.5878	1187.6718	0.084	71	1680	1689	GDFKAAMIYR			Oxidation (M)[7]		Mascot
1187.5878	1187.6718	0.084	71	1680	1689	GDFKAAMIYR			Oxidation (M)[7]		Mascot
1317.5787	1317.6465	0.0678	51	1501	1510	NREEVHMACR			Carbamidomethyl (C)[9], Oxidation (M)[7]		Mascot
1372.7253	1372.6547	-0.0706	-51	1427	1439	EPKATMQGIAQAK					Mascot
1396.7543	1396.7352	-0.0191	-14	1315	1327	ALQSTNTHVLANK					Mascot
1413.7267	1413.6804	-0.0463	-33	276	287	TGNIHEIKSVCR			Carbamidomethyl (C)[11]		Mascot
1413.7267	1413.6804	-0.0463	-33	276	287	TGNIHEIKSVCR			Carbamidomethyl (C)[11]		Mascot
1443.6963	1443.6581	-0.0382	-26	649	661	QEEDVSVPQADVK					Mascot
1474.736	1474.7662	0.0302	20	446	458	HVFETCPLGSISK			Carbamidomethyl (C)[6]		Mascot
1501.8584	1501.7302	-0.1282	-85	1196	1209	ALQSLLSQDASIKK					Mascot

Calc. Mass	Obsrv. Mass	± da	± ppm	Start Seq.	End Seq.	Sequence	Ion Score	C. I. %	Modification	Rank	Result Type
1551.761	1551.7861	0.0251	16	1006	1019	SSSISTVQREDSQK					Mascot
1688.7843	1688.8044	0.0201	12	1503	1516	EEVHMACRQQVASK			Carbamidomethyl (C)[7], Oxidation (M)[5]		Mascot
1704.8915	1704.7454	-0.1461	-86	219	234	GAKELFEAQSLDAIGR					Mascot
1704.8915	1704.7454	-0.1461	-86	219	234	GAKELFEAQSLDAIGR					Mascot
1760.9615	1761.0294	0.0679	39	1118	1133	MVKYQLSSPGAPEILK					Mascot
1838.8782	1838.9222	0.044	24	631	644	WLFETQPMHTLYEK			Oxidation (M)[8]		Mascot
2021.1252	2021.0325	-0.0927	-46	1223	1239	MTLYSLLFHSVQQKVVK					Mascot
2021.1252	2021.0325	-0.0927	-46	1223	1239	MTLYSLLFHSVQQKVVK					Mascot
2046.9512	2047.0533	0.1021	50	846	862	SCTMLFESQPLYAIQDK			Carbamidomethyl (C)[2], Oxidation (M)[4]		Mascot
2133.0605	2132.9612	-0.0993	-47	1463	1481	QSEEKVLGNDLQAAMQSLR			Oxidation (M)[15]		Mascot
2138.05	2138.0662	0.0162	8	459	478	AFEEEISAASTEEVVKGDVK					Mascot
2138.05	2138.0662	0.0162	8	459	478	AFEEEISAASTEEVVKGDVK					Mascot

2 Effector protein hopAB1 OS=Pseudomonas syringae pv. syringae (strain B728a) GN=hopAB1 PE=3 SV=1 HPAB1_PSEU2 57629.2 6.08 15 55 0 5.431

Peptide Information

Calc. Mass	Obsrv. Mass	± da	± ppm	Start Seq.	End Seq.	Sequence	Ion Score	C. I. %	Modification	Rank	Result Type
815.379	815.4124	0.0334	41	478	483	FMDMKK			Oxidation (M)[2]		Mascot
856.5251	856.5144	-0.0107	-12	101	107	IVQELVR					Mascot
866.473	866.4102	-0.0628	-72	78	85	ATPPVEPR					Mascot
981.4611	981.5152	0.0541	55	204	211	FTGWMAPR			Oxidation (M)[5]		Mascot
1027.5063	1027.4902	-0.0161	-16	475	482	ALRFMDMK			Oxidation (M)[5]		Mascot
1031.464	1031.5057	0.0417	40	18	26	ADDEPVTER					Mascot
1122.5538	1122.5563	0.0025	2	86	95	QPPEAQPAER					Mascot
1320.6027	1320.595	-0.0077	-6	249	260	SANQTDTDEALR					Mascot
1321.6899	1321.5924	-0.0975	-74	505	516	NIAGYAFRVVPD					Mascot
1396.6704	1396.7352	0.0648	46	329	341	QATTESSSVFPSR					Mascot
1429.7693	1429.6732	-0.0961	-67	108	120	AGANLNNVRTMLR					Mascot
1467.7074	1467.7855	0.0781	53	415	427	TISKADAESQDFR					Mascot
1476.7037	1476.7444	0.0407	28	249	261	SANQTDTDEALRR					Mascot
1640.7639	1640.927	0.1631	99	1	15	MSGINGAGPSNFFWR					Mascot
1640.7639	1640.927	0.1631	99	1	15	MSGINGAGPSNFFWR					Mascot
1656.7588	1656.7595	0.0007	0	1	15	MSGINGAGPSNFFWR			Oxidation (M)[1]		Mascot
1838.9	1838.9222	0.0222	12	117	132	TMLRNVMDNNAVAFSR					Mascot

3 Tetratricopeptide repeat protein 5 OS=Mus musculus GN=Ttc5 PE=1 SV=2 TTC5_MOUSE 49106.8 5.9 13 55 0 7.421

Peptide Information

Calc. Mass	Obsrv. Mass	± da	± ppm	Start Seq.	End Seq.	Sequence	Ion Score	C. I. %	Modification	Rank	Result Type
868.4159	868.4597	0.0438	50	309	316	YQSASGQK					Mascot
890.5193	890.4373	-0.082	-92	274	281	LTSLLESK					Mascot
960.4421	960.4789	0.0368	38	402	409	DYSFSSVR					Mascot
1145.5586	1145.6022	0.0436	38	400	409	GKDYSFSSVR					Mascot
1223.6128	1223.5138	-0.099	-81	220	230	ASSNPDLHLNR					Mascot
1262.674	1262.6157	-0.0583	-46	264	273	EQQLLEFLSR					Mascot
1263.5522	1263.632	0.0798	63	43	52	QQDVQEEMEK					Mascot
1372.6088	1372.6547	0.0459	33	148	159	QLQTDSGDEHSR					Mascot
1501.8519	1501.7302	-0.1217	-81	135	147	NKVSLQNLSMVLR					Mascot
1507.7751	1507.7378	-0.0373	-25	202	215	ISQQALSAYAQAEK					Mascot
1656.7898	1656.7595	-0.0303	-18	2	15	MADEEEEAKHVLQK					Mascot
2046.9485	2047.0533	0.1048	51	116	134	KGDVASAHTCFSGALTH CK			Carbamidomethyl (C)[10,18]		Mascot
2138.0593	2138.0662	0.0069	3	289	308	LQSMLGSLRPAHLGPCG DGR			Carbamidomethyl (C)[16], Oxidation (M)[4]		Mascot
2138.0593	2138.0662	0.0069	3	289	308	LQSMLGSLRPAHLGPCG DGR			Carbamidomethyl (C)[16], Oxidation (M)[4]		Mascot

4	Pericentriolar material 1 protein OS=Gallus gallus GN=PCM1 PE=2 SV=1	PCM1_CHICK	214133.5	4.86	31	54	0	12.538

Peptide Information

Calc. Mass	Obsrv. Mass	± da	± ppm	Start Seq.	End Seq.	Sequence	Ion Score	C. I. %	Modification	Rank	Result Type
800.4373	800.3734	-0.0639	-80	649	654	LQQQQR					Mascot
804.4573	804.4032	-0.0541	-67	1210	1216	KNSSQLK					Mascot
807.3591	807.3952	0.0361	45	1151	1157	NQSDTSR					Mascot
813.4716	813.4261	-0.0455	-56	1843	1849	VEDLPLK					Mascot
815.3893	815.4124	0.0231	28	175	181	DPQQEAK					Mascot
832.4159	832.4285	0.0126	15	52	59	LGGEAETR					Mascot
847.507	847.4264	-0.0806	-95	1494	1500	QIKAIMK			Oxidation (M)[6]		Mascot
849.4326	849.4046	-0.028	-33	887	892	RQENFR					Mascot
855.4431	855.4562	0.0131	15	618	624	QQPNNVR					Mascot
863.3853	863.397	0.0117	14	776	783	SEGSEAQR					Mascot
890.4651	890.4373	-0.0278	-31	666	672	LMEIQEK					Mascot
914.4617	914.4417	-0.02	-22	642	648	EKFYEAK					Mascot
917.5414	917.5063	-0.0351	-38	625	632	VSTNKLQK					Mascot

Calc. Mass	Obsrv. Mass	± da	± ppm	Start Seq.	End Seq.	Sequence	Ion Score	C. I. %	Modification	Rank	Result Type
960.5109	960.4789	-0.032	-33	51	59	KLGGEAETR					Mascot
963.4602	963.4813	0.0211	22	1151	1158	NQSDTSRR					Mascot
970.4588	970.5104	0.0516	53	1339	1346	HLSENNEK					Mascot
1004.4941	1004.4809	-0.0132	-13	879	886	QQQNISMR					Mascot
1018.5601	1018.5104	-0.0497	-49	665	672	KLMEIQEK					Mascot
1106.4531	1106.5085	0.0554	50	544	553	ASEDSMSSHR					Mascot
1122.4481	1122.5563	0.1082	96	544	553	ASEDSMSSHR			Oxidation (M)[6]		Mascot
1145.5984	1145.6022	0.0038	3	197	205	MLQQQEQLK					Mascot
1175.5725	1175.6577	0.0852	72	130	139	KESGEPLQCK			Carbamidomethyl (C)[9]		Mascot
1262.7103	1262.6157	-0.0946	-75	1328	1338	ALYALQDIVTR					Mascot
1317.6943	1317.6465	-0.0478	-36	196	205	RMLQQQEQLK			Oxidation (M)[2]		Mascot
1443.7227	1443.6581	-0.0646	-45	904	914	EQWQEQINQLK					Mascot
1476.7363	1476.7444	0.0081	5	1587	1599	LMQDLDNNSISVK					Mascot
1522.7418	1522.7518	0.01	7	1525	1537	MVLTLTQQNDESK			Oxidation (M)[1]		Mascot
1703.944	1703.7974	-0.1466	-86	1546	1561	QLGSILQDSLAKFAGR					Mascot
1704.8876	1704.7454	-0.1422	-83	278	292	QAESLSLTREISQSR					Mascot
1704.8876	1704.7454	-0.1422	-83	278	292	QAESLSLTREISQSR					Mascot
1760.896	1761.0294	0.1334	76	1587	1601	LMQDLDNNSISVKQR					Mascot
2016.1263	2016.1077	-0.0186	-9	1843	1860	VEDLPLKLAVYSEADLLK					Mascot
2046.9696	2047.0533	0.0837	41	1508	1524	EHMDEVCSSQLLTSVRR			Carbamidomethyl (C)[7]		Mascot

5	Dual specificity phosphatase 28 OS=Homo sapiens GN=DUSP28 PE=2 SV=1	DUS28_HUMAN	18654.5	8.61	9	54	0	2.498	

Peptide Information

Calc. Mass	Obsrv. Mass	± da	± ppm	Start Seq.	End Seq.	Sequence	Ion Score	C. I. %	Modification	Rank	Result Type
804.3668	804.4032	0.0364	45	1	8	MGPAEAGR			Oxidation (M)[1]		Mascot
813.4326	813.4261	-0.0065	-8	2	9	GPAEAGRR					Mascot
944.473	944.493	0.02	21	1	9	MGPAEAGRR					Mascot
960.468	960.4789	0.0109	11	1	9	MGPAEAGRR			Oxidation (M)[1]		Mascot
1175.6201	1175.6577	0.0376	32	43	53	AGVTLCVNVSR			Carbamidomethyl (C)[6]		Mascot
1308.7345	1308.6566	-0.0779	-60	123	134	GLSLAKAFQMVK			Oxidation (M)[10]		Mascot
1501.725	1501.7302	0.0052	3	108	120	SRSAAVCTAYLMR			Carbamidomethyl (C)[7], Oxidation (M)[12]		Mascot
1551.752	1551.7861	0.0341	22	110	122	SAAVCTAYLMRHR			Carbamidomethyl (C)[5], Oxidation (M)[10]		Mascot
1838.9655	1838.9222	-0.0433	-24	43	59	AGVTLCVNVSRQQPGPR			Carbamidomethyl (C)[6]		Mascot
2114.1353	2113.9597	-0.1756	-83	22	42	VAPSLFLGSARAAGAEE QLAR					Mascot

6	Vinculin OS=Gallus gallus GN=VCL PE=1 SV=4	VINC_CHICK	125052.2	5.43	23	53	0	9.464		

Peptide Information

Calc. Mass	Obsrv. Mass	± da	± ppm	Start Seq.	End Seq.	Sequence	Ion Score	C. I. %	Modification	Rank	Result Type
807.3777	807.3952	0.0175	22	1040	1045	QCTDKR			Carbamidomethyl (C)[2]		Mascot
857.4952	857.4737	-0.0215	-25	521	528	GLVAEGRR					Mascot
866.4036	866.4102	0.0066	8	262	268	DTEAMKR			Oxidation (M)[5]		Mascot
868.4271	868.4597	0.0326	38	904	910	QLHDEAR					Mascot
873.4676	873.473	0.0054	6	301	308	QILDEAGK					Mascot
944.4472	944.493	0.0458	48	816	823	SFLDSGYR					Mascot
993.4855	993.4908	0.0053	5	171	178	MAKMIDER					Mascot
1028.5847	1028.5176	-0.0671	-65	985	994	GNDIIAAAKR					Mascot
1145.5586	1145.6022	0.0436	38	700	708	NQWIDNVEK					Mascot
1175.6154	1175.6577	0.0423	36	721	731	SLLDASEEAIK					Mascot
1308.6584	1308.6566	-0.0018	-1	994	1004	RMALLMAEMSR					Mascot
1317.7009	1317.6465	-0.0544	-41	217	228	NTKSQGIEEALK					Mascot
1321.7012	1321.5924	-0.1088	-82	1119	1129	TDAGFTLRWVR					Mascot
1360.7219	1360.6564	-0.0655	-48	981	993	WSSKGNDIIAAAK					Mascot
1507.7136	1507.7378	0.0242	16	286	300	DPNAPPGDAGEQAIR					Mascot
1522.681	1522.7518	0.0708	46	890	903	AGEAINQPMMMAAR			Oxidation (M)[9,10]		Mascot
1633.8003	1633.8066	0.0063	4	91	105	AAQMLQADPYSVPAR			Oxidation (M)[4]		Mascot
1642.015	1642.0579	0.0429	26	1057	1071	IPTISTQLKILSTVK					Mascot
1688.8749	1688.8044	-0.0705	-42	327	341	TLGQMTDQLADLRAR					Mascot
1704.8698	1704.7454	-0.1244	-73	327	341	TLGQMTDQLADLRAR			Oxidation (M)[5]		Mascot
1704.8698	1704.7454	-0.1244	-73	327	341	TLGQMTDQLADLRAR			Oxidation (M)[5]		Mascot
1720.8397	1720.7665	-0.0732	-43	73	87	DMPPAFIKVENACTK			Carbamidomethyl (C)[13]		Mascot
1761.0129	1761.0294	0.0165	9	480	496	AVANTRPVKAAVHLEGK					Mascot
2114.1638	2113.9597	-0.2041	-97	1048	1065	TNLLQVCERIPTISTQLK			Carbamidomethyl (C)[7]		Mascot
2279.137	2279.2256	0.0886	39	320	339	EILGTCKTLGQMTDQLAD LR			Carbamidomethyl (C)[6], Oxidation (M)[12]		Mascot

7	Kinesin-like protein KIF16B OS=Homo sapiens GN=KIF16B PE=1 SV=2	KI16B_HUMAN	152487.8	5.86	26	53	0	25.123		

Peptide Information

Calc. Mass	Obsrv. Mass	± da	± ppm	Start Seq.	End Seq.	Sequence	Ion Score	C. I. %	Modification	Rank	Result Type
832.441	832.4285	-0.0125	-15	17	23	EKDLEAK					Mascot

847.4243	847.4264	0.0021	2	1224	1229	FREMHK					Mascot
863.4192	863.397	-0.0222	-26	1224	1229	FREMHK			Oxidation (M)[4]		Mascot
873.4617	873.473	0.0113	13	825	830	FFEFKR					Mascot
902.4764	902.4393	-0.0371	-41	1226	1232	EMHKTLK			Oxidation (M)[2]		Mascot
904.4846	904.4449	-0.0397	-44	1036	1044	LASLNSGSR					Mascot
905.5316	905.4454	-0.0862	-95	169	175	TFNLRVR					Mascot
944.5206	944.493	-0.0276	-29	10	16	VRPMNRR			Oxidation (M)[4]		Mascot
960.5659	960.4789	-0.087	-91	1	9	MASVKVAVR					Mascot
981.4822	981.5152	0.033	34	125	132	ICEGLFSR			Carbamidomethyl (C)[2]		Mascot
993.4749	993.4908	0.0159	16	1087	1095	DHHGTLEGK					Mascot
1010.4789	1010.3888	-0.0901	-89	736	743	EKDEQYAK					Mascot
1010.4789	1010.3888	-0.0901	-89	736	743	EKDEQYAK					Mascot
1028.5847	1028.5176	-0.0671	-65	1003	1011	EALERALAR					Mascot
1145.595	1145.6022	0.0072	6	426	434	WNETQNILK					Mascot
1188.5854	1188.6544	0.069	58	714	723	ELNNNEKAEK					Mascot
1375.7217	1375.759	0.0373	27	415	425	VQELTKEWTNK					Mascot
1413.7697	1413.6804	-0.0893	-63	684	694	LREQQEIELQK					Mascot
1413.7697	1413.6804	-0.0893	-63	684	694	LREQQEIELQK					Mascot
1424.7241	1424.6902	-0.0339	-24	1150	1161	DNEKLHNGTIQR					Mascot
1429.6741	1429.6732	-0.0009	-1	1022	1033	HSTLGMEIEEQR					Mascot
1476.8097	1476.7444	-0.0653	-44	1235	1247	YAELAALEFPPKK					Mascot
1501.651	1501.7302	0.0792	53	1136	1149	VISEGCSTSADTMK			Carbamidomethyl (C)[6], Oxidation (M)[13]		Mascot
1688.8412	1688.8044	-0.0368	-22	566	581	SGLLSSFSLSMTDLSK			Oxidation (M)[11]		Mascot
1692.7429	1692.8475	0.1046	62	55	68	TFTYDFSFYSADTK					Mascot
1971.8634	1972.0367	0.1733	88	1136	1153	VISEGCSTSADTMKDNEK			Carbamidomethyl (C)[6]		Mascot
2004.9042	2005.0336	0.1294	65	231	247	FTQAKFDSEMPCETVSK			Carbamidomethyl (C)[12]		Mascot
2020.8992	2021.0325	0.1333	66	231	247	FTQAKFDSEMPCETVSK			Carbamidomethyl (C)[12], Oxidation (M)[10]		Mascot
2020.8992	2021.0325	0.1333	66	231	247	FTQAKFDSEMPCETVSK			Carbamidomethyl (C)[12], Oxidation (M)[10]		Mascot
2113.9285	2113.9597	0.0312	15	89	108	SAFEGYNACVFAYGQTGSGK			Carbamidomethyl (C)[9]		Mascot
2138.0408	2138.0662	0.0254	12	1016	1033	HSALQRHSTLGMEIEEQR			Oxidation (M)[12]		Mascot
2138.0408	2138.0662	0.0254	12	1016	1033	HSALQRHSTLGMEIEEQR			Oxidation (M)[12]		Mascot

8	DNA-directed RNA polymerase subunit beta' (Fragment) OS=Weissella hellenica GN=rpoC PE=3 SV=1	RPOC_WEIHE	117375.3	5.92	24	53	0	9.199

Peptide Information

Calc. Mass	Obsrv. Mass	± da	± ppm	Start Seq.	End Seq.	Sequence	Ion Score	C. I. %	Modification	Rank	Result Type

815.4443	815.4124	-0.0319	-39	249	255	SLSHMLK					Mascot
855.4505	855.4562	0.0057	7	1001	1007	HIEVMAR					Mascot
866.4474	866.4102	-0.0372	-43	492	499	IMMTSVGK					Mascot
885.5013	885.502	0.0007	1	195	201	VINRNNR					Mascot
890.4578	890.4373	-0.0205	-23	624	631	GLITDSER					Mascot
902.4512	902.4393	-0.0119	-13	683	691	GLMAAPNGR			Oxidation (M)[3]		Mascot
914.5166	914.4417	-0.0749	-82	311	318	RDLAGNIR					Mascot
925.5002	925.5018	0.0016	2	464	470	YVHLHTR					Mascot
960.4996	960.4789	-0.0207	-22	1036	1044	DANEGTLLK					Mascot
1027.4579	1027.4902	0.0323	31	579	587	DLGYDESTK					Mascot
1028.5847	1028.5176	-0.0671	-65	312	321	DLAGNIRAAK					Mascot
1107.5317	1107.5645	0.0328	30	785	794	SVFEPQTGDK					Mascot
1134.5646	1134.601	0.0364	32	48	57	MGLVLDMSPR			Oxidation (M)[1]		Mascot
1193.6273	1193.6071	-0.0202	-17	82	90	QLLSEREYR					Mascot
1205.6307	1205.6465	0.0158	13	12	22	CGVEVTSAKVR			Carbamidomethyl (C)[1]		Mascot
1308.6794	1308.6566	-0.0228	-17	602	612	EKPEIIEDAHK					Mascot
1322.6383	1322.6727	0.0344	26	945	955	MRVGEGDHIHR			Oxidation (M)[1]		Mascot
1322.6383	1322.6727	0.0344	26	945	955	MRVGEGDHIHR			Oxidation (M)[1]		Mascot
1396.8271	1396.7352	-0.0919	-66	737	748	RLVDVAQDVIIR					Mascot
1424.6951	1424.6902	-0.0049	-3	670	682	GNISNFTQLAGMR			Oxidation (M)[12]		Mascot
1622.7843	1622.849	0.0647	40	444	457	EGEGMIFKDVNEVR					Mascot
1656.7786	1656.7595	-0.0191	-12	327	340	HDEDVMDVLEDVIK					Mascot
1658.8279	1658.7491	-0.0788	-48	221	235	MLQEAADALVDNGRR					Mascot
1908.9637	1908.8254	-0.1383	-72	703	719	EGLSVLEMFFSTHGARK					Mascot
2047.0819	2047.0533	-0.0286	-14	471	489	IGIQTASLSAEKPFTAEQR					Mascot

9 DNA topoisomerase 1 OS=Mus musculus GN=Top1 PE=1 SV=2 | TOP1_MOUSE | 91275.1 | 9.35 | 22 | 52 | 0 | 9.178

Peptide Information

Calc. Mass	Obsrv. Mass	± da	± ppm	Start Seq.	End Seq.	Sequence	Ion Score	C. I. %	Modification	Rank	Result Type
846.493	846.4564	-0.0366	-43	154	160	IKTEDIK					Mascot
849.4689	849.4046	-0.0643	-76	459	464	IRNQYR					Mascot
866.356	866.4102	0.0542	63	287	293	EMTNDEK					Mascot
890.4366	890.4373	0.0007	1	439	445	GEKDWQK					Mascot
917.5091	917.5063	-0.0028	-3	637	644	APPKTFEK					Mascot

944.4908	944.493	0.0022	2	658	665	DQLADARR					Mascot
970.4332	970.5104	0.0772	80	645	652	SMMNLQSK			Oxidation (M)[2,3]		Mascot
998.5992	998.5615	-0.0377	-38	133	140	EDIKPLKR					Mascot
1010.4459	1010.3888	-0.0571	-57	286	293	KEMTNDEK			Oxidation (M)[3]		Mascot
1010.4459	1010.3888	-0.0571	-57	286	293	KEMTNDEK			Oxidation (M)[3]		Mascot
1058.5265	1058.5524	0.0259	24	535	542	DSIRYYNK					Mascot
1058.5265	1058.5524	0.0259	24	535	542	DSIRYYNK					Mascot
1106.6066	1106.5085	-0.0981	-89	618	626	ILSYNRANR					Mascot
1134.5062	1134.601	0.0948	84	561	569	QPEDDLFDR					Mascot
1317.7009	1317.6465	-0.0544	-41	711	722	EENKQIALGTSK					Mascot
1360.7253	1360.6564	-0.0689	-51	692	702	AVQRLEEQLMK			Oxidation (M)[10]		Mascot
1443.697	1443.6581	-0.0389	-27	641	652	TFEKSMMNLQSK					Mascot
1501.8525	1501.7302	-0.1223	-81	352	364	IANFKIEPPGLFR					Mascot
1507.8115	1507.7378	-0.0737	-49	593	605	TYNASITLQQQLK					Mascot
1522.802	1522.7518	-0.0502	-33	624	636	ANRAVAILCNHQR			Carbamidomethyl (C)[9]		Mascot
1688.933	1688.8044	-0.1286	-76	715	729	QIALGTSKLNYLDPR					Mascot
1720.8065	1720.7665	-0.04	-23	118	132	IKDEPEDDGYFAPPK					Mascot
1797.8411	1797.9178	0.0767	43	336	349	LLKEYGFCVMDNHR			Carbamidomethyl (C)[8], Oxidation (M)[10]		Mascot
1971.8615	1972.0367	0.1752	89	1	17	MSGDHLHNDSQIEADFR					Mascot

10	Regulator of telomere elongation helicase 1 homolog OS=Drosophila erecta GN=GG18780 PE=3 SV=1	RTEL1_DROER	110531.6	8.9	19	52	0	17.724

Peptide Information

Calc. Mass	Obsrv. Mass	± da	± ppm	Start Seq.	End Seq.	Sequence	Ion Score	C. I. %	Modification	Rank	Result Type
819.4207	819.3983	-0.0224	-27	796	803	SANESAIK					Mascot
873.4577	873.473	0.0153	18	431	438	QQGGWLGK					Mascot
885.4577	885.502	0.0443	50	101	108	ANNWGVPK					Mascot
963.5105	963.4813	-0.0292	-30	439	449	GTIAAASGTSK					Mascot
998.4836	998.5615	0.0779	78	128	135	RTAYANMR			Oxidation (M)[7]		Mascot
1004.5332	1004.4809	-0.0523	-52	314	321	EMLLELEK					Mascot
1106.559	1106.5085	-0.0505	-46	786	795	REPGSNATFK					Mascot
1188.579	1188.6544	0.0754	63	115	124	THSQLTQAMR			Oxidation (M)[9]		Mascot
1262.5934	1262.6157	0.0223	18	762	772	ETDAPLETVCK			Carbamidomethyl (C)[10]		Mascot
1263.625	1263.632	0.007	6	260	270	ICEESASVQIK			Carbamidomethyl (C)[2]		Mascot
1396.6526	1396.7352	0.0826	59	699	710	NDYGAILLCDSR			Carbamidomethyl (C)[9]		Mascot
1413.6614	1413.6804	0.019	13	143	153	DQLCIHPEVMR			Carbamidomethyl (C)[4], Oxidation (M)[10]		Mascot

1413.6614	1413.6804	0.019	13	143	153	DQLCIHPEVMR	Carbamidomethyl (C)[4], Oxidation (M)[10]	Mascot
1424.7996	1424.6902	-0.1094	-77	773	785	TEDEPLAAIPKLK		Mascot
1693.016	1692.8475	-0.1685	-100	636	651	NGRAVIITGLPFPPLK		Mascot
1797.884	1797.9178	0.0338	19	406	420	VYASFKVHVQMEESK	Oxidation (M)[11]	Mascot
2005.0713	2005.0336	-0.0377	-19	534	551	DNPKYISSLGQTILNVSR		Mascot
2016.0621	2016.1077	0.0456	23	516	533	IIGTGPDRQQLISNYANR		Mascot
2021.0913	2021.0325	-0.0588	-29	242	259	IELGNTIVILDEAHNIEK		Mascot
2021.0913	2021.0325	-0.0588	-29	242	259	IELGNTIVILDEAHNIEK		Mascot
2279.209	2279.2256	0.0166	7	303	321	DFTLDDLTLLKEMLLELEK		Mascot

Gel Idx/Pos	270/K22	Instr./Gel Origin	BA2151/Sample Project 20140814	Process Status	Analysis Succeeded
Plate [#] Name	[1] Sample Project 20140814	Instrument Sample Name		Spectra	11

Rank	Protein Name	Accession No.	Protein MW	Protein PI	Pep. Count	Protein Score	Protein Score C. I. %	Intensity Matched	Total Ion Score	Total Ion C. I. %	Confirmed
1	Phosphoglycerate kinase, cytosolic OS=Triticum aestivum PE=2 SV=1	PGKY_WHEAT	42152.7	5.64	19	602	100	52.168	479	100	

Peptide Information

Calc. Mass	Obsrv. Mass	± da	± ppm	Start Seq.	End Seq.	Sequence	Ion Score	C. I. %	Modification	Rank	Result Type
1030.6409	1030.6354	-0.0055	-5	75	83	FSLKPLVAR					Mascot
1074.6306	1074.6031	-0.0275	-26	192	202	KPFAAIVGGSK					Mascot
1089.5786	1089.5549	-0.0237	-22	6	16	SVGTLGEADLK					Mascot
1298.6587	1298.6323	-0.0264	-20	24	35	ADLNVPLDDAQK					Mascot
1335.6063	1335.6732	0.0669	50	126	136	EEEKNDPEFAK					Mascot
1388.7421	1388.7196	-0.0225	-16	179	191	ELDYLVGAVANPK					Mascot
1509.8247	1509.8083	-0.0164	-11	166	178	FLRPSVAGFLMQK			Oxidation (M)[11]		Mascot
1509.8247	1509.8083	-0.0164	-11	166	178	FLRPSVAGFLMQK	16	0	Oxidation (M)[11]		Mascot
1573.8433	1573.8103	-0.033	-21	350	366	GVTTIIGGGDSVAAVEK					Mascot
1720.9956	1721.0206	0.025	15	106	122	LAAALPDGGVLLLENVR					Mascot
1720.9956	1721.0206	0.025	15	106	122	LAAALPDGGVLLLENVR	152	100			Mascot
1769.8568	1769.8505	-0.0063	-4	317	331	TVIWNGPMGVFEFEK			Oxidation (M)[8]		Mascot
1802.9762	1802.8641	-0.1121	-62	217	232	VDILILGGGMIFTFYK			Oxidation (M)[10]		Mascot
1919.9611	1919.9871	0.026	14	138	155	LASVADLYVNDAFGTAH R					Mascot
1919.9611	1919.9871	0.026	14	138	155	LASVADLYVNDAFGTAH R	148	100			Mascot
2048.0559	2048.0833	0.0274	13	137	155	KLASVADLYVNDAFGTAH R					Mascot
2048.0559	2048.0833	0.0274	13	137	155	KLASVADLYVNDAFGTAH R	163	100			Mascot
2089.1274	2089.1179	-0.0095	-5	241	259	SLVEEDKLELATSLIETAK					Mascot
2102.1379	2102.135	-0.0029	-1	265	284	LLLPTDVVVADKFAADAE SK					Mascot
2159.2224	2159.1672	-0.0552	-26	106	125	LAAALPDGGVLLLENVRF YK					Mascot
2268.1582	2268.1665	0.0083	4	285	306	IVPATAIPDGWMGLDVGP DSIK			Oxidation (M)[12]		Mascot
2446.2092	2446.2239	0.0147	6	84	105	LSELLGLEVVMAPDCIGE EVEK			Carbamidomethyl (C)[15], Oxidation (M)[11]		Mascot
2878.4866	2878.5269	0.0403	14	373	401	MSHISTGGGASLELLEGK PLPGVLALDEA			Oxidation (M)[1]		Mascot

Rank	Protein Name	Accession No.	Protein MW	Protein PI	Pep. Count	Protein Score	Protein Score C. I. %	Intensity Matched	Total Ion Score	Total Ion C. I. %	Confirmed
2	Phosphoglycerate kinase OS=Trichodesmium erythraeum (strain IMS101) GN=pgk PE=3 SV=1	PGK_TRIEI	42536.1	5.01	7	185	100	4.821	163	100	

Peptide Information

Calc. Mass	Obsrv. Mass	± da	± ppm	Start Seq.	End Seq.	Sequence	Ion Score	C. I. %	Modification	Rank	Result Type
1074.6089	1074.6031	-0.0058	-5	74	82	MRLTLVGER					Mascot
1217.6372	1217.6416	0.0044	4	334	345	GTEAIAQTLADK					Mascot
1573.8507	1573.8103	-0.0404	-26	261	275	GVTMLLPTDVVVADK			Oxidation (M)[4]		Mascot
1702.9349	1702.9895	0.0546	32	219	233	LLLGGGMIFTFYKAR			Oxidation (M)[7]		Mascot
1737.8339	1737.8613	0.0274	16	316	330	TVIWNGPMGVFEMEK					Mascot
1769.8237	1769.8505	0.0268	15	316	330	TVIWNGPMGVFEMEK			Oxidation (M)[8,13]		Mascot
1802.8953	1802.8641	-0.0312	-17	370	387	MSHISTGGGASLELLEGK			Oxidation (M)[1]		Mascot
2048.0195	2048.0833	0.0638	31	136	154	QLASVADLYVNDAFGTA HR					Mascot
2048.0195	2048.0833	0.0638	31	136	154	QLASVADLYVNDAFGTA HR	163	100			Mascot

3	Phosphoglycerate kinase OS=Acaryochloris marina (strain MBIC 11017) GN=pgk PE=3 SV=1	PGK_ACAM1	42006.9	5.23	5	177	100	4.336	163	100

Peptide Information

Calc. Mass	Obsrv. Mass	± da	± ppm	Start Seq.	End Seq.	Sequence	Ion Score	C. I. %	Modification	Rank	Result Type
1702.9349	1702.9895	0.0546	32	220	234	LLIGGGMIFTFYKAR			Oxidation (M)[7]		Mascot
1714.9124	1714.8605	-0.0519	-30	19	34	VLVRADFNVPLDGDGK					Mascot
1801.9922	1801.8727	-0.1195	-66	217	232	VDKLLIGGGMIFTFYK					Mascot
1802.8953	1802.8641	-0.0312	-17	373	390	MSHISTGGGASLELLEGK			Oxidation (M)[1]		Mascot
2048.0195	2048.0833	0.0638	31	137	155	QLASVADLYVNDAFGTA HR					Mascot
2048.0195	2048.0833	0.0638	31	137	155	QLASVADLYVNDAFGTA HR	163	100			Mascot

4	COP9/Signalosome and eIF3 complex-shared subunit 1 OS=Caenorhabditis briggsae GN=cif-1 PE=3 SV=1	EIF3M_CAEBR	44359.2	5.64	15	78	99.236	10.612	

Peptide Information

Calc. Mass	Obsrv. Mass	± da	± ppm	Start Seq.	End Seq.	Sequence	Ion Score	C. I. %	Modification	Rank	Result Type
993.5952	993.5469	-0.0483	-49	171	178	EILRAVHR					Mascot
1056.6664	1056.6548	-0.0116	-11	279	287	VDEVILLKK					Mascot
1056.6664	1056.6548	-0.0116	-11	279	287	VDEVILLKK					Mascot
1532.8717	1532.7784	-0.0933	-61	288	300	IRLLTLMSLAEEK			Oxidation (M)[7]		Mascot
1580.6792	1580.7913	0.1121	71	204	217	DAATARDDAMECVR			Carbamidomethyl (C)[12]		Mascot
1596.6741	1596.7736	0.0995	62	204	217	DAATARDDAMECVR			Carbamidomethyl (C)[12], Oxidation (M)[10]		Mascot

Calc. Mass	Obsrv. Mass	± da	± ppm	Start Seq.	End Seq.	Sequence	Ion Score	C. I. %	Modification	Rank	Result Type
1702.8833	1702.9895	0.1062	62	82	96	CEPVVDAFIKNVSPK			Carbamidomethyl (C)[1]		Mascot
1705.7885	1705.9337	0.1452	85	210	224	DDAMECVRTAVVDPK			Carbamidomethyl (C)[6]		Mascot
1747.951	1747.8907	-0.0603	-34	290	304	LLTLMSLAEEKNEIK			Oxidation (M)[5]		Mascot
1847.9287	1848.0094	0.0807	44	218	233	TAVVDPKSFSFDHLER					Mascot
1901.9576	1901.9785	0.0209	11	375	390	QTHNNVHEVNQRIEAL					Mascot
1911.9845	1911.9336	-0.0509	-27	192	209	VMTALLGTYTEKDAATAR					Mascot
2012.9998	2013.045	0.0452	22	1	17	MADTRELPVFAYIDDIK			Oxidation (M)[1]		Mascot
2048.0342	2048.0833	0.0491	24	337	353	INEMANTLIVSSYQHRR			Oxidation (M)[4]		Mascot
2048.0342	2048.0833	0.0491	24	337	353	INEMANTLIVSSYQHRR	0	0	Oxidation (M)[4]		Mascot
2070.0828	2070.0623	-0.0205	-10	242	260	SSDPLMFTALELFISGTLK					Mascot
2127.0869	2127.0981	0.0112	5	113	130	VLSNLYKGYSNFHTVQE K					Mascot
2382.2988	2382.2368	-0.062	-26	239	260	ALKSSDPLMFTALELFISG TLK					Mascot

5	DNA-directed RNA polymerase subunit beta'' OS=Platanus occidentalis GN=rpoC2 PE=3 SV=1	RPOC2_PLAOC	158301.4	9.42	25	71	95.992	20.923

Peptide Information

Calc. Mass	Obsrv. Mass	± da	± ppm	Start Seq.	End Seq.	Sequence	Ion Score	C. I. %	Modification	Rank	Result Type
806.4077	806.3821	-0.0256	-32	17	24	AIDGTAMK					Mascot
925.4639	925.5118	0.0479	52	1356	1362	DTLFHHR					Mascot
963.5734	963.517	-0.0564	-59	969	976	RDPPIPIR					Mascot
1176.6736	1176.6947	0.0211	18	739	750	HSGILIPPGTGK					Mascot
1198.6539	1198.5521	-0.1018	-85	268	278	NQDIGIGLVNR					Mascot
1416.7516	1416.774	0.0224	16	875	887	MDLVKSTISHTGK					Mascot
1436.8584	1436.746	-0.1124	-78	1219	1230	HIEIIVRQITSK					Mascot
1466.7386	1466.8182	0.0796	54	611	622	RNSILAYFDDPR					Mascot
1532.8907	1532.7784	-0.1123	-73	736	750	ISRHSGILIPPGTGK					Mascot
1573.8584	1573.8103	-0.0481	-31	597	610	SDISIEIPINGIFR					Mascot
1646.828	1646.8221	-0.0059	-4	221	235	RTDCGTLQGISVSPR			Carbamidomethyl (C)[4]		Mascot
1696.8779	1696.806	-0.0719	-42	89	102	HHHYGNVHAVEKLR					Mascot
1758.9133	1758.9395	0.0262	15	849	864	NGSSIKEVHASFVEVR					Mascot
1769.944	1769.8505	-0.0935	-53	263	278	CIAARNQDIGIGLVNR			Carbamidomethyl (C)[1]		Mascot
1773.8668	1773.8256	-0.0412	-23	341	357	TFHTGGVFTGGTAEHVR					Mascot
1773.8668	1773.8256	-0.0412	-23	341	357	TFHTGGVFTGGTAEHVR					Mascot
1901.9076	1901.9785	0.0709	37	1348	1362	NLFEGEMRDTLFHHR					Mascot
1902.9564	1903.0101	0.0537	28	1	16	MEVLMAERADLVFHNK					Mascot
1917.9025	1917.9778	0.0753	39	1348	1362	NLFEGEMRDTLFHHR			Oxidation (M)[7]		Mascot

1918.9658	1918.9615	-0.0043	-2	722	738	IFSGDIHFPGETDKISR					Mascot
1919.9644	1919.9871	0.0227	12	838	854	TCLVLNWDQGKNGSSIK			Carbamidomethyl (C)[2]		Mascot
1919.9644	1919.9871	0.0227	12	838	854	TCLVLNWDQGKNGSSIK			Carbamidomethyl (C)[2]		Mascot
1976.9786	1977.0045	0.0259	13	1017	1032	QTLQVFKYYLMDENGK					Mascot
1992.9736	1992.9662	-0.0074	-4	1017	1032	QTLQVFKYYLMDENGK			Oxidation (M)[11]		Mascot
2087.0039	2087.0811	0.0772	37	1276	1294	ASLNTQSFISEASFQETAR					Mascot
2236.0664	2236.1714	0.105	47	1156	1174	SIDSISMTLENRVEGWNER					Mascot
2292.1072	2292.1704	0.0632	28	222	242	TDCGTLQGISVSPRNGMITER			Carbamidomethyl (C)[3]		Mascot
2382.2009	2382.2368	0.0359	15	1231	1252	VLVSEDGMSNVFSPGELIGLFR			Oxidation (M)[8]		Mascot
2878.3181	2878.5269	0.2088	73	117	141	QEMNPNFRMTNPSNPVHIMSFSGAR			Oxidation (M)[3]		Mascot

6	Probable serine/threonine-protein kinase DDB_G0283301 OS=Dictyostelium discoideum GN=DDB_G0283301 PE=3 SV=1	Y3301_DICDI	78328	8.53	19	69	92.706	35.244

Peptide Information

Calc. Mass	Obsrv. Mass	± da	± ppm	Start Seq.	End Seq.	Sequence	Ion Score	C. I. %	Modification	Rank	Result Type
965.5666	965.5859	0.0193	20	356	363	SKLVEVYK					Mascot
965.5666	965.5859	0.0193	20	356	363	SKLVEVYK					Mascot
1176.6041	1176.6947	0.0906	77	121	130	CLGKETIEAR			Carbamidomethyl (C)[1]		Mascot
1287.6791	1287.6227	-0.0564	-44	110	120	INLESDIENLK					Mascot
1335.6395	1335.6732	0.0337	25	175	185	CLEEGRVDMVK			Carbamidomethyl (C)[1]		Mascot
1406.6394	1406.7185	0.0791	56	467	478	ENSETNENTLQK					Mascot
1426.6532	1426.7292	0.076	53	2	12	TSQNWKNCFNK			Carbamidomethyl (C)[8]		Mascot
1445.821	1445.8306	0.0096	7	609	621	SLLTDSIISQQLK					Mascot
1466.7737	1466.8182	0.0445	30	583	594	LITLKTEYENDK					Mascot
1501.8373	1501.712	-0.1253	-83	302	314	INIYPIGQVSIER					Mascot
1501.8373	1501.712	-0.1253	-83	302	314	INIYPIGQVSIER					Mascot
1532.7454	1532.7784	0.033	22	521	532	MENPFYEFKISK					Mascot
1573.8778	1573.8103	-0.0675	-43	240	252	LFFIQFILGNFSK					Mascot
1702.9586	1702.9895	0.0309	18	607	621	EKSLLTDSIISQQLK					Mascot
1703.969	1703.9712	0.0022	1	288	301	LTEIKPRYLNEISK					Mascot
1720.8977	1721.0206	0.1229	71	316	332	NELGRGGNGTVYSGVLK					Mascot
1720.8977	1721.0206	0.1229	71	316	332	NELGRGGNGTVYSGVLK					Mascot
1882.933	1882.9894	0.0564	30	500	515	SDIYSLGVSFMMLLYK			Oxidation (M)[11]		Mascot
2148.0576	2148.0784	0.0208	10	151	168	IMHELISRNVMFIDDNGK			Oxidation (M)[2]		Mascot

2159.2104	2159.1672	-0.0432	-20	609	627	SLLTDSIISQQLKLMNQVK					Mascot
2413.2546	2413.3105	0.0559	23	500	520	SDIYSLGVSFMMLLYKVV YGK					Mascot
2446.3633	2446.2239	-0.1394	-57	538	559	TVVALENFLVPIVPTFLPD SFK					Mascot

7	50S ribosomal protein L1 OS=Gluconacetobacter diazotrophicus (strain ATCC 49037 / DSM 5601 / PAl5) GN=rplA PE=3 SV=1	RL1_GLUDA	24028.8	9.49	12	66	85.777	6.749	

Peptide Information

Calc. Mass	Obsrv. Mass	± da	± ppm	Start Seq.	End Seq.	Sequence	Ion Score	C. I. %	Modification	Rank	Result Type
963.5179	963.517	-0.0009	-1	141	149	LGTVTMDVK					Mascot
1056.6049	1056.6548	0.0499	47	60	70	GLLSLPNGTGK					Mascot
1056.6049	1056.6548	0.0499	47	60	70	GLLSLPNGTGK	3	0			Mascot
1176.6041	1176.6947	0.0906	77	211	222	AALSSTMGPGIR			Oxidation (M)[7]		Mascot
1201.6648	1201.6372	-0.0276	-23	6	16	RLTAAQATVDR					Mascot
1287.7015	1287.6227	-0.0788	-61	7	18	LTAAQATVDRNK					Mascot
1426.8376	1426.7292	-0.1084	-76	60	73	GLLSLPNGTGKTLR					Mascot
1436.7493	1436.746	-0.0033	-2	150	163	GAVTAAKSGQVEYR					Mascot
1466.6801	1466.8182	0.1381	94	113	125	CIATPDMMALVGR			Carbamidomethyl (C)[1], Oxidation (M)[7,8]		Mascot
1573.8698	1573.8103	-0.0595	-38	184	197	LAENIRAFVDAVQK					Mascot
1748.8856	1748.9803	0.0947	54	113	128	CIATPDMMALVGRLGK			Carbamidomethyl (C)[1], Oxidation (M)[7]		Mascot
1861.043	1860.9657	-0.0773	-42	19	36	AYGLDEAIALVKQVATAK					Mascot
1909.9913	1909.9153	-0.076	-40	53	70	HADQMVRGLLSLPNGTG K			Oxidation (M)[5]		Mascot

8	Flagellum-specific ATP synthase OS=Agrobacterium tumefaciens (strain C58 / ATCC 33970) GN=fliI PE=3 SV=1	FLII_AGRT5	51003.4	6.25	14	65	81.678	2.321	

Peptide Information

Calc. Mass	Obsrv. Mass	± da	± ppm	Start Seq.	End Seq.	Sequence	Ion Score	C. I. %	Modification	Rank	Result Type
963.5543	963.517	-0.0373	-39	194	202	STLLSMLAK					Mascot
1192.5052	1192.5385	0.0333	28	114	123	VSPDESWCGR			Carbamidomethyl (C)[8]		Mascot
1238.6852	1238.679	-0.0062	-5	72	83	SATGIHLGEVVR					Mascot
1320.7284	1320.6322	-0.0962	-73	61	71	HVRLGEFVAHR					Mascot
1626.8407	1626.9022	0.0615	38	194	208	STLLSMLAKADAFDK			Oxidation (M)[6]		Mascot
1705.8789	1705.9337	0.0548	32	236	251	SVAVVATSDESPMLRK			Oxidation (M)[13]		Mascot
1848.0015	1848.0094	0.0079	4	300	315	GYPASVFTELPRLLER					Mascot
1903.0219	1903.0101	-0.0118	-6	166	182	TGVRAIDIFSPLCLGQR			Carbamidomethyl (C)[13]		Mascot

1917.9739	1917.9778	0.0039	2	410	427	LIGGYRPGTDPDLDMAVK						Mascot
1977.0665	1977.0045	-0.062	-31	281	299	FAHAIREVAVASGEPPVAR						Mascot
2070.0979	2070.0623	-0.0356	-17	360	378	SLAEEGRYPPINPLASISR						Mascot
2078.124	2077.9761	-0.1479	-71	124	144	TINALGEPIDGQGPLASGIVR						Mascot
2148.1672	2148.0784	-0.0888	-41	64	83	LGEFVAHRSATGIHLGEVVR						Mascot
2878.4114	2878.5269	0.1155	40	84	108	VEPDICYVCPIEPGEPIGIHDTVIR			Carbamidomethyl (C)[6,9]			Mascot

9	DNA-directed RNA polymerase subunit beta' OS=Gluconobacter oxydans (strain 621H) GN=rpoC PE=3 SV=1	RPOC_GLUOX	160509.6	6.78	26	63	74.708	5.452

Peptide Information

Calc. Mass	Obsrv. Mass	± da	± ppm	Start Seq.	End Seq.	Sequence	Ion Score	C. I. %	Modification	Rank	Result Type
925.4738	925.5118	0.038	41	1253	1261	GDPLVDGPR					Mascot
1030.5428	1030.6354	0.0926	90	982	991	TFHIGGAATR					Mascot
1192.535	1192.5385	0.0035	3	940	949	VGICAHCYGR			Carbamidomethyl (C)[4,7]		Mascot
1238.6304	1238.679	0.0486	39	134	143	DVEPVLYFEK					Mascot
1287.7454	1287.6227	-0.1227	-95	366	376	RPLKSLSDMLK					Mascot
1320.6907	1320.6322	-0.0585	-44	166	175	RDQYLLDEIR					Mascot
1335.7743	1335.6732	-0.1011	-76	1294	1304	INDKHIEVIVR					Mascot
1372.6857	1372.7031	0.0174	13	1241	1252	HVSVQEGDFVQK					Mascot
1406.6879	1406.7185	0.0306	22	780	792	GSPAQMKQLAGMR			Oxidation (M)[6,12]		Mascot
1500.8315	1500.7413	-0.0902	-60	1402	1415	LIPAGTGSVMNRLR			Oxidation (M)[10]		Mascot
1509.8571	1509.8083	-0.0488	-32	1298	1309	HIEVIVRQMLQK			Oxidation (M)[9]		Mascot
1509.8571	1509.8083	-0.0488	-32	1298	1309	HIEVIVRQMLQK			Oxidation (M)[9]		Mascot
1596.7952	1596.7736	-0.0216	-14	48	60	TFKPERDGLFCAR			Carbamidomethyl (C)[11]		Mascot
1626.9174	1626.9022	-0.0152	-9	1387	1401	VDTLNGLKENVIVGR					Mascot
1701.9316	1701.8943	-0.0373	-22	1012	1026	NVVENSQKVLVVMSR					Mascot
1714.9601	1714.8605	-0.0996	-58	415	428	KMALELFKPFIYSK					Mascot
1738.8395	1738.8728	0.0333	19	813	828	EGLSVLDYFTSSHGAR					Mascot
1769.899	1769.8505	-0.0485	-27	1098	1113	MDEVTGLSSKVVVDYK					Mascot
1802.9858	1802.8641	-0.1217	-67	1377	1394	VLTDAATSGKVDTLNGLK					Mascot
1890.959	1890.9968	0.0378	20	992	1009	GAEQSMVEASRDGIVTIK					Mascot
2031.1598	2031.083	-0.0768	-38	891	908	TLSKDVIHPVTQDVILPR					Mascot
2073.0247	2073.0952	0.0705	34	1358	1376	ASLQTQSFISAASFQETTR					Mascot
2102.0271	2102.135	0.1079	51	761	779	QVIGKPTNSVWMMSHSGAR			Oxidation (M)[12]		Mascot

2266.1748	2266.1482	-0.0266	-12	2	21	NELMKILGQTGQSVTFD QIK			Oxidation (M)[4]		Mascot
2270.1848	2270.1543	-0.0305	-13	444	461	MVEKERPEVWDILEEVIR					Mascot
2413.2102	2413.3105	0.1003	42	1	21	MNELMKILGQTGQSVTF DQIK			Oxidation (M)[1,5]		Mascot
2446.2429	2446.2239	-0.019	-8	1020	1040	VLVVMSRNCEILLTDENG VER			Carbamidomethyl (C)[9]		Mascot

10	Serpin-Z2B OS=Triticum aestivum PE=1 SV=1	SPZ2B_WHEAT	43011.4	5.18	4	62	63.442	1.654	54	99.623

Peptide Information

Calc. Mass	Obsrv. Mass	± da	± ppm	Start Seq.	End Seq.	Sequence	Ion Score	C. I. %	Modification	Rank	Result Type
925.5214	925.5118	-0.0096	-10	11	18	LSIAHQTR					Mascot
1192.5382	1192.5385	0.0003	0	182	191	GAWTDQFDPR					Mascot
1372.7068	1372.7031	-0.0037	-3	159	171	DILPAGSIDNTTR					Mascot
1665.8595	1665.8763	0.0168	10	261	274	LSAEPEFLEQHIPR					Mascot
1665.8595	1665.8763	0.0168	10	261	274	LSAEPEFLEQHIPR	54	99.623			Mascot

Gel Idx/Pos	271/K23	Instr./Gel Origin	BA2151/Sample Project 20140814	Process Status	Analysis Succeeded
Plate [#] Name	[1] Sample Project 20140814	Instrument Sample Name		Spectra	11

Rank	Protein Name	Accession No.	Protein MW	Protein PI	Pep. Count	Protein Score	Protein Score C. I. %	Intensity Matched	Total Ion Score	Total Ion C. I. %	Confirmed
1	Beta-amylase OS=Triticum aestivum GN=BMY1 PE=2 SV=1	AMYB_WHEAT	56860.2	5.24	5	142	100	12.516	132	100	

Peptide Information

Calc. Mass	Obsrv. Mass	± da	± ppm	Start Seq.	End Seq.	Sequence	Ion Score	C. I. %	Modification	Rank	Result Type
993.4999	993.4896	-0.0103	-10	27	34	FEKGDEIR					Mascot
1285.6212	1285.6163	-0.0049	-4	61	71	GPKAYDWSAYK					Mascot
1607.6512	1607.7347	0.0835	52	333	345	HHASMNFTCAEMR			Carbamidomethyl (C)[9], Oxidation (M)[5]		Mascot
2087.0557	2087.0823	0.0266	13	129	146	NIEYLTLGVDDQPLFHGR					Mascot
2087.0557	2087.0823	0.0266	13	129	146	NIEYLTLGVDDQPLFHGR	132	100			Mascot
2124.9194	2125.0215	0.1021	48	329	345	MLTRHHASMNFTCAEMR			Carbamidomethyl (C)[13], Oxidation (M)[1,9]		Mascot

Rank	Protein Name	Accession No.	Protein MW	Protein PI	Pep. Count	Protein Score	Protein Score C. I. %	Intensity Matched	Total Ion Score	Total Ion C. I. %	Confirmed
2	UDP-arabinopyranose mutase 3 OS=Oryza sativa subsp. japonica GN=UAM3 PE=1 SV=1	RGP3_ORYSJ	41651.9	6.01	8	110	100	2.598	83	100	

Peptide Information

Calc. Mass	Obsrv. Mass	± da	± ppm	Start Seq.	End Seq.	Sequence	Ion Score	C. I. %	Modification	Rank	Result Type
839.441	839.4368	-0.0042	-5	157	163	GYPFSLR					Mascot
989.5414	989.5106	-0.0308	-31	274	282	ASNPFVNLK					Mascot
1180.6321	1180.6431	0.011	9	121	130	DINALEQHIK					Mascot
1485.7908	1485.7749	-0.0159	-11	2	17	ASSDAAAAQAATPLLK					Mascot
1501.6958	1501.7019	0.0061	4	59	70	VPEGFDYELYNR					Mascot
1501.6958	1501.7019	0.0061	4	59	70	VPEGFDYELYNR	83	100			Mascot
1632.8262	1632.8073	-0.0189	-12	1	17	MASSDAAAAQAATPLLK			Oxidation (M)[1]		Mascot
1761.7758	1761.7963	0.0205	12	207	221	GTLFPMCGMNLAFDR			Carbamidomethyl (C)[7], Oxidation (M)[6,9]		Mascot
2114.9778	2115.0823	0.1045	49	59	75	VPEGFDYELYNRDDINR					Mascot

Rank	Protein Name	Accession No.	Protein MW	Protein PI	Pep. Count	Protein Score	Protein Score C. I. %	Intensity Matched	Total Ion Score	Total Ion C. I. %	Confirmed
3	Alpha-1,4-glucan-protein synthase [UDP-forming] OS=Zea mays GN=UPTG PE=1 SV=2	UPTG_MAIZE	41690.8	5.75	7	105	99.998	2.655	83	100	

Peptide Information

Calc. Mass	Obsrv. Mass	± da	± ppm	Start Seq.	End Seq.	Sequence	Ion Score	C. I. %	Modification	Rank	Result Type
839.441	839.4368	-0.0042	-5	159	165	GYPFSLR					Mascot

Calc. Mass	Obsrv. Mass	± da	± ppm	Start Seq.	End Seq.	Sequence	Ion Score	C. I. %	Modification	Rank	Result Type
989.5414	989.5106	-0.0308	-31	276	284	ASNPFVNLK					Mascot
1180.6321	1180.6431	0.011	9	123	132	DINALEQHIK					Mascot
1501.6958	1501.7019	0.0061	4	61	72	VPEGFDYELYNR					Mascot
1501.6958	1501.7019	0.0061	4	61	72	VPEGFDYELYNR	83	100			Mascot
1712.943	1712.7983	-0.1447	-84	2	19	AGTVTVPGSSTPSTPLLK					Mascot
1761.7758	1761.7963	0.0205	12	209	223	GTLFPMCGMNLAFDR			Carbamidomethyl (C)[7], Oxidation (M)[6,9]		Mascot
2292.1335	2292.1665	0.033	14	133	151	NLLSPSTPFFFNTLYDPY R					Mascot

4	Alpha-1,4-glucan-protein synthase [UDP-forming] 1 OS=Solanum tuberosum GN=UPTG1 PE=1 SV=2	UPTG1_SOLTU	42462.1	6.19	7	105	99.998	2.434	83	100

Peptide Information

Calc. Mass	Obsrv. Mass	± da	± ppm	Start Seq.	End Seq.	Sequence	Ion Score	C. I. %	Modification	Rank	Result Type
989.5414	989.5106	-0.0308	-31	266	274	ASNPFVNLK					Mascot
1180.6321	1180.6431	0.011	9	113	122	DINALEQHIK					Mascot
1501.6958	1501.7019	0.0061	4	51	62	VPEGFDYELYNR					Mascot
1501.6958	1501.7019	0.0061	4	51	62	VPEGFDYELYNR	83	100			Mascot
1507.7938	1507.7085	-0.0853	-57	186	198	NTRYVDAVMTIPK					Mascot
1758.8037	1758.8679	0.0642	37	298	311	ECTTVQQCYLELSK			Carbamidomethyl (C)[2,8]		Mascot
1761.7758	1761.7963	0.0205	12	199	213	GTLFPMCGMNLAFDR			Carbamidomethyl (C)[7], Oxidation (M)[6,9]		Mascot
2049.1953	2048.9988	-0.1965	-96	2	20	AAATPLLKDELDIVIPTIR					Mascot

5	UDP-arabinopyranose mutase 1 OS=Oryza sativa subsp. japonica GN=UAM1 PE=1 SV=1	RGP1_ORYSJ	41834.8	5.82	6	99	99.993	2.525	83	100

Peptide Information

Calc. Mass	Obsrv. Mass	± da	± ppm	Start Seq.	End Seq.	Sequence	Ion Score	C. I. %	Modification	Rank	Result Type
839.441	839.4368	-0.0042	-5	159	165	GYPFSLR					Mascot
989.5414	989.5106	-0.0308	-31	276	284	ASNPFVNLK					Mascot
1180.6321	1180.6431	0.011	9	123	132	DINALEQHIK					Mascot
1501.6958	1501.7019	0.0061	4	61	72	VPEGFDYELYNR					Mascot
1501.6958	1501.7019	0.0061	4	61	72	VPEGFDYELYNR	83	100			Mascot
1761.7758	1761.7963	0.0205	12	209	223	GTLFPMCGMNLAFDR			Carbamidomethyl (C)[7], Oxidation (M)[6,9]		Mascot
2292.1335	2292.1665	0.033	14	133	151	NLLSPSTPFFFNTLYDPY R					Mascot

6	Alpha-1,4-glucan-protein synthase [UDP-forming] OS=Pisum sativum GN=UPTG PE=1 SV=1	UPTG_PEA	42059	5.73	6	99	99.993	2.682	83	100

Peptide Information

Calc. Mass	Obsrv. Mass	± da	± ppm	Start Seq.	End Seq.	Sequence	Ion Score	C. I. %	Modification	Rank	Result Type
837.3771	837.4368	0.0597	71	301	307	DCTSVQK			Carbamidomethyl (C)[2]		Mascot
839.441	839.4368	-0.0042	-5	152	158	GYPFSLR					Mascot
989.5414	989.5106	-0.0308	-31	269	277	ASNPFVNLK					Mascot
1501.6958	1501.7019	0.0061	4	54	65	VPEGFDYELYNR					Mascot
1501.6958	1501.7019	0.0061	4	54	65	VPEGFDYELYNR	83	100			Mascot
1701.8555	1701.8016	-0.0539	-32	111	125	DPTGHEINALEQHIK					Mascot
2292.1335	2292.1665	0.033	14	126	144	NLLSPSTPFFFNTLYDPY R					Mascot

7	Alpha-1,4-glucan-protein synthase [UDP-forming] (Fragments) OS=Phoenix dactylifera PE=1 SV=1	UPTG_PHODC	6899.6	7.82	2	95	99.981	1.731	83	100

Peptide Information

Calc. Mass	Obsrv. Mass	± da	± ppm	Start Seq.	End Seq.	Sequence	Ion Score	C. I. %	Modification	Rank	Result Type
989.5414	989.5106	-0.0308	-31	52	60	ASNPFVNLK					Mascot
1501.6958	1501.7019	0.0061	4	4	15	VPEGFDYELYNR					Mascot
1501.6958	1501.7019	0.0061	4	4	15	VPEGFDYELYNR	83	100			Mascot

8	Insulin-like growth factor 1 receptor OS=Rattus norvegicus GN=Igf1r PE=2 SV=2	IGF1R_RAT	157804.5	5.57	23	72	96.344	9.215	17	0

Peptide Information

Calc. Mass	Obsrv. Mass	± da	± ppm	Start Seq.	End Seq.	Sequence	Ion Score	C. I. %	Modification	Rank	Result Type
837.3672	837.4368	0.0696	83	205	210	CWTTNR			Carbamidomethyl (C)[1]		Mascot
993.4709	993.4896	0.0187	19	1179	1186	WMSPESLK			Oxidation (M)[2]		Mascot
1124.6423	1124.6307	-0.0116	-10	1330	1340	AENGPGVLVLR					Mascot
1237.5372	1237.5508	0.0136	11	1160	1168	DIYETDYYR					Mascot
1237.5372	1237.5508	0.0136	11	1160	1168	DIYETDYYR	17	0			Mascot
1253.5514	1253.5743	0.0229	18	205	213	CWTTNRCQK			Carbamidomethyl (C)[1,7]		Mascot
1287.5964	1287.5964	0	0	195	204	TTINNEYNYR					Mascot
1389.7518	1389.6323	-0.1195	-86	1031	1043	VAIKTVNEAASMR					Mascot
1421.7053	1421.7081	0.0028	2	742	754	DVLQVANTTMSSR					Mascot
1498.7683	1498.6951	-0.0732	-49	584	596	AVTLTMVENDHIR					Mascot
1507.6338	1507.7085	0.0747	50	698	710	GPCCACPKTEAEK			Carbamidomethyl (C)[3,4,6]		Mascot
1543.7533	1543.7749	0.0216	14	1357	1370	ANERALPLPQSSTC			Carbamidomethyl (C)[14]		Mascot

1579.8955	1579.7639	-0.1316	-83	789	801	TVISNLRPFTLYR					Mascot
1593.7921	1593.755	-0.0371	-23	649	660	WQRQPQDGYLFR					Mascot
1607.938	1607.9587	0.0207	13	391	404	IRHSHALVSLSFLK					Mascot
1671.8047	1671.6985	-0.1062	-64	1005	1020	ELGQGSFGMVYEGVAK					Mascot
1671.8047	1671.6985	-0.1062	-64	1005	1020	ELGQGSFGMVYEGVAK					Mascot
1680.8334	1680.8318	-0.0016	-1	742	756	DVLQVANTTMSSRSR			Oxidation (M)[10]		Mascot
1687.7997	1687.7386	-0.0611	-36	1005	1020	ELGQGSFGMVYEGVAK			Oxidation (M)[9]		Mascot
1712.7657	1712.7983	0.0326	19	870	883	YGSQVEDQRECVSR			Carbamidomethyl (C)[11]		Mascot
1754.9219	1754.7466	-0.1753	-100	584	599	AVTLTMVENDHIRGAK					Mascot
1851.8191	1851.9681	0.149	80	1341	1356	ASFDERQPYAHMNGGR			Oxidation (M)[12]		Mascot
1981.9934	1982.0659	0.0725	37	341	358	TIDSVTSAQMLQGCTILK			Carbamidomethyl (C)[14], Oxidation (M)[10]		Mascot
2056.95	2056.9958	0.0458	22	1113	1131	MIQMAGEIADGMAYLNA NK			Oxidation (M)[1]		Mascot
2114.9487	2115.0823	0.1336	63	1152	1168	IGDFGMTRDIYETDYYR					Mascot
2152.9019	2153.0459	0.144	67	177	194	ECGDLCPGTLEEKPMCE K			Carbamidomethyl (C)[2,6,16]		Mascot
2168.8967	2169.0918	0.1951	90	177	194	ECGDLCPGTLEEKPMCE K			Carbamidomethyl (C)[2,6,16], Oxidation (M)[15]		Mascot

9 DNA polymerase epsilon catalytic subunit A OS=Arabidopsis thaliana GN=POL2A PE=1 SV=1 | DPOE1_ARATH | 251873.9 | 6.19 | 36 | 68 | 90.817 | 24.094

Peptide Information

Calc. Mass	Obsrv. Mass	± da	± ppm	Start Seq.	End Seq.	Sequence	Ion Score	C. I. %	Modification	Rank	Result Type
801.4716	801.4733	0.0017	2	918	924	EEGILIK					Mascot
837.4135	837.4368	0.0233	28	1022	1029	SCAVTTAK			Carbamidomethyl (C)[2]		Mascot
884.4948	884.4348	-0.06	-68	582	588	LRDDPIR					Mascot
925.4414	925.5121	0.0707	76	653	659	SDYYHLK					Mascot
929.5666	929.5305	-0.0361	-39	918	925	EEGILIKK					Mascot
993.5146	993.4896	-0.025	-25	1022	1030	SCAVTTAKR			Carbamidomethyl (C)[2]		Mascot
1024.5898	1024.5244	-0.0654	-64	129	137	NHLSGLQKK					Mascot
1237.5782	1237.5508	-0.0274	-22	1595	1604	CAAAFQWLDR			Carbamidomethyl (C)[1]		Mascot
1237.6311	1237.5508	-0.0803	-65	549	559	DLEYAITVEGK					Mascot
1253.6381	1253.5743	-0.0638	-51	2133	2142	MEIFMDIAKR					Mascot
1285.6279	1285.6163	-0.0116	-9	2133	2142	MEIFMDIAKR			Oxidation (M)[1,5]		Mascot
1325.79	1325.7498	-0.0402	-30	705	715	RVLDKPITEVR					Mascot
1352.7031	1352.7662	0.0631	47	1459	1469	AIYVLYCHVSK			Carbamidomethyl (C)[7]		Mascot
1525.7567	1525.7372	-0.0195	-13	96	107	DKMELELEAYLR			Oxidation (M)[3]		Mascot
1541.8646	1541.7656	-0.099	-64	306	318	VTNVKNEVELLQR					Mascot
1553.7881	1553.7815	-0.0066	-4	141	153	ISFDTVQQLMEVK			Oxidation (M)[10]		Mascot

Calc. Mass	Obsrv. Mass	± da	± ppm	Start Seq.	End Seq.	Sequence	Ion Score	C. I. %	Modification	Rank	Result Type
1593.7876	1593.755	-0.0326	-20	1044	1056	GLRCQYIVACEPK			Carbamidomethyl (C)[4,10]		Mascot
1603.7091	1603.7758	0.0667	42	560	572	MRMDSISNYDEVK			Oxidation (M)[1]		Mascot
1607.8826	1607.7347	-0.1479	-92	1939	1952	EQIGSTFINMLVKK					Mascot
1607.8826	1607.9587	0.0761	47	1939	1952	EQIGSTFINMLVKK					Mascot
1609.8545	1609.9105	0.056	35	123	136	EDLDLKNHLSGLQK					Mascot
1623.8774	1623.9006	0.0232	14	1939	1952	EQIGSTFINMLVKK			Oxidation (M)[10]		Mascot
1629.802	1629.7983	-0.0037	-2	723	735	ENPFYVDTVRSFR					Mascot
1632.8666	1632.8073	-0.0593	-36	1064	1078	AVPVAIFTTNPEVMK			Oxidation (M)[14]		Mascot
1646.8636	1646.7966	-0.067	-41	1380	1395	AALLADPGVEGIYETK					Mascot
1646.8636	1646.7966	-0.067	-41	1380	1395	AALLADPGVEGIYETK					Mascot
1657.8505	1657.7937	-0.0568	-34	1962	1977	EINVSDASRVSGQAPK					Mascot
1662.9612	1662.8118	-0.1494	-90	1791	1804	VMQKVFALLLTDLR			Oxidation (M)[2]		Mascot
1666.808	1666.7974	-0.0106	-6	783	795	CILNSFYGYVMRK			Carbamidomethyl (C)[1], Oxidation (M)[11]		Mascot
1668.8494	1668.7944	-0.055	-33	1512	1525	FQVHYVDHPEAAKK					Mascot
1686.8909	1686.7914	-0.0995	-59	18	32	KPKVVNTAEDELESK					Mascot
1696.8364	1696.7971	-0.0393	-23	1338	1351	VPIDEYFQGKCVNK			Carbamidomethyl (C)[11]		Mascot
1700.8966	1700.7888	-0.1078	-63	387	402	DSYLPQGSHGLKAVTK					Mascot
1712.816	1712.7983	-0.0177	-10	1924	1938	GSSESLEAQMIEYLR					Mascot
1728.8109	1728.8325	0.0216	12	1924	1938	GSSESLEAQMIEYLR			Oxidation (M)[10]		Mascot
1998.9623	1999.0538	0.0915	46	2071	2086	IYDREQMESSLLEMVR					Mascot
1999.9978	1999.9877	-0.0101	-5	181	196	REQRPQDCLDSIVDLR			Carbamidomethyl (C)[8]		Mascot
1999.9978	1999.9877	-0.0101	-5	181	196	REQRPQDCLDSIVDLR			Carbamidomethyl (C)[8]		Mascot
2056.958	2056.9958	0.0378	18	716	732	EAGICMRENPFYVDTVR			Carbamidomethyl (C)[5]		Mascot
2077.9834	2077.9719	-0.0115	-6	796	814	GARWYSMEMAGVVTYT GAK					Mascot
3048.47	3048.6245	0.1545	51	612	637	LQPPSIVTDEICTACDFN RPGKTCLR			Carbamidomethyl (C)[12,15,24]		Mascot
3111.4199	3111.5645	0.1446	46	994	1021	DIADSELLDYISESSTMSK SLADYGEQK			Oxidation (M)[17]		Mascot

10	DNA repair and recombination protein RDH54 OS=Saccharomyces cerevisiae (strain ATCC 204508 / S288c) GN=RDH54 PE=1 SV=4	RDH54_YEAST	108847.3	9.27	22	66	86.727	15.425	

Peptide Information

Calc. Mass	Obsrv. Mass	± da	± ppm	Start Seq.	End Seq.	Sequence	Ion Score	C. I. %	Modification	Rank	Result Type
947.4767	947.4902	0.0135	14	911	917	QCLVHYK			Carbamidomethyl (C)[2]		Mascot
947.4767	947.4902	0.0135	14	911	917	QCLVHYK			Carbamidomethyl (C)[2]		Mascot
993.4862	993.4896	0.0034	3	107	113	YFTIMYR					Mascot
1101.5034	1101.4282	-0.0752	-68	138	146	LCFYNEAGK			Carbamidomethyl (C)[2]		Mascot

1176.6405	1176.5907	-0.0498	-42	802	811	QLMKNSLSQK		Mascot
1285.6206	1285.6163	-0.0043	-3	241	251	QTNEHMTTVPK		Mascot
1366.7703	1366.724	-0.0463	-34	295	305	FLRPHQREGVK		Mascot
1579.8326	1579.7639	-0.0687	-43	880	893	STTTWTSALDLQKK		Mascot
1589.7264	1589.6837	-0.0427	-27	133	146	ASSDKLCFYNEAGK	Carbamidomethyl (C)[7]	Mascot
1607.8389	1607.7347	-0.1042	-65	879	892	RSTTTWTSALDLQK		Mascot
1607.8389	1607.9587	0.1198	75	879	892	RSTTTWTSALDLQK		Mascot
1623.8953	1623.9006	0.0053	3	279	294	NAAAEVDVIVDPLLGK		Mascot
1636.8376	1636.8175	-0.0201	-12	911	923	QCLVHYKHIDPAR	Carbamidomethyl (C)[2]	Mascot
1645.8942	1645.7811	-0.1131	-69	788	801	LVTTGCIDEKILQR	Carbamidomethyl (C)[6]	Mascot
1647.8225	1647.7867	-0.0358	-22	832	845	EDLKDLFSVHTDTK		Mascot
1651.7671	1651.8032	0.0361	22	646	659	SHIKDTQSQDSYSR		Mascot
1686.797	1686.7914	-0.0056	-3	122	137	TWSGDGYATLKASSDK		Mascot
1700.8636	1700.7888	-0.0748	-44	237	251	SVTKQTNEHMTTVPK		Mascot
1716.8585	1716.7775	-0.081	-47	237	251	SVTKQTNEHMTTVPK	Oxidation (M)[10]	Mascot
1732.8899	1732.793	-0.0969	-56	414	429	IGVLTLSSRNSPDMDK		Mascot
1851.9204	1851.9681	0.0477	26	464	478	HLIDMLVCDEGHRLK	Carbamidomethyl (C)[8], Oxidation (M)[5]	Mascot
2169.105	2169.0918	-0.0132	-6	261	278	YYPVFDVNKIDNPIVMNK		Mascot
2240.1379	2240.1995	0.0616	27	214	233	NDGGKYQMPLSQLFSLN TVK		Mascot
2297.229	2297.2	-0.029	-13	721	741	DSIVTSFNRNPAIFGFLLS AK		Mascot
3111.4731	3111.5645	0.0914	29	684	710	VVVVSNYTQTLDIIENLM NMAGMSHCR	Carbamidomethyl (C)[26], Oxidation (M)[18]	Mascot

Gel Idx/Pos	272/K24	Instr./Gel Origin	BA2151/Sample Project 20140814	Process Status	Analysis Succeeded
Plate [#] Name	[1] Sample Project 20140814	Instrument Sample Name		Spectra	11

Rank	Protein Name	Accession No.	Protein MW	Protein PI	Pep. Count	Protein Score	Protein Score C. I. %	Intensity Matched	Total Ion Score	Total Ion C. I. %	Confirmed
1	Protein translocase subunit SecA OS=Bacillus subtilis (strain 168) GN=secA PE=1 SV=1	SECA_BACSU	95698.5	5.49	24	69	93.792	38.711			

Peptide Information

Calc. Mass	Obsrv. Mass	± da	± ppm	Start Seq.	End Seq.	Sequence	Ion Score	C. I. %	Modification	Rank	Result Type
800.4083	800.3974	-0.0109	-14	296	302	AHVAMQK			Oxidation (M)[5]		Mascot
804.4647	804.3971	-0.0676	-84	1	7	MLGILNK			Oxidation (M)[1]		Mascot
840.4283	840.4226	-0.0057	-7	410	416	TMEGKFK					Mascot
849.4498	849.4354	-0.0144	-17	367	375	LAGMTGTAK					Mascot
853.3906	853.4456	0.055	64	554	560	TMAMLDR			Oxidation (M)[2]		Mascot
856.4233	856.5088	0.0855	100	410	416	TMEGKFK			Oxidation (M)[2]		Mascot
882.4389	882.449	0.0101	11	187	193	DNMVLYK					Mascot
904.4846	904.4703	-0.0143	-16	577	584	AVESSQKR					Mascot
917.425	917.4637	0.0387	42	249	255	DYTYDIK					Mascot
923.4944	923.4753	-0.0191	-21	15	21	TLNRYEK					Mascot
1008.5724	1008.5222	-0.0502	-50	692	699	IITKYNEK					Mascot
1245.5997	1245.4998	-0.0999	-80	246	255	AEKDYTYDIK					Mascot
1413.6614	1413.6869	0.0255	18	549	560	FGAERTMAMLDR			Oxidation (M)[7]		Mascot
1413.6614	1413.6869	0.0255	18	549	560	FGAERTMAMLDR			Oxidation (M)[7]		Mascot
1435.7461	1435.6493	-0.0968	-67	256	268	TKAVQLTEEGMTK					Mascot
1507.8011	1507.7505	-0.0506	-34	1	13	MLGILNKMFDPTK					Mascot
1522.6764	1522.7584	0.082	54	680	691	EPDEMLELIMDR			Oxidation (M)[5,10]		Mascot
1522.874	1522.7584	-0.1156	-76	233	245	LYVQANAFVRTLK					Mascot
1550.7731	1550.7554	-0.0177	-11	258	271	AVQLTEEGMTKAEK			Oxidation (M)[9]		Mascot
1566.8163	1566.752	-0.0643	-41	269	282	AEKAFGIDNLFDVK					Mascot
1674.8922	1674.774	-0.1182	-71	595	607	QLLQYDDVLRQQR					Mascot
1675.8796	1675.786	-0.0936	-56	478	494	GAVTIATNMAGRGTDIK					Mascot
2047.1071	2047.0742	-0.0329	-16	52	70	LEKGATTDDLLVEAFAVVR					Mascot
2047.1071	2047.0742	-0.0329	-16	52	70	LEKGATTDDLLVEAFAVVR					Mascot
2114.1541	2113.9873	-0.1668	-79	383	400	NIYNMQVVTIPTNRPVVR					Mascot
2120.0981	2120.0352	-0.0629	-30	55	74	GATTDDLLVEAFAVVREA					Mascot

Calc. Mass	Obsrv. Mass	± da	± ppm	Start Seq.	End Seq.	Sequence	Ion Score	C. I. %	Modification	Rank	Result Type
						SR					
2138.0144	2138.0818	0.0674	32	674	691	SDIFGKEPDEMLELIMDR					Mascot
2138.0144	2138.0818	0.0674	32	674	691	SDIFGKEPDEMLELIMDR					Mascot

2	NADP-dependent alkenal double bond reductase P2 OS=Arabidopsis thaliana GN=P2 PE=2 SV=2	P2_ARATH	38249.3	8.09	4	66	87.324	6.979	58	99.835	

Peptide Information

Calc. Mass	Obsrv. Mass	± da	± ppm	Start Seq.	End Seq.	Sequence	Ion Score	C. I. %	Modification	Rank	Result Type
844.5138	844.4587	-0.0551	-65	189	195	EKVDLLK					Mascot
886.4418	886.4405	-0.0013	-1	11	18	DHVSGFPK					Mascot
1223.5369	1223.5114	-0.0255	-21	198	207	FGFDDAFNYK					Mascot
1223.5369	1223.5114	-0.0255	-21	198	207	FGFDDAFNYK	58	99.835			Mascot
1674.7727	1674.774	0.0013	1	44	56	NLYLSCDPYMRSR			Carbamidomethyl (C)[6]		Mascot
1690.7676	1690.7365	-0.0311	-18	44	56	NLYLSCDPYMRSR			Carbamidomethyl (C)[6], Oxidation (M)[10]		Mascot
1690.7676	1690.7365	-0.0311	-18	44	56	NLYLSCDPYMRSR			Carbamidomethyl (C)[6], Oxidation (M)[10]		Mascot

3	(+)-pulegone reductase OS=Mentha piperita PE=1 SV=1	PULR_MENPI	38175.5	6.25	4	66	85.107	1.633	58	99.835	

Peptide Information

Calc. Mass	Obsrv. Mass	± da	± ppm	Start Seq.	End Seq.	Sequence	Ion Score	C. I. %	Modification	Rank	Result Type
802.4417	802.449	0.0073	9	19	25	QSDLALR					Mascot
844.5138	844.4587	-0.0551	-65	188	194	EKVDLLK					Mascot
1004.5598	1004.494	-0.0658	-66	293	300	FLEMVLPR					Mascot
1223.5369	1223.5114	-0.0255	-21	197	206	FGFDDAFNYK					Mascot
1223.5369	1223.5114	-0.0255	-21	197	206	FGFDDAFNYK	58	99.835			Mascot

4	NADP-dependent alkenal double bond reductase P1 OS=Arabidopsis thaliana GN=P1 PE=1 SV=1	P1_ARATH	38394.2	5.81	3	64	76.933	1.555	58	99.835	

Peptide Information

Calc. Mass	Obsrv. Mass	± da	± ppm	Start Seq.	End Seq.	Sequence	Ion Score	C. I. %	Modification	Rank	Result Type
844.5138	844.4587	-0.0551	-65	191	197	EKVDLLK					Mascot
1223.5369	1223.5114	-0.0255	-21	200	209	FGFDDAFNYK					Mascot
1223.5369	1223.5114	-0.0255	-21	200	209	FGFDDAFNYK	58	99.835			Mascot
1232.6481	1232.6123	-0.0358	-29	210	220	EESDLTAALKR					Mascot

5	60 kDa SS-A/Ro ribonucleoprotein OS=Homo sapiens GN=TROVE2 PE=1 SV=2	RO60_HUMAN	61372.5	8.27	17	63	74.119	26.237			

Peptide Information

Calc. Mass	Obsrv. Mass	± da	± ppm	Start Seq.	End Seq.	Sequence	Ion Score	C. I. %	Modification	Rank	Result Type
802.4491	802.449	-0.0001	0	489	495	KMDIPAK					Mascot
810.374	810.4387	0.0647	80	79	85	SFSQEGR					Mascot
818.444	818.4371	-0.0069	-8	489	495	KMDIPAK			Oxidation (M)[2]		Mascot
820.3981	820.4104	0.0123	15	114	120	AVSEVCR			Carbamidomethyl (C)[6]		Mascot
825.4689	825.4537	-0.0152	-18	333	340	TGHGLRGK					Mascot
853.4124	853.4456	0.0332	39	532	538	NFTLDMI					Mascot
866.4288	866.4147	-0.0141	-16	134	140	DLKESMK			Oxidation (M)[6]		Mascot
917.4727	917.4637	-0.009	-10	208	214	EVHELYK					Mascot
1031.5918	1031.5171	-0.0747	-72	160	170	GGMALALAVTK					Mascot
1107.6045	1107.582	-0.0225	-20	360	369	TFKTVEPTGK					Mascot
1205.714	1205.6243	-0.0897	-74	225	234	LLKYLEAVEK					Mascot
1322.7501	1322.6819	-0.0682	-52	160	172	GGMALALAVTKYK					Mascot
1322.7501	1322.6819	-0.0682	-52	160	172	GGMALALAVTKYK					Mascot
1426.8053	1426.7145	-0.0908	-64	341	351	LKWRPDEEILK					Mascot
1550.7996	1550.7554	-0.0442	-29	371	384	FLLAVDVSASMNQR					Mascot
1561.7163	1561.7512	0.0349	22	2	14	EESVNQMQPLNEK			Oxidation (M)[7]		Mascot
1566.7944	1566.752	-0.0424	-27	371	384	FLLAVDVSASMNQR			Oxidation (M)[11]		Mascot
1706.9006	1706.7487	-0.1519	-89	370	384	RFLLAVDVSASMNQR					Mascot
2081.9631	2082.0254	0.0623	30	496	514	LIVCGMTSNGFTIADPDDR			Carbamidomethyl (C)[4]		Mascot
2097.958	2097.9773	0.0193	9	496	514	LIVCGMTSNGFTIADPDDR			Carbamidomethyl (C)[4], Oxidation (M)[6]		Mascot
2097.958	2097.9773	0.0193	9	496	514	LIVCGMTSNGFTIADPDDR	4	0	Carbamidomethyl (C)[4], Oxidation (M)[6]		Mascot
2138.095	2138.0818	-0.0132	-6	151	170	AIADWYNEKGGMALALAVTK			Oxidation (M)[12]		Mascot
2138.095	2138.0818	-0.0132	-6	151	170	AIADWYNEKGGMALALAVTK			Oxidation (M)[12]		Mascot

6	Deoxyribose-phosphate aldolase OS=Chromobacterium violaceum (strain ATCC 12472 / DSM 30191 / JCM 1249 / NBRC 12614 / NCIMB 9131 / NCTC 9757) GN=deoC PE=3 SV=1	DEOC_CHRVO	27027	5.59	11	59	25.358	5.161	

Peptide Information

Calc. Mass	Obsrv. Mass	± da	± ppm	Start Seq.	End Seq.	Sequence	Ion Score	C. I. %	Modification	Rank	Result Type
802.5185	802.449	-0.0695	-87	52	58	FVPIAKK					Mascot
817.4713	817.4036	-0.0677	-83	29	35	VAALCRK			Carbamidomethyl (C)[5]		Mascot

830.473	830.4448	-0.0282	-34	2	9	SALIEAAR					Mascot
904.4734	904.4703	-0.0031	-3	151	158	EASEISIR					Mascot
920.5087	920.4707	-0.038	-41	116	123	DIGFELVK					Mascot
1010.436	1010.4018	-0.0342	-34	189	197	EQGGQCGFK			Carbamidomethyl (C)[6]		Mascot
1145.595	1145.5896	-0.0054	-5	218	227	LLGEDWVSAR					Mascot
1626.9425	1626.939	-0.0035	-2	136	150	VIIESGELKDAALIR					Mascot
1626.9425	1626.939	-0.0035	-2	136	150	VIIESGELKDAALIR					Mascot
1633.873	1633.8252	-0.0478	-29	109	123	ALMAGNRDIGFELVK					Mascot
1674.8633	1674.774	-0.0893	-53	36	51	AKSPDGTVAAVCVFPR			Carbamidomethyl (C)[12]		Mascot
2166.0232	2166.1021	0.0789	36	10	28	RALSLMDLTTLNDDDTDE K					Mascot

7	Protein translocase subunit SecA OS=Bacillus cereus (strain ATCC 14579 / DSM 31) GN=secA PE=3 SV=1	SECA_BACCR	95245.3	5.37	22	58	23.62	6.479	

Peptide Information

Calc. Mass	Obsrv. Mass	± da	± ppm	Start Seq.	End Seq.	Sequence	Ion Score	C. I. %	Modification	Rank	Result Type
802.5219	802.449	-0.0729	-91	1	7	MIGILKK					Mascot
809.4628	809.3958	-0.067	-83	456	462	HNILNAK					Mascot
810.3992	810.4387	0.0395	49	694	699	YNEKEK					Mascot
818.5168	818.4371	-0.0797	-97	1	7	MIGILKK			Oxidation (M)[1]		Mascot
822.4719	822.4258	-0.0461	-56	43	49	GKTLEFK					Mascot
831.4318	831.4265	-0.0053	-6	671	676	EEELRR					Mascot
840.392	840.4226	0.0306	36	187	193	DNMGLYK					Mascot
849.4512	849.4354	-0.0158	-19	296	302	AHVVMHR					Mascot
1032.5472	1032.5051	-0.0421	-41	740	748	AYGQIDPLR					Mascot
1128.6372	1128.549	-0.0882	-78	501	511	NVGLAVIGTER					Mascot
1129.61	1129.574	-0.036	-32	467	477	EADIIAEAGIK					Mascot
1205.6307	1205.6243	-0.0064	-5	572	582	MVSRAVESAQK					Mascot
1236.5525	1236.5498	-0.0027	-2	614	623	QEVMESDNLR			Oxidation (M)[4]		Mascot
1261.5955	1261.4814	-0.1141	-90	810	820	GDQVGRNDLCK			Carbamidomethyl (C)[10]		Mascot
1322.6079	1322.6819	0.074	56	698	707	EKLMPEEQMR			Oxidation (M)[4,9]		Mascot
1322.6079	1322.6819	0.074	56	698	707	EKLMPEEQMR			Oxidation (M)[4,9]		Mascot
1507.8605	1507.7505	-0.11	-73	283	295	HVALLHHINQGLR					Mascot
1566.7291	1566.752	0.0229	15	700	711	LMPEEQMREFEK					Mascot
1674.885	1674.774	-0.111	-66	269	282	AEKAFHIENLFDLK					Mascot
1675.8796	1675.786	-0.0936	-56	478	494	GAVTIATNMAGRGTDIK					Mascot
2079.9871	2080.0752	0.0881	42	614	631	QEVMESDNLRGIIEGMM K					Mascot

2082.0073	2082.0254	0.0181	9	717	733	VVDTKWTEHIDAMDHLR			Oxidation (M)[13]		Mascot
2279.1013	2279.2351	0.1338	59	230	248	STELYMFANAFVRTLENE K			Oxidation (M)[6]		Mascot
2760.4336	2760.4214	-0.0122	-4	19	42	MQKTVEQIDALESSIKPLT DEQLK			Oxidation (M)[1]		Mascot

8 Protein translocase subunit SecA OS=Bacillus cereus (strain B4264) GN=secA PE=3 SV=1 | SECA_BACC4 | 95228.4 | 5.43 | 22 | 58 | 21.84 | 6.548

Peptide Information

Calc. Mass	Obsrv. Mass	± da	± ppm	Start Seq.	End Seq.	Sequence	Ion Score	C. I. %	Modification	Rank	Result Type
802.5219	802.449	-0.0729	-91	1	7	MIGILKK					Mascot
809.4628	809.3958	-0.067	-83	456	462	HNILNAK					Mascot
810.3992	810.4387	0.0395	49	694	699	YNEKEK					Mascot
818.5168	818.4371	-0.0797	-97	1	7	MIGILKK			Oxidation (M)[1]		Mascot
822.4719	822.4258	-0.0461	-56	43	49	GKTLEFK					Mascot
831.4318	831.4265	-0.0053	-6	671	676	EEELRR					Mascot
849.4512	849.4354	-0.0158	-19	296	302	AHVVMHR					Mascot
882.4389	882.449	0.0101	11	187	193	DNMVLYK					Mascot
1032.5472	1032.5051	-0.0421	-41	740	748	AYGQIDPLR					Mascot
1128.6372	1128.549	-0.0882	-78	501	511	NVGLAVIGTER					Mascot
1129.61	1129.574	-0.036	-32	467	477	EADIIAEAGIK					Mascot
1205.6307	1205.6243	-0.0064	-5	572	582	MVSRAVESAQK					Mascot
1236.5525	1236.5498	-0.0027	-2	614	623	QEVMESDNLR			Oxidation (M)[4]		Mascot
1261.5955	1261.4814	-0.1141	-90	810	820	GDQVGRNDLCK			Carbamidomethyl (C)[10]		Mascot
1322.6079	1322.6819	0.074	56	698	707	EKLMPEEQMR			Oxidation (M)[4,9]		Mascot
1322.6079	1322.6819	0.074	56	698	707	EKLMPEEQMR			Oxidation (M)[4,9]		Mascot
1507.8605	1507.7505	-0.11	-73	283	295	HVALLHHINQGLR					Mascot
1566.7291	1566.752	0.0229	15	700	711	LMPEEQMREFEK					Mascot
1674.885	1674.774	-0.111	-66	269	282	AEKAFHIENLFDLK					Mascot
1675.8796	1675.786	-0.0936	-56	478	494	GAVTIATNMAGRGTDIK					Mascot
2079.9871	2080.0752	0.0881	42	614	631	QEVMESDNLRGIIEGMM K					Mascot
2082.0073	2082.0254	0.0181	9	717	733	VVDTKWTEHIDAMDHLR			Oxidation (M)[13]		Mascot
2279.1013	2279.2351	0.1338	59	230	248	STELYMFANAFVRTLENE K			Oxidation (M)[6]		Mascot
2760.4336	2760.4214	-0.0122	-4	19	42	MQKTVEQIDALESSIKPLT DEQLK			Oxidation (M)[1]		Mascot

9 Extracellular matrix-binding protein ebh OS=Staphylococcus aureus (strain JH9) GN=ebh PE=4 SV=1 | EBH_STAA9 | 1144290.2 | 5.88 | 72 | 58 | 21.84 | 64.602

Protein Group

Extracellular matrix-binding protein ebh OS=Staphylococcus aureus (strain JH1) GN=ebh PE=4 SV=1	EBH_STAA2	1144290.2	5.8800 001144 4092

Peptide Information

Calc. Mass	Obsrv. Mass	± da	± ppm	Start Seq.	End Seq.	Sequence	Ion Score	C. I. %	Modification	Rank	Result Type
800.4625	800.3974	-0.0651	-81	9496	9503	VQGNAIAK					Mascot
802.4417	802.449	0.0073	9	3101	3107	QGLTLDR					Mascot
804.421	804.3971	-0.0239	-30	6112	6119	ASQVNSAK					Mascot
806.4003	806.4193	0.019	24	4334	4341	TQGANTSK					Mascot
810.4468	810.4387	-0.0081	-10	8512	8519	AALHGDVK					Mascot
813.3849	813.4233	0.0384	47	3011	3017	SDLDHAR					Mascot
817.4162	817.4036	-0.0126	-15	5994	6001	TALNGDAR					Mascot
818.4618	818.4371	-0.0247	-30	6540	6547	VSGVTDLK					Mascot
822.4315	822.4258	-0.0057	-7	10601	10608	SASKNTSK					Mascot
825.4101	825.4537	0.0436	53	4065	4071	QNAYTTK					Mascot
829.3798	829.4282	0.0484	58	1195	1201	QPEGDQR					Mascot
830.4366	830.4448	0.0082	10	3086	3093	VAEANQAK					Mascot
831.4683	831.4265	-0.0418	-50	10294	10300	KQEAISR					Mascot
832.4159	832.3732	-0.0427	-51	8009	8016	NSLNGEAK					Mascot
836.3745	836.4033	0.0288	34	4712	4719	QTGSNSDK					Mascot
844.4523	844.4587	0.0064	8	500	507	TQGPTVNK					Mascot
846.468	846.4697	0.0017	2	1817	1824	DVVGISTR					Mascot
856.4563	856.5088	0.0525	61	888	894	AVVYYNK					Mascot
860.4584	860.4519	-0.0065	-8	4643	4650	AQVTSAQR					Mascot
870.4468	870.5168	0.07	80	508	515	LYYAAGGR					Mascot
874.4853	874.4361	-0.0492	-56	259	265	SNRIQTR					Mascot
917.4686	917.4637	-0.0049	-5	9177	9184	QNIDQATK					Mascot
937.5465	937.4795	-0.067	-71	1755	1762	TATYKVVR					Mascot
959.5156	959.4689	-0.0467	-49	7513	7521	LADAKQDAK					Mascot
1028.5483	1028.4957	-0.0526	-51	4860	4869	AALNGAENLR					Mascot
1069.5637	1069.4946	-0.0691	-65	4270	4280	AGHVSEVTAAK					Mascot
1129.6827	1129.574	-0.1087	-96	10123	10132	VVEKELSVVK					Mascot
1145.6049	1145.5896	-0.0153	-13	9995	10004	ALKEIDEAEK					Mascot
1188.6219	1188.6204	-0.0015	-1	559	569	VEVSQGNVQTK					Mascot
1205.5645	1205.6243	0.0598	50	121	132	DSATAATTQPDK					Mascot
1210.5732	1210.5837	0.0105	9	5163	5173	NSSQTLNTAMK			Oxidation (M)[10]		Mascot

1223.5651	1223.5114	-0.0537	-44	5565	5576	ASGNYVNADANK		Mascot
1223.5651	1223.5114	-0.0537	-44	5565	5576	ASGNYVNADANK		Mascot
1232.6165	1232.6123	-0.0042	-3	248	257	TMPQRQQTSR		Mascot
1236.6252	1236.5498	-0.0754	-61	6173	6184	ATSLNTAMGNLK	Oxidation (M)[8]	Mascot
1250.678	1250.6512	-0.0268	-21	1451	1461	AQLYLTPYGPK		Mascot
1253.7001	1253.693	-0.0071	-6	885	894	QIRAVVYYNK		Mascot
1296.5815	1296.6487	0.0672	52	5691	5702	AGQNYTDASQNK		Mascot
1317.658	1317.6481	-0.0099	-8	7054	7065	ANNVDAAMDKLR		Mascot
1322.6699	1322.6819	0.012	9	10303	10313	DFSNEKINSIR		Mascot
1322.6699	1322.6819	0.012	9	10303	10313	DFSNEKINSIR		Mascot
1323.6063	1323.6782	0.0719	54	6446	6457	ASENYIDADPTK		Mascot
1396.6704	1396.7373	0.0669	48	9340	9352	GSTNYVNADTQVK		Mascot
1413.7155	1413.6869	-0.0286	-20	8188	8199	AQTLDHAMELLR	Oxidation (M)[8]	Mascot
1413.7155	1413.6869	-0.0286	-20	8188	8199	AQTLDHAMELLR	Oxidation (M)[8]	Mascot
1426.7438	1426.7145	-0.0293	-21	10549	10560	DNEHSPLLFAKR		Mascot
1435.6846	1435.6493	-0.0353	-25	6800	6811	TKAEQLDQAMER	Oxidation (M)[10]	Mascot
1507.7136	1507.7505	0.0369	24	7076	7088	QNQNYTDASPNKK		Mascot
1522.7245	1522.7584	0.0339	22	4557	4569	NGQNYLDATERNK		Mascot
1522.7245	1522.7584	0.0339	22	4557	4569	NGQNYLDATERNK		Mascot
1528.8079	1528.6652	-0.1427	-93	5854	5867	DGVETALQNVQRAK		Mascot
1550.7115	1550.7554	0.0439	28	3586	3599	DQVTEAMNQVNSAK	Oxidation (M)[7]	Mascot
1561.7704	1561.7512	-0.0192	-12	9669	9682	LKEIEASNATDQDK		Mascot
1566.7871	1566.752	-0.0351	-22	3011	3024	SDLDHARQALTPDK		Mascot
1622.8385	1622.869	0.0305	19	1015	1029	ISEDAHPIVLGNTEK		Mascot
1626.9214	1626.939	0.0176	11	2025	2039	NVEVPVKVYPVANAK		Mascot
1626.9214	1626.939	0.0176	11	2025	2039	NVEVPVKVYPVANAK		Mascot
1628.8351	1628.7987	-0.0364	-22	3086	3100	VAEANQAKDQLNTAR		Mascot
1644.8738	1644.73	-0.1438	-87	8940	8954	LAEAKALDQAMQALR	Oxidation (M)[11]	Mascot
1672.8694	1672.7924	-0.077	-46	310	324	GAPYNLPTTPWNTLK		Mascot
1674.8479	1674.774	-0.0739	-44	8814	8828	LTEAQALNQAMEALR	Oxidation (M)[11]	Mascot
1675.7379	1675.786	0.0481	29	139	153	QNESQSANKNGNDNR		Mascot
1706.8556	1706.7487	-0.1069	-63	3465	3481	ATSAVTTNKSALNGDEK		Mascot
2033.9808	2034.0166	0.0358	18	3194	3212	GVIGETTNPTMDVNTVN QK	Oxidation (M)[11]	Mascot
2033.9808	2034.0166	0.0358	18	3194	3212	GVIGETTNPTMDVNTVN QK	Oxidation (M)[11]	Mascot
2046.9285	2047.0742	0.1457	71	2791	2809	SVTTDGMTQSSIQAYEN AK	Oxidation (M)[7]	Mascot
2047.0667	2047.0742	0.0075	4	6654	6673	QSITGQIDSATQVTGVQS		Mascot

						VK					
2082.0825	2082.0254	-0.0571	-27	9742	9759	QVQALIDEIDRNPNLTDK					Mascot
2097.0684	2097.0562	-0.0122	-6	5846	5865	AQGPNTAKDGVETALQN VQR					Mascot
2098.1252	2097.9773	-0.1479	-70	7284	7303	DALKQQVQNATTVAGVN NVK					Mascot
2098.1252	2097.9773	-0.1479	-70	7284	7303	DALKQQVQNATTVAGVN NVK					Mascot
2114.0771	2113.9873	-0.0898	-42	7934	7953	QNAQNVNTAMGNLKQGI ANK					Mascot
2120.0591	2120.0352	-0.0239	-11	8274	8291	QQANNRLDQLDHLNNAQ K					Mascot
2138.1313	2138.0818	-0.0495	-23	9245	9263	QNANKQQVDQALQNILN AK					Mascot
2138.1313	2138.0818	-0.0495	-23	2004	2024	NTVGNTHKTAVVTLPSG QGTR					Mascot
2279.1514	2279.2351	0.0837	37	946	966	NTTITTLPSGWTSNLTKS DNK					Mascot
2301.114	2301.2361	0.1221	53	5029	5050	VSDANNVQHTATELNGA MTALK			Oxidation (M)[18]		Mascot
2759.4243	2759.342	-0.0823	-30	5715	5740	AIIGQTTSPSMIAQEINQA KDQVTAK			Oxidation (M)[11]		Mascot
2760.4487	2760.4214	-0.0273	-10	1309	1332	SLIYRYDATLGTQITTNDI LTMLK			Oxidation (M)[22]		Mascot

10	Protein lin-7 homolog C OS=Gallus gallus GN=LIN7C PE=1 SV=1	LIN7C_CHICK	21947.5	8.52	10	58	20.02	6.064	

Protein Group

Protein lin-7 homolog C OS=Bos taurus GN=LIN7C PE=2 SV=1	LIN7C_BOVIN	21934.5	8.5200 004577 6367
Protein lin-7 homolog C OS=Homo sapiens GN=LIN7C PE=1 SV=1	LIN7C_HUMAN	21934.5	8.5200 004577 6367
Protein lin-7 homolog C OS=Mus musculus GN=Lin7c PE=1 SV=2	LIN7C_MOUSE	21934.5	8.5200 004577 6367
Protein lin-7 homolog C OS=Pongo abelii GN=LIN7C PE=2 SV=1	LIN7C_PONAB	21934.5	8.5200 004577 6367
Protein lin-7 homolog C OS=Rattus norvegicus GN=Lin7c PE=1 SV=1	LIN7C_RAT	21934.5	8.5200 004577 6367

Peptide Information

Calc. Mass	Obsrv. Mass	± da	± ppm	Start Seq.	End Seq.	Sequence	Ion Score	C. I. %	Modification	Rank	Result Type
812.4625	812.4197	-0.0428	-53	2	9	AALGEPVR					Mascot
911.5309	911.4791	-0.0518	-57	122	130	IIPGGIADR					Mascot
959.4979	959.4689	-0.029	-30	1	9	MAALGEPVR			Oxidation (M)[1]		Mascot
1008.4666	1008.5222	0.0556	55	177	184	VLEEMESR			Oxidation (M)[5]		Mascot
1210.6903	1210.5837	-0.1066	-88	2	12	AALGEPVRLER					Mascot

1322.6522	1322.6819	0.0297	22	41	51	VLQSEFCNAVR	Carbamidomethyl (C)[7]	Mascot
1322.6522	1322.6819	0.0297	22	41	51	VLQSEFCNAVR	Carbamidomethyl (C)[7]	Mascot
1396.6777	1396.7373	0.0596	43	177	187	VLEEMESRFEK		Mascot
1550.865	1550.7554	-0.1096	-71	27	40	SGEVPPQKLQALQR		Mascot
2033.9888	2034.0166	0.0278	14	137	155	GDQLLSVNGVSVEGEHH EK		Mascot
2034.0576	2034.0166	-0.041	-20	93	111	VVELPKTEEGLGFNIMGG K	Oxidation (M)[16]	Mascot

Gel Idx/Pos	273/L1	Instr./Gel Origin	BA2151/Sample Project 20140814	Process Status	Analysis Succeeded
Plate [#] Name	[1] Sample Project 20140814	Instrument Sample Name		Spectra	11

Rank	Protein Name	Accession No.	Protein MW	Protein PI	Pep. Count	Protein Score	Protein Score C. I. %	Intensity Matched	Total Ion Score	Total Ion C. I. %	Confirmed
1	Proline--tRNA ligase OS=Metallosphaera sedula (strain ATCC 51363 / DSM 5348) GN=proS PE=3 SV=1	SYP_METS5	55153.6	6.24	17	65	84.042	5.449			

Peptide Information

Calc. Mass	Obsrv. Mass	± da	± ppm	Start Seq.	End Seq.	Sequence	Ion Score	C. I. %	Modification	Rank	Result Type
806.4519	806.3817	-0.0702	-87	419	424	KFLENR					Mascot
818.4366	818.4104	-0.0262	-32	368	374	RDTLEGK					Mascot
820.4312	820.4069	-0.0243	-30	126	132	GYSQLPR					Mascot
830.4155	830.4454	0.0299	36	200	205	RPEWDK					Mascot
837.3472	837.4008	0.0536	64	300	306	SEEDTEK					Mascot
854.473	854.4067	-0.0663	-78	79	85	KEAEHIK					Mascot
944.5523	944.4589	-0.0934	-99	48	55	QNVTTLIR					Mascot
1049.5626	1049.4989	-0.0637	-61	379	386	EELVKEFR					Mascot
1187.6056	1187.6055	-0.0001	0	80	89	EAEHIKGFEK					Mascot
1192.5692	1192.5261	-0.0431	-36	300	309	SEEDTEKINK					Mascot
1228.582	1228.6349	0.0529	43	35	45	GSGVWMPYGFK					Mascot
1228.582	1228.6349	0.0529	43	35	45	GSGVWMPYGFK					Mascot
1273.6383	1273.6464	0.0081	6	387	397	NLEDQISADLR					Mascot
1280.637	1280.6497	0.0127	10	176	186	QVDEAVEIYSK					Mascot
1280.637	1280.6497	0.0127	10	176	186	QVDEAVEIYSK					Mascot
1381.7257	1381.6913	-0.0344	-25	143	153	YETKATRPMIR			Oxidation (M)[9]		Mascot
1513.7621	1513.7412	-0.0209	-14	35	47	GSGVWMPYGFKIR			Oxidation (M)[6]		Mascot
1523.8138	1523.6859	-0.1279	-84	307	319	INKYAMEVESVLK					Mascot
2318.1047	2318.0894	-0.0153	-7	156	175	ELSTFKEAHTVHETFEDAAR					Mascot

Rank	Protein Name	Accession No.	Protein MW	Protein PI	Pep. Count	Protein Score	Protein Score C. I. %	Intensity Matched	Total Ion Score	Total Ion C. I. %	Confirmed
2	Non-structural polyprotein OS=O'nyong-nyong virus (strain SG650) PE=2 SV=1	POLN_ONNVS	283692.7	6.96	37	64	75.846	10.741			

Peptide Information

Calc. Mass	Obsrv. Mass	± da	± ppm	Start Seq.	End Seq.	Sequence	Ion Score	C. I. %	Modification	Rank	Result Type
803.353	803.4163	0.0633	79	86	92	SAEDPER					Mascot
804.3266	804.2931	-0.0335	-42	2188	2193	FTMDMK			Oxidation (M)[3,5]		Mascot

809.4879	809.4114	-0.0765	-95	969	975	ILQNPPK		Mascot
811.3549	811.4044	0.0495	61	71	76	RMMSDR	Oxidation (M)[2]	Mascot
815.3682	815.4351	0.0669	82	1261	1267	AYGYADR		Mascot
816.4937	816.4218	-0.0719	-88	761	767	KLEISAR		Mascot
818.3574	818.4104	0.053	65	585	591	TCTHSGR	Carbamidomethyl (C)[2]	Mascot
820.4709	820.4069	-0.064	-78	225	231	LSIMRGK	Oxidation (M)[4]	Mascot
821.394	821.4147	0.0207	25	1374	1379	WPESFR		Mascot
827.3498	827.4085	0.0587	71	71	76	RMMSDR	Oxidation (M)[2,3]	Mascot
834.3992	834.3798	-0.0194	-23	1478	1483	DKEWEK		Mascot
837.4465	837.4008	-0.0457	-55	1417	1423	ELASVYR		Mascot
848.4083	848.4207	0.0124	15	1010	1016	ANVCWAK	Carbamidomethyl (C)[4]	Mascot
874.4628	874.411	-0.0518	-59	2451	2458	ALADEVTR		Mascot
887.4655	887.4447	-0.0208	-23	810	817	VVLCGDPK	Carbamidomethyl (C)[4]	Mascot
888.4244	888.4429	0.0185	21	1360	1368	GVPGDGVCK	Carbamidomethyl (C)[8]	Mascot
900.5513	900.4651	-0.0862	-96	1630	1637	IEGVQKVK		Mascot
905.4332	905.4537	0.0205	23	1592	1598	TVPCLCR	Carbamidomethyl (C)[4,6]	Mascot
906.468	906.4623	-0.0057	-6	2142	2149	EFASSPIR		Mascot
919.4706	919.4503	-0.0203	-22	1159	1165	MEWLVNK		Mascot
928.4379	928.4558	0.0179	19	2188	2194	FTMDMKR		Mascot
944.4328	944.4589	0.0261	28	2188	2194	FTMDMKR	Oxidation (M)[3]	Mascot
1003.567	1003.5089	-0.0581	-58	2462	2470	TGLITELEK		Mascot
1033.579	1033.5012	-0.0778	-75	2064	2072	ATFNPSKLR		Mascot
1060.5997	1060.5491	-0.0506	-48	100	110	LASAAGKVTDK		Mascot
1107.51	1107.5377	0.0277	25	1792	1800	TINVTCDER	Carbamidomethyl (C)[6]	Mascot
1232.6304	1232.6024	-0.028	-23	1801	1811	EGKILPMASDR	Oxidation (M)[7]	Mascot
1262.6449	1262.6086	-0.0363	-29	1937	1946	CYPPKLDEIK	Carbamidomethyl (C)[1]	Mascot
1320.6464	1320.6154	-0.031	-23	401	411	DMEDEKLLGVR	Oxidation (M)[2]	Mascot
1344.7019	1344.6713	-0.0306	-23	2451	2461	ALADEVTRWQR		Mascot
1554.8163	1554.7832	-0.0331	-21	239	252	VLFSVGSTLYPESR		Mascot
1622.7261	1622.7574	0.0313	19	746	758	KENCQEISNDVMR	Carbamidomethyl (C)[4]	Mascot
1622.7843	1622.7574	-0.0269	-17	1046	1059	AYSPEVALNEICTR	Carbamidomethyl (C)[12]	Mascot
1674.7793	1674.775	-0.0043	-3	1577	1591	QKCPVDDADASFPPK	Carbamidomethyl (C)[3]	Mascot
1684.8555	1684.8519	-0.0036	-2	2073	2086	SYPKQHSYHAPTIR		Mascot
2120.0581	2120.0562	-0.0019	-1	2476	2495	YEVQGITAVITSMATFAS SK	Oxidation (M)[13]	Mascot
2176.1973	2176.1775	-0.0198	-9	1432	1453	LGVSSVAIPLLSTGVYSG GKDR		Mascot
2213.2036	2213.0464	-0.1572	-71	2087	2107	SAVPSPFQNTLQNVLAAA		Mascot

2303.1587	2303.0713	-0.0874	-38	2142	2161	TKR EFASSPIRVTTENLTMYV TK			Oxidation (M)[16]		Mascot

3	Avenin-like b5 OS=Triticum aestivum PE=2 SV=1	AVLB5_WHEAT	33763.4	8.28	4	62	68.884	4.047	52	99.263

Protein Group

Avenin-like b4 OS=Triticum aestivum PE=3 SV=1	AVLB4_WHEAT	33700.4	8.2899 999618 5303

Peptide Information

Calc. Mass	Obsrv. Mass	± da	± ppm	Start Seq.	End Seq.	Sequence	Ion Score	C. I. %	Modification	Rank	Result Type
818.4553	818.4104	-0.0449	-55	248	254	MSLQALR					Mascot
834.4502	834.3798	-0.0704	-84	248	254	MSLQALR			Oxidation (M)[1]		Mascot
1245.6587	1245.6598	0.0011	1	202	211	QLSQIPEQFR					Mascot
1245.6587	1245.6598	0.0011	1	202	211	QLSQIPEQFR	52	99.263			Mascot
1280.6455	1280.6497	0.0042	3	115	124	QERQQQAQHK					Mascot
1280.6455	1280.6497	0.0042	3	115	124	QERQQQAQHK					Mascot
1381.7006	1381.6913	-0.0093	-7	212	223	CQAIHNVAEAIR			Carbamidomethyl (C)[1]		Mascot

4	Avenin-like b10 OS=Triticum aestivum PE=3 SV=1	AVLBA_WHEAT	33381.3	8.12	4	62	68.159	3.192	52	99.263

Peptide Information

Calc. Mass	Obsrv. Mass	± da	± ppm	Start Seq.	End Seq.	Sequence	Ion Score	C. I. %	Modification	Rank	Result Type
818.4553	818.4104	-0.0449	-55	248	254	MSLQALR					Mascot
834.4502	834.3798	-0.0704	-84	248	254	MSLQALR			Oxidation (M)[1]		Mascot
1192.6289	1192.5261	-0.1028	-86	248	257	MSLQALRSMR					Mascot
1245.6587	1245.6598	0.0011	1	202	211	QLSQIPEQFR					Mascot
1245.6587	1245.6598	0.0011	1	202	211	QLSQIPEQFR	52	99.263			Mascot
1381.7006	1381.6913	-0.0093	-7	212	223	CQAIHNVAEAIR			Carbamidomethyl (C)[1]		Mascot

5	Nesprin-1 OS=Mus musculus GN=Syne1 PE=1 SV=2	SYNE1_MOUSE	1016650.4	5.43	76	62	68.159	28.925

Peptide Information

Calc. Mass	Obsrv. Mass	± da	± ppm	Start Seq.	End Seq.	Sequence	Ion Score	C. I. %	Modification	Rank	Result Type
800.4122	800.4175	0.0053	7	6	12	ASSRSHR					Mascot
803.3934	803.4163	0.0229	29	7247	7252	YKDYSK					Mascot
806.3712	806.3817	0.0105	13	5610	5615	EMEELR					Mascot
807.3995	807.4081	0.0086	11	1555	1560	FEENLR					Mascot
809.361	809.4114	0.0504	62	2408	2414	HFSGSMK			Oxidation (M)[6]		Mascot

812.4009	812.4257	0.0248	31	921	927	HVEANSR		Mascot
813.3811	813.4298	0.0487	60	356	361	VQYEMK	Oxidation (M)[5]	Mascot
814.4682	814.4454	-0.0228	-28	4073	4078	QVKHFR		Mascot
815.437	815.4351	-0.0019	-2	4079	4085	ALQEQAR		Mascot
816.4574	816.4218	-0.0356	-44	596	602	NLSVEVR		Mascot
818.4254	818.4104	-0.015	-18	5232	5239	LPGSSTEK		Mascot
819.4393	819.3972	-0.0421	-51	2616	2622	SCQLALK	Carbamidomethyl (C)[2]	Mascot
820.4233	820.4069	-0.0164	-20	195	201	QMGIEVK	Oxidation (M)[2]	Mascot
821.3934	821.4147	0.0213	26	8526	8532	LSQMNGR	Oxidation (M)[4]	Mascot
830.4479	830.4454	-0.0025	-3	8693	8699	STPNRQK		Mascot
834.4501	834.3798	-0.0703	-84	5073	5079	MASLEKR		Mascot
837.4828	837.4008	-0.082	-98	3852	3858	AQLSKYK		Mascot
850.4451	850.4283	-0.0168	-20	5073	5079	MASLEKR	Oxidation (M)[1]	Mascot
854.389	854.4067	0.0177	21	2802	2808	TQDESFK		Mascot
856.4271	856.4946	0.0675	79	8731	8737	SDPRPER		Mascot
863.4985	863.4218	-0.0767	-89	7301	7307	DSLFILR		Mascot
866.4586	866.4195	-0.0391	-45	6063	6069	MSTIRMK		Mascot
872.4584	872.428	-0.0304	-35	6090	6096	QEQALQR		Mascot
874.4628	874.411	-0.0518	-59	7058	7065	NSVENALK		Mascot
887.4556	887.4447	-0.0109	-12	8785	8791	SFHPMLR		Mascot
888.4421	888.4429	0.0008	1	5195	5202	AVAQDQEK		Mascot
900.5513	900.4651	-0.0862	-96	3321	3328	LEALLSVR		Mascot
905.4873	905.4537	-0.0336	-37	8437	8443	MKQNLQK	Oxidation (M)[1]	Mascot
906.5295	906.4623	-0.0672	-74	5122	5129	LSEFAVLK		Mascot
919.4883	919.4503	-0.038	-41	1178	1185	GESLSWLK		Mascot
928.4734	928.4558	-0.0176	-19	954	961	GDPEELLR		Mascot
990.4738	990.4847	0.0109	11	1991	1998	SIEDIEER		Mascot
999.449	999.4688	0.0198	20	8518	8525	ESHDLQDR		Mascot
1033.4871	1033.5012	0.0141	14	3010	3018	AEPMTEDLK		Mascot
1049.4819	1049.4989	0.017	16	3010	3018	AEPMTEDLK	Oxidation (M)[4]	Mascot
1057.5889	1057.5293	-0.0596	-56	6624	6632	EEIQQLLGK		Mascot
1060.5521	1060.5491	-0.003	-3	6538	6547	DLEGGISELK		Mascot
1081.5538	1081.5199	-0.0339	-31	3154	3162	SFHANLHQK		Mascot
1091.5038	1091.5485	0.0447	41	1672	1681	CEAIASSPEK	Carbamidomethyl (C)[1]	Mascot
1107.5826	1107.5377	-0.0449	-41	4493	4502	TCKTAQASLK	Carbamidomethyl (C)[2]	Mascot
1111.563	1111.5903	0.0273	25	2139	2148	ELDSFTSKGK		Mascot

1177.567	1177.5564	-0.0106	-9	8536	8544	VCSLLEDWR			Carbamidomethyl (C)[2]	Mascot
1228.6394	1228.6349	-0.0045	-4	503	511	MEFLELKYR				Mascot
1228.6394	1228.6349	-0.0045	-4	503	511	MEFLELKYR				Mascot
1240.5184	1240.6106	0.0922	74	6431	6440	EMSEEMDKNK				Mascot
1245.662	1245.6598	-0.0022	-2	4409	4419	VMADLGLNERK				Mascot
1245.662	1245.6598	-0.0022	-2	4409	4419	VMADLGLNERK				Mascot
1259.5917	1259.6522	0.0605	48	3575	3583	QDWQVYQHR				Mascot
1262.606	1262.6086	0.0026	2	8526	8535	LSQMNGRWDR				Mascot
1280.7111	1280.6497	-0.0614	-48	890	899	KPWDHTKLQK				Mascot
1280.7111	1280.6497	-0.0614	-48	890	899	KPWDHTKLQK				Mascot
1320.6616	1320.6154	-0.0462	-35	8423	8433	WELLQAQAMSK			Oxidation (M)[9]	Mascot
1324.6281	1324.6381	0.01	8	1353	1363	YLFQTGSSHER				Mascot
1324.6281	1324.6381	0.01	8	1353	1363	YLFQTGSSHER				Mascot
1331.743	1331.6564	-0.0866	-65	8350	8360	FQIQQTANILR				Mascot
1333.6958	1333.6558	-0.04	-30	1315	1326	LESTLTGLEQSR				Mascot
1340.6151	1340.6417	0.0266	20	6993	7004	TDFAEQLGAMNK			Oxidation (M)[10]	Mascot
1344.7019	1344.6713	-0.0306	-23	3235	3246	ARQLWEGQAASK				Mascot
1353.7096	1353.6858	-0.0238	-18	7869	7879	WQHLLDLMAAR				Mascot
1360.6281	1360.7036	0.0755	55	1530	1539	YWEELQEHAR	7	0		Mascot
1360.7695	1360.7036	-0.0659	-48	364	374	QVEHIIQPLQR				Mascot
1364.7242	1364.6757	-0.0485	-36	2558	2569	VEMFLGELLAAR			Oxidation (M)[3]	Mascot
1381.6594	1381.6913	0.0319	23	5799	5810	GYQEQIDSLNSK				Mascot
1404.7086	1404.7167	0.0081	6	5684	5695	MQAVQLCQSALR			Carbamidomethyl (C)[7]	Mascot
1427.7853	1427.7773	-0.008	-6	4298	4309	QKLIEQLGLDDR				Mascot
1430.6945	1430.6456	-0.0489	-34	8705	8718	CSLSQPGPSVSSPK			Carbamidomethyl (C)[1]	Mascot
1486.7319	1486.7845	0.0526	35	1165	1176	HMVDEIRNDITK			Oxidation (M)[2]	Mascot
1507.7462	1507.745	-0.0012	-1	7727	7738	WTDDLTELMLVR			Oxidation (M)[9]	Mascot
1513.8485	1513.7412	-0.1073	-71	6728	6739	LVERTNLYQHLK				Mascot
1523.7013	1523.6859	-0.0154	-10	3501	3512	AFESWLEQEQEK				Mascot
1554.7833	1554.7832	-0.0001	0	2817	2829	TQFQDIMTVAKEK			Oxidation (M)[7]	Mascot
1603.7567	1603.8362	0.0795	50	1096	1109	DTCGACHTALKELK			Carbamidomethyl (C)[3,6]	Mascot
1622.7843	1622.7574	-0.0269	-17	2401	2414	IQDSLEKHFSGSMK			Oxidation (M)[13]	Mascot
1622.7843	1622.7574	-0.0269	-17	2401	2414	IQDSLEKHFSGSMK			Oxidation (M)[13]	Mascot
1714.8494	1714.8402	-0.0092	-5	4798	4812	EQAKVNEETLPAEEK				Mascot
1822.9407	1822.8926	-0.0481	-26	7050	7065	EQHLLGDRNSVENALK				Mascot
1822.9407	1822.8926	-0.0481	-26	7050	7065	EQHLLGDRNSVENALK				Mascot
1835.9175	1835.9231	0.0056	3	6840	6855	DHLSAFLEFSKEVDAK				Mascot

1851.9408	1851.9221	-0.0187	-10	4279	4294	LALALQEEMYAIDDLK			Oxidation (M)[9]		Mascot
1905.96	1906.0371	0.0771	40	5754	5768	QEMKHLQQLIEEAHR			Oxidation (M)[3]		Mascot
1905.96	1906.0371	0.0771	40	5754	5768	QEMKHLQQLIEEAHR			Oxidation (M)[3]		Mascot
2119.9709	2120.0562	0.0853	40	5977	5994	YQDSLQSISTKMEAMEM K					Mascot
2175.071	2175.1843	0.1133	52	8323	8343	GAVGLSGDPSSLESQMR QLDK					Mascot
2176.1438	2176.1775	0.0337	15	5736	5753	HSLIFPPKEAVVQYEQYK					Mascot
2318.1545	2318.0894	-0.0651	-28	6597	6617	ALQDLVDLLDTGQEKMT GDQK					Mascot

6 Dynein heavy chain 5, axonemal OS=Homo sapiens GN=DNAH5 PE=1 SV=3 DYH5_HUMAN 532504 5.79 57 60 49.536 19.73

Peptide Information

Calc. Mass	Obsrv. Mass	± da	± ppm	Start Seq.	End Seq.	Sequence	Ion Score	C. I. %	Modification	Rank	Result Type
800.4454	800.4175	-0.0279	-35	4440	4445	IPAWWK					Mascot
806.4229	806.3817	-0.0412	-51	2773	2778	LWQMTK					Mascot
807.3843	807.4081	0.0238	29	1877	1883	DLSSTER					Mascot
811.3325	811.4044	0.0719	89	253	258	IEDCMK			Carbamidomethyl (C)[4], Oxidation (M)[5]		Mascot
813.4828	813.4298	-0.053	-65	1647	1653	QLPKEAK					Mascot
814.4893	814.4454	-0.0439	-54	30	36	RALLDAR					Mascot
815.3756	815.4351	0.0595	73	1600	1605	YNMPFK			Oxidation (M)[3]		Mascot
816.4573	816.4218	-0.0355	-43	976	982	NTLEAIR					Mascot
818.4447	818.4104	-0.0343	-42	2134	2139	FFTLYK					Mascot
819.3843	819.3972	0.0129	16	1841	1847	DSEEALR					Mascot
820.3406	820.4069	0.0663	81	4002	4007	SWCPDR			Carbamidomethyl (C)[3]		Mascot
823.3945	823.4	0.0055	7	2754	2760	GFSEEVR					Mascot
829.493	829.4117	-0.0813	-98	2504	2509	LELWLR					Mascot
836.405	836.4023	-0.0027	-3	473	478	FETFHR					Mascot
846.4567	846.4349	-0.0218	-26	3223	3229	ELEAKEK					Mascot
848.4559	848.4207	-0.0352	-41	1711	1716	RLCFPR			Carbamidomethyl (C)[3]		Mascot
851.408	851.438	0.03	35	3965	3970	NEKMWK			Oxidation (M)[4]		Mascot
856.525	856.4946	-0.0304	-35	1408	1414	QLNLLQK					Mascot
874.4781	874.411	-0.0671	-77	1751	1757	SVKFHEK					Mascot
905.4575	905.4537	-0.0038	-4	3791	3798	TAEEVTQK					Mascot
919.4996	919.4503	-0.0493	-54	4236	4243	GVSWTTIR					Mascot
928.5403	928.4558	-0.0845	-91	4440	4446	IPAWWKK					Mascot
944.5523	944.4589	-0.0934	-99	976	983	NTLEAIRK					Mascot

1031.5732	1031.5179	-0.0553	-54	3266	3275	AQAIVDSISK		Mascot
1033.571	1033.5012	-0.0698	-68	3203	3211	MNTGLEKLK		Mascot
1037.4755	1037.5135	0.038	37	2103	2111	SVAMMVPDR	Oxidation (M)[4,5]	Mascot
1049.5262	1049.4989	-0.0273	-26	3683	3691	EVDVLDGFR		Mascot
1057.4871	1057.5293	0.0422	40	4015	4023	YIVDSMGEK	Oxidation (M)[6]	Mascot
1091.5579	1091.5485	-0.0094	-9	3212	3222	EASESVAALSK		Mascot
1107.5582	1107.5377	-0.0205	-19	1754	1761	FHEKIYDR		Mascot
1187.6816	1187.6055	-0.0761	-64	4549	4558	NMKLIESKPK		Mascot
1192.5126	1192.5261	0.0135	11	2607	2615	YDPECHMIK	Carbamidomethyl (C)[5]	Mascot
1212.6008	1212.574	-0.0268	-22	522	530	EYNFLDQRK		Mascot
1212.6008	1212.574	-0.0268	-22	521	529	KEYNFLDQR		Mascot
1228.7261	1228.6349	-0.0912	-74	2761	2771	DSVTKLVPLTR		Mascot
1228.7261	1228.6349	-0.0912	-74	2761	2771	DSVTKLVPLTR		Mascot
1232.6919	1232.6024	-0.0895	-73	226	235	CDILELKTLK	Carbamidomethyl (C)[1]	Mascot
1245.5491	1245.6598	0.1107	89	1133	1142	EVITSMDCFK	Carbamidomethyl (C)[8], Oxidation (M)[6]	Mascot
1245.5615	1245.6598	0.0983	79	1490	1498	AMMERHWER		Mascot
1259.5872	1259.6522	0.065	52	2256	2268	HGMMTLGPSGAGK	Oxidation (M)[3]	Mascot
1280.6589	1280.6497	-0.0092	-7	3238	3248	ADMVLKEVTMK	Oxidation (M)[3]	Mascot
1280.6589	1280.6497	-0.0092	-7	3238	3248	ADMVLKEVTMK	Oxidation (M)[3]	Mascot
1302.6147	1302.6412	0.0265	20	4244	4254	YMIGEIQYGGR	Oxidation (M)[2]	Mascot
1353.6831	1353.6858	0.0027	2	3835	3845	LVNEMYQTSLR		Mascot
1367.6663	1367.6431	-0.0232	-17	2470	2482	EQGGEVSQAHLGR		Mascot
1414.7107	1414.7664	0.0557	39	3744	3755	THLMEDVTANKR		Mascot
1427.804	1427.7773	-0.0267	-19	1037	1049	AVECIISVPKGVR	Carbamidomethyl (C)[4]	Mascot
1430.6469	1430.6456	-0.0013	-1	2985	2996	SYNTSNLMEDLK	Oxidation (M)[8]	Mascot
1447.7944	1447.689	-0.1054	-73	1459	1470	ALKDWQAFLDLK		Mascot
1486.7406	1486.7845	0.0439	30	1212	1223	AWMVVIGRHCNK	Carbamidomethyl (C)[10], Oxidation (M)[3]	Mascot
1622.769	1622.7574	-0.0116	-7	329	342	EMDIRITDATNEAK	Oxidation (M)[2]	Mascot
1622.769	1622.7574	-0.0116	-7	329	342	EMDIRITDATNEAK	Oxidation (M)[2]	Mascot
1714.9275	1714.8402	-0.0873	-51	1299	1312	VDTLHYAWEKLLAR		Mascot
1822.8739	1822.8926	0.0187	10	3043	3058	DEIDEINSDLASVMKK	Oxidation (M)[14]	Mascot
1823.0498	1822.8926	-0.1572	-86	2944	2962	VIRTPQGNALLVGVGGS GK		Mascot
1835.9659	1835.9231	-0.0428	-23	4380	4394	LQKMGPFQPMNIFLR	Oxidation (M)[4]	Mascot
1851.9608	1851.9221	-0.0387	-21	4380	4394	LQKMGPFQPMNIFLR	Oxidation (M)[4,10]	Mascot
1905.8608	1906.0371	0.1763	93	1227	1241	SEMENIFMLIEEFNK	Oxidation (M)[3,8]	Mascot
1905.8608	1906.0371	0.1763	93	1227	1241	SEMENIFMLIEEFNK	Oxidation (M)[3,8]	Mascot
2116.9956	2117.0945	0.0989	47	492	510	TYSVLQDSTIEGLEDMAT	Oxidation (M)[16]	Mascot

Calc. Mass	Obsrv. Mass	± da	± ppm	Start Seq.	End Seq.	Sequence	Ion Score	C. I. %	Modification	Rank	Result Type
						K					
2318.0623	2318.0894	0.0271	12	2269	2287	TTCIHTLMRAMTDCGKPHR			Carbamidomethyl (C)[3,14], Oxidation (M)[8,11]		Mascot
2426.1016	2426.1196	0.018	7	3059	3077	EFPRCLPTNENLHDYFMSR			Carbamidomethyl (C)[5]		Mascot

7	Elongation factor Ts OS=Cytophaga hutchinsonii (strain ATCC 33406 / NCIMB 9469) GN=tsf PE=3 SV=1	EFTS_CYTH3	29717.3	6.04	13	60	47.158	4.447

Peptide Information

Calc. Mass	Obsrv. Mass	± da	± ppm	Start Seq.	End Seq.	Sequence	Ion Score	C. I. %	Modification	Rank	Result Type
812.4665	812.4257	-0.0408	-50	234	239	LNKFYK					Mascot
816.4686	816.4218	-0.0468	-57	45	52	GQKVSAAR					Mascot
830.5168	830.4454	-0.0714	-86	162	169	LGVMVALK					Mascot
846.5117	846.4349	-0.0768	-91	162	169	LGVMVALK			Oxidation (M)[4]		Mascot
856.5614	856.4946	-0.0668	-78	229	236	IALGKLNK					Mascot
1033.5525	1033.5012	-0.0513	-50	2	11	STITAAEVNK					Mascot
1262.6199	1262.6086	-0.0113	-9	255	265	SISQMLDGFHK					Mascot
1302.7377	1302.6412	-0.0965	-74	2	13	STITAAEVNKLR					Mascot
1353.7373	1353.6858	-0.0515	-38	123	134	TVQEHIIDLTGK					Mascot
1471.6525	1471.7223	0.0698	47	12	24	LRTMTGAGMMDCK			Carbamidomethyl (C)[12]		Mascot
1674.8553	1674.775	-0.0803	-48	182	197	DVAMQAAAMKPVALDK			Oxidation (M)[4]		Mascot
1684.9302	1684.8519	-0.0783	-46	218	233	AEGKPEAMLEKIALGK					Mascot
1905.9917	1906.0371	0.0454	24	26	43	ALTESGGDFEAAIDILRK					Mascot
1905.9917	1906.0371	0.0454	24	25	42	KALTESGGDFEAAIDILR					Mascot

8	Protein URA2 OS=Saccharomyces cerevisiae (strain ATCC 204508 / S288c) GN=URA2 PE=1 SV=5	PYR1_YEAST	246198.3	5.57	31	60	45.927	11.056

Peptide Information

Calc. Mass	Obsrv. Mass	± da	± ppm	Start Seq.	End Seq.	Sequence	Ion Score	C. I. %	Modification	Rank	Result Type
806.3825	806.3817	-0.0008	-1	153	160	DAGSMLGR					Mascot
814.3802	814.4454	0.0652	80	1497	1503	DAQTSHR					Mascot
815.3682	815.4351	0.0669	82	98	103	YFESNR					Mascot
818.444	818.4104	-0.0336	-41	1734	1741	VDVGMGIK					Mascot
821.3974	821.4147	0.0173	21	872	877	VWEMTR					Mascot
825.4505	825.409	-0.0415	-50	1269	1275	SFPFISK					Mascot
834.439	834.3798	-0.0592	-71	1734	1741	VDVGMGIK			Oxidation (M)[5]		Mascot
837.3923	837.4008	0.0085	10	872	877	VWEMTR			Oxidation (M)[4]		Mascot
850.4053	850.4283	0.023	27	915	921	QLGFDDR					Mascot

872.4407	872.428	-0.0127	-15	2073	2079	TVHSLCR			Carbamidomethyl (C)[6]	Mascot
888.4283	888.4429	0.0146	16	1383	1389	LYNMGYK				Mascot
1031.5732	1031.5179	-0.0553	-54	1374	1382	QELLSSVQK				Mascot
1033.4983	1033.5012	0.0029	3	1181	1189	EIEMDAVAR				Mascot
1049.4932	1049.4989	0.0057	5	1181	1189	EIEMDAVAR			Oxidation (M)[4]	Mascot
1053.5615	1053.4933	-0.0682	-65	940	948	EYGITPFVK				Mascot
1057.6252	1057.5293	-0.0959	-91	1756	1764	LTIDDIVLR				Mascot
1091.5692	1091.5485	-0.0207	-19	161	170	LSLEKSGSDR				Mascot
1107.6409	1107.5377	-0.1032	-93	1362	1371	NILLSIGSYK				Mascot
1141.6187	1141.5264	-0.0923	-81	2194	2202	QMKYGLFVR				Mascot
1177.5961	1177.5564	-0.0397	-34	912	921	QAKQLGFDDR				Mascot
1212.6266	1212.574	-0.0526	-43	150	160	HLRDAGSMLGR				Mascot
1212.6266	1212.574	-0.0526	-43	150	160	HLRDAGSMLGR				Mascot
1228.6216	1228.6349	0.0133	11	150	160	HLRDAGSMLGR			Oxidation (M)[8]	Mascot
1228.6216	1228.6349	0.0133	11	150	160	HLRDAGSMLGR			Oxidation (M)[8]	Mascot
1273.7296	1273.6464	-0.0832	-65	1477	1487	CAKLLIEAISR			Carbamidomethyl (C)[1]	Mascot
1324.65	1324.6381	-0.0119	-9	2165	2175	ENMAIMHPLPR			Oxidation (M)[3]	Mascot
1324.65	1324.6381	-0.0119	-9	2165	2175	ENMAIMHPLPR	4	0	Oxidation (M)[3]	Mascot
1331.7604	1331.6564	-0.104	-78	1661	1672	EDLALIMTVKAK				Mascot
1340.645	1340.6417	-0.0033	-2	2165	2175	ENMAIMHPLPR			Oxidation (M)[3,6]	Mascot
1353.6797	1353.6858	0.0061	5	2140	2150	FNSPEEYARLK				Mascot
1364.7573	1364.6757	-0.0816	-60	1242	1253	ITGPYNIQFIAK				Mascot
1430.7386	1430.6456	-0.093	-65	2024	2037	YSPVPIINGGNGSR				Mascot
1471.7064	1471.7223	0.0159	11	28	41	DGTVLQGYSFGAEK				Mascot
1486.6996	1486.7845	0.0849	57	866	877	GYSVDKVWEMTR			Oxidation (M)[10]	Mascot
1554.8679	1554.7832	-0.0847	-54	495	507	VYFVPVTAEFVRK				Mascot
1603.873	1603.8362	-0.0368	-23	1344	1357	YEAYLKSLLATGFK				Mascot
1655.7992	1655.8416	0.0424	26	1963	1977	TCSSFIAAMERLGGR			Carbamidomethyl (C)[2]	Mascot
1714.9698	1714.8402	-0.1296	-76	1635	1650	TADLASVLLLTSLQNR				Mascot
2318.3125	2318.0894	-0.2231	-96	1883	1903	LMSSRPPRELVAPGAIQN LIR				Mascot

9	Avenin-like b6 OS=Triticum aestivum PE=3 SV=1	AVLB6_WHEAT	33385.3	7.83	3	59	33.475	2.903	52	99.263

Protein Group

Avenin-like b1 OS=Triticum aestivum GN=AVNLB PE=1 SV=1	AVLB1_WHEAT	33788.2	8.0799 999237 0605
Avenin-like b2 OS=Triticum aestivum PE=2 SV=1	AVLB2_WHEAT	33575.2	7.8200 001716

			6138
Avenin-like b3 OS=Triticum aestivum PE=2 SV=1	AVLB3_WHEAT	33442.3	7.8299 999237 0605
Avenin-like b7 OS=Triticum aestivum PE=3 SV=1	AVLB7_WHEAT	33357.2	7.8299 999237 0605
Avenin-like b8 OS=Triticum aestivum PE=3 SV=1	AVLB8_WHEAT	33355.3	7.8299 999237 0605
Avenin-like b9 OS=Triticum aestivum PE=3 SV=1	AVLB9_WHEAT	33411.3	7.8200 001716 6138

Peptide Information

Calc. Mass	Obsrv. Mass	± da	± ppm	Start Seq.	End Seq.	Sequence	Ion Score	C. I. %	Modification	Rank	Result Type
818.4553	818.4104	-0.0449	-55	248	254	MSLQALR					Mascot
834.4502	834.3798	-0.0704	-84	248	254	MSLQALR			Oxidation (M)[1]		Mascot
1245.6587	1245.6598	0.0011	1	202	211	QLSQIPEQFR					Mascot
1245.6587	1245.6598	0.0011	1	202	211	QLSQIPEQFR	52	99.263			Mascot
1381.7006	1381.6913	-0.0093	-7	212	223	CQAIHNVAEAIR			Carbamidomethyl (C)[1]		Mascot

10	Putative DNA helicase ino80 OS=Aspergillus clavatus (strain ATCC 1007 / CBS 513.65 / DSM 816 / NCTC 3887 / NRRL 1) GN=ino80 PE=3 SV=1	INO80_ASPCL	192274.5	9.19	30	59	33.475	11.266	

Peptide Information

Calc. Mass	Obsrv. Mass	± da	± ppm	Start Seq.	End Seq.	Sequence	Ion Score	C. I. %	Modification	Rank	Result Type
806.4366	806.3817	-0.0549	-68	1691	1697	AKTTTER					Mascot
815.4733	815.4351	-0.0382	-47	268	274	KPSAEKR					Mascot
832.3907	832.3245	-0.0662	-80	663	668	NEREER					Mascot
846.5043	846.4349	-0.0694	-82	622	629	KTAQLASK					Mascot
866.473	866.4195	-0.0535	-62	1181	1188	LLFSSSGR					Mascot
887.4581	887.4447	-0.0134	-15	1045	1051	LNEDQLR					Mascot
888.501	888.4429	-0.0581	-65	302	309	VSRASNVR					Mascot
900.4573	900.4651	0.0078	9	1091	1097	AYYTNLR					Mascot
990.4059	990.4847	0.0788	80	655	661	EMMSFWK			Oxidation (M)[2,3]		Mascot
1003.4915	1003.5089	0.0174	17	276	283	RNVDQESR					Mascot
1031.5116	1031.5179	0.0063	6	443	452	ANKANDAAEK					Mascot
1037.5303	1037.5135	-0.0168	-16	18	26	YPVYSPPSK					Mascot
1053.5323	1053.4933	-0.039	-37	1068	1076	HVQQELGDK					Mascot
1057.5161	1057.5293	0.0132	12	1189	1198	LDVAGPDNEK					Mascot

1187.6743	1187.6055	-0.0688	-58	1542	1551	LITRGTIEER		Mascot
1232.6052	1232.6024	-0.0028	-2	777	788	QAAMANAQNAVK	Oxidation (M)[4]	Mascot
1245.6685	1245.6598	-0.0087	-7	673	683	LAEKQEIESAK		Mascot
1245.6838	1245.6598	-0.024	-19	254	264	EPEPLPTPLPR		Mascot
1280.6569	1280.6497	-0.0072	-6	983	992	WKNLLGFSCR	Carbamidomethyl (C)[9]	Mascot
1280.6569	1280.6497	-0.0072	-6	983	992	WKNLLGFSCR	Carbamidomethyl (C)[9]	Mascot
1320.7383	1320.6154	-0.1229	-93	1531	1541	LGQTRQVTVYR		Mascot
1333.7145	1333.6558	-0.0587	-44	1098	1108	NRVSIMDLIEK	Oxidation (M)[6]	Mascot
1367.6372	1367.6431	0.0059	4	380	391	TFEKQANAGMDR		Mascot
1404.7053	1404.7167	0.0114	8	1403	1413	YTHIEVPSMRR	Oxidation (M)[9]	Mascot
1414.8062	1414.7664	-0.0398	-28	1053	1063	LHMILKPFMLR	Oxidation (M)[3]	Mascot
1430.7162	1430.6456	-0.0706	-49	572	584	EPTPQPLSAFESK		Mascot
1554.9124	1554.7832	-0.1292	-83	1053	1064	LHMILKPFMLRR		Mascot
1714.8145	1714.8402	0.0257	15	2	17	TGGPPYNSQSPTQQPR		Mascot
1835.9724	1835.9231	-0.0493	-27	962	976	VKWQYMILDEAQAIK		Mascot
1851.9674	1851.9221	-0.0453	-24	962	976	VKWQYMILDEAQAIK	Oxidation (M)[6]	Mascot
2117.2368	2117.0945	-0.1423	-67	233	253	KATPPPPTAPVPVPAPLP EIK		Mascot
2174.9797	2175.1843	0.2046	94	718	740	GAEADASGDAAVDGSDE TVRPGK		Mascot

Gel Idx/Pos	274/L2	Instr./Gel Origin	BA2151/Sample Project 20140814	Process Status	Analysis Succeeded
Plate [#] Name	[1] Sample Project 20140814	Instrument Sample Name		Spectra	11

Rank	Protein Name	Accession No.	Protein MW	Protein PI	Pep. Count	Protein Score	Protein Score C. I. %	Intensity Matched	Total Ion Score	Total Ion C. I. %	Confirmed
1	Guanine nucleotide-binding protein subunit beta-like protein OS=Brassica napus GN=GB1 PE=2 SV=1	GBLP_BRANA	36157.3	8.05	3	107	99.999	3.282	99	100	

Peptide Information

Calc. Mass	Obsrv. Mass	± da	± ppm	Start Seq.	End Seq.	Sequence	Ion Score	C. I. %	Modification	Rank	Result Type
1335.6903	1335.699	0.0087	7	107	118	DVLSVAFSLDNR					Mascot
1335.6903	1335.699	0.0087	7	107	118	DVLSVAFSLDNR	99	100			Mascot
1422.6707	1422.6573	-0.0134	-9	278	292	AEAEKSDGSGTAATK					Mascot
2193.1221	2193.0671	-0.055	-25	158	177	FSPNTLQPTIVSASCDKTVK			Carbamidomethyl (C)[15]		Mascot

Rank	Protein Name	Accession No.	Protein MW	Protein PI	Pep. Count	Protein Score	Protein Score C. I. %	Intensity Matched	Total Ion Score	Total Ion C. I. %	Confirmed
2	Guanine nucleotide-binding protein subunit beta-like protein OS=Medicago sativa GN=GB1 PE=1 SV=1	GBLP_MEDSA	36043.2	7.07	2	105	99.998	3.202	99	100	

Peptide Information

Calc. Mass	Obsrv. Mass	± da	± ppm	Start Seq.	End Seq.	Sequence	Ion Score	C. I. %	Modification	Rank	Result Type
1335.6903	1335.699	0.0087	7	107	118	DVLSVAFSIDNR					Mascot
1335.6903	1335.699	0.0087	7	107	118	DVLSVAFSIDNR	99	100			Mascot
2134.075	2134.0605	-0.0145	-7	228	246	LYSLDAGSIIHALCFSPNR			Carbamidomethyl (C)[14]		Mascot

Rank	Protein Name	Accession No.	Protein MW	Protein PI	Pep. Count	Protein Score	Protein Score C. I. %	Intensity Matched	Total Ion Score	Total Ion C. I. %	Confirmed
3	Guanine nucleotide-binding protein subunit beta-like protein OS=Glycine max PE=1 SV=1	GBLP_SOYBN	35985.2	7.62	2	104	99.998	3.159	99	100	

Peptide Information

Calc. Mass	Obsrv. Mass	± da	± ppm	Start Seq.	End Seq.	Sequence	Ion Score	C. I. %	Modification	Rank	Result Type
1335.6903	1335.699	0.0087	7	107	118	DVLSVAFSIDNR					Mascot
1335.6903	1335.699	0.0087	7	107	118	DVLSVAFSIDNR	99	100			Mascot
1747.8457	1747.854	0.0083	5	273	290	VDLKTEADATSGGGNANK					Mascot

Rank	Protein Name	Accession No.	Protein MW	Protein PI	Pep. Count	Protein Score	Protein Score C. I. %	Intensity Matched	Total Ion Score	Total Ion C. I. %	Confirmed
4	Guanine nucleotide-binding protein subunit beta-like protein A OS=Arabidopsis thaliana GN=RACK1A PE=1 SV=2	GBLPA_ARATH	36124.4	7.62	1	99	99.994	3.06	99	100	

Peptide Information

Calc. Mass	Obsrv. Mass	± da	± ppm	Start Seq.	End Seq.	Sequence	Ion Score	C. I. %	Modification	Rank	Result Type

1335.6903	1335.699	0.0087	7	107	118	DVLSVAFSLDNR					Mascot
1335.6903	1335.699	0.0087	7	107	118	DVLSVAFSLDNR	99	100			Mascot

5 Nesprin-1 OS=Homo sapiens GN=SYNE1 PE=1 SV=4 SYNE1_HUMAN 1017127.1 5.37 71 64 78.473 58.396

Peptide Information

Calc. Mass	Obsrv. Mass	± da	± ppm	Start Seq.	End Seq.	Sequence	Ion Score	C. I. %	Modification	Rank	Result Type
872.4584	872.4738	0.0154	18	6081	6087	QEQALQR					Mascot
922.412	922.4426	0.0306	33	5304	5310	CLNMQEK			Carbamidomethyl (C)[1]		Mascot
1066.5238	1066.4919	-0.0319	-30	756	764	VPVMDAQYK			Oxidation (M)[4]		Mascot
1075.5742	1075.561	-0.0132	-12	2572	2580	ESLDKLSQR					Mascot
1093.567	1093.5804	0.0134	12	4495	4504	TCKSAQASLK			Carbamidomethyl (C)[2]		Mascot
1152.6008	1152.5258	-0.075	-65	1737	1745	LHLEQLDER					Mascot
1222.5732	1222.5833	0.0101	8	1889	1899	QTVEATNSMNK					Mascot
1248.6803	1248.6611	-0.0192	-15	3334	3343	EIQMKMIVTR					Mascot
1252.6276	1252.6643	0.0367	29	3735	3745	IDKVDTVMMGK			Oxidation (M)[8]		Mascot
1262.6376	1262.6654	0.0278	22	2063	2072	LEVTWDDTKR					Mascot
1264.6752	1264.6726	-0.0026	-2	3334	3343	EIQMKMIVTR			Oxidation (M)[4]		Mascot
1276.6566	1276.6672	0.0106	8	5612	5621	ETEELRQMIK					Mascot
1279.61	1279.6604	0.0504	39	3789	3798	EIHDHMEQLK					Mascot
1280.6702	1280.663	-0.0072	-6	3334	3343	EIQMKMIVTR			Oxidation (M)[4,6]		Mascot
1280.6958	1280.663	-0.0328	-26	1818	1829	GELQSLQGHLAK					Mascot
1294.675	1294.6664	-0.0086	-7	1107	1117	ELRAAIDSTYR					Mascot
1302.5922	1302.6322	0.04	31	8722	8735	GGSDSSLSEPGPGR					Mascot
1320.6583	1320.689	0.0307	23	6837	6847	DHLNAFLEFSK					Mascot
1335.6726	1335.699	0.0264	20	3368	3379	DMWASLLSAGIR			Oxidation (M)[2]		Mascot
1335.6726	1335.699	0.0264	20	3368	3379	DMWASLLSAGIR			Oxidation (M)[2]		Mascot
1401.7737	1401.7839	0.0102	7	7002	7013	SWQILQGLVTEK					Mascot
1403.7562	1403.7765	0.0203	14	3165	3176	ESALENLKIQMK					Mascot
1430.7784	1430.7469	-0.0315	-22	7834	7845	IQLQQMGERLAK			Oxidation (M)[6]		Mascot
1433.7635	1433.7699	0.0064	4	5943	5954	SWETLKNVISEK					Mascot
1443.8165	1443.7639	-0.0526	-36	4186	4198	QASVNTIIEKVNK					Mascot
1444.7179	1444.7787	0.0608	42	7436	7447	HWSLISSQTTER					Mascot
1445.7748	1445.7709	-0.0039	-3	5937	5948	LGDLQRSWETLK					Mascot
1445.79	1445.7709	-0.0191	-13	32	43	TFTKWINSHLAK					Mascot
1455.7955	1455.6965	-0.099	-68	3278	3290	IVAEHNQFSLGIK					Mascot

1461.6501	1461.7517	0.1016	70	1227	1238	MLSNFGDCVQYK	Carbamidomethyl (C)[8]	Mascot
1473.806	1473.7593	-0.0467	-32	6483	6494	LQQILNFQNDLK		Mascot
1481.6842	1481.7261	0.0419	28	7496	7507	AHELFQAEMFSR	Oxidation (M)[9]	Mascot
1494.7812	1494.7688	-0.0124	-8	1737	1747	LHLEQLDERWR		Mascot
1525.7493	1525.7437	-0.0056	-4	3557	3570	EDVIPSGIPQAEDR		Mascot
1602.8309	1602.8632	0.0323	20	900	913	QIADIHVAFQSMVK	Oxidation (M)[12]	Mascot
1618.8071	1618.853	0.0459	28	2059	2071	EINRLEVTWDDTK		Mascot
1630.8184	1630.8601	0.0417	26	2049	2062	DVAFAPEVDREINR		Mascot
1634.7479	1634.8573	0.1094	67	2743	2755	NQLEQWMESVDQK		Mascot
1700.8214	1700.9672	0.1458	86	1063	1076	VFFSDKGPHHLCEK	Carbamidomethyl (C)[12]	Mascot
1717.8677	1717.9836	0.1159	67	7803	7817	VSQNGDILIEEMIEK		Mascot
1717.8677	1717.9836	0.1159	67	7803	7817	VSQNGDILIEEMIEK		Mascot
1729.9265	1729.9792	0.0527	30	4411	4425	VMADLGLNERQVIQK	Oxidation (M)[2]	Mascot
1743.9739	1743.9794	0.0055	3	8469	8483	LELSTDIQTIELQIK		Mascot
1747.8644	1747.854	-0.0104	-6	333	347	DLTRAQMVESNLQDK		Mascot
1850.8781	1850.9246	0.0465	25	6436	6451	NKNLFSQAFPENGDNR		Mascot
1870.9293	1871.1074	0.1781	95	2538	2553	FNTENLGESKQHIPEK		Mascot
1882.0215	1881.9395	-0.082	-44	8594	8608	QLMQIKHELLESQLR	Oxidation (M)[3]	Mascot
1894.9182	1894.9457	0.0275	15	618	634	YGNTVASLQAWLEDAEK		Mascot
1894.9182	1894.9457	0.0275	15	618	634	YGNTVASLQAWLEDAEK		Mascot
1898.9495	1898.9443	-0.0052	-3	7343	7359	QQTSLQAGVLDYETFAK		Mascot
1908.9563	1908.9429	-0.0134	-7	1350	1365	ETVVRYLFQTGSSHER		Mascot
1923.0559	1922.9436	-0.1123	-58	5546	5563	AKVLAHGTIAWNSASQLR		Mascot
1923.929	1923.9412	0.0122	6	1430	1445	LEDTLEEDIKTMEMVK		Mascot
1942.856	1942.9286	0.0726	37	7246	7261	DYSKQCASTVQQQEDR	Carbamidomethyl (C)[6]	Mascot
1965.9586	1965.9661	0.0075	4	3090	3106	CFDIPQNISEVSTSLQK	Carbamidomethyl (C)[1]	Mascot
1966.9539	1966.968	0.0141	7	471	488	SVNGIPVPPDQLEDMAE R		Mascot
1997.9999	1997.9744	-0.0255	-13	6072	6087	LNDQLEEQRQEQALQR		Mascot
1998.9662	1998.9618	-0.0044	-2	8703	8721	CSLSQPGPSVSSPHSRS TK	Carbamidomethyl (C)[1]	Mascot
2102.9521	2103.04	0.0879	42	692	708	ELFMEVKQYAQADEMDR		Mascot
2109.0432	2109.1035	0.0603	29	2577	2596	LSQRGQLLSEEGHGAGQ EGR		Mascot
2118.947	2119.0366	0.0896	42	692	708	ELFMEVKQYAQADEMDR	Oxidation (M)[4]	Mascot
2126.0071	2126.0657	0.0586	28	3894	3911	DKIDQLQSDYQDLCSIGK	Carbamidomethyl (C)[14]	Mascot
2127.0605	2127.1038	0.0433	20	534	551	ESVEQLLQNYVSFIENSK		Mascot
2127.0605	2127.1038	0.0433	20	534	551	ESVEQLLQNYVSFIENSK		Mascot
2177.9736	2178.0469	0.0733	34	2909	2927	QNTTASGCELMHTEMQA	Carbamidomethyl (C)[8]	Mascot

						LR					
2177.9736	2178.0469	0.0733	34	2909	2927	QNTTASGCELMHTEMQA LR			Carbamidomethyl (C)[8]		Mascot
2190.0972	2190.1123	0.0151	7	4536	4553	LQELQSVYDSVLQKCSH R			Carbamidomethyl (C)[15]		Mascot
2193.1453	2193.0671	-0.0782	-36	963	980	HTEFFSQLDQRVLNAFLK					Mascot
2194.0959	2194.0432	-0.0527	-24	1290	1309	RDVQQQIAQAQQGEGGL PDR					Mascot
2209.0627	2209.0549	-0.0078	-4	5326	5343	EMVETQINSVKCWVQET K			Carbamidomethyl (C)[12]		Mascot
2209.9636	2210.0381	0.0745	34	2909	2927	QNTTASGCELMHTEMQA LR			Carbamidomethyl (C)[8], Oxidation (M)[11,15]		Mascot
2226.1045	2226.0239	-0.0806	-36	4426	4445	ALSDAQSHVNCLSDLVG QRR			Carbamidomethyl (C)[11]		Mascot
2280.1216	2280.196	0.0744	33	3173	3191	IQMKDFEVSAEPIQDWLS K			Oxidation (M)[3]		Mascot
2453.1897	2453.3049	0.1152	47	1466	1487	EKELNALETSSSAMDMQI SQIK					Mascot
2479.2498	2479.364	0.1142	46	8497	8518	AIILSINLCSPEFTQADSK ESR			Carbamidomethyl (C)[9]		Mascot
2480.2781	2480.363	0.0849	34	2768	2788	EKFVLLDHLQSILSEAED HTR					Mascot
2921.5217	2921.5732	0.0515	18	799	822	VKECYSPLLYESQQLLIPL EELEK			Carbamidomethyl (C)[4]		Mascot
2923.4143	2923.5444	0.1301	45	3896	3920	IDQLQSDYQDLCSIGKEH VFSLEAK			Carbamidomethyl (C)[12]		Mascot
2935.429	2935.5488	0.1198	41	1591	1617	VLQEHMDLCQALESLSS AITAFSASAR			Carbamidomethyl (C)[9]		Mascot
2939.3999	2939.5347	0.1348	46	4210	4232	EINHLNDQWLDLCRQSN NLCLQR			Carbamidomethyl (C)[13,20]		Mascot
2951.4238	2951.5461	0.1223	41	1591	1617	VLQEHMDLCQALESLSS AITAFSASAR			Carbamidomethyl (C)[9], Oxidation (M)[6]		Mascot

6	30S ribosomal protein S8 OS=Francisella tularensis subsp. novicida (strain U112) GN=rpsH PE=3 SV=1	RS8_FRATN	14486.6	9.38	9	60	47.158	10.425	

Peptide Information

Calc. Mass	Obsrv. Mass	± da	± ppm	Start Seq.	End Seq.	Sequence	Ion Score	C. I. %	Modification	Rank	Result Type
1262.6627	1262.6654	0.0027	2	120	132	DLGVGGEIIGYVA					Mascot
1280.725	1280.663	-0.062	-48	23	33	EFVSVPFSKIK					Mascot
1280.725	1280.663	-0.062	-48	23	33	EFVSVPFSKIK					Mascot
1443.6243	1443.7639	0.1396	97	2	13	SMQDPIADMFTR			Oxidation (M)[2,9]		Mascot
1489.8009	1489.7595	-0.0414	-28	118	132	ARDLGVGGEIIGYVA					Mascot
1615.7997	1615.8309	0.0312	19	52	66	GTTSMGHPSIEIELK			Oxidation (M)[5]		Mascot
1882.0143	1881.9395	-0.0748	-40	32	47	IKMEIANFLVNEGYIK					Mascot
2103.0249	2103.04	0.0151	7	34	51	MEIANFLVNEGYIKSCSK			Carbamidomethyl (C)[16]		Mascot
2119.0198	2119.0366	0.0168	8	34	51	MEIANFLVNEGYIKSCSK			Carbamidomethyl (C)[16], Oxidation (M)[1]		Mascot
2149.1289	2149.0676	-0.0613	-29	90	110	SHADLPKVYGGYGVAIVS TSK					Mascot
2937.5212	2937.5327	0.0115	4	52	78	GTTSMGHPSIEIELKYHA GVPVIEMIK					Mascot

7	Malate dehydrogenase 1 OS=Burkholderia vietnamiensis (strain G4 / LMG 22486) GN=mdh1 PE=3 SV=1	MDH1_BURVG	35802.4	6.24	12	56	0	9.389

Peptide Information

Calc. Mass	Obsrv. Mass	± da	± ppm	Start Seq.	End Seq.	Sequence	Ion Score	C. I. %	Modification	Rank	Result Type
1450.7107	1450.7391	0.0284	20	81	93	DADYAMLVGSRPR					Mascot
1474.7795	1474.7556	-0.0239	-16	168	181	LAARCGVTVDAVSR			Carbamidomethyl (C)[5]		Mascot
1509.7737	1509.7313	-0.0424	-28	215	226	WYLDTFIPEVAR					Mascot
1509.7737	1509.7313	-0.0424	-28	215	226	WYLDTFIPEVAR					Mascot
1537.8698	1537.7664	-0.1034	-67	294	307	VVPDLEIDALARAR					Mascot
1745.8739	1745.9937	0.1198	69	308	323	IDASVAELVDEMQAVR					Mascot
1893.0706	1892.9376	-0.133	-70	7	24	RVAVTGAAGQIAYSLLFR					Mascot
1906.8608	1906.9453	0.0845	44	278	293	GLMFGMPTICSEGRYR			Carbamidomethyl (C)[10], Oxidation (M)[3,6]		Mascot
1908.8521	1908.9429	0.0908	48	182	197	MAVWGNHSPTMFPDYR					Mascot
1923.9858	1923.9412	-0.0446	-23	77	93	VAFRDADYAMLVGSRPR					Mascot
1948.9658	1948.9265	-0.0393	-20	198	214	HALIDQQPAPMRVGDER			Oxidation (M)[11]		Mascot
2036.14	2036.0845	-0.0555	-27	124	142	QVKVLVVGNPANTNAWV AR					Mascot
2178.0356	2178.0469	0.0113	5	236	256	GASSAASAANAAIDQMR DWIR			Oxidation (M)[16]		Mascot
2178.0356	2178.0469	0.0113	5	236	256	GASSAASAANAAIDQMR DWIR			Oxidation (M)[16]		Mascot

8	Uridylate kinase OS=Methanobrevibacter smithii (strain PS / ATCC 35061 / DSM 861) GN=pyrH PE=3 SV=1	PYRH_METS3	24605.6	4.91	9	56	0	3.76

Peptide Information

Calc. Mass	Obsrv. Mass	± da	± ppm	Start Seq.	End Seq.	Sequence	Ion Score	C. I. %	Modification	Rank	Result Type
1444.8007	1444.7787	-0.022	-15	212	225	AINGEEVGTKVISK					Mascot
1878.0405	1877.9895	-0.051	-27	3	19	IVVAIGGSILLKEYDCK			Carbamidomethyl (C)[16]		Mascot
1881.8762	1881.9395	0.0633	34	177	192	AGTYEFFDMTAIQMIK			Oxidation (M)[9]		Mascot
1898.9415	1898.9443	0.0028	1	160	176	EITASEMIEFISGKDTK					Mascot
1909.035	1908.9429	-0.0921	-48	157	173	LIKEITASEMIEFISGK					Mascot
1922.9529	1922.9436	-0.0093	-5	135	151	LINLTSVDGMYDKDPNK					Mascot
2109.0823	2109.1035	0.0212	10	193	211	RSSLETVIANGYDSENLIK					Mascot
2210.0508	2210.0381	-0.0127	-6	174	192	DTKAGTYEFFDMTAIQMI K					Mascot
2226.0457	2226.0239	-0.0218	-10	174	192	DTKAGTYEFFDMTAIQMI K			Oxidation (M)[12]		Mascot
2951.4844	2951.5461	0.0617	21	194	221	SSLETVIANGYDSENLIKA INGEEVGTK					Mascot

9	Trigger factor OS=Methylobacterium radiotolerans (strain ATCC 27329 / DSM 1819 / JCM 2831) GN=tig PE=3 SV=1	TIG_METRJ	52027.6	4.68	13	55	0	9.827

Peptide Information

Calc. Mass	Obsrv. Mass	± da	± ppm	Start Seq.	End Seq.	Sequence	Ion Score	C. I. %	Modification	Rank	Result Type
951.4642	951.4565	-0.0077	-8	285	292	DFEAASRR					Mascot
1093.6365	1093.5804	-0.0561	-51	422	431	VVDHVLGQVK					Mascot
1279.6212	1279.6604	0.0392	31	152	162	MAGQSRPFTER					Mascot
1280.6338	1280.663	0.0292	23	265	275	SMGMESLEKLR					Mascot
1280.6338	1280.663	0.0292	23	265	275	SMGMESLEKLR					Mascot
1433.8489	1433.7699	-0.079	-55	43	55	GFRPGKVPVAHLR					Mascot
1443.7438	1443.7639	0.0201	14	376	388	VSDEEVNQALIAR					Mascot
1487.7999	1487.7015	-0.0984	-66	1	13	MQVTEINAQGLKR					Mascot
1892.9601	1892.9376	-0.0225	-12	240	257	DAEFDVTVTKIQAAGEAK					Mascot
1966.9869	1966.968	-0.0189	-10	174	191	VTIDFVGRIDGEEFQGGK					Mascot
2035.968	2036.0845	0.1165	57	163	181	EEGAEAQSGDRVTIDFV GR					Mascot
2206.1033	2206.0479	-0.0554	-25	57	76	VYGRSVMAEVVQNAVNE ANR					Mascot
2280.168	2280.196	0.028	12	115	135	VALEVMPSFELADLSDVS LTK			Oxidation (M)[6]		Mascot
2937.5891	2937.5327	-0.0564	-19	361	388	LGLVLAQVGESADIKVSD EEVNQALIAR					Mascot

10	Primosomal protein N' OS=Helicobacter pylori (strain ATCC 700392 / 26695) GN=priA PE=3 SV=1	PRIA_HELPY	71171.5	9.01	15	55	0	3.829

Peptide Information

Calc. Mass	Obsrv. Mass	± da	± ppm	Start Seq.	End Seq.	Sequence	Ion Score	C. I. %	Modification	Rank	Result Type
922.5066	922.4426	-0.064	-69	531	537	LCLLEFK			Carbamidomethyl (C)[2]		Mascot
1248.6809	1248.6611	-0.0198	-16	1	10	MFYHLIAPLK			Oxidation (M)[1]		Mascot
1403.7893	1403.7765	-0.0128	-9	562	574	GVTLSNFKAPIEK					Mascot
1450.7029	1450.7391	0.0362	25	375	387	ICSACQSEVLVGK			Carbamidomethyl (C)[2,5]		Mascot
1474.8239	1474.7556	-0.0683	-46	1	12	MFYHLIAPLKNK					Mascot
1481.7999	1481.7261	-0.0738	-50	287	298	YTPTQKNIIFEK					Mascot
1525.826	1525.7437	-0.0823	-54	195	206	QFLEKLYSQEIK					Mascot
1602.9075	1602.8632	-0.0443	-28	22	35	EQHQKGALVNIPLR					Mascot
1618.8799	1618.853	-0.0269	-17	470	484	ALEEGVSLLYQIAGR					Mascot
1747.949	1747.854	-0.095	-54	318	332	NEQAIIFVPTRANFK					Mascot

1906.9434	1906.9453	0.0019	1	221	236	ELGLIIVDEEHDFSYK		Mascot
1922.8922	1922.9436	0.0514	27	342	357	SVQCPFCSVNMSLHLK	Carbamidomethyl (C)[4,7], Oxidation (M)[11]	Mascot
2226.1514	2226.0239	-0.1275	-57	465	484	SNSYRALEEGVSLLYQIA GR		Mascot
2250.0828	2250.0776	-0.0052	-2	342	360	SVQCPFCSVNMSLHLKT NK	Carbamidomethyl (C)[4,7]	Mascot
2939.5757	2939.5347	-0.041	-14	95	120	ECDLVGLEKIEPILNILSQ TQTNALK	Carbamidomethyl (C)[2]	Mascot

Gel Idx/Pos	275/L3	Instr./Gel Origin	BA2151/Sample Project 20140814	Process Status	Analysis Succeeded
Plate [#] Name	[1] Sample Project 20140814	Instrument Sample Name		Spectra	11

Rank	Protein Name	Accession No.	Protein MW	Protein PI	Pep. Count	Protein Score	Protein Score C. I. %	Intensity Matched	Total Ion Score	Total Ion C. I. %	Confirmed
1	ATP synthase subunit alpha, mitochondrial OS=Oryza sativa subsp. indica GN=ATPA PE=2 SV=1	ATPAM_ORYSI	55624	5.85	19	741	100	54.9	641	100	

Protein Group

ATP synthase subunit alpha, mitochondrial OS=Oryza sativa GN=ATPA PE=2 SV=1	ATPAM_ORYSA	55624	5.8499999046 3257
ATP synthase subunit alpha, mitochondrial OS=Oryza sativa subsp. japonica GN=ATPA PE=1 SV=1	ATPAM_ORYSJ	55624	5.8499999046 3257

Peptide Information

Calc. Mass	Obsrv. Mass	± da	± ppm	Start Seq.	End Seq.	Sequence	Ion Score	C. I. %	Modification	Rank	Result Type
815.4621	815.4589	-0.0032	-4	167	173	ELIIGDR					Mascot
860.5022	860.4783	-0.0239	-28	283	289	QMSLLLR					Mascot
876.4971	876.4769	-0.0202	-23	283	289	QMSLLLR			Oxidation (M)[2]		Mascot
884.4221	884.4244	0.0023	3	121	128	GALSDHER					Mascot
892.4886	892.4926	0.004	4	395	401	LELAQYR					Mascot
972.5473	972.5458	-0.0015	-2	33	42	VVSVGDGIAR					Mascot
1026.5942	1026.593	-0.0012	-1	154	163	AVDSLVPIGR					Mascot
1026.5942	1026.593	-0.0012	-1	154	163	AVDSLVPIGR	70	99.99			Mascot
1203.658	1203.6637	0.0057	5	7	17	AAELTTLLESR					Mascot
1242.6147	1242.5919	-0.0228	-18	143	153	SVHEPMQTGLK			Oxidation (M)[6]		Mascot
1300.7471	1300.7046	-0.0425	-33	178	189	TAIAIDTILNQK					Mascot
1341.705	1341.667	-0.038	-28	433	443	QPQYEPLPIEK					Mascot
1438.8489	1438.8525	0.0036	3	363	376	GIRPAINVGLSVSR					Mascot
1438.8489	1438.8525	0.0036	3	363	376	GIRPAINVGLSVSR	54	99.605			Mascot
1537.7434	1537.7633	0.0199	13	295	307	EAFPGDVFYLHSR					Mascot
1537.7434	1537.7633	0.0199	13	295	307	EAFPGDVFYLHSR	109	100			Mascot
1704.8262	1704.8168	-0.0094	-6	262	276	DNGMHALIIYDDLSK					Mascot
1724.8789	1724.9037	0.0248	14	444	458	QIVVIYAAVNGFCDR			Carbamidomethyl (C)[13]		Mascot
1834.8429	1834.8728	0.0299	16	18	32	MTNFYTNFQVDEIGR					Mascot
1834.8429	1834.8728	0.0299	16	18	32	MTNFYTNFQVDEIGR	130	100			Mascot
1850.8378	1850.8463	0.0085	5	18	32	MTNFYTNFQVDEIGR			Oxidation (M)[1]		Mascot
1850.8378	1850.8463	0.0085	5	18	32	MTNFYTNFQVDEIGR	167	100	Oxidation (M)[1]		Mascot

2031.9692	2031.9708	0.0016	1	195	212	GTNESETLYCVYVAIGQK			Carbamidomethyl (C)[10]		Mascot
2141.0583	2141.0762	0.0179	8	43	62	VYGLNEIQAGEMVEFAS GVK					Mascot
2157.0532	2157.0676	0.0144	7	43	62	VYGLNEIQAGEMVEFAS GVK			Oxidation (M)[12]		Mascot
2308.1567	2308.2153	0.0586	25	402	423	EVAAFAQFGSDLDAATQ ALLNR					Mascot
2308.1567	2308.2153	0.0586	25	402	423	EVAAFAQFGSDLDAATQ ALLNR	241	100			Mascot
2373.2661	2373.2607	-0.0054	-2	63	85	GIALNLENENVGIVVFGS DTAIK					Mascot

2	ATP synthase subunit alpha, mitochondrial OS=Phaseolus vulgaris GN=ATPA PE=1 SV=1	ATPAM_PHAVU	55595.1	6.51	16	715	100	53.484	641	100	

Peptide Information

Calc. Mass	Obsrv. Mass	± da	± ppm	Start Seq.	End Seq.	Sequence	Ion Score	C. I. %	Modification	Rank	Result Type
815.4621	815.4589	-0.0032	-4	167	173	ELIIGDR					Mascot
860.5022	860.4783	-0.0239	-28	283	289	QMSLLLR					Mascot
876.4971	876.4769	-0.0202	-23	283	289	QMSLLLR			Oxidation (M)[2]		Mascot
884.4221	884.4244	0.0023	3	121	128	GALSDHER					Mascot
892.4886	892.4926	0.004	4	395	401	LELAQYR					Mascot
972.5473	972.5458	-0.0015	-2	33	42	VVSVGDGIAR					Mascot
1026.5942	1026.593	-0.0012	-1	154	163	AVDSLVPIGR					Mascot
1026.5942	1026.593	-0.0012	-1	154	163	AVDSLVPIGR	70	99.99			Mascot
1203.658	1203.6637	0.0057	5	7	17	AAELTTLLESR					Mascot
1242.6147	1242.5919	-0.0228	-18	143	153	SVHEPMQTGLK			Oxidation (M)[6]		Mascot
1300.7471	1300.7046	-0.0425	-33	178	189	TAIAIDTILNQK					Mascot
1438.8489	1438.8525	0.0036	3	363	376	GIRPAINVGLSVSR					Mascot
1438.8489	1438.8525	0.0036	3	363	376	GIRPAINVGLSVSR	54	99.605			Mascot
1537.7434	1537.7633	0.0199	13	295	307	EAFPGDVFYLHSR					Mascot
1537.7434	1537.7633	0.0199	13	295	307	EAFPGDVFYLHSR	109	100			Mascot
1704.8262	1704.8168	-0.0094	-6	262	276	DNGMHALIIYDDLSK					Mascot
1834.8429	1834.8728	0.0299	16	18	32	MTNFYTNFQVDEIGR					Mascot
1834.8429	1834.8728	0.0299	16	18	32	MTNFYTNFQVDEIGR	130	100			Mascot
1850.8378	1850.8463	0.0085	5	18	32	MTNFYTNFQVDEIGR			Oxidation (M)[1]		Mascot
1850.8378	1850.8463	0.0085	5	18	32	MTNFYTNFQVDEIGR	167	100	Oxidation (M)[1]		Mascot
2141.0583	2141.0762	0.0179	8	43	62	VYGLNEIQAGEMVEFAS GVK					Mascot
2157.0532	2157.0676	0.0144	7	43	62	VYGLNEIQAGEMVEFAS GVK			Oxidation (M)[12]		Mascot
2308.1567	2308.2153	0.0586	25	402	423	EVAAFAQFGSDLDAATQ ALLNR					Mascot

2308.1567	2308.2153	0.0586	25	402	423	EVAAFAQFGSDLDAATQ ALLNR	241	100			Mascot
2373.2661	2373.2607	-0.0054	-2	63	85	GIALNLENENVGIVVFGS DTAIK					Mascot

3	ATP synthase subunit alpha, mitochondrial OS=Zea mays GN=ATPA PE=3 SV=1	ATPAM_MAIZE	55430.9	5.85	19	572	100	47.542	474	100	

Peptide Information

Calc. Mass	Obsrv. Mass	± da	± ppm	Start Seq.	End Seq.	Sequence	Ion Score	C. I. %	Modification	Rank	Result Type
815.4621	815.4589	-0.0032	-4	167	173	ELIIGDR					Mascot
860.5022	860.4783	-0.0239	-28	283	289	QMSLLLR					Mascot
876.4971	876.4769	-0.0202	-23	283	289	QMSLLLR			Oxidation (M)[2]		Mascot
884.4221	884.4244	0.0023	3	121	128	GALSDHER					Mascot
892.4886	892.4926	0.004	4	395	401	LELAQYR					Mascot
972.5473	972.5458	-0.0015	-2	33	42	VVSVGDGIAR					Mascot
1026.5942	1026.593	-0.0012	-1	154	163	AVDSLVPIGR					Mascot
1026.5942	1026.593	-0.0012	-1	154	163	AVDSLVPIGR	70	99.99			Mascot
1203.658	1203.6637	0.0057	5	7	17	AAELTTLLESR					Mascot
1242.6147	1242.5919	-0.0228	-18	143	153	SVHEPMQTGLK			Oxidation (M)[6]		Mascot
1300.7471	1300.7046	-0.0425	-33	178	189	TAIAIDTILNQK					Mascot
1341.705	1341.667	-0.038	-28	433	443	QPQYEPLPIEK					Mascot
1438.8489	1438.8525	0.0036	3	363	376	GIRPAINVGLSVSR					Mascot
1438.8489	1438.8525	0.0036	3	363	376	GIRPAINVGLSVSR	54	99.605			Mascot
1537.7434	1537.7633	0.0199	13	295	307	EAFPGDVFYLHSR					Mascot
1537.7434	1537.7633	0.0199	13	295	307	EAFPGDVFYLHSR	109	100			Mascot
1638.8268	1638.778	-0.0488	-30	388	401	QVCGSSKLELAQYR			Carbamidomethyl (C)[3]		Mascot
1704.8262	1704.8168	-0.0094	-6	262	276	DNGMHALIIYDDLSK					Mascot
1724.8789	1724.9037	0.0248	14	444	458	QIVVIYAAVNGFCDR			Carbamidomethyl (C)[13]		Mascot
2031.9692	2031.9708	0.0016	1	195	212	GTNESETLYCVYVAIGQK			Carbamidomethyl (C)[10]		Mascot
2141.0583	2141.0762	0.0179	8	43	62	VYGLNEIQAGEMVEFAS GVK					Mascot
2157.0532	2157.0676	0.0144	7	43	62	VYGLNEIQAGEMVEFAS GVK			Oxidation (M)[12]		Mascot
2308.1567	2308.2153	0.0586	25	402	423	EVAAFAQFGSDLDAATQ ALLNR					Mascot
2308.1567	2308.2153	0.0586	25	402	423	EVAAFAQFGSDLDAATQ ALLNR	241	100			Mascot
2373.2661	2373.2607	-0.0054	-2	63	85	GIALNLENENVGIVVFGS DTAIK					Mascot

4	ATP synthase subunit alpha, mitochondrial OS=Glycine max GN=ATPA PE=1 SV=1	ATPAM_SOYBN	55581.2	6.23	15	539	100	45.963	474	100	

Peptide Information

Calc. Mass	Obsrv. Mass	± da	± ppm	Start Seq.	End Seq.	Sequence	Ion Score	C. I. %	Modification	Rank	Result Type
815.4621	815.4589	-0.0032	-4	167	173	ELIIGDR					Mascot
860.5022	860.4783	-0.0239	-28	283	289	QMSLLLR					Mascot
876.4971	876.4769	-0.0202	-23	283	289	QMSLLLR			Oxidation (M)[2]		Mascot
884.4221	884.4244	0.0023	3	121	128	GALSDHER					Mascot
892.4886	892.4926	0.004	4	395	401	LELAQYR					Mascot
972.5473	972.5458	-0.0015	-2	33	42	VVSVGDGIAR					Mascot
1026.5942	1026.593	-0.0012	-1	154	163	AVDSLVPIGR					Mascot
1026.5942	1026.593	-0.0012	-1	154	163	AVDSLVPIGR	70	99.99			Mascot
1203.658	1203.6637	0.0057	5	7	17	AAELTTLLESR					Mascot
1242.6147	1242.5919	-0.0228	-18	143	153	SVHEPMQTGLK			Oxidation (M)[6]		Mascot
1300.7471	1300.7046	-0.0425	-33	178	189	TAIAIDTILNQK					Mascot
1438.8489	1438.8525	0.0036	3	363	376	GIRPAINVGLSVSR					Mascot
1438.8489	1438.8525	0.0036	3	363	376	GIRPAINVGLSVSR	54	99.605			Mascot
1537.7434	1537.7633	0.0199	13	295	307	EAFPGDVFYLHSR					Mascot
1537.7434	1537.7633	0.0199	13	295	307	EAFPGDVFYLHSR	109	100			Mascot
1704.8262	1704.8168	-0.0094	-6	262	276	DNGMHALIIYDDLSK					Mascot
2141.0583	2141.0762	0.0179	8	43	62	VYGLNEIQAGEMVEFAS GVK					Mascot
2157.0532	2157.0676	0.0144	7	43	62	VYGLNEIQAGEMVEFAS GVK			Oxidation (M)[12]		Mascot
2308.1567	2308.2153	0.0586	25	402	423	EVAAFAQFGSDLDAATQ ALLNR					Mascot
2308.1567	2308.2153	0.0586	25	402	423	EVAAFAQFGSDLDAATQ ALLNR	241	100			Mascot
2373.2661	2373.2607	-0.0054	-2	63	85	GIALNLENENVGIVVFGS DTAIK					Mascot

5	ATP synthase subunit alpha, mitochondrial OS=Beta vulgaris GN=ATPA PE=3 SV=1	ATPAM_BETVU	55245.8	6.01	14	532	100	45.074	474	100	

Peptide Information

Calc. Mass	Obsrv. Mass	± da	± ppm	Start Seq.	End Seq.	Sequence	Ion Score	C. I. %	Modification	Rank	Result Type
815.4621	815.4589	-0.0032	-4	167	173	ELIIGDR					Mascot
860.5022	860.4783	-0.0239	-28	283	289	QMSLLLR					Mascot
876.4971	876.4769	-0.0202	-23	283	289	QMSLLLR			Oxidation (M)[2]		Mascot
884.4221	884.4244	0.0023	3	121	128	GALSDHER					Mascot
892.4886	892.4926	0.004	4	395	401	LELAQYR					Mascot
972.5473	972.5458	-0.0015	-2	33	42	VVSVGDGIAR					Mascot

1026.5942	1026.593	-0.0012	-1	154	163	AVDSLVPIGR					Mascot
1026.5942	1026.593	-0.0012	-1	154	163	AVDSLVPIGR	70	99.99			Mascot
1242.6147	1242.5919	-0.0228	-18	143	153	SVHEPMQTGLK			Oxidation (M)[6]		Mascot
1300.7471	1300.7046	-0.0425	-33	178	189	TAIAIDTILNQK					Mascot
1438.8489	1438.8525	0.0036	3	363	376	GIRPAINVGLSVSR					Mascot
1438.8489	1438.8525	0.0036	3	363	376	GIRPAINVGLSVSR	54	99.605			Mascot
1537.7434	1537.7633	0.0199	13	295	307	EAFPGDVFYLHSR					Mascot
1537.7434	1537.7633	0.0199	13	295	307	EAFPGDVFYLHSR	109	100			Mascot
1704.8262	1704.8168	-0.0094	-6	262	276	DNGMHALIIYDDLSK					Mascot
2141.0583	2141.0762	0.0179	8	43	62	VYGLNEIQAGEMVEFAS GVK					Mascot
2157.0532	2157.0676	0.0144	7	43	62	VYGLNEIQAGEMVEFAS GVK			Oxidation (M)[12]		Mascot
2308.1567	2308.2153	0.0586	25	402	423	EVAAFAQFGSDLDAATQ ALLNR					Mascot
2308.1567	2308.2153	0.0586	25	402	423	EVAAFAQFGSDLDAATQ ALLNR	241	100			Mascot
2373.2661	2373.2607	-0.0054	-2	63	85	GIALNLENENVGIVVFGS DTAIK					Mascot

6	ATP synthase subunit alpha, mitochondrial OS=Oenothera biennis GN=ATPA PE=3 SV=1	ATPAM_OENBI	55847.3	6.23	14	532	100	45.188	474	100

Peptide Information

Calc. Mass	Obsrv. Mass	± da	± ppm	Start Seq.	End Seq.	Sequence	Ion Score	C. I. %	Modification	Rank	Result Type
815.4621	815.4589	-0.0032	-4	167	173	ELIIGDR					Mascot
860.5022	860.4783	-0.0239	-28	283	289	QMSLLLR					Mascot
876.4971	876.4769	-0.0202	-23	283	289	QMSLLLR			Oxidation (M)[2]		Mascot
892.4886	892.4926	0.004	4	395	401	LELAQYR					Mascot
1010.5126	1010.4449	-0.0677	-67	121	129	GALGDHERR					Mascot
1026.5942	1026.593	-0.0012	-1	154	163	AVDSLVPIGR					Mascot
1026.5942	1026.593	-0.0012	-1	154	163	AVDSLVPIGR	70	99.99			Mascot
1203.658	1203.6637	0.0057	5	7	17	AAELTTLLESR					Mascot
1242.6147	1242.5919	-0.0228	-18	143	153	SVHEPMQTGLK			Oxidation (M)[6]		Mascot
1300.7471	1300.7046	-0.0425	-33	178	189	TAIAIDTILNQK					Mascot
1438.8489	1438.8525	0.0036	3	363	376	GIRPAINVGLSVSR					Mascot
1438.8489	1438.8525	0.0036	3	363	376	GIRPAINVGLSVSR	54	99.605			Mascot
1537.7434	1537.7633	0.0199	13	295	307	EAFPGDVFYLHSR					Mascot
1537.7434	1537.7633	0.0199	13	295	307	EAFPGDVFYLHSR	109	100			Mascot
1704.8262	1704.8168	-0.0094	-6	262	276	DNGMHALIIYDDLSK					Mascot
2141.0583	2141.0762	0.0179	8	43	62	VYGLNEIQAGEMVEFAS GVK					Mascot

2157.0532	2157.0676	0.0144	7	43	62	VYGLNEIQAGEMVEFAS GVK			Oxidation (M)[12]		Mascot
2308.1567	2308.2153	0.0586	25	402	423	EVAAFAQFGSDLDAATQ ALLNR					Mascot
2308.1567	2308.2153	0.0586	25	402	423	EVAAFAQFGSDLDAATQ ALLNR	241	100			Mascot
2373.2661	2373.2607	-0.0054	-2	63	85	GIALNLENENVGIVVFGS DTAIK					Mascot

7	ATP synthase subunit alpha, mitochondrial OS=Pisum sativum GN=ATPA PE=1 SV=2	ATPAM_PEA	55296.1	6.01	13	522	100	45.184	474	100	

Peptide Information

Calc. Mass	Obsrv. Mass	± da	± ppm	Start Seq.	End Seq.	Sequence	Ion Score	C. I. %	Modification	Rank	Result Type
815.4621	815.4589	-0.0032	-4	167	173	ELIIGDR					Mascot
860.5022	860.4783	-0.0239	-28	283	289	QMSLLLR					Mascot
876.4971	876.4769	-0.0202	-23	283	289	QMSLLLR			Oxidation (M)[2]		Mascot
884.4221	884.4244	0.0023	3	121	128	GALSDHER					Mascot
892.4886	892.4926	0.004	4	395	401	LELAQYR					Mascot
972.5473	972.5458	-0.0015	-2	33	42	VVSVGDGIAR					Mascot
1026.5942	1026.593	-0.0012	-1	154	163	AVDSLVPIGR					Mascot
1026.5942	1026.593	-0.0012	-1	154	163	AVDSLVPIGR	70	99.99			Mascot
1203.658	1203.6637	0.0057	5	7	17	AAELTTLLESR					Mascot
1242.6147	1242.5919	-0.0228	-18	143	153	SVHEPMQTGLK			Oxidation (M)[6]		Mascot
1300.7471	1300.7046	-0.0425	-33	178	189	TAIAIDTILNQK					Mascot
1438.8489	1438.8525	0.0036	3	363	376	GIRPAINVGLSVSR					Mascot
1438.8489	1438.8525	0.0036	3	363	376	GIRPAINVGLSVSR	54	99.605			Mascot
1537.7434	1537.7633	0.0199	13	295	307	EAFPGDVFYLHSR					Mascot
1537.7434	1537.7633	0.0199	13	295	307	EAFPGDVFYLHSR	109	100			Mascot
1704.8262	1704.8168	-0.0094	-6	262	276	DNGMHALIIYDDLSK					Mascot
2308.1567	2308.2153	0.0586	25	402	423	EVAAFAQFGSDLDAATQ ALLNR					Mascot
2308.1567	2308.2153	0.0586	25	402	423	EVAAFAQFGSDLDAATQ ALLNR	241	100			Mascot

8	ATP synthase subunit alpha, mitochondrial OS=Brassica napus GN=ATPA PE=3 SV=1	ATPAM_BRANA	55392.9	6.23	12	517	100	45.939	474	100	

Protein Group

ATP synthase subunit alpha, mitochondrial OS=Brassica campestris GN=ATPA PE=3 SV=1	ATPAM_BRACM	55307.8	6.0100 002288 8184
ATP synthase subunit alpha, mitochondrial OS=Raphanus sativus GN=ATPA PE=3 SV=1	ATPAM_RAPSA	55307.8	6.0100 002288 8184

Peptide Information

Calc. Mass	Obsrv. Mass	± da	± ppm	Start Seq.	End Seq.	Sequence	Ion Score	C. I. %	Modification	Rank	Result Type
815.4621	815.4589	-0.0032	-4	167	173	ELLIGDR					Mascot
860.5022	860.4783	-0.0239	-28	283	289	QMSLLLR					Mascot
876.4971	876.4769	-0.0202	-23	283	289	QMSLLLR			Oxidation (M)[2]		Mascot
892.4886	892.4926	0.004	4	395	401	LELAQYR					Mascot
1026.5942	1026.593	-0.0012	-1	154	163	AVDSLVPIGR					Mascot
1026.5942	1026.593	-0.0012	-1	154	163	AVDSLVPIGR	70	99.99			Mascot
1242.6147	1242.5919	-0.0228	-18	143	153	SVHEPMQTGLK			Oxidation (M)[6]		Mascot
1438.8489	1438.8525	0.0036	3	363	376	GIRPAINVGLSVSR					Mascot
1438.8489	1438.8525	0.0036	3	363	376	GIRPAINVGLSVSR	54	99.605			Mascot
1519.8228	1519.7561	-0.0667	-44	7	19	AAELTNLFESRIR					Mascot
1519.8228	1519.7561	-0.0667	-44	7	19	AAELTNLFESRIR					Mascot
1537.7434	1537.7633	0.0199	13	295	307	EAFPGDVFYLHSR					Mascot
1537.7434	1537.7633	0.0199	13	295	307	EAFPGDVFYLHSR	109	100			Mascot
1638.8268	1638.778	-0.0488	-30	388	401	QVCGSSKLELAQYR			Carbamidomethyl (C)[3]		Mascot
1704.8262	1704.8168	-0.0094	-6	262	276	DNGMHALIIYDDLSK					Mascot
1756.9197	1756.97	0.0503	29	104	120	AMLGRVVDAMGVPIDGR					Mascot
2308.1567	2308.2153	0.0586	25	402	423	EVAAFAQFGSDLDAATQ ALLNR					Mascot
2308.1567	2308.2153	0.0586	25	402	423	EVAAFAQFGSDLDAATQ ALLNR	241	100			Mascot

9 ATP synthase subunit alpha, mitochondrial OS=Triticum aestivum GN=ATPA PE=3 SV=1 ATPAM_WHEAT 55514.8 5.7 20 507 100 38.614 400 100

Peptide Information

Calc. Mass	Obsrv. Mass	± da	± ppm	Start Seq.	End Seq.	Sequence	Ion Score	C. I. %	Modification	Rank	Result Type
815.4621	815.4589	-0.0032	-4	167	173	ELIIGDR					Mascot
860.5022	860.4783	-0.0239	-28	283	289	QMSLLLR					Mascot
876.4971	876.4769	-0.0202	-23	283	289	QMSLLLR			Oxidation (M)[2]		Mascot
884.4221	884.4244	0.0023	3	121	128	GALSDHER					Mascot
892.4886	892.4926	0.004	4	395	401	LELAQYR					Mascot
972.5473	972.5458	-0.0015	-2	33	42	VVSVGDGIAR					Mascot
1026.5942	1026.593	-0.0012	-1	154	163	AVDSLVPIGR					Mascot
1026.5942	1026.593	-0.0012	-1	154	163	AVDSLVPIGR	70	99.99			Mascot
1203.658	1203.6637	0.0057	5	7	17	AAELTTLLESR					Mascot
1242.6147	1242.5919	-0.0228	-18	143	153	SVHEPMQTGLK			Oxidation (M)[6]		Mascot
1300.7471	1300.7046	-0.0425	-33	178	189	TAIAIDTILNQK					Mascot

1326.7627	1326.7194	-0.0433	-33	470	481	AILSTINPELQK					Mascot
1341.705	1341.667	-0.038	-28	433	443	QPQYEPLPIEK					Mascot
1438.8489	1438.8525	0.0036	3	363	376	GIRPAINVGLSVSR					Mascot
1438.8489	1438.8525	0.0036	3	363	376	GIRPAINVGLSVSR	54	99.605			Mascot
1537.7434	1537.7633	0.0199	13	295	307	EAFPGDVFYLHSR					Mascot
1537.7434	1537.7633	0.0199	13	295	307	EAFPGDVFYLHSR	109	100			Mascot
1638.8268	1638.778	-0.0488	-30	388	401	QVCGSSKLELAQYR			Carbamidomethyl (C)[3]		Mascot
1704.8262	1704.8168	-0.0094	-6	262	276	DNGMHALIIYDDLSK					Mascot
1724.8789	1724.9037	0.0248	14	444	458	QIVVIYAAVNGFCDR			Carbamidomethyl (C)[13]		Mascot
1834.8429	1834.8728	0.0299	16	18	32	MTNFYTNFQVDEIGR					Mascot
1834.8429	1834.8728	0.0299	16	18	32	MTNFYTNFQVDEIGR	130	100			Mascot
1850.8378	1850.8463	0.0085	5	18	32	MTNFYTNFQVDEIGR			Oxidation (M)[1]		Mascot
1850.8378	1850.8463	0.0085	5	18	32	MTNFYTNFQVDEIGR	167	100	Oxidation (M)[1]		Mascot
2031.9692	2031.9708	0.0016	1	195	212	GTNESETLYCVYVAIGQK			Carbamidomethyl (C)[10]		Mascot
2141.0583	2141.0762	0.0179	8	43	62	VYGLNEIQAGEMVEFAS GVK					Mascot
2157.0532	2157.0676	0.0144	7	43	62	VYGLNEIQAGEMVEFAS GVK			Oxidation (M)[12]		Mascot
2373.2661	2373.2607	-0.0054	-2	63	85	GIALNLENENVGIVVFGS DTAIK					Mascot

10	ATP synthase subunit alpha, mitochondrial OS=Arabidopsis thaliana GN=ATPA PE=1 SV=2	ATPAM_ARATH	55295.9	6.23	9	499	100	45.106	474	100		

Peptide Information

Calc. Mass	Obsrv. Mass	± da	± ppm	Start Seq.	End Seq.	Sequence	Ion Score	C. I. %	Modification	Rank	Result Type
860.5022	860.4783	-0.0239	-28	283	289	QMSLLLR					Mascot
876.4971	876.4769	-0.0202	-23	283	289	QMSLLLR			Oxidation (M)[2]		Mascot
892.4886	892.4926	0.004	4	395	401	LELAQYR					Mascot
1026.5942	1026.593	-0.0012	-1	154	163	AVDSLVPIGR					Mascot
1026.5942	1026.593	-0.0012	-1	154	163	AVDSLVPIGR	70	99.99			Mascot
1242.6147	1242.5919	-0.0228	-18	143	153	SVHEPMQTGLK			Oxidation (M)[6]		Mascot
1438.8489	1438.8525	0.0036	3	363	376	GIRPAINVGLSVSR					Mascot
1438.8489	1438.8525	0.0036	3	363	376	GIRPAINVGLSVSR	54	99.605			Mascot
1519.8228	1519.7561	-0.0667	-44	7	19	AAELTNLFESRIR					Mascot
1519.8228	1519.7561	-0.0667	-44	7	19	AAELTNLFESRIR					Mascot
1537.7434	1537.7633	0.0199	13	295	307	EAFPGDVFYLHSR					Mascot
1537.7434	1537.7633	0.0199	13	295	307	EAFPGDVFYLHSR	109	100			Mascot
1704.8262	1704.8168	-0.0094	-6	262	276	DNGMHALIIYDDLSK					Mascot

2308.1567	2308.2153	0.0586	25	402	423	EVAAFAQFGSDLDAATQ ALLNR			Mascot
2308.1567	2308.2153	0.0586	25	402	423	EVAAFAQFGSDLDAATQ ALLNR	241	100	Mascot

Gel Idx/Pos	276/L4	Instr./Gel Origin	BA2151/Sample Project 20140814	Process Status	Analysis Succeeded
Plate [#] Name	[1] Sample Project 20140814	Instrument Sample Name		Spectra	11

Rank	Protein Name	Accession No.	Protein MW	Protein PI	Pep. Count	Protein Score	Protein Score C. I. %	Intensity Matched	Total Ion Score	Total Ion C. I. %	Confirmed
1	Beta-amylase OS=Hordeum vulgare GN=BMY1 PE=1 SV=1	AMYB_HORVU	59894.5	5.58	9	290	100	19.536	267	100	

Peptide Information

Calc. Mass	Obsrv. Mass	± da	± ppm	Start Seq.	End Seq.	Sequence	Ion Score	C. I. %	Modification	Rank	Result Type
1016.5564	1016.5525	-0.0039	-4	411	418	LFGFTYLR					Mascot
1016.5564	1016.5525	-0.0039	-4	411	418	LFGFTYLR	59	99.858			Mascot
1026.4739	1026.4773	0.0034	3	248	256	DNGTYLSEK					Mascot
1285.6212	1285.5966	-0.0246	-19	61	71	GPKAYDWSAYK					Mascot
1315.5769	1315.5629	-0.014	-11	335	345	ASINFTCAEMR			Carbamidomethyl (C)[7], Oxidation (M)[10]		Mascot
1326.6688	1326.6639	-0.0049	-4	384	394	YDPTAYNTILR					Mascot
1326.6688	1326.6639	-0.0049	-4	384	394	YDPTAYNTILR	81	100			Mascot
1701.7247	1701.7289	0.0042	2	147	160	SAVQMYADYMTSFR			Oxidation (M)[5,10]		Mascot
1705.8181	1705.8896	0.0715	42	243	256	TQFFRDNGTYLSEK					Mascot
1752.8916	1752.8677	-0.0239	-14	419	433	LSNQLVEGQNYVNFK					Mascot
2013.9778	2013.9973	0.0195	10	303	320	VPSHAAELTAGYYNLHDR					Mascot
2013.9778	2013.9973	0.0195	10	303	320	VPSHAAELTAGYYNLHDR	127	100			Mascot

Rank	Protein Name	Accession No.	Protein MW	Protein PI	Pep. Count	Protein Score	Protein Score C. I. %	Intensity Matched	Total Ion Score	Total Ion C. I. %	Confirmed
2	Beta-amylase OS=Hordeum vulgare subsp. spontaneum GN=BMY1 PE=1 SV=1	AMYB_HORVS	59886.4	5.66	8	286	100	20.235	267	100	

Peptide Information

Calc. Mass	Obsrv. Mass	± da	± ppm	Start Seq.	End Seq.	Sequence	Ion Score	C. I. %	Modification	Rank	Result Type
1016.5564	1016.5525	-0.0039	-4	411	418	LFGFTYLR					Mascot
1016.5564	1016.5525	-0.0039	-4	411	418	LFGFTYLR	59	99.858			Mascot
1253.6121	1253.5848	-0.0273	-22	248	258	DNGTYLTEKGR					Mascot
1285.6212	1285.5966	-0.0246	-19	61	71	GPKAYDWSAYK					Mascot
1315.5769	1315.5629	-0.014	-11	335	345	ASINFTCAEMR			Carbamidomethyl (C)[7], Oxidation (M)[10]		Mascot
1326.6688	1326.6639	-0.0049	-4	384	394	YDPTAYNTILR					Mascot
1326.6688	1326.6639	-0.0049	-4	384	394	YDPTAYNTILR	81	100			Mascot
1701.7247	1701.7289	0.0042	2	147	160	SAVQMYADYMTSFR			Oxidation (M)[5,10]		Mascot
1752.8916	1752.8677	-0.0239	-14	419	433	LSNQLVEGQNYVNFK					Mascot

2013.9778	2013.9973	0.0195	10	303	320	VPSHAAELTAGYYNLHDR					Mascot
2013.9778	2013.9973	0.0195	10	303	320	VPSHAAELTAGYYNLHDR	127	100			Mascot

3	Beta-amylase (Fragment) OS=Secale cereale GN=BMY1 PE=2 SV=1	AMYB_SECCE	24561.9	5.08	3	148	100	12.266	140	100

Peptide Information

Calc. Mass	Obsrv. Mass	± da	± ppm	Start Seq.	End Seq.	Sequence	Ion Score	C. I. %	Modification	Rank	Result Type
1016.5564	1016.5525	-0.0039	-4	107	114	LFGFTYLR					Mascot
1016.5564	1016.5525	-0.0039	-4	107	114	LFGFTYLR	59	99.858			Mascot
1326.6688	1326.6639	-0.0049	-4	80	90	YDPTAYNTILR					Mascot
1326.6688	1326.6639	-0.0049	-4	80	90	YDPTAYNTILR	81	100			Mascot
1573.6998	1573.8138	0.114	72	29	41	HHASLNFTCAEMR			Carbamidomethyl (C)[9]		Mascot
1589.6948	1589.7802	0.0854	54	29	41	HHASLNFTCAEMR			Carbamidomethyl (C)[9], Oxidation (M)[12]		Mascot

4	Beta-amylase OS=Triticum aestivum GN=BMY1 PE=2 SV=1	AMYB_WHEAT	56860.2	5.24	6	71	95.605	11.117	59	99.858

Peptide Information

Calc. Mass	Obsrv. Mass	± da	± ppm	Start Seq.	End Seq.	Sequence	Ion Score	C. I. %	Modification	Rank	Result Type
1016.5564	1016.5525	-0.0039	-4	411	418	LFGFTYLR					Mascot
1016.5564	1016.5525	-0.0039	-4	411	418	LFGFTYLR	59	99.858			Mascot
1285.6212	1285.5966	-0.0246	-19	61	71	GPKAYDWSAYK					Mascot
1431.7585	1431.6398	-0.1187	-83	458	469	SKPEMPIEMILK			Oxidation (M)[5]		Mascot
1591.6562	1591.7605	0.1043	66	333	345	HHASMNFTCAEMR			Carbamidomethyl (C)[9]		Mascot
1775.9229	1775.828	-0.0949	-53	404	418	NGPPEHKLFGFTYLR					Mascot
2087.0557	2087.0767	0.021	10	129	146	NIEYLTLGVDDQPLFHGR					Mascot

5	DNA-directed RNA polymerase subunit beta' (Fragment) OS=Oenococcus oeni GN=rpoC PE=3 SV=1	RPOC_OENOE	112294	6.34	23	66	87.324	17.436		

Peptide Information

Calc. Mass	Obsrv. Mass	± da	± ppm	Start Seq.	End Seq.	Sequence	Ion Score	C. I. %	Modification	Rank	Result Type
946.4597	946.4897	0.03	32	287	294	MNQMGLPR					Mascot
981.3578	981.3875	0.0297	30	754	761	EEDCGTDR			Carbamidomethyl (C)[4]		Mascot
1008.536	1008.4733	-0.0627	-62	476	485	IGIAASSFDK					Mascot
1038.5215	1038.4958	-0.0257	-25	584	592	DLGYNISTR					Mascot

Calc. Mass	Obsrv. Mass	± da	± ppm	Start Seq.	End Seq.	Sequence	Ion Score	C. I. %	Modification	Rank	Result Type
1074.5538	1074.5442	-0.0096	-9	628	636	RGLLTDDER					Mascot
1139.6056	1139.6871	0.0815	72	609	618	GEVLKEAHEK					Mascot
1146.6113	1146.585	-0.0263	-23	907	917	GRAEISEVTGK					Mascot
1221.5958	1221.5872	-0.0086	-7	931	941	TVTIEGETDTR					Mascot
1326.7025	1326.6639	-0.0386	-29	486	496	AKPFTDHQRAR					Mascot
1326.7025	1326.6639	-0.0386	-29	486	496	AKPFTDHQRAR					Mascot
1385.6254	1385.642	0.0166	12	450	462	GEGMIFSSADEVK			Oxidation (M)[4]		Mascot
1636.9269	1636.8292	-0.0977	-60	206	220	LLDLNAPGIIVQNEK					Mascot
1637.8381	1637.7708	-0.0673	-41	332	345	HEDVVQDVLEEVVK					Mascot
1651.8762	1651.8462	-0.03	-18	584	598	DLGYNISTRSGLTVR					Mascot
1652.9343	1652.8204	-0.1139	-69	346	359	EHPVLLNRAPTLHR					Mascot
1652.9343	1652.8204	-0.1139	-69	346	359	EHPVLLNRAPTLHR					Mascot
1668.8625	1668.7999	-0.0626	-38	570	583	YQVTRTSVLLDDMK					Mascot
1674.8619	1674.8002	-0.0617	-37	599	613	MSDVTELPEKGEVLK					Mascot
1684.8575	1684.7843	-0.0732	-43	570	583	YQVTRTSVLLDDMK			Oxidation (M)[13]		Mascot
1700.8749	1700.7959	-0.079	-46	222	236	MLQEAVDALIDNGRR					Mascot
1716.8698	1716.775	-0.0948	-55	221	235	RMLQEAVDALIDNGR			Oxidation (M)[2]		Mascot
1752.9379	1752.8677	-0.0702	-40	962	977	SEAINEGPLDPKELIK					Mascot
1832.0099	1831.8981	-0.1118	-61	360	376	LGIQAFEPVLVSGKAMR			Oxidation (M)[16]		Mascot
1835.8779	1836.0403	0.1624	88	708	723	EGLCVLEMFISTHGAR			Carbamidomethyl (C)[4], Oxidation (M)[8]		Mascot
2013.0415	2013.0154	-0.0261	-13	26	42	MGHIELAAPVTHIWYFK					Mascot
2583.2983	2583.3201	0.0218	8	463	485	MALQNHEVELHTRIGIAA SSFDK			Oxidation (M)[1]		Mascot
2583.2983	2583.3201	0.0218	8	463	485	MALQNHEVELHTRIGIAA SSFDK			Oxidation (M)[1]		Mascot

6	tRNA(Ile)-lysidine synthase OS=Oceanobacillus iheyensis (strain DSM 14371 / JCM 11309 / KCTC 3954 / HTE831) GN=tilS PE=3 SV=1	TILS_OCEIH	54675.2	6.96	15	65	83.67	15.69

Peptide Information

Calc. Mass	Obsrv. Mass	± da	± ppm	Start Seq.	End Seq.	Sequence	Ion Score	C. I. %	Modification	Rank	Result Type
866.4077	866.4145	0.0068	8	397	403	MTWDGLK			Oxidation (M)[1]		Mascot
981.4523	981.3875	-0.0648	-66	230	237	EDENYLAK					Mascot
1035.5006	1035.4246	-0.076	-73	197	203	YTRNYYR					Mascot
1035.5006	1035.4246	-0.076	-73	197	203	YTRNYYR					Mascot
1547.8904	1547.7717	-0.1187	-77	217	229	LFITAQRLSETLR					Mascot
1574.738	1574.8074	0.0694	44	81	93	CIAHQVDVGEYQR			Carbamidomethyl (C)[1]		Mascot
1605.7504	1605.7754	0.025	16	181	193	ENEVPFREDQTNK					Mascot

Calc. Mass	Obsrv. Mass	± da	± ppm	Start Seq.	End Seq.	Sequence	Ion Score	C. I. %	Modification	Rank	Result Type
1636.9058	1636.8292	-0.0766	-47	200	212	NYYRNEIIPLLTK					Mascot
1652.8279	1652.8204	-0.0075	-5	305	318	QEGNTYIDFPLSLR					Mascot
1652.8279	1652.8204	-0.0075	-5	305	318	QEGNTYIDFPLSLR					Mascot
1680.8439	1680.8463	0.0024	1	224	237	LSETLREDENYLAK					Mascot
1700.8062	1700.7959	-0.0103	-6	444	458	AFGSNQFCQSGEKIK			Carbamidomethyl (C)[8]		Mascot
1993.0801	1992.9198	-0.1603	-80	156	172	RSFASGQLIRPFLCVNK			Carbamidomethyl (C)[14]		Mascot
1993.0801	1992.9198	-0.1603	-80	156	172	RSFASGQLIRPFLCVNK			Carbamidomethyl (C)[14]		Mascot
2014.0618	2013.9973	-0.0645	-32	256	272	ISFSNQAFIERPHALQR					Mascot
2014.0618	2013.9973	-0.0645	-32	256	272	ISFSNQAFIERPHALQR					Mascot
2155.271	2155.0588	-0.2122	-98	373	391	NNIIIPIDSVALPLHIRTR					Mascot
2225.0542	2225.1389	0.0847	38	238	255	EANRMVEEVIIWDENFSK			Oxidation (M)[5]		Mascot
2255.2983	2255.1514	-0.1469	-65	370	389	NSRNNIIIPIDSVALPLHIR					Mascot

7	ATP-dependent zinc metalloprotease FtsH OS=Atopobium parvulum (strain ATCC 33793 / DSM 20469 / JCM 10300 / VPI 0546) GN=ftsH PE=3 SV=1	FTSH_ATOPD	70604.1	5.51	17	63	72.268	11.28	

Peptide Information

Calc. Mass	Obsrv. Mass	± da	± ppm	Start Seq.	End Seq.	Sequence	Ion Score	C. I. %	Modification	Rank	Result Type
946.4476	946.4897	0.0421	44	185	193	DVAGQEEAK					Mascot
1020.4996	1020.574	0.0744	73	623	631	ENTFAPVDK					Mascot
1028.4531	1028.5205	0.0674	66	72	81	FTTGSGDSEK					Mascot
1072.611	1072.5723	-0.0387	-36	587	595	ALQTLKENR					Mascot
1123.5967	1123.6426	0.0459	41	578	586	RIVEEGHQR					Mascot
1251.6844	1251.6166	-0.0678	-54	598	607	LHEIAHYLQK					Mascot
1262.5392	1262.6199	0.0807	64	532	542	AMVTQYGMSDK			Oxidation (M)[2,8]		Mascot
1326.661	1326.6639	0.0029	2	194	204	ESMQEIVSFLK			Oxidation (M)[3]		Mascot
1326.661	1326.6639	0.0029	2	194	204	ESMQEIVSFLK			Oxidation (M)[3]		Mascot
1549.8445	1549.7682	-0.0763	-49	357	370	GREAVLQIHANDVK					Mascot
1652.8564	1652.8204	-0.036	-22	608	621	KETITGEEFMNILK					Mascot
1652.8564	1652.8204	-0.036	-22	608	621	KETITGEEFMNILK					Mascot
1668.8513	1668.7999	-0.0514	-31	608	621	KETITGEEFMNILK			Oxidation (M)[10]		Mascot
1680.8625	1680.8463	-0.0162	-10	609	622	ETITGEEFMNILKR					Mascot
1684.8541	1684.7843	-0.0698	-41	39	52	QQIQTVSYSEFLNK					Mascot
1975.0594	1975.0963	0.0369	19	411	429	VTTEDLTESVDVVIAGAKK					Mascot
2118.9761	2119.1121	0.136	64	303	320	EQTLNQLLSEMDGFDNHK					Mascot
2166.9971	2167.0732	0.0761	35	506	525	AAEELIFGEMTNGASNDIER					Mascot

2182.9922	2183.0481	0.0559	26	506	525	AAEELIFGEMTNGASNDIER			Oxidation (M)[10]		Mascot
2240.9812	2241.1599	0.1787	80	145	168	AMGDDGPSMNFGGGFGGLGGNLGR					Mascot
2633.3682	2633.4299	0.0617	23	440	464	DVVAYHETGHAIVGAIQKNDAPVTK					Mascot

8	Glutamine--fructose-6-phosphate aminotransferase [isomerizing] OS=Pyrococcus kodakaraensis (strain ATCC BAA-918 / JCM 12380 / KOD1) GN=glmS PE=3 SV=3	GLMS_PYRKO	66483	5.26	17	62	69.592	8.31

Peptide Information

Calc. Mass	Obsrv. Mass	± da	± ppm	Start Seq.	End Seq.	Sequence	Ion Score	C. I. %	Modification	Rank	Result Type
866.473	866.4145	-0.0585	-68	219	225	DSYVVKR					Mascot
917.4515	917.5028	0.0513	56	462	468	DFFYIGR					Mascot
946.539	946.4897	-0.0493	-52	12	19	KACEVIVK			Carbamidomethyl (C)[3]		Mascot
1020.503	1020.574	0.071	70	525	533	MVSNIEEAK					Mascot
1078.5562	1078.484	-0.0722	-67	536	545	GAMIISLSDR			Oxidation (M)[3]		Mascot
1123.5565	1123.6426	0.0861	77	2	11	CGIIGYIGDR			Carbamidomethyl (C)[1]		Mascot
1247.6412	1247.6477	0.0065	5	525	535	MVSNIEEAKAR					Mascot
1251.6515	1251.6166	-0.0349	-28	2	12	CGIIGYIGDRK			Carbamidomethyl (C)[1]		Mascot
1275.658	1275.5962	-0.0618	-48	316	326	VPIVEEASEFR					Mascot
1382.6919	1382.7528	0.0609	44	1	12	MCGIIGYIGDRK			Carbamidomethyl (C)[2]		Mascot
1636.8363	1636.8292	-0.0071	-4	54	67	LTEKLGFLEMEGNR					Mascot
1652.8313	1652.8204	-0.0109	-7	54	67	LTEKLGFLEMEGNR			Oxidation (M)[10]		Mascot
1652.8313	1652.8204	-0.0109	-7	54	67	LTEKLGFLEMEGNR			Oxidation (M)[10]		Mascot
1716.8802	1716.775	-0.1052	-61	484	499	EISYIHAEGLSAGELK					Mascot
1786.0004	1785.9382	-0.0622	-35	361	378	NGAKVLAVVNVVGSMATR					Mascot
1832.0276	1831.8981	-0.1295	-71	436	451	VPELVELALKHDEALR					Mascot
1985.0875	1984.901	-0.1865	-94	429	445	LEDELMKVPELVELALK			Oxidation (M)[6]		Mascot
2026.1141	2026.0259	-0.0882	-44	399	416	TYTTQLTVLTMLAIELAK			Oxidation (M)[11]		Mascot
2183.0789	2183.0481	-0.0308	-14	417	435	VLGTASEDYLEKLEDELMK					Mascot
2199.0737	2199.0496	-0.0241	-11	417	435	VLGTASEDYLEKLEDELMK			Oxidation (M)[18]		Mascot

9	Genome polyprotein OS=Pea seed-borne mosaic virus (strain DPD1) PE=2 SV=1	POLG_PSBMV	367464.9	8.66	40	62	64.274	31.579

Peptide Information

Calc. Mass	Obsrv. Mass	± da	± ppm	Start Seq.	End Seq.	Sequence	Ion Score	C. I. %	Modification	Rank	Result Type

947.5673	947.4861	-0.0812	-86	1533	1540	LLVDRGFK				Mascot
1038.5653	1038.4958	-0.0695	-67	2462	2470	GAFIKDIMK			Oxidation (M)[8]	Mascot
1052.5088	1052.5461	0.0373	35	617	624	FFSEFFTK				Mascot
1057.4772	1057.4255	-0.0517	-49	2786	2793	DVWFMSTR			Oxidation (M)[5]	Mascot
1072.6361	1072.5723	-0.0638	-59	278	286	QVTLIDKQK				Mascot
1078.5527	1078.484	-0.0687	-64	3114	3122	NSTEVYIPR				Mascot
1197.5747	1197.6605	0.0858	72	205	216	LQSTSFDASGGK				Mascot
1206.6008	1206.5842	-0.0166	-14	1088	1098	SQQMLASGGRR			Oxidation (M)[4]	Mascot
1208.6093	1208.5798	-0.0295	-24	729	738	TETGRMWIAK			Oxidation (M)[6]	Mascot
1222.5997	1222.5835	-0.0162	-13	2091	2100	IDLNPHNPMR			Oxidation (M)[9]	Mascot
1234.6539	1234.5884	-0.0655	-53	3113	3122	RNSTEVYIPR				Mascot
1234.6539	1234.5884	-0.0655	-53	3113	3122	RNSTEVYIPR	7	0		Mascot
1251.6515	1251.6166	-0.0349	-28	2297	2308	EGHCGLPAVALK			Carbamidomethyl (C)[4]	Mascot
1253.5977	1253.5848	-0.0129	-10	1716	1725	LRDSSMQLCK			Carbamidomethyl (C)[9], Oxidation (M)[6]	Mascot
1262.6681	1262.6199	-0.0482	-38	2288	2296	YWKHWITTK				Mascot
1264.6644	1264.5583	-0.1061	-84	711	722	NHLVIGNTGDPK				Mascot
1275.6726	1275.5962	-0.0764	-60	113	123	CKINENSIVAK			Carbamidomethyl (C)[1]	Mascot
1290.7456	1290.6512	-0.0944	-73	1758	1768	IPFYLKGVPEK				Mascot
1326.626	1326.6639	0.0379	29	1699	1709	YNGTMHPEIHK				Mascot
1326.626	1326.6639	0.0379	29	1699	1709	YNGTMHPEIHK				Mascot
1342.6208	1342.6393	0.0185	14	1699	1709	YNGTMHPEIHK			Oxidation (M)[5]	Mascot
1365.7672	1365.726	-0.0412	-30	2416	2427	CPGQLVTKHVVK			Carbamidomethyl (C)[1]	Mascot
1382.691	1382.7528	0.0618	45	203	216	GKLQSTSFDASGGK				Mascot
1449.7737	1449.6862	-0.0875	-60	2339	2350	YLLNAEALQWTK				Mascot
1547.8118	1547.7717	-0.0401	-26	2239	2250	DFPPFPQRLQFR				Mascot
1573.6848	1573.8138	0.129	82	777	789	WPTMMDVATACYK			Carbamidomethyl (C)[11]	Mascot
1574.785	1574.8074	0.0224	14	1178	1189	QVTREEFFDYLK				Mascot
1589.6797	1589.7802	0.1005	63	777	789	WPTMMDVATACYK			Carbamidomethyl (C)[11], Oxidation (M)[4]	Mascot
1605.6747	1605.7754	0.1007	63	777	789	WPTMMDVATACYK			Carbamidomethyl (C)[11], Oxidation (M)[4,5]	Mascot
1646.7765	1646.793	0.0165	10	2269	2282	SIQSVITESCMTFK			Carbamidomethyl (C)[10], Oxidation (M)[11]	Mascot
1705.912	1705.8896	-0.0224	-13	994	1009	VSADAELLRDGFVVSK				Mascot
1732.8461	1732.792	-0.0541	-31	2969	2985	SDRDVDAGSSGTITVPR				Mascot
1752.8949	1752.8677	-0.0272	-16	1351	1368	GAVGSGKSTGLPCYLSA K			Carbamidomethyl (C)[13]	Mascot
1797.0017	1796.9437	-0.058	-32	2560	2575	GVWNGSLKAELRPIEK				Mascot
1992.9702	1992.9198	-0.0504	-25	3131	3147	DPSLARYGFDFYEITAK				Mascot
1993.0138	1992.9198	-0.094	-47	1513	1529	KGHNILVYVSSYNEVDR				Mascot
2012.9971	2013.0154	0.0183	9	1855	1871	WMVDHSGENIVKLQNAR			Oxidation (M)[2]	Mascot

Calc. Mass	Obsrv. Mass	± da	± ppm	Start Seq.	End Seq.	Sequence	Ion Score	C. I. %	Modification	Rank	Result Type
2014.1268	2013.9973	-0.1295	-64	1121	1136	FVNMLLVLSMIFKLWK			Oxidation (M)[4,10]		Mascot
2014.1268	2013.9973	-0.1295	-64	1121	1136	FVNMLLVLSMIFKLWK			Oxidation (M)[4,10]		Mascot
2166.9897	2167.0732	0.0835	39	3168	3187	SNSLFGLDGNVGTQEENTER					Mascot
2183.1741	2183.0481	-0.126	-58	258	277	TESIDVLIEQVMTIAGKHAK					Mascot
2199.1228	2199.0496	-0.0732	-33	976	993	TQHTYPIVQNQLDIMIER					Mascot
2239.012	2239.1616	0.1496	67	1782	1802	SDSGFGRMSTASACNVAYTLK			Carbamidomethyl (C)[14], Oxidation (M)[8]		Mascot
2566.2566	2566.2996	0.043	17	1827	1850	SQFDLMSSHVTNSSSISLAGLVNR			Oxidation (M)[6]		Mascot
2633.303	2633.4299	0.1269	48	1026	1047	NSFTDLPYVQQLQQTMSFSRVK			Oxidation (M)[16]		Mascot
2739.3416	2739.4189	0.0773	28	1218	1241	HGEIRFEQTVALMALLAMMFGSDR			Oxidation (M)[13]		Mascot

10	ATP-dependent helicase/nuclease subunit A OS=Desulforudis audaxviator (strain MP104C) GN=addA PE=3 SV=1	ADDA_DESAP	135086.2	5.5	22	62	63.442	9.914	

Peptide Information

Calc. Mass	Obsrv. Mass	± da	± ppm	Start Seq.	End Seq.	Sequence	Ion Score	C. I. %	Modification	Rank	Result Type
900.5261	900.5527	0.0266	30	311	317	ERIQNLK					Mascot
932.4948	932.4709	-0.0239	-26	1057	1065	GLSPQAFGR					Mascot
1197.6416	1197.6605	0.0189	16	276	286	AAFLAFGFTPR					Mascot
1262.5132	1262.6199	0.1067	85	540	551	DTAGTADDEPDR					Mascot
1326.6589	1326.6639	0.005	4	187	197	SNPWPEAWLAR					Mascot
1326.6589	1326.6639	0.005	4	187	197	SNPWPEAWLAR					Mascot
1431.7339	1431.6398	-0.0941	-66	1182	1195	VGPGGEAALADRYR					Mascot
1449.8101	1449.6862	-0.1239	-85	446	457	LAEPALFLEKYR					Mascot
1490.7268	1490.6649	-0.0619	-42	292	304	AGVDQELKDLCSR			Carbamidomethyl (C)[11]		Mascot
1589.8534	1589.7802	-0.0732	-46	220	234	DLVGTGLEAAVFELR					Mascot
1591.7786	1591.7605	-0.0181	-11	424	438	QDAAAPNLFMVGDVK			Oxidation (M)[10]		Mascot
1606.8622	1606.8096	-0.0526	-33	55	69	LLVVTFTNAAAAEMR					Mascot
1700.8656	1700.7959	-0.0697	-41	184	197	FARSNPWPEAWLAR					Mascot
1705.9537	1705.8896	-0.0641	-38	276	291	AAFLAFGFTPRLPAAR					Mascot
1716.8123	1716.775	-0.0373	-22	5	19	NWTGPQEAAIGCREK			Carbamidomethyl (C)[12]		Mascot
1747.781	1747.8706	0.0896	51	138	150	QETLEEFFEEQYR					Mascot
1831.9524	1831.8981	-0.0543	-30	813	827	RFYMPDLNGEVLLHK					Mascot
1984.983	1984.901	-0.082	-41	859	874	EQLAEEMRILYVAMTR			Oxidation (M)[7,14]		Mascot
2043.0474	2043.0416	-0.0058	-3	324	341	RPEELLSEMAALGPAMRR			Oxidation (M)[9]		Mascot
2155.0566	2155.0588	0.0022	1	198	219	AAAFGADPQGLDGDGPL					Mascot

						AWGLR		
2239.1177	2239.1616	0.0439	20	424	443	QDAAAPNLFMVGDVKQS IYR	Oxidation (M)[10]	Mascot
2633.3181	2633.4299	0.1118	42	1000	1023	ALSWHYPWSALASCGAK ISATEVK	Carbamidomethyl (C)[14]	Mascot
2842.4768	2842.3943	-0.0825	-29	43	69	ISDPAAPVDVDRLLVVTF TNAAAAEMR		Mascot

Gel Idx/Pos	277/L5	Instr./Gel Origin	BA2151/Sample Project 20140814	Process Status	Analysis Succeeded
Plate [#] Name	[1] Sample Project 20140814	Instrument Sample Name		Spectra	11

Rank	Protein Name	Accession No.	Protein MW	Protein PI	Pep. Count	Protein Score	Protein Score C. I. %	Intensity Matched	Total Ion Score	Total Ion C. I. %	Confirmed
1	Adenosylhomocysteinase OS=Phalaenopsis sp. GN=SAHH PE=2 SV=1	SAHH_PHASS	53734.3	5.79	11	302	100	12.311	265	100	

Peptide Information

Calc. Mass	Obsrv. Mass	± da	± ppm	Start Seq.	End Seq.	Sequence	Ion Score	C. I. %	Modification	Rank	Result Type
1008.4745	1008.4741	-0.0004	0	18	26	DLSQADFGR					Mascot
1008.4745	1008.4741	-0.0004	0	18	26	DLSQADFGR	75	99.997			Mascot
1041.5146	1041.5033	-0.0113	-11	246	254	HSLPDGLMR			Oxidation (M)[8]		Mascot
1044.4568	1044.4551	-0.0017	-2	238	245	FDNLYGCR			Carbamidomethyl (C)[7]		Mascot
1044.4568	1044.4551	-0.0017	-2	238	245	FDNLYGCR	30	17.525	Carbamidomethyl (C)[7]		Mascot
1071.6157	1071.6138	-0.0019	-2	369	377	ITIKPQTDR					Mascot
1107.547	1107.5271	-0.0199	-18	44	53	AEFGPSQPFK					Mascot
1161.5392	1161.538	-0.0012	-1	329	337	DIIMVDHMR			Oxidation (M)[4,8]		Mascot
1235.6379	1235.6345	-0.0034	-3	16	26	VKDLSQADFGR					Mascot
1259.5837	1259.5884	0.0047	4	236	245	SKFDNLYGCR			Carbamidomethyl (C)[9]		Mascot
1259.5837	1259.5884	0.0047	4	236	245	SKFDNLYGCR	44	96.601	Carbamidomethyl (C)[9]		Mascot
1992.9438	1992.9331	-0.0107	-5	27	43	LEIELAEVEMPGLMACR			Carbamidomethyl (C)[16], Oxidation (M)[10,14]		Mascot
2178.9807	2179.0144	0.0337	15	83	101	WCSCNIFSTQDHAAAAIA R			Carbamidomethyl (C)[2,4]		Mascot
2178.9807	2179.0144	0.0337	15	83	101	WCSCNIFSTQDHAAAAIA R	114	100	Carbamidomethyl (C)[2,4]		Mascot
2740.4915	2740.4712	-0.0203	-7	57	82	ISGSLHMTIQTAVLIETLT ALGAEVR			Oxidation (M)[7]		Mascot

Rank	Protein Name	Accession No.	Protein MW	Protein PI	Pep. Count	Protein Score	Protein Score C. I. %	Intensity Matched	Total Ion Score	Total Ion C. I. %	Confirmed
2	Adenosylhomocysteinase OS=Medicago sativa GN=SAHH PE=2 SV=1	SAHH_MEDSA	53744.2	5.69	10	288	100	12.855	256	100	

Peptide Information

Calc. Mass	Obsrv. Mass	± da	± ppm	Start Seq.	End Seq.	Sequence	Ion Score	C. I. %	Modification	Rank	Result Type
1026.4309	1026.4722	0.0413	40	18	26	DMSQADFGR					Mascot
1041.5146	1041.5033	-0.0113	-11	246	254	HSLPDGLMR			Oxidation (M)[8]		Mascot
1044.4568	1044.4551	-0.0017	-2	238	245	FDNLYGCR			Carbamidomethyl (C)[7]		Mascot
1044.4568	1044.4551	-0.0017	-2	238	245	FDNLYGCR	30	17.525	Carbamidomethyl (C)[7]		Mascot
1071.6157	1071.6138	-0.0019	-2	369	377	ITIKPQTDR					Mascot

Calc. Mass	Obsrv. Mass	± da	± ppm	Start Seq.	End Seq.	Sequence	Ion Score	C. I. %	Modification	Rank	Result Type
1137.5576	1137.5415	-0.0161	-14	44	53	TEFGPSQPFK					Mascot
1259.5837	1259.5884	0.0047	4	236	245	SKFDNLYGCR			Carbamidomethyl (C)[9]		Mascot
1259.5837	1259.5884	0.0047	4	236	245	SKFDNLYGCR	44	96.601	Carbamidomethyl (C)[9]		Mascot
1902.0485	1902.0322	-0.0163	-9	378	394	WVFPETKSGIIVLAEGR					Mascot
1902.0485	1902.0322	-0.0163	-9	378	394	WVFPETKSGIIVLAEGR	67	99.983			Mascot
1992.9438	1992.9331	-0.0107	-5	27	43	LEIELAEVEMPGLMSCR			Carbamidomethyl (C)[16], Oxidation (M)[10]		Mascot
2178.9807	2179.0144	0.0337	15	83	101	WCSCNIFSTQDHAAAAIA R			Carbamidomethyl (C)[2,4]		Mascot
2178.9807	2179.0144	0.0337	15	83	101	WCSCNIFSTQDHAAAAIA R	114	100	Carbamidomethyl (C)[2,4]		Mascot
2510.2959	2510.2471	-0.0488	-19	213	235	LYQMQASGTLLFPAINVN DSVTK					Mascot

3	Adenosylhomocysteinase OS=Mesembryanthemum crystallinum GN=SAHH PE=2 SV=1	SAHH_MESCR	53771.2	5.75	10	220	100	9.959	189	100

Peptide Information

Calc. Mass	Obsrv. Mass	± da	± ppm	Start Seq.	End Seq.	Sequence	Ion Score	C. I. %	Modification	Rank	Result Type
1026.4309	1026.4722	0.0413	40	18	26	DMSQADFGR					Mascot
1041.5146	1041.5033	-0.0113	-11	246	254	HSLPDGLMR			Oxidation (M)[8]		Mascot
1044.4568	1044.4551	-0.0017	-2	238	245	FDNLYGCR			Carbamidomethyl (C)[7]		Mascot
1044.4568	1044.4551	-0.0017	-2	238	245	FDNLYGCR	30	17.525	Carbamidomethyl (C)[7]		Mascot
1071.6157	1071.6138	-0.0019	-2	369	377	ITIKPQTDR					Mascot
1137.5576	1137.5415	-0.0161	-14	44	53	TEFGPSQPFK					Mascot
1161.5392	1161.538	-0.0012	-1	329	337	DIIMVDHMR			Oxidation (M)[4,8]		Mascot
1259.5837	1259.5884	0.0047	4	236	245	SKFDNLYGCR			Carbamidomethyl (C)[9]		Mascot
1259.5837	1259.5884	0.0047	4	236	245	SKFDNLYGCR	44	96.601	Carbamidomethyl (C)[9]		Mascot
1992.9438	1992.9331	-0.0107	-5	27	43	LEIELAEVEMPGLMACR			Carbamidomethyl (C)[16], Oxidation (M)[10,14]		Mascot
2178.9807	2179.0144	0.0337	15	83	101	WCSCNIFSTQDHAAAAIA R			Carbamidomethyl (C)[2,4]		Mascot
2178.9807	2179.0144	0.0337	15	83	101	WCSCNIFSTQDHAAAAIA R	114	100	Carbamidomethyl (C)[2,4]		Mascot
2235.2207	2235.0598	-0.1609	-72	378	397	FVFPETNTGIIVLAEGRLM K					Mascot

4	Adenosylhomocysteinase 1 OS=Arabidopsis thaliana GN=SAHH1 PE=1 SV=1	SAHH1_ARATH	53971.3	5.66	10	219	100	9.946	189	100

Peptide Information

Calc. Mass	Obsrv. Mass	± da	± ppm	Start Seq.	End Seq.	Sequence	Ion Score	C. I. %	Modification	Rank	Result Type
1026.4309	1026.4722	0.0413	40	18	26	DMSQADFGR					Mascot
1041.5146	1041.5033	-0.0113	-11	246	254	HSLPDGLMR			Oxidation (M)[8]		Mascot

1044.4568	1044.4551	-0.0017	-2	238	245	FDNLYGCR			Carbamidomethyl (C)[7]		Mascot
1044.4568	1044.4551	-0.0017	-2	238	245	FDNLYGCR	30	17.525	Carbamidomethyl (C)[7]		Mascot
1071.6157	1071.6138	-0.0019	-2	369	377	ITIKPQTDR					Mascot
1137.5576	1137.5415	-0.0161	-14	44	53	TEFGPSQPFK					Mascot
1161.5392	1161.538	-0.0012	-1	329	337	DIIMVDHMR			Oxidation (M)[4,8]		Mascot
1247.7947	1247.6736	-0.1211	-97	447	458	VALLHLGKLGAR					Mascot
1259.5837	1259.5884	0.0047	4	236	245	SKFDNLYGCR			Carbamidomethyl (C)[9]		Mascot
1259.5837	1259.5884	0.0047	4	236	245	SKFDNLYGCR	44	96.601	Carbamidomethyl (C)[9]		Mascot
1992.9438	1992.9331	-0.0107	-5	27	43	LELELAEVEMPGLMACR			Carbamidomethyl (C)[16], Oxidation (M)[10,14]		Mascot
2178.9807	2179.0144	0.0337	15	83	101	WCSCNIFSTQDHAAAAIA R			Carbamidomethyl (C)[2,4]		Mascot
2178.9807	2179.0144	0.0337	15	83	101	WCSCNIFSTQDHAAAAIA R	114	100	Carbamidomethyl (C)[2,4]		Mascot

5	Adenosylhomocysteinase OS=Triticum aestivum GN=SAHH PE=2 SV=1	SAHH_WHEAT	54086.4	5.65	13	214	100	12.763	167	100	

Peptide Information

Calc. Mass	Obsrv. Mass	± da	± ppm	Start Seq.	End Seq.	Sequence	Ion Score	C. I. %	Modification	Rank	Result Type
827.5713	827.5648	-0.0065	-8	176	182	IVLTIIR					Mascot
1028.6099	1028.6066	-0.0033	-3	385	394	TGIIVLAEGR					Mascot
1028.6099	1028.6066	-0.0033	-3	385	394	TGIIVLAEGR	13	0			Mascot
1041.5146	1041.5033	-0.0113	-11	246	254	HSLPDGLMR			Oxidation (M)[8]		Mascot
1044.4568	1044.4551	-0.0017	-2	238	245	FDNLYGCR			Carbamidomethyl (C)[7]		Mascot
1044.4568	1044.4551	-0.0017	-2	238	245	FDNLYGCR	30	17.525	Carbamidomethyl (C)[7]		Mascot
1071.6157	1071.6138	-0.0019	-2	369	377	ITIKPQTDR					Mascot
1137.5576	1137.5415	-0.0161	-14	44	53	TEFGPSQPFK					Mascot
1161.5392	1161.538	-0.0012	-1	329	337	DIIMVDHMR			Oxidation (M)[4,8]		Mascot
1259.5837	1259.5884	0.0047	4	236	245	SKFDNLYGCR			Carbamidomethyl (C)[9]		Mascot
1259.5837	1259.5884	0.0047	4	236	245	SKFDNLYGCR	44	96.601	Carbamidomethyl (C)[9]		Mascot
1758.7428	1758.7628	0.02	11	111	123	GETLEEYWWCTER			Carbamidomethyl (C)[10]		Mascot
1758.7428	1758.7628	0.02	11	111	123	GETLEEYWWCTER	79	99.999	Carbamidomethyl (C)[10]		Mascot
1916.0641	1916.0217	-0.0424	-22	378	394	WVFPETKTGIIVLAEGR					Mascot
1992.9438	1992.9331	-0.0107	-5	27	43	LELELAEVEMPGLMACR			Carbamidomethyl (C)[16], Oxidation (M)[10,14]		Mascot
2584.2964	2584.322	0.0256	10	213	235	LYQMQESGTLLFPAINVN DSVTK			Oxidation (M)[4]		Mascot
2740.4915	2740.4712	-0.0203	-7	57	82	ISGSLHMTIQTAVLIETLT ALGAEVR			Oxidation (M)[7]		Mascot

6	Adenosylhomocysteinase OS=Nicotiana tabacum GN=SAHH PE=2 SV=1	SAHH_TOBAC	53640.2	5.51	9	214	100	9.802	189	100	

Protein Group

Adenosylhomocysteinase OS=Nicotiana sylvestris GN=SAHH PE=2 SV=1	SAHH_NICSY	53640.2	5.5100 002288 8184

Peptide Information

Calc. Mass	Obsrv. Mass	± da	± ppm	Start Seq.	End Seq.	Sequence	Ion Score	C. I. %	Modification	Rank	Result Type
1026.4309	1026.4722	0.0413	40	18	26	DMSQADFGR					Mascot
1041.5146	1041.5033	-0.0113	-11	246	254	HSLPDGLMR			Oxidation (M)[8]		Mascot
1044.4568	1044.4551	-0.0017	-2	238	245	FDNLYGCR			Carbamidomethyl (C)[7]		Mascot
1044.4568	1044.4551	-0.0017	-2	238	245	FDNLYGCR	30	17.525	Carbamidomethyl (C)[7]		Mascot
1071.6157	1071.6138	-0.0019	-2	369	377	ITIKPQTDR					Mascot
1137.5576	1137.5415	-0.0161	-14	44	53	TEFGPSQPFK					Mascot
1161.5392	1161.538	-0.0012	-1	329	337	DIIMVDHMR			Oxidation (M)[4,8]		Mascot
1259.5837	1259.5884	0.0047	4	236	245	SKFDNLYGCR			Carbamidomethyl (C)[9]		Mascot
1259.5837	1259.5884	0.0047	4	236	245	SKFDNLYGCR	44	96.601	Carbamidomethyl (C)[9]		Mascot
1992.9438	1992.9331	-0.0107	-5	27	43	LEIELAEVEMPGLMACR			Carbamidomethyl (C)[16], Oxidation (M)[10,14]		Mascot
2178.9807	2179.0144	0.0337	15	83	101	WCSCNIFSTQDHAAAAIA R			Carbamidomethyl (C)[2,4]		Mascot
2178.9807	2179.0144	0.0337	15	83	101	WCSCNIFSTQDHAAAAIA R	114	100	Carbamidomethyl (C)[2,4]		Mascot

7	Adenosylhomocysteinase OS=Petroselinum crispum GN=SAHH PE=2 SV=2	SAHH_PETCR	53774.1	5.6	9	214	100	9.378	189	100	

Peptide Information

Calc. Mass	Obsrv. Mass	± da	± ppm	Start Seq.	End Seq.	Sequence	Ion Score	C. I. %	Modification	Rank	Result Type
977.4839	977.5073	0.0234	24	378	385	WVFPDTGR					Mascot
1041.5146	1041.5033	-0.0113	-11	246	254	HSLPDGLMR			Oxidation (M)[8]		Mascot
1044.4568	1044.4551	-0.0017	-2	238	245	FDNLYGCR			Carbamidomethyl (C)[7]		Mascot
1044.4568	1044.4551	-0.0017	-2	238	245	FDNLYGCR	30	17.525	Carbamidomethyl (C)[7]		Mascot
1071.6157	1071.6138	-0.0019	-2	369	377	ITIKPQTDR					Mascot
1137.5576	1137.5415	-0.0161	-14	44	53	TEFGPSQPFK					Mascot
1259.5837	1259.5884	0.0047	4	236	245	SKFDNLYGCR			Carbamidomethyl (C)[9]		Mascot
1259.5837	1259.5884	0.0047	4	236	245	SKFDNLYGCR	44	96.601	Carbamidomethyl (C)[9]		Mascot
1992.9438	1992.9331	-0.0107	-5	27	43	LELELAEVEMPGLMSCR			Carbamidomethyl (C)[16], Oxidation (M)[10]		Mascot
2178.9807	2179.0144	0.0337	15	83	101	WCSCNIFSTQDHAAAAIA R			Carbamidomethyl (C)[2,4]		Mascot
2178.9807	2179.0144	0.0337	15	83	101	WCSCNIFSTQDHAAAAIA R	114	100	Carbamidomethyl (C)[2,4]		Mascot
2740.4915	2740.4712	-0.0203	-7	57	82	ITGSLHMTIQTGVLIETLT			Oxidation (M)[7]		Mascot

ALGAEVR

8	Adenosylhomocysteinase OS=Solanum lycopersicum GN=SAHH PE=2 SV=1	SAHH_SOLLC	53876.2	5.57	8	209	100	9.171	189	100	

Peptide Information

Calc. Mass	Obsrv. Mass	± da	± ppm	Start Seq.	End Seq.	Sequence	Ion Score	C. I. %	Modification	Rank	Result Type
1026.4309	1026.4722	0.0413	40	18	26	DMSQADFGR					Mascot
1041.5146	1041.5033	-0.0113	-11	246	254	HSLPDGLMR			Oxidation (M)[8]		Mascot
1044.4568	1044.4551	-0.0017	-2	238	245	FDNLYGCR			Carbamidomethyl (C)[7]		Mascot
1044.4568	1044.4551	-0.0017	-2	238	245	FDNLYGCR	30	17.525	Carbamidomethyl (C)[7]		Mascot
1071.6157	1071.6138	-0.0019	-2	369	377	ITIKPQTDR					Mascot
1161.5392	1161.538	-0.0012	-1	329	337	DIIMVDHMR			Oxidation (M)[4,8]		Mascot
1259.5837	1259.5884	0.0047	4	236	245	SKFDNLYGCR			Carbamidomethyl (C)[9]		Mascot
1259.5837	1259.5884	0.0047	4	236	245	SKFDNLYGCR	44	96.601	Carbamidomethyl (C)[9]		Mascot
1887.9554	1887.9125	-0.0429	-23	27	43	LEIELAEVEMPGLMASR					Mascot
2178.9807	2179.0144	0.0337	15	83	101	WCSCNIFSTQDHAAAAIA R			Carbamidomethyl (C)[2,4]		Mascot
2178.9807	2179.0144	0.0337	15	83	101	WCSCNIFSTQDHAAAAIA R	114	100	Carbamidomethyl (C)[2,4]		Mascot

9	Adenosylhomocysteinase OS=Lupinus luteus GN=SAHH PE=1 SV=1	SAHH_LUPLU	53805.2	5.64	8	206	100	9.117	188	100	

Peptide Information

Calc. Mass	Obsrv. Mass	± da	± ppm	Start Seq.	End Seq.	Sequence	Ion Score	C. I. %	Modification	Rank	Result Type
1026.4309	1026.4722	0.0413	40	18	26	DMSQADFGR					Mascot
1041.5146	1041.5033	-0.0113	-11	246	254	HSLPDGLMR			Oxidation (M)[8]		Mascot
1044.4568	1044.4551	-0.0017	-2	238	245	FDNLYGCR			Carbamidomethyl (C)[7]		Mascot
1044.4568	1044.4551	-0.0017	-2	238	245	FDNLYGCR	30	17.525	Carbamidomethyl (C)[7]		Mascot
1071.6157	1071.6138	-0.0019	-2	369	377	ITIKPQTDR					Mascot
1259.5837	1259.5884	0.0047	4	236	245	SKFDNLYGCR			Carbamidomethyl (C)[9]		Mascot
1259.5837	1259.5884	0.0047	4	236	245	SKFDNLYGCR	44	96.601	Carbamidomethyl (C)[9]		Mascot
1887.9554	1887.9125	-0.0429	-23	27	43	LEIELAEVEMPGLMASR					Mascot
1916.0277	1916.0217	-0.006	-3	378	394	WVFPETNTGIIILAEGR					Mascot
2178.9807	2179.0144	0.0337	15	83	101	WCSCNIFSTQDHAAAAIA R			Carbamidomethyl (C)[2,4]		Mascot
2178.9807	2179.0144	0.0337	15	83	101	WCSCNIFSTQDHAAAAIA R	114	100	Carbamidomethyl (C)[2,4]		Mascot

10	Adenosylhomocysteinase 2 OS=Arabidopsis thaliana GN=SAHH2 PE=1 SV=1	SAHH2_ARATH	53809.1	5.49	7	204	100	9.01	189	100	

Peptide Information

Calc. Mass	Obsrv. Mass	± da	± ppm	Start Seq.	End Seq.	Sequence	Ion Score	C. I. %	Modification	Rank	Result Type
1026.4309	1026.4722	0.0413	40	18	26	DMSQADFGR					Mascot
1041.5146	1041.5033	-0.0113	-11	246	254	HSLPDGLMR			Oxidation (M)[8]		Mascot
1044.4568	1044.4551	-0.0017	-2	238	245	FDNLYGCR			Carbamidomethyl (C)[7]		Mascot
1044.4568	1044.4551	-0.0017	-2	238	245	FDNLYGCR	30	0	Carbamidomethyl (C)[7]		Mascot
1071.6157	1071.6138	-0.0019	-2	369	377	ITIKPQTDR					Mascot
1161.5392	1161.538	-0.0012	-1	329	337	DIIMVDHMR			Oxidation (M)[4,8]		Mascot
1259.5837	1259.5884	0.0047	4	236	245	SKFDNLYGCR			Carbamidomethyl (C)[9]		Mascot
1259.5837	1259.5884	0.0047	4	236	245	SKFDNLYGCR	44	95.831	Carbamidomethyl (C)[9]		Mascot
2178.9807	2179.0144	0.0337	15	83	101	WCSCNIFSTQDHAAAAIA R			Carbamidomethyl (C)[2,4]		Mascot
2178.9807	2179.0144	0.0337	15	83	101	WCSCNIFSTQDHAAAAIA R	114	100	Carbamidomethyl (C)[2,4]		Mascot

Gel Idx/Pos	278/L6	Instr./Gel Origin	BA2151/Sample Project 20140814	Process Status	Analysis Succeeded
Plate [#] Name	[1] Sample Project 20140814	Instrument Sample Name		Spectra	11

Rank	Protein Name	Accession No.	Protein MW	Protein PI	Pep. Count	Protein Score	Protein Score C. I. %	Intensity Matched	Total Ion Score	Total Ion C. I. %	Confirmed
1	ATP synthase subunit alpha, mitochondrial OS=Oryza sativa subsp. indica GN=ATPA PE=2 SV=1	ATPAM_ORYSI	55624	5.85	19	837	100	47.013	739	100	

Protein Group

ATP synthase subunit alpha, mitochondrial OS=Oryza sativa GN=ATPA PE=2 SV=1	ATPAM_ORYSA	55624	5.8499999046 3257
ATP synthase subunit alpha, mitochondrial OS=Oryza sativa subsp. japonica GN=ATPA PE=1 SV=1	ATPAM_ORYSJ	55624	5.8499999046 3257

Peptide Information

Calc. Mass	Obsrv. Mass	± da	± ppm	Start Seq.	End Seq.	Sequence	Ion Score	C. I. %	Modification	Rank	Result Type
815.4621	815.4484	-0.0137	-17	167	173	ELIIGDR					Mascot
860.5022	860.4489	-0.0533	-62	283	289	QMSLLLR					Mascot
874.4741	874.4857	0.0116	13	487	494	GGLTNERK					Mascot
876.4971	876.4717	-0.0254	-29	283	289	QMSLLLR			Oxidation (M)[2]		Mascot
884.4221	884.4128	-0.0093	-11	121	128	GALSDHER					Mascot
892.4886	892.4791	-0.0095	-11	395	401	LELAQYR					Mascot
972.5473	972.5329	-0.0144	-15	33	42	VVSVGDGIAR					Mascot
972.5473	972.5329	-0.0144	-15	33	42	VVSVGDGIAR	72	99.992			Mascot
1026.5942	1026.5762	-0.018	-18	154	163	AVDSLVPIGR					Mascot
1026.5942	1026.5762	-0.018	-18	154	163	AVDSLVPIGR	73	99.995			Mascot
1137.5756	1137.5282	-0.0474	-42	385	394	AMKQVCGSLK			Carbamidomethyl (C)[6], Oxidation (M)[2]		Mascot
1203.658	1203.6455	-0.0125	-10	7	17	AAELTTLLESR					Mascot
1203.658	1203.6455	-0.0125	-10	7	17	AAELTTLLESR	74	99.996			Mascot
1242.6147	1242.5756	-0.0391	-31	143	153	SVHEPMQTGLK			Oxidation (M)[6]		Mascot
1300.7471	1300.6886	-0.0585	-45	178	189	TAIAIDTILNQK					Mascot
1341.705	1341.649	-0.056	-42	433	443	QPQYEPLPIEK					Mascot
1438.8489	1438.8287	-0.0202	-14	363	376	GIRPAINVGLSVSR					Mascot
1438.8489	1438.8287	-0.0202	-14	363	376	GIRPAINVGLSVSR	45	96.436			Mascot
1537.7434	1537.7349	-0.0085	-6	295	307	EAFPGDVFYLHSR					Mascot
1537.7434	1537.7349	-0.0085	-6	295	307	EAFPGDVFYLHSR	108	100			Mascot
1720.821	1720.9739	0.1529	89	262	276	DNGMHALIIYDDLSK			Oxidation (M)[4]		Mascot
1724.8789	1724.8748	-0.0041	-2	444	458	QIVVIYAAVNGFCDR			Carbamidomethyl (C)[13]		Mascot

1834.8429	1834.851	0.0081	4	18	32	MTNFYTNFQVDEIGR					Mascot
1850.8378	1850.8162	-0.0216	-12	18	32	MTNFYTNFQVDEIGR			Oxidation (M)[1]		Mascot
1850.8378	1850.8162	-0.0216	-12	18	32	MTNFYTNFQVDEIGR	133	100	Oxidation (M)[1]		Mascot
2157.0532	2157.0288	-0.0244	-11	43	62	VYGLNEIQAGEMVEFAS GVK			Oxidation (M)[12]		Mascot
2308.1567	2308.1646	0.0079	3	402	423	EVAAFAQFGSDLDAATQ ALLNR					Mascot
2308.1567	2308.1646	0.0079	3	402	423	EVAAFAQFGSDLDAATQ ALLNR	233	100			Mascot

2	ATP synthase subunit alpha, mitochondrial OS=Phaseolus vulgaris GN=ATPA PE=1 SV=1	ATPAM_PHAVU	55595.1	6.51	16	813	100	45.707	739	100	

Peptide Information

Calc. Mass	Obsrv. Mass	± da	± ppm	Start Seq.	End Seq.	Sequence	Ion Score	C. I. %	Modification	Rank	Result Type
815.4621	815.4484	-0.0137	-17	167	173	ELIIGDR					Mascot
860.5022	860.4489	-0.0533	-62	283	289	QMSLLLR					Mascot
876.4971	876.4717	-0.0254	-29	283	289	QMSLLLR			Oxidation (M)[2]		Mascot
884.4221	884.4128	-0.0093	-11	121	128	GALSDHER					Mascot
892.4886	892.4791	-0.0095	-11	395	401	LELAQYR					Mascot
972.5473	972.5329	-0.0144	-15	33	42	VVSVGDGIAR					Mascot
972.5473	972.5329	-0.0144	-15	33	42	VVSVGDGIAR	72	99.992			Mascot
1026.5942	1026.5762	-0.018	-18	154	163	AVDSLVPIGR					Mascot
1026.5942	1026.5762	-0.018	-18	154	163	AVDSLVPIGR	73	99.995			Mascot
1203.658	1203.6455	-0.0125	-10	7	17	AAELTTLLESR					Mascot
1203.658	1203.6455	-0.0125	-10	7	17	AAELTTLLESR	74	99.996			Mascot
1242.6147	1242.5756	-0.0391	-31	143	153	SVHEPMQTGLK			Oxidation (M)[6]		Mascot
1300.7471	1300.6886	-0.0585	-45	178	189	TAIAIDTILNQK					Mascot
1438.8489	1438.8287	-0.0202	-14	363	376	GIRPAINVGLSVSR					Mascot
1438.8489	1438.8287	-0.0202	-14	363	376	GIRPAINVGLSVSR	45	96.436			Mascot
1537.7434	1537.7349	-0.0085	-6	295	307	EAFPGDVFYLHSR					Mascot
1537.7434	1537.7349	-0.0085	-6	295	307	EAFPGDVFYLHSR	108	100			Mascot
1712.0204	1711.9003	-0.1201	-70	470	484	DILTTIKPELLQSLK					Mascot
1720.821	1720.9739	0.1529	89	262	276	DNGMHALIIYDDLSK			Oxidation (M)[4]		Mascot
1834.8429	1834.851	0.0081	4	18	32	MTNFYTNFQVDEIGR					Mascot
1850.8378	1850.8162	-0.0216	-12	18	32	MTNFYTNFQVDEIGR			Oxidation (M)[1]		Mascot
1850.8378	1850.8162	-0.0216	-12	18	32	MTNFYTNFQVDEIGR	133	100	Oxidation (M)[1]		Mascot
2157.0532	2157.0288	-0.0244	-11	43	62	VYGLNEIQAGEMVEFAS GVK			Oxidation (M)[12]		Mascot
2308.1567	2308.1646	0.0079	3	402	423	EVAAFAQFGSDLDAATQ					Mascot

						ALLNR					
2308.1567	2308.1646	0.0079	3	402	423	EVAAFAQFGSDLDAATQ ALLNR	233	100			Mascot

3	ATP synthase subunit alpha, mitochondrial OS=Zea mays GN=ATPA PE=3 SV=1	ATPAM_MAIZE	55430.9	5.85	17	686	100	43.434	606	100

Peptide Information

Calc. Mass	Obsrv. Mass	± da	± ppm	Start Seq.	End Seq.	Sequence	Ion Score	C. I. %	Modification	Rank	Result Type
815.4621	815.4484	-0.0137	-17	167	173	ELIIGDR					Mascot
860.5022	860.4489	-0.0533	-62	283	289	QMSLLLR					Mascot
874.4741	874.4857	0.0116	13	487	494	GGLTNERK					Mascot
876.4971	876.4717	-0.0254	-29	283	289	QMSLLLR			Oxidation (M)[2]		Mascot
884.4221	884.4128	-0.0093	-11	121	128	GALSDHER					Mascot
892.4886	892.4791	-0.0095	-11	395	401	LELAQYR					Mascot
972.5473	972.5329	-0.0144	-15	33	42	VVSVGDGIAR					Mascot
972.5473	972.5329	-0.0144	-15	33	42	VVSVGDGIAR	72	99.992			Mascot
1026.5942	1026.5762	-0.018	-18	154	163	AVDSLVPIGR					Mascot
1026.5942	1026.5762	-0.018	-18	154	163	AVDSLVPIGR	73	99.995			Mascot
1203.658	1203.6455	-0.0125	-10	7	17	AAELTTLLESR					Mascot
1203.658	1203.6455	-0.0125	-10	7	17	AAELTTLLESR	74	99.996			Mascot
1242.6147	1242.5756	-0.0391	-31	143	153	SVHEPMQTGLK			Oxidation (M)[6]		Mascot
1300.7471	1300.6886	-0.0585	-45	178	189	TAIAIDTILNQK					Mascot
1341.705	1341.649	-0.056	-42	433	443	QPQYEPLPIEK					Mascot
1438.8489	1438.8287	-0.0202	-14	363	376	GIRPAINVGLSVSR					Mascot
1438.8489	1438.8287	-0.0202	-14	363	376	GIRPAINVGLSVSR	45	96.436			Mascot
1537.7434	1537.7349	-0.0085	-6	295	307	EAFPGDVFYLHSR					Mascot
1537.7434	1537.7349	-0.0085	-6	295	307	EAFPGDVFYLHSR	108	100			Mascot
1720.821	1720.9739	0.1529	89	262	276	DNGMHALIIYDDLSK			Oxidation (M)[4]		Mascot
1724.8789	1724.8748	-0.0041	-2	444	458	QIVVIYAAVNGFCDR			Carbamidomethyl (C)[13]		Mascot
2157.0532	2157.0288	-0.0244	-11	43	62	VYGLNEIQAGEMVEFAS GVK			Oxidation (M)[12]		Mascot
2308.1567	2308.1646	0.0079	3	402	423	EVAAFAQFGSDLDAATQ ALLNR					Mascot
2308.1567	2308.1646	0.0079	3	402	423	EVAAFAQFGSDLDAATQ ALLNR	233	100			Mascot

4	ATP synthase subunit alpha, mitochondrial OS=Glycine max GN=ATPA PE=1 SV=1	ATPAM_SOYBN	55581.2	6.23	16	679	100	42.468	606	100

Peptide Information

Calc. Mass	Obsrv. Mass	± da	± ppm	Start Seq.	End Seq.	Sequence	Ion Score	C. I. %	Modification	Rank	Result Type

815.4621	815.4484	-0.0137	-17	167	173	ELIIGDR					Mascot
860.5022	860.4489	-0.0533	-62	283	289	QMSLLLR					Mascot
876.4971	876.4717	-0.0254	-29	283	289	QMSLLLR			Oxidation (M)[2]		Mascot
884.4221	884.4128	-0.0093	-11	121	128	GALSDHER					Mascot
892.4886	892.4791	-0.0095	-11	395	401	LELAQYR					Mascot
972.5473	972.5329	-0.0144	-15	33	42	VVSVGDGIAR					Mascot
972.5473	972.5329	-0.0144	-15	33	42	VVSVGDGIAR	72	99.992			Mascot
1026.5942	1026.5762	-0.018	-18	154	163	AVDSLVPIGR					Mascot
1026.5942	1026.5762	-0.018	-18	154	163	AVDSLVPIGR	73	99.995			Mascot
1137.5756	1137.5282	-0.0474	-42	385	394	AMKQVCGSLK			Carbamidomethyl (C)[6], Oxidation (M)[2]		Mascot
1203.658	1203.6455	-0.0125	-10	7	17	AAELTTLLESR					Mascot
1203.658	1203.6455	-0.0125	-10	7	17	AAELTTLLESR	74	99.996			Mascot
1242.6147	1242.5756	-0.0391	-31	143	153	SVHEPMQTGLK			Oxidation (M)[6]		Mascot
1300.7471	1300.6886	-0.0585	-45	178	189	TAIAIDTILNQK					Mascot
1438.8489	1438.8287	-0.0202	-14	363	376	GIRPAINVGLSVSR					Mascot
1438.8489	1438.8287	-0.0202	-14	363	376	GIRPAINVGLSVSR	45	96.436			Mascot
1537.7434	1537.7349	-0.0085	-6	295	307	EAFPGDVFYLHSR					Mascot
1537.7434	1537.7349	-0.0085	-6	295	307	EAFPGDVFYLHSR	108	100			Mascot
1712.0204	1711.9003	-0.1201	-70	470	484	DILTTIKPELLQSLK					Mascot
1720.821	1720.9739	0.1529	89	262	276	DNGMHALIIYDDLSK			Oxidation (M)[4]		Mascot
2157.0532	2157.0288	-0.0244	-11	43	62	VYGLNEIQAGEMVEFAS GVK			Oxidation (M)[12]		Mascot
2308.1567	2308.1646	0.0079	3	402	423	EVAAFAQFGSDLDAATQ ALLNR					Mascot
2308.1567	2308.1646	0.0079	3	402	423	EVAAFAQFGSDLDAATQ ALLNR	233	100			Mascot

5	ATP synthase subunit alpha, mitochondrial OS=Pisum sativum GN=ATPA PE=1 SV=2	ATPAM_PEA	55296.1	6.01	16	678	100	42.517	606	100

Peptide Information

Calc. Mass	Obsrv. Mass	± da	± ppm	Start Seq.	End Seq.	Sequence	Ion Score	C. I. %	Modification	Rank	Result Type
815.4621	815.4484	-0.0137	-17	167	173	ELIIGDR					Mascot
860.5022	860.4489	-0.0533	-62	283	289	QMSLLLR					Mascot
876.4971	876.4717	-0.0254	-29	283	289	QMSLLLR			Oxidation (M)[2]		Mascot
884.4221	884.4128	-0.0093	-11	121	128	GALSDHER					Mascot
892.4886	892.4791	-0.0095	-11	395	401	LELAQYR					Mascot
972.5473	972.5329	-0.0144	-15	33	42	VVSVGDGIAR					Mascot
972.5473	972.5329	-0.0144	-15	33	42	VVSVGDGIAR	72	99.992			Mascot

1026.5942	1026.5762	-0.018	-18	154	163	AVDSLVPIGR					Mascot
1026.5942	1026.5762	-0.018	-18	154	163	AVDSLVPIGR	73	99.995			Mascot
1137.5756	1137.5282	-0.0474	-42	385	394	AMKQVCGSLK			Carbamidomethyl (C)[6], Oxidation (M)[2]		Mascot
1203.658	1203.6455	-0.0125	-10	7	17	AAELTTLLESR					Mascot
1203.658	1203.6455	-0.0125	-10	7	17	AAELTTLLESR	74	99.996			Mascot
1242.6147	1242.5756	-0.0391	-31	143	153	SVHEPMQTGLK			Oxidation (M)[6]		Mascot
1300.7471	1300.6886	-0.0585	-45	178	189	TAIAIDTILNQK					Mascot
1363.7039	1363.6704	-0.0335	-25	459	469	MPLDKIAQYER					Mascot
1438.8489	1438.8287	-0.0202	-14	363	376	GIRPAINVGLSVSR					Mascot
1438.8489	1438.8287	-0.0202	-14	363	376	GIRPAINVGLSVSR	45	96.436			Mascot
1537.7434	1537.7349	-0.0085	-6	295	307	EAFPGDVFYLHSR					Mascot
1537.7434	1537.7349	-0.0085	-6	295	307	EAFPGDVFYLHSR	108	100			Mascot
1720.821	1720.9739	0.1529	89	262	276	DNGMHALIIYDDLSK			Oxidation (M)[4]		Mascot
1729.0106	1728.8871	-0.1235	-71	470	484	DILSTIKQELLQSLK					Mascot
2308.1567	2308.1646	0.0079	3	402	423	EVAAFAQFGSDLDAATQ ALLNR					Mascot
2308.1567	2308.1646	0.0079	3	402	423	EVAAFAQFGSDLDAATQ ALLNR	233	100			Mascot

6	ATP synthase subunit alpha, mitochondrial OS=Triticum aestivum GN=ATPA PE=3 SV=1	ATPAM_WHEAT	55514.8	5.7	18	593	100	42.72	505	100	

Peptide Information

Calc. Mass	Obsrv. Mass	± da	± ppm	Start Seq.	End Seq.	Sequence	Ion Score	C. I. %	Modification	Rank	Result Type
815.4621	815.4484	-0.0137	-17	167	173	ELIIGDR					Mascot
860.5022	860.4489	-0.0533	-62	283	289	QMSLLLR					Mascot
874.4741	874.4857	0.0116	13	487	494	GGLTNERK					Mascot
876.4971	876.4717	-0.0254	-29	283	289	QMSLLLR			Oxidation (M)[2]		Mascot
884.4221	884.4128	-0.0093	-11	121	128	GALSDHER					Mascot
892.4886	892.4791	-0.0095	-11	395	401	LELAQYR					Mascot
972.5473	972.5329	-0.0144	-15	33	42	VVSVGDGIAR					Mascot
972.5473	972.5329	-0.0144	-15	33	42	VVSVGDGIAR	72	99.992			Mascot
1026.5942	1026.5762	-0.018	-18	154	163	AVDSLVPIGR					Mascot
1026.5942	1026.5762	-0.018	-18	154	163	AVDSLVPIGR	73	99.995			Mascot
1203.658	1203.6455	-0.0125	-10	7	17	AAELTTLLESR					Mascot
1203.658	1203.6455	-0.0125	-10	7	17	AAELTTLLESR	74	99.996			Mascot
1242.6147	1242.5756	-0.0391	-31	143	153	SVHEPMQTGLK			Oxidation (M)[6]		Mascot
1300.7471	1300.6886	-0.0585	-45	178	189	TAIAIDTILNQK					Mascot

1326.7627	1326.7087	-0.054	-41	470	481	AILSTINPELQK					Mascot
1341.705	1341.649	-0.056	-42	433	443	QPQYEPLPIEK					Mascot
1438.8489	1438.8287	-0.0202	-14	363	376	GIRPAINVGLSVSR					Mascot
1438.8489	1438.8287	-0.0202	-14	363	376	GIRPAINVGLSVSR	45	96.436			Mascot
1537.7434	1537.7349	-0.0085	-6	295	307	EAFPGDVFYLHSR					Mascot
1537.7434	1537.7349	-0.0085	-6	295	307	EAFPGDVFYLHSR	108	100			Mascot
1720.821	1720.9739	0.1529	89	262	276	DNGMHALIIYDDLSK			Oxidation (M)[4]		Mascot
1724.8789	1724.8748	-0.0041	-2	444	458	QIVVIYAAVNGFCDR			Carbamidomethyl (C)[13]		Mascot
1834.8429	1834.851	0.0081	4	18	32	MTNFYTNFQVDEIGR					Mascot
1850.8378	1850.8162	-0.0216	-12	18	32	MTNFYTNFQVDEIGR			Oxidation (M)[1]		Mascot
1850.8378	1850.8162	-0.0216	-12	18	32	MTNFYTNFQVDEIGR	133	100	Oxidation (M)[1]		Mascot
2157.0532	2157.0288	-0.0244	-11	43	62	VYGLNEIQAGEMVEFAS GVK			Oxidation (M)[12]		Mascot

7	ATP synthase subunit alpha, mitochondrial OS=Oenothera biennis GN=ATPA PE=3 SV=1	ATPAM_OENBI	55847.3	6.23	14	591	100	40.887	535	100		

Peptide Information

Calc. Mass	Obsrv. Mass	± da	± ppm	Start Seq.	End Seq.	Sequence	Ion Score	C. I. %	Modification	Rank	Result Type
815.4621	815.4484	-0.0137	-17	167	173	ELIIGDR					Mascot
860.5022	860.4489	-0.0533	-62	283	289	QMSLLLR					Mascot
876.4971	876.4717	-0.0254	-29	283	289	QMSLLLR			Oxidation (M)[2]		Mascot
892.4886	892.4791	-0.0095	-11	395	401	LELAQYR					Mascot
1010.5126	1010.4536	-0.059	-58	121	129	GALGDHERR					Mascot
1026.5942	1026.5762	-0.018	-18	154	163	AVDSLVPIGR					Mascot
1026.5942	1026.5762	-0.018	-18	154	163	AVDSLVPIGR	73	99.995			Mascot
1137.5756	1137.5282	-0.0474	-42	385	394	AMKQVCGSLK			Carbamidomethyl (C)[6], Oxidation (M)[2]		Mascot
1203.658	1203.6455	-0.0125	-10	7	17	AAELTTLLESR					Mascot
1203.658	1203.6455	-0.0125	-10	7	17	AAELTTLLESR	74	99.996			Mascot
1242.6147	1242.5756	-0.0391	-31	143	153	SVHEPMQTGLK			Oxidation (M)[6]		Mascot
1300.7471	1300.6886	-0.0585	-45	178	189	TAIAIDTILNQK					Mascot
1438.8489	1438.8287	-0.0202	-14	363	376	GIRPAINVGLSVSR					Mascot
1438.8489	1438.8287	-0.0202	-14	363	376	GIRPAINVGLSVSR	45	96.436			Mascot
1537.7434	1537.7349	-0.0085	-6	295	307	EAFPGDVFYLHSR					Mascot
1537.7434	1537.7349	-0.0085	-6	295	307	EAFPGDVFYLHSR	108	100			Mascot
1720.821	1720.9739	0.1529	89	262	276	DNGMHALIIYDDLSK			Oxidation (M)[4]		Mascot
2157.0532	2157.0288	-0.0244	-11	43	62	VYGLNEIQAGEMVEFAS GVK			Oxidation (M)[12]		Mascot
2308.1567	2308.1646	0.0079	3	402	423	EVAAFAQFGSDLDAATQ					Mascot

						ALLNR					
2308.1567	2308.1646	0.0079	3	402	423	EVAAFAQFGSDLDAATQ ALLNR	233	100			Mascot

8	ATP synthase subunit alpha, mitochondrial OS=Beta vulgaris GN=ATPA PE=3 SV=1	ATPAM_BETVU	55245.8	6.01	14	589	100	40.547	532	100

Peptide Information

Calc. Mass	Obsrv. Mass	± da	± ppm	Start Seq.	End Seq.	Sequence	Ion Score	C. I. %	Modification	Rank	Result Type
815.4621	815.4484	-0.0137	-17	167	173	ELIIGDR					Mascot
860.5022	860.4489	-0.0533	-62	283	289	QMSLLLR					Mascot
876.4971	876.4717	-0.0254	-29	283	289	QMSLLLR			Oxidation (M)[2]		Mascot
884.4221	884.4128	-0.0093	-11	121	128	GALSDHER					Mascot
892.4886	892.4791	-0.0095	-11	395	401	LELAQYR					Mascot
972.5473	972.5329	-0.0144	-15	33	42	VVSVGDGIAR					Mascot
972.5473	972.5329	-0.0144	-15	33	42	VVSVGDGIAR	72	99.992			Mascot
1026.5942	1026.5762	-0.018	-18	154	163	AVDSLVPIGR					Mascot
1026.5942	1026.5762	-0.018	-18	154	163	AVDSLVPIGR	73	99.995			Mascot
1110.5864	1110.549	-0.0374	-34	492	500	KMELDSFLK					Mascot
1242.6147	1242.5756	-0.0391	-31	143	153	SVHEPMQTGLK			Oxidation (M)[6]		Mascot
1300.7471	1300.6886	-0.0585	-45	178	189	TAIAIDTILNQK					Mascot
1438.8489	1438.8287	-0.0202	-14	363	376	GIRPAINVGLSVSR					Mascot
1438.8489	1438.8287	-0.0202	-14	363	376	GIRPAINVGLSVSR	45	96.436			Mascot
1537.7434	1537.7349	-0.0085	-6	295	307	EAFPGDVFYLHSR					Mascot
1537.7434	1537.7349	-0.0085	-6	295	307	EAFPGDVFYLHSR	108	100			Mascot
1720.821	1720.9739	0.1529	89	262	276	DNGMHALIIYDDLSK			Oxidation (M)[4]		Mascot
2157.0532	2157.0288	-0.0244	-11	43	62	VYGLNEIQAGEMVEFAS GVK			Oxidation (M)[12]		Mascot
2308.1567	2308.1646	0.0079	3	402	423	EVAAFAQFGSDLDAATQ ALLNR					Mascot
2308.1567	2308.1646	0.0079	3	402	423	EVAAFAQFGSDLDAATQ ALLNR	233	100			Mascot

9	ATP synthase subunit alpha, mitochondrial OS=Arabidopsis thaliana GN=ATPA PE=1 SV=2	ATPAM_ARATH	55295.9	6.23	14	517	100	40.534	460	100

Peptide Information

Calc. Mass	Obsrv. Mass	± da	± ppm	Start Seq.	End Seq.	Sequence	Ion Score	C. I. %	Modification	Rank	Result Type
860.5022	860.4489	-0.0533	-62	283	289	QMSLLLR					Mascot
874.4741	874.4857	0.0116	13	485	492	GGLTNERK					Mascot
876.4971	876.4717	-0.0254	-29	283	289	QMSLLLR			Oxidation (M)[2]		Mascot

892.4886	892.4791	-0.0095	-11	395	401	LELAQYR					Mascot
1026.5942	1026.5762	-0.018	-18	154	163	AVDSLVPIGR					Mascot
1026.5942	1026.5762	-0.018	-18	154	163	AVDSLVPIGR	73	99.995			Mascot
1110.5864	1110.549	-0.0374	-34	492	500	KMELDAFLK			Oxidation (M)[2]		Mascot
1137.5756	1137.5282	-0.0474	-42	385	394	AMKQVCGSLK			Carbamidomethyl (C)[6], Oxidation (M)[2]		Mascot
1168.5818	1168.563	-0.0188	-16	121	130	GALSDHEQRR					Mascot
1242.6147	1242.5756	-0.0391	-31	143	153	SVHEPMQTGLK			Oxidation (M)[6]		Mascot
1438.8489	1438.8287	-0.0202	-14	363	376	GIRPAINVGLSVSR					Mascot
1438.8489	1438.8287	-0.0202	-14	363	376	GIRPAINVGLSVSR	45	96.436			Mascot
1519.8228	1519.7316	-0.0912	-60	7	19	AAELTNLFESRIR					Mascot
1519.8228	1519.7316	-0.0912	-60	7	19	AAELTNLFESRIR					Mascot
1537.7434	1537.7349	-0.0085	-6	295	307	EAFPGDVFYLHSR					Mascot
1537.7434	1537.7349	-0.0085	-6	295	307	EAFPGDVFYLHSR	108	100			Mascot
1720.821	1720.9739	0.1529	89	262	276	DNGMHALIIYDDLSK			Oxidation (M)[4]		Mascot
1728.9136	1728.8871	-0.0265	-15	104	120	AMLGRVVDAMGVPIDGK					Mascot
2308.1567	2308.1646	0.0079	3	402	423	EVAAFAQFGSDLDAATQ ALLNR					Mascot
2308.1567	2308.1646	0.0079	3	402	423	EVAAFAQFGSDLDAATQ ALLNR	233	100			Mascot

10	ATP synthase subunit alpha, mitochondrial OS=Brassica napus GN=ATPA PE=3 SV=1	ATPAM_BRANA	55392.9	6.23	13	509	100	40.634	460	100	

Protein Group

ATP synthase subunit alpha, mitochondrial OS=Brassica campestris GN=ATPA PE=3 SV=1	ATPAM_BRACM	55307.8	6.0100 002288 8184
ATP synthase subunit alpha, mitochondrial OS=Raphanus sativus GN=ATPA PE=3 SV=1	ATPAM_RAPSA	55307.8	6.0100 002288 8184

Peptide Information

Calc. Mass	Obsrv. Mass	± da	± ppm	Start Seq.	End Seq.	Sequence	Ion Score	C. I. %	Modification	Rank	Result Type
815.4621	815.4484	-0.0137	-17	167	173	ELLIGDR					Mascot
860.5022	860.4489	-0.0533	-62	283	289	QMSLLLR					Mascot
874.4741	874.4857	0.0116	13	485	492	GGLTNERK					Mascot
876.4971	876.4717	-0.0254	-29	283	289	QMSLLLR			Oxidation (M)[2]		Mascot
892.4886	892.4791	-0.0095	-11	395	401	LELAQYR					Mascot
1026.5942	1026.5762	-0.018	-18	154	163	AVDSLVPIGR					Mascot
1026.5942	1026.5762	-0.018	-18	154	163	AVDSLVPIGR	73	99.995			Mascot
1168.5818	1168.563	-0.0188	-16	121	130	GALSDHEQRR					Mascot
1235.6089	1235.6002	-0.0087	-7	493	502	MEPDAFLKER					Mascot

1242.6147	1242.5756	-0.0391	-31	143	153	SVHEPMQTGLK			Oxidation (M)[6]	Mascot
1438.8489	1438.8287	-0.0202	-14	363	376	GIRPAINVGLSVSR				Mascot
1438.8489	1438.8287	-0.0202	-14	363	376	GIRPAINVGLSVSR	45	96.682		Mascot
1519.8228	1519.7316	-0.0912	-60	7	19	AAELTNLFESRIR				Mascot
1519.8228	1519.7316	-0.0912	-60	7	19	AAELTNLFESRIR				Mascot
1537.7434	1537.7349	-0.0085	-6	295	307	EAFPGDVFYLHSR				Mascot
1537.7434	1537.7349	-0.0085	-6	295	307	EAFPGDVFYLHSR	108	100		Mascot
1720.821	1720.9739	0.1529	89	262	276	DNGMHALIIYDDLSK			Oxidation (M)[4]	Mascot
2308.1567	2308.1646	0.0079	3	402	423	EVAAFAQFGSDLDAATQ ALLNR				Mascot
2308.1567	2308.1646	0.0079	3	402	423	EVAAFAQFGSDLDAATQ ALLNR	233	100		Mascot

Gel Idx/Pos	279/L7	Instr./Gel Origin	BA2151/Sample Project 20140814	Process Status	Analysis Succeeded
Plate [#] Name	[1] Sample Project 20140814	Instrument Sample Name		Spectra	11

Rank	Protein Name	Accession No.	Protein MW	Protein PI	Pep. Count	Protein Score	Protein Score C. I. %	Intensity Matched	Total Ion Score	Total Ion C. I. %	Confirmed
1	6-phosphogluconate dehydrogenase, decarboxylating 1 OS=Oryza sativa subsp. japonica GN=G6PGH1 PE=2 SV=1	6PGD1_ORYSJ	52972.9	5.85	14	415	100	32.959	359	100	

Peptide Information

Calc. Mass	Obsrv. Mass	± da	± ppm	Start Seq.	End Seq.	Sequence	Ion Score	C. I. %	Modification	Rank	Result Type
846.4316	846.4282	-0.0034	-4	39	45	VDETVQR					Mascot
908.4142	908.4077	-0.0065	-7	115	121	EKAMEER			Oxidation (M)[4]		Mascot
943.5207	943.5165	-0.0042	-4	320	327	AQLIEDVR					Mascot
1052.5524	1052.5509	-0.0015	-1	26	34	GFPISVYNR					Mascot
1052.5524	1052.5509	-0.0015	-1	26	34	GFPISVYNR	70	99.988			Mascot
1064.5735	1064.5676	-0.0059	-6	292	300	FLSGLKDER					Mascot
1064.5735	1064.5676	-0.0059	-6	292	300	FLSGLKDER	43	94.247			Mascot
1109.6426	1109.641	-0.0016	-1	441	450	LPANLVQAQR					Mascot
1258.5488	1258.5519	0.0031	2	451	460	DYFGAHTYER					Mascot
1258.5488	1258.5519	0.0031	2	451	460	DYFGAHTYER	79	99.999			Mascot
1263.6539	1263.6343	-0.0196	-16	35	45	TTSKVDETVQR					Mascot
1380.7706	1380.7672	-0.0034	-2	439	450	DRLPANLVQAQR					Mascot
1380.7706	1380.7672	-0.0034	-2	439	450	DRLPANLVQAQR	66	99.972			Mascot
1580.7203	1580.7152	-0.0051	-3	461	473	VDMPGSFHTEWFK					Mascot
1665.8265	1665.834	0.0075	5	122	138	GLLYLGMGVSGGEEGAR					Mascot
1681.8214	1681.806	-0.0154	-9	122	138	GLLYLGMGVSGGEEGAR			Oxidation (M)[7]		Mascot
1681.8214	1681.806	-0.0154	-9	122	138	GLLYLGMGVSGGEEGAR	102	100	Oxidation (M)[7]		Mascot
2012.1208	2012.077	-0.0438	-22	6	25	IGLAGLAVMGQNLALNIA EK			Oxidation (M)[9]		Mascot
2443.2463	2443.2715	0.0252	10	269	291	WTVQQAAELSVAAPTIEA SLDSR					Mascot
2808.3333	2808.3503	0.017	6	412	437	VVCLAINNGVSTPGMSAS LAYFDSYR			Carbamidomethyl (C)[3], Oxidation (M)[15]		Mascot

Rank	Protein Name	Accession No.	Protein MW	Protein PI	Pep. Count	Protein Score	Protein Score C. I. %	Intensity Matched	Total Ion Score	Total Ion C. I. %	Confirmed
2	6-phosphogluconate dehydrogenase, decarboxylating 1 OS=Spinacia oleracea GN=pgdC PE=1 SV=1	6PGD1_SPIOL	53496.4	6.04	8	313	100	25.451	294	100	

Peptide Information

Calc. Mass	Obsrv. Mass	± da	± ppm	Start Seq.	End Seq.	Sequence	Ion Score	C. I. %	Modification	Rank	Result Type

Calc. Mass	Obsrv. Mass	± da	± ppm	Start Seq.	End Seq.	Sequence	Ion Score	C. I. %	Modification	Rank	Result Type
1052.5524	1052.5509	-0.0015	-1	27	35	GFPISVYNR					Mascot
1052.5524	1052.5509	-0.0015	-1	27	35	GFPISVYNR	70	99.988			Mascot
1064.5735	1064.5676	-0.0059	-6	292	300	FLSGLKDER					Mascot
1064.5735	1064.5676	-0.0059	-6	292	300	FLSGLKDER	43	94.247			Mascot
1109.6426	1109.641	-0.0016	-1	445	454	LPANLVQAQR					Mascot
1258.5488	1258.5519	0.0031	2	455	464	DYFGAHTYER					Mascot
1258.5488	1258.5519	0.0031	2	455	464	DYFGAHTYER	79	99.999			Mascot
1394.7863	1394.7758	-0.0105	-8	443	454	ERLPANLVQAQR					Mascot
1665.8265	1665.834	0.0075	5	123	139	GLLYLGMGVSGGEEGAR					Mascot
1681.8214	1681.806	-0.0154	-9	123	139	GLLYLGMGVSGGEEGAR			Oxidation (M)[7]		Mascot
1681.8214	1681.806	-0.0154	-9	123	139	GLLYLGMGVSGGEEGAR	102	100	Oxidation (M)[7]		Mascot
2012.1208	2012.077	-0.0438	-22	7	26	IGLAGLAVMGQNLALNIA EK			Oxidation (M)[9]		Mascot
2744.4214	2744.4241	0.0027	1	265	291	GTGKWTVQQAAELSVAA PTIASSLDSR					Mascot

3	6-phosphogluconate dehydrogenase, decarboxylating 3 OS=Arabidopsis thaliana GN=At3g02360 PE=2 SV=1	6GPD3_ARATH	53828.6	7.02	11	223	100	23.691	191	100	

Peptide Information

Calc. Mass	Obsrv. Mass	± da	± ppm	Start Seq.	End Seq.	Sequence	Ion Score	C. I. %	Modification	Rank	Result Type
943.5458	943.5165	-0.0293	-31	156	163	NIEDIVLK					Mascot
1052.5524	1052.5509	-0.0015	-1	28	36	GFPISVYNR					Mascot
1052.5524	1052.5509	-0.0015	-1	28	36	GFPISVYNR	70	99.988			Mascot
1064.5735	1064.5676	-0.0059	-6	294	302	FLSGLKDER					Mascot
1064.5735	1064.5676	-0.0059	-6	294	302	FLSGLKDER	43	94.247			Mascot
1109.6426	1109.641	-0.0016	-1	447	456	LPANLVQAQR					Mascot
1199.6896	1199.6263	-0.0633	-53	359	369	GWGLKLGELAR					Mascot
1258.5488	1258.5519	0.0031	2	457	466	DYFGAHTYER					Mascot
1258.5488	1258.5519	0.0031	2	457	466	DYFGAHTYER	79	99.999			Mascot
1394.7863	1394.7758	-0.0105	-8	445	456	ERLPANLVQAQR					Mascot
1567.9166	1567.7902	-0.1264	-81	156	169	NIEDIVLKVAAQVR					Mascot
2012.1208	2012.077	-0.0438	-22	8	27	IGLAGLAVMGQNLALNIA EK			Oxidation (M)[9]		Mascot
2077.0391	2077.1428	0.1037	50	50	67	KEGNLPLYGFHDPESFV K					Mascot
2880.427	2880.4761	0.0491	17	189	214	MVHNGIEYGDMQLIAEAY DVLKSVGK					Mascot

4	6-phosphogluconate dehydrogenase, decarboxylating 1, chloroplastic OS=Arabidopsis thaliana GN=At1g64190 PE=2 SV=1	6PGD1_ARATH	53686.2	5.34	7	191	100	14.541	172	100	

Peptide Information

Calc. Mass	Obsrv. Mass	± da	± ppm	Start Seq.	End Seq.	Sequence	Ion Score	C. I. %	Modification	Rank	Result Type
1052.5524	1052.5509	-0.0015	-1	29	37	GFPISVYNR					Mascot
1052.5524	1052.5509	-0.0015	-1	29	37	GFPISVYNR	70	99.988			Mascot
1109.6426	1109.641	-0.0016	-1	449	458	LPANLVQAQR					Mascot
1320.6642	1320.6567	-0.0075	-6	316	328	EEIGSASSGIDKK					Mascot
1644.834	1644.684	-0.15	-91	49	64	AAVEGNLPVSGQYSPR					Mascot
1665.8265	1665.834	0.0075	5	125	141	GLLYLGMGVSGGEEGAR					Mascot
1681.8214	1681.806	-0.0154	-9	125	141	GLLYLGMGVSGGEEGAR			Oxidation (M)[7]		Mascot
1681.8214	1681.806	-0.0154	-9	125	141	GLLYLGMGVSGGEEGAR	102	100	Oxidation (M)[7]		Mascot
2012.1208	2012.077	-0.0438	-22	9	28	IGLAGLAVMGQNLALNIAEK			Oxidation (M)[9]		Mascot
2458.2395	2458.2971	0.0576	23	271	293	WTVQQAAELSVAAPTIAASLDCR			Carbamidomethyl (C)[22]		Mascot

5	6-phosphogluconate dehydrogenase, decarboxylating 2, chloroplastic OS=Arabidopsis thaliana GN=At5g41670 PE=1 SV=1	6PGD2_ARATH	53626.3	5.62	7	190	100	14.697	172	100	

Peptide Information

Calc. Mass	Obsrv. Mass	± da	± ppm	Start Seq.	End Seq.	Sequence	Ion Score	C. I. %	Modification	Rank	Result Type
908.4506	908.4077	-0.0429	-47	1	8	MESVALSR			Oxidation (M)[1]		Mascot
1052.5524	1052.5509	-0.0015	-1	29	37	GFPISVYNR					Mascot
1052.5524	1052.5509	-0.0015	-1	29	37	GFPISVYNR	70	99.988			Mascot
1109.6426	1109.641	-0.0016	-1	449	458	LPANLVQAQR					Mascot
1233.6071	1233.6252	0.0181	15	316	327	EDIGSASRGVDK					Mascot
1665.8265	1665.834	0.0075	5	125	141	GLLYLGMGVSGGEEGAR					Mascot
1681.8214	1681.806	-0.0154	-9	125	141	GLLYLGMGVSGGEEGAR			Oxidation (M)[7]		Mascot
1681.8214	1681.806	-0.0154	-9	125	141	GLLYLGMGVSGGEEGAR	102	100	Oxidation (M)[7]		Mascot
1809.9164	1809.8413	-0.0751	-41	124	141	KGLLYLGMGVSGGEEGAR			Oxidation (M)[8]		Mascot
2458.2395	2458.2971	0.0576	23	271	293	WTVQQAAELSVAAPTIAASLDCR			Carbamidomethyl (C)[22]		Mascot

6	6-phosphogluconate dehydrogenase, decarboxylating 2, chloroplastic OS=Oryza sativa subsp. japonica GN=G6PGH2 PE=2 SV=1	6PGD2_ORYSJ	54494	6.09	5	181	100	12.527	172	100	

Peptide Information

Calc. Mass	Obsrv. Mass	± da	± ppm	Start Seq.	End Seq.	Sequence	Ion Score	C. I. %	Modification	Rank	Result Type
1052.5524	1052.5509	-0.0015	-1	44	52	GFPISVYNR					Mascot

1052.5524	1052.5509	-0.0015	-1	44	52	GFPISVYNR	70	99.988			Mascot
1123.6582	1123.5663	-0.0919	-82	466	475	LPANLIQAQR					Mascot
1256.6746	1256.6576	-0.017	-14	378	388	GWNLNLAELAR					Mascot
1665.8265	1665.834	0.0075	5	140	156	GILYLGMGVSGGEEGAR					Mascot
1681.8214	1681.806	-0.0154	-9	140	156	GILYLGMGVSGGEEGAR			Oxidation (M)[7]		Mascot
1681.8214	1681.806	-0.0154	-9	140	156	GILYLGMGVSGGEEGAR	102	100	Oxidation (M)[7]		Mascot
1756.9341	1756.9557	0.0216	12	411	425	NPELANLIVDREFAR					Mascot

7	6-phosphogluconate dehydrogenase, decarboxylating 2, chloroplastic OS=Spinacia oleracea GN=pgdP PE=1 SV=1	6PGD2_SPIOL	58541.7	5.54	8	169	100	20.796	149	100	

Peptide Information

Calc. Mass	Obsrv. Mass	± da	± ppm	Start Seq.	End Seq.	Sequence	Ion Score	C. I. %	Modification	Rank	Result Type
1052.5524	1052.5509	-0.0015	-1	71	79	GFPISVYNR					Mascot
1052.5524	1052.5509	-0.0015	-1	71	79	GFPISVYNR	70	99.988			Mascot
1109.6426	1109.641	-0.0016	-1	491	500	LPANLVQAQR					Mascot
1258.5488	1258.5519	0.0031	2	501	510	DYFGAHTYER					Mascot
1258.5488	1258.5519	0.0031	2	501	510	DYFGAHTYER	79	99.999			Mascot
1286.6699	1286.578	-0.0919	-71	358	369	EEVNAIRGGVDK					Mascot
1439.7642	1439.724	-0.0402	-28	71	83	GFPISVYNRTASK					Mascot
1661.7952	1661.7936	-0.0016	-1	399	413	SAEMGWDLNLGELAR					Mascot
2448.2017	2448.2573	0.0556	23	184	205	FGPSLMPGGDFQAYDNI QHILK					Mascot
2458.2395	2458.2971	0.0576	23	313	335	WTVQQAAELSVAAPTIAA SLDCR			Carbamidomethyl (C)[22]		Mascot

8	6-phosphogluconate dehydrogenase, decarboxylating OS=Staphylococcus aureus (strain Mu50 / ATCC 700699) GN=gnd PE=1 SV=1	6PGD_STAAM	51922.1	5.01	7	100	99.995	18.165	79	99.999	

Protein Group

6-phosphogluconate dehydrogenase, decarboxylating OS=Staphylococcus aureus (strain COL) GN=gnd PE=3 SV=1	6PGD_STAAC	51941.1	5.0199 999809 2651
6-phosphogluconate dehydrogenase, decarboxylating OS=Staphylococcus aureus (strain MRSA252) GN=gnd PE=3 SV=1	6PGD_STAAR	51941.1	5.0199 999809 2651
6-phosphogluconate dehydrogenase, decarboxylating OS=Staphylococcus aureus (strain MSSA476) GN=gnd PE=3 SV=1	6PGD_STAAS	51941.1	5.0199 999809 2651
6-phosphogluconate dehydrogenase, decarboxylating OS=Staphylococcus aureus (strain MW2) GN=gnd PE=3 SV=1	6PGD_STAAW	51941.1	5.0199 999809 2651
6-phosphogluconate dehydrogenase, decarboxylating	6PGD_STAAN	51941.1	5.0199

OS=Staphylococcus aureus (strain N315) GN=gnd PE=1 SV=1				999809 2651					

Peptide Information

Calc. Mass	Obsrv. Mass	± da	± ppm	Start Seq.	End Seq.	Sequence	Ion Score	C. I. %	Modification	Rank	Result Type
1205.5433	1205.6416	0.0983	82	342	351	ASEDNEWNLK					Mascot
1258.5488	1258.5519	0.0031	2	445	454	DYFGAHTYER					Mascot
1258.5488	1258.5519	0.0031	2	445	454	DYFGAHTYER	79	99.999			Mascot
1377.6646	1377.7756	0.111	81	301	313	ELNGPKASFDGDK					Mascot
1380.7594	1380.7672	0.0078	6	432	444	AADLPANLIQAQR					Mascot
1380.7594	1380.7672	0.0078	6	432	444	AADLPANLIQAQR	3	0			Mascot
1386.6437	1386.6377	-0.006	-4	445	455	DYFGAHTYERK					Mascot
1617.7544	1617.8448	0.0904	56	456	468	DKEGVFHTQWIEE					Mascot
1617.7544	1617.8448	0.0904	56	456	468	DKEGVFHTQWIEE					Mascot
2458.2725	2458.2971	0.0246	10	47	67	GKNIHPTYSLEEFVNSLE KPR					Mascot

9	6-phosphogluconate dehydrogenase, decarboxylating OS=Lactococcus lactis subsp. cremoris (strain MG1363) GN=gnd PE=1 SV=3	6PGD_LACLM	52541.5	4.92	6	93	99.973	11.262	79	99.999

Protein Group

6-phosphogluconate dehydrogenase, decarboxylating OS=Lactococcus lactis subsp. lactis (strain IL1403) GN=gnd PE=3 SV=1	6PGD_LACLA	52505.6	5

Peptide Information

Calc. Mass	Obsrv. Mass	± da	± ppm	Start Seq.	End Seq.	Sequence	Ion Score	C. I. %	Modification	Rank	Result Type
1185.5997	1185.6371	0.0374	32	35	44	TTSKTEEVYK					Mascot
1258.5488	1258.5519	0.0031	2	448	457	DYFGAHTYER					Mascot
1258.5488	1258.5519	0.0031	2	448	457	DYFGAHTYER	79	99.999			Mascot
1343.681	1343.7355	0.0545	41	133	146	GALLGPSMMPGGQK					Mascot
1633.8652	1633.811	-0.0542	-33	70	85	IMLMVQAGAATDATIK					Mascot
1665.855	1665.834	-0.021	-13	70	85	IMLMVQAGAATDATIK			Oxidation (M)[2,4]		Mascot
1777.9371	1777.8411	-0.096	-54	147	162	EAYDLVAPIFEQIAAK					Mascot
1805.9612	1805.9734	0.0122	7	69	85	RIMLMVQAGAATDATIK			Oxidation (M)[3]		Mascot

10	6-phosphogluconate dehydrogenase, decarboxylating OS=Synechocystis sp. (strain PCC 6803 / Kazusa) GN=gnd PE=3 SV=1	6PGD_SYNY3	53067.9	5.12	4	87	99.897	10.068	79	99.999

Peptide Information

Calc. Mass	Obsrv. Mass	± da	± ppm	Start	End	Sequence	Ion	C. I. %	Modification	Rank	Result Type

				Seq.	Seq.		Score			
1258.5488	1258.5519	0.0031	2	458	467	DYFGAHTYER				Mascot
1258.5488	1258.5519	0.0031	2	458	467	DYFGAHTYER	79	99.999		Mascot
1338.7489	1338.7576	0.0087	6	446	457	AVLPQNLTQAQR				Mascot
1370.6919	1370.6703	-0.0216	-16	1	11	MQFNVAIMTKR			Oxidation (M)[1,8]	Mascot
2458.3164	2458.2971	-0.0193	-8	277	299	WTVMSGLELGVPIPTIYA AVNAR				Mascot

Gel Idx/Pos	280/L8	Instr./Gel Origin	BA2151/Sample Project 20140814	Process Status	Analysis Succeeded
Plate [#] Name	[1] Sample Project 20140814	Instrument Sample Name		Spectra	11

Rank	Protein Name	Accession No.	Protein MW	Protein PI	Pep. Count	Protein Score	Protein Score C. I. %	Intensity Matched	Total Ion Score	Total Ion C. I. %	Confirmed
1	Granule-bound starch synthase 1, chloroplastic/amyloplastic OS=Triticum aestivum GN=WAXY PE=1 SV=1	SSG1_WHEAT	68506	8.25	22	647	100	28.567	558	100	

Peptide Information

Calc. Mass	Obsrv. Mass	± da	± ppm	Start Seq.	End Seq.	Sequence	Ion Score	C. I. %	Modification	Rank	Result Type
817.46	817.4323	-0.0277	-34	113	119	VMVISPR			Oxidation (M)[2]		Mascot
821.3723	821.3473	-0.025	-30	524	530	TGFHMGR			Oxidation (M)[5]		Mascot
908.5173	908.4616	-0.0557	-61	53	59	QSRKPHR					Mascot
917.3974	917.3704	-0.027	-29	146	151	YFHCYK			Carbamidomethyl (C)[4]		Mascot
971.6036	971.574	-0.0296	-30	405	413	VPLVAFIGR					Mascot
1099.6986	1099.6765	-0.0221	-20	404	413	KVPLVAFIGR					Mascot
1123.4871	1123.4641	-0.023	-20	341	349	GCELDNIMR			Carbamidomethyl (C)[2], Oxidation (M)[8]		Mascot
1346.6774	1346.636	-0.0414	-31	556	567	VVGTPAYHEMVK			Oxidation (M)[10]		Mascot
1390.6825	1390.6514	-0.0311	-22	157	167	VFVDHPCFLEK			Carbamidomethyl (C)[7]		Mascot
1396.7432	1396.7229	-0.0203	-15	391	403	EALQAEVGLPVDR					Mascot
1503.7988	1503.7808	-0.018	-12	310	322	INWMKAGILQADK			Oxidation (M)[4]		Mascot
1503.7988	1503.7808	-0.018	-12	192	204	FSLLCQAALEVPR	88	100	Carbamidomethyl (C)[5]		Mascot
1524.838	1524.7905	-0.0475	-31	391	404	EALQAEVGLPVDRK					Mascot
1564.7611	1564.7469	-0.0142	-9	490	502	FEPCGLIQLQGMR			Carbamidomethyl (C)[4], Oxidation (M)[12]		Mascot
1564.7689	1564.7469	-0.022	-14	264	276	VAFCIHNISYQGR	67	99.981	Carbamidomethyl (C)[4]		Mascot
1684.7966	1684.7849	-0.0117	-7	277	290	FSFDDFAQLNLPDR					Mascot
1684.7966	1684.7849	-0.0117	-7	277	290	FSFDDFAQLNLPDR	119	100			Mascot
1831.8497	1831.8289	-0.0208	-11	293	308	SSFDFIDGYDKPVEGR					Mascot
1831.8497	1831.8289	-0.0208	-11	293	308	SSFDFIDGYDKPVEGR	113	100			Mascot
1841.7937	1841.7733	-0.0204	-11	176	191	IYGPDAGTDYEDNQQR					Mascot
1841.7937	1841.7733	-0.0204	-11	176	191	IYGPDAGTDYEDNQQR	68	99.984			Mascot
1913.9855	1913.9971	0.0116	6	370	386	DKFLTVNYDVTTALEGK					Mascot
1997.0226	1997.0088	-0.0138	-7	323	340	VLTVSPYYAEELISGEAR					Mascot
1997.0226	1997.0088	-0.0138	-7	323	340	VLTVSPYYAEELISGEAR	106	100			Mascot
2098.9312	2098.9155	-0.0157	-7	174	191	EKIYGPDAGTDYEDNQQ R					Mascot
2232.0688	2232.0498	-0.019	-9	205	223	ILDLNNNPHFSGPYAMLC			Carbamidomethyl (C)[18]		Mascot

		R									
2	Granule-bound starch synthase 1, chloroplastic/amyloplastic OS=Hordeum vulgare GN=WAXY PE=1 SV=1	SSG1_HORVU	66853	7.06	16	524	100	27.835	471	100

Peptide Information

Calc. Mass	Obsrv. Mass	± da	± ppm	Start Seq.	End Seq.	Sequence	Ion Score	C. I. %	Modification	Rank	Result Type
821.3723	821.3473	-0.025	-30	512	518	TGFHMGR			Oxidation (M)[5]		Mascot
971.6036	971.574	-0.0296	-30	393	401	VPLVAFIGR					Mascot
1099.6986	1099.6765	-0.0221	-20	392	401	KVPLVAFIGR					Mascot
1123.4871	1123.4641	-0.023	-20	329	337	GCELDNIMR			Carbamidomethyl (C)[2], Oxidation (M)[8]		Mascot
1136.5695	1136.5237	-0.0458	-40	136	144	VADEYERVR					Mascot
1396.7432	1396.7229	-0.0203	-15	379	391	EALQAEVGLPVDR					Mascot
1500.7516	1500.7559	0.0043	3	112	123	VMVVSPRYDQYK			Oxidation (M)[2]		Mascot
1503.7988	1503.7808	-0.018	-12	298	310	INWMKAGILQADK			Oxidation (M)[4]		Mascot
1503.7988	1503.7808	-0.018	-12	298	310	INWMKAGILQADK			Oxidation (M)[4]		Mascot
1524.838	1524.7905	-0.0475	-31	379	392	EALQAEVGLPVDRK					Mascot
1564.7611	1564.7469	-0.0142	-9	478	490	FEPCGLIQLQGMR			Carbamidomethyl (C)[4], Oxidation (M)[12]		Mascot
1564.7689	1564.7469	-0.022	-14	252	264	VAFCIHNISYQGR	67	99.981	Carbamidomethyl (C)[4]		Mascot
1684.7966	1684.7849	-0.0117	-7	265	278	FSFDDFAQLNLPDR					Mascot
1684.7966	1684.7849	-0.0117	-7	265	278	FSFDDFAQLNLPDR	119	100			Mascot
1831.8497	1831.8289	-0.0208	-11	281	296	SSFDFIDGYDKPVEGR					Mascot
1831.8497	1831.8289	-0.0208	-11	281	296	SSFDFIDGYDKPVEGR	113	100			Mascot
1841.7937	1841.7733	-0.0204	-11	175	190	IYGPDAGTDYEDNQQR					Mascot
1841.7937	1841.7733	-0.0204	-11	175	190	IYGPDAGTDYEDNQQR	68	99.984			Mascot
1997.0226	1997.0088	-0.0138	-7	311	328	VLTVSPYYAEELISGEAR					Mascot
1997.0226	1997.0088	-0.0138	-7	311	328	VLTVSPYYAEELISGEAR	106	100			Mascot
2098.9312	2098.9155	-0.0157	-7	173	190	EKIYGPDAGTDYEDNQQ R					Mascot

3	Granule-bound starch synthase 1, chloroplastic/amyloplastic OS=Oryza sativa GN=WAXY PE=2 SV=1	SSG1_ORYSA	66850.9	8.34	9	84	99.804	6.025	66	99.973

Peptide Information

Calc. Mass	Obsrv. Mass	± da	± ppm	Start Seq.	End Seq.	Sequence	Ion Score	C. I. %	Modification	Rank	Result Type
817.46	817.4323	-0.0277	-34	119	125	VMVISPR			Oxidation (M)[2]		Mascot
821.3723	821.3473	-0.025	-30	515	521	TGFHMGR			Oxidation (M)[5]		Mascot
860.4472	860.4199	-0.0273	-32	307	314	AGILESDR					Mascot

908.4584	908.4616	0.0032	4	143	149	VADRYER					Mascot
1123.4871	1123.4641	-0.023	-20	333	341	GCELDNIMR			Carbamidomethyl (C)[2], Oxidation (M)[8]		Mascot
1507.7145	1507.7305	0.016	11	515	527	TGFHMGRLSVDCK			Carbamidomethyl (C)[12]		Mascot
1564.7611	1564.7469	-0.0142	-9	481	493	FEPCGLIQLQGMR			Carbamidomethyl (C)[4], Oxidation (M)[12]		Mascot
1564.7689	1564.7469	-0.022	-14	256	268	VAFCIHNISYQGR	67	99.981	Carbamidomethyl (C)[4]		Mascot
2099.0437	2098.9155	-0.1282	-61	1	21	MSALTTSQLATSATGFGI ADR					Mascot

4	Granule-bound starch synthase 1, chloroplastic/amyloplastic OS=Oryza sativa subsp. japonica GN=WAXY PE=1 SV=1	SSG1_ORYSJ	67004.1	8.34	8	81	99.55	5.859	66	99.973	

Protein Group

Granule-bound starch synthase 1, chloroplastic/amyloplastic OS=Oryza sativa subsp. indica GN=WAXY PE=2 SV=2	SSG1_ORYSI	67004.1	8.3400 001525 8789

Peptide Information

Calc. Mass	Obsrv. Mass	± da	± ppm	Start Seq.	End Seq.	Sequence	Ion Score	C. I. %	Modification	Rank	Result Type
817.46	817.4323	-0.0277	-34	119	125	VMVISPR			Oxidation (M)[2]		Mascot
821.3723	821.3473	-0.025	-30	518	524	TGFHMGR			Oxidation (M)[5]		Mascot
908.4584	908.4616	0.0032	4	143	149	VADRYER					Mascot
1123.4871	1123.4641	-0.023	-20	336	344	GCELDNIMR			Carbamidomethyl (C)[2], Oxidation (M)[8]		Mascot
1507.7145	1507.7305	0.016	11	518	530	TGFHMGRLSVDCK			Carbamidomethyl (C)[12]		Mascot
1564.7611	1564.7469	-0.0142	-9	484	496	FEPCGLIQLQGMR			Carbamidomethyl (C)[4], Oxidation (M)[12]		Mascot
1564.7689	1564.7469	-0.022	-14	259	271	VAFCIHNISYQGR	67	99.981	Carbamidomethyl (C)[4]		Mascot
2099.0437	2098.9155	-0.1282	-61	1	21	MSALTTSQLATSATGFGI ADR					Mascot

5	Granule-bound starch synthase 1, chloroplastic/amyloplastic OS=Oryza glaberrima GN=WAXY PE=1 SV=1	SSG1_ORYGL	67003.1	8.47	7	77	99.016	5.065	66	99.973	

Peptide Information

Calc. Mass	Obsrv. Mass	± da	± ppm	Start Seq.	End Seq.	Sequence	Ion Score	C. I. %	Modification	Rank	Result Type
817.46	817.4323	-0.0277	-34	119	125	VMVISPR			Oxidation (M)[2]		Mascot
821.3723	821.3473	-0.025	-30	518	524	TGFHMGR			Oxidation (M)[5]		Mascot
908.4584	908.4616	0.0032	4	143	149	VADRYER					Mascot
1123.4871	1123.4641	-0.023	-20	336	344	GCELDNIMR			Carbamidomethyl (C)[2], Oxidation (M)[8]		Mascot
1564.7611	1564.7469	-0.0142	-9	484	496	FEPCGLIQLQGMR			Carbamidomethyl (C)[4], Oxidation (M)[12]		Mascot
1564.7689	1564.7469	-0.022	-14	259	271	VAFCIHNISYQGR	67	99.981	Carbamidomethyl (C)[4]		Mascot
2099.0437	2098.9155	-0.1282	-61	1	21	MSALTTSQLATSATGFGI					Mascot

		ADR								
6	Ketol-acid reductoisomerase OS=Saccharophagus degradans (strain 2-40 / ATCC 43961 / DSM 17024) GN=ilvC PE=3 SV=1	ILVC_SACD2	36712.4	5.19	13	67	88.96	5.618		

Peptide Information

Calc. Mass	Obsrv. Mass	± da	± ppm	Start Seq.	End Seq.	Sequence	Ion Score	C. I. %	Modification	Rank	Result Type
817.4713	817.4323	-0.039	-48	273	279	AMRQALK					Mascot
846.4315	846.4318	0.0003	0	265	271	VINEESR					Mascot
962.4288	962.4726	0.0438	46	1	7	MQVYYDK			Oxidation (M)[1]		Mascot
971.5043	971.574	0.0697	72	91	98	EEIEPNIK					Mascot
974.5265	974.4645	-0.062	-64	265	272	VINEESRK					Mascot
995.5408	995.5961	0.0553	56	176	184	SGIIETTFK					Mascot
1148.5616	1148.5261	-0.0355	-31	8	17	DCDLSIIQGK			Carbamidomethyl (C)[2]		Mascot
1276.6566	1276.6259	-0.0307	-24	8	18	DCDLSIIQGKK			Carbamidomethyl (C)[2]		Mascot
1334.6812	1334.6597	-0.0215	-16	307	319	NNAAHPIEQVGGK					Mascot
1507.7396	1507.7305	-0.0091	-6	320	331	LRAMMPWIESNK			Oxidation (M)[4,5]		Mascot
1830.9597	1830.8259	-0.1338	-73	143	161	GGGIPDLIAIFQDASGTAK					Mascot
2013.9487	2013.9863	0.0376	19	289	305	NFILEGQSNYPSMTAWR					Mascot
2994.5215	2994.4067	-0.1148	-38	19	47	VAIIGYGSQGHAHACNLK DSGVDVTVGLR			Carbamidomethyl (C)[15]		Mascot
2994.5215	2994.4067	-0.1148	-38	19	47	VAIIGYGSQGHAHACNLK DSGVDVTVGLR			Carbamidomethyl (C)[15]		Mascot

7	2,3,4,5-tetrahydropyridine-2,6-dicarboxylate N-succinyltransferase OS=Rhizobium etli (strain CIAT 652) GN=dapD PE=3 SV=1	DAPD_RHIE6	30795.4	5.56	12	63	71.622	16.952

Peptide Information

Calc. Mass	Obsrv. Mass	± da	± ppm	Start Seq.	End Seq.	Sequence	Ion Score	C. I. %	Modification	Rank	Result Type
818.473	818.4179	-0.0551	-67	218	224	STKIVDR					Mascot
908.4142	908.4616	0.0474	52	21	28	DNVNMSTK					Mascot
947.4866	947.4777	-0.0089	-9	74	81	LNDMEVVK					Mascot
962.4901	962.4726	-0.0175	-18	270	277	VDEQTRSK					Mascot
1070.5889	1070.493	-0.0959	-90	112	120	AVPNCVVRR			Carbamidomethyl (C)[5]		Mascot
1161.5933	1161.4969	-0.0964	-83	195	204	SEVVEGCIIR			Carbamidomethyl (C)[7]		Mascot
1181.5719	1181.5421	-0.0298	-25	1	11	MSATDLASLEK			Oxidation (M)[1]		Mascot
1181.5719	1181.5421	-0.0298	-25	1	11	MSATDLASLEK			Oxidation (M)[1]		Mascot
1326.5862	1326.6403	0.0541	41	97	106	FENWGENQFR					Mascot
1457.7483	1457.7385	-0.0098	-7	33	46	DAVEAALNLLDEGK					Mascot

1473.7485	1473.7303	-0.0182	-12	53	65	GADGVWTVNQWLK	Mascot
1601.8435	1601.7562	-0.0873	-54	53	66	GADGVWTVNQWLKK	Mascot
1684.8865	1684.7849	-0.1016	-60	33	48	DAVEAALNLLDEGKAR	Mascot
1684.8865	1684.7849	-0.1016	-60	33	48	DAVEAALNLLDEGKAR	Mascot

8 Myosin-1 OS=Homo sapiens GN=MYH1 PE=1 SV=3 MYH1_HUMAN 223975.8 5.59 35 62 68.884 27.238

Peptide Information

Calc. Mass	Obsrv. Mass	± da	± ppm	Start Seq.	End Seq.	Sequence	Ion Score	C. I. %	Modification	Rank	Result Type
818.4617	818.4179	-0.0438	-54	863	869	TKEELAK					Mascot
860.4625	860.4199	-0.0426	-50	253	260	IHFGTTGK					Mascot
930.5618	930.5106	-0.0512	-55	915	922	TKIQLEAK					Mascot
947.4792	947.4777	-0.0015	-2	1694	1701	ATLEQTER					Mascot
974.4975	974.4645	-0.033	-34	676	683	CIIPNETK			Carbamidomethyl (C)[1]		Mascot
1136.631	1136.5237	-0.1073	-94	407	416	VKVGNEYVTK					Mascot
1147.5889	1147.5341	-0.0548	-48	1673	1681	EQLAMVERR			Oxidation (M)[5]		Mascot
1153.6365	1153.5391	-0.0974	-84	717	725	ILYADFKQR					Mascot
1163.5361	1163.5142	-0.0219	-19	1584	1592	DEEIDQMKR					Mascot
1181.5984	1181.5421	-0.0563	-48	803	811	VEYQKMVER					Mascot
1181.5984	1181.5421	-0.0563	-48	803	811	VEYQKMVER					Mascot
1185.5494	1185.5555	0.0061	5	1776	1785	EQDTSAHLER					Mascot
1197.5933	1197.5513	-0.042	-35	803	811	VEYQKMVER			Oxidation (M)[6]		Mascot
1229.6637	1229.5718	-0.0919	-75	553	562	NKLYEQHLGK					Mascot
1258.6903	1258.6033	-0.087	-69	563	573	SNNFQKPKPAK					Mascot
1276.5903	1276.6259	0.0356	28	1825	1835	ELEGEVESEQK					Mascot
1326.6406	1326.6403	-0.0003	0	792	802	TQAMCRGFLAR			Carbamidomethyl (C)[5], Oxidation (M)[4]		Mascot
1334.6951	1334.6597	-0.0354	-27	1310	1320	QAFTQQIEELK					Mascot
1396.6815	1396.7229	0.0414	30	1282	1293	ARLQTESGEYSR					Mascot
1409.665	1409.7198	0.0548	39	1762	1774	AITDAAMMAEELK			Oxidation (M)[7]		Mascot
1503.7649	1503.7808	0.0159	11	171	185	ENQSILITGESGAGK					Mascot
1503.7649	1503.7808	0.0159	11	171	185	ENQSILITGESGAGK					Mascot
1538.7557	1538.8597	0.104	68	1902	1913	RIQHELEEAEER					Mascot
1584.7952	1584.7136	-0.0816	-51	812	823	RESIFCIQYNVR			Carbamidomethyl (C)[6]		Mascot
1601.8633	1601.7562	-0.1071	-67	957	970	DIDDLELTLAKVEK					Mascot
1680.8744	1680.8347	-0.0397	-24	131	144	WLPVYNAEVVTAYR					Mascot
1831.8781	1831.8289	-0.0492	-27	1884	1899	RQAEEAEEQSNVNLSK					Mascot

Calc. Mass	Obsrv. Mass	± da	± ppm	Start Seq.	End Seq.	Sequence	Ion Score	C. I. %	Modification	Rank	Result Type
1831.8781	1831.8289	-0.0492	-27	1884	1899	RQAEEAEEQSNVNLSK					Mascot
1838.0494	1837.9043	-0.1451	-79	1717	1732	VQLLHTQNTSLINTKK					Mascot
1859.887	1859.8765	-0.0105	-6	1031	1046	LEQQVDDLEGSLEQEK					Mascot
1894.9254	1894.8071	-0.1183	-62	1200	1216	HADSVAELGEQIDNLQR					Mascot
1913.8831	1913.9971	0.114	60	1230	1246	MEIDDLASNMETVSKAK			Oxidation (M)[1,10]		Mascot
1990.8885	1990.9467	0.0582	29	1	18	MSSDSEMAIFGEAAPFLR			Oxidation (M)[1,7]		Mascot
1996.9934	1997.0088	0.0154	8	1656	1672	DTQLHLDDALRSQEDLK					Mascot
1997.0298	1997.0088	-0.021	-11	1181	1198	DLEEATLQHEATAATLRK					Mascot
2013.9117	2013.9863	0.0746	37	1622	1638	MEGDLNEMEIQLNHANR					Mascot
2025.036	2025.0175	-0.0185	-9	1180	1197	RDLEEATLQHEATAATLR					Mascot
2086.9937	2087.0305	0.0368	18	1	19	MSSDSEMAIFGEAAPFLR K					Mascot
3191.5195	3191.6616	0.1421	45	881	908	MVTLMQEKNDLQLQVQA EADSLADAEER			Oxidation (M)[1]		Mascot

9 Aspartate aminotransferase, chloroplastic OS=Arabidopsis thaliana GN=ASP5 PE=2 SV=2 | AAT5_ARATH | 49970.6 | 8.18 | 15 | 62 | 65.882 | 12.706

Peptide Information

Calc. Mass	Obsrv. Mass	± da	± ppm	Start Seq.	End Seq.	Sequence	Ion Score	C. I. %	Modification	Rank	Result Type
821.4152	821.3473	-0.0679	-83	186	192	NIFNDAK					Mascot
908.4876	908.4616	-0.026	-29	390	396	DWSFILK					Mascot
962.5417	962.4726	-0.0691	-72	80	88	LNLGVGAYR					Mascot
1180.6361	1180.5587	-0.0774	-66	387	396	SGKDWSFILK					Mascot
1181.6049	1181.5421	-0.0628	-53	375	384	QELYDSLVSK					Mascot
1181.6049	1181.5421	-0.0628	-53	375	384	QELYDSLVSK					Mascot
1314.5851	1314.6147	0.0296	23	361	371	AEMEMMAGRIK			Oxidation (M)[3,5,6]		Mascot
1409.6406	1409.7198	0.0792	56	287	298	GMEFFVAQSYSK			Oxidation (M)[2]		Mascot
1424.7268	1424.7246	-0.0022	-2	375	386	QELYDSLVSKDK					Mascot
1538.7771	1538.8597	0.0826	54	205	218	TIGLDFEGMIADIK			Oxidation (M)[9]		Mascot
1831.9695	1831.8289	-0.1406	-77	307	324	IGAINVVCSSADAATRVK			Carbamidomethyl (C)[8]		Mascot
1831.9695	1831.8289	-0.1406	-77	307	324	IGAINVVCSSADAATRVK			Carbamidomethyl (C)[8]		Mascot
1837.9602	1837.9043	-0.0559	-30	329	344	RIARPMYSNPPVHGAR			Oxidation (M)[6]		Mascot
1869.8324	1869.8092	-0.0232	-12	438	453	CEYLADAIIDSYHNVS			Carbamidomethyl (C)[1]		Mascot
1878.9596	1878.787	-0.1726	-92	111	127	GDNKEYLPIEGLAAFNK					Mascot
2232.1521	2232.0498	-0.1023	-46	390	408	DWSFILKQIGMFSFTGLN K					Mascot
2333.0901	2333.2544	0.1643	70	397	417	QIGMFSFTGLNKAQSDN MTDK					Mascot

10 Methenyltetrahydrofolate synthase domain-containing protein OS=Xenopus laevis GN=mthfsd PE=2 SV=1 | MTHSD_XENLA | 46576.1 | 8.82 | 14 | 60 | 39.329 | 11.1

Peptide Information

Calc. Mass	Obsrv. Mass	± da	± ppm	Start Seq.	End Seq.	Sequence	Ion Score	C. I. %	Modification	Rank	Result Type
817.4778	817.4323	-0.0455	-56	252	259	AGKDVSLK					Mascot
818.4519	818.4179	-0.034	-42	61	66	RTWEVK					Mascot
870.5519	870.5098	-0.0421	-48	78	85	LAALQARK					Mascot
930.4752	930.5106	0.0354	38	244	250	NLQDRER					Mascot
1132.6222	1132.5223	-0.0999	-88	292	301	HLANAQLHTK					Mascot
1314.6471	1314.6147	-0.0324	-25	2	13	EPGIRVDPGCSK			Carbamidomethyl (C)[10]		Mascot
1396.7219	1396.7229	0.001	1	350	361	AFLDYRDVAQAK					Mascot
1412.6838	1412.7625	0.0787	56	279	291	ELGPCGSVLSEHK			Carbamidomethyl (C)[5]		Mascot
1461.6825	1461.7089	0.0264	18	1	13	MEPGIRVDPGCSK			Carbamidomethyl (C)[11], Oxidation (M)[1]		Mascot
1473.767	1473.7303	-0.0367	-25	384	396	TQRPKANGNQGFR					Mascot
1500.8356	1500.7559	-0.0797	-53	221	233	AKPQGIIWSMITR					Mascot
1656.9366	1656.8812	-0.0554	-33	220	233	RAKPQGIIWSMITR					Mascot
1997.1179	1997.0088	-0.1091	-55	135	153	VQVDLVVVGSVAVSVQG WR					Mascot
1997.1179	1997.0088	-0.1091	-55	135	153	VQVDLVVVGSVAVSVQG WR					Mascot
3173.6665	3173.7478	0.0813	26	124	153	DYSVPMGLDAKVQVDLV VVGSVAVSVQGWR					Mascot

Gel Idx/Pos	281/L9	Instr./Gel Origin	BA2151/Sample Project 20140814	Process Status	Analysis Succeeded
Plate [#] Name	[1] Sample Project 20140814	Instrument Sample Name		Spectra	11

Rank	Protein Name	Accession No.	Protein MW	Protein PI	Pep. Count	Protein Score	Protein Score C. I. %	Intensity Matched	Total Ion Score	Total Ion C. I. %	Confirmed
1	Ketol-acid reductoisomerase, chloroplastic OS=Oryza sativa subsp. japonica GN=Os05g0573700 PE=1 SV=1	ILV5_ORYSJ	62679.9	6.01	12	320	100	18.924	279	100	

Peptide Information

Calc. Mass	Obsrv. Mass	± da	± ppm	Start Seq.	End Seq.	Sequence	Ion Score	C. I. %	Modification	Rank	Result Type
823.3945	823.3724	-0.0221	-27	296	302	SDIFGER					Mascot
863.5098	863.4813	-0.0285	-33	242	248	RLYVQGK					Mascot
877.46	877.456	-0.004	-5	423	429	MWKVGEK					Mascot
1099.6833	1099.6649	-0.0184	-17	135	144	SDIVVKIGLR					Mascot
1288.73	1288.6642	-0.0658	-51	95	105	NLFPLLPEAFK					Mascot
1372.7219	1372.7006	-0.0213	-16	80	91	VSLAGHEEYIVR					Mascot
1372.7219	1372.7006	-0.0213	-16	80	91	VSLAGHEEYIVR	102	100			Mascot
1528.6884	1528.6809	-0.0075	-5	491	504	GVAFMVDNCSTTAR			Carbamidomethyl (C)[9]		Mascot
1544.6832	1544.6357	-0.0475	-31	491	504	GVAFMVDNCSTTAR			Carbamidomethyl (C)[9], Oxidation (M)[5]		Mascot
1544.6832	1544.6357	-0.0475	-31	491	504	GVAFMVDNCSTTAR	39	87.21	Carbamidomethyl (C)[9], Oxidation (M)[5]		Mascot
1664.9847	1664.9518	-0.0329	-20	303	318	GILLGAVHGIVEALFR					Mascot
1713.8364	1713.8126	-0.0238	-14	350	364	GMLEVYNSLTEEGKK			Oxidation (M)[2]		Mascot
1980.041	1980.0227	-0.0183	-9	109	127	QIGVIGWGSQGPAQAQNLR					Mascot
1980.041	1980.0227	-0.0183	-9	109	127	QIGVIGWGSQGPAQAQNLR	80	100			Mascot
2085.9949	2085.9739	-0.021	-10	249	268	EINGAGINSSFAVHQDVDGR					Mascot
2085.9949	2085.9739	-0.021	-10	249	268	EINGAGINSSFAVHQDVDGR	59	99.885			Mascot
2947.3892	2947.3635	-0.0257	-9	465	490	GHSYSEIINESVIESVDSLNPFMHAR			Oxidation (M)[23]		Mascot

Rank	Protein Name	Accession No.	Protein MW	Protein PI	Pep. Count	Protein Score	Protein Score C. I. %	Intensity Matched	Total Ion Score	Total Ion C. I. %	Confirmed
2	Ketol-acid reductoisomerase, chloroplastic OS=Pisum sativum GN=PGAAIR PE=1 SV=1	ILV5_PEA	63211.2	6.62	9	133	100	5.315	101	100	

Peptide Information

Calc. Mass	Obsrv. Mass	± da	± ppm	Start Seq.	End Seq.	Sequence	Ion Score	C. I. %	Modification	Rank	Result Type
800.4625	800.406	-0.0565	-71	37	43	LSPQSIR					Mascot
823.3945	823.3724	-0.0221	-27	298	304	SDIFGER					Mascot
863.5098	863.4813	-0.0285	-33	244	250	RLYVQGK					Mascot

1062.5289	1062.4911	-0.0378	-36	410	419	EGLPAFPMGK			Oxidation (M)[8]		Mascot
1544.6832	1544.6357	-0.0475	-31	493	506	GVSFMVDNCSTTAR			Carbamidomethyl (C)[9]		Mascot
1544.6832	1544.6357	-0.0475	-31	493	506	GVSFMVDNCSTTAR	21	0	Carbamidomethyl (C)[9]		Mascot
1684.8766	1684.7802	-0.0964	-57	80	93	ERVNLAGHEEYIVR					Mascot
1684.8766	1684.7802	-0.0964	-57	80	93	ERVNLAGHEEYIVR	8	0			Mascot
1831.8093	1831.8252	0.0159	9	148	164	GSSSFNEAREAGFSEEK					Mascot
1980.041	1980.0227	-0.0183	-9	111	129	QIGVIGWGSQGPAQAQNLR					Mascot
1980.041	1980.0227	-0.0183	-9	111	129	QIGVIGWGSQGPAQAQNLR	80	100			Mascot
2947.3892	2947.3635	-0.0257	-9	467	492	GHSYSEIINESVIESVDSLNPFMHAR			Oxidation (M)[23]		Mascot

3	Ketol-acid reductoisomerase, chloroplastic OS=Arabidopsis thaliana GN=At3g58610 PE=2 SV=2	ILV5_ARATH	64171.5	6.36	9	125	100	4.449	101	100

Peptide Information

Calc. Mass	Obsrv. Mass	± da	± ppm	Start Seq.	End Seq.	Sequence	Ion Score	C. I. %	Modification	Rank	Result Type
808.4233	808.3677	-0.0556	-69	68	75	MVSSSAVK					Mascot
823.3945	823.3724	-0.0221	-27	308	314	SDIFGER					Mascot
863.5098	863.4813	-0.0285	-33	254	260	RLYVQGK					Mascot
1099.6833	1099.6649	-0.0184	-17	147	156	SDIVVKIGLR					Mascot
1544.6832	1544.6357	-0.0475	-31	503	516	GVSFMVDNCSTTAR			Carbamidomethyl (C)[9]		Mascot
1544.6832	1544.6357	-0.0475	-31	503	516	GVSFMVDNCSTTAR	21	0	Carbamidomethyl (C)[9]		Mascot
1980.041	1980.0227	-0.0183	-9	121	139	QIGVIGWGSQGPAQAQNLR					Mascot
1980.041	1980.0227	-0.0183	-9	121	139	QIGVIGWGSQGPAQAQNLR	80	100			Mascot
1992.9542	1992.976	0.0218	11	2	22	AAATSSIAPSLSCPSPSSSSK			Carbamidomethyl (C)[13]		Mascot
2027.9954	2028.0043	0.0089	4	357	375	TISTQGMLAVYNSLSEEGK					Mascot
2947.3892	2947.3635	-0.0257	-9	477	502	GHSYSEIINESVIESVDSLNPFMHAR			Oxidation (M)[23]		Mascot

4	Granule-bound starch synthase 1, chloroplastic/amyloplastic OS=Hordeum vulgare GN=WAXY PE=1 SV=1	SSG1_HORVU	66853	7.06	15	91	99.953	4.504	43	95.081

Peptide Information

Calc. Mass	Obsrv. Mass	± da	± ppm	Start Seq.	End Seq.	Sequence	Ion Score	C. I. %	Modification	Rank	Result Type
881.3999	881.4545	0.0546	62	136	142	VADEYER					Mascot
1099.6986	1099.6649	-0.0337	-31	392	401	KVPLVAFIGR					Mascot
1310.5868	1310.675	0.0882	67	556	565	NCMIQDLSWK			Carbamidomethyl (C)[2], Oxidation (M)[3]		Mascot
1363.6741	1363.7131	0.039	29	124	135	DAWDTSVISEIK					Mascot

1396.7432	1396.699	-0.0442	-32	379	391	EALQAEVGLPVDR					Mascot
1487.804	1487.7142	-0.0898	-60	298	310	INWMKAGILQADK					Mascot
1500.7516	1500.7604	0.0088	6	112	123	VMVVSPRYDQYK			Oxidation (M)[2]		Mascot
1503.7988	1503.7736	-0.0252	-17	298	310	INWMKAGILQADK			Oxidation (M)[4]		Mascot
1544.7626	1544.6357	-0.1269	-82	519	532	LSVDCNVVEPADVK			Carbamidomethyl (C)[5]		Mascot
1544.7626	1544.6357	-0.1269	-82	519	532	LSVDCNVVEPADVK			Carbamidomethyl (C)[5]		Mascot
1564.7611	1564.7455	-0.0156	-10	478	490	FEPCGLIQLQGMR			Carbamidomethyl (C)[4], Oxidation (M)[12]		Mascot
1672.8575	1672.8046	-0.0529	-32	519	533	LSVDCNVVEPADVKK			Carbamidomethyl (C)[5]		Mascot
1684.7966	1684.7802	-0.0164	-10	265	278	FSFDDFAQLNLPDR					Mascot
1684.7966	1684.7802	-0.0164	-10	265	278	FSFDDFAQLNLPDR	43	95.081			Mascot
1827.9381	1827.8527	-0.0854	-47	34	52	NPADAALGMRTIGASAAP K			Oxidation (M)[9]		Mascot
1831.8497	1831.8252	-0.0245	-13	281	296	SSFDFIDGYDKPVEGR					Mascot
1841.7937	1841.7708	-0.0229	-12	175	190	IYGPDAGTDYEDNQQR					Mascot
1976.9971	1977.0291	0.032	16	91	111	TGGLGDVLGGLPPAMAA NGHR			Oxidation (M)[15]		Mascot

5	Granule-bound starch synthase 1, chloroplastic/amyloplastic OS=Triticum aestivum GN=WAXY PE=1 SV=1	SSG1_WHEAT	68506	8.25	14	79	99.303	4.52	43	95.081

Peptide Information

Calc. Mass	Obsrv. Mass	± da	± ppm	Start Seq.	End Seq.	Sequence	Ion Score	C. I. %	Modification	Rank	Result Type
1099.6986	1099.6649	-0.0337	-31	404	413	KVPLVAFIGR					Mascot
1310.5868	1310.675	0.0882	67	568	577	NCMIQDLSWK			Carbamidomethyl (C)[2], Oxidation (M)[3]		Mascot
1363.6741	1363.7131	0.039	29	125	136	DAWDTSVISEIK					Mascot
1390.6825	1390.6663	-0.0162	-12	157	167	VFVDHPCFLEK			Carbamidomethyl (C)[7]		Mascot
1396.7432	1396.699	-0.0442	-32	391	403	EALQAEVGLPVDR					Mascot
1400.7632	1400.7122	-0.051	-36	433	445	EEDVQIVLLGTGK					Mascot
1487.804	1487.7142	-0.0898	-60	310	322	INWMKAGILQADK					Mascot
1503.7988	1503.7736	-0.0252	-17	310	322	INWMKAGILQADK			Oxidation (M)[4]		Mascot
1544.7626	1544.6357	-0.1269	-82	531	544	LSVDCNVVEPADVK			Carbamidomethyl (C)[5]		Mascot
1544.7626	1544.6357	-0.1269	-82	531	544	LSVDCNVVEPADVK			Carbamidomethyl (C)[5]		Mascot
1564.7611	1564.7455	-0.0156	-10	490	502	FEPCGLIQLQGMR			Carbamidomethyl (C)[4], Oxidation (M)[12]		Mascot
1628.8829	1628.8304	-0.0525	-32	553	567	AVKVVGTPAYHEMVK					Mascot
1672.8575	1672.8046	-0.0529	-32	531	545	LSVDCNVVEPADVKK			Carbamidomethyl (C)[5]		Mascot
1684.7966	1684.7802	-0.0164	-10	277	290	FSFDDFAQLNLPDR					Mascot
1684.7966	1684.7802	-0.0164	-10	277	290	FSFDDFAQLNLPDR	43	95.081			Mascot
1831.8497	1831.8252	-0.0245	-13	293	308	SSFDFIDGYDKPVEGR					Mascot

	1841.7937	1841.7708	-0.0229	-12	176	191	IYGPDAGTDYEDNQQR					Mascot

6	Protein translocase subunit SecA OS=Prochlorococcus marinus (strain NATL1A) GN=secA PE=3 SV=1	SECA_PROM1	108033.5	5.48	23	68	90.384	21.286	

Peptide Information

Calc. Mass	Obsrv. Mass	± da	± ppm	Start Seq.	End Seq.	Sequence	Ion Score	C. I. %	Modification	Rank	Result Type
854.4366	854.4697	0.0331	39	43	49	TSEFRSK					Mascot
860.4836	860.4467	-0.0369	-43	731	738	SLEGAQKK					Mascot
877.4665	877.456	-0.0105	-12	904	910	DPLIEYK					Mascot
881.4397	881.4545	0.0148	17	382	390	LSGMTGTAK			Oxidation (M)[4]		Mascot
1099.6106	1099.6649	0.0543	49	241	250	QAAQVVENLK					Mascot
1164.5685	1164.5215	-0.047	-40	416	424	QDWVDQVFK					Mascot
1267.61	1267.585	-0.025	-20	844	853	EQLRNAYDMK					Mascot
1310.7063	1310.675	-0.0313	-24	432	443	AVAKETADIHQK					Mascot
1390.7689	1390.6663	-0.1026	-74	239	250	YKQAAQVVENLK					Mascot
1410.7263	1410.6476	-0.0787	-56	899	910	GYGQKDPLIEYK					Mascot
1497.7479	1497.8195	0.0716	48	854	866	EAKVEQSHPGIMR			Oxidation (M)[12]		Mascot
1508.8472	1508.7236	-0.1236	-82	313	324	ELFIKDVNYIVR					Mascot
1508.8472	1508.7236	-0.1236	-82	313	324	ELFIKDVNYIVR					Mascot
1525.7506	1525.8068	0.0562	37	343	355	RWSDGQHQAIEAK					Mascot
1584.7211	1584.7517	0.0306	19	274	287	SCILTDEGFANTEK			Carbamidomethyl (C)[2]		Mascot
1622.7401	1622.7906	0.0505	31	717	730	VEEDMPIESGMLTR			Oxidation (M)[5]		Mascot
1664.8279	1664.9518	0.1239	74	416	429	QDWVDQVFKTEAAK					Mascot
1684.9229	1684.7802	-0.1427	-85	224	238	TPLIISGQVERSQEK					Mascot
1684.9229	1684.7802	-0.1427	-85	224	238	TPLIISGQVERSQEK					Mascot
1831.9233	1831.8252	-0.0981	-54	927	941	NVIYSMFMFQPAQKK					Mascot
1896.959	1896.9313	-0.0277	-15	572	588	SFLNIFPVSLTEDTDAK					Mascot
1994.9414	1995.0007	0.0593	30	640	655	NEYEEVLSQEETNVRR					Mascot
1994.9414	1995.0007	0.0593	30	640	655	NEYEEVLSQEETNVRR					Mascot
2012.0601	2012.0258	-0.0343	-17	691	707	FFLSLEDNLLRIFGGDR					Mascot
2012.0601	2012.0258	-0.0343	-17	691	707	FFLSLEDNLLRIFGGDR					Mascot
2020.9757	2021.0045	0.0288	14	325	342	NDEAVIVDEFTGRVMPG R			Oxidation (M)[15]		Mascot
3178.5137	3178.4089	-0.1048	-33	788	814	TMEEIVEAYVNEDLPPEE WNLTNLVSK			Oxidation (M)[2]		Mascot

7	Protein phosphatase 1B OS=Bos taurus GN=PPM1B PE=2 SV=2	PPM1B_BOVIN	54023.9	4.98	15	66	86.417	23.71	

Peptide Information

Calc. Mass	Obsrv. Mass	± da	± ppm	Start Seq.	End Seq.	Sequence	Ion Score	C. I. %	Modification	Rank	Result Type
807.3665	807.3904	0.0239	30	301	307	VSDEAMR					Mascot
823.3614	823.3724	0.011	13	301	307	VSDEAMR			Oxidation (M)[6]		Mascot
826.3763	826.391	0.0147	18	113	118	IDEYMR					Mascot
877.4335	877.456	0.0225	26	320	326	VEEIMEK					Mascot
1229.6749	1229.5601	-0.1148	-93	365	374	RHVIEAVYSR					Mascot
1354.6486	1354.6848	0.0362	27	309	319	DSELDKYLESR					Mascot
1372.693	1372.7006	0.0076	6	108	118	TGFLKIDEYMR					Mascot
1372.693	1372.7006	0.0076	6	108	118	TGFLKIDEYMR					Mascot
1461.7306	1461.7062	-0.0244	-17	10	23	TEKHNAHGAGNGLR					Mascot
1503.6785	1503.7736	0.0951	63	201	213	ALGDYDYKCVDGK			Carbamidomethyl (C)[9]		Mascot
1525.7567	1525.8068	0.0501	33	315	326	YLESRVEEIMEK					Mascot
1528.6884	1528.6809	-0.0075	-5	327	340	SGEEGMPDLAHVMR					Mascot
1544.6832	1544.6357	-0.0475	-31	327	340	SGEEGMPDLAHVMR			Oxidation (M)[6]		Mascot
1544.6832	1544.6357	-0.0475	-31	327	340	SGEEGMPDLAHVMR	5	0	Oxidation (M)[6]		Mascot
1827.9236	1827.8527	-0.0709	-39	192	208	VNGSLAVSRALGDYDYK					Mascot
1983.9858	1984.0242	0.0384	19	143	158	HIYFINCGDSRAVLYR			Carbamidomethyl (C)[7]		Mascot
1994.9786	1995.0007	0.0221	11	283	300	GSRDNMSIVLVCFSNAPK			Carbamidomethyl (C)[12]		Mascot
1994.9786	1995.0007	0.0221	11	283	300	GSRDNMSIVLVCFSNAPK			Carbamidomethyl (C)[12]		Mascot
2010.9736	2011.0082	0.0346	17	283	300	GSRDNMSIVLVCFSNAPK			Carbamidomethyl (C)[12], Oxidation (M)[6]		Mascot
2017.8967	2017.9888	0.0921	46	159	175	SGQVCFSTQDHKPCNPR			Carbamidomethyl (C)[5,14]		Mascot
2056.0598	2055.9629	-0.0969	-47	214	231	GPTEQLVSPEPEVYEILR					Mascot

8	Pyruvate kinase PKM OS=Gallus gallus GN=PKM PE=2 SV=2	KPYM_CHICK	58433.9	7.29	18	64 75.846	3.3

Peptide Information

Calc. Mass	Obsrv. Mass	± da	± ppm	Start Seq.	End Seq.	Sequence	Ion Score	C. I. %	Modification	Rank	Result Type
826.4352	826.391	-0.0442	-53	376	382	MQHAIAR					Mascot
919.507	919.4172	-0.0898	-98	467	474	GVFPVLCK			Carbamidomethyl (C)[7]		Mascot
947.4979	947.4842	-0.0137	-14	489	497	VNLGMNVGK			Oxidation (M)[5]		Mascot
1037.4801	1037.4924	0.0123	12	311	318	MMIGRCNR			Carbamidomethyl (C)[6]		Mascot
1062.5579	1062.4911	-0.0668	-63	392	399	QQFEEILR					Mascot
1158.6412	1158.5867	-0.0545	-47	489	499	VNLGMNVGKAR					Mascot
1229.6372	1229.5601	-0.0771	-63	32	42	LDIDSEPTIAR					Mascot

Calc. Mass	Obsrv. Mass	± da	± ppm	Start Seq.	End Seq.	Sequence	Ion Score	C. I. %	Modification	Rank	Result Type
1235.6235	1235.5846	-0.0389	-31	62	72	EMIKSGMNVAR					Mascot
1267.6133	1267.585	-0.0283	-22	62	72	EMIKSGMNVAR			Oxidation (M)[2,7]		Mascot
1394.775	1394.6718	-0.1032	-74	266	277	IISKIENHEGVR					Mascot
1447.8407	1447.7192	-0.1215	-84	173	185	IYVDDGLISLLVK					Mascot
1507.7607	1507.7196	-0.0411	-27	422	435	CLAAALIVMTESGR			Carbamidomethyl (C)[1], Oxidation (M)[9]		Mascot
1525.8445	1525.8068	-0.0377	-25	447	460	APIIAVTRNDQTAR					Mascot
1564.7312	1564.7455	0.0143	9	406	421	EPADAMAAGAVEASFK					Mascot
1622.7557	1622.7906	0.0349	22	475	488	QPAHDAWAEDVDLR					Mascot
1699.7666	1699.7753	0.0087	5	279	293	FDEIMEASDGIMVAR			Oxidation (M)[5]		Mascot
1713.7822	1713.8126	0.0304	18	190	205	DFVMTEVENGGMLGSK					Mascot
1828.0215	1827.8527	-0.1688	-92	294	310	GDLGIEIPAEKVFLAQK					Mascot
1841.8772	1841.7708	-0.1064	-58	190	206	DFVMTEVENGGMLGSKK					Mascot

9	ATP-dependent helicase/nuclease subunit A OS=Streptococcus pyogenes serotype M12 (strain MGAS2096) GN=addA PE=3 SV=2	ADDA_STRPB	140691.8	6.08	23	57	0	13.156	

Peptide Information

Calc. Mass	Obsrv. Mass	± da	± ppm	Start Seq.	End Seq.	Sequence	Ion Score	C. I. %	Modification	Rank	Result Type
809.4226	809.3802	-0.0424	-52	449	455	FMVGDIK					Mascot
826.3512	826.391	0.0398	48	199	205	NFSGNCK			Carbamidomethyl (C)[6]		Mascot
854.5134	854.4697	-0.0437	-51	900	906	KVYFIGK					Mascot
856.4312	856.4808	0.0496	58	473	478	FRDYQK					Mascot
870.5771	870.4969	-0.0802	-92	74	80	ILDKILR					Mascot
877.4778	877.456	-0.0218	-25	2	8	LFNINEK					Mascot
881.455	881.4545	-0.0005	-1	67	73	TFVMVER					Mascot
925.4486	925.4702	0.0216	23	27	33	HLQENER					Mascot
1062.495	1062.4911	-0.0039	-4	1094	1102	EIDSNEEVK					Mascot
1164.626	1164.5215	-0.1045	-90	541	550	AQLLLYNSDK					Mascot
1310.7137	1310.675	-0.0387	-30	889	899	LLYVAMTRAEK			Oxidation (M)[6]		Mascot
1320.7271	1320.6003	-0.1268	-96	540	550	RAQLLLYNSDK					Mascot
1385.757	1385.6582	-0.0988	-71	798	809	QAVNLMTIHKSK			Oxidation (M)[6]		Mascot
1447.7428	1447.7192	-0.0236	-16	1141	1152	VDPISQEEYVLR					Mascot
1500.8169	1500.7604	-0.0565	-38	690	703	GAHPELIHDTLLGK					Mascot
1503.6646	1503.7736	0.109	72	199	211	NFSGNCKDTSAFR			Carbamidomethyl (C)[6]		Mascot
1601.8381	1601.7487	-0.0894	-56	321	336	DVTGLIPSGNDVTVSK					Mascot
1713.8669	1713.8126	-0.0543	-32	810	823	GLQFPYVFILNCDK			Carbamidomethyl (C)[12]		Mascot
1977.1267	1977.0291	-0.0976	-49	846	863	YLADIKVLLGETTLNSVK					Mascot

1984.1074	1984.0242	-0.0832	-42	318	336	LIRDVTGLIPSGNDVTVSK					Mascot
1992.9847	1992.976	-0.0087	-4	150	166	YGYSIGISSQFRIMQDK					Mascot
2012.0668	2012.0258	-0.041	-20	880	896	LATLSEQMRLLYVAMTR			Oxidation (M)[8]		Mascot
2012.0668	2012.0258	-0.041	-20	880	896	LATLSEQMRLLYVAMTR			Oxidation (M)[8]		Mascot
2028.0616	2028.0043	-0.0573	-28	880	896	LATLSEQMRLLYVAMTR			Oxidation (M)[8,15]		Mascot
2947.5557	2947.3635	-0.1922	-65	781	807	VLETQNDLADVEVAAPK QAVNLMTIHK					Mascot

10	Uncharacterized protein YER077C OS=Saccharomyces cerevisiae (strain ATCC 204508 / S288c) GN=YER077C PE=1 SV=1	YEQ7_YEAST	80181.5	9.47	20	56	0	4.248

Peptide Information

Calc. Mass	Obsrv. Mass	± da	± ppm	Start Seq.	End Seq.	Sequence	Ion Score	C. I. %	Modification	Rank	Result Type
818.4366	818.4209	-0.0157	-19	588	594	ASIEDKR					Mascot
863.5389	863.4813	-0.0576	-67	547	553	FLFPIVK					Mascot
1099.5684	1099.6649	0.0965	88	539	546	VYRFNSWK					Mascot
1127.6321	1127.5607	-0.0714	-63	111	119	DQAFHILRK					Mascot
1181.5984	1181.5524	-0.046	-39	28	37	IGVDTCIFTR			Carbamidomethyl (C)[6]		Mascot
1288.7332	1288.6642	-0.069	-54	477	488	VASSAQISITRR					Mascot
1323.6799	1323.6215	-0.0584	-44	327	337	FMVVLNDLMNK					Mascot
1368.7158	1368.583	-0.1328	-97	576	587	SLLNGEFGKEFK					Mascot
1480.744	1480.6848	-0.0592	-40	413	424	HVVGLFMNYCLK			Carbamidomethyl (C)[10]		Mascot
1480.744	1480.6848	-0.0592	-40	413	424	HVVGLFMNYCLK			Carbamidomethyl (C)[10]		Mascot
1500.7805	1500.7604	-0.0201	-13	120	132	FHKINNEALADTK					Mascot
1507.8591	1507.7196	-0.1395	-93	42	54	INTAINLTEHLLR					Mascot
1525.8566	1525.8068	-0.0498	-33	542	553	FNSWKFLFPIVK					Mascot
1622.8901	1622.7906	-0.0995	-61	152	165	LFYTILGVNGEQLR					Mascot
1672.8541	1672.8046	-0.0495	-30	520	534	IRGFIDSVEATYSSK					Mascot
1681.8868	1681.8208	-0.066	-39	504	519	AVINKDASNAEATLHK					Mascot
1732.8357	1732.7723	-0.0634	-37	307	320	DSITYTLMIRSCTR			Carbamidomethyl (C)[12], Oxidation (M)[8]		Mascot
1734.9636	1734.8271	-0.1365	-79	133	148	LGPTSQSDLKFLSLTK					Mascot
1953.0441	1953.0052	-0.0389	-20	152	168	LFYTILGVNGEQLRDSK					Mascot
1992.8983	1992.976	0.0777	39	366	381	QGMGLWALCEYFQFDK			Carbamidomethyl (C)[9]		Mascot
2021.1324	2021.0045	-0.1279	-63	38	54	MLPRINTAINLTEHLLR			Oxidation (M)[1]		Mascot

Gel Idx/Pos	282/L10	Instr./Gel Origin	BA2151/Sample Project 20140814	Process Status	Analysis Succeeded
Plate [#] Name	[1] Sample Project 20140814	Instrument Sample Name		Spectra	11

Rank	Protein Name	Accession No.	Protein MW	Protein PI	Pep. Count	Protein Score	Protein Score C. I. %	Intensity Matched	Total Ion Score	Total Ion C. I. %	Confirmed
1	Granule-bound starch synthase 1, chloroplastic/amyloplastic OS=Hordeum vulgare GN=WAXY PE=1 SV=1	SSG1_HORVU	66853	7.06	15	198	100	9.56	153	100	

Peptide Information

Calc. Mass	Obsrv. Mass	± da	± ppm	Start Seq.	End Seq.	Sequence	Ion Score	C. I. %	Modification	Rank	Result Type
971.6036	971.5504	-0.0532	-55	393	401	VPLVAFIGR					Mascot
1099.6986	1099.6608	-0.0378	-34	392	401	KVPLVAFIGR					Mascot
1107.4922	1107.5118	0.0196	18	329	337	GCELDNIMR			Carbamidomethyl (C)[2]		Mascot
1136.5695	1136.5132	-0.0563	-50	136	144	VADEYERVR					Mascot
1201.5597	1201.5629	0.0032	3	239	248	SNYQSNGIYR					Mascot
1363.6741	1363.7002	0.0261	19	124	135	DAWDTSVISEIK					Mascot
1396.7432	1396.7061	-0.0371	-27	379	391	EALQAEVGLPVDR					Mascot
1500.7516	1500.7078	-0.0438	-29	112	123	VMVVSPRYDQYK			Oxidation (M)[2]		Mascot
1503.7988	1503.7635	-0.0353	-23	298	310	INWMKAGILQADK			Oxidation (M)[4]		Mascot
1544.7626	1544.6477	-0.1149	-74	519	532	LSVDCNVVEPADVK			Carbamidomethyl (C)[5]		Mascot
1564.7611	1564.7291	-0.032	-20	478	490	FEPCGLIQLQGMR			Carbamidomethyl (C)[4], Oxidation (M)[12]		Mascot
1684.7966	1684.7695	-0.0271	-16	265	278	FSFDDFAQLNLPDR					Mascot
1684.7966	1684.7695	-0.0271	-16	265	278	FSFDDFAQLNLPDR	91	100			Mascot
1831.8497	1831.8109	-0.0388	-21	281	296	SSFDFIDGYDKPVEGR					Mascot
1841.7937	1841.7545	-0.0392	-21	175	190	IYGPDAGTDYEDNQQR					Mascot
1997.0226	1996.9845	-0.0381	-19	311	328	VLTVSPYYAEELISGEAR					Mascot
1997.0226	1996.9845	-0.0381	-19	311	328	VLTVSPYYAEELISGEAR	63	99.949			Mascot

Rank	Protein Name	Accession No.	Protein MW	Protein PI	Pep. Count	Protein Score	Protein Score C. I. %	Intensity Matched	Total Ion Score	Total Ion C. I. %	Confirmed
2	Granule-bound starch synthase 1, chloroplastic/amyloplastic OS=Triticum aestivum GN=WAXY PE=1 SV=1	SSG1_WHEAT	68506	8.25	14	190	100	9.538	153	100	

Peptide Information

Calc. Mass	Obsrv. Mass	± da	± ppm	Start Seq.	End Seq.	Sequence	Ion Score	C. I. %	Modification	Rank	Result Type
971.6036	971.5504	-0.0532	-55	405	413	VPLVAFIGR					Mascot
1099.6986	1099.6608	-0.0378	-34	404	413	KVPLVAFIGR					Mascot
1107.4922	1107.5118	0.0196	18	341	349	GCELDNIMR			Carbamidomethyl (C)[2]		Mascot

Calc. Mass	Obsrv. Mass	± da	± ppm	Start Seq.	End Seq.	Sequence	Ion Score	C. I. %	Modification	Rank	Result Type
1201.5597	1201.5629	0.0032	3	251	260	SNYQSNGIYR					Mascot
1363.6741	1363.7002	0.0261	19	125	136	DAWDTSVISEIK					Mascot
1390.6825	1390.6661	-0.0164	-12	157	167	VFVDHPCFLEK			Carbamidomethyl (C)[7]		Mascot
1396.7432	1396.7061	-0.0371	-27	391	403	EALQAEVGLPVDR					Mascot
1503.7988	1503.7635	-0.0353	-23	310	322	INWMKAGILQADK			Oxidation (M)[4]		Mascot
1544.7626	1544.6477	-0.1149	-74	531	544	LSVDCNVVEPADVK			Carbamidomethyl (C)[5]		Mascot
1564.7611	1564.7291	-0.032	-20	490	502	FEPCGLIQLQGMR			Carbamidomethyl (C)[4], Oxidation (M)[12]		Mascot
1684.7966	1684.7695	-0.0271	-16	277	290	FSFDDFAQLNLPDR					Mascot
1684.7966	1684.7695	-0.0271	-16	277	290	FSFDDFAQLNLPDR	91	100			Mascot
1831.8497	1831.8109	-0.0388	-21	293	308	SSFDFIDGYDKPVEGR					Mascot
1841.7937	1841.7545	-0.0392	-21	176	191	IYGPDAGTDYEDNQQR					Mascot
1997.0226	1996.9845	-0.0381	-19	323	340	VLTVSPYYAEELISGEAR					Mascot
1997.0226	1996.9845	-0.0381	-19	323	340	VLTVSPYYAEELISGEAR	63	99.949			Mascot

3	2,3,4,5-tetrahydropyridine-2,6-dicarboxylate N-succinyltransferase OS=Rhizobium etli (strain CIAT 652) GN=dapD PE=3 SV=1	DAPD_RHIE6	30795.4	5.56	13	71	95.502	11.464	

Peptide Information

Calc. Mass	Obsrv. Mass	± da	± ppm	Start Seq.	End Seq.	Sequence	Ion Score	C. I. %	Modification	Rank	Result Type
818.473	818.4026	-0.0704	-86	218	224	STKIVDR					Mascot
947.4866	947.4667	-0.0199	-21	74	81	LNDMEVVK					Mascot
962.4901	962.4623	-0.0278	-29	270	277	VDEQTRSK					Mascot
1070.5889	1070.4917	-0.0972	-91	112	120	AVPNCVVRR			Carbamidomethyl (C)[5]		Mascot
1161.5933	1161.4916	-0.1017	-88	195	204	SEVVEGCIIR			Carbamidomethyl (C)[7]		Mascot
1181.5719	1181.531	-0.0409	-35	1	11	MSATDLASLEK			Oxidation (M)[1]		Mascot
1181.5719	1181.531	-0.0409	-35	1	11	MSATDLASLEK			Oxidation (M)[1]		Mascot
1194.5175	1194.5398	0.0223	19	82	92	GGSGNSTWWDK					Mascot
1326.5862	1326.6208	0.0346	26	97	106	FENWGENQFR					Mascot
1416.7529	1416.7258	-0.0271	-19	107	119	AAGFRAVPNCVVR			Carbamidomethyl (C)[10]		Mascot
1457.7483	1457.7318	-0.0165	-11	33	46	DAVEAALNLLDEGK					Mascot
1473.7485	1473.7223	-0.0262	-18	53	65	GADGVWTVNQWLK					Mascot
1601.8435	1601.7502	-0.0933	-58	53	66	GADGVWTVNQWLKK					Mascot
1684.8865	1684.7695	-0.117	-69	33	48	DAVEAALNLLDEGKAR					Mascot
1684.8865	1684.7695	-0.117	-69	33	48	DAVEAALNLLDEGKAR					Mascot

4	Integrin-linked protein kinase OS=Bos taurus GN=ILK PE=2 SV=1	ILK_BOVIN	51927.1	8.3	16	70	94.206	7.107	

Peptide Information

Calc. Mass	Obsrv. Mass	± da	± ppm	Start Seq.	End Seq.	Sequence	Ion Score	C. I. %	Modification	Rank	Result Type
823.413	823.3699	-0.0431	-52	297	303	FALDMAR					Mascot
1148.6167	1148.5098	-0.1069	-93	47	56	SAVVEMLIMR					Mascot
1160.5841	1160.5685	-0.0156	-13	152	161	AEKMGQNLNR					Mascot
1164.6116	1164.509	-0.1026	-88	47	56	SAVVEMLIMR			Oxidation (M)[6]		Mascot
1164.6116	1164.509	-0.1026	-88	47	56	SAVVEMLIMR			Oxidation (M)[6]		Mascot
1176.579	1176.5525	-0.0265	-23	152	161	AEKMGQNLNR			Oxidation (M)[4]		Mascot
1185.5028	1185.5476	0.0448	38	1	9	MDDIFTQCR			Carbamidomethyl (C)[8]		Mascot
1197.6304	1197.5349	-0.0955	-80	162	170	IPYKDTFWK					Mascot
1201.4977	1201.5629	0.0652	54	1	9	MDDIFTQCR			Carbamidomethyl (C)[8], Oxidation (M)[1]		Mascot
1326.6437	1326.6208	-0.0229	-17	199	209	LNENHSGELWK					Mascot
1333.7046	1333.5802	-0.1244	-93	155	165	MGQNLNRIPYK					Mascot
1446.7773	1446.6991	-0.0782	-54	212	223	WQGNDIVVKMLK			Oxidation (M)[10]		Mascot
1465.6848	1465.8252	0.1404	96	424	435	LMKICMNEDPAK			Carbamidomethyl (C)[5], Oxidation (M)[2]		Mascot
1490.7817	1490.7133	-0.0684	-46	44	56	EGRSAVVEMLIMR					Mascot
1574.8036	1574.7018	-0.1018	-65	350	363	MYAPAWVAPEALQK					Mascot
1601.9083	1601.7502	-0.1581	-99	436	448	RPKFDMIVPILEK			Oxidation (M)[6]		Mascot
1680.7972	1680.8156	0.0184	11	389	403	EVPFADLSNMEIGMK					Mascot
1977.9117	1977.9292	0.0175	9	318	334	HALNSRSVMIDEDMTAR			Oxidation (M)[9,14]		Mascot
2011.9497	2012.0045	0.0548	27	324	341	SVMIDEDMTARISMADVK					Mascot
2431.2075	2431.136	-0.0715	-29	350	370	MYAPAWVAPEALQKKPEDTNR			Oxidation (M)[1]		Mascot

5	Integrin-linked protein kinase OS=Mus musculus GN=Ilk PE=1 SV=2	ILK_MOUSE	51853.1	8.3	16	70	94.071	7.3	

Protein Group

Integrin-linked protein kinase OS=Homo sapiens GN=ILK PE=1 SV=2	ILK_HUMAN	51899.1	8.3000001907 3486
Integrin-linked protein kinase OS=Rattus norvegicus GN=Ilk PE=2 SV=1	ILK_RAT	51853.1	8.3000001907 3486

Peptide Information

Calc. Mass	Obsrv. Mass	± da	± ppm	Start Seq.	End Seq.	Sequence	Ion Score	C. I. %	Modification	Rank	Result Type
823.413	823.3699	-0.0431	-52	297	303	FALDMAR					Mascot
1148.6167	1148.5098	-0.1069	-93	47	56	SAVVEMLIMR					Mascot
1153.5558	1153.5277	-0.0281	-24	132	141	YGEMPVDKAK			Oxidation (M)[4]		Mascot

Calc. Mass	Obsrv. Mass	± da	± ppm	Start Seq.	End Seq.	Sequence	Ion Score	C. I. %	Modification	Rank	Result Type
1160.5841	1160.5685	-0.0156	-13	152	161	AEKMGQNLNR					Mascot
1164.6116	1164.509	-0.1026	-88	47	56	SAVVEMLIMR			Oxidation (M)[6]		Mascot
1164.6116	1164.509	-0.1026	-88	47	56	SAVVEMLIMR			Oxidation (M)[6]		Mascot
1176.579	1176.5525	-0.0265	-23	152	161	AEKMGQNLNR			Oxidation (M)[4]		Mascot
1185.5028	1185.5476	0.0448	38	1	9	MDDIFTQCR			Carbamidomethyl (C)[8]		Mascot
1197.6304	1197.5349	-0.0955	-80	162	170	IPYKDTFWK					Mascot
1201.4977	1201.5629	0.0652	54	1	9	MDDIFTQCR			Carbamidomethyl (C)[8], Oxidation (M)[1]		Mascot
1326.6437	1326.6208	-0.0229	-17	199	209	LNENHSGELWK					Mascot
1333.7046	1333.5802	-0.1244	-93	155	165	MGQNLNRIPYK					Mascot
1465.6848	1465.8252	0.1404	96	424	435	LMKICMNEDPAK			Carbamidomethyl (C)[5], Oxidation (M)[2]		Mascot
1490.7817	1490.7133	-0.0684	-46	44	56	EGRSAVVEMLIMR					Mascot
1574.8036	1574.7018	-0.1018	-65	350	363	MYAPAWVAPEALQK					Mascot
1601.9083	1601.7502	-0.1581	-99	436	448	RPKFDMIVPILEK			Oxidation (M)[6]		Mascot
1680.7972	1680.8156	0.0184	11	389	403	EVPFADLSNMEIGMK					Mascot
1977.9117	1977.9292	0.0175	9	318	334	HALNSRSVMIDEDMTAR			Oxidation (M)[9,14]		Mascot
2011.9497	2012.0045	0.0548	27	324	341	SVMIDEDMTARISMADVK					Mascot
2431.2075	2431.136	-0.0715	-29	350	370	MYAPAWVAPEALQKKPE DTNR			Oxidation (M)[1]		Mascot

6	Thylakoid membrane protein slr1796 OS=Synechocystis sp. (strain PCC 6803 / Kazusa) GN=slr1796 PE=4 SV=1	Y1796_SYNY3	23124.9	5.32	11	66	87.029	2.976

Peptide Information

Calc. Mass	Obsrv. Mass	± da	± ppm	Start Seq.	End Seq.	Sequence	Ion Score	C. I. %	Modification	Rank	Result Type
823.3865	823.3699	-0.0166	-20	182	188	SESMELK					Mascot
962.5305	962.4623	-0.0682	-71	156	164	IFDAQGALK					Mascot
1107.5463	1107.5118	-0.0345	-31	182	190	SESMELKQR					Mascot
1163.6167	1163.5073	-0.1094	-94	72	80	LNLRESFER					Mascot
1361.7069	1361.6495	-0.0574	-42	2	12	IMSVCLPWLAR			Carbamidomethyl (C)[5], Oxidation (M)[2]		Mascot
1508.7423	1508.7075	-0.0348	-23	1	12	MIMSVCLPWLAR			Carbamidomethyl (C)[6], Oxidation (M)[1,3]		Mascot
1614.7395	1614.8405	0.101	63	189	201	QRTYNEFNSELVD					Mascot
1665.9462	1665.807	-0.1392	-84	81	94	KLPVILVYYLDDSK					Mascot
1808.9099	1808.9484	0.0385	21	174	188	DLFDLLPRSESMELK			Oxidation (M)[12]		Mascot
1824.874	1824.9247	0.0507	28	1	14	MIMSVCLPWLARCR			Carbamidomethyl (C)[6,13], Oxidation (M)[1,3]		Mascot
1910.9219	1911.0066	0.0847	44	98	112	QYAFIVSRMQEFYGR			Oxidation (M)[9]		Mascot

7	Integrin-linked protein kinase OS=Pongo abelii GN=ILK PE=2 SV=1	ILK_PONAB	51884.1	8.04	15	65	81.678	7.111

Peptide Information

Calc. Mass	Obsrv. Mass	± da	± ppm	Start Seq.	End Seq.	Sequence	Ion Score	C. I. %	Modification	Rank	Result Type
823.413	823.3699	-0.0431	-52	297	303	FALDMAR					Mascot
1148.6167	1148.5098	-0.1069	-93	47	56	SAVVEMLIMR					Mascot
1153.5558	1153.5277	-0.0281	-24	132	141	YGEMPVDKAK			Oxidation (M)[4]		Mascot
1160.5841	1160.5685	-0.0156	-13	152	161	AEKMGQNLNR					Mascot
1164.6116	1164.509	-0.1026	-88	47	56	SAVVEMLIMR			Oxidation (M)[6]		Mascot
1164.6116	1164.509	-0.1026	-88	47	56	SAVVEMLIMR			Oxidation (M)[6]		Mascot
1176.579	1176.5525	-0.0265	-23	152	161	AEKMGQNLNR			Oxidation (M)[4]		Mascot
1185.5028	1185.5476	0.0448	38	1	9	MDDIFTQCR			Carbamidomethyl (C)[8]		Mascot
1197.6304	1197.5349	-0.0955	-80	162	170	IPYKDTFWK					Mascot
1201.4977	1201.5629	0.0652	54	1	9	MDDIFTQCR			Carbamidomethyl (C)[8], Oxidation (M)[1]		Mascot
1326.6437	1326.6208	-0.0229	-17	199	209	LNENHSGELWK					Mascot
1333.7046	1333.5802	-0.1244	-93	155	165	MGQNLNRIPYK					Mascot
1465.6848	1465.8252	0.1404	96	424	435	LMKICMNEDPAK			Carbamidomethyl (C)[5], Oxidation (M)[2]		Mascot
1490.7817	1490.7133	-0.0684	-46	44	56	EGRSAVVEMLIMR					Mascot
1574.8036	1574.7018	-0.1018	-65	350	363	MYAPAWVAPEALQK					Mascot
1601.9083	1601.7502	-0.1581	-99	436	448	RPKFDMIVPILEK			Oxidation (M)[6]		Mascot
1977.9117	1977.9292	0.0175	9	318	334	HALNSRSVMIDEDMTAR			Oxidation (M)[9,14]		Mascot
2011.9497	2012.0045	0.0548	27	324	341	SVMIDEDMTARISMADVK					Mascot
2431.2625	2431.136	-0.1265	-52	297	317	FALDMARGMAFLHTLEPL IPR			Oxidation (M)[5,9]		Mascot

8	Ketol-acid reductoisomerase OS=Polynucleobacter necessarius subsp. necessarius (strain STIR1) GN=ilvC PE=3 SV=1	ILVC_POLNS	36713.7	5.77	12	64	80.814	3.453

Peptide Information

Calc. Mass	Obsrv. Mass	± da	± ppm	Start Seq.	End Seq.	Sequence	Ion Score	C. I. %	Modification	Rank	Result Type
818.4254	818.4026	-0.0228	-28	332	338	LVDETKN					Mascot
947.4841	947.4667	-0.0174	-18	322	329	AMMPWIAK					Mascot
1185.6548	1185.5476	-0.1072	-90	120	130	ADLDVIMIAPK					Mascot
1189.6221	1189.5219	-0.1002	-84	322	331	AMMPWIAKNK					Mascot
1201.6497	1201.5629	-0.0868	-72	120	130	ADLDVIMIAPK			Oxidation (M)[7]		Mascot
1205.6169	1205.5598	-0.0571	-47	322	331	AMMPWIAKNK			Oxidation (M)[2]		Mascot
1244.6958	1244.5751	-0.1207	-97	37	48	DSGVNVTVGLRK					Mascot
1263.6362	1263.6224	-0.0138	-11	265	275	VVTEDTKNAMR					Mascot

Calc. Mass	Obsrv. Mass	± da	± ppm	Start Seq.	End Seq.	Sequence	Ion Score	C. I. %	Modification	Rank	Result Type
1553.7628	1553.7078	-0.055	-35	276	288	QCLKDIQTGEYAK			Carbamidomethyl (C)[2]		Mascot
1575.8126	1575.7871	-0.0255	-16	49	64	DGASWSKAANAGLTVK					Mascot
1614.8698	1614.8405	-0.0293	-18	56	71	AANAGLTVKEVDEAVK					Mascot
2976.6013	2976.4062	-0.1951	-66	19	47	VTIIGYGSQGHAHALNLK DSGVNVTVGLR					Mascot
3035.5347	3035.5315	-0.0032	-1	92	119	EVHGNIKQGAALAFAHGF NVHYGQVQSR					Mascot
3035.5347	3035.5315	-0.0032	-1	92	119	EVHGNIKQGAALAFAHGF NVHYGQVQSR					Mascot
3056.5068	3056.5515	0.0447	15	72	98	DADVVMMLLPDEQIADV YNKEVHGNIK					Mascot

9	tRNA-dihydrouridine(47) synthase [NAD(P)(+)] OS=Sclerotinia sclerotiorum (strain ATCC 18683 / 1980 / Ss-1) GN=dus3 PE=3 SV=1	DUS3_SCLS1	84990.5	6.77	18	62	65.882	7.459

Peptide Information

Calc. Mass	Obsrv. Mass	± da	± ppm	Start Seq.	End Seq.	Sequence	Ion Score	C. I. %	Modification	Rank	Result Type
823.4421	823.3699	-0.0722	-88	537	542	QQRYTK					Mascot
1136.5695	1136.5132	-0.0563	-50	387	396	AHESEITPPR					Mascot
1235.6487	1235.5657	-0.083	-67	494	505	MGTMTGKPTALK					Mascot
1253.6121	1253.5471	-0.065	-52	402	412	SSIVQNYNNSK					Mascot
1305.6726	1305.6112	-0.0614	-47	319	329	YVEPPFLPSEK					Mascot
1323.6903	1323.6133	-0.077	-58	637	647	SASERLQYIEK					Mascot
1363.6124	1363.7002	0.0878	64	739	750	HKSNSYEIEAEG					Mascot
1393.7369	1393.6663	-0.0706	-51	521	534	LGAPGCAAITLHGR			Carbamidomethyl (C)[6]		Mascot
1461.7737	1461.6838	-0.0899	-62	319	330	YVEPPFLPSEKR					Mascot
1497.7255	1497.7776	0.0521	35	1	13	MTDEIPNPVQPEK					Mascot
1507.6581	1507.7036	0.0455	30	564	576	DDLTDTIMEPDAR			Oxidation (M)[8]		Mascot
1507.6581	1507.7036	0.0455	30	564	576	DDLTDTIMEPDAR			Oxidation (M)[8]		Mascot
1574.8591	1574.7018	-0.1573	-100	668	679	RFLLEWLSFAHR					Mascot
1734.9241	1734.8081	-0.116	-67	494	509	MGTMTGKPTALKTIER					Mascot
1808.8848	1808.9484	0.0636	35	152	167	IKLCNSTSNSPEFSPK			Carbamidomethyl (C)[4]		Mascot
1841.9314	1841.7545	-0.1769	-96	1	16	MTDEIPNPVQPEKSLK			Oxidation (M)[1]		Mascot
2011.9291	2012.0045	0.0754	37	651	668	YGLEAWGSDEMGVGQT RR					Mascot
2431.302	2431.136	-0.166	-68	332	353	IYFGPETPVLAPLTTQGN LPFR					Mascot
2830.3823	2830.2712	-0.1111	-39	355	379	LCVELGAQLTYSEMAMSI PLFQGQK			Carbamidomethyl (C)[2], Oxidation (M)[14]		Mascot

10	Integrin-linked protein kinase OS=Cavia porcellus GN=ILK PE=2 SV=1	ILK_CAVPO	51788	8.3	15	60	42.06	6.956

Peptide Information

Calc. Mass	Obsrv. Mass	± da	± ppm	Start Seq.	End Seq.	Sequence	Ion Score	C. I. %	Modification	Rank	Result Type
823.413	823.3699	-0.0431	-52	297	303	FALDMAR					Mascot
1148.6167	1148.5098	-0.1069	-93	47	56	SAVVEMLIMR					Mascot
1160.5841	1160.5685	-0.0156	-13	152	161	AEKMGQNLNR					Mascot
1164.6116	1164.509	-0.1026	-88	47	56	SAVVEMLIMR			Oxidation (M)[6]		Mascot
1164.6116	1164.509	-0.1026	-88	47	56	SAVVEMLIMR			Oxidation (M)[6]		Mascot
1176.579	1176.5525	-0.0265	-23	152	161	AEKMGQNLNR			Oxidation (M)[4]		Mascot
1185.5278	1185.5476	0.0198	17	132	141	YGEMPMDKAK			Oxidation (M)[4]		Mascot
1197.6304	1197.5349	-0.0955	-80	162	170	IPYKDTFWK					Mascot
1201.5228	1201.5629	0.0401	33	132	141	YGEMPMDKAK			Oxidation (M)[4,6]		Mascot
1326.6437	1326.6208	-0.0229	-17	199	209	LNENHSGELWK					Mascot
1333.7046	1333.5802	-0.1244	-93	155	165	MGQNLNRIPYK					Mascot
1465.6848	1465.8252	0.1404	96	423	434	LMKICMNEDPAK			Carbamidomethyl (C)[5], Oxidation (M)[2]		Mascot
1490.7817	1490.7133	-0.0684	-46	44	56	EGRSAVVEMLIMR					Mascot
1574.8036	1574.7018	-0.1018	-65	350	363	MYAPAWVAPEALQK					Mascot
1601.9083	1601.7502	-0.1581	-99	435	447	RPKFDMIVPILEK			Oxidation (M)[6]		Mascot
1680.7972	1680.8156	0.0184	11	389	403	EVPFADLSNMEIGMK					Mascot
1977.9117	1977.9292	0.0175	9	318	334	HALNSRSVMIDEDMTAR			Oxidation (M)[9,14]		Mascot
2011.9497	2012.0045	0.0548	27	324	341	SVMIDEDMTARISMADVK					Mascot
2431.2075	2431.136	-0.0715	-29	350	370	MYAPAWVAPEALQKKPE DTNR			Oxidation (M)[1]		Mascot

Gel Idx/Pos	283/L11	Instr./Gel Origin	BA2151/Sample Project 20140814	Process Status	Analysis Succeeded
Plate [#] Name	[1] Sample Project 20140814	Instrument Sample Name		Spectra	11

Rank	Protein Name	Accession No.	Protein MW	Protein PI	Pep. Count	Protein Score	Protein Score C. I. %	Intensity Matched	Total Ion Score	Total Ion C. I. %	Confirmed
1	Ketol-acid reductoisomerase, chloroplastic OS=Oryza sativa subsp. japonica GN=Os05g0573700 PE=1 SV=1	ILV5_ORYSJ	62679.9	6.01	11	324	100	21.377	289	100	

Peptide Information

Calc. Mass	Obsrv. Mass	± da	± ppm	Start Seq.	End Seq.	Sequence	Ion Score	C. I. %	Modification	Rank	Result Type
823.3945	823.3763	-0.0182	-22	296	302	SDIFGER					Mascot
863.5098	863.4956	-0.0142	-16	242	248	RLYVQGK					Mascot
877.46	877.4656	0.0056	6	423	429	MWKVGEK					Mascot
1288.73	1288.6733	-0.0567	-44	95	105	NLFPLLPEAFK					Mascot
1372.7219	1372.7041	-0.0178	-13	80	91	VSLAGHEEYIVR					Mascot
1372.7219	1372.7041	-0.0178	-13	80	91	VSLAGHEEYIVR	98	100			Mascot
1528.6884	1528.6823	-0.0061	-4	491	504	GVAFMVDNCSTTAR			Carbamidomethyl (C)[9]		Mascot
1544.6832	1544.6385	-0.0447	-29	491	504	GVAFMVDNCSTTAR			Carbamidomethyl (C)[9], Oxidation (M)[5]		Mascot
1544.6832	1544.6385	-0.0447	-29	491	504	GVAFMVDNCSTTAR	61	99.928	Carbamidomethyl (C)[9], Oxidation (M)[5]		Mascot
1664.9847	1664.9684	-0.0163	-10	303	318	GILLGAVHGIVEALFR					Mascot
1713.8364	1713.8142	-0.0222	-13	350	364	GMLEVYNSLTEEGKK			Oxidation (M)[2]		Mascot
1980.041	1980.0363	-0.0047	-2	109	127	QIGVIGWGSQGPAQAQN LR					Mascot
1980.041	1980.0363	-0.0047	-2	109	127	QIGVIGWGSQGPAQAQN LR	130	100			Mascot
2085.9949	2085.9619	-0.033	-16	249	268	EINGAGINSSFAVHQDVD GR					Mascot
2947.3892	2947.3728	-0.0164	-6	465	490	GHSYSEIINESVIESVDSL NPFMHAR			Oxidation (M)[23]		Mascot

Rank	Protein Name	Accession No.	Protein MW	Protein PI	Pep. Count	Protein Score	Protein Score C. I. %	Intensity Matched	Total Ion Score	Total Ion C. I. %	Confirmed
2	Ketol-acid reductoisomerase, chloroplastic OS=Arabidopsis thaliana GN=At3g58610 PE=2 SV=2	ILV5_ARATH	64171.5	6.36	10	204	100	7.972	174	100	

Peptide Information

Calc. Mass	Obsrv. Mass	± da	± ppm	Start Seq.	End Seq.	Sequence	Ion Score	C. I. %	Modification	Rank	Result Type
808.4233	808.3535	-0.0698	-86	68	75	MVSSSAVK					Mascot
823.3945	823.3763	-0.0182	-22	308	314	SDIFGER					Mascot
863.5098	863.4956	-0.0142	-16	254	260	RLYVQGK					Mascot
1439.7417	1439.7469	0.0052	4	76	88	APVSLDFETSVFK					Mascot
1544.6832	1544.6385	-0.0447	-29	503	516	GVSFMVDNCSTTAR			Carbamidomethyl (C)[9]		Mascot

Calc. Mass	Obsrv. Mass	± da	± ppm	Start Seq.	End Seq.	Sequence	Ion Score	C. I. %	Modification	Rank	Result Type
1544.6832	1544.6385	-0.0447	-29	503	516	GVSFMVDNCSTTAR	44	96.301	Carbamidomethyl (C)[9]		Mascot
1560.6782	1560.6761	-0.0021	-1	503	516	GVSFMVDNCSTTAR			Carbamidomethyl (C)[9], Oxidation (M)[5]		Mascot
1891.0647	1890.9558	-0.1089	-58	31	48	TLALPNIGFLSSSSKSLR					Mascot
1980.041	1980.0363	-0.0047	-2	121	139	QIGVIGWGSQGPAQAQN LR					Mascot
1980.041	1980.0363	-0.0047	-2	121	139	QIGVIGWGSQGPAQAQN LR	130	100			Mascot
1992.9542	1992.9772	0.023	12	2	22	AAATSSIAPSLSCPSPSS SSK			Carbamidomethyl (C)[13]		Mascot
2027.9954	2028.0066	0.0112	6	357	375	TISTQGMLAVYNSLSEEG K					Mascot
2947.3892	2947.3728	-0.0164	-6	477	502	GHSYSEIINESVIESVDSL NPFMHAR			Oxidation (M)[23]		Mascot

3	Ketol-acid reductoisomerase, chloroplastic OS=Pisum sativum GN=PGAAIR PE=1 SV=1	ILV5_PEA	63211.2	6.62	8	195	100	7.872	174	100

Peptide Information

Calc. Mass	Obsrv. Mass	± da	± ppm	Start Seq.	End Seq.	Sequence	Ion Score	C. I. %	Modification	Rank	Result Type
823.3945	823.3763	-0.0182	-22	298	304	SDIFGER					Mascot
863.5098	863.4956	-0.0142	-16	244	250	RLYVQGK					Mascot
1544.6832	1544.6385	-0.0447	-29	493	506	GVSFMVDNCSTTAR			Carbamidomethyl (C)[9]		Mascot
1544.6832	1544.6385	-0.0447	-29	493	506	GVSFMVDNCSTTAR	44	96.301	Carbamidomethyl (C)[9]		Mascot
1560.6782	1560.6761	-0.0021	-1	493	506	GVSFMVDNCSTTAR			Carbamidomethyl (C)[9], Oxidation (M)[5]		Mascot
1684.8766	1684.7736	-0.103	-61	80	93	ERVNLAGHEEYIVR					Mascot
1831.8093	1831.8303	0.021	11	148	164	GSSSFNEAREAGFSEEK					Mascot
1980.041	1980.0363	-0.0047	-2	111	129	QIGVIGWGSQGPAQAQN LR					Mascot
1980.041	1980.0363	-0.0047	-2	111	129	QIGVIGWGSQGPAQAQN LR	130	100			Mascot
2257.1863	2257.1133	-0.073	-32	59	79	VSAPPATHPVSLDFETSV FKK					Mascot
2947.3892	2947.3728	-0.0164	-6	467	492	GHSYSEIINESVIESVDSL NPFMHAR			Oxidation (M)[23]		Mascot

4	Actin-17 OS=Dictyostelium discoideum GN=act17 PE=3 SV=1	ACT17_DICDI	41772.9	5.28	13	67	88.703	13.882	

Peptide Information

Calc. Mass	Obsrv. Mass	± da	± ppm	Start Seq.	End Seq.	Sequence	Ion Score	C. I. %	Modification	Rank	Result Type
976.4483	976.486	0.0377	39	20	29	AGFAGDDAPR					Mascot
1161.6184	1161.5178	-0.1006	-87	315	325	ELTALAPSTMK					Mascot
1177.6133	1177.531	-0.0823	-70	315	325	ELTALAPSTMK			Oxidation (M)[10]		Mascot
1283.6235	1283.561	-0.0625	-49	41	51	YRDVMVGMGQK					Mascot
1354.6234	1354.6958	0.0724	53	52	63	DSYVGDEAQSKR					Mascot

1507.7639	1507.7208	-0.0431	-29	179	192	LDLAGNDLTDYLGK					Mascot
1583.8315	1583.8403	0.0088	6	217	229	LSYITLDFQNEIK					Mascot
1735.9113	1735.7894	-0.1219	-70	230	245	IVSSALEKSYELPDGK					Mascot
1979.9531	1980.0363	0.0832	42	70	85	YPIERGIITNWDDMEK					Mascot
1979.9531	1980.0363	0.0832	42	70	85	YPIERGIITNWDDMEK					Mascot
1995.0433	1995.004	-0.0393	-20	198	214	GYPFISIDETEIVRDIK					Mascot
1995.0433	1995.004	-0.0393	-20	198	214	GYPFISIDETEIVRDIK					Mascot
1995.9481	1996.016	0.0679	34	70	85	YPIERGIITNWDDMEK			Oxidation (M)[14]		Mascot
2034.0867	2033.9788	-0.1079	-53	179	197	LDLAGNDLTDYLGKLLSGR					Mascot
2038.942	2038.9474	0.0054	3	1	19	MECGDVQALVIDNGSSISK			Carbamidomethyl (C)[3], Oxidation (M)[1]		Mascot
2165.1602	2165.106	-0.0542	-25	193	211	LLSGRGYPFISIDETEIVR					Mascot
2257.0806	2257.1133	0.0327	14	291	311	DLYDNVVLSGGTTMFPGIADR			Oxidation (M)[14]		Mascot

5	Heparan sulfate glucosamine 3-O-sulfotransferase 6 OS=Mus musculus GN=Hs3st6 PE=2 SV=1	HS3S6_MOUSE	37676.9	10.55	12	64	78.473	3.238

Peptide Information

Calc. Mass	Obsrv. Mass	± da	± ppm	Start Seq.	End Seq.	Sequence	Ion Score	C. I. %	Modification	Rank	Result Type
1439.7906	1439.7469	-0.0437	-30	320	330	LQAFYRPFNRK					Mascot
1465.7546	1465.8529	0.0983	67	211	224	HGLGPVDTAWSAVR					Mascot
1551.7915	1551.744	-0.0475	-31	154	166	TPSYFVTQEAPRR					Mascot
1664.9517	1664.9684	0.0167	10	167	181	IHGMSPDTKLIVVVR					Mascot
1698.8639	1698.8505	-0.0134	-8	238	251	YFPLSHFLFVSGER					Mascot
1705.8612	1705.7919	-0.0693	-41	139	153	GLMPRTLDGQITMEK			Oxidation (M)[3]		Mascot
1977.1029	1977.005	-0.0979	-50	57	75	APAPVPAPAEPPHTSLRLR					Mascot
1993.0039	1992.9772	-0.0267	-13	112	128	LHPDVRALGSEPHFFDR					Mascot
2000.0812	2000.0134	-0.0678	-34	252	270	LVSDPAGEVGRVQDFLGLK					Mascot
2024.0825	2024.006	-0.0765	-38	206	224	ALAFRHGLGPVDTAWSAVR					Mascot
2086.0273	2085.9619	-0.0654	-31	2	26	AGSGGLGGGAGDLQGAGTGQGTALR					Mascot
2257.3142	2257.1133	-0.2009	-89	30	50	APLALVVLLLSAYCLFALPGR			Carbamidomethyl (C)[14]		Mascot

6	Tuftelin-interacting protein 11 OS=Xenopus tropicalis GN=tfip11 PE=2 SV=2	TFP11_XENTR	95634	5.96	22	64	77.972	16.602

Peptide Information

Calc. Mass	Obsrv. Mass	± da	± ppm	Start Seq.	End Seq.	Sequence	Ion Score	C. I. %	Modification	Rank	Result Type

962.5306	962.4772	-0.0534	-55	802	810	GVVFVQGEK					Mascot
1181.6096	1181.5424	-0.0672	-57	156	166	MGYVQGRGLGK			Oxidation (M)[1]		Mascot
1211.5944	1211.6919	0.0975	80	104	113	ETFPKDFEAK					Mascot
1229.5355	1229.5475	0.012	10	433	443	DPTYGTDVMSK			Oxidation (M)[9]		Mascot
1310.6587	1310.6962	0.0375	29	718	728	ESIAYLTQTER					Mascot
1384.6315	1384.6716	0.0401	29	213	222	EFQKEMSQWR			Oxidation (M)[6]		Mascot
1385.5929	1385.701	0.1081	78	400	409	YYEEYKMSEK			Oxidation (M)[7]		Mascot
1453.615	1453.6378	0.0228	16	91	103	SDSDSDSETQARR					Mascot
1465.7104	1465.8529	0.1425	97	691	702	DKFNEALDIMNR					Mascot
1480.7213	1480.681	-0.0403	-27	703	717	AVSSSVGAYMQPGAR					Mascot
1480.7213	1480.681	-0.0403	-27	703	717	AVSSSVGAYMQPGAR					Mascot
1508.8544	1508.7253	-0.1291	-86	167	180	NAQGIIAPIEAKQR					Mascot
1508.8544	1508.7253	-0.1291	-86	167	180	NAQGIIAPIEAKQR					Mascot
1528.8192	1528.6823	-0.1369	-90	677	690	SMFSDLVLAHPAIK					Mascot
1551.6697	1551.744	0.0743	48	200	212	DFPVVDSEEEEEK					Mascot
1564.714	1564.741	0.027	17	134	146	SNTDFGSWERHTK					Mascot
1705.8829	1705.7919	-0.091	-53	811	825	TWVPTSLQSLIDMAK			Oxidation (M)[13]		Mascot
1771.9412	1771.8419	-0.0993	-56	677	692	SMFSDLVLAHPAIKDK					Mascot
1962.8718	1963.016	0.1442	73	376	391	IQPMCDNPLTLEECAR			Carbamidomethyl (C)[5,14], Oxidation (M)[4]		Mascot
1962.8718	1963.016	0.1442	73	376	391	IQPMCDNPLTLEECAR			Carbamidomethyl (C)[5,14], Oxidation (M)[4]		Mascot
1983.9017	1984.0321	0.1304	66	39	54	HFQTKEEATYGMWAER					Mascot
1994.9277	1995.004	0.0763	38	427	443	DWNPLRDPTYGTDVMSK					Mascot
1994.9277	1995.004	0.0763	38	427	443	DWNPLRDPTYGTDVMSK					Mascot
1999.8967	2000.0134	0.1167	58	39	54	HFQTKEEATYGMWAER			Oxidation (M)[12]		Mascot
2007.9393	2008.0262	0.0869	43	196	212	QSIKDFPVVDSEEEEEK					Mascot
2010.9226	2011.0029	0.0803	40	427	443	DWNPLRDPTYGTDVMSK			Oxidation (M)[15]		Mascot
2018.0223	2018.0063	-0.016	-8	358	374	AIQNLSKVLETVEECER			Carbamidomethyl (C)[15]		Mascot
2083.9343	2084.0059	0.0716	34	200	216	DFPVVDSEEEEEKEFQK					Mascot

7 Myosin-4 OS=Rattus norvegicus GN=Myh4 PE=2 SV=1 MYH4_RAT 223653.3 5.58 31 62 69.592 22.604

Peptide Information

Calc. Mass	Obsrv. Mass	± da	± ppm	Start Seq.	End Seq.	Sequence	Ion Score	C. I. %	Modification	Rank	Result Type
865.4084	865.462	0.0536	62	1789	1795	NMEQTVK			Oxidation (M)[2]		Mascot
962.5153	962.4772	-0.0381	-40	64	73	TEGGATVTVK					Mascot
1161.5997	1161.5178	-0.0819	-71	1255	1264	TLEDQLSEVK					Mascot

1229.6637	1229.5475	-0.1162	-94	553	562	NKLYEQHLGK		Mascot
1267.6641	1267.5898	-0.0743	-59	45	55	ESYVKATVQSR		Mascot
1283.5751	1283.561	-0.0141	-11	1853	1862	ELTYQTEEDR		Mascot
1354.6719	1354.6958	0.0239	18	792	802	TQAVCRGYLMR	Carbamidomethyl (C)[5]	Mascot
1371.613	1371.6715	0.0585	43	846	857	SAETEKEMATMK	Oxidation (M)[8]	Mascot
1390.7424	1390.6732	-0.0692	-50	1255	1266	TLEDQLSEVKTK		Mascot
1465.7784	1465.8529	0.0745	51	261	273	LASADIETYLLEK		Mascot
1490.7961	1490.7202	-0.0759	-51	1310	1321	QAFTQQIEELKR		Mascot
1490.7961	1490.7202	-0.0759	-51	1310	1321	QAFTQQIEELKR		Mascot
1503.7649	1503.7681	0.0032	2	171	185	ENQSILITGESGAGK		Mascot
1525.8262	1525.7972	-0.029	-19	192	205	VIQYFATIAVTGDK		Mascot
1615.7922	1615.7275	-0.0647	-40	1823	1835	VRELENEVENEQK		Mascot
1646.8418	1646.7592	-0.0826	-50	1098	1111	IEDEQALGMQLQKK	Oxidation (M)[9]	Mascot
1684.9229	1684.7736	-0.1493	-89	1565	1578	IQLELNQVKSEIDR		Mascot
1698.7561	1698.8505	0.0944	56	1230	1244	MEIDDLASNMETVSK	Oxidation (M)[1]	Mascot
1705.8263	1705.7919	-0.0344	-20	433	445	AMYEKMFLWMVTR		Mascot
1713.7789	1713.8142	0.0353	21	74	87	EDQVFSMNPPKYDK	Oxidation (M)[7]	Mascot
1960.8779	1960.9724	0.0945	48	1	18	MSSDAEMAVFGEAAPYL R	Oxidation (M)[1]	Mascot
1962.9515	1963.016	0.0645	33	1885	1901	QAEEAEEQSNVNLAKFR		Mascot
1962.9515	1963.016	0.0645	33	1885	1901	QAEEAEEQSNVNLAKFR		Mascot
1967.0193	1967.015	-0.0043	-2	1181	1198	DLEEATLQHEATAAALRK		Mascot
1976.8729	1977.005	0.1321	67	1	18	MSSDAEMAVFGEAAPYL R	Oxidation (M)[1,7]	Mascot
1980.0437	1980.0363	-0.0074	-4	726	743	YKVLNASAIPEGQFIDSK		Mascot
1980.0437	1980.0363	-0.0074	-4	726	743	YKVLNASAIPEGQFIDSK		Mascot
1995.0254	1995.004	-0.0214	-11	1180	1197	RDLEEATLQHEATAAALR		Mascot
1995.0254	1995.004	-0.0214	-11	1180	1197	RDLEEATLQHEATAAALR		Mascot
1996.0134	1996.016	0.0026	1	619	639	TLAFLFSGGQAAEAEGG GGKK		Mascot
2018.0475	2018.0063	-0.0412	-20	981	998	NLTEEMAGLDENIVKLTK		Mascot
2024.0457	2024.006	-0.0397	-20	438	453	MFLWMVTRINQQLDTK		Mascot
2034.0424	2033.9788	-0.0636	-31	981	998	NLTEEMAGLDENIVKLTK	Oxidation (M)[6]	Mascot
2056.0354	2055.9534	-0.082	-40	438	453	MFLWMVTRINQQLDTK	Oxidation (M)[1,5]	Mascot
2086.0298	2085.9619	-0.0679	-33	1114	1130	ELQARIEELEEEIEAER		Mascot
2165.0278	2165.106	0.0782	36	1059	1077	LEGDLKLAQESTMDIEND K	Oxidation (M)[13]	Mascot
2257.0764	2257.1133	0.0369	16	1401	1420	LQDAEEHVEAVNSKCAS LEK	Carbamidomethyl (C)[15]	Mascot
2753.3093	2753.2891	-0.0202	-7	1622	1645	MEGDLNEMEIQLNHANR QAAEAIR		Mascot

8 Myosin-4 OS=Mus musculus GN=Myh4 PE=1 SV=1 MYH4_MOUSE 223632.3 5.58 31 61 56.048 22.617

Peptide Information

Calc. Mass	Obsrv. Mass	± da	± ppm	Start Seq.	End Seq.	Sequence	Ion Score	C. I. %	Modification	Rank	Result Type
865.4084	865.462	0.0536	62	1789	1795	NMEQTVK			Oxidation (M)[2]		Mascot
962.5153	962.4772	-0.0381	-40	64	73	TEGGATVTVK					Mascot
1161.5997	1161.5178	-0.0819	-71	1255	1264	TLEDQLSEVK					Mascot
1229.6637	1229.5475	-0.1162	-94	553	562	NKLYEQHLGK					Mascot
1267.6641	1267.5898	-0.0743	-59	45	55	ESYVKATVQSR					Mascot
1283.5751	1283.561	-0.0141	-11	1853	1862	ELTYQTEEDR					Mascot
1354.6719	1354.6958	0.0239	18	792	802	TQAVCRGYLMR			Carbamidomethyl (C)[5]		Mascot
1371.7478	1371.6715	-0.0763	-56	1801	1813	LDEAEQLALKGGK					Mascot
1384.6083	1384.6716	0.0633	46	846	857	SAETEKEMANMK			Oxidation (M)[8]		Mascot
1390.7424	1390.6732	-0.0692	-50	1255	1266	TLEDQLSEVKTK					Mascot
1400.6031	1400.7194	0.1163	83	846	857	SAETEKEMANMK			Oxidation (M)[8,11]		Mascot
1465.7784	1465.8529	0.0745	51	261	273	LASADIETYLLEK					Mascot
1490.7961	1490.7202	-0.0759	-51	1310	1321	QAFTQQIEELKR					Mascot
1490.7961	1490.7202	-0.0759	-51	1310	1321	QAFTQQIEELKR					Mascot
1503.7649	1503.7681	0.0032	2	171	185	ENQSILITGESGAGK					Mascot
1525.8262	1525.7972	-0.029	-19	192	205	VIQYFATIAVTGDK					Mascot
1615.7922	1615.7275	-0.0647	-40	1823	1835	VRELENEVENEQK					Mascot
1646.8418	1646.7592	-0.0826	-50	1098	1111	IEDEQALGMQLQKK			Oxidation (M)[9]		Mascot
1684.9229	1684.7736	-0.1493	-89	1565	1578	IQLELNQVKSEIDR					Mascot
1698.7561	1698.8505	0.0944	56	1230	1244	MEIDDLASNMETVSK			Oxidation (M)[1]		Mascot
1890.9478	1890.9558	0.008	4	979	995	VKNLTEEMAGLDENIAK			Oxidation (M)[8]		Mascot
1960.8779	1960.9724	0.0945	48	1	18	MSSDAEMAVFGEAAPYL R			Oxidation (M)[1]		Mascot
1962.9515	1963.016	0.0645	33	1885	1901	QAEEAEEQSNVNLAKFR					Mascot
1962.9515	1963.016	0.0645	33	1885	1901	QAEEAEEQSNVNLAKFR					Mascot
1967.0193	1967.015	-0.0043	-2	1181	1198	DLEEATLQHEATAAALRK					Mascot
1976.8729	1977.005	0.1321	67	1	18	MSSDAEMAVFGEAAPYL R			Oxidation (M)[1,7]		Mascot
1980.0437	1980.0363	-0.0074	-4	726	743	YKVLNASAIPEGQFIDSK					Mascot
1980.0437	1980.0363	-0.0074	-4	726	743	YKVLNASAIPEGQFIDSK					Mascot
1995.0254	1995.004	-0.0214	-11	1180	1197	RDLEEATLQHEATAAALR					Mascot
1995.0254	1995.004	-0.0214	-11	1180	1197	RDLEEATLQHEATAAALR					Mascot
1996.0134	1996.016	0.0026	1	619	639	TLAFLFSGGQAAEAEGG GGKK					Mascot

Calc. Mass	Obsrv. Mass	± da	± ppm	Start Seq.	End Seq.	Sequence	Ion Score	C. I. %	Modification	Rank	Result Type
2006.011	2006.0157	0.0047	2	981	998	NLTEEMAGLDENIAKLTK			Oxidation (M)[6]		Mascot
2024.0457	2024.006	-0.0397	-20	438	453	MFLWMVTRINQQLDTK					Mascot
2056.0354	2055.9534	-0.082	-40	438	453	MFLWMVTRINQQLDTK			Oxidation (M)[1,5]		Mascot
2086.0298	2085.9619	-0.0679	-33	1114	1130	ELQARIEELEEEIEAER					Mascot
2165.0278	2165.106	0.0782	36	1059	1077	LEGDLKLAQESTMDIEND K			Oxidation (M)[13]		Mascot
2257.0764	2257.1133	0.0369	16	1401	1420	LQDAEEHVEAVNSKCAS LEK			Carbamidomethyl (C)[15]		Mascot
2753.3093	2753.2891	-0.0202	-7	1622	1645	MEGDLNEMEIQLNHANR QAAEAIR					Mascot

9	Keratin, type I cytoskeletal 18 OS=Scyliorhinus stellaris GN=krt18 PE=1 SV=1	K1C18_SCYST	46819.9	5.24	14	59	36.47	20.983

Peptide Information

Calc. Mass	Obsrv. Mass	± da	± ppm	Start Seq.	End Seq.	Sequence	Ion Score	C. I. %	Modification	Rank	Result Type
1055.6207	1055.5889	-0.0318	-30	137	145	LILQIDNAR					Mascot
1281.611	1281.5565	-0.0545	-43	250	259	NKDDAEIWYK					Mascot
1288.7472	1288.6733	-0.0739	-57	394	405	TIVTTQKVVDGK					Mascot
1310.641	1310.6962	0.0552	42	260	270	NQMDGYKVEVK					Mascot
1326.6359	1326.6599	0.024	18	260	270	NQMDGYKVEVK			Oxidation (M)[3]		Mascot
1328.5787	1328.6801	0.1014	76	126	136	EVYDMTVDNAR			Oxidation (M)[5]		Mascot
1372.7179	1372.7041	-0.0138	-10	163	174	QSVENDINGLRK					Mascot
1372.7179	1372.7041	-0.0138	-10	163	174	QSVENDINGLRK					Mascot
1503.7762	1503.7681	-0.0081	-5	305	317	NSLEGTLRDTELR					Mascot
1615.8538	1615.7275	-0.1263	-78	401	415	VVDGKIVSDETVQIN					Mascot
1735.8545	1735.7894	-0.0651	-38	5	20	GYTSLSSHSGPMSIRR					Mascot
1771.828	1771.8419	0.0139	8	21	37	SMPLQSSASSISGYGQR			Oxidation (M)[2]		Mascot
1980.0079	1980.0363	0.0284	14	332	348	LEADLHQIRGDMQAQVR					Mascot
1980.0079	1980.0363	0.0284	14	332	348	LEADLHQIRGDMQAQVR					Mascot
1996.0029	1996.016	0.0131	7	332	348	LEADLHQIRGDMQAQVR			Oxidation (M)[12]		Mascot
1999.9655	2000.0134	0.0479	24	2	19	TYRGYTSLSSHSGPMSIR					Mascot
2037.9871	2037.9758	-0.0113	-6	46	68	VASLGSSSSSAAGIGMG GVGNQK			Oxidation (M)[16]		Mascot
2576.3413	2576.3042	-0.0371	-14	369	392	RLLDGEDINTLVESTSGV TSQTIK					Mascot

10	Exodeoxyribonuclease 7 large subunit OS=Xylella fastidiosa (strain Temecula1 / ATCC 700964) GN=xseA PE=3 SV=1	EX7L_XYLFT	49922.8	9.92	13	58	12.304	2.462

Protein Group

Exodeoxyribonuclease 7 large subunit OS=Xylella fastidiosa (strain M23) GN=xseA PE=3 SV=1	EX7L_XYLF2	49922.8	9.9200 000762

Peptide Information

Calc. Mass	Obsrv. Mass	± da	± ppm	Start Seq.	End Seq.	Sequence	Ion Score	C. I. %	Modification	Rank	Result Type
865.4778	865.462	-0.0158	-18	84	90	LTLYEAR					Mascot
881.4371	881.4611	0.024	27	58	64	CAMFKPK			Carbamidomethyl (C)[1]		Mascot
962.5339	962.4772	-0.0567	-59	153	160	DILSVLMR			Oxidation (M)[7]		Mascot
1364.7216	1364.6803	-0.0413	-30	301	311	LQARNPEMHLR					Mascot
1439.8693	1439.7469	-0.1224	-85	139	152	RIAVITSPSGAVIR					Mascot
1447.8241	1447.7117	-0.1124	-78	267	278	ALRQHLGQLQQR					Mascot
1649.8251	1649.8036	-0.0215	-13	51	64	DAQAQVRCAMFKPK			Carbamidomethyl (C)[8]		Mascot
1976.9462	1977.005	0.0588	30	204	221	GGGSLEDLWAFNNEQLAR					Mascot
1984.1338	1984.0321	-0.1017	-51	388	406	ALEAVNPLATVARGYALVR					Mascot
1992.0621	1991.9901	-0.072	-36	2	18	QHRDEILTPSQLNTLAR					Mascot
2000.1764	2000.0134	-0.163	-81	382	400	LHGLVRALEAVNPLATVAR					Mascot
2257.1208	2257.1133	-0.0075	-3	91	110	GDYQIVLDHLEESGEGALRR					Mascot
2947.4795	2947.3728	-0.1067	-36	84	109	LTLYEARGDYQIVLDHLEESGEGALR					Mascot

Gel Idx/Pos	284/L12	Instr./Gel Origin	BA2151/Sample Project 20140814	Process Status	Analysis Succeeded
Plate [#] Name	[1] Sample Project 20140814	Instrument Sample Name		Spectra	11

Rank	Protein Name	Accession No.	Protein MW	Protein PI	Pep. Count	Protein Score	Protein Score C. I. %	Intensity Matched	Total Ion Score	Total Ion C. I. %	Confirmed
1	Glucose-1-phosphate adenylyltransferase small subunit, chloroplastic/amyloplastic OS=Hordeum vulgare PE=2 SV=1	GLGS_HORVU	56412.8	6.11	19	761	100	35.022	661	100	

Peptide Information

Calc. Mass	Obsrv. Mass	± da	± ppm	Start Seq.	End Seq.	Sequence	Ion Score	C. I. %	Modification	Rank	Result Type
854.444	854.4305	-0.0135	-16	240	247	ATAFGLMK			Oxidation (M)[7]		Mascot
964.605	964.5187	-0.0863	-89	47	55	GLVARPVPR					Mascot
972.4673	972.4487	-0.0186	-19	488	495	ETDGYFIK					Mascot
1009.5975	1009.5825	-0.015	-15	300	307	HVMLQLLR					Mascot
1017.5952	1017.5868	-0.0084	-8	406	414	IHHSVVGLR					Mascot
1025.5925	1025.5709	-0.0216	-21	300	307	HVMLQLLR			Oxidation (M)[3]		Mascot
1029.5	1029.5046	0.0046	4	150	159	AYGSNIGGYK					Mascot
1032.5472	1032.5397	-0.0075	-7	370	378	SAPIYTQPR					Mascot
1032.5472	1032.5397	-0.0075	-7	370	378	SAPIYTQPR	45	96.929			Mascot
1074.6194	1074.5847	-0.0347	-32	254	262	IIEFAEKPK					Mascot
1256.7109	1256.7004	-0.0105	-8	104	115	AKPAVPLGANYR					Mascot
1256.7109	1256.7004	-0.0105	-8	104	115	AKPAVPLGANYR	29	0			Mascot
1384.6896	1384.6841	-0.0055	-4	359	369	KPIPDFSFYDR					Mascot
1384.6896	1384.6841	-0.0055	-4	359	369	KPIPDFSFYDR	88	100			Mascot
1412.8121	1412.6833	-0.1288	-91	103	115	RAKPAVPLGANYR					Mascot
1625.8646	1625.8612	-0.0034	-2	132	145	IYVLTQFNSASLNR	114	100			Mascot
1733.8489	1733.8354	-0.0135	-8	285	299	EMPYIASMGIYVISK			Oxidation (M)[2,8]		Mascot
1786.9368	1786.9095	-0.0273	-15	116	131	LIDIPVSNCLNSNISK			Carbamidomethyl (C)[9]		Mascot
1875.864	1875.8551	-0.0089	-5	223	239	ETDADITVAALPMDEER					Mascot
1889.9525	1889.9269	-0.0256	-14	385	402	VLDADVTDSVIGEGCVIK			Carbamidomethyl (C)[15]		Mascot
1891.859	1891.8402	-0.0188	-10	223	239	ETDADITVAALPMDEER			Oxidation (M)[13]		Mascot
1970.9965	1970.9867	-0.0098	-5	470	487	IGDNVMIINVDNVQEAAR					Mascot
1970.9965	1970.9867	-0.0098	-5	470	487	IGDNVMIINVDNVQEAAR	164	100			Mascot
1986.9913	1986.968	-0.0233	-12	470	487	IGDNVMIINVDNVQEAAR			Oxidation (M)[6]		Mascot
2368.0874	2368.0959	0.0085	4	308	330	EQFPGANDFGSEVIPGAT STGMR					Mascot
3061.4287	3061.4373	0.0086	3	160	187	NEGFVEVLAAQQSPDNP					Mascot

3061.4287	3061.4373	0.0086	3	160	187	DWFQGTADAVR NEGFVEVLAAQQSPDNP DWFQGTADAVR	221	100			Mascot

2	Glucose-1-phosphate adenylyltransferase small subunit, chloroplastic/amyloplastic OS=Triticum aestivum GN=AGP-S PE=2 SV=1	GLGS_WHEAT	52399.6	5.54	21	673	100	35.931	546	100		

Peptide Information

Calc. Mass	Obsrv. Mass	± da	± ppm	Start Seq.	End Seq.	Sequence	Ion Score	C. I. %	Modification	Rank	Result Type
854.444	854.4305	-0.0135	-16	200	207	ATAFGLMK			Oxidation (M)[7]		Mascot
947.4833	947.4744	-0.0089	-9	9	17	TFPSPSPSK					Mascot
972.4673	972.4487	-0.0186	-19	448	455	ETDGYFIK					Mascot
1009.5975	1009.5825	-0.015	-15	260	267	HVMLQLLR					Mascot
1017.5952	1017.5868	-0.0084	-8	366	374	IHHSVVGLR					Mascot
1025.5925	1025.5709	-0.0216	-21	260	267	HVMLQLLR			Oxidation (M)[3]		Mascot
1029.5	1029.5046	0.0046	4	110	119	AYGSNIGGYK					Mascot
1032.5472	1032.5397	-0.0075	-7	330	338	SAPIYTQPR					Mascot
1032.5472	1032.5397	-0.0075	-7	330	338	SAPIYTQPR	45	96.929			Mascot
1074.6194	1074.5847	-0.0347	-32	214	222	IIEFAEKPK					Mascot
1256.7109	1256.7004	-0.0105	-8	64	75	AKPAVPLGANYR					Mascot
1256.7109	1256.7004	-0.0105	-8	64	75	AKPAVPLGANYR	29	0			Mascot
1384.6896	1384.6841	-0.0055	-4	319	329	KPIPDFSFYDR					Mascot
1384.6896	1384.6841	-0.0055	-4	319	329	KPIPDFSFYDR	88	100			Mascot
1412.8121	1412.6833	-0.1288	-91	63	75	RAKPAVPLGANYR					Mascot
1621.7925	1621.7832	-0.0093	-6	228	242	AMMVDTTILGLDDAR					Mascot
1637.7874	1637.786	-0.0014	-1	228	242	AMMVDTTILGLDDAR			Oxidation (M)[2]		Mascot
1653.7822	1653.77	-0.0122	-7	228	242	AMMVDTTILGLDDAR			Oxidation (M)[2,3]		Mascot
1733.8489	1733.8354	-0.0135	-8	245	259	EMPYIASMGIYVISK			Oxidation (M)[2,8]		Mascot
1786.9368	1786.9095	-0.0273	-15	76	91	LIDIPVSNCLNSNISK			Carbamidomethyl (C)[9]		Mascot
1804.915	1804.9409	0.0259	14	1	17	MDVPLASKTFPSPSPSK			Oxidation (M)[1]		Mascot
1875.864	1875.8551	-0.0089	-5	183	199	ETDADITVAALPMDEER					Mascot
1889.9525	1889.9269	-0.0256	-14	345	362	VLDADVTDSVIGEGCVIK			Carbamidomethyl (C)[15]		Mascot
1891.859	1891.8402	-0.0188	-10	183	199	ETDADITVAALPMDEER			Oxidation (M)[13]		Mascot
1970.9965	1970.9867	-0.0098	-5	430	447	IGDNVMIINVDNVQEAAR					Mascot
1970.9965	1970.9867	-0.0098	-5	430	447	IGDNVMIINVDNVQEAAR	164	100			Mascot
1986.9913	1986.968	-0.0233	-12	430	447	IGDNVMIINVDNVQEAAR			Oxidation (M)[6]		Mascot
2318.2537	2318.1902	-0.0635	-27	76	95	LIDIPVSNCLNSNISKIYVR			Carbamidomethyl (C)[9]		Mascot
2368.0874	2368.0959	0.0085	4	268	290	EQFPGANDFGSEVIPGAT					Mascot

Calc. Mass	Obsrv. Mass	± da	± ppm	Start Seq.	End Seq.	Sequence	Ion Score	C. I. %	Modification	Rank	Result Type
						STGMR					
3061.4287	3061.4373	0.0086	3	120	147	NEGFVEVLAAQQSPDNP DWFQGTADAVR					Mascot
3061.4287	3061.4373	0.0086	3	120	147	NEGFVEVLAAQQSPDNP DWFQGTADAVR	221	100			Mascot

3	Glucose-1-phosphate adenylyltransferase small subunit, chloroplastic OS=Arabidopsis thaliana GN=APS1 PE=2 SV=2	GLGS_ARATH	56957.2	6.13	13	235	100	10.336	188	100	

Peptide Information

Calc. Mass	Obsrv. Mass	± da	± ppm	Start Seq.	End Seq.	Sequence	Ion Score	C. I. %	Modification	Rank	Result Type
854.444	854.4305	-0.0135	-16	247	254	ATAFGLMK			Oxidation (M)[7]		Mascot
972.4673	972.4487	-0.0186	-19	495	502	ETDGYFIK					Mascot
1017.5952	1017.5868	-0.0084	-8	413	421	IHHSVVGLR					Mascot
1032.5472	1032.5397	-0.0075	-7	377	385	SAPIYTQPR					Mascot
1032.5472	1032.5397	-0.0075	-7	377	385	SAPIYTQPR	45	96.929			Mascot
1074.6194	1074.5847	-0.0347	-32	261	269	IIEFAEKPK					Mascot
1256.7109	1256.7004	-0.0105	-8	111	122	AKPAVPLGANYR					Mascot
1256.7109	1256.7004	-0.0105	-8	111	122	AKPAVPLGANYR	29	0			Mascot
1406.8114	1406.7175	-0.0939	-67	454	467	GSVPIGIGKNSHIK					Mascot
1412.8121	1412.6833	-0.1288	-91	110	122	RAKPAVPLGANYR					Mascot
1570.7432	1570.8472	0.104	66	153	166	HLSRAYASNMGGYK			Oxidation (M)[10]		Mascot
1625.8646	1625.8612	-0.0034	-2	139	152	IYVLTQFNSASLNR	114	100			Mascot
1786.9368	1786.9095	-0.0273	-15	123	138	LIDIPVSNCLNSNISK			Carbamidomethyl (C)[9]		Mascot
1921.9246	1922.0004	0.0758	39	392	409	MLDADVTDSVIGEGCVIK			Carbamidomethyl (C)[15]		Mascot
1971.0142	1970.9867	-0.0275	-14	12	31	VPPASTSNSTGKATEAVP TR					Mascot
1971.0142	1970.9867	-0.0275	-14	12	31	VPPASTSNSTGKATEAVP TR					Mascot

4	Glucose-1-phosphate adenylyltransferase small subunit, chloroplastic OS=Brassica napus GN=AGPS1 PE=2 SV=1	GLGS_BRANA	57294.3	5.87	12	228	100	7.086	188	100	

Peptide Information

Calc. Mass	Obsrv. Mass	± da	± ppm	Start Seq.	End Seq.	Sequence	Ion Score	C. I. %	Modification	Rank	Result Type
854.444	854.4305	-0.0135	-16	247	254	ATAFGLMK			Oxidation (M)[7]		Mascot
972.4673	972.4487	-0.0186	-19	495	502	ETDGYFIK					Mascot
1032.5472	1032.5397	-0.0075	-7	377	385	SAPIYTQPR					Mascot
1032.5472	1032.5397	-0.0075	-7	377	385	SAPIYTQPR	45	96.929			Mascot
1074.6194	1074.5847	-0.0347	-32	261	269	IIEFAEKPK					Mascot

1256.7109	1256.7004	-0.0105	-8	111	122	AKPAVPLGANYR					Mascot
1256.7109	1256.7004	-0.0105	-8	111	122	AKPAVPLGANYR	29	0			Mascot
1270.7478	1270.7382	-0.0096	-8	89	102	SVLGIILGGGAGTR					Mascot
1412.8121	1412.6833	-0.1288	-91	110	122	RAKPAVPLGANYR					Mascot
1570.7432	1570.8472	0.104	66	153	166	HLSRAYASNMGGYK			Oxidation (M)[10]		Mascot
1607.9115	1607.848	-0.0635	-39	44	57	LNPTQEIIISNLPR					Mascot
1625.8646	1625.8612	-0.0034	-2	139	152	IYVLTQFNSASLNR	114	100			Mascot
1786.9368	1786.9095	-0.0273	-15	123	138	LIDIPVSNCLNSNISK			Carbamidomethyl (C)[9]		Mascot
1921.9246	1922.0004	0.0758	39	392	409	MLDADVTDSVIGEGCVIK			Carbamidomethyl (C)[15]		Mascot

5	Glucose-1-phosphate adenylyltransferase small subunit, chloroplastic/amyloplastic OS=Solanum tuberosum PE=1 SV=2	GLGS_SOLTU	57603.4	6.73	12	225	100	8.429	188	100	

Peptide Information

Calc. Mass	Obsrv. Mass	± da	± ppm	Start Seq.	End Seq.	Sequence	Ion Score	C. I. %	Modification	Rank	Result Type
854.444	854.4305	-0.0135	-16	248	255	ATAFGLMK			Oxidation (M)[7]		Mascot
972.4673	972.4487	-0.0186	-19	496	503	ETDGYFIK					Mascot
1017.5952	1017.5868	-0.0084	-8	414	422	IHHSVVGLR					Mascot
1032.5472	1032.5397	-0.0075	-7	378	386	SAPIYTQPR					Mascot
1032.5472	1032.5397	-0.0075	-7	378	386	SAPIYTQPR	45	96.929			Mascot
1256.7109	1256.7004	-0.0105	-8	112	123	AKPAVPLGANYR					Mascot
1256.7109	1256.7004	-0.0105	-8	112	123	AKPAVPLGANYR	29	0			Mascot
1270.7478	1270.7382	-0.0096	-8	90	103	SVLGIILGGGAGTR					Mascot
1412.8121	1412.6833	-0.1288	-91	111	123	RAKPAVPLGANYR					Mascot
1570.7432	1570.8472	0.104	66	154	167	HLSRAYASNMGGYK			Oxidation (M)[10]		Mascot
1625.8646	1625.8612	-0.0034	-2	140	153	IYVLTQFNSASLNR	114	100			Mascot
1786.9368	1786.9095	-0.0273	-15	124	139	LIDIPVSNCLNSNISK			Carbamidomethyl (C)[9]		Mascot
1921.9246	1922.0004	0.0758	39	393	410	MLDADVTDSVIGEGCVIK			Carbamidomethyl (C)[15]		Mascot
2366.1445	2366.0994	-0.0451	-19	316	338	DKFPGANDFGSEVIPGAT SLGMR					Mascot

6	Glucose-1-phosphate adenylyltransferase small subunit, chloroplastic OS=Solanum lycopersicum PE=2 SV=1	GLGS_SOLLC	57733.5	6.49	12	223	100	7.79	188	100	

Peptide Information

Calc. Mass	Obsrv. Mass	± da	± ppm	Start Seq.	End Seq.	Sequence	Ion Score	C. I. %	Modification	Rank	Result Type
854.444	854.4305	-0.0135	-16	248	255	ATAFGLMK			Oxidation (M)[7]		Mascot

972.4673	972.4487	-0.0186	-19	496	503	ETDGYFIK					Mascot
1017.5952	1017.5868	-0.0084	-8	414	422	IHHSVVGLR					Mascot
1032.5472	1032.5397	-0.0075	-7	378	386	SAPIYTQPR					Mascot
1032.5472	1032.5397	-0.0075	-7	378	386	SAPIYTQPR	45	96.929			Mascot
1256.7109	1256.7004	-0.0105	-8	112	123	AKPAVPLGANYR					Mascot
1256.7109	1256.7004	-0.0105	-8	112	123	AKPAVPLGANYR	29	0			Mascot
1270.7478	1270.7382	-0.0096	-8	90	103	SVLGIILGGGAGTR					Mascot
1412.8121	1412.6833	-0.1288	-91	111	123	RAKPAVPLGANYR					Mascot
1505.8145	1505.75	-0.0645	-43	455	468	GSVPIGIGKNCLYK			Carbamidomethyl (C)[11]		Mascot
1625.8646	1625.8612	-0.0034	-2	140	153	IYVLTQFNSASLNR	114	100			Mascot
1786.9368	1786.9095	-0.0273	-15	124	139	LIDIPVSNCLNSNISK			Carbamidomethyl (C)[9]		Mascot
1921.9246	1922.0004	0.0758	39	393	410	MLDADVTDSVIGEGCVIK			Carbamidomethyl (C)[15]		Mascot
2366.1445	2366.0994	-0.0451	-19	316	338	DKFPGANDFGSEVIPGAT SLGMR					Mascot

7	Glucose-1-phosphate adenylyltransferase small subunit, chloroplastic/amyloplastic OS=Oryza sativa subsp. japonica GN=AGPS PE=2 SV=4	GLGS_ORYSJ	56467.8	6.58	11	222	100	7.429	188	100

Peptide Information

Calc. Mass	Obsrv. Mass	± da	± ppm	Start Seq.	End Seq.	Sequence	Ion Score	C. I. %	Modification	Rank	Result Type
854.444	854.4305	-0.0135	-16	241	248	ATAFGLMK			Oxidation (M)[7]		Mascot
972.4673	972.4487	-0.0186	-19	489	496	ETDGYFIK					Mascot
1017.5952	1017.5868	-0.0084	-8	407	415	IHHSVVGLR					Mascot
1032.5472	1032.5397	-0.0075	-7	371	379	SAPIYTQPR					Mascot
1032.5472	1032.5397	-0.0075	-7	371	379	SAPIYTQPR	45	96.929			Mascot
1256.7109	1256.7004	-0.0105	-8	105	116	AKPAVPLGANYR					Mascot
1256.7109	1256.7004	-0.0105	-8	105	116	AKPAVPLGANYR	29	0			Mascot
1382.6619	1382.704	0.0421	30	41	55	GVASSSSSSSAGRR					Mascot
1412.8121	1412.6833	-0.1288	-91	104	116	RAKPAVPLGANYR					Mascot
1625.8646	1625.8612	-0.0034	-2	133	146	IYVLTQFNSASLNR	114	100			Mascot
1733.8489	1733.8354	-0.0135	-8	286	300	EMPYIASMGIYVISK			Oxidation (M)[2,8]		Mascot
1786.9368	1786.9095	-0.0273	-15	117	132	LIDIPVSNCLNSNISK			Carbamidomethyl (C)[9]		Mascot
1889.9525	1889.9269	-0.0256	-14	386	403	VLDADVTDSVIGEGCVIK			Carbamidomethyl (C)[15]		Mascot

8	Glucose-1-phosphate adenylyltransferase small subunit 1, chloroplastic OS=Vicia faba GN=AGPC PE=2 SV=1	GLGS1_VICFA	55934.6	6.43	13	195	100	4.164	143	100

Peptide Information

Calc. Mass	Obsrv. Mass	± da	± ppm	Start Seq.	End Seq.	Sequence	Ion Score	C. I. %	Modification	Rank	Result Type

Calc. Mass	Obsrv. Mass	± da	± ppm	Start Seq.	End Seq.	Sequence	Ion Score	C. I. %	Modification	Rank	Result Type
854.444	854.4305	-0.0135	-16	235	242	ATAFGLMK			Oxidation (M)[7]		Mascot
1074.6194	1074.5847	-0.0347	-32	249	257	IIEFAEKPK					Mascot
1256.7109	1256.7004	-0.0105	-8	99	110	AKPAVPLGANYR					Mascot
1256.7109	1256.7004	-0.0105	-8	99	110	AKPAVPLGANYR	29	0			Mascot
1270.7478	1270.7382	-0.0096	-8	77	90	SVLGIILGGGAGTR					Mascot
1406.8114	1406.7175	-0.0939	-67	442	455	GSVPIGIGKNSHIK					Mascot
1412.8121	1412.6833	-0.1288	-91	98	110	RAKPAVPLGANYR					Mascot
1625.8646	1625.8612	-0.0034	-2	127	140	IYVLTQFNSASLNR	114	100			Mascot
1733.9221	1733.8354	-0.0867	-50	365	379	SSPIYTQPRYLPPSK					Mascot
1786.9368	1786.9095	-0.0273	-15	111	126	LIDIPVSNCLNSNISK			Carbamidomethyl (C)[9]		Mascot
1892.0084	1891.8402	-0.1682	-89	2	20	SSIVTSGVINVPRSSSSS K					Mascot
2338.1089	2338.1716	0.0627	27	380	400	MLDADITDSVIGEGCVIKN CK			Carbamidomethyl (C)[15,20]		Mascot
2366.1445	2366.0994	-0.0451	-19	303	325	DKFPGANDFGSEVIPGAT SIGMR					Mascot
2400.1982	2400.0647	-0.1335	-56	354	373	KPVPDFSFYDRSSPIYTQ PR					Mascot

9	Glucose-1-phosphate adenylyltransferase small subunit 2, chloroplastic OS=Vicia faba GN=AGPP PE=2 SV=1	GLGS2_VICFA	56309.7	6.19	13	193	100	4.599	143	100	

Peptide Information

Calc. Mass	Obsrv. Mass	± da	± ppm	Start Seq.	End Seq.	Sequence	Ion Score	C. I. %	Modification	Rank	Result Type
818.4804	818.4702	-0.0102	-12	1	8	MAAIGVLK			Oxidation (M)[1]		Mascot
854.444	854.4305	-0.0135	-16	239	246	ATAFGLMK			Oxidation (M)[7]		Mascot
1017.5952	1017.5868	-0.0084	-8	405	413	IHHSVVGLR					Mascot
1256.7109	1256.7004	-0.0105	-8	103	114	AKPAVPLGANYR					Mascot
1256.7109	1256.7004	-0.0105	-8	103	114	AKPAVPLGANYR	29	0			Mascot
1270.7478	1270.7382	-0.0096	-8	81	94	SVLGIILGGGAGTR					Mascot
1412.8121	1412.6833	-0.1288	-91	102	114	RAKPAVPLGANYR					Mascot
1488.8057	1488.7391	-0.0666	-45	55	68	NPFIVSPKAVSDSK					Mascot
1625.8646	1625.8612	-0.0034	-2	131	144	IYVLTQFNSASLNR	114	100			Mascot
1733.9221	1733.8354	-0.0867	-50	369	383	SSPIYTQPRYLPPSK					Mascot
1786.9368	1786.9095	-0.0273	-15	115	130	LIDIPVSNCLNSNISK			Carbamidomethyl (C)[9]		Mascot
2050.0664	2050.0479	-0.0185	-9	2	23	AAIGVLKVPPSSSSSSSS SSSK					Mascot
2338.1089	2338.1716	0.0627	27	384	404	MLDADITDSVIGEGCVIKN CK			Carbamidomethyl (C)[15,20]		Mascot
2400.1982	2400.0647	-0.1335	-56	358	377	KPVPDFSFYDRSSPIYTQ PR					Mascot

10	Glucose-1-phosphate adenylyltransferase small	GLGS_BETVU	54104.5	5.59	12	187	100	3.932	143	100	

subunit, chloroplastic/amyloplastic (Fragment) OS=Beta vulgaris GN=AGPB1 PE=2 SV=1

Peptide Information

Calc. Mass	Obsrv. Mass	± da	± ppm	Start Seq.	End Seq.	Sequence	Ion Score	C. I. %	Modification	Rank	Result Type
854.444	854.4305	-0.0135	-16	228	235	ATAFGLMK			Oxidation (M)[7]		Mascot
972.4673	972.4487	-0.0186	-19	464	471	ETDGYFIK					Mascot
1074.6194	1074.5847	-0.0347	-32	242	250	IIEFAEKPK					Mascot
1256.7109	1256.7004	-0.0105	-8	92	103	AKPAVPLGANYR					Mascot
1256.7109	1256.7004	-0.0105	-8	92	103	AKPAVPLGANYR	29	0			Mascot
1270.7478	1270.7382	-0.0096	-8	70	83	SVLGIILGGGAGTR					Mascot
1412.8121	1412.6833	-0.1288	-91	91	103	RAKPAVPLGANYR					Mascot
1570.9064	1570.8472	-0.0592	-38	430	445	FLAAKGSVPIGIGNAR					Mascot
1625.8646	1625.8612	-0.0034	-2	120	133	IYVLTQFNSASLNR	114	100			Mascot
1733.9221	1733.8354	-0.0867	-50	358	372	SSPIYTQPRYLPPSK					Mascot
1786.9368	1786.9095	-0.0273	-15	104	119	LIDIPVSNCLNSNISK			Carbamidomethyl (C)[9]		Mascot
2338.1089	2338.1716	0.0627	27	373	393	MLDADITDSVIGEGCVIKNCK			Carbamidomethyl (C)[15,20]		Mascot
2400.1982	2400.0647	-0.1335	-56	347	366	KPVPDFSFYDRSSPIYTQPR					Mascot

Gel Idx/Pos	285/L13	Instr./Gel Origin	BA2151/Sample Project 20140814	Process Status	Analysis Succeeded
Plate [#] Name	[1] Sample Project 20140814	Instrument Sample Name		Spectra	11

Rank	Protein Name	Accession No.	Protein MW	Protein PI	Pep. Count	Protein Score	Protein Score C. I. %	Intensity Matched	Total Ion Score	Total Ion C. I. %	Confirmed
1	2,3-bisphosphoglycerate-independent phosphoglycerate mutase OS=Nicotiana tabacum PE=1 SV=1	PMGI_TOBAC	61257.2	5.98	10	136	100	6.045	117	100	

Peptide Information

Calc. Mass	Obsrv. Mass	± da	± ppm	Start Seq.	End Seq.	Sequence	Ion Score	C. I. %	Modification	Rank	Result Type
801.4464	801.4352	-0.0112	-14	403	409	ALEIAER					Mascot
807.3632	807.3817	0.0185	23	2	9	GSSGDAWK					Mascot
960.4897	960.4462	-0.0435	-45	341	348	HSGEYLVR					Mascot
1128.5208	1128.4897	-0.0311	-28	296	304	ALEYENFDK					Mascot
1323.6328	1323.6229	-0.0099	-7	214	223	YENDWDVVKR					Mascot
1407.7015	1407.6813	-0.0202	-14	224	236	GWDAQVLGEAPHK					Mascot
1481.7074	1481.6864	-0.021	-14	362	373	FGHVTFFWNGNR					Mascot
1563.8027	1563.7573	-0.0454	-29	223	236	RGWDAQVLGEAPHK					Mascot
1731.9501	1731.9226	-0.0275	-16	126	142	GTLHLIGLLSDGGVHSR					Mascot
1731.9501	1731.9226	-0.0275	-16	126	142	GTLHLIGLLSDGGVHSR	117	100			Mascot
2063.9202	2064.1187	0.1985	96	207	222	MYVTMDRYENDWDVVK					Mascot

Rank	Protein Name	Accession No.	Protein MW	Protein PI	Pep. Count	Protein Score	Protein Score C. I. %	Intensity Matched	Total Ion Score	Total Ion C. I. %	Confirmed
2	2,3-bisphosphoglycerate-independent phosphoglycerate mutase OS=Ricinus communis PE=1 SV=2	PMGI_RICCO	61007.9	5.52	5	129	100	6.574	117	100	

Peptide Information

Calc. Mass	Obsrv. Mass	± da	± ppm	Start Seq.	End Seq.	Sequence	Ion Score	C. I. %	Modification	Rank	Result Type
1016.537	1016.5208	-0.0162	-16	184	191	DLENLREK					Mascot
1481.7074	1481.6864	-0.021	-14	359	370	FGHVTFFWNGNR					Mascot
1731.9501	1731.9226	-0.0275	-16	123	139	GTLHLIGLLSDGGVHSR					Mascot
1731.9501	1731.9226	-0.0275	-16	123	139	GTLHLIGLLSDGGVHSR	117	100			Mascot
1985.9169	1985.8885	-0.0284	-14	192	210	GVDAQVASGGGRMYVT MDR			Oxidation (M)[13]		Mascot
2888.417	2888.5979	0.1809	63	451	477	MIIDAIEQVGGIYVVTADH GNAEDMVK					Mascot
2888.417	2888.5979	0.1809	63	451	477	MIIDAIEQVGGIYVVTADH GNAEDMVK					Mascot

Rank	Protein Name	Accession No.	Protein MW	Protein PI	Pep. Count	Protein Score	Protein Score C. I. %	Intensity Matched	Total Ion Score	Total Ion C. I. %	Confirmed
3	Keratin, type II cytoskeletal 6A OS=Homo sapiens	K2C6A_HUMAN	60293.4	8.09	16	79	99.335	7.421	23	0	

GN=KRT6A PE=1 SV=3

Peptide Information

Calc. Mass	Obsrv. Mass	± da	± ppm	Start Seq.	End Seq.	Sequence	Ion Score	C. I. %	Modification	Rank	Result Type
801.4577	801.4352	-0.0225	-28	437	443	AKQDLAR					Mascot
955.5207	955.4658	-0.0549	-57	393	400	SEIDHVKK					Mascot
965.4911	965.4459	-0.0452	-47	16	24	RGFSANSAR					Mascot
989.4534	989.4816	0.0282	29	551	559	YTTTSSSSR					Mascot
1012.5058	1012.4256	-0.0802	-79	31	40	SGFSSVSVSR					Mascot
1012.5058	1012.4256	-0.0802	-79	31	40	SGFSSVSVSR					Mascot
1016.5371	1016.5208	-0.0163	-16	224	232	QLDSIVGER					Mascot
1026.4561	1026.4895	0.0334	33	273	281	DVDAAYMNK					Mascot
1107.543	1107.5179	-0.0251	-23	339	347	AQYEEIAQR					Mascot
1165.5848	1165.5593	-0.0255	-22	360	369	YEELQVTAGR	23	0			Mascot
1357.7322	1357.647	-0.0852	-63	425	436	NKLEGLEDALQK					Mascot
1407.7114	1407.6813	-0.0301	-21	288	299	ADTLTDEINFLR					Mascot
1439.6836	1439.6473	-0.0363	-25	241	252	GMQDLVEDFKNK			Oxidation (M)[2]		Mascot
1455.6863	1455.667	-0.0193	-13	348	359	SRAEAESWYQTK					Mascot
1507.8188	1507.7111	-0.1077	-71	444	455	LLKEYQELMNVK					Mascot
1624.7708	1624.8428	0.072	44	1	15	MASTSTTIRSHSSSR			Oxidation (M)[1]		Mascot
1890.9708	1890.9491	-0.0217	-11	208	222	QNLEPLFEQYINNLR					Mascot

4	Keratin, type II cytoskeletal 6C OS=Homo sapiens GN=KRT6C PE=1 SV=3	K2C6C_HUMAN	60273.4	8.09	15	72	96.888	5.594	23	0	

Peptide Information

Calc. Mass	Obsrv. Mass	± da	± ppm	Start Seq.	End Seq.	Sequence	Ion Score	C. I. %	Modification	Rank	Result Type
801.4577	801.4352	-0.0225	-28	437	443	AKQDLAR					Mascot
955.5207	955.4658	-0.0549	-57	393	400	SEIDHVKK					Mascot
965.4911	965.4459	-0.0452	-47	16	24	RGFSANSAR					Mascot
989.4534	989.4816	0.0282	29	551	559	YTTTSSSSR					Mascot
1016.5371	1016.5208	-0.0163	-16	224	232	QLDSIVGER					Mascot
1026.5215	1026.4895	-0.032	-31	31	40	SGFSSISVSR					Mascot
1107.543	1107.5179	-0.0251	-23	339	347	AQYEEIAQR					Mascot
1165.5848	1165.5593	-0.0255	-22	360	369	YEELQVTAGR	23	0			Mascot
1357.7322	1357.647	-0.0852	-63	425	436	NKLEGLEDALQK					Mascot
1407.7114	1407.6813	-0.0301	-21	288	299	ADTLTDEINFLR					Mascot

1455.6863	1455.667	-0.0193	-13	348	359	SRAEAESWYQTK					Mascot
1462.79	1462.7646	-0.0254	-17	183	194	FLEQQNKVLDTK					Mascot
1507.8188	1507.7111	-0.1077	-71	444	455	LLKEYQELMNVK					Mascot
1624.7708	1624.8428	0.072	44	1	15	MASTSTTIRSHSSSR			Oxidation (M)[1]		Mascot
1890.9708	1890.9491	-0.0217	-11	208	222	QNLEPLFEQYINNLR					Mascot

5	Ribosomal RNA small subunit methyltransferase G OS=Lactobacillus johnsonii (strain CNCM I-12250 / La1 / NCC 533) GN=rsmG PE=3 SV=1	RSMG_LACJO	26848.4	8.24	12	62	65.087	6.832		

Peptide Information

Calc. Mass	Obsrv. Mass	± da	± ppm	Start Seq.	End Seq.	Sequence	Ion Score	C. I. %	Modification	Rank	Result Type
801.508	801.4352	-0.0728	-91	213	219	TLIVVEK					Mascot
807.4359	807.3817	-0.0542	-67	143	149	FDLVTGR					Mascot
965.4761	965.4459	-0.0302	-31	1	8	MNPEIFAK			Oxidation (M)[1]		Mascot
974.4789	974.399	-0.0799	-82	179	187	AQDELAEAK					Mascot
999.4781	999.4669	-0.0112	-11	13	20	YGFELNEK					Mascot
1259.6995	1259.6414	-0.0581	-46	2	12	NPEIFAKELAK					Mascot
1390.7399	1390.657	-0.0829	-60	1	12	MNPEIFAKELAK					Mascot
1490.7737	1490.7073	-0.0664	-45	110	122	FLDELVNDLGLDK					Mascot
1624.8402	1624.8428	0.0026	2	123	137	VTLVHSRAEDAGQNK					Mascot
1731.9528	1731.9226	-0.0302	-17	108	122	LKFLDELVNDLGLDK					Mascot
1731.9528	1731.9226	-0.0302	-17	108	122	LKFLDELVNDLGLDK					Mascot
1765.8796	1765.8262	-0.0534	-30	24	37	QFATYYDKLVEFNK					Mascot
1864.9763	1865.0505	0.0742	40	179	196	AQDELAEAKHAIDVLGGK					Mascot

6	Pyridoxal biosynthesis lyase PdxS OS=Bacillus circulans GN=pdxS PE=3 SV=1	PDXS_BACCI	31560.3	5.79	12	62	61.719	9.251	14	0

Peptide Information

Calc. Mass	Obsrv. Mass	± da	± ppm	Start Seq.	End Seq.	Sequence	Ion Score	C. I. %	Modification	Rank	Result Type
826.3433	826.3253	-0.018	-22	11	17	GMAEMQK			Oxidation (M)[2,5]		Mascot
982.4444	982.4243	-0.0201	-20	10	17	RGMAEMQK			Oxidation (M)[3,6]		Mascot
1036.5059	1036.4984	-0.0075	-7	240	248	SDSPEKFAR					Mascot
1165.6464	1165.5593	-0.0871	-75	259	268	DYKLIAEVSK					Mascot
1186.6976	1186.6573	-0.0403	-34	72	82	VVSIPVMAKAR			Oxidation (M)[7]		Mascot
1186.6976	1186.6573	-0.0403	-34	72	82	VVSIPVMAKAR	14	0	Oxidation (M)[7]		Mascot
1235.6412	1235.5923	-0.0489	-40	138	148	INEGASMLRTK			Oxidation (M)[7]		Mascot

1339.7329	1339.6302	-0.1027	-77	276	287	GIEISKLHASER		Mascot
1357.7509	1357.647	-0.1039	-77	269	281	NLGAPMKGIEISK		Mascot
1445.7417	1445.6167	-0.125	-86	32	46	VAEAAGASAVMALER		Mascot
1462.7029	1462.7646	0.0617	42	18	31	GGVIMDVMNAEQAK		Mascot
1527.8126	1527.7069	-0.1057	-69	147	161	TKGEPGTGNIVEAVR		Mascot
1538.7849	1538.7139	-0.071	-46	249	261	AIVEATTHYKDYK		Mascot

7 Keratin, type I cytoskeletal 16 OS=Homo sapiens GN=KRT16 PE=1 SV=4 | K1C16_HUMAN | 51578.3 | 4.99 | 15 | 57 | 1.603 | 3.746

Peptide Information

Calc. Mass	Obsrv. Mass	± da	± ppm	Start Seq.	End Seq.	Sequence	Ion Score	C. I. %	Modification	Rank	Result Type
807.3995	807.3817	-0.0178	-22	197	203	LAADDFR					Mascot
989.5374	989.4816	-0.0558	-56	331	338	SEVTELRR					Mascot
1021.5676	1021.4713	-0.0963	-94	246	253	EELAYLRK					Mascot
1029.5939	1029.5128	-0.0811	-79	226	234	VLDELTLAR					Mascot
1036.5323	1036.4984	-0.0339	-33	149	155	IRDWYQR					Mascot
1106.5259	1106.4862	-0.0397	-36	119	127	VTMQNLNDR			Oxidation (M)[3]		Mascot
1141.483	1141.4948	0.0118	10	291	299	DQYEQMAEK					Mascot
1241.5942	1241.6284	0.0342	28	254	263	NHEEEMLALR					Mascot
1259.6743	1259.6414	-0.0329	-26	204	213	TKYEHELALR					Mascot
1338.6649	1338.6427	-0.0222	-17	42	55	APSTYGGGLSVSSR					Mascot
1357.7183	1357.647	-0.0713	-53	214	225	QTVEADVNGLRR					Mascot
1379.7278	1379.6923	-0.0355	-26	408	418	TRLEQEIATYR					Mascot
1929.868	1929.865	-0.003	-2	285	299	ILNEMRDQYEQMAEK			Oxidation (M)[5,12]		Mascot
2064.1448	2064.1187	-0.0261	-13	178	196	IIAATIENAQPILQIDNAR					Mascot
2350.0906	2350.0681	-0.0225	-10	420	441	LLEGEDAHLSSQQASGQ SYSSR					Mascot

8 3-ketoacyl-CoA synthase 5 OS=Arabidopsis thaliana GN=KCS5 PE=1 SV=1 | KCS5_ARATH | 56358 | 9.01 | 14 | 55 | 0 | 7.849

Peptide Information

Calc. Mass	Obsrv. Mass	± da	± ppm	Start Seq.	End Seq.	Sequence	Ion Score	C. I. %	Modification	Rank	Result Type
1118.5986	1118.4888	-0.1098	-98	271	281	MGGAAILLSNR			Oxidation (M)[1]		Mascot
1177.6133	1177.5221	-0.0912	-77	327	337	DLMTIAGEALK			Oxidation (M)[3]		Mascot
1193.6354	1193.5529	-0.0825	-69	370	378	WKPYIPDFK					Mascot
1244.6191	1244.5271	-0.092	-74	1	11	MSDFSSSVKLK			Oxidation (M)[1]		Mascot

1323.6151	1323.6229	0.0078	6	100	110	VPFSSFMEHSR					Mascot
1339.61	1339.6302	0.0202	15	100	110	VPFSSFMEHSR			Oxidation (M)[7]		Mascot
1407.7413	1407.6813	-0.06	-43	119	129	SVEFQMRILER					Mascot
1439.7238	1439.6473	-0.0765	-53	400	412	NLQLSGEHVEASR					Mascot
1507.6482	1507.7111	0.0629	42	469	481	TPTDGAWSDCIER			Carbamidomethyl (C)[10]		Mascot
1624.8231	1624.8428	0.0197	12	442	455	NDRVWQIAFGSGFK					Mascot
1792.9785	1792.8931	-0.0854	-48	365	378	IFNPKWKPYIPDFK					Mascot
1792.9785	1792.8931	-0.0854	-48	365	378	IFNPKWKPYIPDFK					Mascot
1886.9027	1886.97	0.0673	36	156	171	NEAQMVIFTAMEDLFK					Mascot
2086.9897	2087.021	0.0313	15	212	231	SYNLSGMGCSASLISVDVAR			Carbamidomethyl (C)[9]		Mascot
2350.2192	2350.0681	-0.1511	-64	261	281	AMLLPNCLFRMGGAAILLSNR			Carbamidomethyl (C)[7], Oxidation (M)[2,11]		Mascot
2657.3313	2657.2178	-0.1135	-43	156	178	NEAQMVIFTAMEDLFKNTGLKPK			Oxidation (M)[5,11]		Mascot

9	Keratin, type II cytoskeletal 80 OS=Homo sapiens GN=KRT80 PE=1 SV=2	K2C80_HUMAN	51007	5.58	15	54	0	6.403			

Peptide Information

Calc. Mass	Obsrv. Mass	± da	± ppm	Start Seq.	End Seq.	Sequence	Ion Score	C. I. %	Modification	Rank	Result Type
1016.537	1016.5208	-0.0162	-16	385	393	KLVEGEEGR					Mascot
1033.5208	1033.4907	-0.0301	-29	357	364	QDMARQLR			Oxidation (M)[3]		Mascot
1040.5082	1040.4762	-0.032	-31	366	373	YQELMNVK			Oxidation (M)[5]		Mascot
1051.4724	1051.5277	0.0553	53	81	88	NQEKEEMK			Oxidation (M)[7]		Mascot
1128.5103	1128.4897	-0.0206	-18	191	199	DLDAECLHR			Carbamidomethyl (C)[6]		Mascot
1165.5519	1165.5593	0.0074	6	233	243	DVSVTVGMDSR					Mascot
1168.603	1168.5851	-0.0179	-15	365	373	KYQELMNVK			Oxidation (M)[6]		Mascot
1367.6625	1367.6041	-0.0584	-43	45	57	SLTGCWSAGTISK			Carbamidomethyl (C)[5]		Mascot
1439.7815	1439.6473	-0.1342	-93	206	217	LKSLESFVELMK			Oxidation (M)[11]		Mascot
1498.7571	1498.6656	-0.0915	-61	244	256	CHIDLSGIVEEVK			Carbamidomethyl (C)[1]		Mascot
1514.7672	1514.6619	-0.1053	-70	178	189	RTDMEFTFVQLK					Mascot
1514.7672	1514.6619	-0.1053	-70	178	189	RTDMEFTFVQLK					Mascot
1536.7177	1536.734	0.0163	11	329	342	TAEEQGELAFQDAK					Mascot
1765.8604	1765.8262	-0.0342	-19	329	344	TAEEQGELAFQDAKTK					Mascot
1890.959	1890.9491	-0.0099	-5	226	243	DLAAQVKDVSVTVGMDSR					Mascot
2054.124	2054.0876	-0.0364	-18	146	163	VSQERGQLEANLLQVLEK					Mascot

10	Keratin, type II cytoskeletal 6B OS=Mus musculus GN=Krt6b PE=1 SV=3	K2C6B_MOUSE	60627.3	8.51	12	54	0	10.147	23	0	

Peptide Information

Calc. Mass	Obsrv. Mass	± da	± ppm	Start Seq.	End Seq.	Sequence	Ion Score	C. I. %	Modification	Rank	Result Type
955.5207	955.4658	-0.0549	-57	390	397	SEIDHVKK					Mascot
1029.4558	1029.5128	0.057	55	270	278	DVDAAYMTK			Oxidation (M)[7]		Mascot
1036.531	1036.4984	-0.0326	-31	260	268	AENEFVTVK					Mascot
1141.5558	1141.4948	-0.061	-53	269	278	KDVDAAYMTK					Mascot
1158.6226	1158.5902	-0.0324	-28	5	14	TTIKSQTSHR					Mascot
1165.5848	1165.5593	-0.0255	-22	357	366	YEELQVTAGR	23	0			Mascot
1186.6539	1186.6573	0.0034	3	220	229	RQLDSIIGER					Mascot
1186.6539	1186.6573	0.0034	3	220	229	RQLDSIIGER					Mascot
1455.6863	1455.667	-0.0193	-13	345	356	SRAEAESWYQTK					Mascot
1462.79	1462.7646	-0.0254	-17	180	191	FLEQQNKVLDTK					Mascot
1507.8188	1507.7111	-0.1077	-71	441	452	LLKEYQELMNVK					Mascot
2054.0125	2054.0876	0.0751	37	205	220	QNLEPMFEQYISNLRR			Oxidation (M)[6]		Mascot
2350.0994	2350.0681	-0.0313	-13	68	92	ISIGGGSCGIGGGYGSRF GGAYGSR			Carbamidomethyl (C)[8]		Mascot

Gel Idx/Pos	286/L14	Instr./Gel Origin	BA2151/Sample Project 20140814	Process Status	Analysis Succeeded
Plate [#] Name	[1] Sample Project 20140814	Instrument Sample Name		Spectra	11

Rank	Protein Name	Accession No.	Protein MW	Protein PI	Pep. Count	Protein Score	Protein Score C. I. %	Intensity Matched	Total Ion Score	Total Ion C. I. %	Confirmed
1	2,3-bisphosphoglycerate-independent phosphoglycerate mutase OS=Mesembryanthemum crystallinum GN=PGM1 PE=1 SV=1	PMGI_MESCR	61316.1	5.39	10	211	100	3.913	191	100	

Peptide Information

Calc. Mass	Obsrv. Mass	± da	± ppm	Start Seq.	End Seq.	Sequence	Ion Score	C. I. %	Modification	Rank	Result Type
947.3961	947.4342	0.0381	40	207	213	MYVTMDR			Oxidation (M)[1,5]		Mascot
1042.4874	1042.4586	-0.0288	-28	353	361	TFACSETVK			Carbamidomethyl (C)[4]		Mascot
1139.5369	1139.5072	-0.0297	-26	214	222	YENDWSVVK					Mascot
1403.6511	1403.5919	-0.0592	-42	315	326	YAGMLQYDGELK			Oxidation (M)[4]		Mascot
1481.7074	1481.6821	-0.0253	-17	362	373	FGHVTFFWNGNR					Mascot
1682.9225	1682.8103	-0.1122	-67	327	340	LPNRYLVSPPEIER					Mascot
1749.8265	1749.843	0.0165	9	1	15	MGSTEFSWKLADHPK			Oxidation (M)[1]		Mascot
1999.9325	1999.9305	-0.002	-1	195	213	GVDAQIASGGGRMYVTMDR			Oxidation (M)[13]		Mascot
2035.9253	2035.931	0.0057	3	207	222	MYVTMDRYENDWSVVK					Mascot
2377.1882	2377.1641	-0.0241	-10	171	192	DVLDGSSVGFVETLENDLAQLR					Mascot
2377.1882	2377.1641	-0.0241	-10	171	192	DVLDGSSVGFVETLENDLAQLR	191	100			Mascot

Rank	Protein Name	Accession No.	Protein MW	Protein PI	Pep. Count	Protein Score	Protein Score C. I. %	Intensity Matched	Total Ion Score	Total Ion C. I. %	Confirmed
2	2,3-bisphosphoglycerate-independent phosphoglycerate mutase OS=Ricinus communis PE=1 SV=2	PMGI_RICCO	61007.9	5.52	9	142	100	11.064	124	100	

Peptide Information

Calc. Mass	Obsrv. Mass	± da	± ppm	Start Seq.	End Seq.	Sequence	Ion Score	C. I. %	Modification	Rank	Result Type
896.4697	896.4312	-0.0385	-43	149	157	GAAEHGAKR					Mascot
947.3961	947.4342	0.0381	40	204	210	MYVTMDR			Oxidation (M)[1,5]		Mascot
1016.537	1016.5164	-0.0206	-20	184	191	DLENLREK					Mascot
1322.6488	1322.6094	-0.0394	-30	211	220	YENDWNVVKR					Mascot
1481.7074	1481.6821	-0.0253	-17	359	370	FGHVTFFWNGNR					Mascot
1731.9501	1731.92	-0.0301	-17	123	139	GTLHLIGLLSDGGVHSR					Mascot
1731.9501	1731.92	-0.0301	-17	123	139	GTLHLIGLLSDGGVHSR	124	100			Mascot
1774.8395	1774.8846	0.0451	25	293	306	ALEYENFDTFDRVR					Mascot

Calc. Mass	Obsrv. Mass	± da	± ppm	Start Seq.	End Seq.	Sequence	Ion Score	C. I. %	Modification	Rank	Result Type
1985.9169	1985.8677	-0.0492	-25	192	210	GVDAQVASGGGRMYVT MDR			Oxidation (M)[13]		Mascot
2888.417	2888.5957	0.1787	62	451	477	MIIDAIEQVGGIYVVTADH GNAEDMVK					Mascot
2888.417	2888.5957	0.1787	62	451	477	MIIDAIEQVGGIYVVTADH GNAEDMVK					Mascot

3	2,3-bisphosphoglycerate-independent phosphoglycerate mutase OS=Nicotiana tabacum PE=1 SV=1	PMGI_TOBAC	61257.2	5.98	9	142	100	7.365	124	100	

Peptide Information

Calc. Mass	Obsrv. Mass	± da	± ppm	Start Seq.	End Seq.	Sequence	Ion Score	C. I. %	Modification	Rank	Result Type
801.4464	801.4344	-0.012	-15	403	409	ALEIAER					Mascot
947.3961	947.4342	0.0381	40	207	213	MYVTMDR			Oxidation (M)[1,5]		Mascot
1042.4874	1042.4586	-0.0288	-28	353	361	TFACSETVK			Carbamidomethyl (C)[4]		Mascot
1407.7015	1407.6548	-0.0467	-33	224	236	GWDAQVLGEAPHK					Mascot
1481.7074	1481.6821	-0.0253	-17	362	373	FGHVTFFWNGNR					Mascot
1563.8027	1563.7623	-0.0404	-26	223	236	RGWDAQVLGEAPHK					Mascot
1682.8167	1682.8103	-0.0064	-4	424	439	VNLPNGDMVGHTGDIK			Oxidation (M)[8]		Mascot
1731.9501	1731.92	-0.0301	-17	126	142	GTLHLIGLLSDGGVHSR					Mascot
1731.9501	1731.92	-0.0301	-17	126	142	GTLHLIGLLSDGGVHSR	124	100			Mascot
1999.9325	1999.9305	-0.002	-1	195	213	GIDAQVASGGGRMYVTM DR			Oxidation (M)[13]		Mascot

4	Pre-mRNA-splicing regulator WTAP OS=Xenopus tropicalis GN=wtap PE=2 SV=1	FL2D_XENTR	44455.9	5.17	14	66	85.446	23.455			

Peptide Information

Calc. Mass	Obsrv. Mass	± da	± ppm	Start Seq.	End Seq.	Sequence	Ion Score	C. I. %	Modification	Rank	Result Type
896.4723	896.4312	-0.0411	-46	192	198	KYSEELK					Mascot
1019.5269	1019.5002	-0.0267	-26	283	291	THSSVGLYR					Mascot
1030.5826	1030.5607	-0.0219	-21	72	79	RENILVMR					Mascot
1186.6136	1186.6544	0.0408	34	1	10	MTNEEPLPKK					Mascot
1186.6136	1186.6544	0.0408	34	1	10	MTNEEPLPKK					Mascot
1317.7162	1317.6008	-0.1154	-88	13	23	LNESDFKVLPR					Mascot
1423.7037	1423.6309	-0.0728	-51	269	282	ILNGPSNGGSSHQR					Mascot
1439.7166	1439.6475	-0.0691	-48	32	43	QFEAYVQALENK					Mascot
1467.7074	1467.6503	-0.0571	-39	44	56	YTDLNSNDVTGLR					Mascot
1538.6816	1538.7056	0.024	16	250	264	TPSSESKDEGETSGK					Mascot
1579.8553	1579.7738	-0.0815	-52	110	123	ATMVDPAINLFFIK					Mascot

1595.8502	1595.7675	-0.0827	-52	110	123	ATMVDPAINLFFIK			Oxidation (M)[3]		Mascot
1646.8102	1646.7578	-0.0524	-32	161	173	CRMLIQENQELGR			Carbamidomethyl (C)[1]		Mascot
2000.0342	1999.9305	-0.1037	-52	163	179	MLIQENQELGRQLSQGR					Mascot
2888.406	2888.5957	0.1897	66	32	56	QFEAYVQALENKYTDLN SNDVTGLR					Mascot
2888.406	2888.5957	0.1897	66	32	56	QFEAYVQALENKYTDLN SNDVTGLR					Mascot
3137.5466	3137.4119	-0.1347	-43	84	109	EQEMQECTTQIQHLKQV QQPSVAQLR			Carbamidomethyl (C)[7]		Mascot
3137.5466	3137.4119	-0.1347	-43	84	109	EQEMQECTTQIQHLKQV QQPSVAQLR			Carbamidomethyl (C)[7]		Mascot

5 Alpha-actinin-4 OS=Gallus gallus GN=ACTN4 PE=1 SV=1 ACTN4_CHICK 104712.2 5.13 21 60 49.536 16.875

Peptide Information

Calc. Mass	Obsrv. Mass	± da	± ppm	Start Seq.	End Seq.	Sequence	Ion Score	C. I. %	Modification	Rank	Result Type
896.52	896.4312	-0.0888	-99	590	597	IADLHSIK					Mascot
924.5625	924.4944	-0.0681	-74	89	96	LPKPERGK					Mascot
947.5043	947.4342	-0.0701	-74	507	514	EALEKTEK					Mascot
1021.4982	1021.4742	-0.024	-23	317	324	TIQEMQQK			Oxidation (M)[5]		Mascot
1143.5753	1143.5586	-0.0167	-15	568	577	ATLPDADRER					Mascot
1174.5739	1174.5229	-0.051	-43	881	892	GPDAAPGALDYK					Mascot
1317.6005	1317.6008	0.0003	0	717	726	HTNYTMEHIR			Oxidation (M)[6]		Mascot
1445.7417	1445.608	-0.1337	-92	313	324	SPQKTIQEMQQK					Mascot
1461.7267	1461.6458	-0.0809	-55	359	371	LSNRPAFMPSEGR					Mascot
1467.7954	1467.6503	-0.1451	-99	401	412	LEPLDHLAEKFR					Mascot
1485.7598	1485.6726	-0.0872	-59	175	186	NVNVQNFHISWK					Mascot
1507.7024	1507.7135	0.0111	7	60	72	AGTQIENIDEDFR					Mascot
1577.8071	1577.7646	-0.0425	-27	389	400	GYEEWLLNEIRR					Mascot
1611.849	1611.788	-0.061	-38	34	46	DLLLDPAWEKQQR					Mascot
1682.7803	1682.8103	0.03	18	751	764	DAKGISQEQMQEFR			Oxidation (M)[10]		Mascot
1730.9119	1730.8828	-0.0291	-17	357	371	LRLSNRPAFMPSEGR					Mascot
1731.8483	1731.92	0.0717	41	862	875	RELPPEQAEYCIAR			Carbamidomethyl (C)[11]		Mascot
1731.8483	1731.92	0.0717	41	862	875	RELPPEQAEYCIAR			Carbamidomethyl (C)[11]		Mascot
1761.7933	1761.8749	0.0816	46	629	642	ALQDEQSRQQCNER			Carbamidomethyl (C)[11]		Mascot
1792.8687	1792.894	0.0253	14	876	892	MAPYRGPDAAPGALDYK					Mascot
1792.8687	1792.894	0.0253	14	876	892	MAPYRGPDAAPGALDYK					Mascot
1985.9386	1985.8677	-0.0709	-36	372	388	MVSDINTGWQHLEQAEK					Mascot
2721.3091	2721.1895	-0.1196	-44	249	273	AIMTYVSSFYHAFSGAQK AETAANR					Mascot

6	tRNA-dihydrouridine(47) synthase [NAD(P)(+)] OS=Meyerozyma guilliermondii (strain ATCC 6260 / CBS 566 / DSM 6381 / JCM 1539 / NBRC 10279 / NRRL Y-324) GN=DUS3 PE=3 SV=2	DUS3_PICGU	69033.8	6.68	17	60	43.378	5.301

Peptide Information

Calc. Mass	Obsrv. Mass	± da	± ppm	Start Seq.	End Seq.	Sequence	Ion Score	C. I. %	Modification	Rank	Result Type
1139.5732	1139.5072	-0.066	-58	589	598	TGDNFTFIPK					Mascot
1306.6638	1306.6625	-0.0013	-1	107	118	STHDVTTYLAAK					Mascot
1317.6509	1317.6008	-0.0501	-38	119	130	APDIDGVCPVFK			Carbamidomethyl (C)[8]		Mascot
1326.6941	1326.6265	-0.0676	-51	187	197	YLFEVSDNVIK					Mascot
1327.6172	1327.6183	0.0011	1	75	84	DMRQQHEEVR					Mascot
1339.5397	1339.6204	0.0807	60	46	57	YVNDDDAEAGDR					Mascot
1407.7716	1407.6548	-0.1168	-83	553	563	INQRPPQWRGR					Mascot
1473.7697	1473.6963	-0.0734	-50	422	434	EVGAVVTEWNNKK					Mascot
1482.7323	1482.6758	-0.0565	-38	564	576	NELETLLGSTDYK					Mascot
1530.7661	1530.674	-0.0921	-60	577	588	DWIKITEMFYGK					Mascot
1646.7567	1646.7578	0.0011	1	528	539	RFMCEFLSFTHR			Carbamidomethyl (C)[4], Oxidation (M)[3]		Mascot
1745.9467	1745.8672	-0.0795	-46	356	372	ILKGMNASSGDIPVTVK			Oxidation (M)[5]		Mascot
1765.8716	1765.8306	-0.041	-23	198	212	YLDSQVQSDENIAKR					Mascot
1825.8602	1825.8073	-0.0529	-29	213	228	EEAKENNASYVEAPFK					Mascot
2190.0398	2190.189	0.1492	68	100	118	FGPEHCRSTHDVTTYLAAK			Carbamidomethyl (C)[6]		Mascot
2377.2439	2377.1641	-0.0798	-34	477	496	GALIKPWIFEEVEAQQYLDK					Mascot
2377.2439	2377.1641	-0.0798	-34	477	496	GALIKPWIFEEVEAQQYLDK					Mascot
2428.1316	2428.0933	-0.0383	-16	508	528	FADFSLEHWGSDEYGVGLSRR					Mascot

7	DNA ligase OS=Rhodopseudomonas palustris (strain HaA2) GN=ligA PE=3 SV=1	DNLJ_RHOP2	78022.5	5.76	17	60	40.71	6.45

Peptide Information

Calc. Mass	Obsrv. Mass	± da	± ppm	Start Seq.	End Seq.	Sequence	Ion Score	C. I. %	Modification	Rank	Result Type
924.5374	924.4944	-0.043	-47	535	541	RTIELHR					Mascot
1002.5215	1002.4324	-0.0891	-89	398	406	EGDTVVVQR					Mascot
1016.6099	1016.5164	-0.0935	-92	668	678	LGAKVAGSVSK					Mascot
1030.535	1030.5607	0.0257	25	178	186	TIPAACELR			Carbamidomethyl (C)[6]		Mascot
1193.5909	1193.5282	-0.0627	-53	307	315	VDRLDYQER					Mascot
1423.7468	1423.6309	-0.1159	-81	294	306	AALPYDIDGVVYK					Mascot

Calc. Mass	Obsrv. Mass	± da	± ppm	Start Seq.	End Seq.	Sequence	Ion Score	C. I. %	Modification	Rank	Result Type
1467.6962	1467.6503	-0.0459	-31	513	525	LEDIEGYGETSVR					Mascot
1481.7206	1481.6821	-0.0385	-26	562	574	HYGTLDAFLAAMR			Oxidation (M)[12]		Mascot
1482.77	1482.6758	-0.0942	-64	310	321	LDYQERLGFVSR					Mascot
1536.7706	1536.7263	-0.0443	-29	422	434	RPADATPYHFPHK					Mascot
1595.8275	1595.7675	-0.06	-38	680	695	TDYVVAGADAGSKLTK					Mascot
1682.9509	1682.8103	-0.1406	-84	1	16	MTKAPKPAPDIATLTK					Mascot
1730.9225	1730.8828	-0.0397	-23	493	507	GWVKEPADIFTLQAR					Mascot
1803.9388	1803.8309	-0.1079	-60	469	484	HFVSRLAFDIDGLGEK					Mascot
1986.0292	1985.8677	-0.1615	-81	64	83	FPELVSASSPTQTVGAAPAR					Mascot
2089.1289	2089.0813	-0.0476	-23	331	349	FAAEQATTVLEKIDIQVGR					Mascot
2190.0535	2190.189	0.1355	62	203	223	QEEAGDTVFANPRNSAAGSLR					Mascot

8	Glutamate--tRNA ligase OS=Bradyrhizobium sp. (strain ORS278) GN=gltX PE=3 SV=1	SYE_BRASO	52666.2	6.34	14	54	0	3.197

Peptide Information

Calc. Mass	Obsrv. Mass	± da	± ppm	Start Seq.	End Seq.	Sequence	Ion Score	C. I. %	Modification	Rank	Result Type
924.5625	924.4944	-0.0681	-74	436	444	LGAVAQPLR					Mascot
1012.521	1012.4288	-0.0922	-91	301	309	SAARFDFAK					Mascot
1139.5903	1139.5072	-0.0831	-73	2	12	TSSTTSSIVTR					Mascot
1196.6635	1196.5817	-0.0818	-68	138	148	EAPEGVKPTIR					Mascot
1214.6389	1214.6692	0.0303	25	248	258	RHGALGVDAYR					Mascot
1413.6501	1413.6185	-0.0316	-22	104	115	CYATAEELVAMR			Carbamidomethyl (C)[1]		Mascot
1467.7855	1467.6503	-0.1352	-92	28	39	TALFNWLYARGR					Mascot
1527.8068	1527.7057	-0.1011	-66	13	27	FAPSPTGFLHIGGAR					Mascot
1536.838	1536.7263	-0.1117	-73	135	148	DPKEAPEGVKPTIR					Mascot
1551.7836	1551.745	-0.0386	-25	90	103	EVAEQLLATGMAYR					Mascot
1730.8378	1730.8828	0.045	26	151	166	APLTGETMVEDQVQGR					Mascot
1825.9807	1825.8073	-0.1734	-95	167	181	VVWQNENLDDLVLLR					Mascot
2000.0229	1999.9305	-0.0924	-46	149	166	LRAPLTGETMVEDQVQGR					Mascot
2657.4521	2657.2217	-0.2304	-87	401	426	ALIGRLHAALSAVEPWSGPATEAALR					Mascot
2657.4521	2657.6619	0.2098	79	401	426	ALIGRLHAALSAVEPWSGPATEAALR					Mascot

9	Protein CSN12 homolog OS=Neosartorya fumigata (strain ATCC MYA-4609 / Af293 / CBS 101355 / FGSC A1100) GN=csn12 PE=3 SV=1	CSN12_ASPFU	51610.6	8.64	13	54	0	2.457

Peptide Information

Calc. Mass	Obsrv. Mass	± da	± ppm	Start Seq.	End Seq.	Sequence	Ion Score	C. I. %	Modification	Rank	Result Type
801.4101	801.4344	0.0243	30	192	198	APIEESR					Mascot
1143.6521	1143.5586	-0.0935	-82	391	401	VPVAEFAAALR					Mascot
1158.5282	1158.6012	0.073	63	183	191	MFTLCLSDR			Carbamidomethyl (C)[5], Oxidation (M)[1]		Mascot
1349.6818	1349.5732	-0.1086	-80	319	328	LFRPLCDCIR			Carbamidomethyl (C)[6,8]		Mascot
1577.8357	1577.7646	-0.0711	-45	439	455	GMVVLSKGGTAFPGTGV					Mascot
1611.8701	1611.788	-0.0821	-51	20	35	LAAALTPIATSQSPDR					Mascot
1646.9014	1646.7578	-0.1436	-87	101	114	VFDAWKNVANTLIR					Mascot
1745.8956	1745.8672	-0.0284	-16	228	244	ALSASSADLPELEAFPK					Mascot
1787.8752	1787.8271	-0.0481	-27	252	265	YYVGVIHFLDENYR					Mascot
1888.8105	1888.8816	0.0711	38	266	280	EAEEHLAYAWNMCHK			Carbamidomethyl (C)[13]		Mascot
2043.8965	2044.0699	0.1734	85	151	169	GAMAFGDQLQDDVAADF EK			Oxidation (M)[3]		Mascot
2100.0034	2099.8513	-0.1521	-72	36	53	LLSFYNFSDSDHISADLR					Mascot
2190.0425	2190.189	0.1465	67	329	348	KGDLVGFDTAMFAGEEE FVK					Mascot

10	DNA ligase OS=Rhodopseudomonas palustris (strain ATCC BAA-98 / CGA009) GN=ligA PE=3 SV=2	DNLJ_RHOPA	78800.7	5.53	16	54	0	19.619	

Peptide Information

Calc. Mass	Obsrv. Mass	± da	± ppm	Start Seq.	End Seq.	Sequence	Ion Score	C. I. %	Modification	Rank	Result Type
801.4577	801.4344	-0.0233	-29	506	512	ARNAELK					Mascot
1002.5215	1002.4324	-0.0891	-89	398	406	EGDTVVVQR					Mascot
1016.6099	1016.5164	-0.0935	-92	668	678	LGAKVAGSVSK					Mascot
1186.6791	1186.6544	-0.0247	-21	226	236	DVTVTASRPLK					Mascot
1186.6791	1186.6544	-0.0247	-21	226	236	DVTVTASRPLK					Mascot
1193.5909	1193.5282	-0.0627	-53	307	315	VDRLDYQER					Mascot
1439.7013	1439.6475	-0.0538	-37	679	692	KTDYVVAGEDAGSK					Mascot
1480.7278	1480.6633	-0.0645	-44	513	525	LEQLEGYGETSVR					Mascot
1481.7206	1481.6821	-0.0385	-26	562	574	HYGTLDAFLSAMR					Mascot
1482.77	1482.6758	-0.0942	-64	310	321	LDYQERLGFVSR					Mascot
1497.7156	1497.6793	-0.0363	-24	562	574	HYGTLDAFLSAMR			Oxidation (M)[12]		Mascot
1595.8428	1595.7675	-0.0753	-47	293	306	RASLPYDIDGVVYK					Mascot
1682.8457	1682.8103	-0.0354	-21	382	397	GIGNDGEPIRDGVDIR					Mascot
1730.9225	1730.8828	-0.0397	-23	493	507	GWVQEPADIFTLKAR					Mascot
1803.9388	1803.8309	-0.1079	-60	469	484	HFVSRLAFDIDGLGEK					Mascot

2036.0295	2035.931	-0.0985	-48	508	525	NAELKLEQLEGYGETSV R	Mascot
2089.1289	2089.0813	-0.0476	-23	331	349	FAAEQATTVLEKIDIQVG R	Mascot
2189.9985	2190.189	0.1905	87	36	54	AYYQDDAPKISDADYDAL R	Mascot

Gel Idx/Pos	287/L15	Instr./Gel Origin	BA2151/Sample Project 20140814	Process Status	Analysis Succeeded
Plate [#] Name	[1] Sample Project 20140814	Instrument Sample Name		Spectra	11

Rank	Protein Name	Accession No.	Protein MW	Protein PI	Pep. Count	Protein Score	Protein Score C. I. %	Intensity Matched	Total Ion Score	Total Ion C. I. %	Confirmed
1	2,3-bisphosphoglycerate-independent phosphoglycerate mutase OS=Nicotiana tabacum PE=1 SV=1	PMGI_TOBAC	61257.2	5.98	10	147	100	5.375	126	100	

Peptide Information

Calc. Mass	Obsrv. Mass	± da	± ppm	Start Seq.	End Seq.	Sequence	Ion Score	C. I. %	Modification	Rank	Result Type
801.4464	801.4226	-0.0238	-30	403	409	ALEIAER					Mascot
947.3961	947.4465	0.0504	53	207	213	MYVTMDR			Oxidation (M)[1,5]		Mascot
1042.4874	1042.4751	-0.0123	-12	353	361	TFACSETVK			Carbamidomethyl (C)[4]		Mascot
1323.6328	1323.6545	0.0217	16	214	223	YENDWDVVKR					Mascot
1481.7074	1481.6935	-0.0139	-9	362	373	FGHVTFFWNGNR					Mascot
1492.7312	1492.7253	-0.0059	-4	440	453	ATIEACKSADEAVK			Carbamidomethyl (C)[6]		Mascot
1682.865	1682.848	-0.017	-10	224	238	GWDAQVLGEAPHKFK					Mascot
1731.9501	1731.9423	-0.0078	-5	126	142	GTLHLIGLLSDGGVHSR					Mascot
1731.9501	1731.9423	-0.0078	-5	126	142	GTLHLIGLLSDGGVHSR	126	100			Mascot
1801.8864	1801.8964	0.01	6	290	304	MVMLAKALEYENFDK					Mascot
1999.9325	1999.953	0.0205	10	195	213	GIDAQVASGGGRMYVTMDR			Oxidation (M)[13]		Mascot

Rank	Protein Name	Accession No.	Protein MW	Protein PI	Pep. Count	Protein Score	Protein Score C. I. %	Intensity Matched	Total Ion Score	Total Ion C. I. %	Confirmed
2	2,3-bisphosphoglycerate-independent phosphoglycerate mutase OS=Ricinus communis PE=1 SV=2	PMGI_RICCO	61007.9	5.52	9	144	100	8.998	126	100	

Peptide Information

Calc. Mass	Obsrv. Mass	± da	± ppm	Start Seq.	End Seq.	Sequence	Ion Score	C. I. %	Modification	Rank	Result Type
896.4697	896.4455	-0.0242	-27	149	157	GAAEHGAKR					Mascot
947.3961	947.4465	0.0504	53	204	210	MYVTMDR			Oxidation (M)[1,5]		Mascot
1016.537	1016.5391	0.0021	2	184	191	DLENLREK					Mascot
1481.7074	1481.6935	-0.0139	-9	359	370	FGHVTFFWNGNR					Mascot
1589.8071	1589.7869	-0.0202	-13	220	233	RGWDAQVLGEAPYK					Mascot
1731.9501	1731.9423	-0.0078	-5	123	139	GTLHLIGLLSDGGVHSR					Mascot
1731.9501	1731.9423	-0.0078	-5	123	139	GTLHLIGLLSDGGVHSR	126	100			Mascot
1774.8395	1774.8988	0.0593	33	293	306	ALEYENFDTFDRVR					Mascot
1985.9169	1985.921	0.0041	2	192	210	GVDAQVASGGGRMYVT			Oxidation (M)[13]		Mascot

						MDR					
2888.417	2888.6157	0.1987	69	451	477	MIIDAIEQVGGIYVVTADHGNAEDMVK					Mascot
2888.417	2888.6157	0.1987	69	451	477	MIIDAIEQVGGIYVVTADHGNAEDMVK					Mascot

3	DNA ligase OS=Rhodopseudomonas palustris (strain HaA2) GN=ligA PE=3 SV=1	DNLJ_RHOP2	78022.5	5.76	18	67	88.439	5.686

Peptide Information

Calc. Mass	Obsrv. Mass	± da	± ppm	Start Seq.	End Seq.	Sequence	Ion Score	C. I. %	Modification	Rank	Result Type
800.4625	800.3853	-0.0772	-96	343	349	IDIQVGR					Mascot
817.4526	817.436	-0.0166	-20	638	645	RDTAVAGK					Mascot
924.5374	924.5121	-0.0253	-27	535	541	RTIELHR					Mascot
1016.6099	1016.5391	-0.0708	-70	668	678	LGAKVAGSVSK					Mascot
1193.5909	1193.5657	-0.0252	-21	307	315	VDRLDYQER					Mascot
1465.7257	1465.6643	-0.0614	-42	562	574	HYGTLDAFLAAMR					Mascot
1467.6962	1467.656	-0.0402	-27	513	525	LEDIEGYGETSVR					Mascot
1481.7206	1481.6935	-0.0271	-18	562	574	HYGTLDAFLAAMR			Oxidation (M)[12]		Mascot
1482.77	1482.6882	-0.0818	-55	310	321	LDYQERLGFVSR					Mascot
1511.8481	1511.7073	-0.1408	-93	485	496	QIVLFHERGWVK					Mascot
1536.7706	1536.7607	-0.0099	-6	422	434	RPADATPYHFPHK					Mascot
1589.7667	1589.7869	0.0202	13	202	215	RQEEAGDTVFANPR					Mascot
1595.8275	1595.7886	-0.0389	-24	680	695	TDYVVAGADAGSKLTK					Mascot
1682.9509	1682.848	-0.1029	-61	1	16	MTKAPKPAPDIATLTK					Mascot
1694.9324	1694.8152	-0.1172	-69	639	655	DTAVAGKTVVFTGSLTK					Mascot
1730.9225	1730.8933	-0.0292	-17	493	507	GWVKEPADIFTLQAR					Mascot
1986.0292	1985.921	-0.1082	-54	64	83	FPELVSASSPTQTVGAAPAR					Mascot
2089.1289	2089.1365	0.0076	4	331	349	FAAEQATTVLEKIDIQVGR					Mascot
2190.0535	2190.2087	0.1552	71	203	223	QEEAGDTVFANPRNSAAGSLR					Mascot

4	Chromodomain-helicase-DNA-binding protein 7 OS=Gallus gallus GN=CHD7 PE=2 SV=1	CHD7_CHICK	339825.6	5.91	36	65	83.67	43.416

Peptide Information

Calc. Mass	Obsrv. Mass	± da	± ppm	Start Seq.	End Seq.	Sequence	Ion Score	C. I. %	Modification	Rank	Result Type
817.405	817.436	0.031	38	923	929	QDIDQAK					Mascot
883.442	883.4014	-0.0406	-46	1331	1336	RYPYER					Mascot
1077.584	1077.6084	0.0244	23	1613	1621	NLLVYGWGR					Mascot

1141.5242	1141.5587	0.0345	30	80	88	MHLMDQPNR				Mascot
1168.5845	1168.6143	0.0298	26	2952	2962	ATDKTEGTAFK				Mascot
1176.6418	1176.5784	-0.0634	-54	1734	1742	HHCNKVLLR			Carbamidomethyl (C)[3]	Mascot
1186.6063	1186.6746	0.0683	58	1539	1549	AELDIDALNGR				Mascot
1186.6063	1186.6746	0.0683	58	1539	1549	AELDIDALNGR				Mascot
1200.5896	1200.5991	0.0095	8	759	767	RYTEDLEFK				Mascot
1320.6287	1320.5984	-0.0303	-23	989	1000	NCILADEMGLGK			Carbamidomethyl (C)[2]	Mascot
1323.5609	1323.6545	0.0936	71	2014	2023	YFNGFVNMCR			Carbamidomethyl (C)[9], Oxidation (M)[8]	Mascot
1339.7151	1339.698	-0.0171	-13	1102	1112	CVVIDEAHRLK			Carbamidomethyl (C)[1]	Mascot
1363.6587	1363.6205	-0.0382	-28	2190	2201	SDIKEETDIADK				Mascot
1483.8228	1483.6877	-0.1351	-91	2705	2718	LLTGPVVREEGASR				Mascot
1499.7601	1499.7074	-0.0527	-35	1507	1521	GSTFAKASFVASGNR				Mascot
1499.7601	1499.7074	-0.0527	-35	1507	1521	GSTFAKASFVASGNR	11	0		Mascot
1501.8705	1501.994	0.1235	82	1179	1190	LQAILKPMMLRR			Oxidation (M)[8,9]	Mascot
1507.7614	1507.7189	-0.0425	-28	1055	1065	RTIQLYEMYFK			Oxidation (M)[8]	Mascot
1595.6919	1595.7886	0.0967	61	2233	2246	SISEKGSEEDEEEK				Mascot
1605.6915	1605.7777	0.0862	54	1867	1880	DEIDEFANSPPEDK				Mascot
1620.8414	1620.808	-0.0334	-21	2498	2512	HLLNGSLIDGEPPMK				Mascot
1647.8119	1647.7604	-0.0515	-31	1633	1645	RQLTEQDVETICR			Carbamidomethyl (C)[12]	Mascot
1706.8894	1706.8812	-0.0082	-5	1319	1331	CLDILEDYLIQRR			Carbamidomethyl (C)[1]	Mascot
1730.8232	1730.8933	0.0701	41	588	602	SDDYLPSVEPQPQQK				Mascot
1731.9568	1731.9423	-0.0145	-8	1001	1014	TIQSITFLYEIYLK				Mascot
1731.9568	1731.9423	-0.0145	-8	1001	1014	TIQSITFLYEIYLK				Mascot
1763.8673	1763.9591	0.0918	52	2599	2613	LHPTYTVDMPSYVPK			Oxidation (M)[9]	Mascot
1787.7878	1787.8606	0.0728	41	198	212	GDFSMQQHGQPQQQR			Oxidation (M)[5]	Mascot
1792.8099	1792.9136	0.1037	58	1157	1171	FPSETTFMQEFGDLK			Oxidation (M)[8]	Mascot
1792.9375	1792.9136	-0.0239	-13	2498	2513	HLLNGSLIDGEPPMKR			Oxidation (M)[14]	Mascot
1801.9589	1801.8964	-0.0625	-35	2482	2497	TQMELLQAGLSRTPTR				Mascot
1888.9263	1888.9054	-0.0209	-11	1056	1070	TIQLYEMYFKDPQGR				Mascot
1891.8879	1891.907	0.0191	10	2170	2187	STEQTESKVEGSENPAAK				Mascot
1913.8416	1913.9198	0.0782	41	457	472	NMQQNRPFMGMSSTPR			Oxidation (M)[2,9]	Mascot
1925.9097	1925.9506	0.0409	21	1812	1828	ADSTLCFLERVGMPDAK			Carbamidomethyl (C)[6], Oxidation (M)[13]	Mascot
1929.8364	1929.8978	0.0614	32	457	472	NMQQNRPFMGMSSTPR			Oxidation (M)[2,9,11]	Mascot
1974.9979	1974.9341	-0.0638	-32	2431	2448	ESQVVSENGQEKVVDLSK				Mascot
1985.975	1985.921	-0.054	-27	1745	1760	MLYYLRQEVIGDQADR			Oxidation (M)[1]	Mascot
1999.9178	1999.953	0.0352	18	940	955	EPEMERVERPPADDWK			Oxidation (M)[4]	Mascot

2036.1023	2035.9226	-0.1797	-88	2571	2589	IPVINLEDGTRLVGEDAPK					Mascot

5 Pentalenene synthase OS=Streptomyces exfoliatus GN=penA PE=1 SV=4 | PENA_STREX | 38206.7 | 5.19 | 13 | 62 | 68.884 | 15.569

Peptide Information

Calc. Mass	Obsrv. Mass	± da	± ppm	Start Seq.	End Seq.	Sequence	Ion Score	C. I. %	Modification	Rank	Result Type
810.3853	810.4101	0.0248	31	15	21	QSPDHAR					Mascot
812.4274	812.4083	-0.0191	-24	139	144	SARHWR					Mascot
1125.5759	1125.5614	-0.0145	-13	38	47	SDAAAERHLR					Mascot
1186.5634	1186.6746	0.1112	94	251	260	SVSHMQNEVR					Mascot
1186.5634	1186.6746	0.1112	94	251	260	SVSHMQNEVR					Mascot
1320.6399	1320.5984	-0.0415	-31	231	241	GEQNNMVMILR			Oxidation (M)[6]		Mascot
1413.7015	1413.6373	-0.0642	-45	251	262	SVSHMQNEVRAR					Mascot
1439.5865	1439.666	0.0795	55	127	138	TCEGMTPAWCAR			Carbamidomethyl (C)[2,10]		Mascot
1445.6914	1445.6254	-0.066	-46	249	260	SRSVSHMQNEVR			Oxidation (M)[7]		Mascot
1492.736	1492.7253	-0.0107	-7	231	242	GEQNNMVMILRR			Oxidation (M)[6,8]		Mascot
1595.6876	1595.7886	0.101	63	126	138	RTCEGMTPAWCAR			Carbamidomethyl (C)[3,11]		Mascot
1605.8557	1605.7777	-0.078	-49	263	275	LEQYLLLESCLPK			Carbamidomethyl (C)[10]		Mascot
1621.8156	1621.7865	-0.0291	-18	1	14	MPQDVDFHIPLPGR					Mascot
1621.8156	1621.7865	-0.0291	-18	1	14	MPQDVDFHIPLPGR					Mascot
1992.0033	1991.9492	-0.0541	-27	276	292	VGEIYQLDTAEREALER					Mascot

6 Dynein heavy chain 1, axonemal OS=Rattus norvegicus GN=Dnah1 PE=2 SV=2 | DYH1_RAT | 519025.4 | 5.64 | 47 | 60 | 45.927 | 20.235

Peptide Information

Calc. Mass	Obsrv. Mass	± da	± ppm	Start Seq.	End Seq.	Sequence	Ion Score	C. I. %	Modification	Rank	Result Type
800.4512	800.3853	-0.0659	-82	1465	1471	QLPVESK					Mascot
854.3746	854.4077	0.0331	39	3784	3790	IMMNEGK			Oxidation (M)[2,3]		Mascot
896.389	896.4455	0.0565	63	4227	4235	SSSMGGQSR					Mascot
947.4792	947.4465	-0.0327	-35	2125	2134	AGAIASDTNK					Mascot
1021.5425	1021.4824	-0.0601	-59	3509	3516	LSDRDFLR					Mascot
1042.4412	1042.4751	0.0339	33	2848	2855	NYGMSEWR					Mascot
1077.5762	1077.6084	0.0322	30	3695	3703	MEYIPVAVR					Mascot
1078.5891	1078.6062	0.0171	16	3996	4004	LIEHINPDK					Mascot
1139.5402	1139.5653	0.0251	22	2730	2739	MEDLGVAFNK			Oxidation (M)[1]		Mascot
1141.5121	1141.5587	0.0466	41	1135	1143	QQHVEDEEK					Mascot

1158.6517	1158.613	-0.0387	-33	4011	4020	LWLTSLPSNK		Mascot
1168.6361	1168.6143	-0.0218	-19	2236	2245	FLESSIAFVR		Mascot
1193.6083	1193.5657	-0.0426	-36	3099	3108	DTLLTMDQIK	Oxidation (M)[6]	Mascot
1200.5645	1200.5991	0.0346	29	4125	4135	YTAGEINYGGR		Mascot
1323.6144	1323.6545	0.0401	30	3964	3974	AEAMMRNSIER	Oxidation (M)[4]	Mascot
1326.7086	1326.6461	-0.0625	-47	1613	1624	ASVHDIIEMAIK		Mascot
1327.6893	1327.637	-0.0523	-39	676	686	EDFTFVSLLTR		Mascot
1338.6947	1338.6641	-0.0306	-23	2929	2940	ANLMAAYTGRVR	Oxidation (M)[4]	Mascot
1339.6093	1339.698	0.0887	66	3964	3974	AEAMMRNSIER	Oxidation (M)[4,5]	Mascot
1349.6882	1349.6195	-0.0687	-51	1262	1272	NVIESFKTMHK	Oxidation (M)[9]	Mascot
1422.741	1422.655	-0.086	-60	739	750	DSWISTLKVAMR	Oxidation (M)[11]	Mascot
1480.7543	1480.6757	-0.0786	-53	2667	2678	SHYTFNLRDLSK		Mascot
1497.7002	1497.7102	0.01	7	620	631	EVNLDYERSMNK		Mascot
1501.8599	1501.994	0.1341	89	550	562	VFTQRVVQANALR		Mascot
1507.7209	1507.7189	-0.002	-1	1147	1158	IQLMDQNNFQEK		Mascot
1513.6952	1513.6871	-0.0081	-5	620	631	EVNLDYERSMNK	Oxidation (M)[10]	Mascot
1514.8075	1514.6705	-0.137	-90	361	374	ALLPTDDALGHVHR		Mascot
1514.8075	1514.6705	-0.137	-90	361	374	ALLPTDDALGHVHR		Mascot
1589.8646	1589.7869	-0.0777	-49	2399	2413	ATLTASRSEAVAWVK		Mascot
1605.8955	1605.7777	-0.1178	-73	4285	4298	LLMVITQTLSDMLK		Mascot
1621.8904	1621.7865	-0.1039	-64	4285	4298	LLMVITQTLSDMLK	Oxidation (M)[3]	Mascot
1621.8904	1621.7865	-0.1039	-64	4285	4298	LLMVITQTLSDMLK	Oxidation (M)[3]	Mascot
1646.8246	1646.7733	-0.0513	-31	303	316	HPDLQQPQTIEQGR		Mascot
1647.8337	1647.7604	-0.0733	-44	1781	1798	FGGAPAGPAGTGKTETT K		Mascot
1652.9054	1652.7756	-0.1298	-79	256	269	VQVPFQVLPGRCPR	Carbamidomethyl (C)[12]	Mascot
1706.8993	1706.8812	-0.0181	-11	2250	2265	EVVASTNSNLTMSLLK		Mascot
1730.8569	1730.8933	0.0364	21	3352	3368	ADKSQSPGQPPGAHPTR		Mascot
1763.8138	1763.9591	0.1453	82	3265	3278	ACTSICQWVRAMHK	Carbamidomethyl (C)[2,6], Oxidation (M)[12]	Mascot
1774.8574	1774.8988	0.0414	23	1962	1975	RENPTMNEELICLR	Carbamidomethyl (C)[12]	Mascot
1801.9816	1801.8964	-0.0852	-47	2789	2803	LKLVLFVDAMSHICR	Carbamidomethyl (C)[14]	Mascot
1873.9048	1873.9302	0.0254	14	2566	2583	NLVDINFVCAMGPPGGG R	Carbamidomethyl (C)[9]	Mascot
1888.9586	1888.9054	-0.0532	-28	1276	1291	QFKDIPACQDVALDIR	Carbamidomethyl (C)[8]	Mascot
1974.9735	1974.9341	-0.0394	-20	1963	1978	ENPTMNEELICLRAIR	Carbamidomethyl (C)[11], Oxidation (M)[5]	Mascot
1999.8815	1999.953	0.0715	36	1932	1948	LSSEQLSSQDHYDFGMR		Mascot
2002.9579	2003.0692	0.1113	56	2593	2608	HFNYLSFIEMDEVSKK	Oxidation (M)[10]	Mascot
2036.0481	2035.9226	-0.1255	-62	379	397	QVSFSKCINTGISLPEAG	Carbamidomethyl (C)[7]	Mascot

						K					
2087.092	2087.0459	-0.0461	-22	658	675	VPNQGLVSVPEYPFREQ K					Mascot
2089.0466	2089.1365	0.0899	43	3774	3790	LMFAFLLCVRIMMNEGK			Carbamidomethyl (C)[8], Oxidation (M)[2]		Mascot
2657.2051	2657.2825	0.0774	29	755	775	DMSKGWYNLYETNWEV YLMSK					Mascot
2721.4583	2721.22	-0.2383	-88	1316	1338	HWEVLSNEININVRPKAN LTFAR					Mascot
2888.3992	2888.6157	0.2165	75	1799	1824	DLGKALAIQTVVFNCSDQ LDFMAMGK			Carbamidomethyl (C)[15], Oxidation (M)[22]		Mascot
2888.3992	2888.6157	0.2165	75	1799	1824	DLGKALAIQTVVFNCSDQ LDFMAMGK			Carbamidomethyl (C)[15], Oxidation (M)[22]		Mascot
3137.6016	3137.4365	-0.1651	-53	2521	2547	NFIFFIDDLNMPALETYGA QPPIELLR					Mascot
3137.6016	3137.4365	-0.1651	-53	2521	2547	NFIFFIDDLNMPALETYGA QPPIELLR					Mascot

7	Scarecrow-like protein 9 OS=Arabidopsis thaliana GN=SCL9 PE=2 SV=1	SCL9_ARATH	81605	5.99	17	60	45.927	16.748	

Peptide Information

Calc. Mass	Obsrv. Mass	± da	± ppm	Start Seq.	End Seq.	Sequence	Ion Score	C. I. %	Modification	Rank	Result Type
1016.5119	1016.5391	0.0272	27	217	224	REIEEANR					Mascot
1021.5312	1021.4824	-0.0488	-48	645	652	VERPETYK					Mascot
1141.5485	1141.5587	0.0102	9	549	558	AENLHDESVK					Mascot
1171.5929	1171.4989	-0.094	-80	472	481	FSMYGSPKVR					Mascot
1186.6725	1186.6746	0.0021	2	362	371	CAGQLLKQIR			Carbamidomethyl (C)[1]		Mascot
1186.6725	1186.6746	0.0021	2	362	371	CAGQLLKQIR			Carbamidomethyl (C)[1]		Mascot
1480.6584	1480.6757	0.0173	12	252	263	DEICVEEERSSK			Carbamidomethyl (C)[4]		Mascot
1482.8356	1482.6882	-0.1474	-99	511	523	LFGVPFEYKAIAK					Mascot
1497.7961	1497.7102	-0.0859	-57	675	686	TSLHKVHTFYHK					Mascot
1620.8016	1620.808	0.0064	4	225	237	FNPEENELIVNFR					Mascot
1646.8285	1646.7733	-0.0552	-34	122	135	YPPSPERNLAFAER					Mascot
1647.8247	1647.7604	-0.0643	-39	420	432	AHQLFLACCPFRK			Carbamidomethyl (C)[8,9]		Mascot
1763.8302	1763.9591	0.1289	73	98	112	VCMLQESLDLEAAER			Carbamidomethyl (C)[2]		Mascot
1775.0175	1774.8988	-0.1187	-67	396	412	LAGTGSQIYKGIVSKPR					Mascot
1875.9819	1875.9926	0.0107	6	658	674	AMRSGLVQVPFDPSIMK					Mascot
1891.9769	1891.907	-0.0699	-37	658	674	AMRSGLVQVPFDPSIMK			Oxidation (M)[2]		Mascot
1926.0001	1925.9506	-0.0495	-26	275	292	SDVVDKILVHVPGGESM K			Oxidation (M)[17]		Mascot
1992.0001	1991.9492	-0.0509	-26	1	18	MITEPSLTGISGMVNRNR			Oxidation (M)[1]		Mascot
2377.2083	2377.1819	-0.0264	-11	384	405	LAHCFANGLEARLAGTG SQIYK			Carbamidomethyl (C)[4]		Mascot

8	DNA ligase OS=Rhodopseudomonas palustris (strain ATCC BAA-98 / CGA009) GN=ligA PE=3 SV=2	DNLJ_RHOPA	78800.7	5.53	17	59	37.916	16.752	

Peptide Information

Calc. Mass	Obsrv. Mass	± da	± ppm	Start Seq.	End Seq.	Sequence	Ion Score	C. I. %	Modification	Rank	Result Type
800.4625	800.3853	-0.0772	-96	343	349	IDIQVGR					Mascot
801.4577	801.4226	-0.0351	-44	506	512	ARNAELK					Mascot
817.4526	817.436	-0.0166	-20	638	645	RDTAVAGK					Mascot
1016.6099	1016.5391	-0.0708	-70	668	678	LGAKVAGSVSK					Mascot
1186.6791	1186.6746	-0.0045	-4	226	236	DVTVTASRPLK					Mascot
1186.6791	1186.6746	-0.0045	-4	226	236	DVTVTASRPLK					Mascot
1193.5909	1193.5657	-0.0252	-21	307	315	VDRLDYQER					Mascot
1439.7013	1439.666	-0.0353	-25	679	692	KTDYVVAGEDAGSK					Mascot
1480.7278	1480.6757	-0.0521	-35	513	525	LEQLEGYGETSVR					Mascot
1481.7206	1481.6935	-0.0271	-18	562	574	HYGTLDAFLSAMR					Mascot
1482.77	1482.6882	-0.0818	-55	310	321	LDYQERLGFVSR					Mascot
1497.7156	1497.7102	-0.0054	-4	562	574	HYGTLDAFLSAMR			Oxidation (M)[12]		Mascot
1595.8428	1595.7886	-0.0542	-34	293	306	RASLPYDIDGVVYK					Mascot
1682.8457	1682.848	0.0023	1	382	397	GIGNDGEPIRDGVDIR					Mascot
1730.9225	1730.8933	-0.0292	-17	493	507	GWVQEPADIFTLKAR					Mascot
1991.9346	1991.9492	0.0146	7	28	44	LEIEGHDKAYYQDDAPK					Mascot
2036.0295	2035.9226	-0.1069	-53	508	525	NAELKLEQLEGYGETSV R					Mascot
2089.1289	2089.1365	0.0076	4	331	349	FAAEQATTVLEKIDIQVG R					Mascot
2189.9985	2190.2087	0.2102	96	36	54	AYYQDDAPKISDADYDAL R					Mascot

9	Glutamate--tRNA ligase 2 OS=Novosphingobium aromaticivorans (strain DSM 12444) GN=gltX2 PE=3 SV=1	SYE2_NOVAD	53228.5	5.49	13	57	0	10.098	

Peptide Information

Calc. Mass	Obsrv. Mass	± da	± ppm	Start Seq.	End Seq.	Sequence	Ion Score	C. I. %	Modification	Rank	Result Type
972.522	972.4341	-0.0879	-90	124	131	RELAQAER					Mascot
1158.5287	1158.613	0.0843	73	216	225	GDDHINNAFR					Mascot
1200.6624	1200.5991	-0.0633	-53	349	360	LATLAPGFAPDK					Mascot
1214.6389	1214.6959	0.057	47	262	272	RHGALGVDAYR					Mascot
1320.639	1320.5984	-0.0406	-31	2	15	ASATDTVSAGSTPR					Mascot
1467.6744	1467.656	-0.0184	-13	1	15	MASATDTVSAGSTPR			Oxidation (M)[1]		Mascot
1509.7478	1509.7305	-0.0173	-11	113	124	CYLTQEELAARR			Carbamidomethyl (C)[1]		Mascot

1527.8068	1527.7321	-0.0747	-49	22	36	FAPSPTGFLHIGGAR					Mascot
1694.8014	1694.8152	0.0138	8	1	17	MASATDTVSAGSTPRDK					Mascot
1801.9457	1801.8964	-0.0493	-27	97	112	HAEVAHKLLEAGHAYR					Mascot
2089.1541	2089.1365	-0.0176	-8	455	475	ASLTGQTTSPGIFDVLALL GK					Mascot
2888.5452	2888.6157	0.0705	24	374	400	VRAADINELAAGSVFLFA QRPLAMAEK					Mascot
2888.5452	2888.6157	0.0705	24	374	400	VRAADINELAAGSVFLFA QRPLAMAEK					Mascot
3137.5989	3137.4365	-0.1624	-52	232	261	GMHAIEGGWPDPVYAHI PLIHGADGAKLSK					Mascot
3137.5989	3137.4365	-0.1624	-52	232	261	GMHAIEGGWPDPVYAHI PLIHGADGAKLSK					Mascot

10 Lysine--tRNA ligase OS=Oryza sativa subsp. japonica GN=Os03g0586800 PE=2 SV=1 | SYK_ORYSJ | 68913.8 | 5.87 | 16 | 56 | 0 | 10.107

Peptide Information

Calc. Mass	Obsrv. Mass	± da	± ppm	Start Seq.	End Seq.	Sequence	Ion Score	C. I. %	Modification	Rank	Result Type
801.473	801.4226	-0.0504	-63	269	274	HIFKTR					Mascot
896.4876	896.4455	-0.0421	-47	511	517	FELFVNK					Mascot
953.4761	953.4969	0.0208	22	447	454	YLIDACAK			Carbamidomethyl (C)[6]		Mascot
1077.5687	1077.6084	0.0397	37	535	542	QRFEEQLK					Mascot
1139.6208	1139.5653	-0.0555	-49	342	350	VYEIGKQFR					Mascot
1141.5848	1141.5587	-0.0261	-23	56	68	AAATAAASGEPPK					Mascot
1403.724	1403.6001	-0.1239	-88	591	602	EVLLFPAMKPQD			Oxidation (M)[8]		Mascot
1509.8497	1509.7305	-0.1192	-79	321	332	LYMRIAPELYLK					Mascot
1536.7648	1536.7607	-0.0041	-3	420	432	KIDMIEELEAMAK			Oxidation (M)[4]		Mascot
1646.9113	1646.7733	-0.138	-84	333	347	ELVVGGLDRVYEIGK					Mascot
1744.8534	1744.8921	0.0387	22	13	29	LAGLSAGCGEEPQQLSK			Carbamidomethyl (C)[8]		Mascot
1787.9578	1787.8606	-0.0972	-54	105	119	FLANITVADYIEKYK					Mascot
1792.9705	1792.9136	-0.0569	-32	503	517	SRPGLTERFELFVNK					Mascot
1792.9705	1792.9136	-0.0569	-32	503	517	SRPGLTERFELFVNK					Mascot
1875.9487	1875.9926	0.0439	23	143	159	AQSSKLLFYDLYGGGEK					Mascot
1914.0055	1913.9198	-0.0857	-45	258	272	YLDLMVNHEVRHIFK					Mascot
1930.0004	1929.8978	-0.1026	-53	258	272	YLDLMVNHEVRHIFK			Oxidation (M)[5]		Mascot
2100.0178	2099.8896	-0.1282	-61	225	244	SEGSAVPTPWAPGMGR NIEK			Oxidation (M)[14]		Mascot

Gel Idx/Pos	288/L16	Instr./Gel Origin	BA2151/Sample Project 20140814	Process Status	Analysis Succeeded
Plate [#] Name	[1] Sample Project 20140814	Instrument Sample Name		Spectra	11

Rank	Protein Name	Accession No.	Protein MW	Protein PI	Pep. Count	Protein Score	Protein Score C. I. %	Intensity Matched	Total Ion Score	Total Ion C. I. %	Confirmed
1	Alanine aminotransferase 2 OS=Hordeum vulgare PE=1 SV=1	ALA2_HORVU	53357.3	5.93	14	320	100	23.979	260	100	

Peptide Information

Calc. Mass	Obsrv. Mass	± da	± ppm	Start Seq.	End Seq.	Sequence	Ion Score	C. I. %	Modification	Rank	Result Type
915.5258	915.5275	0.0017	2	365	373	DGILASLAR					Mascot
960.5109	960.5087	-0.0022	-2	123	132	DAIASGIASR					Mascot
1022.5741	1022.5752	0.0011	1	23	31	GEIVIHAQR					Mascot
1023.5258	1023.5353	0.0095	9	419	427	APDAFYALR					Mascot
1023.5258	1023.5353	0.0095	9	419	427	APDAFYALR	64	99.967			Mascot
1089.4783	1089.488	0.0097	9	300	308	GYYGECGKR			Carbamidomethyl (C)[6]		Mascot
1096.5634	1096.5681	0.0047	4	87	96	TLFSADSISR					Mascot
1197.7136	1197.5985	-0.1151	-96	97	107	AKQILAMIPGR					Mascot
1213.7086	1213.5964	-0.1122	-92	97	107	AKQILAMIPGR			Oxidation (M)[7]		Mascot
1243.7004	1243.7025	0.0021	2	362	373	AEKDGILASLAR					Mascot
1407.7379	1407.7445	0.0066	5	415	427	AANKAPDAFYALR					Mascot
1500.7482	1500.7115	-0.0367	-24	309	322	GGYFEITGFSAPVR					Mascot
1500.7482	1500.7115	-0.0367	-24	309	322	GGYFEITGFSAPVR	61	99.935			Mascot
1656.8494	1656.8071	-0.0423	-26	308	322	RGGYFEITGFSAPVR					Mascot
1778.7843	1778.8208	0.0365	21	469	482	FTVFHEAFMSEYRD					Mascot
2640.3933	2640.4429	0.0496	19	428	452	LLESTGIVVVPGSGFGQV PGTWHFR					Mascot
2640.3933	2640.4429	0.0496	19	428	452	LLESTGIVVVPGSGFGQV PGTWHFR	134	100			Mascot
3146.5286	3146.6016	0.073	23	133	161	DGFPANADDIFLTDGASP GVHLMMQLLIR			Oxidation (M)[23,24]		Mascot

Rank	Protein Name	Accession No.	Protein MW	Protein PI	Pep. Count	Protein Score	Protein Score C. I. %	Intensity Matched	Total Ion Score	Total Ion C. I. %	Confirmed
2	Alanine aminotransferase 2 OS=Panicum miliaceum PE=1 SV=1	ALA2_PANMI	53276.1	5.82	7	154	100	14.486	138	100	

Peptide Information

Calc. Mass	Obsrv. Mass	± da	± ppm	Start Seq.	End Seq.	Sequence	Ion Score	C. I. %	Modification	Rank	Result Type
1023.5258	1023.5353	0.0095	9	419	427	APDAFYALR					Mascot
1023.5258	1023.5353	0.0095	9	419	427	APDAFYALR	64	99.967			Mascot

1089.4783	1089.488	0.0097	9	300	308	GYYGECGKR			Carbamidomethyl (C)[6]		Mascot
1373.7094	1373.7268	0.0174	13	1	13	MAATVAVENLNPK			Oxidation (M)[1]		Mascot
1407.7379	1407.7445	0.0066	5	415	427	AANKAPDAFYALR					Mascot
1484.7203	1484.7324	0.0121	8	309	322	GGYMEITGFSAPVR					Mascot
1500.7152	1500.7115	-0.0037	-2	309	322	GGYMEITGFSAPVR			Oxidation (M)[4]		Mascot
1500.7152	1500.7115	-0.0037	-2	309	322	GGYMEITGFSAPVR	74	99.996	Oxidation (M)[4]		Mascot
1640.8214	1640.8143	-0.0071	-4	308	322	RGGYMEITGFSAPVR					Mascot
1656.8163	1656.8071	-0.0092	-6	308	322	RGGYMEITGFSAPVR			Oxidation (M)[5]		Mascot
1714.8986	1714.823	-0.0756	-44	393	407	AEGAMYLFPQIHLPK					Mascot

3	Minor spike protein OS=Enterobacteria phage G4 GN=H PE=3 SV=1	VGH_BPG4	35684.7	6.86	11	62	68.884	19.752

Peptide Information

Calc. Mass	Obsrv. Mass	± da	± ppm	Start Seq.	End Seq.	Sequence	Ion Score	C. I. %	Modification	Rank	Result Type
915.4894	915.5275	0.0381	42	250	258	VGADIDAVR					Mascot
963.5179	963.6027	0.0848	88	95	103	LMEAVGLSK			Oxidation (M)[2]		Mascot
963.5179	963.6027	0.0848	88	95	103	LMEAVGLSK			Oxidation (M)[2]		Mascot
1435.7461	1435.7279	-0.0182	-13	95	108	LMEAVGLSKSASDK					Mascot
1677.8993	1677.8005	-0.0988	-59	2	20	FGSIAGGIASALAGGLMGK					Mascot
1693.8942	1693.7909	-0.1033	-61	2	20	FGSIAGGIASALAGGLMGK			Oxidation (M)[17]		Mascot
1791.8701	1791.8958	0.0257	14	114	128	DYLAAAFPELNPWER					Mascot
1808.9398	1808.8041	-0.1357	-75	1	20	MFGSIAGGIASALAGGLMGK					Mascot
1808.9398	1808.8041	-0.1357	-75	1	20	MFGSIAGGIASALAGGLMGK					Mascot
1824.9347	1824.8032	-0.1315	-72	1	20	MFGSIAGGIASALAGGLMGK			Oxidation (M)[1]		Mascot
1835.9531	1835.8195	-0.1336	-73	200	216	ESMSRVGAILENTSLTK					Mascot
1851.9481	1851.957	0.0089	5	200	216	ESMSRVGAILENTSLTK			Oxidation (M)[3]		Mascot
2186.0547	2185.9954	-0.0593	-27	226	244	QMLTQAQTAGQYFTNDQIK					Mascot
2656.3425	2656.4346	0.0921	35	54	81	SAIQGSTVPNSQEAAPSAISGILADSGK					Mascot
2656.3425	2656.4346	0.0921	35	54	81	SAIQGSTVPNSQEAAPSAISGILADSGK					Mascot
2685.3301	2685.4236	0.0935	35	226	248	QMLTQAQTAGQYFTNDQIKELTR					Mascot
3146.5349	3146.6016	0.0667	21	21	53	LFGGGQSADSTGIQGNVLASDNNVVGANDAGIK					Mascot

4	DNA-directed RNA polymerase subunit beta' OS=Mycoplasma genitalium (strain ATCC 33530 / G-37 / NCTC 10195) GN=rpoC PE=3 SV=1	RPOC_MYCGE	146195.7	9.35	24	62	63.442	17.217

Peptide Information

Calc. Mass	Obsrv. Mass	± da	± ppm	Start Seq.	End Seq.	Sequence	Ion Score	C. I. %	Modification	Rank	Result Type
838.4305	838.3538	-0.0767	-91	41	47	AETINYK					Mascot
870.3696	870.3408	-0.0288	-33	879	886	GMTDTAMK			Oxidation (M)[2]		Mascot
1023.6098	1023.5353	-0.0745	-73	184	191	RVIGYIFR					Mascot
1023.6098	1023.5353	-0.0745	-73	184	191	RVIGYIFR					Mascot
1170.5493	1170.6429	0.0936	80	879	889	GMTDTAMKTAK			Oxidation (M)[2]		Mascot
1350.6318	1350.7031	0.0713	53	805	817	DNSIVVMADSGAR			Oxidation (M)[7]		Mascot
1484.7314	1484.7324	0.001	1	818	830	GNISNFTQLFGMR					Mascot
1500.7264	1500.7115	-0.0149	-10	818	830	GNISNFTQLFGMR			Oxidation (M)[12]		Mascot
1500.7264	1500.7115	-0.0149	-10	818	830	GNISNFTQLFGMR			Oxidation (M)[12]		Mascot
1516.8516	1516.7238	-0.1278	-84	970	982	QICATSIKEVLVR			Carbamidomethyl (C)[3]		Mascot
1522.8047	1522.7615	-0.0432	-28	978	989	EVLVRSVIYCER			Carbamidomethyl (C)[10]		Mascot
1555.7388	1555.8936	0.1548	100	748	760	QNYFDSADQQVLK					Mascot
1561.8698	1561.7623	-0.1075	-69	929	941	SLIESLFDRIVNR					Mascot
1693.8392	1693.7909	-0.0483	-29	361	375	LLQESVDALFDNSSR					Mascot
1726.9222	1726.9022	-0.02	-12	344	359	ILEDGTVPSIVVNNEK					Mascot
1778.8848	1778.8208	-0.064	-36	728	743	YSTFSSTTVSVFDIPK					Mascot
1788.9603	1788.9532	-0.0071	-4	250	264	IDLNLEFSRLNDALR					Mascot
1790.9324	1790.9417	0.0093	5	475	489	NQDDLIWGIVYDVIK					Mascot
1801.9192	1801.863	-0.0562	-31	452	467	FDENGNEIRPIAASIR					Mascot
1849.9403	1849.8336	-0.1067	-58	360	375	RLLQESVDALFDNSSR					Mascot
1867.9913	1867.9723	-0.019	-10	590	606	KGQLGEGIIFSTVYEAR					Mascot
1902.9994	1902.9763	-0.0231	-12	1209	1226	MPVIAINQVFGIDEAASK					Mascot
1908.9823	1908.9388	-0.0435	-23	102	118	MGHIALVSPVAHIWMSK			Oxidation (M)[1,15]		Mascot
1973.0702	1973.001	-0.0692	-35	938	954	IVNRYSITPIVDPETQK					Mascot
2622.312	2622.4661	0.1541	59	468	489	QAEDMIKNQDDLIWGIVY DVIK			Oxidation (M)[5]		Mascot
2655.3083	2655.437	0.1287	48	567	589	DGRPIVTPTQDMVLGNY YLTTER			Oxidation (M)[12]		Mascot
2688.3291	2688.4185	0.0894	33	1268	1292	IPAGTGFLTDEELTFLGS KTVAEEY					Mascot

5	Glutamate 5-kinase OS=Bradyrhizobium japonicum (strain USDA 110) GN=proB PE=3 SV=1	PROB_BRAJA	39573	6.28	12	56	0	2.058	

Peptide Information

Calc. Mass	Obsrv. Mass	± da	± ppm	Start Seq.	End Seq.	Sequence	Ion Score	C. I. %	Modification	Rank	Result Type

Calc. Mass	Obsrv. Mass	± da	± ppm	Start Seq.	End Seq.	Sequence	Ion Score	C. I. %	Modification	Rank	Result Type
806.4155	806.3668	-0.0487	-60	308	314	VEGQFAR					Mascot
1096.6725	1096.5681	-0.1044	-95	296	307	AGASLLPAGVIK					Mascot
1170.663	1170.6429	-0.0201	-17	272	281	RWIAGTLEPK					Mascot
1408.6777	1408.7418	0.0641	46	31	43	TSWLAALADDMAK			Oxidation (M)[11]		Mascot
1473.7731	1473.8744	0.1013	69	229	243	IATTGGTHMLIASGK			Oxidation (M)[9]		Mascot
1516.8231	1516.7238	-0.0993	-65	308	321	VEGQFARGDAVIVR					Mascot
1714.9447	1714.823	-0.1217	-71	47	63	EGRDVLVVSSGSIALGR					Mascot
1786.9156	1786.837	-0.0786	-44	31	46	TSWLAALADDMAKLHK			Oxidation (M)[11]		Mascot
1791.9211	1791.8958	-0.0253	-14	257	271	CTWFLTPANPITSRK			Carbamidomethyl (C)[1]		Mascot
1849.9113	1849.8336	-0.0777	-42	27	43	GEVRTSWLAALADDMAK			Oxidation (M)[15]		Mascot
2093.1714	2093.0232	-0.1482	-71	71	91	GPLKLEESQAAAAVGQIA LAR					Mascot
2186.1057	2185.9954	-0.1103	-50	347	366	SSPDVMTILGISGRAEMI HR			Oxidation (M)[6]		Mascot

6	Translation factor GUF1, mitochondrial OS=Bos taurus GN=GUF1 PE=2 SV=1	GUF1_BOVIN	75175.5	8.86	16	55	0	15.691

Peptide Information

Calc. Mass	Obsrv. Mass	± da	± ppm	Start Seq.	End Seq.	Sequence	Ion Score	C. I. %	Modification	Rank	Result Type
1022.4943	1022.5752	0.0809	79	488	495	IMMLCQAR			Carbamidomethyl (C)[5]		Mascot
1023.5265	1023.5353	0.0088	9	248	255	AHCRNPLR			Carbamidomethyl (C)[3]		Mascot
1023.5265	1023.5353	0.0088	9	248	255	AHCRNPLR			Carbamidomethyl (C)[3]		Mascot
1170.6113	1170.6429	0.0316	27	196	205	IDLKNADPER					Mascot
1407.6627	1407.7445	0.0818	58	1	11	MWTLAGQGWWR			Oxidation (M)[1]		Mascot
1473.7499	1473.8744	0.1245	84	2	13	WTLAGQGWWRGR					Mascot
1484.6509	1484.7324	0.0815	55	58	69	IDMSCFPVENTR			Carbamidomethyl (C)[5], Oxidation (M)[3]		Mascot
1626.7727	1626.801	0.0283	17	497	509	AVQKNMMYIDQNR			Oxidation (M)[6]		Mascot
1691.8673	1691.8809	0.0136	8	213	227	VFDIPGDECIKISAK			Carbamidomethyl (C)[9]		Mascot
1700.9218	1700.8652	-0.0566	-33	451	465	EITIINPAQFPDKSK					Mascot
1742.9324	1742.814	-0.1184	-68	449	463	EKEITIINPAQFPDK					Mascot
1788.9855	1788.9532	-0.0323	-18	268	285	GVIANVALFDGVVSKGDK					Mascot
1790.8994	1790.9417	0.0423	24	209	223	QIEKVFDIPGDECIK			Carbamidomethyl (C)[13]		Mascot
1868.0237	1867.9723	-0.0514	-28	374	390	SAIEKLTLNDSSVVVHR					Mascot
1903.0297	1902.9763	-0.0534	-28	26	43	GLLWPHLAPARGTAAES R					Mascot
2368.2793	2368.1907	-0.0886	-37	554	574	MDILLNGSIVEELVTVVHK DK			Oxidation (M)[1]		Mascot
2652.4019	2652.4846	0.0827	31	464	487	SKVTEYLEPVVLGTIITPD EYTGK					Mascot

7	Valine--tRNA ligase OS=Lactobacillus acidophilus	SYV_LACAC	101735.4	5.44	19	55	0	17.928

(strain ATCC 700396 / NCK56 / N2 / NCFM) GN=valS
PE=3 SV=1

Peptide Information

Calc. Mass	Obsrv. Mass	± da	± ppm	Start Seq.	End Seq.	Sequence	Ion Score	C. I. %	Modification	Rank	Result Type
915.5509	915.5275	-0.0234	-26	320	327	EALVKDLK					Mascot
960.536	960.5087	-0.0273	-28	872	879	IQDLKESK					Mascot
1021.6153	1021.5305	-0.0848	-83	507	515	DVVLHGLIR					Mascot
1023.5469	1023.5353	-0.0116	-11	134	142	LGLSLDYSR					Mascot
1023.5469	1023.5353	-0.0116	-11	134	142	LGLSLDYSR	2	0			Mascot
1197.6085	1197.5985	-0.01	-8	692	701	LWLSMPHEGK					Mascot
1213.6034	1213.5964	-0.007	-6	692	701	LWLSMPHEGK			Oxidation (M)[5]		Mascot
1225.567	1225.5969	0.0299	24	565	574	MDAAWNFINK			Oxidation (M)[1]		Mascot
1350.6722	1350.7031	0.0309	23	702	713	SIMTASYPVTHK			Oxidation (M)[3]		Mascot
1373.6484	1373.7268	0.0784	57	622	633	LFGEYQFGEAGR					Mascot
1408.6954	1408.7418	0.0464	33	421	433	KTGETYVGEEAPK					Mascot
1435.7369	1435.7279	-0.009	-6	110	120	EAFVKQVWDWK					Mascot
1640.7697	1640.8143	0.0446	27	858	871	MADYESQLSGVRER					Mascot
1656.7646	1656.8071	0.0425	26	858	871	MADYESQLSGVRER			Oxidation (M)[1]		Mascot
1818.0272	1817.9269	-0.1003	-55	34	50	KAHPYSIVIPPPNVTGK					Mascot
1824.9603	1824.8032	-0.1571	-86	837	853	LSNKGFVDHAPEAVVNK					Mascot
1835.8785	1835.8195	-0.059	-32	560	574	FSYTKMDAAWNFINK					Mascot
1851.8827	1851.957	0.0743	40	522	538	MSKSLGNGIDPMDVIDK			Oxidation (M)[1,12]		Mascot
1909.0154	1908.9388	-0.0766	-40	492	506	MIFQSLHFTKERPFK					Mascot
2538.2117	2538.3054	0.0937	37	70	92	RMEGYDTLYLPGMDHAG IATQAK					Mascot
2652.2566	2652.4846	0.228	86	739	761	MEVNAPMSSEIDIMIKLD DLNNK			Oxidation (M)[1,7]		Mascot
2656.3506	2656.4346	0.084	32	171	193	GEYIINWDPTLETALSDIE VIHK					Mascot
2656.3506	2656.4346	0.084	32	171	193	GEYIINWDPTLETALSDIE VIHK					Mascot
2668.2515	2668.373	0.1215	46	739	761	MEVNAPMSSEIDIMIKLD DLNNK			Oxidation (M)[1,7,14]		Mascot

8 Median body protein OS=Giardia intestinalis PE=2 SV=1 MEDB_GIAIN 100578.8 5.31 22 52 0 28.053

Peptide Information

Calc. Mass	Obsrv. Mass	± da	± ppm	Start Seq.	End Seq.	Sequence	Ion Score	C. I. %	Modification	Rank	Result Type
1022.5265	1022.5752	0.0487	48	467	474	YDQLAREK					Mascot

Calc. Mass	Obsrv. Mass	± da	± ppm	Start	End	Sequence	Ion	C. I. %	Modification	Rank	Result Type
1023.4523	1023.5353	0.083	81	306	314	AQEMSASQR			Oxidation (M)[4]		Mascot
1023.4523	1023.5353	0.083	81	306	314	AQEMSASQR			Oxidation (M)[4]		Mascot
1116.5645	1116.5822	0.0177	16	351	361	ADAGEAALRDK					Mascot
1192.6064	1192.5736	-0.0328	-28	662	671	VMAMELTELR					Mascot
1213.6722	1213.5964	-0.0758	-62	542	551	VMRTELLHSK					Mascot
1229.6671	1229.5897	-0.0774	-63	542	551	VMRTELLHSK			Oxidation (M)[2]		Mascot
1231.6464	1231.5696	-0.0768	-62	686	695	ARDVEMLELR					Mascot
1247.6412	1247.5634	-0.0778	-62	686	695	ARDVEMLELR			Oxidation (M)[6]		Mascot
1408.6703	1408.7418	0.0715	51	315	326	YSANQAQELQEK					Mascot
1464.7476	1464.7604	0.0128	9	611	623	TATVRETEMSALR					Mascot
1465.7389	1465.7679	0.029	20	662	673	VMAMELTELREK			Oxidation (M)[2]		Mascot
1500.8016	1500.7115	-0.0901	-60	824	836	NTTDNLLTRDIPK					Mascot
1500.8016	1500.7115	-0.0901	-60	824	836	NTTDNLLTRDIPK					Mascot
1522.7544	1522.7615	0.0071	5	214	225	NLPHMDDLQRQR					Mascot
1571.7772	1571.8849	0.1077	69	191	203	QDTNSRLPQEIDR					Mascot
1618.7854	1618.8442	0.0588	36	239	253	GNTSGVMAEIENIQR					Mascot
1672.8977	1672.8159	-0.0818	-49	602	615	ITELEQQQRTATVR					Mascot
1674.8956	1674.8202	-0.0754	-45	841	856	LIERPAGTTTMGRSGK					Mascot
1758.7889	1758.8611	0.0722	41	254	269	QIDDANSSASSEHELR					Mascot
1790.8813	1790.9417	0.0604	34	238	253	RGNTSGVMAEIENIQR			Oxidation (M)[8]		Mascot
1792.8245	1792.8221	-0.0024	-1	792	809	AMTSTGMGVSAASPAFYK			Oxidation (M)[2]		Mascot
1792.8245	1792.8221	-0.0024	-1	792	809	AMTSTGMGVSAASPAFYK			Oxidation (M)[2]		Mascot
1808.8195	1808.8041	-0.0154	-9	792	809	AMTSTGMGVSAASPAFYK			Oxidation (M)[2,7]		Mascot
1808.8195	1808.8041	-0.0154	-9	792	809	AMTSTGMGVSAASPAFYK			Oxidation (M)[2,7]		Mascot
1849.9515	1849.8336	-0.1179	-64	343	359	QLHEALERADAGEAALR					Mascot
2093.0042	2093.0232	0.019	9	789	809	ATKAMTSTGMGVSAASPAFYK			Oxidation (M)[5]		Mascot
2186.0144	2185.9954	-0.019	-9	2	19	SEAMVFSKIDEYTLFMSR			Oxidation (M)[4,16]		Mascot
2678.3091	2678.3438	0.0347	13	315	337	YSANQAQELQEKAMQAEELLQQK					Mascot

9	50S ribosomal protein L3 OS=Acidovorax sp. (strain JS42) GN=rplC PE=3 SV=1	RL3_ACISJ	23374.3	10.13	8	52	0	12.73	6	0

Protein Group

50S ribosomal protein L3 OS=Acidovorax ebreus (strain TPSY) GN=rplC PE=3 SV=1	RL3_ACIET	23374.3	10.130000114409

Peptide Information

Calc. Mass	Obsrv. Mass	± da	± ppm	Start	End	Sequence	Ion	C. I. %	Modification	Rank	Result Type

				Seq.	Seq.		Score				
1116.6372	1116.5822	-0.055	-49	2	12	SLSNSLGLLGR					Mascot
1247.6776	1247.5634	-0.1142	-92	1	12	MSLSNSLGLLGR					Mascot
1464.8281	1464.7604	-0.0677	-46	62	75	ASRVTKPEAGHLAK					Mascot
1622.7704	1622.7867	0.0163	10	147	162	SHNVPGSIGMAQDPGR					Mascot
1677.9171	1677.8005	-0.1166	-69	115	131	VDVQGTSIGKGFAGTIK					Mascot
1726.9963	1726.9022	-0.0941	-54	199	216	GAVPGSKGGFVTVRPAV K					Mascot
2640.3411	2640.4429	0.1018	39	169	192	MTGHLGDVTVTTQNLDV VRIDEAR					Mascot
2640.3411	2640.4429	0.1018	39	169	192	MTGHLGDVTVTTQNLDV VRIDEAR					Mascot
2656.3359	2656.4346	0.0987	37	169	192	MTGHLGDVTVTTQNLDV VRIDEAR			Oxidation (M)[1]		Mascot
2656.3359	2656.4346	0.0987	37	169	192	MTGHLGDVTVTTQNLDV VRIDEAR	6	0	Oxidation (M)[1]		Mascot
2688.384	2688.4185	0.0345	13	19	43	LFTDDGDAVPVTVVDVS NNRVTQVK					Mascot

10	Tetraacyldisaccharide 4'-kinase OS=Rickettsia peacockii (strain Rustic) GN=lpxK PE=3 SV=1	LPXK_RICPU	36242.5	9.22	10	52	0	10.202

Peptide Information

Calc. Mass	Obsrv. Mass	± da	± ppm	Start Seq.	End Seq.	Sequence	Ion Score	C. I. %	Modification	Rank	Result Type
1407.672	1407.7445	0.0725	52	47	60	VICVGNCSVGGTGK			Carbamidomethyl (C)[3,7]		Mascot
1436.8545	1436.7386	-0.1159	-81	61	72	TQIVMYLAKLLK			Oxidation (M)[5]		Mascot
1436.8545	1436.7386	-0.1159	-81	61	72	TQIVMYLAKLLK			Oxidation (M)[5]		Mascot
1464.825	1464.7604	-0.0646	-44	2	12	IKLLYPEFWQK					Mascot
1561.8698	1561.7623	-0.1075	-69	210	223	LINAQIVPSHNIDK					Mascot
1700.8391	1700.8652	0.0261	15	224	238	TKNYFAFSGIGNPER					Mascot
1791.0123	1790.9417	-0.0706	-39	210	225	LINAQIVPSHNIDKTK					Mascot
1849.9847	1849.8336	-0.1511	-82	239	254	FFATLKNYGLNITGYK					Mascot
2652.564	2652.4846	-0.0794	-30	13	34	RNIIAYLLLPISLIYQFLGY LR					Mascot
2656.4097	2656.4346	0.0249	9	35	60	ASLARPIMLPAKVICVGN CSVGGTGK			Carbamidomethyl (C)[15,19]		Mascot
2656.4097	2656.4346	0.0249	9	35	60	ASLARPIMLPAKVICVGN CSVGGTGK			Carbamidomethyl (C)[15,19]		Mascot
2672.4045	2672.4204	0.0159	6	35	60	ASLARPIMLPAKVICVGN CSVGGTGK			Carbamidomethyl (C)[15,19], Oxidation (M)[8]		Mascot
2672.4045	2672.4204	0.0159	6	35	60	ASLARPIMLPAKVICVGN CSVGGTGK			Carbamidomethyl (C)[15,19], Oxidation (M)[8]		Mascot
2719.4568	2719.3882	-0.0686	-25	152	176	DFTIVSVDSQRLFGNGFLI PAGPLR					Mascot

Gel Idx/Pos	289/L17	Instr./Gel Origin	BA2151/Sample Project 20140814	Process Status	Analysis Succeeded
Plate [#] Name	[1] Sample Project 20140814	Instrument Sample Name		Spectra	11

Rank	Protein Name	Accession No.	Protein MW	Protein PI	Pep. Count	Protein Score	Protein Score C. I. %	Intensity Matched	Total Ion Score	Total Ion C. I. %	Confirmed
1	Ribulose bisphosphate carboxylase large chain OS=Triticum aestivum GN=rbcL PE=1 SV=2	RBL_WHEAT	53444.6	6.22	28	858	100	58.535	661	100	

Peptide Information

Calc. Mass	Obsrv. Mass	± da	± ppm	Start Seq.	End Seq.	Sequence	Ion Score	C. I. %	Modification	Rank	Result Type
830.4366	830.4283	-0.0083	-10	440	446	EGNEIIR					Mascot
898.41	898.4123	0.0023	3	306	312	NHGMHFR					Mascot
912.472	912.4646	-0.0074	-8	296	303	AMHAVIDR					Mascot
914.405	914.3882	-0.0168	-18	306	312	NHGMHFR			Oxidation (M)[4]		Mascot
928.4669	928.441	-0.0259	-28	296	303	AMHAVIDR			Oxidation (M)[2]		Mascot
971.4073	971.4023	-0.005	-5	188	194	ACYECLR			Carbamidomethyl (C)[2,5]		Mascot
971.4073	971.4023	-0.005	-5	188	194	ACYECLR	24	0	Carbamidomethyl (C)[2,5]		Mascot
1021.5312	1021.5198	-0.0114	-11	33	41	DTDILAAFR					Mascot
1021.5312	1021.5198	-0.0114	-11	33	41	DTDILAAFR	56	99.763			Mascot
1037.4899	1037.4851	-0.0048	-5	351	358	DDFIEKDR					Mascot
1116.583	1116.5812	-0.0018	-2	422	431	VALEACVQAR			Carbamidomethyl (C)[6]		Mascot
1170.5586	1170.6375	0.0789	67	304	312	QKNHGMHFR			Oxidation (M)[6]		Mascot
1170.5586	1170.6375	0.0789	67	304	312	QKNHGMHFR			Oxidation (M)[6]		Mascot
1187.6644	1187.6611	-0.0033	-3	286	295	DNGLLLHIHR					Mascot
1247.6129	1247.5988	-0.0141	-11	218	227	FVFCAEAIYK			Carbamidomethyl (C)[4]		Mascot
1285.6859	1285.5776	-0.1083	-84	436	446	DLAREGNEIIR					Mascot
1293.6871	1293.6863	-0.0008	-1	340	350	EMTLGFVDLLR	90	100			Mascot
1309.6821	1309.6604	-0.0217	-17	340	350	EMTLGFVDLLR			Oxidation (M)[2]		Mascot
1325.6896	1325.6711	-0.0185	-14	306	316	NHGMHFRVLAK			Oxidation (M)[4]		Mascot
1339.6417	1339.6206	-0.0211	-16	467	477	FEFEPVDTIDK					Mascot
1407.6678	1407.6458	-0.022	-16	22	32	LTYYTPEYETK					Mascot
1451.6219	1451.6394	0.0175	12	202	213	DDENVNSQPFMR					Mascot
1465.7546	1465.765	0.0104	7	147	159	TFQGPPHGIQVER					Mascot
1465.7546	1465.765	0.0104	7	147	159	TFQGPPHGIQVER	102	100			Mascot
1502.8512	1502.849	-0.0022	-1	165	177	YGRPLLGCTIKPK			Carbamidomethyl (C)[8]		Mascot
1502.8512	1502.849	-0.0022	-1	165	177	YGRPLLGCTIKPK	6	0	Carbamidomethyl (C)[8]		Mascot

1518.741	1518.7408	-0.0002	0	216	227	DRFVFCAEAIYK			Carbamidomethyl (C)[6]		Mascot
1546.736	1546.7114	-0.0246	-16	451	463	WSPELAAACEVWK			Carbamidomethyl (C)[9]		Mascot
1651.8578	1651.8324	-0.0254	-15	464	477	AIKFEFEPVDTIDK					Mascot
1893.9739	1893.9507	-0.0232	-12	335	350	LEGEREMTLGFVDLLR			Oxidation (M)[7]		Mascot
1950.916	1950.9291	0.0131	7	237	253	GHYLNATAGTCEEMIKR			Carbamidomethyl (C)[11]		Mascot
1966.911	1966.9098	-0.0012	-1	237	253	GHYLNATAGTCEEMIKR			Carbamidomethyl (C)[11], Oxidation (M)[14]		Mascot
2041.0311	2041.0352	0.0041	2	340	356	EMTLGFVDLLRDDFIEK					Mascot
2057.0261	2057.0132	-0.0129	-6	340	356	EMTLGFVDLLRDDFIEK			Oxidation (M)[2]		Mascot
2169.9871	2169.999	0.0119	5	195	213	GGLDFTKDDENVNSQPF MR					Mascot
2169.9871	2169.999	0.0119	5	195	213	GGLDFTKDDENVNSQPF MR	93	100			Mascot
2185.9819	2185.9814	-0.0005	0	195	213	GGLDFTKDDENVNSQPF MR			Oxidation (M)[18]		Mascot
2252.1824	2252.1777	-0.0047	-2	140	159	IPPTYSKTFQGPPHGIQV ER					Mascot
3036.4707	3036.5229	0.0522	17	259	285	ELGVPIVMHDYLTGGFTA NTTLAHYCR			Carbamidomethyl (C)[26]		Mascot
3036.4707	3036.5229	0.0522	17	259	285	ELGVPIVMHDYLTGGFTA NTTLAHYCR	159	100	Carbamidomethyl (C)[26]		Mascot
3052.4656	3052.5239	0.0583	19	259	285	ELGVPIVMHDYLTGGFTA NTTLAHYCR			Carbamidomethyl (C)[26], Oxidation (M)[8]		Mascot
3840.8564	3840.968	0.1116	29	42	79	VSPQPGVPPEEAGAAVA AESSTGTWTTVWTDGLT SLDR					Mascot
3840.8564	3840.968	0.1116	29	42	79	VSPQPGVPPEEAGAAVA AESSTGTWTTVWTDGLT SLDR	131	100			Mascot

2	Ribulose bisphosphate carboxylase large chain OS=Hordeum vulgare GN=rbcL PE=3 SV=2	RBL_HORVU	53671.8	6.22	28	856	100	58.535	661	100

Peptide Information

Calc. Mass	Obsrv. Mass	± da	± ppm	Start Seq.	End Seq.	Sequence	Ion Score	C. I. %	Modification	Rank	Result Type
830.4366	830.4283	-0.0083	-10	440	446	EGNEIIR					Mascot
898.41	898.4123	0.0023	3	306	312	NHGMHFR					Mascot
912.472	912.4646	-0.0074	-8	296	303	AMHAVIDR					Mascot
914.405	914.3882	-0.0168	-18	306	312	NHGMHFR			Oxidation (M)[4]		Mascot
928.4669	928.441	-0.0259	-28	296	303	AMHAVIDR			Oxidation (M)[2]		Mascot
971.4073	971.4023	-0.005	-5	188	194	ACYECLR			Carbamidomethyl (C)[2,5]		Mascot
971.4073	971.4023	-0.005	-5	188	194	ACYECLR	24	0	Carbamidomethyl (C)[2,5]		Mascot
1021.5312	1021.5198	-0.0114	-11	33	41	DTDILAAFR					Mascot
1021.5312	1021.5198	-0.0114	-11	33	41	DTDILAAFR	56	99.763			Mascot
1037.4899	1037.4851	-0.0048	-5	351	358	DDFIEKDR					Mascot
1116.583	1116.5812	-0.0018	-2	422	431	VALEACVQAR			Carbamidomethyl (C)[6]		Mascot

1170.5586	1170.6375	0.0789	67	304	312	QKNHGMHFR			Oxidation (M)[6]	Mascot
1170.5586	1170.6375	0.0789	67	304	312	QKNHGMHFR			Oxidation (M)[6]	Mascot
1187.6644	1187.6611	-0.0033	-3	286	295	DNGLLLHIHR				Mascot
1247.6129	1247.5988	-0.0141	-11	218	227	FVFCAEAIYK			Carbamidomethyl (C)[4]	Mascot
1285.6859	1285.5776	-0.1083	-84	436	446	DLAREGNEIIR				Mascot
1293.6871	1293.6863	-0.0008	-1	340	350	EMTLGFVDLLR	90	100		Mascot
1309.6821	1309.6604	-0.0217	-17	340	350	EMTLGFVDLLR			Oxidation (M)[2]	Mascot
1325.6896	1325.6711	-0.0185	-14	306	316	NHGMHFRVLAK			Oxidation (M)[4]	Mascot
1339.6417	1339.6206	-0.0211	-16	467	477	FEFEPVDTIDK				Mascot
1407.6678	1407.6458	-0.022	-16	22	32	LTYYTPEYETK				Mascot
1451.6219	1451.6394	0.0175	12	202	213	DDENVNSQPFMR				Mascot
1465.7546	1465.765	0.0104	7	147	159	TFQGPPHGIQVER				Mascot
1465.7546	1465.765	0.0104	7	147	159	TFQGPPHGIQVER	102	100		Mascot
1502.8512	1502.849	-0.0022	-1	165	177	YGRPLLGCTIKPK			Carbamidomethyl (C)[8]	Mascot
1502.8512	1502.849	-0.0022	-1	165	177	YGRPLLGCTIKPK	6	0	Carbamidomethyl (C)[8]	Mascot
1518.741	1518.7408	-0.0002	0	216	227	DRFVFCAEAIYK			Carbamidomethyl (C)[6]	Mascot
1546.736	1546.7114	-0.0246	-16	451	463	WSPELAAACEVWK			Carbamidomethyl (C)[9]	Mascot
1651.8578	1651.8324	-0.0254	-15	464	477	AIKFEFEPVDTIDK				Mascot
1893.9739	1893.9507	-0.0232	-12	335	350	LEGEREMTLGFVDLLR			Oxidation (M)[7]	Mascot
1950.916	1950.9291	0.0131	7	237	253	GHYLNATAGTCEEMIKR			Carbamidomethyl (C)[11]	Mascot
1966.911	1966.9098	-0.0012	-1	237	253	GHYLNATAGTCEEMIKR			Carbamidomethyl (C)[11], Oxidation (M)[14]	Mascot
2041.0311	2041.0352	0.0041	2	340	356	EMTLGFVDLLRDDFIEK				Mascot
2057.0261	2057.0132	-0.0129	-6	340	356	EMTLGFVDLLRDDFIEK			Oxidation (M)[2]	Mascot
2169.9871	2169.999	0.0119	5	195	213	GGLDFTKDDENVNSQPFMR				Mascot
2169.9871	2169.999	0.0119	5	195	213	GGLDFTKDDENVNSQPFMR	93	100		Mascot
2185.9819	2185.9814	-0.0005	0	195	213	GGLDFTKDDENVNSQPFMR			Oxidation (M)[18]	Mascot
2252.1824	2252.1777	-0.0047	-2	140	159	IPPTYSKTFQGPPHGIQVER				Mascot
3036.4707	3036.5229	0.0522	17	259	285	ELGVPIVMHDYLTGGFTANTTLAHYCR			Carbamidomethyl (C)[26]	Mascot
3036.4707	3036.5229	0.0522	17	259	285	ELGVPIVMHDYLTGGFTANTTLAHYCR	159	100	Carbamidomethyl (C)[26]	Mascot
3052.4656	3052.5239	0.0583	19	259	285	ELGVPIVMHDYLTGGFTANTTLAHYCR			Carbamidomethyl (C)[26], Oxidation (M)[8]	Mascot
3840.8564	3840.968	0.1116	29	42	79	VSPQPGVPPEEAGAAVAAESSTGTWTTVWTDGLTSLDR				Mascot
3840.8564	3840.968	0.1116	29	42	79	VSPQPGVPPEEAGAAVAAESSTGTWTTVWTDGLTSLDR	131	100		Mascot

3	Ribulose bisphosphate carboxylase large chain OS=Brachypodium distachyon GN=rbcL PE=3 SV=1	RBL_BRADI	53326.6	6.22	25	731	100	57.42	569	100

Peptide Information

Calc. Mass	Obsrv. Mass	± da	± ppm	Start Seq.	End Seq.	Sequence	Ion Score	C. I. %	Modification	Rank	Result Type
830.4366	830.4283	-0.0083	-10	440	446	EGNEIIR					Mascot
898.41	898.4123	0.0023	3	306	312	NHGMHFR					Mascot
912.472	912.4646	-0.0074	-8	296	303	AMHAVIDR					Mascot
914.405	914.3882	-0.0168	-18	306	312	NHGMHFR			Oxidation (M)[4]		Mascot
928.4669	928.441	-0.0259	-28	296	303	AMHAVIDR			Oxidation (M)[2]		Mascot
963.5622	963.5828	0.0206	21	9	18	ASVGFKAGVK					Mascot
971.4073	971.4023	-0.005	-5	188	194	ACYECLR			Carbamidomethyl (C)[2,5]		Mascot
971.4073	971.4023	-0.005	-5	188	194	ACYECLR	24	0	Carbamidomethyl (C)[2,5]		Mascot
1021.5312	1021.5198	-0.0114	-11	33	41	DTDILAAFR					Mascot
1021.5312	1021.5198	-0.0114	-11	33	41	DTDILAAFR	56	99.763			Mascot
1037.4899	1037.4851	-0.0048	-5	351	358	DDFIEKDR					Mascot
1116.583	1116.5812	-0.0018	-2	422	431	VALEACVQAR			Carbamidomethyl (C)[6]		Mascot
1170.5586	1170.6375	0.0789	67	304	312	QKNHGMHFR			Oxidation (M)[6]		Mascot
1170.5586	1170.6375	0.0789	67	304	312	QKNHGMHFR			Oxidation (M)[6]		Mascot
1187.6644	1187.6611	-0.0033	-3	286	295	DNGLLLHIHR					Mascot
1247.6129	1247.5988	-0.0141	-11	218	227	FVFCAEAIYK			Carbamidomethyl (C)[4]		Mascot
1275.7307	1275.6725	-0.0582	-46	340	350	EITLGFVDLLR					Mascot
1285.6859	1285.5776	-0.1083	-84	436	446	DLAREGNEIIR					Mascot
1309.6947	1309.6604	-0.0343	-26	306	316	NHGMHFRVLAK					Mascot
1325.6896	1325.6711	-0.0185	-14	306	316	NHGMHFRVLAK			Oxidation (M)[4]		Mascot
1407.6678	1407.6458	-0.022	-16	22	32	LTYYTPEYETK					Mascot
1451.6219	1451.6394	0.0175	12	202	213	DDENVNSQPFMR					Mascot
1465.7546	1465.765	0.0104	7	147	159	TFQGPPHGIQVER	102	100			Mascot
1465.7573	1465.765	0.0077	5	464	476	AIKFEFAPVDTID					Mascot
1502.8512	1502.849	-0.0022	-1	165	177	YGRPLLGCTIKPK			Carbamidomethyl (C)[8]		Mascot
1502.8512	1502.849	-0.0022	-1	165	177	YGRPLLGCTIKPK	6	0	Carbamidomethyl (C)[8]		Mascot
1518.741	1518.7408	-0.0002	0	216	227	DRFVFCAEAIYK			Carbamidomethyl (C)[6]		Mascot
1546.736	1546.7114	-0.0246	-16	451	463	WSPELAAACEVWK			Carbamidomethyl (C)[9]		Mascot
2169.9871	2169.999	0.0119	5	195	213	GGLDFTKDDENVNSQPFMR					Mascot
2169.9871	2169.999	0.0119	5	195	213	GGLDFTKDDENVNSQPFMR	93	100			Mascot
2185.9819	2185.9814	-0.0005	0	195	213	GGLDFTKDDENVNSQPFMR			Oxidation (M)[18]		Mascot
2252.1824	2252.1777	-0.0047	-2	140	159	IPPTYSKTFQGPPHGIQVER					Mascot

Calc. Mass	Obsrv. Mass	± da	± ppm	Start Seq.	End Seq.	Sequence	Ion Score	C. I. %	Modification	Rank	Result Type
3036.4707	3036.5229	0.0522	17	259	285	ELGVPIVMHDYLTGGFTA NTTLAHYCR			Carbamidomethyl (C)[26]		Mascot
3036.4707	3036.5229	0.0522	17	259	285	ELGVPIVMHDYLTGGFTA NTTLAHYCR	159	100	Carbamidomethyl (C)[26]		Mascot
3052.4656	3052.5239	0.0583	19	259	285	ELGVPIVMHDYLTGGFTA NTTLAHYCR			Carbamidomethyl (C)[26], Oxidation (M)[8]		Mascot
3840.8564	3840.968	0.1116	29	42	79	VSPQPGVPPEEAGAAVA AESSTGTWTTVWTDGLT SLDR					Mascot
3840.8564	3840.968	0.1116	29	42	79	VSPQPGVPPEEAGAAVA AESSTGTWTTVWTDGLT SLDR	131	100			Mascot

4	Ribulose bisphosphate carboxylase large chain OS=Agrostis stolonifera GN=rbcL PE=3 SV=1	RBL_AGRST	53412.6	6.22	27	682	100	54.16	500	100

Peptide Information

Calc. Mass	Obsrv. Mass	± da	± ppm	Start Seq.	End Seq.	Sequence	Ion Score	C. I. %	Modification	Rank	Result Type
830.4366	830.4283	-0.0083	-10	440	446	EGNEIIR					Mascot
898.41	898.4123	0.0023	3	306	312	NHGMHFR					Mascot
912.472	912.4646	-0.0074	-8	296	303	AMHAVIDR					Mascot
914.405	914.3882	-0.0168	-18	306	312	NHGMHFR			Oxidation (M)[4]		Mascot
928.4669	928.441	-0.0259	-28	296	303	AMHAVIDR			Oxidation (M)[2]		Mascot
963.5622	963.5828	0.0206	21	9	18	ASVGFKAGVK					Mascot
971.4073	971.4023	-0.005	-5	188	194	ACYECLR			Carbamidomethyl (C)[2,5]		Mascot
971.4073	971.4023	-0.005	-5	188	194	ACYECLR	24	0	Carbamidomethyl (C)[2,5]		Mascot
1021.5312	1021.5198	-0.0114	-11	33	41	DTDILAAFR					Mascot
1021.5312	1021.5198	-0.0114	-11	33	41	DTDILAAFR	56	99.763			Mascot
1037.4899	1037.4851	-0.0048	-5	351	358	DDFIEKDR					Mascot
1116.583	1116.5812	-0.0018	-2	422	431	VALEACVQAR			Carbamidomethyl (C)[6]		Mascot
1170.5586	1170.6375	0.0789	67	304	312	QKNHGMHFR			Oxidation (M)[6]		Mascot
1170.5586	1170.6375	0.0789	67	304	312	QKNHGMHFR			Oxidation (M)[6]		Mascot
1187.6644	1187.6611	-0.0033	-3	286	295	DNGLLLHIHR					Mascot
1247.6129	1247.5988	-0.0141	-11	218	227	FVFCAEAIYK			Carbamidomethyl (C)[4]		Mascot
1285.6859	1285.5776	-0.1083	-84	436	446	DLAREGNEIIR					Mascot
1293.6871	1293.6863	-0.0008	-1	340	350	EMTLGFVDLLR	90	100			Mascot
1309.6821	1309.6604	-0.0217	-17	340	350	EMTLGFVDLLR			Oxidation (M)[2]		Mascot
1325.6896	1325.6711	-0.0185	-14	306	316	NHGMHFRVLAK			Oxidation (M)[4]		Mascot
1339.6417	1339.6206	-0.0211	-16	467	477	FEFEPVDTIDK					Mascot
1407.6678	1407.6458	-0.022	-16	22	32	LTYYTPEYETK					Mascot
1451.6219	1451.6394	0.0175	12	202	213	DDENVNSQPFMR					Mascot
1465.7546	1465.765	0.0104	7	147	159	TFQGPPHGIQVER					Mascot

1465.7546	1465.765	0.0104	7	147	159	TFQGPPHGIQVER	102	100			Mascot
1502.8512	1502.849	-0.0022	-1	165	177	YGRPLLGCTIKPK			Carbamidomethyl (C)[8]		Mascot
1502.8512	1502.849	-0.0022	-1	165	177	YGRPLLGCTIKPK	6	0	Carbamidomethyl (C)[8]		Mascot
1518.741	1518.7408	-0.0002	0	216	227	DRFVFCAEAIYK			Carbamidomethyl (C)[6]		Mascot
1546.736	1546.7114	-0.0246	-16	451	463	WSPELAAACEVWK			Carbamidomethyl (C)[9]		Mascot
1651.8578	1651.8324	-0.0254	-15	464	477	AIKFEFEPVDTIDK					Mascot
1893.9739	1893.9507	-0.0232	-12	335	350	LEGEREMTLGFVDLLR			Oxidation (M)[7]		Mascot
1950.916	1950.9291	0.0131	7	237	253	GHYLNATAGTCEEMIKR			Carbamidomethyl (C)[11]		Mascot
1966.911	1966.9098	-0.0012	-1	237	253	GHYLNATAGTCEEMIKR			Carbamidomethyl (C)[11], Oxidation (M)[14]		Mascot
2041.0311	2041.0352	0.0041	2	340	356	EMTLGFVDLLRDDFIEK					Mascot
2057.0261	2057.0132	-0.0129	-6	340	356	EMTLGFVDLLRDDFIEK			Oxidation (M)[2]		Mascot
2169.9871	2169.999	0.0119	5	195	213	GGLDFTKDDENVNSQPF MR					Mascot
2169.9871	2169.999	0.0119	5	195	213	GGLDFTKDDENVNSQPF MR	93	100			Mascot
2185.9819	2185.9814	-0.0005	0	195	213	GGLDFTKDDENVNSQPF MR			Oxidation (M)[18]		Mascot
3840.8564	3840.968	0.1116	29	42	79	VSPQPGVPPEEAGAAVA AESSTGTWTTVWTDGLT SLDR					Mascot
3840.8564	3840.968	0.1116	29	42	79	VSPQPGVPPEEAGAAVA AESSTGTWTTVWTDGLT SLDR	131	100			Mascot

5	Ribulose bisphosphate carboxylase large chain (Fragment) OS=Bauera rubioides GN=rbcL PE=3 SV=2	RBL_BAURU	51965.2	6.23	20	664	100	48.925	546	100	

Peptide Information

Calc. Mass	Obsrv. Mass	± da	± ppm	Start Seq.	End Seq.	Sequence	Ion Score	C. I. %	Modification	Rank	Result Type
830.4366	830.4283	-0.0083	-10	430	436	EGNEIIR					Mascot
880.4536	880.4	-0.0536	-61	296	302	NHGIHFR					Mascot
912.472	912.4646	-0.0074	-8	286	293	AMHAVIDR					Mascot
928.4669	928.441	-0.0259	-28	286	293	AMHAVIDR			Oxidation (M)[2]		Mascot
1021.5312	1021.5198	-0.0114	-11	23	31	DTDILAAFR					Mascot
1021.5312	1021.5198	-0.0114	-11	23	31	DTDILAAFR	56	99.763			Mascot
1037.4899	1037.4851	-0.0048	-5	341	348	DDFIEKDR					Mascot
1116.583	1116.5812	-0.0018	-2	412	421	VALEACVQAR			Carbamidomethyl (C)[6]		Mascot
1187.6644	1187.6611	-0.0033	-3	276	285	DNGLLLHIHR					Mascot
1227.6443	1227.6686	0.0243	20	208	217	LLFCAEAIYK			Carbamidomethyl (C)[4]		Mascot
1245.6433	1245.6863	0.043	35	430	440	EGNEIIREASK					Mascot
1245.6433	1245.6863	0.043	35	430	440	EGNEIIREASK					Mascot
1261.7151	1261.6537	-0.0614	-49	330	340	DITLGFVDLLR					Mascot

Calc. Mass	Obsrv. Mass	± da	± ppm	Start Seq.	End Seq.	Sequence	Ion Score	C. I. %	Modification	Rank	Result Type
1285.6859	1285.5776	-0.1083	-84	426	436	DLAREGNEIIR					Mascot
1291.7383	1291.6506	-0.0877	-68	296	306	NHGIHFRVLAK					Mascot
1451.6219	1451.6394	0.0175	12	192	203	DDENVNSQPFMR					Mascot
1456.7029	1456.6587	-0.0442	-30	454	465	EIKFEFPAMDTL			Oxidation (M)[9]		Mascot
1465.7546	1465.765	0.0104	7	137	149	TFQGPPHGIQVER					Mascot
1465.7546	1465.765	0.0104	7	137	149	TFQGPPHGIQVER	102	100			Mascot
1502.8512	1502.849	-0.0022	-1	155	167	YGRPLLGCTIKPK			Carbamidomethyl (C)[8]		Mascot
1502.8512	1502.849	-0.0022	-1	155	167	YGRPLLGCTIKPK	6	0	Carbamidomethyl (C)[8]		Mascot
1546.736	1546.7114	-0.0246	-16	441	453	WSPELAAACEVWK			Carbamidomethyl (C)[9]		Mascot
2169.9871	2169.999	0.0119	5	185	203	GGLDFTKDDENVNSQPF MR					Mascot
2169.9871	2169.999	0.0119	5	185	203	GGLDFTKDDENVNSQPF MR	93	100			Mascot
2185.9819	2185.9814	-0.0005	0	185	203	GGLDFTKDDENVNSQPF MR			Oxidation (M)[18]		Mascot
3036.4707	3036.5229	0.0522	17	249	275	ELGVPIVMHDYLTGGFTA NTTLAHYCR			Carbamidomethyl (C)[26]		Mascot
3036.4707	3036.5229	0.0522	17	249	275	ELGVPIVMHDYLTGGFTA NTTLAHYCR	159	100	Carbamidomethyl (C)[26]		Mascot
3052.4656	3052.5239	0.0583	19	249	275	ELGVPIVMHDYLTGGFTA NTTLAHYCR			Carbamidomethyl (C)[26], Oxidation (M)[8]		Mascot
3840.8564	3840.968	0.1116	29	32	69	VSPQPGVPPEEAGAAVA AESSTGTWTTVWTDGLT SLDR					Mascot
3840.8564	3840.968	0.1116	29	32	69	VSPQPGVPPEEAGAAVA AESSTGTWTTVWTDGLT SLDR	131	100			Mascot

6	Ribulose bisphosphate carboxylase large chain OS=Ceratophyllum demersum GN=rbcL PE=3 SV=2	RBL_CERDE	53105.6	6	17	629	100	46.98	548	100		

Peptide Information

Calc. Mass	Obsrv. Mass	± da	± ppm	Start Seq.	End Seq.	Sequence	Ion Score	C. I. %	Modification	Rank	Result Type
880.4536	880.4	-0.0536	-61	306	312	NHGIHFR					Mascot
912.472	912.4646	-0.0074	-8	296	303	AMHAVIDR					Mascot
928.4669	928.441	-0.0259	-28	296	303	AMHAVIDR			Oxidation (M)[2]		Mascot
1021.5312	1021.5198	-0.0114	-11	33	41	DTDILAAFR					Mascot
1021.5312	1021.5198	-0.0114	-11	33	41	DTDILAAFR	56	99.763			Mascot
1037.4899	1037.4851	-0.0048	-5	351	358	DDFIEKDR					Mascot
1116.6194	1116.5812	-0.0382	-34	422	431	VALEACVKAR			Carbamidomethyl (C)[6]		Mascot
1187.6644	1187.6611	-0.0033	-3	286	295	DNGLLLHIHR					Mascot
1247.6129	1247.5988	-0.0141	-11	218	227	FVFCAEAIYK			Carbamidomethyl (C)[4]		Mascot
1261.7151	1261.6537	-0.0614	-49	340	350	DITLGFVDLLR					Mascot
1291.7383	1291.6506	-0.0877	-68	306	316	NHGIHFRVLAK					Mascot

1451.6219	1451.6394	0.0175	12	202	213	DDENVNSQPFMR					Mascot
1465.7546	1465.765	0.0104	7	147	159	TFQGPPHGIQVER					Mascot
1465.7546	1465.765	0.0104	7	147	159	TFQGPPHGIQVER	102	100			Mascot
1502.8512	1502.849	-0.0022	-1	165	177	YGRPLLGCTIKPK			Carbamidomethyl (C)[8]		Mascot
1502.8512	1502.849	-0.0022	-1	165	177	YGRPLLGCTIKPK	6	0	Carbamidomethyl (C)[8]		Mascot
1518.741	1518.7408	-0.0002	0	216	227	DRFVFCAEAIYK			Carbamidomethyl (C)[6]		Mascot
1546.736	1546.7114	-0.0246	-16	451	463	WSPELAAACEVWK			Carbamidomethyl (C)[9]		Mascot
2169.9871	2169.999	0.0119	5	195	213	GGLDFTKDDENVNSQPF MR					Mascot
2169.9871	2169.999	0.0119	5	195	213	GGLDFTKDDENVNSQPF MR	93	100			Mascot
2185.9819	2185.9814	-0.0005	0	195	213	GGLDFTKDDENVNSQPF MR			Oxidation (M)[18]		Mascot
3036.4707	3036.5229	0.0522	17	259	285	ELGVPIVMHDYLTGGFTA NTTLAHYCR			Carbamidomethyl (C)[26]		Mascot
3036.4707	3036.5229	0.0522	17	259	285	ELGVPIVMHDYLTGGFTA NTTLAHYCR	159	100	Carbamidomethyl (C)[26]		Mascot
3052.4656	3052.5239	0.0583	19	259	285	ELGVPIVMHDYLTGGFTA NTTLAHYCR			Carbamidomethyl (C)[26], Oxidation (M)[8]		Mascot
3840.8564	3840.968	0.1116	29	42	79	VSPQPGVPPEEAGAAVA AESSTGTWTTVWTDGLT SLDR					Mascot
3840.8564	3840.968	0.1116	29	42	79	VSPQPGVPPEEAGAAVA AESSTGTWTTVWTDGLT SLDR	131	100			Mascot

7	Ribulose bisphosphate carboxylase large chain (Fragment) OS=Gladiolus guienzii GN=rbcL PE=3 SV=1	RBL_GLAGU	51225.5	6.23	20	619	100	52.48	505	100	

Peptide Information

Calc. Mass	Obsrv. Mass	± da	± ppm	Start Seq.	End Seq.	Sequence	Ion Score	C. I. %	Modification	Rank	Result Type
830.4366	830.4283	-0.0083	-10	433	439	EGNEIIR					Mascot
898.41	898.4123	0.0023	3	299	305	NHGMHFR					Mascot
912.472	912.4646	-0.0074	-8	289	296	AMHAVIDR					Mascot
914.405	914.3882	-0.0168	-18	299	305	NHGMHFR			Oxidation (M)[4]		Mascot
928.4669	928.441	-0.0259	-28	289	296	AMHAVIDR			Oxidation (M)[2]		Mascot
1021.5312	1021.5198	-0.0114	-11	26	34	DTDILAAFR					Mascot
1021.5312	1021.5198	-0.0114	-11	26	34	DTDILAAFR	56	99.763			Mascot
1116.583	1116.5812	-0.0018	-2	415	424	VALEACVQAR			Carbamidomethyl (C)[6]		Mascot
1170.5586	1170.6375	0.0789	67	297	305	QKNHGMHFR			Oxidation (M)[6]		Mascot
1170.5586	1170.6375	0.0789	67	297	305	QKNHGMHFR			Oxidation (M)[6]		Mascot
1187.6644	1187.6611	-0.0033	-3	279	288	DNGLLLHIHR					Mascot
1247.6129	1247.5988	-0.0141	-11	211	220	FVFCAEAIYK			Carbamidomethyl (C)[4]		Mascot
1285.6859	1285.5776	-0.1083	-84	429	439	DLAREGNEIIR					Mascot

1293.6871	1293.6863	-0.0008	-1	333	343	EMTLGFVDLLR	90	100			Mascot
1309.6821	1309.6604	-0.0217	-17	333	343	EMTLGFVDLLR			Oxidation (M)[2]		Mascot
1325.6896	1325.6711	-0.0185	-14	299	309	NHGMHFRVLAK			Oxidation (M)[4]		Mascot
1407.6678	1407.6458	-0.022	-16	15	25	LTYYTPEYETK					Mascot
1451.6219	1451.6394	0.0175	12	195	206	DDENVNSQPFMR					Mascot
1465.7546	1465.765	0.0104	7	140	152	TFQGPPHGIQVER					Mascot
1465.7546	1465.765	0.0104	7	140	152	TFQGPPHGIQVER	102	100			Mascot
1502.8512	1502.849	-0.0022	-1	158	170	YGRPLLGCTIKPK			Carbamidomethyl (C)[8]		Mascot
1502.8512	1502.849	-0.0022	-1	158	170	YGRPLLGCTIKPK	6	0	Carbamidomethyl (C)[8]		Mascot
1518.741	1518.7408	-0.0002	0	209	220	DRFVFCAEAIYK			Carbamidomethyl (C)[6]		Mascot
1893.9739	1893.9507	-0.0232	-12	328	343	LEGEREMTLGFVDLLR			Oxidation (M)[7]		Mascot
2057.0261	2057.0132	-0.0129	-6	333	349	EMTLGFVDLLRDDYIEK					Mascot
2169.9871	2169.999	0.0119	5	188	206	GGLDFTKDDENVNSQPFMR					Mascot
2169.9871	2169.999	0.0119	5	188	206	GGLDFTKDDENVNSQPFMR	93	100			Mascot
2185.9819	2185.9814	-0.0005	0	188	206	GGLDFTKDDENVNSQPFMR			Oxidation (M)[18]		Mascot
3036.4707	3036.5229	0.0522	17	252	278	ELGVPIVMHDYLTGGFTANTTLAHYCR			Carbamidomethyl (C)[26]		Mascot
3036.4707	3036.5229	0.0522	17	252	278	ELGVPIVMHDYLTGGFTANTTLAHYCR	159	100	Carbamidomethyl (C)[26]		Mascot
3052.4656	3052.5239	0.0583	19	252	278	ELGVPIVMHDYLTGGFTANTTLAHYCR			Carbamidomethyl (C)[26], Oxidation (M)[8]		Mascot

8	Ribulose bisphosphate carboxylase large chain OS=Dioscorea elephantipes GN=rbcL PE=3 SV=1	RBL_DIOEL	53420.7	6.13	23	615	100	50.696	476	100	

Peptide Information

Calc. Mass	Obsrv. Mass	± da	± ppm	Start Seq.	End Seq.	Sequence	Ion Score	C. I. %	Modification	Rank	Result Type
830.4366	830.4283	-0.0083	-10	440	446	EGNEIIR					Mascot
898.41	898.4123	0.0023	3	306	312	NHGMHFR					Mascot
912.472	912.4646	-0.0074	-8	296	303	AMHAVIDR					Mascot
914.405	914.3882	-0.0168	-18	306	312	NHGMHFR			Oxidation (M)[4]		Mascot
928.4669	928.441	-0.0259	-28	296	303	AMHAVIDR			Oxidation (M)[2]		Mascot
963.5622	963.5828	0.0206	21	9	18	ASVGFKAGVK					Mascot
1021.5312	1021.5198	-0.0114	-11	33	41	DTDILAAFR					Mascot
1021.5312	1021.5198	-0.0114	-11	33	41	DTDILAAFR	56	99.763			Mascot
1037.4899	1037.4851	-0.0048	-5	351	358	DDFIEKDR					Mascot
1116.583	1116.5812	-0.0018	-2	422	431	VALEACVQAR			Carbamidomethyl (C)[6]		Mascot
1170.5586	1170.6375	0.0789	67	304	312	QKNHGMHFR			Oxidation (M)[6]		Mascot
1170.5586	1170.6375	0.0789	67	304	312	QKNHGMHFR			Oxidation (M)[6]		Mascot

Calc. Mass	Obsrv. Mass	± da	± ppm	Start Seq.	End Seq.	Sequence	Ion Score	C. I. %	Modification	Rank	Result Type
1187.6644	1187.6611	-0.0033	-3	286	295	DNGLLLHIHR					Mascot
1261.6285	1261.6537	0.0252	20	218	227	FLFCAEAIYK			Carbamidomethyl (C)[4]		Mascot
1285.6859	1285.5776	-0.1083	-84	436	446	DLAREGNEIIR					Mascot
1293.6871	1293.6863	-0.0008	-1	340	350	EMTLGFVDLLR	90	100			Mascot
1309.6821	1309.6604	-0.0217	-17	340	350	EMTLGFVDLLR			Oxidation (M)[2]		Mascot
1325.6896	1325.6711	-0.0185	-14	306	316	NHGMHFRVLAK			Oxidation (M)[4]		Mascot
1421.7635	1421.748	-0.0155	-11	135	146	LEDLRIPTSYSK					Mascot
1451.6219	1451.6394	0.0175	12	202	213	DDENVNSQPFMR					Mascot
1465.7546	1465.765	0.0104	7	147	159	TFQGPPHGIQVER					Mascot
1465.7546	1465.765	0.0104	7	147	159	TFQGPPHGIQVER	102	100			Mascot
1502.8512	1502.849	-0.0022	-1	165	177	YGRPLLGCTIKPK			Carbamidomethyl (C)[8]		Mascot
1502.8512	1502.849	-0.0022	-1	165	177	YGRPLLGCTIKPK	6	0	Carbamidomethyl (C)[8]		Mascot
1546.736	1546.7114	-0.0246	-16	451	463	WSPELAAACEVWK			Carbamidomethyl (C)[9]		Mascot
1893.9739	1893.9507	-0.0232	-12	335	350	LEGEREMTLGFVDLLR			Oxidation (M)[7]		Mascot
1950.916	1950.9291	0.0131	7	237	253	GHYLNATAGTCEEMIKR			Carbamidomethyl (C)[11]		Mascot
1966.911	1966.9098	-0.0012	-1	237	253	GHYLNATAGTCEEMIKR			Carbamidomethyl (C)[11], Oxidation (M)[14]		Mascot
2041.0311	2041.0352	0.0041	2	340	356	EMTLGFVDLLRDDFIEK					Mascot
2057.0261	2057.0132	-0.0129	-6	340	356	EMTLGFVDLLRDDFIEK			Oxidation (M)[2]		Mascot
2169.9871	2169.999	0.0119	5	195	213	GGLDFTKDDENVNSQPF MR					Mascot
2169.9871	2169.999	0.0119	5	195	213	GGLDFTKDDENVNSQPF MR	93	100			Mascot
2185.9819	2185.9814	-0.0005	0	195	213	GGLDFTKDDENVNSQPF MR			Oxidation (M)[18]		Mascot
3840.8564	3840.968	0.1116	29	42	79	VSPQPGVPPEEAGAAVA AESSTGTWTTVWTDGLT SLDR					Mascot
3840.8564	3840.968	0.1116	29	42	79	VSPQPGVPPEEAGAAVA AESSTGTWTTVWTDGLT SLDR	131	100			Mascot

9	Ribulose bisphosphate carboxylase large chain (Fragment) OS=Isophysis tasmanica GN=rbcL PE=3 SV=1	RBL_ISOTA	52248	5.87	18	603	100	51.653	506	100	

Peptide Information

Calc. Mass	Obsrv. Mass	± da	± ppm	Start Seq.	End Seq.	Sequence	Ion Score	C. I. %	Modification	Rank	Result Type
830.4366	830.4283	-0.0083	-10	431	437	EGNEIIR					Mascot
898.41	898.4123	0.0023	3	297	303	NHGMHFR					Mascot
912.472	912.4646	-0.0074	-8	287	294	AMHAVIDR					Mascot
914.405	914.3882	-0.0168	-18	297	303	NHGMHFR			Oxidation (M)[4]		Mascot
928.4669	928.441	-0.0259	-28	287	294	AMHAVIDR			Oxidation (M)[2]		Mascot

1021.5312	1021.5198	-0.0114	-11	24	32	DTDILAAFR					Mascot
1021.5312	1021.5198	-0.0114	-11	24	32	DTDILAAFR	56	99.763			Mascot
1116.583	1116.5812	-0.0018	-2	413	422	VALEACVQAR			Carbamidomethyl (C)[6]		Mascot
1170.5586	1170.6375	0.0789	67	295	303	QKNHGMHFR			Oxidation (M)[6]		Mascot
1170.5586	1170.6375	0.0789	67	295	303	QKNHGMHFR			Oxidation (M)[6]		Mascot
1187.6644	1187.6611	-0.0033	-3	277	286	DNGLLLHIHR					Mascot
1261.6285	1261.6537	0.0252	20	209	218	FLFCAEAIYK			Carbamidomethyl (C)[4]		Mascot
1285.6859	1285.5776	-0.1083	-84	427	437	DLAREGNEIIR					Mascot
1293.6871	1293.6863	-0.0008	-1	331	341	EMTLGFVDLLR	90	100			Mascot
1309.6821	1309.6604	-0.0217	-17	331	341	EMTLGFVDLLR			Oxidation (M)[2]		Mascot
1325.6896	1325.6711	-0.0185	-14	297	307	NHGMHFRVLAK			Oxidation (M)[4]		Mascot
1451.6219	1451.6394	0.0175	12	193	204	DDENVNSQPFMR					Mascot
1465.7546	1465.765	0.0104	7	138	150	TFQGPPHGIQVER					Mascot
1465.7546	1465.765	0.0104	7	138	150	TFQGPPHGIQVER	102	100			Mascot
1502.8512	1502.849	-0.0022	-1	156	168	YGRPLLGCTIKPK			Carbamidomethyl (C)[8]		Mascot
1502.8512	1502.849	-0.0022	-1	156	168	YGRPLLGCTIKPK	6	0	Carbamidomethyl (C)[8]		Mascot
1950.916	1950.9291	0.0131	7	228	244	GHYLNATAGTCEEMIKR			Carbamidomethyl (C)[11]		Mascot
1966.911	1966.9098	-0.0012	-1	228	244	GHYLNATAGTCEEMIKR			Carbamidomethyl (C)[11], Oxidation (M)[14]		Mascot
2057.0261	2057.0132	-0.0129	-6	331	347	EMTLGFVDLLRDDYIEK					Mascot
2169.9871	2169.999	0.0119	5	186	204	GGLDFTKDDENVNSQPF MR					Mascot
2169.9871	2169.999	0.0119	5	186	204	GGLDFTKDDENVNSQPF MR	93	100			Mascot
2185.9819	2185.9814	-0.0005	0	186	204	GGLDFTKDDENVNSQPF MR			Oxidation (M)[18]		Mascot
3036.4707	3036.5229	0.0522	17	250	276	ELGVPIVMHDYLTGGFTA NTTLAHYCR			Carbamidomethyl (C)[26]		Mascot
3036.4707	3036.5229	0.0522	17	250	276	ELGVPIVMHDYLTGGFTA NTTLAHYCR	159	100	Carbamidomethyl (C)[26]		Mascot
3052.4656	3052.5239	0.0583	19	250	276	ELGVPIVMHDYLTGGFTA NTTLAHYCR			Carbamidomethyl (C)[26], Oxidation (M)[8]		Mascot

10	Ribulose bisphosphate carboxylase large chain (Fragment) OS=Watsonia angusta GN=rbcL PE=3 SV=1	RBL_WATAN	49704.7	6.34	17	595	100	51.321	506	100	

Peptide Information

Calc. Mass	Obsrv. Mass	± da	± ppm	Start Seq.	End Seq.	Sequence	Ion Score	C. I. %	Modification	Rank	Result Type
898.41	898.4123	0.0023	3	299	305	NHGMHFR					Mascot
912.472	912.4646	-0.0074	-8	289	296	AMHAVIDR					Mascot
914.405	914.3882	-0.0168	-18	299	305	NHGMHFR			Oxidation (M)[4]		Mascot
928.4669	928.441	-0.0259	-28	289	296	AMHAVIDR			Oxidation (M)[2]		Mascot

1021.5312	1021.5198	-0.0114	-11	26	34	DTDILAAFR				Mascot
1021.5312	1021.5198	-0.0114	-11	26	34	DTDILAAFR	56	99.755		Mascot
1116.583	1116.5812	-0.0018	-2	415	424	VALEACVQAR			Carbamidomethyl (C)[6]	Mascot
1170.5586	1170.6375	0.0789	67	297	305	QKNHGMHFR			Oxidation (M)[6]	Mascot
1170.5586	1170.6375	0.0789	67	297	305	QKNHGMHFR			Oxidation (M)[6]	Mascot
1187.6644	1187.6611	-0.0033	-3	279	288	DNGLLLHIHR				Mascot
1261.6285	1261.6537	0.0252	20	211	220	FLFCAEALYK			Carbamidomethyl (C)[4]	Mascot
1293.6871	1293.6863	-0.0008	-1	333	343	EMTLGFVDLLR	90	100		Mascot
1309.6821	1309.6604	-0.0217	-17	333	343	EMTLGFVDLLR			Oxidation (M)[2]	Mascot
1325.6896	1325.6711	-0.0185	-14	299	309	NHGMHFRVLAK			Oxidation (M)[4]	Mascot
1451.6219	1451.6394	0.0175	12	195	206	DDENVNSQPFMR				Mascot
1465.7546	1465.765	0.0104	7	140	152	TFQGPPHGIQVER				Mascot
1465.7546	1465.765	0.0104	7	140	152	TFQGPPHGIQVER	102	100		Mascot
1502.8512	1502.849	-0.0022	-1	158	170	YGRPLLGCTIKPK			Carbamidomethyl (C)[8]	Mascot
1502.8512	1502.849	-0.0022	-1	158	170	YGRPLLGCTIKPK	6	0	Carbamidomethyl (C)[8]	Mascot
1893.9739	1893.9507	-0.0232	-12	328	343	LEGEREMTLGFVDLLR			Oxidation (M)[7]	Mascot
1950.916	1950.9291	0.0131	7	230	246	GHYLNATAGTCEEMIKR			Carbamidomethyl (C)[11]	Mascot
1966.911	1966.9098	-0.0012	-1	230	246	GHYLNATAGTCEEMIKR			Carbamidomethyl (C)[11], Oxidation (M)[14]	Mascot
2057.0261	2057.0132	-0.0129	-6	333	349	EMTLGFVDLLRDDYIEK				Mascot
2169.9871	2169.999	0.0119	5	188	206	GGLDFTKDDENVNSQPF MR				Mascot
2169.9871	2169.999	0.0119	5	188	206	GGLDFTKDDENVNSQPF MR	93	100		Mascot
2185.9819	2185.9814	-0.0005	0	188	206	GGLDFTKDDENVNSQPF MR			Oxidation (M)[18]	Mascot
3036.4707	3036.5229	0.0522	17	252	278	ELGVPIVMHDYLTGGFTA NTTLAHYCR			Carbamidomethyl (C)[26]	Mascot
3036.4707	3036.5229	0.0522	17	252	278	ELGVPIVMHDYLTGGFTA NTTLAHYCR	159	100	Carbamidomethyl (C)[26]	Mascot
3052.4656	3052.5239	0.0583	19	252	278	ELGVPIVMHDYLTGGFTA NTTLAHYCR			Carbamidomethyl (C)[26], Oxidation (M)[8]	Mascot

Gel Idx/Pos	290/L18	Instr./Gel Origin	BA2151/Sample Project 20140814	Process Status	Analysis Succeeded
Plate [#] Name	[1] Sample Project 20140814	Instrument Sample Name		Spectra	11

Rank	Protein Name	Accession No.	Protein MW	Protein PI	Pep. Count	Protein Score	Protein Score C. I. %	Intensity Matched	Total Ion Score	Total Ion C. I. %	Confirmed
1	Glucose-1-phosphate adenylyltransferase large subunit, chloroplastic/amyloplastic OS=Triticum aestivum GN=AGP-L PE=2 SV=2	GLGL2_WHEAT	58399.1	6.12	29	1,140	100	63.89	955	100	

Peptide Information

Calc. Mass	Obsrv. Mass	± da	± ppm	Start Seq.	End Seq.	Sequence	Ion Score	C. I. %	Modification	Rank	Result Type
802.424	802.4251	0.0011	1	16	22	ACISPVR			Carbamidomethyl (C)[2]		Mascot
820.4159	820.4044	-0.0115	-14	33	40	VGDSSSIR					Mascot
847.452	847.4308	-0.0212	-25	426	433	LNSGSELK					Mascot
866.4618	866.4388	-0.023	-27	251	258	ASEYGLVK					Mascot
927.5873	927.5493	-0.038	-41	311	318	DVLLNLLK					Mascot
945.4352	945.4153	-0.0199	-21	373	379	FEFYDPK					Mascot
952.4887	952.4767	-0.012	-13	380	387	TPFFTSPR					Mascot
952.4887	952.4767	-0.012	-13	380	387	TPFFTSPR	51	99.189			Mascot
1013.5626	1013.5337	-0.0289	-29	458	467	VPIGVGENTK					Mascot
1023.5945	1023.5873	-0.0072	-7	415	423	IEHSIIGVR					Mascot
1023.5945	1023.5873	-0.0072	-7	415	423	IEHSIIGVR	53	99.446			Mascot
1061.595	1061.5651	-0.0299	-28	31	40	LKVGDSSSIR					Mascot
1170.7205	1170.6298	-0.0907	-77	311	320	DVLLNLLKSR					Mascot
1261.6359	1261.6375	0.0016	1	111	122	ATPAVPIGGCYR			Carbamidomethyl (C)[10]		Mascot
1261.6359	1261.6375	0.0016	1	111	122	ATPAVPIGGCYR	76	99.998	Carbamidomethyl (C)[10]		Mascot
1302.6624	1302.6641	0.0017	1	401	411	EAIISHGCFLR			Carbamidomethyl (C)[8]		Mascot
1302.6624	1302.6641	0.0017	1	401	411	EAIISHGCFLR	83	100	Carbamidomethyl (C)[8]		Mascot
1322.6191	1322.5989	-0.0202	-15	468	478	ISNCIIDMNAR			Carbamidomethyl (C)[4], Oxidation (M)[8]		Mascot
1322.6191	1322.5989	-0.0202	-15	468	478	ISNCIIDMNAR	37	79.715	Carbamidomethyl (C)[4], Oxidation (M)[8]		Mascot
1503.7882	1503.7552	-0.033	-22	195	205	KFIWVLEDYYK					Mascot
1553.7709	1553.7338	-0.0371	-24	297	309	YPYIASMGVYVFK			Oxidation (M)[7]		Mascot
1643.821	1643.8031	-0.0179	-11	139	152	IFVMTQFNSASLNR			Oxidation (M)[4]		Mascot
1646.8174	1646.8174	0	0	321	334	YAELHDFGSEILPR					Mascot
1646.8174	1646.8174	0	0	321	334	YAELHDFGSEILPR	108	100			Mascot
1652.8167	1652.7986	-0.0181	-11	282	296	VDTSFLNFAIDDPAK					Mascot
1674.8732	1674.8409	-0.0323	-19	452	467	LMSEGKVPIGVGENTK			Oxidation (M)[2]		Mascot

Calc. Mass	Obsrv. Mass	± da	± ppm	Start Seq.	End Seq.	Sequence	Ion Score	C. I. %	Modification	Rank	Result Type
1709.8719	1709.8036	-0.0683	-40	297	310	YPYIASMGVYVFKR			Oxidation (M)[7]		Mascot
1756.9592	1756.9644	0.0052	3	208	222	SIEHILILSGDQLYR					Mascot
1756.9592	1756.9644	0.0052	3	208	222	SIEHILILSGDQLYR	110	100			Mascot
1770.7826	1770.7554	-0.0272	-15	358	372	SFFDANMALCEQPPK			Carbamidomethyl (C)[10], Oxidation (M)[7]		Mascot
1838.8776	1838.8567	-0.0209	-11	123	138	LIDIPMSNCFNSGINK			Carbamidomethyl (C)[9], Oxidation (M)[6]		Mascot
1910.8879	1910.8832	-0.0047	-2	489	504	EGVQEADRPEEGYYIR					Mascot
2055.9399	2055.9438	0.0039	2	232	250	HVDDNADITLSCAPVGES R			Carbamidomethyl (C)[12]		Mascot
2371.1924	2371.2029	0.0105	4	54	77	GPAATGAQCVLTSDASP ADTLVLR			Carbamidomethyl (C)[9]		Mascot
2371.1924	2371.2029	0.0105	4	54	77	GPAATGAQCVLTSDASP ADTLVLR	198	100	Carbamidomethyl (C)[9]		Mascot
2763.3162	2763.3372	0.021	8	335	357	ALHDHNVQAYVFTDYWE DIGTIR					Mascot
2862.4634	2862.4819	0.0185	6	83	110	NYADPNEVAAVILGGGT GTQLFPLTSTR					Mascot
2862.4634	2862.4819	0.0185	6	83	110	NYADPNEVAAVILGGGT GTQLFPLTSTR	239	100			Mascot

2	Glucose-1-phosphate adenylyltransferase large subunit 1, chloroplastic/amyloplastic OS=Hordeum vulgare PE=2 SV=2	GLGL1_HORVU	58580.1	6.17	27	1,120	100	62.895	955	100

Peptide Information

Calc. Mass	Obsrv. Mass	± da	± ppm	Start Seq.	End Seq.	Sequence	Ion Score	C. I. %	Modification	Rank	Result Type
808.3254	808.3248	-0.0006	-1	24	30	EGSACER			Carbamidomethyl (C)[5]		Mascot
847.452	847.4308	-0.0212	-25	427	434	LNSGSELK					Mascot
866.4618	866.4388	-0.023	-27	252	259	ASEYGLVK					Mascot
927.5873	927.5493	-0.038	-41	312	319	DVLLNLLK					Mascot
945.4352	945.4153	-0.0199	-21	374	380	FEFYDPK					Mascot
952.4887	952.4767	-0.012	-13	381	388	TPFFTSPR					Mascot
952.4887	952.4767	-0.012	-13	381	388	TPFFTSPR	51	99.189			Mascot
1013.5626	1013.5337	-0.0289	-29	459	468	VPIGVGENTK					Mascot
1023.5945	1023.5873	-0.0072	-7	416	424	IEHSIIGVR					Mascot
1023.5945	1023.5873	-0.0072	-7	416	424	IEHSIIGVR	53	99.446			Mascot
1170.7205	1170.6298	-0.0907	-77	312	321	DVLLNLLKSR					Mascot
1261.6359	1261.6375	0.0016	1	112	123	ATPAVPIGGCYR			Carbamidomethyl (C)[10]		Mascot
1261.6359	1261.6375	0.0016	1	112	123	ATPAVPIGGCYR	76	99.998	Carbamidomethyl (C)[10]		Mascot
1302.6624	1302.6641	0.0017	1	402	412	EAIISHGCFLR			Carbamidomethyl (C)[8]		Mascot
1302.6624	1302.6641	0.0017	1	402	412	EAIISHGCFLR	83	100	Carbamidomethyl (C)[8]		Mascot
1322.6191	1322.5989	-0.0202	-15	469	479	ISNCIIDMNAR			Carbamidomethyl (C)[4], Oxidation (M)[8]		Mascot
1322.6191	1322.5989	-0.0202	-15	469	479	ISNCIIDMNAR	37	79.715	Carbamidomethyl (C)[4], Oxidation (M)[8]		Mascot

1503.7882	1503.7552	-0.033	-22	196	206	KFIWVLEDYYK					Mascot
1553.7709	1553.7338	-0.0371	-24	298	310	YPYIASMGVYVFK			Oxidation (M)[7]		Mascot
1643.821	1643.8031	-0.0179	-11	140	153	IFVMTQFNSASLNR			Oxidation (M)[4]		Mascot
1646.8174	1646.8174	0	0	322	335	YAELHDFGSEILPR					Mascot
1646.8174	1646.8174	0	0	322	335	YAELHDFGSEILPR	108	100			Mascot
1652.8167	1652.7986	-0.0181	-11	283	297	VDTSFLNFAIDDPAK					Mascot
1674.8732	1674.8409	-0.0323	-19	453	468	LMSEGKVPIGVGENTK			Oxidation (M)[2]		Mascot
1709.8719	1709.8036	-0.0683	-40	298	311	YPYIASMGVYVFKR			Oxidation (M)[7]		Mascot
1756.9592	1756.9644	0.0052	3	209	223	SIEHILILSGDQLYR					Mascot
1756.9592	1756.9644	0.0052	3	209	223	SIEHILILSGDQLYR	110	100			Mascot
1770.7826	1770.7554	-0.0272	-15	359	373	SFFDANMALCEQPPK			Carbamidomethyl (C)[10], Oxidation (M)[7]		Mascot
1838.8776	1838.8567	-0.0209	-11	124	139	LIDIPMSNCFNSGINK			Carbamidomethyl (C)[9], Oxidation (M)[6]		Mascot
1910.8879	1910.8832	-0.0047	-2	490	505	EGVQEADRPEEGYYIR					Mascot
2055.9399	2055.9438	0.0039	2	233	251	HVDDNADITLSCAPVGES R			Carbamidomethyl (C)[12]		Mascot
2371.1924	2371.2029	0.0105	4	55	78	GPAATGAQCVLTSDASP ADTLVLR			Carbamidomethyl (C)[9]		Mascot
2371.1924	2371.2029	0.0105	4	55	78	GPAATGAQCVLTSDASP ADTLVLR	198	100	Carbamidomethyl (C)[9]		Mascot
2763.3162	2763.3372	0.021	8	336	358	ALHDHNVQAYVFTDYWE DIGTIR					Mascot
2862.4634	2862.4819	0.0185	6	84	111	NYADPNEVAAVILGGGT GTQLFPLTSTR					Mascot
2862.4634	2862.4819	0.0185	6	84	111	NYADPNEVAAVILGGGT GTQLFPLTSTR	239	100			Mascot

3	Glucose-1-phosphate adenylyltransferase large subunit, chloroplastic/amyloplastic (Fragment) OS=Triticum aestivum GN=AGA.7 PE=2 SV=1	GLGL3_WHEAT	56037.9	6.61	22	496	100	47.287	383	100

Peptide Information

Calc. Mass	Obsrv. Mass	± da	± ppm	Start Seq.	End Seq.	Sequence	Ion Score	C. I. %	Modification	Rank	Result Type
847.452	847.4308	-0.0212	-25	404	411	LNSGSELK					Mascot
866.4618	866.4388	-0.023	-27	232	239	ASEYGLVK					Mascot
908.5424	908.4543	-0.0881	-97	10	17	APLRAPQR					Mascot
927.5873	927.5493	-0.038	-41	292	299	DVLLNLLK					Mascot
945.4352	945.4153	-0.0199	-21	354	360	FEFYDPK					Mascot
952.4887	952.4767	-0.012	-13	361	368	TPFFTSPR					Mascot
952.4887	952.4767	-0.012	-13	361	368	TPFFTSPR	51	99.189			Mascot
1013.5626	1013.5337	-0.0289	-29	436	445	VPIGVGENTK					Mascot
1061.5626	1061.5651	0.0025	2	246	254	VVQFSEQPK					Mascot
1170.7205	1170.6298	-0.0907	-77	292	301	DVLLNLLKSR					Mascot

Calc. Mass	Obsrv. Mass	± da	± ppm	Start Seq.	End Seq.	Sequence	Ion Score	C. I. %	Modification	Rank	Result Type
1261.6359	1261.6375	0.0016	1	92	103	ATPAVPIGGCYR			Carbamidomethyl (C)[10]		Mascot
1261.6359	1261.6375	0.0016	1	92	103	ATPAVPIGGCYR	76	99.998	Carbamidomethyl (C)[10]		Mascot
1322.6191	1322.5989	-0.0202	-15	446	456	ISNCIIDMNAR			Carbamidomethyl (C)[4], Oxidation (M)[8]		Mascot
1322.6191	1322.5989	-0.0202	-15	446	456	ISNCIIDMNAR	37	79.715	Carbamidomethyl (C)[4], Oxidation (M)[8]		Mascot
1553.7709	1553.7338	-0.0371	-24	278	290	YPYIASMGVYVFK			Oxidation (M)[7]		Mascot
1643.821	1643.8031	-0.0179	-11	120	133	IFVMTQFNSASLNR			Oxidation (M)[4]		Mascot
1646.8174	1646.8174	0	0	302	315	YAELHDFGSEILPR					Mascot
1646.8174	1646.8174	0	0	302	315	YAELHDFGSEILPR	108	100			Mascot
1652.8167	1652.7986	-0.0181	-11	263	277	VDTSFLNFAIDDPAK					Mascot
1674.8732	1674.8409	-0.0323	-19	430	445	LMSEGKVPIGVGENTK			Oxidation (M)[2]		Mascot
1709.8719	1709.8036	-0.0683	-40	278	291	YPYIASMGVYVFKR			Oxidation (M)[7]		Mascot
1756.9592	1756.9644	0.0052	3	189	203	SIEHILILSGDQLYR					Mascot
1756.9592	1756.9644	0.0052	3	189	203	SIEHILILSGDQLYR	110	100			Mascot
1838.8776	1838.8567	-0.0209	-11	104	119	LIDIPMSNCFNSGINK			Carbamidomethyl (C)[9], Oxidation (M)[6]		Mascot
1910.8879	1910.8832	-0.0047	-2	467	482	EGVQEADRPEEGYYIR					Mascot
2055.9399	2055.9438	0.0039	2	213	231	HVDDNADITLSCAPVGES R			Carbamidomethyl (C)[12]		Mascot
2763.3162	2763.3372	0.021	8	316	338	ALHDHNVQAYVFTDYWE DIGTIR					Mascot

4	Glucose-1-phosphate adenylyltransferase large subunit 2, chloroplastic/amyloplastic OS=Zea mays GN=AGP2 PE=2 SV=1	GLGL2_MAIZE	58863.4	8.37	14	419	100	25.454	367	100	

Peptide Information

Calc. Mass	Obsrv. Mass	± da	± ppm	Start Seq.	End Seq.	Sequence	Ion Score	C. I. %	Modification	Rank	Result Type
820.3804	820.4044	0.024	29	13	19	ACMSPVR			Carbamidomethyl (C)[2]		Mascot
945.4352	945.4153	-0.0199	-21	371	377	FEFYDPK					Mascot
952.4887	952.4767	-0.012	-13	378	385	TPFFTSPR					Mascot
952.4887	952.4767	-0.012	-13	378	385	TPFFTSPR	51	99.189			Mascot
1013.5626	1013.5337	-0.0289	-29	456	465	VPIGVGENTK					Mascot
1204.6572	1204.6145	-0.0427	-35	2	12	QFSSVLPLEGK					Mascot
1261.6359	1261.6375	0.0016	1	108	119	ATPAVPIGGCYR			Carbamidomethyl (C)[10]		Mascot
1261.6359	1261.6375	0.0016	1	108	119	ATPAVPIGGCYR	76	99.998	Carbamidomethyl (C)[10]		Mascot
1503.7882	1503.7552	-0.033	-22	192	202	KFIWVLEDYYK					Mascot
1553.774	1553.7338	-0.0402	-26	410	423	ECAIEHSIVGVPSR			Carbamidomethyl (C)[2]		Mascot
1643.821	1643.8031	-0.0179	-11	136	149	IFVMTQFNSASLNR			Oxidation (M)[4]		Mascot
1738.771	1738.9294	0.1584	91	466	479	ISNCIIDMNCQGWK			Carbamidomethyl (C)[4,10]		Mascot
1770.7826	1770.7554	-0.0272	-15	356	370	SFFDANMALCEQPPK			Carbamidomethyl (C)[10], Oxidation (M)[7]		Mascot

	1838.8776	1838.8567	-0.0209	-11	120	135	LIDIPMSNCFNSGINK			Carbamidomethyl (C)[9], Oxidation (M)[6]		Mascot
	2055.9399	2055.9438	0.0039	2	229	247	HVDDNADITLSCAPVGES R			Carbamidomethyl (C)[12]		Mascot
	2862.4634	2862.4819	0.0185	6	80	107	NYADPNEVAAVILGGGT GTQLFPLTSTR					Mascot
	2862.4634	2862.4819	0.0185	6	80	107	NYADPNEVAAVILGGGT GTQLFPLTSTR	239	100			Mascot

5	Multifunctional CCA protein OS=Nitrosospira multiformis (strain ATCC 25196 / NCIMB 11849) GN=cca PE=3 SV=1	CCA_NITMU	46448.8	6.36	16	77	98.921	9.364	

Peptide Information

Calc. Mass	Obsrv. Mass	± da	± ppm	Start Seq.	End Seq.	Sequence	Ion Score	C. I. %	Modification	Rank	Result Type
901.489	901.4371	-0.0519	-58	174	180	VWQEIAR					Mascot
951.5258	951.4418	-0.084	-88	3	11	TYLVGGSVR					Mascot
970.4774	970.5287	0.0513	53	285	292	INPPNEMR					Mascot
974.5087	974.4543	-0.0544	-56	277	284	LVQGLCER			Carbamidomethyl (C)[6]		Mascot
1061.5851	1061.5651	-0.02	-19	360	368	LREAFQAAR					Mascot
1114.658	1114.5627	-0.0953	-85	91	100	RDLTINAIAK					Mascot
1169.5586	1169.6031	0.0445	38	257	266	GTTPPEEWPR					Mascot
1210.6613	1210.6331	-0.0282	-23	1	11	MKTYLVGGSVR					Mascot
1261.5953	1261.6375	0.0422	33	378	388	EMARNVSDPSR					Mascot
1261.5953	1261.6375	0.0422	33	378	388	EMARNVSDPSR					Mascot
1277.6572	1277.6416	-0.0156	-12	186	195	HPSRMFYALR					Mascot
1316.7103	1316.6576	-0.0527	-40	274	284	SVRLVQGLCER			Carbamidomethyl (C)[9]		Mascot
1318.6783	1318.6398	-0.0385	-29	369	381	SVDAGAIAKEMAR					Mascot
1338.6802	1338.6141	-0.0661	-49	123	134	HIGPAFTEDPVR					Mascot
1455.8279	1455.7698	-0.0581	-40	389	401	LPVAINTRVSETR					Mascot
1503.7737	1503.7552	-0.0185	-12	174	185	VWQEIARGLMER			Oxidation (M)[10]		Mascot
1706.9337	1706.7755	-0.1582	-93	123	137	HIGPAFTEDPVRVLR					Mascot

6	E3 ubiquitin-protein ligase IAP-3 OS=Orgyia pseudotsugata multicapsid polyhedrosis virus GN=IAP3 PE=1 SV=1	IAP3_NPVOP	30968.8	6.65	14	76	98.761	11.648	

Peptide Information

Calc. Mass	Obsrv. Mass	± da	± ppm	Start Seq.	End Seq.	Sequence	Ion Score	C. I. %	Modification	Rank	Result Type
866.4553	866.4388	-0.0165	-19	262	268	AVRMYQV					Mascot
906.4468	906.4552	0.0084	9	117	123	TFAEWPR					Mascot
1023.5543	1023.5873	0.033	32	175	182	CEYVLLVK			Carbamidomethyl (C)[1]		Mascot

1023.5543	1023.5873	0.033	32	175	182	CEYVLLVK			Carbamidomethyl (C)[1]		Mascot
1169.5182	1169.6031	0.0849	73	67	76	GDDPETDHKR					Mascot
1176.5142	1176.6185	0.1043	89	86	95	NNAHDTPHDR					Mascot
1187.5725	1187.6322	0.0597	50	5	16	AIGAPQEGADMK					Mascot
1204.6473	1204.6145	-0.0328	-27	117	126	TFAEWPRGLK					Mascot
1316.6681	1316.6576	-0.0105	-8	76	85	RWAPQCPFVR			Carbamidomethyl (C)[6]		Mascot
1643.8251	1643.8031	-0.022	-13	171	182	WYDRCEYVLLVK			Carbamidomethyl (C)[5]		Mascot
1668.7949	1668.8027	0.0078	5	86	100	NNAHDTPHDRAPPAR					Mascot
1709.8462	1709.8036	-0.0426	-25	185	198	DFVQRVMTEACVVR			Carbamidomethyl (C)[11]		Mascot
1807.8433	1807.8961	0.0528	29	38	53	MAASGFYYLGRGDEVR			Oxidation (M)[1]		Mascot
1892.8776	1892.9161	0.0385	20	245	261	CAAGVTTCPVCRGQLDK			Carbamidomethyl (C)[1,8,11]		Mascot
2371.0276	2371.2029	0.1753	74	157	174	DWEPDDAPWQQHARWYDR					Mascot
2371.0276	2371.2029	0.1753	74	157	174	DWEPDDAPWQQHARWYDR					Mascot

7 Heat-inducible transcription repressor HrcA OS=Bacillus clausii (strain KSM-K16) GN=hrcA PE=3 SV=1 | HRCA_BACSK | 38881.1 | 5.93 | 13 | 63 | 70.285 | 10.7

Peptide Information

Calc. Mass	Obsrv. Mass	± da	± ppm	Start Seq.	End Seq.	Sequence	Ion Score	C. I. %	Modification	Rank	Result Type
802.4053	802.4251	0.0198	25	20	27	SAEPVGSR					Mascot
934.4741	934.4881	0.014	15	65	72	VPSQQGYR					Mascot
1170.6842	1170.6298	-0.0544	-46	185	195	LVGTPLSSLQR					Mascot
1176.6624	1176.6185	-0.0439	-37	197	206	LNTELAQLFK					Mascot
1204.6783	1204.6145	-0.0638	-53	325	335	VVDSLSKDLTK					Mascot
1217.6484	1217.6367	-0.0117	-10	20	31	SAEPVGSRSISK					Mascot
1261.6423	1261.6375	-0.0048	-4	33	43	EDIPYSPATIR					Mascot
1261.6423	1261.6375	-0.0048	-4	33	43	EDIPYSPATIR					Mascot
1553.7264	1553.7338	0.0074	5	100	112	LQAMEEVFQESAR			Oxidation (M)[4]		Mascot
1577.8944	1577.8142	-0.0802	-51	134	146	LRHIQIIPMSNQK					Mascot
1641.8959	1641.822	-0.0739	-45	257	269	ALEEENLLQKWLR					Mascot
1703.9578	1703.8396	-0.1182	-69	253	266	LLYKALEEENLLQK					Mascot
2353.1956	2353.2478	0.0522	22	113	133	ILSEMTSYVSIVLGPESINER			Oxidation (M)[5]		Mascot
2763.3342	2763.3372	0.003	1	207	230	AHINNYEHILHMLQQSMVPASGEK			Oxidation (M)[12]		Mascot

8 S-adenosylmethionine decarboxylase proenzyme OS=Clostridium botulinum (strain Eklund 17B / Type B) GN=speD PE=3 SV=1 | SPED_CLOBB | 31765 | 5.06 | 11 | 60 | 49.536 | 4.306

Peptide Information

Calc. Mass	Obsrv. Mass	± da	± ppm	Start Seq.	End Seq.	Sequence	Ion Score	C. I. %	Modification	Rank	Result Type
804.4283	804.4122	-0.0161	-20	2	8	MLGLENK					Mascot
820.4233	820.4044	-0.0189	-23	2	8	MLGLENK			Oxidation (M)[1]		Mascot
951.4637	951.4418	-0.0219	-23	1	8	MMLGLENK			Oxidation (M)[1]		Mascot
994.4952	994.4808	-0.0144	-14	179	187	GFTRDVDGK					Mascot
1061.6024	1061.5651	-0.0373	-35	2	10	MLGLENKLK			Oxidation (M)[1]		Mascot
1176.6293	1176.6185	-0.0108	-9	53	62	LTNILCDVTK			Carbamidomethyl (C)[6]		Mascot
1284.7019	1284.6494	-0.0525	-41	250	259	QRLEIENNLR					Mascot
1503.7584	1503.7552	-0.0032	-2	100	112	SCNLGQIDILNTR			Carbamidomethyl (C)[2]		Mascot
1652.8378	1652.7986	-0.0392	-24	195	208	ITSIQDYIDENTLK					Mascot
1778.7867	1778.9137	0.127	71	39	52	DYIAYIDEQYNSER					Mascot
2411.2605	2411.2905	0.03	12	231	249	EIELQNYLFNRDVYEIKPK					Mascot
2862.4619	2862.4819	0.02	7	74	99	QDYDPQGASVTILISEETL AVKEIDK					Mascot
2862.4619	2862.4819	0.02	7	74	99	QDYDPQGASVTILISEETL AVKEIDK					Mascot

9	30S ribosomal protein S7 OS=Methanosarcina mazei (strain ATCC BAA-159 / DSM 3647 / Goe1 / Go1 / JCM 11833 / OCM 88) GN=rps7 PE=3 SV=1	RS7_METMA	21124.4	10.08	11	59	34.99	2.999

Peptide Information

Calc. Mass	Obsrv. Mass	± da	± ppm	Start Seq.	End Seq.	Sequence	Ion Score	C. I. %	Modification	Rank	Result Type
820.4563	820.4044	-0.0519	-63	115	122	YGGISVPK					Mascot
847.4454	847.4308	-0.0146	-17	57	63	LANNLMR			Oxidation (M)[6]		Mascot
851.4733	851.4301	-0.0432	-51	172	178	SFSINRK					Mascot
1013.5486	1013.5337	-0.0149	-15	123	131	AVDTAPQRR					Mascot
1061.6354	1061.5651	-0.0703	-66	113	122	LKYGGISVPK					Mascot
1553.8687	1553.7338	-0.1349	-87	26	40	YVSLAPVIVPHSSGK					Mascot
1575.8167	1575.7378	-0.0789	-50	7	19	IFGKWDPTEVEVR					Mascot
1577.8282	1577.8142	-0.014	-9	44	56	QQFNKSEISIVER					Mascot
1578.7904	1578.8844	0.094	60	57	70	LANNLMRTETNTGK			Oxidation (M)[6]		Mascot
1674.8479	1674.8409	-0.007	-4	153	168	SVAECLATELIGAANR			Carbamidomethyl (C)[5]		Mascot
1709.9697	1709.8036	-0.1661	-97	25	40	RYVSLAPVIVPHSSGK					Mascot

10	Ribulose bisphosphate carboxylase small chain, chloroplastic OS=Silene pratensis GN=RBCS PE=2 SV=1	RBS_SILPR	19969.9	8.57	9	57	0	8.43

Peptide Information

Calc. Mass	Obsrv. Mass	± da	± ppm	Start Seq.	End Seq.	Sequence	Ion Score	C. I. %	Modification	Rank	Result Type
914.4229	914.4911	0.0682	75	122	127	YWTMWK					Mascot
951.4894	951.4418	-0.0476	-50	30	38	STSAFPVSR					Mascot
962.5417	962.4715	-0.0702	-73	157	164	IIGFDNKR					Mascot
1042.5065	1042.5739	0.0674	65	148	156	NAYPDAHVR					Mascot
1302.6083	1302.6641	0.0558	43	54	63	VNCMQVWPPR			Carbamidomethyl (C)[3], Oxidation (M)[4]		Mascot
1302.6083	1302.6641	0.0558	43	54	63	VNCMQVWPPR			Carbamidomethyl (C)[3], Oxidation (M)[4]		Mascot
1641.8352	1641.822	-0.0132	-8	54	66	VNCMQVWPPRNLK			Carbamidomethyl (C)[3]		Mascot
1738.8945	1738.9294	0.0349	20	164	177	RQVQCISFIAYKPE			Carbamidomethyl (C)[5]		Mascot
2015.9797	2015.9988	0.0191	9	93	109	GWVPCLEFEVGPAHVYR			Carbamidomethyl (C)[5]		Mascot
2839.415	2839.3291	-0.0859	-30	1	29	MASLMSNAAVVTASTAA QANMVAPFSGLK					Mascot

Gel Idx/Pos	291/L19	Instr./Gel Origin	BA2151/Sample Project 20140814	Process Status	Analysis Succeeded
Plate [#] Name	[1] Sample Project 20140814	Instrument Sample Name		Spectra	11

Rank	Protein Name	Accession No.	Protein MW	Protein PI	Pep. Count	Protein Score	Protein Score C. I. %	Intensity Matched	Total Ion Score	Total Ion C. I. %	Confirmed
1	ATP synthase subunit alpha, chloroplastic OS=Triticum aestivum GN=atpA PE=3 SV=2	ATPA_WHEAT	55318.3	6.11	27	932	100	63.84	754	100	

Peptide Information

Calc. Mass	Obsrv. Mass	± da	± ppm	Start Seq.	End Seq.	Sequence	Ion Score	C. I. %	Modification	Rank	Result Type
815.4621	815.4515	-0.0106	-13	166	172	ELIIGDR					Mascot
822.4104	822.4001	-0.0103	-13	17	22	IEQYNR					Mascot
857.4839	857.4806	-0.0033	-4	24	31	VGIENIGR					Mascot
876.4971	876.4763	-0.0208	-24	273	279	QMSLLLR			Oxidation (M)[2]		Mascot
917.4799	917.4731	-0.0068	-7	406	413	TSQNQLAR					Mascot
920.52	920.5038	-0.0162	-18	457	463	FLDELRK					Mascot
961.4949	961.4859	-0.009	-9	120	128	GEIIASESR					Mascot
985.5789	985.571	-0.0079	-8	23	31	KVGIENIGR					Mascot
1013.5739	1013.5659	-0.008	-8	32	41	VVQVGDGIAR					Mascot
1107.5542	1107.5417	-0.0125	-11	15	22	ERIEQYNR					Mascot
1115.5691	1115.5665	-0.0026	-2	492	500	EAIQEQLER					Mascot
1115.5691	1115.5665	-0.0026	-2	492	500	EAIQEQLER	52	99.389			Mascot
1274.6951	1274.6492	-0.0459	-36	177	188	TAVATDTILNQK					Mascot
1282.7366	1282.7344	-0.0022	-2	129	140	LIESPAPSIISR					Mascot
1282.7366	1282.7344	-0.0022	-2	129	140	LIESPAPSIISR	72	99.993			Mascot
1292.7097	1292.6729	-0.0368	-28	481	491	TFTEQAEILLK					Mascot
1317.705	1317.6711	-0.0339	-26	256	266	HTLIIYDDLSK					Mascot
1416.7845	1416.7905	0.006	4	95	107	IAQIPVSEAYLGR					Mascot
1416.7845	1416.7905	0.006	4	95	107	IAQIPVSEAYLGR	124	100			Mascot
1438.8376	1438.7535	-0.0841	-58	129	141	LIESPAPSIISRR					Mascot
1507.7639	1507.7301	-0.0338	-22	444	456	GYLDSLEIEQVNK					Mascot
1507.7639	1507.7301	-0.0338	-22	444	456	GYLDSLEIEQVNK	66	99.974			Mascot
1519.8051	1519.8083	0.0032	2	189	202	GQGVICVYVAIGQR			Carbamidomethyl (C)[6]		Mascot
1519.8051	1519.8083	0.0032	2	189	202	GQGVICVYVAIGQR	55	99.679	Carbamidomethyl (C)[6]		Mascot
1553.7384	1553.7443	0.0059	4	285	297	EAYPGDVFYLHSR					Mascot
1553.7384	1553.7443	0.0059	4	285	297	EAYPGDVFYLHSR	103	100			Mascot

Calc. Mass	Obsrv. Mass	± da	± ppm	Start Seq.	End Seq.	Sequence	Ion Score	C. I. %	Modification	Rank	Result Type
1593.853	1593.7144	-0.1386	-87	267	279	QAQAYRQMSLLLR			Oxidation (M)[8]		Mascot
1607.8275	1607.8008	-0.0267	-17	467	480	DTKPQFQEIISSSK					Mascot
2121.0896	2121.1057	0.0161	8	42	61	IIGLGEIMSGELVEFAEGTR					Mascot
2137.0845	2137.0872	0.0027	1	42	61	IIGLGEIMSGELVEFAEGTR			Oxidation (M)[8]		Mascot
2137.0845	2137.0872	0.0027	1	42	61	IIGLGEIMSGELVEFAEGTR	132	100	Oxidation (M)[8]		Mascot
2225.2136	2225.2644	0.0508	23	120	140	GEIIASESRLIESPAPSIISR					Mascot
2289.2158	2289.2336	0.0178	8	142	162	SVYEPLQTGLIAIDSMIPIGR			Oxidation (M)[16]		Mascot
2316.1831	2316.2073	0.0242	10	423	443	QSQANPLPVEEQIATIYTGTR					Mascot
2316.1831	2316.2073	0.0242	10	423	443	QSQANPLPVEEQIATIYTGTR	148	100			Mascot
2445.3171	2445.3088	-0.0083	-3	141	162	RSVYEPLQTGLIAIDSMIPIGR			Oxidation (M)[17]		Mascot

2	ATP synthase subunit alpha, chloroplastic OS=Lolium perenne GN=atpA PE=3 SV=1	ATPA_LOLPR	55761.4	6.11	25	855	100	61.03	699	100	

Peptide Information

Calc. Mass	Obsrv. Mass	± da	± ppm	Start Seq.	End Seq.	Sequence	Ion Score	C. I. %	Modification	Rank	Result Type
815.4621	815.4515	-0.0106	-13	166	172	ELIIGDR					Mascot
822.4104	822.4001	-0.0103	-13	17	22	IEQYNR					Mascot
857.4839	857.4806	-0.0033	-4	24	31	VGIENIGR					Mascot
876.4971	876.4763	-0.0208	-24	273	279	QMSLLLR			Oxidation (M)[2]		Mascot
917.4799	917.4731	-0.0068	-7	406	413	TSQNQLAR					Mascot
961.4949	961.4859	-0.009	-9	120	128	GEIIASESR					Mascot
985.5789	985.571	-0.0079	-8	23	31	KVGIENIGR					Mascot
1013.5739	1013.5659	-0.008	-8	32	41	VVQVGDGIAR					Mascot
1107.5542	1107.5417	-0.0125	-11	15	22	ERIEQYNR					Mascot
1115.5691	1115.5665	-0.0026	-2	492	500	EAIQEQLER					Mascot
1115.5691	1115.5665	-0.0026	-2	492	500	EAIQEQLER	52	99.389			Mascot
1274.6951	1274.6492	-0.0459	-36	177	188	TAVATDTILNQK					Mascot
1282.7366	1282.7344	-0.0022	-2	129	140	LIESPAPSIISR					Mascot
1282.7366	1282.7344	-0.0022	-2	129	140	LIESPAPSIISR	72	99.993			Mascot
1292.7097	1292.6729	-0.0368	-28	481	491	TFTEQAEILLK					Mascot
1317.705	1317.6711	-0.0339	-26	256	266	HTLIIYDDLSK					Mascot
1416.7845	1416.7905	0.006	4	95	107	IAQIPVSEAYLGR					Mascot
1416.7845	1416.7905	0.006	4	95	107	IAQIPVSEAYLGR	124	100			Mascot
1438.8376	1438.7535	-0.0841	-58	129	141	LIESPAPSIISRR					Mascot
1507.7639	1507.7301	-0.0338	-22	444	456	GYLDSLEIEQVNK					Mascot

Calc. Mass	Obsrv. Mass	± da	± ppm	Start Seq.	End Seq.	Sequence	Ion Score	C. I. %	Modification	Rank	Result Type
1507.7639	1507.7301	-0.0338	-22	444	456	GYLDSLEIEQVNK	66	99.974			Mascot
1553.7384	1553.7443	0.0059	4	285	297	EAYPGDVFYLHSR					Mascot
1553.7384	1553.7443	0.0059	4	285	297	EAYPGDVFYLHSR	103	100			Mascot
1593.853	1593.7144	-0.1386	-87	267	279	QAQAYRQMSLLLR			Oxidation (M)[8]		Mascot
1607.8275	1607.8008	-0.0267	-17	467	480	DTKPQFQEILSSSK					Mascot
2121.0896	2121.1057	0.0161	8	42	61	IIGLGEIMSGELVEFAEGTR					Mascot
2137.0845	2137.0872	0.0027	1	42	61	IIGLGEIMSGELVEFAEGTR			Oxidation (M)[8]		Mascot
2137.0845	2137.0872	0.0027	1	42	61	IIGLGEIMSGELVEFAEGTR	132	100	Oxidation (M)[8]		Mascot
2225.2136	2225.2644	0.0508	23	120	140	GEIIASESRLIESPAPSIISR					Mascot
2289.2158	2289.2336	0.0178	8	142	162	SVYEPLQTGLIAIDSMIPIGR			Oxidation (M)[16]		Mascot
2316.1831	2316.2073	0.0242	10	423	443	QSQANPLPVEEQIATIYTGTR					Mascot
2316.1831	2316.2073	0.0242	10	423	443	QSQANPLPVEEQIATIYTGTR	148	100			Mascot
2445.3171	2445.3088	-0.0083	-3	141	162	RSVYEPLQTGLIAIDSMIPIGR			Oxidation (M)[17]		Mascot

3	ATP synthase subunit alpha, chloroplastic OS=Hordeum vulgare GN=atpA PE=3 SV=1	ATPA_HORVU	55317.3	6.32	26	787	100	60.526	622	100

Peptide Information

Calc. Mass	Obsrv. Mass	± da	± ppm	Start Seq.	End Seq.	Sequence	Ion Score	C. I. %	Modification	Rank	Result Type
815.4621	815.4515	-0.0106	-13	166	172	ELIIGDR					Mascot
822.4104	822.4001	-0.0103	-13	17	22	IEQYNR					Mascot
857.4839	857.4806	-0.0033	-4	24	31	VGIENIGR					Mascot
876.4971	876.4763	-0.0208	-24	273	279	QMSLLLR			Oxidation (M)[2]		Mascot
917.4799	917.4731	-0.0068	-7	406	413	TSQNQLAR					Mascot
920.52	920.5038	-0.0162	-18	457	463	FLDELRK					Mascot
961.4949	961.4859	-0.009	-9	120	128	GEIIASESR					Mascot
985.5789	985.571	-0.0079	-8	23	31	KVGIENIGR					Mascot
1013.5739	1013.5659	-0.008	-8	32	41	VVQVGDGIAR					Mascot
1107.5542	1107.5417	-0.0125	-11	15	22	ERIEQYNR					Mascot
1115.5691	1115.5665	-0.0026	-2	492	500	EAIQEQLER					Mascot
1115.5691	1115.5665	-0.0026	-2	492	500	EAIQEQLER	52	99.389			Mascot
1274.6951	1274.6492	-0.0459	-36	177	188	TAVATDTILNQK					Mascot
1282.7366	1282.7344	-0.0022	-2	129	140	LIESPAPSIISR					Mascot
1282.7366	1282.7344	-0.0022	-2	129	140	LIESPAPSIISR	72	99.993			Mascot
1292.7097	1292.6729	-0.0368	-28	481	491	TFTEQAEILLK					Mascot

Calc. Mass	Obsrv. Mass	± da	± ppm	Start Seq.	End Seq.	Sequence	Ion Score	C. I. %	Modification	Rank	Result Type
1317.705	1317.6711	-0.0339	-26	256	266	HTLIIYDDLSK					Mascot
1416.7845	1416.7905	0.006	4	95	107	IAQIPVSEAYLGR					Mascot
1416.7845	1416.7905	0.006	4	95	107	IAQIPVSEAYLGR	124	100			Mascot
1438.8376	1438.7535	-0.0841	-58	129	141	LIESPAPSIISRR					Mascot
1507.7639	1507.7301	-0.0338	-22	444	456	GYLDSLEIEQVNK					Mascot
1507.7639	1507.7301	-0.0338	-22	444	456	GYLDSLEIEQVNK	66	99.974			Mascot
1519.8051	1519.8083	0.0032	2	189	202	GQGVICVYVAIGQR			Carbamidomethyl (C)[6]		Mascot
1519.8051	1519.8083	0.0032	2	189	202	GQGVICVYVAIGQR	55	99.679	Carbamidomethyl (C)[6]		Mascot
1553.7384	1553.7443	0.0059	4	285	297	EAYPGDVFYLHSR					Mascot
1553.7384	1553.7443	0.0059	4	285	297	EAYPGDVFYLHSR	103	100			Mascot
1593.853	1593.7144	-0.1386	-87	267	279	QAQAYRQMSLLLR			Oxidation (M)[8]		Mascot
1607.8275	1607.8008	-0.0267	-17	467	480	DTKPQFQEIISSSK					Mascot
2225.2136	2225.2644	0.0508	23	120	140	GEIIASESRLIESPAPSIIS R					Mascot
2289.2158	2289.2336	0.0178	8	142	162	SVYEPLQTGLIAIDSMIPI GR			Oxidation (M)[16]		Mascot
2316.1831	2316.2073	0.0242	10	423	443	QSQANPLPVEEQIATIYT GTR					Mascot
2316.1831	2316.2073	0.0242	10	423	443	QSQANPLPVEEQIATIYT GTR	148	100			Mascot
2445.3171	2445.3088	-0.0083	-3	141	162	RSVYEPLQTGLIAIDSMIPI GR			Oxidation (M)[17]		Mascot

4	ATP synthase subunit alpha, chloroplastic OS=Agrostis stolonifera GN=atpA PE=3 SV=1	ATPA_AGRST	55491.4	6.11	23	761	100	58.911	627	100	

Peptide Information

Calc. Mass	Obsrv. Mass	± da	± ppm	Start Seq.	End Seq.	Sequence	Ion Score	C. I. %	Modification	Rank	Result Type
815.4621	815.4515	-0.0106	-13	166	172	ELIIGDR					Mascot
822.4104	822.4001	-0.0103	-13	17	22	IEQYNR					Mascot
857.4839	857.4806	-0.0033	-4	24	31	VGIENIGR					Mascot
876.4971	876.4763	-0.0208	-24	273	279	QMSLLLR			Oxidation (M)[2]		Mascot
917.4799	917.4731	-0.0068	-7	406	413	TSQNQLAR					Mascot
920.52	920.5038	-0.0162	-18	457	463	FLDELRK					Mascot
961.4949	961.4859	-0.009	-9	120	128	GEIIASESR					Mascot
985.5789	985.571	-0.0079	-8	23	31	KVGIENIGR					Mascot
1013.5739	1013.5659	-0.008	-8	32	41	VVQVGDGIAR					Mascot
1107.5542	1107.5417	-0.0125	-11	15	22	ERIEQYNR					Mascot
1115.5691	1115.5665	-0.0026	-2	492	500	EAIQEQLER					Mascot
1115.5691	1115.5665	-0.0026	-2	492	500	EAIQEQLER	52	99.389			Mascot
1274.6951	1274.6492	-0.0459	-36	177	188	TAVATDTILNQK					Mascot

Calc. Mass	Obsrv. Mass	± da	± ppm	Start Seq.	End Seq.	Sequence	Ion Score	C. I. %	Modification	Rank	Result Type
1292.7097	1292.6729	-0.0368	-28	481	491	TFTEQAEILLK					Mascot
1317.705	1317.6711	-0.0339	-26	256	266	HTLIIYDDLSK					Mascot
1416.7845	1416.7905	0.006	4	95	107	IAQIPVSEAYLGR					Mascot
1416.7845	1416.7905	0.006	4	95	107	IAQIPVSEAYLGR	124	100			Mascot
1507.7639	1507.7301	-0.0338	-22	444	456	GYLDSLEIEQVNK					Mascot
1507.7639	1507.7301	-0.0338	-22	444	456	GYLDSLEIEQVNK	66	99.974			Mascot
1553.7384	1553.7443	0.0059	4	285	297	EAYPGDVFYLHSR					Mascot
1553.7384	1553.7443	0.0059	4	285	297	EAYPGDVFYLHSR	103	100			Mascot
1593.853	1593.7144	-0.1386	-87	267	279	QAQAYRQMSLLLR			Oxidation (M)[8]		Mascot
1607.8275	1607.8008	-0.0267	-17	467	480	DTKPQFQEIISSSK					Mascot
2121.0896	2121.1057	0.0161	8	42	61	IIGLGEIMSGELVEFAEGT R					Mascot
2137.0845	2137.0872	0.0027	1	42	61	IIGLGEIMSGELVEFAEGT R			Oxidation (M)[8]		Mascot
2137.0845	2137.0872	0.0027	1	42	61	IIGLGEIMSGELVEFAEGT R	132	100	Oxidation (M)[8]		Mascot
2289.2158	2289.2336	0.0178	8	142	162	SVYEPLQTGLIAIDSMIPI GR			Oxidation (M)[16]		Mascot
2316.1831	2316.2073	0.0242	10	423	443	QSQANPLPVEEQIATIYT GTR					Mascot
2316.1831	2316.2073	0.0242	10	423	443	QSQANPLPVEEQIATIYT GTR	148	100			Mascot
2445.3171	2445.3088	-0.0083	-3	141	162	RSVYEPLQTGLIAIDSMIPI GR			Oxidation (M)[17]		Mascot

5	ATP synthase subunit alpha, chloroplastic OS=Oryza sativa GN=atpA PE=2 SV=1	ATPA_ORYSA	55687.5	5.95	20	516	100	46.528	412	100	

Protein Group

ATP synthase subunit alpha, chloroplastic OS=Oryza nivara GN=atpA PE=3 SV=1	ATPA_ORYNI	55687.5	5.9499 998092 6514
ATP synthase subunit alpha, chloroplastic OS=Oryza sativa subsp. indica GN=atpA PE=2 SV=1	ATPA_ORYSI	55687.5	5.9499 998092 6514
ATP synthase subunit alpha, chloroplastic OS=Oryza sativa subsp. japonica GN=atpA PE=2 SV=1	ATPA_ORYSJ	55687.5	5.9499 998092 6514

Peptide Information

Calc. Mass	Obsrv. Mass	± da	± ppm	Start Seq.	End Seq.	Sequence	Ion Score	C. I. %	Modification	Rank	Result Type
815.4621	815.4515	-0.0106	-13	166	172	ELIIGDR					Mascot
822.4104	822.4001	-0.0103	-13	17	22	IEQYNR					Mascot
857.4839	857.4806	-0.0033	-4	24	31	VGIENIGR					Mascot
876.4971	876.4763	-0.0208	-24	273	279	QMSLLLR			Oxidation (M)[2]		Mascot
917.4799	917.4731	-0.0068	-7	406	413	TSQNQLAR					Mascot
920.52	920.5038	-0.0162	-18	457	463	FLDELRK					Mascot

985.5789	985.571	-0.0079	-8	23	31	KVGIENIGR					Mascot
1013.5739	1013.5659	-0.008	-8	32	41	VVQVGDGIAR					Mascot
1107.5542	1107.5417	-0.0125	-11	15	22	ERIEQYNR					Mascot
1115.5691	1115.5665	-0.0026	-2	492	500	EAIQEQLER					Mascot
1115.5691	1115.5665	-0.0026	-2	492	500	EAIQEQLER	52	99.389			Mascot
1274.6951	1274.6492	-0.0459	-36	177	188	TAVATDTILNQK					Mascot
1317.705	1317.6711	-0.0339	-26	256	266	HTLIIYDDLSK					Mascot
1321.6998	1321.611	-0.0888	-67	444	455	GYLDSLEIGQVK					Mascot
1416.7845	1416.7905	0.006	4	95	107	IAQIPVSEAYLGR					Mascot
1416.7845	1416.7905	0.006	4	95	107	IAQIPVSEAYLGR	124	100			Mascot
1553.7384	1553.7443	0.0059	4	285	297	EAYPGDVFYLHSR					Mascot
1553.7384	1553.7443	0.0059	4	285	297	EAYPGDVFYLHSR	103	100			Mascot
1593.853	1593.7144	-0.1386	-87	267	279	QAQAYRQMSLLLR			Oxidation (M)[8]		Mascot
1607.8275	1607.8008	-0.0267	-17	467	480	DTKPQFQEIISSSK					Mascot
2121.0896	2121.1057	0.0161	8	42	61	IIGLGEIMSGELVEFAEGT R					Mascot
2137.0845	2137.0872	0.0027	1	42	61	IIGLGEIMSGELVEFAEGT R			Oxidation (M)[8]		Mascot
2137.0845	2137.0872	0.0027	1	42	61	IIGLGEIMSGELVEFAEGT R	132	100	Oxidation (M)[8]		Mascot
2289.2158	2289.2336	0.0178	8	142	162	SVYEPLQTGLIAIDSMIPI GR			Oxidation (M)[16]		Mascot
2445.3171	2445.3088	-0.0083	-3	141	162	RSVYEPLQTGLIAIDSMIPI GR			Oxidation (M)[17]		Mascot

6	ATP synthase subunit alpha, chloroplastic OS=Saccharum officinarum GN=atpA PE=3 SV=1	ATPA_SACOF	55713.4	5.87	19	508	100	46.425	412	100	

Protein Group

ATP synthase subunit alpha, chloroplastic OS=Saccharum hybrid GN=atpA PE=2 SV=2	ATPA_SACHY	55773.4	5.8699 998855 5908
ATP synthase subunit alpha, chloroplastic OS=Sorghum bicolor GN=atpA PE=3 SV=1	ATPA_SORBI	55713.4	5.8699 998855 5908

Peptide Information

Calc. Mass	Obsrv. Mass	± da	± ppm	Start Seq.	End Seq.	Sequence	Ion Score	C. I. %	Modification	Rank	Result Type
815.4621	815.4515	-0.0106	-13	166	172	ELIIGDR					Mascot
822.4104	822.4001	-0.0103	-13	17	22	IEQYNR					Mascot
857.4839	857.4806	-0.0033	-4	24	31	VGIENIGR					Mascot
876.4971	876.4763	-0.0208	-24	273	279	QMSLLLR			Oxidation (M)[2]		Mascot
917.4799	917.4731	-0.0068	-7	406	413	TSQNQLAR					Mascot
920.52	920.5038	-0.0162	-18	457	463	FLDELRK					Mascot

985.5789	985.571	-0.0079	-8	23	31	KVGIENIGR					Mascot
1013.5739	1013.5659	-0.008	-8	32	41	VVQVGDGIAR					Mascot
1107.5542	1107.5417	-0.0125	-11	15	22	ERIEQYNR					Mascot
1115.5691	1115.5665	-0.0026	-2	492	500	EAIQEQLER					Mascot
1115.5691	1115.5665	-0.0026	-2	492	500	EAIQEQLER	52	99.389			Mascot
1274.6951	1274.6492	-0.0459	-36	177	188	TAVATDTILNQK					Mascot
1317.705	1317.6711	-0.0339	-26	256	266	HTLIIYDDLSK					Mascot
1416.7845	1416.7905	0.006	4	95	107	IAQIPVSEAYLGR					Mascot
1416.7845	1416.7905	0.006	4	95	107	IAQIPVSEAYLGR	124	100			Mascot
1553.7384	1553.7443	0.0059	4	285	297	EAYPGDVFYLHSR					Mascot
1553.7384	1553.7443	0.0059	4	285	297	EAYPGDVFYLHSR	103	100			Mascot
1593.853	1593.7144	-0.1386	-87	267	279	QAQAYRQMSLLLR			Oxidation (M)[8]		Mascot
1607.8275	1607.8008	-0.0267	-17	467	480	DTKPQFQEIISSSK					Mascot
2121.0896	2121.1057	0.0161	8	42	61	IIGLGEIMSGELVEFAEGT R					Mascot
2137.0845	2137.0872	0.0027	1	42	61	IIGLGEIMSGELVEFAEGT R			Oxidation (M)[8]		Mascot
2137.0845	2137.0872	0.0027	1	42	61	IIGLGEIMSGELVEFAEGT R	132	100	Oxidation (M)[8]		Mascot
2289.2158	2289.2336	0.0178	8	142	162	SVYEPLQTGLIAIDSMIPI GR			Oxidation (M)[16]		Mascot
2445.3171	2445.3088	-0.0083	-3	141	162	RSVYEPLQTGLIAIDSMIPI GR			Oxidation (M)[17]		Mascot

7	ATP synthase subunit alpha, chloroplastic OS=Zea mays GN=atpA PE=3 SV=1	ATPA_MAIZE	55729.4	5.87	19	404	100	33.835	309	100	

Peptide Information

Calc. Mass	Obsrv. Mass	± da	± ppm	Start Seq.	End Seq.	Sequence	Ion Score	C. I. %	Modification	Rank	Result Type
815.4621	815.4515	-0.0106	-13	166	172	ELIIGDR					Mascot
822.4104	822.4001	-0.0103	-13	17	22	IEQYNR					Mascot
857.4839	857.4806	-0.0033	-4	24	31	VGIENIGR					Mascot
876.4971	876.4763	-0.0208	-24	273	279	QMSLLLR			Oxidation (M)[2]		Mascot
917.4799	917.4731	-0.0068	-7	406	413	TSQNQLAR					Mascot
920.52	920.5038	-0.0162	-18	457	463	FLDELRK					Mascot
985.5789	985.571	-0.0079	-8	23	31	KVGIENIGR					Mascot
1013.5739	1013.5659	-0.008	-8	32	41	VVQVGDGIAR					Mascot
1107.5542	1107.5417	-0.0125	-11	15	22	ERIEQYNR					Mascot
1115.5691	1115.5665	-0.0026	-2	492	500	EAIQEQLER					Mascot
1115.5691	1115.5665	-0.0026	-2	492	500	EAIQEQLER	52	99.389			Mascot
1274.6951	1274.6492	-0.0459	-36	177	188	TAVATDTILNQK					Mascot

1317.705	1317.6711	-0.0339	-26	256	266	HTLIIYDDLSK					Mascot
1416.7845	1416.7905	0.006	4	95	107	IAQIPVSEAYLGR					Mascot
1416.7845	1416.7905	0.006	4	95	107	IAQIPVSEAYLGR	124	100			Mascot
1569.7697	1569.7413	-0.0284	-18	285	297	EAYLGDVFYLHSR					Mascot
1593.853	1593.7144	-0.1386	-87	267	279	QAQAYRQMSLLLR			Oxidation (M)[8]		Mascot
1607.8275	1607.8008	-0.0267	-17	467	480	DTKPQFQEIISSSK					Mascot
2121.0896	2121.1057	0.0161	8	42	61	IIGLGEIMSGELVEFAEGT R					Mascot
2137.0845	2137.0872	0.0027	1	42	61	IIGLGEIMSGELVEFAEGT R			Oxidation (M)[8]		Mascot
2137.0845	2137.0872	0.0027	1	42	61	IIGLGEIMSGELVEFAEGT R	132	100	Oxidation (M)[8]		Mascot
2289.2158	2289.2336	0.0178	8	142	162	SVYEPLQTGLIAIDSMIPI GR			Oxidation (M)[16]		Mascot
2445.3171	2445.3088	-0.0083	-3	141	162	RSVYEPLQTGLIAIDSMIPI GR			Oxidation (M)[17]		Mascot

8	ATP synthase subunit alpha, chloroplastic OS=Populus trichocarpa GN=atpA PE=3 SV=1	ATPA_POPTR	55345	5.2	15	291	100	35.469	228	100	

Protein Group

ATP synthase subunit alpha, chloroplastic OS=Populus alba GN=atpA PE=3 SV=1	ATPA_POPAL	55345	5.1999 998092 6514

Peptide Information

Calc. Mass	Obsrv. Mass	± da	± ppm	Start Seq.	End Seq.	Sequence	Ion Score	C. I. %	Modification	Rank	Result Type
815.4621	815.4515	-0.0106	-13	166	172	ELIIGDR					Mascot
822.4104	822.4001	-0.0103	-13	17	22	IEQYNR					Mascot
876.4825	876.4763	-0.0062	-7	501	507	FLLQEQV					Mascot
1107.5542	1107.5417	-0.0125	-11	15	22	ERIEQYNR					Mascot
1251.6467	1251.6694	0.0227	18	481	491	TFTEEAEALLK					Mascot
1281.6686	1281.678	0.0094	7	470	480	TQFQEIISSTK					Mascot
1317.705	1317.6711	-0.0339	-26	256	266	HTLIIYDDLSK					Mascot
1416.7845	1416.7905	0.006	4	95	107	IAQIPVSEAYLGR					Mascot
1416.7845	1416.7905	0.006	4	95	107	IAQIPVSEAYLGR	124	100			Mascot
1471.7864	1471.7582	-0.0282	-19	203	216	ASSVAQVVNALQER					Mascot
1553.7384	1553.7443	0.0059	4	285	297	EAYPGDVFYLHSR					Mascot
1553.7384	1553.7443	0.0059	4	285	297	EAYPGDVFYLHSR	103	100			Mascot
1593.853	1593.7144	-0.1386	-87	267	279	QAQAYRQMSLLLR			Oxidation (M)[8]		Mascot
1646.8894	1646.8112	-0.0782	-47	1	14	MVTIRADEISNIIR			Oxidation (M)[1]		Mascot
1990.9902	1990.9777	-0.0125	-6	492	507	EAIQEQMERFLLQEQV					Mascot
2289.2158	2289.2336	0.0178	8	142	162	SVYEPLQTGLIAIDSMIPI			Oxidation (M)[16]		Mascot

						GR					
2445.3171	2445.3088	-0.0083	-3	141	162	RSVYEPLQTGLIAIDSMIPI GR			Oxidation (M)[17]		Mascot

9	ATP synthase subunit alpha, chloroplastic OS=Nymphaea alba GN=atpA PE=3 SV=1	ATPA_NYMAL	55539.3	5.58	14	284	100	37.216	228	100	

Peptide Information

Calc. Mass	Obsrv. Mass	± da	± ppm	Start Seq.	End Seq.	Sequence	Ion Score	C. I. %	Modification	Rank	Result Type
815.4621	815.4515	-0.0106	-13	166	172	ELIIGDR					Mascot
822.4104	822.4001	-0.0103	-13	17	22	IEQYNR					Mascot
876.4971	876.4763	-0.0208	-24	273	279	QMSLLLR			Oxidation (M)[2]		Mascot
1044.5684	1044.5809	0.0125	12	6	14	AEEISNIIR					Mascot
1107.5542	1107.5417	-0.0125	-11	15	22	ERIEQYNR					Mascot
1274.6951	1274.6492	-0.0459	-36	177	188	TAVATDTILNQK					Mascot
1282.7743	1282.7344	-0.0399	-31	457	466	FIVQLRTHLR					Mascot
1282.7743	1282.7344	-0.0399	-31	457	466	FIVQLRTHLR					Mascot
1317.705	1317.6711	-0.0339	-26	256	266	HTLIIYDDLSK					Mascot
1416.7845	1416.7905	0.006	4	95	107	IAQIPVSEAYLGR					Mascot
1416.7845	1416.7905	0.006	4	95	107	IAQIPVSEAYLGR	124	100			Mascot
1553.7384	1553.7443	0.0059	4	285	297	EAYPGDVFYLHSR					Mascot
1553.7384	1553.7443	0.0059	4	285	297	EAYPGDVFYLHSR	103	100			Mascot
1593.853	1593.7144	-0.1386	-87	267	279	QAQAYRQMSLLLR			Oxidation (M)[8]		Mascot
2271.2344	2271.2744	0.04	18	120	140	GEILASEYRLIESPAPGIIS R					Mascot
2289.2158	2289.2336	0.0178	8	142	162	SVYEPLQTGLIAIDSMIPI GR			Oxidation (M)[16]		Mascot
2445.3171	2445.3088	-0.0083	-3	141	162	RSVYEPLQTGLIAIDSMIPI GR			Oxidation (M)[17]		Mascot

10	ATP synthase subunit alpha, chloroplastic OS=Atropa belladonna GN=atpA PE=3 SV=1	ATPA_ATRBE	55476.1	5.26	14	284	100	36.229	228	100	

Peptide Information

Calc. Mass	Obsrv. Mass	± da	± ppm	Start Seq.	End Seq.	Sequence	Ion Score	C. I. %	Modification	Rank	Result Type
815.4621	815.4515	-0.0106	-13	166	172	ELIIGDR					Mascot
822.4104	822.4001	-0.0103	-13	17	22	IEQYNR					Mascot
876.4971	876.4763	-0.0208	-24	273	279	QMSLLLR			Oxidation (M)[2]		Mascot
1107.5542	1107.5417	-0.0125	-11	15	22	ERIEQYNR					Mascot
1251.6467	1251.6694	0.0227	18	481	491	TFTEEAEALLK					Mascot
1281.7566	1281.678	-0.0786	-61	457	466	FLVELRTYLK					Mascot

1317.705	1317.6711	-0.0339	-26	256	266	HTLIIYDDLSK				Mascot
1416.7845	1416.7905	0.006	4	95	107	IAQIPVSEAYLGR				Mascot
1416.7845	1416.7905	0.006	4	95	107	IAQIPVSEAYLGR	124	100		Mascot
1553.7384	1553.7443	0.0059	4	285	297	EAYPGDVFYLHSR				Mascot
1553.7384	1553.7443	0.0059	4	285	297	EAYPGDVFYLHSR	103	100		Mascot
1593.853	1593.7144	-0.1386	-87	267	279	QAQAYRQMSLLLR			Oxidation (M)[8]	Mascot
1646.8894	1646.8112	-0.0782	-47	1	14	MVTIRADEISNIIR			Oxidation (M)[1]	Mascot
2225.1006	2225.2644	0.1638	74	481	499	TFTEEAEALLKEAIQEQMK			Oxidation (M)[18]	Mascot
2289.2158	2289.2336	0.0178	8	142	162	SVYEPLQTGLIAIDSMIPIGR			Oxidation (M)[16]	Mascot
2445.3171	2445.3088	-0.0083	-3	141	162	RSVYEPLQTGLIAIDSMIPIGR			Oxidation (M)[17]	Mascot

Gel Idx/Pos	292/L20	Instr./Gel Origin	BA2151/Sample Project 20140814	Process Status	Analysis Succeeded
Plate [#] Name	[1] Sample Project 20140814	Instrument Sample Name		Spectra	11

Rank	Protein Name	Accession No.	Protein MW	Protein PI	Pep. Count	Protein Score	Protein Score C. I. %	Intensity Matched	Total Ion Score	Total Ion C. I. %	Confirmed
1	4-hydroxy-tetrahydrodipicolinate reductase OS=Thermobifida fusca (strain YX) GN=dapB PE=3 SV=1	DAPB_THEFY	25958.5	5.62	9	60	40.71	21.235			

Peptide Information

Calc. Mass	Obsrv. Mass	± da	± ppm	Start Seq.	End Seq.	Sequence	Ion Score	C. I. %	Modification	Rank	Result Type
875.5098	875.4527	-0.0571	-65	4	12	VGVFGALGR					Mascot
885.5152	885.5341	0.0189	21	228	235	KVASLPDR					Mascot
1263.7242	1263.7505	0.0263	21	1	12	MIKVGVFGALGR			Oxidation (M)[1]		Mascot
1263.7242	1263.7505	0.0263	21	1	12	MIKVGVFGALGR	10	0	Oxidation (M)[1]		Mascot
1320.6981	1320.7614	0.0633	48	216	227	ESFMPGVLLGVR			Oxidation (M)[4]		Mascot
1890.9668	1891.0233	0.0565	30	136	154	ADAPSGTAYRTAELIGAA R					Mascot
1906.9254	1907.0146	0.0892	47	155	174	AHAGLGDAPDATTSELP GAR					Mascot
2045.0056	2045.01	0.0044	2	210	227	HDSMNRESFMPGVLLGV R					Mascot
2061.0005	2061.0049	0.0044	2	210	227	HDSMNRESFMPGVLLGV R			Oxidation (M)[4]		Mascot
2194.104	2194.0408	-0.0632	-29	63	82	WLIEHGIHAVVGTTGFDD AR					Mascot
2194.104	2194.0408	-0.0632	-29	63	82	WLIEHGIHAVVGTTGFDD AR					Mascot
2216.0679	2216.0227	-0.0452	-20	20	41	AVEAADDTELVAGIDVGD DRGK					Mascot

Rank	Protein Name	Accession No.	Protein MW	Protein PI	Pep. Count	Protein Score	Protein Score C. I. %	Intensity Matched	Total Ion Score	Total Ion C. I. %	Confirmed
2	Probable uridylyltransferase SAR2262 OS=Staphylococcus aureus (strain MRSA252) GN=SAR2262 PE=3 SV=1	URTF_STAAR	45077.8	4.95	12	55	0	7.489			

Peptide Information

Calc. Mass	Obsrv. Mass	± da	± ppm	Start Seq.	End Seq.	Sequence	Ion Score	C. I. %	Modification	Rank	Result Type
923.5421	923.4944	-0.0477	-52	130	136	QLKELHR					Mascot
929.5414	929.4495	-0.0919	-99	2	9	LDKNQLAK					Mascot
1116.6525	1116.5741	-0.0784	-70	308	317	LGFILNAVNR					Mascot
1116.6525	1116.5741	-0.0784	-70	308	317	LGFILNAVNR					Mascot
1262.6475	1262.7115	0.064	51	55	66	TIDDVSAVSEVK					Mascot
1284.7271	1284.6344	-0.0927	-72	80	90	RLEEQGLQAIK					Mascot

1363.6675	1363.7332	0.0657	48	198	210	IMETPNGNGGVFK					Mascot
1438.8417	1438.7573	-0.0844	-59	318	329	ELPYHLAIKNLK					Mascot
1534.7417	1534.8468	0.1051	68	21	33	IMSNNEKEALEEK					Mascot
1634.8319	1634.7838	-0.0481	-29	91	106	EGQFAVLLMAGGQGTR					Mascot
1891.0059	1891.0233	0.0174	9	28	44	EALEEKVASLDLDFIAK					Mascot
2112.0906	2111.9329	-0.1577	-75	91	110	EGQFAVLLMAGGQGTRLGYK			Oxidation (M)[9]		Mascot
3527.791	3527.626	-0.165	-47	178	210	QDNIVALSEAGQLILNQQGRIMETPNGNGGVFK			Oxidation (M)[22]		Mascot

3 Exodeoxyribonuclease 7 large subunit OS=Campylobacter lari (strain RM2100 / D67 / ATCC BAA-1060) GN=xseA PE=3 SV=1 | EX7L_CAMLR | 44155.3 | 8.78 | 14 | 53 | 0 | 2.336

Peptide Information

Calc. Mass	Obsrv. Mass	± da	± ppm	Start Seq.	End Seq.	Sequence	Ion Score	C. I. %	Modification	Rank	Result Type
826.3763	826.3635	-0.0128	-15	155	160	EYNLCK			Carbamidomethyl (C)[5]		Mascot
842.5094	842.4449	-0.0645	-77	351	357	NLINLQK					Mascot
885.4465	885.5341	0.0876	99	344	350	NFFDKSK					Mascot
1003.5418	1003.4968	-0.045	-45	358	366	DGKNISLEK					Mascot
1058.501	1058.5083	0.0073	7	47	55	SSIACVMFK			Carbamidomethyl (C)[5], Oxidation (M)[7]		Mascot
1245.6772	1245.7174	0.0402	32	272	281	FKSQMLNHLK					Mascot
1261.6722	1261.7255	0.0533	42	272	281	FKSQMLNHLK			Oxidation (M)[5]		Mascot
1547.8792	1547.7542	-0.125	-81	319	331	SVLNLKLLNYENK					Mascot
1666.837	1666.7762	-0.0608	-36	274	286	SQMLNHLKFYQNK			Oxidation (M)[3]		Mascot
1890.9668	1891.0233	0.0565	30	184	201	ANEYDLDAIILARGGGSR					Mascot
1953.0725	1952.9351	-0.1374	-70	132	149	KIGIITSFTSAALQDMLK			Oxidation (M)[16]		Mascot
2045.0437	2045.01	-0.0337	-16	12	29	SLLEFHLDDIELSGEISK					Mascot
2046.0079	2046.0138	0.0059	3	30	46	ITIHGSGHWYFDLKDEK					Mascot
2101.0383	2100.8896	-0.1487	-71	67	85	VGDMLDLRGYVSLYEASGR					Mascot
2107.0608	2106.9263	-0.1345	-64	332	348	LNNFEELLAQHKNFFDK					Mascot

4 Probable dual-specificity RNA methyltransferase RlmN OS=Kocuria rhizophila (strain ATCC 9341 / DSM 348 / NBRC 103217 / DC2201) GN=rlmN PE=3 SV=1 | RLMN_KOCRD | 42071.5 | 7.67 | 12 | 52 | 0 | 24.688

Peptide Information

Calc. Mass	Obsrv. Mass	± da	± ppm	Start Seq.	End Seq.	Sequence	Ion Score	C. I. %	Modification	Rank	Result Type
842.4301	842.4449	0.0148	18	204	211	ATMGAVHR					Mascot
866.3924	866.4651	0.0727	84	99	106	ADEGMTVK			Oxidation (M)[5]		Mascot

Calc. Mass	Obsrv. Mass	± da	± ppm	Start Seq.	End Seq.	Sequence	Ion Score	C. I. %	Modification	Rank	Result Type
1003.4592	1003.4968	0.0376	37	301	308	DINDQGWR					Mascot
1116.507	1116.5741	0.0671	60	283	291	DYYDATGRR					Mascot
1116.507	1116.5741	0.0671	60	283	291	DYYDATGRR					Mascot
1219.7157	1219.7335	0.0178	15	291	300	RVSIEYALIR					Mascot
1263.6362	1263.7505	0.1143	90	96	106	QLRADEGMTVK			Oxidation (M)[8]		Mascot
1263.6362	1263.7505	0.1143	90	96	106	QLRADEGMTVK			Oxidation (M)[8]		Mascot
1340.6078	1340.655	0.0472	35	174	186	DGAVGDPEEAPQR					Mascot
1416.788	1416.8582	0.0702	50	226	239	GLTMSTVGLVPGIR			Oxidation (M)[4]		Mascot
1438.8337	1438.7573	-0.0764	-53	80	92	EELVAKAMPPLLK					Mascot
1930.9402	1930.9395	-0.0007	0	111	126	LFDNAMVESVLMRYDK					Mascot
1946.9351	1946.9208	-0.0143	-7	111	126	LFDNAMVESVLMRYDK			Oxidation (M)[6]		Mascot
2045.0676	2045.01	-0.0576	-28	339	355	WTASRPGVEQNFVERLR					Mascot
2248.1868	2247.9897	-0.1971	-88	204	225	ATMGAVHRIIDPSPEGLGI SAR					Mascot

5	Olfactomedin-like protein 1 OS=Homo sapiens GN=OLFML1 PE=1 SV=2	OLFL1_HUMAN	46377.5	8.29	12	51	0	5.631	

Peptide Information

Calc. Mass	Obsrv. Mass	± da	± ppm	Start Seq.	End Seq.	Sequence	Ion Score	C. I. %	Modification	Rank	Result Type
1212.626	1212.5336	-0.0924	-76	98	106	EIDYIQYLR					Mascot
1284.7383	1284.6344	-0.1039	-81	83	94	SAVGNLALRVER					Mascot
1363.6754	1363.7332	0.0578	42	183	193	NNTVWEFANIR					Mascot
1632.8374	1632.7489	-0.0885	-54	41	54	VLEQGLEKCTQATR			Carbamidomethyl (C)[9]		Mascot
1666.8344	1666.7762	-0.0582	-35	364	376	RPRSHSMIHYNPR			Oxidation (M)[7]		Mascot
1696.8066	1696.7806	-0.026	-15	135	149	TLLNASCDNMLMGIK			Carbamidomethyl (C)[7], Oxidation (M)[10]		Mascot
1965.9918	1965.986	-0.0058	-3	133	149	IRTLLNASCDNMLMGIK			Carbamidomethyl (C)[9], Oxidation (M)[12]		Mascot
2061.0071	2061.0049	-0.0022	-1	74	91	CQTYTSEYKSAVGNLAL R			Carbamidomethyl (C)[1]		Mascot
2106.9915	2106.9263	-0.0652	-31	49	65	CTQATRAYIQEFQEFSK			Carbamidomethyl (C)[1]		Mascot
2129.9089	2129.9443	0.0354	17	157	174	MMDTHGSWMKDAVYNS PK			Oxidation (M)[1,2]		Mascot
2145.9038	2146.085	0.1812	84	157	174	MMDTHGSWMKDAVYNS PK			Oxidation (M)[1,2,9]		Mascot
2145.9038	2146.085	0.1812	84	157	174	MMDTHGSWMKDAVYNS PK			Oxidation (M)[1,2,9]		Mascot
2210.1604	2210.0166	-0.1438	-65	379	396	QLYAWNEGNQIIYKLQTK					Mascot
2210.1604	2210.0166	-0.1438	-65	379	396	QLYAWNEGNQIIYKLQTK					Mascot
2265.2139	2265.0088	-0.2051	-91	175	193	VYLLIGSRNNTVWEFANI R					Mascot

6	Polymerase cofactor VP35 OS=Lake Victoria marburgvirus (strain Musoke-80) GN=VP35 PE=1 SV=1	VP35_MABVM	36348	9.03	10	51	0	2.267	

Peptide Information

Calc. Mass	Obsrv. Mass	± da	± ppm	Start Seq.	End Seq.	Sequence	Ion Score	C. I. %	Modification	Rank	Result Type
866.411	866.4651	0.0541	62	123	130	GMSEMLAK					Mascot
906.4163	906.4699	0.0536	59	183	191	NATTDAADK					Mascot
1301.6962	1301.6947	-0.0015	-1	288	298	NFQTVPRPSQK					Mascot
1438.6843	1438.7573	0.073	51	178	191	GTMVKNATTDAADK			Oxidation (M)[3]		Mascot
1677.8654	1677.7709	-0.0945	-56	82	96	QISDIQSTLSEVTTR					Mascot
1890.9598	1891.0233	0.0635	34	38	54	KPKGTVGLQCSPCLMSK			Carbamidomethyl (C)[10,13]		Mascot
1906.9548	1907.0146	0.0598	31	38	54	KPKGTVGLQCSPCLMSK			Carbamidomethyl (C)[10,13], Oxidation (M)[15]		Mascot
1931.0637	1930.9395	-0.1242	-64	19	35	VPIDQVFGANPLEKLYK					Mascot
1946.8623	1946.9208	0.0585	30	2	18	WDSSYMQQVSEGLMTGK					Mascot
3347.8879	3347.646	-0.2419	-72	212	241	DLALLLFTHLPGNNTPFHILAQVLSKIAYK					Mascot
3601.7012	3601.72	0.0188	5	1	32	MWDSSYMQQVSEGLMTGKVPIDQVFGANPLEK			Oxidation (M)[1]		Mascot

7	Argininosuccinate synthase OS=Burkholderia phytofirmans (strain DSM 17436 / PsJN) GN=argG PE=3 SV=1	ASSY_BURPP	45951.6	5.83	12	51	0	2.113	

Peptide Information

Calc. Mass	Obsrv. Mass	± da	± ppm	Start Seq.	End Seq.	Sequence	Ion Score	C. I. %	Modification	Rank	Result Type
885.5264	885.5341	0.0077	9	104	110	RQIEIAR					Mascot
1003.5418	1003.4968	-0.045	-45	293	301	GIESITLDR					Mascot
1058.5128	1058.5083	-0.0045	-4	74	81	DFVFPMFR					Mascot
1262.6376	1262.7115	0.0739	59	59	68	QDNIFIEDLR					Mascot
1363.6886	1363.7332	0.0446	33	242	253	LSAAEMLTELNR			Oxidation (M)[6]		Mascot
1634.9364	1634.7838	-0.1526	-93	7	22	VVLAYSGGLDTSVILK					Mascot
1922.9607	1922.9498	-0.0109	-6	59	73	QDNIFIEDLREEFVR					Mascot
1966.0465	1965.986	-0.0605	-31	105	124	QIEIARATGAQAVSHGATGK					Mascot
2060.9978	2061.0049	0.0071	3	314	329	YASLIYNGYWWSPERR					Mascot
2106.1455	2105.9856	-0.1599	-76	330	347	AIQVLIDHTQEKVNGWVR					Mascot
2179.0237	2179.175	0.1513	69	176	195	QGGAPYSMDANLLHISFEGR			Oxidation (M)[8]		Mascot
3527.6714	3527.626	-0.0454	-13	211	241	WTVSPEQAPDQAEYIDIEYEHGDPVAINGKR					Mascot

8	Xylose isomerase OS=Agrobacterium radiobacter (strain K84 / ATCC BAA-868) GN=xylA PE=3 SV=1	XYLA_AGRRK	49081.3	5.55	12	49	0	2.436	

Peptide Information

Calc. Mass	Obsrv. Mass	± da	± ppm	Start Seq.	End Seq.	Sequence	Ion Score	C. I. %	Modification	Rank	Result Type
979.3972	979.4737	0.0765	78	171	178	TCMDATHK			Carbamidomethyl (C)[2], Oxidation (M)[3]		Mascot
1269.6838	1269.6855	0.0017	1	2	13	STGFFGDIAKVK					Mascot
1301.6848	1301.6947	0.0099	8	27	37	HYNKDEVVLGK					Mascot
1320.6696	1320.7614	0.0918	70	179	190	LGGENYVLWGGR					Mascot
1416.7192	1416.8582	0.139	98	1	13	MSTGFFGDIAKVK			Oxidation (M)[1]		Mascot
1547.8441	1547.7542	-0.0899	-58	136	148	LLWGTANLFSNRR					Mascot
1946.9647	1946.9208	-0.0439	-23	240	255	HQYDYDVATVYGFLKK					Mascot
1966.1372	1965.986	-0.1512	-77	222	239	IGFKGAILIEPKPQEPTK					Mascot
2091.0981	2090.9734	-0.1247	-60	129	147	QADTGVKLLWGTANLFSNR					Mascot
2091.0981	2090.9734	-0.1247	-60	129	147	QADTGVKLLWGTANLFSNR					Mascot
2119.1182	2119.031	-0.0872	-41	397	414	LLRGEYSLDQIAQWVEAK					Mascot
2258.1089	2258.0017	-0.1072	-47	110	128	NFAENTKNLNEIVDYFAQK					Mascot
2265.054	2265.0088	-0.0452	-20	171	190	TCMDATHKLGGENYVLWGGR			Carbamidomethyl (C)[2]		Mascot

9	Nuclear distribution protein PAC1 OS=Pyrenophora tritici-repentis (strain Pt-1C-BFP) GN=pac1 PE=3 SV=1	LIS1_PYRTR	50977	8.43	12	49	0	13.916	

Peptide Information

Calc. Mass	Obsrv. Mass	± da	± ppm	Start Seq.	End Seq.	Sequence	Ion Score	C. I. %	Modification	Rank	Result Type
875.5098	875.4527	-0.0571	-65	58	64	KWTSVVR					Mascot
906.4713	906.4699	-0.0014	-2	1	8	MSAILTDR					Mascot
922.4662	922.4631	-0.0031	-3	1	8	MSAILTDR			Oxidation (M)[1]		Mascot
979.5458	979.4737	-0.0721	-74	51	58	YEGLLEKK					Mascot
1116.6525	1116.5741	-0.0784	-70	59	67	WTSVVRLQK					Mascot
1116.6525	1116.5741	-0.0784	-70	59	67	WTSVVRLQK					Mascot
1212.6121	1212.5336	-0.0785	-65	351	360	TLTGHDNWIR					Mascot
1381.7322	1381.7372	0.005	4	370	381	YLLSASDDKTIR					Mascot
1381.7322	1381.7372	0.005	4	370	381	YLLSASDDKTIR					Mascot
1534.7432	1534.8468	0.1036	68	379	390	TIRCWDLTQEGR			Carbamidomethyl (C)[4]		Mascot
1666.7667	1666.7762	0.0095	6	35	49	EEANVSANFDDATRK					Mascot
1760.9177	1760.7662	-0.1515	-86	319	335	KPPPLSSSAEYIATGSR					Mascot
2135.1145	2134.9883	-0.1262	-59	351	369	TLTGHDNWIRSLVFHPGGK					Mascot
2178.228	2178.0657	-0.1623	-75	9	28	QAEELHKAIIAYLGVINAP					Mascot

						K					
2178.228	2178.314	0.086	39	9	28	QAEELHKAIIAYLGVINAPK					Mascot
3353.7021	3353.6753	-0.0268	-8	288	318	CTFIGHEHVVECVTIAPPVSYANLASLAGLK			Carbamidomethyl (C)[1,12]		Mascot

10	DNA mismatch repair protein MutS OS=Magnetospirillum magneticum (strain AMB-1 / ATCC 700264) GN=mutS PE=3 SV=2	MUTS_MAGSA	95491.6	5.8	16	49	0	4.179	

Peptide Information

Calc. Mass	Obsrv. Mass	± da	± ppm	Start Seq.	End Seq.	Sequence	Ion Score	C. I. %	Modification	Rank	Result Type
922.4047	922.4631	0.0584	63	819	827	SDQASGMAR					Mascot
1098.6517	1098.5603	-0.0914	-83	708	717	ALVILDEIGR					Mascot
1138.5376	1138.553	0.0154	14	588	597	VDDSTAFDIR					Mascot
1262.6852	1262.7115	0.0263	21	601	612	HPVVEAALEAAR					Mascot
1291.6252	1291.7385	0.1133	88	25	34	AHPDCLLFYR			Carbamidomethyl (C)[5]		Mascot
1380.7118	1380.7087	-0.0031	-2	469	480	RYQDETGIASLK					Mascot
1416.7273	1416.8582	0.1309	92	759	770	LAALSCHMMRVK			Carbamidomethyl (C)[6]		Mascot
1547.9016	1547.7542	-0.1474	-95	423	436	LQRALAPDLPLNAR					Mascot
1664.8102	1664.807	-0.0032	-2	335	348	LDMVGFFVEHESVR					Mascot
1678.8186	1678.8494	0.0308	18	755	768	LTSRLAALSCHMMR			Carbamidomethyl (C)[10], Oxidation (M)[12,13]		Mascot
1696.7563	1696.7806	0.0243	14	35	48	MGDFYELFFDDAVK					Mascot
1891.0259	1891.0233	-0.0026	-1	629	645	LWLVTGPNMAGKSTFLR					Mascot
1907.0209	1907.0146	-0.0063	-3	629	645	LWLVTGPNMAGKSTFLR			Oxidation (M)[9]		Mascot
1922.9165	1922.9498	0.0333	17	88	105	SGFKVAIGEQMEDPAEAK			Oxidation (M)[11]		Mascot
1953.1128	1952.9351	-0.1777	-91	171	189	TLAAALARLSPGELLVSDR					Mascot
3601.9126	3601.72	-0.1926	-53	708	740	ALVILDEIGRGTATFDGLSIAWAVVEHLHEVNR					Mascot
3618.7898	3618.6265	-0.1633	-45	136	170	SHNYLAAVAEAQGTLGLAWVDVSTGDFAMQAIAPK			Oxidation (M)[29]		Mascot
3618.7898	3618.6265	-0.1633	-45	136	170	SHNYLAAVAEAQGTLGLAWVDVSTGDFAMQAIAPK			Oxidation (M)[29]		Mascot

Gel Idx/Pos	293/L21	Instr./Gel Origin	BA2151/Sample Project 20140814	Process Status	Analysis Succeeded
Plate [#] Name	[1] Sample Project 20140814	Instrument Sample Name		Spectra	11

Rank	Protein Name	Accession No.	Protein MW	Protein PI	Pep. Count	Protein Score	Protein Score C. I. %	Intensity Matched	Total Ion Score	Total Ion C. I. %	Confirmed
1	50S ribosomal protein L2 OS=Shewanella woodyi (strain ATCC 51908 / MS32) GN=rplB PE=3 SV=1	RL2_SHEWM	30188.3	10.87	13	63	70.285	12.562			

Peptide Information

Calc. Mass	Obsrv. Mass	± da	± ppm	Start Seq.	End Seq.	Sequence	Ion Score	C. I. %	Modification	Rank	Result Type
906.4315	906.4564	0.0249	27	81	87	LEYDPNR					Mascot
1204.6473	1204.6118	-0.0355	-29	244	254	HPVSPWGVPTK					Mascot
1219.6582	1219.7087	0.0505	41	60	68	QHYRIIDFK					Mascot
1275.6454	1275.7289	0.0835	65	53	63	HVGGGHKQHYR					Mascot
1301.7358	1301.6593	-0.0765	-59	2	13	AIIKCKPTSAGR			Carbamidomethyl (C)[5]		Mascot
1381.7145	1381.718	0.0035	3	103	115	YILAAKGMQAGDK			Oxidation (M)[8]		Mascot
1381.7145	1381.718	0.0035	3	103	115	YILAAKGMQAGDK			Oxidation (M)[8]		Mascot
1432.7764	1432.6985	-0.0779	-54	1	13	MAIIKCKPTSAGR			Carbamidomethyl (C)[6]		Mascot
1547.6941	1547.7727	0.0786	51	190	203	ATMGEVGNAEHMLR			Oxidation (M)[3,12]		Mascot
1661.897	1661.7749	-0.1221	-73	88	102	TANIALVLYADGERR					Mascot
1760.8848	1760.764	-0.1208	-69	109	125	GMQAGDKIQSGIDAEIK					Mascot
1776.8796	1776.7695	-0.1101	-62	109	125	GMQAGDKIQSGIDAEIK			Oxidation (M)[2]		Mascot
1908.1066	1908.0073	-0.0993	-52	19	36	VVNTDLHKGKPFAGLLAK					Mascot
2159.0159	2158.9331	-0.0828	-38	222	243	GVAMNPIDHPHGGGEGR TSGGR					Mascot
2242.0525	2242.0107	-0.0418	-19	184	203	VPVDCRATMGEVGNAEH MLR			Carbamidomethyl (C)[5]		Mascot
2242.0525	2242.0107	-0.0418	-19	184	203	VPVDCRATMGEVGNAEH MLR			Carbamidomethyl (C)[5]		Mascot
2258.0476	2258.0088	-0.0388	-17	184	203	VPVDCRATMGEVGNAEH MLR			Carbamidomethyl (C)[5], Oxidation (M)[9]		Mascot

Rank	Protein Name	Accession No.	Protein MW	Protein PI	Pep. Count	Protein Score	Protein Score C. I. %	Intensity Matched	Total Ion Score	Total Ion C. I. %	Confirmed
2	Nuclear distribution protein PAC1 OS=Pyrenophora tritici-repentis (strain Pt-1C-BFP) GN=pac1 PE=3 SV=1	LIS1_PYRTR	50977	8.43	14	62	66.659	18.061			

Peptide Information

Calc. Mass	Obsrv. Mass	± da	± ppm	Start Seq.	End Seq.	Sequence	Ion Score	C. I. %	Modification	Rank	Result Type
840.4111	840.4207	0.0096	11	250	256	GHADWVR					Mascot
875.5098	875.459	-0.0508	-58	58	64	KWTSVVR					Mascot
904.4556	904.4637	0.0081	9	69	75	VMELEQR					Mascot

Calc. Mass	Obsrv. Mass	± da	± ppm	Start Seq.	End Seq.	Sequence	Ion Score	C. I. %	Modification	Rank	Result Type
906.4713	906.4564	-0.0149	-16	1	8	MSAILTDR					Mascot
922.4662	922.4489	-0.0173	-19	1	8	MSAILTDR			Oxidation (M)[1]		Mascot
979.5458	979.4662	-0.0796	-81	51	58	YEGLLEKK					Mascot
1116.6525	1116.5555	-0.097	-87	59	67	WTSVVRLQK					Mascot
1116.6525	1116.5555	-0.097	-87	59	67	WTSVVRLQK					Mascot
1381.7322	1381.718	-0.0142	-10	370	381	YLLSASDDKTIR					Mascot
1381.7322	1381.718	-0.0142	-10	370	381	YLLSASDDKTIR					Mascot
1534.7432	1534.8329	0.0897	58	379	390	TIRCWDLTQEGR			Carbamidomethyl (C)[4]		Mascot
1666.7667	1666.7633	-0.0034	-2	35	49	EEANVSANFDDATRK					Mascot
1760.9177	1760.764	-0.1537	-87	319	335	KPPPLSSSAEYIATGSR					Mascot
2004.9202	2004.9366	0.0164	8	391	407	CVKTVTDAHGHFVSCMR			Carbamidomethyl (C)[1,15]		Mascot
2178.228	2178.0786	-0.1494	-69	9	28	QAEELHKAIIAYLGVINAP K					Mascot
2178.228	2178.4072	0.1792	82	9	28	QAEELHKAIIAYLGVINAP K					Mascot
2214.0059	2214.019	0.0131	6	29	48	TAEAFREEANVSANFDD ATR					Mascot
3353.7021	3353.7017	-0.0004	0	288	318	CTFIGHEHVVECVTIAPP VSYANLASLAGLK			Carbamidomethyl (C)[1,12]		Mascot

3	Exodeoxyribonuclease 7 large subunit OS=Campylobacter lari (strain RM2100 / D67 / ATCC BAA-1060) GN=xseA PE=3 SV=1	EX7L_CAMLR	44155.3	8.78	15	61	60.827	3.202

Peptide Information

Calc. Mass	Obsrv. Mass	± da	± ppm	Start Seq.	End Seq.	Sequence	Ion Score	C. I. %	Modification	Rank	Result Type
826.3763	826.3522	-0.0241	-29	155	160	EYNLCK			Carbamidomethyl (C)[5]		Mascot
842.5094	842.463	-0.0464	-55	351	357	NLINLQK					Mascot
885.4465	885.5197	0.0732	83	344	350	NFFDKSK					Mascot
1003.5418	1003.5305	-0.0113	-11	358	366	DGKNISLEK					Mascot
1245.6772	1245.6768	-0.0004	0	272	281	FKSQMLNHLK					Mascot
1261.6722	1261.6919	0.0197	16	272	281	FKSQMLNHLK			Oxidation (M)[5]		Mascot
1376.7421	1376.693	-0.0491	-36	112	123	LEKEGLFDINAK					Mascot
1423.7097	1423.7504	0.0407	29	92	104	SMQKTSLGDLEAK			Oxidation (M)[2]		Mascot
1547.8792	1547.7727	-0.1065	-69	319	331	SVLNLKLLNYENK					Mascot
1650.842	1650.7726	-0.0694	-42	274	286	SQMLNHLKFYQNK					Mascot
1666.837	1666.7633	-0.0737	-44	274	286	SQMLNHLKFYQNK			Oxidation (M)[3]		Mascot
1890.9668	1890.9977	0.0309	16	184	201	ANEYDLDAIILARGGGSR					Mascot
1937.0776	1936.9377	-0.1399	-72	132	149	KIGIITSFTSAALQDMLK					Mascot
2045.0437	2044.9952	-0.0485	-24	12	29	SLLEFHLDDIELSGEISK					Mascot
2046.0079	2046.0022	-0.0057	-3	30	46	ITIHGSGHWYFDLKDEK					Mascot

2101.0383	2100.8921	-0.1462	-70	67	85	VGDMLDLRGYVSLYEASGR						Mascot
2107.0608	2106.9568	-0.104	-49	332	348	LNNFEELLAQHKNFFDK						Mascot

4 S-methyl-5'-thioadenosine phosphorylase OS=Mus musculus GN=Mtap PE=2 SV=1 | MTAP_MOUSE | 31612 | 6.71 | 11 | 61 | 58.026 | 3.091

Peptide Information

Calc. Mass	Obsrv. Mass	± da	± ppm	Start Seq.	End Seq.	Sequence	Ion Score	C. I. %	Modification	Rank	Result Type
951.4564	951.4338	-0.0226	-24	239	246	TMKENANK			Oxidation (M)[2]		Mascot
1098.4918	1098.5452	0.0534	49	1	11	MASGSACTAVK			Carbamidomethyl (C)[7], Oxidation (M)[1]		Mascot
1275.6515	1275.7289	0.0774	61	272	282	NMAQFSVLPPR			Oxidation (M)[2]		Mascot
1291.6688	1291.7158	0.047	36	61	71	HGRQHTIMPSK					Mascot
1409.743	1409.7151	-0.0279	-20	52	63	NVDCVLLARHGR			Carbamidomethyl (C)[4]		Mascot
2017.99	2017.9053	-0.0847	-42	100	116	EEIQPGDMVIIDQFIDR					Mascot
2046.0576	2046.0022	-0.0554	-27	188	206	TWGADVVNMTTVPEVVLAK			Oxidation (M)[9]		Mascot
2062.1584	2062.0437	-0.1147	-56	33	51	YVDTPFGKPSDALILGKIK					Mascot
2179.1646	2179.3655	0.2009	92	30	49	TEKYVDTPFGKPSDALILGK					Mascot
2240.9297	2241.0149	0.0852	38	207	225	EAGICYASIAMATDYDCWK			Carbamidomethyl (C)[5,17], Oxidation (M)[11]		Mascot
2242.1802	2242.0107	-0.1695	-76	64	82	QHTIMPSKVNYQANIWALK					Mascot
2242.1802	2242.0107	-0.1695	-76	64	82	QHTIMPSKVNYQANIWALK					Mascot
2258.175	2258.0088	-0.1662	-74	64	82	QHTIMPSKVNYQANIWALK			Oxidation (M)[5]		Mascot

5 Uncharacterized protein Lmo1967 OS=Listeria monocytogenes serovar 1/2a (strain ATCC BAA-679 / EGD-e) GN=lmo1967 PE=3 SV=1 | Y1967_LISMO | 45463.6 | 5.32 | 14 | 55 | 0 | 2.64

Peptide Information

Calc. Mass	Obsrv. Mass	± da	± ppm	Start Seq.	End Seq.	Sequence	Ion Score	C. I. %	Modification	Rank	Result Type
875.4581	875.459	0.0009	1	333	340	TNAIETAR					Mascot
929.5163	929.4438	-0.0725	-78	369	376	IQQEGRAK					Mascot
1219.6239	1219.7087	0.0848	70	383	392	ELVTMEQELK					Mascot
1246.7002	1246.7515	0.0513	41	146	156	VKQSINEITSK					Mascot
1261.6635	1261.6919	0.0284	23	315	325	QVSETTNELLK					Mascot
1291.6926	1291.7158	0.0232	18	55	65	LVDMLTETNKK					Mascot
1403.6874	1403.6797	-0.0077	-5	333	344	TNAIETARENER					Mascot
1438.7537	1438.7299	-0.0238	-17	148	159	QSINEITSKYQK					Mascot

Calc. Mass	Obsrv. Mass	± da	± ppm	Start Seq.	End Seq.	Sequence	Ion Score	C. I. %	Modification	Rank	Result Type
1547.7847	1547.7727	-0.012	-8	327	340	NADMLKTNAIETAR					Mascot
1664.7797	1664.7537	-0.026	-16	1	14	MTENKPSEQTNELK			Oxidation (M)[1]		Mascot
1733.899	1733.8439	-0.0551	-32	378	392	AVAEKELVTMEQELK			Oxidation (M)[10]		Mascot
1760.8048	1760.764	-0.0408	-23	178	191	LMEDNSFLEQLYDK			Oxidation (M)[2]		Mascot
2192.2622	2192.0518	-0.2104	-96	295	314	NQVAIALTLLRQQQAVAA QR					Mascot
2242.1787	2242.0107	-0.168	-75	306	325	QQQAVAAQRQVSETTNE LLK					Mascot
2242.1787	2242.0107	-0.168	-75	306	325	QQQAVAAQRQVSETTNE LLK					Mascot

6	Nod factor export ATP-binding protein I OS=Bradyrhizobium sp. (strain SNU001) GN=nodI PE=3 SV=1	NODI_BRASS	34429.9	6.41	10	54	0	22.745

Peptide Information

Calc. Mass	Obsrv. Mass	± da	± ppm	Start Seq.	End Seq.	Sequence	Ion Score	C. I. %	Modification	Rank	Result Type
979.4877	979.4662	-0.0215	-22	298	305	LTGREMEK			Oxidation (M)[6]		Mascot
1263.6224	1263.7356	0.1132	90	52	61	MLLGMIWPDR			Oxidation (M)[1,5]		Mascot
1263.6224	1263.7356	0.1132	90	52	61	MLLGMIWPDR	15	0	Oxidation (M)[1,5]		Mascot
1318.7148	1318.6046	-0.1102	-84	131	143	ADVRVSLLGGGMK			Oxidation (M)[12]		Mascot
1376.6627	1376.693	0.0303	22	33	46	GECFGLLGPNGAGK			Carbamidomethyl (C)[3]		Mascot
1416.749	1416.8495	0.1005	71	52	63	MLLGMIWPDRGK					Mascot
1432.744	1432.6985	-0.0455	-32	52	63	MLLGMIWPDRGK			Oxidation (M)[1]		Mascot
1438.722	1438.7299	0.0079	5	268	279	CPDEISVHLRGR			Carbamidomethyl (C)[1]		Mascot
1547.8032	1547.7727	-0.0305	-20	2	15	NMSNMAIDLVGVRK					Mascot
1579.7931	1579.7299	-0.0632	-40	2	15	NMSNMAIDLVGVRK			Oxidation (M)[2,5]		Mascot
1678.8438	1678.8386	-0.0052	-3	1	15	MNMSNMAIDLVGVRK					Mascot
1875.9634	1875.9746	0.0112	6	187	202	GKTILLTTHFMEEAER					Mascot
2159.1245	2158.9331	-0.1914	-89	79	97	RGVGVVPQFDNLEPEFT VR					Mascot

7	4-hydroxy-tetrahydrodipicolinate reductase OS=Thermobifida fusca (strain YX) GN=dapB PE=3 SV=1	DAPB_THEFY	25958.5	5.62	8	53	0	23.388

Peptide Information

Calc. Mass	Obsrv. Mass	± da	± ppm	Start Seq.	End Seq.	Sequence	Ion Score	C. I. %	Modification	Rank	Result Type
875.5098	875.459	-0.0508	-58	4	12	VGVFGALGR					Mascot
885.5152	885.5197	0.0045	5	228	235	KVASLPDR					Mascot
1263.7242	1263.7356	0.0114	9	1	12	MIKVGVFGALGR			Oxidation (M)[1]		Mascot
1263.7242	1263.7356	0.0114	9	1	12	MIKVGVFGALGR	14	0	Oxidation (M)[1]		Mascot

Calc. Mass	Obsrv. Mass	± da	± ppm	Start Seq.	End Seq.	Sequence	Ion Score	C. I. %	Modification	Rank	Result Type
1320.6981	1320.7546	0.0565	43	216	227	ESFMPGVLLGVR			Oxidation (M)[4]		Mascot
1432.7981	1432.6985	-0.0996	-70	216	228	ESFMPGVLLGVRK					Mascot
1890.9668	1890.9977	0.0309	16	136	154	ADAPSGTAYRTAELIGAAR					Mascot
2045.0056	2044.9952	-0.0104	-5	210	227	HDSMNRESFMPGVLLGVR					Mascot
2194.104	2194.0378	-0.0662	-30	63	82	WLIEHGIHAVVGTTGFDDAR					Mascot

8	Elongation factor 4 OS=Chlorobium phaeobacteroides (strain BS1) GN=lepA PE=3 SV=1	LEPA_CHLPB	67730	5.31	15	52	0	3.149	

Peptide Information

Calc. Mass	Obsrv. Mass	± da	± ppm	Start Seq.	End Seq.	Sequence	Ion Score	C. I. %	Modification	Rank	Result Type
823.3978	823.4287	0.0309	38	548	554	ETISAMR			Oxidation (M)[6]		Mascot
842.4301	842.463	0.0329	39	63	69	SHAIQMR					Mascot
951.4927	951.4338	-0.0589	-62	548	555	ETISAMRK			Oxidation (M)[6]		Mascot
1098.5062	1098.5452	0.039	36	438	446	NTDYLDTTR					Mascot
1246.6936	1246.7515	0.0579	46	544	554	VIARETISAMR					Mascot
1262.6886	1262.6959	0.0073	6	544	554	VIARETISAMR			Oxidation (M)[10]		Mascot
1393.7396	1393.7166	-0.023	-17	238	250	VFLADEIGTMGLK					Mascot
1409.7345	1409.7151	-0.0194	-14	238	250	VFLADEIGTMGLK			Oxidation (M)[10]		Mascot
1416.7158	1416.8495	0.1337	94	200	211	ALIFDSVFDAYR					Mascot
1661.9408	1661.7749	-0.1659	-100	212	226	GAIVYLRIVDGMLNK					Mascot
1937.0161	1936.9377	-0.0784	-40	256	273	TLGAGDVGYLICSIKDVR			Carbamidomethyl (C)[12]		Mascot
2004.9008	2004.9366	0.0358	18	468	484	SVSKGYASMDYEYIGYR			Oxidation (M)[9]		Mascot
2019.0944	2018.9275	-0.1669	-83	252	270	QPVKTLGAGDVGYLICSIK			Carbamidomethyl (C)[16]		Mascot
2119.1328	2119.0391	-0.0937	-44	525	543	SIIPRQMYEVAIQAAIGSR			Oxidation (M)[7]		Mascot
2130.094	2129.9331	-0.1609	-76	232	250	FFANEKVFLADEIGTMGLK					Mascot
2146.0889	2146.0811	-0.0078	-4	232	250	FFANEKVFLADEIGTMGLK			Oxidation (M)[16]		Mascot
2214.1521	2214.019	-0.1331	-60	347	364	CGFLGLLHMEIIQERLER			Carbamidomethyl (C)[1]		Mascot
3527.8052	3527.6382	-0.167	-47	397	427	MPDAGFIEKIEEPYVSVQIITLAEYIGNIMK			Oxidation (M)[1]		Mascot

9	Glycine--tRNA ligase beta subunit OS=Actinobacillus pleuropneumoniae serotype 3 (strain JL03) GN=glyS PE=3 SV=1	SYGB_ACTPJ	76275	5.2	15	52	0	3.158	

Peptide Information

Calc. Mass	Obsrv. Mass	± da	± ppm	Start Seq.	End Seq.	Sequence	Ion Score	C. I. %	Modification	Rank	Result Type

Calc. Mass	Obsrv. Mass	± da	± ppm	Start Seq.	End Seq.	Sequence	Ion Score	C. I. %	Modification	Rank	Result Type
808.4199	808.3516	-0.0683	-84	507	514	SAALFGDK					Mascot
923.4226	923.4773	0.0547	59	147	153	MMRWGDK					Mascot
936.5149	936.4298	-0.0851	-91	506	514	KSAALFGDK					Mascot
1245.615	1245.6768	0.0618	50	287	296	YFPIYDKDGK					Mascot
1269.7889	1269.6744	-0.1145	-90	483	494	AALGSLRIIVEK					Mascot
1364.6805	1364.7455	0.065	48	211	222	EKGSVIADFNER					Mascot
1403.7059	1403.6797	-0.0262	-19	655	666	VMVNAEDANLRR			Oxidation (M)[2]		Mascot
1733.8488	1733.8439	-0.0049	-3	97	112	GCGISVEQAERLATDK			Carbamidomethyl (C)[2]		Mascot
1879.09	1879.0388	-0.0512	-27	490	505	IIVEKNLPLDLEDLVR					Mascot
1946.9137	1946.8938	-0.0199	-10	90	107	AAEGWARGCGISVEQAE R			Carbamidomethyl (C)[9]		Mascot
1966.1332	1965.9669	-0.1663	-85	120	137	AVIEGQPTKNLLVDIISR					Mascot
2019.0104	2018.9275	-0.0829	-41	1	18	MTTQNFLAEIGTEELPPK					Mascot
2091.0791	2090.9656	-0.1135	-54	438	457	FAGDELPKSLVACSVALA DK			Carbamidomethyl (C)[13]		Mascot
2091.0791	2090.9656	-0.1135	-54	438	457	FAGDELPKSLVACSVALA DK			Carbamidomethyl (C)[13]		Mascot
2130.094	2129.9331	-0.1609	-76	265	282	FEERFLAVPAEALVYTMK			Oxidation (M)[17]		Mascot
3601.9055	3601.7256	-0.1799	-50	150	181	WGDKTEQFVRPVHTVTL FFGGELIEGEILGVK					Mascot

10	Glycine--tRNA ligase beta subunit OS=Actinobacillus pleuropneumoniae serotype 7 (strain AP76) GN=glyS PE=3 SV=1	SYGB_ACTP7	75768.8	5.26	15	52	0	3.144

Peptide Information

Calc. Mass	Obsrv. Mass	± da	± ppm	Start Seq.	End Seq.	Sequence	Ion Score	C. I. %	Modification	Rank	Result Type
808.4199	808.3516	-0.0683	-84	507	514	SAALFGDK					Mascot
923.4226	923.4773	0.0547	59	147	153	MMRWGDK					Mascot
936.5149	936.4298	-0.0851	-91	506	514	KSAALFGDK					Mascot
1245.615	1245.6768	0.0618	50	287	296	YFPIYDKDGK					Mascot
1269.7889	1269.6744	-0.1145	-90	483	494	AALGSLRIIVEK					Mascot
1301.6709	1301.6593	-0.0116	-9	553	563	RPTRPADFDAR					Mascot
1364.6805	1364.7455	0.065	48	211	222	EKGSVIADFNER					Mascot
1733.8488	1733.8439	-0.0049	-3	97	112	GCGISVEQAERLATDK			Carbamidomethyl (C)[2]		Mascot
1879.09	1879.0388	-0.0512	-27	490	505	IIVEKNLPLDLEDLVR					Mascot
1946.9137	1946.8938	-0.0199	-10	90	107	AAEGWARGCGISVEQAE R			Carbamidomethyl (C)[9]		Mascot
1966.1332	1965.9669	-0.1663	-85	120	137	AVIEGQPTKNLLVDIISR					Mascot
2019.0104	2018.9275	-0.0829	-41	1	18	MTTQNFLAEIGTEELPPK					Mascot
2091.0791	2090.9656	-0.1135	-54	438	457	FAGDELPKSLVACSVALA DK			Carbamidomethyl (C)[13]		Mascot

2091.0791	2090.9656	-0.1135	-54	438	457	FAGDELPKSLVACSVALA DK	Carbamidomethyl (C)[13]	Mascot
2130.094	2129.9331	-0.1609	-76	265	282	FEERFLAVPAEALVYTMK	Oxidation (M)[17]	Mascot
3601.9055	3601.7256	-0.1799	-50	150	181	WGDKTEQFVRPVHTVTL FFGGELIEGEILGVK		Mascot

Gel Idx/Pos	294/L22	Instr./Gel Origin	BA2151/Sample Project 20140814	Process Status	Analysis Succeeded
Plate [#] Name	[1] Sample Project 20140814	Instrument Sample Name		Spectra	11

Rank	Protein Name	Accession No.	Protein MW	Protein PI	Pep. Count	Protein Score	Protein Score C. I. %	Intensity Matched	Total Ion Score	Total Ion C. I. %	Confirmed
1	Putative ciliary rootlet coiled-coil protein-like 3 protein OS=Homo sapiens PE=5 SV=2	CROL3_HUMAN	250066.2	5.64	35	70	94.206	14.022			

Peptide Information

Calc. Mass	Obsrv. Mass	± da	± ppm	Start Seq.	End Seq.	Sequence	Ion Score	C. I. %	Modification	Rank	Result Type
804.3846	804.4183	0.0337	42	1021	1027	SEGVEQR					Mascot
811.5148	811.4601	-0.0547	-67	274	280	GLQIKPR					Mascot
814.4781	814.5592	0.0811	100	1663	1669	EVLGLQR					Mascot
817.405	817.4589	0.0539	66	479	485	LEAAEER					Mascot
819.3843	819.433	0.0487	59	1164	1170	DALSEER					Mascot
823.3581	823.4265	0.0684	83	46	52	DFSPSDR					Mascot
832.4159	832.4409	0.025	30	1937	1943	QALEESR					Mascot
835.4091	835.4343	0.0252	30	172	179	SAVQMGSR					Mascot
845.4515	845.4951	0.0436	52	850	856	ELWAAQK					Mascot
851.404	851.4108	0.0068	8	172	179	SAVQMGSR			Oxidation (M)[5]		Mascot
858.4427	858.5036	0.0609	71	1261	1267	QVAQQER					Mascot
863.404	863.4628	0.0588	68	180	187	TEACVGAR			Carbamidomethyl (C)[4]		Mascot
864.3879	864.4678	0.0799	92	1858	1864	LSEAECR			Carbamidomethyl (C)[6]		Mascot
932.4618	932.4739	0.0121	13	881	887	LEVQQCR			Carbamidomethyl (C)[6]		Mascot
988.517	988.5034	-0.0136	-14	1937	1944	QALEESRR					Mascot
1001.5527	1001.5231	-0.0296	-30	849	856	RELWAAQK					Mascot
1033.5525	1033.5222	-0.0303	-29	1415	1423	SLLSEELSR					Mascot
1037.5262	1037.5398	0.0136	13	1558	1566	AEQEKASFK					Mascot
1053.5687	1053.5669	-0.0018	-2	333	342	QASPTPVPTR					Mascot
1053.5687	1053.5669	-0.0018	-2	333	342	QASPTPVPTR					Mascot
1087.5643	1087.604	0.0397	37	533	541	WAQRQPSSK					Mascot
1115.6307	1115.6151	-0.0156	-14	2186	2195	VLEEQVASLK					Mascot
1118.5623	1118.5353	-0.027	-24	653	662	AGEMLQLQGR			Oxidation (M)[4]		Mascot
1155.6732	1155.6075	-0.0657	-57	1400	1410	IGQPTQALSLK					Mascot
1164.5677	1164.5743	0.0066	6	513	523	ELAGMTGSVQR			Oxidation (M)[5]		Mascot
1216.6168	1216.5809	-0.0359	-30	2095	2105	EEDVARLGAEK					Mascot

1216.6644	1216.5809	-0.0835	-69	454	463	LETTEAQLRR					Mascot
1260.6907	1260.6345	-0.0562	-45	1415	1425	SLLSEELSRAR					Mascot
1302.6899	1302.6438	-0.0461	-35	309	320	LEDTILSPTASR					Mascot
1340.703	1340.7075	0.0045	3	2225	2236	HVGNISTNRSQK					Mascot
1384.7795	1384.764	-0.0155	-11	590	602	QAVVLGTDLAELR					Mascot
1384.7795	1384.764	-0.0155	-11	590	602	QAVVLGTDLAELR					Mascot
1415.7173	1415.7048	-0.0125	-9	1095	1106	GLQQACGRLEQR			Carbamidomethyl (C)[6]		Mascot
1458.766	1458.7604	-0.0056	-4	608	621	GLADLQADTARTAR					Mascot
1479.722	1479.7737	0.0517	35	1845	1857	DDSRIQMATLSSR					Mascot
1507.7289	1507.7626	0.0337	22	46	59	DFSPSDRVAGGPFR					Mascot
1507.7289	1507.7626	0.0337	22	46	59	DFSPSDRVAGGPFR					Mascot
1699.793	1699.8628	0.0698	41	63	80	VGNSAGSQGKGGPMGS HR			Oxidation (M)[14]		Mascot
2564.2927	2564.2705	-0.0222	-9	298	320	KPFAGSMGWQRLEDTIL SPTASR			Oxidation (M)[7]		Mascot

2	Microtubule-actin cross-linking factor 1 OS=Mus musculus GN=Macf1 PE=1 SV=2	MACF1_MOUSE	836892.8	5.3	74	69	93.193	29.987

Peptide Information

Calc. Mass	Obsrv. Mass	± da	± ppm	Start Seq.	End Seq.	Sequence	Ion Score	C. I. %	Modification	Rank	Result Type
800.3719	800.4361	0.0642	80	5570	5575	EVMEHR					Mascot
804.421	804.4183	-0.0027	-3	6501	6507	SLDDARK					Mascot
807.4108	807.4246	0.0138	17	4585	4590	EFEARR					Mascot
811.4018	811.4601	0.0583	72	3770	3775	QYELMK					Mascot
814.5032	814.5592	0.056	69	6472	6478	QDVVLIK					Mascot
815.4985	815.5005	0.002	2	1158	1164	TIDVIVR					Mascot
817.4638	817.4589	-0.0049	-6	7187	7193	RSKPSSR					Mascot
821.4298	821.4269	-0.0029	-4	2918	2924	QGRMVSK			Oxidation (M)[4]		Mascot
827.3967	827.4335	0.0368	44	3770	3775	QYELMK			Oxidation (M)[5]		Mascot
829.4665	829.4321	-0.0344	-41	6151	6157	EPLTELK					Mascot
831.457	831.471	0.014	17	2999	3006	GKEAGIEK					Mascot
832.4384	832.4409	0.0025	3	4855	4861	TANRQSR					Mascot
837.4465	837.4022	-0.0443	-53	1405	1411	YISDALR					Mascot
839.4733	839.431	-0.0423	-50	3449	3455	HKELQGK					Mascot
845.5091	845.4951	-0.014	-17	2170	2176	EQVKTIK					Mascot
846.468	846.5141	0.0461	54	4523	4530	ATEVTVAR					Mascot
849.4465	849.4623	0.0158	19	3942	3948	YTTLHSK					Mascot
851.4872	851.4108	-0.0764	-90	1048	1054	VYISELK					Mascot

853.489	853.4554	-0.0336	-39	3662	3668	EKLHQAK		Mascot
858.4791	858.5036	0.0245	29	4122	4128	QDIARQK		Mascot
864.4098	864.4678	0.058	67	6690	6696	DDTTWVK		Mascot
870.5229	870.5388	0.0159	18	5653	5660	LMALGPIR		Mascot
890.4618	890.4818	0.02	22	4628	4634	WVELTDK		Mascot
893.4727	893.4639	-0.0088	-10	2506	2513	LSVDNAFK		Mascot
908.4506	908.4781	0.0275	30	3344	3350	DIEMRTK	Oxidation (M)[4]	Mascot
921.4346	921.4852	0.0506	55	4911	4918	AMLNEAEK	Oxidation (M)[2]	Mascot
922.4485	922.4679	0.0194	21	4862	4868	LKDCMQK	Carbamidomethyl (C)[4]	Mascot
932.4683	932.4739	0.0056	6	1412	1418	RLEEEEK		Mascot
946.4952	946.5076	0.0124	13	3245	3252	TVQSENIR		Mascot
963.453	963.4975	0.0445	46	2628	2635	IDTESGWR		Mascot
988.517	988.5034	-0.0136	-14	2806	2815	AGIRGSNGEK		Mascot
991.4989	991.5438	0.0449	45	5172	5179	QCGKLTER	Carbamidomethyl (C)[2]	Mascot
993.5475	993.5377	-0.0098	-10	1405	1412	YISDALRR		Mascot
1001.5349	1001.5231	-0.0118	-12	228	235	MFNALIHR		Mascot
1004.5081	1004.5174	0.0093	9	3609	3617	SSLLCAEPK	Carbamidomethyl (C)[5]	Mascot
1014.569	1014.5282	-0.0408	-40	68	76	AVVRVADER		Mascot
1021.5499	1021.5029	-0.047	-46	4577	4584	QQVQFMLK		Mascot
1023.5622	1023.606	0.0438	43	83	90	TFTKWVNK		Mascot
1027.5143	1027.5166	0.0023	2	1027	1034	AHFQHLMK	Oxidation (M)[7]	Mascot
1037.5449	1037.5398	-0.0051	-5	4577	4584	QQVQFMLK	Oxidation (M)[6]	Mascot
1053.5034	1053.5669	0.0635	60	4195	4202	LQQFMENK	Oxidation (M)[5]	Mascot
1053.5034	1053.5669	0.0635	60	4195	4202	LQQFMENK	Oxidation (M)[5]	Mascot
1087.5854	1087.604	0.0186	17	1082	1090	DARQDITLR		Mascot
1106.5034	1106.5123	0.0089	8	1018	1026	IEEEVEACK	Carbamidomethyl (C)[8]	Mascot
1109.5958	1109.5552	-0.0406	-37	2315	2323	LMGKLNMFR		Mascot
1127.4819	1127.5483	0.0664	59	3493	3501	DMKQSMAER	Oxidation (M)[2,6]	Mascot
1141.5857	1141.4982	-0.0875	-77	2315	2323	LMGKLNMFR	Oxidation (M)[2,7]	Mascot
1155.6045	1155.6075	0.003	3	4976	4984	LKEFQESFK		Mascot
1158.6154	1158.6189	0.0035	3	341	350	SFPQNPVELK		Mascot
1189.5881	1189.5776	-0.0105	-9	1058	1066	LLLEECEQR	Carbamidomethyl (C)[6]	Mascot
1193.5797	1193.6187	0.039	33	3669	3677	EQYEVLQER		Mascot
1199.6742	1199.646	-0.0282	-24	5457	5466	ALNEEIINRK		Mascot
1212.6947	1212.5834	-0.1113	-92	2466	2476	APLTVLQSIDR		Mascot
1216.5804	1216.5809	0.0005	0	57	67	SQDSVLDPAER		Mascot

1216.5804	1216.5809	0.0005	0	57	67	SQDSVLDPAER					Mascot
1232.6117	1232.623	0.0113	9	4685	4694	QQLEETSEIR					Mascot
1260.6682	1260.6345	-0.0337	-27	2177	2188	DIPSETGTSLIK					Mascot
1289.6519	1289.6633	0.0114	9	5165	5175	ELEALNKQCGK			Carbamidomethyl (C)[9]		Mascot
1320.7271	1320.6062	-0.1209	-92	3899	3910	GDLRFVTISGQK					Mascot
1320.7271	1320.6062	-0.1209	-92	3899	3910	GDLRFVTISGQK					Mascot
1366.759	1366.6744	-0.0846	-62	1487	1498	TSQIFLAKHGHK					Mascot
1366.759	1366.6744	-0.0846	-62	1487	1498	TSQIFLAKHGHK					Mascot
1372.6566	1372.6788	0.0222	16	6904	6915	SVEPTHAPFMEK					Mascot
1384.7794	1384.764	-0.0154	-11	3652	3663	LSPQELTALREK					Mascot
1384.7981	1384.764	-0.0341	-25	2892	2903	VVTQIKMVSHVK			Oxidation (M)[7]		Mascot
1407.7261	1407.715	-0.0111	-8	6354	6367	LMLLSRGDSGSGSK					Mascot
1415.7278	1415.7048	-0.023	-16	97	107	KHINDLYEDLR					Mascot
1458.7985	1458.7604	-0.0381	-26	3044	3056	TVNLCSILKPGEK			Carbamidomethyl (C)[5]		Mascot
1461.7333	1461.7346	0.0013	1	4771	4782	TWLDEKQSQQAK					Mascot
1463.774	1463.7532	-0.0208	-14	6340	6351	AKEEIYNQLLDK					Mascot
1479.7551	1479.7737	0.0186	13	4417	4428	ERQESLQTVFSR					Mascot
1482.7435	1482.7701	0.0266	18	1419	1430	VVEEEKQEHVEK					Mascot
1516.7465	1516.7252	-0.0213	-14	6903	6915	KSVEPTHAPFMEK			Oxidation (M)[11]		Mascot
1661.8237	1661.824	0.0003	0	6092	6106	LCTMPPVGTDLNTVK			Carbamidomethyl (C)[2], Oxidation (M)[4]		Mascot
1707.964	1707.7964	-0.1676	-98	108	123	DGHNLISLLEVLSGIK					Mascot
1838.8412	1838.9429	0.1017	55	6874	6888	ALITEHQSFMEEMTR			Oxidation (M)[10]		Mascot
2224.2349	2224.0476	-0.1873	-84	6945	6962	INQLSARWQQVWLLALE R					Mascot
2246.116	2246.2185	0.1025	46	246	264	VQVQSNRENLEQAFEVA ER					Mascot
2723.2417	2723.3296	0.0879	32	2963	2987	EAAVLSEEESDQEVTIGD EPDSFVK					Mascot
2839.458	2839.3608	-0.0972	-34	2740	2766	LISPELAAMIQVDPLAEQ GGTGVCELK			Carbamidomethyl (C)[24]		Mascot
2839.458	2839.3608	-0.0972	-34	2740	2766	LISPELAAMIQVDPLAEQ GGTGVCELK			Carbamidomethyl (C)[24]		Mascot
3133.4492	3133.6768	0.2276	73	1517	1543	TYHDLCDGSANQLQQLQ SELAQQTEQK			Carbamidomethyl (C)[6]		Mascot

3	Pre-mRNA-splicing factor 18 OS=Mus musculus GN=Prpf18 PE=1 SV=1	PRP18_MOUSE	40036	8.19	19	69	92.536	7.775

Peptide Information

Calc. Mass	Obsrv. Mass	± da	± ppm	Start Seq.	End Seq.	Sequence	Ion Score	C. I. %	Modification	Rank	Result Type
800.4737	800.4361	-0.0376	-47	82	87	QEVIRR					Mascot
809.4152	809.4311	0.0159	20	207	212	EDYVKR					Mascot

815.4733	815.5005	0.0272	33	122	128	GLRNDLK					Mascot
817.46	817.4589	-0.0011	-1	75	81	LPMTLSR					Mascot
829.4778	829.4321	-0.0457	-55	19	25	NLLVENK					Mascot
835.423	835.4343	0.0113	14	184	190	DMDIITK					Mascot
849.5192	849.4623	-0.0569	-67	313	319	KYIQGLK					Mascot
851.4178	851.4108	-0.007	-8	184	190	DMDIITK			Oxidation (M)[2]		Mascot
856.4635	856.5267	0.0632	74	90	96	ERGEPIR					Mascot
868.3869	868.4468	0.0599	69	336	342	CVEYNAL			Carbamidomethyl (C)[1]		Mascot
893.4583	893.4639	0.0056	6	321	327	LMTICQK			Carbamidomethyl (C)[5]		Mascot
993.5186	993.5377	0.0191	19	45	52	CGYKIQPK			Carbamidomethyl (C)[1]		Mascot
1049.5594	1049.5669	0.0075	7	320	327	RLMTICQK			Carbamidomethyl (C)[6]		Mascot
1155.662	1155.6075	-0.0545	-47	112	121	IEILTPEVNK					Mascot
1216.5481	1216.5809	0.0328	27	36	44	KEEEAYYER					Mascot
1216.5481	1216.5809	0.0328	27	36	44	KEEEAYYER					Mascot
1260.6907	1260.6345	-0.0562	-45	213	224	SVQGKLNSATQK					Mascot
1458.8097	1458.7604	-0.0493	-34	75	86	LPMTLSRQEVIR			Oxidation (M)[3]		Mascot
1461.6646	1461.7346	0.07	48	97	108	LFGETDYDAFQR					Mascot
1516.8635	1516.7252	-0.1383	-91	194	206	FLLGVWAKELNAR					Mascot
1818.8877	1818.9351	0.0474	26	321	335	LMTICQKHFPTDPSK			Carbamidomethyl (C)[5], Oxidation (M)[2]		Mascot

4	Acetyl-coenzyme A carboxylase carboxyl transferase subunit beta OS=Prochlorococcus marinus (strain SARG / CCMP1375 / SS120) GN=accD PE=3 SV=1	ACCD_PROMA	32714.1	8.99	14	68	92.002	5.445

Peptide Information

Calc. Mass	Obsrv. Mass	± da	± ppm	Start Seq.	End Seq.	Sequence	Ion Score	C. I. %	Modification	Rank	Result Type
804.4727	804.4183	-0.0544	-68	229	236	ALIGFAGR					Mascot
821.4515	821.4269	-0.0246	-30	12	18	KDQFVGK					Mascot
858.5043	858.5036	-0.0007	-1	238	244	VIEQTLR					Mascot
864.4686	864.4678	-0.0008	-1	96	102	AYADRLR					Mascot
960.5737	960.5273	-0.0464	-48	229	237	ALIGFAGRR					Mascot
988.5421	988.5034	-0.0387	-39	61	68	INSEERIK					Mascot
1014.6054	1014.5282	-0.0772	-76	237	244	RVIEQTLR					Mascot
1115.642	1115.6151	-0.0269	-24	238	246	VIEQTLREK					Mascot
1189.6536	1189.5776	-0.076	-64	101	111	LRESQASTGLK					Mascot
1415.689	1415.7048	0.0158	11	1	11	MSLFDWFAARR			Oxidation (M)[1]		Mascot
1455.7955	1455.7073	-0.0882	-61	82	94	NLAPIDPLGFKDR					Mascot
2224.0957	2224.0476	-0.0481	-22	173	192	MQEGMLSLMQMAKISGA					Mascot

						LER				
2564.2849	2564.2705	-0.0144	-6	112	134	DGVTTGVCRVEEIPLALA VMDFR			Carbamidomethyl (C)[8], Oxidation (M)[20]	Mascot
2839.3025	2839.3608	0.0583	21	19	42	VIQETEESDGLWGKCPE CGQVVYR			Carbamidomethyl (C)[15,18]	Mascot
2839.3025	2839.3608	0.0583	21	19	42	VIQETEESDGLWGKCPE CGQVVYR			Carbamidomethyl (C)[15,18]	Mascot

5	227 kDa spindle- and centromere-associated protein OS=Parascaris univalens GN=PUMA1 PE=2 SV=1	PUMA_PARUN	227763.9	5.47	47	67	89.931	18.138

Peptide Information

Calc. Mass	Obsrv. Mass	± da	± ppm	Start Seq.	End Seq.	Sequence	Ion Score	C. I. %	Modification	Rank	Result Type
800.4737	800.4361	-0.0376	-47	879	884	IVERQR					Mascot
804.4461	804.4183	-0.0278	-35	716	722	LTTLNDK					Mascot
807.3665	807.4246	0.0581	72	536	542	NDLGCTK			Carbamidomethyl (C)[5]		Mascot
815.4846	815.5005	0.0159	19	1940	1946	SRPATRK					Mascot
817.4162	817.4589	0.0427	52	157	162	LDEERR					Mascot
829.489	829.4321	-0.0569	-69	430	436	QLVDAKR					Mascot
832.3907	832.4409	0.0502	60	1397	1402	QRDEER					Mascot
837.4464	837.4022	-0.0442	-53	105	110	KIEEYR					Mascot
845.4839	845.4951	0.0112	13	1337	1343	DSLNRLK					Mascot
846.4315	846.5141	0.0826	98	1511	1517	DDAERLK					Mascot
847.4091	847.481	0.0719	85	1286	1293	GALNDAMR					Mascot
851.4621	851.4108	-0.0513	-60	77	83	DLNSFKK					Mascot
863.4039	863.4628	0.0589	68	1286	1293	GALNDAMR			Oxidation (M)[7]		Mascot
868.4635	868.4468	-0.0167	-19	1577	1583	AELRDHK					Mascot
890.4876	890.4818	-0.0058	-7	677	683	EVMALRR			Oxidation (M)[3]		Mascot
921.3982	921.4852	0.087	94	751	757	LEQECDK			Carbamidomethyl (C)[5]		Mascot
960.536	960.5273	-0.0087	-9	1190	1197	LIVTETER					Mascot
963.4815	963.4975	0.016	17	1656	1663	IVQMESEK					Mascot
991.5128	991.5438	0.031	31	903	911	AEMATVELK					Mascot
1004.5986	1004.5174	-0.0812	-81	1127	1135	IVQTTTITK					Mascot
1021.481	1021.5029	0.0219	21	1045	1052	ELNSHEHR					Mascot
1033.4585	1033.5222	0.0637	62	1892	1899	ESYTYQSR					Mascot
1037.5295	1037.5398	0.0103	10	726	734	EKQSIMSSK					Mascot
1049.4932	1049.5669	0.0737	70	750	757	KLEQECDK			Carbamidomethyl (C)[6]		Mascot
1053.5211	1053.5669	0.0458	43	304	311	KYEETVER					Mascot
1053.5245	1053.5669	0.0424	40	726	734	EKQSIMSSK			Oxidation (M)[6]		Mascot
1066.5635	1066.55	-0.0135	-13	1381	1389	LSSMKEMLK					Mascot

Calc. Mass	Obsrv. Mass	± da	± ppm	Start Seq.	End Seq.	Sequence	Ion Score	C. I. %	Modification	Rank	Result Type
1101.5219	1101.5842	0.0623	57	1803	1811	ARSHQMSER					Mascot
1107.5714	1107.5498	-0.0216	-20	1656	1664	IVQMESEKK			Oxidation (M)[4]		Mascot
1109.5698	1109.5552	-0.0146	-13	1228	1237	ALDELANAHR					Mascot
1118.5549	1118.5353	-0.0196	-18	1262	1270	SRLENSEQR					Mascot
1155.6005	1155.6075	0.007	6	1875	1884	EAYRTSSTIK					Mascot
1158.5862	1158.6189	0.0327	28	444	453	AVDDANRELR					Mascot
1164.5453	1164.5743	0.029	25	792	801	DAMTEVEELK					Mascot
1189.6172	1189.5776	-0.0396	-33	1633	1643	AQLSTAADERK					Mascot
1216.5991	1216.5809	-0.0182	-15	1104	1113	HQIDTIMNTK			Oxidation (M)[7]		Mascot
1216.5991	1216.5809	-0.0182	-15	1104	1113	HQIDTIMNTK			Oxidation (M)[7]		Mascot
1232.6304	1232.623	-0.0074	-6	1533	1542	NLVEQTEIMR					Mascot
1260.698	1260.6345	-0.0635	-50	901	911	LRAEMATVELK					Mascot
1289.6631	1289.6633	0.0002	0	1010	1019	NLERVCETLR			Carbamidomethyl (C)[6]		Mascot
1302.6172	1302.6438	0.0266	20	1665	1675	DVDNQLEEVNK					Mascot
1312.6678	1312.634	-0.0338	-26	999	1009	KDVHMAQNELK					Mascot
1316.6805	1316.5812	-0.0993	-75	683	693	RLNDELDTNVK					Mascot
1357.6383	1357.6873	0.049	36	547	557	NVEFEYNSTVR					Mascot
1366.5791	1366.6744	0.0953	70	2	13	EGTCSEEENLAK			Carbamidomethyl (C)[4]		Mascot
1366.5791	1366.6744	0.0953	70	2	13	EGTCSEEENLAK			Carbamidomethyl (C)[4]		Mascot
1455.7222	1455.7073	-0.0149	-10	941	952	ISFMEKMLEEAK					Mascot
1479.7186	1479.7737	0.0551	37	350	363	LGDLAAGSYRGDER					Mascot
1482.7699	1482.7701	0.0002	0	202	213	NLAYYLNAEQRK					Mascot
1516.6986	1516.7252	0.0266	18	1433	1445	VQAELENSQNDNR					Mascot
2246.0288	2246.2185	0.1897	84	1725	1743	NCAQLQQQIAQLEMDNG ER			Carbamidomethyl (C)[2]		Mascot

6 Protein MGF 505-2R OS=African swine fever virus (isolate Tick/Malawi/Lil 20-1/1983) GN=Mal-035 PE=3 SV=1 | 5052R_ASFM2 | 62459.6 | 9.02 | 20 | 65 | 84.405 | 7.378

Peptide Information

Calc. Mass	Obsrv. Mass	± da	± ppm	Start Seq.	End Seq.	Sequence	Ion Score	C. I. %	Modification	Rank	Result Type
823.4924	823.4265	-0.0659	-80	518	524	VISYKTI					Mascot
829.493	829.4321	-0.0609	-73	415	421	VIHGIYK					Mascot
845.4702	845.4951	0.0249	29	431	437	VMFHLAK					Mascot
847.4407	847.481	0.0403	48	502	508	VITEEEK					Mascot
853.4751	853.4554	-0.0197	-23	33	39	RHGSLQR					Mascot
868.4597	868.4468	-0.0129	-15	398	404	NMVYTIK					Mascot

893.4695	893.4639	-0.0056	-6	369	375	MAIQKMR			Oxidation (M)[1]		Mascot
946.5754	946.5076	-0.0678	-72	366	373	VIKMAIQK			Oxidation (M)[4]		Mascot
1037.5262	1037.5398	0.0136	13	151	158	YQEELSIR					Mascot
1051.5895	1051.6747	0.0852	81	40	48	IGDDHILIR					Mascot
1101.6528	1101.5842	-0.0686	-62	458	466	LDVARFKPR					Mascot
1109.6136	1109.5552	-0.0584	-53	142	150	HNMLPILQK			Oxidation (M)[3]		Mascot
1118.6027	1118.5353	-0.0674	-60	429	437	EKVMFHLAK			Oxidation (M)[4]		Mascot
1216.6783	1216.5809	-0.0974	-80	50	60	DLILSTNEALK					Mascot
1216.6783	1216.5809	-0.0974	-80	50	60	DLILSTNEALK					Mascot
1372.7794	1372.6788	-0.1006	-73	49	60	RDLILSTNEALK					Mascot
1433.8264	1433.7382	-0.0882	-62	265	275	LLDYFLRQLPR					Mascot
1482.741	1482.7701	0.0291	20	398	409	NMVYTIKYEHGK					Mascot
1661.8528	1661.824	-0.0288	-17	352	365	VRTIMDELSISPER			Oxidation (M)[5]		Mascot
1839.0626	1838.9429	-0.1197	-65	312	326	LLRYVIEYESTLVIK					Mascot
2224.1697	2224.0476	-0.1221	-55	431	449	VMFHLAKLYVAQNAATQ FR			Oxidation (M)[2]		Mascot

7	DNA gyrase subunit A OS=Rickettsia typhi (strain ATCC VR-144 / Wilmington) GN=gyrA PE=3 SV=1	GYRA_RICTY	101375.9	5.49	25	63	72.899	9.092

Peptide Information

Calc. Mass	Obsrv. Mass	± da	± ppm	Start Seq.	End Seq.	Sequence	Ion Score	C. I. %	Modification	Rank	Result Type
800.4447	800.4361	-0.0086	-11	128	134	MSKVAHK					Mascot
807.3665	807.4246	0.0581	72	408	413	QELMDR			Oxidation (M)[4]		Mascot
818.444	818.4578	0.0138	17	70	77	IVGDVMGK					Mascot
821.4515	821.4269	-0.0246	-30	543	549	VPLSSYR					Mascot
831.4458	831.471	0.0252	30	135	141	LVEDIDK					Mascot
837.4352	837.4022	-0.033	-39	652	658	SDLLDFK					Mascot
847.4672	847.481	0.0138	16	700	706	FPVESLR					Mascot
858.5294	858.5036	-0.0258	-30	495	501	EELIKVK					Mascot
863.4515	863.4628	0.0113	13	557	564	GRSGLSMR					Mascot
864.5189	864.4678	-0.0511	-59	366	372	TIYLLNK					Mascot
891.4716	891.4639	-0.0077	-9	850	856	CKLESVR			Carbamidomethyl (C)[1]		Mascot
921.5265	921.4852	-0.0413	-45	40	47	DGLKPVHR					Mascot
946.5138	946.5076	-0.0062	-7	431	438	VMLNERGK					Mascot
963.5179	963.4975	-0.0204	-21	277	284	IAEMVKEK			Oxidation (M)[4]		Mascot
993.5363	993.5377	0.0014	1	651	658	RSDLLDFK					Mascot
1001.472	1001.5231	0.0511	51	123	130	YTESRMSK					Mascot

1101.6449	1101.5842	-0.0607	-55	448	456	AILEMKLQR					Mascot
1115.6494	1115.6151	-0.0343	-31	823	833	TGLVVGVMPVK			Oxidation (M)[8]		Mascot
1164.6372	1164.5743	-0.0629	-54	543	552	VPLSSYRSQK					Mascot
1340.84	1340.7075	-0.1325	-99	489	499	LLEILKEELIK					Mascot
1357.7838	1357.6873	-0.0965	-71	595	606	LYKLPLSNPQGK					Mascot
1482.6815	1482.7701	0.0886	60	834	846	MDDELMLITNSGK			Oxidation (M)[1]		Mascot
1549.8506	1549.7593	-0.0913	-59	721	734	LAKEDSVISMTVLK			Oxidation (M)[10]		Mascot
1707.9349	1707.7964	-0.1385	-81	439	453	LSFTEVQAKAILEMK					Mascot
1818.9015	1818.9351	0.0336	18	806	822	ITDRGGSGIINMDINDK					Mascot

8	DNA gyrase subunit A OS=Rickettsia bellii (strain RML369-C) GN=gyrA PE=3 SV=1	GYRA_RICBR	100958.2	5.15	24	63	72.268	8.751

Peptide Information

Calc. Mass	Obsrv. Mass	± da	± ppm	Start Seq.	End Seq.	Sequence	Ion Score	C. I. %	Modification	Rank	Result Type
800.4447	800.4361	-0.0086	-11	128	134	MAKVAHK			Oxidation (M)[1]		Mascot
818.444	818.4578	0.0138	17	70	77	IVGDVMGK					Mascot
821.4515	821.4269	-0.0246	-30	543	549	VPLSSYR					Mascot
829.5002	829.4321	-0.0681	-82	239	246	GSIAIRGR					Mascot
837.4352	837.4022	-0.033	-39	652	658	SDLLDFK					Mascot
845.4614	845.4951	0.0337	40	135	141	LIEDIDK					Mascot
847.4672	847.481	0.0138	16	700	706	FPVESLR					Mascot
849.4101	849.4623	0.0522	61	502	508	EEFATPR					Mascot
858.5294	858.5036	-0.0258	-30	495	501	EELIKVK					Mascot
863.4515	863.4628	0.0113	13	557	564	GRSGLSMR					Mascot
864.5189	864.4678	-0.0511	-59	366	372	TIYLLNK					Mascot
891.424	891.4639	0.0399	45	431	438	AMLNEEGK					Mascot
921.5265	921.4852	-0.0413	-45	40	47	DGLKPVHR					Mascot
963.5179	963.4975	-0.0204	-21	277	284	IAEMVKEK			Oxidation (M)[4]		Mascot
988.5309	988.5034	-0.0275	-28	463	470	NKLEEDLK					Mascot
993.5363	993.5377	0.0014	1	651	658	RSDLLDFK					Mascot
1001.472	1001.5231	0.0511	51	123	130	YTESRMAK			Oxidation (M)[6]		Mascot
1115.6494	1115.6151	-0.0343	-31	823	833	TGLVVGVMPVK			Oxidation (M)[8]		Mascot
1340.84	1340.7075	-0.1325	-99	489	499	LLEILKEELIK					Mascot
1357.7838	1357.6873	-0.0965	-71	595	606	LYKLPLSNPQGK					Mascot
1461.7253	1461.7346	0.0093	6	426	438	LVDDKAMLNEEGK					Mascot
1482.6815	1482.7701	0.0886	60	834	846	MDDELMLITNSGK			Oxidation (M)[1]		Mascot

Calc. Mass	Obsrv. Mass	± da	± ppm	Start Seq.	End Seq.	Sequence	Ion Score	C. I. %	Modification	Rank	Result Type
1628.7598	1628.8672	0.1074	66	49	62	IIYSMHEAGNHANR			Oxidation (M)[5]		Mascot
1818.9015	1818.9351	0.0336	18	806	822	ITDRGGSGIINMDINDK					Mascot

9	Serine--tRNA ligase OS=Deinococcus radiodurans (strain ATCC 13939 / DSM 20539 / JCM 16871 / LMG 4051 / NBRC 15346 / NCIMB 9279 / R1 / VKM B-1422) GN=serS PE=3 SV=1	SYS_DEIRA	47876.9	5.2	17	63	71.622	7.79

Peptide Information

Calc. Mass	Obsrv. Mass	± da	± ppm	Start Seq.	End Seq.	Sequence	Ion Score	C. I. %	Modification	Rank	Result Type
800.3897	800.4361	0.0464	58	9	15	ENPDAVR					Mascot
815.4522	815.5005	0.0483	59	274	279	VHEFRK					Mascot
819.4094	819.433	0.0236	29	75	81	DLSEDLK					Mascot
831.4795	831.471	-0.0085	-10	152	159	VARVSGSR					Mascot
832.4047	832.4409	0.0362	43	89	95	EQEDALK					Mascot
963.4564	963.4975	0.0411	43	44	51	VEAMQTER					Mascot
1004.5258	1004.5174	-0.0084	-8	73	81	GKDLSEDLK					Mascot
1051.5969	1051.6747	0.0778	74	1	8	MLDLKFIR			Oxidation (M)[1]		Mascot
1066.535	1066.55	0.015	14	280	287	VEQYVLCR			Carbamidomethyl (C)[7]		Mascot
1109.6313	1109.5552	-0.0761	-69	155	164	VSGSRSYLLK					Mascot
1216.6433	1216.5809	-0.0624	-51	6	15	FIRENPDAVR					Mascot
1216.6433	1216.5809	-0.0624	-51	6	15	FIRENPDAVR					Mascot
1260.5815	1260.6345	0.053	42	114	123	DEDDNVELRR					Mascot
1366.609	1366.6744	0.0654	48	317	329	VIQNCTGDMGAGK			Carbamidomethyl (C)[5], Oxidation (M)[9]		Mascot
1366.609	1366.6744	0.0654	48	317	329	VIQNCTGDMGAGK			Carbamidomethyl (C)[5], Oxidation (M)[9]		Mascot
1384.7432	1384.764	0.0208	15	22	33	NVALDLDDLLQR					Mascot
1384.7432	1384.764	0.0208	15	22	33	NVALDLDDLLQR					Mascot
1455.8165	1455.7073	-0.1092	-75	89	100	EQEDALKQLLLR					Mascot
1461.7114	1461.7346	0.0232	16	44	56	VEAMQTERNANAK					Mascot
1507.7177	1507.7626	0.0449	30	288	299	ADQEEGLKWFER					Mascot
1507.7177	1507.7626	0.0449	30	288	299	ADQEEGLKWFER	1	0			Mascot

10	DNA gyrase subunit A OS=Rickettsia prowazekii (strain Madrid E) GN=gyrA PE=3 SV=1	GYRA_RICPR	101301.9	5.42	24	62	68.159	8.626

Peptide Information

Calc. Mass	Obsrv. Mass	± da	± ppm	Start Seq.	End Seq.	Sequence	Ion Score	C. I. %	Modification	Rank	Result Type
800.4447	800.4361	-0.0086	-11	128	134	MAKVAHK			Oxidation (M)[1]		Mascot
818.444	818.4578	0.0138	17	70	77	IVGDVMGK					Mascot

821.4515	821.4269	-0.0246	-30	543	549	VPLSSYR		Mascot
831.4458	831.471	0.0252	30	135	141	LVEDIDK		Mascot
835.3944	835.4343	0.0399	48	502	508	EEFASPR		Mascot
837.4352	837.4022	-0.033	-39	652	658	SDLLDFK		Mascot
847.4672	847.481	0.0138	16	700	706	FPVESLR		Mascot
858.5294	858.5036	-0.0258	-30	495	501	EELIKVK		Mascot
863.4515	863.4628	0.0113	13	557	564	GRSGLSMR		Mascot
864.5189	864.4678	-0.0511	-59	366	372	TIYLLNK		Mascot
891.4716	891.4639	-0.0077	-9	850	856	CKLESVR	Carbamidomethyl (C)[1]	Mascot
921.5265	921.4852	-0.0413	-45	40	47	DGLKPVHR		Mascot
963.5179	963.4975	-0.0204	-21	277	284	IAEMVKEK	Oxidation (M)[4]	Mascot
993.5363	993.5377	0.0014	1	651	658	RSDLLDFK		Mascot
1001.472	1001.5231	0.0511	51	123	130	YTESRMAK	Oxidation (M)[6]	Mascot
1101.6449	1101.5842	-0.0607	-55	448	456	AILEMKLQR		Mascot
1115.6494	1115.6151	-0.0343	-31	823	833	TGLVVGVMPVK	Oxidation (M)[8]	Mascot
1164.6372	1164.5743	-0.0629	-54	543	552	VPLSSYRSQK		Mascot
1340.84	1340.7075	-0.1325	-99	489	499	LLEILKEELIK		Mascot
1357.7838	1357.6873	-0.0965	-71	595	606	LYKLPLSNPQGK		Mascot
1482.6815	1482.7701	0.0886	60	834	846	MDDELMLITNSGK	Oxidation (M)[1]	Mascot
1549.8506	1549.7593	-0.0913	-59	721	734	LAKEDSVISMTVLK	Oxidation (M)[10]	Mascot
1661.8275	1661.824	-0.0035	-2	399	413	ASNDTNLAKQELMAR		Mascot
1818.9015	1818.9351	0.0336	18	806	822	ITDRGGSGIINMDINDK		Mascot

Gel Idx/Pos	295/L23	Instr./Gel Origin	BA2151/Sample Project 20140814	Process Status	Analysis Succeeded
Plate [#] Name	[1] Sample Project 20140814	Instrument Sample Name		Spectra	11

Rank	Protein Name	Accession No.	Protein MW	Protein PI	Pep. Count	Protein Score	Protein Score C. I. %	Intensity Matched	Total Ion Score	Total Ion C. I. %	Confirmed
1	Keratin, type I cytoskeletal 9 OS=Homo sapiens GN=KRT9 PE=1 SV=3	K1C9_HUMAN	62254.9	5.14	12	89	99.935	3.988	48	98.295	

Peptide Information

Calc. Mass	Obsrv. Mass	± da	± ppm	Start Seq.	End Seq.	Sequence	Ion Score	C. I. %	Modification	Rank	Result Type
913.4084	913.4289	0.0205	22	234	240	MTLDDFR			Oxidation (M)[1]		Mascot
982.4337	982.4589	0.0252	26	35	46	FSSSGGGGGGGR					Mascot
1060.5634	1060.5697	0.0063	6	225	233	TLLDIDNTR					Mascot
1065.4994	1065.5458	0.0464	44	155	163	STMQELNSR					Mascot
1081.4943	1081.5223	0.028	26	155	163	STMQELNSR			Oxidation (M)[3]		Mascot
1095.547	1095.5701	0.0231	21	185	192	IQDWYDKK					Mascot
1232.5979	1232.6223	0.0244	20	14	29	SGGGGGGGLGSGGSIR					Mascot
1235.5288	1235.6146	0.0858	69	47	59	FSSSSGYGGGSSR					Mascot
1323.6726	1323.6731	0.0005	0	241	250	IKFEMEQNLR			Oxidation (M)[5]		Mascot
1323.6726	1323.6731	0.0005	0	241	250	IKFEMEQNLR	4	0	Oxidation (M)[5]		Mascot
1605.7432	1605.7699	0.0267	17	200	212	NYSPYYNTIDDLK					Mascot
1791.7278	1791.7716	0.0438	24	491	513	GGSGGSYGGGGSGGGY GGGSGSR	48	98.295			Mascot
1812.8584	1812.8986	0.0402	22	14	34	SGGGGGGGLGSGGSIRS SYSR					Mascot
1837.9655	1837.955	-0.0105	-6	375	390	HGVQELEIELQSQLSK					Mascot

Rank	Protein Name	Accession No.	Protein MW	Protein PI	Pep. Count	Protein Score	Protein Score C. I. %	Intensity Matched	Total Ion Score	Total Ion C. I. %	Confirmed
2	Intraflagellar transport protein 172 homolog OS=Danio rerio GN=ift172 PE=2 SV=1	IF172_DANRE	199080.5	5.61	37	86	99.861	14.009			

Peptide Information

Calc. Mass	Obsrv. Mass	± da	± ppm	Start Seq.	End Seq.	Sequence	Ion Score	C. I. %	Modification	Rank	Result Type
807.3995	807.4188	0.0193	24	815	821	AGDLFER					Mascot
810.4542	810.4192	-0.035	-43	2	7	QLKYMK					Mascot
811.3766	811.4166	0.04	49	828	833	ALECYR			Carbamidomethyl (C)[4]		Mascot
812.4876	812.4122	-0.0754	-93	1654	1660	VEQVLPK					Mascot
816.4686	816.4243	-0.0443	-54	135	141	VRQANTK					Mascot
819.4723	819.4213	-0.051	-62	1219	1225	AEAFLLR					Mascot
823.3766	823.4351	0.0585	71	1498	1503	MWSDLR			Oxidation (M)[1]		Mascot

824.4083	824.441	0.0327	40	1060	1066	AAVNMYR		Mascot
826.4491	826.4271	-0.022	-27	2	7	QLKYMK	Oxidation (M)[5]	Mascot
831.457	831.4301	-0.0269	-32	1097	1105	SLGGEAAVK		Mascot
833.3999	833.372	-0.0279	-33	1038	1044	ELEAESR		Mascot
835.4746	835.4055	-0.0691	-83	558	564	VTMFPLK		Mascot
865.4414	865.4575	0.0161	19	659	665	FLNETNK		Mascot
877.46	877.4401	-0.0199	-23	782	789	AAWLAMSK		Mascot
927.5258	927.4841	-0.0417	-45	1376	1383	VAKELDPR		Mascot
1006.4476	1006.4911	0.0435	43	273	281	GTWDESSPK		Mascot
1013.5891	1013.5228	-0.0663	-65	1433	1440	QNFKVLHK		Mascot
1023.5469	1023.628	0.0811	79	1563	1571	HTELIPADK		Mascot
1025.6102	1025.5587	-0.0515	-50	1226	1234	AQRPELAIK		Mascot
1033.5579	1033.5253	-0.0326	-32	265	272	VFNWSPRK		Mascot
1064.5483	1064.5533	0.005	5	815	823	AGDLFERTR		Mascot
1067.519	1067.5596	0.0406	38	647	656	CFAALGDVSK	Carbamidomethyl (C)[1]	Mascot
1095.5867	1095.5701	-0.0166	-15	688	696	LAMLDKNFK	Oxidation (M)[3]	Mascot
1114.6104	1114.5632	-0.0472	-42	8	18	TLLTPQDGAAK		Mascot
1139.4899	1139.5697	0.0798	70	1489	1497	DRDCAEAFR	Carbamidomethyl (C)[4]	Mascot
1187.7107	1187.6211	-0.0896	-75	340	350	NLSTGTRVVLK		Mascot
1193.6096	1193.6241	0.0145	12	1173	1182	EAVLMHVHNK	Oxidation (M)[5]	Mascot
1201.5341	1201.6339	0.0998	83	1010	1018	NKMYDDMIR	Oxidation (M)[3]	Mascot
1212.6583	1212.6089	-0.0494	-41	1654	1663	VEQVLPKDER		Mascot
1302.6649	1302.6768	0.0119	9	565	576	GDIVDLERSNGK		Mascot
1320.6803	1320.6177	-0.0626	-47	1310	1320	CWMKAAELAIK	Carbamidomethyl (C)[1]	Mascot
1323.6222	1323.6731	0.0509	38	824	833	NNQRALECYR	Carbamidomethyl (C)[8]	Mascot
1323.6222	1323.6731	0.0509	38	824	833	NNQRALECYR	Carbamidomethyl (C)[8]	Mascot
1363.6675	1363.7101	0.0426	31	1643	1653	EWVLTVSMDQR		Mascot
1379.6625	1379.7222	0.0597	43	1643	1653	EWVLTVSMDQR	Oxidation (M)[8]	Mascot
1384.6566	1384.7765	0.1199	87	1706	1716	DDWNKFLMATK	Oxidation (M)[8]	Mascot
1384.6566	1384.7765	0.1199	87	1706	1716	DDWNKFLMATK	Oxidation (M)[8]	Mascot
1443.7413	1443.7413	0	0	433	444	TEFMNPHLISVR		Mascot
1507.8228	1507.7723	-0.0505	-33	634	646	LSLEAQQLHIAER		Mascot
1507.8228	1507.7723	-0.0505	-33	634	646	LSLEAQQLHIAER		Mascot
1584.9109	1584.7711	-0.1398	-88	841	855	AVDLARVAFPAEVVK		Mascot
1657.8618	1657.8401	-0.0217	-13	326	339	FEMTYVGLSQVIVR	Oxidation (M)[3]	Mascot

3	Phosphoribosylaminoimidazole-succinocarboxamide	PUR7_MANSM	32381.8	5.34	13	70	94.071	3.418

synthase OS=Mannheimia succiniciproducens (strain MBEL55E) GN=purC PE=3 SV=1

Peptide Information

Calc. Mass	Obsrv. Mass	± da	± ppm	Start Seq.	End Seq.	Sequence	Ion Score	C. I. %	Modification	Rank	Result Type
807.428	807.4188	-0.0092	-11	1	7	MAELSLK			Oxidation (M)[1]		Mascot
864.4825	864.4208	-0.0617	-71	178	185	DAAIALYK					Mascot
937.4989	937.4431	-0.0558	-60	186	194	EAAAYALTK					Mascot
1010.4677	1010.5079	0.0402	40	16	23	DLYEIDDK					Mascot
1110.5394	1110.5577	0.0183	16	24	32	RMLMVASDR			Oxidation (M)[2,4]		Mascot
1193.6412	1193.6241	-0.0171	-14	277	286	YQEALDLLTK					Mascot
1212.7059	1212.6089	-0.097	-80	167	177	QIGKELAAQVR					Mascot
1262.5801	1262.642	0.0619	49	249	258	DWLEQSGWNK					Mascot
1265.6372	1265.6455	0.0083	7	14	23	VRDLYEIDDK					Mascot
1487.858	1487.7836	-0.0744	-50	259	272	QPPAPKVPADVIQK					Mascot
1584.8633	1584.7711	-0.0922	-58	33	46	LSAFDVILEDPIPR					Mascot
1621.797	1621.8745	0.0775	48	235	248	EGTNPPSFDKQFVR					Mascot
1837.9728	1837.955	-0.0178	-10	186	202	EAAAYALTKGIIICDTK			Carbamidomethyl (C)[14]		Mascot

4	Keratin, type II cytoskeletal 73 OS=Bos taurus GN=KRT73 PE=2 SV=1	K2C73_BOVIN	59539.8	6.96	15	69	93.792	4.189	19	0

Peptide Information

Calc. Mass	Obsrv. Mass	± da	± ppm	Start Seq.	End Seq.	Sequence	Ion Score	C. I. %	Modification	Rank	Result Type
814.3842	814.4427	0.0585	72	68	75	AGGYGFSR					Mascot
837.4213	837.4028	-0.0185	-22	38	46	GLSGGFGSR					Mascot
999.4451	999.4961	0.051	51	242	250	DVDAAYMSK					Mascot
1015.4401	1015.516	0.0759	75	242	250	DVDAAYMSK			Oxidation (M)[7]		Mascot
1026.5038	1026.5347	0.0309	30	47	55	SLYSLGCAR			Carbamidomethyl (C)[7]		Mascot
1060.5244	1060.5697	0.0453	43	1	8	MSRQFTYK					Mascot
1064.5042	1064.5533	0.0491	46	193	201	QLEMLSGDR			Oxidation (M)[4]		Mascot
1106.5623	1106.5417	-0.0206	-19	204	212	LDSELRSMR					Mascot
1127.5402	1127.5436	0.0034	3	241	250	KDVDAAYMSK					Mascot
1171.6106	1171.5842	-0.0264	-23	4	14	QFTYKSGAAAK					Mascot
1199.5474	1199.6605	0.1131	94	56	67	SVSFNMASGSGR					Mascot
1265.6736	1265.6455	-0.0281	-22	308	318	AQYEDIALKSK					Mascot
1277.71	1277.7323	0.0223	17	425	435	LALDIEIATYR	19	0			Mascot

1338.6899	1338.6897	-0.0002	0	317	328	SKAEAEALYQTK					Mascot
1360.7682	1360.7316	-0.0366	-27	515	527	LGSTSEIKDLLGK					Mascot
1367.7318	1367.7141	-0.0177	-13	138	149	ALNNKFASFIDK					Mascot

5 Hemolysin OS=Serratia marcescens GN=shlA PE=1 SV=1 HLYA_SERMA 165095.3 8.55 29 66 86.101 10.213

Peptide Information

Calc. Mass	Obsrv. Mass	± da	± ppm	Start Seq.	End Seq.	Sequence	Ion Score	C. I. %	Modification	Rank	Result Type
814.4166	814.4427	0.0261	32	461	467	NHTSSLR					Mascot
820.4047	820.4149	0.0102	12	1420	1427	VADATTDK					Mascot
830.4843	830.432	-0.0523	-63	527	534	TANAKAVR					Mascot
835.3792	835.4055	0.0263	31	298	305	VDDASSNK					Mascot
836.4108	836.4198	0.009	11	720	726	DSEKTTR					Mascot
864.4938	864.4208	-0.073	-84	287	293	VKAYDIR					Mascot
865.4638	865.4575	-0.0063	-7	1428	1434	YNSVARR					Mascot
877.4625	877.4401	-0.0224	-26	819	827	AITSGSDVK					Mascot
918.4527	918.4467	-0.006	-7	1350	1358	VDADSVQGK					Mascot
948.5261	948.4582	-0.0679	-72	1400	1408	VGTPRYAGK					Mascot
982.47	982.4589	-0.0111	-11	453	460	SSERGYQR					Mascot
1015.553	1015.516	-0.037	-36	423	432	LLGANVSADR					Mascot
1032.5573	1032.5116	-0.0457	-44	414	422	LISTQEDVK					Mascot
1037.5626	1037.5352	-0.0274	-26	693	702	TALTVNGYAK					Mascot
1068.5433	1068.5432	-0.0001	0	1359	1368	VGGDLHVESR					Mascot
1101.6011	1101.616	0.0149	14	828	837	GNLTINARDK					Mascot
1114.5487	1114.5632	0.0145	13	715	723	IEHTRDSEK					Mascot
1139.5692	1139.5697	0.0005	0	1092	1103	ASAKGGFTADSK					Mascot
1187.6267	1187.6211	-0.0056	-5	275	286	LAGSLNAGDELK					Mascot
1302.7013	1302.6768	-0.0245	-19	571	583	LNGQQGVTITGSK					Mascot
1316.6958	1316.5952	-0.1006	-76	1274	1285	DNWSLGIKANAK					Mascot
1323.6387	1323.6731	0.0344	26	727	740	TENSASSLSGGSVK					Mascot
1323.7267	1323.6731	-0.0536	-40	441	452	DVHLAGLVEKDK					Mascot
1360.7068	1360.7316	0.0248	18	754	767	LVADKGDASVSGNK					Mascot
1363.6448	1363.7101	0.0653	48	1074	1085	LDQASDKQSESR					Mascot
1366.7213	1366.6907	-0.0306	-22	746	758	DVTFSGSKLVADK					Mascot
1366.7213	1366.6907	-0.0306	-22	746	758	DVTFSGSKLVADK					Mascot
1463.7601	1463.802	0.0419	29	1048	1061	KDAIYQGTALNGGR					Mascot

1837.8563	1837.955	0.0987	54	471	486	WSNSDESESLKASELR					Mascot
3133.4678	3133.6973	0.2295	73	233	261	VARDGTVQASQQMPTAL DSYYLGSMQAGR			Oxidation (M)[13,25]		Mascot

6	Filamin-A-interacting protein 1 OS=Rattus norvegicus GN=Filip1 PE=1 SV=1	FLIP1_RAT	138294.6	8.25	35	65	82.901	11.488	

Peptide Information

Calc. Mass	Obsrv. Mass	± da	± ppm	Start Seq.	End Seq.	Sequence	Ion Score	C. I. %	Modification	Rank	Result Type
802.4417	802.4499	0.0082	10	518	523	IKQEER					Mascot
810.3661	810.4192	0.0531	66	1187	1193	AETQSMK			Oxidation (M)[6]		Mascot
812.364	812.4122	0.0482	59	512	517	KNMMEK			Oxidation (M)[3,4]		Mascot
816.4825	816.4243	-0.0582	-71	767	773	EVLNLTK					Mascot
824.4261	824.441	0.0149	18	157	162	QKETYR					Mascot
831.4207	831.4301	0.0094	11	31	38	GPSEDAKK					Mascot
837.4022	837.4028	0.0006	1	539	545	VMDVTEK			Oxidation (M)[2]		Mascot
884.4658	884.438	-0.0278	-31	730	736	IHELMNK					Mascot
891.479	891.4423	-0.0367	-41	1159	1166	IPMSKGMK					Mascot
909.4532	909.4614	0.0082	9	513	519	NMMEKIK			Oxidation (M)[2]		Mascot
909.4532	909.4614	0.0082	9	513	519	NMMEKIK			Oxidation (M)[2]		Mascot
913.3971	913.4289	0.0318	35	754	760	FMEEETK					Mascot
918.4639	918.4467	-0.0172	-19	578	584	LRSEEER					Mascot
927.5118	927.4841	-0.0277	-30	784	792	ALRPSGNGR					Mascot
948.5374	948.4582	-0.0792	-83	905	912	QGQPLHIR					Mascot
995.5996	995.639	0.0394	40	1043	1051	QTVPAPVRK					Mascot
995.5996	995.639	0.0394	40	1043	1051	QTVPAPVRK					Mascot
1015.5782	1015.516	-0.0622	-61	419	426	ELRLEVEK					Mascot
1016.5047	1016.5214	0.0167	16	298	305	LEVDFEHK					Mascot
1025.5262	1025.5587	0.0325	32	564	571	EYSLTKER					Mascot
1068.5181	1068.5432	0.0251	23	320	328	LANQESHNR					Mascot
1114.6102	1114.5632	-0.047	-42	718	727	DLQAEVQALK					Mascot
1141.6034	1141.5438	-0.0596	-52	728	736	EKIHELMNK					Mascot
1171.53	1171.5842	0.0542	46	754	762	FMEEETKNK			Oxidation (M)[2]		Mascot
1187.6704	1187.6211	-0.0493	-42	164	173	MLEQLLLAEK					Mascot
1201.6722	1201.6339	-0.0383	-32	839	848	QVGLKKPMER			Oxidation (M)[8]		Mascot
1212.5929	1212.6089	0.016	13	502	511	SFTVMLVDER			Oxidation (M)[5]		Mascot
1232.6733	1232.6223	-0.051	-41	489	499	AELSLKDDLTK					Mascot
1262.6198	1262.642	0.0222	18	855	865	YPPAANELTMR					Mascot

1277.677	1277.7323	0.0553	43	1187	1197	AETQSMKIELK			Mascot
1290.6835	1290.6887	0.0052	4	763	773	NMGREVLNLTK		Oxidation (M)[2]	Mascot
1323.6461	1323.6731	0.027	20	44	55	KEEDVMASGTIK		Oxidation (M)[6]	Mascot
1323.6461	1323.6731	0.027	20	44	55	KEEDVMASGTIK		Oxidation (M)[6]	Mascot
1338.6471	1338.6897	0.0426	32	1183	1193	FQPRAETQSMK		Oxidation (M)[10]	Mascot
1379.6471	1379.7222	0.0751	54	478	488	ELECSESRLEK		Carbamidomethyl (C)[4]	Mascot
1493.7932	1493.7603	-0.0329	-22	1110	1123	SPRNHLSSRPGASK			Mascot
1584.7574	1584.7711	0.0137	9	431	443	MSELEKLEEAFSR		Oxidation (M)[1]	Mascot
1657.8942	1657.8401	-0.0541	-33	641	654	RLQQLEVVEGDLMK			Mascot

7	Aspartate--tRNA ligase OS=Anaeromyxobacter dehalogenans (strain 2CP-1 / ATCC BAA-258) GN=aspS PE=3 SV=1	SYD_ANAD2	68390	6.26	18	63	70.961	7.091

Peptide Information

Calc. Mass	Obsrv. Mass	± da	± ppm	Start Seq.	End Seq.	Sequence	Ion Score	C. I. %	Modification	Rank	Result Type
814.4457	814.4427	-0.003	-4	565	571	DVVAWPK					Mascot
820.4709	820.4149	-0.056	-68	147	153	TLMTRAK					Mascot
835.4785	835.4055	-0.073	-87	136	141	YLDLRR					Mascot
836.4658	836.4198	-0.046	-55	147	153	TLMTRAK			Oxidation (M)[3]		Mascot
948.4897	948.4582	-0.0315	-33	336	344	VPASANFSR					Mascot
1023.5873	1023.628	0.0407	40	529	537	FGFLLDALK					Mascot
1032.4779	1032.5116	0.0337	33	11	19	THSCGELTK			Carbamidomethyl (C)[4]		Mascot
1064.5483	1064.5533	0.005	5	505	513	LHDPEVQAR					Mascot
1110.4998	1110.5577	0.0579	52	225	232	CFRDEDLR			Carbamidomethyl (C)[1]		Mascot
1126.6328	1126.5381	-0.0947	-84	84	94	VVSRGGNVNPK					Mascot
1171.647	1171.5842	-0.0628	-54	565	574	DVVAWPKTQK					Mascot
1199.6168	1199.6605	0.0437	36	37	47	DHGGAVFIDLR					Mascot
1302.745	1302.6768	-0.0682	-52	553	564	LVMLLTGAESLR					Mascot
1323.621	1323.6731	0.0521	39	517	528	AMGITDEEARSK			Oxidation (M)[2]		Mascot
1323.621	1323.6731	0.0521	39	517	528	AMGITDEEARSK			Oxidation (M)[2]		Mascot
1366.7101	1366.6907	-0.0194	-14	345	355	TEIDKLEEYVK					Mascot
1366.7101	1366.6907	-0.0194	-14	345	355	TEIDKLEEYVK					Mascot
1475.6796	1475.7709	0.0913	62	575	589	GTDLMTGAPGDVDAR					Mascot
1493.7206	1493.7603	0.0397	27	427	440	MGLIPEYGSGGAWR					Mascot
1507.7653	1507.7723	0.007	5	154	165	VNHLTRNYFTDK					Mascot
1507.7653	1507.7723	0.007	5	154	165	VNHLTRNYFTDK					Mascot
1657.8545	1657.8401	-0.0144	-9	365	380	AKVGEGGEWTQSPLAK					Mascot

8	43 kDa receptor-associated protein of the synapse OS=Torpedo californica GN=RAPSN PE=1 SV=3	RAPSN_TORCA	47733.3	7.13	16	62	68.159	5.69

Peptide Information

Calc. Mass	Obsrv. Mass	± da	± ppm	Start Seq.	End Seq.	Sequence	Ion Score	C. I. %	Modification	Rank	Result Type
807.3665	807.4188	0.0523	65	1	7	MGQDQTK					Mascot
812.4373	812.4122	-0.0251	-31	258	264	HRSDIGK					Mascot
823.3615	823.4351	0.0736	89	1	7	MGQDQTK			Oxidation (M)[1]		Mascot
826.3763	826.4271	0.0508	61	59	64	YEDMLR					Mascot
832.3618	832.3424	-0.0194	-23	75	81	QMGDPER					Mascot
836.4512	836.4198	-0.0314	-38	405	412	SSVKPGYV					Mascot
854.3535	854.4364	0.0829	97	295	300	CWMTEK			Carbamidomethyl (C)[1]		Mascot
891.3923	891.4423	0.05	56	398	404	GCPNCKR			Carbamidomethyl (C)[2,5]		Mascot
982.4563	982.4589	0.0026	3	210	217	YHMAAAYR					Mascot
1049.5375	1049.5485	0.011	10	65	74	FAVAQSEAAR					Mascot
1064.5042	1064.5533	0.0491	46	338	346	EMGSDQLLR			Oxidation (M)[2]		Mascot
1110.5513	1110.5577	0.0064	6	210	218	YHMAAAYRK					Mascot
1126.5463	1126.5381	-0.0082	-7	210	218	YHMAAAYRK			Oxidation (M)[3]		Mascot
1259.6128	1259.6743	0.0615	49	149	159	ALRYAHGNDDK					Mascot
1302.6649	1302.6768	0.0119	9	2	12	GQDQTKQQIEK					Mascot
1325.6307	1325.6694	0.0387	29	328	337	AHCLYETIYR			Carbamidomethyl (C)[3]		Mascot
1443.662	1443.7413	0.0793	55	206	217	AMSRYHMAAAYR			Oxidation (M)[2]		Mascot
1487.7345	1487.7836	0.0491	33	45	58	ALGCLITAHSEMGK			Carbamidomethyl (C)[4]		Mascot

9	Probable exosome complex RNA-binding protein 1 OS=Pyrococcus abyssi (strain GE5 / Orsay) GN=PYRAB06090 PE=3 SV=1	ECR1_PYRAB	29296.9	9.08	14	62	64.274	6.313

Peptide Information

Calc. Mass	Obsrv. Mass	± da	± ppm	Start Seq.	End Seq.	Sequence	Ion Score	C. I. %	Modification	Rank	Result Type
802.4781	802.4499	-0.0282	-35	217	223	GLTDRIK					Mascot
807.422	807.4188	-0.0032	-4	25	31	NGRGTFR					Mascot
870.5658	870.5538	-0.012	-14	201	208	LAIEAILK					Mascot
884.5087	884.438	-0.0707	-80	237	244	GVIEEIPK					Mascot
909.4537	909.4614	0.0077	8	28	35	GTFREGSR					Mascot
909.4537	909.4614	0.0077	8	28	35	GTFREGSR					Mascot
982.4696	982.4589	-0.0107	-11	166	174	GGSMINMIK			Oxidation (M)[4,7]		Mascot

1033.5135	1033.5253	0.0118	11	138	147	GMPFNGGPLK			Oxidation (M)[2]		Mascot
1110.5646	1110.5577	-0.0069	-6	166	175	GGSMINMIKK			Oxidation (M)[4,7]		Mascot
1114.658	1114.5632	-0.0948	-85	102	110	IDLLRTDLR					Mascot
1141.6464	1141.5438	-0.1026	-90	235	244	EKGVIEEIPK					Mascot
1171.5814	1171.5842	0.0028	2	212	221	ESHTRGLTDR					Mascot
1407.7448	1407.7302	-0.0146	-10	162	174	VIGRGGSMINMIK			Oxidation (M)[8,11]		Mascot
1584.8997	1584.7711	-0.1286	-81	10	24	ELVVPGTLLAQGPYK					Mascot
1605.8483	1605.7699	-0.0784	-49	94	106	IQDFTDEKIDLLR					Mascot

10	Aspartate--tRNA ligase OS=Shewanella sp. (strain MR-7) GN=aspS PE=3 SV=1	SYD_SHESR	66232.6	5.25	17	62	61.719	7.115

Peptide Information

Calc. Mass	Obsrv. Mass	± da	± ppm	Start Seq.	End Seq.	Sequence	Ion Score	C. I. %	Modification	Rank	Result Type
807.4069	807.4188	0.0119	15	550	556	DVMAFPK					Mascot
813.4505	813.408	-0.0425	-52	211	216	YYQIVK					Mascot
823.4019	823.4351	0.0332	40	550	556	DVMAFPK			Oxidation (M)[3]		Mascot
831.5046	831.4301	-0.0745	-90	144	151	AKVTSAVR					Mascot
849.4465	849.4384	-0.0081	-10	177	183	DYLVPSR					Mascot
854.4771	854.4364	-0.0407	-48	342	349	FVGIYGAK					Mascot
1010.5153	1010.5079	-0.0074	-7	334	341	KQIDDYTK					Mascot
1015.4335	1015.516	0.0825	81	272	279	MTYNEAMR					Mascot
1065.6091	1065.5458	-0.0633	-59	514	522	FGFLLEALR					Mascot
1110.4998	1110.5577	0.0579	52	217	224	CFRDEDLR			Carbamidomethyl (C)[1]		Mascot
1171.5347	1171.5842	0.0495	42	272	280	MTYNEAMRR					Mascot
1187.5297	1187.6211	0.0914	77	272	280	MTYNEAMRR			Oxidation (M)[1]		Mascot
1235.5586	1235.6146	0.056	45	2	11	RSHYCGDVNK			Carbamidomethyl (C)[5]		Mascot
1308.7014	1308.6779	-0.0235	-18	538	549	IIMLMTGASSIR			Oxidation (M)[3]		Mascot
1360.6638	1360.7316	0.0678	50	77	88	ARPDSQVNAQMK			Oxidation (M)[11]		Mascot
1366.5991	1366.6907	0.0916	67	1	11	MRSHYCGDVNK			Carbamidomethyl (C)[6]		Mascot
1366.5991	1366.6907	0.0916	67	1	11	MRSHYCGDVNK			Carbamidomethyl (C)[6]		Mascot
1367.8118	1367.7141	-0.0977	-71	320	333	VAALRIPGGASLSR					Mascot
1475.7061	1475.7709	0.0648	44	490	501	IHNQEMQSAVFR			Oxidation (M)[6]		Mascot
1837.8352	1837.955	0.1198	65	303	319	NVEFAVFSGPANDEEGR					Mascot

Gel Idx/Pos	296/L24	Instr./Gel Origin	BA2151/Sample Project 20140814	Process Status	Analysis Succeeded
Plate [#] Name	[1] Sample Project 20140814	Instrument Sample Name		Spectra	11

Rank	Protein Name	Accession No.	Protein MW	Protein PI	Pep. Count	Protein Score	Protein Score C. I. %	Intensity Matched	Total Ion Score	Total Ion C. I. %	Confirmed
1	5-methyltetrahydropteroyltriglutamate--homocysteine methyltransferase OS=Mesembryanthemum crystallinum GN=METE PE=2 SV=1	METE_MESCR	85053.8	5.9	15	298	100	15.247	248	100	

Peptide Information

Calc. Mass	Obsrv. Mass	± da	± ppm	Start Seq.	End Seq.	Sequence	Ion Score	C. I. %	Modification	Rank	Result Type
1096.5786	1096.6073	0.0287	26	287	296	YLFAGVVDGR					Mascot
1096.5786	1096.6073	0.0287	26	287	296	YLFAGVVDGR	86	100			Mascot
1132.6613	1132.5739	-0.0874	-77	174	183	SFDLLSLLPK					Mascot
1199.5732	1199.6453	0.0721	60	19	28	FALESFWDGK					Mascot
1308.7046	1308.7032	-0.0014	-1	458	468	ISEEEYVKAIK					Mascot
1404.7733	1404.7596	-0.0137	-10	274	286	TLDLVKAEFPSGK					Mascot
1540.739	1540.7914	0.0524	34	373	387	DEAFFSANAAALASR					Mascot
1540.739	1540.7914	0.0524	34	373	387	DEAFFSANAAALASR	40	91.868			Mascot
1790.9623	1790.9556	-0.0067	-4	556	571	GMLTGPVTILNWSFVR					Mascot
1806.9572	1807.0002	0.043	24	556	571	GMLTGPVTILNWSFVR			Oxidation (M)[2]		Mascot
1839.0375	1839.0028	-0.0347	-19	254	270	TLISLKGVTGYGFDLVR					Mascot
1860.1245	1860.0032	-0.1213	-65	174	189	SFDLLSLLPKILPIYK					Mascot
1860.1245	1860.0032	-0.1213	-65	174	189	SFDLLSLLPKILPIYK					Mascot
1991.0081	1991.0825	0.0744	37	477	493	LQEELDIDVLVHGEPER					Mascot
1991.0081	1991.0825	0.0744	37	477	493	LQEELDIDVLVHGEPER	123	100			Mascot
2032.0645	2032.1113	0.0468	23	740	758	YGEVNPALSNMVAAAKQLR					Mascot
2125.0674	2125.1477	0.0803	38	367	387	ALSGQKDEAFFSANAAALASR					Mascot
2286.1985	2286.0615	-0.137	-60	717	736	KMLAVLESNVLWVNPDCGLK			Carbamidomethyl (C)[17]		Mascot
2310.3069	2310.3999	0.093	40	427	447	LNLPILPTTTIGSFPQTVELR					Mascot
2438.4019	2438.5037	0.1018	42	426	447	KLNLPILPTTTIGSFPQTVELR					Mascot

Rank	Protein Name	Accession No.	Protein MW	Protein PI	Pep. Count	Protein Score	Protein Score C. I. %	Intensity Matched	Total Ion Score	Total Ion C. I. %	Confirmed
2	5-methyltetrahydropteroyltriglutamate--homocysteine methyltransferase OS=Solenostemon scutellarioides GN=MET PE=1 SV=2	METE_SOLSC	84821.9	6.09	17	269	100	11.676	210	100	

Peptide Information

Calc. Mass	Obsrv. Mass	± da	± ppm	Start	End	Sequence	Ion	C. I. %	Modification	Rank	Result Type

				Seq.	Seq.		Score				
944.5411	944.5458	0.0047	5	465	472	AIKEEINK					Mascot
1096.5786	1096.6073	0.0287	26	286	295	YLFAGVVDGR					Mascot
1096.5786	1096.6073	0.0287	26	286	295	YLFAGVVDGR	86	100			Mascot
1194.7028	1194.6112	-0.0916	-77	37	46	CQLILGIHLK			Carbamidomethyl (C)[1]		Mascot
1199.5732	1199.6453	0.0721	60	19	28	FALESFWDGK					Mascot
1308.7046	1308.7032	-0.0014	-1	457	467	ISEEEYVKAIK					Mascot
1456.7505	1456.765	0.0145	10	576	587	FETCYQIALAIK			Carbamidomethyl (C)[4]		Mascot
1485.7908	1485.7977	0.0069	5	704	716	IPSTEEIADRINK					Mascot
1754.896	1754.9259	0.0299	17	451	464	EFKPTRISEEEYVK					Mascot
1790.9623	1790.9556	-0.0067	-4	555	570	GMLTGPVTILNWSFVR					Mascot
1806.9572	1807.0002	0.043	24	555	570	GMLTGPVTILNWSFVR			Oxidation (M)[2]		Mascot
1864.8766	1864.9762	0.0996	53	617	631	SEHAFYLDWAVHSFR					Mascot
1874.1401	1874.02	-0.1201	-64	173	188	TFPLLSLLDKILPIYK					Mascot
1991.0081	1991.0825	0.0744	37	476	492	LQEELDIDVLVHGEPER					Mascot
1991.0081	1991.0825	0.0744	37	476	492	LQEELDIDVLVHGEPER	123	100			Mascot
2025.0984	2025.0925	-0.0059	-3	37	54	CQLILGIHLKQMSDAGIK			Carbamidomethyl (C)[1]		Mascot
2041.0933	2041.0894	-0.0039	-2	37	54	CQLILGIHLKQMSDAGIK			Carbamidomethyl (C)[1], Oxidation (M)[12]		Mascot
2054.095	2054.1929	0.0979	48	29	46	SSAEDLEKCQLILGIHLK			Carbamidomethyl (C)[9]		Mascot
2067.0327	2067.125	0.0923	45	571	587	NDQPRFETCYQIALAIK			Carbamidomethyl (C)[9]		Mascot
2310.3069	2310.3999	0.093	40	426	446	LNLPILPTTTIGSFPQTVELR					Mascot
2438.4019	2438.5037	0.1018	42	425	446	KLNLPILPTTTIGSFPQTVELR					Mascot

3	5-methyltetrahydropteroyltriglutamate--homocysteine methyltransferase OS=Catharanthus roseus GN=METE PE=2 SV=1	METE_CATRO	85089	6.1	10	231	100	12.571	208	100

Peptide Information

Calc. Mass	Obsrv. Mass	± da	± ppm	Start Seq.	End Seq.	Sequence	Ion Score	C. I. %	Modification	Rank	Result Type
1096.5786	1096.6073	0.0287	26	287	296	YLFAGVVDGR					Mascot
1096.5786	1096.6073	0.0287	26	287	296	YLFAGVVDGR	86	100			Mascot
1456.7505	1456.765	0.0145	10	577	588	FETCYQIALAIK			Carbamidomethyl (C)[4]		Mascot
1485.7908	1485.7977	0.0069	5	705	717	IPSTEEIADRINK					Mascot
1790.9623	1790.9556	-0.0067	-4	556	571	GMLTGPVTILNWSFVR					Mascot
1806.9572	1807.0002	0.043	24	556	571	GMLTGPVTILNWSFVR			Oxidation (M)[2]		Mascot
1860.1245	1860.0032	-0.1213	-65	174	189	TFPLLSLLDKILPVYK					Mascot
1860.1245	1860.0032	-0.1213	-65	174	189	TFPLLSLLDKILPVYK					Mascot

Calc. Mass	Obsrv. Mass	± da	± ppm	Start Seq.	End Seq.	Sequence	Ion Score	C. I. %	Modification	Rank	Result Type
1864.9836	1864.9762	-0.0074	-4	739	755	KYAEVKPALENMVSAAK			Oxidation (M)[12]		Mascot
1991.0081	1991.0825	0.0744	37	477	493	LQEELDIDVLVHGEPER					Mascot
1991.0081	1991.0825	0.0744	37	477	493	LQEELDIDVLVHGEPER	123	100			Mascot
2067.0327	2067.125	0.0923	45	572	588	NDQPRFETCYQIALAIK			Carbamidomethyl (C)[9]		Mascot
2310.3069	2310.3999	0.093	40	427	447	LNLPVLPTTTIGSFPQTLE LR					Mascot
2438.4019	2438.5037	0.1018	42	426	447	KLNLPVLPTTTIGSFPQTL ELR					Mascot

4	5-methyltetrahydropteroyltriglutamate--homocysteine methyltransferase OS=Arabidopsis thaliana GN=CIMS PE=1 SV=1	METE_ARATH	84645.6	6.09	9	101	99.996	7.577	84	100	

Peptide Information

Calc. Mass	Obsrv. Mass	± da	± ppm	Start Seq.	End Seq.	Sequence	Ion Score	C. I. %	Modification	Rank	Result Type
1096.5786	1096.6073	0.0287	26	287	296	YLFAGVVDGR	86	100			Mascot
1096.5858	1096.6073	0.0215	20	401	411	AAAALKGSDHR					Mascot
1199.5732	1199.6453	0.0721	60	19	28	FALESFWDGK					Mascot
1399.7904	1399.7325	-0.0579	-41	393	406	VTNEGVQKAAAALK					Mascot
1790.9623	1790.9556	-0.0067	-4	556	571	GMLTGPVTILNWSFVR					Mascot
1806.9572	1807.0002	0.043	24	556	571	GMLTGPVTILNWSFVR			Oxidation (M)[2]		Mascot
1864.8766	1864.9762	0.0996	53	618	632	SEHAFYLDWAVHSFR					Mascot
1874.1401	1874.02	-0.1201	-64	174	189	SFELLSLLPKILPIYK					Mascot
2310.3069	2310.3999	0.093	40	427	447	LNLPILPTTTIGSFPQTVE LR					Mascot
2438.4019	2438.5037	0.1018	42	426	447	KLNLPILPTTTIGSFPQTV ELR					Mascot

5	5-methyltetrahydropteroyltriglutamate--homocysteine methyltransferase (Fragments) OS=Populus euphratica PE=1 SV=1	METE_POPEU	2244.2	5.91	1	94	99.98	4.711	86	100	

Peptide Information

Calc. Mass	Obsrv. Mass	± da	± ppm	Start Seq.	End Seq.	Sequence	Ion Score	C. I. %	Modification	Rank	Result Type
1096.5786	1096.6073	0.0287	26	12	21	YLFAGVVDGR					Mascot
1096.5786	1096.6073	0.0287	26	12	21	YLFAGVVDGR	86	100			Mascot

6	Keratin, type I cytoskeletal 14 OS=Homo sapiens GN=KRT14 PE=1 SV=4	K1C14_HUMAN	51871.5	5.09	17	81	99.529	5.435	

Peptide Information

Calc. Mass	Obsrv. Mass	± da	± ppm	Start Seq.	End Seq.	Sequence	Ion Score	C. I. %	Modification	Rank	Result Type

807.3995	807.4216	0.0221	27	195	201	LAADDFR					Mascot
989.5374	989.5598	0.0224	23	329	336	SEISELRR					Mascot
993.5615	993.515	-0.0465	-47	244	251	EELAYLKK					Mascot
1036.5422	1036.5507	0.0085	8	195	203	LAADDFRTK					Mascot
1106.5624	1106.559	-0.0034	-3	31	41	ISSVLAGGSCR			Carbamidomethyl (C)[10]		Mascot
1122.579	1122.6044	0.0254	23	408	416	LEQEIATYR					Mascot
1141.5194	1141.5367	0.0173	15	289	297	DQYEKMAEK					Mascot
1379.7278	1379.7571	0.0293	21	406	416	TRLEQEIATYR					Mascot
1425.6969	1425.7517	0.0548	38	42	56	APSTYGGGLSVSSSR					Mascot
1556.8279	1556.7858	-0.0421	-27	133	146	VRALEEANADLEVK					Mascot
1754.7506	1754.9259	0.1753	100	1	15	MTTCSRQFTSSSSMK			Carbamidomethyl (C)[4], Oxidation (M)[1]		Mascot
1880.9535	1880.959	0.0055	3	117	132	VTMQNLNDRLASYLDK					Mascot
1896.9484	1896.9429	-0.0055	-3	117	132	VTMQNLNDRLASYLDK			Oxidation (M)[3]		Mascot
1908.9406	1908.8901	-0.0505	-26	337	352	TMQNLEIELQSQLSMK			Oxidation (M)[2]		Mascot
2054.124	2054.1929	0.0689	34	176	194	ILTATVDNANVLLQIDNAR					Mascot
2101.9819	2102.0679	0.086	41	262	282	GQVGGDVNVEMDAAPG VDLSR			Oxidation (M)[11]		Mascot
2296.262	2296.3796	0.1176	51	174	194	NKILTATVDNANVLLQIDN AR					Mascot
2296.262	2296.3796	0.1176	51	174	194	NKILTATVDNANVLLQIDN AR					Mascot
2309.064	2309.1641	0.1001	43	418	439	LLEGEDAHLSSSQFSSG SQSSR					Mascot

7	Uncharacterized ATP-binding protein MJ0169 OS=Methanocaldococcus jannaschii (strain ATCC 43067 / DSM 2661 / JAL-1 / JCM 10045 / NBRC 100440) GN=MJ0169 PE=4 SV=1	Y169_METJA	28931.4	5.78	14	78	99.163	14.204	

Peptide Information

Calc. Mass	Obsrv. Mass	± da	± ppm	Start Seq.	End Seq.	Sequence	Ion Score	C. I. %	Modification	Rank	Result Type
1041.5398	1041.574	0.0342	33	211	218	MSVIEYRK			Oxidation (M)[1]		Mascot
1041.5398	1041.574	0.0342	33	210	217	KMSVIEYR			Oxidation (M)[2]		Mascot
1096.5092	1096.6073	0.0981	89	219	228	NSPASQAYMK					Mascot
1096.5092	1096.6073	0.0981	89	219	228	NSPASQAYMK					Mascot
1199.6089	1199.6453	0.0364	30	126	136	EMATHLAIADK					Mascot
1340.7317	1340.7307	-0.001	-1	1	12	MIFMIITIASGK			Oxidation (M)[1]		Mascot
1582.8435	1582.8448	0.0013	1	191	204	GKVLVEVPEDENVR					Mascot
1754.9543	1754.9259	-0.0284	-16	1	17	MIFMIITIASGKGGVGK			Oxidation (M)[1,4]		Mascot
1774.8827	1775.0265	0.1438	81	156	172	ESAEMAGTPLMGVVLNR					Mascot
1790.8776	1790.9556	0.078	44	156	172	ESAEMAGTPLMGVVLNR			Oxidation (M)[5]		Mascot

Calc. Mass	Obsrv. Mass	± da	± ppm	Start Seq.	End Seq.	Sequence	Ion Score	C. I. %	Modification	Rank	Result Type
1806.8724	1807.0002	0.1278	71	156	172	ESAEMAGTPLMGVVLNR			Oxidation (M)[5,11]		Mascot
1855.0212	1855.0111	-0.0101	-5	79	95	TGVYVLPTSLSLEGYKK					Mascot
2032.0565	2032.1113	0.0548	27	154	172	LKESAEMAGTPLMGVVL NR			Oxidation (M)[7]		Mascot
2296.1562	2296.3796	0.2234	97	35	55	VLAIDGDISMANLGILFNM EK			Oxidation (M)[10,19]		Mascot
2296.1562	2296.3796	0.2234	97	35	55	VLAIDGDISMANLGILFNM EK			Oxidation (M)[10,19]		Mascot
2424.2512	2424.4856	0.2344	97	35	56	VLAIDGDISMANLGILFNM EKK			Oxidation (M)[10,19]		Mascot
2424.2512	2424.4856	0.2344	97	34	55	KVLAIDGDISMANLGILFN MEK			Oxidation (M)[11,20]		Mascot
2937.5278	2937.5366	0.0088	3	219	245	NSPASQAYMKLASIIAGV PIYIEDEIK			Oxidation (M)[9]		Mascot

8	Coiled-coil domain-containing protein 171 OS=Homo sapiens GN=CCDC171 PE=2 SV=1	CC171_HUMAN	154083	6.37	23	62	69.592	10.586	24	0

Peptide Information

Calc. Mass	Obsrv. Mass	± da	± ppm	Start Seq.	End Seq.	Sequence	Ion Score	C. I. %	Modification	Rank	Result Type
851.4039	851.3958	-0.0081	-10	630	636	NKSDTMR					Mascot
1053.5398	1053.5461	0.0063	6	907	915	NSFAKLMDK					Mascot
1111.5742	1111.5896	0.0154	14	1111	1119	HLTQLEQDK					Mascot
1163.6089	1163.641	0.0321	28	1090	1099	SLSEAKMELR					Mascot
1194.6589	1194.6112	-0.0477	-40	106	115	LAEAHRIQEK					Mascot
1231.6616	1231.5936	-0.068	-55	621	629	IRHLEYICK			Carbamidomethyl (C)[8]		Mascot
1325.6332	1325.6797	0.0465	35	255	265	QTSELEFSTQR					Mascot
1373.7424	1373.7046	-0.0378	-28	446	457	DKPPSFSVVLER					Mascot
1404.7192	1404.7596	0.0404	29	1180	1191	LHLETFAMEGLK			Oxidation (M)[8]		Mascot
1407.6459	1407.7499	0.104	74	2	14	NLNTSSNTGDTQR					Mascot
1417.7897	1417.8048	0.0151	11	771	783	TLAQALSTVEEKK					Mascot
1507.7244	1507.7881	0.0637	42	1217	1228	IMTLEKEMTSHR			Oxidation (M)[2,8]		Mascot
1538.6864	1538.8311	0.1447	94	1	14	MNLNTSSNTGDTQR					Mascot
1657.8401	1657.8494	0.0093	6	916	929	ISLVMECIPLHSSR			Carbamidomethyl (C)[7], Oxidation (M)[5]		Mascot
1699.9313	1699.8865	-0.0448	-26	896	911	ICGHLLIGAAKNSFAK			Carbamidomethyl (C)[2]		Mascot
1790.9396	1790.9556	0.016	9	77	92	GEALRQSLEYDLAVAR					Mascot
1806.9644	1807.0002	0.0358	20	1229	1244	SHIAALKSELHTACLR			Carbamidomethyl (C)[14]		Mascot
1838.9242	1839.0028	0.0786	43	283	298	LEENIEAERAAHLESK					Mascot
1908.9167	1908.8901	-0.0266	-14	1141	1156	ECVANHMRAVENTLHK			Carbamidomethyl (C)[2]		Mascot
1990.9287	1991.0825	0.1538	77	192	207	NEMESHIRETALEEFR					Mascot
1990.9287	1991.0825	0.1538	77	192	207	NEMESHIRETALEEFR	1	0			Mascot
2165.0618	2165.1357	0.0739	34	1205	1222	SFMDVYQLASTRIMTLEK			Oxidation (M)[3,14]		Mascot

2268.1328	2268.2661	0.1333	59	1245	1265	ENASLQSIGSRDHSNLSIPSR					Mascot
2268.1328	2268.2661	0.1333	59	1245	1265	ENASLQSIGSRDHSNLSIPSR	24	0			Mascot
2286.1548	2286.0615	-0.0933	-41	219	237	ELQFIVQEQDTAVQNMHKK					Mascot

9	Keratin, type I cytoskeletal 16 OS=Homo sapiens GN=KRT16 PE=1 SV=4	K1C16_HUMAN	51578.3	4.99	15	62	69.592	8.277

Peptide Information

Calc. Mass	Obsrv. Mass	± da	± ppm	Start Seq.	End Seq.	Sequence	Ion Score	C. I. %	Modification	Rank	Result Type
807.3995	807.4216	0.0221	27	197	203	LAADDFR					Mascot
857.4839	857.4821	-0.0018	-2	156	162	QRPSEIK					Mascot
989.5374	989.5598	0.0224	23	331	338	SEVTELRR					Mascot
1036.5422	1036.5507	0.0085	8	197	205	LAADDFRTK					Mascot
1096.531	1096.6073	0.0763	70	303	311	DAETWFLSK					Mascot
1096.531	1096.6073	0.0763	70	303	311	DAETWFLSK					Mascot
1106.5624	1106.559	-0.0034	-3	31	41	ISSVLAGGSCR			Carbamidomethyl (C)[10]		Mascot
1122.579	1122.6044	0.0254	23	410	418	LEQEIATYR					Mascot
1141.483	1141.5367	0.0537	47	291	299	DQYEQMAEK					Mascot
1201.6172	1201.6487	0.0315	26	214	224	QTVEADVNGLR					Mascot
1379.7278	1379.7571	0.0293	21	408	418	TRLEQEIATYR					Mascot
1556.8279	1556.7858	-0.0421	-27	135	148	VRALEEANADLEVK					Mascot
1754.7506	1754.9259	0.1753	100	1	15	MTTCSRQFTSSSSMK			Carbamidomethyl (C)[4], Oxidation (M)[1]		Mascot
1880.9535	1880.959	0.0055	3	119	134	VTMQNLNDRLASYLDK					Mascot
1896.9484	1896.9429	-0.0055	-3	119	134	VTMQNLNDRLASYLDK			Oxidation (M)[3]		Mascot
2064.1448	2064.2173	0.0725	35	178	196	IIAATIENAQPILQIDNAR					Mascot
2350.0906	2350.1877	0.0971	41	420	441	LLEGEDAHLSSQQASGQSYSSR					Mascot

10	N-(5'-phosphoribosyl)anthranilate isomerase OS=Bacillus cereus (strain AH187) GN=trpF PE=3 SV=1	TRPF_BACC7	23016.1	8.76	10	58	23.62	4.04

Peptide Information

Calc. Mass	Obsrv. Mass	± da	± ppm	Start Seq.	End Seq.	Sequence	Ion Score	C. I. %	Modification	Rank	Result Type
1027.4956	1027.5306	0.035	34	126	134	ERFYGGNGK					Mascot
1163.5977	1163.641	0.0433	37	99	109	ALGVTSEIDMK					Mascot
1492.7617	1492.7697	0.008	5	136	147	FSWELLAHMSKK			Oxidation (M)[9]		Mascot
1838.9316	1839.0028	0.0712	39	99	115	ALGVTSEIDMKNAQAYK					Mascot

1854.9266	1855.0111	0.0845	46	99	115	ALGVTSEIDMKNAQAYK	Oxidation (M)[10]	Mascot
1873.9331	1874.02	0.0869	46	110	125	NAQAYKTDYILFDSPK		Mascot
1953.9587	1954.0707	0.112	57	169	186	TVRPYMVDVSSGVETEG K		Mascot
1990.9327	1991.0825	0.1498	75	17	34	ACEYGADALGFVFAESK R	Carbamidomethyl (C)[2]	Mascot
1990.9327	1991.0825	0.1498	75	16	33	RACEYGADALGFVFAES K	Carbamidomethyl (C)[3]	Mascot
2025.1339	2025.0925	-0.0414	-20	150	168	EKTILAGGLNALNIEEAIR		Mascot
2082.0537	2082.0764	0.0227	11	169	187	TVRPYMVDVSSGVETEG KK		Mascot

Rank	Protein Name	Accession No.	Protein MW	Protein PI	Pep. Count	Protein Score	Protein Score C. I. %	Intensity Matched	Total Ion Score	Total Ion C. I. %	Confirmed
1	Chaperone protein ClpB1 OS=Oryza sativa subsp. japonica GN=CLPB1 PE=2 SV=1	CLPB1_ORYSJ	101062.4	5.9	25	188	100	12.96	105	100	

Peptide Information

Calc. Mass	Obsrv. Mass	± da	± ppm	Start Seq.	End Seq.	Sequence	Ion Score	C. I. %	Modification	Rank	Result Type
842.557	842.4762	-0.0808	-96	193	199	VVRILSR					Mascot
929.5203	929.4902	-0.0301	-32	545	552	WTGIPVTR					Mascot
954.454	954.4502	-0.0038	-4	360	367	YEGHHGVR					Mascot
959.4905	959.4772	-0.0133	-14	553	560	LGQNDKER					Mascot
1005.5034	1005.4828	-0.0206	-20	742	749	DLVMQEVR			Oxidation (M)[4]		Mascot
1043.5845	1043.5747	-0.0098	-9	216	225	TAVVEGLAQR					Mascot
1085.5011	1085.5	-0.0011	-1	254	261	YRGEFEER					Mascot
1098.663	1098.6564	-0.0066	-6	372	382	ALVVAAQLSAR					Mascot
1181.6538	1181.6431	-0.0107	-9	751	759	HFRPELLNR					Mascot
1184.6271	1184.6323	0.0052	4	229	239	GDVPSNLLDVR					Mascot
1315.7151	1315.6771	-0.038	-29	739	749	VARDLVMQEVR					Mascot
1440.7152	1440.7169	0.0017	1	313	324	CIGATTLEEYRK			Carbamidomethyl (C)[1]		Mascot
1445.7053	1445.7189	0.0136	9	393	405	AIDLVDEACANVR			Carbamidomethyl (C)[9]		Mascot
1492.7278	1492.74	0.0122	8	483	494	EELQFTLQEAER					Mascot
1594.8475	1594.8578	0.0103	6	671	683	RPYSVILFDEVEK					Mascot
1648.829	1648.8375	0.0085	5	483	495	EELQFTLQEAERR					Mascot
1734.9684	1734.8088	-0.1596	-92	372	387	ALVVAAQLSARYIMGR			Oxidation (M)[14]		Mascot
1745.9069	1745.9255	0.0186	11	619	633	ALAEQLFDDENLLVR					Mascot
1745.9069	1745.9255	0.0186	11	619	633	ALAEQLFDDENLLVR	75	99.997			Mascot
1750.9486	1750.9218	-0.0268	-15	670	683	RRPYSVILFDEVEK					Mascot
1765.0371	1765.0576	0.0205	12	276	291	VILFIDEIHLVLGAGR					Mascot
1784.8661	1784.8901	0.024	13	406	420	VQLDSQPEEIDNLER					Mascot
1868.0026	1868.0242	0.0216	12	684	700	AHVAVFNTLLQVLDDGR					Mascot
2285.2402	2285.2891	0.0489	21	591	613	AGLGRPQQPTGSFLFLG PTGVGK					Mascot
2306.1743	2306.1892	0.0149	6	292	312	TEGSMDAANLFKPMLAR GQLR					Mascot
2307.1729	2307.2207	0.0478	21	648	669	LIGAPPGYVGHEEGGQL					Mascot

Calc. Mass	Obsrv. Mass	± da	± ppm	Start Seq.	End Seq.	Sequence	Ion Score	C. I. %	Modification	Rank	Result Type
						TEQVR					
2307.1729	2307.2207	0.0478	21	648	669	LIGAPPGYVGHEEGGQL TEQVR	30	21.14			Mascot

2	Elongation factor 2 OS=Beta vulgaris PE=2 SV=1	EF2_BETVU	94707.7	5.93	11	153	100	6.32	136	100

Peptide Information

Calc. Mass	Obsrv. Mass	± da	± ppm	Start Seq.	End Seq.	Sequence	Ion Score	C. I. %	Modification	Rank	Result Type
890.5095	890.5068	-0.0027	-3	483	490	FSVSPVVR					Mascot
1039.6622	1039.6492	-0.013	-13	151	159	IRPVLTVNK					Mascot
1135.5049	1135.5085	0.0036	3	674	683	EGALAEENMR			Oxidation (M)[9]		Mascot
1204.6031	1204.6097	0.0066	5	411	421	IMGPNYVPGEK					Mascot
1220.598	1220.6019	0.0039	3	411	421	IMGPNYVPGEK			Oxidation (M)[2]		Mascot
1236.677	1236.5984	-0.0786	-64	480	490	AMKFSVSPVVR			Oxidation (M)[2]		Mascot
1543.7727	1543.7751	0.0024	2	269	280	RGFVQFCYEPIK			Carbamidomethyl (C)[7]		Mascot
1767.8469	1767.9607	0.1138	64	828	842	GLKEQMTPLSEFEDK			Oxidation (M)[6]		Mascot
2132.0593	2132.0872	0.0279	13	753	770	GHVFEEMQRPGTPLYNI K			Oxidation (M)[7]		Mascot
2178.0061	2178.1887	0.1826	84	163	180	CFLELQVDGEEAYTTFQK			Carbamidomethyl (C)[1]		Mascot
2257.2034	2257.2478	0.0444	20	33	55	STLTDSLVAAAGIIAQEVA GDVR					Mascot
2257.2034	2257.2478	0.0444	20	33	55	STLTDSLVAAAGIIAQEVA GDVR	136	100			Mascot
2646.3479	2646.4104	0.0625	24	121	144	ITDGALVVVDCIEGVCVQ TETVLR			Carbamidomethyl (C)[11,16]		Mascot

3	Chaperone protein ClpB1 OS=Arabidopsis thaliana GN=CLPB1 PE=1 SV=2	CLPB1_ARATH	101460.2	5.81	22	130	100	11.744	75	99.997

Peptide Information

Calc. Mass	Obsrv. Mass	± da	± ppm	Start Seq.	End Seq.	Sequence	Ion Score	C. I. %	Modification	Rank	Result Type
842.557	842.4762	-0.0808	-96	191	197	VVRILSR					Mascot
856.5363	856.5551	0.0188	22	440	446	ARLIEVR					Mascot
929.5203	929.4902	-0.0301	-32	544	551	WTGIPVTR					Mascot
945.5112	945.4409	-0.0703	-74	885	893	RSDAAQAVK					Mascot
954.454	954.4502	-0.0038	-4	358	365	YEGHHGVR					Mascot
1043.5845	1043.5747	-0.0098	-9	214	223	TAVVEGLAQR					Mascot
1085.5011	1085.5	-0.0011	-1	252	259	YRGEFEER					Mascot
1172.5907	1172.6288	0.0381	32	227	237	GDVPNSLTDVR					Mascot
1181.6538	1181.6431	-0.0107	-9	750	758	HFRPELLNR					Mascot
1199.6532	1199.5935	-0.0597	-50	381	390	YITGRHLPDK					Mascot
1440.7152	1440.7169	0.0017	1	311	322	CIGATTLEEYRK			Carbamidomethyl (C)[1]		Mascot

1445.7053	1445.7189	0.0136	9	391	403	AIDLVDEACANVR			Carbamidomethyl (C)[9]		Mascot
1484.8431	1484.7329	-0.1102	-74	125	138	DLLNEVGVATARVK					Mascot
1484.8431	1484.7329	-0.1102	-74	125	138	DLLNEVGVATARVK					Mascot
1512.8381	1512.759	-0.0791	-52	224	237	IVKGDVPNSLTDVR					Mascot
1594.8071	1594.8578	0.0507	32	151	165	KVESASGDTNFQALK					Mascot
1745.9069	1745.9255	0.0186	11	618	632	ALAEQLFDDENLLVR					Mascot
1745.9069	1745.9255	0.0186	11	618	632	ALAEQLFDDENLLVR	75	99.997			Mascot
1767.9712	1767.9607	-0.0105	-6	571	587	VVGQNQAVNAVSEAILR					Mascot
1784.8661	1784.8901	0.024	13	404	418	VQLDSQPEEIDNLER					Mascot
1868.0026	1868.0242	0.0216	12	683	699	AHVAVFNTLLQVLDDGR					Mascot
2285.2402	2285.2891	0.0489	21	590	612	AGLGRPQQPTGSFLFLG PTGVGK					Mascot
2306.1743	2306.1892	0.0149	6	290	310	TEGSMDAANLFKPMLAR GQLR					Mascot
2406.2524	2406.2437	-0.0087	-4	647	669	LIGAPPGYVGHEEGGQL TEAVRR					Mascot

4	Plectin (Fragment) OS=Cricetulus griseus GN=PLEC PE=1 SV=1	PLEC_CRIGR	510476.5	5.58	52	69	93.647	27.076

Peptide Information

Calc. Mass	Obsrv. Mass	± da	± ppm	Start Seq.	End Seq.	Sequence	Ion Score	C. I. %	Modification	Rank	Result Type
865.4778	865.4394	-0.0384	-44	1262	1268	FISETLR					Mascot
871.4996	871.4256	-0.074	-85	1107	1113	VNQLLER					Mascot
890.4876	890.5068	0.0192	22	4118	4124	SMIIDRR					Mascot
929.4911	929.4902	-0.0009	-1	1480	1487	QAQAERAR					Mascot
945.4636	945.4409	-0.0227	-24	1469	1476	LQAEEAER					Mascot
955.4917	955.5226	0.0309	32	3873	3880	LSVYQAMK			Oxidation (M)[7]		Mascot
959.5269	959.4772	-0.0497	-52	3928	3935	DRLLSAER					Mascot
961.4949	961.4987	0.0038	4	3266	3274	DLSEVGSVR					Mascot
1001.5262	1001.5527	0.0265	26	955	964	AQAEVEGLGK					Mascot
1021.5789	1021.5178	-0.0611	-60	1262	1269	FISETLRR					Mascot
1043.5303	1043.5747	0.0444	43	4218	4226	VSITEAMHR					Mascot
1085.4607	1085.5	0.0393	36	2365	2373	QQSDHDAER					Mascot
1135.6106	1135.5085	-0.1021	-90	3341	3351	AGVVGPELHEK					Mascot
1148.5729	1148.5072	-0.0657	-57	2199	2208	MKQVAEEAAR			Oxidation (M)[1]		Mascot
1172.627	1172.6288	0.0018	2	1447	1457	VKAQAEAAQEK					Mascot
1181.558	1181.6431	0.0851	72	1877	1886	IRSSAEDTMR			Oxidation (M)[9]		Mascot
1188.6219	1188.5979	-0.024	-20	1582	1592	SLAQADAEKQK					Mascot

1199.6742	1199.5935	-0.0807	-67	2374	2383	LREAIAELER		Mascot
1204.5739	1204.6097	0.0358	30	2463	2471	QQRQMEQEK		Mascot
1220.5688	1220.6019	0.0331	27	2463	2471	QQRQMEQEK	Oxidation (M)[5]	Mascot
1252.5652	1252.6116	0.0464	37	1700	1710	AEEESRSTSEK		Mascot
1298.7216	1298.6329	-0.0887	-68	1114	1124	WQAVLAQIDVR		Mascot
1315.6852	1315.6771	-0.0081	-6	1286	1297	ERLAEGEAALEK		Mascot
1440.7917	1440.7169	-0.0748	-52	1570	1581	LRLQAEEVAQQR		Mascot
1445.8112	1445.7189	-0.0923	-64	29	40	LQNVQIALDYLR		Mascot
1492.837	1492.74	-0.097	-65	523	535	LDLQYAKLLNSSK		Mascot
1501.8584	1501.8257	-0.0327	-22	1760	1773	VLTEKLAAISEATR		Mascot
1512.7554	1512.759	0.0036	2	3560	3571	QQNLASYDYVRR		Mascot
1543.8439	1543.7751	-0.0688	-45	2106	2118	QKAQVEQELTTLR		Mascot
1631.8309	1631.8627	0.0318	19	3059	3074	LLDAQLSTGGMVDPSK		Mascot
1648.7959	1648.8375	0.0416	25	4374	4388	DALDRSMVEEGTGLR		Mascot
1739.936	1739.916	-0.02	-11	3456	3470	CVEDPETGLRLLPLK	Carbamidomethyl (C)[1]	Mascot
1767.9276	1767.9607	0.0331	19	3033	3048	DPYSGQSVSLFQALKK		Mascot
1783.9589	1783.9109	-0.048	-27	3583	3597	IISLETYNLFREGTK		Mascot
1786.9044	1786.9601	0.0557	31	3364	3379	DPYSGTTISLFQAMKK		Mascot
1786.9044	1786.9601	0.0557	31	3364	3379	DPYSGTTISLFQAMKK		Mascot
1799.0314	1798.9045	-0.1269	-71	3648	3664	LLLEAQAATGFLLDPVK		Mascot
2031.0981	2031.1051	0.007	3	2731	2749	LLEAQIATGGIIDTVHSHR		Mascot
2068.0417	2068.0713	0.0296	14	2598	2615	LEQGHTTVAELSQREDV R		Mascot
2093.0542	2093.0884	0.0342	16	4379	4398	SMVEEGTGLRLLEAAAQ SSK	Oxidation (M)[2]	Mascot
2115.0605	2115.0623	0.0018	1	3771	3788	SYVDPSTDERLSYTQLLK		Mascot
2188.9705	2189.0391	0.0686	31	3418	3436	GYFDEEMSRILADPSDDT K		Mascot
2204.9653	2205.0281	0.0628	28	3418	3436	GYFDEEMSRILADPSDDT K	Oxidation (M)[7]	Mascot
2237.1118	2237.1768	0.065	29	2150	2168	SQVEEELFSVRVQMEEL GK		Mascot
2257.1055	2257.2478	0.1423	63	1646	1664	AETEQGEQQRQLLEEEL AR		Mascot
2257.1055	2257.2478	0.1423	63	1646	1664	AETEQGEQQRQLLEEEL AR		Mascot
2264.054	2264.1226	0.0686	30	3176	3193	TMTVWELISSEYFTQEQR	Oxidation (M)[2]	Mascot
2276.2104	2276.1965	-0.0139	-6	1156	1175	QEQIQAVPIPNSQAAREQ LR		Mascot
2285.1846	2285.2891	0.1045	46	3850	3870	TLQKFLEGTSCIAGVFVD ATK	Carbamidomethyl (C)[11]	Mascot
2292.2195	2292.2676	0.0481	21	1	21	DGHNLISLLEVLSGDSLP REK		Mascot
2307.1404	2307.2207	0.0803	35	3437	3455	GFFDPNTHENLTYLQLLE R		Mascot

Calc. Mass	Obsrv. Mass	± da	± ppm	Start Seq.	End Seq.	Sequence	Ion Score	C. I. %	Modification	Rank	Result Type
2307.1404	2307.2207	0.0803	35	2778	2796	GFFDPNTHENLTYLQLLER					Mascot
2325.0552	2325.2378	0.1826	79	3995	4014	RGLFDEEMNEILTDPSDDTK					Mascot
2358.1506	2358.2539	0.1033	44	4150	4172	AGTLSITEFADMLSGNAGGFRSR					Mascot
2366.2058	2366.3059	0.1001	42	2354	2373	VTLVQTLEIQRQQSDHDAER					Mascot
2422.2109	2422.2366	0.0257	11	464	485	RIDSAEWGVDLPSVEAQLGSHR					Mascot
2646.2324	2646.4104	0.178	67	890	910	HYQQLLQSLEQGEQEESRCQR			Carbamidomethyl (C)[19]		Mascot

5 Adenylate kinase OS=Alkalilimnicola ehrlichei (strain MLHE-1) GN=adk PE=3 SV=1 KAD_ALHEH 23896.2 4.96 10 62 62.59 6.912

Peptide Information

Calc. Mass	Obsrv. Mass	± da	± ppm	Start Seq.	End Seq.	Sequence	Ion Score	C. I. %	Modification	Rank	Result Type
865.4751	865.4394	-0.0357	-41	124	131	RVHPGSGR					Mascot
970.5316	970.4643	-0.0673	-69	41	50	AGTPLGQQAK					Mascot
1098.6266	1098.6564	0.0298	27	41	51	AGTPLGQQAKK					Mascot
1492.7465	1492.74	-0.0065	-4	24	36	FDIPQISTGDMLR					Mascot
1671.8185	1671.9025	0.084	50	142	156	EEGKDDVTGEPLVQR					Mascot
1909.978	1910.0229	0.0449	24	125	141	VHPGSGRVYHVEHNPPK					Mascot
1909.978	1910.0229	0.0449	24	125	141	VHPGSGRVYHVEHNPPK					Mascot
2065.0557	2065.0598	0.0041	2	51	69	KVMDAGELVSDDIIMGLVK			Oxidation (M)[3,15]		Mascot
2065.1223	2065.0598	-0.0625	-30	3	23	VILLGPPGAGKGTQAAGICER			Carbamidomethyl (C)[19]		Mascot
2206.1094	2206.0483	-0.0611	-28	52	71	VMDAGELVSDDIIMGLVKER			Oxidation (M)[2]		Mascot
2206.1094	2206.0483	-0.0611	-28	52	71	VMDAGELVSDDIIMGLVKER			Oxidation (M)[2]		Mascot
2408.2305	2408.2285	-0.002	-1	98	119	AEGINVDAVVEIQVPDEDIVQR					Mascot

6 Plectin OS=Homo sapiens GN=PLEC PE=1 SV=3 PLEC_HUMAN 533461.8 5.74 49 58 6.032 22.366

Peptide Information

Calc. Mass	Obsrv. Mass	± da	± ppm	Start Seq.	End Seq.	Sequence	Ion Score	C. I. %	Modification	Rank	Result Type
865.4778	865.4394	-0.0384	-44	1473	1479	FISETLR					Mascot
890.4876	890.5068	0.0192	22	4329	4335	SMIIDRR					Mascot
945.4636	945.4409	-0.0227	-24	2111	2118	QLAAEEER					Mascot
955.4917	955.5226	0.0309	32	4084	4091	LSVYQAMK			Oxidation (M)[7]		Mascot
959.5156	959.4772	-0.0384	-40	1596	1603	LQLEATER					Mascot
961.5247	961.4987	-0.026	-27	2524	2531	LRVAEMSR					Mascot

1001.5374	1001.5527	0.0153	15	1526	1533	EAKELQQR		Mascot
1005.5033	1005.4828	-0.0205	-20	2685	2693	LVASMEEAR		Mascot
1021.4991	1021.5178	0.0187	18	1	9	MVAGMLMPR	Oxidation (M)[1]	Mascot
1043.5303	1043.5747	0.0444	43	4429	4437	VSITEAMHR		Mascot
1085.4607	1085.5	0.0393	36	2576	2584	QQSDHDAER		Mascot
1148.4678	1148.5072	0.0394	34	3629	3637	GYFSEEMNR	Oxidation (M)[7]	Mascot
1164.5896	1164.6117	0.0221	19	3784	3793	LTAEDLFEAR		Mascot
1172.6786	1172.6288	-0.0498	-42	299	307	EKLLLWSQR		Mascot
1199.6742	1199.5935	-0.0807	-67	2585	2594	LREAIAELER		Mascot
1204.6606	1204.6097	-0.0509	-42	2372	2381	VQMEELSKLK		Mascot
1220.6344	1220.6019	-0.0325	-27	4130	4140	MGIVGPEFKDK		Mascot
1236.6293	1236.5984	-0.0309	-25	4130	4140	MGIVGPEFKDK	Oxidation (M)[1]	Mascot
1252.6241	1252.6116	-0.0125	-10	2402	2411	FLQEEAEKMK		Mascot
1292.7573	1292.6599	-0.0974	-75	3839	3849	QTLSIYQALKK		Mascot
1298.7314	1298.6329	-0.0985	-76	3925	3935	KELIPTEEALR		Mascot
1315.7086	1315.6771	-0.0315	-24	102	112	RPVAMVMPARR	Oxidation (M)[5,7]	Mascot
1445.8112	1445.7189	-0.0923	-64	237	248	LQNVQIALDYLR		Mascot
1492.837	1492.74	-0.097	-65	734	746	LDLQYAKLLNSSK		Mascot
1501.7356	1501.8257	0.0901	60	4564	4576	DVGAYSKYLTCPK	Carbamidomethyl (C)[11]	Mascot
1512.6925	1512.759	0.0665	44	3969	3981	DTHDQLSEPSEVR		Mascot
1527.8993	1527.7686	-0.1307	-86	3424	3436	ILITIVEEVETLR		Mascot
1535.7999	1535.6783	-0.1216	-79	3739	3752	AQLMADFQAGRVTK		Mascot
1543.8439	1543.7751	-0.0688	-45	2317	2329	QKAQVEQELTTLR		Mascot
1648.7959	1648.8375	0.0416	25	4585	4599	DALDRSMVEEGTGLR		Mascot
1739.936	1739.916	-0.02	-11	3667	3681	CVEDPETGLRLLPLK	Carbamidomethyl (C)[1]	Mascot
1767.9963	1767.9607	-0.0356	-20	3116	3131	SLVPAAELLESRVIDR		Mascot
1783.9185	1783.9109	-0.0076	-4	2744	2761	AALAHSEEVTASQVAATK		Mascot
1784.896	1784.8901	-0.0059	-3	1367	1382	QEQIQAMPLADSQAVR		Mascot
1799.0314	1798.9045	-0.1269	-71	3859	3875	LLLEAQAATGFLLDPVK		Mascot
1868.1467	1868.0242	-0.1225	-66	3421	3436	VIKILITIVEEVETLR		Mascot
1909.8708	1910.0229	0.1521	80	2488	2503	EQMAQQLAEETQGFQR	Oxidation (M)[3]	Mascot
1909.8708	1910.0229	0.1521	80	2488	2503	EQMAQQLAEETQGFQR	Oxidation (M)[3]	Mascot
2115.0176	2115.0623	0.0447	21	4361	4381	AGTLSITEFADMLSGNAG GFR		Mascot
2178.1626	2178.1887	0.0261	12	1726	1744	TAQLERSLQEEHVAVAQ LR		Mascot
2189.2375	2189.0391	-0.1984	-91	329	346	LFNAIIHRHKPLLIDMNK	Oxidation (M)[16]	Mascot
2205.9805	2206.0483	0.0678	31	308	325	MVEGYQGLRCDNFTSS WR	Carbamidomethyl (C)[10]	Mascot

2205.9805	2206.0483	0.0678	31	308	325	MVEGYQGLRCDNFTSSWR			Carbamidomethyl (C)[10]		Mascot
2257.1055	2257.2478	0.1423	63	1857	1875	AETEQGEQQRQLLEEELAR					Mascot
2257.1055	2257.2478	0.1423	63	1857	1875	AETEQGEQQRQLLEEELAR					Mascot
2292.2195	2292.2676	0.0481	21	209	229	DGHNLISLLEVLSGDSLPREK					Mascot
2298.1799	2298.324	0.1441	63	4061	4081	NLQKFLEGTSCIAGVFVDATK			Carbamidomethyl (C)[11]		Mascot
2307.1404	2307.2207	0.0803	35	2989	3007	GFFDPNTHENLTYLQLLER					Mascot
2307.1404	2307.2207	0.0803	35	2989	3007	GFFDPNTHENLTYLQLLER					Mascot
2325.0552	2325.2378	0.1826	79	4206	4225	RGLFDEEMNEILTDPSDDTK					Mascot
2358.1506	2358.2539	0.1033	44	4361	4383	AGTLSITEFADMLSGNAGGFRSR					Mascot
2366.2058	2366.3059	0.1001	42	2565	2584	VTLVQTLEIQRQQSDHDAER					Mascot
2422.2686	2422.2366	-0.032	-13	2744	2767	AALAHSEEVTASQVAATKTLPNGR					Mascot
2933.5037	2933.4939	-0.0098	-3	3184	3212	DLLPSDMAVALLEAQAGTGHIIDPATSAR					Mascot

7	DNA-directed RNA polymerase subunit beta' OS=Granulibacter bethesdensis (strain ATCC BAA-1260 / CGDNIH1) GN=rpoC PE=3 SV=1	RPOC_GRABC	155113.4	7.78	25	56	0	10.694	

Peptide Information

Calc. Mass	Obsrv. Mass	± da	± ppm	Start Seq.	End Seq.	Sequence	Ion Score	C. I. %	Modification	Rank	Result Type
804.3734	804.3645	-0.0089	-11	1380	1387	LSGQEAAE					Mascot
865.5254	865.4394	-0.086	-99	1244	1250	HIEVIVR					Mascot
890.473	890.5068	0.0338	38	389	396	YGHATTIK					Mascot
1085.6062	1085.5	-0.1062	-98	1123	1132	TRDITGGLPR					Mascot
1135.5742	1135.5085	-0.0657	-58	1275	1283	LEFEAENRK					Mascot
1172.6093	1172.6288	0.0195	17	22	31	IQMASPEQIR					Mascot
1188.6041	1188.5979	-0.0062	-5	22	31	IQMASPEQIR			Oxidation (M)[3]		Mascot
1199.4965	1199.5935	0.097	81	169	178	QDEFGDDAFR					Mascot
1236.6318	1236.5984	-0.0334	-27	204	214	AELKETTSETK					Mascot
1315.7482	1315.6771	-0.0711	-54	1133	1143	VAELFEARRPK					Mascot
1484.8367	1484.7329	-0.1038	-70	1348	1361	LIPAGTGSVMNRLR					Mascot
1484.8367	1484.7329	-0.1038	-70	1348	1361	LIPAGTGSVMNRLR					Mascot
1529.8282	1529.7177	-0.1105	-72	997	1010	LLTDEGAQVTRAQK					Mascot
1535.7371	1535.6783	-0.0588	-38	818	832	AVMDGGEVVSSLSER					Mascot
1594.7531	1594.8578	0.1047	66	299	312	MLQESVDALFDNGR					Mascot
1742.8668	1742.8557	-0.0111	-6	1363	1379	IASGQDEAKGVGQETPR					Mascot

1750.8541	1750.9218	0.0677	39	299	313	MLQESVDALFDNGRR						Mascot
1765.0518	1765.0576	0.0058	3	571	586	VVTTPGRMLIAQILPR						Mascot
1783.9147	1783.9109	-0.0038	-2	1044	1059	MDEVTGLTSKVVVDYK						Mascot
1813.8927	1813.8201	-0.0726	-40	938	955	GAEQSSVEASHDGIVTVK						Mascot
2188.0386	2188.0952	0.0566	26	707	725	QELGRPINSVWMMSHSG AR			Oxidation (M)[12,13]			Mascot
2189.2441	2189.0391	-0.205	-94	427	446	APTLHRLGIQAFEPVLIEG K						Mascot
2206.0115	2206.0483	0.0368	17	794	812	LVDVAQDCIIVENDCGTE R			Carbamidomethyl (C)[8,15]			Mascot
2206.1536	2206.0483	-0.1053	-48	2	21	NELMKILGQGGQSLSFD QIK						Mascot
2285.3315	2285.2891	-0.0424	-19	578	597	MLIAQILPRHPNVPFALIN K						Mascot
2286.1799	2286.2839	0.104	45	401	418	MVEKERPEVWDILEEVIR			Oxidation (M)[1]			Mascot
2292.1731	2292.2676	0.0945	41	1187	1207	HVSVQEGDFVRLGDPLV DGPR						Mascot

8	Uncharacterized protein YabB OS=Bacillus subtilis (strain 168) GN=yabB PE=4 SV=1	YABB_BACSU	28435	6.46	10	55	0	5.599

Peptide Information

Calc. Mass	Obsrv. Mass	± da	± ppm	Start Seq.	End Seq.	Sequence	Ion Score	C. I. %	Modification		Rank	Result Type
970.4589	970.4643	0.0054	6	2	9	VSLHDDER						Mascot
1346.7764	1346.7725	-0.0039	-3	164	176	QGGKAALVHRPGR						Mascot
1725.8337	1725.9689	0.1352	78	128	141	TPKQTEQNMNEHLR						Mascot
1739.8606	1739.916	0.0554	32	131	144	QTEQNMNEHLRIAR						Mascot
1910.0706	1910.0229	-0.0477	-25	204	221	EANTILVEGIKGGRPDLK						Mascot
1910.0706	1910.0229	-0.0477	-25	204	221	EANTILVEGIKGGRPDLK						Mascot
2065.0859	2065.0598	-0.0261	-13	69	86	ADILGVEIQERLHDMAVR						Mascot
2065.0859	2065.0598	-0.0261	-13	69	86	ADILGVEIQERLHDMAVR						Mascot
2093.2053	2093.0884	-0.1169	-56	168	185	AALVHRPGRLLEIFELMK						Mascot
2178.0383	2178.1887	0.1504	69	2	19	VSLHDDERLDYLLAEDM K			Oxidation (M)[17]			Mascot
2286.1611	2286.2839	0.1228	54	87	105	SVEYNKLDDQIQIIHDDLK						Mascot
2325.0737	2325.2378	0.1641	71	1	19	MVSLHDDERLDYLLAED MK			Oxidation (M)[1,18]			Mascot

9	Tyrosine recombinase XerD-like OS=Streptococcus thermophilus (strain ATCC BAA-250 / LMG 18311) GN=stu0259 PE=3 SV=1	XERDL_STRT2	28894.4	8.92	10	54	0	4.237

Protein Group

Tyrosine recombinase XerD-like OS=Streptococcus thermophilus (strain ATCC BAA-491 / LMD-9) GN=STER_0306 PE=3 SV=1	XERDL_STRTD	28894.4	8.9200 000762 9395
Tyrosine recombinase XerD-like OS=Streptococcus	XERDL_STRT1	28894.4	8.9200

thermophilus (strain CNRZ 1066) GN=str0259 PE=3 SV=1		000762 9395	

Peptide Information

Calc. Mass	Obsrv. Mass	± da	± ppm	Start Seq.	End Seq.	Sequence	Ion Score	C. I. %	Modification	Rank	Result Type
865.4625	865.4394	-0.0231	-27	21	28	KTISDSSK					Mascot
921.4788	921.4047	-0.0741	-80	229	237	GTGAFELAR					Mascot
941.4761	941.4935	0.0174	18	1	8	MTISSFQK					Mascot
1001.5414	1001.5527	0.0113	11	60	67	EWKPSVQK					Mascot
1188.6219	1188.5979	-0.024	-20	179	189	NDNSQGLILSK					Mascot
1445.8475	1445.7189	-0.1286	-89	229	242	GTGAFELARLLGLK					Mascot
1742.9589	1742.8557	-0.1032	-59	70	83	RSAVNQFLLYLYQK					Mascot
2065.0496	2065.0598	0.0102	5	199	218	QIQSYVGGCGLSGVTAQ ALR			Carbamidomethyl (C)[9]		Mascot
2065.0496	2065.0598	0.0102	5	199	218	QIQSYVGGCGLSGVTAQ ALR			Carbamidomethyl (C)[9]		Mascot
2284.3237	2284.2947	-0.029	-13	123	142	LACLFILELGLLPSEILELK			Carbamidomethyl (C)[3]		Mascot
2344.2329	2344.2454	0.0125	5	1	20	MTISSFQKQLTTQITNFLA R			Oxidation (M)[1]		Mascot

10	Nucleoside-triphosphatase THEP1 OS=Methanocaldococcus jannaschii (strain ATCC 43067 / DSM 2661 / JAL-1 / JCM 10045 / NBRC 100440) GN=MJ1559 PE=3 SV=2	NTPTH_METJA	19076.5	9.1	9	54	0	3.55

Peptide Information

Calc. Mass	Obsrv. Mass	± da	± ppm	Start Seq.	End Seq.	Sequence	Ion Score	C. I. %	Modification	Rank	Result Type
1005.5615	1005.4828	-0.0787	-78	157	164	LFEEILNK					Mascot
1135.618	1135.5085	-0.1095	-96	3	13	IFITGMPGVGK			Oxidation (M)[6]		Mascot
1164.6147	1164.6117	-0.003	-3	114	123	FSEVVDEVIK					Mascot
1292.7097	1292.6599	-0.0498	-39	113	123	KFSEVVDEVIK					Mascot
1406.7648	1406.84	0.0752	53	1	13	MRIFITGMPGVGK					Mascot
2050.0967	2050.0938	-0.0029	-1	74	91	YAVFIENLDNVGVEAIKR					Mascot
2065.0701	2065.0598	-0.0103	-5	50	68	IITLDTNEETILAYVGDGK					Mascot
2065.0701	2065.0598	-0.0103	-5	50	68	IITLDTNEETILAYVGDGK					Mascot
2178.1804	2178.1887	0.0083	4	71	90	VGKYAVFIENLDNVGVEA IK					Mascot
2306.249	2306.1892	-0.0598	-26	50	70	IITLDTNEETILAYVGDGKI K					Mascot

Gel Idx/Pos	300/M2	Instr./Gel Origin	BA2151/Sample Project 20140814	Process Status	Analysis Succeeded
Plate [#] Name	[1] Sample Project 20140814	Instrument Sample Name		Spectra	11

Rank	Protein Name	Accession No.	Protein MW	Protein PI	Pep. Count	Protein Score	Protein Score C. I. %	Intensity Matched	Total Ion Score	Total Ion C. I. %	Confirmed
1	Keratin, type II cytoskeletal 5 OS=Mus musculus GN=Krt5 PE=1 SV=1	K2C5_MOUSE	61956.7	7.59	25	127	100	16.753			

Peptide Information

Calc. Mass	Obsrv. Mass	± da	± ppm	Start Seq.	End Seq.	Sequence	Ion Score	C. I. %	Modification	Rank	Result Type
804.4097	804.4075	-0.0022	-3	392	398	SEIDNVK					Mascot
816.4872	816.4263	-0.0609	-75	386	391	MIQRLR					Mascot
832.4821	832.4073	-0.0748	-90	386	391	MIQRLR			Oxidation (M)[1]		Mascot
870.4316	870.4857	0.0541	62	30	37	TSFSSVSR					Mascot
972.5472	972.5155	-0.0317	-33	415	423	GELALKDAR					Mascot
986.5266	986.4811	-0.0455	-46	223	231	QLDGVLGER					Mascot
1015.4625	1015.4745	0.012	12	378	385	HEISEMNR					Mascot
1015.4625	1015.4745	0.012	12	378	385	HEISEMNR					Mascot
1053.5436	1053.46	-0.0836	-79	2	10	SRQSSVSFR					Mascot
1055.5229	1055.5291	0.0062	6	369	377	HGDDLRNTK					Mascot
1055.5229	1055.5291	0.0062	6	369	377	HGDDLRNTK					Mascot
1127.5804	1127.545	-0.0354	-31	567	576	FVSTTSSSRR					Mascot
1155.535	1155.6088	0.0738	64	446	454	EYQELMNTK					Mascot
1161.6376	1161.6108	-0.0268	-23	180	188	VRFLEQQNK					Mascot
1171.53	1171.5474	0.0174	15	446	454	EYQELMNTK			Oxidation (M)[6]		Mascot
1173.6263	1173.5146	-0.1117	-95	194	203	WALLQEQGTK					Mascot
1374.6431	1374.7126	0.0695	51	375	385	NTKHEISEMNR			Oxidation (M)[9]		Mascot
1380.7118	1380.6337	-0.0781	-57	16	29	SFSAASAITPSVSR					Mascot
1382.6482	1382.6498	0.0016	1	46	60	ISLGGACGAGGYGSR			Carbamidomethyl (C)[7]		Mascot
1382.6482	1382.6498	0.0016	1	46	60	ISLGGACGAGGYGSR			Carbamidomethyl (C)[7]		Mascot
1410.7297	1410.649	-0.0807	-57	260	271	TTAENEFVMLKK					Mascot
1426.7246	1426.6411	-0.0835	-59	260	271	TTAENEFVMLKK			Oxidation (M)[9]		Mascot
1438.736	1438.6431	-0.0929	-65	259	270	RTTAENEFVMLK					Mascot
1441.6702	1441.6471	-0.0231	-16	287	298	VDALMDEINFMK			Oxidation (M)[5]		Mascot
1441.6702	1441.6471	-0.0231	-16	287	298	VDALMDEINFMK			Oxidation (M)[5]		Mascot
1454.7308	1454.6672	-0.0636	-44	259	270	RTTAENEFVMLK			Oxidation (M)[10]		Mascot

1455.6936	1455.6631	-0.0305	-21	30	45	TSFSSVSRSGGGGGGR					Mascot
1515.853	1515.8472	-0.0058	-4	194	206	WALLQEQGTKTIK					Mascot
1575.7366	1575.8531	0.1165	74	378	389	HEISEMNRMIQR			Oxidation (M)[6,9]		Mascot
1843.908	1843.9823	0.0743	40	399	414	KQCANLQNAIAEAEQR			Carbamidomethyl (C)[3]		Mascot
1876.9552	1877.0045	0.0493	26	207	221	QNLDPLFEQYINNLR					Mascot
1887.8944	1888.0312	0.1368	72	359	374	YEELQQTAGRHGDDLR					Mascot
1967.9379	1968.0066	0.0687	35	234	249	LDSELRNMQDLVEDYK					Mascot

2	Keratin, type II cytoskeletal 5 OS=Rattus norvegicus GN=Krt5 PE=1 SV=1	K2C5_RAT	61958.7	7.6	21	88	99.912	14.059	

Peptide Information

Calc. Mass	Obsrv. Mass	± da	± ppm	Start Seq.	End Seq.	Sequence	Ion Score	C. I. %	Modification	Rank	Result Type
804.4097	804.4075	-0.0022	-3	394	400	SEIDNVK					Mascot
816.4872	816.4263	-0.0609	-75	388	393	MIQRLR					Mascot
832.4821	832.4073	-0.0748	-90	388	393	MIQRLR			Oxidation (M)[1]		Mascot
972.5472	972.5155	-0.0317	-33	417	425	GELALKDAR					Mascot
1015.4625	1015.4745	0.012	12	380	387	HEISEMNR					Mascot
1015.4625	1015.4745	0.012	12	380	387	HEISEMNR					Mascot
1053.5436	1053.46	-0.0836	-79	2	10	SRQSSVSFR					Mascot
1055.5229	1055.5291	0.0062	6	371	379	HGDDLRNTK					Mascot
1055.5229	1055.5291	0.0062	6	371	379	HGDDLRNTK					Mascot
1127.5804	1127.545	-0.0354	-31	563	572	FVSTTSSSRR					Mascot
1155.535	1155.6088	0.0738	64	448	456	EYQELMNTK					Mascot
1161.6376	1161.6108	-0.0268	-23	182	190	VRFLEQQNK					Mascot
1171.53	1171.5474	0.0174	15	448	456	EYQELMNTK			Oxidation (M)[6]		Mascot
1176.579	1176.5924	0.0134	11	224	233	RQLDGVMGER			Oxidation (M)[7]		Mascot
1374.6431	1374.7126	0.0695	51	377	387	NTKHEISEMNR			Oxidation (M)[9]		Mascot
1380.7118	1380.6337	-0.0781	-57	16	29	SFSAASAITPSVSR					Mascot
1410.7297	1410.649	-0.0807	-57	262	273	TTAENEFVMLKK					Mascot
1412.6877	1412.6342	-0.0535	-38	30	44	TTFSSVSRSGGGGGR					Mascot
1426.7246	1426.6411	-0.0835	-59	262	273	TTAENEFVMLKK			Oxidation (M)[9]		Mascot
1438.736	1438.6431	-0.0929	-65	261	272	RTTAENEFVMLK					Mascot
1441.6702	1441.6471	-0.0231	-16	289	300	VDALMDEINFMK			Oxidation (M)[5]		Mascot
1441.6702	1441.6471	-0.0231	-16	289	300	VDALMDEINFMK			Oxidation (M)[5]		Mascot
1454.7308	1454.6672	-0.0636	-44	261	272	RTTAENEFVMLK			Oxidation (M)[10]		Mascot
1575.7366	1575.8531	0.1165	74	380	391	HEISEMNRMIQR			Oxidation (M)[6,9]		Mascot

1843.908	1843.9823	0.0743	40	401	416	KQCANLQNAIAEAEQR			Carbamidomethyl (C)[3]		Mascot
1876.9552	1877.0045	0.0493	26	209	223	QNLDPLFEQYINNLR					Mascot
1887.8944	1888.0312	0.1368	72	361	376	YEELQQTAGRHGDDLR					Mascot
1967.9379	1968.0066	0.0687	35	236	251	LDSELRNMQDLVEDYK					Mascot

3	Chlorophyll a-b binding protein 1, chloroplastic OS=Sinapis alba GN=CAB1 PE=3 SV=1	CB21_SINAL	28328.2	5.17	7	72	96.742	6.232	42	93.478

Peptide Information

Calc. Mass	Obsrv. Mass	± da	± ppm	Start Seq.	End Seq.	Sequence	Ion Score	C. I. %	Modification	Rank	Result Type
983.4985	983.468	-0.0305	-31	125	132	FGEAVWFK					Mascot
1055.5303	1055.5291	-0.0012	-1	96	103	ELEVIHCR			Carbamidomethyl (C)[7]		Mascot
1055.5303	1055.5291	-0.0012	-1	96	103	ELEVIHCR	42	93.478	Carbamidomethyl (C)[7]		Mascot
1325.6743	1325.6754	0.0011	1	94	103	NRELEVIHCR			Carbamidomethyl (C)[9]		Mascot
1409.7094	1409.6514	-0.058	-41	2	16	AASTMALSSPAFAGK					Mascot
1575.849	1575.8531	0.0041	3	17	32	AVKLSPGASEVFGTGR					Mascot
1870.9402	1871.043	0.1028	55	1	19	MAASTMALSSPAFAGKA VK			Oxidation (M)[1,6]		Mascot
1919.9871	1920.0138	0.0267	14	104	120	WAMLGALGCVFPELLAR			Carbamidomethyl (C)[9], Oxidation (M)[3]		Mascot

4	Chlorophyll a-b binding protein 40, chloroplastic OS=Nicotiana tabacum GN=CAB40 PE=2 SV=1	CB24_TOBAC	28450.2	5.48	7	70	94.206	7.206	42	93.478

Peptide Information

Calc. Mass	Obsrv. Mass	± da	± ppm	Start Seq.	End Seq.	Sequence	Ion Score	C. I. %	Modification	Rank	Result Type
983.4985	983.468	-0.0305	-31	127	134	FGEAVWFK					Mascot
1055.5303	1055.5291	-0.0012	-1	98	105	ELEVIHCR			Carbamidomethyl (C)[7]		Mascot
1055.5303	1055.5291	-0.0012	-1	98	105	ELEVIHCR	42	93.478	Carbamidomethyl (C)[7]		Mascot
1325.6743	1325.6754	0.0011	1	96	105	NRELEVIHCR			Carbamidomethyl (C)[9]		Mascot
1408.6493	1408.6495	0.0002	0	44	56	TVSSGSPWYGPDR					Mascot
1441.6992	1441.6471	-0.0521	-36	2	16	AASTMALSSPSFAGK			Oxidation (M)[5]		Mascot
1441.6992	1441.6471	-0.0521	-36	2	16	AASTMALSSPSFAGK			Oxidation (M)[5]		Mascot
1870.9402	1871.043	0.1028	55	1	19	MAASTMALSSPSFAGKA VK			Oxidation (M)[1]		Mascot
1919.9871	1920.0138	0.0267	14	106	122	WAMLGALGCVFPELLAR			Carbamidomethyl (C)[9], Oxidation (M)[3]		Mascot

5	Keratin, type II cytoskeletal 5 OS=Bos taurus GN=KRT5 PE=1 SV=1	K2C5_BOVIN	63069.4	7.62	19	68	92.184	13.723

Peptide Information

Calc. Mass	Obsrv. Mass	± da	± ppm	Start Seq.	End Seq.	Sequence	Ion Score	C. I. %	Modification	Rank	Result Type

Calc. Mass	Obsrv. Mass	± da	± ppm	Start Seq.	End Seq.	Sequence	Ion Score	C. I. %	Modification	Rank	Result Type
804.4097	804.4075	-0.0022	-3	399	405	SEIDNVK					Mascot
816.4872	816.4263	-0.0609	-75	393	398	MIQRLR					Mascot
832.4821	832.4073	-0.0748	-90	393	398	MIQRLR			Oxidation (M)[1]		Mascot
972.5472	972.5155	-0.0317	-33	422	430	GELALKDAR					Mascot
986.5266	986.4811	-0.0455	-46	230	238	QLDGIVGER					Mascot
1015.4625	1015.4745	0.012	12	385	392	HEISEMNR					Mascot
1015.4625	1015.4745	0.012	12	385	392	HEISEMNR					Mascot
1051.5531	1051.4957	-0.0574	-55	74	84	ISISASGGGFR					Mascot
1055.5229	1055.5291	0.0062	6	376	384	HGDDLRNTK					Mascot
1055.5229	1055.5291	0.0062	6	376	384	HGDDLRNTK					Mascot
1155.535	1155.6088	0.0738	64	453	461	EYQELMNTK					Mascot
1161.6376	1161.6108	-0.0268	-23	187	195	VRFLEQQNK					Mascot
1171.53	1171.5474	0.0174	15	453	461	EYQELMNTK			Oxidation (M)[6]		Mascot
1173.6263	1173.5146	-0.1117	-95	201	210	WALLQEQGTK					Mascot
1355.6703	1355.6799	0.0096	7	49	63	VSLGGAYGAGGFGSR					Mascot
1374.6431	1374.7126	0.0695	51	382	392	NTKHEISEMNR			Oxidation (M)[9]		Mascot
1410.7224	1410.649	-0.0734	-52	16	29	SFSTASAITPSVSR					Mascot
1426.7246	1426.6411	-0.0835	-59	267	278	TTAENEFVMLKK			Oxidation (M)[9]		Mascot
1438.736	1438.6431	-0.0929	-65	266	277	RTTAENEFVMLK					Mascot
1441.6702	1441.6471	-0.0231	-16	294	305	VDALMDEINFMK			Oxidation (M)[5]		Mascot
1441.6702	1441.6471	-0.0231	-16	294	305	VDALMDEINFMK			Oxidation (M)[5]		Mascot
1454.7308	1454.6672	-0.0636	-44	266	277	RTTAENEFVMLK			Oxidation (M)[10]		Mascot
1575.7366	1575.8531	0.1165	74	385	396	HEISEMNRMIQR			Oxidation (M)[6,9]		Mascot
1887.8944	1888.0312	0.1368	72	366	381	YEELQQTAGRHGDDLR					Mascot
1951.943	1951.9948	0.0518	27	241	256	LDSELRNMQDLVEDFK					Mascot
1967.9379	1968.0066	0.0687	35	241	256	LDSELRNMQDLVEDFK			Oxidation (M)[8]		Mascot

6	Chlorophyll a-b binding protein 91R, chloroplastic OS=Petunia sp. GN=CAB91R PE=3 SV=1	CB25_PETSP	28536.2	5.31	6	65	83.29	6.017	42	93.478	

Peptide Information

Calc. Mass	Obsrv. Mass	± da	± ppm	Start Seq.	End Seq.	Sequence	Ion Score	C. I. %	Modification	Rank	Result Type
983.4985	983.468	-0.0305	-31	127	134	FGEAVWFK					Mascot
1055.5303	1055.5291	-0.0012	-1	98	105	ELEVIHCR			Carbamidomethyl (C)[7]		Mascot
1055.5303	1055.5291	-0.0012	-1	98	105	ELEVIHCR	42	93.478	Carbamidomethyl (C)[7]		Mascot
1325.6743	1325.6754	0.0011	1	96	105	NRELEVIHCR			Carbamidomethyl (C)[9]		Mascot

Calc. Mass	Obsrv. Mass	± da	± ppm	Start Seq.	End Seq.	Sequence	Ion Score	C. I. %	Modification	Rank	Result Type
1409.7094	1409.6514	-0.058	-41	2	16	AAATMALSSPSFAGK					Mascot
1870.9402	1871.043	0.1028	55	1	19	MAAATMALSSPSFAGKA VK			Oxidation (M)[1,6]		Mascot
1919.9871	1920.0138	0.0267	14	106	122	WAMLGALGCVFPELLAR			Carbamidomethyl (C)[9], Oxidation (M)[3]		Mascot

7	Chlorophyll a-b binding protein E, chloroplastic OS=Nicotiana plumbaginifolia GN=CABE PE=3 SV=1	CB25_NICPL	28430.2	5.32	6	65	82.095	6.356	42	93.478	

Protein Group

Chlorophyll a-b binding protein 16, chloroplastic OS=Nicotiana tabacum GN=CAB16 PE=2 SV=1	CB21_TOBAC	28331.2	5.4800 000190 7349

Peptide Information

Calc. Mass	Obsrv. Mass	± da	± ppm	Start Seq.	End Seq.	Sequence	Ion Score	C. I. %	Modification	Rank	Result Type
983.4985	983.468	-0.0305	-31	126	133	FGEAVWFK					Mascot
1055.5303	1055.5291	-0.0012	-1	97	104	ELEVIHCR			Carbamidomethyl (C)[7]		Mascot
1055.5303	1055.5291	-0.0012	-1	97	104	ELEVIHCR	42	93.478	Carbamidomethyl (C)[7]		Mascot
1308.6794	1308.6891	0.0097	7	2	15	AASTTALSSPFAGK					Mascot
1325.6743	1325.6754	0.0011	1	95	104	NRELEVIHCR			Carbamidomethyl (C)[9]		Mascot
1439.72	1439.6439	-0.0761	-53	1	15	MAASTTALSSPFAGK					Mascot
1455.7148	1455.6631	-0.0517	-36	1	15	MAASTTALSSPFAGK			Oxidation (M)[1]		Mascot
1919.9871	1920.0138	0.0267	14	105	121	WAMLGALGCVFPELLAR			Carbamidomethyl (C)[9], Oxidation (M)[3]		Mascot

8	Chromosome partition protein Smc OS=Caulobacter crescentus (strain NA1000 / CB15N) GN=smc PE=1 SV=1	SMC_CAUCN	123426.9	5.43	24	64	77.972	10.628			

Peptide Information

Calc. Mass	Obsrv. Mass	± da	± ppm	Start Seq.	End Seq.	Sequence	Ion Score	C. I. %	Modification	Rank	Result Type
804.421	804.4075	-0.0135	-17	317	323	LSNDLSR					Mascot
816.4322	816.4263	-0.0059	-7	324	330	IDADRAR					Mascot
830.4553	830.4332	-0.0221	-27	593	599	ELKPGMR					Mascot
846.4502	846.4493	-0.0009	-1	593	599	ELKPGMR			Oxidation (M)[6]		Mascot
876.4057	876.4464	0.0407	46	800	807	AESLEGDR					Mascot
897.5152	897.5043	-0.0109	-12	354	362	ALVAAAPER					Mascot
904.4734	904.4707	-0.0027	-3	763	770	TALDVETR					Mascot
1021.5095	1021.4806	-0.0289	-28	959	966	LETMRSER					Mascot
1037.5044	1037.507	0.0026	3	959	966	LETMRSER			Oxidation (M)[4]		Mascot
1037.5044	1037.507	0.0026	3	959	966	LETMRSER			Oxidation (M)[4]		Mascot
1055.5731	1055.5291	-0.044	-42	345	353	IAPELEEVR					Mascot

1055.5731	1055.5291	-0.044	-42	345	353	IAPELEEVR						Mascot
1189.6172	1189.6564	0.0392	33	763	772	TALDVETRER						Mascot
1337.6453	1337.6776	0.0323	24	46	57	WVMGANSAKAMR				Oxidation (M)[3]		Mascot
1374.6971	1374.7126	0.0155	11	317	328	LSNDLSRIDADR						Mascot
1380.6754	1380.6337	-0.0417	-30	506	519	SGHAPALDSVSPDK						Mascot
1409.7019	1409.6514	-0.0505	-36	427	441	AAVGPEVDPAAADAR						Mascot
1423.7653	1423.6481	-0.1172	-82	674	686	GPPDAERLLTQAR						Mascot
1441.6926	1441.6471	-0.0455	-32	1117	1128	MDRLFGVTMAER				Oxidation (M)[1]		Mascot
1441.7506	1441.6471	-0.1035	-72	468	480	AAEQEAQARQLAR						Mascot
1452.7805	1452.6401	-0.1404	-97	163	176	ILEEAAGVSGLHTR						Mascot
1463.7336	1463.714	-0.0196	-13	843	856	AKASDALETAETTR						Mascot
1486.7836	1486.6707	-0.1129	-76	1104	1116	FIAITHNPVTMSR						Mascot
1568.8102	1568.7498	-0.0604	-39	22	37	IEPGLTGIVGPNGCGK				Carbamidomethyl (C)[14]		Mascot
1794.9933	1794.9221	-0.0712	-40	146	161	QGQISELIGAKPQNRR						Mascot
1797.0116	1797.011	-0.0006	0	284	300	EEATIAQAILGQLAIQK						Mascot
1934.0706	1934.0508	-0.0198	-10	345	362	IAPELEEVRALVAAAPER						Mascot
1968.0219	1968.0066	-0.0153	-8	264	283	ASAAAQVAITEAEAAMPP LR						Mascot

9 Zinc finger Ran-binding domain-containing protein 2 OS=Sus scrofa GN=ZRANB2 PE=2 SV=1 | ZRAB2_PIG | 37545 | 9.91 | 15 | 62 | 68.884 | 5.312

Peptide Information

Calc. Mass	Obsrv. Mass	± da	± ppm	Start Seq.	End Seq.	Sequence	Ion Score	C. I. %	Modification	Rank	Result Type
804.4573	804.4075	-0.0498	-62	55	61	TLAEKSR					Mascot
816.407	816.4263	0.0193	24	256	262	SSSRSHR					Mascot
1021.4908	1021.4806	-0.0102	-10	288	297	SRSSSTGDPK					Mascot
1037.4971	1037.507	0.0099	10	217	226	SSSPSSSRSR					Mascot
1037.4971	1037.507	0.0099	10	217	226	SSSPSSSRSR					Mascot
1051.5055	1051.4957	-0.0098	-9	110	117	ENVEYIER					Mascot
1065.5259	1065.5117	-0.0142	-13	19	27	KCGNVNFAR			Carbamidomethyl (C)[2]		Mascot
1122.5646	1122.5731	0.0085	8	44	54	MMKAGGTEIGK					Mascot
1173.5018	1173.5146	0.0128	11	271	281	SYSSSSSSPER					Mascot
1325.5944	1325.6754	0.081	61	62	72	GLFSANDWQCK			Carbamidomethyl (C)[10]		Mascot
1396.5767	1396.6475	0.0708	51	82	92	RSECNMCNTPK			Carbamidomethyl (C)[4,7]		Mascot
1412.5715	1412.6342	0.0627	44	82	92	RSECNMCNTPK			Carbamidomethyl (C)[4,7], Oxidation (M)[6]		Mascot
1419.6573	1419.7238	0.0665	47	8	19	VSDGDWICPDKK			Carbamidomethyl (C)[8]		Mascot
1424.6361	1424.6302	-0.0059	-4	312	326	SSSGSSHSGSRSSSK					Mascot

1455.8054	1455.6631	-0.1423	-98	138	151	AVGPASILKEVEDK					Mascot
1465.6639	1465.6833	0.0194	13	309	322	HHRSSSGSSHSGSR					Mascot
1568.7275	1568.7498	0.0223	14	60	72	SRGLFSANDWQCK			Carbamidomethyl (C)[12]		Mascot

10	Keratin, type II cytoskeletal 5 OS=Pan troglodytes GN=KRT5 PE=2 SV=1	K2C5_PANTR	62728.1	6.89	17	60	44.667	13.988	

Peptide Information

Calc. Mass	Obsrv. Mass	± da	± ppm	Start Seq.	End Seq.	Sequence	Ion Score	C. I. %	Modification	Rank	Result Type
816.4872	816.4263	-0.0609	-75	394	399	MIQRLR					Mascot
832.4821	832.4073	-0.0748	-90	394	399	MIQRLR			Oxidation (M)[1]		Mascot
972.5472	972.5155	-0.0317	-33	423	431	GELALKDAR					Mascot
1015.4625	1015.4745	0.012	12	386	393	HEISEMNR					Mascot
1015.4625	1015.4745	0.012	12	386	393	HEISEMNR					Mascot
1053.5436	1053.46	-0.0836	-79	2	10	SRQSSVSFR					Mascot
1055.5229	1055.5291	0.0062	6	377	385	HGDDLRNTK					Mascot
1055.5229	1055.5291	0.0062	6	377	385	HGDDLRNTK					Mascot
1155.535	1155.6088	0.0738	64	454	462	EYQELMNTK					Mascot
1161.6376	1161.6108	-0.0268	-23	188	196	VRFLEQQNK					Mascot
1171.53	1171.5474	0.0174	15	454	462	EYQELMNTK			Oxidation (M)[6]		Mascot
1374.6431	1374.7126	0.0695	51	383	393	NTKHEISEMNR			Oxidation (M)[9]		Mascot
1396.6638	1396.6475	-0.0163	-12	49	63	VSLGGACGVGGYGSR			Carbamidomethyl (C)[7]		Mascot
1410.7224	1410.649	-0.0734	-52	16	29	SFSTASAITPSVSR					Mascot
1426.7246	1426.6411	-0.0835	-59	268	279	TTAENEFVMLKK			Oxidation (M)[9]		Mascot
1438.736	1438.6431	-0.0929	-65	267	278	RTTAENEFVMLK					Mascot
1439.7125	1439.6439	-0.0686	-48	562	578	GLGVGFGSGGGSSSSVK					Mascot
1441.6702	1441.6471	-0.0231	-16	295	306	VDALMDEINFMK			Oxidation (M)[5]		Mascot
1441.6702	1441.6471	-0.0231	-16	295	306	VDALMDEINFMK			Oxidation (M)[5]		Mascot
1454.7308	1454.6672	-0.0636	-44	267	278	RTTAENEFVMLK			Oxidation (M)[10]		Mascot
1575.7366	1575.8531	0.1165	74	386	397	HEISEMNRMIQR			Oxidation (M)[6,9]		Mascot
1887.8944	1888.0312	0.1368	72	367	382	YEELQQTAGRHGDDLR					Mascot
1951.943	1951.9948	0.0518	27	242	257	LDSELRNMQDLVEDFK					Mascot
1967.9379	1968.0066	0.0687	35	242	257	LDSELRNMQDLVEDFK			Oxidation (M)[8]		Mascot

Gel Idx/Pos	301/M3	Instr./Gel Origin	BA2151/Sample Project 20140814	Process Status	Analysis Succeeded
Plate [#] Name	[1] Sample Project 20140814	Instrument Sample Name		Spectra	11

Rank	Protein Name	Accession No.	Protein MW	Protein PI	Pep. Count	Protein Score	Protein Score C. I. %	Intensity Matched	Total Ion Score	Total Ion C. I. %	Confirmed
1	Chlorophyll a-b binding protein, chloroplastic OS=Spinacia oleracea PE=1 SV=1	CB2A_SPIOL	28577.4	5.31	8	159	100	10.651	122	100	

Peptide Information

Calc. Mass	Obsrv. Mass	± da	± ppm	Start Seq.	End Seq.	Sequence	Ion Score	C. I. %	Modification	Rank	Result Type
983.4985	983.4614	-0.0371	-38	127	134	FGEAVWFK					Mascot
1055.5303	1055.5221	-0.0082	-8	98	105	ELEVIHCR			Carbamidomethyl (C)[7]		Mascot
1055.5303	1055.5221	-0.0082	-8	98	105	ELEVIHCR	58	99.854	Carbamidomethyl (C)[7]		Mascot
1325.6743	1325.6655	-0.0088	-7	96	105	NRELEVIHCR			Carbamidomethyl (C)[9]		Mascot
1325.6743	1325.6655	-0.0088	-7	96	105	NRELEVIHCR	64	99.957	Carbamidomethyl (C)[9]		Mascot
1423.7097	1423.6359	-0.0738	-52	2	16	ASSTMALSSPSLAGK			Oxidation (M)[5]		Mascot
1554.7502	1554.734	-0.0162	-10	1	16	MASSTMALSSPSLAGK			Oxidation (M)[1]		Mascot
1597.8909	1597.7753	-0.1156	-72	17	32	AVKLGPTASEIIGEGR					Mascot
1705.9153	1705.9233	0.008	5	2	19	ASSTMALSSPSLAGKAVK					Mascot
1903.9922	1904.0045	0.0123	6	106	122	WAMLGALGCVFPELLAR			Carbamidomethyl (C)[9]		Mascot
1919.9871	1919.9934	0.0063	3	106	122	WAMLGALGCVFPELLAR			Carbamidomethyl (C)[9], Oxidation (M)[3]		Mascot

Rank	Protein Name	Accession No.	Protein MW	Protein PI	Pep. Count	Protein Score	Protein Score C. I. %	Intensity Matched	Total Ion Score	Total Ion C. I. %	Confirmed
2	Chlorophyll a-b binding protein 40, chloroplastic OS=Nicotiana tabacum GN=CAB40 PE=2 SV=1	CB24_TOBAC	28450.2	5.48	7	150	100	10.613	122	100	

Peptide Information

Calc. Mass	Obsrv. Mass	± da	± ppm	Start Seq.	End Seq.	Sequence	Ion Score	C. I. %	Modification	Rank	Result Type
983.4985	983.4614	-0.0371	-38	127	134	FGEAVWFK					Mascot
1055.5303	1055.5221	-0.0082	-8	98	105	ELEVIHCR			Carbamidomethyl (C)[7]		Mascot
1055.5303	1055.5221	-0.0082	-8	98	105	ELEVIHCR	58	99.854	Carbamidomethyl (C)[7]		Mascot
1325.6743	1325.6655	-0.0088	-7	96	105	NRELEVIHCR			Carbamidomethyl (C)[9]		Mascot
1325.6743	1325.6655	-0.0088	-7	96	105	NRELEVIHCR	64	99.957	Carbamidomethyl (C)[9]		Mascot
1441.6992	1441.6422	-0.057	-40	2	16	AASTMALSSPSFAGK			Oxidation (M)[5]		Mascot
1607.7812	1607.7842	0.003	2	42	56	AKTVSSGSPWYGPDR					Mascot
1870.9402	1871.0072	0.067	36	1	19	MAASTMALSSPSFAGKA VK			Oxidation (M)[1]		Mascot
1903.9922	1904.0045	0.0123	6	106	122	WAMLGALGCVFPELLAR			Carbamidomethyl (C)[9]		Mascot
1919.9871	1919.9934	0.0063	3	106	122	WAMLGALGCVFPELLAR			Carbamidomethyl (C)[9], Oxidation (M)[3]		Mascot

3	Chlorophyll a-b binding protein 50, chloroplastic OS=Nicotiana tabacum GN=CAB50 PE=2 SV=1	CB25_TOBAC	28500.3	5.84	6	146	100	10.192	122	100	

Peptide Information

Calc. Mass	Obsrv. Mass	± da	± ppm	Start Seq.	End Seq.	Sequence	Ion Score	C. I. %	Modification	Rank	Result Type
983.4985	983.4614	-0.0371	-38	127	134	FGEAVWFK					Mascot
1055.5303	1055.5221	-0.0082	-8	98	105	ELEVIHCR			Carbamidomethyl (C)[7]		Mascot
1055.5303	1055.5221	-0.0082	-8	98	105	ELEVIHCR	58	99.854	Carbamidomethyl (C)[7]		Mascot
1325.6743	1325.6655	-0.0088	-7	96	105	NRELEVIHCR			Carbamidomethyl (C)[9]		Mascot
1325.6743	1325.6655	-0.0088	-7	96	105	NRELEVIHCR	64	99.957	Carbamidomethyl (C)[9]		Mascot
1697.8892	1697.7871	-0.1021	-60	2	19	AAATMALSSSSFAGKAVK					Mascot
1828.9296	1828.9736	0.044	24	1	19	MAAATMALSSSSFAGKAVK					Mascot
1860.9194	1860.9775	0.0581	31	1	19	MAAATMALSSSSFAGKAVK			Oxidation (M)[1,6]		Mascot
1903.9922	1904.0045	0.0123	6	106	122	WAMLGALGCVFPELLAR			Carbamidomethyl (C)[9]		Mascot
1919.9871	1919.9934	0.0063	3	106	122	WAMLGALGCVFPELLAR			Carbamidomethyl (C)[9], Oxidation (M)[3]		Mascot

4	Chlorophyll a-b binding protein 1, chloroplastic OS=Sinapis alba GN=CAB1 PE=3 SV=1	CB21_SINAL	28328.2	5.17	6	146	100	9.939	122	100	

Peptide Information

Calc. Mass	Obsrv. Mass	± da	± ppm	Start Seq.	End Seq.	Sequence	Ion Score	C. I. %	Modification	Rank	Result Type
983.4985	983.4614	-0.0371	-38	125	132	FGEAVWFK					Mascot
1055.5303	1055.5221	-0.0082	-8	96	103	ELEVIHCR			Carbamidomethyl (C)[7]		Mascot
1055.5303	1055.5221	-0.0082	-8	96	103	ELEVIHCR	58	99.854	Carbamidomethyl (C)[7]		Mascot
1325.6743	1325.6655	-0.0088	-7	94	103	NRELEVIHCR			Carbamidomethyl (C)[9]		Mascot
1325.6743	1325.6655	-0.0088	-7	94	103	NRELEVIHCR	64	99.957	Carbamidomethyl (C)[9]		Mascot
1575.849	1575.8107	-0.0383	-24	17	32	AVKLSPGASEVFGTGR					Mascot
1870.9402	1871.0072	0.067	36	1	19	MAASTMALSSPAFAGKAVK			Oxidation (M)[1,6]		Mascot
1903.9922	1904.0045	0.0123	6	104	120	WAMLGALGCVFPELLAR			Carbamidomethyl (C)[9]		Mascot
1919.9871	1919.9934	0.0063	3	104	120	WAMLGALGCVFPELLAR			Carbamidomethyl (C)[9], Oxidation (M)[3]		Mascot

5	Chlorophyll a-b binding protein 91R, chloroplastic OS=Petunia sp. GN=CAB91R PE=3 SV=1	CB25_PETSP	28536.2	5.31	6	145	100	10.001	122	100	

Peptide Information

Calc. Mass	Obsrv. Mass	± da	± ppm	Start Seq.	End Seq.	Sequence	Ion Score	C. I. %	Modification	Rank	Result Type

Calc. Mass	Obsrv. Mass	± da	± ppm	Start Seq.	End Seq.	Sequence	Ion Score	C. I. %	Modification	Rank	Result Type
983.4985	983.4614	-0.0371	-38	127	134	FGEAVWFK					Mascot
1055.5303	1055.5221	-0.0082	-8	98	105	ELEVIHCR			Carbamidomethyl (C)[7]		Mascot
1055.5303	1055.5221	-0.0082	-8	98	105	ELEVIHCR	58	99.854	Carbamidomethyl (C)[7]		Mascot
1325.6743	1325.6655	-0.0088	-7	96	105	NRELEVIHCR			Carbamidomethyl (C)[9]		Mascot
1325.6743	1325.6655	-0.0088	-7	96	105	NRELEVIHCR	64	99.957	Carbamidomethyl (C)[9]		Mascot
1608.8229	1608.7917	-0.0312	-19	17	32	AVKFSPSSSEITGNGK					Mascot
1870.9402	1871.0072	0.067	36	1	19	MAAATMALSSPSFAGKA VK			Oxidation (M)[1,6]		Mascot
1903.9922	1904.0045	0.0123	6	106	122	WAMLGALGCVFPELLAR			Carbamidomethyl (C)[9]		Mascot
1919.9871	1919.9934	0.0063	3	106	122	WAMLGALGCVFPELLAR			Carbamidomethyl (C)[9], Oxidation (M)[3]		Mascot

6	Chlorophyll a-b binding protein 3C, chloroplastic OS=Solanum lycopersicum GN=CAB3C PE=3 SV=1	CB2G_SOLLC	28478.2	5.47	6	145	100	9.982	122	100	

Peptide Information

Calc. Mass	Obsrv. Mass	± da	± ppm	Start Seq.	End Seq.	Sequence	Ion Score	C. I. %	Modification	Rank	Result Type
983.4985	983.4614	-0.0371	-38	127	134	FGEAVWFK					Mascot
1055.5303	1055.5221	-0.0082	-8	98	105	ELEVIHCR			Carbamidomethyl (C)[7]		Mascot
1055.5303	1055.5221	-0.0082	-8	98	105	ELEVIHCR	58	99.854	Carbamidomethyl (C)[7]		Mascot
1325.6743	1325.6655	-0.0088	-7	96	105	NRELEVIHCR			Carbamidomethyl (C)[9]		Mascot
1325.6743	1325.6655	-0.0088	-7	96	105	NRELEVIHCR	64	99.957	Carbamidomethyl (C)[9]		Mascot
1575.7551	1575.8107	0.0556	35	42	56	AKPASSGSPWYGPDR					Mascot
1903.9922	1904.0045	0.0123	6	106	122	WAMLGALGCVFPELLAR			Carbamidomethyl (C)[9]		Mascot
1904.9457	1905.0001	0.0544	29	1	19	MATSTMALSSSTFAGKA VK			Oxidation (M)[1]		Mascot
1919.9871	1919.9934	0.0063	3	106	122	WAMLGALGCVFPELLAR			Carbamidomethyl (C)[9], Oxidation (M)[3]		Mascot

7	Chlorophyll a-b binding protein 1B, chloroplastic OS=Solanum lycopersicum GN=CAB1B PE=3 SV=1	CB2B_SOLLC	28225.1	5.15	6	145	100	9.894	122	100	

Peptide Information

Calc. Mass	Obsrv. Mass	± da	± ppm	Start Seq.	End Seq.	Sequence	Ion Score	C. I. %	Modification	Rank	Result Type
983.4985	983.4614	-0.0371	-38	125	132	FGEAVWFK					Mascot
1055.5303	1055.5221	-0.0082	-8	96	103	ELEVIHCR			Carbamidomethyl (C)[7]		Mascot
1055.5303	1055.5221	-0.0082	-8	96	103	ELEVIHCR	58	99.854	Carbamidomethyl (C)[7]		Mascot
1325.6743	1325.6655	-0.0088	-7	94	103	NRELEVIHCR			Carbamidomethyl (C)[9]		Mascot
1325.6743	1325.6655	-0.0088	-7	94	103	NRELEVIHCR	64	99.957	Carbamidomethyl (C)[9]		Mascot
1406.6335	1406.6436	0.0101	7	42	54	SAPSSSPWYGPDR					Mascot
1870.9038	1871.0072	0.1034	55	1	19	MAAATMALSSPSFAGQA VK			Oxidation (M)[1,6]		Mascot

1903.9922	1904.0045	0.0123	6	104	120	WAMLGALGCVFPELLAR			Carbamidomethyl (C)[9]		Mascot
1919.9871	1919.9934	0.0063	3	104	120	WAMLGALGCVFPELLAR			Carbamidomethyl (C)[9], Oxidation (M)[3]		Mascot

8	Chlorophyll a-b binding protein, chloroplastic OS=Zea mays PE=2 SV=1	CB22_MAIZE	28148.2	5.29	6	145	100	9.934	122	100	

Peptide Information

Calc. Mass	Obsrv. Mass	± da	± ppm	Start Seq.	End Seq.	Sequence	Ion Score	C. I. %	Modification	Rank	Result Type
983.4985	983.4614	-0.0371	-38	125	132	FGEAVWFK					Mascot
1055.5303	1055.5221	-0.0082	-8	96	103	ELEVIHCR			Carbamidomethyl (C)[7]		Mascot
1055.5303	1055.5221	-0.0082	-8	96	103	ELEVIHCR	58	99.854	Carbamidomethyl (C)[7]		Mascot
1320.6543	1320.6302	-0.0241	-18	17	29	AVNVPSSSFGEAR					Mascot
1325.6743	1325.6655	-0.0088	-7	94	103	NRELEVIHCR			Carbamidomethyl (C)[9]		Mascot
1325.6743	1325.6655	-0.0088	-7	94	103	NRELEVIHCR	64	99.957	Carbamidomethyl (C)[9]		Mascot
1592.7295	1592.7844	0.0549	34	1	16	MASSTMALSSTAFAGK			Oxidation (M)[1,6]		Mascot
1903.9922	1904.0045	0.0123	6	104	120	WAMLGALGCVFPELLAR			Carbamidomethyl (C)[9]		Mascot
1919.9871	1919.9934	0.0063	3	104	120	WAMLGALGCVFPELLAR			Carbamidomethyl (C)[9], Oxidation (M)[3]		Mascot

9	Chlorophyll a-b binding protein E, chloroplastic OS=Nicotiana plumbaginifolia GN=CABE PE=3 SV=1	CB25_NICPL	28430.2	5.32	6	145	100	10.243	122	100	

Protein Group

Chlorophyll a-b binding protein 16, chloroplastic OS=Nicotiana tabacum GN=CAB16 PE=2 SV=1	CB21_TOBAC	28331.2	5.4800 000190 7349

Peptide Information

Calc. Mass	Obsrv. Mass	± da	± ppm	Start Seq.	End Seq.	Sequence	Ion Score	C. I. %	Modification	Rank	Result Type
983.4985	983.4614	-0.0371	-38	126	133	FGEAVWFK					Mascot
1055.5303	1055.5221	-0.0082	-8	97	104	ELEVIHCR			Carbamidomethyl (C)[7]		Mascot
1055.5303	1055.5221	-0.0082	-8	97	104	ELEVIHCR	58	99.854	Carbamidomethyl (C)[7]		Mascot
1308.6794	1308.704	0.0246	19	2	15	AASTTALSSPFAGK					Mascot
1325.6743	1325.6655	-0.0088	-7	95	104	NRELEVIHCR			Carbamidomethyl (C)[9]		Mascot
1325.6743	1325.6655	-0.0088	-7	95	104	NRELEVIHCR	64	99.957	Carbamidomethyl (C)[9]		Mascot
1439.72	1439.6511	-0.0689	-48	1	15	MAASTTALSSPFAGK					Mascot
1903.9922	1904.0045	0.0123	6	105	121	WAMLGALGCVFPELLAR			Carbamidomethyl (C)[9]		Mascot
1919.9871	1919.9934	0.0063	3	105	121	WAMLGALGCVFPELLAR			Carbamidomethyl (C)[9], Oxidation (M)[3]		Mascot

10	Chlorophyll a-b binding protein C, chloroplastic OS=Nicotiana plumbaginifolia GN=CABC PE=3 SV=1	CB23_NICPL	28522.3	5.69	5	139	100	10.052	122	100	

Peptide Information

Calc. Mass	Obsrv. Mass	± da	± ppm	Start Seq.	End Seq.	Sequence	Ion Score	C. I. %	Modification	Rank	Result Type
983.4985	983.4614	-0.0371	-38	127	134	FGEAVWFK					Mascot
1055.5303	1055.5221	-0.0082	-8	98	105	ELEVIHCR			Carbamidomethyl (C)[7]		Mascot
1055.5303	1055.5221	-0.0082	-8	98	105	ELEVIHCR	58	99.844	Carbamidomethyl (C)[7]		Mascot
1325.6743	1325.6655	-0.0088	-7	96	105	NRELEVIHCR			Carbamidomethyl (C)[9]		Mascot
1325.6743	1325.6655	-0.0088	-7	96	105	NRELEVIHCR	64	99.954	Carbamidomethyl (C)[9]		Mascot
1860.9194	1860.9775	0.0581	31	1	19	MAASTMALSSSSFAGKAVK			Oxidation (M)[1]		Mascot
1876.9143	1876.9902	0.0759	40	1	19	MAASTMALSSSSFAGKAVK			Oxidation (M)[1,6]		Mascot
1903.9922	1904.0045	0.0123	6	106	122	WAMLGALGCVFPELLAR			Carbamidomethyl (C)[9]		Mascot
1919.9871	1919.9934	0.0063	3	106	122	WAMLGALGCVFPELLAR			Carbamidomethyl (C)[9], Oxidation (M)[3]		Mascot

Gel Idx/Pos	302/M4	Instr./Gel Origin	BA2151/Sample Project 20140814	Process Status	Analysis Succeeded
Plate [#] Name	[1] Sample Project 20140814	Instrument Sample Name		Spectra	11

Rank	Protein Name	Accession No.	Protein MW	Protein PI	Pep. Count	Protein Score	Protein Score C. I. %	Intensity Matched	Total Ion Score	Total Ion C. I. %	Confirmed
1	Chlorophyll a-b binding protein, chloroplastic OS=Zea mays PE=2 SV=1	CB22_MAIZE	28148.2	5.29	6	96	99.985	9.878	73	99.994	

Peptide Information

Calc. Mass	Obsrv. Mass	± da	± ppm	Start Seq.	End Seq.	Sequence	Ion Score	C. I. %	Modification	Rank	Result Type
983.4985	983.463	-0.0355	-36	125	132	FGEAVWFK					Mascot
983.4985	983.463	-0.0355	-36	125	132	FGEAVWFK	25	0			Mascot
1055.5303	1055.5171	-0.0132	-13	96	103	ELEVIHCR			Carbamidomethyl (C)[7]		Mascot
1055.5303	1055.5171	-0.0132	-13	96	103	ELEVIHCR	48	98.071	Carbamidomethyl (C)[7]		Mascot
1320.6543	1320.611	-0.0433	-33	17	29	AVNVPSSSFGEAR					Mascot
1325.6743	1325.652	-0.0223	-17	94	103	NRELEVIHCR			Carbamidomethyl (C)[9]		Mascot
1576.7346	1576.7382	0.0036	2	1	16	MASSTMALSSTAFAGK			Oxidation (M)[1]		Mascot
1919.9871	1919.9635	-0.0236	-12	104	120	WAMLGALGCVFPELLAR			Carbamidomethyl (C)[9], Oxidation (M)[3]		Mascot

Rank	Protein Name	Accession No.	Protein MW	Protein PI	Pep. Count	Protein Score	Protein Score C. I. %	Intensity Matched	Total Ion Score	Total Ion C. I. %	Confirmed
2	Chlorophyll a-b binding protein, chloroplastic OS=Spinacia oleracea PE=1 SV=1	CB2A_SPIOL	28577.4	5.31	5	90	99.947	28.26	73	99.994	

Peptide Information

Calc. Mass	Obsrv. Mass	± da	± ppm	Start Seq.	End Seq.	Sequence	Ion Score	C. I. %	Modification	Rank	Result Type
983.4985	983.463	-0.0355	-36	127	134	FGEAVWFK					Mascot
983.4985	983.463	-0.0355	-36	127	134	FGEAVWFK	25	0			Mascot
1055.5303	1055.5171	-0.0132	-13	98	105	ELEVIHCR			Carbamidomethyl (C)[7]		Mascot
1055.5303	1055.5171	-0.0132	-13	98	105	ELEVIHCR	48	98.071	Carbamidomethyl (C)[7]		Mascot
1325.6743	1325.652	-0.0223	-17	96	105	NRELEVIHCR			Carbamidomethyl (C)[9]		Mascot
1407.7148	1407.6229	-0.0919	-65	2	16	ASSTMALSSPSLAGK					Mascot
1407.7148	1407.6229	-0.0919	-65	2	16	ASSTMALSSPSLAGK					Mascot
1423.7097	1423.6178	-0.0919	-65	2	16	ASSTMALSSPSLAGK			Oxidation (M)[5]		Mascot
1423.7097	1423.6178	-0.0919	-65	2	16	ASSTMALSSPSLAGK			Oxidation (M)[5]		Mascot
1919.9871	1919.9635	-0.0236	-12	106	122	WAMLGALGCVFPELLAR			Carbamidomethyl (C)[9], Oxidation (M)[3]		Mascot

Rank	Protein Name	Accession No.	Protein MW	Protein PI	Pep. Count	Protein Score	Protein Score C. I. %	Intensity Matched	Total Ion Score	Total Ion C. I. %	Confirmed
3	Chlorophyll a-b binding protein E, chloroplastic OS=Nicotiana plumbaginifolia GN=CABE PE=3 SV=1	CB25_NICPL	28430.2	5.32	5	90	99.946	18.913	73	99.994	

Protein Group

	Chlorophyll a-b binding protein 16, chloroplastic OS=Nicotiana tabacum GN=CAB16 PE=2 SV=1	CB21_TOBAC	28331.2	5.4800 000190 7349						

Peptide Information

Calc. Mass	Obsrv. Mass	± da	± ppm	Start Seq.	End Seq.	Sequence	Ion Score	C. I. %	Modification	Rank	Result Type
983.4985	983.463	-0.0355	-36	126	133	FGEAVWFK					Mascot
983.4985	983.463	-0.0355	-36	126	133	FGEAVWFK	25	0			Mascot
1055.5303	1055.5171	-0.0132	-13	97	104	ELEVIHCR			Carbamidomethyl (C)[7]		Mascot
1055.5303	1055.5171	-0.0132	-13	97	104	ELEVIHCR	48	98.071	Carbamidomethyl (C)[7]		Mascot
1325.6743	1325.652	-0.0223	-17	95	104	NRELEVIHCR			Carbamidomethyl (C)[9]		Mascot
1439.72	1439.6124	-0.1076	-75	1	15	MAASTTALSSPFAGK					Mascot
1439.72	1439.6124	-0.1076	-75	1	15	MAASTTALSSPFAGK					Mascot
1455.7148	1455.6071	-0.1077	-74	1	15	MAASTTALSSPFAGK			Oxidation (M)[1]		Mascot
1919.9871	1919.9635	-0.0236	-12	105	121	WAMLGALGCVFPELLAR			Carbamidomethyl (C)[9], Oxidation (M)[3]		Mascot

4	Chlorophyll a-b binding protein 22R, chloroplastic OS=Petunia sp. GN=CAB22R PE=3 SV=1	CB23_PETSP	28560.3	5.48	5	89	99.935	9.993	73	99.994

Peptide Information

Calc. Mass	Obsrv. Mass	± da	± ppm	Start Seq.	End Seq.	Sequence	Ion Score	C. I. %	Modification	Rank	Result Type
983.4985	983.463	-0.0355	-36	127	134	FGEAVWFK					Mascot
983.4985	983.463	-0.0355	-36	127	134	FGEAVWFK	25	0			Mascot
1055.5303	1055.5171	-0.0132	-13	98	105	ELEVIHCR			Carbamidomethyl (C)[7]		Mascot
1055.5303	1055.5171	-0.0132	-13	98	105	ELEVIHCR	48	98.071	Carbamidomethyl (C)[7]		Mascot
1325.6743	1325.652	-0.0223	-17	96	105	NRELEVIHCR			Carbamidomethyl (C)[9]		Mascot
1395.7419	1395.6245	-0.1174	-84	123	134	NGIKFGEAVWFK					Mascot
1576.7346	1576.7382	0.0036	2	1	16	MAATTMALSSSSFAGK			Oxidation (M)[1]		Mascot

5	Chlorophyll a-b binding protein type I, chloroplastic OS=Pinus thunbergii PE=2 SV=1	CB21_PINTH	28753.5	5.31	5	89	99.925	10.307	73	99.994

Peptide Information

Calc. Mass	Obsrv. Mass	± da	± ppm	Start Seq.	End Seq.	Sequence	Ion Score	C. I. %	Modification	Rank	Result Type
983.4985	983.463	-0.0355	-36	126	133	FGEAVWFK					Mascot
983.4985	983.463	-0.0355	-36	126	133	FGEAVWFK	25	0			Mascot
1055.5303	1055.5171	-0.0132	-13	97	104	ELEVIHCR			Carbamidomethyl (C)[7]		Mascot
1055.5303	1055.5171	-0.0132	-13	97	104	ELEVIHCR	48	98.071	Carbamidomethyl (C)[7]		Mascot
1219.6576	1219.653	-0.0046	-4	29	39	VGTAQARITMR			Oxidation (M)[10]		Mascot

1325.6743	1325.652	-0.0223	-17	95	104	NRELEVIHCR			Carbamidomethyl (C)[9]		Mascot
1395.7419	1395.6245	-0.1174	-84	122	133	NGLKFGEAVWFK					Mascot

6	Chlorophyll a-b binding protein 4, chloroplastic OS=Solanum lycopersicum GN=CAB4 PE=2 SV=1	CB24_SOLLC	28984.4	5.33	5	88	99.916	9.756	73	99.994

Peptide Information

Calc. Mass	Obsrv. Mass	± da	± ppm	Start Seq.	End Seq.	Sequence	Ion Score	C. I. %	Modification	Rank	Result Type
963.5007	963.4922	-0.0085	-9	26	34	KVGNFGEGR					Mascot
983.4985	983.463	-0.0355	-36	125	132	FGEAVWFK					Mascot
983.4985	983.463	-0.0355	-36	125	132	FGEAVWFK	25	0			Mascot
1021.5425	1021.4512	-0.0913	-89	19	26	SQNEFIRK					Mascot
1055.5303	1055.5171	-0.0132	-13	96	103	ELEVIHCR			Carbamidomethyl (C)[7]		Mascot
1055.5303	1055.5171	-0.0132	-13	96	103	ELEVIHCR	48	98.071	Carbamidomethyl (C)[7]		Mascot
1325.6743	1325.652	-0.0223	-17	94	103	NRELEVIHCR			Carbamidomethyl (C)[9]		Mascot

7	Chlorophyll a-b binding protein 36, chloroplastic OS=Nicotiana tabacum GN=CAB36 PE=3 SV=1	CB23_TOBAC	28734.4	5.33	5	88	99.912	9.687	73	99.994

Peptide Information

Calc. Mass	Obsrv. Mass	± da	± ppm	Start Seq.	End Seq.	Sequence	Ion Score	C. I. %	Modification	Rank	Result Type
807.4108	807.3736	-0.0372	-46	27	34	IGSFNGGR					Mascot
845.4475	845.4431	-0.0044	-5	19	25	SQNELVR					Mascot
983.4985	983.463	-0.0355	-36	125	132	FGEAVWFK					Mascot
983.4985	983.463	-0.0355	-36	125	132	FGEAVWFK	25	0			Mascot
1055.5303	1055.5171	-0.0132	-13	96	103	ELEVIHCR			Carbamidomethyl (C)[7]		Mascot
1055.5303	1055.5171	-0.0132	-13	96	103	ELEVIHCR	48	98.071	Carbamidomethyl (C)[7]		Mascot
1325.6743	1325.652	-0.0223	-17	94	103	NRELEVIHCR			Carbamidomethyl (C)[9]		Mascot

8	Chlorophyll a-b binding protein 25, chloroplastic OS=Petunia sp. GN=CAB25 PE=3 SV=1	CB24_PETSP	28320.1	5.45	4	85	99.84	9.502	73	99.994

Peptide Information

Calc. Mass	Obsrv. Mass	± da	± ppm	Start Seq.	End Seq.	Sequence	Ion Score	C. I. %	Modification	Rank	Result Type
983.4985	983.463	-0.0355	-36	126	133	FGEAVWFK					Mascot
983.4985	983.463	-0.0355	-36	126	133	FGEAVWFK	25	0			Mascot
1055.5303	1055.5171	-0.0132	-13	97	104	ELEVIHCR			Carbamidomethyl (C)[7]		Mascot
1055.5303	1055.5171	-0.0132	-13	97	104	ELEVIHCR	48	98.071	Carbamidomethyl (C)[7]		Mascot

	1325.6743	1325.652	-0.0223	-17	95	104	NRELEVIHCR			Carbamidomethyl (C)[9]		Mascot
	2336.2043	2336.0491	-0.1552	-66	105	125	WAMLGALGCVFPELFAR NGVK			Carbamidomethyl (C)[9]		Mascot

9	Chlorophyll a-b binding protein 1, chloroplastic OS=Sinapis alba GN=CAB1 PE=3 SV=1	CB21_SINAL	28328.2	5.17	4	85	99.821	9.512	73	99.994	

Peptide Information

Calc. Mass	Obsrv. Mass	± da	± ppm	Start Seq.	End Seq.	Sequence	Ion Score	C. I. %	Modification	Rank	Result Type
983.4985	983.463	-0.0355	-36	125	132	FGEAVWFK					Mascot
983.4985	983.463	-0.0355	-36	125	132	FGEAVWFK	25	0			Mascot
1055.5303	1055.5171	-0.0132	-13	96	103	ELEVIHCR			Carbamidomethyl (C)[7]		Mascot
1055.5303	1055.5171	-0.0132	-13	96	103	ELEVIHCR	48	98.071	Carbamidomethyl (C)[7]		Mascot
1325.6743	1325.652	-0.0223	-17	94	103	NRELEVIHCR			Carbamidomethyl (C)[9]		Mascot
1919.9611	1919.9635	0.0024	1	37	54	KTVKPTGPSGSPWYGSD R					Mascot

10	Chlorophyll a-b binding protein 1B, chloroplastic OS=Solanum lycopersicum GN=CAB1B PE=3 SV=1	CB2B_SOLLC	28225.1	5.15	4	85	99.817	9.512	73	99.994	

Protein Group

Chlorophyll a-b binding protein 21, chloroplastic OS=Nicotiana tabacum GN=CAB21 PE=2 SV=1	CB22_TOBAC	28279.2	5.1500 000953 6743

Peptide Information

Calc. Mass	Obsrv. Mass	± da	± ppm	Start Seq.	End Seq.	Sequence	Ion Score	C. I. %	Modification	Rank	Result Type
983.4985	983.463	-0.0355	-36	125	132	FGEAVWFK					Mascot
983.4985	983.463	-0.0355	-36	125	132	FGEAVWFK	25	0			Mascot
1055.5303	1055.5171	-0.0132	-13	96	103	ELEVIHCR			Carbamidomethyl (C)[7]		Mascot
1055.5303	1055.5171	-0.0132	-13	96	103	ELEVIHCR	48	98.071	Carbamidomethyl (C)[7]		Mascot
1325.6743	1325.652	-0.0223	-17	94	103	NRELEVIHCR			Carbamidomethyl (C)[9]		Mascot
1919.9871	1919.9635	-0.0236	-12	104	120	WAMLGALGCVFPELLAR			Carbamidomethyl (C)[9], Oxidation (M)[3]		Mascot

Gel Idx/Pos	303/M5	Instr./Gel Origin	BA2151/Sample Project 20140814	Process Status	Analysis Succeeded
Plate [#] Name	[1] Sample Project 20140814	Instrument Sample Name		Spectra	11

Rank	Protein Name	Accession No.	Protein MW	Protein PI	Pep. Count	Protein Score	Protein Score C. I. %	Intensity Matched	Total Ion Score	Total Ion C. I. %	Confirmed
1	Acetylglutamate kinase OS=Pelobacter propionicus (strain DSM 2379) GN=argB PE=3 SV=1	ARGB_PELPD	31383.6	5.6	10	56	0	25.714			

Peptide Information

Calc. Mass	Obsrv. Mass	± da	± ppm	Start Seq.	End Seq.	Sequence	Ion Score	C. I. %	Modification	Rank	Result Type
890.5015	890.4451	-0.0564	-63	1	7	MEKLIEK					Mascot
1351.6311	1351.6569	0.0258	19	30	41	YGGHAMSDEKLK			Oxidation (M)[6]		Mascot
1550.8611	1550.7734	-0.0877	-57	40	52	LKESFALDVIMLR			Oxidation (M)[11]		Mascot
1584.8163	1584.7068	-0.1095	-69	222	236	AGQLLSSIAVADMHR			Oxidation (M)[13]		Mascot
1811.9432	1811.9572	0.014	8	220	236	DKAGQLLSSIAVADMHR					Mascot
1811.9432	1811.9572	0.014	8	220	236	DKAGQLLSSIAVADMHR					Mascot
1941.0302	1940.9358	-0.0944	-49	107	125	EVVGYLNQHGGRAVGLS GK					Mascot
1951.0906	1950.9327	-0.1579	-81	222	239	AGQLLSSIAVADMHRLIR					Mascot
1951.0906	1950.9327	-0.1579	-81	222	239	AGQLLSSIAVADMHRLIR					Mascot
1967.0854	1966.9016	-0.1838	-93	222	239	AGQLLSSIAVADMHRLIR			Oxidation (M)[13]		Mascot
1967.0854	1966.9016	-0.1838	-93	222	239	AGQLLSSIAVADMHRLIR	2	0	Oxidation (M)[13]		Mascot
2592.2354	2592.1523	-0.0831	-32	240	263	EEAITGGMIPKVVCCADA LEEGVK			Carbamidomethyl (C)[14,15], Oxidation (M)[8]		Mascot
3435.72	3435.7095	-0.0105	-3	135	166	LLQDVVGDDGTTEQVDM GYVGDVVKVNTDLLK					Mascot
3458.7407	3458.6831	-0.0576	-17	87	118	VTDSETMAVVEMVLVGQ VNKEVVGYLNQHGGR					Mascot

Rank	Protein Name	Accession No.	Protein MW	Protein PI	Pep. Count	Protein Score	Protein Score C. I. %	Intensity Matched	Total Ion Score	Total Ion C. I. %	Confirmed
2	26S proteasome regulatory subunit rpn-1 OS=Neurospora crassa (strain ATCC 24698 / 74-OR23-1A / CBS 708.71 / DSM 1257 / FGSC 987) GN=rpn-1 PE=3 SV=2	RPN1_NEUCR	100529.8	4.86	16	48	0	12.987			

Peptide Information

Calc. Mass	Obsrv. Mass	± da	± ppm	Start Seq.	End Seq.	Sequence	Ion Score	C. I. %	Modification	Rank	Result Type
820.3988	820.3776	-0.0212	-26	402	407	DSWVWK					Mascot
853.4414	853.3951	-0.0463	-54	698	704	VYDTLSR					Mascot
918.4791	918.4658	-0.0133	-14	559	565	LNDKWSR					Mascot
945.5516	945.4924	-0.0592	-63	255	262	VAHSIYKK					Mascot
1105.5558	1105.525	-0.0308	-28	57	65	SELEMLVER					Mascot

1144.527	1144.5598	0.0328	29	224	232	FLDENTYSR					Mascot
1891.8668	1891.9006	0.0338	18	306	321	QGIPLEFERSNDDDEK					Mascot
1902.9684	1902.9875	0.0191	10	806	820	SHYLLYWIVTAMHPR			Oxidation (M)[12]		Mascot
1902.9684	1902.9875	0.0191	10	806	820	SHYLLYWIVTAMHPR			Oxidation (M)[12]		Mascot
1910.035	1909.8667	-0.1683	-88	680	697	RAVPLAMGLISPSNPQM K					Mascot
1921.9364	1921.9122	-0.0242	-13	322	336	IYECLSNQKLSEYFK			Carbamidomethyl (C)[4]		Mascot
1956.0835	1955.9598	-0.1237	-63	821	837	MLVTLDEDLKPLTVNVR					Mascot
1980.9192	1980.912	-0.0072	-4	356	373	SHLESSRVAGMTNFDSA R			Oxidation (M)[11]		Mascot
2592.3767	2592.1523	-0.2244	-87	872	894	AELEDDQYISLSSTLEGL VILRK					Mascot
3290.592	3290.5535	-0.0385	-12	408	437	TKDEGMMSTVASLGTLLL WDVENGLDHVDK			Oxidation (M)[6]		Mascot
3417.8887	3417.7549	-0.1338	-39	821	852	MLVTLDEDLKPLTVNVRV GQAVDVVGQAGRPK					Mascot
3433.8835	3433.7078	-0.1757	-51	821	852	MLVTLDEDLKPLTVNVRV GQAVDVVGQAGRPK			Oxidation (M)[1]		Mascot
3476.7415	3476.7288	-0.0127	-4	233	261	VCLYMVSMVNLLTYPDN ETFLRVAHSIYK			Carbamidomethyl (C)[2]		Mascot

3	Schlafen family member 5 OS=Mus musculus GN=Slfn5 PE=2 SV=2	SLFN5_MOUSE	102236.6	6.49	16	47	0	27.482

Peptide Information

Calc. Mass	Obsrv. Mass	± da	± ppm	Start Seq.	End Seq.	Sequence	Ion Score	C. I. %	Modification	Rank	Result Type
820.3981	820.3776	-0.0205	-25	792	797	DIEKCR			Carbamidomethyl (C)[5]		Mascot
945.4676	945.4924	0.0248	26	305	312	APDSWEIK					Mascot
959.5706	959.4997	-0.0709	-74	798	805	DKLLLAMR					Mascot
1138.5232	1138.5187	-0.0045	-4	346	355	SMLNTTPCSK			Carbamidomethyl (C)[8]		Mascot
1281.4908	1281.5505	0.0597	47	29	38	EEMDTTHCMK			Carbamidomethyl (C)[8]		Mascot
1517.6777	1517.7465	0.0688	45	258	269	MPVYHFCGQNHK			Carbamidomethyl (C)[7]		Mascot
1786.9368	1786.8433	-0.0935	-52	808	823	SMSQLAEEPSLLVQVR					Mascot
1851.9092	1851.8918	-0.0174	-9	346	361	SMLNTTPCSKTVFTHK			Carbamidomethyl (C)[8]		Mascot
1900.8721	1900.9346	0.0625	33	297	312	FCCAAFAKAPDSWEIK			Carbamidomethyl (C)[2,3]		Mascot
1966.9327	1966.9016	-0.0311	-16	365	380	CVEDLQKDYFPVSPNR			Carbamidomethyl (C)[1]		Mascot
1966.9327	1966.9016	-0.0311	-16	365	380	CVEDLQKDYFPVSPNR			Carbamidomethyl (C)[1]		Mascot
2683.2935	2683.1504	-0.1431	-53	628	649	TFMKNTFDNVQHIIVDEA QNFR			Oxidation (M)[3]		Mascot
3163.6033	3163.5439	-0.0594	-19	84	110	NHLDEMLYGKLFLIYVSS WDVAASHVR					Mascot
3163.6033	3163.5439	-0.0594	-19	84	110	NHLDEMLYGKLFLIYVSS WDVAASHVR					Mascot
3265.5764	3265.5474	-0.029	-9	328	355	MIEINPDLSSFPQMIPWK SMLNTTPCSK			Carbamidomethyl (C)[26]		Mascot

3401.7021	3401.7397	0.0376	11	317	345	KPLTANDWASRMIEINPD LSSFPQMIPWK			Oxidation (M)[12]		Mascot
3417.697	3417.7549	0.0579	17	317	345	KPLTANDWASRMIEINPD LSSFPQMIPWK			Oxidation (M)[12,25]		Mascot
3435.7124	3435.7095	-0.0029	-1	401	429	NLINMEMRCFSQGILIFSH SWAVDLGLQR			Carbamidomethyl (C)[9]		Mascot
3447.6123	3447.7361	0.1238	36	29	59	EEMDTTHCMKQNEIISQA VCALLNSGGGVVR			Carbamidomethyl (C)[8,20]		Mascot
3447.6123	3447.7361	0.1238	36	29	59	EEMDTTHCMKQNEIISQA VCALLNSGGGVVR			Carbamidomethyl (C)[8,20]		Mascot

4	GFP-like non-fluorescent chromoprotein OS=Montipora efflorescens PE=1 SV=2	NFCP_MONEF	25180.2	7.63	8	45	0	11.173

Peptide Information

Calc. Mass	Obsrv. Mass	± da	± ppm	Start Seq.	End Seq.	Sequence	Ion Score	C. I. %	Modification	Rank	Result Type
1226.5549	1226.5923	0.0374	30	136	145	TQGWEPHSER					Mascot
1263.6328	1263.6095	-0.0233	-18	31	41	GRPYEGEQTVK					Mascot
1272.6327	1272.5884	-0.0443	-35	1	11	MSVIATQMTYK					Mascot
1281.6045	1281.5505	-0.054	-42	185	194	MPGYHYVDRK			Oxidation (M)[1]		Mascot
1884.9823	1884.9646	-0.0177	-9	146	162	LFARGGMLIGNNFMALK			Oxidation (M)[7,14]		Mascot
1902.9055	1902.9875	0.082	43	163	178	LEGGGHYLCEFKTTYK			Carbamidomethyl (C)[9]		Mascot
1902.9055	1902.9875	0.082	43	163	178	LEGGGHYLCEFKTTYK			Carbamidomethyl (C)[9]		Mascot
2592.2358	2592.1523	-0.0835	-32	195	216	LDVTNHNKDYTSVEQCEI SIAR			Carbamidomethyl (C)[16]		Mascot
3374.5378	3374.6655	0.1277	38	1	30	MSVIATQMTYKVYMSGT VNGHYFEVEGDGK			Oxidation (M)[1,8]		Mascot

5	Protein strawberry notch OS=Drosophila melanogaster GN=sno PE=1 SV=1	SBNO_DROME	201426.5	8.3	22	44	0	44.315

Peptide Information

Calc. Mass	Obsrv. Mass	± da	± ppm	Start Seq.	End Seq.	Sequence	Ion Score	C. I. %	Modification	Rank	Result Type
853.405	853.3951	-0.0099	-12	1536	1542	DYNNISK					Mascot
890.371	890.4451	0.0741	83	1086	1093	GSNNNDNR					Mascot
1078.5674	1078.5051	-0.0623	-58	1787	1795	SNSKMQVIR			Oxidation (M)[5]		Mascot
1208.5398	1208.5289	-0.0109	-9	524	534	GNNSMMEAVQK					Mascot
1351.6418	1351.6569	0.0151	11	885	897	GVGAMEIVAMDMK					Mascot
1507.743	1507.702	-0.041	-27	884	897	RGVGAMEIVAMDMK					Mascot
1550.6958	1550.7734	0.0776	50	959	970	TMWGQFWSSHQR					Mascot
1678.7908	1678.7651	-0.0257	-15	958	970	KTMWGQFWSSHQR					Mascot
1786.9156	1786.8433	-0.0723	-40	1561	1575	YFTDTMTAIIQQAKR					Mascot
1851.9923	1851.8918	-0.1005	-54	732	748	DLSDIGATRIEVHALNK					Mascot

Calc. Mass	Obsrv. Mass	± da	± ppm	Start Seq.	End Seq.	Sequence	Ion Score	C. I. %	Modification	Rank	Result Type
1875.9065	1875.8892	-0.0173	-9	468	484	RHKPGCMSYAGGASQIR			Carbamidomethyl (C)[6]		Mascot
1875.9065	1875.8892	-0.0173	-9	468	484	RHKPGCMSYAGGASQIR			Carbamidomethyl (C)[6]		Mascot
1891.9014	1891.9006	-0.0008	0	468	484	RHKPGCMSYAGGASQIR			Carbamidomethyl (C)[6], Oxidation (M)[7]		Mascot
1918.9717	1918.9216	-0.0501	-26	1370	1388	DVAIISEAASSGISLQSDR					Mascot
1941.0818	1940.9358	-0.146	-75	1762	1778	QRLYHVLAGSVLSVWGR					Mascot
1956.0186	1955.9598	-0.0588	-30	764	781	RGVIFSTYSALIGESNNK					Mascot
1966.9943	1966.9016	-0.0927	-47	927	942	KIYDQSVELWVEAMQK					Mascot
1966.9943	1966.9016	-0.0927	-47	927	942	KIYDQSVELWVEAMQK					Mascot
1982.9893	1982.8955	-0.0938	-47	927	942	KIYDQSVELWVEAMQK			Oxidation (M)[14]		Mascot
1982.9893	1982.8955	-0.0938	-47	927	942	KIYDQSVELWVEAMQK			Oxidation (M)[14]		Mascot
1988.9225	1988.8542	-0.0683	-34	959	973	TMWGQFWSSHQRFFK			Oxidation (M)[2]		Mascot
2004.7538	2004.8306	0.0768	38	1144	1160	SDIDDEDEDHDVDSDQR					Mascot
3271.5828	3271.5298	-0.053	-16	928	955	IYDQSVELWVEAMQKFT EAAELIDAESR					Mascot
3278.5874	3278.5632	-0.0242	-7	855	883	NMAYMVRLGLWGQGTA FGNFNDFITAVER					Mascot
3387.6104	3387.6821	0.0717	21	1622	1649	GMIWQEAIDKYADLFNEN EGFYLSHQLR					Mascot
3394.7854	3394.697	-0.0884	-26	1510	1542	DIAGALVGVGIIVNSESHP GVLSLDKDYNNISK					Mascot
3419.6538	3419.7341	0.0803	23	1480	1509	QALETVMRTIMGYESPLV PPPTDYSGEFFK			Oxidation (M)[7]		Mascot
3419.6538	3419.7341	0.0803	23	1480	1509	QALETVMRTIMGYESPLV PPPTDYSGEFFK			Oxidation (M)[7]		Mascot
3435.6487	3435.7095	0.0608	18	1480	1509	QALETVMRTIMGYESPLV PPPTDYSGEFFK			Oxidation (M)[7,11]		Mascot

6	Beta-glucosidase 44 OS=Arabidopsis thaliana GN=BGLU44 PE=2 SV=1	BGL44_ARATH	59117	9.44	10	43	0	2.433

Peptide Information

Calc. Mass	Obsrv. Mass	± da	± ppm	Start Seq.	End Seq.	Sequence	Ion Score	C. I. %	Modification	Rank	Result Type
800.4301	800.4221	-0.008	-10	260	265	KYYQAK					Mascot
1517.7344	1517.7465	0.0121	8	86	98	NATAEITVDQYHR					Mascot
1916.8813	1916.9415	0.0602	31	363	378	DLGYQQDWNVEFGFAK					Mascot
1995.0117	1994.9181	-0.0936	-47	208	226	VVAALGYDNGIFAPGRCS K			Carbamidomethyl (C)[17]		Mascot
2004.8433	2004.8306	-0.0127	-6	181	196	DFADYAEFCYKTFGDR			Carbamidomethyl (C)[9]		Mascot
2683.3855	2683.1504	-0.2351	-88	379	401	LGKPIGPRAYSSWLYNVP WGMYK					Mascot
3233.4839	3233.54	0.0561	17	410	439	YGNPTMILSENGMDDPG NVTLAQGLHDTTR			Oxidation (M)[6]		Mascot
3249.4788	3249.533	0.0542	17	410	439	YGNPTMILSENGMDDPG NVTLAQGLHDTTR			Oxidation (M)[6,13]		Mascot
3249.4788	3249.533	0.0542	17	410	439	YGNPTMILSENGMDDPG NVTLAQGLHDTTR			Oxidation (M)[6,13]		Mascot
3265.7832	3265.5474	-0.2358	-72	2	31	RHLSSPPWPLLLLLLLSS					Mascot

						FTSGESSLSAEK						
3396.8235	3396.6963	-0.1272	-37	1	31	MRHLSSPPWPLLLLLLLS SFTSGESSLSAEK						Mascot
3458.668	3458.6831	0.0151	4	410	441	YGNPTMILSENGMDDPG NVTLAQGLHDTTRIK						Mascot

7 Deoxyribodipyrimidine photo-lyase OS=Methanothermobacter thermautotrophicus (strain ATCC 29096 / DSM 1053 / JCM 10044 / NBRC 100330 / Delta H) GN=phr PE=3 SV=2 | PHR_METTH | 52013.9 | 5.59 | 13 | 43 | 0 | 1.902

Peptide Information

Calc. Mass	Obsrv. Mass	± da	± ppm	Start Seq.	End Seq.	Sequence	Ion Score	C. I. %	Modification	Rank	Result Type
810.3385	810.3385	0	0	352	357	MHGYMR			Oxidation (M)[1]		Mascot
812.4124	812.4256	0.0132	16	358	363	MYWGKK					Mascot
836.4512	836.3863	-0.0649	-78	117	123	GYLDIQK					Mascot
853.3872	853.3951	0.0079	9	232	237	LECFER			Carbamidomethyl (C)[3]		Mascot
859.4744	859.395	-0.0794	-92	79	85	DVRSNLR					Mascot
918.5342	918.4658	-0.0684	-74	172	178	RFMVPLR					Mascot
959.4468	959.4997	0.0529	55	321	327	EYEYSLR					Mascot
1065.5697	1065.5365	-0.0332	-31	173	180	FMVPLRMR			Oxidation (M)[2]		Mascot
1256.6157	1256.5941	-0.0216	-17	155	165	EEYSAGTFKPK					Mascot
1507.6279	1507.702	0.0741	49	352	362	MHGYMRMYWGK			Oxidation (M)[1,5,7]		Mascot
1919.8691	1919.9287	0.0596	31	184	200	MDSLDLEPGPEFEDAVR					Mascot
1948.9321	1948.9728	0.0407	21	268	284	ASEAGECPEFLEELIVR			Carbamidomethyl (C)[7]		Mascot
2683.2935	2683.1504	-0.1431	-53	9	31	NLNGEEPDLRGSYVVYW MQASVR					Mascot

8 Uncharacterized 22.3 kDa protein in LEF3-IAP2 intergenic region OS=Autographa californica nuclear polyhedrosis virus PE=4 SV=1 | Y068_NPVAC | 22376.6 | 9.51 | 7 | 43 | 0 | 11.478

Peptide Information

Calc. Mass	Obsrv. Mass	± da	± ppm	Start Seq.	End Seq.	Sequence	Ion Score	C. I. %	Modification	Rank	Result Type
1138.5925	1138.5187	-0.0738	-65	1	9	MVFIIDTQR			Oxidation (M)[1]		Mascot
1256.6304	1256.5941	-0.0363	-29	125	134	TKTLTMYNER					Mascot
1272.6252	1272.5884	-0.0368	-29	125	134	TKTLTMYNER			Oxidation (M)[6]		Mascot
1902.9644	1902.9875	0.0231	12	49	64	SFGRHIAVVDMWESIR					Mascot
1902.9644	1902.9875	0.0231	12	49	64	SFGRHIAVVDMWESIR					Mascot
1917.0077	1916.9415	-0.0662	-35	86	102	ELIINVASNTPLDNTFR					Mascot
1918.9592	1918.9216	-0.0376	-20	49	64	SFGRHIAVVDMWESIR			Oxidation (M)[11]		Mascot
1921.9403	1921.9122	-0.0281	-15	65	80	WQILNGDEIEVSPEHR					Mascot

Calc. Mass	Obsrv. Mass	± da	± ppm	Start Seq.	End Seq.	Sequence	Ion Score	C. I. %	Modification	Rank	Result Type
3374.6587	3374.6655	0.0068	2	53	80	HIAVVDMWESIRWQILNG DEIEVSPEHR			Oxidation (M)[7]		Mascot
3445.7341	3445.6912	-0.0429	-12	10	39	HFAHQFTTNPFVIFSNLL GATGHSHAFYKR					Mascot

9	Glutamate--tRNA ligase 1 OS=Ehrlichia canis (strain Jake) GN=gltX1 PE=3 SV=1	SYE1_EHRCJ	54288	6.6	11	43	0	4.365

Peptide Information

Calc. Mass	Obsrv. Mass	± da	± ppm	Start Seq.	End Seq.	Sequence	Ion Score	C. I. %	Modification	Rank	Result Type
820.4046	820.3776	-0.027	-33	110	116	EKEESAK					Mascot
959.4905	959.4997	0.0092	10	131	139	SAPNSNITR					Mascot
1226.6569	1226.5923	-0.0646	-53	24	33	TALFNWLYAK					Mascot
1351.7229	1351.6569	-0.066	-49	242	252	RHQAIGIHEYK					Mascot
1517.7972	1517.7465	-0.0507	-33	9	23	FAPSPTGHLHLGGAR					Mascot
1851.9819	1851.8918	-0.0901	-49	441	456	MNAPSIIEIMINLQHK					Mascot
1934.9893	1934.9537	-0.0356	-18	51	67	SSQELIDSIINAMSWLK					Mascot
1941.0262	1940.9358	-0.0904	-47	401	416	ISEKDWNHNILMSIIK					Mascot
1950.9841	1950.9327	-0.0514	-26	51	67	SSQELIDSIINAMSWLK			Oxidation (M)[13]		Mascot
1950.9841	1950.9327	-0.0514	-26	51	67	SSQELIDSIINAMSWLK			Oxidation (M)[13]		Mascot
3233.47	3233.54	0.07	22	84	109	HVEIANQLMLNNKAYYCY CSEEEINK			Carbamidomethyl (C)[17,19]		Mascot
3249.4648	3249.533	0.0682	21	84	109	HVEIANQLMLNNKAYYCY CSEEEINK			Carbamidomethyl (C)[17,19], Oxidation (M)[9]		Mascot
3249.4648	3249.533	0.0682	21	84	109	HVEIANQLMLNNKAYYCY CSEEEINK			Carbamidomethyl (C)[17,19], Oxidation (M)[9]		Mascot
3290.7771	3290.5535	-0.2236	-68	357	385	NLVNLAKESLFYVEDIPISI DQESITVIK					Mascot
3372.7395	3372.6665	-0.073	-22	304	331	LEFLNNHYINITDNETILN MIIPIIEEK			Oxidation (M)[20]		Mascot

10	Formamidopyrimidine-DNA glycosylase OS=Photobacterium profundum GN=mutM PE=3 SV=3	FPG_PHOPR	30132.8	8.78	8	43	0	1.757

Peptide Information

Calc. Mass	Obsrv. Mass	± da	± ppm	Start Seq.	End Seq.	Sequence	Ion Score	C. I. %	Modification	Rank	Result Type
820.406	820.3776	-0.0284	-35	105	110	YNDPRR					Mascot
895.4342	895.4035	-0.0307	-34	156	162	QFIMDNK					Mascot
1925.038	1924.9072	-0.1308	-68	204	221	SVLAFAIEQGGTTLKDFK					Mascot
1935.0334	1934.9537	-0.0797	-41	35	50	WPIPEEIQQIEGQVIR					Mascot
1981.1086	1980.912	-0.1966	-99	12	29	MGITPHMVGGQTVTKIIVR					Mascot
2635.3582	2635.1743	-0.1839	-70	2	25	PELPEVEVSRMGITPHMV GQTVTK					Mascot
2651.3533	2651.157	-0.1963	-74	2	25	PELPEVEVSRMGITPHMV			Oxidation (M)[11]		Mascot

						GQTVTK		
3163.6245	3163.5439	-0.0806	-25	156	184	QFIMDNKVVVGVGNIYAN ESLFSAGIHPK	Oxidation (M)[4]	Mascot
3163.6245	3163.5439	-0.0806	-25	156	184	QFIMDNKVVVGVGNIYAN ESLFSAGIHPK	Oxidation (M)[4]	Mascot
3476.8975	3476.7288	-0.1687	-49	58	89	YLLLHTDVGYAIVHLGMS GSLRILPASIPPEK	Oxidation (M)[17]	Mascot

Gel Idx/Pos	304/M6	Instr./Gel Origin	BA2151/Sample Project 20140814	Process Status	Analysis Succeeded
Plate [#] Name	[1] Sample Project 20140814	Instrument Sample Name		Spectra	11

Rank	Protein Name	Accession No.	Protein MW	Protein PI	Pep. Count	Protein Score	Protein Score C. I. %	Intensity Matched	Total Ion Score	Total Ion C. I. %	Confirmed
1	CAP-Gly domain-containing linker protein 1 OS=Gallus gallus GN=CLIP1 PE=2 SV=1	CLIP1_CHICK	161612.8	5.27	34	66	86.417	35.793			

Peptide Information

Calc. Mass	Obsrv. Mass	± da	± ppm	Start Seq.	End Seq.	Sequence	Ion Score	C. I. %	Modification	Rank	Result Type
905.5203	905.5419	0.0216	24	117	124	GIFTRPSK					Mascot
933.5251	933.4335	-0.0916	-98	1266	1273	TLESDKLK					Mascot
943.4479	943.4584	0.0105	11	1182	1189	DEVTSHQK					Mascot
954.4448	954.4504	0.0056	6	1016	1023	ETSEMITK			Oxidation (M)[5]		Mascot
960.5248	960.4383	-0.0865	-90	689	696	LLEVTEEK					Mascot
960.5248	960.4383	-0.0865	-90	689	696	LLEVTEEK					Mascot
972.4819	972.4381	-0.0438	-45	403	410	HVLEMEAK			Oxidation (M)[5]		Mascot
974.5153	974.4619	-0.0534	-55	697	704	EQTLENLK					Mascot
976.5422	976.4539	-0.0883	-90	493	501	VATVSEKSR					Mascot
988.5057	988.4509	-0.0548	-55	1062	1070	QAEQAKADK					Mascot
998.4512	998.4162	-0.035	-35	265	271	YFQCQPR			Carbamidomethyl (C)[4]		Mascot
1033.5459	1033.5018	-0.0441	-43	500	507	SRIMELER					Mascot
1034.5299	1034.4974	-0.0325	-31	931	938	MNDELRLK			Oxidation (M)[1]		Mascot
1102.5739	1102.5439	-0.03	-27	793	801	QIQNLETEK					Mascot
1112.5194	1112.5409	0.0215	19	109	116	YFQCEPLR			Carbamidomethyl (C)[4]		Mascot
1126.5045	1126.5062	0.0017	2	920	930	SSGDSSAQLMK			Oxidation (M)[10]		Mascot
1135.5413	1135.5653	0.024	21	416	425	AMVEAADREK			Oxidation (M)[2]		Mascot
1141.4865	1141.4979	0.0114	10	988	996	MQDQLTDMK			Oxidation (M)[1,8]		Mascot
1193.5944	1193.5908	-0.0036	-3	580	590	TLSASNERMGK					Mascot
1232.6157	1232.5913	-0.0244	-20	605	614	ENSDVIELWK					Mascot
1235.6519	1235.5559	-0.096	-78	469	478	ELEQSLLFEK					Mascot
1240.6281	1240.6077	-0.0204	-16	671	680	QENEKSQHLK					Mascot
1320.6794	1320.576	-0.1034	-78	638	650	GVGAQTAEFAELK					Mascot
1374.6458	1374.6755	0.0297	22	1013	1023	YEKETSEMITK			Oxidation (M)[8]		Mascot
1490.807	1490.7255	-0.0815	-55	1	14	MSMLKPSGLKAPSK			Oxidation (M)[1]		Mascot
1497.7656	1497.7782	0.0126	8	454	466	GDLETQTKLEHAR					Mascot

1505.72	1505.6194	-0.1006	-67	411	423	MDQLRAMVEAADR					Mascot
1507.7599	1507.7115	-0.0484	-32	194	209	TASGSVSNLSEAGSLK					Mascot
1588.7312	1588.7958	0.0646	41	242	255	GEWCGVELDEPLGK			Carbamidomethyl (C)[4]		Mascot
1635.8549	1635.8245	-0.0304	-19	194	210	TASGSVSNLSEAGSLKK					Mascot
1827.8541	1827.8322	-0.0219	-12	998	1012	QMETSQNQYKDLQAK			Oxidation (M)[2]		Mascot
1827.8541	1827.8322	-0.0219	-12	998	1012	QMETSQNQYKDLQAK			Oxidation (M)[2]		Mascot
1884.8427	1884.8516	0.0089	5	920	936	SSGDSSAQLMKMNDELR			Oxidation (M)[10]		Mascot
2007.0128	2006.9666	-0.0462	-23	1225	1242	SISDTSALITQKDEELEK					Mascot
2040.1084	2040.0092	-0.0992	-49	1154	1172	LAAAQKSQQLAALQEEN VK					Mascot
2586.1228	2586.197	0.0742	29	37	60	APSSEKSSSTTTADAHD DFVDDFR					Mascot

2	Kinesin-like protein KIF22 OS=Danio rerio GN=kif22 PE=2 SV=1	KIF22_DANRE	70777.2	9.6	18	61	52.904	8.15

Peptide Information

Calc. Mass	Obsrv. Mass	± da	± ppm	Start Seq.	End Seq.	Sequence	Ion Score	C. I. %	Modification	Rank	Result Type
905.5124	905.5419	0.0295	33	438	444	KEIQMLK			Oxidation (M)[5]		Mascot
928.3685	928.4445	0.076	82	417	424	MMMGSAER			Oxidation (M)[1]		Mascot
943.5359	943.4584	-0.0775	-82	50	56	LEIINWR					Mascot
960.3583	960.4383	0.08	83	417	424	MMMGSAER			Oxidation (M)[1,2,3]		Mascot
960.3583	960.4383	0.08	83	417	424	MMMGSAER			Oxidation (M)[1,2,3]		Mascot
988.5058	988.4509	-0.0549	-56	5	15	VAVNDGAAGSK					Mascot
1034.5551	1034.4974	-0.0577	-56	439	446	EIQMLKEK			Oxidation (M)[4]		Mascot
1129.5961	1129.5293	-0.0668	-59	283	294	VVDALNTGAGGR					Mascot
1232.6998	1232.5913	-0.1085	-88	129	139	EVFNLVGAQKK					Mascot
1374.6794	1374.6755	-0.0039	-3	37	49	SEGSCVRGLGPQK			Carbamidomethyl (C)[5]		Mascot
1438.6995	1438.7139	0.0144	10	26	36	LRPYMDKQDEK			Oxidation (M)[5]		Mascot
1490.738	1490.7255	-0.0125	-8	1	15	MAQRVAVNDGAAGSK			Oxidation (M)[1]		Mascot
1507.7275	1507.7115	-0.016	-11	598	610	EINGDFTQVEDLK					Mascot
1620.834	1620.7412	-0.0928	-57	246	259	LYLVDLAGSEDNRR					Mascot
1629.8669	1629.8071	-0.0598	-37	620	634	FSSFIKANILSSMGK					Mascot
1635.8225	1635.8245	0.002	1	598	611	EINGDFTQVEDLKK					Mascot
1644.8817	1644.7634	-0.1183	-72	283	298	VVDALNTGAGGRVPYR					Mascot
1657.7897	1657.7754	-0.0143	-9	328	341	YYFDTFSALNFAAK					Mascot
2007.0142	2006.9666	-0.0476	-24	241	258	QQTGKLYLVDLAGSEDN R					Mascot
2340.1216	2340.272	0.1504	64	389	410	AEHSPSPPLHPQSSPDS SVLDR					Mascot

3	Iron sulfur cluster assembly protein 1, mitochondrial OS=Kluyveromyces lactis (strain ATCC 8585 / CBS 2359 / DSM 70799 / NBRC 1267 / NRRL Y-1140 / WM37) GN=ISU1 PE=3 SV=1	ISU1_KLULA	19251.9	9.57	9	55	0	25.967

Peptide Information

Calc. Mass	Obsrv. Mass	± da	± ppm	Start Seq.	End Seq.	Sequence	Ion Score	C. I. %	Modification	Rank	Result Type
932.5047	932.4376	-0.0671	-72	128	136	GKTLEDAAK					Mascot
944.5523	944.4809	-0.0714	-76	137	144	IKNTEIAR					Mascot
952.4669	952.4574	-0.0095	-10	4	11	GQMFAVQR			Oxidation (M)[3]		Mascot
1320.5784	1320.576	-0.0024	-2	12	25	MGSALMHGGGGGTR			Oxidation (M)[1,6]		Mascot
1490.7268	1490.7255	-0.0013	-1	34	49	ATAPTVGSSPSMASAR					Mascot
1786.9025	1786.8097	-0.0928	-52	50	63	FYHPKVIDHYTNPR					Mascot
1827.9348	1827.8322	-0.1026	-56	55	70	VIDHYTNPRNVGSLDK					Mascot
1827.9348	1827.8322	-0.1026	-56	55	70	VIDHYTNPRNVGSLDK					Mascot
2036.9417	2037.0267	0.085	42	109	127	TFGCGSAIASSSYMTELV R			Carbamidomethyl (C)[4]		Mascot
2040.059	2040.0092	-0.0498	-24	12	33	MGSALMHGGGGGTRIIG VAAAR					Mascot

4	UPF0061 protein VV1_0039 OS=Vibrio vulnificus (strain CMCP6) GN=VV1_0039 PE=3 SV=1	Y039_VIBVU	56155.6	5.24	13	54	0	7.237

Peptide Information

Calc. Mass	Obsrv. Mass	± da	± ppm	Start Seq.	End Seq.	Sequence	Ion Score	C. I. %	Modification	Rank	Result Type
905.4839	905.5419	0.058	64	113	121	GAGLTPFSR					Mascot
974.52	974.4619	-0.0581	-60	122	130	MGDGRAVLR					Mascot
1152.6194	1152.5459	-0.0735	-64	329	337	VFSQLMRQK			Oxidation (M)[6]		Mascot
1232.5874	1232.5913	0.0039	3	421	430	CEQMRAVNPK			Carbamidomethyl (C)[1]		Mascot
1302.6471	1302.6364	-0.0107	-8	165	175	EQVEQGACLIR			Carbamidomethyl (C)[8]		Mascot
1592.7803	1592.705	-0.0753	-47	378	390	EDEQTVIDLFIDR					Mascot
1592.7803	1592.705	-0.0753	-47	378	390	EDEQTVIDLFIDR					Mascot
1782.869	1782.8169	-0.0521	-29	400	415	YLERVAMEQTASGEAK					Mascot
1865.8566	1865.7625	-0.0941	-50	76	92	YAGHQFGVYNPDLGDGR					Mascot
1885.0178	1884.8516	-0.1662	-88	445	461	AQQGDFSEVHTLAKLLK					Mascot
1914.8936	1914.9117	0.0181	9	135	151	EYLCSEAMAALGIETTR			Carbamidomethyl (C)[4]		Mascot
1930.8885	1930.8867	-0.0018	-1	135	151	EYLCSEAMAALGIETTR			Carbamidomethyl (C)[4], Oxidation (M)[8]		Mascot
2355.1914	2355.3279	0.1365	58	93	112	GLLLGEMQNQQGQWFDI HLK					Mascot
2372.1584	2372.2554	0.097	41	131	151	STLREYLCSEAMAALGIE TTR			Carbamidomethyl (C)[8]		Mascot

2586.2583	2586.197	-0.0613	-24	378	399	EDEQTVIDLFIDRDAAHGWLSR						Mascot

5 Orotidine 5'-phosphate decarboxylase OS=Pachysolen tannophilus GN=URA3 PE=3 SV=1 | PYRF_PACTA | 29779.4 | 5.69 | 11 | 54 | 0 | 4.78

Peptide Information

Calc. Mass	Obsrv. Mass	± da	± ppm	Start Seq.	End Seq.	Sequence	Ion Score	C. I. %	Modification	Rank	Result Type
909.475	909.4286	-0.0464	-51	23	29	LFELMEK					Mascot
1081.571	1081.5464	-0.0246	-23	22	29	RLFELMEK			Oxidation (M)[6]		Mascot
1126.5964	1126.5062	-0.0902	-80	243	252	GRDPVLQGER					Mascot
1202.5688	1202.6294	0.0606	50	159	169	GSLAYGEYTNK					Mascot
1232.6382	1232.5913	-0.0469	-38	245	254	DPVLQGERYR					Mascot
1438.736	1438.7139	-0.0221	-15	1	13	MAVLDVSYGERAK					Mascot
1497.7697	1497.7782	0.0085	6	96	108	FADIGNTVKNQYK					Mascot
1786.9546	1786.8097	-0.1449	-81	221	237	TVDQVIDSGSDIIIVGR					Mascot
1843.9436	1843.7957	-0.1479	-80	159	175	GSLAYGEYTNKTVEIAK					Mascot
2080.0823	2080.0234	-0.0589	-28	114	133	IAQWADITNAHGVTGEGIVK					Mascot
2372.2742	2372.2554	-0.0188	-8	137	158	EAALETTLEPRGLLMLAELSSK					Mascot

6 Chaperone protein DnaJ 1 OS=Mycobacterium leprae (strain TN) GN=dnaJ1 PE=3 SV=2 | DNAJ1_MYCLE | 41738 | 8.75 | 12 | 54 | 0 | 2.569

Peptide Information

Calc. Mass	Obsrv. Mass	± da	± ppm	Start Seq.	End Seq.	Sequence	Ion Score	C. I. %	Modification	Rank	Result Type
940.437	940.4492	0.0122	13	68	74	KEYDETR					Mascot
968.4432	968.4633	0.0201	21	69	75	EYDETRR					Mascot
974.5377	974.4619	-0.0758	-78	324	332	GTSDGRILR					Mascot
1033.5579	1033.5018	-0.0561	-54	76	85	LFAGGWLGGR					Mascot
1057.5273	1057.4988	-0.0285	-27	244	253	IPSGVEDGQR					Mascot
1240.712	1240.6077	-0.1043	-84	254	265	IRLAGQGEAGLR					Mascot
1768.9592	1768.8688	-0.0904	-51	51	66	FKVVSEAHNVLSDPVK					Mascot
1809.8878	1809.8578	-0.03	-17	367	383	ALEAYAAAERSSGFNPR					Mascot
1810.9447	1810.8842	-0.0605	-33	266	282	GAPSGDLYVTVHVRPDK					Mascot
1930.9043	1930.8867	-0.0176	-9	36	52	YLHPDANPDNSAGERFK					Mascot
1995.9631	1995.965	0.0019	1	33	50	LARYLHPDANPDNSAGER					Mascot
2355.2666	2355.3279	0.0613	26	354	376	VAVPLNLEGAAQKALEAYAAAER					Mascot

7 Peptide chain release factor 1 OS=Cyanothece sp. | RF1_CYAP4 | 41152 | 5.24 | 13 | 53 | 0 | 6.642

(strain PCC 7425 / ATCC 29141) GN=prfA PE=3 SV=1

Peptide Information

Calc. Mass	Obsrv. Mass	± da	± ppm	Start Seq.	End Seq.	Sequence	Ion Score	C. I. %	Modification	Rank	Result Type
904.492	904.5524	0.0604	67	115	121	NIMLEIR			Oxidation (M)[3]		Mascot
954.4349	954.4504	0.0155	16	259	265	IFCTEER			Carbamidomethyl (C)[3]		Mascot
988.4807	988.4509	-0.0298	-30	233	243	SGGAGGQNVNK					Mascot
1081.6113	1081.5464	-0.0649	-60	94	102	SAHLEQRLK					Mascot
1135.5702	1135.5653	-0.0049	-4	300	310	SQVGTGSRSEK					Mascot
1152.6121	1152.5459	-0.0662	-57	58	67	TVQQHLAEAR					Mascot
1270.6863	1270.6686	-0.0177	-14	189	200	VQRVPATEASGR					Mascot
1487.7965	1487.7212	-0.0753	-51	12	23	SVELTFHELTRR					Mascot
1588.7635	1588.7958	0.0323	20	109	121	DPNDDKNIMLEIR			Oxidation (M)[9]		Mascot
1606.8323	1606.801	-0.0313	-19	166	179	EAILEIQGEQVYSK					Mascot
1606.8323	1606.801	-0.0313	-19	166	179	EAILEIQGEQVYSK					Mascot
1620.8928	1620.7412	-0.1516	-94	58	71	TVQQHLAEARQIAR					Mascot
1657.7592	1657.7754	0.0162	10	44	57	ASLEETVDTYDTWK					Mascot
1884.8975	1884.8516	-0.0459	-24	42	57	ARASLEETVDTYDTWK					Mascot

8	Putative sporulation transcription regulator WhiA OS=Pediococcus pentosaceus (strain ATCC 25745 / 183-1w) GN=whiA PE=3 SV=1	WHIA_PEDPA	35520.8	9.27	11	53	0	8.883	12	0

Peptide Information

Calc. Mass	Obsrv. Mass	± da	± ppm	Start Seq.	End Seq.	Sequence	Ion Score	C. I. %	Modification	Rank	Result Type
837.4069	837.4003	-0.0066	-8	212	217	IMRDMR			Oxidation (M)[2]		Mascot
932.5233	932.4376	-0.0857	-92	23	30	AELMALIR			Oxidation (M)[4]		Mascot
942.5295	942.4451	-0.0844	-90	179	186	SGYIVYLK					Mascot
943.5319	943.4584	-0.0735	-78	261	268	LREVAEAR					Mascot
952.5435	952.4574	-0.0861	-90	291	298	SGINHRLR					Mascot
1081.5459	1081.5464	0.0005	0	207	214	FEDVRIMR			Oxidation (M)[7]		Mascot
1193.6749	1193.5908	-0.0841	-70	263	272	EVAEARLIHR					Mascot
1497.7908	1497.7782	-0.0126	-8	10	22	ELTSLEVHEKNAK					Mascot
1606.782	1606.801	0.019	12	130	145	GAFLASGSVNNPETSR					Mascot
1606.782	1606.801	0.019	12	130	145	GAFLASGSVNNPETSR					Mascot
1825.9879	1825.8855	-0.1024	-56	41	56	QLTLNTQTENPAIARR					Mascot
1991.0444	1991.0398	-0.0046	-2	113	129	VPEGLLTEESELRSYLR					Mascot

1991.0444	1991.0398	-0.0046	-2	113	129	VPEGLLTEESELRSYLR	12	0			Mascot

9	Titin OS=Mus musculus GN=Ttn PE=1 SV=1	TITIN_MOUSE	3933362.2	5.91	119	53	0	76.564

Peptide Information

Calc. Mass	Obsrv. Mass	± da	± ppm	Start Seq.	End Seq.	Sequence	Ion Score	C. I. %	Modification	Rank	Result Type
822.4355	822.3881	-0.0474	-58	21945	21952	YGVGEGLK					Mascot
837.377	837.4003	0.0233	28	10081	10086	RMEEEK			Oxidation (M)[2]		Mascot
856.5363	856.5048	-0.0315	-37	24578	24584	RLIGELR					Mascot
870.5659	870.5151	-0.0508	-58	19305	19312	LIEGLVVK					Mascot
904.525	904.5524	0.0274	30	18631	18638	YSGKPKPK					Mascot
905.5203	905.5419	0.0216	24	7948	7955	LRSAPAYK					Mascot
909.4281	909.4286	0.0005	1	29495	29502	MVCSSVAR			Carbamidomethyl (C)[3]		Mascot
928.4231	928.4445	0.0214	23	5997	6005	SVDNGHSGR					Mascot
932.4796	932.4376	-0.042	-45	33897	33903	TEEERLR					Mascot
933.5152	933.4335	-0.0817	-88	33178	33185	IIEFGQAR					Mascot
940.4887	940.4492	-0.0395	-42	14945	14952	GGVEYLFR					Mascot
942.5367	942.4451	-0.0916	-97	4941	4949	DGRPLVASK					Mascot
943.4302	943.4584	0.0282	30	32368	32375	YKMSSDGR					Mascot
944.4108	944.4809	0.0701	74	29513	29519	DSEYQFR					Mascot
952.4734	952.4574	-0.016	-17	24250	24258	KPTYDGGSK					Mascot
954.4051	954.4504	0.0453	47	25850	25857	EEGEFTDK					Mascot
960.4996	960.4383	-0.0613	-64	34620	34628	TAESPEAKK					Mascot
960.4996	960.4383	-0.0613	-64	19104	19111	DLEVSEIR					Mascot
968.4796	968.4633	-0.0163	-17	16572	16580	VENDHGVAK					Mascot
972.4342	972.4381	0.0039	4	34009	34016	SELDYMSK					Mascot
974.5054	974.4619	-0.0435	-45	22335	22343	VSAVNHYGK					Mascot
976.4945	976.4539	-0.0406	-42	414	421	EVKQETDK					Mascot
982.484	982.4176	-0.0664	-68	26563	26571	TDGGQYTLK					Mascot
988.4291	988.4509	0.0218	22	34009	34016	SELDYMSK			Oxidation (M)[6]		Mascot
998.436	998.4162	-0.0198	-20	25300	25307	NSECYVAR			Carbamidomethyl (C)[4]		Mascot
999.4927	999.4207	-0.072	-72	16648	16655	NCKYSVTK			Carbamidomethyl (C)[2]		Mascot
1033.5161	1033.5018	-0.0143	-14	23287	23296	DGKELEGTGK					Mascot
1034.5112	1034.4974	-0.0138	-13	17240	17248	EEVSRSEAK					Mascot
1057.4694	1057.4988	0.0294	28	18062	18069	ECMYTIPK			Carbamidomethyl (C)[2], Oxidation (M)[3]		Mascot
1068.5321	1068.4933	-0.0388	-36	6880	6889	LSDHSVEPGK					Mascot
1078.531	1078.5182	-0.0128	-12	28738	28748	NAMGSASATIR					Mascot

1081.571	1081.5464	-0.0246	-23	10985	10994	MEAPPPKAPK	Oxidation (M)[1]	Mascot
1102.5415	1102.5439	0.0024	2	34280	34289	TEAYAVSSFK		Mascot
1112.5234	1112.5409	0.0175	16	3621	3629	GPCPPYFFK	Carbamidomethyl (C)[3]	Mascot
1126.5562	1126.5062	-0.05	-44	15724	15732	MPVKDTTYR	Oxidation (M)[1]	Mascot
1129.5347	1129.5293	-0.0054	-5	32884	32893	DAGFYVVCAK	Carbamidomethyl (C)[8]	Mascot
1130.6052	1130.5435	-0.0617	-55	13889	13897	LDIEDREIK		Mascot
1130.6052	1130.5435	-0.0617	-55	13889	13897	LDIEDREIK		Mascot
1135.563	1135.5653	0.0023	2	22920	22930	SDSGPYPLTAK		Mascot
1141.5042	1141.4979	-0.0063	-6	3605	3614	STEMESGEKK	Oxidation (M)[4]	Mascot
1145.5797	1145.5424	-0.0373	-33	27615	27624	DGVEIEERAK		Mascot
1152.5929	1152.5459	-0.047	-41	34832	34841	TLSTQMNITK	Oxidation (M)[6]	Mascot
1168.5732	1168.517	-0.0562	-48	31594	31603	DTGEYTLELK		Mascot
1193.5869	1193.5908	0.0039	3	34046	34056	AQRESSSSVSR		Mascot
1202.6165	1202.6294	0.0129	11	16776	16786	FSPPSDPKTAR		Mascot
1232.6382	1232.5913	-0.0469	-38	21316	21325	RETQAVNWTK		Mascot
1235.6783	1235.5559	-0.1224	-99	20845	20854	LIPGQEYIFR		Mascot
1240.6321	1240.6077	-0.0244	-20	29905	29914	LITGNEYQFR		Mascot
1270.6791	1270.6686	-0.0105	-8	2040	2049	TKDELLHWTK		Mascot
1302.71	1302.6364	-0.0736	-56	16120	16129	WMRVNSRPIK	Oxidation (M)[2]	Mascot
1320.6115	1320.576	-0.0355	-27	28112	28122	AWVSVTNNCNR	Carbamidomethyl (C)[9]	Mascot
1323.6726	1323.6266	-0.046	-35	29423	29433	YTVILDNAVCR	Carbamidomethyl (C)[10]	Mascot
1374.7111	1374.6755	-0.0356	-26	9512	9522	LEIEELEKSER		Mascot
1412.6362	1412.6117	-0.0245	-17	14182	14192	FECEISKENEK	Carbamidomethyl (C)[3]	Mascot
1438.7424	1438.7139	-0.0285	-20	12747	12758	KPEEEEPKVEPK		Mascot
1481.7246	1481.7222	-0.0024	-2	33820	33830	WEQFYVMPLPR	Oxidation (M)[7]	Mascot
1487.7278	1487.7212	-0.0066	-4	19851	19862	ANLLANNEYYFR		Mascot
1490.7446	1490.7255	-0.0191	-13	3977	3990	STGQDVALRTEEGK		Mascot
1497.7948	1497.7782	-0.0166	-11	25211	25225	EPGPPGTPFVTAISK		Mascot
1505.5884	1505.6194	0.031	21	13518	13530	GEYVCDCGTDTTK	Carbamidomethyl (C)[5,7]	Mascot
1507.6482	1507.7115	0.0633	42	17719	17731	LSWKMPDDDGGDR	Oxidation (M)[5]	Mascot
1535.7563	1535.7308	-0.0255	-17	31387	31399	ISGLVEGTMYYFR		Mascot
1574.7849	1574.7878	0.0029	2	25965	25977	VENLTEGAIYYFR		Mascot
1578.7793	1578.7988	0.0195	12	7637	7651	DTSATLGASVVLECR	Carbamidomethyl (C)[14]	Mascot
1590.707	1590.8219	0.1149	72	10812	10823	EEYVHEEEYVHK		Mascot
1592.7512	1592.705	-0.0462	-29	34004	34016	LSEYKSELDYMSK		Mascot
1592.7949	1592.705	-0.0899	-56	5826	5840	NDAGTASCSIELKVK	Carbamidomethyl (C)[8]	Mascot

1604.8378	1604.8212	-0.0166	-10	9912	9926	ASEIIDVSSKAEEVK		Mascot
1605.7611	1605.7703	0.0092	6	7240	7253	DMCSAQLSVKEPPK	Carbamidomethyl (C)[3], Oxidation (M)[2]	Mascot
1606.7571	1606.801	0.0439	27	2107	2119	VVGKPDPECEWYK	Carbamidomethyl (C)[9]	Mascot
1606.7571	1606.801	0.0439	27	2107	2119	VVGKPDPECEWYK	Carbamidomethyl (C)[9]	Mascot
1607.8025	1607.8101	0.0076	5	20724	20738	GDDEVVTSSHLAIHK		Mascot
1620.8051	1620.7412	-0.0639	-39	31188	31201	NNEFTVPDLKMGQK		Mascot
1625.7628	1625.6965	-0.0663	-41	18862	18874	VPDLLEGCQYEFR	Carbamidomethyl (C)[8]	Mascot
1628.7704	1628.797	0.0266	16	14461	14473	WFRNEEAIFDSSK		Mascot
1629.8152	1629.8071	-0.0081	-5	26891	26904	MEAPEIELDADLRK		Mascot
1635.8589	1635.8245	-0.0344	-21	15234	15248	TISAYAELVISPSER		Mascot
1644.8414	1644.7634	-0.078	-47	16418	16431	CNEHLVPVLTYTAK	Carbamidomethyl (C)[1]	Mascot
1646.8418	1646.7828	-0.059	-36	7586	7600	NLASLKIPSAEMNDK	Oxidation (M)[12]	Mascot
1657.8391	1657.7754	-0.0637	-38	34817	34831	TSQASEEVKTHAEIK		Mascot
1736.8451	1736.8196	-0.0255	-15	25084	25100	DDGGQYGITVSNVVGQK		Mascot
1736.9065	1736.8196	-0.0869	-50	18501	18515	DPITVPERPEDLEVK		Mascot
1768.8964	1768.8688	-0.0276	-16	31589	31603	ETTIRDTGEYTLELK		Mascot
1782.8868	1782.8169	-0.0699	-39	19365	19381	KDTGEYQLTVSNAAGTK		Mascot
1786.868	1786.8097	-0.0583	-33	22629	22644	YDTGKFVMTIENPAGK	Oxidation (M)[8]	Mascot
1809.8575	1809.8578	0.0003	0	17913	17928	EFMEVEEGTDVNIVAK		Mascot
1811.0208	1810.8842	-0.1366	-75	2357	2372	MKPRPIAILQGLSDQK	Oxidation (M)[1]	Mascot
1818.8909	1818.8171	-0.0738	-41	24189	24203	FKTTGLEEGIEYEFR		Mascot
1825.8749	1825.8855	0.0106	6	4150	4164	NILMAEDPRIHEEDK	Oxidation (M)[4]	Mascot
1827.0276	1826.8615	-0.1661	-91	27203	27218	IDIPFKGRPQATVAWK		Mascot
1827.9236	1827.8322	-0.0914	-50	30502	30518	GDSGRYFLTLENTAGVK		Mascot
1827.9698	1827.8322	-0.1376	-75	185	202	ATSTAELVVQGEEVVPAK		Mascot
1838.8987	1838.9059	0.0072	4	8576	8592	LKETNGLSGSSVVMECK	Carbamidomethyl (C)[16]	Mascot
1841.9103	1841.8361	-0.0742	-40	329	345	TQTTTMATGPEVPPPWK		Mascot
1843.8457	1843.7957	-0.05	-27	5532	5547	KDSGEYTFEVQNDVGR		Mascot
1849.9403	1849.7875	-0.1528	-83	28399	28415	RETSTAVWSEVSATVAR		Mascot
1855.947	1855.8387	-0.1083	-58	20388	20403	EILEPPEIDLDASMRK		Mascot
1865.8552	1865.7625	-0.0927	-50	18639	18653	IDWFKDEADVLEDDR		Mascot
1884.8835	1884.8516	-0.0319	-17	23852	23868	NSVSLSWEKPEHDGGSR		Mascot
1885.9338	1885.8545	-0.0793	-42	9611	9627	DQGNYRLVCGPHIASAK	Carbamidomethyl (C)[9]	Mascot
1892.9501	1892.8965	-0.0536	-28	21929	21944	LHEGIEYTFRVSAENK		Mascot
1893.0117	1892.8965	-0.1152	-61	13542	13558	VEKPLYGVEVFVGETAR		Mascot
1908.9385	1908.8068	-0.1317	-69	19650	19665	QCAFRVPGLIEGNEYR	Carbamidomethyl (C)[2]	Mascot
1914.9841	1914.9117	-0.0724	-38	13822	13838	DEVILQCEISKADAPVK	Carbamidomethyl (C)[7]	Mascot

Calc. Mass	Obsrv. Mass	± da	± ppm	Start Seq.	End Seq.	Sequence	Ion Score	C. I. %	Modification	Rank	Result Type
1930.9076	1930.8867	-0.0209	-11	27340	27355	LTTGSEYQFRVCAENR			Carbamidomethyl (C)[12]		Mascot
1980.8967	1980.9403	0.0436	22	14442	14458	EQQEVVFNCEVNTEGAK			Carbamidomethyl (C)[9]		Mascot
1990.0638	1989.9581	-0.1057	-53	3967	3985	ILMASADTLKSTGQDVALR					Mascot
1991.0671	1991.0398	-0.0273	-14	4458	4474	FLCTVSGTPVIEIIWQK			Carbamidomethyl (C)[3]		Mascot
1991.0671	1991.0398	-0.0273	-14	4458	4474	FLCTVSGTPVIEIIWQK			Carbamidomethyl (C)[3]		Mascot
1991.1899	1991.3247	0.1348	68	21854	21873	EGTLSIKVVGKPGIPTGPIK					Mascot
1996.1115	1995.965	-0.1465	-73	28850	28868	NAFVTPGPPSIPEVTKITK					Mascot
2006.9753	2006.9666	-0.0087	-4	21241	21256	LIEGHEYQFRICAENK			Carbamidomethyl (C)[12]		Mascot
2037.0739	2037.0267	-0.0472	-23	3264	3280	FCAVISGRPQPKISWYK			Carbamidomethyl (C)[2]		Mascot
2038.9386	2038.9896	0.051	25	7851	7868	NEYPLSQSPNCGITTTEK			Carbamidomethyl (C)[11]		Mascot
2039.9669	2040.0092	0.0423	21	20619	20635	IDGYIISYREEDQPADR					Mascot
2055.0547	2054.9504	-0.1043	-51	13559	13575	FEIELSEPDVHGQWKLK					Mascot
2055.0547	2054.9504	-0.1043	-51	13559	13575	FEIELSEPDVHGQWKLK					Mascot
2071.0818	2070.9636	-0.1182	-57	23718	23736	YILTVENSSGSKSAFVNVR					Mascot
2079.9905	2080.0234	0.0329	16	25978	25997	VMAENEFGVGVPTETSDAVK					Mascot
2119.0989	2118.9736	-0.1253	-59	23979	23997	QTTRVNAESTENNSLLTIK					Mascot
2372.2166	2372.2554	0.0388	16	1143	1163	LVISMTFADDAGEYTIVIRNK			Oxidation (M)[5]		Mascot
2586.3562	2586.197	-0.1592	-62	30691	30714	VTGLVEGLEYQFRTYALNAAGVSK					Mascot
2717.2512	2717.0793	-0.1719	-63	1234	1255	DTVMVRTFVEDQEFHISSFEER			Oxidation (M)[4]		Mascot
3312.5842	3312.3125	-0.2717	-82	30311	30340	VSAENEYGVGEPYEMPEPIVATEQPAPPRR					Mascot

10	Cone cGMP-specific 3',5'-cyclic phosphodiesterase subunit alpha' OS=Homo sapiens GN=PDE6C PE=1 SV=2	PDE6C_HUMAN	99767.4	5.5	20	53	0	10.888

Peptide Information

Calc. Mass	Obsrv. Mass	± da	± ppm	Start Seq.	End Seq.	Sequence	Ion Score	C. I. %	Modification	Rank	Result Type
904.4669	904.5524	0.0855	95	442	448	MNKLENR					Mascot
909.4788	909.4286	-0.0502	-55	152	159	THNVPDVK					Mascot
942.5003	942.4451	-0.0552	-59	105	113	NGIPEVASR					Mascot
999.5081	999.4207	-0.0874	-87	548	554	WMYTVRK			Oxidation (M)[2]		Mascot
1033.5929	1033.5018	-0.0911	-88	30	38	VEVLGEIFK					Mascot
1068.5143	1068.4933	-0.021	-20	608	616	GTNNLYQMK					Mascot
1193.5984	1193.5908	-0.0076	-6	240	249	SQILMWSANK			Oxidation (M)[5]		Mascot
1202.6814	1202.6294	-0.052	-43	189	199	EVLAVIMAVNK			Oxidation (M)[7]		Mascot

1232.5333	1232.5913	0.058	47	96	104	CSMFLCRSR	Carbamidomethyl (C)[1,6], Oxidation (M)[3]	Mascot
1270.5984	1270.6686	0.0702	55	814	824	SLADEYDAKMK		Mascot
1588.7822	1588.7958	0.0136	9	754	766	TVLQQQPIPMMDR	Oxidation (M)[10,11]	Mascot
1606.9163	1606.801	-0.1153	-72	489	502	QLVAILKEDLPDPR		Mascot
1606.9163	1606.801	-0.1153	-72	489	502	QLVAILKEDLPDPR		Mascot
1644.8163	1644.7634	-0.0529	-32	264	276	ALYTVRSYLNCER	Carbamidomethyl (C)[11]	Mascot
1843.8708	1843.7957	-0.0751	-41	200	215	VNASEFSKQDEEVFSK		Mascot
1884.9749	1884.8516	-0.1233	-65	794	809	FHKEITPMLSGLQNNR		Mascot
1980.922	1980.9403	0.0183	9	473	488	FQEKLNVDVIDDCEEK	Carbamidomethyl (C)[13]	Mascot
1990.0215	1989.9581	-0.0634	-32	511	527	FSDFPLTEHGLIKCGIR	Carbamidomethyl (C)[14]	Mascot
1990.9773	1991.0398	0.0625	31	87	102	LAHLLQADRCSMFLCR	Carbamidomethyl (C)[10,15]	Mascot
1990.9773	1991.0398	0.0625	31	87	102	LAHLLQADRCSMFLCR	Carbamidomethyl (C)[10,15]	Mascot
2006.9722	2006.9666	-0.0056	-3	87	102	LAHLLQADRCSMFLCR	Carbamidomethyl (C)[10,15], Oxidation (M)[12]	Mascot
2080.1589	2080.0234	-0.1355	-65	133	151	EVVFPLDIGIVGWAAHTK K		Mascot
2355.1843	2355.3279	0.1436	61	775	793	LQVGFIDFVCTFVYKEFS R	Carbamidomethyl (C)[10]	Mascot

Rank	Protein Name	Accession No.	Protein MW	Protein PI	Pep. Count	Protein Score	Protein Score C. I. %	Intensity Matched	Total Ion Score	Total Ion C. I. %	Confirmed
1	GRIP and coiled-coil domain-containing protein 2 OS=Mus musculus GN=Gcc2 PE=1 SV=2	GCC2_MOUSE	195294.5	5.07	34	76	98.732	16.788			

Peptide Information

Calc. Mass	Obsrv. Mass	± da	± ppm	Start Seq.	End Seq.	Sequence	Ion Score	C. I. %	Modification	Rank	Result Type
944.4908	944.5034	0.0126	13	1598	1604	LERNQER					Mascot
956.4544	956.4509	-0.0035	-4	1274	1281	TSAEQHQR					Mascot
979.4268	979.4794	0.0526	54	369	375	DEFFHER					Mascot
1008.5221	1008.4695	-0.0526	-52	1432	1439	TSFRDQVR					Mascot
1034.5001	1034.498	-0.0021	-2	103	111	QEVEDSVTK					Mascot
1057.4368	1057.4866	0.0498	47	1047	1054	NSTCQYER			Carbamidomethyl (C)[4]		Mascot
1112.5293	1112.5459	0.0166	15	496	504	ISQEFETMK					Mascot
1129.6324	1129.5466	-0.0858	-76	615	623	EQRLSVELR					Mascot
1145.6348	1145.5505	-0.0843	-74	721	729	KLQLMVEER					Mascot
1168.6433	1168.5311	-0.1122	-96	689	698	LHSEKAQLSR					Mascot
1300.7219	1300.6162	-0.1057	-81	1290	1301	VVALQEESRAAK					Mascot
1384.7141	1384.6962	-0.0179	-13	1343	1353	EHLEMLIDQLK			Oxidation (M)[5]		Mascot
1481.7091	1481.7145	0.0054	4	624	637	EAAGQAAQHNSELR					Mascot
1507.7244	1507.7048	-0.0196	-13	85	96	AETEQQCLCLKK			Carbamidomethyl (C)[7,9]		Mascot
1519.8552	1519.7643	-0.0909	-60	446	458	EKLALMFEIQGLK					Mascot
1535.8502	1535.7336	-0.1166	-76	446	458	EKLALMFEIQGLK			Oxidation (M)[6]		Mascot
1587.8774	1587.7635	-0.1139	-72	1128	1140	QLQKTMQELELVK					Mascot
1603.7698	1603.7729	0.0031	2	759	772	ASILEENEEEDVVK					Mascot
1606.7993	1606.8026	0.0033	2	1576	1589	ETEATNAILMEQIK			Oxidation (M)[10]		Mascot
1606.7993	1606.8026	0.0033	2	1576	1589	ETEATNAILMEQIK			Oxidation (M)[10]		Mascot
1607.7911	1607.8123	0.0212	13	1242	1255	GELEASQQQVEVYK					Mascot
1634.7616	1634.7471	-0.0145	-9	167	180	EQLKSQSDSEDNVR					Mascot
1635.8007	1635.8202	0.0195	12	653	666	EKSQNDQSITVQMK					Mascot
1693.8081	1693.7559	-0.0522	-31	1665	1679	SSGWASYLHSWSGLR					Mascot
1694.8153	1694.7759	-0.0394	-23	249	263	EEVTELMSQIETSAK					Mascot
1706.8708	1706.7218	-0.149	-87	520	534	SAFNEKDALLETVNR					Mascot

Calc. Mass	Obsrv. Mass	± da	± ppm	Start Seq.	End Seq.	Sequence	Ion Score	C. I. %	Modification	Rank	Result Type
1736.7578	1736.8229	0.0651	37	808	821	CLQEDSAVQCEELR			Carbamidomethyl (C)[1,10]		Mascot
1736.7578	1736.8229	0.0651	37	808	821	CLQEDSAVQCEELR			Carbamidomethyl (C)[1,10]		Mascot
1765.8688	1765.9045	0.0357	20	624	639	EAAGQAAQHNSELRQR					Mascot
1768.8534	1768.8822	0.0288	16	558	573	NLQADNSMYLASLGQK			Oxidation (M)[8]		Mascot
1781.8296	1781.8245	-0.0051	-3	480	495	EMMEILQTELGESAGK			Oxidation (M)[2]		Mascot
1825.998	1825.8992	-0.0988	-54	1633	1648	LLPVIDTMLQLSPEEK					Mascot
1827.011	1826.8519	-0.1591	-87	542	557	LLSQQELVPELESTIK					Mascot
1841.9929	1841.821	-0.1719	-93	1633	1648	LLPVIDTMLQLSPEEK			Oxidation (M)[8]		Mascot
1888.9685	1888.8826	-0.0859	-45	846	861	EALQLDLLEMKNTNEK					Mascot
1892.927	1892.8944	-0.0326	-17	1167	1182	LTNKNSTIEDLEQEMK					Mascot
1904.9634	1904.8815	-0.0819	-43	846	861	EALQLDLLEMKNTNEK			Oxidation (M)[10]		Mascot
1945.9171	1945.9729	0.0558	29	1	20	MEDSAPDAVAAAPSGTP KSK			Oxidation (M)[1]		Mascot
2252.2861	2252.085	-0.2011	-89	1066	1085	IETLQANAKLLEAQILEVQ K					Mascot
2252.2861	2252.085	-0.2011	-89	1066	1085	IETLQANAKLLEAQILEVQ K					Mascot

2	Elongation factor Ts OS=Hahella chejuensis (strain KCTC 2396) GN=tsf PE=3 SV=1	EFTS_HAHCH	30625.8	4.94	13	64	77.972	5.873

Peptide Information

Calc. Mass	Obsrv. Mass	± da	± ppm	Start Seq.	End Seq.	Sequence	Ion Score	C. I. %	Modification	Rank	Result Type
837.4464	837.4064	-0.04	-48	198	203	EREIYK					Mascot
944.5411	944.5034	-0.0377	-40	56	65	VSADGVVAVK					Mascot
1008.5216	1008.4695	-0.0521	-52	1	10	MAAISASMVK					Mascot
1129.6511	1129.5466	-0.1045	-93	252	262	KAGAQVVAMVR					Mascot
1145.646	1145.5505	-0.0955	-83	252	262	KAGAQVVAMVR			Oxidation (M)[9]		Mascot
1438.7393	1438.6042	-0.1351	-94	1	13	MAAISASMVKELR			Oxidation (M)[1,8]		Mascot
1519.7639	1519.7643	0.0004	0	272	285	EEVDFAAEVAAQLK					Mascot
1607.9043	1607.8123	-0.092	-57	227	240	FLAEISLLEQAFVK					Mascot
1677.8364	1677.8026	-0.0338	-20	106	121	KSADVAALMESGLEEK					Mascot
1693.8313	1693.7559	-0.0754	-45	106	121	KSADVAALMESGLEEK			Oxidation (M)[9]		Mascot
1705.8425	1705.7797	-0.0628	-37	107	122	SADVAALMESGLEEKR					Mascot
1768.9164	1768.8822	-0.0342	-19	172	187	DIAMHIAAVNPQFVSR					Mascot
1892.892	1892.8944	0.0024	1	138	155	ASMLSSDVVGAYVHGNN R			Oxidation (M)[3]		Mascot
2048.9929	2049.0408	0.0479	23	137	155	RASMLSSDVVGAYVHGN NR			Oxidation (M)[4]		Mascot
2131.0449	2131.0413	-0.0036	-2	204	223	AQADQSGKPAEIVDKMV DGR			Oxidation (M)[16]		Mascot

3	50S ribosomal protein L1 OS=Borrelia turicatae (strain	RL1_BORT9	24919.2	9.52	12	63	72.899	7.952

91E135) GN=rplA PE=3 SV=1

Peptide Information

Calc. Mass	Obsrv. Mass	± da	± ppm	Start Seq.	End Seq.	Sequence	Ion Score	C. I. %	Modification	Rank	Result Type
1034.563	1034.498	-0.065	-63	165	174	NGVINFSVGK					Mascot
1057.4836	1057.4866	0.003	3	184	191	ENYDEFIK					Mascot
1347.7379	1347.6158	-0.1221	-91	162	174	ANKNGVINFSVGK					Mascot
1578.7646	1578.7942	0.0296	19	86	100	EAGAVYVGDDDLINK					Mascot
1592.8102	1592.7111	-0.0991	-62	58	70	DTVVLPNQFMREK			Oxidation (M)[10]		Mascot
1606.8323	1606.8026	-0.0297	-18	38	51	FDETIDVSVNLNLK					Mascot
1606.8323	1606.8026	-0.0297	-18	38	51	FDETIDVSVNLNLK					Mascot
1694.9211	1694.7759	-0.1452	-86	20	34	SYSIDDAISLLKEIK					Mascot
1770.9419	1770.8192	-0.1227	-69	132	147	GLMPNPKTQTITNDLK					Mascot
1786.9368	1786.8242	-0.1126	-63	132	147	GLMPNPKTQTITNDLK			Oxidation (M)[3]		Mascot
1810.8752	1810.8756	0.0004	0	165	181	NGVINFSVGKSSMDNNK					Mascot
1815.8292	1815.8209	-0.0083	-5	103	119	GGFSDFDVVVATPDMMK					Mascot
1826.8701	1826.8519	-0.0182	-10	165	181	NGVINFSVGKSSMDNNK			Oxidation (M)[13]		Mascot
1892.877	1892.8944	0.0174	9	203	220	GTFVDSVYMSSTMGPSV K					Mascot
2042.9927	2042.972	-0.0207	-10	101	119	VKGGFSDFDVVVATPDM MK					Mascot
2058.9875	2058.9429	-0.0446	-22	101	119	VKGGFSDFDVVVATPDM MK			Oxidation (M)[17]		Mascot

4 Chromosome partition protein MukB OS=Haemophilus influenzae (strain ATCC 51907 / DSM 11121 / KW20 / Rd) GN=mukB PE=3 SV=1 MUKB_HAEIN 173749.5 5.06 27 63 72.268 30.478

Peptide Information

Calc. Mass	Obsrv. Mass	± da	± ppm	Start Seq.	End Seq.	Sequence	Ion Score	C. I. %	Modification	Rank	Result Type
846.4501	846.407	-0.0431	-51	715	721	LNMLAER					Mascot
870.5155	870.5077	-0.0078	-9	1218	1224	KPENKVR					Mascot
944.4982	944.5034	0.0052	6	1433	1440	GKDIVPCR			Carbamidomethyl (C)[7]		Mascot
1078.531	1078.5234	-0.0076	-7	680	687	ERELTMQR			Oxidation (M)[6]		Mascot
1167.5463	1167.5629	0.0166	14	271	280	AFQDMESALR					Mascot
1507.7784	1507.7048	-0.0736	-49	284	296	MTLEAIKVTQSDR			Oxidation (M)[1]		Mascot
1519.7281	1519.7643	0.0362	24	1032	1043	LEQMQAQRDTQR			Oxidation (M)[4]		Mascot
1578.854	1578.7942	-0.0598	-38	1225	1236	FFIAVYQHLRER					Mascot
1622.8132	1622.7886	-0.0246	-15	1198	1211	TAVADNEYLRDSLR					Mascot

1634.8359	1634.7471	-0.0888	-54	813	826	FPEIPLFGCAAREK			Carbamidomethyl (C)[9]		Mascot
1635.8346	1635.8202	-0.0144	-9	518	531	AYQLVCKIAGEMPR			Carbamidomethyl (C)[6]		Mascot
1644.9067	1644.7655	-0.1412	-86	256	269	SLRDYLLPENLGVR					Mascot
1645.9271	1645.7777	-0.1494	-91	453	467	ALQYQQAIAALEKAK					Mascot
1736.8813	1736.8229	-0.0584	-34	390	403	ITRYQEDIAELTER					Mascot
1736.8813	1736.8229	-0.0584	-34	390	403	ITRYQEDIAELTER					Mascot
1765.9304	1765.9045	-0.0259	-15	340	354	AEQNLSQHRLVDLSR					Mascot
1768.8389	1768.8822	0.0433	24	941	954	EQLDIAEQDEYFIR					Mascot
1783.8949	1783.8257	-0.0692	-39	810	824	YSRFPEIPLFGCAAR			Carbamidomethyl (C)[12]		Mascot
1815.9447	1815.8209	-0.1238	-68	349	364	LVDLSREVAELAESER					Mascot
1827.8793	1827.8257	-0.0536	-29	1176	1191	ELAYLSADELRSMSDK					Mascot
1827.8872	1827.8257	-0.0615	-34	836	851	DEVAEQHAQIAFDVQK					Mascot
1833.964	1833.8317	-0.1323	-72	122	137	LRPGVCYAVLDTINSR			Carbamidomethyl (C)[6]		Mascot
1841.981	1841.821	-0.16	-87	48	62	FRSLTLINWNGFFAR					Mascot
1843.8743	1843.7876	-0.0867	-47	1176	1191	ELAYLSADELRSMSDK			Oxidation (M)[13]		Mascot
1843.8743	1843.7876	-0.0867	-47	1176	1191	ELAYLSADELRSMSDK			Oxidation (M)[13]		Mascot
1915.0032	1914.9091	-0.0941	-49	544	559	EYPSQKLQAQQTPQLR					Mascot
1927.9984	1927.9122	-0.0862	-45	562	577	LHELEQRYAQQQSAVK					Mascot
1946.0641	1945.9729	-0.0912	-47	1294	1310	IRMLNQGLQNIAFGQVK			Oxidation (M)[3]		Mascot
1990.9274	1990.9972	0.0698	35	1244	1260	TDDPIDAIEQMEIELSR			Oxidation (M)[11]		Mascot
2003.0856	2003.072	-0.0136	-7	1296	1313	MLNQGLQNIAFGQVKSVR					Mascot

5	DNA polymerase epsilon catalytic subunit A OS=Arabidopsis thaliana GN=POL2A PE=1 SV=1	DPOE1_ARATH	251873.9	6.19	33	62	65.882	12.985	

Peptide Information

Calc. Mass	Obsrv. Mass	± da	± ppm	Start Seq.	End Seq.	Sequence	Ion Score	C. I. %	Modification	Rank	Result Type
837.4135	837.4064	-0.0071	-8	1022	1029	SCAVTTAK			Carbamidomethyl (C)[2]		Mascot
846.4753	846.407	-0.0683	-81	910	917	AMIIPASK			Oxidation (M)[2]		Mascot
870.4719	870.5077	0.0358	41	1331	1337	VFYINSK					Mascot
960.4608	960.441	-0.0198	-21	698	704	YCQKAYK			Carbamidomethyl (C)[2]		Mascot
1129.5269	1129.5466	0.0197	17	2133	2141	MEIFMDIAK			Oxidation (M)[1,5]		Mascot
1152.5354	1152.5452	0.0098	9	1434	1442	TTTECHYLK			Carbamidomethyl (C)[5]		Mascot
1195.5994	1195.5596	-0.0398	-33	1338	1347	VPIDEYFQGK					Mascot
1195.5994	1195.5596	-0.0398	-33	1338	1347	VPIDEYFQGK					Mascot
1300.5726	1300.6162	0.0436	34	562	572	MDSISNYDEVK					Mascot
1438.7358	1438.6042	-0.1316	-91	98	108	MELELEAYLRR			Oxidation (M)[1]		Mascot

1519.7573	1519.7643	0.007	5	1556	1568	EGIKALDDFPCVR			Carbamidomethyl (C)[11]		Mascot
1587.7141	1587.7635	0.0494	31	560	572	MRMDSISNYDEVK					Mascot
1603.7091	1603.7729	0.0638	40	560	572	MRMDSISNYDEVK			Oxidation (M)[1]		Mascot
1607.8826	1607.8123	-0.0703	-44	1939	1952	EQIGSTFINMLVKK					Mascot
1628.9292	1628.8	-0.1292	-79	910	924	AMIIPASKEEGILIK			Oxidation (M)[2]		Mascot
1629.802	1629.8051	0.0031	2	723	735	ENPFYVDTVRSFR					Mascot
1634.8822	1634.7471	-0.1351	-83	243	256	VCAFDIETTKLPLK			Carbamidomethyl (C)[2]		Mascot
1693.8942	1693.7559	-0.1383	-82	141	154	ISFDTVQQLMEVKR					Mascot
1735.8466	1735.8351	-0.0115	-7	2075	2088	EQMESSLLEMVRQR					Mascot
1736.803	1736.8229	0.0199	11	2089	2101	ERMYHMQDVVCIR			Carbamidomethyl (C)[11]		Mascot
1736.803	1736.8229	0.0199	11	2089	2101	ERMYHMQDVVCIR			Carbamidomethyl (C)[11]		Mascot
1768.7928	1768.8822	0.0894	51	2089	2101	ERMYHMQDVVCIR			Carbamidomethyl (C)[11], Oxidation (M)[3,6]		Mascot
1783.865	1783.8257	-0.0393	-22	45	59	LGWLLTFSSSSWEDR					Mascot
1785.8212	1785.843	0.0218	12	562	576	MDSISNYDEVKDEIK					Mascot
1809.8186	1809.8734	0.0548	30	799	814	WYSMEMAGVVTYTGAK			Oxidation (M)[4]		Mascot
1817.8483	1817.8241	-0.0242	-13	469	484	GSGTLCEMLLMVEAYK			Carbamidomethyl (C)[6], Oxidation (M)[8]		Mascot
1822.9442	1822.9509	0.0067	4	859	874	LTISYPCVMLNVDVAK			Carbamidomethyl (C)[7]		Mascot
1825.8636	1825.8992	0.0356	19	661	677	QIESEFVDAGANIMSSK					Mascot
1826.976	1826.8519	-0.1241	-68	1443	1458	RSIPLVYLYNSTSTGR					Mascot
1833.8431	1833.8317	-0.0114	-6	469	484	GSGTLCEMLLMVEAYK			Carbamidomethyl (C)[6], Oxidation (M)[8,11]		Mascot
1841.8586	1841.821	-0.0376	-20	661	677	QIESEFVDAGANIMSSK			Oxidation (M)[14]		Mascot
1843.8967	1843.7876	-0.1091	-59	182	196	EQRPQDCLDSIVDLR			Carbamidomethyl (C)[7]		Mascot
1843.8967	1843.7876	-0.1091	-59	182	196	EQRPQDCLDSIVDLR			Carbamidomethyl (C)[7]		Mascot
1855.9094	1855.8334	-0.076	-41	1590	1604	IAMFRCAAAFQWLDR			Carbamidomethyl (C)[6]		Mascot
1888.7953	1888.8826	0.0873	46	2060	2074	EWSCADTQCGKIYDR			Carbamidomethyl (C)[4,9]		Mascot
1904.9462	1904.8815	-0.0647	-34	875	890	NNTNDQYQTLVDPVRK					Mascot
1945.9689	1945.9729	0.004	2	403	419	AKLGYDPLEVNPEDMVR					Mascot
1961.9637	1961.9678	0.0041	2	403	419	AKLGYDPLEVNPEDMVR			Oxidation (M)[15]		Mascot
2003.0413	2003.072	0.0307	15	706	722	VLDKPITEVREAGICMR			Carbamidomethyl (C)[15], Oxidation (M)[16]		Mascot
2077.9834	2078.0337	0.0503	24	796	814	GARWYSMEMAGVVTYT GAK					Mascot
2108.0195	2108.0623	0.0428	20	197	213	EYDVPYHVRFAIDNDVR					Mascot

6	Uncharacterized protein M1249L OS=African swine fever virus (isolate Warthog/Namibia/Wart80/1980) GN=War-070 PE=3 SV=1	VF249_ASFWA	145470.2	5.6	25	62	62.59	49.815

Peptide Information

Calc. Mass	Obsrv. Mass	± da	± ppm	Start Seq.	End Seq.	Sequence	Ion Score	C. I. %	Modification	Rank	Result Type

Calc. Mass	Obsrv. Mass	± da	± ppm	Start Seq.	End Seq.	Sequence	Ion Score	C. I. %	Modification	Rank	Result Type
956.52	956.4509	-0.0691	-72	836	842	TRLFEYK					Mascot
960.4785	960.441	-0.0375	-39	392	398	QWELQEK					Mascot
1112.5881	1112.5459	-0.0422	-38	222	230	LAEEMVHRK					Mascot
1195.618	1195.5596	-0.0584	-49	527	535	IFIWQETMK					Mascot
1195.618	1195.5596	-0.0584	-49	527	535	IFIWQETMK					Mascot
1202.5325	1202.6251	0.0926	77	804	813	TYENAYGEQK					Mascot
1211.6129	1211.5498	-0.0631	-52	527	535	IFIWQETMK			Oxidation (M)[8]		Mascot
1300.6644	1300.6162	-0.0482	-37	887	897	IDNHYALADLR					Mascot
1428.7594	1428.6866	-0.0728	-51	886	897	KIDNHYALADLR					Mascot
1507.838	1507.7048	-0.1332	-88	741	752	YRPDFTQLARLK					Mascot
1519.7275	1519.7643	0.0368	24	1102	1113	HLSYEEKEDLEK					Mascot
1535.7596	1535.7336	-0.026	-17	68	80	LPDLSMPMEAYIR					Mascot
1592.8578	1592.7111	-0.1467	-92	696	709	IDGSIPMHKPRTPK			Oxidation (M)[7]		Mascot
1634.8723	1634.7471	-0.1252	-77	536	549	ACTFIHFGKLVDVK			Carbamidomethyl (C)[2]		Mascot
1635.8081	1635.8202	0.0121	7	311	323	EKEQMILQQVDMK			Oxidation (M)[5]		Mascot
1645.8584	1645.7777	-0.0807	-49	289	302	AGYLEVLWNPVEQK					Mascot
1694.8431	1694.7759	-0.0672	-40	1085	1098	HVSTFNKPPMHVER			Oxidation (M)[10]		Mascot
1705.8789	1705.7797	-0.0992	-58	345	359	VQSDAIREILTMEGK			Oxidation (M)[12]		Mascot
1735.9113	1735.8351	-0.0762	-44	1167	1181	EFALTELNTIIQSEK					Mascot
1769.0055	1768.8822	-0.1233	-70	81	96	QLLVDSDVVPIVSEKK					Mascot
1827.8265	1827.8257	-0.0008	0	434	448	NNTTNWNLCKACGFK			Carbamidomethyl (C)[9,12]		Mascot
1827.8265	1827.8257	-0.0008	0	434	448	NNTTNWNLCKACGFK			Carbamidomethyl (C)[9,12]		Mascot
1884.9126	1884.8418	-0.0708	-38	788	803	YLQNAPNFNSELAEFK					Mascot
1892.8848	1892.8944	0.0096	5	483	497	EKENNQGLIYSYFCK			Carbamidomethyl (C)[14]		Mascot
1977.0957	1977.0253	-0.0704	-36	47	62	LIDFLFTLKDFVNHVR					Mascot
2021.0056	2021.0204	0.0148	7	898	914	CSVCHVLQHEVGQLNIK			Carbamidomethyl (C)[1,4]		Mascot
2021.0056	2021.0204	0.0148	7	898	914	CSVCHVLQHEVGQLNIK			Carbamidomethyl (C)[1,4]		Mascot
2035.0906	2034.975	-0.1156	-57	1085	1101	HVSTFNKPPMHVERLVK			Oxidation (M)[10]		Mascot
2108.0454	2108.0623	0.0169	8	212	229	SGGHSWLINRLAEEMVHR			Oxidation (M)[15]		Mascot

7 Elongation factor G OS=Nitrosospira multiformis (strain ATCC 25196 / NCIMB 11849) GN=fusA PE=3 SV=1 EFG_NITMU 77422 5.35 19 60 50.685 8.831

Peptide Information

Calc. Mass	Obsrv. Mass	± da	± ppm	Start Seq.	End Seq.	Sequence	Ion Score	C. I. %	Modification	Rank	Result Type
944.5411	944.5034	-0.0377	-40	687	695	NVAEAIISK					Mascot

1006.455	1006.4622	0.0072	7	672	679	ATYTMEFK			Oxidation (M)[5]		Mascot
1168.535	1168.5311	-0.0039	-3	144	153	MDRAGANFMR					Mascot
1202.5721	1202.6251	0.053	44	206	216	DIPANMVEDAK					Mascot
1572.803	1572.736	-0.067	-43	505	518	QSGGRGQYGHVWIK					Mascot
1606.7531	1606.8026	0.0495	31	666	679	SATQGRATYTMEFK			Oxidation (M)[11]		Mascot
1606.8588	1606.8026	-0.0562	-35	527	541	GFEFIDAIKGGTVPR					Mascot
1645.7573	1645.7777	0.0204	12	188	201	MKAIHWDDASQGMR					Mascot
1677.7472	1677.8026	0.0554	33	188	201	MKAIHWDDASQGMR			Oxidation (M)[1,13]		Mascot
1706.8497	1706.7218	-0.1279	-75	474	489	EFGVEANVGAPQVAYR					Mascot
1760.9034	1760.8884	-0.015	-9	634	649	GMIQGMDDLPGLKVVR			Oxidation (M)[2,6]		Mascot
1770.8732	1770.8192	-0.054	-30	650	665	AEVPLAEMFGYATSLR			Oxidation (M)[8]		Mascot
1785.8701	1785.843	-0.0271	-15	147	161	AGANFMRVYEQIQSR			Oxidation (M)[6]		Mascot
1786.8469	1786.8242	-0.0227	-13	672	686	ATYTMEFKHYAEAPK					Mascot
1817.9327	1817.8241	-0.1086	-60	429	444	MGIALNRLAQEDPSFR					Mascot
1826.0535	1825.8992	-0.1543	-84	162	178	LKAHPVPIQLPIGAEDK					Mascot
1833.9276	1833.8317	-0.0959	-52	429	444	MGIALNRLAQEDPSFR			Oxidation (M)[1]		Mascot
1914.9702	1914.9091	-0.0611	-32	12	29	NIGVMAHIDAGKTTTTER					Mascot
1930.9652	1930.8986	-0.0666	-34	12	29	NIGVMAHIDAGKTTTTER			Oxidation (M)[5]		Mascot
1990.944	1990.9972	0.0532	27	510	526	GQYGHVWIKMEQNEAGK			Oxidation (M)[10]		Mascot
2020.0718	2019.9525	-0.1193	-59	254	271	IRTLANEIVPMLCGSAFK			Carbamidomethyl (C)[13]		Mascot
2109.1162	2109.0498	-0.0664	-31	647	665	VVRAEVPLAEMFGYATSLR					Mascot

8 Rootletin OS=Homo sapiens GN=CROCC PE=1 SV=1 CROCC_HUMAN 228786.8 5.45 32 60 47.158 40.108

Peptide Information

Calc. Mass	Obsrv. Mass	± da	± ppm	Start Seq.	End Seq.	Sequence	Ion Score	C. I. %	Modification	Rank	Result Type
837.4135	837.4064	-0.0071	-8	1626	1632	MKANETK			Oxidation (M)[1]		Mascot
944.5047	944.5034	-0.0013	-1	667	674	QLEQLEGK					Mascot
1057.5636	1057.4866	-0.077	-73	1412	1421	AQGLEAELAR					Mascot
1102.5487	1102.5476	-0.0011	-1	775	784	QAEQEATVAR					Mascot
1112.5807	1112.5459	-0.0348	-31	1890	1898	LRSHEDTVR					Mascot
1129.5597	1129.5466	-0.0131	-12	1676	1685	EAQAQALQDR					Mascot
1130.6165	1130.5438	-0.0727	-64	1255	1265	TAVGKEAGELR					Mascot
1130.6416	1130.5438	-0.0978	-86	1234	1243	ISLKLANEDK					Mascot
1135.5378	1135.4432	-0.0946	-83	1352	1360	LQEQEGEFR					Mascot
1300.6968	1300.6162	-0.0806	-62	1770	1781	LDAARQALSEAR					Mascot

1384.6743	1384.6962	0.0219	16	2004	2017	SSAPFSPPSGPPEK					Mascot
1412.7969	1412.703	-0.0939	-66	1428	1441	AAEAQLGGLRSALR					Mascot
1412.7969	1412.703	-0.0939	-66	1428	1441	AAEAQLGGLRSALR					Mascot
1481.7091	1481.7145	0.0054	4	986	997	EQRAAHEEDLQR					Mascot
1507.7751	1507.7048	-0.0703	-47	105	117	DELAIKYNAVSER					Mascot
1519.7057	1519.7643	0.0586	39	380	392	DLAQQQMQSDLDK					Mascot
1535.7007	1535.7336	0.0329	21	380	392	DLAQQQMQSDLDK			Oxidation (M)[7]		Mascot
1572.8591	1572.736	-0.1231	-78	953	966	QQIIATQEKASLDK					Mascot
1578.8235	1578.7942	-0.0293	-19	540	553	GRYEASQDLLGTLR					Mascot
1589.7991	1589.8545	0.0554	35	476	491	TADASNGSLRGLSGQR					Mascot
1644.8075	1644.7655	-0.042	-26	1036	1049	EELSEEIAALQQER					Mascot
1693.781	1693.7559	-0.0251	-15	1538	1552	QLAEMEAERDSATSR					Mascot
1705.8716	1705.7797	-0.0919	-54	1730	1746	GLTEALAQSSASLNSTR					Mascot
1770.9093	1770.8192	-0.0901	-51	1670	1685	LGLSDREAQAQALQDR					Mascot
1782.9708	1782.8263	-0.1445	-81	360	374	QALLQAQLEEQLRDK					Mascot
1822.9518	1822.9509	-0.0009	0	827	841	HERSQLQEQLAQLSR					Mascot
1825.8973	1825.8992	0.0019	1	1755	1769	ALTACEHDRQVLQER			Carbamidomethyl (C)[5]		Mascot
1827.9308	1827.8257	-0.1051	-57	1676	1691	EAQAQALQDRVDSLQR					Mascot
1827.9308	1827.8257	-0.1051	-57	1676	1691	EAQAQALQDRVDSLQR					Mascot
1843.8781	1843.7876	-0.0905	-49	643	658	LEEEQEDAVQDGARVR					Mascot
1843.8781	1843.7876	-0.0905	-49	643	658	LEEEQEDAVQDGARVR					Mascot
1885.0138	1884.8418	-0.172	-91	1574	1590	LSGVQAELALQEESVRR					Mascot
1928.056	1927.9122	-0.1438	-75	334	352	AVQEAGLGLSTGLRLAES R					Mascot
1976.9893	1977.0253	0.036	18	1078	1094	LMGTRHSLATISLEMER			Oxidation (M)[2,15]		Mascot
2108.0981	2108.0623	-0.0358	-17	118	135	LEQALRLEPGELETQEPR					Mascot
2252.2244	2252.085	-0.1394	-62	353	372	AEAALEKQALLQAQLEEQ LR					Mascot
2252.2244	2252.085	-0.1394	-62	353	372	AEAALEKQALLQAQLEEQ LR					Mascot

9	UPF0415 protein C7orf25 homolog OS=Mus musculus PE=2 SV=1	CG025_MOUSE	46740.3	6.35	14	60	44.667	12.285

Peptide Information

Calc. Mass	Obsrv. Mass	± da	± ppm	Start Seq.	End Seq.	Sequence	Ion Score	C. I. %	Modification	Rank	Result Type
944.5887	944.5034	-0.0853	-90	345	353	ALRLVASSK					Mascot
1034.5663	1034.498	-0.0683	-66	181	189	LKDMGISVR			Oxidation (M)[4]		Mascot
1057.575	1057.4866	-0.0884	-84	225	233	VDRDNVLAR					Mascot
1130.6106	1130.5438	-0.0668	-59	390	398	FSVFIHQPR					Mascot

1130.6106	1130.5438	-0.0668	-59	390	398	FSVFIHQPR					Mascot
1202.6012	1202.6251	0.0239	20	275	284	VLTEQAEQER					Mascot
1396.6777	1396.6927	0.015	11	298	309	DKELFACESAVK			Carbamidomethyl (C)[7]		Mascot
1507.7864	1507.7048	-0.0816	-54	61	73	ESHLQSTNLTHLK					Mascot
1572.8744	1572.736	-0.1384	-88	228	241	DNVLARVAFPTEIK					Mascot
1587.787	1587.7635	-0.0235	-15	2	15	SAYSMLSDRIVMAK			Oxidation (M)[5]		Mascot
1603.7819	1603.7729	-0.009	-6	2	15	SAYSMLSDRIVMAK			Oxidation (M)[5,12]		Mascot
1606.8071	1606.8026	-0.0045	-3	141	154	SIIEQAEDFLQASR					Mascot
1606.8071	1606.8026	-0.0045	-3	141	154	SIIEQAEDFLQASR					Mascot
1722.9385	1722.7915	-0.147	-85	354	369	INSRSLTIFGTGDTLK					Mascot
1822.9811	1822.9509	-0.0302	-17	102	118	QTLVVDVVANGGHTWVK					Mascot
1825.8967	1825.8992	0.0025	1	405	421	EAFAVPLPKDLTDDSAH					Mascot
1927.9773	1927.9122	-0.0651	-34	124	140	AEALHNIWLGRGQYGDK					Mascot

10	Alanine--tRNA ligase OS=Caulobacter crescentus (strain ATCC 19089 / CB15) GN=alaS PE=3 SV=1	SYA_CAUCR	93812.8	5.35	18	60	40.71	26.334	

Peptide Information

Calc. Mass	Obsrv. Mass	± da	± ppm	Start Seq.	End Seq.	Sequence	Ion Score	C. I. %	Modification	Rank	Result Type
872.4836	872.4827	-0.0009	-1	739	746	VDALIADR					Mascot
944.5159	944.5034	-0.0125	-13	142	150	KIAGLGDDR					Mascot
1006.5098	1006.4622	-0.0476	-47	61	69	AASSQKCVR			Carbamidomethyl (C)[7]		Mascot
1396.6968	1396.6927	-0.0041	-3	48	60	NVFTGAETPAHPR					Mascot
1519.7323	1519.7643	0.032	21	667	680	AYSVELCGGTHVAR			Carbamidomethyl (C)[7]		Mascot
1629.9171	1629.8051	-0.112	-69	801	817	KQLASGVVALVGTSDGK					Mascot
1722.8293	1722.7915	-0.0378	-22	282	298	SSSFLLADGVSPSNEGR					Mascot
1781.8739	1781.8245	-0.0494	-28	408	424	AKGLTVDTDAFDAAMQK					Mascot
1785.9341	1785.843	-0.0911	-51	344	358	AEALIVETLRQEEER					Mascot
1827.9573	1827.8257	-0.1316	-72	265	281	AEGAQAPSHRVIADHLR					Mascot
1827.9573	1827.8257	-0.1316	-72	265	281	AEGAQAPSHRVIADHLR					Mascot
1835.0062	1834.8837	-0.1225	-67	713	729	FLLDQAGVAKALADQFK					Mascot
1866.9015	1866.8588	-0.0427	-23	410	426	GLTVDTDAFDAAMQKQR					Mascot
1945.939	1945.9729	0.0339	17	452	470	AGATDFVGYETTETTGV VK					Mascot
1976.9634	1977.0253	0.0619	31	629	647	EMAPQEAIEAGAVALFGE K			Oxidation (M)[2]		Mascot
1991.038	1990.9972	-0.0408	-20	832	853	FSAADLAKAAVIAMGGQ GAGGK					Mascot
2018.0052	2017.9011	-0.1041	-52	1	17	MPSLNEIRSTFLDYFGK					Mascot

2077.9761	2078.0337	0.0576	28	593	610	MRFDFSHGGPLTAEELD<br>R	Mascot
2147.0251	2147.0247	-0.0004	0	854	875	ADFAQGGAPDDSKAEEG<br>LAAVK	Mascot

Gel Idx/Pos	306/M8	Instr./Gel Origin	BA2151/Sample Project 20140814	Process Status	Analysis Succeeded
Plate [#] Name	[1] Sample Project 20140814	Instrument Sample Name		Spectra	11

Rank	Protein Name	Accession No.	Protein MW	Protein PI	Pep. Count	Protein Score	Protein Score C. I. %	Intensity Matched	Total Ion Score	Total Ion C. I. %	Confirmed
1	Oxygen-evolving enhancer protein 2, chloroplastic OS=Triticum aestivum GN=PSBP PE=2 SV=1	PSBP_WHEAT	27423.9	8.84	15	525	100	50.016	411	100	

Peptide Information

Calc. Mass	Obsrv. Mass	± da	± ppm	Start Seq.	End Seq.	Sequence	Ion Score	C. I. %	Modification	Rank	Result Type
849.3585	849.3956	0.0371	44	208	216	TADGDEGGK					Mascot
895.4706	895.4012	-0.0694	-78	28	34	TQLFVCK			Carbamidomethyl (C)[6]		Mascot
945.5152	945.4871	-0.0281	-30	114	121	EFPGQVLR					Mascot
945.5152	945.4871	-0.0281	-30	114	121	EFPGQVLR	59	99.868			Mascot
1230.6589	1230.6284	-0.0305	-25	112	121	EREFPGQVLR					Mascot
1243.6682	1243.6417	-0.0265	-21	198	207	QYYSITVLTR					Mascot
1243.6682	1243.6417	-0.0265	-21	198	207	QYYSITVLTR	67	99.979			Mascot
1253.6848	1253.6121	-0.0727	-58	217	228	HQLITATVADGK					Mascot
1326.6688	1326.6298	-0.039	-29	246	258	KFVENAAGSFSVA					Mascot
1434.6537	1434.5995	-0.0542	-38	88	100	NTDFVAYSGEGFK					Mascot
1434.6537	1434.5995	-0.0542	-38	88	100	NTDFVAYSGEGFK	91	100			Mascot
1562.7487	1562.6932	-0.0555	-36	87	100	KNTDFVAYSGEGFK					Mascot
1562.7487	1562.6932	-0.0555	-36	87	100	KNTDFVAYSGEGFK	82	100			Mascot
2084.0254	2083.97	-0.0554	-27	208	228	TADGDEGGKHQLITATVA DGK					Mascot
2242.0874	2242.0212	-0.0662	-30	122	141	YEDNFDATSNLSVIINPTT K					Mascot
2242.0874	2242.0212	-0.0662	-30	122	141	YEDNFDATSNLSVIINPTT K	112	100			Mascot
2752.2795	2752.23	-0.0495	-18	170	197	TDSEGGFESDAVATANV LESSAPVVDGK					Mascot
2921.4204	2921.3787	-0.0417	-14	143	169	TITDYGSPEEFLSQVGFL LGQQSYGGK					Mascot
2943.6301	2943.3691	-0.261	-89	53	84	AALSLLAGAAAIAVKVSP AAAAYGEAANVFGK					Mascot
3049.5154	3049.4673	-0.0481	-16	142	169	KTITDYGSPEEFLSQVGF LLGQQSYGGK					Mascot

Rank	Protein Name	Accession No.	Protein MW	Protein PI	Pep. Count	Protein Score	Protein Score C. I. %	Intensity Matched	Total Ion Score	Total Ion C. I. %	Confirmed
2	Oxygen-evolving enhancer protein 2, chloroplastic OS=Spinacia oleracea GN=PSBP PE=1 SV=1	PSBP_SPIOL	28614.4	8.58	3	134	100	41.76	127	100	

Peptide Information

Calc. Mass	Obsrv. Mass	± da	± ppm	Start Seq.	End Seq.	Sequence	Ion Score	C. I. %	Modification	Rank	Result Type

Calc. Mass	Obsrv. Mass	± da	± ppm	Start Seq.	End Seq.	Sequence	Ion Score	C. I. %	Modification	Rank	Result Type
849.3585	849.3956	0.0371	44	216	224	TADGDEGGK					Mascot
945.5152	945.4871	-0.0281	-30	122	129	EFPGQVLR					Mascot
945.5152	945.4871	-0.0281	-30	122	129	EFPGQVLR	59	99.868			Mascot
1243.6682	1243.6417	-0.0265	-21	206	215	QYYSITVLTR					Mascot
1243.6682	1243.6417	-0.0265	-21	206	215	QYYSITVLTR	67	99.979			Mascot

3	Uncharacterized protein YgaU OS=Escherichia coli (strain K12) GN=ygaU PE=1 SV=2	YGAU_ECOLI	16053.3	5.71	7	78	99.143	27.497	37	78.218

Protein Group

Uncharacterized protein YgaU OS=Escherichia coli O6:H1 (strain CFT073 / ATCC 700928 / UPEC) GN=ygaU PE=3 SV=2	YGAU_ECOL6	16053.3	5.7100<br>000381<br>4697

Peptide Information

Calc. Mass	Obsrv. Mass	± da	± ppm	Start Seq.	End Seq.	Sequence	Ion Score	C. I. %	Modification	Rank	Result Type
816.4097	816.4324	0.0227	28	38	45	TGIPDADK					Mascot
867.4683	867.4064	-0.0619	-71	31	37	VQEHLNK					Mascot
945.5516	945.4871	-0.0645	-68	138	145	IYPGQVLR					Mascot
945.5516	945.4871	-0.0645	-68	138	145	IYPGQVLR	37	78.218			Mascot
957.5363	957.459	-0.0773	-81	46	54	VNIQIADGK					Mascot
971.5019	971.4952	-0.0067	-7	1	8	MGLFNFVK			Oxidation (M)[1]		Mascot
1413.7737	1413.6572	-0.1165	-82	138	149	IYPGQVLRIPEE					Mascot
1826.8668	1826.7615	-0.1053	-58	14	29	LWDAVTGQHDKDDQAK					Mascot

4	Oxygen-evolving enhancer protein 2, chloroplastic OS=Cucumis sativus GN=PSBP PE=2 SV=1	PSBP_CUCSA	28292.3	8.61	5	74	98.081	29.89	59	99.868

Peptide Information

Calc. Mass	Obsrv. Mass	± da	± ppm	Start Seq.	End Seq.	Sequence	Ion Score	C. I. %	Modification	Rank	Result Type
807.4611	807.3864	-0.0747	-93	161	167	VDYLLGK					Mascot
849.3585	849.3956	0.0371	44	212	220	TADGDEGGK					Mascot
945.5152	945.4871	-0.0281	-30	118	125	EFPGQVLR					Mascot
945.5152	945.4871	-0.0281	-30	118	125	EFPGQVLR	59	99.868			Mascot
1230.6589	1230.6284	-0.0305	-25	116	125	EREFPGQVLR					Mascot
1255.5841	1255.6243	0.0402	32	150	160	DFGSPEEFLSK					Mascot

5	Phosphatidylinositol 4-phosphate 3-kinase C2 domain-containing subunit beta OS=Homo sapiens GN=PIK3C2B PE=1 SV=2	P3C2B_HUMAN	186817.5	6.95	29	71	95.898	58.777		

Peptide Information

Calc. Mass	Obsrv. Mass	± da	± ppm	Start Seq.	End Seq.	Sequence	Ion Score	C. I. %	Modification	Rank	Result Type
816.4686	816.4324	-0.0362	-44	943	949	RAVSDLR					Mascot
856.4887	856.4432	-0.0455	-53	572	579	DPSVLAVR					Mascot
872.5022	872.4561	-0.0461	-53	565	571	MQPKIQK					Mascot
906.5043	906.4324	-0.0719	-79	452	458	KFDIDIR					Mascot
927.4279	927.4816	0.0537	58	42	48	HDKEENR					Mascot
927.4279	927.4816	0.0537	58	42	48	HDKEENR					Mascot
945.4887	945.4871	-0.0016	-2	1613	1620	ELDLAQEK					Mascot
945.4887	945.4871	-0.0016	-2	1613	1620	ELDLAQEK					Mascot
961.5248	961.4619	-0.0629	-65	745	752	QVLTCGRK			Carbamidomethyl (C)[5]		Mascot
1145.6273	1145.5449	-0.0824	-72	268	278	DTSGKPVARSK					Mascot
1145.6273	1145.5449	-0.0824	-72	268	278	DTSGKPVARSK					Mascot
1199.5626	1199.6095	0.0469	39	1028	1037	QFFALNGSCR			Carbamidomethyl (C)[9]		Mascot
1225.7263	1225.6605	-0.0658	-54	569	579	IQKDPSVLAVR					Mascot
1230.5498	1230.6284	0.0786	64	2	12	SSTQGNGEHWK					Mascot
1241.6637	1241.6112	-0.0525	-42	1153	1162	DRPLADWLQK					Mascot
1243.6868	1243.6417	-0.0451	-36	1384	1393	GYIYVVKVMR			Oxidation (M)[9]		Mascot
1243.7191	1243.6417	-0.0774	-62	459	468	LQLMEQKVVR					Mascot
1248.6439	1248.5892	-0.0547	-44	1090	1099	QDMLTLQMIR					Mascot
1253.6671	1253.6121	-0.055	-44	277	287	SKTMPPQVPPR			Oxidation (M)[4]		Mascot
1255.6754	1255.6243	-0.0511	-41	572	582	DPSVLAVRENR					Mascot
1265.6484	1265.587	-0.0614	-49	960	970	DGLKDSQFSIR					Mascot
1279.7257	1279.6042	-0.1215	-95	1504	1515	VGGEVKLSISYK					Mascot
1389.6766	1389.668	-0.0086	-6	1114	1124	MVIFRCFSTGR			Carbamidomethyl (C)[6], Oxidation (M)[1]		Mascot
1396.7655	1396.7544	-0.0111	-8	1003	1015	LAQQVREAAPSAR					Mascot
1434.6583	1434.5995	-0.0588	-41	1205	1216	TTGHMFHIDFGR			Oxidation (M)[5]		Mascot
1434.6583	1434.5995	-0.0588	-41	1205	1216	TTGHMFHIDFGR			Oxidation (M)[5]		Mascot
1474.8376	1474.7225	-0.1151	-78	1350	1362	LTLSFASRTHTLK					Mascot
1476.7185	1476.7363	0.0178	12	1127	1139	GMVEMIPNAETLR			Oxidation (M)[2]		Mascot
1489.7546	1489.7549	0.0003	0	1621	1634	TGWFALGSRSHGTL					Mascot
1489.7546	1489.7549	0.0003	0	1621	1634	TGWFALGSRSHGTL					Mascot
1584.8533	1584.7068	-0.1465	-92	1418	1431	LLFPSSHLPSFPSR					Mascot
1996.9249	1996.9503	0.0254	13	1084	1099	CGDDLRQDMLTLQMIR			Carbamidomethyl (C)[1], Oxidation (M)[9,14]		Mascot
2242.0842	2242.0212	-0.063	-28	23	41	ELAMAEALQMEYDALSR LR			Oxidation (M)[4,10]		Mascot

2242.0842	2242.0212	-0.063	-28	23	41	ELAMAEALQMEYDALSR LR			Oxidation (M)[4,10]		Mascot
2839.4878	2839.2737	-0.2141	-75	1528	1552	GLQLLQDGNDPDPYVKIY LLPDPQK					Mascot

6	Oxygen-evolving enhancer protein 2, chloroplastic OS=Pisum sativum GN=PSBP PE=1 SV=1	PSBP_PEA	28201.3	8.29	4	70	94.593	29.793	59	99.868

Peptide Information

Calc. Mass	Obsrv. Mass	± da	± ppm	Start Seq.	End Seq.	Sequence	Ion Score	C. I. %	Modification	Rank	Result Type
807.4611	807.3864	-0.0747	-93	157	163	VDYLLGK					Mascot
849.3585	849.3956	0.0371	44	208	216	TADGDEGGK					Mascot
945.5152	945.4871	-0.0281	-30	114	121	EFPGQVLR					Mascot
945.5152	945.4871	-0.0281	-30	114	121	EFPGQVLR	59	99.868			Mascot
1230.6589	1230.6284	-0.0305	-25	112	121	EREFPGQVLR					Mascot

7	Protein translocase subunit SecA OS=Mycoplasma mobile (strain ATCC 43663 / 163K / NCTC 11711) GN=secA PE=3 SV=1	SECA_MYCMO	98154.3	6.71	25	59	30.34	20.869

Peptide Information

Calc. Mass	Obsrv. Mass	± da	± ppm	Start Seq.	End Seq.	Sequence	Ion Score	C. I. %	Modification	Rank	Result Type
807.428	807.3864	-0.0416	-52	14	20	LAESMLK			Oxidation (M)[5]		Mascot
835.4421	835.399	-0.0431	-52	570	576	AFDKAQR					Mascot
867.4241	867.4064	-0.0177	-20	362	370	LSGMTGTGK			Oxidation (M)[4]		Mascot
899.4509	899.4482	-0.0027	-3	552	558	EIFAEYK					Mascot
901.4414	901.4521	0.0107	12	634	640	YGELYTR					Mascot
943.5319	943.4584	-0.0735	-78	513	520	IDNQLKGR					Mascot
959.5408	959.4599	-0.0809	-84	713	721	AIISALDEK					Mascot
987.5291	987.4987	-0.0304	-31	772	780	IIMNNPSAK					Mascot
1050.6167	1050.5128	-0.1039	-99	285	293	LQNALRAHK					Mascot
1078.6143	1078.5118	-0.1025	-95	410	418	YKAVVEEIK					Mascot
1115.642	1115.5532	-0.0888	-80	712	721	RAIISALDEK					Mascot
1126.5562	1126.4995	-0.0567	-50	39	47	NMTNVFKEK			Oxidation (M)[2]		Mascot
1126.5562	1126.4995	-0.0567	-50	39	47	NMTNVFKEK			Oxidation (M)[2]		Mascot
1145.6426	1145.5449	-0.0977	-85	704	712	LPTNLFERR					Mascot
1145.6426	1145.5449	-0.0977	-85	704	712	LPTNLFERR					Mascot
1212.5201	1212.6169	0.0968	80	183	192	DNMVNSFEEK					Mascot
1213.6252	1213.6421	0.0169	14	550	558	WKEIFAEYK					Mascot
1220.6271	1220.5409	-0.0862	-71	588	597	NVLNYDDVIR					Mascot

1243.6641	1243.6417	-0.0224	-18	458	468	QNKNEADIIAK					Mascot
1243.6641	1243.6417	-0.0224	-18	458	468	QNKNEADIIAK					Mascot
1255.6066	1255.6243	0.0177	14	578	587	IEGFNYDNRK					Mascot
1298.631	1298.6427	0.0117	9	722	731	WQNHINLMDK					Mascot
1306.6863	1306.5751	-0.1112	-85	598	607	QQRDIIYSQR					Mascot
1348.7219	1348.6049	-0.117	-87	587	597	KNVLNYDDVIR					Mascot
1461.7981	1461.7522	-0.0459	-31	14	26	LAESMLKSINDLK					Mascot
1474.7689	1474.7225	-0.0464	-31	346	357	TLATITYQNFFR					Mascot
1876.0062	1875.8774	-0.1288	-69	832	847	VFEKNILSDLNLVDEK					Mascot
2943.459	2943.3691	-0.0899	-31	76	102	RPYDVQMIGGLILDFGSV AEMKTGEGK			Oxidation (M)[7,21]		Mascot

8 DNA ligase OS=Pelodictyon phaeoclathratiforme (strain DSM 5477 / BU-1) GN=ligA PE=3 SV=1 DNLJ_PELPB 76046.7 5.61 19 59 25.358 32.48

Peptide Information

Calc. Mass	Obsrv. Mass	± da	± ppm	Start Seq.	End Seq.	Sequence	Ion Score	C. I. %	Modification	Rank	Result Type
811.3944	811.3817	-0.0127	-16	512	517	EKSYER					Mascot
817.4778	817.4125	-0.0653	-80	305	312	EKLGATAK					Mascot
830.4553	830.3892	-0.0661	-80	492	498	LERMGPK					Mascot
866.4553	866.3989	-0.0564	-65	183	189	GEVFMRK					Mascot
867.4907	867.4064	-0.0843	-97	526	533	HVGRATAR					Mascot
870.5407	870.4938	-0.0469	-54	156	163	TVATIPLR					Mascot
872.52	872.4561	-0.0639	-73	380	386	DRVIIEK					Mascot
945.5476	945.4871	-0.0605	-64	630	639	GGRVVGSVSK					Mascot
945.5476	945.4871	-0.0605	-64	630	639	GGRVVGSVSK					Mascot
983.5057	983.4235	-0.0822	-84	72	79	EFPAVQHR					Mascot
1098.6378	1098.6064	-0.0314	-29	7	16	AAEAIAQLRR					Mascot
1212.5797	1212.6169	0.0372	31	256	265	LGFFTGDHYR					Mascot
1224.5969	1224.5914	-0.0055	-4	228	237	SMSFVAYYLK			Oxidation (M)[2]		Mascot
1230.6624	1230.6284	-0.034	-28	495	505	MGPKSAQNILR			Oxidation (M)[1]		Mascot
1242.7164	1242.6104	-0.106	-85	499	509	SAQNILRALEK					Mascot
1306.6936	1306.5751	-0.1185	-91	178	188	AIEVRGEVFMR					Mascot
1346.7175	1346.6908	-0.0267	-20	205	217	FANPRNATAGSLK					Mascot
1389.7234	1389.668	-0.0554	-40	238	249	GLKDESTPHIHR					Mascot
1786.9558	1786.8795	-0.0763	-43	241	255	DESTPHIHRLELLAR					Mascot
2264.2397	2264.0291	-0.2106	-93	121	141	FDGVAISLIYQDGILVRGA TR					Mascot

9	Rod cGMP-specific 3',5'-cyclic phosphodiesterase subunit alpha OS=Homo sapiens GN=PDE6A PE=1 SV=4	PDE6A_HUMAN	100338.1	5.48	20	57	0	26.652

Peptide Information

Calc. Mass	Obsrv. Mass	± da	± ppm	Start Seq.	End Seq.	Sequence	Ion Score	C. I. %	Modification	Rank	Result Type
817.4526	817.4125	-0.0401	-49	828	834	QKQQSAK					Mascot
867.4471	867.4064	-0.0407	-47	630	636	HHLEFGK					Mascot
895.4091	895.4012	-0.0079	-9	268	274	AFLNCDR			Carbamidomethyl (C)[5]		Mascot
927.4426	927.4816	0.039	42	77	83	CIFNVMK			Carbamidomethyl (C)[1], Oxidation (M)[6]		Mascot
927.4426	927.4816	0.039	42	77	83	CIFNVMK			Carbamidomethyl (C)[1], Oxidation (M)[6]		Mascot
944.5159	944.4563	-0.0596	-63	103	111	NGIAELATR					Mascot
1126.5813	1126.4995	-0.0818	-73	275	284	YSVGLLDMTK					Mascot
1126.5813	1126.4995	-0.0818	-73	275	284	YSVGLLDMTK					Mascot
1188.6656	1188.6014	-0.0642	-54	187	197	DVVAIIMAVNK			Oxidation (M)[7]		Mascot
1220.5914	1220.5409	-0.0505	-41	94	102	MSLFMYRTR			Oxidation (M)[1]		Mascot
1226.6602	1226.618	-0.0422	-34	214	223	YLNFANLIMK					Mascot
1226.6602	1226.618	-0.0422	-34	214	223	YLNFANLIMK					Mascot
1242.6552	1242.6104	-0.0448	-36	214	223	YLNFANLIMK			Oxidation (M)[9]		Mascot
1320.614	1320.6151	0.0011	1	373	383	EPLDESGWMIK			Oxidation (M)[9]		Mascot
1389.7042	1389.668	-0.0362	-26	460	470	CDNEEIQKILK			Carbamidomethyl (C)[1]		Mascot
1438.6849	1438.6021	-0.0828	-58	808	819	EWKALADEYDAK					Mascot
1562.7268	1562.6932	-0.0336	-22	456	467	YHVKCDNEEIQK			Carbamidomethyl (C)[5]		Mascot
1562.7268	1562.6932	-0.0336	-22	456	467	YHVKCDNEEIQK			Carbamidomethyl (C)[5]		Mascot
1822.9116	1822.9395	0.0279	15	605	620	GTNNLYQMKSQNPLAK			Oxidation (M)[8]		Mascot
1826.8628	1826.7615	-0.1013	-55	835	855	SAAAGNQPGGNPSPGG ATTSK					Mascot
1902.9015	1902.9313	0.0298	16	791	806	FHEEITPMLDGITNNR			Oxidation (M)[8]		Mascot
1946.0454	1945.9672	-0.0782	-40	637	652	TLLRDESLNIFQNLNR					Mascot
2322.1265	2322.114	-0.0125	-5	287	306	EFFDVWPVLMGEVPPYS GPR					Mascot
2369.0061	2369.0166	0.0105	4	690	707	TYESEQEWTQYMMLEQT R			Oxidation (M)[12]		Mascot
2752.2971	2752.23	-0.0671	-24	582	604	RYFTDLEALAMVTAAFCH DIDHR			Carbamidomethyl (C)[17]		Mascot

10	Ribulose bisphosphate carboxylase large chain OS=Physcomitrella patens subsp. patens GN=rbcL PE=1 SV=2	RBL_PHYPA	53184.7	6.29	14	56	0	2.356

Peptide Information

Calc. Mass	Obsrv. Mass	± da	± ppm	Start	End	Sequence	Ion	C. I. %	Modification	Rank	Result Type

				Seq.	Seq.		Score		
808.4312	808.3707	-0.0605	-75	15	21	AGVKDYR			Mascot
830.4366	830.3892	-0.0474	-57	440	446	EGNEIIR			Mascot
898.41	898.4531	0.0431	48	306	312	NHGMHFR			Mascot
910.4451	910.4152	-0.0299	-33	188	194	AVYECLR		Carbamidomethyl (C)[5]	Mascot
912.472	912.4209	-0.0511	-56	296	303	AMHAVLDR			Mascot
957.5186	957.459	-0.0596	-62	1	8	MSPRPEIK			Mascot
1078.6003	1078.5118	-0.0885	-82	178	187	LGLSAKNYGR			Mascot
1154.5636	1154.6008	0.0372	32	304	312	QKNHGMHFR			Mascot
1325.6896	1325.6447	-0.0449	-34	306	316	NHGMHFRVLAK		Oxidation (M)[4]	Mascot
1456.7207	1456.5995	-0.1212	-83	464	475	EIKFEFDTVDTL			Mascot
1467.6169	1467.6929	0.076	52	202	213	DDENVNSQPFMR		Oxidation (M)[11]	Mascot
1826.8596	1826.7615	-0.0981	-54	19	32	DYRLTYYTPDYQTK			Mascot
1945.9476	1945.9672	0.0196	10	447	463	EAAKWSPELAAACEVWK		Carbamidomethyl (C)[13]	Mascot
1966.911	1966.8835	-0.0275	-14	237	253	GHYLNATAGTCEEMIKR		Carbamidomethyl (C)[11], Oxidation (M)[14]	Mascot

Gel Idx/Pos	307/M9	Instr./Gel Origin	BA2151/Sample Project 20140814	Process Status	Analysis Succeeded
Plate [#] Name	[1] Sample Project 20140814	Instrument Sample Name		Spectra	11

Rank	Protein Name	Accession No.	Protein MW	Protein PI	Pep. Count	Protein Score	Protein Score C. I. %	Intensity Matched	Total Ion Score	Total Ion C. I. %	Confirmed
1	Lebercilin OS=Homo sapiens GN=LCA5 PE=1 SV=2	LCA5_HUMAN	80676.3	7.31	18	53	0	5.255			

Peptide Information

Calc. Mass	Obsrv. Mass	± da	± ppm	Start Seq.	End Seq.	Sequence	Ion Score	C. I. %	Modification	Rank	Result Type
807.3995	807.3825	-0.017	-21	567	573	SNPFSQK					Mascot
858.5043	858.4273	-0.077	-90	310	316	EKELALR					Mascot
870.5043	870.5071	0.0028	3	302	309	LPKSSPNK					Mascot
945.5363	945.5183	-0.018	-19	423	430	EKASLLER					Mascot
952.4767	952.437	-0.0397	-42	582	589	NSMEKLSK			Oxidation (M)[3]		Mascot
1027.5181	1027.4468	-0.0713	-69	664	671	SFNPNRHR					Mascot
1102.5851	1102.4987	-0.0864	-78	477	485	ELQDSRNLK					Mascot
1145.5546	1145.5316	-0.023	-20	5	15	AGSPGTDQERK					Mascot
1163.6281	1163.522	-0.1061	-91	81	90	VGFRSQSLNR					Mascot
1517.8251	1517.7074	-0.1177	-78	486	498	YPVLPLLPDFESK					Mascot
1590.7281	1590.8569	0.1288	81	408	419	LEDEWEREELDK					Mascot
1590.7281	1590.8569	0.1288	81	408	419	LEDEWEREELDK					Mascot
1642.8646	1642.7039	-0.1607	-98	112	125	INELQNEVSELQVK					Mascot
1701.8694	1701.8138	-0.0556	-33	389	402	EEKFVTDEELHVVK					Mascot
1838.9065	1838.8755	-0.031	-17	376	391	HGEAGILNPIMEREEK			Oxidation (M)[11]		Mascot
1909.7836	1909.8557	0.0721	38	648	663	DQEHDEDEGFFLSEGR					Mascot
2002.9546	2002.8453	-0.1093	-55	544	562	SPASPNEFAFGSYVPSFA K					Mascot
2010.894	2011.001	0.107	53	19	35	HHYSYLSDFETPQSSGR					Mascot
2839.3872	2839.2639	-0.1233	-43	518	543	LFNGHHLQDISFSTPKGE GQNSGNVR					Mascot

Rank	Protein Name	Accession No.	Protein MW	Protein PI	Pep. Count	Protein Score	Protein Score C. I. %	Intensity Matched	Total Ion Score	Total Ion C. I. %	Confirmed
2	UPF0403 protein BLi02323/BL05224 OS=Bacillus licheniformis (strain DSM 13 / ATCC 14580) GN=BLi02323 PE=3 SV=1	Y2323_BACLD	16128.7	4.64	8	53	0	1.455			

Peptide Information

Calc. Mass	Obsrv. Mass	± da	± ppm	Start Seq.	End Seq.	Sequence	Ion Score	C. I. %	Modification	Rank	Result Type
822.4138	822.3672	-0.0466	-57	88	94	EATAKMR			Oxidation (M)[6]		Mascot
856.5073	856.49	-0.0173	-20	11	17	QLVLPMR					Mascot

1019.552	1019.4836	-0.0684	-67	18	26	QELVQAGFK					Mascot
1149.4882	1149.5146	0.0264	23	2	10	STAYEEYMR					Mascot
1491.7075	1491.649	-0.0585	-39	74	87	GPDETVTVFAGQDR					Mascot
1947.9044	1947.9255	0.0211	11	95	111	EYFEGYEPSSPSMALLK					Mascot
2002.9725	2002.8453	-0.1272	-64	2	17	STAYEEYMRQLVLPMR			Oxidation (M)[8]		Mascot
2166.0029	2166.1221	0.1192	55	1	17	MSTAYEEYMRQLVLPMR			Oxidation (M)[1,9,16]		Mascot

3	Vinculin OS=Rattus norvegicus GN=Vcl PE=1 SV=1	VINC_RAT	117112.5	5.83	22	50	0	22.094	

Protein Group

Vinculin OS=Mus musculus GN=Vcl PE=1 SV=4	VINC_MOUSE	117214.5	5.7699<br>999809<br>2651

Peptide Information

Calc. Mass	Obsrv. Mass	± da	± ppm	Start Seq.	End Seq.	Sequence	Ion Score	C. I. %	Modification	Rank	Result Type
807.3777	807.3825	0.0048	6	971	976	QCTDKR			Carbamidomethyl (C)[2]		Mascot
817.4236	817.3801	-0.0435	-53	163	170	NLGPGMTK					Mascot
822.4144	822.3672	-0.0472	-57	1061	1066	KTPWYQ					Mascot
824.4625	824.4399	-0.0226	-27	489	496	AAVHLEGK					Mascot
893.4396	893.3693	-0.0703	-79	374	381	LEAMTNSK					Mascot
993.4855	993.4419	-0.0436	-44	171	178	MAKMIDER					Mascot
1074.5765	1074.5298	-0.0467	-43	72	80	RDMPPAFIK					Mascot
1102.5024	1102.4987	-0.0037	-3	630	639	AANFENHSGR					Mascot
1145.5586	1145.5316	-0.027	-24	700	708	NQWIDNVEK					Mascot
1163.5912	1163.522	-0.0692	-59	163	173	NLGPGMTKMAK			Oxidation (M)[6]		Mascot
1184.5472	1184.5255	-0.0217	-18	926	935	MALLMAEMSR			Oxidation (M)[1,5]		Mascot
1247.6478	1247.6019	-0.0459	-37	781	792	EAVKAASDELSK					Mascot
1271.6703	1271.6239	-0.0464	-36	220	230	NQGIEEALKNR					Mascot
1590.8823	1590.8569	-0.0254	-16	513	528	GVGQAAIRGLVAEGHR					Mascot
1590.8823	1590.8569	-0.0254	-16	513	528	GVGQAAIRGLVAEGHR					Mascot
1701.9381	1701.8138	-0.1243	-73	429	444	DDILRSLGEIAALTSK					Mascot
1909.9246	1909.8557	-0.0689	-36	785	802	AASDELSKTISPMVMDAK			Oxidation (M)[13]		Mascot
1965.9196	1965.8628	-0.0568	-29	174	188	MIDERQQELTHQEHR			Oxidation (M)[1]		Mascot
1965.9196	1965.8628	-0.0568	-29	174	188	MIDERQQELTHQEHR			Oxidation (M)[1]		Mascot
2002.995	2002.8453	-0.1497	-75	521	538	GLVAEGHRLANVMMGPY<br>R			Oxidation (M)[13,14]		Mascot
2352.1426	2352.0896	-0.053	-23	286	308	DPNASPGDAGEQAIRQIL<br>DEAGK					Mascot
2362.2041	2362.1111	-0.093	-39	737	758	CKVAMANIQPQMLVAGA<br>TSIAR			Carbamidomethyl (C)[1], Oxidation (M)[5,12]		Mascot

2380.1165	2380.0464	-0.0701	-29	388	409	IDAAQNWLADPNGGPEG EEQIR					Mascot
2380.1165	2380.0464	-0.0701	-29	388	409	IDAAQNWLADPNGGPEG EEQIR					Mascot
2447.1877	2447.1404	-0.0473	-19	833	853	EAFQPQEPDFPPPPPDL EQLR					Mascot

4	Malonyl-[acyl-carrier protein] O-methyltransferase OS=Anoxybacillus flavithermus (strain DSM 21510 / WK1) GN=bioC PE=3 SV=1	BIOC_ANOFW	30159.2	6.8	11	48	0	4.82

Peptide Information

Calc. Mass	Obsrv. Mass	± da	± ppm	Start Seq.	End Seq.	Sequence	Ion Score	C. I. %	Modification	Rank	Result Type
817.4058	817.3801	-0.0257	-31	32	38	MAHALMK			Oxidation (M)[1]		Mascot
878.4651	878.4049	-0.0602	-69	2	8	VEKMIDK			Oxidation (M)[4]		Mascot
945.5008	945.5183	0.0175	19	31	38	KMAHALMK			Oxidation (M)[2]		Mascot
956.52	956.5038	-0.0162	-17	60	67	LLHDAYPK					Mascot
956.52	956.5038	-0.0162	-17	60	67	LLHDAYPK					Mascot
993.5107	993.4419	-0.0688	-69	1	8	MVEKMIDK					Mascot
1184.5881	1184.5255	-0.0626	-53	223	231	YCQRPSVFK			Carbamidomethyl (C)[2]		Mascot
1210.4979	1210.5563	0.0584	48	232	240	AMMHEYEQR			Oxidation (M)[2]		Mascot
1245.5858	1245.6169	0.0311	25	15	24	FSERATTYDR					Mascot
1353.6508	1353.6449	-0.0059	-4	249	259	ATYHCIFIDIT			Carbamidomethyl (C)[5]		Mascot
1756.9514	1756.835	-0.1164	-66	68	84	AELTAVDIAPGMIEKAK					Mascot
2266.1145	2266.0044	-0.1101	-49	212	231	HIGATNSTSGRYCQRPS VFK			Carbamidomethyl (C)[13]		Mascot
2266.1145	2266.0044	-0.1101	-49	212	231	HIGATNSTSGRYCQRPS VFK			Carbamidomethyl (C)[13]		Mascot

5	Polar flagellar hook-associated protein 2 OS=Vibrio parahaemolyticus serotype O3:K6 (strain RIMD 2210633) GN=fliDP PE=3 SV=2	FLIDP_VIBPA	73204.8	4.72	15	48	0	4.209

Peptide Information

Calc. Mass	Obsrv. Mass	± da	± ppm	Start Seq.	End Seq.	Sequence	Ion Score	C. I. %	Modification	Rank	Result Type
878.44	878.4049	-0.0351	-40	637	643	MEGLEKR			Oxidation (M)[1]		Mascot
956.5159	956.5038	-0.0121	-13	396	403	AKLDPQER					Mascot
956.5159	956.5038	-0.0121	-13	396	403	AKLDPQER					Mascot
1010.4901	1010.4216	-0.0685	-68	618	625	SLTEQNYR					Mascot
1027.5419	1027.4468	-0.0951	-93	543	551	IDQAPENLK					Mascot
1069.4983	1069.5214	0.0231	22	648	657	FTAMQDATGK					Mascot
1145.6049	1145.5316	-0.0733	-64	406	415	LEKIDEAEAK					Mascot

Calc. Mass	Obsrv. Mass	± da	± ppm	Start Seq.	End Seq.	Sequence	Ion Score	C. I. %	Modification	Rank	Result Type
1259.6478	1259.6461	-0.0017	-1	369	379	IADEKQELDAK					Mascot
1286.6448	1286.5712	-0.0736	-57	626	636	LNDDQAALDRR					Mascot
1410.7336	1410.663	-0.0706	-50	183	194	DHQIKVSVEAER					Mascot
1701.7935	1701.8138	0.0203	12	52	65	ESLDTMKNLMANFR			Oxidation (M)[6,10]		Mascot
1774.9447	1774.8676	-0.0771	-43	125	139	LQISLGDDRFNIEVR					Mascot
1910.0454	1909.8557	-0.1897	-99	598	615	IEDAIHGITGITGSIRTR					Mascot
2061.896	2061.8323	-0.0637	-31	1	20	MSLGPVGMSGGMDINSM VSK			Oxidation (M)[1,8,12,17]		Mascot
2352.2405	2352.0896	-0.1509	-64	448	469	LSSHNNVIEDAIEGVDLTL KGK					Mascot
3117.457	3117.1565	-0.3005	-96	419	447	AQSSFEEYLGMTEVQAG QDSEVLLDGVAK			Oxidation (M)[11]		Mascot

6	Uncharacterized protein ZK353.3 OS=Caenorhabditis elegans GN=ZK353.3 PE=4 SV=1	YOJ3_CAEEL	18001.9	8.64	8	47	0	4.093	

Peptide Information

Calc. Mass	Obsrv. Mass	± da	± ppm	Start Seq.	End Seq.	Sequence	Ion Score	C. I. %	Modification	Rank	Result Type
805.3798	805.3553	-0.0245	-30	30	36	DAKNSDR					Mascot
856.4089	856.49	0.0811	95	154	160	MTMKSMK					Mascot
1068.5143	1068.4587	-0.0556	-52	39	47	YDNLAMLGR			Oxidation (M)[6]		Mascot
1433.6764	1433.6365	-0.0399	-28	69	81	NTVDSQMMLGPPK			Oxidation (M)[7]		Mascot
1458.7257	1458.7021	-0.0236	-16	20	32	LMIGPENNEKDAK					Mascot
1577.7662	1577.6318	-0.1344	-85	69	82	NTVDSQMMLGPPKK			Oxidation (M)[7,8]		Mascot
1948.0784	1947.9255	-0.1529	-78	1	19	MGIIISLVAPTVSSTISSR			Oxidation (M)[1]		Mascot
2266.1794	2266.0044	-0.175	-77	61	81	APVARPTRNTVDSQMML GPPK					Mascot
2266.1794	2266.0044	-0.175	-77	61	81	APVARPTRNTVDSQMML GPPK					Mascot

7	4-hydroxy-3-methylbut-2-en-1-yl diphosphate synthase OS=Rhodopseudomonas palustris (strain HaA2) GN=ispG PE=3 SV=1	ISPG_RHOP2	46016.8	5.97	12	46	0	3.943	

Peptide Information

Calc. Mass	Obsrv. Mass	± da	± ppm	Start Seq.	End Seq.	Sequence	Ion Score	C. I. %	Modification	Rank	Result Type
856.4774	856.49	0.0126	15	202	209	AEEIGLPK					Mascot
878.473	878.4049	-0.0681	-78	327	333	SIQDFIR					Mascot
945.5152	945.5183	0.0031	3	124	132	INPGNVGFK					Mascot
950.4578	950.3837	-0.0741	-78	396	404	GENIAGDFK					Mascot
1149.5721	1149.5146	-0.0575	-50	334	342	VEMPSWKTR			Oxidation (M)[3]		Mascot
1163.6167	1163.522	-0.0947	-81	75	85	EEAAAAVPHIR					Mascot

1219.6681	1219.5609	-0.1072	-88	405	414	ALVIDYIEQR	Mascot
1302.6107	1302.6205	0.0098	8	170	181	LMDENAASAQPR	Mascot
1320.6907	1320.5996	-0.0911	-69	393	404	TLRGENIAGDFK	Mascot
1458.7845	1458.7021	-0.0824	-56	56	69	QVAALARAGSEMVR	Mascot
1590.8235	1590.8569	0.0334	21	4	18	LENPLQNDVAGPAPR	Mascot
1590.8235	1590.8569	0.0334	21	4	18	LENPLQNDVAGPAPR	Mascot
1672.8071	1672.782	-0.0251	-15	170	184	LMDENAASAQPRDVR	Mascot

8	NADH-quinone oxidoreductase subunit B OS=Photorhabdus luminescens subsp. laumondii (strain TT01) GN=nuoB PE=3 SV=1	NUOB_PHOLL	25797.7	6.31	9	46	0	3.298	

Peptide Information

Calc. Mass	Obsrv. Mass	± da	± ppm	Start Seq.	End Seq.	Sequence	Ion Score	C. I. %	Modification	Rank	Result Type
1029.4596	1029.4346	-0.025	-24	8	16	IDPNGENDR					Mascot
1577.7642	1577.6318	-0.1324	-84	42	54	LEHALHDMVNWGR					Mascot
1590.7291	1590.8569	0.1278	80	96	109	QADFMVVAGTCFTK			Carbamidomethyl (C)[11], Oxidation (M)[5]		Mascot
1590.7291	1590.8569	0.1278	80	96	109	QADFMVVAGTCFTK			Carbamidomethyl (C)[11], Oxidation (M)[5]		Mascot
1631.8654	1631.7579	-0.1075	-66	185	198	RPLSWVVGDQGVYR					Mascot
1909.8708	1909.8557	-0.0151	-8	1	16	MDYTLTRIDPNGENDR					Mascot
1948.0031	1947.9255	-0.0776	-40	110	125	MAPVIQRLYDQMLEPK			Oxidation (M)[1]		Mascot
2001.9521	2001.8856	-0.0665	-33	92	109	ASPRQADFMVVAGTCFT K			Carbamidomethyl (C)[15], Oxidation (M)[9]		Mascot
2001.9521	2001.8856	-0.0665	-33	92	109	ASPRQADFMVVAGTCFT K			Carbamidomethyl (C)[15], Oxidation (M)[9]		Mascot
2352.1765	2352.0896	-0.0869	-37	22	41	QEIVADPLEQHVHRSVY MGK			Oxidation (M)[18]		Mascot
2839.2737	2839.2639	-0.0098	-3	126	151	WVISMGACANSGGMYDI YSVVQGVDK			Carbamidomethyl (C)[8], Oxidation (M)[5,14]		Mascot

9	Allergen Bla g 4 (Fragment) OS=Blattella germanica PE=1 SV=1	BLG4_BLAGE	21199.4	6.47	8	46	0	2.541	

Peptide Information

Calc. Mass	Obsrv. Mass	± da	± ppm	Start Seq.	End Seq.	Sequence	Ion Score	C. I. %	Modification	Rank	Result Type
817.4301	817.3801	-0.05	-61	162	168	AIEEDLK					Mascot
941.5203	941.4282	-0.0921	-98	138	145	FHPKLGDK					Mascot
945.5251	945.5183	-0.0068	-7	161	168	KAIEEDLK					Mascot
1068.507	1068.4587	-0.0483	-45	65	73	YTDSQGKNR					Mascot
1590.7435	1590.8569	0.1134	71	82	94	FEGNKFTIDYNDK					Mascot
1590.7435	1590.8569	0.1134	71	82	94	FEGNKFTIDYNDK					Mascot

1774.8871	1774.8676	-0.0195	-11	19	32	HESLVPNLDYERFR					Mascot
1795.9225	1795.8834	-0.0391	-22	33	49	GSWIIAAGTSEALTQYK					Mascot
1874.8741	1874.7969	-0.0772	-41	50	64	CWIDRFSYDDALVSK			Carbamidomethyl (C)[1]		Mascot

10	UDP-N-acetylmuramoyl-L-alanyl-D-glutamate--2,6-diaminopimelate ligase OS=Chlorobium tepidum (strain ATCC 49652 / DSM 12025 / TLS) GN=murE PE=3 SV=1	MURE_CHLTE	54894.6	5.53	13	45	0	2.646

Peptide Information

Calc. Mass	Obsrv. Mass	± da	± ppm	Start Seq.	End Seq.	Sequence	Ion Score	C. I. %	Modification	Rank	Result Type
800.4413	800.3631	-0.0782	-98	173	178	LHDLFR					Mascot
811.3766	811.3616	-0.015	-18	255	261	AEFMAAR			Oxidation (M)[4]		Mascot
814.4053	814.3696	-0.0357	-44	166	172	TTPEPNR					Mascot
858.4679	858.4273	-0.0406	-47	415	422	IAAELADR					Mascot
952.3975	952.437	0.0395	41	179	186	QMVDAGCR			Carbamidomethyl (C)[7], Oxidation (M)[2]		Mascot
993.4934	993.4419	-0.0515	-52	354	360	MERIWSR			Oxidation (M)[1]		Mascot
1102.5276	1102.4987	-0.0289	-26	480	488	GHEAYQEIR					Mascot
1149.5834	1149.5146	-0.0688	-60	394	404	LAVVFGCGGNR			Carbamidomethyl (C)[7]		Mascot
1163.6168	1163.522	-0.0948	-81	156	165	IGNHDIPLER					Mascot
1219.6715	1219.5609	-0.1106	-91	117	128	LMLIGVTGTNGK			Oxidation (M)[2]		Mascot
1447.7322	1447.666	-0.0662	-46	343	356	SLAAADAIAGRMER			Oxidation (M)[12]		Mascot
1684.8541	1684.756	-0.0981	-58	101	116	ALAELSKAFYGNASDK					Mascot
2165.9314	2166.1221	0.1907	88	492	508	HHFSDRECLEACFAQMK			Carbamidomethyl (C)[8,12]		Mascot

Rank	Protein Name	Accession No.	Protein MW	Protein PI	Pep. Count	Protein Score	Protein Score C. I. %	Intensity Matched	Total Ion Score	Total Ion C. I. %	Confirmed
1	Keratin, type II cytoskeletal 1 OS=Homo sapiens GN=KRT1 PE=1 SV=6	K2C1_HUMAN	66170.1	8.15	16	221	100	3.893	159	100	

Peptide Information

Calc. Mass	Obsrv. Mass	± da	± ppm	Start Seq.	End Seq.	Sequence	Ion Score	C. I. %	Modification	Rank	Result Type
998.4836	998.435	-0.0486	-49	1	8	MSRQFSSR					Mascot
1006.4297	1006.3874	-0.0423	-42	589	602	GGSGGGGGGSSGGR					Mascot
1014.4785	1014.4041	-0.0744	-73	1	8	MSRQFSSR			Oxidation (M)[1]		Mascot
1033.516	1033.4742	-0.0418	-40	484	492	TLLEGEESR					Mascot
1065.5211	1065.4583	-0.0628	-59	356	364	AQYEDIAQK					Mascot
1125.5422	1125.4442	-0.098	-87	367	376	AEAESLYQSK					Mascot
1127.5402	1127.4773	-0.0629	-56	289	298	KDVDGAYMTK					Mascot
1141.5194	1141.504	-0.0154	-13	464	472	DYQELMNTK					Mascot
1265.6372	1265.583	-0.0542	-43	278	288	TNAENEFVTIK					Mascot
1277.71	1277.6653	-0.0447	-35	473	483	LALDLEIATYR	72	99.994			Mascot
1332.5195	1332.5144	-0.0051	-4	258	267	NMQDMVEDYR			Oxidation (M)[2,5]		Mascot
1383.6903	1383.6151	-0.0752	-54	186	197	SLNNQFASFIDK					Mascot
1475.7853	1475.7074	-0.0779	-53	200	211	FLEQQNQVLQTK	35	67.488			Mascot
1657.793	1657.7584	-0.0346	-21	13	29	SGGGFSSGSAGIINYQR					Mascot
1716.8511	1716.8031	-0.048	-28	418	432	QISNLQQSISDAEQR	56	99.751			Mascot
2121.0107	2121.0425	0.0318	15	9	29	SGYRSGGGFSSGSAGIIN YQR					Mascot
3312.3083	3312.2603	-0.048	-14	550	588	GSYGSGGSSYGSGGGS YGSGGGGGGHGSYGSG SSSGGYR					Mascot

Rank	Protein Name	Accession No.	Protein MW	Protein PI	Pep. Count	Protein Score	Protein Score C. I. %	Intensity Matched	Total Ion Score	Total Ion C. I. %	Confirmed
2	Keratin, type II cytoskeletal 1 OS=Pan troglodytes GN=KRT1 PE=1 SV=1	K2C1_PANTR	65620.8	7.62	14	211	100	3.365	161	100	

Peptide Information

Calc. Mass	Obsrv. Mass	± da	± ppm	Start Seq.	End Seq.	Sequence	Ion Score	C. I. %	Modification	Rank	Result Type
998.4836	998.435	-0.0486	-49	1	8	MSRQFSSR					Mascot
1014.4785	1014.4041	-0.0744	-73	1	8	MSRQFSSR			Oxidation (M)[1]		Mascot
1033.516	1033.4742	-0.0418	-40	479	487	TLLEGEESR					Mascot

Calc. Mass	Obsrv. Mass	± da	± ppm	Start Seq.	End Seq.	Sequence	Ion Score	C. I. %	Modification	Rank	Result Type
1065.5211	1065.4583	-0.0628	-59	351	359	AQYEDIAQK					Mascot
1125.5422	1125.4442	-0.098	-87	362	371	AEAESLYQSK					Mascot
1127.5402	1127.4773	-0.0629	-56	284	293	KDVDGAYMTK					Mascot
1141.5194	1141.504	-0.0154	-13	459	467	DYQELMNTK					Mascot
1265.6372	1265.583	-0.0542	-43	273	283	TNAENEFVTIK					Mascot
1277.71	1277.6653	-0.0447	-35	468	478	LALDLEIATYR	72	99.994			Mascot
1383.6903	1383.6151	-0.0752	-54	181	192	SLNNQFASFIDK					Mascot
1475.7853	1475.7074	-0.0779	-53	195	206	FLEQQNQVLQTK	35	67.488			Mascot
1657.793	1657.7584	-0.0346	-21	13	29	SGGGFSSGSAGIINYQR					Mascot
1716.8511	1716.8031	-0.048	-28	413	427	QISNLQQSISDAEQR	56	99.751			Mascot
2121.0107	2121.0425	0.0318	15	9	29	SGYRSGGGFSSGSAGIIN YQR					Mascot
3312.3083	3312.2603	-0.048	-14	545	583	GSYGSGGSSYGSGGGS YGSGGGGGGHGSYGSG SSSGGYR					Mascot

3	Keratin, type I cytoskeletal 10 OS=Homo sapiens GN=KRT10 PE=1 SV=6	K1C10_HUMAN	59019.8	5.13	11	187	100	2.535	157	100	

Peptide Information

Calc. Mass	Obsrv. Mass	± da	± ppm	Start Seq.	End Seq.	Sequence	Ion Score	C. I. %	Modification	Rank	Result Type
807.3995	807.3381	-0.0614	-76	229	235	LAADDFR					Mascot
993.4999	993.4412	-0.0587	-59	238	245	YENEVALR					Mascot
1060.5092	1060.511	0.0018	2	1	9	MSVRYSSSK			Oxidation (M)[1]		Mascot
1106.5259	1106.4727	-0.0532	-48	148	156	VTMQNLNDR			Oxidation (M)[3]		Mascot
1118.5087	1118.4744	-0.0343	-31	185	194	HGNSHQGEPR					Mascot
1201.6172	1201.5573	-0.0599	-50	246	256	QSVEADINGLR					Mascot
1262.5972	1262.5723	-0.0249	-20	451	464	SLLEGEGSSGGGGR					Mascot
1390.6809	1390.6479	-0.033	-24	387	399	QSLEASLAETEGR					Mascot
1434.7699	1434.7098	-0.0601	-42	440	450	IRLENEIQTYR	52	99.411			Mascot
1493.7343	1493.6898	-0.0445	-30	323	334	SQYEQLAEQNRK	15	0			Mascot
1707.7722	1707.7307	-0.0415	-24	41	59	GSLGGGFSSGGFSGGSF SR	90	100			Mascot

4	Keratin, type I cytoskeletal 9 OS=Homo sapiens GN=KRT9 PE=1 SV=3	K1C9_HUMAN	62254.9	5.14	12	169	100	4.224	131	100	

Peptide Information

Calc. Mass	Obsrv. Mass	± da	± ppm	Start Seq.	End Seq.	Sequence	Ion Score	C. I. %	Modification	Rank	Result Type
913.4084	913.3467	-0.0617	-68	234	240	MTLDDFR			Oxidation (M)[1]		Mascot
982.4337	982.403	-0.0307	-31	35	46	FSSSGGGGGGGR					Mascot

1060.5634	1060.511	-0.0524	-49	225	233	TLLDIDNTR					Mascot
1065.4994	1065.4583	-0.0411	-39	155	163	STMQELNSR					Mascot
1232.5979	1232.5566	-0.0413	-34	14	29	SGGGGGGGLGSGGSIR					Mascot
1235.5288	1235.562	0.0332	27	47	59	FSSSSGYGGGSSR					Mascot
1323.6726	1323.6052	-0.0674	-51	241	250	IKFEMEQNLR			Oxidation (M)[5]		Mascot
1605.7432	1605.6647	-0.0785	-49	200	212	NYSPYYNTIDDLK					Mascot
1707.7504	1707.7307	-0.0197	-12	47	63	FSSSSGYGGGSSRVCGR			Carbamidomethyl (C)[15]		Mascot
1791.7278	1791.6755	-0.0523	-29	491	513	GGSGGSYGGGSGGGY GGGSGSR	131	100			Mascot
1837.9655	1837.8822	-0.0833	-45	375	390	HGVQELEIELQSQLSK					Mascot
2510.1318	2510.0449	-0.0869	-35	450	472	EIETYHNLLEGGQEDFES SGAGK					Mascot

5	Keratin, type I cytoskeletal 10 OS=Canis familiaris GN=KRT10 PE=2 SV=1	K1C10_CANFA	57847.2	5.09	7	155	100	1.692	142	100

Peptide Information

Calc. Mass	Obsrv. Mass	± da	± ppm	Start Seq.	End Seq.	Sequence	Ion Score	C. I. %	Modification	Rank	Result Type
807.3995	807.3381	-0.0614	-76	219	225	LAADDFR					Mascot
993.4999	993.4412	-0.0587	-59	228	235	YENEVALR					Mascot
1060.5092	1060.511	0.0018	2	1	9	MSVRYSSSK			Oxidation (M)[1]		Mascot
1106.5259	1106.4727	-0.0532	-48	138	146	VTMQNLNDR			Oxidation (M)[3]		Mascot
1201.6172	1201.5573	-0.0599	-50	236	246	QSVEADINGLR					Mascot
1434.7699	1434.7098	-0.0601	-42	430	440	IRLENEIQTYR	52	99.411			Mascot
1707.7722	1707.7307	-0.0415	-24	35	53	GSIGGGFSSGGFSGGSF SR	90	100			Mascot

6	Keratin, type II cytoskeletal 2 epidermal OS=Homo sapiens GN=KRT2 PE=1 SV=2	K22E_HUMAN	65678.3	8.07	11	137	100	4.708	105	100

Peptide Information

Calc. Mass	Obsrv. Mass	± da	± ppm	Start Seq.	End Seq.	Sequence	Ion Score	C. I. %	Modification	Rank	Result Type
1037.5262	1037.4777	-0.0485	-47	239	247	YLDGLTAER					Mascot
1107.543	1107.5071	-0.0359	-32	354	362	AQYEEIAQR					Mascot
1125.514	1125.4442	-0.0698	-62	2	10	SCQISCKSR			Carbamidomethyl (C)[2,6]		Mascot
1193.6161	1193.5719	-0.0442	-37	375	384	YEELQVTVGR					Mascot
1263.6943	1263.6121	-0.0822	-65	471	481	LALDVEIATYR					Mascot
1320.5829	1320.5498	-0.0331	-25	46	61	HGGGGGGFGGGGFGSR					Mascot
1320.5829	1320.5498	-0.0331	-25	46	61	HGGGGGGFGGGGFGSR	71	99.992			Mascot
1460.7996	1460.704	-0.0956	-65	303	314	VDLLNQEIEFLK					Mascot

1475.7853	1475.7074	-0.0779	-53	198	209	FLEQQNQVLQTK	35	67.488			Mascot
1740.7057	1740.6633	-0.0424	-24	525	544	GSSSGGGYSSGSSSYGS GGR					Mascot
1838.9144	1838.8708	-0.0436	-24	71	92	SISISVAGGGGGFGAAG GFGGR					Mascot
1890.8578	1890.8998	0.042	22	573	595	YGSGGGSKGGSISGGGY GSGGGK					Mascot

7	Keratin, type II cytoskeletal 73 OS=Mus musculus GN=Krt73 PE=1 SV=1	K2C73_MOUSE	59501.9	8.36	7	119	100	1.415	105	100	

Peptide Information

Calc. Mass	Obsrv. Mass	± da	± ppm	Start Seq.	End Seq.	Sequence	Ion Score	C. I. %	Modification	Rank	Result Type
1033.5459	1033.4742	-0.0717	-69	407	414	EELARMLR			Oxidation (M)[6]		Mascot
1060.5997	1060.511	-0.0887	-84	359	367	LRSEIESVK					Mascot
1127.5402	1127.4773	-0.0629	-56	240	249	KDVDAAYMSK					Mascot
1194.6226	1194.5653	-0.0573	-48	33	45	AAGKGLSGGFSSR					Mascot
1265.6736	1265.583	-0.0906	-72	307	317	AQYEDIALKSK					Mascot
1277.71	1277.6653	-0.0447	-35	424	434	LALDIEIATYR	72	99.994			Mascot
1475.7853	1475.7074	-0.0779	-53	151	162	FLEQQNQVLQTK	35	67.488			Mascot

8	Keratin, type II cytoskeletal 73 OS=Rattus norvegicus GN=Krt73 PE=1 SV=1	K2C73_RAT	60976.6	8.17	6	117	100	1.174	106	100	

Peptide Information

Calc. Mass	Obsrv. Mass	± da	± ppm	Start Seq.	End Seq.	Sequence	Ion Score	C. I. %	Modification	Rank	Result Type
1033.5459	1033.4742	-0.0717	-69	407	414	EELARMLR			Oxidation (M)[6]		Mascot
1060.5997	1060.511	-0.0887	-84	359	367	LRSEIESVK					Mascot
1127.5402	1127.4773	-0.0629	-56	240	249	KDVDAAYMSK					Mascot
1265.6736	1265.583	-0.0906	-72	307	317	AQYEDIALKSK					Mascot
1277.71	1277.6653	-0.0447	-35	424	434	LALDIEIATYR	72	99.994			Mascot
1475.7853	1475.7074	-0.0779	-53	151	162	FLEQQNQVLQTK	35	67.488			Mascot

9	Glial fibrillary acidic protein OS=Bos taurus GN=GFAP PE=2 SV=2	GFAP_BOVIN	49538.4	5.36	11	97	99.989	3.592	72	99.994	

Peptide Information

Calc. Mass	Obsrv. Mass	± da	± ppm	Start Seq.	End Seq.	Sequence	Ion Score	C. I. %	Modification	Rank	Result Type
848.422	848.3391	-0.0829	-98	60	66	ETRASER					Mascot
905.4323	905.3986	-0.0337	-37	297	304	GTNESLER					Mascot

1060.4919	1060.511	0.0191	18	276	283	HEANDYRR					Mascot
1140.4659	1140.5194	0.0535	47	67	75	AEMMELNDR			Oxidation (M)[3,4]		Mascot
1259.6339	1259.6371	0.0032	3	149	158	QKLQDETNQR					Mascot
1263.6328	1263.6121	-0.0207	-16	159	169	LEAENNLAAYR					Mascot
1276.6566	1276.5819	-0.0747	-59	408	418	TVEMRDGEVIK					Mascot
1277.71	1277.6653	-0.0447	-35	353	363	LALDIEIATYR	72	99.994			Mascot
1320.6324	1320.5498	-0.0826	-63	297	307	GTNESLERQMR					Mascot
1320.6324	1320.5498	-0.0826	-63	297	307	GTNESLERQMR					Mascot
1373.6366	1373.6219	-0.0147	-11	13	25	SYVSSSEMVVGGR			Oxidation (M)[8]		Mascot
1434.825	1434.7098	-0.1152	-80	33	45	LSLARMPPPLPAR			Oxidation (M)[6]		Mascot

10	Keratin, type II cytoskeletal 7 OS=Pan troglodytes GN=KRT7 PE=2 SV=1	K2C7_PANTR	51439.4	5.41	10	92	99.97	2.325	72	99.994

Peptide Information

Calc. Mass	Obsrv. Mass	± da	± ppm	Start Seq.	End Seq.	Sequence	Ion Score	C. I. %	Modification	Rank	Result Type
1033.5208	1033.4742	-0.0466	-45	366	373	QDMARQLR			Oxidation (M)[3]		Mascot
1060.5634	1060.511	-0.0524	-49	394	402	KLLEGEESR					Mascot
1104.5797	1104.491	-0.0887	-80	53	64	SAYGGPVGAGIR					Mascot
1127.5402	1127.4773	-0.0629	-56	199	208	KDVDAAYMSK					Mascot
1201.606	1201.5573	-0.0487	-41	332	342	LEAAIAEAEER					Mascot
1277.71	1277.6653	-0.0447	-35	383	393	LALDIEIATYR	72	99.994			Mascot
1379.6332	1379.6514	0.0182	13	303	313	NTRNEISEMNR			Oxidation (M)[9]		Mascot
1460.7274	1460.704	-0.0234	-16	306	317	NEISEMNRAIQR					Mascot
1490.8213	1490.7118	-0.1095	-73	111	122	FLEQQNKLLETK					Mascot
2139.0273	2138.9399	-0.0874	-41	162	179	LEAELQSMQDVVEDFKN K			Oxidation (M)[8]		Mascot

Gel Idx/Pos	309/M11	Instr./Gel Origin	BA2151/Sample Project 20140814	Process Status	Analysis Succeeded
Plate [#] Name	[1] Sample Project 20140814	Instrument Sample Name		Spectra	11

Rank	Protein Name	Accession No.	Protein MW	Protein PI	Pep. Count	Protein Score	Protein Score C. I. %	Intensity Matched	Total Ion Score	Total Ion C. I. %	Confirmed
1	Keratin, type I cytoskeletal 9 OS=Homo sapiens GN=KRT9 PE=1 SV=3	K1C9_HUMAN	62254.9	5.14	10	197	100	4.024	172	100	

Peptide Information

Calc. Mass	Obsrv. Mass	± da	± ppm	Start Seq.	End Seq.	Sequence	Ion Score	C. I. %	Modification	Rank	Result Type
913.4084	913.3546	-0.0538	-59	234	240	MTLDDFR			Oxidation (M)[1]		Mascot
982.4337	982.396	-0.0377	-38	35	46	FSSSGGGGGGGR					Mascot
1060.5634	1060.5035	-0.0599	-56	225	233	TLLDIDNTR					Mascot
1074.5215	1074.4745	-0.047	-44	5	13	QFSSSYLSR					Mascot
1157.5909	1157.53	-0.0609	-53	251	261	QGVDADINGLR					Mascot
1232.5979	1232.553	-0.0449	-36	14	29	SGGGGGGGLGSGGSIR					Mascot
1235.5288	1235.4944	-0.0344	-28	47	59	FSSSSGYGGGSSR					Mascot
1235.5288	1235.4944	-0.0344	-28	47	59	FSSSSGYGGGSSR	23	0			Mascot
1323.6726	1323.5927	-0.0799	-60	241	250	IKFEMEQNLR			Oxidation (M)[5]		Mascot
1605.7432	1605.6782	-0.065	-40	200	212	NYSPYYNTIDDLK					Mascot
1791.7278	1791.6697	-0.0581	-32	491	513	GGSGGSYGGGGSGGGY GGGSGSR	149	100			Mascot

Rank	Protein Name	Accession No.	Protein MW	Protein PI	Pep. Count	Protein Score	Protein Score C. I. %	Intensity Matched	Total Ion Score	Total Ion C. I. %	Confirmed
2	Alpha-1-purothionin (Fragment) OS=Triticum aestivum GN=THI1.1 PE=1 SV=2	THN1_WHEAT	14371.6	4.76	3	197	100	6.536	183	100	

Peptide Information

Calc. Mass	Obsrv. Mass	± da	± ppm	Start Seq.	End Seq.	Sequence	Ion Score	C. I. %	Modification	Rank	Result Type
835.3913	835.3531	-0.0382	-46	40	46	LCAGVCR			Carbamidomethyl (C)[2,6]		Mascot
999.4135	999.3727	-0.0408	-41	27	33	NCYNLCR			Carbamidomethyl (C)[2,6]		Mascot
999.4135	999.3727	-0.0408	-41	27	33	NCYNLCR	41	93.926	Carbamidomethyl (C)[2,6]		Mascot
2456.0706	2455.999	-0.0716	-29	62	82	LALESNSDEPDTIEYCNL GCR			Carbamidomethyl (C)[16,20]		Mascot
2456.0706	2455.999	-0.0716	-29	62	82	LALESNSDEPDTIEYCNL GCR	142	100	Carbamidomethyl (C)[16,20]		Mascot

Rank	Protein Name	Accession No.	Protein MW	Protein PI	Pep. Count	Protein Score	Protein Score C. I. %	Intensity Matched	Total Ion Score	Total Ion C. I. %	Confirmed
3	Alpha-2-purothionin OS=Triticum aestivum GN=THI1.2 PE=2 SV=1	THN2_WHEAT	15403.1	5.13	2	192	100	5.567	183	100	

Peptide Information

Calc. Mass	Obsrv. Mass	± da	± ppm	Start Seq.	End Seq.	Sequence	Ion Score	C. I. %	Modification	Rank	Result Type

Calc. Mass	Obsrv. Mass	± da	± ppm	Start Seq.	End Seq.	Sequence	Ion Score	C. I. %	Modification	Rank	Result Type
999.4135	999.3727	-0.0408	-41	38	44	NCYNLCR			Carbamidomethyl (C)[2,6]		Mascot
999.4135	999.3727	-0.0408	-41	38	44	NCYNLCR	41	93.926	Carbamidomethyl (C)[2,6]		Mascot
2456.0706	2455.999	-0.0716	-29	73	93	LALESNSDEPDTIEYCNL GCR			Carbamidomethyl (C)[16,20]		Mascot
2456.0706	2455.999	-0.0716	-29	73	93	LALESNSDEPDTIEYCNL GCR	142	100	Carbamidomethyl (C)[16,20]		Mascot

4	Keratin, type II cytoskeletal 1 OS=Homo sapiens GN=KRT1 PE=1 SV=6	K2C1_HUMAN	66170.1	8.15	15	190	100	7.69	139	100

Peptide Information

Calc. Mass	Obsrv. Mass	± da	± ppm	Start Seq.	End Seq.	Sequence	Ion Score	C. I. %	Modification	Rank	Result Type
802.4417	802.3693	-0.0724	-90	180	185	EREQIK					Mascot
932.5047	932.4337	-0.071	-76	410	417	SEIDNVKK					Mascot
973.5312	973.4794	-0.0518	-53	396	403	IEISELNR					Mascot
999.4452	999.3727	-0.0725	-73	290	298	DVDGAYMTK					Mascot
999.4452	999.3727	-0.0725	-73	290	298	DVDGAYMTK					Mascot
1006.4297	1006.4298	0.0001	0	589	602	GGSGGGGGGSSGGR					Mascot
1033.516	1033.4652	-0.0508	-49	484	492	TLLEGEESR					Mascot
1127.5402	1127.4724	-0.0678	-60	289	298	KDVDGAYMTK					Mascot
1141.5194	1141.4908	-0.0286	-25	464	472	DYQELMNTK					Mascot
1152.5531	1152.4904	-0.0627	-54	268	276	NKYEDEINK					Mascot
1157.5143	1157.53	0.0157	14	464	472	DYQELMNTK			Oxidation (M)[6]		Mascot
1265.6372	1265.5684	-0.0688	-54	278	288	TNAENEFVTIK					Mascot
1277.71	1277.6562	-0.0538	-42	473	483	LALDLEIATYR	56	99.785			Mascot
1383.6903	1383.6099	-0.0804	-58	186	197	SLNNQFASFIDK					Mascot
1657.793	1657.7355	-0.0575	-35	13	29	SGGGFSSGSAGIINYQR					Mascot
1716.8511	1716.7841	-0.067	-39	418	432	QISNLQQSISDAEQR	85	100			Mascot
3312.3083	3312.2419	-0.0664	-20	550	588	GSYGSGGSSYGSGGGS YGSGGGGGGHGSYGSG SSSGGYR					Mascot

5	Keratin, type II cytoskeletal 1 OS=Pan troglodytes GN=KRT1 PE=1 SV=1	K2C1_PANTR	65620.8	7.62	14	183	100	7.378	140	100

Peptide Information

Calc. Mass	Obsrv. Mass	± da	± ppm	Start Seq.	End Seq.	Sequence	Ion Score	C. I. %	Modification	Rank	Result Type
802.4417	802.3693	-0.0724	-90	175	180	EREQIK					Mascot
932.5047	932.4337	-0.071	-76	405	412	SEIDNVKK					Mascot
973.5312	973.4794	-0.0518	-53	391	398	IEISELNR					Mascot

999.4452	999.3727	-0.0725	-73	285	293	DVDGAYMTK					Mascot
999.4452	999.3727	-0.0725	-73	285	293	DVDGAYMTK					Mascot
1033.516	1033.4652	-0.0508	-49	479	487	TLLEGEESR					Mascot
1127.5402	1127.4724	-0.0678	-60	284	293	KDVDGAYMTK					Mascot
1141.5194	1141.4908	-0.0286	-25	459	467	DYQELMNTK					Mascot
1152.5531	1152.4904	-0.0627	-54	263	271	NKYEDEINK					Mascot
1157.5143	1157.53	0.0157	14	459	467	DYQELMNTK			Oxidation (M)[6]		Mascot
1265.6372	1265.5684	-0.0688	-54	273	283	TNAENEFVTIK					Mascot
1277.71	1277.6562	-0.0538	-42	468	478	LALDLEIATYR	56	99.785			Mascot
1383.6903	1383.6099	-0.0804	-58	181	192	SLNNQFASFIDK					Mascot
1657.793	1657.7355	-0.0575	-35	13	29	SGGGFSSGSAGIINYQR					Mascot
1716.8511	1716.7841	-0.067	-39	413	427	QISNLQQSISDAEQR	85	100			Mascot
3312.3083	3312.2419	-0.0664	-20	545	583	GSYGSGGSSYGSGGGS YGSGGGGGGHGSYGSG SSSGGYR					Mascot

6	Keratin, type II cytoskeletal 8 OS=Homo sapiens GN=KRT8 PE=1 SV=7	K2C8_HUMAN	53671.1	5.52	16	97	99.99	6.033	56	99.785

Peptide Information

Calc. Mass	Obsrv. Mass	± da	± ppm	Start Seq.	End Seq.	Sequence	Ion Score	C. I. %	Modification	Rank	Result Type
818.4366	818.3601	-0.0765	-93	466	472	IETRDGK					Mascot
827.4297	827.3799	-0.0498	-60	102	108	FASFIDK					Mascot
835.4091	835.3531	-0.056	-67	363	369	AKQDMAR			Oxidation (M)[5]		Mascot
870.4316	870.4943	0.0627	72	33	40	ISSSSFSR					Mascot
932.4683	932.4337	-0.0346	-37	394	401	LLEGEESR					Mascot
1030.568	1030.5055	-0.0625	-61	123	130	WSLLQQQK					Mascot
1033.5208	1033.4652	-0.0556	-54	365	372	QDMARQLR			Oxidation (M)[3]		Mascot
1045.5525	1045.5063	-0.0462	-44	150	158	QLETLGQEK					Mascot
1060.5634	1060.5035	-0.0599	-56	393	401	KLLEGEESR					Mascot
1152.5531	1152.4904	-0.0627	-54	177	185	NKYEDEINK					Mascot
1153.5558	1153.4781	-0.0777	-67	373	381	EYQELMNVK					Mascot
1201.6536	1201.5728	-0.0808	-67	149	158	RQLETLGQEK					Mascot
1208.594	1208.537	-0.057	-47	303	312	TKTEISEMNR					Mascot
1277.71	1277.6562	-0.0538	-42	382	392	LALDIEIATYR	56	99.785			Mascot
1320.6716	1320.5657	-0.1059	-80	253	264	SLDMDSIIAEVK					Mascot
1507.688	1507.6849	-0.0031	-2	402	414	LESGMQNMSIHTK			Oxidation (M)[5,8]		Mascot

7	Keratin, type II cytoskeletal 8 OS=Rattus norvegicus	K2C8_RAT	53985.3	5.83	16	94	99.981	6.281	56	99.785

GN=Krt8 PE=1 SV=3

Peptide Information

Calc. Mass	Obsrv. Mass	± da	± ppm	Start Seq.	End Seq.	Sequence	Ion Score	C. I. %	Modification	Rank	Result Type
818.4366	818.3601	-0.0765	-93	466	472	IETRDGK					Mascot
827.4297	827.3799	-0.0498	-60	102	108	FASFIDK					Mascot
835.4091	835.3531	-0.056	-67	363	369	AKQDMAR			Oxidation (M)[5]		Mascot
870.4316	870.4943	0.0627	72	33	40	ISSSSFSR					Mascot
913.4374	913.3546	-0.0828	-91	41	49	VGSSSSSFR					Mascot
932.4683	932.4337	-0.0346	-37	394	401	LLEGEESR					Mascot
1030.568	1030.5055	-0.0625	-61	123	130	WSLLQQQK					Mascot
1033.5208	1033.4652	-0.0556	-54	365	372	QDMARQLR			Oxidation (M)[3]		Mascot
1053.5323	1053.4763	-0.056	-53	226	233	QIHEEEIR					Mascot
1060.5634	1060.5035	-0.0599	-56	393	401	KLLEGEESR					Mascot
1152.5531	1152.4904	-0.0627	-54	177	185	NKYEDEINK					Mascot
1153.5558	1153.4781	-0.0777	-67	373	381	EYQELMNVK					Mascot
1194.5784	1194.5454	-0.033	-28	303	312	SKTEISEMNR					Mascot
1277.71	1277.6562	-0.0538	-42	382	392	LALDIEIATYR	56	99.785			Mascot
1507.688	1507.6849	-0.0031	-2	402	414	LESGMQNMSIHTK			Oxidation (M)[5,8]		Mascot
1699.8722	1699.7703	-0.1019	-60	326	341	GQRATLEAAIADAEQR					Mascot

8	Keratin, type II cytoskeletal 68 kDa, component IA (Fragment) OS=Bos taurus PE=2 SV=1	K2CA_BOVIN	18295	8.54	7	93	99.972	2.548	56	99.785	

Peptide Information

Calc. Mass	Obsrv. Mass	± da	± ppm	Start Seq.	End Seq.	Sequence	Ion Score	C. I. %	Modification	Rank	Result Type
846.454	846.3753	-0.0787	-93	118	126	LGSGGRGSR					Mascot
1106.5146	1106.4612	-0.0534	-48	57	65	TLLEGEECR			Carbamidomethyl (C)[8]		Mascot
1107.5389	1107.4855	-0.0534	-48	168	177	VTQSSSQSQR					Mascot
1153.5558	1153.4781	-0.0777	-67	37	45	EYQELMNVK					Mascot
1277.71	1277.6562	-0.0538	-42	46	56	LALDIEIATYR	56	99.785			Mascot
1507.8188	1507.6849	-0.1339	-89	34	45	LLKEYQELMNVK					Mascot
2365.2068	2364.9919	-0.2149	-91	46	65	LALDIEIATYRTLLEGEEC R			Carbamidomethyl (C)[19]		Mascot

9	Glial fibrillary acidic protein OS=Bos taurus GN=GFAP PE=2 SV=2	GFAP_BOVIN	49538.4	5.36	13	92	99.962	3.464	56	99.785	

Peptide Information

Calc. Mass	Obsrv. Mass	± da	± ppm	Start Seq.	End Seq.	Sequence	Ion Score	C. I. %	Modification	Rank	Result Type
848.422	848.3549	-0.0671	-79	60	66	ETRASER					Mascot
856.4523	856.4671	0.0148	17	394	401	SVSEGHLK					Mascot
1003.4803	1003.4233	-0.057	-57	151	158	LQDETNQR					Mascot
1060.4919	1060.5035	0.0116	11	276	283	HEANDYRR					Mascot
1063.5167	1063.4937	-0.023	-22	257	266	FADLNDAAAR					Mascot
1140.4659	1140.4991	0.0332	29	67	75	AEMMELNDR			Oxidation (M)[3,4]		Mascot
1208.5906	1208.537	-0.0536	-44	316	326	EAASYQEALAR					Mascot
1259.6339	1259.6295	-0.0044	-3	149	158	QKLQDETNQR					Mascot
1263.6328	1263.6216	-0.0112	-9	159	169	LEAENNLAAYR					Mascot
1277.71	1277.6562	-0.0538	-42	353	363	LALDIEIATYR	56	99.785			Mascot
1320.6324	1320.5657	-0.0667	-51	297	307	GTNESLERQMR					Mascot
1434.825	1434.6969	-0.1281	-89	33	45	LSLARMPPPLPAR			Oxidation (M)[6]		Mascot
1471.7864	1471.671	-0.1154	-78	138	150	DNLAQDLGTLRQK					Mascot

10	Keratin, type II cytoskeletal 8 OS=Xenopus laevis PE=2 SV=1	K2C8_XENLA	55644.9	5.3	14	89	99.938	4.618	56	99.785

Peptide Information

Calc. Mass	Obsrv. Mass	± da	± ppm	Start Seq.	End Seq.	Sequence	Ion Score	C. I. %	Modification	Rank	Result Type
824.4294	824.369	-0.0604	-73	1	7	MSVRSTK			Oxidation (M)[1]		Mascot
827.4297	827.3799	-0.0498	-60	110	116	FASFIDK					Mascot
835.4091	835.3531	-0.056	-67	371	377	AKQDMSR					Mascot
851.4039	851.3754	-0.0285	-33	371	377	AKQDMSR			Oxidation (M)[5]		Mascot
932.4683	932.4337	-0.0346	-37	402	409	LLEGEESR					Mascot
973.4949	973.4794	-0.0155	-16	158	166	QLDGLGQDK					Mascot
1033.5208	1033.4652	-0.0556	-54	373	380	QDMSRQLR					Mascot
1037.4899	1037.4746	-0.0153	-15	273	281	AQYEDVANK					Mascot
1053.5211	1053.4763	-0.0448	-43	304	312	YGDDLKNTK					Mascot
1060.5634	1060.5035	-0.0599	-56	401	409	KLLEGEESR					Mascot
1139.5402	1139.4666	-0.0736	-65	381	389	DYQELMNVK					Mascot
1152.5645	1152.4904	-0.0741	-64	294	303	YQELQTSAGR					Mascot
1155.535	1155.5016	-0.0334	-29	381	389	DYQELMNVK			Oxidation (M)[6]		Mascot
1208.6382	1208.537	-0.1012	-84	8	18	VTYRTSSAAPR					Mascot
1277.71	1277.6562	-0.0538	-42	390	400	LALDIEIATYR	56	99.785			Mascot
1490.8213	1490.6932	-0.1281	-86	194	205	RTELENEFVLLK					Mascot

Gel Idx/Pos	310/M12	Instr./Gel Origin	BA2151/Sample Project 20140814	Process Status	Analysis Succeeded
Plate [#] Name	[1] Sample Project 20140814	Instrument Sample Name		Spectra	11

Rank	Protein Name	Accession No.	Protein MW	Protein PI	Pep. Count	Protein Score	Protein Score C. I. %	Intensity Matched	Total Ion Score	Total Ion C. I. %	Confirmed
1	Alpha-amylase/trypsin inhibitor CM2 OS=Triticum aestivum PE=1 SV=2	IAAC2_WHEAT	16019.7	6.86	5	312	100	31.26	282	100	

Peptide Information

Calc. Mass	Obsrv. Mass	± da	± ppm	Start Seq.	End Seq.	Sequence	Ion Score	C. I. %	Modification	Rank	Result Type
814.3876	814.3585	-0.0291	-36	107	113	DLPGCPR			Carbamidomethyl (C)[5]		Mascot
1017.5211	1017.4822	-0.0389	-38	97	106	TSDPNSGVLK					Mascot
1278.5532	1278.514	-0.0392	-31	77	86	ELYDASQHCR			Carbamidomethyl (C)[9]		Mascot
1278.5532	1278.514	-0.0392	-31	77	86	ELYDASQHCR	83	100	Carbamidomethyl (C)[9]		Mascot
2703.3044	2703.2476	-0.0568	-21	46	71	EYVAQQTCGVGIVGSPV STEPGNTPR			Carbamidomethyl (C)[8]		Mascot
2703.3044	2703.2476	-0.0568	-21	46	71	EYVAQQTCGVGIVGSPV STEPGNTPR	199	100	Carbamidomethyl (C)[8]		Mascot
2726.3101	2726.219	-0.0911	-33	122	145	VLVTPGHCNVMTVHNTP YCLGLDI			Carbamidomethyl (C)[8,19], Oxidation (M)[11]		Mascot

Rank	Protein Name	Accession No.	Protein MW	Protein PI	Pep. Count	Protein Score	Protein Score C. I. %	Intensity Matched	Total Ion Score	Total Ion C. I. %	Confirmed
2	Chymotrypsin inhibitor WCI OS=Triticum aestivum PE=1 SV=1	ICIW2_WHEAT	13505.5	7.42	3	144	100	4.549	129	100	

Peptide Information

Calc. Mass	Obsrv. Mass	± da	± ppm	Start Seq.	End Seq.	Sequence	Ion Score	C. I. %	Modification	Rank	Result Type
889.456	889.452	-0.004	-4	87	93	CPTEVKR			Carbamidomethyl (C)[1]		Mascot
1120.5415	1120.5042	-0.0373	-33	47	56	ELAAISSNCR			Carbamidomethyl (C)[9]		Mascot
1986.0193	1985.9745	-0.0448	-23	67	86	AFPPSQSQGGGPPQPPL APR					Mascot
1986.0193	1985.9745	-0.0448	-23	67	86	AFPPSQSQGGGPPQPPL APR	129	100			Mascot

Rank	Protein Name	Accession No.	Protein MW	Protein PI	Pep. Count	Protein Score	Protein Score C. I. %	Intensity Matched	Total Ion Score	Total Ion C. I. %	Confirmed
3	Alpha-amylase/trypsin inhibitor CM1 OS=Triticum aestivum PE=1 SV=1	IAAC1_WHEAT	16076.8	7.49	6	118	100	24.495	83	100	

Peptide Information

Calc. Mass	Obsrv. Mass	± da	± ppm	Start Seq.	End Seq.	Sequence	Ion Score	C. I. %	Modification	Rank	Result Type
811.4573	811.4189	-0.0384	-47	92	97	YFIGRR					Mascot
814.3876	814.3585	-0.0291	-36	107	113	DLPGCPR			Carbamidomethyl (C)[5]		Mascot
1102.5852	1102.5223	-0.0629	-57	97	106	RSDPNSSVLK					Mascot
1278.5532	1278.514	-0.0392	-31	77	86	ELYDASQHCR			Carbamidomethyl (C)[9]		Mascot

1278.5532	1278.514	-0.0392	-31	77	86	ELYDASQHCR	83	100	Carbamidomethyl (C)[9]		Mascot
1741.8538	1741.7435	-0.1103	-63	98	113	SDPNSSVLKDLPGCPR			Carbamidomethyl (C)[14]		Mascot
2709.2786	2709.2214	-0.0572	-21	46	71	EYVAQQTCGISISGSAVS TEPGNTPR			Carbamidomethyl (C)[8]		Mascot

4	Trypsin/alpha-amylase inhibitor CMX2 OS=Triticum aestivum PE=2 SV=1	IACX2_WHEAT	14279.3	9.08	4	78	99.2	4.195	67	99.985

Protein Group

Trypsin/alpha-amylase inhibitor CMX1/CMX3 OS=Triticum aestivum PE=2 SV=1	IACX1_WHEAT	14221.3	9.2299 995422 3633

Peptide Information

Calc. Mass	Obsrv. Mass	± da	± ppm	Start Seq.	End Seq.	Sequence	Ion Score	C. I. %	Modification	Rank	Result Type
845.3934	845.4031	0.0097	11	27	33	EQCVPGR			Carbamidomethyl (C)[3]		Mascot
961.5465	961.514	-0.0325	-34	112	120	EFIAGIVGR					Mascot
961.5465	961.514	-0.0325	-34	112	120	EFIAGIVGR	67	99.985			Mascot
1347.5999	1347.5624	-0.0375	-28	50	60	QTCGYYLSAER			Carbamidomethyl (C)[3]		Mascot
1351.6964	1351.5908	-0.1056	-78	34	44	EITYESLNARR					Mascot
1351.6964	1351.5908	-0.1056	-78	34	44	EITYESLNARR					Mascot

5	Glucose-6-phosphate 1-dehydrogenase OS=Streptococcus pneumoniae serotype 4 (strain ATCC BAA-334 / TIGR4) GN=zwf PE=3 SV=2	G6PD_STRPN	56811.5	5.45	14	58	3.843	2.653

Peptide Information

Calc. Mass	Obsrv. Mass	± da	± ppm	Start Seq.	End Seq.	Sequence	Ion Score	C. I. %	Modification	Rank	Result Type
835.4342	835.3749	-0.0593	-71	290	297	SGKIDGMK					Mascot
845.4515	845.4031	-0.0484	-57	175	181	IDHYLGK					Mascot
869.4669	869.4252	-0.0417	-48	330	336	GVPFFFR					Mascot
889.4162	889.452	0.0358	40	105	111	YQAEHNK					Mascot
1186.5488	1186.5447	-0.0041	-3	223	233	GGYYDQSGALR					Mascot
1193.6348	1193.55	-0.0848	-71	182	191	EMIQSIFAVR					Mascot
1350.6649	1350.5801	-0.0848	-63	131	142	SENIVDGKGFER					Mascot
1358.6587	1358.5756	-0.0831	-61	270	280	NLYHPTDEELK					Mascot
1494.774	1494.6927	-0.0813	-54	192	203	FANLIFENVWNK					Mascot
1566.7468	1566.7344	-0.0124	-8	461	475	AGSMGPQASFDLLEK			Oxidation (M)[4]		Mascot
1851.8872	1851.8739	-0.0133	-7	386	401	QVGEEFNLAPNSLDYR					Mascot
1869.9089	1869.8026	-0.1063	-57	217	233	LGVEERGGYYDQSGALR					Mascot
2685.3857	2685.25	-0.1357	-51	182	203	EMIQSIFAVRFANLIFENV			Oxidation (M)[2]		Mascot

2717.4001	2717.1875	-0.2126	-78	234	257	WNK DMVQNHTLQLLSLLAMD KPASFTK			Oxidation (M)[2]		Mascot

6	Mediator of RNA polymerase II transcription subunit 4 OS=Cryptococcus neoformans var. neoformans serotype D (strain B-3501A) GN=MED4 PE=3 SV=1	MED4_CRYNB	26174.2	5.61	10	50	0	2.461

Peptide Information

Calc. Mass	Obsrv. Mass	± da	± ppm	Start Seq.	End Seq.	Sequence	Ion Score	C. I. %	Modification	Rank	Result Type
832.441	832.3661	-0.0749	-90	116	122	SLEDIQK					Mascot
845.4363	845.4031	-0.0332	-39	188	195	GVVGEEQK					Mascot
1162.5885	1162.5494	-0.0391	-34	82	91	KNEVAGLEMR			Oxidation (M)[9]		Mascot
1186.6249	1186.5447	-0.0802	-68	132	142	TLMAHAQSLSK					Mascot
1225.5709	1225.5933	0.0224	18	67	76	EVGHHQEAYR					Mascot
1276.5839	1276.5112	-0.0727	-57	105	115	ELEGMIDQGER					Mascot
1292.5787	1292.5277	-0.051	-39	105	115	ELEGMIDQGER			Oxidation (M)[5]		Mascot
1507.8115	1507.6821	-0.1294	-86	143	157	HSSAPVSSLLAPVDK					Mascot
1599.6931	1599.7561	0.063	39	158	170	AQYAPWPTEMSMR			Oxidation (M)[10,12]		Mascot
2685.2681	2685.25	-0.0181	-7	171	195	MGLLFQLEGSMSGMGE RGVVGEEQK			Oxidation (M)[1]		Mascot
2691.4136	2691.2273	-0.1863	-69	132	157	TLMAHAQSLSKHSSAPV SSLLAPVDK			Oxidation (M)[3]		Mascot
2717.2581	2717.1875	-0.0706	-26	171	195	MGLLFQLEGSMSGMGE RGVVGEEQK			Oxidation (M)[1,11,14]		Mascot

7	Ubiquitin domain-containing protein 2 OS=Homo sapiens GN=UBTD2 PE=1 SV=2	UBTD2_HUMAN	26401.2	5.45	9	50	0	5.363

Peptide Information

Calc. Mass	Obsrv. Mass	± da	± ppm	Start Seq.	End Seq.	Sequence	Ion Score	C. I. %	Modification	Rank	Result Type
1002.5618	1002.5029	-0.0589	-59	68	75	KEIWDALK					Mascot
1003.5822	1003.5386	-0.0436	-43	207	214	FEELKIPK					Mascot
1221.6045	1221.4999	-0.1046	-86	170	179	STDTVFHMKR					Mascot
1350.676	1350.5801	-0.0959	-71	181	193	LHAAEGVEPGSQR					Mascot
1353.6951	1353.5784	-0.1167	-86	194	204	WFFSGRPLTDK					Mascot
1612.7424	1612.7052	-0.0372	-23	39	51	WKSDYPMTDGQLR			Oxidation (M)[7]		Mascot
2178.0974	2177.9639	-0.1335	-61	116	133	YQLPVYCLAPPINMIEEK			Carbamidomethyl (C)[7]		Mascot
2178.0974	2177.9639	-0.1335	-61	116	133	YQLPVYCLAPPINMIEEK			Carbamidomethyl (C)[7]		Mascot
2178.0974	2178.3064	0.209	96	116	133	YQLPVYCLAPPINMIEEK			Carbamidomethyl (C)[7]		Mascot
2225.145	2224.9895	-0.1555	-70	215	234	DYVVQVIVSQPVQNPTPV EN					Mascot
2685.3533	2685.25	-0.1033	-38	181	204	LHAAEGVEPGSQRWFFS					Mascot

GRPLTDK

8	Sulfotransferase 1C2 OS=Rattus norvegicus GN=Sult1c2 PE=2 SV=1	ST1C2_RAT	34961.4	8.17	12	50	0	8.624

Peptide Information

Calc. Mass	Obsrv. Mass	± da	± ppm	Start Seq.	End Seq.	Sequence	Ion Score	C. I. %	Modification	Rank	Result Type
814.3981	814.3585	-0.0396	-49	277	282	FDEIYK					Mascot
856.4886	856.4896	0.001	1	2	9	ALAPELSR					Mascot
1003.524	1003.5386	0.0146	15	1	9	MALAPELSR			Oxidation (M)[1]		Mascot
1120.5238	1120.5042	-0.0196	-17	233	241	MKENPMTNR					Mascot
1225.6833	1225.5933	-0.09	-73	97	107	ANAMPAPRILR			Oxidation (M)[4]		Mascot
1344.7303	1344.611	-0.1193	-89	1	12	MALAPELSRQTK					Mascot
1347.6481	1347.5624	-0.0857	-64	168	178	VSWGSWFDHVK					Mascot
1373.6842	1373.5896	-0.0946	-69	235	246	ENPMTNRSTVPK					Mascot
1375.5691	1375.5874	0.0183	13	285	296	MGGTSLNFCMEL			Carbamidomethyl (C)[9], Oxidation (M)[1]		Mascot
1507.7938	1507.6821	-0.1117	-74	247	259	SVLDQSISPFMRK					Mascot
1869.8047	1869.8026	-0.0021	-1	132	145	NAKDCMVSYYHFYR			Carbamidomethyl (C)[5], Oxidation (M)[6]		Mascot
2709.4373	2709.2214	-0.2159	-80	74	96	TIIQHRHPFIEWARPPQP SGVDK					Mascot

9	DNA polymerase III PolC-type OS=Staphylococcus haemolyticus (strain JCSC1435) GN=polC PE=3 SV=1	DPO3_STAHJ	163645.5	5.12	22	48	0	11.459

Peptide Information

Calc. Mass	Obsrv. Mass	± da	± ppm	Start Seq.	End Seq.	Sequence	Ion Score	C. I. %	Modification	Rank	Result Type
807.4247	807.3776	-0.0471	-58	984	990	VLFGEDK					Mascot
834.3839	834.3725	-0.0114	-14	676	682	DQSEVEK					Mascot
837.3771	837.4197	0.0426	51	2	8	AMTNQEK			Oxidation (M)[2]		Mascot
858.4104	858.4349	0.0245	29	459	465	FSNPHEK					Mascot
870.4389	870.498	0.0591	68	1324	1330	YMDLGKK			Oxidation (M)[2]		Mascot
889.405	889.452	0.047	53	283	289	DDLDHFK					Mascot
961.5023	961.514	0.0117	12	1351	1358	MQPISLEK			Oxidation (M)[1]		Mascot
961.5499	961.514	-0.0359	-37	1028	1036	LVKGCTGVK	2	0	Carbamidomethyl (C)[5]		Mascot
982.4662	982.4185	-0.0477	-49	152	159	KCGFDIDK			Carbamidomethyl (C)[2]		Mascot
1050.452	1050.5215	0.0695	66	818	826	MDGANEEIR			Oxidation (M)[1]		Mascot
1221.6111	1221.4999	-0.1112	-91	449	458	DGEIIDKFER					Mascot
1232.6191	1232.5374	-0.0817	-66	1102	1112	MLQDLSGIDPK			Oxidation (M)[1]		Mascot
1253.5752	1253.547	-0.0282	-22	1234	1244	GLTEEMIDAMK			Oxidation (M)[6]		Mascot

Calc. Mass	Obsrv. Mass	± da	± ppm	Start Seq.	End Seq.	Sequence	Ion Score	C. I. %	Modification	Rank	Result Type
1309.6747	1309.61	-0.0647	-49	874	885	KSLDDGYLVGSR					Mascot
1511.7523	1511.7312	-0.0211	-14	1089	1101	LDILGHDDPTMIR			Oxidation (M)[11]		Mascot
1571.7854	1571.7396	-0.0458	-29	1142	1156	TGTFGVPEFGTGFVR					Mascot
1571.7854	1571.7396	-0.0458	-29	1142	1156	TGTFGVPEFGTGFVR					Mascot
1599.7314	1599.7561	0.0247	15	311	323	DLVMMMTDIEEIK			Oxidation (M)[4,5]		Mascot
1985.026	1984.951	-0.075	-38	1102	1119	MLQDLSGIDPKTIPVDDK					Mascot
2242.0117	2241.9055	-0.1062	-47	1206	1224	DDIMVYLMYAGLEPSMAFK			Oxidation (M)[4,8,16]		Mascot
2242.0117	2241.9055	-0.1062	-47	1206	1224	DDIMVYLMYAGLEPSMAFK			Oxidation (M)[4,8,16]		Mascot
2709.3586	2709.2214	-0.1372	-51	1089	1112	LDILGHDDPTMIRMLQDLSGIDPK			Oxidation (M)[11]		Mascot
2717.3127	2717.1875	-0.1252	-46	772	794	TTDEMLDEFHFLGEEKAHEIVVK					Mascot
2726.3533	2726.219	-0.1343	-49	723	748	AGESTGIPVIATGNAHYLYEHDAIAR					Mascot

10	Minor spike protein H OS=Enterobacteria phage phiX174 (Isolate Sanger) GN=H PE=1 SV=1	H_BPPHS	34398	5.14	10	47	0	16.895	

Peptide Information

Calc. Mass	Obsrv. Mass	± da	± ppm	Start Seq.	End Seq.	Sequence	Ion Score	C. I. %	Modification	Rank	Result Type
1306.6671	1306.7229	0.0558	43	210	221	VASIMENTNLSK					Mascot
1320.61	1320.5359	-0.0741	-56	164	174	EIAEMQNETQK					Mascot
1322.6621	1322.6128	-0.0493	-37	210	221	VASIMENTNLSK			Oxidation (M)[5]		Mascot
1347.6937	1347.5624	-0.1313	-97	153	163	ELTKMQLDNQK					Mascot
1351.6429	1351.5908	-0.0521	-39	304	314	AVADTWNNFWK					Mascot
1351.6429	1351.5908	-0.0521	-39	304	314	AVADTWNNFWK					Mascot
1796.9033	1796.7699	-0.1334	-74	1	20	MFGAIAGGIASALAGGAMSK			Oxidation (M)[1]		Mascot
1851.9308	1851.8739	-0.0569	-31	255	270	VSAEVDLVHQQTQNQR					Mascot
2178.0166	2177.9639	-0.0527	-24	157	174	MQLDNQKEIAEMQNETQK					Mascot
2178.0166	2177.9639	-0.0527	-24	157	174	MQLDNQKEIAEMQNETQK					Mascot
2612.4507	2612.2429	-0.2078	-80	82	108	GLLEGTLQAGTSAVSDKLLDLVGLGGK					Mascot
2703.2866	2703.2476	-0.039	-14	231	253	QMLTQAQTAGGQYFTNDQIKEMTR					Mascot
2703.2866	2703.2476	-0.039	-14	231	253	QMLTQAQTAGGQYFTNDQIKEMTR					Mascot
2717.3198	2717.1875	-0.1323	-49	54	81	SAIQGSNVPNPDEAAPSFVSGAMAKAGK			Oxidation (M)[23]		Mascot

Rank	Protein Name	Accession No.	Protein MW	Protein PI	Pep. Count	Protein Score	Protein Score C. I. %	Intensity Matched	Total Ion Score	Total Ion C. I. %	Confirmed
1	Alpha-amylase/trypsin inhibitor CM2 OS=Triticum aestivum PE=1 SV=2	IAAC2_WHEAT	16019.7	6.86	4	302	100	17.919	282	100	

Peptide Information

Calc. Mass	Obsrv. Mass	± da	± ppm	Start Seq.	End Seq.	Sequence	Ion Score	C. I. %	Modification	Rank	Result Type
814.3876	814.3555	-0.0321	-39	107	113	DLPGCPR			Carbamidomethyl (C)[5]		Mascot
1017.5211	1017.4763	-0.0448	-44	97	106	TSDPNSGVLK					Mascot
1278.5532	1278.5017	-0.0515	-40	77	86	ELYDASQHCR			Carbamidomethyl (C)[9]		Mascot
1278.5532	1278.5017	-0.0515	-40	77	86	ELYDASQHCR	78	99.999	Carbamidomethyl (C)[9]		Mascot
2703.3044	2703.2388	-0.0656	-24	46	71	EYVAQQTCGVGIVGSPV STEPGNTPR			Carbamidomethyl (C)[8]		Mascot
2703.3044	2703.2388	-0.0656	-24	46	71	EYVAQQTCGVGIVGSPV STEPGNTPR	204	100	Carbamidomethyl (C)[8]		Mascot

Rank	Protein Name	Accession No.	Protein MW	Protein PI	Pep. Count	Protein Score	Protein Score C. I. %	Intensity Matched	Total Ion Score	Total Ion C. I. %	Confirmed
2	Keratin, type I cytoskeletal 9 OS=Homo sapiens GN=KRT9 PE=1 SV=3	K1C9_HUMAN	62254.9	5.14	11	125	100	3.045	95	100	

Peptide Information

Calc. Mass	Obsrv. Mass	± da	± ppm	Start Seq.	End Seq.	Sequence	Ion Score	C. I. %	Modification	Rank	Result Type
809.4403	809.4215	-0.0188	-23	164	170	LASYLDK					Mascot
913.4084	913.3705	-0.0379	-41	234	240	MTLDDFR			Oxidation (M)[1]		Mascot
982.4337	982.4032	-0.0305	-31	35	46	FSSSGGGGGGGR					Mascot
1060.5634	1060.5112	-0.0522	-49	225	233	TLLDIDNTR					Mascot
1232.5979	1232.5525	-0.0454	-37	14	29	SGGGGGGGLGSGGSIR					Mascot
1235.5288	1235.5521	0.0233	19	47	59	FSSSSGYGGGSSR					Mascot
1323.6726	1323.6008	-0.0718	-54	241	250	IKFEMEQNLR			Oxidation (M)[5]		Mascot
1605.7432	1605.6605	-0.0827	-52	200	212	NYSPYYNTIDDLK					Mascot
1791.7278	1791.6689	-0.0589	-33	491	513	GGSGGSYGGGGSGGGY GGGSGSR	95	100			Mascot
1837.9655	1837.8745	-0.091	-50	375	390	HGVQELEIELQSQLSK					Mascot
1867.9219	1867.8192	-0.1027	-55	322	336	TLNDMRQEYEQLIAK			Oxidation (M)[5]		Mascot

Rank	Protein Name	Accession No.	Protein MW	Protein PI	Pep. Count	Protein Score	Protein Score C. I. %	Intensity Matched	Total Ion Score	Total Ion C. I. %	Confirmed
3	Alpha-amylase/trypsin inhibitor CM1 OS=Triticum aestivum PE=1 SV=1	IAAC1_WHEAT	16076.8	7.49	4	98	99.991	11.162	78	99.999	

Peptide Information

Calc. Mass	Obsrv. Mass	± da	± ppm	Start Seq.	End Seq.	Sequence	Ion Score	C. I. %	Modification	Rank	Result Type
811.4573	811.3989	-0.0584	-72	92	97	YFIGRR					Mascot
814.3876	814.3555	-0.0321	-39	107	113	DLPGCPR			Carbamidomethyl (C)[5]		Mascot
1278.5532	1278.5017	-0.0515	-40	77	86	ELYDASQHCR			Carbamidomethyl (C)[9]		Mascot
1278.5532	1278.5017	-0.0515	-40	77	86	ELYDASQHCR	78	99.999	Carbamidomethyl (C)[9]		Mascot
2709.2786	2709.21	-0.0686	-25	46	71	EYVAQQTCGISISGSAVS TEPGNTPR			Carbamidomethyl (C)[8]		Mascot

4	Chymotrypsin inhibitor WCI OS=Triticum aestivum PE=1 SV=1	ICIW2_WHEAT	13505.5	7.42	1	96	99.985	1.031	90	100		

Peptide Information

Calc. Mass	Obsrv. Mass	± da	± ppm	Start Seq.	End Seq.	Sequence	Ion Score	C. I. %	Modification	Rank	Result Type
1986.0193	1985.9674	-0.0519	-26	67	86	AFPPSQSQGGGPPQPPL APR					Mascot
1986.0193	1985.9674	-0.0519	-26	67	86	AFPPSQSQGGGPPQPPL APR	90	100			Mascot

5	Alpha-amylase inhibitor 0.28 OS=Triticum aestivum GN=IMA1 PE=1 SV=3	IAA2_WHEAT	17358.3	7.45	6	56	0	2.933	26	0

Peptide Information

Calc. Mass	Obsrv. Mass	± da	± ppm	Start Seq.	End Seq.	Sequence	Ion Score	C. I. %	Modification	Rank	Result Type
830.4189	830.3638	-0.0551	-66	107	113	EVLPGCR			Carbamidomethyl (C)[6]		Mascot
863.4404	863.4083	-0.0321	-37	45	52	VSALTGCR			Carbamidomethyl (C)[7]		Mascot
1050.5579	1050.5188	-0.0391	-37	95	103	SVYQELGVR					Mascot
1050.5579	1050.5188	-0.0391	-37	95	103	SVYQELGVR	26	0			Mascot
1308.6763	1308.6606	-0.0157	-12	45	56	VSALTGCRAMVK			Carbamidomethyl (C)[7], Oxidation (M)[10]		Mascot
1555.8262	1555.7734	-0.0528	-34	57	70	LQCVGSQVPEAVLR			Carbamidomethyl (C)[3]		Mascot
1677.855	1677.755	-0.1	-60	115	129	EVMKLTAASVPEVCK			Carbamidomethyl (C)[14], Oxidation (M)[3]		Mascot

6	C-type lectin domain family 4 member G OS=Mus musculus GN=Clec4g PE=2 SV=1	CLC4G_MOUSE	33989.7	7.52	11	51	0	4.875	

Peptide Information

Calc. Mass	Obsrv. Mass	± da	± ppm	Start Seq.	End Seq.	Sequence	Ion Score	C. I. %	Modification	Rank	Result Type
802.4166	802.4082	-0.0084	-10	140	145	DRENIR					Mascot
806.3461	806.3808	0.0347	43	86	92	DDIGACR			Carbamidomethyl (C)[6]		Mascot
825.3737	825.4016	0.0279	34	2	8	NTGEYNK					Mascot

864.4727	864.4855	0.0128	15	220	226	GYWLGLR					Mascot
1060.5634	1060.5112	-0.0522	-49	9	18	LGSAIEEVSR					Mascot
1106.5762	1106.4797	-0.0965	-87	116	124	LMEQESILK			Oxidation (M)[2]		Mascot
1390.7689	1390.6445	-0.1244	-89	146	157	SELFQALEAVKR					Mascot
1553.7839	1553.6785	-0.1054	-68	72	85	TNASEQKMTLSSLK			Oxidation (M)[8]		Mascot
1566.7615	1566.7317	-0.0298	-19	79	92	MTLSSLKDDIGACR			Carbamidomethyl (C)[13]		Mascot
1571.85	1571.7329	-0.1171	-74	9	23	LGSAIEEVSRGQLGR					Mascot
1571.85	1571.7329	-0.1171	-74	9	23	LGSAIEEVSRGQLGR					Mascot
1699.8623	1699.777	-0.0853	-50	205	219	GLNEQGFLSQHTRGR					Mascot

7	Brefeldin A-inhibited guanine nucleotide-exchange protein 2 OS=Rattus norvegicus GN=Arfgef2 PE=1 SV=1	BIG2_RAT	204297	6.08	27	50	0	8.652	

Peptide Information

Calc. Mass	Obsrv. Mass	± da	± ppm	Start Seq.	End Seq.	Sequence	Ion Score	C. I. %	Modification	Rank	Result Type
806.4155	806.3808	-0.0347	-43	1179	1185	GELANFR					Mascot
836.4546	836.4036	-0.051	-61	834	840	IAMKETK			Oxidation (M)[3]		Mascot
856.4635	856.491	0.0275	32	405	411	SHELRSK					Mascot
867.3876	867.3876	0	0	1	7	MQESQTK			Oxidation (M)[1]		Mascot
869.4515	869.4216	-0.0299	-34	735	741	TFLEGFR					Mascot
905.4686	905.4251	-0.0435	-48	1517	1524	QSLSSIDR					Mascot
942.3986	942.4232	0.0246	26	1676	1682	MYVDENR			Oxidation (M)[1]		Mascot
948.4857	948.3963	-0.0894	-94	273	281	ERGSSISGR					Mascot
959.552	959.4924	-0.0596	-62	972	979	QKNIDTIK					Mascot
973.4697	973.478	0.0083	9	1445	1452	QDNEQLAR					Mascot
982.4952	982.4032	-0.092	-94	1509	1516	HLDVDLDR					Mascot
1002.5578	1002.4931	-0.0647	-65	175	183	NLINQTTAK					Mascot
1050.4998	1050.5188	0.019	18	807	815	MNRGINDSK			Oxidation (M)[1]		Mascot
1050.4998	1050.5188	0.019	18	807	815	MNRGINDSK			Oxidation (M)[1]		Mascot
1118.5226	1118.4751	-0.0475	-42	1684	1692	DSWGEIQQR					Mascot
1140.5507	1140.5129	-0.0378	-33	1612	1620	FMSSQHLFK			Oxidation (M)[2]		Mascot
1187.5746	1187.5034	-0.0712	-60	1376	1383	HWWQDLFR					Mascot
1232.598	1232.5525	-0.0455	-37	165	174	TCYNIYLASK			Carbamidomethyl (C)[2]		Mascot
1323.7056	1323.6008	-0.1048	-79	1175	1185	FLEKGELANFR					Mascot
1353.6654	1353.5713	-0.0941	-70	938	948	IACIFGMQLER			Carbamidomethyl (C)[3], Oxidation (M)[7]		Mascot
1417.678	1417.6937	0.0157	11	1123	1133	IVEISYYNMNR			Oxidation (M)[9]		Mascot
1425.7593	1425.6516	-0.1077	-76	1356	1367	TRGLTVMFEIMK					Mascot

Calc. Mass	Obsrv. Mass	± da	± ppm	Start Seq.	End Seq.	Sequence	Ion Score	C. I. %	Modification	Rank	Result Type
1507.8302	1507.6919	-0.1383	-92	184	196	ATLTQMLNVIFTR					Mascot
1507.8302	1507.6919	-0.1383	-92	184	196	ATLTQMLNVIFTR					Mascot
1566.8171	1566.7317	-0.0854	-55	475	486	MHLKMQIEVFFK			Oxidation (M)[1]		Mascot
1584.7725	1584.6654	-0.1071	-68	258	272	SSLGKVNSENGEAHR					Mascot
1838.9396	1838.8647	-0.0749	-41	94	112	LIAYGHITGNAPDSGAPG K					Mascot
1867.9225	1867.8192	-0.1033	-55	1775	1791	IWVPEEPSQVPAASTAW					Mascot
2685.28	2685.2361	-0.0439	-16	1612	1633	FMSSQHLFKLLDCLQES HSFSK			Carbamidomethyl (C)[13], Oxidation (M)[2]		Mascot

8	Desmin OS=Bos taurus GN=DES PE=2 SV=3	DESM_BOVIN	53556.2	5.21	15	50	0	3.015

Peptide Information

Calc. Mass	Obsrv. Mass	± da	± ppm	Start Seq.	End Seq.	Sequence	Ion Score	C. I. %	Modification	Rank	Result Type
847.4744	847.4078	-0.0666	-79	71	78	ASRLGSTR					Mascot
867.4166	867.3876	-0.029	-33	44	52	GSSSSVTSR					Mascot
999.504	999.4235	-0.0805	-81	376	383	HLKDEMAR					Mascot
1002.5214	1002.4931	-0.0283	-28	213	222	ADVDAATLAR					Mascot
1016.5483	1016.4762	-0.0721	-71	59	70	TSGGAGGLGALR					Mascot
1027.5103	1027.454	-0.0563	-55	379	386	DEMARHLR					Mascot
1039.5531	1039.4548	-0.0983	-95	241	248	KVHEEEIR					Mascot
1060.5634	1060.5112	-0.0522	-49	407	415	KLLEGEESR					Mascot
1263.5964	1263.579	-0.0174	-14	202	212	EEAENNLAAFR					Mascot
1308.6147	1308.6606	0.0459	35	340	350	GTNDSLMRQMR					Mascot
1340.6046	1340.6097	0.0051	4	340	350	GTNDSLMRQMR			Oxidation (M)[7,10]		Mascot
1425.7406	1425.6516	-0.089	-62	396	407	MALDVEIATYRK			Oxidation (M)[1]		Mascot
1584.7612	1584.6654	-0.0958	-60	430	443	ETSPEQRGSEVHTK					Mascot
1599.8086	1599.7491	-0.0595	-37	44	58	GSSSSVTSRVYQVSR					Mascot
1605.7616	1605.6605	-0.1011	-63	2	15	SQAYSSSQRVSSYR					Mascot
1699.861	1699.777	-0.084	-49	176	189	VDVERDNLLDDLQR					Mascot

9	Parvalbumin alpha OS=Homo sapiens GN=PVALB PE=1 SV=2	PRVA_HUMAN	12051	4.98	9	49	0	2.244

Peptide Information

Calc. Mass	Obsrv. Mass	± da	± ppm	Start Seq.	End Seq.	Sequence	Ion Score	C. I. %	Modification	Rank	Result Type
836.4005	836.4036	0.0031	4	85	92	MLMAAGDK					Mascot
891.4781	891.41	-0.0681	-76	77	84	DLSAKETK					Mascot
905.455	905.4251	-0.0299	-33	47	53	VFHMLDK			Oxidation (M)[4]		Mascot

1017.555	1017.4763	-0.0787	-77	46	53	KVFHMLDK					Mascot
1033.5499	1033.4762	-0.0737	-71	46	53	KVFHMLDK			Oxidation (M)[5]		Mascot
1194.5857	1194.5549	-0.0308	-26	82	92	ETKMLMAAGDK					Mascot
1263.6328	1263.579	-0.0538	-43	70	81	GFSPDARDLSAK					Mascot
1308.5923	1308.6606	0.0683	52	85	97	MLMAAGDKDGDGK					Mascot
1340.5822	1340.6097	0.0275	21	85	97	MLMAAGDKDGDGK			Oxidation (M)[1,3]		Mascot
1480.7021	1480.722	0.0199	13	1	13	MSMTDLLNAEDIK					Mascot
1838.8655	1838.8647	-0.0008	0	93	110	DGDGKIGVDEFSTLVAES					Mascot

10	Ubiquitin carboxyl-terminal hydrolase 7 OS=Rattus norvegicus GN=Usp7 PE=1 SV=1	UBP7_RAT	129432.7	5.31	22	49	0	10.193

Peptide Information

Calc. Mass	Obsrv. Mass	± da	± ppm	Start Seq.	End Seq.	Sequence	Ion Score	C. I. %	Modification	Rank	Result Type
802.4417	802.4082	-0.0335	-42	545	550	LQEEKR					Mascot
809.3676	809.4215	0.0539	67	183	189	GFIDDDK					Mascot
816.4138	816.3528	-0.061	-75	1092	1097	YTYLEK					Mascot
825.4213	825.4016	-0.0197	-24	848	855	DGPGNPLR					Mascot
827.4297	827.3748	-0.0549	-66	1035	1040	EFEKFK					Mascot
1002.5731	1002.4931	-0.08	-80	210	218	KHTGYVGLK					Mascot
1016.4869	1016.4762	-0.0107	-11	818	825	MNYFQVAK			Oxidation (M)[1]		Mascot
1039.4803	1039.4548	-0.0255	-25	337	344	EVDYRSDR					Mascot
1050.5327	1050.5188	-0.0139	-13	886	893	ITDFENRR					Mascot
1050.5327	1050.5188	-0.0139	-13	886	893	ITDFENRR					Mascot
1118.5333	1118.4751	-0.0582	-52	716	724	DLLPVMCDR			Carbamidomethyl (C)[7]		Mascot
1187.5957	1187.5034	-0.0923	-78	106	114	FYPDRPHQK					Mascot
1236.5995	1236.517	-0.0825	-67	906	915	EEEITLYPDK					Mascot
1320.665	1320.5447	-0.1203	-91	312	323	MKGTCVEGTIPK			Carbamidomethyl (C)[5]		Mascot
1490.7672	1490.6761	-0.0911	-61	82	94	LSESVLSPPCFVR			Carbamidomethyl (C)[10]		Mascot
1507.7614	1507.6919	-0.0695	-46	680	691	FDKDHDVMLFLK					Mascot
1507.7614	1507.6919	-0.0695	-46	680	691	FDKDHDVMLFLK					Mascot
1553.7238	1553.6785	-0.0453	-29	1	12	MNHQQQQQQQK					Mascot
1555.8049	1555.7734	-0.0315	-20	818	830	MNYFQVAKTVAQR					Mascot
1571.7952	1571.7329	-0.0623	-40	346	358	EDYYDIQLSIKGK					Mascot
1571.7999	1571.7329	-0.067	-43	818	830	MNYFQVAKTVAQR	2	0	Oxidation (M)[1]		Mascot
1584.7839	1584.6654	-0.1185	-75	986	998	ENEMLITVAHFHK			Oxidation (M)[4]		Mascot
1795.8834	1795.7258	-0.1576	-88	848	863	DGPGNPLRHNYEGTLR					Mascot

2612.4194	2612.2397	-0.1797	-69	256	278	SVPLALQRVFYELQHSDK PVGTK		Mascot
2731.2854	2731.2498	-0.0356	-13	1041	1062	FAIVMMGRHQYINEDEYE VNLK	Oxidation (M)[5,6]	Mascot

Gel Idx/Pos	312/M14	Instr./Gel Origin	BA2151/Sample Project 20140814	Process Status	Analysis Succeeded
Plate [#] Name	[1] Sample Project 20140814	Instrument Sample Name		Spectra	11

Rank	Protein Name	Accession No.	Protein MW	Protein PI	Pep. Count	Protein Score	Protein Score C. I. %	Intensity Matched	Total Ion Score	Total Ion C. I. %	Confirmed
1	Minor spike protein OS=Enterobacteria phage S13 GN=H PE=3 SV=1	VGH_BPS13	34384	5.25	10	44	0	12.097			

Peptide Information

Calc. Mass	Obsrv. Mass	± da	± ppm	Start Seq.	End Seq.	Sequence	Ion Score	C. I. %	Modification	Rank	Result Type
1289.6444	1289.761	0.1166	90	315	327	DGKADGIGSNLSR					Mascot
1306.6671	1306.7219	0.0548	42	210	221	VASIMENTNLSK					Mascot
1306.6671	1306.7219	0.0548	42	210	221	VASIMENTNLSK					Mascot
1320.61	1320.7251	0.1151	87	164	174	EIAEMQNETQK					Mascot
1363.6886	1363.5753	-0.1133	-83	153	163	ELTKMQLDNQK			Oxidation (M)[5]		Mascot
1603.8398	1603.7332	-0.1066	-66	175	189	EIAGIQSATSRQNTK					Mascot
1677.8993	1677.7567	-0.1426	-85	2	20	FGAIAGGIASALAGGVMSK					Mascot
1779.8508	1779.722	-0.1288	-72	134	152	AGAGASSSAIVDAGFENQK					Mascot
1828.8534	1828.7395	-0.1139	-62	190	204	DQVYAQNEMLAYQQK					Mascot
1844.8484	1844.7651	-0.0833	-45	190	204	DQVYAQNEMLAYQQK			Oxidation (M)[9]		Mascot
1844.8484	1844.7651	-0.0833	-45	190	204	DQVYAQNEMLAYQQK			Oxidation (M)[9]		Mascot
1866.9226	1866.7467	-0.1759	-94	205	221	ESTARVASIMENTNLSK			Oxidation (M)[10]		Mascot
2703.2866	2703.2512	-0.0354	-13	231	253	QMLTQAQTAGGQYFTNDQIKEMTR					Mascot

Rank	Protein Name	Accession No.	Protein MW	Protein PI	Pep. Count	Protein Score	Protein Score C. I. %	Intensity Matched	Total Ion Score	Total Ion C. I. %	Confirmed
2	Minor spike protein H OS=Enterobacteria phage phiX174 (Isolate Sanger) GN=H PE=1 SV=1	H_BPPHS	34398	5.14	10	43	0	11.822			

Peptide Information

Calc. Mass	Obsrv. Mass	± da	± ppm	Start Seq.	End Seq.	Sequence	Ion Score	C. I. %	Modification	Rank	Result Type
1289.6444	1289.761	0.1166	90	315	327	DGKADGIGSNLSR					Mascot
1306.6671	1306.7219	0.0548	42	210	221	VASIMENTNLSK					Mascot
1306.6671	1306.7219	0.0548	42	210	221	VASIMENTNLSK					Mascot
1320.61	1320.7251	0.1151	87	164	174	EIAEMQNETQK					Mascot
1363.6886	1363.5753	-0.1133	-83	153	163	ELTKMQLDNQK			Oxidation (M)[5]		Mascot
1603.8398	1603.7332	-0.1066	-66	175	189	EIAGIQSATSRQNTK					Mascot
1812.8983	1812.7216	-0.1767	-97	1	20	MFGAIAGGIASALAGGAMSK			Oxidation (M)[1,18]		Mascot

Calc. Mass	Obsrv. Mass	± da	± ppm	Start Seq.	End Seq.	Sequence	Ion Score	C. I. %	Modification	Rank	Result Type
1825.8021	1825.7484	-0.0537	-29	134	152	AGADASSAGMVDAGFENQK					Mascot
1828.8534	1828.7395	-0.1139	-62	190	204	DQVYAQNEMLAYQQK					Mascot
1841.7971	1841.7227	-0.0744	-40	134	152	AGADASSAGMVDAGFENQK			Oxidation (M)[10]		Mascot
1844.8484	1844.7651	-0.0833	-45	190	204	DQVYAQNEMLAYQQK			Oxidation (M)[9]		Mascot
1844.8484	1844.7651	-0.0833	-45	190	204	DQVYAQNEMLAYQQK			Oxidation (M)[9]		Mascot
1866.9226	1866.7467	-0.1759	-94	205	221	ESTARVASIMENTNLSK			Oxidation (M)[10]		Mascot
2703.2866	2703.2512	-0.0354	-13	231	253	QMLTQAQTAGQYFTNDQIKEMTR					Mascot

3	Myosin light chain 3, skeletal muscle isoform OS=Liza ramada PE=1 SV=1	MLE3_LIZRA	16358	4.37	7	41	0	1.322

Peptide Information

Calc. Mass	Obsrv. Mass	± da	± ppm	Start Seq.	End Seq.	Sequence	Ion Score	C. I. %	Modification	Rank	Result Type
959.5771	959.5172	-0.0599	-62	109	117	IVLSTLGEK					Mascot
1249.5841	1249.6886	0.1045	84	97	108	EGNGTVMGAELR			Oxidation (M)[7]		Mascot
1480.7981	1480.7294	-0.0687	-46	63	74	RLNFDAFLPMLK			Oxidation (M)[10]		Mascot
1480.7981	1480.7294	-0.0687	-46	63	74	RLNFDAFLPMLK			Oxidation (M)[10]		Mascot
1738.8429	1738.6759	-0.167	-96	93	108	VFDKEGNGTVMGAELR			Oxidation (M)[11]		Mascot
1779.8695	1779.722	-0.1475	-83	21	36	IGDSQVAFNQVADIMR			Oxidation (M)[15]		Mascot
2039.9921	2039.8225	-0.1696	-83	75	92	EVDALPKGTYDDYVEGLR					Mascot
2703.3518	2703.2512	-0.1006	-37	21	45	IGDSQVAFNQVADIMRALGQNPTNK			Oxidation (M)[15]		Mascot

4	Zinc finger protein 44 OS=Homo sapiens GN=ZNF44 PE=2 SV=3	ZNF44_HUMAN	78982.4	9	15	40	0	13.007

Peptide Information

Calc. Mass	Obsrv. Mass	± da	± ppm	Start Seq.	End Seq.	Sequence	Ion Score	C. I. %	Modification	Rank	Result Type
1363.6311	1363.5753	-0.0558	-41	573	583	THTAEKPYECK			Carbamidomethyl (C)[10]		Mascot
1506.6689	1506.5835	-0.0854	-57	430	442	RHMMAHTGDGPHK			Oxidation (M)[3,4]		Mascot
1555.75	1555.7504	0.0004	0	486	498	KHETTHTGEQPYK					Mascot
1593.7229	1593.674	-0.0489	-31	1	13	MALCYGTFWGYPK			Carbamidomethyl (C)[4]		Mascot
1634.7349	1634.6515	-0.0834	-51	375	388	HMIMHSGDGPHKCK			Carbamidomethyl (C)[13]		Mascot
1705.8367	1705.6868	-0.1499	-88	277	290	QCSKAFPVYSSYLR			Carbamidomethyl (C)[2]		Mascot
1823.8052	1823.7432	-0.062	-34	629	643	THTGEKPYECKECGK			Carbamidomethyl (C)[10,13]		Mascot
1841.8851	1841.7227	-0.1624	-88	402	416	IHEGTHTLEKPYECK			Carbamidomethyl (C)[14]		Mascot
1852.8317	1852.7368	-0.0949	-51	266	280	THTGEKPYECKQCSK			Carbamidomethyl (C)[10,13]		Mascot
1855.7949	1855.7257	-0.0692	-37	545	559	THSEEKSYECQICGK			Carbamidomethyl (C)[10,13]		Mascot

	1858.8575	1858.7217	-0.1358	-73	443	458	CTVCGKAFDSPSVFQR			Carbamidomethyl (C)[1,4]		Mascot
	1886.8813	1886.705	-0.1763	-93	569	583	THERTHTAEKPYECK			Carbamidomethyl (C)[14]		Mascot
	1900.8607	1900.7272	-0.1335	-70	625	639	EHERTHTGEKPYECK			Carbamidomethyl (C)[14]		Mascot
	1902.8585	1902.7205	-0.138	-73	262	276	MHERTHTGEKPYECK			Carbamidomethyl (C)[14]		Mascot
	1902.8585	1902.7205	-0.138	-73	262	276	MHERTHTGEKPYECK			Carbamidomethyl (C)[14]		Mascot
	2023.8718	2023.8278	-0.044	-22	431	448	HMMAHTGDGPHKCTVCGK			Carbamidomethyl (C)[13,16]		Mascot
	2039.8667	2039.8225	-0.0442	-22	431	448	HMMAHTGDGPHKCTVCGK			Carbamidomethyl (C)[13,16], Oxidation (M)[2]		Mascot

5 Glutathione S-transferase Mu 5 (Fragments) OS=Mesocricetus auratus GN=GSTM5 PE=1 SV=1 | GSTM5_MESAU | 16875.3 | 5.51 | 8 | 39 | 0 | 13.807

Peptide Information

Calc. Mass	Obsrv. Mass	± da	± ppm	Start Seq.	End Seq.	Sequence	Ion Score	C. I. %	Modification	Rank	Result Type
1514.6693	1514.7104	0.0411	27	25	36	RYICGEAPDYDR			Carbamidomethyl (C)[4]		Mascot
1526.6979	1526.7119	0.014	9	74	85	VDIMENQIMDFR			Oxidation (M)[4]		Mascot
1621.7302	1621.6829	-0.0473	-29	12	24	MLLEFTDTSYEEK			Oxidation (M)[1]		Mascot
1767.8445	1767.7109	-0.1336	-76	111	124	MFEPKCLDEFPNLK			Carbamidomethyl (C)[6]		Mascot
1777.8313	1777.7118	-0.1195	-67	12	25	MLLEFTDTSYEEKR			Oxidation (M)[1]		Mascot
1777.8313	1777.7118	-0.1195	-67	11	24	RMLLEFTDTSYEEK			Oxidation (M)[2]		Mascot
1779.8881	1779.722	-0.1661	-93	72	85	IRVDIMENQIMDFR					Mascot
1783.8394	1783.6949	-0.1445	-81	111	124	MFEPKCLDEFPNLK			Carbamidomethyl (C)[6], Oxidation (M)[1]		Mascot
1795.8829	1795.713	-0.1699	-95	72	85	IRVDIMENQIMDFR			Oxidation (M)[6]		Mascot
1795.8829	1795.713	-0.1699	-95	72	85	IRVDIMENQIMDFR			Oxidation (M)[6]		Mascot
2042.0304	2041.8759	-0.1545	-76	44	60	FKLDLDFPNLPYLMDGK			Oxidation (M)[14]		Mascot

6 Lysine--tRNA ligase OS=Porphyromonas gingivalis (strain ATCC BAA-308 / W83) GN=lysS PE=3 SV=1 | SYK_PORGI | 66842.3 | 5.23 | 14 | 39 | 0 | 5.053

Peptide Information

Calc. Mass	Obsrv. Mass	± da	± ppm	Start Seq.	End Seq.	Sequence	Ion Score	C. I. %	Modification	Rank	Result Type
959.4792	959.5172	0.038	40	405	412	TNPELTER					Mascot
1249.7991	1249.6886	-0.1105	-88	143	153	AIRPLPVVKEK					Mascot
1363.7073	1363.5753	-0.132	-97	546	556	VMQQLMDINKK			Oxidation (M)[2]		Mascot
1480.7352	1480.7294	-0.0058	-4	370	381	LIDEIFGEKCEK			Carbamidomethyl (C)[10]		Mascot
1480.7352	1480.7294	-0.0058	-4	370	381	LIDEIFGEKCEK			Carbamidomethyl (C)[10]		Mascot
1599.8054	1599.7566	-0.0488	-31	382	394	NYIQPTFITDYPK					Mascot
1599.8054	1599.7566	-0.0488	-31	382	394	NYIQPTFITDYPK					Mascot

Calc. Mass	Obsrv. Mass	± da	± ppm	Start Seq.	End Seq.	Sequence	Ion Score	C. I. %	Modification	Rank	Result Type
1611.6923	1611.5731	-0.1192	-74	154	167	DGEVFDGFTDPEQR					Mascot
1812.8619	1812.7216	-0.1403	-77	68	83	IMGKATFMELQDAEGR			Oxidation (M)[2]		Mascot
1828.8568	1828.7395	-0.1173	-64	68	83	IMGKATFMELQDAEGR			Oxidation (M)[2,8]		Mascot
1844.863	1844.7651	-0.0979	-53	352	367	QVCDKLGVEHNETMGK			Carbamidomethyl (C)[3]		Mascot
1844.863	1844.7651	-0.0979	-53	352	367	QVCDKLGVEHNETMGK			Carbamidomethyl (C)[3]		Mascot
1846.9156	1846.7316	-0.184	-100	109	124	CTDIGDFIGVKGYVFR			Carbamidomethyl (C)[1]		Mascot
1868.8298	1868.7123	-0.1175	-63	152	167	EKDGEVFDGFTDPEQR					Mascot
1882.8496	1882.7079	-0.1417	-75	463	480	ALEYGMPPTSGMGIGMD R					Mascot
1898.8446	1898.719	-0.1256	-66	463	480	ALEYGMPPTSGMGIGMD R			Oxidation (M)[6]		Mascot
1913.9103	1913.7522	-0.1581	-83	271	286	THNPEFTAMEIYVAYK					Mascot
1914.8395	1914.7145	-0.125	-65	463	480	ALEYGMPPTSGMGIGMD R			Oxidation (M)[6,12]		Mascot
1929.9052	1929.7545	-0.1507	-78	271	286	THNPEFTAMEIYVAYK			Oxidation (M)[9]		Mascot
2023.8347	2023.8278	-0.0069	-3	287	301	DYNWMMNFTEQMLER			Oxidation (M)[5]		Mascot
2039.8296	2039.8225	-0.0071	-3	287	301	DYNWMMNFTEQMLER			Oxidation (M)[5,6]		Mascot
2703.3269	2703.2512	-0.0757	-28	120	142	GYVFRTQMGEISVHVQE MTFLSK			Oxidation (M)[8]		Mascot

7	Lysine--tRNA ligase OS=Porphyromonas gingivalis (strain ATCC 33277 / DSM 20709 / JCM 12257) GN=lysS PE=3 SV=1	SYK_PORG3	66912.4	5.23	14	39	0	4.809

Peptide Information

Calc. Mass	Obsrv. Mass	± da	± ppm	Start Seq.	End Seq.	Sequence	Ion Score	C. I. %	Modification	Rank	Result Type
959.4792	959.5172	0.038	40	405	412	TNPELTER					Mascot
1249.7991	1249.6886	-0.1105	-88	143	153	AIRPLPVVKEK					Mascot
1363.7073	1363.5753	-0.132	-97	546	556	VMQQLMDINKK			Oxidation (M)[2]		Mascot
1480.7352	1480.7294	-0.0058	-4	370	381	LIDEIFGEKCEK			Carbamidomethyl (C)[10]		Mascot
1480.7352	1480.7294	-0.0058	-4	370	381	LIDEIFGEKCEK			Carbamidomethyl (C)[10]		Mascot
1599.8054	1599.7566	-0.0488	-31	382	394	NYIQPTFITDYPK					Mascot
1599.8054	1599.7566	-0.0488	-31	382	394	NYIQPTFITDYPK					Mascot
1793.9137	1793.738	-0.1757	-98	302	317	ICMDVLGTTQVKVGEK			Carbamidomethyl (C)[2], Oxidation (M)[3]		Mascot
1812.8619	1812.7216	-0.1403	-77	68	83	IMGKATFMELQDAEGR			Oxidation (M)[2]		Mascot
1828.8568	1828.7395	-0.1173	-64	68	83	IMGKATFMELQDAEGR			Oxidation (M)[2,8]		Mascot
1844.863	1844.7651	-0.0979	-53	352	367	QVCDKLGVEHNETMGK			Carbamidomethyl (C)[3]		Mascot
1844.863	1844.7651	-0.0979	-53	352	367	QVCDKLGVEHNETMGK			Carbamidomethyl (C)[3]		Mascot
1846.9156	1846.7316	-0.184	-100	109	124	CTDIGDFIGVKGYVFR			Carbamidomethyl (C)[1]		Mascot
1858.8091	1858.7217	-0.0874	-47	152	167	EKDGEVFDGFTDSEQR					Mascot
1882.8496	1882.7079	-0.1417	-75	463	480	ALEYGMPPTSGMGIGMD					Mascot

Calc. Mass	Obsrv. Mass	± da	± ppm	Start Seq.	End Seq.	Sequence	Ion Score	C. I. %	Modification	Rank	Result Type
						R					
1898.8446	1898.719	-0.1256	-66	463	480	ALEYGMPPTSGMGIGMD R			Oxidation (M)[6]		Mascot
1913.9103	1913.7522	-0.1581	-83	271	286	THNPEFTAMEIYVAYK					Mascot
1914.8395	1914.7145	-0.125	-65	463	480	ALEYGMPPTSGMGIGMD R			Oxidation (M)[6,12]		Mascot
1929.9052	1929.7545	-0.1507	-78	271	286	THNPEFTAMEIYVAYK			Oxidation (M)[9]		Mascot
2023.8347	2023.8278	-0.0069	-3	287	301	DYNWMMNFTEQMLER			Oxidation (M)[5]		Mascot
2039.8296	2039.8225	-0.0071	-3	287	301	DYNWMMNFTEQMLER			Oxidation (M)[5,6]		Mascot
2703.3269	2703.2512	-0.0757	-28	120	142	GYVFRTQMGEISVHVQE MTFLSK			Oxidation (M)[8]		Mascot

8	Multifunctional CCA protein OS=Cupriavidus necator (strain ATCC 17699 / H16 / DSM 428 / Stanier 337) GN=cca PE=3 SV=1	CCA_CUPNH	45302.2	6.29	11	38	0	2.287	

Peptide Information

Calc. Mass	Obsrv. Mass	± da	± ppm	Start Seq.	End Seq.	Sequence	Ion Score	C. I. %	Modification	Rank	Result Type
869.509	869.4312	-0.0778	-89	210	216	LLPELER					Mascot
1289.6519	1289.761	0.1091	85	281	291	SAQLLEDVCVR			Carbamidomethyl (C)[9]		Mascot
1585.7891	1585.7378	-0.0513	-32	166	180	EMVAAGEVDALVPER					Mascot
1699.9351	1699.7842	-0.1509	-89	300	314	DLALVVAREHGNIHR					Mascot
1706.8279	1706.6899	-0.138	-81	339	353	FAEALQACEADLRGR			Carbamidomethyl (C)[8]		Mascot
1724.9078	1724.8496	-0.0582	-34	129	143	HVSDAFAEDPVRILR					Mascot
1758.8922	1758.7494	-0.1428	-81	126	140	LFRHVSDAFAEDPVR					Mascot
1810.8728	1810.7228	-0.15	-83	151	165	FHEFGVAAETMRLMR			Oxidation (M)[11]		Mascot
1855.8909	1855.7257	-0.1652	-89	147	162	FAARFHEFGVAAETMR			Oxidation (M)[15]		Mascot
1986.0146	1985.9648	-0.0498	-25	163	180	LMREMVAAGEVDALVPE R					Mascot
2039.9828	2039.8225	-0.1603	-79	308	325	EHGNIHRSQEFSAAAVM R					Mascot

9	Multifunctional CCA protein OS=Cupriavidus taiwanensis (strain R1 / LMG 19424) GN=cca PE=3 SV=1	CCA_CUPTR	46135.7	6.3	11	38	0	15.925	

Peptide Information

Calc. Mass	Obsrv. Mass	± da	± ppm	Start Seq.	End Seq.	Sequence	Ion Score	C. I. %	Modification	Rank	Result Type
842.5094	842.4742	-0.0352	-42	300	307	DLAVVVAR					Mascot
869.509	869.4312	-0.0778	-89	210	216	LLPELER					Mascot
1306.7339	1306.7219	-0.012	-9	397	408	VHAARVEAVAQR					Mascot
1306.7339	1306.7219	-0.012	-9	397	408	VHAARVEAVAQR					Mascot
1585.7891	1585.7378	-0.0513	-32	166	180	EMVAAGEVDALVPER					Mascot

1724.9078	1724.8496	-0.0582	-34	129	143	HVSDAFAEDPVRILR					Mascot
1758.8922	1758.7494	-0.1428	-81	126	140	LFRHVSDAFAEDPVR					Mascot
1823.9255	1823.7432	-0.1823	-100	188	202	GLMEARPSRMFEVLR			Oxidation (M)[3,10]		Mascot
1869.8735	1869.787	-0.0865	-46	151	165	FHDFNVAAETMRLMR			Oxidation (M)[11,14]		Mascot
1869.8735	1869.787	-0.0865	-46	151	165	FHDFNVAAETMRLMR			Oxidation (M)[11,14]		Mascot
1898.8966	1898.719	-0.1776	-94	147	162	FAARFHDFNVAAETMR			Oxidation (M)[15]		Mascot
1901.9617	1901.7717	-0.19	-100	355	371	GFEHSDYPQAARLLAAR					Mascot
1986.0146	1985.9648	-0.0498	-25	163	180	LMREMVAAGEVDALVPER					Mascot

10	Dauer abnormal formation protein 25 OS=Caenorhabditis elegans GN=daf-25 PE=1 SV=2	DAF25_CAEEL	44387.6	7.1	10	38	0	4.504	

Peptide Information

Calc. Mass	Obsrv. Mass	± da	± ppm	Start Seq.	End Seq.	Sequence	Ion Score	C. I. %	Modification	Rank	Result Type
1023.4663	1023.4334	-0.0329	-32	1	9	MTTTEEAPK			Oxidation (M)[1]		Mascot
1050.5135	1050.5122	-0.0013	-1	209	217	ESNEIMSLK					Mascot
1304.6515	1304.7002	0.0487	37	60	70	AIELKCDVNDK			Carbamidomethyl (C)[6]		Mascot
1479.7325	1479.7424	0.0099	7	237	249	EKSPEEASLQYAK					Mascot
1520.7308	1520.5796	-0.1512	-99	88	100	QDVCRLLMDSGAR			Carbamidomethyl (C)[4]		Mascot
1785.9092	1785.7401	-0.1691	-95	71	87	TDGTLYTPLMFAALSGK					Mascot
1861.8507	1861.7217	-0.129	-69	314	330	IAAVCQFCSVCGHPGAK			Carbamidomethyl (C)[5,8,11]		Mascot
1869.9562	1869.787	-0.1692	-90	93	109	LLMDSGARMYLVNGIGK			Oxidation (M)[3,9]		Mascot
1869.9562	1869.787	-0.1692	-90	93	109	LLMDSGARMYLVNGIGK			Oxidation (M)[3,9]		Mascot
1989.9457	1989.984	0.0383	19	314	331	IAAVCQFCSVCGHPGAKK			Carbamidomethyl (C)[5,8,11]		Mascot
2023.9893	2023.8278	-0.1615	-80	1	18	MTTTEEAPKSPLFEAIDK			Oxidation (M)[1]		Mascot

Gel Idx/Pos	313/M15	Instr./Gel Origin	BA2151/Sample Project 20140814	Process Status	Analysis Succeeded
Plate [#] Name	[1] Sample Project 20140814	Instrument Sample Name		Spectra	11

Rank	Protein Name	Accession No.	Protein MW	Protein PI	Pep. Count	Protein Score	Protein Score C. I. %	Intensity Matched	Total Ion Score	Total Ion C. I. %	Confirmed
1	Alpha-amylase inhibitor 0.28 OS=Triticum aestivum GN=IMA1 PE=1 SV=3	IAA2_WHEAT	17358.3	7.45	7	355	100	58.353	317	100	

Peptide Information

Calc. Mass	Obsrv. Mass	± da	± ppm	Start Seq.	End Seq.	Sequence	Ion Score	C. I. %	Modification	Rank	Result Type
830.4189	830.389	-0.0299	-36	107	113	EVLPGCR			Carbamidomethyl (C)[6]		Mascot
863.4404	863.4152	-0.0252	-29	45	52	VSALTGCR			Carbamidomethyl (C)[7]		Mascot
1050.5579	1050.522	-0.0359	-34	95	103	SVYQELGVR					Mascot
1050.5579	1050.522	-0.0359	-34	95	103	SVYQELGVR	72	99.995			Mascot
1054.4657	1054.4099	-0.0558	-53	86	94	CGDLSSMLR			Carbamidomethyl (C)[1], Oxidation (M)[7]		Mascot
1174.6136	1174.5415	-0.0721	-61	119	129	LTAASVPEVCK			Carbamidomethyl (C)[10]		Mascot
1555.8262	1555.7826	-0.0436	-28	57	70	LQCVGSQVPEAVLR			Carbamidomethyl (C)[3]		Mascot
1555.8262	1555.7826	-0.0436	-28	57	70	LQCVGSQVPEAVLR	113	100	Carbamidomethyl (C)[3]		Mascot
1981.795	1981.7411	-0.0539	-27	71	85	DCCQQLADINNEWCR			Carbamidomethyl (C)[2,3,14]		Mascot
1981.795	1981.7411	-0.0539	-27	71	85	DCCQQLADINNEWCR	132	100	Carbamidomethyl (C)[2,3,14]		Mascot

Rank	Protein Name	Accession No.	Protein MW	Protein PI	Pep. Count	Protein Score	Protein Score C. I. %	Intensity Matched	Total Ion Score	Total Ion C. I. %	Confirmed
2	Kinesin light chain OS=Caenorhabditis elegans GN=klc-2 PE=2 SV=2	KLC_CAEEL	60529.3	5.59	15	58	12.304	4.356			

Peptide Information

Calc. Mass	Obsrv. Mass	± da	± ppm	Start Seq.	End Seq.	Sequence	Ion Score	C. I. %	Modification	Rank	Result Type
806.4519	806.3986	-0.0533	-66	461	467	NLGALYR					Mascot
821.4376	821.4019	-0.0357	-43	90	95	YQAQRR					Mascot
988.488	988.4697	-0.0183	-19	308	315	DAEPLCKR			Carbamidomethyl (C)[6]		Mascot
1020.5208	1020.4742	-0.0466	-46	106	114	DELSSTQIK					Mascot
1078.5602	1078.55	-0.0102	-9	222	230	YEVAVPLCK			Carbamidomethyl (C)[8]		Mascot
1078.5602	1078.55	-0.0102	-9	222	230	YEVAVPLCK			Carbamidomethyl (C)[8]		Mascot
1154.6052	1154.6152	0.01	9	43	52	EDEAPLPKQK					Mascot
1371.6421	1371.7531	0.111	81	517	529	NSMTTSTSQTGLK			Oxidation (M)[3]		Mascot
1492.8005	1492.807	0.0065	4	392	403	EAEELYKQILTR					Mascot
1554.6887	1554.7639	0.0752	48	1	14	MSNMSQDDVTTGLR					Mascot
1570.6836	1570.78	0.0964	61	1	14	MSNMSQDDVTTGLR			Oxidation (M)[1]		Mascot

1597.785	1597.801	0.016	10	517	531	NSMTTSTSQTGLKNK						Mascot
1599.8602	1599.7595	-0.1007	-63	208	221	TLHNLVIQYASQGR						Mascot
1659.8085	1659.6447	-0.1638	-99	433	449	GEGATANEQAGWAKAA K						Mascot
1674.8181	1674.6683	-0.1498	-89	25	39	EEHSTISNTLETSVK						Mascot
1919.9015	1919.7676	-0.1339	-70	115	130	LQQSEQMVAQLEEENK			Oxidation (M)[7]			Mascot
1950.875	1950.7714	-0.1036	-53	134	150	YMASIKQFDDGTQSDTK			Oxidation (M)[2]			Mascot

3	Nucleoid-associated protein RC1337 OS=Rickettsia conorii (strain ATCC VR-613 / Malish 7) GN=RC1337 PE=3 SV=1	Y1337_RICCN	11878.9	4.99	8	46	0	2.695	

Protein Group

Nucleoid-associated protein RAF_ORF1224 OS=Rickettsia africae (strain ESF-5) GN=RAF_ORF1224 PE=3 SV=1	Y1224_RICAE	11878.9	4.9899 997711 1816
Nucleoid-associated protein RPR_07555 OS=Rickettsia peacockii (strain Rustic) GN=RPR_07555 PE=3 SV=1	Y7555_RICPU	11878.9	4.9899 997711 1816

Peptide Information

Calc. Mass	Obsrv. Mass	± da	± ppm	Start Seq.	End Seq.	Sequence	Ion Score	C. I. %	Modification	Rank	Result Type
820.3981	820.396	-0.0021	-3	10	16	QAQSMQK					Mascot
964.488	964.3998	-0.0882	-91	10	17	QAQSMQKK			Oxidation (M)[5]		Mascot
990.5176	990.4412	-0.0764	-77	65	72	EMLEDLIK					Mascot
1017.5826	1017.49	-0.0926	-91	52	60	ISIDESLLK					Mascot
1020.5109	1020.4742	-0.0367	-36	73	81	VAFNDAQQK					Mascot
1033.554	1033.5048	-0.0492	-48	99	107	LPPGFKMPF					Mascot
1049.549	1049.5134	-0.0356	-34	99	107	LPPGFKMPF			Oxidation (M)[7]		Mascot
1869.7701	1869.762	-0.0081	-4	82	98	CDEDSQNSLSGALNGMR			Carbamidomethyl (C)[1], Oxidation (M)[16]		Mascot
1887.8324	1887.7812	-0.0512	-27	18	33	MQEAQEQMANARYTGK			Oxidation (M)[1,8]		Mascot
1887.8324	1887.7812	-0.0512	-27	18	33	MQEAQEQMANARYTGK			Oxidation (M)[1,8]		Mascot

4	O-methyltransferase 12 OS=Dictyostelium discoideum GN=omt12 PE=1 SV=1	OMT12_DICDI	43014.4	6.31	11	44	0	7.991	

Peptide Information

Calc. Mass	Obsrv. Mass	± da	± ppm	Start Seq.	End Seq.	Sequence	Ion Score	C. I. %	Modification	Rank	Result Type
863.4257	863.4152	-0.0105	-12	42	48	HYSEISK					Mascot
972.5585	972.4767	-0.0818	-84	93	101	NGTLKNLGR					Mascot
988.5032	988.4697	-0.0335	-34	147	154	QLFHNAMK					Mascot
991.3937	991.4374	0.0437	44	54	60	NESYCYR			Carbamidomethyl (C)[5]		Mascot

1078.4987	1078.55	0.0513	48	361	369	NSLPQFVNC			Carbamidomethyl (C)[9]		Mascot
1078.4987	1078.55	0.0513	48	361	369	NSLPQFVNC			Carbamidomethyl (C)[9]		Mascot
1232.6497	1232.5414	-0.1083	-88	61	69	LMRYFVPYK			Oxidation (M)[2]		Mascot
1282.6652	1282.6215	-0.0437	-34	216	225	NENFQHPRLK					Mascot
1492.808	1492.807	-0.001	-1	111	123	IFESLPKTLEMGK					Mascot
1659.787	1659.6447	-0.1423	-86	292	304	DILMMQYFDAKER					Mascot
1922.9219	1922.7317	-0.1902	-99	305	319	SINEWHQLFEKCGFK			Carbamidomethyl (C)[12]		Mascot
1922.9219	1922.7317	-0.1902	-99	305	319	SINEWHQLFEKCGFK			Carbamidomethyl (C)[12]		Mascot
1964.9497	1964.8093	-0.1404	-71	287	302	EAVFKDILMMQYFDAK			Oxidation (M)[9]		Mascot

5	V-type proton ATPase subunit E OS=Sulfolobus islandicus (strain Y.N.15.51 / Yellowstone #2) GN=atpE PE=3 SV=1	VATE_SULIN	22525.9	5.76	11	43	0	22.456		

Peptide Information

Calc. Mass	Obsrv. Mass	± da	± ppm	Start Seq.	End Seq.	Sequence	Ion Score	C. I. %	Modification	Rank	Result Type
911.4944	911.4611	-0.0333	-37	32	39	LLNEGHTK					Mascot
1001.5626	1001.5239	-0.0387	-39	104	112	DAIQSILNK					Mascot
1033.5525	1033.5048	-0.0477	-46	143	151	NVTIKTDDK					Mascot
1035.5834	1035.4921	-0.0913	-88	85	92	VYERVLEK					Mascot
1050.5579	1050.522	-0.0359	-34	40	47	IIQEYTQR					Mascot
1050.5579	1050.522	-0.0359	-34	40	47	IIQEYTQR					Mascot
1062.5314	1062.5221	-0.0093	-9	55	63	TKEEIEGEK					Mascot
1154.5398	1154.6152	0.0754	65	1	9	MDFEQLLDK			Oxidation (M)[1]		Mascot
1174.6427	1174.5415	-0.1012	-86	132	142	STVEKVVGNNK					Mascot
1371.7842	1371.7531	-0.0311	-23	104	115	DAIQSILNKEIK					Mascot
1537.8043	1537.7701	-0.0342	-22	173	185	LILDQVFDSMRGK			Oxidation (M)[10]		Mascot
1667.8962	1667.8032	-0.093	-56	25	39	SLDEAIKLLNEGHTK					Mascot

6	Kinesin-like protein vab-8 OS=Caenorhabditis elegans GN=vab-8 PE=1 SV=3	KI26L_CAEEL	119163.5	9.04	11	43	0	8.003	29	5.308

Peptide Information

Calc. Mass	Obsrv. Mass	± da	± ppm	Start Seq.	End Seq.	Sequence	Ion Score	C. I. %	Modification	Rank	Result Type
812.3276	812.3777	0.0501	62	1	7	MEACSSK			Carbamidomethyl (C)[4]		Mascot
819.3955	819.3602	-0.0353	-43	97	103	TNGKDER					Mascot
845.4978	845.4467	-0.0511	-60	748	754	ITELEIK					Mascot
1001.5989	1001.5239	-0.075	-75	748	755	ITELEIKR					Mascot

1021.4731	1021.4128	-0.0603	-59	1020	1028	MIANSSDQR					Mascot
1037.468	1037.3872	-0.0808	-78	1020	1028	MIANSSDQR			Oxidation (M)[1]		Mascot
1037.468	1037.3872	-0.0808	-78	1020	1028	MIANSSDQR	29	5.308	Oxidation (M)[1]		Mascot
1077.5358	1077.4968	-0.039	-36	280	288	DSSVCQLIR			Carbamidomethyl (C)[5]		Mascot
1078.6368	1078.55	-0.0868	-80	588	596	DHPLRILSK					Mascot
1078.6368	1078.55	-0.0868	-80	588	596	DHPLRILSK					Mascot
1667.8646	1667.8032	-0.0614	-37	275	288	HIPSRDSSVCQLIR			Carbamidomethyl (C)[10]		Mascot
1921.9722	1921.8041	-0.1681	-87	1035	1050	TTMIDAMLQENRILEK			Oxidation (M)[3]		Mascot
1950.9379	1950.7714	-0.1665	-85	891	906	EELQWRSPYAQMTSPK					Mascot
1964.8978	1964.8093	-0.0885	-45	1011	1028	AIGQEDDAKMIANSSDQR			Oxidation (M)[10]		Mascot

7	V-type proton ATPase subunit E OS=Sulfolobus islandicus (strain M.16.4 / Kamchatka #3) GN=atpE PE=3 SV=1	VATE_SULIK	22543.9	5.76	11	42	0	22.456

Protein Group

V-type proton ATPase subunit E OS=Sulfolobus islandicus (strain L.S.2.15 / Lassen #1) GN=atpE PE=3 SV=1	VATE_SULIL	22543.9	5.7600 002288 8184
V-type proton ATPase subunit E OS=Sulfolobus islandicus (strain M.14.25 / Kamchatka #1) GN=atpE PE=3 SV=1	VATE_SULIM	22543.9	5.7600 002288 8184
V-type proton ATPase subunit E OS=Sulfolobus islandicus (strain M.16.27) GN=atpE PE=3 SV=1	VATE_SULIA	22543.9	5.7600 002288 8184
V-type proton ATPase subunit E OS=Sulfolobus islandicus (strain Y.G.57.14 / Yellowstone #1) GN=atpE PE=3 SV=1	VATE_SULIY	22543.9	5.7600 002288 8184

Peptide Information

Calc. Mass	Obsrv. Mass	± da	± ppm	Start Seq.	End Seq.	Sequence	Ion Score	C. I. %	Modification	Rank	Result Type
911.4944	911.4611	-0.0333	-37	32	39	LLNEGHTK					Mascot
1001.5626	1001.5239	-0.0387	-39	104	112	DAIQSILNK					Mascot
1033.5525	1033.5048	-0.0477	-46	143	151	NVTIKTDDK					Mascot
1035.5834	1035.4921	-0.0913	-88	85	92	VYERVLEK					Mascot
1050.5579	1050.522	-0.0359	-34	40	47	IIQEYTQR					Mascot
1050.5579	1050.522	-0.0359	-34	40	47	IIQEYTQR					Mascot
1062.5314	1062.5221	-0.0093	-9	55	63	TKEEIEGEK					Mascot
1154.5398	1154.6152	0.0754	65	1	9	MDFEQLLDK			Oxidation (M)[1]		Mascot
1174.6427	1174.5415	-0.1012	-86	132	142	STVEKVVGNNK					Mascot
1371.7842	1371.7531	-0.0311	-23	104	115	DAIQSILNKEIK					Mascot
1537.8043	1537.7701	-0.0342	-22	173	185	LILDQVFDSMRGK			Oxidation (M)[10]		Mascot
1667.8962	1667.8032	-0.093	-56	25	39	SLDEAIKLLNEGHTK					Mascot

8	Transcriptional repressor scratch 2 OS=Mus musculus GN=Scrt2 PE=2 SV=2	SCRT2_MOUSE	33499	9.23	10	41	0	1.302		

Peptide Information

Calc. Mass	Obsrv. Mass	± da	± ppm	Start Seq.	End Seq.	Sequence	Ion Score	C. I. %	Modification	Rank	Result Type
806.438	806.3986	-0.0394	-49	179	184	HKQTHR					Mascot
850.391	850.3801	-0.0109	-13	193	199	KCPTCGK			Carbamidomethyl (C)[2,5]		Mascot
866.3607	866.4229	0.0622	72	276	281	CRQCDK			Carbamidomethyl (C)[1,4]		Mascot
870.4177	870.4953	0.0776	89	151	160	SGATAGGGHR					Mascot
1017.5687	1017.49	-0.0787	-77	185	193	SLDSQLARK					Mascot
1049.5487	1049.5134	-0.0353	-34	254	262	AFADRSNLR					Mascot
1154.5774	1154.6152	0.0378	33	148	160	AGRSGATAGGGHR					Mascot
1627.8123	1627.7465	-0.0658	-40	259	272	SNLRAHMQTHSAFK					Mascot
1971.8613	1971.6902	-0.1711	-87	122	147	RAGAGGDAAGAGDAGG GGGGGGGGER					Mascot
2012.8914	2012.7578	-0.1336	-66	161	178	HACAECGKTYATSSNLS R			Carbamidomethyl (C)[3,6]		Mascot

9	Acetyl-coenzyme A synthetase OS=Ralstonia metallidurans (strain CH34 / ATCC 43123 / DSM 2839) GN=acsA PE=3 SV=1	ACSA_RALME	72865.9	6.03	6	41	0	21.954	33	56.817

Peptide Information

Calc. Mass	Obsrv. Mass	± da	± ppm	Start Seq.	End Seq.	Sequence	Ion Score	C. I. %	Modification	Rank	Result Type
973.4738	973.4255	-0.0483	-50	486	493	TIWGDPER					Mascot
1050.583	1050.522	-0.061	-58	114	122	VSYQELLAK					Mascot
1050.583	1050.522	-0.061	-58	114	122	VSYQELLAK	33	56.817			Mascot
1077.5728	1077.4968	-0.076	-71	13	22	VFNPPASFAK					Mascot
1174.5562	1174.5415	-0.0147	-13	519	528	ETGYFTIMGR					Mascot
1282.6315	1282.6215	-0.01	-8	64	74	VLDESNAPFYK					Mascot
1964.9924	1964.8093	-0.1831	-93	95	113	GLGDKTAIVFEADDGTVT R					Mascot

10	4-hydroxy-tetrahydrodipicolinate reductase OS=Geobacter uraniireducens (strain Rf4) GN=dapB PE=3 SV=1	DAPB_GEOUR	28641.7	6	10	41	0	5.297		

Peptide Information

Calc. Mass	Obsrv. Mass	± da	± ppm	Start Seq.	End Seq.	Sequence	Ion Score	C. I. %	Modification	Rank	Result Type
820.3618	820.396	0.0342	42	191	197	EGICGER			Carbamidomethyl (C)[4]		Mascot

1017.5323	1017.49	-0.0423	-42	160	169	KDSPSGTAVR		Mascot
1049.5044	1049.5134	0.009	9	191	199	EGICGERTK	Carbamidomethyl (C)[4]	Mascot
1054.5099	1054.4099	-0.1	-95	237	245	DMFSRGSVR		Mascot
1062.5249	1062.5221	-0.0028	-3	200	208	DEIGMQTIR		Mascot
1078.5198	1078.55	0.0302	28	200	208	DEIGMQTIR	Oxidation (M)[5]	Mascot
1078.5198	1078.55	0.0302	28	200	208	DEIGMQTIR	Oxidation (M)[5]	Mascot
1174.6249	1174.5415	-0.0834	-71	84	93	NLEVCGLNKK	Carbamidomethyl (C)[5]	Mascot
1232.6603	1232.5414	-0.1189	-96	1	12	MVKIAVCGAAGR	Carbamidomethyl (C)[7]	Mascot
1492.7643	1492.807	0.0427	29	94	107	SIVIGSTGFTPEER		Mascot
1667.8058	1667.8032	-0.0026	-2	170	184	MGEVVAEALGRDYNK	Oxidation (M)[1]	Mascot
1952.9171	1952.7462	-0.1709	-88	209	226	GGDIVGEHTVYFIGMGER	Oxidation (M)[15]	Mascot
