# Supplementary material for: Proteomic analysis of middle and late stages of bread wheat (Triticum aestivum L.) grain development
Source: Front Plant Sci. 2015 Sep 15;6:735. doi: 10.3389/fpls.2015.00735 (PMC4569854; doi:10.3389/fpls.2015.00735)
Supplement: Supplementary file 8 [file DataSheet7.PDF]

Analysis Information

Report Type	Protein-Peptide Summary by Spot	Analysis Type	Combined (MS+MS/MS)
Sample Set Name	Sample set_20140814	Database	NCBI_VPlant
Analysis Name	R14026-4-VP	Creation Date	09/22/2014 14:05:21
Reported By	09/30/2014 15:00:53 - admin	Last Modified	09/22/2014 14:17:32

MS Acq. : Proc. Methods	(Unspecified) : (Unspecified)
Interpretation Method	(Unspecified)

**Gel Idx/Pos**	110/E9	**Instr./Gel Origin**	BA2151/Sample Project 20140814	**Process Status**	Analysis Succeeded
**Plate [#] Name**	[1] Sample Project 20140814	**Instrument Sample Name**		**Spectra**	11

Rank	Protein Name	Accession No.	Protein MW	Protein PI	Pep. Count	Protein Score	Protein Score C. I. %	Intensity Matched	Total Ion Score	Total Ion C. I. %	Confirmed
1	Os07g0296000, partial [Oryza sativa Japonica Group]	gi|113610945	11738.2	11.31	3	24	0	.397			

Peptide Information

Calc. Mass	Obsrv. Mass	± da	± ppm	Start Seq.	End Seq.	Sequence	Ion Score	C. I. %	Modification	Rank	Result Type
833.4111	833.363	-0.0481	-58	42	48	KEENTGR					Mascot
2724.5168	2724.4023	-0.1145	-42	14	39	VAADVVVSRPPGHRRPF SPSPVLAGR					Mascot
2840.3892	2840.448	0.0588	21	1	27	TTASLDACCSGTRVAAD VVVSRPPGHR			Carbamidomethyl (C)[8,9]		Mascot

Rank	Protein Name	Accession No.	Protein MW	Protein PI	Pep. Count	Protein Score	Protein Score C. I. %	Intensity Matched	Total Ion Score	Total Ion C. I. %	Confirmed
2	Os03g0740833 [Oryza sativa Japonica Group]	gi|255674882	16857.9	10.21	5	23	0	1.984			

Peptide Information

Calc. Mass	Obsrv. Mass	± da	± ppm	Start Seq.	End Seq.	Sequence	Ion Score	C. I. %	Modification	Rank	Result Type
833.425	833.363	-0.062	-74	57	63	SELVEEK					Mascot
848.436	848.4734	0.0374	44	31	37	TQETIEK					Mascot
1269.5892	1269.6726	0.0834	66	90	101	FSSISAPSCSAR			Carbamidomethyl (C)[9]		Mascot
1376.738	1376.8007	0.0627	46	26	37	ITSARTQETIEK					Mascot
1816.9164	1816.9352	0.0188	10	105	120	FFPHPMGGEVVVNSRK			Oxidation (M)[6]		Mascot

Rank	Protein Name	Accession No.	Protein MW	Protein PI	Pep. Count	Protein Score	Protein Score C. I. %	Intensity Matched	Total Ion Score	Total Ion C. I. %	Confirmed
3	Os04g0379500 [Oryza sativa Japonica Group]	gi|255675389	4834.2	4.14	3	23	0	1.373			

Peptide Information

Calc. Mass	Obsrv. Mass	± da	± ppm	Start Seq.	End Seq.	Sequence	Ion Score	C. I. %	Modification	Rank	Result Type
849.4386	849.4496	0.011	13	5	11	ELMSEIK					Mascot
1182.5105	1182.6244	0.1139	96	12	20	VMDMVWNCK			Carbamidomethyl (C)[8]		Mascot
1269.6064	1269.6726	0.0662	52	2	11	MEKELMSEIK			Oxidation (M)[1,6]		Mascot

4 putative MYB DNA-binding domain superfamily protein [Zea mays] gi|413946659 10755.4 7.85 4 23 0 1.622

Protein Group

RADIALIS [Zea mays] gi|226531820 10755.4 7.8499999046 3257

Peptide Information

Calc. Mass	Obsrv. Mass	± da	± ppm	Start Seq.	End Seq.	Sequence	Ion Score	C. I. %	Modification	Rank	Result Type
818.4115	818.3299	-0.0816	-100	51	57	SADDVRR					Mascot
849.4617	849.4496	-0.0121	-14	74	80	VPFPAYR					Mascot
1182.5784	1182.6244	0.046	39	2	13	ASLSMTTSAAAR			Oxidation (M)[5]		Mascot
1269.6157	1269.6726	0.0569	45	39	50	WHNIACAVGGGK			Carbamidomethyl (C)[6]		Mascot

5 uncharacterized protein [Arabidopsis thaliana] gi|18415763 13234.8 10.83 4 23 0 1.887

Protein Group

uncharacterized protein AT4G21780 [Arabidopsis thaliana] gi|332659102 13234.8 10.829999923 7061

Peptide Information

Calc. Mass	Obsrv. Mass	± da	± ppm	Start Seq.	End Seq.	Sequence	Ion Score	C. I. %	Modification	Rank	Result Type
933.4384	933.3732	-0.0652	-70	38	45	RGNSEENK					Mascot
942.493	942.4318	-0.0612	-65	19	25	KEIDYFK					Mascot
1800.016	1799.9218	-0.0942	-52	1	18	MAAPIAIGTRGTIGSLVR			Oxidation (M)[1]		Mascot
1816.7993	1816.9352	0.1359	75	81	96	FFPSMCSAVEVSGENR			Carbamidomethyl (C)[6]		Mascot

6 Phytosulfokine receptor 1 [Aegilops tauschii] gi|475528543 16842.6 5.95 4 23 0 1.081

Peptide Information

Calc. Mass	Obsrv. Mass	± da	± ppm	Start Seq.	End Seq.	Sequence	Ion Score	C. I. %	Modification	Rank	Result Type
848.4625	848.4734	0.0109	13	5	12	LGDFGLAR					Mascot
917.4839	917.4018	-0.0821	-89	76	83	DVTAWAVR					Mascot
1182.6477	1182.6244	-0.0233	-20	134	145	LDAVAGAAPEIR					Mascot

2840.4395	2840.448	0.0085	3	109	133	VLELACACVSEDPKARPT AQQLVER			Carbamidomethyl (C)[6,8]		Mascot

7	hypothetical protein CARUB_v10014916mg [Capsella rubella]	gi|482567521	14767.3	9.47	4	22	0	.697

Peptide Information

Calc. Mass	Obsrv. Mass	± da	± ppm	Start Seq.	End Seq.	Sequence	Ion Score	C. I. %	Modification	Rank	Result Type
848.5096	848.4734	-0.0362	-43	1	7	MIKMVVK					Mascot
942.4098	942.4318	0.022	23	40	47	SACYGTQR			Carbamidomethyl (C)[3]		Mascot
1331.7101	1331.8112	0.1011	76	88	98	TVDVRVVDLCR			Carbamidomethyl (C)[10]		Mascot
2840.5203	2840.448	-0.0723	-25	4	29	MVVKFVVVAMVFAQIFSP IAEAAQGR			Oxidation (M)[1,10]		Mascot

8	hypothetical protein ZEAMMB73_436424 [Zea mays]	gi|413944874	8281.9	6.7	3	21	0	1.705

Peptide Information

Calc. Mass	Obsrv. Mass	± da	± ppm	Start Seq.	End Seq.	Sequence	Ion Score	C. I. %	Modification	Rank	Result Type
833.3821	833.363	-0.0191	-23	62	69	ASPLATNC			Carbamidomethyl (C)[8]		Mascot
917.408	917.4018	-0.0062	-7	1	8	MAAATCHR			Carbamidomethyl (C)[6]		Mascot
933.4029	933.3732	-0.0297	-32	1	8	MAAATCHR			Carbamidomethyl (C)[6], Oxidation (M)[1]		Mascot
942.4686	942.4318	-0.0368	-39	2	9	AAATCHRR			Carbamidomethyl (C)[5]		Mascot

9	Speckle-type POZ protein-like protein [Aegilops tauschii]	gi|475595178	41285.4	5.5	6	20	0	2.691

Peptide Information

Calc. Mass	Obsrv. Mass	± da	± ppm	Start Seq.	End Seq.	Sequence	Ion Score	C. I. %	Modification	Rank	Result Type
834.429	834.3561	-0.0729	-87	83	88	WMLVNR			Oxidation (M)[2]		Mascot
850.4669	850.4095	-0.0574	-67	168	175	IGADVTFK					Mascot
856.4887	856.5733	0.0846	99	93	100	SIVVHSSK					Mascot
959.3896	959.3871	-0.0025	-3	1	7	MGICFCR			Carbamidomethyl (C)[4,6], Oxidation (M)[1]		Mascot
1182.5428	1182.6244	0.0816	69	285	293	RICEGMLCK			Carbamidomethyl (C)[3,8], Oxidation (M)[6]		Mascot
1799.9585	1799.9218	-0.0367	-20	176	191	VQGEVFSAHKNLLAMR					Mascot

10	PREDICTED: xaa-Pro aminopeptidase 1-like [Fragaria vesca subsp. vesca]	gi|470142623	7306.7	9.18	3	20	0	3.694

Peptide Information

Calc. Mass	Obsrv. Mass	± da	± ppm	Start Seq.	End Seq.	Sequence	Ion Score	C. I. %	Modification	Rank	Result Type

822.4355	822.375	-0.0605	-74	57	63	SFLQAEK		Mascot
889.4526	889.3731	-0.0795	-89	49	56	AALWTDGR		Mascot
1959.9998	1960.1753	0.1755	90	2	18	SELALGFQVLSFCYGLR	Carbamidomethyl (C)[13]	Mascot

Gel Idx/Pos	111/E10	Instr./Gel Origin	BA2151/Sample Project 20140814	Process Status	Analysis Succeeded
Plate [#] Name	[1] Sample Project 20140814	Instrument Sample Name		Spectra	11

Rank	Protein Name	Accession No.	Protein MW	Protein PI	Pep. Count	Protein Score	Protein Score C. I. %	Intensity Matched	Total Ion Score	Total Ion C. I. %	Confirmed
1	TPA: hypothetical protein ZEAMMB73_268898 [Zea mays]	gi|414879246	40179.3	8.85	14	63	48.917	10.394			

Peptide Information

Calc. Mass	Obsrv. Mass	± da	± ppm	Start Seq.	End Seq.	Sequence	Ion Score	C. I. %	Modification	Rank	Result Type
842.5094	842.567	0.0576	68	21	27	LVRPETK					Mascot
897.3957	897.4689	0.0732	82	153	159	CPYLGCK			Carbamidomethyl (C)[1,6]		Mascot
948.4931	948.4907	-0.0024	-3	316	324	SGLRDAAMK					Mascot
982.5316	982.4957	-0.0359	-37	76	83	EAEIHQKK					Mascot
1106.6027	1106.5803	-0.0224	-20	183	191	TNPVYLMLR					Mascot
1183.5698	1183.6428	0.073	62	160	169	DATKMMLDVK			Oxidation (M)[5,6]		Mascot
1194.5685	1194.655	0.0865	72	255	265	IHSPMQGDPGR					Mascot
1300.6896	1300.6179	-0.0717	-55	192	204	TSFGFSAKGTGLK					Mascot
1307.7029	1307.7587	0.0558	43	34	44	IQIQLTGFMEK					Mascot
1320.5923	1320.6729	0.0806	61	320	330	DAAMKHLEDMK			Oxidation (M)[4,10]		Mascot
1323.6978	1323.7312	0.0334	25	34	44	IQIQLTGFMEK			Oxidation (M)[9]		Mascot
1323.6978	1323.7312	0.0334	25	34	44	IQIQLTGFMEK			Oxidation (M)[9]		Mascot
1440.6359	1440.7537	0.1178	82	340	352	NAGGGYLCMINDR			Carbamidomethyl (C)[8]		Mascot
1456.6307	1456.7714	0.1407	97	340	352	NAGGGYLCMINDR			Carbamidomethyl (C)[8], Oxidation (M)[9]		Mascot
1638.8044	1638.9562	0.1518	93	229	242	ECSVLTAEVIFQDK			Carbamidomethyl (C)[2]		Mascot
1791.8364	1791.8446	0.0082	5	289	305	RPGAGSDDPLGTCTMIK			Carbamidomethyl (C)[13], Oxidation (M)[15]		Mascot
1791.8364	1791.8446	0.0082	5	289	305	RPGAGSDDPLGTCTMIK			Carbamidomethyl (C)[13], Oxidation (M)[15]		Mascot
1947.9998	1948.0273	0.0275	14	183	199	TNPVYLMLRTSFGFSAK			Oxidation (M)[7]		Mascot

Rank	Protein Name	Accession No.	Protein MW	Protein PI	Pep. Count	Protein Score	Protein Score C. I. %	Intensity Matched	Total Ion Score	Total Ion C. I. %	Confirmed
2	PREDICTED: geraniol 8-hydroxylase-like [Solanum lycopersicum]	gi|460372485	57397.6	8.27	15	60	9.16	3.895			

Peptide Information

Calc. Mass	Obsrv. Mass	± da	± ppm	Start Seq.	End Seq.	Sequence	Ion Score	C. I. %	Modification	Rank	Result Type
807.4723	807.4536	-0.0187	-23	484	490	FGLTLTR					Mascot
897.4061	897.4689	0.0628	70	97	104	QDSAFSSR					Mascot
948.501	948.4907	-0.0103	-11	147	154	LDASHHLR					Mascot

Calc. Mass	Obsrv. Mass	± da	± ppm	Start Seq.	End Seq.	Sequence	Ion Score	C. I. %	Modification	Rank	Result Type
1006.5792	1006.492	-0.0872	-87	234	242	IVHPQGLSR					Mascot
1140.6888	1140.6317	-0.0571	-50	395	404	VLINVWAIGR					Mascot
1265.7511	1265.688	-0.0631	-50	442	452	RICPGLPLAIR			Carbamidomethyl (C)[3]		Mascot
1277.6824	1277.7864	0.104	81	366	376	MHPPAPFLVPR			Oxidation (M)[1]		Mascot
1297.7046	1297.728	0.0234	18	64	75	LADSHGAVMRLK					Mascot
1405.7223	1405.7604	0.0381	27	429	441	GQNFELIPFGAGR					Mascot
1493.7708	1493.8334	0.0626	42	92	104	QVLQKQDSAFSSR					Mascot
1497.7982	1497.8582	0.06	40	184	196	TMLNLLSNTLFSK			Oxidation (M)[2]		Mascot
1605.8231	1605.8174	-0.0057	-4	168	183	SSQIGEAVDIGGAVFR					Mascot
1891.0569	1891.0789	0.022	12	74	91	LKLGQITTVVISSSDMAK					Mascot
2687.3232	2687.3975	0.0743	28	184	207	TMLNLLSNTLFSKDLADP YENSGK			Oxidation (M)[2]		Mascot
3223.5784	3223.5027	-0.0757	-23	105	131	SIPDIVKEENFHMFSVGW LPASHPQWR			Oxidation (M)[13]		Mascot

3	hypothetical protein SORBIDRAFT_09g008636 [Sorghum bicolor]	gi|241944793	15932.6	9.58	11	60	0	15.005			

Peptide Information

Calc. Mass	Obsrv. Mass	± da	± ppm	Start Seq.	End Seq.	Sequence	Ion Score	C. I. %	Modification	Rank	Result Type
897.3948	897.4689	0.0741	83	55	62	TYENDAGK					Mascot
1018.4727	1018.5725	0.0998	98	109	116	YYDESLTK					Mascot
1033.5459	1033.5781	0.0322	31	88	96	AACTDIVKR			Carbamidomethyl (C)[3]		Mascot
1106.6205	1106.5803	-0.0402	-36	35	44	LASETGIFLR					Mascot
1141.6398	1141.6199	-0.0199	-17	1	10	MGHGLEKLLK			Oxidation (M)[1]		Mascot
1179.5681	1179.6729	0.1048	89	131	139	QEWVNLVEY					Mascot
1252.6606	1252.6274	-0.0332	-27	117	127	EQLLAMEPPPK					Mascot
1277.7212	1277.7864	0.0652	51	12	23	GHKLPIEVAEGK					Mascot
1300.6743	1300.6179	-0.0564	-43	76	87	LDVDIKNDGPSK					Mascot
1307.6631	1307.7587	0.0956	73	130	139	KQEWVNLVEY					Mascot
1475.7159	1475.8389	0.123	83	82	95	NDGPSKAACTDIVK			Carbamidomethyl (C)[9]		Mascot
1475.7159	1475.8389	0.123	83	82	95	NDGPSKAACTDIVK			Carbamidomethyl (C)[9]		Mascot

4	PREDICTED: aspartate carbamoyltransferase 2, chloroplastic [Solanum lycopersicum]	gi|460409001	42637.9	6.43	11	59	0	11.789	28	0	

Peptide Information

Calc. Mass	Obsrv. Mass	± da	± ppm	Start Seq.	End Seq.	Sequence	Ion Score	C. I. %	Modification	Rank	Result Type
973.5425	973.5909	0.0484	50	96	104	IEKNSIGGR	29	0			Mascot

973.5862	973.5909	0.0047	5	13	20	ILMSPLRK			Oxidation (M)[3]		Mascot
1033.5161	1033.5781	0.062	60	154	162	GETLEDTIR					Mascot
1057.4983	1057.5587	0.0604	57	268	276	DYLTSMGVR			Oxidation (M)[6]		Mascot
1179.6005	1179.6729	0.0724	61	307	316	VDLYEEARGK					Mascot
1201.5881	1201.6703	0.0822	68	124	133	TRLSFESSMK			Oxidation (M)[9]		Mascot
1232.6304	1232.6776	0.0472	38	1	12	MTISATLSSHGK					Mascot
1259.6591	1259.7482	0.0891	71	135	146	LGGEVLTTENAR					Mascot
1307.7504	1307.7587	0.0083	6	361	371	NGLYIRMALLK			Oxidation (M)[7]		Mascot
1497.744	1497.8582	0.1142	76	317	329	YIVDMSVVNAMQK					Mascot
1513.7389	1513.8529	0.114	75	317	329	YIVDMSVVNAMQK			Oxidation (M)[5]		Mascot
1698.8553	1698.9609	0.1056	62	315	329	GKYIVDMSVVNAMQK			Oxidation (M)[7]		Mascot

5	unnamed protein product [Vitis vinifera]	gi|302143321	31358.2	6.87	12	59	0	5.199	

Peptide Information

Calc. Mass	Obsrv. Mass	± da	± ppm	Start Seq.	End Seq.	Sequence	Ion Score	C. I. %	Modification	Rank	Result Type
1057.6616	1057.5587	-0.1029	-97	5	13	EILQKISVK					Mascot
1068.5797	1068.5731	-0.0066	-6	251	260	HIKDAQSAAK					Mascot
1074.6041	1074.5909	-0.0132	-12	111	119	KTDIEILDK					Mascot
1092.5532	1092.5721	0.0189	17	14	25	VGLGSSTADTGK					Mascot
1139.5692	1139.6287	0.0595	52	161	170	QLSVDHEPSK					Mascot
1277.6697	1277.7864	0.1167	91	14	27	VGLGSSTADTGKGK					Mascot
1323.6614	1323.7312	0.0698	53	273	283	DDISCIVVKFQ			Carbamidomethyl (C)[5]		Mascot
1323.6614	1323.7312	0.0698	53	273	283	DDISCIVVKFQ			Carbamidomethyl (C)[5]		Mascot
1393.645	1393.7693	0.1243	89	239	250	VMSNQEAVDCIK			Carbamidomethyl (C)[10]		Mascot
1458.7911	1458.812	0.0209	14	126	140	GGSTAVTAILINGER					Mascot
1605.7544	1605.8174	0.063	39	94	106	EHTFWTETENAIK					Mascot
1638.8082	1638.9562	0.148	90	156	170	NGEAKQLSVDHEPSK					Mascot
2687.3464	2687.3975	0.0511	19	85	106	THLFDNILKEHTFWTETE NAIK					Mascot

6	SNO glutamine amidotransferase [Bathycoccus prasinos]	gi|412985545	29916	5.45	12	58	0	2.374	

Peptide Information

Calc. Mass	Obsrv. Mass	± da	± ppm	Start Seq.	End Seq.	Sequence	Ion Score	C. I. %	Modification	Rank	Result Type
948.4026	948.4907	0.0881	93	23	29	EHCSMIR			Carbamidomethyl (C)[3], Oxidation (M)[5]		Mascot
951.4717	951.5168	0.0451	47	221	228	GCKPFQSK			Carbamidomethyl (C)[2]		Mascot

Calc. Mass	Obsrv. Mass	± da	± ppm	Start Seq.	End Seq.	Sequence	Ion Score	C. I. %	Modification	Rank	Result Type
1018.5237	1018.5725	0.0488	48	255	263	DQVIADVMK					Mascot
1057.5889	1057.5587	-0.0302	-29	160	169	VGENVEVLAK					Mascot
1107.4987	1107.6064	0.1077	97	1	9	MAIEQQEDK			Oxidation (M)[1]		Mascot
1201.6423	1201.6703	0.028	23	2	11	AIEQQEDKIK					Mascot
1297.6392	1297.728	0.0888	68	218	228	MSKGCKPFQSK			Carbamidomethyl (C)[5]		Mascot
1332.6827	1332.6359	-0.0468	-35	1	11	MAIEQQEDKIK					Mascot
1490.7771	1490.8395	0.0624	42	179	192	EMGVDIESVVVAVK			Oxidation (M)[2]		Mascot
1641.8556	1641.9426	0.087	53	104	119	QGGQELLGGINVDVSR					Mascot
1738.8405	1738.8689	0.0284	16	83	97	CVWGTCAGLIFLADR			Carbamidomethyl (C)[1,6]		Mascot
1813.9443	1813.8425	-0.1018	-56	195	210	NLLATSFHPELTSDLR					Mascot

7	hypothetical protein F775_26998 [Aegilops tauschii]	gi|475624490	66872.3	5.46	15	58	0	16.116	

Peptide Information

Calc. Mass	Obsrv. Mass	± da	± ppm	Start Seq.	End Seq.	Sequence	Ion Score	C. I. %	Modification	Rank	Result Type
807.4393	807.4536	0.0143	18	155	161	MQATTKK					Mascot
832.4498	832.5322	0.0824	99	92	97	FIHMLR			Oxidation (M)[4]		Mascot
982.572	982.4957	-0.0763	-78	568	576	SGFVFGKLK					Mascot
1006.5833	1006.492	-0.0913	-91	342	349	IFSGWRLK					Mascot
1065.4994	1065.5627	0.0633	59	463	470	EETVERMR			Oxidation (M)[7]		Mascot
1252.6208	1252.6274	0.0066	5	162	171	WYTNILEADK					Mascot
1265.6888	1265.688	-0.0008	-1	183	193	LYTPAAVDLFR					Mascot
1307.743	1307.7587	0.0157	12	7	17	TSFVTLNRTIR					Mascot
1421.7438	1421.7313	-0.0125	-9	336	347	FQPGARIFSGWR					Mascot
1716.8085	1716.9579	0.1494	87	469	481	MRLDEEVLMDFFR			Oxidation (M)[1]		Mascot
1716.8085	1716.9579	0.1494	87	469	481	MRLDEEVLMDFFR			Oxidation (M)[1]		Mascot
1838.0323	1838.0459	0.0136	7	320	335	SLLSFLFYVHGVKATR					Mascot
1851.9634	1852.033	0.0696	38	76	91	KATAACELLVNLYTER			Carbamidomethyl (C)[6]		Mascot
1994.1475	1994.1018	-0.0457	-23	315	332	VGSIKSLLSFLFYVHGVK					Mascot
1994.1475	1994.1018	-0.0457	-23	315	332	VGSIKSLLSFLFYVHGVK					Mascot
2705.1787	2705.3398	0.1611	60	405	427	DWMDGMVTEYLVATFAD YFGDVK			Oxidation (M)[3,6]		Mascot
2705.1787	2705.3398	0.1611	60	405	427	DWMDGMVTEYLVATFAD YFGDVK			Oxidation (M)[3,6]		Mascot
3223.4639	3223.5027	0.0388	12	401	427	VYQKDWMDGMVTEYLV ATFADYFGDVK			Oxidation (M)[7,10]		Mascot

8	PREDICTED: disease resistance protein RPM1-like isoform X2 [Setaria italica]	gi|514794453	139405.8	8.66	24	58	0	18.631	

Peptide Information

Calc. Mass	Obsrv. Mass	± da	± ppm	Start Seq.	End Seq.	Sequence	Ion Score	C. I. %	Modification	Rank	Result Type
842.4916	842.567	0.0754	89	1024	1030	HAMKVLK			Oxidation (M)[3]		Mascot
948.536	948.4907	-0.0453	-48	132	140	LIKDASSSK					Mascot
951.4564	951.5168	0.0604	63	427	434	MISTAEQR			Oxidation (M)[1]		Mascot
1018.5276	1018.5725	0.0449	44	118	126	ARVEDVSSR					Mascot
1036.5344	1036.5798	0.0454	44	1	10	MEATVVSVGK			Oxidation (M)[1]		Mascot
1057.6266	1057.5587	-0.0679	-64	863	870	HLRYLSLR					Mascot
1065.5649	1065.5627	-0.0022	-2	895	903	DTFIMTLPK					Mascot
1068.5758	1068.5731	-0.0027	-3	829	837	SFLISDKMK					Mascot
1081.5598	1081.5435	-0.0163	-15	895	903	DTFIMTLPK			Oxidation (M)[5]		Mascot
1082.5365	1082.5487	0.0122	11	200	208	VYDDESVKK					Mascot
1127.5004	1127.5874	0.087	77	234	242	QFYENSPDK					Mascot
1194.6664	1194.655	-0.0114	-10	1160	1168	LQLCYTRLK			Carbamidomethyl (C)[4]		Mascot
1201.6688	1201.6703	0.0015	1	1013	1023	TLSVVNVAWGR					Mascot
1365.6184	1365.7278	0.1094	80	1196	1205	EQCLALRCCR			Carbamidomethyl (C)[3,8,9]		Mascot
1376.709	1376.7865	0.0775	56	163	175	EAMVNLADLVSSK					Mascot
1475.7887	1475.8389	0.0502	34	422	434	AEVIKMISTAEQR					Mascot
1475.7887	1475.8389	0.0502	34	422	434	AEVIKMISTAEQR					Mascot
1490.7421	1490.8395	0.0974	65	874	886	GVTQLPECFGNLR			Carbamidomethyl (C)[8]		Mascot
1491.7849	1491.833	0.0481	32	969	981	LLHAGMNRHDISK					Mascot
1507.7798	1507.8231	0.0433	29	969	981	LLHAGMNRHDISK			Oxidation (M)[6]		Mascot
1507.7798	1507.8231	0.0433	29	969	981	LLHAGMNRHDISK			Oxidation (M)[6]		Mascot
1657.8942	1657.8982	0.004	2	22	36	SMLAEEIALQLGVQR					Mascot
1657.8942	1657.8982	0.004	2	22	36	SMLAEEIALQLGVQR	2	0			Mascot
1716.7938	1716.9579	0.1641	96	688	701	SSFLYMSIFPEDHK			Oxidation (M)[6]		Mascot
1716.7938	1716.9579	0.1641	96	688	701	SSFLYMSIFPEDHK			Oxidation (M)[6]		Mascot
1738.9335	1738.8689	-0.0646	-37	246	262	GHEGETVGATALVKIEK					Mascot
1891.0358	1891.0789	0.0431	23	895	910	DTFIMTLPKTIVNLER					Mascot
3223.5723	3223.5027	-0.0696	-22	307	335	NGSRIVVSAQQAEVASLC TEQPYQMSELK			Carbamidomethyl (C)[18]		Mascot
3312.7031	3312.5403	-0.1628	-49	528	556	ELAGLLQEPECLIVLDDV SSTLEWDAIKR			Carbamidomethyl (C)[11]		Mascot
3312.7031	3312.5403	-0.1628	-49	527	555	RELAGLLQEPECLIVLDD VSSTLEWDAIK			Carbamidomethyl (C)[12]		Mascot

9 PREDICTED: uncharacterized protein LOC101305028 [Fragaria vesca subsp. vesca] gi|470108652 205439.9 4.97 31 58 0 20.27

Peptide Information

Calc. Mass	Obsrv. Mass	± da	± ppm	Start	End	Sequence	Ion	C. I. %	Modification	Rank	Result Type

				Seq.	Seq.		Score		
948.4495	948.4907	0.0412	43	58	64	AEMYYKK		Oxidation (M)[3]	Mascot
951.5331	951.5168	-0.0163	-17	959	966	MGLYQVLK			Mascot
1018.5601	1018.5725	0.0124	12	1478	1486	AIEKAMVEK			Mascot
1036.5343	1036.5798	0.0455	44	922	931	LLGASTMSEK			Mascot
1060.5885	1060.6218	0.0333	31	1207	1215	SEDELKLVK			Mascot
1092.6049	1092.5721	-0.0328	-30	1358	1366	ETLFEGKIR			Mascot
1140.5757	1140.6317	0.056	49	850	859	QNHASSVELR			Mascot
1183.7157	1183.6428	-0.0729	-62	1726	1735	IGRLQLEVQK			Mascot
1201.6423	1201.6703	0.028	23	380	389	LLDVEEEAKR			Mascot
1227.6943	1227.6412	-0.0531	-43	585	594	DLQDEILILR			Mascot
1259.7028	1259.7482	0.0454	36	681	690	LEIMQKLQEK			Mascot
1265.6008	1265.688	0.0872	69	46	56	LIEEDADSFAR			Mascot
1307.7504	1307.7587	0.0083	6	956	966	ALRMGLYQVLK		Oxidation (M)[4]	Mascot
1332.6941	1332.6359	-0.0582	-44	870	881	ISGLEAEGICRK		Carbamidomethyl (C)[10]	Mascot
1379.739	1379.7948	0.0558	40	1071	1082	VVEGEHREGVLR			Mascot
1393.6991	1393.7693	0.0702	50	1379	1390	SISKALENEQMK		Oxidation (M)[11]	Mascot
1405.6628	1405.7604	0.0976	69	967	979	TVDIDANLGCAEK		Carbamidomethyl (C)[10]	Mascot
1421.7019	1421.7313	0.0294	21	46	57	LIEEDADSFARR			Mascot
1458.7184	1458.812	0.0936	64	1216	1229	SVNDQLNGDIANAK			Mascot
1475.8468	1475.8389	-0.0079	-5	891	903	SVTAQIEIFVLQK			Mascot
1475.8468	1475.8389	-0.0079	-5	891	903	SVTAQIEIFVLQK			Mascot
1490.752	1490.8395	0.0875	59	368	379	QCLEKISNLEEK		Carbamidomethyl (C)[2]	Mascot
1491.7551	1491.833	0.0779	52	180	193	QLNDLFGSGEGRAK			Mascot
1507.6945	1507.8231	0.1286	85	1267	1279	SDDAKMVLEDQEK			Mascot
1507.6945	1507.8231	0.1286	85	1267	1279	SDDAKMVLEDQEK			Mascot
1605.7504	1605.8174	0.067	42	1037	1049	DTLDHEFRTQSEK			Mascot
1698.9636	1698.9609	-0.0027	-2	585	598	DLQDEILILRETIK			Mascot
1738.7683	1738.8689	0.1006	58	12	24	MYSWWWDSHISPK		Oxidation (M)[1]	Mascot
1790.8741	1790.8622	-0.0119	-7	42	56	HMIKLIEEDADSFAR		Oxidation (M)[2]	Mascot
1847.0306	1846.8806	-0.15	-81	922	938	LLGASTMSEKLISVLER			Mascot
1993.9569	1994.1018	0.1449	73	904	919	CVEDLEEKNLSLMIER		Carbamidomethyl (C)[1], Oxidation (M)[13]	Mascot
1993.9569	1994.1018	0.1449	73	904	919	CVEDLEEKNLSLMIER		Carbamidomethyl (C)[1], Oxidation (M)[13]	Mascot
2023.0892	2023.017	-0.0722	-36	1185	1201	ILEGQLEVIQMENLHLK		Oxidation (M)[11]	Mascot
2510.2661	2510.2524	-0.0137	-5	883	903	EFEEEQDKSVTAQIEIFVL QK			Mascot

10	HCF152 [Arabidopsis lyrata subsp. lyrata]	gi|297328472	87102	6.55	16	57	0	17.14	

Peptide Information

Calc. Mass	Obsrv. Mass	± da	± ppm	Start Seq.	End Seq.	Sequence	Ion Score	C. I. %	Modification	Rank	Result Type
948.4785	948.4907	0.0122	13	89	96	KTDEAWAK					Mascot
1057.6477	1057.5587	-0.089	-84	128	136	AQSILTRLR					Mascot
1235.5573	1235.6145	0.0572	46	338	347	ECNAEDLKEK			Carbamidomethyl (C)[2]		Mascot
1265.7101	1265.688	-0.0221	-17	159	170	SGQTLYAVSVIK					Mascot
1379.677	1379.7948	0.1178	85	424	435	VADTARMLEAMR			Oxidation (M)[7]		Mascot
1458.7985	1458.812	0.0135	9	144	158	LDANSLGLLAMAAAK					Mascot
1641.8669	1641.9426	0.0757	46	403	416	VFAPDSRIYTTLMK					Mascot
1657.7592	1657.8982	0.139	84	372	385	DEVSEEGFEDVFKK					Mascot
1657.8618	1657.8982	0.0364	22	403	416	VFAPDSRIYTTLMK			Oxidation (M)[13]		Mascot
1674.8414	1674.8341	-0.0073	-4	467	481	QVLAEMARMGVPANR			Oxidation (M)[6,9]		Mascot
1707.7942	1707.8805	0.0863	51	570	583	LANRVFDEMMNDPR					Mascot
1715.8534	1715.9122	0.0588	34	97	111	YVQSTHLPGPTCLSR			Carbamidomethyl (C)[12]		Mascot
1846.8799	1846.8806	0.0007	0	752	766	FWLGLPNSYYGSEWK					Mascot
1993.9866	1994.1018	0.1152	58	441	458	NSHPDEVTYTTVVSAFVK					Mascot
1993.9866	1994.1018	0.1152	58	441	458	NSHPDEVTYTTVVSAFVK					Mascot
2705.4907	2705.3398	-0.1509	-56	144	170	LDANSLGLLAMAAAKSG QTLYAVSVIK					Mascot
2705.4907	2705.3398	-0.1509	-56	144	170	LDANSLGLLAMAAAKSG QTLYAVSVIK					Mascot
2717.2844	2717.262	-0.0224	-8	297	320	VCMTTMHSLVAAYVGFG DLRTAER			Carbamidomethyl (C)[2], Oxidation (M)[3,6]		Mascot
3312.561	3312.5403	-0.0207	-6	659	689	EAPSDSSSDPSPPMLKP DEGLLDTLADICVR			Carbamidomethyl (C)[29]		Mascot
3312.561	3312.5403	-0.0207	-6	659	689	EAPSDSSSDPSPPMLKP DEGLLDTLADICVR			Carbamidomethyl (C)[29]		Mascot

Gel Idx/Pos	112/E11	Instr./Gel Origin	BA2151/Sample Project 20140814	Process Status	Analysis Succeeded
Plate [#] Name	[1] Sample Project 20140814	Instrument Sample Name		Spectra	11

Rank	Protein Name	Accession No.	Protein MW	Protein PI	Pep. Count	Protein Score	Protein Score C. I. %	Intensity Matched	Total Ion Score	Total Ion C. I. %	Confirmed
1	hypothetical protein TRIUR3_16950 [Triticum urartu]	gi|473794549	27510.1	6.25	7	355	100	10.755	315	100	

Peptide Information

Calc. Mass	Obsrv. Mass	± da	± ppm	Start Seq.	End Seq.	Sequence	Ion Score	C. I. %	Modification	Rank	Result Type
815.3795	815.4232	0.0437	54	99	104	TDTWHR					Mascot
827.4985	827.499	0.0005	1	65	72	ADVPGLKK					Mascot
974.5417	974.5937	0.052	53	116	123	FRLPENAK					Mascot
974.5417	974.5937	0.052	53	116	123	FRLPENAK	18	0			Mascot
1057.5314	1057.5701	0.0387	37	56	64	ETPEAHVFK					Mascot
1154.5437	1154.6094	0.0657	57	40	51	TSSDTAAFAGAR					Mascot
1154.5437	1154.6094	0.0657	57	40	51	TSSDTAAFAGAR	72	99.988			Mascot
1657.8392	1657.9315	0.0923	56	77	91	VEVEDGNILQISGER					Mascot
1657.8392	1657.9315	0.0923	56	77	91	VEVEDGNILQISGER	139	100			Mascot
2143.0876	2143.2131	0.1255	59	73	91	EEVKVEVEDGNILQISGE R					Mascot
2143.0876	2143.2131	0.1255	59	73	91	EEVKVEVEDGNILQISGE R	104	100			Mascot

Rank	Protein Name	Accession No.	Protein MW	Protein PI	Pep. Count	Protein Score	Protein Score C. I. %	Intensity Matched	Total Ion Score	Total Ion C. I. %	Confirmed
2	heat shock protein 17.4 [Arabidopsis thaliana]	gi|332644617	17428.8	5.21	6	288	100	8.076	244	100	

Protein Group

RecName: Full=17.4 kDa class I heat shock protein; AltName: Full=17.4 kDa heat shock protein 1; Short=AtHsp17.4A	gi|21431764	17428.8	5.2100 000381 4697
heat shock protein 17.4 [Arabidopsis thaliana]	gi|15231372	17428.8	5.2100 000381 4697

Peptide Information

Calc. Mass	Obsrv. Mass	± da	± ppm	Start Seq.	End Seq.	Sequence	Ion Score	C. I. %	Modification	Rank	Result Type
827.4985	827.499	0.0005	1	64	71	ADVPGLKK					Mascot
974.5417	974.5937	0.052	53	115	122	FRLPENAK					Mascot
974.5417	974.5937	0.052	53	115	122	FRLPENAK	18	0			Mascot
1057.5314	1057.5701	0.0387	37	55	63	ETPEAHVFK					Mascot
1066.5681	1066.5667	-0.0014	-1	2	11	SLVPSFFGGR					Mascot
1657.8392	1657.9315	0.0923	56	76	90	VEVEDGNILQISGER					Mascot

Calc. Mass	Obsrv. Mass	± da	± ppm	Start Seq.	End Seq.	Sequence	Ion Score	C. I. %	Modification	Rank	Result Type
1657.8392	1657.9315	0.0923	56	76	90	VEVEDGNILQISGER	139	100			Mascot
2143.0876	2143.2131	0.1255	59	72	90	EEVKVEVEDGNILQISGER					Mascot
2143.0876	2143.2131	0.1255	59	72	90	EEVKVEVEDGNILQISGER	104	100			Mascot

3	hypothetical protein TRIUR3_24152 [Triticum urartu]	gi|474174891	12287.5	6.75	5	286	100	8.228	244	100	

Peptide Information

Calc. Mass	Obsrv. Mass	± da	± ppm	Start Seq.	End Seq.	Sequence	Ion Score	C. I. %	Modification	Rank	Result Type
815.3795	815.4232	0.0437	54	53	58	TDTWHR					Mascot
827.4985	827.499	0.0005	1	19	26	ADVPGLKK					Mascot
974.5417	974.5937	0.052	53	70	77	FRLPENAK					Mascot
974.5417	974.5937	0.052	53	70	77	FRLPENAK	18	0			Mascot
1657.8392	1657.9315	0.0923	56	31	45	VEVEDGNILQISGER					Mascot
1657.8392	1657.9315	0.0923	56	31	45	VEVEDGNILQISGER	139	100			Mascot
2143.0876	2143.2131	0.1255	59	27	45	EEVKVEVEDGNILQISGER					Mascot
2143.0876	2143.2131	0.1255	59	27	45	EEVKVEVEDGNILQISGER	104	100			Mascot

4	hypothetical protein CARUB_v10018169mg [Capsella rubella]	gi|482560687	17573.9	5.56	6	286	100	9.717	244	100	

Peptide Information

Calc. Mass	Obsrv. Mass	± da	± ppm	Start Seq.	End Seq.	Sequence	Ion Score	C. I. %	Modification	Rank	Result Type
974.5417	974.5937	0.052	53	116	123	FRLPENAK					Mascot
974.5417	974.5937	0.052	53	116	123	FRLPENAK	18	0			Mascot
1057.5314	1057.5701	0.0387	37	56	64	ETPEAHVFK					Mascot
1193.6348	1193.6835	0.0487	41	1	11	MSLIPSIFGGR			Oxidation (M)[1]		Mascot
1493.7339	1493.8207	0.0868	58	129	142	ASMENGVLSVMVPK			Oxidation (M)[3,11]		Mascot
1493.7339	1493.8207	0.0868	58	129	142	ASMENGVLSVMVPK			Oxidation (M)[3,11]		Mascot
1657.8392	1657.9315	0.0923	56	77	91	VEVEDGNILQISGER					Mascot
1657.8392	1657.9315	0.0923	56	77	91	VEVEDGNILQISGER	139	100			Mascot
2143.0876	2143.2131	0.1255	59	73	91	EEVKVEVEDGNILQISGER					Mascot
2143.0876	2143.2131	0.1255	59	73	91	EEVKVEVEDGNILQISGER	104	100			Mascot

5	hypothetical protein ARALYDRAFT_485004 [Arabidopsis lyrata subsp. lyrata]	gi|297323303	17466.9	5.36	5	284	100	7.775	244	100	

Peptide Information

Calc. Mass	Obsrv. Mass	± da	± ppm	Start	End	Sequence	Ion	C. I. %	Modification	Rank	Result Type

				Seq.	Seq.		Score				
974.5417	974.5937	0.052	53	115	122	FRLPENAK					Mascot
974.5417	974.5937	0.052	53	115	122	FRLPENAK	18	0			Mascot
1057.5314	1057.5701	0.0387	37	55	63	ETPEAHVFK					Mascot
1584.8479	1584.8104	-0.0375	-24	142	156	VPESKPEVKSIDISG					Mascot
1657.8392	1657.9315	0.0923	56	76	90	VEVEDGNILQISGER					Mascot
1657.8392	1657.9315	0.0923	56	76	90	VEVEDGNILQISGER	139	100			Mascot
2143.0876	2143.2131	0.1255	59	72	90	EEVKVEVEDGNILQISGE R					Mascot
2143.0876	2143.2131	0.1255	59	72	90	EEVKVEVEDGNILQISGE R	104	100			Mascot

6	HSP20-like chaperone [Arabidopsis thaliana]	gi|332194833	17593	5.36	5	282	100	8.37	244	100	

Protein Group

HSP20-like chaperone [Arabidopsis thaliana]	gi|15220832	17593	5.3600 001335 144
RecName: Full=17.6 kDa class I heat shock protein 3; AltName: Full=17.6 kDa heat shock protein 3; Short=AtHsp17.6C	gi|21264442	17593	5.3600 001335 144

Peptide Information

Calc. Mass	Obsrv. Mass	± da	± ppm	Start Seq.	End Seq.	Sequence	Ion Score	C. I. %	Modification	Rank	Result Type
974.5417	974.5937	0.052	53	116	123	FRLPENAK					Mascot
974.5417	974.5937	0.052	53	116	123	FRLPENAK	18	0			Mascot
1057.5314	1057.5701	0.0387	37	56	64	ETPEAHVFK					Mascot
1193.6348	1193.6835	0.0487	41	1	11	MSLIPSIFGGR			Oxidation (M)[1]		Mascot
1657.8392	1657.9315	0.0923	56	77	91	VEVEDGNILQISGER					Mascot
1657.8392	1657.9315	0.0923	56	77	91	VEVEDGNILQISGER	139	100			Mascot
2143.0876	2143.2131	0.1255	59	73	91	EEVKVEVEDGNILQISGE R					Mascot
2143.0876	2143.2131	0.1255	59	73	91	EEVKVEVEDGNILQISGE R	104	100			Mascot

7	17.6 kDa class I small heat shock protein [Arabidopsis lyrata subsp. lyrata]	gi|297323309	17488	5.56	5	281	100	8.37	244	100	

Protein Group

hypothetical protein CARUB_v10018594mg [Capsella rubella]	gi|482561088	17548	5.8099 999427 7954

Peptide Information

Calc. Mass	Obsrv. Mass	± da	± ppm	Start Seq.	End Seq.	Sequence	Ion Score	C. I. %	Modification	Rank	Result Type

974.5417	974.5937	0.052	53	116	123	FRLPENAK					Mascot
974.5417	974.5937	0.052	53	116	123	FRLPENAK	18	0			Mascot
1057.5314	1057.5701	0.0387	37	56	64	ETPEAHVFK					Mascot
1193.6348	1193.6835	0.0487	41	1	11	MSLIPSIFGGR			Oxidation (M)[1]		Mascot
1657.8392	1657.9315	0.0923	56	77	91	VEVEDGNILQISGER					Mascot
1657.8392	1657.9315	0.0923	56	77	91	VEVEDGNILQISGER	139	100			Mascot
2143.0876	2143.2131	0.1255	59	73	91	EEVKVEVEDGNILQISGE R					Mascot
2143.0876	2143.2131	0.1255	59	73	91	EEVKVEVEDGNILQISGE R	104	100			Mascot

8	hypothetical protein F775_26328 [Aegilops tauschii]	gi|475535143	13066.7	6.17	5	268	100	7.409	244	100	

Peptide Information

Calc. Mass	Obsrv. Mass	± da	± ppm	Start Seq.	End Seq.	Sequence	Ion Score	C. I. %	Modification	Rank	Result Type
815.3795	815.4232	0.0437	54	60	65	TDTWHR					Mascot
827.4985	827.499	0.0005	1	26	33	ADVPGLKK					Mascot
1057.5889	1057.5701	-0.0188	-18	104	112	EEAKKPEVK					Mascot
1657.8392	1657.9315	0.0923	56	38	52	VEVEDGNILQISGER					Mascot
1657.8392	1657.9315	0.0923	56	38	52	VEVEDGNILQISGER	139	100			Mascot
2143.0876	2143.2131	0.1255	59	34	52	EEVKVEVEDGNILQISGE R					Mascot
2143.0876	2143.2131	0.1255	59	34	52	EEVKVEVEDGNILQISGE R	104	100			Mascot

9	17.6 kDa class I small heat shock protein [Arabidopsis lyrata subsp. lyrata]	gi|297337598	17575	5.56	4	263	100	7.309	244	100	

Peptide Information

Calc. Mass	Obsrv. Mass	± da	± ppm	Start Seq.	End Seq.	Sequence	Ion Score	C. I. %	Modification	Rank	Result Type
1057.5314	1057.5701	0.0387	37	56	64	ETPEAHVFK					Mascot
1193.6348	1193.6835	0.0487	41	1	11	MSLIPSIFGGR			Oxidation (M)[1]		Mascot
1657.8392	1657.9315	0.0923	56	77	91	VEVEDGNILQISGER					Mascot
1657.8392	1657.9315	0.0923	56	77	91	VEVEDGNILQISGER	139	100			Mascot
2143.0876	2143.2131	0.1255	59	73	91	EEVKVEVEDGNILQISGE R					Mascot
2143.0876	2143.2131	0.1255	59	73	91	EEVKVEVEDGNILQISGE R	104	100			Mascot

10	ACD-ScHsp26-like protein [Tamarix hispida]	gi|409905493	18397.2	5.71	5	136	100	5.804	121	100	

Peptide Information

Calc. Mass	Obsrv. Mass	± da	± ppm	Start Seq.	End Seq.	Sequence	Ion Score	C. I. %	Modification	Rank	Result Type

827.4985	827.499	0.0005	1	67	74	ADLPGVKK				Mascot
905.4323	905.5045	0.0722	80	94	100	SREQEEK				Mascot
1037.5626	1037.5718	0.0092	9	58	66	ETPEAHIIK				Mascot
1232.6191	1232.671	0.0519	42	120	130	LPEGAKMEDVK			Oxidation (M)[7]	Mascot
1657.8756	1657.9315	0.0559	34	79	93	VEVEDGKVLQISGER				Mascot
1657.8756	1657.9315	0.0559	34	79	93	VEVEDGKVLQISGER	121	100		Mascot

Gel Idx/Pos	113/E12	Instr./Gel Origin	BA2151/Sample Project 20140814	Process Status	Analysis Succeeded
Plate [#] Name	[1] Sample Project 20140814	Instrument Sample Name		Spectra	11

Rank	Protein Name	Accession No.	Protein MW	Protein PI	Pep. Count	Protein Score	Protein Score C. I. %	Intensity Matched	Total Ion Score	Total Ion C. I. %	Confirmed
1	hypothetical protein OsI_22349 [Oryza sativa Indica Group]	gi|218197884	12868.7	5.74	3	16	0	1.795			

Peptide Information

Calc. Mass	Obsrv. Mass	± da	± ppm	Start Seq.	End Seq.	Sequence	Ion Score	C. I. %	Modification	Rank	Result Type
842.5458	842.5699	0.0241	29	17	23	QLLSIIR					Mascot
889.4625	889.3856	-0.0769	-86	102	110	AGSDLDALK					Mascot
931.4479	931.3613	-0.0866	-93	65	72	EGAEQELR					Mascot

Rank	Protein Name	Accession No.	Protein MW	Protein PI	Pep. Count	Protein Score	Protein Score C. I. %	Intensity Matched	Total Ion Score	Total Ion C. I. %	Confirmed
2	unnamed protein product [Vitis vinifera]	gi|297738127	7065.9	10.25	2	15	0	.283			

Peptide Information

Calc. Mass	Obsrv. Mass	± da	± ppm	Start Seq.	End Seq.	Sequence	Ion Score	C. I. %	Modification	Rank	Result Type
856.4927	856.5657	0.073	85	22	28	FLSFKSK					Mascot
1961.0671	1961.2361	0.169	86	29	46	ALTCMISLAPGTLVTRTR			Carbamidomethyl (C)[4]		Mascot

Rank	Protein Name	Accession No.	Protein MW	Protein PI	Pep. Count	Protein Score	Protein Score C. I. %	Intensity Matched	Total Ion Score	Total Ion C. I. %	Confirmed
3	unnamed protein product [Vitis vinifera]	gi|297735569	7437	9.3	2	14	0	.283			

Peptide Information

Calc. Mass	Obsrv. Mass	± da	± ppm	Start Seq.	End Seq.	Sequence	Ion Score	C. I. %	Modification	Rank	Result Type
856.5614	856.5657	0.0043	5	22	28	LNLLKQK					Mascot
1961.1543	1961.2361	0.0818	42	9	26	NIVKPDSGALHTKLNLLK					Mascot

Rank	Protein Name	Accession No.	Protein MW	Protein PI	Pep. Count	Protein Score	Protein Score C. I. %	Intensity Matched	Total Ion Score	Total Ion C. I. %	Confirmed
4	hypothetical protein LOC_Os11g31169 [Oryza sativa Japonica Group]	gi|77551132	6083.9	5.09	2	14	0	.54			

Peptide Information

Calc. Mass	Obsrv. Mass	± da	± ppm	Start Seq.	End Seq.	Sequence	Ion Score	C. I. %	Modification	Rank	Result Type
818.4002	818.3382	-0.062	-76	32	37	EREQEK					Mascot
832.3907	832.3728	-0.0179	-22	28	33	ENERER					Mascot

Rank	Protein Name	Accession No.	Protein MW	Protein PI	Pep. Count	Protein Score	Protein Score C. I. %	Intensity Matched	Total Ion Score	Total Ion C. I. %	Confirmed
5	hypothetical protein ZEAMMB73_113124 [Zea mays]	gi|413955044	6127.1	5.04	2	14	0	.947			

Peptide Information

Calc. Mass	Obsrv. Mass	± da	± ppm	Start Seq.	End Seq.	Sequence	Ion Score	C. I. %	Modification	Rank	Result Type
889.4025	889.3856	-0.0169	-19	29	34	SYMWFR					Mascot
931.4228	931.3613	-0.0615	-66	2	9	NGDLNQDR					Mascot

6	Os08g0518050 [Oryza sativa Japonica Group]	gi|255678584	5946.7	9.38	2	14	0	.756

Peptide Information

Calc. Mass	Obsrv. Mass	± da	± ppm	Start Seq.	End Seq.	Sequence	Ion Score	C. I. %	Modification	Rank	Result Type
832.3184	832.3728	0.0544	65	2	7	MMMMTR			Oxidation (M)[1,2]		Mascot
931.369	931.3613	-0.0077	-8	1	7	MMMMMTR					Mascot

7	hypothetical protein M569_11088, partial [Genlisea aurea]	gi|527193818	17270	6.04	3	14	0	1.388

Peptide Information

Calc. Mass	Obsrv. Mass	± da	± ppm	Start Seq.	End Seq.	Sequence	Ion Score	C. I. %	Modification	Rank	Result Type
818.4002	818.3382	-0.062	-76	89	95	AEDAEKR					Mascot
832.441	832.3728	-0.0682	-82	16	22	IEEINSK					Mascot
842.5458	842.5699	0.0241	29	1	7	LKLEIAR					Mascot

8	RecName: Full=Histone H2B.9	gi|152032521	16246	10.05	3	14	0	1.688

Protein Group

Os05g0574300 [Oryza sativa Japonica Group]	gi|255676595	16246	10.050 000190 7349
RecName: Full=Histone H2B.9	gi|75122571	16246	10.050 000190 7349
hypothetical protein OsI_21083 [Oryza sativa Indica Group]	gi|125553414	16246	10.050 000190 7349

Peptide Information

Calc. Mass	Obsrv. Mass	± da	± ppm	Start Seq.	End Seq.	Sequence	Ion Score	C. I. %	Modification	Rank	Result Type
832.4523	832.3728	-0.0795	-96	121	127	EIQTSVR					Mascot
842.5094	842.5699	0.0605	72	5	12	AEKKPAAK					Mascot
889.4374	889.3856	-0.0518	-58	42	51	GEKGGAGEGK					Mascot

9	conserved hypothetical protein [Ricinus communis]	gi|223526367	6581.2	8.31	2	13	0	.841

Peptide Information

Calc. Mass	Obsrv. Mass	± da	± ppm	Start Seq.	End Seq.	Sequence	Ion Score	C. I. %	Modification	Rank	Result Type
832.4457	832.3728	-0.0729	-88	1	6	MLQRER					Mascot
889.4639	889.3856	-0.0783	-88	29	36	WRATNGGK					Mascot

10	predicted protein [Arabidopsis lyrata subsp. lyrata]	gi|297324307	6918.5	4.76	2	13	0	.841

Peptide Information

Calc. Mass	Obsrv. Mass	± da	± ppm	Start Seq.	End Seq.	Sequence	Ion Score	C. I. %	Modification	Rank	Result Type
832.4345	832.3728	-0.0617	-74	54	60	ISPNMVR			Oxidation (M)[5]		Mascot
889.401	889.3856	-0.0154	-17	2	11	ADAGGDAGQK					Mascot

Gel Idx/Pos	114/E13	Instr./Gel Origin	BA2151/Sample Project 20140814	Process Status	Analysis Succeeded
Plate [#] Name	[1] Sample Project 20140814	Instrument Sample Name		Spectra	11

Rank	Protein Name	Accession No.	Protein MW	Protein PI	Pep. Count	Protein Score	Protein Score C. I. %	Intensity Matched	Total Ion Score	Total Ion C. I. %	Confirmed
1	Globulin-1 S allele [Triticum urartu]	gi|474411419	57108.4	9.1	8	479	100	53.021	458	100	

Peptide Information

Calc. Mass	Obsrv. Mass	± da	± ppm	Start Seq.	End Seq.	Sequence	Ion Score	C. I. %	Modification	Rank	Result Type
906.468	906.5256	0.0576	64	457	463	EVQEVFR					Mascot
1018.5098	1018.5989	0.0891	87	40	47	SLQQCVQR			Carbamidomethyl (C)[5]		Mascot
1376.7169	1376.7614	0.0445	32	258	268	DTYNLLEQRPK					Mascot
1822.8752	1823.0076	0.1324	73	411	426	GSSNLQVVCFEINAER			Carbamidomethyl (C)[9]		Mascot
1822.8752	1823.0076	0.1324	73	411	426	GSSNLQVVCFEINAER	149	100	Carbamidomethyl (C)[9]		Mascot
1906.0182	1906.1533	0.1351	71	392	410	GSAFVVPPGHPVVEIASS R					Mascot
1906.0182	1906.1533	0.1351	71	392	410	GSAFVVPPGHPVVEIASS R	147	100			Mascot
1960.0035	1959.9902	-0.0133	-7	66	82	SDHGFVKALRPFDEVSR					Mascot
2089.9058	2090.0564	0.1506	72	466	483	DQQDEGFVAGPEQQEQ ER					Mascot
2089.9058	2090.0564	0.1506	72	466	483	DQQDEGFVAGPEQQEQ ER	162	100			Mascot
2289.0378	2289.2073	0.1695	74	464	483	AKDQQDEGFVAGPEQQ EQER					Mascot

Rank	Protein Name	Accession No.	Protein MW	Protein PI	Pep. Count	Protein Score	Protein Score C. I. %	Intensity Matched	Total Ion Score	Total Ion C. I. %	Confirmed
2	Globulin-1 S allele [Aegilops tauschii]	gi|475588764	62687.5	9.44	3	87	99.806	9.393	87	100	

Peptide Information

Calc. Mass	Obsrv. Mass	± da	± ppm	Start Seq.	End Seq.	Sequence	Ion Score	C. I. %	Modification	Rank	Result Type
906.468	906.5256	0.0576	64	523	529	EVQEVFR					Mascot
1376.7169	1376.7614	0.0445	32	342	352	DTYNLLEQRPK					Mascot
1684.8766	1684.9836	0.107	64	508	522	LDNPAQELTFGRPAR					Mascot
1684.8766	1684.9836	0.107	64	508	522	LDNPAQELTFGRPAR	87	100			Mascot

Rank	Protein Name	Accession No.	Protein MW	Protein PI	Pep. Count	Protein Score	Protein Score C. I. %	Intensity Matched	Total Ion Score	Total Ion C. I. %	Confirmed
3	ribosomal protein S4 [Nicotiana tabacum]	gi|57014001	41690.3	10.8	12	52	0	5.24			

Peptide Information

Calc. Mass	Obsrv. Mass	± da	± ppm	Start Seq.	End Seq.	Sequence	Ion Score	C. I. %	Modification	Rank	Result Type
832.4523	832.3812	-0.0711	-85	151	157	TRGEEIK					Mascot

870.4904	870.5731	0.0827	95	259	265	NRNIPTR					Mascot
1126.5906	1126.558	-0.0326	-29	173	180	FLDHPWRR					Mascot
1376.6376	1376.7614	0.1238	90	227	238	VCLGSSFAEHNR			Carbamidomethyl (C)[2]		Mascot
1475.7537	1475.833	0.0793	54	12	23	TCRLLSGNVWNR			Carbamidomethyl (C)[2]		Mascot
1804.8945	1805.0304	0.1359	75	121	135	RVCVNNGMVNITHFK			Carbamidomethyl (C)[3], Oxidation (M)[8]		Mascot
1844.9767	1844.9812	0.0045	2	315	331	AVVSYGPNIGHIPHDIR					Mascot
1912.0763	1912.158	0.0817	43	15	30	LLSGNVWNRELTIIQR					Mascot
1920.0048	1920.1335	0.1287	67	64	79	KLSLFYGDLPITEMHR					Mascot
2073.0359	2073.1521	0.1162	56	47	63	IYSRENLNSYIQSQTTR					Mascot
2090.0713	2090.0564	-0.0149	-7	104	120	LHFCETIPQARQPISHR			Carbamidomethyl (C)[4]		Mascot
2090.0713	2090.0564	-0.0149	-7	104	120	LHFCETIPQARQPISHR			Carbamidomethyl (C)[4]		Mascot
2317.2551	2317.2341	-0.021	-9	84	103	TSYIPFLLNPETRSDVIPV R					Mascot
2317.2551	2317.2341	-0.021	-9	84	103	TSYIPFLLNPETRSDVIPV R					Mascot

4	hypothetical protein OsI_18613 [Oryza sativa Indica Group]	gi|125550983	71021.4	4.75	16	51	0	3.341

Peptide Information

Calc. Mass	Obsrv. Mass	± da	± ppm	Start Seq.	End Seq.	Sequence	Ion Score	C. I. %	Modification	Rank	Result Type
878.4512	878.509	0.0578	66	4	10	MQKTQSR					Mascot
888.4785	888.5235	0.045	51	256	262	DEIDRLK					Mascot
1018.5462	1018.5989	0.0527	52	122	129	MSQKQKPR			Oxidation (M)[1]		Mascot
1132.6144	1132.6508	0.0364	32	549	558	LMAQADLARK			Oxidation (M)[2]		Mascot
1136.6058	1136.6324	0.0266	23	78	87	YGNVTGELRK					Mascot
1513.8333	1513.8973	0.064	42	489	501	EISRLNQELANVK					Mascot
1528.8582	1528.8854	0.0272	18	242	255	SEKEALEAVVLVNK					Mascot
1721.8414	1721.8431	0.0017	1	28	41	WLSDNLEVMETQIK			Oxidation (M)[9]		Mascot
1730.8286	1730.9679	0.1393	80	11	24	KSSSWWWDSHISPK					Mascot
1792.8997	1792.9319	0.0322	18	573	588	EVEMQKLVISESAEGK			Oxidation (M)[4]		Mascot
1821.9065	1821.9874	0.0809	44	590	603	EAIRQLCFSLEHYR			Carbamidomethyl (C)[7]		Mascot
1904.98	1905.1296	0.1496	79	57	72	AGVLITHVQNFQQMYR					Mascot
1947.9594	1948.1257	0.1663	85	263	279	ESMVSAAKQFEVELAHR			Oxidation (M)[3]		Mascot
2073.1074	2073.1521	0.0447	22	454	473	LSEANASLEAKLASVEAE LK					Mascot
2117.9954	2118.0901	0.0947	45	532	548	MKQMDDQLNQLQLEHSK			Oxidation (M)[1,4]		Mascot
2271.085	2271.2261	0.1411	62	381	400	VLEFEQLLGDFENSGME VAK			Oxidation (M)[16]		Mascot

5	uncharacterized protein LOC100192728 [Zea mays]	gi|212723576	29050.2	9.61	10	50	0	10.412

Peptide Information

Calc. Mass	Obsrv. Mass	± da	± ppm	Start Seq.	End Seq.	Sequence	Ion Score	C. I. %	Modification	Rank	Result Type
906.4614	906.5256	0.0642	71	173	179	GSLCWRK			Carbamidomethyl (C)[4]		Mascot
1179.6807	1179.6738	-0.0069	-6	117	126	AVDMVLFIKK			Oxidation (M)[4]		Mascot
1565.7563	1565.736	-0.0203	-13	1	14	MKGSGMHLFGESLR			Oxidation (M)[1]		Mascot
1565.7563	1565.736	-0.0203	-13	1	14	MKGSGMHLFGESLR			Oxidation (M)[1]		Mascot
1641.8306	1641.9535	0.1229	75	127	141	TALPLAVDSFMSFSR					Mascot
1667.9691	1668.0333	0.0642	38	32	47	AEVESALLLVNLGLAR					Mascot
1727.9586	1727.9558	-0.0028	-2	69	85	IRGGLVPTTAAVNGVMR			Oxidation (M)[16]		Mascot
1821.8524	1821.9874	0.135	74	15	30	TAFLCGTHCFLAPNGR			Carbamidomethyl (C)[5,9]		Mascot
1848.9637	1849.1047	0.141	76	209	224	LLLRGGLFDTGEELCR			Carbamidomethyl (C)[15]		Mascot
1949.9474	1950.1167	0.1693	87	15	31	TAFLCGTHCFLAPNGRK			Carbamidomethyl (C)[5,9]		Mascot
2317.2664	2317.2341	-0.0323	-14	181	199	LQPNHRVDELVWQEVTL LK					Mascot
2317.2664	2317.2341	-0.0323	-14	181	199	LQPNHRVDELVWQEVTL LK					Mascot

6 50S ribosomal protein L25 [Aegilops tauschii] gi|475566626 16348.7 7.71 8 50 0 1.393

Peptide Information

Calc. Mass	Obsrv. Mass	± da	± ppm	Start Seq.	End Seq.	Sequence	Ion Score	C. I. %	Modification	Rank	Result Type
830.4618	830.4728	0.011	13	141	148	VLSSKPAE					Mascot
928.5323	928.5038	-0.0285	-31	15	22	LQVRAGER					Mascot
1666.8986	1667.0117	0.1131	68	1	14	MLKQSPYFLSTPVR					Mascot
1790.9912	1790.9825	-0.0087	-5	4	18	QSPYFLSTPVRLQVR					Mascot
1805.8772	1806.0344	0.1572	87	133	148	NETMPVCKVLSSKPAE			Carbamidomethyl (C)[7], Oxidation (M)[4]		Mascot
1822.0182	1821.9874	-0.0308	-17	19	36	AGERSTAVVHSGTVLPIK					Mascot
1918.1195	1918.1418	0.0223	12	116	132	VLMQDIPVHPSLKLLSK					Mascot
1934.1144	1934.167	0.0526	27	116	132	VLMQDIPVHPSLKLLSK			Oxidation (M)[3]		Mascot
1994.0892	1994.0939	0.0047	2	86	102	IRTSLVYLCPAEHIPPK			Carbamidomethyl (C)[9]		Mascot

7 hypothetical protein ArthMp074 [Arabidopsis thaliana] gi|13449359 12112.5 10.93 7 50 0 18.655

Protein Group

RecName: Full=Uncharacterized mitochondrial protein AtMg00850; AltName: Full=ORF107e	gi|45477044	12112.5	10.930 000305 1758

Peptide Information

Calc. Mass	Obsrv. Mass	± da	± ppm	Start Seq.	End Seq.	Sequence	Ion Score	C. I. %	Modification	Rank	Result Type

1475.7676	1475.833	0.0654	44	45	56	LKNWLGEMLEAR			Oxidation (M)[8]		Mascot
1678.8218	1678.9332	0.1114	66	10	24	DYPQNVAGNMLSTLR					Mascot
1822.0182	1821.9874	-0.0308	-17	26	41	EQNQLTSVLTGIHILR					Mascot
1822.9116	1823.0076	0.096	53	10	25	DYPQNVAGNMLSTLRK			Oxidation (M)[10]		Mascot
1822.9116	1823.0076	0.096	53	9	24	KDYPQNVAGNMLSTLR			Oxidation (M)[11]		Mascot
1950.1132	1950.1167	0.0035	2	25	41	KEQNQLTSVLTGIHILR					Mascot
2090.0891	2090.0564	-0.0327	-16	76	95	DGGWPTARGLPSLLQAH GTR					Mascot
2090.0891	2090.0564	-0.0327	-16	76	95	DGGWPTARGLPSLLQAH GTR					Mascot

8 Os05g0168800 [Oryza sativa Japonica Group] gi|113578308 71004.5 4.78 16 49 0 3.341

Peptide Information

Calc. Mass	Obsrv. Mass	± da	± ppm	Start Seq.	End Seq.	Sequence	Ion Score	C. I. %	Modification	Rank	Result Type
878.4512	878.509	0.0578	66	4	10	MQKTQSR					Mascot
888.4785	888.5235	0.045	51	256	262	DEIDRLK					Mascot
1018.5462	1018.5989	0.0527	52	122	129	MSQKQKPR			Oxidation (M)[1]		Mascot
1132.6144	1132.6508	0.0364	32	549	558	LMAQADLARK			Oxidation (M)[2]		Mascot
1136.6058	1136.6324	0.0266	23	78	87	YGNVTGELRK					Mascot
1513.8333	1513.8973	0.064	42	489	501	EISRLNQELANVK					Mascot
1528.8582	1528.8854	0.0272	18	242	255	SEKEALEAVVLVNK					Mascot
1721.8414	1721.8431	0.0017	1	28	41	WLSDNLEVMETQIK			Oxidation (M)[9]		Mascot
1730.8286	1730.9679	0.1393	80	11	24	KSSSWWWDSHISPK					Mascot
1792.8997	1792.9319	0.0322	18	573	588	EVEMQKLVISESAEGK			Oxidation (M)[4]		Mascot
1821.9065	1821.9874	0.0809	44	590	603	EAIRQLCFSLEHYR			Carbamidomethyl (C)[7]		Mascot
1904.98	1905.1296	0.1496	79	57	72	AGVLITHVQNFQQMYR					Mascot
1947.9594	1948.1257	0.1663	85	263	279	ESMVSAAKQFEVELAHR			Oxidation (M)[3]		Mascot
2073.1074	2073.1521	0.0447	22	454	473	LSEANASLEAKLASVEAE LK					Mascot
2117.9954	2118.0901	0.0947	45	532	548	MKQMDDQLNQLQLEHSK			Oxidation (M)[1,4]		Mascot
2271.085	2271.2261	0.1411	62	381	400	VLEFEQLLGDFENSGME VAK			Oxidation (M)[16]		Mascot

9 Regulatory protein NPR1 [Triticum urartu] gi|473786885 171630 6.24 23 49 0 17.816

Peptide Information

Calc. Mass	Obsrv. Mass	± da	± ppm	Start Seq.	End Seq.	Sequence	Ion Score	C. I. %	Modification	Rank	Result Type
886.5972	886.5752	-0.022	-25	1280	1287	ILVSLLTK					Mascot

888.4646	888.5235	0.0589	66	1020	1027	RASVEGNR					Mascot
905.4761	905.5005	0.0244	27	628	635	IASDVCIK			Carbamidomethyl (C)[6]		Mascot
906.4536	906.5256	0.072	79	1155	1161	CLEMVVR			Carbamidomethyl (C)[1]		Mascot
1059.5867	1059.6454	0.0587	55	791	799	ELGNLCILK			Carbamidomethyl (C)[6]		Mascot
1136.6423	1136.6324	-0.0099	-9	736	746	NIVVSHGPSVK					Mascot
1179.6368	1179.6738	0.037	31	313	322	TTLANVLYER					Mascot
1359.6937	1359.7504	0.0567	42	1329	1339	LCIEVLEQAER			Carbamidomethyl (C)[2]		Mascot
1382.6886	1382.7468	0.0582	42	1442	1452	FFPRCSNVLDK			Carbamidomethyl (C)[5]		Mascot
1528.8119	1528.8854	0.0735	48	193	206	QIAFIAQVSEHGTK					Mascot
1704.92	1704.912	-0.008	-5	1209	1223	ALDSSDVELVRMLLK			Oxidation (M)[12]		Mascot
1721.8197	1721.8431	0.0234	14	642	656	GLQCLETLDVMDATR			Carbamidomethyl (C)[4]		Mascot
1749.9421	1749.9315	-0.0106	-6	1186	1201	LSLGLVSPEDKGFPYK					Mascot
1838.892	1839.0151	0.1231	67	1313	1328	DYFGITEEGKPSPKDR					Mascot
1844.9866	1844.9812	-0.0054	-3	193	209	QIAFIAQVSEHGTKTSK					Mascot
1860.924	1860.9644	0.0404	22	1311	1326	HRDYFGITEEGKPSPK					Mascot
1891.0323	1891.1592	0.1269	67	756	771	VIISWDDLEPPPLLQR					Mascot
1902.8981	1903.0487	0.1506	79	1474	1489	FHDVHDSLQKAFSEDK					Mascot
1918.0182	1918.1418	0.1236	64	898	915	EISVKFGGAAAHIEYAVR					Mascot
1958.0106	1957.9847	-0.0259	-13	1004	1019	YMLSVRSPFLHAFFAR			Oxidation (M)[2]		Mascot
2215.1646	2215.1089	-0.0557	-25	1038	1056	VEVGYEALQLVLEYLYSA R					Mascot
2271.1714	2271.2261	0.0547	24	46	67	NVLIDNPTTATVSDPVIES SAK					Mascot
2289.1003	2289.2073	0.107	47	313	330	TTLANVLYERIEMQFECR			Carbamidomethyl (C)[17], Oxidation (M)[13]		Mascot

10 PREDICTED: uncharacterized protein LOC101211164 [Cucumis sativus] gi|449446480 103901.4 8.36 11 48 0 36.968 29 0

Peptide Information

Calc. Mass	Obsrv. Mass	± da	± ppm	Start Seq.	End Seq.	Sequence	Ion Score	C. I. %	Modification	Rank	Result Type
805.46	805.4813	0.0213	26	93	100	ASLKGMAK					Mascot
905.4985	905.5005	0.002	2	753	759	LRMVQSR			Oxidation (M)[3]		Mascot
1059.623	1059.6454	0.0224	21	838	846	QIIANMLIK			Oxidation (M)[6]		Mascot
1132.6321	1132.6508	0.0187	17	150	160	INATSTIGTVR					Mascot
1360.8232	1360.7731	-0.0501	-37	763	774	IIVISTSLLVMR			Oxidation (M)[11]		Mascot
1360.8232	1360.7731	-0.0501	-37	763	774	IIVISTSLLVMR			Oxidation (M)[11]		Mascot
1565.7112	1565.736	0.0248	16	1	14	MALGLDGEEESDRK			Oxidation (M)[1]		Mascot
1565.7112	1565.736	0.0248	16	1	14	MALGLDGEEESDRK	29	0	Oxidation (M)[1]		Mascot
1666.8469	1667.0117	0.1648	99	328	342	GVAQQLELFMAQTSK			Oxidation (M)[10]		Mascot

1685.0068	1684.9836	-0.0232	-14	150	165	INATSTIGTVRALLVR	Mascot
1685.0068	1684.9836	-0.0232	-14	150	165	INATSTIGTVRALLVR	Mascot
1790.884	1790.9825	0.0985	55	47	63	LMESKAAPSTAEDIEAK	Mascot
1822.9043	1823.0076	0.1033	57	345	361	LEGDNGNKAHDAQVNLK	Mascot
1822.9043	1823.0076	0.1033	57	345	361	LEGDNGNKAHDAQVNLK	Mascot
1920.0371	1920.1335	0.0964	50	328	344	GVAQQLELFMAQTSKLR	Mascot

Gel Idx/Pos	115/E14	Instr./Gel Origin	BA2151/Sample Project 20140814	Process Status	Analysis Succeeded
Plate [#] Name	[1] Sample Project 20140814	Instrument Sample Name		Spectra	11

Rank	Protein Name	Accession No.	Protein MW	Protein PI	Pep. Count	Protein Score	Protein Score C. I. %	Intensity Matched	Total Ion Score	Total Ion C. I. %	Confirmed
1	RecName: Full=Alpha-amylase/trypsin inhibitor CM3; AltName: Full=Chloroform/methanol-soluble protein CM3; Flags: Precursor	gi|123957	18893.3	7.44	8	503	100	57.66	450	100	

Peptide Information

Calc. Mass	Obsrv. Mass	± da	± ppm	Start Seq.	End Seq.	Sequence	Ion Score	C. I. %	Modification	Rank	Result Type
1010.52	1010.6013	0.0813	80	37	44	TNLLPHCR			Carbamidomethyl (C)[7]		Mascot
1010.52	1010.6013	0.0813	80	37	44	TNLLPHCR	42	90.819	Carbamidomethyl (C)[7]		Mascot
1110.5038	1110.5927	0.0889	80	133	140	EMQWDFVR					Mascot
1126.4987	1126.579	0.0803	71	133	140	EMQWDFVR			Oxidation (M)[2]		Mascot
1126.4987	1126.579	0.0803	71	133	140	EMQWDFVR	18	0	Oxidation (M)[2]		Mascot
1698.9214	1699.0631	0.1417	83	101	115	YFIALPVPSQPVDPR					Mascot
1698.9214	1699.0631	0.1417	83	101	115	YFIALPVPSQPVDPR	90	100			Mascot
1727.8381	1727.9855	0.1474	85	116	132	SGNVGESGLIDLPGCPR			Carbamidomethyl (C)[15]		Mascot
1727.8381	1727.9855	0.1474	85	116	132	SGNVGESGLIDLPGCPR	97	100	Carbamidomethyl (C)[15]		Mascot
1801.8427	1801.9651	0.1224	68	45	60	DYVLQQTCGTFTPGSK			Carbamidomethyl (C)[8]		Mascot
1876.0222	1876.1882	0.166	88	141	157	LLVAPGQCNLATIHNVR			Carbamidomethyl (C)[8]		Mascot
1876.0222	1876.1882	0.166	88	141	157	LLVAPGQCNLATIHNVR	108	100	Carbamidomethyl (C)[8]		Mascot
1957.8564	1958.0225	0.1661	85	81	95	LYCCQELAEISQQCR			Carbamidomethyl (C)[3,4,14]		Mascot
1957.8564	1958.0225	0.1661	85	81	95	LYCCQELAEISQQCR	96	100	Carbamidomethyl (C)[3,4,14]		Mascot
2255.1416	2255.3093	0.1677	74	61	80	LPEWMTSASIYSPGKPYLAK			Oxidation (M)[5]		Mascot

Rank	Protein Name	Accession No.	Protein MW	Protein PI	Pep. Count	Protein Score	Protein Score C. I. %	Intensity Matched	Total Ion Score	Total Ion C. I. %	Confirmed
2	Alpha-amylase/trypsin inhibitor CM3 [Aegilops tauschii]	gi|475546433	25087.4	8.16	6	322	100	42.399	294	100	

Peptide Information

Calc. Mass	Obsrv. Mass	± da	± ppm	Start Seq.	End Seq.	Sequence	Ion Score	C. I. %	Modification	Rank	Result Type
1125.5146	1125.5833	0.0687	61	186	193	QMQWDFVR			Oxidation (M)[2]		Mascot
1698.9214	1699.0631	0.1417	83	154	168	YFIALPVPSQPVDPR					Mascot
1698.9214	1699.0631	0.1417	83	154	168	YFIALPVPSQPVDPR	90	100			Mascot
1727.8381	1727.9855	0.1474	85	169	185	SGNVGESGLIDLPGCPR			Carbamidomethyl (C)[15]		Mascot
1727.8381	1727.9855	0.1474	85	169	185	SGNVGESGLIDLPGCPR	97	100	Carbamidomethyl (C)[15]		Mascot
1801.8427	1801.9651	0.1224	68	98	113	DYVLQQTCGTFTPGSK			Carbamidomethyl (C)[8]		Mascot

Calc. Mass	Obsrv. Mass	± da	± ppm	Start Seq.	End Seq.	Sequence	Ion Score	C. I. %	Modification	Rank	Result Type
1876.0222	1876.1882	0.166	88	194	210	LLVAPGQCNLATIHNVR			Carbamidomethyl (C)[8]		Mascot
1876.0222	1876.1882	0.166	88	194	210	LLVAPGQCNLATIHNVR	108	100	Carbamidomethyl (C)[8]		Mascot
1967.8772	1968.0417	0.1645	84	134	148	LYCCQELAEIPQQCR			Carbamidomethyl (C)[3,4,14]		Mascot

3	RecName: Full=Alpha-amylase/trypsin inhibitor CMd; AltName: Full=Chloroform/methanol-soluble protein CMd; Flags: Precursor	gi|585291	19140.3	6.07	2	116	100	20.247	108	100

Peptide Information

Calc. Mass	Obsrv. Mass	± da	± ppm	Start Seq.	End Seq.	Sequence	Ion Score	C. I. %	Modification	Rank	Result Type
1876.0222	1876.1882	0.166	88	144	160	LLVAPGQCNLATIHNVR			Carbamidomethyl (C)[8]		Mascot
1876.0222	1876.1882	0.166	88	144	160	LLVAPGQCNLATIHNVR	108	100	Carbamidomethyl (C)[8]		Mascot
1967.8772	1968.0417	0.1645	84	84	98	LYCCQELAEIPQQCR			Carbamidomethyl (C)[3,4,14]		Mascot

4	hypothetical chloroplast RF21 (chloroplast) [Bowiea volubilis]	gi|372480575	268181.4	8.96	31	49	0	10.747		

Peptide Information

Calc. Mass	Obsrv. Mass	± da	± ppm	Start Seq.	End Seq.	Sequence	Ion Score	C. I. %	Modification	Rank	Result Type
1037.599	1037.6582	0.0592	57	1775	1784	VDPALIAPNK					Mascot
1061.5851	1061.62	0.0349	33	2264	2272	HLIHVTGER					Mascot
1094.5775	1094.588	0.0105	10	466	474	QMNNHLLPK					Mascot
1110.5942	1110.5927	-0.0015	-1	1037	1045	FPFSVEKTR					Mascot
1114.5641	1114.5619	-0.0022	-2	486	494	SIRSFFSDR					Mascot
1125.5687	1125.5833	0.0146	13	646	654	FLNEVFNSR					Mascot
1142.6892	1142.5779	-0.1113	-97	1625	1636	GILVIGSIGTGR					Mascot
1146.5685	1146.6616	0.0931	81	166	175	IMPESNRGSR					Mascot
1162.6216	1162.6398	0.0182	16	518	527	KQDVSFVPSR					Mascot
1164.6049	1164.6622	0.0573	49	2153	2162	DPPFISVFSR					Mascot
1174.6791	1174.578	-0.1011	-86	1852	1861	KSIIETNTIR					Mascot
1179.6157	1179.6722	0.0565	48	271	280	RPIEGEGFFK					Mascot
1190.6277	1190.6523	0.0246	21	519	528	QDVSFVPSRR					Mascot
1612.8438	1612.8833	0.0395	24	1374	1386	SLMIPSYMIELRK			Oxidation (M)[3,8]		Mascot
1670.8439	1670.9711	0.1272	76	581	593	NPFLDLFHLFHDR					Mascot
1709.9329	1709.9904	0.0575	34	1372	1385	IKSLMIPSYMIELR			Oxidation (M)[5]		Mascot
1722.848	1723.0195	0.1715	100	1421	1439	GSTSGGNMLLGGGPAY GVK					Mascot
1725.9277	1726.0176	0.0899	52	1372	1385	IKSLMIPSYMIELR			Oxidation (M)[5,10]		Mascot
1742.004	1741.9941	-0.0099	-6	948	962	FLINGGTIFPFLFKK					Mascot

1744.8939	1744.963	0.0691	40	1514	1527	EFLVQFSTLMTEKR			Oxidation (M)[10]		Mascot
1748.9429	1748.9598	0.0169	10	191	206	IANETVAGIEISFKEK					Mascot
1755.8881	1756.0088	0.1207	69	1302	1315	CLQNLLLSEEMIHR			Carbamidomethyl (C)[1]		Mascot
1775.9651	1775.9758	0.0107	6	2174	2189	GLITSQTNPPTSIYKR					Mascot
1831.9855	1832.1522	0.1667	91	884	899	SLPFFFVSIGNTPIHR					Mascot
1839.9097	1839.9568	0.0471	26	495	510	WSELHLGSNPTERSAR					Mascot
1867.08	1867.0121	-0.0679	-36	1447	1462	YLNINLISIIPNPINR					Mascot
1889.014	1889.145	0.131	69	1316	1331	NNESPVPLIWAHLRSR					Mascot
1895.042	1895.0681	0.0261	14	1758	1774	CSTINILVIASTHIPQK			Carbamidomethyl (C)[1]		Mascot
1913.9182	1914.0844	0.1662	87	29	44	FDSVGSFTPIFFHQER					Mascot
1956.1376	1956.0698	-0.0678	-35	1835	1852	DLVALINEALSISITQKK					Mascot
1971.9164	1972.0355	0.1191	60	1817	1834	KMFHTNGFGSITMGSNA R			Oxidation (M)[2]		Mascot
1982.0382	1981.9698	-0.0684	-35	1387	1402	LLDRYPTSELNSFWLK					Mascot

5 hypothetical chloroplast RF21 (chloroplast) [Eriospermum cervicorne] gi|372480593 268721.5 8.77 30 48 0 9.829

Peptide Information

Calc. Mass	Obsrv. Mass	± da	± ppm	Start Seq.	End Seq.	Sequence	Ion Score	C. I. %	Modification	Rank	Result Type
1037.599	1037.6582	0.0592	57	1778	1787	VDPALIAPNK					Mascot
1061.5851	1061.62	0.0349	33	2269	2277	HLIHVTGER					Mascot
1079.5521	1079.6042	0.0521	48	326	334	QDLFVSWGK					Mascot
1094.5775	1094.588	0.0105	10	470	478	QMNNHLLPK					Mascot
1110.5942	1110.5927	-0.0015	-1	1032	1040	FPFSVEKTR					Mascot
1114.5641	1114.5619	-0.0022	-2	490	498	SIRSFFSDR					Mascot
1125.5687	1125.5833	0.0146	13	650	658	FLNEVFNSR					Mascot
1142.6892	1142.5779	-0.1113	-97	1623	1634	GILVIGSIGTGR					Mascot
1150.5344	1150.5441	0.0097	8	168	176	KCIMPESNR			Carbamidomethyl (C)[2], Oxidation (M)[4]		Mascot
1162.6216	1162.6398	0.0182	16	522	531	KQDVSFVPSR					Mascot
1174.6791	1174.578	-0.1011	-86	1855	1864	KSILETNTIR					Mascot
1179.6157	1179.6722	0.0565	48	275	284	RPIEGEGFFK					Mascot
1181.595	1181.5985	0.0035	3	2158	2167	DQPFVSVFSR					Mascot
1190.6277	1190.6523	0.0246	21	523	532	QDVSFVPSRR					Mascot
1612.8438	1612.8833	0.0395	24	1369	1381	SLMIPSYMIELRK			Oxidation (M)[3,8]		Mascot
1709.9329	1709.9904	0.0575	34	1367	1380	IKSLMIPSYMIELR			Oxidation (M)[5]		Mascot
1725.9277	1726.0176	0.0899	52	1367	1380	IKSLMIPSYMIELR			Oxidation (M)[5,10]		Mascot
1742.004	1741.9941	-0.0099	-6	943	957	FLINGGTIFPFLFKK					Mascot

1744.8939	1744.963	0.0691	40	1512	1525	EFLVQFSTLMTEKR			Oxidation (M)[10]		Mascot
1748.9429	1748.9598	0.0169	10	195	210	IANETVAGIEISFKEK					Mascot
1755.8881	1756.0088	0.1207	69	1297	1310	CLQNLLLSEEMIHR			Carbamidomethyl (C)[1]		Mascot
1775.9651	1775.9758	0.0107	6	2179	2194	GLITSQTNPPTSIYKR					Mascot
1831.9855	1832.1522	0.1667	91	879	894	SLPFFFVSIGNTPIHR					Mascot
1839.9097	1839.9568	0.0471	26	2212	2227	QRWLSTNSSLSNGSFR					Mascot
1867.1124	1867.0121	-0.1003	-54	1616	1634	LALSPSRGILVIGSIGTGR					Mascot
1889.014	1889.145	0.131	69	1311	1326	NNESPVPLIWAHLRSR					Mascot
1894.9963	1895.0681	0.0718	38	1805	1819	HFFILSYTRGFHLEK					Mascot
1956.1376	1956.0698	-0.0678	-35	1838	1855	DLVALINEALSISITQKK					Mascot
1967.895	1968.0417	0.1467	75	2020	2036	NPLDMMQNGSFSIVDQR			Oxidation (M)[5]		Mascot
1971.9164	1972.0355	0.1191	60	1820	1837	KMFHTNGFGSITMGSNA R			Oxidation (M)[2]		Mascot
1982.0382	1981.9698	-0.0684	-35	1382	1397	LLDRYPTSELNSFWLK					Mascot

6 retrotransposon protein, putative, Ty3-gypsy subclass [Oryza sativa Japonica Group] gi|77555355 147356.8 8.78 24 48 0 41.624

Peptide Information

Calc. Mass	Obsrv. Mass	± da	± ppm	Start Seq.	End Seq.	Sequence	Ion Score	C. I. %	Modification	Rank	Result Type
842.5458	842.5818	0.036	43	716	722	LALEKLR					Mascot
977.5336	977.602	0.0684	70	1046	1053	ELKDTIMK					Mascot
992.5047	992.5699	0.0652	66	216	223	DFQQLVDK					Mascot
1008.5659	1008.5524	-0.0135	-13	1174	1182	VVCLHGVPK			Carbamidomethyl (C)[3]		Mascot
1044.5071	1044.5792	0.0721	69	684	691	VFMEYLDK					Mascot
1078.4912	1078.5892	0.098	91	708	715	EEHEEHLR					Mascot
1091.5402	1091.6376	0.0974	89	552	561	MAANELAEVK			Oxidation (M)[1]		Mascot
1114.6216	1114.5619	-0.0597	-54	562	570	RQVDDLLQK					Mascot
1136.6609	1136.6466	-0.0143	-13	1174	1183	VVCLHGVPKK			Carbamidomethyl (C)[3]		Mascot
1136.6609	1136.6466	-0.0143	-13	1174	1183	VVCLHGVPKK			Carbamidomethyl (C)[3]		Mascot
1146.5942	1146.6616	0.0674	59	776	785	SFLGLAGYYR					Mascot
1670.9404	1670.9711	0.0307	18	692	705	FVVVFIDDILIYSK					Mascot
1686.8783	1687.0203	0.142	84	241	255	AAQFKAQQGSNQRPR					Mascot
1709.9155	1709.9904	0.0749	44	787	800	FIENFSKIARPMTR					Mascot
1720.8766	1721.033	0.1564	91	168	181	SVTEYLHDFNRLAR					Mascot
1725.9105	1726.0176	0.1071	62	787	800	FIENFSKIARPMTR			Oxidation (M)[12]		Mascot
1727.8456	1727.9855	0.1399	81	596	609	MCVDYRALNEVTIK			Carbamidomethyl (C)[2], Oxidation (M)[1]		Mascot
1727.8456	1727.9855	0.1399	81	596	609	MCVDYRALNEVTIK			Carbamidomethyl (C)[2], Oxidation (M)[1]		Mascot

Calc. Mass	Obsrv. Mass	± da	± ppm	Start Seq.	End Seq.	Sequence	Ion Score	C. I. %	Modification	Rank	Result Type
1741.8942	1741.9941	0.0999	57	910	923	SLKYIFTQPDLNMR			Oxidation (M)[13]		Mascot
1831.9701	1832.1522	0.1821	99	770	785	TVSEIRSFLGLAGYYR					Mascot
1839.9535	1839.9568	0.0033	2	453	467	DLDIILGMDWLSRHR					Mascot
1866.9967	1867.0121	0.0154	8	294	310	NMVARPTATLAQNQPVR					Mascot
1870.868	1871.0168	0.1488	80	128	143	AEGEPITWQEFMAAFK			Oxidation (M)[12]		Mascot
1876.0552	1876.1882	0.133	71	536	551	IDLVPGTNPIHKRPYR					Mascot
1876.0552	1876.1882	0.133	71	536	551	IDLVPGTNPIHKRPYR					Mascot
1891.9913	1892.1689	0.1776	94	985	1001	GFVATLEAQPTLFDQVR					Mascot
1939.9	1940.0775	0.1775	91	1084	1098	REIEEYVALCDVCQR			Carbamidomethyl (C)[10,13]		Mascot
1956.0558	1956.0698	0.014	7	1166	1182	LAELYMARVVCLHGVPK			Carbamidomethyl (C)[11]		Mascot
1972.0508	1972.0355	-0.0153	-8	1166	1182	LAELYMARVVCLHGVPK			Carbamidomethyl (C)[11], Oxidation (M)[6]		Mascot

7 hypothetical protein ARALYDRAFT_909888 [Arabidopsis lyrata subsp. lyrata] gi|297317669 66078.4 6.21 14 47 0 2.928

Peptide Information

Calc. Mass	Obsrv. Mass	± da	± ppm	Start Seq.	End Seq.	Sequence	Ion Score	C. I. %	Modification	Rank	Result Type
1008.4996	1008.5524	0.0528	52	544	551	ELFEVDTR					Mascot
1061.6102	1061.62	0.0098	9	129	138	KVSAPSFGLR					Mascot
1079.5885	1079.6042	0.0157	15	201	209	KGETWALFK					Mascot
1106.5994	1106.62	0.0206	19	446	454	IGDLWAIYR					Mascot
1114.5739	1114.5619	-0.012	-11	75	84	GEESVQIQPK					Mascot
1146.5862	1146.6616	0.0754	66	168	177	LGQSQNTKDR					Mascot
1748.9066	1748.9598	0.0532	30	537	551	VIDGDLKELFEVDTR					Mascot
1749.7974	1749.9546	0.1572	90	180	194	FSHLIQCNEGSNTSR			Carbamidomethyl (C)[7]		Mascot
1775.8851	1775.9758	0.0907	51	151	165	ELQWFEEDLPVSVGK					Mascot
1801.8702	1801.9651	0.0949	53	485	500	VLELEPVSVSNEDDEK					Mascot
1839.9388	1839.9568	0.018	10	85	100	ISEFVGPKFNDFGNLR					Mascot
1870.8467	1871.0168	0.1701	91	210	224	NWDINWSSEPDSHRK					Mascot
1972.0386	1972.0355	-0.0031	-2	75	92	GEESVQIQPKISEFVGPK					Mascot
1985.9862	1986.0487	0.0625	31	260	277	MGTGDAEISRIPPHGLYR			Oxidation (M)[1]		Mascot

8 PREDICTED: pentatricopeptide repeat-containing protein At5g15280-like [Solanum lycopersicum] gi|460376279 142913.1 8.16 23 47 0 28.132

Peptide Information

Calc. Mass	Obsrv. Mass	± da	± ppm	Start Seq.	End Seq.	Sequence	Ion Score	C. I. %	Modification	Rank	Result Type

977.5275	977.602	0.0745	76	1219	1226	QNGRFLSR					Mascot
1044.5222	1044.5792	0.057	55	491	498	DNDIRFHK					Mascot
1076.6133	1076.5725	-0.0408	-38	896	905	GKVSTALCLK			Carbamidomethyl (C)[8]		Mascot
1093.6001	1093.6249	0.0248	23	877	886	NLGISIASYR					Mascot
1110.6517	1110.5927	-0.059	-53	71	80	GEILNNPIIK					Mascot
1125.5786	1125.5833	0.0047	4	967	976	DLSSATQYLK					Mascot
1126.6831	1126.579	-0.1041	-92	866	875	VEELLGVVIR					Mascot
1126.6831	1126.579	-0.1041	-92	866	875	VEELLGVVIR					Mascot
1136.6133	1136.6466	0.0333	29	1	9	MLQALLEFR			Oxidation (M)[1]		Mascot
1136.6133	1136.6466	0.0333	29	1	9	MLQALLEFR			Oxidation (M)[1]		Mascot
1146.5474	1146.6616	0.1142	100	1067	1075	FCQHGRVDK			Carbamidomethyl (C)[2]		Mascot
1158.6664	1158.5685	-0.0979	-84	796	805	AVALKDICLR			Carbamidomethyl (C)[8]		Mascot
1158.6664	1158.5685	-0.0979	-84	796	805	AVALKDICLR			Carbamidomethyl (C)[8]		Mascot
1162.6184	1162.6398	0.0214	18	887	895	NIVRLMCTR			Carbamidomethyl (C)[7]		Mascot
1179.6368	1179.6722	0.0354	30	790	800	SSNFDKAVALK					Mascot
1190.631	1190.6523	0.0213	18	1181	1190	ASELLRSMQR					Mascot
1550.7917	1550.9136	0.1219	79	1073	1085	VDKSVDLMDIMLR			Oxidation (M)[8]		Mascot
1692.9432	1693.1073	0.1641	97	17	31	QVGSLLFNLSSFPRK					Mascot
1710.9935	1710.974	-0.0195	-11	148	162	HLIKASEIIATMLVR			Oxidation (M)[12]		Mascot
1727.8826	1727.9855	0.1029	60	128	141	KIESLWGIYMWASK			Oxidation (M)[10]		Mascot
1727.8826	1727.9855	0.1029	60	128	141	KIESLWGIYMWASK			Oxidation (M)[10]		Mascot
1736.8822	1736.9894	0.1072	62	757	771	IFREAAVVCDNMLAK			Carbamidomethyl (C)[9]		Mascot
1749.8412	1749.9546	0.1134	65	446	460	HFDEVNTMVSKMVGR					Mascot
1751.9724	1751.9327	-0.0397	-23	152	167	ASEIIATMLVRSGLFK			Oxidation (M)[8]		Mascot
1823.8553	1823.9805	0.1252	69	967	981	DLSSATQYLKYMMEK			Oxidation (M)[12]		Mascot
1839.8503	1839.9568	0.1065	58	967	981	DLSSATQYLKYMMEK			Oxidation (M)[12,13]		Mascot
1866.8759	1867.0121	0.1362	73	635	650	LDSETHTALMMGLCKK			Carbamidomethyl (C)[14], Oxidation (M)[10,11]		Mascot
1901.0062	1900.9973	-0.0089	-5	801	816	DICLRDEPPAVLPLHR			Carbamidomethyl (C)[3]		Mascot

9	Disease resistance protein RPM1 [Triticum urartu]	gi|474086298	129568	6.87	20	46	0	18.416

Peptide Information

Calc. Mass	Obsrv. Mass	± da	± ppm	Start Seq.	End Seq.	Sequence	Ion Score	C. I. %	Modification	Rank	Result Type
1091.6824	1091.6376	-0.0448	-41	641	650	YLGLVKTAVK					Mascot
1098.579	1098.5846	0.0056	5	87	96	VEGNEPTKPK					Mascot
1142.6317	1142.5779	-0.0538	-47	895	904	KDAASLVPWR					Mascot
1146.6882	1146.6616	-0.0266	-23	193	204	IVSIVGFGGLGK					Mascot

Calc. Mass	Obsrv. Mass	± da	± ppm	Start Seq.	End Seq.	Sequence	Ion Score	C. I. %	Modification	Rank	Result Type
1150.6102	1150.5441	-0.0661	-57	651	660	IPEEIGHDLK					Mascot
1158.5175	1158.5685	0.051	44	519	528	WIDPEDNGGR					Mascot
1158.5175	1158.5685	0.051	44	519	528	WIDPEDNGGR					Mascot
1198.6619	1198.5872	-0.0747	-62	278	286	IYHFLKYSK					Mascot
1670.8094	1670.9711	0.1617	97	732	746	VLNIFFGEMEESAGK					Mascot
1710.8619	1710.974	0.1121	66	703	716	LTCLEELQLYSVDK			Carbamidomethyl (C)[3]		Mascot
1722.9901	1723.0195	0.0294	17	802	816	VPSWINQLTVPLLSR					Mascot
1775.8997	1775.9758	0.0761	43	251	265	ATETMAEWQLINQLK					Mascot
1785.0116	1785.1614	0.1498	84	236	250	VLRDILLELDTELSR					Mascot
1785.0116	1785.1614	0.1498	84	236	250	VLRDILLELDTELSR					Mascot
1802.0463	1801.9651	-0.0812	-45	835	849	LPSLLFLLLWSKDEK					Mascot
1895.1212	1895.0681	-0.0531	-28	162	178	KLSDLVGIDKPINELIK					Mascot
1898.1144	1898.1403	0.0259	14	403	421	KCGGVPLAITTIASLLVGK			Carbamidomethyl (C)[2]		Mascot
1900.9084	1900.9973	0.0889	47	1104	1119	GFWLGPAENKMYLCAK			Carbamidomethyl (C)[14], Oxidation (M)[11]		Mascot
1915.9617	1915.9886	0.0269	14	669	686	GGMISELPPSVGELMNL R			Oxidation (M)[3]		Mascot
1939.9946	1940.0775	0.0829	43	730	746	LRVLNIFFGEMEESAGK					Mascot
1955.9895	1956.0698	0.0803	41	730	746	LRVLNIFFGEMEESAGK			Oxidation (M)[11]		Mascot
1958.093	1958.0225	-0.0705	-36	937	954	QASGSRPNAVYLNLRIAK					Mascot
1958.093	1958.0225	-0.0705	-36	937	954	QASGSRPNAVYLNLRIAK					Mascot
1986.059	1986.0487	-0.0103	-5	341	357	LAFMDSHPQSRLIITTR					Mascot

10 PREDICTED: protein DCL, chloroplastic-like [Setaria italica] gi|514727731 20883.7 6.38 8 46 0 3.573

Peptide Information

Calc. Mass	Obsrv. Mass	± da	± ppm	Start Seq.	End Seq.	Sequence	Ion Score	C. I. %	Modification	Rank	Result Type
1077.5687	1077.5859	0.0172	16	178	186	SSEPFRVQK					Mascot
1126.6355	1126.579	-0.0565	-50	69	78	DVEPVVELVK					Mascot
1126.6355	1126.579	-0.0565	-50	69	78	DVEPVVELVK					Mascot
1152.6082	1152.5813	-0.0269	-23	153	161	CLQAYIKEK			Carbamidomethyl (C)[1]		Mascot
1154.6793	1154.5759	-0.1034	-90	169	177	FLQKHLVNR					Mascot
1164.6306	1164.6622	0.0316	27	132	140	QSRCLFVVR			Carbamidomethyl (C)[4]		Mascot
1704.9392	1705.0746	0.1354	79	17	36	GGGGVPPAASAAAVPGI SLR					Mascot
1888.995	1889.145	0.15	79	99	114	VVVEKLLSHHPCVDEK			Carbamidomethyl (C)[12]		Mascot
1971.9529	1972.0355	0.0826	42	115	131	IGCGLDGIMVDRHPEFR			Carbamidomethyl (C)[3]		Mascot

Gel Idx/Pos	116/E15	Instr./Gel Origin	BA2151/Sample Project 20140814	Process Status	Analysis Succeeded
Plate [#] Name	[1] Sample Project 20140814	Instrument Sample Name		Spectra	11

Rank	Protein Name	Accession No.	Protein MW	Protein PI	Pep. Count	Protein Score	Protein Score C. I. %	Intensity Matched	Total Ion Score	Total Ion C. I. %	Confirmed
1	Nucleoside diphosphate kinase 1 [Triticum urartu]	gi|474369382	16578.6	6.3	10	516	100	50.406	440	100	

Peptide Information

Calc. Mass	Obsrv. Mass	± da	± ppm	Start Seq.	End Seq.	Sequence	Ion Score	C. I. %	Modification	Rank	Result Type
943.5571	943.6226	0.0655	69	17	25	GLIGEVISR					Mascot
943.5571	943.6226	0.0655	69	17	25	GLIGEVISR	64	99.941			Mascot
949.4738	949.5352	0.0614	65	104	112	GDFAVDIGR					Mascot
949.4738	949.5352	0.0614	65	104	112	GDFAVDIGR	47	97.544			Mascot
1234.5487	1234.5994	0.0507	41	141	150	SSQHNWIYEA					Mascot
1370.6659	1370.7637	0.0978	71	113	125	NVIHGSDSVESAR					Mascot
1370.6659	1370.7637	0.0978	71	113	125	NVIHGSDSVESAR	106	100			Mascot
1498.7609	1498.7765	0.0156	10	113	126	NVIHGSDSVESARK					Mascot
1609.8909	1610.0118	0.1209	75	88	103	IIGATNPLASEPGTIR					Mascot
1609.8909	1610.0118	0.1209	75	88	103	IIGATNPLASEPGTIR	102	100			Mascot
1716.8744	1717.0065	0.1321	77	127	140	EIALWFPEGIAEWR					Mascot
1716.8744	1717.0065	0.1321	77	127	140	EIALWFPEGIAEWR	91	100			Mascot
1732.9052	1733.0016	0.0964	56	2	16	AEQTFIMIKPDGVQR					Mascot
1737.9858	1738.1172	0.1314	76	87	103	KIIGATNPLASEPGTIR					Mascot
1737.9858	1738.1172	0.1314	76	87	103	KIIGATNPLASEPGTIR	30	0			Mascot
1748.9	1748.9937	0.0937	54	2	16	AEQTFIMIKPDGVQR			Oxidation (M)[7]		Mascot
1844.9694	1845.1071	0.1377	75	126	140	KEIALWFPEGIAEWR					Mascot

Rank	Protein Name	Accession No.	Protein MW	Protein PI	Pep. Count	Protein Score	Protein Score C. I. %	Intensity Matched	Total Ion Score	Total Ion C. I. %	Confirmed
2	Nucleoside diphosphate kinase 1 [Aegilops tauschii]	gi|475549973	17034.8	6.85	9	504	100	48.761	440	100	

Peptide Information

Calc. Mass	Obsrv. Mass	± da	± ppm	Start Seq.	End Seq.	Sequence	Ion Score	C. I. %	Modification	Rank	Result Type
943.5571	943.6226	0.0655	69	18	26	GLIGEVISR					Mascot
943.5571	943.6226	0.0655	69	18	26	GLIGEVISR	64	99.941			Mascot
949.4738	949.5352	0.0614	65	108	116	GDFAVDIGR					Mascot
949.4738	949.5352	0.0614	65	108	116	GDFAVDIGR	47	97.544			Mascot
1234.5487	1234.5994	0.0507	41	145	154	SSQHNWIYEA					Mascot

1370.6659	1370.7637	0.0978	71	117	129	NVIHGSDSVESAR						Mascot
1370.6659	1370.7637	0.0978	71	117	129	NVIHGSDSVESAR	106	100				Mascot
1498.7609	1498.7765	0.0156	10	117	130	NVIHGSDSVESARK						Mascot
1609.8909	1610.0118	0.1209	75	92	107	IIGATNPLASEPGTIR						Mascot
1609.8909	1610.0118	0.1209	75	92	107	IIGATNPLASEPGTIR	102	100				Mascot
1716.8744	1717.0065	0.1321	77	131	144	EIALWFPEGIAEWR						Mascot
1716.8744	1717.0065	0.1321	77	131	144	EIALWFPEGIAEWR	91	100				Mascot
1737.9858	1738.1172	0.1314	76	91	107	KIIGATNPLASEPGTIR						Mascot
1737.9858	1738.1172	0.1314	76	91	107	KIIGATNPLASEPGTIR	30	0				Mascot
1844.9694	1845.1071	0.1377	75	130	144	KEIALWFPEGIAEWR						Mascot

3	TPA: hypothetical protein ZEAMMB73_585316 [Zea mays]	gi|414867766	7035.6	5.52	3	201	100	30.753	179	100		

Protein Group

TPA: hypothetical protein ZEAMMB73_585316 [Zea mays]	gi|414867765	7035.6	5.5199 999809 2651
TPA: hypothetical protein ZEAMMB73_585316 [Zea mays]	gi|414867767	7035.6	5.5199 999809 2651

Peptide Information

Calc. Mass	Obsrv. Mass	± da	± ppm	Start Seq.	End Seq.	Sequence	Ion Score	C. I. %	Modification	Rank	Result Type
949.4738	949.5352	0.0614	65	31	39	GDFAVDIGR					Mascot
949.4738	949.5352	0.0614	65	31	39	GDFAVDIGR	47	97.544			Mascot
1609.8909	1610.0118	0.1209	75	15	30	IIGATNPLASEPGTIR					Mascot
1609.8909	1610.0118	0.1209	75	15	30	IIGATNPLASEPGTIR	102	100			Mascot
1737.9858	1738.1172	0.1314	76	14	30	KIIGATNPLASEPGTIR					Mascot
1737.9858	1738.1172	0.1314	76	14	30	KIIGATNPLASEPGTIR	30	0			Mascot

4	PREDICTED: nucleoside diphosphate kinase 1-like [Setaria italica]	gi|514817826	16671.7	6.9	4	199	100	30.833	179	100

Peptide Information

Calc. Mass	Obsrv. Mass	± da	± ppm	Start Seq.	End Seq.	Sequence	Ion Score	C. I. %	Modification	Rank	Result Type
949.4738	949.5352	0.0614	65	103	111	GDFAVDIGR					Mascot
949.4738	949.5352	0.0614	65	103	111	GDFAVDIGR	47	97.544			Mascot
1609.8909	1610.0118	0.1209	75	87	102	IIGATNPLASEPGTIR					Mascot
1609.8909	1610.0118	0.1209	75	87	102	IIGATNPLASEPGTIR	102	100			Mascot
1670.8683	1670.9674	0.0991	59	2	15	EHTFIMIKPDGVQR					Mascot

1737.9858	1738.1172	0.1314	76	86	102	KIIGATNPLASEPGTIR					Mascot
1737.9858	1738.1172	0.1314	76	86	102	KIIGATNPLASEPGTIR	30	0			Mascot

5	TPA: nucleoside diphosphate kinase [Zea mays]	gi|414867768	16530.6	6.3	3	193	100	30.753	179	100	

Peptide Information

Calc. Mass	Obsrv. Mass	± da	± ppm	Start Seq.	End Seq.	Sequence	Ion Score	C. I. %	Modification	Rank	Result Type
949.4738	949.5352	0.0614	65	103	111	GDFAVDIGR					Mascot
949.4738	949.5352	0.0614	65	103	111	GDFAVDIGR	47	97.544			Mascot
1609.8909	1610.0118	0.1209	75	87	102	IIGATNPLASEPGTIR					Mascot
1609.8909	1610.0118	0.1209	75	87	102	IIGATNPLASEPGTIR	102	100			Mascot
1737.9858	1738.1172	0.1314	76	86	102	KIIGATNPLASEPGTIR					Mascot
1737.9858	1738.1172	0.1314	76	86	102	KIIGATNPLASEPGTIR	30	0			Mascot

6	TPA: hypothetical protein ZEAMMB73_585316 [Zea mays]	gi|414867769	17688.1	5.65	3	192	100	30.753	179	100	

Peptide Information

Calc. Mass	Obsrv. Mass	± da	± ppm	Start Seq.	End Seq.	Sequence	Ion Score	C. I. %	Modification	Rank	Result Type
949.4738	949.5352	0.0614	65	113	121	GDFAVDIGR					Mascot
949.4738	949.5352	0.0614	65	113	121	GDFAVDIGR	47	97.544			Mascot
1609.8909	1610.0118	0.1209	75	97	112	IIGATNPLASEPGTIR					Mascot
1609.8909	1610.0118	0.1209	75	97	112	IIGATNPLASEPGTIR	102	100			Mascot
1737.9858	1738.1172	0.1314	76	96	112	KIIGATNPLASEPGTIR					Mascot
1737.9858	1738.1172	0.1314	76	96	112	KIIGATNPLASEPGTIR	30	0			Mascot

7	RecName: Full=Nucleoside diphosphate kinase 1; AltName: Full=Nucleoside diphosphate kinase I; Short=NDK I; Short=NDP kinase I; Short=NDPK I	gi|223635304	16834.6	6.3	7	179	100	14.111	138	100	

Peptide Information

Calc. Mass	Obsrv. Mass	± da	± ppm	Start Seq.	End Seq.	Sequence	Ion Score	C. I. %	Modification	Rank	Result Type
943.5571	943.6226	0.0655	69	16	24	GLIGDIISR					Mascot
943.5571	943.6226	0.0655	69	16	24	GLIGDIISR	47	97.561			Mascot
955.5182	955.5574	0.0392	41	29	36	GFFLRGMK					Mascot
1647.8523	1647.944	0.0917	56	2	15	EQSFIMIKPDGVQR					Mascot
1708.9493	1708.913	-0.0363	-21	87	102	IIGATRPWEAAPGTIR					Mascot
1716.8744	1717.0065	0.1321	77	126	139	EIALWFPEGLAEWR					Mascot

1716.8744	1717.0065	0.1321	77	126	139	EIALWFPEGLAEWR	91	100			Mascot
1794.8877	1795.0537	0.166	92	1	15	MEQSFIMIKPDGVQR			Oxidation (M)[1]		Mascot
1810.8827	1811.0403	0.1576	87	1	15	MEQSFIMIKPDGVQR			Oxidation (M)[1,7]		Mascot
1844.9694	1845.1071	0.1377	75	125	139	KEIALWFPEGLAEWR					Mascot

8	RecName: Full=Nucleoside diphosphate kinase 1; AltName: Full=Nucleoside diphosphate kinase I; Short=NDK I; Short=NDP kinase I; Short=NDPK I	gi|6225750	16340.6	6.3	4	179	100	33.411	132	100	

Peptide Information

Calc. Mass	Obsrv. Mass	± da	± ppm	Start Seq.	End Seq.	Sequence	Ion Score	C. I. %	Modification	Rank	Result Type
943.5571	943.6226	0.0655	69	16	24	GLVGEIISR					Mascot
943.5571	943.6226	0.0655	69	16	24	GLVGEIISR	27	0			Mascot
1609.8909	1610.0118	0.1209	75	87	102	IIGATNPLASEPGTIR					Mascot
1609.8909	1610.0118	0.1209	75	87	102	IIGATNPLASEPGTIR	102	100			Mascot
1647.9218	1647.944	0.0222	13	34	47	ALKFINVDRPFAEK					Mascot
1737.9858	1738.1172	0.1314	76	86	102	KIIGATNPLASEPGTIR					Mascot
1737.9858	1738.1172	0.1314	76	86	102	KIIGATNPLASEPGTIR	30	0			Mascot

9	hypothetical protein OsI_34670 [Oryza sativa Indica Group]	gi|125532976	16768.8	6.85	7	178	100	12.913	138	100	

Protein Group

Os10g0563700 [Oryza sativa Japonica Group]	gi|113639936	16768.8	6.8499 999046 3257

Peptide Information

Calc. Mass	Obsrv. Mass	± da	± ppm	Start Seq.	End Seq.	Sequence	Ion Score	C. I. %	Modification	Rank	Result Type
949.4738	949.5352	0.0614	65	105	113	GDFAVDIGR					Mascot
949.4738	949.5352	0.0614	65	105	113	GDFAVDIGR	47	97.544			Mascot
973.5175	973.4818	-0.0357	-37	31	38	GFYLKAMK			Oxidation (M)[7]		Mascot
1272.6008	1272.593	-0.0078	-6	142	151	SNQHPWIYEV					Mascot
1579.8802	1579.9607	0.0805	51	89	104	LVGATNPLAAEPGTIR					Mascot
1716.8744	1717.0065	0.1321	77	128	141	EIALWFPEGIAEWR					Mascot
1716.8744	1717.0065	0.1321	77	128	141	EIALWFPEGIAEWR	91	100			Mascot
1844.9694	1845.1071	0.1377	75	127	141	KEIALWFPEGIAEWR					Mascot
1861.9841	1862.0841	0.1	54	2	17	ALEQTFIMIKPDGVQR			Oxidation (M)[8]		Mascot

10	RecName: Full=Nucleoside diphosphate kinase 1; AltName: Full=Nucleoside diphosphate kinase I; Short=NDK I; Short=NDP kinase I; Short=NDPK I	gi|400404	16279.5	6.42	3	172	100	33.299	132	100	

Peptide Information

Calc. Mass	Obsrv. Mass	± da	± ppm	Start Seq.	End Seq.	Sequence	Ion Score	C. I. %	Modification	Rank	Result Type
943.5571	943.6226	0.0655	69	16	24	GLVGEIISR					Mascot
943.5571	943.6226	0.0655	69	16	24	GLVGEIISR	27	0			Mascot
1609.8909	1610.0118	0.1209	75	87	102	LIGATNPLASEPGTIR					Mascot
1609.8909	1610.0118	0.1209	75	87	102	LIGATNPLASEPGTIR	102	100			Mascot
1737.9858	1738.1172	0.1314	76	86	102	KLIGATNPLASEPGTIR					Mascot
1737.9858	1738.1172	0.1314	76	86	102	KLIGATNPLASEPGTIR	30	0			Mascot

Gel Idx/Pos	117/E16	Instr./Gel Origin	BA2151/Sample Project 20140814	Process Status	Analysis Succeeded
Plate [#] Name	[1] Sample Project 20140814	Instrument Sample Name		Spectra	11

Rank	Protein Name	Accession No.	Protein MW	Protein PI	Pep. Count	Protein Score	Protein Score C. I. %	Intensity Matched	Total Ion Score	Total Ion C. I. %	Confirmed
1	Chain D, 0.19 Alpha-Amylase Inhibitor From Wheat	gi|3318684	13898.6	6.66	5	376	100	38.784	345	100	

Protein Group

Protein Name	Accession No.	Protein MW	Protein PI
Chain A, 0.19 Alpha-Amylase Inhibitor From Wheat	gi|3318681	13898.6	6.6599 998474 1211
Chain B, 0.19 Alpha-Amylase Inhibitor From Wheat	gi|3318682	13898.6	6.6599 998474 1211
Chain C, 0.19 Alpha-Amylase Inhibitor From Wheat	gi|3318683	13898.6	6.6599 998474 1211
RecName: Full=Alpha-amylase inhibitor 0.19; AltName: Full=0.19 alpha-AI; Short=0.19 AI	gi|123963	13898.6	6.6599 998474 1211

Peptide Information

Calc. Mass	Obsrv. Mass	± da	± ppm	Start Seq.	End Seq.	Sequence	Ion Score	C. I. %	Modification	Rank	Result Type
1162.6249	1162.7083	0.0834	72	90	100	LTAASITAVCR			Carbamidomethyl (C)[10]		Mascot
1162.6249	1162.7083	0.0834	72	90	100	LTAASITAVCR	73	99.992	Carbamidomethyl (C)[10]		Mascot
1570.8007	1570.9241	0.1234	79	26	39	LQCNGSQVPEAVLR			Carbamidomethyl (C)[3]		Mascot
1612.7463	1612.8733	0.127	79	67	82	EHGAQEGQAGTGAFPR					Mascot
1612.7463	1612.8733	0.127	79	67	82	EHGAQEGQAGTGAFPR	138	100			Mascot
1663.8361	1663.9354	0.0993	60	101	116	LPIVVDASGDGAYVCK			Carbamidomethyl (C)[15]		Mascot
1862.7731	1862.9274	0.1543	83	40	53	DCCQQLAHISEWCR			Carbamidomethyl (C)[2,3,13]		Mascot
1862.7731	1862.9274	0.1543	83	40	53	DCCQQLAHISEWCR	135	100	Carbamidomethyl (C)[2,3,13]		Mascot

Rank	Protein Name	Accession No.	Protein MW	Protein PI	Pep. Count	Protein Score	Protein Score C. I. %	Intensity Matched	Total Ion Score	Total Ion C. I. %	Confirmed
2	dimeric alpha-amylase inhibitor, partial [Aegilops kotschyi]	gi|386877048	14198.8	6.66	5	375	100	38.784	345	100	

Protein Group

Protein Name	Accession No.	Protein MW	Protein PI
dimeric alpha-amylase inhibitor, partial [Aegilops geniculata]	gi|386877062	14542.9	7.0500 001907 3486
dimeric alpha-amylase inhibitor, partial [Aegilops geniculata]	gi|452055912	14198.8	6.6599 998474 1211
dimeric alpha-amylase inhibitor, partial [Aegilops tauschii]	gi|386877046	14670	6.0799 999237 0605
dimeric alpha-amylase inhibitor, partial [Aegilops	gi|386877050	14826.1	6.8600

tauschii]			001335<br>144
dimeric alpha-amylase inhibitor, partial [Aegilops tauschii]	gi|386877044	14805	6.0999<br>999046<br>3257

Peptide Information

Calc. Mass	Obsrv. Mass	± da	± ppm	Start Seq.	End Seq.	Sequence	Ion Score	C. I. %	Modification	Rank	Result Type
1162.6249	1162.7083	0.0834	72	93	103	LTAASITAVCR			Carbamidomethyl (C)[10]		Mascot
1162.6249	1162.7083	0.0834	72	93	103	LTAASITAVCR	73	99.992	Carbamidomethyl (C)[10]		Mascot
1570.8007	1570.9241	0.1234	79	29	42	LQCNGSQVPEAVLR			Carbamidomethyl (C)[3]		Mascot
1612.7463	1612.8733	0.127	79	70	85	EHGAQEGQAGTGAFPR					Mascot
1612.7463	1612.8733	0.127	79	70	85	EHGAQEGQAGTGAFPR	138	100			Mascot
1663.8361	1663.9354	0.0993	60	104	119	LPIVVDASGDGAYVCK			Carbamidomethyl (C)[15]		Mascot
1862.7731	1862.9274	0.1543	83	43	56	DCCQQLAHISEWCR			Carbamidomethyl (C)[2,3,13]		Mascot
1862.7731	1862.9274	0.1543	83	43	56	DCCQQLAHISEWCR	135	100	Carbamidomethyl (C)[2,3,13]		Mascot

3	dimeric alpha-amylase inhibitor, partial [Aegilops longissima]	gi|386877060	14954.2	7.64	5	374	100	38.784	345	100

Peptide Information

Calc. Mass	Obsrv. Mass	± da	± ppm	Start Seq.	End Seq.	Sequence	Ion Score	C. I. %	Modification	Rank	Result Type
1162.6249	1162.7083	0.0834	72	99	109	LTAASITAVCR			Carbamidomethyl (C)[10]		Mascot
1162.6249	1162.7083	0.0834	72	99	109	LTAASITAVCR	73	99.992	Carbamidomethyl (C)[10]		Mascot
1570.8007	1570.9241	0.1234	79	35	48	LQCNGSQVPEAVLR			Carbamidomethyl (C)[3]		Mascot
1612.7463	1612.8733	0.127	79	76	91	EHGAQEGQAGTGAFPR					Mascot
1612.7463	1612.8733	0.127	79	76	91	EHGAQEGQAGTGAFPR	138	100			Mascot
1663.8361	1663.9354	0.0993	60	110	125	LPIVVDASGDGAYVCK			Carbamidomethyl (C)[15]		Mascot
1862.7731	1862.9274	0.1543	83	49	62	DCCQQLAHISEWCR			Carbamidomethyl (C)[2,3,13]		Mascot
1862.7731	1862.9274	0.1543	83	49	62	DCCQQLAHISEWCR	135	100	Carbamidomethyl (C)[2,3,13]		Mascot

4	dimeric alpha-amylase inhibitor [Triticum aestivum]	gi|386877038	15702.5	5.58	5	373	100	38.784	345	100

Peptide Information

Calc. Mass	Obsrv. Mass	± da	± ppm	Start Seq.	End Seq.	Sequence	Ion Score	C. I. %	Modification	Rank	Result Type
1162.6249	1162.7083	0.0834	72	107	117	LTAASITAVCR			Carbamidomethyl (C)[10]		Mascot
1162.6249	1162.7083	0.0834	72	107	117	LTAASITAVCR	73	99.992	Carbamidomethyl (C)[10]		Mascot
1570.8007	1570.9241	0.1234	79	43	56	LQCNGSQVPEAVLR			Carbamidomethyl (C)[3]		Mascot
1612.7463	1612.8733	0.127	79	84	99	EHGAQEGQAGTGAFPR					Mascot

1612.7463	1612.8733	0.127	79	84	99	EHGAQEGQAGTGAFPR	138	100			Mascot
1663.8361	1663.9354	0.0993	60	118	133	LPIVVDASGDGAYVCK			Carbamidomethyl (C)[15]		Mascot
1862.7731	1862.9274	0.1543	83	57	70	DCCQQLAHISEWCR			Carbamidomethyl (C)[2,3,13]		Mascot
1862.7731	1862.9274	0.1543	83	57	70	DCCQQLAHISEWCR	135	100	Carbamidomethyl (C)[2,3,13]		Mascot

5	Alpha-amylase inhibitor 0.19 [Aegilops tauschii]	gi|475613321	17198.2	6.06	5	372	100	38.784	345	100	

Peptide Information

Calc. Mass	Obsrv. Mass	± da	± ppm	Start Seq.	End Seq.	Sequence	Ion Score	C. I. %	Modification	Rank	Result Type
1162.6249	1162.7083	0.0834	72	120	130	LTAASITAVCR			Carbamidomethyl (C)[10]		Mascot
1162.6249	1162.7083	0.0834	72	120	130	LTAASITAVCR	73	99.992	Carbamidomethyl (C)[10]		Mascot
1570.8007	1570.9241	0.1234	79	56	69	LQCNGSQVPEAVLR			Carbamidomethyl (C)[3]		Mascot
1612.7463	1612.8733	0.127	79	97	112	EHGAQEGQAGTGAFPR					Mascot
1612.7463	1612.8733	0.127	79	97	112	EHGAQEGQAGTGAFPR	138	100			Mascot
1663.8361	1663.9354	0.0993	60	131	146	LPIVVDASGDGAYVCK			Carbamidomethyl (C)[15]		Mascot
1862.7731	1862.9274	0.1543	83	70	83	DCCQQLAHISEWCR			Carbamidomethyl (C)[2,3,13]		Mascot
1862.7731	1862.9274	0.1543	83	70	83	DCCQQLAHISEWCR	135	100	Carbamidomethyl (C)[2,3,13]		Mascot

6	dimeric alpha-amylase inhibitor, partial [Aegilops geniculata]	gi|452055914	14748	6.09	4	366	100	37.992	345	100	

Protein Group

dimeric alpha-amylase inhibitor, partial [Triticum aestivum]	gi|386877068	14415.8	6.8800 001144 4092

Peptide Information

Calc. Mass	Obsrv. Mass	± da	± ppm	Start Seq.	End Seq.	Sequence	Ion Score	C. I. %	Modification	Rank	Result Type
1162.6249	1162.7083	0.0834	72	97	107	LTAASITAVCR			Carbamidomethyl (C)[10]		Mascot
1162.6249	1162.7083	0.0834	72	97	107	LTAASITAVCR	73	99.992	Carbamidomethyl (C)[10]		Mascot
1570.8007	1570.9241	0.1234	79	33	46	LQCNGSQVPEAVLR			Carbamidomethyl (C)[3]		Mascot
1612.7463	1612.8733	0.127	79	74	89	EHGAQEGQAGTGAFPR					Mascot
1612.7463	1612.8733	0.127	79	74	89	EHGAQEGQAGTGAFPR	138	100			Mascot
1862.7731	1862.9274	0.1543	83	47	60	DCCQQLAHISEWCR			Carbamidomethyl (C)[2,3,13]		Mascot
1862.7731	1862.9274	0.1543	83	47	60	DCCQQLAHISEWCR	135	100	Carbamidomethyl (C)[2,3,13]		Mascot

7	dimeric alpha-amylase inhibitor, partial [Aegilops longissima]	gi|386877056	14792	5.28	4	232	100	26.236	211	100	

Protein Group

dimeric alpha-amylase inhibitor, partial [Aegilops	gi|386877058	14718.9	4.9899

	longissima]	997711 1816									

Peptide Information

Calc. Mass	Obsrv. Mass	± da	± ppm	Start Seq.	End Seq.	Sequence	Ion Score	C. I. %	Modification	Rank	Result Type
1162.6249	1162.7083	0.0834	72	98	108	LTAASITAVCR			Carbamidomethyl (C)[10]		Mascot
1162.6249	1162.7083	0.0834	72	98	108	LTAASITAVCR	73	99.992	Carbamidomethyl (C)[10]		Mascot
1570.8007	1570.9241	0.1234	79	34	47	LQCNGSQVPEAVLR			Carbamidomethyl (C)[3]		Mascot
1612.7463	1612.8733	0.127	79	75	90	EHGAQEGQAGTGAFPR					Mascot
1612.7463	1612.8733	0.127	79	75	90	EHGAQEGQAGTGAFPR	138	100			Mascot
1663.8361	1663.9354	0.0993	60	109	124	LPIVVDASGDGAYVCK			Carbamidomethyl (C)[15]		Mascot

8	dimeric alpha-amylase inhibitor, partial [Aegilops peregrina]	gi|386877054	14145.7	5.26	3	225	100	22.795	211	100	

Peptide Information

Calc. Mass	Obsrv. Mass	± da	± ppm	Start Seq.	End Seq.	Sequence	Ion Score	C. I. %	Modification	Rank	Result Type
1162.6249	1162.7083	0.0834	72	92	102	LTAASITAVCR			Carbamidomethyl (C)[10]		Mascot
1162.6249	1162.7083	0.0834	72	92	102	LTAASITAVCR	73	99.992	Carbamidomethyl (C)[10]		Mascot
1612.7463	1612.8733	0.127	79	69	84	EHGAQEGQAGTGAFPR					Mascot
1612.7463	1612.8733	0.127	79	69	84	EHGAQEGQAGTGAFPR	138	100			Mascot
1663.8361	1663.9354	0.0993	60	103	118	LPIVVDASGDGAYVCK			Carbamidomethyl (C)[15]		Mascot

9	dimeric alpha-amylase inhibitor, partial [Triticum aestivum]	gi|386877040	15014.1	5.28	3	224	100	25.443	211	100	

Peptide Information

Calc. Mass	Obsrv. Mass	± da	± ppm	Start Seq.	End Seq.	Sequence	Ion Score	C. I. %	Modification	Rank	Result Type
1162.6249	1162.7083	0.0834	72	101	111	LTAASITAVCR			Carbamidomethyl (C)[10]		Mascot
1162.6249	1162.7083	0.0834	72	101	111	LTAASITAVCR	73	99.992	Carbamidomethyl (C)[10]		Mascot
1570.8007	1570.9241	0.1234	79	37	50	LQCNGSQVPEAVLR			Carbamidomethyl (C)[3]		Mascot
1612.7463	1612.8733	0.127	79	78	93	EHGAQEGQAGTGAFPR					Mascot
1612.7463	1612.8733	0.127	79	78	93	EHGAQEGQAGTGAFPR	138	100			Mascot

10	Alpha-amylase inhibitor 0.19 [Aegilops tauschii]	gi|475613322	17655.3	6.78	3	220	100	18.586	208	100	

Peptide Information

Calc. Mass	Obsrv. Mass	± da	± ppm	Start Seq.	End Seq.	Sequence	Ion Score	C. I. %	Modification	Rank	Result Type
1162.6249	1162.7083	0.0834	72	120	130	LTAASITAVCR			Carbamidomethyl (C)[10]		Mascot

1162.6249	1162.7083	0.0834	72	120	130	LTAASITAVCR	73	99.993	Carbamidomethyl (C)[10]	Mascot
1570.8007	1570.9241	0.1234	79	56	69	LQCNGSQVPEAVLR			Carbamidomethyl (C)[3]	Mascot
1862.7731	1862.9274	0.1543	83	70	83	DCCQQLAHISEWCR			Carbamidomethyl (C)[2,3,13]	Mascot
1862.7731	1862.9274	0.1543	83	70	83	DCCQQLAHISEWCR	135	100	Carbamidomethyl (C)[2,3,13]	Mascot

Gel Idx/Pos	118/E17	Instr./Gel Origin	BA2151/Sample Project 20140814	Process Status	Analysis Succeeded
Plate [#] Name	[1] Sample Project 20140814	Instrument Sample Name		Spectra	11

Rank	Protein Name	Accession No.	Protein MW	Protein PI	Pep. Count	Protein Score	Protein Score C. I. %	Intensity Matched	Total Ion Score	Total Ion C. I. %	Confirmed
1	hypothetical protein F775_31562 [Aegilops tauschii]	gi|475458859	16753.8	6.19	6	324	100	27.362	290	100	

Protein Group

RecName: Full=16.9 kDa class I heat shock protein 1; AltName: Full=HSP 16.9; AltName: Full=Heat shock protein 16.9A; AltName: Full=Heat shock protein 17; AltName: Full=Low molecular weight heat shock protein	gi|123545	16867.8	5.8299 999237 0605
hypothetical protein TRIUR3_03549 [Triticum urartu]	gi|474071007	16824.8	6.1900 000572 2046

Peptide Information

Calc. Mass	Obsrv. Mass	± da	± ppm	Start Seq.	End Seq.	Sequence	Ion Score	C. I. %	Modification	Rank	Result Type
975.5258	975.5861	0.0603	62	110	117	FRLPEDAK					Mascot
975.5258	975.5861	0.0603	62	110	117	FRLPEDAK	30	0			Mascot
1027.6146	1027.5669	-0.0477	-46	137	145	AEVKKPEVK					Mascot
1057.5314	1057.5671	0.0357	34	50	58	ETPEAHVFK					Mascot
1600.8177	1600.9133	0.0956	60	71	85	VEVEDGNVLVVSGER					Mascot
1600.8177	1600.9133	0.0956	60	71	85	VEVEDGNVLVVSGER	97	100			Mascot
1905.9666	1906.083	0.1164	61	26	45	SIVPAISGGSSETAAFAN AR					Mascot
1905.9666	1906.083	0.1164	61	26	45	SIVPAISGGSSETAAFAN AR	164	100			Mascot
2086.0664	2086.1868	0.1204	58	67	85	EEVKVEVEDGNVLVVSG ER					Mascot
2086.0664	2086.1868	0.1204	58	67	85	EEVKVEVEDGNVLVVSG ER					Mascot

Rank	Protein Name	Accession No.	Protein MW	Protein PI	Pep. Count	Protein Score	Protein Score C. I. %	Intensity Matched	Total Ion Score	Total Ion C. I. %	Confirmed
2	hypothetical protein F775_32310 [Aegilops tauschii]	gi|475614915	16783.8	5.83	5	289	100	25.701	261	100	

Peptide Information

Calc. Mass	Obsrv. Mass	± da	± ppm	Start Seq.	End Seq.	Sequence	Ion Score	C. I. %	Modification	Rank	Result Type
1027.6146	1027.5669	-0.0477	-46	137	145	AEVKKPEVK					Mascot
1057.5314	1057.5671	0.0357	34	50	58	ETPEAHVFK					Mascot
1600.8177	1600.9133	0.0956	60	71	85	VEVEDGNVLVVSGER					Mascot
1600.8177	1600.9133	0.0956	60	71	85	VEVEDGNVLVVSGER	97	100			Mascot
1905.9666	1906.083	0.1164	61	26	45	SIVPAISGGSSETAAFAN AR					Mascot
1905.9666	1906.083	0.1164	61	26	45	SIVPAISGGSSETAAFAN	164	100			Mascot

						AR				
2086.0664	2086.1868	0.1204	58	67	85	EEVKVEVEDGNVLVVSG ER				Mascot
2086.0664	2086.1868	0.1204	58	67	85	EEVKVEVEDGNVLVVSG ER				Mascot

3	hypothetical protein F775_43851 [Aegilops tauschii]	gi|475614913	13371.1	5.62	4	147	100	5.564	127	100		

Peptide Information

Calc. Mass	Obsrv. Mass	± da	± ppm	Start Seq.	End Seq.	Sequence	Ion Score	C. I. %	Modification	Rank	Result Type
975.5258	975.5861	0.0603	62	81	88	FRLPEDAK					Mascot
975.5258	975.5861	0.0603	62	81	88	FRLPEDAK	30	0			Mascot
1027.6146	1027.5669	-0.0477	-46	108	116	AEVKKPEVK					Mascot
1600.8177	1600.9133	0.0956	60	42	56	VEVEDGNVLVVSGER					Mascot
1600.8177	1600.9133	0.0956	60	42	56	VEVEDGNVLVVSGER	97	100			Mascot
2086.0664	2086.1868	0.1204	58	38	56	EEVKVEVEDGNVLVVSG ER					Mascot
2086.0664	2086.1868	0.1204	58	38	56	EEVKVEVEDGNVLVVSG ER					Mascot

4	hypothetical protein TRIUR3_03466 [Triticum urartu]	gi|474305544	16122.4	6.18	4	146	100	5.622	127	100

Peptide Information

Calc. Mass	Obsrv. Mass	± da	± ppm	Start Seq.	End Seq.	Sequence	Ion Score	C. I. %	Modification	Rank	Result Type
900.4951	900.465	-0.0301	-33	96	101	WHRFVR					Mascot
975.5258	975.5861	0.0603	62	103	110	FRLPEDAK					Mascot
975.5258	975.5861	0.0603	62	103	110	FRLPEDAK	30	0			Mascot
1600.8177	1600.9133	0.0956	60	71	85	VEVEDGNVLVVSGER					Mascot
1600.8177	1600.9133	0.0956	60	71	85	VEVEDGNVLVVSGER	97	100			Mascot
2086.0664	2086.1868	0.1204	58	67	85	EEVKVEVEDGNVLVVSG ER					Mascot
2086.0664	2086.1868	0.1204	58	67	85	EEVKVEVEDGNVLVVSG ER					Mascot

5	small heat shock protein [Triticum aestivum]	gi|388429141	16869.8	6.77	4	146	100	5.771	127	100

Protein Group

hypothetical protein F775_32308 [Aegilops tauschii]	gi|475614912	16849.8	6.3400 001525 8789
hypothetical protein F775_32309 [Aegilops tauschii]	gi|475614914	16868.8	6.7699 999809 2651

Peptide Information

Calc. Mass	Obsrv. Mass	± da	± ppm	Start Seq.	End Seq.	Sequence	Ion Score	C. I. %	Modification	Rank	Result Type

Calc. Mass	Obsrv. Mass	± da	± ppm	Start Seq.	End Seq.	Sequence	Ion Score	C. I. %	Modification	Rank	Result Type
975.5258	975.5861	0.0603	62	110	117	FRLPEDAK					Mascot
975.5258	975.5861	0.0603	62	110	117	FRLPEDAK	30	0			Mascot
1057.5314	1057.5671	0.0357	34	50	58	ETPEAHVFK					Mascot
1600.8177	1600.9133	0.0956	60	71	85	VEVEDGNVLVVSGER					Mascot
1600.8177	1600.9133	0.0956	60	71	85	VEVEDGNVLVVSGER	97	100			Mascot
2086.0664	2086.1868	0.1204	58	67	85	EEVKVEVEDGNVLVVSG ER					Mascot
2086.0664	2086.1868	0.1204	58	67	85	EEVKVEVEDGNVLVVSG ER					Mascot

6	hypothetical protein TRIUR3_03467 [Triticum urartu]	gi|474305545	18321.7	9.15	4	145	100	5.771	127	100	

Peptide Information

Calc. Mass	Obsrv. Mass	± da	± ppm	Start Seq.	End Seq.	Sequence	Ion Score	C. I. %	Modification	Rank	Result Type
975.5258	975.5861	0.0603	62	122	129	FRLPEDAK					Mascot
975.5258	975.5861	0.0603	62	122	129	FRLPEDAK	30	0			Mascot
1057.5314	1057.5671	0.0357	34	50	58	ETPEAHVFK					Mascot
1600.8177	1600.9133	0.0956	60	71	85	VEVEDGNVLVVSGER					Mascot
1600.8177	1600.9133	0.0956	60	71	85	VEVEDGNVLVVSGER	97	100			Mascot
2086.0664	2086.1868	0.1204	58	67	85	EEVKVEVEDGNVLVVSG ER					Mascot
2086.0664	2086.1868	0.1204	58	67	85	EEVKVEVEDGNVLVVSG ER					Mascot

7	Chain D, Crystal Structure And Assembly Of An Eukaryotic Small Heat Shock Protein	gi|17942919	16842.8	6.19	5	124	100	4.484	97	100	

Protein Group

Chain A, Crystal Structure And Assembly Of An Eukaryotic Small Heat Shock Protein	gi|17942916	16842.8	6.1900 000572 2046
Chain B, Crystal Structure And Assembly Of An Eukaryotic Small Heat Shock Protein	gi|17942917	16842.8	6.1900 000572 2046
Chain C, Crystal Structure And Assembly Of An Eukaryotic Small Heat Shock Protein	gi|17942918	16842.8	6.1900 000572 2046
RecName: Full=16.9 kDa class I heat shock protein 2; AltName: Full=Heat shock protein 16.9B	gi|75282260	16856.8	6.1900 000572 2046

Peptide Information

Calc. Mass	Obsrv. Mass	± da	± ppm	Start Seq.	End Seq.	Sequence	Ion Score	C. I. %	Modification	Rank	Result Type
1027.6146	1027.5669	-0.0477	-46	137	145	AEVKKPEVK					Mascot
1057.5314	1057.5671	0.0357	34	50	58	ETPEAHVFK					Mascot

1600.8177	1600.9133	0.0956	60	71	85	VEVEDGNVLVVSGER					Mascot
1600.8177	1600.9133	0.0956	60	71	85	VEVEDGNVLVVSGER	97	100			Mascot
1875.9559	1876.0944	0.1385	74	26	45	SIVPAISGGGSETAAFAN AR					Mascot
2086.0664	2086.1868	0.1204	58	67	85	EEVKVEVEDGNVLVVSG ER					Mascot
2086.0664	2086.1868	0.1204	58	67	85	EEVKVEVEDGNVLVVSG ER					Mascot

8	hypothetical protein F775_08697 [Aegilops tauschii]	gi|475323471	17117.9	6.19	4	116	100	4.302	97	100	

Peptide Information

Calc. Mass	Obsrv. Mass	± da	± ppm	Start Seq.	End Seq.	Sequence	Ion Score	C. I. %	Modification	Rank	Result Type
1027.6146	1027.5669	-0.0477	-46	139	147	AEVKKPEVK					Mascot
1057.5314	1057.5671	0.0357	34	52	60	ETPEAHVFK					Mascot
1600.8177	1600.9133	0.0956	60	73	87	VEVEDGNVLVVSGER					Mascot
1600.8177	1600.9133	0.0956	60	73	87	VEVEDGNVLVVSGER	97	100			Mascot
2086.0664	2086.1868	0.1204	58	69	87	EEVKVEVEDGNVLVVSG ER					Mascot
2086.0664	2086.1868	0.1204	58	69	87	EEVKVEVEDGNVLVVSG ER					Mascot

9	Chain K, Negative Stain Em Reconstruction Of M.Tuberculosis Acr1(Hsp 16.3) Fitted With Wheat Shsp Dimer	gi|75766427	11406	6.97	3	114	100	4.109	97	100	

Protein Group

Chain A, Negative Stain Em Reconstruction Of M.Tuberculosis Acr1(Hsp 16.3) Fitted With Wheat Shsp Dimer	gi|75766417	11406	6.9699 997901 9165
Chain B, Negative Stain Em Reconstruction Of M.Tuberculosis Acr1(Hsp 16.3) Fitted With Wheat Shsp Dimer	gi|75766418	11406	6.9699 997901 9165
Chain C, Negative Stain Em Reconstruction Of M.Tuberculosis Acr1(Hsp 16.3) Fitted With Wheat Shsp Dimer	gi|75766419	11406	6.9699 997901 9165
Chain D, Negative Stain Em Reconstruction Of M.Tuberculosis Acr1(Hsp 16.3) Fitted With Wheat Shsp Dimer	gi|75766420	11406	6.9699 997901 9165
Chain E, Negative Stain Em Reconstruction Of M.Tuberculosis Acr1(Hsp 16.3) Fitted With Wheat Shsp Dimer	gi|75766421	11406	6.9699 997901 9165
Chain F, Negative Stain Em Reconstruction Of M.Tuberculosis Acr1(Hsp 16.3) Fitted With Wheat Shsp Dimer	gi|75766422	11406	6.9699 997901 9165
Chain G, Negative Stain Em Reconstruction Of M.Tuberculosis Acr1(Hsp 16.3) Fitted With Wheat Shsp Dimer	gi|75766423	11406	6.9699 997901 9165
Chain H, Negative Stain Em Reconstruction Of M.Tuberculosis Acr1(Hsp 16.3) Fitted With Wheat Shsp	gi|75766424	11406	6.9699 997901

Dimer			9165
Chain I, Negative Stain Em Reconstruction Of M.Tuberculosis Acr1(Hsp 16.3) Fitted With Wheat Shsp Dimer	gi|75766425	11406	6.9699997901 9165
Chain J, Negative Stain Em Reconstruction Of M.Tuberculosis Acr1(Hsp 16.3) Fitted With Wheat Shsp Dimer	gi|75766426	11406	6.9699997901 9165
Chain L, Negative Stain Em Reconstruction Of M.Tuberculosis Acr1(Hsp 16.3) Fitted With Wheat Shsp Dimer	gi|75766428	11406	6.9699997901 9165

Peptide Information

Calc. Mass	Obsrv. Mass	± da	± ppm	Start Seq.	End Seq.	Sequence	Ion Score	C. I. %	Modification	Rank	Result Type
1057.5314	1057.5671	0.0357	34	8	16	ETPEAHVFK					Mascot
1600.8177	1600.9133	0.0956	60	29	43	VEVEDGNVLVVSGER					Mascot
1600.8177	1600.9133	0.0956	60	29	43	VEVEDGNVLVVSGER	97	100			Mascot
2086.0664	2086.1868	0.1204	58	25	43	EEVKVEVEDGNVLVVSGER					Mascot
2086.0664	2086.1868	0.1204	58	25	43	EEVKVEVEDGNVLVVSGER					Mascot

10	PREDICTED: uncharacterized protein LOC101766737 [Setaria italica]	gi|514743928	50028.6	6.68	15	72	94.135	4.01

Peptide Information

Calc. Mass	Obsrv. Mass	± da	± ppm	Start Seq.	End Seq.	Sequence	Ion Score	C. I. %	Modification	Rank	Result Type
804.4938	804.492	-0.0018	-2	95	101	SSTIKIR					Mascot
828.4825	828.4824	-0.0001	0	329	335	KVIDPEK					Mascot
832.416	832.3781	-0.0379	-46	166	173	VSTTPDGR					Mascot
837.4352	837.4843	0.0491	59	415	421	FLLDDSK					Mascot
849.4716	849.504	0.0324	38	317	323	EVDLFVK					Mascot
874.4893	874.5138	0.0245	28	102	108	WASLKNR					Mascot
887.5057	887.4702	-0.0355	-40	336	342	QEVLRSR					Mascot
948.5513	948.5442	-0.0071	-7	65	72	TILEFGLR					Mascot
1140.63	1140.6416	0.0116	10	380	389	VFYDATLALK					Mascot
1699.8982	1699.9316	0.0334	20	345	358	SEMLHPLRVMIVSR			Oxidation (M)[3,10]		Mascot
1994.0304	1994.104	0.0736	37	174	190	VVDLFFITDGMELLHTK			Oxidation (M)[11]		Mascot
2440.3115	2440.2034	-0.1081	-44	353	375	VMIVSRGPDTELLVANPVELSGK			Oxidation (M)[2]		Mascot
2705.2295	2705.3423	0.1128	42	109	132	LMSMCPSSYSIPFYPDISQPGPSK			Carbamidomethyl (C)[5], Oxidation (M)[2]		Mascot
2807.4285	2807.5007	0.0722	26	166	190	VSTTPDGRVVDLFFITDGMELLHTK			Oxidation (M)[19]		Mascot
2811.3384	2811.4961	0.1577	56	76	99	GDVSTDGHWCFVVFWVMPRSSTIK			Carbamidomethyl (C)[10]		Mascot

Gel Idx/Pos	119/E18	Instr./Gel Origin	BA2151/Sample Project 20140814	Process Status	Analysis Succeeded
Plate [#] Name	[1] Sample Project 20140814	Instrument Sample Name		Spectra	11

Rank	Protein Name	Accession No.	Protein MW	Protein PI	Pep. Count	Protein Score	Protein Score C. I. %	Intensity Matched	Total Ion Score	Total Ion C. I. %	Confirmed
1	Chain D, 0.19 Alpha-Amylase Inhibitor From Wheat	gi|3318684	13898.6	6.66	6	191	100	7.628	148	100	

Protein Group

Protein Name	Accession No.	Protein MW	Protein PI
Chain A, 0.19 Alpha-Amylase Inhibitor From Wheat	gi|3318681	13898.6	6.6599 998474 1211
Chain B, 0.19 Alpha-Amylase Inhibitor From Wheat	gi|3318682	13898.6	6.6599 998474 1211
Chain C, 0.19 Alpha-Amylase Inhibitor From Wheat	gi|3318683	13898.6	6.6599 998474 1211
RecName: Full=Alpha-amylase inhibitor 0.19; AltName: Full=0.19 alpha-AI; Short=0.19 AI	gi|123963	13898.6	6.6599 998474 1211

Peptide Information

Calc. Mass	Obsrv. Mass	± da	± ppm	Start Seq.	End Seq.	Sequence	Ion Score	C. I. %	Modification	Rank	Result Type
1162.6249	1162.7152	0.0903	78	90	100	LTAASITAVCR			Carbamidomethyl (C)[10]		Mascot
1162.6249	1162.7152	0.0903	78	90	100	LTAASITAVCR			Carbamidomethyl (C)[10]		Mascot
1570.8007	1570.8739	0.0732	47	26	39	LQCNGSQVPEAVLR			Carbamidomethyl (C)[3]		Mascot
1570.8007	1570.8739	0.0732	47	26	39	LQCNGSQVPEAVLR	44	94.642	Carbamidomethyl (C)[3]		Mascot
1612.7463	1612.8792	0.1329	82	67	82	EHGAQEGQAGTGAFPR					Mascot
1612.7463	1612.8792	0.1329	82	67	82	EHGAQEGQAGTGAFPR	104	100			Mascot
1663.8361	1663.931	0.0949	57	101	116	LPIVVDASGDGAYVCK			Carbamidomethyl (C)[15]		Mascot
1862.7731	1862.9211	0.148	79	40	53	DCCQQLAHISEWCR			Carbamidomethyl (C)[2,3,13]		Mascot
1862.7731	1862.9211	0.148	79	40	53	DCCQQLAHISEWCR			Carbamidomethyl (C)[2,3,13]		Mascot
2807.4431	2807.5427	0.0996	35	90	116	LTAASITAVCRLPIVVDAS GDGAYVCK			Carbamidomethyl (C)[10,26]		Mascot

Rank	Protein Name	Accession No.	Protein MW	Protein PI	Pep. Count	Protein Score	Protein Score C. I. %	Intensity Matched	Total Ion Score	Total Ion C. I. %	Confirmed
2	dimeric alpha-amylase inhibitor, partial [Aegilops kotschyi]	gi|386877048	14198.8	6.66	6	190	100	7.628	148	100	

Protein Group

Protein Name	Accession No.	Protein MW	Protein PI
dimeric alpha-amylase inhibitor, partial [Aegilops geniculata]	gi|386877062	14542.9	7.0500 001907 3486
dimeric alpha-amylase inhibitor, partial [Aegilops geniculata]	gi|452055912	14198.8	6.6599 998474 1211

Peptide Information

Calc. Mass	Obsrv. Mass	± da	± ppm	Start Seq.	End Seq.	Sequence	Ion Score	C. I. %	Modification	Rank	Result Type
1162.6249	1162.7152	0.0903	78	93	103	LTAASITAVCR			Carbamidomethyl (C)[10]		Mascot
1162.6249	1162.7152	0.0903	78	93	103	LTAASITAVCR			Carbamidomethyl (C)[10]		Mascot
1570.8007	1570.8739	0.0732	47	29	42	LQCNGSQVPEAVLR			Carbamidomethyl (C)[3]		Mascot
1570.8007	1570.8739	0.0732	47	29	42	LQCNGSQVPEAVLR	44	94.642	Carbamidomethyl (C)[3]		Mascot
1612.7463	1612.8792	0.1329	82	70	85	EHGAQEGQAGTGAFPR					Mascot
1612.7463	1612.8792	0.1329	82	70	85	EHGAQEGQAGTGAFPR	104	100			Mascot
1663.8361	1663.931	0.0949	57	104	119	LPIVVDASGDGAYVCK			Carbamidomethyl (C)[15]		Mascot
1862.7731	1862.9211	0.148	79	43	56	DCCQQLAHISEWCR			Carbamidomethyl (C)[2,3,13]		Mascot
1862.7731	1862.9211	0.148	79	43	56	DCCQQLAHISEWCR			Carbamidomethyl (C)[2,3,13]		Mascot
2807.4431	2807.5427	0.0996	35	93	119	LTAASITAVCRLPIVVDASGDGAYVCK			Carbamidomethyl (C)[10,26]		Mascot

3 dimeric alpha-amylase inhibitor, partial [Aegilops tauschii] gi|386877050 14826.1 6.86 6 189 100 7.628 148 100

Protein Group

dimeric alpha-amylase inhibitor, partial [Aegilops longissima] gi|386877060 14954.2 7.6399998664856

dimeric alpha-amylase inhibitor, partial [Aegilops tauschii] gi|386877044 14805 6.0999999046325 7

dimeric alpha-amylase inhibitor, partial [Aegilops tauschii] gi|386877046 14670 6.0799999237060 5

Peptide Information

Calc. Mass	Obsrv. Mass	± da	± ppm	Start Seq.	End Seq.	Sequence	Ion Score	C. I. %	Modification	Rank	Result Type
1162.6249	1162.7152	0.0903	78	98	108	LTAASITAVCR			Carbamidomethyl (C)[10]		Mascot
1162.6249	1162.7152	0.0903	78	98	108	LTAASITAVCR			Carbamidomethyl (C)[10]		Mascot
1570.8007	1570.8739	0.0732	47	34	47	LQCNGSQVPEAVLR			Carbamidomethyl (C)[3]		Mascot
1570.8007	1570.8739	0.0732	47	34	47	LQCNGSQVPEAVLR	44	94.642	Carbamidomethyl (C)[3]		Mascot
1612.7463	1612.8792	0.1329	82	75	90	EHGAQEGQAGTGAFPR					Mascot
1612.7463	1612.8792	0.1329	82	75	90	EHGAQEGQAGTGAFPR	104	100			Mascot
1663.8361	1663.931	0.0949	57	109	124	LPIVVDASGDGAYVCK			Carbamidomethyl (C)[15]		Mascot
1862.7731	1862.9211	0.148	79	48	61	DCCQQLAHISEWCR			Carbamidomethyl (C)[2,3,13]		Mascot
1862.7731	1862.9211	0.148	79	48	61	DCCQQLAHISEWCR			Carbamidomethyl (C)[2,3,13]		Mascot
2807.4431	2807.5427	0.0996	35	98	124	LTAASITAVCRLPIVVDASGDGAYVCK			Carbamidomethyl (C)[10,26]		Mascot

4 dimeric alpha-amylase inhibitor [Triticum aestivum] gi|386877038 15702.5 5.58 6 188 100 7.628 148 100

Peptide Information

Calc. Mass	Obsrv. Mass	± da	± ppm	Start Seq.	End Seq.	Sequence	Ion Score	C. I. %	Modification	Rank	Result Type
1162.6249	1162.7152	0.0903	78	107	117	LTAASITAVCR			Carbamidomethyl (C)[10]		Mascot
1162.6249	1162.7152	0.0903	78	107	117	LTAASITAVCR			Carbamidomethyl (C)[10]		Mascot
1570.8007	1570.8739	0.0732	47	43	56	LQCNGSQVPEAVLR			Carbamidomethyl (C)[3]		Mascot
1570.8007	1570.8739	0.0732	47	43	56	LQCNGSQVPEAVLR	44	94.642	Carbamidomethyl (C)[3]		Mascot
1612.7463	1612.8792	0.1329	82	84	99	EHGAQEGQAGTGAFPR					Mascot
1612.7463	1612.8792	0.1329	82	84	99	EHGAQEGQAGTGAFPR	104	100			Mascot
1663.8361	1663.931	0.0949	57	118	133	LPIVVDASGDGAYVCK			Carbamidomethyl (C)[15]		Mascot
1862.7731	1862.9211	0.148	79	57	70	DCCQQLAHISEWCR			Carbamidomethyl (C)[2,3,13]		Mascot
1862.7731	1862.9211	0.148	79	57	70	DCCQQLAHISEWCR			Carbamidomethyl (C)[2,3,13]		Mascot
2807.4431	2807.5427	0.0996	35	107	133	LTAASITAVCRLPIVVDAS GDGAYVCK			Carbamidomethyl (C)[10,26]		Mascot

5	Alpha-amylase inhibitor 0.19 [Aegilops tauschii]	gi|475613321	17198.2	6.06	6	186	100	7.628	148	100	

Peptide Information

Calc. Mass	Obsrv. Mass	± da	± ppm	Start Seq.	End Seq.	Sequence	Ion Score	C. I. %	Modification	Rank	Result Type
1162.6249	1162.7152	0.0903	78	120	130	LTAASITAVCR			Carbamidomethyl (C)[10]		Mascot
1162.6249	1162.7152	0.0903	78	120	130	LTAASITAVCR			Carbamidomethyl (C)[10]		Mascot
1570.8007	1570.8739	0.0732	47	56	69	LQCNGSQVPEAVLR			Carbamidomethyl (C)[3]		Mascot
1570.8007	1570.8739	0.0732	47	56	69	LQCNGSQVPEAVLR	44	94.642	Carbamidomethyl (C)[3]		Mascot
1612.7463	1612.8792	0.1329	82	97	112	EHGAQEGQAGTGAFPR					Mascot
1612.7463	1612.8792	0.1329	82	97	112	EHGAQEGQAGTGAFPR	104	100			Mascot
1663.8361	1663.931	0.0949	57	131	146	LPIVVDASGDGAYVCK			Carbamidomethyl (C)[15]		Mascot
1862.7731	1862.9211	0.148	79	70	83	DCCQQLAHISEWCR			Carbamidomethyl (C)[2,3,13]		Mascot
1862.7731	1862.9211	0.148	79	70	83	DCCQQLAHISEWCR			Carbamidomethyl (C)[2,3,13]		Mascot
2807.4431	2807.5427	0.0996	35	120	146	LTAASITAVCRLPIVVDAS GDGAYVCK			Carbamidomethyl (C)[10,26]		Mascot

6	dimeric alpha-amylase inhibitor, partial [Aegilops longissima]	gi|386877056	14792	5.28	5	180	100	6.641	148	100	

Protein Group

dimeric alpha-amylase inhibitor, partial [Aegilops longissima]	gi|386877058	14718.9	4.9899 997711 1816

Peptide Information

Calc. Mass	Obsrv. Mass	± da	± ppm	Start Seq.	End Seq.	Sequence	Ion Score	C. I. %	Modification	Rank	Result Type

1162.6249	1162.7152	0.0903	78	98	108	LTAASITAVCR			Carbamidomethyl (C)[10]		Mascot
1162.6249	1162.7152	0.0903	78	98	108	LTAASITAVCR			Carbamidomethyl (C)[10]		Mascot
1570.8007	1570.8739	0.0732	47	34	47	LQCNGSQVPEAVLR			Carbamidomethyl (C)[3]		Mascot
1570.8007	1570.8739	0.0732	47	34	47	LQCNGSQVPEAVLR	44	94.642	Carbamidomethyl (C)[3]		Mascot
1612.7463	1612.8792	0.1329	82	75	90	EHGAQEGQAGTGAFPR					Mascot
1612.7463	1612.8792	0.1329	82	75	90	EHGAQEGQAGTGAFPR	104	100			Mascot
1663.8361	1663.931	0.0949	57	109	124	LPIVVDASGDGAYVCK			Carbamidomethyl (C)[15]		Mascot
2807.4431	2807.5427	0.0996	35	98	124	LTAASITAVCRLPIVVDAS GDGAYVCK			Carbamidomethyl (C)[10,26]		Mascot

7	Globulin-1 S allele [Triticum urartu]	gi|474323981	55586	7.77	4	176	100	59.944	169	100		

Peptide Information

Calc. Mass	Obsrv. Mass	± da	± ppm	Start Seq.	End Seq.	Sequence	Ion Score	C. I. %	Modification	Rank	Result Type
842.5206	842.5801	0.0595	71	395	401	QVRAQIK					Mascot
1001.5527	1001.6293	0.0766	76	457	465	ALAFPQQAR					Mascot
1001.5527	1001.6293	0.0766	76	457	465	ALAFPQQAR	53	99.226			Mascot
1641.8344	1641.9668	0.1324	81	474	488	AQPESVFVAGPQQQR					Mascot
1641.8344	1641.9668	0.1324	81	474	488	AQPESVFVAGPQQQR	116	100			Mascot
1723.8762	1724.0227	0.1465	85	86	101	GSIGDYRVAYLDAAPR					Mascot

8	dimeric alpha-amylase inhibitor, partial [Aegilops geniculata]	gi|452055914	14748	6.09	4	170	100	7.033	148	100

Protein Group

dimeric alpha-amylase inhibitor, partial [Triticum aestivum]	gi|386877068	14415.8	6.8800 001144 4092

Peptide Information

Calc. Mass	Obsrv. Mass	± da	± ppm	Start Seq.	End Seq.	Sequence	Ion Score	C. I. %	Modification	Rank	Result Type
1162.6249	1162.7152	0.0903	78	97	107	LTAASITAVCR			Carbamidomethyl (C)[10]		Mascot
1162.6249	1162.7152	0.0903	78	97	107	LTAASITAVCR			Carbamidomethyl (C)[10]		Mascot
1570.8007	1570.8739	0.0732	47	33	46	LQCNGSQVPEAVLR			Carbamidomethyl (C)[3]		Mascot
1570.8007	1570.8739	0.0732	47	33	46	LQCNGSQVPEAVLR	44	94.642	Carbamidomethyl (C)[3]		Mascot
1612.7463	1612.8792	0.1329	82	74	89	EHGAQEGQAGTGAFPR					Mascot
1612.7463	1612.8792	0.1329	82	74	89	EHGAQEGQAGTGAFPR	104	100			Mascot
1862.7731	1862.9211	0.148	79	47	60	DCCQQLAHISEWCR			Carbamidomethyl (C)[2,3,13]		Mascot
1862.7731	1862.9211	0.148	79	47	60	DCCQQLAHISEWCR			Carbamidomethyl (C)[2,3,13]		Mascot

9	dimeric alpha-amylase inhibitor, partial [Triticum aestivum]	gi|386877040	15014.1	5.28	3	162	100	6.046	148	100

Peptide Information

Calc. Mass	Obsrv. Mass	± da	± ppm	Start Seq.	End Seq.	Sequence	Ion Score	C. I. %	Modification	Rank	Result Type
1162.6249	1162.7152	0.0903	78	101	111	LTAASITAVCR			Carbamidomethyl (C)[10]		Mascot
1162.6249	1162.7152	0.0903	78	101	111	LTAASITAVCR			Carbamidomethyl (C)[10]		Mascot
1570.8007	1570.8739	0.0732	47	37	50	LQCNGSQVPEAVLR			Carbamidomethyl (C)[3]		Mascot
1570.8007	1570.8739	0.0732	47	37	50	LQCNGSQVPEAVLR	44	94.642	Carbamidomethyl (C)[3]		Mascot
1612.7463	1612.8792	0.1329	82	78	93	EHGAQEGQAGTGAFPR					Mascot
1612.7463	1612.8792	0.1329	82	78	93	EHGAQEGQAGTGAFPR	104	100			Mascot

10	dimeric alpha-amylase inhibitor, partial [Aegilops peregrina]	gi|386877054	14145.7	5.26	4	128	100	4.793	104	100

Peptide Information

Calc. Mass	Obsrv. Mass	± da	± ppm	Start Seq.	End Seq.	Sequence	Ion Score	C. I. %	Modification	Rank	Result Type
1162.6249	1162.7152	0.0903	78	92	102	LTAASITAVCR			Carbamidomethyl (C)[10]		Mascot
1162.6249	1162.7152	0.0903	78	92	102	LTAASITAVCR			Carbamidomethyl (C)[10]		Mascot
1612.7463	1612.8792	0.1329	82	69	84	EHGAQEGQAGTGAFPR					Mascot
1612.7463	1612.8792	0.1329	82	69	84	EHGAQEGQAGTGAFPR	104	100			Mascot
1663.8361	1663.931	0.0949	57	103	118	LPIVVDASGDGAYVCK			Carbamidomethyl (C)[15]		Mascot
2807.4431	2807.5427	0.0996	35	92	118	LTAASITAVCRLPIVVDAS GDGAYVCK			Carbamidomethyl (C)[10,26]		Mascot

Rank	Protein Name	Accession No.	Protein MW	Protein PI	Pep. Count	Protein Score	Protein Score C. I. %	Intensity Matched	Total Ion Score	Total Ion C. I. %	Confirmed
1	RecName: Full=Alpha-amylase/trypsin inhibitor CM3; AltName: Full=Chloroform/methanol-soluble protein CM3; Flags: Precursor	gi|123957	18893.3	7.44	8	583	100	57.249	531	100	

Peptide Information

Calc. Mass	Obsrv. Mass	± da	± ppm	Start Seq.	End Seq.	Sequence	Ion Score	C. I. %	Modification	Rank	Result Type
1010.52	1010.5914	0.0714	71	37	44	TNLLPHCR			Carbamidomethyl (C)[7]		Mascot
1010.52	1010.5914	0.0714	71	37	44	TNLLPHCR	42	89.572	Carbamidomethyl (C)[7]		Mascot
1110.5038	1110.5851	0.0813	73	133	140	EMQWDFVR					Mascot
1126.4987	1126.5664	0.0677	60	133	140	EMQWDFVR			Oxidation (M)[2]		Mascot
1126.4987	1126.5664	0.0677	60	133	140	EMQWDFVR	57	99.678	Oxidation (M)[2]		Mascot
1698.9214	1699.0544	0.133	78	101	115	YFIALPVPSQPVDPR					Mascot
1698.9214	1699.0544	0.133	78	101	115	YFIALPVPSQPVDPR	88	100			Mascot
1727.8381	1727.9695	0.1314	76	116	132	SGNVGESGLIDLPGCPR			Carbamidomethyl (C)[15]		Mascot
1727.8381	1727.9695	0.1314	76	116	132	SGNVGESGLIDLPGCPR	122	100	Carbamidomethyl (C)[15]		Mascot
1801.8427	1801.9408	0.0981	54	45	60	DYVLQQTCGTFTPGSK			Carbamidomethyl (C)[8]		Mascot
1876.0222	1876.1837	0.1615	86	141	157	LLVAPGQCNLATIHNVR			Carbamidomethyl (C)[8]		Mascot
1876.0222	1876.1837	0.1615	86	141	157	LLVAPGQCNLATIHNVR	98	100	Carbamidomethyl (C)[8]		Mascot
1957.8564	1958.0208	0.1644	84	81	95	LYCCQELAEISQQCR			Carbamidomethyl (C)[3,4,14]		Mascot
1957.8564	1958.0208	0.1644	84	81	95	LYCCQELAEISQQCR	124	100	Carbamidomethyl (C)[3,4,14]		Mascot
2255.1416	2255.2786	0.137	61	61	80	LPEWMTSASIYSPGKPYLAK			Oxidation (M)[5]		Mascot

Rank	Protein Name	Accession No.	Protein MW	Protein PI	Pep. Count	Protein Score	Protein Score C. I. %	Intensity Matched	Total Ion Score	Total Ion C. I. %	Confirmed
2	Alpha-amylase/trypsin inhibitor CM3 [Aegilops tauschii]	gi|475546433	25087.4	8.16	6	336	100	41.162	308	100	

Peptide Information

Calc. Mass	Obsrv. Mass	± da	± ppm	Start Seq.	End Seq.	Sequence	Ion Score	C. I. %	Modification	Rank	Result Type
1109.5198	1109.5797	0.0599	54	186	193	QMQWDFVR					Mascot
1125.5146	1125.5731	0.0585	52	186	193	QMQWDFVR			Oxidation (M)[2]		Mascot
1698.9214	1699.0544	0.133	78	154	168	YFIALPVPSQPVDPR					Mascot
1698.9214	1699.0544	0.133	78	154	168	YFIALPVPSQPVDPR	88	100			Mascot
1727.8381	1727.9695	0.1314	76	169	185	SGNVGESGLIDLPGCPR			Carbamidomethyl (C)[15]		Mascot
1727.8381	1727.9695	0.1314	76	169	185	SGNVGESGLIDLPGCPR	122	100	Carbamidomethyl (C)[15]		Mascot

Calc. Mass	Obsrv. Mass	± da	± ppm	Start Seq.	End Seq.	Sequence	Ion Score	C. I. %	Modification	Rank	Result Type
1801.8427	1801.9408	0.0981	54	98	113	DYVLQQTCGTFTPGSK			Carbamidomethyl (C)[8]		Mascot
1876.0222	1876.1837	0.1615	86	194	210	LLVAPGQCNLATIHNVR			Carbamidomethyl (C)[8]		Mascot
1876.0222	1876.1837	0.1615	86	194	210	LLVAPGQCNLATIHNVR	98	100	Carbamidomethyl (C)[8]		Mascot
1967.8772	1968.0333	0.1561	79	134	148	LYCCQELAEIPQQCR			Carbamidomethyl (C)[3,4,14]		Mascot

3 RecName: Full=Alpha-amylase/trypsin inhibitor CMd; AltName: Full=Chloroform/methanol-soluble protein CMd; Flags: Precursor — gi|585291 — 19140.3 — 6.07 — 2 — 106 — 99.998 — 17.475 — 98 — 100

Peptide Information

Calc. Mass	Obsrv. Mass	± da	± ppm	Start Seq.	End Seq.	Sequence	Ion Score	C. I. %	Modification	Rank	Result Type
1876.0222	1876.1837	0.1615	86	144	160	LLVAPGQCNLATIHNVR			Carbamidomethyl (C)[8]		Mascot
1876.0222	1876.1837	0.1615	86	144	160	LLVAPGQCNLATIHNVR	98	100	Carbamidomethyl (C)[8]		Mascot
1967.8772	1968.0333	0.1561	79	84	98	LYCCQELAEIPQQCR			Carbamidomethyl (C)[3,4,14]		Mascot

4 PREDICTED: uncharacterized protein LOC101781929 isoform X1 [Setaria italica] — gi|514717921 — 51781.4 — 8.94 — 13 — 58 — 0 — 14.134

Peptide Information

Calc. Mass	Obsrv. Mass	± da	± ppm	Start Seq.	End Seq.	Sequence	Ion Score	C. I. %	Modification	Rank	Result Type
1008.5109	1008.5428	0.0319	32	290	297	LPEHVEER					Mascot
1012.5422	1012.5822	0.04	40	8	18	KGDASAAPAPK					Mascot
1140.6484	1140.5767	-0.0717	-63	444	454	VRGPSVPVSSR					Mascot
1162.6216	1162.6079	-0.0137	-12	236	245	GRFTQELSPK					Mascot
1173.619	1173.5831	-0.0359	-31	171	179	LFLYDFDLK					Mascot
1710.8293	1710.9543	0.125	73	9	27	GDASAAPAPKADAPGSA EK					Mascot
1752.9504	1752.9395	-0.0109	-6	290	304	LPEHVEERGIPIHAR					Mascot
1755.8984	1755.9916	0.0932	53	58	77	VEASPASAAAAGNGGVA LSR					Mascot
1801.9291	1801.9408	0.0117	6	37	56	LKAAAAESAGTAEASASA PK					Mascot
1883.9934	1884.0436	0.0502	27	58	78	VEASPASAAAAGNGGVA LSRK					Mascot
1898.0131	1898.1085	0.0954	50	377	394	DPLLARDYGALPGELAAR					Mascot
1911.9995	1912.0106	0.0111	6	57	77	RVEASPASAAAAGNGGV ALSR					Mascot
1958.0052	1958.0208	0.0156	8	180	197	LMYGVYKADTSGGLDLV R					Mascot
1958.0052	1958.0208	0.0156	8	180	197	LMYGVYKADTSGGLDLV R					Mascot
1974.0001	1973.9926	-0.0075	-4	180	197	LMYGVYKADTSGGLDLV R			Oxidation (M)[2]		Mascot

5 PREDICTED: uncharacterized protein LOC101754677 — gi|514806940 — 62103.4 — 9.19 — 17 — 57 — 0 — 9.598

[Setaria italica]

Peptide Information

Calc. Mass	Obsrv. Mass	± da	± ppm	Start Seq.	End Seq.	Sequence	Ion Score	C. I. %	Modification	Rank	Result Type
1010.491	1010.5914	0.1004	99	13	20	AYMPNMRK					Mascot
1010.491	1010.5914	0.1004	99	12	19	KAYMPNMR					Mascot
1078.5527	1078.5765	0.0238	22	296	305	KDQPSGYVGK					Mascot
1085.6143	1085.6533	0.039	36	442	450	LVAWPSVWK					Mascot
1114.6104	1114.5723	-0.0381	-34	68	78	AGQSSDVLPLK					Mascot
1124.631	1124.5582	-0.0728	-65	429	438	SLPPVVEVER					Mascot
1130.635	1130.563	-0.072	-64	243	251	EISRPLKCK			Carbamidomethyl (C)[8]		Mascot
1174.6176	1174.5701	-0.0475	-40	150	160	DGSNTNLGLRK					Mascot
1571.8387	1571.9039	0.0652	41	68	83	AGQSSDVLPLKNGASK					Mascot
1670.8279	1670.9606	0.1327	79	184	198	NADLELMAKSAHQAR			Oxidation (M)[7]		Mascot
1682.8643	1682.9562	0.0919	55	199	213	NVDLELMAKSAHQAR					Mascot
1709.8528	1709.9583	0.1055	62	256	271	GSNIPFASETLKASCK			Carbamidomethyl (C)[15]		Mascot
1714.8951	1715.0264	0.1313	77	516	528	YYLWAVFKPREDK					Mascot
1867.0323	1867.0076	-0.0247	-13	423	438	VWELSKSLPPVVEVER					Mascot
1883.9167	1884.0436	0.1269	67	274	290	NCAIVSSKDVAQSEFTK			Carbamidomethyl (C)[2]		Mascot
1970.1031	1970.0323	-0.0708	-36	490	507	AVVNEAEMLIFPSVLLPK					Mascot
1971.9407	1972.0272	0.0865	44	132	149	ELENPTPFHSKDGSNAT K					Mascot
1986.098	1986.0424	-0.0556	-28	490	507	AVVNEAEMLIFPSVLLPK			Oxidation (M)[8]		Mascot

6	Mitochondrial ribosomal protein S11, putative [Ricinus communis]	gi|223542167	25304	10.37	6	51	0	6.111	30	0	

Peptide Information

Calc. Mass	Obsrv. Mass	± da	± ppm	Start Seq.	End Seq.	Sequence	Ion Score	C. I. %	Modification	Rank	Result Type
1008.5407	1008.5428	0.0021	2	184	191	RQAIMSFR					Mascot
1045.6041	1045.6168	0.0127	12	44	53	LGSLPNFGIK					Mascot
1060.5746	1060.5947	0.0201	19	133	145	ATSGAKAVGGGGK					Mascot
1107.5979	1107.644	0.0461	42	1	9	MWSLSSLRK					Mascot
1142.5663	1142.5651	-0.0012	-1	79	87	SYKPMDFVR					Mascot
1158.5613	1158.5547	-0.0066	-6	79	87	SYKPMDFVR			Oxidation (M)[5]		Mascot
1158.5613	1158.5547	-0.0066	-6	79	87	SYKPMDFVR	30	0	Oxidation (M)[5]		Mascot
1916.0713	1916.0728	0.0015	1	100	115	HNIEQNPDIVLINLKR					Mascot

7	maturase K, partial (chloroplast) [Drimia altissima]	gi|409053287	33635.4	9.54	10	50	0	1.463

Peptide Information

Calc. Mass	Obsrv. Mass	± da	± ppm	Start Seq.	End Seq.	Sequence	Ion Score	C. I. %	Modification	Rank	Result Type
815.4443	815.4954	0.0511	63	147	153	GTHLLMK			Oxidation (M)[6]		Mascot
1060.5575	1060.5947	0.0372	35	129	136	EPFIHYVR					Mascot
1078.6255	1078.5765	-0.049	-45	137	146	YQGKAILASK					Mascot
1140.6049	1140.5767	-0.0282	-25	88	97	LTSFGAFLER					Mascot
1715.0354	1715.0264	-0.009	-5	220	235	FDTIVPVILLIGSLSK					Mascot
1873.9596	1874.1099	0.1503	80	88	103	LTSFGAFLERTHFYGK					Mascot
1874.976	1875.1484	0.1724	92	82	97	QSSYLRLTSFGAFLER					Mascot
1883.0004	1883.1118	0.1114	59	123	136	TLWFFKEPFIHYVR					Mascot
1914.1675	1914.045	-0.1225	-64	220	237	FDTIVPVILLIGSLSKAK					Mascot
1941.015	1941.0542	0.0392	20	204	219	NQMLENFFLIDTVTKK					Mascot

8	dynamin, putative [Ricinus communis]	gi|223531786	68883.2	8.69	15	49	0	10.267

Peptide Information

Calc. Mass	Obsrv. Mass	± da	± ppm	Start Seq.	End Seq.	Sequence	Ion Score	C. I. %	Modification	Rank	Result Type
1045.5571	1045.6168	0.0597	57	255	263	NVDMIAARR					Mascot
1061.5521	1061.6239	0.0718	68	255	263	NVDMIAARR			Oxidation (M)[4]		Mascot
1076.6211	1076.5583	-0.0628	-58	100	108	RIADFSLVR					Mascot
1092.6273	1092.592	-0.0353	-32	384	392	HLSPQNIRK					Mascot
1126.6144	1126.5664	-0.048	-43	414	423	LIDGAISYFK					Mascot
1126.6144	1126.5664	-0.048	-43	414	423	LIDGAISYFK					Mascot
1140.5757	1140.5767	0.001	1	506	516	VGNPIANNADR					Mascot
1146.6113	1146.6166	0.0053	5	444	453	SVGETQELKR					Mascot
1158.6113	1158.5547	-0.0566	-49	223	233	GTNALDILEGR					Mascot
1158.6113	1158.5547	-0.0566	-49	223	233	GTNALDILEGR					Mascot
1670.937	1670.9606	0.0236	14	2	16	AAMESLIGLVNRIQR					Mascot
1741.8789	1741.9609	0.082	47	575	589	QLAQLLDEDPALMER					Mascot
1752.8453	1752.9395	0.0942	54	267	281	EFFASSPDYRHLAGR					Mascot
1801.9775	1801.9408	-0.0367	-20	1	16	MAAMESLIGLVNRIQR					Mascot
1873.9689	1874.1099	0.141	75	217	233	LDLMDKGTNALDILEGR					Mascot
1897.98	1898.1085	0.1285	68	575	590	QLAQLLDEDPALMERR					Mascot
1913.975	1914.045	0.07	37	575	590	QLAQLLDEDPALMERR			Oxidation (M)[13]		Mascot

		1916.0123	1916.0728	0.0605	32	454	471	FPTLQAEIAIAASDALER					Mascot
		1940.9858	1941.0542	0.0684	35	524	541	RIGSNVSSYVGMVSEAL R			Oxidation (M)[12]		Mascot

9	Putative disease resistance protein RXW24L [Aegilops tauschii]	gi|475506332	110535	7.95	20	49	0	39.452

Peptide Information

Calc. Mass	Obsrv. Mass	± da	± ppm	Start Seq.	End Seq.	Sequence	Ion Score	C. I. %	Modification	Rank	Result Type
815.5098	815.4954	-0.0144	-18	260	266	QILTGKR					Mascot
832.4457	832.3792	-0.0665	-80	887	892	EICVRR			Carbamidomethyl (C)[3]		Mascot
919.4917	919.5724	0.0807	88	435	441	IKCEELK			Carbamidomethyl (C)[3]		Mascot
1008.5043	1008.5428	0.0385	38	84	91	FMDRLGNR					Mascot
1045.6517	1045.6168	-0.0349	-33	643	651	LVRLFVGNK					Mascot
1060.5997	1060.5947	-0.005	-5	349	357	DVSTRILEK					Mascot
1092.5474	1092.592	0.0446	41	444	452	WVAEGFLDR					Mascot
1109.511	1109.5797	0.0687	62	679	688	FAEEVNESGK					Mascot
1176.6082	1176.6429	0.0347	29	693	702	YLHTGEVMVK					Mascot
1176.6293	1176.6429	0.0136	12	869	878	VKALECSIEK			Carbamidomethyl (C)[6]		Mascot
1699.0516	1699.0544	0.0028	2	901	914	DLLDLFLLLKLNIR					Mascot
1699.0516	1699.0544	0.0028	2	901	914	DLLDLFLLLKLNIR					Mascot
1728.0089	1727.9695	-0.0394	-23	187	204	VVPIVGSGGLGKTTLAMK					Mascot
1728.0089	1727.9695	-0.0394	-23	187	204	VVPIVGSGGLGKTTLAMK					Mascot
1744.0038	1743.9325	-0.0713	-41	187	204	VVPIVGSGGLGKTTLAMK			Oxidation (M)[17]		Mascot
1801.8749	1801.9408	0.0659	37	457	471	LDEVAENCINELINR			Carbamidomethyl (C)[8]		Mascot
1851.9958	1852.1643	0.1685	91	896	910	DQMMKDLLDLFLLLK			Oxidation (M)[3]		Mascot
1870.9327	1871.0052	0.0725	39	154	170	ITTLHEEVNGLVAMDGR			Oxidation (M)[14]		Mascot
1873.9762	1874.1099	0.1337	71	171	186	VKHITALLMDESMELK			Oxidation (M)[9]		Mascot
1875.0409	1875.1484	0.1075	57	358	375	CGGLPLAIITFASLLSNK			Carbamidomethyl (C)[1]		Mascot
1889.9711	1890.1569	0.1858	98	171	186	VKHITALLMDESMELK			Oxidation (M)[9,13]		Mascot
1897.9888	1898.1085	0.1197	63	911	925	LNIRIWLDACTVMHR			Carbamidomethyl (C)[10]		Mascot
1900.9707	1900.9803	0.0096	5	915	929	IWLDACTVMHRLVMR			Carbamidomethyl (C)[6]		Mascot
1913.9836	1914.045	0.0614	32	911	925	LNIRIWLDACTVMHR			Carbamidomethyl (C)[10], Oxidation (M)[13]		Mascot
1957.8379	1958.0208	0.1829	93	302	319	ITDVANGCCSNSSGQPY K			Carbamidomethyl (C)[8,9]		Mascot
1957.8379	1958.0208	0.1829	93	302	319	ITDVANGCCSNSSGQPY K			Carbamidomethyl (C)[8,9]		Mascot

10	PREDICTED: uncharacterized protein LOC101781929 isoform X3 [Setaria italica]	gi|514717928	46191.5	8.51	11	49	0	13.652

Peptide Information

Calc. Mass	Obsrv. Mass	± da	± ppm	Start Seq.	End Seq.	Sequence	Ion Score	C. I. %	Modification	Rank	Result Type
1008.5109	1008.5428	0.0319	32	290	297	LPEHVEER					Mascot
1012.5422	1012.5822	0.04	40	8	18	KGDASAAPAPK					Mascot
1162.6216	1162.6079	-0.0137	-12	236	245	GRFTQELSPK					Mascot
1173.619	1173.5831	-0.0359	-31	171	179	LFLYDFDLK					Mascot
1710.8293	1710.9543	0.125	73	9	27	GDASAAPAPKADAPGSAEK					Mascot
1752.9504	1752.9395	-0.0109	-6	290	304	LPEHVEERGIPIHAR					Mascot
1755.8984	1755.9916	0.0932	53	58	77	VEASPASAAAAGNGGVALSR					Mascot
1801.9291	1801.9408	0.0117	6	37	56	LKAAAAESAGTAEASASAPK					Mascot
1883.9934	1884.0436	0.0502	27	58	78	VEASPASAAAAGNGGVALSRK					Mascot
1911.9995	1912.0106	0.0111	6	57	77	RVEASPASAAAAGNGGVALSR					Mascot
1958.0052	1958.0208	0.0156	8	180	197	LMYGVYKADTSGGLDLVR					Mascot
1958.0052	1958.0208	0.0156	8	180	197	LMYGVYKADTSGGLDLVR					Mascot
1974.0001	1973.9926	-0.0075	-4	180	197	LMYGVYKADTSGGLDLVR			Oxidation (M)[2]		Mascot

Gel Idx/Pos	121/E20	Instr./Gel Origin	BA2151/Sample Project 20140814	Process Status	Analysis Succeeded
Plate [#] Name	[1] Sample Project 20140814	Instrument Sample Name		Spectra	11

Rank	Protein Name	Accession No.	Protein MW	Protein PI	Pep. Count	Protein Score	Protein Score C. I. %	Intensity Matched	Total Ion Score	Total Ion C. I. %	Confirmed
1	Chain D, 0.19 Alpha-Amylase Inhibitor From Wheat	gi|3318684	13898.6	6.66	6	283	100	27.661	243	100	

Protein Group

Protein Name	Accession No.	Protein MW	Protein PI
Chain A, 0.19 Alpha-Amylase Inhibitor From Wheat	gi|3318681	13898.6	6.65999984741211
Chain B, 0.19 Alpha-Amylase Inhibitor From Wheat	gi|3318682	13898.6	6.65999984741211
Chain C, 0.19 Alpha-Amylase Inhibitor From Wheat	gi|3318683	13898.6	6.65999984741211
RecName: Full=Alpha-amylase inhibitor 0.19; AltName: Full=0.19 alpha-AI; Short=0.19 AI	gi|123963	13898.6	6.65999984741211

Peptide Information

Calc. Mass	Obsrv. Mass	± da	± ppm	Start Seq.	End Seq.	Sequence	Ion Score	C. I. %	Modification	Rank	Result Type
1162.6249	1162.7305	0.1056	91	90	100	LTAASITAVCR			Carbamidomethyl (C)[10]		Mascot
1570.8007	1570.9443	0.1436	91	26	39	LQCNGSQVPEAVLR			Carbamidomethyl (C)[3]		Mascot
1612.7463	1612.8998	0.1535	95	67	82	EHGAQEGQAGTGAFPR					Mascot
1612.7463	1612.8998	0.1535	95	67	82	EHGAQEGQAGTGAFPR	141	100			Mascot
1617.8993	1617.8964	-0.0029	-2	86	100	EVVKLTAASITAVCR			Carbamidomethyl (C)[14]		Mascot
1663.8361	1663.9633	0.1272	76	101	116	LPIVVDASGDGAYVCK			Carbamidomethyl (C)[15]		Mascot
1862.7731	1862.9509	0.1778	95	40	53	DCCQQLAHISEWCR			Carbamidomethyl (C)[2,3,13]		Mascot
1862.7731	1862.9509	0.1778	95	40	53	DCCQQLAHISEWCR	102	100	Carbamidomethyl (C)[2,3,13]		Mascot

Rank	Protein Name	Accession No.	Protein MW	Protein PI	Pep. Count	Protein Score	Protein Score C. I. %	Intensity Matched	Total Ion Score	Total Ion C. I. %	Confirmed
2	dimeric alpha-amylase inhibitor, partial [Aegilops kotschyi]	gi|386877048	14198.8	6.66	6	282	100	27.661	243	100	

Protein Group

Protein Name	Accession No.	Protein MW	Protein PI
dimeric alpha-amylase inhibitor, partial [Aegilops geniculata]	gi|386877062	14542.9	7.05000019073486
dimeric alpha-amylase inhibitor, partial [Aegilops geniculata]	gi|452055912	14198.8	6.65999984741211

Peptide Information

Calc. Mass	Obsrv. Mass	± da	± ppm	Start Seq.	End Seq.	Sequence	Ion Score	C. I. %	Modification	Rank	Result Type

Calc. Mass	Obsrv. Mass	± da	± ppm	Start Seq.	End Seq.	Sequence	Ion Score	C. I. %	Modification	Rank	Result Type
1162.6249	1162.7305	0.1056	91	93	103	LTAASITAVCR			Carbamidomethyl (C)[10]		Mascot
1570.8007	1570.9443	0.1436	91	29	42	LQCNGSQVPEAVLR			Carbamidomethyl (C)[3]		Mascot
1612.7463	1612.8998	0.1535	95	70	85	EHGAQEGQAGTGAFPR					Mascot
1612.7463	1612.8998	0.1535	95	70	85	EHGAQEGQAGTGAFPR	141	100			Mascot
1617.8993	1617.8964	-0.0029	-2	89	103	EVVKLTAASITAVCR			Carbamidomethyl (C)[14]		Mascot
1663.8361	1663.9633	0.1272	76	104	119	LPIVVDASGDGAYVCK			Carbamidomethyl (C)[15]		Mascot
1862.7731	1862.9509	0.1778	95	43	56	DCCQQLAHISEWCR			Carbamidomethyl (C)[2,3,13]		Mascot
1862.7731	1862.9509	0.1778	95	43	56	DCCQQLAHISEWCR	102	100	Carbamidomethyl (C)[2,3,13]		Mascot

3	dimeric alpha-amylase inhibitor, partial [Aegilops tauschii]	gi|386877050	14826.1	6.86	6	281	100	27.661	243	100	

Protein Group

dimeric alpha-amylase inhibitor, partial [Aegilops longissima]	gi|386877060	14954.2	7.6399998664856
dimeric alpha-amylase inhibitor, partial [Aegilops tauschii]	gi|386877044	14805	6.0999999046325 7
dimeric alpha-amylase inhibitor, partial [Aegilops tauschii]	gi|386877046	14670	6.0799999237060 5

Peptide Information

Calc. Mass	Obsrv. Mass	± da	± ppm	Start Seq.	End Seq.	Sequence	Ion Score	C. I. %	Modification	Rank	Result Type
1162.6249	1162.7305	0.1056	91	98	108	LTAASITAVCR			Carbamidomethyl (C)[10]		Mascot
1570.8007	1570.9443	0.1436	91	34	47	LQCNGSQVPEAVLR			Carbamidomethyl (C)[3]		Mascot
1612.7463	1612.8998	0.1535	95	75	90	EHGAQEGQAGTGAFPR					Mascot
1612.7463	1612.8998	0.1535	95	75	90	EHGAQEGQAGTGAFPR	141	100			Mascot
1617.8993	1617.8964	-0.0029	-2	94	108	EVVKLTAASITAVCR			Carbamidomethyl (C)[14]		Mascot
1663.8361	1663.9633	0.1272	76	109	124	LPIVVDASGDGAYVCK			Carbamidomethyl (C)[15]		Mascot
1862.7731	1862.9509	0.1778	95	48	61	DCCQQLAHISEWCR			Carbamidomethyl (C)[2,3,13]		Mascot
1862.7731	1862.9509	0.1778	95	48	61	DCCQQLAHISEWCR	102	100	Carbamidomethyl (C)[2,3,13]		Mascot

4	dimeric alpha-amylase inhibitor [Triticum aestivum]	gi|386877038	15702.5	5.58	6	280	100	27.661	243	100	

Peptide Information

Calc. Mass	Obsrv. Mass	± da	± ppm	Start Seq.	End Seq.	Sequence	Ion Score	C. I. %	Modification	Rank	Result Type
1162.6249	1162.7305	0.1056	91	107	117	LTAASITAVCR			Carbamidomethyl (C)[10]		Mascot
1570.8007	1570.9443	0.1436	91	43	56	LQCNGSQVPEAVLR			Carbamidomethyl (C)[3]		Mascot
1612.7463	1612.8998	0.1535	95	84	99	EHGAQEGQAGTGAFPR					Mascot

Calc. Mass	Obsrv. Mass	± da	± ppm	Start Seq.	End Seq.	Sequence	Ion Score	C. I. %	Modification	Rank	Result Type
1612.7463	1612.8998	0.1535	95	84	99	EHGAQEGQAGTGAFPR	141	100			Mascot
1617.8993	1617.8964	-0.0029	-2	103	117	EVVKLTAASITAVCR			Carbamidomethyl (C)[14]		Mascot
1663.8361	1663.9633	0.1272	76	118	133	LPIVVDASGDGAYVCK			Carbamidomethyl (C)[15]		Mascot
1862.7731	1862.9509	0.1778	95	57	70	DCCQQLAHISEWCR			Carbamidomethyl (C)[2,3,13]		Mascot
1862.7731	1862.9509	0.1778	95	57	70	DCCQQLAHISEWCR	102	100	Carbamidomethyl (C)[2,3,13]		Mascot

5	Alpha-amylase inhibitor 0.19 [Aegilops tauschii]	gi|475613321	17198.2	6.06	6	278	100	27.661	243	100

Peptide Information

Calc. Mass	Obsrv. Mass	± da	± ppm	Start Seq.	End Seq.	Sequence	Ion Score	C. I. %	Modification	Rank	Result Type
1162.6249	1162.7305	0.1056	91	120	130	LTAASITAVCR			Carbamidomethyl (C)[10]		Mascot
1570.8007	1570.9443	0.1436	91	56	69	LQCNGSQVPEAVLR			Carbamidomethyl (C)[3]		Mascot
1612.7463	1612.8998	0.1535	95	97	112	EHGAQEGQAGTGAFPR					Mascot
1612.7463	1612.8998	0.1535	95	97	112	EHGAQEGQAGTGAFPR	141	100			Mascot
1617.8993	1617.8964	-0.0029	-2	116	130	EVVKLTAASITAVCR			Carbamidomethyl (C)[14]		Mascot
1663.8361	1663.9633	0.1272	76	131	146	LPIVVDASGDGAYVCK			Carbamidomethyl (C)[15]		Mascot
1862.7731	1862.9509	0.1778	95	70	83	DCCQQLAHISEWCR			Carbamidomethyl (C)[2,3,13]		Mascot
1862.7731	1862.9509	0.1778	95	70	83	DCCQQLAHISEWCR	102	100	Carbamidomethyl (C)[2,3,13]		Mascot

6	dimeric alpha-amylase inhibitor, partial [Aegilops geniculata]	gi|452055914	14748	6.09	5	272	100	27.2	243	100

Protein Group

dimeric alpha-amylase inhibitor, partial [Triticum aestivum]	gi|386877068	14415.8	6.8800 001144 4092

Peptide Information

Calc. Mass	Obsrv. Mass	± da	± ppm	Start Seq.	End Seq.	Sequence	Ion Score	C. I. %	Modification	Rank	Result Type
1162.6249	1162.7305	0.1056	91	97	107	LTAASITAVCR			Carbamidomethyl (C)[10]		Mascot
1570.8007	1570.9443	0.1436	91	33	46	LQCNGSQVPEAVLR			Carbamidomethyl (C)[3]		Mascot
1612.7463	1612.8998	0.1535	95	74	89	EHGAQEGQAGTGAFPR					Mascot
1612.7463	1612.8998	0.1535	95	74	89	EHGAQEGQAGTGAFPR	141	100			Mascot
1617.8993	1617.8964	-0.0029	-2	93	107	EVVKLTAASITAVCR			Carbamidomethyl (C)[14]		Mascot
1862.7731	1862.9509	0.1778	95	47	60	DCCQQLAHISEWCR			Carbamidomethyl (C)[2,3,13]		Mascot
1862.7731	1862.9509	0.1778	95	47	60	DCCQQLAHISEWCR	102	100	Carbamidomethyl (C)[2,3,13]		Mascot

7	dimeric alpha-amylase inhibitor, partial [Aegilops longissima]	gi|386877056	14792	5.28	5	170	100	21.243	141	100

Protein Group

	dimeric alpha-amylase inhibitor, partial [Aegilops longissima]	gi|386877058	14718.9	4.9899 997711 1816						

Peptide Information

Calc. Mass	Obsrv. Mass	± da	± ppm	Start Seq.	End Seq.	Sequence	Ion Score	C. I. %	Modification	Rank	Result Type
1162.6249	1162.7305	0.1056	91	98	108	LTAASITAVCR			Carbamidomethyl (C)[10]		Mascot
1570.8007	1570.9443	0.1436	91	34	47	LQCNGSQVPEAVLR			Carbamidomethyl (C)[3]		Mascot
1612.7463	1612.8998	0.1535	95	75	90	EHGAQEGQAGTGAFPR					Mascot
1612.7463	1612.8998	0.1535	95	75	90	EHGAQEGQAGTGAFPR	141	100			Mascot
1617.8993	1617.8964	-0.0029	-2	94	108	EVVKLTAASITAVCR			Carbamidomethyl (C)[14]		Mascot
1663.8361	1663.9633	0.1272	76	109	124	LPIVVDASGDGAYVCK			Carbamidomethyl (C)[15]		Mascot

8	dimeric alpha-amylase inhibitor, partial [Aegilops peregrina]	gi|386877054	14145.7	5.26	4	163	100	19.485	141	100	

Peptide Information

Calc. Mass	Obsrv. Mass	± da	± ppm	Start Seq.	End Seq.	Sequence	Ion Score	C. I. %	Modification	Rank	Result Type
1162.6249	1162.7305	0.1056	91	92	102	LTAASITAVCR			Carbamidomethyl (C)[10]		Mascot
1612.7463	1612.8998	0.1535	95	69	84	EHGAQEGQAGTGAFPR					Mascot
1612.7463	1612.8998	0.1535	95	69	84	EHGAQEGQAGTGAFPR	141	100			Mascot
1617.8993	1617.8964	-0.0029	-2	88	102	EVVKLTAASITAVCR			Carbamidomethyl (C)[14]		Mascot
1663.8361	1663.9633	0.1272	76	103	118	LPIVVDASGDGAYVCK			Carbamidomethyl (C)[15]		Mascot

9	dimeric alpha-amylase inhibitor, partial [Triticum aestivum]	gi|386877040	15014.1	5.28	4	161	100	20.782	141	100	

Peptide Information

Calc. Mass	Obsrv. Mass	± da	± ppm	Start Seq.	End Seq.	Sequence	Ion Score	C. I. %	Modification	Rank	Result Type
1162.6249	1162.7305	0.1056	91	101	111	LTAASITAVCR			Carbamidomethyl (C)[10]		Mascot
1570.8007	1570.9443	0.1436	91	37	50	LQCNGSQVPEAVLR			Carbamidomethyl (C)[3]		Mascot
1612.7463	1612.8998	0.1535	95	78	93	EHGAQEGQAGTGAFPR					Mascot
1612.7463	1612.8998	0.1535	95	78	93	EHGAQEGQAGTGAFPR	141	100			Mascot
1617.8993	1617.8964	-0.0029	-2	97	111	EVVKLTAASITAVCR			Carbamidomethyl (C)[14]		Mascot

10	dimeric alpha-amylase inhibitor, partial [Triticum dicoccoides]	gi|488508057	15698.5	7.62	1	145	100	17.548	141	100	

Peptide Information

Calc. Mass	Obsrv. Mass	± da	± ppm	Start Seq.	End Seq.	Sequence	Ion Score	C. I. %	Modification	Rank	Result Type

1612.7463	1612.8998	0.1535	95	82	97	EHGAQEGQAGTGAFPR			Mascot
1612.7463	1612.8998	0.1535	95	82	97	EHGAQEGQAGTGAFPR	141	100	Mascot

Gel Idx/Pos	122/E21	Instr./Gel Origin	BA2151/Sample Project 20140814	Process Status	Analysis Succeeded
Plate [#] Name	[1] Sample Project 20140814	Instrument Sample Name		Spectra	11

Rank	Protein Name	Accession No.	Protein MW	Protein PI	Pep. Count	Protein Score	Protein Score C. I. %	Intensity Matched	Total Ion Score	Total Ion C. I. %	Confirmed
1	Ribulose bisphosphate carboxylase small chain, chloroplastic [Triticum urartu]	gi|474416311	15090.5	5.85	14	338	100	37.781	212	100	

Peptide Information

Calc. Mass	Obsrv. Mass	± da	± ppm	Start Seq.	End Seq.	Sequence	Ion Score	C. I. %	Modification	Rank	Result Type
906.5043	906.5795	0.0752	83	29	35	QVDYLIR					Mascot
914.4229	914.481	0.0581	64	65	70	YWTMWK					Mascot
930.4178	930.4788	0.061	66	65	70	YWTMWK			Oxidation (M)[4]		Mascot
965.4873	965.5659	0.0786	81	100	107	IIGFDNMR					Mascot
965.4873	965.5659	0.0786	81	100	107	IIGFDNMR	25	0			Mascot
981.4822	981.5511	0.0689	70	100	107	IIGFDNMR			Oxidation (M)[7]		Mascot
981.4822	981.5511	0.0689	70	100	107	IIGFDNMR	15	0	Oxidation (M)[7]		Mascot
1012.4734	1012.5549	0.0815	80	92	99	EYPDAYVR					Mascot
1012.4734	1012.5549	0.0815	80	92	99	EYPDAYVR	59	99.748			Mascot
1140.5684	1140.6562	0.0878	77	91	99	KEYPDAYVR					Mascot
1165.571	1165.6488	0.0778	67	38	46	WVPCLEFSK			Carbamidomethyl (C)[4]		Mascot
1165.571	1165.6488	0.0778	67	38	46	WVPCLEFSK	29	0	Carbamidomethyl (C)[4]		Mascot
1197.6991	1197.6556	-0.0435	-36	2	11	QVWPIEGIKK					Mascot
1365.5819	1365.696	0.1141	84	53	64	EHNASPGYYDGR	100	100			Mascot
1922.0521	1922.1836	0.1315	68	12	28	FETLSYLPPLSTEALLK					Mascot
2050.147	2050.2974	0.1504	73	11	28	KFETLSYLPPLSTEALLK					Mascot
2268.0789	2268.2593	0.1804	80	108	127	QVQCVSFIAFKPPGCEES GK			Carbamidomethyl (C)[4,15]		Mascot
2296.0837	2296.2825	0.1988	87	71	90	LPMFGCTDATQVINEVEE VK			Carbamidomethyl (C)[6], Oxidation (M)[3]		Mascot
2339.116	2339.302	0.186	80	108	128	QVQCVSFIAFKPPGCEES GKA			Carbamidomethyl (C)[4,15]		Mascot
2408.1836	2408.375	0.1914	79	71	91	LPMFGCTDATQVINEVEE VKK			Carbamidomethyl (C)[6]		Mascot
2424.1785	2424.363	0.1845	76	71	91	LPMFGCTDATQVINEVEE VKK			Carbamidomethyl (C)[6], Oxidation (M)[3]		Mascot

Rank	Protein Name	Accession No.	Protein MW	Protein PI	Pep. Count	Protein Score	Protein Score C. I. %	Intensity Matched	Total Ion Score	Total Ion C. I. %	Confirmed
2	hypothetical protein F775_43781 [Aegilops tauschii]	gi|475517356	19762.9	8.8	15	333	100	39.046	212	100	

Peptide Information

Calc. Mass	Obsrv. Mass	± da	± ppm	Start Seq.	End Seq.	Sequence	Ion Score	C. I. %	Modification	Rank	Result Type

Calc. Mass	Obsrv. Mass	± da	± ppm	Start Seq.	End Seq.	Sequence	Ion Score	C. I. %	Modification	Rank	Result Type
906.5043	906.5795	0.0752	83	76	82	QVDYLIR					Mascot
914.4229	914.481	0.0581	64	112	117	YWTMWK					Mascot
930.4178	930.4788	0.061	66	112	117	YWTMWK			Oxidation (M)[4]		Mascot
965.4873	965.5659	0.0786	81	147	154	IIGFDNMR					Mascot
965.4873	965.5659	0.0786	81	147	154	IIGFDNMR	25	0			Mascot
981.4822	981.5511	0.0689	70	147	154	IIGFDNMR			Oxidation (M)[7]		Mascot
981.4822	981.5511	0.0689	70	147	154	IIGFDNMR	15	0	Oxidation (M)[7]		Mascot
1012.4734	1012.5549	0.0815	80	139	146	EYPDAYVR					Mascot
1012.4734	1012.5549	0.0815	80	139	146	EYPDAYVR	59	99.748			Mascot
1140.5684	1140.6562	0.0878	77	138	146	KEYPDAYVR					Mascot
1165.571	1165.6488	0.0778	67	85	93	WVPCLEFSK			Carbamidomethyl (C)[4]		Mascot
1165.571	1165.6488	0.0778	67	85	93	WVPCLEFSK	29	0	Carbamidomethyl (C)[4]		Mascot
1262.6084	1262.7251	0.1167	92	31	44	SNGASLGSVSNGGR					Mascot
1262.6084	1262.7251	0.1167	92	31	44	SNGASLGSVSNGGR					Mascot
1365.5819	1365.696	0.1141	84	100	111	EHNASPGYYDGR	100	100			Mascot
1862.9681	1862.9376	-0.0305	-16	2	20	APTVMASSATSVAPFQGLK					Mascot
1922.0521	1922.1836	0.1315	68	59	75	FETLSYLPPLSTEALLK					Mascot
2050.147	2050.2974	0.1504	73	58	75	KFETLSYLPPLSTEALLK					Mascot
2268.0789	2268.2593	0.1804	80	155	174	QVQCVSFIAFKPPGCEESGK			Carbamidomethyl (C)[4,15]		Mascot
2296.0837	2296.2825	0.1988	87	118	137	LPMFGCTDATQVINEVEEVK			Carbamidomethyl (C)[6], Oxidation (M)[3]		Mascot
2339.116	2339.302	0.186	80	155	175	QVQCVSFIAFKPPGCEESGKA			Carbamidomethyl (C)[4,15]		Mascot
2408.1836	2408.375	0.1914	79	118	138	LPMFGCTDATQVINEVEEVKK			Carbamidomethyl (C)[6]		Mascot
2424.1785	2424.363	0.1845	76	118	138	LPMFGCTDATQVINEVEEVKK			Carbamidomethyl (C)[6], Oxidation (M)[3]		Mascot

3	Ribulose bisphosphate carboxylase small chain, chloroplastic [Triticum urartu]	gi|473882355	18742.4	8.65	15	332	100	37.854	212	100	

Peptide Information

Calc. Mass	Obsrv. Mass	± da	± ppm	Start Seq.	End Seq.	Sequence	Ion Score	C. I. %	Modification	Rank	Result Type
906.5043	906.5795	0.0752	83	65	71	QVDYLIR					Mascot
914.4229	914.481	0.0581	64	101	106	YWTMWK					Mascot
928.5323	928.5342	0.0019	2	21	29	STAGLPARR					Mascot
930.4178	930.4788	0.061	66	101	106	YWTMWK			Oxidation (M)[4]		Mascot
965.4873	965.5659	0.0786	81	136	143	IIGFDNMR					Mascot
965.4873	965.5659	0.0786	81	136	143	IIGFDNMR	25	0			Mascot
981.4822	981.5511	0.0689	70	136	143	IIGFDNMR			Oxidation (M)[7]		Mascot

981.4822	981.5511	0.0689	70	136	143	IIGFDNMR	15	0	Oxidation (M)[7]		Mascot
1012.4734	1012.5549	0.0815	80	128	135	EYPDAYVR					Mascot
1012.4734	1012.5549	0.0815	80	128	135	EYPDAYVR	59	99.748			Mascot
1140.5684	1140.6562	0.0878	77	127	135	KEYPDAYVR					Mascot
1165.571	1165.6488	0.0778	67	74	82	WVPCLEFSK			Carbamidomethyl (C)[4]		Mascot
1165.571	1165.6488	0.0778	67	74	82	WVPCLEFSK	29	0	Carbamidomethyl (C)[4]		Mascot
1365.5819	1365.696	0.1141	84	89	100	EHNASPGYYDGR	100	100			Mascot
1862.9681	1862.9376	-0.0305	-16	2	20	APTVMASSATSVAPFQG LK					Mascot
1922.0521	1922.1836	0.1315	68	48	64	FETLSYLPPLSTEALLK					Mascot
2050.147	2050.2974	0.1504	73	47	64	KFETLSYLPPLSTEALLK					Mascot
2268.0789	2268.2593	0.1804	80	144	163	QVQCVSFIAFKPPGCEES GK			Carbamidomethyl (C)[4,15]		Mascot
2296.0837	2296.2825	0.1988	87	107	126	LPMFGCTDATQVINEVEE VK			Carbamidomethyl (C)[6], Oxidation (M)[3]		Mascot
2339.116	2339.302	0.186	80	144	164	QVQCVSFIAFKPPGCEES GKA			Carbamidomethyl (C)[4,15]		Mascot
2408.1836	2408.375	0.1914	79	107	127	LPMFGCTDATQVINEVEE VKK			Carbamidomethyl (C)[6]		Mascot
2424.1785	2424.363	0.1845	76	107	127	LPMFGCTDATQVINEVEE VKK			Carbamidomethyl (C)[6], Oxidation (M)[3]		Mascot

4 Ribulose bisphosphate carboxylase small chain PWS4.3, chloroplastic [Aegilops tauschii] gi|475604963 19161.6 8.58 14 327 100 37.682 212 100

Peptide Information

Calc. Mass	Obsrv. Mass	± da	± ppm	Start Seq.	End Seq.	Sequence	Ion Score	C. I. %	Modification	Rank	Result Type
906.5043	906.5795	0.0752	83	71	77	QVDYLIR					Mascot
914.4229	914.481	0.0581	64	107	112	YWTMWK					Mascot
930.4178	930.4788	0.061	66	107	112	YWTMWK			Oxidation (M)[4]		Mascot
965.4873	965.5659	0.0786	81	142	149	IIGFDNMR					Mascot
965.4873	965.5659	0.0786	81	142	149	IIGFDNMR	25	0			Mascot
981.4822	981.5511	0.0689	70	142	149	IIGFDNMR			Oxidation (M)[7]		Mascot
981.4822	981.5511	0.0689	70	142	149	IIGFDNMR	15	0	Oxidation (M)[7]		Mascot
1012.4734	1012.5549	0.0815	80	134	141	EYPDAYVR					Mascot
1012.4734	1012.5549	0.0815	80	134	141	EYPDAYVR	59	99.748			Mascot
1140.5684	1140.6562	0.0878	77	133	141	KEYPDAYVR					Mascot
1165.571	1165.6488	0.0778	67	80	88	WVPCLEFSK			Carbamidomethyl (C)[4]		Mascot
1165.571	1165.6488	0.0778	67	80	88	WVPCLEFSK	29	0	Carbamidomethyl (C)[4]		Mascot
1363.7217	1363.6809	-0.0408	-30	2	15	ASSATSVAPFQGLK					Mascot
1365.5819	1365.696	0.1141	84	95	106	EHNASPGYYDGR	100	100			Mascot
1922.0521	1922.1836	0.1315	68	54	70	FETLSYLPPLSTEALLK					Mascot

Calc. Mass	Obsrv. Mass	± da	± ppm	Start Seq.	End Seq.	Sequence	Ion Score	C. I. %	Modification	Rank	Result Type
2050.147	2050.2974	0.1504	73	53	70	KFETLSYLPPLSTEALLK					Mascot
2268.0789	2268.2593	0.1804	80	150	169	QVQCVSFIAFKPPGCEESGK			Carbamidomethyl (C)[4,15]		Mascot
2296.0837	2296.2825	0.1988	87	113	132	LPMFGCTDATQVINEVEEVK			Carbamidomethyl (C)[6], Oxidation (M)[3]		Mascot
2339.116	2339.302	0.186	80	150	170	QVQCVSFIAFKPPGCEESGKA			Carbamidomethyl (C)[4,15]		Mascot
2408.1836	2408.375	0.1914	79	113	133	LPMFGCTDATQVINEVEEVKK			Carbamidomethyl (C)[6]		Mascot
2424.1785	2424.363	0.1845	76	113	133	LPMFGCTDATQVINEVEEVKK			Carbamidomethyl (C)[6], Oxidation (M)[3]		Mascot

5	RecName: Full=Ribulose bisphosphate carboxylase small chain clone 512; Short=RuBisCO small subunit	gi|132107	13274.5	5.84	11	309	100	36.132	212	100	

Peptide Information

Calc. Mass	Obsrv. Mass	± da	± ppm	Start Seq.	End Seq.	Sequence	Ion Score	C. I. %	Modification	Rank	Result Type
906.5043	906.5795	0.0752	83	14	20	QVDYLIR					Mascot
914.4229	914.481	0.0581	64	50	55	YWTMWK					Mascot
930.4178	930.4788	0.061	66	50	55	YWTMWK			Oxidation (M)[4]		Mascot
965.4873	965.5659	0.0786	81	85	92	IIGFDNMR					Mascot
965.4873	965.5659	0.0786	81	85	92	IIGFDNMR	25	0			Mascot
981.4822	981.5511	0.0689	70	85	92	IIGFDNMR			Oxidation (M)[7]		Mascot
981.4822	981.5511	0.0689	70	85	92	IIGFDNMR	15	0	Oxidation (M)[7]		Mascot
1012.4734	1012.5549	0.0815	80	77	84	EYPDAYVR					Mascot
1012.4734	1012.5549	0.0815	80	77	84	EYPDAYVR	59	99.748			Mascot
1140.5684	1140.6562	0.0878	77	76	84	KEYPDAYVR					Mascot
1165.571	1165.6488	0.0778	67	23	31	WVPCLEFSK			Carbamidomethyl (C)[4]		Mascot
1165.571	1165.6488	0.0778	67	23	31	WVPCLEFSK	29	0	Carbamidomethyl (C)[4]		Mascot
1365.5819	1365.696	0.1141	84	38	49	EHNASPGYYDGR	100	100			Mascot
2268.0789	2268.2593	0.1804	80	93	112	QVQCVSFIAFKPPGCEESGK			Carbamidomethyl (C)[4,15]		Mascot
2296.0837	2296.2825	0.1988	87	56	75	LPMFGCTDATQVINEVEEVK			Carbamidomethyl (C)[6], Oxidation (M)[3]		Mascot
2339.116	2339.302	0.186	80	93	113	QVQCVSFIAFKPPGCEESGKA			Carbamidomethyl (C)[4,15]		Mascot
2408.1836	2408.375	0.1914	79	56	76	LPMFGCTDATQVINEVEEVKK			Carbamidomethyl (C)[6]		Mascot
2424.1785	2424.363	0.1845	76	56	76	LPMFGCTDATQVINEVEEVKK			Carbamidomethyl (C)[6], Oxidation (M)[3]		Mascot

6	RecName: Full=Ribulose bisphosphate carboxylase small chain, chloroplastic; Short=RuBisCO small subunit; Flags: Precursor	gi|3914588	19692.8	8.98	12	297	100	36.261	212	100	

Peptide Information

Calc. Mass	Obsrv. Mass	± da	± ppm	Start	End	Sequence	Ion	C. I. %	Modification	Rank	Result Type

Calc. Mass	Obsrv. Mass	± da	± ppm	Start Seq.	End Seq.	Sequence	Ion Score	C. I. %	Modification	Rank	Result Type
906.5043	906.5795	0.0752	83	75	81	QVDYLIR					Mascot
914.4229	914.481	0.0581	64	111	116	YWTMWK					Mascot
930.4178	930.4788	0.061	66	111	116	YWTMWK			Oxidation (M)[4]		Mascot
965.4873	965.5659	0.0786	81	146	153	IIGFDNMR					Mascot
965.4873	965.5659	0.0786	81	146	153	IIGFDNMR	25	0			Mascot
981.4822	981.5511	0.0689	70	146	153	IIGFDNMR			Oxidation (M)[7]		Mascot
981.4822	981.5511	0.0689	70	146	153	IIGFDNMR	15	0	Oxidation (M)[7]		Mascot
1012.4734	1012.5549	0.0815	80	138	145	EYPDAYVR					Mascot
1012.4734	1012.5549	0.0815	80	138	145	EYPDAYVR	59	99.748			Mascot
1140.5684	1140.6562	0.0878	77	137	145	KEYPDAYVR					Mascot
1165.571	1165.6488	0.0778	67	84	92	WVPCLEFSK			Carbamidomethyl (C)[4]		Mascot
1165.571	1165.6488	0.0778	67	84	92	WVPCLEFSK	29	0	Carbamidomethyl (C)[4]		Mascot
1365.5819	1365.696	0.1141	84	99	110	EHNASPGYYDGR	100	100			Mascot
1862.9681	1862.9376	-0.0305	-16	2	20	APTVMASSATSVAPFQG LK					Mascot
1922.0521	1922.1836	0.1315	68	58	74	FETLSYLPPLSTEALLK					Mascot
2050.147	2050.2974	0.1504	73	57	74	KFETLSYLPPLSTEALLK					Mascot
2296.0837	2296.2825	0.1988	87	117	136	LPMFGCTDATQVLNEVE EVK			Carbamidomethyl (C)[6], Oxidation (M)[3]		Mascot
2408.1836	2408.375	0.1914	79	117	137	LPMFGCTDATQVLNEVE EVKK			Carbamidomethyl (C)[6]		Mascot
2424.1785	2424.363	0.1845	76	117	137	LPMFGCTDATQVLNEVE EVKK			Carbamidomethyl (C)[6], Oxidation (M)[3]		Mascot

7	Ribulose bisphosphate carboxylase small chain PW9, chloroplastic [Aegilops tauschii]	gi|475591676	15088.5	5.85	12	188	100	25.988	87	100	

Peptide Information

Calc. Mass	Obsrv. Mass	± da	± ppm	Start Seq.	End Seq.	Sequence	Ion Score	C. I. %	Modification	Rank	Result Type
906.5043	906.5795	0.0752	83	29	35	QVDYLIR					Mascot
914.4229	914.481	0.0581	64	65	70	YWTMWK					Mascot
930.4178	930.4788	0.061	66	65	70	YWTMWK			Oxidation (M)[4]		Mascot
933.5152	933.5845	0.0693	74	100	107	VIGFDNLR					Mascot
1012.4734	1012.5549	0.0815	80	92	99	EYPDAYVR					Mascot
1012.4734	1012.5549	0.0815	80	92	99	EYPDAYVR	59	99.748			Mascot
1140.5684	1140.6562	0.0878	77	91	99	KEYPDAYVR					Mascot
1165.571	1165.6488	0.0778	67	38	46	WVPCLEFSK			Carbamidomethyl (C)[4]		Mascot
1165.571	1165.6488	0.0778	67	38	46	WVPCLEFSK	29	0	Carbamidomethyl (C)[4]		Mascot
1197.6991	1197.6556	-0.0435	-36	2	11	QVWPIEGIKK					Mascot

Calc. Mass	Obsrv. Mass	± da	± ppm	Start Seq.	End Seq.	Sequence	Ion Score	C. I. %	Modification	Rank	Result Type
1381.5768	1381.6897	0.1129	82	53	64	EHNSSPGYYDGR					Mascot
1922.0521	1922.1836	0.1315	68	12	28	FETLSYLPPLSTEALLK					Mascot
2050.147	2050.2974	0.1504	73	11	28	KFETLSYLPPLSTEALLK					Mascot
2296.085	2296.2825	0.1975	86	108	127	QVQCVSFIAFRPPGCEES GK			Carbamidomethyl (C)[4,15]		Mascot
2408.1836	2408.375	0.1914	79	71	91	LPMFGCTDATQVLNEVE EVKK			Carbamidomethyl (C)[6]		Mascot
2424.1785	2424.363	0.1845	76	71	91	LPMFGCTDATQVLNEVE EVKK			Carbamidomethyl (C)[6], Oxidation (M)[3]		Mascot

8	Ribulose bisphosphate carboxylase small chain PW9, chloroplastic [Triticum urartu]	gi|473721334	19806.9	8.81	13	182	100	26.354	87	100

Peptide Information

Calc. Mass	Obsrv. Mass	± da	± ppm	Start Seq.	End Seq.	Sequence	Ion Score	C. I. %	Modification	Rank	Result Type
906.5043	906.5795	0.0752	83	76	82	QVDYLIR					Mascot
914.4229	914.481	0.0581	64	112	117	YWTMWK					Mascot
930.4178	930.4788	0.061	66	112	117	YWTMWK			Oxidation (M)[4]		Mascot
951.4717	951.5367	0.065	68	147	154	VIGFDNMR					Mascot
1012.4734	1012.5549	0.0815	80	139	146	EYPDAYVR					Mascot
1012.4734	1012.5549	0.0815	80	139	146	EYPDAYVR	59	99.748			Mascot
1140.5684	1140.6562	0.0878	77	138	146	KEYPDAYVR					Mascot
1165.571	1165.6488	0.0778	67	85	93	WVPCLEFSK			Carbamidomethyl (C)[4]		Mascot
1165.571	1165.6488	0.0778	67	85	93	WVPCLEFSK	29	0	Carbamidomethyl (C)[4]		Mascot
1262.6084	1262.7251	0.1167	92	31	44	SGSAGLSNVSNGGR					Mascot
1262.6084	1262.7251	0.1167	92	31	44	SGSAGLSNVSNGGR					Mascot
1381.5768	1381.6897	0.1129	82	100	111	EHNSSPGYYDGR					Mascot
1862.9681	1862.9376	-0.0305	-16	2	20	APAVMASSATTVAPFQG LK			Oxidation (M)[5]		Mascot
1922.0521	1922.1836	0.1315	68	59	75	FETLSYLPPLSTEALLK					Mascot
2050.147	2050.2974	0.1504	73	58	75	KFETLSYLPPLSTEALLK					Mascot
2296.085	2296.2825	0.1975	86	155	174	QVQCVSFIAFRPPGCEES GK			Carbamidomethyl (C)[4,15]		Mascot
2408.1836	2408.375	0.1914	79	118	138	LPMFGCTDATQVLNEVE EVKK			Carbamidomethyl (C)[6]		Mascot
2424.1785	2424.363	0.1845	76	118	138	LPMFGCTDATQVLNEVE EVKK			Carbamidomethyl (C)[6], Oxidation (M)[3]		Mascot

9	Ribulose bisphosphate carboxylase small chain PW9, chloroplastic [Aegilops tauschii]	gi|475591674	17979.8	5.1	12	181	100	24.985	87	100

Peptide Information

Calc. Mass	Obsrv. Mass	± da	± ppm	Start Seq.	End Seq.	Sequence	Ion Score	C. I. %	Modification	Rank	Result Type

906.5043	906.5795	0.0752	83	57	63	QVDYLIR					Mascot
914.4229	914.481	0.0581	64	93	98	YWTMWK					Mascot
930.4178	930.4788	0.061	66	93	98	YWTMWK			Oxidation (M)[4]		Mascot
951.4717	951.5367	0.065	68	128	135	VIGFDNMR					Mascot
1012.4734	1012.5549	0.0815	80	120	127	EYPDAYVR					Mascot
1012.4734	1012.5549	0.0815	80	120	127	EYPDAYVR	59	99.748			Mascot
1140.5684	1140.6562	0.0878	77	119	127	KEYPDAYVR					Mascot
1165.571	1165.6488	0.0778	67	66	74	WVPCLEFSK			Carbamidomethyl (C)[4]		Mascot
1165.571	1165.6488	0.0778	67	66	74	WVPCLEFSK	29	0	Carbamidomethyl (C)[4]		Mascot
1381.5768	1381.6897	0.1129	82	81	92	EHNSSPGYYDGR					Mascot
1922.0521	1922.1836	0.1315	68	40	56	FETLSYLPPLSTEALLK					Mascot
2050.147	2050.2974	0.1504	73	39	56	KFETLSYLPPLSTEALLK					Mascot
2290.1245	2290.2556	0.1311	57	1	23	MAQAIDDAVADPEPRPG GLGPGR					Mascot
2296.085	2296.2825	0.1975	86	136	155	QVQCVSFIAFRPPGCEES GK			Carbamidomethyl (C)[4,15]		Mascot
2408.1836	2408.375	0.1914	79	99	119	LPMFGCTDATQVLNEVE EVKK			Carbamidomethyl (C)[6]		Mascot
2424.1785	2424.363	0.1845	76	99	119	LPMFGCTDATQVLNEVE EVKK			Carbamidomethyl (C)[6], Oxidation (M)[3]		Mascot

10	Ribulose bisphosphate carboxylase small chain PWS4.3, chloroplastic [Triticum urartu]	gi|473868752	20787.4	8.98	13	180	100	25.244	87	100	

Peptide Information

Calc. Mass	Obsrv. Mass	± da	± ppm	Start Seq.	End Seq.	Sequence	Ion Score	C. I. %	Modification	Rank	Result Type
906.5043	906.5795	0.0752	83	87	93	QVDYLIR					Mascot
914.4229	914.481	0.0581	64	123	128	YWTMWK					Mascot
930.4178	930.4788	0.061	66	123	128	YWTMWK			Oxidation (M)[4]		Mascot
951.4717	951.5367	0.065	68	158	165	VIGFDNMR					Mascot
1012.4734	1012.5549	0.0815	80	150	157	EYPDAYVR					Mascot
1012.4734	1012.5549	0.0815	80	150	157	EYPDAYVR	59	99.748			Mascot
1140.5684	1140.6562	0.0878	77	149	157	KEYPDAYVR					Mascot
1165.571	1165.6488	0.0778	67	96	104	WVPCLEFSK			Carbamidomethyl (C)[4]		Mascot
1165.571	1165.6488	0.0778	67	96	104	WVPCLEFSK	29	0	Carbamidomethyl (C)[4]		Mascot
1187.6168	1187.6545	0.0377	32	21	32	STPGPPANPPPR					Mascot
1381.5768	1381.6897	0.1129	82	111	122	EHNSSPGYYDGR					Mascot
1862.9681	1862.9376	-0.0305	-16	2	20	APAVMASSATTVAPFQG LK			Oxidation (M)[5]		Mascot
1922.0521	1922.1836	0.1315	68	70	86	FETLSYLPPLSTEALLK					Mascot
2050.147	2050.2974	0.1504	73	69	86	KFETLSYLPPLSTEALLK					Mascot

2296.085	2296.2825	0.1975	86	166	185	QVQCVSFIAFRPPGCEES GK	Carbamidomethyl (C)[4,15]	Mascot
2408.1836	2408.375	0.1914	79	129	149	LPMFGCTDATQVLNEVE EVKK	Carbamidomethyl (C)[6]	Mascot
2424.1785	2424.363	0.1845	76	129	149	LPMFGCTDATQVLNEVE EVKK	Carbamidomethyl (C)[6], Oxidation (M)[3]	Mascot

Gel Idx/Pos	123/E22	Instr./Gel Origin	BA2151/Sample Project 20140814	Process Status	Analysis Succeeded
Plate [#] Name	[1] Sample Project 20140814	Instrument Sample Name		Spectra	11

Rank	Protein Name	Accession No.	Protein MW	Protein PI	Pep. Count	Protein Score	Protein Score C. I. %	Intensity Matched	Total Ion Score	Total Ion C. I. %	Confirmed
1	hypothetical protein F775_29044 [Aegilops tauschii]	gi|475615325	15440.7	5.92	12	472	100	38.477	369	100	

Peptide Information

Calc. Mass	Obsrv. Mass	± da	± ppm	Start Seq.	End Seq.	Sequence	Ion Score	C. I. %	Modification	Rank	Result Type
916.5032	916.5186	0.0154	17	1	9	MASGAVRPK					Mascot
935.4251	935.5002	0.0751	80	124	131	DCDGITVR			Carbamidomethyl (C)[2]		Mascot
1128.6122	1128.667	0.0548	49	10	18	LAYIVCYVK			Carbamidomethyl (C)[6]		Mascot
1400.7268	1400.8	0.0732	52	63	75	ETDALTGEVQLPK					Mascot
1495.7751	1495.8451	0.07	47	99	113	AVENGAVPVSAPEQK					Mascot
1524.7118	1524.8359	0.1241	81	23	36	SAAFYADAFGYSVR					Mascot
1524.7118	1524.8359	0.1241	81	23	36	SAAFYADAFGYSVR	131	100			Mascot
1584.7911	1584.8219	0.0308	19	124	137	DCDGITVRLGSHVR			Carbamidomethyl (C)[2]		Mascot
1651.8762	1651.9879	0.1117	68	98	113	RAVENGAVPVSAPEQK					Mascot
1895.0571	1894.9302	-0.1269	-67	2	18	ASGAVRPKLAYIVCYVK			Carbamidomethyl (C)[14]		Mascot
2004.8645	2005.0277	0.1632	81	81	97	GPVEICFDYDDVDAAYR			Carbamidomethyl (C)[6]		Mascot
2004.8645	2005.0277	0.1632	81	81	97	GPVEICFDYDDVDAAYR	58	99.725	Carbamidomethyl (C)[6]		Mascot
2144.0771	2144.2583	0.1812	85	44	62	WAELDTGSTTIAFTPLHQ R					Mascot
2144.0771	2144.2583	0.1812	85	44	62	WAELDTGSTTIAFTPLHQ R	180	100			Mascot
2290.0081	2290.1846	0.1765	77	79	97	ERGPVEICFDYDDVDAAY R			Carbamidomethyl (C)[8]		Mascot

Rank	Protein Name	Accession No.	Protein MW	Protein PI	Pep. Count	Protein Score	Protein Score C. I. %	Intensity Matched	Total Ion Score	Total Ion C. I. %	Confirmed
2	hypothetical protein F775_43781 [Aegilops tauschii]	gi|475517356	19762.9	8.8	8	171	100	3.502	128	100	

Peptide Information

Calc. Mass	Obsrv. Mass	± da	± ppm	Start Seq.	End Seq.	Sequence	Ion Score	C. I. %	Modification	Rank	Result Type
906.5043	906.5706	0.0663	73	76	82	QVDYLIR					Mascot
914.4229	914.4921	0.0692	76	112	117	YWTMWK					Mascot
965.4873	965.5562	0.0689	71	147	154	IIGFDNMR					Mascot
981.4822	981.5389	0.0567	58	147	154	IIGFDNMR			Oxidation (M)[7]		Mascot
1012.4734	1012.5547	0.0813	80	139	146	EYPDAYVR					Mascot
1012.4734	1012.5547	0.0813	80	139	146	EYPDAYVR	26	0			Mascot
1165.571	1165.6528	0.0818	70	85	93	WVPCLEFSK			Carbamidomethyl (C)[4]		Mascot

Calc. Mass	Obsrv. Mass	± da	± ppm	Start Seq.	End Seq.	Sequence	Ion Score	C. I. %	Modification	Rank	Result Type
1262.6084	1262.6996	0.0912	72	31	44	SNGASLGSVSNGGR					Mascot
1365.5819	1365.6908	0.1089	80	100	111	EHNASPGYYDGR	103	100			Mascot
1862.9681	1862.929	-0.0391	-21	2	20	APTVMASSATSVAPFQG LK					Mascot

3	RecName: Full=Ribulose bisphosphate carboxylase small chain clone 512; Short=RuBisCO small subunit	gi|132107	13274.5	5.84	6	165	100	3.098	128	100	

Peptide Information

Calc. Mass	Obsrv. Mass	± da	± ppm	Start Seq.	End Seq.	Sequence	Ion Score	C. I. %	Modification	Rank	Result Type
906.5043	906.5706	0.0663	73	14	20	QVDYLIR					Mascot
914.4229	914.4921	0.0692	76	50	55	YWTMWK					Mascot
965.4873	965.5562	0.0689	71	85	92	IIGFDNMR					Mascot
981.4822	981.5389	0.0567	58	85	92	IIGFDNMR			Oxidation (M)[7]		Mascot
1012.4734	1012.5547	0.0813	80	77	84	EYPDAYVR					Mascot
1012.4734	1012.5547	0.0813	80	77	84	EYPDAYVR	26	0			Mascot
1165.571	1165.6528	0.0818	70	23	31	WVPCLEFSK			Carbamidomethyl (C)[4]		Mascot
1365.5819	1365.6908	0.1089	80	38	49	EHNASPGYYDGR	103	100			Mascot

4	RecName: Full=Ribulose bisphosphate carboxylase small chain, chloroplastic; Short=RuBisCO small subunit; Flags: Precursor	gi|3914588	19692.8	8.98	7	162	100	3.382	128	100	

Peptide Information

Calc. Mass	Obsrv. Mass	± da	± ppm	Start Seq.	End Seq.	Sequence	Ion Score	C. I. %	Modification	Rank	Result Type
906.5043	906.5706	0.0663	73	75	81	QVDYLIR					Mascot
914.4229	914.4921	0.0692	76	111	116	YWTMWK					Mascot
965.4873	965.5562	0.0689	71	146	153	IIGFDNMR					Mascot
981.4822	981.5389	0.0567	58	146	153	IIGFDNMR			Oxidation (M)[7]		Mascot
1012.4734	1012.5547	0.0813	80	138	145	EYPDAYVR					Mascot
1012.4734	1012.5547	0.0813	80	138	145	EYPDAYVR	26	0			Mascot
1165.571	1165.6528	0.0818	70	84	92	WVPCLEFSK			Carbamidomethyl (C)[4]		Mascot
1365.5819	1365.6908	0.1089	80	99	110	EHNASPGYYDGR	103	100			Mascot
1862.9681	1862.929	-0.0391	-21	2	20	APTVMASSATSVAPFQG LK					Mascot

5	Ribulose bisphosphate carboxylase small chain, chloroplastic [Triticum urartu]	gi|473882355	18742.4	8.65	7	160	100	3.382	128	100	

Peptide Information

Calc. Mass	Obsrv. Mass	± da	± ppm	Start Seq.	End Seq.	Sequence	Ion Score	C. I. %	Modification	Rank	Result Type

Calc. Mass	Obsrv. Mass	± da	± ppm	Start Seq.	End Seq.	Sequence	Ion Score	C. I. %	Modification	Rank	Result Type
906.5043	906.5706	0.0663	73	65	71	QVDYLIR					Mascot
914.4229	914.4921	0.0692	76	101	106	YWTMWK					Mascot
965.4873	965.5562	0.0689	71	136	143	IIGFDNMR					Mascot
981.4822	981.5389	0.0567	58	136	143	IIGFDNMR			Oxidation (M)[7]		Mascot
1012.4734	1012.5547	0.0813	80	128	135	EYPDAYVR					Mascot
1012.4734	1012.5547	0.0813	80	128	135	EYPDAYVR	26	0			Mascot
1165.571	1165.6528	0.0818	70	74	82	WVPCLEFSK			Carbamidomethyl (C)[4]		Mascot
1365.5819	1365.6908	0.1089	80	89	100	EHNASPGYYDGR	103	100			Mascot
1862.9681	1862.929	-0.0391	-21	2	20	APTVMASSATSVAPFQG LK					Mascot

6	Ribulose bisphosphate carboxylase small chain, chloroplastic [Triticum urartu]	gi|474416311	15090.5	5.85	6	160	100	3.098	128	100	

Peptide Information

Calc. Mass	Obsrv. Mass	± da	± ppm	Start Seq.	End Seq.	Sequence	Ion Score	C. I. %	Modification	Rank	Result Type
906.5043	906.5706	0.0663	73	29	35	QVDYLIR					Mascot
914.4229	914.4921	0.0692	76	65	70	YWTMWK					Mascot
965.4873	965.5562	0.0689	71	100	107	IIGFDNMR					Mascot
981.4822	981.5389	0.0567	58	100	107	IIGFDNMR			Oxidation (M)[7]		Mascot
1012.4734	1012.5547	0.0813	80	92	99	EYPDAYVR					Mascot
1012.4734	1012.5547	0.0813	80	92	99	EYPDAYVR	26	0			Mascot
1165.571	1165.6528	0.0818	70	38	46	WVPCLEFSK			Carbamidomethyl (C)[4]		Mascot
1365.5819	1365.6908	0.1089	80	53	64	EHNASPGYYDGR	103	100			Mascot

7	Ribulose bisphosphate carboxylase small chain PWS4.3, chloroplastic [Aegilops tauschii]	gi|475604963	19161.6	8.58	6	157	100	3.098	128	100	

Peptide Information

Calc. Mass	Obsrv. Mass	± da	± ppm	Start Seq.	End Seq.	Sequence	Ion Score	C. I. %	Modification	Rank	Result Type
906.5043	906.5706	0.0663	73	71	77	QVDYLIR					Mascot
914.4229	914.4921	0.0692	76	107	112	YWTMWK					Mascot
965.4873	965.5562	0.0689	71	142	149	IIGFDNMR					Mascot
981.4822	981.5389	0.0567	58	142	149	IIGFDNMR			Oxidation (M)[7]		Mascot
1012.4734	1012.5547	0.0813	80	134	141	EYPDAYVR					Mascot
1012.4734	1012.5547	0.0813	80	134	141	EYPDAYVR	26	0			Mascot
1165.571	1165.6528	0.0818	70	80	88	WVPCLEFSK			Carbamidomethyl (C)[4]		Mascot
1365.5819	1365.6908	0.1089	80	95	106	EHNASPGYYDGR	103	100			Mascot

8	Chain D, 0.19 Alpha-Amylase Inhibitor From Wheat	gi|3318684	13898.6	6.66	5	133	100	4.086	103	100	

Protein Group

Chain A, 0.19 Alpha-Amylase Inhibitor From Wheat	gi|3318681	13898.6	6.6599998474 1211
Chain B, 0.19 Alpha-Amylase Inhibitor From Wheat	gi|3318682	13898.6	6.6599998474 1211
Chain C, 0.19 Alpha-Amylase Inhibitor From Wheat	gi|3318683	13898.6	6.6599998474 1211
RecName: Full=Alpha-amylase inhibitor 0.19; AltName: Full=0.19 alpha-AI; Short=0.19 AI	gi|123963	13898.6	6.6599998474 1211
dimeric alpha-amylase inhibitor, partial [Aegilops geniculata]	gi|452055912	14198.8	6.6599998474 1211
dimeric alpha-amylase inhibitor, partial [Aegilops kotschyi]	gi|386877048	14198.8	6.6599998474 1211

Peptide Information

Calc. Mass	Obsrv. Mass	± da	± ppm	Start Seq.	End Seq.	Sequence	Ion Score	C. I. %	Modification	Rank	Result Type
1162.6249	1162.7157	0.0908	78	90	100	LTAASITAVCR			Carbamidomethyl (C)[10]		Mascot
1570.8007	1570.91	0.1093	70	26	39	LQCNGSQVPEAVLR			Carbamidomethyl (C)[3]		Mascot
1612.7463	1612.8781	0.1318	82	67	82	EHGAQEGQAGTGAFPR					Mascot
1612.7463	1612.8781	0.1318	82	67	82	EHGAQEGQAGTGAFPR	103	100			Mascot
1663.8361	1663.9304	0.0943	57	101	116	LPIVVDASGDGAYVCK			Carbamidomethyl (C)[15]		Mascot
1862.7731	1862.929	0.1559	84	40	53	DCCQQLAHISEWCR			Carbamidomethyl (C)[2,3,13]		Mascot

9	dimeric alpha-amylase inhibitor, partial [Aegilops tauschii]	gi|386877046	14670	6.08	5	132	100	4.086	103	100	

Protein Group

dimeric alpha-amylase inhibitor, partial [Aegilops geniculata]	gi|386877062	14542.9	7.0500001907 3486
dimeric alpha-amylase inhibitor, partial [Aegilops longissima]	gi|386877060	14954.2	7.6399998664 856
dimeric alpha-amylase inhibitor, partial [Aegilops tauschii]	gi|386877050	14826.1	6.8600001335 144
dimeric alpha-amylase inhibitor, partial [Aegilops tauschii]	gi|386877044	14805	6.0999999046 3257

Peptide Information

Calc. Mass	Obsrv. Mass	± da	± ppm	Start Seq.	End Seq.	Sequence	Ion Score	C. I. %	Modification	Rank	Result Type
1162.6249	1162.7157	0.0908	78	97	107	LTAASITAVCR			Carbamidomethyl (C)[10]		Mascot
1570.8007	1570.91	0.1093	70	33	46	LQCNGSQVPEAVLR			Carbamidomethyl (C)[3]		Mascot
1612.7463	1612.8781	0.1318	82	74	89	EHGAQEGQAGTGAFPR					Mascot
1612.7463	1612.8781	0.1318	82	74	89	EHGAQEGQAGTGAFPR	103	100			Mascot
1663.8361	1663.9304	0.0943	57	108	123	LPIVVDASGDGAYVCK			Carbamidomethyl (C)[15]		Mascot
1862.7731	1862.929	0.1559	84	47	60	DCCQQLAHISEWCR			Carbamidomethyl (C)[2,3,13]		Mascot

10 dimeric alpha-amylase inhibitor [Triticum aestivum] gi|386877038 15702.5 5.58 5 131 100 4.086 103 100

Peptide Information

Calc. Mass	Obsrv. Mass	± da	± ppm	Start Seq.	End Seq.	Sequence	Ion Score	C. I. %	Modification	Rank	Result Type
1162.6249	1162.7157	0.0908	78	107	117	LTAASITAVCR			Carbamidomethyl (C)[10]		Mascot
1570.8007	1570.91	0.1093	70	43	56	LQCNGSQVPEAVLR			Carbamidomethyl (C)[3]		Mascot
1612.7463	1612.8781	0.1318	82	84	99	EHGAQEGQAGTGAFPR					Mascot
1612.7463	1612.8781	0.1318	82	84	99	EHGAQEGQAGTGAFPR	103	100			Mascot
1663.8361	1663.9304	0.0943	57	118	133	LPIVVDASGDGAYVCK			Carbamidomethyl (C)[15]		Mascot
1862.7731	1862.929	0.1559	84	57	70	DCCQQLAHISEWCR			Carbamidomethyl (C)[2,3,13]		Mascot

Rank	Protein Name	Accession No.	Protein MW	Protein PI	Pep. Count	Protein Score	Protein Score C. I. %	Intensity Matched	Total Ion Score	Total Ion C. I. %	Confirmed
1	Globulin-1 S allele [Triticum urartu]	gi|474411419	57108.4	9.1	6	281	100	41.908	269	100	

Peptide Information

Calc. Mass	Obsrv. Mass	± da	± ppm	Start Seq.	End Seq.	Sequence	Ion Score	C. I. %	Modification	Rank	Result Type
906.468	906.5512	0.0832	92	457	463	EVQEVFR					Mascot
959.4476	959.4211	-0.0265	-28	48	54	CQQDRPR			Carbamidomethyl (C)[1]		Mascot
1105.6001	1105.6703	0.0702	63	457	465	EVQEVFRAK					Mascot
1164.444	1164.4379	-0.0061	-5	349	357	WGEEEEDDR					Mascot
1822.8752	1823.0492	0.174	95	411	426	GSSNLQVVCFEINAER			Carbamidomethyl (C)[9]		Mascot
1822.8752	1823.0492	0.174	95	411	426	GSSNLQVVCFEINAER	119	100	Carbamidomethyl (C)[9]		Mascot
1906.0182	1906.2008	0.1826	96	392	410	GSAFVVPPGHPVVEIASSR					Mascot
1906.0182	1906.2008	0.1826	96	392	410	GSAFVVPPGHPVVEIASSR	150	100			Mascot

Rank	Protein Name	Accession No.	Protein MW	Protein PI	Pep. Count	Protein Score	Protein Score C. I. %	Intensity Matched	Total Ion Score	Total Ion C. I. %	Confirmed
2	GLK8 G2-like transcription factor, partial [Zea mays subsp. mays]	gi|407232728	38577.1	5.23	5	44	0	6.707	30	8.557	

Protein Group

putative MYB DNA-binding domain superfamily protein [Zea mays]	gi|413932974	38705.2	5.32999992370605
putative MYB DNA-binding domain superfamily protein [Zea mays]	gi|413932973	38577.1	5.23000001907349
uncharacterized protein LOC100280246 [Zea mays]	gi|226508792	38705.2	5.32999992370605

Peptide Information

Calc. Mass	Obsrv. Mass	± da	± ppm	Start Seq.	End Seq.	Sequence	Ion Score	C. I. %	Modification	Rank	Result Type
888.4897	888.5419	0.0522	59	44	50	SLEVERR					Mascot
898.4152	898.4913	0.0761	85	352	359	SEDGYSLK					Mascot
1765.8578	1766.0272	0.1694	96	97	111	KWMSTAQLWVDSDAK					Mascot
1818.8949	1818.995	0.1001	55	25	43	SAGGMAKGAAPAGIQSCIR			Carbamidomethyl (C)[17], Oxidation (M)[5]		Mascot
1989.9984	1990.1107	0.1123	56	1	19	MGLDVGEIGMGLDLGLDLR			Oxidation (M)[1]		Mascot
1989.9984	1990.1107	0.1123	56	1	19	MGLDVGEIGMGLDLGLD	32	34.059	Oxidation (M)[10]		Mascot

LR

3	predicted protein [Arabidopsis lyrata subsp. lyrata]	gi|297320530	10653.6	10.29	6	39	0	.413

Protein Group

predicted protein [Arabidopsis lyrata subsp. lyrata]	gi|297323545	10653.6	10.289 999961 853

Peptide Information

Calc. Mass	Obsrv. Mass	± da	± ppm	Start Seq.	End Seq.	Sequence	Ion Score	C. I. %	Modification	Rank	Result Type
922.5468	922.5396	-0.0072	-8	52	61	GAGLAPLPAR					Mascot
944.4724	944.5067	0.0343	36	74	81	YSTGIFEK					Mascot
959.5098	959.4211	-0.0887	-92	2	8	VWVDVWR					Mascot
1765.9307	1766.0272	0.0965	55	37	51	VCQWKLASLFTPSTK			Carbamidomethyl (C)[2]		Mascot
1968.1277	1968.1108	-0.0169	-9	42	61	LASLFTPSTKGAGLAPLP AR					Mascot
1995.9528	1996.1115	0.1587	80	18	34	FGPNSMNKQVSFHALMR			Oxidation (M)[6,16]		Mascot

4	hypothetical protein M569_12410, partial [Genlisea aurea]	gi|527191628	74062.9	8.37	10	38	0	2.153

Peptide Information

Calc. Mass	Obsrv. Mass	± da	± ppm	Start Seq.	End Seq.	Sequence	Ion Score	C. I. %	Modification	Rank	Result Type
807.4029	807.4763	0.0734	91	366	372	SMESVVR					Mascot
864.4971	864.493	-0.0041	-5	306	312	SLITKMR			Oxidation (M)[6]		Mascot
876.4574	876.5278	0.0704	80	342	349	FPLDASAR					Mascot
955.4955	955.5062	0.0107	11	374	381	HEKELGSR					Mascot
955.4955	955.5062	0.0107	11	374	381	HEKELGSR	19	0			Mascot
959.425	959.4211	-0.0039	-4	446	454	SAAEDMHAK					Mascot
1105.6034	1105.6703	0.0669	61	363	372	AVKSMESVVR					Mascot
1805.9678	1806.074	0.1062	59	172	187	SMQETVKLIVEQVSSK					Mascot
1815.0045	1814.9138	-0.0907	-50	382	397	LLLSSTEKNVVSIMHK			Oxidation (M)[14]		Mascot
1821.9932	1822.0182	0.025	14	112	126	SPVMELEHLNWLLIK					Mascot
1997.9525	1997.9628	0.0103	5	38	54	TMTSVYLKYFETAQDGK			Oxidation (M)[2]		Mascot

5	RNA binding protein [Arabidopsis lyrata subsp. lyrata]	gi|297319699	89738.9	8.33	9	35	0	26.699

Peptide Information

Calc. Mass	Obsrv. Mass	± da	± ppm	Start Seq.	End Seq.	Sequence	Ion Score	C. I. %	Modification	Rank	Result Type
931.4414	931.3854	-0.056	-60	1	8	MGSSRHEK					Mascot

Calc. Mass	Obsrv. Mass	± da	± ppm	Start Seq.	End Seq.	Sequence	Ion Score	C. I. %	Modification	Rank	Result Type
944.5087	944.5067	-0.002	-2	629	636	YYTVLASK					Mascot
955.4744	955.5062	0.0318	33	21	27	HESWLQR					Mascot
955.4744	955.5062	0.0318	33	21	27	HESWLQR	19	0			Mascot
1333.7322	1333.7976	0.0654	49	36	48	VSSSAQKTGEVIK					Mascot
1333.7322	1333.7976	0.0654	49	36	48	VSSSAQKTGEVIK					Mascot
1361.7457	1361.8301	0.0844	62	480	491	MLETISAIKNNK					Mascot
1822.886	1823.0492	0.1632	90	611	624	EIMRVLVECCLQEK			Carbamidomethyl (C)[9,10], Oxidation (M)[3]		Mascot
1822.886	1823.0492	0.1632	90	611	624	EIMRVLVECCLQEK			Carbamidomethyl (C)[9,10], Oxidation (M)[3]		Mascot
1846.8385	1846.9899	0.1514	82	424	439	VDAFTITTVLDCCGMK			Carbamidomethyl (C)[12,13], Oxidation (M)[15]		Mascot
1921.01	1921.1929	0.1829	95	442	458	SDDPLAMKTFIISIQNK					Mascot
1997.9849	1997.9628	-0.0221	-11	304	321	MAESNVETITAELASIYR					Mascot
2013.9797	2014.0593	0.0796	40	304	321	MAESNVETITAELASIYR			Oxidation (M)[1]		Mascot

6	hypothetical protein, partial [Hordeum vulgare subsp. vulgare]	gi|425856134	10623.6	9.43	5	35	0	.619	

Peptide Information

Calc. Mass	Obsrv. Mass	± da	± ppm	Start Seq.	End Seq.	Sequence	Ion Score	C. I. %	Modification	Rank	Result Type
898.4418	898.4913	0.0495	55	90	96	WLHTVDQ					Mascot
967.5684	967.5065	-0.0619	-64	19	27	HVTATVALR					Mascot
1681.996	1681.96	-0.036	-21	28	45	VGAAVASLAAAVLVVTNR					Mascot
1804.9474	1805.0872	0.1398	77	2	18	VELESQNAATMKVASVK					Mascot
1967.9777	1968.1108	0.1331	68	1	18	MVELESQNAATMKVASVK			Oxidation (M)[1,12]		Mascot

7	hypothetical protein OsI_20846 [Oryza sativa Indica Group]	gi|218197204	71825.3	5.36	8	35	0	1.953	

Peptide Information

Calc. Mass	Obsrv. Mass	± da	± ppm	Start Seq.	End Seq.	Sequence	Ion Score	C. I. %	Modification	Rank	Result Type
864.5189	864.493	-0.0259	-30	189	195	IQYSLIK					Mascot
922.5104	922.5396	0.0292	32	344	351	AKQVHPDK					Mascot
934.5356	934.582	0.0464	50	621	629	LGTIFQGAK					Mascot
955.4414	955.5062	0.0648	68	34	42	EHGVPMGGR			Oxidation (M)[6]		Mascot
955.4414	955.5062	0.0648	68	34	42	EHGVPMGGR	19	0	Oxidation (M)[6]		Mascot
959.4429	959.4211	-0.0218	-23	114	122	LSDTHGSDK					Mascot
1818.8148	1818.995	0.1802	99	5	24	RGSGGAGADQEGGVPMGGSR			Oxidation (M)[16]		Mascot
1953.9409	1954.1312	0.1903	97	235	250	NYDMVPTCIVSLEEKR			Carbamidomethyl (C)[8]		Mascot

2026.0176	2026.0072	-0.0104	-5	296	317	AGHVAATGAGEIQGGGF PAMVK					Mascot

8	MIF4G domain and MA3 domain-containing protein [Arabidopsis thaliana]	gi|79515670	90577.2	6.83	9	34	0	19.583	

Protein Group

MIF4G domain and MA3 domain-containing protein [Arabidopsis thaliana]	gi|332005105	90577.2	6.8299 999237 0605

Peptide Information

Calc. Mass	Obsrv. Mass	± da	± ppm	Start Seq.	End Seq.	Sequence	Ion Score	C. I. %	Modification	Rank	Result Type
864.4421	864.493	0.0509	59	36	44	VSSSASAQK					Mascot
898.5217	898.4913	-0.0304	-34	104	110	DLNRPRK					Mascot
931.4414	931.3854	-0.056	-60	1	8	MGSSRHEK					Mascot
955.4744	955.5062	0.0318	33	21	27	HESWLQR					Mascot
955.4744	955.5062	0.0318	33	21	27	HESWLQR	19	0			Mascot
1361.7457	1361.8301	0.0844	62	488	499	MLETISAIKNNK					Mascot
1822.886	1823.0492	0.1632	90	619	632	EIMRVLVECCLQEK			Carbamidomethyl (C)[9,10], Oxidation (M)[3]		Mascot
1822.886	1823.0492	0.1632	90	619	632	EIMRVLVECCLQEK			Carbamidomethyl (C)[9,10], Oxidation (M)[3]		Mascot
1846.8385	1846.9899	0.1514	82	432	447	VDAFTITTVLDCCGMK			Carbamidomethyl (C)[12,13], Oxidation (M)[15]		Mascot
1921.01	1921.1929	0.1829	95	450	466	SDDPLAMKTFIISIQNK					Mascot
1989.9586	1990.1107	0.1521	76	334	350	SVSSQIFCEEVLTTYAR			Carbamidomethyl (C)[8]		Mascot
1989.9586	1990.1107	0.1521	76	334	350	SVSSQIFCEEVLTTYAR			Carbamidomethyl (C)[8]		Mascot

9	PREDICTED: uncharacterized protein LOC101219885 [Cucumis sativus]	gi|449449288	78080	5.01	10	34	0	16.146	

Peptide Information

Calc. Mass	Obsrv. Mass	± da	± ppm	Start Seq.	End Seq.	Sequence	Ion Score	C. I. %	Modification	Rank	Result Type
934.5104	934.582	0.0716	77	447	453	KQQQQFK					Mascot
979.4955	979.4661	-0.0294	-30	650	658	HHVSLDGSK					Mascot
1333.7079	1333.7976	0.0897	67	437	447	QQMQAMLAIRK			Oxidation (M)[3]		Mascot
1333.7726	1333.7976	0.025	19	522	532	LQEIISKLDFK					Mascot
1345.7191	1345.8385	0.1194	89	436	446	RQQMQAMLAIR					Mascot
1349.7029	1349.7954	0.0925	69	437	447	QQMQAMLAIRK			Oxidation (M)[3,6]		Mascot
1361.714	1361.8301	0.1161	85	436	446	RQQMQAMLAIR			Oxidation (M)[4]		Mascot
1765.9457	1766.0272	0.0815	46	349	362	HRQLQGLEPLQYQR					Mascot
1790.9105	1790.9614	0.0509	28	184	199	DLGNSPMKLFPTVESR					Mascot

1790.9105	1790.9614	0.0509	28	184	199	DLGNSPMKLFPTVESR	10	0			Mascot
1967.9202	1968.1108	0.1906	97	632	649	ADLKSSPMECLPYNASG K			Carbamidomethyl (C)[10]		Mascot
2017.9574	2018.1365	0.1791	89	101	118	GTEVSHELTGNREYNSP K					Mascot
2840.3843	2840.5842	0.1999	70	454	478	NQVSTSSQSISPKCPQEI QSQHIEK			Carbamidomethyl (C)[14]		Mascot

10	uncharacterized protein [Arabidopsis thaliana]	gi|18399392	50021.1	5.77	3	34	0	5.76	27	0

Protein Group

uncharacterized protein AT3G11880 [Arabidopsis thaliana]	gi|332641591	50021.1	5.7699 999809 2651

Peptide Information

Calc. Mass	Obsrv. Mass	± da	± ppm	Start Seq.	End Seq.	Sequence	Ion Score	C. I. %	Modification	Rank	Result Type
944.5775	944.5067	-0.0708	-75	346	354	EASVAVLKK					Mascot
1120.4542	1120.4333	-0.0209	-19	159	167	EDEYQGHDK					Mascot
1990.1307	1990.1107	-0.02	-10	127	144	TTSKFHVAVFVALAMVLR					Mascot
1990.1307	1990.1107	-0.02	-10	127	144	TTSKFHVAVFVALAMVLR	27	0			Mascot

Gel Idx/Pos	125/E24	Instr./Gel Origin	BA2151/Sample Project 20140814	Process Status	Analysis Succeeded
Plate [#] Name	[1] Sample Project 20140814	Instrument Sample Name		Spectra	11

Rank	Protein Name	Accession No.	Protein MW	Protein PI	Pep. Count	Protein Score	Protein Score C. I. %	Intensity Matched	Total Ion Score	Total Ion C. I. %	Confirmed
1	hypothetical protein OsI_15081 [Oryza sativa Indica Group]	gi|218194450	214506.6	7.87	31	62	41.348	10.642			

Peptide Information

Calc. Mass	Obsrv. Mass	± da	± ppm	Start Seq.	End Seq.	Sequence	Ion Score	C. I. %	Modification	Rank	Result Type
807.4108	807.4669	0.0561	69	1554	1561	GAQYGVGR					Mascot
813.4689	813.5146	0.0457	56	1725	1731	QGIPRSR					Mascot
815.4621	815.5014	0.0393	48	170	176	ELIIGDR					Mascot
819.4658	819.4697	0.0039	5	1678	1683	FPRMLR					Mascot
826.457	826.5173	0.0603	73	1975	1981	RWLASPP					Mascot
830.4771	830.4927	0.0156	19	844	850	ISSIPWK					Mascot
837.4941	837.4911	-0.003	-4	737	743	HTPLITR					Mascot
842.5206	842.5815	0.0609	72	1633	1639	VDAIIRR					Mascot
850.4529	850.5135	0.0606	71	1497	1503	ALPQHER					Mascot
865.5618	865.515	-0.0468	-54	1916	1923	LRPPAALK					Mascot
888.5261	888.5284	0.0023	3	580	587	TRQAAITK					Mascot
973.5789	973.6044	0.0255	26	207	215	ASSIKNVVR					Mascot
1033.5215	1033.5986	0.0771	75	1403	1411	AGATWVWSR					Mascot
1036.5535	1036.6017	0.0482	47	1554	1563	GAQYGVGRTK					Mascot
1060.5457	1060.6432	0.0975	92	157	166	SIDAMVPVGR			Oxidation (M)[5]		Mascot
1073.6102	1073.6378	0.0276	26	1455	1463	VRPFADLQK					Mascot
1234.6652	1234.7744	0.1092	88	1494	1503	DLRALPQHER					Mascot
1259.7318	1259.7604	0.0286	23	775	786	VTVLSDTAIRGK					Mascot
1300.5839	1300.682	0.0981	75	1621	1632	AYSGLTDAEMAR			Oxidation (M)[10]		Mascot
1307.681	1307.785	0.104	80	1542	1553	ALGVEGMMLKAR			Oxidation (M)[7,8]		Mascot
1323.6614	1323.7491	0.0877	66	616	626	DQMPKVYDALK			Oxidation (M)[3]		Mascot
1329.658	1329.7509	0.0929	70	1713	1724	QAIPMTSVSHSR			Oxidation (M)[5]		Mascot
1383.8108	1383.7797	-0.0311	-22	1155	1167	VFVTHGSVAVLVR					Mascot
1427.8409	1427.8966	0.0557	39	1298	1309	LVYFKLITGAFR					Mascot
1493.7782	1493.8571	0.0789	53	193	206	GQGVTCIYVAIGQK			Carbamidomethyl (C)[6]		Mascot
1699.9048	1699.9738	0.069	41	659	675	GLMVSNTGNPITVPVGK			Oxidation (M)[3]		Mascot

1791.0123	1790.9393	-0.073	-41	112	129	VVNALGQPIDGKGPINAK					Mascot
1839.0752	1839.0751	-0.0001	0	1155	1170	VFVTHGSVAVLVRWLR					Mascot
1926.9855	1927.046	0.0605	31	1275	1291	GLAPDALEPMLRAQWSR			Oxidation (M)[10]		Mascot
1990.0604	1990.0985	0.0381	19	561	579	AATDNAGNVIGELKLVYNK					Mascot
2152.1584	2152.2019	0.0435	20	1084	1103	LLQTALVVAPPSAQGTPWMR			Oxidation (M)[19]		Mascot

2 Globulin-1 S allele [Triticum urartu] gi|474411419 57108.4 9.1 9 58 0 4.188 35 56.059

Peptide Information

Calc. Mass	Obsrv. Mass	± da	± ppm	Start Seq.	End Seq.	Sequence	Ion Score	C. I. %	Modification	Rank	Result Type
823.4645	823.5065	0.042	51	269	275	IANRHGR					Mascot
837.4101	837.4911	0.081	97	276	282	LYEADAR					Mascot
906.468	906.5355	0.0675	74	457	463	EVQEVFR					Mascot
990.5214	990.5675	0.0461	47	195	203	AALKTSDER					Mascot
1390.7285	1390.797	0.0685	49	199	211	TSDERLGSLLGSR					Mascot
1699.9418	1699.9738	0.032	19	176	190	FQYFSAKPLLASLSK					Mascot
1791.8984	1791.8975	-0.0009	-1	254	268	GDSRDTYNLLEQRPK					Mascot
1822.8752	1823.0372	0.162	89	411	426	GSSNLQVVCFEINAER			Carbamidomethyl (C)[9]		Mascot
1906.0182	1906.1669	0.1487	78	392	410	GSAFVVPPGHPVVEIASSR					Mascot
1906.0182	1906.1669	0.1487	78	392	410	GSAFVVPPGHPVVEIASSR	35	56.059			Mascot

3 TPA: hypothetical protein ZEAMMB73_529703 [Zea mays] gi|414590076 60892.4 9.27 13 56 0 5.239

Peptide Information

Calc. Mass	Obsrv. Mass	± da	± ppm	Start Seq.	End Seq.	Sequence	Ion Score	C. I. %	Modification	Rank	Result Type
807.4103	807.4669	0.0566	70	132	138	DMAIVMK					Mascot
833.3887	833.39	0.0013	2	32	38	EEEVEAK					Mascot
857.5203	857.5762	0.0559	65	101	107	RVQLVDK					Mascot
900.5261	900.6081	0.082	91	269	276	QGRVLEAK					Mascot
905.4686	905.5175	0.0489	54	471	478	KSLSAEDR					Mascot
973.52	973.6044	0.0844	87	143	151	AEEAIEAIK					Mascot
1068.587	1068.6014	0.0144	13	1	10	MPPATSVLPR					Mascot
1232.5978	1232.6953	0.0975	79	460	470	AQSIDENSGRR					Mascot
1329.7372	1329.7509	0.0137	10	143	154	AEEAIEAIKSLR					Mascot
1333.678	1333.7825	0.1045	78	303	313	AQEMLRDLETK					Mascot
1365.6614	1365.7504	0.089	65	132	142	DMAIVMKQQNR			Oxidation (M)[2,6]		Mascot

1365.6614	1365.7504	0.089	65	132	142	DMAIVMKQQNR	18	0	Oxidation (M)[2,6]		Mascot
1513.885	1513.8662	-0.0188	-12	429	442	KKPAPVHDPLGNLK					Mascot
1716.8512	1716.9916	0.1404	82	453	469	AGAVVDKAQSIDENSGR					Mascot
1716.8512	1716.9916	0.1404	82	453	469	AGAVVDKAQSIDENSGR					Mascot

4	conserved hypothetical protein [Ricinus communis]	gi|223512446	9835.2	11.4	8	56	0	1.855

Peptide Information

Calc. Mass	Obsrv. Mass	± da	± ppm	Start Seq.	End Seq.	Sequence	Ion Score	C. I. %	Modification	Rank	Result Type
800.4737	800.4716	-0.0021	-3	11	17	TPSARIR					Mascot
815.4482	815.5014	0.0532	65	18	24	TPSQRAR					Mascot
857.4952	857.5762	0.081	94	16	22	IRTPSQR					Mascot
1073.6466	1073.6378	-0.0088	-8	44	54	IAFAAGVVAVR					Mascot
1383.7526	1383.7797	0.0271	20	74	86	LAGRADVAVVPCR			Carbamidomethyl (C)[12]		Mascot
1427.7866	1427.8966	0.11	77	65	77	VAFPQQQGRLAGR					Mascot
1584.8051	1584.8693	0.0642	41	78	92	ADVAVVPCRVDIAAE			Carbamidomethyl (C)[8]		Mascot
1699.8181	1699.9738	0.1557	92	1	15	MGLNTLDHDRTPSAR			Oxidation (M)[1]		Mascot

5	PREDICTED: mitochondrial adenine nucleotide transporter ADNT1-like [Solanum lycopersicum]	gi|460400027	38662.2	9.42	12	55	0	3.783

Peptide Information

Calc. Mass	Obsrv. Mass	± da	± ppm	Start Seq.	End Seq.	Sequence	Ion Score	C. I. %	Modification	Rank	Result Type
813.4828	813.5146	0.0318	39	323	330	GLVPNSVK					Mascot
973.5101	973.6044	0.0943	97	314	322	HEGVGALYK					Mascot
1033.5314	1033.5986	0.0672	65	194	202	TVFVEEGPR					Mascot
1107.5463	1107.6311	0.0848	77	1	10	MASEDVVGKR			Oxidation (M)[1]		Mascot
1193.6426	1193.7139	0.0713	60	90	98	YIWRTEGLR					Mascot
1329.7274	1329.7509	0.0235	18	311	322	TVRHEGVGALYK					Mascot
1379.8005	1379.8333	0.0328	24	69	80	ILLQVQNSHSIK					Mascot
1657.8439	1657.9591	0.1152	69	103	118	GNGTNCARIVPNSAVK			Carbamidomethyl (C)[6]		Mascot
1800.8585	1800.9937	0.1352	75	295	310	TNAPVEYSGMVDAFRK			Oxidation (M)[10]		Mascot
1909.9689	1909.9952	0.0263	14	236	252	TRPFGLAQDTELSVMTK			Oxidation (M)[15]		Mascot
1989.9885	1990.0985	0.11	55	27	45	MASEGVKAPSHAAIFSVC K			Carbamidomethyl (C)[18]		Mascot
2384.2505	2384.1689	-0.0816	-34	34	58	APSHAAIFSVCKSLAAGG IAGGVSR			Carbamidomethyl (C)[11]		Mascot

6	unnamed protein product [Vitis vinifera]	gi|297742663	86320.3	6.08	20	55	0	7.551

Peptide Information

Calc. Mass	Obsrv. Mass	± da	± ppm	Start Seq.	End Seq.	Sequence	Ion Score	C. I. %	Modification	Rank	Result Type
819.4141	819.4697	0.0556	68	14	20	GNMKVDR					Mascot
858.493	858.5594	0.0664	77	429	435	LEIQLDK					Mascot
863.4985	863.5358	0.0373	43	243	249	SFVELLR					Mascot
874.4781	874.499	0.0209	24	559	565	SQVVQWK					Mascot
905.5302	905.5175	-0.0127	-14	21	29	LVSLSSATK					Mascot
943.5724	943.55	-0.0224	-24	301	308	TFKPPVVR					Mascot
990.5652	990.5675	0.0023	2	410	417	VENMLKIK			Oxidation (M)[4]		Mascot
1033.5386	1033.5986	0.06	58	49	59	RNSTGSVGGAK					Mascot
1157.6525	1157.6715	0.019	16	312	322	LVVVDLAGSER					Mascot
1232.6383	1232.6953	0.057	46	502	512	HGSERTTFGLK					Mascot
1300.7219	1300.682	-0.0399	-31	566	577	RIEAAGNSEILK					Mascot
1320.5776	1320.7004	0.1228	93	480	489	YQEDYMQSIK			Oxidation (M)[6]		Mascot
1320.5776	1320.7004	0.1228	93	480	489	YQEDYMQSIK			Oxidation (M)[6]		Mascot
1365.6328	1365.7504	0.1176	86	1	13	MAASGGYRNGTHK			Oxidation (M)[1]		Mascot
1365.6328	1365.7504	0.1176	86	1	13	MAASGGYRNGTHK			Oxidation (M)[1]		Mascot
1407.8054	1407.8213	0.0159	11	21	34	LVSLSSATKSSSLK					Mascot
1493.7595	1493.8571	0.0976	65	756	768	TLNSSPTFQTELR					Mascot
1513.8585	1513.8662	0.0077	5	312	325	LVVVDLAGSERIDK					Mascot
1600.7814	1600.9283	0.1469	92	323	337	IDKSGSEGHTLEEAK					Mascot
1791.8	1791.8975	0.0975	54	285	300	DSALLSENCNSSHMVK			Carbamidomethyl (C)[9]		Mascot
1927.0244	1927.046	0.0216	11	225	242	TGDVSLPGATLVEIRDQR					Mascot
2705.2949	2705.4072	0.1123	42	625	649	VLGNLDSFMSQIRHSQLS DSGNGDK					Mascot

7	putative histone-lysine N-methyltransferase [Arabidopsis thaliana]	gi|332658165	269117.4	6.44	23	54	0	13.45	

Protein Group

RecName: Full=Probable histone-lysine N-methyltransferase ATXR3; AltName: Full=Protein SET DOMAIN GROUP 2; AltName: Full=Trithorax-related protein 3; Short=TRX-related protein 3	gi|229488102	269117.4	6.4400 000572 2046
putative histone-lysine N-methyltransferase [Arabidopsis thaliana]	gi|186511821	269117.4	6.4400 000572 2046

Peptide Information

Calc. Mass	Obsrv. Mass	± da	± ppm	Start Seq.	End Seq.	Sequence	Ion Score	C. I. %	Modification	Rank	Result Type

815.441	815.5014	0.0604	74	1775	1780	YVSYRK					Mascot
819.4108	819.4697	0.0589	72	1012	1018	SHSSVFR					Mascot
823.4607	823.5065	0.0458	56	2057	2062	VRYVMR					Mascot
832.441	832.3934	-0.0476	-57	1434	1440	VSQIEEK					Mascot
833.4152	833.39	-0.0252	-30	236	242	GEFIPDR					Mascot
844.5251	844.5616	0.0365	43	887	894	ASLLSVVR					Mascot
868.5073	868.5245	0.0172	20	1089	1095	LHQLVMK					Mascot
905.4799	905.5175	0.0376	42	297	304	SGLDRTTR					Mascot
906.468	906.5355	0.0675	74	717	723	EIFVENR					Mascot
990.485	990.5675	0.0825	83	959	967	TDTSVNTPR					Mascot
1033.5347	1033.5986	0.0639	62	1033	1041	SPETIAMLR			Oxidation (M)[7]		Mascot
1036.5687	1036.6017	0.033	32	2279	2286	QPQRPWPK					Mascot
1090.6104	1090.6218	0.0114	10	1329	1338	SLKQTTDVAK					Mascot
1107.5714	1107.6311	0.0597	54	1434	1442	VSQIEEKMK			Oxidation (M)[8]		Mascot
1165.575	1165.6805	0.1055	91	1443	1452	NGYYVSHGLR					Mascot
1165.575	1165.6805	0.1055	91	1443	1452	NGYYVSHGLR	18	0			Mascot
1193.679	1193.7139	0.0349	29	1996	2004	LVRFINFER					Mascot
1300.7372	1300.682	-0.0552	-42	2136	2145	SLLWLRDEIR					Mascot
1307.693	1307.785	0.092	70	1202	1211	VFHMLRYDVK					Mascot
1320.6332	1320.7004	0.0672	51	284	293	FVNEDIYHQR					Mascot
1320.6332	1320.7004	0.0672	51	284	293	FVNEDIYHQR					Mascot
1323.6879	1323.7491	0.0612	46	1202	1211	VFHMLRYDVK			Oxidation (M)[4]		Mascot
1365.7638	1365.7504	-0.0134	-10	1941	1951	VLKDWHGLLER					Mascot
1365.7638	1365.7504	-0.0134	-10	1941	1951	VLKDWHGLLER	18	0			Mascot
1493.7635	1493.8571	0.0936	63	2021	2032	KYFSDIHLDVEK					Mascot
1699.8181	1699.9738	0.1557	92	920	935	CPSRPARPSPASSDSK			Carbamidomethyl (C)[1]		Mascot
1791.9496	1791.8975	-0.0521	-29	829	843	KIVLNDGFPLCLMQK			Carbamidomethyl (C)[11], Oxidation (M)[13]		Mascot

8	uncharacterized protein LOC100384832 [Zea mays]	gi|293333705	56613.2	9.35	11	51	0	4.764

Peptide Information

Calc. Mass	Obsrv. Mass	± da	± ppm	Start Seq.	End Seq.	Sequence	Ion Score	C. I. %	Modification	Rank	Result Type
807.4103	807.4669	0.0566	70	94	100	DMAIVMK					Mascot
857.5203	857.5762	0.0559	65	63	69	RVQLVDK					Mascot
900.5261	900.6081	0.082	91	231	238	QGRVLEAK					Mascot
905.4686	905.5175	0.0489	54	433	440	KSLSAEDR					Mascot

973.52	973.6044	0.0844	87	105	113	AEEAIEAIK					Mascot
1232.5978	1232.6953	0.0975	79	422	432	AQSIDENSGRR					Mascot
1329.7372	1329.7509	0.0137	10	105	116	AEEAIEAIKSLR					Mascot
1333.678	1333.7825	0.1045	78	265	275	AQEMLRDLETK					Mascot
1365.6614	1365.7504	0.089	65	94	104	DMAIVMKQQNR			Oxidation (M)[2,6]		Mascot
1365.6614	1365.7504	0.089	65	94	104	DMAIVMKQQNR	18	0	Oxidation (M)[2,6]		Mascot
1513.885	1513.8662	-0.0188	-12	391	404	KKPAPVHDPLGNLK					Mascot
1716.8512	1716.9916	0.1404	82	415	431	AGAVVDKAQSIDENSGR					Mascot
1716.8512	1716.9916	0.1404	82	415	431	AGAVVDKAQSIDENSGR					Mascot

9 hypothetical protein OsI_05619 [Oryza sativa Indica Group] gi|218189935 26167.2 10.86 7 50 0 2.107

Peptide Information

Calc. Mass	Obsrv. Mass	± da	± ppm	Start Seq.	End Seq.	Sequence	Ion Score	C. I. %	Modification	Rank	Result Type
815.4257	815.5014	0.0757	93	149	155	IAETPER					Mascot
1090.5891	1090.6218	0.0327	30	78	87	FELTGPSIAR					Mascot
1157.6572	1157.6715	0.0143	12	67	77	VAVCLVGGARR			Carbamidomethyl (C)[4]		Mascot
1165.6801	1165.6805	0.0004	0	131	142	AAPPGSRLAAVR					Mascot
1165.6801	1165.6805	0.0004	0	131	142	AAPPGSRLAAVR	18	0			Mascot
1308.7747	1308.761	-0.0137	-10	125	137	LSLLARAAPPGSR					Mascot
1584.8639	1584.8693	0.0054	3	1	16	MKPGGAASSVLPAARR			Oxidation (M)[1]		Mascot
1910.1069	1909.9952	-0.1117	-58	158	175	VLTASNSPNGIQVQKIIK					Mascot

10 SET domain-containing protein [Arabidopsis lyrata subsp. lyrata] gi|297316093 272060.8 6.18 21 49 0 7.735

Peptide Information

Calc. Mass	Obsrv. Mass	± da	± ppm	Start Seq.	End Seq.	Sequence	Ion Score	C. I. %	Modification	Rank	Result Type
815.441	815.5014	0.0604	74	1805	1810	YVSYRK					Mascot
819.4108	819.4697	0.0589	72	1042	1048	SHSSVFR					Mascot
823.4607	823.5065	0.0458	56	2087	2092	VRYVMR					Mascot
832.441	832.3934	-0.0476	-57	1464	1470	VSQIEEK					Mascot
833.4152	833.39	-0.0252	-30	239	245	GEFIPDR					Mascot
868.5073	868.5245	0.0172	20	1119	1125	LHQLVMK					Mascot
905.5302	905.5175	-0.0127	-14	1520	1527	LSKTVTEK					Mascot
955.4479	955.5417	0.0938	98	332	339	EYSSTVNR					Mascot
1036.5687	1036.6017	0.033	32	2309	2316	QPQRPWPK					Mascot

1107.5714	1107.6311	0.0597	54	1464	1472	VSQIEEKMK			Oxidation (M)[8]	Mascot
1118.5477	1118.6057	0.058	52	391	399	LYRDSYSSK				Mascot
1165.575	1165.6805	0.1055	91	1473	1482	NGYYVSHGLR				Mascot
1165.575	1165.6805	0.1055	91	1473	1482	NGYYVSHGLR	18	0		Mascot
1182.6375	1182.6561	0.0186	16	788	797	SIGKCLMFVK			Carbamidomethyl (C)[5]	Mascot
1182.6375	1182.6561	0.0186	16	788	797	SIGKCLMFVK			Carbamidomethyl (C)[5]	Mascot
1193.679	1193.7139	0.0349	29	2026	2034	LVRFINFER				Mascot
1300.7372	1300.682	-0.0552	-42	2166	2175	SLLWLRDEIR				Mascot
1365.7638	1365.7504	-0.0134	-10	1971	1981	VLKDWHGLLER				Mascot
1365.7638	1365.7504	-0.0134	-10	1971	1981	VLKDWHGLLER	18	0		Mascot
1487.8217	1487.8589	0.0372	25	736	749	IANLLEGYTIAPGR				Mascot
1493.7635	1493.8571	0.0936	63	2051	2062	KYFSDIHLDVEK				Mascot
1507.7607	1507.8632	0.1025	68	1789	1800	MCQVVLKEIESR			Carbamidomethyl (C)[2], Oxidation (M)[1]	Mascot
1699.8181	1699.9738	0.1557	92	950	965	CPSRPARPSPASSDSK			Carbamidomethyl (C)[1]	Mascot
1791.9496	1791.8975	-0.0521	-29	859	873	KIVLNDGFPLCLMQK			Carbamidomethyl (C)[11], Oxidation (M)[13]	Mascot

Gel Idx/Pos	126/F1	Instr./Gel Origin	BA2151/Sample Project 20140814	Process Status	Analysis Succeeded
Plate [#] Name	[1] Sample Project 20140814	Instrument Sample Name		Spectra	11

Rank	Protein Name	Accession No.	Protein MW	Protein PI	Pep. Count	Protein Score	Protein Score C. I. %	Intensity Matched	Total Ion Score	Total Ion C. I. %	Confirmed
1	Putative prefoldin subunit 2 [Aegilops tauschii]	gi|475621959	16264.4	5.85	11	95	99.971	8.75	40	80.678	

Peptide Information

Calc. Mass	Obsrv. Mass	± da	± ppm	Start Seq.	End Seq.	Sequence	Ion Score	C. I. %	Modification	Rank	Result Type
920.5312	920.5696	0.0384	42	74	81	EVLPAVHR					Mascot
920.5312	920.5696	0.0384	42	74	81	EVLPAVHR	28	0			Mascot
973.5499	973.568	0.0181	19	62	70	MIGGVLVER					Mascot
989.5448	989.5548	0.01	10	62	70	MIGGVLVER			Oxidation (M)[1]		Mascot
1001.5262	1001.5497	0.0235	23	84	92	EGLEEVVAR					Mascot
1042.4762	1042.5468	0.0706	68	103	110	EMTEFELK			Oxidation (M)[2]		Mascot
1143.535	1143.5796	0.0446	39	24	32	TEMNQLYTK			Oxidation (M)[3]		Mascot
1154.5762	1154.6056	0.0294	25	102	110	KEMTEFELK					Mascot
1154.5762	1154.6056	0.0294	25	102	110	KEMTEFELK					Mascot
1162.6692	1162.6561	-0.0131	-11	74	83	EVLPAVHRNK					Mascot
1243.6641	1243.714	0.0499	40	82	92	NKEGLEEVVAR					Mascot
1243.6641	1243.714	0.0499	40	82	92	NKEGLEEVVAR	12	0			Mascot
1262.7579	1262.6655	-0.0924	-73	71	81	TIKEVLPAVHR					Mascot
1331.7716	1331.7196	-0.052	-39	62	73	MIGGVLVERTIK			Oxidation (M)[1]		Mascot
1333.6344	1333.7375	0.1031	77	103	112	EMTEFELKYK			Oxidation (M)[2]		Mascot
1333.6344	1333.7375	0.1031	77	103	112	EMTEFELKYK			Oxidation (M)[2]		Mascot

Rank	Protein Name	Accession No.	Protein MW	Protein PI	Pep. Count	Protein Score	Protein Score C. I. %	Intensity Matched	Total Ion Score	Total Ion C. I. %	Confirmed
2	putative prefoldin subunit 2 [Triticum urartu]	gi|473960164	16389.4	5.83	8	68	83.846	2.893	40	80.678	

Protein Group

Putative prefoldin subunit 2 [Aegilops tauschii]	gi|475552446	16389.4	5.8299 999237 0605

Peptide Information

Calc. Mass	Obsrv. Mass	± da	± ppm	Start Seq.	End Seq.	Sequence	Ion Score	C. I. %	Modification	Rank	Result Type
892.4556	892.5085	0.0529	59	96	102	MKEALER			Oxidation (M)[1]		Mascot
920.5312	920.5696	0.0384	42	77	84	EVLPAVHR					Mascot
920.5312	920.5696	0.0384	42	77	84	EVLPAVHR	28	0			Mascot

Calc. Mass	Obsrv. Mass	± da	± ppm	Start Seq.	End Seq.	Sequence	Ion Score	C. I. %	Modification	Rank	Result Type
973.5499	973.568	0.0181	19	65	73	MIGGVLVER					Mascot
989.5448	989.5548	0.01	10	65	73	MIGGVLVER			Oxidation (M)[1]		Mascot
1001.5262	1001.5497	0.0235	23	87	95	EGLEEVVAR					Mascot
1136.5834	1136.5999	0.0165	15	105	113	QEITEFELK					Mascot
1143.535	1143.5796	0.0446	39	27	35	TEMNQLYTK			Oxidation (M)[3]		Mascot
1162.6692	1162.6561	-0.0131	-11	77	86	EVLPAVHRNK					Mascot
1243.6641	1243.714	0.0499	40	85	95	NKEGLEEVVAR					Mascot
1243.6641	1243.714	0.0499	40	85	95	NKEGLEEVVAR	12	0			Mascot

3	hypothetical protein CARUB_v10000308mg, partial [Capsella rubella]	gi|482555843	83015.9	5.4	19	66	76.107	10.576

Peptide Information

Calc. Mass	Obsrv. Mass	± da	± ppm	Start Seq.	End Seq.	Sequence	Ion Score	C. I. %	Modification	Rank	Result Type
802.4417	802.4725	0.0308	38	267	273	IGNLETR					Mascot
807.3665	807.4438	0.0773	96	565	570	EQQMQK			Oxidation (M)[4]		Mascot
820.4523	820.4497	-0.0026	-3	27	34	ATANSTKK					Mascot
821.4515	821.4584	0.0069	8	731	737	YRLLGEA					Mascot
834.5195	834.4493	-0.0702	-84	315	321	LYALARK					Mascot
836.4698	836.4729	0.0031	4	687	693	LFMALNK					Mascot
886.5179	886.4849	-0.033	-37	465	472	LCPVKAAK			Carbamidomethyl (C)[2]		Mascot
982.4741	982.5057	0.0316	32	541	548	NTVGYWSR					Mascot
1115.5449	1115.5979	0.053	48	475	483	CGCLHVLTR			Carbamidomethyl (C)[1,3]		Mascot
1118.5801	1118.5729	-0.0072	-6	155	164	SSQDRPLTSK					Mascot
1165.5526	1165.6305	0.0779	67	484	492	MVMEQCIVR			Carbamidomethyl (C)[6]		Mascot
1201.6536	1201.6705	0.0169	14	144	154	SDRPSTVPKSK					Mascot
1223.6532	1223.6602	0.007	6	539	548	LKNTVGYWSR					Mascot
1243.6399	1243.714	0.0741	60	474	483	KCGCLHVLTR			Carbamidomethyl (C)[2,4]		Mascot
1243.6399	1243.714	0.0741	60	474	483	KCGCLHVLTR			Carbamidomethyl (C)[2,4]		Mascot
1262.6926	1262.6655	-0.0271	-21	493	503	LDVAMFNAILR					Mascot
1331.7026	1331.7196	0.017	13	155	166	SSQDRPLTSKGR					Mascot
1333.707	1333.7375	0.0305	23	153	164	SKSSQDRPLTSK					Mascot
1333.707	1333.7375	0.0305	23	153	164	SKSSQDRPLTSK					Mascot
1757.8593	1757.906	0.0467	27	211	227	AFSSASAVDSVLFEEAK					Mascot
1883.0597	1882.9701	-0.0896	-48	267	282	IGNLETRIVVLEEELR					Mascot

4	Aldehyde dehydrogenase family 2 member C4 [Aegilops tauschii]	gi|475538858	53811.4	5.53	15	65	67.018	18.52

Peptide Information

Calc. Mass	Obsrv. Mass	± da	± ppm	Start Seq.	End Seq.	Sequence	Ion Score	C. I. %	Modification	Rank	Result Type
811.4421	811.45	0.0079	10	133	139	IHGESLR					Mascot
815.437	815.4153	-0.0217	-27	429	435	NLDIANR					Mascot
890.4764	890.5004	0.024	27	246	253	LIMEASAR					Mascot
906.4713	906.5106	0.0393	43	246	253	LIMEASAR			Oxidation (M)[3]		Mascot
934.4411	934.5311	0.09	96	293	301	GEVCVAGSR			Carbamidomethyl (C)[4]		Mascot
1006.5931	1006.5109	-0.0822	-82	419	428	YGLAAGIITK					Mascot
1033.5525	1033.5587	0.0062	6	356	366	SEGATLLTGGK					Mascot
1057.5637	1057.5532	-0.0105	-10	52	62	ADVDLAVGAAR					Mascot
1182.6589	1182.6128	-0.0461	-39	133	143	IHGESLRVSGK					Mascot
1182.6589	1182.6128	-0.0461	-39	133	143	IHGESLRVSGK					Mascot
1243.6682	1243.714	0.0458	37	140	150	VSGKYQGYTLK					Mascot
1243.6682	1243.714	0.0458	37	140	150	VSGKYQGYTLK					Mascot
1332.7303	1332.7048	-0.0255	-19	246	257	LIMEASARSNLK					Mascot
1333.649	1333.7375	0.0885	66	2	13	GSNEMVMVPEIK					Mascot
1333.649	1333.7375	0.0885	66	2	13	GSNEMVMVPEIK					Mascot
1349.644	1349.7333	0.0893	66	2	13	GSNEMVMVPEIK			Oxidation (M)[5]		Mascot
1355.7716	1355.7042	-0.0674	-50	113	124	IIDIPAAVQMLR			Oxidation (M)[10]		Mascot
1657.7747	1657.9304	0.1557	94	470	483	DQGMMAIDKYMQVK					Mascot
1657.7747	1657.9304	0.1557	94	470	483	DQGMMAIDKYMQVK					Mascot
1707.8661	1707.8776	0.0115	7	36	51	DPRTGDVLAHIAEADK					Mascot
1805.8375	1805.9062	0.0687	38	325	341	VGDPFDVATNMGPQVDK			Oxidation (M)[11]		Mascot

5 pentatricopeptide repeat-containing protein, putative [Ricinus communis] gi|223539233 47139.8 8.72 14 63 54.472 2.951

Peptide Information

Calc. Mass	Obsrv. Mass	± da	± ppm	Start Seq.	End Seq.	Sequence	Ion Score	C. I. %	Modification	Rank	Result Type
853.4488	853.4913	0.0425	50	317	323	LIDMYAK					Mascot
939.5444	939.5062	-0.0382	-41	99	106	QVHALMIK					Mascot
942.5255	942.4948	-0.0307	-33	107	116	AGTDLGPIAK					Mascot
981.5152	981.5219	0.0067	7	236	243	VFSSLSWR					Mascot
1001.4938	1001.5497	0.0559	56	126	134	YGYLGDSVK					Mascot
1057.5234	1057.5532	0.0298	28	117	125	TALIDMYSK			Oxidation (M)[6]		Mascot
1107.6093	1107.6112	0.0019	2	163	172	ALGIFGAMRR			Oxidation (M)[8]		Mascot

Calc. Mass	Obsrv. Mass	± da	± ppm	Start Seq.	End Seq.	Sequence	Ion Score	C. I. %	Modification	Rank	Result Type
1331.7028	1331.7196	0.0168	13	1	11	MYSSLLVIAYR			Oxidation (M)[1]		Mascot
1417.6417	1417.7399	0.0982	69	305	316	FGFTSDTQLCNK			Carbamidomethyl (C)[10]		Mascot
1507.838	1507.7996	-0.0384	-25	327	339	ILNARSVFDGIFR					Mascot
1615.7422	1615.8756	0.1334	83	341	354	DVVSWTSMIDAYGR			Oxidation (M)[8]		Mascot
1636.8694	1636.8833	0.0139	8	143	156	DVVTWNSLLSSFLR					Mascot
1900.9474	1900.9791	0.0317	17	172	187	REGVEFSEFTLCSVLK			Carbamidomethyl (C)[12]		Mascot
1919.0459	1918.8678	-0.1781	-93	1	16	MYSSLLVIAYRNFLTK					Mascot
1919.0459	1918.8678	-0.1781	-93	1	16	MYSSLLVIAYRNFLTK					Mascot

6	RNA polymerase beta subunit-2 (chloroplast) [Salvia miltiorrhiza]	gi|401879732	159216.8	9.4	28	63	46.509	10.216

Protein Group

RNA polymerase beta subunit-2 (chloroplast) [Salvia miltiorrhiza]	gi|459014483	159216.8	9.3999<br>996185<br>3027

Peptide Information

Calc. Mass	Obsrv. Mass	± da	± ppm	Start Seq.	End Seq.	Sequence	Ion Score	C. I. %	Modification	Rank	Result Type
806.4883	806.4321	-0.0562	-70	1210	1215	IQKVYR					Mascot
814.4781	814.4446	-0.0335	-41	358	365	APSNGKIK					Mascot
821.4515	821.4584	0.0069	8	857	863	ASFVEIR					Mascot
834.439	834.4493	0.0103	12	17	24	VIDGTAMK					Mascot
847.4341	847.448	0.0139	16	2	8	EVLMAER					Mascot
856.5615	856.5681	0.0066	8	1274	1281	ALLLGITR					Mascot
920.5676	920.5696	0.002	2	1085	1092	NAPHLKLK					Mascot
920.5676	920.5696	0.002	2	1085	1092	NAPHLKLK					Mascot
934.455	934.5311	0.0761	81	1346	1353	SPLEMETK					Mascot
939.4716	939.5062	0.0346	37	660	666	YQMKVDR					Mascot
942.5156	942.4948	-0.0208	-22	9	16	ANLVFHNK					Mascot
944.5524	944.4956	-0.0568	-60	191	199	KGVVDTAVR					Mascot
1006.535	1006.5109	-0.0241	-24	17	25	VIDGTAMKR			Oxidation (M)[7]		Mascot
1010.4645	1010.5422	0.0777	77	1	8	MEVLMAER			Oxidation (M)[1,5]		Mascot
1012.4669	1012.5197	0.0528	52	761	767	NWMYVQR			Oxidation (M)[3]		Mascot
1060.6514	1060.5878	-0.0636	-60	243	251	IFIQTLIGR					Mascot
1078.5449	1078.5739	0.029	27	1346	1354	SPLEMETKK			Oxidation (M)[5]		Mascot
1084.6038	1084.6068	0.003	3	1371	1379	KLFDSLFSK					Mascot
1115.5157	1115.5979	0.0822	74	528	536	FFTSHFSDK					Mascot
1154.7045	1154.6056	-0.0989	-86	870	878	HFLRIDLLK					Mascot

Calc. Mass	Obsrv. Mass	± da	± ppm	Start Seq.	End Seq.	Sequence	Ion Score	C. I. %	Modification	Rank	Result Type
1154.7045	1154.6056	-0.0989	-86	870	878	HFLRIDLLK					Mascot
1162.658	1162.6561	-0.0019	-2	739	750	HSGVLIPPGTGK					Mascot
1227.6117	1227.676	0.0643	52	366	375	FNEDLVHPTR					Mascot
1243.6107	1243.714	0.1033	83	528	537	FFTSHFSDKK					Mascot
1243.6107	1243.714	0.1033	83	528	537	FFTSHFSDKK					Mascot
1332.6471	1332.7048	0.0577	43	1029	1038	FQYYLIDENK					Mascot
1436.8584	1436.7327	-0.1257	-87	1225	1236	HIEIIVRQITSK					Mascot
1436.8584	1436.7327	-0.1257	-87	1225	1236	HIEIIVRQITSK					Mascot
1562.7487	1562.8428	0.0941	60	722	735	IFSGDIHFPGETDK					Mascot
1657.7711	1657.9304	0.1593	96	498	511	DQDQINAHSCSVKR			Carbamidomethyl (C)[10]		Mascot
1657.7711	1657.9304	0.1593	96	498	511	DQDQINAHSCSVKR			Carbamidomethyl (C)[10]		Mascot
1757.9368	1757.906	-0.0308	-18	9	24	ANLVFHNKVIDGTAMK					Mascot
1900.9124	1900.9791	0.0667	35	953	968	MGPFNDVKYHNHNAIK			Oxidation (M)[1]		Mascot

7 PREDICTED: uncharacterized protein LOC101783793 [Setaria italica] gi|514782348 31606.6 11.81 13 62 45.263 4.359

Peptide Information

Calc. Mass	Obsrv. Mass	± da	± ppm	Start Seq.	End Seq.	Sequence	Ion Score	C. I. %	Modification	Rank	Result Type
886.4676	886.4849	0.0173	20	138	144	LPQGCRR			Carbamidomethyl (C)[5]		Mascot
892.5073	892.5085	0.0012	1	271	277	CLFIIAR			Carbamidomethyl (C)[1]		Mascot
897.5013	897.4681	-0.0332	-37	130	137	ALTSGHRR					Mascot
934.441	934.5311	0.0901	96	1	8	MLAADQNR			Oxidation (M)[1]		Mascot
992.4717	992.5588	0.0871	88	243	252	SMDASPALGK			Oxidation (M)[2]		Mascot
1078.627	1078.5739	-0.0531	-49	74	82	HPPPLPKHR					Mascot
1105.6589	1105.6265	-0.0324	-29	86	95	VVVHSRPVGR					Mascot
1105.6589	1105.6265	-0.0324	-29	86	95	VVVHSRPVGR	3	0			Mascot
1232.627	1232.6476	0.0206	17	161	171	QPLLYGDEAAR					Mascot
1308.7634	1308.7041	-0.0593	-45	100	113	SSLPPAAVAGGLLR					Mascot
1332.7971	1332.7048	-0.0923	-69	84	95	ARVVVHSRPVGR					Mascot
1507.8955	1507.7996	-0.0959	-64	100	115	SSLPPAAVAGGLLRAK					Mascot
1691.9916	1691.913	-0.0786	-46	96	113	GLGRSSLPPAAVAGGLL R					Mascot
1900.9512	1900.9791	0.0279	15	25	42	EDGGGAVRVSIPSLWSD R					Mascot

8 PREDICTED: transformation/transcription domain-associated protein-like [Fragaria vesca subsp. vesca] gi|470102756 441673.5 6.58 46 62 42.684 11.156

Peptide Information

Calc. Mass	Obsrv. Mass	± da	± ppm	Start Seq.	End Seq.	Sequence	Ion Score	C. I. %	Modification	Rank	Result Type
802.4669	802.4725	0.0056	7	967	973	TQLLAEK					Mascot
811.4052	811.45	0.0448	55	493	499	TLVMGMK			Oxidation (M)[4,6]		Mascot
815.4596	815.4153	-0.0443	-54	1933	1938	MPIWIR					Mascot
820.3981	820.4497	0.0516	63	3411	3417	ELSGVCR			Carbamidomethyl (C)[6]		Mascot
821.5131	821.4584	-0.0547	-67	1181	1187	SLVYVLK					Mascot
829.376	829.4451	0.0691	83	2122	2128	EASTMYK					Mascot
836.4108	836.4729	0.0621	74	1046	1053	NSSSSNLK					Mascot
846.4866	846.5065	0.0199	24	237	244	MVAAISVR					Mascot
847.4341	847.448	0.0139	16	3347	3353	DIMEALR					Mascot
854.411	854.4635	0.0525	61	2213	2219	SLCSMLK			Carbamidomethyl (C)[3], Oxidation (M)[5]		Mascot
864.476	864.5106	0.0346	40	2465	2471	QFMLGLR					Mascot
868.4927	868.5136	0.0209	24	661	668	FIFGAVSK					Mascot
880.4709	880.4979	0.027	31	2465	2471	QFMLGLR			Oxidation (M)[3]		Mascot
939.5622	939.5062	-0.056	-60	3521	3529	VGADIPIVR					Mascot
963.551	963.5128	-0.0382	-40	2409	2416	EIVSFLQK					Mascot
975.5002	975.5643	0.0641	66	2349	2356	VMLVPECK			Carbamidomethyl (C)[7]		Mascot
992.4651	992.5588	0.0937	94	1098	1105	QADVLMCR			Carbamidomethyl (C)[7]		Mascot
993.4855	993.5475	0.062	62	25	32	LQMAMEVR			Oxidation (M)[3]		Mascot
1001.6102	1001.5497	-0.0605	-60	1344	1352	VATSLNKLR					Mascot
1012.459	1012.5197	0.0607	60	3572	3579	VMNQMFDK					Mascot
1028.454	1028.532	0.078	76	3572	3579	VMNQMFDK			Oxidation (M)[2]		Mascot
1042.4952	1042.5468	0.0516	49	898	906	NGGIDTFYR					Mascot
1057.5691	1057.5532	-0.0159	-15	1858	1865	FGWNHLKR					Mascot
1060.5786	1060.5878	0.0092	9	245	254	GPESVPPHLK					Mascot
1066.5527	1066.5559	0.0032	3	3658	3666	LQAYSDITR					Mascot
1078.5562	1078.5739	0.0177	16	3347	3355	DIMEALRSK			Oxidation (M)[3]		Mascot
1082.4935	1082.551	0.0575	53	3418	3427	ACFSADAVNK			Carbamidomethyl (C)[2]		Mascot
1084.6223	1084.6068	-0.0155	-14	1378	1386	AKIISMFFK					Mascot
1107.6093	1107.6112	0.0019	2	2465	2473	QFMLGLRAR			Oxidation (M)[3]		Mascot
1115.515	1115.5979	0.0829	74	2302	2312	EMGPSSGSHVK					Mascot
1201.6688	1201.6705	0.0017	1	2933	2941	DILETWRLR					Mascot
1223.6605	1223.6602	-0.0003	0	1933	1941	MPIWIRYTK			Oxidation (M)[1]		Mascot
1227.5674	1227.676	0.1086	88	2708	2717	CSESLAELYR			Carbamidomethyl (C)[1]		Mascot
1243.6641	1243.714	0.0499	40	2327	2339	QGADAGAVISNLK					Mascot

1243.6641	1243.714	0.0499	40	2327	2339	QGADAGAVISNLK					Mascot
1248.6881	1248.6755	-0.0126	-10	2462	2471	VERQFMLGLR					Mascot
1314.6185	1314.7166	0.0981	75	2	12	SPVQDFNQHSR					Mascot
1331.7101	1331.7196	0.0095	7	1249	1260	NVQSCLALLASR			Carbamidomethyl (C)[5]		Mascot
1332.6729	1332.7048	0.0319	24	2579	2589	KHAQFLNEMSK					Mascot
1349.7246	1349.7333	0.0087	6	1339	1350	FMNPKVATSLNK					Mascot
1355.7529	1355.7042	-0.0487	-36	3869	3881	GVTELVEAALTPR					Mascot
1379.6888	1379.6986	0.0098	7	1706	1715	CFLNYLRHEK			Carbamidomethyl (C)[1]		Mascot
1417.7686	1417.7399	-0.0287	-20	3477	3488	FPAVLKLEEESR					Mascot
1435.7648	1435.7716	0.0068	5	2207	2219	LLDAGKSLCSMLK			Carbamidomethyl (C)[9]		Mascot
1562.8762	1562.8428	-0.0334	-21	3831	3843	QKVINNVEHVINR					Mascot
1600.855	1600.8834	0.0284	18	2344	2356	LINERVMLVPECK			Carbamidomethyl (C)[12]		Mascot
1819.9589	1819.9797	0.0208	11	3147	3162	VLYLLSFDTPNEPVGR					Mascot
1882.8752	1882.9701	0.0949	50	277	291	SFADYIRQHEESICK			Carbamidomethyl (C)[14]		Mascot
2384.2722	2384.0923	-0.1799	-75	3142	3162	SHLARVLYLLSFDTPNEP VGR					Mascot

9	PREDICTED: unconventional myosin-Va-like [Solanum lycopersicum]	gi|460396333	174273.4	8.51	33	62	35.69	13.353

Peptide Information

Calc. Mass	Obsrv. Mass	± da	± ppm	Start Seq.	End Seq.	Sequence	Ion Score	C. I. %	Modification	Rank	Result Type
802.5185	802.4725	-0.046	-57	48	54	IAKVFPK					Mascot
811.4494	811.45	0.0006	1	747	752	TYIMRK					Mascot
820.4675	820.4497	-0.0178	-22	904	909	LQFEKR					Mascot
821.4515	821.4584	0.0069	8	776	781	LYEQLR					Mascot
827.4443	827.478	0.0337	41	747	752	TYIMRK			Oxidation (M)[4]		Mascot
846.4679	846.5065	0.0386	46	1016	1022	LETAERK					Mascot
878.5094	878.4912	-0.0182	-21	752	758	KEFVSLR					Mascot
886.4893	886.4849	-0.0044	-5	171	178	YLAHLGGR					Mascot
888.4421	888.5007	0.0586	66	935	942	QVEEANAK					Mascot
911.4581	911.4946	0.0365	40	1213	1221	SSSASLFGR					Mascot
928.4557	928.4855	0.0298	32	708	715	GYQMGKTK			Oxidation (M)[4]		Mascot
981.5186	981.5219	0.0033	3	705	713	GLKGYQMGK					Mascot
1028.5524	1028.532	-0.0204	-20	601	609	FSSIGSRFK					Mascot
1033.5525	1033.5587	0.0062	6	38	47	TSDGKEVVAK					Mascot
1037.5739	1037.5587	-0.0152	-15	235	242	TYLLERSR					Mascot
1057.575	1057.5532	-0.0218	-21	943	951	VVQEREAAR					Mascot

1060.5786	1060.5878	0.0092	9	896	903	KVEELTWR					Mascot
1066.6143	1066.5559	-0.0584	-55	953	962	AIEEAPPVIK					Mascot
1126.5596	1126.571	0.0114	10	162	170	TETTKMLMR			Oxidation (M)[6]		Mascot
1165.6477	1165.6305	-0.0172	-15	1337	1346	SNHVPPFLVR					Mascot
1205.6381	1205.6478	0.0097	8	695	704	VACQMILDKK			Carbamidomethyl (C)[3]		Mascot
1262.6045	1262.6655	0.061	48	1114	1123	QQENQDMLIK			Oxidation (M)[7]		Mascot
1331.7001	1331.7196	0.0195	15	856	866	AAIITQCGWRR			Carbamidomethyl (C)[7]		Mascot
1332.6981	1332.7048	0.0067	5	351	361	FHLNMTAELLK			Oxidation (M)[5]		Mascot
1333.6648	1333.7375	0.0727	55	1517	1527	ENSGFVFLHQR					Mascot
1333.6648	1333.7375	0.0727	55	1517	1527	ENSGFVFLHQR					Mascot
1417.7555	1417.7399	-0.0156	-11	167	178	MLMRYLAHLGGR					Mascot
1435.6846	1435.7716	0.087	61	1037	1048	LSNMESENQVLR			Oxidation (M)[4]		Mascot
1436.8333	1436.7327	-0.1006	-70	830	842	QTKAVTILQAHAR					Mascot
1436.8333	1436.7327	-0.1006	-70	830	842	QTKAVTILQAHAR					Mascot
1507.7289	1507.7996	0.0707	47	1517	1529	ENSGFVFLHQRSS					Mascot
1527.7659	1527.791	0.0251	16	770	781	AMLSCKLYEQLR			Carbamidomethyl (C)[5], Oxidation (M)[2]		Mascot
1691.8082	1691.913	0.1048	62	336	350	GEEIDSSVIKDEQSR					Mascot
1805.835	1805.9062	0.0712	39	107	120	LPHLYDTHMMEQYK					Mascot
1918.9539	1918.8678	-0.0861	-45	1033	1048	LEEKLSNMESENQVLR					Mascot
1918.9539	1918.8678	-0.0861	-45	1033	1048	LEEKLSNMESENQVLR					Mascot
2384.249	2384.0923	-0.1567	-66	375	396	VMVTPEEVITRTLDPEAA LGSR					Mascot

10 Riboflavin biosynthesis protein ribBA, chloroplastic [Aegilops tauschii] gi|475607284 75864.9 6.78 19 60 13.248 12.486

Peptide Information

Calc. Mass	Obsrv. Mass	± da	± ppm	Start Seq.	End Seq.	Sequence	Ion Score	C. I. %	Modification	Rank	Result Type
806.4631	806.4321	-0.031	-38	62	68	RASAFVR					Mascot
827.441	827.478	0.037	45	84	89	YKFVDR					Mascot
888.4608	888.5007	0.0399	45	622	629	LMTNNPAK					Mascot
906.5156	906.5106	-0.005	-6	396	403	YRNGGVLK					Mascot
944.4319	944.4956	0.0637	67	213	220	NDPLDENK					Mascot
963.504	963.5128	0.0088	9	614	621	DIGVRTMR			Oxidation (M)[7]		Mascot
982.441	982.5057	0.0647	66	203	211	CYAIGDAGR			Carbamidomethyl (C)[1]		Mascot
989.5084	989.5548	0.0464	47	45	53	ADQMVQLGK					Mascot
1012.5462	1012.5197	-0.0265	-26	76	83	LFTELAYR					Mascot
1078.5562	1078.5739	0.0177	16	313	323	NGSGIISVGMK			Oxidation (M)[10]		Mascot

1152.5895	1152.6266	0.0371	32	653	661	ENQKYLETK		Mascot
1193.6321	1193.6578	0.0257	22	10	19	HAAHRVSMLR	Oxidation (M)[8]	Mascot
1308.6399	1308.7041	0.0642	49	619	629	TMRLMTNNPAK	Oxidation (M)[2,5]	Mascot
1331.7278	1331.7196	-0.0082	-6	357	370	VGISTGVSAADRAK		Mascot
1349.6948	1349.7333	0.0385	29	277	288	EGKFVIAVDDEK		Mascot
1355.7543	1355.7042	-0.0501	-37	558	569	GVLVYLRGHEGR		Mascot
1435.842	1435.7716	-0.0704	-49	630	643	FVGLKGYGLAVVGR		Mascot
1507.8335	1507.7996	-0.0339	-22	15	27	VSMLRTMVSQLVK	Oxidation (M)[3]	Mascot
1883.0597	1882.9701	-0.0896	-48	369	386	AKTILALASPDSKPSDLR		Mascot

Gel Idx/Pos	127/F2	Instr./Gel Origin	BA2151/Sample Project 20140814	Process Status	Analysis Succeeded
Plate [#] Name	[1] Sample Project 20140814	Instrument Sample Name		Spectra	11

Rank	Protein Name	Accession No.	Protein MW	Protein PI	Pep. Count	Protein Score	Protein Score C. I. %	Intensity Matched	Total Ion Score	Total Ion C. I. %	Confirmed
1	hypothetical protein TRIUR3_03549 [Triticum urartu]	gi|474071007	16824.8	6.19	7	233	100	26.074	157	100	

Protein Group

hypothetical protein F775_31562 [Aegilops tauschii]	gi|475458859	16753.8	6.1900 000572 2046

Peptide Information

Calc. Mass	Obsrv. Mass	± da	± ppm	Start Seq.	End Seq.	Sequence	Ion Score	C. I. %	Modification	Rank	Result Type
827.4985	827.4829	-0.0156	-19	59	66	ADLPGVKK					Mascot
975.5258	975.5746	0.0488	50	110	117	FRLPEDAK					Mascot
975.5258	975.5746	0.0488	50	110	117	FRLPEDAK	24	0			Mascot
1057.5314	1057.5516	0.0202	19	50	58	ETPEAHVFK					Mascot
1057.5314	1057.5516	0.0202	19	50	58	ETPEAHVFK	14	0			Mascot
1600.8177	1600.8934	0.0757	47	71	85	VEVEDGNVLVVSGER					Mascot
1600.8177	1600.8934	0.0757	47	71	85	VEVEDGNVLVVSGER	9	0			Mascot
1905.9666	1906.0603	0.0937	49	26	45	SIVPAISGGSSETAAFAN AR					Mascot
1905.9666	1906.0603	0.0937	49	26	45	SIVPAISGGSSETAAFAN AR	142	100			Mascot
2086.0664	2086.155	0.0886	42	67	85	EEVKVEVEDGNVLVVSG ER					Mascot
2260.0347	2260.1248	0.0901	40	7	25	SNVFDPFADLWADPFDT FR					Mascot

Rank	Protein Name	Accession No.	Protein MW	Protein PI	Pep. Count	Protein Score	Protein Score C. I. %	Intensity Matched	Total Ion Score	Total Ion C. I. %	Confirmed
2	RecName: Full=16.9 kDa class I heat shock protein 1; AltName: Full=HSP 16.9; AltName: Full=Heat shock protein 16.9A; AltName: Full=Heat shock protein 17; AltName: Full=Low molecular weight heat shock protein	gi|123545	16867.8	5.83	6	226	100	25.752	157	100	

Peptide Information

Calc. Mass	Obsrv. Mass	± da	± ppm	Start Seq.	End Seq.	Sequence	Ion Score	C. I. %	Modification	Rank	Result Type
975.5258	975.5746	0.0488	50	110	117	FRLPEDAK					Mascot
975.5258	975.5746	0.0488	50	110	117	FRLPEDAK	24	0			Mascot
1057.5314	1057.5516	0.0202	19	50	58	ETPEAHVFK					Mascot
1057.5314	1057.5516	0.0202	19	50	58	ETPEAHVFK	14	0			Mascot
1600.8177	1600.8934	0.0757	47	71	85	VEVEDGNVLVVSGER					Mascot
1600.8177	1600.8934	0.0757	47	71	85	VEVEDGNVLVVSGER	9	0			Mascot

Calc. Mass	Obsrv. Mass	± da	± ppm	Start Seq.	End Seq.	Sequence	Ion Score	C. I. %	Modification	Rank	Result Type
1905.9666	1906.0603	0.0937	49	26	45	SIVPAISGGSSETAAFANAR					Mascot
1905.9666	1906.0603	0.0937	49	26	45	SIVPAISGGSSETAAFANAR	142	100			Mascot
2086.0664	2086.155	0.0886	42	67	85	EEVKVEVEDGNVLVVSGER					Mascot
2260.0347	2260.1248	0.0901	40	7	25	SNVFDPFADLWADPFDTFR					Mascot

3	hypothetical protein F775_32309 [Aegilops tauschii]	gi|475614914	16868.8	6.77	7	213	100	12.481	138	100	

Peptide Information

Calc. Mass	Obsrv. Mass	± da	± ppm	Start Seq.	End Seq.	Sequence	Ion Score	C. I. %	Modification	Rank	Result Type
827.4985	827.4829	-0.0156	-19	59	66	ADLPGVKK					Mascot
975.5258	975.5746	0.0488	50	110	117	FRLPEDAK					Mascot
975.5258	975.5746	0.0488	50	110	117	FRLPEDAK	24	0			Mascot
1057.5314	1057.5516	0.0202	19	50	58	ETPEAHVFK					Mascot
1057.5314	1057.5516	0.0202	19	50	58	ETPEAHVFK	14	0			Mascot
1600.8177	1600.8934	0.0757	47	71	85	VEVEDGNVLVVSGER					Mascot
1600.8177	1600.8934	0.0757	47	71	85	VEVEDGNVLVVSGER	9	0			Mascot
1932.9774	1933.0692	0.0918	47	26	45	SIVPAISGGNSETAAFANAR					Mascot
1932.9774	1933.0692	0.0918	47	26	45	SIVPAISGGNSETAAFANAR	124	100			Mascot
2086.0664	2086.155	0.0886	42	67	85	EEVKVEVEDGNVLVVSGER					Mascot
2260.0347	2260.1248	0.0901	40	7	25	SNVFDPFADLWADPFDTFR					Mascot

4	hypothetical protein F775_32310 [Aegilops tauschii]	gi|475614915	16783.8	5.83	5	193	100	23.738	157	100	

Peptide Information

Calc. Mass	Obsrv. Mass	± da	± ppm	Start Seq.	End Seq.	Sequence	Ion Score	C. I. %	Modification	Rank	Result Type
827.4985	827.4829	-0.0156	-19	59	66	ADLPGVKK					Mascot
1057.5314	1057.5516	0.0202	19	50	58	ETPEAHVFK					Mascot
1057.5314	1057.5516	0.0202	19	50	58	ETPEAHVFK	14	0			Mascot
1600.8177	1600.8934	0.0757	47	71	85	VEVEDGNVLVVSGER					Mascot
1600.8177	1600.8934	0.0757	47	71	85	VEVEDGNVLVVSGER	9	0			Mascot
1905.9666	1906.0603	0.0937	49	26	45	SIVPAISGGSSETAAFANAR					Mascot
1905.9666	1906.0603	0.0937	49	26	45	SIVPAISGGSSETAAFANAR	142	100			Mascot
2086.0664	2086.155	0.0886	42	67	85	EEVKVEVEDGNVLVVSGER					Mascot

5	hypothetical protein TRIUR3_16950 [Triticum urartu]	gi|473794549	27510.1	6.25	8	76	97.665	7.284	34	33.159	

Peptide Information

Calc. Mass	Obsrv. Mass	± da	± ppm	Start Seq.	End Seq.	Sequence	Ion Score	C. I. %	Modification	Rank	Result Type
815.3795	815.4125	0.033	40	99	104	TDTWHR					Mascot
827.4985	827.4829	-0.0156	-19	65	72	ADVPGLKK					Mascot
904.437	904.4432	0.0062	7	92	98	NKEQEEK					Mascot
974.5417	974.5853	0.0436	45	116	123	FRLPENAK					Mascot
1057.5314	1057.5516	0.0202	19	56	64	ETPEAHVFK					Mascot
1057.5314	1057.5516	0.0202	19	56	64	ETPEAHVFK	14	0			Mascot
1154.5437	1154.5985	0.0548	47	40	51	TSSDTAAFAGAR					Mascot
1154.5437	1154.5985	0.0548	47	40	51	TSSDTAAFAGAR	20	0			Mascot
1657.8392	1657.9052	0.066	40	77	91	VEVEDGNILQISGER					Mascot
1657.8392	1657.9052	0.066	40	77	91	VEVEDGNILQISGER	17	0			Mascot
2143.0876	2143.1763	0.0887	41	73	91	EEVKVEVEDGNILQISGER					Mascot

6	hypothetical protein TRIUR3_03467 [Triticum urartu]	gi|474305545	18321.7	9.15	6	76	97.611	4.703	14	0

Peptide Information

Calc. Mass	Obsrv. Mass	± da	± ppm	Start Seq.	End Seq.	Sequence	Ion Score	C. I. %	Modification	Rank	Result Type
827.4985	827.4829	-0.0156	-19	59	66	ADLPGVKK					Mascot
975.5258	975.5746	0.0488	50	122	129	FRLPEDAK					Mascot
975.5258	975.5746	0.0488	50	122	129	FRLPEDAK	24	0			Mascot
1057.5314	1057.5516	0.0202	19	50	58	ETPEAHVFK					Mascot
1057.5314	1057.5516	0.0202	19	50	58	ETPEAHVFK	14	0			Mascot
1600.8177	1600.8934	0.0757	47	71	85	VEVEDGNVLVVSGER					Mascot
1600.8177	1600.8934	0.0757	47	71	85	VEVEDGNVLVVSGER	9	0			Mascot
2086.0664	2086.155	0.0886	42	67	85	EEVKVEVEDGNVLVVSGER					Mascot
2260.0347	2260.1248	0.0901	40	7	25	SNVFDPFADLWADPFDTFR					Mascot

7	hypothetical protein CARUB_v10018594mg [Capsella rubella]	gi|482561088	17548	5.81	9	74	96.466	4.186	14	0

Peptide Information

Calc. Mass	Obsrv. Mass	± da	± ppm	Start Seq.	End Seq.	Sequence	Ion Score	C. I. %	Modification	Rank	Result Type
812.4083	812.4328	0.0245	30	108	114	SSGKFMR					Mascot
822.3475	822.4102	0.0627	76	92	98	SSENEEK					Mascot

828.4111	828.4686	0.0575	69	99	104	SDKWHR					Mascot
974.5417	974.5853	0.0436	45	116	123	FRLPENAK					Mascot
1053.6302	1053.5323	-0.0979	-93	143	151	VPEKKPEVK					Mascot
1057.5314	1057.5516	0.0202	19	56	64	ETPEAHVFK					Mascot
1057.5314	1057.5516	0.0202	19	56	64	ETPEAHVFK	14	0			Mascot
1492.7544	1492.7943	0.0399	27	43	55	DVAAFTNAKVDWR					Mascot
1657.8392	1657.9052	0.066	40	77	91	VEVEDGNILQISGER					Mascot
1657.8392	1657.9052	0.066	40	77	91	VEVEDGNILQISGER	17	0			Mascot
2143.0876	2143.1763	0.0887	41	73	91	EEVKVEVEDGNILQISGE R					Mascot

8 hypothetical protein CARUB_v10018169mg [Capsella rubella] gi|482560687 17573.9 5.56 9 74 96.035 4.401 14 0

Peptide Information

Calc. Mass	Obsrv. Mass	± da	± ppm	Start Seq.	End Seq.	Sequence	Ion Score	C. I. %	Modification	Rank	Result Type
812.4083	812.4328	0.0245	30	108	114	SSGKFMR					Mascot
822.3475	822.4102	0.0627	76	92	98	SSENEEK					Mascot
828.4111	828.4686	0.0575	69	99	104	SDKWHR					Mascot
974.5417	974.5853	0.0436	45	116	123	FRLPENAK					Mascot
1057.5314	1057.5516	0.0202	19	56	64	ETPEAHVFK					Mascot
1057.5314	1057.5516	0.0202	19	56	64	ETPEAHVFK	14	0			Mascot
1492.7544	1492.7943	0.0399	27	43	55	DVAAFTNAKVDWR					Mascot
1657.8392	1657.9052	0.066	40	77	91	VEVEDGNILQISGER					Mascot
1657.8392	1657.9052	0.066	40	77	91	VEVEDGNILQISGER	17	0			Mascot
1943.0089	1943.0315	0.0226	12	129	146	ASMENGVLSVMVPKVPE R					Mascot
2143.0876	2143.1763	0.0887	41	73	91	EEVKVEVEDGNILQISGE R					Mascot

9 heat shock protein 17.4 [Arabidopsis thaliana] gi|332644617 17428.8 5.21 8 71 91.715 4.425 14 0

Protein Group

RecName: Full=17.4 kDa class I heat shock protein; AltName: Full=17.4 kDa heat shock protein 1; Short=AtHsp17.4A	gi|21431764	17428.8	5.2100 000381 4697
heat shock protein 17.4 [Arabidopsis thaliana]	gi|15231372	17428.8	5.2100 000381 4697

Peptide Information

Calc. Mass	Obsrv. Mass	± da	± ppm	Start Seq.	End Seq.	Sequence	Ion Score	C. I. %	Modification	Rank	Result Type
812.4083	812.4328	0.0245	30	107	113	SSGKFMR					Mascot

Calc. Mass	Obsrv. Mass	± da	± ppm	Start Seq.	End Seq.	Sequence	Ion Score	C. I. %	Modification	Rank	Result Type
822.3475	822.4102	0.0627	76	91	97	SSENEEK					Mascot
827.4985	827.4829	-0.0156	-19	64	71	ADVPGLKK					Mascot
828.4033	828.4686	0.0653	79	107	113	SSGKFMR			Oxidation (M)[6]		Mascot
974.5417	974.5853	0.0436	45	115	122	FRLPENAK					Mascot
1057.5314	1057.5516	0.0202	19	55	63	ETPEAHVFK					Mascot
1057.5314	1057.5516	0.0202	19	55	63	ETPEAHVFK	14	0			Mascot
1492.7544	1492.7943	0.0399	27	42	54	DVAAFTNAKVDWR					Mascot
1657.8392	1657.9052	0.066	40	76	90	VEVEDGNILQISGER					Mascot
1657.8392	1657.9052	0.066	40	76	90	VEVEDGNILQISGER	17	0			Mascot
2143.0876	2143.1763	0.0887	41	72	90	EEVKVEVEDGNILQISGER					Mascot

10 hypothetical protein F775_32308 [Aegilops tauschii] gi|475614912 16849.8 6.34 5 68 85.93 4.39 14 0

Peptide Information

Calc. Mass	Obsrv. Mass	± da	± ppm	Start Seq.	End Seq.	Sequence	Ion Score	C. I. %	Modification	Rank	Result Type
827.4985	827.4829	-0.0156	-19	60	67	ADLPGVKK					Mascot
975.5258	975.5746	0.0488	50	111	118	FRLPEDAK					Mascot
975.5258	975.5746	0.0488	50	111	118	FRLPEDAK	24	0			Mascot
1057.5314	1057.5516	0.0202	19	51	59	ETPEAHVFK					Mascot
1057.5314	1057.5516	0.0202	19	51	59	ETPEAHVFK	14	0			Mascot
1600.8177	1600.8934	0.0757	47	72	86	VEVEDGNVLVVSGER					Mascot
1600.8177	1600.8934	0.0757	47	72	86	VEVEDGNVLVVSGER	9	0			Mascot
2086.0664	2086.155	0.0886	42	68	86	EEVKVEVEDGNVLVVSGER					Mascot

Gel Idx/Pos	128/F3	Instr./Gel Origin	BA2151/Sample Project 20140814	Process Status	Analysis Succeeded
Plate [#] Name	[1] Sample Project 20140814	Instrument Sample Name		Spectra	11

Rank	Protein Name	Accession No.	Protein MW	Protein PI	Pep. Count	Protein Score	Protein Score C. I. %	Intensity Matched	Total Ion Score	Total Ion C. I. %	Confirmed
1	Chain D, 0.19 Alpha-Amylase Inhibitor From Wheat	gi|3318684	13898.6	6.66	6	350	100	35.685	310	100	

Protein Group

Chain A, 0.19 Alpha-Amylase Inhibitor From Wheat	gi|3318681	13898.6	6.6599998474 1211
Chain B, 0.19 Alpha-Amylase Inhibitor From Wheat	gi|3318682	13898.6	6.6599998474 1211
Chain C, 0.19 Alpha-Amylase Inhibitor From Wheat	gi|3318683	13898.6	6.6599998474 1211
RecName: Full=Alpha-amylase inhibitor 0.19; AltName: Full=0.19 alpha-AI; Short=0.19 AI	gi|123963	13898.6	6.6599998474 1211

Peptide Information

Calc. Mass	Obsrv. Mass	± da	± ppm	Start Seq.	End Seq.	Sequence	Ion Score	C. I. %	Modification	Rank	Result Type
1162.6249	1162.6868	0.0619	53	90	100	LTAASITAVCR			Carbamidomethyl (C)[10]		Mascot
1162.6249	1162.6868	0.0619	53	90	100	LTAASITAVCR	36	55.669	Carbamidomethyl (C)[10]		Mascot
1570.8007	1570.892	0.0913	58	26	39	LQCNGSQVPEAVLR			Carbamidomethyl (C)[3]		Mascot
1612.7463	1612.8384	0.0921	57	67	82	EHGAQEGQAGTGAFPR					Mascot
1612.7463	1612.8384	0.0921	57	67	82	EHGAQEGQAGTGAFPR	144	100			Mascot
1617.8993	1617.8442	-0.0551	-34	86	100	EVVKLTAASITAVCR			Carbamidomethyl (C)[14]		Mascot
1663.8361	1663.8826	0.0465	28	101	116	LPIVVDASGDGAYVCK			Carbamidomethyl (C)[15]		Mascot
1862.7731	1862.8889	0.1158	62	40	53	DCCQQLAHISEWCR			Carbamidomethyl (C)[2,3,13]		Mascot
1862.7731	1862.8889	0.1158	62	40	53	DCCQQLAHISEWCR	130	100	Carbamidomethyl (C)[2,3,13]		Mascot

Rank	Protein Name	Accession No.	Protein MW	Protein PI	Pep. Count	Protein Score	Protein Score C. I. %	Intensity Matched	Total Ion Score	Total Ion C. I. %	Confirmed
2	dimeric alpha-amylase inhibitor, partial [Aegilops kotschyi]	gi|386877048	14198.8	6.66	6	349	100	35.685	310	100	

Protein Group

dimeric alpha-amylase inhibitor, partial [Aegilops geniculata]	gi|386877062	14542.9	7.0500001907 3486
dimeric alpha-amylase inhibitor, partial [Aegilops geniculata]	gi|452055912	14198.8	6.6599998474 1211
dimeric alpha-amylase inhibitor, partial [Aegilops tauschii]	gi|386877046	14670	6.0799999237 0605

	dimeric alpha-amylase inhibitor, partial [Aegilops tauschii]	gi|386877050	14826.1	6.8600001335144							
	dimeric alpha-amylase inhibitor, partial [Aegilops tauschii]	gi|386877044	14805	6.0999999046 3257							

Peptide Information

Calc. Mass	Obsrv. Mass	± da	± ppm	Start Seq.	End Seq.	Sequence	Ion Score	C. I. %	Modification	Rank	Result Type
1162.6249	1162.6868	0.0619	53	93	103	LTAASITAVCR			Carbamidomethyl (C)[10]		Mascot
1162.6249	1162.6868	0.0619	53	93	103	LTAASITAVCR	36	55.669	Carbamidomethyl (C)[10]		Mascot
1570.8007	1570.892	0.0913	58	29	42	LQCNGSQVPEAVLR			Carbamidomethyl (C)[3]		Mascot
1612.7463	1612.8384	0.0921	57	70	85	EHGAQEGQAGTGAFPR					Mascot
1612.7463	1612.8384	0.0921	57	70	85	EHGAQEGQAGTGAFPR	144	100			Mascot
1617.8993	1617.8442	-0.0551	-34	89	103	EVVKLTAASITAVCR			Carbamidomethyl (C)[14]		Mascot
1663.8361	1663.8826	0.0465	28	104	119	LPIVVDASGDGAYVCK			Carbamidomethyl (C)[15]		Mascot
1862.7731	1862.8889	0.1158	62	43	56	DCCQQLAHISEWCR			Carbamidomethyl (C)[2,3,13]		Mascot
1862.7731	1862.8889	0.1158	62	43	56	DCCQQLAHISEWCR	130	100	Carbamidomethyl (C)[2,3,13]		Mascot

3	dimeric alpha-amylase inhibitor, partial [Aegilops longissima]	gi|386877060	14954.2	7.64	6	348	100	35.685	310	100	

Peptide Information

Calc. Mass	Obsrv. Mass	± da	± ppm	Start Seq.	End Seq.	Sequence	Ion Score	C. I. %	Modification	Rank	Result Type
1162.6249	1162.6868	0.0619	53	99	109	LTAASITAVCR			Carbamidomethyl (C)[10]		Mascot
1162.6249	1162.6868	0.0619	53	99	109	LTAASITAVCR	36	55.669	Carbamidomethyl (C)[10]		Mascot
1570.8007	1570.892	0.0913	58	35	48	LQCNGSQVPEAVLR			Carbamidomethyl (C)[3]		Mascot
1612.7463	1612.8384	0.0921	57	76	91	EHGAQEGQAGTGAFPR					Mascot
1612.7463	1612.8384	0.0921	57	76	91	EHGAQEGQAGTGAFPR	144	100			Mascot
1617.8993	1617.8442	-0.0551	-34	95	109	EVVKLTAASITAVCR			Carbamidomethyl (C)[14]		Mascot
1663.8361	1663.8826	0.0465	28	110	125	LPIVVDASGDGAYVCK			Carbamidomethyl (C)[15]		Mascot
1862.7731	1862.8889	0.1158	62	49	62	DCCQQLAHISEWCR			Carbamidomethyl (C)[2,3,13]		Mascot
1862.7731	1862.8889	0.1158	62	49	62	DCCQQLAHISEWCR	130	100	Carbamidomethyl (C)[2,3,13]		Mascot

4	dimeric alpha-amylase inhibitor [Triticum aestivum]	gi|386877038	15702.5	5.58	6	347	100	35.685	310	100	

Peptide Information

Calc. Mass	Obsrv. Mass	± da	± ppm	Start Seq.	End Seq.	Sequence	Ion Score	C. I. %	Modification	Rank	Result Type
1162.6249	1162.6868	0.0619	53	107	117	LTAASITAVCR			Carbamidomethyl (C)[10]		Mascot

Calc. Mass	Obsrv. Mass	± da	± ppm	Start Seq.	End Seq.	Sequence	Ion Score	C. I. %	Modification	Rank	Result Type
1162.6249	1162.6868	0.0619	53	107	117	LTAASITAVCR	36	55.669	Carbamidomethyl (C)[10]		Mascot
1570.8007	1570.892	0.0913	58	43	56	LQCNGSQVPEAVLR			Carbamidomethyl (C)[3]		Mascot
1612.7463	1612.8384	0.0921	57	84	99	EHGAQEGQAGTGAFPR					Mascot
1612.7463	1612.8384	0.0921	57	84	99	EHGAQEGQAGTGAFPR	144	100			Mascot
1617.8993	1617.8442	-0.0551	-34	103	117	EVVKLTAASITAVCR			Carbamidomethyl (C)[14]		Mascot
1663.8361	1663.8826	0.0465	28	118	133	LPIVVDASGDGAYVCK			Carbamidomethyl (C)[15]		Mascot
1862.7731	1862.8889	0.1158	62	57	70	DCCQQLAHISEWCR			Carbamidomethyl (C)[2,3,13]		Mascot
1862.7731	1862.8889	0.1158	62	57	70	DCCQQLAHISEWCR	130	100	Carbamidomethyl (C)[2,3,13]		Mascot

5	Alpha-amylase inhibitor 0.19 [Aegilops tauschii]	gi|475613321	17198.2	6.06	6	345	100	35.685	310	100	

Peptide Information

Calc. Mass	Obsrv. Mass	± da	± ppm	Start Seq.	End Seq.	Sequence	Ion Score	C. I. %	Modification	Rank	Result Type
1162.6249	1162.6868	0.0619	53	120	130	LTAASITAVCR			Carbamidomethyl (C)[10]		Mascot
1162.6249	1162.6868	0.0619	53	120	130	LTAASITAVCR	36	55.669	Carbamidomethyl (C)[10]		Mascot
1570.8007	1570.892	0.0913	58	56	69	LQCNGSQVPEAVLR			Carbamidomethyl (C)[3]		Mascot
1612.7463	1612.8384	0.0921	57	97	112	EHGAQEGQAGTGAFPR					Mascot
1612.7463	1612.8384	0.0921	57	97	112	EHGAQEGQAGTGAFPR	144	100			Mascot
1617.8993	1617.8442	-0.0551	-34	116	130	EVVKLTAASITAVCR			Carbamidomethyl (C)[14]		Mascot
1663.8361	1663.8826	0.0465	28	131	146	LPIVVDASGDGAYVCK			Carbamidomethyl (C)[15]		Mascot
1862.7731	1862.8889	0.1158	62	70	83	DCCQQLAHISEWCR			Carbamidomethyl (C)[2,3,13]		Mascot
1862.7731	1862.8889	0.1158	62	70	83	DCCQQLAHISEWCR	130	100	Carbamidomethyl (C)[2,3,13]		Mascot

6	dimeric alpha-amylase inhibitor, partial [Triticum aestivum]	gi|386877068	14415.8	6.88	5	340	100	34.955	310	100	

Peptide Information

Calc. Mass	Obsrv. Mass	± da	± ppm	Start Seq.	End Seq.	Sequence	Ion Score	C. I. %	Modification	Rank	Result Type
1162.6249	1162.6868	0.0619	53	95	105	LTAASITAVCR			Carbamidomethyl (C)[10]		Mascot
1162.6249	1162.6868	0.0619	53	95	105	LTAASITAVCR	36	55.669	Carbamidomethyl (C)[10]		Mascot
1570.8007	1570.892	0.0913	58	31	44	LQCNGSQVPEAVLR			Carbamidomethyl (C)[3]		Mascot
1612.7463	1612.8384	0.0921	57	72	87	EHGAQEGQAGTGAFPR					Mascot
1612.7463	1612.8384	0.0921	57	72	87	EHGAQEGQAGTGAFPR	144	100			Mascot
1617.8993	1617.8442	-0.0551	-34	91	105	EVVKLTAASITAVCR			Carbamidomethyl (C)[14]		Mascot
1862.7731	1862.8889	0.1158	62	45	58	DCCQQLAHISEWCR			Carbamidomethyl (C)[2,3,13]		Mascot
1862.7731	1862.8889	0.1158	62	45	58	DCCQQLAHISEWCR	130	100	Carbamidomethyl (C)[2,3,13]		Mascot

7	dimeric alpha-amylase inhibitor, partial [Aegilops geniculata]	gi|452055914	14748	6.09	5	339	100	34.955	310	100	

Peptide Information

Calc. Mass	Obsrv. Mass	± da	± ppm	Start Seq.	End Seq.	Sequence	Ion Score	C. I. %	Modification	Rank	Result Type
1162.6249	1162.6868	0.0619	53	97	107	LTAASITAVCR			Carbamidomethyl (C)[10]		Mascot
1162.6249	1162.6868	0.0619	53	97	107	LTAASITAVCR	36	55.669	Carbamidomethyl (C)[10]		Mascot
1570.8007	1570.892	0.0913	58	33	46	LQCNGSQVPEAVLR			Carbamidomethyl (C)[3]		Mascot
1612.7463	1612.8384	0.0921	57	74	89	EHGAQEGQAGTGAFPR					Mascot
1612.7463	1612.8384	0.0921	57	74	89	EHGAQEGQAGTGAFPR	144	100			Mascot
1617.8993	1617.8442	-0.0551	-34	93	107	EVVKLTAASITAVCR			Carbamidomethyl (C)[14]		Mascot
1862.7731	1862.8889	0.1158	62	47	60	DCCQQLAHISEWCR			Carbamidomethyl (C)[2,3,13]		Mascot
1862.7731	1862.8889	0.1158	62	47	60	DCCQQLAHISEWCR	130	100	Carbamidomethyl (C)[2,3,13]		Mascot

8	dimeric alpha-amylase inhibitor, partial [Aegilops longissima]	gi|386877056	14792	5.28	5	209	100	28.475	180	100	

Protein Group

dimeric alpha-amylase inhibitor, partial [Aegilops longissima]	gi|386877058	14718.9	4.9899997711816

Peptide Information

Calc. Mass	Obsrv. Mass	± da	± ppm	Start Seq.	End Seq.	Sequence	Ion Score	C. I. %	Modification	Rank	Result Type
1162.6249	1162.6868	0.0619	53	98	108	LTAASITAVCR			Carbamidomethyl (C)[10]		Mascot
1162.6249	1162.6868	0.0619	53	98	108	LTAASITAVCR	36	55.669	Carbamidomethyl (C)[10]		Mascot
1570.8007	1570.892	0.0913	58	34	47	LQCNGSQVPEAVLR			Carbamidomethyl (C)[3]		Mascot
1612.7463	1612.8384	0.0921	57	75	90	EHGAQEGQAGTGAFPR					Mascot
1612.7463	1612.8384	0.0921	57	75	90	EHGAQEGQAGTGAFPR	144	100			Mascot
1617.8993	1617.8442	-0.0551	-34	94	108	EVVKLTAASITAVCR			Carbamidomethyl (C)[14]		Mascot
1663.8361	1663.8826	0.0465	28	109	124	LPIVVDASGDGAYVCK			Carbamidomethyl (C)[15]		Mascot

9	dimeric alpha-amylase inhibitor, partial [Aegilops peregrina]	gi|386877054	14145.7	5.26	4	202	100	26.444	180	100	

Peptide Information

Calc. Mass	Obsrv. Mass	± da	± ppm	Start Seq.	End Seq.	Sequence	Ion Score	C. I. %	Modification	Rank	Result Type
1162.6249	1162.6868	0.0619	53	92	102	LTAASITAVCR			Carbamidomethyl (C)[10]		Mascot
1162.6249	1162.6868	0.0619	53	92	102	LTAASITAVCR	36	55.669	Carbamidomethyl (C)[10]		Mascot
1612.7463	1612.8384	0.0921	57	69	84	EHGAQEGQAGTGAFPR					Mascot
1612.7463	1612.8384	0.0921	57	69	84	EHGAQEGQAGTGAFPR	144	100			Mascot

1617.8993	1617.8442	-0.0551	-34	88	102	EVVKLTAASITAVCR			Carbamidomethyl (C)[14]		Mascot
1663.8361	1663.8826	0.0465	28	103	118	LPIVVDASGDGAYVCK			Carbamidomethyl (C)[15]		Mascot

10	dimeric alpha-amylase inhibitor, partial [Triticum aestivum]	gi|386877040	15014.1	5.28	4	201	100	27.744	180	100	

Peptide Information

Calc. Mass	Obsrv. Mass	± da	± ppm	Start Seq.	End Seq.	Sequence	Ion Score	C. I. %	Modification	Rank	Result Type
1162.6249	1162.6868	0.0619	53	101	111	LTAASITAVCR			Carbamidomethyl (C)[10]		Mascot
1162.6249	1162.6868	0.0619	53	101	111	LTAASITAVCR	36	55.669	Carbamidomethyl (C)[10]		Mascot
1570.8007	1570.892	0.0913	58	37	50	LQCNGSQVPEAVLR			Carbamidomethyl (C)[3]		Mascot
1612.7463	1612.8384	0.0921	57	78	93	EHGAQEGQAGTGAFPR					Mascot
1612.7463	1612.8384	0.0921	57	78	93	EHGAQEGQAGTGAFPR	144	100			Mascot
1617.8993	1617.8442	-0.0551	-34	97	111	EVVKLTAASITAVCR			Carbamidomethyl (C)[14]		Mascot

Gel Idx/Pos	129/F4	Instr./Gel Origin	BA2151/Sample Project 20140814	Process Status	Analysis Succeeded
Plate [#] Name	[1] Sample Project 20140814	Instrument Sample Name		Spectra	11

Rank	Protein Name	Accession No.	Protein MW	Protein PI	Pep. Count	Protein Score	Protein Score C. I. %	Intensity Matched	Total Ion Score	Total Ion C. I. %	Confirmed
1	Chain D, 0.19 Alpha-Amylase Inhibitor From Wheat	gi|3318684	13898.6	6.66	6	317	100	36.012	278	100	

Protein Group

Chain A, 0.19 Alpha-Amylase Inhibitor From Wheat	gi|3318681	13898.6	6.6599 998474 1211
Chain B, 0.19 Alpha-Amylase Inhibitor From Wheat	gi|3318682	13898.6	6.6599 998474 1211
Chain C, 0.19 Alpha-Amylase Inhibitor From Wheat	gi|3318683	13898.6	6.6599 998474 1211
RecName: Full=Alpha-amylase inhibitor 0.19; AltName: Full=0.19 alpha-AI; Short=0.19 AI	gi|123963	13898.6	6.6599 998474 1211
dimeric alpha-amylase inhibitor, partial [Aegilops geniculata]	gi|452055912	14198.8	6.6599 998474 1211
dimeric alpha-amylase inhibitor, partial [Aegilops kotschyi]	gi|386877048	14198.8	6.6599 998474 1211

Peptide Information

Calc. Mass	Obsrv. Mass	± da	± ppm	Start Seq.	End Seq.	Sequence	Ion Score	C. I. %	Modification	Rank	Result Type
1162.6249	1162.6742	0.0493	42	90	100	LTAASITAVCR			Carbamidomethyl (C)[10]		Mascot
1570.8007	1570.8644	0.0637	41	26	39	LQCNGSQVPEAVLR			Carbamidomethyl (C)[3]		Mascot
1612.7463	1612.8197	0.0734	46	67	82	EHGAQEGQAGTGAFPR					Mascot
1612.7463	1612.8197	0.0734	46	67	82	EHGAQEGQAGTGAFPR	147	100			Mascot
1617.8993	1617.8162	-0.0831	-51	86	100	EVVKLTAASITAVCR			Carbamidomethyl (C)[14]		Mascot
1663.8361	1663.8524	0.0163	10	101	116	LPIVVDASGDGAYVCK			Carbamidomethyl (C)[15]		Mascot
1862.7731	1862.8665	0.0934	50	40	53	DCCQQLAHISEWCR			Carbamidomethyl (C)[2,3,13]		Mascot
1862.7731	1862.8665	0.0934	50	40	53	DCCQQLAHISEWCR	131	100	Carbamidomethyl (C)[2,3,13]		Mascot

Rank	Protein Name	Accession No.	Protein MW	Protein PI	Pep. Count	Protein Score	Protein Score C. I. %	Intensity Matched	Total Ion Score	Total Ion C. I. %	Confirmed
2	dimeric alpha-amylase inhibitor, partial [Aegilops tauschii]	gi|386877046	14670	6.08	6	316	100	36.012	278	100	

Protein Group

dimeric alpha-amylase inhibitor, partial [Aegilops geniculata]	gi|386877062	14542.9	7.0500 001907 3486
dimeric alpha-amylase inhibitor, partial [Aegilops	gi|386877060	14954.2	7.6399

	longissima]			998664856
	dimeric alpha-amylase inhibitor, partial [Aegilops tauschii]	gi|386877050	14826.1	6.8600001335144
	dimeric alpha-amylase inhibitor, partial [Aegilops tauschii]	gi|386877044	14805	6.09999990463257

Peptide Information

Calc. Mass	Obsrv. Mass	± da	± ppm	Start Seq.	End Seq.	Sequence	Ion Score	C. I. %	Modification	Rank	Result Type
1162.6249	1162.6742	0.0493	42	97	107	LTAASITAVCR			Carbamidomethyl (C)[10]		Mascot
1570.8007	1570.8644	0.0637	41	33	46	LQCNGSQVPEAVLR			Carbamidomethyl (C)[3]		Mascot
1612.7463	1612.8197	0.0734	46	74	89	EHGAQEGQAGTGAFPR					Mascot
1612.7463	1612.8197	0.0734	46	74	89	EHGAQEGQAGTGAFPR	147	100			Mascot
1617.8993	1617.8162	-0.0831	-51	93	107	EVVKLTAASITAVCR			Carbamidomethyl (C)[14]		Mascot
1663.8361	1663.8524	0.0163	10	108	123	LPIVVDASGDGAYVCK			Carbamidomethyl (C)[15]		Mascot
1862.7731	1862.8665	0.0934	50	47	60	DCCQQLAHISEWCR			Carbamidomethyl (C)[2,3,13]		Mascot
1862.7731	1862.8665	0.0934	50	47	60	DCCQQLAHISEWCR	131	100	Carbamidomethyl (C)[2,3,13]		Mascot

3	dimeric alpha-amylase inhibitor [Triticum aestivum]	gi|386877038	15702.5	5.58	6	314	100	36.012	278	100

Peptide Information

Calc. Mass	Obsrv. Mass	± da	± ppm	Start Seq.	End Seq.	Sequence	Ion Score	C. I. %	Modification	Rank	Result Type
1162.6249	1162.6742	0.0493	42	107	117	LTAASITAVCR			Carbamidomethyl (C)[10]		Mascot
1570.8007	1570.8644	0.0637	41	43	56	LQCNGSQVPEAVLR			Carbamidomethyl (C)[3]		Mascot
1612.7463	1612.8197	0.0734	46	84	99	EHGAQEGQAGTGAFPR					Mascot
1612.7463	1612.8197	0.0734	46	84	99	EHGAQEGQAGTGAFPR	147	100			Mascot
1617.8993	1617.8162	-0.0831	-51	103	117	EVVKLTAASITAVCR			Carbamidomethyl (C)[14]		Mascot
1663.8361	1663.8524	0.0163	10	118	133	LPIVVDASGDGAYVCK			Carbamidomethyl (C)[15]		Mascot
1862.7731	1862.8665	0.0934	50	57	70	DCCQQLAHISEWCR			Carbamidomethyl (C)[2,3,13]		Mascot
1862.7731	1862.8665	0.0934	50	57	70	DCCQQLAHISEWCR	131	100	Carbamidomethyl (C)[2,3,13]		Mascot

4	Alpha-amylase inhibitor 0.19 [Aegilops tauschii]	gi|475613321	17198.2	6.06	6	312	100	36.012	278	100

Peptide Information

Calc. Mass	Obsrv. Mass	± da	± ppm	Start Seq.	End Seq.	Sequence	Ion Score	C. I. %	Modification	Rank	Result Type
1162.6249	1162.6742	0.0493	42	120	130	LTAASITAVCR			Carbamidomethyl (C)[10]		Mascot
1570.8007	1570.8644	0.0637	41	56	69	LQCNGSQVPEAVLR			Carbamidomethyl (C)[3]		Mascot

1612.7463	1612.8197	0.0734	46	97	112	EHGAQEGQAGTGAFPR					Mascot
1612.7463	1612.8197	0.0734	46	97	112	EHGAQEGQAGTGAFPR	147	100			Mascot
1617.8993	1617.8162	-0.0831	-51	116	130	EVVKLTAASITAVCR			Carbamidomethyl (C)[14]		Mascot
1663.8361	1663.8524	0.0163	10	131	146	LPIVVDASGDGAYVCK			Carbamidomethyl (C)[15]		Mascot
1862.7731	1862.8665	0.0934	50	70	83	DCCQQLAHISEWCR			Carbamidomethyl (C)[2,3,13]		Mascot
1862.7731	1862.8665	0.0934	50	70	83	DCCQQLAHISEWCR	131	100	Carbamidomethyl (C)[2,3,13]		Mascot

5	dimeric alpha-amylase inhibitor, partial [Aegilops geniculata]	gi|452055914	14748	6.09	5	307	100	35.74	278	100	

Protein Group

dimeric alpha-amylase inhibitor, partial [Triticum aestivum]	gi|386877068	14415.8	6.8800 001144 4092

Peptide Information

Calc. Mass	Obsrv. Mass	± da	± ppm	Start Seq.	End Seq.	Sequence	Ion Score	C. I. %	Modification	Rank	Result Type
1162.6249	1162.6742	0.0493	42	97	107	LTAASITAVCR			Carbamidomethyl (C)[10]		Mascot
1570.8007	1570.8644	0.0637	41	33	46	LQCNGSQVPEAVLR			Carbamidomethyl (C)[3]		Mascot
1612.7463	1612.8197	0.0734	46	74	89	EHGAQEGQAGTGAFPR					Mascot
1612.7463	1612.8197	0.0734	46	74	89	EHGAQEGQAGTGAFPR	147	100			Mascot
1617.8993	1617.8162	-0.0831	-51	93	107	EVVKLTAASITAVCR			Carbamidomethyl (C)[14]		Mascot
1862.7731	1862.8665	0.0934	50	47	60	DCCQQLAHISEWCR			Carbamidomethyl (C)[2,3,13]		Mascot
1862.7731	1862.8665	0.0934	50	47	60	DCCQQLAHISEWCR	131	100	Carbamidomethyl (C)[2,3,13]		Mascot

6	RecName: Full=Alpha-amylase/trypsin inhibitor CM3; AltName: Full=Chloroform/methanol-soluble protein CM3; Flags: Precursor	gi|123957	18893.3	7.44	7	236	100	13.203	195	100	

Peptide Information

Calc. Mass	Obsrv. Mass	± da	± ppm	Start Seq.	End Seq.	Sequence	Ion Score	C. I. %	Modification	Rank	Result Type
1010.52	1010.5569	0.0369	37	37	44	TNLLPHCR			Carbamidomethyl (C)[7]		Mascot
1110.5038	1110.5485	0.0447	40	133	140	EMQWDFVR					Mascot
1126.4987	1126.5249	0.0262	23	133	140	EMQWDFVR			Oxidation (M)[2]		Mascot
1698.9214	1698.9817	0.0603	35	101	115	YFIALPVPSQPVDPR					Mascot
1698.9214	1698.9817	0.0603	35	101	115	YFIALPVPSQPVDPR	75	99.995			Mascot
1727.8381	1727.9132	0.0751	43	116	132	SGNVGESGLIDLPGCPR			Carbamidomethyl (C)[15]		Mascot
1801.8427	1801.869	0.0263	15	45	60	DYVLQQTCGTFTPGSK			Carbamidomethyl (C)[8]		Mascot
1876.0222	1876.1145	0.0923	49	141	157	LLVAPGQCNLATIHNVR			Carbamidomethyl (C)[8]		Mascot
1876.0222	1876.1145	0.0923	49	141	157	LLVAPGQCNLATIHNVR	35	46.426	Carbamidomethyl (C)[8]		Mascot

Calc. Mass	Obsrv. Mass	± da	± ppm	Start Seq.	End Seq.	Sequence	Ion Score	C. I. %	Modification	Rank	Result Type
1957.8564	1957.9537	0.0973	50	81	95	LYCCQELAEISQQCR			Carbamidomethyl (C)[3,4,14]		Mascot
1957.8564	1957.9537	0.0973	50	81	95	LYCCQELAEISQQCR	85	100	Carbamidomethyl (C)[3,4,14]		Mascot

7	dimeric alpha-amylase inhibitor, partial [Aegilops longissima]	gi|386877056	14792	5.28	5	176	100	22.941	147	100

Protein Group

dimeric alpha-amylase inhibitor, partial [Aegilops longissima]	gi|386877058	14718.9	4.9899 997711 1816

Peptide Information

Calc. Mass	Obsrv. Mass	± da	± ppm	Start Seq.	End Seq.	Sequence	Ion Score	C. I. %	Modification	Rank	Result Type
1162.6249	1162.6742	0.0493	42	98	108	LTAASITAVCR			Carbamidomethyl (C)[10]		Mascot
1570.8007	1570.8644	0.0637	41	34	47	LQCNGSQVPEAVLR			Carbamidomethyl (C)[3]		Mascot
1612.7463	1612.8197	0.0734	46	75	90	EHGAQEGQAGTGAFPR					Mascot
1612.7463	1612.8197	0.0734	46	75	90	EHGAQEGQAGTGAFPR	147	100			Mascot
1617.8993	1617.8162	-0.0831	-51	94	108	EVVKLTAASITAVCR			Carbamidomethyl (C)[14]		Mascot
1663.8361	1663.8524	0.0163	10	109	124	LPIVVDASGDGAYVCK			Carbamidomethyl (C)[15]		Mascot

8	dimeric alpha-amylase inhibitor, partial [Aegilops peregrina]	gi|386877054	14145.7	5.26	4	169	100	21.581	147	100

Peptide Information

Calc. Mass	Obsrv. Mass	± da	± ppm	Start Seq.	End Seq.	Sequence	Ion Score	C. I. %	Modification	Rank	Result Type
1162.6249	1162.6742	0.0493	42	92	102	LTAASITAVCR			Carbamidomethyl (C)[10]		Mascot
1612.7463	1612.8197	0.0734	46	69	84	EHGAQEGQAGTGAFPR					Mascot
1612.7463	1612.8197	0.0734	46	69	84	EHGAQEGQAGTGAFPR	147	100			Mascot
1617.8993	1617.8162	-0.0831	-51	88	102	EVVKLTAASITAVCR			Carbamidomethyl (C)[14]		Mascot
1663.8361	1663.8524	0.0163	10	103	118	LPIVVDASGDGAYVCK			Carbamidomethyl (C)[15]		Mascot

9	dimeric alpha-amylase inhibitor, partial [Triticum aestivum]	gi|386877040	15014.1	5.28	4	168	100	22.669	147	100

Peptide Information

Calc. Mass	Obsrv. Mass	± da	± ppm	Start Seq.	End Seq.	Sequence	Ion Score	C. I. %	Modification	Rank	Result Type
1162.6249	1162.6742	0.0493	42	101	111	LTAASITAVCR			Carbamidomethyl (C)[10]		Mascot
1570.8007	1570.8644	0.0637	41	37	50	LQCNGSQVPEAVLR			Carbamidomethyl (C)[3]		Mascot
1612.7463	1612.8197	0.0734	46	78	93	EHGAQEGQAGTGAFPR					Mascot
1612.7463	1612.8197	0.0734	46	78	93	EHGAQEGQAGTGAFPR	147	100			Mascot
1617.8993	1617.8162	-0.0831	-51	97	111	EVVKLTAASITAVCR			Carbamidomethyl (C)[14]		Mascot

10	dimeric alpha-amylase inhibitor, partial [Triticum dicoccoides]	gi|488508057	15698.5	7.62	1	151	100	20.246	147	100	

Peptide Information

Calc. Mass	Obsrv. Mass	± da	± ppm	Start Seq.	End Seq.	Sequence	Ion Score	C. I. %	Modification	Rank	Result Type
1612.7463	1612.8197	0.0734	46	82	97	EHGAQEGQAGTGAFPR					Mascot
1612.7463	1612.8197	0.0734	46	82	97	EHGAQEGQAGTGAFPR	147	100			Mascot

Gel Idx/Pos	130/F5	Instr./Gel Origin	BA2151/Sample Project 20140814	Process Status	Analysis Succeeded
Plate [#] Name	[1] Sample Project 20140814	Instrument Sample Name		Spectra	11

Rank	Protein Name	Accession No.	Protein MW	Protein PI	Pep. Count	Protein Score	Protein Score C. I. %	Intensity Matched	Total Ion Score	Total Ion C. I. %	Confirmed
1	hypothetical protein F775_31562 [Aegilops tauschii]	gi|475458859	16753.8	6.19	5	49	0	3.871	26	0	

Peptide Information

Calc. Mass	Obsrv. Mass	± da	± ppm	Start Seq.	End Seq.	Sequence	Ion Score	C. I. %	Modification	Rank	Result Type
827.4985	827.458	-0.0405	-49	59	66	ADLPGVKK					Mascot
975.5258	975.5727	0.0469	48	110	117	FRLPEDAK					Mascot
1027.6146	1027.5535	-0.0611	-59	137	145	AEVKKPEVK					Mascot
1184.6521	1184.6407	-0.0114	-10	112	122	LPEDAKVGEVK					Mascot
1905.9666	1906.0742	0.1076	56	26	45	SIVPAISGGSSETAAFAN AR					Mascot
1905.9666	1906.0742	0.1076	56	26	45	SIVPAISGGSSETAAFAN AR	26	0			Mascot

Rank	Protein Name	Accession No.	Protein MW	Protein PI	Pep. Count	Protein Score	Protein Score C. I. %	Intensity Matched	Total Ion Score	Total Ion C. I. %	Confirmed
2	TPA: hypothetical protein ZEAMMB73_051641 [Zea mays]	gi|414586135	9956	9.49	7	46	0	4.869			

Peptide Information

Calc. Mass	Obsrv. Mass	± da	± ppm	Start Seq.	End Seq.	Sequence	Ion Score	C. I. %	Modification	Rank	Result Type
959.4475	959.3898	-0.0577	-60	2	9	ERHGMSAR			Oxidation (M)[5]		Mascot
1106.483	1106.5709	0.0879	79	1	9	MERHGMSAR			Oxidation (M)[1,6]		Mascot
1109.5256	1109.5765	0.0509	46	49	59	EATTTTMAGAR					Mascot
1193.5732	1193.691	0.1178	99	82	92	MVPQGSNPLHN					Mascot
1365.6692	1365.7216	0.0524	38	81	92	RMVPQGSNPLHN			Oxidation (M)[2]		Mascot
1475.702	1475.8262	0.1242	84	46	59	HGREATTTTMAGAR			Oxidation (M)[10]		Mascot
1475.7336	1475.8262	0.0926	63	68	81	ADLDTEAALGESKR					Mascot

Rank	Protein Name	Accession No.	Protein MW	Protein PI	Pep. Count	Protein Score	Protein Score C. I. %	Intensity Matched	Total Ion Score	Total Ion C. I. %	Confirmed
3	hypothetical protein M569_13739, partial [Genlisea aurea]	gi|527189384	13084.2	11.13	8	43	0	5.912			

Peptide Information

Calc. Mass	Obsrv. Mass	± da	± ppm	Start Seq.	End Seq.	Sequence	Ion Score	C. I. %	Modification	Rank	Result Type
802.5032	802.4953	-0.0079	-10	92	98	AIKTIEK					Mascot
827.4846	827.458	-0.0266	-32	48	54	KPNKANR					Mascot
831.4934	831.4162	-0.0772	-93	108	115	AGIDLSKK					Mascot

Calc. Mass	Obsrv. Mass	± da	± ppm	Start Seq.	End Seq.	Sequence	Ion Score	C. I. %	Modification	Rank	Result Type
889.4566	889.3767	-0.0799	-90	75	81	IWWEAGK					Mascot
973.5829	973.5884	0.0055	6	18	25	YPKISPLR					Mascot
1179.6368	1179.6708	0.034	29	1	12	VGSISSQFGGLK					Mascot
1258.7002	1258.6863	-0.0139	-11	95	106	TIEKNGLDAVAK					Mascot
1277.6671	1277.7864	0.1193	93	41	51	TCPFTGKKPNK			Carbamidomethyl (C)[2]		Mascot

4	hypothetical protein TRIUR3_03549 [Triticum urartu]	gi|474071007	16824.8	6.19	4	43	0	3.766	26	0	

Peptide Information

Calc. Mass	Obsrv. Mass	± da	± ppm	Start Seq.	End Seq.	Sequence	Ion Score	C. I. %	Modification	Rank	Result Type
827.4985	827.458	-0.0405	-49	59	66	ADLPGVKK					Mascot
975.5258	975.5727	0.0469	48	110	117	FRLPEDAK					Mascot
1027.6146	1027.5535	-0.0611	-59	137	145	AEVKKPEVK					Mascot
1905.9666	1906.0742	0.1076	56	26	45	SIVPAISGGSSETAAFANAR					Mascot
1905.9666	1906.0742	0.1076	56	26	45	SIVPAISGGSSETAAFANAR	26	0			Mascot

5	RecName: Full=16.9 kDa class I heat shock protein 1; AltName: Full=HSP 16.9; AltName: Full=Heat shock protein 16.9A; AltName: Full=Heat shock protein 17; AltName: Full=Low molecular weight heat shock protein	gi|123545	16867.8	5.83	3	40	0	3.592	26	0	

Peptide Information

Calc. Mass	Obsrv. Mass	± da	± ppm	Start Seq.	End Seq.	Sequence	Ion Score	C. I. %	Modification	Rank	Result Type
975.5258	975.5727	0.0469	48	110	117	FRLPEDAK					Mascot
1027.6146	1027.5535	-0.0611	-59	137	145	AEVKKPEVK					Mascot
1905.9666	1906.0742	0.1076	56	26	45	SIVPAISGGSSETAAFANAR					Mascot
1905.9666	1906.0742	0.1076	56	26	45	SIVPAISGGSSETAAFANAR	26	0			Mascot

6	hypothetical protein F775_32310 [Aegilops tauschii]	gi|475614915	16783.8	5.83	3	40	0	3.447	26	0	

Peptide Information

Calc. Mass	Obsrv. Mass	± da	± ppm	Start Seq.	End Seq.	Sequence	Ion Score	C. I. %	Modification	Rank	Result Type
827.4985	827.458	-0.0405	-49	59	66	ADLPGVKK					Mascot
1027.6146	1027.5535	-0.0611	-59	137	145	AEVKKPEVK					Mascot
1905.9666	1906.0742	0.1076	56	26	45	SIVPAISGGSSETAAFANAR					Mascot
1905.9666	1906.0742	0.1076	56	26	45	SIVPAISGGSSETAAFANAR	26	0			Mascot

7	Os02g0246300 [Oryza sativa Japonica Group]	gi|255670760	15085.1	9.37	3	36	0	3.865	21	0	

Peptide Information

Calc. Mass	Obsrv. Mass	± da	± ppm	Start Seq.	End Seq.	Sequence	Ion Score	C. I. %	Modification	Rank	Result Type
831.4934	831.4162	-0.0772	-93	31	37	IQKDISK					Mascot
1090.5375	1090.5977	0.0602	55	108	116	DLEEKQNSK					Mascot
1905.927	1906.0742	0.1472	77	1	19	MAAAAASSSTPAAVREM QR					Mascot
1905.927	1906.0742	0.1472	77	1	19	MAAAAASSSTPAAVREM QR	21	0			Mascot

8	hypothetical protein F775_05844 [Aegilops tauschii]	gi|475543142	29795	6.75	4	36	0	7.634	21	0

Peptide Information

Calc. Mass	Obsrv. Mass	± da	± ppm	Start Seq.	End Seq.	Sequence	Ion Score	C. I. %	Modification	Rank	Result Type
889.4486	889.3767	-0.0719	-81	2	9	VEGRGSER					Mascot
1365.6394	1365.7216	0.0822	60	211	223	NPQASDPSVDHAK					Mascot
1838.859	1839.0247	0.1657	90	259	278	MDTAPAATVPGPPSNAT PSG					Mascot
1838.859	1839.0247	0.1657	90	259	278	MDTAPAATVPGPPSNAT PSG					Mascot
1905.9528	1906.0742	0.1214	64	224	239	NPHFLVLLSESFMEAR			Oxidation (M)[13]		Mascot
1905.9528	1906.0742	0.1214	64	224	239	NPHFLVLLSESFMEAR	21	0	Oxidation (M)[13]		Mascot

9	glutathione peroxidase 1 [Pinus tabuliformis]	gi|532528680	18908.6	5.5	6	36	0	4.305

Peptide Information

Calc. Mass	Obsrv. Mass	± da	± ppm	Start Seq.	End Seq.	Sequence	Ion Score	C. I. %	Modification	Rank	Result Type
1308.6794	1308.7278	0.0484	37	22	33	GNDVDLSIYKGK					Mascot
1320.6794	1320.6605	-0.0189	-14	105	117	VEVNGSSAAPVYK					Mascot
1390.7325	1390.7526	0.0201	14	138	149	FLVDKDGNVVER					Mascot
1507.7751	1507.8104	0.0353	23	19	31	DIRGNDVDLSIYK					Mascot
1708.9269	1708.844	-0.0829	-49	105	120	VEVNGSSAAPVYKFLK					Mascot
1838.8767	1839.0247	0.148	80	2	18	TGTSSEQYSSVHDLTVK					Mascot
1838.8767	1839.0247	0.148	80	2	18	TGTSSEQYSSVHDLTVK					Mascot

10	PREDICTED: uncharacterized protein LOC101293157 [Fragaria vesca subsp. vesca]	gi|470147723	19874.2	4.63	7	36	0	4.697

Peptide Information

Calc. Mass	Obsrv. Mass	± da	± ppm	Start Seq.	End Seq.	Sequence	Ion Score	C. I. %	Modification	Rank	Result Type

847.4407	847.5054	0.0647	76	143	149	ITEEVEK		Mascot
1003.5306	1003.5864	0.0558	56	31	39	EEAVVVETK		Mascot
1033.516	1033.5718	0.0558	54	162	170	VAKENSEEK		Mascot
1090.5739	1090.5977	0.0238	22	53	62	KIEGSDNTVK		Mascot
1263.6548	1263.7386	0.0838	66	1	12	MGGCATKPKVSK	Carbamidomethyl (C)[4]	Mascot
1365.6646	1365.7216	0.057	42	74	85	SLSNLFQNEEGK		Mascot
1493.7594	1493.825	0.0656	44	73	85	KSLSNLFQNEEGK		Mascot

Gel Idx/Pos	131/F6	Instr./Gel Origin	BA2151/Sample Project 20140814	Process Status	Analysis Succeeded
Plate [#] Name	[1] Sample Project 20140814	Instrument Sample Name		Spectra	11

Rank	Protein Name	Accession No.	Protein MW	Protein PI	Pep. Count	Protein Score	Protein Score C. I. %	Intensity Matched	Total Ion Score	Total Ion C. I. %	Confirmed
1	PREDICTED: E3 ubiquitin-protein ligase BRE1-like 1-like isoform X1 [Setaria italica]	gi|514816085	102563.9	8	26	76	97.555	13.632			

Peptide Information

Calc. Mass	Obsrv. Mass	± da	± ppm	Start Seq.	End Seq.	Sequence	Ion Score	C. I. %	Modification	Rank	Result Type
815.4733	815.4041	-0.0692	-85	301	306	LEEIRR					Mascot
817.4526	817.4223	-0.0303	-37	801	807	ANSLREK					Mascot
831.3591	831.4349	0.0758	91	522	528	HESTDSR					Mascot
835.3693	835.4144	0.0451	54	216	222	DSSAWNR					Mascot
1002.5578	1002.6257	0.0679	68	808	816	KDNSAVLQK					Mascot
1002.5578	1002.6257	0.0679	68	808	816	KDNSAVLQK					Mascot
1060.5634	1060.6061	0.0427	40	266	275	LGNKDVSAEK					Mascot
1066.5891	1066.559	-0.0301	-28	35	43	LAEQLEVHK					Mascot
1092.5797	1092.5649	-0.0148	-14	529	536	QLIEYRDR					Mascot
1118.5874	1118.5629	-0.0245	-22	402	410	DVLKLCNEK			Carbamidomethyl (C)[6]		Mascot
1139.6168	1139.6016	-0.0152	-13	456	465	DASLQLHSLR					Mascot
1141.6688	1141.5803	-0.0885	-78	710	718	IVRLEDQLR					Mascot
1184.5955	1184.6274	0.0319	27	1	9	MIYFNPMLR					Mascot
1193.7365	1193.6676	-0.0689	-58	594	603	DLVRLSHILK					Mascot
1194.5671	1194.6404	0.0733	61	443	453	EMGAVQSELSK			Oxidation (M)[2]		Mascot
1262.6951	1262.6583	-0.0368	-29	466	477	AEVSSLSSILTR					Mascot
1265.593	1265.6681	0.0751	59	790	800	IEDDLDLMSSK					Mascot
1332.6907	1332.6061	-0.0846	-63	178	189	QLHSATSNIFSK					Mascot
1345.7396	1345.7233	-0.0163	-12	77	88	LLADLDLVSVCK			Carbamidomethyl (C)[11]		Mascot
1390.79	1390.746	-0.044	-32	466	478	AEVSSLSSILTRK					Mascot
1407.6533	1407.7469	0.0936	66	478	488	KEQEIEQTSCR			Carbamidomethyl (C)[10]		Mascot
1458.8163	1458.8102	-0.0061	-4	574	587	LATSEAKIAELGQK					Mascot
1497.7544	1497.8158	0.0614	41	347	359	SQAELDDHQTLLK					Mascot
1657.8909	1657.8805	-0.0104	-6	374	387	QFNQKVDLAEIPQK					Mascot
1837.8936	1838.0188	0.1252	68	695	709	NLQHASMLMDLYNKK			Oxidation (M)[7,9]		Mascot
1838.8735	1839.0095	0.136	74	755	770	LMQSMDELQAKVGSNR			Oxidation (M)[2,5]		Mascot

1993.9608	1994.0825	0.1217	61	727	744	LSEDGMQQSISLGNSQRK			Oxidation (M)[6]		Mascot
1993.9608	1994.0825	0.1217	61	727	744	LSEDGMQQSISLGNSQRK			Oxidation (M)[6]		Mascot

2	PREDICTED: E3 ubiquitin-protein ligase BRE1-like 1-like isoform X8 [Setaria italica]	gi|514816099	99477.4	8.01	26	76	97.44	13.632

Peptide Information

Calc. Mass	Obsrv. Mass	± da	± ppm	Start Seq.	End Seq.	Sequence	Ion Score	C. I. %	Modification	Rank	Result Type
815.4733	815.4041	-0.0692	-85	301	306	LEEIRR					Mascot
817.4526	817.4223	-0.0303	-37	777	783	ANSLREK					Mascot
831.3591	831.4349	0.0758	91	522	528	HESTDSR					Mascot
835.3693	835.4144	0.0451	54	216	222	DSSAWNR					Mascot
1002.5578	1002.6257	0.0679	68	784	792	KDNSAVLQK					Mascot
1002.5578	1002.6257	0.0679	68	784	792	KDNSAVLQK					Mascot
1060.5634	1060.6061	0.0427	40	266	275	LGNKDVSAEK					Mascot
1066.5891	1066.559	-0.0301	-28	35	43	LAEQLEVHK					Mascot
1092.5797	1092.5649	-0.0148	-14	529	536	QLIEYRDR					Mascot
1118.5874	1118.5629	-0.0245	-22	402	410	DVLKLCNEK			Carbamidomethyl (C)[6]		Mascot
1139.6168	1139.6016	-0.0152	-13	456	465	DASLQLHSLR					Mascot
1141.6688	1141.5803	-0.0885	-78	686	694	IVRLEDQLR					Mascot
1184.5955	1184.6274	0.0319	27	1	9	MIYFNPMLR					Mascot
1193.7365	1193.6676	-0.0689	-58	594	603	DLVRLSHILK					Mascot
1194.5671	1194.6404	0.0733	61	443	453	EMGAVQSELSK			Oxidation (M)[2]		Mascot
1262.6951	1262.6583	-0.0368	-29	466	477	AEVSSLSSILTR					Mascot
1265.593	1265.6681	0.0751	59	766	776	IEDDLDLMSSK					Mascot
1332.6907	1332.6061	-0.0846	-63	178	189	QLHSATSNIFSK					Mascot
1345.7396	1345.7233	-0.0163	-12	77	88	LLADLDLVSVCK			Carbamidomethyl (C)[11]		Mascot
1390.79	1390.746	-0.044	-32	466	478	AEVSSLSSILTRK					Mascot
1407.6533	1407.7469	0.0936	66	478	488	KEQEIEQTSCR			Carbamidomethyl (C)[10]		Mascot
1458.8163	1458.8102	-0.0061	-4	574	587	LATSEAKIAELGQK					Mascot
1497.7544	1497.8158	0.0614	41	347	359	SQAELDDHQTLLK					Mascot
1657.8909	1657.8805	-0.0104	-6	374	387	QFNQKVDLAEIPQK					Mascot
1837.8936	1838.0188	0.1252	68	671	685	NLQHASMLMDLYNKK			Oxidation (M)[7,9]		Mascot
1838.8735	1839.0095	0.136	74	731	746	LMQSMDELQAKVGSNR			Oxidation (M)[2,5]		Mascot
1993.9608	1994.0825	0.1217	61	703	720	LSEDGMQQSISLGNSQRK			Oxidation (M)[6]		Mascot
1993.9608	1994.0825	0.1217	61	703	720	LSEDGMQQSISLGNSQRK			Oxidation (M)[6]		Mascot

3	PREDICTED: uncharacterized protein LOC101305028 [Fragaria vesca subsp. vesca]	gi|470108652	205439.9	4.97	36	75	96.85	15.795

Peptide Information

Calc. Mass	Obsrv. Mass	± da	± ppm	Start Seq.	End Seq.	Sequence	Ion Score	C. I. %	Modification	Rank	Result Type
817.405	817.4223	0.0173	21	439	445	AEEEALR					Mascot
829.3686	829.3665	-0.0021	-3	1312	1318	SHEEAEK					Mascot
831.4683	831.4349	-0.0334	-40	1715	1721	LSEQAKR					Mascot
832.441	832.3676	-0.0734	-88	373	379	ISNLEEK					Mascot
951.5331	951.5015	-0.0316	-33	959	966	MGLYQVLK					Mascot
1016.5258	1016.5536	0.0278	27	600	607	LEEEVELR					Mascot
1027.5242	1027.5396	0.0154	15	127	135	TPEMPAPIR			Oxidation (M)[4]		Mascot
1032.4745	1032.5415	0.067	65	1037	1044	DTLDHEFR					Mascot
1036.5343	1036.5635	0.0292	28	922	931	LLGASTMSEK					Mascot
1060.5885	1060.6061	0.0176	17	1207	1215	SEDELKLVK					Mascot
1092.6049	1092.5649	-0.04	-37	1358	1366	ETLFEGKIR					Mascot
1118.6317	1118.5629	-0.0688	-62	1050	1059	FLVLQSGAQR					Mascot
1140.5757	1140.6119	0.0362	32	850	859	QNHASSVELR					Mascot
1193.6348	1193.6676	0.0328	27	1677	1686	LFDANNKLMK					Mascot
1201.6423	1201.6404	-0.0019	-2	380	389	LLDVEEEAKR					Mascot
1259.7028	1259.6901	-0.0127	-10	681	690	LEIMQKLQEK					Mascot
1263.625	1263.7197	0.0947	75	31	41	ENLTDMDAKVK					Mascot
1265.6008	1265.6681	0.0673	53	46	56	LIEEDADSFAR					Mascot
1308.6827	1308.7308	0.0481	37	920	931	QKLLGASTMSEK			Oxidation (M)[9]		Mascot
1332.6941	1332.6061	-0.088	-66	870	881	ISGLEAEGICRK			Carbamidomethyl (C)[10]		Mascot
1345.6899	1345.7233	0.0334	25	71	80	LVEEFYRAYR					Mascot
1379.739	1379.7723	0.0333	24	1071	1082	VVEGEHREGVLR					Mascot
1405.6628	1405.7343	0.0715	51	967	979	TVDIDANLGCAEK			Carbamidomethyl (C)[10]		Mascot
1407.6582	1407.7469	0.0887	63	1782	1793	KAPFCACMPPTK			Carbamidomethyl (C)[5,7]		Mascot
1458.7184	1458.8102	0.0918	63	1216	1229	SVNDQLNGDIANAK					Mascot
1475.8468	1475.8168	-0.03	-20	891	903	SVTAQIEIFVLQK					Mascot
1490.752	1490.7974	0.0454	30	368	379	QCLEKISNLEEK			Carbamidomethyl (C)[2]		Mascot
1491.7551	1491.8051	0.05	34	180	193	QLNDLFGSGEGRAK					Mascot
1507.6945	1507.8065	0.112	74	1267	1279	SDDAKMVLEDQEK					Mascot
1605.7504	1605.7875	0.0371	23	1037	1049	DTLDHEFRTQSEK					Mascot
1738.7683	1738.9048	0.1365	79	12	24	MYSWWWDSHISPK			Oxidation (M)[1]		Mascot

1873.9066	1874.0637	0.1571	84	1587	1602	YPSSESLVEKEYSIDK					Mascot
1908.9232	1908.9504	0.0272	14	1284	1299	LHEDSDLHSKEIGCLR			Carbamidomethyl (C)[14]		Mascot
1993.9569	1994.0825	0.1256	63	904	919	CVEDLEEKNLSLMIER			Carbamidomethyl (C)[1], Oxidation (M)[13]		Mascot
1993.9569	1994.0825	0.1256	63	904	919	CVEDLEEKNLSLMIER			Carbamidomethyl (C)[1], Oxidation (M)[13]		Mascot
2023.0892	2022.9906	-0.0986	-49	1185	1201	ILEGQLEVIQMENLHLK			Oxidation (M)[11]		Mascot
2510.2661	2510.239	-0.0271	-11	883	903	EFEEEQDKSVTAQIEIFVL QK					Mascot

4 PREDICTED: uncharacterized protein LOC101222879 [Cucumis sativus] gi|449446736 49694 8.67 17 73 95.008 5.882

Peptide Information

Calc. Mass	Obsrv. Mass	± da	± ppm	Start Seq.	End Seq.	Sequence	Ion Score	C. I. %	Modification	Rank	Result Type
817.405	817.4223	0.0173	21	257	264	DAENAVAK					Mascot
835.3978	835.4144	0.0166	20	336	343	QAEMSAAK					Mascot
836.4009	836.4019	0.001	1	227	234	DGPPHASR					Mascot
864.4574	864.4551	-0.0023	-3	208	214	LQNFSQK					Mascot
952.4231	952.4902	0.0671	70	25	32	QGQDHDPR					Mascot
993.5211	993.5444	0.0233	23	399	408	LEAGKSSSSK					Mascot
1066.5416	1066.559	0.0174	16	33	42	FEATVTVDGK					Mascot
1092.5645	1092.5649	0.0004	0	351	360	EGSQSISTRK					Mascot
1106.5663	1106.5614	-0.0049	-4	197	205	LTGMQHLYK			Oxidation (M)[4]		Mascot
1139.5706	1139.6016	0.031	27	227	236	DGPPHASRFR					Mascot
1184.6205	1184.6274	0.0069	6	4	12	TKLQELCHR			Carbamidomethyl (C)[7]		Mascot
1306.6638	1306.6534	-0.0104	-8	143	153	HEVSYKSSELK					Mascot
1332.7092	1332.6061	-0.1031	-77	197	207	LTGMQHLYKNK					Mascot
1497.7843	1497.8158	0.0315	21	361	373	RAPSCDLALEIPR			Carbamidomethyl (C)[5]		Mascot
1657.8618	1657.8805	0.0187	11	336	350	QAEMSAAKVAYFTIK					Mascot
1699.7513	1699.9058	0.1545	91	154	168	ESSKDSPIMSNMDVK			Oxidation (M)[9,12]		Mascot
1716.8925	1716.9358	0.0433	25	410	423	IFVCPRQPNMTIPK			Carbamidomethyl (C)[4], Oxidation (M)[10]		Mascot

5 PREDICTED: E3 ubiquitin-protein ligase BRE1-like 1-like isoform X7 [Setaria italica] gi|514816097 100200.8 8.04 25 71 91.715 13.446

Peptide Information

Calc. Mass	Obsrv. Mass	± da	± ppm	Start Seq.	End Seq.	Sequence	Ion Score	C. I. %	Modification	Rank	Result Type
815.4733	815.4041	-0.0692	-85	283	288	LEEIRR					Mascot
817.4526	817.4223	-0.0303	-37	783	789	ANSLREK					Mascot
831.3591	831.4349	0.0758	91	504	510	HESTDSR					Mascot

835.3693	835.4144	0.0451	54	198	204	DSSAWNR		Mascot
1002.5578	1002.6257	0.0679	68	790	798	KDNSAVLQK		Mascot
1002.5578	1002.6257	0.0679	68	790	798	KDNSAVLQK		Mascot
1060.5634	1060.6061	0.0427	40	248	257	LGNKDVSAEK		Mascot
1066.5891	1066.559	-0.0301	-28	17	25	LAEQLEVHK		Mascot
1092.5797	1092.5649	-0.0148	-14	511	518	QLIEYRDR		Mascot
1118.5874	1118.5629	-0.0245	-22	384	392	DVLKLCNEK	Carbamidomethyl (C)[6]	Mascot
1139.6168	1139.6016	-0.0152	-13	438	447	DASLQLHSLR		Mascot
1141.6688	1141.5803	-0.0885	-78	692	700	IVRLEDQLR		Mascot
1193.7365	1193.6676	-0.0689	-58	576	585	DLVRLSHILK		Mascot
1194.5671	1194.6404	0.0733	61	425	435	EMGAVQSELSK	Oxidation (M)[2]	Mascot
1262.6951	1262.6583	-0.0368	-29	448	459	AEVSSLSSILTR		Mascot
1265.593	1265.6681	0.0751	59	772	782	IEDDLDLMSSK		Mascot
1332.6907	1332.6061	-0.0846	-63	160	171	QLHSATSNIFSK		Mascot
1345.7396	1345.7233	-0.0163	-12	59	70	LLADLDLVSVCK	Carbamidomethyl (C)[11]	Mascot
1390.79	1390.746	-0.044	-32	448	460	AEVSSLSSILTRK		Mascot
1407.6533	1407.7469	0.0936	66	460	470	KEQEIEQTSCR	Carbamidomethyl (C)[10]	Mascot
1458.8163	1458.8102	-0.0061	-4	556	569	LATSEAKIAELGQK		Mascot
1497.7544	1497.8158	0.0614	41	329	341	SQAELDDHQTLLK		Mascot
1657.8909	1657.8805	-0.0104	-6	356	369	QFNQKVDLAEIPQK		Mascot
1837.8936	1838.0188	0.1252	68	677	691	NLQHASMLMDLYNKK	Oxidation (M)[7,9]	Mascot
1838.8735	1839.0095	0.136	74	737	752	LMQSMDELQAKVGSNR	Oxidation (M)[2,5]	Mascot
1993.9608	1994.0825	0.1217	61	709	726	LSEDGMQQSISLGNSQR K	Oxidation (M)[6]	Mascot
1993.9608	1994.0825	0.1217	61	709	726	LSEDGMQQSISLGNSQR K	Oxidation (M)[6]	Mascot

6	PREDICTED: E3 ubiquitin-protein ligase BRE1-like 1-like isoform X5 [Setaria italica]	gi|514816093	101694.5	8.17	25	70	90.266	13.446

Protein Group

PREDICTED: E3 ubiquitin-protein ligase BRE1-like 1-like isoform X2 [Setaria italica]	gi|514816087	101694.5	8.1700 000762 9395
PREDICTED: E3 ubiquitin-protein ligase BRE1-like 1-like isoform X3 [Setaria italica]	gi|514816089	101694.5	8.1700 000762 9395
PREDICTED: E3 ubiquitin-protein ligase BRE1-like 1-like isoform X4 [Setaria italica]	gi|514816091	101694.5	8.1700 000762 9395
PREDICTED: E3 ubiquitin-protein ligase BRE1-like 1-like isoform X6 [Setaria italica]	gi|514816095	101694.5	8.1700 000762 9395

Peptide Information

Calc. Mass	Obsrv. Mass	± da	± ppm	Start Seq.	End Seq.	Sequence	Ion Score	C. I. %	Modification	Rank	Result Type
815.4733	815.4041	-0.0692	-85	297	302	LEEIRR					Mascot
817.4526	817.4223	-0.0303	-37	797	803	ANSLREK					Mascot
831.3591	831.4349	0.0758	91	518	524	HESTDSR					Mascot
835.3693	835.4144	0.0451	54	212	218	DSSAWNR					Mascot
1002.5578	1002.6257	0.0679	68	804	812	KDNSAVLQK					Mascot
1002.5578	1002.6257	0.0679	68	804	812	KDNSAVLQK					Mascot
1060.5634	1060.6061	0.0427	40	262	271	LGNKDVSAEK					Mascot
1066.5891	1066.559	-0.0301	-28	31	39	LAEQLEVHK					Mascot
1092.5797	1092.5649	-0.0148	-14	525	532	QLIEYRDR					Mascot
1118.5874	1118.5629	-0.0245	-22	398	406	DVLKLCNEK			Carbamidomethyl (C)[6]		Mascot
1139.6168	1139.6016	-0.0152	-13	452	461	DASLQLHSLR					Mascot
1141.6688	1141.5803	-0.0885	-78	706	714	IVRLEDQLR					Mascot
1193.7365	1193.6676	-0.0689	-58	590	599	DLVRLSHILK					Mascot
1194.5671	1194.6404	0.0733	61	439	449	EMGAVQSELSK			Oxidation (M)[2]		Mascot
1262.6951	1262.6583	-0.0368	-29	462	473	AEVSSLSSILTR					Mascot
1265.593	1265.6681	0.0751	59	786	796	IEDDLDLMSSK					Mascot
1332.6907	1332.6061	-0.0846	-63	174	185	QLHSATSNIFSK					Mascot
1345.7396	1345.7233	-0.0163	-12	73	84	LLADLDLVSVCK			Carbamidomethyl (C)[11]		Mascot
1390.79	1390.746	-0.044	-32	462	474	AEVSSLSSILTRK					Mascot
1407.6533	1407.7469	0.0936	66	474	484	KEQEIEQTSCR			Carbamidomethyl (C)[10]		Mascot
1458.8163	1458.8102	-0.0061	-4	570	583	LATSEAKIAELGQK					Mascot
1497.7544	1497.8158	0.0614	41	343	355	SQAELDDHQTLLK					Mascot
1657.8909	1657.8805	-0.0104	-6	370	383	QFNQKVDLAEIPQK					Mascot
1837.8936	1838.0188	0.1252	68	691	705	NLQHASMLMDLYNKK			Oxidation (M)[7,9]		Mascot
1838.8735	1839.0095	0.136	74	751	766	LMQSMDELQAKVGSNR			Oxidation (M)[2,5]		Mascot
1993.9608	1994.0825	0.1217	61	723	740	LSEDGMQQSISLGNSQR K			Oxidation (M)[6]		Mascot
1993.9608	1994.0825	0.1217	61	723	740	LSEDGMQQSISLGNSQR K			Oxidation (M)[6]		Mascot

7 uncharacterized protein LOC100276431 [Zea mays] gi|226531372 44446 6.44 17 69 87.168 8.623

Peptide Information

Calc. Mass	Obsrv. Mass	± da	± ppm	Start Seq.	End Seq.	Sequence	Ion Score	C. I. %	Modification	Rank	Result Type
806.4553	806.4064	-0.0489	-61	314	320	MTSIKAR					Mascot
808.4597	808.426	-0.0337	-42	312	318	TKMTSIK					Mascot

Calc. Mass	Obsrv. Mass	± da	± ppm	Start Seq.	End Seq.	Sequence	Ion Score	C. I. %	Modification	Rank	Result Type
836.3931	836.4019	0.0088	11	99	105	MQSDISR					Mascot
847.4341	847.4191	-0.015	-18	113	120	MDAQLAAK					Mascot
849.3697	849.4355	0.0658	77	90	96	ESTNDQR					Mascot
863.4291	863.4727	0.0436	50	113	120	MDAQLAAK			Oxidation (M)[1]		Mascot
1060.5569	1060.6061	0.0492	46	155	163	MVIGNQQVR			Oxidation (M)[1]		Mascot
1118.5623	1118.5629	0.0006	1	113	122	MDAQLAAKDR					Mascot
1232.6844	1232.6542	-0.0302	-24	123	133	ELATLTRTEAK					Mascot
1263.6436	1263.7197	0.0761	60	198	208	SGMEIMNLLQK					Mascot
1277.6519	1277.765	0.1131	89	99	109	MQSDISRLEAK					Mascot
1320.6543	1320.6505	-0.0038	-3	145	154	LQQERDEFQK					Mascot
1407.697	1407.7469	0.0499	35	353	364	SIIQEQACIMSK			Carbamidomethyl (C)[8]		Mascot
1490.7268	1490.7974	0.0706	47	237	248	KQELMQENADLR			Oxidation (M)[5]		Mascot
1605.8087	1605.7875	-0.0212	-13	198	211	SGMEIMNLLQKEGR					Mascot
1707.8483	1707.861	0.0127	7	150	163	DEFQKMVIGNQQVR			Oxidation (M)[6]		Mascot
1707.8483	1707.861	0.0127	7	150	163	DEFQKMVIGNQQVR			Oxidation (M)[6]		Mascot
1738.8462	1738.9048	0.0586	34	194	208	ESSRSGMEIMNLLQK			Oxidation (M)[7]		Mascot
2717.2988	2717.2246	-0.0742	-27	279	303	QESGSPQSPLGGKTDVF DLPFHMAR			Oxidation (M)[23]		Mascot

8	annexin [Populus tomentosa]	gi|429326384	36225.7	6.18	15	68	86.251	11.556

Peptide Information

Calc. Mass	Obsrv. Mass	± da	± ppm	Start Seq.	End Seq.	Sequence	Ion Score	C. I. %	Modification	Rank	Result Type
833.4152	833.4118	-0.0034	-4	114	120	SSHDLFK					Mascot
849.3771	849.4355	0.0584	69	272	278	AEVDMER					Mascot
982.4476	982.4804	0.0328	33	68	75	ELSSDFER					Mascot
1036.5323	1036.5635	0.0312	30	123	130	QAYHARYK					Mascot
1060.5535	1060.6061	0.0526	50	114	122	SSHDLFKAR					Mascot
1081.516	1081.5455	0.0295	27	183	191	AYSDDEIIR					Mascot
1082.6205	1082.5272	-0.0933	-86	174	182	ILHEKISDK					Mascot
1106.551	1106.5614	0.0104	9	272	280	AEVDMERIK			Oxidation (M)[5]		Mascot
1126.5925	1126.5674	-0.0251	-22	306	316	MLLALIGHGDA			Oxidation (M)[1]		Mascot
1323.6978	1323.703	0.0052	4	234	244	ATIKCLTYPEK			Carbamidomethyl (C)[5]		Mascot
1405.7104	1405.7343	0.0239	17	267	278	VVTTRAEVDMER					Mascot
1427.8257	1427.8231	-0.0026	-2	76	87	VVLLWTLDLAER					Mascot
1497.7584	1497.8158	0.0574	38	51	63	QVYAEAYGQDLLK					Mascot
1994.0706	1994.0825	0.0119	6	47	63	NLIRQVYAEAYGQDLLK					Mascot

Calc. Mass	Obsrv. Mass	± da	± ppm	Start Seq.	End Seq.	Sequence	Ion Score	C. I. %	Modification	Rank	Result Type
1994.0706	1994.0825	0.0119	6	47	63	NLIRQVYAEAYGQDLLK					Mascot
2384.2166	2384.0759	-0.1407	-59	1	22	MASLKVPASVPPPYEDA EQLNK					Mascot

9 hypothetical protein ZEAMMB73_503203 [Zea mays] gi|413922118 44347 6.7 17 67 79.19 8.623

Peptide Information

Calc. Mass	Obsrv. Mass	± da	± ppm	Start Seq.	End Seq.	Sequence	Ion Score	C. I. %	Modification	Rank	Result Type
806.4553	806.4064	-0.0489	-61	314	320	MTSIKAR					Mascot
808.4597	808.426	-0.0337	-42	312	318	TKMTSIK					Mascot
836.3931	836.4019	0.0088	11	99	105	MQSDISR					Mascot
847.4341	847.4191	-0.015	-18	113	120	MDAQLAAK					Mascot
849.3697	849.4355	0.0658	77	90	96	ESTNDQR					Mascot
863.4291	863.4727	0.0436	50	113	120	MDAQLAAK			Oxidation (M)[1]		Mascot
1060.5569	1060.6061	0.0492	46	155	163	MVIGNQQVR			Oxidation (M)[1]		Mascot
1118.5623	1118.5629	0.0006	1	113	122	MDAQLAAKDR					Mascot
1232.6844	1232.6542	-0.0302	-24	123	133	ELATLTRTEAK					Mascot
1263.6436	1263.7197	0.0761	60	198	208	SGMEIMNLLQK					Mascot
1277.6519	1277.765	0.1131	89	99	109	MQSDISRLEAK					Mascot
1320.6543	1320.6505	-0.0038	-3	145	154	LQQERDEFQK					Mascot
1407.697	1407.7469	0.0499	35	353	364	SIIQEQACIMSK			Carbamidomethyl (C)[8]		Mascot
1490.7268	1490.7974	0.0706	47	237	248	KQELMQENADLR			Oxidation (M)[5]		Mascot
1605.8087	1605.7875	-0.0212	-13	198	211	SGMEIMNLLQKEGR					Mascot
1707.8483	1707.861	0.0127	7	150	163	DEFQKMVIGNQQVR			Oxidation (M)[6]		Mascot
1707.8483	1707.861	0.0127	7	150	163	DEFQKMVIGNQQVR			Oxidation (M)[6]		Mascot
1738.8462	1738.9048	0.0586	34	194	208	ESSRSGMEIMNLLQK			Oxidation (M)[7]		Mascot
2717.2988	2717.2246	-0.0742	-27	279	303	QESGSPQSPLGGKTDVF DLPFHMAR			Oxidation (M)[23]		Mascot

10 hypothetical protein ZEAMMB73_503203 [Zea mays] gi|413922119 44491.1 6.7 17 66 77.701 8.623

Peptide Information

Calc. Mass	Obsrv. Mass	± da	± ppm	Start Seq.	End Seq.	Sequence	Ion Score	C. I. %	Modification	Rank	Result Type
806.4553	806.4064	-0.0489	-61	314	320	MTSIKAR					Mascot
808.4597	808.426	-0.0337	-42	312	318	TKMTSIK					Mascot
836.3931	836.4019	0.0088	11	99	105	MQSDISR					Mascot
847.4341	847.4191	-0.015	-18	113	120	MDAQLAAK					Mascot
849.3697	849.4355	0.0658	77	90	96	ESTNDQR					Mascot
863.4291	863.4727	0.0436	50	113	120	MDAQLAAK			Oxidation (M)[1]		Mascot

1060.5569	1060.6061	0.0492	46	155	163	MVIGNQQVR	Oxidation (M)[1]	Mascot
1118.5623	1118.5629	0.0006	1	113	122	MDAQLAAKDR		Mascot
1232.6844	1232.6542	-0.0302	-24	123	133	ELATLTRTEAK		Mascot
1263.6436	1263.7197	0.0761	60	198	208	SGMEIMNLLQK		Mascot
1277.6519	1277.765	0.1131	89	99	109	MQSDISRLEAK		Mascot
1320.6543	1320.6505	-0.0038	-3	145	154	LQQERDEFQK		Mascot
1407.697	1407.7469	0.0499	35	353	364	SIIQEQACIMSK	Carbamidomethyl (C)[8]	Mascot
1490.7268	1490.7974	0.0706	47	237	248	KQELMQENADLR	Oxidation (M)[5]	Mascot
1605.8087	1605.7875	-0.0212	-13	198	211	SGMEIMNLLQKEGR		Mascot
1707.8483	1707.861	0.0127	7	150	163	DEFQKMVIGNQQVR	Oxidation (M)[6]	Mascot
1707.8483	1707.861	0.0127	7	150	163	DEFQKMVIGNQQVR	Oxidation (M)[6]	Mascot
1738.8462	1738.9048	0.0586	34	194	208	ESSRSGMEIMNLLQK	Oxidation (M)[7]	Mascot
2717.2988	2717.2246	-0.0742	-27	279	303	QESGSPQSPLGGKTDVF DLPFHMAR	Oxidation (M)[23]	Mascot

Gel Idx/Pos	132/F7	Instr./Gel Origin	BA2151/Sample Project 20140814	Process Status	Analysis Succeeded
Plate [#] Name	[1] Sample Project 20140814	Instrument Sample Name		Spectra	11

Rank	Protein Name	Accession No.	Protein MW	Protein PI	Pep. Count	Protein Score	Protein Score C. I. %	Intensity Matched	Total Ion Score	Total Ion C. I. %	Confirmed
1	Globulin-1 S allele [Triticum urartu]	gi|474411419	57108.4	9.1	9	336	100	36.867	312	100	

Peptide Information

Calc. Mass	Obsrv. Mass	± da	± ppm	Start Seq.	End Seq.	Sequence	Ion Score	C. I. %	Modification	Rank	Result Type
906.468	906.5155	0.0475	52	457	463	EVQEVFR					Mascot
1320.5452	1320.6606	0.1154	87	349	358	WGEEEEDDRR					Mascot
1390.7285	1390.7463	0.0178	13	199	211	TSDERLGSLLGSR					Mascot
1473.802	1473.7731	-0.0289	-20	220	232	SISIVRASEEQVR					Mascot
1685.8606	1685.9408	0.0802	48	442	456	LDDPAQELTFGRPAR					Mascot
1685.8606	1685.9408	0.0802	48	442	456	LDDPAQELTFGRPAR	22	0			Mascot
1699.9418	1699.9164	-0.0254	-15	176	190	FQYFSAKPLLASLSK					Mascot
1791.8984	1791.8416	-0.0568	-32	254	268	GDSRDTYNLLEQRPK					Mascot
1822.8752	1822.9807	0.1055	58	411	426	GSSNLQVVCFEINAER			Carbamidomethyl (C)[9]		Mascot
1822.8752	1822.9807	0.1055	58	411	426	GSSNLQVVCFEINAER	145	100	Carbamidomethyl (C)[9]		Mascot
1906.0182	1906.1257	0.1075	56	392	410	GSAFVVPPGHPVVEIASSR					Mascot
1906.0182	1906.1257	0.1075	56	392	410	GSAFVVPPGHPVVEIASSR	145	100			Mascot

Rank	Protein Name	Accession No.	Protein MW	Protein PI	Pep. Count	Protein Score	Protein Score C. I. %	Intensity Matched	Total Ion Score	Total Ion C. I. %	Confirmed
2	PREDICTED: C-terminal processing peptidase, chloroplastic-like [Fragaria vesca subsp. vesca]	gi|470123883	59729	9.36	16	68	84.214	6.946			

Peptide Information

Calc. Mass	Obsrv. Mass	± da	± ppm	Start Seq.	End Seq.	Sequence	Ion Score	C. I. %	Modification	Rank	Result Type
1037.5667	1037.5734	0.0067	6	221	228	FLEPEKFK					Mascot
1082.5729	1082.5405	-0.0324	-30	201	209	EETYTAIKK					Mascot
1126.5562	1126.5715	0.0153	14	169	177	MIDRSYVDK					Mascot
1165.6252	1165.627	0.0018	2	461	471	AVLFGEPTFGK					Mascot
1165.6252	1165.627	0.0018	2	461	471	AVLFGEPTFGK					Mascot
1181.5984	1181.6407	0.0423	36	407	416	GVIVYICDSR			Carbamidomethyl (C)[7]		Mascot
1427.7828	1427.8638	0.081	57	36	47	WKCLPLGVVEAR			Carbamidomethyl (C)[3]		Mascot
1457.6914	1457.7926	0.1012	69	189	200	ENALRNEPMNNR					Mascot
1473.6863	1473.7731	0.0868	59	189	200	ENALRNEPMNNR			Oxidation (M)[9]		Mascot

Calc. Mass	Obsrv. Mass	± da	± ppm	Start Seq.	End Seq.	Sequence	Ion Score	C. I. %	Modification	Rank	Result Type
1493.7893	1493.8148	0.0255	17	407	419	GVIVYICDSRGVR			Carbamidomethyl (C)[7]		Mascot
1565.8468	1565.7141	-0.1327	-85	112	125	LAGSLHKVINCPEK			Carbamidomethyl (C)[11]		Mascot
1593.8707	1593.7484	-0.1223	-77	69	83	SDGSSKHNLLLGPIR					Mascot
1759.9449	1760.0138	0.0689	39	372	386	TLRDNNVNAFVLDLR					Mascot
1791.96	1791.8416	-0.1184	-66	355	371	LTTFNQNASGAVKEAIK					Mascot
1910.9139	1910.9396	0.0257	13	1	17	MEVFATSASCPHFLVSK			Carbamidomethyl (C)[10]		Mascot
1926.9089	1927.0072	0.0983	51	1	17	MEVFATSASCPHFLVSK			Carbamidomethyl (C)[10], Oxidation (M)[1]		Mascot
2252.2861	2252.1042	-0.1819	-81	434	456	EPLAVLVNKGTASASEIL AGALK					Mascot
2255.2656	2255.124	-0.1416	-63	130	150	VFVRFVVGVMVVMSVSV SVSK					Mascot
2705.3755	2705.3333	-0.0422	-16	151	172	VPSWALTEENLLFLEAW RMIDR			Oxidation (M)[19]		Mascot
2705.3755	2705.3333	-0.0422	-16	151	172	VPSWALTEENLLFLEAW RMIDR			Oxidation (M)[19]		Mascot

3 PREDICTED: uncharacterized protein At4g28440-like [Fragaria vesca subsp. vesca] gi|470106609 16689.8 9.48 9 63 53.411 3.521

Peptide Information

Calc. Mass	Obsrv. Mass	± da	± ppm	Start Seq.	End Seq.	Sequence	Ion Score	C. I. %	Modification	Rank	Result Type
886.488	886.5641	0.0761	86	123	130	IEVTEPAK					Mascot
982.5026	982.4886	-0.014	-14	103	110	NAKIDMFK			Oxidation (M)[6]		Mascot
1197.6448	1197.5919	-0.0529	-44	59	68	SVSQHLRQSR					Mascot
1359.7883	1359.7566	-0.0317	-23	123	134	IEVTEPAKFVVK					Mascot
1637.9221	1637.9283	0.0062	4	31	46	VSDLKPGTSGHTLVVK					Mascot
1873.9689	1874.0587	0.0898	48	87	102	NEQVDLMKPDSTVIIR			Oxidation (M)[7]		Mascot
2255.262	2255.124	-0.138	-61	9	30	AGTASAQQSNAKPGLRK PVFVK					Mascot
2367.1973	2367.4001	0.2028	86	66	86	QSRISECLIGDETGTILFT AR			Carbamidomethyl (C)[7]		Mascot
2705.5562	2705.3333	-0.2229	-82	31	56	VSDLKPGTSGHTLVVKVI NATTVLQK					Mascot
2705.5562	2705.3333	-0.2229	-82	31	56	VSDLKPGTSGHTLVVKVI NATTVLQK					Mascot

4 PREDICTED: uncharacterized protein LOC101255127 [Solanum lycopersicum] gi|460365851 177796.3 8.83 27 58 0 31.927

Peptide Information

Calc. Mass	Obsrv. Mass	± da	± ppm	Start Seq.	End Seq.	Sequence	Ion Score	C. I. %	Modification	Rank	Result Type
807.4433	807.4484	0.0051	6	1103	1109	VMPFGLK			Oxidation (M)[2]		Mascot
832.4523	832.3693	-0.083	-100	145	151	SLEQSIR					Mascot
888.54	888.5143	-0.0257	-29	991	998	TDISVIIK					Mascot

1037.5701	1037.5734	0.0033	3	1534	1542	FVINMLIAT			Oxidation (M)[5]		Mascot
1066.5714	1066.5596	-0.0118	-11	1141	1149	LLGFMVSQR			Oxidation (M)[5]		Mascot
1101.6626	1101.6012	-0.0614	-56	325	335	SGKIVSQAALK					Mascot
1107.5826	1107.5984	0.0158	14	143	151	MKSLEQSIR			Oxidation (M)[1]		Mascot
1139.5692	1139.6292	0.06	53	99	107	RDQSYTLEK					Mascot
1141.6576	1141.6035	-0.0541	-47	462	471	TLNVLSPIER					Mascot
1158.583	1158.6248	0.0418	36	1264	1272	EWGIYYLSK					Mascot
1165.6252	1165.627	0.0018	2	826	835	DVKFIGPDFK					Mascot
1165.6252	1165.627	0.0018	2	826	835	DVKFIGPDFK					Mascot
1313.7173	1313.7223	0.005	4	354	365	REDVATIVSAPR					Mascot
1320.6538	1320.6606	0.0068	5	576	587	MMPLGAESVLEK			Oxidation (M)[1]		Mascot
1323.6475	1323.7111	0.0636	48	200	210	YCNQLRGVEGK			Carbamidomethyl (C)[2]		Mascot
1353.6984	1353.7109	0.0125	9	1318	1328	YIFQKPMPTGR			Oxidation (M)[7]		Mascot
1365.7777	1365.713	-0.0647	-47	588	599	LSPSNTLFLFVK					Mascot
1565.72	1565.7141	-0.0059	-4	1032	1043	VRMCVDYHDLNK			Carbamidomethyl (C)[4], Oxidation (M)[3]		Mascot
1627.8182	1627.8843	0.0661	41	1118	1131	SMTTMYNLKLNPAK			Oxidation (M)[2]		Mascot
1638.8561	1638.9734	0.1173	72	1186	1199	FIAQLATTCEPIFK			Carbamidomethyl (C)[9]		Mascot
1655.8396	1655.9286	0.089	54	1103	1117	VMPFGLKNAGATYMR					Mascot
1655.8396	1655.9286	0.089	54	1103	1117	VMPFGLKNAGATYMR					Mascot
1685.901	1685.9408	0.0398	24	546	559	VHEEFASPYKPILR					Mascot
1685.901	1685.9408	0.0398	24	546	559	VHEEFASPYKPILR					Mascot
1716.7786	1716.9432	0.1646	96	783	797	ATESYQSSFPEPVMK			Oxidation (M)[14]		Mascot
1716.7786	1716.9432	0.1646	96	783	797	ATESYQSSFPEPVMK			Oxidation (M)[14]		Mascot
1759.9636	1760.0138	0.0502	29	722	737	GLGICLQGRAYPVSLR			Carbamidomethyl (C)[5]		Mascot
1844.9905	1844.9774	-0.0131	-7	1298	1312	LKHYLSSYTTYLISR					Mascot
1906.0103	1906.1257	0.1154	61	1141	1157	LLGFMVSQRGIELDPSK			Oxidation (M)[5]		Mascot
1906.0103	1906.1257	0.1154	61	1141	1157	LLGFMVSQRGIELDPSK			Oxidation (M)[5]		Mascot
1927.9219	1928.0828	0.1609	83	781	797	ARATESYQSSFPEPVMK					Mascot
1943.9167	1944.0605	0.1438	74	781	797	ARATESYQSSFPEPVMK			Oxidation (M)[16]		Mascot
1994.0165	1994.092	0.0755	38	1048	1064	DNFPLPNIHILLDNCAK			Carbamidomethyl (C)[15]		Mascot

5 hypothetical protein PRUPE_ppa009637mg [Prunus persica] gi|462419119 31594.5 9.45 11 58 0 5.103

Peptide Information

Calc. Mass	Obsrv. Mass	± da	± ppm	Start Seq.	End Seq.	Sequence	Ion Score	C. I. %	Modification	Rank	Result Type
975.5734	975.5868	0.0134	14	33	41	SHGIPVLPR					Mascot

Calc. Mass	Obsrv. Mass	± da	± ppm	Start Seq.	End Seq.	Sequence	Ion Score	C. I. %	Modification	Rank	Result Type
1037.5449	1037.5734	0.0285	27	258	266	GVAMLLYDR					Mascot
1320.7059	1320.6606	-0.0453	-34	93	103	ENPPPIHTLFR					Mascot
1359.7228	1359.7566	0.0338	25	246	257	VEVRSVDSDVVR					Mascot
1627.7997	1627.8843	0.0846	52	1	14	MELSFSTKNTTPQK			Oxidation (M)[1]		Mascot
1655.7734	1655.9286	0.1552	94	108	122	MPPLDFDGSYTGLSR					Mascot
1655.7734	1655.9286	0.1552	94	108	122	MPPLDFDGSYTGLSR	2	0			Mascot
1699.9126	1699.9164	0.0038	2	139	154	NPAVAGLHPDTPILER					Mascot
1822.0181	1821.9591	-0.059	-32	155	170	IPLGRNELDDAINLLR					Mascot
1910.9641	1910.9396	-0.0245	-13	250	266	SVDSDVVRGVAMLLYDR			Oxidation (M)[12]		Mascot
1927.0508	1927.0072	-0.0436	-23	137	154	ARNPAVAGLHPDTPILER					Mascot
2369.1443	2369.1567	0.0124	5	108	129	MPPLDFDGSYTGLSRAA QEAVK			Oxidation (M)[1]		Mascot

6 zeaxanthin epoxidase, putative [Ricinus communis] gi|223548701 52005.7 8.47 14 57 0 4.859

Peptide Information

Calc. Mass	Obsrv. Mass	± da	± ppm	Start Seq.	End Seq.	Sequence	Ion Score	C. I. %	Modification	Rank	Result Type
1082.5881	1082.5405	-0.0476	-44	52	60	GFDVKIFEK					Mascot
1101.6151	1101.6012	-0.0139	-13	173	182	VTVILEDGQK					Mascot
1107.6633	1107.5984	-0.0649	-59	416	425	ITKPSVHVAR					Mascot
1193.6525	1193.6736	0.0211	18	57	66	IFEKDLSSVR					Mascot
1359.6434	1359.7566	0.1132	83	61	72	DLSSVRGEGMHR			Oxidation (M)[10]		Mascot
1373.7471	1373.7445	-0.0026	-2	377	388	IFRVSMVHAASR					Mascot
1390.693	1390.7463	0.0533	38	380	392	VSMVHAASRMASK			Oxidation (M)[3]		Mascot
1593.7908	1593.7484	-0.0424	-27	107	121	INGLADGVSGEWFTK					Mascot
1837.9232	1837.9916	0.0684	37	302	316	DIYDRDVIYPWGIGR					Mascot
1876.2106	1876.0709	-0.1397	-74	30	49	LRILIAGGGIGGLVLALAA K					Mascot
1888.0361	1888.1119	0.0758	40	142	158	MALQDILLNAVGFDIVR					Mascot
1904.031	1904.0781	0.0471	25	142	158	MALQDILLNAVGFDIVR			Oxidation (M)[1]		Mascot
1927.9913	1928.0828	0.0915	47	234	251	VFLGLNQYFVASDVGNG K					Mascot
2255.1113	2255.124	0.0127	6	163	182	VVDFMEDSSKVTVILEDG QK			Oxidation (M)[5]		Mascot
2705.3618	2705.3333	-0.0285	-11	393	415	VLTTYQPYIHFQSGPMSH LSSRR					Mascot
2705.3618	2705.3333	-0.0285	-11	393	415	VLTTYQPYIHFQSGPMSH LSSRR					Mascot

7 PREDICTED: myosin-9-like [Cicer arietinum] gi|502182590 145079.1 5.08 24 57 0 21.269

Peptide Information

Calc. Mass	Obsrv. Mass	± da	± ppm	Start	End	Sequence	Ion	C. I. %	Modification	Rank	Result Type

				Seq.	Seq.		Score				
975.5656	975.5868	0.0212	22	88	96	LIVAMGSLR			Oxidation (M)[5]		Mascot
1092.5167	1092.5701	0.0534	49	1085	1094	SELNASNTEK					Mascot
1141.6365	1141.6035	-0.033	-29	25	34	ALQVPKGWDK					Mascot
1181.5797	1181.6407	0.061	52	943	952	LSAAYDEKER					Mascot
1193.6268	1193.6736	0.0468	39	313	322	KLMGDLEMLK			Oxidation (M)[3]		Mascot
1277.7101	1277.7743	0.0642	50	35	46	LFVSVVSVENGK					Mascot
1329.7261	1329.7091	-0.017	-13	431	442	LEIENLSSLPSK					Mascot
1331.7529	1331.728	-0.0249	-19	396	407	ESTANLSLQLKK					Mascot
1331.7529	1331.728	-0.0249	-19	396	407	ESTANLSLQLKK					Mascot
1359.7114	1359.7566	0.0452	33	462	472	QQIEQLEESKK					Mascot
1379.7278	1379.7778	0.05	36	159	170	AINENNHEVIVK					Mascot
1493.7152	1493.8148	0.0996	67	591	604	DGSASEELLSNMLK					Mascot
1507.688	1507.8145	0.1265	84	848	860	LELEDNCSLMGAR			Carbamidomethyl (C)[7]		Mascot
1565.7	1565.7141	0.0141	9	895	908	DLTSDMDGILDENK					Mascot
1605.8483	1605.7992	-0.0491	-31	459	471	VFKQQIEQLEESK					Mascot
1637.8203	1637.9283	0.108	66	332	346	LAGLEMDLSAAYVER					Mascot
1699.9337	1699.9164	-0.0173	-10	951	965	ERIASNALLEVSELR					Mascot
1804.9109	1805.0232	0.1123	62	1140	1154	ASIEERIMQLENDLK			Oxidation (M)[8]		Mascot
1822.8375	1822.9807	0.1432	79	893	908	EKDLTSDMDGILDENK					Mascot
1822.8375	1822.9807	0.1432	79	893	908	EKDLTSDMDGILDENK					Mascot
1837.9291	1837.9916	0.0625	34	443	458	LSTLEKSFQLSEEGNR					Mascot
1838.8324	1839.0139	0.1815	99	893	908	EKDLTSDMDGILDENK			Oxidation (M)[8]		Mascot
1844.9977	1844.9774	-0.0203	-11	155	170	SHLKAINENNHEVIVK					Mascot
1856.9059	1857.0701	0.1642	88	987	1001	TEVNTMQTQYEQKLK			Oxidation (M)[6]		Mascot
1911.0294	1910.9396	-0.0898	-47	352	368	EIEQLTLSSGHTIARQK					Mascot
1928.0084	1928.0828	0.0744	39	1173	1188	INSQLQQTIQQLEQEK					Mascot
2252.071	2252.1042	0.0332	15	1075	1094	QSENEIMALKSELNASNT EK			Oxidation (M)[7]		Mascot

8 Os04g0583150 [Oryza sativa Japonica Group] gi|255675722 69066.2 9.31 19 56 0 16.731

Peptide Information

Calc. Mass	Obsrv. Mass	± da	± ppm	Start Seq.	End Seq.	Sequence	Ion Score	C. I. %	Modification	Rank	Result Type
888.4897	888.5143	0.0246	28	273	281	AGRASDALK					Mascot
974.5703	974.5925	0.0222	23	346	354	VEKIMGVAK					Mascot
1006.5098	1006.4913	-0.0185	-18	448	456	ISRASDAMR					Mascot

1033.5459	1033.564	0.0181	18	276	284	ASDALKCLR			Carbamidomethyl (C)[7]		Mascot
1066.5276	1066.5596	0.032	30	218	226	LSRDADAYR					Mascot
1101.5609	1101.6012	0.0403	37	43	52	SCASPLLEPK			Carbamidomethyl (C)[2]		Mascot
1107.5212	1107.5984	0.0772	70	565	573	DMQGTRLDR			Oxidation (M)[2]		Mascot
1126.6038	1126.5715	-0.0323	-29	336	345	VIVMHVEAGR			Oxidation (M)[4]		Mascot
1323.7379	1323.7111	-0.0268	-20	582	593	SLRDAGLEVHVK					Mascot
1473.7744	1473.7731	-0.0013	-1	31	42	RQLASFVLHCSR			Carbamidomethyl (C)[10]		Mascot
1507.819	1507.8145	-0.0045	-3	538	552	VDTALAGLMVGVFSK					Mascot
1523.8138	1523.8939	0.0801	53	538	552	VDTALAGLMVGVFSK			Oxidation (M)[9]		Mascot
1600.7537	1600.8983	0.1446	90	95	106	QTEQLAFECYRR			Carbamidomethyl (C)[9]		Mascot
1628.7955	1628.9452	0.1497	92	594	606	WLQTNFSFVEEKT					Mascot
1655.8064	1655.9286	0.1222	74	132	144	QWSSLEFLVEDFR					Mascot
1655.8064	1655.9286	0.1222	74	132	144	QWSSLEFLVEDFR					Mascot
1699.8109	1699.9164	0.1055	62	183	199	GAPAAVAFSSAMQAYNK			Oxidation (M)[12]		Mascot
1821.9893	1821.9591	-0.0302	-17	363	378	VTDCILSTIVNGFVKR			Carbamidomethyl (C)[4]		Mascot
1822.9368	1822.9807	0.0439	24	556	570	FNELIELLKDMQGTR			Oxidation (M)[11]		Mascot
1822.9368	1822.9807	0.0439	24	556	570	FNELIELLKDMQGTR			Oxidation (M)[11]		Mascot
1910.9828	1910.9396	-0.0432	-23	538	555	VDTALAGLMVGVFSKCSR			Carbamidomethyl (C)[16]		Mascot
1926.9777	1927.0072	0.0295	15	538	555	VDTALAGLMVGVFSKCSR			Carbamidomethyl (C)[16], Oxidation (M)[9]		Mascot
2384.1638	2384.0955	-0.0683	-29	183	204	GAPAAVAFSSAMQAYNKLHMYR					Mascot

9 unnamed protein product [Vitis vinifera] gi|296088147 100791.5 7.59 19 55 0 22.335

Peptide Information

Calc. Mass	Obsrv. Mass	± da	± ppm	Start Seq.	End Seq.	Sequence	Ion Score	C. I. %	Modification	Rank	Result Type
807.4215	807.4484	0.0269	33	602	607	MLERMK					Mascot
888.5778	888.5143	-0.0635	-71	424	430	AFLLLRR					Mascot
906.5189	906.5155	-0.0034	-4	1	7	MLLRTTR			Oxidation (M)[1]		Mascot
1060.5455	1060.6169	0.0714	67	561	570	LNEANAMLGK					Mascot
1139.6129	1139.6292	0.0163	14	669	678	AFQIVSTMVK			Oxidation (M)[8]		Mascot
1158.5862	1158.6248	0.0386	33	821	830	NEGRVQEAQK					Mascot
1235.5072	1235.6135	0.1063	86	790	798	YDNCLEFMK			Carbamidomethyl (C)[4], Oxidation (M)[8]		Mascot
1320.6616	1320.6606	-0.001	-1	776	786	AISSIIEHYCK			Carbamidomethyl (C)[10]		Mascot
1359.7301	1359.7566	0.0265	19	491	503	LEQANGILGSMVK					Mascot
1365.6943	1365.713	0.0187	14	62	74	SLASHMTPHLAGK			Oxidation (M)[6]		Mascot
1487.8251	1487.8179	-0.0072	-5	491	504	LEQANGILGSMVKK					Mascot

1507.8302	1507.8145	-0.0157	-10	666	678	LDRAFQIVSTMVK					Mascot
1523.8251	1523.8939	0.0688	45	666	678	LDRAFQIVSTMVK			Oxidation (M)[11]		Mascot
1565.7047	1565.7141	0.0094	6	289	301	QEMVEKGCQPSTR			Carbamidomethyl (C)[8], Oxidation (M)[3]		Mascot
1685.9368	1685.9408	0.004	2	488	503	LGRLEQANGILGSMVK					Mascot
1685.9368	1685.9408	0.004	2	488	503	LGRLEQANGILGSMVK					Mascot
1699.8142	1699.9164	0.1022	60	407	420	TYNELMEGLCRVSK			Carbamidomethyl (C)[10]		Mascot
1707.921	1707.8672	-0.0538	-32	59	74	TLKSLASHMTPHLAGK			Oxidation (M)[9]		Mascot
1822.9408	1822.9807	0.0399	22	383	398	EGWVVSAFQLLSVMEK					Mascot
1822.9408	1822.9807	0.0399	22	383	398	EGWVVSAFQLLSVMEK					Mascot
1838.9358	1839.0139	0.0781	42	383	398	EGWVVSAFQLLSVMEK			Oxidation (M)[14]		Mascot
1934.0858	1934.1437	0.0579	30	75	91	IIGLQSNNVELGVRFFK					Mascot
2426.1843	2426.2397	0.0554	23	735	755	CGVPTEDLYNFLVVGLCK EGR			Carbamidomethyl (C)[1,17]		Mascot
2426.1843	2426.2397	0.0554	23	735	755	CGVPTEDLYNFLVVGLCK EGR			Carbamidomethyl (C)[1,17]		Mascot

10	RNA-binding family protein isoform 1 [Theobroma cacao]	gi|508709444	33786	5.07	11	55	0	7.532

Peptide Information

Calc. Mass	Obsrv. Mass	± da	± ppm	Start Seq.	End Seq.	Sequence	Ion Score	C. I. %	Modification	Rank	Result Type
1165.563	1165.627	0.064	55	219	230	ASSAGTAIMSNR					Mascot
1165.563	1165.627	0.064	55	219	230	ASSAGTAIMSNR					Mascot
1179.6368	1179.6605	0.0237	20	208	218	TKSAFAVAEQK					Mascot
1181.558	1181.6407	0.0827	70	219	230	ASSAGTAIMSNR			Oxidation (M)[9]		Mascot
1307.6511	1307.7468	0.0957	73	249	260	AAEDVGMLTKEK			Oxidation (M)[7]		Mascot
1323.6647	1323.7111	0.0464	35	131	142	KAEDVMSTMLAK					Mascot
1353.6355	1353.7109	0.0754	56	197	207	EMNEIFQVSEK					Mascot
1417.7832	1417.7914	0.0082	6	184	196	LSIGTAVVNEKMR					Mascot
1473.8384	1473.7731	-0.0653	-44	33	46	TVKVSNISLAASQR					Mascot
1655.8387	1655.9286	0.0899	54	65	78	RETENAQVAYVTFK					Mascot
1655.8387	1655.9286	0.0899	54	65	78	RETENAQVAYVTFK					Mascot
1791.9521	1791.8416	-0.1105	-62	178	194	MGLSEKLSIGTAVVNEK			Oxidation (M)[1]		Mascot
1821.9276	1821.9591	0.0315	17	1	17	MSVPLDHTNQSLGAVPR					Mascot
1837.9226	1837.9916	0.069	38	1	17	MSVPLDHTNQSLGAVPR			Oxidation (M)[1]		Mascot
2227.0376	2227.0925	0.0549	25	47	64	DIKEFFSFSGDIQYVEMR			Oxidation (M)[17]		Mascot

Gel Idx/Pos	133/F8	Instr./Gel Origin	BA2151/Sample Project 20140814	Process Status	Analysis Succeeded
Plate [#] Name	[1] Sample Project 20140814	Instrument Sample Name		Spectra	11

Rank	Protein Name	Accession No.	Protein MW	Protein PI	Pep. Count	Protein Score	Protein Score C. I. %	Intensity Matched	Total Ion Score	Total Ion C. I. %	Confirmed
1	Globulin-1 S allele [Aegilops tauschii]	gi|475588764	62687.5	9.44	8	206	100	12.138	183	100	

Peptide Information

Calc. Mass	Obsrv. Mass	± da	± ppm	Start Seq.	End Seq.	Sequence	Ion Score	C. I. %	Modification	Rank	Result Type
837.4101	837.3646	-0.0455	-54	360	366	LYEADAR					Mascot
906.468	906.5266	0.0586	65	523	529	EVQEVFR					Mascot
1477.7581	1477.835	0.0769	52	173	184	NYRVAIMEVNPR			Oxidation (M)[7]		Mascot
1684.8766	1684.9712	0.0946	56	508	522	LDNPAQELTFGRPAR					Mascot
1684.8766	1684.9712	0.0946	56	508	522	LDNPAQELTFGRPAR	12	0			Mascot
1996.7963	1996.918	0.1217	61	430	447	GSGSESESESEEQQDQ QR					Mascot
1996.7963	1996.918	0.1217	61	430	447	GSGSESESESEEQQDQ QR	58	99.747			Mascot
2123.0549	2123.115	0.0601	28	222	242	EGDVIVAPAGSIMHLANT DGR					Mascot
2152.8975	2153.009	0.1115	52	429	447	RGSGSESESESEEQQDQ QR					Mascot
3681.833	3682.1257	0.2927	79	458	492	GSAFVVPPGHPVVEIASS QGSSNLQVVCFEINAER			Carbamidomethyl (C)[28]		Mascot
3681.833	3682.1257	0.2927	79	458	492	GSAFVVPPGHPVVEIASS QGSSNLQVVCFEINAER	114	100	Carbamidomethyl (C)[28]		Mascot

Rank	Protein Name	Accession No.	Protein MW	Protein PI	Pep. Count	Protein Score	Protein Score C. I. %	Intensity Matched	Total Ion Score	Total Ion C. I. %	Confirmed
2	Globulin-1 S allele [Triticum urartu]	gi|474411419	57108.4	9.1	10	144	100	20.578	106	100	

Peptide Information

Calc. Mass	Obsrv. Mass	± da	± ppm	Start Seq.	End Seq.	Sequence	Ion Score	C. I. %	Modification	Rank	Result Type
837.4101	837.3646	-0.0455	-54	276	282	LYEADAR					Mascot
906.468	906.5266	0.0586	65	457	463	EVQEVFR					Mascot
1477.7581	1477.835	0.0769	52	89	100	NYRVAIMEVNPR			Oxidation (M)[7]		Mascot
1822.8752	1822.9938	0.1186	65	411	426	GSSNLQVVCFEINAER			Carbamidomethyl (C)[9]		Mascot
1906.0182	1906.1251	0.1069	56	392	410	GSAFVVPPGHPVVEIASS R					Mascot
1958.9396	1958.993	0.0534	27	40	54	SLQQCVQRCQQDRPR			Carbamidomethyl (C)[5,9]		Mascot
2089.9058	2090.0396	0.1338	64	466	483	DQQDEGFVAGPEQQEQ ER					Mascot
2089.9058	2090.0396	0.1338	64	466	483	DQQDEGFVAGPEQQEQ ER	106	100			Mascot
2289.0378	2289.1877	0.1499	65	464	483	AKDQQDEGFVAGPEQQ EQER					Mascot

2663.3457	2663.5601	0.2144	81	316	339	LAVVLEGEGEVEIVCPHL GRDSER			Carbamidomethyl (C)[15]		Mascot
3709.8755	3710.1519	0.2764	75	392	426	GSAFVVPPGHPVVEIASS RGSSNLQVVCFEINAER			Carbamidomethyl (C)[28]		Mascot

3 Phosphoribosylamine--glycine ligase, chloroplastic [Aegilops tauschii] gi|475535472 55786 5.63 14 56 0 8.342

Peptide Information

Calc. Mass	Obsrv. Mass	± da	± ppm	Start Seq.	End Seq.	Sequence	Ion Score	C. I. %	Modification	Rank	Result Type
802.4318	802.3547	-0.0771	-96	506	511	RDIGWR					Mascot
847.4169	847.3521	-0.0648	-76	63	68	NSERWR					Mascot
849.4689	849.3922	-0.0767	-90	56	62	HSHLTVR					Mascot
906.5043	906.5266	0.0223	25	371	377	LIEYNVR					Mascot
958.5468	958.5798	0.033	34	507	514	DIGWRALK					Mascot
1059.598	1059.6349	0.0369	35	86	96	MTVLVIGGGGR					Mascot
1388.7897	1388.8042	0.0145	10	366	377	SGLPKLIEYNVR					Mascot
1388.7897	1388.8042	0.0145	10	366	377	SGLPKLIEYNVR					Mascot
1439.7968	1439.8177	0.0209	15	352	364	FVGVLYAGLMIEK					Mascot
1477.7402	1477.835	0.0948	64	1	13	MACAAYSIRGHLK			Carbamidomethyl (C)[3]		Mascot
1641.8054	1641.9208	0.1154	70	197	209	LCDKYNIPTAQYR			Carbamidomethyl (C)[2]		Mascot
1684.8501	1684.9712	0.1211	72	69	85	SIPKASPEDGTAVAEGR					Mascot
1684.8501	1684.9712	0.1211	72	69	85	SIPKASPEDGTAVAEGR					Mascot
2274.1846	2274.2271	0.0425	19	86	106	MTVLVIGGGGREHALCY ALTR			Carbamidomethyl (C)[16]		Mascot
2300.1665	2300.25	0.0835	36	73	96	ASPEDGTAVAEGRMTVL VIGGGGR					Mascot
2311.1274	2311.1843	0.0569	25	173	195	AGIPTFGPSSEAAALEGS KDFMK					Mascot
2327.1223	2327.1411	0.0188	8	173	195	AGIPTFGPSSEAAALEGS KDFMK			Oxidation (M)[22]		Mascot

4 hypothetical protein F775_25019 [Aegilops tauschii] gi|475614586 60239.9 4.93 14 45 0 17.061

Peptide Information

Calc. Mass	Obsrv. Mass	± da	± ppm	Start Seq.	End Seq.	Sequence	Ion Score	C. I. %	Modification	Rank	Result Type
802.4206	802.3547	-0.0659	-82	205	211	SGHFLNK					Mascot
817.4414	817.3932	-0.0482	-59	172	178	TEQQLAK					Mascot
849.4577	849.3922	-0.0655	-77	52	59	GLGFVSNR					Mascot
854.3639	854.3523	-0.0116	-14	265	271	DTADYNR					Mascot
889.5142	889.5203	0.0061	7	291	297	LLDPYLR					Mascot
958.5217	958.5798	0.0581	61	204	211	RSGHFLNK					Mascot

Calc. Mass	Obsrv. Mass	± da	± ppm	Start Seq.	End Seq.	Sequence	Ion Score	C. I. %	Modification	Rank	Result Type
1360.7948	1360.7733	-0.0215	-16	356	368	FLGSVQATILGVR					Mascot
1360.7948	1360.7733	-0.0215	-16	356	368	FLGSVQATILGVR					Mascot
1641.8749	1641.9208	0.0459	28	249	264	GGFKFPSSVYGVVAAR					Mascot
1678.8119	1678.943	0.1311	78	272	284	HLLFFCDRNNSQK			Carbamidomethyl (C)[6]		Mascot
1838.9257	1839.0537	0.128	70	419	434	DGRRPANSNGYLYLSR					Mascot
1996.9467	1996.918	-0.0287	-14	494	510	LASDMEMLAAIERDFER					Mascot
1996.9467	1996.918	-0.0287	-14	494	510	LASDMEMLAAIERDFER					Mascot
2090.0071	2090.0396	0.0325	16	384	402	VACSPVSRELVEDSDGE LK			Carbamidomethyl (C)[3]		Mascot
2090.0071	2090.0396	0.0325	16	384	402	VACSPVSRELVEDSDGE LK			Carbamidomethyl (C)[3]		Mascot
2122.9077	2123.115	0.2073	98	188	203	DMFDFFRHDWETAFSR			Oxidation (M)[2]		Mascot
2272.1167	2272.2039	0.0872	38	17	35	ADLADELEFFTELRNTLM K			Oxidation (M)[18]		Mascot

5	E3 ubiquitin-protein ligase UPL5 [Arabidopsis thaliana]	gi|332657749	101156	6.25	17	45	0	8.611

Protein Group

E3 ubiquitin-protein ligase UPL5 [Arabidopsis thaliana]	gi|15235410	101156	6.25
RecName: Full=E3 ubiquitin-protein ligase UPL5; Short=Ubiquitin-protein ligase 5	gi|75207797	101156	6.25

Peptide Information

Calc. Mass	Obsrv. Mass	± da	± ppm	Start Seq.	End Seq.	Sequence	Ion Score	C. I. %	Modification	Rank	Result Type
817.4665	817.3932	-0.0733	-90	634	640	ISLEDIK					Mascot
847.4308	847.3521	-0.0787	-93	584	591	FSPNPASK					Mascot
874.4451	874.4152	-0.0299	-34	285	291	KVCPDQK			Carbamidomethyl (C)[3]		Mascot
915.4062	915.3291	-0.0771	-84	645	651	IMYNSCK			Carbamidomethyl (C)[6]		Mascot
995.607	995.6909	0.0839	84	606	614	VIALALMHK					Mascot
1301.7576	1301.6923	-0.0653	-50	711	721	RFATPILEQVK					Mascot
1382.7283	1382.7384	0.0101	7	95	106	LQIFVRMMSGGK			Oxidation (M)[7]		Mascot
1388.709	1388.8042	0.0952	69	682	693	DTIELCPDGKLK			Carbamidomethyl (C)[6]		Mascot
1388.7169	1388.8042	0.0873	63	860	872	LIAQDHVSSSFGK					Mascot
1439.8192	1439.8177	-0.0015	-1	493	504	RHLAMLLFPDVK					Mascot
1997.03	1996.918	-0.112	-56	204	221	IVTFFAMIPVESDESIAK					Mascot
1997.03	1996.918	-0.112	-56	204	221	IVTFFAMIPVESDESIAK					Mascot
2271.0823	2271.1941	0.1118	49	51	70	QEIDADHMAASAQQTLIS WR					Mascot
2300.1404	2300.25	0.1096	48	148	167	ENSLTYYSIEQDASLQLV AR					Mascot
2311.2729	2311.1843	-0.0886	-38	369	389	VAFPIPIVLPMQSTALEAE			Oxidation (M)[11]		Mascot

						IR					
2345.0237	2345.2429	0.2192	93	6	28	SSADDSTNNANRSYSAV AGTDNK					Mascot
2617.3271	2617.3359	0.0088	3	557	576	EWFYLVCQEIFNPKNTLF LR			Carbamidomethyl (C)[7]		Mascot
3681.7776	3682.1257	0.3481	95	417	449	EVGNSETMSSSWSQYLS ILKIINSMSNIYQGAK			Oxidation (M)[8]		Mascot
3681.7776	3682.1257	0.3481	95	417	449	EVGNSETMSSSWSQYLS ILKIINSMSNIYQGAK			Oxidation (M)[8]		Mascot

6	WD-40 repeat family protein / notchless protein, putative [Arabidopsis thaliana]	gi|332008882	53434.1	8.63	11	45	0	7.355

Protein Group

RecName: Full=Notchless protein homolog	gi|75334042	53434.1	8.6300 001144 4092
WD-40 repeat family protein / notchless protein, putative [Arabidopsis thaliana]	gi|15237273	53434.1	8.6300 001144 4092

Peptide Information

Calc. Mass	Obsrv. Mass	± da	± ppm	Start Seq.	End Seq.	Sequence	Ion Score	C. I. %	Modification	Rank	Result Type
817.4526	817.3932	-0.0594	-73	459	466	VVSGGKDR					Mascot
888.5189	888.52	0.0011	1	225	231	IWDITLK					Mascot
889.4374	889.5203	0.0829	93	324	331	TKGDSPER					Mascot
1182.575	1182.6188	0.0438	37	214	224	FVTSSKDGDAR					Mascot
1958.9211	1958.993	0.0719	37	165	181	HLVSGSKSGEICCWNPK			Carbamidomethyl (C)[12,13]		Mascot
2025.0011	2024.9546	-0.0465	-23	196	212	WITGISWEPVHLSSPCR			Carbamidomethyl (C)[16]		Mascot
2153.0962	2153.009	-0.0872	-40	195	212	KWITGISWEPVHLSSPCR			Carbamidomethyl (C)[17]		Mascot
2300.335	2300.25	-0.085	-37	82	100	VLTIVYQQQAVFRIRPVN R					Mascot
2312.0466	2312.1604	0.1138	49	438	458	QDLPGHADEVFAVDWSP DGEK					Mascot
2839.3535	2839.4954	0.1419	50	438	464	QDLPGHADEVFAVDWSP DGEKVVSGGK					Mascot
3681.792	3682.1257	0.3337	91	46	76	FLDNEEMLPYSFYVSDEE LLVPVGTYLEKNK					Mascot
3681.792	3682.1257	0.3337	91	46	76	FLDNEEMLPYSFYVSDEE LLVPVGTYLEKNK					Mascot

7	Putative signal peptidase complex subunit 3 [Aegilops tauschii]	gi|475622770	18572.6	8.71	7	43	0	1.512

Peptide Information

Calc. Mass	Obsrv. Mass	± da	± ppm	Start Seq.	End Seq.	Sequence	Ion Score	C. I. %	Modification	Rank	Result Type
874.3988	874.4152	0.0164	19	1	7	MHSWGTR					Mascot
1344.7118	1344.7631	0.0513	38	109	120	DQANVQVEVKSK					Mascot

Calc. Mass	Obsrv. Mass	± da	± ppm	Start Seq.	End Seq.	Sequence	Ion Score	C. I. %	Modification	Rank	Result Type
1477.8009	1477.835	0.0341	23	119	131	SKYPLIDQGTSLR					Mascot
2118.1099	2118.073	-0.0369	-17	135	152	VQLVLHWHIMPNAGAMIR			Oxidation (M)[10,16]		Mascot
2118.1099	2118.073	-0.0369	-17	135	152	VQLVLHWHIMPNAGAMIR			Oxidation (M)[10,16]		Mascot
2230.21	2230.3457	0.1357	61	134	152	KVQLVLHWHIMPNAGAMIR			Oxidation (M)[11]		Mascot
2271.2366	2271.1941	-0.0425	-19	135	154	VQLVLHWHIMPNAGAMIRGK					Mascot
2272.186	2272.2039	0.0179	8	59	78	VTLTFSLSANLESLFTWNTK					Mascot

8	unnamed protein product [Ostreococcus tauri]	gi|308808488	19102.6	5.87	5	43	0	1.123	

Peptide Information

Calc. Mass	Obsrv. Mass	± da	± ppm	Start Seq.	End Seq.	Sequence	Ion Score	C. I. %	Modification	Rank	Result Type
807.4247	807.3519	-0.0728	-90	71	77	EVFDGIK					Mascot
930.5115	930.5588	0.0473	51	18	26	VVARSADGR					Mascot
930.5115	930.5588	0.0473	51	18	26	VVARSADGR	22	0			Mascot
1908.8539	1909.0396	0.1857	97	1	17	MPRDATDANSEVSCALR			Carbamidomethyl (C)[14], Oxidation (M)[1]		Mascot
1959.062	1958.993	-0.069	-35	129	146	LVSVLELPFVAMGEEAVR					Mascot
2122.967	2123.115	0.148	70	97	116	ALSEDGDGRLSVLSCESDGR			Carbamidomethyl (C)[15]		Mascot

9	hypothetical protein PRUPE_ppa011572mg [Prunus persica]	gi|462414883	23600.8	5.83	6	42	0	3.028	

Peptide Information

Calc. Mass	Obsrv. Mass	± da	± ppm	Start Seq.	End Seq.	Sequence	Ion Score	C. I. %	Modification	Rank	Result Type
834.4105	834.3815	-0.029	-35	36	42	GFEPTQR					Mascot
1359.6283	1359.7456	0.1173	86	16	26	MIDFMSSVLER			Oxidation (M)[1,5]		Mascot
1641.8339	1641.9208	0.0869	53	16	29	MIDFMSSVLERVAK			Oxidation (M)[1]		Mascot
2112.9768	2113.0164	0.0396	19	188	205	LHCSFNEDESAHQKQLAV			Carbamidomethyl (C)[3]		Mascot
2118.1377	2118.073	-0.0647	-31	166	184	EMLLQSPLHVAESPLNLAR					Mascot
2118.1377	2118.073	-0.0647	-31	166	184	EMLLQSPLHVAESPLNLAR	13	0			Mascot
2274.2388	2274.2271	-0.0117	-5	166	185	EMLLQSPLHVAESPLNLARR					Mascot

10	hypothetical protein PRUPE_ppa009287mg [Prunus persica]	gi|462405147	31732.3	9.63	9	42	0	11.973	

Peptide Information

Calc. Mass	Obsrv. Mass	± da	± ppm	Start Seq.	End Seq.	Sequence	Ion Score	C. I. %	Modification	Rank	Result Type

807.4207	807.3519	-0.0688	-85	273	280	LQASSSSK		Mascot
1344.7886	1344.7631	-0.0255	-19	207	218	VLSSFLQNPVLK		Mascot
1360.769	1360.7733	0.0043	3	233	243	LENKMLQMILK		Mascot
1360.769	1360.7733	0.0043	3	233	243	LENKMLQMILK		Mascot
1477.7281	1477.835	0.1069	72	193	206	GSPVDTPFGSRTEK		Mascot
1667.9301	1668.0143	0.0842	50	12	27	SGLVVANERRPSGGIR		Mascot
1684.9493	1684.9712	0.0219	13	21	35	RPSGGIRIENPFTLK		Mascot
1684.9493	1684.9712	0.0219	13	21	35	RPSGGIRIENPFTLK		Mascot
1908.9258	1909.0396	0.1138	60	2	20	EASSSNSSSKSGLVVAN ER		Mascot
2025.007	2024.9546	-0.0524	-26	244	259	HQQVIEELMEENEKLR		Mascot
2113.0859	2113.0164	-0.0695	-33	97	118	NIEAGIGCGVGIGHGFGV GITK	Carbamidomethyl (C)[8]	Mascot

Gel Idx/Pos	134/F9	Instr./Gel Origin	BA2151/Sample Project 20140814	Process Status	Analysis Succeeded
Plate [#] Name	[1] Sample Project 20140814	Instrument Sample Name		Spectra	11

Rank	Protein Name	Accession No.	Protein MW	Protein PI	Pep. Count	Protein Score	Protein Score C. I. %	Intensity Matched	Total Ion Score	Total Ion C. I. %	Confirmed
1	Globulin-1 S allele [Triticum urartu]	gi|474411419	57108.4	9.1	8	141	100	27.186	116	100	

Peptide Information

Calc. Mass	Obsrv. Mass	± da	± ppm	Start Seq.	End Seq.	Sequence	Ion Score	C. I. %	Modification	Rank	Result Type
818.4003	818.3643	-0.036	-44	226	232	ASEEQVR					Mascot
906.468	906.5375	0.0695	77	457	463	EVQEVFR					Mascot
1164.444	1164.4258	-0.0182	-16	349	357	WGEEEEDDR					Mascot
1791.8984	1791.8669	-0.0315	-18	254	268	GDSRDTYNLLEQRPK					Mascot
1822.8752	1823.0137	0.1385	76	411	426	GSSNLQVVCFEINAER			Carbamidomethyl (C)[9]		Mascot
1822.8752	1823.0137	0.1385	76	411	426	GSSNLQVVCFEINAER	21	0	Carbamidomethyl (C)[9]		Mascot
1906.0182	1906.1636	0.1454	76	392	410	GSAFVVPPGHPVVEIASSR					Mascot
1906.0182	1906.1636	0.1454	76	392	410	GSAFVVPPGHPVVEIASSR	95	100			Mascot
1958.9396	1959.0035	0.0639	33	40	54	SLQQCVQRCQQDRPR			Carbamidomethyl (C)[5,9]		Mascot
2289.0378	2289.1997	0.1619	71	464	483	AKDQQDEGFVAGPEQQEQER					Mascot

Rank	Protein Name	Accession No.	Protein MW	Protein PI	Pep. Count	Protein Score	Protein Score C. I. %	Intensity Matched	Total Ion Score	Total Ion C. I. %	Confirmed
2	PREDICTED: methyltransferase-like protein 6-like [Cicer arietinum]	gi|502116710	37588.3	6.28	5	41	0	6.241	21	0	

Peptide Information

Calc. Mass	Obsrv. Mass	± da	± ppm	Start Seq.	End Seq.	Sequence	Ion Score	C. I. %	Modification	Rank	Result Type
1169.6174	1169.7087	0.0913	78	59	68	LFHTRHSSGK					Mascot
1565.7806	1565.7477	-0.0329	-21	131	145	ATDIINANDVASSFK					Mascot
1822.8541	1823.0137	0.1596	88	263	277	SDGTRSYFFCLNTVR			Carbamidomethyl (C)[10]		Mascot
1822.8541	1823.0137	0.1596	88	263	277	SDGTRSYFFCLNTVR	21	0	Carbamidomethyl (C)[10]		Mascot
2262.082	2262.1145	0.0325	14	278	296	DLFLGAGFIELELDYCCVK			Carbamidomethyl (C)[16,17]		Mascot
2718.3267	2718.3132	-0.0135	-5	278	300	DLFLGAGFIELELDYCCVKSVNR			Carbamidomethyl (C)[16,17]		Mascot

Rank	Protein Name	Accession No.	Protein MW	Protein PI	Pep. Count	Protein Score	Protein Score C. I. %	Intensity Matched	Total Ion Score	Total Ion C. I. %	Confirmed
3	hypothetical protein ARALYDRAFT_475877 [Arabidopsis lyrata subsp. lyrata]	gi|297332981	36022	9.47	8	41	0	22.586			

Peptide Information

Calc. Mass	Obsrv. Mass	± da	± ppm	Start Seq.	End Seq.	Sequence	Ion Score	C. I. %	Modification	Rank	Result Type

Calc. Mass	Obsrv. Mass	± da	± ppm	Start Seq.	End Seq.	Sequence	Ion Score	C. I. %	Modification	Rank	Result Type
805.405	805.4794	0.0744	92	90	96	DVSESIR					Mascot
906.4713	906.5375	0.0662	73	309	316	GLQKDSMK					Mascot
1791.9388	1791.8669	-0.0719	-40	49	65	RYGSWAIITGPTDGIGK					Mascot
1906.0104	1906.1636	0.1532	80	226	242	KSGIDVQCQVPLYVATK			Carbamidomethyl (C)[8]		Mascot
1906.0104	1906.1636	0.1532	80	226	242	KSGIDVQCQVPLYVATK			Carbamidomethyl (C)[8]		Mascot
1930.9368	1931.1014	0.1646	85	211	225	TYVDQFTKCLHVEYK			Carbamidomethyl (C)[9]		Mascot
2461.1804	2461.2722	0.0918	37	99	120	YSQTQILTVVMDFSGDID EGVK			Oxidation (M)[11]		Mascot
2705.3643	2705.3823	0.018	7	184	210	GAIINMGSGAAALIPSYPF YSVYAGAK			Oxidation (M)[6]		Mascot
2718.3984	2718.3132	-0.0852	-31	147	169	YFHEVDEELLNNLIKINVE GTTK					Mascot

4 hypothetical protein TRIUR3_30438 [Triticum urartu] gi|473944792 22130 10.47 4 40 0 9.704 21 0

Peptide Information

Calc. Mass	Obsrv. Mass	± da	± ppm	Start Seq.	End Seq.	Sequence	Ion Score	C. I. %	Modification	Rank	Result Type
842.5206	842.5831	0.0625	74	55	62	AIRGLQGK					Mascot
1320.7634	1320.6969	-0.0665	-50	58	69	GLQGKIVYSLSR					Mascot
1822.9303	1823.0137	0.0834	46	10	25	VVNSAACKMFPLNSLR			Carbamidomethyl (C)[7], Oxidation (M)[9]		Mascot
1822.9303	1823.0137	0.0834	46	10	25	VVNSAACKMFPLNSLR	21	0	Carbamidomethyl (C)[7], Oxidation (M)[9]		Mascot
1958.9066	1959.0035	0.0969	49	126	142	MPPGYYTGSPQLEEHPR					Mascot

5 TPA: hypothetical protein ZEAMMB73_041554 [Zea mays] gi|414585990 7860.1 8.93 1 38 0 5.318 32 59.238

Peptide Information

Calc. Mass	Obsrv. Mass	± da	± ppm	Start Seq.	End Seq.	Sequence	Ion Score	C. I. %	Modification	Rank	Result Type
1360.7008	1360.7939	0.0931	68	53	63	EVNYSVHLFPR					Mascot
1360.7008	1360.7939	0.0931	68	53	63	EVNYSVHLFPR	32	59.238			Mascot

6 Delta(24)-sterol reductase [Triticum urartu] gi|474169153 56396.9 8.64 8 38 0 6.639 16 0

Peptide Information

Calc. Mass	Obsrv. Mass	± da	± ppm	Start Seq.	End Seq.	Sequence	Ion Score	C. I. %	Modification	Rank	Result Type
891.4352	891.3884	-0.0468	-52	82	89	DGLVCTAR			Carbamidomethyl (C)[5]		Mascot
1342.7147	1342.7579	0.0432	32	130	141	VEPLVNMGQISR					Mascot
1360.6855	1360.7939	0.1084	80	249	261	EVAQAYADAVAPR					Mascot
1360.6855	1360.7939	0.1084	80	249	261	EVAQAYADAVAPR	16	0			Mascot

Calc. Mass	Obsrv. Mass	± da	± ppm	Start Seq.	End Seq.	Sequence	Ion Score	C. I. %	Modification	Rank	Result Type
1475.7754	1475.8553	0.0799	54	105	116	VRHFEVDLSAFR					Mascot
1792.0231	1791.8669	-0.1562	-87	361	375	FLFGWLMPPKVSLLK			Oxidation (M)[7]		Mascot
1930.015	1930.1239	0.1089	56	82	98	DGLVCTARKPWIAVGMR			Carbamidomethyl (C)[5]		Mascot
1958.9396	1959.0035	0.0639	33	324	338	GEFVEYIPTREYYHR					Mascot
2262.1013	2262.1145	0.0132	6	384	401	NYYHDNHVIQDMLVPLYK					Mascot

7 beta-ketoacyl reductase 1 [Arabidopsis thaliana] gi|332196567 36024 9.51 7 35 0 22.461

Protein Group

RecName: Full=Very-long-chain 3-oxoacyl-CoA reductase 1; AltName: Full=Beta-ketoacyl reductase 1; Short=AtKCR1; AltName: Full=Protein GLOSSY 8; Short=gl8At	gi|75301204	36024	9.5100 002288 8184
beta-ketoacyl reductase 1 [Arabidopsis thaliana]	gi|18408847	36024	9.5100 002288 8184

Peptide Information

Calc. Mass	Obsrv. Mass	± da	± ppm	Start Seq.	End Seq.	Sequence	Ion Score	C. I. %	Modification	Rank	Result Type
906.4713	906.5375	0.0662	73	309	316	GLQKDSMK					Mascot
1791.9388	1791.8669	-0.0719	-40	49	65	RYGSWAIITGPTDGIGK					Mascot
1906.0104	1906.1636	0.1532	80	226	242	KSGIDVQCQVPLYVATK			Carbamidomethyl (C)[8]		Mascot
1906.0104	1906.1636	0.1532	80	226	242	KSGIDVQCQVPLYVATK			Carbamidomethyl (C)[8]		Mascot
1930.9368	1931.1014	0.1646	85	211	225	TYVDQFTKCLHVEYK			Carbamidomethyl (C)[9]		Mascot
2461.1804	2461.2722	0.0918	37	99	120	YSQTQILTVVMDFSGDID EGVK			Oxidation (M)[11]		Mascot
2705.3643	2705.3823	0.018	7	184	210	GAIINMGSGAAALIPSYPF YSVYAGAK			Oxidation (M)[6]		Mascot
2718.3984	2718.3132	-0.0852	-31	147	169	YFHEVDEELINNLIKINVE GTTK					Mascot

8 uncharacterized protein, partial [Silene latifolia] gi|520770713 19816.4 5.38 6 35 0 2.302

Peptide Information

Calc. Mass	Obsrv. Mass	± da	± ppm	Start Seq.	End Seq.	Sequence	Ion Score	C. I. %	Modification	Rank	Result Type
805.4566	805.4794	0.0228	28	79	85	FADALLR					Mascot
818.4002	818.3643	-0.0359	-44	159	165	ERDEAAK					Mascot
889.4737	889.3947	-0.079	-89	161	168	DEAAKISR					Mascot
1475.8468	1475.8553	0.0085	6	97	110	IYGLGTGPKVETLK					Mascot
1838.9072	1839.0477	0.1405	76	21	35	EWGENREFLVDLFGK					Mascot
2289.1736	2289.1997	0.0261	11	111	129	KLQNQPEHQGLTLHFVE DR					Mascot

9 Cell elongation protein DIMINUTO [Aegilops tauschii] gi|475570458 65125.2 8.26 8 35 0 6.639 16 0

Peptide Information

Calc. Mass	Obsrv. Mass	± da	± ppm	Start Seq.	End Seq.	Sequence	Ion Score	C. I. %	Modification	Rank	Result Type
891.4352	891.3884	-0.0468	-52	82	89	DGLVCTAR			Carbamidomethyl (C)[5]		Mascot
1342.7147	1342.7579	0.0432	32	130	141	VEPLVNMGQISR					Mascot
1360.6855	1360.7939	0.1084	80	249	261	EVAQAYADAVAPR					Mascot
1360.6855	1360.7939	0.1084	80	249	261	EVAQAYADAVAPR	16	0			Mascot
1475.7754	1475.8553	0.0799	54	105	116	VRHFEVDLSAFR					Mascot
1792.0231	1791.8669	-0.1562	-87	361	375	FLFGWLMPPKVSLLK			Oxidation (M)[7]		Mascot
1930.015	1930.1239	0.1089	56	82	98	DGLVCTARKPWIAVGMR			Carbamidomethyl (C)[5]		Mascot
1958.9396	1959.0035	0.0639	33	324	338	GEFVEYIPTREYYHR					Mascot
2262.1013	2262.1145	0.0132	6	384	401	NYYHDNHVIQDMLVPLYK					Mascot

10	ARF-type transcription factor, partial [Zea mays subsp. mays]	gi|408690390	75595.3	5.93	4	34	0	24.036	20	0		

Protein Group

auxin response factor 1 [Zea mays]	gi|413937283	75595.3	5.9299 998283 3862

Peptide Information

Calc. Mass	Obsrv. Mass	± da	± ppm	Start Seq.	End Seq.	Sequence	Ion Score	C. I. %	Modification	Rank	Result Type
1342.7876	1342.7579	-0.0297	-22	71	81	ILCKVVNVELR			Carbamidomethyl (C)[3]		Mascot
1684.9528	1684.9957	0.0429	25	550	565	VIMQGVAVGRAVDLTR					Mascot
1684.9528	1684.9957	0.0429	25	550	565	VIMQGVAVGRAVDLTR					Mascot
1822.8865	1823.0137	0.1272	70	1	19	MAEAGVARGSGSAGDAL FR					Mascot
1822.8865	1823.0137	0.1272	70	1	19	MAEAGVARGSGSAGDAL FR	20	0			Mascot
1838.8813	1839.0477	0.1664	90	1	19	MAEAGVARGSGSAGDAL FR			Oxidation (M)[1]		Mascot
1905.9778	1906.1636	0.1858	97	125	142	TLTASDTSTHGGFSVLRR					Mascot
1905.9778	1906.1636	0.1858	97	125	142	TLTASDTSTHGGFSVLRR	5	0			Mascot

Gel Idx/Pos	135/F10	Instr./Gel Origin	BA2151/Sample Project 20140814	Process Status	Analysis Succeeded
Plate [#] Name	[1] Sample Project 20140814	Instrument Sample Name		Spectra	11

Rank	Protein Name	Accession No.	Protein MW	Protein PI	Pep. Count	Protein Score	Protein Score C. I. %	Intensity Matched	Total Ion Score	Total Ion C. I. %	Confirmed
1	Globulin-1 S allele [Triticum urartu]	gi|474411419	57108.4	9.1	8	257	100	50.95	238	100	

Peptide Information

Calc. Mass	Obsrv. Mass	± da	± ppm	Start Seq.	End Seq.	Sequence	Ion Score	C. I. %	Modification	Rank	Result Type
906.468	906.5305	0.0625	69	457	463	EVQEVFR					Mascot
1105.6001	1105.587	-0.0131	-12	457	465	EVQEVFRAK					Mascot
1320.5452	1320.672	0.1268	96	349	358	WGEEEEDDRR					Mascot
1685.8606	1685.9565	0.0959	57	442	456	LDDPAQELTFGRPAR					Mascot
1791.8984	1791.864	-0.0344	-19	254	268	GDSRDTYNLLEQRPK					Mascot
1822.8752	1823.0027	0.1275	70	411	426	GSSNLQVVCFEINAER			Carbamidomethyl (C)[9]		Mascot
1822.8752	1823.0027	0.1275	70	411	426	GSSNLQVVCFEINAER	89	100	Carbamidomethyl (C)[9]		Mascot
1906.0182	1906.1558	0.1376	72	392	410	GSAFVVPPGHPVVEIASS R					Mascot
1906.0182	1906.1558	0.1376	72	392	410	GSAFVVPPGHPVVEIASS R	150	100			Mascot
1958.9396	1959.004	0.0644	33	40	54	SLQQCVQRCQQDRPR			Carbamidomethyl (C)[5,9]		Mascot

Rank	Protein Name	Accession No.	Protein MW	Protein PI	Pep. Count	Protein Score	Protein Score C. I. %	Intensity Matched	Total Ion Score	Total Ion C. I. %	Confirmed
2	cell envelope integrity inner membrane protein TolA [Zea mays]	gi|413946720	32095.4	9.04	10	51	0	11.861			

Peptide Information

Calc. Mass	Obsrv. Mass	± da	± ppm	Start Seq.	End Seq.	Sequence	Ion Score	C. I. %	Modification	Rank	Result Type
819.3954	819.4332	0.0378	46	225	230	RTEEER					Mascot
829.489	829.4873	-0.0017	-2	140	148	QVAGAKGAK					Mascot
957.5629	957.5659	0.003	3	40	47	IHLSGFRK					Mascot
982.4588	982.4962	0.0374	38	149	156	SEPEQQHK					Mascot
1345.6707	1345.8041	0.1334	99	214	225	ATDPTESLAERR					Mascot
1822.9043	1823.0027	0.0984	54	8	26	QGGGSSKPPAAAGQELD PR					Mascot
1822.9043	1823.0027	0.0984	54	8	26	QGGGSSKPPAAAGQELD PR	1	0			Mascot
1922.0746	1922.1403	0.0657	34	278	296	EVIAVAVAAFSLGVFVSS R					Mascot
1927.9719	1928.0995	0.1276	66	126	145	KPEDADADAAAATKQVA GAK					Mascot
2425.3604	2425.2205	-0.1399	-58	278	300	EVIAVAVAAFSLGVFVSS RLFSR					Mascot

2454.22	2454.2939	0.0739	30	27	46	YEWEENASSFILRIHLSG FR						Mascot

3 PREDICTED: uncharacterized protein LOC101228103 [Cucumis sativus] gi|449483949 51424.5 9.72 14 47 0 5.116

Peptide Information

Calc. Mass	Obsrv. Mass	± da	± ppm	Start Seq.	End Seq.	Sequence	Ion Score	C. I. %	Modification	Rank	Result Type
800.4988	800.4455	-0.0533	-67	167	173	NKVVVNK					Mascot
847.452	847.496	0.044	52	357	363	QSKEDIK					Mascot
854.5345	854.5865	0.052	61	58	65	ISSILPPK					Mascot
916.521	916.5445	0.0235	26	248	255	LKSAQQNK					Mascot
930.4825	930.5034	0.0209	22	66	73	NKCNPGIK			Carbamidomethyl (C)[3]		Mascot
982.4874	982.4962	0.0088	9	83	91	TSISTMSQK					Mascot
1475.7601	1475.8414	0.0813	55	194	208	GAAGPSFAEQGSKIR					Mascot
1593.8483	1593.7775	-0.0708	-44	258	271	VLVTNQDYIVTNSK					Mascot
1649.7734	1649.9337	0.1603	97	68	82	CNPGIKTASADVCTR			Carbamidomethyl (C)[1,13]		Mascot
1655.8024	1655.9434	0.141	85	190	206	AYSKGAAGPSFAEQGSK					Mascot
1655.8024	1655.9434	0.141	85	190	206	AYSKGAAGPSFAEQGSK					Mascot
1765.9166	1765.9445	0.0279	16	444	457	FLKEHLLNQHPMSR			Oxidation (M)[12]		Mascot
1943.9161	1944.0599	0.1438	74	74	91	TASADVCTRTSISTMSQK			Carbamidomethyl (C)[7]		Mascot
1993.9747	1994.1165	0.1418	71	14	31	DSDELTLKSMPSNSKPTK			Oxidation (M)[10]		Mascot
2426.1113	2426.2661	0.1548	64	209	231	AFSGNLQSQGRGNDQG MNVNTSK			Oxidation (M)[17]		Mascot
2426.1113	2426.2661	0.1548	64	209	231	AFSGNLQSQGRGNDQG MNVNTSK			Oxidation (M)[17]		Mascot

4 Ribulose bisphosphate carboxylase small chain PWS4.3, chloroplastic [Triticum urartu] gi|473721335 19632.8 8.81 8 47 0 6.767

Peptide Information

Calc. Mass	Obsrv. Mass	± da	± ppm	Start Seq.	End Seq.	Sequence	Ion Score	C. I. %	Modification	Rank	Result Type
887.4945	887.5483	0.0538	61	21	29	STAGLPVSR					Mascot
906.5043	906.5305	0.0262	29	75	81	QVDYLIR					Mascot
930.4178	930.5034	0.0856	92	111	116	YWTMWK			Oxidation (M)[4]		Mascot
1320.6615	1320.672	0.0105	8	30	43	RSSGSLGSVSNGGR					Mascot
1848.9524	1849.1241	0.1717	93	2	20	APAVMASSASTVAPFQG LK			Oxidation (M)[5]		Mascot
1922.0521	1922.1403	0.0882	46	58	74	FETLSYLPPLSTEALLK					Mascot
1926.9708	1927.012	0.0412	21	138	153	EYPDAYVRVIGFDNLR					Mascot
2408.1836	2408.2317	0.0481	20	117	137	LPMFGCTDATQVLNEVE			Carbamidomethyl (C)[6]		Mascot

			EVKK						
5	hypothetical protein M569_10486, partial [Genlisea aurea]	gi|527194806	68550.5	6.02	14	47	0	3.024	

Peptide Information

Calc. Mass	Obsrv. Mass	± da	± ppm	Start Seq.	End Seq.	Sequence	Ion Score	C. I. %	Modification	Rank	Result Type
847.4632	847.496	0.0328	39	418	425	GTGTDKLR					Mascot
856.5363	856.5854	0.0491	57	275	281	LNRLNVK					Mascot
870.5268	870.5939	0.0671	77	2	8	ALNLRQR					Mascot
887.5057	887.5483	0.0426	48	93	100	RLVADASR					Mascot
905.4509	905.5063	0.0554	61	9	15	QTDCIIR			Carbamidomethyl (C)[4]		Mascot
933.4709	933.3954	-0.0755	-81	517	525	AVDALMEGK					Mascot
988.5197	988.59	0.0703	71	173	180	EIEEIVEK					Mascot
1543.8228	1543.9767	0.1539	100	504	516	NLLSYDHQLALTR					Mascot
1649.8639	1649.9337	0.0698	42	465	480	IKALNASLASASSMNR			Oxidation (M)[14]		Mascot
1721.9908	1721.8359	-0.1549	-90	368	383	QVIDKHTNIATALLGK					Mascot
1838.9794	1839.0417	0.0623	34	352	367	HLMTAVNSLPELTERK					Mascot
1844.863	1844.9637	0.1007	55	200	216	CPQGGPAEMVASLLDQR			Carbamidomethyl (C)[1], Oxidation (M)[9]		Mascot
2454.2188	2454.2939	0.0751	31	138	158	VHDQYLEFVSLEDNLFSL ASK					Mascot
2564.2185	2564.3748	0.1563	61	159	180	NCYVQLNDPSAGEKEIEE IVEK			Carbamidomethyl (C)[2]		Mascot

6	PREDICTED: uncharacterized protein LOC101244885 [Solanum lycopersicum]	gi|460394951	76246.9	9.16	18	46	0	9.509

Peptide Information

Calc. Mass	Obsrv. Mass	± da	± ppm	Start Seq.	End Seq.	Sequence	Ion Score	C. I. %	Modification	Rank	Result Type
807.4433	807.4564	0.0131	16	37	43	VMPFGLK			Oxidation (M)[2]		Mascot
829.5142	829.4873	-0.0269	-32	261	269	GVGAVLVSK					Mascot
906.4614	906.5305	0.0691	76	593	599	MTRAFHK			Oxidation (M)[1]		Mascot
916.5284	916.5445	0.0161	18	225	233	AMPTGKLAK					Mascot
1060.615	1060.5781	-0.0369	-35	96	104	YNLKLNPAK					Mascot
1105.5095	1105.587	0.0775	70	270	279	SGQHYPMAAK			Oxidation (M)[7]		Mascot
1313.7423	1313.7593	0.017	13	568	578	RIDQLALIDEK					Mascot
1471.7549	1471.8213	0.0664	45	52	63	AMKTIFHDMIHK					Mascot
1475.72	1475.8414	0.1214	82	618	629	DTMREVYSFALK			Oxidation (M)[3]		Mascot
1638.8561	1639.001	0.1449	88	159	172	FIAQSTVVCEPIFK			Carbamidomethyl (C)[9]		Mascot
1655.8396	1655.9434	0.1038	63	37	51	VMPFGLKNAGATYMR					Mascot

1655.8396	1655.9434	0.1038	63	37	51	VMPFGLKNAGATYMR					Mascot
1679.7914	1679.9193	0.1279	76	603	617	MALCGPDLSTQMLSR			Carbamidomethyl (C)[4]		Mascot
1721.8468	1721.8359	-0.0109	-6	507	520	GWHEMLPYALLGYR			Oxidation (M)[5]		Mascot
1791.9939	1791.864	-0.1299	-72	105	121	CAFGVPAGKLLGFIVSR			Carbamidomethyl (C)[1]		Mascot
1838.9946	1839.0417	0.0471	26	419	433	MTLNLFLNGEVLYRR					Mascot
1927.09	1927.012	-0.078	-40	535	552	VYGTEAVVPVEVVIPSLR					Mascot
1958.9091	1959.004	0.0949	48	397	413	RYLESGTYPEDATSNQK					Mascot
2254.939	2255.1506	0.2116	94	473	489	VLRADYFWMTMENDCCK			Carbamidomethyl (C)[15,16], Oxidation (M)[9]		Mascot

7	PREDICTED: mitochondrial import inner membrane translocase subunit TIM23-2-like isoform X2 [Cicer arietinum]	gi|502102216	10704	4.93	5	46	0	4.669	

Peptide Information

Calc. Mass	Obsrv. Mass	± da	± ppm	Start Seq.	End Seq.	Sequence	Ion Score	C. I. %	Modification	Rank	Result Type
982.4476	982.4962	0.0486	49	80	88	AFESNDTAK					Mascot
1331.7206	1331.7468	0.0262	20	22	32	DLDIPIQNLYK					Mascot
1331.7206	1331.7468	0.0262	20	22	32	DLDIPIQNLYK	5	0			Mascot
2408.0837	2408.2317	0.148	61	1	21	MAHNGSNSDPNPQARFY NPYK					Mascot
2953.4829	2953.5752	0.0923	31	22	46	DLDIPIQNLYKLSTSPEFL FDEEAR					Mascot
3312.563	3312.5615	-0.0015	0	49	79	RSSWGENLTFYTGCGYL GGSIAGVGFVDDVK			Carbamidomethyl (C)[14]		Mascot

8	GDSL esterase/lipase EXL6 [Arabidopsis thaliana]	gi|332197657	38970.2	9.49	11	46	0	2.8

Protein Group

GDSL esterase/lipase EXL6 [Arabidopsis thaliana]	gi|15222904	38970.2	9.4899 997711 1816
RecName: Full=GDSL esterase/lipase EXL6; AltName: Full=Family II extracellular lipase 6; Short=Family II lipase EXL6; Flags: Precursor	gi|75163376	38970.2	9.4899 997711 1816

Peptide Information

Calc. Mass	Obsrv. Mass	± da	± ppm	Start Seq.	End Seq.	Sequence	Ion Score	C. I. %	Modification	Rank	Result Type
807.4182	807.4564	0.0382	47	193	198	MVVWTR			Oxidation (M)[1]		Mascot
916.5098	916.5445	0.0347	38	127	135	VLSAGDQVK					Mascot
922.4628	922.531	0.0682	74	203	210	DLYDLGAR					Mascot
928.4774	928.5101	0.0327	35	136	142	DFKDYLK					Mascot
1721.9836	1721.8359	-0.1477	-86	326	340	AYEVISKPIVYQIAK					Mascot
1791.9238	1791.864	-0.0598	-33	270	284	FVYVDIYGTLMDLVK			Oxidation (M)[11]		Mascot

Calc. Mass	Obsrv. Mass	± da	± ppm	Start Seq.	End Seq.	Sequence	Ion Score	C. I. %	Modification	Rank	Result Type
1844.8901	1844.9637	0.0736	40	228	243	ASFGGVFGWCNFLLNR			Carbamidomethyl (C)[10]		Mascot
1928.0068	1928.0995	0.0927	48	211	227	KFAVMGVMPVGCLPIHR			Carbamidomethyl (C)[12], Oxidation (M)[5]		Mascot
1944.0017	1944.0599	0.0582	30	211	227	KFAVMGVMPVGCLPIHR			Carbamidomethyl (C)[12], Oxidation (M)[5,8]		Mascot
1963.1263	1963.1896	0.0633	32	326	343	AYEVISKPIVYQIAKGLA					Mascot
2823.3381	2823.5266	0.1885	67	228	251	ASFGGVFGWCNFLLNRIT EDFNMK			Carbamidomethyl (C)[10]		Mascot
2839.3333	2839.5134	0.1801	63	228	251	ASFGGVFGWCNFLLNRIT EDFNMK			Carbamidomethyl (C)[10], Oxidation (M)[23]		Mascot
2921.5408	2921.5774	0.0366	13	156	182	EIVSNAVFLISEGNNDLG YFVAPALLR					Mascot

9	PREDICTED: conserved oligomeric Golgi complex subunit 4-like isoform X1 [Setaria italica]	gi|514712032	84466.9	5.32	14	45	0	9.041

Peptide Information

Calc. Mass	Obsrv. Mass	± da	± ppm	Start Seq.	End Seq.	Sequence	Ion Score	C. I. %	Modification	Rank	Result Type
872.52	872.5765	0.0565	65	189	195	DLLDIKR					Mascot
906.454	906.5305	0.0765	84	126	133	HLDAAHSR					Mascot
988.517	988.59	0.073	74	141	149	AEAALDRSR					Mascot
1179.6224	1179.6788	0.0564	48	665	673	LEVIMMQKR			Oxidation (M)[5,6]		Mascot
1313.7535	1313.7593	0.0058	4	66	77	ALDARLDSLLAR					Mascot
1638.8997	1639.001	0.1013	62	394	408	GLRDVKPELGPQAMK					Mascot
1655.8309	1655.9434	0.1125	68	567	581	SCLSELGEISASFKK			Carbamidomethyl (C)[2]		Mascot
1655.8309	1655.9434	0.1125	68	567	581	SCLSELGEISASFKK			Carbamidomethyl (C)[2]		Mascot
1716.9214	1716.9664	0.045	26	582	597	ILHSGMEHLVASVAPR					Mascot
1844.9324	1844.9637	0.0313	17	705	719	FSRLSQMSTILNFER			Oxidation (M)[7]		Mascot
1944.0801	1944.0599	-0.0202	-10	279	295	LFKDIVLAVEENDAVLR					Mascot
2408.1299	2408.2317	0.1018	42	545	564	HEIEELCAEQVFHAPADR EK			Carbamidomethyl (C)[7]		Mascot
2454.2734	2454.2939	0.0205	8	156	179	RALAADDLAAAATAAHEF LTIDAR					Mascot
2839.4414	2839.5134	0.072	25	720	744	VSEILDFWGDNAGHLTW LLTPAEVR					Mascot
3312.7031	3312.5615	-0.1416	-43	512	542	LFLGGVGVQKTGEEIATA LNNMDISSEYVLK			Oxidation (M)[22]		Mascot

10	PREDICTED: uncharacterized protein LOC101215899 [Cucumis sativus]	gi|449438327	72700.6	10.91	15	44	0	7.782

Peptide Information

Calc. Mass	Obsrv. Mass	± da	± ppm	Start Seq.	End Seq.	Sequence	Ion Score	C. I. %	Modification	Rank	Result Type
800.4625	800.4455	-0.017	-21	192	198	ATPLRDK					Mascot
821.4111	821.464	0.0529	64	15	21	TSTTQQR					Mascot

847.438	847.496	0.058	68	112	118	SQSVDRR		Mascot
854.5206	854.5865	0.0659	77	511	517	HTVTLKR		Mascot
868.6342	868.5492	-0.085	-98	518	524	IKLLLLR		Mascot
916.5251	916.5445	0.0194	21	86	93	FPSPLLSR		Mascot
922.5469	922.531	-0.0159	-17	461	468	IVDAHVLR		Mascot
1182.6702	1182.6387	-0.0315	-27	45	54	DNGVLQRKPR		Mascot
1232.678	1232.6791	0.0011	1	243	254	KVNGIGSGMVVR	Oxidation (M)[9]	Mascot
1331.7417	1331.7468	0.0051	4	273	284	LSLDLNSSELIK		Mascot
1331.7417	1331.7468	0.0051	4	273	284	LSLDLNSSELIK		Mascot
1543.8439	1543.9767	0.1328	86	120	133	TTTPRSITPVLDSR		Mascot
1655.8323	1655.9434	0.1111	67	479	492	FVNARADATFMLQR	Oxidation (M)[11]	Mascot
1655.8323	1655.9434	0.1111	67	479	492	FVNARADATFMLQR	Oxidation (M)[11]	Mascot
2440.0894	2440.2507	0.1613	66	62	85	YMSPSPSTSTSTSSTTSS SASSRR		Mascot
2839.415	2839.5134	0.0984	35	625	648	ILLQQCEDFLSTLAAMQV KDCSLR	Carbamidomethyl (C)[6,21]	Mascot
3312.6121	3312.5615	-0.0506	-15	574	605	AIADIQNLKDAVGSAVDV MQAMASSICSLSSK	Carbamidomethyl (C)[27], Oxidation (M)[19,22]	Mascot

Gel Idx/Pos	136/F11	Instr./Gel Origin	BA2151/Sample Project 20140814	Process Status	Analysis Succeeded
Plate [#] Name	[1] Sample Project 20140814	Instrument Sample Name		Spectra	11

Rank	Protein Name	Accession No.	Protein MW	Protein PI	Pep. Count	Protein Score	Protein Score C. I. %	Intensity Matched	Total Ion Score	Total Ion C. I. %	Confirmed
1	dimeric alpha-amylase inhibitor, partial [Triticum durum]	gi|386877064	14502.8	4.78	4	184	100	21.903	160	100	

Peptide Information

Calc. Mass	Obsrv. Mass	± da	± ppm	Start Seq.	End Seq.	Sequence	Ion Score	C. I. %	Modification	Rank	Result Type
1134.6188	1134.6531	0.0343	30	96	106	LTAASITAVCK			Carbamidomethyl (C)[10]		Mascot
1570.8007	1570.9113	0.1106	70	32	45	LQCNGSQVPEAVLR			Carbamidomethyl (C)[3]		Mascot
1677.8517	1677.9301	0.0784	47	107	122	LPIVIDASGDGAYVCK			Carbamidomethyl (C)[15]		Mascot
1887.8403	1887.9731	0.1328	70	73	90	EHGVQEGGQAGTGAFPSCR			Carbamidomethyl (C)[17]		Mascot
1887.8403	1887.9731	0.1328	70	73	90	EHGVQEGGQAGTGAFPSCR	160	100	Carbamidomethyl (C)[17]		Mascot

Rank	Protein Name	Accession No.	Protein MW	Protein PI	Pep. Count	Protein Score	Protein Score C. I. %	Intensity Matched	Total Ion Score	Total Ion C. I. %	Confirmed
2	dimeric alpha-amylase inhibitor [Kengyilia tahelacana]	gi|534313994	15728.5	4.88	4	62	39.982	10.416	40	89.457	

Protein Group

dimeric alpha-amylase inhibitor [Agropyron cristatum]	gi|534313874	15712.5	4.8699998555908
dimeric alpha-amylase inhibitor [Kengyilia alatavica]	gi|534313889	15731.5	4.88000011444092
dimeric alpha-amylase inhibitor [Kengyilia kaschgarica]	gi|534313925	15731.5	4.88000011444092
dimeric alpha-amylase inhibitor [Kengyilia zhaosuensis]	gi|534314015	15731.5	4.88000011444092

Peptide Information

Calc. Mass	Obsrv. Mass	± da	± ppm	Start Seq.	End Seq.	Sequence	Ion Score	C. I. %	Modification	Rank	Result Type
1571.7847	1571.8998	0.1151	73	43	56	LQCNGSEVPEAVLR	41	92.397	Carbamidomethyl (C)[3]		Mascot
1571.8826	1571.8998	0.0172	11	103	117	EVVKLTAASVPAVCK			Carbamidomethyl (C)[14]		Mascot
1597.6696	1597.8231	0.1535	96	71	83	CGALYSMLDNMYK			Carbamidomethyl (C)[1], Oxidation (M)[7,11]		Mascot
1677.8517	1677.9301	0.0784	47	118	133	LPIVIDASGDGAYVCK			Carbamidomethyl (C)[15]		Mascot

Rank	Protein Name	Accession No.	Protein MW	Protein PI	Pep. Count	Protein Score	Protein Score C. I. %	Intensity Matched	Total Ion Score	Total Ion C. I. %	Confirmed
3	dimeric alpha-amylase inhibitor [Kengyilia batalinii]	gi|534313896	15685.5	4.72	3	56	0	10.416	41	92.397	

Protein Group

dimeric alpha-amylase inhibitor [Kengyilia pamirica]	gi|534313976	15643.5	5.0700001716

Peptide Information

Calc. Mass	Obsrv. Mass	± da	± ppm	Start Seq.	End Seq.	Sequence	Ion Score	C. I. %	Modification	Rank	Result Type
1571.7847	1571.8998	0.1151	73	43	56	LQCNGSEVPEAVLR			Carbamidomethyl (C)[3]		Mascot
1571.7847	1571.8998	0.1151	73	43	56	LQCNGSEVPEAVLR	41	92.397	Carbamidomethyl (C)[3]		Mascot
1597.6696	1597.8231	0.1535	96	71	83	CGALYSMLDNMYK			Carbamidomethyl (C)[1], Oxidation (M)[7,11]		Mascot
1677.8517	1677.9301	0.0784	47	118	133	LPIVIDASGDGAYVCK			Carbamidomethyl (C)[15]		Mascot

4	dimeric alpha-amylase inhibitor [Kengyilia batalinii var. nana]	gi|534313967	15703.5	4.88	3	55	0	9.654	40	89.457		

Peptide Information

Calc. Mass	Obsrv. Mass	± da	± ppm	Start Seq.	End Seq.	Sequence	Ion Score	C. I. %	Modification	Rank	Result Type
1571.7847	1571.8998	0.1151	73	43	56	LQCNGSEVPEAVLR	41	92.397	Carbamidomethyl (C)[3]		Mascot
1571.8826	1571.8998	0.0172	11	103	117	EVVKLTAASVPAVCK			Carbamidomethyl (C)[14]		Mascot
1597.6696	1597.8231	0.1535	96	71	83	CGALYSMLDNMYK			Carbamidomethyl (C)[1], Oxidation (M)[7,11]		Mascot

5	dimeric alpha-amylase inhibitor [Kengyilia gobicola]	gi|534313905	15659.5	4.88	2	50	0	9.654	41	92.397		

Peptide Information

Calc. Mass	Obsrv. Mass	± da	± ppm	Start Seq.	End Seq.	Sequence	Ion Score	C. I. %	Modification	Rank	Result Type
1571.7847	1571.8998	0.1151	73	43	56	LQCNGSEVPEAVLR			Carbamidomethyl (C)[3]		Mascot
1571.7847	1571.8998	0.1151	73	43	56	LQCNGSEVPEAVLR	41	92.397	Carbamidomethyl (C)[3]		Mascot
1597.6696	1597.8231	0.1535	96	71	83	CGALYSMLDNMYK			Carbamidomethyl (C)[1], Oxidation (M)[7,11]		Mascot

6	hypothetical protein TRIUR3_26670 [Triticum urartu]	gi|474396533	38032.2	8.37	9	42	0	7.423	

Peptide Information

Calc. Mass	Obsrv. Mass	± da	± ppm	Start Seq.	End Seq.	Sequence	Ion Score	C. I. %	Modification	Rank	Result Type
959.4734	959.3876	-0.0858	-89	150	157	FDVGWAHK					Mascot
1001.5812	1001.6186	0.0374	37	234	242	VQACGLLLK			Carbamidomethyl (C)[4]		Mascot
1598.8571	1598.8875	0.0304	19	276	290	IIIHGVKEGEAAMSK			Oxidation (M)[13]		Mascot
1784.9541	1784.9056	-0.0485	-27	42	58	IQFPLSIDPLAAETGGR					Mascot
1829.0248	1828.963	-0.0618	-34	234	249	VQACGLLLKSLSMRPR			Carbamidomethyl (C)[4]		Mascot
1845.8987	1845.9434	0.0447	24	74	88	RTNCVYCLASPLTYK			Carbamidomethyl (C)[4,7]		Mascot
1853.0208	1852.8817	-0.1391	-75	219	233	LILFQDLLAFFLDER					Mascot
1854.8989	1854.8948	-0.0041	-2	89	102	KVYSHIELNCCYIR			Carbamidomethyl (C)[10,11]		Mascot

Calc. Mass	Obsrv. Mass	± da	± ppm	Start Seq.	End Seq.	Sequence	Ion Score	C. I. %	Modification	Rank	Result Type
1854.8989	1854.8948	-0.0041	-2	89	102	KVYSHIELNCCYIR			Carbamidomethyl (C)[10,11]		Mascot
1886.8644	1886.9426	0.0782	41	150	164	FDVGWAHKFDVCSYR			Carbamidomethyl (C)[12]		Mascot

7	TPA: hypothetical protein ZEAMMB73_130777 [Zea mays]	gi|414869158	11506.2	10.17	6	38	0	1.372

Peptide Information

Calc. Mass	Obsrv. Mass	± da	± ppm	Start Seq.	End Seq.	Sequence	Ion Score	C. I. %	Modification	Rank	Result Type
832.4159	832.377	-0.0389	-47	54	60	EDQQGKK					Mascot
856.5251	856.5931	0.068	79	27	34	VGDVVLVR					Mascot
933.4359	933.389	-0.0469	-50	2	10	GAHMASGFR					Mascot
1594.9237	1594.8427	-0.081	-51	69	82	VTLLCNLPVPDILK			Carbamidomethyl (C)[5]		Mascot
1599.8966	1599.9204	0.0238	15	12	26	VAGVSLTINHFRSAK					Mascot
1763.9552	1763.873	-0.0822	-47	35	50	AAPLHVGRSTQVWDVK					Mascot

8	hypothetical protein CARUB_v10010837mg, partial [Capsella rubella]	gi|482572210	26162.7	9.56	7	37	0	21.361

Peptide Information

Calc. Mass	Obsrv. Mass	± da	± ppm	Start Seq.	End Seq.	Sequence	Ion Score	C. I. %	Modification	Rank	Result Type
832.4563	832.377	-0.0793	-95	189	195	WESIAVK					Mascot
1170.5902	1170.7004	0.1102	94	54	62	LDRSYDFVR					Mascot
1480.7465	1480.8875	0.141	95	41	53	ITMQSPETLAAFR			Oxidation (M)[3]		Mascot
1778.9105	1778.9586	0.0481	27	38	53	EGKITMQSPETLAAFR					Mascot
1854.9457	1854.8948	-0.0509	-27	196	210	NQLHKSLETSWQLDR					Mascot
1854.9457	1854.8948	-0.0509	-27	196	210	NQLHKSLETSWQLDR					Mascot
1887.855	1887.9731	0.1181	63	151	165	NADKLMYHIYMMGPR			Oxidation (M)[6,11,12]		Mascot
1888.0872	1887.9731	-0.1141	-60	172	188	VRGILMILQIVGSTTMR					Mascot
1904.082	1903.9358	-0.1462	-77	172	188	VRGILMILQIVGSTTMR			Oxidation (M)[6]		Mascot

9	predicted protein [Arabidopsis lyrata subsp. lyrata]	gi|297340059	27640.1	9.08	9	36	0	11.307

Peptide Information

Calc. Mass	Obsrv. Mass	± da	± ppm	Start Seq.	End Seq.	Sequence	Ion Score	C. I. %	Modification	Rank	Result Type
1023.5543	1023.5453	-0.009	-9	91	98	VQYMILEK					Mascot
1179.6554	1179.6804	0.025	21	90	98	RVQYMILEK					Mascot
1569.7942	1569.874	0.0798	51	91	103	VQYMILEKATDSR			Oxidation (M)[4]		Mascot
1571.7478	1571.8998	0.152	97	18	30	MCKATVLDEITMK			Carbamidomethyl (C)[2], Oxidation (M)[1,12]		Mascot

1571.7478	1571.8998	0.152	97	18	30	MCKATVLDEITMK			Carbamidomethyl (C)[2], Oxidation (M)[1,12]		Mascot
1587.8126	1587.8307	0.0181	11	218	231	ESFHDGSVILTNIR					Mascot
1597.7648	1597.8231	0.0583	36	121	133	VMCRVAAFNELCK			Carbamidomethyl (C)[3,12]		Mascot
1763.9646	1763.873	-0.0916	-52	21	35	ATVLDEITMKICLIK			Carbamidomethyl (C)[12], Oxidation (M)[9]		Mascot
1859.9531	1859.8984	-0.0547	-29	39	55	ALLDADVATVLVNNMER			Oxidation (M)[15]		Mascot
1885.861	1885.9412	0.0802	43	2	17	AFHNDLSDQISHSMQR					Mascot
1901.856	1901.965	0.109	57	2	17	AFHNDLSDQISHSMQR			Oxidation (M)[14]		Mascot

10	telomere repeat binding factor 2 [Arabidopsis thaliana]	gi|332010980	33050.4	9.87	8	34	0	1.755

Protein Group

RecName: Full=Telomere repeat-binding factor 2; Short=AtTRB2; AltName: Full=MYB transcription factor; AltName: Full=Telomere-binding protein 3; Short=AtTBP3	gi|75333972	33050.4	9.8699998855 5908
telomere repeat binding factor 2 [Arabidopsis thaliana]	gi|332010979	33050.4	9.8699998855 5908
telomere repeat binding factor 2 [Arabidopsis thaliana]	gi|30698320	33050.4	9.8699998855 5908
telomere repeat binding factor 2 [Arabidopsis thaliana]	gi|15240783	33050.4	9.8699998855 5908

Peptide Information

Calc. Mass	Obsrv. Mass	± da	± ppm	Start Seq.	End Seq.	Sequence	Ion Score	C. I. %	Modification	Rank	Result Type
832.4271	832.377	-0.0501	-60	138	144	ELRGSDR					Mascot
1055.5845	1055.6667	0.0822	78	171	180	HLSSNGTLVK					Mascot
1479.8306	1479.8748	0.0442	30	30	42	TILSDTEFSLILK					Mascot
1587.9218	1587.8307	-0.0911	-57	128	140	IIFEAITNLRELR					Mascot
1641.8846	1641.9323	0.0477	29	8	22	WTPEEEAALKAGVLK					Mascot
1749.884	1749.881	-0.003	-2	272	288	AEAEAEAAQIFAKAAMK					Mascot
1859.903	1859.8984	-0.0046	-2	100	119	AKPTSPGGSGGGSPRTC ASK			Carbamidomethyl (C)[17]		Mascot
1903.9508	1903.9358	-0.015	-8	212	228	DPTKPEENGANSLTKFR					Mascot

Gel Idx/Pos	137/F12	Instr./Gel Origin	BA2151/Sample Project 20140814	Process Status	Analysis Succeeded
Plate [#] Name	[1] Sample Project 20140814	Instrument Sample Name		Spectra	11

Rank	Protein Name	Accession No.	Protein MW	Protein PI	Pep. Count	Protein Score	Protein Score C. I. %	Intensity Matched	Total Ion Score	Total Ion C. I. %	Confirmed
1	RecName: Full=Alpha-amylase inhibitor 0.53	gi|123968	13689.5	5.23	4	344	100	41.573	322	100	

Peptide Information

Calc. Mass	Obsrv. Mass	± da	± ppm	Start Seq.	End Seq.	Sequence	Ion Score	C. I. %	Modification	Rank	Result Type
1162.6249	1162.7124	0.0875	75	90	100	LTAASITAVCR			Carbamidomethyl (C)[10]		Mascot
1162.6249	1162.7124	0.0875	75	90	100	LTAASITAVCR	58	99.788	Carbamidomethyl (C)[10]		Mascot
1570.8007	1570.9186	0.1179	75	26	39	LQCNGSQVPEAVLR			Carbamidomethyl (C)[3]		Mascot
1663.8361	1663.9296	0.0935	56	101	116	LPIVVDASGDGAYVCK			Carbamidomethyl (C)[15]		Mascot
1663.8361	1663.9296	0.0935	56	101	116	LPIVVDASGDGAYVCK	104	100	Carbamidomethyl (C)[15]		Mascot
1846.8137	1846.9543	0.1406	76	67	84	EHGVSEGGQAGTGAFPSC R			Carbamidomethyl (C)[17]		Mascot
1846.8137	1846.9543	0.1406	76	67	84	EHGVSEGGQAGTGAFPSC R	159	100	Carbamidomethyl (C)[17]		Mascot

Rank	Protein Name	Accession No.	Protein MW	Protein PI	Pep. Count	Protein Score	Protein Score C. I. %	Intensity Matched	Total Ion Score	Total Ion C. I. %	Confirmed
2	Chain D, 0.19 Alpha-Amylase Inhibitor From Wheat	gi|3318684	13898.6	6.66	5	193	100	16.352	163	100	

Protein Group

Chain A, 0.19 Alpha-Amylase Inhibitor From Wheat	gi|3318681	13898.6	6.6599 998474 1211
Chain B, 0.19 Alpha-Amylase Inhibitor From Wheat	gi|3318682	13898.6	6.6599 998474 1211
Chain C, 0.19 Alpha-Amylase Inhibitor From Wheat	gi|3318683	13898.6	6.6599 998474 1211
RecName: Full=Alpha-amylase inhibitor 0.19; AltName: Full=0.19 alpha-AI; Short=0.19 AI	gi|123963	13898.6	6.6599 998474 1211
dimeric alpha-amylase inhibitor, partial [Aegilops geniculata]	gi|452055912	14198.8	6.6599 998474 1211
dimeric alpha-amylase inhibitor, partial [Aegilops kotschyi]	gi|386877048	14198.8	6.6599 998474 1211

Peptide Information

Calc. Mass	Obsrv. Mass	± da	± ppm	Start Seq.	End Seq.	Sequence	Ion Score	C. I. %	Modification	Rank	Result Type
1162.6249	1162.7124	0.0875	75	90	100	LTAASITAVCR			Carbamidomethyl (C)[10]		Mascot
1162.6249	1162.7124	0.0875	75	90	100	LTAASITAVCR	58	99.788	Carbamidomethyl (C)[10]		Mascot

Calc. Mass	Obsrv. Mass	± da	± ppm	Start Seq.	End Seq.	Sequence	Ion Score	C. I. %	Modification	Rank	Result Type
1570.8007	1570.9186	0.1179	75	26	39	LQCNGSQVPEAVLR			Carbamidomethyl (C)[3]		Mascot
1612.7463	1612.8715	0.1252	78	67	82	EHGAQEGQAGTGAFPR					Mascot
1663.8361	1663.9296	0.0935	56	101	116	LPIVVDASGDGAYVCK			Carbamidomethyl (C)[15]		Mascot
1663.8361	1663.9296	0.0935	56	101	116	LPIVVDASGDGAYVCK	104	100	Carbamidomethyl (C)[15]		Mascot
1862.7731	1862.9368	0.1637	88	40	53	DCCQQLAHISEWCR			Carbamidomethyl (C)[2,3,13]		Mascot

3	dimeric alpha-amylase inhibitor, partial [Aegilops tauschii]	gi|386877046	14670	6.08	5	192	100	16.352	163	100	

Protein Group

dimeric alpha-amylase inhibitor, partial [Aegilops geniculata]	gi|386877062	14542.9	7.0500 001907 3486
dimeric alpha-amylase inhibitor, partial [Aegilops longissima]	gi|386877060	14954.2	7.6399 998664 856
dimeric alpha-amylase inhibitor, partial [Aegilops tauschii]	gi|386877050	14826.1	6.8600 001335 144
dimeric alpha-amylase inhibitor, partial [Aegilops tauschii]	gi|386877044	14805	6.0999 999046 3257

Peptide Information

Calc. Mass	Obsrv. Mass	± da	± ppm	Start Seq.	End Seq.	Sequence	Ion Score	C. I. %	Modification	Rank	Result Type
1162.6249	1162.7124	0.0875	75	97	107	LTAASITAVCR			Carbamidomethyl (C)[10]		Mascot
1162.6249	1162.7124	0.0875	75	97	107	LTAASITAVCR	58	99.788	Carbamidomethyl (C)[10]		Mascot
1570.8007	1570.9186	0.1179	75	33	46	LQCNGSQVPEAVLR			Carbamidomethyl (C)[3]		Mascot
1612.7463	1612.8715	0.1252	78	74	89	EHGAQEGQAGTGAFPR					Mascot
1663.8361	1663.9296	0.0935	56	108	123	LPIVVDASGDGAYVCK			Carbamidomethyl (C)[15]		Mascot
1663.8361	1663.9296	0.0935	56	108	123	LPIVVDASGDGAYVCK	104	100	Carbamidomethyl (C)[15]		Mascot
1862.7731	1862.9368	0.1637	88	47	60	DCCQQLAHISEWCR			Carbamidomethyl (C)[2,3,13]		Mascot

4	dimeric alpha-amylase inhibitor, partial [Aegilops longissima]	gi|386877056	14792	5.28	5	192	100	20.617	163	100	

Protein Group

dimeric alpha-amylase inhibitor, partial [Aegilops longissima]	gi|386877058	14718.9	4.9899 997711 1816

Peptide Information

Calc. Mass	Obsrv. Mass	± da	± ppm	Start Seq.	End Seq.	Sequence	Ion Score	C. I. %	Modification	Rank	Result Type
1162.6249	1162.7124	0.0875	75	98	108	LTAASITAVCR			Carbamidomethyl (C)[10]		Mascot
1162.6249	1162.7124	0.0875	75	98	108	LTAASITAVCR	58	99.788	Carbamidomethyl (C)[10]		Mascot

Calc. Mass	Obsrv. Mass	± da	± ppm	Start Seq.	End Seq.	Sequence	Ion Score	C. I. %	Modification	Rank	Result Type
1570.8007	1570.9186	0.1179	75	34	47	LQCNGSQVPEAVLR			Carbamidomethyl (C)[3]		Mascot
1612.7463	1612.8715	0.1252	78	75	90	EHGAQEGQAGTGAFPR					Mascot
1663.8361	1663.9296	0.0935	56	109	124	LPIVVDASGDGAYVCK			Carbamidomethyl (C)[15]		Mascot
1663.8361	1663.9296	0.0935	56	109	124	LPIVVDASGDGAYVCK	104	100	Carbamidomethyl (C)[15]		Mascot
1840.7412	1840.8787	0.1375	75	48	61	DCCQQLADISEWCR			Carbamidomethyl (C)[2,3,13]		Mascot

5	dimeric alpha-amylase inhibitor [Triticum aestivum]	gi|386877038	15702.5	5.58	5	191	100	16.352	163	100

Peptide Information

Calc. Mass	Obsrv. Mass	± da	± ppm	Start Seq.	End Seq.	Sequence	Ion Score	C. I. %	Modification	Rank	Result Type
1162.6249	1162.7124	0.0875	75	107	117	LTAASITAVCR			Carbamidomethyl (C)[10]		Mascot
1162.6249	1162.7124	0.0875	75	107	117	LTAASITAVCR	58	99.788	Carbamidomethyl (C)[10]		Mascot
1570.8007	1570.9186	0.1179	75	43	56	LQCNGSQVPEAVLR			Carbamidomethyl (C)[3]		Mascot
1612.7463	1612.8715	0.1252	78	84	99	EHGAQEGQAGTGAFPR					Mascot
1663.8361	1663.9296	0.0935	56	118	133	LPIVVDASGDGAYVCK			Carbamidomethyl (C)[15]		Mascot
1663.8361	1663.9296	0.0935	56	118	133	LPIVVDASGDGAYVCK	104	100	Carbamidomethyl (C)[15]		Mascot
1862.7731	1862.9368	0.1637	88	57	70	DCCQQLAHISEWCR			Carbamidomethyl (C)[2,3,13]		Mascot

6	Alpha-amylase inhibitor 0.19 [Aegilops tauschii]	gi|475613321	17198.2	6.06	5	189	100	16.352	163	100

Peptide Information

Calc. Mass	Obsrv. Mass	± da	± ppm	Start Seq.	End Seq.	Sequence	Ion Score	C. I. %	Modification	Rank	Result Type
1162.6249	1162.7124	0.0875	75	120	130	LTAASITAVCR			Carbamidomethyl (C)[10]		Mascot
1162.6249	1162.7124	0.0875	75	120	130	LTAASITAVCR	58	99.788	Carbamidomethyl (C)[10]		Mascot
1570.8007	1570.9186	0.1179	75	56	69	LQCNGSQVPEAVLR			Carbamidomethyl (C)[3]		Mascot
1612.7463	1612.8715	0.1252	78	97	112	EHGAQEGQAGTGAFPR					Mascot
1663.8361	1663.9296	0.0935	56	131	146	LPIVVDASGDGAYVCK			Carbamidomethyl (C)[15]		Mascot
1663.8361	1663.9296	0.0935	56	131	146	LPIVVDASGDGAYVCK	104	100	Carbamidomethyl (C)[15]		Mascot
1862.7731	1862.9368	0.1637	88	70	83	DCCQQLAHISEWCR			Carbamidomethyl (C)[2,3,13]		Mascot

7	dimeric alpha-amylase inhibitor, partial [Triticum aestivum]	gi|386877042	11899.6	6.13	4	188	100	17.67	163	100

Peptide Information

Calc. Mass	Obsrv. Mass	± da	± ppm	Start Seq.	End Seq.	Sequence	Ion Score	C. I. %	Modification	Rank	Result Type
1162.6249	1162.7124	0.0875	75	73	83	LTAASITAVCR			Carbamidomethyl (C)[10]		Mascot
1162.6249	1162.7124	0.0875	75	73	83	LTAASITAVCR	58	99.788	Carbamidomethyl (C)[10]		Mascot

1570.8007	1570.9186	0.1179	75	9	22	LQCNGSQVPEAVLR			Carbamidomethyl (C)[3]		Mascot
1663.8361	1663.9296	0.0935	56	84	99	LPIVVDASGDGAYVCK			Carbamidomethyl (C)[15]		Mascot
1663.8361	1663.9296	0.0935	56	84	99	LPIVVDASGDGAYVCK	104	100	Carbamidomethyl (C)[15]		Mascot
2395.1487	2395.2461	0.0974	41	84	106	LPIVVDASGDGAYVCKDV AAYPD			Carbamidomethyl (C)[15]		Mascot
2395.1487	2395.2461	0.0974	41	84	106	LPIVVDASGDGAYVCKDV AAYPD			Carbamidomethyl (C)[15]		Mascot

8	dimeric alpha-amylase inhibitor, partial [Aegilops peregrina]	gi|386877054	14145.7	5.26	4	184	100	10.012	163	100

Peptide Information

Calc. Mass	Obsrv. Mass	± da	± ppm	Start Seq.	End Seq.	Sequence	Ion Score	C. I. %	Modification	Rank	Result Type
1162.6249	1162.7124	0.0875	75	92	102	LTAASITAVCR			Carbamidomethyl (C)[10]		Mascot
1162.6249	1162.7124	0.0875	75	92	102	LTAASITAVCR	58	99.788	Carbamidomethyl (C)[10]		Mascot
1612.7463	1612.8715	0.1252	78	69	84	EHGAQEGQAGTGAFPR					Mascot
1663.8361	1663.9296	0.0935	56	103	118	LPIVVDASGDGAYVCK			Carbamidomethyl (C)[15]		Mascot
1663.8361	1663.9296	0.0935	56	103	118	LPIVVDASGDGAYVCK	104	100	Carbamidomethyl (C)[15]		Mascot
1840.7412	1840.8787	0.1375	75	42	55	DCCQQLADISEWCR			Carbamidomethyl (C)[2,3,13]		Mascot

9	dimeric alpha-amylase inhibitor, partial [Aegilops peregrina]	gi|386877052	13953.6	6.49	4	184	100	20.542	163	100

Peptide Information

Calc. Mass	Obsrv. Mass	± da	± ppm	Start Seq.	End Seq.	Sequence	Ion Score	C. I. %	Modification	Rank	Result Type
1162.6249	1162.7124	0.0875	75	90	100	LTAASITAVCR			Carbamidomethyl (C)[10]		Mascot
1162.6249	1162.7124	0.0875	75	90	100	LTAASITAVCR	58	99.788	Carbamidomethyl (C)[10]		Mascot
1570.8007	1570.9186	0.1179	75	26	39	LQCNGSQVPEAVLR			Carbamidomethyl (C)[3]		Mascot
1663.8361	1663.9296	0.0935	56	101	116	LPIVVDASGDGAYVCK			Carbamidomethyl (C)[15]		Mascot
1663.8361	1663.9296	0.0935	56	101	116	LPIVVDASGDGAYVCK	104	100	Carbamidomethyl (C)[15]		Mascot
1840.7412	1840.8787	0.1375	75	40	53	DCCQQLADISEWCR			Carbamidomethyl (C)[2,3,13]		Mascot

10	dimeric alpha-amylase inhibitor, partial [Triticum aestivum]	gi|386877068	14415.8	6.88	4	80	98.908	15.391	58	99.788

Peptide Information

Calc. Mass	Obsrv. Mass	± da	± ppm	Start Seq.	End Seq.	Sequence	Ion Score	C. I. %	Modification	Rank	Result Type
1162.6249	1162.7124	0.0875	75	95	105	LTAASITAVCR			Carbamidomethyl (C)[10]		Mascot
1162.6249	1162.7124	0.0875	75	95	105	LTAASITAVCR	58	99.788	Carbamidomethyl (C)[10]		Mascot
1570.8007	1570.9186	0.1179	75	31	44	LQCNGSQVPEAVLR			Carbamidomethyl (C)[3]		Mascot

1612.7463	1612.8715	0.1252	78	72	87	EHGAQEGQAGTGAFPR		Mascot
1862.7731	1862.9368	0.1637	88	45	58	DCCQQLAHISEWCR	Carbamidomethyl (C)[2,3,13]	Mascot

Gel Idx/Pos	138/F13	Instr./Gel Origin	BA2151/Sample Project 20140814	Process Status	Analysis Succeeded
Plate [#] Name	[1] Sample Project 20140814	Instrument Sample Name		Spectra	11

Rank	Protein Name	Accession No.	Protein MW	Protein PI	Pep. Count	Protein Score	Protein Score C. I. %	Intensity Matched	Total Ion Score	Total Ion C. I. %	Confirmed
1	RecName: Full=Alpha-amylase/trypsin inhibitor CMb; AltName: Full=Chloroform/methanol-soluble protein CMb; Flags: Precursor	gi|585290	17199.2	5.77	6	302	100	37.252	268	100	

Peptide Information

Calc. Mass	Obsrv. Mass	± da	± ppm	Start Seq.	End Seq.	Sequence	Ion Score	C. I. %	Modification	Rank	Result Type
801.4076	801.4696	0.062	77	86	91	FFMGRK			Oxidation (M)[3]		Mascot
1023.4928	1023.5602	0.0674	66	108	115	EVQMDFVR					Mascot
1023.4928	1023.5602	0.0674	66	108	115	EVQMDFVR	20	0			Mascot
1039.4878	1039.5409	0.0531	51	108	115	EVQMDFVR			Oxidation (M)[4]		Mascot
1039.4878	1039.5409	0.0531	51	108	115	EVQMDFVR	36	46.837	Oxidation (M)[4]		Mascot
1168.5052	1168.5846	0.0794	68	46	54	DYVEQQACR			Carbamidomethyl (C)[8]		Mascot
1168.5052	1168.5846	0.0794	68	46	54	DYVEQQACR	74	99.992	Carbamidomethyl (C)[8]		Mascot
1285.6293	1285.7178	0.0885	69	81	90	CQALRFFMGR			Carbamidomethyl (C)[1]		Mascot
1799.8528	1799.9772	0.1244	69	92	107	SRPDQSGLMELPGCPR			Carbamidomethyl (C)[14]		Mascot
1815.8477	1815.9524	0.1047	58	92	107	SRPDQSGLMELPGCPR			Carbamidomethyl (C)[14], Oxidation (M)[9]		Mascot
1815.8477	1815.9524	0.1047	58	92	107	SRPDQSGLMELPGCPR	46	95.475	Carbamidomethyl (C)[14], Oxidation (M)[9]		Mascot
1861.8102	1861.9398	0.1296	70	66	80	QQCCGELANIPQQCR			Carbamidomethyl (C)[3,4,14]		Mascot
1861.8102	1861.9398	0.1296	70	66	80	QQCCGELANIPQQCR	112	100	Carbamidomethyl (C)[3,4,14]		Mascot

Rank	Protein Name	Accession No.	Protein MW	Protein PI	Pep. Count	Protein Score	Protein Score C. I. %	Intensity Matched	Total Ion Score	Total Ion C. I. %	Confirmed
2	RecName: Full=Alpha-amylase/trypsin inhibitor CM16; AltName: Full=Chloroform/methanol-soluble protein CM16; Flags: Precursor	gi|123958	16398.8	5.31	5	296	100	37.2	268	100	

Peptide Information

Calc. Mass	Obsrv. Mass	± da	± ppm	Start Seq.	End Seq.	Sequence	Ion Score	C. I. %	Modification	Rank	Result Type
1023.4928	1023.5602	0.0674	66	108	115	EVQMDFVR					Mascot
1023.4928	1023.5602	0.0674	66	108	115	EVQMDFVR	20	0			Mascot
1039.4878	1039.5409	0.0531	51	108	115	EVQMDFVR			Oxidation (M)[4]		Mascot
1039.4878	1039.5409	0.0531	51	108	115	EVQMDFVR	36	46.837	Oxidation (M)[4]		Mascot
1168.5052	1168.5846	0.0794	68	46	54	DYVEQQACR			Carbamidomethyl (C)[8]		Mascot
1168.5052	1168.5846	0.0794	68	46	54	DYVEQQACR	74	99.992	Carbamidomethyl (C)[8]		Mascot
1175.6307	1175.6776	0.0469	40	55	65	IETPGSPYLAK					Mascot

Calc. Mass	Obsrv. Mass	± da	± ppm	Start Seq.	End Seq.	Sequence	Ion Score	C. I. %	Modification	Rank	Result Type
1799.8528	1799.9772	0.1244	69	92	107	SRPDQSGLMELPGCPR			Carbamidomethyl (C)[14]		Mascot
1815.8477	1815.9524	0.1047	58	92	107	SRPDQSGLMELPGCPR			Carbamidomethyl (C)[14], Oxidation (M)[9]		Mascot
1815.8477	1815.9524	0.1047	58	92	107	SRPDQSGLMELPGCPR	46	95.475	Carbamidomethyl (C)[14], Oxidation (M)[9]		Mascot
1861.8102	1861.9398	0.1296	70	66	80	QQCCGELANIPQQCR			Carbamidomethyl (C)[3,4,14]		Mascot
1861.8102	1861.9398	0.1296	70	66	80	QQCCGELANIPQQCR	112	100	Carbamidomethyl (C)[3,4,14]		Mascot

3	Alpha-amylase/trypsin inhibitor CM16 [Aegilops tauschii]	gi|475546435	16476.8	5.07	2	68	83.47	19.614	46	95.475	

Peptide Information

Calc. Mass	Obsrv. Mass	± da	± ppm	Start Seq.	End Seq.	Sequence	Ion Score	C. I. %	Modification	Rank	Result Type
1168.5051	1168.5846	0.0795	68	46	54	NYVEEQACR			Carbamidomethyl (C)[8]		Mascot
1168.5051	1168.5846	0.0795	68	46	54	NYVEEQACR	14	0	Carbamidomethyl (C)[8]		Mascot
1799.8528	1799.9772	0.1244	69	92	107	SRPDQSGLMELPGCPR			Carbamidomethyl (C)[14]		Mascot
1815.8477	1815.9524	0.1047	58	92	107	SRPDQSGLMELPGCPR			Carbamidomethyl (C)[14], Oxidation (M)[9]		Mascot
1815.8477	1815.9524	0.1047	58	92	107	SRPDQSGLMELPGCPR	46	95.475	Carbamidomethyl (C)[14], Oxidation (M)[9]		Mascot

4	Os03g0225500 [Oryza sativa Japonica Group]	gi|113547904	145413.4	5.96	23	63	54.472	17.606	

Peptide Information

Calc. Mass	Obsrv. Mass	± da	± ppm	Start Seq.	End Seq.	Sequence	Ion Score	C. I. %	Modification	Rank	Result Type
801.4002	801.4696	0.0694	87	647	652	HWTTTR					Mascot
908.4221	908.4642	0.0421	46	167	175	WGSSGASTR					Mascot
1126.5562	1126.5582	0.002	2	185	194	SSTGFILCNK			Carbamidomethyl (C)[8]		Mascot
1179.7208	1179.6786	-0.0422	-36	426	436	APSQVIIPKAR					Mascot
1285.7474	1285.7178	-0.0296	-23	949	960	EVTSPILATAKR					Mascot
1321.6052	1321.713	0.1078	82	540	551	KGSCNEAVAEEK			Carbamidomethyl (C)[4]		Mascot
1424.7202	1424.7662	0.046	32	98	111	SFSGKNMLAGGIDK					Mascot
1587.8068	1587.8656	0.0588	37	1150	1162	AFEVFAWTRASFR					Mascot
1605.7789	1605.8197	0.0408	25	338	353	IGSQEIVGSDGDMGIK					Mascot
1661.8237	1661.9397	0.116	70	136	150	DCLVLEVPSSLMGNK			Carbamidomethyl (C)[2]		Mascot
1662.7938	1662.9066	0.1128	68	692	705	QRTAMLTVMEHGEK			Oxidation (M)[5,9]		Mascot
1696.9058	1696.9159	0.0101	6	1145	1158	ELSLKAFEVFAWTR					Mascot
1724.8953	1724.9501	0.0548	32	653	668	EAEFLASTIVSSLTEK					Mascot
1733.8739	1733.9413	0.0674	39	338	354	IGSQEIVGSDGDMGIKK					Mascot
1751.9261	1752.0166	0.0905	52	844	858	SGIWRVASFAMELLR			Oxidation (M)[11]		Mascot
1751.9261	1752.0166	0.0905	52	844	858	SGIWRVASFAMELLR			Oxidation (M)[11]		Mascot

Calc. Mass	Obsrv. Mass	± da	± ppm	Start Seq.	End Seq.	Sequence	Ion Score	C. I. %	Modification	Rank	Result Type
1753.7837	1753.9288	0.1451	83	1275	1290	ELPYTVSTAEPVDMDS					Mascot
1754.853	1754.9159	0.0629	36	620	635	AAGAFDKEGEMNIFVR					Mascot
1761.9745	1761.8676	-0.1069	-61	944	959	ESIFREVTSPILATAK					Mascot
1769.7787	1769.9449	0.1662	94	1275	1290	ELPYTVSTAEPVDMDS			Oxidation (M)[14]		Mascot
1770.848	1770.9294	0.0814	46	620	635	AAGAFDKEGEMNIFVR			Oxidation (M)[11]		Mascot
1770.848	1770.9294	0.0814	46	620	635	AAGAFDKEGEMNIFVR			Oxidation (M)[11]		Mascot
1773.8628	1773.9258	0.063	36	167	183	WGSSGASTRSSGDILHR					Mascot
1846.8501	1846.9163	0.0662	36	897	911	GDEHGVLIQEYCDRR			Carbamidomethyl (C)[12]		Mascot
1872.8905	1872.9381	0.0476	25	1258	1274	DFPDAGKLMMTAVIMGK			Oxidation (M)[9,10,15]		Mascot
2373.1321	2373.2698	0.1377	58	1213	1233	LCYSPDAVVYDGSFEDV LPVK			Carbamidomethyl (C)[2]		Mascot
2452.1609	2452.1951	0.0342	14	154	175	SLCGNQWAVCIVRWGSS GASTR			Carbamidomethyl (C)[3,10]		Mascot

5	pfkB-type carbohydrate kinase family protein [Arabidopsis lyrata subsp. lyrata]	gi|297335423	37881.4	5.85	12	58	0	4.422

Peptide Information

Calc. Mass	Obsrv. Mass	± da	± ppm	Start Seq.	End Seq.	Sequence	Ion Score	C. I. %	Modification	Rank	Result Type
810.3661	810.4111	0.045	56	1	8	MASSTGEK					Mascot
908.3971	908.4642	0.0671	74	109	114	EFMFYR			Oxidation (M)[3]		Mascot
975.5734	975.5636	-0.0098	-10	54	63	LGGRAAFVGK					Mascot
991.5934	991.5533	-0.0401	-40	95	103	TALAFVTLR					Mascot
1321.6019	1321.713	0.1111	84	80	91	NGVDDQGINFDK					Mascot
1605.7616	1605.8197	0.0581	36	80	94	NGVDDQGINFDKGAR					Mascot
1661.8931	1661.9397	0.0466	28	187	200	TQIMSIWDKADIIK					Mascot
1733.9769	1733.9413	-0.0356	-21	39	57	APGGAPANVAIAVSRLG GR					Mascot
1773.8953	1773.9258	0.0305	17	64	79	LGDDDFGHMLAGILRK			Oxidation (M)[9]		Mascot
1843.0034	1842.9612	-0.0422	-23	230	245	LLLVTLGEKGCTYFTK			Carbamidomethyl (C)[11]		Mascot
1887.9092	1887.9604	0.0512	27	214	229	TMDDKTAMSLWHPNLK					Mascot
2063.0906	2063.1348	0.0442	21	196	213	ADIIKVSDVELEFLTENK					Mascot

6	hypothetical protein ZEAMMB73_621808 [Zea mays]	gi|413924510	63178.1	9.38	15	56	0	10.25

Peptide Information

Calc. Mass	Obsrv. Mass	± da	± ppm	Start Seq.	End Seq.	Sequence	Ion Score	C. I. %	Modification	Rank	Result Type
800.4625	800.5052	0.0427	53	442	448	EVAVVQR					Mascot
813.4941	813.4748	-0.0193	-24	258	266	AAALAALGR					Mascot
956.5636	956.5419	-0.0217	-23	442	449	EVAVVQRR					Mascot

974.5516	974.5739	0.0223	23	559	567	KALSEVEAK					Mascot
1005.4782	1005.5474	0.0692	69	26	34	SASMTSLHR			Oxidation (M)[4]		Mascot
1021.5214	1021.5346	0.0132	13	250	257	RPAYWSNK					Mascot
1428.7554	1428.7439	-0.0115	-8	150	164	NLGNLNAGATGTAVR					Mascot
1734.8593	1734.9948	0.1355	78	180	196	KAPPNGYASSGMGNIVR			Oxidation (M)[12]		Mascot
1815.9977	1815.9524	-0.0453	-25	250	266	RPAYWSNKAAALAALGR					Mascot
1815.9977	1815.9524	-0.0453	-25	250	266	RPAYWSNKAAALAALGR	4	0			Mascot
1827.8694	1827.9731	0.1037	57	466	482	AAKFVEACAAYGEGLDR			Carbamidomethyl (C)[8]		Mascot
1837.9114	1837.9198	0.0084	5	506	522	AVEDCSGALVVRPSYSK			Carbamidomethyl (C)[5]		Mascot
1872.9531	1872.9381	-0.015	-8	133	149	GTTQLVRATSGNMMLHR					Mascot
2044.9733	2045.1367	0.1634	80	6	23	DPPTGCAMFGIYSGLFRR			Carbamidomethyl (C)[6]		Mascot
2063.0151	2063.1348	0.1197	58	304	322	AIYHLKQSSNESASADVSR					Mascot
2389.1267	2389.271	0.1443	60	377	399	HDEADSLLSSAGAPRFGVDESTK					Mascot

7	PREDICTED: presequence protease 1, chloroplastic/mitochondrial-like [Solanum lycopersicum]	gi|460369945	121030.8	5.56	20	55	0	7.956	

Peptide Information

Calc. Mass	Obsrv. Mass	± da	± ppm	Start Seq.	End Seq.	Sequence	Ion Score	C. I. %	Modification	Rank	Result Type
856.5363	856.5789	0.0426	50	2	8	ERAVLLR					Mascot
858.5196	858.4742	-0.0454	-53	114	120	AVLYKHK					Mascot
974.5557	974.5739	0.0182	19	171	178	EPFVELLK					Mascot
991.4744	991.5533	0.0789	80	306	313	FYHPSNAR					Mascot
1021.4697	1021.5346	0.0649	64	487	495	ENNTGSFPR					Mascot
1126.6005	1126.5582	-0.0423	-38	754	762	FKQFVSQSR					Mascot
1445.7159	1445.7721	0.0562	39	957	969	TLDVYDGTSSFLK					Mascot
1475.8508	1475.8292	-0.0216	-15	167	178	YPLKEPFVELLK					Mascot
1566.801	1566.777	-0.024	-15	1020	1032	EEILSTSLEDFRK					Mascot
1661.8527	1661.9397	0.087	52	579	593	VKASMTQEDLAELAR					Mascot
1679.9215	1679.9097	-0.0118	-7	625	640	EPVLVPTEIGDINGVK					Mascot
1751.8964	1752.0166	0.1202	69	878	893	SNEAFVVPTQVNYVGK					Mascot
1751.8964	1752.0166	0.1202	69	878	893	SNEAFVVPTQVNYVGK					Mascot
1791.917	1791.8873	-0.0297	-17	487	502	ENNTGSFPRGLALMLR			Oxidation (M)[14]		Mascot
1795.8398	1795.907	0.0672	37	314	327	IWFYGDDDPNERLR					Mascot
1803.9634	1803.9585	-0.0049	-3	828	844	SLLSKNGCLINLTADGK			Carbamidomethyl (C)[8]		Mascot
1872.9272	1872.9381	0.0109	6	727	741	AMSQRTEDLFYLINR			Oxidation (M)[2]		Mascot

Calc. Mass	Obsrv. Mass	± da	± ppm	Start Seq.	End Seq.	Sequence	Ion Score	C. I. %	Modification	Rank	Result Type
1875.9698	1875.9397	-0.0301	-16	981	998	AIIGTIGDVDSYQLPDAK					Mascot
1883.9709	1883.8893	-0.0816	-43	1042	1060	DKGVVVAVASPDDVEAA NK					Mascot
2400.1501	2400.3511	0.201	84	122	143	TGAEVMSVSNDDENKVF GVVFR					Mascot
2452.1926	2452.1951	0.0025	1	326	346	LRILSEYLNMFDASSAPH ESR			Oxidation (M)[10]		Mascot

8	photosystem I assembly protein ycf4 (chloroplast) [Pinus patula]	gi|356998861	21544.3	9.87	10	55	0	12.248	

Peptide Information

Calc. Mass	Obsrv. Mass	± da	± ppm	Start Seq.	End Seq.	Sequence	Ion Score	C. I. %	Modification	Rank	Result Type
911.4907	911.5076	0.0169	19	141	147	VLYMEIK			Oxidation (M)[4]		Mascot
1023.5696	1023.5602	-0.0094	-9	116	123	IFPQFLMK					Mascot
1023.5696	1023.5602	-0.0094	-9	116	123	IFPQFLMK					Mascot
1039.5646	1039.5409	-0.0237	-23	116	123	IFPQFLMK			Oxidation (M)[7]		Mascot
1039.5646	1039.5409	-0.0237	-23	116	123	IFPQFLMK			Oxidation (M)[7]		Mascot
1055.5957	1055.5548	-0.0409	-39	148	156	GRQDIPLTR					Mascot
1175.5725	1175.6776	0.1051	89	130	139	MEIQEGISPR			Oxidation (M)[1]		Mascot
1179.6708	1179.6786	0.0078	7	115	123	RIFPQFLMK					Mascot
1515.7762	1515.7802	0.004	3	157	169	TGDNVNLREIEQK					Mascot
1572.8533	1572.8451	-0.0082	-5	5	17	SKWLWIEPITGSR					Mascot
1751.9587	1752.0166	0.0579	33	116	129	IFPQFLMKDIQMIK					Mascot
1751.9587	1752.0166	0.0579	33	116	129	IFPQFLMKDIQMIK					Mascot
1767.9536	1767.9447	-0.0089	-5	116	129	IFPQFLMKDIQMIK			Oxidation (M)[7]		Mascot
1783.9485	1783.9905	0.042	24	116	129	IFPQFLMKDIQMIK			Oxidation (M)[7,12]		Mascot
1791.9476	1791.8873	-0.0603	-34	100	114	GIVCLFRWGFPGINR			Carbamidomethyl (C)[4]		Mascot
1887.9667	1887.9604	-0.0063	-3	124	139	DIQMIKMEIQEGISPR					Mascot

9	hypothetical protein SORBIDRAFT_04g030000 [Sorghum bicolor]	gi|241932482	67850.6	6.79	15	54	0	15.204	

Peptide Information

Calc. Mass	Obsrv. Mass	± da	± ppm	Start Seq.	End Seq.	Sequence	Ion Score	C. I. %	Modification	Rank	Result Type
800.4512	800.5052	0.054	67	72	79	TAVVPDAK					Mascot
832.441	832.3814	-0.0596	-72	10	16	ELSEVQK					Mascot
908.4108	908.4642	0.0534	59	2	9	DSDYGVPR					Mascot
1023.5945	1023.5602	-0.0343	-34	80	88	VISEHLAVR					Mascot
1023.5945	1023.5602	-0.0343	-34	80	88	VISEHLAVR					Mascot

1039.4514	1039.5409	0.0895	86	1	9	MDSDYGVPR					Mascot
1039.4514	1039.5409	0.0895	86	1	9	MDSDYGVPR					Mascot
1126.4946	1126.5582	0.0636	56	149	158	NQGGYDMLGR			Oxidation (M)[7]		Mascot
1355.6373	1355.7325	0.0952	70	149	160	NQGGYDMLGRTK			Oxidation (M)[7]		Mascot
1508.7383	1508.8304	0.0921	61	479	492	CGAAPISSMMTVKR			Carbamidomethyl (C)[1]		Mascot
1572.8527	1572.8451	-0.0076	-5	398	410	LLAQLVETEMNRR					Mascot
1724.7942	1724.9501	0.1559	90	165	180	TTEQVNGAMASCQALK			Carbamidomethyl (C)[12], Oxidation (M)[9]		Mascot
1758.8534	1758.9313	0.0779	44	420	433	KFNAICHFFGYQAR			Carbamidomethyl (C)[6]		Mascot
1770.9783	1770.9294	-0.0489	-28	208	224	CATKVVGVPVTLNGDLK			Carbamidomethyl (C)[1]		Mascot
1770.9783	1770.9294	-0.0489	-28	208	224	CATKVVGVPVTLNGDLK			Carbamidomethyl (C)[1]		Mascot
1805.028	1804.9165	-0.1115	-62	72	88	TAVVPDAKVISEHLAVR					Mascot
1912.0586	1911.9377	-0.1209	-63	80	96	VISEHLAVRVGIVFCGR			Carbamidomethyl (C)[15]		Mascot
2452.2766	2452.1951	-0.0815	-33	493	515	WSRGPSATQIGKPAVHM ASVDLK			Oxidation (M)[17]		Mascot

10 PREDICTED: uncharacterized protein LOC101208083 [Cucumis sativus] gi|449455180 61258.3 7.35 14 53 0 10.115

Peptide Information

Calc. Mass	Obsrv. Mass	± da	± ppm	Start Seq.	End Seq.	Sequence	Ion Score	C. I. %	Modification	Rank	Result Type
974.5266	974.5739	0.0473	49	537	545	DPSRVSVSK					Mascot
1021.5411	1021.5346	-0.0065	-6	204	212	TLTETVTEK					Mascot
1111.5135	1111.558	0.0445	40	556	563	VRQFNCCK			Carbamidomethyl (C)[6,7]		Mascot
1126.6466	1126.5582	-0.0884	-78	367	377	AEKAPELVAAK					Mascot
1190.631	1190.559	-0.072	-60	319	328	VREAVNSMLR			Oxidation (M)[8]		Mascot
1210.7267	1210.6742	-0.0525	-43	451	462	AKAAPQAILATR					Mascot
1679.8962	1679.9097	0.0135	8	131	146	ADPVIAESHTLANTIK					Mascot
1790.0283	1789.9257	-0.1026	-57	433	450	AAPQAILATNLAAKPSPR					Mascot
1791.7864	1791.8873	0.1009	56	558	571	QFNCCKQMFITDIS			Carbamidomethyl (C)[4,5]		Mascot
1795.7955	1795.907	0.1115	62	282	295	EYLMHKFEPGEDER			Oxidation (M)[4]		Mascot
1832.0123	1831.9338	-0.0785	-43	381	398	AESTLQSLAAKPSSSVKK					Mascot
1832.0123	1831.9338	-0.0785	-43	381	398	AESTLQSLAAKPSSSVKK					Mascot
1844.9753	1844.9202	-0.0551	-30	213	230	LAPVYSTVTDATHAIASK					Mascot
1844.9753	1844.9202	-0.0551	-30	213	230	LAPVYSTVTDATHAIASK					Mascot
1846.9043	1846.9163	0.012	6	524	540	SSSSAPIFTTTHRDPSR					Mascot
2496.3933	2496.2991	-0.0942	-38	402	426	AVAAKSSSHAEVAPQAIL AVHLTPK					Mascot

Gel Idx/Pos	139/F14	Instr./Gel Origin	BA2151/Sample Project 20140814	Process Status	Analysis Succeeded
Plate [#] Name	[1] Sample Project 20140814	Instrument Sample Name		Spectra	11

Rank	Protein Name	Accession No.	Protein MW	Protein PI	Pep. Count	Protein Score	Protein Score C. I. %	Intensity Matched	Total Ion Score	Total Ion C. I. %	Confirmed
1	RecName: Full=Alpha-amylase/trypsin inhibitor CMb; AltName: Full=Chloroform/methanol-soluble protein CMb; Flags: Precursor	gi|585290	17199.2	5.77	6	185	100	17.133	133	100	

Peptide Information

Calc. Mass	Obsrv. Mass	± da	± ppm	Start Seq.	End Seq.	Sequence	Ion Score	C. I. %	Modification	Rank	Result Type
801.4076	801.452	0.0444	55	86	91	FFMGRK			Oxidation (M)[3]		Mascot
1023.4928	1023.5446	0.0518	51	108	115	EVQMDFVR					Mascot
1039.4878	1039.5559	0.0681	66	108	115	EVQMDFVR			Oxidation (M)[4]		Mascot
1039.4878	1039.5559	0.0681	66	108	115	EVQMDFVR	8	0	Oxidation (M)[4]		Mascot
1168.5052	1168.6005	0.0953	82	46	54	DYVEQQACR			Carbamidomethyl (C)[8]		Mascot
1168.5052	1168.6005	0.0953	82	46	54	DYVEQQACR	57	99.643	Carbamidomethyl (C)[8]		Mascot
1285.6293	1285.7179	0.0886	69	81	90	CQALRFFMGR			Carbamidomethyl (C)[1]		Mascot
1799.8528	1800.002	0.1492	83	92	107	SRPDQSGLMELPGCPR			Carbamidomethyl (C)[14]		Mascot
1815.8477	1815.9718	0.1241	68	92	107	SRPDQSGLMELPGCPR			Carbamidomethyl (C)[14], Oxidation (M)[9]		Mascot
1815.8477	1815.9718	0.1241	68	92	107	SRPDQSGLMELPGCPR	10	0	Carbamidomethyl (C)[14], Oxidation (M)[9]		Mascot
1861.8102	1861.9623	0.1521	82	66	80	QQCCGELANIPQQCR			Carbamidomethyl (C)[3,4,14]		Mascot
1861.8102	1861.9623	0.1521	82	66	80	QQCCGELANIPQQCR	76	99.996	Carbamidomethyl (C)[3,4,14]		Mascot

Rank	Protein Name	Accession No.	Protein MW	Protein PI	Pep. Count	Protein Score	Protein Score C. I. %	Intensity Matched	Total Ion Score	Total Ion C. I. %	Confirmed
2	RecName: Full=Alpha-amylase/trypsin inhibitor CM16; AltName: Full=Chloroform/methanol-soluble protein CM16; Flags: Precursor	gi|123958	16398.8	5.31	5	178	100	16.726	133	100	

Peptide Information

Calc. Mass	Obsrv. Mass	± da	± ppm	Start Seq.	End Seq.	Sequence	Ion Score	C. I. %	Modification	Rank	Result Type
1023.4928	1023.5446	0.0518	51	108	115	EVQMDFVR					Mascot
1039.4878	1039.5559	0.0681	66	108	115	EVQMDFVR			Oxidation (M)[4]		Mascot
1039.4878	1039.5559	0.0681	66	108	115	EVQMDFVR	8	0	Oxidation (M)[4]		Mascot
1168.5052	1168.6005	0.0953	82	46	54	DYVEQQACR			Carbamidomethyl (C)[8]		Mascot
1168.5052	1168.6005	0.0953	82	46	54	DYVEQQACR	57	99.643	Carbamidomethyl (C)[8]		Mascot
1175.6307	1175.6857	0.055	47	55	65	IETPGSPYLAK					Mascot
1799.8528	1800.002	0.1492	83	92	107	SRPDQSGLMELPGCPR			Carbamidomethyl (C)[14]		Mascot
1815.8477	1815.9718	0.1241	68	92	107	SRPDQSGLMELPGCPR			Carbamidomethyl (C)[14], Oxidation (M)[9]		Mascot

Calc. Mass	Obsrv. Mass	± da	± ppm	Start Seq.	End Seq.	Sequence	Ion Score	C. I. %	Modification	Rank	Result Type
1815.8477	1815.9718	0.1241	68	92	107	SRPDQSGLMELPGCPR	10	0	Carbamidomethyl (C)[14], Oxidation (M)[9]		Mascot
1861.8102	1861.9623	0.1521	82	66	80	QQCCGELANIPQQCR			Carbamidomethyl (C)[3,4,14]		Mascot
1861.8102	1861.9623	0.1521	82	66	80	QQCCGELANIPQQCR	76	99.996	Carbamidomethyl (C)[3,4,14]		Mascot

3 Disease resistance protein RPM1 [Triticum urartu] gi|474431373 108524.2 8.73 25 73 94.892 17.444

Peptide Information

Calc. Mass	Obsrv. Mass	± da	± ppm	Start Seq.	End Seq.	Sequence	Ion Score	C. I. %	Modification	Rank	Result Type
801.4213	801.452	0.0307	38	133	139	AQQIGER					Mascot
804.425	804.4014	-0.0236	-29	438	444	SAAEWLK					Mascot
806.4016	806.3947	-0.0069	-9	62	68	AQTHGHR					Mascot
807.4835	807.406	-0.0775	-96	644	649	YLSLRR					Mascot
817.4163	817.4033	-0.013	-16	53	61	DLTAGGAGR					Mascot
818.4366	818.3757	-0.0609	-74	300	306	GETKLDR					Mascot
833.4111	833.3818	-0.0293	-35	583	589	RANDTEK					Mascot
864.4461	864.4405	-0.0056	-6	448	455	SLFPESGK					Mascot
870.5407	870.5858	0.0451	52	654	661	QLPKSIGK					Mascot
872.4407	872.4678	0.0271	31	590	596	MNLSHVR			Oxidation (M)[1]		Mascot
1001.5513	1001.5838	0.0325	32	672	680	ETSVVELPK					Mascot
1021.4486	1021.5485	0.0999	98	230	237	FGDQFDHR					Mascot
1126.5198	1126.5883	0.0685	61	184	193	DPVGVEDHMK					Mascot
1162.6063	1162.6938	0.0875	75	574	583	LSLQESDSKR					Mascot
1365.6753	1365.7491	0.0738	54	1	13	MELVVGASEATMK					Mascot
1679.8608	1679.9415	0.0807	48	535	548	CIVHDMVLEHIVAK			Carbamidomethyl (C)[1], Oxidation (M)[6]		Mascot
1791.9608	1791.9139	-0.0469	-26	534	548	KCIVHDMVLEHIVAK			Carbamidomethyl (C)[2]		Mascot
1812.9023	1813.017	0.1147	63	614	629	FGIVQVLDLEGCMGFK			Carbamidomethyl (C)[12]		Mascot
1815.9198	1815.9718	0.052	29	741	756	LAIYKLSTMSDDPSFK					Mascot
1815.9198	1815.9718	0.052	29	741	756	LAIYKLSTMSDDPSFK					Mascot
1831.9147	1831.9639	0.0492	27	741	756	LAIYKLSTMSDDPSFK			Oxidation (M)[9]		Mascot
1846.991	1846.9449	-0.0461	-25	867	884	LSSDGEITIPNAGFKGLK					Mascot
1862.1302	1861.9623	-0.1679	-90	885	900	LLRFFAPLLPVLTFSK					Mascot
1862.1302	1861.9623	-0.1679	-90	885	900	LLRFFAPLLPVLTFSK					Mascot
1883.9275	1883.9255	-0.002	-1	1	18	MELVVGASEATMKSVMGK			Oxidation (M)[1]		Mascot
1887.9845	1887.9917	0.0072	4	672	687	ETSVVELPKTVCQLER			Carbamidomethyl (C)[12]		Mascot
1887.9845	1887.9917	0.0072	4	672	687	ETSVVELPKTVCQLER			Carbamidomethyl (C)[12]		Mascot
1926.8644	1926.9982	0.1338	69	281	299	GTSALAAKCCSAGASEETR			Carbamidomethyl (C)[9,10]		Mascot

2384.1074	2384.1584	0.051	21	184	203	DPVGVEDHMKELEEWLT NDK		Mascot

4 hypothetical protein F775_03872 [Aegilops tauschii] gi|475598543 288026.1 8.73 39 68 84.214 16.699

Peptide Information

Calc. Mass	Obsrv. Mass	± da	± ppm	Start Seq.	End Seq.	Sequence	Ion Score	C. I. %	Modification	Rank	Result Type
807.4029	807.406	0.0031	4	2368	2375	SVTGMQGK					Mascot
808.3618	808.39	0.0282	35	1001	1007	TCESGVR			Carbamidomethyl (C)[2]		Mascot
814.4781	814.4435	-0.0346	-42	2329	2335	LAINDLR					Mascot
817.4162	817.4033	-0.0129	-16	609	614	DLEERR					Mascot
822.3596	822.3979	0.0383	47	2007	2013	TPSCAMR			Carbamidomethyl (C)[4]		Mascot
828.4686	828.434	-0.0346	-42	404	410	LHSSRTK					Mascot
831.4795	831.4185	-0.061	-73	1232	1238	RVNLSSR					Mascot
833.3498	833.3818	0.032	38	1458	1463	YTFDCK			Carbamidomethyl (C)[5]		Mascot
837.4213	837.4123	-0.009	-11	2175	2181	EHELPGR					Mascot
849.4135	849.4266	0.0131	15	2504	2510	LCTAEQK			Carbamidomethyl (C)[2]		Mascot
853.493	853.4349	-0.0581	-68	1595	1601	VFVYGLR					Mascot
869.4628	869.5018	0.039	45	1179	1185	QHVFNPK					Mascot
906.4866	906.4583	-0.0283	-31	1200	1206	CYVLPVR			Carbamidomethyl (C)[1]		Mascot
974.588	974.564	-0.024	-25	1804	1812	ILGSSEKLK					Mascot
1001.603	1001.5838	-0.0192	-19	2495	2503	IIGIPYTPK					Mascot
1011.5179	1011.5402	0.0223	22	1189	1197	MYILADGTK					Mascot
1024.5283	1024.5574	0.0291	28	2296	2303	SHDEKRPR					Mascot
1077.5762	1077.5876	0.0114	11	281	289	KQMLAFPDK					Mascot
1077.5762	1077.5876	0.0114	11	281	289	KQMLAFPDK					Mascot
1126.6144	1126.5883	-0.0261	-23	1269	1277	IVLYFNETK					Mascot
1175.6267	1175.6857	0.059	50	1500	1510	TTTLPSQGKDK					Mascot
1232.725	1232.6945	-0.0305	-25	290	300	VSFSLPKLDVK					Mascot
1285.6747	1285.7179	0.0432	34	673	685	TASITSEPPGIGR					Mascot
1365.6614	1365.7491	0.0877	64	386	397	LGGTQCNLMLSR			Carbamidomethyl (C)[6], Oxidation (M)[9]		Mascot
1445.7141	1445.7733	0.0592	41	398	408	LMPWMRLHSSR			Oxidation (M)[2,5]		Mascot
1570.8184	1570.9159	0.0975	62	414	427	LSKANSHQEISQTK					Mascot
1571.8752	1571.9174	0.0422	27	860	875	IGVLSEGLSVSLNGAR					Mascot
1571.8752	1571.9174	0.0422	27	860	875	IGVLSEGLSVSLNGAR					Mascot
1707.9138	1707.9233	0.0095	6	282	296	QMLAFPDKVSFSLPK					Mascot
1753.9783	1753.9495	-0.0288	-16	2360	2375	HIIWGVLKSVTGMQGK					Mascot
1769.9731	1770.0162	0.0431	24	2360	2375	HIIWGVLKSVTGMQGK			Oxidation (M)[13]		Mascot

1799.86	1800.002	0.142	79	1520	1534	HEDGFLLSSDYFTIR					Mascot
1819.8419	1819.9672	0.1253	69	1685	1700	SSDIAVKYGMFDDLDK			Oxidation (M)[10]		Mascot
1831.9987	1831.9639	-0.0348	-19	2495	2510	IIGIPYTPKLCTAEQK			Carbamidomethyl (C)[11]		Mascot
1839.0586	1839.0522	-0.0064	-3	91	106	DPKVQLLISDLEIVTR					Mascot
1843.9847	1843.9946	0.0099	5	2329	2343	LAINDLRLLMDTFHR			Oxidation (M)[10]		Mascot
1844.933	1844.9467	0.0137	7	765	779	WEPDAHLALFETFIR					Mascot
1844.933	1844.9467	0.0137	7	765	779	WEPDAHLALFETFIR					Mascot
1888.0004	1887.9917	-0.0087	-5	1263	1277	YYIHGKIVLYFNETK					Mascot
1888.0902	1887.9917	-0.0985	-52	1136	1153	LYGSDIALLAGSLVIQVR					Mascot
1992.9841	1993.0671	0.083	42	380	397	AEIDAKLGGTQCNLMLSR			Carbamidomethyl (C)[12], Oxidation (M)[15]		Mascot
2180.0596	2180.1455	0.0859	39	386	403	LGGTQCNLMLSRLMPWMR			Carbamidomethyl (C)[6], Oxidation (M)[9]		Mascot
2384.1187	2384.1584	0.0397	17	541	560	VSLDWGYREIEVQDMAETSR					Mascot

5	hypothetical protein CARUB_v10017214mg [Capsella rubella]	gi|482559809	52982.3	5.33	15	67	78.705	4.737

Peptide Information

Calc. Mass	Obsrv. Mass	± da	± ppm	Start Seq.	End Seq.	Sequence	Ion Score	C. I. %	Modification	Rank	Result Type
804.4396	804.4014	-0.0382	-47	1	6	MEQKLR					Mascot
818.3639	818.3757	0.0118	14	16	22	DAVNEDR					Mascot
820.4345	820.3961	-0.0384	-47	1	6	MEQKLR			Oxidation (M)[1]		Mascot
872.5087	872.4678	-0.0409	-47	271	278	ALELVEAK					Mascot
906.4276	906.4583	0.0307	34	7	15	TDGLGSSNR					Mascot
975.5331	975.5757	0.0426	44	55	62	SLWTMLPK					Mascot
1054.6157	1054.5491	-0.0666	-63	311	319	VPTGKIFHR					Mascot
1106.6569	1106.589	-0.0679	-61	96	105	APVLESLHLK					Mascot
1162.6256	1162.6938	0.0682	59	180	188	LYLDQVHFK					Mascot
1175.6128	1175.6857	0.0729	62	5	15	LRTDGLGSSNR					Mascot
1232.7072	1232.6945	-0.0127	-10	55	64	SLWTMLPKLK			Oxidation (M)[5]		Mascot
1707.8549	1707.9233	0.0684	40	395	409	GDEKEVATYILENAR					Mascot
1796.8813	1796.9917	0.1104	61	236	253	SEGGYGNGSYVINAPALK					Mascot
1813.0219	1813.017	-0.0049	-3	346	360	LQILKLTDVYLHNDK					Mascot
1926.9669	1926.9982	0.0313	16	351	366	LTDVYLHNDKQNPDVR					Mascot
2384.1592	2384.1584	-0.0008	0	327	345	TYEREWWNLLSIMLDSSPK			Oxidation (M)[13]		Mascot

6	uncharacterized protein [Arabidopsis thaliana]	gi|15240233	72672.4	5.18	19	61	20.881	9.808

Protein Group

uncharacterized protein AT5G37410 [Arabidopsis thaliana]	gi|332006800	72672.4	5.1799 998283 3862

Peptide Information

Calc. Mass	Obsrv. Mass	± da	± ppm	Start Seq.	End Seq.	Sequence	Ion Score	C. I. %	Modification	Rank	Result Type
804.4461	804.4014	-0.0447	-56	609	615	TADEKIK					Mascot
818.4254	818.3757	-0.0497	-61	590	596	GVKEEEK					Mascot
842.5094	842.5838	0.0744	88	583	589	INVIIDR					Mascot
860.4658	860.4125	-0.0533	-62	527	534	GMEVGLVR					Mascot
862.4563	862.3859	-0.0704	-82	353	359	SREMAIR					Mascot
884.4876	884.4411	-0.0465	-53	175	181	SFIDKFK					Mascot
975.5721	975.5757	0.0036	4	334	342	SLSSLEVLK					Mascot
1054.5892	1054.5491	-0.0401	-38	287	296	IEGVGTHLTK					Mascot
1077.5687	1077.5876	0.0189	18	247	254	EREEFLVR					Mascot
1077.5687	1077.5876	0.0189	18	247	254	EREEFLVR					Mascot
1126.6943	1126.5883	-0.106	-94	583	592	INVIIDRGVK					Mascot
1162.6136	1162.6938	0.0802	69	1	10	MAESLNLQLK			Oxidation (M)[1]		Mascot
1175.5765	1175.6857	0.1092	93	266	274	EMNLYKYSK					Mascot
1232.7515	1232.6945	-0.057	-46	561	570	GLLWRLYAIK					Mascot
1815.9786	1815.9718	-0.0068	-4	127	142	IVSCVAYNVVELHKGK			Carbamidomethyl (C)[4]		Mascot
1815.9786	1815.9718	-0.0068	-4	127	142	IVSCVAYNVVELHKGK			Carbamidomethyl (C)[4]		Mascot
1883.891	1883.9255	0.0345	18	499	515	LIEDPGYGDDVSEIAYK					Mascot
2044.957	2045.1516	0.1946	95	597	613	ELPQNESDWLNRTADEK					Mascot
2063.04	2063.1641	0.1241	60	105	122	GVLISCLTMEEPKESEIK			Carbamidomethyl (C)[6]		Mascot
2180.054	2180.1455	0.0915	42	304	321	MLVTEPEKPQDDDLKYS R			Oxidation (M)[1]		Mascot
2384.2927	2384.1584	-0.1343	-56	373	392	VEIGIEEMRELQPLLITCL K			Carbamidomethyl (C)[18]		Mascot

7	pentatricopeptide repeat-containing protein [Arabidopsis lyrata subsp. lyrata]	gi|297312241	59825.7	9.45	18	60	0	14.056	

Peptide Information

Calc. Mass	Obsrv. Mass	± da	± ppm	Start Seq.	End Seq.	Sequence	Ion Score	C. I. %	Modification	Rank	Result Type
807.4723	807.406	-0.0663	-82	170	177	YKGIAGAK					Mascot
808.4199	808.39	-0.0299	-37	275	282	GGFEIGTK					Mascot
860.4658	860.4125	-0.0533	-62	404	410	ILCGIER			Carbamidomethyl (C)[3]		Mascot
872.4836	872.4678	-0.0158	-18	489	495	RETLPEK					Mascot

906.4965	906.4583	-0.0382	-42	162	169	GMLEIISK			Oxidation (M)[2]		Mascot
1077.5398	1077.5876	0.0478	44	411	419	LEHAMSVFK			Oxidation (M)[5]		Mascot
1077.5398	1077.5876	0.0478	44	411	419	LEHAMSVFK			Oxidation (M)[5]		Mascot
1106.5034	1106.589	0.0856	77	374	383	IGEGDEMIDK					Mascot
1168.5667	1168.6005	0.0338	29	339	348	TEEAMTLFAR					Mascot
1168.5667	1168.6005	0.0338	29	339	348	TEEAMTLFAR					Mascot
1184.5616	1184.6152	0.0536	45	339	348	TEEAMTLFAR			Oxidation (M)[5]		Mascot
1365.6389	1365.7491	0.1102	81	374	385	IGEGDEMIDKMK					Mascot
1445.7933	1445.7733	-0.02	-14	310	321	VLLEMEFRGVPR					Mascot
1570.8523	1570.9159	0.0636	40	505	517	QINMSFVKKPHNK					Mascot
1612.9058	1612.8801	-0.0257	-16	34	48	LISSKPSFSPLPPSR					Mascot
1679.8422	1679.9415	0.0993	59	322	335	NTETFNVLINNLCK			Carbamidomethyl (C)[13]		Mascot
1753.8473	1753.9495	0.1022	58	440	454	MCANNQLTRANGLYK			Carbamidomethyl (C)[2]		Mascot
1769.8422	1770.0162	0.174	98	440	454	MCANNQLTRANGLYK			Carbamidomethyl (C)[2], Oxidation (M)[1]		Mascot
1812.9087	1813.017	0.1083	60	17	33	TFISSSSSVNTLQAQSR					Mascot
1815.8373	1815.9718	0.1345	74	283	296	AYNMILDCVCKLCR			Carbamidomethyl (C)[8,10,13]		Mascot
1815.8373	1815.9718	0.1345	74	283	296	AYNMILDCVCKLCR			Carbamidomethyl (C)[8,10,13]		Mascot
1831.8322	1831.9639	0.1317	72	283	296	AYNMILDCVCKLCR			Carbamidomethyl (C)[8,10,13], Oxidation (M)[4]		Mascot
1859.9935	1859.9629	-0.0306	-16	303	317	LQPEVEKVLLEMEFR					Mascot
2044.9613	2045.1516	0.1903	93	432	448	TYDLLMGKMCANNQLTR			Carbamidomethyl (C)[10], Oxidation (M)[6]		Mascot

8	unnamed protein product [Vitis vinifera]	gi|296086640	33834.9	9.9	13	56	0	13.507

Peptide Information

Calc. Mass	Obsrv. Mass	± da	± ppm	Start Seq.	End Seq.	Sequence	Ion Score	C. I. %	Modification	Rank	Result Type
801.4036	801.452	0.0484	60	66	73	GPLNGMGR					Mascot
812.3818	812.3626	-0.0192	-24	8	15	SSLSGSMK			Oxidation (M)[7]		Mascot
817.3985	817.4033	0.0048	6	66	73	GPLNGMGR			Oxidation (M)[6]		Mascot
826.4206	826.4165	-0.0041	-5	280	287	GGFATFAR					Mascot
834.4355	834.3803	-0.0552	-66	304	311	EFAGLNAI					Mascot
864.4938	864.4405	-0.0533	-62	98	105	SVLYGGLR					Mascot
906.493	906.4583	-0.0347	-38	106	113	LGLYEPSK					Mascot
1001.556	1001.5838	0.0278	28	57	65	LQMQLVGGR					Mascot
1021.4917	1021.5485	0.0568	56	161	169	RGAIGEMCK			Carbamidomethyl (C)[8]		Mascot
1037.4867	1037.5526	0.0659	64	161	169	RGAIGEMCK			Carbamidomethyl (C)[8], Oxidation (M)[7]		Mascot
1751.9691	1752.0042	0.0351	20	98	113	SVLYGGLRLGLYEPSK					Mascot

1799.9513	1800.002	0.0507	28	114	128	YVCKWAFGSTNLLLK			Carbamidomethyl (C)[3]		Mascot
1815.9316	1815.9718	0.0402	22	57	73	LQMQLVGGRGPLNGMG R			Oxidation (M)[3,15]		Mascot
1815.9316	1815.9718	0.0402	22	57	73	LQMQLVGGRGPLNGMG R			Oxidation (M)[3,15]		Mascot
1861.9014	1861.9623	0.0609	33	260	275	NGFHCAYQVILTEGPR			Carbamidomethyl (C)[5]		Mascot
1861.9014	1861.9623	0.0609	33	260	275	NGFHCAYQVILTEGPR			Carbamidomethyl (C)[5]		Mascot
2180.2261	2180.1455	-0.0806	-37	86	105	SLYLGLMPALTRSVLYGG LR					Mascot

9	ATP binding protein, putative isoform 3 [Theobroma cacao]	gi|508706274	312336.1	5.04	46	56	0	29.272	

Peptide Information

Calc. Mass	Obsrv. Mass	± da	± ppm	Start Seq.	End Seq.	Sequence	Ion Score	C. I. %	Modification	Rank	Result Type
801.4828	801.452	-0.0308	-38	2398	2404	LEKNVAK					Mascot
803.4622	803.4114	-0.0508	-63	2623	2629	TEVVLSR					Mascot
806.4003	806.3947	-0.0056	-7	322	328	DSTTNLR					Mascot
807.4029	807.406	0.0031	4	1141	1146	LEDRMK			Oxidation (M)[5]		Mascot
808.4563	808.39	-0.0663	-82	1996	2002	LFSTVNK					Mascot
814.3698	814.4435	0.0737	90	1348	1353	CMGFRK			Carbamidomethyl (C)[1], Oxidation (M)[2]		Mascot
818.4155	818.3757	-0.0398	-49	855	861	SNSHFVK					Mascot
819.3995	819.3775	-0.022	-27	884	890	DHPPPEK					Mascot
820.3981	820.3961	-0.002	-2	2316	2321	RCLDEK			Carbamidomethyl (C)[2]		Mascot
822.4389	822.3979	-0.041	-50	729	735	TMLNSLK			Oxidation (M)[2]		Mascot
830.4479	830.4372	-0.0107	-13	1288	1294	SPERSVR					Mascot
831.4029	831.4185	0.0156	19	905	911	MHASLEK			Oxidation (M)[1]		Mascot
833.4086	833.3818	-0.0268	-32	1277	1283	AHVGMYR					Mascot
834.3853	834.3803	-0.005	-6	113	119	STHGYNR					Mascot
837.3593	837.4123	0.053	63	1672	1677	ELEMCR			Carbamidomethyl (C)[5]		Mascot
845.4224	845.4466	0.0242	29	2281	2287	GEAERQR					Mascot
847.4631	847.4274	-0.0357	-42	1402	1408	QSKESLR					Mascot
849.4036	849.4266	0.023	27	1277	1283	AHVGMYR			Oxidation (M)[5]		Mascot
853.4535	853.4349	-0.0186	-22	569	574	MMLRFR					Mascot
860.4724	860.4125	-0.0599	-70	1763	1769	DKDELLK					Mascot
864.4131	864.4405	0.0274	32	2560	2566	TENEMLK					Mascot
869.4484	869.5018	0.0534	61	569	574	MMLRFR			Oxidation (M)[1]		Mascot
872.5087	872.4678	-0.0409	-47	784	790	IILQEEK					Mascot
884.4328	884.4411	0.0083	9	566	572	SSKMMLR			Oxidation (M)[4,5]		Mascot
975.4993	975.5757	0.0764	78	1394	1401	EEIQLSEK					Mascot

1001.5738	1001.5838	0.01	10	285	293	LLTQGSLNR					Mascot
1077.547	1077.5876	0.0406	38	2	10	TNMTPRVSR			Oxidation (M)[3]		Mascot
1077.547	1077.5876	0.0406	38	2	10	TNMTPRVSR			Oxidation (M)[3]		Mascot
1106.551	1106.589	0.038	34	1007	1015	LEMKDGELR			Oxidation (M)[3]		Mascot
1165.5704	1165.6744	0.104	89	1092	1100	RSELECMLK			Carbamidomethyl (C)[6]		Mascot
1175.6631	1175.6857	0.0226	19	1135	1144	TSIITKLEDR					Mascot
1232.5729	1232.6945	0.1216	99	2010	2019	MFQELHDNAK					Mascot
1365.6865	1365.7491	0.0626	46	1093	1103	SELECMLKSLR			Carbamidomethyl (C)[5]		Mascot
1445.8112	1445.7733	-0.0379	-26	655	666	LRDQLLQFLDGK					Mascot
1475.8138	1475.8579	0.0441	30	1947	1959	VNTLMEEKALLSK					Mascot
1641.9421	1641.9644	0.0223	14	1070	1083	ILSEKELLIEELGR					Mascot
1707.9276	1707.9233	-0.0043	-3	2288	2301	LEREELELELHAVK					Mascot
1770.0007	1770.0162	0.0155	9	814	829	LLLLSKQVEDANGELK					Mascot
1771.051	1770.9562	-0.0948	-54	2667	2682	VQLAQKLLGLCTSVLK			Carbamidomethyl (C)[11]		Mascot
1771.051	1770.9562	-0.0948	-54	2667	2682	VQLAQKLLGLCTSVLK			Carbamidomethyl (C)[11]		Mascot
1791.868	1791.9139	0.0459	26	1198	1213	DSILIDQAAMIEEAEK			Oxidation (M)[10]		Mascot
1815.8872	1815.9718	0.0846	47	123	138	QENSQSIAWIGQPETK					Mascot
1815.9634	1815.9718	0.0084	5	1714	1728	KISDLQVQEEVMLQR					Mascot
1820.0024	1819.9672	-0.0352	-19	2323	2337	KDLQQALDHIQILER					Mascot
1831.9583	1831.9639	0.0056	3	1714	1728	KISDLQVQEEVMLQR			Oxidation (M)[12]		Mascot
1838.8875	1839.0522	0.1647	90	1182	1197	DKDICLAESAEMILSK			Carbamidomethyl (C)[5], Oxidation (M)[12]		Mascot
1843.8743	1843.9946	0.1203	65	1250	1264	LEDMEENDILKTHEK					Mascot
1844.8881	1844.9467	0.0586	32	1921	1936	EVQHSQSIIMEDLGMK					Mascot
1844.8881	1844.9467	0.0586	32	1921	1936	EVQHSQSIIMEDLGMK					Mascot
1846.9845	1846.9449	-0.0396	-21	2407	2424	GSGSPFKCIGLGLAQQV K			Carbamidomethyl (C)[8]		Mascot
1859.8691	1859.9629	0.0938	50	1250	1264	LEDMEENDILKTHEK			Oxidation (M)[4]		Mascot
1861.896	1861.9623	0.0663	36	2604	2619	EENNMLKIQNEDLGAK			Oxidation (M)[5]		Mascot
1861.896	1861.9623	0.0663	36	2604	2619	EENNMLKIQNEDLGAK			Oxidation (M)[5]		Mascot
1887.8832	1887.9917	0.1085	57	669	684	QHSYPSSDDQLQEVVR					Mascot
1887.8832	1887.9917	0.1085	57	669	684	QHSYPSSDDQLQEVVR					Mascot

10	Os10g0389100 [Oryza sativa Japonica Group]	gi|113639108	19082.8	4.95	9	55	0	4.015	

Peptide Information

Calc. Mass	Obsrv. Mass	± da	± ppm	Start Seq.	End Seq.	Sequence	Ion Score	C. I. %	Modification	Rank	Result Type
800.4876	800.411	-0.0766	-96	158	164	VIAEIQK					Mascot

806.4189	806.3947	-0.0242	-30	124	129	ENMIKR	Oxidation (M)[3]	Mascot
830.5029	830.4372	-0.0657	-79	9	14	RLCLIR	Carbamidomethyl (C)[3]	Mascot
845.4839	845.4466	-0.0373	-44	130	136	DQIVRSK		Mascot
869.4767	869.5018	0.0251	29	22	28	DFFLLSK		Mascot
1023.4564	1023.5446	0.0882	86	149	157	MFGPDVADR	Oxidation (M)[1]	Mascot
1126.6144	1126.5883	-0.0261	-23	22	30	DFFLLSKEK		Mascot
1751.8456	1752.0042	0.1586	91	29	42	EKIDFMLQSSLHCK	Carbamidomethyl (C)[13], Oxidation (M)[6]	Mascot
1804.9263	1804.9391	0.0128	7	149	164	MFGPDVADRVIAEIQK	Oxidation (M)[1]	Mascot
