# Supplementary material for: Proteomic analysis of middle and late stages of bread wheat (Triticum aestivum L.) grain development
Source: Front Plant Sci. 2015 Sep 15;6:735. doi: 10.3389/fpls.2015.00735 (PMC4569854; doi:10.3389/fpls.2015.00735)
Supplement: Supplementary file 9 [file DataSheet8.PDF]

Analysis Information

Report Type	Protein-Peptide Summary by Spot	Analysis Type	Combined (MS+MS/MS)
Sample Set Name	Sample set_20140814	Database	NCBI_VPlant
Analysis Name	R14026-4-VP1	Creation Date	09/29/2014 15:00:22
Reported By	09/30/2014 14:57:51 - admin	Last Modified	09/29/2014 15:29:13

MS Acq. : Proc. Methods	(Unspecified) : (Unspecified)
Interpretation Method	(Unspecified)

**Gel Idx/Pos**	151/G2	**Instr./Gel Origin**	BA2151/Sample Project 20140814	**Process Status**	Analysis Succeeded
**Plate [#] Name**	[1] Sample Project 20140814	**Instrument Sample Name**		**Spectra**	11

Rank	Protein Name	Accession No.	Protein MW	Protein PI	Pep. Count	Protein Score	Protein Score C. I. %	Intensity Matched	Total Ion Score	Total Ion C. I. %	Confirmed
1	RecName: Full=Alpha-amylase inhibitor 0.53	gi|123968	13689.5	5.23	5	299	100	21.61	268	100	

Peptide Information

Calc. Mass	Obsrv. Mass	± da	± ppm	Start Seq.	End Seq.	Sequence	Ion Score	C. I. %	Modification	Rank	Result Type
1162.6249	1162.6165	-0.0084	-7	90	100	LTAASITAVCR			Carbamidomethyl (C)[10]		Mascot
1162.6249	1162.6165	-0.0084	-7	90	100	LTAASITAVCR	49	98.135	Carbamidomethyl (C)[10]		Mascot
1554.6637	1554.7523	0.0886	57	54	66	CGALYSMLDSMYK			Carbamidomethyl (C)[1], Oxidation (M)[7]		Mascot
1570.8007	1570.7888	-0.0119	-8	26	39	LQCNGSQVPEAVLR			Carbamidomethyl (C)[3]		Mascot
1663.8361	1663.7917	-0.0444	-27	101	116	LPIVVDASGDGAYVCK			Carbamidomethyl (C)[15]		Mascot
1663.8361	1663.7917	-0.0444	-27	101	116	LPIVVDASGDGAYVCK	86	100	Carbamidomethyl (C)[15]		Mascot
1846.8137	1846.8	-0.0137	-7	67	84	EHGVSEGGQAGTGAFPSCR			Carbamidomethyl (C)[17]		Mascot
1846.8137	1846.8	-0.0137	-7	67	84	EHGVSEGGQAGTGAFPSCR	134	100	Carbamidomethyl (C)[17]		Mascot

Rank	Protein Name	Accession No.	Protein MW	Protein PI	Pep. Count	Protein Score	Protein Score C. I. %	Intensity Matched	Total Ion Score	Total Ion C. I. %	Confirmed
2	Chain D, 0.19 Alpha-Amylase Inhibitor From Wheat	gi|3318684	13898.6	6.66	6	174	100	5.722	135	100	

Protein Group

Chain A, 0.19 Alpha-Amylase Inhibitor From Wheat	gi|3318681	13898.6	6.6599998474 1211
Chain B, 0.19 Alpha-Amylase Inhibitor From Wheat	gi|3318682	13898.6	6.6599998474 1211
Chain C, 0.19 Alpha-Amylase Inhibitor From Wheat	gi|3318683	13898.6	6.6599

			998474 1211
RecName: Full=Alpha-amylase inhibitor 0.19; AltName: Full=0.19 alpha-AI; Short=0.19 AI	gi|123963	13898.6	6.6599 998474 1211
dimeric alpha-amylase inhibitor, partial [Aegilops geniculata]	gi|452055912	14198.8	6.6599 998474 1211
dimeric alpha-amylase inhibitor, partial [Aegilops kotschyi]	gi|386877048	14198.8	6.6599 998474 1211

Peptide Information

Calc. Mass	Obsrv. Mass	± da	± ppm	Start Seq.	End Seq.	Sequence	Ion Score	C. I. %	Modification	Rank	Result Type
1162.6249	1162.6165	-0.0084	-7	90	100	LTAASITAVCR			Carbamidomethyl (C)[10]		Mascot
1162.6249	1162.6165	-0.0084	-7	90	100	LTAASITAVCR	49	98.135	Carbamidomethyl (C)[10]		Mascot
1554.6637	1554.7523	0.0886	57	54	66	CGALYSMLDSMYK			Carbamidomethyl (C)[1], Oxidation (M)[7]		Mascot
1570.8007	1570.7888	-0.0119	-8	26	39	LQCNGSQVPEAVLR			Carbamidomethyl (C)[3]		Mascot
1612.7463	1612.728	-0.0183	-11	67	82	EHGAQEGQAGTGAFPR					Mascot
1663.8361	1663.7917	-0.0444	-27	101	116	LPIVVDASGDGAYVCK			Carbamidomethyl (C)[15]		Mascot
1663.8361	1663.7917	-0.0444	-27	101	116	LPIVVDASGDGAYVCK	86	100	Carbamidomethyl (C)[15]		Mascot
1862.7731	1862.7512	-0.0219	-12	40	53	DCCQQLAHISEWCR			Carbamidomethyl (C)[2,3,13]		Mascot

3	dimeric alpha-amylase inhibitor, partial [Aegilops tauschii]	gi|386877046	14670	6.08	6	173	100	5.722	135	100	

Protein Group

dimeric alpha-amylase inhibitor, partial [Aegilops geniculata]	gi|386877062	14542.9	7.0500 001907 3486
dimeric alpha-amylase inhibitor, partial [Aegilops longissima]	gi|386877060	14954.2	7.6399 998664 856
dimeric alpha-amylase inhibitor, partial [Aegilops tauschii]	gi|386877050	14826.1	6.8600 001335 144
dimeric alpha-amylase inhibitor, partial [Aegilops tauschii]	gi|386877044	14805	6.0999 999046 3257

Peptide Information

Calc. Mass	Obsrv. Mass	± da	± ppm	Start Seq.	End Seq.	Sequence	Ion Score	C. I. %	Modification	Rank	Result Type
1162.6249	1162.6165	-0.0084	-7	97	107	LTAASITAVCR			Carbamidomethyl (C)[10]		Mascot
1162.6249	1162.6165	-0.0084	-7	97	107	LTAASITAVCR	49	98.135	Carbamidomethyl (C)[10]		Mascot
1554.6637	1554.7523	0.0886	57	61	73	CGALYSMLDSMYK			Carbamidomethyl (C)[1], Oxidation (M)[7]		Mascot
1570.8007	1570.7888	-0.0119	-8	33	46	LQCNGSQVPEAVLR			Carbamidomethyl (C)[3]		Mascot

Calc. Mass	Obsrv. Mass	± da	± ppm	Start Seq.	End Seq.	Sequence	Ion Score	C. I. %	Modification	Rank	Result Type
1612.7463	1612.728	-0.0183	-11	74	89	EHGAQEGQAGTGAFPR					Mascot
1663.8361	1663.7917	-0.0444	-27	108	123	LPIVVDASGDGAYVCK			Carbamidomethyl (C)[15]		Mascot
1663.8361	1663.7917	-0.0444	-27	108	123	LPIVVDASGDGAYVCK	86	100	Carbamidomethyl (C)[15]		Mascot
1862.7731	1862.7512	-0.0219	-12	47	60	DCCQQLAHISEWCR			Carbamidomethyl (C)[2,3,13]		Mascot

4	dimeric alpha-amylase inhibitor [Triticum aestivum]	gi|386877038	15702.5	5.58	6	171	100	5.722	135	100	

Peptide Information

Calc. Mass	Obsrv. Mass	± da	± ppm	Start Seq.	End Seq.	Sequence	Ion Score	C. I. %	Modification	Rank	Result Type
1162.6249	1162.6165	-0.0084	-7	107	117	LTAASITAVCR			Carbamidomethyl (C)[10]		Mascot
1162.6249	1162.6165	-0.0084	-7	107	117	LTAASITAVCR	49	98.135	Carbamidomethyl (C)[10]		Mascot
1554.6637	1554.7523	0.0886	57	71	83	CGALYSMLDSMYK			Carbamidomethyl (C)[1], Oxidation (M)[7]		Mascot
1570.8007	1570.7888	-0.0119	-8	43	56	LQCNGSQVPEAVLR			Carbamidomethyl (C)[3]		Mascot
1612.7463	1612.728	-0.0183	-11	84	99	EHGAQEGQAGTGAFPR					Mascot
1663.8361	1663.7917	-0.0444	-27	118	133	LPIVVDASGDGAYVCK			Carbamidomethyl (C)[15]		Mascot
1663.8361	1663.7917	-0.0444	-27	118	133	LPIVVDASGDGAYVCK	86	100	Carbamidomethyl (C)[15]		Mascot
1862.7731	1862.7512	-0.0219	-12	57	70	DCCQQLAHISEWCR			Carbamidomethyl (C)[2,3,13]		Mascot

5	Alpha-amylase inhibitor 0.19 [Aegilops tauschii]	gi|475613321	17198.2	6.06	6	169	100	5.722	135	100	

Peptide Information

Calc. Mass	Obsrv. Mass	± da	± ppm	Start Seq.	End Seq.	Sequence	Ion Score	C. I. %	Modification	Rank	Result Type
1162.6249	1162.6165	-0.0084	-7	120	130	LTAASITAVCR			Carbamidomethyl (C)[10]		Mascot
1162.6249	1162.6165	-0.0084	-7	120	130	LTAASITAVCR	49	98.135	Carbamidomethyl (C)[10]		Mascot
1554.6637	1554.7523	0.0886	57	84	96	CGALYSMLDSMYK			Carbamidomethyl (C)[1], Oxidation (M)[7]		Mascot
1570.8007	1570.7888	-0.0119	-8	56	69	LQCNGSQVPEAVLR			Carbamidomethyl (C)[3]		Mascot
1612.7463	1612.728	-0.0183	-11	97	112	EHGAQEGQAGTGAFPR					Mascot
1663.8361	1663.7917	-0.0444	-27	131	146	LPIVVDASGDGAYVCK			Carbamidomethyl (C)[15]		Mascot
1663.8361	1663.7917	-0.0444	-27	131	146	LPIVVDASGDGAYVCK	86	100	Carbamidomethyl (C)[15]		Mascot
1862.7731	1862.7512	-0.0219	-12	70	83	DCCQQLAHISEWCR			Carbamidomethyl (C)[2,3,13]		Mascot

6	dimeric alpha-amylase inhibitor, partial [Aegilops longissima]	gi|386877056	14792	5.28	5	164	100	11.99	135	100	

Protein Group

dimeric alpha-amylase inhibitor, partial [Aegilops longissima]	gi|386877058	14718.9	4.9899 997711 1816

Peptide Information

Calc. Mass	Obsrv. Mass	± da	± ppm	Start	End	Sequence	Ion	C. I. %	Modification	Rank	Result Type

				Seq.	Seq.		Score				
1162.6249	1162.6165	-0.0084	-7	98	108	LTAASITAVCR			Carbamidomethyl (C)[10]		Mascot
1162.6249	1162.6165	-0.0084	-7	98	108	LTAASITAVCR	49	98.135	Carbamidomethyl (C)[10]		Mascot
1570.8007	1570.7888	-0.0119	-8	34	47	LQCNGSQVPEAVLR			Carbamidomethyl (C)[3]		Mascot
1612.7463	1612.728	-0.0183	-11	75	90	EHGAQEGQAGTGAFPR					Mascot
1663.8361	1663.7917	-0.0444	-27	109	124	LPIVVDASGDGAYVCK			Carbamidomethyl (C)[15]		Mascot
1663.8361	1663.7917	-0.0444	-27	109	124	LPIVVDASGDGAYVCK	86	100	Carbamidomethyl (C)[15]		Mascot
1840.7412	1840.7278	-0.0134	-7	48	61	DCCQQLADISEWCR			Carbamidomethyl (C)[2,3,13]		Mascot

7	dimeric alpha-amylase inhibitor, partial [Triticum aestivum]	gi|386877042	11899.6	6.13	4	159	100	5.194	135	100	

Peptide Information

Calc. Mass	Obsrv. Mass	± da	± ppm	Start Seq.	End Seq.	Sequence	Ion Score	C. I. %	Modification	Rank	Result Type
1162.6249	1162.6165	-0.0084	-7	73	83	LTAASITAVCR			Carbamidomethyl (C)[10]		Mascot
1162.6249	1162.6165	-0.0084	-7	73	83	LTAASITAVCR	49	98.135	Carbamidomethyl (C)[10]		Mascot
1554.6637	1554.7523	0.0886	57	37	49	CGALYSMLDSMYK			Carbamidomethyl (C)[1], Oxidation (M)[7]		Mascot
1570.8007	1570.7888	-0.0119	-8	9	22	LQCNGSQVPEAVLR			Carbamidomethyl (C)[3]		Mascot
1663.8361	1663.7917	-0.0444	-27	84	99	LPIVVDASGDGAYVCK			Carbamidomethyl (C)[15]		Mascot
1663.8361	1663.7917	-0.0444	-27	84	99	LPIVVDASGDGAYVCK	86	100	Carbamidomethyl (C)[15]		Mascot

8	dimeric alpha-amylase inhibitor, partial [Aegilops peregrina]	gi|386877054	14145.7	5.26	4	157	100	9.885	135	100	

Peptide Information

Calc. Mass	Obsrv. Mass	± da	± ppm	Start Seq.	End Seq.	Sequence	Ion Score	C. I. %	Modification	Rank	Result Type
1162.6249	1162.6165	-0.0084	-7	92	102	LTAASITAVCR			Carbamidomethyl (C)[10]		Mascot
1162.6249	1162.6165	-0.0084	-7	92	102	LTAASITAVCR	49	98.135	Carbamidomethyl (C)[10]		Mascot
1612.7463	1612.728	-0.0183	-11	69	84	EHGAQEGQAGTGAFPR					Mascot
1663.8361	1663.7917	-0.0444	-27	103	118	LPIVVDASGDGAYVCK			Carbamidomethyl (C)[15]		Mascot
1663.8361	1663.7917	-0.0444	-27	103	118	LPIVVDASGDGAYVCK	86	100	Carbamidomethyl (C)[15]		Mascot
1840.7412	1840.7278	-0.0134	-7	42	55	DCCQQLADISEWCR			Carbamidomethyl (C)[2,3,13]		Mascot

9	dimeric alpha-amylase inhibitor, partial [Aegilops peregrina]	gi|386877052	13953.6	6.49	4	157	100	11.723	135	100	

Peptide Information

Calc. Mass	Obsrv. Mass	± da	± ppm	Start Seq.	End Seq.	Sequence	Ion Score	C. I. %	Modification	Rank	Result Type

Calc. Mass	Obsrv. Mass	± da	± ppm	Start Seq.	End Seq.	Sequence	Ion Score	C. I. %	Modification	Rank	Result Type
1162.6249	1162.6165	-0.0084	-7	90	100	LTAASITAVCR			Carbamidomethyl (C)[10]		Mascot
1162.6249	1162.6165	-0.0084	-7	90	100	LTAASITAVCR	49	98.135	Carbamidomethyl (C)[10]		Mascot
1570.8007	1570.7888	-0.0119	-8	26	39	LQCNGSQVPEAVLR			Carbamidomethyl (C)[3]		Mascot
1663.8361	1663.7917	-0.0444	-27	101	116	LPIVVDASGDGAYVCK			Carbamidomethyl (C)[15]		Mascot
1663.8361	1663.7917	-0.0444	-27	101	116	LPIVVDASGDGAYVCK	86	100	Carbamidomethyl (C)[15]		Mascot
1840.7412	1840.7278	-0.0134	-7	40	53	DCCQQLADISEWCR			Carbamidomethyl (C)[2,3,13]		Mascot

10	dimeric alpha-amylase inhibitor, partial [Triticum aestivum]	gi|386877068	14415.8	6.88	5	78	98.527	4.709	49	98.135

Peptide Information

Calc. Mass	Obsrv. Mass	± da	± ppm	Start Seq.	End Seq.	Sequence	Ion Score	C. I. %	Modification	Rank	Result Type
1162.6249	1162.6165	-0.0084	-7	95	105	LTAASITAVCR			Carbamidomethyl (C)[10]		Mascot
1162.6249	1162.6165	-0.0084	-7	95	105	LTAASITAVCR	49	98.135	Carbamidomethyl (C)[10]		Mascot
1554.6637	1554.7523	0.0886	57	59	71	CGALYSMLDSMYK			Carbamidomethyl (C)[1], Oxidation (M)[7]		Mascot
1570.8007	1570.7888	-0.0119	-8	31	44	LQCNGSQVPEAVLR			Carbamidomethyl (C)[3]		Mascot
1612.7463	1612.728	-0.0183	-11	72	87	EHGAQEGQAGTGAFPR					Mascot
1862.7731	1862.7512	-0.0219	-12	45	58	DCCQQLAHISEWCR			Carbamidomethyl (C)[2,3,13]		Mascot

Gel Idx/Pos	152/G3	Instr./Gel Origin	BA2151/Sample Project 20140814	Process Status	Analysis Succeeded
Plate [#] Name	[1] Sample Project 20140814	Instrument Sample Name		Spectra	11

Rank	Protein Name	Accession No.	Protein MW	Protein PI	Pep. Count	Protein Score	Protein Score C. I. %	Intensity Matched	Total Ion Score	Total Ion C. I. %	Confirmed
1	RecName: Full=Alpha-amylase inhibitor 0.53	gi|123968	13689.5	5.23	6	420	100	36.586	378	100	

Peptide Information

Calc. Mass	Obsrv. Mass	± da	± ppm	Start Seq.	End Seq.	Sequence	Ion Score	C. I. %	Modification	Rank	Result Type
1162.6249	1162.6138	-0.0111	-10	90	100	LTAASITAVCR			Carbamidomethyl (C)[10]		Mascot
1162.6249	1162.6138	-0.0111	-10	90	100	LTAASITAVCR	70	99.985	Carbamidomethyl (C)[10]		Mascot
1554.6637	1554.6335	-0.0302	-19	54	66	CGALYSMLDSMYK			Carbamidomethyl (C)[1], Oxidation (M)[7]		Mascot
1570.8007	1570.7839	-0.0168	-11	26	39	LQCNGSQVPEAVLR			Carbamidomethyl (C)[3]		Mascot
1663.8361	1663.7701	-0.066	-40	101	116	LPIVVDASGDGAYVCK			Carbamidomethyl (C)[15]		Mascot
1663.8361	1663.7701	-0.066	-40	101	116	LPIVVDASGDGAYVCK	141	100	Carbamidomethyl (C)[15]		Mascot
1846.8137	1846.7911	-0.0226	-12	67	84	EHGVSEGGQAGTGAFPSC R			Carbamidomethyl (C)[17]		Mascot
1846.8137	1846.7911	-0.0226	-12	67	84	EHGVSEGGQAGTGAFPSC R	167	100	Carbamidomethyl (C)[17]		Mascot
2807.4431	2807.3105	-0.1326	-47	90	116	LTAASITAVCRLPIVVDAS GDGAYVCK			Carbamidomethyl (C)[10,26]		Mascot

Rank	Protein Name	Accession No.	Protein MW	Protein PI	Pep. Count	Protein Score	Protein Score C. I. %	Intensity Matched	Total Ion Score	Total Ion C. I. %	Confirmed
2	dimeric alpha-amylase inhibitor, partial [Triticum aestivum]	gi|386877042	11899.6	6.13	6	258	100	13.628	211	100	

Peptide Information

Calc. Mass	Obsrv. Mass	± da	± ppm	Start Seq.	End Seq.	Sequence	Ion Score	C. I. %	Modification	Rank	Result Type
1162.6249	1162.6138	-0.0111	-10	73	83	LTAASITAVCR			Carbamidomethyl (C)[10]		Mascot
1162.6249	1162.6138	-0.0111	-10	73	83	LTAASITAVCR	70	99.985	Carbamidomethyl (C)[10]		Mascot
1554.6637	1554.6335	-0.0302	-19	37	49	CGALYSMLDSMYK			Carbamidomethyl (C)[1], Oxidation (M)[7]		Mascot
1570.8007	1570.7839	-0.0168	-11	9	22	LQCNGSQVPEAVLR			Carbamidomethyl (C)[3]		Mascot
1663.8361	1663.7701	-0.066	-40	84	99	LPIVVDASGDGAYVCK			Carbamidomethyl (C)[15]		Mascot
1663.8361	1663.7701	-0.066	-40	84	99	LPIVVDASGDGAYVCK	141	100	Carbamidomethyl (C)[15]		Mascot
2395.1487	2395.0366	-0.1121	-47	84	106	LPIVVDASGDGAYVCKDV AAYPD			Carbamidomethyl (C)[15]		Mascot
2395.1487	2395.0366	-0.1121	-47	84	106	LPIVVDASGDGAYVCKDV AAYPD			Carbamidomethyl (C)[15]		Mascot
2807.4431	2807.3105	-0.1326	-47	73	99	LTAASITAVCRLPIVVDAS GDGAYVCK			Carbamidomethyl (C)[10,26]		Mascot

Rank	Protein Name	Accession No.	Protein MW	Protein PI	Pep. Count	Protein Score	Protein Score C. I. %	Intensity Matched	Total Ion Score	Total Ion C. I. %	Confirmed
3	Chain D, 0.19 Alpha-Amylase Inhibitor From Wheat	gi|3318684	13898.6	6.66	6	253	100	12.85	211	100	

Protein Group

Chain A, 0.19 Alpha-Amylase Inhibitor From Wheat	gi|3318681	13898.6	6.6599998474 1211
Chain B, 0.19 Alpha-Amylase Inhibitor From Wheat	gi|3318682	13898.6	6.6599998474 1211
Chain C, 0.19 Alpha-Amylase Inhibitor From Wheat	gi|3318683	13898.6	6.6599998474 1211
RecName: Full=Alpha-amylase inhibitor 0.19; AltName: Full=0.19 alpha-AI; Short=0.19 AI	gi|123963	13898.6	6.6599998474 1211

Peptide Information

Calc. Mass	Obsrv. Mass	± da	± ppm	Start Seq.	End Seq.	Sequence	Ion Score	C. I. %	Modification	Rank	Result Type
1162.6249	1162.6138	-0.0111	-10	90	100	LTAASITAVCR			Carbamidomethyl (C)[10]		Mascot
1162.6249	1162.6138	-0.0111	-10	90	100	LTAASITAVCR	70	99.985	Carbamidomethyl (C)[10]		Mascot
1554.6637	1554.6335	-0.0302	-19	54	66	CGALYSMLDSMYK			Carbamidomethyl (C)[1], Oxidation (M)[7]		Mascot
1570.8007	1570.7839	-0.0168	-11	26	39	LQCNGSQVPEAVLR			Carbamidomethyl (C)[3]		Mascot
1663.8361	1663.7701	-0.066	-40	101	116	LPIVVDASGDGAYVCK			Carbamidomethyl (C)[15]		Mascot
1663.8361	1663.7701	-0.066	-40	101	116	LPIVVDASGDGAYVCK	141	100	Carbamidomethyl (C)[15]		Mascot
1862.7731	1862.7668	-0.0063	-3	40	53	DCCQQLAHISEWCR			Carbamidomethyl (C)[2,3,13]		Mascot
2807.4431	2807.3105	-0.1326	-47	90	116	LTAASITAVCRLPIVVDASGDGAYVCK			Carbamidomethyl (C)[10,26]		Mascot

4	dimeric alpha-amylase inhibitor, partial [Aegilops kotschyi]	gi|386877048	14198.8	6.66	6	252	100	12.85	211	100	

Protein Group

dimeric alpha-amylase inhibitor, partial [Aegilops geniculata]	gi|452055912	14198.8	6.6599998474 1211

Peptide Information

Calc. Mass	Obsrv. Mass	± da	± ppm	Start Seq.	End Seq.	Sequence	Ion Score	C. I. %	Modification	Rank	Result Type
1162.6249	1162.6138	-0.0111	-10	93	103	LTAASITAVCR			Carbamidomethyl (C)[10]		Mascot
1162.6249	1162.6138	-0.0111	-10	93	103	LTAASITAVCR	70	99.985	Carbamidomethyl (C)[10]		Mascot
1554.6637	1554.6335	-0.0302	-19	57	69	CGALYSMLDSMYK			Carbamidomethyl (C)[1], Oxidation (M)[7]		Mascot
1570.8007	1570.7839	-0.0168	-11	29	42	LQCNGSQVPEAVLR			Carbamidomethyl (C)[3]		Mascot
1663.8361	1663.7701	-0.066	-40	104	119	LPIVVDASGDGAYVCK			Carbamidomethyl (C)[15]		Mascot
1663.8361	1663.7701	-0.066	-40	104	119	LPIVVDASGDGAYVCK	141	100	Carbamidomethyl (C)[15]		Mascot
1862.7731	1862.7668	-0.0063	-3	43	56	DCCQQLAHISEWCR			Carbamidomethyl (C)[2,3,13]		Mascot
2807.4431	2807.3105	-0.1326	-47	93	119	LTAASITAVCRLPIVVDAS			Carbamidomethyl (C)[10,26]		Mascot

GDGAYVCK

5	dimeric alpha-amylase inhibitor, partial [Aegilops tauschii]	gi|386877046	14670	6.08	6	251	100	12.85	211	100

Protein Group

dimeric alpha-amylase inhibitor, partial [Aegilops geniculata]	gi|386877062	14542.9	7.0500 001907 3486
dimeric alpha-amylase inhibitor, partial [Aegilops longissima]	gi|386877060	14954.2	7.6399 998664 856
dimeric alpha-amylase inhibitor, partial [Aegilops tauschii]	gi|386877050	14826.1	6.8600 001335 144
dimeric alpha-amylase inhibitor, partial [Aegilops tauschii]	gi|386877044	14805	6.0999 999046 3257

Peptide Information

Calc. Mass	Obsrv. Mass	± da	± ppm	Start Seq.	End Seq.	Sequence	Ion Score	C. I. %	Modification	Rank	Result Type
1162.6249	1162.6138	-0.0111	-10	97	107	LTAASITAVCR			Carbamidomethyl (C)[10]		Mascot
1162.6249	1162.6138	-0.0111	-10	97	107	LTAASITAVCR	70	99.985	Carbamidomethyl (C)[10]		Mascot
1554.6637	1554.6335	-0.0302	-19	61	73	CGALYSMLDSMYK			Carbamidomethyl (C)[1], Oxidation (M)[7]		Mascot
1570.8007	1570.7839	-0.0168	-11	33	46	LQCNGSQVPEAVLR			Carbamidomethyl (C)[3]		Mascot
1663.8361	1663.7701	-0.066	-40	108	123	LPIVVDASGDGAYVCK			Carbamidomethyl (C)[15]		Mascot
1663.8361	1663.7701	-0.066	-40	108	123	LPIVVDASGDGAYVCK	141	100	Carbamidomethyl (C)[15]		Mascot
1862.7731	1862.7668	-0.0063	-3	47	60	DCCQQLAHISEWCR			Carbamidomethyl (C)[2,3,13]		Mascot
2807.4431	2807.3105	-0.1326	-47	97	123	LTAASITAVCRLPIVVDAS GDGAYVCK			Carbamidomethyl (C)[10,26]		Mascot

6	dimeric alpha-amylase inhibitor [Triticum aestivum]	gi|386877038	15702.5	5.58	6	250	100	12.85	211	100

Peptide Information

Calc. Mass	Obsrv. Mass	± da	± ppm	Start Seq.	End Seq.	Sequence	Ion Score	C. I. %	Modification	Rank	Result Type
1162.6249	1162.6138	-0.0111	-10	107	117	LTAASITAVCR			Carbamidomethyl (C)[10]		Mascot
1162.6249	1162.6138	-0.0111	-10	107	117	LTAASITAVCR	70	99.985	Carbamidomethyl (C)[10]		Mascot
1554.6637	1554.6335	-0.0302	-19	71	83	CGALYSMLDSMYK			Carbamidomethyl (C)[1], Oxidation (M)[7]		Mascot
1570.8007	1570.7839	-0.0168	-11	43	56	LQCNGSQVPEAVLR			Carbamidomethyl (C)[3]		Mascot
1663.8361	1663.7701	-0.066	-40	118	133	LPIVVDASGDGAYVCK			Carbamidomethyl (C)[15]		Mascot
1663.8361	1663.7701	-0.066	-40	118	133	LPIVVDASGDGAYVCK	141	100	Carbamidomethyl (C)[15]		Mascot
1862.7731	1862.7668	-0.0063	-3	57	70	DCCQQLAHISEWCR			Carbamidomethyl (C)[2,3,13]		Mascot
2807.4431	2807.3105	-0.1326	-47	107	133	LTAASITAVCRLPIVVDAS GDGAYVCK			Carbamidomethyl (C)[10,26]		Mascot

7	Alpha-amylase inhibitor 0.19 [Aegilops tauschii]	gi|475613321	17198.2	6.06	6	248	100	12.85	211	100	

Peptide Information

Calc. Mass	Obsrv. Mass	± da	± ppm	Start Seq.	End Seq.	Sequence	Ion Score	C. I. %	Modification	Rank	Result Type
1162.6249	1162.6138	-0.0111	-10	120	130	LTAASITAVCR			Carbamidomethyl (C)[10]		Mascot
1162.6249	1162.6138	-0.0111	-10	120	130	LTAASITAVCR	70	99.985	Carbamidomethyl (C)[10]		Mascot
1554.6637	1554.6335	-0.0302	-19	84	96	CGALYSMLDSMYK			Carbamidomethyl (C)[1], Oxidation (M)[7]		Mascot
1570.8007	1570.7839	-0.0168	-11	56	69	LQCNGSQVPEAVLR			Carbamidomethyl (C)[3]		Mascot
1663.8361	1663.7701	-0.066	-40	131	146	LPIVVDASGDGAYVCK			Carbamidomethyl (C)[15]		Mascot
1663.8361	1663.7701	-0.066	-40	131	146	LPIVVDASGDGAYVCK	141	100	Carbamidomethyl (C)[15]		Mascot
1862.7731	1862.7668	-0.0063	-3	70	83	DCCQQLAHISEWCR			Carbamidomethyl (C)[2,3,13]		Mascot
2807.4431	2807.3105	-0.1326	-47	120	146	LTAASITAVCRLPIVVDAS GDGAYVCK			Carbamidomethyl (C)[10,26]		Mascot

8	dimeric alpha-amylase inhibitor, partial [Aegilops peregrina]	gi|386877052	13953.6	6.49	5	244	100	14.52	211	100	

Peptide Information

Calc. Mass	Obsrv. Mass	± da	± ppm	Start Seq.	End Seq.	Sequence	Ion Score	C. I. %	Modification	Rank	Result Type
1162.6249	1162.6138	-0.0111	-10	90	100	LTAASITAVCR			Carbamidomethyl (C)[10]		Mascot
1162.6249	1162.6138	-0.0111	-10	90	100	LTAASITAVCR	70	99.985	Carbamidomethyl (C)[10]		Mascot
1570.8007	1570.7839	-0.0168	-11	26	39	LQCNGSQVPEAVLR			Carbamidomethyl (C)[3]		Mascot
1663.8361	1663.7701	-0.066	-40	101	116	LPIVVDASGDGAYVCK			Carbamidomethyl (C)[15]		Mascot
1663.8361	1663.7701	-0.066	-40	101	116	LPIVVDASGDGAYVCK	141	100	Carbamidomethyl (C)[15]		Mascot
1840.7412	1840.724	-0.0172	-9	40	53	DCCQQLADISEWCR			Carbamidomethyl (C)[2,3,13]		Mascot
2807.4431	2807.3105	-0.1326	-47	90	116	LTAASITAVCRLPIVVDAS GDGAYVCK			Carbamidomethyl (C)[10,26]		Mascot

9	dimeric alpha-amylase inhibitor, partial [Aegilops longissima]	gi|386877056	14792	5.28	5	242	100	14.52	211	100	

Protein Group

dimeric alpha-amylase inhibitor, partial [Aegilops longissima]	gi|386877058	14718.9	4.9899 997711 1816

Peptide Information

Calc. Mass	Obsrv. Mass	± da	± ppm	Start Seq.	End Seq.	Sequence	Ion Score	C. I. %	Modification	Rank	Result Type
1162.6249	1162.6138	-0.0111	-10	98	108	LTAASITAVCR			Carbamidomethyl (C)[10]		Mascot
1162.6249	1162.6138	-0.0111	-10	98	108	LTAASITAVCR	70	99.985	Carbamidomethyl (C)[10]		Mascot
1570.8007	1570.7839	-0.0168	-11	34	47	LQCNGSQVPEAVLR			Carbamidomethyl (C)[3]		Mascot

1663.8361	1663.7701	-0.066	-40	109	124	LPIVVDASGDGAYVCK			Carbamidomethyl (C)[15]		Mascot
1663.8361	1663.7701	-0.066	-40	109	124	LPIVVDASGDGAYVCK	141	100	Carbamidomethyl (C)[15]		Mascot
1840.7412	1840.724	-0.0172	-9	48	61	DCCQQLADISEWCR			Carbamidomethyl (C)[2,3,13]		Mascot
2807.4431	2807.3105	-0.1326	-47	98	124	LTAASITAVCRLPIVVDAS GDGAYVCK			Carbamidomethyl (C)[10,26]		Mascot

10	dimeric alpha-amylase inhibitor, partial [Aegilops peregrina]	gi|386877054	14145.7	5.26	4	235	100	9.29	211	100

Peptide Information

Calc. Mass	Obsrv. Mass	± da	± ppm	Start Seq.	End Seq.	Sequence	Ion Score	C. I. %	Modification	Rank	Result Type
1162.6249	1162.6138	-0.0111	-10	92	102	LTAASITAVCR			Carbamidomethyl (C)[10]		Mascot
1162.6249	1162.6138	-0.0111	-10	92	102	LTAASITAVCR	70	99.985	Carbamidomethyl (C)[10]		Mascot
1663.8361	1663.7701	-0.066	-40	103	118	LPIVVDASGDGAYVCK			Carbamidomethyl (C)[15]		Mascot
1663.8361	1663.7701	-0.066	-40	103	118	LPIVVDASGDGAYVCK	141	100	Carbamidomethyl (C)[15]		Mascot
1840.7412	1840.724	-0.0172	-9	42	55	DCCQQLADISEWCR			Carbamidomethyl (C)[2,3,13]		Mascot
2807.4431	2807.3105	-0.1326	-47	92	118	LTAASITAVCRLPIVVDAS GDGAYVCK			Carbamidomethyl (C)[10,26]		Mascot

Gel Idx/Pos	153/G4	Instr./Gel Origin	BA2151/Sample Project 20140814	Process Status	Analysis Succeeded
Plate [#] Name	[1] Sample Project 20140814	Instrument Sample Name		Spectra	11

Rank	Protein Name	Accession No.	Protein MW	Protein PI	Pep. Count	Protein Score	Protein Score C. I. %	Intensity Matched	Total Ion Score	Total Ion C. I. %	Confirmed
1	Alpha-amylase/trypsin inhibitor CM16 [Aegilops tauschii]	gi|475546435	16476.8	5.07	4	237	100	44.455	218	100	

Peptide Information

Calc. Mass	Obsrv. Mass	± da	± ppm	Start Seq.	End Seq.	Sequence	Ion Score	C. I. %	Modification	Rank	Result Type
1168.5051	1168.486	-0.0191	-16	46	54	NYVEEQACR			Carbamidomethyl (C)[8]		Mascot
1168.5051	1168.486	-0.0191	-16	46	54	NYVEEQACR	48	98.054	Carbamidomethyl (C)[8]		Mascot
1215.6443	1215.6046	-0.0397	-33	55	65	IEMPGPPYLAK					Mascot
1231.6392	1231.6036	-0.0356	-29	55	65	IEMPGPPYLAK			Oxidation (M)[3]		Mascot
1799.8528	1799.8123	-0.0405	-23	92	107	SRPDQSGLMELPGCPR			Carbamidomethyl (C)[14]		Mascot
1799.8528	1799.8123	-0.0405	-23	92	107	SRPDQSGLMELPGCPR	41	90.424	Carbamidomethyl (C)[14]		Mascot
1815.8477	1815.7909	-0.0568	-31	92	107	SRPDQSGLMELPGCPR			Carbamidomethyl (C)[14], Oxidation (M)[9]		Mascot
1815.8477	1815.7909	-0.0568	-31	92	107	SRPDQSGLMELPGCPR	28	0	Carbamidomethyl (C)[14], Oxidation (M)[9]		Mascot
1933.8314	1933.7983	-0.0331	-17	66	80	QECCEQLANIPQQCR			Carbamidomethyl (C)[3,4,14]		Mascot
1933.8314	1933.7983	-0.0331	-17	66	80	QECCEQLANIPQQCR	128	100	Carbamidomethyl (C)[3,4,14]		Mascot

Rank	Protein Name	Accession No.	Protein MW	Protein PI	Pep. Count	Protein Score	Protein Score C. I. %	Intensity Matched	Total Ion Score	Total Ion C. I. %	Confirmed
2	RecName: Full=Alpha-amylase/trypsin inhibitor CMb; AltName: Full=Chloroform/methanol-soluble protein CMb; Flags: Precursor	gi|585290	17199.2	5.77	6	95	99.966	13.807	60	99.877	

Peptide Information

Calc. Mass	Obsrv. Mass	± da	± ppm	Start Seq.	End Seq.	Sequence	Ion Score	C. I. %	Modification	Rank	Result Type
801.4076	801.3926	-0.015	-19	86	91	FFMGRK			Oxidation (M)[3]		Mascot
1023.4928	1023.462	-0.0308	-30	108	115	EVQMDFVR					Mascot
1039.4878	1039.4647	-0.0231	-22	108	115	EVQMDFVR			Oxidation (M)[4]		Mascot
1168.5052	1168.486	-0.0192	-16	46	54	DYVEQQACR			Carbamidomethyl (C)[8]		Mascot
1168.5052	1168.486	-0.0192	-16	46	54	DYVEQQACR	19	0	Carbamidomethyl (C)[8]		Mascot
1799.8528	1799.8123	-0.0405	-23	92	107	SRPDQSGLMELPGCPR			Carbamidomethyl (C)[14]		Mascot
1799.8528	1799.8123	-0.0405	-23	92	107	SRPDQSGLMELPGCPR	41	90.424	Carbamidomethyl (C)[14]		Mascot
1815.8477	1815.7909	-0.0568	-31	92	107	SRPDQSGLMELPGCPR			Carbamidomethyl (C)[14], Oxidation (M)[9]		Mascot
1815.8477	1815.7909	-0.0568	-31	92	107	SRPDQSGLMELPGCPR	28	0	Carbamidomethyl (C)[14], Oxidation (M)[9]		Mascot
1861.8102	1861.7733	-0.0369	-20	66	80	QQCCGELANIPQQCR			Carbamidomethyl (C)[3,4,14]		Mascot

Calc. Mass	Obsrv. Mass	± da	± ppm	Start Seq.	End Seq.	Sequence	Ion Score	C. I. %	Modification	Rank	Result Type
1927.9478	1927.7932	-0.1546	-80	91	107	KSRPDQSGLMELPGCPR			Carbamidomethyl (C)[15]		Mascot

3	RecName: Full=Alpha-amylase/trypsin inhibitor CM16; AltName: Full=Chloroform/methanol-soluble protein CM16; Flags: Precursor	gi|123958	16398.8	5.31	4	80	98.908	13.591	60	99.877

Peptide Information

Calc. Mass	Obsrv. Mass	± da	± ppm	Start Seq.	End Seq.	Sequence	Ion Score	C. I. %	Modification	Rank	Result Type
1023.4928	1023.462	-0.0308	-30	108	115	EVQMDFVR					Mascot
1039.4878	1039.4647	-0.0231	-22	108	115	EVQMDFVR			Oxidation (M)[4]		Mascot
1168.5052	1168.486	-0.0192	-16	46	54	DYVEQQACR			Carbamidomethyl (C)[8]		Mascot
1168.5052	1168.486	-0.0192	-16	46	54	DYVEQQACR	19	0	Carbamidomethyl (C)[8]		Mascot
1799.8528	1799.8123	-0.0405	-23	92	107	SRPDQSGLMELPGCPR			Carbamidomethyl (C)[14]		Mascot
1799.8528	1799.8123	-0.0405	-23	92	107	SRPDQSGLMELPGCPR	41	90.424	Carbamidomethyl (C)[14]		Mascot
1815.8477	1815.7909	-0.0568	-31	92	107	SRPDQSGLMELPGCPR			Carbamidomethyl (C)[14], Oxidation (M)[9]		Mascot
1815.8477	1815.7909	-0.0568	-31	92	107	SRPDQSGLMELPGCPR	28	0	Carbamidomethyl (C)[14], Oxidation (M)[9]		Mascot
1861.8102	1861.7733	-0.0369	-20	66	80	QQCCGELANIPQQCR			Carbamidomethyl (C)[3,4,14]		Mascot

4	TPA: putative jumonji-like transcription factor family protein [Zea mays]	gi|414885308	100913	6.83	18	52	0	5.449		

Peptide Information

Calc. Mass	Obsrv. Mass	± da	± ppm	Start Seq.	End Seq.	Sequence	Ion Score	C. I. %	Modification	Rank	Result Type
805.3985	805.3469	-0.0516	-64	188	193	CLNKDR			Carbamidomethyl (C)[1]		Mascot
807.3301	807.3323	0.0022	3	295	300	DQMEER					Mascot
1099.5266	1099.5369	0.0103	9	398	407	ETGLSSYQSK					Mascot
1151.5336	1151.4944	-0.0392	-34	249	258	MNNVSAMWAK					Mascot
1167.5286	1167.6178	0.0892	76	249	258	MNNVSAMWAK			Oxidation (M)[1]		Mascot
1215.7129	1215.6046	-0.1083	-89	445	454	LIKLEMEALR					Mascot
1231.7079	1231.6036	-0.1043	-85	445	454	LIKLEMEALR			Oxidation (M)[6]		Mascot
1734.8706	1734.7881	-0.0825	-48	583	595	MYHNLWPEMLKLK			Oxidation (M)[1,9]		Mascot
1751.7363	1751.8036	0.0673	38	540	554	MWAEVHDANTSSEMK			Oxidation (M)[1]		Mascot
1751.7363	1751.8036	0.0673	38	540	554	MWAEVHDANTSSEMK			Oxidation (M)[1]		Mascot
1773.8112	1773.7592	-0.052	-29	106	124	EEGENVAPSTSGRGGG GGR					Mascot
1798.9375	1798.8446	-0.0929	-52	613	627	TYINSLPFQPYTNLK					Mascot
1857.7612	1857.774	0.0128	7	174	187	EQCPFCRGLCNCTR			Carbamidomethyl (C)[3,6,10,12]		Mascot
1927.7112	1927.7932	0.082	43	342	356	SCSNCSYELCISCCK			Carbamidomethyl (C)[2,5,10,13,14]		Mascot
1955.895	1955.771	-0.124	-63	249	265	MNNVSAMWAKVDTSDV			Oxidation (M)[1,7]		Mascot

Calc. Mass	Obsrv. Mass	± da	± ppm	Start Seq.	End Seq.	Sequence	Ion Score	C. I. %	Modification	Rank	Result Type
						R					
2133.8975	2133.9463	0.0488	23	164	180	YAMMSPDAVREQCPFCR			Carbamidomethyl (C)[13,16], Oxidation (M)[3]		Mascot
2186.1453	2186.0232	-0.1221	-56	308	326	GLQLSELIVEKAVSWNDER					Mascot
2189.0947	2188.9839	-0.1108	-51	644	662	LDMGPKSYIAYGYAEELIR					Mascot
2203.9495	2203.9839	0.0344	16	379	397	GIDYMHGGDCKPPNLENDR			Carbamidomethyl (C)[10], Oxidation (M)[5]		Mascot
2401.1638	2401.0813	-0.0825	-34	238	258	EIGLSFVTTNRMNNVSAMWAK			Oxidation (M)[12,18]		Mascot
2405.0537	2405.0984	0.0447	19	535	554	WEPEKMWAEVHDANTSSEMK					Mascot

5	ATP synthase beta chain, partial (chloroplast) [Chlamydomonas pseudogloeogama]	gi|425895750	43407.7	5.49	11	50	0	3.428	

Peptide Information

Calc. Mass	Obsrv. Mass	± da	± ppm	Start Seq.	End Seq.	Sequence	Ion Score	C. I. %	Modification	Rank	Result Type
801.4689	801.3926	-0.0763	-95	354	360	QTVARAR					Mascot
1215.5198	1215.6046	0.0848	70	143	152	EGNDLYTEMK			Oxidation (M)[9]		Mascot
1321.6239	1321.595	-0.0289	-22	11	22	AVSMQPTDGLMR			Oxidation (M)[4]		Mascot
1788.9136	1788.7603	-0.1533	-86	182	196	MRVALTALTMAEYFR			Oxidation (M)[1]		Mascot
1888.952	1888.8032	-0.1488	-79	167	183	VALVYGQMNEPPGARMR					Mascot
1904.947	1904.8231	-0.1239	-65	167	183	VALVYGQMNEPPGARMR			Oxidation (M)[8]		Mascot
1944.9801	1944.8005	-0.1796	-92	383	400	YTSLAESIDGFTKILTGE					Mascot
2157.092	2156.9851	-0.1069	-50	335	353	ELQDIIAILGLDELSEDDR					Mascot
2236.0737	2235.9932	-0.0805	-36	227	247	MPSAVGYQPTLATEMGGLQER					Mascot
2246.1235	2245.9817	-0.1418	-63	161	181	NLSDSKVALVYGQMNEPPGAR					Mascot
2262.1184	2261.9968	-0.1216	-54	161	181	NLSDSKVALVYGQMNEPPGAR			Oxidation (M)[14]		Mascot
2448.2505	2448.0723	-0.1782	-73	333	353	YKELQDIIAILGLDELSEDDR					Mascot
3039.604	3039.4082	-0.1958	-64	11	39	AVSMQPTDGLMRGMEVIDTGKPLIVPVGK					Mascot

6	TPA: annexin A4 [Zea mays]	gi|414589450	36018.5	8.75	11	49	0	2.784	

Protein Group

LOC100283225 [Zea mays]	gi|226508140	36018.5	8.75

Peptide Information

Calc. Mass	Obsrv. Mass	± da	± ppm	Start Seq.	End Seq.	Sequence	Ion Score	C. I. %	Modification	Rank	Result Type
801.3889	801.3926	0.0037	5	123	128	QTYYAR					Mascot

1345.6934	1345.6583	-0.0351	-26	76	87	AMSLWILDPAGR			Oxidation (M)[2]		Mascot
1770.8545	1770.7838	-0.0707	-40	158	173	YEGPEVDPTIVTHDAK					Mascot
1797.9381	1797.8259	-0.1122	-62	223	238	SETSGNFEFALLTILR					Mascot
1827.9421	1827.7761	-0.166	-91	114	128	TPSQLQIMKQTYYAR					Mascot
1889.9386	1889.812	-0.1266	-67	88	104	DATVLREALNGDTMDLR					Mascot
2133.9561	2133.9463	-0.0098	-5	198	215	SWAHLASVSSAYHHMYD R			Oxidation (M)[15]		Mascot
2236.0696	2235.9932	-0.0764	-34	94	113	EALNGDTMDLRAATEIIC SR			Carbamidomethyl (C)[18]		Mascot
2246.0559	2245.9817	-0.0742	-33	198	216	SWAHLASVSSAYHHMYD RK					Mascot
2260.0928	2259.9941	-0.0987	-44	47	64	GLIQQEYRAMYHEELSH R					Mascot
2262.051	2261.9968	-0.0542	-24	198	216	SWAHLASVSSAYHHMYD RK			Oxidation (M)[15]		Mascot
2276.0876	2276.0083	-0.0793	-35	47	64	GLIQQEYRAMYHEELSH R			Oxidation (M)[10]		Mascot
2290.1238	2290.0178	-0.106	-46	158	177	YEGPEVDPTIVTHDAKDL YK					Mascot

7	RecName: Full=Cysteine-rich receptor-like protein kinase 37; Short=Cysteine-rich RLK37; Flags: Precursor	gi|75338510	74191.3	7.15	13	46	0	2.076

Protein Group

cysteine-rich receptor-like protein kinase 37 [Arabidopsis thaliana]	gi|15233524	74191.3	7.1500 000953 6743
cysteine-rich receptor-like protein kinase 37 [Arabidopsis thaliana]	gi|332656995	74191.3	7.1500 000953 6743

Peptide Information

Calc. Mass	Obsrv. Mass	± da	± ppm	Start Seq.	End Seq.	Sequence	Ion Score	C. I. %	Modification	Rank	Result Type
820.4312	820.3605	-0.0707	-86	255	261	AFDNVVR					Mascot
1039.4514	1039.4647	0.0133	13	221	228	ECVNDFQK			Carbamidomethyl (C)[2]		Mascot
1768.9229	1768.7838	-0.1391	-79	262	278	VPAPPPQASSTIIDYGR					Mascot
1771.9259	1771.7704	-0.1555	-88	470	485	DLKASNILLDAEMNPK					Mascot
1831.8678	1831.7776	-0.0902	-49	87	101	QACKTCLEHVIEDTK			Carbamidomethyl (C)[3,6]		Mascot
1873.8572	1873.8174	-0.0398	-21	486	501	VADFGMARLFDMDETR					Mascot
1875.9231	1875.7806	-0.1425	-76	528	544	SDVYSFGVMLLEMISGK					Mascot
1889.8521	1889.812	-0.0401	-21	486	501	VADFGMARLFDMDETR			Oxidation (M)[6]		Mascot
2262.1248	2261.9968	-0.128	-57	171	191	TLEAASTAENSSVLKYYS ATR					Mascot
2263.0652	2263	-0.0652	-29	549	566	LEKEEEEEEELPAFVWK					Mascot
2276.0725	2276.0083	-0.0642	-28	313	332	QSHTIINDVFDSNNGQSM LR					Mascot
2279.116	2279.0071	-0.1089	-48	473	493	ASNILLDAEMNPKVADFG MAR			Oxidation (M)[10]		Mascot

2807.353	2807.2622	-0.0908	-32	313	336	QSHTIINDVFDSNNGQSMLRFDLR					Mascot
3029.4165	3029.4021	-0.0144	-5	192	217	TEFTQISDVYALMQCVPDLSPGNCKR			Carbamidomethyl (C)[15,24]		Mascot

8	hypothetical protein TRIUR3_13738 [Triticum urartu]	gi|474140435	67934.1	8.83	13	46	0	13.001		

Peptide Information

Calc. Mass	Obsrv. Mass	± da	± ppm	Start Seq.	End Seq.	Sequence	Ion Score	C. I. %	Modification	Rank	Result Type
809.4152	809.3411	-0.0741	-92	366	371	YETQLR					Mascot
820.3696	820.3605	-0.0091	-11	608	614	SQQHPHS					Mascot
1023.5543	1023.462	-0.0923	-90	439	447	ITIEAMAFK					Mascot
1039.5493	1039.4647	-0.0846	-81	439	447	ITIEAMAFK			Oxidation (M)[6]		Mascot
1345.6594	1345.6583	-0.0011	-1	284	295	ELTEVAEDNVAR					Mascot
1544.7738	1544.6913	-0.0825	-53	351	365	VHAVVVGSDMSAQTK			Oxidation (M)[10]		Mascot
1782.9055	1782.859	-0.0465	-26	310	325	SEDLLFAMINSVSRGK			Oxidation (M)[8]		Mascot
1815.9244	1815.7909	-0.1335	-74	418	432	VHACKEMLLDIPYAR			Carbamidomethyl (C)[4]		Mascot
1815.9244	1815.7909	-0.1335	-74	418	432	VHACKEMLLDIPYAR			Carbamidomethyl (C)[4]		Mascot
1831.9193	1831.7776	-0.1417	-77	418	432	VHACKEMLLDIPYAR			Carbamidomethyl (C)[4], Oxidation (M)[7]		Mascot
1842.9353	1842.7883	-0.147	-80	588	602	FMEHHMAERIAVVLK			Oxidation (M)[2,6]		Mascot
1842.9353	1842.7883	-0.147	-80	588	602	FMEHHMAERIAVVLK			Oxidation (M)[2,6]		Mascot
1915.9789	1915.8358	-0.1431	-75	222	235	ILWWIHEMRGHYFK					Mascot
1931.9738	1931.7888	-0.185	-96	222	235	ILWWIHEMRGHYFK			Oxidation (M)[8]		Mascot
1994.9713	1994.9395	-0.0318	-16	83	102	GGPVGPCDTRGDPVDVVAAR			Carbamidomethyl (C)[7]		Mascot
2173.1072	2172.9834	-0.1238	-57	93	115	GDPVDVVAARAGGAASSPLGFMK					Mascot
2189.1021	2188.9839	-0.1182	-54	93	115	GDPVDVVAARAGGAASSPLGFMK			Oxidation (M)[22]		Mascot
2280.1846	2279.9844	-0.2002	-88	241	260	HLPLVAGAMIDSHITVEYWK					Mascot
2280.1846	2279.9844	-0.2002	-88	241	260	HLPLVAGAMIDSHITVEYWK					Mascot
2290.2166	2290.0178	-0.1988	-87	213	230	DNVPQVLPKILWWIHEMR			Oxidation (M)[17]		Mascot

9	uncharacterized protein LOC100305776 [Glycine max]	gi|351723607	15952.5	8.04	4	44	0	9.099	22	0

Peptide Information

Calc. Mass	Obsrv. Mass	± da	± ppm	Start Seq.	End Seq.	Sequence	Ion Score	C. I. %	Modification	Rank	Result Type
1788.8698	1788.7603	-0.1095	-61	19	35	SHMGNFPSSPVSLLSGR			Oxidation (M)[3]		Mascot
1904.9965	1904.8231	-0.1734	-91	129	145	EELFQILNKSASDILGQ					Mascot
1916.9647	1916.7744	-0.1903	-99	18	35	KSHMGNFPSSPVSLLSGR			Oxidation (M)[4]		Mascot

1916.9647	1916.7744	-0.1903	-99	18	35	KSHMGNFPSSPVSLLSG R	22	0	Oxidation (M)[4]		Mascot
2290.1814	2290.0178	-0.1636	-71	39	60	VSFVVKASGESSESSTTL TVFK					Mascot

10	PREDICTED: kinesin-like protein KIN12B-like [Setaria italica]	gi|514815485	125574.2	5.29	19	43	0	43.826	

Peptide Information

Calc. Mass	Obsrv. Mass	± da	± ppm	Start Seq.	End Seq.	Sequence	Ion Score	C. I. %	Modification	Rank	Result Type
805.3773	805.3469	-0.0304	-38	934	940	AMYGHAR					Mascot
851.4225	851.3623	-0.0602	-71	1	6	MEMLRR			Oxidation (M)[1]		Mascot
986.5377	986.469	-0.0687	-70	1005	1012	AQLKEQNR					Mascot
1168.5303	1168.486	-0.0443	-38	883	891	QKWMESESK			Oxidation (M)[4]		Mascot
1168.5303	1168.486	-0.0443	-38	883	891	QKWMESESK			Oxidation (M)[4]		Mascot
1321.6707	1321.595	-0.0757	-57	643	654	NSEVRSSLQSSK					Mascot
1751.8317	1751.8036	-0.0281	-16	394	407	VTFLCSISSEHRCR			Carbamidomethyl (C)[5,13]		Mascot
1751.8317	1751.8036	-0.0281	-16	394	407	VTFLCSISSEHRCR			Carbamidomethyl (C)[5,13]		Mascot
1773.8549	1773.7592	-0.0957	-54	627	642	LRVSESPGDGNVEVCR			Carbamidomethyl (C)[15]		Mascot
1781.7502	1781.8518	0.1016	57	908	921	MHAEKTEMELCNEK			Carbamidomethyl (C)[11], Oxidation (M)[1,8]		Mascot
1782.9093	1782.859	-0.0503	-28	853	868	HQTNVSTNGSLLDQIR					Mascot
1822.8309	1822.7942	-0.0367	-20	772	786	REMELEALCEEQAAK			Carbamidomethyl (C)[9], Oxidation (M)[3]		Mascot
1825.9331	1825.7653	-0.1678	-92	371	385	DDLPYDQSRLTYVLK					Mascot
1876.8607	1876.7734	-0.0873	-47	456	473	SGGTTTCKAGYFSAQNA R			Carbamidomethyl (C)[7]		Mascot
1889.8835	1889.812	-0.0715	-38	568	584	EVANNTNADEDLVSDRK					Mascot
1933.9628	1933.7983	-0.1645	-85	464	480	AGYFSAQNARESLHNLR					Mascot
1933.9628	1933.7983	-0.1645	-85	464	480	AGYFSAQNARESLHNLR					Mascot
1955.9314	1955.771	-0.1604	-82	1070	1085	HEMELETMKVHLAESR			Oxidation (M)[3]		Mascot
2236.0776	2235.9932	-0.0844	-38	386	405	DTLGGNSRVTFLCSISSE HR			Carbamidomethyl (C)[13]		Mascot
2276.1155	2276.0083	-0.1072	-47	112	133	TAPESVAVGDRSFAVDG VLDDR					Mascot
2807.2876	2807.2622	-0.0254	-9	170	195	TYTMWGPLGAMVDSGS DHADRGVVPR			Oxidation (M)[4,11]		Mascot
3039.4954	3039.4082	-0.0872	-29	222	246	CSFLEVHNDQINDLLEPS QRDLQIR			Carbamidomethyl (C)[1]		Mascot

Gel Idx/Pos	154/G5	Instr./Gel Origin	BA2151/Sample Project 20140814	Process Status	Analysis Succeeded
Plate [#] Name	[1] Sample Project 20140814	Instrument Sample Name		Spectra	11

Rank	Protein Name	Accession No.	Protein MW	Protein PI	Pep. Count	Protein Score	Protein Score C. I. %	Intensity Matched	Total Ion Score	Total Ion C. I. %	Confirmed
1	Alpha-amylase/trypsin inhibitor CM16 [Aegilops tauschii]	gi|475546435	16476.8	5.07	4	284	100	27.881	264	100	

Peptide Information

Calc. Mass	Obsrv. Mass	± da	± ppm	Start Seq.	End Seq.	Sequence	Ion Score	C. I. %	Modification	Rank	Result Type
1168.5051	1168.489	-0.0161	-14	46	54	NYVEEQACR			Carbamidomethyl (C)[8]		Mascot
1168.5051	1168.489	-0.0161	-14	46	54	NYVEEQACR	62	99.918	Carbamidomethyl (C)[8]		Mascot
1231.6392	1231.59	-0.0492	-40	55	65	IEMPGPPYLAK			Oxidation (M)[3]		Mascot
1231.6392	1231.59	-0.0492	-40	55	65	IEMPGPPYLAK	34	38.18	Oxidation (M)[3]		Mascot
1799.8528	1799.8337	-0.0191	-11	92	107	SRPDQSGLMELPGCPR			Carbamidomethyl (C)[14]		Mascot
1815.8477	1815.7843	-0.0634	-35	92	107	SRPDQSGLMELPGCPR			Carbamidomethyl (C)[14], Oxidation (M)[9]		Mascot
1815.8477	1815.7843	-0.0634	-35	92	107	SRPDQSGLMELPGCPR	61	99.873	Carbamidomethyl (C)[14], Oxidation (M)[9]		Mascot
1933.8314	1933.8021	-0.0293	-15	66	80	QECCEQLANIPQQCR			Carbamidomethyl (C)[3,4,14]		Mascot
1933.8314	1933.8021	-0.0293	-15	66	80	QECCEQLANIPQQCR	107	100	Carbamidomethyl (C)[3,4,14]		Mascot

Rank	Protein Name	Accession No.	Protein MW	Protein PI	Pep. Count	Protein Score	Protein Score C. I. %	Intensity Matched	Total Ion Score	Total Ion C. I. %	Confirmed
2	RecName: Full=Alpha-amylase/trypsin inhibitor CMb; AltName: Full=Chloroform/methanol-soluble protein CMb; Flags: Precursor	gi|585290	17199.2	5.77	5	111	100	20.323	86	100	

Peptide Information

Calc. Mass	Obsrv. Mass	± da	± ppm	Start Seq.	End Seq.	Sequence	Ion Score	C. I. %	Modification	Rank	Result Type
801.4076	801.3785	-0.0291	-36	86	91	FFMGRK			Oxidation (M)[3]		Mascot
1039.4878	1039.4535	-0.0343	-33	108	115	EVQMDFVR			Oxidation (M)[4]		Mascot
1168.5052	1168.489	-0.0162	-14	46	54	DYVEQQACR			Carbamidomethyl (C)[8]		Mascot
1168.5052	1168.489	-0.0162	-14	46	54	DYVEQQACR	25	0	Carbamidomethyl (C)[8]		Mascot
1799.8528	1799.8337	-0.0191	-11	92	107	SRPDQSGLMELPGCPR			Carbamidomethyl (C)[14]		Mascot
1815.8477	1815.7843	-0.0634	-35	92	107	SRPDQSGLMELPGCPR			Carbamidomethyl (C)[14], Oxidation (M)[9]		Mascot
1815.8477	1815.7843	-0.0634	-35	92	107	SRPDQSGLMELPGCPR	61	99.873	Carbamidomethyl (C)[14], Oxidation (M)[9]		Mascot
1861.8102	1861.7701	-0.0401	-22	66	80	QQCCGELANIPQQCR			Carbamidomethyl (C)[3,4,14]		Mascot

Rank	Protein Name	Accession No.	Protein MW	Protein PI	Pep. Count	Protein Score	Protein Score C. I. %	Intensity Matched	Total Ion Score	Total Ion C. I. %	Confirmed
3	RecName: Full=Alpha-amylase/trypsin inhibitor CM16; AltName: Full=Chloroform/methanol-soluble protein CM16; Flags: Precursor	gi|123958	16398.8	5.31	4	105	99.997	20.146	86	100	

Peptide Information

Calc. Mass	Obsrv. Mass	± da	± ppm	Start Seq.	End Seq.	Sequence	Ion Score	C. I. %	Modification	Rank	Result Type
1039.4878	1039.4535	-0.0343	-33	108	115	EVQMDFVR			Oxidation (M)[4]		Mascot
1168.5052	1168.489	-0.0162	-14	46	54	DYVEQQACR			Carbamidomethyl (C)[8]		Mascot
1168.5052	1168.489	-0.0162	-14	46	54	DYVEQQACR	25	0	Carbamidomethyl (C)[8]		Mascot
1799.8528	1799.8337	-0.0191	-11	92	107	SRPDQSGLMELPGCPR			Carbamidomethyl (C)[14]		Mascot
1815.8477	1815.7843	-0.0634	-35	92	107	SRPDQSGLMELPGCPR			Carbamidomethyl (C)[14], Oxidation (M)[9]		Mascot
1815.8477	1815.7843	-0.0634	-35	92	107	SRPDQSGLMELPGCPR	61	99.873	Carbamidomethyl (C)[14], Oxidation (M)[9]		Mascot
1861.8102	1861.7701	-0.0401	-22	66	80	QQCCGELANIPQQCR			Carbamidomethyl (C)[3,4,14]		Mascot

4	PREDICTED: putative B3 domain-containing protein Os04g0347400-like [Setaria italica]	gi|514806063	27610.9	7.11	10	48	0	2.291	

Peptide Information

Calc. Mass	Obsrv. Mass	± da	± ppm	Start Seq.	End Seq.	Sequence	Ion Score	C. I. %	Modification	Rank	Result Type
856.5112	856.5094	-0.0018	-2	37	43	RQGQVLR					Mascot
993.5701	993.475	-0.0951	-96	38	45	QGQVLRHR					Mascot
1068.5547	1068.4811	-0.0736	-69	93	101	VFPKDFMGK					Mascot
1106.5702	1106.4965	-0.0737	-67	2	12	TSPAAAAQHPR					Mascot
1107.4888	1107.5171	0.0283	26	180	188	VFEPDGCQR			Carbamidomethyl (C)[7]		Mascot
1234.6096	1234.6041	-0.0055	-4	194	203	DIKMQQSEQK					Mascot
1253.6056	1253.5657	-0.0399	-32	1	12	MTSPAAAAQHPR			Oxidation (M)[1]		Mascot
1734.9432	1734.7842	-0.159	-92	1	16	MTSPAAAAQHPRVLLR			Oxidation (M)[1]		Mascot
2246.0903	2245.98	-0.1103	-49	222	242	MNVLEASSSNTCHNGIAAIKL			Carbamidomethyl (C)[12], Oxidation (M)[1]		Mascot
2276.0476	2275.9841	-0.0635	-28	170	188	YEGNMVFTVKVFEPDGCQR			Carbamidomethyl (C)[17]		Mascot

5	hypothetical protein TRIUR3_08536 [Triticum urartu]	gi|474407060	49568.3	5.63	14	48	0	6.649	

Peptide Information

Calc. Mass	Obsrv. Mass	± da	± ppm	Start Seq.	End Seq.	Sequence	Ion Score	C. I. %	Modification	Rank	Result Type
808.3505	808.3455	-0.005	-6	217	223	EMAEQGK			Oxidation (M)[2]		Mascot
836.3632	836.3044	-0.0588	-70	428	435	GEGTEDTK					Mascot
890.3962	890.371	-0.0252	-28	341	349	EATGDAGNR					Mascot
908.4142	908.384	-0.0302	-33	359	367	DATGAMAQK			Oxidation (M)[6]		Mascot
909.4676	909.3979	-0.0697	-77	368	376	AGDTAAYIK					Mascot
1039.4547	1039.4535	-0.0012	-1	268	276	DMTAQTMNK					Mascot

Calc. Mass	Obsrv. Mass	± da	± ppm	Start Seq.	End Seq.	Sequence	Ion Score	C. I. %	Modification	Rank	Result Type
1054.447	1054.4471	0.0001	0	330	338	DTTEQTMGR			Oxidation (M)[7]		Mascot
1193.5468	1193.5889	0.0421	35	377	389	DSVMGAAGGAVDK			Oxidation (M)[4]		Mascot
1253.5791	1253.5657	-0.0134	-11	330	340	DTTEQTMGRAK			Oxidation (M)[7]		Mascot
1797.847	1797.8691	0.0221	12	350	367	TGSMAAQVKDATGAMA QK			Oxidation (M)[4,15]		Mascot
1827.8575	1827.7661	-0.0914	-50	288	305	TGSMAAQVKDTTGAMAQ K			Oxidation (M)[4,15]		Mascot
1842.8903	1842.7971	-0.0932	-51	297	314	DTTGAMAQKATDTAAYV K					Mascot
1842.9154	1842.7971	-0.1183	-64	119	135	AKEVTLTTGEMTAEYAK					Mascot
2157.0745	2156.989	-0.0855	-40	121	140	EVTLTTGEMTAEYAKQA AVK			Oxidation (M)[9]		Mascot

6	conserved hypothetical protein [Ricinus communis]	gi|223545807	27687.7	5.22	9	46	0	9.756

Peptide Information

Calc. Mass	Obsrv. Mass	± da	± ppm	Start Seq.	End Seq.	Sequence	Ion Score	C. I. %	Modification	Rank	Result Type
823.3832	823.3573	-0.0259	-31	68	74	YEPESAK					Mascot
906.4832	906.4412	-0.042	-46	239	245	VLRGEWF					Mascot
1105.5233	1105.4919	-0.0314	-28	185	196	ATDGSADAGKGR					Mascot
1320.6688	1320.5688	-0.1	-76	1	12	MEQSRLAAAATR			Oxidation (M)[1]		Mascot
1770.9246	1770.7762	-0.1484	-84	92	108	GTEALTPPRPPHASSPR					Mascot
1831.8192	1831.7676	-0.0516	-28	76	91	DVESEAETEYRTTSSK					Mascot
1905.9076	1905.9261	0.0185	10	51	67	ELKPAVYSGDPEETENK					Mascot
1933.757	1933.8021	0.0451	23	149	163	EKGEEEDEDENEYYR					Mascot
1933.757	1933.8021	0.0451	23	149	163	EKGEEEDEDENEYYR					Mascot
2246.0645	2245.98	-0.0845	-38	197	215	DVIGWLPEQLETAEETME R					Mascot

7	Ubiquitin carboxyl-terminal hydrolase isozyme L5 [Aegilops tauschii]	gi|475606717	44680.1	5.74	12	45	0	4.522

Peptide Information

Calc. Mass	Obsrv. Mass	± da	± ppm	Start Seq.	End Seq.	Sequence	Ion Score	C. I. %	Modification	Rank	Result Type
856.4886	856.5094	0.0208	24	118	124	DERPVIK					Mascot
1099.5929	1099.5061	-0.0868	-79	252	260	MVQPVIQER					Mascot
1106.5762	1106.4965	-0.0797	-72	332	340	IIMEEEKAK			Oxidation (M)[3]		Mascot
1118.5782	1118.4944	-0.0838	-75	2	9	KGWWWEVK					Mascot
1182.63	1182.5389	-0.0911	-77	271	280	FSVMAITKNR			Oxidation (M)[4]		Mascot
1231.6464	1231.59	-0.0564	-46	178	188	GLAIVNCESIR			Carbamidomethyl (C)[7]		Mascot
1231.6464	1231.59	-0.0564	-46	178	188	GLAIVNCESIR			Carbamidomethyl (C)[7]		Mascot

1265.6136	1265.582	-0.0316	-25	1	9	MKGWWWEVK			Oxidation (M)[1]		Mascot
1320.6075	1320.5688	-0.0387	-29	240	251	CPGGIGEMGWLK			Carbamidomethyl (C)[1], Oxidation (M)[8]		Mascot
1649.9122	1649.8076	-0.1046	-63	112	124	WRPPEKDERPVIK					Mascot
1770.9208	1770.7762	-0.1446	-82	264	278	FSQNEIRFSVMAITK					Mascot
2098.042	2097.9436	-0.0984	-47	178	196	GLAIVNCESIRMTSNSFA K			Carbamidomethyl (C)[7]		Mascot
2248.2007	2248.0044	-0.1963	-87	64	82	ELAMDRGVFTELLQQLQ LK			Oxidation (M)[4]		Mascot

8	PREDICTED: villin-4-like [Fragaria vesca subsp. vesca]	gi|470120775	107398.8	5.87	18	44	0	24.86

Peptide Information

Calc. Mass	Obsrv. Mass	± da	± ppm	Start Seq.	End Seq.	Sequence	Ion Score	C. I. %	Modification	Rank	Result Type
801.3923	801.3785	-0.0138	-17	927	933	EHLGMAK			Oxidation (M)[5]		Mascot
828.3226	828.3355	0.0129	16	866	872	ETCMGSK			Carbamidomethyl (C)[3], Oxidation (M)[4]		Mascot
870.4791	870.5258	0.0467	54	844	850	KENNIPR					Mascot
1107.4987	1107.5171	0.0184	17	208	217	CDIASIEDGK			Carbamidomethyl (C)[1]		Mascot
1127.5878	1127.4869	-0.1009	-89	802	811	NLSTPPPMVR			Oxidation (M)[8]		Mascot
1168.5898	1168.489	-0.1008	-86	63	71	HDIHYWLGK					Mascot
1168.5898	1168.489	-0.1008	-86	63	71	HDIHYWLGK					Mascot
1193.5117	1193.5889	0.0772	65	723	732	SNMHGNSFQR			Oxidation (M)[3]		Mascot
1265.6736	1265.582	-0.0916	-72	831	843	SSAIAALTAGFEK					Mascot
1785.8589	1785.7701	-0.0888	-50	26	41	IENFCPVSVPQSSHGK			Carbamidomethyl (C)[5]		Mascot
1799.8857	1799.8337	-0.052	-29	2	18	AVSMRDLDPAFHGAGQK					Mascot
1815.8807	1815.7843	-0.0964	-53	2	18	AVSMRDLDPAFHGAGQK			Oxidation (M)[4]		Mascot
1815.8807	1815.7843	-0.0964	-53	2	18	AVSMRDLDPAFHGAGQK			Oxidation (M)[4]		Mascot
1823.9248	1823.8304	-0.0944	-52	42	57	FFMGDSYVILKTTASK			Oxidation (M)[3]		Mascot
1825.8352	1825.7783	-0.0569	-31	884	899	EGEAEDEGLPVHPYQR					Mascot
1825.9192	1825.7783	-0.1409	-77	176	191	IFQFNGSNSSIQERAK					Mascot
1841.9141	1841.8524	-0.0617	-33	174	189	SKIFQFNGSNSSIQER					Mascot
1853.8552	1853.7834	-0.0718	-39	508	523	EVPDDTYQEDGVALFR					Mascot
1915.9971	1915.83	-0.1671	-87	264	281	AEPVGADSLTRELLETSK					Mascot
1962.9161	1962.8292	-0.0869	-44	1	18	MAVSMRDLDPAFHGAG QK			Oxidation (M)[1,5]		Mascot
2264.189	2263.9922	-0.1968	-87	451	472	ASAISLASNMVASMKFLP AQAR					Mascot
2280.1838	2279.9905	-0.1933	-85	451	472	ASAISLASNMVASMKFLP AQAR			Oxidation (M)[10]		Mascot
2280.1838	2279.9905	-0.1933	-85	451	472	ASAISLASNMVASMKFLP AQAR			Oxidation (M)[10]		Mascot

9	hypothetical protein PRUPE_ppa017833mg [Prunus persica]	gi|462422012	80635	8.01	15	44	0	5.796

Peptide Information

Calc. Mass	Obsrv. Mass	± da	± ppm	Start Seq.	End Seq.	Sequence	Ion Score	C. I. %	Modification	Rank	Result Type
906.405	906.4412	0.0362	40	445	451	EKEDEEK					Mascot
908.389	908.384	-0.005	-6	403	411	ASCSGGDVR			Carbamidomethyl (C)[3]		Mascot
1105.5857	1105.4919	-0.0938	-85	60	69	GMSTMPRVVK					Mascot
1150.5641	1150.4761	-0.088	-76	186	195	AQWDAFVASR					Mascot
1771.7778	1771.7725	-0.0053	-3	574	588	TDGEQIFMMPYNPGR			Oxidation (M)[8]		Mascot
1838.884	1838.8573	-0.0267	-15	456	472	VIEVGDYSNMEAPSSLK					Mascot
1859.932	1859.7787	-0.1533	-82	473	487	TLCRFLETTHLPEDK			Carbamidomethyl (C)[3]		Mascot
1905.9303	1905.9261	-0.0042	-2	658	673	DIIMDPSLGFEYKYAK			Oxidation (M)[4]		Mascot
1961.95	1961.8278	-0.1222	-62	655	670	FMRDIIMDPSLGFEYK					Mascot
1964.9495	1964.808	-0.1415	-72	284	302	VDGSNDVLTMALGPEHP GR					Mascot
2157.0105	2156.989	-0.0215	-10	571	588	LQKTDGEQIFMMPYNPG R			Oxidation (M)[11,12]		Mascot
2188.1292	2187.9866	-0.1426	-65	87	106	GVGQAHIEMQSYIGVLAR SR			Oxidation (M)[9]		Mascot
2188.9729	2188.9819	0.009	4	36	56	FASSSAETEPTSQTTISD DSK					Mascot
2264.0752	2263.9922	-0.083	-37	224	243	GYAGLEDQLEETMPVVEI DR					Mascot
2276.1809	2275.9841	-0.1968	-86	477	495	FLETTHLPEDKTLQFTIDK					Mascot
2280.0701	2279.9905	-0.0796	-35	224	243	GYAGLEDQLEETMPVVEI DR			Oxidation (M)[13]		Mascot
2280.0701	2279.9905	-0.0796	-35	224	243	GYAGLEDQLEETMPVVEI DR			Oxidation (M)[13]		Mascot

10	root border cell-specific protein [Zea mays]	gi|226531694	37097	8.73	10	44	0	9.215

Peptide Information

Calc. Mass	Obsrv. Mass	± da	± ppm	Start Seq.	End Seq.	Sequence	Ion Score	C. I. %	Modification	Rank	Result Type
800.3533	800.3393	-0.014	-17	262	268	DHANDTK					Mascot
909.4676	909.3979	-0.0697	-77	296	304	AGYDGSVLK					Mascot
914.4941	914.4219	-0.0722	-79	93	100	LSPAEEIR					Mascot
993.5146	993.475	-0.0396	-40	101	108	TIMDRSVR			Oxidation (M)[3]		Mascot
1118.6028	1118.4944	-0.1084	-97	1	10	MPFLLTPGAR			Oxidation (M)[1]		Mascot
1932.9413	1932.7833	-0.158	-82	279	295	VDFAYMLDVDSLGFNVK					Mascot
1933.9535	1933.8021	-0.1514	-78	321	338	TLIVEMLQAARASSSDPE			Oxidation (M)[6]		Mascot
1933.9535	1933.8021	-0.1514	-78	321	338	TLIVEMLQAARASSSDPE			Oxidation (M)[6]		Mascot
2098.1477	2097.9436	-0.2041	-97	1	20	MPFLLTPGARAVPSPSTL SR					Mascot

2246.1123	2245.98	-0.1323	-59	242	261	EAKVDPISQFSTPITSHM NK	Oxidation (M)[18]	Mascot
2839.386	2839.2646	-0.1214	-43	279	304	VDFAYMLDVDSLGFNVK AGYDGSVLK	Oxidation (M)[6]	Mascot

Gel Idx/Pos	155/G6	Instr./Gel Origin	BA2151/Sample Project 20140814	Process Status	Analysis Succeeded
Plate [#] Name	[1] Sample Project 20140814	Instrument Sample Name		Spectra	11

Rank	Protein Name	Accession No.	Protein MW	Protein PI	Pep. Count	Protein Score	Protein Score C. I. %	Intensity Matched	Total Ion Score	Total Ion C. I. %	Confirmed
1	hypothetical protein ZEAMMB73_025977 [Zea mays]	gi|413945715	26473.3	5.43	5	24	0	.684			

Peptide Information

Calc. Mass	Obsrv. Mass	± da	± ppm	Start Seq.	End Seq.	Sequence	Ion Score	C. I. %	Modification	Rank	Result Type
807.4247	807.3845	-0.0402	-50	165	171	FAAIEEK					Mascot
1308.6001	1308.64	0.0399	30	51	61	QGMDHYANKTK			Oxidation (M)[3]		Mascot
1320.6113	1320.5625	-0.0488	-37	49	59	TRQGMDHYANK					Mascot
1838.8596	1838.8875	0.0279	15	29	43	SFPYYDDLSKLYDGR					Mascot
1927.9042	1927.8556	-0.0486	-25	1	17	MMVEAPDSVWASFAARK			Oxidation (M)[1,2]		Mascot

Rank	Protein Name	Accession No.	Protein MW	Protein PI	Pep. Count	Protein Score	Protein Score C. I. %	Intensity Matched	Total Ion Score	Total Ion C. I. %	Confirmed
2	hypothetical protein ZEAMMB73_937976 [Zea mays]	gi|413921014	7138.3	8.5	3	22	0	.36			

Peptide Information

Calc. Mass	Obsrv. Mass	± da	± ppm	Start Seq.	End Seq.	Sequence	Ion Score	C. I. %	Modification	Rank	Result Type
959.3896	959.3253	-0.0643	-67	1	7	MYCCGLR			Carbamidomethyl (C)[3,4]		Mascot
1194.5712	1194.6129	0.0417	35	48	57	MQFDSLDLIP			Oxidation (M)[1]		Mascot
1927.85	1927.8556	0.0056	3	43	57	CWCSRMQFDSLDLIP			Carbamidomethyl (C)[1,3]		Mascot

Rank	Protein Name	Accession No.	Protein MW	Protein PI	Pep. Count	Protein Score	Protein Score C. I. %	Intensity Matched	Total Ion Score	Total Ion C. I. %	Confirmed
3	ribosomal protein S15 [Vitis vinifera]	gi|224365635	10495	11.42	3	21	0	.663			

Protein Group

RecName: Full=30S ribosomal protein S15, chloroplastic	gi|118595719	10495	11.420 000076 2939
ribosomal protein S15 [Vitis vinifera]	gi|91984050	10495	11.420 000076 2939

Peptide Information

Calc. Mass	Obsrv. Mass	± da	± ppm	Start Seq.	End Seq.	Sequence	Ion Score	C. I. %	Modification	Rank	Result Type
1107.5906	1107.5387	-0.0519	-47	44	52	DYLSQRGLR					Mascot
1618.8911	1619.0524	0.1613	100	4	17	NLFISVISQKEANR					Mascot
1959.9559	1960.0977	0.1418	72	14	30	EANRGSVEFQVFSFTNK					Mascot

Rank	Protein Name	Accession No.	Protein MW	Protein PI	Pep. Count	Protein Score	Protein Score C. I. %	Intensity Matched	Total Ion Score	Total Ion C. I. %	Confirmed
4	uncharacterized protein [Arabidopsis thaliana]	gi|186510515	6685.5	8.64	3	20	0	.471			

Protein Group

uncharacterized protein AT3G27968 [Arabidopsis thaliana]	gi|332643866	6685.5	8.6400 003433 2275

Peptide Information

Calc. Mass	Obsrv. Mass	± da	± ppm	Start Seq.	End Seq.	Sequence	Ion Score	C. I. %	Modification	Rank	Result Type
1194.6915	1194.6129	-0.0786	-66	2	12	IVVSYGLGKMK					Mascot
1838.9357	1838.8875	-0.0482	-26	17	30	LYEMIEDYLLKQHK			Oxidation (M)[4]		Mascot
1927.8062	1927.8556	0.0494	26	42	56	RNCNTLGYEDLECHF			Carbamidomethyl (C)[3,13]		Mascot

5	PREDICTED: uncharacterized protein LOC101290864 [Fragaria vesca subsp. vesca]	gi|470127683	43108.1	9.19	6	20	0	8.197	

Peptide Information

Calc. Mass	Obsrv. Mass	± da	± ppm	Start Seq.	End Seq.	Sequence	Ion Score	C. I. %	Modification	Rank	Result Type
807.3366	807.3845	0.0479	59	275	281	EDESAEK					Mascot
856.4457	856.5172	0.0715	83	1	7	MPKSHTR					Mascot
889.321	889.3167	-0.0043	-5	376	382	FNYSDED					Mascot
931.3647	931.2901	-0.0746	-80	92	98	YSEMCNK			Carbamidomethyl (C)[5]		Mascot
931.3647	931.2901	-0.0746	-80	92	98	YSEMCNK			Carbamidomethyl (C)[5]		Mascot
1107.5165	1107.5387	0.0222	20	167	176	DVGDSETEKK					Mascot
1320.5914	1320.5625	-0.0289	-22	242	253	ISDVDATDNENK					Mascot

6	ribosomal protein S15 (chloroplast) [Trochodendron aralioides]	gi|479279353	10809.1	10.88	3	20	0	.903	

Protein Group

ribosomal protein S15 (chloroplast) [Trochodendron aralioides]	gi|511348588	10809.1	10.880 000114 4409

Peptide Information

Calc. Mass	Obsrv. Mass	± da	± ppm	Start Seq.	End Seq.	Sequence	Ion Score	C. I. %	Modification	Rank	Result Type
870.5043	870.5399	0.0356	41	82	88	LDIREPK					Mascot
1107.5906	1107.5387	-0.0519	-47	46	54	DYLSQRGLR					Mascot
1618.9163	1619.0524	0.1361	84	2	15	VKNAFITIISQEQK					Mascot

7	RecName: Full=Desiccation stress protein DSP-22, chloroplastic; Flags: Precursor	gi|461965	22091.2	8.61	4	19	0	.982	

Peptide Information

Calc. Mass	Obsrv. Mass	± da	± ppm	Start	End	Sequence	Ion	C. I. %	Modification	Rank	Result Type

				Seq.	Seq.		Score				
917.4323	917.3455	-0.0868	-95	59	65	EEQQQQK					Mascot
1308.6729	1308.64	-0.0329	-25	34	43	QSYELPLMRR			Oxidation (M)[8]		Mascot
1788.9604	1789.0601	0.0997	56	177	192	FAMIGLVALAFTEYVK			Oxidation (M)[3]		Mascot
1838.754	1838.8875	0.1335	73	1	16	MASSTCYATIPAMSCR			Carbamidomethyl (C)[6,15], Oxidation (M)[1,13]		Mascot

8	hypothetical protein ZEAMMB73_777913 [Zea mays]	gi|413942918	4388.2	8.76	2	19	0	.218

Peptide Information

Calc. Mass	Obsrv. Mass	± da	± ppm	Start Seq.	End Seq.	Sequence	Ion Score	C. I. %	Modification	Rank	Result Type
1107.5901	1107.5387	-0.0514	-46	19	28	SCMIATILAK			Carbamidomethyl (C)[2]		Mascot
2399.1697	2399.0247	-0.145	-60	19	38	SCMIATILAKYSCTLYFDIL			Carbamidomethyl (C)[2,13], Oxidation (M)[3]		Mascot

9	RecName: Full=Bifunctional chitinase/lysozyme; Includes: RecName: Full=Chitinase; Includes: RecName: Full=Lysozyme	gi|3121859	2875.5	9.98	2	19	0	.633

Peptide Information

Calc. Mass	Obsrv. Mass	± da	± ppm	Start Seq.	End Seq.	Sequence	Ion Score	C. I. %	Modification	Rank	Result Type
999.4637	999.4119	-0.0518	-52	9	16	SMFDQMLK					Mascot
1308.6188	1308.64	0.0212	16	9	18	SMFDQMLKHR			Oxidation (M)[2]		Mascot

10	RecName: Full=Putative defensin-like protein 237; AltName: Full=Putative S locus cysteine-rich-like protein 21; Short=Protein SCRL21; Short=SCR-like protein 21; Flags: Precursor	gi|254763282	11641.6	9.2	3	19	0	.519

Peptide Information

Calc. Mass	Obsrv. Mass	± da	± ppm	Start Seq.	End Seq.	Sequence	Ion Score	C. I. %	Modification	Rank	Result Type
807.3777	807.3845	0.0068	8	40	46	CGTDRAK			Carbamidomethyl (C)[1]		Mascot
1107.4484	1107.5387	0.0903	82	71	79	CDDDRQGNK			Carbamidomethyl (C)[1]		Mascot
1838.812	1838.8875	0.0755	41	61	75	QVLNTISSCRCDDDR			Carbamidomethyl (C)[9,11]		Mascot

Gel Idx/Pos	156/G7	Instr./Gel Origin	BA2151/Sample Project 20140814	Process Status	Analysis Succeeded
Plate [#] Name	[1] Sample Project 20140814	Instrument Sample Name		Spectra	11

Rank	Protein Name	Accession No.	Protein MW	Protein PI	Pep. Count	Protein Score	Protein Score C. I. %	Intensity Matched	Total Ion Score	Total Ion C. I. %	Confirmed
1	hypothetical protein ARALYDRAFT_901556 [Arabidopsis lyrata subsp. lyrata]	gi|297324883	248110.8	6.27	35	69	87.168	14.899			

Peptide Information

Calc. Mass	Obsrv. Mass	± da	± ppm	Start Seq.	End Seq.	Sequence	Ion Score	C. I. %	Modification	Rank	Result Type
822.4542	822.3892	-0.065	-79	1223	1228	WKMTLK			Oxidation (M)[3]		Mascot
826.4821	826.4093	-0.0728	-88	1059	1064	KFYLQK					Mascot
829.4526	829.3951	-0.0575	-69	1164	1170	ENRPSVK					Mascot
848.3897	848.4283	0.0386	45	1399	1405	NTQDGWK					Mascot
852.3702	852.399	0.0288	34	697	703	EAGICMR			Carbamidomethyl (C)[5], Oxidation (M)[6]		Mascot
856.4345	856.5104	0.0759	89	1725	1731	LLHDACK			Carbamidomethyl (C)[6]		Mascot
870.4719	870.5199	0.048	55	1310	1316	VFYINSK					Mascot
886.488	886.4082	-0.0798	-90	2029	2036	DPALLTEK					Mascot
888.4727	888.4403	-0.0324	-36	620	625	LEWVWR					Mascot
896.4836	896.4196	-0.064	-71	1068	1075	ISSYTGIR					Mascot
982.5278	982.4394	-0.0884	-90	1922	1929	FISMIVEK			Oxidation (M)[4]		Mascot
1027.5571	1027.4839	-0.0732	-71	1871	1878	HLPEYIQK					Mascot
1058.5518	1058.5005	-0.0513	-48	720	727	YEYKTLNK					Mascot
1141.5498	1141.4553	-0.0945	-83	1397	1405	HRNTQDGWK					Mascot
1229.6677	1229.5732	-0.0945	-77	67	76	FRPYLYAATK					Mascot
1323.6614	1323.6398	-0.0216	-16	1450	1461	MSVVVVNPYGDK			Oxidation (M)[1]		Mascot
1365.7195	1365.6191	-0.1004	-74	1011	1022	RLADFLGDTMVK					Mascot
1381.7145	1381.6163	-0.0982	-71	1011	1022	RLADFLGDTMVK			Oxidation (M)[10]		Mascot
1479.6931	1479.7128	0.0197	13	2052	2063	EQMENSLVQMVR			Oxidation (M)[3]		Mascot
1493.7272	1493.7051	-0.0221	-15	1209	1220	NVDYQGWLELEK					Mascot
1529.821	1529.7037	-0.1173	-77	1045	1058	AVPVAIFKTDDPEK					Mascot
1542.8275	1542.7236	-0.1039	-67	91	103	YQTQVADIQIVHK					Mascot
1628.9292	1628.8151	-0.1141	-70	891	905	AMIIPASKEEGILIK			Oxidation (M)[2]		Mascot
1738.868	1738.8005	-0.0675	-39	1130	1145	IVDMFSSANKDGVLDK					Mascot
1796.8612	1796.92	0.0588	33	353	366	AKFVCHLDCFSWVK			Carbamidomethyl (C)[5,9]		Mascot
1801.8533	1801.845	-0.0083	-5	450	465	GSGTLCEMLLMVEAYK			Carbamidomethyl (C)[6]		Mascot

1801.8533	1801.845	-0.0083	-5	450	465	GSGTLCEMLLMVEAYK			Carbamidomethyl (C)[6]		Mascot
1805.948	1805.8442	-0.1038	-57	1569	1583	IAVFRCASAIQWLDR			Carbamidomethyl (C)[6]		Mascot
1817.8483	1817.8295	-0.0188	-10	450	465	GSGTLCEMLLMVEAYK			Carbamidomethyl (C)[6], Oxidation (M)[8]		Mascot
1829.8811	1829.8708	-0.0103	-6	163	177	EQRPQDCLDSVVDLR			Carbamidomethyl (C)[7]		Mascot
1833.8431	1833.8386	-0.0045	-2	450	465	GSGTLCEMLLMVEAYK			Carbamidomethyl (C)[6], Oxidation (M)[8,11]		Mascot
1833.8431	1833.8386	-0.0045	-2	450	465	GSGTLCEMLLMVEAYK			Carbamidomethyl (C)[6], Oxidation (M)[8,11]		Mascot
1838.9722	1838.8856	-0.0866	-47	840	855	FTISYPCVILNVDVAK			Carbamidomethyl (C)[7]		Mascot
1890.9304	1890.8982	-0.0322	-17	856	871	NNSNDQYQTLVDPVRK					Mascot
1927.0219	1926.8374	-0.1845	-96	1750	1766	SPSSKLHDPALHLMLHK			Oxidation (M)[14]		Mascot
1929.9482	1929.907	-0.0412	-21	449	465	KGSGTLCEMLLMVEAYK			Carbamidomethyl (C)[7]		Mascot
1961.9381	1961.9165	-0.0216	-11	449	465	KGSGTLCEMLLMVEAYK			Carbamidomethyl (C)[7], Oxidation (M)[9,12]		Mascot
1961.9637	1961.9165	-0.0472	-24	384	400	AKLGYDPLEVNPEDMVR			Oxidation (M)[15]		Mascot
1967.0671	1966.9036	-0.1635	-83	839	855	KFTISYPCVILNVDVAK			Carbamidomethyl (C)[8]		Mascot
2717.2771	2717.0457	-0.2314	-85	1930	1954	IGNIRSHMMDINVSDASW ASGQAPK			Oxidation (M)[8,9]		Mascot
3052.5125	3052.573	0.0605	20	807	833	IGKPLELDTDGIWCALPG SFPENFTFK			Carbamidomethyl (C)[14]		Mascot

2 predicted protein [Arabidopsis lyrata subsp. lyrata] gi|297329688 71528.3 5.24 20 68 83.846 4.795

Peptide Information

Calc. Mass	Obsrv. Mass	± da	± ppm	Start Seq.	End Seq.	Sequence	Ion Score	C. I. %	Modification	Rank	Result Type
835.4494	835.4028	-0.0466	-56	476	481	LKNMWK			Oxidation (M)[4]		Mascot
841.4526	841.4343	-0.0183	-22	322	328	LHSSEIR					Mascot
847.4341	847.4329	-0.0012	-1	144	150	LGEIEMR					Mascot
859.4883	859.4316	-0.0567	-66	315	321	NVEEKLK					Mascot
863.4291	863.429	-0.0001	0	144	150	LGEIEMR			Oxidation (M)[6]		Mascot
914.4703	914.3859	-0.0844	-92	289	295	HHHSQLR					Mascot
1033.5525	1033.4937	-0.0588	-57	311	319	SISKNVEEK					Mascot
1058.5841	1058.5005	-0.0836	-79	422	430	NILQVTNEK					Mascot
1068.5758	1068.4973	-0.0785	-73	514	522	MKVEYAAIK			Oxidation (M)[1]		Mascot
1201.6423	1201.5826	-0.0597	-50	164	174	LNDTLQADLAK					Mascot
1316.6328	1316.5868	-0.046	-35	2	12	DLNLDENENLK					Mascot
1357.7434	1357.6669	-0.0765	-56	163	174	RLNDTLQADLAK					Mascot
1460.7228	1460.7269	0.0041	3	375	388	GDTSEALAQALQEK					Mascot
1475.7522	1475.7263	-0.0259	-18	361	374	LAEASIANIMAESR					Mascot
1493.7496	1493.7051	-0.0445	-30	483	494	SYINRWIDPSSR					Mascot
1543.7711	1543.7606	-0.0105	-7	2	14	DLNLDENENLKAR					Mascot

Calc. Mass	Obsrv. Mass	± da	± ppm	Start Seq.	End Seq.	Sequence	Ion Score	C. I. %	Modification	Rank	Result Type
1674.8115	1674.7028	-0.1087	-65	1	14	MDLNLDENENLKAR					Mascot
1699.8762	1699.7977	-0.0785	-46	75	88	HNLQEELAEAYRVK					Mascot
1707.8912	1707.7456	-0.1456	-85	175	188	LEEQTRTYAQVIEK					Mascot
1799.9188	1799.8578	-0.061	-34	108	123	QVRFFQGSVAAAFSER					Mascot
2904.3914	2904.3218	-0.0696	-24	28	52	DIEQLCMQQGGPSILGVA TRMHFQR			Carbamidomethyl (C)[6], Oxidation (M)[7,21]		Mascot

3	hypothetical protein F775_00962 [Aegilops tauschii]	gi|475540576	66614.5	5.22	17	65	69.92	6.257	

Peptide Information

Calc. Mass	Obsrv. Mass	± da	± ppm	Start Seq.	End Seq.	Sequence	Ion Score	C. I. %	Modification	Rank	Result Type
812.4083	812.3741	-0.0342	-42	263	270	TGMPPGPR					Mascot
863.4046	863.429	0.0244	28	254	259	TWEWNK					Mascot
1027.532	1027.4839	-0.0481	-47	126	134	SAHQTVAWK					Mascot
1037.5303	1037.499	-0.0313	-30	157	164	DFWTLDLK					Mascot
1104.6201	1104.5208	-0.0993	-90	316	323	WYPLELRK					Mascot
1106.5775	1106.5032	-0.0743	-67	181	189	SGHRMVLYK			Oxidation (M)[5]		Mascot
1141.5387	1141.4553	-0.0834	-73	221	229	IYMYGGYFK					Mascot
1193.5645	1193.5961	0.0316	26	230	240	EVSSDKNASEK					Mascot
1316.6581	1316.5868	-0.0713	-54	34	45	VGEEDDIDAILK					Mascot
1507.7209	1507.7136	-0.0073	-5	494	506	TPTPGESLRDFCK			Carbamidomethyl (C)[12]		Mascot
1707.8813	1707.7456	-0.1357	-79	111	125	NEWKLVSSPNSPPPR	4	0			Mascot
1799.9386	1799.8578	-0.0808	-45	34	49	VGEEDDIDAILKNIQK					Mascot
1805.8593	1805.8442	-0.0151	-8	81	96	DTELILYGGEFYNGSK					Mascot
1829.8608	1829.8708	0.01	5	263	279	TGMPPGPRAGFSMCVHK			Carbamidomethyl (C)[14]		Mascot
1989.876	1989.9128	0.0368	18	135	151	NNVYMFGGEFTSPNQER					Mascot
2369.0549	2368.9724	-0.0825	-35	507	525	RTNMYWQMAAYEHTQH TGK			Oxidation (M)[4]		Mascot
2904.4263	2904.3218	-0.1045	-36	70	96	SNGSLTINPSKDTELILYG GEFYNGSK					Mascot

4	TPA: hypothetical protein ZEAMMB73_719057 [Zea mays]	gi|414881404	89975.2	5.39	18	63	52.326	7.767	

Peptide Information

Calc. Mass	Obsrv. Mass	± da	± ppm	Start Seq.	End Seq.	Sequence	Ion Score	C. I. %	Modification	Rank	Result Type
802.4305	802.4217	-0.0088	-11	372	379	SVDIGSPK					Mascot
834.3853	834.3375	-0.0478	-57	122	128	QSSGHYR					Mascot
869.4224	869.426	0.0036	4	302	308	QDSVHQR					Mascot
888.3767	888.4403	0.0636	72	509	515	YESMNTK			Oxidation (M)[4]		Mascot

1027.4666	1027.4839	0.0173	17	707	714	EFNYPMAR					Mascot
1163.5804	1163.4836	-0.0968	-83	196	206	GSVASSLYSHR					Mascot
1262.6052	1262.5918	-0.0134	-11	625	636	SGDSSFFAGFLK					Mascot
1321.5995	1321.6213	0.0218	16	784	794	GFGMHVPEEFR			Oxidation (M)[4]		Mascot
1321.5995	1321.6213	0.0218	16	784	794	GFGMHVPEEFR			Oxidation (M)[4]		Mascot
1390.7002	1390.6545	-0.0457	-33	625	637	SGDSSFFAGFLKK					Mascot
1475.7139	1475.7263	0.0124	8	122	133	QSSGHYRQYPPR					Mascot
1542.7329	1542.7236	-0.0093	-6	14	26	IGVEERPQCSDPR			Carbamidomethyl (C)[9]		Mascot
1799.8407	1799.8578	0.0171	10	386	402	DKDSNVEPSSDGPLSPR					Mascot
1805.7097	1805.8442	0.1345	74	438	453	GEYDANDEYDGDVSTR					Mascot
1833.9342	1833.8386	-0.0956	-52	362	379	AEGLSFSPSRSVDIGSPK					Mascot
1833.9342	1833.8386	-0.0956	-52	362	379	AEGLSFSPSRSVDIGSPK					Mascot
1838.9396	1838.8856	-0.054	-29	27	43	GGADWAALQQDPVELLR					Mascot
1967.0345	1966.9036	-0.1309	-67	27	44	GGADWAALQQDPVELLR K					Mascot
2904.2402	2904.3218	0.0816	28	438	463	GEYDANDEYDGDVSTRS KPESNVNDK					Mascot
3052.3105	3052.573	0.2625	86	534	560	VEDDENCVLGAESISNNC DENNKDAIK			Carbamidomethyl (C)[7,18]		Mascot

5 hypothetical protein OsI_10115 [Oryza sativa Indica Group] gi|218192137 291771.2 4.93 41 63 46.509 13.818

Peptide Information

Calc. Mass	Obsrv. Mass	± da	± ppm	Start Seq.	End Seq.	Sequence	Ion Score	C. I. %	Modification	Rank	Result Type
812.342	812.3741	0.0321	40	2242	2248	YDADNSK					Mascot
829.4778	829.3951	-0.0827	-100	1185	1191	NVQILDK					Mascot
838.3941	838.3813	-0.0128	-15	1369	1375	NAYDVEK					Mascot
848.4658	848.4283	-0.0375	-44	2381	2388	QKMVGASK					Mascot
852.421	852.399	-0.022	-26	372	378	QYAQSQK					Mascot
856.4709	856.5104	0.0395	46	292	298	AHMELKK					Mascot
874.4417	874.4199	-0.0218	-25	1693	1699	ETFNHVK					Mascot
879.3876	879.4272	0.0396	45	867	873	SQEEVCK			Carbamidomethyl (C)[6]		Mascot
992.5081	992.464	-0.0441	-44	653	661	TVAELNSMK					Mascot
999.4816	999.4437	-0.0379	-38	1033	1040	DMSVIQYK			Oxidation (M)[2]		Mascot
1003.4803	1003.5164	0.0361	36	1999	2007	GAIQDNETR					Mascot
1068.4956	1068.4973	0.0017	2	2240	2248	QKYDADNSK					Mascot
1141.5228	1141.4553	-0.0675	-59	687	695	SEMEKMVQK			Oxidation (M)[3,6]		Mascot
1168.5337	1168.5148	-0.0189	-16	927	936	ACDLMSQLSK			Carbamidomethyl (C)[2], Oxidation (M)[5]		Mascot

1229.6736	1229.5732	-0.1004	-82	476	486	LNLENTVGELK					Mascot
1308.5923	1308.619	0.0267	20	383	393	LTLDMEMANDR					Mascot
1336.5872	1336.6111	0.0239	18	361	371	QAEDALCCLEK			Carbamidomethyl (C)[7,8]		Mascot
1357.7686	1357.6669	-0.1017	-75	476	487	LNLENTVGELKK					Mascot
1373.6794	1373.6379	-0.0415	-30	1973	1985	DLEVAVEDAEGVK					Mascot
1381.6338	1381.6163	-0.0175	-13	298	309	KLSDDMAMEVDK					Mascot
1390.7325	1390.6545	-0.078	-56	1953	1963	VDEKVQFLQER					Mascot
1427.6505	1427.7582	0.1077	75	1938	1949	SSMTQLMQKDDK			Oxidation (M)[3]		Mascot
1460.7777	1460.7269	-0.0508	-35	550	561	LNLENTVCELKK			Carbamidomethyl (C)[8]		Mascot
1475.7523	1475.7263	-0.026	-18	1902	1914	CTLENLLEALGSR			Carbamidomethyl (C)[1]		Mascot
1479.7261	1479.7128	-0.0133	-9	1611	1622	DKNLALFNECQK			Carbamidomethyl (C)[10]		Mascot
1493.7166	1493.7051	-0.0115	-8	1877	1888	HELDHEVTMLNR					Mascot
1513.7129	1513.7131	0.0002	0	734	746	LEDLHTNSQEEAK					Mascot
1543.8765	1543.7606	-0.1159	-75	1778	1790	EEVLKIEILGLCK			Carbamidomethyl (C)[12]		Mascot
1674.7024	1674.7028	0.0004	0	508	521	EDADTMHAQLQDER			Oxidation (M)[6]		Mascot
1699.861	1699.7977	-0.0633	-37	1112	1125	SQEEVNRLTPEIER					Mascot
1738.9221	1738.8005	-0.1216	-70	1018	1032	SSEYKNTILLLNSEK					Mascot
1799.8414	1799.8578	0.0164	9	851	866	MHAEAALSMVENLHSK			Oxidation (M)[1,9]		Mascot
1805.8586	1805.8442	-0.0144	-8	1079	1094	EVVETMQASLQDEAQK					Mascot
1817.8763	1817.8295	-0.0468	-26	1198	1213	EEVDSLQVSLNEEAQK					Mascot
1833.8937	1833.8386	-0.0551	-30	234	249	QSLEEQISSESQRANK					Mascot
1833.8937	1833.8386	-0.0551	-30	234	249	QSLEEQISSESQRANK					Mascot
1838.884	1838.8856	0.0016	1	959	974	SEMVDFLQLSLQDEGK					Mascot
1908.9008	1908.7762	-0.1246	-65	1827	1843	YNALDDENAAVIAECIK			Carbamidomethyl (C)[15]		Mascot
1934.027	1933.8875	-0.1395	-72	2467	2481	IQYVLLKLEEEHEYK					Mascot
1961.9597	1961.9165	-0.0432	-22	1079	1095	EVVETMQASLQDEAQKR					Mascot
1961.9597	1961.9165	-0.0432	-22	1078	1094	REVVETMQASLQDEAQK					Mascot
1966.9327	1966.9036	-0.0291	-15	344	359	EIESFHFSLQDEMAKR					Mascot

6 predicted protein [Arabidopsis lyrata subsp. lyrata] gi|297337983 142588.2 8.42 27 61 24.442 7.612

Peptide Information

Calc. Mass	Obsrv. Mass	± da	± ppm	Start Seq.	End Seq.	Sequence	Ion Score	C. I. %	Modification	Rank	Result Type
812.4149	812.3741	-0.0408	-50	191	197	NVSTYTK					Mascot
826.4278	826.4093	-0.0185	-22	1020	1026	QNGSRHK					Mascot
856.525	856.5104	-0.0146	-17	1236	1244	AQIALAAAK					Mascot
859.452	859.4316	-0.0204	-24	453	459	NEDQILK					Mascot

870.4614	870.5199	0.0585	67	1055	1061	HMADRLK					Mascot
874.488	874.4199	-0.0681	-78	757	764	DLGSEIIK					Mascot
886.4563	886.4082	-0.0481	-54	1055	1061	HMADRLK			Oxidation (M)[2]		Mascot
891.4054	891.4424	0.037	42	878	886	EEGSSAPSK					Mascot
933.4822	933.4267	-0.0555	-59	1280	1287	LAMESINR					Mascot
992.5258	992.464	-0.0618	-62	181	190	VGSSDLSSLK					Mascot
1026.4924	1026.4803	-0.0121	-12	1126	1135	DMGAAALAYK			Oxidation (M)[2]		Mascot
1027.5167	1027.4839	-0.0328	-32	164	173	ISSSAPSNHK					Mascot
1184.6423	1184.5457	-0.0966	-82	55	64	LENVLGHFQK					Mascot
1193.5797	1193.5961	0.0164	14	627	637	FSTLDQPGSNK					Mascot
1263.6176	1263.624	0.0064	5	920	930	VSLESNKEDSR					Mascot
1286.6157	1286.6093	-0.0064	-5	1049	1059	EATDLKHMADR					Mascot
1302.6107	1302.6377	0.027	21	1049	1059	EATDLKHMADR			Oxidation (M)[8]		Mascot
1308.6464	1308.619	-0.0274	-21	650	661	TMITQASAENVK			Oxidation (M)[2]		Mascot
1323.625	1323.6398	0.0148	11	1263	1273	ALDLNFQDMEK					Mascot
1357.7434	1357.6669	-0.0765	-56	757	768	DLGSEIIKNNVR					Mascot
1365.541	1365.6191	0.0781	57	862	873	CPGENCIEGDSK			Carbamidomethyl (C)[1,6]		Mascot
1390.6697	1390.6545	-0.0152	-11	277	289	STPDDLVVSDSQK					Mascot
1456.6658	1456.698	0.0322	22	331	342	ESCEELVSKTMK			Carbamidomethyl (C)[3], Oxidation (M)[11]		Mascot
1479.7261	1479.7128	-0.0133	-9	1262	1273	RALDLNFQDMEK					Mascot
1485.6138	1485.7278	0.114	77	1113	1123	LCEFCAHEYEK			Carbamidomethyl (C)[2,5]		Mascot
1657.7527	1657.7573	0.0046	3	844	857	AYGEDCSIENFKPK			Carbamidomethyl (C)[6]		Mascot
1738.8607	1738.8005	-0.0602	-35	818	833	DGEAYSTIDRPGTTKK					Mascot
1926.8975	1926.8374	-0.0601	-31	816	832	CRDGEAYSTIDRPGTTK			Carbamidomethyl (C)[1]		Mascot
1983.9805	1983.9072	-0.0733	-37	191	208	NVSTYTKEGLNMLPSAS R			Oxidation (M)[12]		Mascot

7	WRKY32 [Catharanthus roseus]	gi|389595896	56786.4	5.93	15	60	11.227	3.095	

Peptide Information

Calc. Mass	Obsrv. Mass	± da	± ppm	Start Seq.	End Seq.	Sequence	Ion Score	C. I. %	Modification	Rank	Result Type
835.3904	835.4028	0.0124	15	46	52	DSDKQSR					Mascot
856.4709	856.5104	0.0395	46	406	412	KHIEMAK					Mascot
869.4549	869.426	-0.0289	-33	380	386	YGQKMVK			Oxidation (M)[5]		Mascot
874.4628	874.4199	-0.0429	-49	118	125	QGDLKDAK					Mascot
891.5298	891.4424	-0.0874	-98	243	249	VIEIVYR					Mascot
896.3414	896.4196	0.0782	87	1	8	MDDGNNSK			Oxidation (M)[1]		Mascot

Calc. Mass	Obsrv. Mass	± da	± ppm	Start Seq.	End Seq.	Sequence	Ion Score	C. I. %	Modification	Rank	Result Type
1091.4939	1091.5054	0.0115	11	426	434	HDHDMPVPK			Oxidation (M)[5]		Mascot
1104.4813	1104.5208	0.0395	36	224	232	CTYSKCSAK			Carbamidomethyl (C)[1,6]		Mascot
1332.639	1332.5303	-0.1087	-82	2	14	DDGNNSKGEVGLK					Mascot
1456.6849	1456.698	0.0131	9	299	311	DPGREAPPVMESR			Oxidation (M)[10]		Mascot
1479.6744	1479.7128	0.0384	26	1	14	MDDGNNSKGEVGLK			Oxidation (M)[1]		Mascot
1605.7603	1605.7351	-0.0252	-16	480	495	EGGELTSKPSETGGEK					Mascot
1707.8987	1707.7456	-0.1531	-90	502	517	TLLSIGFEIKPCSGSV			Carbamidomethyl (C)[12]		Mascot
1966.9651	1966.9036	-0.0615	-31	234	249	IECSDNSNRVIEIVYR			Carbamidomethyl (C)[3]		Mascot
2369.1841	2368.9724	-0.2117	-89	496	517	AMESARTLLSIGFEIKPCS GSV			Carbamidomethyl (C)[18], Oxidation (M)[2]		Mascot

8 PREDICTED: uncharacterized protein LOC101305274 [Fragaria vesca subsp. vesca] gi|470131854 22495.8 9.45 11 60 0 5.784

Peptide Information

Calc. Mass	Obsrv. Mass	± da	± ppm	Start Seq.	End Seq.	Sequence	Ion Score	C. I. %	Modification	Rank	Result Type
814.3399	814.4	0.0601	74	132	137	EYSMER					Mascot
826.4682	826.4093	-0.0589	-71	189	194	KLSHWR					Mascot
863.5137	863.429	-0.0847	-98	110	116	FLPLFAR					Mascot
1026.5363	1026.4803	-0.056	-55	71	78	VYLMLPMK			Oxidation (M)[4,7]		Mascot
1182.6373	1182.5424	-0.0949	-80	71	79	VYLMLPMKR			Oxidation (M)[4,7]		Mascot
1286.7103	1286.6093	-0.101	-78	172	182	ATWKPNLDTIK					Mascot
1515.7333	1515.7372	0.0039	3	1	15	MGNSASNKQVHGTGK					Mascot
1515.7333	1515.7372	0.0039	3	1	15	MGNSASNKQVHGTGK					Mascot
1529.757	1529.7037	-0.0533	-35	117	130	ICPAGDAHAFVFPK			Carbamidomethyl (C)[2]		Mascot
1543.8479	1543.7606	-0.0873	-57	172	184	ATWKPNLDTIKEK					Mascot
1657.8519	1657.7573	-0.0946	-57	117	131	ICPAGDAHAFVFPKK			Carbamidomethyl (C)[2]		Mascot
1800.0015	1799.8578	-0.1437	-80	167	182	QLSGKATWKPNLDTIK					Mascot

9 Os03g0152900 [Oryza sativa Japonica Group] gi|255674213 111029 6.83 24 59 0 8.94

Peptide Information

Calc. Mass	Obsrv. Mass	± da	± ppm	Start Seq.	End Seq.	Sequence	Ion Score	C. I. %	Modification	Rank	Result Type
807.4545	807.3857	-0.0688	-85	873	879	ALLGMFR					Mascot
810.3741	810.3807	0.0066	8	413	420	DSFGGTAR					Mascot
822.3961	822.3892	-0.0069	-8	703	709	ALCGMVR			Carbamidomethyl (C)[3], Oxidation (M)[5]		Mascot
838.3546	838.3813	0.0267	32	672	677	MQQEMR			Oxidation (M)[1]		Mascot
888.5036	888.4403	-0.0633	-71	651	658	IADLISEK					Mascot

1033.571	1033.4937	-0.0773	-75	532	539	RLMLDLEK			Oxidation (M)[3]		Mascot
1106.551	1106.5032	-0.0478	-43	488	496	NSEKMQLEK					Mascot
1286.5616	1286.6093	0.0477	37	892	902	GMANFAKCESR			Carbamidomethyl (C)[8], Oxidation (M)[2]		Mascot
1308.7019	1308.619	-0.0829	-63	880	891	TGHNEVIAQIAR					Mascot
1365.7234	1365.6191	-0.1043	-76	678	688	HRQGLEDEILR					Mascot
1373.6519	1373.6379	-0.014	-10	105	116	VSGFSLFVDSCR			Carbamidomethyl (C)[11]		Mascot
1427.6254	1427.7582	0.1328	93	667	677	STQEKMQQEMR			Oxidation (M)[6,10]		Mascot
1460.7526	1460.7269	-0.0257	-18	624	635	NIACELEKQLSR			Carbamidomethyl (C)[4]		Mascot
1475.6796	1475.7263	0.0467	32	206	219	LGNDDPSEGGIMVR			Oxidation (M)[12]		Mascot
1513.8016	1513.7131	-0.0885	-58	1	14	MAANGRASVRPVER					Mascot
1515.7577	1515.7372	-0.0205	-14	456	467	IKEEVDYESLYK					Mascot
1515.7577	1515.7372	-0.0205	-14	456	467	IKEEVDYESLYK					Mascot
1529.7531	1529.7037	-0.0494	-32	104	116	RVSGFSLFVDSCR			Carbamidomethyl (C)[12]		Mascot
1628.8214	1628.8151	-0.0063	-4	105	118	VSGFSLFVDSCRVR			Carbamidomethyl (C)[11]		Mascot
1657.723	1657.7573	0.0343	21	510	523	VTSNMQIENMAMEK			Oxidation (M)[5,10]		Mascot
1829.8191	1829.8708	0.0517	28	510	524	VTSNMQIENMAMEKR			Oxidation (M)[5,10,12]		Mascot
1838.8701	1838.8856	0.0155	8	468	482	KVEHEVDHLTSEMER					Mascot
1941.9851	1941.8958	-0.0893	-46	281	296	DLEHVFQLLQIGEMNR					Mascot
1989.9158	1989.9128	-0.003	-2	433	449	HFSETSSTIMFGQRAMK			Oxidation (M)[10,16]		Mascot
2031.0717	2030.9117	-0.16	-79	563	580	QKQLENISNTNILADTTK					Mascot

10	hypothetical protein M569_13662 [Genlisea aurea]	gi|527189511	126975.5	5.25	31	58	0	12.236	

Peptide Information

Calc. Mass	Obsrv. Mass	± da	± ppm	Start Seq.	End Seq.	Sequence	Ion Score	C. I. %	Modification	Rank	Result Type
807.4141	807.3857	-0.0284	-35	142	147	SLREMR			Oxidation (M)[5]		Mascot
810.3992	810.3807	-0.0185	-23	90	95	YEELTR					Mascot
812.3164	812.3741	0.0577	71	667	672	EMEEMK			Oxidation (M)[2]		Mascot
817.446	817.3896	-0.0564	-69	257	262	RMLNQR					Mascot
832.3795	832.3041	-0.0754	-91	843	849	QESEPSR					Mascot
834.4203	834.3375	-0.0828	-99	401	407	LEQSTEK					Mascot
847.438	847.4329	-0.0051	-6	894	901	GRGSVSER					Mascot
848.3745	848.4283	0.0538	63	298	304	EDDINSR					Mascot
869.4111	869.426	0.0149	17	245	251	LHEAEDR					Mascot
888.4672	888.4403	-0.0269	-30	340	346	EEIELQK					Mascot
914.4288	914.3859	-0.0429	-47	589	596	ETFAATMK			Oxidation (M)[7]		Mascot
992.4643	992.464	-0.0003	0	225	233	ETNDASISR					Mascot

1026.5215	1026.4803	-0.0412	-40	373	380	HEEELKNK		Mascot
1027.5419	1027.4839	-0.058	-56	779	789	GGVESSPPAVK		Mascot
1033.5413	1033.4937	-0.0476	-46	466	474	SDTEDLLLK		Mascot
1068.4956	1068.4973	0.0017	2	416	424	ENDFNSKSK		Mascot
1091.5262	1091.5054	-0.0208	-19	258	265	MLNQREER	Oxidation (M)[1]	Mascot
1104.532	1104.5208	-0.0112	-10	236	243	EDLREWEK		Mascot
1184.5834	1184.5457	-0.0377	-32	544	552	EYDLFIEQK		Mascot
1201.5947	1201.5826	-0.0121	-10	968	977	LEDPELTEQK		Mascot
1308.6252	1308.619	-0.0062	-5	589	599	ETFAATMKHEK	Oxidation (M)[7]	Mascot
1316.6945	1316.5868	-0.1077	-82	702	712	DIEELIELSQK		Mascot
1357.6958	1357.6669	-0.0289	-21	968	978	LEDPELTEQKR		Mascot
1365.6646	1365.6191	-0.0455	-33	651	661	ESELNDINYLR		Mascot
1507.7275	1507.7136	-0.0139	-9	766	778	AEDYLKESAQPEK		Mascot
1515.7802	1515.7372	-0.043	-28	175	188	SLEVEAKFHAADAK		Mascot
1515.7802	1515.7372	-0.043	-28	175	188	SLEVEAKFHAADAK		Mascot
1796.9025	1796.92	0.0175	10	772	789	ESAQPEKGGVESSPPAV K		Mascot
1801.8563	1801.845	-0.0113	-6	833	849	SLDAATDAPKQESEPSR		Mascot
1801.9291	1801.845	-0.0841	-47	553	567	SQLEKQIQSQEENLK		Mascot
1927.0093	1926.8374	-0.1719	-89	580	596	ELSILELSKETFAATMK	Oxidation (M)[16]	Mascot
2717.1816	2717.0457	-0.1359	-50	866	893	GAETDDALSIDHDGNSQ NSGMAKGGGGR		Mascot

Gel Idx/Pos	157/G8	Instr./Gel Origin	BA2151/Sample Project 20140814	Process Status	Analysis Succeeded
Plate [#] Name	[1] Sample Project 20140814	Instrument Sample Name		Spectra	11

Rank	Protein Name	Accession No.	Protein MW	Protein PI	Pep. Count	Protein Score	Protein Score C. I. %	Intensity Matched	Total Ion Score	Total Ion C. I. %	Confirmed
1	PREDICTED: dehydration-responsive element-binding protein 2E-like [Setaria italica]	gi|514740358	24326.2	10.31	5	57	0	5.01	36	54.504	

Peptide Information

Calc. Mass	Obsrv. Mass	± da	± ppm	Start Seq.	End Seq.	Sequence	Ion Score	C. I. %	Modification	Rank	Result Type
831.4431	831.4418	-0.0013	-2	42	48	DSAQRVR					Mascot
903.4279	903.4227	-0.0052	-6	2	10	AAQQESGGR					Mascot
1515.8544	1515.7192	-0.1352	-89	156	169	AAKHAVHPSFVVPR					Mascot
1515.8544	1515.7192	-0.1352	-89	156	169	AAKHAVHPSFVVPR	36	54.504			Mascot
1838.9219	1838.8655	-0.0564	-31	115	132	ATACAGVGEQFALAAVF R			Carbamidomethyl (C)[4]		Mascot
1944.9304	1944.8307	-0.0997	-51	2	21	AAQQESGGRAATATAAP ACR			Carbamidomethyl (C)[19]		Mascot

Rank	Protein Name	Accession No.	Protein MW	Protein PI	Pep. Count	Protein Score	Protein Score C. I. %	Intensity Matched	Total Ion Score	Total Ion C. I. %	Confirmed
2	hypothetical protein ZEAMMB73_548145 [Zea mays]	gi|413956164	60307.6	6.41	13	56	0	6.934			

Protein Group

uncharacterized protein LOC100280228 [Zea mays]	gi|226498010	60307.6	6.4099 998474 1211

Peptide Information

Calc. Mass	Obsrv. Mass	± da	± ppm	Start Seq.	End Seq.	Sequence	Ion Score	C. I. %	Modification	Rank	Result Type
803.4158	803.367	-0.0488	-61	473	478	HDRTFK					Mascot
848.4584	848.3998	-0.0586	-69	136	144	GSGSSVARK					Mascot
858.4693	858.4929	0.0236	27	243	249	GFVRSHR					Mascot
908.4294	908.3735	-0.0559	-62	286	292	WMELTGR			Oxidation (M)[2]		Mascot
1321.6423	1321.6199	-0.0224	-17	231	242	LGDPQFPFSADK					Mascot
1321.6423	1321.6199	-0.0224	-17	231	242	LGDPQFPFSADK					Mascot
1338.6471	1338.6079	-0.0392	-29	1	11	MLVERFGGEER			Oxidation (M)[1]		Mascot
1475.8074	1475.703	-0.1044	-71	459	471	VSCISVGRMPLLK			Carbamidomethyl (C)[3], Oxidation (M)[9]		Mascot
1479.6945	1479.7188	0.0243	16	219	230	MQLRSFMNGHSR			Oxidation (M)[1]		Mascot
1817.9902	1817.822	-0.1682	-93	450	466	NTLTNGIVKVSCISVGR			Carbamidomethyl (C)[12]		Mascot
1817.9902	1817.822	-0.1682	-93	450	466	NTLTNGIVKVSCISVGR	6	0	Carbamidomethyl (C)[12]		Mascot
1929.7251	1929.9089	0.1838	95	371	388	DSSPSATEEDCSNSNSD K			Carbamidomethyl (C)[11]		Mascot

1941.8613	1941.8744	0.0131	7	293	309	DANFSIPAEASDFESWR					Mascot
2717.1431	2717.0105	-0.1326	-49	389	411	AQDMDVSHTFEPSWMN DFTGVMR			Oxidation (M)[4]		Mascot
2840.3179	2840.269	-0.0489	-17	149	171	NDSIWGAWFFFTHYFKP VMSTDK			Oxidation (M)[19]		Mascot

3	PREDICTED: putative uncharacterized hydrolase C1020.07-like [Setaria italica]	gi|514808766	34017.4	6.36	7	50	0	6.223	27	0	

Peptide Information

Calc. Mass	Obsrv. Mass	± da	± ppm	Start Seq.	End Seq.	Sequence	Ion Score	C. I. %	Modification	Rank	Result Type
858.493	858.4929	-0.0001	0	179	186	LIDEALGK					Mascot
890.4077	890.3884	-0.0193	-22	126	132	MTAYFNK			Oxidation (M)[1]		Mascot
955.5359	955.4586	-0.0773	-81	133	141	TGWPAKAPK					Mascot
1194.6439	1194.5687	-0.0752	-63	158	167	TELFMALIEK					Mascot
1338.7338	1338.6079	-0.1259	-94	158	168	TELFMALIEKK			Oxidation (M)[5]		Mascot
1515.853	1515.7192	-0.1338	-88	214	227	AEKITIFAGDVVPR					Mascot
1515.853	1515.7192	-0.1338	-88	214	227	AEKITIFAGDVVPR	27	0			Mascot
1929.9698	1929.9089	-0.0609	-32	1	19	MAATASSTLLQGSKFSSA R			Oxidation (M)[1]		Mascot

4	hypothetical protein OsI_12279 [Oryza sativa Indica Group]	gi|218193159	34137.7	8.36	7	46	0	6.764	27	0	

Protein Group

Os03g0565200 [Oryza sativa Japonica Group]	gi|113548970	34137.7	8.3599 996566 7725

Peptide Information

Calc. Mass	Obsrv. Mass	± da	± ppm	Start Seq.	End Seq.	Sequence	Ion Score	C. I. %	Modification	Rank	Result Type
847.4019	847.3937	-0.0082	-10	131	137	MTAYFSK					Mascot
858.493	858.4929	-0.0001	0	184	191	LIDEALGK					Mascot
1194.6439	1194.5687	-0.0752	-63	163	172	TELFMALIEK					Mascot
1338.7338	1338.6079	-0.1259	-94	163	173	TELFMALIEKK			Oxidation (M)[5]		Mascot
1497.7406	1497.6967	-0.0439	-29	305	316	FDLEFCANLLQK			Carbamidomethyl (C)[6]		Mascot
1515.853	1515.7192	-0.1338	-88	219	232	AEKITIFAGDVVPR					Mascot
1515.853	1515.7192	-0.1338	-88	219	232	AEKITIFAGDVVPR	27	0			Mascot
1958.9681	1958.8148	-0.1533	-78	305	320	FDLEFCANLLQKQFVS			Carbamidomethyl (C)[6]		Mascot

5	hypothetical protein TRIUR3_14206 [Triticum urartu]	gi|474314641	18385.3	4.93	7	46	0	6.464			

Peptide Information

Calc. Mass	Obsrv. Mass	± da	± ppm	Start	End	Sequence	Ion	C. I. %	Modification	Rank	Result Type

				Seq.	Seq.		Score				
801.4213	801.4565	0.0352	44	75	82	GAPRSDAK					Mascot
831.4683	831.4418	-0.0265	-32	125	132	ATGVRAEK					Mascot
1193.5281	1193.5756	0.0475	40	64	74	SGDESEAETIR					Mascot
1349.6292	1349.6318	0.0026	2	63	74	RSGDESEAETIR					Mascot
1515.7802	1515.7192	-0.061	-40	113	124	LEKAWQEVEQQK					Mascot
1515.7802	1515.7192	-0.061	-40	113	124	LEKAWQEVEQQK					Mascot
2222.116	2222.094	-0.022	-10	144	162	DQARVLEVEETLNGVHQER					Mascot
2239.0774	2239.0542	-0.0232	-10	86	105	VGLSFNANAELFYMTAVMTK			Oxidation (M)[14,18]		Mascot

6 hypothetical protein ZEAMMB73_548145 [Zea mays] gi|413956163 58861.9 6.49 12 45 0 6.408

Peptide Information

Calc. Mass	Obsrv. Mass	± da	± ppm	Start Seq.	End Seq.	Sequence	Ion Score	C. I. %	Modification	Rank	Result Type
803.4158	803.367	-0.0488	-61	460	465	HDRTFK					Mascot
848.4584	848.3998	-0.0586	-69	123	131	GSGSSVARK					Mascot
858.4693	858.4929	0.0236	27	230	236	GFVRSHR					Mascot
908.4294	908.3735	-0.0559	-62	273	279	WMELTGR			Oxidation (M)[2]		Mascot
1321.6423	1321.6199	-0.0224	-17	218	229	LGDPQFPFSADK					Mascot
1321.6423	1321.6199	-0.0224	-17	218	229	LGDPQFPFSADK					Mascot
1475.8074	1475.703	-0.1044	-71	446	458	VSCISVGRMPLLK			Carbamidomethyl (C)[3], Oxidation (M)[9]		Mascot
1479.6945	1479.7188	0.0243	16	206	217	MQLRSFMNGHSR			Oxidation (M)[1]		Mascot
1817.9902	1817.822	-0.1682	-93	437	453	NTLTNGIVKVSCISVGR			Carbamidomethyl (C)[12]		Mascot
1817.9902	1817.822	-0.1682	-93	437	453	NTLTNGIVKVSCISVGR	6	0	Carbamidomethyl (C)[12]		Mascot
1929.7251	1929.9089	0.1838	95	358	375	DSSPSATEEDCSNSNSDK			Carbamidomethyl (C)[11]		Mascot
1941.8613	1941.8744	0.0131	7	280	296	DANFSIPAEASDFESWR					Mascot
2717.1431	2717.0105	-0.1326	-49	376	398	AQDMDVSHTFEPSWMNDFTGVMR			Oxidation (M)[4]		Mascot
2840.3179	2840.269	-0.0489	-17	136	158	NDSIWGAWFFFTHYFKPVMSTDK			Oxidation (M)[19]		Mascot

7 Protein cbbY [Aegilops tauschii] gi|475514518 42890.3 8.22 6 45 0 5.656 27 0

Peptide Information

Calc. Mass	Obsrv. Mass	± da	± ppm	Start Seq.	End Seq.	Sequence	Ion Score	C. I. %	Modification	Rank	Result Type
858.493	858.4929	-0.0001	0	181	188	LIDEALGK					Mascot
890.4077	890.3884	-0.0193	-22	128	134	MTAYFNK			Oxidation (M)[1]		Mascot

Calc. Mass	Obsrv. Mass	± da	± ppm	Start Seq.	End Seq.	Sequence	Ion Score	C. I. %	Modification	Rank	Result Type
1194.6439	1194.5687	-0.0752	-63	160	169	TELFMALIEK					Mascot
1338.7338	1338.6079	-0.1259	-94	160	170	TELFMALIEKK			Oxidation (M)[5]		Mascot
1497.7406	1497.6967	-0.0439	-29	312	323	FDLEFCANLLQK			Carbamidomethyl (C)[6]		Mascot
1515.853	1515.7192	-0.1338	-88	216	229	AEKITIFAGDVVPR					Mascot
1515.853	1515.7192	-0.1338	-88	216	229	AEKITIFAGDVVPR	27	0			Mascot

8	PREDICTED: nucleolar protein 58-like [Cicer arietinum]	gi|502127226	46346.4	9.44	13	45	0	8.66			

Peptide Information

Calc. Mass	Obsrv. Mass	± da	± ppm	Start Seq.	End Seq.	Sequence	Ion Score	C. I. %	Modification	Rank	Result Type
810.3516	810.3676	0.016	20	276	281	FEEEEK					Mascot
848.4512	848.3998	-0.0514	-61	157	164	SPSPSFVK					Mascot
948.4996	948.4483	-0.0513	-54	315	323	GEKESLASK					Mascot
1058.4823	1058.4834	0.0011	1	342	349	DQMYTKEK			Oxidation (M)[3]		Mascot
1194.6035	1194.5687	-0.0348	-29	354	364	EMATSTSQIVK					Mascot
1308.5923	1308.5863	-0.006	-5	283	293	MPCESSKVDQK			Carbamidomethyl (C)[3]		Mascot
1321.6821	1321.6199	-0.0622	-47	97	108	FDNGTLIVAMPK			Oxidation (M)[10]		Mascot
1321.6821	1321.6199	-0.0622	-47	97	108	FDNGTLIVAMPK			Oxidation (M)[10]		Mascot
1338.686	1338.6079	-0.0781	-58	133	145	GTTPSKSTTTTTR					Mascot
1738.8469	1738.7531	-0.0938	-54	294	307	YVDHNMFLEGKEIK			Oxidation (M)[6]		Mascot
1793.0167	1792.9717	-0.045	-25	52	67	ITYVVSSQTLKIVGER					Mascot
1793.0167	1792.9717	-0.045	-25	52	67	ITYVVSSQTLKIVGER					Mascot
1838.8741	1838.8655	-0.0086	-5	290	304	VDQKYVDHNMFLEGK			Oxidation (M)[10]		Mascot
2258.0295	2258.1106	0.0811	36	75	92	WSRFNQSYNVPENCEVT K			Carbamidomethyl (C)[14]		Mascot
2368.1973	2368.2007	0.0034	1	31	49	ENEEAYFLHIYLPGFIKER					Mascot

9	PREDICTED: protein FRIGIDA-like [Setaria italica]	gi|514728183	60554.2	6.47	9	44	0	10.634	25	0	

Peptide Information

Calc. Mass	Obsrv. Mass	± da	± ppm	Start Seq.	End Seq.	Sequence	Ion Score	C. I. %	Modification	Rank	Result Type
847.452	847.3937	-0.0583	-69	90	96	ELTSLER					Mascot
864.4938	864.4283	-0.0655	-76	376	382	AYLRDVK					Mascot
890.4479	890.3884	-0.0595	-67	459	467	ANGSAFAPR					Mascot
903.4352	903.4227	-0.0125	-14	325	331	QTPELCR			Carbamidomethyl (C)[6]		Mascot
993.5033	993.4468	-0.0565	-57	31	39	QSAVSMNLK			Oxidation (M)[6]		Mascot
1491.7512	1491.6871	-0.0641	-43	251	263	FSLEGHMLTTDIK					Mascot
1507.7462	1507.7032	-0.043	-29	251	263	FSLEGHMLTTDIK			Oxidation (M)[7]		Mascot

Calc. Mass	Obsrv. Mass	± da	± ppm	Start Seq.	End Seq.	Sequence	Ion Score	C. I. %	Modification	Rank	Result Type
1515.8376	1515.7192	-0.1184	-78	83	96	EAAVVAKELTSLER					Mascot
1515.8376	1515.7192	-0.1184	-78	83	96	EAAVVAKELTSLER	25	0			Mascot
1792.8899	1792.9717	0.0818	46	251	265	FSLEGHMLTTDIKER			Oxidation (M)[7]		Mascot
1792.8899	1792.9717	0.0818	46	251	265	FSLEGHMLTTDIKER			Oxidation (M)[7]		Mascot
1801.8195	1801.8271	0.0076	4	1	17	MSDLESVAALMESTSSK			Oxidation (M)[1]		Mascot
1817.8143	1817.822	0.0077	4	1	17	MSDLESVAALMESTSSK			Oxidation (M)[1,11]		Mascot
1817.8143	1817.822	0.0077	4	1	17	MSDLESVAALMESTSSK			Oxidation (M)[1,11]		Mascot

10	WRKY DNA-binding protein 32 [Arabidopsis thaliana]	gi|332660432	52132.8	5.75	11	44	0	6.909

Protein Group

RecName: Full=Probable WRKY transcription factor 32; AltName: Full=WRKY DNA-binding protein 32	gi|29839502	52132.8	5.75
WRKY DNA-binding protein 32 [Arabidopsis thaliana]	gi|42567286	52132.8	5.75

Peptide Information

Calc. Mass	Obsrv. Mass	± da	± ppm	Start Seq.	End Seq.	Sequence	Ion Score	C. I. %	Modification	Rank	Result Type
801.4213	801.4565	0.0352	44	57	63	DQVQGVR					Mascot
810.3529	810.3676	0.0147	18	169	174	DGYNWR					Mascot
822.4105	822.408	-0.0025	-3	26	32	DGLSQFR					Mascot
835.4155	835.3668	-0.0487	-58	433	439	ESEKQSK					Mascot
850.4781	850.3942	-0.0839	-99	176	182	YGQKQVK					Mascot
1253.5203	1253.6033	0.083	66	1	11	MEEDTGIDEAK			Oxidation (M)[1]		Mascot
1320.5382	1320.5499	0.0117	9	193	202	CTYTECCAKK			Carbamidomethyl (C)[1,6,7]		Mascot
1323.6176	1323.6218	0.0042	3	26	36	DGLSQFRDEEK					Mascot
1792.8436	1792.9717	0.1281	71	319	335	FVVHAAGDVGICGDGYR			Carbamidomethyl (C)[12]		Mascot
1792.8436	1792.9717	0.1281	71	319	335	FVVHAAGDVGICGDGYR			Carbamidomethyl (C)[12]		Mascot
1930.0645	1929.9089	-0.1556	-81	366	382	HIETAVENTKAVIITYK					Mascot
1958.8899	1958.8148	-0.0751	-38	1	17	MEEDTGIDEAKTYTVEK					Mascot

Gel Idx/Pos	158/G9	Instr./Gel Origin	BA2151/Sample Project 20140814	Process Status	Analysis Succeeded
Plate [#] Name	[1] Sample Project 20140814	Instrument Sample Name		Spectra	11

Rank	Protein Name	Accession No.	Protein MW	Protein PI	Pep. Count	Protein Score	Protein Score C. I. %	Intensity Matched	Total Ion Score	Total Ion C. I. %	Confirmed
1	Late embryogenesis abundant protein Lea14-A [Triticum urartu]	gi|474110039	16319.3	4.79	6	105	99.997	3.827	71	99.984	

Peptide Information

Calc. Mass	Obsrv. Mass	± da	± ppm	Start Seq.	End Seq.	Sequence	Ion Score	C. I. %	Modification	Rank	Result Type
905.4979	905.4672	-0.0307	-34	144	151	LPTLSDLF					Mascot
905.4979	905.4672	-0.0307	-34	144	151	LPTLSDLF	45	93.82			Mascot
1259.6703	1259.6483	-0.022	-17	37	48	DGATLSGRLDVR					Mascot
1327.6967	1327.6471	-0.0496	-37	96	106	VPYDFLMSLVK			Oxidation (M)[7]		Mascot
1403.7781	1403.6831	-0.095	-68	139	151	AGELKLPTLSDLF					Mascot
1629.6849	1629.6421	-0.0428	-26	107	119	DAGKDWDLDYEMR			Oxidation (M)[12]		Mascot
2022.0001	2021.9229	-0.0772	-38	49	65	NPYSHTIPICEISYSLK			Carbamidomethyl (C)[10]		Mascot
2022.0001	2021.9229	-0.0772	-38	49	65	NPYSHTIPICEISYSLK	26	0	Carbamidomethyl (C)[10]		Mascot

Rank	Protein Name	Accession No.	Protein MW	Protein PI	Pep. Count	Protein Score	Protein Score C. I. %	Intensity Matched	Total Ion Score	Total Ion C. I. %	Confirmed
2	conserved hypothetical protein [Ricinus communis]	gi|223532945	8689	4.67	7	70	89.57	2.083	29	0	

Peptide Information

Calc. Mass	Obsrv. Mass	± da	± ppm	Start Seq.	End Seq.	Sequence	Ion Score	C. I. %	Modification	Rank	Result Type
835.423	835.3513	-0.0717	-86	1	7	MLLEDSK					Mascot
851.4178	851.3828	-0.035	-41	1	7	MLLEDSK			Oxidation (M)[1]		Mascot
1037.4283	1037.5099	0.0816	79	31	38	NNENDEFR					Mascot
1126.4318	1126.4785	0.0467	41	66	75	NDDNNSTMSI			Oxidation (M)[8]		Mascot
1165.5232	1165.5651	0.0419	36	30	38	KNNENDEFR	29	0			Mascot
1193.5294	1193.5924	0.063	53	31	39	NNENDEFRR					Mascot
1201.479	1201.5695	0.0905	75	21	30	GSDNCDDYKK			Carbamidomethyl (C)[5]		Mascot
1699.865	1699.7897	-0.0753	-44	51	65	GWKAESIEDIIHSSK					Mascot

Rank	Protein Name	Accession No.	Protein MW	Protein PI	Pep. Count	Protein Score	Protein Score C. I. %	Intensity Matched	Total Ion Score	Total Ion C. I. %	Confirmed
3	hypothetical protein OsI_19530 [Oryza sativa Indica Group]	gi|125551895	75666.6	6.38	20	68	82.691	7.216			

Peptide Information

Calc. Mass	Obsrv. Mass	± da	± ppm	Start Seq.	End Seq.	Sequence	Ion Score	C. I. %	Modification	Rank	Result Type

829.4202	829.3697	-0.0505	-61	402	407	KYEAYR					Mascot
858.4791	858.4236	-0.0555	-65	303	309	DVAERLR					Mascot
894.468	894.4447	-0.0233	-26	178	185	LSDFGISR					Mascot
912.4244	912.3962	-0.0282	-31	71	77	YICQNSK			Carbamidomethyl (C)[3]		Mascot
927.4352	927.4594	0.0242	26	605	612	SGRAMFDK			Oxidation (M)[5]		Mascot
1033.5215	1033.4933	-0.0282	-27	322	329	TGQWSLWR					Mascot
1103.4786	1103.5062	0.0276	25	314	321	MHREEEEK			Oxidation (M)[1]		Mascot
1201.6801	1201.5695	-0.1106	-92	436	445	LTVVSRFSHR					Mascot
1320.673	1320.5637	-0.1093	-83	193	203	MQYTIHVIGSR			Oxidation (M)[1]		Mascot
1358.6846	1358.6504	-0.0342	-25	297	307	QRPEMKDVAER					Mascot
1379.6914	1379.6575	-0.0339	-25	377	388	ATNNYSLDNVIR					Mascot
1403.7312	1403.6831	-0.0481	-34	408	419	GRLEDNMQVVVK			Oxidation (M)[7]		Mascot
1427.7465	1427.7651	0.0186	13	18	29	AKWMPINSHSIK			Oxidation (M)[4]		Mascot
1475.7635	1475.7231	-0.0404	-27	236	249	AKACENGLSTGLVR			Carbamidomethyl (C)[4]		Mascot
1479.7811	1479.7106	-0.0705	-48	446	457	NIIKLLGCCFNK			Carbamidomethyl (C)[8,9]		Mascot
1507.7864	1507.7065	-0.0799	-53	376	388	KATNNYSLDNVIR					Mascot
1627.7671	1627.6421	-0.125	-77	334	348	VNKQDTGFSSSNSTR					Mascot
1838.0059	1837.8918	-0.1141	-62	220	235	NDVYSFGVVLLELITR					Mascot
1868.0238	1867.8401	-0.1837	-98	569	584	DVYNFGIVLLELITMK					Mascot
2022.0729	2021.9229	-0.15	-74	410	426	LEDNMQVVVKWVTFVSK					Mascot
2022.0729	2021.9229	-0.15	-74	410	426	LEDNMQVVVKWVTFVSK					Mascot

4 Myb family transcription factor APL [Aegilops tauschii] gi|475571352 32787.7 5.94 11 58 0 22.142

Peptide Information

Calc. Mass	Obsrv. Mass	± da	± ppm	Start Seq.	End Seq.	Sequence	Ion Score	C. I. %	Modification	Rank	Result Type
817.4526	817.3734	-0.0792	-97	256	262	VRSQEAK					Mascot
820.3869	820.3835	-0.0034	-4	263	269	SVECTPK			Carbamidomethyl (C)[4]		Mascot
858.5043	858.4236	-0.0807	-94	122	129	ADLSAIIR					Mascot
1201.647	1201.5695	-0.0775	-64	171	180	LQMRIEAQGR					Mascot
1287.7015	1287.6256	-0.0759	-59	118	129	DTGRADLSAIIR					Mascot
1308.6808	1308.6294	-0.0514	-39	29	38	LRWTPDLHDR					Mascot
1403.7198	1403.6831	-0.0367	-26	160	170	ELCEQLEVQKK			Carbamidomethyl (C)[3]		Mascot
2021.9523	2021.9229	-0.0294	-15	189	207	AQENISFDANGSVGLENT R					Mascot
2021.9523	2021.9229	-0.0294	-15	189	207	AQENISFDANGSVGLENT R					Mascot
2235.0459	2235.1289	0.083	37	2	26	EGMSYGGGAAGAGAGA AALSPRDPK			Oxidation (M)[3]		Mascot

2235.1868	2235.1289	-0.0579	-26	270	289	TEDLLLLDLNIKGGYDLSSR					Mascot
2262.9834	2263.1274	0.144	64	238	255	HMSDDNLHDNNLDFQLYR			Oxidation (M)[2]		Mascot

5 late embryogenesis abundant protein lea14-a [Allium sativum] gi|371721826 16833.7 5.16 3 57 0 2.256 45 93.82

Peptide Information

Calc. Mass	Obsrv. Mass	± da	± ppm	Start Seq.	End Seq.	Sequence	Ion Score	C. I. %	Modification	Rank	Result Type
905.4979	905.4672	-0.0307	-34	144	151	LPTLSDLF					Mascot
905.4979	905.4672	-0.0307	-34	144	151	LPTLSDLF	45	93.82			Mascot
1327.6967	1327.6471	-0.0496	-37	96	106	VPYDFLISVMK			Oxidation (M)[10]		Mascot
1349.7134	1349.6364	-0.077	-57	140	151	GQMKLPTLSDLF					Mascot

6 RecName: Full=Late embryogenesis abundant protein Lea14-A gi|1170745 16526.6 4.84 3 56 0 1.283 45 93.82

Peptide Information

Calc. Mass	Obsrv. Mass	± da	± ppm	Start Seq.	End Seq.	Sequence	Ion Score	C. I. %	Modification	Rank	Result Type
864.4495	864.4537	0.0042	5	1	7	MSQLLEK			Oxidation (M)[1]		Mascot
905.4979	905.4672	-0.0307	-34	144	151	LPTLSDIF					Mascot
905.4979	905.4672	-0.0307	-34	144	151	LPTLSDIF	45	93.82			Mascot
1287.6791	1287.6256	-0.0535	-42	21	32	KPEASVSDVDLK					Mascot

7 PREDICTED: probable galacturonosyltransferase 6-like isoform X6 [Cicer arietinum] gi|502109873 72621.5 7.39 17 56 0 4.612

Peptide Information

Calc. Mass	Obsrv. Mass	± da	± ppm	Start Seq.	End Seq.	Sequence	Ion Score	C. I. %	Modification	Rank	Result Type
810.4178	810.384	-0.0338	-42	556	561	IMYLDR					Mascot
820.4047	820.3835	-0.0212	-26	155	161	NTDEITK					Mascot
822.3774	822.3696	-0.0078	-9	128	134	ELCSTGR			Carbamidomethyl (C)[3]		Mascot
982.5138	982.4162	-0.0976	-99	556	562	IMYLDRR			Oxidation (M)[2]		Mascot
1193.558	1193.5924	0.0344	29	128	137	ELCSTGRDQK			Carbamidomethyl (C)[3]		Mascot
1194.5646	1194.5737	0.0091	8	260	270	AFPDCIAMAAK			Carbamidomethyl (C)[5]		Mascot
1201.5518	1201.5695	0.0177	15	225	235	EMEHAVGEATK					Mascot
1210.5596	1210.5928	0.0332	27	260	270	AFPDCIAMAAK			Carbamidomethyl (C)[5], Oxidation (M)[8]		Mascot
1259.6161	1259.6483	0.0322	26	249	259	HMEASLSKANR			Oxidation (M)[2]		Mascot
1277.6583	1277.683	0.0247	19	155	165	NTDEITKVTEK					Mascot

1358.6118	1358.6504	0.0386	28	273	283	AMHQNTEEQVR			Oxidation (M)[2]		Mascot
1479.7448	1479.7106	-0.0342	-23	260	272	AFPDCIAMAAKLR			Carbamidomethyl (C)[5], Oxidation (M)[8]		Mascot
1486.7318	1486.7281	-0.0037	-2	223	235	IREMEHAVGEATK			Oxidation (M)[4]		Mascot
1627.797	1627.6421	-0.1549	-95	271	283	LRAMHQNTEEQVR			Oxidation (M)[4]		Mascot
1707.7758	1707.7434	-0.0324	-19	484	497	MDMFINFSDPFIAK			Oxidation (M)[1,3]		Mascot
1838.9066	1838.8928	-0.0138	-8	467	483	VIAGVGTCQEGETSFRK			Carbamidomethyl (C)[8]		Mascot
1890.8499	1890.9318	0.0819	43	225	241	EMEHAVGEATKDSDLSR			Oxidation (M)[2]		Mascot
1930.9771	1930.8529	-0.1242	-64	562	578	RWHILGLGYDSGIDSNK					Mascot

8 hypothetical protein M569_02673, partial [Genlisea aurea] gi|527206555 33294.2 5.47 11 55 0 4.319

Peptide Information

Calc. Mass	Obsrv. Mass	± da	± ppm	Start Seq.	End Seq.	Sequence	Ion Score	C. I. %	Modification	Rank	Result Type
810.3628	810.384	0.0212	26	107	112	DEYEVR					Mascot
827.4297	827.4014	-0.0283	-34	235	241	FASLDFK					Mascot
927.4352	927.4594	0.0242	26	175	182	TGTFTMNR					Mascot
938.4578	938.4265	-0.0313	-33	106	112	KDEYEVR					Mascot
1033.5314	1033.4933	-0.0381	-37	209	217	SDIAWKDAK					Mascot
1037.4899	1037.5099	0.02	19	227	234	DEFELTQR					Mascot
1126.5674	1126.4785	-0.0889	-79	96	105	HASGLLPEMR			Oxidation (M)[9]		Mascot
1287.6903	1287.6256	-0.0647	-50	1	13	TDAASATVSIPVR					Mascot
1873.9556	1873.9125	-0.0431	-23	25	40	SFVDQNKQNFIPPPSR					Mascot
2021.9961	2021.9229	-0.0732	-36	157	174	QVDGIVAEFTDINRGMEK					Mascot
2021.9961	2021.9229	-0.0732	-36	157	174	QVDGIVAEFTDINRGMEK					Mascot
2044.0797	2043.9326	-0.1471	-72	32	48	QNFIPPPSRMPNYVVLR			Oxidation (M)[10]		Mascot

9 PREDICTED: probable galacturonosyltransferase 6-like isoform X4 [Cicer arietinum] gi|502109865 72749.6 7.7 17 54 0 4.612

Peptide Information

Calc. Mass	Obsrv. Mass	± da	± ppm	Start Seq.	End Seq.	Sequence	Ion Score	C. I. %	Modification	Rank	Result Type
810.4178	810.384	-0.0338	-42	557	562	IMYLDR					Mascot
820.4047	820.3835	-0.0212	-26	156	162	NTDEITK					Mascot
822.3774	822.3696	-0.0078	-9	128	134	ELCSTGR			Carbamidomethyl (C)[3]		Mascot
982.5138	982.4162	-0.0976	-99	557	563	IMYLDRR			Oxidation (M)[2]		Mascot
1193.558	1193.5924	0.0344	29	128	137	ELCSTGRDQK			Carbamidomethyl (C)[3]		Mascot
1194.5646	1194.5737	0.0091	8	261	271	AFPDCIAMAAK			Carbamidomethyl (C)[5]		Mascot

1201.5518	1201.5695	0.0177	15	226	236	EMEHAVGEATK						Mascot
1210.5596	1210.5928	0.0332	27	261	271	AFPDCIAMAAK			Carbamidomethyl (C)[5], Oxidation (M)[8]			Mascot
1259.6161	1259.6483	0.0322	26	250	260	HMEASLSKANR			Oxidation (M)[2]			Mascot
1277.6583	1277.683	0.0247	19	156	166	NTDEITKVTEK						Mascot
1358.6118	1358.6504	0.0386	28	274	284	AMHQNTEEQVR			Oxidation (M)[2]			Mascot
1479.7448	1479.7106	-0.0342	-23	261	273	AFPDCIAMAAKLR			Carbamidomethyl (C)[5], Oxidation (M)[8]			Mascot
1486.7318	1486.7281	-0.0037	-2	224	236	IREMEHAVGEATK			Oxidation (M)[4]			Mascot
1627.797	1627.6421	-0.1549	-95	272	284	LRAMHQNTEEQVR			Oxidation (M)[4]			Mascot
1707.7758	1707.7434	-0.0324	-19	485	498	MDMFINFSDPFIAK			Oxidation (M)[1,3]			Mascot
1838.9066	1838.8928	-0.0138	-8	468	484	VIAGVGTCQEGETSFRK			Carbamidomethyl (C)[8]			Mascot
1890.8499	1890.9318	0.0819	43	226	242	EMEHAVGEATKDSDLSR			Oxidation (M)[2]			Mascot
1930.9771	1930.8529	-0.1242	-64	563	579	RWHILGLGYDSGIDSNK						Mascot

10	PREDICTED: uncharacterized protein LOC101256605 [Solanum lycopersicum]	gi|460396155	170456.7	4.74	28	52	0	8.956

Peptide Information

Calc. Mass	Obsrv. Mass	± da	± ppm	Start Seq.	End Seq.	Sequence	Ion Score	C. I. %	Modification		Rank	Result Type
806.3461	806.3558	0.0097	12	507	513	CAAAEER			Carbamidomethyl (C)[1]			Mascot
817.4301	817.3734	-0.0567	-69	1075	1081	ILEAEDK						Mascot
820.441	820.3835	-0.0575	-70	819	825	VEETSKK						Mascot
822.4355	822.3696	-0.0659	-80	1264	1271	VSAAFAEK						Mascot
851.3815	851.3828	0.0013	2	959	965	ESEVMEK						Mascot
858.3522	858.4236	0.0714	83	598	604	CAEHEGR			Carbamidomethyl (C)[1]			Mascot
878.473	878.4086	-0.0644	-73	237	243	KALDFER						Mascot
938.4465	938.4265	-0.02	-21	756	762	LQEYEEK						Mascot
1033.5161	1033.4933	-0.0228	-22	302	310	SQVQDIESK						Mascot
1081.483	1081.4514	-0.0316	-29	1382	1390	EEKEAMSNK			Oxidation (M)[6]			Mascot
1165.5405	1165.5651	0.0246	21	1170	1179	ESEGKELMDK						Mascot
1182.6365	1182.5366	-0.0999	-84	1217	1226	HLESVVEELK						Mascot
1182.6365	1182.5366	-0.0999	-84	1217	1226	HLESVVEELK						Mascot
1193.6008	1193.5924	-0.0084	-7	1400	1410	SSSEELQAKSK						Mascot
1262.6838	1262.5579	-0.1259	-100	882	893	SSSLEVVLAETK						Mascot
1263.6063	1263.642	0.0357	28	1061	1072	LASSETDNEGLK						Mascot
1302.6396	1302.6357	-0.0039	-3	968	978	SAEEQLERQGR						Mascot
1358.6118	1358.6504	0.0386	28	593	604	SVADKCAEHEGR			Carbamidomethyl (C)[6]			Mascot
1403.7264	1403.6831	-0.0433	-31	633	644	VSDLEQLLETEK						Mascot

1475.77	1475.7231	-0.0469	-32	108	121	VAGSLKDTESQNVK	Mascot
1479.7438	1479.7106	-0.0332	-22	912	923	NLEDVYRNSIEK	Mascot
1627.7963	1627.6421	-0.1542	-95	187	200	EAFDRLGLEFESSK	Mascot
1657.8214	1657.7297	-0.0917	-55	1385	1399	EAMSNKGLEHEATLK	Mascot
1699.9476	1699.7897	-0.1579	-93	840	853	IQELEEQISILEKK	Mascot
1891.0171	1890.9318	-0.0853	-45	838	852	YRIQELEEQISILEK	Mascot
2044.0597	2043.9326	-0.1271	-62	756	772	LQEYEEKIAHLDSELVK	Mascot
2218.1812	2218.1646	-0.0166	-7	337	356	ENISSLELLISSTKEDLQAK	Mascot
2257.1572	2257.0898	-0.0674	-30	1347	1365	SQLEVFQAEIHQKSQLESR	Mascot
2263.2026	2263.1274	-0.0752	-33	330	350	ASESQVKENISSLELLISSTK	Mascot

Gel Idx/Pos	159/G10	Instr./Gel Origin	BA2151/Sample Project 20140814	Process Status	Analysis Succeeded
Plate [#] Name	[1] Sample Project 20140814	Instrument Sample Name		Spectra	11

Rank	Protein Name	Accession No.	Protein MW	Protein PI	Pep. Count	Protein Score	Protein Score C. I. %	Intensity Matched	Total Ion Score	Total Ion C. I. %	Confirmed
1	predicted protein [Bathycoccus prasinos]	gi|412985790	70296.5	4.99	20	87	99.81	6.846			

Peptide Information

Calc. Mass	Obsrv. Mass	± da	± ppm	Start Seq.	End Seq.	Sequence	Ion Score	C. I. %	Modification	Rank	Result Type
802.424	802.4233	-0.0007	-1	85	91	KHVGMSK			Oxidation (M)[5]		Mascot
993.5186	993.476	-0.0426	-43	193	200	SFPICTLR			Carbamidomethyl (C)[5]		Mascot
1033.4545	1033.4932	0.0387	37	408	416	EEGEGKNDR					Mascot
1037.4899	1037.4971	0.0072	7	245	254	ENEAGTAFAK					Mascot
1066.5164	1066.4847	-0.0317	-30	330	338	GDRISEFDK					Mascot
1081.5525	1081.4749	-0.0776	-72	97	106	ESVEKFAGSK					Mascot
1158.6306	1158.5542	-0.0764	-66	255	263	RVFAYVFEK					Mascot
1193.5909	1193.5891	-0.0018	-2	245	255	ENEAGTAFAKR					Mascot
1287.6368	1287.578	-0.0588	-46	121	130	EERFGLDFFK					Mascot
1308.7271	1308.6404	-0.0867	-66	397	407	ILNAQKHLDEK					Mascot
1343.7417	1343.6594	-0.0823	-61	281	292	KPEALDSETLLK					Mascot
1379.5719	1379.6708	0.0989	72	355	365	CANYGIEYMSR			Carbamidomethyl (C)[1], Oxidation (M)[9]		Mascot
1497.7042	1497.7174	0.0132	9	310	321	WTMEEASEIFVR					Mascot
1513.6992	1513.7052	0.006	4	310	321	WTMEEASEIFVR			Oxidation (M)[3]		Mascot
1563.722	1563.7153	-0.0067	-4	267	278	DLLEREDMWANR			Oxidation (M)[8]		Mascot
1707.8185	1707.7317	-0.0868	-51	339	354	DDDDAVAFVTATAQLR					Mascot
1716.928	1716.8074	-0.1206	-70	131	145	KFDIVLNGLDNLEAR					Mascot
1856.8848	1856.9265	0.0417	22	310	325	WTMEEASEIFVRSAGK			Oxidation (M)[3]		Mascot
2717.2708	2717.0269	-0.2439	-90	179	200	YCSCFECAPKPVPKSFPI CTLR			Carbamidomethyl (C)[2,4,7,19]		Mascot
3052.3774	3052.5518	0.1744	57	339	365	DDDDAVAFVTATAQLRC ANYGIEYMSR			Carbamidomethyl (C)[17]		Mascot
3311.9414	3312.2476	0.3062	92	370	402	GVAGNIVHAVATTNAIISG LIVIEALKILNAQK					Mascot

Rank	Protein Name	Accession No.	Protein MW	Protein PI	Pep. Count	Protein Score	Protein Score C. I. %	Intensity Matched	Total Ion Score	Total Ion C. I. %	Confirmed
2	unnamed protein product [Vitis vinifera]	gi|297742497	21341.2	10.03	13	64	63.836	7.998			

Peptide Information

Calc. Mass	Obsrv. Mass	± da	± ppm	Start Seq.	End Seq.	Sequence	Ion Score	C. I. %	Modification	Rank	Result Type
847.4785	847.439	-0.0395	-47	112	119	LAFLGNGR					Mascot

Calc. Mass	Obsrv. Mass	± da	± ppm	Start Seq.	End Seq.	Sequence	Ion Score	C. I. %	Modification	Rank	Result Type
1003.5795	1003.5281	-0.0514	-51	112	120	LAFLGNGRR					Mascot
1065.5476	1065.4773	-0.0703	-66	177	184	IIYHSNYR					Mascot
1201.4897	1201.5873	0.0976	81	43	53	EMSSETMMAGK					Mascot
1262.6271	1262.5753	-0.0518	-41	1	11	MVSSSPRTNQR					Mascot
1306.7267	1306.6328	-0.0939	-72	175	184	LKIIYHSNYR					Mascot
1357.5908	1357.6211	0.0303	22	42	53	REMSSETMMAGK					Mascot
1373.5858	1373.6257	0.0399	29	42	53	REMSSETMMAGK			Oxidation (M)[3]		Mascot
1405.5757	1405.6198	0.0441	31	42	53	REMSSETMMAGK			Oxidation (M)[3,8,9]		Mascot
1487.8005	1487.7064	-0.0941	-63	29	41	SPEGWPVGLVYRK					Mascot
1491.7084	1491.7074	-0.001	-1	86	97	LFQRCDEPAMPK			Carbamidomethyl (C)[5]		Mascot
1507.7032	1507.7042	0.001	1	86	97	LFQRCDEPAMPK			Carbamidomethyl (C)[5], Oxidation (M)[10]		Mascot
1507.7032	1507.7042	0.001	1	86	97	LFQRCDEPAMPK			Carbamidomethyl (C)[5], Oxidation (M)[10]		Mascot
1527.6792	1527.679	-0.0002	0	64	76	SSNQTCPRMSFGR			Carbamidomethyl (C)[6]		Mascot
1584.6815	1584.6841	0.0026	2	155	170	ACAAMTPMAASNSTGK			Carbamidomethyl (C)[2], Oxidation (M)[5]		Mascot
1699.7743	1699.7893	0.015	9	121	137	ETEGGGATSRGAGEHQ R					Mascot
2510.0276	2510.0449	0.0173	7	147	170	CQDGDHGRACAAMTPM AASNSTGK			Carbamidomethyl (C)[1,10], Oxidation (M)[13]		Mascot

3	PREDICTED: wall-associated receptor kinase 2-like [Setaria italica]	gi|514774448	86554.4	5.81	12	61	17.153	2.896	32	0

Peptide Information

Calc. Mass	Obsrv. Mass	± da	± ppm	Start Seq.	End Seq.	Sequence	Ion Score	C. I. %	Modification	Rank	Result Type
973.5312	973.508	-0.0232	-24	669	676	ELLDTQVR	32	0			Mascot
1118.5371	1118.4799	-0.0572	-51	264	273	NVANCDAAKR			Carbamidomethyl (C)[5]		Mascot
1140.5388	1140.5468	0.008	7	471	480	SKMMEAAQTK			Oxidation (M)[3]		Mascot
1277.6008	1277.6824	0.0816	64	642	652	ALYLDGPEEDR					Mascot
1405.6958	1405.6198	-0.076	-54	641	652	KALYLDGPEEDR					Mascot
1412.6661	1412.5449	-0.1212	-86	473	484	MMEAAQTKEFAR					Mascot
1584.6815	1584.6841	0.0026	2	694	706	CLSMNGEERPTMK			Carbamidomethyl (C)[1], Oxidation (M)[4,12]		Mascot
1716.8956	1716.8074	-0.0882	-51	249	263	TDDFAVPVVLDWAIR					Mascot
1796.9794	1796.9375	-0.0419	-23	624	639	SDVYSFGVVVLELLTR					Mascot
1890.9644	1890.9237	-0.0407	-22	481	495	EFAREMFILSQINHR					Mascot
2369.0913	2368.9504	-0.1409	-59	50	68	CARDFGYELVCNHSYKP PR			Carbamidomethyl (C)[1,11]		Mascot
2510.1882	2510.0449	-0.1433	-57	404	427	FFEQNGGVILQQQMHSG GGAGGFK			Oxidation (M)[14]		Mascot

4	unnamed protein product [Ostreococcus tauri]	gi|308807913	23734.2	5.44	12	60	9.16	4.819

Peptide Information

Calc. Mass	Obsrv. Mass	± da	± ppm	Start Seq.	End Seq.	Sequence	Ion Score	C. I. %	Modification	Rank	Result Type
1081.5385	1081.4749	-0.0636	-59	105	114	ASARSAYEAR					Mascot
1106.551	1106.496	-0.055	-50	95	104	TMAAELEAVR			Oxidation (M)[2]		Mascot
1107.455	1107.5167	0.0617	56	117	126	ENEDADSTAR					Mascot
1193.6017	1193.5891	-0.0126	-11	1	10	MTVDQIAIMR			Oxidation (M)[1]		Mascot
1262.6521	1262.5753	-0.0768	-61	94	104	RTMAAELEAVR			Oxidation (M)[3]		Mascot
1263.556	1263.6522	0.0962	76	116	126	RENEDADSTAR					Mascot
1390.7358	1390.644	-0.0918	-66	162	173	MQLARLSDALEK			Oxidation (M)[1]		Mascot
1475.7635	1475.7178	-0.0457	-31	95	108	TMAAELEAVRASAR					Mascot
1491.7584	1491.7074	-0.051	-34	95	108	TMAAELEAVRASAR			Oxidation (M)[2]		Mascot
1563.7366	1563.7153	-0.0213	-14	40	53	ALNAMCAERAGLDR			Carbamidomethyl (C)[6], Oxidation (M)[5]		Mascot
1605.7657	1605.675	-0.0907	-56	13	26	VDVFGFQHVDDATR					Mascot
1856.946	1856.9265	-0.0195	-11	64	82	AADADATVEALAGRAGE LR					Mascot
2369.1223	2368.9504	-0.1719	-73	28	48	FALDVSEDTTVRALNAM CAER			Carbamidomethyl (C)[18]		Mascot

5 Os03g0322200 [Oryza sativa Japonica Group] gi|113548437 12035.7 8.82 8 59 0 2.684

Peptide Information

Calc. Mass	Obsrv. Mass	± da	± ppm	Start Seq.	End Seq.	Sequence	Ion Score	C. I. %	Modification	Rank	Result Type
973.5248	973.508	-0.0168	-17	28	36	AVGQLGCIR			Carbamidomethyl (C)[7]		Mascot
1037.5011	1037.4971	-0.004	-4	18	27	YGQASSPTAR					Mascot
1194.5242	1194.5734	0.0492	41	1	11	MNGGSPVMTER			Oxidation (M)[1]		Mascot
1332.6324	1332.5005	-0.1319	-99	2	13	NGGSPVMTERER					Mascot
1427.7026	1427.7507	0.0481	34	15	27	AYRYGQASSPTAR					Mascot
1479.6678	1479.7141	0.0463	31	1	13	MNGGSPVMTERER			Oxidation (M)[1]		Mascot
1529.6616	1529.671	0.0094	6	38	52	SWSNDSLSSYGGGGR					Mascot
1657.7566	1657.7561	-0.0005	0	37	52	KSWSNDSLSSYGGGGR					Mascot

6 hypothetical protein OsI_11356 [Oryza sativa Indica Group] gi|125543671 12234.8 8.82 8 59 0 2.684

Peptide Information

Calc. Mass	Obsrv. Mass	± da	± ppm	Start Seq.	End Seq.	Sequence	Ion Score	C. I. %	Modification	Rank	Result Type
973.5248	973.508	-0.0168	-17	28	36	AVGQLGCIR			Carbamidomethyl (C)[7]		Mascot
1037.5011	1037.4971	-0.004	-4	18	27	YGQASSPTAR					Mascot

1194.5242	1194.5734	0.0492	41	1	11	MNGGSPVMTER			Oxidation (M)[1]		Mascot
1332.6324	1332.5005	-0.1319	-99	2	13	NGGSPVMTERER					Mascot
1427.7026	1427.7507	0.0481	34	15	27	AYRYGQASSPTAR					Mascot
1479.6678	1479.7141	0.0463	31	1	13	MNGGSPVMTERER			Oxidation (M)[1]		Mascot
1529.6616	1529.671	0.0094	6	38	52	SWSNDSLSSYGGGGR					Mascot
1657.7566	1657.7561	-0.0005	0	37	52	KSWSNDSLSSYGGGGR					Mascot

7	phosphoenolpyruvate carboxylase 4 [Arabidopsis thaliana]	gi|332196714	117254.4	6.68	21	58	0	7.264

Protein Group

RecName: Full=Phosphoenolpyruvate carboxylase 4; Short=AtPPC4; Short=PEPC 4; Short=PEPCase 4	gi|73917652	117254.4	6.6799 998283 3862
phosphoenolpyruvate carboxylase 4 [Arabidopsis thaliana]	gi|30697740	117254.4	6.6799 998283 3862

Peptide Information

Calc. Mass	Obsrv. Mass	± da	± ppm	Start Seq.	End Seq.	Sequence	Ion Score	C. I. %	Modification	Rank	Result Type
905.4938	905.4398	-0.054	-60	72	79	QLTSEISK					Mascot
1003.4803	1003.5281	0.0478	48	1007	1014	DEDNNKLR					Mascot
1037.5851	1037.4971	-0.088	-85	111	119	VHNVTQLAR					Mascot
1065.5728	1065.4773	-0.0955	-90	670	677	LLSIDWYR					Mascot
1066.4874	1066.4847	-0.0027	-3	35	43	EVGNPFMEK			Oxidation (M)[7]		Mascot
1140.6008	1140.5468	-0.054	-47	210	219	QKPTPVDEAR					Mascot
1193.6677	1193.5891	-0.0786	-66	669	677	KLLSIDWYR					Mascot
1259.6664	1259.629	-0.0374	-30	80	90	MPLEEALTLAR			Oxidation (M)[1]		Mascot
1262.6448	1262.5753	-0.0695	-55	395	406	IDNGSRSGLTSR					Mascot
1306.6096	1306.6328	0.0232	18	899	909	GHADDLKEMYK					Mascot
1357.7474	1357.6211	-0.1263	-93	220	231	AGLNIVEQSLWK					Mascot
1427.8409	1427.7507	-0.0902	-63	880	893	FVLPAWLGVGAGLK					Mascot
1475.7794	1475.7178	-0.0616	-42	868	879	AIPWVFAWTQTR					Mascot
1490.7485	1490.6838	-0.0647	-43	197	208	EITSLWQTDELR					Mascot
1527.759	1527.679	-0.08	-52	700	712	DAGRFTAAWELYK					Mascot
1838.8966	1838.8805	-0.0161	-9	351	366	AHLPACIDFGESRHTK			Carbamidomethyl (C)[6]		Mascot
1838.8966	1838.8805	-0.0161	-9	351	366	AHLPACIDFGESRHTK			Carbamidomethyl (C)[6]		Mascot
1908.8657	1908.7643	-0.1014	-53	261	278	FGSWMGGDRDGNPNVTAK					Mascot
1977.0361	1976.9419	-0.0942	-48	955	971	ELMTTEKYVLVISGHEK					Mascot
2003.9742	2003.9404	-0.0338	-17	928	944	ADIPMTKHYDEQLVSEK					Mascot

Calc. Mass	Obsrv. Mass	± da	± ppm	Start Seq.	End Seq.	Sequence	Ion Score	C. I. %	Modification	Rank	Result Type
2054.1394	2054.0728	-0.0666	-32	650	668	VVPLFETVNDLRAAGPSI R					Mascot
2510.1477	2510.0449	-0.1028	-41	678	699	EHIQKNHNGHQEVMVGY SDSGK			Oxidation (M)[14]		Mascot

8	PREDICTED: armadillo repeat-containing protein 6-like [Setaria italica]	gi|514718472	49316.2	4.99	14	57	0	4.915	

Peptide Information

Calc. Mass	Obsrv. Mass	± da	± ppm	Start Seq.	End Seq.	Sequence	Ion Score	C. I. %	Modification	Rank	Result Type
847.4957	847.439	-0.0567	-67	161	167	IVMDILK			Oxidation (M)[3]		Mascot
948.4897	948.411	-0.0787	-83	153	160	FRQSEGPK					Mascot
1033.4918	1033.4932	0.0014	1	435	443	AKVMHGSCK			Carbamidomethyl (C)[8], Oxidation (M)[4]		Mascot
1073.5837	1073.5188	-0.0649	-60	421	430	TILLNDGAEK					Mascot
1473.7982	1473.6688	-0.1294	-88	155	167	QSEGPKIVMDILK			Oxidation (M)[9]		Mascot
1483.7573	1483.7032	-0.0541	-36	50	64	VPGEAAAAEVSPMVR					Mascot
1485.8384	1485.6992	-0.1392	-94	325	338	LAASDANKSVIIQR					Mascot
1487.7635	1487.7064	-0.0571	-38	112	126	NGGVEALVALCASAR			Carbamidomethyl (C)[11]		Mascot
1497.8094	1497.7174	-0.092	-61	202	213	VDELILHVMREK			Oxidation (M)[9]		Mascot
1605.7472	1605.675	-0.0722	-45	437	451	VMHGSCKDAASSALR			Carbamidomethyl (C)[6], Oxidation (M)[2]		Mascot
1628.8313	1628.8247	-0.0066	-4	263	278	EQVAPSSLPSACAALK			Carbamidomethyl (C)[12]		Mascot
1851.9204	1851.8669	-0.0535	-29	395	410	FPSSGQTQKQACLMIR			Carbamidomethyl (C)[12]		Mascot
2252.167	2251.9436	-0.2234	-99	227	246	VLLTPDDNRVVASQVYG YSR					Mascot
2510.2126	2510.0449	-0.1677	-67	372	394	SPENAARAMEIGYGTLAI QAMQR			Oxidation (M)[9,21]		Mascot

9	DHHC-type zinc finger family protein [Arabidopsis thaliana]	gi|332658152	79657.8	8.79	17	57	0	7.202	

Protein Group

DHHC-type zinc finger family protein [Arabidopsis thaliana]	gi|22328644	79657.8	8.7899 999618 5303
RecName: Full=Probable protein S-acyltransferase 19; AltName: Full=Probable palmitoyltransferase At4g15080; AltName: Full=Zinc finger DHHC domain-containing protein At4g15080	gi|75245666	79657.8	8.7899 999618 5303

Peptide Information

Calc. Mass	Obsrv. Mass	± da	± ppm	Start Seq.	End Seq.	Sequence	Ion Score	C. I. %	Modification	Rank	Result Type
847.4706	847.439	-0.0316	-37	689	695	MTLTLPR			Oxidation (M)[1]		Mascot
1033.5525	1033.4932	-0.0593	-57	115	124	TSTLPNSSVK					Mascot
1066.4912	1066.4847	-0.0065	-6	481	490	NSYAPSQGSR					Mascot

1073.564	1073.5188	-0.0452	-42	5	13	HGWQLPAHK					Mascot
1106.5146	1106.496	-0.0186	-17	250	258	DMETEIVNR					Mascot
1201.6589	1201.5873	-0.0716	-60	4	13	KHGWQLPAHK					Mascot
1259.5256	1259.629	0.1034	82	471	480	NNDPMLSHCR			Carbamidomethyl (C)[9], Oxidation (M)[5]		Mascot
1287.6652	1287.578	-0.0872	-68	125	137	GSVGDAQRVEAAK					Mascot
1320.6794	1320.5592	-0.1202	-91	594	605	YISVPATTSEPR					Mascot
1405.5195	1405.6198	0.1003	71	196	205	CVDCFDHHCR			Carbamidomethyl (C)[1,4,9]		Mascot
1432.7213	1432.6693	-0.052	-36	684	695	EGQDRMTLTLPR			Oxidation (M)[6]		Mascot
1490.6979	1490.6838	-0.0141	-9	64	77	CTAINPADPGIMSK			Carbamidomethyl (C)[1], Oxidation (M)[12]		Mascot
1657.7534	1657.7561	0.0027	2	468	480	EIRNNDPMLSHCR			Carbamidomethyl (C)[12], Oxidation (M)[8]		Mascot
1837.8861	1837.8904	0.0043	2	250	265	DMETEIVNRLGNGFSR					Mascot
1987.939	1987.9645	0.0255	13	377	395	MVPSTVDPDAAETAERGNK					Mascot
2003.934	2003.9404	0.0064	3	377	395	MVPSTVDPDAAETAERGNK			Oxidation (M)[1]		Mascot
2023.0303	2022.8824	-0.1479	-73	447	467	SGTISVVSSVSTEANGATLSR					Mascot
2367.0024	2367.2104	0.208	88	140	158	SCFNPLAICCGVFVYEDCR			Carbamidomethyl (C)[2,9,10,18]		Mascot

10	PREDICTED: mitogen-activated protein kinase kinase kinase kinase 2-like [Solanum lycopersicum]	gi|460398205	79555.8	6.17	17	57	0	6.873

Peptide Information

Calc. Mass	Obsrv. Mass	± da	± ppm	Start Seq.	End Seq.	Sequence	Ion Score	C. I. %	Modification	Rank	Result Type
973.5676	973.508	-0.0596	-61	540	548	STANLVQIK					Mascot
982.4774	982.413	-0.0644	-66	688	694	YMQLQQR			Oxidation (M)[2]		Mascot
1106.4895	1106.496	0.0065	6	51	59	CNSNLDDIR			Carbamidomethyl (C)[1]		Mascot
1262.5907	1262.5753	-0.0154	-12	51	60	CNSNLDDIRR			Carbamidomethyl (C)[1]		Mascot
1323.625	1323.6371	0.0121	9	569	580	TASVGDWIMESK					Mascot
1323.625	1323.6371	0.0121	9	569	580	TASVGDWIMESK					Mascot
1327.6125	1327.6224	0.0099	7	420	431	LDSDHQEEGGLK					Mascot
1432.7828	1432.6693	-0.1135	-79	302	314	MLQLKDAAQLASK			Oxidation (M)[1]		Mascot
1458.7153	1458.6808	-0.0345	-24	248	259	SFKEMVAMCLVK			Carbamidomethyl (C)[9], Oxidation (M)[5]		Mascot
1516.7854	1516.7207	-0.0647	-43	433	446	NSSKTELPPLTSDK					Mascot
1584.743	1584.6841	-0.0589	-37	251	263	EMVAMCLVKDQTK			Carbamidomethyl (C)[6], Oxidation (M)[2,5]		Mascot
1707.7504	1707.7317	-0.0187	-11	695	708	LNSMSNQGEDWDRR					Mascot
1837.9583	1837.8904	-0.0679	-37	104	120	ISYPDGFEEAVIGSILK					Mascot
1890.963	1890.9237	-0.0393	-21	225	241	VLLMTINSAPPGLDYDR			Oxidation (M)[4]		Mascot
1908.8644	1908.7643	-0.1001	-52	345	360	FQASMVQDDDEIPEIR			Oxidation (M)[5]		Mascot

2020.983	2020.9701	-0.0129	-6	569	586	TASVGDWIMESKLMPPS R	Oxidation (M)[9]	Mascot
2689.2634	2689.3337	0.0703	26	463	487	SQSGPLMPGVELSHSAS ERSANFER	Oxidation (M)[7]	Mascot
2717.2375	2717.0269	-0.2106	-78	174	197	NTFTGTPCWMAPEVLQP GTGYDFK	Carbamidomethyl (C)[8]	Mascot

Gel Idx/Pos	160/G11	Instr./Gel Origin	BA2151/Sample Project 20140814	Process Status	Analysis Succeeded
Plate [#] Name	[1] Sample Project 20140814	Instrument Sample Name		Spectra	11

Rank	Protein Name	Accession No.	Protein MW	Protein PI	Pep. Count	Protein Score	Protein Score C. I. %	Intensity Matched	Total Ion Score	Total Ion C. I. %	Confirmed
1	hypothetical protein TRIUR3_27725 [Triticum urartu]	gi|473741089	18823.3	4.55	11	459	100	78.457	383	100	

Peptide Information

Calc. Mass	Obsrv. Mass	± da	± ppm	Start Seq.	End Seq.	Sequence	Ion Score	C. I. %	Modification	Rank	Result Type
890.4553	890.4107	-0.0446	-50	86	92	QFISHMK					Mascot
896.4546	896.4383	-0.0163	-18	1	7	MLVYQDK					Mascot
906.4502	906.4373	-0.0129	-14	86	92	QFISHMK			Oxidation (M)[6]		Mascot
912.4495	912.4365	-0.013	-14	1	7	MLVYQDK			Oxidation (M)[1]		Mascot
912.4495	912.4365	-0.013	-14	1	7	MLVYQDK	25	0	Oxidation (M)[1]		Mascot
1046.5564	1046.5015	-0.0549	-52	86	93	QFISHMKR					Mascot
1062.5514	1062.5211	-0.0303	-29	86	93	QFISHMKR			Oxidation (M)[6]		Mascot
1063.5784	1063.5646	-0.0138	-13	67	75	VVDIVDTFR					Mascot
1063.5784	1063.5646	-0.0138	-13	67	75	VVDIVDTFR	72	99.986			Mascot
1075.5419	1075.5221	-0.0198	-18	76	84	LQEQPAFDK					Mascot
1122.5314	1122.5162	-0.0152	-14	102	111	LEGDDLDAFK					Mascot
1203.6368	1203.6132	-0.0236	-20	76	85	LQEQPAFDKK					Mascot
1203.6368	1203.6132	-0.0236	-20	76	85	LQEQPAFDKK	48	96.445			Mascot
1250.6263	1250.6012	-0.0251	-20	102	112	LEGDDLDAFKK					Mascot
1598.7697	1598.7454	-0.0243	-15	8	21	LSGDELLSDSFPYR					Mascot
1598.7697	1598.7454	-0.0243	-15	8	21	LSGDELLSDSFPYR	139	100			Mascot
1736.8643	1736.8264	-0.0379	-22	149	164	EGAADPTFLYFAHGLK					Mascot
1736.8643	1736.8264	-0.0379	-22	149	164	EGAADPTFLYFAHGLK	99	100			Mascot
2781.323	2781.2561	-0.0669	-24	125	148	LKDLQFFVGESMHDDGG VVFAYYK			Oxidation (M)[12]		Mascot

Rank	Protein Name	Accession No.	Protein MW	Protein PI	Pep. Count	Protein Score	Protein Score C. I. %	Intensity Matched	Total Ion Score	Total Ion C. I. %	Confirmed
2	RecName: Full=Translationally-controlled tumor protein homolog; Short=TCTP	gi|75246527	18851.3	4.55	11	459	100	78.453	383	100	

Peptide Information

Calc. Mass	Obsrv. Mass	± da	± ppm	Start Seq.	End Seq.	Sequence	Ion Score	C. I. %	Modification	Rank	Result Type
890.4553	890.4107	-0.0446	-50	86	92	QFISHMK					Mascot
896.4546	896.4383	-0.0163	-18	1	7	MLVYQDK					Mascot

906.4502	906.4373	-0.0129	-14	86	92	QFISHMK			Oxidation (M)[6]		Mascot
912.4495	912.4365	-0.013	-14	1	7	MLVYQDK			Oxidation (M)[1]		Mascot
912.4495	912.4365	-0.013	-14	1	7	MLVYQDK	25	0	Oxidation (M)[1]		Mascot
1046.5564	1046.5015	-0.0549	-52	86	93	QFISHMKR					Mascot
1062.5514	1062.5211	-0.0303	-29	86	93	QFISHMKR			Oxidation (M)[6]		Mascot
1063.5784	1063.5646	-0.0138	-13	67	75	VVDIVDTFR					Mascot
1063.5784	1063.5646	-0.0138	-13	67	75	VVDIVDTFR	72	99.986			Mascot
1075.5419	1075.5221	-0.0198	-18	76	84	LQEQPAFDK					Mascot
1150.5627	1150.5693	0.0066	6	102	111	LEGDDLDVFK					Mascot
1203.6368	1203.6132	-0.0236	-20	76	85	LQEQPAFDKK					Mascot
1203.6368	1203.6132	-0.0236	-20	76	85	LQEQPAFDKK	48	96.445			Mascot
1278.6577	1278.6486	-0.0091	-7	102	112	LEGDDLDVFKK					Mascot
1598.7697	1598.7454	-0.0243	-15	8	21	LSGDELLSDSFPYR					Mascot
1598.7697	1598.7454	-0.0243	-15	8	21	LSGDELLSDSFPYR	139	100			Mascot
1736.8643	1736.8264	-0.0379	-22	149	164	EGAADPTFLYFAHGLK					Mascot
1736.8643	1736.8264	-0.0379	-22	149	164	EGAADPTFLYFAHGLK	99	100			Mascot
2781.323	2781.2561	-0.0669	-24	125	148	LKDLQFFVGESMHDDGG VVFAYYK			Oxidation (M)[12]		Mascot

3 RecName: Full=Translationally-controlled tumor protein homolog; Short=TCTP; AltName: Full=HTP gi|20140865 18929.4 4.53 8 429 100 77.799 383 100

Peptide Information

Calc. Mass	Obsrv. Mass	± da	± ppm	Start Seq.	End Seq.	Sequence	Ion Score	C. I. %	Modification	Rank	Result Type
896.4546	896.4383	-0.0163	-18	1	7	MLVYQDK					Mascot
912.4495	912.4365	-0.013	-14	1	7	MLVYQDK			Oxidation (M)[1]		Mascot
912.4495	912.4365	-0.013	-14	1	7	MLVYQDK	25	0	Oxidation (M)[1]		Mascot
1063.5784	1063.5646	-0.0138	-13	67	75	VVDIVDTFR					Mascot
1063.5784	1063.5646	-0.0138	-13	67	75	VVDIVDTFR	72	99.986			Mascot
1075.5419	1075.5221	-0.0198	-18	76	84	LQEQPAFDK					Mascot
1150.5626	1150.5693	0.0067	6	102	111	LEGEELDAFK					Mascot
1203.6368	1203.6132	-0.0236	-20	76	85	LQEQPAFDKK					Mascot
1203.6368	1203.6132	-0.0236	-20	76	85	LQEQPAFDKK	48	96.445			Mascot
1278.6576	1278.6486	-0.009	-7	102	112	LEGEELDAFKK					Mascot
1598.7697	1598.7454	-0.0243	-15	8	21	LSGDELLSDSFPYR					Mascot
1598.7697	1598.7454	-0.0243	-15	8	21	LSGDELLSDSFPYR	139	100			Mascot
1736.8643	1736.8264	-0.0379	-22	149	164	EGAADPTFLYFAHGLK					Mascot
1736.8643	1736.8264	-0.0379	-22	149	164	EGAADPTFLYFAHGLK	99	100			Mascot

4	hypothetical protein F775_43961 [Aegilops tauschii]	gi|475625225	23152.4	4.77	11	288	100	39.046	219	100

Peptide Information

Calc. Mass	Obsrv. Mass	± da	± ppm	Start Seq.	End Seq.	Sequence	Ion Score	C. I. %	Modification	Rank	Result Type
890.4553	890.4107	-0.0446	-50	133	139	QFISHMK					Mascot
906.4502	906.4373	-0.0129	-14	133	139	QFISHMK			Oxidation (M)[6]		Mascot
1046.5564	1046.5015	-0.0549	-52	133	140	QFISHMKR					Mascot
1062.5514	1062.5211	-0.0303	-29	133	140	QFISHMKR			Oxidation (M)[6]		Mascot
1063.5784	1063.5646	-0.0138	-13	114	122	VVDIVDTFR					Mascot
1063.5784	1063.5646	-0.0138	-13	114	122	VVDIVDTFR	72	99.986			Mascot
1073.5449	1073.4961	-0.0488	-45	1	9	MAVDWNIPK					Mascot
1075.5419	1075.5221	-0.0198	-18	123	131	LQEQPAFDK					Mascot
1150.5627	1150.5693	0.0066	6	149	158	LEGDDLDVFK					Mascot
1182.5833	1182.5425	-0.0408	-35	34	42	MVVLMTMWR			Oxidation (M)[1]		Mascot
1182.5833	1182.5425	-0.0408	-35	34	42	MVVLMTMWR			Oxidation (M)[1]		Mascot
1203.6368	1203.6132	-0.0236	-20	123	132	LQEQPAFDKK					Mascot
1203.6368	1203.6132	-0.0236	-20	123	132	LQEQPAFDKK	48	96.445			Mascot
1278.6577	1278.6486	-0.0091	-7	149	159	LEGDDLDVFKK					Mascot
1736.8643	1736.8264	-0.0379	-22	185	200	EGAADPTFLYFAHGLK					Mascot
1736.8643	1736.8264	-0.0379	-22	185	200	EGAADPTFLYFAHGLK	99	100			Mascot
1838.9065	1838.8792	-0.0273	-15	48	63	NEITHDKMLPPTEASR					Mascot

5	translationally controlled tumor protein, partial [Cyrtandra longifolia]	gi|494115166	12959.5	4.88	5	146	100	28.119	119	100

Protein Group

translationally controlled tumor protein, partial [Cyrtandra kauaiensis]	gi|494115170	12959.5	4.8800 001144 4092
translationally controlled tumor protein, partial [Cyrtandra paludosa var. paludosa]	gi|494115164	12959.5	4.8800 001144 4092

Peptide Information

Calc. Mass	Obsrv. Mass	± da	± ppm	Start Seq.	End Seq.	Sequence	Ion Score	C. I. %	Modification	Rank	Result Type
1063.5784	1063.5646	-0.0138	-13	17	25	VVDIVDTFR					Mascot
1063.5784	1063.5646	-0.0138	-13	17	25	VVDIVDTFR	72	99.986			Mascot
1075.5419	1075.5221	-0.0198	-18	26	34	LQEQPAFDK					Mascot

Calc. Mass	Obsrv. Mass	± da	± ppm	Start Seq.	End Seq.	Sequence	Ion Score	C. I. %	Modification	Rank	Result Type
1203.6368	1203.6132	-0.0236	-20	26	35	LQEQPAFDKK					Mascot
1203.6368	1203.6132	-0.0236	-20	26	35	LQEQPAFDKK	48	96.445			Mascot
1320.7046	1320.5889	-0.1157	-88	52	62	LEGEELEVFKK					Mascot
1718.9575	1718.8237	-0.1338	-78	47	61	LLTAKLEGEELEVFK					Mascot
1718.9575	1718.8237	-0.1338	-78	47	61	LLTAKLEGEELEVFK					Mascot

6	translationally controlled tumor protein, partial [Cyrtandra cordifolia]	gi|494115168	12228.3	5.34	4	137	100	26.996	119	100	

Peptide Information

Calc. Mass	Obsrv. Mass	± da	± ppm	Start Seq.	End Seq.	Sequence	Ion Score	C. I. %	Modification	Rank	Result Type
1063.5784	1063.5646	-0.0138	-13	11	19	VVDIVDTFR					Mascot
1063.5784	1063.5646	-0.0138	-13	11	19	VVDIVDTFR	72	99.986			Mascot
1075.5419	1075.5221	-0.0198	-18	20	28	LQEQPAFDK					Mascot
1203.6368	1203.6132	-0.0236	-20	20	29	LQEQPAFDKK					Mascot
1203.6368	1203.6132	-0.0236	-20	20	29	LQEQPAFDKK	48	96.445			Mascot
1320.7046	1320.5889	-0.1157	-88	46	56	LEGEELEVFKK					Mascot

7	PREDICTED: translationally-controlled tumor protein homolog [Setaria italica]	gi|514810173	18946.5	4.49	4	136	100	26.914	119	100	

Peptide Information

Calc. Mass	Obsrv. Mass	± da	± ppm	Start Seq.	End Seq.	Sequence	Ion Score	C. I. %	Modification	Rank	Result Type
1063.5784	1063.5646	-0.0138	-13	67	75	VVDIVDTFR					Mascot
1063.5784	1063.5646	-0.0138	-13	67	75	VVDIVDTFR	72	99.986			Mascot
1075.5419	1075.5221	-0.0198	-18	76	84	LQEQPAFDK					Mascot
1203.6368	1203.6132	-0.0236	-20	76	85	LQEQPAFDKK					Mascot
1203.6368	1203.6132	-0.0236	-20	76	85	LQEQPAFDKK	48	96.445			Mascot
1752.8593	1752.8281	-0.0312	-18	149	164	DGATDPTFLYFAHGLK					Mascot

8	translationally controlled tumor protein [Elaeis guineensis]	gi|192910898	19187.5	4.53	4	136	100	28.014	119	100	

Peptide Information

Calc. Mass	Obsrv. Mass	± da	± ppm	Start Seq.	End Seq.	Sequence	Ion Score	C. I. %	Modification	Rank	Result Type
1063.5784	1063.5646	-0.0138	-13	67	75	VVDIVDTFR					Mascot
1063.5784	1063.5646	-0.0138	-13	67	75	VVDIVDTFR	72	99.986			Mascot
1075.5419	1075.5221	-0.0198	-18	76	84	LQEQPAFDK					Mascot

1203.6368	1203.6132	-0.0236	-20	76	85	LQEQPAFDKK						Mascot
1203.6368	1203.6132	-0.0236	-20	76	85	LQEQPAFDKK	48	96.445				Mascot
1758.8949	1758.8143	-0.0806	-46	149	164	EGATDPTFLYLAYGLK						Mascot

9	RecName: Full=Translationally-controlled tumor protein homolog; Short=TCTP	gi|20140683	19097.5	4.57	4	135	100	26.93	119	100	

Protein Group

translationally-controlled tumor protein homolog [Glycine max]	gi|351724251	19097.5	4.5700 001716 6138

Peptide Information

Calc. Mass	Obsrv. Mass	± da	± ppm	Start Seq.	End Seq.	Sequence	Ion Score	C. I. %	Modification	Rank	Result Type
1063.5784	1063.5646	-0.0138	-13	67	75	VVDIVDTFR					Mascot
1063.5784	1063.5646	-0.0138	-13	67	75	VVDIVDTFR	72	99.986			Mascot
1075.5419	1075.5221	-0.0198	-18	76	84	LQEQPAFDK					Mascot
1203.6368	1203.6132	-0.0236	-20	76	85	LQEQPAFDKK					Mascot
1203.6368	1203.6132	-0.0236	-20	76	85	LQEQPAFDKK	48	96.445			Mascot
1348.7107	1348.644	-0.0667	-49	102	112	LDAEQQELFKK					Mascot

10	PREDICTED: translationally-controlled tumor protein homolog [Solanum lycopersicum]	gi|460368783	18977.5	4.54	4	135	100	26.892	119	100	

Protein Group

RecName: Full=Translationally-controlled tumor protein homolog; Short=TCTP; AltName: Full=p23	gi|115502466	18891.5	4.5799 999237 0605

Peptide Information

Calc. Mass	Obsrv. Mass	± da	± ppm	Start Seq.	End Seq.	Sequence	Ion Score	C. I. %	Modification	Rank	Result Type
890.4941	890.4107	-0.0834	-94	112	119	KNIESATK					Mascot
1063.5784	1063.5646	-0.0138	-13	67	75	VVDIVDTFR					Mascot
1063.5784	1063.5646	-0.0138	-13	67	75	VVDIVDTFR	72	99.986			Mascot
1075.5419	1075.5221	-0.0198	-18	76	84	LQEQPAFDK					Mascot
1203.6368	1203.6132	-0.0236	-20	76	85	LQEQPAFDKK					Mascot
1203.6368	1203.6132	-0.0236	-20	76	85	LQEQPAFDKK	48	96.445			Mascot

Gel Idx/Pos	161/G12	Instr./Gel Origin	BA2151/Sample Project 20140814	Process Status	Analysis Succeeded
Plate [#] Name	[1] Sample Project 20140814	Instrument Sample Name		Spectra	11

Rank	Protein Name	Accession No.	Protein MW	Protein PI	Pep. Count	Protein Score	Protein Score C. I. %	Intensity Matched	Total Ion Score	Total Ion C. I. %	Confirmed
1	RecName: Full=2-Cys peroxiredoxin BAS1, chloroplastic; AltName: Full=Thiol-specific antioxidant protein; Flags: Precursor	gi|2829687	23426.2	5.71	6	208	100	50.838	146	100	

Protein Group

Protein Name	Accession No.	Protein MW	Protein PI
RecName: Full=2-Cys peroxiredoxin BAS1, chloroplastic; AltName: Full=Thiol-specific antioxidant protein; Flags: Precursor	gi|2499477	23398.1	5.4800 000190 7349

Peptide Information

Calc. Mass	Obsrv. Mass	± da	± ppm	Start Seq.	End Seq.	Sequence	Ion Score	C. I. %	Modification	Rank	Result Type
805.4818	805.4572	-0.0246	-31	141	147	GLFIIDK					Mascot
819.4207	819.4034	-0.0173	-21	164	170	SVDETLR					Mascot
1021.5565	1021.5298	-0.0267	-26	114	122	YPLVSDVTK					Mascot
1360.7107	1360.6843	-0.0264	-19	11	23	AAAEYDLPLVGNK					Mascot
1485.8424	1485.8036	-0.0388	-26	127	140	SFGVLIPDQGIALR					Mascot
1485.8424	1485.8036	-0.0388	-26	127	140	SFGVLIPDQGIALR	109	100			Mascot
1707.9137	1707.8635	-0.0502	-29	148	163	EGVIQHSTINNLGIGR					Mascot
1707.9137	1707.8635	-0.0502	-29	148	163	EGVIQHSTINNLGIGR	37	68.442			Mascot

Rank	Protein Name	Accession No.	Protein MW	Protein PI	Pep. Count	Protein Score	Protein Score C. I. %	Intensity Matched	Total Ion Score	Total Ion C. I. %	Confirmed
2	putative 2-Cys peroxiredoxin BAS1, chloroplastic [Aegilops tauschii]	gi|475600934	22967.7	4.92	5	195	100	50.317	146	100	

Peptide Information

Calc. Mass	Obsrv. Mass	± da	± ppm	Start Seq.	End Seq.	Sequence	Ion Score	C. I. %	Modification	Rank	Result Type
805.4818	805.4572	-0.0246	-31	136	142	GLFIIDK					Mascot
819.4207	819.4034	-0.0173	-21	159	165	SVDETLR					Mascot
1021.5565	1021.5298	-0.0267	-26	109	117	YPLVSDVTK					Mascot
1485.8424	1485.8036	-0.0388	-26	122	135	SFGVLIPDQGIALR					Mascot
1485.8424	1485.8036	-0.0388	-26	122	135	SFGVLIPDQGIALR	109	100			Mascot
1707.9137	1707.8635	-0.0502	-29	143	158	EGVIQHSTINNLGIGR					Mascot
1707.9137	1707.8635	-0.0502	-29	143	158	EGVIQHSTINNLGIGR	37	68.442			Mascot

Rank	Protein Name	Accession No.	Protein MW	Protein PI	Pep. Count	Protein Score	Protein Score C. I. %	Intensity Matched	Total Ion Score	Total Ion C. I. %	Confirmed
3	2-Cysteine peroxiredoxin [Arabidopsis thaliana]	gi|332003616	29932.3	5.55	3	168	100	44.981	146	100	

Protein Group

2-Cysteine peroxiredoxin [Arabidopsis thaliana]	gi|18415155	29932.3	5.5500 001907 3486
RecName: Full=2-Cys peroxiredoxin BAS1-like, chloroplastic; Short=2-Cys Prx B; Short=2-Cys peroxiredoxin B; AltName: Full=Thiol-specific antioxidant protein B; Flags: Precursor	gi|334302930	29932.3	5.5500 001907 3486
hypothetical protein ARALYDRAFT_908552 [Arabidopsis lyrata subsp. lyrata]	gi|297317047	29109.1	5.8200 001716 6138

Peptide Information

Calc. Mass	Obsrv. Mass	± da	± ppm	Start Seq.	End Seq.	Sequence	Ion Score	C. I. %	Modification	Rank	Result Type
805.4818	805.4572	-0.0246	-31	203	209	GLFIIDK					Mascot
1485.8424	1485.8036	-0.0388	-26	189	202	SFGVLIPDQGIALR					Mascot
1485.8424	1485.8036	-0.0388	-26	189	202	SFGVLIPDQGIALR	109	100			Mascot
1707.9137	1707.8635	-0.0502	-29	210	225	EGVIQHSTINNLGIGR					Mascot
1707.9137	1707.8635	-0.0502	-29	210	225	EGVIQHSTINNLGIGR	37	68.442			Mascot

4	Os02g0537700 [Oryza sativa Japonica Group]	gi|113536581	28307.5	5.67	3	131	100	33.691	109	100	

Protein Group

RecName: Full=2-Cys peroxiredoxin BAS1, chloroplastic; AltName: Full=Thiol-specific antioxidant protein; Flags: Precursor	gi|75323389	28307.5	5.6700 000762 9395

Peptide Information

Calc. Mass	Obsrv. Mass	± da	± ppm	Start Seq.	End Seq.	Sequence	Ion Score	C. I. %	Modification	Rank	Result Type
805.4818	805.4572	-0.0246	-31	191	197	GLFIIDK					Mascot
1021.5385	1021.5298	-0.0087	-9	44	53	LSASSSRSAR					Mascot
1485.8424	1485.8036	-0.0388	-26	177	190	SFGVLIPDQGIALR					Mascot
1485.8424	1485.8036	-0.0388	-26	177	190	SFGVLIPDQGIALR	109	100			Mascot

5	PREDICTED: 2-Cys peroxiredoxin BAS1, chloroplastic-like [Setaria italica]	gi|514713083	28203.6	5.97	3	130	100	33.691	109	100	

Peptide Information

Calc. Mass	Obsrv. Mass	± da	± ppm	Start Seq.	End Seq.	Sequence	Ion Score	C. I. %	Modification	Rank	Result Type
805.4818	805.4572	-0.0246	-31	190	196	GLFIIDK					Mascot
1021.5565	1021.5298	-0.0267	-26	163	171	YPLVSDVTK					Mascot

Calc. Mass	Obsrv. Mass	± da	± ppm	Start Seq.	End Seq.	Sequence	Ion Score	C. I. %	Modification	Rank	Result Type
1485.8424	1485.8036	-0.0388	-26	176	189	SFGVLIPDQGIALR					Mascot
1485.8424	1485.8036	-0.0388	-26	176	189	SFGVLIPDQGIALR	109	100			Mascot

6	hypothetical protein OsI_07554 [Oryza sativa Indica Group]	gi|218190919	28481.5	5.67	3	130	100	33.691	109	100

Peptide Information

Calc. Mass	Obsrv. Mass	± da	± ppm	Start Seq.	End Seq.	Sequence	Ion Score	C. I. %	Modification	Rank	Result Type
805.4818	805.4572	-0.0246	-31	193	199	GLFIIDK					Mascot
1021.5385	1021.5298	-0.0087	-9	46	55	LSASSSRSAR					Mascot
1485.8424	1485.8036	-0.0388	-26	179	192	SFGVLIPDQGIALR					Mascot
1485.8424	1485.8036	-0.0388	-26	179	192	SFGVLIPDQGIALR	109	100			Mascot

7	thioredoxin peroxidase [Elaeis guineensis]	gi|192910848	31894.5	7.66	2	121	100	32.729	109	100

Peptide Information

Calc. Mass	Obsrv. Mass	± da	± ppm	Start Seq.	End Seq.	Sequence	Ion Score	C. I. %	Modification	Rank	Result Type
805.4818	805.4572	-0.0246	-31	203	209	GLFIIDK					Mascot
1485.8424	1485.8036	-0.0388	-26	189	202	SFGVLIPDQGIALR					Mascot
1485.8424	1485.8036	-0.0388	-26	189	202	SFGVLIPDQGIALR	109	100			Mascot

8	PREDICTED: 2-Cys peroxiredoxin BAS1-like, chloroplastic-like [Solanum lycopersicum]	gi|460407951	29728.2	6	3	58	0	18.631	37	68.442

Peptide Information

Calc. Mass	Obsrv. Mass	± da	± ppm	Start Seq.	End Seq.	Sequence	Ion Score	C. I. %	Modification	Rank	Result Type
805.4818	805.4572	-0.0246	-31	197	203	GLFIIDK					Mascot
819.4207	819.4034	-0.0173	-21	220	226	SVDETLR					Mascot
1707.9137	1707.8635	-0.0502	-29	204	219	EGVIQHSTINNLGIGR					Mascot
1707.9137	1707.8635	-0.0502	-29	204	219	EGVIQHSTINNLGIGR	37	68.442			Mascot

9	thioredoxin peroxidase, partial [Nicotiana tabacum]	gi|407907615	29972.4	8.25	3	58	0	18.631	37	68.442

Peptide Information

Calc. Mass	Obsrv. Mass	± da	± ppm	Start Seq.	End Seq.	Sequence	Ion Score	C. I. %	Modification	Rank	Result Type
805.4818	805.4572	-0.0246	-31	200	206	GLFIIDK					Mascot
819.4207	819.4034	-0.0173	-21	223	229	SVDETLR					Mascot
1707.9137	1707.8635	-0.0502	-29	207	222	EGVIQHSTINNLGIGR					Mascot

1707.9137	1707.8635	-0.0502	-29	207	222	EGVIQHSTINNLGIGR	37	68.442			Mascot

10	RecName: Full=2-Cys peroxiredoxin BAS1, chloroplastic; Short=2-Cys Prx A; Short=2-Cys peroxiredoxin A; AltName: Full=Thiol-specific antioxidant protein A; Flags: Precursor	gi|14916972	29188	6.92	2	50	0	14.257	37	68.442	

Protein Group

2-Cys peroxiredoxin BAS1 [Arabidopsis thaliana]	gi|332641556	29188	6.9200 000762 9395
2-Cys peroxiredoxin BAS1 [Arabidopsis thaliana]	gi|15229806	29188	6.9200 000762 9395
2-cys peroxiredoxin, chloroplast [Arabidopsis lyrata subsp. lyrata]	gi|297328580	29142.9	6.1100 001335 144
RecName: Full=2-Cys peroxiredoxin BAS1, chloroplastic; AltName: Full=Thiol-specific antioxidant protein; Flags: Precursor	gi|3121825	29048.9	7.6999 998092 6514
hypothetical protein CARUB_v10014399mg [Capsella rubella]	gi|482567043	29220	6.8499 999046 3257

Peptide Information

Calc. Mass	Obsrv. Mass	± da	± ppm	Start Seq.	End Seq.	Sequence	Ion Score	C. I. %	Modification	Rank	Result Type
805.4818	805.4572	-0.0246	-31	196	202	GLFIIDK					Mascot
1707.9137	1707.8635	-0.0502	-29	203	218	EGVIQHSTINNLGIGR					Mascot
1707.9137	1707.8635	-0.0502	-29	203	218	EGVIQHSTINNLGIGR	37	68.442			Mascot

Gel Idx/Pos	162/G13	Instr./Gel Origin	BA2151/Sample Project 20140814	Process Status	Analysis Succeeded
Plate [#] Name	[1] Sample Project 20140814	Instrument Sample Name		Spectra	11

Rank	Protein Name	Accession No.	Protein MW	Protein PI	Pep. Count	Protein Score	Protein Score C. I. %	Intensity Matched	Total Ion Score	Total Ion C. I. %	Confirmed
1	Serpin-Z2B [Aegilops tauschii]	gi|475621781	43026.4	5.18	12	631	100	44.5	571	100	

Protein Group

RecName: Full=Serpin-Z2B; AltName: Full=TriaeZ2b; AltName: Full=WSZ2b; AltName: Full=WZS3	gi|75279909	43011.4	5.1799 998283 3862

Peptide Information

Calc. Mass	Obsrv. Mass	± da	± ppm	Start Seq.	End Seq.	Sequence	Ion Score	C. I. %	Modification	Rank	Result Type
925.5214	925.5009	-0.0205	-22	11	18	LSIAHQTR					Mascot
925.5214	925.5009	-0.0205	-22	11	18	LSIAHQTR	47	96.159			Mascot
947.5156	947.4725	-0.0431	-45	2	10	ATTLATDVR					Mascot
1137.6667	1137.6322	-0.0345	-30	172	181	LVLGNALYFK					Mascot
1137.6667	1137.6322	-0.0345	-30	172	181	LVLGNALYFK	56	99.49			Mascot
1192.5382	1192.5111	-0.0271	-23	182	191	GAWTDQFDPR					Mascot
1192.5382	1192.5111	-0.0271	-23	182	191	GAWTDQFDPR	71	99.986			Mascot
1223.5903	1223.5408	-0.0495	-40	127	137	AEAQSVDFQTK					Mascot
1372.7068	1372.6772	-0.0296	-22	159	171	DILPAGSIDNTTR					Mascot
1372.7068	1372.6772	-0.0296	-22	159	171	DILPAGSIDNTTR	91	100			Mascot
1446.7965	1446.7487	-0.0478	-33	11	22	LSIAHQTRFAFR					Mascot
1514.7485	1514.7039	-0.0446	-29	125	137	YKAEAQSVDFQTK					Mascot
1514.7485	1514.7039	-0.0446	-29	125	137	YKAEAQSVDFQTK	106	100			Mascot
1531.7751	1531.7251	-0.05	-33	138	151	AAEVTAQVNSWVEK					Mascot
2085.155	2085.105	-0.05	-24	152	171	VTTGLIKDILPAGSIDNTT R					Mascot
2085.155	2085.105	-0.05	-24	152	171	VTTGLIKDILPAGSIDNTT R	200	100			Mascot
2838.4858	2838.4041	-0.0817	-29	99	124	VAFANGVFVDASLQLKPS FQELAVCK			Carbamidomethyl (C)[25]		Mascot
3751.9614	3751.9146	-0.0468	-12	23	61	LASAISSNPESTVNNAAF SPVSLHVALSLITAGAGG ATR					Mascot

Rank	Protein Name	Accession No.	Protein MW	Protein PI	Pep. Count	Protein Score	Protein Score C. I. %	Intensity Matched	Total Ion Score	Total Ion C. I. %	Confirmed
2	Serpin-Z2B [Triticum urartu]	gi|473793747	45225.7	6.03	9	312	100	30.998	280	100	

Peptide Information

Calc. Mass	Obsrv. Mass	± da	± ppm	Start Seq.	End Seq.	Sequence	Ion Score	C. I. %	Modification	Rank	Result Type

Calc. Mass	Obsrv. Mass	± da	± ppm	Start Seq.	End Seq.	Sequence	Ion Score	C. I. %	Modification	Rank	Result Type
925.5214	925.5009	-0.0205	-22	11	18	LSIAHQTR					Mascot
925.5214	925.5009	-0.0205	-22	11	18	LSIAHQTR	47	96.159			Mascot
947.5156	947.4725	-0.0431	-45	2	10	ATTLATDVR					Mascot
1137.6667	1137.6322	-0.0345	-30	189	198	LVLGNALYFK					Mascot
1137.6667	1137.6322	-0.0345	-30	189	198	LVLGNALYFK	56	99.49			Mascot
1192.5382	1192.5111	-0.0271	-23	199	208	GAWTDQFDPR					Mascot
1192.5382	1192.5111	-0.0271	-23	199	208	GAWTDQFDPR	71	99.986			Mascot
1223.5903	1223.5408	-0.0495	-40	127	137	AEAQSVDFQTK					Mascot
1446.7965	1446.7487	-0.0478	-33	11	22	LSIAHQTRFAFR					Mascot
1514.7485	1514.7039	-0.0446	-29	125	137	YKAEAQSVDFQTK					Mascot
1514.7485	1514.7039	-0.0446	-29	125	137	YKAEAQSVDFQTK	106	100			Mascot
1531.7751	1531.7251	-0.05	-33	155	168	AAEVTAQVNSWVEK					Mascot
2838.4858	2838.4041	-0.0817	-29	99	124	VAFANGVFVDASLQLKPS FQELAVCK			Carbamidomethyl (C)[25]		Mascot

3	RecName: Full=Serpin-Z2A; AltName: Full=TriaeZ2a; AltName: Full=WSZ2a	gi|75313847	43341.5	5.46	7	228	100	18.112	209	100	

Peptide Information

Calc. Mass	Obsrv. Mass	± da	± ppm	Start Seq.	End Seq.	Sequence	Ion Score	C. I. %	Modification	Rank	Result Type
925.5214	925.5009	-0.0205	-22	11	18	LSIAHQTR					Mascot
925.5214	925.5009	-0.0205	-22	11	18	LSIAHQTR	47	96.159			Mascot
947.5156	947.4725	-0.0431	-45	2	10	ATTLATDVR					Mascot
1137.6667	1137.6322	-0.0345	-30	172	181	LVLGNALYFK					Mascot
1137.6667	1137.6322	-0.0345	-30	172	181	LVLGNALYFK	56	99.49			Mascot
1182.5175	1182.5314	0.0139	12	182	191	GAWTDQFDSR					Mascot
1223.5903	1223.5408	-0.0495	-40	127	137	AEAQSVDFQTK					Mascot
1514.7485	1514.7039	-0.0446	-29	125	137	YKAEAQSVDFQTK					Mascot
1514.7485	1514.7039	-0.0446	-29	125	137	YKAEAQSVDFQTK	106	100			Mascot
1531.7751	1531.7251	-0.05	-33	138	151	AAEVTAQVNSWVEK					Mascot

4	RecName: Full=Serpin-ZX; AltName: Full=BSZx; AltName: Full=HorvuZx	gi|75281963	42920.3	6.77	6	117	100	12.358	102	100	

Peptide Information

Calc. Mass	Obsrv. Mass	± da	± ppm	Start Seq.	End Seq.	Sequence	Ion Score	C. I. %	Modification	Rank	Result Type
868.5138	868.5022	-0.0116	-13	357	364	SLPVEPVK					Mascot
925.5214	925.5009	-0.0205	-22	8	15	LSIAHQTR					Mascot

925.5214	925.5009	-0.0205	-22	8	15	LSIAHQTR	47	96.159			Mascot
1137.6667	1137.6322	-0.0345	-30	171	180	LVLGNALYFK					Mascot
1137.6667	1137.6322	-0.0345	-30	171	180	LVLGNALYFK	56	99.49			Mascot
1345.6958	1345.619	-0.0768	-57	158	170	EILPAGSVDSTTR					Mascot
1513.7645	1513.7068	-0.0577	-38	137	150	APEVAGQVNSWVEK					Mascot
1558.7869	1558.6805	-0.1064	-68	270	282	HMPMQKVPVGQFK			Oxidation (M)[2,4]		Mascot

5 Serpin-ZX [Aegilops tauschii] gi|475620098 39259.2 5.25 5 114 100 12.801 102 100

Peptide Information

Calc. Mass	Obsrv. Mass	± da	± ppm	Start Seq.	End Seq.	Sequence	Ion Score	C. I. %	Modification	Rank	Result Type
925.5214	925.5009	-0.0205	-22	8	15	LSIAHQTR					Mascot
925.5214	925.5009	-0.0205	-22	8	15	LSIAHQTR	47	96.159			Mascot
1137.6667	1137.6322	-0.0345	-30	174	183	LVLGNALYFK					Mascot
1137.6667	1137.6322	-0.0345	-30	174	183	LVLGNALYFK	56	99.49			Mascot
1159.6583	1159.6261	-0.0322	-28	239	248	HPPTEKVPVR					Mascot
1262.6012	1262.5374	-0.0638	-51	129	139	AETHSVDFQTK					Mascot
1553.7595	1553.7238	-0.0357	-23	127	139	YKAETHSVDFQTK					Mascot

6 PREDICTED: serpin-ZXA-like [Setaria italica] gi|514815360 70604.3 4.99 6 112 100 12.288 101 100

Peptide Information

Calc. Mass	Obsrv. Mass	± da	± ppm	Start Seq.	End Seq.	Sequence	Ion Score	C. I. %	Modification	Rank	Result Type
825.3737	825.3939	0.0202	24	444	450	FDASETR					Mascot
925.5214	925.5009	-0.0205	-22	8	15	LSIAHQTR					Mascot
925.5214	925.5009	-0.0205	-22	8	15	LSIAHQTR	47	96.159			Mascot
1137.6667	1137.6322	-0.0345	-30	428	437	LVLGNALYFK					Mascot
1137.6667	1137.6322	-0.0345	-30	176	185	LVLGNALYFK	56	99.49			Mascot
1175.5692	1175.5488	-0.0204	-17	131	140	AETHSIDFQK					Mascot
1345.7474	1345.619	-0.1284	-95	231	242	VLKLPYQQGGDK					Mascot
1552.8555	1552.71	-0.1455	-94	2	15	ATADIRLSIAHQTR					Mascot

7 RecName: Full=Serpin-Z7; AltName: Full=BSZ7; AltName: Full=HorvuZ7 gi|75282567 42851.2 5.45 4 110 100 11.913 102 100

Peptide Information

Calc. Mass	Obsrv. Mass	± da	± ppm	Start Seq.	End Seq.	Sequence	Ion Score	C. I. %	Modification	Rank	Result Type

925.5214	925.5009	-0.0205	-22	11	18	LSIAHQTR					Mascot
925.5214	925.5009	-0.0205	-22	11	18	LSIAHQTR	47	96.159			Mascot
1137.6667	1137.6322	-0.0345	-30	175	184	LVLGNALYFK					Mascot
1137.6667	1137.6322	-0.0345	-30	175	184	LVLGNALYFK	56	99.49			Mascot
1320.6431	1320.5927	-0.0504	-38	264	274	LSTEPDFLENR					Mascot
1496.7969	1496.707	-0.0899	-60	230	242	VLKLPYQHGGDNR					Mascot

8	Serpin-ZX [Aegilops tauschii]	gi|475616371	28932	5.2	3	81	99.295	6.164	74	99.992		

Peptide Information

Calc. Mass	Obsrv. Mass	± da	± ppm	Start Seq.	End Seq.	Sequence	Ion Score	C. I. %	Modification	Rank	Result Type
868.5138	868.5022	-0.0116	-13	222	229	SLPVEPVK					Mascot
1137.6667	1137.6322	-0.0345	-30	38	47	LVLGNALYFK					Mascot
1137.6667	1137.6322	-0.0345	-30	38	47	LVLGNALYFK	56	99.49			Mascot
1372.7068	1372.6772	-0.0296	-22	25	37	EILPAGSVDNTTR					Mascot
1372.7068	1372.6772	-0.0296	-22	25	37	EILPAGSVDNTTR	18	0			Mascot

9	Serpin-ZX [Triticum urartu]	gi|474139641	42666.2	5.89	3	80	98.957	6.164	74	99.992

Peptide Information

Calc. Mass	Obsrv. Mass	± da	± ppm	Start Seq.	End Seq.	Sequence	Ion Score	C. I. %	Modification	Rank	Result Type
868.5138	868.5022	-0.0116	-13	358	365	SLPVEPVK					Mascot
1137.6667	1137.6322	-0.0345	-30	174	183	LVLGNALYFK					Mascot
1137.6667	1137.6322	-0.0345	-30	174	183	LVLGNALYFK	56	99.49			Mascot
1372.7068	1372.6772	-0.0296	-22	161	173	EILPAGSVDNTTR					Mascot
1372.7068	1372.6772	-0.0296	-22	161	173	EILPAGSVDNTTR	18	0			Mascot

10	RecName: Full=Serpin-ZXA; AltName: Full=OrysaZxa	gi|75294978	42113.9	5.75	7	75	96.625	3.833	56	99.49

Peptide Information

Calc. Mass	Obsrv. Mass	± da	± ppm	Start Seq.	End Seq.	Sequence	Ion Score	C. I. %	Modification	Rank	Result Type
893.4727	893.4134	-0.0593	-66	116	124	TFGDVAVGK					Mascot
906.5043	906.4158	-0.0885	-98	276	283	QVTVGQFK					Mascot
1137.6667	1137.6322	-0.0345	-30	172	181	LVLGNALYFK					Mascot
1137.6667	1137.6322	-0.0345	-30	172	181	LVLGNALYFK	56	99.49			Mascot
1182.5824	1182.5314	-0.051	-43	205	215	SVQAPFMSTSK					Mascot
1262.6012	1262.5374	-0.0638	-51	127	137	AETHSVDFQTK					Mascot

1345.7474	1345.619	-0.1284	-95	227	238	VLKLPYQQGGDK	Mascot
1553.7595	1553.7238	-0.0357	-23	125	137	YKAETHSVDFQTK	Mascot

Gel Idx/Pos	163/G14	Instr./Gel Origin	BA2151/Sample Project 20140814	Process Status	Analysis Succeeded
Plate [#] Name	[1] Sample Project 20140814	Instrument Sample Name		Spectra	11

Rank	Protein Name	Accession No.	Protein MW	Protein PI	Pep. Count	Protein Score	Protein Score C. I. %	Intensity Matched	Total Ion Score	Total Ion C. I. %	Confirmed
1	putative 2-Cys peroxiredoxin BAS1, chloroplastic [Aegilops tauschii]	gi|475600934	22967.7	4.92	11	281	100	68.878	186	100	

Peptide Information

Calc. Mass	Obsrv. Mass	± da	± ppm	Start Seq.	End Seq.	Sequence	Ion Score	C. I. %	Modification	Rank	Result Type
805.4818	805.4611	-0.0207	-26	136	142	GLFIIDK					Mascot
818.4077	818.3916	-0.0161	-20	191	197	SMKPDPK			Oxidation (M)[2]		Mascot
819.4207	819.4026	-0.0181	-22	159	165	SVDETLR					Mascot
1021.5565	1021.5464	-0.0101	-10	109	117	YPLVSDVTK					Mascot
1485.8424	1485.8185	-0.0239	-16	122	135	SFGVLIPDQGIALR					Mascot
1485.8424	1485.8185	-0.0239	-16	122	135	SFGVLIPDQGIALR	134	100			Mascot
1707.9137	1707.8865	-0.0272	-16	143	158	EGVIQHSTINNLGIGR					Mascot
1707.9137	1707.8865	-0.0272	-16	143	158	EGVIQHSTINNLGIGR	17	0			Mascot
1748.943	1748.8667	-0.0763	-44	101	117	SGGLGDLKYPLVSDVTK					Mascot
2010.9808	2010.9224	-0.0584	-29	19	36	APDFAAEAVFDQEFINVK					Mascot
2010.9808	2010.9224	-0.0584	-29	19	36	APDFAAEAVFDQEFINVK					Mascot
2494.3777	2494.3333	-0.0444	-18	136	158	GLFIIDKEGVIQHSTINNL GIGR					Mascot
2494.3777	2494.3333	-0.0444	-18	136	158	GLFIIDKEGVIQHSTINNL GIGR	34	26.689			Mascot
2700.3992	2700.3516	-0.0476	-18	76	99	INTEILGVSVDSVFSHLA WVQTER					Mascot
2700.3992	2700.3516	-0.0476	-18	76	99	INTEILGVSVDSVFSHLA WVQTER					Mascot
2857.3826	2857.3193	-0.0633	-22	166	190	TLQALQYVQENPDEVCP AGWKPGEK			Carbamidomethyl (C)[16]		Mascot

Rank	Protein Name	Accession No.	Protein MW	Protein PI	Pep. Count	Protein Score	Protein Score C. I. %	Intensity Matched	Total Ion Score	Total Ion C. I. %	Confirmed
2	RecName: Full=2-Cys peroxiredoxin BAS1, chloroplastic; AltName: Full=Thiol-specific antioxidant protein; Flags: Precursor	gi|2829687	23426.2	5.71	11	278	100	68.743	186	100	

Protein Group

Protein Name	Accession No.	Protein MW	Protein PI
RecName: Full=2-Cys peroxiredoxin BAS1, chloroplastic; AltName: Full=Thiol-specific antioxidant protein; Flags: Precursor	gi|2499477	23398.1	5.4800 000190 7349

Peptide Information

Calc. Mass	Obsrv. Mass	± da	± ppm	Start Seq.	End Seq.	Sequence	Ion Score	C. I. %	Modification	Rank	Result Type

Calc. Mass	Obsrv. Mass	± da	± ppm	Start Seq.	End Seq.	Sequence	Ion Score	C. I. %	Modification	Rank	Result Type
805.4818	805.4611	-0.0207	-26	141	147	GLFIIDK					Mascot
818.4077	818.3916	-0.0161	-20	195	201	SMKPDPK			Oxidation (M)[2]		Mascot
819.4207	819.4026	-0.0181	-22	164	170	SVDETLR					Mascot
1021.5565	1021.5464	-0.0101	-10	114	122	YPLVSDVTK					Mascot
1360.7107	1360.6747	-0.036	-26	11	23	AAAEYDLPLVGNK					Mascot
1485.8424	1485.8185	-0.0239	-16	127	140	SFGVLIPDQGIALR					Mascot
1485.8424	1485.8185	-0.0239	-16	127	140	SFGVLIPDQGIALR	134	100			Mascot
1707.9137	1707.8865	-0.0272	-16	148	163	EGVIQHSTINNLGIGR					Mascot
1707.9137	1707.8865	-0.0272	-16	148	163	EGVIQHSTINNLGIGR	17	0			Mascot
1748.943	1748.8667	-0.0763	-44	106	122	SGGLGDLKYPLVSDVTK					Mascot
2010.9808	2010.9224	-0.0584	-29	24	41	APDFAAEAVFDQEFINVK					Mascot
2010.9808	2010.9224	-0.0584	-29	24	41	APDFAAEAVFDQEFINVK					Mascot
2494.3777	2494.3333	-0.0444	-18	141	163	GLFIIDKEGVIQHSTINNL GIGR					Mascot
2494.3777	2494.3333	-0.0444	-18	141	163	GLFIIDKEGVIQHSTINNL GIGR	34	26.689			Mascot
2700.3992	2700.3516	-0.0476	-18	81	104	INTEILGVSVDSVFSHLA WVQTER					Mascot
2700.3992	2700.3516	-0.0476	-18	81	104	INTEILGVSVDSVFSHLA WVQTER					Mascot

3	RecName: Full=2-Cys peroxiredoxin BAS1-like, chloroplastic; Short=2-Cys Prx B; Short=2-Cys peroxiredoxin B; AltName: Full=Thiol-specific antioxidant protein B; Flags: Precursor	gi|334302930	29932.3	5.55	8	237	100	61.384	186	100	

Protein Group

2-Cysteine peroxiredoxin [Arabidopsis thaliana]	gi|18415155	29932.3	5.5500 001907 3486
2-Cysteine peroxiredoxin [Arabidopsis thaliana]	gi|332003616	29932.3	5.5500 001907 3486

Peptide Information

Calc. Mass	Obsrv. Mass	± da	± ppm	Start Seq.	End Seq.	Sequence	Ion Score	C. I. %	Modification	Rank	Result Type
805.4818	805.4611	-0.0207	-26	203	209	GLFIIDK					Mascot
818.4077	818.3916	-0.0161	-20	258	264	SMKPDPK			Oxidation (M)[2]		Mascot
1485.8424	1485.8185	-0.0239	-16	189	202	SFGVLIPDQGIALR					Mascot
1485.8424	1485.8185	-0.0239	-16	189	202	SFGVLIPDQGIALR	134	100			Mascot
1707.9137	1707.8865	-0.0272	-16	210	225	EGVIQHSTINNLGIGR					Mascot
1707.9137	1707.8865	-0.0272	-16	210	225	EGVIQHSTINNLGIGR	17	0			Mascot
1748.9066	1748.8667	-0.0399	-23	168	184	SGGLGDLNYPLVSDITK					Mascot

Calc. Mass	Obsrv. Mass	± da	± ppm	Start Seq.	End Seq.	Sequence	Ion Score	C. I. %	Modification	Rank	Result Type
1935.8998	1935.8794	-0.0204	-11	1	19	MSMASIASSSSTTLLSSSR			Oxidation (M)[1,3]		Mascot
2494.3777	2494.3333	-0.0444	-18	203	225	GLFIIDKEGVIQHSTINNLGIGR					Mascot
2494.3777	2494.3333	-0.0444	-18	203	225	GLFIIDKEGVIQHSTINNLGIGR	34	26.689			Mascot
2857.3826	2857.3193	-0.0633	-22	233	257	TLQALQYVQENPDEVCPAGWKPGEK			Carbamidomethyl (C)[16]		Mascot

4	hypothetical protein ARALYDRAFT_908552 [Arabidopsis lyrata subsp. lyrata]	gi|297317047	29109.1	5.82	7	229	100	61.157	186	100

Peptide Information

Calc. Mass	Obsrv. Mass	± da	± ppm	Start Seq.	End Seq.	Sequence	Ion Score	C. I. %	Modification	Rank	Result Type
805.4818	805.4611	-0.0207	-26	194	200	GLFIIDK					Mascot
818.4077	818.3916	-0.0161	-20	249	255	SMKPDPK			Oxidation (M)[2]		Mascot
1485.8424	1485.8185	-0.0239	-16	180	193	SFGVLIPDQGIALR					Mascot
1485.8424	1485.8185	-0.0239	-16	180	193	SFGVLIPDQGIALR	134	100			Mascot
1707.9137	1707.8865	-0.0272	-16	201	216	EGVIQHSTINNLGIGR					Mascot
1707.9137	1707.8865	-0.0272	-16	201	216	EGVIQHSTINNLGIGR	17	0			Mascot
1748.9066	1748.8667	-0.0399	-23	159	175	SGGLGDLNYPLVSDITK					Mascot
2494.3777	2494.3333	-0.0444	-18	194	216	GLFIIDKEGVIQHSTINNLGIGR					Mascot
2494.3777	2494.3333	-0.0444	-18	194	216	GLFIIDKEGVIQHSTINNLGIGR	34	26.689			Mascot
2857.3826	2857.3193	-0.0633	-22	224	248	TLQALQYVQENPDEVCPAGWKPGEK			Carbamidomethyl (C)[16]		Mascot

5	Os02g0537700 [Oryza sativa Japonica Group]	gi|113536581	28307.5	5.67	7	175	100	53.175	134	100

Protein Group

RecName: Full=2-Cys peroxiredoxin BAS1, chloroplastic; AltName: Full=Thiol-specific antioxidant protein; Flags: Precursor	gi|75323389	28307.5	5.6700000762 9395

Peptide Information

Calc. Mass	Obsrv. Mass	± da	± ppm	Start Seq.	End Seq.	Sequence	Ion Score	C. I. %	Modification	Rank	Result Type
805.4818	805.4611	-0.0207	-26	191	197	GLFIIDK					Mascot
818.4077	818.3916	-0.0161	-20	246	252	SMKPDPK			Oxidation (M)[2]		Mascot
1021.5385	1021.5464	0.0079	8	44	53	LSASSSRSAR					Mascot
1485.8424	1485.8185	-0.0239	-16	177	190	SFGVLIPDQGIALR					Mascot
1485.8424	1485.8185	-0.0239	-16	177	190	SFGVLIPDQGIALR	134	100			Mascot
1539.8715	1539.7283	-0.1432	-93	36	50	APAARPLRLSASSSR					Mascot
2054.9707	2054.9089	-0.0618	-30	74	91	APDFDAEAVFDQEFINVK					Mascot

	2700.3992	2700.3516	-0.0476	-18	131	154	LNTEILGVSIDSVFSHLAW VQTDR						Mascot
	2700.3992	2700.3516	-0.0476	-18	131	154	LNTEILGVSIDSVFSHLAW VQTDR						Mascot

6	hypothetical protein OsI_07554 [Oryza sativa Indica Group]	gi|218190919	28481.5	5.67	6	166	100	53.104	134	100	

Peptide Information

Calc. Mass	Obsrv. Mass	± da	± ppm	Start Seq.	End Seq.	Sequence	Ion Score	C. I. %	Modification	Rank	Result Type
805.4818	805.4611	-0.0207	-26	193	199	GLFIIDK					Mascot
818.4077	818.3916	-0.0161	-20	248	254	SMKPDPK			Oxidation (M)[2]		Mascot
1021.5385	1021.5464	0.0079	8	46	55	LSASSSRSAR					Mascot
1485.8424	1485.8185	-0.0239	-16	179	192	SFGVLIPDQGIALR					Mascot
1485.8424	1485.8185	-0.0239	-16	179	192	SFGVLIPDQGIALR	134	100			Mascot
2054.9707	2054.9089	-0.0618	-30	76	93	APDFDAEAVFDQEFINVK					Mascot
2700.3992	2700.3516	-0.0476	-18	133	156	LNTEILGVSIDSVFSHLAW VQTDR					Mascot
2700.3992	2700.3516	-0.0476	-18	133	156	LNTEILGVSIDSVFSHLAW VQTDR					Mascot

7	PREDICTED: 2-Cys peroxiredoxin BAS1, chloroplastic-like [Setaria italica]	gi|514713083	28203.6	5.97	6	166	100	52.822	134	100	

Peptide Information

Calc. Mass	Obsrv. Mass	± da	± ppm	Start Seq.	End Seq.	Sequence	Ion Score	C. I. %	Modification	Rank	Result Type
805.4818	805.4611	-0.0207	-26	190	196	GLFIIDK					Mascot
818.4077	818.3916	-0.0161	-20	245	251	SMKPDPK			Oxidation (M)[2]		Mascot
1021.5565	1021.5464	-0.0101	-10	163	171	YPLVSDVTK					Mascot
1485.8424	1485.8185	-0.0239	-16	176	189	SFGVLIPDQGIALR					Mascot
1485.8424	1485.8185	-0.0239	-16	176	189	SFGVLIPDQGIALR	134	100			Mascot
1748.943	1748.8667	-0.0763	-44	155	171	SGGLGDLKYPLVSDVTK					Mascot
2700.3992	2700.3516	-0.0476	-18	130	153	LNTEILGVSIDSVFSHLAW VQTDR					Mascot
2700.3992	2700.3516	-0.0476	-18	130	153	LNTEILGVSIDSVFSHLAW VQTDR					Mascot

8	thioredoxin peroxidase [Elaeis guineensis]	gi|192910848	31894.5	7.66	3	144	100	49.063	134	100	

Peptide Information

Calc. Mass	Obsrv. Mass	± da	± ppm	Start Seq.	End Seq.	Sequence	Ion Score	C. I. %	Modification	Rank	Result Type
805.4818	805.4611	-0.0207	-26	203	209	GLFIIDK					Mascot
818.4077	818.3916	-0.0161	-20	258	264	SMKPDPK			Oxidation (M)[2]		Mascot

1485.8424	1485.8185	-0.0239	-16	189	202	SFGVLIPDQGIALR					Mascot
1485.8424	1485.8185	-0.0239	-16	189	202	SFGVLIPDQGIALR	134	100			Mascot

9	PREDICTED: 2-Cys peroxiredoxin BAS1-like, chloroplastic-like [Solanum lycopersicum]	gi|460407951	29728.2	6	7	93	99.948	15.224	51	98.574	

Peptide Information

Calc. Mass	Obsrv. Mass	± da	± ppm	Start Seq.	End Seq.	Sequence	Ion Score	C. I. %	Modification	Rank	Result Type
805.4818	805.4611	-0.0207	-26	197	203	GLFIIDK					Mascot
818.4077	818.3916	-0.0161	-20	252	258	SMKPDPK			Oxidation (M)[2]		Mascot
819.4207	819.4026	-0.0181	-22	220	226	SVDETLR					Mascot
1707.9137	1707.8865	-0.0272	-16	204	219	EGVIQHSTINNLGIGR					Mascot
1707.9137	1707.8865	-0.0272	-16	204	219	EGVIQHSTINNLGIGR	17	0			Mascot
1748.9066	1748.8667	-0.0399	-23	162	178	SGGLGDLNYPLISDVTK					Mascot
2494.3777	2494.3333	-0.0444	-18	197	219	GLFIIDKEGVIQHSTINNL GIGR					Mascot
2494.3777	2494.3333	-0.0444	-18	197	219	GLFIIDKEGVIQHSTINNL GIGR	34	26.689			Mascot
2857.3826	2857.3193	-0.0633	-22	227	251	TLQALQYVQENPDEVCP AGWKPGEK			Carbamidomethyl (C)[16]		Mascot

10	2-Cys peroxiredoxin BAS1 [Arabidopsis thaliana]	gi|332641556	29188	6.92	7	92	99.943	13.2	51	98.574	

Protein Group

2-Cys peroxiredoxin BAS1 [Arabidopsis thaliana]	gi|15229806	29188	6.9200 000762 9395
RecName: Full=2-Cys peroxiredoxin BAS1, chloroplastic; Short=2-Cys Prx A; Short=2-Cys peroxiredoxin A; AltName: Full=Thiol-specific antioxidant protein A; Flags: Precursor	gi|14916972	29188	6.9200 000762 9395

Peptide Information

Calc. Mass	Obsrv. Mass	± da	± ppm	Start Seq.	End Seq.	Sequence	Ion Score	C. I. %	Modification	Rank	Result Type
805.4818	805.4611	-0.0207	-26	196	202	GLFIIDK					Mascot
818.4077	818.3916	-0.0161	-20	251	257	SMKPDPK			Oxidation (M)[2]		Mascot
1550.8021	1550.8054	0.0033	2	2	17	ASVASSTTLISSPSSR					Mascot
1694.8821	1694.8005	-0.0816	-48	35	51	TLSSPSASASLRSGFAR					Mascot
1707.9137	1707.8865	-0.0272	-16	203	218	EGVIQHSTINNLGIGR					Mascot
1707.9137	1707.8865	-0.0272	-16	203	218	EGVIQHSTINNLGIGR	17	0			Mascot
1748.9066	1748.8667	-0.0399	-23	161	177	SGGLGDLNYPLISDVTK					Mascot
2494.3777	2494.3333	-0.0444	-18	196	218	GLFIIDKEGVIQHSTINNL GIGR					Mascot

2494.3777	2494.3333	-0.0444	-18	196	218	GLFIIDKEGVIQHSTINNL GIGR	34	26.689	Mascot

Gel Idx/Pos	164/G15	Instr./Gel Origin	BA2151/Sample Project 20140814	Process Status	Analysis Succeeded
Plate [#] Name	[1] Sample Project 20140814	Instrument Sample Name		Spectra	11

Rank	Protein Name	Accession No.	Protein MW	Protein PI	Pep. Count	Protein Score	Protein Score C. I. %	Intensity Matched	Total Ion Score	Total Ion C. I. %	Confirmed
1	RecName: Full=Serpin-Z1A; AltName: Full=TriaeZ1a; AltName: Full=WSZ1a; Short=WSZ1; AltName: Full=WSZCI	gi|75282265	43262.2	5.6	7	534	100	51.094	508	100	

Peptide Information

Calc. Mass	Obsrv. Mass	± da	± ppm	Start Seq.	End Seq.	Sequence	Ion Score	C. I. %	Modification	Rank	Result Type
1176.5896	1176.5861	-0.0035	-3	261	270	LSAEPDFLER					Mascot
1176.5896	1176.5861	-0.0035	-3	261	270	LSAEPDFLER	94	100			Mascot
1292.7097	1292.6851	-0.0246	-19	289	300	ISFGIEASDLLK					Mascot
1292.7097	1292.6851	-0.0246	-19	289	300	ISFGIEASDLLK	83	100			Mascot
1567.873	1567.8324	-0.0406	-26	287	300	FKISFGIEASDLLK					Mascot
1679.8864	1679.8699	-0.0165	-10	261	274	LSAEPDFLERHIPR					Mascot
2113.0999	2113.052	-0.0479	-23	379	398	EDISGVVLFMGHVVNPLLSS					Mascot
2113.0999	2113.052	-0.0479	-23	379	398	EDISGVVLFMGHVVNPLLSS	63	99.918			Mascot
2129.0947	2129.0298	-0.0649	-30	379	398	EDISGVVLFMGHVVNPLLSS			Oxidation (M)[10]		Mascot
2129.0947	2129.0298	-0.0649	-30	379	398	EDISGVVLFMGHVVNPLLSS	73	99.992	Oxidation (M)[10]		Mascot
2720.3525	2720.3323	-0.0202	-7	328	353	VSSVFHQAFVEVNEQGTEAAASTAIK					Mascot
2720.3525	2720.3323	-0.0202	-7	328	353	VSSVFHQAFVEVNEQGTEAAASTAIK	259	100			Mascot
2944.5642	2944.7695	0.2053	70	101	126	FANGVFVDASLLLKPSFQEIAVCKYK			Carbamidomethyl (C)[23]		Mascot

Rank	Protein Name	Accession No.	Protein MW	Protein PI	Pep. Count	Protein Score	Protein Score C. I. %	Intensity Matched	Total Ion Score	Total Ion C. I. %	Confirmed
2	Serpin-Z1B [Aegilops tauschii]	gi|475546073	43257.1	5.44	7	520	100	51.078	495	100	

Peptide Information

Calc. Mass	Obsrv. Mass	± da	± ppm	Start Seq.	End Seq.	Sequence	Ion Score	C. I. %	Modification	Rank	Result Type
1176.5896	1176.5861	-0.0035	-3	262	271	LSAEPDFLER					Mascot
1176.5896	1176.5861	-0.0035	-3	262	271	LSAEPDFLER	94	100			Mascot
1292.7097	1292.6851	-0.0246	-19	290	301	ISFGIEASDLLK					Mascot
1292.7097	1292.6851	-0.0246	-19	290	301	ISFGIEASDLLK	83	100			Mascot
1567.873	1567.8324	-0.0406	-26	288	301	FKISFGIEASDLLK					Mascot
1679.8864	1679.8699	-0.0165	-10	262	275	LSAEPDFLERHIPR					Mascot
2267.1628	2267.1016	-0.0612	-27	241	261	QFSMYILLPEAPGGLSSL			Oxidation (M)[4]		Mascot

Calc. Mass	Obsrv. Mass	± da	± ppm	Start Seq.	End Seq.	Sequence	Ion Score	C. I. %	Modification	Rank	Result Type
						AEK					
2720.3525	2720.3323	-0.0202	-7	329	354	VSSVFHQAFVEVNEQGT EAAASTAIK					Mascot
2720.3525	2720.3323	-0.0202	-7	329	354	VSSVFHQAFVEVNEQGT EAAASTAIK	259	100			Mascot
3039.3896	3039.376	-0.0136	-4	302	328	CLGLQLPFSDEADFSEM VDSPMPQGLR			Carbamidomethyl (C)[1]		Mascot
3055.3845	3055.3569	-0.0276	-9	302	328	CLGLQLPFSDEADFSEM VDSPMPQGLR			Carbamidomethyl (C)[1], Oxidation (M)[17]		Mascot
3071.3796	3071.3579	-0.0217	-7	302	328	CLGLQLPFSDEADFSEM VDSPMPQGLR			Carbamidomethyl (C)[1], Oxidation (M)[17,22]		Mascot
3071.3796	3071.3579	-0.0217	-7	302	328	CLGLQLPFSDEADFSEM VDSPMPQGLR	60	99.843	Carbamidomethyl (C)[1], Oxidation (M)[17,22]		Mascot

3	RecName: Full=Serpin-Z1B; AltName: Full=TriaeZ1b; AltName: Full=WSZ1b; AltName: Full=WZS2	gi|75279910	43119.9	5.44	6	506	100	46.058	486	100	

Peptide Information

Calc. Mass	Obsrv. Mass	± da	± ppm	Start Seq.	End Seq.	Sequence	Ion Score	C. I. %	Modification	Rank	Result Type
1176.5896	1176.5861	-0.0035	-3	262	271	LSAEPDFLER					Mascot
1176.5896	1176.5861	-0.0035	-3	262	271	LSAEPDFLER	94	100			Mascot
1679.8864	1679.8699	-0.0165	-10	262	275	LSAEPDFLERHIPR					Mascot
2113.0999	2113.052	-0.0479	-23	380	399	EDISGVVLFMGHVVNPLL SS					Mascot
2113.0999	2113.052	-0.0479	-23	380	399	EDISGVVLFMGHVVNPLL SS	63	99.918			Mascot
2129.0947	2129.0298	-0.0649	-30	380	399	EDISGVVLFMGHVVNPLL SS			Oxidation (M)[10]		Mascot
2129.0947	2129.0298	-0.0649	-30	380	399	EDISGVVLFMGHVVNPLL SS	73	99.992	Oxidation (M)[10]		Mascot
2267.1628	2267.1016	-0.0612	-27	241	261	QFSMYILLPEAPGGLSSL AEK			Oxidation (M)[4]		Mascot
2720.3525	2720.3323	-0.0202	-7	329	354	VSSVFHQAFVEVNEQGT EAAASTAIK					Mascot
2720.3525	2720.3323	-0.0202	-7	329	354	VSSVFHQAFVEVNEQGT EAAASTAIK	259	100			Mascot
3039.3896	3039.376	-0.0136	-4	302	328	CLGLQLPFSDEADFSEM VDSPMPQGLR			Carbamidomethyl (C)[1]		Mascot
3055.3845	3055.3569	-0.0276	-9	302	328	CLGLQLPFSDEADFSEM VDSPMPQGLR			Carbamidomethyl (C)[1], Oxidation (M)[17]		Mascot
3071.3796	3071.3579	-0.0217	-7	302	328	CLGLQLPFSDEADFSEM VDSPMPQGLR			Carbamidomethyl (C)[1], Oxidation (M)[17,22]		Mascot
3071.3796	3071.3579	-0.0217	-7	302	328	CLGLQLPFSDEADFSEM VDSPMPQGLR	60	99.843	Carbamidomethyl (C)[1], Oxidation (M)[17,22]		Mascot

4	Serpin-Z1C [Triticum urartu]	gi|474075261	42956	5.62	6	447	100	44.544	426	100	

Peptide Information

Calc. Mass	Obsrv. Mass	± da	± ppm	Start Seq.	End Seq.	Sequence	Ion Score	C. I. %	Modification	Rank	Result Type
1176.5896	1176.5861	-0.0035	-3	261	270	LSAEPDFLER					Mascot
1176.5896	1176.5861	-0.0035	-3	261	270	LSAEPDFLER	94	100			Mascot

1679.8864	1679.8699	-0.0165	-10	261	274	LSAEPDFLERHIPR					Mascot
2113.0999	2113.052	-0.0479	-23	379	398	EDISGVVLFMGHVVNPLL SS					Mascot
2113.0999	2113.052	-0.0479	-23	379	398	EDISGVVLFMGHVVNPLL SS	63	99.918			Mascot
2129.0947	2129.0298	-0.0649	-30	379	398	EDISGVVLFMGHVVNPLL SS			Oxidation (M)[10]		Mascot
2129.0947	2129.0298	-0.0649	-30	379	398	EDISGVVLFMGHVVNPLL SS	73	99.992	Oxidation (M)[10]		Mascot
2267.1628	2267.1016	-0.0612	-27	240	260	QFSMYILLPEAPGGLSSL AEK			Oxidation (M)[4]		Mascot
2720.3525	2720.3323	-0.0202	-7	328	353	VSSVFHQAFVEVNEQGT EAAASTAIK					Mascot
2720.3525	2720.3323	-0.0202	-7	328	353	VSSVFHQAFVEVNEQGT EAAASTAIK	259	100			Mascot
3037.3853	3037.4165	0.0312	10	301	327	CLGLQLPFSNEADFSEM VDSPMAHGLR			Carbamidomethyl (C)[1], Oxidation (M)[17]		Mascot

5	RecName: Full=Serpin-Z1C; AltName: Full=TriaeZ1c; AltName: Full=WSZ1c	gi|75313848	42969	5.62	5	442	100	44.052	426	100

Peptide Information

Calc. Mass	Obsrv. Mass	± da	± ppm	Start Seq.	End Seq.	Sequence	Ion Score	C. I. %	Modification	Rank	Result Type
1176.5896	1176.5861	-0.0035	-3	261	270	LSAEPDFLER					Mascot
1176.5896	1176.5861	-0.0035	-3	261	270	LSAEPDFLER	94	100			Mascot
1679.8864	1679.8699	-0.0165	-10	261	274	LSAEPDFLERHIPR					Mascot
2113.0999	2113.052	-0.0479	-23	379	398	EDISGVVLFMGHVVNPLL SS					Mascot
2113.0999	2113.052	-0.0479	-23	379	398	EDISGVVLFMGHVVNPLL SS	63	99.918			Mascot
2129.0947	2129.0298	-0.0649	-30	379	398	EDISGVVLFMGHVVNPLL SS			Oxidation (M)[10]		Mascot
2129.0947	2129.0298	-0.0649	-30	379	398	EDISGVVLFMGHVVNPLL SS	73	99.992	Oxidation (M)[10]		Mascot
2720.3525	2720.3323	-0.0202	-7	328	353	VSSVFHQAFVEVNEQGT EAAASTAIK					Mascot
2720.3525	2720.3323	-0.0202	-7	328	353	VSSVFHQAFVEVNEQGT EAAASTAIK	259	100			Mascot
3037.3853	3037.4165	0.0312	10	301	327	CLGLQLPFSNEADFSEM VDSPMAHGLR			Carbamidomethyl (C)[1], Oxidation (M)[17]		Mascot

6	Serpin-Z2B [Triticum urartu]	gi|473793747	45225.7	6.03	6	96	99.978	7.418	83	100

Peptide Information

Calc. Mass	Obsrv. Mass	± da	± ppm	Start Seq.	End Seq.	Sequence	Ion Score	C. I. %	Modification	Rank	Result Type
1154.6278	1154.5731	-0.0547	-47	146	154	YVMQLFLPK			Oxidation (M)[3]		Mascot
1192.5382	1192.5776	0.0394	33	199	208	GAWTDQFDPR					Mascot
1292.7097	1292.6851	-0.0246	-19	306	317	ISFGIEASDLLK					Mascot
1292.7097	1292.6851	-0.0246	-19	306	317	ISFGIEASDLLK	83	100			Mascot

1385.7019	1385.7474	0.0455	33	176	188	DILPAGSIDNNTR					Mascot
1567.873	1567.8324	-0.0406	-26	304	317	FKISFGIEASDLLK					Mascot
1665.8595	1665.8348	-0.0247	-15	278	291	LSAEPEFLEQHIPR					Mascot

7	RecName: Full=Serpin-Z2A; AltName: Full=TriaeZ2a; AltName: Full=WSZ2a	gi|75313847	43341.5	5.46	3	88	99.849	7.327	83	100	

Peptide Information

Calc. Mass	Obsrv. Mass	± da	± ppm	Start Seq.	End Seq.	Sequence	Ion Score	C. I. %	Modification	Rank	Result Type
1182.5175	1182.5504	0.0329	28	182	191	GAWTDQFDSR					Mascot
1292.7097	1292.6851	-0.0246	-19	289	300	ISFGIEASDLLK					Mascot
1292.7097	1292.6851	-0.0246	-19	289	300	ISFGIEASDLLK	83	100			Mascot
1567.873	1567.8324	-0.0406	-26	287	300	FKISFGIEASDLLK					Mascot

8	RecName: Full=14-3-3-like protein A; Short=14-3-3A	gi|112684	29447.8	4.83	12	66	74.398	1.994			

Peptide Information

Calc. Mass	Obsrv. Mass	± da	± ppm	Start Seq.	End Seq.	Sequence	Ion Score	C. I. %	Modification	Rank	Result Type
819.4458	819.4134	-0.0324	-40	95	101	IETELSK					Mascot
922.4199	922.4544	0.0345	37	130	136	MKGDYHR			Oxidation (M)[1]		Mascot
1051.5419	1051.516	-0.0259	-25	80	89	GNEAYVASIK					Mascot
1198.5334	1198.5574	0.024	20	251	262	EAASKPEGEGHS					Mascot
1336.7107	1336.6428	-0.0679	-51	148	159	KEAAENTLVAYK					Mascot
1616.849	1616.8638	0.0148	9	34	48	VAKTADVGELTVEER					Mascot
1632.7648	1632.84	0.0752	46	24	36	YEEMVEFMEKVAK					Mascot
1699.7955	1699.7889	-0.0066	-4	2	16	STAEATREENVYMAK					Mascot
1846.8309	1846.7903	-0.0406	-22	1	16	MSTAEATREENVYMAK			Oxidation (M)[1]		Mascot
2131.9675	2132.0564	0.0889	42	17	33	LAEQAERYEEMVEFMEK					Mascot
2289.249	2289.0933	-0.1557	-68	109	129	LLDSHLVPSATAAESKVF YLK					Mascot
3008.5688	3008.7617	0.1929	64	149	176	EAAENTLVAYKSAQDIAL ADLPTTHPIR					Mascot

9	RecName: Full=Protein HIRA; AltName: Full=Histone regulator protein	gi|122211829	106526.1	7.81	19	63	47.727	12.436			

Protein Group

protein HIRA [Zea mays]	gi|162463112	106526.1	7.8099 999427 7954

Peptide Information

Calc. Mass	Obsrv. Mass	± da	± ppm	Start Seq.	End Seq.	Sequence	Ion Score	C. I. %	Modification	Rank	Result Type
821.4338	821.426	-0.0078	-9	190	195	TVMIWR			Oxidation (M)[3]		Mascot
855.4683	855.4509	-0.0174	-20	573	579	LSIEHTR					Mascot
885.4213	885.4675	0.0462	52	269	275	FNNSTFR					Mascot
908.4948	908.4161	-0.0787	-87	241	248	HSAPVLER					Mascot
958.484	958.52	0.036	38	885	894	VGSASPTDPK					Mascot
1063.5531	1063.5066	-0.0465	-44	640	648	GTKTLWSDR					Mascot
1130.5988	1130.5557	-0.0431	-38	756	766	CGSPLVALASR			Carbamidomethyl (C)[1]		Mascot
1232.594	1232.6211	0.0271	22	913	923	EDILPSMASNR					Mascot
1292.6515	1292.6851	0.0336	26	618	631	GAGDMIGVGALSTK			Oxidation (M)[5]		Mascot
1292.6515	1292.6851	0.0336	26	618	631	GAGDMIGVGALSTK			Oxidation (M)[5]		Mascot
1575.7485	1575.8232	0.0747	47	64	76	DHFGSVNCVRWAK			Carbamidomethyl (C)[8]		Mascot
1711.847	1711.8552	0.0082	5	563	579	ASAGAGNDGRLSIEHTR					Mascot
1846.9269	1846.7903	-0.1366	-74	190	204	TVMIWRTSDWSLAHK			Oxidation (M)[3]		Mascot
2113.0781	2113.052	-0.0261	-12	580	599	SMAPSSLTPCSALSIHVINK			Carbamidomethyl (C)[10]		Mascot
2113.0781	2113.052	-0.0261	-12	580	599	SMAPSSLTPCSALSIHVINK			Carbamidomethyl (C)[10]		Mascot
2129.073	2129.0298	-0.0432	-20	580	599	SMAPSSLTPCSALSIHVINK			Carbamidomethyl (C)[10], Oxidation (M)[2]		Mascot
2129.073	2129.0298	-0.0432	-20	580	599	SMAPSSLTPCSALSIHVINK			Carbamidomethyl (C)[10], Oxidation (M)[2]		Mascot
2131.9131	2132.0564	0.1433	67	524	543	MNGTKPSYGSNSNSNNCGVK			Carbamidomethyl (C)[17], Oxidation (M)[1]		Mascot
2150.9626	2151.033	0.0704	33	96	116	KAGSGTSEFGSGEPPDAENWK					Mascot
2702.3745	2702.4214	0.0469	17	388	413	YGDVGGRQSNLAESPAQLLLEQASAK					Mascot
2742.3616	2742.3228	-0.0388	-14	721	747	ACILHDSLASLVASPDESSAKDAGTVK			Carbamidomethyl (C)[2]		Mascot
3006.6523	3006.4106	-0.2417	-80	2	27	ILEKPSWIRHEGLQIFSDIQTGGLR					Mascot
3037.532	3037.4165	-0.1155	-38	249	275	GEWAATFDFLGHNAPIVVVKFNNSTFR					Mascot

10	hypothetical protein ARALYDRAFT_907545 [Arabidopsis lyrata subsp. lyrata]	gi|297322380	189426	7.02	22	59	0	38.497	

Peptide Information

Calc. Mass	Obsrv. Mass	± da	± ppm	Start Seq.	End Seq.	Sequence	Ion Score	C. I. %	Modification	Rank	Result Type
800.4625	800.4015	-0.061	-76	255	261	SPITNIR					Mascot
922.4047	922.4544	0.0497	54	1360	1368	GPSMNSSAR			Oxidation (M)[4]		Mascot
1016.5015	1016.4904	-0.0111	-11	1249	1256	IMHACIQK			Carbamidomethyl (C)[5], Oxidation (M)[2]		Mascot
1161.5746	1161.5653	-0.0093	-8	899	909	LETADNSVNAK					Mascot

1176.5426	1176.5861	0.0435	37	990	999	RPSNMAVDDR			Oxidation (M)[5]	Mascot
1176.5426	1176.5861	0.0435	37	990	999	RPSNMAVDDR	18	0	Oxidation (M)[5]	Mascot
1232.5753	1232.6211	0.0458	37	1512	1523	TSDPSPEVSSAR				Mascot
1274.6951	1274.6344	-0.0607	-48	712	722	LVERSVISDEK				Mascot
1314.6549	1314.6804	0.0255	19	1054	1063	NTPDLERWQR				Mascot
1320.5736	1320.6599	0.0863	65	182	193	TSSAPPNMDEQK			Oxidation (M)[8]	Mascot
1354.6454	1354.696	0.0506	37	1301	1311	MLSCKQELSSR			Carbamidomethyl (C)[4], Oxidation (M)[1]	Mascot
1575.7697	1575.8232	0.0535	34	990	1002	RPSNMAVDDRWTK				Mascot
1616.8602	1616.8638	0.0036	2	586	600	SSVETAPVATTEIRR				Mascot
1632.7347	1632.84	0.1053	64	1205	1218	VAEEGQVEQTEEER				Mascot
1864.9573	1864.8802	-0.0771	-41	716	732	SVISDEKLETMSGVLEK				Mascot
2063.0315	2063.0562	0.0247	12	1071	1088	GLFPSPHTPMQVMHKAE R				Mascot
2143.0125	2142.8479	-0.1646	-77	1257	1274	LLGYNQDPHEENIEALCK			Carbamidomethyl (C)[17]	Mascot
2186.0474	2186.0989	0.0515	24	106	127	SQGVSGEPVVGGPANPT ESFNR				Mascot
2748.3647	2748.3564	-0.0083	-3	1670	1696	EGGEEPGSLIEFGLGGD VLGSVLEMIK			Oxidation (M)[25]	Mascot
2990.5552	2990.4666	-0.0886	-30	1419	1447	VVPMPQRSVGEEPITLGP QGGLGQGMSIR				Mascot
3006.55	3006.4106	-0.1394	-46	1419	1447	VVPMPQRSVGEEPITLGP QGGLGQGMSIR			Oxidation (M)[4]	Mascot
3022.4841	3022.3948	-0.0893	-30	1033	1060	MQGPIISRPMQPVGPMG GMGRNTPDLER				Mascot
3023.5671	3023.3804	-0.1867	-62	1003	1032	NQGSLPAGYGGNLGFRP GQGGNLGVLRNPR				Mascot
3054.4741	3054.3555	-0.1186	-39	1033	1060	MQGPIISRPMQPVGPMG GMGRNTPDLER			Oxidation (M)[1,10]	Mascot
3054.4741	3054.3555	-0.1186	-39	1033	1060	MQGPIISRPMQPVGPMG GMGRNTPDLER			Oxidation (M)[1,10]	Mascot
3070.469	3070.3481	-0.1209	-39	1033	1060	MQGPIISRPMQPVGPMG GMGRNTPDLER			Oxidation (M)[1,10,16]	Mascot
3071.5923	3071.3579	-0.2344	-76	1000	1029	WTKNQGSLPAGYGGNL GFRPGQGGNLGVLR				Mascot
3071.5923	3071.3579	-0.2344	-76	1000	1029	WTKNQGSLPAGYGGNL GFRPGQGGNLGVLR				Mascot

Gel Idx/Pos	165/G16	Instr./Gel Origin	BA2151/Sample Project 20140814	Process Status	Analysis Succeeded
Plate [#] Name	[1] Sample Project 20140814	Instrument Sample Name		Spectra	11

Rank	Protein Name	Accession No.	Protein MW	Protein PI	Pep. Count	Protein Score	Protein Score C. I. %	Intensity Matched	Total Ion Score	Total Ion C. I. %	Confirmed
1	14-3-3 protein [Triticum aestivum]	gi|431822520	29360.7	4.83	22	693	100	68.468	515	100	

Peptide Information

Calc. Mass	Obsrv. Mass	± da	± ppm	Start Seq.	End Seq.	Sequence	Ion Score	C. I. %	Modification	Rank	Result Type
816.421	816.4076	-0.0134	-16	17	23	LAEQAER					Mascot
907.5247	907.4792	-0.0455	-50	49	56	NLLSVAYK					Mascot
917.5302	917.5087	-0.0215	-23	68	75	IISSIEQK					Mascot
922.4199	922.4067	-0.0132	-14	130	136	MKGDYHR			Oxidation (M)[1]		Mascot
1051.5419	1051.522	-0.0199	-19	80	89	GNEAYVASIK					Mascot
1076.5946	1076.5731	-0.0215	-20	93	101	TRIETELSK					Mascot
1189.6609	1189.6497	-0.0112	-9	222	231	DSTLIMQLLR					Mascot
1189.6609	1189.6497	-0.0112	-9	222	231	DSTLIMQLLR	87	100			Mascot
1205.6559	1205.6342	-0.0217	-18	222	231	DSTLIMQLLR			Oxidation (M)[6]		Mascot
1205.6559	1205.6342	-0.0217	-18	222	231	DSTLIMQLLR	35	48.365	Oxidation (M)[6]		Mascot
1208.6157	1208.611	-0.0047	-4	149	159	EAAENTLVAYK					Mascot
1318.6486	1318.6364	-0.0122	-9	37	48	TADVGELTVEER					Mascot
1318.6486	1318.6364	-0.0122	-9	37	48	TADVGELTVEER	118	100			Mascot
1334.5643	1334.542	-0.0223	-17	24	33	YEEMVEFMEK					Mascot
1336.7107	1336.6345	-0.0762	-57	148	159	KEAAENTLVAYK					Mascot
1350.5592	1350.5243	-0.0349	-26	24	33	YEEMVEFMEK			Oxidation (M)[4]		Mascot
1366.5542	1366.511	-0.0432	-32	24	33	YEEMVEFMEK			Oxidation (M)[4,8]		Mascot
1418.7485	1418.7345	-0.014	-10	68	79	IISSIEQKEESR					Mascot
1517.8799	1517.8373	-0.0426	-28	49	62	NLLSVAYKNVIGAR					Mascot
1552.7601	1552.745	-0.0151	-10	76	89	EESRGNEAYVASIK					Mascot
1818.9708	1818.9503	-0.0205	-11	160	176	SAQDIALADLPTTHPIR					Mascot
1818.9708	1818.9503	-0.0205	-11	160	176	SAQDIALADLPTTHPIR	140	100			Mascot
1846.8309	1846.968	0.1371	74	1	16	MSTAEATREENVYMAK			Oxidation (M)[1]		Mascot
2106.9463	2106.8904	-0.0559	-27	232	250	DNLTLWTSDNAEEGGDEIK					Mascot
2114.9763	2114.9133	-0.063	-30	203	221	QAFDEAIAELDSLGEESYK					Mascot
2131.9675	2131.9492	-0.0183	-9	17	33	LAEQAERYEEMVEFMEK					Mascot
2131.9675	2131.9492	-0.0183	-9	17	33	LAEQAERYEEMVEFMEK	34	30.667			Mascot

2147.9624	2147.9199	-0.0425	-20	17	33	LAEQAERYEEMVEFMEK			Oxidation (M)[11]		Mascot
2147.9624	2147.9199	-0.0425	-20	17	33	LAEQAERYEEMVEFMEK	19	0	Oxidation (M)[15]		Mascot
2163.9573	2163.8999	-0.0574	-27	17	33	LAEQAERYEEMVEFMEK			Oxidation (M)[11,15]		Mascot
2163.9573	2163.8999	-0.0574	-27	17	33	LAEQAERYEEMVEFMEK	44	93.24	Oxidation (M)[11,15]		Mascot
2331.2019	2331.1804	-0.0215	-9	177	196	LGLALNFSVFYYEILNSPDR					Mascot
2331.2019	2331.1804	-0.0215	-9	177	196	LGLALNFSVFYYEILNSPDR	125	100			Mascot
3199.4297	3199.406	-0.0237	-7	232	261	DNLTLWTSDNAEEGGDEIKEAASKPEGEGH					Mascot
3285.6196	3285.6016	-0.018	-5	203	231	QAFDEAIAELDSLGEESYKDSTLIMQLLR					Mascot
3301.6145	3301.5984	-0.0161	-5	203	231	QAFDEAIAELDSLGEESYKDSTLIMQLLR			Oxidation (M)[25]		Mascot

2	RecName: Full=14-3-3-like protein A; Short=14-3-3A	gi|112684	29447.8	4.83	21	677	100	67.885	515	100	

Peptide Information

Calc. Mass	Obsrv. Mass	± da	± ppm	Start Seq.	End Seq.	Sequence	Ion Score	C. I. %	Modification	Rank	Result Type
816.421	816.4076	-0.0134	-16	17	23	LAEQAER					Mascot
907.5247	907.4792	-0.0455	-50	49	56	NLLSVAYK					Mascot
917.5302	917.5087	-0.0215	-23	68	75	IISSIEQK					Mascot
922.4199	922.4067	-0.0132	-14	130	136	MKGDYHR			Oxidation (M)[1]		Mascot
1051.5419	1051.522	-0.0199	-19	80	89	GNEAYVASIK					Mascot
1076.5946	1076.5731	-0.0215	-20	93	101	TRIETELSK					Mascot
1189.6609	1189.6497	-0.0112	-9	222	231	DSTLIMQLLR					Mascot
1189.6609	1189.6497	-0.0112	-9	222	231	DSTLIMQLLR	87	100			Mascot
1205.6559	1205.6342	-0.0217	-18	222	231	DSTLIMQLLR			Oxidation (M)[6]		Mascot
1205.6559	1205.6342	-0.0217	-18	222	231	DSTLIMQLLR	35	48.365	Oxidation (M)[6]		Mascot
1208.6157	1208.611	-0.0047	-4	149	159	EAAENTLVAYK					Mascot
1318.6486	1318.6364	-0.0122	-9	37	48	TADVGELTVEER					Mascot
1318.6486	1318.6364	-0.0122	-9	37	48	TADVGELTVEER	118	100			Mascot
1334.5643	1334.542	-0.0223	-17	24	33	YEEMVEFMEK					Mascot
1336.7107	1336.6345	-0.0762	-57	148	159	KEAAENTLVAYK					Mascot
1350.5592	1350.5243	-0.0349	-26	24	33	YEEMVEFMEK			Oxidation (M)[4]		Mascot
1366.5542	1366.511	-0.0432	-32	24	33	YEEMVEFMEK			Oxidation (M)[4,8]		Mascot
1418.7485	1418.7345	-0.014	-10	68	79	IISSIEQKEESR					Mascot
1517.8799	1517.8373	-0.0426	-28	49	62	NLLSVAYKNVIGAR					Mascot
1552.7601	1552.745	-0.0151	-10	76	89	EESRGNEAYVASIK					Mascot
1818.9708	1818.9503	-0.0205	-11	160	176	SAQDIALADLPTTHPIR					Mascot

Calc. Mass	Obsrv. Mass	± da	± ppm	Start Seq.	End Seq.	Sequence	Ion Score	C. I. %	Modification	Rank	Result Type
1818.9708	1818.9503	-0.0205	-11	160	176	SAQDIALADLPTTHPIR	140	100			Mascot
1846.8309	1846.968	0.1371	74	1	16	MSTAEATREENVYMAK			Oxidation (M)[1]		Mascot
2106.9463	2106.8904	-0.0559	-27	232	250	DNLTLWTSDNAEEGGDE IK					Mascot
2114.9763	2114.9133	-0.063	-30	203	221	QAFDEAIAELDSLGEESY K					Mascot
2131.9675	2131.9492	-0.0183	-9	17	33	LAEQAERYEEMVEFMEK					Mascot
2131.9675	2131.9492	-0.0183	-9	17	33	LAEQAERYEEMVEFMEK	34	30.667			Mascot
2147.9624	2147.9199	-0.0425	-20	17	33	LAEQAERYEEMVEFMEK			Oxidation (M)[11]		Mascot
2147.9624	2147.9199	-0.0425	-20	17	33	LAEQAERYEEMVEFMEK	19	0	Oxidation (M)[15]		Mascot
2163.9573	2163.8999	-0.0574	-27	17	33	LAEQAERYEEMVEFMEK			Oxidation (M)[11,15]		Mascot
2163.9573	2163.8999	-0.0574	-27	17	33	LAEQAERYEEMVEFMEK	44	93.24	Oxidation (M)[11,15]		Mascot
2331.2019	2331.1804	-0.0215	-9	177	196	LGLALNFSVFYYEILNSPD R					Mascot
2331.2019	2331.1804	-0.0215	-9	177	196	LGLALNFSVFYYEILNSPD R	125	100			Mascot
3285.6196	3285.6016	-0.018	-5	203	231	QAFDEAIAELDSLGEESY KDSTLIMQLLR					Mascot
3301.6145	3301.5984	-0.0161	-5	203	231	QAFDEAIAELDSLGEESY KDSTLIMQLLR			Oxidation (M)[25]		Mascot

3	uncharacterized protein [Phleum pratense]	gi|409972305	29275.7	4.82	20	664	100	67.446	515	100	

Protein Group

uncharacterized protein [Phleum pratense]	gi|409972067	29275.7	4.8200 001716 6138
uncharacterized protein [Phleum pratense]	gi|409971647	29275.7	4.8200 001716 6138
uncharacterized protein [Phleum pratense]	gi|409972513	29275.7	4.8200 001716 6138

Peptide Information

Calc. Mass	Obsrv. Mass	± da	± ppm	Start Seq.	End Seq.	Sequence	Ion Score	C. I. %	Modification	Rank	Result Type
816.421	816.4076	-0.0134	-16	17	23	LAEQAER					Mascot
907.5247	907.4792	-0.0455	-50	49	56	NLLSVAYK					Mascot
917.5302	917.5087	-0.0215	-23	68	75	IISSIEQK					Mascot
922.4199	922.4067	-0.0132	-14	130	136	MKGDYHR			Oxidation (M)[1]		Mascot
1051.5419	1051.522	-0.0199	-19	80	89	GNEAYVASIK					Mascot
1076.5946	1076.5731	-0.0215	-20	93	101	TRIETELSK					Mascot
1189.6609	1189.6497	-0.0112	-9	222	231	DSTLIMQLLR					Mascot
1189.6609	1189.6497	-0.0112	-9	222	231	DSTLIMQLLR	87	100			Mascot

1205.6559	1205.6342	-0.0217	-18	222	231	DSTLIMQLLR			Oxidation (M)[6]		Mascot
1205.6559	1205.6342	-0.0217	-18	222	231	DSTLIMQLLR	35	48.365	Oxidation (M)[6]		Mascot
1208.6157	1208.611	-0.0047	-4	149	159	EAAENTLVAYK					Mascot
1318.6486	1318.6364	-0.0122	-9	37	48	TADVGELTVEER					Mascot
1318.6486	1318.6364	-0.0122	-9	37	48	TADVGELTVEER	118	100			Mascot
1334.5643	1334.542	-0.0223	-17	24	33	YEEMVEFMEK					Mascot
1336.7107	1336.6345	-0.0762	-57	148	159	KEAAENTLVAYK					Mascot
1350.5592	1350.5243	-0.0349	-26	24	33	YEEMVEFMEK			Oxidation (M)[4]		Mascot
1366.5542	1366.511	-0.0432	-32	24	33	YEEMVEFMEK			Oxidation (M)[4,8]		Mascot
1418.7485	1418.7345	-0.014	-10	68	79	IISSIEQKEESR					Mascot
1517.8799	1517.8373	-0.0426	-28	49	62	NLLSVAYKNVIGAR					Mascot
1552.7601	1552.745	-0.0151	-10	76	89	EESRGNEAYVASIK					Mascot
1818.9708	1818.9503	-0.0205	-11	160	176	SAQDIALADLPTTHPIR					Mascot
1818.9708	1818.9503	-0.0205	-11	160	176	SAQDIALADLPTTHPIR	140	100			Mascot
1846.8309	1846.968	0.1371	74	1	16	MSTAEATREENVYMAK			Oxidation (M)[1]		Mascot
2114.9763	2114.9133	-0.063	-30	203	221	QAFDEAIAELDSLGEESY K					Mascot
2131.9675	2131.9492	-0.0183	-9	17	33	LAEQAERYEEMVEFMEK					Mascot
2131.9675	2131.9492	-0.0183	-9	17	33	LAEQAERYEEMVEFMEK	34	30.667			Mascot
2147.9624	2147.9199	-0.0425	-20	17	33	LAEQAERYEEMVEFMEK			Oxidation (M)[11]		Mascot
2147.9624	2147.9199	-0.0425	-20	17	33	LAEQAERYEEMVEFMEK	19	0	Oxidation (M)[15]		Mascot
2163.9573	2163.8999	-0.0574	-27	17	33	LAEQAERYEEMVEFMEK			Oxidation (M)[11,15]		Mascot
2163.9573	2163.8999	-0.0574	-27	17	33	LAEQAERYEEMVEFMEK	44	93.24	Oxidation (M)[11,15]		Mascot
2331.2019	2331.1804	-0.0215	-9	177	196	LGLALNFSVFYYEILNSPD R					Mascot
2331.2019	2331.1804	-0.0215	-9	177	196	LGLALNFSVFYYEILNSPD R	125	100			Mascot
3285.6196	3285.6016	-0.018	-5	203	231	QAFDEAIAELDSLGEESY KDSTLIMQLLR					Mascot
3301.6145	3301.5984	-0.0161	-5	203	231	QAFDEAIAELDSLGEESY KDSTLIMQLLR			Oxidation (M)[25]		Mascot

4 14-3-3-like protein A [Aegilops tauschii] gi|475611352 32785.4 4.77 20 656 100 67.446 515 100

Peptide Information

Calc. Mass	Obsrv. Mass	± da	± ppm	Start Seq.	End Seq.	Sequence	Ion Score	C. I. %	Modification	Rank	Result Type
816.421	816.4076	-0.0134	-16	17	23	LAEQAER					Mascot
907.5247	907.4792	-0.0455	-50	49	56	NLLSVAYK					Mascot
917.5302	917.5087	-0.0215	-23	68	75	IISSIEQK					Mascot
922.4199	922.4067	-0.0132	-14	130	136	MKGDYHR			Oxidation (M)[1]		Mascot

1051.5419	1051.522	-0.0199	-19	80	89	GNEAYVASIK					Mascot
1076.5946	1076.5731	-0.0215	-20	93	101	TRIETELSK					Mascot
1189.6609	1189.6497	-0.0112	-9	222	231	DSTLIMQLLR					Mascot
1189.6609	1189.6497	-0.0112	-9	222	231	DSTLIMQLLR	87	100			Mascot
1205.6559	1205.6342	-0.0217	-18	222	231	DSTLIMQLLR			Oxidation (M)[6]		Mascot
1205.6559	1205.6342	-0.0217	-18	222	231	DSTLIMQLLR	35	48.365	Oxidation (M)[6]		Mascot
1208.6157	1208.611	-0.0047	-4	149	159	EAAENTLVAYK					Mascot
1318.6486	1318.6364	-0.0122	-9	37	48	TADVGELTVEER					Mascot
1318.6486	1318.6364	-0.0122	-9	37	48	TADVGELTVEER	118	100			Mascot
1334.5643	1334.542	-0.0223	-17	24	33	YEEMVEFMEK					Mascot
1336.7107	1336.6345	-0.0762	-57	148	159	KEAAENTLVAYK					Mascot
1350.5592	1350.5243	-0.0349	-26	24	33	YEEMVEFMEK			Oxidation (M)[4]		Mascot
1366.5542	1366.511	-0.0432	-32	24	33	YEEMVEFMEK			Oxidation (M)[4,8]		Mascot
1418.7485	1418.7345	-0.014	-10	68	79	IISSIEQKEESR					Mascot
1517.8799	1517.8373	-0.0426	-28	49	62	NLLSVAYKNVIGAR					Mascot
1552.7601	1552.745	-0.0151	-10	76	89	EESRGNEAYVASIK					Mascot
1818.9708	1818.9503	-0.0205	-11	160	176	SAQDIALADLPTTHPIR					Mascot
1818.9708	1818.9503	-0.0205	-11	160	176	SAQDIALADLPTTHPIR	140	100			Mascot
1846.8309	1846.968	0.1371	74	1	16	MSTAEATREENVYMAK			Oxidation (M)[1]		Mascot
2114.9763	2114.9133	-0.063	-30	203	221	QAFDEAIAELDSLGEESY K					Mascot
2131.9675	2131.9492	-0.0183	-9	17	33	LAEQAERYEEMVEFMEK					Mascot
2131.9675	2131.9492	-0.0183	-9	17	33	LAEQAERYEEMVEFMEK	34	30.667			Mascot
2147.9624	2147.9199	-0.0425	-20	17	33	LAEQAERYEEMVEFMEK			Oxidation (M)[11]		Mascot
2147.9624	2147.9199	-0.0425	-20	17	33	LAEQAERYEEMVEFMEK	19	0	Oxidation (M)[15]		Mascot
2163.9573	2163.8999	-0.0574	-27	17	33	LAEQAERYEEMVEFMEK			Oxidation (M)[11,15]		Mascot
2163.9573	2163.8999	-0.0574	-27	17	33	LAEQAERYEEMVEFMEK	44	93.24	Oxidation (M)[11,15]		Mascot
2331.2019	2331.1804	-0.0215	-9	177	196	LGLALNFSVFYYEILNSPD R					Mascot
2331.2019	2331.1804	-0.0215	-9	177	196	LGLALNFSVFYYEILNSPD R	125	100			Mascot
3285.6196	3285.6016	-0.018	-5	203	231	QAFDEAIAELDSLGEESY KDSTLIMQLLR					Mascot
3301.6145	3301.5984	-0.0161	-5	203	231	QAFDEAIAELDSLGEESY KDSTLIMQLLR			Oxidation (M)[25]		Mascot

5 uncharacterized protein, partial [Phleum pratense] gi|409972289 27447.9 4.91 19 648 100 67.206 515 100

Peptide Information

Calc. Mass	Obsrv. Mass	± da	± ppm	Start Seq.	End Seq.	Sequence	Ion Score	C. I. %	Modification	Rank	Result Type
816.421	816.4076	-0.0134	-16	5	11	LAEQAER					Mascot

907.5247	907.4792	-0.0455	-50	37	44	NLLSVAYK				Mascot
917.5302	917.5087	-0.0215	-23	56	63	IISSIEQK				Mascot
922.4199	922.4067	-0.0132	-14	118	124	MKGDYHR			Oxidation (M)[1]	Mascot
1051.5419	1051.522	-0.0199	-19	68	77	GNEAYVASIK				Mascot
1076.5946	1076.5731	-0.0215	-20	81	89	TRIETELSK				Mascot
1189.6609	1189.6497	-0.0112	-9	210	219	DSTLIMQLLR				Mascot
1189.6609	1189.6497	-0.0112	-9	210	219	DSTLIMQLLR	87	100		Mascot
1205.6559	1205.6342	-0.0217	-18	210	219	DSTLIMQLLR			Oxidation (M)[6]	Mascot
1205.6559	1205.6342	-0.0217	-18	210	219	DSTLIMQLLR	35	48.365	Oxidation (M)[6]	Mascot
1208.6157	1208.611	-0.0047	-4	137	147	EAAENTLVAYK				Mascot
1318.6486	1318.6364	-0.0122	-9	25	36	TADVGELTVEER				Mascot
1318.6486	1318.6364	-0.0122	-9	25	36	TADVGELTVEER	118	100		Mascot
1334.5643	1334.542	-0.0223	-17	12	21	YEEMVEFMEK				Mascot
1336.7107	1336.6345	-0.0762	-57	136	147	KEAAENTLVAYK				Mascot
1350.5592	1350.5243	-0.0349	-26	12	21	YEEMVEFMEK			Oxidation (M)[4]	Mascot
1366.5542	1366.511	-0.0432	-32	12	21	YEEMVEFMEK			Oxidation (M)[4,8]	Mascot
1418.7485	1418.7345	-0.014	-10	56	67	IISSIEQKEESR				Mascot
1517.8799	1517.8373	-0.0426	-28	37	50	NLLSVAYKNVIGAR				Mascot
1552.7601	1552.745	-0.0151	-10	64	77	EESRGNEAYVASIK				Mascot
1818.9708	1818.9503	-0.0205	-11	148	164	SAQDIALADLPTTHPIR				Mascot
1818.9708	1818.9503	-0.0205	-11	148	164	SAQDIALADLPTTHPIR	140	100		Mascot
2114.9763	2114.9133	-0.063	-30	191	209	QAFDEAIAELDSLGEESYK				Mascot
2131.9675	2131.9492	-0.0183	-9	5	21	LAEQAERYEEMVEFMEK				Mascot
2131.9675	2131.9492	-0.0183	-9	5	21	LAEQAERYEEMVEFMEK	34	30.667		Mascot
2147.9624	2147.9199	-0.0425	-20	5	21	LAEQAERYEEMVEFMEK			Oxidation (M)[11]	Mascot
2147.9624	2147.9199	-0.0425	-20	5	21	LAEQAERYEEMVEFMEK	19	0	Oxidation (M)[15]	Mascot
2163.9573	2163.8999	-0.0574	-27	5	21	LAEQAERYEEMVEFMEK			Oxidation (M)[11,15]	Mascot
2163.9573	2163.8999	-0.0574	-27	5	21	LAEQAERYEEMVEFMEK	44	93.24	Oxidation (M)[11,15]	Mascot
2331.2019	2331.1804	-0.0215	-9	165	184	LGLALNFSVFYYEILNSPDR				Mascot
2331.2019	2331.1804	-0.0215	-9	165	184	LGLALNFSVFYYEILNSPDR	125	100		Mascot
3285.6196	3285.6016	-0.018	-5	191	219	QAFDEAIAELDSLGEESYKDSTLIMQLLR				Mascot
3301.6145	3301.5984	-0.0161	-5	191	219	QAFDEAIAELDSLGEESYKDSTLIMQLLR			Oxidation (M)[25]	Mascot

6	Os03g0710800 [Oryza sativa Japonica Group]	gi|113549522	29273.7	4.81	17	514	100	56.838	396	100		

Protein Group

RecName: Full=14-3-3-like protein GF14-F; AltName:	gi|76789644	29273.7	4.8099

Full=14-3-3-like protein S94; AltName: Full=G-box factor 14-3-3 homolog F; AltName: Full=OsGF14a; AltName: Full=Stress-regulated 14-3-3 protein; Short=SR14-3-3			999427 7954
hypothetical protein OsI_13240 [Oryza sativa Indica Group]	gi|125545466	29273.7	4.8099 999427 7954

Peptide Information

Calc. Mass	Obsrv. Mass	± da	± ppm	Start Seq.	End Seq.	Sequence	Ion Score	C. I. %	Modification	Rank	Result Type
816.421	816.4076	-0.0134	-16	17	23	LAEQAER					Mascot
907.5247	907.4792	-0.0455	-50	49	56	NLLSVAYK					Mascot
917.5302	917.5087	-0.0215	-23	68	75	IISSIEQK					Mascot
922.4199	922.4067	-0.0132	-14	130	136	MKGDYHR			Oxidation (M)[1]		Mascot
1051.5419	1051.522	-0.0199	-19	80	89	GNEAYVASIK					Mascot
1189.6609	1189.6497	-0.0112	-9	222	231	DSTLIMQLLR					Mascot
1189.6609	1189.6497	-0.0112	-9	222	231	DSTLIMQLLR	87	100			Mascot
1205.6559	1205.6342	-0.0217	-18	222	231	DSTLIMQLLR			Oxidation (M)[6]		Mascot
1205.6559	1205.6342	-0.0217	-18	222	231	DSTLIMQLLR	35	48.365	Oxidation (M)[6]		Mascot
1208.6157	1208.611	-0.0047	-4	149	159	EAAENTLVAYK					Mascot
1334.5643	1334.542	-0.0223	-17	24	33	YEEMVEFMEK					Mascot
1336.7107	1336.6345	-0.0762	-57	148	159	KEAAENTLVAYK					Mascot
1350.5592	1350.5243	-0.0349	-26	24	33	YEEMVEFMEK			Oxidation (M)[4]		Mascot
1366.5542	1366.511	-0.0432	-32	24	33	YEEMVEFMEK			Oxidation (M)[4,8]		Mascot
1418.7485	1418.7345	-0.014	-10	68	79	IISSIEQKEESR					Mascot
1517.8799	1517.8373	-0.0426	-28	49	62	NLLSVAYKNVIGAR					Mascot
1552.7601	1552.745	-0.0151	-10	76	89	EESRGNEAYVASIK					Mascot
1818.9708	1818.9503	-0.0205	-11	160	176	SAQDIALADLPTTHPIR					Mascot
1818.9708	1818.9503	-0.0205	-11	160	176	SAQDIALADLPTTHPIR	140	100			Mascot
2114.9766	2114.9133	-0.0633	-30	203	221	QAFDDAIAELDTLGEESY K					Mascot
2131.9675	2131.9492	-0.0183	-9	17	33	LAEQAERYEEMVEFMEK					Mascot
2131.9675	2131.9492	-0.0183	-9	17	33	LAEQAERYEEMVEFMEK	34	30.667			Mascot
2147.9624	2147.9199	-0.0425	-20	17	33	LAEQAERYEEMVEFMEK			Oxidation (M)[11]		Mascot
2147.9624	2147.9199	-0.0425	-20	17	33	LAEQAERYEEMVEFMEK	19	0	Oxidation (M)[15]		Mascot
2163.9573	2163.8999	-0.0574	-27	17	33	LAEQAERYEEMVEFMEK			Oxidation (M)[11,15]		Mascot
2163.9573	2163.8999	-0.0574	-27	17	33	LAEQAERYEEMVEFMEK	44	93.24	Oxidation (M)[11,15]		Mascot
2331.2019	2331.1804	-0.0215	-9	177	196	LGLALNFSVFYYEILNSPD R					Mascot
2331.2019	2331.1804	-0.0215	-9	177	196	LGLALNFSVFYYEILNSPD	125	100			Mascot

						R					
3285.6196	3285.6016	-0.018	-5	203	231	QAFDDAIAELDTLGEESY KDSTLIMQLLR					Mascot
3301.6145	3301.5984	-0.0161	-5	203	231	QAFDDAIAELDTLGEESY KDSTLIMQLLR			Oxidation (M)[25]		Mascot

7	uncharacterized protein, partial [Phleum pratense]	gi|409971719	12623.2	4.36	7	409	100	44.137	352	100	

Peptide Information

Calc. Mass	Obsrv. Mass	± da	± ppm	Start Seq.	End Seq.	Sequence	Ion Score	C. I. %	Modification	Rank	Result Type
1189.6609	1189.6497	-0.0112	-9	76	85	DSTLIMQLLR					Mascot
1189.6609	1189.6497	-0.0112	-9	76	85	DSTLIMQLLR	87	100			Mascot
1205.6559	1205.6342	-0.0217	-18	76	85	DSTLIMQLLR			Oxidation (M)[6]		Mascot
1205.6559	1205.6342	-0.0217	-18	76	85	DSTLIMQLLR	35	48.365	Oxidation (M)[6]		Mascot
1208.6157	1208.611	-0.0047	-4	3	13	EAAENTLVAYK					Mascot
1336.7107	1336.6345	-0.0762	-57	2	13	KEAAENTLVAYK					Mascot
1818.9708	1818.9503	-0.0205	-11	14	30	SAQDIALADLPTTHPIR					Mascot
1818.9708	1818.9503	-0.0205	-11	14	30	SAQDIALADLPTTHPIR	140	100			Mascot
2114.9763	2114.9133	-0.063	-30	57	75	QAFDEAIAELDSLGEESY K					Mascot
2331.2019	2331.1804	-0.0215	-9	31	50	LGLALNFSVFYYEILNSPD R					Mascot
2331.2019	2331.1804	-0.0215	-9	31	50	LGLALNFSVFYYEILNSPD R	125	100			Mascot
3285.6196	3285.6016	-0.018	-5	57	85	QAFDEAIAELDSLGEESY KDSTLIMQLLR					Mascot
3301.6145	3301.5984	-0.0161	-5	57	85	QAFDEAIAELDSLGEESY KDSTLIMQLLR			Oxidation (M)[25]		Mascot

8	uncharacterized protein, partial [Phleum pratense]	gi|409971903	12880.3	4.33	7	408	100	44.137	352	100	

Peptide Information

Calc. Mass	Obsrv. Mass	± da	± ppm	Start Seq.	End Seq.	Sequence	Ion Score	C. I. %	Modification	Rank	Result Type
1189.6609	1189.6497	-0.0112	-9	79	88	DSTLIMQLLR					Mascot
1189.6609	1189.6497	-0.0112	-9	79	88	DSTLIMQLLR	87	100			Mascot
1205.6559	1205.6342	-0.0217	-18	79	88	DSTLIMQLLR			Oxidation (M)[6]		Mascot
1205.6559	1205.6342	-0.0217	-18	79	88	DSTLIMQLLR	35	48.365	Oxidation (M)[6]		Mascot
1208.6157	1208.611	-0.0047	-4	6	16	EAAENTLVAYK					Mascot
1336.7107	1336.6345	-0.0762	-57	5	16	KEAAENTLVAYK					Mascot
1818.9708	1818.9503	-0.0205	-11	17	33	SAQDIALADLPTTHPIR					Mascot
1818.9708	1818.9503	-0.0205	-11	17	33	SAQDIALADLPTTHPIR	140	100			Mascot
2114.9763	2114.9133	-0.063	-30	60	78	QAFDEAIAELDSLGEESY K					Mascot
2331.2019	2331.1804	-0.0215	-9	34	53	LGLALNFSVFYYEILNSPD					Mascot

Calc. Mass	Obsrv. Mass	± da	± ppm	Start Seq.	End Seq.	Sequence	Ion Score	C. I. %	Modification	Rank	Result Type
						R					
2331.2019	2331.1804	-0.0215	-9	34	53	LGLALNFSVFYYEILNSPD R	125	100			Mascot
3285.6196	3285.6016	-0.018	-5	60	88	QAFDEAIAELDSLGEESY KDSTLIMQLLR					Mascot
3301.6145	3301.5984	-0.0161	-5	60	88	QAFDEAIAELDSLGEESY KDSTLIMQLLR			Oxidation (M)[25]		Mascot

9	PREDICTED: 14-3-3-like protein-like [Fragaria vesca subsp. vesca]	gi|470136735	29802.9	4.75	13	383	100	34.441	311	100	

Peptide Information

Calc. Mass	Obsrv. Mass	± da	± ppm	Start Seq.	End Seq.	Sequence	Ion Score	C. I. %	Modification	Rank	Result Type
816.421	816.4076	-0.0134	-16	17	23	LAEQAER					Mascot
907.5247	907.4792	-0.0455	-50	49	56	NLLSVAYK					Mascot
917.5302	917.5087	-0.0215	-23	68	75	IISSIEQK					Mascot
922.4199	922.4067	-0.0132	-14	130	136	MKGDYHR			Oxidation (M)[1]		Mascot
1189.6609	1189.6497	-0.0112	-9	222	231	DSTLIMQLLR					Mascot
1189.6609	1189.6497	-0.0112	-9	222	231	DSTLIMQLLR	87	100			Mascot
1205.6559	1205.6342	-0.0217	-18	222	231	DSTLIMQLLR			Oxidation (M)[6]		Mascot
1205.6559	1205.6342	-0.0217	-18	222	231	DSTLIMQLLR	35	48.365	Oxidation (M)[6]		Mascot
1334.5643	1334.542	-0.0223	-17	24	33	YEEMVEFMEK					Mascot
1350.5592	1350.5243	-0.0349	-26	24	33	YEEMVEFMEK			Oxidation (M)[4]		Mascot
1366.5542	1366.511	-0.0432	-32	24	33	YEEMVEFMEK			Oxidation (M)[4,8]		Mascot
1418.7485	1418.7345	-0.014	-10	68	79	IISSIEQKEESR					Mascot
1517.8799	1517.8373	-0.0426	-28	49	62	NLLSVAYKNVIGAR					Mascot
1559.7812	1559.8628	0.0816	52	80	92	GNEDHVSIIKEYR					Mascot
1741.8062	1741.7874	-0.0188	-11	2	16	SPTESTREENVYMAK					Mascot
1741.8062	1741.7874	-0.0188	-11	2	16	SPTESTREENVYMAK	53	99.09			Mascot
1757.801	1757.7686	-0.0324	-18	2	16	SPTESTREENVYMAK			Oxidation (M)[13]		Mascot
1757.801	1757.7686	-0.0324	-18	2	16	SPTESTREENVYMAK	55	99.485	Oxidation (M)[13]		Mascot
2121.9824	2121.9568	-0.0256	-12	232	250	DNLTLWTSDITDDAGDEI K					Mascot
2131.9675	2131.9492	-0.0183	-9	17	33	LAEQAERYEEMVEFMEK					Mascot
2131.9675	2131.9492	-0.0183	-9	17	33	LAEQAERYEEMVEFMEK	34	30.667			Mascot
2147.9624	2147.9199	-0.0425	-20	17	33	LAEQAERYEEMVEFMEK			Oxidation (M)[11]		Mascot
2147.9624	2147.9199	-0.0425	-20	17	33	LAEQAERYEEMVEFMEK	19	0	Oxidation (M)[15]		Mascot
2163.9573	2163.8999	-0.0574	-27	17	33	LAEQAERYEEMVEFMEK			Oxidation (M)[11,15]		Mascot
2163.9573	2163.8999	-0.0574	-27	17	33	LAEQAERYEEMVEFMEK	44	93.24	Oxidation (M)[11,15]		Mascot
2331.2019	2331.1804	-0.0215	-9	177	196	LGLALNFSVFYYEILNSPD R					Mascot
2331.2019	2331.1804	-0.0215	-9	177	196	LGLALNFSVFYYEILNSPD	125	100			Mascot

R

10	hypothetical protein PRUPE_ppa010141mg [Prunus persica]	gi|462395986	29570.8	4.78	13	337	100	30.73	256	100	

Peptide Information

Calc. Mass	Obsrv. Mass	± da	± ppm	Start Seq.	End Seq.	Sequence	Ion Score	C. I. %	Modification	Rank	Result Type
816.421	816.4076	-0.0134	-16	16	22	LAEQAER					Mascot
907.5247	907.4792	-0.0455	-50	49	56	NLLSVAYK					Mascot
922.4199	922.4067	-0.0132	-14	130	136	MKGDYHR			Oxidation (M)[1]		Mascot
1189.6609	1189.6497	-0.0112	-9	222	231	DSTLIMQLLR					Mascot
1189.6609	1189.6497	-0.0112	-9	222	231	DSTLIMQLLR	87	100			Mascot
1205.6559	1205.6342	-0.0217	-18	222	231	DSTLIMQLLR			Oxidation (M)[6]		Mascot
1205.6559	1205.6342	-0.0217	-18	222	231	DSTLIMQLLR	35	48.365	Oxidation (M)[6]		Mascot
1334.5643	1334.542	-0.0223	-17	23	32	YEEMVEFMEK					Mascot
1350.5592	1350.5243	-0.0349	-26	23	32	YEEMVEFMEK			Oxidation (M)[4]		Mascot
1366.5542	1366.511	-0.0432	-32	23	32	YEEMVEFMEK			Oxidation (M)[4,8]		Mascot
1517.8799	1517.8373	-0.0426	-28	49	62	NLLSVAYKNVIGAR					Mascot
1559.8098	1559.8628	0.053	34	95	108	IENELSNICGGILK			Carbamidomethyl (C)[9]		Mascot
1786.948	1786.9465	-0.0015	-1	93	108	ARIENELSNICGGILK			Carbamidomethyl (C)[11]		Mascot
2114.9766	2114.9133	-0.0633	-30	203	221	QAFDEAIAELDTLGEDSY K					Mascot
2116.1318	2115.9209	-0.2109	-100	95	113	IENELSNICGGILKLLDSK			Carbamidomethyl (C)[9]		Mascot
2131.9675	2131.9492	-0.0183	-9	16	32	LAEQAERYEEMVEFMEK					Mascot
2131.9675	2131.9492	-0.0183	-9	16	32	LAEQAERYEEMVEFMEK	34	30.667			Mascot
2147.9624	2147.9199	-0.0425	-20	16	32	LAEQAERYEEMVEFMEK			Oxidation (M)[11]		Mascot
2147.9624	2147.9199	-0.0425	-20	16	32	LAEQAERYEEMVEFMEK	19	0	Oxidation (M)[15]		Mascot
2163.9573	2163.8999	-0.0574	-27	16	32	LAEQAERYEEMVEFMEK			Oxidation (M)[11,15]		Mascot
2163.9573	2163.8999	-0.0574	-27	16	32	LAEQAERYEEMVEFMEK	44	93.24	Oxidation (M)[11,15]		Mascot
2331.2019	2331.1804	-0.0215	-9	177	196	LGLALNFSVFYYEILNSPD R					Mascot
2331.2019	2331.1804	-0.0215	-9	177	196	LGLALNFSVFYYEILNSPD R	125	100			Mascot
3285.6196	3285.6016	-0.018	-5	203	231	QAFDEAIAELDTLGEDSY KDSTLIMQLLR					Mascot
3301.6145	3301.5984	-0.0161	-5	203	231	QAFDEAIAELDTLGEDSY KDSTLIMQLLR			Oxidation (M)[25]		Mascot

Gel Idx/Pos	166/G17	Instr./Gel Origin	BA2151/Sample Project 20140814	Process Status	Analysis Succeeded
Plate [#] Name	[1] Sample Project 20140814	Instrument Sample Name		Spectra	11

Rank	Protein Name	Accession No.	Protein MW	Protein PI	Pep. Count	Protein Score	Protein Score C. I. %	Intensity Matched	Total Ion Score	Total Ion C. I. %	Confirmed
1	14-3-3-like protein GF14-B [Triticum urartu]	gi|474147722	30043.1	4.69	21	513	100	44.306	365	100	

Protein Group

14-3-3-like protein GF14-B [Aegilops tauschii]	gi|475549223	30043.1	4.6900 000572 2046

Peptide Information

Calc. Mass	Obsrv. Mass	± da	± ppm	Start Seq.	End Seq.	Sequence	Ion Score	C. I. %	Modification	Rank	Result Type
816.421	816.4134	-0.0076	-9	18	24	LAEQAER					Mascot
818.444	818.4239	-0.0201	-25	103	109	ICDGILK			Carbamidomethyl (C)[2]		Mascot
844.4523	844.4604	0.0081	10	2	9	TAPAELSR					Mascot
907.5247	907.4822	-0.0425	-47	50	57	NLLSVAYK					Mascot
917.5302	917.5153	-0.0149	-16	69	76	IISSIEQK					Mascot
932.4294	932.4268	-0.0026	-3	131	137	MKGDYYR					Mascot
948.4244	948.4129	-0.0115	-12	131	137	MKGDYYR			Oxidation (M)[1]		Mascot
999.4451	999.4496	0.0045	5	10	17	EENVYMAK			Oxidation (M)[6]		Mascot
1144.6321	1144.6272	-0.0049	-4	81	90	GNEDRVTLIK					Mascot
1189.6609	1189.6603	-0.0006	-1	223	232	DSTLIMQLLR					Mascot
1189.6609	1189.6603	-0.0006	-1	223	232	DSTLIMQLLR	22	0			Mascot
1205.6559	1205.6425	-0.0134	-11	223	232	DSTLIMQLLR			Oxidation (M)[6]		Mascot
1205.6559	1205.6425	-0.0134	-11	223	232	DSTLIMQLLR	23	0	Oxidation (M)[6]		Mascot
1212.5565	1212.6031	0.0466	38	150	160	DAAENTMVAYK					Mascot
1228.5514	1228.5819	0.0305	25	150	160	DAAENTMVAYK			Oxidation (M)[7]		Mascot
1366.5542	1366.5227	-0.0315	-23	25	34	YEEMVEFMEK			Oxidation (M)[4,8]		Mascot
1406.6646	1406.6599	-0.0047	-3	38	49	TVDSEELTVEER					Mascot
1406.6646	1406.6599	-0.0047	-3	38	49	TVDSEELTVEER	109	100			Mascot
1418.7485	1418.7394	-0.0091	-6	69	80	IISSIEQKEESR					Mascot
1418.7485	1418.7394	-0.0091	-6	69	80	IISSIEQKEESR	69	99.978			Mascot
1708.9116	1708.85	-0.0616	-36	110	125	LLETHLVPSSTAPESK					Mascot
1786.9811	1786.9723	-0.0088	-5	161	177	AAQDIALAELAPTHPIR					Mascot
1786.9811	1786.9723	-0.0088	-5	161	177	AAQDIALAELAPTHPIR	156	100			Mascot
1808.8848	1808.9418	0.057	32	2	17	TAPAELSREENVYMAK					Mascot

Calc. Mass	Obsrv. Mass	± da	± ppm	Start Seq.	End Seq.	Sequence	Ion Score	C. I. %	Modification	Rank	Result Type
1824.8796	1824.8773	-0.0023	-1	2	17	TAPAELSREENVYMAK			Oxidation (M)[14]		Mascot
2147.9624	2147.9368	-0.0256	-12	18	34	LAEQAERYEEMVEFMEK			Oxidation (M)[11]		Mascot
2163.9573	2163.9104	-0.0469	-22	18	34	LAEQAERYEEMVEFMEK			Oxidation (M)[11,15]		Mascot
2163.9573	2163.9104	-0.0469	-22	18	34	LAEQAERYEEMVEFMEK	8	0	Oxidation (M)[11,15]		Mascot
2174.9976	2174.946	-0.0516	-24	204	222	QAFDEAISELDTLSEESYK					Mascot
2331.2019	2331.2019	0	0	178	197	LGLALNFSVFYYEILNSPDR					Mascot
2351.0886	2351.0681	-0.0205	-9	233	252	DNLTLWTSDITEDTAEEEIR					Mascot
2776.3159	2776.304	-0.0119	-4	233	256	DNLTLWTSDITEDTAEEEIREAPK					Mascot

2	14-3-3-like protein GF14-6 [Zea mays]	gi|262359935	29758	4.76	17	451	100	41.96	350	100

Protein Group

RecName: Full=14-3-3-like protein GF14-6	gi|1345587	29758	4.76000022888184
TPA: general regulatory factor1 isoform 1 [Zea mays]	gi|414586860	29758	4.76000022888184
TPA: general regulatory factor1 isoform 2 [Zea mays]	gi|414586861	29758	4.76000022888184

Peptide Information

Calc. Mass	Obsrv. Mass	± da	± ppm	Start Seq.	End Seq.	Sequence	Ion Score	C. I. %	Modification	Rank	Result Type
816.421	816.4134	-0.0076	-9	17	23	LAEQAER					Mascot
818.444	818.4239	-0.0201	-25	102	108	ICDGILK			Carbamidomethyl (C)[2]		Mascot
907.5247	907.4822	-0.0425	-47	49	56	NLLSVAYK					Mascot
917.5302	917.5153	-0.0149	-16	68	75	IISSIEQK					Mascot
932.4294	932.4268	-0.0026	-3	130	136	MKGDYYR					Mascot
948.4244	948.4129	-0.0115	-12	130	136	MKGDYYR			Oxidation (M)[1]		Mascot
999.4451	999.4496	0.0045	5	9	16	EENVYMAK			Oxidation (M)[6]		Mascot
1144.6321	1144.6272	-0.0049	-4	80	89	GNEDRVTLIK					Mascot
1189.6609	1189.6603	-0.0006	-1	222	231	DSTLIMQLLR					Mascot
1189.6609	1189.6603	-0.0006	-1	222	231	DSTLIMQLLR	22	0			Mascot
1205.6559	1205.6425	-0.0134	-11	222	231	DSTLIMQLLR			Oxidation (M)[6]		Mascot
1205.6559	1205.6425	-0.0134	-11	222	231	DSTLIMQLLR	23	0	Oxidation (M)[6]		Mascot
1212.5565	1212.6031	0.0466	38	149	159	DAAENTMVAYK					Mascot
1228.5514	1228.5819	0.0305	25	149	159	DAAENTMVAYK			Oxidation (M)[7]		Mascot

1366.5542	1366.5227	-0.0315	-23	24	33	YEEMVEFMEK			Oxidation (M)[4,8]		Mascot
1388.738	1388.7262	-0.0118	-8	68	79	IISSIEQKEEGR					Mascot
1388.738	1388.7262	-0.0118	-8	68	79	IISSIEQKEEGR	54	99.292			Mascot
1406.6646	1406.6599	-0.0047	-3	37	48	TVDSEELTVEER					Mascot
1406.6646	1406.6599	-0.0047	-3	37	48	TVDSEELTVEER	109	100			Mascot
1708.9116	1708.85	-0.0616	-36	109	124	LLETHLVPSSTAPESK					Mascot
1786.9811	1786.9723	-0.0088	-5	160	176	AAQDIALAELAPTHPIR					Mascot
1786.9811	1786.9723	-0.0088	-5	160	176	AAQDIALAELAPTHPIR	156	100			Mascot
2147.9624	2147.9368	-0.0256	-12	17	33	LAEQAERYEEMVEFMEK			Oxidation (M)[11]		Mascot
2163.9573	2163.9104	-0.0469	-22	17	33	LAEQAERYEEMVEFMEK			Oxidation (M)[11,15]		Mascot
2163.9573	2163.9104	-0.0469	-22	17	33	LAEQAERYEEMVEFMEK	8	0	Oxidation (M)[11,15]		Mascot
2174.9976	2174.946	-0.0516	-24	203	221	QAFDEAISELDTLSEESY K					Mascot
2331.2019	2331.2019	0	0	177	196	LGLALNFSVFYYEILNSPD R					Mascot

3	TPA: general regulatory factor1 [Zea mays]	gi|414586863	31835.1	4.9	17	445	100	41.96	350	100		

Peptide Information

Calc. Mass	Obsrv. Mass	± da	± ppm	Start Seq.	End Seq.	Sequence	Ion Score	C. I. %	Modification	Rank	Result Type
816.421	816.4134	-0.0076	-9	34	40	LAEQAER					Mascot
818.444	818.4239	-0.0201	-25	119	125	ICDGILK			Carbamidomethyl (C)[2]		Mascot
907.5247	907.4822	-0.0425	-47	66	73	NLLSVAYK					Mascot
917.5302	917.5153	-0.0149	-16	85	92	IISSIEQK					Mascot
932.4294	932.4268	-0.0026	-3	147	153	MKGDYYR					Mascot
948.4244	948.4129	-0.0115	-12	147	153	MKGDYYR			Oxidation (M)[1]		Mascot
999.4451	999.4496	0.0045	5	26	33	EENVYMAK			Oxidation (M)[6]		Mascot
1144.6321	1144.6272	-0.0049	-4	97	106	GNEDRVTLIK					Mascot
1189.6609	1189.6603	-0.0006	-1	239	248	DSTLIMQLLR					Mascot
1189.6609	1189.6603	-0.0006	-1	239	248	DSTLIMQLLR	22	0			Mascot
1205.6559	1205.6425	-0.0134	-11	239	248	DSTLIMQLLR			Oxidation (M)[6]		Mascot
1205.6559	1205.6425	-0.0134	-11	239	248	DSTLIMQLLR	23	0	Oxidation (M)[6]		Mascot
1212.5565	1212.6031	0.0466	38	166	176	DAAENTMVAYK					Mascot
1228.5514	1228.5819	0.0305	25	166	176	DAAENTMVAYK			Oxidation (M)[7]		Mascot
1366.5542	1366.5227	-0.0315	-23	41	50	YEEMVEFMEK			Oxidation (M)[4,8]		Mascot
1388.738	1388.7262	-0.0118	-8	85	96	IISSIEQKEEGR					Mascot
1388.738	1388.7262	-0.0118	-8	85	96	IISSIEQKEEGR	54	99.292			Mascot
1406.6646	1406.6599	-0.0047	-3	54	65	TVDSEELTVEER					Mascot

1406.6646	1406.6599	-0.0047	-3	54	65	TVDSEELTVEER	109	100			Mascot
1708.9116	1708.85	-0.0616	-36	126	141	LLETHLVPSSTAPESK					Mascot
1786.9811	1786.9723	-0.0088	-5	177	193	AAQDIALAELAPTHPIR					Mascot
1786.9811	1786.9723	-0.0088	-5	177	193	AAQDIALAELAPTHPIR	156	100			Mascot
2147.9624	2147.9368	-0.0256	-12	34	50	LAEQAERYEEMVEFMEK			Oxidation (M)[11]		Mascot
2163.9573	2163.9104	-0.0469	-22	34	50	LAEQAERYEEMVEFMEK			Oxidation (M)[11,15]		Mascot
2163.9573	2163.9104	-0.0469	-22	34	50	LAEQAERYEEMVEFMEK	8	0	Oxidation (M)[11,15]		Mascot
2174.9976	2174.946	-0.0516	-24	220	238	QAFDEAISELDTLSEESY K					Mascot
2331.2019	2331.2019	0	0	194	213	LGLALNFSVFYYEILNSPD R					Mascot

4 14-3-3-like protein GF14-12 [Zea mays] gi|413918561 29724.9 4.75 16 442 100 41.508 350 100

Peptide Information

Calc. Mass	Obsrv. Mass	± da	± ppm	Start Seq.	End Seq.	Sequence	Ion Score	C. I. %	Modification	Rank	Result Type
816.421	816.4134	-0.0076	-9	17	23	LAEQAER					Mascot
818.444	818.4239	-0.0201	-25	102	108	ICDGILK			Carbamidomethyl (C)[2]		Mascot
907.5247	907.4822	-0.0425	-47	49	56	NLLSVAYK					Mascot
917.5302	917.5153	-0.0149	-16	68	75	IISSIEQK					Mascot
932.4294	932.4268	-0.0026	-3	130	136	MKGDYYR					Mascot
948.4244	948.4129	-0.0115	-12	130	136	MKGDYYR			Oxidation (M)[1]		Mascot
999.4451	999.4496	0.0045	5	9	16	EENVYMAK			Oxidation (M)[6]		Mascot
1144.6321	1144.6272	-0.0049	-4	80	89	GNEDRVTLIK					Mascot
1189.6609	1189.6603	-0.0006	-1	222	231	DSTLIMQLLR					Mascot
1189.6609	1189.6603	-0.0006	-1	222	231	DSTLIMQLLR	22	0			Mascot
1205.6559	1205.6425	-0.0134	-11	222	231	DSTLIMQLLR			Oxidation (M)[6]		Mascot
1205.6559	1205.6425	-0.0134	-11	222	231	DSTLIMQLLR	23	0	Oxidation (M)[6]		Mascot
1212.5565	1212.6031	0.0466	38	149	159	DAAENTMVAYK					Mascot
1228.5514	1228.5819	0.0305	25	149	159	DAAENTMVAYK			Oxidation (M)[7]		Mascot
1366.5542	1366.5227	-0.0315	-23	24	33	YEEMVEFMEK			Oxidation (M)[4,8]		Mascot
1388.738	1388.7262	-0.0118	-8	68	79	IISSIEQKEEGR					Mascot
1388.738	1388.7262	-0.0118	-8	68	79	IISSIEQKEEGR	54	99.292			Mascot
1406.6646	1406.6599	-0.0047	-3	37	48	TVDSEELTVEER					Mascot
1406.6646	1406.6599	-0.0047	-3	37	48	TVDSEELTVEER	109	100			Mascot
1786.9811	1786.9723	-0.0088	-5	160	176	AAQDIALAELAPTHPIR					Mascot
1786.9811	1786.9723	-0.0088	-5	160	176	AAQDIALAELAPTHPIR	156	100			Mascot
2147.9624	2147.9368	-0.0256	-12	17	33	LAEQAERYEEMVEFMEK			Oxidation (M)[11]		Mascot

2163.9573	2163.9104	-0.0469	-22	17	33	LAEQAERYEEMVEFMEK			Oxidation (M)[11,15]		Mascot
2163.9573	2163.9104	-0.0469	-22	17	33	LAEQAERYEEMVEFMEK	8	0	Oxidation (M)[11,15]		Mascot
2174.9976	2174.946	-0.0516	-24	203	221	QAFDEAISELDTLSEESYK					Mascot
2331.2019	2331.2019	0	0	177	196	LGLALNFSVFYYEILNSPDR					Mascot

5	hypothetical protein SORBIDRAFT_06g019100 [Sorghum bicolor]	gi|241937809	29744	4.76	16	440	100	41.508	350	100

Peptide Information

Calc. Mass	Obsrv. Mass	± da	± ppm	Start Seq.	End Seq.	Sequence	Ion Score	C. I. %	Modification	Rank	Result Type
816.421	816.4134	-0.0076	-9	17	23	LAEQAER					Mascot
818.444	818.4239	-0.0201	-25	102	108	ICDGILK			Carbamidomethyl (C)[2]		Mascot
907.5247	907.4822	-0.0425	-47	49	56	NLLSVAYK					Mascot
917.5302	917.5153	-0.0149	-16	68	75	IISSIEQK					Mascot
932.4294	932.4268	-0.0026	-3	130	136	MKGDYYR					Mascot
948.4244	948.4129	-0.0115	-12	130	136	MKGDYYR			Oxidation (M)[1]		Mascot
999.4451	999.4496	0.0045	5	9	16	EENVYMAK			Oxidation (M)[6]		Mascot
1144.6321	1144.6272	-0.0049	-4	80	89	GNEDRVTLIK					Mascot
1189.6609	1189.6603	-0.0006	-1	222	231	DSTLIMQLLR					Mascot
1189.6609	1189.6603	-0.0006	-1	222	231	DSTLIMQLLR	22	0			Mascot
1205.6559	1205.6425	-0.0134	-11	222	231	DSTLIMQLLR			Oxidation (M)[6]		Mascot
1205.6559	1205.6425	-0.0134	-11	222	231	DSTLIMQLLR	23	0	Oxidation (M)[6]		Mascot
1212.5565	1212.6031	0.0466	38	149	159	DAAENTMVAYK					Mascot
1228.5514	1228.5819	0.0305	25	149	159	DAAENTMVAYK			Oxidation (M)[7]		Mascot
1366.5542	1366.5227	-0.0315	-23	24	33	YEEMVEFMEK			Oxidation (M)[4,8]		Mascot
1388.738	1388.7262	-0.0118	-8	68	79	IISSIEQKEEGR					Mascot
1388.738	1388.7262	-0.0118	-8	68	79	IISSIEQKEEGR	54	99.292			Mascot
1406.6646	1406.6599	-0.0047	-3	37	48	TVDSEELTVEER					Mascot
1406.6646	1406.6599	-0.0047	-3	37	48	TVDSEELTVEER	109	100			Mascot
1786.9811	1786.9723	-0.0088	-5	160	176	AAQDIALAELAPTHPIR					Mascot
1786.9811	1786.9723	-0.0088	-5	160	176	AAQDIALAELAPTHPIR	156	100			Mascot
2147.9624	2147.9368	-0.0256	-12	17	33	LAEQAERYEEMVEFMEK			Oxidation (M)[11]		Mascot
2163.9573	2163.9104	-0.0469	-22	17	33	LAEQAERYEEMVEFMEK			Oxidation (M)[11,15]		Mascot
2163.9573	2163.9104	-0.0469	-22	17	33	LAEQAERYEEMVEFMEK	8	0	Oxidation (M)[11,15]		Mascot
2174.9976	2174.946	-0.0516	-24	203	221	QAFDEAISELDTLSEESYK					Mascot
2331.2019	2331.2019	0	0	177	196	LGLALNFSVFYYEILNSPDR					Mascot

6	RecName: Full=14-3-3-like protein GF14-12	gi|1345588	29731.9	4.75	15	409	100	37.347	328	100	

Peptide Information

Calc. Mass	Obsrv. Mass	± da	± ppm	Start Seq.	End Seq.	Sequence	Ion Score	C. I. %	Modification	Rank	Result Type
816.421	816.4134	-0.0076	-9	17	23	LAEQAER					Mascot
818.444	818.4239	-0.0201	-25	102	108	ICDGILK			Carbamidomethyl (C)[2]		Mascot
907.5247	907.4822	-0.0425	-47	49	56	NLLSVAYK					Mascot
917.5302	917.5153	-0.0149	-16	68	75	IISSIEQK					Mascot
932.4294	932.4268	-0.0026	-3	130	136	MKGDYYR					Mascot
948.4244	948.4129	-0.0115	-12	130	136	MKGDYYR			Oxidation (M)[1]		Mascot
999.4451	999.4496	0.0045	5	9	16	EENVYMAK			Oxidation (M)[6]		Mascot
1144.6321	1144.6272	-0.0049	-4	80	89	GNEDRVTLIK					Mascot
1212.5565	1212.6031	0.0466	38	149	159	DAAENTMVAYK					Mascot
1228.5514	1228.5819	0.0305	25	149	159	DAAENTMVAYK			Oxidation (M)[7]		Mascot
1366.5542	1366.5227	-0.0315	-23	24	33	YEEMVEFMEK			Oxidation (M)[4,8]		Mascot
1388.738	1388.7262	-0.0118	-8	68	79	IISSIEQKEEGR					Mascot
1388.738	1388.7262	-0.0118	-8	68	79	IISSIEQKEEGR	54	99.292			Mascot
1406.6646	1406.6599	-0.0047	-3	37	48	TVDSEELTVEER					Mascot
1406.6646	1406.6599	-0.0047	-3	37	48	TVDSEELTVEER	109	100			Mascot
1786.9811	1786.9723	-0.0088	-5	160	176	AAQDIALAELAPTHPIR					Mascot
1786.9811	1786.9723	-0.0088	-5	160	176	AAQDIALAELAPTHPIR	156	100			Mascot
2147.9624	2147.9368	-0.0256	-12	17	33	LAEQAERYEEMVEFMEK			Oxidation (M)[11]		Mascot
2163.9573	2163.9104	-0.0469	-22	17	33	LAEQAERYEEMVEFMEK			Oxidation (M)[11,15]		Mascot
2163.9573	2163.9104	-0.0469	-22	17	33	LAEQAERYEEMVEFMEK	8	0	Oxidation (M)[11,15]		Mascot
2174.9976	2174.946	-0.0516	-24	203	221	QAFDEAISELDTLSEESY K					Mascot
2331.2019	2331.2019	0	0	177	196	LGLALNFSVFYYEILNSPD R					Mascot

7	FUSICOCCIN receptor protein s [Zea mays]	gi|413918562	98275.5	6.45	20	402	100	44.066	350	100	

Peptide Information

Calc. Mass	Obsrv. Mass	± da	± ppm	Start Seq.	End Seq.	Sequence	Ion Score	C. I. %	Modification	Rank	Result Type
816.421	816.4134	-0.0076	-9	17	23	LAEQAER					Mascot
818.444	818.4239	-0.0201	-25	102	108	ICDGILK			Carbamidomethyl (C)[2]		Mascot
907.5247	907.4822	-0.0425	-47	49	56	NLLSVAYK					Mascot
913.4308	913.433	0.0022	2	374	381	SSMAHPQR					Mascot
917.5302	917.5153	-0.0149	-16	68	75	IISSIEQK					Mascot

Calc. Mass	Obsrv. Mass	± da	± ppm	Start Seq.	End Seq.	Sequence	Ion Score	C. I. %	Modification	Rank	Result Type
932.4294	932.4268	-0.0026	-3	130	136	MKGDYYR					Mascot
948.4244	948.4129	-0.0115	-12	130	136	MKGDYYR			Oxidation (M)[1]		Mascot
999.4451	999.4496	0.0045	5	9	16	EENVYMAK			Oxidation (M)[6]		Mascot
1144.6321	1144.6272	-0.0049	-4	80	89	GNEDRVTLIK					Mascot
1189.6609	1189.6603	-0.0006	-1	234	243	DSTLIMQLLR					Mascot
1189.6609	1189.6603	-0.0006	-1	234	243	DSTLIMQLLR	22	0			Mascot
1205.6559	1205.6425	-0.0134	-11	234	243	DSTLIMQLLR			Oxidation (M)[6]		Mascot
1205.6559	1205.6425	-0.0134	-11	234	243	DSTLIMQLLR	23	0	Oxidation (M)[6]		Mascot
1212.5565	1212.6031	0.0466	38	161	171	DAAENTMVAYK					Mascot
1221.5819	1221.6437	0.0618	51	502	512	DTRSSQSPTSR					Mascot
1228.5514	1228.5819	0.0305	25	161	171	DAAENTMVAYK			Oxidation (M)[7]		Mascot
1366.5542	1366.5227	-0.0315	-23	24	33	YEEMVEFMEK			Oxidation (M)[4,8]		Mascot
1388.738	1388.7262	-0.0118	-8	68	79	IISSIEQKEEGR					Mascot
1388.738	1388.7262	-0.0118	-8	68	79	IISSIEQKEEGR	54	99.292			Mascot
1406.6646	1406.6599	-0.0047	-3	37	48	TVDSEELTVEER					Mascot
1406.6646	1406.6599	-0.0047	-3	37	48	TVDSEELTVEER	109	100			Mascot
1558.7393	1558.7302	-0.0091	-6	137	148	YYDCMNPIVVIR			Carbamidomethyl (C)[4], Oxidation (M)[5]		Mascot
1558.7393	1558.7302	-0.0091	-6	137	148	YYDCMNPIVVIR			Carbamidomethyl (C)[4], Oxidation (M)[5]		Mascot
1786.9811	1786.9723	-0.0088	-5	172	188	AAQDIALAELAPTHPIR					Mascot
1786.9811	1786.9723	-0.0088	-5	172	188	AAQDIALAELAPTHPIR	156	100			Mascot
2147.9624	2147.9368	-0.0256	-12	17	33	LAEQAERYEEMVEFMEK			Oxidation (M)[11]		Mascot
2163.9573	2163.9104	-0.0469	-22	17	33	LAEQAERYEEMVEFMEK			Oxidation (M)[11,15]		Mascot
2163.9573	2163.9104	-0.0469	-22	17	33	LAEQAERYEEMVEFMEK	8	0	Oxidation (M)[11,15]		Mascot
2174.9976	2174.946	-0.0516	-24	215	233	QAFDEAISELDTLSEESY K					Mascot
2331.2019	2331.2019	0	0	189	208	LGLALNFSVFYYEILNSPD R					Mascot
2383.2729	2383.0728	-0.2001	-84	551	575	RAASIQAAVPSVNSAPAV TSGGPFK					Mascot

8	14-3-3-like protein GF14-12 [Zea mays]	gi|262399364	28252.3	4.8	14	396	100	37.172	328	100

Peptide Information

Calc. Mass	Obsrv. Mass	± da	± ppm	Start Seq.	End Seq.	Sequence	Ion Score	C. I. %	Modification	Rank	Result Type
816.421	816.4134	-0.0076	-9	4	10	LAEQAER					Mascot
818.444	818.4239	-0.0201	-25	89	95	ICDGILK			Carbamidomethyl (C)[2]		Mascot
907.5247	907.4822	-0.0425	-47	36	43	NLLSVAYK					Mascot
917.5302	917.5153	-0.0149	-16	55	62	IISSIEQK					Mascot
932.4294	932.4268	-0.0026	-3	117	123	MKGDYYR					Mascot

Calc. Mass	Obsrv. Mass	± da	± ppm	Start Seq.	End Seq.	Sequence	Ion Score	C. I. %	Modification	Rank	Result Type
948.4244	948.4129	-0.0115	-12	117	123	MKGDYYR			Oxidation (M)[1]		Mascot
1144.6321	1144.6272	-0.0049	-4	67	76	GNEDRVTLIK					Mascot
1212.5565	1212.6031	0.0466	38	136	146	DAAENTMVAYK					Mascot
1228.5514	1228.5819	0.0305	25	136	146	DAAENTMVAYK			Oxidation (M)[7]		Mascot
1366.5542	1366.5227	-0.0315	-23	11	20	YEEMVEFMEK			Oxidation (M)[4,8]		Mascot
1388.738	1388.7262	-0.0118	-8	55	66	IISSIEQKEEGR					Mascot
1388.738	1388.7262	-0.0118	-8	55	66	IISSIEQKEEGR	54	99.292			Mascot
1406.6646	1406.6599	-0.0047	-3	24	35	TVDSEELTVEER					Mascot
1406.6646	1406.6599	-0.0047	-3	24	35	TVDSEELTVEER	109	100			Mascot
1786.9811	1786.9723	-0.0088	-5	147	163	AAQDIALAELAPTHPIR					Mascot
1786.9811	1786.9723	-0.0088	-5	147	163	AAQDIALAELAPTHPIR	156	100			Mascot
2147.9624	2147.9368	-0.0256	-12	4	20	LAEQAERYEEMVEFMEK			Oxidation (M)[11]		Mascot
2163.9573	2163.9104	-0.0469	-22	4	20	LAEQAERYEEMVEFMEK			Oxidation (M)[11,15]		Mascot
2163.9573	2163.9104	-0.0469	-22	4	20	LAEQAERYEEMVEFMEK	8	0	Oxidation (M)[11,15]		Mascot
2174.9976	2174.946	-0.0516	-24	190	208	QAFDEAISELDTLSEESYK					Mascot
2331.2019	2331.2019	0	0	164	183	LGLALNFSVFYYEILNSPDR					Mascot

9	14-3-3-like protein GF14-D [Aegilops tauschii]	gi|475557630	37202.4	4.94	14	334	100	13.485	269	100

Peptide Information

Calc. Mass	Obsrv. Mass	± da	± ppm	Start Seq.	End Seq.	Sequence	Ion Score	C. I. %	Modification	Rank	Result Type
816.421	816.4134	-0.0076	-9	17	23	LAEQAER					Mascot
907.5247	907.4822	-0.0425	-47	53	60	NLLSVAYK					Mascot
917.5302	917.5153	-0.0149	-16	72	79	IISSIEQK					Mascot
1025.5123	1025.5107	-0.0016	-2	84	93	GNDAHAATIR					Mascot
1189.6609	1189.6603	-0.0006	-1	226	235	DSTLIMQLLR					Mascot
1189.6609	1189.6603	-0.0006	-1	226	235	DSTLIMQLLR	22	0			Mascot
1205.6559	1205.6425	-0.0134	-11	226	235	DSTLIMQLLR			Oxidation (M)[6]		Mascot
1205.6559	1205.6425	-0.0134	-11	226	235	DSTLIMQLLR	23	0	Oxidation (M)[6]		Mascot
1358.6256	1358.7042	0.0786	58	152	163	KEAAESTMNAYK			Oxidation (M)[8]		Mascot
1388.738	1388.7262	-0.0118	-8	72	83	IISSIEQKEEGR					Mascot
1388.738	1388.7262	-0.0118	-8	72	83	IISSIEQKEEGR	54	99.292			Mascot
1394.5603	1394.526	-0.0343	-25	24	33	YEEMVEFMER			Oxidation (M)[4,8]		Mascot
1558.7344	1558.7302	-0.0042	-3	37	52	ATGGAGPGEELSVEER					Mascot
1558.7344	1558.7302	-0.0042	-3	37	52	ATGGAGPGEELSVEER	86	100			Mascot
1660.771	1660.8453	0.0743	45	24	36	YEEMVEFMERVAK					Mascot

1696.7847	1696.7582	-0.0265	-16	2	16	SPAEPTRDESVYMAK			Oxidation (M)[13]		Mascot
1772.9653	1772.9572	-0.0081	-5	164	180	AAQDIALADLAPTHPIR					Mascot
1772.9653	1772.9572	-0.0081	-5	164	180	AAQDIALADLAPTHPIR	106	100			Mascot
2191.9634	2191.9226	-0.0408	-19	17	33	LAEQAERYEEMVEFMER			Oxidation (M)[11,15]		Mascot
2331.2019	2331.2019	0	0	181	200	LGLALNFSVFYYEILNSPDR					Mascot

10	Os04g0462500 [Oryza sativa Japonica Group]	gi|113564574	29959.1	4.76	15	287	100	17.853	209	100		

Protein Group

RecName: Full=14-3-3-like protein GF14-B; AltName: Full=G-box factor 14-3-3 homolog B	gi|75296478	29959.1	4.7600002288184
hypothetical protein OsI_16185 [Oryza sativa Indica Group]	gi|125548594	29959.1	4.7600002288184

Peptide Information

Calc. Mass	Obsrv. Mass	± da	± ppm	Start Seq.	End Seq.	Sequence	Ion Score	C. I. %	Modification	Rank	Result Type
816.421	816.4134	-0.0076	-9	18	24	LAEQAER					Mascot
818.444	818.4239	-0.0201	-25	103	109	ICDGILK			Carbamidomethyl (C)[2]		Mascot
907.5247	907.4822	-0.0425	-47	50	57	NLLSVAYK					Mascot
917.5302	917.5153	-0.0149	-16	69	76	IISSIEQK					Mascot
932.4294	932.4268	-0.0026	-3	131	137	MKGDYYR					Mascot
948.4244	948.4129	-0.0115	-12	131	137	MKGDYYR			Oxidation (M)[1]		Mascot
999.4451	999.4496	0.0045	5	10	17	EENVYMAK			Oxidation (M)[6]		Mascot
1144.6321	1144.6272	-0.0049	-4	81	90	GNEDRVTLIK					Mascot
1189.6609	1189.6603	-0.0006	-1	223	232	DSTLIMQLLR					Mascot
1189.6609	1189.6603	-0.0006	-1	223	232	DSTLIMQLLR	22	0			Mascot
1205.6559	1205.6425	-0.0134	-11	223	232	DSTLIMQLLR			Oxidation (M)[6]		Mascot
1205.6559	1205.6425	-0.0134	-11	223	232	DSTLIMQLLR	23	0	Oxidation (M)[6]		Mascot
1212.5565	1212.6031	0.0466	38	150	160	DAAENTMVAYK					Mascot
1228.5514	1228.5819	0.0305	25	150	160	DAAENTMVAYK			Oxidation (M)[7]		Mascot
1366.5542	1366.5227	-0.0315	-23	25	34	YEEMVEFMEK			Oxidation (M)[4,8]		Mascot
1406.6646	1406.6599	-0.0047	-3	38	49	TVDSEELTVEER					Mascot
1406.6646	1406.6599	-0.0047	-3	38	49	TVDSEELTVEER	109	100			Mascot
1418.7485	1418.7394	-0.0091	-6	69	80	IISSIEQKEESR					Mascot
1418.7485	1418.7394	-0.0091	-6	69	80	IISSIEQKEESR	69	99.978			Mascot
2147.9624	2147.9368	-0.0256	-12	18	34	LAEQAERYEEMVEFMEK			Oxidation (M)[11]		Mascot

2163.9573	2163.9104	-0.0469	-22	18	34	LAEQAERYEEMVEFMEK			Oxidation (M)[11,15]	Mascot
2163.9573	2163.9104	-0.0469	-22	18	34	LAEQAERYEEMVEFMEK	8	0	Oxidation (M)[11,15]	Mascot
2174.9976	2174.946	-0.0516	-24	204	222	QAFDEAISELDTLSEESYK				Mascot
2331.2019	2331.2019	0	0	178	197	LGLALNFSVFYYEILNSPDR				Mascot

Gel Idx/Pos	167/G18	Instr./Gel Origin	BA2151/Sample Project 20140814	Process Status	Analysis Succeeded
Plate [#] Name	[1] Sample Project 20140814	Instrument Sample Name		Spectra	11

Rank	Protein Name	Accession No.	Protein MW	Protein PI	Pep. Count	Protein Score	Protein Score C. I. %	Intensity Matched	Total Ion Score	Total Ion C. I. %	Confirmed
1	14-3-3 protein [Triticum aestivum]	gi|431822520	29360.7	4.83	19	346	100	41.698	206	100	

Peptide Information

Calc. Mass	Obsrv. Mass	± da	± ppm	Start Seq.	End Seq.	Sequence	Ion Score	C. I. %	Modification	Rank	Result Type
816.421	816.4305	0.0095	12	17	23	LAEQAER					Mascot
819.4458	819.4396	-0.0062	-8	95	101	IETELSK					Mascot
906.425	906.4412	0.0162	18	130	136	MKGDYHR					Mascot
907.5247	907.4802	-0.0445	-49	49	56	NLLSVAYK					Mascot
917.5302	917.5102	-0.02	-22	68	75	IISSIEQK					Mascot
922.4199	922.43	0.0101	11	130	136	MKGDYHR			Oxidation (M)[1]		Mascot
922.4199	922.43	0.0101	11	130	136	MKGDYHR			Oxidation (M)[1]		Mascot
928.5324	928.4614	-0.071	-76	125	131	VFYLKMK					Mascot
1051.5419	1051.551	0.0091	9	80	89	GNEAYVASIK					Mascot
1051.5419	1051.551	0.0091	9	80	89	GNEAYVASIK					Mascot
1076.5946	1076.6132	0.0186	17	93	101	TRIETELSK					Mascot
1076.5946	1076.6132	0.0186	17	93	101	TRIETELSK					Mascot
1111.5015	1111.4783	-0.0232	-21	251	261	EAASKPEGEGH					Mascot
1189.6609	1189.6735	0.0126	11	222	231	DSTLIMQLLR					Mascot
1205.6559	1205.6677	0.0118	10	222	231	DSTLIMQLLR			Oxidation (M)[6]		Mascot
1205.6559	1205.6677	0.0118	10	222	231	DSTLIMQLLR	13	0	Oxidation (M)[6]		Mascot
1208.6157	1208.6475	0.0318	26	149	159	EAAENTLVAYK					Mascot
1318.6486	1318.6693	0.0207	16	37	48	TADVGELTVEER					Mascot
1318.6486	1318.6693	0.0207	16	37	48	TADVGELTVEER	89	100			Mascot
1336.7107	1336.722	0.0113	8	148	159	KEAAENTLVAYK					Mascot
1366.5542	1366.5656	0.0114	8	24	33	YEEMVEFMEK			Oxidation (M)[4,8]		Mascot
1418.7485	1418.7694	0.0209	15	68	79	IISSIEQKEESR					Mascot
1418.7485	1418.7694	0.0209	15	68	79	IISSIEQKEESR	28	0			Mascot
1552.7601	1552.7839	0.0238	15	76	89	EESRGNEAYVASIK					Mascot
1664.7546	1664.8337	0.0791	48	24	36	YEEMVEFMEKVAK			Oxidation (M)[4,8]		Mascot
1818.9708	1818.9923	0.0215	12	160	176	SAQDIALADLPTTHPIR					Mascot
1818.9708	1818.9923	0.0215	12	160	176	SAQDIALADLPTTHPIR	90	100			Mascot

2163.9573	2163.9624	0.0051	2	17	33	LAEQAERYEEMVEFMEK			Oxidation (M)[11,15]		Mascot

2	14-3-3-like protein A [Aegilops tauschii]	gi|475611352	32785.4	4.77	19	340	100	41.698	206	100		

Peptide Information

Calc. Mass	Obsrv. Mass	± da	± ppm	Start Seq.	End Seq.	Sequence	Ion Score	C. I. %	Modification	Rank	Result Type
816.421	816.4305	0.0095	12	17	23	LAEQAER					Mascot
819.4458	819.4396	-0.0062	-8	95	101	IETELSK					Mascot
906.425	906.4412	0.0162	18	130	136	MKGDYHR					Mascot
907.5247	907.4802	-0.0445	-49	49	56	NLLSVAYK					Mascot
917.5302	917.5102	-0.02	-22	68	75	IISSIEQK					Mascot
922.4199	922.43	0.0101	11	130	136	MKGDYHR			Oxidation (M)[1]		Mascot
922.4199	922.43	0.0101	11	130	136	MKGDYHR			Oxidation (M)[1]		Mascot
928.5324	928.4614	-0.071	-76	125	131	VFYLKMK					Mascot
1051.5419	1051.551	0.0091	9	80	89	GNEAYVASIK					Mascot
1051.5419	1051.551	0.0091	9	80	89	GNEAYVASIK					Mascot
1076.5946	1076.6132	0.0186	17	93	101	TRIETELSK					Mascot
1076.5946	1076.6132	0.0186	17	93	101	TRIETELSK					Mascot
1111.5015	1111.4783	-0.0232	-21	282	292	EAASKPEGEGH					Mascot
1189.6609	1189.6735	0.0126	11	222	231	DSTLIMQLLR					Mascot
1205.6559	1205.6677	0.0118	10	222	231	DSTLIMQLLR			Oxidation (M)[6]		Mascot
1205.6559	1205.6677	0.0118	10	222	231	DSTLIMQLLR	13	0	Oxidation (M)[6]		Mascot
1208.6157	1208.6475	0.0318	26	149	159	EAAENTLVAYK					Mascot
1318.6486	1318.6693	0.0207	16	37	48	TADVGELTVEER					Mascot
1318.6486	1318.6693	0.0207	16	37	48	TADVGELTVEER	89	100			Mascot
1336.7107	1336.722	0.0113	8	148	159	KEAAENTLVAYK					Mascot
1366.5542	1366.5656	0.0114	8	24	33	YEEMVEFMEK			Oxidation (M)[4,8]		Mascot
1418.7485	1418.7694	0.0209	15	68	79	IISSIEQKEESR					Mascot
1418.7485	1418.7694	0.0209	15	68	79	IISSIEQKEESR	28	0			Mascot
1552.7601	1552.7839	0.0238	15	76	89	EESRGNEAYVASIK					Mascot
1664.7546	1664.8337	0.0791	48	24	36	YEEMVEFMEKVAK			Oxidation (M)[4,8]		Mascot
1818.9708	1818.9923	0.0215	12	160	176	SAQDIALADLPTTHPIR					Mascot
1818.9708	1818.9923	0.0215	12	160	176	SAQDIALADLPTTHPIR	90	100			Mascot
2163.9573	2163.9624	0.0051	2	17	33	LAEQAERYEEMVEFMEK			Oxidation (M)[11,15]		Mascot

3	uncharacterized protein [Phleum pratense]	gi|409972305	29275.7	4.82	18	334	100	41.338	206	100		

Protein Group

RecName: Full=14-3-3-like protein A; Short=14-3-3A	gi|112684	29447.8	4.8299<br>999237<br>0605
uncharacterized protein [Phleum pratense]	gi|409972067	29275.7	4.8200<br>001716<br>6138
uncharacterized protein [Phleum pratense]	gi|409972513	29275.7	4.8200<br>001716<br>6138
uncharacterized protein [Phleum pratense]	gi|409971647	29275.7	4.8200<br>001716<br>6138

Peptide Information

Calc. Mass	Obsrv. Mass	± da	± ppm	Start Seq.	End Seq.	Sequence	Ion Score	C. I. %	Modification	Rank	Result Type
816.421	816.4305	0.0095	12	17	23	LAEQAER					Mascot
819.4458	819.4396	-0.0062	-8	95	101	IETELSK					Mascot
906.425	906.4412	0.0162	18	130	136	MKGDYHR					Mascot
907.5247	907.4802	-0.0445	-49	49	56	NLLSVAYK					Mascot
917.5302	917.5102	-0.02	-22	68	75	IISSIEQK					Mascot
922.4199	922.43	0.0101	11	130	136	MKGDYHR			Oxidation (M)[1]		Mascot
922.4199	922.43	0.0101	11	130	136	MKGDYHR			Oxidation (M)[1]		Mascot
928.5324	928.4614	-0.071	-76	125	131	VFYLKMK					Mascot
1051.5419	1051.551	0.0091	9	80	89	GNEAYVASIK					Mascot
1051.5419	1051.551	0.0091	9	80	89	GNEAYVASIK					Mascot
1076.5946	1076.6132	0.0186	17	93	101	TRIETELSK					Mascot
1076.5946	1076.6132	0.0186	17	93	101	TRIETELSK					Mascot
1189.6609	1189.6735	0.0126	11	222	231	DSTLIMQLLR					Mascot
1205.6559	1205.6677	0.0118	10	222	231	DSTLIMQLLR			Oxidation (M)[6]		Mascot
1205.6559	1205.6677	0.0118	10	222	231	DSTLIMQLLR	13	0	Oxidation (M)[6]		Mascot
1208.6157	1208.6475	0.0318	26	149	159	EAAENTLVAYK					Mascot
1318.6486	1318.6693	0.0207	16	37	48	TADVGELTVEER					Mascot
1318.6486	1318.6693	0.0207	16	37	48	TADVGELTVEER	89	100			Mascot
1336.7107	1336.722	0.0113	8	148	159	KEAAENTLVAYK					Mascot
1366.5542	1366.5656	0.0114	8	24	33	YEEMVEFMEK			Oxidation (M)[4,8]		Mascot
1418.7485	1418.7694	0.0209	15	68	79	IISSIEQKEESR					Mascot
1418.7485	1418.7694	0.0209	15	68	79	IISSIEQKEESR	28	0			Mascot
1552.7601	1552.7839	0.0238	15	76	89	EESRGNEAYVASIK					Mascot

Calc. Mass	Obsrv. Mass	± da	± ppm	Start Seq.	End Seq.	Sequence	Ion Score	C. I. %	Modification	Rank	Result Type
1664.7546	1664.8337	0.0791	48	24	36	YEEMVEFMEKVAK			Oxidation (M)[4,8]		Mascot
1818.9708	1818.9923	0.0215	12	160	176	SAQDIALADLPTTHPIR					Mascot
1818.9708	1818.9923	0.0215	12	160	176	SAQDIALADLPTTHPIR	90	100			Mascot
2163.9573	2163.9624	0.0051	2	17	33	LAEQAERYEEMVEFMEK			Oxidation (M)[11,15]		Mascot

4	uncharacterized protein, partial [Phleum pratense]	gi|409972289	27447.9	4.91	18	327	100	41.338	206	100

Peptide Information

Calc. Mass	Obsrv. Mass	± da	± ppm	Start Seq.	End Seq.	Sequence	Ion Score	C. I. %	Modification	Rank	Result Type
816.421	816.4305	0.0095	12	5	11	LAEQAER					Mascot
819.4458	819.4396	-0.0062	-8	83	89	IETELSK					Mascot
906.425	906.4412	0.0162	18	118	124	MKGDYHR					Mascot
907.5247	907.4802	-0.0445	-49	37	44	NLLSVAYK					Mascot
917.5302	917.5102	-0.02	-22	56	63	IISSIEQK					Mascot
922.4199	922.43	0.0101	11	118	124	MKGDYHR			Oxidation (M)[1]		Mascot
922.4199	922.43	0.0101	11	118	124	MKGDYHR			Oxidation (M)[1]		Mascot
928.5324	928.4614	-0.071	-76	113	119	VFYLKMK					Mascot
1051.5419	1051.551	0.0091	9	68	77	GNEAYVASIK					Mascot
1051.5419	1051.551	0.0091	9	68	77	GNEAYVASIK					Mascot
1076.5946	1076.6132	0.0186	17	81	89	TRIETELSK					Mascot
1076.5946	1076.6132	0.0186	17	81	89	TRIETELSK					Mascot
1189.6609	1189.6735	0.0126	11	210	219	DSTLIMQLLR					Mascot
1205.6559	1205.6677	0.0118	10	210	219	DSTLIMQLLR			Oxidation (M)[6]		Mascot
1205.6559	1205.6677	0.0118	10	210	219	DSTLIMQLLR	13	0	Oxidation (M)[6]		Mascot
1208.6157	1208.6475	0.0318	26	137	147	EAAENTLVAYK					Mascot
1318.6486	1318.6693	0.0207	16	25	36	TADVGELTVEER					Mascot
1318.6486	1318.6693	0.0207	16	25	36	TADVGELTVEER	89	100			Mascot
1336.7107	1336.722	0.0113	8	136	147	KEAAENTLVAYK					Mascot
1366.5542	1366.5656	0.0114	8	12	21	YEEMVEFMEK			Oxidation (M)[4,8]		Mascot
1418.7485	1418.7694	0.0209	15	56	67	IISSIEQKEESR					Mascot
1418.7485	1418.7694	0.0209	15	56	67	IISSIEQKEESR	28	0			Mascot
1552.7601	1552.7839	0.0238	15	64	77	EESRGNEAYVASIK					Mascot
1664.7546	1664.8337	0.0791	48	12	24	YEEMVEFMEKVAK			Oxidation (M)[4,8]		Mascot
1818.9708	1818.9923	0.0215	12	148	164	SAQDIALADLPTTHPIR					Mascot
1818.9708	1818.9923	0.0215	12	148	164	SAQDIALADLPTTHPIR	90	100			Mascot
2163.9573	2163.9624	0.0051	2	5	21	LAEQAERYEEMVEFMEK			Oxidation (M)[11,15]		Mascot

5	uncharacterized protein, partial [Phleum pratense]	gi|409972463	18302.3	5.62	16	226	100	21.953	117	100

Peptide Information

Calc. Mass	Obsrv. Mass	± da	± ppm	Start Seq.	End Seq.	Sequence	Ion Score	C. I. %	Modification	Rank	Result Type
816.421	816.4305	0.0095	12	17	23	LAEQAER					Mascot
819.4458	819.4396	-0.0062	-8	95	101	IETELSK					Mascot
906.425	906.4412	0.0162	18	130	136	MKGDYHR					Mascot
907.5247	907.4802	-0.0445	-49	49	56	NLLSVAYK					Mascot
917.5302	917.5102	-0.02	-22	68	75	IISSIEQK					Mascot
922.4199	922.43	0.0101	11	130	136	MKGDYHR			Oxidation (M)[1]		Mascot
922.4199	922.43	0.0101	11	130	136	MKGDYHR			Oxidation (M)[1]		Mascot
928.5324	928.4614	-0.071	-76	125	131	VFYLKMK					Mascot
1051.5419	1051.551	0.0091	9	80	89	GNEAYVASIK					Mascot
1051.5419	1051.551	0.0091	9	80	89	GNEAYVASIK					Mascot
1076.5946	1076.6132	0.0186	17	93	101	TRIETELSK					Mascot
1076.5946	1076.6132	0.0186	17	93	101	TRIETELSK					Mascot
1208.6157	1208.6475	0.0318	26	149	159	EAAENTLVAYK					Mascot
1318.6486	1318.6693	0.0207	16	37	48	TADVGELTVEER					Mascot
1318.6486	1318.6693	0.0207	16	37	48	TADVGELTVEER	89	100			Mascot
1336.7107	1336.722	0.0113	8	148	159	KEAAENTLVAYK					Mascot
1366.5542	1366.5656	0.0114	8	24	33	YEEMVEFMEK			Oxidation (M)[4,8]		Mascot
1418.7485	1418.7694	0.0209	15	68	79	IISSIEQKEESR					Mascot
1418.7485	1418.7694	0.0209	15	68	79	IISSIEQKEESR	28	0			Mascot
1552.7601	1552.7839	0.0238	15	76	89	EESRGNEAYVASIK					Mascot
1664.7546	1664.8337	0.0791	48	24	36	YEEMVEFMEKVAK			Oxidation (M)[4,8]		Mascot
2163.9573	2163.9624	0.0051	2	17	33	LAEQAERYEEMVEFMEK			Oxidation (M)[11,15]		Mascot

6	Os03g0710800 [Oryza sativa Japonica Group]	gi|113549522	29273.7	4.81	16	226	100	32.281	117	100		

Protein Group

RecName: Full=14-3-3-like protein GF14-F; AltName: Full=14-3-3-like protein S94; AltName: Full=G-box factor 14-3-3 homolog F; AltName: Full=OsGF14a; AltName: Full=Stress-regulated 14-3-3 protein; Short=SR14-3-3	gi|76789644	29273.7	4.8099 999427 7954
hypothetical protein OsI_13240 [Oryza sativa Indica Group]	gi|125545466	29273.7	4.8099 999427 7954

Peptide Information

Calc. Mass	Obsrv. Mass	± da	± ppm	Start Seq.	End Seq.	Sequence	Ion Score	C. I. %	Modification	Rank	Result Type

Calc. Mass	Obsrv. Mass	± da	± ppm	Start Seq.	End Seq.	Sequence	Ion Score	C. I. %	Modification	Rank	Result Type
816.421	816.4305	0.0095	12	17	23	LAEQAER					Mascot
819.4458	819.4396	-0.0062	-8	95	101	IETELSK					Mascot
906.425	906.4412	0.0162	18	130	136	MKGDYHR					Mascot
907.5247	907.4802	-0.0445	-49	49	56	NLLSVAYK					Mascot
917.5302	917.5102	-0.02	-22	68	75	IISSIEQK					Mascot
922.4199	922.43	0.0101	11	130	136	MKGDYHR			Oxidation (M)[1]		Mascot
922.4199	922.43	0.0101	11	130	136	MKGDYHR			Oxidation (M)[1]		Mascot
928.5324	928.4614	-0.071	-76	125	131	VFYLKMK					Mascot
1051.5419	1051.551	0.0091	9	80	89	GNEAYVASIK					Mascot
1051.5419	1051.551	0.0091	9	80	89	GNEAYVASIK					Mascot
1189.6609	1189.6735	0.0126	11	222	231	DSTLIMQLLR					Mascot
1205.6559	1205.6677	0.0118	10	222	231	DSTLIMQLLR			Oxidation (M)[6]		Mascot
1205.6559	1205.6677	0.0118	10	222	231	DSTLIMQLLR	13	0	Oxidation (M)[6]		Mascot
1208.6157	1208.6475	0.0318	26	149	159	EAAENTLVAYK					Mascot
1336.7107	1336.722	0.0113	8	148	159	KEAAENTLVAYK					Mascot
1366.5542	1366.5656	0.0114	8	24	33	YEEMVEFMEK			Oxidation (M)[4,8]		Mascot
1418.7485	1418.7694	0.0209	15	68	79	IISSIEQKEESR					Mascot
1418.7485	1418.7694	0.0209	15	68	79	IISSIEQKEESR	28	0			Mascot
1552.7601	1552.7839	0.0238	15	76	89	EESRGNEAYVASIK					Mascot
1664.7546	1664.8337	0.0791	48	24	36	YEEMVEFMEKVAK			Oxidation (M)[4,8]		Mascot
1818.9708	1818.9923	0.0215	12	160	176	SAQDIALADLPTTHPIR					Mascot
1818.9708	1818.9923	0.0215	12	160	176	SAQDIALADLPTTHPIR	90	100			Mascot
2163.9573	2163.9624	0.0051	2	17	33	LAEQAERYEEMVEFMEK			Oxidation (M)[11,15]		Mascot

7 uncharacterized protein, partial [Phleum pratense] gi|409971621 16541.4 5.57 14 210 100 21.432 117 100

Protein Group

uncharacterized protein, partial [Phleum pratense]	gi|409971855	16413.3	5.5700 001716 6138

Peptide Information

Calc. Mass	Obsrv. Mass	± da	± ppm	Start Seq.	End Seq.	Sequence	Ion Score	C. I. %	Modification	Rank	Result Type
816.421	816.4305	0.0095	12	17	23	LAEQAER					Mascot
819.4458	819.4396	-0.0062	-8	95	101	IETELSK					Mascot
906.425	906.4412	0.0162	18	130	136	MKGDYHR					Mascot
907.5247	907.4802	-0.0445	-49	49	56	NLLSVAYK					Mascot

917.5302	917.5102	-0.02	-22	68	75	IISSIEQK					Mascot
922.4199	922.43	0.0101	11	130	136	MKGDYHR			Oxidation (M)[1]		Mascot
922.4199	922.43	0.0101	11	130	136	MKGDYHR			Oxidation (M)[1]		Mascot
928.5324	928.4614	-0.071	-76	125	131	VFYLKMK					Mascot
1051.5419	1051.551	0.0091	9	80	89	GNEAYVASIK					Mascot
1051.5419	1051.551	0.0091	9	80	89	GNEAYVASIK					Mascot
1076.5946	1076.6132	0.0186	17	93	101	TRIETELSK					Mascot
1076.5946	1076.6132	0.0186	17	93	101	TRIETELSK					Mascot
1318.6486	1318.6693	0.0207	16	37	48	TADVGELTVEER					Mascot
1318.6486	1318.6693	0.0207	16	37	48	TADVGELTVEER	89	100			Mascot
1366.5542	1366.5656	0.0114	8	24	33	YEEMVEFMEK			Oxidation (M)[4,8]		Mascot
1418.7485	1418.7694	0.0209	15	68	79	IISSIEQKEESR					Mascot
1418.7485	1418.7694	0.0209	15	68	79	IISSIEQKEESR	28	0			Mascot
1552.7601	1552.7839	0.0238	15	76	89	EESRGNEAYVASIK					Mascot
1664.7546	1664.8337	0.0791	48	24	36	YEEMVEFMEKVAK			Oxidation (M)[4,8]		Mascot
2163.9573	2163.9624	0.0051	2	17	33	LAEQAERYEEMVEFMEK			Oxidation (M)[11,15]		Mascot

8	uncharacterized protein, partial [Phleum pratense]	gi|409971935	17807	5.04	10	151	100	23.62	90	100	

Peptide Information

Calc. Mass	Obsrv. Mass	± da	± ppm	Start Seq.	End Seq.	Sequence	Ion Score	C. I. %	Modification	Rank	Result Type
807.3917	807.4189	0.0272	34	152	158	DSTLIMQ					Mascot
819.4458	819.4396	-0.0062	-8	25	31	IETELSK					Mascot
906.425	906.4412	0.0162	18	60	66	MKGDYHR					Mascot
922.4199	922.43	0.0101	11	60	66	MKGDYHR			Oxidation (M)[1]		Mascot
922.4199	922.43	0.0101	11	60	66	MKGDYHR			Oxidation (M)[1]		Mascot
928.5324	928.4614	-0.071	-76	55	61	VFYLKMK					Mascot
1051.5419	1051.551	0.0091	9	10	19	GNEAYVASIK					Mascot
1051.5419	1051.551	0.0091	9	10	19	GNEAYVASIK					Mascot
1076.5946	1076.6132	0.0186	17	23	31	TRIETELSK					Mascot
1076.5946	1076.6132	0.0186	17	23	31	TRIETELSK					Mascot
1208.6157	1208.6475	0.0318	26	79	89	EAAENTLVAYK					Mascot
1336.7107	1336.722	0.0113	8	78	89	KEAAENTLVAYK					Mascot
1552.7601	1552.7839	0.0238	15	6	19	EESRGNEAYVASIK					Mascot
1818.9708	1818.9923	0.0215	12	90	106	SAQDIALADLPTTHPIR					Mascot
1818.9708	1818.9923	0.0215	12	90	106	SAQDIALADLPTTHPIR	90	100			Mascot

9	14-3-3-like protein GF14-B [Triticum urartu]	gi|474147722	30043.1	4.69	17	135	100	13.93	28	0	

Protein Group

14-3-3-like protein GF14-B [Aegilops tauschii]	gi|475549223	30043.1	4.6900 000572 2046

Peptide Information

Calc. Mass	Obsrv. Mass	± da	± ppm	Start Seq.	End Seq.	Sequence	Ion Score	C. I. %	Modification	Rank	Result Type
816.421	816.4305	0.0095	12	18	24	LAEQAER					Mascot
819.4458	819.4396	-0.0062	-8	96	102	IETELSK					Mascot
844.4523	844.4958	0.0435	52	2	9	TAPAELSR					Mascot
907.5247	907.4802	-0.0445	-49	50	57	NLLSVAYK					Mascot
917.5302	917.5102	-0.02	-22	69	76	IISSIEQK					Mascot
928.5324	928.4614	-0.071	-76	126	132	VFYLKMK					Mascot
948.4244	948.4443	0.0199	21	131	137	MKGDYYR			Oxidation (M)[1]		Mascot
1091.4712	1091.5502	0.079	72	77	85	EESRGNEDR					Mascot
1189.6609	1189.6735	0.0126	11	223	232	DSTLIMQLLR					Mascot
1205.6559	1205.6677	0.0118	10	223	232	DSTLIMQLLR			Oxidation (M)[6]		Mascot
1205.6559	1205.6677	0.0118	10	223	232	DSTLIMQLLR	13	0	Oxidation (M)[6]		Mascot
1212.5565	1212.6271	0.0706	58	150	160	DAAENTMVAYK					Mascot
1228.5514	1228.6252	0.0738	60	150	160	DAAENTMVAYK			Oxidation (M)[7]		Mascot
1340.6515	1340.6578	0.0063	5	149	160	KDAAENTMVAYK					Mascot
1356.6464	1356.6136	-0.0328	-24	149	160	KDAAENTMVAYK			Oxidation (M)[8]		Mascot
1366.5542	1366.5656	0.0114	8	25	34	YEEMVEFMEK			Oxidation (M)[4,8]		Mascot
1406.6646	1406.6847	0.0201	14	38	49	TVDSEELTVEER					Mascot
1418.7485	1418.7694	0.0209	15	69	80	IISSIEQKEESR					Mascot
1418.7485	1418.7694	0.0209	15	69	80	IISSIEQKEESR	28	0			Mascot
1664.7546	1664.8337	0.0791	48	25	37	YEEMVEFMEKVAK			Oxidation (M)[4,8]		Mascot
1786.9811	1787.0072	0.0261	15	161	177	AAQDIALAELAPTHPIR					Mascot
2163.9573	2163.9624	0.0051	2	18	34	LAEQAERYEEMVEFMEK			Oxidation (M)[11,15]		Mascot

10	uncharacterized protein, partial [Phleum pratense]	gi|409971903	12880.3	4.33	4	125	100	19.906	90	100	

Protein Group

uncharacterized protein, partial [Phleum pratense]	gi|409971719	12623.2	4.3600 001335 144

Peptide Information

Calc. Mass	Obsrv. Mass	± da	± ppm	Start Seq.	End Seq.	Sequence	Ion Score	C. I. %	Modification	Rank	Result Type

1189.6609	1189.6735	0.0126	11	79	88	DSTLIMQLLR				Mascot
1205.6559	1205.6677	0.0118	10	79	88	DSTLIMQLLR			Oxidation (M)[6]	Mascot
1205.6559	1205.6677	0.0118	10	79	88	DSTLIMQLLR	13	0	Oxidation (M)[6]	Mascot
1208.6157	1208.6475	0.0318	26	6	16	EAAENTLVAYK				Mascot
1336.7107	1336.722	0.0113	8	5	16	KEAAENTLVAYK				Mascot
1818.9708	1818.9923	0.0215	12	17	33	SAQDIALADLPTTHPIR				Mascot
1818.9708	1818.9923	0.0215	12	17	33	SAQDIALADLPTTHPIR	90	100		Mascot

Gel Idx/Pos	168/G19	Instr./Gel Origin	BA2151/Sample Project 20140814	Process Status	Analysis Succeeded
Plate [#] Name	[1] Sample Project 20140814	Instrument Sample Name		Spectra	11

Rank	Protein Name	Accession No.	Protein MW	Protein PI	Pep. Count	Protein Score	Protein Score C. I. %	Intensity Matched	Total Ion Score	Total Ion C. I. %	Confirmed
1	14-3-3-like protein GF14-B [Triticum urartu]	gi|474147722	30043.1	4.69	20	684	100	62.719	529	100	

Protein Group

14-3-3-like protein GF14-B [Aegilops tauschii]	gi|475549223	30043.1	4.6900 000572 2046

Peptide Information

Calc. Mass	Obsrv. Mass	± da	± ppm	Start Seq.	End Seq.	Sequence	Ion Score	C. I. %	Modification	Rank	Result Type
816.421	816.4236	0.0026	3	18	24	LAEQAER					Mascot
844.4523	844.465	0.0127	15	2	9	TAPAELSR					Mascot
907.5247	907.5185	-0.0062	-7	50	57	NLLSVAYK					Mascot
917.5302	917.5294	-0.0008	-1	69	76	IISSIEQK					Mascot
917.5302	917.5294	-0.0008	-1	69	76	IISSIEQK	43	90.214			Mascot
932.4294	932.4377	0.0083	9	131	137	MKGDYYR					Mascot
948.4244	948.4241	-0.0003	0	131	137	MKGDYYR			Oxidation (M)[1]		Mascot
948.4244	948.4241	-0.0003	0	131	137	MKGDYYR	15	0	Oxidation (M)[1]		Mascot
999.4451	999.4538	0.0087	9	10	17	EENVYMAK			Oxidation (M)[6]		Mascot
1091.4712	1091.5469	0.0757	69	77	85	EESRGNEDR					Mascot
1189.6609	1189.6698	0.0089	7	223	232	DSTLIMQLLR					Mascot
1189.6609	1189.6698	0.0089	7	223	232	DSTLIMQLLR	80	99.998			Mascot
1205.6559	1205.6586	0.0027	2	223	232	DSTLIMQLLR			Oxidation (M)[6]		Mascot
1205.6559	1205.6586	0.0027	2	223	232	DSTLIMQLLR	37	60.226	Oxidation (M)[6]		Mascot
1212.5565	1212.5978	0.0413	34	150	160	DAAENTMVAYK					Mascot
1228.5514	1228.6074	0.056	46	150	160	DAAENTMVAYK			Oxidation (M)[7]		Mascot
1366.5542	1366.5485	-0.0057	-4	25	34	YEEMVEFMEK			Oxidation (M)[4,8]		Mascot
1366.5542	1366.5485	-0.0057	-4	25	34	YEEMVEFMEK	1	0	Oxidation (M)[4,8]		Mascot
1406.6646	1406.6774	0.0128	9	38	49	TVDSEELTVEER					Mascot
1406.6646	1406.6774	0.0128	9	38	49	TVDSEELTVEER	94	100			Mascot
1418.7485	1418.7595	0.011	8	69	80	IISSIEQKEESR					Mascot
1708.9116	1708.8749	-0.0367	-21	110	125	LLETHLVPSSTAPESK					Mascot
1786.9811	1786.9965	0.0154	9	161	177	AAQDIALAELAPTHPIR					Mascot
1786.9811	1786.9965	0.0154	9	161	177	AAQDIALAELAPTHPIR	156	100			Mascot

1808.8848	1808.9656	0.0808	45	2	17	TAPAELSREENVYMAK					Mascot
1824.8796	1824.9293	0.0497	27	2	17	TAPAELSREENVYMAK			Oxidation (M)[14]		Mascot
2163.9573	2163.936	-0.0213	-10	18	34	LAEQAERYEEMVEFMEK			Oxidation (M)[11,15]		Mascot
2174.9976	2174.9839	-0.0137	-6	204	222	QAFDEAISELDTLSEESY K					Mascot
2331.2019	2331.2153	0.0134	6	178	197	LGLALNFSVFYYEILNSPD R					Mascot
2351.0886	2351.105	0.0164	7	233	252	DNLTLWTSDITEDTAEEEI R					Mascot
2351.0886	2351.105	0.0164	7	233	252	DNLTLWTSDITEDTAEEEI R	155	100			Mascot
2776.3159	2776.3403	0.0244	9	233	256	DNLTLWTSDITEDTAEEEI REAPK					Mascot

2	14-3-3-like protein GF14-6 [Zea mays]	gi|262359935	29758	4.76	16	483	100	58.969	374	100

Protein Group

RecName: Full=14-3-3-like protein GF14-6	gi|1345587	29758	4.7600 002288 8184
TPA: general regulatory factor1 isoform 1 [Zea mays]	gi|414586860	29758	4.7600 002288 8184
TPA: general regulatory factor1 isoform 2 [Zea mays]	gi|414586861	29758	4.7600 002288 8184

Peptide Information

Calc. Mass	Obsrv. Mass	± da	± ppm	Start Seq.	End Seq.	Sequence	Ion Score	C. I. %	Modification	Rank	Result Type
816.421	816.4236	0.0026	3	17	23	LAEQAER					Mascot
907.5247	907.5185	-0.0062	-7	49	56	NLLSVAYK					Mascot
917.5302	917.5294	-0.0008	-1	68	75	IISSIEQK					Mascot
917.5302	917.5294	-0.0008	-1	68	75	IISSIEQK	43	90.214			Mascot
932.4294	932.4377	0.0083	9	130	136	MKGDYYR					Mascot
948.4244	948.4241	-0.0003	0	130	136	MKGDYYR			Oxidation (M)[1]		Mascot
948.4244	948.4241	-0.0003	0	130	136	MKGDYYR	15	0	Oxidation (M)[1]		Mascot
999.4451	999.4538	0.0087	9	9	16	EENVYMAK			Oxidation (M)[6]		Mascot
1189.6609	1189.6698	0.0089	7	222	231	DSTLIMQLLR					Mascot
1189.6609	1189.6698	0.0089	7	222	231	DSTLIMQLLR	80	99.998			Mascot
1205.6559	1205.6586	0.0027	2	222	231	DSTLIMQLLR			Oxidation (M)[6]		Mascot
1205.6559	1205.6586	0.0027	2	222	231	DSTLIMQLLR	37	60.226	Oxidation (M)[6]		Mascot
1212.5565	1212.5978	0.0413	34	149	159	DAAENTMVAYK					Mascot
1228.5514	1228.6074	0.056	46	149	159	DAAENTMVAYK			Oxidation (M)[7]		Mascot

1366.5542	1366.5485	-0.0057	-4	24	33	YEEMVEFMEK			Oxidation (M)[4,8]		Mascot
1366.5542	1366.5485	-0.0057	-4	24	33	YEEMVEFMEK	1	0	Oxidation (M)[4,8]		Mascot
1388.738	1388.7393	0.0013	1	68	79	IISSIEQKEEGR					Mascot
1406.6646	1406.6774	0.0128	9	37	48	TVDSEELTVEER					Mascot
1406.6646	1406.6774	0.0128	9	37	48	TVDSEELTVEER	94	100			Mascot
1708.9116	1708.8749	-0.0367	-21	109	124	LLETHLVPSSTAPESK					Mascot
1786.9811	1786.9965	0.0154	9	160	176	AAQDIALAELAPTHPIR					Mascot
1786.9811	1786.9965	0.0154	9	160	176	AAQDIALAELAPTHPIR	156	100			Mascot
1828.8568	1829.0045	0.1477	81	1	16	MASAELSREENVYMAK					Mascot
2163.9573	2163.936	-0.0213	-10	17	33	LAEQAERYEEMVEFMEK			Oxidation (M)[11,15]		Mascot
2174.9976	2174.9839	-0.0137	-6	203	221	QAFDEAISELDTLSEESY K					Mascot
2331.2019	2331.2153	0.0134	6	177	196	LGLALNFSVFYYEILNSPD R					Mascot

3	TPA: general regulatory factor1 [Zea mays]	gi|414586863	31835.1	4.9	16	479	100	58.969	374	100	

Peptide Information

Calc. Mass	Obsrv. Mass	± da	± ppm	Start Seq.	End Seq.	Sequence	Ion Score	C. I. %	Modification	Rank	Result Type
816.421	816.4236	0.0026	3	34	40	LAEQAER					Mascot
907.5247	907.5185	-0.0062	-7	66	73	NLLSVAYK					Mascot
917.5302	917.5294	-0.0008	-1	85	92	IISSIEQK					Mascot
917.5302	917.5294	-0.0008	-1	85	92	IISSIEQK	43	90.214			Mascot
932.4294	932.4377	0.0083	9	147	153	MKGDYYR					Mascot
948.4244	948.4241	-0.0003	0	147	153	MKGDYYR			Oxidation (M)[1]		Mascot
948.4244	948.4241	-0.0003	0	147	153	MKGDYYR	15	0	Oxidation (M)[1]		Mascot
999.4451	999.4538	0.0087	9	26	33	EENVYMAK			Oxidation (M)[6]		Mascot
1189.6609	1189.6698	0.0089	7	239	248	DSTLIMQLLR					Mascot
1189.6609	1189.6698	0.0089	7	239	248	DSTLIMQLLR	80	99.998			Mascot
1205.6559	1205.6586	0.0027	2	239	248	DSTLIMQLLR			Oxidation (M)[6]		Mascot
1205.6559	1205.6586	0.0027	2	239	248	DSTLIMQLLR	37	60.226	Oxidation (M)[6]		Mascot
1212.5565	1212.5978	0.0413	34	166	176	DAAENTMVAYK					Mascot
1228.5514	1228.6074	0.056	46	166	176	DAAENTMVAYK			Oxidation (M)[7]		Mascot
1366.5542	1366.5485	-0.0057	-4	41	50	YEEMVEFMEK			Oxidation (M)[4,8]		Mascot
1366.5542	1366.5485	-0.0057	-4	41	50	YEEMVEFMEK	1	0	Oxidation (M)[4,8]		Mascot
1388.738	1388.7393	0.0013	1	85	96	IISSIEQKEEGR					Mascot
1406.6646	1406.6774	0.0128	9	54	65	TVDSEELTVEER					Mascot
1406.6646	1406.6774	0.0128	9	54	65	TVDSEELTVEER	94	100			Mascot

1708.9116	1708.8749	-0.0367	-21	126	141	LLETHLVPSSTAPESK					Mascot
1786.9811	1786.9965	0.0154	9	177	193	AAQDIALAELAPTHPIR					Mascot
1786.9811	1786.9965	0.0154	9	177	193	AAQDIALAELAPTHPIR	156	100			Mascot
1828.8568	1829.0045	0.1477	81	18	33	MASAELSREENVYMAK					Mascot
2163.9573	2163.936	-0.0213	-10	34	50	LAEQAERYEEMVEFMEK			Oxidation (M)[11,15]		Mascot
2174.9976	2174.9839	-0.0137	-6	220	238	QAFDEAISELDTLSEESYK					Mascot
2331.2019	2331.2153	0.0134	6	194	213	LGLALNFSVFYYEILNSPDR					Mascot

4	14-3-3-like protein GF14-12 [Zea mays]	gi|413918561	29724.9	4.75	15	474	100	58.683	374	100

Peptide Information

Calc. Mass	Obsrv. Mass	± da	± ppm	Start Seq.	End Seq.	Sequence	Ion Score	C. I. %	Modification	Rank	Result Type
816.421	816.4236	0.0026	3	17	23	LAEQAER					Mascot
907.5247	907.5185	-0.0062	-7	49	56	NLLSVAYK					Mascot
917.5302	917.5294	-0.0008	-1	68	75	IISSIEQK					Mascot
917.5302	917.5294	-0.0008	-1	68	75	IISSIEQK	43	90.214			Mascot
932.4294	932.4377	0.0083	9	130	136	MKGDYYR					Mascot
948.4244	948.4241	-0.0003	0	130	136	MKGDYYR			Oxidation (M)[1]		Mascot
948.4244	948.4241	-0.0003	0	130	136	MKGDYYR	15	0	Oxidation (M)[1]		Mascot
999.4451	999.4538	0.0087	9	9	16	EENVYMAK			Oxidation (M)[6]		Mascot
1189.6609	1189.6698	0.0089	7	222	231	DSTLIMQLLR					Mascot
1189.6609	1189.6698	0.0089	7	222	231	DSTLIMQLLR	80	99.998			Mascot
1205.6559	1205.6586	0.0027	2	222	231	DSTLIMQLLR			Oxidation (M)[6]		Mascot
1205.6559	1205.6586	0.0027	2	222	231	DSTLIMQLLR	37	60.226	Oxidation (M)[6]		Mascot
1212.5565	1212.5978	0.0413	34	149	159	DAAENTMVAYK					Mascot
1228.5514	1228.6074	0.056	46	149	159	DAAENTMVAYK			Oxidation (M)[7]		Mascot
1366.5542	1366.5485	-0.0057	-4	24	33	YEEMVEFMEK			Oxidation (M)[4,8]		Mascot
1366.5542	1366.5485	-0.0057	-4	24	33	YEEMVEFMEK	1	0	Oxidation (M)[4,8]		Mascot
1388.738	1388.7393	0.0013	1	68	79	IISSIEQKEEGR					Mascot
1406.6646	1406.6774	0.0128	9	37	48	TVDSEELTVEER					Mascot
1406.6646	1406.6774	0.0128	9	37	48	TVDSEELTVEER	94	100			Mascot
1786.9811	1786.9965	0.0154	9	160	176	AAQDIALAELAPTHPIR					Mascot
1786.9811	1786.9965	0.0154	9	160	176	AAQDIALAELAPTHPIR	156	100			Mascot
1828.8568	1829.0045	0.1477	81	1	16	MASAELSREENVYMAK					Mascot
2163.9573	2163.936	-0.0213	-10	17	33	LAEQAERYEEMVEFMEK			Oxidation (M)[11,15]		Mascot
2174.9976	2174.9839	-0.0137	-6	203	221	QAFDEAISELDTLSEESYK					Mascot

2331.2019	2331.2153	0.0134	6	177	196	LGLALNFSVFYYEILNSPDR					Mascot

5	hypothetical protein SORBIDRAFT_06g019100 [Sorghum bicolor]	gi|241937809	29744	4.76	15	473	100	58.683	374	100	

Peptide Information

Calc. Mass	Obsrv. Mass	± da	± ppm	Start Seq.	End Seq.	Sequence	Ion Score	C. I. %	Modification	Rank	Result Type
816.421	816.4236	0.0026	3	17	23	LAEQAER					Mascot
907.5247	907.5185	-0.0062	-7	49	56	NLLSVAYK					Mascot
917.5302	917.5294	-0.0008	-1	68	75	IISSIEQK					Mascot
917.5302	917.5294	-0.0008	-1	68	75	IISSIEQK	43	90.214			Mascot
932.4294	932.4377	0.0083	9	130	136	MKGDYYR					Mascot
948.4244	948.4241	-0.0003	0	130	136	MKGDYYR			Oxidation (M)[1]		Mascot
948.4244	948.4241	-0.0003	0	130	136	MKGDYYR	15	0	Oxidation (M)[1]		Mascot
999.4451	999.4538	0.0087	9	9	16	EENVYMAK			Oxidation (M)[6]		Mascot
1189.6609	1189.6698	0.0089	7	222	231	DSTLIMQLLR					Mascot
1189.6609	1189.6698	0.0089	7	222	231	DSTLIMQLLR	80	99.998			Mascot
1205.6559	1205.6586	0.0027	2	222	231	DSTLIMQLLR			Oxidation (M)[6]		Mascot
1205.6559	1205.6586	0.0027	2	222	231	DSTLIMQLLR	37	60.226	Oxidation (M)[6]		Mascot
1212.5565	1212.5978	0.0413	34	149	159	DAAENTMVAYK					Mascot
1228.5514	1228.6074	0.056	46	149	159	DAAENTMVAYK			Oxidation (M)[7]		Mascot
1366.5542	1366.5485	-0.0057	-4	24	33	YEEMVEFMEK			Oxidation (M)[4,8]		Mascot
1366.5542	1366.5485	-0.0057	-4	24	33	YEEMVEFMEK	1	0	Oxidation (M)[4,8]		Mascot
1388.738	1388.7393	0.0013	1	68	79	IISSIEQKKEEGR					Mascot
1406.6646	1406.6774	0.0128	9	37	48	TVDSEELTVEER					Mascot
1406.6646	1406.6774	0.0128	9	37	48	TVDSEELTVEER	94	100			Mascot
1786.9811	1786.9965	0.0154	9	160	176	AAQDIALAELAPTHPIR					Mascot
1786.9811	1786.9965	0.0154	9	160	176	AAQDIALAELAPTHPIR	156	100			Mascot
1828.8568	1829.0045	0.1477	81	1	16	MASAELSREENVYMAK					Mascot
2163.9573	2163.936	-0.0213	-10	17	33	LAEQAERYEEMVEFMEK			Oxidation (M)[11,15]		Mascot
2174.9976	2174.9839	-0.0137	-6	203	221	QAFDEAISELDTLSEESYK					Mascot
2331.2019	2331.2153	0.0134	6	177	196	LGLALNFSVFYYEILNSPDR					Mascot

6	FUSICOCCIN receptor protein s [Zea mays]	gi|413918562	98275.5	6.45	20	445	100	60.564	372	100	

Peptide Information

Calc. Mass	Obsrv. Mass	± da	± ppm	Start Seq.	End Seq.	Sequence	Ion Score	C. I. %	Modification	Rank	Result Type

816.421	816.4236	0.0026	3	17	23	LAEQAER					Mascot
907.5247	907.5185	-0.0062	-7	49	56	NLLSVAYK					Mascot
917.5302	917.5294	-0.0008	-1	68	75	IISSIEQK					Mascot
917.5302	917.5294	-0.0008	-1	68	75	IISSIEQK	43	90.214			Mascot
929.4258	929.4907	0.0649	70	374	381	SSMAHPQR			Oxidation (M)[3]		Mascot
932.4294	932.4377	0.0083	9	130	136	MKGDYYR					Mascot
948.4244	948.4241	-0.0003	0	130	136	MKGDYYR			Oxidation (M)[1]		Mascot
948.4244	948.4241	-0.0003	0	130	136	MKGDYYR	15	0	Oxidation (M)[1]		Mascot
999.4451	999.4538	0.0087	9	9	16	EENVYMAK			Oxidation (M)[6]		Mascot
1189.6609	1189.6698	0.0089	7	234	243	DSTLIMQLLR					Mascot
1189.6609	1189.6698	0.0089	7	234	243	DSTLIMQLLR	80	99.998			Mascot
1205.6559	1205.6586	0.0027	2	234	243	DSTLIMQLLR			Oxidation (M)[6]		Mascot
1205.6559	1205.6586	0.0027	2	234	243	DSTLIMQLLR	37	60.226	Oxidation (M)[6]		Mascot
1212.5565	1212.5978	0.0413	34	161	171	DAAENTMVAYK					Mascot
1221.5819	1221.6547	0.0728	60	502	512	DTRSSQSPTSR					Mascot
1228.5514	1228.6074	0.056	46	161	171	DAAENTMVAYK			Oxidation (M)[7]		Mascot
1366.5542	1366.5485	-0.0057	-4	24	33	YEEMVEFMEK			Oxidation (M)[4,8]		Mascot
1366.5542	1366.5485	-0.0057	-4	24	33	YEEMVEFMEK	1	0	Oxidation (M)[4,8]		Mascot
1388.738	1388.7393	0.0013	1	68	79	IISSIEQKEEGR					Mascot
1406.6646	1406.6774	0.0128	9	37	48	TVDSEELTVEER					Mascot
1406.6646	1406.6774	0.0128	9	37	48	TVDSEELTVEER	94	100			Mascot
1558.7393	1558.7461	0.0068	4	137	148	YYDCMNPIVVIR			Carbamidomethyl (C)[4], Oxidation (M)[5]		Mascot
1786.9811	1786.9965	0.0154	9	172	188	AAQDIALAELAPTHPIR					Mascot
1786.9811	1786.9965	0.0154	9	172	188	AAQDIALAELAPTHPIR	156	100			Mascot
1828.8568	1829.0045	0.1477	81	1	16	MASAELSREENVYMAK					Mascot
2163.9573	2163.936	-0.0213	-10	17	33	LAEQAERYEEMVEFMEK			Oxidation (M)[11,15]		Mascot
2174.9976	2174.9839	-0.0137	-6	215	233	QAFDEAISELDTLSEESY K					Mascot
2197.0205	2196.9714	-0.0491	-22	132	148	GDYYRYYDCMNPIVVIR			Carbamidomethyl (C)[9]		Mascot
2331.2019	2331.2153	0.0134	6	189	208	LGLALNFSVFYYEILNSPD R					Mascot
2383.2729	2383.0979	-0.175	-73	551	575	RAASIQAAVPSVNSAPAV TSGGPFK					Mascot
2383.2729	2383.0979	-0.175	-73	551	575	RAASIQAAVPSVNSAPAV TSGGPFK					Mascot

7	RecName: Full=14-3-3-like protein GF14-12	gi|1345588	29731.9	4.75	14	384	100	48.539	293	100	

Peptide Information

Calc. Mass	Obsrv. Mass	± da	± ppm	Start Seq.	End Seq.	Sequence	Ion Score	C. I. %	Modification	Rank	Result Type

816.421	816.4236	0.0026	3	17	23	LAEQAER					Mascot
907.5247	907.5185	-0.0062	-7	49	56	NLLSVAYK					Mascot
917.5302	917.5294	-0.0008	-1	68	75	IISSIEQK					Mascot
917.5302	917.5294	-0.0008	-1	68	75	IISSIEQK	43	90.214			Mascot
932.4294	932.4377	0.0083	9	130	136	MKGDYYR					Mascot
948.4244	948.4241	-0.0003	0	130	136	MKGDYYR			Oxidation (M)[1]		Mascot
948.4244	948.4241	-0.0003	0	130	136	MKGDYYR	15	0	Oxidation (M)[1]		Mascot
999.4451	999.4538	0.0087	9	9	16	EENVYMAK			Oxidation (M)[6]		Mascot
1212.5565	1212.5978	0.0413	34	149	159	DAAENTMVAYK					Mascot
1228.5514	1228.6074	0.056	46	149	159	DAAENTMVAYK			Oxidation (M)[7]		Mascot
1366.5542	1366.5485	-0.0057	-4	24	33	YEEMVEFMEK			Oxidation (M)[4,8]		Mascot
1366.5542	1366.5485	-0.0057	-4	24	33	YEEMVEFMEK	1	0	Oxidation (M)[4,8]		Mascot
1388.738	1388.7393	0.0013	1	68	79	IISSIEQKEEGR					Mascot
1406.6646	1406.6774	0.0128	9	37	48	TVDSEELTVEER					Mascot
1406.6646	1406.6774	0.0128	9	37	48	TVDSEELTVEER	94	100			Mascot
1786.9811	1786.9965	0.0154	9	160	176	AAQDIALAELAPTHPIR					Mascot
1786.9811	1786.9965	0.0154	9	160	176	AAQDIALAELAPTHPIR	156	100			Mascot
1828.8568	1829.0045	0.1477	81	1	16	MASAELSREENVYMAK					Mascot
2163.9573	2163.936	-0.0213	-10	17	33	LAEQAERYEEMVEFMEK			Oxidation (M)[11,15]		Mascot
2174.9976	2174.9839	-0.0137	-6	203	221	QAFDEAISELDTLSEESYK					Mascot
2331.2019	2331.2153	0.0134	6	177	196	LGLALNFSVFYYEILNSPDR					Mascot

8	14-3-3-like protein GF14-12 [Zea mays]	gi|262399364	28252.3	4.8	12	364	100	48.259	293	100

Peptide Information

Calc. Mass	Obsrv. Mass	± da	± ppm	Start Seq.	End Seq.	Sequence	Ion Score	C. I. %	Modification	Rank	Result Type
816.421	816.4236	0.0026	3	4	10	LAEQAER					Mascot
907.5247	907.5185	-0.0062	-7	36	43	NLLSVAYK					Mascot
917.5302	917.5294	-0.0008	-1	55	62	IISSIEQK					Mascot
917.5302	917.5294	-0.0008	-1	55	62	IISSIEQK	43	90.214			Mascot
932.4294	932.4377	0.0083	9	117	123	MKGDYYR					Mascot
948.4244	948.4241	-0.0003	0	117	123	MKGDYYR			Oxidation (M)[1]		Mascot
948.4244	948.4241	-0.0003	0	117	123	MKGDYYR	15	0	Oxidation (M)[1]		Mascot
1212.5565	1212.5978	0.0413	34	136	146	DAAENTMVAYK					Mascot
1228.5514	1228.6074	0.056	46	136	146	DAAENTMVAYK			Oxidation (M)[7]		Mascot
1366.5542	1366.5485	-0.0057	-4	11	20	YEEMVEFMEK			Oxidation (M)[4,8]		Mascot

1366.5542	1366.5485	-0.0057	-4	11	20	YEEMVEFMEK	1	0	Oxidation (M)[4,8]		Mascot
1388.738	1388.7393	0.0013	1	55	66	IISSIEQKEEGR					Mascot
1406.6646	1406.6774	0.0128	9	24	35	TVDSEELTVEER					Mascot
1406.6646	1406.6774	0.0128	9	24	35	TVDSEELTVEER	94	100			Mascot
1786.9811	1786.9965	0.0154	9	147	163	AAQDIALAELAPTHPIR					Mascot
1786.9811	1786.9965	0.0154	9	147	163	AAQDIALAELAPTHPIR	156	100			Mascot
2163.9573	2163.936	-0.0213	-10	4	20	LAEQAERYEEMVEFMEK			Oxidation (M)[11,15]		Mascot
2174.9976	2174.9839	-0.0137	-6	190	208	QAFDEAISELDTLSEESYK					Mascot
2331.2019	2331.2153	0.0134	6	164	183	LGLALNFSVFYYEILNSPDR					Mascot

9	PREDICTED: 14-3-3-like protein GF14-12-like [Setaria italica]	gi|514801939	29750.9	4.75	14	307	100	18.661	217	100	

Peptide Information

Calc. Mass	Obsrv. Mass	± da	± ppm	Start Seq.	End Seq.	Sequence	Ion Score	C. I. %	Modification	Rank	Result Type
816.421	816.4236	0.0026	3	17	23	LAEQAER					Mascot
907.5247	907.5185	-0.0062	-7	49	56	NLLSVAYK					Mascot
917.5302	917.5294	-0.0008	-1	68	75	IISSIEQK					Mascot
917.5302	917.5294	-0.0008	-1	68	75	IISSIEQK	43	90.214			Mascot
932.4294	932.4377	0.0083	9	130	136	MKGDYYR					Mascot
948.4244	948.4241	-0.0003	0	130	136	MKGDYYR			Oxidation (M)[1]		Mascot
948.4244	948.4241	-0.0003	0	130	136	MKGDYYR	15	0	Oxidation (M)[1]		Mascot
999.4451	999.4538	0.0087	9	9	16	EENVYMAK			Oxidation (M)[6]		Mascot
1189.6609	1189.6698	0.0089	7	222	231	DSTLIMQLLR					Mascot
1189.6609	1189.6698	0.0089	7	222	231	DSTLIMQLLR	80	99.998			Mascot
1205.6559	1205.6586	0.0027	2	222	231	DSTLIMQLLR			Oxidation (M)[6]		Mascot
1205.6559	1205.6586	0.0027	2	222	231	DSTLIMQLLR	37	60.226	Oxidation (M)[6]		Mascot
1212.5565	1212.5978	0.0413	34	149	159	DAAENTMVAYK					Mascot
1228.5514	1228.6074	0.056	46	149	159	DAAENTMVAYK			Oxidation (M)[7]		Mascot
1366.5542	1366.5485	-0.0057	-4	24	33	YEEMVEFMEK			Oxidation (M)[4,8]		Mascot
1366.5542	1366.5485	-0.0057	-4	24	33	YEEMVEFMEK	1	0	Oxidation (M)[4,8]		Mascot
1388.738	1388.7393	0.0013	1	68	79	IISSIEQKEEGR					Mascot
1406.6646	1406.6774	0.0128	9	37	48	TVDSEELTVEER					Mascot
1406.6646	1406.6774	0.0128	9	37	48	TVDSEELTVEER	94	100			Mascot
1828.8568	1829.0045	0.1477	81	1	16	MASAELSREENVYMAK					Mascot
2163.9573	2163.936	-0.0213	-10	17	33	LAEQAERYEEMVEFMEK			Oxidation (M)[11,15]		Mascot
2174.9976	2174.9839	-0.0137	-6	203	221	QAFDEAISELDTLSEESY					Mascot

2331.2019	2331.2153	0.0134	6	177	196	K LGLALNFSVFYYEILNSPD R					Mascot

10	Os02g0580300 [Oryza sativa Japonica Group]	gi|113536765	29844.9	4.71	14	305	100	18.856	217	100

Protein Group

RecName: Full=14-3-3-like protein GF14-E; AltName: Full=G-box factor 14-3-3 homolog E	gi|75290255	29844.9	4.7100 000381 4697
hypothetical protein OsI_07806 [Oryza sativa Indica Group]	gi|125540033	29844.9	4.7100 000381 4697

Peptide Information

Calc. Mass	Obsrv. Mass	± da	± ppm	Start Seq.	End Seq.	Sequence	Ion Score	C. I. %	Modification	Rank	Result Type
816.421	816.4236	0.0026	3	18	24	LAEQAER					Mascot
907.5247	907.5185	-0.0062	-7	50	57	NLLSVAYK					Mascot
917.5302	917.5294	-0.0008	-1	69	76	IISSIEQK					Mascot
917.5302	917.5294	-0.0008	-1	69	76	IISSIEQK	43	90.214			Mascot
932.4294	932.4377	0.0083	9	131	137	MKGDYYR					Mascot
948.4244	948.4241	-0.0003	0	131	137	MKGDYYR			Oxidation (M)[1]		Mascot
948.4244	948.4241	-0.0003	0	131	137	MKGDYYR	15	0	Oxidation (M)[1]		Mascot
999.4451	999.4538	0.0087	9	10	17	EENVYMAK			Oxidation (M)[6]		Mascot
1091.4712	1091.5469	0.0757	69	77	85	EESRGNEDR					Mascot
1189.6609	1189.6698	0.0089	7	223	232	DSTLIMQLLR					Mascot
1189.6609	1189.6698	0.0089	7	223	232	DSTLIMQLLR	80	99.998			Mascot
1205.6559	1205.6586	0.0027	2	223	232	DSTLIMQLLR			Oxidation (M)[6]		Mascot
1205.6559	1205.6586	0.0027	2	223	232	DSTLIMQLLR	37	60.226	Oxidation (M)[6]		Mascot
1212.5565	1212.5978	0.0413	34	150	160	DAAENTMVAYK					Mascot
1228.5514	1228.6074	0.056	46	150	160	DAAENTMVAYK			Oxidation (M)[7]		Mascot
1366.5542	1366.5485	-0.0057	-4	25	34	YEEMVEFMEK			Oxidation (M)[4,8]		Mascot
1366.5542	1366.5485	-0.0057	-4	25	34	YEEMVEFMEK	1	0	Oxidation (M)[4,8]		Mascot
1406.6646	1406.6774	0.0128	9	38	49	TVDSEELTVEER					Mascot
1406.6646	1406.6774	0.0128	9	38	49	TVDSEELTVEER	94	100			Mascot
1418.7485	1418.7595	0.011	8	69	80	IISSIEQKEESR					Mascot
2163.9573	2163.936	-0.0213	-10	18	34	LAEQAERYEEMVEFMEK			Oxidation (M)[11,15]		Mascot
2174.9976	2174.9839	-0.0137	-6	204	222	QAFDEAISELDTLSEESY K					Mascot
2331.2019	2331.2153	0.0134	6	178	197	LGLALNFSVFYYEILNSPD R					Mascot

Gel Idx/Pos	169/G20	Instr./Gel Origin	BA2151/Sample Project 20140814	Process Status	Analysis Succeeded
Plate [#] Name	[1] Sample Project 20140814	Instrument Sample Name		Spectra	11

Rank	Protein Name	Accession No.	Protein MW	Protein PI	Pep. Count	Protein Score	Protein Score C. I. %	Intensity Matched	Total Ion Score	Total Ion C. I. %	Confirmed
1	14-3-3-like protein GF14-B [Triticum urartu]	gi|474147722	30043.1	4.69	24	529	100	67.813	337	100	

Protein Group

14-3-3-like protein GF14-B [Aegilops tauschii]	gi|475549223	30043.1	4.6900 000572 2046

Peptide Information

Calc. Mass	Obsrv. Mass	± da	± ppm	Start Seq.	End Seq.	Sequence	Ion Score	C. I. %	Modification	Rank	Result Type
816.421	816.4315	0.0105	13	18	24	LAEQAER					Mascot
818.444	818.4407	-0.0033	-4	103	109	ICDGILK			Carbamidomethyl (C)[2]		Mascot
819.4458	819.4365	-0.0093	-11	96	102	IETELSK					Mascot
844.4523	844.4795	0.0272	32	2	9	TAPAELSR					Mascot
907.5247	907.5327	0.008	9	50	57	NLLSVAYK					Mascot
917.5302	917.538	0.0078	9	69	76	IISSIEQK					Mascot
917.5302	917.538	0.0078	9	69	76	IISSIEQK	25	0			Mascot
932.4294	932.4701	0.0407	44	131	137	MKGDYYR					Mascot
948.4244	948.4344	0.01	11	131	137	MKGDYYR			Oxidation (M)[1]		Mascot
948.4244	948.4344	0.01	11	131	137	MKGDYYR	15	0	Oxidation (M)[1]		Mascot
999.4451	999.4573	0.0122	12	10	17	EENVYMAK			Oxidation (M)[6]		Mascot
1004.5622	1004.5597	-0.0025	-2	94	102	GKIETELSK					Mascot
1091.4712	1091.5339	0.0627	57	77	85	EESRGNEDR					Mascot
1144.6321	1144.6542	0.0221	19	81	90	GNEDRVTLIK					Mascot
1189.6609	1189.678	0.0171	14	223	232	DSTLIMQLLR					Mascot
1205.6559	1205.6694	0.0135	11	223	232	DSTLIMQLLR			Oxidation (M)[6]		Mascot
1205.6559	1205.6694	0.0135	11	223	232	DSTLIMQLLR	18	0	Oxidation (M)[6]		Mascot
1212.5565	1212.6193	0.0628	52	150	160	DAAENTMVAYK					Mascot
1228.5514	1228.6051	0.0537	44	150	160	DAAENTMVAYK			Oxidation (M)[7]		Mascot
1366.5542	1366.5502	-0.004	-3	25	34	YEEMVEFMEK			Oxidation (M)[4,8]		Mascot
1406.6646	1406.6904	0.0258	18	38	49	TVDSEELTVEER					Mascot
1406.6646	1406.6904	0.0258	18	38	49	TVDSEELTVEER	101	100			Mascot
1418.7485	1418.777	0.0285	20	69	80	IISSIEQKEESR					Mascot
1418.7485	1418.777	0.0285	20	69	80	IISSIEQKEESR	33	3.669			Mascot

Calc. Mass	Obsrv. Mass	± da	± ppm	Start Seq.	End Seq.	Sequence	Ion Score	C. I. %	Modification	Rank	Result Type
1708.9116	1708.9102	-0.0014	-1	110	125	LLETHLVPSSTAPESK					Mascot
1786.9811	1787.0148	0.0337	19	161	177	AAQDIALAELAPTHPIR					Mascot
1786.9811	1787.0148	0.0337	19	161	177	AAQDIALAELAPTHPIR	146	100			Mascot
1808.8848	1808.9774	0.0926	51	2	17	TAPAELSREENVYMAK					Mascot
1824.8796	1824.9255	0.0459	25	2	17	TAPAELSREENVYMAK			Oxidation (M)[14]		Mascot
2163.9573	2163.9636	0.0063	3	18	34	LAEQAERYEEMVEFMEK			Oxidation (M)[11,15]		Mascot
2163.9573	2163.9636	0.0063	3	18	34	LAEQAERYEEMVEFMEK	13	0	Oxidation (M)[11,15]		Mascot
2174.9976	2175.0115	0.0139	6	204	222	QAFDEAISELDTLSEESYK					Mascot
2331.2019	2331.2488	0.0469	20	178	197	LGLALNFSVFYYEILNSPDR					Mascot
2351.0886	2351.1287	0.0401	17	233	252	DNLTLWTSDITEDTAEEEIR					Mascot
2776.3159	2776.377	0.0611	22	233	256	DNLTLWTSDITEDTAEEEIREAPK					Mascot

2	14-3-3-like protein GF14-6 [Zea mays]	gi|262359935	29758	4.76	17	418	100	63.572	304	100

Protein Group

RecName: Full=14-3-3-like protein GF14-6	gi|1345587	29758	4.7600002288184
TPA: general regulatory factor1 isoform 1 [Zea mays]	gi|414586860	29758	4.7600002288184
TPA: general regulatory factor1 isoform 2 [Zea mays]	gi|414586861	29758	4.7600002288184

Peptide Information

Calc. Mass	Obsrv. Mass	± da	± ppm	Start Seq.	End Seq.	Sequence	Ion Score	C. I. %	Modification	Rank	Result Type
816.421	816.4315	0.0105	13	17	23	LAEQAER					Mascot
818.444	818.4407	-0.0033	-4	102	108	ICDGILK			Carbamidomethyl (C)[2]		Mascot
907.5247	907.5327	0.008	9	49	56	NLLSVAYK					Mascot
917.5302	917.538	0.0078	9	68	75	IISSIEQK					Mascot
917.5302	917.538	0.0078	9	68	75	IISSIEQK	25	0			Mascot
932.4294	932.4701	0.0407	44	130	136	MKGDYYR					Mascot
948.4244	948.4344	0.01	11	130	136	MKGDYYR			Oxidation (M)[1]		Mascot
948.4244	948.4344	0.01	11	130	136	MKGDYYR	15	0	Oxidation (M)[1]		Mascot
999.4451	999.4573	0.0122	12	9	16	EENVYMAK			Oxidation (M)[6]		Mascot
1144.6321	1144.6542	0.0221	19	80	89	GNEDRVTLIK					Mascot
1189.6609	1189.678	0.0171	14	222	231	DSTLIMQLLR					Mascot

1205.6559	1205.6694	0.0135	11	222	231	DSTLIMQLLR			Oxidation (M)[6]		Mascot
1205.6559	1205.6694	0.0135	11	222	231	DSTLIMQLLR	18	0	Oxidation (M)[6]		Mascot
1212.5565	1212.6193	0.0628	52	149	159	DAAENTMVAYK					Mascot
1228.5514	1228.6051	0.0537	44	149	159	DAAENTMVAYK			Oxidation (M)[7]		Mascot
1366.5542	1366.5502	-0.004	-3	24	33	YEEMVEFMEK			Oxidation (M)[4,8]		Mascot
1388.738	1388.7384	0.0004	0	68	79	IISSIEQKEEGR					Mascot
1406.6646	1406.6904	0.0258	18	37	48	TVDSEELTVEER					Mascot
1406.6646	1406.6904	0.0258	18	37	48	TVDSEELTVEER	101	100			Mascot
1708.9116	1708.9102	-0.0014	-1	109	124	LLETHLVPSSTAPESK					Mascot
1786.9811	1787.0148	0.0337	19	160	176	AAQDIALAELAPTHPIR					Mascot
1786.9811	1787.0148	0.0337	19	160	176	AAQDIALAELAPTHPIR	146	100			Mascot
2163.9573	2163.9636	0.0063	3	17	33	LAEQAERYEEMVEFMEK			Oxidation (M)[11,15]		Mascot
2163.9573	2163.9636	0.0063	3	17	33	LAEQAERYEEMVEFMEK	13	0	Oxidation (M)[11,15]		Mascot
2174.9976	2175.0115	0.0139	6	203	221	QAFDEAISELDTLSEESY K					Mascot
2331.2019	2331.2488	0.0469	20	177	196	LGLALNFSVFYYEILNSPD R					Mascot

3 TPA: general regulatory factor1 [Zea mays] gi|414586863 31835.1 4.9 17 413 100 63.572 304 100

Peptide Information

Calc. Mass	Obsrv. Mass	± da	± ppm	Start Seq.	End Seq.	Sequence	Ion Score	C. I. %	Modification	Rank	Result Type
816.421	816.4315	0.0105	13	34	40	LAEQAER					Mascot
818.444	818.4407	-0.0033	-4	119	125	ICDGILK			Carbamidomethyl (C)[2]		Mascot
907.5247	907.5327	0.008	9	66	73	NLLSVAYK					Mascot
917.5302	917.538	0.0078	9	85	92	IISSIEQK					Mascot
917.5302	917.538	0.0078	9	85	92	IISSIEQK	25	0			Mascot
932.4294	932.4701	0.0407	44	147	153	MKGDYYR					Mascot
948.4244	948.4344	0.01	11	147	153	MKGDYYR			Oxidation (M)[1]		Mascot
948.4244	948.4344	0.01	11	147	153	MKGDYYR	15	0	Oxidation (M)[1]		Mascot
999.4451	999.4573	0.0122	12	26	33	EENVYMAK			Oxidation (M)[6]		Mascot
1144.6321	1144.6542	0.0221	19	97	106	GNEDRVTLIK					Mascot
1189.6609	1189.678	0.0171	14	239	248	DSTLIMQLLR					Mascot
1205.6559	1205.6694	0.0135	11	239	248	DSTLIMQLLR			Oxidation (M)[6]		Mascot
1205.6559	1205.6694	0.0135	11	239	248	DSTLIMQLLR	18	0	Oxidation (M)[6]		Mascot
1212.5565	1212.6193	0.0628	52	166	176	DAAENTMVAYK					Mascot
1228.5514	1228.6051	0.0537	44	166	176	DAAENTMVAYK			Oxidation (M)[7]		Mascot
1366.5542	1366.5502	-0.004	-3	41	50	YEEMVEFMEK			Oxidation (M)[4,8]		Mascot

1388.738	1388.7384	0.0004	0	85	96	IISSIEQKEEGR					Mascot
1406.6646	1406.6904	0.0258	18	54	65	TVDSEELTVEER					Mascot
1406.6646	1406.6904	0.0258	18	54	65	TVDSEELTVEER	101	100			Mascot
1708.9116	1708.9102	-0.0014	-1	126	141	LLETHLVPSSTAPESK					Mascot
1786.9811	1787.0148	0.0337	19	177	193	AAQDIALAELAPTHPIR					Mascot
1786.9811	1787.0148	0.0337	19	177	193	AAQDIALAELAPTHPIR	146	100			Mascot
2163.9573	2163.9636	0.0063	3	34	50	LAEQAERYEEMVEFMEK			Oxidation (M)[11,15]		Mascot
2163.9573	2163.9636	0.0063	3	34	50	LAEQAERYEEMVEFMEK	13	0	Oxidation (M)[11,15]		Mascot
2174.9976	2175.0115	0.0139	6	220	238	QAFDEAISELDTLSEESY K					Mascot
2331.2019	2331.2488	0.0469	20	194	213	LGLALNFSVFYYEILNSPD R					Mascot

4	14-3-3-like protein GF14-12 [Zea mays]	gi|413918561	29724.9	4.75	16	409	100	63.114	304	100	

Peptide Information

Calc. Mass	Obsrv. Mass	± da	± ppm	Start Seq.	End Seq.	Sequence	Ion Score	C. I. %	Modification	Rank	Result Type
816.421	816.4315	0.0105	13	17	23	LAEQAER					Mascot
818.444	818.4407	-0.0033	-4	102	108	ICDGILK			Carbamidomethyl (C)[2]		Mascot
907.5247	907.5327	0.008	9	49	56	NLLSVAYK					Mascot
917.5302	917.538	0.0078	9	68	75	IISSIEQK					Mascot
917.5302	917.538	0.0078	9	68	75	IISSIEQK	25	0			Mascot
932.4294	932.4701	0.0407	44	130	136	MKGDYYR					Mascot
948.4244	948.4344	0.01	11	130	136	MKGDYYR			Oxidation (M)[1]		Mascot
948.4244	948.4344	0.01	11	130	136	MKGDYYR	15	0	Oxidation (M)[1]		Mascot
999.4451	999.4573	0.0122	12	9	16	EENVYMAK			Oxidation (M)[6]		Mascot
1144.6321	1144.6542	0.0221	19	80	89	GNEDRVTLIK					Mascot
1189.6609	1189.678	0.0171	14	222	231	DSTLIMQLLR					Mascot
1205.6559	1205.6694	0.0135	11	222	231	DSTLIMQLLR			Oxidation (M)[6]		Mascot
1205.6559	1205.6694	0.0135	11	222	231	DSTLIMQLLR	18	0	Oxidation (M)[6]		Mascot
1212.5565	1212.6193	0.0628	52	149	159	DAAENTMVAYK					Mascot
1228.5514	1228.6051	0.0537	44	149	159	DAAENTMVAYK			Oxidation (M)[7]		Mascot
1366.5542	1366.5502	-0.004	-3	24	33	YEEMVEFMEK			Oxidation (M)[4,8]		Mascot
1388.738	1388.7384	0.0004	0	68	79	IISSIEQKEEGR					Mascot
1406.6646	1406.6904	0.0258	18	37	48	TVDSEELTVEER					Mascot
1406.6646	1406.6904	0.0258	18	37	48	TVDSEELTVEER	101	100			Mascot
1786.9811	1787.0148	0.0337	19	160	176	AAQDIALAELAPTHPIR					Mascot
1786.9811	1787.0148	0.0337	19	160	176	AAQDIALAELAPTHPIR	146	100			Mascot

2163.9573	2163.9636	0.0063	3	17	33	LAEQAERYEEMVEFMEK			Oxidation (M)[11,15]		Mascot
2163.9573	2163.9636	0.0063	3	17	33	LAEQAERYEEMVEFMEK	13	0	Oxidation (M)[11,15]		Mascot
2174.9976	2175.0115	0.0139	6	203	221	QAFDEAISELDTLSEESY K					Mascot
2331.2019	2331.2488	0.0469	20	177	196	LGLALNFSVFYYEILNSPD R					Mascot

5	hypothetical protein SORBIDRAFT_06g019100 [Sorghum bicolor]	gi|241937809	29744	4.76	16	408	100	63.114	304	100	

Peptide Information

Calc. Mass	Obsrv. Mass	± da	± ppm	Start Seq.	End Seq.	Sequence	Ion Score	C. I. %	Modification	Rank	Result Type
816.421	816.4315	0.0105	13	17	23	LAEQAER					Mascot
818.444	818.4407	-0.0033	-4	102	108	ICDGILK			Carbamidomethyl (C)[2]		Mascot
907.5247	907.5327	0.008	9	49	56	NLLSVAYK					Mascot
917.5302	917.538	0.0078	9	68	75	IISSIEQK					Mascot
917.5302	917.538	0.0078	9	68	75	IISSIEQK	25	0			Mascot
932.4294	932.4701	0.0407	44	130	136	MKGDYYR					Mascot
948.4244	948.4344	0.01	11	130	136	MKGDYYR			Oxidation (M)[1]		Mascot
948.4244	948.4344	0.01	11	130	136	MKGDYYR	15	0	Oxidation (M)[1]		Mascot
999.4451	999.4573	0.0122	12	9	16	EENVYMAK			Oxidation (M)[6]		Mascot
1144.6321	1144.6542	0.0221	19	80	89	GNEDRVTLIK					Mascot
1189.6609	1189.678	0.0171	14	222	231	DSTLIMQLLR					Mascot
1205.6559	1205.6694	0.0135	11	222	231	DSTLIMQLLR			Oxidation (M)[6]		Mascot
1205.6559	1205.6694	0.0135	11	222	231	DSTLIMQLLR	18	0	Oxidation (M)[6]		Mascot
1212.5565	1212.6193	0.0628	52	149	159	DAAENTMVAYK					Mascot
1228.5514	1228.6051	0.0537	44	149	159	DAAENTMVAYK			Oxidation (M)[7]		Mascot
1366.5542	1366.5502	-0.004	-3	24	33	YEEMVEFMEK			Oxidation (M)[4,8]		Mascot
1388.738	1388.7384	0.0004	0	68	79	IISSIEQKEEGR					Mascot
1406.6646	1406.6904	0.0258	18	37	48	TVDSEELTVEER					Mascot
1406.6646	1406.6904	0.0258	18	37	48	TVDSEELTVEER	101	100			Mascot
1786.9811	1787.0148	0.0337	19	160	176	AAQDIALAELAPTHPIR					Mascot
1786.9811	1787.0148	0.0337	19	160	176	AAQDIALAELAPTHPIR	146	100			Mascot
2163.9573	2163.9636	0.0063	3	17	33	LAEQAERYEEMVEFMEK			Oxidation (M)[11,15]		Mascot
2163.9573	2163.9636	0.0063	3	17	33	LAEQAERYEEMVEFMEK	13	0	Oxidation (M)[11,15]		Mascot
2174.9976	2175.0115	0.0139	6	203	221	QAFDEAISELDTLSEESY K					Mascot
2331.2019	2331.2488	0.0469	20	177	196	LGLALNFSVFYYEILNSPD R					Mascot

6	FUSICOCCIN receptor protein s [Zea mays]	gi|413918562	98275.5	6.45	20	385	100	65.264	304	100	

Peptide Information

Calc. Mass	Obsrv. Mass	± da	± ppm	Start Seq.	End Seq.	Sequence	Ion Score	C. I. %	Modification	Rank	Result Type
816.421	816.4315	0.0105	13	17	23	LAEQAER					Mascot
818.444	818.4407	-0.0033	-4	102	108	ICDGILK			Carbamidomethyl (C)[2]		Mascot
907.5247	907.5327	0.008	9	49	56	NLLSVAYK					Mascot
913.4308	913.4915	0.0607	66	374	381	SSMAHPQR					Mascot
917.5302	917.538	0.0078	9	68	75	IISSIEQK					Mascot
917.5302	917.538	0.0078	9	68	75	IISSIEQK	25	0			Mascot
932.4294	932.4701	0.0407	44	130	136	MKGDYYR					Mascot
948.4244	948.4344	0.01	11	130	136	MKGDYYR			Oxidation (M)[1]		Mascot
948.4244	948.4344	0.01	11	130	136	MKGDYYR	15	0	Oxidation (M)[1]		Mascot
999.4451	999.4573	0.0122	12	9	16	EENVYMAK			Oxidation (M)[6]		Mascot
1144.6321	1144.6542	0.0221	19	80	89	GNEDRVTLIK					Mascot
1189.6609	1189.678	0.0171	14	234	243	DSTLIMQLLR					Mascot
1205.6559	1205.6694	0.0135	11	234	243	DSTLIMQLLR			Oxidation (M)[6]		Mascot
1205.6559	1205.6694	0.0135	11	234	243	DSTLIMQLLR	18	0	Oxidation (M)[6]		Mascot
1212.5565	1212.6193	0.0628	52	161	171	DAAENTMVAYK					Mascot
1221.5819	1221.6707	0.0888	73	502	512	DTRSSQSPTSR					Mascot
1221.5819	1221.6707	0.0888	73	502	512	DTRSSQSPTSR	15	0			Mascot
1228.5514	1228.6051	0.0537	44	161	171	DAAENTMVAYK			Oxidation (M)[7]		Mascot
1366.5542	1366.5502	-0.004	-3	24	33	YEEMVEFMEK			Oxidation (M)[4,8]		Mascot
1388.738	1388.7384	0.0004	0	68	79	IISSIEQKEEGR					Mascot
1406.6646	1406.6904	0.0258	18	37	48	TVDSEELTVEER					Mascot
1406.6646	1406.6904	0.0258	18	37	48	TVDSEELTVEER	101	100			Mascot
1558.7393	1558.7654	0.0261	17	137	148	YYDCMNPIVVIR			Carbamidomethyl (C)[4], Oxidation (M)[5]		Mascot
1786.9811	1787.0148	0.0337	19	172	188	AAQDIALAELAPTHPIR					Mascot
1786.9811	1787.0148	0.0337	19	172	188	AAQDIALAELAPTHPIR	146	100			Mascot
2163.9573	2163.9636	0.0063	3	17	33	LAEQAERYEEMVEFMEK			Oxidation (M)[11,15]		Mascot
2163.9573	2163.9636	0.0063	3	17	33	LAEQAERYEEMVEFMEK	13	0	Oxidation (M)[11,15]		Mascot
2174.9976	2175.0115	0.0139	6	215	233	QAFDEAISELDTLSEESYK					Mascot
2331.2019	2331.2488	0.0469	20	189	208	LGLALNFSVFYYEILNSPDR					Mascot
2383.2729	2383.1218	-0.1511	-63	551	575	RAASIQAAVPSVNSAPAVTSGGPFK					Mascot

7	RecName: Full=14-3-3-like protein GF14-12	gi|1345588	29731.9	4.75	15	382	100	57.527	286	100	

Peptide Information

Calc. Mass	Obsrv. Mass	± da	± ppm	Start Seq.	End Seq.	Sequence	Ion Score	C. I. %	Modification	Rank	Result Type
816.421	816.4315	0.0105	13	17	23	LAEQAER					Mascot
818.444	818.4407	-0.0033	-4	102	108	ICDGILK			Carbamidomethyl (C)[2]		Mascot
907.5247	907.5327	0.008	9	49	56	NLLSVAYK					Mascot
917.5302	917.538	0.0078	9	68	75	IISSIEQK					Mascot
917.5302	917.538	0.0078	9	68	75	IISSIEQK	25	0			Mascot
932.4294	932.4701	0.0407	44	130	136	MKGDYYR					Mascot
948.4244	948.4344	0.01	11	130	136	MKGDYYR			Oxidation (M)[1]		Mascot
948.4244	948.4344	0.01	11	130	136	MKGDYYR	15	0	Oxidation (M)[1]		Mascot
999.4451	999.4573	0.0122	12	9	16	EENVYMAK			Oxidation (M)[6]		Mascot
1144.6321	1144.6542	0.0221	19	80	89	GNEDRVTLIK					Mascot
1212.5565	1212.6193	0.0628	52	149	159	DAAENTMVAYK					Mascot
1228.5514	1228.6051	0.0537	44	149	159	DAAENTMVAYK			Oxidation (M)[7]		Mascot
1366.5542	1366.5502	-0.004	-3	24	33	YEEMVEFMEK			Oxidation (M)[4,8]		Mascot
1388.738	1388.7384	0.0004	0	68	79	IISSIEQKEEGR					Mascot
1406.6646	1406.6904	0.0258	18	37	48	TVDSEELTVEER					Mascot
1406.6646	1406.6904	0.0258	18	37	48	TVDSEELTVEER	101	100			Mascot
1786.9811	1787.0148	0.0337	19	160	176	AAQDIALAELAPTHPIR					Mascot
1786.9811	1787.0148	0.0337	19	160	176	AAQDIALAELAPTHPIR	146	100			Mascot
2163.9573	2163.9636	0.0063	3	17	33	LAEQAERYEEMVEFMEK			Oxidation (M)[11,15]		Mascot
2163.9573	2163.9636	0.0063	3	17	33	LAEQAERYEEMVEFMEK	13	0	Oxidation (M)[11,15]		Mascot
2174.9976	2175.0115	0.0139	6	203	221	QAFDEAISELDTLSEESYK					Mascot
2331.2019	2331.2488	0.0469	20	177	196	LGLALNFSVFYYEILNSPDR					Mascot

8	14-3-3-like protein GF14-12 [Zea mays]	gi|262399364	28252.3	4.8	14	369	100	57.387	286	100	

Peptide Information

Calc. Mass	Obsrv. Mass	± da	± ppm	Start Seq.	End Seq.	Sequence	Ion Score	C. I. %	Modification	Rank	Result Type
816.421	816.4315	0.0105	13	4	10	LAEQAER					Mascot
818.444	818.4407	-0.0033	-4	89	95	ICDGILK			Carbamidomethyl (C)[2]		Mascot
907.5247	907.5327	0.008	9	36	43	NLLSVAYK					Mascot
917.5302	917.538	0.0078	9	55	62	IISSIEQK					Mascot
917.5302	917.538	0.0078	9	55	62	IISSIEQK	25	0			Mascot
932.4294	932.4701	0.0407	44	117	123	MKGDYYR					Mascot
948.4244	948.4344	0.01	11	117	123	MKGDYYR			Oxidation (M)[1]		Mascot

948.4244	948.4344	0.01	11	117	123	MKGDYYR	15	0	Oxidation (M)[1]		Mascot
1144.6321	1144.6542	0.0221	19	67	76	GNEDRVTLIK					Mascot
1212.5565	1212.6193	0.0628	52	136	146	DAAENTMVAYK					Mascot
1228.5514	1228.6051	0.0537	44	136	146	DAAENTMVAYK			Oxidation (M)[7]		Mascot
1366.5542	1366.5502	-0.004	-3	11	20	YEEMVEFMEK			Oxidation (M)[4,8]		Mascot
1388.738	1388.7384	0.0004	0	55	66	IISSIEQKEEGR					Mascot
1406.6646	1406.6904	0.0258	18	24	35	TVDSEELTVEER					Mascot
1406.6646	1406.6904	0.0258	18	24	35	TVDSEELTVEER	101	100			Mascot
1786.9811	1787.0148	0.0337	19	147	163	AAQDIALAELAPTHPIR					Mascot
1786.9811	1787.0148	0.0337	19	147	163	AAQDIALAELAPTHPIR	146	100			Mascot
2163.9573	2163.9636	0.0063	3	4	20	LAEQAERYEEMVEFMEK			Oxidation (M)[11,15]		Mascot
2163.9573	2163.9636	0.0063	3	4	20	LAEQAERYEEMVEFMEK	13	0	Oxidation (M)[11,15]		Mascot
2174.9976	2175.0115	0.0139	6	190	208	QAFDEAISELDTLSEESY K					Mascot
2331.2019	2331.2488	0.0469	20	164	183	LGLALNFSVFYYEILNSPD R					Mascot

9	Os02g0580300 [Oryza sativa Japonica Group]	gi|113536765	29844.9	4.71	17	300	100	22.977	190	100		

Protein Group

RecName: Full=14-3-3-like protein GF14-E; AltName: Full=G-box factor 14-3-3 homolog E	gi|75290255	29844.9	4.7100 000381 4697
hypothetical protein OsI_07806 [Oryza sativa Indica Group]	gi|125540033	29844.9	4.7100 000381 4697

Peptide Information

Calc. Mass	Obsrv. Mass	± da	± ppm	Start Seq.	End Seq.	Sequence	Ion Score	C. I. %	Modification	Rank	Result Type
816.421	816.4315	0.0105	13	18	24	LAEQAER					Mascot
818.444	818.4407	-0.0033	-4	103	109	ICDGILK			Carbamidomethyl (C)[2]		Mascot
819.4458	819.4365	-0.0093	-11	96	102	IETELSK					Mascot
907.5247	907.5327	0.008	9	50	57	NLLSVAYK					Mascot
917.5302	917.538	0.0078	9	69	76	IISSIEQK					Mascot
917.5302	917.538	0.0078	9	69	76	IISSIEQK	25	0			Mascot
932.4294	932.4701	0.0407	44	131	137	MKGDYYR					Mascot
948.4244	948.4344	0.01	11	131	137	MKGDYYR			Oxidation (M)[1]		Mascot
948.4244	948.4344	0.01	11	131	137	MKGDYYR	15	0	Oxidation (M)[1]		Mascot
999.4451	999.4573	0.0122	12	10	17	EENVYMAK			Oxidation (M)[6]		Mascot
1004.5622	1004.5597	-0.0025	-2	94	102	GKIETELSK					Mascot

1091.4712	1091.5339	0.0627	57	77	85	EESRGNEDR					Mascot
1189.6609	1189.678	0.0171	14	223	232	DSTLIMQLLR					Mascot
1205.6559	1205.6694	0.0135	11	223	232	DSTLIMQLLR			Oxidation (M)[6]		Mascot
1205.6559	1205.6694	0.0135	11	223	232	DSTLIMQLLR	18	0	Oxidation (M)[6]		Mascot
1212.5565	1212.6193	0.0628	52	150	160	DAAENTMVAYK					Mascot
1228.5514	1228.6051	0.0537	44	150	160	DAAENTMVAYK			Oxidation (M)[7]		Mascot
1366.5542	1366.5502	-0.004	-3	25	34	YEEMVEFMEK			Oxidation (M)[4,8]		Mascot
1406.6646	1406.6904	0.0258	18	38	49	TVDSEELTVEER					Mascot
1406.6646	1406.6904	0.0258	18	38	49	TVDSEELTVEER	101	100			Mascot
1418.7485	1418.777	0.0285	20	69	80	IISSIEQKEESR					Mascot
1418.7485	1418.777	0.0285	20	69	80	IISSIEQKEESR	33	3.669			Mascot
2163.9573	2163.9636	0.0063	3	18	34	LAEQAERYEEMVEFMEK			Oxidation (M)[11,15]		Mascot
2163.9573	2163.9636	0.0063	3	18	34	LAEQAERYEEMVEFMEK	13	0	Oxidation (M)[11,15]		Mascot
2174.9976	2175.0115	0.0139	6	204	222	QAFDEAISELDTLSEESY K					Mascot
2331.2595	2331.2488	-0.0107	-5	110	130	LLDSHLVPSSTAPESKVF YLK					Mascot

10	Os04g0462500 [Oryza sativa Japonica Group]	gi|113564574	29959.1	4.76	16	292	100	23.018	191	100		

Protein Group

RecName: Full=14-3-3-like protein GF14-B; AltName: Full=G-box factor 14-3-3 homolog B	gi|75296478	29959.1	4.7600 002288 8184
hypothetical protein OsI_16185 [Oryza sativa Indica Group]	gi|125548594	29959.1	4.7600 002288 8184

Peptide Information

Calc. Mass	Obsrv. Mass	± da	± ppm	Start Seq.	End Seq.	Sequence	Ion Score	C. I. %	Modification	Rank	Result Type
816.421	816.4315	0.0105	13	18	24	LAEQAER					Mascot
818.444	818.4407	-0.0033	-4	103	109	ICDGILK			Carbamidomethyl (C)[2]		Mascot
907.5247	907.5327	0.008	9	50	57	NLLSVAYK					Mascot
917.5302	917.538	0.0078	9	69	76	IISSIEQK					Mascot
917.5302	917.538	0.0078	9	69	76	IISSIEQK	25	0			Mascot
932.4294	932.4701	0.0407	44	131	137	MKGDYYR					Mascot
948.4244	948.4344	0.01	11	131	137	MKGDYYR			Oxidation (M)[1]		Mascot
948.4244	948.4344	0.01	11	131	137	MKGDYYR	15	0	Oxidation (M)[1]		Mascot
999.4451	999.4573	0.0122	12	10	17	EENVYMAK			Oxidation (M)[6]		Mascot
1091.4712	1091.5339	0.0627	57	77	85	EESRGNEDR					Mascot

1144.6321	1144.6542	0.0221	19	81	90	GNEDRVTLIK				Mascot
1189.6609	1189.678	0.0171	14	223	232	DSTLIMQLLR				Mascot
1205.6559	1205.6694	0.0135	11	223	232	DSTLIMQLLR			Oxidation (M)[6]	Mascot
1205.6559	1205.6694	0.0135	11	223	232	DSTLIMQLLR	18	0	Oxidation (M)[6]	Mascot
1212.5565	1212.6193	0.0628	52	150	160	DAAENTMVAYK				Mascot
1228.5514	1228.6051	0.0537	44	150	160	DAAENTMVAYK			Oxidation (M)[7]	Mascot
1366.5542	1366.5502	-0.004	-3	25	34	YEEMVEFMEK			Oxidation (M)[4,8]	Mascot
1406.6646	1406.6904	0.0258	18	38	49	TVDSEELTVEER				Mascot
1406.6646	1406.6904	0.0258	18	38	49	TVDSEELTVEER	101	100		Mascot
1418.7485	1418.777	0.0285	20	69	80	IISSIEQKEESR				Mascot
1418.7485	1418.777	0.0285	20	69	80	IISSIEQKEESR	33	28.43		Mascot
2163.9573	2163.9636	0.0063	3	18	34	LAEQAERYEEMVEFMEK			Oxidation (M)[11,15]	Mascot
2163.9573	2163.9636	0.0063	3	18	34	LAEQAERYEEMVEFMEK	13	0	Oxidation (M)[11,15]	Mascot
2174.9976	2175.0115	0.0139	6	204	222	QAFDEAISELDTLSEESY K				Mascot
2331.2019	2331.2488	0.0469	20	178	197	LGLALNFSVFYYEILNSPD R				Mascot

Gel Idx/Pos	170/G21	Instr./Gel Origin	BA2151/Sample Project 20140814	Process Status	Analysis Succeeded
Plate [#] Name	[1] Sample Project 20140814	Instrument Sample Name		Spectra	11

Rank	Protein Name	Accession No.	Protein MW	Protein PI	Pep. Count	Protein Score	Protein Score C. I. %	Intensity Matched	Total Ion Score	Total Ion C. I. %	Confirmed
1	14-3-3 protein [Triticum aestivum]	gi|431822518	29843.9	4.71	15	134	100	16.02	38	75.693	

Protein Group

14-3-3-like protein B [Aegilops tauschii]	gi|475569230	29843.9	4.7100 000381 4697

Peptide Information

Calc. Mass	Obsrv. Mass	± da	± ppm	Start Seq.	End Seq.	Sequence	Ion Score	C. I. %	Modification	Rank	Result Type
816.421	816.4322	0.0112	14	18	24	LAEQAER					Mascot
818.444	818.4481	0.0041	5	103	109	ICDGILK			Carbamidomethyl (C)[2]		Mascot
843.4934	843.517	0.0236	28	161	168	AAQEIALK					Mascot
907.5247	907.5406	0.0159	18	50	57	NLLSVAYK					Mascot
917.5302	917.5402	0.01	11	69	76	IISSIEQK					Mascot
932.4294	932.4441	0.0147	16	131	137	MKGDYYR					Mascot
948.4244	948.4377	0.0133	14	131	137	MKGDYYR			Oxidation (M)[1]		Mascot
999.4451	999.462	0.0169	17	10	17	EENVYMAK			Oxidation (M)[6]		Mascot
1016.5986	1016.6001	0.0015	1	94	102	GKIEVELTK					Mascot
1059.5946	1059.6146	0.02	19	169	177	ELPPTHPIR					Mascot
1059.5946	1059.6146	0.02	19	169	177	ELPPTHPIR	38	75.693			Mascot
1189.6609	1189.6863	0.0254	21	223	232	DSTLIMQLLR					Mascot
1189.6609	1189.6863	0.0254	21	223	232	DSTLIMQLLR					Mascot
1205.6559	1205.6742	0.0183	15	223	232	DSTLIMQLLR			Oxidation (M)[6]		Mascot
1366.5542	1366.5648	0.0106	8	25	34	YEEMVEFMEK			Oxidation (M)[4,8]		Mascot
1406.6646	1406.691	0.0264	19	38	49	TVDSEELTVEER					Mascot
1406.6646	1406.691	0.0264	19	38	49	TVDSEELTVEER					Mascot
1418.7485	1418.7678	0.0193	14	69	80	IISSIEQKEESR					Mascot
2131.9675	2132.0005	0.033	15	18	34	LAEQAERYEEMVEFMEK					Mascot
2163.9573	2163.9697	0.0124	6	18	34	LAEQAERYEEMVEFMEK			Oxidation (M)[11,15]		Mascot
2331.2019	2331.2139	0.012	5	178	197	LGLALNFSVFYYEILNSPD R					Mascot

Rank	Protein Name	Accession No.	Protein MW	Protein PI	Pep. Count	Protein Score	Protein Score C. I. %	Intensity Matched	Total Ion Score	Total Ion C. I. %	Confirmed
2	14-3-3-like protein B [Triticum urartu]	gi|474253094	29786.9	4.67	14	87	99.797	11.057			

Protein Group

RecName: Full=14-3-3-like protein B; Short=14-3-3B	gi|2492487	29786.9	4.6700000762 9395

Peptide Information

Calc. Mass	Obsrv. Mass	± da	± ppm	Start Seq.	End Seq.	Sequence	Ion Score	C. I. %	Modification	Rank	Result Type
816.421	816.4322	0.0112	14	18	24	LAEQAER					Mascot
818.444	818.4481	0.0041	5	103	109	ICDGILK			Carbamidomethyl (C)[2]		Mascot
907.5247	907.5406	0.0159	18	50	57	NLLSVAYK					Mascot
917.5302	917.5402	0.01	11	69	76	IISSIEQK					Mascot
932.4294	932.4441	0.0147	16	131	137	MKGDYYR					Mascot
948.4244	948.4377	0.0133	14	131	137	MKGDYYR			Oxidation (M)[1]		Mascot
999.4451	999.462	0.0169	17	10	17	EENVYMAK			Oxidation (M)[6]		Mascot
1016.5986	1016.6001	0.0015	1	94	102	GKIEVELTK					Mascot
1189.6609	1189.6863	0.0254	21	223	232	DSTLIMQLLR					Mascot
1189.6609	1189.6863	0.0254	21	223	232	DSTLIMQLLR					Mascot
1205.6559	1205.6742	0.0183	15	223	232	DSTLIMQLLR			Oxidation (M)[6]		Mascot
1366.5542	1366.5648	0.0106	8	25	34	YEEMVEFMEK			Oxidation (M)[4,8]		Mascot
1406.6646	1406.691	0.0264	19	38	49	TVDSEELTVEER					Mascot
1406.6646	1406.691	0.0264	19	38	49	TVDSEELTVEER					Mascot
1418.7485	1418.7678	0.0193	14	69	80	IISSIEQKEESR					Mascot
1827.0123	1827.0411	0.0288	16	161	177	AAQEIALAELPPTHPIR					Mascot
2131.9675	2132.0005	0.033	15	18	34	LAEQAERYEEMVEFMEK					Mascot
2163.9573	2163.9697	0.0124	6	18	34	LAEQAERYEEMVEFMEK			Oxidation (M)[11,15]		Mascot
2331.2019	2331.2139	0.012	5	178	197	LGLALNFSVFYYEILNSPDR					Mascot

3	PREDICTED: 14-3-3-like protein GF14-A-like [Setaria italica]	gi|514797180	28713.3	4.83	8	76	97.718	11.06	38	75.693

Peptide Information

Calc. Mass	Obsrv. Mass	± da	± ppm	Start Seq.	End Seq.	Sequence	Ion Score	C. I. %	Modification	Rank	Result Type
816.421	816.4322	0.0112	14	18	24	LAEQAER					Mascot
907.5247	907.5406	0.0159	18	50	57	NLLSVAYK					Mascot
1059.5946	1059.6146	0.02	19	169	177	ELPPTHPIR					Mascot
1059.5946	1059.6146	0.02	19	169	177	ELPPTHPIR	38	75.693			Mascot
1189.6609	1189.6863	0.0254	21	223	232	DSTLIMQLLR					Mascot

1189.6609	1189.6863	0.0254	21	223	232	DSTLIMQLLR						Mascot
1205.6559	1205.6742	0.0183	15	223	232	DSTLIMQLLR			Oxidation (M)[6]			Mascot
1366.5542	1366.5648	0.0106	8	25	34	YEEMVEFMEK			Oxidation (M)[4,8]			Mascot
1418.7485	1418.7678	0.0193	14	69	80	IVSSIEQKEETR						Mascot
2131.9675	2132.0005	0.033	15	18	34	LAEQAERYEEMVEFMEK						Mascot
2163.9573	2163.9697	0.0124	6	18	34	LAEQAERYEEMVEFMEK			Oxidation (M)[11,15]			Mascot
2331.2019	2331.2139	0.012	5	178	197	LGLALNFSVFYYEILNSPD R						Mascot

4	14-3-3-like protein GF14-B [Triticum urartu]	gi|474147722	30043.1	4.69	13	76	97.611	10.837

Protein Group

14-3-3-like protein GF14-B [Aegilops tauschii]	gi|475549223	30043.1	4.6900 000572 2046

Peptide Information

Calc. Mass	Obsrv. Mass	± da	± ppm	Start Seq.	End Seq.	Sequence	Ion Score	C. I. %	Modification	Rank	Result Type
816.421	816.4322	0.0112	14	18	24	LAEQAER					Mascot
818.444	818.4481	0.0041	5	103	109	ICDGILK			Carbamidomethyl (C)[2]		Mascot
844.4523	844.4928	0.0405	48	2	9	TAPAELSR					Mascot
907.5247	907.5406	0.0159	18	50	57	NLLSVAYK					Mascot
917.5302	917.5402	0.01	11	69	76	IISSIEQK					Mascot
932.4294	932.4441	0.0147	16	131	137	MKGDYYR					Mascot
948.4244	948.4377	0.0133	14	131	137	MKGDYYR			Oxidation (M)[1]		Mascot
999.4451	999.462	0.0169	17	10	17	EENVYMAK			Oxidation (M)[6]		Mascot
1189.6609	1189.6863	0.0254	21	223	232	DSTLIMQLLR					Mascot
1189.6609	1189.6863	0.0254	21	223	232	DSTLIMQLLR					Mascot
1205.6559	1205.6742	0.0183	15	223	232	DSTLIMQLLR			Oxidation (M)[6]		Mascot
1366.5542	1366.5648	0.0106	8	25	34	YEEMVEFMEK			Oxidation (M)[4,8]		Mascot
1406.6646	1406.691	0.0264	19	38	49	TVDSEELTVEER					Mascot
1406.6646	1406.691	0.0264	19	38	49	TVDSEELTVEER					Mascot
1418.7485	1418.7678	0.0193	14	69	80	IISSIEQKEESR					Mascot
2131.9675	2132.0005	0.033	15	18	34	LAEQAERYEEMVEFMEK					Mascot
2163.9573	2163.9697	0.0124	6	18	34	LAEQAERYEEMVEFMEK			Oxidation (M)[11,15]		Mascot
2331.2019	2331.2139	0.012	5	178	197	LGLALNFSVFYYEILNSPD R					Mascot

5	RecName: Full=14-3-3-like protein 2	gi|205686167	7715.8	4.28	7	72	94.526	7.445

Peptide Information

Calc. Mass	Obsrv. Mass	± da	± ppm	Start Seq.	End Seq.	Sequence	Ion Score	C. I. %	Modification	Rank	Result Type
816.421	816.4322	0.0112	14	1	7	LAEQAER					Mascot
907.5247	907.5406	0.0159	18	18	25	NLLSVAYK					Mascot
917.5302	917.5402	0.01	11	26	33	IISSIEQK					Mascot
1189.6609	1189.6863	0.0254	21	57	66	DSTLIMQLLR					Mascot
1189.6609	1189.6863	0.0254	21	57	66	DSTLIMQLLR					Mascot
1205.6559	1205.6742	0.0183	15	57	66	DSTLIMQLLR			Oxidation (M)[6]		Mascot
1366.5542	1366.5648	0.0106	8	8	17	YEEMVEYMEK			Oxidation (M)[4]		Mascot
1418.7485	1418.7678	0.0193	14	26	37	IISSIEQKEESR					Mascot
2163.9573	2163.9697	0.0124	6	1	17	LAEQAERYEEMVEYMEK			Oxidation (M)[11]		Mascot

6	14-3-3-like protein A [Triticum urartu]	gi|474293618	28778.5	5.2	7	72	93.715	10.674	38	75.693

Peptide Information

Calc. Mass	Obsrv. Mass	± da	± ppm	Start Seq.	End Seq.	Sequence	Ion Score	C. I. %	Modification	Rank	Result Type
816.421	816.4322	0.0112	14	17	23	LAEQAER					Mascot
907.5247	907.5406	0.0159	18	49	56	NLLSVAYK					Mascot
1059.5946	1059.6146	0.02	19	168	176	ELPPTHPIR					Mascot
1059.5946	1059.6146	0.02	19	168	176	ELPPTHPIR	38	75.693			Mascot
1189.6609	1189.6863	0.0254	21	222	231	DSTLIMQLLR					Mascot
1189.6609	1189.6863	0.0254	21	222	231	DSTLIMQLLR					Mascot
1205.6559	1205.6742	0.0183	15	222	231	DSTLIMQLLR			Oxidation (M)[6]		Mascot
1366.5542	1366.5648	0.0106	8	24	33	YEEMVEFMEK			Oxidation (M)[4,8]		Mascot
2131.9675	2132.0005	0.033	15	17	33	LAEQAERYEEMVEFMEK					Mascot
2163.9573	2163.9697	0.0124	6	17	33	LAEQAERYEEMVEFMEK			Oxidation (M)[11,15]		Mascot
2331.2019	2331.2139	0.012	5	177	196	LGLALNFSVFYYEILNSPDR					Mascot

7	Os02g0580300 [Oryza sativa Japonica Group]	gi|113536765	29844.9	4.71	12	68	83.846	10.534

Protein Group

RecName: Full=14-3-3-like protein GF14-E; AltName: Full=G-box factor 14-3-3 homolog E	gi|75290255	29844.9	4.7100000381 4697
hypothetical protein OsI_07806 [Oryza sativa Indica Group]	gi|125540033	29844.9	4.7100000381 4697

Peptide Information

Calc. Mass	Obsrv. Mass	± da	± ppm	Start	End Sequence	Ion	C. I. %	Modification	Rank	Result Type

				Seq.	Seq.		Score				
816.421	816.4322	0.0112	14	18	24	LAEQAER					Mascot
818.444	818.4481	0.0041	5	103	109	ICDGILK			Carbamidomethyl (C)[2]		Mascot
907.5247	907.5406	0.0159	18	50	57	NLLSVAYK					Mascot
917.5302	917.5402	0.01	11	69	76	IISSIEQK					Mascot
932.4294	932.4441	0.0147	16	131	137	MKGDYYR					Mascot
948.4244	948.4377	0.0133	14	131	137	MKGDYYR			Oxidation (M)[1]		Mascot
999.4451	999.462	0.0169	17	10	17	EENVYMAK			Oxidation (M)[6]		Mascot
1189.6609	1189.6863	0.0254	21	223	232	DSTLIMQLLR					Mascot
1189.6609	1189.6863	0.0254	21	223	232	DSTLIMQLLR					Mascot
1205.6559	1205.6742	0.0183	15	223	232	DSTLIMQLLR			Oxidation (M)[6]		Mascot
1366.5542	1366.5648	0.0106	8	25	34	YEEMVEFMEK			Oxidation (M)[4,8]		Mascot
1406.6646	1406.691	0.0264	19	38	49	TVDSEELTVEER					Mascot
1406.6646	1406.691	0.0264	19	38	49	TVDSEELTVEER					Mascot
1418.7485	1418.7678	0.0193	14	69	80	IISSIEQKEESR					Mascot
2131.9675	2132.0005	0.033	15	18	34	LAEQAERYEEMVEFMEK					Mascot
2163.9573	2163.9697	0.0124	6	18	34	LAEQAERYEEMVEFMEK			Oxidation (M)[11,15]		Mascot
2331.2019	2331.2139	0.012	5	178	197	LGLALNFSVFYYEILNSPDR					Mascot

8	Os04g0462500 [Oryza sativa Japonica Group]	gi|113564574	29959.1	4.76	12	67	81.021	10.534

Protein Group

RecName: Full=14-3-3-like protein GF14-B; AltName: Full=G-box factor 14-3-3 homolog B	gi|75296478	29959.1	4.7600 002288 8184
hypothetical protein OsI_16185 [Oryza sativa Indica Group]	gi|125548594	29959.1	4.7600 002288 8184

Peptide Information

Calc. Mass	Obsrv. Mass	± da	± ppm	Start Seq.	End Seq.	Sequence	Ion Score	C. I. %	Modification	Rank	Result Type
816.421	816.4322	0.0112	14	18	24	LAEQAER					Mascot
818.444	818.4481	0.0041	5	103	109	ICDGILK			Carbamidomethyl (C)[2]		Mascot
907.5247	907.5406	0.0159	18	50	57	NLLSVAYK					Mascot
917.5302	917.5402	0.01	11	69	76	IISSIEQK					Mascot
932.4294	932.4441	0.0147	16	131	137	MKGDYYR					Mascot
948.4244	948.4377	0.0133	14	131	137	MKGDYYR			Oxidation (M)[1]		Mascot
999.4451	999.462	0.0169	17	10	17	EENVYMAK			Oxidation (M)[6]		Mascot

Calc. Mass	Obsrv. Mass	± da	± ppm	Start Seq.	End Seq.	Sequence	Ion Score	C. I. %	Modification	Rank	Result Type
1189.6609	1189.6863	0.0254	21	223	232	DSTLIMQLLR					Mascot
1189.6609	1189.6863	0.0254	21	223	232	DSTLIMQLLR					Mascot
1205.6559	1205.6742	0.0183	15	223	232	DSTLIMQLLR			Oxidation (M)[6]		Mascot
1366.5542	1366.5648	0.0106	8	25	34	YEEMVEFMEK			Oxidation (M)[4,8]		Mascot
1406.6646	1406.691	0.0264	19	38	49	TVDSEELTVEER					Mascot
1406.6646	1406.691	0.0264	19	38	49	TVDSEELTVEER					Mascot
1418.7485	1418.7678	0.0193	14	69	80	IISSIEQKEESR					Mascot
2131.9675	2132.0005	0.033	15	18	34	LAEQAERYEEMVEFMEK					Mascot
2163.9573	2163.9697	0.0124	6	18	34	LAEQAERYEEMVEFMEK			Oxidation (M)[11,15]		Mascot
2331.2019	2331.2139	0.012	5	178	197	LGLALNFSVFYYEILNSPDR					Mascot

9	hypothetical protein CARUB_v10014407mg [Capsella rubella]	gi|482567046	29888.1	4.82	11	64	62.993	14.151

Peptide Information

Calc. Mass	Obsrv. Mass	± da	± ppm	Start Seq.	End Seq.	Sequence	Ion Score	C. I. %	Modification	Rank	Result Type
816.421	816.4322	0.0112	14	14	20	LAEQAER					Mascot
907.5247	907.5406	0.0159	18	46	53	NLLSVAYK					Mascot
917.5302	917.5402	0.01	11	65	72	IISSIEQK					Mascot
1059.5793	1059.6146	0.0353	33	90	98	GKIETELNR					Mascot
1059.5793	1059.6146	0.0353	33	90	98	GKIETELNR					Mascot
1189.6609	1189.6863	0.0254	21	219	228	DSTLIMQLLR					Mascot
1189.6609	1189.6863	0.0254	21	219	228	DSTLIMQLLR					Mascot
1205.6559	1205.6742	0.0183	15	219	228	DSTLIMQLLR			Oxidation (M)[6]		Mascot
1366.5542	1366.5648	0.0106	8	21	30	YEEMVEFMEK			Oxidation (M)[4,8]		Mascot
1406.6646	1406.691	0.0264	19	34	45	TVDTDELTVEER					Mascot
1406.6646	1406.691	0.0264	19	34	45	TVDTDELTVEER					Mascot
1418.7485	1418.7678	0.0193	14	65	76	IISSIEQKEESR					Mascot
1788.9603	1789.1007	0.1404	78	157	173	SAQDIALADLAPTHPIR					Mascot
2131.9675	2132.0005	0.033	15	14	30	LAEQAERYEEMVEFMEK					Mascot
2163.9573	2163.9697	0.0124	6	14	30	LAEQAERYEEMVEFMEK			Oxidation (M)[11,15]		Mascot
2331.2019	2331.2139	0.012	5	174	193	LGLALNFSVFYYEILNSPDR					Mascot

10	RecName: Full=14-3-3-like protein	gi|12229593	29348.8	4.79	11	62	31.09	9.253

Peptide Information

Calc. Mass	Obsrv. Mass	± da	± ppm	Start Seq.	End Seq.	Sequence	Ion Score	C. I. %	Modification	Rank	Result Type

816.421	816.4322	0.0112	14	17	23	LAEQAER		Mascot
818.444	818.4481	0.0041	5	102	108	ICDGILK	Carbamidomethyl (C)[2]	Mascot
907.5247	907.5406	0.0159	18	49	56	NLLSVAYK		Mascot
917.5302	917.5402	0.01	11	68	75	IISSIEQK		Mascot
999.4451	999.462	0.0169	17	9	16	EENVYMAK	Oxidation (M)[6]	Mascot
1189.6609	1189.6863	0.0254	21	222	231	DSTLIMQLLR		Mascot
1189.6609	1189.6863	0.0254	21	222	231	DSTLIMQLLR		Mascot
1205.6559	1205.6742	0.0183	15	222	231	DSTLIMQLLR	Oxidation (M)[6]	Mascot
1366.5542	1366.5648	0.0106	8	24	33	YEEMVEFMEK	Oxidation (M)[4,8]	Mascot
1418.7485	1418.7678	0.0193	14	68	79	IISSIEQKEESR		Mascot
1420.6803	1420.7113	0.031	22	37	48	TVDTEELTVEER		Mascot
1420.6803	1420.7113	0.031	22	37	48	TVDTEELTVEER		Mascot
2131.9675	2132.0005	0.033	15	17	33	LAEQAERYEEMVEFMEK		Mascot
2163.9573	2163.9697	0.0124	6	17	33	LAEQAERYEEMVEFMEK	Oxidation (M)[11,15]	Mascot
2331.2019	2331.2139	0.012	5	177	196	LGLALNFSVFYYEILNSPD R		Mascot

Gel Idx/Pos	171/G22	Instr./Gel Origin	BA2151/Sample Project 20140814	Process Status	Analysis Succeeded
Plate [#] Name	[1] Sample Project 20140814	Instrument Sample Name		Spectra	11

Rank	Protein Name	Accession No.	Protein MW	Protein PI	Pep. Count	Protein Score	Protein Score C. I. %	Intensity Matched	Total Ion Score	Total Ion C. I. %	Confirmed
1	14-3-3-like protein B [Triticum urartu]	gi|474253094	29786.9	4.67	22	416	100	72.463	248	100	

Protein Group

RecName: Full=14-3-3-like protein B; Short=14-3-3B	gi|2492487	29786.9	4.6700 000762 9395

Peptide Information

Calc. Mass	Obsrv. Mass	± da	± ppm	Start Seq.	End Seq.	Sequence	Ion Score	C. I. %	Modification	Rank	Result Type
816.421	816.4395	0.0185	23	18	24	LAEQAER					Mascot
831.4822	831.5016	0.0194	23	96	102	IEVELTK					Mascot
907.5247	907.5403	0.0156	17	50	57	NLLSVAYK					Mascot
917.5302	917.5472	0.017	19	69	76	IISSIEQK					Mascot
932.4294	932.4494	0.02	21	131	137	MKGDYYR					Mascot
948.4244	948.4431	0.0187	20	131	137	MKGDYYR			Oxidation (M)[1]		Mascot
948.4244	948.4431	0.0187	20	131	137	MKGDYYR	15	0	Oxidation (M)[1]		Mascot
999.4451	999.4665	0.0214	21	10	17	EENVYMAK			Oxidation (M)[6]		Mascot
1016.5986	1016.6107	0.0121	12	94	102	GKIEVELTK					Mascot
1091.4712	1091.5564	0.0852	78	77	85	EESRGNEDR					Mascot
1189.6609	1189.6909	0.03	25	223	232	DSTLIMQLLR					Mascot
1205.6559	1205.6827	0.0268	22	223	232	DSTLIMQLLR			Oxidation (M)[6]		Mascot
1205.6559	1205.6827	0.0268	22	223	232	DSTLIMQLLR	32	0	Oxidation (M)[6]		Mascot
1212.5565	1212.6049	0.0484	40	150	160	DAAENTMVAYK					Mascot
1228.5514	1228.5693	0.0179	15	150	160	DAAENTMVAYK			Oxidation (M)[7]		Mascot
1300.6532	1300.6512	-0.002	-2	138	148	YLAEFKSGTER					Mascot
1356.6464	1356.6509	0.0045	3	149	160	KDAAENTMVAYK			Oxidation (M)[8]		Mascot
1366.5542	1366.5667	0.0125	9	25	34	YEEMVEFMEK			Oxidation (M)[4,8]		Mascot
1366.5542	1366.5667	0.0125	9	25	34	YEEMVEFMEK			Oxidation (M)[4,8]		Mascot
1406.6646	1406.7013	0.0367	26	38	49	TVDSEELTVEER					Mascot
1406.6646	1406.7013	0.0367	26	38	49	TVDSEELTVEER	102	100			Mascot
1418.7485	1418.7878	0.0393	28	69	80	IISSIEQKEESR					Mascot
1517.8799	1517.9158	0.0359	24	50	63	NLLSVAYKNVIGAR					Mascot
1680.8804	1680.9084	0.028	17	110	125	LLDSHLVPSSTAPESK					Mascot

1827.0123	1827.0615	0.0492	27	161	177	AAQEIALAELPPTHPIR					Mascot
1827.0123	1827.0615	0.0492	27	161	177	AAQEIALAELPPTHPIR	114	100			Mascot
2160.9819	2160.9971	0.0152	7	204	222	QAFDEAISELDSLSEESY K					Mascot
2163.9573	2163.9719	0.0146	7	18	34	LAEQAERYEEMVEFMEK			Oxidation (M)[11,15]		Mascot
2331.2595	2331.269	0.0095	4	110	130	LLDSHLVPSSTAPESKVF YLK					Mascot
2595.1768	2595.2207	0.0439	17	233	255	DNLTLWTSDISEDAAEEM KDAPK			Oxidation (M)[18]		Mascot

2	14-3-3 protein [Triticum aestivum]	gi|431822518	29843.9	4.71	23	352	100	31.194	175	100		

Protein Group

14-3-3-like protein B [Aegilops tauschii]	gi|475569230	29843.9	4.7100 000381 4697

Peptide Information

Calc. Mass	Obsrv. Mass	± da	± ppm	Start Seq.	End Seq.	Sequence	Ion Score	C. I. %	Modification	Rank	Result Type
816.421	816.4395	0.0185	23	18	24	LAEQAER					Mascot
831.4822	831.5016	0.0194	23	96	102	IEVELTK					Mascot
907.5247	907.5403	0.0156	17	50	57	NLLSVAYK					Mascot
917.5302	917.5472	0.017	19	69	76	IISSIEQK					Mascot
932.4294	932.4494	0.02	21	131	137	MKGDYYR					Mascot
948.4244	948.4431	0.0187	20	131	137	MKGDYYR			Oxidation (M)[1]		Mascot
948.4244	948.4431	0.0187	20	131	137	MKGDYYR	15	0	Oxidation (M)[1]		Mascot
999.4451	999.4665	0.0214	21	10	17	EENVYMAK			Oxidation (M)[6]		Mascot
1016.5986	1016.6107	0.0121	12	94	102	GKIEVELTK					Mascot
1059.5946	1059.6219	0.0273	26	169	177	ELPPTHPIR					Mascot
1059.5946	1059.6219	0.0273	26	169	177	ELPPTHPIR	41	85.76			Mascot
1091.4712	1091.5564	0.0852	78	77	85	EESRGNEDR					Mascot
1189.6609	1189.6909	0.03	25	223	232	DSTLIMQLLR					Mascot
1205.6559	1205.6827	0.0268	22	223	232	DSTLIMQLLR			Oxidation (M)[6]		Mascot
1205.6559	1205.6827	0.0268	22	223	232	DSTLIMQLLR	32	0	Oxidation (M)[6]		Mascot
1212.5565	1212.6049	0.0484	40	150	160	DAAENTMVAYK					Mascot
1228.5514	1228.5693	0.0179	15	150	160	DAAENTMVAYK			Oxidation (M)[7]		Mascot
1300.6532	1300.6512	-0.002	-2	138	148	YLAEFKSGTER					Mascot
1356.6464	1356.6509	0.0045	3	149	160	KDAAENTMVAYK			Oxidation (M)[8]		Mascot
1366.5542	1366.5667	0.0125	9	25	34	YEEMVEFMEK			Oxidation (M)[4,8]		Mascot
1366.5542	1366.5667	0.0125	9	25	34	YEEMVEFMEK			Oxidation (M)[4,8]		Mascot

Calc. Mass	Obsrv. Mass	± da	± ppm	Start Seq.	End Seq.	Sequence	Ion Score	C. I. %	Modification	Rank	Result Type
1406.6646	1406.7013	0.0367	26	38	49	TVDSEELTVEER					Mascot
1406.6646	1406.7013	0.0367	26	38	49	TVDSEELTVEER	102	100			Mascot
1418.7485	1418.7878	0.0393	28	69	80	IISSIEQKEESR					Mascot
1517.8799	1517.9158	0.0359	24	50	63	NLLSVAYKNVIGAR					Mascot
1680.8804	1680.9084	0.028	17	110	125	LLDSHLVPSSTAPESK					Mascot
1884.0702	1884.0795	0.0093	5	161	177	AAQEIALKELPPTHPIR					Mascot
2160.9819	2160.9971	0.0152	7	204	222	QAFDEAISELDSLSEESY K					Mascot
2163.9573	2163.9719	0.0146	7	18	34	LAEQAERYEEMVEFMEK			Oxidation (M)[11,15]		Mascot
2331.2595	2331.269	0.0095	4	110	130	LLDSHLVPSSTAPESKVF YLK					Mascot
2595.1768	2595.2207	0.0439	17	233	255	DNLTLWTSDISEDAAEEM KDAPK			Oxidation (M)[18]		Mascot

3	Os02g0580300 [Oryza sativa Japonica Group]	gi|113536765	29844.9	4.71	17	247	100	28.234	132	100	

Protein Group

RecName: Full=14-3-3-like protein GF14-E; AltName: Full=G-box factor 14-3-3 homolog E	gi|75290255	29844.9	4.7100 000381 4697
hypothetical protein OsI_07806 [Oryza sativa Indica Group]	gi|125540033	29844.9	4.7100 000381 4697

Peptide Information

Calc. Mass	Obsrv. Mass	± da	± ppm	Start Seq.	End Seq.	Sequence	Ion Score	C. I. %	Modification	Rank	Result Type
816.421	816.4395	0.0185	23	18	24	LAEQAER					Mascot
907.5247	907.5403	0.0156	17	50	57	NLLSVAYK					Mascot
917.5302	917.5472	0.017	19	69	76	IISSIEQK					Mascot
932.4294	932.4494	0.02	21	131	137	MKGDYYR					Mascot
948.4244	948.4431	0.0187	20	131	137	MKGDYYR			Oxidation (M)[1]		Mascot
948.4244	948.4431	0.0187	20	131	137	MKGDYYR	15	0	Oxidation (M)[1]		Mascot
999.4451	999.4665	0.0214	21	10	17	EENVYMAK			Oxidation (M)[6]		Mascot
1091.4712	1091.5564	0.0852	78	77	85	EESRGNEDR					Mascot
1189.6609	1189.6909	0.03	25	223	232	DSTLIMQLLR					Mascot
1205.6559	1205.6827	0.0268	22	223	232	DSTLIMQLLR			Oxidation (M)[6]		Mascot
1205.6559	1205.6827	0.0268	22	223	232	DSTLIMQLLR	32	0	Oxidation (M)[6]		Mascot
1212.5565	1212.6049	0.0484	40	150	160	DAAENTMVAYK					Mascot
1228.5514	1228.5693	0.0179	15	150	160	DAAENTMVAYK			Oxidation (M)[7]		Mascot
1356.6464	1356.6509	0.0045	3	149	160	KDAAENTMVAYK			Oxidation (M)[8]		Mascot
1366.5542	1366.5667	0.0125	9	25	34	YEEMVEFMEK			Oxidation (M)[4,8]		Mascot

Calc. Mass	Obsrv. Mass	± da	± ppm	Start Seq.	End Seq.	Sequence	Ion Score	C. I. %	Modification	Rank	Result Type
1366.5542	1366.5667	0.0125	9	25	34	YEEMVEFMEK			Oxidation (M)[4,8]		Mascot
1406.6646	1406.7013	0.0367	26	38	49	TVDSEELTVEER					Mascot
1406.6646	1406.7013	0.0367	26	38	49	TVDSEELTVEER	102	100			Mascot
1418.7485	1418.7878	0.0393	28	69	80	IISSIEQKEESR					Mascot
1517.8799	1517.9158	0.0359	24	50	63	NLLSVAYKNVIGAR					Mascot
1680.8804	1680.9084	0.028	17	110	125	LLDSHLVPSSTAPESK					Mascot
1867.8855	1867.9791	0.0936	50	2	17	SQPAELSREENVYMAK			Oxidation (M)[14]		Mascot
2163.9573	2163.9719	0.0146	7	18	34	LAEQAERYEEMVEFMEK			Oxidation (M)[11,15]		Mascot
2331.2595	2331.269	0.0095	4	110	130	LLDSHLVPSSTAPESKVF YLK					Mascot

4 14-3-3-like protein GF14-B [Triticum urartu] gi|474147722 30043.1 4.69 17 246 100 28.463 133 100

Protein Group

14-3-3-like protein GF14-B [Aegilops tauschii] gi|475549223 30043.1 4.6900 000572 2046

Peptide Information

Calc. Mass	Obsrv. Mass	± da	± ppm	Start Seq.	End Seq.	Sequence	Ion Score	C. I. %	Modification	Rank	Result Type
816.421	816.4395	0.0185	23	18	24	LAEQAER					Mascot
844.4523	844.4822	0.0299	35	2	9	TAPAELSR					Mascot
907.5247	907.5403	0.0156	17	50	57	NLLSVAYK					Mascot
917.5302	917.5472	0.017	19	69	76	IISSIEQK					Mascot
932.4294	932.4494	0.02	21	131	137	MKGDYYR					Mascot
948.4244	948.4431	0.0187	20	131	137	MKGDYYR			Oxidation (M)[1]		Mascot
948.4244	948.4431	0.0187	20	131	137	MKGDYYR	15	0	Oxidation (M)[1]		Mascot
999.4451	999.4665	0.0214	21	10	17	EENVYMAK			Oxidation (M)[6]		Mascot
1091.4712	1091.5564	0.0852	78	77	85	EESRGNEDR					Mascot
1189.6609	1189.6909	0.03	25	223	232	DSTLIMQLLR					Mascot
1205.6559	1205.6827	0.0268	22	223	232	DSTLIMQLLR			Oxidation (M)[6]		Mascot
1205.6559	1205.6827	0.0268	22	223	232	DSTLIMQLLR	32	0	Oxidation (M)[6]		Mascot
1212.5565	1212.6049	0.0484	40	150	160	DAAENTMVAYK					Mascot
1228.5514	1228.5693	0.0179	15	150	160	DAAENTMVAYK			Oxidation (M)[7]		Mascot
1356.6464	1356.6509	0.0045	3	149	160	KDAAENTMVAYK			Oxidation (M)[8]		Mascot
1366.5542	1366.5667	0.0125	9	25	34	YEEMVEFMEK			Oxidation (M)[4,8]		Mascot
1366.5542	1366.5667	0.0125	9	25	34	YEEMVEFMEK			Oxidation (M)[4,8]		Mascot
1406.6646	1406.7013	0.0367	26	38	49	TVDSEELTVEER					Mascot
1406.6646	1406.7013	0.0367	26	38	49	TVDSEELTVEER	102	100			Mascot

1418.7485	1418.7878	0.0393	28	69	80	IISSIEQKEESR					Mascot
1517.8799	1517.9158	0.0359	24	50	63	NLLSVAYKNVIGAR					Mascot
1824.8796	1825.0389	0.1593	87	2	17	TAPAELSREENVYMAK			Oxidation (M)[14]		Mascot
2163.9573	2163.9719	0.0146	7	18	34	LAEQAERYEEMVEFMEK			Oxidation (M)[11,15]		Mascot
2331.2019	2331.269	0.0671	29	178	197	LGLALNFSVFYYEILNSPDR					Mascot

5	Os04g0462500 [Oryza sativa Japonica Group]	gi|113564574	29959.1	4.76	15	226	100	27.713	133	100	

Protein Group

RecName: Full=14-3-3-like protein GF14-B; AltName: Full=G-box factor 14-3-3 homolog B	gi|75296478	29959.1	4.76000022888184
hypothetical protein OsI_16185 [Oryza sativa Indica Group]	gi|125548594	29959.1	4.76000022888184

Peptide Information

Calc. Mass	Obsrv. Mass	± da	± ppm	Start Seq.	End Seq.	Sequence	Ion Score	C. I. %	Modification	Rank	Result Type
816.421	816.4395	0.0185	23	18	24	LAEQAER					Mascot
907.5247	907.5403	0.0156	17	50	57	NLLSVAYK					Mascot
917.5302	917.5472	0.017	19	69	76	IISSIEQK					Mascot
932.4294	932.4494	0.02	21	131	137	MKGDYYR					Mascot
948.4244	948.4431	0.0187	20	131	137	MKGDYYR			Oxidation (M)[1]		Mascot
948.4244	948.4431	0.0187	20	131	137	MKGDYYR	15	0	Oxidation (M)[1]		Mascot
999.4451	999.4665	0.0214	21	10	17	EENVYMAK			Oxidation (M)[6]		Mascot
1091.4712	1091.5564	0.0852	78	77	85	EESRGNEDR					Mascot
1189.6609	1189.6909	0.03	25	223	232	DSTLIMQLLR					Mascot
1205.6559	1205.6827	0.0268	22	223	232	DSTLIMQLLR			Oxidation (M)[6]		Mascot
1205.6559	1205.6827	0.0268	22	223	232	DSTLIMQLLR	32	0	Oxidation (M)[6]		Mascot
1212.5565	1212.6049	0.0484	40	150	160	DAAENTMVAYK					Mascot
1228.5514	1228.5693	0.0179	15	150	160	DAAENTMVAYK			Oxidation (M)[7]		Mascot
1356.6464	1356.6509	0.0045	3	149	160	KDAAENTMVAYK			Oxidation (M)[8]		Mascot
1366.5542	1366.5667	0.0125	9	25	34	YEEMVEFMEK			Oxidation (M)[4,8]		Mascot
1366.5542	1366.5667	0.0125	9	25	34	YEEMVEFMEK			Oxidation (M)[4,8]		Mascot
1406.6646	1406.7013	0.0367	26	38	49	TVDSEELTVEER					Mascot
1406.6646	1406.7013	0.0367	26	38	49	TVDSEELTVEER	102	100			Mascot
1418.7485	1418.7878	0.0393	28	69	80	IISSIEQKEESR					Mascot
1517.8799	1517.9158	0.0359	24	50	63	NLLSVAYKNVIGAR					Mascot

2163.9573	2163.9719	0.0146	7	18	34	LAEQAERYEEMVEFMEK			Oxidation (M)[11,15]		Mascot
2331.2019	2331.269	0.0671	29	178	197	LGLALNFSVFYYEILNSPD R					Mascot

6	14-3-3-like protein GF14-12 [Zea mays]	gi|413918561	29724.9	4.75	13	212	100	27.23	133	100

Protein Group

14-3-3 protein [Setaria italica]	gi|357529948	29778.9	4.75
PREDICTED: 14-3-3-like protein GF14-12-like [Setaria italica]	gi|514801939	29750.9	4.75

Peptide Information

Calc. Mass	Obsrv. Mass	± da	± ppm	Start Seq.	End Seq.	Sequence	Ion Score	C. I. %	Modification	Rank	Result Type
816.421	816.4395	0.0185	23	17	23	LAEQAER					Mascot
907.5247	907.5403	0.0156	17	49	56	NLLSVAYK					Mascot
917.5302	917.5472	0.017	19	68	75	IISSIEQK					Mascot
932.4294	932.4494	0.02	21	130	136	MKGDYYR					Mascot
948.4244	948.4431	0.0187	20	130	136	MKGDYYR			Oxidation (M)[1]		Mascot
948.4244	948.4431	0.0187	20	130	136	MKGDYYR	15	0	Oxidation (M)[1]		Mascot
999.4451	999.4665	0.0214	21	9	16	EENVYMAK			Oxidation (M)[6]		Mascot
1189.6609	1189.6909	0.03	25	222	231	DSTLIMQLLR					Mascot
1205.6559	1205.6827	0.0268	22	222	231	DSTLIMQLLR			Oxidation (M)[6]		Mascot
1205.6559	1205.6827	0.0268	22	222	231	DSTLIMQLLR	32	0	Oxidation (M)[6]		Mascot
1212.5565	1212.6049	0.0484	40	149	159	DAAENTMVAYK					Mascot
1228.5514	1228.5693	0.0179	15	149	159	DAAENTMVAYK			Oxidation (M)[7]		Mascot
1356.6464	1356.6509	0.0045	3	148	159	KDAAENTMVAYK			Oxidation (M)[8]		Mascot
1366.5542	1366.5667	0.0125	9	24	33	YEEMVEFMEK			Oxidation (M)[4,8]		Mascot
1366.5542	1366.5667	0.0125	9	24	33	YEEMVEFMEK			Oxidation (M)[4,8]		Mascot
1406.6646	1406.7013	0.0367	26	37	48	TVDSEELTVEER					Mascot
1406.6646	1406.7013	0.0367	26	37	48	TVDSEELTVEER	102	100			Mascot
1517.8799	1517.9158	0.0359	24	49	62	NLLSVAYKNVIGAR					Mascot
2163.9573	2163.9719	0.0146	7	17	33	LAEQAERYEEMVEFMEK			Oxidation (M)[11,15]		Mascot
2331.2019	2331.269	0.0671	29	177	196	LGLALNFSVFYYEILNSPD R					Mascot

7	14-3-3-like protein GF14-6 [Zea mays]	gi|262359935	29758	4.76	13	211	100	27.23	133	100

Protein Group

RecName: Full=14-3-3-like protein GF14-6	gi|1345587	29758	4.7600

			002288 8184
TPA: general regulatory factor1 isoform 1 [Zea mays]	gi|414586860	29758	4.7600 002288 8184
TPA: general regulatory factor1 isoform 2 [Zea mays]	gi|414586861	29758	4.7600 002288 8184
hypothetical protein SORBIDRAFT_06g019100 [Sorghum bicolor]	gi|241937809	29744	4.7600 002288 8184

Peptide Information

Calc. Mass	Obsrv. Mass	± da	± ppm	Start Seq.	End Seq.	Sequence	Ion Score	C. I. %	Modification	Rank	Result Type
816.421	816.4395	0.0185	23	17	23	LAEQAER					Mascot
907.5247	907.5403	0.0156	17	49	56	NLLSVAYK					Mascot
917.5302	917.5472	0.017	19	68	75	IISSIEQK					Mascot
932.4294	932.4494	0.02	21	130	136	MKGDYYR					Mascot
948.4244	948.4431	0.0187	20	130	136	MKGDYYR			Oxidation (M)[1]		Mascot
948.4244	948.4431	0.0187	20	130	136	MKGDYYR	15	0	Oxidation (M)[1]		Mascot
999.4451	999.4665	0.0214	21	9	16	EENVYMAK			Oxidation (M)[6]		Mascot
1189.6609	1189.6909	0.03	25	222	231	DSTLIMQLLR					Mascot
1205.6559	1205.6827	0.0268	22	222	231	DSTLIMQLLR			Oxidation (M)[6]		Mascot
1205.6559	1205.6827	0.0268	22	222	231	DSTLIMQLLR	32	0	Oxidation (M)[6]		Mascot
1212.5565	1212.6049	0.0484	40	149	159	DAAENTMVAYK					Mascot
1228.5514	1228.5693	0.0179	15	149	159	DAAENTMVAYK			Oxidation (M)[7]		Mascot
1356.6464	1356.6509	0.0045	3	148	159	KDAAENTMVAYK			Oxidation (M)[8]		Mascot
1366.5542	1366.5667	0.0125	9	24	33	YEEMVEFMEK			Oxidation (M)[4,8]		Mascot
1366.5542	1366.5667	0.0125	9	24	33	YEEMVEFMEK			Oxidation (M)[4,8]		Mascot
1406.6646	1406.7013	0.0367	26	37	48	TVDSEELTVEER					Mascot
1406.6646	1406.7013	0.0367	26	37	48	TVDSEELTVEER	102	100			Mascot
1517.8799	1517.9158	0.0359	24	49	62	NLLSVAYKNVIGAR					Mascot
2163.9573	2163.9719	0.0146	7	17	33	LAEQAERYEEMVEFMEK			Oxidation (M)[11,15]		Mascot
2331.2019	2331.269	0.0671	29	177	196	LGLALNFSVFYYEILNSPD R					Mascot

8	TPA: general regulatory factor1 [Zea mays]	gi|414586863	31835.1	4.9	13	208	100	27.23	133	100	

Peptide Information

Calc. Mass	Obsrv. Mass	± da	± ppm	Start	End	Sequence	Ion	C. I. %	Modification	Rank	Result Type

				Seq.	Seq.		Score				
816.421	816.4395	0.0185	23	34	40	LAEQAER					Mascot
907.5247	907.5403	0.0156	17	66	73	NLLSVAYK					Mascot
917.5302	917.5472	0.017	19	85	92	IISSIEQK					Mascot
932.4294	932.4494	0.02	21	147	153	MKGDYYR					Mascot
948.4244	948.4431	0.0187	20	147	153	MKGDYYR			Oxidation (M)[1]		Mascot
948.4244	948.4431	0.0187	20	147	153	MKGDYYR	15	0	Oxidation (M)[1]		Mascot
999.4451	999.4665	0.0214	21	26	33	EENVYMAK			Oxidation (M)[6]		Mascot
1189.6609	1189.6909	0.03	25	239	248	DSTLIMQLLR					Mascot
1205.6559	1205.6827	0.0268	22	239	248	DSTLIMQLLR			Oxidation (M)[6]		Mascot
1205.6559	1205.6827	0.0268	22	239	248	DSTLIMQLLR	32	0	Oxidation (M)[6]		Mascot
1212.5565	1212.6049	0.0484	40	166	176	DAAENTMVAYK					Mascot
1228.5514	1228.5693	0.0179	15	166	176	DAAENTMVAYK			Oxidation (M)[7]		Mascot
1356.6464	1356.6509	0.0045	3	165	176	KDAAENTMVAYK			Oxidation (M)[8]		Mascot
1366.5542	1366.5667	0.0125	9	41	50	YEEMVEFMEK			Oxidation (M)[4,8]		Mascot
1366.5542	1366.5667	0.0125	9	41	50	YEEMVEFMEK			Oxidation (M)[4,8]		Mascot
1406.6646	1406.7013	0.0367	26	54	65	TVDSEELTVEER					Mascot
1406.6646	1406.7013	0.0367	26	54	65	TVDSEELTVEER	102	100			Mascot
1517.8799	1517.9158	0.0359	24	66	79	NLLSVAYKNVIGAR					Mascot
2163.9573	2163.9719	0.0146	7	34	50	LAEQAERYEEMVEFMEK			Oxidation (M)[11,15]		Mascot
2331.2019	2331.269	0.0671	29	194	213	LGLALNFSVFYYEILNSPD R					Mascot

9 hypothetical protein CARUB_v10014407mg [Capsella rubella] gi|482567046 29888.1 4.82 13 177 100 27.176 116 100

Peptide Information

Calc. Mass	Obsrv. Mass	± da	± ppm	Start Seq.	End Seq.	Sequence	Ion Score	C. I. %	Modification	Rank	Result Type
816.421	816.4395	0.0185	23	14	20	LAEQAER					Mascot
907.5247	907.5403	0.0156	17	46	53	NLLSVAYK					Mascot
917.5302	917.5472	0.017	19	65	72	IISSIEQK					Mascot
1059.5793	1059.6219	0.0426	40	90	98	GKIETELNR					Mascot
1059.5793	1059.6219	0.0426	40	90	98	GKIETELNR					Mascot
1189.6609	1189.6909	0.03	25	219	228	DSTLIMQLLR					Mascot
1205.6559	1205.6827	0.0268	22	219	228	DSTLIMQLLR			Oxidation (M)[6]		Mascot
1205.6559	1205.6827	0.0268	22	219	228	DSTLIMQLLR	32	0	Oxidation (M)[6]		Mascot
1302.6802	1302.6305	-0.0497	-38	79	89	GDHVSIIKDYR					Mascot

Calc. Mass	Obsrv. Mass	± da	± ppm	Start Seq.	End Seq.	Sequence	Ion Score	C. I. %	Modification	Rank	Result Type
1366.5542	1366.5667	0.0125	9	21	30	YEEMVEFMEK			Oxidation (M)[4,8]		Mascot
1366.5542	1366.5667	0.0125	9	21	30	YEEMVEFMEK			Oxidation (M)[4,8]		Mascot
1382.691	1382.5564	-0.1346	-97	2	13	SSSREENVYLAK					Mascot
1406.6646	1406.7013	0.0367	26	34	45	TVDTDELTVEER					Mascot
1406.6646	1406.7013	0.0367	26	34	45	TVDTDELTVEER	84	100			Mascot
1418.7485	1418.7878	0.0393	28	65	76	IISSIEQKEESR					Mascot
1517.8799	1517.9158	0.0359	24	46	59	NLLSVAYKNVIGAR					Mascot
2163.9573	2163.9719	0.0146	7	14	30	LAEQAERYEEMVEFMEK			Oxidation (M)[11,15]		Mascot
2331.2019	2331.269	0.0671	29	174	193	LGLALNFSVFYYEILNSPDR					Mascot

10 FUSICOCCIN receptor protein s [Zea mays] gi|413918562 98275.5 6.45 15 175 100 35.012 133 100

Peptide Information

Calc. Mass	Obsrv. Mass	± da	± ppm	Start Seq.	End Seq.	Sequence	Ion Score	C. I. %	Modification	Rank	Result Type
816.421	816.4395	0.0185	23	17	23	LAEQAER					Mascot
907.5247	907.5403	0.0156	17	49	56	NLLSVAYK					Mascot
913.4308	913.499	0.0682	75	374	381	SSMAHPQR					Mascot
913.4308	913.499	0.0682	75	374	381	SSMAHPQR					Mascot
917.5302	917.5472	0.017	19	68	75	IISSIEQK					Mascot
932.4294	932.4494	0.02	21	130	136	MKGDYYR					Mascot
948.4244	948.4431	0.0187	20	130	136	MKGDYYR			Oxidation (M)[1]		Mascot
948.4244	948.4431	0.0187	20	130	136	MKGDYYR	15	0	Oxidation (M)[1]		Mascot
999.4451	999.4665	0.0214	21	9	16	EENVYMAK			Oxidation (M)[6]		Mascot
1189.6609	1189.6909	0.03	25	234	243	DSTLIMQLLR					Mascot
1205.6559	1205.6827	0.0268	22	234	243	DSTLIMQLLR			Oxidation (M)[6]		Mascot
1205.6559	1205.6827	0.0268	22	234	243	DSTLIMQLLR	32	0	Oxidation (M)[6]		Mascot
1212.5565	1212.6049	0.0484	40	161	171	DAAENTMVAYK					Mascot
1221.5819	1221.6818	0.0999	82	502	512	DTRSSQSPTSR					Mascot
1221.5819	1221.6818	0.0999	82	502	512	DTRSSQSPTSR	5	0			Mascot
1228.5514	1228.5693	0.0179	15	161	171	DAAENTMVAYK			Oxidation (M)[7]		Mascot
1356.6464	1356.6509	0.0045	3	160	171	KDAAENTMVAYK			Oxidation (M)[8]		Mascot
1366.5542	1366.5667	0.0125	9	24	33	YEEMVEFMEK			Oxidation (M)[4,8]		Mascot
1366.5542	1366.5667	0.0125	9	24	33	YEEMVEFMEK			Oxidation (M)[4,8]		Mascot
1406.6646	1406.7013	0.0367	26	37	48	TVDSEELTVEER					Mascot
1406.6646	1406.7013	0.0367	26	37	48	TVDSEELTVEER	102	100			Mascot
1517.8799	1517.9158	0.0359	24	49	62	NLLSVAYKNVIGAR					Mascot
2163.9573	2163.9719	0.0146	7	17	33	LAEQAERYEEMVEFMEK			Oxidation (M)[11,15]		Mascot

2331.2019	2331.269	0.0671	29	189	208	LGLALNFSVFYYEILNSPD R	Mascot

Gel Idx/Pos	172/G23	Instr./Gel Origin	BA2151/Sample Project 20140814	Process Status	Analysis Succeeded
Plate [#] Name	[1] Sample Project 20140814	Instrument Sample Name		Spectra	11

Rank	Protein Name	Accession No.	Protein MW	Protein PI	Pep. Count	Protein Score	Protein Score C. I. %	Intensity Matched	Total Ion Score	Total Ion C. I. %	Confirmed
1	14-3-3-like protein B [Triticum urartu]	gi|474253094	29786.9	4.67	12	206	100	7.351	153	100	

Protein Group

RecName: Full=14-3-3-like protein B; Short=14-3-3B	gi|2492487	29786.9	4.6700 000762 9395

Peptide Information

Calc. Mass	Obsrv. Mass	± da	± ppm	Start Seq.	End Seq.	Sequence	Ion Score	C. I. %	Modification	Rank	Result Type
816.421	816.4482	0.0272	33	18	24	LAEQAER					Mascot
818.444	818.468	0.024	29	103	109	ICDGILK			Carbamidomethyl (C)[2]		Mascot
917.5302	917.5295	-0.0007	-1	69	76	IISSIEQK					Mascot
948.4244	948.4635	0.0391	41	131	137	MKGDYYR			Oxidation (M)[1]		Mascot
1018.4985	1018.5401	0.0416	41	1	9	MAQPAELSR			Oxidation (M)[1]		Mascot
1189.6609	1189.7001	0.0392	33	223	232	DSTLIMQLLR					Mascot
1205.6559	1205.6904	0.0345	29	223	232	DSTLIMQLLR			Oxidation (M)[6]		Mascot
1212.5565	1212.6239	0.0674	56	150	160	DAAENTMVAYK					Mascot
1212.5565	1212.6239	0.0674	56	150	160	DAAENTMVAYK					Mascot
1228.5514	1228.6569	0.1055	86	150	160	DAAENTMVAYK			Oxidation (M)[7]		Mascot
1366.5542	1366.6586	0.1044	76	25	34	YEEMVEFMEK			Oxidation (M)[4,8]		Mascot
1406.6646	1406.7145	0.0499	35	38	49	TVDSEELTVEER					Mascot
1406.6646	1406.7145	0.0499	35	38	49	TVDSEELTVEER	49	97.546			Mascot
1418.7485	1418.7648	0.0163	11	69	80	IISSIEQKEESR					Mascot
1827.0123	1827.0712	0.0589	32	161	177	AAQEIALAELPPTHPIR					Mascot
1827.0123	1827.0712	0.0589	32	161	177	AAQEIALAELPPTHPIR	104	100			Mascot
2183.9648	2184.0884	0.1236	57	233	251	DNLTLWTSDISEDAAEEM K			Oxidation (M)[18]		Mascot

Rank	Protein Name	Accession No.	Protein MW	Protein PI	Pep. Count	Protein Score	Protein Score C. I. %	Intensity Matched	Total Ion Score	Total Ion C. I. %	Confirmed
2	hypothetical protein F775_15742 [Aegilops tauschii]	gi|475618593	21703	8.78	7	176	100	3.601	137	100	

Peptide Information

Calc. Mass	Obsrv. Mass	± da	± ppm	Start Seq.	End Seq.	Sequence	Ion Score	C. I. %	Modification	Rank	Result Type
834.3886	834.3536	-0.035	-42	32	38	MSQGQQR					Mascot

Calc. Mass	Obsrv. Mass	± da	± ppm	Start Seq.	End Seq.	Sequence	Ion Score	C. I. %	Modification	Rank	Result Type
850.3835	850.4597	0.0762	90	32	38	MSQGQQR			Oxidation (M)[1]		Mascot
858.425	858.4931	0.0681	79	101	109	GGPAAAMQR					Mascot
874.4199	874.4664	0.0465	53	101	109	GGPAAAMQR			Oxidation (M)[7]		Mascot
1201.6172	1201.6555	0.0383	32	110	121	SDAAAIQAAEVR					Mascot
1201.6172	1201.6555	0.0383	32	110	121	SDAAAIQAAEVR	50	98.326			Mascot
1467.8391	1467.8503	0.0112	8	193	208	TVRPGGIAASVAAAAR					Mascot
1501.7493	1501.8018	0.0525	35	167	180	LVADKEVEADDAAR					Mascot
2183.0298	2183.0886	0.0588	27	39	62	RPSSDQAQAGGGGQGQ GQGGGAVR					Mascot
2183.0298	2183.0886	0.0588	27	39	62	RPSSDQAQAGGGGQGQ GQGGGAVR	87	100			Mascot
2680.3438	2680.4312	0.0874	33	122	149	ATGLDVNIPGGLAAQAQS AADANAWAAR					Mascot

3	14-3-3 protein [Triticum aestivum]	gi|431822518	29843.9	4.71	12	98	99.986	4.922	49	97.546	

Protein Group

14-3-3-like protein B [Aegilops tauschii]	gi|475569230	29843.9	4.7100 000381 4697

Peptide Information

Calc. Mass	Obsrv. Mass	± da	± ppm	Start Seq.	End Seq.	Sequence	Ion Score	C. I. %	Modification	Rank	Result Type
816.421	816.4482	0.0272	33	18	24	LAEQAER					Mascot
818.444	818.468	0.024	29	103	109	ICDGILK			Carbamidomethyl (C)[2]		Mascot
917.5302	917.5295	-0.0007	-1	69	76	IISSIEQK					Mascot
948.4244	948.4635	0.0391	41	131	137	MKGDYYR			Oxidation (M)[1]		Mascot
1018.4985	1018.5401	0.0416	41	1	9	MAQPAELSR			Oxidation (M)[1]		Mascot
1059.5946	1059.6129	0.0183	17	169	177	ELPPTHPIR					Mascot
1189.6609	1189.7001	0.0392	33	223	232	DSTLIMQLLR					Mascot
1205.6559	1205.6904	0.0345	29	223	232	DSTLIMQLLR			Oxidation (M)[6]		Mascot
1212.5565	1212.6239	0.0674	56	150	160	DAAENTMVAYK					Mascot
1212.5565	1212.6239	0.0674	56	150	160	DAAENTMVAYK					Mascot
1228.5514	1228.6569	0.1055	86	150	160	DAAENTMVAYK			Oxidation (M)[7]		Mascot
1366.5542	1366.6586	0.1044	76	25	34	YEEMVEFMEK			Oxidation (M)[4,8]		Mascot
1406.6646	1406.7145	0.0499	35	38	49	TVDSEELTVEER					Mascot
1406.6646	1406.7145	0.0499	35	38	49	TVDSEELTVEER	49	97.546			Mascot
1418.7485	1418.7648	0.0163	11	69	80	IISSIEQKEESR					Mascot
2183.9648	2184.0884	0.1236	57	233	251	DNLTLWTSDISEDAAEEM K			Oxidation (M)[18]		Mascot

4	Os02g0580300 [Oryza sativa Japonica Group]	gi|113536765	29844.9	4.71	12	96	99.974	4.62	49	97.546	

Protein Group

RecName: Full=14-3-3-like protein GF14-E; AltName: Full=G-box factor 14-3-3 homolog E	gi|75290255	29844.9	4.7100 000381 4697
hypothetical protein OsI_07806 [Oryza sativa Indica Group]	gi|125540033	29844.9	4.7100 000381 4697

Peptide Information

Calc. Mass	Obsrv. Mass	± da	± ppm	Start Seq.	End Seq.	Sequence	Ion Score	C. I. %	Modification	Rank	Result Type
816.421	816.4482	0.0272	33	18	24	LAEQAER					Mascot
818.444	818.468	0.024	29	103	109	ICDGILK			Carbamidomethyl (C)[2]		Mascot
819.4458	819.4619	0.0161	20	96	102	IETELSK					Mascot
887.4581	887.4862	0.0281	32	2	9	SQPAELSR					Mascot
917.5302	917.5295	-0.0007	-1	69	76	IISSIEQK					Mascot
948.4244	948.4635	0.0391	41	131	137	MKGDYYR			Oxidation (M)[1]		Mascot
1018.4985	1018.5401	0.0416	41	1	9	MSQPAELSR					Mascot
1189.6609	1189.7001	0.0392	33	223	232	DSTLIMQLLR					Mascot
1205.6559	1205.6904	0.0345	29	223	232	DSTLIMQLLR			Oxidation (M)[6]		Mascot
1212.5565	1212.6239	0.0674	56	150	160	DAAENTMVAYK					Mascot
1212.5565	1212.6239	0.0674	56	150	160	DAAENTMVAYK					Mascot
1228.5514	1228.6569	0.1055	86	150	160	DAAENTMVAYK			Oxidation (M)[7]		Mascot
1366.5542	1366.6586	0.1044	76	25	34	YEEMVEFMEK			Oxidation (M)[4,8]		Mascot
1406.6646	1406.7145	0.0499	35	38	49	TVDSEELTVEER					Mascot
1406.6646	1406.7145	0.0499	35	38	49	TVDSEELTVEER	49	97.546			Mascot
1418.7485	1418.7648	0.0163	11	69	80	IISSIEQKEESR					Mascot

5	RecName: Full=Kinesin-like protein NACK2; AltName: Full=NPK1-activating kinesin 2	gi|75303645	108131.8	8.46	26	95	99.971	8.834	

Peptide Information

Calc. Mass	Obsrv. Mass	± da	± ppm	Start Seq.	End Seq.	Sequence	Ion Score	C. I. %	Modification	Rank	Result Type
802.4781	802.4758	-0.0023	-3	27	33	TTPSKIR					Mascot
812.4009	812.4586	0.0577	71	278	284	EGSHINR					Mascot
818.4254	818.468	0.0426	52	907	914	DSAEVVAK					Mascot
822.4104	822.4509	0.0405	49	96	102	VYEQGAR					Mascot
823.456	823.4778	0.0218	26	722	728	AYVTELK					Mascot
828.505	828.467	-0.038	-46	14	20	IVRTPSR					Mascot

829.4315	829.4429	0.0114	14	304	310	SGHIPYR					Mascot
864.4356	864.499	0.0634	73	296	303	LSCSGGKR			Carbamidomethyl (C)[3]		Mascot
870.5519	870.5682	0.0163	19	888	894	RLQLVNK					Mascot
913.4634	913.5018	0.0384	42	705	711	MQTMFKK					Mascot
977.5778	977.5485	-0.0293	-30	731	738	VAKLQYQK					Mascot
993.4595	993.5327	0.0732	74	266	275	ASQTSADGTR					Mascot
1201.6536	1201.6555	0.0019	2	379	388	EVSRLEAELR					Mascot
1201.6536	1201.6555	0.0019	2	379	388	EVSRLEAELR					Mascot
1211.6168	1211.6366	0.0198	16	877	886	WDIPPDGKQR					Mascot
1212.7198	1212.6239	-0.0959	-79	2	13	VIGTPVTTPLSK					Mascot
1212.7198	1212.6239	-0.0959	-79	2	13	VIGTPVTTPLSK					Mascot
1301.6696	1301.6438	-0.0258	-20	424	434	DLAQSQLELER					Mascot
1301.6696	1301.6438	-0.0258	-20	424	434	DLAQSQLELER	11	0			Mascot
1324.5872	1324.6782	0.091	69	412	421	MEEEMNELKR			Oxidation (M)[1]		Mascot
1324.5872	1324.6782	0.091	69	412	421	MEEEMNELKR			Oxidation (M)[1]		Mascot
1333.6594	1333.6329	-0.0265	-20	578	589	SQESNIASLEQK					Mascot
1472.7314	1472.7424	0.011	7	895	906	LWSDPLNMQNVR					Mascot
1619.8309	1619.8855	0.0546	34	356	370	EVTTTAQVNMVVAEK					Mascot
1635.8701	1635.8854	0.0153	9	132	146	GITESAVNDIYGRIK					Mascot
1909.0614	1908.8799	-0.1815	-95	278	294	EGSHINRSLLTVTNVIR					Mascot
2182.9856	2183.0886	0.103	47	596	614	SIDELVMHLPSCHESADSR			Carbamidomethyl (C)[12]		Mascot
2182.9856	2183.0886	0.103	47	596	614	SIDELVMHLPSCHESADSR			Carbamidomethyl (C)[12]		Mascot
2184.1594	2184.0884	-0.071	-33	889	906	LQLVNKLWSDPLNMQNVR			Oxidation (M)[14]		Mascot
2208.9543	2209.1475	0.1932	87	77	95	HTGPYSFDYVFDPTCSTSK			Carbamidomethyl (C)[15]		Mascot
2680.3538	2680.4312	0.0774	29	250	275	SFLATLNLVDLAGSERASQTSADGTR					Mascot

6	14-3-3-like protein GF14-B [Triticum urartu]	gi|474147722	30043.1	4.69	11	92	99.941	4.588	49	97.546	

Protein Group

14-3-3-like protein GF14-B [Aegilops tauschii]	gi|475549223	30043.1	4.6900000572046

Peptide Information

Calc. Mass	Obsrv. Mass	± da	± ppm	Start Seq.	End Seq.	Sequence	Ion Score	C. I. %	Modification	Rank	Result Type
816.421	816.4482	0.0272	33	18	24	LAEQAER					Mascot
818.444	818.468	0.024	29	103	109	ICDGILK			Carbamidomethyl (C)[2]		Mascot

Calc. Mass	Obsrv. Mass	± da	± ppm	Start Seq.	End Seq.	Sequence	Ion Score	C. I. %	Modification	Rank	Result Type
819.4458	819.4619	0.0161	20	96	102	IETELSK					Mascot
917.5302	917.5295	-0.0007	-1	69	76	IISSIEQK					Mascot
948.4244	948.4635	0.0391	41	131	137	MKGDYYR			Oxidation (M)[1]		Mascot
1189.6609	1189.7001	0.0392	33	223	232	DSTLIMQLLR					Mascot
1205.6559	1205.6904	0.0345	29	223	232	DSTLIMQLLR			Oxidation (M)[6]		Mascot
1212.5565	1212.6239	0.0674	56	150	160	DAAENTMVAYK					Mascot
1212.5565	1212.6239	0.0674	56	150	160	DAAENTMVAYK					Mascot
1228.5514	1228.6569	0.1055	86	150	160	DAAENTMVAYK			Oxidation (M)[7]		Mascot
1366.5542	1366.6586	0.1044	76	25	34	YEEMVEFMEK			Oxidation (M)[4,8]		Mascot
1406.6646	1406.7145	0.0499	35	38	49	TVDSEELTVEER					Mascot
1406.6646	1406.7145	0.0499	35	38	49	TVDSEELTVEER	49	97.546			Mascot
1418.7485	1418.7648	0.0163	11	69	80	IISSIEQKEESR					Mascot
1786.9811	1787.038	0.0569	32	161	177	AAQDIALAELAPTHPIR					Mascot

7	14-3-3-like protein GF14-12 [Zea mays]	gi|413918561	29724.9	4.75	11	92	99.94	4.589	49	97.546	

Peptide Information

Calc. Mass	Obsrv. Mass	± da	± ppm	Start Seq.	End Seq.	Sequence	Ion Score	C. I. %	Modification	Rank	Result Type
816.421	816.4482	0.0272	33	17	23	LAEQAER					Mascot
818.444	818.468	0.024	29	102	108	ICDGILK			Carbamidomethyl (C)[2]		Mascot
864.4243	864.499	0.0747	86	1	8	MASAELSR					Mascot
917.5302	917.5295	-0.0007	-1	68	75	IISSIEQK					Mascot
948.4244	948.4635	0.0391	41	130	136	MKGDYYR			Oxidation (M)[1]		Mascot
1018.5779	1018.5401	-0.0378	-37	93	101	GKIETELTK					Mascot
1189.6609	1189.7001	0.0392	33	222	231	DSTLIMQLLR					Mascot
1205.6559	1205.6904	0.0345	29	222	231	DSTLIMQLLR			Oxidation (M)[6]		Mascot
1212.5565	1212.6239	0.0674	56	149	159	DAAENTMVAYK					Mascot
1212.5565	1212.6239	0.0674	56	149	159	DAAENTMVAYK					Mascot
1228.5514	1228.6569	0.1055	86	149	159	DAAENTMVAYK			Oxidation (M)[7]		Mascot
1366.5542	1366.6586	0.1044	76	24	33	YEEMVEFMEK			Oxidation (M)[4,8]		Mascot
1406.6646	1406.7145	0.0499	35	37	48	TVDSEELTVEER					Mascot
1406.6646	1406.7145	0.0499	35	37	48	TVDSEELTVEER	49	97.546			Mascot
1786.9811	1787.038	0.0569	32	160	176	AAQDIALAELAPTHPIR					Mascot

8	14-3-3-like protein GF14-6 [Zea mays]	gi|262359935	29758	4.76	11	91	99.921	4.589	49	97.546	

Protein Group

RecName: Full=14-3-3-like protein GF14-6	gi|1345587	29758	4.7600

			002288 8184
TPA: general regulatory factor1 isoform 1 [Zea mays]	gi|414586860	29758	4.7600 002288 8184
TPA: general regulatory factor1 isoform 2 [Zea mays]	gi|414586861	29758	4.7600 002288 8184
hypothetical protein SORBIDRAFT_06g019100 [Sorghum bicolor]	gi|241937809	29744	4.7600 002288 8184

Peptide Information

Calc. Mass	Obsrv. Mass	± da	± ppm	Start Seq.	End Seq.	Sequence	Ion Score	C. I. %	Modification	Rank	Result Type
816.421	816.4482	0.0272	33	17	23	LAEQAER					Mascot
818.444	818.468	0.024	29	102	108	ICDGILK			Carbamidomethyl (C)[2]		Mascot
864.4243	864.499	0.0747	86	1	8	MASAELSR					Mascot
917.5302	917.5295	-0.0007	-1	68	75	IISSIEQK					Mascot
948.4244	948.4635	0.0391	41	130	136	MKGDYYR			Oxidation (M)[1]		Mascot
1018.5779	1018.5401	-0.0378	-37	93	101	GKIETELTK					Mascot
1189.6609	1189.7001	0.0392	33	222	231	DSTLIMQLLR					Mascot
1205.6559	1205.6904	0.0345	29	222	231	DSTLIMQLLR			Oxidation (M)[6]		Mascot
1212.5565	1212.6239	0.0674	56	149	159	DAAENTMVAYK					Mascot
1212.5565	1212.6239	0.0674	56	149	159	DAAENTMVAYK					Mascot
1228.5514	1228.6569	0.1055	86	149	159	DAAENTMVAYK			Oxidation (M)[7]		Mascot
1366.5542	1366.6586	0.1044	76	24	33	YEEMVEFMEK			Oxidation (M)[4,8]		Mascot
1406.6646	1406.7145	0.0499	35	37	48	TVDSEELTVEER					Mascot
1406.6646	1406.7145	0.0499	35	37	48	TVDSEELTVEER	49	97.546			Mascot
1786.9811	1787.038	0.0569	32	160	176	AAQDIALAELAPTHPIR					Mascot

9	TPA: general regulatory factor1 [Zea mays]	gi|414586863	31835.1	4.9	11	88	99.838	4.589	49	97.546	

Peptide Information

Calc. Mass	Obsrv. Mass	± da	± ppm	Start Seq.	End Seq.	Sequence	Ion Score	C. I. %	Modification	Rank	Result Type
816.421	816.4482	0.0272	33	34	40	LAEQAER					Mascot
818.444	818.468	0.024	29	119	125	ICDGILK			Carbamidomethyl (C)[2]		Mascot
864.4243	864.499	0.0747	86	18	25	MASAELSR					Mascot
917.5302	917.5295	-0.0007	-1	85	92	IISSIEQK					Mascot

948.4244	948.4635	0.0391	41	147	153	MKGDYYR			Oxidation (M)[1]		Mascot
1018.5779	1018.5401	-0.0378	-37	110	118	GKIETELTK					Mascot
1189.6609	1189.7001	0.0392	33	239	248	DSTLIMQLLR					Mascot
1205.6559	1205.6904	0.0345	29	239	248	DSTLIMQLLR			Oxidation (M)[6]		Mascot
1212.5565	1212.6239	0.0674	56	166	176	DAAENTMVAYK					Mascot
1212.5565	1212.6239	0.0674	56	166	176	DAAENTMVAYK					Mascot
1228.5514	1228.6569	0.1055	86	166	176	DAAENTMVAYK			Oxidation (M)[7]		Mascot
1366.5542	1366.6586	0.1044	76	41	50	YEEMVEFMEK			Oxidation (M)[4,8]		Mascot
1406.6646	1406.7145	0.0499	35	54	65	TVDSEELTVEER					Mascot
1406.6646	1406.7145	0.0499	35	54	65	TVDSEELTVEER	49	97.546			Mascot
1786.9811	1787.038	0.0569	32	177	193	AAQDIALAELAPTHPIR					Mascot

10 FUSICOCCIN receptor protein s [Zea mays] gi|413918562 98275.5 6.45 17 87 99.806 7.517 49 97.546

Peptide Information

Calc. Mass	Obsrv. Mass	± da	± ppm	Start Seq.	End Seq.	Sequence	Ion Score	C. I. %	Modification	Rank	Result Type
816.421	816.4482	0.0272	33	17	23	LAEQAER					Mascot
818.444	818.468	0.024	29	102	108	ICDGILK			Carbamidomethyl (C)[2]		Mascot
828.5189	828.467	-0.0519	-63	734	741	GVVVVLDK					Mascot
839.4774	839.4791	0.0017	2	727	733	GFKFNVK					Mascot
849.4061	849.4724	0.0663	78	505	512	SSQSPTSR					Mascot
864.4243	864.499	0.0747	86	1	8	MASAELSR					Mascot
913.4308	913.5018	0.071	78	374	381	SSMAHPQR					Mascot
917.5302	917.5295	-0.0007	-1	68	75	IISSIEQK					Mascot
948.4244	948.4635	0.0391	41	130	136	MKGDYYR			Oxidation (M)[1]		Mascot
1018.5779	1018.5401	-0.0378	-37	93	101	GKIETELTK					Mascot
1189.6609	1189.7001	0.0392	33	234	243	DSTLIMQLLR					Mascot
1205.6559	1205.6904	0.0345	29	234	243	DSTLIMQLLR			Oxidation (M)[6]		Mascot
1212.5565	1212.6239	0.0674	56	161	171	DAAENTMVAYK					Mascot
1212.5565	1212.6239	0.0674	56	161	171	DAAENTMVAYK					Mascot
1228.5514	1228.6569	0.1055	86	161	171	DAAENTMVAYK			Oxidation (M)[7]		Mascot
1366.5542	1366.6586	0.1044	76	24	33	YEEMVEFMEK			Oxidation (M)[4,8]		Mascot
1406.6646	1406.7145	0.0499	35	37	48	TVDSEELTVEER					Mascot
1406.6646	1406.7145	0.0499	35	37	48	TVDSEELTVEER	49	97.546			Mascot
1558.7393	1558.7932	0.0539	35	137	148	YYDCMNPIVVIR			Carbamidomethyl (C)[4], Oxidation (M)[5]		Mascot
1786.9811	1787.038	0.0569	32	172	188	AAQDIALAELAPTHPIR					Mascot

2117.106	2117.1597	0.0537	25	533	550	SSLLERMHVLDEANYLVK	Mascot
2117.106	2117.1597	0.0537	25	533	550	SSLLERMHVLDEANYLVK	Mascot

Gel Idx/Pos	173/G24	Instr./Gel Origin	BA2151/Sample Project 20140814	Process Status	Analysis Succeeded
Plate [#] Name	[1] Sample Project 20140814	Instrument Sample Name		Spectra	11

Rank	Protein Name	Accession No.	Protein MW	Protein PI	Pep. Count	Protein Score	Protein Score C. I. %	Intensity Matched	Total Ion Score	Total Ion C. I. %	Confirmed
1	Alpha-soluble NSF attachment protein [Aegilops tauschii]	gi|475620929	35156.5	4.96	18	757	100	49.687	636	100	

Peptide Information

Calc. Mass	Obsrv. Mass	± da	± ppm	Start Seq.	End Seq.	Sequence	Ion Score	C. I. %	Modification	Rank	Result Type
853.405	853.4409	0.0359	42	155	161	ASDYLER					Mascot
855.4505	855.4409	-0.0096	-11	83	89	IANCHLK			Carbamidomethyl (C)[4]		Mascot
871.3615	871.397	0.0355	41	280	286	EFDGMTR			Oxidation (M)[5]		Mascot
1051.5571	1051.5884	0.0313	30	19	28	LSGWGLFGSK					Mascot
1178.6052	1178.6536	0.0484	41	195	204	ATEIFEEIAR					Mascot
1178.6052	1178.6536	0.0484	41	195	204	ATEIFEEIAR	86	100			Mascot
1202.5212	1202.5748	0.0536	45	29	38	YEDAADLYDK					Mascot
1286.6699	1286.7242	0.0543	42	231	242	ADAVAIQNSLER					Mascot
1286.6699	1286.7242	0.0543	42	231	242	ADAVAIQNSLER	97	100			Mascot
1413.6646	1413.7217	0.0571	40	243	254	YQEIDPTFSGTR					Mascot
1413.6646	1413.7217	0.0571	40	243	254	YQEIDPTFSGTR	112	100			Mascot
1459.7792	1459.8174	0.0382	26	182	194	VAEIAAQLEQYPK					Mascot
1459.7792	1459.8174	0.0382	26	182	194	VAEIAAQLEQYPK	73	99.99			Mascot
1487.7821	1487.844	0.0619	42	218	230	GILLNAGICQLCR			Carbamidomethyl (C)[9,12]		Mascot
1487.7821	1487.844	0.0619	42	218	230	GILLNAGICQLCR	96	100	Carbamidomethyl (C)[9,12]		Mascot
1530.7257	1530.813	0.0873	57	274	286	FTDAIKEFDGMTR					Mascot
1546.7207	1546.7697	0.049	32	274	286	FTDAIKEFDGMTR			Oxidation (M)[11]		Mascot
1620.7786	1620.8264	0.0478	29	258	273	LLADLAASMDDGDVAK			Oxidation (M)[9]		Mascot
1683.7432	1683.7976	0.0544	32	94	108	HEAASAYVEAANCYK			Carbamidomethyl (C)[13]		Mascot
1707.8073	1707.8562	0.0489	29	141	154	DIGEIYQQEQDLEK	52	98.826			Mascot
1811.8381	1811.9164	0.0783	43	94	109	HEAASAYVEAANCYKK			Carbamidomethyl (C)[13]		Mascot
1911.8931	1911.9473	0.0542	28	162	179	AADLFDSEGQTSQSNTIK					Mascot
1911.8931	1911.9473	0.0542	28	162	179	AADLFDSEGQTSQSNTIK	120	100			Mascot
2070.9185	2070.9846	0.0661	32	90	108	GDSKHEAASAYVEAANCYK			Carbamidomethyl (C)[17]		Mascot
2417.2458	2417.3477	0.1019	42	110	131	FSPQEAAQALDQAVNLFLEIGR					Mascot

Rank	Protein Name	Accession No.	Protein MW	Protein PI	Pep. Count	Protein Score	Protein Score C. I. %	Intensity Matched	Total Ion Score	Total Ion C. I. %	Confirmed
2	Alpha-soluble NSF attachment protein [Triticum urartu]	gi|474212649	42706.3	6.36	18	738	100	49.687	636	100	

Peptide Information

Calc. Mass	Obsrv. Mass	± da	± ppm	Start Seq.	End Seq.	Sequence	Ion Score	C. I. %	Modification	Rank	Result Type
853.405	853.4409	0.0359	42	220	226	ASDYLER					Mascot
855.4505	855.4409	-0.0096	-11	148	154	IANCHLK			Carbamidomethyl (C)[4]		Mascot
871.3615	871.397	0.0355	41	345	351	EFDGMTR			Oxidation (M)[5]		Mascot
1051.5571	1051.5884	0.0313	30	19	28	LSGWGLFGSK					Mascot
1178.6052	1178.6536	0.0484	41	260	269	ATEIFEEIAR					Mascot
1178.6052	1178.6536	0.0484	41	260	269	ATEIFEEIAR	86	100			Mascot
1202.5212	1202.5748	0.0536	45	29	38	YEDAADLYDK					Mascot
1286.6699	1286.7242	0.0543	42	296	307	ADAVAIQNSLER					Mascot
1286.6699	1286.7242	0.0543	42	296	307	ADAVAIQNSLER	97	100			Mascot
1413.6646	1413.7217	0.0571	40	308	319	YQEIDPTFSGTR					Mascot
1413.6646	1413.7217	0.0571	40	308	319	YQEIDPTFSGTR	112	100			Mascot
1459.7792	1459.8174	0.0382	26	247	259	VAEIAAQLEQYPK					Mascot
1459.7792	1459.8174	0.0382	26	247	259	VAEIAAQLEQYPK	73	99.99			Mascot
1487.7821	1487.844	0.0619	42	283	295	GILLNAGICQLCR			Carbamidomethyl (C)[9,12]		Mascot
1487.7821	1487.844	0.0619	42	283	295	GILLNAGICQLCR	96	100	Carbamidomethyl (C)[9,12]		Mascot
1530.7257	1530.813	0.0873	57	339	351	FTDAIKEFDGMTR					Mascot
1546.7207	1546.7697	0.049	32	339	351	FTDAIKEFDGMTR			Oxidation (M)[11]		Mascot
1620.7786	1620.8264	0.0478	29	323	338	LLADLAASMDDGDVAK			Oxidation (M)[9]		Mascot
1683.7432	1683.7976	0.0544	32	159	173	HEAASAYVEAANCYK			Carbamidomethyl (C)[13]		Mascot
1707.8073	1707.8562	0.0489	29	206	219	DIGEIYQQEQDLEK	52	98.826			Mascot
1811.8381	1811.9164	0.0783	43	159	174	HEAASAYVEAANCYKK			Carbamidomethyl (C)[13]		Mascot
1911.8931	1911.9473	0.0542	28	227	244	AADLFDSEGQTSQSNTIK					Mascot
1911.8931	1911.9473	0.0542	28	227	244	AADLFDSEGQTSQSNTIK	120	100			Mascot
2070.9185	2070.9846	0.0661	32	155	173	GDSKHEAASAYVEAANC YK			Carbamidomethyl (C)[17]		Mascot
2417.2458	2417.3477	0.1019	42	175	196	FSPQEAAQALDQAVNLFL EIGR					Mascot

3	alpha-soluble NSF attachment protein isoform 2 [Zea mays]	gi|413921832	32605.3	5.04	11	389	100	26.445	333	100		

Protein Group

alpha-soluble NSF attachment protein [Zea mays]	gi|226529563	32605.3	5.0399 999618 5303
alpha-soluble NSF attachment protein isoform 1 [Zea mays]	gi|413921831	32605.3	5.0399 999618

Peptide Information

Calc. Mass	Obsrv. Mass	± da	± ppm	Start Seq.	End Seq.	Sequence	Ion Score	C. I. %	Modification	Rank	Result Type
855.4505	855.4409	-0.0096	-11	59	65	IANCHLK			Carbamidomethyl (C)[4]		Mascot
1051.5571	1051.5884	0.0313	30	19	28	LSGWGLFGSK					Mascot
1413.6646	1413.7217	0.0571	40	219	230	YQEIDPTFSGTR					Mascot
1413.6646	1413.7217	0.0571	40	219	230	YQEIDPTFSGTR	112	100			Mascot
1459.7792	1459.8174	0.0382	26	158	170	VAEIAAQLEQYPK					Mascot
1459.7792	1459.8174	0.0382	26	158	170	VAEIAAQLEQYPK	73	99.99			Mascot
1487.7821	1487.844	0.0619	42	194	206	GILLNAGICQLCR			Carbamidomethyl (C)[9,12]		Mascot
1487.7821	1487.844	0.0619	42	194	206	GILLNAGICQLCR	96	100	Carbamidomethyl (C)[9,12]		Mascot
1560.7363	1560.7832	0.0469	30	250	262	FTDAIKEFDSMTR					Mascot
1683.7432	1683.7976	0.0544	32	70	84	HEAASAYVEAANCYK			Carbamidomethyl (C)[13]		Mascot
1707.8073	1707.8562	0.0489	29	117	130	DIGEIYQQEQDLEK	52	98.826			Mascot
1811.8381	1811.9164	0.0783	43	70	85	HEAASAYVEAANCYKK			Carbamidomethyl (C)[13]		Mascot
2070.9185	2070.9846	0.0661	32	66	84	GDSKHEAASAYVEAANC YK			Carbamidomethyl (C)[17]		Mascot
2219.0657	2219.1252	0.0595	27	19	38	LSGWGLFGSKYEDAADL FDK					Mascot

4	hypothetical protein SORBIDRAFT_07g018430 [Sorghum bicolor]	gi|241940600	32649.3	5.04	9	373	100	26.056	333	100	

Protein Group

PREDICTED: alpha-soluble NSF attachment protein-like [Setaria italica]	gi|514794867	32573.3	5.0399 999618 5303

Peptide Information

Calc. Mass	Obsrv. Mass	± da	± ppm	Start Seq.	End Seq.	Sequence	Ion Score	C. I. %	Modification	Rank	Result Type
855.4505	855.4409	-0.0096	-11	59	65	IANCHLK			Carbamidomethyl (C)[4]		Mascot
1413.6646	1413.7217	0.0571	40	219	230	YQEIDPTFSGTR					Mascot
1413.6646	1413.7217	0.0571	40	219	230	YQEIDPTFSGTR	112	100			Mascot
1459.7792	1459.8174	0.0382	26	158	170	VAEIAAQLEQYPK					Mascot
1459.7792	1459.8174	0.0382	26	158	170	VAEIAAQLEQYPK	73	99.99			Mascot
1487.7821	1487.844	0.0619	42	194	206	GILLNAGICQLCR			Carbamidomethyl (C)[9,12]		Mascot
1487.7821	1487.844	0.0619	42	194	206	GILLNAGICQLCR	96	100	Carbamidomethyl (C)[9,12]		Mascot
1560.7363	1560.7832	0.0469	30	250	262	FTDAIKEFDSMTR					Mascot
1683.7432	1683.7976	0.0544	32	70	84	HEAASAYVEAANCYK			Carbamidomethyl (C)[13]		Mascot

1707.8073	1707.8562	0.0489	29	117	130	DIGEIYQQEQDLEK	52	98.826			Mascot
1811.8381	1811.9164	0.0783	43	70	85	HEAASAYVEAANCYKK			Carbamidomethyl (C)[13]		Mascot
2070.9185	2070.9846	0.0661	32	66	84	GDSKHEAASAYVEAANC YK			Carbamidomethyl (C)[17]		Mascot

5	alpha-soluble NSF attachment protein [Elaeis guineensis]	gi|192912984	32899.4	5.07	7	232	100	24.156	208	100

Peptide Information

Calc. Mass	Obsrv. Mass	± da	± ppm	Start Seq.	End Seq.	Sequence	Ion Score	C. I. %	Modification	Rank	Result Type
1051.5571	1051.5884	0.0313	30	19	28	LSGWGLFGSK					Mascot
1132.6361	1132.574	-0.0621	-55	171	180	AIEIFEAIAR					Mascot
1413.6646	1413.7217	0.0571	40	219	230	YQELDPTFSGTR					Mascot
1413.6646	1413.7217	0.0571	40	219	230	YQELDPTFSGTR	112	100			Mascot
1487.7821	1487.844	0.0619	42	194	206	GILLNAGICQLCR			Carbamidomethyl (C)[9,12]		Mascot
1487.7821	1487.844	0.0619	42	194	206	GILLNAGICQLCR	96	100	Carbamidomethyl (C)[9,12]		Mascot
1530.8461	1530.813	-0.0331	-22	52	65	AGAVYIKLASCHLK			Carbamidomethyl (C)[11]		Mascot
1547.7999	1547.7852	-0.0147	-9	181	193	HSMNNNLLKYSVK					Mascot
1620.7574	1620.8264	0.069	43	250	262	FTDVIKEYDSMTR			Oxidation (M)[11]		Mascot

6	alpha-soluble NSF attachment protein [Elaeis guineensis]	gi|192912986	32933.4	5	6	228	100	24.543	208	100

Peptide Information

Calc. Mass	Obsrv. Mass	± da	± ppm	Start Seq.	End Seq.	Sequence	Ion Score	C. I. %	Modification	Rank	Result Type
855.4505	855.4409	-0.0096	-11	59	65	LANCHLK			Carbamidomethyl (C)[4]		Mascot
1202.5212	1202.5748	0.0536	45	29	38	YEDAADLYDK					Mascot
1206.663	1206.6838	0.0208	17	18	28	KLNGWGLFGSK					Mascot
1413.6646	1413.7217	0.0571	40	219	230	YQELDPTFSGTR					Mascot
1413.6646	1413.7217	0.0571	40	219	230	YQELDPTFSGTR	112	100			Mascot
1487.7821	1487.844	0.0619	42	194	206	GILLNAGICQLCR			Carbamidomethyl (C)[9,12]		Mascot
1487.7821	1487.844	0.0619	42	194	206	GILLNAGICQLCR	96	100	Carbamidomethyl (C)[9,12]		Mascot
1620.7574	1620.8264	0.069	43	250	262	FTDVIKEYDSMTR			Oxidation (M)[11]		Mascot

7	PREDICTED: alpha-soluble NSF attachment protein-like [Setaria italica]	gi|514815509	32714.3	5.01	6	202	100	26.366	183	100

Peptide Information

Calc. Mass	Obsrv. Mass	± da	± ppm	Start Seq.	End Seq.	Sequence	Ion Score	C. I. %	Modification	Rank	Result Type

Calc. Mass	Obsrv. Mass	± da	± ppm	Start Seq.	End Seq.	Sequence	Ion Score	C. I. %	Modification	Rank	Result Type
1178.6052	1178.6536	0.0484	41	171	180	ATELFEEIAR					Mascot
1178.6052	1178.6536	0.0484	41	171	180	ATELFEEIAR	86	100			Mascot
1211.6321	1211.7211	0.089	73	19	28	LSGWGFFRNK					Mascot
1411.7118	1411.7406	0.0288	20	15	26	ADQKLSGWGFFR					Mascot
1487.7821	1487.844	0.0619	42	194	206	GILLNAGICQLCR			Carbamidomethyl (C)[9,12]		Mascot
1487.7821	1487.844	0.0619	42	194	206	GILLNAGICQLCR	96	100	Carbamidomethyl (C)[9,12]		Mascot
1683.7432	1683.7976	0.0544	32	70	84	HEAASAYVEAANCYK			Carbamidomethyl (C)[13]		Mascot
1811.8381	1811.9164	0.0783	43	70	85	HEAASAYVEAANCYKK			Carbamidomethyl (C)[13]		Mascot

8	RecName: Full=Actin	gi|461465	41940	5.46	14	158	100	7.987	88	100	

Peptide Information

Calc. Mass	Obsrv. Mass	± da	± ppm	Start Seq.	End Seq.	Sequence	Ion Score	C. I. %	Modification	Rank	Result Type
976.4483	976.4879	0.0396	41	21	30	AGFAGDDAPR					Mascot
1132.527	1132.574	0.047	42	199	208	GYSFTTTAER					Mascot
1182.5273	1182.6241	0.0968	82	53	63	DAYVGDEAQSK					Mascot
1198.7056	1198.7493	0.0437	36	31	41	AVFPSIVGRPR					Mascot
1459.6813	1459.8174	0.1361	93	362	374	AEYDESGPSIVHR					Mascot
1459.6813	1459.8174	0.1361	93	362	374	AEYDESGPSIVHR					Mascot
1493.7703	1493.8156	0.0453	30	315	328	MSKEITALAPSSMK					Mascot
1509.7651	1509.8357	0.0706	47	315	328	MSKEITALAPSSMK			Oxidation (M)[1]		Mascot
1515.7491	1515.8109	0.0618	41	87	97	IWHHTFYNELR					Mascot
1525.76	1525.7906	0.0306	20	315	328	MSKEITALAPSSMK			Oxidation (M)[1,13]		Mascot
1600.8363	1600.8148	-0.0215	-13	186	198	DLTDHLMKILTER			Oxidation (M)[7]		Mascot
1620.8414	1620.8264	-0.015	-9	299	314	IVLSGGSTMFPGIADR					Mascot
1747.8861	1747.9551	0.069	39	241	256	SYELPDGQVITIGAER					Mascot
1747.8861	1747.9551	0.069	39	241	256	SYELPDGQVITIGAER	88	100			Mascot
1919.8943	1920.0375	0.1432	75	218	233	LSYIALDFEQEMETSK			Oxidation (M)[12]		Mascot
1948.8746	1948.9633	0.0887	46	71	86	YPIEHGIVSNWDDMEK			Oxidation (M)[14]		Mascot
1954.0645	1954.1154	0.0509	26	98	115	VAPEEHPVLLTEAPLNPK					Mascot
3151.6423	3151.7866	0.1443	46	150	179	TTGIVLDSGDGVSHTVPI YEGYALPHAILR					Mascot

9	actin, partial [Hemarthria compressa]	gi|405132025	28611.6	5.56	11	150	100	6.54	88	100	

Peptide Information

Calc. Mass	Obsrv. Mass	± da	± ppm	Start Seq.	End Seq.	Sequence	Ion Score	C. I. %	Modification	Rank	Result Type

853.4236	853.4409	0.0173	20	2	9	FNGTGMVK					Mascot
976.4483	976.4879	0.0396	41	10	19	AGFAGDDAPR					Mascot
1132.527	1132.574	0.047	42	188	197	GYSFTTTAER					Mascot
1182.5273	1182.6241	0.0968	82	42	52	DAYVGDEAQSK					Mascot
1198.7056	1198.7493	0.0437	36	20	30	AVFPSIVGRPR					Mascot
1515.7491	1515.8109	0.0618	41	76	86	IWHHTFYNELR					Mascot
1547.8098	1547.7852	-0.0246	-16	169	182	LDLAGRDLTDSLMK					Mascot
1747.8861	1747.9551	0.069	39	230	245	SYELPDGQVITIGAER					Mascot
1747.8861	1747.9551	0.069	39	230	245	SYELPDGQVITIGAER	88	100			Mascot
1948.8746	1948.9633	0.0887	46	60	75	YPIEHGIVSNWDDMEK			Oxidation (M)[14]		Mascot
1954.0645	1954.1154	0.0509	26	87	104	VAPEEHPVLLTEAPLNPK					Mascot
3151.6423	3151.7866	0.1443	46	139	168	TTGIVLDSGDGVSHTVPI YEGYALPHAILR					Mascot

10	RecName: Full=Actin-58	gi|231496	41987.2	5.46	13	150	100	7.226	88	100

Peptide Information

Calc. Mass	Obsrv. Mass	± da	± ppm	Start Seq.	End Seq.	Sequence	Ion Score	C. I. %	Modification	Rank	Result Type
976.4483	976.4879	0.0396	41	21	30	AGFAGDDAPR					Mascot
1132.527	1132.574	0.047	42	199	208	GYSFTTTAER					Mascot
1182.5273	1182.6241	0.0968	82	53	63	DAYVGDEAQSK					Mascot
1459.6813	1459.8174	0.1361	93	362	374	AEYDESGPSIVHR					Mascot
1459.6813	1459.8174	0.1361	93	362	374	AEYDESGPSIVHR					Mascot
1493.7703	1493.8156	0.0453	30	315	328	MSKELTALAPSSMK					Mascot
1509.7651	1509.8357	0.0706	47	315	328	MSKELTALAPSSMK			Oxidation (M)[1]		Mascot
1515.7491	1515.8109	0.0618	41	87	97	IWHHTFYNELR					Mascot
1525.76	1525.7906	0.0306	20	315	328	MSKELTALAPSSMK			Oxidation (M)[1,13]		Mascot
1546.7761	1546.7697	-0.0064	-4	21	35	AGFAGDDAPRAVFPR					Mascot
1600.8363	1600.8148	-0.0215	-13	186	198	DLTDHLMKILTER			Oxidation (M)[7]		Mascot
1747.8861	1747.9551	0.069	39	241	256	SYELPDGQVITIGAER					Mascot
1747.8861	1747.9551	0.069	39	241	256	SYELPDGQVITIGAER	88	100			Mascot
1822.9891	1822.9315	-0.0576	-32	36	52	IVGRPRHTGVMVGMGQK					Mascot
1948.8746	1948.9633	0.0887	46	71	86	YPIEHGIVSNWDDMEK			Oxidation (M)[14]		Mascot
1954.0645	1954.1154	0.0509	26	98	115	VAPEEHPVLLTEAPLNPK					Mascot
3151.6423	3151.7866	0.1443	46	150	179	TTGIVLDSGDGVSHTVPI YEGYALPHAILR					Mascot

Gel Idx/Pos	174/H1	Instr./Gel Origin	BA2151/Sample Project 20140814	Process Status	Analysis Succeeded
Plate [#] Name	[1] Sample Project 20140814	Instrument Sample Name		Spectra	11

Rank	Protein Name	Accession No.	Protein MW	Protein PI	Pep. Count	Protein Score	Protein Score C. I. %	Intensity Matched	Total Ion Score	Total Ion C. I. %	Confirmed
1	Spermidine synthase 1 [Triticum urartu]	gi|473890074	35788.8	4.99	11	787	100	49.629	727	100	

Peptide Information

Calc. Mass	Obsrv. Mass	± da	± ppm	Start Seq.	End Seq.	Sequence	Ion Score	C. I. %	Modification	Rank	Result Type
850.3975	850.4307	0.0332	39	1	8	MEAETAAK					Mascot
1211.7107	1211.7185	0.0078	6	112	124	VLVIGGGDGGVLR					Mascot
1211.7107	1211.7185	0.0078	6	112	124	VLVIGGGDGGVLR	126	100			Mascot
1339.8057	1339.8129	0.0072	5	111	124	KVLVIGGGDGGVLR					Mascot
1339.8057	1339.8129	0.0072	5	111	124	KVLVIGGGDGGVLR	94	100			Mascot
1355.7682	1355.7615	-0.0067	-5	164	176	VSLHIGDGVAFLK					Mascot
1355.7682	1355.7615	-0.0067	-5	164	176	VSLHIGDGVAFLK	90	100			Mascot
1440.8422	1440.8477	0.0055	4	77	89	VLVLDGVIQVTER					Mascot
1440.8422	1440.8477	0.0055	4	77	89	VLVLDGVIQVTER	92	100			Mascot
1672.7847	1672.7766	-0.0081	-5	129	142	HSSVEQIDICEIDK			Carbamidomethyl (C)[10]		Mascot
1673.8435	1673.8464	0.0029	2	150	163	QFFPHLALGFEDPR					Mascot
1673.8435	1673.8464	0.0029	2	150	163	QFFPHLALGFEDPR	79	99.998			Mascot
1835.8811	1835.8411	-0.04	-22	61	76	SDYQNVLVFQSSTYGK					Mascot
2180.9858	2181.0007	0.0149	7	299	316	FYNSEFHTASFCLPSFAR			Carbamidomethyl (C)[12]		Mascot
2180.9858	2181.0007	0.0149	7	299	316	FYNSEFHTASFCLPSFAR	121	100	Carbamidomethyl (C)[12]		Mascot
2447.1792	2447.1609	-0.0183	-7	129	149	HSSVEQIDICEIDKMVVD VSK			Carbamidomethyl (C)[10], Oxidation (M)[15]		Mascot
3797.8181	3797.9089	0.0908	24	177	211	NAPEGTYDAVIVDSSDPV GPAQELFEKPFFESVSR					Mascot
3797.8181	3797.9089	0.0908	24	177	211	NAPEGTYDAVIVDSSDPV GPAQELFEKPFFESVSR	125	100			Mascot

Rank	Protein Name	Accession No.	Protein MW	Protein PI	Pep. Count	Protein Score	Protein Score C. I. %	Intensity Matched	Total Ion Score	Total Ion C. I. %	Confirmed
2	Spermidine synthase 1 [Aegilops tauschii]	gi|475617456	32925.4	5.68	10	738	100	49.22	687	100	

Peptide Information

Calc. Mass	Obsrv. Mass	± da	± ppm	Start Seq.	End Seq.	Sequence	Ion Score	C. I. %	Modification	Rank	Result Type
850.3975	850.4307	0.0332	39	1	8	MEAETAAK					Mascot
1211.7107	1211.7185	0.0078	6	112	124	VLVIGGGDGGVLR					Mascot
1211.7107	1211.7185	0.0078	6	112	124	VLVIGGGDGGVLR	126	100			Mascot
1339.8057	1339.8129	0.0072	5	111	124	KVLVIGGGDGGVLR					Mascot

Calc. Mass	Obsrv. Mass	± da	± ppm	Start Seq.	End Seq.	Sequence	Ion Score	C. I. %	Modification	Rank	Result Type
1339.8057	1339.8129	0.0072	5	111	124	KVLVIGGGDGGVLR	94	100			Mascot
1355.7682	1355.7615	-0.0067	-5	164	176	VSLHIGDGVAFLK					Mascot
1355.7682	1355.7615	-0.0067	-5	164	176	VSLHIGDGVAFLK	90	100			Mascot
1440.8422	1440.8477	0.0055	4	77	89	VLVLDGVIQVTER					Mascot
1440.8422	1440.8477	0.0055	4	77	89	VLVLDGVIQVTER	92	100			Mascot
1672.7847	1672.7766	-0.0081	-5	129	142	HSSVEQIDICEIDK			Carbamidomethyl (C)[10]		Mascot
1673.8435	1673.8464	0.0029	2	150	163	QFFPHLALGFEDPR					Mascot
1673.8435	1673.8464	0.0029	2	150	163	QFFPHLALGFEDPR	79	99.998			Mascot
1836.8651	1836.8328	-0.0323	-18	61	76	SDYQDVLVFQSSTYGK					Mascot
1836.8651	1836.8328	-0.0323	-18	61	76	SDYQDVLVFQSSTYGK	85	100			Mascot
2180.9858	2181.0007	0.0149	7	271	288	FYNSEFHTASFCLPSFAR			Carbamidomethyl (C)[12]		Mascot
2180.9858	2181.0007	0.0149	7	271	288	FYNSEFHTASFCLPSFAR	121	100	Carbamidomethyl (C)[12]		Mascot
2447.1792	2447.1609	-0.0183	-7	129	149	HSSVEQIDICEIDKMVVD VSK			Carbamidomethyl (C)[10], Oxidation (M)[15]		Mascot

3	Spermidine synthase 1 [Aegilops tauschii]	gi|475620447	29048.4	5.21	7	542	100	45.374	510	100	

Peptide Information

Calc. Mass	Obsrv. Mass	± da	± ppm	Start Seq.	End Seq.	Sequence	Ion Score	C. I. %	Modification	Rank	Result Type
1211.7107	1211.7185	0.0078	6	46	58	VLVIGGGDGGVLR					Mascot
1211.7107	1211.7185	0.0078	6	46	58	VLVIGGGDGGVLR	126	100			Mascot
1339.8057	1339.8129	0.0072	5	45	58	KVLVIGGGDGGVLR					Mascot
1339.8057	1339.8129	0.0072	5	45	58	KVLVIGGGDGGVLR	94	100			Mascot
1355.7682	1355.7615	-0.0067	-5	98	110	VSLHIGDGVAFLK					Mascot
1355.7682	1355.7615	-0.0067	-5	98	110	VSLHIGDGVAFLK	90	100			Mascot
1672.7847	1672.7766	-0.0081	-5	63	76	HSSVEQIDICEIDK			Carbamidomethyl (C)[10]		Mascot
1673.8435	1673.8464	0.0029	2	84	97	QFFPHLALGFEDPR					Mascot
1673.8435	1673.8464	0.0029	2	84	97	QFFPHLALGFEDPR	79	99.998			Mascot
2180.9858	2181.0007	0.0149	7	233	250	FYNSEFHTASFCLPSFAR			Carbamidomethyl (C)[12]		Mascot
2180.9858	2181.0007	0.0149	7	233	250	FYNSEFHTASFCLPSFAR	121	100	Carbamidomethyl (C)[12]		Mascot
2447.1792	2447.1609	-0.0183	-7	63	83	HSSVEQIDICEIDKMVVD VSK			Carbamidomethyl (C)[10], Oxidation (M)[15]		Mascot

4	RecName: Full=Spermidine synthase 1; Short=SPDSY 1; AltName: Full=Putrescine aminopropyltransferase 1	gi|12229998	35522.7	5.23	7	428	100	17.641	402	100	

Protein Group

Os07g0408700 [Oryza sativa Japonica Group]	gi|113610974	35522.7	5.2300 000190 7349

TPA: spermidine synthase 1 [Zea mays]	gi|414588850	35254.6	5.1500 000953 6743

Peptide Information

Calc. Mass	Obsrv. Mass	± da	± ppm	Start Seq.	End Seq.	Sequence	Ion Score	C. I. %	Modification	Rank	Result Type
976.488	976.5054	0.0174	18	1	9	MEAEAAAKR					Mascot
1211.7107	1211.7185	0.0078	6	111	123	VLVIGGGDGGVLR					Mascot
1211.7107	1211.7185	0.0078	6	111	123	VLVIGGGDGGVLR	126	100			Mascot
1339.8057	1339.8129	0.0072	5	110	123	KVLVIGGGDGGVLR					Mascot
1339.8057	1339.8129	0.0072	5	110	123	KVLVIGGGDGGVLR	94	100			Mascot
1355.7682	1355.7615	-0.0067	-5	163	175	VSLHIGDGVAFLK					Mascot
1355.7682	1355.7615	-0.0067	-5	163	175	VSLHIGDGVAFLK	90	100			Mascot
1440.8422	1440.8477	0.0055	4	76	88	VLVLDGVIQVTER					Mascot
1440.8422	1440.8477	0.0055	4	76	88	VLVLDGVIQVTER	92	100			Mascot
1672.7847	1672.7766	-0.0081	-5	128	141	HSSVEQIDICEIDK			Carbamidomethyl (C)[10]		Mascot
2447.1792	2447.1609	-0.0183	-7	128	148	HSSVEQIDICEIDKMVVD VSK			Carbamidomethyl (C)[10], Oxidation (M)[15]		Mascot

5	LOC100282908 [Zea mays]	gi|226492509	39859.9	5.66	6	421	100	17.392	402	100

Peptide Information

Calc. Mass	Obsrv. Mass	± da	± ppm	Start Seq.	End Seq.	Sequence	Ion Score	C. I. %	Modification	Rank	Result Type
1211.7107	1211.7185	0.0078	6	149	161	VLVIGGGDGGVLR					Mascot
1211.7107	1211.7185	0.0078	6	149	161	VLVIGGGDGGVLR	126	100			Mascot
1339.8057	1339.8129	0.0072	5	148	161	KVLVIGGGDGGVLR					Mascot
1339.8057	1339.8129	0.0072	5	148	161	KVLVIGGGDGGVLR	94	100			Mascot
1355.7682	1355.7615	-0.0067	-5	201	213	VSLHIGDGVAFLK					Mascot
1355.7682	1355.7615	-0.0067	-5	201	213	VSLHIGDGVAFLK	90	100			Mascot
1440.8422	1440.8477	0.0055	4	114	126	VLVLDGVIQVTER					Mascot
1440.8422	1440.8477	0.0055	4	114	126	VLVLDGVIQVTER	92	100			Mascot
1672.7847	1672.7766	-0.0081	-5	166	179	HSSVEQIDICEIDK			Carbamidomethyl (C)[10]		Mascot
2447.1792	2447.1609	-0.0183	-7	166	186	HSSVEQIDICEIDKMVVD VSK			Carbamidomethyl (C)[10], Oxidation (M)[15]		Mascot

6	hypothetical protein OsI_25717 [Oryza sativa Indica Group]	gi|218199468	49545.9	5.39	6	417	100	17.392	402	100

Peptide Information

Calc. Mass	Obsrv. Mass	± da	± ppm	Start Seq.	End Seq.	Sequence	Ion Score	C. I. %	Modification	Rank	Result Type

1211.7107	1211.7185	0.0078	6	245	257	VLVIGGGDGGVLR					Mascot
1211.7107	1211.7185	0.0078	6	245	257	VLVIGGGDGGVLR	126	100			Mascot
1339.8057	1339.8129	0.0072	5	244	257	KVLVIGGGDGGVLR					Mascot
1339.8057	1339.8129	0.0072	5	244	257	KVLVIGGGDGGVLR	94	100			Mascot
1355.7682	1355.7615	-0.0067	-5	297	309	VSLHIGDGVAFLK					Mascot
1355.7682	1355.7615	-0.0067	-5	297	309	VSLHIGDGVAFLK	90	100			Mascot
1440.8422	1440.8477	0.0055	4	210	222	VLVLDGVIQVTER					Mascot
1440.8422	1440.8477	0.0055	4	210	222	VLVLDGVIQVTER	92	100			Mascot
1672.7847	1672.7766	-0.0081	-5	262	275	HSSVEQIDICEIDK			Carbamidomethyl (C)[10]		Mascot
2447.1792	2447.1609	-0.0183	-7	262	282	HSSVEQIDICEIDKMVVD VSK			Carbamidomethyl (C)[10], Oxidation (M)[15]		Mascot

7	TPA: hypothetical protein ZEAMMB73_606346 [Zea mays]	gi|414884328	36567.3	5.05	5	415	100	16.61	402	100

Protein Group

uncharacterized protein LOC100272866 [Zea mays]	gi|226529177	36567.3	5.0500 001907 3486

Peptide Information

Calc. Mass	Obsrv. Mass	± da	± ppm	Start Seq.	End Seq.	Sequence	Ion Score	C. I. %	Modification	Rank	Result Type
976.488	976.5054	0.0174	18	1	9	MEAEAAAKR					Mascot
1211.7107	1211.7185	0.0078	6	121	133	VLVIGGGDGGVLR					Mascot
1211.7107	1211.7185	0.0078	6	121	133	VLVIGGGDGGVLR	126	100			Mascot
1339.8057	1339.8129	0.0072	5	120	133	KVLVIGGGDGGVLR					Mascot
1339.8057	1339.8129	0.0072	5	120	133	KVLVIGGGDGGVLR	94	100			Mascot
1355.7682	1355.7615	-0.0067	-5	173	185	VSLHIGDGVAFLK					Mascot
1355.7682	1355.7615	-0.0067	-5	173	185	VSLHIGDGVAFLK	90	100			Mascot
1440.8422	1440.8477	0.0055	4	86	98	VLVLDGVIQVTER					Mascot
1440.8422	1440.8477	0.0055	4	86	98	VLVLDGVIQVTER	92	100			Mascot

8	TPA: hypothetical protein ZEAMMB73_606346 [Zea mays]	gi|414884326	31549.8	5.36	4	413	100	16.361	402	100

Peptide Information

Calc. Mass	Obsrv. Mass	± da	± ppm	Start Seq.	End Seq.	Sequence	Ion Score	C. I. %	Modification	Rank	Result Type
1211.7107	1211.7185	0.0078	6	70	82	VLVIGGGDGGVLR					Mascot
1211.7107	1211.7185	0.0078	6	70	82	VLVIGGGDGGVLR	126	100			Mascot

Calc. Mass	Obsrv. Mass	± da	± ppm	Start Seq.	End Seq.	Sequence	Ion Score	C. I. %	Modification	Rank	Result Type
1339.8057	1339.8129	0.0072	5	69	82	KVLVIGGGDGGVLR					Mascot
1339.8057	1339.8129	0.0072	5	69	82	KVLVIGGGDGGVLR	94	100			Mascot
1355.7682	1355.7615	-0.0067	-5	122	134	VSLHIGDGVAFLK					Mascot
1355.7682	1355.7615	-0.0067	-5	122	134	VSLHIGDGVAFLK	90	100			Mascot
1440.8422	1440.8477	0.0055	4	35	47	VLVLDGVIQVTER					Mascot
1440.8422	1440.8477	0.0055	4	35	47	VLVLDGVIQVTER	92	100			Mascot

9	spermidine synthase-like [Cucumis sativus]	gi|525507343	34785.5	4.95	5	372	100	15.434	357	100	

Peptide Information

Calc. Mass	Obsrv. Mass	± da	± ppm	Start Seq.	End Seq.	Sequence	Ion Score	C. I. %	Modification	Rank	Result Type
1211.7107	1211.7185	0.0078	6	108	120	VLVIGGGDGGVLR					Mascot
1211.7107	1211.7185	0.0078	6	108	120	VLVIGGGDGGVLR	126	100			Mascot
1339.8057	1339.8129	0.0072	5	107	120	KVLVIGGGDGGVLR					Mascot
1339.8057	1339.8129	0.0072	5	107	120	KVLVIGGGDGGVLR	94	100			Mascot
1355.7682	1355.7615	-0.0067	-5	160	172	VTLHVGDGVAFLK					Mascot
1355.7682	1355.7615	-0.0067	-5	160	172	VTLHVGDGVAFLK	53	99.251			Mascot
1695.849	1695.8082	-0.0408	-24	146	159	EFFPRVAIGYEDPR					Mascot
1836.8651	1836.8328	-0.0323	-18	57	72	SDYQDVLVFQSSTYGK					Mascot
1836.8651	1836.8328	-0.0323	-18	57	72	SDYQDVLVFQSSTYGK	85	100			Mascot

10	PREDICTED: spermidine synthase 1-like [Setaria italica]	gi|514726599	36445.1	4.93	6	331	100	15.414	311	100	

Peptide Information

Calc. Mass	Obsrv. Mass	± da	± ppm	Start Seq.	End Seq.	Sequence	Ion Score	C. I. %	Modification	Rank	Result Type
976.488	976.5054	0.0174	18	1	9	MEAEAAAKR					Mascot
1211.7107	1211.7185	0.0078	6	121	133	VLVIGGGDGGVLR					Mascot
1211.7107	1211.7185	0.0078	6	121	133	VLVIGGGDGGVLR	126	100			Mascot
1339.8057	1339.8129	0.0072	5	120	133	KVLVIGGGDGGVLR					Mascot
1339.8057	1339.8129	0.0072	5	120	133	KVLVIGGGDGGVLR	94	100			Mascot
1440.8422	1440.8477	0.0055	4	86	98	VLVLDGVIQVTER					Mascot
1440.8422	1440.8477	0.0055	4	86	98	VLVLDGVIQVTER	92	100			Mascot
1672.7847	1672.7766	-0.0081	-5	138	151	HSSVEQIDICEIDK			Carbamidomethyl (C)[10]		Mascot
2447.1792	2447.1609	-0.0183	-7	138	158	HSSVEQIDICEIDKMVVDVSK			Carbamidomethyl (C)[10], Oxidation (M)[15]		Mascot

Gel Idx/Pos	175/H2	Instr./Gel Origin	BA2151/Sample Project 20140814	Process Status	Analysis Succeeded
Plate [#] Name	[1] Sample Project 20140814	Instrument Sample Name		Spectra	11

Rank	Protein Name	Accession No.	Protein MW	Protein PI	Pep. Count	Protein Score	Protein Score C. I. %	Intensity Matched	Total Ion Score	Total Ion C. I. %	Confirmed
1	Oxygen-evolving enhancer protein 1, chloroplastic [Triticum urartu]	gi|474352688	34635.5	5.75	18	749	100	50.833	624	100	

Protein Group

Protein Name	Accession No.	Protein MW	Protein PI
Oxygen-evolving enhancer protein 1, chloroplastic [Aegilops tauschii]	gi|475627843	34635.5	5.75

Peptide Information

Calc. Mass	Obsrv. Mass	± da	± ppm	Start Seq.	End Seq.	Sequence	Ion Score	C. I. %	Modification	Rank	Result Type
850.4305	850.4356	0.0051	6	232	239	GSSFLDPK					Mascot
930.468	930.4703	0.0023	2	147	154	NEPPAFQK					Mascot
950.571	950.5679	-0.0031	-3	203	210	VPFLFTVK					Mascot
950.571	950.5679	-0.0031	-3	203	210	VPFLFTVK	59	99.772			Mascot
1080.5573	1080.563	0.0057	5	86	94	LTFDEIQSK					Mascot
1159.6106	1159.5874	-0.0232	-20	147	156	NEPPAFQKTK					Mascot
1236.6583	1236.6716	0.0133	11	85	94	RLTFDEIQSK					Mascot
1236.6583	1236.6716	0.0133	11	85	94	RLTFDEIQSK	34	35.369			Mascot
1328.6555	1328.6542	-0.0013	-1	130	140	FCLEPTSFTVK			Carbamidomethyl (C)[2]		Mascot
1456.7505	1456.749	-0.0015	-1	129	140	KFCLEPTSFTVK			Carbamidomethyl (C)[3]		Mascot
1506.7655	1506.7395	-0.026	-17	1	15	MAASLQAAATLMPAK			Oxidation (M)[1,12]		Mascot
1562.7559	1562.7727	0.0168	11	242	258	GGSTGYDNAVALPAGGR					Mascot
1562.7559	1562.7727	0.0168	11	242	258	GGSTGYDNAVALPAGGR	145	100			Mascot
1742.9581	1742.8796	-0.0785	-45	2	19	AASLQAAATLMPAKIGGR			Oxidation (M)[11]		Mascot
1760.8813	1760.9	0.0187	11	186	202	DGIDYAAVTVQLPGGER					Mascot
1760.8813	1760.9	0.0187	11	186	202	DGIDYAAVTVQLPGGER	136	100			Mascot
1775.8784	1775.8879	0.0095	5	240	258	GRGGSTGYDNAVALPAG GR					Mascot
2168.9917	2168.9822	-0.0095	-4	101	121	GTGTANQCPTIDGGVDS FPFK			Carbamidomethyl (C)[8]		Mascot
2168.9917	2168.9822	-0.0095	-4	101	121	GTGTANQCPTIDGGVDS FPFK	103	100	Carbamidomethyl (C)[8]		Mascot
2280.2024	2280.2263	0.0239	10	211	231	QLVATGKPESFSGPFLVP SYR					Mascot
2280.2024	2280.2263	0.0239	10	211	231	QLVATGKPESFSGPFLVP SYR	147	100			Mascot
2294.1299	2294.1667	0.0368	16	182	202	FEEKDGIDYAAVTVQLPG					Mascot

						GER					
2434.1482	2434.1724	0.0242	10	242	266	GGSTGYDNAVALPAGGR GDEEELAK					Mascot
2590.2883	2590.2869	-0.0014	-1	286	310	SKPETGEVIGVFESVQPS DTDLGAK					Mascot

2	PREDICTED: oxygen-evolving enhancer protein 1, chloroplastic-like [Setaria italica]	gi|514777415	35110.8	5.74	11	497	100	22.62	443	100	

Peptide Information

Calc. Mass	Obsrv. Mass	± da	± ppm	Start Seq.	End Seq.	Sequence	Ion Score	C. I. %	Modification	Rank	Result Type
850.4305	850.4356	0.0051	6	237	244	GSSFLDPK					Mascot
930.468	930.4703	0.0023	2	152	159	NAPPEFQK					Mascot
950.571	950.5679	-0.0031	-3	208	215	VPFLFTVK					Mascot
950.571	950.5679	-0.0031	-3	208	215	VPFLFTVK	59	99.772			Mascot
1159.6106	1159.5874	-0.0232	-20	152	161	NAPPEFQKTK					Mascot
1328.6555	1328.6542	-0.0013	-1	135	145	FCLEPTSFTVK			Carbamidomethyl (C)[2]		Mascot
1456.7505	1456.749	-0.0015	-1	134	145	KFCLEPTSFTVK			Carbamidomethyl (C)[3]		Mascot
1562.7559	1562.7727	0.0168	11	247	263	GGSTGYDNAVALPAGGR					Mascot
1562.7559	1562.7727	0.0168	11	247	263	GGSTGYDNAVALPAGGR	145	100			Mascot
1760.8813	1760.9	0.0187	11	191	207	DGIDYAAVTVQLPGGER					Mascot
1760.8813	1760.9	0.0187	11	191	207	DGIDYAAVTVQLPGGER	136	100			Mascot
1775.8784	1775.8879	0.0095	5	245	263	GRGGSTGYDNAVALPAG GR					Mascot
2168.9917	2168.9822	-0.0095	-4	106	126	GTGTANQCPTIDGGVDS FPFK			Carbamidomethyl (C)[8]		Mascot
2168.9917	2168.9822	-0.0095	-4	106	126	GTGTANQCPTIDGGVDS FPFK	103	100	Carbamidomethyl (C)[8]		Mascot
2294.1299	2294.1667	0.0368	16	187	207	FEEKDGIDYAAVTVQLPG GER					Mascot

3	collinsiaXIII-like protein, partial [Collinsia rattanii]	gi|471272162	17934.1	5.03	12	433	100	21.31	340	100	

Peptide Information

Calc. Mass	Obsrv. Mass	± da	± ppm	Start Seq.	End Seq.	Sequence	Ion Score	C. I. %	Modification	Rank	Result Type
850.4305	850.4356	0.0051	6	104	111	GSSFLDPK					Mascot
930.468	930.4703	0.0023	2	19	26	NAPPEFQK					Mascot
950.571	950.5679	-0.0031	-3	75	82	VPFLFTVK					Mascot
950.571	950.5679	-0.0031	-3	75	82	VPFLFTVK	59	99.772			Mascot
1159.6106	1159.5874	-0.0232	-20	19	28	NAPPEFQKTK					Mascot
1328.6555	1328.6542	-0.0013	-1	2	12	FCLEPTSFTVK			Carbamidomethyl (C)[2]		Mascot
1456.7505	1456.749	-0.0015	-1	1	12	KFCLEPTSFTVK			Carbamidomethyl (C)[3]		Mascot
1562.7559	1562.7727	0.0168	11	114	130	GGSTGYDNAVALPAGGR					Mascot

1562.7559	1562.7727	0.0168	11	114	130	GGSTGYDNAVALPAGGR	145	100			Mascot
1760.8813	1760.9	0.0187	11	58	74	DGIDYAAVTVQLPGGER					Mascot
1760.8813	1760.9	0.0187	11	58	74	DGIDYAAVTVQLPGGER	136	100			Mascot
1773.0005	1772.9404	-0.0601	-34	151	167	ITLSVTKSKPETGEIIG					Mascot
1775.8784	1775.8879	0.0095	5	112	130	GRGGSTGYDNAVALPAG GR					Mascot
2294.1299	2294.1667	0.0368	16	54	74	FEEKDGIDYAAVTVQLPG GER					Mascot
2296.123	2296.1807	0.0577	25	33	53	LTYTLDEIEGPFEVSPDG SVK					Mascot

4	hypothetical protein OsI_02088 [Oryza sativa Indica Group]	gi|218188287	35097.8	6.1	17	426	100	45.31	315	100		

Protein Group

Os01g0501800 [Oryza sativa Japonica Group]	gi|113532665	35067.8	6.0999 999046 3257

Peptide Information

Calc. Mass	Obsrv. Mass	± da	± ppm	Start Seq.	End Seq.	Sequence	Ion Score	C. I. %	Modification	Rank	Result Type
811.413	811.3917	-0.0213	-26	129	135	AGKYNMK					Mascot
850.4305	850.4356	0.0051	6	239	246	GSSFLDPK					Mascot
930.468	930.4703	0.0023	2	154	161	NAPPEFQK					Mascot
1080.5573	1080.563	0.0057	5	93	101	LTFDEIQSK					Mascot
1159.6106	1159.5874	-0.0232	-20	154	163	NAPPEFQKTK					Mascot
1176.5677	1176.597	0.0293	25	58	68	EVANKCADAAK			Carbamidomethyl (C)[6]		Mascot
1236.6583	1236.6716	0.0133	11	92	101	RLTFDEIQSK					Mascot
1236.6583	1236.6716	0.0133	11	92	101	RLTFDEIQSK	34	35.369			Mascot
1328.6555	1328.6542	-0.0013	-1	137	147	FCLEPTSFTVK			Carbamidomethyl (C)[2]		Mascot
1456.7505	1456.749	-0.0015	-1	136	147	KFCLEPTSFTVK			Carbamidomethyl (C)[3]		Mascot
1562.7559	1562.7727	0.0168	11	249	265	GGSTGYDNAVALPAGGR					Mascot
1562.7559	1562.7727	0.0168	11	249	265	GGSTGYDNAVALPAGGR	145	100			Mascot
1593.8456	1593.7844	-0.0612	-38	21	36	ASSAALPSRPSSHVAR					Mascot
1760.8813	1760.9	0.0187	11	193	209	DGIDYAAVTVQLPGGER					Mascot
1760.8813	1760.9	0.0187	11	193	209	DGIDYAAVTVQLPGGER	136	100			Mascot
1775.8784	1775.8879	0.0095	5	247	265	GRGGSTGYDNAVALPAG GR					Mascot
2280.1494	2280.2263	0.0769	34	168	188	LTYTLDEIEGPLEVSSDG TIK					Mascot
2280.1494	2280.2263	0.0769	34	168	188	LTYTLDEIEGPLEVSSDG TIK					Mascot
2294.1299	2294.1667	0.0368	16	189	209	FEEKDGIDYAAVTVQLPG					Mascot

						GER						
2434.1482	2434.1724	0.0242	10	249	273	GGSTGYDNAVALPAGGR GDEEELAK						Mascot
2590.2883	2590.2869	-0.0014	-1	293	317	SKPETGEVIGVFESVQPS DTDLGAK						Mascot

5 collinsiaXIII-like protein, partial [Collinsia rattanii] gi|471272202 17806 4.91 11 423 100 20.718 340 100

Protein Group

collinsiaXIII-like protein, partial [Collinsia linearis]	gi|471272198	17806	4.9099 998474 1211
collinsiaXIII-like protein, partial [Collinsia linearis]	gi|471272196	17806	4.9099 998474 1211
collinsiaXIII-like protein, partial [Collinsia rattanii]	gi|471272200	17806	4.9099 998474 1211
collinsiaXIII-like protein, partial [Collinsia rattanii]	gi|471272204	17806	4.9099 998474 1211

Peptide Information

Calc. Mass	Obsrv. Mass	± da	± ppm	Start Seq.	End Seq.	Sequence	Ion Score	C. I. %	Modification	Rank	Result Type
850.4305	850.4356	0.0051	6	103	110	GSSFLDPK					Mascot
930.468	930.4703	0.0023	2	18	25	NAPPEFQK					Mascot
950.571	950.5679	-0.0031	-3	74	81	VPFLFTVK					Mascot
950.571	950.5679	-0.0031	-3	74	81	VPFLFTVK	59	99.772			Mascot
1159.6106	1159.5874	-0.0232	-20	18	27	NAPPEFQKTK					Mascot
1328.6555	1328.6542	-0.0013	-1	1	11	FCLEPTSFTVK			Carbamidomethyl (C)[2]		Mascot
1562.7559	1562.7727	0.0168	11	113	129	GGSTGYDNAVALPAGGR					Mascot
1562.7559	1562.7727	0.0168	11	113	129	GGSTGYDNAVALPAGGR	145	100			Mascot
1760.8813	1760.9	0.0187	11	57	73	DGIDYAAVTVQLPGGER					Mascot
1760.8813	1760.9	0.0187	11	57	73	DGIDYAAVTVQLPGGER	136	100			Mascot
1773.0005	1772.9404	-0.0601	-34	150	166	ITLSVTKSKPETGEIIG					Mascot
1775.8784	1775.8879	0.0095	5	111	129	GRGGSTGYDNAVALPAG GR					Mascot
2294.1299	2294.1667	0.0368	16	53	73	FEEKDGIDYAAVTVQLPG GER					Mascot
2296.123	2296.1807	0.0577	25	32	52	LTYTLDEIEGPFEVSPDG SVK					Mascot

6 collinsiaXIII-like protein, partial [Collinsia linearis] gi|471272208 16888.6 5 10 414 100 19.969 340 100

Peptide Information

Calc. Mass	Obsrv. Mass	± da	± ppm	Start Seq.	End Seq.	Sequence	Ion Score	C. I. %	Modification	Rank	Result Type
850.4305	850.4356	0.0051	6	97	104	GSSFLDPK					Mascot
930.468	930.4703	0.0023	2	12	19	NAPPEFQK					Mascot
950.571	950.5679	-0.0031	-3	68	75	VPFLFTVK					Mascot
950.571	950.5679	-0.0031	-3	68	75	VPFLFTVK	59	99.772			Mascot
1159.6106	1159.5874	-0.0232	-20	12	21	NAPPEFQKTK					Mascot
1182.6365	1182.5724	-0.0641	-54	1	11	SFTVKAESVSK					Mascot
1562.7559	1562.7727	0.0168	11	107	123	GGSTGYDNAVALPAGGR					Mascot
1562.7559	1562.7727	0.0168	11	107	123	GGSTGYDNAVALPAGGR	145	100			Mascot
1760.8813	1760.9	0.0187	11	51	67	DGIDYAAVTVQLPGGER					Mascot
1760.8813	1760.9	0.0187	11	51	67	DGIDYAAVTVQLPGGER	136	100			Mascot
1775.8784	1775.8879	0.0095	5	105	123	GRGGSTGYDNAVALPAG GR					Mascot
2294.1299	2294.1667	0.0368	16	47	67	FEEKDGIDYAAVTVQLPG GER					Mascot
2296.123	2296.1807	0.0577	25	26	46	LTYTLDEIEGPFEVSPDG SVK					Mascot

7 collinsiaXIII-like protein, partial [Collinsia rattanii] gi|471272164 17235.7 5.04 10 413 100 20.613 340 100

Protein Group

collinsiaXIII-like protein, partial [Collinsia rattanii] gi|471272166 17235.7 5.0399 999618 5303

Peptide Information

Calc. Mass	Obsrv. Mass	± da	± ppm	Start Seq.	End Seq.	Sequence	Ion Score	C. I. %	Modification	Rank	Result Type
850.4305	850.4356	0.0051	6	103	110	GSSFLDPK					Mascot
930.468	930.4703	0.0023	2	18	25	NAPPEFQK					Mascot
950.571	950.5679	-0.0031	-3	74	81	VPFLFTVK					Mascot
950.571	950.5679	-0.0031	-3	74	81	VPFLFTVK	59	99.772			Mascot
1159.6106	1159.5874	-0.0232	-20	18	27	NAPPEFQKTK					Mascot
1328.6555	1328.6542	-0.0013	-1	1	11	FCLEPTSFTVK			Carbamidomethyl (C)[2]		Mascot
1562.7559	1562.7727	0.0168	11	113	129	GGSTGYDNAVALPAGGR					Mascot
1562.7559	1562.7727	0.0168	11	113	129	GGSTGYDNAVALPAGGR	145	100			Mascot
1760.8813	1760.9	0.0187	11	57	73	DGIDYAAVTVQLPGGER					Mascot
1760.8813	1760.9	0.0187	11	57	73	DGIDYAAVTVQLPGGER	136	100			Mascot
1775.8784	1775.8879	0.0095	5	111	129	GRGGSTGYDNAVALPAG GR					Mascot
2294.1299	2294.1667	0.0368	16	53	73	FEEKDGIDYAAVTVQLPG GER					Mascot

	2296.123	2296.1807	0.0577	25	32	52	LTYTLDEIEGPFEVSPDG SVK						Mascot
8	collinsiaXIII-like protein, partial [Collinsia linearis]	gi|471272192	17635.9	4.91	10	412	100	20.613	340	100			

Protein Group

collinsiaXIII-like protein, partial [Collinsia linearis]	gi|471272190	17635.9	4.9099 998474 1211
collinsiaXIII-like protein, partial [Collinsia linearis]	gi|471272188	17635.9	4.9099 998474 1211
collinsiaXIII-like protein, partial [Collinsia linearis]	gi|471272186	17635.9	4.9099 998474 1211
collinsiaXIII-like protein, partial [Collinsia linearis]	gi|471272184	17635.9	4.9099 998474 1211
collinsiaXIII-like protein, partial [Collinsia linearis]	gi|471272194	17635.9	4.9099 998474 1211
collinsiaXIII-like protein, partial [Collinsia linearis]	gi|471272182	17635.9	4.9099 998474 1211
collinsiaXIII-like protein, partial [Collinsia linearis]	gi|471272180	17635.9	4.9099 998474 1211
collinsiaXIII-like protein, partial [Collinsia rattanii]	gi|471272178	17635.9	4.9099 998474 1211
collinsiaXIII-like protein, partial [Collinsia rattanii]	gi|471272176	17635.9	4.9099 998474 1211
collinsiaXIII-like protein, partial [Collinsia rattanii]	gi|471272174	17635.9	4.9099 998474 1211
collinsiaXIII-like protein, partial [Collinsia rattanii]	gi|471272172	17635.9	4.9099 998474 1211
collinsiaXIII-like protein, partial [Collinsia rattanii]	gi|471272170	17635.9	4.9099 998474 1211
collinsiaXIII-like protein, partial [Collinsia rattanii]	gi|471272168	17635.9	4.9099

998474
1211

Peptide Information

Calc. Mass	Obsrv. Mass	± da	± ppm	Start Seq.	End Seq.	Sequence	Ion Score	C. I. %	Modification	Rank	Result Type
850.4305	850.4356	0.0051	6	103	110	GSSFLDPK					Mascot
930.468	930.4703	0.0023	2	18	25	NAPPEFQK					Mascot
950.571	950.5679	-0.0031	-3	74	81	VPFLFTVK					Mascot
950.571	950.5679	-0.0031	-3	74	81	VPFLFTVK	59	99.772			Mascot
1159.6106	1159.5874	-0.0232	-20	18	27	NAPPEFQKTK					Mascot
1328.6555	1328.6542	-0.0013	-1	1	11	FCLEPTSFTVK			Carbamidomethyl (C)[2]		Mascot
1562.7559	1562.7727	0.0168	11	113	129	GGSTGYDNAVALPAGGR					Mascot
1562.7559	1562.7727	0.0168	11	113	129	GGSTGYDNAVALPAGGR	145	100			Mascot
1760.8813	1760.9	0.0187	11	57	73	DGIDYAAVTVQLPGGER					Mascot
1760.8813	1760.9	0.0187	11	57	73	DGIDYAAVTVQLPGGER	136	100			Mascot
1775.8784	1775.8879	0.0095	5	111	129	GRGGSTGYDNAVALPAG GR					Mascot
2294.1299	2294.1667	0.0368	16	53	73	FEEKDGIDYAAVTVQLPG GER					Mascot
2296.123	2296.1807	0.0577	25	32	52	LTYTLDEIEGPFEVSPDG SVK					Mascot

9 collinsiaXIII-like protein, partial [Collinsia linearis] gi|471272206 17905.1 4.91 10 411 100 20.613 340 100

Peptide Information

Calc. Mass	Obsrv. Mass	± da	± ppm	Start Seq.	End Seq.	Sequence	Ion Score	C. I. %	Modification	Rank	Result Type
850.4305	850.4356	0.0051	6	103	110	GSSFLDPK					Mascot
930.468	930.4703	0.0023	2	18	25	NAPPEFQK					Mascot
950.571	950.5679	-0.0031	-3	74	81	VPFLFTVK					Mascot
950.571	950.5679	-0.0031	-3	74	81	VPFLFTVK	59	99.772			Mascot
1159.6106	1159.5874	-0.0232	-20	18	27	NAPPEFQKTK					Mascot
1328.6555	1328.6542	-0.0013	-1	1	11	FCLEPTSFTVK			Carbamidomethyl (C)[2]		Mascot
1562.7559	1562.7727	0.0168	11	113	129	GGSTGYDNAVALPAGGR					Mascot
1562.7559	1562.7727	0.0168	11	113	129	GGSTGYDNAVALPAGGR	145	100			Mascot
1760.8813	1760.9	0.0187	11	57	73	DGIDYAAVTVQLPGGER					Mascot
1760.8813	1760.9	0.0187	11	57	73	DGIDYAAVTVQLPGGER	136	100			Mascot
1775.8784	1775.8879	0.0095	5	111	129	GRGGSTGYDNAVALPAG GR					Mascot
2294.1299	2294.1667	0.0368	16	53	73	FEEKDGIDYAAVTVQLPG GER					Mascot
2296.123	2296.1807	0.0577	25	32	52	LTYTLDEIEGPFEVSPDG					Mascot

SVK

10	RecName: Full=Oxygen-evolving enhancer protein 1, chloroplastic; Short=OEE1; AltName: Full=33 kDa subunit of oxygen evolving system of photosystem II; AltName: Full=33 kDa thylakoid membrane protein; AltName: Full=OEC 33 kDa subunit; Flags: Precurso	gi|11134054	35377.1	5.89	14	394	100	26.49	314	100	

Peptide Information

Calc. Mass	Obsrv. Mass	± da	± ppm	Start Seq.	End Seq.	Sequence	Ion Score	C. I. %	Modification	Rank	Result Type
850.4305	850.4356	0.0051	6	238	245	GSSFLDPK					Mascot
988.521	988.5354	0.0144	15	37	46	AFGVEPAAAR					Mascot
1080.5573	1080.563	0.0057	5	92	100	LTFDEIQSK					Mascot
1236.6583	1236.6716	0.0133	11	91	100	RLTFDEIQSK					Mascot
1236.6583	1236.6716	0.0133	11	91	100	RLTFDEIQSK	34	35.369			Mascot
1328.6555	1328.6542	-0.0013	-1	136	146	FCLEPTSFTVK			Carbamidomethyl (C)[2]		Mascot
1444.7867	1444.7297	-0.057	-39	24	36	NNLQLRSAQSVSK					Mascot
1456.7505	1456.749	-0.0015	-1	135	146	KFCLEPTSFTVK			Carbamidomethyl (C)[3]		Mascot
1544.7704	1544.7468	-0.0236	-15	147	160	AESVNKNAPPDFQK					Mascot
1562.7559	1562.7727	0.0168	11	248	264	GGSTGYDNAVALPAGGR					Mascot
1562.7559	1562.7727	0.0168	11	248	264	GGSTGYDNAVALPAGGR	145	100			Mascot
1664.8346	1664.8987	0.0641	39	1	16	MAASLQAAATLMQPTK			Oxidation (M)[1,12]		Mascot
1760.8813	1760.9	0.0187	11	192	208	DGIDYAAVTVQLPGGER					Mascot
1760.8813	1760.9	0.0187	11	192	208	DGIDYAAVTVQLPGGER	136	100			Mascot
1775.8784	1775.8879	0.0095	5	246	264	GRGGSTGYDNAVALPAG GR					Mascot
2152.1907	2152.1355	-0.0552	-26	2	23	AASLQAAATLMQPTKVG VAPAR					Mascot
2294.1299	2294.1667	0.0368	16	188	208	FEEKDGIDYAAVTVQLPG GER					Mascot

Gel Idx/Pos	176/H3	Instr./Gel Origin	BA2151/Sample Project 20140814	Process Status	Analysis Succeeded
Plate [#] Name	[1] Sample Project 20140814	Instrument Sample Name		Spectra	11

Rank	Protein Name	Accession No.	Protein MW	Protein PI	Pep. Count	Protein Score	Protein Score C. I. %	Intensity Matched	Total Ion Score	Total Ion C. I. %	Confirmed
1	ADP glucose pyrophosphorylase [Triticum aestivum]	gi|469952290	52399.6	5.54	20	465	100	40.291	348	100	

Protein Group

Protein Name	Accession No.	Protein MW	Protein PI
RecName: Full=Glucose-1-phosphate adenylyltransferase small subunit, chloroplastic/amyloplastic; AltName: Full=ADP-glucose pyrophosphorylase; AltName: Full=ADP-glucose synthase; AltName: Full=AGPase B; AltName: Full=Alpha-D-glucose-1-phosphate adeny	gi|232172	52399.6	5.5399999618 5303

Peptide Information

Calc. Mass	Obsrv. Mass	± da	± ppm	Start Seq.	End Seq.	Sequence	Ion Score	C. I. %	Modification	Rank	Result Type
854.444	854.4503	0.0063	7	200	207	ATAFGLMK			Oxidation (M)[7]		Mascot
972.4673	972.473	0.0057	6	448	455	ETDGYFIK					Mascot
1009.5975	1009.6042	0.0067	7	260	267	HVMLQLLR					Mascot
1017.5952	1017.603	0.0078	8	366	374	IHHSVVGLR					Mascot
1017.5952	1017.603	0.0078	8	366	374	IHHSVVGLR	69	99.975			Mascot
1025.5925	1025.5934	0.0009	1	260	267	HVMLQLLR			Oxidation (M)[3]		Mascot
1025.5925	1025.5934	0.0009	1	260	267	HVMLQLLR			Oxidation (M)[3]		Mascot
1032.5472	1032.5532	0.006	6	330	338	SAPIYTQPR					Mascot
1032.5472	1032.5532	0.006	6	330	338	SAPIYTQPR	43	89.703			Mascot
1074.6194	1074.6204	0.001	1	214	222	IIEFAEKPK					Mascot
1256.5801	1256.6061	0.026	21	18	27	REQCNIDGHK			Carbamidomethyl (C)[4]		Mascot
1384.6896	1384.7012	0.0116	8	319	329	KPIPDFSFYDR					Mascot
1384.6896	1384.7012	0.0116	8	319	329	KPIPDFSFYDR	103	100			Mascot
1390.8165	1390.6974	-0.1191	-86	407	420	GGIPIGIGKNSHIK					Mascot
1489.67	1489.7454	0.0754	51	19	31	EQCNIDGHKSSSK			Carbamidomethyl (C)[3]		Mascot
1522.7761	1522.7578	-0.0183	-12	106	119	HLSRAYGSNIGGYK					Mascot
1553.7628	1553.8525	0.0897	58	200	213	ATAFGLMKIDEEGR			Oxidation (M)[7]		Mascot
1621.7925	1621.7865	-0.006	-4	228	242	AMMVDTTILGLDDAR					Mascot
1637.7874	1637.7786	-0.0088	-5	228	242	AMMVDTTILGLDDAR			Oxidation (M)[2]		Mascot
1653.7822	1653.774	-0.0082	-5	228	242	AMMVDTTILGLDDAR			Oxidation (M)[2,3]		Mascot
1657.8796	1657.8049	-0.0747	-45	2	17	DVPLASKTFPSPSPSK					Mascot
1786.9368	1786.9772	0.0404	23	76	91	LIDIPVSNCLNSNISK			Carbamidomethyl (C)[9]		Mascot

1889.9525	1889.9342	-0.0183	-10	345	362	VLDADVTDSVIGEGCVIK			Carbamidomethyl (C)[15]		Mascot
1970.9965	1971.0115	0.015	8	430	447	IGDNVMIINVDNVQEAAR					Mascot
1970.9965	1971.0115	0.015	8	430	447	IGDNVMIINVDNVQEAAR	133	100			Mascot
1986.9913	1986.9746	-0.0167	-8	430	447	IGDNVMIINVDNVQEAAR			Oxidation (M)[6]		Mascot
1986.9913	1986.9746	-0.0167	-8	430	447	IGDNVMIINVDNVQEAAR	96	100	Oxidation (M)[6]		Mascot
2318.2537	2318.1223	-0.1314	-57	76	95	LIDIPVSNCLNSNISKIYVR			Carbamidomethyl (C)[9]		Mascot
2368.0874	2368.1069	0.0195	8	268	290	EQFPGANDFGSEVIPGAT STGMR					Mascot
2398.219	2398.1899	-0.0291	-12	319	338	KPIPDFSFYDRSAPIYTQP R					Mascot

2	Glucose-1-phosphate adenylyltransferase small subunit, chloroplastic/amyloplastic [Aegilops tauschii]	gi|475605779	52345.5	5.53	18	446	100	39.872	348	100	

Peptide Information

Calc. Mass	Obsrv. Mass	± da	± ppm	Start Seq.	End Seq.	Sequence	Ion Score	C. I. %	Modification	Rank	Result Type
854.444	854.4503	0.0063	7	200	207	ATAFGLMK			Oxidation (M)[7]		Mascot
972.4673	972.473	0.0057	6	448	455	ETDGYFIK					Mascot
1009.5975	1009.6042	0.0067	7	260	267	HVMLQLLR					Mascot
1017.5952	1017.603	0.0078	8	366	374	IHHSVVGLR					Mascot
1017.5952	1017.603	0.0078	8	366	374	IHHSVVGLR	69	99.975			Mascot
1025.5925	1025.5934	0.0009	1	260	267	HVMLQLLR			Oxidation (M)[3]		Mascot
1025.5925	1025.5934	0.0009	1	260	267	HVMLQLLR			Oxidation (M)[3]		Mascot
1032.5472	1032.5532	0.006	6	330	338	SAPIYTQPR					Mascot
1032.5472	1032.5532	0.006	6	330	338	SAPIYTQPR	43	89.703			Mascot
1074.6194	1074.6204	0.001	1	214	222	IIEFAEKPK					Mascot
1256.7109	1256.6061	-0.1048	-83	64	75	AKPAVPLGANYR					Mascot
1384.6896	1384.7012	0.0116	8	319	329	KPIPDFSFYDR					Mascot
1384.6896	1384.7012	0.0116	8	319	329	KPIPDFSFYDR	103	100			Mascot
1390.8165	1390.6974	-0.1191	-86	407	420	GGIPIGIGKNSHIK					Mascot
1475.6544	1475.7617	0.1073	73	19	31	EQCNVDGHKSSSK			Carbamidomethyl (C)[3]		Mascot
1522.7761	1522.7578	-0.0183	-12	106	119	HLSRAYGSNIGGYK					Mascot
1553.7628	1553.8525	0.0897	58	200	213	ATAFGLMKIDEEGR			Oxidation (M)[7]		Mascot
1621.7925	1621.7865	-0.006	-4	228	242	AMMVDTTILGLDDAR					Mascot
1637.7874	1637.7786	-0.0088	-5	228	242	AMMVDTTILGLDDAR			Oxidation (M)[2]		Mascot
1653.7822	1653.774	-0.0082	-5	228	242	AMMVDTTILGLDDAR			Oxidation (M)[2,3]		Mascot
1786.9368	1786.9772	0.0404	23	76	91	LIDIPVSNCLNSNISK			Carbamidomethyl (C)[9]		Mascot
1889.9525	1889.9342	-0.0183	-10	345	362	VLDADVTDSVIGEGCVIK			Carbamidomethyl (C)[15]		Mascot
1970.9965	1971.0115	0.015	8	430	447	IGDNVMIINVDNVQEAAR					Mascot

1970.9965	1971.0115	0.015	8	430	447	IGDNVMIINVDNVQEAAR	133	100			Mascot
1986.9913	1986.9746	-0.0167	-8	430	447	IGDNVMIINVDNVQEAAR			Oxidation (M)[6]		Mascot
1986.9913	1986.9746	-0.0167	-8	430	447	IGDNVMIINVDNVQEAAR	96	100	Oxidation (M)[6]		Mascot
2368.0874	2368.1069	0.0195	8	268	290	EQFPGANDFGSEVIPGAT STGMR					Mascot
2398.219	2398.1899	-0.0291	-12	319	338	KPIPDFSFYDRSAPIYTQP R					Mascot

3	RecName: Full=Glucose-1-phosphate adenylyltransferase small subunit, chloroplastic/amyloplastic; AltName: Full=ADP-glucose pyrophosphorylase; AltName: Full=ADP-glucose synthase; AltName: Full=AGPase B; AltName: Full=Alpha-D-glucose-1-phosphate adeny	gi|1707940	56412.8	6.11	17	431	100	39.071	348	100	

Peptide Information

Calc. Mass	Obsrv. Mass	± da	± ppm	Start Seq.	End Seq.	Sequence	Ion Score	C. I. %	Modification	Rank	Result Type
854.444	854.4503	0.0063	7	240	247	ATAFGLMK			Oxidation (M)[7]		Mascot
972.4673	972.473	0.0057	6	488	495	ETDGYFIK					Mascot
1009.5975	1009.6042	0.0067	7	300	307	HVMLQLLR					Mascot
1017.5952	1017.603	0.0078	8	406	414	IHHSVVGLR					Mascot
1017.5952	1017.603	0.0078	8	406	414	IHHSVVGLR	69	99.975			Mascot
1025.5925	1025.5934	0.0009	1	300	307	HVMLQLLR			Oxidation (M)[3]		Mascot
1025.5925	1025.5934	0.0009	1	300	307	HVMLQLLR			Oxidation (M)[3]		Mascot
1032.5472	1032.5532	0.006	6	370	378	SAPIYTQPR					Mascot
1032.5472	1032.5532	0.006	6	370	378	SAPIYTQPR	43	89.703			Mascot
1074.6194	1074.6204	0.001	1	254	262	IIEFAEKPK					Mascot
1256.7109	1256.6061	-0.1048	-83	104	115	AKPAVPLGANYR					Mascot
1384.6896	1384.7012	0.0116	8	359	369	KPIPDFSFYDR					Mascot
1384.6896	1384.7012	0.0116	8	359	369	KPIPDFSFYDR	103	100			Mascot
1390.8165	1390.6974	-0.1191	-86	447	460	GGIPIGIGKNSHIK					Mascot
1522.7761	1522.7578	-0.0183	-12	146	159	HLSRAYGSNIGGYK					Mascot
1553.7628	1553.8525	0.0897	58	240	253	ATAFGLMKIDEEGR			Oxidation (M)[7]		Mascot
1786.9368	1786.9772	0.0404	23	116	131	LIDIPVSNCLNSNISK			Carbamidomethyl (C)[9]		Mascot
1889.9525	1889.9342	-0.0183	-10	385	402	VLDADVTDSVIGEGCVIK			Carbamidomethyl (C)[15]		Mascot
1894.0038	1894.0343	0.0305	16	1	18	MAMAAAASPSKILIPPHR			Oxidation (M)[1,3]		Mascot
1970.9965	1971.0115	0.015	8	470	487	IGDNVMIINVDNVQEAAR					Mascot
1970.9965	1971.0115	0.015	8	470	487	IGDNVMIINVDNVQEAAR	133	100			Mascot
1986.9913	1986.9746	-0.0167	-8	470	487	IGDNVMIINVDNVQEAAR			Oxidation (M)[6]		Mascot
1986.9913	1986.9746	-0.0167	-8	470	487	IGDNVMIINVDNVQEAAR	96	100	Oxidation (M)[6]		Mascot

Calc. Mass	Obsrv. Mass	± da	± ppm	Start Seq.	End Seq.	Sequence	Ion Score	C. I. %	Modification	Rank	Result Type
2368.0874	2368.1069	0.0195	8	308	330	EQFPGANDFGSEVIPGAT STGMR					Mascot
2398.219	2398.1899	-0.0291	-12	359	378	KPIPDFSFYDRSAPIYTQP R					Mascot

4	Glucose-1-phosphate adenylyltransferase small subunit, chloroplastic/amyloplastic [Triticum urartu]	gi|474108293	65309.1	7.9	17	291	100	35.534	214	100	

Peptide Information

Calc. Mass	Obsrv. Mass	± da	± ppm	Start Seq.	End Seq.	Sequence	Ion Score	C. I. %	Modification	Rank	Result Type
854.444	854.4503	0.0063	7	241	248	ATAFGLMK			Oxidation (M)[7]		Mascot
1009.5975	1009.6042	0.0067	7	301	308	HVMLQLLR					Mascot
1017.5952	1017.603	0.0078	8	407	415	IHHSVVGLR					Mascot
1017.5952	1017.603	0.0078	8	407	415	IHHSVVGLR	69	99.975			Mascot
1025.5925	1025.5934	0.0009	1	301	308	HVMLQLLR			Oxidation (M)[3]		Mascot
1025.5925	1025.5934	0.0009	1	301	308	HVMLQLLR			Oxidation (M)[3]		Mascot
1032.5472	1032.5532	0.006	6	371	379	SAPIYTQPR					Mascot
1032.5472	1032.5532	0.006	6	371	379	SAPIYTQPR	43	89.703			Mascot
1074.6194	1074.6204	0.001	1	255	263	IIEFAEKPK					Mascot
1256.7109	1256.6061	-0.1048	-83	105	116	AKPAVPLGANYR					Mascot
1384.6896	1384.7012	0.0116	8	360	370	KPIPDFSFYDR					Mascot
1384.6896	1384.7012	0.0116	8	360	370	KPIPDFSFYDR	103	100			Mascot
1390.8165	1390.6974	-0.1191	-86	448	461	GGIPIGIGKNSHIK					Mascot
1522.7761	1522.7578	-0.0183	-12	147	160	HLSRAYGSNIGGYK					Mascot
1553.7628	1553.8525	0.0897	58	241	254	ATAFGLMKIDEEGR			Oxidation (M)[7]		Mascot
1621.7925	1621.7865	-0.006	-4	269	283	AMMVDTTILGLDDAR					Mascot
1637.7874	1637.7786	-0.0088	-5	269	283	AMMVDTTILGLDDAR			Oxidation (M)[2]		Mascot
1653.7822	1653.774	-0.0082	-5	269	283	AMMVDTTILGLDDAR			Oxidation (M)[2,3]		Mascot
1786.9368	1786.9772	0.0404	23	117	132	LIDIPVSNCLNSNISK			Carbamidomethyl (C)[9]		Mascot
1889.9525	1889.9342	-0.0183	-10	386	403	VLDADVTDSVIGEGCVIK			Carbamidomethyl (C)[15]		Mascot
1894.0038	1894.0343	0.0305	16	1	18	MAMAAAASPSKILIPPHR			Oxidation (M)[1,3]		Mascot
2366.2246	2366.0886	-0.136	-57	12	34	ILIPPHRASAATAAASTSC DSLR			Carbamidomethyl (C)[19]		Mascot
2368.0874	2368.1069	0.0195	8	309	331	EQFPGANDFGSEVIPGAT STGMR					Mascot
2398.219	2398.1899	-0.0291	-12	360	379	KPIPDFSFYDRSAPIYTQP R					Mascot

5	TPA: glucose-1-phosphate adenylyltransferase small subunit (ADP-glucose pyrophosphorylase) isoform 1 [Zea mays]	gi|414870680	56789	6.48	12	256	100	32.208	214	100	

Protein Group

TPA: glucose-1-phosphate adenylyltransferase small subunit (ADP-glucose pyrophosphorylase) isoform 2 [Zea mays]	gi|414870681	56789	6.4800 000190 7349

Peptide Information

Calc. Mass	Obsrv. Mass	± da	± ppm	Start Seq.	End Seq.	Sequence	Ion Score	C. I. %	Modification	Rank	Result Type
854.444	854.4503	0.0063	7	244	251	ATAFGLMK			Oxidation (M)[7]		Mascot
972.4673	972.473	0.0057	6	492	499	ETDGYFIK					Mascot
1017.5952	1017.603	0.0078	8	410	418	IHHSVVGLR					Mascot
1017.5952	1017.603	0.0078	8	410	418	IHHSVVGLR	69	99.975			Mascot
1032.5472	1032.5532	0.006	6	374	382	SAPIYTQPR					Mascot
1032.5472	1032.5532	0.006	6	374	382	SAPIYTQPR	43	89.703			Mascot
1074.6194	1074.6204	0.001	1	258	266	IIEFAEKPK					Mascot
1256.7109	1256.6061	-0.1048	-83	108	119	AKPAVPLGANYR					Mascot
1384.6896	1384.7012	0.0116	8	363	373	KPIPDFSFYDR					Mascot
1384.6896	1384.7012	0.0116	8	363	373	KPIPDFSFYDR	103	100			Mascot
1553.7628	1553.8525	0.0897	58	244	257	ATAFGLMKIDEEGR			Oxidation (M)[7]		Mascot
1738.9633	1738.8987	-0.0646	-37	2	18	AMAAIASPSSRTLIPPR					Mascot
1786.9368	1786.9772	0.0404	23	120	135	LIDIPVSNCLNSNISK			Carbamidomethyl (C)[9]		Mascot
1889.9525	1889.9342	-0.0183	-10	389	406	VLDADVTDSVIGEGCVIK			Carbamidomethyl (C)[15]		Mascot
2398.219	2398.1899	-0.0291	-12	363	382	KPIPDFSFYDRSAPIYTQP R					Mascot

6	TPA: glucose-1-phosphate adenylyltransferase small subunit (ADP-glucose pyrophosphorylase) [Zea mays]	gi|414870682	34950.8	4.94	9	252	100	31.538	214	100	

Peptide Information

Calc. Mass	Obsrv. Mass	± da	± ppm	Start Seq.	End Seq.	Sequence	Ion Score	C. I. %	Modification	Rank	Result Type
854.444	854.4503	0.0063	7	42	49	ATAFGLMK			Oxidation (M)[7]		Mascot
972.4673	972.473	0.0057	6	290	297	ETDGYFIK					Mascot
1017.5952	1017.603	0.0078	8	208	216	IHHSVVGLR					Mascot
1017.5952	1017.603	0.0078	8	208	216	IHHSVVGLR	69	99.975			Mascot
1032.5472	1032.5532	0.006	6	172	180	SAPIYTQPR					Mascot
1032.5472	1032.5532	0.006	6	172	180	SAPIYTQPR	43	89.703			Mascot
1074.6194	1074.6204	0.001	1	56	64	IIEFAEKPK					Mascot
1384.6896	1384.7012	0.0116	8	161	171	KPIPDFSFYDR					Mascot
1384.6896	1384.7012	0.0116	8	161	171	KPIPDFSFYDR	103	100			Mascot

1553.7628	1553.8525	0.0897	58	42	55	ATAFGLMKIDEEGR			Oxidation (M)[7]		Mascot
1889.9525	1889.9342	-0.0183	-10	187	204	VLDADVTDSVIGEGCVIK			Carbamidomethyl (C)[15]		Mascot
2398.219	2398.1899	-0.0291	-12	161	180	KPIPDFSFYDRSAPIYTQP R					Mascot

7	PREDICTED: glucose-1-phosphate adenylyltransferase small subunit, chloroplastic/amyloplastic-like [Setaria italica]	gi|514795259	56535.9	6.62	11	249	100	31.498	214	100	

Peptide Information

Calc. Mass	Obsrv. Mass	± da	± ppm	Start Seq.	End Seq.	Sequence	Ion Score	C. I. %	Modification	Rank	Result Type
854.444	854.4503	0.0063	7	242	249	ATAFGLMK			Oxidation (M)[7]		Mascot
972.4673	972.473	0.0057	6	490	497	ETDGYFIK					Mascot
1017.5952	1017.603	0.0078	8	408	416	IHHSVVGLR					Mascot
1017.5952	1017.603	0.0078	8	408	416	IHHSVVGLR	69	99.975			Mascot
1032.5472	1032.5532	0.006	6	372	380	SAPIYTQPR					Mascot
1032.5472	1032.5532	0.006	6	372	380	SAPIYTQPR	43	89.703			Mascot
1074.6194	1074.6204	0.001	1	256	264	IIEFAEKPK					Mascot
1256.7109	1256.6061	-0.1048	-83	106	117	AKPAVPLGANYR					Mascot
1384.6896	1384.7012	0.0116	8	361	371	KPIPDFSFYDR					Mascot
1384.6896	1384.7012	0.0116	8	361	371	KPIPDFSFYDR	103	100			Mascot
1522.7761	1522.7578	-0.0183	-12	148	161	HLSRAYGSNIGGYK					Mascot
1553.7628	1553.8525	0.0897	58	242	255	ATAFGLMKIDEEGR			Oxidation (M)[7]		Mascot
1786.9368	1786.9772	0.0404	23	118	133	LIDIPVSNCLNSNISK			Carbamidomethyl (C)[9]		Mascot
2398.219	2398.1899	-0.0291	-12	361	380	KPIPDFSFYDRSAPIYTQP R					Mascot

8	glucose-1-phosphate adenylyltransferase, putative [Ricinus communis]	gi|223536136	57474.1	6.48	13	218	100	27.796	172	100	

Peptide Information

Calc. Mass	Obsrv. Mass	± da	± ppm	Start Seq.	End Seq.	Sequence	Ion Score	C. I. %	Modification	Rank	Result Type
854.444	854.4503	0.0063	7	248	255	ATAFGLMK			Oxidation (M)[7]		Mascot
972.4673	972.473	0.0057	6	496	503	ETDGYFIK					Mascot
1017.5952	1017.603	0.0078	8	414	422	IHHSVVGLR					Mascot
1017.5952	1017.603	0.0078	8	414	422	IHHSVVGLR	69	99.975			Mascot
1074.6194	1074.6204	0.001	1	262	270	IIEFAEKPK					Mascot
1107.5901	1107.5511	-0.039	-35	1	11	MASMAAIGVLK			Oxidation (M)[1]		Mascot
1232.6667	1232.6057	-0.061	-49	308	317	NVMLDLLRDK			Oxidation (M)[3]		Mascot

Calc. Mass	Obsrv. Mass	± da	± ppm	Start Seq.	End Seq.	Sequence	Ion Score	C. I. %	Modification	Rank	Result Type
1256.7109	1256.6061	-0.1048	-83	112	123	AKPAVPLGANYR					Mascot
1384.6896	1384.7012	0.0116	8	367	377	KPIPDFSFYDR					Mascot
1384.6896	1384.7012	0.0116	8	367	377	KPIPDFSFYDR	103	100			Mascot
1475.6299	1475.7617	0.1318	89	51	63	SSSFSGHYNYNGR					Mascot
1487.7887	1487.7383	-0.0504	-34	64	77	TPMIVSPKAVSDSR					Mascot
1553.7628	1553.8525	0.0897	58	248	261	ATAFGLMKIDEEGR			Oxidation (M)[7]		Mascot
1760.7505	1760.8923	0.1418	81	12	28	VPSASSSSFSNSSNCSR			Carbamidomethyl (C)[15]		Mascot
1786.9368	1786.9772	0.0404	23	124	139	LIDIPVSNCLNSNISK			Carbamidomethyl (C)[9]		Mascot

9	ADP glucose pyrophosphorylase [Actinidia chinensis]	gi|398363561	55762.7	7.62	11	162	100	13.9	125	100	

Peptide Information

Calc. Mass	Obsrv. Mass	± da	± ppm	Start Seq.	End Seq.	Sequence	Ion Score	C. I. %	Modification	Rank	Result Type
854.444	854.4503	0.0063	7	232	239	ATAFGLMK			Oxidation (M)[7]		Mascot
972.4673	972.473	0.0057	6	480	487	ETDGYFIK					Mascot
1017.5952	1017.603	0.0078	8	398	406	IHHSVVGLR					Mascot
1017.5952	1017.603	0.0078	8	398	406	IHHSVVGLR	69	99.975			Mascot
1032.5472	1032.5532	0.006	6	362	370	SAPIYTQPR					Mascot
1032.5472	1032.5532	0.006	6	362	370	SAPIYTQPR	43	89.703			Mascot
1074.6194	1074.6204	0.001	1	246	254	IIEFAEKPK					Mascot
1232.6667	1232.6057	-0.061	-49	292	301	DVMLNLLRDK			Oxidation (M)[3]		Mascot
1256.7109	1256.6061	-0.1048	-83	96	107	AKPAVPLGANYR					Mascot
1553.7628	1553.8525	0.0897	58	232	245	ATAFGLMKIDEEGR			Oxidation (M)[7]		Mascot
1786.9368	1786.9772	0.0404	23	108	123	LIDIPVSNCLNSNISK			Carbamidomethyl (C)[9]		Mascot
1971.0142	1971.0115	-0.0027	-1	462	479	IGDDVKIINSDNIQEAAR					Mascot
1971.0142	1971.0115	-0.0027	-1	462	479	IGDDVKIINSDNIQEAAR	14	0			Mascot
2366.1445	2366.0886	-0.0559	-24	300	322	DKFPGANDFGSEVIPGAT SIGMR					Mascot
2382.1394	2382.1074	-0.032	-13	300	322	DKFPGANDFGSEVIPGAT SIGMR			Oxidation (M)[22]		Mascot

10	hypothetical protein CARUB_v10026231mg [Capsella rubella]	gi|482549012	56911.9	5.68	11	147	100	10.805	112	100	

Peptide Information

Calc. Mass	Obsrv. Mass	± da	± ppm	Start Seq.	End Seq.	Sequence	Ion Score	C. I. %	Modification	Rank	Result Type
854.444	854.4503	0.0063	7	247	254	ATAFGLMK			Oxidation (M)[7]		Mascot
1017.5952	1017.603	0.0078	8	413	421	IHHSVVGLR					Mascot
1017.5952	1017.603	0.0078	8	413	421	IHHSVVGLR	69	99.975			Mascot

1032.5472	1032.5532	0.006	6	377	385	SAPIYTQPR				Mascot
1032.5472	1032.5532	0.006	6	377	385	SAPIYTQPR	43	90.961		Mascot
1060.6038	1060.5721	-0.0317	-30	261	269	IVEFAEKPK				Mascot
1140.5354	1140.5737	0.0383	34	12	22	VPASSSFTGCK			Carbamidomethyl (C)[10]	Mascot
1228.6896	1228.6472	-0.0424	-35	60	70	ETRTPTIVSPK				Mascot
1256.7109	1256.6061	-0.1048	-83	111	122	AKPAVPLGANYR				Mascot
1553.7628	1553.8525	0.0897	58	247	260	ATAFGLMKIDEEGR			Oxidation (M)[7]	Mascot
1731.8445	1731.8688	0.0243	14	292	306	EMPYIASMGIYVVSR			Oxidation (M)[2]	Mascot
1786.9368	1786.9772	0.0404	23	123	138	LIDIPVSNCLNSNISK			Carbamidomethyl (C)[9]	Mascot
2717.3711	2717.1138	-0.2573	-95	292	314	EMPYIASMGIYVVSRDVM LELLR			Oxidation (M)[2,8]	Mascot

Gel Idx/Pos	177/H4	Instr./Gel Origin	BA2151/Sample Project 20140814	Process Status	Analysis Succeeded
Plate [#] Name	[1] Sample Project 20140814	Instrument Sample Name		Spectra	11

Rank	Protein Name	Accession No.	Protein MW	Protein PI	Pep. Count	Protein Score	Protein Score C. I. %	Intensity Matched	Total Ion Score	Total Ion C. I. %	Confirmed
1	Oxygen-evolving enhancer protein 1, chloroplastic [Triticum urartu]	gi|474352688	34635.5	5.75	16	667	100	48.012	560	100	

Protein Group

Protein Name	Accession No.	Protein MW	Protein PI
Oxygen-evolving enhancer protein 1, chloroplastic [Aegilops tauschii]	gi|475627843	34635.5	5.75

Peptide Information

Calc. Mass	Obsrv. Mass	± da	± ppm	Start Seq.	End Seq.	Sequence	Ion Score	C. I. %	Modification	Rank	Result Type
930.468	930.4618	-0.0062	-7	147	154	NEPPAFQK					Mascot
950.571	950.5623	-0.0087	-9	203	210	VPFLFTVK					Mascot
950.571	950.5623	-0.0087	-9	203	210	VPFLFTVK	58	99.747			Mascot
1080.5573	1080.5521	-0.0052	-5	86	94	LTFDEIQSK					Mascot
1236.6583	1236.6652	0.0069	6	85	94	RLTFDEIQSK					Mascot
1236.6583	1236.6652	0.0069	6	85	94	RLTFDEIQSK	21	0			Mascot
1328.6555	1328.6475	-0.008	-6	130	140	FCLEPTSFTVK			Carbamidomethyl (C)[2]		Mascot
1360.6591	1360.7015	0.0424	31	259	270	GDEEELAKENVK					Mascot
1456.7505	1456.7262	-0.0243	-17	129	140	KFCLEPTSFTVK			Carbamidomethyl (C)[3]		Mascot
1562.7559	1562.765	0.0091	6	242	258	GGSTGYDNAVALPAGGR					Mascot
1562.7559	1562.765	0.0091	6	242	258	GGSTGYDNAVALPAGGR	107	100			Mascot
1742.9581	1742.8563	-0.1018	-58	2	19	AASLQAAATLMPAKIGGR			Oxidation (M)[11]		Mascot
1760.8813	1760.8878	0.0065	4	186	202	DGIDYAAVTVQLPGGER					Mascot
1760.8813	1760.8878	0.0065	4	186	202	DGIDYAAVTVQLPGGER	140	100			Mascot
2168.9917	2168.9763	-0.0154	-7	101	121	GTGTANQCPTIDGGVDS FPFK			Carbamidomethyl (C)[8]		Mascot
2268.0952	2268.1057	0.0105	5	161	181	LTYTLDEMEGPLEVGAD GTLK			Oxidation (M)[8]		Mascot
2280.2024	2280.2117	0.0093	4	211	231	QLVATGKPESFSGPFLVP SYR					Mascot
2280.2024	2280.2117	0.0093	4	211	231	QLVATGKPESFSGPFLVP SYR	157	100			Mascot
2294.1299	2294.1372	0.0073	3	182	202	FEEKDGIDYAAVTVQLPG GER					Mascot
2434.1482	2434.1592	0.011	5	242	266	GGSTGYDNAVALPAGGR GDEEELAK					Mascot

Calc. Mass	Obsrv. Mass	± da	± ppm	Start Seq.	End Seq.	Sequence	Ion Score	C. I. %	Modification	Rank	Result Type
2434.1482	2434.1592	0.011	5	242	266	GGSTGYDNAVALPAGGR GDEEELAK	18	0			Mascot
2590.2883	2590.271	-0.0173	-7	286	310	SKPETGEVIGVFESVQPS DTDLGAK					Mascot
2590.2883	2590.271	-0.0173	-7	286	310	SKPETGEVIGVFESVQPS DTDLGAK	58	99.742			Mascot

2	hypothetical protein OsI_02088 [Oryza sativa Indica Group]	gi|218188287	35097.8	6.1	12	411	100	45.54	345	100

Protein Group

Os01g0501800 [Oryza sativa Japonica Group]	gi|113532665	35067.8	6.0999 999046 3257

Peptide Information

Calc. Mass	Obsrv. Mass	± da	± ppm	Start Seq.	End Seq.	Sequence	Ion Score	C. I. %	Modification	Rank	Result Type
930.468	930.4618	-0.0062	-7	154	161	NAPPEFQK					Mascot
1080.5573	1080.5521	-0.0052	-5	93	101	LTFDEIQSK					Mascot
1236.6583	1236.6652	0.0069	6	92	101	RLTFDEIQSK					Mascot
1236.6583	1236.6652	0.0069	6	92	101	RLTFDEIQSK	21	0			Mascot
1328.6555	1328.6475	-0.008	-6	137	147	FCLEPTSFTVK			Carbamidomethyl (C)[2]		Mascot
1360.6591	1360.7015	0.0424	31	266	277	GDEEELAKENVK					Mascot
1456.7505	1456.7262	-0.0243	-17	136	147	KFCLEPTSFTVK			Carbamidomethyl (C)[3]		Mascot
1562.7559	1562.765	0.0091	6	249	265	GGSTGYDNAVALPAGGR					Mascot
1562.7559	1562.765	0.0091	6	249	265	GGSTGYDNAVALPAGGR	107	100			Mascot
1760.8813	1760.8878	0.0065	4	193	209	DGIDYAAVTVQLPGGER					Mascot
1760.8813	1760.8878	0.0065	4	193	209	DGIDYAAVTVQLPGGER	140	100			Mascot
2280.1494	2280.2117	0.0623	27	168	188	LTYTLDEIEGPLEVSSDG TIK					Mascot
2280.1494	2280.2117	0.0623	27	168	188	LTYTLDEIEGPLEVSSDG TIK					Mascot
2294.1299	2294.1372	0.0073	3	189	209	FEEKDGIDYAAVTVQLPG GER					Mascot
2434.1482	2434.1592	0.011	5	249	273	GGSTGYDNAVALPAGGR GDEEELAK					Mascot
2434.1482	2434.1592	0.011	5	249	273	GGSTGYDNAVALPAGGR GDEEELAK	18	0			Mascot
2590.2883	2590.271	-0.0173	-7	293	317	SKPETGEVIGVFESVQPS DTDLGAK					Mascot
2590.2883	2590.271	-0.0173	-7	293	317	SKPETGEVIGVFESVQPS DTDLGAK	58	99.742			Mascot

3	Oxygen-evolving enhancer protein 1, chloroplast precursor, putative [Ricinus communis]	gi|223538464	35454.1	5.58	12	351	100	44.623	286	100

Peptide Information

Calc. Mass	Obsrv. Mass	± da	± ppm	Start Seq.	End Seq.	Sequence	Ion Score	C. I. %	Modification	Rank	Result Type
1080.5573	1080.5521	-0.0052	-5	93	101	LTFDEIQSK					Mascot
1205.6121	1205.6165	0.0044	4	274	285	ENTKNAASSVGK					Mascot
1236.6583	1236.6652	0.0069	6	92	101	RLTFDEIQSK					Mascot
1236.6583	1236.6652	0.0069	6	92	101	RLTFDEIQSK	21	0			Mascot
1328.6555	1328.6475	-0.008	-6	137	147	FCLEPTSFTVK			Carbamidomethyl (C)[2]		Mascot
1456.7505	1456.7262	-0.0243	-17	136	147	KFCLEPTSFTVK			Carbamidomethyl (C)[3]		Mascot
1562.7559	1562.765	0.0091	6	249	265	GGSTGYDNAVALPAGGR					Mascot
1562.7559	1562.765	0.0091	6	249	265	GGSTGYDNAVALPAGGR	107	100			Mascot
1649.8534	1649.8151	-0.0383	-23	321	334	DVKIQGIWYAQLES					Mascot
1664.8346	1664.8744	0.0398	24	1	16	MAASLQAAATLMQPTK			Oxidation (M)[1,12]		Mascot
1760.8813	1760.8878	0.0065	4	193	209	DGIDYAAVTVQLPGGER					Mascot
1760.8813	1760.8878	0.0065	4	193	209	DGIDYAAVTVQLPGGER	140	100			Mascot
2280.1282	2280.2117	0.0835	37	168	188	LTYTLDEIEGPFEVGPDG TVK					Mascot
2280.1282	2280.2117	0.0835	37	168	188	LTYTLDEIEGPFEVGPDG TVK					Mascot
2294.1299	2294.1372	0.0073	3	189	209	FEEKDGIDYAAVTVQLPG GER					Mascot
2434.1482	2434.1592	0.011	5	249	273	GGSTGYDNAVALPAGGR GDEEELAK					Mascot
2434.1482	2434.1592	0.011	5	249	273	GGSTGYDNAVALPAGGR GDEEELAK	18	0			Mascot

4	collinsiaXIII-like protein, partial [Collinsia rattanii]	gi|471272162	17934.1	5.03	7	347	100	23.269	306	100

Peptide Information

Calc. Mass	Obsrv. Mass	± da	± ppm	Start Seq.	End Seq.	Sequence	Ion Score	C. I. %	Modification	Rank	Result Type
930.468	930.4618	-0.0062	-7	19	26	NAPPEFQK					Mascot
950.571	950.5623	-0.0087	-9	75	82	VPFLFTVK					Mascot
950.571	950.5623	-0.0087	-9	75	82	VPFLFTVK	58	99.747			Mascot
1328.6555	1328.6475	-0.008	-6	2	12	FCLEPTSFTVK			Carbamidomethyl (C)[2]		Mascot
1456.7505	1456.7262	-0.0243	-17	1	12	KFCLEPTSFTVK			Carbamidomethyl (C)[3]		Mascot
1562.7559	1562.765	0.0091	6	114	130	GGSTGYDNAVALPAGGR					Mascot
1562.7559	1562.765	0.0091	6	114	130	GGSTGYDNAVALPAGGR	107	100			Mascot
1760.8813	1760.8878	0.0065	4	58	74	DGIDYAAVTVQLPGGER					Mascot
1760.8813	1760.8878	0.0065	4	58	74	DGIDYAAVTVQLPGGER	140	100			Mascot
2294.1299	2294.1372	0.0073	3	54	74	FEEKDGIDYAAVTVQLPG GER					Mascot

5	collinsiaXIII-like protein, partial [Collinsia linearis]	gi|471272208	16888.6	5	6	340	100	23.01	306	100

Peptide Information

Calc. Mass	Obsrv. Mass	± da	± ppm	Start Seq.	End Seq.	Sequence	Ion Score	C. I. %	Modification	Rank	Result Type
930.468	930.4618	-0.0062	-7	12	19	NAPPEFQK					Mascot
950.571	950.5623	-0.0087	-9	68	75	VPFLFTVK					Mascot
950.571	950.5623	-0.0087	-9	68	75	VPFLFTVK	58	99.747			Mascot
1182.6365	1182.5691	-0.0674	-57	1	11	SFTVKAESVSK					Mascot
1562.7559	1562.765	0.0091	6	107	123	GGSTGYDNAVALPAGGR					Mascot
1562.7559	1562.765	0.0091	6	107	123	GGSTGYDNAVALPAGGR	107	100			Mascot
1760.8813	1760.8878	0.0065	4	51	67	DGIDYAAVTVQLPGGER					Mascot
1760.8813	1760.8878	0.0065	4	51	67	DGIDYAAVTVQLPGGER	140	100			Mascot
2294.1299	2294.1372	0.0073	3	47	67	FEEKDGIDYAAVTVQLPG GER					Mascot

6	collinsiaXIII-like protein, partial [Collinsia rattanii]	gi|471272164	17235.7	5.04	6	340	100	23.086	306	100	

Protein Group

collinsiaXIII-like protein, partial [Collinsia rattanii]	gi|471272166	17235.7	5.0399 999618 5303

Peptide Information

Calc. Mass	Obsrv. Mass	± da	± ppm	Start Seq.	End Seq.	Sequence	Ion Score	C. I. %	Modification	Rank	Result Type
930.468	930.4618	-0.0062	-7	18	25	NAPPEFQK					Mascot
950.571	950.5623	-0.0087	-9	74	81	VPFLFTVK					Mascot
950.571	950.5623	-0.0087	-9	74	81	VPFLFTVK	58	99.747			Mascot
1328.6555	1328.6475	-0.008	-6	1	11	FCLEPTSFTVK			Carbamidomethyl (C)[2]		Mascot
1562.7559	1562.765	0.0091	6	113	129	GGSTGYDNAVALPAGGR					Mascot
1562.7559	1562.765	0.0091	6	113	129	GGSTGYDNAVALPAGGR	107	100			Mascot
1760.8813	1760.8878	0.0065	4	57	73	DGIDYAAVTVQLPGGER					Mascot
1760.8813	1760.8878	0.0065	4	57	73	DGIDYAAVTVQLPGGER	140	100			Mascot
2294.1299	2294.1372	0.0073	3	53	73	FEEKDGIDYAAVTVQLPG GER					Mascot

7	collinsiaXIII-like protein, partial [Collinsia linearis]	gi|471272192	17635.9	4.91	6	339	100	23.086	306	100	

Protein Group

collinsiaXIII-like protein, partial [Collinsia linearis]	gi|471272190	17635.9	4.9099 998474 1211

collinsiaXIII-like protein, partial [Collinsia linearis]	gi|471272188	17635.9	4.9099<br>998474<br>1211
collinsiaXIII-like protein, partial [Collinsia linearis]	gi|471272186	17635.9	4.9099<br>998474<br>1211
collinsiaXIII-like protein, partial [Collinsia linearis]	gi|471272184	17635.9	4.9099<br>998474<br>1211
collinsiaXIII-like protein, partial [Collinsia linearis]	gi|471272198	17806	4.9099<br>998474<br>1211
collinsiaXIII-like protein, partial [Collinsia linearis]	gi|471272206	17905.1	4.9099<br>998474<br>1211
collinsiaXIII-like protein, partial [Collinsia linearis]	gi|471272194	17635.9	4.9099<br>998474<br>1211
collinsiaXIII-like protein, partial [Collinsia linearis]	gi|471272196	17806	4.9099<br>998474<br>1211
collinsiaXIII-like protein, partial [Collinsia linearis]	gi|471272182	17635.9	4.9099<br>998474<br>1211
collinsiaXIII-like protein, partial [Collinsia linearis]	gi|471272180	17635.9	4.9099<br>998474<br>1211

collinsiaXIII-like protein, partial [Collinsia rattanii]	gi|471272178	17635.9	4.9099<br>998474<br>1211
collinsiaXIII-like protein, partial [Collinsia rattanii]	gi|471272176	17635.9	4.9099<br>998474<br>1211
collinsiaXIII-like protein, partial [Collinsia rattanii]	gi|471272174	17635.9	4.9099<br>998474<br>1211
collinsiaXIII-like protein, partial [Collinsia rattanii]	gi|471272172	17635.9	4.9099<br>998474<br>1211
collinsiaXIII-like protein, partial [Collinsia rattanii]	gi|471272170	17635.9	4.9099<br>998474<br>1211
collinsiaXIII-like protein, partial [Collinsia rattanii]	gi|471272168	17635.9	4.9099<br>998474<br>1211
collinsiaXIII-like protein, partial [Collinsia rattanii]	gi|471272204	17806	4.9099<br>998474<br>1211
collinsiaXIII-like protein, partial [Collinsia rattanii]	gi|471272202	17806	4.9099<br>998474<br>1211
collinsiaXIII-like protein, partial [Collinsia rattanii]	gi|471272200	17806	4.9099<br>998474<br>1211

Peptide Information

Calc. Mass	Obsrv. Mass	± da	± ppm	Start	End	Sequence	Ion	C. I. %	Modification	Rank	Result Type

				Seq.	Seq.		Score				
930.468	930.4618	-0.0062	-7	18	25	NAPPEFQK					Mascot
950.571	950.5623	-0.0087	-9	74	81	VPFLFTVK					Mascot
950.571	950.5623	-0.0087	-9	74	81	VPFLFTVK	58	99.747			Mascot
1328.6555	1328.6475	-0.008	-6	1	11	FCLEPTSFTVK			Carbamidomethyl (C)[2]		Mascot
1562.7559	1562.765	0.0091	6	113	129	GGSTGYDNAVALPAGGR					Mascot
1562.7559	1562.765	0.0091	6	113	129	GGSTGYDNAVALPAGGR	107	100			Mascot
1760.8813	1760.8878	0.0065	4	57	73	DGIDYAAVTVQLPGGER					Mascot
1760.8813	1760.8878	0.0065	4	57	73	DGIDYAAVTVQLPGGER	140	100			Mascot
2294.1299	2294.1372	0.0073	3	53	73	FEEKDGIDYAAVTVQLPG GER					Mascot

8	PREDICTED: oxygen-evolving enhancer protein 1, chloroplastic-like [Setaria italica]	gi|514777415	35110.8	5.74	8	338	100	24.021	306	100

Peptide Information

Calc. Mass	Obsrv. Mass	± da	± ppm	Start Seq.	End Seq.	Sequence	Ion Score	C. I. %	Modification	Rank	Result Type
930.468	930.4618	-0.0062	-7	152	159	NAPPEFQK					Mascot
950.571	950.5623	-0.0087	-9	208	215	VPFLFTVK					Mascot
950.571	950.5623	-0.0087	-9	208	215	VPFLFTVK	58	99.747			Mascot
1328.6555	1328.6475	-0.008	-6	135	145	FCLEPTSFTVK			Carbamidomethyl (C)[2]		Mascot
1456.7505	1456.7262	-0.0243	-17	134	145	KFCLEPTSFTVK			Carbamidomethyl (C)[3]		Mascot
1562.7559	1562.765	0.0091	6	247	263	GGSTGYDNAVALPAGGR					Mascot
1562.7559	1562.765	0.0091	6	247	263	GGSTGYDNAVALPAGGR	107	100			Mascot
1760.8813	1760.8878	0.0065	4	191	207	DGIDYAAVTVQLPGGER					Mascot
1760.8813	1760.8878	0.0065	4	191	207	DGIDYAAVTVQLPGGER	140	100			Mascot
2168.9917	2168.9763	-0.0154	-7	106	126	GTGTANQCPTIDGGVDS FPFK			Carbamidomethyl (C)[8]		Mascot
2294.1299	2294.1372	0.0073	3	187	207	FEEKDGIDYAAVTVQLPG GER					Mascot

9	RecName: Full=Oxygen-evolving enhancer protein 1, chloroplastic; Short=OEE1; AltName: Full=33 kDa subunit of oxygen evolving system of photosystem II; AltName: Full=33 kDa thylakoid membrane protein; AltName: Full=OEC 33 kDa subunit; Flags: Precurso	gi|11134054	35377.1	5.89	12	330	100	28.877	267	100

Peptide Information

Calc. Mass	Obsrv. Mass	± da	± ppm	Start Seq.	End Seq.	Sequence	Ion Score	C. I. %	Modification	Rank	Result Type
988.521	988.5256	0.0046	5	37	46	AFGVEPAAAR					Mascot
1080.5573	1080.5521	-0.0052	-5	92	100	LTFDEIQSK					Mascot

1236.6583	1236.6652	0.0069	6	91	100	RLTFDEIQSK					Mascot
1236.6583	1236.6652	0.0069	6	91	100	RLTFDEIQSK	21	0			Mascot
1328.6555	1328.6475	-0.008	-6	136	146	FCLEPTSFTVK			Carbamidomethyl (C)[2]		Mascot
1456.7505	1456.7262	-0.0243	-17	135	146	KFCLEPTSFTVK			Carbamidomethyl (C)[3]		Mascot
1544.7704	1544.7515	-0.0189	-12	147	160	AESVNKNAPPDFQK					Mascot
1562.7559	1562.765	0.0091	6	248	264	GGSTGYDNAVALPAGGR					Mascot
1562.7559	1562.765	0.0091	6	248	264	GGSTGYDNAVALPAGGR	107	100			Mascot
1664.8346	1664.8744	0.0398	24	1	16	MAASLQAAATLMQPTK			Oxidation (M)[1,12]		Mascot
1747.9259	1747.8792	-0.0467	-27	47	61	LTCSLQTELKDLAQK			Carbamidomethyl (C)[3]		Mascot
1760.8813	1760.8878	0.0065	4	192	208	DGIDYAAVTVQLPGGER					Mascot
1760.8813	1760.8878	0.0065	4	192	208	DGIDYAAVTVQLPGGER	140	100			Mascot
2152.1907	2152.1318	-0.0589	-27	2	23	AASLQAAATLMQPTKVG VAPAR					Mascot
2294.1299	2294.1372	0.0073	3	188	208	FEEKDGIDYAAVTVQLPG GER					Mascot

10	PREDICTED: oxygen-evolving enhancer protein 1, chloroplastic-like [Cucumis sativus]	gi|449448384	35109.9	6.24	9	327	100	29.214	286	100	

Protein Group

PREDICTED: oxygen-evolving enhancer protein 1, chloroplastic-like [Cucumis sativus]	gi|449497717	35109.9	6.2399 997711 1816

Peptide Information

Calc. Mass	Obsrv. Mass	± da	± ppm	Start Seq.	End Seq.	Sequence	Ion Score	C. I. %	Modification	Rank	Result Type
1080.5573	1080.5521	-0.0052	-5	91	99	LTFDEIQSK					Mascot
1236.6583	1236.6652	0.0069	6	90	99	RLTFDEIQSK					Mascot
1236.6583	1236.6652	0.0069	6	90	99	RLTFDEIQSK	21	0			Mascot
1328.6555	1328.6475	-0.008	-6	135	145	FCLEPTSFTVK			Carbamidomethyl (C)[2]		Mascot
1456.7505	1456.7262	-0.0243	-17	134	145	KFCLEPTSFTVK			Carbamidomethyl (C)[3]		Mascot
1544.8101	1544.7515	-0.0586	-38	2	17	AASVQAAAATLMQPSK					Mascot
1560.8051	1560.731	-0.0741	-47	2	17	AASVQAAAATLMQPSK			Oxidation (M)[12]		Mascot
1562.7559	1562.765	0.0091	6	247	263	GGSTGYDNAVALPAGGR					Mascot
1562.7559	1562.765	0.0091	6	247	263	GGSTGYDNAVALPAGGR	107	100			Mascot
1760.8813	1760.8878	0.0065	4	191	207	DGIDYAAVTVQLPGGER					Mascot
1760.8813	1760.8878	0.0065	4	191	207	DGIDYAAVTVQLPGGER	140	100			Mascot
2294.1299	2294.1372	0.0073	3	187	207	FEEKDGIDYAAVTVQLPG GER					Mascot

2434.1482	2434.1592	0.011	5	247	271	GGSTGYDNAVALPAGGR GDEEELAK			Mascot
2434.1482	2434.1592	0.011	5	247	271	GGSTGYDNAVALPAGGR GDEEELAK	18	0	Mascot

Gel Idx/Pos	178/H5	Instr./Gel Origin	BA2151/Sample Project 20140814	Process Status	Analysis Succeeded
Plate [#] Name	[1] Sample Project 20140814	Instrument Sample Name		Spectra	11

Rank	Protein Name	Accession No.	Protein MW	Protein PI	Pep. Count	Protein Score	Protein Score C. I. %	Intensity Matched	Total Ion Score	Total Ion C. I. %	Confirmed
1	L-ascorbate peroxidase 2, cytosolic [Aegilops tauschii]	gi|475538008	27983.9	5.1	13	731	100	39.839	640	100	

Peptide Information

Calc. Mass	Obsrv. Mass	± da	± ppm	Start Seq.	End Seq.	Sequence	Ion Score	C. I. %	Modification	Rank	Result Type
911.4291	911.4346	0.0055	6	54	62	TGGPFGTMK			Oxidation (M)[8]		Mascot
974.491	974.4979	0.0069	7	32	39	NCAPLMLR			Carbamidomethyl (C)[2]		Mascot
990.4859	990.4873	0.0014	1	32	39	NCAPLMLR			Carbamidomethyl (C)[2], Oxidation (M)[6]		Mascot
1113.615	1113.6182	0.0032	3	201	210	EGLLQLPTDK					Mascot
1249.6172	1249.6227	0.0055	4	121	131	QDKPEPPPEGR					Mascot
1249.6172	1249.6227	0.0055	4	121	131	QDKPEPPPEGR	70	99.984			Mascot
1309.6495	1309.6583	0.0088	7	132	143	LPDATQGSDHLR					Mascot
1309.6495	1309.6583	0.0088	7	132	143	LPDATQGSDHLR	112	100			Mascot
1503.7592	1503.7513	-0.0079	-5	40	53	LAWHSAGTFDVATK					Mascot
1585.8949	1585.8997	0.0048	3	211	224	TLLTDPAFRPLVDK					Mascot
1585.8949	1585.8997	0.0048	3	211	224	TLLTDPAFRPLVDK	65	99.951			Mascot
1686.8043	1686.7753	-0.029	-17	5	19	CYPTVSDEYLAAVAK			Carbamidomethyl (C)[1]		Mascot
1834.9229	1834.9248	0.0019	1	63	80	CPAELAHGANAGLDIAVR	143	100	Carbamidomethyl (C)[1]		Mascot
2046.908	2046.8977	-0.0103	-5	225	242	YAADEDAFFADYAEAHLK					Mascot
2046.908	2046.8977	-0.0103	-5	225	242	YAADEDAFFADYAEAHLK	149	100			Mascot
2633.2988	2633.3027	0.0039	1	144	168	QVFSTQMGLSDQDIVALS GGHTLGR			Oxidation (M)[7]		Mascot
2633.2988	2633.3027	0.0039	1	144	168	QVFSTQMGLSDQDIVALS GGHTLGR	101	100	Oxidation (M)[7]		Mascot
2993.4204	2993.4258	0.0054	2	174	200	SGFEGAWTANPLIFDNSY FTELLSGEK					Mascot
3689.864	3689.927	0.063	17	87	120	EQFPILSYADFYQLAGVV AVEVTGGPEVPFHPGR					Mascot

Rank	Protein Name	Accession No.	Protein MW	Protein PI	Pep. Count	Protein Score	Protein Score C. I. %	Intensity Matched	Total Ion Score	Total Ion C. I. %	Confirmed
2	ascorbate peroxidase [Saccharum hybrid cultivar GT28]	gi|397702109	27256.7	5.18	10	500	100	44.966	432	100	

Peptide Information

Calc. Mass	Obsrv. Mass	± da	± ppm	Start Seq.	End Seq.	Sequence	Ion Score	C. I. %	Modification	Rank	Result Type
911.4291	911.4346	0.0055	6	53	61	TGGPFGTMK			Oxidation (M)[8]		Mascot
974.491	974.4979	0.0069	7	31	38	NCAPLMLR			Carbamidomethyl (C)[2]		Mascot

990.4859	990.4873	0.0014	1	31	38	NCAPLMLR			Carbamidomethyl (C)[2], Oxidation (M)[6]		Mascot
1249.6172	1249.6227	0.0055	4	120	130	QDKPEPPPEGR					Mascot
1249.6172	1249.6227	0.0055	4	120	130	QDKPEPPPEGR	70	99.984			Mascot
1309.6495	1309.6583	0.0088	7	131	142	LPDATQGSDHLR					Mascot
1309.6495	1309.6583	0.0088	7	131	142	LPDATQGSDHLR	112	100			Mascot
1503.7592	1503.7513	-0.0079	-5	39	52	LAWHSAGTFDVATK					Mascot
1557.8635	1557.7363	-0.1272	-82	210	223	ALLSDPSFRPLVDK					Mascot
1585.8552	1585.8997	0.0445	28	25	38	GLIAEKNCAPLMLR			Carbamidomethyl (C)[8]		Mascot
1585.8552	1585.8997	0.0445	28	25	38	GLIAEKNCAPLMLR			Carbamidomethyl (C)[8]		Mascot
1601.8502	1601.8862	0.036	22	25	38	GLIAEKNCAPLMLR			Carbamidomethyl (C)[8], Oxidation (M)[12]		Mascot
1817.9253	1817.9055	-0.0198	-11	62	79	NPAEQAHGANAGLEIAV R					Mascot
1817.9253	1817.9055	-0.0198	-11	62	79	NPAEQAHGANAGLEIAV R	10	0			Mascot
2046.908	2046.8977	-0.0103	-5	224	241	YAADEDAFFADYAEAHLK					Mascot
2046.908	2046.8977	-0.0103	-5	224	241	YAADEDAFFADYAEAHLK	149	100			Mascot
2633.2988	2633.3027	0.0039	1	143	167	QVFSTQMGLSDQDIVALS GGHTLGR			Oxidation (M)[7]		Mascot
2633.2988	2633.3027	0.0039	1	143	167	QVFSTQMGLSDQDIVALS GGHTLGR	101	100	Oxidation (M)[7]		Mascot

3	TPA: APx2-Cytosolic Ascorbate Peroxidase [Zea mays]	gi|414591286	31014.7	5.77	11	495	100	39.547	432	100	

Protein Group

TPA: hypothetical protein ZEAMMB73_314819 [Zea mays]	gi|414591285	30596.5	5.9200 000762 9395

Peptide Information

Calc. Mass	Obsrv. Mass	± da	± ppm	Start Seq.	End Seq.	Sequence	Ion Score	C. I. %	Modification	Rank	Result Type
911.4291	911.4346	0.0055	6	89	97	TGGPFGTMK			Oxidation (M)[8]		Mascot
974.491	974.4979	0.0069	7	67	74	NCAPLMLR			Carbamidomethyl (C)[2]		Mascot
990.4859	990.4873	0.0014	1	67	74	NCAPLMLR			Carbamidomethyl (C)[2], Oxidation (M)[6]		Mascot
1249.6172	1249.6227	0.0055	4	156	166	QDKPEPPPEGR					Mascot
1249.6172	1249.6227	0.0055	4	156	166	QDKPEPPPEGR	70	99.984			Mascot
1309.6495	1309.6583	0.0088	7	167	178	LPDATQGSDHLR					Mascot
1309.6495	1309.6583	0.0088	7	167	178	LPDATQGSDHLR	112	100			Mascot
1503.7592	1503.7513	-0.0079	-5	75	88	LAWHSAGTFDVATK					Mascot
1557.8635	1557.7363	-0.1272	-82	246	259	ALLSDPSFRPLVDK					Mascot
1585.8552	1585.8997	0.0445	28	61	74	GLIAEKNCAPLMLR			Carbamidomethyl (C)[8]		Mascot

Calc. Mass	Obsrv. Mass	± da	± ppm	Start Seq.	End Seq.	Sequence	Ion Score	C. I. %	Modification	Rank	Result Type
1585.8552	1585.8997	0.0445	28	61	74	GLIAEKNCAPLMLR			Carbamidomethyl (C)[8]		Mascot
1601.8502	1601.8862	0.036	22	61	74	GLIAEKNCAPLMLR			Carbamidomethyl (C)[8], Oxidation (M)[12]		Mascot
1831.9409	1831.8876	-0.0533	-29	98	115	NPAEQAHGANAGLEIAIR					Mascot
2046.908	2046.8977	-0.0103	-5	260	277	YAADEDAFFADYAEAHLK					Mascot
2046.908	2046.8977	-0.0103	-5	260	277	YAADEDAFFADYAEAHLK	149	100			Mascot
2551.189	2551.406	0.217	85	207	228	DRSGFEGAWTSNPLIFD NSYFK					Mascot
2633.2988	2633.3027	0.0039	1	179	203	QVFSTQMGLSDQDIVALS GGHTLGR			Oxidation (M)[7]		Mascot
2633.2988	2633.3027	0.0039	1	179	203	QVFSTQMGLSDQDIVALS GGHTLGR	101	100	Oxidation (M)[7]		Mascot

4 ascorbate peroxidase2 [Zea mays] gi|162457709 27408.9 5.28 10 491 100 39.138 432 100

Peptide Information

Calc. Mass	Obsrv. Mass	± da	± ppm	Start Seq.	End Seq.	Sequence	Ion Score	C. I. %	Modification	Rank	Result Type
911.4291	911.4346	0.0055	6	53	61	TGGPFGTMK			Oxidation (M)[8]		Mascot
974.491	974.4979	0.0069	7	31	38	NCAPLMLR			Carbamidomethyl (C)[2]		Mascot
990.4859	990.4873	0.0014	1	31	38	NCAPLMLR			Carbamidomethyl (C)[2], Oxidation (M)[6]		Mascot
1249.6172	1249.6227	0.0055	4	120	130	QDKPEPPPEGR					Mascot
1249.6172	1249.6227	0.0055	4	120	130	QDKPEPPPEGR	70	99.984			Mascot
1309.6495	1309.6583	0.0088	7	131	142	LPDATQGSDHLR					Mascot
1309.6495	1309.6583	0.0088	7	131	142	LPDATQGSDHLR	112	100			Mascot
1557.8635	1557.7363	-0.1272	-82	210	223	ALLSDPSFRPLVDK					Mascot
1585.8552	1585.8997	0.0445	28	25	38	GLIAEKNCAPLMLR			Carbamidomethyl (C)[8]		Mascot
1585.8552	1585.8997	0.0445	28	25	38	GLIAEKNCAPLMLR			Carbamidomethyl (C)[8]		Mascot
1601.8502	1601.8862	0.036	22	25	38	GLIAEKNCAPLMLR			Carbamidomethyl (C)[8], Oxidation (M)[12]		Mascot
1686.8407	1686.7753	-0.0654	-39	1	14	MVKAYPTVNEDYLK			Oxidation (M)[1]		Mascot
2046.908	2046.8977	-0.0103	-5	224	241	YAADEDAFFADYAEAHLK					Mascot
2046.908	2046.8977	-0.0103	-5	224	241	YAADEDAFFADYAEAHLK	149	100			Mascot
2551.189	2551.406	0.217	85	171	192	DRSGFEGAWTSNPLIFD NSYFK					Mascot
2633.2988	2633.3027	0.0039	1	143	167	QVFSTQMGLSDQDIVALS GGHTLGR			Oxidation (M)[7]		Mascot
2633.2988	2633.3027	0.0039	1	143	167	QVFSTQMGLSDQDIVALS GGHTLGR	101	100	Oxidation (M)[7]		Mascot

5 TPA: hypothetical protein ZEAMMB73_314819 [Zea mays] gi|414591283 20838.4 4.69 7 474 100 24 432 100

Peptide Information

Calc. Mass	Obsrv. Mass	± da	± ppm	Start Seq.	End Seq.	Sequence	Ion Score	C. I. %	Modification	Rank	Result Type

Calc. Mass	Obsrv. Mass	± da	± ppm	Start Seq.	End Seq.	Sequence	Ion Score	C. I. %	Modification	Rank	Result Type
1249.6172	1249.6227	0.0055	4	61	71	QDKPEPPPEGR					Mascot
1249.6172	1249.6227	0.0055	4	61	71	QDKPEPPPEGR	70	99.984			Mascot
1309.6495	1309.6583	0.0088	7	72	83	LPDATQGSDHLR					Mascot
1309.6495	1309.6583	0.0088	7	72	83	LPDATQGSDHLR	112	100			Mascot
1557.8635	1557.7363	-0.1272	-82	151	164	ALLSDPSFRPLVDK					Mascot
1831.9409	1831.8876	-0.0533	-29	3	20	NPAEQAHGANAGLEIAIR					Mascot
2046.908	2046.8977	-0.0103	-5	165	182	YAADEDAFFADYAEAHLK					Mascot
2046.908	2046.8977	-0.0103	-5	165	182	YAADEDAFFADYAEAHLK	149	100			Mascot
2551.189	2551.406	0.217	85	112	133	DRSGFEGAWTSNPLIFD NSYFK					Mascot
2633.2988	2633.3027	0.0039	1	84	108	QVFSTQMGLSDQDIVALS GGHTLGR			Oxidation (M)[7]		Mascot
2633.2988	2633.3027	0.0039	1	84	108	QVFSTQMGLSDQDIVALS GGHTLGR	101	100	Oxidation (M)[7]		Mascot

6 PREDICTED: L-ascorbate peroxidase 2, cytosolic-like isoform X2 [Setaria italica] gi|514737330 27257.8 5.18 8 474 100 38.409 432 100

Peptide Information

Calc. Mass	Obsrv. Mass	± da	± ppm	Start Seq.	End Seq.	Sequence	Ion Score	C. I. %	Modification	Rank	Result Type
911.4291	911.4346	0.0055	6	53	61	TGGPFGTMK			Oxidation (M)[8]		Mascot
974.491	974.4979	0.0069	7	31	38	NCAPLMLR			Carbamidomethyl (C)[2]		Mascot
990.4859	990.4873	0.0014	1	31	38	NCAPLMLR			Carbamidomethyl (C)[2], Oxidation (M)[6]		Mascot
1249.6172	1249.6227	0.0055	4	120	130	QDKPEPPPEGR					Mascot
1249.6172	1249.6227	0.0055	4	120	130	QDKPEPPPEGR	70	99.984			Mascot
1309.6495	1309.6583	0.0088	7	131	142	LPDATQGSDHLR					Mascot
1309.6495	1309.6583	0.0088	7	131	142	LPDATQGSDHLR	112	100			Mascot
1585.8552	1585.8997	0.0445	28	25	38	GLIAEKNCAPLMLR			Carbamidomethyl (C)[8]		Mascot
1585.8552	1585.8997	0.0445	28	25	38	GLIAEKNCAPLMLR			Carbamidomethyl (C)[8]		Mascot
1601.8502	1601.8862	0.036	22	25	38	GLIAEKNCAPLMLR			Carbamidomethyl (C)[8], Oxidation (M)[12]		Mascot
2046.908	2046.8977	-0.0103	-5	224	241	YAADEDAFFADYAEAHLK					Mascot
2046.908	2046.8977	-0.0103	-5	224	241	YAADEDAFFADYAEAHLK	149	100			Mascot
2551.189	2551.406	0.217	85	171	192	DRSGFEGAWTSNPLIFD NSYFK					Mascot
2633.2988	2633.3027	0.0039	1	143	167	QVFSTQMGLSDQDIVALS GGHTLGR			Oxidation (M)[7]		Mascot
2633.2988	2633.3027	0.0039	1	143	167	QVFSTQMGLSDQDIVALS GGHTLGR	101	100	Oxidation (M)[7]		Mascot

7 uncharacterized protein, partial [Phleum pratense] gi|409972491 27716.9 5.1 9 404 100 22.138 356 100

Protein Group

uncharacterized protein, partial [Phleum pratense] gi|409971785 28089.1 5.0999 999046

			3257	
	uncharacterized protein, partial [Phleum pratense]	gi|409972079	27687.9	5.0999 999046 3257

Peptide Information

Calc. Mass	Obsrv. Mass	± da	± ppm	Start Seq.	End Seq.	Sequence	Ion Score	C. I. %	Modification	Rank	Result Type
974.491	974.4979	0.0069	7	30	37	NCAPLMLR			Carbamidomethyl (C)[2]		Mascot
990.4859	990.4873	0.0014	1	30	37	NCAPLMLR			Carbamidomethyl (C)[2], Oxidation (M)[6]		Mascot
1113.615	1113.6182	0.0032	3	199	208	EGLLQLPTDK					Mascot
1503.7592	1503.7513	-0.0079	-5	38	51	IAWHSAGTFDVATK					Mascot
1585.8949	1585.8997	0.0048	3	209	222	TLLTDPAFRPLVDK					Mascot
1585.8949	1585.8997	0.0048	3	209	222	TLLTDPAFRPLVDK	65	99.951			Mascot
1601.8502	1601.8862	0.036	22	24	37	GLIAEKNCAPLMLR			Carbamidomethyl (C)[8], Oxidation (M)[12]		Mascot
1686.8043	1686.7753	-0.029	-17	3	17	CYPTVSDEYLAAVAK			Carbamidomethyl (C)[1]		Mascot
1834.9229	1834.9248	0.0019	1	61	78	CPAELAHGANAGLDIAVR	143	100	Carbamidomethyl (C)[1]		Mascot
2046.908	2046.8977	-0.0103	-5	223	240	YAADEDAFFADYAEAHLK					Mascot
2046.908	2046.8977	-0.0103	-5	223	240	YAADEDAFFADYAEAHLK	149	100			Mascot
2601.3091	2601.3193	0.0102	4	142	166	QVFTAQMGLSDQDIVALS GGHTLGR					Mascot

8	uncharacterized protein, partial [Phleum pratense]	gi|409971705	28709.4	5.18	9	403	100	22.138	356	100	

Peptide Information

Calc. Mass	Obsrv. Mass	± da	± ppm	Start Seq.	End Seq.	Sequence	Ion Score	C. I. %	Modification	Rank	Result Type
974.491	974.4979	0.0069	7	40	47	NCAPLMLR			Carbamidomethyl (C)[2]		Mascot
990.4859	990.4873	0.0014	1	40	47	NCAPLMLR			Carbamidomethyl (C)[2], Oxidation (M)[6]		Mascot
1113.615	1113.6182	0.0032	3	209	218	EGLLQLPTDK					Mascot
1503.7592	1503.7513	-0.0079	-5	48	61	IAWHSAGTFDVATK					Mascot
1585.8949	1585.8997	0.0048	3	219	232	TLLTDPAFRPLVDK					Mascot
1585.8949	1585.8997	0.0048	3	219	232	TLLTDPAFRPLVDK	65	99.951			Mascot
1601.8502	1601.8862	0.036	22	34	47	GLIAEKNCAPLMLR			Carbamidomethyl (C)[8], Oxidation (M)[12]		Mascot
1686.8043	1686.7753	-0.029	-17	13	27	CYPTVSDEYLAAVAK			Carbamidomethyl (C)[1]		Mascot
1834.9229	1834.9248	0.0019	1	71	88	CPAELAHGANAGLDIAVR	143	100	Carbamidomethyl (C)[1]		Mascot
2046.908	2046.8977	-0.0103	-5	233	250	YAADEDAFFADYAEAHLK					Mascot

2046.908	2046.8977	-0.0103	-5	233	250	YAADEDAFFADYAEAHLK	149	100			Mascot
2601.3091	2601.3193	0.0102	4	152	176	QVFTAQMGLSDQDIVALS GGHTLGR					Mascot

9	Os07g0694700 [Oryza sativa Japonica Group]	gi|113612277	27214.6	5.21	11	399	100	34.091	331	100	

Protein Group

RecName: Full=L-ascorbate peroxidase 2, cytosolic; AltName: Full=APXb; AltName: Full=OsAPx02	gi|75308965	27214.6	5.2100 000381 4697
hypothetical protein OsI_27453 [Oryza sativa Indica Group]	gi|218200316	27214.6	5.2100 000381 4697

Peptide Information

Calc. Mass	Obsrv. Mass	± da	± ppm	Start Seq.	End Seq.	Sequence	Ion Score	C. I. %	Modification	Rank	Result Type
911.4291	911.4346	0.0055	6	54	62	TGGPFGTMK			Oxidation (M)[8]		Mascot
974.491	974.4979	0.0069	7	32	39	NCAPLMLR			Carbamidomethyl (C)[2]		Mascot
990.4859	990.4873	0.0014	1	32	39	NCAPLMLR			Carbamidomethyl (C)[2], Oxidation (M)[6]		Mascot
1249.6172	1249.6227	0.0055	4	121	131	QDKPEPPPEGR					Mascot
1249.6172	1249.6227	0.0055	4	121	131	QDKPEPPPEGR	70	99.984			Mascot
1309.6495	1309.6583	0.0088	7	132	143	LPDATQGSDHLR					Mascot
1309.6495	1309.6583	0.0088	7	132	143	LPDATQGSDHLR	112	100			Mascot
1533.7445	1533.7635	0.019	12	40	53	LAWHSAGTFDVSSR					Mascot
1557.8458	1557.7363	-0.1095	-70	211	224	ALMADPAFRPLVEK					Mascot
1585.8552	1585.8997	0.0445	28	26	39	GLIAEKNCAPLMLR			Carbamidomethyl (C)[8]		Mascot
1585.8552	1585.8997	0.0445	28	26	39	GLIAEKNCAPLMLR			Carbamidomethyl (C)[8]		Mascot
1601.8502	1601.8862	0.036	22	26	39	GLIAEKNCAPLMLR			Carbamidomethyl (C)[8], Oxidation (M)[12]		Mascot
1798.9222	1798.8157	-0.1065	-59	5	21	SYPTVSDEYLAAVGKAK					Mascot
2046.908	2046.8977	-0.0103	-5	225	242	YAADEDAFFADYAEAHLK					Mascot
2046.908	2046.8977	-0.0103	-5	225	242	YAADEDAFFADYAEAHLK	149	100			Mascot
2601.2727	2601.3193	0.0466	18	132	155	LPDATQGSDHLRQVFSA QMGLSDK					Mascot
2712.3159	2712.3928	0.0769	28	54	80	TGGPFGTMKNPGEQSHA ANAGLDIAVR			Oxidation (M)[8]		Mascot

10	uncharacterized protein, partial [Phleum pratense]	gi|409972069	26190.2	5.17	8	398	100	21.565	356	100	

Peptide Information

Calc. Mass	Obsrv. Mass	± da	± ppm	Start	End	Sequence	Ion	C. I. %	Modification	Rank	Result Type

				Seq.	Seq.		Score			
974.491	974.4979	0.0069	7	16	23	NCAPLMLR			Carbamidomethyl (C)[2]	Mascot
990.4859	990.4873	0.0014	1	16	23	NCAPLMLR			Carbamidomethyl (C)[2], Oxidation (M)[6]	Mascot
1113.615	1113.6182	0.0032	3	185	194	EGLLQLPTDK				Mascot
1503.7592	1503.7513	-0.0079	-5	24	37	IAWHSAGTFDVATK				Mascot
1585.8949	1585.8997	0.0048	3	195	208	TLLTDPAFRPLVDK				Mascot
1585.8949	1585.8997	0.0048	3	195	208	TLLTDPAFRPLVDK	65	99.948		Mascot
1601.8502	1601.8862	0.036	22	10	23	GLIAEKNCAPLMLR			Carbamidomethyl (C)[8], Oxidation (M)[12]	Mascot
1834.9229	1834.9248	0.0019	1	47	64	CPAELAHGANAGLDIAVR	143	100	Carbamidomethyl (C)[1]	Mascot
2046.908	2046.8977	-0.0103	-5	209	226	YAADEDAFFADYAEAHLK				Mascot
2046.908	2046.8977	-0.0103	-5	209	226	YAADEDAFFADYAEAHLK	149	100		Mascot
2601.3091	2601.3193	0.0102	4	128	152	QVFTAQMGLSDQDIVALS GGHTLGR				Mascot

Gel Idx/Pos	179/H6	Instr./Gel Origin	BA2151/Sample Project 20140814	Process Status	Analysis Succeeded
Plate [#] Name	[1] Sample Project 20140814	Instrument Sample Name		Spectra	11

Rank	Protein Name	Accession No.	Protein MW	Protein PI	Pep. Count	Protein Score	Protein Score C. I. %	Intensity Matched	Total Ion Score	Total Ion C. I. %	Confirmed
1	L-ascorbate peroxidase 2, cytosolic [Aegilops tauschii]	gi|475538008	27983.9	5.1	11	568	100	48.165	503	100	

Peptide Information

Calc. Mass	Obsrv. Mass	± da	± ppm	Start Seq.	End Seq.	Sequence	Ion Score	C. I. %	Modification	Rank	Result Type
911.4291	911.4317	0.0026	3	54	62	TGGPFGTMK			Oxidation (M)[8]		Mascot
974.491	974.4974	0.0064	7	32	39	NCAPLMLR			Carbamidomethyl (C)[2]		Mascot
990.4859	990.4855	-0.0004	0	32	39	NCAPLMLR			Carbamidomethyl (C)[2], Oxidation (M)[6]		Mascot
1113.615	1113.6061	-0.0089	-8	201	210	EGLLQLPTDK					Mascot
1249.6172	1249.6228	0.0056	4	121	131	QDKPEPPPEGR					Mascot
1249.6172	1249.6228	0.0056	4	121	131	QDKPEPPPEGR	67	99.966			Mascot
1309.6495	1309.656	0.0065	5	132	143	LPDATQGSDHLR					Mascot
1309.6495	1309.656	0.0065	5	132	143	LPDATQGSDHLR	110	100			Mascot
1503.7592	1503.7535	-0.0057	-4	40	53	LAWHSAGTFDVATK					Mascot
1585.8949	1585.8948	-0.0001	0	211	224	TLLTDPAFRPLVDK					Mascot
1585.8949	1585.8948	-0.0001	0	211	224	TLLTDPAFRPLVDK	59	99.766			Mascot
1686.8043	1686.7644	-0.0399	-24	5	19	CYPTVSDEYLAAVAK			Carbamidomethyl (C)[1]		Mascot
1834.9229	1834.9218	-0.0011	-1	63	80	CPAELAHGANAGLDIAVR	153	100	Carbamidomethyl (C)[1]		Mascot
2046.908	2046.9011	-0.0069	-3	225	242	YAADEDAFFADYAEAHLK					Mascot
2617.304	2617.2988	-0.0052	-2	144	168	QVFSTQMGLSDQDIVALS GGHTLGR					Mascot
2633.2988	2633.3018	0.003	1	144	168	QVFSTQMGLSDQDIVALS GGHTLGR			Oxidation (M)[7]		Mascot
2633.2988	2633.3018	0.003	1	144	168	QVFSTQMGLSDQDIVALS GGHTLGR	114	100	Oxidation (M)[7]		Mascot

Rank	Protein Name	Accession No.	Protein MW	Protein PI	Pep. Count	Protein Score	Protein Score C. I. %	Intensity Matched	Total Ion Score	Total Ion C. I. %	Confirmed
2	ascorbate peroxidase [Saccharum hybrid cultivar GT28]	gi|397702109	27256.7	5.18	11	369	100	54.532	291	100	

Peptide Information

Calc. Mass	Obsrv. Mass	± da	± ppm	Start Seq.	End Seq.	Sequence	Ion Score	C. I. %	Modification	Rank	Result Type
899.5672	899.4792	-0.088	-98	23	30	LRGLIAEK					Mascot
911.4291	911.4317	0.0026	3	53	61	TGGPFGTMK			Oxidation (M)[8]		Mascot
974.491	974.4974	0.0064	7	31	38	NCAPLMLR			Carbamidomethyl (C)[2]		Mascot
990.4859	990.4855	-0.0004	0	31	38	NCAPLMLR			Carbamidomethyl (C)[2], Oxidation (M)[6]		Mascot
1249.6172	1249.6228	0.0056	4	120	130	QDKPEPPPEGR					Mascot

Calc. Mass	Obsrv. Mass	± da	± ppm	Start Seq.	End Seq.	Sequence	Ion Score	C. I. %	Modification	Rank	Result Type
1249.6172	1249.6228	0.0056	4	120	130	QDKPEPPPEGR	67	99.966			Mascot
1309.6495	1309.656	0.0065	5	131	142	LPDATQGSDHLR					Mascot
1309.6495	1309.656	0.0065	5	131	142	LPDATQGSDHLR	110	100			Mascot
1503.7592	1503.7535	-0.0057	-4	39	52	LAWHSAGTFDVATK					Mascot
1557.8635	1557.7429	-0.1206	-77	210	223	ALLSDPSFRPLVDK					Mascot
1585.8552	1585.8948	0.0396	25	25	38	GLIAEKNCAPLMLR			Carbamidomethyl (C)[8]		Mascot
1585.8552	1585.8948	0.0396	25	25	38	GLIAEKNCAPLMLR			Carbamidomethyl (C)[8]		Mascot
1601.8502	1601.8773	0.0271	17	25	38	GLIAEKNCAPLMLR			Carbamidomethyl (C)[8], Oxidation (M)[12]		Mascot
1817.9253	1817.907	-0.0183	-10	62	79	NPAEQAHGANAGLEIAV R					Mascot
1817.9253	1817.907	-0.0183	-10	62	79	NPAEQAHGANAGLEIAV R	11	0			Mascot
2046.908	2046.9011	-0.0069	-3	224	241	YAADEDAFFADYAEAHLK					Mascot
2617.304	2617.2988	-0.0052	-2	143	167	QVFSTQMGLSDQDIVALS GGHTLGR					Mascot
2633.2988	2633.3018	0.003	1	143	167	QVFSTQMGLSDQDIVALS GGHTLGR			Oxidation (M)[7]		Mascot
2633.2988	2633.3018	0.003	1	143	167	QVFSTQMGLSDQDIVALS GGHTLGR	114	100	Oxidation (M)[7]		Mascot

3	TPA: hypothetical protein ZEAMMB73_314819 [Zea mays]	gi|414591285	30596.5	5.92	12	362	100	48.179	291	100	

Peptide Information

Calc. Mass	Obsrv. Mass	± da	± ppm	Start Seq.	End Seq.	Sequence	Ion Score	C. I. %	Modification	Rank	Result Type
899.5672	899.4792	-0.088	-98	59	66	LRGLIAEK					Mascot
911.4291	911.4317	0.0026	3	89	97	TGGPFGTMK			Oxidation (M)[8]		Mascot
974.491	974.4974	0.0064	7	67	74	NCAPLMLR			Carbamidomethyl (C)[2]		Mascot
990.4859	990.4855	-0.0004	0	67	74	NCAPLMLR			Carbamidomethyl (C)[2], Oxidation (M)[6]		Mascot
1249.6172	1249.6228	0.0056	4	156	166	QDKPEPPPEGR					Mascot
1249.6172	1249.6228	0.0056	4	156	166	QDKPEPPPEGR	67	99.966			Mascot
1309.6495	1309.656	0.0065	5	167	178	LPDATQGSDHLR					Mascot
1309.6495	1309.656	0.0065	5	167	178	LPDATQGSDHLR	110	100			Mascot
1503.7592	1503.7535	-0.0057	-4	75	88	LAWHSAGTFDVATK					Mascot
1557.8635	1557.7429	-0.1206	-77	246	259	ALLSDPSFRPLVDK					Mascot
1585.8552	1585.8948	0.0396	25	61	74	GLIAEKNCAPLMLR			Carbamidomethyl (C)[8]		Mascot
1585.8552	1585.8948	0.0396	25	61	74	GLIAEKNCAPLMLR			Carbamidomethyl (C)[8]		Mascot
1601.8502	1601.8773	0.0271	17	61	74	GLIAEKNCAPLMLR			Carbamidomethyl (C)[8], Oxidation (M)[12]		Mascot
1831.9409	1831.8951	-0.0458	-25	98	115	NPAEQAHGANAGLEIAIR					Mascot
1856.0011	1855.8724	-0.1287	-69	229	245	ELLSGEKEGLLQLPSDK					Mascot
2046.908	2046.9011	-0.0069	-3	260	277	YAADEDAFFADYAEAHLK					Mascot

Calc. Mass	Obsrv. Mass	± da	± ppm	Start Seq.	End Seq.	Sequence	Ion Score	C. I. %	Modification	Rank	Result Type
2617.304	2617.2988	-0.0052	-2	179	203	QVFSTQMGLSDQDIVALS GGHTLGR					Mascot
2633.2988	2633.3018	0.003	1	179	203	QVFSTQMGLSDQDIVALS GGHTLGR			Oxidation (M)[7]		Mascot
2633.2988	2633.3018	0.003	1	179	203	QVFSTQMGLSDQDIVALS GGHTLGR	114	100	Oxidation (M)[7]		Mascot

4	TPA: APx2-Cytosolic Ascorbate Peroxidase [Zea mays]	gi|414591286	31014.7	5.77	12	361	100	48.179	291	100

Peptide Information

Calc. Mass	Obsrv. Mass	± da	± ppm	Start Seq.	End Seq.	Sequence	Ion Score	C. I. %	Modification	Rank	Result Type
899.5672	899.4792	-0.088	-98	59	66	LRGLIAEK					Mascot
911.4291	911.4317	0.0026	3	89	97	TGGPFGTMK			Oxidation (M)[8]		Mascot
974.491	974.4974	0.0064	7	67	74	NCAPLMLR			Carbamidomethyl (C)[2]		Mascot
990.4859	990.4855	-0.0004	0	67	74	NCAPLMLR			Carbamidomethyl (C)[2], Oxidation (M)[6]		Mascot
1249.6172	1249.6228	0.0056	4	156	166	QDKPEPPPEGR					Mascot
1249.6172	1249.6228	0.0056	4	156	166	QDKPEPPPEGR	67	99.966			Mascot
1309.6495	1309.656	0.0065	5	167	178	LPDATQGSDHLR					Mascot
1309.6495	1309.656	0.0065	5	167	178	LPDATQGSDHLR	110	100			Mascot
1503.7592	1503.7535	-0.0057	-4	75	88	LAWHSAGTFDVATK					Mascot
1557.8635	1557.7429	-0.1206	-77	246	259	ALLSDPSFRPLVDK					Mascot
1585.8552	1585.8948	0.0396	25	61	74	GLIAEKNCAPLMLR			Carbamidomethyl (C)[8]		Mascot
1585.8552	1585.8948	0.0396	25	61	74	GLIAEKNCAPLMLR			Carbamidomethyl (C)[8]		Mascot
1601.8502	1601.8773	0.0271	17	61	74	GLIAEKNCAPLMLR			Carbamidomethyl (C)[8], Oxidation (M)[12]		Mascot
1831.9409	1831.8951	-0.0458	-25	98	115	NPAEQAHGANAGLEIAIR					Mascot
1856.0011	1855.8724	-0.1287	-69	229	245	ELLSGEKEGLLQLPSDK					Mascot
2046.908	2046.9011	-0.0069	-3	260	277	YAADEDAFFADYAEAHLK					Mascot
2617.304	2617.2988	-0.0052	-2	179	203	QVFSTQMGLSDQDIVALS GGHTLGR					Mascot
2633.2988	2633.3018	0.003	1	179	203	QVFSTQMGLSDQDIVALS GGHTLGR			Oxidation (M)[7]		Mascot
2633.2988	2633.3018	0.003	1	179	203	QVFSTQMGLSDQDIVALS GGHTLGR	114	100	Oxidation (M)[7]		Mascot

5	ascorbate peroxidase2 [Zea mays]	gi|162457709	27408.9	5.28	11	357	100	47.42	291	100

Peptide Information

Calc. Mass	Obsrv. Mass	± da	± ppm	Start Seq.	End Seq.	Sequence	Ion Score	C. I. %	Modification	Rank	Result Type
899.5672	899.4792	-0.088	-98	23	30	LRGLIAEK					Mascot
911.4291	911.4317	0.0026	3	53	61	TGGPFGTMK			Oxidation (M)[8]		Mascot
974.491	974.4974	0.0064	7	31	38	NCAPLMLR			Carbamidomethyl (C)[2]		Mascot

990.4859	990.4855	-0.0004	0	31	38	NCAPLMLR			Carbamidomethyl (C)[2], Oxidation (M)[6]		Mascot
1249.6172	1249.6228	0.0056	4	120	130	QDKPEPPPEGR					Mascot
1249.6172	1249.6228	0.0056	4	120	130	QDKPEPPPEGR	67	99.966			Mascot
1309.6495	1309.656	0.0065	5	131	142	LPDATQGSDHLR					Mascot
1309.6495	1309.656	0.0065	5	131	142	LPDATQGSDHLR	110	100			Mascot
1557.8635	1557.7429	-0.1206	-77	210	223	ALLSDPSFRPLVDK					Mascot
1585.8552	1585.8948	0.0396	25	25	38	GLIAEKNCAPLMLR			Carbamidomethyl (C)[8]		Mascot
1585.8552	1585.8948	0.0396	25	25	38	GLIAEKNCAPLMLR			Carbamidomethyl (C)[8]		Mascot
1601.8502	1601.8773	0.0271	17	25	38	GLIAEKNCAPLMLR			Carbamidomethyl (C)[8], Oxidation (M)[12]		Mascot
1686.8407	1686.7644	-0.0763	-45	1	14	MVKAYPTVNEDYLK			Oxidation (M)[1]		Mascot
1856.0011	1855.8724	-0.1287	-69	193	209	ELLSGEKEGLLQLPSDK					Mascot
2046.908	2046.9011	-0.0069	-3	224	241	YAADEDAFFADYAEAHLK					Mascot
2617.304	2617.2988	-0.0052	-2	143	167	QVFSTQMGLSDQDIVALS GGHTLGR					Mascot
2633.2988	2633.3018	0.003	1	143	167	QVFSTQMGLSDQDIVALS GGHTLGR			Oxidation (M)[7]		Mascot
2633.2988	2633.3018	0.003	1	143	167	QVFSTQMGLSDQDIVALS GGHTLGR	114	100	Oxidation (M)[7]		Mascot

6	PREDICTED: L-ascorbate peroxidase 2, cytosolic-like isoform X2 [Setaria italica]	gi|514737330	27257.8	5.18	10	349	100	46.705	291	100	

Peptide Information

Calc. Mass	Obsrv. Mass	± da	± ppm	Start Seq.	End Seq.	Sequence	Ion Score	C. I. %	Modification	Rank	Result Type
899.5672	899.4792	-0.088	-98	23	30	LRGLIAEK					Mascot
911.4291	911.4317	0.0026	3	53	61	TGGPFGTMK			Oxidation (M)[8]		Mascot
974.491	974.4974	0.0064	7	31	38	NCAPLMLR			Carbamidomethyl (C)[2]		Mascot
990.4859	990.4855	-0.0004	0	31	38	NCAPLMLR			Carbamidomethyl (C)[2], Oxidation (M)[6]		Mascot
1249.6172	1249.6228	0.0056	4	120	130	QDKPEPPPEGR					Mascot
1249.6172	1249.6228	0.0056	4	120	130	QDKPEPPPEGR	67	99.966			Mascot
1309.6495	1309.656	0.0065	5	131	142	LPDATQGSDHLR					Mascot
1309.6495	1309.656	0.0065	5	131	142	LPDATQGSDHLR	110	100			Mascot
1585.8552	1585.8948	0.0396	25	25	38	GLIAEKNCAPLMLR			Carbamidomethyl (C)[8]		Mascot
1585.8552	1585.8948	0.0396	25	25	38	GLIAEKNCAPLMLR			Carbamidomethyl (C)[8]		Mascot
1601.8502	1601.8773	0.0271	17	25	38	GLIAEKNCAPLMLR			Carbamidomethyl (C)[8], Oxidation (M)[12]		Mascot
1789.9192	1789.922	0.0028	2	62	79	DPAELAHGANAGLDIAVR					Mascot
1856.0011	1855.8724	-0.1287	-69	193	209	ELLSGEKEGLLQLPSDK					Mascot
2046.908	2046.9011	-0.0069	-3	224	241	YAADEDAFFADYAEAHLK					Mascot
2617.304	2617.2988	-0.0052	-2	143	167	QVFSTQMGLSDQDIVALS GGHTLGR					Mascot

Calc. Mass	Obsrv. Mass	± da	± ppm	Start Seq.	End Seq.	Sequence	Ion Score	C. I. %	Modification	Rank	Result Type
2633.2988	2633.3018	0.003	1	143	167	QVFSTQMGLSDQDIVALS GGHTLGR			Oxidation (M)[7]		Mascot
2633.2988	2633.3018	0.003	1	143	167	QVFSTQMGLSDQDIVALS GGHTLGR	114	100	Oxidation (M)[7]		Mascot

7	PREDICTED: L-ascorbate peroxidase 2, cytosolic-like isoform X1 [Setaria italica]	gi|514737328	24992.8	5.88	9	342	100	45.323	291	100	

Peptide Information

Calc. Mass	Obsrv. Mass	± da	± ppm	Start Seq.	End Seq.	Sequence	Ion Score	C. I. %	Modification	Rank	Result Type
899.5672	899.4792	-0.088	-98	23	30	LRGLIAEK					Mascot
911.4291	911.4317	0.0026	3	53	61	TGGPFGTMK			Oxidation (M)[8]		Mascot
974.491	974.4974	0.0064	7	31	38	NCAPLMLR			Carbamidomethyl (C)[2]		Mascot
990.4859	990.4855	-0.0004	0	31	38	NCAPLMLR			Carbamidomethyl (C)[2], Oxidation (M)[6]		Mascot
1249.6172	1249.6228	0.0056	4	120	130	QDKPEPPPEGR					Mascot
1249.6172	1249.6228	0.0056	4	120	130	QDKPEPPPEGR	67	99.966			Mascot
1309.6495	1309.656	0.0065	5	131	142	LPDATQGSDHLR					Mascot
1309.6495	1309.656	0.0065	5	131	142	LPDATQGSDHLR	110	100			Mascot
1585.8552	1585.8948	0.0396	25	25	38	GLIAEKNCAPLMLR			Carbamidomethyl (C)[8]		Mascot
1585.8552	1585.8948	0.0396	25	25	38	GLIAEKNCAPLMLR			Carbamidomethyl (C)[8]		Mascot
1601.8502	1601.8773	0.0271	17	25	38	GLIAEKNCAPLMLR			Carbamidomethyl (C)[8], Oxidation (M)[12]		Mascot
1789.9192	1789.922	0.0028	2	62	79	DPAELAHGANAGLDIAVR					Mascot
1856.0011	1855.8724	-0.1287	-69	193	209	ELLSGEKEGLLQLPSDK					Mascot
2617.304	2617.2988	-0.0052	-2	143	167	QVFSTQMGLSDQDIVALS GGHTLGR					Mascot
2633.2988	2633.3018	0.003	1	143	167	QVFSTQMGLSDQDIVALS GGHTLGR			Oxidation (M)[7]		Mascot
2633.2988	2633.3018	0.003	1	143	167	QVFSTQMGLSDQDIVALS GGHTLGR	114	100	Oxidation (M)[7]		Mascot

8	TPA: hypothetical protein ZEAMMB73_314819 [Zea mays]	gi|414591283	20838.4	4.69	7	332	100	26.426	291	100	

Peptide Information

Calc. Mass	Obsrv. Mass	± da	± ppm	Start Seq.	End Seq.	Sequence	Ion Score	C. I. %	Modification	Rank	Result Type
1249.6172	1249.6228	0.0056	4	61	71	QDKPEPPPEGR					Mascot
1249.6172	1249.6228	0.0056	4	61	71	QDKPEPPPEGR	67	99.966			Mascot
1309.6495	1309.656	0.0065	5	72	83	LPDATQGSDHLR					Mascot
1309.6495	1309.656	0.0065	5	72	83	LPDATQGSDHLR	110	100			Mascot
1557.8635	1557.7429	-0.1206	-77	151	164	ALLSDPSFRPLVDK					Mascot
1831.9409	1831.8951	-0.0458	-25	3	20	NPAEQAHGANAGLEIAIR					Mascot
1856.0011	1855.8724	-0.1287	-69	134	150	ELLSGEKEGLLQLPSDK					Mascot

Calc. Mass	Obsrv. Mass	± da	± ppm	Start Seq.	End Seq.	Sequence	Ion Score	C. I. %	Modification	Rank	Result Type
2046.908	2046.9011	-0.0069	-3	165	182	YAADEDAFFADYAEAHLK					Mascot
2617.304	2617.2988	-0.0052	-2	84	108	QVFSTQMGLSDQDIVALS GGHTLGR					Mascot
2633.2988	2633.3018	0.003	1	84	108	QVFSTQMGLSDQDIVALS GGHTLGR			Oxidation (M)[7]		Mascot
2633.2988	2633.3018	0.003	1	84	108	QVFSTQMGLSDQDIVALS GGHTLGR	114	100	Oxidation (M)[7]		Mascot

9	hypothetical protein ZEAMMB73_022439 [Zea mays]	gi|413956093	21012.6	6.6	5	320	100	20.145	299	100

Peptide Information

Calc. Mass	Obsrv. Mass	± da	± ppm	Start Seq.	End Seq.	Sequence	Ion Score	C. I. %	Modification	Rank	Result Type
911.4291	911.4317	0.0026	3	53	61	TGGPFGTMK			Oxidation (M)[8]		Mascot
1249.6172	1249.6228	0.0056	4	120	130	EDKPQPPPEGR					Mascot
1249.6172	1249.6228	0.0056	4	120	130	EDKPQPPPEGR	36	51.042			Mascot
1309.6859	1309.656	-0.0299	-23	131	142	LPDATKGSDHLR					Mascot
1309.6859	1309.656	-0.0299	-23	131	142	LPDATKGSDHLR	110	100			Mascot
1686.7858	1686.7644	-0.0214	-13	4	18	NYPTVSAEYSEAVEK					Mascot
1834.9229	1834.9218	-0.0011	-1	62	79	CPAELAHGANAGLDIAVR	153	100	Carbamidomethyl (C)[1]		Mascot

10	hypothetical protein ZEAMMB73_022439 [Zea mays]	gi|413956091	23689.7	7	5	318	100	20.145	299	100

Peptide Information

Calc. Mass	Obsrv. Mass	± da	± ppm	Start Seq.	End Seq.	Sequence	Ion Score	C. I. %	Modification	Rank	Result Type
911.4291	911.4317	0.0026	3	53	61	TGGPFGTMK			Oxidation (M)[8]		Mascot
1249.6172	1249.6228	0.0056	4	120	130	EDKPQPPPEGR					Mascot
1249.6172	1249.6228	0.0056	4	120	130	EDKPQPPPEGR	36	51.042			Mascot
1309.6859	1309.656	-0.0299	-23	131	142	LPDATKGSDHLR					Mascot
1309.6859	1309.656	-0.0299	-23	131	142	LPDATKGSDHLR	110	100			Mascot
1686.7858	1686.7644	-0.0214	-13	4	18	NYPTVSAEYSEAVEK					Mascot
1834.9229	1834.9218	-0.0011	-1	62	79	CPAELAHGANAGLDIAVR	153	100	Carbamidomethyl (C)[1]		Mascot

Gel Idx/Pos	180/H7	Instr./Gel Origin	BA2151/Sample Project 20140814	Process Status	Analysis Succeeded
Plate [#] Name	[1] Sample Project 20140814	Instrument Sample Name		Spectra	11

Rank	Protein Name	Accession No.	Protein MW	Protein PI	Pep. Count	Protein Score	Protein Score C. I. %	Intensity Matched	Total Ion Score	Total Ion C. I. %	Confirmed
1	1,2-dihydroxy-3-keto-5-methylthiopentene dioxygenase 2 [Aegilops tauschii]	gi|475567867	64127.3	5.72	19	466	100	33.706	395	100	

Peptide Information

Calc. Mass	Obsrv. Mass	± da	± ppm	Start Seq.	End Seq.	Sequence	Ion Score	C. I. %	Modification	Rank	Result Type
808.3618	808.3902	0.0284	35	500	506	KGMEGDR			Oxidation (M)[3]		Mascot
817.4818	817.4586	-0.0232	-28	35	41	EFIPLAK					Mascot
834.4355	834.4135	-0.022	-26	85	91	LPNYEAK					Mascot
1013.5639	1013.5618	-0.0021	-2	27	34	LPHHREPK					Mascot
1088.512	1088.5109	-0.0011	-1	120	127	DQNEQWIR					Mascot
1088.512	1088.5109	-0.0011	-1	120	127	DQNEQWIR	54	99.126			Mascot
1140.4773	1140.569	0.0917	80	399	407	ADMRAMEER			Oxidation (M)[3,6]		Mascot
1194.6122	1194.6138	0.0016	1	415	424	QLQMMGFLAR					Mascot
1215.5892	1215.5856	-0.0036	-3	146	155	FTLDSDNYIK					Mascot
1232.6304	1232.5762	-0.0542	-44	258	267	EKIVECELGR			Carbamidomethyl (C)[6]		Mascot
1351.6019	1351.6625	0.0606	45	392	402	QEQQSSRADMR			Oxidation (M)[10]		Mascot
1369.6819	1369.6958	0.0139	10	286	298	VSDANGVRPDSPR					Mascot
1417.7079	1417.6674	-0.0405	-29	133	145	GGMIVLPAGMYHR			Oxidation (M)[3]		Mascot
1433.7029	1433.6815	-0.0214	-15	133	145	GGMIVLPAGMYHR			Oxidation (M)[3,10]		Mascot
1433.7029	1433.6815	-0.0214	-15	133	145	GGMIVLPAGMYHR			Oxidation (M)[3,10]		Mascot
1465.6417	1465.6401	-0.0016	-1	108	119	YCLEGSGYFDVR			Carbamidomethyl (C)[2]		Mascot
1465.6417	1465.6401	-0.0016	-1	108	119	YCLEGSGYFDVR	103	100	Carbamidomethyl (C)[2]		Mascot
1561.7979	1561.755	-0.0429	-27	132	145	KGGMIVLPAGMYHR			Oxidation (M)[4,11]		Mascot
1573.7679	1573.7462	-0.0217	-14	146	158	FTLDSDNYIKAMR					Mascot
1589.7628	1589.777	0.0142	9	146	158	FTLDSDNYIKAMR			Oxidation (M)[12]		Mascot
1704.7245	1704.6937	-0.0308	-18	71	84	GYSYVDICDVCPEK			Carbamidomethyl (C)[8,11]		Mascot
1704.7245	1704.6937	-0.0308	-18	71	84	GYSYVDICDVCPEK	98	100	Carbamidomethyl (C)[8,11]		Mascot
1815.8297	1815.8312	0.0015	1	94	107	NFFEEHLHTDEEIR					Mascot
1815.8297	1815.8312	0.0015	1	94	107	NFFEEHLHTDEEIR	140	100			Mascot
2057.0088	2057.0054	-0.0034	-2	92	107	LKNFFEEHLHTDEEIR					Mascot
2520.1421	2520.1194	-0.0227	-9	71	91	GYSYVDICDVCPEKLPNYEAK			Carbamidomethyl (C)[8,11]		Mascot

2	PREDICTED: 1,2-dihydroxy-3-keto-5-methylthiopentene dioxygenase 2-like [Setaria italica]	gi|514821864	23719.6	4.93	12	416	100	26.778	341	100

Peptide Information

Calc. Mass	Obsrv. Mass	± da	± ppm	Start Seq.	End Seq.	Sequence	Ion Score	C. I. %	Modification	Rank	Result Type
834.4355	834.4135	-0.022	-26	85	91	LPNYEAK					Mascot
1013.5639	1013.5618	-0.0021	-2	27	34	LPHHREPK					Mascot
1074.4963	1074.5231	0.0268	25	120	127	DQNDQWIR					Mascot
1177.6324	1177.5857	-0.0467	-40	181	189	EYVDRIINR					Mascot
1215.662	1215.5856	-0.0764	-63	32	41	EPKEFIPLDK					Mascot
1417.7079	1417.6674	-0.0405	-29	133	145	GGMIVLPAGMYHR			Oxidation (M)[3]		Mascot
1433.7029	1433.6815	-0.0214	-15	133	145	GGMIVLPAGMYHR			Oxidation (M)[3,10]		Mascot
1433.7029	1433.6815	-0.0214	-15	133	145	GGMIVLPAGMYHR			Oxidation (M)[3,10]		Mascot
1465.6417	1465.6401	-0.0016	-1	108	119	YCLEGSGYFDVR			Carbamidomethyl (C)[2]		Mascot
1465.6417	1465.6401	-0.0016	-1	108	119	YCLEGSGYFDVR	103	100	Carbamidomethyl (C)[2]		Mascot
1561.7979	1561.755	-0.0429	-27	132	145	KGGMIVLPAGMYHR			Oxidation (M)[4,11]		Mascot
1704.7245	1704.6937	-0.0308	-18	71	84	GYSYVDICDVCPEK			Carbamidomethyl (C)[8,11]		Mascot
1704.7245	1704.6937	-0.0308	-18	71	84	GYSYVDICDVCPEK	98	100	Carbamidomethyl (C)[8,11]		Mascot
1815.8297	1815.8312	0.0015	1	94	107	NFFEEHLHTDEEIR					Mascot
1815.8297	1815.8312	0.0015	1	94	107	NFFEEHLHTDEEIR	140	100			Mascot
2057.0088	2057.0054	-0.0034	-2	92	107	IKNFFEEHLHTDEEIR					Mascot
2520.1421	2520.1194	-0.0227	-9	71	91	GYSYVDICDVCPEKLPNY EAK			Carbamidomethyl (C)[8,11]		Mascot

3	RecName: Full=1,2-dihydroxy-3-keto-5-methylthiopentene dioxygenase 2; AltName: Full=Acireductone dioxygenase (Fe(2+)-requiring) 2; Short=ARD 2; Short=Fe-ARD 2; AltName: Full=Submergence-induced protein 2A	gi|122247504	23654.5	5.08	10	294	100	25.074	243	100

Protein Group

Os03g0161800 [Oryza sativa Japonica Group]	gi|113547517	23654.5	5.0799 999237 0605
RecName: Full=1,2-dihydroxy-3-keto-5-methylthiopentene dioxygenase 2; AltName: Full=Acireductone dioxygenase (Fe(2+)-requiring) 2; Short=ARD 2; Short=Fe-ARD 2; AltName: Full=Submergence-induced protein 2A	gi|148886787	23654.5	5.0799 999237 0605
hypothetical protein OsI_10122 [Oryza sativa Indica	gi|218192139	23654.5	5.0799

Group] 999237 0605

Peptide Information

Calc. Mass	Obsrv. Mass	± da	± ppm	Start Seq.	End Seq.	Sequence	Ion Score	C. I. %	Modification	Rank	Result Type
834.4355	834.4135	-0.022	-26	85	91	LPNYEAK					Mascot
1013.5639	1013.5618	-0.0021	-2	27	34	LPHHREPK					Mascot
1074.4963	1074.5231	0.0268	25	120	127	DQNDQWIR					Mascot
1215.5892	1215.5856	-0.0036	-3	146	155	FTLDSDNYIK					Mascot
1417.7079	1417.6674	-0.0405	-29	133	145	GGMIVLPAGMYHR			Oxidation (M)[3]		Mascot
1433.7029	1433.6815	-0.0214	-15	133	145	GGMIVLPAGMYHR			Oxidation (M)[3,10]		Mascot
1433.7029	1433.6815	-0.0214	-15	133	145	GGMIVLPAGMYHR			Oxidation (M)[3,10]		Mascot
1465.6417	1465.6401	-0.0016	-1	108	119	YCLEGSGYFDVR			Carbamidomethyl (C)[2]		Mascot
1465.6417	1465.6401	-0.0016	-1	108	119	YCLEGSGYFDVR	103	100	Carbamidomethyl (C)[2]		Mascot
1561.7979	1561.755	-0.0429	-27	132	145	KGGMIVLPAGMYHR			Oxidation (M)[4,11]		Mascot
1573.7679	1573.7462	-0.0217	-14	146	158	FTLDSDNYIKAMR					Mascot
1589.7628	1589.777	0.0142	9	146	158	FTLDSDNYIKAMR			Oxidation (M)[12]		Mascot
1815.8297	1815.8312	0.0015	1	94	107	NFFEEHLHTDEEIR					Mascot
1815.8297	1815.8312	0.0015	1	94	107	NFFEEHLHTDEEIR	140	100			Mascot
2057.0088	2057.0054	-0.0034	-2	92	107	LKNFFEEHLHTDEEIR					Mascot

4	TPA: 1,2-dihydroxy-3-keto-5-methylthiopentene dioxygenase 2 [Zea mays]	gi|414864926	23622.5	5.07	10	294	100	25.076	243	100	

Peptide Information

Calc. Mass	Obsrv. Mass	± da	± ppm	Start Seq.	End Seq.	Sequence	Ion Score	C. I. %	Modification	Rank	Result Type
834.4355	834.4135	-0.022	-26	85	91	LPNYEAK					Mascot
1013.5639	1013.5618	-0.0021	-2	27	34	LPHHREPK					Mascot
1075.4803	1075.5006	0.0203	19	120	127	DQDDQWIR					Mascot
1215.5892	1215.5856	-0.0036	-3	146	155	FTLDSDNYIK					Mascot
1417.7079	1417.6674	-0.0405	-29	133	145	GGMIVLPAGMYHR			Oxidation (M)[3]		Mascot
1433.7029	1433.6815	-0.0214	-15	133	145	GGMIVLPAGMYHR			Oxidation (M)[3,10]		Mascot
1433.7029	1433.6815	-0.0214	-15	133	145	GGMIVLPAGMYHR			Oxidation (M)[3,10]		Mascot
1465.6417	1465.6401	-0.0016	-1	108	119	YCLEGSGYFDVR			Carbamidomethyl (C)[2]		Mascot
1465.6417	1465.6401	-0.0016	-1	108	119	YCLEGSGYFDVR	103	100	Carbamidomethyl (C)[2]		Mascot
1561.7979	1561.755	-0.0429	-27	132	145	KGGMIVLPAGMYHR			Oxidation (M)[4,11]		Mascot

1573.7679	1573.7462	-0.0217	-14	146	158	FTLDSDNYIKAMR					Mascot
1589.7628	1589.777	0.0142	9	146	158	FTLDSDNYIKAMR			Oxidation (M)[12]		Mascot
1815.8297	1815.8312	0.0015	1	94	107	NFFEEHLHTDEEIR					Mascot
1815.8297	1815.8312	0.0015	1	94	107	NFFEEHLHTDEEIR	140	100			Mascot
2057.0088	2057.0054	-0.0034	-2	92	107	IKNFFEEHLHTDEEIR					Mascot

5	TPA: hypothetical protein ZEAMMB73_401091 [Zea mays]	gi|414864927	20942	4.78	9	289	100	24.674	243	100	

Peptide Information

Calc. Mass	Obsrv. Mass	± da	± ppm	Start Seq.	End Seq.	Sequence	Ion Score	C. I. %	Modification	Rank	Result Type
834.4355	834.4135	-0.022	-26	85	91	LPNYEAK					Mascot
1013.5639	1013.5618	-0.0021	-2	27	34	LPHHREPK					Mascot
1075.4803	1075.5006	0.0203	19	120	127	DQDDQWIR					Mascot
1215.5892	1215.5856	-0.0036	-3	146	155	FTLDSDNYIK					Mascot
1417.7079	1417.6674	-0.0405	-29	133	145	GGMIVLPAGMYHR			Oxidation (M)[3]		Mascot
1433.7029	1433.6815	-0.0214	-15	133	145	GGMIVLPAGMYHR			Oxidation (M)[3,10]		Mascot
1433.7029	1433.6815	-0.0214	-15	133	145	GGMIVLPAGMYHR			Oxidation (M)[3,10]		Mascot
1465.6417	1465.6401	-0.0016	-1	108	119	YCLEGSGYFDVR			Carbamidomethyl (C)[2]		Mascot
1465.6417	1465.6401	-0.0016	-1	108	119	YCLEGSGYFDVR	103	100	Carbamidomethyl (C)[2]		Mascot
1561.7979	1561.755	-0.0429	-27	132	145	KGGMIVLPAGMYHR			Oxidation (M)[4,11]		Mascot
1815.8297	1815.8312	0.0015	1	94	107	NFFEEHLHTDEEIR					Mascot
1815.8297	1815.8312	0.0015	1	94	107	NFFEEHLHTDEEIR	140	100			Mascot
2057.0088	2057.0054	-0.0034	-2	92	107	IKNFFEEHLHTDEEIR					Mascot

6	RecName: Full=1,2-dihydroxy-3-keto-5-methylthiopentene dioxygenase 1; AltName: Full=Acireductone dioxygenase (Fe(2+)-requiring) 1; Short=ARD 1; Short=Fe-ARD 1	gi|363805494	21606.7	5.21	8	282	100	23.655	243	100	

Peptide Information

Calc. Mass	Obsrv. Mass	± da	± ppm	Start Seq.	End Seq.	Sequence	Ion Score	C. I. %	Modification	Rank	Result Type
1075.4803	1075.5006	0.0203	19	103	110	DENDQWIR					Mascot
1417.7079	1417.6674	-0.0405	-29	116	128	GGMIVLPAGMYHR			Oxidation (M)[3]		Mascot
1433.7029	1433.6815	-0.0214	-15	116	128	GGMIVLPAGMYHR			Oxidation (M)[3,10]		Mascot
1433.7029	1433.6815	-0.0214	-15	116	128	GGMIVLPAGMYHR			Oxidation (M)[3,10]		Mascot
1465.6417	1465.6401	-0.0016	-1	91	102	YCLEGSGYFDVR			Carbamidomethyl (C)[2]		Mascot
1465.6417	1465.6401	-0.0016	-1	91	102	YCLEGSGYFDVR	103	100	Carbamidomethyl (C)[2]		Mascot

Calc. Mass	Obsrv. Mass	± da	± ppm	Start Seq.	End Seq.	Sequence	Ion Score	C. I. %	Modification	Rank	Result Type
1561.7979	1561.755	-0.0429	-27	115	128	KGGMIVLPAGMYHR			Oxidation (M)[4,11]		Mascot
1600.7238	1600.7271	0.0033	2	35	47	LNPDNWENDENLK					Mascot
1815.8297	1815.8312	0.0015	1	77	90	NFFEEHLHTDEEIR					Mascot
1815.8297	1815.8312	0.0015	1	77	90	NFFEEHLHTDEEIR	140	100			Mascot
1959.0546	1958.8679	-0.1867	-95	164	180	EYLDKLLKPEGQAVEAR					Mascot
2057.0088	2057.0054	-0.0034	-2	75	90	IKNFFEEHLHTDEEIR					Mascot

7	PREDICTED: 1,2-dihydroxy-3-keto-5-methylthiopentene dioxygenase 2-like [Setaria italica]	gi|514816677	23835.7	5.09	9	281	100	23.972	243	100

Peptide Information

Calc. Mass	Obsrv. Mass	± da	± ppm	Start Seq.	End Seq.	Sequence	Ion Score	C. I. %	Modification	Rank	Result Type
834.4355	834.4135	-0.022	-26	85	91	LPNYEAK					Mascot
953.4211	953.4387	0.0176	18	2	9	ETEFQDGK					Mascot
1013.5639	1013.5618	-0.0021	-2	27	34	LPHHREPK					Mascot
1075.5167	1075.5006	-0.0161	-15	120	127	DENDKWIR					Mascot
1417.7079	1417.6674	-0.0405	-29	133	145	GGMIVLPAGMYHR			Oxidation (M)[3]		Mascot
1433.7029	1433.6815	-0.0214	-15	133	145	GGMIVLPAGMYHR			Oxidation (M)[3,10]		Mascot
1433.7029	1433.6815	-0.0214	-15	133	145	GGMIVLPAGMYHR			Oxidation (M)[3,10]		Mascot
1465.6417	1465.6401	-0.0016	-1	108	119	YCLEGSGYFDVR			Carbamidomethyl (C)[2]		Mascot
1465.6417	1465.6401	-0.0016	-1	108	119	YCLEGSGYFDVR	103	100	Carbamidomethyl (C)[2]		Mascot
1561.7979	1561.755	-0.0429	-27	132	145	KGGMIVLPAGMYHR			Oxidation (M)[4,11]		Mascot
1815.8297	1815.8312	0.0015	1	94	107	NFFEEHLHTDEEIR					Mascot
1815.8297	1815.8312	0.0015	1	94	107	NFFEEHLHTDEEIR	140	100			Mascot
2057.0088	2057.0054	-0.0034	-2	92	107	IKNFFEEHLHTDEEIR					Mascot

8	aci-reductone dioxygenase-like protein [Elaeis guineensis]	gi|192913044	23806.5	4.78	6	267	100	21.492	243	100

Peptide Information

Calc. Mass	Obsrv. Mass	± da	± ppm	Start Seq.	End Seq.	Sequence	Ion Score	C. I. %	Modification	Rank	Result Type
1107.4524	1107.5133	0.0609	55	120	127	DENDCWIR			Carbamidomethyl (C)[5]		Mascot
1136.5582	1136.5194	-0.0388	-34	190	199	EVDGYVVEAR					Mascot
1159.647	1159.6044	-0.0426	-37	42	51	LSELGIVSWR					Mascot
1465.6417	1465.6401	-0.0016	-1	108	119	YCLEGSGYFDVR			Carbamidomethyl (C)[2]		Mascot
1465.6417	1465.6401	-0.0016	-1	108	119	YCLEGSGYFDVR	103	100	Carbamidomethyl (C)[2]		Mascot
1815.8297	1815.8312	0.0015	1	94	107	NFFEEHLHTDEEIR					Mascot

Calc. Mass	Obsrv. Mass	± da	± ppm	Start Seq.	End Seq.	Sequence	Ion Score	C. I. %	Modification	Rank	Result Type
1815.8297	1815.8312	0.0015	1	94	107	NFFEEHLHTDEEIR	140	100			Mascot
2057.0088	2057.0054	-0.0034	-2	92	107	IKNFFEEHLHTDEEIR					Mascot

9	hypothetical protein OsI_33604 [Oryza sativa Indica Group]	gi|218184531	25781.5	4.87	7	230	100	17.267	201	100	

Peptide Information

Calc. Mass	Obsrv. Mass	± da	± ppm	Start Seq.	End Seq.	Sequence	Ion Score	C. I. %	Modification	Rank	Result Type
989.4786	989.5115	0.0329	33	18	26	EILEGEDGK					Mascot
1013.5639	1013.5618	-0.0021	-2	44	51	LPHHREPK					Mascot
1074.4963	1074.5231	0.0268	25	137	144	DQNDQWIR					Mascot
1417.7079	1417.6674	-0.0405	-29	150	162	GGMIVLPAGMYHR			Oxidation (M)[3]		Mascot
1433.7029	1433.6815	-0.0214	-15	150	162	GGMIVLPAGMYHR			Oxidation (M)[3,10]		Mascot
1433.7029	1433.6815	-0.0214	-15	150	162	GGMIVLPAGMYHR			Oxidation (M)[3,10]		Mascot
1465.6417	1465.6401	-0.0016	-1	125	136	YCLEGSGYFDVR			Carbamidomethyl (C)[2]		Mascot
1465.6417	1465.6401	-0.0016	-1	125	136	YCLEGSGYFDVR	103	100	Carbamidomethyl (C)[2]		Mascot
1561.7979	1561.755	-0.0429	-27	149	162	KGGMIVLPAGMYHR			Oxidation (M)[4,11]		Mascot
1704.7245	1704.6937	-0.0308	-18	88	101	GYSYVDICDVCPEK			Carbamidomethyl (C)[8,11]		Mascot
1704.7245	1704.6937	-0.0308	-18	88	101	GYSYVDICDVCPEK	98	100	Carbamidomethyl (C)[8,11]		Mascot

10	RecName: Full=1,2-dihydroxy-3-keto-5-methylthiopentene dioxygenase 1; AltName: Full=Acireductone dioxygenase (Fe(2+)-requiring) 1; Short=ARD 1; Short=Fe-ARD 1; AltName: Full=Submergence-induced protein 2	gi|148886785	23854.6	5.07	6	224	100	17.096	201	100	

Protein Group

Os10g0419400 [Oryza sativa Japonica Group]	gi|113639227	23854.6	5.0700 001716 6138
RecName: Full=1,2-dihydroxy-3-keto-5-methylthiopentene dioxygenase 1; AltName: Full=Acireductone dioxygenase (Fe(2+)-requiring) 1; Short=ARD 1; Short=Fe-ARD 1; AltName: Full=OsIDI1; AltName: Full=Submergence-induced protein 2	gi|148886786	23854.6	5.0700 001716 6138

Peptide Information

Calc. Mass	Obsrv. Mass	± da	± ppm	Start Seq.	End Seq.	Sequence	Ion Score	C. I. %	Modification	Rank	Result Type
1013.5639	1013.5618	-0.0021	-2	27	34	LPHHREPK					Mascot
1074.4963	1074.5231	0.0268	25	120	127	DQNDQWIR					Mascot

1417.7079	1417.6674	-0.0405	-29	133	145	GGMIVLPAGMYHR			Oxidation (M)[3]		Mascot
1433.7029	1433.6815	-0.0214	-15	133	145	GGMIVLPAGMYHR			Oxidation (M)[3,10]		Mascot
1433.7029	1433.6815	-0.0214	-15	133	145	GGMIVLPAGMYHR			Oxidation (M)[3,10]		Mascot
1465.6417	1465.6401	-0.0016	-1	108	119	YCLEGSGYFDVR			Carbamidomethyl (C)[2]		Mascot
1465.6417	1465.6401	-0.0016	-1	108	119	YCLEGSGYFDVR	103	100	Carbamidomethyl (C)[2]		Mascot
1561.7979	1561.755	-0.0429	-27	132	145	KGGMIVLPAGMYHR			Oxidation (M)[4,11]		Mascot
1704.7245	1704.6937	-0.0308	-18	71	84	GYSYVDICDVCPEK			Carbamidomethyl (C)[8,11]		Mascot
1704.7245	1704.6937	-0.0308	-18	71	84	GYSYVDICDVCPEK	98	100	Carbamidomethyl (C)[8,11]		Mascot

Gel Idx/Pos	181/H8	Instr./Gel Origin	BA2151/Sample Project 20140814	Process Status	Analysis Succeeded
Plate [#] Name	[1] Sample Project 20140814	Instrument Sample Name		Spectra	11

Rank	Protein Name	Accession No.	Protein MW	Protein PI	Pep. Count	Protein Score	Protein Score C. I. %	Intensity Matched	Total Ion Score	Total Ion C. I. %	Confirmed
1	hypothetical protein CARUB_v10010775mg [Capsella rubella]	gi|482573055	9280.7	9.7	8	68	85.93	3.336			

Protein Group

hypothetical protein CARUB_v10010775mg [Capsella rubella]	gi|482573056	9280.7	9.6999998092 6514

Peptide Information

Calc. Mass	Obsrv. Mass	± da	± ppm	Start Seq.	End Seq.	Sequence	Ion Score	C. I. %	Modification	Rank	Result Type
804.4151	804.3704	-0.0447	-56	57	62	FQAWPR					Mascot
806.4366	806.3979	-0.0387	-48	2	10	ATSSGGKAK					Mascot
937.4771	937.3888	-0.0883	-94	1	10	MATSSGGKAK					Mascot
1165.5161	1165.5382	0.0221	19	48	56	EWWTATDEK					Mascot
1217.6637	1217.5461	-0.1176	-97	76	85	QNFIVKSRPE					Mascot
1278.6689	1278.6652	-0.0037	-3	35	47	IFGGTTPGTVSNK					Mascot
1354.7148	1354.6846	-0.0302	-22	63	75	TAGPPVVMNPISR			Oxidation (M)[8]		Mascot
2011.0576	2011.0222	-0.0354	-18	11	28	YIIGALFGSFGISYIFDK					Mascot

Rank	Protein Name	Accession No.	Protein MW	Protein PI	Pep. Count	Protein Score	Protein Score C. I. %	Intensity Matched	Total Ion Score	Total Ion C. I. %	Confirmed
2	Protein VERNALIZATION INSENSITIVE 3 [Triticum urartu]	gi|474019031	84609.7	8.03	17	59	0	12.624			

Peptide Information

Calc. Mass	Obsrv. Mass	± da	± ppm	Start Seq.	End Seq.	Sequence	Ion Score	C. I. %	Modification	Rank	Result Type
803.3682	803.3351	-0.0331	-41	652	658	FEDAGHK					Mascot
821.4073	821.3494	-0.0579	-70	17	23	LMSVDEK					Mascot
832.3795	832.3132	-0.0663	-80	640	647	EPGNSSNK					Mascot
837.4022	837.4219	0.0197	24	17	23	LMSVDEK			Oxidation (M)[2]		Mascot
852.4435	852.399	-0.0445	-52	698	704	ATPHDRR					Mascot
906.46	906.3746	-0.0854	-94	149	156	AILSMEDK					Mascot
921.402	921.4919	0.0899	98	464	472	TNSGGQSDR					Mascot
921.402	921.4919	0.0899	98	464	472	TNSGGQSDR					Mascot

1060.5457	1060.5209	-0.0248	-23	207	216	TGILPSGQCK			Carbamidomethyl (C)[9]		Mascot
1182.6378	1182.5597	-0.0781	-66	230	238	QHDLLRSWK					Mascot
1182.6378	1182.5597	-0.0781	-66	230	238	QHDLLRSWK					Mascot
1309.5437	1309.6454	0.1017	78	564	574	ESSNSMEQNQR					Mascot
1419.7994	1419.7095	-0.0899	-63	270	281	YSVLHKFVDIAK					Mascot
1518.8679	1518.7317	-0.1362	-90	257	269	IFLGHKVLFSTEK					Mascot
1556.7487	1556.7242	-0.0245	-16	284	299	LEAEVGSVAGHGSMGR					Mascot
1650.9174	1650.7777	-0.1397	-85	78	93	VVTGKSSGPVVHVQEK					Mascot
1812.9021	1812.8462	-0.0559	-31	282	299	QKLEAEVGSVAGHGSMG R					Mascot
1901.8521	1901.9127	0.0606	32	174	189	DPTIWLSCSSDHPMQK			Carbamidomethyl (C)[8]		Mascot
2011.0204	2011.0222	0.0018	1	441	460	ESDLKGLAPGGAGLVDQ NNR					Mascot
2239.0737	2239.1106	0.0369	16	620	639	SFNTKPSDNIFQNGSSKP DR					Mascot

3	Os01g0811100 [Oryza sativa Japonica Group]	gi|113534119	27505.9	5.75	10	56	0	3.968

Protein Group

RecName: Full=Proteasome subunit alpha type-3; AltName: Full=20S proteasome alpha subunit G; AltName: Full=20S proteasome subunit alpha-7	gi|12229919	27505.9	5.75
hypothetical protein OsI_04163 [Oryza sativa Indica Group]	gi|218189257	27505.9	5.75

Peptide Information

Calc. Mass	Obsrv. Mass	± da	± ppm	Start Seq.	End Seq.	Sequence	Ion Score	C. I. %	Modification	Rank	Result Type
884.3995	884.4624	0.0629	71	94	101	SEAASYEK					Mascot
937.423	937.3888	-0.0342	-36	58	65	MMLEGSNR					Mascot
1119.619	1119.516	-0.103	-92	179	187	LKLSELTCR			Carbamidomethyl (C)[8]		Mascot
1217.6559	1217.5461	-0.1098	-90	42	52	CKDGIVLGVEK			Carbamidomethyl (C)[1]		Mascot
1349.589	1349.6361	0.0471	35	237	249	VAAQAALEEMDAD			Oxidation (M)[10]		Mascot
1447.7574	1447.6946	-0.0628	-43	30	43	AVDNSGTVVGIKCK			Carbamidomethyl (C)[13]		Mascot
1497.74	1497.7069	-0.0331	-22	53	65	LVTSKMMLEGSNR			Oxidation (M)[6,7]		Mascot
1799.8521	1799.8931	0.041	23	144	159	DGPQLYMIEPSGVSYK			Oxidation (M)[7]		Mascot
1959.9294	1959.9055	-0.0239	-12	2	20	SSIGTGYDLSVTTFSPDG R					Mascot
2225.1814	2225.0032	-0.1782	-80	21	41	VFQVEYATKAVDNSGTV VGIK					Mascot

4	RIN4d protein [Glycine max]	gi|351724035	27193.9	8.7	11	56	0	5.972

Peptide Information

Calc. Mass	Obsrv. Mass	± da	± ppm	Start Seq.	End Seq.	Sequence	Ion Score	C. I. %	Modification	Rank	Result Type
856.4523	856.5198	0.0675	79	75	81	TTHELQK					Mascot
903.4166	903.3887	-0.0279	-31	82	89	SREDGDPK					Mascot
909.3922	909.4176	0.0254	28	98	106	HGGGDSSHR					Mascot
921.4424	921.4919	0.0495	54	90	97	QFTDSPAR					Mascot
921.4424	921.4919	0.0495	54	90	97	QFTDSPAR					Mascot
932.4506	932.3976	-0.053	-57	37	44	MINPNDTK					Mascot
1027.499	1027.4686	-0.0304	-30	1	9	MAQHSNVPK			Oxidation (M)[1]		Mascot
1034.5854	1034.4829	-0.1025	-99	165	172	SRLRPSYR					Mascot
1255.6754	1255.5903	-0.0851	-68	71	81	GSVRTTHELQK					Mascot
1316.623	1316.5839	-0.0391	-30	141	152	TPGRDSPSWEGK					Mascot
1354.671	1354.6846	0.0136	10	62	74	VHSEDPSGKGSVR					Mascot
1492.7061	1492.7777	0.0716	48	31	44	GQSGSKMINPNDTK			Oxidation (M)[7]		Mascot

5	RecName: Full=Ribulose bisphosphate carboxylase small chain, chloroplastic; Short=RuBisCO small subunit; Flags: Precursor	gi|132161	20253.3	8.86	9	54	0	7.63

Peptide Information

Calc. Mass	Obsrv. Mass	± da	± ppm	Start Seq.	End Seq.	Sequence	Ion Score	C. I. %	Modification	Rank	Result Type
893.4727	893.4232	-0.0495	-55	30	38	SSAGFPVTK					Mascot
921.504	921.4919	-0.0121	-13	84	90	EVEYLLR					Mascot
921.504	921.4919	-0.0121	-13	84	90	EVEYLLR					Mascot
958.424	958.4549	0.0309	32	121	126	YWTMWR			Oxidation (M)[4]		Mascot
958.424	958.4549	0.0309	32	121	126	YWTMWR			Oxidation (M)[4]		Mascot
1021.5676	1021.4678	-0.0998	-98	30	39	SSAGFPVTKK					Mascot
1447.7937	1447.6946	-0.0991	-68	1	14	MALISSAAVTTINR					Mascot
1492.7141	1492.7777	0.0636	43	92	103	GWVPCVEFELEK			Carbamidomethyl (C)[5]		Mascot
1527.853	1527.7109	-0.1421	-93	15	29	APVQANLATPFTGLK					Mascot
1527.853	1527.7109	-0.1421	-93	15	29	APVQANLATPFTGLK					Mascot
1542.792	1542.7349	-0.0571	-37	53	65	VNCMQVWPPVGKK			Carbamidomethyl (C)[3]		Mascot
2002.9155	2003.0315	0.116	58	104	120	GFVHRQYNSSPGYYDGR					Mascot

6	TPA: proteasome subunit alpha type [Zea mays]	gi|414591027	27534.9	5.93	10	54	0	4.408

Protein Group

uncharacterized protein LOC100383475 [Zea mays]	gi|293331913	27534.9	5.9299 998283 3862

Peptide Information

Calc. Mass	Obsrv. Mass	± da	± ppm	Start Seq.	End Seq.	Sequence	Ion Score	C. I. %	Modification	Rank	Result Type
884.3995	884.4624	0.0629	71	94	101	SEAASYEK					Mascot
937.423	937.3888	-0.0342	-36	58	65	MMLEGSNR					Mascot
1119.619	1119.516	-0.103	-92	179	187	LKLSELTCR			Carbamidomethyl (C)[8]		Mascot
1217.6559	1217.5461	-0.1098	-90	42	52	CKDGIVLGVEK			Carbamidomethyl (C)[1]		Mascot
1321.5576	1321.6331	0.0755	57	237	249	AAAQAALEEMDAD			Oxidation (M)[10]		Mascot
1447.7574	1447.6946	-0.0628	-43	30	43	AVDNSGTVVGIKCK			Carbamidomethyl (C)[13]		Mascot
1497.74	1497.7069	-0.0331	-22	53	65	LVTSKMMLEGSNR			Oxidation (M)[6,7]		Mascot
1799.8521	1799.8931	0.041	23	144	159	DGPQLYMIEPSGVSYK			Oxidation (M)[7]		Mascot
1959.9294	1959.9055	-0.0239	-12	2	20	SSIGTGYDLSVTTFSPDG R					Mascot
2225.1814	2225.0032	-0.1782	-80	21	41	VFQVEYATKAVDNSGTV VGIK					Mascot

7	hypothetical protein CARUB_v10004182mg, partial [Capsella rubella]	gi|482551858	89092.2	5.02	21	54	0	11.983

Peptide Information

Calc. Mass	Obsrv. Mass	± da	± ppm	Start Seq.	End Seq.	Sequence	Ion Score	C. I. %	Modification	Rank	Result Type
820.4159	820.4221	0.0062	8	625	631	ESSTQLR					Mascot
834.3475	834.3089	-0.0386	-46	301	307	DAEEENK					Mascot
891.4418	891.3674	-0.0744	-83	247	254	EATSDQLK					Mascot
906.4639	906.3746	-0.0893	-99	536	543	RDSSSQVK					Mascot
907.4631	907.3831	-0.08	-88	235	241	RFQEAEK					Mascot
925.4738	925.4077	-0.0661	-71	188	195	TTALSYNR					Mascot
941.4794	941.3932	-0.0862	-92	660	667	TMSMKISK			Oxidation (M)[2]		Mascot
957.4744	957.3837	-0.0907	-95	660	667	TMSMKISK			Oxidation (M)[2,4]		Mascot
1060.5885	1060.5209	-0.0676	-64	454	462	LSELETQLK					Mascot
1062.5361	1062.5435	0.0074	7	433	440	ERELSCLR			Carbamidomethyl (C)[6]		Mascot
1119.6256	1119.516	-0.1096	-98	143	152	GKLTTTIEEK					Mascot
1193.6195	1193.5845	-0.035	-29	577	586	ISEMSTEIKR					Mascot
1321.6383	1321.6331	-0.0052	-4	236	246	FQEAEKVAEDR					Mascot

1366.6882	1366.6445	-0.0437	-32	308	319	AISSKNLETMEK			Oxidation (M)[10]		Mascot
1461.718	1461.7311	0.0131	9	242	254	VAEDREATSDQLK					Mascot
1491.736	1491.7024	-0.0336	-23	668	680	TSDELEQAQIMVK					Mascot
1597.8431	1597.7969	-0.0462	-29	129	142	QQIETVNLEIADPK					Mascot
1650.8732	1650.7777	-0.0955	-58	572	585	LLSQRISEMSTEIK			Oxidation (M)[9]		Mascot
1838.8688	1838.8862	0.0174	9	587	602	AESTIQELMSESEQLK			Oxidation (M)[9]		Mascot
1844.9712	1844.9204	-0.0508	-28	741	757	DIGSKTTAVEQLEALNR					Mascot
1902.0178	1901.9127	-0.1051	-55	498	514	QAQSKVQELLTELAESK					Mascot
1942.7858	1942.8658	0.08	41	92	110	GETSSSSSSDSDSDHSSKR					Mascot

8	TPA: proteasome subunit alpha type isoform 2 [Zea mays]	gi|414887869	27549	5.92	10	53	0	4.495	

Protein Group

TPA: proteasome subunit alpha type isoform 1 [Zea mays]	gi|414887868	27549	5.9200000762 9395
proteasome subunit alpha type 3 [Zea mays]	gi|226528124	27549	5.9200000762 9395

Peptide Information

Calc. Mass	Obsrv. Mass	± da	± ppm	Start Seq.	End Seq.	Sequence	Ion Score	C. I. %	Modification	Rank	Result Type
884.3995	884.4624	0.0629	71	94	101	SEAASYEK					Mascot
937.423	937.3888	-0.0342	-36	58	65	MMLEGSNR					Mascot
1119.619	1119.516	-0.103	-92	179	187	LKLSELTCR			Carbamidomethyl (C)[8]		Mascot
1217.6559	1217.5461	-0.1098	-90	42	52	CKDGIVLGVEK			Carbamidomethyl (C)[1]		Mascot
1321.5576	1321.6331	0.0755	57	237	249	AAAQAALEEMDAD			Oxidation (M)[10]		Mascot
1461.7731	1461.7311	-0.042	-29	30	43	AVDNSGTIVGIKCK			Carbamidomethyl (C)[13]		Mascot
1497.74	1497.7069	-0.0331	-22	53	65	LVTSKMMLEGSNR			Oxidation (M)[6,7]		Mascot
1799.8521	1799.8931	0.041	23	144	159	DGPQLYMIEPSGVSYK			Oxidation (M)[7]		Mascot
1959.9294	1959.9055	-0.0239	-12	2	20	SSIGTGYDLSVTTFSPDGR					Mascot
2239.197	2239.1106	-0.0864	-39	21	41	VFQVEYATKAVDNSGTIVGIK					Mascot

9	PREDICTED: uncharacterized protein LOC101248911 [Solanum lycopersicum]	gi|460366870	184893.5	8.95	27	52	0	15.153

Peptide Information

Calc. Mass	Obsrv. Mass	± da	± ppm	Start Seq.	End Seq.	Sequence	Ion Score	C. I. %	Modification	Rank	Result Type
804.4322	804.3704	-0.0618	-77	138	145	KNGTAASR					Mascot
806.3726	806.3979	0.0253	31	654	658	CERWR			Carbamidomethyl (C)[1]		Mascot
810.3563	810.3364	-0.0199	-25	1	7	MSWGSSR					Mascot
820.4159	820.4221	0.0062	8	1323	1329	SIQTESR					Mascot
826.3512	826.3533	0.0021	3	1	7	MSWGSSR			Oxidation (M)[1]		Mascot
838.4087	838.3524	-0.0563	-67	551	557	SSMKDVR			Oxidation (M)[3]		Mascot
856.4886	856.5198	0.0312	36	795	802	SNAQIPVK					Mascot
863.4217	863.3525	-0.0692	-80	484	492	DSSGAASLR					Mascot
870.468	870.5333	0.0653	75	381	390	GAGGAPEAIK					Mascot
912.3693	912.3717	0.0024	3	832	838	ESDQYDR					Mascot
912.3693	912.3717	0.0024	3	832	838	ESDQYDR					Mascot
921.4709	921.4919	0.021	23	338	345	NDTMSLLK					Mascot
921.4709	921.4919	0.021	23	338	345	NDTMSLLK					Mascot
937.4659	937.3888	-0.0771	-82	338	345	NDTMSLLK			Oxidation (M)[4]		Mascot
1027.4625	1027.4686	0.0061	6	1183	1191	RAAYCSDGK			Carbamidomethyl (C)[5]		Mascot
1060.5131	1060.5209	0.0078	7	591	600	GQHSSSSKSR					Mascot
1068.4705	1068.4905	0.02	19	832	839	ESDQYDRR					Mascot
1119.5099	1119.516	0.0061	5	818	827	MANGDENPKK			Oxidation (M)[1]		Mascot
1216.6685	1216.5658	-0.1027	-84	1197	1206	KHVSVLNEYK					Mascot
1316.6554	1316.5839	-0.0715	-54	102	113	TPQISQNQGTSR					Mascot
1374.7377	1374.6411	-0.0966	-70	1198	1209	HVSVLNEYKSAK					Mascot
1492.722	1492.7777	0.0557	37	80	92	FGGYGSFLPTYQR					Mascot
1497.8173	1497.7069	-0.1104	-74	555	567	DVRVAHVFDAQLK					Mascot
1542.8486	1542.7349	-0.1137	-74	795	809	SNAQIPVKSSDVLGK					Mascot
1582.8951	1582.7416	-0.1535	-97	659	672	LLPYGTKPAQLPER					Mascot
1582.8951	1582.7416	-0.1535	-97	659	672	LLPYGTKPAQLPER					Mascot
1650.8368	1650.7777	-0.0591	-36	1015	1028	VQSRLTMEDIDSLK			Oxidation (M)[7]		Mascot
1799.7913	1799.8931	0.1018	57	1381	1397	DPGIGVCQNASHNSMGR			Carbamidomethyl (C)[7]		Mascot
1812.8796	1812.8462	-0.0334	-18	1596	1611	LLDFTKDVNSAMEASR			Oxidation (M)[12]		Mascot
1842.8489	1842.8755	0.0266	14	673	686	WICSMLYWLPGMNR			Carbamidomethyl (C)[3], Oxidation (M)[5]		Mascot
2003.0292	2003.0315	0.0023	1	1229	1246	LEENISDQLGSKEVLNSK					Mascot
3166.301	3166.5615	0.2605	82	1	26	MSWGSSRVQMMEEHDL EEGEACYYNK			Carbamidomethyl (C)[22]		Mascot

10	TPA: hypothetical protein ZEAMMB73_943597 [Zea	gi|414887867	27806.1	6.11	10	51	0	4.495	

Peptide Information

Calc. Mass	Obsrv. Mass	± da	± ppm	Start Seq.	End Seq.	Sequence	Ion Score	C. I. %	Modification	Rank	Result Type
884.3995	884.4624	0.0629	71	94	101	SEAASYEK					Mascot
937.423	937.3888	-0.0342	-36	58	65	MMLEGSNR					Mascot
1119.619	1119.516	-0.103	-92	181	189	LKLSELTCR			Carbamidomethyl (C)[8]		Mascot
1217.6559	1217.5461	-0.1098	-90	42	52	CKDGIVLGVEK			Carbamidomethyl (C)[1]		Mascot
1321.5576	1321.6331	0.0755	57	239	251	AAAQAALEEMDAD			Oxidation (M)[10]		Mascot
1461.7731	1461.7311	-0.042	-29	30	43	AVDNSGTIVGIKCK			Carbamidomethyl (C)[13]		Mascot
1497.74	1497.7069	-0.0331	-22	53	65	LVTSKMMLEGSNR			Oxidation (M)[6,7]		Mascot
1799.8521	1799.8931	0.041	23	146	161	DGPQLYMIEPSGVSYK			Oxidation (M)[7]		Mascot
1959.9294	1959.9055	-0.0239	-12	2	20	SSIGTGYDLSVTTFSPDG R					Mascot
2239.197	2239.1106	-0.0864	-39	21	41	VFQVEYATKAVDNSGTIV GIK					Mascot

Gel Idx/Pos	182/H9	Instr./Gel Origin	BA2151/Sample Project 20140814	Process Status	Analysis Succeeded
Plate [#] Name	[1] Sample Project 20140814	Instrument Sample Name		Spectra	11

Rank	Protein Name	Accession No.	Protein MW	Protein PI	Pep. Count	Protein Score	Protein Score C. I. %	Intensity Matched	Total Ion Score	Total Ion C. I. %	Confirmed
1	Chitinase 2 [Triticum urartu]	gi|474441224	24930.5	4.95	9	572	100	33.181	518	100	

Peptide Information

Calc. Mass	Obsrv. Mass	± da	± ppm	Start Seq.	End Seq.	Sequence	Ion Score	C. I. %	Modification	Rank	Result Type
816.3886	816.3838	-0.0048	-6	212	217	FTYETR					Mascot
916.5462	916.502	-0.0442	-48	160	168	VLASLQTGK					Mascot
996.4897	996.4883	-0.0014	-1	151	159	QTGFYPGAR					Mascot
996.4897	996.4883	-0.0014	-1	151	159	QTGFYPGAR	67	99.967			Mascot
1454.7314	1454.7075	-0.0239	-16	194	206	LPGFFIWSADSSK					Mascot
1813.9542	1813.8994	-0.0548	-30	169	186	TTEELGLLSPDQGIAAAK					Mascot
1869.8865	1869.8793	-0.0072	-4	55	70	LINEYGLDGVDVDYER					Mascot
1869.8865	1869.8793	-0.0072	-4	55	70	LINEYGLDGVDVDYER	133	100			Mascot
2134.0928	2134.092	-0.0008	0	34	54	VSFAPASVDSWVANAVA SLSR					Mascot
2134.0928	2134.092	-0.0008	0	34	54	VSFAPASVDSWVANAVA SLSR	146	100			Mascot
2193.0823	2193.0747	-0.0076	-3	93	112	AAFPNITTSIAPFEDDTVQ R					Mascot
2193.0823	2193.0747	-0.0076	-3	93	112	AAFPNITTSIAPFEDDTVQ R	171	100			Mascot
3580.6042	3580.6394	0.0352	10	121	150	YSGVIDYVNFQFYGYGA NTDVPTYVMFYDR			Oxidation (M)[26]		Mascot

Rank	Protein Name	Accession No.	Protein MW	Protein PI	Pep. Count	Protein Score	Protein Score C. I. %	Intensity Matched	Total Ion Score	Total Ion C. I. %	Confirmed
2	Chitinase 2 [Aegilops tauschii]	gi|475498798	31802	5.24	11	487	100	31.779	428	100	

Peptide Information

Calc. Mass	Obsrv. Mass	± da	± ppm	Start Seq.	End Seq.	Sequence	Ion Score	C. I. %	Modification	Rank	Result Type
816.3886	816.3838	-0.0048	-6	276	281	FTYETR					Mascot
870.4468	870.4536	0.0068	8	2	8	TNGYLFR					Mascot
916.5462	916.502	-0.0442	-48	224	232	VLASLQTGK					Mascot
1001.4873	1001.4518	-0.0355	-35	1	8	MTNGYLFR					Mascot
1086.5731	1086.59	0.0169	16	177	184	YYRALWSK					Mascot
1180.5957	1180.597	0.0013	1	9	19	EYLGAQSTGVR					Mascot
1180.5957	1180.597	0.0013	1	9	19	EYLGAQSTGVR	109	100			Mascot
1454.7314	1454.7075	-0.0239	-16	258	270	LPGFFIWSADSSK					Mascot

Calc. Mass	Obsrv. Mass	± da	± ppm	Start Seq.	End Seq.	Sequence	Ion Score	C. I. %	Modification	Rank	Result Type
1813.9542	1813.8994	-0.0548	-30	233	250	TTEELGLLSPDQGIAAAK					Mascot
1869.8865	1869.8793	-0.0072	-4	119	134	LINEYGLDGVDVDYER					Mascot
1869.8865	1869.8793	-0.0072	-4	119	134	LINEYGLDGVDVDYER	133	100			Mascot
2150.1274	2150.0847	-0.0427	-20	76	97	AAHPNVSVMAGLGGDSV LDIVK					Mascot
2166.1223	2166.0786	-0.0437	-20	76	97	AAHPNVSVMAGLGGDSV LDIVK			Oxidation (M)[9]		Mascot
2166.1223	2166.0786	-0.0437	-20	76	97	AAHPNVSVMAGLGGDSV LDIVK	15	0	Oxidation (M)[9]		Mascot
2193.0823	2193.0747	-0.0076	-3	157	176	AAFPNITTSIAPFEDDTVQ R					Mascot
2193.0823	2193.0747	-0.0076	-3	157	176	AAFPNITTSIAPFEDDTVQ R	171	100			Mascot

3	hypothetical protein F775_31937 [Aegilops tauschii]	gi|475582132	31327.1	5.38	11	250	100	6.789	191	100

Peptide Information

Calc. Mass	Obsrv. Mass	± da	± ppm	Start Seq.	End Seq.	Sequence	Ion Score	C. I. %	Modification	Rank	Result Type
870.4791	870.4536	-0.0255	-29	208	214	DRPNIQK					Mascot
965.505	965.4653	-0.0397	-41	55	62	NYKGLQDK					Mascot
995.523	995.4653	-0.0577	-58	215	222	FIEIVSCK			Carbamidomethyl (C)[7]		Mascot
1086.5983	1086.59	-0.0083	-8	193	201	FQIFFSGIK					Mascot
1340.7209	1340.6887	-0.0322	-24	254	264	LDPQFLLEHTK					Mascot
1468.8159	1468.7197	-0.0962	-65	254	265	LDPQFLLEHTKK					Mascot
1540.7366	1540.7316	-0.005	-3	38	49	LYVAYHCPYAQR			Carbamidomethyl (C)[7]		Mascot
1540.7366	1540.7316	-0.005	-3	38	49	LYVAYHCPYAQR	69	99.979	Carbamidomethyl (C)[7]		Mascot
1630.8951	1630.8925	-0.0026	-2	65	78	IIGIDLADRPAWYK					Mascot
1937.9127	1937.8662	-0.0465	-24	106	123	YIDSNFDGPALLPDDSAK					Mascot
2138.0752	2138.0918	0.0166	8	38	54	LYVAYHCPYAQRAWIAR			Carbamidomethyl (C)[7]		Mascot
2356.1667	2356.1624	-0.0043	-2	16	37	ENLPPSLTSTSEPPPLFD GTTR					Mascot
2356.1667	2356.1624	-0.0043	-2	16	37	ENLPPSLTSTSEPPPLFD GTTR	122	100			Mascot

4	Protein IN2-1-like protein B [Triticum urartu]	gi|473887484	41166.5	8.84	11	240	100	5.834	191	100

Peptide Information

Calc. Mass	Obsrv. Mass	± da	± ppm	Start Seq.	End Seq.	Sequence	Ion Score	C. I. %	Modification	Rank	Result Type
870.4791	870.4536	-0.0255	-29	208	214	DRPNIQK					Mascot
965.505	965.4653	-0.0397	-41	55	62	NYKGLQDK					Mascot
1086.5983	1086.59	-0.0083	-8	193	201	FQIFFSGIK					Mascot
1340.7209	1340.6887	-0.0322	-24	230	240	LDPQFLLEHTK					Mascot
1468.8159	1468.7197	-0.0962	-65	230	241	LDPQFLLEHTKK					Mascot

1492.7908	1492.7394	-0.0514	-34	325	337	IFLDIRDPGSFGR					Mascot
1540.7366	1540.7316	-0.005	-3	38	49	LYVAYHCPYAQR			Carbamidomethyl (C)[7]		Mascot
1540.7366	1540.7316	-0.005	-3	38	49	LYVAYHCPYAQR	69	99.979	Carbamidomethyl (C)[7]		Mascot
1798.9222	1798.8801	-0.0421	-23	215	229	FIEEVNKIDAYTQTK					Mascot
1937.9127	1937.8662	-0.0465	-24	106	123	YIDSNFDGPALLPDDSAK					Mascot
2138.0752	2138.0918	0.0166	8	38	54	LYVAYHCPYAQRAWIAR			Carbamidomethyl (C)[7]		Mascot
2356.1667	2356.1624	-0.0043	-2	16	37	ENLPPSLTSTSEPPPLFD GTTR					Mascot
2356.1667	2356.1624	-0.0043	-2	16	37	ENLPPSLTSTSEPPPLFD GTTR	122	100			Mascot

5	hypothetical protein OsI_11052 [Oryza sativa Indica Group]	gi|218192567	29138.7	4.78	11	128	100	4.691	69	99.979	

Peptide Information

Calc. Mass	Obsrv. Mass	± da	± ppm	Start Seq.	End Seq.	Sequence	Ion Score	C. I. %	Modification	Rank	Result Type
812.4737	812.415	-0.0587	-72	206	212	GRPNLQK					Mascot
925.4989	925.4219	-0.077	-83	138	146	ASYSSIVAK					Mascot
962.4941	962.4642	-0.0299	-31	220	227	IHAYTETK					Mascot
965.505	965.4653	-0.0397	-41	53	60	NYKGLQDK					Mascot
1086.5983	1086.59	-0.0083	-8	191	199	FQIFFSGIK					Mascot
1106.6093	1106.5179	-0.0914	-83	250	258	ELVQYIVDK					Mascot
1297.6747	1297.6273	-0.0474	-37	228	238	QDPRLPEDVTK					Mascot
1458.7336	1458.7119	-0.0217	-15	220	231	IHAYTETKQDPR					Mascot
1540.7366	1540.7316	-0.005	-3	36	47	LYVAYHCPYAQR			Carbamidomethyl (C)[7]		Mascot
1540.7366	1540.7316	-0.005	-3	36	47	LYVAYHCPYAQR	69	99.979	Carbamidomethyl (C)[7]		Mascot
1630.8951	1630.8925	-0.0026	-2	63	76	IVAIDLADRPAWYK					Mascot
2138.0752	2138.0918	0.0166	8	36	52	LYVAYHCPYAQRAWIAR			Carbamidomethyl (C)[7]		Mascot

6	RecName: Full=Protein IN2-1 homolog B; AltName: Full=Glutathione S-transferase GSTZ5	gi|223635219	27458.1	5.35	9	115	100	4.485	69	99.979	

Protein Group

Os03g0283100 [Oryza sativa Japonica Group]	gi|113548222	27458.1	5.3499 999046 3257
RecName: Full=Protein IN2-1 homolog B; AltName: Full=Glutathione S-transferase GSTZ5	gi|75153256	27458.1	5.3499 999046 3257

Peptide Information

Calc. Mass	Obsrv. Mass	± da	± ppm	Start Seq.	End Seq.	Sequence	Ion Score	C. I. %	Modification	Rank	Result Type
812.4737	812.415	-0.0587	-72	206	212	GRPNLQK					Mascot
925.4989	925.4219	-0.077	-83	138	146	ASYSSIVAK					Mascot
962.4941	962.4642	-0.0299	-31	220	227	IHAYTETK					Mascot
965.505	965.4653	-0.0397	-41	53	60	NYKGLQDK					Mascot
1086.5983	1086.59	-0.0083	-8	191	199	FQIFFSGIK					Mascot
1540.7366	1540.7316	-0.005	-3	36	47	LYVAYHCPYAQR			Carbamidomethyl (C)[7]		Mascot
1540.7366	1540.7316	-0.005	-3	36	47	LYVAYHCPYAQR	69	99.979	Carbamidomethyl (C)[7]		Mascot
1630.8951	1630.8925	-0.0026	-2	63	76	IVAIDLADRPAWYK					Mascot
2075.0325	2075.0427	0.0102	5	147	166	GDVCDEAVAALDKIEAAL SK			Carbamidomethyl (C)[4]		Mascot
2138.0752	2138.0918	0.0166	8	36	52	LYVAYHCPYAQRAWIAR			Carbamidomethyl (C)[7]		Mascot

7	TPA: hypothetical protein ZEAMMB73_052208 [Zea mays]	gi|414866201	19391	9.14	6	102	99.994	2.629	69	99.979	

Peptide Information

Calc. Mass	Obsrv. Mass	± da	± ppm	Start Seq.	End Seq.	Sequence	Ion Score	C. I. %	Modification	Rank	Result Type
965.505	965.4653	-0.0397	-41	111	118	NYKGLQDK					Mascot
1508.7717	1508.7002	-0.0715	-47	38	49	QEDAAHYHLRLR					Mascot
1540.7366	1540.7316	-0.005	-3	94	105	LYVAYHCPYAQR			Carbamidomethyl (C)[7]		Mascot
1540.7366	1540.7316	-0.005	-3	94	105	LYVAYHCPYAQR	69	99.979	Carbamidomethyl (C)[7]		Mascot
1652.7711	1652.8206	0.0495	30	35	47	CPRQEDAAHYHLR			Carbamidomethyl (C)[1]		Mascot
2138.0752	2138.0918	0.0166	8	94	110	LYVAYHCPYAQRAWIAR			Carbamidomethyl (C)[7]		Mascot
2147.1125	2147.0427	-0.0698	-33	50	71	DRSSSQTVIVAMAAAAPA SSVK					Mascot

8	TPA: hypothetical protein ZEAMMB73_052208 [Zea mays]	gi|414866204	23207.8	8.27	6	98	99.984	2.629	69	99.979	

Peptide Information

Calc. Mass	Obsrv. Mass	± da	± ppm	Start Seq.	End Seq.	Sequence	Ion Score	C. I. %	Modification	Rank	Result Type
965.505	965.4653	-0.0397	-41	111	118	NYKGLQDK					Mascot
1508.7717	1508.7002	-0.0715	-47	38	49	QEDAAHYHLRLR					Mascot
1540.7366	1540.7316	-0.005	-3	94	105	LYVAYHCPYAQR			Carbamidomethyl (C)[7]		Mascot
1540.7366	1540.7316	-0.005	-3	94	105	LYVAYHCPYAQR	69	99.979	Carbamidomethyl (C)[7]		Mascot

Calc. Mass	Obsrv. Mass	± da	± ppm	Start Seq.	End Seq.	Sequence	Ion Score	C. I. %	Modification	Rank	Result Type
1652.7711	1652.8206	0.0495	30	35	47	CPRQEDAAHYHLR			Carbamidomethyl (C)[1]		Mascot
2138.0752	2138.0918	0.0166	8	94	110	LYVAYHCPYAQRAWIAR			Carbamidomethyl (C)[7]		Mascot
2147.1125	2147.0427	-0.0698	-33	50	71	DRSSSQTVIVAMAAAAPASSVK					Mascot

9	TPA: hypothetical protein ZEAMMB73_052208 [Zea mays]	gi|414866203	23757.1	6.9	6	97	99.982	2.629	69	99.979

Peptide Information

Calc. Mass	Obsrv. Mass	± da	± ppm	Start Seq.	End Seq.	Sequence	Ion Score	C. I. %	Modification	Rank	Result Type
965.505	965.4653	-0.0397	-41	111	118	NYKGLQDK					Mascot
1508.7717	1508.7002	-0.0715	-47	38	49	QEDAAHYHLRLR					Mascot
1540.7366	1540.7316	-0.005	-3	94	105	LYVAYHCPYAQR			Carbamidomethyl (C)[7]		Mascot
1540.7366	1540.7316	-0.005	-3	94	105	LYVAYHCPYAQR	69	99.979	Carbamidomethyl (C)[7]		Mascot
1652.7711	1652.8206	0.0495	30	35	47	CPRQEDAAHYHLR			Carbamidomethyl (C)[1]		Mascot
2138.0752	2138.0918	0.0166	8	94	110	LYVAYHCPYAQRAWIAR			Carbamidomethyl (C)[7]		Mascot
2147.1125	2147.0427	-0.0698	-33	50	71	DRSSSQTVIVAMAAAAPASSVK					Mascot

10	PREDICTED: protein IN2-1 homolog B-like isoform X2 [Setaria italica]	gi|514820205	33515.3	8.49	7	96	99.978	2.556	69	99.979

Peptide Information

Calc. Mass	Obsrv. Mass	± da	± ppm	Start Seq.	End Seq.	Sequence	Ion Score	C. I. %	Modification	Rank	Result Type
812.4737	812.415	-0.0587	-72	259	265	GRPNLQK					Mascot
965.505	965.4653	-0.0397	-41	106	113	NYKGLQDK					Mascot
1540.7366	1540.7316	-0.005	-3	89	100	LYVAYHCPYAQR			Carbamidomethyl (C)[7]		Mascot
1540.7366	1540.7316	-0.005	-3	89	100	LYVAYHCPYAQR	69	99.979	Carbamidomethyl (C)[7]		Mascot
1646.8901	1646.8737	-0.0164	-10	116	129	IVAIDLADRPSWYK					Mascot
1798.9222	1798.8801	-0.0421	-23	266	280	FIEEVNKIDAYTQTK					Mascot
2132.0605	2132.0559	-0.0046	-2	200	219	EDVSEESVAALDKIEEALGK					Mascot
2138.0752	2138.0918	0.0166	8	89	105	LYVAYHCPYAQRAWIAR			Carbamidomethyl (C)[7]		Mascot

Gel Idx/Pos	183/H10	Instr./Gel Origin	BA2151/Sample Project 20140814	Process Status	Analysis Succeeded
Plate [#] Name	[1] Sample Project 20140814	Instrument Sample Name		Spectra	11

Rank	Protein Name	Accession No.	Protein MW	Protein PI	Pep. Count	Protein Score	Protein Score C. I. %	Intensity Matched	Total Ion Score	Total Ion C. I. %	Confirmed
1	Chitinase 2 [Triticum urartu]	gi|474441224	24930.5	4.95	5	144	100	4.64	124	100	

Peptide Information

Calc. Mass	Obsrv. Mass	± da	± ppm	Start Seq.	End Seq.	Sequence	Ion Score	C. I. %	Modification	Rank	Result Type
816.3886	816.3824	-0.0062	-8	212	217	FTYETR					Mascot
875.5171	875.4537	-0.0634	-72	1	7	MAVKWLK					Mascot
996.4897	996.4843	-0.0054	-5	151	159	QTGFYPGAR					Mascot
996.4897	996.4843	-0.0054	-5	151	159	QTGFYPGAR	15	0			Mascot
1869.8865	1869.8718	-0.0147	-8	55	70	LINEYGLDGVDVDYER					Mascot
1869.8865	1869.8718	-0.0147	-8	55	70	LINEYGLDGVDVDYER	31	0			Mascot
2193.0823	2193.0718	-0.0105	-5	93	112	AAFPNITTSIAPFEDDTVQR					Mascot
2193.0823	2193.0718	-0.0105	-5	93	112	AAFPNITTSIAPFEDDTVQR	78	99.997			Mascot

Rank	Protein Name	Accession No.	Protein MW	Protein PI	Pep. Count	Protein Score	Protein Score C. I. %	Intensity Matched	Total Ion Score	Total Ion C. I. %	Confirmed
2	Chitinase 2 [Aegilops tauschii]	gi|475498798	31802	5.24	7	138	100	5.739	109	100	

Peptide Information

Calc. Mass	Obsrv. Mass	± da	± ppm	Start Seq.	End Seq.	Sequence	Ion Score	C. I. %	Modification	Rank	Result Type
816.3886	816.3824	-0.0062	-8	276	281	FTYETR					Mascot
870.4468	870.5117	0.0649	75	2	8	TNGYLFR					Mascot
968.4836	968.4766	-0.007	-7	215	223	QTGFYPGAK					Mascot
1180.5957	1180.5895	-0.0062	-5	9	19	EYLGAQSTGVR					Mascot
1869.8865	1869.8718	-0.0147	-8	119	134	LINEYGLDGVDVDYER					Mascot
1869.8865	1869.8718	-0.0147	-8	119	134	LINEYGLDGVDVDYER	31	0			Mascot
2166.1223	2166.0857	-0.0366	-17	76	97	AAHPNVSVMAGLGGDSVLDIVK			Oxidation (M)[9]		Mascot
2166.1223	2166.0857	-0.0366	-17	76	97	AAHPNVSVMAGLGGDSVLDIVK			Oxidation (M)[9]		Mascot
2193.0823	2193.0718	-0.0105	-5	157	176	AAFPNITTSIAPFEDDTVQR					Mascot
2193.0823	2193.0718	-0.0105	-5	157	176	AAFPNITTSIAPFEDDTVQR	78	99.997			Mascot

Rank	Protein Name	Accession No.	Protein MW	Protein PI	Pep. Count	Protein Score	Protein Score C. I. %	Intensity Matched	Total Ion Score	Total Ion C. I. %	Confirmed
3	Os05g0569100 [Oryza sativa Japonica Group]	gi|255676587	51833.9	8.73	15	65	71.274	5.516			

Peptide Information

Calc. Mass	Obsrv. Mass	± da	± ppm	Start Seq.	End Seq.	Sequence	Ion Score	C. I. %	Modification	Rank	Result Type
805.4427	805.4028	-0.0399	-50	42	47	KLHHDR					Mascot
847.4632	847.4567	-0.0065	-8	16	23	ASTKDVAR					Mascot
858.5519	858.4796	-0.0723	-84	418	424	SLLRTLR					Mascot
863.4006	863.4581	0.0575	67	184	191	GAESTGWR					Mascot
889.456	889.4669	0.0109	12	89	96	TATAPICR			Carbamidomethyl (C)[7]		Mascot
979.4955	979.4592	-0.0363	-37	337	344	EGNNVYRK					Mascot
991.5571	991.5018	-0.0553	-56	305	312	FKVVDDIR					Mascot
1021.452	1021.4922	0.0402	39	97	104	VNHMEGYR			Oxidation (M)[4]		Mascot
1064.5446	1064.5117	-0.0329	-31	361	369	VVIMSWDSK					Mascot
1068.5685	1068.4873	-0.0812	-76	345	354	YVGTTTATVR					Mascot
1349.6705	1349.6477	-0.0228	-17	215	226	ICMATIGPAPYR			Carbamidomethyl (C)[2]		Mascot
1506.6788	1506.6841	0.0053	4	431	444	CRPSPTSAGCTNAK			Carbamidomethyl (C)[1,10]		Mascot
1738.9739	1738.8165	-0.1574	-91	2	15	ETIPVELWQEILLR					Mascot
1870.0143	1869.8718	-0.1425	-76	1	15	METIPVELWQEILLR					Mascot
1870.0143	1869.8718	-0.1425	-76	1	15	METIPVELWQEILLR					Mascot
2010.9525	2010.9916	0.0391	19	105	122	IANICNGFLCFASHSTAK			Carbamidomethyl (C)[5,10]		Mascot

4	hypothetical protein OsI_11131 [Oryza sativa Indica Group]	gi|218192609	46804.9	9.22	17	61	22.682	8.038

Peptide Information

Calc. Mass	Obsrv. Mass	± da	± ppm	Start Seq.	End Seq.	Sequence	Ion Score	C. I. %	Modification	Rank	Result Type
849.4135	849.4452	0.0317	37	300	306	MQALQDK			Oxidation (M)[1]		Mascot
863.4985	863.4581	-0.0404	-47	398	404	KAFQELK					Mascot
883.4268	883.4626	0.0358	41	319	326	DSASGRYK					Mascot
889.5287	889.4669	-0.0618	-69	346	353	MASLKLAR					Mascot
1021.5676	1021.4922	-0.0754	-74	217	225	VEVKNAAYK					Mascot
1023.5806	1023.5582	-0.0224	-22	32	39	QLRHQVSR					Mascot
1052.5306	1052.4878	-0.0428	-41	351	358	LAREFMNR			Oxidation (M)[6]		Mascot
1182.6055	1182.5619	-0.0436	-37	253	261	HFPNWPEKK					Mascot
1182.6055	1182.5619	-0.0436	-37	253	261	HFPNWPEKK					Mascot
1201.6801	1201.6447	-0.0354	-29	97	106	QQFSRAPLVR					Mascot
1320.7092	1320.6069	-0.1023	-77	354	364	EFMNRVVNALK					Mascot
1426.7173	1426.7455	0.0282	20	60	72	KLDNSSTGNSYLK					Mascot
1506.6456	1506.6841	0.0385	26	277	289	NLESEASSFHDDR					Mascot

Calc. Mass	Obsrv. Mass	± da	± ppm	Start Seq.	End Seq.	Sequence	Ion Score	C. I. %	Modification	Rank	Result Type
1537.7284	1537.8041	0.0757	49	1	13	MMREGDACVALLR			Carbamidomethyl (C)[8], Oxidation (M)[1]		Mascot
1537.7284	1537.8041	0.0757	49	1	13	MMREGDACVALLR	8	0	Carbamidomethyl (C)[8], Oxidation (M)[1]		Mascot
1549.6257	1549.762	0.1363	88	84	96	EAMDLNSTSCYSR			Carbamidomethyl (C)[10], Oxidation (M)[3]		Mascot
1553.7233	1553.7684	0.0451	29	1	13	MMREGDACVALLR			Carbamidomethyl (C)[8], Oxidation (M)[1,2]		Mascot
1553.7233	1553.7684	0.0451	29	1	13	MMREGDACVALLR			Carbamidomethyl (C)[8], Oxidation (M)[1,2]		Mascot
1594.7748	1594.9049	0.1301	82	236	248	WLDGELSYLVDER					Mascot
1676.8021	1676.8383	0.0362	22	174	190	NDAKSGSMGIPAATNSR					Mascot
2011.0026	2010.9916	-0.011	-5	300	316	MQALQDKIEQGIHNTER					Mascot

5	hypothetical protein F775_52423 [Aegilops tauschii]	gi|475562810	21925.1	6	6	60	4.878	3.458	33	.866

Peptide Information

Calc. Mass	Obsrv. Mass	± da	± ppm	Start Seq.	End Seq.	Sequence	Ion Score	C. I. %	Modification	Rank	Result Type
1201.6536	1201.6447	-0.0089	-7	179	190	EDAVAVATAAKR					Mascot
1211.5652	1211.566	0.0008	1	49	59	VTETDEPHAGR					Mascot
1367.6663	1367.6545	-0.0118	-9	49	60	VTETDEPHAGRR					Mascot
1527.7188	1527.7136	-0.0052	-3	10	24	TGDVYPPSAAAHDAR					Mascot
1527.7188	1527.7136	-0.0052	-3	10	24	TGDVYPPSAAAHDAR	33	0.866			Mascot
1594.9275	1594.9049	-0.0226	-14	159	174	LRDVLGSAAAVLPANK					Mascot
1739.8308	1739.8219	-0.0089	-5	26	39	QRDEVLTHDDQQQK					Mascot

6	Protein kinase APK1B, chloroplast precursor, putative [Ricinus communis]	gi|223527043	45894.4	9.5	13	59	0	9.826	

Peptide Information

Calc. Mass	Obsrv. Mass	± da	± ppm	Start Seq.	End Seq.	Sequence	Ion Score	C. I. %	Modification	Rank	Result Type
812.3719	812.3954	0.0235	29	2	8	GSCFSVR			Carbamidomethyl (C)[3]		Mascot
850.4669	850.418	-0.0489	-57	220	227	LSDFGLAK					Mascot
968.3989	968.4766	0.0777	80	388	396	STAETCDGK			Carbamidomethyl (C)[6]		Mascot
979.5029	979.4592	-0.0437	-45	306	313	IFQVMDAR					Mascot
1184.5916	1184.5519	-0.0397	-34	1	10	MGSCFSVRIK			Carbamidomethyl (C)[4]		Mascot
1265.6484	1265.6163	-0.0321	-25	207	217	ASNILLDSNYR					Mascot
1492.7643	1492.7629	-0.0014	-1	220	234	LSDFGLAKDGPTGSK					Mascot
1527.8026	1527.7136	-0.089	-58	9	22	IKAESPLHHGADPR					Mascot
1527.8026	1527.7136	-0.089	-58	9	22	IKAESPLHHGADPR					Mascot
1553.8104	1553.7684	-0.042	-27	326	339	VANLAVQCISPEPR			Carbamidomethyl (C)[8]		Mascot
1553.8104	1553.7684	-0.042	-27	326	339	VANLAVQCISPEPR			Carbamidomethyl (C)[8]		Mascot

1597.7566	1597.8176	0.061	38	27	43	GGHDVSGSSSAVPSTPR					Mascot
2192.999	2193.0718	0.0728	33	241	260	VMGTYGYAAPEYMATGH LTK			Oxidation (M)[2,13]		Mascot
2193.1526	2193.0718	-0.0808	-37	149	166	LLVYEFMPKGSLENHLFR					Mascot
2385.177	2385.0212	-0.1558	-65	64	85	AATRNFRPDSVLGEGGF GCVFK			Carbamidomethyl (C)[19]		Mascot

7	predicted protein [Arabidopsis lyrata subsp. lyrata]	gi|297310638	66189	9.75	15	58	0	9.682	

Peptide Information

Calc. Mass	Obsrv. Mass	± da	± ppm	Start Seq.	End Seq.	Sequence	Ion Score	C. I. %	Modification	Rank	Result Type
806.4366	806.4187	-0.0179	-22	338	345	SSLATTAR					Mascot
827.3642	827.4335	0.0693	84	395	401	DHNENAK					Mascot
838.424	838.4058	-0.0182	-22	182	188	CNAFKAK			Carbamidomethyl (C)[1]		Mascot
847.4341	847.4567	0.0226	27	29	35	ELDLMAR					Mascot
863.4291	863.4581	0.029	34	29	35	ELDLMAR			Oxidation (M)[5]		Mascot
909.4901	909.4121	-0.078	-86	549	555	LSTYRNR					Mascot
979.5393	979.4592	-0.0801	-82	179	186	LVKCNAFK			Carbamidomethyl (C)[4]		Mascot
1052.5558	1052.4878	-0.068	-65	258	265	CLKIFESR			Carbamidomethyl (C)[1]		Mascot
1105.5208	1105.5255	0.0047	4	478	487	FVGSSQHGMR					Mascot
1320.6907	1320.6069	-0.0838	-63	312	324	YPSGSRVASTPAK					Mascot
1353.658	1353.5756	-0.0824	-61	17	28	LGDRDTFTMAAR					Mascot
1474.8047	1474.7167	-0.088	-60	8	20	QNMSVLLTKLGDR					Mascot
1490.7996	1490.6664	-0.1332	-89	8	20	QNMSVLLTKLGDR			Oxidation (M)[3]		Mascot
1490.7996	1490.6664	-0.1332	-89	8	20	QNMSVLLTKLGDR	5	0	Oxidation (M)[3]		Mascot
1537.7679	1537.8041	0.0362	24	243	255	LATMERNELGEFK					Mascot
1537.7679	1537.8041	0.0362	24	243	255	LATMERNELGEFK					Mascot
1549.881	1549.762	-0.119	-77	357	370	KTSLTAPHTKPNVR					Mascot
1553.7628	1553.7684	0.0056	4	243	255	LATMERNELGEFK			Oxidation (M)[4]		Mascot
1553.7628	1553.7684	0.0056	4	243	255	LATMERNELGEFK			Oxidation (M)[4]		Mascot
1722.8125	1722.8147	0.0022	1	272	285	AVREVMNQMMEAWK					Mascot
1738.8074	1738.8165	0.0091	5	272	285	AVREVMNQMMEAWK			Oxidation (M)[6]		Mascot
2193.1372	2193.0718	-0.0654	-30	216	234	GLVECLLSFLVSEDWAA RK			Carbamidomethyl (C)[5]		Mascot
2193.1372	2193.0718	-0.0654	-30	216	234	GLVECLLSFLVSEDWAA RK			Carbamidomethyl (C)[5]		Mascot

8	hypothetical protein M569_17127, partial [Genlisea aurea]	gi|527183144	28759.6	7.19	11	58	0	4.312	

Peptide Information

Calc. Mass	Obsrv. Mass	± da	± ppm	Start	End	Sequence	Ion	C. I. %	Modification	Rank	Result Type

				Seq.	Seq.		Score				
822.3992	822.4144	0.0152	18	142	147	EELEFR					Mascot
858.4832	858.4796	-0.0036	-4	70	76	ALNNWLK					Mascot
871.5247	871.4719	-0.0528	-61	184	191	QIAVEIAK					Mascot
889.4414	889.4669	0.0255	29	171	177	HFTEVEK					Mascot
925.427	925.4169	-0.0101	-11	2	9	ATMWGTMK					Mascot
967.5207	967.4658	-0.0549	-57	25	33	LLDASHSPK					Mascot
1037.4932	1037.5133	0.0201	19	150	158	CAESSKDLK			Carbamidomethyl (C)[1]		Mascot
1105.6001	1105.5255	-0.0746	-67	124	134	SAINNFAAAVK					Mascot
1161.5032	1161.532	0.0288	25	34	42	ETTDHHHER					Mascot
2034.9202	2034.9753	0.0551	27	2	17	ATMWGTMKFYHENQFK			Oxidation (M)[3]		Mascot
2165.9607	2166.0857	0.125	58	1	17	MATMWGTMKFYHENQF K			Oxidation (M)[1]		Mascot
2165.9607	2166.0857	0.125	58	1	17	MATMWGTMKFYHENQF K			Oxidation (M)[1]		Mascot

9 hypothetical protein TRIUR3_26563 [Triticum urartu] gi|474400087 13679.8 6.29 5 57 0 3.057 33 .866

Peptide Information

Calc. Mass	Obsrv. Mass	± da	± ppm	Start Seq.	End Seq.	Sequence	Ion Score	C. I. %	Modification	Rank	Result Type
1201.6536	1201.6447	-0.0089	-7	86	97	EDAVAVATAAKR					Mascot
1211.5652	1211.566	0.0008	1	45	55	VTETDEPHAGR					Mascot
1367.6663	1367.6545	-0.0118	-9	45	56	VTETDEPHAGRR					Mascot
1527.7188	1527.7136	-0.0052	-3	10	24	TGDVYPPSAAAHDAR					Mascot
1527.7188	1527.7136	-0.0052	-3	10	24	TGDVYPPSAAAHDAR	33	0.866			Mascot
1609.7928	1609.7908	-0.002	-1	42	55	ELRVTETDEPHAGR					Mascot

10 RD22-like protein precursor [Vitis vinifera] gi|526117780 39502.3 9.05 12 57 0 4.67

Peptide Information

Calc. Mass	Obsrv. Mass	± da	± ppm	Start Seq.	End Seq.	Sequence	Ion Score	C. I. %	Modification	Rank	Result Type
838.4529	838.4058	-0.0471	-56	63	71	GGVNVHAGK					Mascot
858.4832	858.4796	-0.0036	-4	120	127	HVYVGVGK					Mascot
919.4844	919.434	-0.0504	-55	74	84	SGGGTTVGVGK					Mascot
925.485	925.4169	-0.0681	-74	108	117	GGVSVNAGHK					Mascot
1023.5694	1023.5582	-0.0112	-11	63	73	GGVNVHAGKGK					Mascot
1323.6614	1323.6246	-0.0368	-28	305	317	AYMVPLVGADGSK			Oxidation (M)[3]		Mascot
1518.8197	1518.7103	-0.1094	-72	248	261	LGKGVQMISTEVEK					Mascot

1553.8395	1553.7684	-0.0711	-46	55	71	GTSVSVGKGGVNVHAGK	Mascot
1553.8395	1553.7684	-0.0711	-46	55	71	GTSVSVGKGGVNVHAGK	Mascot
1594.7959	1594.9049	0.109	68	262	275	ETPEQQYTITTGVK	Mascot
1698.9207	1698.8364	-0.0843	-50	41	54	AIRDILRPDLMEEK	Mascot
1722.8909	1722.8147	-0.0762	-44	262	276	ETPEQQYTITTGVKK	Mascot
2035.1044	2034.9753	-0.1291	-63	85	107	GTGVNVHAGKGKPGGG TTVGVGK	Mascot

Gel Idx/Pos	184/H11	Instr./Gel Origin	BA2151/Sample Project 20140814	Process Status	Analysis Succeeded
Plate [#] Name	[1] Sample Project 20140814	Instrument Sample Name		Spectra	11

Rank	Protein Name	Accession No.	Protein MW	Protein PI	Pep. Count	Protein Score	Protein Score C. I. %	Intensity Matched	Total Ion Score	Total Ion C. I. %	Confirmed
1	Coatomer subunit epsilon-1 [Aegilops tauschii]	gi|475617191	31742.1	5.22	4	104	99.996	1.86	88	100	

Peptide Information

Calc. Mass	Obsrv. Mass	± da	± ppm	Start Seq.	End Seq.	Sequence	Ion Score	C. I. %	Modification	Rank	Result Type
1473.7261	1473.7142	-0.0119	-8	185	196	EAYLIFQDFAEK					Mascot
1643.8387	1643.842	0.0033	2	91	105	EWLSDSAIGSNPVLR					Mascot
2215.1982	2215.1887	-0.0095	-4	124	143	HTHTGGTLDLHALNVQIF LK					Mascot
2955.4119	2955.4148	0.0029	1	12	39	NLFYLGAYQSAINNSDVP GLDADAAAER					Mascot
2955.4119	2955.4148	0.0029	1	12	39	NLFYLGAYQSAINNSDVP GLDADAAAER	90	100			Mascot

Rank	Protein Name	Accession No.	Protein MW	Protein PI	Pep. Count	Protein Score	Protein Score C. I. %	Intensity Matched	Total Ion Score	Total Ion C. I. %	Confirmed
2	Chitinase 2 [Triticum urartu]	gi|474441224	24930.5	4.95	5	74	96.466	2.344	55	99.362	

Peptide Information

Calc. Mass	Obsrv. Mass	± da	± ppm	Start Seq.	End Seq.	Sequence	Ion Score	C. I. %	Modification	Rank	Result Type
816.3886	816.393	0.0044	5	212	217	FTYETR					Mascot
881.4475	881.3793	-0.0682	-77	218	225	AQEIVANH					Mascot
996.4897	996.4911	0.0014	1	151	159	QTGFYPGAR					Mascot
1869.8865	1869.8965	0.01	5	55	70	LINEYGLDGVDVDYER					Mascot
1869.8865	1869.8965	0.01	5	55	70	LINEYGLDGVDVDYER	55	99.362			Mascot
2193.0823	2193.0918	0.0095	4	93	112	AAFPNITTSIAPFEDDTVQ R					Mascot

Rank	Protein Name	Accession No.	Protein MW	Protein PI	Pep. Count	Protein Score	Protein Score C. I. %	Intensity Matched	Total Ion Score	Total Ion C. I. %	Confirmed
3	Chitinase 2 [Aegilops tauschii]	gi|475498798	31802	5.24	5	72	93.715	2.451	55	99.362	

Peptide Information

Calc. Mass	Obsrv. Mass	± da	± ppm	Start Seq.	End Seq.	Sequence	Ion Score	C. I. %	Modification	Rank	Result Type
816.3886	816.393	0.0044	5	276	281	FTYETR					Mascot
881.4475	881.3793	-0.0682	-77	282	289	AQEIVANH					Mascot
1866.0121	1865.8778	-0.1343	-72	215	232	QTGFYPGAKVLASLQTG K					Mascot
1869.8865	1869.8965	0.01	5	119	134	LINEYGLDGVDVDYER					Mascot
1869.8865	1869.8965	0.01	5	119	134	LINEYGLDGVDVDYER	55	99.362			Mascot

Calc. Mass	Obsrv. Mass	± da	± ppm	Start Seq.	End Seq.	Sequence	Ion Score	C. I. %	Modification	Rank	Result Type
2193.0823	2193.0918	0.0095	4	157	176	AAFPNITTSIAPFEDDTVQR					Mascot

4 expressed protein [Chlorella variabilis] gi|307110034 60717.3 9.45 17 67 82.288 5.701

Peptide Information

Calc. Mass	Obsrv. Mass	± da	± ppm	Start Seq.	End Seq.	Sequence	Ion Score	C. I. %	Modification	Rank	Result Type
810.3628	810.3497	-0.0131	-16	79	84	EYDVER					Mascot
818.4478	818.371	-0.0768	-94	265	271	SEARSIR					Mascot
820.4271	820.3516	-0.0755	-92	269	275	SIRSDSR					Mascot
870.4791	870.5472	0.0681	78	391	399	SPAAGSPRK					Mascot
893.4323	893.4317	-0.0006	-1	442	450	SLSADSTGR					Mascot
1064.5042	1064.4967	-0.0075	-7	3	13	IAECGSGGSVK			Carbamidomethyl (C)[4]		Mascot
1167.5026	1167.5627	0.0601	51	350	359	EGEGDREYGR					Mascot
1184.6132	1184.5742	-0.039	-33	451	463	GGAASTPGRASPR					Mascot
1192.5991	1192.5551	-0.044	-37	2	13	KIAECGSGGSVK			Carbamidomethyl (C)[5]		Mascot
1323.6395	1323.6639	0.0244	18	1	13	MKIAECGSGGSVK			Carbamidomethyl (C)[6]		Mascot
1323.6395	1323.6639	0.0244	18	1	13	MKIAECGSGGSVK			Carbamidomethyl (C)[6]		Mascot
1479.7186	1479.7594	0.0408	28	437	450	SAHYKSLSADSTGR					Mascot
1537.7098	1537.7924	0.0826	54	85	97	EVVQRNAEGTMCK			Carbamidomethyl (C)[12], Oxidation (M)[11]		Mascot
1605.7649	1605.7428	-0.0221	-14	279	293	GRMADAELSSNALDR					Mascot
1869.8938	1869.8965	0.0027	1	399	418	KGEGEGGGVPAAPDSDVATR					Mascot
1870.0228	1869.8965	-0.1263	-68	124	140	YLHGASMAVRTALRPAR					Mascot
2151.0869	2151.0779	-0.009	-4	190	206	TDTAYLQDVLLDWQRWK					Mascot
2215.1367	2215.1887	0.052	23	207	227	SPADDFIHVAAPPASLARHSR					Mascot

5 unnamed protein product [Vitis vinifera] gi|297741339 309452.2 5.24 44 67 82.288 24.602

Peptide Information

Calc. Mass	Obsrv. Mass	± da	± ppm	Start Seq.	End Seq.	Sequence	Ion Score	C. I. %	Modification	Rank	Result Type
806.4003	806.3951	-0.0052	-6	438	444	DSTTNLR					Mascot
816.4574	816.393	-0.0644	-79	68	74	VDRTPTK					Mascot
818.4254	818.371	-0.0544	-66	935	941	DELKDAK					Mascot
832.3907	832.321	-0.0697	-84	164	171	IDGNGSNR					Mascot
845.4224	845.3984	-0.024	-28	2170	2176	GEAERQR					Mascot
849.4135	849.3749	-0.0386	-45	1014	1020	MQNSLEK					Mascot
852.4284	852.3857	-0.0427	-50	1457	1463	MVVFNDK					Mascot

853.4535	853.4216	-0.0319	-37	675	680	MMLRFR		Mascot
854.4254	854.3849	-0.0405	-47	2134	2140	KTYAEDK		Mascot
864.3781	864.368	-0.0101	-12	229	235	SMHGYNR		Mascot
865.4084	865.3809	-0.0275	-32	1014	1020	MQNSLEK	Oxidation (M)[1]	Mascot
891.4207	891.4144	-0.0063	-7	604	610	FPEDQQK		Mascot
955.4521	955.4329	-0.0192	-20	672	678	CTKMMLR	Carbamidomethyl (C)[1], Oxidation (M)[4]	Mascot
963.5145	963.4689	-0.0456	-47	2643	2651	GFAIDALEK		Mascot
977.5699	977.4943	-0.0756	-77	1853	1860	LLKTEVMK	Oxidation (M)[7]	Mascot
989.501	989.4658	-0.0352	-36	333	340	IRAEEESR		Mascot
996.4965	996.4911	-0.0054	-5	615	622	CIVRMSSK	Carbamidomethyl (C)[1], Oxidation (M)[5]	Mascot
1006.5217	1006.4384	-0.0833	-83	100	107	NRFGWAQK		Mascot
1033.5234	1033.5221	-0.0013	-1	2465	2473	VMELEGEVK		Mascot
1060.5521	1060.5587	0.0066	6	1467	1476	SLELEVGDAK		Mascot
1107.5503	1107.5435	-0.0068	-6	966	974	SHYMELLSK		Mascot
1118.594	1118.5259	-0.0681	-61	1396	1405	EIESALESLK		Mascot
1125.5898	1125.5221	-0.0677	-60	2213	2222	ALQEASEHIK		Mascot
1167.5245	1167.5627	0.0382	33	1198	1206	RSDMENMLR	Oxidation (M)[4]	Mascot
1192.5692	1192.5551	-0.0141	-12	2047	2056	SSIEEELTER		Mascot
1201.6423	1201.6628	0.0205	17	2327	2337	IEELEALAASR		Mascot
1201.6423	1201.6628	0.0205	17	2327	2337	IEELEALAASR		Mascot
1211.5647	1211.5796	0.0149	12	1084	1093	NMMLLETEAK	Oxidation (M)[2,3]	Mascot
1217.6385	1217.6083	-0.0302	-25	1163	1172	RSWISEQVGR		Mascot
1265.5878	1265.6313	0.0435	34	229	238	SMHGYNRCLK	Carbamidomethyl (C)[8]	Mascot
1323.6647	1323.6639	-0.0008	-1	1083	1093	KNMMLLETEAK	Oxidation (M)[3]	Mascot
1323.6647	1323.6639	-0.0008	-1	1083	1093	KNMMLLETEAK	Oxidation (M)[3]	Mascot
1372.743	1372.6571	-0.0859	-63	2146	2156	LLERSVEELER		Mascot
1473.7544	1473.7142	-0.0402	-27	2548	2559	ETEEERLQLAQK		Mascot
1475.655	1475.7523	0.0973	66	741	751	RFQDFYEEGER		Mascot
1490.7268	1490.6857	-0.0411	-28	1252	1264	NIQLSESEGMNLR		Mascot
1535.7007	1535.705	0.0043	3	1265	1277	KDDLLQDQESCGK	Carbamidomethyl (C)[11]	Mascot
1643.8599	1643.842	-0.0179	-11	2495	2508	NNLLKIENEDLSNK		Mascot
1657.8909	1657.8033	-0.0876	-53	1099	1112	LYHVTQDNKILGEK		Mascot
1665.7207	1665.8556	0.1349	81	1863	1875	TECCNVLQDLEER	Carbamidomethyl (C)[3,4]	Mascot
1791.7749	1791.7368	-0.0381	-21	2191	2205	HQMQNVESSDADMKR	Oxidation (M)[3]	Mascot
1833.8073	1833.84	0.0327	18	1033	1047	AFQISNEEEMDEVHR		Mascot
1908.967	1908.8296	-0.1374	-72	1772	1786	MRLMIQVQELEAEYR		Mascot
1927.0066	1926.8954	-0.1112	-58	1235	1251	SQLSEMNLNHVSALKQK		Mascot

1943.0015	1942.8795	-0.122	-63	1235	1251	SQLSEMNLNHVSALKQK			Oxidation (M)[6]		Mascot
2193.1396	2193.0918	-0.0478	-22	1983	2002	GLLFDLSLLQESASNSKD QK					Mascot
2717.3198	2717.0981	-0.2217	-82	2262	2286	ALEAMVEQVKPEGFSTH VQNSSSNK					Mascot
2955.3962	2955.4148	0.0186	6	2059	2085	VIDSLEADIFEMSNALGQ MNDSIDSLK					Mascot
2955.3962	2955.4148	0.0186	6	2059	2085	VIDSLEADIFEMSNALGQ MNDSIDSLK					Mascot

6	DNA repair protein RAD50, putative [Ricinus communis] gi|223525882	156507.6	4.98	28	57	0	9.98

Peptide Information

Calc. Mass	Obsrv. Mass	± da	± ppm	Start Seq.	End Seq.	Sequence	Ion Score	C. I. %	Modification	Rank	Result Type
805.3872	805.3314	-0.0558	-69	360	366	DMGNLQK					Mascot
806.389	806.3951	0.0061	8	305	311	ELSDQSK					Mascot
813.3811	813.3849	0.0038	5	914	920	GFSDMLK			Oxidation (M)[5]		Mascot
816.4574	816.393	-0.0644	-79	255	262	SVASSPLR					Mascot
831.4683	831.4468	-0.0215	-26	240	246	SSLQIQR					Mascot
845.4363	845.3984	-0.0379	-45	921	927	EVEALER					Mascot
853.4274	853.4216	-0.0058	-7	1225	1231	ANNELHR					Mascot
889.4261	889.3993	-0.0268	-30	367	373	ELEDEVR					Mascot
905.476	905.4269	-0.0491	-54	412	419	MEIANLSK					Mascot
906.4349	906.4423	0.0074	8	1110	1116	EEICSLR			Carbamidomethyl (C)[4]		Mascot
921.4709	921.5189	0.048	52	412	419	MEIANLSK			Oxidation (M)[1]		Mascot
921.4709	921.5189	0.048	52	412	419	MEIANLSK			Oxidation (M)[1]		Mascot
925.46	925.4243	-0.0357	-39	1333	1339	YFHMSLK					Mascot
941.4549	941.4062	-0.0487	-52	1333	1339	YFHMSLK			Oxidation (M)[4]		Mascot
979.4149	979.4709	0.056	57	1340	1347	CAEVESER			Carbamidomethyl (C)[1]		Mascot
989.4897	989.4658	-0.0239	-24	784	791	ENIELSER					Mascot
1006.4874	1006.4384	-0.049	-49	575	582	IQCTDLEK			Carbamidomethyl (C)[3]		Mascot
1060.4695	1060.5587	0.0892	84	164	173	DFSFSASGSR					Mascot
1064.5371	1064.4967	-0.0404	-38	624	632	QQLESFQGK					Mascot
1178.6085	1178.5804	-0.0281	-24	412	421	MEIANLSKEK			Oxidation (M)[1]		Mascot
1491.755	1491.733	-0.022	-15	1134	1145	KSLNEVQFENQR					Mascot
1539.8239	1539.7926	-0.0313	-20	1015	1028	ADRATLEASLHTVR					Mascot
1585.8254	1585.8348	0.0094	6	646	658	DIMISHKEILENK			Oxidation (M)[3]		Mascot
1643.8599	1643.842	-0.0179	-11	666	679	SDNLLKEQEVEALR					Mascot
1675.7955	1675.8267	0.0312	19	360	373	DMGNLQKELEDEVR					Mascot
1699.9337	1699.8229	-0.1108	-65	684	697	QLETQISILQNEKR					Mascot

Calc. Mass	Obsrv. Mass	± da	± ppm	Start Seq.	End Seq.	Sequence	Ion Score	C. I. %	Modification	Rank	Result Type
1791.8984	1791.7368	-0.1616	-90	247	262	DEFNQVSRSVASSPLR					Mascot
2151.0498	2151.0779	0.0281	13	186	204	ETYSPLNNLTGIMNNQIGR			Oxidation (M)[13]		Mascot
2193.1472	2193.0918	-0.0554	-25	1275	1293	LQLLETKLAEALEANDMYK					Mascot
2717.0728	2717.0981	0.0253	9	709	731	RGMMSSSCLDDSNNEIMMFNSSR			Carbamidomethyl (C)[8], Oxidation (M)[3,4,17]		Mascot
2955.4226	2955.4148	-0.0078	-3	845	868	LQEMQNMWLEAQSENEYLKIANLK			Oxidation (M)[4,7]		Mascot
2955.4226	2955.4148	-0.0078	-3	845	868	LQEMQNMWLEAQSENEYLKIANLK			Oxidation (M)[4,7]		Mascot

7 PREDICTED: expansin-like A3-like [Cucumis sativus] gi|449521407 29900.6 8.57 11 56 0 4.383

Peptide Information

Calc. Mass	Obsrv. Mass	± da	± ppm	Start Seq.	End Seq.	Sequence	Ion Score	C. I. %	Modification	Rank	Result Type
807.4359	807.3985	-0.0374	-46	103	109	ADFVLSR					Mascot
854.444	854.3849	-0.0591	-69	111	118	AFSAMALK			Oxidation (M)[5]		Mascot
893.4662	893.4317	-0.0345	-39	136	142	RIPCGYK			Carbamidomethyl (C)[4]		Mascot
979.5029	979.4709	-0.032	-33	137	144	IPCGYKNK			Carbamidomethyl (C)[3]		Mascot
982.539	982.4441	-0.0949	-97	110	118	KAFSAMALK			Oxidation (M)[6]		Mascot
1006.5098	1006.4384	-0.0714	-71	83	91	RLCNTAGSK			Carbamidomethyl (C)[3]		Mascot
1167.543	1167.5627	0.0197	17	192	201	NYGAVWDTNK					Mascot
1309.6383	1309.6417	0.0034	3	92	102	VVLTDQNYDSR					Mascot
1323.644	1323.6639	0.0199	15	191	201	RNYGAVWDTNK					Mascot
1323.644	1323.6639	0.0199	15	191	201	RNYGAVWDTNK					Mascot
1491.759	1491.733	-0.026	-17	192	204	NYGAVWDTNKVPK					Mascot
1833.9706	1833.84	-0.1306	-71	121	136	GQELLNTGIVDVEYKR					Mascot

8 hypothetical protein F775_23308 [Aegilops tauschii] gi|475545912 224213.6 4.68 29 55 0 10.676

Peptide Information

Calc. Mass	Obsrv. Mass	± da	± ppm	Start Seq.	End Seq.	Sequence	Ion Score	C. I. %	Modification	Rank	Result Type
807.4029	807.3985	-0.0044	-5	254	259	DLREMK			Oxidation (M)[5]		Mascot
813.3737	813.3849	0.0112	14	926	932	DAEDVHK					Mascot
820.3682	820.3516	-0.0166	-20	1542	1548	EEQASEK					Mascot
849.4424	849.3749	-0.0675	-79	548	556	SLSASAGTR					Mascot
865.4414	865.3809	-0.0605	-70	753	759	LAVDEYR					Mascot
870.5083	870.5472	0.0389	45	601	607	YKGVYLK					Mascot
884.52	884.4672	-0.0528	-60	1330	1337	IGVKENPK					Mascot
891.4166	891.4144	-0.0022	-2	1482	1488	QQTTEER					Mascot

893.3959	893.4317	0.0358	40	1705	1712	TTDENTGR					Mascot
918.4639	918.3832	-0.0807	-88	1750	1757	VSSQNQQK					Mascot
921.5152	921.5189	0.0037	4	1261	1267	EVYQVKR					Mascot
921.5152	921.5189	0.0037	4	1261	1267	EVYQVKR					Mascot
941.4686	941.4062	-0.0624	-66	926	933	DAEDVHKK					Mascot
951.4617	951.4586	-0.0031	-3	182	189	AHHAAWMK					Mascot
958.5316	958.4507	-0.0809	-84	1673	1680	ISLQNQQK					Mascot
977.4356	977.4943	0.0587	60	479	487	AAMEAEAER					Mascot
1064.5735	1064.4967	-0.0768	-72	751	759	AKLAVDEYR					Mascot
1081.5129	1081.5175	0.0046	4	1982	1989	TRMEMEIR			Oxidation (M)[3]		Mascot
1178.6272	1178.5804	-0.0468	-40	1984	1992	MEMEIRILK			Oxidation (M)[1]		Mascot
1184.6521	1184.5742	-0.0779	-66	1617	1626	EPVVLEKQDK					Mascot
1192.5514	1192.5551	0.0037	3	1050	1059	NLMKEDADEK					Mascot
1223.5184	1223.6138	0.0954	78	1608	1616	NQWDLCMEK			Carbamidomethyl (C)[6]		Mascot
1479.8167	1479.7594	-0.0573	-39	1918	1931	HGALEPVSVSKDIK					Mascot
1527.7649	1527.722	-0.0429	-28	1196	1209	ADINNEAPIEKDAK					Mascot
1657.9385	1657.8033	-0.1352	-82	238	253	GDRVALGPAVLASLYR					Mascot
1870.0393	1869.8965	-0.1428	-76	548	565	SLSASAGTRDDIVLLVPR					Mascot
1870.0393	1869.8965	-0.1428	-76	548	565	SLSASAGTRDDIVLLVPR					Mascot
1911.9117	1912.0369	0.1252	65	1412	1427	VDDHKTLDMHEEVSIK			Oxidation (M)[9]		Mascot
2258.1716	2258.2048	0.0332	15	39	60	APGPPPFRPPPPPSAATT AEFR					Mascot
2717.1926	2717.0981	-0.0945	-35	1499	1522	KMAMLGDDITLGCCEFQ ADSPETK			Carbamidomethyl (C)[13,14]		Mascot
2955.3936	2955.4148	0.0212	7	2	28	SSSSDELLLVQELPMDM HSDGGAPQRR					Mascot
2955.3936	2955.4148	0.0212	7	2	28	SSSSDELLLVQELPMDM HSDGGAPQRR					Mascot

9 PREDICTED: uncharacterized protein LOC101261498 [Solanum lycopersicum] gi|460414433 68900.7 9.53 18 55 0 5.74

Peptide Information

Calc. Mass	Obsrv. Mass	± da	± ppm	Start Seq.	End Seq.	Sequence	Ion Score	C. I. %	Modification	Rank	Result Type
813.4213	813.3849	-0.0364	-45	622	629	VANGAEPR					Mascot
849.4498	849.3749	-0.0749	-88	518	524	ACKLTEK			Carbamidomethyl (C)[2]		Mascot
852.4322	852.3857	-0.0465	-55	161	167	QNYSGKR					Mascot
893.4435	893.4317	-0.0118	-13	110	117	NSTSNSKR					Mascot
906.3911	906.4423	0.0512	56	440	447	NEDASTNR					Mascot
909.4029	909.4251	0.0222	24	512	520	GGGGMRACK			Carbamidomethyl (C)[8], Oxidation (M)[5]		Mascot

958.4775	958.4507	-0.0268	-28	229	236	CVPSPKDR			Carbamidomethyl (C)[1]		Mascot
1033.491	1033.5221	0.0311	30	280	288	NDGDKVETR					Mascot
1064.479	1064.4967	0.0177	17	276	284	LSCRNDGDK			Carbamidomethyl (C)[3]		Mascot
1118.5735	1118.5259	-0.0476	-43	507	517	KPSQKGGGGMR			Oxidation (M)[10]		Mascot
1178.5544	1178.5804	0.026	22	120	129	IMQMDAPVNK			Oxidation (M)[2,4]		Mascot
1309.6351	1309.6417	0.0066	5	387	397	LSTNKMVCQGR			Carbamidomethyl (C)[8], Oxidation (M)[6]		Mascot
1507.7421	1507.7452	0.0031	2	136	149	GLSTEISANLTNCK			Carbamidomethyl (C)[13]		Mascot
1535.6577	1535.705	0.0473	31	263	275	SVQMQHCSNVDSK			Carbamidomethyl (C)[7], Oxidation (M)[4]		Mascot
1699.9299	1699.8229	-0.107	-63	1	16	MEALKGPVLVDVDVSK					Mascot
1833.8899	1833.84	-0.0499	-27	205	220	SDVMDVTKDVDDLPLR			Oxidation (M)[4]		Mascot
1837.9088	1837.9287	0.0199	11	305	320	VHSVPTMCHAPLYTPK			Carbamidomethyl (C)[8]		Mascot
1870.0532	1869.8965	-0.1567	-84	326	343	LALAPSKDLDSLLADTVK					Mascot
1870.0532	1869.8965	-0.1567	-84	326	343	LALAPSKDLDSLLADTVK					Mascot

10	hypothetical protein CARUB_v10004182mg, partial [Capsella rubella]	gi|482551858	89092.2	5.02	22	55	0	7.193	

Peptide Information

Calc. Mass	Obsrv. Mass	± da	± ppm	Start Seq.	End Seq.	Sequence	Ion Score	C. I. %	Modification	Rank	Result Type
816.4461	816.393	-0.0531	-65	632	638	DLEAQLK					Mascot
820.4159	820.3516	-0.0643	-78	625	631	ESSTQLR					Mascot
834.3475	834.3265	-0.021	-25	301	307	DAEEENK					Mascot
864.4131	864.368	-0.0451	-52	313	319	NLETMEK					Mascot
891.4418	891.4144	-0.0274	-31	247	254	EATSDQLK					Mascot
906.4639	906.4423	-0.0216	-24	536	543	RDSSSQVK					Mascot
925.4738	925.4243	-0.0495	-53	188	195	TTALSYNR					Mascot
941.4794	941.4062	-0.0732	-78	660	667	TMSMKISK			Oxidation (M)[2]		Mascot
963.4993	963.4689	-0.0304	-32	29	36	TKTEIDEK					Mascot
977.4911	977.4943	0.0032	3	441	448	GIHETHQR					Mascot
1037.5183	1037.5251	0.0068	7	577	585	ISEMSTEIK					Mascot
1060.5885	1060.5587	-0.0298	-28	454	462	LSELETQLK					Mascot
1140.6511	1140.5684	-0.0827	-73	697	706	EGELLLLPEK					Mascot
1193.6195	1193.6221	0.0026	2	577	586	ISEMSTEIKR					Mascot
1320.6278	1320.5967	-0.0311	-24	301	312	DAEEENKAISSK					Mascot
1479.7173	1479.7594	0.0421	28	639	652	SSEQGVSELSESLK					Mascot
1491.736	1491.733	-0.003	-2	668	680	TSDELEQAQIMVK					Mascot
1507.7308	1507.7452	0.0144	10	668	680	TSDELEQAQIMVK			Oxidation (M)[11]		Mascot

1537.7466	1537.7924	0.0458	30	441	453	GIHETHQRESSTR		Mascot
1643.8599	1643.842	-0.0179	-11	549	562	VESAEKLVEELNQR		Mascot
1838.8688	1838.9227	0.0539	29	587	602	AESTIQELMSESEQLK	Oxidation (M)[9]	Mascot
1844.9712	1844.9404	-0.0308	-17	741	757	DIGSKTTAVEQLEALNR		Mascot
1942.7858	1942.8795	0.0937	48	92	110	GETSSSSSSDSDSDHSS KR		Mascot

Rank	Protein Name	Accession No.	Protein MW	Protein PI	Pep. Count	Protein Score	Protein Score C. I. %	Intensity Matched	Total Ion Score	Total Ion C. I. %	Confirmed
1	hypothetical protein F775_26756 [Aegilops tauschii]	gi|475500309	26436.2	5.51	9	401	100	36.361	352	100	

Peptide Information

Calc. Mass	Obsrv. Mass	± da	± ppm	Start Seq.	End Seq.	Sequence	Ion Score	C. I. %	Modification	Rank	Result Type
830.4366	830.4372	0.0006	1	132	138	DLEQVAR					Mascot
1127.5845	1127.5596	-0.0249	-22	184	192	FSVSFQKER					Mascot
1187.5957	1187.5911	-0.0046	-4	143	151	TVHFWQVDR					Mascot
1187.5957	1187.5911	-0.0046	-4	143	151	TVHFWQVDR	72	99.984			Mascot
1213.6172	1213.6096	-0.0076	-6	222	233	EVDLPAANTGAR					Mascot
1433.6842	1433.678	-0.0062	-4	71	83	QCLIFDGPEAGAR			Carbamidomethyl (C)[2]		Mascot
1433.6842	1433.678	-0.0062	-4	71	83	QCLIFDGPEAGAR	102	100	Carbamidomethyl (C)[2]		Mascot
1513.8196	1513.8158	-0.0038	-3	116	130	GGVLFMPGVPGVVER					Mascot
1529.8146	1529.7975	-0.0171	-11	116	130	GGVLFMPGVPGVVER			Oxidation (M)[6]		Mascot
1529.8146	1529.7975	-0.0171	-11	116	130	GGVLFMPGVPGVVER	58	99.611	Oxidation (M)[6]		Mascot
1812.9711	1812.901	-0.0701	-39	152	168	GDALPLGLPQIMMALTR			Oxidation (M)[12]		Mascot
1828.9659	1828.9332	-0.0327	-18	152	168	GDALPLGLPQIMMALTR			Oxidation (M)[12,13]		Mascot
1828.9659	1828.9332	-0.0327	-18	152	168	GDALPLGLPQIMMALTR			Oxidation (M)[12,13]		Mascot
1878.928	1878.8839	-0.0441	-23	196	214	AYMSGPAHGIHPLANAA GK			Oxidation (M)[3]		Mascot
1961.9036	1961.8901	-0.0135	-7	55	70	QVEAHHFCAHLNEDVR			Carbamidomethyl (C)[8]		Mascot
1961.9036	1961.8901	-0.0135	-7	55	70	QVEAHHFCAHLNEDVR	120	100	Carbamidomethyl (C)[8]		Mascot

Rank	Protein Name	Accession No.	Protein MW	Protein PI	Pep. Count	Protein Score	Protein Score C. I. %	Intensity Matched	Total Ion Score	Total Ion C. I. %	Confirmed
2	hypothetical protein TRIUR3_05104 [Triticum urartu]	gi|474427757	29228.5	5.54	6	275	100	27.561	250	100	

Peptide Information

Calc. Mass	Obsrv. Mass	± da	± ppm	Start Seq.	End Seq.	Sequence	Ion Score	C. I. %	Modification	Rank	Result Type
1187.5957	1187.5911	-0.0046	-4	168	176	TVHFWQVDR					Mascot
1187.5957	1187.5911	-0.0046	-4	168	176	TVHFWQVDR	72	99.984			Mascot
1213.6172	1213.6096	-0.0076	-6	247	258	EVDLPAANTGAR					Mascot
1513.8196	1513.8158	-0.0038	-3	141	155	GGVLFMPGVPGVVER					Mascot
1529.8146	1529.7975	-0.0171	-11	141	155	GGVLFMPGVPGVVER			Oxidation (M)[6]		Mascot
1529.8146	1529.7975	-0.0171	-11	141	155	GGVLFMPGVPGVVER	58	99.611	Oxidation (M)[6]		Mascot

1812.9711	1812.901	-0.0701	-39	177	193	GDALPLGLPQIMMALTR			Oxidation (M)[12]		Mascot
1828.9659	1828.9332	-0.0327	-18	177	193	GDALPLGLPQIMMALTR			Oxidation (M)[12,13]		Mascot
1828.9659	1828.9332	-0.0327	-18	177	193	GDALPLGLPQIMMALTR			Oxidation (M)[12,13]		Mascot
1878.928	1878.8839	-0.0441	-23	221	239	AYMSGPAHGIHPLANAA GK			Oxidation (M)[3]		Mascot
1961.9036	1961.8901	-0.0135	-7	80	95	QVEAHHFCAHLNEDVR			Carbamidomethyl (C)[8]		Mascot
1961.9036	1961.8901	-0.0135	-7	80	95	QVEAHHFCAHLNEDVR	120	100	Carbamidomethyl (C)[8]		Mascot

3	PREDICTED: uncharacterized protein LOC101776375 [Setaria italica]	gi|514783322	26612.3	5.72	4	264	100	25.495	250	100	

Peptide Information

Calc. Mass	Obsrv. Mass	± da	± ppm	Start Seq.	End Seq.	Sequence	Ion Score	C. I. %	Modification	Rank	Result Type
1187.5957	1187.5911	-0.0046	-4	143	151	TVHFWQVDR					Mascot
1187.5957	1187.5911	-0.0046	-4	143	151	TVHFWQVDR	72	99.984			Mascot
1513.8196	1513.8158	-0.0038	-3	116	130	GGVLFMPGVPGVVER					Mascot
1529.8146	1529.7975	-0.0171	-11	116	130	GGVLFMPGVPGVVER			Oxidation (M)[6]		Mascot
1529.8146	1529.7975	-0.0171	-11	116	130	GGVLFMPGVPGVVER	58	99.611	Oxidation (M)[6]		Mascot
1812.9711	1812.901	-0.0701	-39	152	168	GDALPLGLPQIMMALTR			Oxidation (M)[12]		Mascot
1828.9659	1828.9332	-0.0327	-18	152	168	GDALPLGLPQIMMALTR			Oxidation (M)[12,13]		Mascot
1828.9659	1828.9332	-0.0327	-18	152	168	GDALPLGLPQIMMALTR			Oxidation (M)[12,13]		Mascot
1961.9036	1961.8901	-0.0135	-7	55	70	QVEAHHFCAHLNEDVR			Carbamidomethyl (C)[8]		Mascot
1961.9036	1961.8901	-0.0135	-7	55	70	QVEAHHFCAHLNEDVR	120	100	Carbamidomethyl (C)[8]		Mascot

4	lipoprotein [Zea mays]	gi|413923448	26549.3	5.87	3	187	100	15.505	178	100	

Protein Group

uncharacterized protein LOC100193701 [Zea mays]	gi|212722552	26549.3	5.8699 998855 5908

Peptide Information

Calc. Mass	Obsrv. Mass	± da	± ppm	Start Seq.	End Seq.	Sequence	Ion Score	C. I. %	Modification	Rank	Result Type
1201.6113	1201.5857	-0.0256	-21	143	151	TIHFWQVDR					Mascot
1513.8196	1513.8158	-0.0038	-3	116	130	GGVLFMPGVPGVVER					Mascot
1529.8146	1529.7975	-0.0171	-11	116	130	GGVLFMPGVPGVVER			Oxidation (M)[6]		Mascot
1529.8146	1529.7975	-0.0171	-11	116	130	GGVLFMPGVPGVVER	58	99.611	Oxidation (M)[6]		Mascot
1961.9036	1961.8901	-0.0135	-7	55	70	QVEAHHFCAHLNEDVR			Carbamidomethyl (C)[8]		Mascot

Calc. Mass	Obsrv. Mass	± da	± ppm	Start Seq.	End Seq.	Sequence	Ion Score	C. I. %	Modification	Rank	Result Type
1961.9036	1961.8901	-0.0135	-7	55	70	QVEAHHFCAHLNEDVR	120	100	Carbamidomethyl (C)[8]		Mascot

5 hypothetical protein SORBIDRAFT_04g031810 [Sorghum bicolor] gi|241932585 26471.2 5.88 3 130 100 8.21 120 100

Peptide Information

Calc. Mass	Obsrv. Mass	± da	± ppm	Start Seq.	End Seq.	Sequence	Ion Score	C. I. %	Modification	Rank	Result Type
1201.6113	1201.5857	-0.0256	-21	143	151	TIHFWQVDR					Mascot
1812.9711	1812.901	-0.0701	-39	152	168	GDALPLGIPQIMMALTR			Oxidation (M)[12]		Mascot
1828.9659	1828.9332	-0.0327	-18	152	168	GDALPLGIPQIMMALTR			Oxidation (M)[12,13]		Mascot
1828.9659	1828.9332	-0.0327	-18	152	168	GDALPLGIPQIMMALTR			Oxidation (M)[12,13]		Mascot
1961.9036	1961.8901	-0.0135	-7	55	70	QVEAHHFCAHLNEDVR			Carbamidomethyl (C)[8]		Mascot
1961.9036	1961.8901	-0.0135	-7	55	70	QVEAHHFCAHLNEDVR	120	100	Carbamidomethyl (C)[8]		Mascot

6 hypothetical protein PRUPE_ppa019607mg [Prunus persica] gi|462423663 34754 9.53 17 75 96.922 26.161

Peptide Information

Calc. Mass	Obsrv. Mass	± da	± ppm	Start Seq.	End Seq.	Sequence	Ion Score	C. I. %	Modification	Rank	Result Type
849.3771	849.3477	-0.0294	-35	102	109	SSNAMPDK					Mascot
864.4421	864.3787	-0.0634	-73	266	273	DASTQSKK					Mascot
893.337	893.4068	0.0698	78	18	24	EEEEDDK					Mascot
1145.6161	1145.5809	-0.0352	-31	132	141	GSEVIQEQKK					Mascot
1182.6477	1182.5507	-0.097	-82	249	259	KPSTPVPSNQK					Mascot
1209.5858	1209.579	-0.0068	-6	172	181	DPPDERDPLR					Mascot
1217.5944	1217.5725	-0.0219	-18	223	233	SQQQNLGSPMK					Mascot
1219.5988	1219.5808	-0.018	-15	102	112	SSNAMPDKELK					Mascot
1219.5988	1219.5808	-0.018	-15	102	112	SSNAMPDKELK	1	0			Mascot
1235.5936	1235.5739	-0.0197	-16	102	112	SSNAMPDKELK			Oxidation (M)[5]		Mascot
1244.649	1244.6057	-0.0433	-35	25	35	MSLGTIMLNHK					Mascot
1249.5431	1249.5618	0.0187	15	15	24	EVKEEEEDDK					Mascot
1260.6439	1260.651	0.0071	6	25	35	MSLGTIMLNHK			Oxidation (M)[1]		Mascot
1325.5413	1325.6569	0.1156	87	118	127	MEEEEEEEKK			Oxidation (M)[1]		Mascot
1373.6954	1373.6248	-0.0706	-51	222	233	RSQQQNLGSPMK					Mascot
1529.7887	1529.7975	0.0088	6	182	193	IFYETLYEQVPK					Mascot
1529.7887	1529.7975	0.0088	6	182	193	IFYETLYEQVPK					Mascot
1764.8293	1764.9802	0.1509	86	41	57	IMNNANGNSTSAAREAK			Oxidation (M)[2]		Mascot
1942.9717	1942.8739	-0.0978	-50	61	77	EEQLDNDSDKPTKAKPK					Mascot

Calc. Mass	Obsrv. Mass	± da	± ppm	Start Seq.	End Seq.	Sequence	Ion Score	C. I. %	Modification	Rank	Result Type
1961.8806	1961.8901	0.0095	5	194	209	SEMAQFWLMECGLLSK			Carbamidomethyl (C)[11], Oxidation (M)[3,9]		Mascot
1961.8806	1961.8901	0.0095	5	194	209	SEMAQFWLMECGLLSK			Carbamidomethyl (C)[11], Oxidation (M)[3,9]		Mascot
2011.0536	2011.0291	-0.0245	-12	178	193	DPLRIFYETLYEQVPK					Mascot

7 Os05g0569500 [Oryza sativa Japonica Group] gi|113579915 26739.2 5.58 4 73 95.233 15.267 58 99.611

Peptide Information

Calc. Mass	Obsrv. Mass	± da	± ppm	Start Seq.	End Seq.	Sequence	Ion Score	C. I. %	Modification	Rank	Result Type
1201.6113	1201.5857	-0.0256	-21	143	151	TIHFWQVDR					Mascot
1513.8196	1513.8158	-0.0038	-3	116	130	GGVLFMPGVPGVVER					Mascot
1529.8146	1529.7975	-0.0171	-11	116	130	GGVLFMPGVPGVVER			Oxidation (M)[6]		Mascot
1529.8146	1529.7975	-0.0171	-11	116	130	GGVLFMPGVPGVVER	58	99.611	Oxidation (M)[6]		Mascot
1812.9711	1812.901	-0.0701	-39	152	168	GDALPLGLPQIMMALTR			Oxidation (M)[12]		Mascot
1828.9659	1828.9332	-0.0327	-18	152	168	GDALPLGLPQIMMALTR			Oxidation (M)[12,13]		Mascot
1828.9659	1828.9332	-0.0327	-18	152	168	GDALPLGLPQIMMALTR			Oxidation (M)[12,13]		Mascot
2322.1045	2322.1433	0.0388	17	193	214	ENRAYMSGPDHGIHPLA NAAGK			Oxidation (M)[6]		Mascot

8 hypothetical protein SORBIDRAFT_04g023030 [Sorghum bicolor] gi|241932118 41243.2 9.12 14 68 83.846 4.745

Peptide Information

Calc. Mass	Obsrv. Mass	± da	± ppm	Start Seq.	End Seq.	Sequence	Ion Score	C. I. %	Modification	Rank	Result Type
810.3628	810.3467	-0.0161	-20	146	152	ENGFSEK					Mascot
1155.6555	1155.5795	-0.076	-66	15	25	SGAISIVHIMK					Mascot
1195.6001	1195.6018	0.0017	1	277	288	AHGMPSGGPTKR					Mascot
1213.6899	1213.6096	-0.0803	-66	135	145	IDGPLNISKTR					Mascot
1231.5525	1231.5842	0.0317	26	173	182	MVPDYNEHAR					Mascot
1342.6057	1342.6772	0.0715	53	33	45	GMPSQPPPGSTDR			Oxidation (M)[2]		Mascot
1359.5482	1359.6335	0.0853	63	313	324	EYVGSEDSTCGR			Carbamidomethyl (C)[10]		Mascot
1511.7489	1511.7952	0.0463	31	2	14	DSQIWPIPPDTSR					Mascot
1514.8359	1514.8027	-0.0332	-22	15	28	SGAISIVHIMKSQK			Oxidation (M)[10]		Mascot
1557.7261	1557.8049	0.0788	51	327	341	SGKVHGMPSSGPMNR			Oxidation (M)[7]		Mascot
1591.7581	1591.8108	0.0527	33	330	344	VHGMPSSGPMNRGHK					Mascot
1743.849	1743.834	-0.015	-9	159	172	WDFPEQWQGLVNPK					Mascot
1812.8221	1812.901	0.0789	44	120	134	LGYCTIESEDGYHIR			Carbamidomethyl (C)[4]		Mascot
1942.9941	1942.8739	-0.1202	-62	294	311	KIQHNQGATSTTTADLTR					Mascot

9 hypothetical protein TRIUR3_17666 [Triticum urartu] gi|474387985 26438.1 5.82 3 66 78.209 12.514 58 99.611

Peptide Information

Calc. Mass	Obsrv. Mass	± da	± ppm	Start Seq.	End Seq.	Sequence	Ion Score	C. I. %	Modification	Rank	Result Type
1127.5845	1127.5596	-0.0249	-22	184	192	FSVSFQKER					Mascot
1213.6172	1213.6096	-0.0076	-6	222	233	EVDLPAANTGAR					Mascot
1513.8196	1513.8158	-0.0038	-3	116	130	GGVLFMPGVPGVVER					Mascot
1529.8146	1529.7975	-0.0171	-11	116	130	GGVLFMPGVPGVVER			Oxidation (M)[6]		Mascot
1529.8146	1529.7975	-0.0171	-11	116	130	GGVLFMPGVPGVVER	58	99.611	Oxidation (M)[6]		Mascot

10 Ankyrin-2 [Triticum urartu] gi|474347049 58755.6 6.69 15 64 58.478 18.984

Peptide Information

Calc. Mass	Obsrv. Mass	± da	± ppm	Start Seq.	End Seq.	Sequence	Ion Score	C. I. %	Modification	Rank	Result Type
800.3897	800.3456	-0.0441	-55	510	516	HEGSDKK					Mascot
820.3618	820.3426	-0.0192	-23	253	258	QEDCLR			Carbamidomethyl (C)[4]		Mascot
858.4427	858.4106	-0.0321	-37	530	538	QAAAAAEAR					Mascot
1032.5321	1032.4746	-0.0575	-56	300	309	SGTIPLSSDR					Mascot
1187.6572	1187.5911	-0.0661	-56	4	14	LGFAAPWVQAK					Mascot
1187.6572	1187.5911	-0.0661	-56	4	14	LGFAAPWVQAK	2	0			Mascot
1191.662	1191.5863	-0.0757	-64	94	104	GFVDVVDTLVK					Mascot
1194.6953	1194.5979	-0.0974	-82	82	93	VAVHALVSAATR					Mascot
1219.6178	1219.5808	-0.037	-30	25	36	GYPATAAAREGR					Mascot
1219.6178	1219.5808	-0.037	-30	25	36	GYPATAAAREGR	5	0			Mascot
1231.6504	1231.5842	-0.0662	-54	540	550	GVPFKPGICEK			Carbamidomethyl (C)[9]		Mascot
1259.7219	1259.5978	-0.1241	-99	288	299	AGFERAVLGAIR					Mascot
1359.7454	1359.6335	-0.1119	-82	540	551	GVPFKPGICEKK			Carbamidomethyl (C)[9]		Mascot
1444.7948	1444.6819	-0.1129	-78	2	14	EKLGFAAPWVQAK					Mascot
1591.8302	1591.8108	-0.0194	-12	1	14	MEKLGFAAPWVQAK			Oxidation (M)[1]		Mascot
1661.7919	1661.7654	-0.0265	-16	115	129	LGAWSWDAATGEELR					Mascot
1828.9705	1828.9332	-0.0373	-20	4	21	LGFAAPWVQAKGADVNGK					Mascot
1828.9705	1828.9332	-0.0373	-20	4	21	LGFAAPWVQAKGADVNGK					Mascot

Gel Idx/Pos	186/H13	Instr./Gel Origin	BA2151/Sample Project 20140814	Process Status	Analysis Succeeded
Plate [#] Name	[1] Sample Project 20140814	Instrument Sample Name		Spectra	11

Rank	Protein Name	Accession No.	Protein MW	Protein PI	Pep. Count	Protein Score	Protein Score C. I. %	Intensity Matched	Total Ion Score	Total Ion C. I. %	Confirmed
1	hypothetical protein TRIUR3_05104 [Triticum urartu]	gi|474427757	29228.5	5.54	8	320	100	34.243	277	100	

Peptide Information

Calc. Mass	Obsrv. Mass	± da	± ppm	Start Seq.	End Seq.	Sequence	Ion Score	C. I. %	Modification	Rank	Result Type
1187.5957	1187.5876	-0.0081	-7	168	176	TVHFWQVDR					Mascot
1187.5957	1187.5876	-0.0081	-7	168	176	TVHFWQVDR	62	99.865			Mascot
1213.6172	1213.6056	-0.0116	-10	247	258	EVDLPAANTGAR					Mascot
1405.653	1405.6421	-0.0109	-8	96	108	QCLVFDGPDAGAR			Carbamidomethyl (C)[2]		Mascot
1405.653	1405.6421	-0.0109	-8	96	108	QCLVFDGPDAGAR	106	100	Carbamidomethyl (C)[2]		Mascot
1513.8196	1513.7732	-0.0464	-31	141	155	GGVLFMPGVPGVVER					Mascot
1513.8196	1513.7732	-0.0464	-31	141	155	GGVLFMPGVPGVVER					Mascot
1529.8146	1529.7788	-0.0358	-23	141	155	GGVLFMPGVPGVVER			Oxidation (M)[6]		Mascot
1660.7959	1660.7781	-0.0178	-11	194	207	EGQLRQDLADCVEK			Carbamidomethyl (C)[11]		Mascot
1796.9762	1796.9534	-0.0228	-13	177	193	GDALPLGLPQIMMALTR					Mascot
1812.9711	1812.9301	-0.041	-23	177	193	GDALPLGLPQIMMALTR			Oxidation (M)[12]		Mascot
1828.9659	1828.9254	-0.0405	-22	177	193	GDALPLGLPQIMMALTR			Oxidation (M)[12,13]		Mascot
1828.9659	1828.9254	-0.0405	-22	177	193	GDALPLGLPQIMMALTR	3	0	Oxidation (M)[12,13]		Mascot
1878.928	1878.8932	-0.0348	-19	221	239	AYMSGPAHGIHPLANAA GK			Oxidation (M)[3]		Mascot
1961.9036	1961.8876	-0.016	-8	80	95	QVEAHHFCAHLNEDVR			Carbamidomethyl (C)[8]		Mascot
1961.9036	1961.8876	-0.016	-8	80	95	QVEAHHFCAHLNEDVR	109	100	Carbamidomethyl (C)[8]		Mascot

Rank	Protein Name	Accession No.	Protein MW	Protein PI	Pep. Count	Protein Score	Protein Score C. I. %	Intensity Matched	Total Ion Score	Total Ion C. I. %	Confirmed
2	hypothetical protein F775_26756 [Aegilops tauschii]	gi|475500309	26436.2	5.51	10	233	100	28.552	171	100	

Peptide Information

Calc. Mass	Obsrv. Mass	± da	± ppm	Start Seq.	End Seq.	Sequence	Ion Score	C. I. %	Modification	Rank	Result Type
830.4366	830.4315	-0.0051	-6	132	138	DLEQVAR					Mascot
842.4407	842.4792	0.0385	46	184	190	FSVSFQK					Mascot
1187.5957	1187.5876	-0.0081	-7	143	151	TVHFWQVDR					Mascot
1187.5957	1187.5876	-0.0081	-7	143	151	TVHFWQVDR	62	99.865			Mascot
1213.6172	1213.6056	-0.0116	-10	222	233	EVDLPAANTGAR					Mascot
1433.6842	1433.6738	-0.0104	-7	71	83	QCLIFDGPEAGAR			Carbamidomethyl (C)[2]		Mascot

1513.8196	1513.7732	-0.0464	-31	116	130	GGVLFMPGVPGVVER					Mascot
1513.8196	1513.7732	-0.0464	-31	116	130	GGVLFMPGVPGVVER					Mascot
1529.8146	1529.7788	-0.0358	-23	116	130	GGVLFMPGVPGVVER			Oxidation (M)[6]		Mascot
1660.7959	1660.7781	-0.0178	-11	169	182	EGQLRQDLADCVEK			Carbamidomethyl (C)[11]		Mascot
1796.9762	1796.9534	-0.0228	-13	152	168	GDALPLGLPQIMMALTR					Mascot
1812.9711	1812.9301	-0.041	-23	152	168	GDALPLGLPQIMMALTR			Oxidation (M)[12]		Mascot
1828.9659	1828.9254	-0.0405	-22	152	168	GDALPLGLPQIMMALTR			Oxidation (M)[12,13]		Mascot
1828.9659	1828.9254	-0.0405	-22	152	168	GDALPLGLPQIMMALTR	3	0	Oxidation (M)[12,13]		Mascot
1878.928	1878.8932	-0.0348	-19	196	214	AYMSGPAHGIHPLANAA GK			Oxidation (M)[3]		Mascot
1961.9036	1961.8876	-0.016	-8	55	70	QVEAHHFCAHLNEDVR			Carbamidomethyl (C)[8]		Mascot
1961.9036	1961.8876	-0.016	-8	55	70	QVEAHHFCAHLNEDVR	109	100	Carbamidomethyl (C)[8]		Mascot

3	PREDICTED: uncharacterized protein LOC101776375 [Setaria italica]	gi|514783322	26612.3	5.72	6	201	100	24.85	171	100	

Peptide Information

Calc. Mass	Obsrv. Mass	± da	± ppm	Start Seq.	End Seq.	Sequence	Ion Score	C. I. %	Modification	Rank	Result Type
1187.5957	1187.5876	-0.0081	-7	143	151	TVHFWQVDR					Mascot
1187.5957	1187.5876	-0.0081	-7	143	151	TVHFWQVDR	62	99.865			Mascot
1372.7068	1372.6777	-0.0291	-21	222	235	EVDIPATTTAGAAR					Mascot
1513.8196	1513.7732	-0.0464	-31	116	130	GGVLFMPGVPGVVER					Mascot
1513.8196	1513.7732	-0.0464	-31	116	130	GGVLFMPGVPGVVER					Mascot
1529.8146	1529.7788	-0.0358	-23	116	130	GGVLFMPGVPGVVER			Oxidation (M)[6]		Mascot
1660.7959	1660.7781	-0.0178	-11	169	182	EGQLRQDLADCVEK			Carbamidomethyl (C)[11]		Mascot
1796.9762	1796.9534	-0.0228	-13	152	168	GDALPLGLPQIMMALTR					Mascot
1812.9711	1812.9301	-0.041	-23	152	168	GDALPLGLPQIMMALTR			Oxidation (M)[12]		Mascot
1828.9659	1828.9254	-0.0405	-22	152	168	GDALPLGLPQIMMALTR			Oxidation (M)[12,13]		Mascot
1828.9659	1828.9254	-0.0405	-22	152	168	GDALPLGLPQIMMALTR	3	0	Oxidation (M)[12,13]		Mascot
1961.9036	1961.8876	-0.016	-8	55	70	QVEAHHFCAHLNEDVR			Carbamidomethyl (C)[8]		Mascot
1961.9036	1961.8876	-0.016	-8	55	70	QVEAHHFCAHLNEDVR	109	100	Carbamidomethyl (C)[8]		Mascot

4	hypothetical protein SORBIDRAFT_04g031810 [Sorghum bicolor]	gi|241932585	26471.2	5.88	4	145	100	19.355	127	100	

Peptide Information

Calc. Mass	Obsrv. Mass	± da	± ppm	Start Seq.	End Seq.	Sequence	Ion Score	C. I. %	Modification	Rank	Result Type
1201.6113	1201.5734	-0.0379	-32	143	151	TIHFWQVDR					Mascot
1561.8196	1561.7587	-0.0609	-39	116	130	GGFLFMPGVPGVVER					Mascot

Calc. Mass	Obsrv. Mass	± da	± ppm	Start Seq.	End Seq.	Sequence	Ion Score	C. I. %	Modification	Rank	Result Type
1577.8146	1577.7471	-0.0675	-43	116	130	GGFLFMPGVPGVVER			Oxidation (M)[6]		Mascot
1577.8146	1577.7471	-0.0675	-43	116	130	GGFLFMPGVPGVVER	17	0	Oxidation (M)[6]		Mascot
1796.9762	1796.9534	-0.0228	-13	152	168	GDALPLGIPQIMMALTR					Mascot
1812.9711	1812.9301	-0.041	-23	152	168	GDALPLGIPQIMMALTR			Oxidation (M)[12]		Mascot
1828.9659	1828.9254	-0.0405	-22	152	168	GDALPLGIPQIMMALTR			Oxidation (M)[12,13]		Mascot
1828.9659	1828.9254	-0.0405	-22	152	168	GDALPLGIPQIMMALTR	3	0	Oxidation (M)[12,13]		Mascot
1961.9036	1961.8876	-0.016	-8	55	70	QVEAHHFCAHLNEDVR			Carbamidomethyl (C)[8]		Mascot
1961.9036	1961.8876	-0.016	-8	55	70	QVEAHHFCAHLNEDVR	109	100	Carbamidomethyl (C)[8]		Mascot

5	lipoprotein [Zea mays]	gi|413923448	26549.3	5.87	5	129	100	13.314	109	100	

Protein Group

uncharacterized protein LOC100193701 [Zea mays]	gi|212722552	26549.3	5.8699 998855 5908

Peptide Information

Calc. Mass	Obsrv. Mass	± da	± ppm	Start Seq.	End Seq.	Sequence	Ion Score	C. I. %	Modification	Rank	Result Type
1201.6113	1201.5734	-0.0379	-32	143	151	TIHFWQVDR					Mascot
1513.8196	1513.7732	-0.0464	-31	116	130	GGVLFMPGVPGVVER					Mascot
1513.8196	1513.7732	-0.0464	-31	116	130	GGVLFMPGVPGVVER					Mascot
1529.8146	1529.7788	-0.0358	-23	116	130	GGVLFMPGVPGVVER			Oxidation (M)[6]		Mascot
1660.7959	1660.7781	-0.0178	-11	169	182	EGQLRQDLADCVEK			Carbamidomethyl (C)[11]		Mascot
1961.9036	1961.8876	-0.016	-8	55	70	QVEAHHFCAHLNEDVR			Carbamidomethyl (C)[8]		Mascot
1961.9036	1961.8876	-0.016	-8	55	70	QVEAHHFCAHLNEDVR	109	100	Carbamidomethyl (C)[8]		Mascot
1966.944	1966.8827	-0.0613	-31	196	214	AYMSGPEHGIHPLANAT GK			Oxidation (M)[3]		Mascot

6	Chaperone DnaJ-domain superfamily protein isoform 2 [Theobroma cacao]	gi|508726657	12048.3	10.19	8	58	0	8.695	

Peptide Information

Calc. Mass	Obsrv. Mass	± da	± ppm	Start Seq.	End Seq.	Sequence	Ion Score	C. I. %	Modification	Rank	Result Type
907.4917	907.5023	0.0106	12	99	106	DVMLGKTK			Oxidation (M)[3]		Mascot
1217.6559	1217.5684	-0.0875	-72	94	104	INEAKDVMLGK					Mascot
1233.6508	1233.5664	-0.0844	-68	94	104	INEAKDVMLGK			Oxidation (M)[8]		Mascot
1433.6519	1433.6738	0.0219	15	40	51	FYEGGFQPTMTR					Mascot
1447.7594	1447.6998	-0.0596	-41	20	31	YGIHAWQAFKAR					Mascot

1449.6467	1449.7527	0.106	73	40	51	FYEGGFQPTMTR			Oxidation (M)[10]		Mascot
1561.7468	1561.7587	0.0119	8	39	51	KFYEGGFQPTMTR					Mascot
1577.7417	1577.7471	0.0054	3	39	51	KFYEGGFQPTMTR			Oxidation (M)[11]		Mascot
1577.7417	1577.7471	0.0054	3	39	51	KFYEGGFQPTMTR	8	0	Oxidation (M)[11]		Mascot
1605.7479	1605.7603	0.0124	8	40	52	FYEGGFQPTMTRR			Oxidation (M)[10]		Mascot
1853.8964	1853.8694	-0.027	-15	76	93	VMVANHPDAGGSHYLASK					Mascot
1981.9913	1981.9564	-0.0349	-18	75	93	KVMVANHPDAGGSHYLASK					Mascot

7	unnamed protein product [Vitis vinifera]	gi|297735028	112647.9	5.92	22	57	0	16.786	

Peptide Information

Calc. Mass	Obsrv. Mass	± da	± ppm	Start Seq.	End Seq.	Sequence	Ion Score	C. I. %	Modification	Rank	Result Type
804.3482	804.2753	-0.0729	-91	178	183	EENEQR					Mascot
805.3872	805.4	0.0128	16	866	872	SMQPQSK					Mascot
871.4091	871.425	0.0159	18	296	302	QHLECGK			Carbamidomethyl (C)[5]		Mascot
1126.4503	1126.5515	0.1012	90	712	720	DNMEMDLAR			Oxidation (M)[3,5]		Mascot
1191.5422	1191.5807	0.0385	32	175	183	CVKEENEQR			Carbamidomethyl (C)[1]		Mascot
1213.6787	1213.6056	-0.0731	-60	206	215	IVDLDQELLR					Mascot
1217.6307	1217.5684	-0.0623	-51	304	313	IAKLEAECQR			Carbamidomethyl (C)[8]		Mascot
1233.6395	1233.5664	-0.0731	-59	734	743	EMEQLLTELK					Mascot
1314.7263	1314.6304	-0.0959	-73	91	101	ILEDQVKDLNK					Mascot
1465.7659	1465.7864	0.0205	14	950	962	SPISPSHHHNKPK					Mascot
1545.769	1545.7767	0.0077	5	809	820	CEDLQEQLQRVK			Carbamidomethyl (C)[1]		Mascot
1561.7209	1561.7587	0.0378	24	401	413	NHELQTSRSMCAK			Carbamidomethyl (C)[11]		Mascot
1577.7159	1577.7471	0.0312	20	401	413	NHELQTSRSMCAK			Carbamidomethyl (C)[11], Oxidation (M)[10]		Mascot
1577.7159	1577.7471	0.0312	20	401	413	NHELQTSRSMCAK			Carbamidomethyl (C)[11], Oxidation (M)[10]		Mascot
1593.8608	1593.7478	-0.113	-71	950	963	SPISPSHHHNKPKK					Mascot
1748.9276	1748.9541	0.0265	15	539	554	ISIILESQTTDTDKGK					Mascot
1788.8507	1788.8773	0.0266	15	754	768	SLAETQLKCMTESYK			Carbamidomethyl (C)[9]		Mascot
1796.9025	1796.9534	0.0509	28	45	61	ASLASVASLSDKENYNK					Mascot
1829.0935	1828.9254	-0.1681	-92	653	668	LSLVEFFLGLSHILIK					Mascot
1829.0935	1828.9254	-0.1681	-92	653	668	LSLVEFFLGLSHILIK					Mascot
1871.0597	1870.8809	-0.1788	-96	143	158	LQLESITLLRLTAEDR					Mascot
1903.9436	1903.8823	-0.0613	-32	637	652	NIEDFSTSVDKFLFNK					Mascot
1966.9928	1966.8827	-0.1101	-56	36	56	TAATSGSDKASLASVASLSDK					Mascot
2166.1289	2166.1816	0.0527	24	769	787	SLELHAQELEAEVISLQEK					Mascot

Calc. Mass	Obsrv. Mass	± da	± ppm	Start Seq.	End Seq.	Sequence	Ion Score	C. I. %	Modification	Rank	Result Type
2644.457	2644.2983	-0.1587	-60	596	619	EHVINQNLVAAVSQIHQF VLSLGK					Mascot

8	hypothetical protein OsI_32610 [Oryza sativa Indica Group]	gi|125531006	32660	8.57	11	56	0	11.491

Peptide Information

Calc. Mass	Obsrv. Mass	± da	± ppm	Start Seq.	End Seq.	Sequence	Ion Score	C. I. %	Modification	Rank	Result Type
1185.674	1185.5723	-0.1017	-86	159	170	GVPAAVFGQALR					Mascot
1261.6899	1261.6027	-0.0872	-69	171	182	VFDIVSAANLGR					Mascot
1314.538	1314.6304	0.0924	70	75	84	ESEADERCYR			Carbamidomethyl (C)[8]		Mascot
1545.8306	1545.7767	-0.0539	-35	20	33	VTADNLAVEMLTIR					Mascot
1557.8748	1557.7686	-0.1062	-68	230	243	YVAARLPVDGVELR					Mascot
1561.8254	1561.7587	-0.0667	-43	20	33	VTADNLAVEMLTIR			Oxidation (M)[10]		Mascot
1605.9071	1605.7603	-0.1468	-91	249	264	VARVLGAPAAAVEEPR					Mascot
1618.87	1618.7729	-0.0971	-60	220	234	GHLGVVYDAKYVAAR					Mascot
1903.9331	1903.8823	-0.0508	-27	200	217	MLDGGRPLPETAEGFAS R					Mascot
1961.9857	1961.8876	-0.0981	-50	1	18	MTTPAMSAMQPVSEVLI R					Mascot
1961.9857	1961.8876	-0.0981	-50	1	18	MTTPAMSAMQPVSEVLI R					Mascot
2019.0363	2018.9503	-0.086	-43	2	19	TTPAMSAMQPVSEVLIRR			Oxidation (M)[5,8]		Mascot
2166.0715	2166.1816	0.1101	51	1	19	MTTPAMSAMQPVSEVLI RR			Oxidation (M)[1,6,9]		Mascot

9	conserved hypothetical protein [Ricinus communis]	gi|223550018	48299	9.15	15	55	0	18.044

Peptide Information

Calc. Mass	Obsrv. Mass	± da	± ppm	Start Seq.	End Seq.	Sequence	Ion Score	C. I. %	Modification	Rank	Result Type
907.5247	907.5023	-0.0224	-25	159	165	QEVKYLK					Mascot
1209.6296	1209.5538	-0.0758	-63	173	183	SFDGVVSMLVR					Mascot
1217.6273	1217.5684	-0.0589	-48	354	363	SFEQQNLVPR					Mascot
1231.6641	1231.5624	-0.1017	-83	21	31	NESISIEGVRK					Mascot
1233.674	1233.5664	-0.1076	-87	154	162	IFWQRQEVK					Mascot
1372.7543	1372.6777	-0.0766	-56	19	30	LRNESISIEGVR					Mascot
1447.641	1447.6998	0.0588	41	242	253	DLVDMFFESNSK			Oxidation (M)[5]		Mascot
1449.689	1449.7527	0.0637	44	118	130	EMEELSTLEGGVR					Mascot
1465.684	1465.7864	0.1024	70	118	130	EMEELSTLEGGVR			Oxidation (M)[2]		Mascot
1577.7839	1577.7471	-0.0368	-23	118	131	EMEELSTLEGGVRK					Mascot
1577.7839	1577.7471	-0.0368	-23	118	131	EMEELSTLEGGVRK					Mascot

1593.7789	1593.7478	-0.0311	-20	118	131	EMEELSTLEGGVRK			Oxidation (M)[2]		Mascot
1605.8571	1605.7603	-0.0968	-60	147	158	IMDLQQKIFWQR					Mascot
1815.0527	1814.9299	-0.1228	-68	192	208	IKLVFGIGPGYPTSLPR					Mascot
1879.0687	1878.8932	-0.1755	-93	364	379	TNVLLLQTLFFANKEK					Mascot
1903.9409	1903.8823	-0.0586	-31	349	363	WQSERSFEQQNLVPR					Mascot
1962.0695	1961.8876	-0.1819	-93	380	396	TEAAITELLVGLNYIWR					Mascot
1962.0695	1961.8876	-0.1819	-93	380	396	TEAAITELLVGLNYIWR					Mascot
1973.9161	1973.8999	-0.0162	-8	237	253	LEGDKDLVDMFFESNSK					Mascot
1989.911	1989.915	0.004	2	237	253	LEGDKDLVDMFFESNSK			Oxidation (M)[10]		Mascot

10	PREDICTED: F-box protein At2g32560-like [Solanum lycopersicum]	gi|460410995	46869.1	6.89	13	55	0	29.587

Peptide Information

Calc. Mass	Obsrv. Mass	± da	± ppm	Start Seq.	End Seq.	Sequence	Ion Score	C. I. %	Modification	Rank	Result Type
870.4792	870.535	0.0558	64	349	355	TTHIDRK					Mascot
882.4693	882.4167	-0.0526	-60	248	254	YPPHGRR					Mascot
1213.6113	1213.6056	-0.0057	-5	144	152	EWLWHISSR					Mascot
1219.6808	1219.578	-0.1028	-84	133	143	WGRVLGPAAHR					Mascot
1219.6808	1219.578	-0.1028	-84	133	143	WGRVLGPAAHR					Mascot
1513.6554	1513.7732	0.1178	78	359	372	EEGNEADGFYGGIR					Mascot
1513.6554	1513.7732	0.1178	78	359	372	EEGNEADGFYGGIR					Mascot
1529.7529	1529.7788	0.0259	17	373	384	KLHSNEEISMWR					Mascot
1545.7557	1545.7767	0.021	14	241	253	TDTFQARYPPHGR					Mascot
1557.759	1557.7686	0.0096	6	374	385	LHSNEEISMWRR					Mascot
1618.837	1618.7729	-0.0641	-40	255	268	AMAIETGVTWDRLR					Mascot
1700.8346	1700.894	0.0594	35	52	66	GFLESLMSSNVMTLR			Oxidation (M)[7]		Mascot
1812.9347	1812.9301	-0.0046	-3	51	66	KGFLESLMSSNVMTLR					Mascot
1828.9296	1828.9254	-0.0042	-2	51	66	KGFLESLMSSNVMTLR			Oxidation (M)[8]		Mascot
1828.9296	1828.9254	-0.0042	-2	51	66	KGFLESLMSSNVMTLR	5	0	Oxidation (M)[8]		Mascot
1844.9246	1844.9261	0.0015	1	51	66	KGFLESLMSSNVMTLR			Oxidation (M)[8,13]		Mascot
1927.0146	1926.8472	-0.1674	-87	165	180	GLMTYLSQFWPISIVR			Oxidation (M)[3]		Mascot
2166.1787	2166.1816	0.0029	1	34	50	LLSIWFWNDFLILNPFK					Mascot

Gel Idx/Pos	187/H14	Instr./Gel Origin	BA2151/Sample Project 20140814	Process Status	Analysis Succeeded
Plate [#] Name	[1] Sample Project 20140814	Instrument Sample Name		Spectra	11

Rank	Protein Name	Accession No.	Protein MW	Protein PI	Pep. Count	Protein Score	Protein Score C. I. %	Intensity Matched	Total Ion Score	Total Ion C. I. %	Confirmed
1	Triosephosphate isomerase, cytosolic [Triticum urartu]	gi|473927006	16951.5	5.08	4	350	100	19.054	330	100	

Peptide Information

Calc. Mass	Obsrv. Mass	± da	± ppm	Start Seq.	End Seq.	Sequence	Ion Score	C. I. %	Modification	Rank	Result Type
1577.8031	1577.8138	0.0107	7	55	69	VASPAQAQEVHDGLR					Mascot
1577.8031	1577.8138	0.0107	7	55	69	VASPAQAQEVHDGLR	99	100			Mascot
1607.8289	1607.8387	0.0098	6	71	85	WLHANVGPAVAESTR					Mascot
1607.8289	1607.8387	0.0098	6	71	85	WLHANVGPAVAESTR	138	100			Mascot
1705.8981	1705.9072	0.0091	5	55	70	VASPAQAQEVHDGLRK					Mascot
1705.8981	1705.9072	0.0091	5	55	70	VASPAQAQEVHDGLRK	94	100			Mascot
1735.9238	1735.9298	0.006	3	70	85	KWLHANVGPAVAESTR					Mascot

Rank	Protein Name	Accession No.	Protein MW	Protein PI	Pep. Count	Protein Score	Protein Score C. I. %	Intensity Matched	Total Ion Score	Total Ion C. I. %	Confirmed
2	uncharacterized protein, partial [Phleum pratense]	gi|409971955	28971.9	5.61	11	304	100	20.985	239	100	

Protein Group

uncharacterized protein, partial [Phleum pratense]	gi|409971807	28971.9	5.6100<br>001335<br>144
uncharacterized protein, partial [Phleum pratense]	gi|409972411	28999.9	5.6100<br>001335<br>144

Peptide Information

Calc. Mass	Obsrv. Mass	± da	± ppm	Start Seq.	End Seq.	Sequence	Ion Score	C. I. %	Modification	Rank	Result Type
954.4832	954.4868	0.0036	4	20	27	FFVGGNWK					Mascot
1033.6041	1033.5706	-0.0335	-32	129	138	VAYALAQGLK					Mascot
1082.5782	1082.5771	-0.0011	-1	19	27	KFFVGGNWK					Mascot
1388.7202	1388.7308	0.0106	8	139	150	VIACIGETLEQR			Carbamidomethyl (C)[4]		Mascot
1388.7202	1388.7308	0.0106	8	139	150	VIACIGETLEQR	101	100	Carbamidomethyl (C)[4]		Mascot
1607.8289	1607.8387	0.0098	6	207	221	WLHANVGPAVAESTR					Mascot
1607.8289	1607.8387	0.0098	6	207	221	WLHANVGPAVAESTR	138	100			Mascot

1735.9238	1735.9298	0.006	3	206	221	KWLHANVGPAVAESTR					Mascot
1964.0005	1963.9949	-0.0056	-3	151	169	EAGTTMEVVAAQTKAIAEK			Oxidation (M)[6]		Mascot
1970.925	1971.0166	0.0916	46	1	18	PHTASDSPHRADQPMAPR					Mascot
1987.0331	1987.0134	-0.0197	-10	69	85	ALLRPDFAVAAQNCWVR			Carbamidomethyl (C)[14]		Mascot
2831.4978	2831.2188	-0.279	-99	165	190	AIAEKISDWTNVVLAYEPVWAIGTGK					Mascot
2842.5237	2842.5278	0.0041	1	235	261	ELAAQPDLDGFLVGGASLKPEFVDIIK					Mascot

3	uncharacterized protein [Phleum pratense]	gi|409972183	27533.2	5.52	10	297	100	20.819	239	100

Peptide Information

Calc. Mass	Obsrv. Mass	± da	± ppm	Start Seq.	End Seq.	Sequence	Ion Score	C. I. %	Modification	Rank	Result Type
954.4832	954.4868	0.0036	4	6	13	FFVGGNWK					Mascot
1033.6041	1033.5706	-0.0335	-32	115	124	VAYALAQGLK					Mascot
1082.5782	1082.5771	-0.0011	-1	5	13	KFFVGGNWK					Mascot
1388.7202	1388.7308	0.0106	8	125	136	VIACIGETLEQR			Carbamidomethyl (C)[4]		Mascot
1388.7202	1388.7308	0.0106	8	125	136	VIACIGETLEQR	101	100	Carbamidomethyl (C)[4]		Mascot
1607.8289	1607.8387	0.0098	6	193	207	WLHANVGPAVAESTR					Mascot
1607.8289	1607.8387	0.0098	6	193	207	WLHANVGPAVAESTR	138	100			Mascot
1735.9238	1735.9298	0.006	3	192	207	KWLHANVGPAVAESTR					Mascot
1964.0005	1963.9949	-0.0056	-3	137	155	EAGTTMEVVAAQTKAIAEK			Oxidation (M)[6]		Mascot
1987.0331	1987.0134	-0.0197	-10	55	71	ALLRPDFAVAAQNCWVR			Carbamidomethyl (C)[14]		Mascot
2831.4978	2831.2188	-0.279	-99	151	176	AIAEKISDWTNVVLAYEPVWAIGTGK					Mascot
2842.5237	2842.5278	0.0041	1	221	247	ELAAQPDLDGFLVGGASLKPEFVDIIK					Mascot

4	uncharacterized protein, partial [Phleum pratense]	gi|409971755	23002	5.97	8	297	100	24.577	248	100

Peptide Information

Calc. Mass	Obsrv. Mass	± da	± ppm	Start Seq.	End Seq.	Sequence	Ion Score	C. I. %	Modification	Rank	Result Type
1033.6041	1033.5706	-0.0335	-32	74	83	VAYALAQGLK					Mascot
1388.7202	1388.7308	0.0106	8	84	95	VIACIGETLEQR			Carbamidomethyl (C)[4]		Mascot
1388.7202	1388.7308	0.0106	8	84	95	VIACIGETLEQR	101	100	Carbamidomethyl (C)[4]		Mascot
1607.8289	1607.8387	0.0098	6	152	166	WLHANVGPAVAESTR					Mascot
1607.8289	1607.8387	0.0098	6	152	166	WLHANVGPAVAESTR	138	100			Mascot
1735.9238	1735.9298	0.006	3	151	166	KWLHANVGPAVAESTR					Mascot
1964.0005	1963.9949	-0.0056	-3	96	114	EAGTTMEVVAAQTKAIAEK			Oxidation (M)[6]		Mascot

Calc. Mass	Obsrv. Mass	± da	± ppm	Start Seq.	End Seq.	Sequence	Ion Score	C. I. %	Modification	Rank	Result Type
1973.0175	1973.0276	0.0101	5	14	30	ALLRPDFAVAGQNCWVR			Carbamidomethyl (C)[14]		Mascot
1973.0175	1973.0276	0.0101	5	14	30	ALLRPDFAVAGQNCWVR	9	0	Carbamidomethyl (C)[14]		Mascot
2831.4978	2831.2188	-0.279	-99	110	135	AIAEKISDWTNVVLAYEPVWAIGTGK					Mascot
2842.5237	2842.5278	0.0041	1	180	206	ELAAQPDLDGFLVGGASLKPEFVDIIK					Mascot

5	uncharacterized protein, partial [Phleum pratense]	gi|409971617	29186	5.8	10	295	100	20.819	239	100	

Protein Group

uncharacterized protein, partial [Phleum pratense]	gi|409971939	29156	5.80000019073486

Peptide Information

Calc. Mass	Obsrv. Mass	± da	± ppm	Start Seq.	End Seq.	Sequence	Ion Score	C. I. %	Modification	Rank	Result Type
954.4832	954.4868	0.0036	4	21	28	FFVGGNWK					Mascot
1033.6041	1033.5706	-0.0335	-32	130	139	VAYALAQGLK					Mascot
1082.5782	1082.5771	-0.0011	-1	20	28	KFFVGGNWK					Mascot
1388.7202	1388.7308	0.0106	8	140	151	VIACIGETLEQR			Carbamidomethyl (C)[4]		Mascot
1388.7202	1388.7308	0.0106	8	140	151	VIACIGETLEQR	101	100	Carbamidomethyl (C)[4]		Mascot
1607.8289	1607.8387	0.0098	6	208	222	WLHANVGPAVAESTR					Mascot
1607.8289	1607.8387	0.0098	6	208	222	WLHANVGPAVAESTR	138	100			Mascot
1735.9238	1735.9298	0.006	3	207	222	KWLHANVGPAVAESTR					Mascot
1964.0005	1963.9949	-0.0056	-3	152	170	EAGTTMEVVAAQTKAIAEK			Oxidation (M)[6]		Mascot
1987.0331	1987.0134	-0.0197	-10	70	86	ALLRPDFAVAAQNCWVR			Carbamidomethyl (C)[14]		Mascot
2831.4978	2831.2188	-0.279	-99	166	191	AIAEKISDWTNVVLAYEPVWAIGTGK					Mascot
2842.5237	2842.5278	0.0041	1	236	262	ELAAQPDLDGFLVGGASLKPEFVDIIK					Mascot

6	uncharacterized protein, partial [Phleum pratense]	gi|409971953	26113.5	5.08	8	284	100	20.088	239	100	

Peptide Information

Calc. Mass	Obsrv. Mass	± da	± ppm	Start Seq.	End Seq.	Sequence	Ion Score	C. I. %	Modification	Rank	Result Type
1033.6041	1033.5706	-0.0335	-32	103	112	VAYALAQGLK					Mascot
1388.7202	1388.7308	0.0106	8	113	124	VIACIGETLEQR			Carbamidomethyl (C)[4]		Mascot
1388.7202	1388.7308	0.0106	8	113	124	VIACIGETLEQR	101	100	Carbamidomethyl (C)[4]		Mascot
1607.8289	1607.8387	0.0098	6	181	195	WLHANVGPAVAESTR					Mascot
1607.8289	1607.8387	0.0098	6	181	195	WLHANVGPAVAESTR	138	100			Mascot

Calc. Mass	Obsrv. Mass	± da	± ppm	Start Seq.	End Seq.	Sequence	Ion Score	C. I. %	Modification	Rank	Result Type
1735.9238	1735.9298	0.006	3	180	195	KWLHANVGPAVAESTR					Mascot
1964.0005	1963.9949	-0.0056	-3	125	143	EAGTTMEVVAAQTKAIAEK			Oxidation (M)[6]		Mascot
1987.0331	1987.0134	-0.0197	-10	43	59	ALLRPDFAVAAQNCWVR			Carbamidomethyl (C)[14]		Mascot
2831.4978	2831.2188	-0.279	-99	139	164	AIAEKISDWTNVVLAYEPVWAIGTGK					Mascot
2842.5237	2842.5278	0.0041	1	209	235	ELAAQPDLDGFLVGGASLKPEFVDIIK					Mascot

7 uncharacterized protein, partial [Phleum pratense] gi|409972439 13715 5.35 5 173 100 7.97 138 100

Peptide Information

Calc. Mass	Obsrv. Mass	± da	± ppm	Start Seq.	End Seq.	Sequence	Ion Score	C. I. %	Modification	Rank	Result Type
1607.8289	1607.8387	0.0098	6	67	81	WLHANVGPAVAESTR					Mascot
1607.8289	1607.8387	0.0098	6	67	81	WLHANVGPAVAESTR	138	100			Mascot
1735.9238	1735.9298	0.006	3	66	81	KWLHANVGPAVAESTR					Mascot
1964.0005	1963.9949	-0.0056	-3	11	29	EAGTTMEVVAAQTKAIAEK			Oxidation (M)[6]		Mascot
2831.4978	2831.2188	-0.279	-99	25	50	AIAEKISDWTNVVLAYEPVWAIGTGK					Mascot
2842.5237	2842.5278	0.0041	1	95	121	ELAAQPDLDGFLVGGASLKPEFVDIIK					Mascot

8 triosephosphate isomerase [Oryza sativa Indica Group] gi|399144539 27484.3 5.58 9 163 100 15.556 115 100

Protein Group

Os01g0841600 [Oryza sativa Japonica Group]	gi|113534299	27484.3	5.57999992370605
hypothetical protein OsI_04384 [Oryza sativa Indica Group]	gi|125528336	27415.2	5.3899998664856

Peptide Information

Calc. Mass	Obsrv. Mass	± da	± ppm	Start Seq.	End Seq.	Sequence	Ion Score	C. I. %	Modification	Rank	Result Type
954.4832	954.4868	0.0036	4	6	13	FFVGGNWK					Mascot
1082.5782	1082.5771	-0.0011	-1	5	13	KFFVGGNWK					Mascot
1107.51	1107.5504	0.0404	36	14	23	CNGTGEDVKK			Carbamidomethyl (C)[1]		Mascot
1388.7202	1388.7308	0.0106	8	125	136	VIACIGETLEQR			Carbamidomethyl (C)[4]		Mascot
1388.7202	1388.7308	0.0106	8	125	136	VIACIGETLEQR	101	100	Carbamidomethyl (C)[4]		Mascot
1591.8187	1591.8215	0.0028	2	177	191	VATPAQAQEVHDGLR					Mascot

Calc. Mass	Obsrv. Mass	± da	± ppm	Start Seq.	End Seq.	Sequence	Ion Score	C. I. %	Modification	Rank	Result Type
1629.8595	1629.8319	-0.0276	-17	193	207	WLVTNVSPAVAESTR					Mascot
1964.0005	1963.9949	-0.0056	-3	137	155	EAGTTMEVVAAQTKAIAE K			Oxidation (M)[6]		Mascot
1989.0123	1989.0219	0.0096	5	55	71	GLLRPDFSVAAQNCWVR			Carbamidomethyl (C)[14]		Mascot
1989.0123	1989.0219	0.0096	5	55	71	GLLRPDFSVAAQNCWVR	14	0	Carbamidomethyl (C)[14]		Mascot
2831.4978	2831.2188	-0.279	-99	151	176	AIAEKISDWTNVVLAYEP VWAIGTGK					Mascot

9	triosephosphate isomerase [Glycine max]	gi|351721638	27441.3	5.87	10	156	100	21.268	101	100	

Peptide Information

Calc. Mass	Obsrv. Mass	± da	± ppm	Start Seq.	End Seq.	Sequence	Ion Score	C. I. %	Modification	Rank	Result Type
954.4832	954.4868	0.0036	4	5	12	FFVGGNWK					Mascot
1037.4569	1037.5319	0.075	72	13	21	CNGTTEEVK			Carbamidomethyl (C)[1]		Mascot
1082.5782	1082.5771	-0.0011	-1	4	12	KFFVGGNWK					Mascot
1388.7202	1388.7308	0.0106	8	124	135	VIACIGETLEQR			Carbamidomethyl (C)[4]		Mascot
1388.7202	1388.7308	0.0106	8	124	135	VIACIGETLEQR	101	100	Carbamidomethyl (C)[4]		Mascot
1605.8344	1605.8469	0.0125	8	176	190	VATPAQAQEVHADLR					Mascot
1639.8187	1639.8257	0.007	4	192	206	WVHDNVSAEVAASVR					Mascot
1639.8187	1639.8257	0.007	4	192	206	WVHDNVSAEVAASVR					Mascot
1674.881	1674.8303	-0.0507	-30	100	113	RQLLNELNEFVGDK					Mascot
1733.9293	1733.9089	-0.0204	-12	176	191	VATPAQAQEVHADLRK					Mascot
1767.9137	1767.9196	0.0059	3	191	206	KWVHDNVSAEVAASVR					Mascot
1972.9222	1973.0276	0.1054	53	5	21	FFVGGNWKCNGTTEEVK			Carbamidomethyl (C)[9]		Mascot
1972.9222	1973.0276	0.1054	53	5	21	FFVGGNWKCNGTTEEVK			Carbamidomethyl (C)[9]		Mascot

10	Triosephosphate isomerase isoform 1 [Theobroma cacao]	gi|508726238	32445.8	8.05	9	143	100	13.772	101	100	

Peptide Information

Calc. Mass	Obsrv. Mass	± da	± ppm	Start Seq.	End Seq.	Sequence	Ion Score	C. I. %	Modification	Rank	Result Type
954.4832	954.4868	0.0036	4	5	12	FFVGGNWK					Mascot
1082.5782	1082.5771	-0.0011	-1	4	12	KFFVGGNWK					Mascot
1098.5078	1098.5739	0.0661	60	171	178	FVGYFMYR			Oxidation (M)[6]		Mascot
1353.6831	1353.6589	-0.0242	-18	249	261	IIYGGSVTAANCK			Carbamidomethyl (C)[12]		Mascot
1388.7202	1388.7308	0.0106	8	124	135	VIACIGETLEQR			Carbamidomethyl (C)[4]		Mascot
1388.7202	1388.7308	0.0106	8	124	135	VIACIGETLEQR	101	100	Carbamidomethyl (C)[4]		Mascot
1635.845	1635.8441	-0.0009	-1	218	232	VATPAQAQEVHSELR					Mascot
1763.9399	1763.9388	-0.0011	-1	218	233	VATPAQAQEVHSELRK					Mascot

1971.9382	1972.0454	0.1072	54	5	21	FFVGGNWKCNGTTEQVK	Carbamidomethyl (C)[9]	Mascot
2842.5237	2842.5278	0.0041	1	262	288	ELAAQPDVDGFLVGGAS LKPEFIDIIK		Mascot

Gel Idx/Pos	188/H15	Instr./Gel Origin	BA2151/Sample Project 20140814	Process Status	Analysis Succeeded
Plate [#] Name	[1] Sample Project 20140814	Instrument Sample Name		Spectra	11

Rank	Protein Name	Accession No.	Protein MW	Protein PI	Pep. Count	Protein Score	Protein Score C. I. %	Intensity Matched	Total Ion Score	Total Ion C. I. %	Confirmed
1	hypothetical protein TRIUR3_31593 [Triticum urartu]	gi|473979984	19793.1	5.63	12	372	100	20.517	305	100	

Peptide Information

Calc. Mass	Obsrv. Mass	± da	± ppm	Start Seq.	End Seq.	Sequence	Ion Score	C. I. %	Modification	Rank	Result Type
807.3645	807.3835	0.019	24	86	91	GDHWHR					Mascot
888.4421	888.4516	0.0095	11	63	69	VEIEENR					Mascot
926.5669	926.5472	-0.0197	-21	129	136	KLAPEQIK					Mascot
1108.6473	1108.6488	0.0015	1	130	139	LAPEQIKGPR					Mascot
1203.5601	1203.5669	0.0068	6	155	167	TIGDAGAAGGEER					Mascot
1203.5601	1203.5669	0.0068	6	155	167	TIGDAGAAGGEER	33	0.377			Mascot
1247.6453	1247.6178	-0.0275	-22	6	16	MDVALLADPFR					Mascot
1329.6951	1329.6992	0.0041	3	17	27	ILEHVPFGFDR	20	0			Mascot
1331.655	1331.6841	0.0291	22	154	167	KTIGDAGAAGGEER					Mascot
1373.6907	1373.6976	0.0069	5	59	69	EDLKVEIEENR					Mascot
1770.8328	1770.8367	0.0039	2	42	57	ETSDSHEIVVDVPGMR					Mascot
1786.8276	1786.8215	-0.0061	-3	42	57	ETSDSHEIVVDVPGMR			Oxidation (M)[15]		Mascot
2226.1614	2226.1665	0.0051	2	105	126	LPDNADLDSIAASLDAGV LTVR					Mascot
2226.1614	2226.1665	0.0051	2	105	126	LPDNADLDSIAASLDAGV LTVR	241	100			Mascot
2421.1689	2421.1802	0.0113	5	17	37	ILEHVPFGFDRDDVAMVS MAR			Oxidation (M)[16]		Mascot
2437.1638	2437.1423	-0.0215	-9	17	37	ILEHVPFGFDRDDVAMVS MAR			Oxidation (M)[16,19]		Mascot
2437.1638	2437.1423	-0.0215	-9	17	37	ILEHVPFGFDRDDVAMVS MAR	12	0	Oxidation (M)[16,19]		Mascot

Rank	Protein Name	Accession No.	Protein MW	Protein PI	Pep. Count	Protein Score	Protein Score C. I. %	Intensity Matched	Total Ion Score	Total Ion C. I. %	Confirmed
2	hypothetical protein F775_30396 [Aegilops tauschii]	gi|475584601	13170.8	8.01	5	263	100	16.607	241	100	

Peptide Information

Calc. Mass	Obsrv. Mass	± da	± ppm	Start Seq.	End Seq.	Sequence	Ion Score	C. I. %	Modification	Rank	Result Type
807.3645	807.3835	0.019	24	31	36	GDHWHR					Mascot
926.5669	926.5472	-0.0197	-21	74	81	KLAPEQIK					Mascot
1108.6473	1108.6488	0.0015	1	75	84	LAPEQIKGPR					Mascot
1231.5913	1231.608	0.0167	14	100	112	TIGDVGAAGGEER					Mascot

Calc. Mass	Obsrv. Mass	± da	± ppm	Start Seq.	End Seq.	Sequence	Ion Score	C. I. %	Modification	Rank	Result Type
2226.1614	2226.1665	0.0051	2	50	71	LPDNADLDSIAASLDAGV LTVR					Mascot
2226.1614	2226.1665	0.0051	2	50	71	LPDNADLDSIAASLDAGV LTVR	241	100			Mascot

3	22.0 kDa class IV heat shock protein precursor [Zea mays]	gi|226509936	22928.8	6.01	7	86	99.777	2.401	57	99.605

Peptide Information

Calc. Mass	Obsrv. Mass	± da	± ppm	Start Seq.	End Seq.	Sequence	Ion Score	C. I. %	Modification	Rank	Result Type
807.3645	807.3835	0.019	24	123	128	GDHWHR					Mascot
926.5669	926.5472	-0.0197	-21	166	173	KLAPEQIK					Mascot
1108.6473	1108.6488	0.0015	1	167	176	LAPEQIKGPR					Mascot
1329.6951	1329.6992	0.0041	3	55	65	ILEHVPFGFDR	20	0			Mascot
1357.6343	1357.7228	0.0885	65	191	204	ESIGTGAGDGHQTK					Mascot
2226.1978	2226.1665	-0.0313	-14	142	163	LPENADLXSVAASLDSGV LTVR	25	0			Mascot
2421.1689	2421.1802	0.0113	5	55	75	ILEHVPFGFDRDDVAMVS MAR			Oxidation (M)[16]		Mascot
2437.1638	2437.1423	-0.0215	-9	55	75	ILEHVPFGFDRDDVAMVS MAR			Oxidation (M)[16,19]		Mascot
2437.1638	2437.1423	-0.0215	-9	55	75	ILEHVPFGFDRDDVAMVS MAR	12	0	Oxidation (M)[16,19]		Mascot

4	hypothetical protein SORBIDRAFT_06g017850 [Sorghum bicolor]	gi|241937725	25084.6	5.62	7	76	97.257	9.3	48	97.224

Peptide Information

Calc. Mass	Obsrv. Mass	± da	± ppm	Start Seq.	End Seq.	Sequence	Ion Score	C. I. %	Modification	Rank	Result Type
807.3645	807.3835	0.019	24	126	131	GDHWHR					Mascot
926.5669	926.5472	-0.0197	-21	169	176	KLAPEQIK					Mascot
1108.6473	1108.6488	0.0015	1	170	179	LAPEQIKGPR					Mascot
1287.6177	1287.6305	0.0128	10	180	194	VVGIAGGDGDGGDAK					Mascot
1329.6951	1329.6992	0.0041	3	58	68	ILEHVPFGFDR	20	0			Mascot
2313.1833	2313.186	0.0027	1	35	57	GGRDEAAAVSPLSDVGL LADPFR					Mascot
2313.1833	2313.186	0.0027	1	35	57	GGRDEAAAVSPLSDVGL LADPFR	16	0			Mascot
2421.1689	2421.1802	0.0113	5	58	78	ILEHVPFGFDRDDVAMVS MAR			Oxidation (M)[16]		Mascot
2437.1638	2437.1423	-0.0215	-9	58	78	ILEHVPFGFDRDDVAMVS MAR			Oxidation (M)[16,19]		Mascot
2437.1638	2437.1423	-0.0215	-9	58	78	ILEHVPFGFDRDDVAMVS MAR	12	0	Oxidation (M)[16,19]		Mascot

5	PREDICTED: 23.2 kDa heat shock protein-like [Setaria italica]	gi|514801671	23632	5.52	8	66	76.65	2.851	32	0

Peptide Information

Calc. Mass	Obsrv. Mass	± da	± ppm	Start Seq.	End Seq.	Sequence	Ion Score	C. I. %	Modification	Rank	Result Type
807.3645	807.3835	0.019	24	128	133	GDHWHR					Mascot
926.5669	926.5472	-0.0197	-21	171	178	KLAPEQIK					Mascot
1108.6473	1108.6488	0.0015	1	172	181	LAPEQIKGPR					Mascot
1329.6951	1329.6992	0.0041	3	60	70	ILEHVPFGFDR	20	0			Mascot
1358.6912	1358.6569	-0.0343	-25	182	196	VVGIAGGDGDGDAKK					Mascot
1794.8691	1794.7769	-0.0922	-51	85	100	ETPEAHEIVVDVPGMR			Oxidation (M)[15]		Mascot
2335.1499	2335.158	0.0081	3	81	100	VDWRETPEAHEIVVDVP GMR					Mascot
2421.1689	2421.1802	0.0113	5	60	80	ILEHVPFGFDRDDVAMVS MAR			Oxidation (M)[16]		Mascot
2437.1638	2437.1423	-0.0215	-9	60	80	ILEHVPFGFDRDDVAMVS MAR			Oxidation (M)[16,19]		Mascot
2437.1638	2437.1423	-0.0215	-9	60	80	ILEHVPFGFDRDDVAMVS MAR	12	0	Oxidation (M)[16,19]		Mascot

6 hypothetical protein OsI_24498 [Oryza sativa Indica Group] gi|218198899 47969.9 9.16 16 66 73.191 28.769

Peptide Information

Calc. Mass	Obsrv. Mass	± da	± ppm	Start Seq.	End Seq.	Sequence	Ion Score	C. I. %	Modification	Rank	Result Type
910.4265	910.4223	-0.0042	-5	169	176	SSVDFEAR					Mascot
923.425	923.3699	-0.0551	-60	203	211	EMGGSSISR					Mascot
934.4662	934.4159	-0.0503	-54	363	370	DAMDINKK					Mascot
1089.4921	1089.5977	0.1056	97	74	88	GGGGGGGGGSSASPK					Mascot
1182.5532	1182.5615	0.0083	7	203	213	EMGGSSISRSR			Oxidation (M)[2]		Mascot
1233.5966	1233.6416	0.045	36	237	246	NLMKPMNEEK					Mascot
1233.5966	1233.6416	0.045	36	237	246	NLMKPMNEEK					Mascot
1249.5916	1249.6362	0.0446	36	237	246	NLMKPMNEEK			Oxidation (M)[3]		Mascot
1265.5864	1265.6317	0.0453	36	237	246	NLMKPMNEEK			Oxidation (M)[3,6]		Mascot
1265.5864	1265.6317	0.0453	36	237	246	NLMKPMNEEK			Oxidation (M)[3,6]		Mascot
1303.6787	1303.6538	-0.0249	-19	135	145	MLDPNTSRTLR					Mascot
1319.6737	1319.6538	-0.0199	-15	135	145	MLDPNTSRTLR			Oxidation (M)[1]		Mascot
1336.6227	1336.6696	0.0469	35	96	108	VETSEDTSGGVQK					Mascot
1336.6227	1336.6696	0.0469	35	96	108	VETSEDTSGGVQK					Mascot
1371.6095	1371.7052	0.0957	70	371	384	SSSASSRSTDGSSR					Mascot
1407.6804	1407.702	0.0216	15	247	256	DHWYFNKIER					Mascot
1782.9315	1782.9763	0.0448	25	259	273	LISCDLNFVMAIMKK			Carbamidomethyl (C)[4]		Mascot

Calc. Mass	Obsrv. Mass	± da	± ppm	Start Seq.	End Seq.	Sequence	Ion Score	C. I. %	Modification	Rank	Result Type
1786.9194	1786.8215	-0.0979	-55	344	359	EAANRPIVHSHEDLAK					Mascot
1897.8903	1897.9833	0.093	49	24	39	FGCPVWAYDADIISQR			Carbamidomethyl (C)[3]		Mascot
1926.985	1926.8667	-0.1183	-61	257	272	QKLISCDLNFVMAIMK			Carbamidomethyl (C)[6], Oxidation (M)[12]		Mascot
2313.1577	2313.186	0.0283	12	113	134	LLPEGAGSPMDVLCQVG LDGIR			Carbamidomethyl (C)[14], Oxidation (M)[10]		Mascot
2313.1577	2313.186	0.0283	12	113	134	LLPEGAGSPMDVLCQVG LDGIR			Carbamidomethyl (C)[14], Oxidation (M)[10]		Mascot
2355.2275	2355.1719	-0.0556	-24	339	359	LNNAREAANRPIVHSHED LAK					Mascot

7 hypothetical protein LOC_Os12g24060 [Oryza sativa Japonica Group] gi|77555175 11523.8 8.96 8 65 70.605 1.781

Peptide Information

Calc. Mass	Obsrv. Mass	± da	± ppm	Start Seq.	End Seq.	Sequence	Ion Score	C. I. %	Modification	Rank	Result Type
1219.6025	1219.6005	-0.002	-2	52	62	GQALEESSSRR					Mascot
1348.5911	1348.6592	0.0681	50	22	34	DSGVLCGQNGGER			Carbamidomethyl (C)[6]		Mascot
1371.6903	1371.7052	0.0149	11	94	105	IFNHEISIPSTN					Mascot
2020.9393	2021.0273	0.088	44	44	61	DGKPDMWKGQALEESS SR					Mascot
2034.9994	2034.9897	-0.0097	-5	2	21	AGRCKPVVAATSSCLGG GMR			Carbamidomethyl (C)[4,14]		Mascot
2166.04	2166.1382	0.0982	45	1	21	MAGRCKPVVAATSSCLG GGMR			Carbamidomethyl (C)[5,15]		Mascot
2373.2449	2373.1953	-0.0496	-21	63	83	AALESSIHEVLFQIINFAN EK					Mascot
2501.3398	2501.2498	-0.09	-36	63	84	AALESSIHEVLFQIINFAN EKK					Mascot

8 hypothetical protein CHLNCDRAFT_8690, partial [Chlorella variabilis] gi|307102657 43928 9.06 15 63 46.509 9.056

Peptide Information

Calc. Mass	Obsrv. Mass	± da	± ppm	Start Seq.	End Seq.	Sequence	Ion Score	C. I. %	Modification	Rank	Result Type
816.4574	816.4616	0.0042	5	309	316	GVGVVTER					Mascot
849.4577	849.4256	-0.0321	-38	29	36	TGQFIGAR					Mascot
888.4574	888.4516	-0.0058	-7	279	285	QLPDPYR					Mascot
942.4904	942.4316	-0.0588	-62	178	184	QFQLNHR					Mascot
1089.5034	1089.5977	0.0943	87	325	333	LAYGMFDTR			Oxidation (M)[5]		Mascot
1259.7358	1259.6282	-0.1076	-85	286	297	AAFAQLVLALDK					Mascot
1329.6686	1329.6992	0.0306	23	334	346	GSVDPFAPDSPIK					Mascot
1335.7267	1335.6426	-0.0841	-63	266	278	GGKIGLIDYGQSK					Mascot
1342.6208	1342.6519	0.0311	23	161	170	LEFDFCREAR			Carbamidomethyl (C)[6]		Mascot
1348.6678	1348.6592	-0.0086	-6	37	47	GDFVPEQICRK			Carbamidomethyl (C)[9]		Mascot

Calc. Mass	Obsrv. Mass	± da	± ppm	Start Seq.	End Seq.	Sequence	Ion Score	C. I. %	Modification	Rank	Result Type
1541.8105	1541.8086	-0.0019	-1	185	198	IDVPRSVPGMVTDR					Mascot
1897.9703	1897.9833	0.013	7	347	362	AMGIEKFPPDMFFVLR					Mascot
1958.9893	1958.8638	-0.1255	-64	334	352	GSVDPFAPDSPIKAMGIEK					Mascot
1967.9644	1967.9647	0.0003	0	317	333	EEAPLQARLAYGMFDTR					Mascot
2313.2925	2313.186	-0.1065	-46	286	308	AAFAQLVLALDKGDNAAIATALR					Mascot
2313.2925	2313.186	-0.1065	-46	286	308	AAFAQLVLALDKGDNAAIATALR					Mascot

9 PREDICTED: uncharacterized protein LOC101221655, partial [Cucumis sativus] gi|449467938 65353.1 8.79 18 62 42.684 22.744

Peptide Information

Calc. Mass	Obsrv. Mass	± da	± ppm	Start Seq.	End Seq.	Sequence	Ion Score	C. I. %	Modification	Rank	Result Type
814.5032	814.457	-0.0462	-57	444	451	DVVLGALK					Mascot
849.4498	849.4256	-0.0242	-28	456	462	LAQDKMK			Oxidation (M)[6]		Mascot
979.5206	979.4996	-0.021	-21	55	62	NELYANKK					Mascot
1247.6743	1247.6178	-0.0565	-45	71	81	VDHLGHIISEK					Mascot
1302.6147	1302.6642	0.0495	38	322	332	ISGELHWEGMK			Oxidation (M)[10]		Mascot
1304.6998	1304.6792	-0.0206	-16	107	117	GFLGLTSYYRK					Mascot
1304.6998	1304.6792	-0.0206	-16	107	117	GFLGLTSYYRK					Mascot
1308.665	1308.6771	0.0121	9	212	222	ELMTVVMSVQR			Oxidation (M)[3]		Mascot
1332.6219	1332.6824	0.0605	45	140	150	WSEEAHEAFVK					Mascot
1336.7412	1336.6696	-0.0716	-54	396	406	FSKYGHFLPLK					Mascot
1336.7412	1336.6696	-0.0716	-54	396	406	FSKYGHFLPLK					Mascot
1346.6434	1346.6832	0.0398	30	430	441	DTPNSSLDEQLK					Mascot
1541.9302	1541.8086	-0.1216	-79	232	245	FLTAPNIIDVVVIK					Mascot
1770.8256	1770.8367	0.0111	6	368	382	VWEDISMDFVEGLSK			Oxidation (M)[7]		Mascot
1822.8759	1822.9637	0.0878	48	135	150	TGGFKWSEEAHEAFVK					Mascot
1826.947	1826.769	-0.178	-97	383	398	ANGMEVIFVVVDRFSK			Oxidation (M)[4]		Mascot
2011.9318	2012.0647	0.1329	66	490	505	ENKDEIPMLTESYEWK					Mascot
2166.1409	2166.1382	-0.0027	-1	205	222	AKPIYERELMTVVMSVQR			Oxidation (M)[10]		Mascot
2421.2417	2421.1802	-0.0615	-25	212	231	ELMTVVMSVQRWHSYLLGQK			Oxidation (M)[3]		Mascot
2437.2366	2437.1423	-0.0943	-39	212	231	ELMTVVMSVQRWHSYLLGQK			Oxidation (M)[3,7]		Mascot
2437.2366	2437.1423	-0.0943	-39	212	231	ELMTVVMSVQRWHSYLLGQK			Oxidation (M)[3,7]		Mascot
2501.2395	2501.2498	0.0103	4	293	314	LTPMILHTYHDSVFGGHSGFLR			Oxidation (M)[4]		Mascot

10 PREDICTED: pentatricopeptide repeat-containing protein At2g20710, mitochondrial-like [Cucumis sativus] gi|449438086 56583.6 8.44 16 62 38.584 28.26

Peptide Information

Calc. Mass	Obsrv. Mass	± da	± ppm	Start Seq.	End Seq.	Sequence	Ion Score	C. I. %	Modification	Rank	Result Type
934.5026	934.4159	-0.0867	-93	155	162	KAEAIMQK			Oxidation (M)[6]		Mascot
1001.5626	1001.5844	0.0218	22	268	276	KAEQLIGDK					Mascot
1249.6456	1249.6362	-0.0094	-8	256	267	AGLSENSISMLK					Mascot
1265.6406	1265.6317	-0.0089	-7	256	267	AGLSENSISMLK			Oxidation (M)[10]		Mascot
1265.6406	1265.6317	-0.0089	-7	256	267	AGLSENSISMLK			Oxidation (M)[10]		Mascot
1302.5565	1302.6642	0.1077	83	197	207	EMEEMGIGHNR					Mascot
1318.5515	1318.66	0.1085	82	197	207	EMEEMGIGHNR			Oxidation (M)[2]		Mascot
1334.5464	1334.6627	0.1163	87	197	207	EMEEMGIGHNR			Oxidation (M)[2,5]		Mascot
1357.7322	1357.7228	-0.0094	-7	326	337	LDDIDGAERILK					Mascot
1361.6808	1361.6946	0.0138	10	389	401	LASGYHSNGLTNK					Mascot
1393.7356	1393.6891	-0.0465	-33	256	268	AGLSENSISMLKK			Oxidation (M)[10]		Mascot
1770.9684	1770.8367	-0.1317	-74	101	116	CPSPGHIAIQLHLISK			Carbamidomethyl (C)[1]		Mascot
1786.9447	1786.8215	-0.1232	-69	50	64	TSIVRVLDQWVEEGR					Mascot
1945.9589	1946.0093	0.0504	26	136	151	DHKVYGALLHCYVENK			Carbamidomethyl (C)[11]		Mascot
1997.0201	1997.0109	-0.0092	-5	417	432	WKPNYDILAACLEYLK			Carbamidomethyl (C)[11]		Mascot
2013.1062	2013.0306	-0.0756	-38	99	116	NKCPSPGHIAIQLHLISK			Carbamidomethyl (C)[3]		Mascot
2013.1062	2013.0306	-0.0756	-38	99	116	NKCPSPGHIAIQLHLISK			Carbamidomethyl (C)[3]		Mascot
2029.0747	2029.0111	-0.0636	-31	433	450	TNGNVELAEEIIGLLCKR			Carbamidomethyl (C)[16]		Mascot
2035.0358	2034.9897	-0.0461	-23	279	295	WLAYQYLMTLYAAIGNK			Oxidation (M)[8]		Mascot
2040.9783	2041.047	0.0687	34	2	17	VKLHCSQSWLFCSNFK			Carbamidomethyl (C)[5,12]		Mascot
2271.1365	2271.2261	0.0896	39	55	73	VLDQWVEEGRQVNQSD LQK					Mascot

Gel Idx/Pos	189/H16	Instr./Gel Origin	BA2151/Sample Project 20140814	Process Status	Analysis Succeeded
Plate [#] Name	[1] Sample Project 20140814	Instrument Sample Name		Spectra	11

Rank	Protein Name	Accession No.	Protein MW	Protein PI	Pep. Count	Protein Score	Protein Score C. I. %	Intensity Matched	Total Ion Score	Total Ion C. I. %	Confirmed
1	hypothetical protein F775_30396 [Aegilops tauschii]	gi|475584601	13170.8	8.01	5	246	100	3.747	223	100	

Peptide Information

Calc. Mass	Obsrv. Mass	± da	± ppm	Start Seq.	End Seq.	Sequence	Ion Score	C. I. %	Modification	Rank	Result Type
807.3645	807.4138	0.0493	61	31	36	GDHWHR					Mascot
926.5669	926.511	-0.0559	-60	74	81	KLAPEQIK					Mascot
1108.6473	1108.6591	0.0118	11	75	84	LAPEQIKGPR					Mascot
1231.5913	1231.6066	0.0153	12	100	112	TIGDVGAAGGEER					Mascot
2226.1614	2226.1792	0.0178	8	50	71	LPDNADLDSIAASLDAGV LTVR					Mascot
2226.1614	2226.1792	0.0178	8	50	71	LPDNADLDSIAASLDAGV LTVR	223	100			Mascot

Rank	Protein Name	Accession No.	Protein MW	Protein PI	Pep. Count	Protein Score	Protein Score C. I. %	Intensity Matched	Total Ion Score	Total Ion C. I. %	Confirmed
2	hypothetical protein TRIUR3_31593 [Triticum urartu]	gi|473979984	19793.1	5.63	6	245	100	4.387	223	100	

Peptide Information

Calc. Mass	Obsrv. Mass	± da	± ppm	Start Seq.	End Seq.	Sequence	Ion Score	C. I. %	Modification	Rank	Result Type
807.3645	807.4138	0.0493	61	86	91	GDHWHR					Mascot
926.5669	926.511	-0.0559	-60	129	136	KLAPEQIK					Mascot
1108.6473	1108.6591	0.0118	11	130	139	LAPEQIKGPR					Mascot
1203.5601	1203.5775	0.0174	14	155	167	TIGDAGAAGGEER					Mascot
1786.8276	1786.8252	-0.0024	-1	42	57	ETSDSHEIVVDVPGMR			Oxidation (M)[15]		Mascot
2226.1614	2226.1792	0.0178	8	105	126	LPDNADLDSIAASLDAGV LTVR					Mascot
2226.1614	2226.1792	0.0178	8	105	126	LPDNADLDSIAASLDAGV LTVR	223	100			Mascot

Rank	Protein Name	Accession No.	Protein MW	Protein PI	Pep. Count	Protein Score	Protein Score C. I. %	Intensity Matched	Total Ion Score	Total Ion C. I. %	Confirmed
3	Glycine-rich protein 2 [Triticum urartu]	gi|474091493	15630.7	4.66	1	98	99.985	1.041	93	100	

Peptide Information

Calc. Mass	Obsrv. Mass	± da	± ppm	Start Seq.	End Seq.	Sequence	Ion Score	C. I. %	Modification	Rank	Result Type
2109.957	2109.9753	0.0183	9	44	62	SLNENDTVEFEVITGDDG R					Mascot
2109.957	2109.9753	0.0183	9	44	62	SLNENDTVEFEVITGDDG R	93	100			Mascot

Rank	Protein Name	Accession No.	Protein MW	Protein PI	Pep. Count	Protein Score	Protein Score C. I. %	Intensity Matched	Total Ion Score	Total Ion C. I. %	Confirmed
4	Vicilin-like antimicrobial peptides 2-2 [Triticum urartu]	gi|473890163	75298.3	5.79	12	90	99.913	6.306	66	99.961	

Peptide Information

Calc. Mass	Obsrv. Mass	± da	± ppm	Start Seq.	End Seq.	Sequence	Ion Score	C. I. %	Modification	Rank	Result Type
807.4359	807.4138	-0.0221	-27	660	667	KGAVFQSA					Mascot
847.3904	847.4501	0.0597	70	607	612	EEEERR					Mascot
849.4941	849.432	-0.0621	-73	360	366	NSVFRVK					Mascot
1231.5186	1231.6066	0.088	71	602	611	GDDPREEEER					Mascot
1232.5753	1232.6047	0.0294	24	458	468	KAEQEEQEGGK					Mascot
1233.5342	1233.6423	0.1081	88	551	559	EQEEEEERR					Mascot
1490.7485	1490.7582	0.0097	7	202	214	SPQSIITYNPDQK					Mascot
1875.8403	1875.9401	0.0998	53	348	364	GGDHGQEGVECKNSVF R			Carbamidomethyl (C)[11]		Mascot
1982.9666	1982.9745	0.0079	4	102	119	VTYIQEGGSETSSLEVQR					Mascot
2021.1251	2021.0497	-0.0754	-37	315	334	ATEIAIVTHGSGIVQVGG RR					Mascot
2027.9194	2027.8228	-0.0966	-48	441	458	SGSTIMACVSCAEELERK			Carbamidomethyl (C)[8,11]		Mascot
2027.9194	2027.8228	-0.0966	-48	441	458	SGSTIMACVSCAEELERK			Carbamidomethyl (C)[8,11]		Mascot
2501.2307	2501.2637	0.033	13	120	141	GDVYNLEQGSILYIQSYP NATR					Mascot
2501.2307	2501.2637	0.033	13	120	141	GDVYNLEQGSILYIQSYP NATR	66	99.961			Mascot

5	hypothetical protein CARUB_v10009732mg [Capsella rubella]	gi|482574064	36510.6	9.31	10	67	79.663	7.062	27	0

Peptide Information

Calc. Mass	Obsrv. Mass	± da	± ppm	Start Seq.	End Seq.	Sequence	Ion Score	C. I. %	Modification	Rank	Result Type
806.4883	806.431	-0.0573	-71	19	25	FRSGLVK					Mascot
834.3951	834.3316	-0.0635	-76	283	289	ESIDNTR					Mascot
1133.5797	1133.6221	0.0424	37	304	315	NLPSTVGSGSSK					Mascot
1201.694	1201.5886	-0.1054	-88	70	79	EVSIYKIPPR					Mascot
1319.6074	1319.6652	0.0578	44	134	145	ENSVEPSLDSSR					Mascot
1320.6655	1320.6492	-0.0163	-12	33	43	SHTSFSETRLR					Mascot
1371.7478	1371.7228	-0.025	-18	292	303	NEPLSDLSQLKK					Mascot
1475.7085	1475.7623	0.0538	36	133	145	RENSVEPSLDSSR	27	0			Mascot
2013.0876	2013.0465	-0.0411	-20	256	275	AAGITRSPLPPPPNDPVA SR					Mascot
2013.0876	2013.0465	-0.0411	-20	256	275	AAGITRSPLPPPPNDPVA SR					Mascot
2226.0244	2226.1792	0.1548	70	114	133	LEDSNSGDLFAACFVDP GRR			Carbamidomethyl (C)[13]		Mascot
2226.0244	2226.1792	0.1548	70	114	133	LEDSNSGDLFAACFVDP GRR			Carbamidomethyl (C)[13]		Mascot

6	hypothetical protein ZEAMMB73_809415 [Zea mays]	gi|413935325	39703.6	11.82	14	62	45.263	4.914

Peptide Information

Calc. Mass	Obsrv. Mass	± da	± ppm	Start Seq.	End Seq.	Sequence	Ion Score	C. I. %	Modification	Rank	Result Type
864.4686	864.4284	-0.0402	-47	48	54	DSFRALR					Mascot
1068.5658	1068.526	-0.0398	-37	171	180	ATRANHSGVR					Mascot
1308.6827	1308.6833	0.0006	0	219	231	AEMSGITAGLSKK			Oxidation (M)[3]		Mascot
1316.6012	1316.573	-0.0282	-21	156	167	RIEASDHCSGGK			Carbamidomethyl (C)[8]		Mascot
1348.7584	1348.7158	-0.0426	-32	181	192	VNLLEVSSGRFK					Mascot
1371.7451	1371.7228	-0.0223	-16	281	293	TRRPASATGELGR					Mascot
1415.7788	1415.656	-0.1228	-87	18	30	RVGIGMGNIVLDR			Oxidation (M)[6]		Mascot
1522.7775	1522.6498	-0.1277	-84	134	147	GHHAARGGFWSALR					Mascot
1786.829	1786.8252	-0.0038	-2	198	213	VDGRSSTWMSGHVDPR					Mascot
1794.9569	1794.8135	-0.1434	-80	174	190	ANHSGVRVNLLEVSSGR					Mascot
1818.9419	1818.8596	-0.0823	-45	1	17	MEFAALQPSPAALTSLR			Oxidation (M)[1]		Mascot
1828.0076	1827.8334	-0.1742	-95	2	18	EFAALQPSPAALTSLRR					Mascot
1859.9069	1859.9205	0.0136	7	202	218	SSTWMSGHVDPRAALTK			Oxidation (M)[5]		Mascot
1959.0481	1958.8876	-0.1605	-82	1	18	MEFAALQPSPAALTSLRR					Mascot

7	PREDICTED: uncharacterized protein LOC101758684 [Setaria italica]	gi|514788348	119401.7	8.5	21	60	13.248	12.185

Peptide Information

Calc. Mass	Obsrv. Mass	± da	± ppm	Start Seq.	End Seq.	Sequence	Ion Score	C. I. %	Modification	Rank	Result Type
856.4675	856.3848	-0.0827	-97	918	924	KATFFSR					Mascot
856.5251	856.5345	0.0094	11	610	617	LDGLLLGR					Mascot
1108.6837	1108.6591	-0.0246	-22	508	516	LLTRILHDK					Mascot
1133.5872	1133.6221	0.0349	31	1077	1085	MEEVLEVLR			Oxidation (M)[1]		Mascot
1182.6841	1182.5759	-0.1082	-91	512	521	ILHDKTNTLK					Mascot
1201.6133	1201.5886	-0.0247	-21	267	277	GDLGMEIPIEK					Mascot
1203.6879	1203.5775	-0.1104	-92	36	45	SVEMISRLLR					Mascot
1265.7437	1265.6492	-0.0945	-75	723	736	LAPAAAAAGRAVAR					Mascot
1304.5861	1304.6917	0.1056	81	121	131	GDEKMISMSYK			Oxidation (M)[5]		Mascot
1308.7345	1308.6833	-0.0512	-39	209	219	IDMIALSFVRK			Oxidation (M)[3]		Mascot
1320.5811	1320.6492	0.0681	52	121	131	GDEKMISMSYK			Oxidation (M)[5,8]		Mascot
1371.7858	1371.7228	-0.063	-46	278	288	IFFAQKVMIFK					Mascot

1379.6848	1379.6825	-0.0023	-2	798	811	HGPTAQMAAAAPTR					Mascot
1465.7546	1465.6221	-0.1325	-90	596	609	VVAVAAGDLDGWHR					Mascot
1475.7775	1475.7623	-0.0152	-10	1074	1085	DVKMEEVLEVLR			Oxidation (M)[4]		Mascot
1812.8334	1812.8584	0.025	14	1	19	MAAAAAGGEPAWGEEP AAR					Mascot
1837.894	1837.9329	0.0389	21	2	20	AAAAAGGEPAWGEEPAA RR					Mascot
1859.8667	1859.9205	0.0538	29	353	368	ICLQAESCVDYGAVFK			Carbamidomethyl (C)[2,8]		Mascot
1868.0786	1867.8969	-0.1817	-97	447	464	QSLIVRGVIPMLSAANAK					Mascot
1875.9779	1875.9401	-0.0378	-20	26	42	IVCTLGPASRSVEMISR			Carbamidomethyl (C)[3]		Mascot
2012.9447	2013.0465	0.1018	51	465	483	AFDSEATEEALGFAIENA K					Mascot
2012.9447	2013.0465	0.1018	51	465	483	AFDSEATEEALGFAIENA K					Mascot
2109.9731	2109.9753	0.0022	1	647	668	HAMLGGGGAASGADEE APAERR					Mascot
2109.9731	2109.9753	0.0022	1	647	668	HAMLGGGGAASGADEE APAERR					Mascot

8 hypothetical protein F775_22273 [Aegilops tauschii] gi|475598498 70646.2 6.7 19 60 9.16 8.803

Peptide Information

Calc. Mass	Obsrv. Mass	± da	± ppm	Start Seq.	End Seq.	Sequence	Ion Score	C. I. %	Modification	Rank	Result Type
806.4407	806.431	-0.0097	-12	412	418	FVQGLDK					Mascot
826.3763	826.4028	0.0265	32	1	6	MEDLFR			Oxidation (M)[1]		Mascot
864.4574	864.4284	-0.029	-34	2	8	EDLFRGK					Mascot
906.5043	906.4753	-0.029	-32	497	504	VAAEFLTR					Mascot
925.4295	925.4399	0.0104	11	488	496	ATTMGSTEK					Mascot
1033.5863	1033.5269	-0.0594	-57	559	566	LQMWSKLK					Mascot
1060.5382	1060.5751	0.0369	35	183	191	ILDERSDGR					Mascot
1107.5576	1107.5616	0.004	4	147	156	LLDMSSGGRR			Oxidation (M)[4]		Mascot
1108.6184	1108.6591	0.0407	37	403	411	FRLVEMVAK			Oxidation (M)[6]		Mascot
1193.6096	1193.6261	0.0165	14	169	179	GSTAFRPGLCK			Carbamidomethyl (C)[10]		Mascot
1203.5793	1203.5775	-0.0018	-1	435	443	WEEFKEAHK					Mascot
1319.6776	1319.6652	-0.0124	-9	211	222	WSMEVGISAALR					Mascot
1371.7129	1371.7228	0.0099	7	547	558	VHGVQHDVIDPR					Mascot
1501.7856	1501.8094	0.0238	16	101	114	AAPSDESSRLDLLK					Mascot
1507.7177	1507.7338	0.0161	11	428	439	ADELQGRWEEFK					Mascot
1592.8717	1592.7451	-0.1266	-79	405	418	LVEMVAKFVQGLDK			Oxidation (M)[4]		Mascot
1592.8717	1592.7451	-0.1266	-79	405	418	LVEMVAKFVQGLDK			Oxidation (M)[4]		Mascot
1812.916	1812.8584	-0.0576	-32	488	504	ATTMGSTEKVAAEFLTR					Mascot
1837.8749	1837.9329	0.058	32	568	582	EVPLMFRQSEETESR					Mascot

Calc. Mass	Obsrv. Mass	± da	± ppm	Start Seq.	End Seq.	Sequence	Ion Score	C. I. %	Modification	Rank	Result Type
2226.0681	2226.1792	0.1111	50	497	515	VAAEFLTREMGFMLNEAHK			Oxidation (M)[10,13]		Mascot
2226.0681	2226.1792	0.1111	50	497	515	VAAEFLTREMGFMLNEAHK			Oxidation (M)[10,13]		Mascot

9	predicted protein [Bathycoccus prasinos]	gi|412990761	76307.4	9.45	21	58	0	9.576		

Peptide Information

Calc. Mass	Obsrv. Mass	± da	± ppm	Start Seq.	End Seq.	Sequence	Ion Score	C. I. %	Modification	Rank	Result Type
816.4686	816.4059	-0.0627	-77	657	663	RILGSDR					Mascot
824.3567	824.4014	0.0447	54	2	8	SMNNNTK			Oxidation (M)[2]		Mascot
826.3611	826.4028	0.0417	50	438	444	ENMTTSK			Oxidation (M)[3]		Mascot
847.4156	847.4501	0.0345	41	83	90	GDANLETK					Mascot
867.3876	867.4467	0.0591	68	353	359	MSKNEDK			Oxidation (M)[1]		Mascot
925.4738	925.4399	-0.0339	-37	487	495	LGSTFSTGR					Mascot
928.4563	928.4812	0.0249	27	587	593	WTSFYPK					Mascot
1078.5641	1078.5558	-0.0083	-8	102	111	SRPFTTANGK					Mascot
1106.5953	1106.5249	-0.0704	-64	29	37	FSNLKVENR					Mascot
1301.5944	1301.6255	0.0311	24	180	190	SPDFAYCKGTR			Carbamidomethyl (C)[7]		Mascot
1323.6593	1323.6796	0.0203	15	130	140	LFGEAHQAHWK					Mascot
1348.7583	1348.7158	-0.0425	-32	23	33	LQTLERFSNLK					Mascot
1371.7703	1371.7228	-0.0475	-35	219	231	ALQTERVALASGR					Mascot
1465.6425	1465.6221	-0.0204	-14	635	647	CTKHGCNGGTFAR			Carbamidomethyl (C)[1,6]		Mascot
1472.7704	1472.7251	-0.0453	-31	658	670	ILGSDRLEDIADR					Mascot
1501.7533	1501.8094	0.0561	37	241	253	IITSSQTYNPYSK					Mascot
1522.7167	1522.6498	-0.0669	-44	34	47	VENRVVSGASMDDK			Oxidation (M)[11]		Mascot
1794.9307	1794.8135	-0.1172	-65	164	179	FSVSIEDPSQMIKIGK			Oxidation (M)[11]		Mascot
1812.9451	1812.8584	-0.0867	-48	398	414	ALLGNQELKASDPNNTK					Mascot
1967.0134	1966.9479	-0.0655	-33	124	140	SELSVKLFGEAHQAHWK					Mascot
2013.0006	2013.0465	0.0459	23	144	160	AGMLYALLDCKPYLCPK			Carbamidomethyl (C)[10,15]		Mascot
2013.0006	2013.0465	0.0459	23	144	160	AGMLYALLDCKPYLCPK			Carbamidomethyl (C)[10,15]		Mascot

10	hypothetical protein SORBIDRAFT_03g043060 [Sorghum bicolor]	gi|241930914	132914.4	6.69	21	58	0	8.947	17	0

Peptide Information

Calc. Mass	Obsrv. Mass	± da	± ppm	Start Seq.	End Seq.	Sequence	Ion Score	C. I. %	Modification	Rank	Result Type
816.4825	816.4059	-0.0766	-94	675	681	KLDELAK					Mascot
828.4799	828.4328	-0.0471	-57	588	594	VQQAARR					Mascot

870.4679	870.553	0.0851	98	423	429	HSEKELK				Mascot
1068.507	1068.526	0.019	18	780	788	TYGTEREGR				Mascot
1106.5841	1106.5249	-0.0592	-53	579	587	IQTYTIPDR				Mascot
1145.5334	1145.5854	0.052	45	220	229	WANGNAELDR				Mascot
1201.6536	1201.5886	-0.065	-54	247	257	DGALSDVKQIR				Mascot
1304.658	1304.6917	0.0337	26	366	376	AEELTKELDEK				Mascot
1319.7053	1319.6652	-0.0401	-30	843	854	AQITSLSETLEK				Mascot
1323.6539	1323.6796	0.0257	19	77	88	ATVSIVFDNSDR				Mascot
1381.653	1381.6605	0.0075	5	630	641	NMDAAKEVAFNR			Oxidation (M)[2]	Mascot
1406.7196	1406.6732	-0.0464	-33	924	935	DCSSIVDKLLEK			Carbamidomethyl (C)[2]	Mascot
1522.7053	1522.6498	-0.0555	-36	313	325	ETSVMNNQEETLK				Mascot
1590.7734	1590.6875	-0.0859	-54	616	629	NAVAYVFGSTFVCR			Carbamidomethyl (C)[13]	Mascot
1592.8644	1592.7451	-0.1193	-75	1159	1173	FVDGVSTVTRTVPSK				Mascot
1592.8644	1592.7451	-0.1193	-75	1159	1173	FVDGVSTVTRTVPSK				Mascot
1679.6755	1679.7848	0.1093	65	949	962	SGTDYDFHSCEPHK			Carbamidomethyl (C)[10]	Mascot
1838.8516	1838.9313	0.0797	43	864	879	QDYDQAESELNVGRSK				Mascot
2011.9125	2012.0151	0.1026	51	313	329	ETSVMNNQEETLKSEEK			Oxidation (M)[5]	Mascot
2094.0867	2093.9812	-0.1055	-50	18	38	TVVSGFDPLFNAITGLNG SGK				Mascot
2094.0867	2093.9812	-0.1055	-50	18	38	TVVSGFDPLFNAITGLNG SGK	17	0		Mascot
2313.2119	2313.1821	-0.0298	-13	306	325	LSHVLIKETSVMNNQEET LK				Mascot
3263.5471	3263.3474	-0.1997	-61	260	288	IDELDENTETIKADIQEMD NNISTLAAEK				Mascot

Gel Idx/Pos	190/H17	Instr./Gel Origin	BA2151/Sample Project 20140814	Process Status	Analysis Succeeded
Plate [#] Name	[1] Sample Project 20140814	Instrument Sample Name		Spectra	11

Rank	Protein Name	Accession No.	Protein MW	Protein PI	Pep. Count	Protein Score	Protein Score C. I. %	Intensity Matched	Total Ion Score	Total Ion C. I. %	Confirmed
1	Serpin-Z2B [Aegilops tauschii]	gi|475621781	43026.4	5.18	5	105	99.997	8.133	93	100	

Protein Group

RecName: Full=Serpin-Z2B; AltName: Full=TriaeZ2b; AltName: Full=WSZ2b; AltName: Full=WZS3	gi|75279909	43011.4	5.1799 998283 3862

Peptide Information

Calc. Mass	Obsrv. Mass	± da	± ppm	Start Seq.	End Seq.	Sequence	Ion Score	C. I. %	Modification	Rank	Result Type
925.5214	925.5336	0.0122	13	11	18	LSIAHQTR					Mascot
925.5214	925.5336	0.0122	13	11	18	LSIAHQTR					Mascot
1005.5363	1005.5119	-0.0244	-24	230	238	LPYKQGGDK					Mascot
1223.5903	1223.6508	0.0605	49	127	137	AEAQSVDFQTK					Mascot
1258.7253	1258.7123	-0.013	-10	289	300	ISLGIEASDLLK					Mascot
1665.8595	1665.8937	0.0342	21	261	274	LSAEPEFLEQHIPR					Mascot
1665.8595	1665.8937	0.0342	21	261	274	LSAEPEFLEQHIPR	93	100			Mascot

Rank	Protein Name	Accession No.	Protein MW	Protein PI	Pep. Count	Protein Score	Protein Score C. I. %	Intensity Matched	Total Ion Score	Total Ion C. I. %	Confirmed
2	Serpin-Z2B [Triticum urartu]	gi|473793747	45225.7	6.03	4	102	99.994	7.758	93	100	

Peptide Information

Calc. Mass	Obsrv. Mass	± da	± ppm	Start Seq.	End Seq.	Sequence	Ion Score	C. I. %	Modification	Rank	Result Type
925.5214	925.5336	0.0122	13	11	18	LSIAHQTR					Mascot
925.5214	925.5336	0.0122	13	11	18	LSIAHQTR					Mascot
1005.5363	1005.5119	-0.0244	-24	247	255	LPYKQGGDK					Mascot
1223.5903	1223.6508	0.0605	49	127	137	AEAQSVDFQTK					Mascot
1665.8595	1665.8937	0.0342	21	278	291	LSAEPEFLEQHIPR					Mascot
1665.8595	1665.8937	0.0342	21	278	291	LSAEPEFLEQHIPR	93	100			Mascot

Rank	Protein Name	Accession No.	Protein MW	Protein PI	Pep. Count	Protein Score	Protein Score C. I. %	Intensity Matched	Total Ion Score	Total Ion C. I. %	Confirmed
3	Myosin class II heavy chain (ISS) [Ostreococcus tauri]	gi|308801757	304318.4	5.04	54	79	98.687	18.475			

Peptide Information

Calc. Mass	Obsrv. Mass	± da	± ppm	Start Seq.	End Seq.	Sequence	Ion Score	C. I. %	Modification	Rank	Result Type

802.4781	802.4518	-0.0263	-33	459	465	AKISEVR		Mascot
803.437	803.4647	0.0277	34	2377	2382	RLETER		Mascot
810.4105	810.4369	0.0264	33	2165	2172	SSGGIFSR		Mascot
814.4893	814.4571	-0.0322	-40	366	372	GLIRAER		Mascot
816.3846	816.4369	0.0523	64	586	593	ATSASHDK		Mascot
829.4778	829.4441	-0.0337	-41	1887	1894	AVAELQAK		Mascot
832.4159	832.3348	-0.0811	-97	1718	1724	EAEEAKR		Mascot
834.4138	834.3451	-0.0687	-82	1337	1343	QLEASMR		Mascot
844.4523	844.5013	0.049	58	2662	2669	SQVSAPQK		Mascot
846.4567	846.4658	0.0091	11	2232	2239	ISADEIAK		Mascot
848.4294	848.4423	0.0129	15	272	279	GVELGAMR	Oxidation (M)[7]	Mascot
874.4603	874.4697	0.0094	11	132	139	HPPPPMAK		Mascot
878.473	878.4695	-0.0035	-4	2034	2040	ELDAFKR		Mascot
905.5124	905.5015	-0.0109	-12	2224	2231	MKLAELGK	Oxidation (M)[1]	Mascot
911.4581	911.4898	0.0317	35	2210	2216	LHEDELR		Mascot
916.4846	916.4991	0.0145	16	2186	2192	DVEERIR		Mascot
929.5163	929.4803	-0.036	-39	2690	2698	GKVAEAQAR		Mascot
972.5109	972.4764	-0.0345	-35	2138	2146	LQGELDAAR		Mascot
1009.5425	1009.5217	-0.0208	-21	2163	2172	AKSSGGIFSR		Mascot
1015.5279	1015.5372	0.0093	9	2072	2079	QRDALQER		Mascot
1021.487	1021.5103	0.0233	23	495	503	ALLDSEMDK		Mascot
1036.5092	1036.5175	0.0083	8	901	909	SMSDTQLVR		Mascot
1041.467	1041.554	0.087	84	261	268	TLNDEFMR	Oxidation (M)[7]	Mascot
1065.5912	1065.5699	-0.0213	-20	2024	2032	LRHDVAQAR		Mascot
1066.5164	1066.5413	0.0249	23	2511	2518	KYEDDLQR		Mascot
1088.631	1088.587	-0.044	-40	2269	2277	QITELQKTK		Mascot
1103.5804	1103.594	0.0136	12	1072	1080	RDSETQLVR		Mascot
1107.5649	1107.5663	0.0014	1	270	279	MKGVELGAMR	Oxidation (M)[1]	Mascot
1136.5695	1136.5609	-0.0086	-8	1785	1793	DEVSVERFR		Mascot
1145.5797	1145.5726	-0.0071	-6	1862	1870	DDEQIERLK		Mascot
1155.5753	1155.6353	0.06	52	552	560	KTHNEELER		Mascot
1184.6998	1184.5907	-0.1091	-92	2659	2669	LVKSQVSAPQK		Mascot
1189.6172	1189.6564	0.0392	33	694	703	TELERALSDR		Mascot
1193.6022	1193.6523	0.0501	42	2585	2594	ARQEYTAQAR		Mascot
1205.626	1205.6394	0.0134	11	935	945	LETLLSDEASK		Mascot
1223.6123	1223.6508	0.0385	31	666	675	MLLEAISQMR	Oxidation (M)[1,9]	Mascot

Calc. Mass	Obsrv. Mass	± da	± ppm	Start Seq.	End Seq.	Sequence	Ion Score	C. I. %	Modification	Rank	Result Type
1232.5978	1232.6149	0.0171	14	704	714	ASVSDAQRNER					Mascot
1258.689	1258.7123	0.0233	19	1825	1835	LAQLESELVEK					Mascot
1304.6945	1304.691	-0.0035	-3	946	957	VTELSSVVDDLK					Mascot
1308.7046	1308.699	-0.0056	-4	854	864	DAYDTKIIELK					Mascot
1318.671	1318.6818	0.0108	8	757	768	AESARQLEASTR					Mascot
1323.6573	1323.6802	0.0229	17	650	660	SQMREEVTSLK			Oxidation (M)[3]		Mascot
1340.7897	1340.6893	-0.1004	-75	1883	1894	ELLRAVAELQAK					Mascot
1347.6686	1347.7191	0.0505	37	639	649	LEMNARLESER					Mascot
1349.6981	1349.7034	0.0053	4	492	503	ALKALLDSEMDK			Oxidation (M)[10]		Mascot
1507.8591	1507.7797	-0.0794	-53	573	585	IRALEEAHQISLK					Mascot
1576.833	1576.8002	-0.0328	-21	2528	2541	TLAYNEEVQAAVLR					Mascot
1605.8418	1605.7922	-0.0496	-31	1296	1309	AAQIDFEQRVVAMK					Mascot
1687.9113	1687.8617	-0.0496	-29	1481	1494	TLLEEQSKLLEEQK					Mascot
1703.8745	1703.8182	-0.0563	-33	1138	1152	AAVECDLQRQVSTVK			Carbamidomethyl (C)[5]		Mascot
1818.8903	1818.9581	0.0678	37	2419	2434	EDNDEAVAMVAEILRK			Oxidation (M)[9]		Mascot
1838.8516	1838.9561	0.1045	57	444	460	EAVDNNSSTAEAEFRAK					Mascot
1860.0186	1859.8619	-0.1567	-84	1738	1755	IAALSAASSEDTSIRLVR					Mascot
2444.3396	2444.1562	-0.1834	-75	1153	1174	AELEALRNAPKPEPIIIEPT DK					Mascot

4 PREDICTED: presequence protease 1, chloroplastic/mitochondrial-like [Cucumis sativus] gi|449438420 122932.4 5.45 27 72 93.569 9.072

Peptide Information

Calc. Mass	Obsrv. Mass	± da	± ppm	Start Seq.	End Seq.	Sequence	Ion Score	C. I. %	Modification	Rank	Result Type
808.4563	808.439	-0.0173	-21	861	867	SEKFIGK					Mascot
810.4468	810.4369	-0.0099	-12	37	43	SSFVSRK					Mascot
831.4934	831.4246	-0.0688	-83	857	863	NLIKSEK					Mascot
834.425	834.3451	-0.0799	-96	777	782	MENRLR			Oxidation (M)[1]		Mascot
847.4672	847.47	0.0028	3	364	370	LFSEPVR					Mascot
848.4009	848.4423	0.0414	49	880	886	NSTWNAR					Mascot
870.5308	870.5579	0.0271	31	126	132	AVLFRHK					Mascot
878.5094	878.4695	-0.0399	-45	2	8	EKSVFLR					Mascot
905.4839	905.5015	0.0176	19	78	85	QFSSLAPR					Mascot
974.5557	974.515	-0.0407	-42	183	190	EPFVELLK					Mascot
989.4655	989.5588	0.0933	94	729	736	ACTHMVVR			Carbamidomethyl (C)[2], Oxidation (M)[5]		Mascot
989.4655	989.5588	0.0933	94	729	736	ACTHMVVR			Carbamidomethyl (C)[2], Oxidation (M)[5]		Mascot
1003.5822	1003.5573	-0.0249	-25	549	557	AVFSPLIEK					Mascot

1009.5499	1009.5217	-0.0282	-28	1	8	MEKSVFLR					Mascot
1021.4697	1021.5103	0.0406	40	499	507	ENNTGSFPR					Mascot
1033.5691	1033.5393	-0.0298	-29	20	27	IFFRSAHR					Mascot
1066.5641	1066.5413	-0.0228	-21	768	776	QFVSQSKSR					Mascot
1136.5582	1136.5609	0.0027	2	375	385	YPSGDGGDLKK					Mascot
1205.6776	1205.6394	-0.0382	-32	465	474	VEELILNTFK					Mascot
1302.6246	1302.686	0.0614	47	566	576	VTIEMQPDPEK			Oxidation (M)[5]		Mascot
1318.675	1318.6818	0.0068	5	269	280	GVYSQPDNILGR					Mascot
1347.7015	1347.7191	0.0176	13	1044	1055	NFADALEAVRNK					Mascot
1412.6726	1412.6868	0.0142	10	114	125	VSEEFIGECKSK			Carbamidomethyl (C)[9]		Mascot
1493.6788	1493.7631	0.0843	56	593	605	ESMTEEDLAELAR					Mascot
1703.8898	1703.8182	-0.0716	-42	28	42	LCPSTLPPRSSFVSR			Carbamidomethyl (C)[2]		Mascot
1738.8547	1738.8406	-0.0141	-8	191	205	GSLHTFLNAFTYPDR					Mascot
1739.7753	1739.9204	0.1451	83	133	148	KTGAEVMSVSNDDENK			Oxidation (M)[7]		Mascot
1859.9255	1859.8619	-0.0636	-34	9	23	SLTCSSLVCNRIFFR			Carbamidomethyl (C)[4,9]		Mascot

5	PREDICTED: LOW QUALITY PROTEIN: zinc finger CCCH domain-containing protein 16-like [Setaria italica]	gi|514713515	86788.7	8.49	21	72	93.109	13.078	

Peptide Information

Calc. Mass	Obsrv. Mass	± da	± ppm	Start Seq.	End Seq.	Sequence	Ion Score	C. I. %	Modification	Rank	Result Type
822.3853	822.4337	0.0484	59	495	501	HHGQDTK					Mascot
835.4169	835.4909	0.074	89	519	524	HKHEER					Mascot
882.4904	882.46	-0.0304	-34	432	438	LQHSNRK					Mascot
905.4515	905.5015	0.05	55	245	251	VHFYPDK					Mascot
922.441	922.4858	0.0448	49	1	10	MSASATAAGR					Mascot
925.4778	925.5336	0.0558	60	299	306	FGELANFK					Mascot
925.4778	925.5336	0.0558	60	299	306	FGELANFK					Mascot
972.4857	972.4764	-0.0093	-10	605	613	SAGKEHSTR					Mascot
1009.5036	1009.5217	0.0181	18	225	232	AHCPFHLK			Carbamidomethyl (C)[3]		Mascot
1026.4739	1026.5063	0.0324	32	576	584	SSSKYDDPK					Mascot
1066.5276	1066.5413	0.0137	13	442	450	SSSDRYVPR					Mascot
1088.5582	1088.587	0.0288	26	116	126	AAEASAPEKSK					Mascot
1245.627	1245.6302	0.0032	3	307	316	VCRNGSFHLR			Carbamidomethyl (C)[2]		Mascot
1308.6154	1308.699	0.0836	64	242	251	CSRVHFYPDK			Carbamidomethyl (C)[1]		Mascot
1323.6726	1323.6802	0.0076	6	345	355	QITCEFVAVTR			Carbamidomethyl (C)[4]		Mascot
1340.678	1340.6893	0.0113	8	299	309	FGELANFKVCR			Carbamidomethyl (C)[10]		Mascot

1347.6461	1347.7191	0.073	54	199	210	EPSMSAQEVLEK					Mascot
1400.645	1400.6951	0.0501	36	356	366	WKASICGEYMR			Carbamidomethyl (C)[6]		Mascot
1665.8669	1665.8937	0.0268	16	245	258	VHFYPDKSSTLLMK					Mascot
1665.8669	1665.8937	0.0268	16	245	258	VHFYPDKSSTLLMK					Mascot
1687.8473	1687.8617	0.0144	9	407	422	KMVALFGPAADATHDK			Oxidation (M)[2]		Mascot
1838.8125	1838.9561	0.1436	78	677	693	GDAHSDAEAQHQRSSSR					Mascot
1926.9515	1926.903	-0.0485	-25	104	124	AAAAAAAAEEARAAEAS APEK					Mascot

6	hypothetical protein CARUB_v10025889mg [Capsella rubella]	gi|482548719	95281.1	9.42	28	71	92.949	16.238	

Peptide Information

Calc. Mass	Obsrv. Mass	± da	± ppm	Start Seq.	End Seq.	Sequence	Ion Score	C. I. %	Modification	Rank	Result Type
802.4491	802.4518	0.0027	3	824	830	ALPNKIM			Oxidation (M)[7]		Mascot
803.441	803.4647	0.0237	29	804	809	LSEWIR					Mascot
807.4835	807.4244	-0.0591	-73	822	828	HKALPNK					Mascot
826.4893	826.465	-0.0243	-29	168	174	NKPVVNR					Mascot
844.4788	844.5013	0.0225	27	776	782	QGGKLWR					Mascot
846.4567	846.4658	0.0091	11	692	698	EAEKELK					Mascot
914.4479	914.4769	0.029	32	112	119	DGAHVPYR					Mascot
925.4448	925.5336	0.0888	96	671	677	EMKDQFK					Mascot
925.4448	925.5336	0.0888	96	671	677	EMKDQFK					Mascot
929.5064	929.4803	-0.0261	-28	633	639	GRWNQLR					Mascot
1005.4921	1005.5119	0.0198	20	175	183	DPVSSEMLK					Mascot
1021.487	1021.5103	0.0233	23	175	183	DPVSSEMLK			Oxidation (M)[7]		Mascot
1033.5314	1033.5393	0.0079	8	748	757	GPAAFIDTNK					Mascot
1060.5521	1060.5763	0.0242	23	339	347	KIAEVEDEK					Mascot
1066.5752	1066.5413	-0.0339	-32	306	313	ELHELNRR					Mascot
1088.5582	1088.587	0.0288	26	340	348	IAEVEDEKR					Mascot
1107.5463	1107.5663	0.02	18	483	492	TEEAAMATKR					Mascot
1155.6732	1155.6353	-0.0379	-33	554	564	AALAEELAVLR					Mascot
1182.5573	1182.5828	0.0255	22	433	441	MKQEAEQFR			Oxidation (M)[1]		Mascot
1182.5573	1182.5828	0.0255	22	433	441	MKQEAEQFR			Oxidation (M)[1]		Mascot
1263.6371	1263.6604	0.0233	18	759	769	MVPMGQVSMRK					Mascot
1308.6287	1308.699	0.0703	54	175	185	DPVSSEMLKMR			Oxidation (M)[7]		Mascot
1340.5859	1340.6893	0.1034	77	13	25	ATGSTNMNNTSSR					Mascot
1349.6597	1349.7034	0.0437	32	435	444	QEAEQFRQWK					Mascot

1400.7744	1400.6951	-0.0793	-57	415	426	LQDEIQSIKAQK					Mascot
1412.822	1412.6868	-0.1352	-96	678	689	EIVGLLRQSELR					Mascot
1576.833	1576.8002	-0.0328	-21	186	198	QQVEYLQAELSLR					Mascot
1665.7715	1665.8937	0.1222	73	40	55	INTDSPENGGYNGSLK					Mascot
1665.7715	1665.8937	0.1222	73	40	55	INTDSPENGGYNGSLK					Mascot
1673.7952	1673.7948	-0.0004	0	293	305	EWEHKLLQNSMDK			Oxidation (M)[11]		Mascot
1699.8473	1699.8533	0.006	4	646	659	NLLQYMFNSLAETR					Mascot
1828.9586	1828.8724	-0.0862	-47	168	183	NKPVVNRDPVSSEMLK			Oxidation (M)[14]		Mascot
1828.9586	1828.8724	-0.0862	-47	168	183	NKPVVNRDPVSSEMLK			Oxidation (M)[14]		Mascot

7	hypothetical protein CHLNCDRAFT_54773 [Chlorella variabilis]	gi|307103656	96039.6	5.78	23	69	87.168	15.771	

Peptide Information

Calc. Mass	Obsrv. Mass	± da	± ppm	Start Seq.	End Seq.	Sequence	Ion Score	C. I. %	Modification	Rank	Result Type
800.4009	800.4326	0.0317	40	846	853	ATATSGHR					Mascot
806.3825	806.4419	0.0594	74	855	863	QMGGSGAAK					Mascot
822.3774	822.4337	0.0563	68	855	863	QMGGSGAAK			Oxidation (M)[2]		Mascot
834.4203	834.3451	-0.0752	-90	244	251	LADTESAK					Mascot
835.4091	835.4909	0.0818	98	794	802	GGSALSGMR					Mascot
844.4635	844.5013	0.0378	45	639	647	STAKPAGGR					Mascot
872.4836	872.4808	-0.0028	-3	62	70	LGEAQGAVK					Mascot
874.488	874.4697	-0.0183	-21	413	419	VELEKEK					Mascot
905.421	905.5015	0.0805	89	184	191	SQADLEDK					Mascot
925.5577	925.5336	-0.0241	-26	682	690	ALVPANGKR					Mascot
925.5577	925.5336	-0.0241	-26	682	690	ALVPANGKR					Mascot
972.5585	972.4764	-0.0821	-84	785	793	QTAGSKKPR					Mascot
1015.5279	1015.5372	0.0093	9	844	853	SKATATSGHR					Mascot
1016.5119	1016.5201	0.0082	8	215	222	EREGLQER					Mascot
1021.5095	1021.5103	0.0008	1	855	865	QMGGSGAAKSK					Mascot
1065.5358	1065.5699	0.0341	32	133	142	TALANAMTTR			Oxidation (M)[7]		Mascot
1109.5442	1109.6252	0.081	73	1	10	MATTQAAMLR			Oxidation (M)[1]		Mascot
1109.5442	1109.6252	0.081	73	1	10	MATTQAAMLR			Oxidation (M)[1]		Mascot
1144.6433	1144.5996	-0.0437	-38	639	650	STAKPAGGRATK					Mascot
1182.5242	1182.5828	0.0586	50	401	410	LGSTMQEMNR			Oxidation (M)[5]		Mascot
1182.5242	1182.5828	0.0586	50	401	410	LGSTMQEMNR			Oxidation (M)[5]		Mascot
1232.6528	1232.6149	-0.0379	-31	791	802	KPRGGSALSGMR			Oxidation (M)[11]		Mascot

Calc. Mass	Obsrv. Mass	± da	± ppm	Start Seq.	End Seq.	Sequence	Ion Score	C. I. %	Modification	Rank	Result Type
1308.6763	1308.699	0.0227	17	1	12	MATTQAAMLRAK			Oxidation (M)[1]		Mascot
1493.7594	1493.7631	0.0037	2	47	59	EQIQSEASVALYR					Mascot
1605.8013	1605.7922	-0.0091	-6	128	142	AEAERTALANAMTTR					Mascot
1665.829	1665.8937	0.0647	39	236	251	ELSGTSTRLADTESAK					Mascot
1665.829	1665.8937	0.0647	39	236	251	ELSGTSTRLADTESAK					Mascot
1828.9552	1828.8724	-0.0828	-45	264	280	EARQLAADLASAWEVAK					Mascot
1828.9552	1828.8724	-0.0828	-45	264	280	EARQLAADLASAWEVAK					Mascot

8	hypothetical protein CHLNCDRAFT_58634 [Chlorella variabilis]	gi|307104981	82129.6	5.01	21	68	85.267	6.197

Peptide Information

Calc. Mass	Obsrv. Mass	± da	± ppm	Start Seq.	End Seq.	Sequence	Ion Score	C. I. %	Modification	Rank	Result Type
800.4625	800.4326	-0.0299	-37	670	677	LLNNAGAK					Mascot
831.4431	831.4246	-0.0185	-22	608	614	TAEAQRR					Mascot
874.4675	874.4697	0.0022	3	44	50	CRQALAR			Carbamidomethyl (C)[1]		Mascot
886.5356	886.4823	-0.0533	-60	698	704	LRDELLK					Mascot
888.4785	888.4644	-0.0141	-16	700	706	DELLKDR					Mascot
972.5109	972.4764	-0.0345	-35	337	345	LQGEVEAAR					Mascot
974.465	974.515	0.05	51	262	270	AEGEARDAR					Mascot
1015.5167	1015.5372	0.0205	20	99	108	AAAQAELDAR					Mascot
1041.6415	1041.554	-0.0875	-84	670	679	LLNNAGAKIK					Mascot
1119.551	1119.5428	-0.0082	-7	133	143	AMRGMQAAAGR					Mascot
1136.5695	1136.5609	-0.0086	-8	328	336	QYGKQVEER					Mascot
1171.6178	1171.5927	-0.0251	-21	99	109	AAAQAELDARR					Mascot
1205.5804	1205.6394	0.059	49	120	131	AAQASGGATRCR			Carbamidomethyl (C)[11]		Mascot
1302.6284	1302.686	0.0576	44	597	607	DLEGQLQESQR					Mascot
1400.7128	1400.6951	-0.0177	-13	174	186	LAEADRADALAER					Mascot
1455.7299	1455.7954	0.0655	45	247	261	AAEAAQAAAQAAQQR					Mascot
1576.6908	1576.8002	0.1094	69	194	207	QAADEACQQAIDEK			Carbamidomethyl (C)[7]		Mascot
1687.7882	1687.8617	0.0735	44	420	433	DQLERELENAADER					Mascot
1739.8895	1739.9204	0.0309	18	245	261	QRAAEAAQAAAQAAQQR					Mascot
1818.9344	1818.9581	0.0237	13	210	226	FLAQKSAVEVAAAENDR					Mascot
1926.8975	1926.903	0.0055	3	435	451	QLQEDADAVQAQMQAHK			Oxidation (M)[13]		Mascot

9	phosphoglucan phosphatase LSF1 [Arabidopsis thaliana]	gi|332640157	66269.8	9.17	18	67	80.579	6.512

Protein Group

RecName: Full=Phosphoglucan phosphatase LSF1, chloroplastic; AltName: Full=Phosphoglucan phosphatase like sex Four1; AltName: Full=Protein LIKE SEX4 1; Flags: Precursor	gi|387942514	66269.8	9.1700 000762 9395
phosphoglucan phosphatase LSF1 [Arabidopsis thaliana]	gi|18395843	66269.8	9.1700 000762 9395

Peptide Information

Calc. Mass	Obsrv. Mass	± da	± ppm	Start Seq.	End Seq.	Sequence	Ion Score	C. I. %	Modification	Rank	Result Type
808.4199	808.439	0.0191	24	89	96	FALSADGK					Mascot
810.3992	810.4369	0.0377	47	136	142	DFGDTKK					Mascot
836.4625	836.4257	-0.0368	-44	38	45	GIAYLGSR					Mascot
897.4577	897.4619	0.0042	5	46	52	EKFGFNR					Mascot
916.521	916.4991	-0.0219	-24	190	197	NLLSSNLR					Mascot
989.5699	989.5588	-0.0111	-11	113	121	IIMVGDTLK					Mascot
989.5699	989.5588	-0.0111	-11	113	121	IIMVGDTLK					Mascot
1005.5649	1005.5119	-0.053	-53	113	121	IIMVGDTLK			Oxidation (M)[3]		Mascot
1036.5211	1036.5175	-0.0036	-3	508	515	YIINGDWR					Mascot
1066.5164	1066.5413	0.0249	23	359	367	DADSFDLRK					Mascot
1117.665	1117.6404	-0.0246	-22	113	122	IIMVGDTLKK					Mascot
1136.6058	1136.5609	-0.0449	-40	562	570	VLTESERFR					Mascot
1193.6426	1193.6523	0.0097	8	180	189	GRVSFVTWNK					Mascot
1232.7031	1232.6149	-0.0882	-72	111	121	ARIIMVGDTLK			Oxidation (M)[5]		Mascot
1263.6427	1263.6604	0.0177	14	123	135	ASDSSGGTLVEIK					Mascot
1302.6147	1302.686	0.0713	55	386	396	VFVTCTTGFDR			Carbamidomethyl (C)[5]		Mascot
1347.6475	1347.7191	0.0716	53	280	291	GELSYNHALGMR					Mascot
1605.714	1605.7922	0.0782	49	198	214	ASSQGSGNSGYAAFSSK					Mascot
1828.8429	1828.8724	0.0295	16	16	32	SCPSIMIGSSFRSGNGR			Carbamidomethyl (C)[2], Oxidation (M)[6]		Mascot
1828.8429	1828.8724	0.0295	16	16	32	SCPSIMIGSSFRSGNGR			Carbamidomethyl (C)[2], Oxidation (M)[6]		Mascot
1926.9292	1926.903	-0.0262	-14	123	141	ASDSSGGTLVEIKDFGDTK					Mascot

10	hypothetical protein F775_09910 [Aegilops tauschii]	gi|475570667	176931.3	7.19	31	67	78.705	14.973

Peptide Information

Calc. Mass	Obsrv. Mass	± da	± ppm	Start Seq.	End Seq.	Sequence	Ion Score	C. I. %	Modification	Rank	Result Type

802.4094	802.4518	0.0424	53	1377	1382	STLYYR		Mascot
803.3893	803.4647	0.0754	94	967	973	LGDDDLR		Mascot
806.3825	806.4419	0.0594	74	1029	1034	ERDMQK		Mascot
822.3774	822.4337	0.0563	68	1029	1034	ERDMQK	Oxidation (M)[4]	Mascot
829.4315	829.4441	0.0126	15	497	502	HFEQLR		Mascot
832.406	832.3348	-0.0712	-86	1383	1389	TGHSFQR		Mascot
835.4672	835.4909	0.0237	28	219	225	EFSILAR		Mascot
836.4108	836.4257	0.0149	18	360	367	TKNSSDGK		Mascot
848.3995	848.4423	0.0428	50	292	299	DEADASLK		Mascot
856.5251	856.5348	0.0097	11	726	733	VGLELVAR		Mascot
870.5308	870.5579	0.0271	31	641	647	AFKHVIR		Mascot
874.488	874.4697	-0.0183	-21	1270	1276	LIDEEKK		Mascot
922.4628	922.4858	0.023	25	1201	1208	GTSKSEWK		Mascot
943.5207	943.4969	-0.0238	-25	443	450	KLENTPNK		Mascot
972.5359	972.4764	-0.0595	-61	1548	1556	AAELPKSEK		Mascot
982.5316	982.4998	-0.0318	-32	556	564	SLEAGLHQK		Mascot
1036.4882	1036.5175	0.0293	28	347	355	HCGYTATVK	Carbamidomethyl (C)[2]	Mascot
1041.5323	1041.554	0.0217	21	988	996	ALEQQEAPR		Mascot
1060.6765	1060.5763	-0.1002	-94	1493	1501	QILLSFIVK		Mascot
1065.5211	1065.5699	0.0488	46	781	790	LDDAVNYGAK		Mascot
1066.4535	1066.5413	0.0878	82	471	480	DASVSEDSEK		Mascot
1109.6677	1109.6252	-0.0425	-38	282	291	VGDLHITVKK		Mascot
1109.6677	1109.6252	-0.0425	-38	282	291	VGDLHITVKK		Mascot
1117.6464	1117.6404	-0.006	-5	336	346	DSSTLGVVIVK		Mascot
1171.6041	1171.5927	-0.0114	-10	795	804	VIAVCGPYHR	Carbamidomethyl (C)[5]	Mascot
1182.6226	1182.5828	-0.0398	-34	444	453	LENTPNKNPR		Mascot
1182.6226	1182.5828	-0.0398	-34	444	453	LENTPNKNPR		Mascot
1184.6534	1184.5907	-0.0627	-53	493	502	GLGKHFEQLR		Mascot
1223.7107	1223.6508	-0.0599	-49	554	564	LKSLEAGLHQK		Mascot
1232.6733	1232.6149	-0.0584	-47	508	518	ESTIEDAKIVK		Mascot
1302.6722	1302.686	0.0138	11	565	575	SLEELTKMAHK	Oxidation (M)[8]	Mascot
1393.7474	1393.6858	-0.0616	-44	691	701	LKWVESFLSER		Mascot
1703.7871	1703.8182	0.0311	18	1055	1069	ENFQHDSGSLLEAEK		Mascot
1738.837	1738.8406	0.0036	2	702	714	YCWTLKDEFAHLR	Carbamidomethyl (C)[2]	Mascot

Gel Idx/Pos	191/H18	Instr./Gel Origin	BA2151/Sample Project 20140814	Process Status	Analysis Succeeded
Plate [#] Name	[1] Sample Project 20140814	Instrument Sample Name		Spectra	11

Rank	Protein Name	Accession No.	Protein MW	Protein PI	Pep. Count	Protein Score	Protein Score C. I. %	Intensity Matched	Total Ion Score	Total Ion C. I. %	Confirmed
1	Vicilin-like antimicrobial peptides 2-2 [Aegilops tauschii]	gi|475617685	54616.7	6.23	11	242	100	14.689	199	100	

Peptide Information

Calc. Mass	Obsrv. Mass	± da	± ppm	Start Seq.	End Seq.	Sequence	Ion Score	C. I. %	Modification	Rank	Result Type
805.4818	805.4856	0.0038	5	51	57	GLFLLDK					Mascot
1048.5674	1048.5896	0.0222	21	202	211	TSVLAGFEPK					Mascot
1161.6879	1161.6694	-0.0185	-16	51	60	GLFLLDKVEK					Mascot
1353.7122	1353.746	0.0338	25	61	73	VVESEGGSVHVVR					Mascot
1353.7122	1353.746	0.0338	25	61	73	VVESEGGSVHVVR	89	100			Mascot
1618.7968	1618.791	-0.0058	-4	99	112	EGLMHIGFITMEPK			Oxidation (M)[4]		Mascot
1634.7917	1634.7892	-0.0025	-2	99	112	EGLMHIGFITMEPK			Oxidation (M)[4,11]		Mascot
1634.7917	1634.7892	-0.0025	-2	99	112	EGLMHIGFITMEPK	0	0	Oxidation (M)[4,11]		Mascot
1860.8035	1860.8376	0.0341	18	26	40	WEEGEGEWRPEEEAK					Mascot
2145.9473	2146.0002	0.0529	25	24	40	ERWEEGEGEWRPEEEAK					Mascot
2166.1958	2166.2502	0.0544	25	113	130	TLFVPQYIDSNLILFVQR					Mascot
2166.1958	2166.2502	0.0544	25	113	130	TLFVPQYIDSNLILFVQR	110	100			Mascot
2179.0046	2178.9937	-0.0109	-5	150	169	MGDVLQIDAGSTFYMVNTGK			Oxidation (M)[1,15]		Mascot
2565.1511	2565.2175	0.0664	26	74	98	GLPASGVPEAPWQHGGWSAGCGACR			Carbamidomethyl (C)[21,24]		Mascot
2931.3982	2931.4702	0.072	25	173	200	LHIICSIDASDSAGFAPYQSFYLGGGGK			Carbamidomethyl (C)[5]		Mascot

Rank	Protein Name	Accession No.	Protein MW	Protein PI	Pep. Count	Protein Score	Protein Score C. I. %	Intensity Matched	Total Ion Score	Total Ion C. I. %	Confirmed
2	Vicilin-like antimicrobial peptides 2-2 [Triticum urartu]	gi|474404647	43446.1	6.74	4	123	100	8.332	110	100	

Peptide Information

Calc. Mass	Obsrv. Mass	± da	± ppm	Start Seq.	End Seq.	Sequence	Ion Score	C. I. %	Modification	Rank	Result Type
869.386	869.4692	0.0832	96	144	151	EHGQGGER					Mascot
2166.1958	2166.2502	0.0544	25	5	22	TLFVPQYIDSNLILFVQR					Mascot
2166.1958	2166.2502	0.0544	25	5	22	TLFVPQYIDSNLILFVQR	110	100			Mascot
2565.4077	2565.2175	-0.1902	-74	5	26	TLFVPQYIDSNLILFVQRGDVK					Mascot
2931.3982	2931.4702	0.072	25	65	92	LHIICSIDASDSAGFAPYQSFYLGGGGK			Carbamidomethyl (C)[5]		Mascot

Rank	Protein Name	Accession No.	Protein MW	Protein PI	Pep. Count	Protein Score	Protein Score C. I. %	Intensity Matched	Total Ion Score	Total Ion C. I. %	Confirmed
3	Structural maintenance of chromosomes (SMC) family	gi|508785945	139800.6	6.16	29	73	95.233	14.746			

protein isoform 1 [Theobroma cacao]

Peptide Information

Calc. Mass	Obsrv. Mass	± da	± ppm	Start Seq.	End Seq.	Sequence	Ion Score	C. I. %	Modification	Rank	Result Type
801.4577	801.4104	-0.0473	-59	94	99	EEVRLR					Mascot
806.5134	806.4398	-0.0736	-91	1082	1088	YIGVKVK					Mascot
817.4566	817.4421	-0.0145	-18	392	397	DKWLQK					Mascot
824.4182	824.429	0.0108	13	273	279	MSKDVTK			Oxidation (M)[1]		Mascot
850.4338	850.4501	0.0163	19	269	275	ELDKMSK					Mascot
860.4472	860.4551	0.0079	9	893	899	DEKNNLK					Mascot
861.4611	861.4595	-0.0016	-2	1143	1150	TAVGNMIR					Mascot
876.4421	876.3741	-0.068	-78	1019	1025	KDESIER					Mascot
878.44	878.4639	0.0239	27	364	370	GIMEREK			Oxidation (M)[3]		Mascot
904.4921	904.4666	-0.0255	-28	329	335	QLQMLQK			Oxidation (M)[4]		Mascot
992.5775	992.5404	-0.0371	-37	371	378	QLSILYQK					Mascot
1033.5571	1033.5256	-0.0315	-30	1143	1151	TAVGNMIRR			Oxidation (M)[6]		Mascot
1161.5681	1161.6694	0.1013	87	27	38	VNCVVGANGSGK			Carbamidomethyl (C)[3]		Mascot
1333.7587	1333.7	-0.0587	-44	371	381	QLSILYQKQGR					Mascot
1346.7162	1346.7493	0.0331	25	820	831	AELETNLTTNLK					Mascot
1353.7526	1353.746	-0.0066	-5	5	15	QIIIEGFKSYR					Mascot
1353.7526	1353.746	-0.0066	-5	5	15	QIIIEGFKSYR					Mascot
1474.8112	1474.7936	-0.0176	-12	819	831	KAELETNLTTNLK					Mascot
1556.7479	1556.7747	0.0268	17	939	952	ELGPLSSDAFETYK					Mascot
1747.9337	1747.9246	-0.0091	-5	705	719	ITAFVTEQQQLDAKR					Mascot
1757.928	1757.9246	-0.0034	-2	301	315	KQTALDLDVIDLEER					Mascot
1927.0226	1926.9143	-0.1083	-56	602	618	LNFSPKFTPAFAQVFGR					Mascot
1965.9222	1965.9501	0.0279	14	772	788	HAEMGTELIDHLTPEEK			Oxidation (M)[4]		Mascot
1965.9222	1965.9501	0.0279	14	772	788	HAEMGTELIDHLTPEEK			Oxidation (M)[4]		Mascot
2146.0808	2146.0002	-0.0806	-38	398	415	EIDDLQRVLSSNLMQEQK					Mascot
2158.0784	2158.0427	-0.0357	-17	1036	1054	EVFSELVQGGHGHLVMM KK			Oxidation (M)[16,17]		Mascot
2194.1543	2193.9834	-0.1709	-78	536	555	FFTAVEVTAGNSLFHVVV EK					Mascot
2219.0972	2219.2107	0.1135	51	329	346	QLQMLQKEIQDSTEELNR			Oxidation (M)[4]		Mascot
2512.1587	2512.2041	0.0454	18	1188	1209	VNVVSKEDALDFIEHDQS HNTD					Mascot
2581.3718	2581.2151	-0.1567	-61	682	704	EEELKGVGSELQNILLPS QLEQK					Mascot
2709.3374	2709.2837	-0.0537	-20	513	535	ICREYNIGGVFGPIIELLN CDEK			Carbamidomethyl (C)[2,20]		Mascot

4	Structural maintenance of chromosomes (SMC) family protein isoform 2 [Theobroma cacao]	gi|508785946	139163.2	6.21	28	67	79.19	14.476	

Peptide Information

Calc. Mass	Obsrv. Mass	± da	± ppm	Start Seq.	End Seq.	Sequence	Ion Score	C. I. %	Modification	Rank	Result Type
801.4577	801.4104	-0.0473	-59	94	99	EEVRLR					Mascot
806.5134	806.4398	-0.0736	-91	1076	1082	YIGVKVK					Mascot
817.4414	817.4421	0.0007	1	687	694	GVGSELQK					Mascot
824.4182	824.429	0.0108	13	273	279	MSKDVTK			Oxidation (M)[1]		Mascot
850.4338	850.4501	0.0163	19	269	275	ELDKMSK					Mascot
860.4472	860.4551	0.0079	9	887	893	DEKNNLK					Mascot
861.4611	861.4595	-0.0016	-2	1137	1144	TAVGNMIR					Mascot
876.4421	876.3741	-0.068	-78	1013	1019	KDESIER					Mascot
878.44	878.4639	0.0239	27	364	370	GIMEREK			Oxidation (M)[3]		Mascot
904.4921	904.4666	-0.0255	-28	329	335	QLQMLQK			Oxidation (M)[4]		Mascot
992.5775	992.5404	-0.0371	-37	371	378	QLSILYQK					Mascot
1033.5571	1033.5256	-0.0315	-30	1137	1145	TAVGNMIRR			Oxidation (M)[6]		Mascot
1161.5681	1161.6694	0.1013	87	27	38	VNCVVGANGSGK			Carbamidomethyl (C)[3]		Mascot
1333.7587	1333.7	-0.0587	-44	371	381	QLSILYQKQGR					Mascot
1346.7162	1346.7493	0.0331	25	814	825	AELETNLTTNLK					Mascot
1353.7526	1353.746	-0.0066	-5	5	15	QIIIEGFKSYR					Mascot
1353.7526	1353.746	-0.0066	-5	5	15	QIIIEGFKSYR					Mascot
1474.8112	1474.7936	-0.0176	-12	813	825	KAELETNLTTNLK					Mascot
1556.7479	1556.7747	0.0268	17	933	946	ELGPLSSDAFETYK					Mascot
1747.9337	1747.9246	-0.0091	-5	699	713	ITAFVTEQQQLDAKR					Mascot
1757.928	1757.9246	-0.0034	-2	301	315	KQTALDLDVIDLEER					Mascot
1927.0226	1926.9143	-0.1083	-56	602	618	LNFSPKFTPAFAQVFGR					Mascot
1965.9222	1965.9501	0.0279	14	766	782	HAEMGTELIDHLTPEEK			Oxidation (M)[4]		Mascot
1965.9222	1965.9501	0.0279	14	766	782	HAEMGTELIDHLTPEEK			Oxidation (M)[4]		Mascot
2146.0808	2146.0002	-0.0806	-38	398	415	EIDDLQRVLSSNLMQEQK					Mascot
2158.0784	2158.0427	-0.0357	-17	1030	1048	EVFSELVQGGHGHLVMMKK			Oxidation (M)[16,17]		Mascot
2194.1543	2193.9834	-0.1709	-78	536	555	FFTAVEVTAGNSLFHVVVEK					Mascot
2219.0972	2219.2107	0.1135	51	329	346	QLQMLQKEIQDSTEELNR			Oxidation (M)[4]		Mascot
2512.1587	2512.2041	0.0454	18	1182	1203	VNVVSKEDALDFIEHDQSHNTD					Mascot
2709.3374	2709.2837	-0.0537	-20	513	535	ICREYNIGGVFGPIIELLNCDEK			Carbamidomethyl (C)[2,20]		Mascot

5 Alanyl-tRNA synthetase [Triticum urartu] gi|474423501 208125.5 8.47 31 63 46.509 15.39

Peptide Information

Calc. Mass	Obsrv. Mass	± da	± ppm	Start Seq.	End Seq.	Sequence	Ion Score	C. I. %	Modification	Rank	Result Type
800.4485	800.3987	-0.0498	-62	1716	1724	RGGAGGVAR					Mascot
809.4264	809.4132	-0.0132	-16	634	639	VDYTRR					Mascot
810.3596	810.4244	0.0648	80	1277	1283	MISCAGR			Carbamidomethyl (C)[4], Oxidation (M)[1]		Mascot
813.5053	813.4472	-0.0581	-71	1015	1021	AIANRLR					Mascot
818.3613	818.433	0.0717	88	1328	1333	NSMHWK			Oxidation (M)[3]		Mascot
850.4893	850.4501	-0.0392	-46	1160	1166	GIRVSYR					Mascot
860.4724	860.4551	-0.0173	-20	832	839	LEGATLEK					Mascot
881.4298	881.4735	0.0437	50	1343	1350	EKGGMGFR					Mascot
884.4083	884.4766	0.0683	77	1397	1403	FSSAMWR					Mascot
897.4247	897.459	0.0343	38	1343	1350	EKGGMGFR			Oxidation (M)[5]		Mascot
963.4781	963.4902	0.0121	13	1425	1432	SISIWDDK					Mascot
1026.5844	1026.4967	-0.0877	-85	1753	1761	NGVLFAHLR					Mascot
1033.5459	1033.5256	-0.0203	-20	921	929	EAVVKAMNR			Oxidation (M)[7]		Mascot
1048.584	1048.5896	0.0056	5	1552	1558	VRVFWWR					Mascot
1068.5619	1068.5385	-0.0234	-22	1510	1518	SAYRALMTR					Mascot
1161.6263	1161.6694	0.0431	37	274	285	LAGAGIQPYSGK					Mascot
1210.6613	1210.6152	-0.0461	-38	1104	1113	CVTSIKFSIR			Carbamidomethyl (C)[1]		Mascot
1210.6613	1210.6152	-0.0461	-38	1104	1113	CVTSIKFSIR			Carbamidomethyl (C)[1]		Mascot
1353.6168	1353.746	0.1292	95	380	391	DEEASFENTLAK					Mascot
1353.6168	1353.746	0.1292	95	380	391	DEEASFENTLAK					Mascot
1368.6292	1368.5964	-0.0328	-24	1490	1502	GGGDDVVAWAHER					Mascot
1375.7944	1375.6714	-0.123	-89	703	713	KIEYIVNQQIK					Mascot
1391.7543	1391.7126	-0.0417	-30	1616	1626	RPELHPLTWSR					Mascot
1474.7723	1474.7936	0.0213	14	1433	1445	WIPGTLSMTPTVR			Oxidation (M)[8]		Mascot
1480.7135	1480.8369	0.1234	83	804	817	ITAVTAECASQAMK			Carbamidomethyl (C)[8]		Mascot
1656.9166	1656.8723	-0.0443	-27	887	903	AVKIAIDAAEAALSEGK					Mascot
1748.033	1747.9246	-0.1084	-62	1783	1797	HNVRAIVAPILFEIR					Mascot
1757.8615	1757.9246	0.0631	36	1328	1342	NSMHWKAWMALASPK					Mascot
1959.0599	1959.0446	-0.0153	-8	1798	1813	EHVIYFKSFVIQHVGR					Mascot
2158.2424	2158.0427	-0.1997	-93	1787	1804	AIVAPILFEIREHVIYFK					Mascot
2266.1697	2266.1055	-0.0642	-28	639	657	RTLIAPNHTCTHMLNFAL R			Carbamidomethyl (C)[10]		Mascot
2266.1697	2266.1055	-0.0642	-28	639	657	RTLIAPNHTCTHMLNFAL			Carbamidomethyl (C)[10]		Mascot

Calc. Mass	Obsrv. Mass	± da	± ppm	Start Seq.	End Seq.	Sequence	Ion Score	C. I. %	Modification	Rank	Result Type
						R					
2380.1892	2380.1516	-0.0376	-16	307	328	TLSFAIADGSQPGNEGRE YVLR					Mascot
2380.1892	2380.1516	-0.0376	-16	307	328	TLSFAIADGSQPGNEGRE YVLR					Mascot
2701.4277	2701.3364	-0.0913	-34	1559	1582	VLHGILPAESTLMHRHITT IGTCK			Carbamidomethyl (C)[23], Oxidation (M)[13]		Mascot
2709.4797	2709.2837	-0.196	-72	992	1014	FPQSITQYRPISLCPVLYK IASK			Carbamidomethyl (C)[14]		Mascot

6 PREDICTED: uncharacterized protein LOC101207654 [Cucumis sativus] gi|449443456 19980.2 9.08 10 60 2.663 5.388

Peptide Information

Calc. Mass	Obsrv. Mass	± da	± ppm	Start Seq.	End Seq.	Sequence	Ion Score	C. I. %	Modification	Rank	Result Type
834.4138	834.343	-0.0708	-85	24	30	AQCNTLK			Carbamidomethyl (C)[3]		Mascot
906.4461	906.4791	0.033	36	133	139	EISRNCK			Carbamidomethyl (C)[6]		Mascot
921.4523	921.538	0.0857	93	143	150	KESVEDSK					Mascot
1033.5459	1033.5256	-0.0203	-20	24	32	AQCNTLKAK			Carbamidomethyl (C)[3]		Mascot
1048.6071	1048.5896	-0.0175	-17	105	113	KEMVSTIIK					Mascot
1570.8475	1570.8223	-0.0252	-16	114	127	ETNSKFFTIATALK					Mascot
1634.8611	1634.7892	-0.0719	-44	119	132	FFTIATALKFSTCK			Carbamidomethyl (C)[13]		Mascot
1634.8611	1634.7892	-0.0719	-44	119	132	FFTIATALKFSTCK			Carbamidomethyl (C)[13]		Mascot
1757.948	1757.9246	-0.0234	-13	89	104	CIAKLLGGSHQLFASR			Carbamidomethyl (C)[1]		Mascot
2146.177	2146.0002	-0.1768	-82	6	23	LFISPFFMFIVLIASTQR			Oxidation (M)[8]		Mascot
2380.0618	2380.1516	0.0898	38	33	52	ISCLDCQSNYDFSGNLIM VK			Carbamidomethyl (C)[3,6], Oxidation (M)[18]		Mascot
2380.0618	2380.1516	0.0898	38	33	52	ISCLDCQSNYDFSGNLIM VK			Carbamidomethyl (C)[3,6], Oxidation (M)[18]		Mascot

7 d-lactate dehydrogenase, putative [Ricinus communis] gi|223526119 60328.8 6 12 60 0 4.539 19 0

Peptide Information

Calc. Mass	Obsrv. Mass	± da	± ppm	Start Seq.	End Seq.	Sequence	Ion Score	C. I. %	Modification	Rank	Result Type
809.4304	809.4132	-0.0172	-21	309	315	GFYLGPR					Mascot
810.4355	810.4244	-0.0111	-14	373	379	AKSDYVK					Mascot
850.424	850.4501	0.0261	31	1	7	MVNFPAR			Oxidation (M)[1]		Mascot
860.4724	860.4551	-0.0173	-20	136	143	VSVDLEAK					Mascot
925.4989	925.4429	-0.056	-61	300	308	TTGISATFK					Mascot
1132.6473	1132.5575	-0.0898	-79	98	106	EAFLEIRVR					Mascot
1368.6254	1368.5964	-0.029	-21	219	229	EMGEDVFWAIR			Oxidation (M)[2]		Mascot
1480.7253	1480.8369	0.1116	75	218	229	KEMGEDVFWAIR					Mascot
1568.7454	1568.8074	0.062	40	454	465	EFYYLMTPFVSR			Oxidation (M)[6]		Mascot

Calc. Mass	Obsrv. Mass	± da	± ppm	Start Seq.	End Seq.	Sequence	Ion Score	C. I. %	Modification	Rank	Result Type
2184.0173	2184.0283	0.011	5	469	487	AAYINYMDFDIGAMGMIK K			Oxidation (M)[7,14]		Mascot
2581.4197	2581.2151	-0.2046	-79	69	91	FADLEMPKPLAIVIPETVE ELVK					Mascot
2597.4148	2597.2502	-0.1646	-63	69	91	FADLEMPKPLAIVIPETVE ELVK			Oxidation (M)[6]		Mascot
2743.3662	2743.3516	-0.0146	-5	400	424	GYVILDPYGGVMHNISSE AIAFPHR					Mascot
2759.3611	2759.3423	-0.0188	-7	400	424	GYVILDPYGGVMHNISSE AIAFPHR			Oxidation (M)[12]		Mascot
2759.3611	2759.3423	-0.0188	-7	400	424	GYVILDPYGGVMHNISSE AIAFPHR	19	0	Oxidation (M)[12]		Mascot

8	Speckle-type POZ protein-like protein [Aegilops tauschii]	gi|475559238	59666.2	6.25	15	59	0	7.82	

Peptide Information

Calc. Mass	Obsrv. Mass	± da	± ppm	Start Seq.	End Seq.	Sequence	Ion Score	C. I. %	Modification	Rank	Result Type
807.4359	807.4217	-0.0142	-18	376	382	TLSPFSR					Mascot
821.3635	821.4166	0.0531	65	363	369	DEEVSSR					Mascot
826.3954	826.4537	0.0583	71	57	63	VGGHDWR					Mascot
861.4723	861.4595	-0.0128	-15	456	462	GCTVRIR			Carbamidomethyl (C)[2]		Mascot
881.4363	881.4735	0.0372	42	50	56	IESETFR					Mascot
963.5291	963.4902	-0.0389	-40	1	9	MSALVSSLR					Mascot
992.5193	992.5404	0.0211	21	443	451	AATIGEMKR			Oxidation (M)[7]		Mascot
1161.6626	1161.6694	0.0068	6	34	42	IEQFKQIQK					Mascot
1177.5444	1177.6122	0.0678	58	363	372	DEEVSSRAER					Mascot
1568.7737	1568.8074	0.0337	21	43	56	MLGNGAKIESETFR			Oxidation (M)[1]		Mascot
1741.8248	1741.9788	0.154	88	178	193	YRDGMASTTTMVPPSK					Mascot
1757.8197	1757.9246	0.1049	60	178	193	YRDGMASTTTMVPPSK			Oxidation (M)[5]		Mascot
1860.8943	1860.8376	-0.0567	-30	180	196	DGMASTTTMVPPSKLHR			Oxidation (M)[3,9]		Mascot
1966.1294	1965.9501	-0.1793	-91	512	529	VDVPLVLMLIQVGKEPSL			Oxidation (M)[8]		Mascot
1966.1294	1965.9501	-0.1793	-91	512	529	VDVPLVLMLIQVGKEPSL			Oxidation (M)[8]		Mascot
2565.166	2565.2175	0.0515	20	197	219	EPTEVPSESKEGSDPYM EIEVGR					Mascot
2581.1611	2581.2151	0.054	21	197	219	EPTEVPSESKEGSDPYM EIEVGR			Oxidation (M)[17]		Mascot
2709.3772	2709.2837	-0.0935	-35	155	177	EHLKDGCLIIVCDVTVLD MHTIK			Carbamidomethyl (C)[7,12]		Mascot

9	RecName: Full=Cytochrome c	gi|118010	12158.1	9.79	8	58	0	3.016	

Peptide Information

Calc. Mass	Obsrv. Mass	± da	± ppm	Start Seq.	End Seq.	Sequence	Ion Score	C. I. %	Modification	Rank	Result Type

806.4771	806.4398	-0.0373	-46	81	87	KYIPGTK					Mascot
807.4433	807.4217	-0.0216	-27	88	94	MVFPGLK			Oxidation (M)[1]		Mascot
875.4257	875.4569	0.0312	36	64	70	AVNWEEK					Mascot
906.5294	906.4791	-0.0503	-55	100	107	ABLLAYLK					Mascot
1429.7985	1429.7078	-0.0907	-63	88	99	MVFPGLKKPZZR					Mascot
1536.6925	1536.6725	-0.02	-13	1	16	ASFBZAPAPAGBSASGEK					Mascot
1536.6925	1536.6725	-0.02	-13	1	16	ASFBZAPAPAGBSASGEK					Mascot
1568.6693	1568.8074	0.1381	88	22	35	CAZCHTVBZGAGHK			Carbamidomethyl (C)[1,4]		Mascot
1926.908	1926.9143	0.0063	3	1	19	ASFBZAPAPAGBSASGEKIFK					Mascot

10 Os09g0502500 [Oryza sativa Japonica Group] gi|255679034 35137.4 9.51 12 57 0 3.771

Peptide Information

Calc. Mass	Obsrv. Mass	± da	± ppm	Start Seq.	End Seq.	Sequence	Ion Score	C. I. %	Modification	Rank	Result Type
818.3791	818.433	0.0539	66	316	322	AEEGWAR					Mascot
824.4043	824.429	0.0247	30	1	7	MATSSRR			Oxidation (M)[1]		Mascot
875.404	875.4569	0.0529	60	323	331	SMAGHATGK			Oxidation (M)[2]		Mascot
893.4952	893.438	-0.0572	-64	270	276	QRVTFSR					Mascot
1010.5087	1010.4754	-0.0333	-33	8	15	MRAVQYDK					Mascot
1026.5038	1026.4967	-0.0071	-7	8	15	MRAVQYDK			Oxidation (M)[1]		Mascot
1033.4871	1033.5256	0.0385	37	332	340	IIVEMGDEQ					Mascot
1132.5933	1132.5575	-0.0358	-32	57	66	GVARPFMPNK			Oxidation (M)[7]		Mascot
1375.7217	1375.6714	-0.0503	-37	208	220	VLDYATPEGAALR					Mascot
1568.8068	1568.8074	0.0006	0	10	24	AVQYDKYGGGAQALK					Mascot
1747.8717	1747.9246	0.0529	30	289	304	EDMELVAGMVAEGKLR					Mascot
1887.9706	1887.8765	-0.0941	-50	188	207	VTATCGARNAGLVGGLGADK			Carbamidomethyl (C)[5]		Mascot
1905.8682	1905.9642	0.096	50	323	340	SMAGHATGKIIVEMGDEQ			Oxidation (M)[2,14]		Mascot

Gel Idx/Pos	192/H19	Instr./Gel Origin	BA2151/Sample Project 20140814	Process Status	Analysis Succeeded
Plate [#] Name	[1] Sample Project 20140814	Instrument Sample Name		Spectra	11

Rank	Protein Name	Accession No.	Protein MW	Protein PI	Pep. Count	Protein Score	Protein Score C. I. %	Intensity Matched	Total Ion Score	Total Ion C. I. %	Confirmed
1	RecName: Full=Triosephosphate isomerase, cytosolic; Short=TIM; Short=Triose-phosphate isomerase	gi|2507469	26948	5.39	9	442	100	51.484	394	100	

Peptide Information

Calc. Mass	Obsrv. Mass	± da	± ppm	Start Seq.	End Seq.	Sequence	Ion Score	C. I. %	Modification	Rank	Result Type
954.4832	954.5059	0.0227	24	5	12	FFVGGNWK					Mascot
954.4832	954.5059	0.0227	24	5	12	FFVGGNWK	63	99.919			Mascot
1033.6041	1033.6255	0.0214	21	114	123	VAYALAQGLK					Mascot
1289.6332	1289.6741	0.0409	32	195	206	TNVSPEVAESTR					Mascot
1289.6332	1289.6741	0.0409	32	195	206	TNVSPEVAESTR	53	99.186			Mascot
1312.6566	1312.6898	0.0332	25	207	219	IIYGGSVTGASCK			Carbamidomethyl (C)[12]		Mascot
1374.7046	1374.7457	0.0411	30	124	135	VIACVGETLEQR			Carbamidomethyl (C)[4]		Mascot
1374.7046	1374.7457	0.0411	30	124	135	VIACVGETLEQR	94	100	Carbamidomethyl (C)[4]		Mascot
1604.8503	1604.8995	0.0492	31	176	190	VATPAQAQEVHANLR					Mascot
1604.8503	1604.8995	0.0492	31	176	190	VATPAQAQEVHANLR	88	100			Mascot
1811.9585	1812.0146	0.0561	31	56	70	LRPEIQVAAQNCWVK			Carbamidomethyl (C)[12]		Mascot
1811.9585	1812.0146	0.0561	31	56	70	LRPEIQVAAQNCWVK	95	100	Carbamidomethyl (C)[12]		Mascot
2011.0906	2011.0872	-0.0034	-2	54	70	AKLRPEIQVAAQNCWVK			Carbamidomethyl (C)[14]		Mascot
3053.5625	3053.6904	0.1279	42	72	100	GGAFTGEVSAEMLANLG VPWVILGHSERR					Mascot
3069.5576	3069.6748	0.1172	38	72	100	GGAFTGEVSAEMLANLG VPWVILGHSERR			Oxidation (M)[12]		Mascot

Rank	Protein Name	Accession No.	Protein MW	Protein PI	Pep. Count	Protein Score	Protein Score C. I. %	Intensity Matched	Total Ion Score	Total Ion C. I. %	Confirmed
2	uncharacterized protein, partial [Phleum pratense]	gi|409972141	25636.3	5.18	6	364	100	49.398	340	100	

Peptide Information

Calc. Mass	Obsrv. Mass	± da	± ppm	Start Seq.	End Seq.	Sequence	Ion Score	C. I. %	Modification	Rank	Result Type
954.4832	954.5059	0.0227	24	11	18	FFVGGNWK					Mascot
954.4832	954.5059	0.0227	24	11	18	FFVGGNWK	63	99.919			Mascot
1033.6041	1033.6255	0.0214	21	120	129	VAYALAQGLK					Mascot
1351.6741	1351.6884	0.0143	11	107	119	ALLGESSEFVGDK					Mascot
1374.7046	1374.7457	0.0411	30	130	141	VIACVGETLEQR			Carbamidomethyl (C)[4]		Mascot
1374.7046	1374.7457	0.0411	30	130	141	VIACVGETLEQR	94	100	Carbamidomethyl (C)[4]		Mascot

Calc. Mass	Obsrv. Mass	± da	± ppm	Start Seq.	End Seq.	Sequence	Ion Score	C. I. %	Modification	Rank	Result Type
1604.8503	1604.8995	0.0492	31	182	196	VATPAQAQEVHANLR					Mascot
1604.8503	1604.8995	0.0492	31	182	196	VATPAQAQEVHANLR	88	100			Mascot
1811.9585	1812.0146	0.0561	31	62	76	LRPEIQVAAQNCWVK			Carbamidomethyl (C)[12]		Mascot
1811.9585	1812.0146	0.0561	31	62	76	LRPEIQVAAQNCWVK	95	100	Carbamidomethyl (C)[12]		Mascot

3	RecName: Full=Triosephosphate isomerase, cytosolic; Short=TIM; Short=Triose-phosphate isomerase	gi|1174749	27138.1	5.24	7	337	100	34.433	306	100

Peptide Information

Calc. Mass	Obsrv. Mass	± da	± ppm	Start Seq.	End Seq.	Sequence	Ion Score	C. I. %	Modification	Rank	Result Type
954.4832	954.5059	0.0227	24	5	12	FFVGGNWK					Mascot
954.4832	954.5059	0.0227	24	5	12	FFVGGNWK	63	99.919			Mascot
1033.6041	1033.6255	0.0214	21	114	123	VAYALAQGLK					Mascot
1289.6332	1289.6741	0.0409	32	195	206	TNVSPEVAESTR					Mascot
1289.6332	1289.6741	0.0409	32	195	206	TNVSPEVAESTR	53	99.186			Mascot
1312.6566	1312.6898	0.0332	25	207	219	IIYGGSVTGASCK			Carbamidomethyl (C)[12]		Mascot
1374.7046	1374.7457	0.0411	30	124	135	VIACVGETLEQR			Carbamidomethyl (C)[4]		Mascot
1374.7046	1374.7457	0.0411	30	124	135	VIACVGETLEQR	94	100	Carbamidomethyl (C)[4]		Mascot
1811.9585	1812.0146	0.0561	31	56	70	LRPEIQVAAQNCWVK			Carbamidomethyl (C)[12]		Mascot
1811.9585	1812.0146	0.0561	31	56	70	LRPEIQVAAQNCWVK	95	100	Carbamidomethyl (C)[12]		Mascot
2346.2493	2346.2766	0.0273	12	155	175	IKDWTNVVVAYEPVWAIG TGK					Mascot

4	uncharacterized protein, partial [Phleum pratense]	gi|409972323	22917.1	6.22	6	307	100	47.756	277	100

Peptide Information

Calc. Mass	Obsrv. Mass	± da	± ppm	Start Seq.	End Seq.	Sequence	Ion Score	C. I. %	Modification	Rank	Result Type
1033.6041	1033.6255	0.0214	21	75	84	VAYALAQGLK					Mascot
1351.6741	1351.6884	0.0143	11	62	74	ALLGESSEFVGDK					Mascot
1374.7046	1374.7457	0.0411	30	85	96	VIACVGETLEQR			Carbamidomethyl (C)[4]		Mascot
1374.7046	1374.7457	0.0411	30	85	96	VIACVGETLEQR	94	100	Carbamidomethyl (C)[4]		Mascot
1604.8503	1604.8995	0.0492	31	137	151	VATPAQAQEVHANLR					Mascot
1604.8503	1604.8995	0.0492	31	137	151	VATPAQAQEVHANLR	88	100			Mascot
1811.9585	1812.0146	0.0561	31	17	31	LRPEIQVAAQNCWVK			Carbamidomethyl (C)[12]		Mascot
1811.9585	1812.0146	0.0561	31	17	31	LRPEIQVAAQNCWVK	95	100	Carbamidomethyl (C)[12]		Mascot
3069.5212	3069.6748	0.1536	50	33	61	GGAFTGEVSAEMLANLG VPWDILGHSERR					Mascot
3085.5161	3085.6816	0.1655	54	33	61	GGAFTGEVSAEMLANLG VPWDILGHSERR			Oxidation (M)[12]		Mascot

5	Triosephosphate isomerase, cytosolic [Aegilops	gi|475538000	33826.6	8.29	11	304	100	37.213	243	100

tauschii]

Peptide Information

Calc. Mass	Obsrv. Mass	± da	± ppm	Start Seq.	End Seq.	Sequence	Ion Score	C. I. %	Modification	Rank	Result Type
1033.6041	1033.6255	0.0214	21	172	181	VAYALAQGLK					Mascot
1289.6332	1289.6741	0.0409	32	253	264	TNVSPEVAESTR					Mascot
1289.6332	1289.6741	0.0409	32	253	264	TNVSPEVAESTR	53	99.186			Mascot
1312.6566	1312.6898	0.0332	25	265	277	IIYGGSVTGASCK			Carbamidomethyl (C)[12]		Mascot
1374.7046	1374.7457	0.0411	30	182	193	VIACVGETLEQR			Carbamidomethyl (C)[4]		Mascot
1374.7046	1374.7457	0.0411	30	182	193	VIACVGETLEQR	94	100	Carbamidomethyl (C)[4]		Mascot
1415.6359	1415.6653	0.0294	21	159	171	SLMGESSEFVGEK			Oxidation (M)[3]		Mascot
1571.7371	1571.7865	0.0494	31	158	171	RSLMGESSEFVGEK			Oxidation (M)[4]		Mascot
1811.9585	1812.0146	0.0561	31	114	128	LRPEIQVAAQNCWVK			Carbamidomethyl (C)[12]		Mascot
1811.9585	1812.0146	0.0561	31	114	128	LRPEIQVAAQNCWVK	95	100	Carbamidomethyl (C)[12]		Mascot
1997.075	1997.0447	-0.0303	-15	112	128	GKLRPEIQVAAQNCWVK			Carbamidomethyl (C)[14]		Mascot
1997.075	1997.0447	-0.0303	-15	112	128	GKLRPEIQVAAQNCWVK			Carbamidomethyl (C)[14]		Mascot
2346.2493	2346.2766	0.0273	12	213	233	IKDWTNVVVAYEPVWAIG TGK					Mascot
2925.4929	2925.5884	0.0955	33	130	157	GGAFTGEVSAEMLVNLG VPWVILGHSER					Mascot
2941.4878	2941.5771	0.0893	30	130	157	GGAFTGEVSAEMLVNLG VPWVILGHSER			Oxidation (M)[12]		Mascot
3053.5876	3053.6904	0.1028	34	129	157	KGGAFTGEVSAEMLVNL GVPWVILGHSER					Mascot
3069.5828	3069.6748	0.092	30	129	157	KGGAFTGEVSAEMLVNL GVPWVILGHSER			Oxidation (M)[13]		Mascot

6 uncharacterized protein, partial [Phleum pratense] gi|409971625 21091.1 5.76 5 273 100 32.619 253 100

Peptide Information

Calc. Mass	Obsrv. Mass	± da	± ppm	Start Seq.	End Seq.	Sequence	Ion Score	C. I. %	Modification	Rank	Result Type
954.4832	954.5059	0.0227	24	12	19	FFVGGNWK					Mascot
954.4832	954.5059	0.0227	24	12	19	FFVGGNWK	63	99.919			Mascot
1033.6041	1033.6255	0.0214	21	121	130	VAYALAQGLK					Mascot
1351.6741	1351.6884	0.0143	11	108	120	ALLGESSEFVGDK					Mascot
1374.7046	1374.7457	0.0411	30	131	142	VIACVGETLEQR			Carbamidomethyl (C)[4]		Mascot
1374.7046	1374.7457	0.0411	30	131	142	VIACVGETLEQR	94	100	Carbamidomethyl (C)[4]		Mascot
1811.9585	1812.0146	0.0561	31	63	77	LRPEIQVAAQNCWVK			Carbamidomethyl (C)[12]		Mascot
1811.9585	1812.0146	0.0561	31	63	77	LRPEIQVAAQNCWVK	95	100	Carbamidomethyl (C)[12]		Mascot

7 Triosephosphate isomerase, cytosolic [Triticum urartu] gi|474302100 17829.2 5.71 6 251 100 28.905 212 100

Peptide Information

Calc. Mass	Obsrv. Mass	± da	± ppm	Start Seq.	End Seq.	Sequence	Ion Score	C. I. %	Modification	Rank	Result Type
954.4832	954.5059	0.0227	24	5	12	FFVGGNWK					Mascot
954.4832	954.5059	0.0227	24	5	12	FFVGGNWK	63	99.919			Mascot
1289.6332	1289.6741	0.0409	32	119	130	TNVSPEVAESTR					Mascot
1289.6332	1289.6741	0.0409	32	119	130	TNVSPEVAESTR	53	99.186			Mascot
1811.9585	1812.0146	0.0561	31	56	70	LRPEIQVAAQNCWVK			Carbamidomethyl (C)[12]		Mascot
1811.9585	1812.0146	0.0561	31	56	70	LRPEIQVAAQNCWVK	95	100	Carbamidomethyl (C)[12]		Mascot
1997.075	1997.0447	-0.0303	-15	54	70	GKLRPEIQVAAQNCWVK			Carbamidomethyl (C)[14]		Mascot
1997.075	1997.0447	-0.0303	-15	54	70	GKLRPEIQVAAQNCWVK			Carbamidomethyl (C)[14]		Mascot
2925.4929	2925.5884	0.0955	33	72	99	GGAFTGEVSAEMLVNLG VPWVILGHSER					Mascot
2941.4878	2941.5771	0.0893	30	72	99	GGAFTGEVSAEMLVNLG VPWVILGHSER			Oxidation (M)[12]		Mascot
3053.5876	3053.6904	0.1028	34	71	99	KGGAFTGEVSAEMLVNL GVPWVILGHSER					Mascot
3069.5828	3069.6748	0.092	30	71	99	KGGAFTGEVSAEMLVNL GVPWVILGHSER			Oxidation (M)[13]		Mascot

8 uncharacterized protein, partial [Phleum pratense] gi|409972101 17263 5.04 4 207 100 30.556 189 100

Peptide Information

Calc. Mass	Obsrv. Mass	± da	± ppm	Start Seq.	End Seq.	Sequence	Ion Score	C. I. %	Modification	Rank	Result Type
1033.6041	1033.6255	0.0214	21	86	95	VAYALAQGLK					Mascot
1351.6741	1351.6884	0.0143	11	73	85	ALLGESSEFVGDK					Mascot
1374.7046	1374.7457	0.0411	30	96	107	VIACVGETLEQR			Carbamidomethyl (C)[4]		Mascot
1374.7046	1374.7457	0.0411	30	96	107	VIACVGETLEQR	94	100	Carbamidomethyl (C)[4]		Mascot
1811.9585	1812.0146	0.0561	31	28	42	LRPEIQVAAQNCWVK			Carbamidomethyl (C)[12]		Mascot
1811.9585	1812.0146	0.0561	31	28	42	LRPEIQVAAQNCWVK	95	100	Carbamidomethyl (C)[12]		Mascot

9 uncharacterized protein, partial [Phleum pratense] gi|409971759 16855.6 4.69 4 199 100 26.312 182 100

Peptide Information

Calc. Mass	Obsrv. Mass	± da	± ppm	Start Seq.	End Seq.	Sequence	Ion Score	C. I. %	Modification	Rank	Result Type
1033.6041	1033.6255	0.0214	21	32	41	VAYALAQGLK					Mascot
1351.6741	1351.6884	0.0143	11	19	31	ALLGESSEFVGDK					Mascot
1374.7046	1374.7457	0.0411	30	42	53	VIACVGETLEQR			Carbamidomethyl (C)[4]		Mascot
1374.7046	1374.7457	0.0411	30	42	53	VIACVGETLEQR	94	100	Carbamidomethyl (C)[4]		Mascot
1604.8503	1604.8995	0.0492	31	94	108	VATPAQAQEVHANLR					Mascot

1604.8503	1604.8995	0.0492	31	94	108	VATPAQAQEVHANLR	88	100			Mascot

10	uncharacterized protein, partial [Phleum pratense]	gi|409971969	14974.7	4.69	3	194	100	26.178	182	100

Peptide Information

Calc. Mass	Obsrv. Mass	± da	± ppm	Start Seq.	End Seq.	Sequence	Ion Score	C. I. %	Modification	Rank	Result Type
1033.6041	1033.6255	0.0214	21	4	13	VAYALAQGLK					Mascot
1374.7046	1374.7457	0.0411	30	14	25	VIACVGETLEQR			Carbamidomethyl (C)[4]		Mascot
1374.7046	1374.7457	0.0411	30	14	25	VIACVGETLEQR	94	100	Carbamidomethyl (C)[4]		Mascot
1604.8503	1604.8995	0.0492	31	66	80	VATPAQAQEVHANLR					Mascot
1604.8503	1604.8995	0.0492	31	66	80	VATPAQAQEVHANLR	88	100			Mascot

Gel Idx/Pos	193/H20	Instr./Gel Origin	BA2151/Sample Project 20140814	Process Status	Analysis Succeeded
Plate [#] Name	[1] Sample Project 20140814	Instrument Sample Name		Spectra	11

Rank	Protein Name	Accession No.	Protein MW	Protein PI	Pep. Count	Protein Score	Protein Score C. I. %	Intensity Matched	Total Ion Score	Total Ion C. I. %	Confirmed
1	Disease resistance protein RPP13 [Triticum urartu]	gi|473786130	115619.3	8.15	24	69	86.87	4.106			

Peptide Information

Calc. Mass	Obsrv. Mass	± da	± ppm	Start Seq.	End Seq.	Sequence	Ion Score	C. I. %	Modification	Rank	Result Type
807.3526	807.4201	0.0675	84	508	513	NRSDCR			Carbamidomethyl (C)[5]		Mascot
810.4104	810.4314	0.021	26	957	963	EQSPPPR					Mascot
906.4791	906.4875	0.0084	9	105	111	FRDEIAR					Mascot
954.4172	954.484	0.0668	70	209	216	AWATMACK			Carbamidomethyl (C)[7], Oxidation (M)[5]		Mascot
960.4931	960.5721	0.079	82	949	956	SCQVTPLR			Carbamidomethyl (C)[2]		Mascot
1036.5786	1036.5469	-0.0317	-31	434	442	RLSAEGYIK					Mascot
1043.5343	1043.5565	0.0222	21	273	280	MELWHAIK			Oxidation (M)[1]		Mascot
1106.6205	1106.5507	-0.0698	-63	177	188	VVSIVGFGGSGK					Mascot
1141.5969	1141.5421	-0.0548	-48	675	683	LMVHMNQLR					Mascot
1182.6552	1182.6012	-0.054	-46	915	926	VVAPAEAMAPVK					Mascot
1193.6022	1193.6482	0.046	39	447	456	RSAQQVAYDR					Mascot
1210.5708	1210.6221	0.0513	42	209	218	AWATMACKQK			Carbamidomethyl (C)[7], Oxidation (M)[5]		Mascot
1338.7012	1338.707	0.0058	4	250	260	ELEDNISHLLR					Mascot
1357.7369	1357.7451	0.0082	6	946	956	LQRSCQVTPLR			Carbamidomethyl (C)[5]		Mascot
1374.6934	1374.729	0.0356	26	1011	1023	TVPSIMPNGSKEV			Oxidation (M)[6]		Mascot
1444.7941	1444.7816	-0.0125	-9	261	272	TNRCLVVIDNIK			Carbamidomethyl (C)[4]		Mascot
1455.7366	1455.7355	-0.0011	-1	754	766	DDPTFLGYLSSLK					Mascot
1491.8026	1491.7732	-0.0294	-20	464	476	NIIRPIDAHNNSK					Mascot
1633.692	1633.845	0.153	94	204	216	EFSCRAWATMACK			Carbamidomethyl (C)[4,12], Oxidation (M)[10]		Mascot
2011.0317	2011.0259	-0.0058	-3	328	345	LSVHGCSLEVEWGTPIVK			Carbamidomethyl (C)[6]		Mascot
2064.0066	2064.2024	0.1958	95	147	164	TYEACPAVGIEQAKEELR			Carbamidomethyl (C)[5]		Mascot
2278.3469	2278.2344	-0.1125	-49	835	854	LCLVVQKPIFPTIRQGALP K			Carbamidomethyl (C)[2]		Mascot
2344.3057	2344.1826	-0.1231	-53	583	603	LLHIKYLTLGSSVSRPLD GMK			Oxidation (M)[20]		Mascot
2353.2068	2353.2222	0.0154	7	541	562	SLTVFGSAGEAVSELKSC ELLR			Carbamidomethyl (C)[18]		Mascot

Rank	Protein Name	Accession No.	Protein MW	Protein PI	Pep. Count	Protein Score	Protein Score C. I. %	Intensity Matched	Total Ion Score	Total Ion C. I. %	Confirmed
2	Vicilin-like antimicrobial peptides 2-2 [Triticum urartu]	gi|473890163	75298.3	5.79	4	63	48.917	1.283	58	99.738	

Peptide Information

Calc. Mass	Obsrv. Mass	± da	± ppm	Start Seq.	End Seq.	Sequence	Ion Score	C. I. %	Modification	Rank	Result Type
807.4359	807.4201	-0.0158	-20	660	667	KGAVFQSA					Mascot
847.3904	847.4603	0.0699	82	607	612	EEEERR					Mascot
1085.595	1085.6261	0.0311	29	31	42	AGAAVGGQVVEK					Mascot
1982.9666	1983.0228	0.0562	28	102	119	VTYIQEGGSETSSLEVQR					Mascot
1982.9666	1983.0228	0.0562	28	102	119	VTYIQEGGSETSSLEVQR	58	99.738			Mascot

3	transcription initiation factor brf1, putative [Ricinus communis]	gi|223534976	71159.4	4.95	18	62	45.263	13.292

Peptide Information

Calc. Mass	Obsrv. Mass	± da	± ppm	Start Seq.	End Seq.	Sequence	Ion Score	C. I. %	Modification	Rank	Result Type
906.4276	906.4875	0.0599	66	58	66	SIEGGNSSR					Mascot
929.4323	929.4902	0.0579	62	575	583	NDDLGPGNK					Mascot
954.405	954.484	0.079	83	587	594	EEDAEAYK					Mascot
960.536	960.5721	0.0361	38	375	382	DEQLLSKK					Mascot
993.5251	993.5272	0.0021	2	536	543	INYDVLEK					Mascot
1084.5496	1084.6506	0.101	93	307	315	IPYAYGLCK			Carbamidomethyl (C)[8]		Mascot
1106.5299	1106.5507	0.0208	19	443	450	IIWEEMNR			Oxidation (M)[6]		Mascot
1182.6881	1182.6012	-0.0869	-73	171	180	LLDPSIFIHK					Mascot
1325.5856	1325.7107	0.1251	94	584	594	NEKEEDAEAYK					Mascot
1325.5856	1325.7107	0.1251	94	584	594	NEKEEDAEAYK					Mascot
1373.6842	1373.7097	0.0255	19	291	302	GSSSKELLCQHK			Carbamidomethyl (C)[9]		Mascot
1444.743	1444.7816	0.0386	27	460	473	EAVAAAAKEAWEAK					Mascot
1757.8777	1757.8977	0.02	11	507	524	AAEAKNSVPAQSASEAA R					Mascot
1838.9	1838.9741	0.0741	40	113	127	RTEQVQAACLYIACR			Carbamidomethyl (C)[9,14]		Mascot
1952.9559	1953.0179	0.062	32	274	290	AEELRESSTDQSNFVLK					Mascot
2025.0917	2025.0688	-0.0229	-11	128	143	ENRKPYLLIDFSNFLR					Mascot
2025.0917	2025.0688	-0.0229	-11	128	143	ENRKPYLLIDFSNFLR					Mascot
2304.0457	2304.2551	0.2094	91	82	101	NGLDMGENLAIVDQAMM YYR					Mascot
2344.1272	2344.1826	0.0554	24	476	497	DCPEEMQAARELEAAVA AALAK			Carbamidomethyl (C)[2]		Mascot
2831.2974	2831.2942	-0.0032	-1	341	365	KENLSAMNNNNDSNSVS TMPFLFEK					Mascot

4	PREDICTED: disease resistance protein RPM1-like [Fragaria vesca subsp. vesca]	gi|470131535	105347.9	8.63	21	62	34.192	22.458

Peptide Information

Calc. Mass	Obsrv. Mass	± da	± ppm	Start Seq.	End Seq.	Sequence	Ion Score	C. I. %	Modification	Rank	Result Type
942.5632	942.531	-0.0322	-34	64	70	VWVRQVR					Mascot
948.4819	948.4719	-0.01	-11	242	249	DMVQQLSK					Mascot
960.5659	960.5721	0.0062	6	685	692	LGIVQMRK			Oxidation (M)[6]		Mascot
972.5771	972.5209	-0.0562	-58	684	691	RLGIVQMR					Mascot
1066.5891	1066.5424	-0.0467	-44	841	850	VPSGIEHLSK					Mascot
1182.5249	1182.6012	0.0763	65	633	642	SGPYMDFHTK					Mascot
1201.6974	1201.6433	-0.0541	-45	193	205	IVVSLAGMGGIGK					Mascot
1308.6794	1308.6979	0.0185	14	516	526	DQNFTTVVKEK					Mascot
1320.6365	1320.627	-0.0095	-7	391	400	RIDEWEMVAR			Oxidation (M)[7]		Mascot
1373.7271	1373.7097	-0.0174	-13	401	413	SLGAEIEGNDKLK					Mascot
1427.8329	1427.8096	-0.0233	-16	115	127	IASQLQAINSRVK					Mascot
1487.7523	1487.7484	-0.0039	-3	255	268	VPGVVANMDNSQLK			Oxidation (M)[8]		Mascot
1627.8585	1627.9191	0.0606	37	254	268	RVPGVVANMDNSQLK					Mascot
1633.8115	1633.845	0.0335	21	179	192	NQLVGWLCKDTSGR			Carbamidomethyl (C)[8]		Mascot
1719.8595	1719.8928	0.0333	19	539	552	LSMHNSVQYVQKNR			Oxidation (M)[3]		Mascot
1719.8595	1719.8928	0.0333	19	539	552	LSMHNSVQYVQKNR			Oxidation (M)[3]		Mascot
1725.021	1724.9025	-0.1185	-69	748	761	WIPSLHSLVRLYLK					Mascot
1983.1056	1983.0228	-0.0828	-42	365	382	DILSRCEGLPLAIVTIGR			Carbamidomethyl (C)[6]		Mascot
1983.1056	1983.0228	-0.0828	-42	365	382	DILSRCEGLPLAIVTIGR			Carbamidomethyl (C)[6]		Mascot
1997.0096	1997.0375	0.0279	14	349	364	VFQLNKCPPHLQDICK			Carbamidomethyl (C)[7,15]		Mascot
1997.0096	1997.0375	0.0279	14	349	364	VFQLNKCPPHLQDICK			Carbamidomethyl (C)[7,15]		Mascot
2278.0657	2278.2344	0.1687	74	392	411	IDEWEMVARSLGAEIEGN DK			Oxidation (M)[6]		Mascot
2478.1685	2478.3159	0.1474	59	140	160	FIKAEQGLGFDSVEDTW QDHR					Mascot
3875.0315	3874.905	-0.1265	-33	417	448	MVLLLSFNDLPYFLKSCF LYLSIFPEGHLIER			Carbamidomethyl (C)[17]		Mascot
3875.0315	3874.905	-0.1265	-33	417	448	MVLLLSFNDLPYFLKSCF LYLSIFPEGHLIER			Carbamidomethyl (C)[17]		Mascot

5	cytochrome c biogenesis C (mitochondrion) [Liriodendron tulipifera]	gi|484759969	26445.1	10.72	10	62	34.192	3.024	

Protein Group

cytochrome c biogenesis C (mitochondrion) [Liriodendron tulipifera]	gi|480541935	26445.1	10.720 000267 0288

Peptide Information

Calc. Mass	Obsrv. Mass	± da	± ppm	Start	End	Sequence	Ion	C. I. %	Modification	Rank	Result Type

				Seq.	Seq.		Score				
1036.6401	1036.5469	-0.0932	-90	156	165	AGPIDIPIIK					Mascot
1107.5518	1107.5734	0.0216	20	112	120	GRPMWGTFR					Mascot
1122.6418	1122.6133	-0.0285	-25	53	62	IPYVHVPAAR					Mascot
1338.6761	1338.707	0.0309	23	40	52	VAPPDLQQGGNSR					Mascot
1475.804	1475.7908	-0.0132	-9	27	39	LFLTAMAIHSSLR			Oxidation (M)[6]		Mascot
1633.7965	1633.845	0.0485	30	1	14	MSVSLLQPSFFMSK			Oxidation (M)[1,12]		Mascot
1743.9099	1743.9038	-0.0061	-3	2	16	SVSLLQPSFFMSKTR			Oxidation (M)[11]		Mascot
1890.9453	1891.0214	0.0761	40	1	16	MSVSLLQPSFFMSKTR			Oxidation (M)[1,12]		Mascot
1890.9453	1891.0214	0.0761	40	1	16	MSVSLLQPSFFMSKTR	6	0	Oxidation (M)[1,12]		Mascot
1952.9363	1953.0179	0.0816	42	166	182	SPVNWWNTSHQPGSISR					Mascot
2025.054	2025.0688	0.0148	7	216	233	LPIPSFPESPLTEEIEAR					Mascot
2025.054	2025.0688	0.0148	7	216	233	LPIPSFPESPLTEEIEAR					Mascot

6 hypothetical protein ARALYDRAFT_492917 [Arabidopsis lyrata subsp. lyrata] gi|297313772 93270.5 8.57 20 61 29.485 11.022

Peptide Information

Calc. Mass	Obsrv. Mass	± da	± ppm	Start Seq.	End Seq.	Sequence	Ion Score	C. I. %	Modification	Rank	Result Type
807.4029	807.4201	0.0172	21	311	316	EKMVER			Oxidation (M)[3]		Mascot
948.4381	948.4719	0.0338	36	755	763	DGNVTEASR					Mascot
1043.5632	1043.5565	-0.0067	-6	9	16	TSYLFTRR					Mascot
1066.4551	1066.5424	0.0873	82	303	310	YDEAFMFK			Oxidation (M)[6]		Mascot
1210.6943	1210.6221	-0.0722	-60	478	487	HSKAVELWLK					Mascot
1308.673	1308.6979	0.0249	19	522	532	EILGRGFVMDR			Oxidation (M)[9]		Mascot
1320.6477	1320.627	-0.0207	-16	732	742	EMHSKNVHPNK					Mascot
1323.5925	1323.6747	0.0822	62	303	312	YDEAFMFKEK			Oxidation (M)[6]		Mascot
1336.6427	1336.7051	0.0624	47	732	742	EMHSKNVHPNK			Oxidation (M)[2]		Mascot
1390.7723	1390.7231	-0.0492	-35	219	230	TTCNILLTSLVR			Carbamidomethyl (C)[3]		Mascot
1475.7775	1475.7908	0.0133	9	688	699	VEEAKLLLEEMR			Oxidation (M)[11]		Mascot
1743.8442	1743.9038	0.0596	34	755	769	DGNVTEASRLLHEMR			Oxidation (M)[14]		Mascot
1890.9341	1891.0214	0.0873	46	181	195	KMSDLLIEVYCTQFK			Carbamidomethyl (C)[11], Oxidation (M)[2]		Mascot
1890.9341	1891.0214	0.0873	46	181	195	KMSDLLIEVYCTQFK			Carbamidomethyl (C)[11], Oxidation (M)[2]		Mascot
2006.0264	2005.9977	-0.0287	-14	387	404	GLSLTSSTYNTLIKGYCK			Carbamidomethyl (C)[17]		Mascot
2034.059	2034.0125	-0.0465	-23	737	754	NVHPNKITYTVMIGGYAR					Mascot
2054.0774	2054.1418	0.0644	31	213	230	GMFPSKTTCNILLTSLVR			Carbamidomethyl (C)[9], Oxidation (M)[2]		Mascot
2064.1409	2064.2024	0.0615	30	313	330	MVERGVEPTLITYSILVK			Oxidation (M)[1]		Mascot

2278.0415	2278.2344	0.1929	85	527	546	GFVMDRVSYNTLISGCC GNK			Carbamidomethyl (C)[16,17]		Mascot
2344.2449	2344.1826	-0.0623	-27	72	90	QLITVLSPHEFDRLFPEF R					Mascot
3670.7273	3670.8557	0.1284	35	417	448	EMLSIGFNVNQGSFTSVI CLLCSHHMFDSALR			Carbamidomethyl (C)[19,22]		Mascot
3671.7068	3671.8105	0.1037	28	278	310	MEEAGVVPNVVTYNTVID GLGMSGRYDEAFMFK			Oxidation (M)[1,22]		Mascot
3671.7068	3671.8105	0.1037	28	278	310	MEEAGVVPNVVTYNTVID GLGMSGRYDEAFMFK			Oxidation (M)[1,22]		Mascot
3687.7017	3687.8135	0.1118	30	278	310	MEEAGVVPNVVTYNTVID GLGMSGRYDEAFMFK			Oxidation (M)[1,22,31]		Mascot

7 PREDICTED: putative GDP-L-fucose synthase 2-like [Setaria italica] gi|514765239 36505.9 8.61 12 61 27.843 1.875

Peptide Information

Calc. Mass	Obsrv. Mass	± da	± ppm	Start Seq.	End Seq.	Sequence	Ion Score	C. I. %	Modification	Rank	Result Type
834.3814	834.3493	-0.0321	-38	305	311	AMGWEPK			Oxidation (M)[2]		Mascot
847.4341	847.4603	0.0262	31	268	274	ELAEMVR					Mascot
1085.6215	1085.6261	0.0046	4	24	33	SAKVFLAGHR					Mascot
1338.7021	1338.707	0.0049	4	163	173	IVGIKMCQAYR			Carbamidomethyl (C)[7]		Mascot
1347.6573	1347.6825	0.0252	19	58	69	AELDLTCEAAVR			Carbamidomethyl (C)[7]		Mascot
1475.7523	1475.7908	0.0385	26	58	70	AELDLTCEAAVRK			Carbamidomethyl (C)[7]		Mascot
1517.7531	1517.7731	0.02	13	283	295	VVWDTSRPDGVMR					Mascot
1604.8062	1604.8911	0.0849	53	56	69	TRAELDLTCEAAVR			Carbamidomethyl (C)[9]		Mascot
2010.9702	2011.0259	0.0557	28	232	249	EFTHAHDAADAVVLLMD R					Mascot
2072.0229	2072.0522	0.0293	14	87	106	VGGLHASAAAPVDFMTE NLR			Oxidation (M)[15]		Mascot
2304.2361	2304.2551	0.019	8	210	231	FHHAKAAGAAEVVVWGS GLQLR					Mascot
2478.2009	2478.3159	0.115	46	2	26	PSHGTPPHATANDGTAS FLADKSAK					Mascot

8 Xyloglucan galactosyltransferase KATAMARI1-like protein [Triticum urartu] gi|474371812 65845.6 6.9 15 59 0 12.475

Peptide Information

Calc. Mass	Obsrv. Mass	± da	± ppm	Start Seq.	End Seq.	Sequence	Ion Score	C. I. %	Modification	Rank	Result Type
807.4148	807.4201	0.0053	7	112	117	IAWDFR					Mascot
906.5117	906.4875	-0.0242	-27	130	137	LLFMPAAK			Oxidation (M)[4]		Mascot
1201.5895	1201.6433	0.0538	45	94	103	RPEWNVMGGR					Mascot
1320.6041	1320.627	0.0229	17	71	80	YLWGYNTTMR			Oxidation (M)[9]		Mascot
1325.6995	1325.7107	0.0112	8	310	320	SIHPDVVKQMR			Oxidation (M)[10]		Mascot

Calc. Mass	Obsrv. Mass	± da	± ppm	Start Seq.	End Seq.	Sequence	Ion Score	C. I. %	Modification	Rank	Result Type
1325.6995	1325.7107	0.0112	8	310	320	SIHPDVVKQMR			Oxidation (M)[10]		Mascot
1351.6158	1351.7251	0.1093	81	509	521	CTSADELGQSGVK			Carbamidomethyl (C)[1]		Mascot
1633.8406	1633.845	0.0044	3	486	500	VPKASHVHLSDSSNR					Mascot
1688.8656	1688.8695	0.0039	2	104	117	DHFLVGGRIAWDFR					Mascot
1699.7891	1699.8739	0.0848	50	202	215	GQLIDQCRTSSFCK			Carbamidomethyl (C)[7,13]		Mascot
1873.8671	1874.0049	0.1378	74	210	225	TSSFCKLLECDLGESK			Carbamidomethyl (C)[5,10]		Mascot
2011.0437	2011.0259	-0.0178	-9	390	406	TIGPHEWDPFFSKPKPK					Mascot
2072.0554	2072.0522	-0.0032	-2	540	560	QSDGGVASKQGIQSVPI MGGR					Mascot
2181.0532	2181.1062	0.053	24	119	137	LTDEESDWGNKLLFMPA AK			Oxidation (M)[15]		Mascot
2321.0803	2321.1953	0.115	50	235	253	MFQSSLFCLQPQGDSYT RR			Carbamidomethyl (C)[8]		Mascot
3442.6389	3442.7466	0.1077	31	254	283	SAFDSMLAGCIPVFFHPG SAYVQYTWHLPK			Carbamidomethyl (C)[10], Oxidation (M)[6]		Mascot

9 hypothetical protein TRIUR3_19508 [Triticum urartu] gi|474413405 35530.8 9.03 14 59 0 2.156

Peptide Information

Calc. Mass	Obsrv. Mass	± da	± ppm	Start Seq.	End Seq.	Sequence	Ion Score	C. I. %	Modification	Rank	Result Type
804.4396	804.3704	-0.0692	-86	1	7	MNAALLR			Oxidation (M)[1]		Mascot
807.4319	807.4201	-0.0118	-15	148	155	STRSTAGK					Mascot
960.5261	960.5721	0.046	48	173	180	TWGQKNVK					Mascot
993.4611	993.5272	0.0661	67	262	268	IWWSNCK			Carbamidomethyl (C)[6]		Mascot
1193.6017	1193.6482	0.0465	39	178	187	NVKIDLMCGK			Carbamidomethyl (C)[8], Oxidation (M)[7]		Mascot
1259.7583	1259.6898	-0.0685	-54	270	279	SLQLQLRFVR					Mascot
1308.6902	1308.6979	0.0077	6	292	302	LMQMELAIISK			Oxidation (M)[2,4]		Mascot
1336.6791	1336.7051	0.026	19	140	150	GLTCWLSRSTR			Carbamidomethyl (C)[4]		Mascot
1338.7311	1338.707	-0.0241	-18	159	169	KPQHERAMTLK					Mascot
1407.7222	1407.7445	0.0223	16	292	303	LMQMELAIISKD			Oxidation (M)[2]		Mascot
1756.8977	1756.939	0.0413	24	2	16	NAALLRVAEDDLDWR					Mascot
1979.0526	1979.025	-0.0276	-14	123	139	IYKLMLPLLPCGNATMK			Carbamidomethyl (C)[11], Oxidation (M)[5]		Mascot
2011.0537	2011.0259	-0.0278	-14	74	90	LFLKQALNMEMAIVMAR			Oxidation (M)[9,11]		Mascot
2185.0046	2185.0598	0.0552	25	78	96	QALNMEMAIVMARESSA ER			Oxidation (M)[5,7,11]		Mascot

10 Cytochrome P450 71D7 [Triticum urartu] gi|473954864 84793.5 7.25 18 58 0 3.567

Peptide Information

Calc. Mass	Obsrv. Mass	± da	± ppm	Start Seq.	End Seq.	Sequence	Ion Score	C. I. %	Modification	Rank	Result Type
906.5295	906.4875	-0.042	-46	635	642	VLGYDVLK					Mascot

929.4938	929.4902	-0.0036	-4	268	276	GGEESILPK		Mascot
954.4648	954.484	0.0192	20	205	211	ACCKFLR	Carbamidomethyl (C)[2,3]	Mascot
1033.6154	1033.5406	-0.0748	-72	609	616	LVIRETFR		Mascot
1126.6038	1126.5538	-0.05	-44	65	76	KPVAGAAAGPCK	Carbamidomethyl (C)[11]	Mascot
1253.6848	1253.5927	-0.0921	-73	268	279	GGEESILPKAPR		Mascot
1259.6968	1259.6898	-0.007	-6	517	526	GIIHEHLERR		Mascot
1357.7257	1357.7451	0.0194	14	575	587	NPAAMVKATAEVR		Mascot
1363.7581	1363.7045	-0.0536	-39	441	453	ALLSALISDGTFR		Mascot
1373.7206	1373.7097	-0.0109	-8	575	587	NPAAMVKATAEVR	Oxidation (M)[5]	Mascot
1379.6954	1379.7577	0.0623	45	684	696	GTDFELLPFGAGR		Mascot
1475.8403	1475.7908	-0.0495	-34	601	612	LGELPYMRLVIR	Oxidation (M)[7]	Mascot
1487.6584	1487.7484	0.09	60	218	230	AVVSTNGFDDFCR	Carbamidomethyl (C)[12]	Mascot
2029.0502	2029.0277	-0.0225	-11	478	496	LATGLNTADLWPSSWLAGR		Mascot
2054.2485	2054.1418	-0.1067	-52	747	765	ANLLLRPSLRVPLPTSPPL		Mascot
2321.0359	2321.1953	0.1594	69	218	237	AVVSTNGFDDFCRSCPTLMK	Carbamidomethyl (C)[12,15], Oxidation (M)[19]	Mascot
2533.2942	2533.2239	-0.0703	-28	397	419	LTATMGVLTYGGRDMIFAPYAIR	Oxidation (M)[5]	Mascot
3525.6951	3525.7358	0.0407	12	543	574	IHKDGVIDMDIFSAGSETSATTLEWVMAELMK		Mascot
3874.8257	3874.905	0.0793	20	546	581	DGVIDMDIFSAGSETSATTLEWVMAELMKNPAAMVK	Oxidation (M)[6]	Mascot
3874.8257	3874.905	0.0793	20	546	581	DGVIDMDIFSAGSETSATTLEWVMAELMKNPAAMVK	Oxidation (M)[6]	Mascot

Gel Idx/Pos	194/H21	Instr./Gel Origin	BA2151/Sample Project 20140814	Process Status	Analysis Succeeded
Plate [#] Name	[1] Sample Project 20140814	Instrument Sample Name		Spectra	11

Rank	Protein Name	Accession No.	Protein MW	Protein PI	Pep. Count	Protein Score	Protein Score C. I. %	Intensity Matched	Total Ion Score	Total Ion C. I. %	Confirmed
1	Vicilin-like antimicrobial peptides 2-2 [Triticum urartu]	gi|473890163	75298.3	5.79	7	193	100	25.518	178	100	

Peptide Information

Calc. Mass	Obsrv. Mass	± da	± ppm	Start Seq.	End Seq.	Sequence	Ion Score	C. I. %	Modification	Rank	Result Type
1085.595	1085.6288	0.0338	31	31	42	AGAAVGGQVVEK					Mascot
1370.7386	1370.7841	0.0455	33	31	44	AGAAVGGQVVEKER					Mascot
1844.0389	1844.0031	-0.0358	-19	182	198	ILRQGFGVSAEVVEAIR					Mascot
1982.9666	1983.0311	0.0645	33	102	119	VTYIQEGGSETSSLEVQR					Mascot
1982.9666	1983.0311	0.0645	33	102	119	VTYIQEGGSETSSLEVQR	178	100			Mascot
2168.083	2168.1536	0.0706	33	100	119	GKVTYIQEGGSETSSLEV QR					Mascot
2351.0828	2351.2314	0.1486	63	219	238	SNWTEEIFDALWGDESP LNK					Mascot
3258.677	3258.8091	0.1321	41	71	99	LQFITMDPGALFLPVQLH ADAVFYVHSGR			Oxidation (M)[6]		Mascot

Rank	Protein Name	Accession No.	Protein MW	Protein PI	Pep. Count	Protein Score	Protein Score C. I. %	Intensity Matched	Total Ion Score	Total Ion C. I. %	Confirmed
2	RecName: Full=Triosephosphate isomerase, cytosolic; Short=TIM; Short=Triose-phosphate isomerase	gi|2507469	26948	5.39	8	99	99.987	4.893	60	99.879	

Peptide Information

Calc. Mass	Obsrv. Mass	± da	± ppm	Start Seq.	End Seq.	Sequence	Ion Score	C. I. %	Modification	Rank	Result Type
954.4832	954.5046	0.0214	22	5	12	FFVGGNWK					Mascot
1033.6041	1033.6332	0.0291	28	114	123	VAYALAQGLK					Mascot
1289.6332	1289.6774	0.0442	34	195	206	TNVSPEVAESTR					Mascot
1374.7046	1374.7511	0.0465	34	124	135	VIACVGETLEQR			Carbamidomethyl (C)[4]		Mascot
1604.8503	1604.9038	0.0535	33	176	190	VATPAQAQEVHANLR					Mascot
1604.8503	1604.9038	0.0535	33	176	190	VATPAQAQEVHANLR	60	99.879			Mascot
1811.9585	1812.0214	0.0629	35	56	70	LRPEIQVAAQNCWVK			Carbamidomethyl (C)[12]		Mascot
2011.0906	2011.0569	-0.0337	-17	54	70	AKLRPEIQVAAQNCWVK			Carbamidomethyl (C)[14]		Mascot
2835.3862	2835.3923	0.0061	2	124	149	VIACVGETLEQREAGSTM EVVAEQTK			Carbamidomethyl (C)[4]		Mascot

Rank	Protein Name	Accession No.	Protein MW	Protein PI	Pep. Count	Protein Score	Protein Score C. I. %	Intensity Matched	Total Ion Score	Total Ion C. I. %	Confirmed
3	uncharacterized protein, partial [Phleum pratense]	gi|409972141	25636.3	5.18	7	91	99.926	3.765	60	99.879	

Peptide Information

Calc. Mass	Obsrv. Mass	± da	± ppm	Start Seq.	End Seq.	Sequence	Ion Score	C. I. %	Modification	Rank	Result Type

Calc. Mass	Obsrv. Mass	± da	± ppm	Start Seq.	End Seq.	Sequence	Ion Score	C. I. %	Modification	Rank	Result Type
954.4832	954.5046	0.0214	22	11	18	FFVGGNWK					Mascot
1033.6041	1033.6332	0.0291	28	120	129	VAYALAQGLK					Mascot
1351.6741	1351.7256	0.0515	38	107	119	ALLGESSEFVGDK					Mascot
1374.7046	1374.7511	0.0465	34	130	141	VIACVGETLEQR			Carbamidomethyl (C)[4]		Mascot
1604.8503	1604.9038	0.0535	33	182	196	VATPAQAQEVHANLR					Mascot
1604.8503	1604.9038	0.0535	33	182	196	VATPAQAQEVHANLR	60	99.879			Mascot
1811.9585	1812.0214	0.0629	35	62	76	LRPEIQVAAQNCWVK			Carbamidomethyl (C)[12]		Mascot
1965.9797	1966.057	0.0773	39	142	160	EAGSTMTVVAEQTKAIADK			Oxidation (M)[6]		Mascot

4	uncharacterized protein, partial [Phleum pratense]	gi|409972323	22917.1	6.22	6	86	99.767	3.558	60	99.879	

Peptide Information

Calc. Mass	Obsrv. Mass	± da	± ppm	Start Seq.	End Seq.	Sequence	Ion Score	C. I. %	Modification	Rank	Result Type
1033.6041	1033.6332	0.0291	28	75	84	VAYALAQGLK					Mascot
1351.6741	1351.7256	0.0515	38	62	74	ALLGESSEFVGDK					Mascot
1374.7046	1374.7511	0.0465	34	85	96	VIACVGETLEQR			Carbamidomethyl (C)[4]		Mascot
1604.8503	1604.9038	0.0535	33	137	151	VATPAQAQEVHANLR					Mascot
1604.8503	1604.9038	0.0535	33	137	151	VATPAQAQEVHANLR	60	99.879			Mascot
1811.9585	1812.0214	0.0629	35	17	31	LRPEIQVAAQNCWVK			Carbamidomethyl (C)[12]		Mascot
1965.9797	1966.057	0.0773	39	97	115	EAGSTMTVVAEQTKAIADK			Oxidation (M)[6]		Mascot

5	uncharacterized protein, partial [Phleum pratense]	gi|409971759	16855.6	4.69	5	86	99.726	2.904	60	99.879	

Peptide Information

Calc. Mass	Obsrv. Mass	± da	± ppm	Start Seq.	End Seq.	Sequence	Ion Score	C. I. %	Modification	Rank	Result Type
1033.6041	1033.6332	0.0291	28	32	41	VAYALAQGLK					Mascot
1351.6741	1351.7256	0.0515	38	19	31	ALLGESSEFVGDK					Mascot
1374.7046	1374.7511	0.0465	34	42	53	VIACVGETLEQR			Carbamidomethyl (C)[4]		Mascot
1604.8503	1604.9038	0.0535	33	94	108	VATPAQAQEVHANLR					Mascot
1604.8503	1604.9038	0.0535	33	94	108	VATPAQAQEVHANLR	60	99.879			Mascot
1965.9797	1966.057	0.0773	39	54	72	EAGSTMTVVAEQTKAIADK			Oxidation (M)[6]		Mascot

6	uncharacterized protein, partial [Phleum pratense]	gi|409971969	14974.7	4.69	4	80	99.004	2.6	60	99.879	

Peptide Information

Calc. Mass	Obsrv. Mass	± da	± ppm	Start Seq.	End Seq.	Sequence	Ion Score	C. I. %	Modification	Rank	Result Type

Calc. Mass	Obsrv. Mass	± da	± ppm	Start Seq.	End Seq.	Sequence	Ion Score	C. I. %	Modification	Rank	Result Type
1033.6041	1033.6332	0.0291	28	4	13	VAYALAQGLK					Mascot
1374.7046	1374.7511	0.0465	34	14	25	VIACVGETLEQR			Carbamidomethyl (C)[4]		Mascot
1604.8503	1604.9038	0.0535	33	66	80	VATPAQAQEVHANLR					Mascot
1604.8503	1604.9038	0.0535	33	66	80	VATPAQAQEVHANLR	60	99.879			Mascot
1965.9797	1966.057	0.0773	39	26	44	EAGSTMTVVAEQTKAIAD K			Oxidation (M)[6]		Mascot

7 uncharacterized protein, partial [Phleum pratense] gi|409971967 12322.3 4.58 3 76 97.44 2.501 60 99.879

Peptide Information

Calc. Mass	Obsrv. Mass	± da	± ppm	Start Seq.	End Seq.	Sequence	Ion Score	C. I. %	Modification	Rank	Result Type
1374.7046	1374.7511	0.0465	34	9	20	VIACVGETLEQR			Carbamidomethyl (C)[4]		Mascot
1604.8503	1604.9038	0.0535	33	61	75	VATPAQAQEVHANLR					Mascot
1604.8503	1604.9038	0.0535	33	61	75	VATPAQAQEVHANLR	60	99.879			Mascot
1965.9797	1966.057	0.0773	39	21	39	EAGSTMTVVAEQTKAIAD K			Oxidation (M)[6]		Mascot

8 uncharacterized protein, partial [Phleum pratense] gi|409972453 13958.2 4.66 3 74 96.466 2.501 60 99.879

Peptide Information

Calc. Mass	Obsrv. Mass	± da	± ppm	Start Seq.	End Seq.	Sequence	Ion Score	C. I. %	Modification	Rank	Result Type
1374.7046	1374.7511	0.0465	34	4	15	VIACVGETLEQR			Carbamidomethyl (C)[4]		Mascot
1604.8503	1604.9038	0.0535	33	56	70	VATPAQAQEVHANLR					Mascot
1604.8503	1604.9038	0.0535	33	56	70	VATPAQAQEVHANLR	60	99.879			Mascot
1965.9797	1966.057	0.0773	39	16	34	EAGSTMTVVAEQTKAIAD K			Oxidation (M)[6]		Mascot

9 PREDICTED: putative ribonuclease H protein At1g65750-like [Solanum lycopersicum] gi|460398755 117874.7 9.32 18 62 32.659 31.661

Peptide Information

Calc. Mass	Obsrv. Mass	± da	± ppm	Start Seq.	End Seq.	Sequence	Ion Score	C. I. %	Modification	Rank	Result Type
1358.7777	1358.7045	-0.0732	-54	905	916	IILEVDSELLSK					Mascot
1688.7511	1688.8789	0.1278	76	2	16	NDNGEWIQGDDNIAK					Mascot
1688.7511	1688.8789	0.1278	76	2	16	NDNGEWIQGDDNIAK					Mascot
1719.8735	1719.8934	0.0199	12	928	941	CQPTIYQIQDIVNK			Carbamidomethyl (C)[1]		Mascot
1752.8989	1753.0168	0.1179	67	372	387	TSPINYLGCPLYIGGK			Carbamidomethyl (C)[9]		Mascot
1754.98	1754.9846	0.0046	3	702	717	GKDDILHILITGNFAK					Mascot
1819.7916	1819.9508	0.1592	87	1	16	MNDNGEWIQGDDNIAK					Mascot
1819.7916	1819.9508	0.1592	87	1	16	MNDNGEWIQGDDNIAK					Mascot

1827.8728	1828.0031	0.1303	71	65	83	VVMSMNPNSAPGPDGIG GK					Mascot
1835.7865	1835.9595	0.173	94	1	16	MNDNGEWIQGDDNIAK			Oxidation (M)[1]		Mascot
1843.8678	1844.0031	0.1353	73	65	83	VVMSMNPNSAPGPDGIG GK			Oxidation (M)[3]		Mascot
1938.928	1938.9795	0.0515	27	889	904	AALYGLSWCEQHGYKR			Carbamidomethyl (C)[9]		Mascot
1966.0757	1966.057	-0.0187	-10	132	148	DFRPISLSNFSNKIISK					Mascot
1997.0127	1997.0426	0.0299	15	444	461	NINKVIADFFWGSDSVGK					Mascot
2011.1223	2011.0569	-0.0654	-33	262	280	QGDPLSPALFILGVEVLS R					Mascot
2164.1406	2164.0935	-0.0471	-22	112	129	YMTHACLVLLPKIEHPNK			Carbamidomethyl (C)[6]		Mascot
2309.3228	2309.2227	-0.1001	-43	259	280	GLKQGDPLSPALFILGVE VLSR					Mascot
2353.1282	2353.2297	0.1015	43	463	483	YHWASLETMAYPISEGGI GVR			Oxidation (M)[9]		Mascot
2353.1282	2353.2297	0.1015	43	463	483	YHWASLETMAYPISEGGI GVR			Oxidation (M)[9]		Mascot
2442.4331	2442.228	-0.2051	-84	601	622	QLVPPLLVPNILDTVIQAK NEK					Mascot
2835.2732	2835.3923	0.1191	42	550	573	WNIHTGNCSFWWDNWI GDGAVATK			Carbamidomethyl (C)[8]		Mascot
3509.7266	3509.7595	0.0329	9	94	123	DDLLAAVQDFFNGEIMPR YMTHACLVLLPK			Carbamidomethyl (C)[24], Oxidation (M)[16,20]		Mascot
3509.7266	3509.7595	0.0329	9	94	123	DDLLAAVQDFFNGEIMPR YMTHACLVLLPK			Carbamidomethyl (C)[24], Oxidation (M)[16,20]		Mascot
3654.7085	3654.8074	0.0989	27	24	55	DMFSGSSLRVNEEILQCI PNMVTADQNDVLDK			Carbamidomethyl (C)[17], Oxidation (M)[2]		Mascot
3670.7034	3670.8213	0.1179	32	24	55	DMFSGSSLRVNEEILQCI PNMVTADQNDVLDK			Carbamidomethyl (C)[17], Oxidation (M)[2,21]		Mascot

10	ribosomal protein L2, partial (chloroplast) [Alocasia sp. ASPNZ01]	gi|387625115	7674.2	11.87	6	56	0	1.361

Protein Group

ribosomal protein L2, partial (chloroplast) [Remusatia vivipara]	gi|387625121	7674.2	11.869 999885 5591

Peptide Information

Calc. Mass	Obsrv. Mass	± da	± ppm	Start Seq.	End Seq.	Sequence	Ion Score	C. I. %	Modification	Rank	Result Type
1270.7202	1270.7268	0.0066	5	4	13	CWLGKRPVVR			Carbamidomethyl (C)[1]		Mascot
1471.7805	1471.7883	0.0078	5	37	49	RPTTPWGYPALGR					Mascot
1627.8816	1627.8811	-0.0005	0	37	50	RPTTPWGYPALGRR					Mascot
1730.8029	1730.8875	0.0846	49	14	30	GVVMNPVDHPHGGGEG R			Oxidation (M)[4]		Mascot
2225.0994	2225.1672	0.0678	30	14	35	GVVMNPVDHPHGGGEG RAPIGR			Oxidation (M)[4]		Mascot
2322.1997	2322.2332	0.0335	14	9	30	RPVVRGVVMNPVDHPH					Mascot

GGGEGR

Gel Idx/Pos	195/H22	Instr./Gel Origin	BA2151/Sample Project 20140814	Process Status	Analysis Succeeded
Plate [#] Name	[1] Sample Project 20140814	Instrument Sample Name		Spectra	11

Rank	Protein Name	Accession No.	Protein MW	Protein PI	Pep. Count	Protein Score	Protein Score C. I. %	Intensity Matched	Total Ion Score	Total Ion C. I. %	Confirmed
1	Glutathione S-transferase DHAR2 [Triticum urartu]	gi|474023258	45260	8.3	15	503	100	30.216	431	100	

Peptide Information

Calc. Mass	Obsrv. Mass	± da	± ppm	Start Seq.	End Seq.	Sequence	Ion Score	C. I. %	Modification	Rank	Result Type
909.4862	909.5072	0.021	23	78	84	KVPYQMK			Oxidation (M)[6]		Mascot
944.5662	944.5848	0.0186	20	70	77	VLLTLEEK					Mascot
951.4683	951.5133	0.045	47	339	346	AYDAAVWR					Mascot
1005.5	1005.5252	0.0252	25	104	113	VPVYNGGDGK					Mascot
1023.4589	1023.4998	0.0409	40	157	166	SKDASDGSEK					Mascot
1098.5942	1098.6393	0.0451	41	245	254	ENLIAGWAPK					Mascot
1128.5909	1128.6136	0.0227	20	2	10	GSWPQRELR					Mascot
1202.682	1202.7219	0.0399	33	147	156	IFSTFVTFLK					Mascot
1202.682	1202.7219	0.0399	33	147	156	IFSTFVTFLK	78	99.997			Mascot
1497.8213	1497.8668	0.0455	30	202	213	LYHLQVALEHFK					Mascot
1497.8213	1497.8668	0.0455	30	202	213	LYHLQVALEHFK	90	100			Mascot
1574.8578	1574.9065	0.0487	31	85	97	LIDVSNKPDWFLK					Mascot
1607.8639	1607.9105	0.0466	29	167	180	ALVDELQALEEHLK					Mascot
1827.8444	1827.9144	0.07	38	53	69	AAVGHPDTLGDCPFSQR			Carbamidomethyl (C)[12]		Mascot
1827.8444	1827.9144	0.07	38	53	69	AAVGHPDTLGDCPFSQR	137	100	Carbamidomethyl (C)[12]		Mascot
1866.9484	1866.9868	0.0384	21	129	146	YPTPSLVTPAEYASVGSK					Mascot
2021.0338	2021.1149	0.0811	40	217	234	VPETLTSVHAYTEALFSR					Mascot
2021.0338	2021.1149	0.0811	40	217	234	VPETLTSVHAYTEALFSR	126	100			Mascot
2108.1135	2108.1692	0.0557	26	181	201	AHGPYINGANISAVDLSL APK					Mascot

Rank	Protein Name	Accession No.	Protein MW	Protein PI	Pep. Count	Protein Score	Protein Score C. I. %	Intensity Matched	Total Ion Score	Total Ion C. I. %	Confirmed
2	hypothetical protein TRIUR3_28410 [Triticum urartu]	gi|474060617	28387.9	5.53	7	302	100	24.585	273	100	

Peptide Information

Calc. Mass	Obsrv. Mass	± da	± ppm	Start Seq.	End Seq.	Sequence	Ion Score	C. I. %	Modification	Rank	Result Type
930.4389	930.4752	0.0363	39	194	200	FEAYICK			Carbamidomethyl (C)[6]		Mascot
971.5673	971.6036	0.0363	37	51	58	FVTNHLLK					Mascot
971.5673	971.6036	0.0363	37	51	58	FVTNHLLK	34	10.733			Mascot

Calc. Mass	Obsrv. Mass	± da	± ppm	Start Seq.	End Seq.	Sequence	Ion Score	C. I. %	Modification	Rank	Result Type
1078.535	1078.5786	0.0436	40	207	215	GYPLLEACR			Carbamidomethyl (C)[8]		Mascot
1078.535	1078.5786	0.0436	40	207	215	GYPLLEACR	55	99.304	Carbamidomethyl (C)[8]		Mascot
1145.5698	1145.615	0.0452	39	156	165	GHNLSLEYGR					Mascot
1145.5698	1145.615	0.0452	39	156	165	GHNLSLEYGR	85	100			Mascot
1406.6271	1406.6857	0.0586	42	130	139	HREWESCFQK			Carbamidomethyl (C)[7]		Mascot
1796.9075	1796.9415	0.034	19	113	127	VHLGFIYCVSDLVMK			Carbamidomethyl (C)[8], Oxidation (M)[14]		Mascot
1927.9371	1928.0159	0.0788	41	35	50	VHVAIYYESLCPYSAR			Carbamidomethyl (C)[11]		Mascot
1927.9371	1928.0159	0.0788	41	35	50	VHVAIYYESLCPYSAR	99	100	Carbamidomethyl (C)[11]		Mascot

3	Os05g0116100 [Oryza sativa Japonica Group]	gi|113578021	23726.3	5.81	6	255	100	19.257	227	100	

Peptide Information

Calc. Mass	Obsrv. Mass	± da	± ppm	Start Seq.	End Seq.	Sequence	Ion Score	C. I. %	Modification	Rank	Result Type
944.5662	944.5848	0.0186	20	26	33	VLLTLEEK					Mascot
1405.7587	1405.7078	-0.0509	-36	201	213	EHLIAGWAPKVNA					Mascot
1497.8213	1497.8668	0.0455	30	158	169	LYHLQVALEHFK					Mascot
1497.8213	1497.8668	0.0455	30	158	169	LYHLQVALEHFK	90	100			Mascot
1607.9003	1607.9105	0.0102	6	123	136	ALLTELQALEEHLK					Mascot
1827.8444	1827.9144	0.07	38	9	25	AAVGHPDTLGDCPFSQR			Carbamidomethyl (C)[12]		Mascot
1827.8444	1827.9144	0.07	38	9	25	AAVGHPDTLGDCPFSQR	137	100	Carbamidomethyl (C)[12]		Mascot
1892.9641	1893.01	0.0459	24	85	102	YPTPSLVTPPEYASVGSK					Mascot

4	hypothetical protein OsI_18213 [Oryza sativa Indica Group]	gi|218195985	17371.9	5.56	5	164	100	14.733	137	100	

Peptide Information

Calc. Mass	Obsrv. Mass	± da	± ppm	Start Seq.	End Seq.	Sequence	Ion Score	C. I. %	Modification	Rank	Result Type
944.5662	944.5848	0.0186	20	26	33	VLLTLEEK					Mascot
1490.8076	1490.8031	-0.0045	-3	103	114	IFSCFITFLKSK			Carbamidomethyl (C)[4]		Mascot
1607.9003	1607.9105	0.0102	6	123	136	ALLTELQALEEHLK					Mascot
1827.8444	1827.9144	0.07	38	9	25	AAVGHPDTLGDCPFSQR			Carbamidomethyl (C)[12]		Mascot
1827.8444	1827.9144	0.07	38	9	25	AAVGHPDTLGDCPFSQR	137	100	Carbamidomethyl (C)[12]		Mascot
1892.9641	1893.01	0.0459	24	85	102	YPTPSLVTPPEYASVGSK					Mascot

5	TIR-NBS-LRR type disease resistance protein [Glycine max]	gi|351723791	48093.9	4.52	15	66	74.398	10.345			

Peptide Information

Calc. Mass	Obsrv. Mass	± da	± ppm	Start Seq.	End Seq.	Sequence	Ion Score	C. I. %	Modification	Rank	Result Type

Calc. Mass	Obsrv. Mass	± da	± ppm	Start Seq.	End Seq.	Sequence	Ion Score	C. I. %	Modification	Rank	Result Type
807.4723	807.4291	-0.0432	-54	305	310	YIVREK					Mascot
849.4498	849.457	0.0072	8	228	234	SIMDVLR			Oxidation (M)[3]		Mascot
971.5632	971.6036	0.0404	42	142	149	DQQILKAR					Mascot
971.5632	971.6036	0.0404	42	142	149	DQQILKAR					Mascot
1088.6133	1088.5938	-0.0195	-18	20	31	VVGITGMGGIGK					Mascot
1094.5411	1094.5826	0.0415	38	114	122	QLNMFTANR					Mascot
1130.6052	1130.6245	0.0193	17	326	335	DLNIVSLDNK					Mascot
1130.6052	1130.6245	0.0193	17	326	335	DLNIVSLDNK					Mascot
1165.6324	1165.6284	-0.004	-3	32	41	STLGRALYER					Mascot
1182.546	1182.5983	0.0523	44	1	10	MESHFSTLSK			Oxidation (M)[1]		Mascot
1193.6195	1193.6403	0.0208	17	225	234	ESKSIMDVLR			Oxidation (M)[6]		Mascot
1238.631	1238.636	0.005	4	114	123	QLNMFTANRK			Oxidation (M)[4]		Mascot
1320.7271	1320.6687	-0.0584	-44	58	69	LYRLEGSAGVQK					Mascot
1578.8163	1578.8715	0.0552	35	271	284	GFNPEYGLQVLVDK					Mascot
1602.8997	1602.8824	-0.0173	-11	20	36	VVGITGMGGIGKSTLGR					Mascot
1607.7523	1607.9105	0.1582	98	172	184	NAFKNNYIMSDFK			Oxidation (M)[9]		Mascot
1796.9177	1796.9415	0.0238	13	212	227	DVSYWGSALVSLRESK					Mascot

6 Os08g0260800 [Oryza sativa Japonica Group] gi|255678300 99819.5 8.63 23 65 67.769 28.172

Peptide Information

Calc. Mass	Obsrv. Mass	± da	± ppm	Start Seq.	End Seq.	Sequence	Ion Score	C. I. %	Modification	Rank	Result Type
807.4359	807.4291	-0.0068	-8	708	713	NLYELR					Mascot
849.4689	849.457	-0.0119	-14	447	453	NSFGRIR					Mascot
906.4825	906.4809	-0.0016	-2	845	852	TITGRACK			Carbamidomethyl (C)[7]		Mascot
930.5367	930.4752	-0.0615	-66	599	607	GLGNLTSLR					Mascot
1088.6462	1088.5938	-0.0524	-48	2	11	AEAVILFAVR					Mascot
1097.679	1097.6506	-0.0284	-26	241	250	QGVLRNLIGK					Mascot
1126.6984	1126.6205	-0.0779	-69	274	282	LHLFLVEKK					Mascot
1145.5409	1145.615	0.0741	65	853	862	NQTAFLHMAP			Oxidation (M)[8]		Mascot
1145.5409	1145.615	0.0741	65	853	862	NQTAFLHMAP			Oxidation (M)[8]		Mascot
1152.5201	1152.606	0.0859	75	352	361	SDTDDKCLAK			Carbamidomethyl (C)[7]		Mascot
1169.5303	1169.564	0.0337	29	587	595	NFRCCTGVR			Carbamidomethyl (C)[4,5]		Mascot
1233.666	1233.6422	-0.0238	-19	770	779	WLVVQKDAMK			Oxidation (M)[9]		Mascot
1315.6654	1315.662	-0.0034	-3	580	589	LHDATWRNFR					Mascot
1347.7817	1347.6643	-0.1174	-87	1	12	MAEAVILFAVRK					Mascot

Calc. Mass	Obsrv. Mass	± da	± ppm	Start Seq.	End Seq.	Sequence	Ion Score	C. I. %	Modification	Rank	Result Type
1467.7108	1467.7849	0.0741	50	837	849	DSETSCIKTITGR			Carbamidomethyl (C)[6]		Mascot
1497.806	1497.8668	0.0608	41	129	141	EAEVNLVHLFQAK					Mascot
1497.806	1497.8668	0.0608	41	129	141	EAEVNLVHLFQAK					Mascot
1519.7509	1519.8446	0.0937	62	49	59	ELRLMHEFLCR			Carbamidomethyl (C)[10], Oxidation (M)[5]		Mascot
1582.7894	1582.8146	0.0252	16	32	45	FVTQLTELQGSMGR			Oxidation (M)[12]		Mascot
1606.7651	1606.9036	0.1385	86	52	63	LMHEFLCRMDVR			Carbamidomethyl (C)[7]		Mascot
1607.905	1607.9105	0.0055	3	434	446	ELVHRNMLQLVQK					Mascot
1768.9341	1768.9301	-0.004	-2	129	143	EAEVNLVHLFQAKDR					Mascot
1833.8086	1833.9481	0.1395	76	471	485	RECFGITYEDGHHGR			Carbamidomethyl (C)[3]		Mascot
1851.9746	1851.8506	-0.124	-67	32	47	FVTQLTELQGSMGRIR			Oxidation (M)[12]		Mascot
2109.2068	2109.1589	-0.0479	-23	123	141	IASLVKEAEVNLVHLFQAK					Mascot
2109.2068	2109.1589	-0.0479	-23	123	141	IASLVKEAEVNLVHLFQAK					Mascot

7	PREDICTED: glutathione S-transferase DHAR2-like [Fragaria vesca subsp. vesca]	gi|470143488	23824.6	6.67	4	64	64.659	2.57	50	97.7	

Peptide Information

Calc. Mass	Obsrv. Mass	± da	± ppm	Start Seq.	End Seq.	Sequence	Ion Score	C. I. %	Modification	Rank	Result Type
848.4546	848.496	0.0414	49	1	8	MALEVAAK			Oxidation (M)[1]		Mascot
944.5662	944.5848	0.0186	20	26	33	VLLTLEEK					Mascot
1590.8163	1590.8862	0.0699	44	182	194	YTELLFSRESFAK					Mascot
2109.1338	2109.1589	0.0251	12	136	156	AHGPYIAGEKVTAADLSLAPK					Mascot
2109.1338	2109.1589	0.0251	12	136	156	AHGPYIAGEKVTAADLSLAPK	50	97.7			Mascot

8	dehydroascorbate reductase [Rosa roxburghii]	gi|514996321	23765.6	6.31	4	64	64.659	6.759	50	97.7	

Peptide Information

Calc. Mass	Obsrv. Mass	± da	± ppm	Start Seq.	End Seq.	Sequence	Ion Score	C. I. %	Modification	Rank	Result Type
848.4546	848.496	0.0414	49	1	8	MALEVAAK			Oxidation (M)[1]		Mascot
944.5662	944.5848	0.0186	20	26	33	VLLTLEEK					Mascot
1497.7485	1497.8668	0.1183	79	170	181	WTVPESLTHYHK					Mascot
1497.7485	1497.8668	0.1183	79	170	181	WTVPESLTHYHK					Mascot
2109.1338	2109.1589	0.0251	12	136	156	AHGPYIAGEKVTAADLSLAPK					Mascot
2109.1338	2109.1589	0.0251	12	136	156	AHGPYIAGEKVTAADLSLAPK	50	97.7			Mascot

9	hypothetical protein F775_31970 [Aegilops tauschii]	gi|475578125	25615.9	6.35	3	64	58.478	3.731	55	99.304	

Peptide Information

Calc. Mass	Obsrv. Mass	± da	± ppm	Start Seq.	End Seq.	Sequence	Ion Score	C. I. %	Modification	Rank	Result Type
1078.535	1078.5786	0.0436	40	207	215	GYPLLEACR			Carbamidomethyl (C)[8]		Mascot
1078.535	1078.5786	0.0436	40	207	215	GYPLLEACR	55	99.304	Carbamidomethyl (C)[8]		Mascot
1331.7583	1331.6748	-0.0835	-63	54	64	FVANHLLKAYR					Mascot
1796.9075	1796.9415	0.034	19	116	130	VHLGFIYCVSDLVMK			Carbamidomethyl (C)[8], Oxidation (M)[14]		Mascot

10 predicted protein [Bathycoccus prasinos] gi|412993083 235807 5.33 33 63 46.509 28.107

Peptide Information

Calc. Mass	Obsrv. Mass	± da	± ppm	Start Seq.	End Seq.	Sequence	Ion Score	C. I. %	Modification	Rank	Result Type
832.4159	832.3475	-0.0684	-82	824	830	ESDNIVR					Mascot
866.4296	866.447	0.0174	20	122	129	AAMLMAMK					Mascot
870.5771	870.5741	-0.003	-3	2109	2116	KPGKISLK					Mascot
929.5778	929.5046	-0.0732	-79	1048	1055	RLIEALSK					Mascot
1023.4961	1023.4998	0.0037	4	554	562	LCMLNASAK			Carbamidomethyl (C)[2], Oxidation (M)[3]		Mascot
1034.5518	1034.5588	0.007	7	1926	1933	FEEILVER					Mascot
1078.5792	1078.5786	-0.0006	-1	906	914	HVSLFNHPK					Mascot
1078.5792	1078.5786	-0.0006	-1	906	914	HVSLFNHPK					Mascot
1088.6462	1088.5938	-0.0524	-48	915	923	KPVLTDFLR					Mascot
1098.6993	1098.6393	-0.06	-55	53	63	VATVALKVAAR					Mascot
1102.658	1102.6025	-0.0555	-50	203	213	KIGTTASVGIR					Mascot
1112.5371	1112.6166	0.0795	71	296	305	YATNPQFSGK					Mascot
1114.6466	1114.6252	-0.0214	-19	171	180	NVLNLIAETK					Mascot
1145.6287	1145.615	-0.0137	-12	1402	1411	ANNHPRLPAR					Mascot
1145.6287	1145.615	-0.0137	-12	1402	1411	ANNHPRLPAR					Mascot
1165.6324	1165.6284	-0.004	-3	1056	1065	ISLNIDPTHR					Mascot
1169.6057	1169.564	-0.0417	-36	1475	1484	GLQMMSFLVK			Oxidation (M)[4]		Mascot
1173.6085	1173.6523	0.0438	37	1579	1588	LHACIVDFAK			Carbamidomethyl (C)[4]		Mascot
1228.6355	1228.5991	-0.0364	-30	1295	1305	SSSKFGSMLLR			Oxidation (M)[8]		Mascot
1233.5681	1233.6422	0.0741	60	477	486	FPLTMHNSDR			Oxidation (M)[5]		Mascot
1238.5577	1238.636	0.0783	63	1683	1692	QMLDNMIMDK					Mascot
1278.7239	1278.6633	-0.0606	-47	1299	1309	FGSMLLRDIVK					Mascot
1320.6543	1320.6687	0.0144	11	1725	1735	VSDTQQWSTLR					Mascot
1380.749	1380.7648	0.0158	11	1473	1484	ARGLQMMSFLVK					Mascot
1412.7389	1412.7742	0.0353	25	1473	1484	ARGLQMMSFLVK			Oxidation (M)[6,7]		Mascot

1422.6902	1422.6721	-0.0181	-13	658	669	LMLGSLLCACER	Carbamidomethyl (C)[8,10]	Mascot
1438.6852	1438.6798	-0.0054	-4	658	669	LMLGSLLCACER	Carbamidomethyl (C)[8,10], Oxidation (M)[2]	Mascot
1467.7325	1467.7849	0.0524	36	764	776	LTSLSSFLDEAER		Mascot
1490.7744	1490.8031	0.0287	19	2051	2064	MALDASRLSNAAVR	Oxidation (M)[1]	Mascot
1613.7667	1613.8188	0.0521	32	1441	1455	ASDSPNAYLPTHGQR		Mascot
1768.7914	1768.9301	0.1387	78	1678	1692	EGSEKQMLDNMIMDK		Mascot
1783.9232	1783.8997	-0.0235	-13	1146	1161	AMNVAAIIRDVQSHSR	Oxidation (M)[2]	Mascot
1836.9459	1837.0265	0.0806	44	689	704	LLLHLRAMSAYASCSK	Carbamidomethyl (C)[14], Oxidation (M)[8]	Mascot
1892.9825	1893.01	0.0275	15	1559	1577	LAAHALSAKVHGSDDSV SK		Mascot
1902.9742	1903.0134	0.0392	21	1456	1472	CFQIAYSLSTASASKLR	Carbamidomethyl (C)[1]	Mascot
1980.967	1981.1085	0.1415	71	471	486	LEMFVKFPLTMHNSDR	Oxidation (M)[3]	Mascot
2108.0659	2108.1692	0.1033	49	831	849	KDVFPFAATVVDQIDEAS R		Mascot

Gel Idx/Pos	196/H23	Instr./Gel Origin	BA2151/Sample Project 20140814	Process Status	Analysis Succeeded
Plate [#] Name	[1] Sample Project 20140814	Instrument Sample Name		Spectra	11

Rank	Protein Name	Accession No.	Protein MW	Protein PI	Pep. Count	Protein Score	Protein Score C. I. %	Intensity Matched	Total Ion Score	Total Ion C. I. %	Confirmed
1	hypothetical protein TRIUR3_31593 [Triticum urartu]	gi|473979984	19793.1	5.63	13	289	100	13.744	220	100	

Peptide Information

Calc. Mass	Obsrv. Mass	± da	± ppm	Start Seq.	End Seq.	Sequence	Ion Score	C. I. %	Modification	Rank	Result Type
817.4162	817.4504	0.0342	42	79	84	REVEER					Mascot
888.4421	888.4931	0.051	57	63	69	VEIEENR					Mascot
926.5669	926.6085	0.0416	45	129	136	KLAPEQIK					Mascot
935.4595	935.5031	0.0436	47	85	91	KGDHWHR					Mascot
939.4617	939.5032	0.0415	44	99	104	FWRQMR			Oxidation (M)[5]		Mascot
943.4785	943.5114	0.0329	35	95	101	SYGKFWR					Mascot
1108.6473	1108.7036	0.0563	51	130	139	LAPEQIKGPR					Mascot
1329.6951	1329.762	0.0669	50	17	27	ILEHVPFGFDR					Mascot
1373.6907	1373.7559	0.0652	47	59	69	EDLKVEIEENR					Mascot
1770.8328	1770.8926	0.0598	34	42	57	ETSDSHEIVVDVPGMR					Mascot
2002.9626	2003.1459	0.1833	92	1	16	MEHWRMDVALLADPFR			Oxidation (M)[1]		Mascot
2226.1614	2226.271	0.1096	49	105	126	LPDNADLDSIAASLDAGV LTVR					Mascot
2226.1614	2226.271	0.1096	49	105	126	LPDNADLDSIAASLDAGV LTVR	220	100			Mascot
2421.1689	2421.2732	0.1043	43	17	37	ILEHVPFGFDRDDVAMVS MAR			Oxidation (M)[16]		Mascot
2437.1638	2437.2385	0.0747	31	17	37	ILEHVPFGFDRDDVAMVS MAR			Oxidation (M)[16,19]		Mascot

Rank	Protein Name	Accession No.	Protein MW	Protein PI	Pep. Count	Protein Score	Protein Score C. I. %	Intensity Matched	Total Ion Score	Total Ion C. I. %	Confirmed
2	hypothetical protein F775_30396 [Aegilops tauschii]	gi|475584601	13170.8	8.01	8	262	100	10.841	220	100	

Peptide Information

Calc. Mass	Obsrv. Mass	± da	± ppm	Start Seq.	End Seq.	Sequence	Ion Score	C. I. %	Modification	Rank	Result Type
926.5669	926.6085	0.0416	45	74	81	KLAPEQIK					Mascot
935.4595	935.5031	0.0436	47	30	36	KGDHWHR					Mascot
939.4617	939.5032	0.0415	44	44	49	FWRQMR			Oxidation (M)[5]		Mascot
943.4785	943.5114	0.0329	35	40	46	SYGKFWR					Mascot
1108.6473	1108.7036	0.0563	51	75	84	LAPEQIKGPR					Mascot
1231.5913	1231.6536	0.0623	51	100	112	TIGDVGAAGGEER					Mascot

1359.6863	1359.7513	0.065	48	99	112	KTIGDVGAAGGEER					Mascot
2226.1614	2226.271	0.1096	49	50	71	LPDNADLDSIAASLDAGV LTVR					Mascot
2226.1614	2226.271	0.1096	49	50	71	LPDNADLDSIAASLDAGV LTVR	220	100			Mascot

3 Proteasome subunit beta type-6 [Triticum urartu] gi|474432171 24640.6 6.06 9 209 100 9.35 161 100

Peptide Information

Calc. Mass	Obsrv. Mass	± da	± ppm	Start Seq.	End Seq.	Sequence	Ion Score	C. I. %	Modification	Rank	Result Type
817.4163	817.4504	0.0341	42	144	152	DGASGGVVR					Mascot
828.5302	828.579	0.0488	59	136	143	VVSLAIAR					Mascot
849.4828	849.4609	-0.0219	-26	62	68	LLAYQNK					Mascot
943.4744	943.5114	0.037	39	163	170	RSFHPGDK					Mascot
1245.6798	1245.7362	0.0564	45	153	163	TVTINEEGVKR					Mascot
1467.7261	1467.7992	0.0731	50	11	22	ISQLTDNVYVCR			Carbamidomethyl (C)[11]		Mascot
1467.7261	1467.7992	0.0731	50	11	22	ISQLTDNVYVCR	43	92.653	Carbamidomethyl (C)[11]		Mascot
1582.7708	1582.8483	0.0775	49	23	37	SGSAADTQIISDYVR					Mascot
1822.9698	1823.0623	0.0925	51	83	99	YEGGQIYSVPLGGTILR					Mascot
1822.9698	1823.0623	0.0925	51	83	99	YEGGQIYSVPLGGTILR	118	100			Mascot
1981.0654	1981.1412	0.0758	38	38	54	YFLHQHTIQLGQPATVK					Mascot

4 Proteasome subunit beta type-6 [Aegilops tauschii] gi|475537960 25801 6.2 8 199 100 9.452 161 100

Peptide Information

Calc. Mass	Obsrv. Mass	± da	± ppm	Start Seq.	End Seq.	Sequence	Ion Score	C. I. %	Modification	Rank	Result Type
817.4163	817.4504	0.0341	42	183	191	DGASGGVVR					Mascot
828.5302	828.579	0.0488	59	175	182	VVSLAIAR					Mascot
849.4828	849.4609	-0.0219	-26	101	107	LLAYQNK					Mascot
943.4744	943.5114	0.037	39	202	209	RSFHPGDK					Mascot
1467.7261	1467.7992	0.0731	50	50	61	ISQLTDNVYVCR			Carbamidomethyl (C)[11]		Mascot
1467.7261	1467.7992	0.0731	50	50	61	ISQLTDNVYVCR	43	92.653	Carbamidomethyl (C)[11]		Mascot
1568.7551	1568.8354	0.0803	51	62	76	SGSAADTQVISDYVR					Mascot
1822.9698	1823.0623	0.0925	51	122	138	YEGGQIYSVPLGGTILR					Mascot
1822.9698	1823.0623	0.0925	51	122	138	YEGGQIYSVPLGGTILR	118	100			Mascot
1981.0654	1981.1412	0.0758	38	77	93	YFLHQHTIQLGQPATVK					Mascot

5 Proteasome subunit beta type-6 [Triticum urartu] gi|473930301 26187 5.32 8 199 100 9.11 161 100

Peptide Information

Calc. Mass	Obsrv. Mass	± da	± ppm	Start Seq.	End Seq.	Sequence	Ion Score	C. I. %	Modification	Rank	Result Type
817.4163	817.4504	0.0341	42	190	198	DGASGGVVR					Mascot
828.5302	828.579	0.0488	59	182	189	VVSLAIAR					Mascot
849.4828	849.4609	-0.0219	-26	108	114	LLAYQNK					Mascot
943.4744	943.5114	0.037	39	209	216	RSFHPGDK					Mascot
1467.7261	1467.7992	0.0731	50	57	68	ISQLTDNVYVCR			Carbamidomethyl (C)[11]		Mascot
1467.7261	1467.7992	0.0731	50	57	68	ISQLTDNVYVCR	43	92.653	Carbamidomethyl (C)[11]		Mascot
1582.7708	1582.8483	0.0775	49	69	83	SGSAADTQIISDYVR					Mascot
1822.9698	1823.0623	0.0925	51	129	145	YEGGQIYSVPLGGTILR					Mascot
1822.9698	1823.0623	0.0925	51	129	145	YEGGQIYSVPLGGTILR	118	100			Mascot
1981.0654	1981.1412	0.0758	38	84	100	YFLHQHTIQLGQPATVK					Mascot

6	Proteasome subunit beta type-6 [Aegilops tauschii]	gi|475510325	26720.2	5.33	8	198	100	9.452	161	100	

Peptide Information

Calc. Mass	Obsrv. Mass	± da	± ppm	Start Seq.	End Seq.	Sequence	Ion Score	C. I. %	Modification	Rank	Result Type
817.4163	817.4504	0.0341	42	192	200	DGASGGVVR					Mascot
828.5302	828.579	0.0488	59	184	191	VVSLAIAR					Mascot
849.4828	849.4609	-0.0219	-26	110	116	LLAYQNK					Mascot
943.4744	943.5114	0.037	39	215	222	RSFHPGDK					Mascot
1467.7261	1467.7992	0.0731	50	59	70	ISQLTDNVYVCR			Carbamidomethyl (C)[11]		Mascot
1467.7261	1467.7992	0.0731	50	59	70	ISQLTDNVYVCR	43	92.653	Carbamidomethyl (C)[11]		Mascot
1568.7551	1568.8354	0.0803	51	71	85	SGSAADTQVISDYVR					Mascot
1822.9698	1823.0623	0.0925	51	131	147	YEGGQIYSVPLGGTILR					Mascot
1822.9698	1823.0623	0.0925	51	131	147	YEGGQIYSVPLGGTILR	118	100			Mascot
1981.0654	1981.1412	0.0758	38	86	102	YFLHQHTIQLGQPATVK					Mascot

7	PREDICTED: uncharacterized protein LOC101308150 [Fragaria vesca subsp. vesca]	gi|470149402	36700.7	6.66	13	71	91.715	7.946	

Peptide Information

Calc. Mass	Obsrv. Mass	± da	± ppm	Start Seq.	End Seq.	Sequence	Ion Score	C. I. %	Modification	Rank	Result Type
820.4675	820.467	-0.0005	-1	18	24	QFALVSR					Mascot
948.5625	948.5124	-0.0501	-53	18	25	QFALVSRK					Mascot
971.5771	971.5494	-0.0277	-29	137	144	ELENLILK					Mascot
1236.71	1236.802	0.092	74	235	244	LSRFPQVYVK					Mascot

Calc. Mass	Obsrv. Mass	± da	± ppm	Start Seq.	End Seq.	Sequence	Ion Score	C. I. %	Modification	Rank	Result Type
1245.6838	1245.7362	0.0524	42	104	114	FDHSLVTSVLK					Mascot
1336.6685	1336.7333	0.0648	48	152	162	FNPYLWPSGQK					Mascot
1336.6685	1336.7333	0.0648	48	152	162	FNPYLWPSGQK					Mascot
1358.6443	1358.731	0.0867	64	163	174	MTNEVGKAMFSK			Oxidation (M)[1]		Mascot
1373.7788	1373.7559	-0.0229	-17	104	115	FDHSLVTSVLKK					Mascot
1467.6793	1467.7992	0.1199	82	175	188	AGELGAPVGFMCMK			Carbamidomethyl (C)[12]		Mascot
1467.6793	1467.7992	0.1199	82	175	188	AGELGAPVGFMCMK			Carbamidomethyl (C)[12]		Mascot
1794.8149	1794.8594	0.0445	25	120	136	FVGCCLANPAEDGTGVK			Carbamidomethyl (C)[4,5]		Mascot
2002.9982	2003.1459	0.1477	74	47	64	VIDSHLHVWASPQEAAD K					Mascot
2248.0566	2248.2319	0.1753	78	277	296	VMWGSDFPFVVAECGYK GAK			Carbamidomethyl (C)[14]		Mascot
3234.6829	3234.6953	0.0124	4	85	114	SMEEAGVDGALIVQPINH KFDHSLVTSVLK					Mascot

8 hypothetical protein TRIUR3_02653 [Triticum urartu] gi|473895929 88455.4 5.43 23 62 37.154 18.749

Peptide Information

Calc. Mass	Obsrv. Mass	± da	± ppm	Start Seq.	End Seq.	Sequence	Ion Score	C. I. %	Modification	Rank	Result Type
805.4124	805.4517	0.0393	49	648	654	VMEAVEK					Mascot
828.5302	828.579	0.0488	59	232	238	RIIEGLK					Mascot
830.4618	830.5016	0.0398	48	12	18	VEELNVK					Mascot
832.441	832.3593	-0.0817	-98	483	489	ADLEKEK					Mascot
888.4785	888.4931	0.0146	16	690	697	VITAQDNK					Mascot
906.4349	906.4817	0.0468	52	455	461	KELDDMR					Mascot
935.4985	935.5031	0.0046	5	182	189	FGGILDWK					Mascot
963.4564	963.5076	0.0512	53	552	559	LACDEVTR			Carbamidomethyl (C)[3]		Mascot
1120.5997	1120.6395	0.0398	36	212	220	RFIAEEVEK					Mascot
1179.6256	1179.6649	0.0393	33	213	222	FIAEEVEKSK					Mascot
1196.6127	1196.6122	-0.0005	0	440	449	VRSMVSVQMK			Oxidation (M)[4,9]		Mascot
1228.6	1228.6025	0.0025	2	776	785	SIAMMFMNRK					Mascot
1253.6593	1253.6095	-0.0498	-40	442	452	SMVSVQMKLAK			Oxidation (M)[2,7]		Mascot
1259.6478	1259.6926	0.0448	36	755	767	ADTIATTAAAEPK					Mascot
1320.6941	1320.7295	0.0354	27	221	231	SKVLQELCSTR			Carbamidomethyl (C)[8]		Mascot
1347.705	1347.7017	-0.0033	-2	516	526	TMRELSVVQER					Mascot
1358.7349	1358.731	-0.0039	-3	527	538	TEAAKMPIELQK					Mascot
1582.7681	1582.8483	0.0802	51	132	144	EQQQISRHNSAER					Mascot
1754.8014	1754.92	0.1186	68	102	116	NFSEDIGSLTINECR			Carbamidomethyl (C)[14]		Mascot
1770.8076	1770.8926	0.085	48	117	131	ANKVEENCHDQLEGK			Carbamidomethyl (C)[8]		Mascot

1927.9971	1928.0287	0.0316	16	2	18	EELNVEGAQKVEELNVK					Mascot
2012.9304	2013.1245	0.1941	96	618	635	GSDKSMMVPLEDYDALN K					Mascot
2012.9304	2013.1245	0.1941	96	618	635	GSDKSMMVPLEDYDALN K					Mascot
2035.0568	2035.0688	0.012	6	497	514	DKVHHATVSVSSLQEEL R					Mascot

9	Mitochondrial transcription termination factor family protein, putative isoform 1 [Theobroma cacao]	gi|508712975	51008.1	9.32	14	61	26.162	4.508	

Protein Group

Mitochondrial transcription termination factor family protein, putative isoform 1 [Theobroma cacao]	gi|508712974	51008.1	9.3199 996948 2422
Mitochondrial transcription termination factor family protein, putative isoform 1 [Theobroma cacao]	gi|508712973	51008.1	9.3199 996948 2422
Mitochondrial transcription termination factor family protein, putative isoform 1 [Theobroma cacao]	gi|508712976	51008.1	9.3199 996948 2422

Peptide Information

Calc. Mass	Obsrv. Mass	± da	± ppm	Start Seq.	End Seq.	Sequence	Ion Score	C. I. %	Modification	Rank	Result Type
817.4567	817.4504	-0.0063	-8	161	167	VLEGFPR					Mascot
849.4611	849.4609	-0.0002	0	9	15	ATTLKCR			Carbamidomethyl (C)[6]		Mascot
866.4651	866.4438	-0.0213	-25	1	8	MAVTSLTK			Oxidation (M)[1]		Mascot
948.4567	948.5124	0.0557	59	173	179	ENEICRK			Carbamidomethyl (C)[5]		Mascot
1179.6045	1179.6649	0.0604	51	134	143	FQIDPDGFLK					Mascot
1233.7413	1233.6908	-0.0505	-41	2	13	AVTSLTKATTLK					Mascot
1318.7478	1318.7152	-0.0326	-25	34	44	YQISLANLLQR					Mascot
1335.7056	1335.7086	0.003	2	133	143	RFQIDPDGFLK					Mascot
1364.7817	1364.7406	-0.0411	-30	1	13	MAVTSLTKATTLK					Mascot
1507.7621	1507.799	0.0369	24	283	294	VDCLCKHGLIHR			Carbamidomethyl (C)[3,5]		Mascot
1754.8895	1754.92	0.0305	17	14	28	CRLSSTQFFSTVPPK			Carbamidomethyl (C)[1]		Mascot
1875.9966	1876.0045	0.0079	4	239	255	VLGMALGEMSRCLGLLR			Carbamidomethyl (C)[12]		Mascot
1953.1492	1953.0508	-0.0984	-50	359	377	SNGALGLEIGLKSLIKPSR					Mascot
2248.186	2248.2319	0.0459	20	212	230	LKPLLEEFVELGFSENEV					Mascot

R

10 Disease resistance RPP13-like protein 4 [Aegilops tauschii] gi|475571627 101261.1 6.27 20 60 0 17.149

Peptide Information

Calc. Mass	Obsrv. Mass	± da	± ppm	Start Seq.	End Seq.	Sequence	Ion Score	C. I. %	Modification	Rank	Result Type
828.5553	828.579	0.0237	29	654	660	EIVKLVK					Mascot
849.4036	849.4609	0.0573	67	490	495	KYHCNK			Carbamidomethyl (C)[4]		Mascot
935.4251	935.5031	0.078	83	27	34	EGVCESVR			Carbamidomethyl (C)[4]		Mascot
948.4567	948.5124	0.0557	59	135	143	MEAANGSLR					Mascot
963.4451	963.5076	0.0625	65	880	888	ETNGIMEGI					Mascot
1196.5729	1196.6122	0.0393	33	644	653	ACHNLEDLPK			Carbamidomethyl (C)[2]		Mascot
1233.6069	1233.6908	0.0839	68	101	112	DLKTAEGGSEAR					Mascot
1245.6798	1245.7362	0.0564	45	423	433	LKDSINGLETR					Mascot
1253.7253	1253.6095	-0.1158	-92	144	154	IVDHVFSPVLK					Mascot
1265.6749	1265.6963	0.0214	17	43	52	WQRSLGVTYR					Mascot
1329.7009	1329.762	0.0611	46	314	324	DVLQKVQENEK					Mascot
1336.6526	1336.7333	0.0807	60	27	38	EGVCESVRSVSK			Carbamidomethyl (C)[4]		Mascot
1336.6526	1336.7333	0.0807	60	27	38	EGVCESVRSVSK			Carbamidomethyl (C)[4]		Mascot
1358.6621	1358.731	0.0689	51	53	65	NASGAPEPLEVMK			Oxidation (M)[12]		Mascot
1733.8892	1734.0138	0.1246	72	242	256	DGIDHLVHVLDMIEK					Mascot
1822.8899	1823.0623	0.1724	95	119	134	SGMQCINDVLATVRSR			Carbamidomethyl (C)[5], Oxidation (M)[3]		Mascot
1822.8899	1823.0623	0.1724	95	119	134	SGMQCINDVLATVRSR			Carbamidomethyl (C)[5], Oxidation (M)[3]		Mascot
1875.9707	1876.0045	0.0338	18	436	451	QCLLCLAVFPEDAVIK			Carbamidomethyl (C)[2,5]		Mascot
1908.9662	1909.0807	0.1145	60	715	731	TLSIRIGNSVDSDQFEK					Mascot
2297.0615	2297.2886	0.2271	99	574	592	TCLSTVQLGRWQDSDYDPR			Carbamidomethyl (C)[2]		Mascot
2373.208	2373.2878	0.0798	34	658	677	LVKLEYLDVSECYLLSGMPK			Carbamidomethyl (C)[12], Oxidation (M)[18]		Mascot
2421.2046	2421.2732	0.0686	28	370	390	EISNIWGKVDDSFNVQDVSIR					Mascot

Gel Idx/Pos	197/H24	Instr./Gel Origin	BA2151/Sample Project 20140814	Process Status	Analysis Succeeded
Plate [#] Name	[1] Sample Project 20140814	Instrument Sample Name		Spectra	11

Rank	Protein Name	Accession No.	Protein MW	Protein PI	Pep. Count	Protein Score	Protein Score C. I. %	Intensity Matched	Total Ion Score	Total Ion C. I. %	Confirmed
1	alpha-amylase inhibitor 1 [Aegilops tauschii]	gi|442614142	22813.2	6.2	4	174	100	18.147	152	100	

Protein Group

hypothetical protein F775_28748 [Aegilops tauschii]	gi|475569529	22813.2	6.1999998092 6514

Peptide Information

Calc. Mass	Obsrv. Mass	± da	± ppm	Start Seq.	End Seq.	Sequence	Ion Score	C. I. %	Modification	Rank	Result Type
1950.9413	1951.0499	0.1086	56	60	76	DALLQQCSPVADMSFLR			Carbamidomethyl (C)[7]		Mascot
1966.9362	1967.0344	0.0982	50	60	76	DALLQQCSPVADMSFLR			Carbamidomethyl (C)[7], Oxidation (M)[13]		Mascot
1966.9362	1967.0344	0.0982	50	60	76	DALLQQCSPVADMSFLR	111	100	Carbamidomethyl (C)[7], Oxidation (M)[13]		Mascot
2635.1885	2635.3235	0.135	51	77	97	SQVVQHSSCLVMWEQC CQQLK			Carbamidomethyl (C)[9,16,17]		Mascot
2651.1833	2651.3323	0.149	56	77	97	SQVVQHSSCLVMWEQC CQQLK			Carbamidomethyl (C)[9,16,17], Oxidation (M)[12]		Mascot
3060.4521	3060.6226	0.1705	56	77	101	SQVVQHSSCLVMWEQC CQQLKAIPK			Carbamidomethyl (C)[9,16,17], Oxidation (M)[12]		Mascot
3217.5198	3217.7222	0.2024	63	27	54	SAWEPQHPSSPEHQPTP QPQEHPVPHQK					Mascot
3217.5198	3217.7222	0.2024	63	27	54	SAWEPQHPSSPEHQPTP QPQEHPVPHQK	41	88.683			Mascot

Rank	Protein Name	Accession No.	Protein MW	Protein PI	Pep. Count	Protein Score	Protein Score C. I. %	Intensity Matched	Total Ion Score	Total Ion C. I. %	Confirmed
2	Avenin-3 [Triticum urartu]	gi|474329936	22657.1	6.35	4	132	100	17.231	111	100	

Peptide Information

Calc. Mass	Obsrv. Mass	± da	± ppm	Start Seq.	End Seq.	Sequence	Ion Score	C. I. %	Modification	Rank	Result Type
1950.9413	1951.0499	0.1086	56	60	76	DALLQQCSPVADMSFLR			Carbamidomethyl (C)[7]		Mascot
1966.9362	1967.0344	0.0982	50	60	76	DALLQQCSPVADMSFLR			Carbamidomethyl (C)[7], Oxidation (M)[13]		Mascot
1966.9362	1967.0344	0.0982	50	60	76	DALLQQCSPVADMSFLR	111	100	Carbamidomethyl (C)[7], Oxidation (M)[13]		Mascot
2635.1885	2635.3235	0.135	51	77	97	SQVVQHSSCLVMWEQC CQQLK			Carbamidomethyl (C)[9,16,17]		Mascot
2651.1833	2651.3323	0.149	56	77	97	SQVVQHSSCLVMWEQC CQQLK			Carbamidomethyl (C)[9,16,17], Oxidation (M)[12]		Mascot
3060.4521	3060.6226	0.1705	56	77	101	SQVVQHSSCLVMWEQC CQQLKAIPK			Carbamidomethyl (C)[9,16,17], Oxidation (M)[12]		Mascot
3222.5352	3222.7131	0.1779	55	27	54	TAWEPHHPSSPEQQPTP QPQEQPVPHQK					Mascot

3	Carbonic anhydrase, chloroplastic [Aegilops tauschii]	gi|475583417	22653.3	5.97	8	102	99.994	5.782	57	99.716

Peptide Information

Calc. Mass	Obsrv. Mass	± da	± ppm	Start Seq.	End Seq.	Sequence	Ion Score	C. I. %	Modification	Rank	Result Type
856.4563	856.4152	-0.0411	-48	10	16	TGFEKFK					Mascot
1120.6038	1120.6555	0.0517	46	23	31	KPDFFEPLK					Mascot
1219.5234	1219.6055	0.0821	67	38	47	YMVFACADSR			Carbamidomethyl (C)[6]		Mascot
1235.5184	1235.5841	0.0657	53	38	47	YMVFACADSR			Carbamidomethyl (C)[6], Oxidation (M)[2]		Mascot
1278.6478	1278.718	0.0702	55	185	196	LVGGHYDFVSGK					Mascot
1794.7719	1794.8651	0.0932	52	117	131	DGADDSFHFVEDWVR					Mascot
1794.7719	1794.8651	0.0932	52	117	131	DGADDSFHFVEDWVR	41	88.991			Mascot
1855.9476	1856.0358	0.0882	48	17	31	TEVYDKKPDFFEPLK					Mascot
1946.0052	1946.1089	0.1037	53	48	65	VCPSVTLGLEPGEAFTIR			Carbamidomethyl (C)[2]		Mascot
1946.0052	1946.1089	0.1037	53	48	65	VCPSVTLGLEPGEAFTIR	16	0	Carbamidomethyl (C)[2]		Mascot
3239.5898	3239.7078	0.118	36	48	76	VCPSVTLGLEPGEAFTIR NIANMVPSYCK			Carbamidomethyl (C)[2,28], Oxidation (M)[23]		Mascot

4	RecName: Full=Carbonic anhydrase, chloroplastic; AltName: Full=Carbonate dehydratase; Flags: Precursor	gi|729003	35736.1	8.93	8	83	99.523	5.659	57	99.716

Peptide Information

Calc. Mass	Obsrv. Mass	± da	± ppm	Start Seq.	End Seq.	Sequence	Ion Score	C. I. %	Modification	Rank	Result Type
856.4563	856.4152	-0.0411	-48	132	138	TGFEKFK					Mascot
1120.6038	1120.6555	0.0517	46	145	153	KPDFFEPLK					Mascot
1187.6685	1187.6923	0.0238	20	13	22	SPVFVFAHKR					Mascot
1219.5234	1219.6055	0.0821	67	160	169	YMVFACADSR			Carbamidomethyl (C)[6]		Mascot
1235.5184	1235.5841	0.0657	53	160	169	YMVFACADSR			Carbamidomethyl (C)[6], Oxidation (M)[2]		Mascot
1278.6478	1278.718	0.0702	55	307	318	LVGGHYDFVSGK					Mascot
1794.7719	1794.8651	0.0932	52	239	253	DGADDSFHFVEDWVR					Mascot
1794.7719	1794.8651	0.0932	52	239	253	DGADDSFHFVEDWVR	41	88.991			Mascot
1855.9476	1856.0358	0.0882	48	139	153	TEVYDKKPDFFEPLK					Mascot
1946.0052	1946.1089	0.1037	53	170	187	VCPSVTLGLEPGEAFTIR			Carbamidomethyl (C)[2]		Mascot
1946.0052	1946.1089	0.1037	53	170	187	VCPSVTLGLEPGEAFTIR	16	0	Carbamidomethyl (C)[2]		Mascot

5	hypothetical protein CARUB_v10012171mg, partial [Capsella rubella]	gi|482575015	91497.8	6.08	18	57	0	6.889

Peptide Information

Calc. Mass	Obsrv. Mass	± da	± ppm	Start	End	Sequence	Ion	C. I. %	Modification	Rank	Result Type

				Seq.	Seq.		Score				
849.4135	849.4711	0.0576	68	29	35	AMQELNK			Oxidation (M)[2]		Mascot
1120.6473	1120.6555	0.0082	7	123	132	HIQTPIGLNK					Mascot
1182.6154	1182.6144	-0.001	-1	518	527	LVDISGEFFR					Mascot
1219.6934	1219.6055	-0.0879	-72	229	239	FVLLLDDLGSK					Mascot
1278.6577	1278.718	0.0603	47	702	713	EVLPSTSSPSFK					Mascot
1380.6981	1380.7871	0.089	64	450	461	EMALWIATNFGK					Mascot
1406.7526	1406.7052	-0.0474	-34	701	713	KEVLPSTSSPSFK					Mascot
1493.821	1493.8135	-0.0075	-5	343	356	TALNLTSGEFLLSK					Mascot
1647.8411	1647.8419	0.0008	0	688	700	ILDIMIDWESKER					Mascot
1818.9874	1818.9391	-0.0483	-27	758	773	GISIRNAHPHFVAPFR					Mascot
1822.8562	1822.8593	0.0031	2	264	279	SEEVCKDMEAVGIEVK			Carbamidomethyl (C)[5]		Mascot
1902.0226	1902.057	0.0344	18	645	661	IQGVDRLGSSIQSLCLR			Carbamidomethyl (C)[15]		Mascot
1954.9514	1955.0426	0.0912	47	133	147	RVEMVLCYHQLYAEK			Carbamidomethyl (C)[7], Oxidation (M)[4]		Mascot
1956.1675	1956.0698	-0.0977	-50	590	607	LGSIVGIVMSLRNLQVLK			Oxidation (M)[9]		Mascot
1997.0273	1997.0676	0.0403	20	602	618	NLQVLKFYNSGVCIDAR			Carbamidomethyl (C)[13]		Mascot
2560.3406	2560.2871	-0.0535	-21	334	356	TVQEWSHAKTALNLTSG EFLLSK					Mascot
2651.48	2651.3323	-0.1477	-56	419	443	EGLDIINLLVSSHLLMNG GVTTKVK					Mascot
3268.6921	3268.7007	0.0086	3	728	755	DLTWLAFAQNLVYLDVM RSSSIEEIINK					Mascot

6	RNA polymerase beta'' chain [Adiantum capillus-veneris]	gi|30352024	161476.5	9.48	23	57	0	15.723

Peptide Information

Calc. Mass	Obsrv. Mass	± da	± ppm	Start Seq.	End Seq.	Sequence	Ion Score	C. I. %	Modification	Rank	Result Type
832.4271	832.3571	-0.07	-84	997	1004	ASSNLGQR					Mascot
856.4312	856.4152	-0.016	-19	448	454	GEIHWSK					Mascot
889.5036	889.4626	-0.041	-46	696	703	SGGLIRMR					Mascot
905.4873	905.4216	-0.0657	-73	13	20	TIDRAAMK					Mascot
906.4276	906.4916	0.064	71	935	942	NNSVTDTR					Mascot
1142.563	1142.6293	0.0663	58	1397	1405	DFNPSFFIR					Mascot
1208.6456	1208.6232	-0.0224	-19	291	300	SPLTCKSIFR			Carbamidomethyl (C)[5]		Mascot
1208.6456	1208.6232	-0.0224	-19	291	300	SPLTCKSIFR			Carbamidomethyl (C)[5]		Mascot
1380.7369	1380.7871	0.0502	36	171	182	EGLSLTEYIISR					Mascot
1490.8228	1490.8168	-0.006	-4	1206	1219	LIGNPWSHLLGAGR					Mascot
1493.7747	1493.8135	0.0388	26	552	563	SNYILSNVWLER					Mascot

1706.8708	1706.8285	-0.0423	-25	414	428	TGQYVESQQVIAEVR				Mascot
1811.9552	1811.973	0.0178	10	440	454	KPIYPNSRGEIHWSK				Mascot
1811.9552	1811.973	0.0178	10	440	454	KPIYPNSRGEIHWSK				Mascot
1902.8871	1903.0526	0.1655	87	121	137	MIDPLNPVHMMSVSGAR			Oxidation (M)[1,10,11]	Mascot
1903.0297	1903.0526	0.0229	12	549	563	RPRSNYILSNVWLER				Mascot
1922.931	1923.0242	0.0932	48	564	579	AELENSVSLLMERCQK			Carbamidomethyl (C)[14], Oxidation (M)[11]	Mascot
1931.9504	1932.0483	0.0979	51	634	650	LQLDGGTGNKSSRPWC R			Carbamidomethyl (C)[16]	Mascot
1952.0159	1952.0452	0.0293	15	26	43	LVVCFGIASTTNISDQVK			Carbamidomethyl (C)[4]	Mascot
1954.9691	1955.0426	0.0735	38	97	112	LRQSIEAWYATSECLK			Carbamidomethyl (C)[14]	Mascot
1990.1008	1990.0015	-0.0993	-50	714	730	ILPGYIYNPEKQINISK				Mascot
2635.22	2635.3235	0.1035	39	1039	1060	GFVDYSIDKSEHQDFYLI DESK				Mascot
3215.6624	3215.7126	0.0502	16	655	683	GNFFPIPEEVYLTHEPSS SILVTNNAIVK				Mascot
3230.6038	3230.7385	0.1347	42	765	793	TFVPMTPVSEYNLSSDSL AQVASRFDKPK			Oxidation (M)[5]	Mascot
3243.6877	3243.696	0.0083	3	1206	1233	LIGNPWSHLLGAGRSME HCQLILIDQIR			Carbamidomethyl (C)[19], Oxidation (M)[16]	Mascot

7 hypothetical protein F775_52633 [Aegilops tauschii] gi|475617695 45217.8 8.77 12 55 0 22.9

Peptide Information

Calc. Mass	Obsrv. Mass	± da	± ppm	Start Seq.	End Seq.	Sequence	Ion Score	C. I. %	Modification	Rank	Result Type
849.5305	849.4711	-0.0594	-70	201	208	VKIGHAPK					Mascot
906.4792	906.4916	0.0124	14	255	262	FGVRNDAK					Mascot
1145.519	1145.6301	0.1111	97	1	10	MAGELMQHGR			Oxidation (M)[1]		Mascot
1490.8247	1490.8168	-0.0079	-5	241	254	TMVSSVTVLALDVR					Mascot
1826.9661	1826.8625	-0.1036	-57	11	25	HLDDRHEIFVPPVPR					Mascot
1909.9954	1910.0164	0.021	11	186	202	LINLEGWTPHGGCTKVK			Carbamidomethyl (C)[13]		Mascot
1946.074	1946.1089	0.0349	18	237	254	VSPRTMVSSVTVLALDVR			Oxidation (M)[6]		Mascot
1946.074	1946.1089	0.0349	18	237	254	VSPRTMVSSVTVLALDVR			Oxidation (M)[6]		Mascot
1950.0841	1950.0786	-0.0055	-3	241	258	TMVSSVTVLALDVRFGV R					Mascot
1955.0444	1955.0426	-0.0018	-1	339	357	QVVILLSAGSTSAEEVHS K					Mascot
1966.8899	1967.0344	0.1445	73	295	310	FWHDSGTVECVRSCVK			Carbamidomethyl (C)[10,14]		Mascot
1966.8899	1967.0344	0.1445	73	295	310	FWHDSGTVECVRSCVK			Carbamidomethyl (C)[10,14]		Mascot
1989.0182	1989.0048	-0.0134	-7	363	381	RAGEISVALVTGCSDLQG R			Carbamidomethyl (C)[13]		Mascot
2635.4817	2635.3235	-0.1582	-60	209	232	LQLLAYLQLDAGNHVLEV GNTIIK					Mascot

8 Ycf1, partial (chloroplast) [Horminum pyrenaicum] gi|323403917 182260 9.92 24 54 0 7.375

Peptide Information

Calc. Mass	Obsrv. Mass	± da	± ppm	Start Seq.	End Seq.	Sequence	Ion Score	C. I. %	Modification	Rank	Result Type
871.5108	871.5052	-0.0056	-6	1111	1117	NQNVKIR					Mascot
889.5353	889.4626	-0.0727	-82	1310	1317	ITISSIQK					Mascot
1142.6416	1142.6293	-0.0123	-11	187	196	NALIENIIDK					Mascot
1144.6936	1144.6077	-0.0859	-75	1330	1339	NLTLSELVKK					Mascot
1187.642	1187.6923	0.0503	42	547	556	NWLTDGIQIK					Mascot
1235.6056	1235.5841	-0.0215	-17	929	938	LPGYETNQWK					Mascot
1325.6705	1325.7621	0.0916	69	482	492	GCMLITQSIFR			Carbamidomethyl (C)[2]		Mascot
1380.8573	1380.7871	-0.0702	-51	1340	1352	GVLIVEPLRLSGK					Mascot
1490.8214	1490.8168	-0.0046	-3	274	286	YLLTKIVTDPDGR					Mascot
1557.8271	1557.953	0.1259	81	1098	1110	VDIEAWIIIDTNR					Mascot
1647.8894	1647.8419	-0.0475	-29	1408	1421	HFGFLVPENILSFK					Mascot
1706.873	1706.8285	-0.0445	-26	1084	1095	YFDWKIFHFYLK					Mascot
1909.865	1910.0164	0.1514	79	1494	1508	YWFDTNNGSRFSMLR			Oxidation (M)[13]		Mascot
1917.9236	1918.0648	0.1412	74	912	928	ASFERQGMVHSELANNK					Mascot
1927.0085	1926.9918	-0.0167	-9	939	953	NWLKGHFQYHLSQIR					Mascot
1946.0344	1946.1089	0.0745	38	76	92	ISFTFSPSLSIFVEMIK					Mascot
1946.0344	1946.1089	0.0745	38	76	92	ISFTFSPSLSIFVEMIK					Mascot
1962.0293	1962.0658	0.0365	19	76	92	ISFTFSPSLSIFVEMIK			Oxidation (M)[15]		Mascot
1964.9282	1965.0521	0.1239	63	1451	1467	NVTNSSRVSHDNNHLDR					Mascot
1989.0037	1989.0048	0.0011	1	244	262	ESGSPNLNLKGPSLFSE GR					Mascot
1990.0354	1990.0015	-0.0339	-17	1354	1370	DGQFIMYQTIGISLLYK					Mascot
2004.9847	2004.9845	-0.0002	0	1130	1146	NLFYLMIPETNSPNSHK					Mascot
2322.3472	2322.283	-0.0642	-28	792	810	IFIEKIYTEIFLSIINIPR					Mascot
2560.2249	2560.2871	0.0622	24	917	938	QGMVHSELANNKLPGYE TNQWK			Oxidation (M)[3]		Mascot
3060.4812	3060.6226	0.1414	46	859	883	ISNIKENSHIFYDLSYMSQ AYVFYK					Mascot
3247.5576	3247.7231	0.1655	51	696	724	DSMISNQIIHESFSQIASP SWTNSPLTEK					Mascot

9 TPA: hypothetical protein ZEAMMB73_723278 [Zea mays] gi|414872512 33120.4 8.59 12 52 0 29.35

Peptide Information

Calc. Mass	Obsrv. Mass	± da	± ppm	Start Seq.	End Seq.	Sequence	Ion Score	C. I. %	Modification	Rank	Result Type
867.4756	867.4846	0.009	10	1	7	MTFLISR					Mascot

Calc. Mass	Obsrv. Mass	± da	± ppm	Start Seq.	End Seq.	Sequence	Ion Score	C. I. %	Modification	Rank	Result Type
883.4706	883.4744	0.0038	4	1	7	MTFLISR			Oxidation (M)[1]		Mascot
906.4899	906.4916	0.0017	2	126	132	LLDRMMK					Mascot
938.4797	938.516	0.0363	39	126	132	LLDRMMK			Oxidation (M)[5,6]		Mascot
1092.6412	1092.6106	-0.0306	-28	160	169	KYGIAATLQK					Mascot
1145.679	1145.6301	-0.0489	-43	140	151	RGAAFGLAGVVK					Mascot
1187.5831	1187.6923	0.1092	92	288	297	LTEVYDTISF					Mascot
1219.6174	1219.6055	-0.0119	-10	230	241	AMMSQLTGPGVK					Mascot
1228.7083	1228.5996	-0.1087	-88	20	30	MINAGILIIDR					Mascot
1235.6123	1235.5841	-0.0282	-23	230	241	AMMSQLTGPGVK			Oxidation (M)[2]		Mascot
1297.5907	1297.6373	0.0466	36	51	61	ASDEETYDLVR					Mascot
1406.7018	1406.7052	0.0034	2	103	115	AVSDCLSPLMVSK			Carbamidomethyl (C)[5]		Mascot
1918.0215	1918.0648	0.0433	23	1	17	MTFLISRALADPNVDVR					Mascot
1923.0262	1923.0242	-0.002	-1	34	49	ENVPLLFPIFESYLNK					Mascot
3222.8	3222.7131	-0.0869	-27	194	221	LGKLFEPYVIQMLPFLLVS FSDQVLAVR					Mascot

10 PREDICTED: myosin-J heavy chain-like isoform X1 [Setaria italica] gi|514816760 173960.1 8.83 22 52 0 22.675

Peptide Information

Calc. Mass	Obsrv. Mass	± da	± ppm	Start Seq.	End Seq.	Sequence	Ion Score	C. I. %	Modification	Rank	Result Type
856.3869	856.4152	0.0283	33	1112	1118	DDMGFKK			Oxidation (M)[3]		Mascot
865.3945	865.4634	0.0689	80	1092	1098	SRMNSDR					Mascot
871.4883	871.5052	0.0169	19	379	386	TPEGNIIK					Mascot
905.4761	905.4216	-0.0545	-60	1354	1361	SGLCVLEK			Carbamidomethyl (C)[4]		Mascot
1092.6082	1092.6106	0.0024	2	164	172	TETTKLIMR					Mascot
1219.6868	1219.6055	-0.0813	-67	1119	1129	GKPVAACIIYK			Carbamidomethyl (C)[7]		Mascot
1297.6787	1297.6373	-0.0414	-32	219	229	FVEIQFDKSGK					Mascot
1493.7272	1493.8135	0.0863	58	87	98	YTLNEIYTYTGR					Mascot
1811.9512	1811.973	0.0218	12	733	748	GEILDNASRIVQGHFR					Mascot
1811.9512	1811.973	0.0218	12	733	748	GEILDNASRIVQGHFR					Mascot
1822.9269	1822.8593	-0.0676	-37	516	530	STHETFAMKLFQNLR					Mascot
1860.0338	1860.0336	-0.0002	0	654	671	SGGVLEAIRISLAGYPTR					Mascot
1876.0579	1876.0226	-0.0353	-19	1142	1157	TTIFDFIIHTINSILK					Mascot
1876.0579	1876.0226	-0.0353	-19	1142	1157	TTIFDFIIHTINSILK	5	0			Mascot
1909.094	1909.0375	-0.0565	-30	1256	1272	EISPLLSLCIQAPKLAR			Carbamidomethyl (C)[9]		Mascot
1910.043	1910.0164	-0.0266	-14	71	86	LVYLHEPGVLCNLARR			Carbamidomethyl (C)[11]		Mascot
1931.938	1932.0483	0.1103	57	53	70	VLPRDTEADLGGVDDMT K					Mascot

1949.9386	1950.0786	0.14	72	1187	1204	GFIMAPSRSSSDTHLSEK		Mascot
1967.0386	1967.0344	-0.0042	-2	403	418	TVYARLFDWLVDNINK		Mascot
1967.0386	1967.0344	-0.0042	-2	403	418	TVYARLFDWLVDNINK		Mascot
1972.9078	1973.0416	0.1338	68	1220	1235	TCMSHIDARYPAMLFK	Carbamidomethyl (C)[2], Oxidation (M)[3,13]	Mascot
1989.9487	1990.0015	0.0528	27	681	696	FGLLVPEHMDERFDER		Mascot
2560.3811	2560.2871	-0.094	-37	87	108	YTLNEIYTYTGRILIAVNPF AK		Mascot
3217.5505	3217.7222	0.1717	53	57	85	DTEADLGGVDDMTKLVY LHEPGVLCNLAR	Carbamidomethyl (C)[25], Oxidation (M)[12]	Mascot
3217.5505	3217.7222	0.1717	53	57	85	DTEADLGGVDDMTKLVY LHEPGVLCNLAR	Carbamidomethyl (C)[25], Oxidation (M)[12]	Mascot
3373.7766	3373.814	0.0374	11	1130	1157	CLLHWGVFEAERTTIFDFI IHTINSILK	Carbamidomethyl (C)[1]	Mascot

Gel Idx/Pos	201/I1	Instr./Gel Origin	BA2151/Sample Project 20140814	Process Status	Analysis Succeeded
Plate [#] Name	[1] Sample Project 20140814	Instrument Sample Name		Spectra	11

Rank	Protein Name	Accession No.	Protein MW	Protein PI	Pep. Count	Protein Score	Protein Score C. I. %	Intensity Matched	Total Ion Score	Total Ion C. I. %	Confirmed
1	Avenin-3 [Triticum urartu]	gi|474329936	22657.1	6.35	4	136	100	18.408	115	100	

Peptide Information

Calc. Mass	Obsrv. Mass	± da	± ppm	Start Seq.	End Seq.	Sequence	Ion Score	C. I. %	Modification	Rank	Result Type
1950.9413	1950.929	-0.0123	-6	60	76	DALLQQCSPVADMSFLR			Carbamidomethyl (C)[7]		Mascot
1966.9362	1966.9025	-0.0337	-17	60	76	DALLQQCSPVADMSFLR			Carbamidomethyl (C)[7], Oxidation (M)[13]		Mascot
1966.9362	1966.9025	-0.0337	-17	60	76	DALLQQCSPVADMSFLR	115	100	Carbamidomethyl (C)[7], Oxidation (M)[13]		Mascot
2591.2527	2591.2151	-0.0376	-15	55	76	LNPCRDALLQQCSPVAD MSFLR			Carbamidomethyl (C)[4,12]		Mascot
2635.1885	2635.1636	-0.0249	-9	77	97	SQVVQHSSCLVMWEQC CQQLK			Carbamidomethyl (C)[9,16,17]		Mascot
2651.1833	2651.147	-0.0363	-14	77	97	SQVVQHSSCLVMWEQC CQQLK			Carbamidomethyl (C)[9,16,17], Oxidation (M)[12]		Mascot
2651.1833	2651.147	-0.0363	-14	77	97	SQVVQHSSCLVMWEQC CQQLK			Carbamidomethyl (C)[9,16,17], Oxidation (M)[12]		Mascot
3222.5352	3222.5293	-0.0059	-2	27	54	TAWEPHHPSSPEQQPTP QPQEQPVPHQK					Mascot

Rank	Protein Name	Accession No.	Protein MW	Protein PI	Pep. Count	Protein Score	Protein Score C. I. %	Intensity Matched	Total Ion Score	Total Ion C. I. %	Confirmed
2	alpha-amylase inhibitor 1 [Aegilops tauschii]	gi|442614142	22813.2	6.2	4	136	100	18.353	115	100	

Protein Group

hypothetical protein F775_28748 [Aegilops tauschii]	gi|475569529	22813.2	6.1999998092 6514

Peptide Information

Calc. Mass	Obsrv. Mass	± da	± ppm	Start Seq.	End Seq.	Sequence	Ion Score	C. I. %	Modification	Rank	Result Type
1950.9413	1950.929	-0.0123	-6	60	76	DALLQQCSPVADMSFLR			Carbamidomethyl (C)[7]		Mascot
1966.9362	1966.9025	-0.0337	-17	60	76	DALLQQCSPVADMSFLR			Carbamidomethyl (C)[7], Oxidation (M)[13]		Mascot
1966.9362	1966.9025	-0.0337	-17	60	76	DALLQQCSPVADMSFLR	115	100	Carbamidomethyl (C)[7], Oxidation (M)[13]		Mascot
2591.2527	2591.2151	-0.0376	-15	55	76	LNPCRDALLQQCSPVAD MSFLR			Carbamidomethyl (C)[4,12]		Mascot
2635.1885	2635.1636	-0.0249	-9	77	97	SQVVQHSSCLVMWEQC CQQLK			Carbamidomethyl (C)[9,16,17]		Mascot
2651.1833	2651.147	-0.0363	-14	77	97	SQVVQHSSCLVMWEQC CQQLK			Carbamidomethyl (C)[9,16,17], Oxidation (M)[12]		Mascot
2651.1833	2651.147	-0.0363	-14	77	97	SQVVQHSSCLVMWEQC CQQLK			Carbamidomethyl (C)[9,16,17], Oxidation (M)[12]		Mascot
3217.5198	3217.5295	0.0097	3	27	54	SAWEPQHPSSPEHQPTP					Mascot

			QPQEHPVPHQK							
3	Os03g0267600 [Oryza sativa Japonica Group]	gi|113548137	99710	8.92	17	59	0	20.159		

Peptide Information

Calc. Mass	Obsrv. Mass	± da	± ppm	Start Seq.	End Seq.	Sequence	Ion Score	C. I. %	Modification	Rank	Result Type
849.4247	849.4175	-0.0072	-8	874	881	IMAASQGR			Oxidation (M)[2]		Mascot
920.4948	920.4496	-0.0452	-49	2	9	PTKGQFSR					Mascot
1097.5198	1097.5914	0.0716	65	812	820	HAFSMVSFR			Oxidation (M)[5]		Mascot
1187.6016	1187.6215	0.0199	17	67	78	EPRSADAGGSLK					Mascot
1347.6475	1347.599	-0.0485	-36	97	107	HMSNVPSYLQR			Oxidation (M)[2]		Mascot
1507.7183	1507.6649	-0.0534	-35	267	278	NIEEAVHHCSRR			Carbamidomethyl (C)[9]		Mascot
1875.9481	1875.8912	-0.0569	-30	238	255	NISPLSDPLLTATGSSMR			Oxidation (M)[17]		Mascot
1875.9481	1875.8912	-0.0569	-30	238	255	NISPLSDPLLTATGSSMR			Oxidation (M)[17]		Mascot
1903.0298	1902.9384	-0.0914	-48	882	899	AQASYVPHRPPLSPVGR A					Mascot
1903.0298	1902.9384	-0.0914	-48	882	899	AQASYVPHRPPLSPVGR A					Mascot
1996.9611	1996.9279	-0.0332	-17	675	692	EFAAFISAVSQQEPETSR					Mascot
2120.1611	2120.0249	-0.1362	-64	203	222	ARYSPSPNSAVLNLLPVH GK					Mascot
2252.0574	2252.0735	0.0161	7	349	368	IPYSCPLPIMDSSEELGTS R			Carbamidomethyl (C)[5]		Mascot
2323.158	2323.1211	-0.0369	-16	328	348	NASLSPVGFSKDIGQHHE FPR					Mascot
2501.3147	2501.2114	-0.1033	-41	543	563	YLQQDIDSTTTRHALLQL AWK					Mascot
2590.9902	2591.2151	0.2249	87	756	779	SGGACDCGGWDEGCML SVLSDDAR			Carbamidomethyl (C)[5,7,14], Oxidation (M)[15]		Mascot
3218.4941	3218.5266	0.0325	10	238	266	NISPLSDPLLTATGSSMR HEMVDDEETTR			Oxidation (M)[17]		Mascot
3234.489	3234.5254	0.0364	11	238	266	NISPLSDPLLTATGSSMR HEMVDDEETTR			Oxidation (M)[17,21]		Mascot
3249.5618	3249.5183	-0.0435	-13	527	554	TETNTNQPPQVLNSERY LQQDIDSTTTR					Mascot
3249.5618	3249.5183	-0.0435	-13	527	554	TETNTNQPPQVLNSERY LQQDIDSTTTR					Mascot
3272.4016	3272.4976	0.096	29	693	721	HSSSQHSSRSMSTPTDC SCPLGNFHPNTR			Carbamidomethyl (C)[17,19]		Mascot

4	PREDICTED: phospholipase D delta-like [Solanum lycopersicum]	gi|460373442	99555.9	6.87	18	58	0	5.544

Peptide Information

Calc. Mass	Obsrv. Mass	± da	± ppm	Start Seq.	End Seq.	Sequence	Ion Score	C. I. %	Modification	Rank	Result Type
856.4312	856.3511	-0.0801	-94	767	773	GQIYGYR					Mascot
1074.5249	1074.4609	-0.064	-60	790	798	EPEALTCVR			Carbamidomethyl (C)[7]		Mascot

1145.5586	1145.559	0.0004	0	800	809	VNEVAEGNWK					Mascot
1187.5427	1187.6215	0.0788	66	501	511	DSTAIPEDDPK					Mascot
1281.578	1281.5437	-0.0343	-27	129	140	DDDVFGAQVMGK					Mascot
1412.6805	1412.7021	0.0216	15	417	429	DDVHQPTFPAGTK					Mascot
1443.7009	1443.7377	0.0368	25	372	384	CVLVDTQAPGNNR			Carbamidomethyl (C)[1]		Mascot
1676.8942	1676.8063	-0.0879	-52	335	348	FFKHSSVICVLSPR			Carbamidomethyl (C)[9]		Mascot
1811.9296	1811.9285	-0.0011	-1	114	128	IPLAHPMDCLDFRVK			Carbamidomethyl (C)[9]		Mascot
1811.9296	1811.9285	-0.0011	-1	114	128	IPLAHPMDCLDFRVK			Carbamidomethyl (C)[9]		Mascot
1860.9855	1860.9155	-0.07	-38	148	166	IASGEVVSGWFPVIGASG K					Mascot
1901.8633	1901.9995	0.1362	72	473	487	TMSHWHDDAMLKIER			Oxidation (M)[2,10]		Mascot
1924.8859	1924.931	0.0451	23	317	333	FFINTAGVMGTHDEETR					Mascot
1927.9357	1927.906	-0.0297	-15	49	67	KPQTGSTADDGNGELPN VK					Mascot
1951.0317	1950.929	-0.1027	-53	556	572	NLMVDKSIEAAYIQAIR			Oxidation (M)[3]		Mascot
2587.1704	2587.1665	-0.0039	-2	665	685	SMQILDSHPQDYLNFYCL GNR			Carbamidomethyl (C)[17], Oxidation (M)[2]		Mascot
3247.5493	3247.5312	-0.0181	-6	245	270	CWEDICYAITEAHHLIYIV GWSVFHK			Carbamidomethyl (C)[1,6]		Mascot
3262.6743	3262.5371	-0.1372	-42	836	866	VGPLAEYENFPDVGGRIL GNHAPTIPDVLTT					Mascot
3403.6462	3403.6716	0.0254	7	99	126	VIPNSQNPVWDEHFRIPL AHPMDCLDFR			Carbamidomethyl (C)[24]		Mascot
3419.6414	3419.696	0.0546	16	99	126	VIPNSQNPVWDEHFRIPL AHPMDCLDFR			Carbamidomethyl (C)[24], Oxidation (M)[22]		Mascot

5	hypothetical protein M569_05984, partial [Genlisea aurea]	gi|527201790	57172.9	5.83	14	52	0	31.261	

Peptide Information

Calc. Mass	Obsrv. Mass	± da	± ppm	Start Seq.	End Seq.	Sequence	Ion Score	C. I. %	Modification	Rank	Result Type
821.3635	821.3973	0.0338	41	424	431	GGSDTEQK					Mascot
867.4207	867.4254	0.0047	5	343	349	LDESTFR					Mascot
895.4277	895.4161	-0.0116	-13	158	163	VCRWMK			Carbamidomethyl (C)[2], Oxidation (M)[5]		Mascot
1037.4987	1037.4581	-0.0406	-39	475	482	RGWFSPCK			Carbamidomethyl (C)[7]		Mascot
1060.5382	1060.5123	-0.0259	-24	312	320	SNLTVNEQR					Mascot
1097.5222	1097.5914	0.0692	63	62	71	KTEGAYADSR					Mascot
1145.6096	1145.559	-0.0506	-44	271	281	KLGDANMIGAR					Mascot
1298.598	1298.5768	-0.0212	-16	332	342	SPLMFGGDMRR			Oxidation (M)[4,9]		Mascot
1637.7112	1637.774	0.0628	38	483	496	SDLNQMWELTENGT					Mascot
1901.8997	1901.9995	0.0998	52	161	177	WMKNGFMSVNTTLGAG R			Oxidation (M)[2,7]		Mascot
1927.9807	1927.906	-0.0747	-39	164	181	NGFMSVNTTLGAGRAFL R			Oxidation (M)[4]		Mascot

2501.1943	2501.2114	0.0171	7	251	271	ITDDDWDSWTDILGHVDI TRK						Mascot
2839.3357	2839.3015	-0.0342	-12	285	310	GKSWPDSDMLPLGFLTD QGSNTGPYR						Mascot
3218.5178	3218.5266	0.0088	3	17	43	GWNSYDSFTWIISEEEFV QNARNVAQK						Mascot

6	PREDICTED: probable carotenoid cleavage dioxygenase 4, chloroplastic-like [Cicer arietinum]	gi|502161858	63639.6	7.19	12	51	0	2.607

Peptide Information

Calc. Mass	Obsrv. Mass	± da	± ppm	Start Seq.	End Seq.	Sequence	Ion Score	C. I. %	Modification	Rank	Result Type
823.4382	823.4039	-0.0343	-42	534	540	FIVMDAK					Mascot
1097.5773	1097.5914	0.0141	13	163	171	ATLCSRYVK			Carbamidomethyl (C)[4]		Mascot
1145.6412	1145.559	-0.0822	-72	541	551	TASLEVVAEVK					Mascot
1860.8884	1860.9155	0.0271	15	261	276	HDFNGKLFMSMTAHPK					Mascot
1910.0093	1909.8794	-0.1299	-68	463	480	YVYAAVGDPMPKISGVV K			Oxidation (M)[10]		Mascot
1950.0616	1949.9401	-0.1215	-62	534	551	FIVMDAKTASLEVVAEVK					Mascot
1981.0253	1980.9288	-0.0965	-49	556	572	VPYGFHGLFVKESELMK					Mascot
1997.0201	1996.9279	-0.0922	-46	556	572	VPYGFHGLFVKESELMK			Oxidation (M)[16]		Mascot
2839.355	2839.3015	-0.0535	-19	277	300	IDADTGECFAFRYGPVPP FLTYFR			Carbamidomethyl (C)[8]		Mascot
3231.5669	3231.5159	-0.051	-16	301	329	FDSNGVKNSDVPVFSMT TPTFLHDFAITK			Oxidation (M)[16]		Mascot
3233.7026	3233.5259	-0.1767	-55	203	234	GSLTAARVITGQYNPSNG IGLANTSLALFGNR					Mascot
3272.5925	3272.4976	-0.0949	-29	331	362	YAVFGDIQIGMNPLGMIS GGSPVGSDSSKISR			Oxidation (M)[11,16]		Mascot
3406.7754	3406.6287	-0.1467	-43	177	209	IENEAGYPLIPNVFSGFN SLIASAARGSLTAAR					Mascot

7	hypothetical protein CHLNCDRAFT_34770 [Chlorella variabilis]	gi|307109320	54789.1	6.07	13	51	0	4.632

Peptide Information

Calc. Mass	Obsrv. Mass	± da	± ppm	Start Seq.	End Seq.	Sequence	Ion Score	C. I. %	Modification	Rank	Result Type
849.4359	849.4175	-0.0184	-22	369	375	QATRMAR			Oxidation (M)[5]		Mascot
865.4665	865.4085	-0.058	-67	45	51	EIAEYLK					Mascot
1097.6313	1097.5914	-0.0399	-36	193	202	QVAVTLPDVR					Mascot
1208.5654	1208.5465	-0.0189	-16	140	150	TNWESSGGSRK					Mascot
1637.6903	1637.774	0.0837	51	334	347	QQMMAHIDVCMGGK			Carbamidomethyl (C)[10], Oxidation (M)[3,4]		Mascot
1664.7124	1664.7518	0.0394	24	373	386	MARHMVVDCGMSDR			Carbamidomethyl (C)[9]		Mascot
1680.7074	1680.7386	0.0312	19	373	386	MARHMVVDCGMSDR			Carbamidomethyl (C)[9], Oxidation (M)[1]		Mascot
1910.0535	1909.8794	-0.1741	-91	78	95	TLLARAVAGEAGVPFFYK					Mascot

Calc. Mass	Obsrv. Mass	± da	± ppm	Start Seq.	End Seq.	Sequence	Ion Score	C. I. %	Modification	Rank	Result Type
1921.951	1921.9042	-0.0468	-24	123	139	APCIIFIDEIDAMGGKR			Carbamidomethyl (C)[3], Oxidation (M)[13]		Mascot
1942.8387	1942.8536	0.0149	8	456	480	DGAELPGGGGGGGGGG GGGGSGGWR					Mascot
1951.9542	1951.9275	-0.0267	-14	402	418	QAVDDEVQAMLKAAYQR			Oxidation (M)[10]		Mascot
1951.9681	1951.9275	-0.0406	-21	35	51	GCDEAIAELKEIAEYLK			Carbamidomethyl (C)[2]		Mascot
2591.4038	2591.2151	-0.1887	-73	427	449	EGELHRLAQALLQDETLT LAEIK					Mascot
2839.5676	2839.3015	-0.2661	-94	203	228	GRQQILELYLAGKPVAAD VDTELLAR					Mascot

8	PREDICTED: expansin-like A1-like [Cucumis sativus]	gi|449454269	29960.7	8.24	9	50	0	7.202	

Protein Group

PREDICTED: expansin-like A1-like [Cucumis sativus]	gi|449470768	29960.7	8.2399 997711 1816

Peptide Information

Calc. Mass	Obsrv. Mass	± da	± ppm	Start Seq.	End Seq.	Sequence	Ion Score	C. I. %	Modification	Rank	Result Type
823.4308	823.4039	-0.0269	-33	103	109	TDFVVSR					Mascot
1037.5514	1037.4581	-0.0933	-90	127	135	TGIVDIEYK					Mascot
1138.5925	1138.4963	-0.0962	-84	110	120	KAFSAMALDGK					Mascot
1507.7614	1507.6649	-0.0965	-64	222	234	GIMINYALPADWK			Oxidation (M)[3]		Mascot
1818.932	1818.828	-0.104	-57	219	234	NGRGIMINYALPADWK					Mascot
1875.96	1875.8912	-0.0688	-37	192	208	NYGAIWDTNKVPEGAIK					Mascot
1875.96	1875.8912	-0.0688	-37	192	208	NYGAIWDTNKVPEGAIK					Mascot
2651.2771	2651.147	-0.1301	-49	235	257	TGEIYDTGIQIKDIATEAC NPWR			Carbamidomethyl (C)[19]		Mascot
2651.2771	2651.147	-0.1301	-49	235	257	TGEIYDTGIQIKDIATEAC NPWR			Carbamidomethyl (C)[19]		Mascot
3218.707	3218.5266	-0.1804	-56	137	162	IPCEYNKNLLIQVVEWSH KPYYLAIK			Carbamidomethyl (C)[3]		Mascot
3234.5273	3234.5254	-0.0019	-1	163	191	FLYQGGQTDITAVDLATQ DGSGGWQYMRR					Mascot
3250.5222	3250.5164	-0.0058	-2	163	191	FLYQGGQTDITAVDLATQ DGSGGWQYMRR			Oxidation (M)[27]		Mascot

9	hypothetical protein OsI_09468 [Oryza sativa Indica Group]	gi|218191830	20941.7	5.98	8	50	0	5.186	

Protein Group

Os02g0819200 [Oryza sativa Japonica Group]	gi|113538063	20941.7	5.9800 000190 7349

Peptide Information

Calc. Mass	Obsrv. Mass	± da	± ppm	Start Seq.	End Seq.	Sequence	Ion Score	C. I. %	Modification	Rank	Result Type
920.4142	920.4496	0.0354	38	182	188	RMIDQEE					Mascot
938.5054	938.453	-0.0524	-56	164	172	QAEVIGGHK					Mascot
1335.6361	1335.6204	-0.0157	-12	118	128	LENLAAECYPR			Carbamidomethyl (C)[8]		Mascot
1875.9634	1875.8912	-0.0722	-38	147	163	AGVTKMPSIQLWSDSQK					Mascot
1875.9634	1875.8912	-0.0722	-38	147	163	AGVTKMPSIQLWSDSQK					Mascot
1891.9583	1891.8856	-0.0727	-38	147	163	AGVTKMPSIQLWSDSQK			Oxidation (M)[6]		Mascot
1921.9954	1921.9042	-0.0912	-47	131	146	FYCVDVNAVPQKLVNR			Carbamidomethyl (C)[3]		Mascot
1952.0898	1951.9275	-0.1623	-83	24	41	RVLAGGRPPPPCISFLSK			Carbamidomethyl (C)[12]		Mascot
1952.0898	1951.9275	-0.1623	-83	24	41	RVLAGGRPPPPCISFLSK			Carbamidomethyl (C)[12]		Mascot
1964.9672	1964.9341	-0.0331	-17	2	23	ATSAYAATVAGEAAAAAA SPSR					Mascot
2252.1089	2252.0735	-0.0354	-16	1	24	MATSAYAATVAGEAAAA AASPSRR					Mascot

10	Ubiquitin carboxyl-terminal hydrolase family protein isoform 1 [Theobroma cacao]	gi|508702012	48629.4	8.99	12	50	0	9.111

Protein Group

Ubiquitin carboxyl-terminal hydrolase family protein isoform 1 [Theobroma cacao]	gi|508702013	48629.4	8.9899 997711 1816

Peptide Information

Calc. Mass	Obsrv. Mass	± da	± ppm	Start Seq.	End Seq.	Sequence	Ion Score	C. I. %	Modification	Rank	Result Type
849.3889	849.4175	0.0286	34	187	192	YSNYFR					Mascot
920.5352	920.4496	-0.0856	-93	241	248	LPAGFFLR					Mascot
1037.5527	1037.4581	-0.0946	-91	233	240	VRPNFNYK					Mascot
1182.5977	1182.5458	-0.0519	-44	325	334	HSGIFYMSLK					Mascot
1507.6217	1507.6649	0.0432	29	397	409	NEGLNDLSCDEEL			Carbamidomethyl (C)[9]		Mascot
1876.0514	1875.8912	-0.1602	-85	284	299	MVGVFHELLSLSLFKR					Mascot
1876.0514	1875.8912	-0.1602	-85	283	298	RMVGVFHELLSLSLFK					Mascot
1892.0463	1891.8856	-0.1607	-85	284	299	MVGVFHELLSLSLFKR			Oxidation (M)[1]		Mascot
1949.9028	1949.9401	0.0373	19	95	110	TFINNNPGLFDTYYDR					Mascot
2619.3857	2619.1821	-0.2036	-78	17	38	FDFDSFLNPKFTFQIKPIS SLK					Mascot
2651.2261	2651.147	-0.0791	-30	257	278	DWLELDYISPYEDVSHLD QASR					Mascot

2651.2261	2651.147	-0.0791	-30	257	278	DWLELDYISPYEDVSHLD QASR	Mascot
3221.5176	3221.5283	0.0107	3	253	278	EWVRDWLELDYISPYED VSHLDQASR	Mascot
3221.5176	3221.5283	0.0107	3	253	278	EWVRDWLELDYISPYED VSHLDQASR	Mascot
3247.627	3247.5312	-0.0958	-29	193	221	LTGSPGEGQSFLELVEW NPEFAKSVIEQR	Mascot

Rank	Protein Name	Accession No.	Protein MW	Protein PI	Pep. Count	Protein Score	Protein Score C. I. %	Intensity Matched	Total Ion Score	Total Ion C. I. %	Confirmed
1	Glutathione S-transferase 1 [Triticum urartu]	gi|474049299	26837.7	5.8	3	78	98.56	8.566	68	99.967	

Peptide Information

Calc. Mass	Obsrv. Mass	± da	± ppm	Start Seq.	End Seq.	Sequence	Ion Score	C. I. %	Modification	Rank	Result Type
1343.7318	1343.7368	0.005	4	170	181	NVLAVYEAHLSK					Mascot
1380.7271	1380.7369	0.0098	7	6	17	LYGATLSWNVTR					Mascot
1380.7271	1380.7369	0.0098	7	6	17	LYGATLSWNVTR	68	99.967			Mascot
2732.3237	2732.3357	0.012	4	18	42	CVAALEEAGVEYELVPIN FGTGEHK			Carbamidomethyl (C)[1]		Mascot

Rank	Protein Name	Accession No.	Protein MW	Protein PI	Pep. Count	Protein Score	Protein Score C. I. %	Intensity Matched	Total Ion Score	Total Ion C. I. %	Confirmed
2	hypothetical protein SORBIDRAFT_03g005520 [Sorghum bicolor]	gi|241927148	72683	5.17	20	78	98.457	10.359			

Peptide Information

Calc. Mass	Obsrv. Mass	± da	± ppm	Start Seq.	End Seq.	Sequence	Ion Score	C. I. %	Modification	Rank	Result Type
832.3795	832.3247	-0.0548	-66	517	523	QDDAEVR					Mascot
887.3927	887.4387	0.046	52	51	57	MSTQNYK			Oxidation (M)[1]		Mascot
1120.611	1120.5863	-0.0247	-22	2	11	SSWLRSAVSK					Mascot
1129.6073	1129.5697	-0.0376	-33	12	23	AVEAGGRSGVAR					Mascot
1131.6117	1131.6042	-0.0075	-7	7	18	SAVSKAVEAGGR					Mascot
1131.6117	1131.6042	-0.0075	-7	7	18	SAVSKAVEAGGR	8	0			Mascot
1145.6273	1145.5773	-0.05	-44	65	74	RLEEVAVSSR					Mascot
1177.6423	1177.5775	-0.0648	-55	271	282	VLATSLANSSSK					Mascot
1185.5933	1185.5563	-0.037	-31	51	60	MSTQNYKSVK					Mascot
1207.6682	1207.5801	-0.0881	-73	524	533	TLFSDIDKLR					Mascot
1211.6453	1211.5697	-0.0756	-62	421	430	CTSNFLKLTK			Carbamidomethyl (C)[1]		Mascot
1247.5862	1247.6451	0.0589	47	411	420	ENKTNDELER					Mascot
1267.6464	1267.6483	0.0019	1	1	11	MSSWLRSAVSK			Oxidation (M)[1]		Mascot
1378.7325	1378.7106	-0.0219	-16	449	460	TYVDNLALLNSR					Mascot
1418.8213	1418.6959	-0.1254	-88	269	282	LKVLATSLANSSSK					Mascot
1467.6884	1467.7151	0.0267	18	596	608	SELMLDTDSELAK			Oxidation (M)[4]		Mascot
1503.8013	1503.6906	-0.1107	-74	314	326	DLTTEISALEKQR					Mascot

Calc. Mass	Obsrv. Mass	± da	± ppm	Start Seq.	End Seq.	Sequence	Ion Score	C. I. %	Modification	Rank	Result Type
1505.817	1505.6841	-0.1329	-88	271	285	VLATSLANSSSKAEK					Mascot
1827.8735	1827.8459	-0.0276	-15	122	137	VLFYDADIGGPPMNFR			Oxidation (M)[13]		Mascot
2268.1316	2268.1216	-0.01	-4	596	615	SELMLDTDSELAKLELEFGK					Mascot
2732.2732	2732.3357	0.0625	23	355	377	DQFHEANNQMIFSLHAKENDLSK			Oxidation (M)[10]		Mascot

3	hypothetical protein F775_30949 [Aegilops tauschii]	gi|475622260	29468.1	6.14	3	78	98.385	8.566	68	99.967	

Peptide Information

Calc. Mass	Obsrv. Mass	± da	± ppm	Start Seq.	End Seq.	Sequence	Ion Score	C. I. %	Modification	Rank	Result Type
1343.7318	1343.7368	0.005	4	191	202	NVLAVYEAHLSK					Mascot
1380.7271	1380.7369	0.0098	7	6	17	LYGATLSWNVTR					Mascot
1380.7271	1380.7369	0.0098	7	6	17	LYGATLSWNVTR	68	99.967			Mascot
2732.3237	2732.3357	0.012	4	18	42	CVAALEEAGVEYELVPINFGTGEHK			Carbamidomethyl (C)[1]		Mascot

4	PWWP domain-containing protein [Arabidopsis lyrata subsp. lyrata]	gi|297328473	78524.1	5.25	19	78	98.385	38.266			

Peptide Information

Calc. Mass	Obsrv. Mass	± da	± ppm	Start Seq.	End Seq.	Sequence	Ion Score	C. I. %	Modification	Rank	Result Type
1088.5582	1088.5459	-0.0123	-11	661	670	KEAANIADEK					Mascot
1107.5352	1107.5505	0.0153	14	552	561	LIPCSDSTSK			Carbamidomethyl (C)[4]		Mascot
1120.6725	1120.5863	-0.0862	-77	529	539	KSFGIGASILK					Mascot
1143.5463	1143.5775	0.0312	27	653	661	EQMVHEDKK					Mascot
1145.507	1145.5773	0.0703	61	313	323	EDSSTIHGGDK					Mascot
1159.5413	1159.5918	0.0505	44	653	661	EQMVHEDKK			Oxidation (M)[3]		Mascot
1177.6576	1177.5775	-0.0801	-68	447	456	TLAEFIAEKR					Mascot
1349.6179	1349.6985	0.0806	60	136	147	VSDSEDLGEDRK					Mascot
1366.8165	1366.6991	-0.1174	-86	301	312	IKTQNIINPGIR					Mascot
1380.6364	1380.7369	0.1005	73	460	471	HNRNTSHENSGK					Mascot
1380.6364	1380.7369	0.1005	73	460	471	HNRNTSHENSGK					Mascot
1392.6172	1392.6808	0.0636	46	690	701	QQPNKNCSDSSK			Carbamidomethyl (C)[7]		Mascot
1401.6362	1401.7177	0.0815	58	540	551	VANQMHCSTPTR			Carbamidomethyl (C)[7]		Mascot
1430.7737	1430.6981	-0.0756	-53	398	410	SSLVEVSDLEPKK					Mascot
1432.6373	1432.696	0.0587	41	649	660	DSAKEQMVHEDK			Oxidation (M)[7]		Mascot
1827.9528	1827.8459	-0.1069	-58	324	340	VSSAVFFEPADLVGYVK					Mascot
2138.0652	2138.0757	0.0105	5	371	390	GYTDLPEFVTLQGSVESAPK					Mascot
2252.1082	2252.1233	0.0151	7	263	282	QSSLPDFIDAIDFALEEVS					Mascot

Calc. Mass	Obsrv. Mass	± da	± ppm	Start Seq.	End Seq.	Sequence	Ion Score	C. I. %	Modification	Rank	Result Type
						R					
2252.1267	2252.1233	-0.0034	-2	303	323	TQNIINPGIREDSSTIHGG DK					Mascot
2274.1145	2274.0916	-0.0229	-10	284	302	IEFGLACSCISEEVYQKIK			Carbamidomethyl (C)[7,9]		Mascot
2754.3904	2754.3176	-0.0728	-26	579	605	AEALSAREISPSTNETLS SPHAASVTK					Mascot

5	PREDICTED: myosin-11-like [Setaria italica]	gi|514758641	51420.2	4.73	21	76	97.665	8.642	

Peptide Information

Calc. Mass	Obsrv. Mass	± da	± ppm	Start Seq.	End Seq.	Sequence	Ion Score	C. I. %	Modification	Rank	Result Type
887.4944	887.4387	-0.0557	-63	39	45	VQDLERK					Mascot
913.4335	913.5012	0.0677	74	299	305	MEYDLVK			Oxidation (M)[1]		Mascot
1060.5634	1060.5676	0.0042	4	224	232	VEELEGTRK					Mascot
1078.5198	1078.5381	0.0183	17	160	168	EMELGDTRK					Mascot
1079.5116	1079.552	0.0404	37	431	439	DFVRGENDK					Mascot
1085.6565	1085.5514	-0.1051	-97	289	298	ELEAGVIVKK					Mascot
1104.5532	1104.5519	-0.0013	-1	128	136	VEELDDTRK					Mascot
1107.4735	1107.5505	0.077	70	264	272	AQNVEMDER			Oxidation (M)[6]		Mascot
1131.6005	1131.6042	0.0037	3	222	231	AKVEELEGTR					Mascot
1131.6005	1131.6042	0.0037	3	222	231	AKVEELEGTR					Mascot
1161.5746	1161.5846	0.01	9	190	199	AKAEELEDTR					Mascot
1175.5903	1175.588	-0.0023	-2	126	135	AKVEELDDTR					Mascot
1177.563	1177.5775	0.0145	12	158	167	AREMELGDTR					Mascot
1190.6263	1190.5598	-0.0665	-56	311	321	LGTEVSTVEQK					Mascot
1192.5845	1192.5831	-0.0014	-1	399	409	EAVADAFDVQK					Mascot
1193.558	1193.5875	0.0295	25	158	167	AREMELGDTR			Oxidation (M)[4]		Mascot
1336.6049	1336.6594	0.0545	41	410	420	EENMKESNDLK					Mascot
1401.7156	1401.7177	0.0021	1	66	78	AAHVLSNQVSTMK			Oxidation (M)[12]		Mascot
1467.7843	1467.7151	-0.0692	-47	423	434	VEEVYAIKDFVR					Mascot
1498.7094	1498.8478	0.1384	92	299	310	MEYDLVKVENDK			Oxidation (M)[1]		Mascot
1836.8359	1836.932	0.0961	52	8	23	ESSSQSQGWQLEEVSR					Mascot
1927.9027	1927.9574	0.0547	28	334	349	MEEIMEAVLKEFDAEK			Oxidation (M)[1]		Mascot
1955.9531	1955.9537	0.0006	0	105	122	AGLVFMDAAGLYQEVAE R			Oxidation (M)[6]		Mascot

6	uncharacterized protein, partial [Phleum pratense]	gi|409972015	18297.5	9.06	2	74	96.546	7.274	68	99.967

Peptide Information

Calc. Mass	Obsrv. Mass	± da	± ppm	Start Seq.	End Seq.	Sequence	Ion Score	C. I. %	Modification	Rank	Result Type

1336.7518	1336.6594	-0.0924	-69	42	53	VHQISARMAPVK					Mascot
1380.7271	1380.7369	0.0098	7	54	65	LYGATLSWNVTR					Mascot
1380.7271	1380.7369	0.0098	7	54	65	LYGATLSWNVTR	68	99.967			Mascot

7	PREDICTED: uncharacterized protein LOC101212971 [Cucumis sativus]	gi|449457917	158329.2	5.23	27	74	96.384	34.626

Peptide Information

Calc. Mass	Obsrv. Mass	± da	± ppm	Start Seq.	End Seq.	Sequence	Ion Score	C. I. %	Modification	Rank	Result Type
834.4025	834.3307	-0.0718	-86	142	148	DVNLMDK					Mascot
907.4553	907.4711	0.0158	17	443	450	LEATMQSK					Mascot
1076.5809	1076.5511	-0.0298	-28	1350	1358	FKLPMPSEK					Mascot
1099.4803	1099.572	0.0917	83	897	904	RDDDPYYR					Mascot
1104.5242	1104.5519	0.0277	25	1366	1375	MESEPLPSSK					Mascot
1107.455	1107.5505	0.0955	86	841	849	DSDDKQDER					Mascot
1120.519	1120.5863	0.0673	60	1366	1375	MESEPLPSSK			Oxidation (M)[1]		Mascot
1143.5291	1143.5775	0.0484	42	1165	1174	HAANAFDNQR					Mascot
1145.5698	1145.5773	0.0075	7	1044	1051	EEWLQRER					Mascot
1147.5742	1147.5962	0.022	19	1016	1024	DEVFEPQKR					Mascot
1147.5742	1147.5962	0.022	19	1016	1024	DEVFEPQKR					Mascot
1151.6532	1151.5947	-0.0585	-51	523	532	LPSIDTRPPR					Mascot
1192.5879	1192.5831	-0.0048	-4	142	151	DVNLMDKDVK			Oxidation (M)[5]		Mascot
1209.5165	1209.5621	0.0456	38	611	621	TSMNSPSDNTR					Mascot
1227.5237	1227.5518	0.0281	23	914	923	REYDDETGSR					Mascot
1366.6783	1366.6991	0.0208	15	433	442	EYCKQLEQLR			Carbamidomethyl (C)[3]		Mascot
1371.8333	1371.7085	-0.1248	-91	499	511	GVPRVRPPLPAGR					Mascot
1394.6223	1394.7161	0.0938	67	973	983	YENADSYYNKK					Mascot
1425.6605	1425.7273	0.0668	47	1189	1202	EVDGSDHNALGPSK					Mascot
1447.754	1447.7092	-0.0448	-31	993	1004	EHVEKEEILHGK					Mascot
1592.8027	1592.7312	-0.0715	-45	1302	1315	ATETVDNHHLAEKK					Mascot
1607.7673	1607.827	0.0597	37	1086	1100	SGHGAEEKAWGSHVR					Mascot
1818.8981	1818.8566	-0.0415	-23	641	657	GNTPAYSAQNLGIVEER					Mascot
1827.8654	1827.8459	-0.0195	-11	1204	1218	SQENQNSYRSQMVLK			Oxidation (M)[12]		Mascot
1927.7964	1927.9574	0.161	84	366	383	GFHSGFGMPGWSNNMGGR			Oxidation (M)[8,15]		Mascot
2235.0579	2235.1545	0.0966	43	865	883	DWDPSLAHQHPLKTDGFDR					Mascot
2249.9575	2250.0808	0.1233	55	1281	1301	EIENNNGGSSEANKNPDDSMK					Mascot

2252.0249	2252.1233	0.0984	44	1295	1314	NPDDSMKATETVDNHHL AEK						Mascot
2252.0249	2252.1233	0.0984	44	1295	1314	NPDDSMKATETVDNHHL AEK						Mascot
2264.0144	2264.1145	0.1001	44	413	432	STGVDVSDFFNFGLNED SWK						Mascot
2268.0198	2268.1216	0.1018	45	1295	1314	NPDDSMKATETVDNHHL AEK			Oxidation (M)[6]			Mascot

8	Dynein 1-alpha heavy chain, flagellar inner arm (IC) [Ostreococcus tauri]	gi|308805562	573822.4	5.63	55	72	94.135	65.625

Peptide Information

Calc. Mass	Obsrv. Mass	± da	± ppm	Start Seq.	End Seq.	Sequence	Ion Score	C. I. %	Modification	Rank	Result Type
887.4833	887.4387	-0.0446	-50	4533	4540	VLTQPSDK					Mascot
906.46	906.4601	0.0001	0	1353	1360	IDLESMAK					Mascot
907.4302	907.4711	0.0409	45	2019	2025	EIKCDSR			Carbamidomethyl (C)[4]		Mascot
1078.5449	1078.5381	-0.0068	-6	1479	1488	IMDITAAAEK			Oxidation (M)[2]		Mascot
1079.5779	1079.552	-0.0259	-24	1470	1478	MKLHSHAQK					Mascot
1085.5851	1085.5514	-0.0337	-31	183	190	HIREFIDR					Mascot
1104.5684	1104.5519	-0.0165	-15	705	713	AKTWNDEIK					Mascot
1118.6569	1118.5621	-0.0948	-85	60	68	DEVLVLKFR					Mascot
1120.603	1120.5863	-0.0167	-15	1797	1807	AISAGDKLAMK			Oxidation (M)[10]		Mascot
1135.5413	1135.5942	0.0529	47	4128	4137	LSEDVLGSCR			Carbamidomethyl (C)[9]		Mascot
1136.5592	1136.5918	0.0326	29	4032	4042	FMALGQGMGPK					Mascot
1147.647	1147.5962	-0.0508	-44	50	59	AVLEFLDKGR					Mascot
1147.647	1147.5962	-0.0508	-44	50	59	AVLEFLDKGR					Mascot
1151.5621	1151.5947	0.0326	28	3280	3288	VCECVMILK			Carbamidomethyl (C)[2,4]		Mascot
1167.6191	1167.5881	-0.031	-27	3841	3850	LALSFQMTTR					Mascot
1183.614	1183.5747	-0.0393	-33	3841	3850	LALSFQMTTR			Oxidation (M)[7]		Mascot
1185.6151	1185.5563	-0.0588	-50	862	872	ISGASFFDITK					Mascot
1187.5184	1187.5979	0.0795	67	3300	3309	GMMADTNFLR			Oxidation (M)[2,3]		Mascot
1190.6093	1190.5598	-0.0495	-42	962	971	SAIYDYAIFK					Mascot
1191.6079	1191.5857	-0.0222	-19	3498	3508	SLGVPMSEPFK					Mascot
1199.6816	1199.5828	-0.0988	-82	4541	4550	CLVSELPILR			Carbamidomethyl (C)[1]		Mascot
1207.6028	1207.5801	-0.0227	-19	3498	3508	SLGVPMSEPFK			Oxidation (M)[6]		Mascot
1236.6656	1236.571	-0.0946	-76	3964	3974	VPMAVSNYIVK			Oxidation (M)[3]		Mascot
1247.6115	1247.6451	0.0336	27	3221	3230	EEELIKDSER					Mascot
1269.6521	1269.6287	-0.0234	-18	2649	2658	GRDLNWLHMK					Mascot
1283.7583	1283.6488	-0.1095	-85	4381	4390	HNYLKIVIQR					Mascot

1284.7172	1284.6481	-0.0691	-54	2770	2779	NANELLRLWR				Mascot
1299.7267	1299.649	-0.0777	-60	69	79	ERVIDEVKPSK				Mascot
1306.6903	1306.6305	-0.0598	-46	2098	2108	SHYDFGLRALK				Mascot
1343.6089	1343.7368	0.1279	95	4435	4444	DVQSWMTWYK				Mascot
1349.6995	1349.6985	-0.001	-1	1628	1638	FILDICRAEGR			Carbamidomethyl (C)[6]	Mascot
1378.7438	1378.7106	-0.0332	-24	3016	3027	EDLHANKVPVTR				Mascot
1380.7556	1380.7369	-0.0187	-14	2197	2207	IIQLYETMLTR				Mascot
1380.7556	1380.7369	-0.0187	-14	2197	2207	IIQLYETMLTR	3	0		Mascot
1392.6763	1392.6808	0.0045	3	5032	5042	CLATLVYEMHR			Carbamidomethyl (C)[1]	Mascot
1394.7097	1394.7161	0.0064	5	318	329	VCGVLESFQSLR			Carbamidomethyl (C)[2]	Mascot
1396.7505	1396.7318	-0.0187	-13	2197	2207	IIQLYETMLTR			Oxidation (M)[8]	Mascot
1396.7505	1396.7318	-0.0187	-13	2197	2207	IIQLYETMLTR	2	0	Oxidation (M)[8]	Mascot
1401.7882	1401.7177	-0.0705	-50	3947	3957	LSELERLCLLR			Carbamidomethyl (C)[8]	Mascot
1408.6711	1408.7352	0.0641	46	5032	5042	CLATLVYEMHR			Carbamidomethyl (C)[1], Oxidation (M)[9]	Mascot
1410.741	1410.7175	-0.0235	-17	3839	3850	DKLALSFQMTTR				Mascot
1412.7169	1412.7311	0.0142	10	2854	2865	VRVYQDVGDYAK				Mascot
1412.7169	1412.7311	0.0142	10	2854	2865	VRVYQDVGDYAK				Mascot
1418.7737	1418.6959	-0.0778	-55	572	584	SSSDALELVKELK				Mascot
1425.7416	1425.7273	-0.0143	-10	1243	1254	AMMMIPTFSKLR				Mascot
1432.7716	1432.696	-0.0756	-53	1479	1491	IMDITAAAEKELK				Mascot
1447.6821	1447.7092	0.0271	19	2376	2386	HREPLTCMFEK			Carbamidomethyl (C)[7]	Mascot
1452.7118	1452.7034	-0.0084	-6	3646	3658	LPNPHYDPDVTGK				Mascot
1467.7764	1467.7151	-0.0613	-42	2720	2731	ELIVNMTLDLYK			Oxidation (M)[6]	Mascot
1503.736	1503.6906	-0.0454	-30	2120	2132	DSPELSEEVVLMR				Mascot
1505.7457	1505.6841	-0.0616	-41	3614	3626	SFYYAPNGAKMIK			Oxidation (M)[11]	Mascot
1592.7084	1592.7312	0.0228	14	1011	1023	WMDGTCLEVPEQK			Carbamidomethyl (C)[6]	Mascot
1819.0073	1818.8566	-0.1507	-83	914	930	VEEIVHGSSTGKVVHLK				Mascot
1836.9823	1836.932	-0.0503	-27	3040	3056	ANLHIVLAMSPVGDMLR				Mascot
1864.9586	1864.9832	0.0246	13	287	302	QLEDPGMQVVLDVLHR			Oxidation (M)[7]	Mascot
2020.9176	2021.0447	0.1271	63	80	98	ALTDTTNACEPMRDVGG GR			Carbamidomethyl (C)[9]	Mascot
2233.9597	2234.1318	0.1721	77	5047	5068	TGMFSCCVGSGMGVAAL VSVED			Carbamidomethyl (C)[6,7]	Mascot
2233.9597	2234.1318	0.1721	77	5047	5068	TGMFSCCVGSGMGVAAL VSVED			Carbamidomethyl (C)[6,7]	Mascot
2249.9546	2250.0808	0.1262	56	5047	5068	TGMFSCCVGSGMGVAAL VSVED			Carbamidomethyl (C)[6,7], Oxidation (M)[3]	Mascot
2252.0952	2252.1233	0.0281	12	3289	3309	GLPNVSWSGAKGMMAD TNFLR				Mascot
2252.0952	2252.1233	0.0281	12	3289	3309	GLPNVSWSGAKGMMAD TNFLR				Mascot

2264.1235	2264.1145	-0.009	-4	3280	3299	VCECVMILKGLPNVSWS GAK			Carbamidomethyl (C)[2,4], Oxidation (M)[6]	Mascot
2268.0901	2268.1216	0.0315	14	3289	3309	GLPNVSWSGAKGMMAD TNFLR			Oxidation (M)[13]	Mascot
2280.1475	2280.1387	-0.0088	-4	603	621	MLNNQIIGKMQDVLEQFS R			Oxidation (M)[1]	Mascot
2286.2122	2286.0735	-0.1387	-61	2208	2229	HTTMLVGETGGGKSVILE TIAR			Oxidation (M)[4]	Mascot
2714.2766	2714.3528	0.0762	28	1158	1180	WIEEIGAATYNVDVELMN NVYDR				Mascot
2715.2351	2715.3311	0.096	35	2387	2410	YMDKMTAFCYEGADLDG SVTALIK			Carbamidomethyl (C)[9], Oxidation (M)[2]	Mascot
2731.23	2731.304	0.074	27	2387	2410	YMDKMTAFCYEGADLDG SVTALIK			Carbamidomethyl (C)[9], Oxidation (M)[2,5]	Mascot

9 multi-sensor hybrid histidine kinase [Bathycoccus prasinos] gi|412990839 117830.8 6.44 26 71 92.444 43.422

Peptide Information

Calc. Mass	Obsrv. Mass	± da	± ppm	Start Seq.	End Seq.	Sequence	Ion Score	C. I. %	Modification	Rank	Result Type
867.4393	867.4488	0.0095	11	631	638	LGNFSAMK					Mascot
1058.5114	1058.5356	0.0242	23	484	493	SHELSEATGK					Mascot
1078.5133	1078.5381	0.0248	23	979	987	CGGLECVRK			Carbamidomethyl (C)[1,6]		Mascot
1135.5742	1135.5942	0.02	18	988	996	IREQYPDSK					Mascot
1145.6525	1145.5773	-0.0752	-66	1034	1043	SLVDDIVTRK					Mascot
1175.6783	1175.588	-0.0903	-77	730	739	ELLAFAQKQK					Mascot
1185.6726	1185.5563	-0.1163	-98	949	959	DGVVALELIEK					Mascot
1191.5422	1191.5857	0.0435	37	649	659	VNMSIGDENGR					Mascot
1192.6321	1192.5831	-0.049	-41	555	566	FDGSGLGLAISR					Mascot
1195.614	1195.5868	-0.0272	-23	389	398	FTEKGDIMVR					Mascot
1195.614	1195.5868	-0.0272	-23	389	398	FTEKGDIMVR					Mascot
1207.5372	1207.5801	0.0429	36	649	659	VNMSIGDENGR			Oxidation (M)[3]		Mascot
1209.5028	1209.5621	0.0593	49	939	948	CGCDDVVWAK			Carbamidomethyl (C)[1,3]		Mascot
1211.6089	1211.5697	-0.0392	-32	389	398	FTEKGDIMVR			Oxidation (M)[8]		Mascot
1247.6412	1247.6451	0.0039	3	527	538	DTGIGLSANMIR					Mascot
1269.7526	1269.6287	-0.1239	-98	76	85	LQEEILQLRK					Mascot
1320.7271	1320.6163	-0.1108	-84	555	567	FDGSGLGLAISRK					Mascot
1349.7173	1349.6985	-0.0188	-14	2	14	VVVVPDSNNAHAK					Mascot
1351.7368	1351.6703	-0.0665	-49	727	737	FERELLAFAQK					Mascot
1380.6503	1380.7369	0.0866	63	703	714	DVAAHPEDEKNR					Mascot
1380.6503	1380.7369	0.0866	63	703	714	DVAAHPEDEKNR					Mascot
1408.6664	1408.7352	0.0688	49	760	774	SGSVARSTDGGSAEK					Mascot
1422.7476	1422.7004	-0.0472	-33	740	753	LVSEDGSTFLGAVK					Mascot

1424.7322	1424.7264	-0.0058	-4	1017	1028	DGFTGWLTKPFR						Mascot
1425.777	1425.7273	-0.0497	-35	919	930	ILLAEDHLINMK			Oxidation (M)[11]			Mascot
1428.7958	1428.7225	-0.0733	-51	775	786	KPKEHTPVEVHK						Mascot
1440.573	1440.6863	0.1133	79	595	606	CQVANENDMTDK			Carbamidomethyl (C)[1], Oxidation (M)[9]			Mascot
2250.0676	2250.0808	0.0132	6	997	1016	IPIVAVTADAMTESRDNC MR			Carbamidomethyl (C)[18]			Mascot
2252.0225	2252.1233	0.1008	45	430	448	LAHMCPHFSPKDGEQPQ EK			Carbamidomethyl (C)[5], Oxidation (M)[4]			Mascot
2252.0225	2252.1233	0.1008	45	430	448	LAHMCPHFSPKDGEQPQ EK			Carbamidomethyl (C)[5], Oxidation (M)[4]			Mascot
2286.1548	2286.0735	-0.0813	-36	354	374	LNVGYHVDEGVPKTVLG DAMR			Oxidation (M)[20]			Mascot

10	PREDICTED: protein translocase subunit SECA1, chloroplastic-like [Fragaria vesca subsp. vesca]	gi|470106151	115016.6	6.72	25	70	89.327	17.901	

Peptide Information

Calc. Mass	Obsrv. Mass	± da	± ppm	Start Seq.	End Seq.	Sequence	Ion Score	C. I. %	Modification	Rank	Result Type
907.4995	907.4711	-0.0284	-31	878	884	EAYLQKR					Mascot
1076.5582	1076.5511	-0.0071	-7	101	109	LSDSELREK					Mascot
1107.6093	1107.5505	-0.0588	-53	737	745	IQGLMRAFR			Oxidation (M)[5]		Mascot
1118.6061	1118.5621	-0.044	-39	573	581	EMLMPRVVK			Oxidation (M)[2]		Mascot
1131.5946	1131.6042	0.0096	8	33	42	TSFFAGKAFR					Mascot
1131.5946	1131.6042	0.0096	8	33	42	TSFFAGKAFR					Mascot
1145.6273	1145.5773	-0.05	-44	43	52	LPETSRISSR					Mascot
1177.6324	1177.5775	-0.0549	-47	108	116	EKTLQFQQR					Mascot
1207.6868	1207.5801	-0.1067	-88	660	669	LRSAFLEIMK					Mascot
1267.7369	1267.6483	-0.0886	-70	650	661	GPALDDVIAKLR					Mascot
1284.7094	1284.6481	-0.0613	-48	479	489	AVVVEISRMHK			Oxidation (M)[9]		Mascot
1378.7261	1378.7106	-0.0155	-11	731	742	IFGGDRIQGLMR			Oxidation (M)[11]		Mascot
1380.7594	1380.7369	-0.0225	-16	682	695	VVSAGGLHVVGTER					Mascot
1380.7594	1380.7369	-0.0225	-16	682	695	VVSAGGLHVVGTER					Mascot
1384.7981	1384.7305	-0.0676	-49	451	462	VTIVPTNKPMIR			Oxidation (M)[10]		Mascot
1401.6692	1401.7177	0.0485	35	201	211	RDCEWVGQVPR			Carbamidomethyl (C)[3]		Mascot
1424.7017	1424.7264	0.0247	17	464	476	DESDVVFRATTGK					Mascot
1443.7988	1443.6909	-0.1079	-75	884	896	RTIIESQAPGLMK					Mascot
1452.7482	1452.7034	-0.0448	-31	929	940	GYAQRDPLIEYK					Mascot
1503.7584	1503.6906	-0.0678	-45	217	229	VGLIQQNMTSEQR					Mascot
1592.8656	1592.7312	-0.1344	-84	920	933	FVQQAVGLRGYAQR					Mascot
1837.0443	1836.932	-0.1123	-61	913	928	EHLQALKFVQQAVGLR					Mascot

1864.9586	1864.9832	0.0246	13	374	389	EVLIVDEFTGRVMQGR	Oxidation (M)[13]	Mascot
1955.9167	1955.9537	0.037	19	303	318	MASVFERDIHYTVDEK	Oxidation (M)[1]	Mascot
2264.3062	2264.1145	-0.1917	-85	140	159	RVLGLRPFDVQLIGGMVL HK	Oxidation (M)[16]	Mascot
2285.0754	2285.1548	0.0794	35	263	281	NFNYCVIDEVDSILIDEAR	Carbamidomethyl (C)[5]	Mascot
2288.188	2288.0762	-0.1118	-49	119	139	QGESLDSLLPEAFAVIRE ASR		Mascot

Gel Idx/Pos	203/I3	Instr./Gel Origin	BA2151/Sample Project 20140814	Process Status	Analysis Succeeded
Plate [#] Name	[1] Sample Project 20140814	Instrument Sample Name		Spectra	11

Rank	Protein Name	Accession No.	Protein MW	Protein PI	Pep. Count	Protein Score	Protein Score C. I. %	Intensity Matched	Total Ion Score	Total Ion C. I. %	Confirmed
1	hypothetical protein TRIUR3_28410 [Triticum urartu]	gi|474060617	28387.9	5.53	9	250	100	17.641	202	100	

Peptide Information

Calc. Mass	Obsrv. Mass	± da	± ppm	Start Seq.	End Seq.	Sequence	Ion Score	C. I. %	Modification	Rank	Result Type
930.4389	930.4361	-0.0028	-3	194	200	FEAYICK			Carbamidomethyl (C)[6]		Mascot
971.5673	971.5276	-0.0397	-41	51	58	FVTNHLLK					Mascot
1078.535	1078.5316	-0.0034	-3	207	215	GYPLLEACR			Carbamidomethyl (C)[8]		Mascot
1078.535	1078.5316	-0.0034	-3	207	215	GYPLLEACR	55	99.436	Carbamidomethyl (C)[8]		Mascot
1145.5698	1145.5648	-0.005	-4	156	165	GHNLSLEYGR					Mascot
1145.5698	1145.5648	-0.005	-4	156	165	GHNLSLEYGR	79	99.997			Mascot
1406.6271	1406.6199	-0.0072	-5	130	139	HREWESCFQK			Carbamidomethyl (C)[7]		Mascot
1406.6271	1406.6199	-0.0072	-5	130	139	HREWESCFQK			Carbamidomethyl (C)[7]		Mascot
1796.9075	1796.84	-0.0675	-38	113	127	VHLGFIYCVSDLVMK			Carbamidomethyl (C)[8], Oxidation (M)[14]		Mascot
1845.9594	1845.8855	-0.0739	-40	62	79	DGLLDAADLTLVPYGNAK					Mascot
1927.9371	1927.9288	-0.0083	-4	35	50	VHVAIYYESLCPYSAR			Carbamidomethyl (C)[11]		Mascot
1927.9371	1927.9288	-0.0083	-4	35	50	VHVAIYYESLCPYSAR	68	99.969	Carbamidomethyl (C)[11]		Mascot
3402.8162	3402.6677	-0.1485	-44	1	34	MARGLHHLLLLAALLQQL SATSAGDVATGSGGEK			Oxidation (M)[1]		Mascot

Rank	Protein Name	Accession No.	Protein MW	Protein PI	Pep. Count	Protein Score	Protein Score C. I. %	Intensity Matched	Total Ion Score	Total Ion C. I. %	Confirmed
2	Avenin-3 [Triticum urartu]	gi|474329936	22657.1	6.35	3	116	100	18.253	102	100	

Peptide Information

Calc. Mass	Obsrv. Mass	± da	± ppm	Start Seq.	End Seq.	Sequence	Ion Score	C. I. %	Modification	Rank	Result Type
1950.9413	1950.9283	-0.013	-7	60	76	DALLQQCSPVADMSFLR			Carbamidomethyl (C)[7]		Mascot
1966.9362	1966.8981	-0.0381	-19	60	76	DALLQQCSPVADMSFLR			Carbamidomethyl (C)[7], Oxidation (M)[13]		Mascot
1966.9362	1966.8981	-0.0381	-19	60	76	DALLQQCSPVADMSFLR	102	100	Carbamidomethyl (C)[7], Oxidation (M)[13]		Mascot
2635.1885	2635.1633	-0.0252	-10	77	97	SQVVQHSSCLVMWEQC CQQLK			Carbamidomethyl (C)[9,16,17]		Mascot
2651.1833	2651.1372	-0.0461	-17	77	97	SQVVQHSSCLVMWEQC CQQLK			Carbamidomethyl (C)[9,16,17], Oxidation (M)[12]		Mascot
3222.5352	3222.5425	0.0073	2	27	54	TAWEPHHPSSPEQQPTP QPQEQPVPHQK					Mascot

Rank	Protein Name	Accession No.	Protein MW	Protein PI	Pep. Count	Protein Score	Protein Score C. I. %	Intensity Matched	Total Ion Score	Total Ion C. I. %	Confirmed
3	alpha-amylase inhibitor 1 [Aegilops tauschii]	gi|442614142	22813.2	6.2	2	109	99.999	18.089	102	100	

Protein Group

	hypothetical protein F775_28748 [Aegilops tauschii]	gi|475569529	22813.2	6.1999 998092 6514

Peptide Information

Calc. Mass	Obsrv. Mass	± da	± ppm	Start Seq.	End Seq.	Sequence	Ion Score	C. I. %	Modification	Rank	Result Type
1950.9413	1950.9283	-0.013	-7	60	76	DALLQQCSPVADMSFLR			Carbamidomethyl (C)[7]		Mascot
1966.9362	1966.8981	-0.0381	-19	60	76	DALLQQCSPVADMSFLR			Carbamidomethyl (C)[7], Oxidation (M)[13]		Mascot
1966.9362	1966.8981	-0.0381	-19	60	76	DALLQQCSPVADMSFLR	102	100	Carbamidomethyl (C)[7], Oxidation (M)[13]		Mascot
2635.1885	2635.1633	-0.0252	-10	77	97	SQVVQHSSCLVMWEQC CQQLK			Carbamidomethyl (C)[9,16,17]		Mascot
2651.1833	2651.1372	-0.0461	-17	77	97	SQVVQHSSCLVMWEQC CQQLK			Carbamidomethyl (C)[9,16,17], Oxidation (M)[12]		Mascot

4	hypothetical protein F775_31970 [Aegilops tauschii]	gi|475578125	25615.9	6.35	4	69	88.297	3.735	55	99.436

Peptide Information

Calc. Mass	Obsrv. Mass	± da	± ppm	Start Seq.	End Seq.	Sequence	Ion Score	C. I. %	Modification	Rank	Result Type
1078.535	1078.5316	-0.0034	-3	207	215	GYPLLEACR			Carbamidomethyl (C)[8]		Mascot
1078.535	1078.5316	-0.0034	-3	207	215	GYPLLEACR	55	99.436	Carbamidomethyl (C)[8]		Mascot
1796.9075	1796.84	-0.0675	-38	116	130	VHLGFIYCVSDLVMK			Carbamidomethyl (C)[8], Oxidation (M)[14]		Mascot
1845.9594	1845.8855	-0.0739	-40	65	82	DGLLDAADLTLVPYGNAK					Mascot
1955.9684	1955.9403	-0.0281	-14	38	53	VHVAIYYESLCPYSVR			Carbamidomethyl (C)[11]		Mascot

5	hypothetical protein CARUB_v10001394mg [Capsella rubella]	gi|482556862	36134.2	5.78	12	62	32.659	11.035

Peptide Information

Calc. Mass	Obsrv. Mass	± da	± ppm	Start Seq.	End Seq.	Sequence	Ion Score	C. I. %	Modification	Rank	Result Type
987.5693	987.5199	-0.0494	-50	64	72	SGARIQLSR					Mascot
1078.5011	1078.5316	0.0305	28	325	334	VESATERATD					Mascot
1078.5011	1078.5316	0.0305	28	325	334	VESATERATD					Mascot
1138.574	1138.5322	-0.0418	-37	53	63	GGSTITEFQAK					Mascot
1182.575	1182.547	-0.028	-24	294	304	GDFISGTSDRK					Mascot
1208.6521	1208.5541	-0.098	-81	145	155	SFIEESKAGIK					Mascot
1380.661	1380.7098	0.0488	35	276	287	NIMEITQMTGAR			Oxidation (M)[3]		Mascot
1613.7806	1613.7089	-0.0717	-44	156	169	ISPLDNTYYGLSDR					Mascot
1845.8059	1845.8855	0.0796	43	1	17	MESTESYAAGSPEELTK			Oxidation (M)[1]		Mascot

Calc. Mass	Obsrv. Mass	± da	± ppm	Start Seq.	End Seq.	Sequence	Ion Score	C. I. %	Modification	Rank	Result Type
1908.9563	1908.8411	-0.1152	-60	68	83	IQLSRNQEFFPGTTDR					Mascot
1928.0634	1927.9288	-0.1346	-70	125	144	LVVPNSSCGGIIGKGGATI K			Carbamidomethyl (C)[8]		Mascot
1928.0634	1927.9288	-0.1346	-70	125	144	LVVPNSSCGGIIGKGGATI K			Carbamidomethyl (C)[8]		Mascot
1983.0182	1982.8906	-0.1276	-64	152	169	AGIKISPLDNTYYGLSDR					Mascot
2592.3376	2592.1531	-0.1845	-71	251	275	EEPSNTVTIGVSDEHIGL VLGRGGR					Mascot

6 PREDICTED: oxysterol-binding protein-related protein 1C-like [Solanum lycopersicum] gi|460394838 88876 6.7 19 58 0 19.506

Peptide Information

Calc. Mass	Obsrv. Mass	± da	± ppm	Start Seq.	End Seq.	Sequence	Ion Score	C. I. %	Modification	Rank	Result Type
848.4108	848.4304	0.0196	23	180	186	AETKEDR					Mascot
849.4465	849.4261	-0.0204	-24	389	395	LPEPHEK					Mascot
867.457	867.432	-0.025	-29	167	174	FSINTGTK					Mascot
930.5043	930.4361	-0.0682	-73	496	503	GLHFISEK					Mascot
1106.5841	1106.5463	-0.0378	-34	389	397	LPEPHEKEK					Mascot
1187.5739	1187.6111	0.0372	31	725	733	MQERGWQPR					Mascot
1195.6106	1195.5608	-0.0498	-42	522	531	FWGDTNLKSK					Mascot
1380.7131	1380.7098	-0.0033	-2	603	614	NPHQVHGVIHDK					Mascot
1412.6653	1412.7141	0.0488	35	292	303	DRQTYSEASLDK					Mascot
1517.8145	1517.7177	-0.0968	-64	398	411	GVGLWSMIKDNIGK					Mascot
1550.8398	1550.7809	-0.0589	-38	686	698	LPPTDSRLRPDQR					Mascot
1647.6843	1647.7654	0.0811	49	699	712	FLENGEYDMADSEK					Mascot
1663.6792	1663.7589	0.0797	48	699	712	FLENGEYDMADSEK			Oxidation (M)[9]		Mascot
1811.8534	1811.9325	0.0791	44	759	773	WISCPHIFGEISQHT			Carbamidomethyl (C)[4]		Mascot
1818.8309	1818.8202	-0.0107	-6	504	518	VSHHPMILACHCQGR			Carbamidomethyl (C)[10,12], Oxidation (M)[6]		Mascot
1851.9369	1851.8595	-0.0774	-42	111	127	IVTNSETEKGSMIIGEK			Oxidation (M)[12]		Mascot
1918.8688	1918.905	0.0362	19	572	586	LYCDHYGTMYIRGNR			Carbamidomethyl (C)[3]		Mascot
2684.0691	2684.1558	0.0867	32	626	649	WDESLHYSMGDNPSDD VGQDSDSK					Mascot
3222.4185	3222.5425	0.124	38	432	457	CFEEFEYSYLLDQAYEW GKTGNSVMR			Carbamidomethyl (C)[1]		Mascot
3238.4133	3238.552	0.1387	43	432	457	CFEEFEYSYLLDQAYEW GKTGNSVMR			Carbamidomethyl (C)[1], Oxidation (M)[25]		Mascot
3448.5022	3448.7388	0.2366	69	626	655	WDESLHYSMGDNPSDD VGQDSDSKSHLIWK					Mascot

7 PREDICTED: expansin-like A1-like [Cucumis sativus] gi|449454269 29960.7 8.24 10 57 0 5.431

Protein Group

PREDICTED: expansin-like A1-like [Cucumis sativus] gi|449470768 29960.7 8.2399

997711
1816

Peptide Information

Calc. Mass	Obsrv. Mass	± da	± ppm	Start Seq.	End Seq.	Sequence	Ion Score	C. I. %	Modification	Rank	Result Type
823.4308	823.4077	-0.0231	-28	103	109	TDFVVSR					Mascot
1037.5514	1037.463	-0.0884	-85	127	135	TGIVDIEYK					Mascot
1138.5925	1138.5322	-0.0603	-53	110	120	KAFSAMALDGK					Mascot
1337.6597	1337.6547	-0.005	-4	191	201	RNYGAIWDTNK					Mascot
1507.7614	1507.6714	-0.09	-60	222	234	GIMINYALPADWK			Oxidation (M)[3]		Mascot
1818.932	1818.8202	-0.1118	-61	219	234	NGRGIMINYALPADWK					Mascot
1875.96	1875.8877	-0.0723	-39	192	208	NYGAIWDTNKVPEGAIK					Mascot
1875.96	1875.8877	-0.0723	-39	192	208	NYGAIWDTNKVPEGAIK					Mascot
2651.2771	2651.1372	-0.1399	-53	235	257	TGEIYDTGIQIKDIATEAC NPWR			Carbamidomethyl (C)[19]		Mascot
3218.707	3218.5381	-0.1689	-52	137	162	IPCEYNKNLLIQVVEWSH KPYYLAIK			Carbamidomethyl (C)[3]		Mascot
3250.5222	3250.5249	0.0027	1	163	191	FLYQGGQTDITAVDLATQ DGSGGWQYMRR			Oxidation (M)[27]		Mascot

8 hypothetical protein CARUB_v10001394mg [Capsella rubella] gi|482556861 30514.3 5.55 10 52 0 8.104

Peptide Information

Calc. Mass	Obsrv. Mass	± da	± ppm	Start Seq.	End Seq.	Sequence	Ion Score	C. I. %	Modification	Rank	Result Type
987.5693	987.5199	-0.0494	-50	64	72	SGARIQLSR					Mascot
1138.574	1138.5322	-0.0418	-37	53	63	GGSTITEFQAK					Mascot
1208.6521	1208.5541	-0.098	-81	145	155	SFIEESKAGIK					Mascot
1380.661	1380.7098	0.0488	35	255	266	NIMEITQMTGAR			Oxidation (M)[3]		Mascot
1613.7806	1613.7089	-0.0717	-44	156	169	ISPLDNTYYGLSDR					Mascot
1845.8059	1845.8855	0.0796	43	1	17	MESTESYAAGSPEELTK			Oxidation (M)[1]		Mascot
1908.9563	1908.8411	-0.1152	-60	68	83	IQLSRNQEFFPGTTDR					Mascot
1928.0634	1927.9288	-0.1346	-70	125	144	LVVPNSSCGGIIGKGGATI K			Carbamidomethyl (C)[8]		Mascot
1928.0634	1927.9288	-0.1346	-70	125	144	LVVPNSSCGGIIGKGGATI K			Carbamidomethyl (C)[8]		Mascot
1983.0182	1982.8906	-0.1276	-64	152	169	AGIKISPLDNTYYGLSDR					Mascot
2592.3376	2592.1531	-0.1845	-71	230	254	EEPSNTVTIGVSDEHIGL VLGRGGR					Mascot

9 uncharacterized protein [Arabidopsis thaliana] gi|334184987 9377.8 9.51 6 52 0 6.342

Protein Group

uncharacterized protein AT2G48121 [Arabidopsis thaliana]	gi|330255847	9377.8	9.5100 002288 8184

Peptide Information

Calc. Mass	Obsrv. Mass	± da	± ppm	Start Seq.	End Seq.	Sequence	Ion Score	C. I. %	Modification	Rank	Result Type
971.488	971.5276	0.0396	41	4	10	FCGRYLR			Carbamidomethyl (C)[2]		Mascot
1926.9783	1926.8879	-0.0904	-47	61	75	RLMFEVFDFEQIQPK					Mascot
1927.9773	1927.9288	-0.0485	-25	13	27	RLHNFIIYSEEVHDR					Mascot
1927.9773	1927.9288	-0.0485	-25	13	27	RLHNFIIYSEEVHDR					Mascot
1942.9731	1942.8854	-0.0877	-45	61	75	RLMFEVFDFEQIQPK			Oxidation (M)[3]		Mascot
1956.9777	1956.9514	-0.0263	-13	62	77	LMFEVFDFEQIQPKAV			Oxidation (M)[2]		Mascot
2684.4155	2684.1558	-0.2597	-97	36	60	NPATTAVQQAIHGLAYTI YGKPDVR					Mascot
2840.5166	2840.3169	-0.1997	-70	36	61	NPATTAVQQAIHGLAYTI YGKPDVRR					Mascot

10	predicted protein [Bathycoccus prasinos]	gi|412986644	29267.6	6.72	10	52	0	3.075	

Peptide Information

Calc. Mass	Obsrv. Mass	± da	± ppm	Start Seq.	End Seq.	Sequence	Ion Score	C. I. %	Modification	Rank	Result Type
866.4189	866.4011	-0.0178	-21	212	219	IGYMPGGR			Oxidation (M)[4]		Mascot
1046.5114	1046.5054	-0.006	-6	153	161	QVSEEEGLR					Mascot
1195.6066	1195.5608	-0.0458	-38	245	256	ERAAGTYGSVGK					Mascot
1380.7305	1380.7098	-0.0207	-15	44	54	SCLLLQFTDKR			Carbamidomethyl (C)[2]		Mascot
1550.7598	1550.7809	0.0211	14	81	93	LQIWDTAGQESFR					Mascot
1796.7909	1796.84	0.0491	27	116	129	ETFENLNAWLEDCR			Carbamidomethyl (C)[13]		Mascot
1851.9891	1851.8595	-0.1296	-70	2	17	SYAYLFKYIIIGDTGK					Mascot
1860.9603	1860.9138	-0.0465	-25	98	114	SYYRGAAGALLVYDVSR					Mascot
1983.0297	1982.8906	-0.1391	-70	1	17	MSYAYLFKYIIIGDTGK					Mascot
3373.6404	3373.6201	-0.0203	-6	165	195	ENGLLFVETSAMTSANV DGAFIETARVICNK			Carbamidomethyl (C)[29], Oxidation (M)[12]		Mascot

Gel Idx/Pos	204/I4	Instr./Gel Origin	BA2151/Sample Project 20140814	Process Status	Analysis Succeeded
Plate [#] Name	[1] Sample Project 20140814	Instrument Sample Name		Spectra	11

Rank	Protein Name	Accession No.	Protein MW	Protein PI	Pep. Count	Protein Score	Protein Score C. I. %	Intensity Matched	Total Ion Score	Total Ion C. I. %	Confirmed
1	L-ascorbate peroxidase 1, cytosolic [Triticum urartu]	gi|474311703	27561.1	5.85	12	417	100	29.858	330	100	

Protein Group

L-ascorbate peroxidase 1, cytosolic [Aegilops tauschii]	gi|475626297	27561.1	5.8499 999046 3257

Peptide Information

Calc. Mass	Obsrv. Mass	± da	± ppm	Start Seq.	End Seq.	Sequence	Ion Score	C. I. %	Modification	Rank	Result Type
895.4342	895.4674	0.0332	37	53	61	TGGPFGTMK					Mascot
911.4291	911.429	-0.0001	0	53	61	TGGPFGTMK			Oxidation (M)[8]		Mascot
919.4196	919.4172	-0.0024	-3	230	236	AFFEDYK					Mascot
1006.4808	1006.4757	-0.0051	-5	31	38	NCSPLMLR			Carbamidomethyl (C)[2], Oxidation (M)[6]		Mascot
1036.4847	1036.4836	-0.0011	-1	173	181	SGFEGPWTR					Mascot
1036.4847	1036.4836	-0.0011	-1	173	181	SGFEGPWTR	25	0			Mascot
1249.6172	1249.6107	-0.0065	-5	120	130	EDKPQPPPEGR					Mascot
1249.6172	1249.6107	-0.0065	-5	120	130	EDKPQPPPEGR	58	99.591			Mascot
1505.7384	1505.7271	-0.0113	-8	39	52	LAWHSAGTFDVSSK					Mascot
1525.7434	1525.7363	-0.0071	-5	230	241	AFFEDYKEAHLR					Mascot
1525.7434	1525.7363	-0.0071	-5	230	241	AFFEDYKEAHLR	44	88.624			Mascot
1627.9418	1627.9352	-0.0066	-4	210	223	TLLTDPVFRPLVEK					Mascot
1627.9418	1627.9352	-0.0066	-4	210	223	TLLTDPVFRPLVEK	52	98.419			Mascot
1712.8378	1712.8055	-0.0323	-19	4	18	TYPVVSAEYQEAVEK					Mascot
1831.9773	1831.9725	-0.0048	-3	62	79	KPAEQAHAANAGLDIAVR					Mascot
1831.9773	1831.9725	-0.0048	-3	62	79	KPAEQAHAANAGLDIAVR	152	100			Mascot
2071.0237	2071.0107	-0.013	-6	148	167	QMGLSDQDIVALSGGHT LGR			Oxidation (M)[2]		Mascot
2559.3711	2559.2598	-0.1113	-43	62	85	KPAEQAHAANAGLDIAVR MLEPIK			Oxidation (M)[19]		Mascot

Rank	Protein Name	Accession No.	Protein MW	Protein PI	Pep. Count	Protein Score	Protein Score C. I. %	Intensity Matched	Total Ion Score	Total Ion C. I. %	Confirmed
2	uncharacterized protein, partial [Phleum pratense]	gi|409971721	19776	5.13	5	181	100	15.936	154	100	

Protein Group

uncharacterized protein, partial [Phleum pratense]	gi|409972081	19705	5.1300 001144

Peptide Information

Calc. Mass	Obsrv. Mass	± da	± ppm	Start Seq.	End Seq.	Sequence	Ion Score	C. I. %	Modification	Rank	Result Type
919.4196	919.4172	-0.0024	-3	160	166	AFFEDYK					Mascot
1249.6172	1249.6107	-0.0065	-5	50	60	EDKPQPPPEGR					Mascot
1249.6172	1249.6107	-0.0065	-5	50	60	EDKPQPPPEGR	58	99.591			Mascot
1525.7434	1525.7363	-0.0071	-5	160	171	AFFEDYKEAHLR					Mascot
1525.7434	1525.7363	-0.0071	-5	160	171	AFFEDYKEAHLR	44	88.624			Mascot
1627.9418	1627.9352	-0.0066	-4	140	153	TLLTDPVFRPLVEK					Mascot
1627.9418	1627.9352	-0.0066	-4	140	153	TLLTDPVFRPLVEK	52	98.419			Mascot
2071.0237	2071.0107	-0.013	-6	78	97	QMGLSDQDIVALSGGHT LGR			Oxidation (M)[2]		Mascot

3 uncharacterized protein, partial [Phleum pratense] gi|409971619 13234.6 5.15 5 131 100 13.184 96 100

Protein Group

uncharacterized protein, partial [Phleum pratense] gi|409972029 13234.6 5.1500 000953 6743

Peptide Information

Calc. Mass	Obsrv. Mass	± da	± ppm	Start Seq.	End Seq.	Sequence	Ion Score	C. I. %	Modification	Rank	Result Type
919.4196	919.4172	-0.0024	-3	97	103	AFFEDYK					Mascot
1050.5327	1050.4817	-0.051	-49	1	9	PQEGVDHLR					Mascot
1525.7434	1525.7363	-0.0071	-5	97	108	AFFEDYKEAHLR					Mascot
1525.7434	1525.7363	-0.0071	-5	97	108	AFFEDYKEAHLR	44	88.624			Mascot
1627.9418	1627.9352	-0.0066	-4	77	90	TLLTDPVFRPLVEK					Mascot
1627.9418	1627.9352	-0.0066	-4	77	90	TLLTDPVFRPLVEK	52	98.419			Mascot
2071.0237	2071.0107	-0.013	-6	15	34	QMGLSDQDIVALSGGHT LGR			Oxidation (M)[2]		Mascot

4 uncharacterized protein, partial [Phleum pratense] gi|409971665 14973.5 5.51 4 120 100 12.96 96 100

Peptide Information

Calc. Mass	Obsrv. Mass	± da	± ppm	Start Seq.	End Seq.	Sequence	Ion Score	C. I. %	Modification	Rank	Result Type
919.4196	919.4172	-0.0024	-3	113	119	AFFEDYK					Mascot

Calc. Mass	Obsrv. Mass	± da	± ppm	Start Seq.	End Seq.	Sequence	Ion Score	C. I. %	Modification	Rank	Result Type
1525.7434	1525.7363	-0.0071	-5	113	124	AFFEDYKEAHLR					Mascot
1525.7434	1525.7363	-0.0071	-5	113	124	AFFEDYKEAHLR	44	88.624			Mascot
1627.9418	1627.9352	-0.0066	-4	93	106	TLLTDPVFRPLVEK					Mascot
1627.9418	1627.9352	-0.0066	-4	93	106	TLLTDPVFRPLVEK	52	98.419			Mascot
2071.0237	2071.0107	-0.013	-6	31	50	QMGLSDQDIVALSGGHT LGR			Oxidation (M)[2]		Mascot

5 uncharacterized protein, partial [Phleum pratense] gi|409972493 10286.2 5.31 3 115 100 11.595 96 100

Peptide Information

Calc. Mass	Obsrv. Mass	± da	± ppm	Start Seq.	End Seq.	Sequence	Ion Score	C. I. %	Modification	Rank	Result Type
919.4196	919.4172	-0.0024	-3	70	76	AFFEDYK					Mascot
1525.7434	1525.7363	-0.0071	-5	70	81	AFFEDYKEAHLR					Mascot
1525.7434	1525.7363	-0.0071	-5	70	81	AFFEDYKEAHLR	44	88.624			Mascot
1627.9418	1627.9352	-0.0066	-4	50	63	TLLTDPVFRPLVEK					Mascot
1627.9418	1627.9352	-0.0066	-4	50	63	TLLTDPVFRPLVEK	52	98.419			Mascot

6 hypothetical protein ZEAMMB73_022439 [Zea mays] gi|413956090 32615.5 6.51 6 83 99.465 5.494 58 99.591

Peptide Information

Calc. Mass	Obsrv. Mass	± da	± ppm	Start Seq.	End Seq.	Sequence	Ion Score	C. I. %	Modification	Rank	Result Type
895.4454	895.4674	0.022	25	9	16	CASFLAAR			Carbamidomethyl (C)[1]		Mascot
911.4291	911.429	-0.0001	0	106	114	TGGPFGTMK			Oxidation (M)[8]		Mascot
1096.5997	1096.5054	-0.0943	-86	290	299	EAHLKLSELG					Mascot
1249.6172	1249.6107	-0.0065	-5	173	183	EDKPQPPPEGR					Mascot
1249.6172	1249.6107	-0.0065	-5	173	183	EDKPQPPPEGR	58	99.591			Mascot
1655.7992	1655.9353	0.1361	82	2	16	VQSCASKCASFLAAR			Carbamidomethyl (C)[4,8]		Mascot
2071.0237	2071.0107	-0.013	-6	201	220	QMGLSDQDIVALSGGHT LGR			Oxidation (M)[2]		Mascot

7 ascorbate peroxidase [Eleusine coracana] gi|338760827 27561 5.79 5 80 98.957 6.086 58 99.591

Peptide Information

Calc. Mass	Obsrv. Mass	± da	± ppm	Start Seq.	End Seq.	Sequence	Ion Score	C. I. %	Modification	Rank	Result Type
895.4342	895.4674	0.0332	37	53	61	TGGPFGTMK					Mascot
911.4291	911.429	-0.0001	0	53	61	TGGPFGTMK			Oxidation (M)[8]		Mascot
919.4196	919.4172	-0.0024	-3	230	236	AFFEDYK					Mascot
1249.6172	1249.6107	-0.0065	-5	120	130	EDKPQPPPEGR					Mascot
1249.6172	1249.6107	-0.0065	-5	120	130	EDKPQPPPEGR	58	99.591			Mascot

Calc. Mass	Obsrv. Mass	± da	± ppm	Start Seq.	End Seq.	Sequence	Ion Score	C. I. %	Modification	Rank	Result Type
1743.8073	1743.8441	0.0368	21	4	18	NYPSVSAEYQETVEK					Mascot
2071.0237	2071.0107	-0.013	-6	148	167	QMGLSDQDIVALSGGHT LGR			Oxidation (M)[2]		Mascot

8 hypothetical protein ZEAMMB73_022439 [Zea mays] gi|413956093 21012.6 6.6 4 78 98.347 5.906 58 99.591

Peptide Information

Calc. Mass	Obsrv. Mass	± da	± ppm	Start Seq.	End Seq.	Sequence	Ion Score	C. I. %	Modification	Rank	Result Type
895.4342	895.4674	0.0332	37	53	61	TGGPFGTMK					Mascot
911.4291	911.429	-0.0001	0	53	61	TGGPFGTMK			Oxidation (M)[8]		Mascot
1249.6172	1249.6107	-0.0065	-5	120	130	EDKPQPPPEGR					Mascot
1249.6172	1249.6107	-0.0065	-5	120	130	EDKPQPPPEGR	58	99.591			Mascot
1885.9178	1885.8928	-0.025	-13	2	18	AKNYPTVSAEYSEAVEK					Mascot
2071.0237	2071.0107	-0.013	-6	148	167	QMGLSDQDIVALSGGHT LGR			Oxidation (M)[2]		Mascot

9 hypothetical protein ZEAMMB73_022439 [Zea mays] gi|413956091 23689.7 7 4 76 97.665 5.906 58 99.591

Peptide Information

Calc. Mass	Obsrv. Mass	± da	± ppm	Start Seq.	End Seq.	Sequence	Ion Score	C. I. %	Modification	Rank	Result Type
895.4342	895.4674	0.0332	37	53	61	TGGPFGTMK					Mascot
911.4291	911.429	-0.0001	0	53	61	TGGPFGTMK			Oxidation (M)[8]		Mascot
1249.6172	1249.6107	-0.0065	-5	120	130	EDKPQPPPEGR					Mascot
1249.6172	1249.6107	-0.0065	-5	120	130	EDKPQPPPEGR	58	99.591			Mascot
1885.9178	1885.8928	-0.025	-13	2	18	AKNYPTVSAEYSEAVEK					Mascot
2071.0237	2071.0107	-0.013	-6	148	167	QMGLSDQDIVALSGGHT LGR			Oxidation (M)[2]		Mascot

10 APx1 - Cytosolic Ascorbate Peroxidase [Zea mays] gi|226504576 27481.9 5.65 4 74 96.546 5.906 58 99.591

Protein Group

APx1 - Cytosolic Ascorbate Peroxidase [Zea mays]	gi|226530305	27461.9	5.5500 001907 3486
APx1-Cytosolic Ascorbate Peroxidase [Zea mays]	gi|413956092	27461.9	5.5500 001907 3486

Peptide Information

Calc. Mass	Obsrv. Mass	± da	± ppm	Start	End	Sequence	Ion	C. I. %	Modification	Rank	Result Type

				Seq.	Seq.		Score			
895.4342	895.4674	0.0332	37	53	61	TGGPFGTMK				Mascot
911.4291	911.429	-0.0001	0	53	61	TGGPFGTMK			Oxidation (M)[8]	Mascot
1249.6172	1249.6107	-0.0065	-5	120	130	EDKPQPPPEGR				Mascot
1249.6172	1249.6107	-0.0065	-5	120	130	EDKPQPPPEGR	58	99.78		Mascot
1885.9178	1885.8928	-0.025	-13	2	18	AKNYPTVSAEYSEAVEK				Mascot
2071.0237	2071.0107	-0.013	-6	148	167	QMGLSDQDIVALSGGHT LGR			Oxidation (M)[2]	Mascot

Rank	Protein Name	Accession No.	Protein MW	Protein PI	Pep. Count	Protein Score	Protein Score C. I. %	Intensity Matched	Total Ion Score	Total Ion C. I. %	Confirmed
1	Avenin-3 [Triticum urartu]	gi|474329936	22657.1	6.35	1	69	88.297	14.6	69	99.984	

Protein Group

Protein Name	Accession No.	Protein MW	Protein PI
alpha-amylase inhibitor 1 [Aegilops tauschii]	gi|442614142	22813.2	6.1999998092 6514
hypothetical protein F775_28748 [Aegilops tauschii]	gi|475569529	22813.2	6.1999998092 6514

Peptide Information

Calc. Mass	Obsrv. Mass	± da	± ppm	Start Seq.	End Seq.	Sequence	Ion Score	C. I. %	Modification	Rank	Result Type
1966.9362	1966.9166	-0.0196	-10	60	76	DALLQQCSPVADMSFLR			Carbamidomethyl (C)[7], Oxidation (M)[13]		Mascot
1966.9362	1966.9166	-0.0196	-10	60	76	DALLQQCSPVADMSFLR	69	99.984	Carbamidomethyl (C)[7], Oxidation (M)[13]		Mascot

Rank	Protein Name	Accession No.	Protein MW	Protein PI	Pep. Count	Protein Score	Protein Score C. I. %	Intensity Matched	Total Ion Score	Total Ion C. I. %	Confirmed
2	maturase K, partial (chloroplast) [Petiveria alliacea]	gi|384584729	30843.1	9.75	9	50	0	8.545			

Protein Group

Protein Name	Accession No.	Protein MW	Protein PI
maturase K, partial (chloroplast) [Petiveria alliacea]	gi|384584727	30843.1	9.75
maturase K, partial (chloroplast) [Petiveria alliacea]	gi|384584725	30843.1	9.75
maturase K, partial (chloroplast) [Petiveria alliacea]	gi|384584723	30843.1	9.75
maturase K, partial (chloroplast) [Petiveria alliacea]	gi|384584721	30843.1	9.75

maturase K, partial (chloroplast) [Petiveria alliacea]	gi|384584719	30843.1	9.75
maturase K, partial (chloroplast) [Petiveria alliacea]	gi|384584717	30843.1	9.75
maturase K, partial (chloroplast) [Petiveria alliacea]	gi|384584731	30843.1	9.75

Peptide Information

Calc. Mass	Obsrv. Mass	± da	± ppm	Start Seq.	End Seq.	Sequence	Ion Score	C. I. %	Modification	Rank	Result Type
1078.6255	1078.5385	-0.087	-81	108	117	YQGKAILASK					Mascot
1226.6602	1226.5571	-0.1031	-84	118	127	GTSLLMYKWK					Mascot
1699.004	1698.8789	-0.1251	-74	191	206	FDTIVPIIPLVGSLSK					Mascot
1734.0353	1733.9216	-0.1137	-66	86	99	ILTNNFLVILWLFK					Mascot
1921.9596	1921.9318	-0.0278	-14	64	79	STSFGVFFERIYFHGK					Mascot
1951.9509	1951.9303	-0.0206	-11	220	236	SVWADLSDSDIIDRFGR					Mascot
1998.9781	1998.9249	-0.0532	-27	57	73	NQSSHLRSTSFGVFFER					Mascot
1998.9781	1999.1576	0.1795	90	57	73	NQSSHLRSTSFGVFFER					Mascot
3233.6123	3233.4875	-0.1248	-39	147	174	IYINQLSSHSLDFMGFLSS MQLNFSVVR					Mascot
3249.6072	3249.5203	-0.0869	-27	147	174	IYINQLSSHSLDFMGFLSS MQLNFSVVR			Oxidation (M)[14]		Mascot
3389.7134	3389.6125	-0.1009	-30	146	174	RIYINQLSSHSLDFMGFL SSMQLNFSVVR					Mascot
3405.7083	3405.6138	-0.0945	-28	146	174	RIYINQLSSHSLDFMGFL SSMQLNFSVVR			Oxidation (M)[15]		Mascot

3	Zinc finger protein 7 [Triticum urartu]	gi|474142506	12280.2	9.18	6	50	0	4.279

Peptide Information

Calc. Mass	Obsrv. Mass	± da	± ppm	Start Seq.	End Seq.	Sequence	Ion Score	C. I. %	Modification	Rank	Result Type
1097.5586	1097.6028	0.0442	40	101	109	DHELDLSLR					Mascot
1210.6427	1210.552	-0.0907	-75	101	110	DHELDLSLRL					Mascot

1637.8256	1637.806	-0.0196	-12	35	49	FRSSQALGGHQNAHK					Mascot
1925.9062	1925.8889	-0.0173	-9	73	92	AAGAVWVSEESAAAGG MAYK					Mascot
1941.9011	1941.9039	0.0028	1	73	92	AAGAVWVSEESAAAGG MAYK			Oxidation (M)[17]		Mascot
3417.6282	3417.6863	0.0581	17	59	92	ETAVATTHAWNPGRAAG AVWVSEESAAAGGMAYK					Mascot
3433.623	3433.6763	0.0533	16	59	92	ETAVATTHAWNPGRAAG AVWVSEESAAAGGMAYK			Oxidation (M)[31]		Mascot
3477.7183	3477.6982	-0.0201	-6	2	32	EHAQDELSLELTLATAVV APAPGFFLCVYCR			Carbamidomethyl (C)[27,30]		Mascot

4	Uncharacterized protein isoform 1 [Theobroma cacao]	gi|508782149	196678.4	8.67	24	49	0	26.783	

Protein Group

Uncharacterized protein isoform 1 [Theobroma cacao]	gi|508782148	196678.4	8.6700 000762 9395
Uncharacterized protein isoform 1 [Theobroma cacao]	gi|508782147	196678.4	8.6700 000762 9395
Uncharacterized protein isoform 1 [Theobroma cacao]	gi|508782145	196678.4	8.6700 000762 9395
Uncharacterized protein isoform 1 [Theobroma cacao]	gi|508782144	196678.4	8.6700 000762 9395
Uncharacterized protein isoform 1 [Theobroma cacao]	gi|508782150	196678.4	8.6700 000762 9395

Peptide Information

Calc. Mass	Obsrv. Mass	± da	± ppm	Start Seq.	End Seq.	Sequence	Ion Score	C. I. %	Modification	Rank	Result Type
867.4723	867.4315	-0.0408	-47	1695	1701	VVFFAER					Mascot
1067.5303	1067.608	0.0777	73	787	796	SAAFIGVSCR			Carbamidomethyl (C)[9]		Mascot
1089.6051	1089.561	-0.0441	-40	956	965	FGKIPEVSGR					Mascot
1139.5076	1139.5272	0.0196	17	1635	1643	RENEYESGR					Mascot

1147.6106	1147.6825	0.0719	63	1033	1042	FATDELRPAK					Mascot
1208.6382	1208.5667	-0.0715	-59	491	501	EDRHGAIVPSK					Mascot
1210.5575	1210.552	-0.0055	-5	1457	1465	FHPWCAHQK			Carbamidomethyl (C)[5]		Mascot
1224.5314	1224.5448	0.0134	11	271	280	TFHCESNTTK			Carbamidomethyl (C)[4]		Mascot
1534.7432	1534.7936	0.0504	33	613	625	SHVTSKEQFGVCR			Carbamidomethyl (C)[12]		Mascot
1665.8517	1665.8008	-0.0509	-31	1616	1630	GEMVVEYVGEIVGLR			Oxidation (M)[3]		Mascot
1749.8589	1749.933	0.0741	42	989	1003	TVEKSYNSNAVHCIK			Carbamidomethyl (C)[13]		Mascot
1810.8212	1810.8914	0.0702	39	1017	1031	DRPIVCGEYGEICSR			Carbamidomethyl (C)[6,13]		Mascot
1813.0331	1812.9271	-0.106	-58	1033	1048	FATDELRPAKIVPLSR					Mascot
1845.8258	1845.8774	0.0516	28	1136	1150	SEKYCCIPDGIAYNR			Carbamidomethyl (C)[5,6]		Mascot
1857.8107	1857.9448	0.1341	72	1412	1428	CPNVDTMSAFDVSGVSR			Carbamidomethyl (C)[1], Oxidation (M)[7]		Mascot
1885.9655	1885.9272	-0.0383	-20	971	988	AGAFDSFESLGTSKSILR					Mascot
1935.8722	1935.925	0.0528	27	57	73	MECAVTRSSTMSTFVGR			Carbamidomethyl (C)[3], Oxidation (M)[1]		Mascot
1951.8671	1951.9303	0.0632	32	57	73	MECAVTRSSTMSTFVGR			Carbamidomethyl (C)[3], Oxidation (M)[1,11]		Mascot
1971.9164	1971.9203	0.0039	2	1139	1154	YCCIPDGIAYNRSNIR			Carbamidomethyl (C)[2,3]		Mascot
1990.907	1990.8903	-0.0167	-8	474	490	TSNPNVQHCLMESSKSR			Carbamidomethyl (C)[9], Oxidation (M)[11]		Mascot
2014.9513	2014.9128	-0.0385	-19	456	473	QHATSSVGMSHELGRFDR					Mascot
3373.4954	3373.6094	0.114	34	686	717	INPSAEVLDASGCAEDQHGDAGMRCIGSAVNR			Carbamidomethyl (C)[13,25], Oxidation (M)[23]		Mascot
3373.4954	3373.6094	0.114	34	686	717	INPSAEVLDASGCAEDQHGDAGMRCIGSAVNR			Carbamidomethyl (C)[13,25], Oxidation (M)[23]		Mascot
3405.5337	3405.6138	0.0801	24	340	370	NVTGVVPGFSAVHGMDSCQSSNIHSDRFDER			Carbamidomethyl (C)[18]		Mascot
3406.4927	3406.6189	0.1262	37	162	190	FGTVVDPPKSCYPEPMIHHANFCGEEESR			Carbamidomethyl (C)[11,23], Oxidation (M)[16]		Mascot
3419.5491	3419.6731	0.124	36	1695	1722	VVFFAERDIYPGEEITYDYHFNHEDEGK					Mascot
3419.5491	3419.6731	0.124	36	1695	1722	VVFFAERDIYPGEEITYDYHFNHEDEGK					Mascot

5 Uncharacterized protein TCM_002883 [Theobroma cacao] gi|508701991 56896.1 6.68 14 48 0 22.169

Peptide Information

Calc. Mass	Obsrv. Mass	± da	± ppm	Start Seq.	End Seq.	Sequence	Ion Score	C. I. %	Modification	Rank	Result Type
1078.531	1078.5385	0.0075	7	177	185	DTCSRAQLK			Carbamidomethyl (C)[3]		Mascot
1208.598	1208.5667	-0.0313	-26	203	212	NEMLFAQLDK					Mascot
1211.5977	1211.5553	-0.0424	-35	436	446	SISTPMSPFTK			Oxidation (M)[6]		Mascot
1224.5929	1224.5448	-0.0481	-39	203	212	NEMLFAQLDK			Oxidation (M)[3]		Mascot
1637.7008	1637.806	0.1052	64	385	398	SFEKGEFEMSMMGK					Mascot
1733.8462	1733.9216	0.0754	43	199	212	HMTRNEMLFAQLDK					Mascot
1749.8411	1749.933	0.0919	53	199	212	HMTRNEMLFAQLDK			Oxidation (M)[2]		Mascot

Calc. Mass	Obsrv. Mass	± da	± ppm	Start Seq.	End Seq.	Sequence	Ion Score	C. I. %	Modification	Rank	Result Type
1765.8361	1765.9094	0.0733	42	199	212	HMTRNEMLFAQLDK			Oxidation (M)[2,7]		Mascot
1810.8827	1810.8914	0.0087	5	2	16	FKMKPGEDITNMLDR			Oxidation (M)[3]		Mascot
1845.8939	1845.8774	-0.0165	-9	370	384	ETCEFTTFISQIELK			Carbamidomethyl (C)[3]		Mascot
1887.1174	1887.3033	0.1859	99	24	39	LNQLGKPILEHEIVKR					Mascot
1925.9282	1925.8889	-0.0393	-20	1	16	MFKMKPGEDITNMLDR					Mascot
1935.9327	1935.925	-0.0077	-4	158	174	ANLSLMARDDESEVELK			Oxidation (M)[6]		Mascot
1941.9231	1941.9039	-0.0192	-10	1	16	MFKMKPGEDITNMLDR			Oxidation (M)[1]		Mascot
1967.0419	1966.9166	-0.1253	-64	412	427	DGIFLNQERYIQVMLK					Mascot
1967.0419	1966.9166	-0.1253	-64	412	427	DGIFLNQERYIQVMLK					Mascot
1983.0369	1982.9031	-0.1338	-67	412	427	DGIFLNQERYIQVMLK			Oxidation (M)[14]		Mascot
1983.0369	1982.9031	-0.1338	-67	412	427	DGIFLNQERYIQVMLK			Oxidation (M)[14]		Mascot
2015.0485	2014.9128	-0.1357	-67	265	282	FDAKSDEAIFLGYALNLK					Mascot
3249.55	3249.5203	-0.0297	-9	97	125	SIALKANILEEELDSLSCD DDEELAMVAR			Carbamidomethyl (C)[18]		Mascot
3433.832	3433.6763	-0.1557	-45	52	81	VIAICEAKDLNIITLDEICG PLLTHELELK			Carbamidomethyl (C)[5,19]		Mascot

6 PREDICTED: uncharacterized protein LOC101514722 isoform X4 [Cicer arietinum] gi|502150368 23952.2 5.55 8 48 0 17.28

Peptide Information

Calc. Mass	Obsrv. Mass	± da	± ppm	Start Seq.	End Seq.	Sequence	Ion Score	C. I. %	Modification	Rank	Result Type
1097.6313	1097.6028	-0.0285	-26	19	28	TPEGLPRISK					Mascot
1147.5854	1147.6825	0.0971	85	110	120	LDAAGHAHIDK					Mascot
1226.5756	1226.5571	-0.0185	-15	141	151	ESSLMMSQSVK					Mascot
1698.8037	1698.8789	0.0752	44	141	155	ESSLMMSQSVKNTEK					Mascot
1733.899	1733.9216	0.0226	13	123	137	KLQDDLTDEMVVLAK			Oxidation (M)[10]		Mascot
1893.8712	1893.9132	0.042	22	58	73	DFESNVKENPSEIEEK					Mascot
1967.0081	1966.9166	-0.0915	-47	9	25	EQLEQLAEEKTPEGLPR					Mascot
1967.0081	1966.9166	-0.0915	-47	9	25	EQLEQLAEEKTPEGLPR	10	0			Mascot
3164.6455	3164.499	-0.1465	-46	188	213	TSCLTWLVMFVMTCVFV MVILLIRVT			Carbamidomethyl (C)[3,14], Oxidation (M)[9,12]		Mascot

7 unnamed protein product [Vitis vinifera] gi|296082274 55017.7 9.12 12 47 0 9.881

Peptide Information

Calc. Mass	Obsrv. Mass	± da	± ppm	Start Seq.	End Seq.	Sequence	Ion Score	C. I. %	Modification	Rank	Result Type
1089.496	1089.561	0.065	60	361	369	QDWSDVPSR					Mascot
1281.5933	1281.5422	-0.0511	-40	431	440	WEAMPFESLR			Oxidation (M)[4]		Mascot

Calc. Mass	Obsrv. Mass	± da	± ppm	Start Seq.	End Seq.	Sequence	Ion Score	C. I. %	Modification	Rank	Result Type
1490.7935	1490.7404	-0.0531	-36	2	14	RPLHPSSRPSSNR					Mascot
1534.7352	1534.7936	0.0584	38	458	470	MDGNKLDDLVCVR			Carbamidomethyl (C)[11]		Mascot
1550.7302	1550.7864	0.0562	36	458	470	MDGNKLDDLVCVR			Carbamidomethyl (C)[11], Oxidation (M)[1]		Mascot
1637.8289	1637.806	-0.0229	-14	1	14	MRPLHPSSRPSSNR			Oxidation (M)[1]		Mascot
1665.7286	1665.8008	0.0722	43	385	400	GQADQAGDLGQFGNCK			Carbamidomethyl (C)[15]		Mascot
1903.0913	1902.928	-0.1633	-86	271	286	SIQNPVYLFQGIVLRR					Mascot
1903.0913	1902.928	-0.1633	-86	271	286	SIQNPVYLFQGIVLRR					Mascot
1908.9756	1908.891	-0.0846	-44	1	16	MRPLHPSSRPSSNRMR					Mascot
1936.0837	1935.925	-0.1587	-82	71	87	GFGITAHIIDHCKLILK			Carbamidomethyl (C)[12]		Mascot
1990.8787	1990.8903	0.0116	6	442	457	EARDHYFQMEGVSMYK					Mascot
3438.8184	3438.6631	-0.1553	-45	17	46	FTLFHLICAAVFFSLFVFG IQSSLFAGSRR			Carbamidomethyl (C)[8]		Mascot
3463.6799	3463.6655	-0.0144	-4	182	212	QFQTCAVVGNSGDLLKT EFGEEIDSHDVVIR			Carbamidomethyl (C)[5]		Mascot

8 hypothetical protein OsI_23568 [Oryza sativa Indica Group] gi|125555928 16347.4 9.21 7 47 0 17.22

Peptide Information

Calc. Mass	Obsrv. Mass	± da	± ppm	Start Seq.	End Seq.	Sequence	Ion Score	C. I. %	Modification	Rank	Result Type
1313.5063	1313.5408	0.0345	26	51	63	MFAEGEGGDGDGR			Oxidation (M)[1]		Mascot
1765.9008	1765.9094	0.0086	5	64	79	LELLPFSDGTENGFVK					Mascot
1810.8177	1810.8914	0.0737	41	81	98	GDAAELGAYMASFHASG R					Mascot
1922.0018	1921.9318	-0.07	-36	64	80	LELLPFSDGTENGFVKR					Mascot
1966.9188	1966.9166	-0.0022	-1	81	99	GDAAELGAYMASFHASG RR					Mascot
1967.0709	1966.9166	-0.1543	-78	30	49	LLAAAPDALVTFSTAAAA HR					Mascot
1982.9137	1982.9031	-0.0106	-5	81	99	GDAAELGAYMASFHASG RR			Oxidation (M)[10]		Mascot
1982.9137	1982.9031	-0.0106	-5	80	98	RGDAAELGAYMASFHAS GR			Oxidation (M)[11]		Mascot

9 hypothetical protein PRUPE_ppa002000mg [Prunus persica] gi|462418901 83368.9 8.84 14 47 0 10.485

Peptide Information

Calc. Mass	Obsrv. Mass	± da	± ppm	Start Seq.	End Seq.	Sequence	Ion Score	C. I. %	Modification	Rank	Result Type
867.4611	867.4315	-0.0296	-34	613	619	EAPLYFK					Mascot
1019.5269	1019.5901	0.0632	62	279	286	DTSRHYLK					Mascot
1097.5474	1097.6028	0.0554	50	393	401	HEEAEELLK					Mascot
1139.599	1139.5272	-0.0718	-63	287	296	DVSLGHPKMR					Mascot

1147.6656	1147.6825	0.0169	15	1	10	MLAGVKFIPR			Oxidation (M)[1]		Mascot
1518.7911	1518.7906	-0.0005	0	228	242	WGSLGQLTVSAASNK					Mascot
1637.7775	1637.806	0.0285	17	687	699	GMLQLPEEDMYRR					Mascot
1809.9229	1810.0663	0.1434	79	393	407	HEEAEELLKEVENIK					Mascot
1876.011	1875.913	-0.098	-52	582	597	EVVFLETVMGLAQQRR					Mascot
1876.011	1875.913	-0.098	-52	582	597	EVVFLETVMGLAQQRR					Mascot
1891.8165	1891.9205	0.104	55	92	108	NRSHGSLEDEEFSDGGR					Mascot
1892.0059	1891.9205	-0.0854	-45	582	597	EVVFLETVMGLAQQRR			Oxidation (M)[9]		Mascot
1972.0255	1971.9203	-0.1052	-53	597	612	RHCIVECIPLPHEIAK			Carbamidomethyl (C)[3,7]		Mascot
2875.3667	2875.3521	-0.0146	-5	318	345	DSGLIAEAVSSLNTFSND GSFMSEVLGK					Mascot
3405.6367	3405.6138	-0.0229	-7	313	345	DVSTKDSGLIAEAVSSLN TFSNDGSFMSEVLGK					Mascot
3438.5205	3438.6631	0.1426	41	169	202	DDGSGYPEDTDEPKAGA DQLLSSSVVGDGGASWR					Mascot

10	TPA: hypothetical protein ZEAMMB73_093311 [Zea mays]	gi|414871942	221401.2	8.75	24	46	0	38.415

Peptide Information

Calc. Mass	Obsrv. Mass	± da	± ppm	Start Seq.	End Seq.	Sequence	Ion Score	C. I. %	Modification	Rank	Result Type
1089.467	1089.561	0.094	86	1510	1517	QQYTFDCK			Carbamidomethyl (C)[7]		Mascot
1106.6317	1106.542	-0.0897	-81	166	176	IAGPNHTLGVK					Mascot
1212.6372	1212.5419	-0.0953	-79	1679	1689	AFEPPKPSPSR					Mascot
1281.5892	1281.5422	-0.047	-37	250	260	CGYAIANIDER			Carbamidomethyl (C)[1]		Mascot
1298.6562	1298.5831	-0.0731	-56	1920	1929	QFQVMFDVLR			Oxidation (M)[5]		Mascot
1490.6846	1490.7404	0.0558	37	1226	1236	ERSLPWWDDMR					Mascot
1534.7537	1534.7936	0.0399	26	1254	1266	FLATTNPYEHVDK					Mascot
1550.803	1550.7864	-0.0166	-11	1155	1169	VTMLRSASGTTPAMK					Mascot
1765.8756	1765.9094	0.0338	19	1577	1591	HEDGFLLSSDYITIR					Mascot
1807.9082	1807.8781	-0.0301	-17	684	699	FSASLVISHLSMCIDK			Carbamidomethyl (C)[13]		Mascot
1859.8811	1859.9421	0.061	33	1479	1495	HAPLSEDDDPAFGLTFK					Mascot
1902.016	1901.8928	-0.1232	-65	1237	1251	YYLHGKIILYFNETK					Mascot
1902.9015	1902.928	0.0265	14	1113	1129	DYTSPLFSSLSGKCQGR			Carbamidomethyl (C)[14]		Mascot
1902.9015	1902.928	0.0265	14	1113	1129	DYTSPLFSSLSGKCQGR			Carbamidomethyl (C)[14]		Mascot
1921.9767	1921.9318	-0.0449	-23	1577	1592	HEDGFLLSSDYITIRR					Mascot
1925.9274	1925.8889	-0.0385	-20	1267	1282	LQIVSEYMEIQQTDGR			Oxidation (M)[8]		Mascot
1956.0087	1955.9845	-0.0242	-12	1336	1352	NPLNHYLHALPVEGEPR					Mascot
1966.9269	1966.9166	-0.0103	-5	1228	1242	SLPWWDDMRYYLHGK					Mascot
1966.9269	1966.9166	-0.0103	-5	1228	1242	SLPWWDDMRYYLHGK					Mascot

1982.9219	1982.9031	-0.0188	-9	1228	1242	SLPWWDDMRYYLHGK	Oxidation (M)[8]	Mascot
1982.9219	1982.9031	-0.0188	-9	1228	1242	SLPWWDDMRYYLHGK	Oxidation (M)[8]	Mascot
1999.1263	1999.1576	0.0313	16	384	401	VFTIASLDVPVDPFLPIR		Mascot
2015.0444	2014.9128	-0.1316	-65	1028	1045	VLYNGVEVDIHDTTALQK		Mascot
3164.5535	3164.499	-0.0545	-17	346	372	SLPQDDFEEATPHFDVQI DLSEIHLIR		Mascot
3373.4998	3373.6094	0.1096	32	1396	1425	GIMSGSPCKMTDDDFPT MNLGAHDLAWVFK	Carbamidomethyl (C)[8], Oxidation (M)[3,10]	Mascot
3373.4998	3373.6094	0.1096	32	1396	1425	GIMSGSPCKMTDDDFPT MNLGAHDLAWVFK	Carbamidomethyl (C)[8], Oxidation (M)[3,10]	Mascot
3389.4946	3389.6125	0.1179	35	1396	1425	GIMSGSPCKMTDDDFPT MNLGAHDLAWVFK	Carbamidomethyl (C)[8], Oxidation (M)[3,10,18]	Mascot
3433.4995	3433.6763	0.1768	51	1057	1087	SYYVSCQNMVLAEGSGA CSEGFQAGFRQSSR	Carbamidomethyl (C)[6,18]	Mascot
3445.6692	3445.6343	-0.0349	-10	1707	1740	VPQDSSSSIHVGSPSVQ HLDASGSSSSLHSKANR		Mascot
3477.541	3477.6982	0.1572	45	1053	1083	QAFRSYYVSCQNMVLAE GSGACSEGFQAGFR	Carbamidomethyl (C)[10,22]	Mascot

Gel Idx/Pos	206/I6	Instr./Gel Origin	BA2151/Sample Project 20140814	Process Status	Analysis Succeeded
Plate [#] Name	[1] Sample Project 20140814	Instrument Sample Name		Spectra	11

Rank	Protein Name	Accession No.	Protein MW	Protein PI	Pep. Count	Protein Score	Protein Score C. I. %	Intensity Matched	Total Ion Score	Total Ion C. I. %	Confirmed
1	hypothetical protein TRIUR3_28410 [Triticum urartu]	gi|474060617	28387.9	5.53	7	86	99.744	10.984	52	99.021	

Peptide Information

Calc. Mass	Obsrv. Mass	± da	± ppm	Start Seq.	End Seq.	Sequence	Ion Score	C. I. %	Modification	Rank	Result Type
971.5673	971.5522	-0.0151	-16	51	58	FVTNHLLK					Mascot
1078.535	1078.5322	-0.0028	-3	207	215	GYPLLEACR			Carbamidomethyl (C)[8]		Mascot
1145.5698	1145.5636	-0.0062	-5	156	165	GHNLSLEYGR					Mascot
1145.5698	1145.5636	-0.0062	-5	156	165	GHNLSLEYGR	24	0			Mascot
1406.6271	1406.6254	-0.0017	-1	130	139	HREWESCFQK			Carbamidomethyl (C)[7]		Mascot
1534.7571	1534.7842	0.0271	18	140	152	QGLDPKPVTECYK			Carbamidomethyl (C)[11]		Mascot
1927.9371	1927.9271	-0.01	-5	35	50	VHVAIYYESLCPYSAR			Carbamidomethyl (C)[11]		Mascot
1927.9371	1927.9271	-0.01	-5	35	50	VHVAIYYESLCPYSAR	28	0	Carbamidomethyl (C)[11]		Mascot
3402.8162	3402.6047	-0.2115	-62	1	34	MARGLHHLLLLAALLQQL SATSAGDVATGSGGEK			Oxidation (M)[1]		Mascot

Rank	Protein Name	Accession No.	Protein MW	Protein PI	Pep. Count	Protein Score	Protein Score C. I. %	Intensity Matched	Total Ion Score	Total Ion C. I. %	Confirmed
2	Avenin-3 [Triticum urartu]	gi|474329936	22657.1	6.35	1	68	83.846	18.83	68	99.975	

Protein Group

alpha-amylase inhibitor 1 [Aegilops tauschii]	gi|442614142	22813.2	6.1999 998092 6514
hypothetical protein F775_28748 [Aegilops tauschii]	gi|475569529	22813.2	6.1999 998092 6514

Peptide Information

Calc. Mass	Obsrv. Mass	± da	± ppm	Start Seq.	End Seq.	Sequence	Ion Score	C. I. %	Modification	Rank	Result Type
1950.9413	1950.9211	-0.0202	-10	60	76	DALLQQCSPVADMSFLR			Carbamidomethyl (C)[7]		Mascot
1966.9362	1966.9214	-0.0148	-8	60	76	DALLQQCSPVADMSFLR			Carbamidomethyl (C)[7], Oxidation (M)[13]		Mascot
1966.9362	1966.9214	-0.0148	-8	60	76	DALLQQCSPVADMSFLR	68	99.975	Carbamidomethyl (C)[7], Oxidation (M)[13]		Mascot

Rank	Protein Name	Accession No.	Protein MW	Protein PI	Pep. Count	Protein Score	Protein Score C. I. %	Intensity Matched	Total Ion Score	Total Ion C. I. %	Confirmed
3	catalytic, putative [Ricinus communis]	gi|223536510	27443.9	5.76	9	55	0	5.152			

Peptide Information

Calc. Mass	Obsrv. Mass	± da	± ppm	Start Seq.	End Seq.	Sequence	Ion Score	C. I. %	Modification	Rank	Result Type
1315.6498	1315.6089	-0.0409	-31	105	115	CPPITAVELCR			Carbamidomethyl (C)[1,10]		Mascot
1523.682	1523.78	0.098	64	228	242	RIADSSATDDLGESS					Mascot
1600.7935	1600.7502	-0.0433	-27	105	117	CPPITAVELCRER			Carbamidomethyl (C)[1,10]		Mascot
1700.8823	1700.816	-0.0663	-39	101	115	SGLKCPPITAVELCR			Carbamidomethyl (C)[5,14]		Mascot
1812.8619	1812.9358	0.0739	41	213	227	EFGNCELRSMVLVDK			Carbamidomethyl (C)[5], Oxidation (M)[10]		Mascot
1980.0886	1979.9556	-0.133	-67	21	38	ILHLVRHAQGTHNVAGEK					Mascot
2652.3813	2652.1565	-0.2248	-85	190	212	FLQHTLNALTIDSHPSVK TEICK			Carbamidomethyl (C)[22]		Mascot
2842.4993	2842.3843	-0.115	-40	2	26	DNTTTTTTVQCLHPLGHS KILHLVR			Carbamidomethyl (C)[11]		Mascot
3410.8179	3410.6309	-0.187	-55	178	207	QEVEIAIVTHNRFLQHTLN ALTIDSHPSVK					Mascot

4	hypothetical protein RCOM_0991200 [Ricinus communis]	gi|223539804	88125.8	9.07	17	54	0	6.239	

Peptide Information

Calc. Mass	Obsrv. Mass	± da	± ppm	Start Seq.	End Seq.	Sequence	Ion Score	C. I. %	Modification	Rank	Result Type
971.4792	971.5522	0.073	75	91	99	SPLSESHSK					Mascot
1106.5034	1106.5375	0.0341	31	562	571	EESSPSPVMK			Oxidation (M)[9]		Mascot
1266.6549	1266.5482	-0.1067	-84	389	400	QGRLGSDSPPPR					Mascot
1308.5671	1308.6357	0.0686	52	262	272	AMDPKENNAMR			Oxidation (M)[2,10]		Mascot
1491.6857	1491.7422	0.0565	38	254	266	DQTTNNTRAMDPK					Mascot
1507.6805	1507.7377	0.0572	38	254	266	DQTTNNTRAMDPK			Oxidation (M)[10]		Mascot
1518.6959	1518.798	0.1021	67	195	206	LPEWEEETEQTK					Mascot
1590.7654	1590.8092	0.0438	28	377	391	SATAPSTPMTNRQGR			Oxidation (M)[9]		Mascot
1600.7748	1600.7502	-0.0246	-15	61	73	TMESPLEKHQQTR			Oxidation (M)[2]		Mascot
1631.8711	1631.8994	0.0283	17	150	165	EIRTNAAILSASSGNK					Mascot
1812.8948	1812.9358	0.041	23	455	471	VSNNNNNSRASPVQSPK					Mascot
1839.0375	1838.8999	-0.1376	-75	100	115	LPLPLQVFELKEGNNK					Mascot
1948.9658	1948.9332	-0.0326	-17	74	90	SMPSPDNLKHQQNSPLR					Mascot
1959.9805	1959.8427	-0.1378	-70	2	18	GTGMVQEQQNLEKQIEK					Mascot
1979.9413	1979.9556	0.0143	7	697	713	ERDASDDLLEVICGMLK			Carbamidomethyl (C)[13], Oxidation (M)[15]		Mascot
2652.2722	2652.1565	-0.1157	-44	672	694	ASICISSSGEQTPLQQIW SEFQR			Carbamidomethyl (C)[4]		Mascot
3393.634	3393.6404	0.0064	2	715	744	DLAGDSINGWGDCPIEM SETVLDIERLIFK			Carbamidomethyl (C)[13]		Mascot
3408.7065	3408.6533	-0.0532	-16	532	561	CDKLLHSIAEMTATELQP			Carbamidomethyl (C)[1], Oxidation (M)[11]		Mascot

SPVSVLDSLFYK

5	PREDICTED: glycine-rich RNA-binding protein-like [Solanum lycopersicum]	gi|460406533	16573.6	9.2	8	53	0	3.803

Peptide Information

Calc. Mass	Obsrv. Mass	± da	± ppm	Start Seq.	End Seq.	Sequence	Ion Score	C. I. %	Modification	Rank	Result Type
1037.4396	1037.5201	0.0805	78	121	132	EGGNGGGYGGGR					Mascot
1078.5138	1078.5322	0.0184	17	87	99	GSGGGGGFGGGRR					Mascot
1193.5406	1193.6127	0.0721	60	120	132	REGGNGGGYGGGR					Mascot
1308.5676	1308.6357	0.0681	52	121	134	EGGNGGGYGGGRDR					Mascot
1490.7275	1490.7395	0.012	8	47	59	SRGFGFVTFTDEK					Mascot
1518.6965	1518.798	0.1015	67	63	76	NAIEAMNGQNLDGR			Oxidation (M)[6]		Mascot
1617.6791	1617.7544	0.0753	47	100	119	GGGGFGGGGGGYNGG GGYGR					Mascot
1892.8701	1892.9152	0.0451	24	60	76	SMRNAIEAMNGQNLDGR			Oxidation (M)[2]		Mascot
1908.8651	1908.8383	-0.0268	-14	60	76	SMRNAIEAMNGQNLDGR			Oxidation (M)[2,9]		Mascot

6	Os02g0810300 [Oryza sativa Japonica Group]	gi|113538000	33062.8	6.09	10	52	0	9.4

Peptide Information

Calc. Mass	Obsrv. Mass	± da	± ppm	Start Seq.	End Seq.	Sequence	Ion Score	C. I. %	Modification	Rank	Result Type
1107.4524	1107.5276	0.0752	68	1	10	MAGGEDEGWR					Mascot
1138.6514	1138.5516	-0.0998	-88	59	69	ILAGKHMVGGR					Mascot
1170.5472	1170.5618	0.0146	12	284	292	SSRHMAYYR					Mascot
1263.5536	1263.6754	0.1218	96	1	11	MAGGEDEGWRR					Mascot
1266.6226	1266.5482	-0.0744	-59	273	283	RSSPFDASPFR					Mascot
1315.7369	1315.6089	-0.128	-97	244	254	NLLSVVESWLR					Mascot
1813.0317	1812.9358	-0.0959	-53	169	184	VLLLDEITVDLDVVTR					Mascot
1859.8303	1859.9141	0.0838	45	185	198	MDLLDFFKEECEQR			Carbamidomethyl (C)[11]		Mascot
1875.8252	1875.9167	0.0915	49	185	198	MDLLDFFKEECEQR			Carbamidomethyl (C)[11], Oxidation (M)[1]		Mascot
1875.8252	1875.9167	0.0915	49	185	198	MDLLDFFKEECEQR			Carbamidomethyl (C)[11], Oxidation (M)[1]		Mascot
3411.6743	3411.6301	-0.0442	-13	199	228	EATIVYATHIFDGLESWA TDIAYIQEGELR					Mascot
3422.6548	3422.6599	0.0051	1	70	100	DVVRVLNGSAFHDTQLV CNGDLSYLGGSWSR			Carbamidomethyl (C)[18]		Mascot

7	PREDICTED: uncharacterized protein LOC101294551 [Fragaria vesca subsp. vesca]	gi|470122985	17442.5	6.28	8	52	0	7.351

Peptide Information

Calc. Mass	Obsrv. Mass	± da	± ppm	Start	End	Sequence	Ion	C. I. %	Modification	Rank	Result Type

				Seq.	Seq.		Score				
1170.63	1170.5618	-0.0682	-58	84	92	HLDMKIIER			Oxidation (M)[4]		Mascot
1315.5988	1315.6089	0.0101	8	44	54	DFLANFDCSVK			Carbamidomethyl (C)[8]		Mascot
1406.5862	1406.6254	0.0392	28	94	105	SSAMAVTNCYMR			Carbamidomethyl (C)[9], Oxidation (M)[4]		Mascot
1422.5811	1422.6089	0.0278	20	94	105	SSAMAVTNCYMR			Carbamidomethyl (C)[9], Oxidation (M)[4,11]		Mascot
1518.6862	1518.798	0.1118	74	93	105	KSSAMAVTNCYMR			Carbamidomethyl (C)[10]		Mascot
1534.7761	1534.7842	0.0081	5	32	43	STRWDTYHITVR					Mascot
1550.676	1550.7969	0.1209	78	93	105	KSSAMAVTNCYMR			Carbamidomethyl (C)[10], Oxidation (M)[5,12]		Mascot
1901.9612	1901.9048	-0.0564	-30	73	88	QTCFAPLPSLKHLDMK			Carbamidomethyl (C)[3], Oxidation (M)[15]		Mascot
3377.5342	3377.6091	0.0749	22	44	72	DFLANFDCSVKLSIYAFG SEDMLFSEDTR			Carbamidomethyl (C)[8]		Mascot
3393.5291	3393.6404	0.1113	33	44	72	DFLANFDCSVKLSIYAFG SEDMLFSEDTR			Carbamidomethyl (C)[8], Oxidation (M)[22]		Mascot
3402.6304	3402.6047	-0.0257	-8	3	34	WNGAATPMEDAGVVDA APNLCEATIIIDKSTR			Carbamidomethyl (C)[21], Oxidation (M)[8]		Mascot

8 TMV resistance protein N [Theobroma cacao] gi|508778601 154012.2 6.92 22 51 0 13.426

Peptide Information

Calc. Mass	Obsrv. Mass	± da	± ppm	Start Seq.	End Seq.	Sequence	Ion Score	C. I. %	Modification	Rank	Result Type
971.4839	971.5522	0.0683	70	292	298	ETICHRR			Carbamidomethyl (C)[4]		Mascot
987.5985	987.5455	-0.053	-54	1220	1228	NFIGIPKAK					Mascot
991.5822	991.5373	-0.0449	-45	598	605	LLQLDYVK					Mascot
1078.5198	1078.5322	0.0124	11	751	759	DLQNMNSLK			Oxidation (M)[5]		Mascot
1106.559	1106.5375	-0.0215	-19	565	574	YGKTATNHSK					Mascot
1107.5107	1107.5276	0.0169	15	505	512	LMMHQMIR			Oxidation (M)[2,3,6]		Mascot
1144.6685	1144.5614	-0.1071	-94	495	504	SLLVVSERNK					Mascot
1315.7402	1315.6089	-0.1313	-100	214	227	VGIATICGIGGIGK			Carbamidomethyl (C)[7]		Mascot
1347.6791	1347.6012	-0.0779	-58	1123	1133	VQIYNDLEEPK					Mascot
1430.7368	1430.7245	-0.0123	-9	1229	1238	NKIFWFSFWR					Mascot
1438.7173	1438.6182	-0.0991	-69	277	289	ANKIYNSDDGITK					Mascot
1490.8101	1490.7395	-0.0706	-47	1103	1114	EIELKVNEEFLK					Mascot
1518.7161	1518.798	0.0819	54	505	516	LMMHQMIRDMGR					Mascot
1534.7109	1534.7842	0.0733	48	505	516	LMMHQMIRDMGR			Oxidation (M)[2]		Mascot
1550.7059	1550.7969	0.091	59	505	516	LMMHQMIRDMGR			Oxidation (M)[2,3]		Mascot
1811.9836	1811.9358	-0.0478	-26	862	877	LPMSGLHVITSQPLYR					Mascot
1811.9836	1811.9358	-0.0478	-26	862	877	LPMSGLHVITSQPLYR					Mascot
1844.7942	1844.9073	0.1131	61	133	146	HEESFKYEMDMVQR			Oxidation (M)[9]		Mascot
1858.9691	1858.8917	-0.0774	-42	149	165	TALREVADLGGMLLEDR					Mascot

1860.7892	1860.9288	0.1396	75	133	146	HEESFKYEMDMVQR			Oxidation (M)[9,11]		Mascot
1874.9641	1874.9512	-0.0129	-7	149	165	TALREVADLGGMLLEDR			Oxidation (M)[12]		Mascot
1928.0237	1927.9271	-0.0966	-50	166	181	HESQFIQDIIKQVQSK					Mascot
1928.0237	1927.9271	-0.0966	-50	166	181	HESQFIQDIIKQVQSK					Mascot
1935.8099	1935.9364	0.1265	65	345	360	QMFDLEESSNYGDLCK			Carbamidomethyl (C)[15]		Mascot
1951.8048	1951.9337	0.1289	66	345	360	QMFDLEESSNYGDLCK			Carbamidomethyl (C)[15], Oxidation (M)[2]		Mascot
1951.8048	1951.9337	0.1289	66	345	360	QMFDLEESSNYGDLCK			Carbamidomethyl (C)[15], Oxidation (M)[2]		Mascot
2238.332	2238.1487	-0.1833	-82	697	716	LIEVDQSIAEIKTLVVLNLK					Mascot
2668.4233	2668.1592	-0.2641	-99	1181	1203	IGYLNLGVILLDEDDKIFD FLSR					Mascot
3124.6348	3124.4956	-0.1392	-45	476	502	ILDGCDYYTVIGIENLINR SLLVVSER			Carbamidomethyl (C)[5]		Mascot
3402.5823	3402.6047	0.0224	7	1140	1168	VFYDCGIISMYDINGFPK GWYNHHAVGNK			Carbamidomethyl (C)[5]		Mascot

9	retrotransposon protein, putative, unclassified [Oryza sativa Japonica Group]	gi|77549906	151895.4	8.49	22	51	0	34.328	

Peptide Information

Calc. Mass	Obsrv. Mass	± da	± ppm	Start Seq.	End Seq.	Sequence	Ion Score	C. I. %	Modification	Rank	Result Type
808.3948	808.3493	-0.0455	-56	459	466	FSDGVGAR					Mascot
1106.6093	1106.5375	-0.0718	-65	382	390	QEFIIESLK					Mascot
1107.4922	1107.5276	0.0354	32	862	870	NCDLVEMAR			Carbamidomethyl (C)[2]		Mascot
1158.5538	1158.5764	0.0226	20	841	850	GLEHEFSSPR					Mascot
1170.5394	1170.5618	0.0224	19	354	363	NISGVCFMAR			Carbamidomethyl (C)[6], Oxidation (M)[8]		Mascot
1343.7101	1343.7344	0.0243	18	196	206	LKTHEMNVLSR			Oxidation (M)[6]		Mascot
1406.7135	1406.6254	-0.0881	-63	454	466	NSLQRFSDGVGAR					Mascot
1517.8574	1517.7848	-0.0726	-48	613	625	FELKNVALVEDLK					Mascot
1534.7649	1534.7842	0.0193	13	943	956	STDGLFLGYPAHTR					Mascot
1550.8285	1550.7969	-0.0316	-20	68	81	NILLSGISRSGYDR					Mascot
1590.7152	1590.8092	0.094	59	1203	1216	FQASPCASHCQAVK			Carbamidomethyl (C)[6,10]		Mascot
1836.7932	1836.9183	0.1251	68	26	39	MQSYIMAEDYDIWR			Oxidation (M)[1]		Mascot
1838.9647	1838.8999	-0.0648	-35	41	57	VSHPYVIPEAINTAAEK					Mascot
1852.7881	1852.8778	0.0897	48	26	39	MQSYIMAEDYDIWR			Oxidation (M)[1,6]		Mascot
1857.9414	1857.9287	-0.0127	-7	513	529	SSSSNNVSTQREILAHK					Mascot
1874.8385	1874.9512	0.1127	60	273	288	GGPNRCFECGSIDHLR			Carbamidomethyl (C)[6,9]		Mascot
1902.9841	1902.9312	-0.0529	-28	479	496	TVFKSIGIMSTVNTSSSK			Oxidation (M)[9]		Mascot
1902.9841	1902.9312	-0.0529	-28	479	496	TVFKSIGIMSTVNTSSSK			Oxidation (M)[9]		Mascot
1943.9432	1943.8976	-0.0456	-23	881	896	KFWAEAINTACYISNR			Carbamidomethyl (C)[11]		Mascot

1948.8932	1948.9332	0.04	21	26	40	MQSYIMAEDYDIWRK					Mascot
1967.0227	1966.9214	-0.1013	-51	483	501	SIGIMSTVNTSSSKSNVV R					Mascot
1967.0597	1966.9214	-0.1383	-70	40	57	KVSHPYVIPEAINTAAEK					Mascot
1983.0176	1982.9019	-0.1157	-58	483	501	SIGIMSTVNTSSSKSNVV R			Oxidation (M)[5]		Mascot
1983.0176	1982.9019	-0.1157	-58	483	501	SIGIMSTVNTSSSKSNVV R			Oxidation (M)[5]		Mascot
2588.3218	2588.1743	-0.1475	-57	992	1013	LYHMDVKSAFLNGFIQAE VYVK			Oxidation (M)[4]		Mascot
3452.8142	3452.657	-0.1572	-46	734	766	HAKIVSTSHALIVSVMTDA PGQLLHMDTVGPAR					Mascot

10 uncharacterized protein [Arabidopsis thaliana] gi|15219597 30261.2 9.95 10 51 0 5.016

Protein Group

uncharacterized protein AT1G66190 [Arabidopsis thaliana]	gi|332196352	30261.2	9.9499 998092 6514

Peptide Information

Calc. Mass	Obsrv. Mass	± da	± ppm	Start Seq.	End Seq.	Sequence	Ion Score	C. I. %	Modification	Rank	Result Type
1107.5735	1107.5276	-0.0459	-41	51	59	AAAWAWYLR					Mascot
1208.5614	1208.5538	-0.0076	-6	184	194	RNGSDSNTTTR					Mascot
1491.6533	1491.7422	0.0889	60	197	209	TNNDKSYNGGFMK			Oxidation (M)[12]		Mascot
1518.7944	1518.798	0.0036	2	85	97	IEATKNMILSENR					Mascot
1534.7893	1534.7842	-0.0051	-3	85	97	IEATKNMILSENR			Oxidation (M)[7]		Mascot
1812.9226	1812.9358	0.0132	7	142	158	LNIDDSSFSTSLSSVLK					Mascot
1852.9045	1852.8778	-0.0267	-14	32	50	RAMMVYGGGGGGDDLA VVK					Mascot
1935.9602	1935.9364	-0.0238	-12	215	232	SLWKGMIVMGPGSTVCG R			Carbamidomethyl (C)[16]		Mascot
1948.9362	1948.9332	-0.003	-2	1	17	MLPFANYYISSPSVSEK			Oxidation (M)[1]		Mascot
1951.9552	1951.9337	-0.0215	-11	215	232	SLWKGMIVMGPGSTVCG R			Carbamidomethyl (C)[16], Oxidation (M)[6]		Mascot
1951.9552	1951.9337	-0.0215	-11	215	232	SLWKGMIVMGPGSTVCG R			Carbamidomethyl (C)[16], Oxidation (M)[6]		Mascot
2440.1013	2440.2778	0.1765	72	219	242	GMIVMGPGSTVCGRSDD VASQAGR			Carbamidomethyl (C)[12], Oxidation (M)[2,5]		Mascot
2842.2061	2842.3843	0.1782	63	159	182	HNGDDHHNHNRNDDYG FDDHGLLK					Mascot

Gel Idx/Pos	207/I7	Instr./Gel Origin	BA2151/Sample Project 20140814	Process Status	Analysis Succeeded
Plate [#] Name	[1] Sample Project 20140814	Instrument Sample Name		Spectra	11

Rank	Protein Name	Accession No.	Protein MW	Protein PI	Pep. Count	Protein Score	Protein Score C. I. %	Intensity Matched	Total Ion Score	Total Ion C. I. %	Confirmed
1	hypothetical protein F775_31970 [Aegilops tauschii]	gi|475578125	25615.9	6.35	9	349	100	42.845	295	100	

Peptide Information

Calc. Mass	Obsrv. Mass	± da	± ppm	Start Seq.	End Seq.	Sequence	Ion Score	C. I. %	Modification	Rank	Result Type
941.5567	941.5405	-0.0162	-17	54	61	FVANHLLK					Mascot
1078.535	1078.5276	-0.0074	-7	207	215	GYPLLEACR			Carbamidomethyl (C)[8]		Mascot
1078.535	1078.5276	-0.0074	-7	207	215	GYPLLEACR	66	99.949	Carbamidomethyl (C)[8]		Mascot
1117.5637	1117.5453	-0.0184	-16	159	168	GHNLSLEYGK					Mascot
1562.7632	1562.7413	-0.0219	-14	143	155	QGLDPRPVTECYK			Carbamidomethyl (C)[11]		Mascot
1796.9075	1796.8546	-0.0529	-29	116	130	VHLGFIYCVSDLVMK			Carbamidomethyl (C)[8], Oxidation (M)[14]		Mascot
1796.9075	1796.8546	-0.0529	-29	116	130	VHLGFIYCVSDLVMK	84	100	Carbamidomethyl (C)[8], Oxidation (M)[14]		Mascot
1845.9594	1845.8875	-0.0719	-39	65	82	DGLLDAADLTLVPYGNAK					Mascot
1955.9684	1955.9551	-0.0133	-7	38	53	VHVAIYYESLCPYSVR			Carbamidomethyl (C)[11]		Mascot
1955.9684	1955.9551	-0.0133	-7	38	53	VHVAIYYESLCPYSVR	128	100	Carbamidomethyl (C)[11]		Mascot
2236.1609	2236.1377	-0.0232	-10	62	82	AYRDGLLDAADLTLVPYG NAK					Mascot
2236.1609	2236.1377	-0.0232	-10	62	82	AYRDGLLDAADLTLVPYG NAK	17	0			Mascot
3399.7524	3399.7383	-0.0141	-4	169	198	QTAALVPPHQFVPWVVV DGKPLYNDYGNFK					Mascot

Rank	Protein Name	Accession No.	Protein MW	Protein PI	Pep. Count	Protein Score	Protein Score C. I. %	Intensity Matched	Total Ion Score	Total Ion C. I. %	Confirmed
2	hypothetical protein TRIUR3_28410 [Triticum urartu]	gi|474060617	28387.9	5.53	8	207	100	18.142	168	100	

Peptide Information

Calc. Mass	Obsrv. Mass	± da	± ppm	Start Seq.	End Seq.	Sequence	Ion Score	C. I. %	Modification	Rank	Result Type
1074.5062	1074.5544	0.0482	45	232	241	ADGDAIDEIR					Mascot
1078.535	1078.5276	-0.0074	-7	207	215	GYPLLEACR			Carbamidomethyl (C)[8]		Mascot
1078.535	1078.5276	-0.0074	-7	207	215	GYPLLEACR	66	99.949	Carbamidomethyl (C)[8]		Mascot
1113.467	1113.4602	-0.0068	-6	132	139	EWESCFQK			Carbamidomethyl (C)[5]		Mascot
1406.6271	1406.6171	-0.01	-7	130	139	HREWESCFQK			Carbamidomethyl (C)[7]		Mascot
1406.6271	1406.6171	-0.01	-7	130	139	HREWESCFQK			Carbamidomethyl (C)[7]		Mascot
1796.9075	1796.8546	-0.0529	-29	113	127	VHLGFIYCVSDLVMK			Carbamidomethyl (C)[8], Oxidation (M)[14]		Mascot
1796.9075	1796.8546	-0.0529	-29	113	127	VHLGFIYCVSDLVMK	84	100	Carbamidomethyl (C)[8], Oxidation (M)[14]		Mascot
1845.9594	1845.8875	-0.0719	-39	62	79	DGLLDAADLTLVPYGNAK					Mascot

Calc. Mass	Obsrv. Mass	± da	± ppm	Start Seq.	End Seq.	Sequence	Ion Score	C. I. %	Modification	Rank	Result Type
1899.7782	1899.9275	0.1493	79	216	231	RPGMEAENMCTADFDR			Carbamidomethyl (C)[10]		Mascot
2236.1609	2236.1377	-0.0232	-10	59	79	AYRDGLLDAADLTLVPYG NAK					Mascot
2236.1609	2236.1377	-0.0232	-10	59	79	AYRDGLLDAADLTLVPYG NAK	17	0			Mascot

3	Os02g0494400 [Oryza sativa Japonica Group]	gi|255670913	40328.2	6.07	13	61	24.442		6.504

Peptide Information

Calc. Mass	Obsrv. Mass	± da	± ppm	Start Seq.	End Seq.	Sequence	Ion Score	C. I. %	Modification	Rank	Result Type
1060.5997	1060.5416	-0.0581	-55	247	256	ATELKSLGNK					Mascot
1110.5248	1110.5111	-0.0137	-12	300	308	ALLDAYECR			Carbamidomethyl (C)[8]		Mascot
1159.559	1159.5585	-0.0005	0	234	244	NISDNGEPVSK					Mascot
1238.6198	1238.5703	-0.0495	-40	300	309	ALLDAYECRK			Carbamidomethyl (C)[8]		Mascot
1315.6602	1315.6038	-0.0564	-43	234	245	NISDNGEPVSKR					Mascot
1438.7294	1438.6074	-0.122	-85	43	58	GAPMGGMGALHLAAGK					Mascot
1438.7294	1438.6074	-0.122	-85	43	58	GAPMGGMGALHLAAGK					Mascot
1462.6478	1462.6199	-0.0279	-19	344	356	LDPGNSEMEDALR			Oxidation (M)[8]		Mascot
1497.8246	1497.7302	-0.0944	-63	317	329	AYYRQGAALMLLK					Mascot
1651.8519	1651.7041	-0.1478	-89	43	60	GAPMGGMGALHLAAGK GR					Mascot
1867.9045	1867.917	0.0125	7	100	116	YLLDHGADVNKASHDGR					Mascot
1910.0131	1909.9011	-0.112	-59	94	110	HLSTVKYLLDHGADVNK					Mascot
1954.0315	1953.9353	-0.0962	-49	176	194	MVDGHLTPLATAITAGEL K			Oxidation (M)[1]		Mascot
2204.1533	2204.1172	-0.0361	-16	1	19	MVEKLLFDAAHNGDLYIV R					Mascot
2220.1482	2220.1172	-0.031	-14	1	19	MVEKLLFDAAHNGDLYIV R			Oxidation (M)[1]		Mascot

4	hypothetical protein SORBIDRAFT_06g016540 [Sorghum bicolor]	gi|241939010	28466.5	4.92	11	56		0	29.712

Peptide Information

Calc. Mass	Obsrv. Mass	± da	± ppm	Start Seq.	End Seq.	Sequence	Ion Score	C. I. %	Modification	Rank	Result Type
1046.4062	1046.4946	0.0884	84	79	87	DDYDGGYNK					Mascot
1113.4443	1113.4602	0.0159	14	88	97	SGTNDDEYGR					Mascot
1117.5498	1117.5453	-0.0045	-4	210	218	DPEHAQRHK					Mascot
1145.4052	1145.4414	0.0362	32	168	176	DDDDSEMYR					Mascot
1145.4052	1145.4414	0.0362	32	168	176	DDDDSEMYR					Mascot
1230.5498	1230.5654	0.0156	13	2	12	ADEYGRSGYGR					Mascot
1230.5498	1230.5654	0.0156	13	2	12	ADEYGRSGYGR					Mascot

1273.5001	1273.5621	0.062	49	168	177	DDDDSEMYRK					Mascot
1377.5852	1377.6057	0.0205	15	1	12	MADEYGRSGYGR			Oxidation (M)[1]		Mascot
1385.5928	1385.6241	0.0313	23	154	167	KSGGADDGEYGSSR					Mascot
1549.6401	1549.7284	0.0883	57	108	123	SSGDDAYTGGAGYNSK					Mascot
1635.677	1635.7319	0.0549	34	124	139	SSGDDDAYTGVGYNSK					Mascot
1955.8003	1955.9551	0.1548	79	8	26	SGYGRSGAGDDYDSGY NSK					Mascot
1955.8003	1955.9551	0.1548	79	8	26	SGYGRSGAGDDYDSGY NSK					Mascot

5	predicted protein [Arabidopsis lyrata subsp. lyrata]	gi|297324601	44026.3	8.31	12	54	0	7.184	

Peptide Information

Calc. Mass	Obsrv. Mass	± da	± ppm	Start Seq.	End Seq.	Sequence	Ion Score	C. I. %	Modification	Rank	Result Type
812.4083	812.3837	-0.0246	-30	26	31	FRTTCK			Carbamidomethyl (C)[5]		Mascot
1060.4518	1060.5416	0.0898	85	181	189	DYGVFCSGR			Carbamidomethyl (C)[6]		Mascot
1110.5215	1110.5111	-0.0104	-9	193	202	GNTYWVAGDK					Mascot
1438.7325	1438.6074	-0.1251	-87	190	202	SLKGNTYWVAGDK					Mascot
1438.7325	1438.6074	-0.1251	-87	190	202	SLKGNTYWVAGDK					Mascot
1441.7686	1441.6571	-0.1115	-77	319	330	TRVYVVGEDIYK					Mascot
1490.6329	1490.7308	0.0979	66	148	158	SCYYENDQKQR			Carbamidomethyl (C)[2]		Mascot
1490.6329	1490.7308	0.0979	66	148	158	SCYYENDQKQR			Carbamidomethyl (C)[2]		Mascot
1864.9148	1864.9388	0.024	13	100	116	GNTSLVVWDSSTGETRR					Mascot
1867.9623	1867.917	-0.0453	-24	251	265	ILDFSNEMKIWLTNK			Oxidation (M)[8]		Mascot
1900.0175	1899.9275	-0.09	-47	2	17	KISHLPYDLESEILSR					Mascot
1905.9926	1905.8704	-0.1222	-64	84	99	ISKIFHCEGLILCSTK			Carbamidomethyl (C)[7,13]		Mascot
1954.9117	1954.9359	0.0242	12	174	189	VLDYFTRDYGVFCSGR			Carbamidomethyl (C)[13]		Mascot
2236.1284	2236.1377	0.0093	4	221	240	FPLPFESFNPEDTAALSV VR					Mascot
2236.1284	2236.1377	0.0093	4	221	240	FPLPFESFNPEDTAALSV VR					Mascot

6	hypothetical protein CARUB_v10017645mg [Capsella rubella]	gi|482560205	36582	5.97	13	54	0	4.135	

Peptide Information

Calc. Mass	Obsrv. Mass	± da	± ppm	Start Seq.	End Seq.	Sequence	Ion Score	C. I. %	Modification	Rank	Result Type
847.507	847.4771	-0.0299	-35	2	9	ASLKMIGK					Mascot
1060.5431	1060.5416	-0.0015	-1	150	157	LPMFLHMR			Oxidation (M)[3]		Mascot
1100.563	1100.4924	-0.0706	-64	265	273	GRNEPCLVR			Carbamidomethyl (C)[6]		Mascot

1238.6263	1238.5703	-0.056	-45	255	264	EKYDQESLVK					Mascot
1409.6366	1409.6029	-0.0337	-24	158	169	AAAEDFCEIVER			Carbamidomethyl (C)[7]		Mascot
1632.6954	1632.8257	0.1303	80	224	236	MMIETDSPYCEIK			Carbamidomethyl (C)[10], Oxidation (M)[1]		Mascot
1651.7744	1651.7041	-0.0703	-43	158	171	AAAEDFCEIVERNK			Carbamidomethyl (C)[7]		Mascot
1732.9381	1732.8788	-0.0593	-34	96	110	HYQALFSLAKEGIQK					Mascot
1883.8705	1883.873	0.0025	1	174	191	FTGGVAHSFTGSAMDRDK					Mascot
1899.8655	1899.9275	0.062	33	174	191	FTGGVAHSFTGSAMDRDK			Oxidation (M)[14]		Mascot
1905.9086	1905.8704	-0.0382	-20	6	22	MIGKSDIAVNFTDGMFK			Oxidation (M)[1,15]		Mascot
1926.8876	1926.8663	-0.0213	-11	172	189	NRFTGGVAHSFTGSAMDR			Oxidation (M)[16]		Mascot
1944.9558	1944.8699	-0.0859	-44	192	208	LLAFDQMYLGVNGCSLK			Carbamidomethyl (C)[14], Oxidation (M)[7]		Mascot
2188.0776	2188.1265	0.0489	22	190	208	DKLLAFDQMYLGVNGCSLK			Carbamidomethyl (C)[16], Oxidation (M)[9]		Mascot

7	PREDICTED: uncharacterized protein DDB_G0271670-like [Setaria italica]	gi|514772152	39548.8	10.24	13	54	0	17.626

Peptide Information

Calc. Mass	Obsrv. Mass	± da	± ppm	Start Seq.	End Seq.	Sequence	Ion Score	C. I. %	Modification	Rank	Result Type
812.3818	812.3837	0.0019	2	156	162	STSDKMK			Oxidation (M)[6]		Mascot
816.4323	816.4512	0.0189	23	260	267	GQVQTAGR					Mascot
922.5104	922.4555	-0.0549	-60	117	126	AGVAPAPPSR					Mascot
1078.6116	1078.5276	-0.084	-78	116	126	RAGVAPAPPSR					Mascot
1078.6116	1078.5276	-0.084	-78	116	126	RAGVAPAPPSR					Mascot
1106.6139	1106.5508	-0.0631	-57	22	30	MFRPASLLR			Oxidation (M)[1]		Mascot
1187.5474	1187.6162	0.0688	58	60	71	APPPASACSSSR			Carbamidomethyl (C)[8]		Mascot
1347.5819	1347.5969	0.015	11	1	14	MDHGGSGGGRSSSR					Mascot
1402.8165	1402.7367	-0.0798	-57	337	348	LLRSYLSLNAPR					Mascot
1490.6581	1490.7308	0.0727	49	202	214	LGESAETFFCSSR			Carbamidomethyl (C)[10]		Mascot
1490.6581	1490.7308	0.0727	49	202	214	LGESAETFFCSSR			Carbamidomethyl (C)[10]		Mascot
1732.8436	1732.8788	0.0352	20	285	299	DGFRPVVCAAEEQVR			Carbamidomethyl (C)[8]		Mascot
1864.9916	1864.9388	-0.0528	-28	117	135	AGVAPAPPSRFSVDAPPTK					Mascot
1971.9771	1971.9398	-0.0373	-19	95	112	DFASLVARTESFSTAVDR					Mascot
2210.1345	2210.1372	0.0027	1	60	82	APPPASACSSSRALLAADAAVAR			Carbamidomethyl (C)[8]		Mascot

8	hypothetical protein PRUPE_ppa002755mg [Prunus persica]	gi|462399759	72717.1	8.9	15	54	0	6.391

Peptide Information

Calc. Mass	Obsrv. Mass	± da	± ppm	Start	End	Sequence	Ion	C. I. %	Modification	Rank	Result Type

				Seq.	Seq.		Score				
872.4182	872.4965	0.0783	90	517	523	EYMKSSK					Mascot
1100.5106	1100.4924	-0.0182	-17	137	145	TEKTDESYK					Mascot
1106.5518	1106.5508	-0.001	-1	36	44	KFYFSTADK					Mascot
1132.5481	1132.4417	-0.1064	-94	116	125	NAEDISVQEK					Mascot
1147.5776	1147.5221	-0.0555	-48	1	10	MDIAGNIEIR			Oxidation (M)[1]		Mascot
1252.5701	1252.5615	-0.0086	-7	298	307	SYDLMDHMLK					Mascot
1268.5649	1268.5273	-0.0376	-30	298	307	SYDLMDHMLK			Oxidation (M)[5]		Mascot
1590.7759	1590.7792	0.0033	2	102	115	NLTLDENFPVGTDR					Mascot
1675.8246	1675.705	-0.1196	-71	146	162	ADNGPKTSSGLTVSEGR					Mascot
1796.9137	1796.8546	-0.0591	-33	454	470	SVENPLQDLGGKPASER					Mascot
1796.9137	1796.8546	-0.0591	-33	454	470	SVENPLQDLGGKPASER					Mascot
1865.0378	1864.9388	-0.099	-53	576	591	DIPNLRDILLSIPEEK					Mascot
1944.9524	1944.8699	-0.0825	-42	326	342	GIYASEGWFMKLVEGNK			Oxidation (M)[10]		Mascot
1953.8912	1953.9353	0.0441	23	401	417	TEGADHFLVACHDWAPK			Carbamidomethyl (C)[11]		Mascot
2009.9691	2009.8527	-0.1164	-58	615	629	YDLFHMILHSVWYNR			Oxidation (M)[6]		Mascot
2013.0698	2012.9529	-0.1169	-58	229	245	WNPRPTSLSQMNALLLR			Oxidation (M)[11]		Mascot
2234.1792	2234.1152	-0.064	-29	308	325	VYIYKEGEKPVFHQPLMR					Mascot
2250.1741	2250.0649	-0.1092	-49	308	325	VYIYKEGEKPVFHQPLMR			Oxidation (M)[17]		Mascot

9	calcium ion binding protein, putative [Ricinus communis] gi|223528939	57226.9	7.7	14	53	0	9.692

Peptide Information

Calc. Mass	Obsrv. Mass	± da	± ppm	Start Seq.	End Seq.	Sequence	Ion Score	C. I. %	Modification	Rank	Result Type
805.376	805.3926	0.0166	21	148	154	MTSPPEK			Oxidation (M)[1]		Mascot
941.4727	941.5405	0.0678	72	384	390	ITFEEFR					Mascot
1074.5613	1074.5544	-0.0069	-6	146	154	IRMTSPPEK			Oxidation (M)[3]		Mascot
1116.5837	1116.5048	-0.0789	-71	138	145	IFFNYEKR					Mascot
1182.575	1182.549	-0.026	-22	126	135	NLTNISDSYR					Mascot
1230.6624	1230.5654	-0.097	-79	17	26	VLSNQQLCIR			Carbamidomethyl (C)[8]		Mascot
1230.6624	1230.5654	-0.097	-79	17	26	VLSNQQLCIR			Carbamidomethyl (C)[8]		Mascot
1438.6267	1438.6074	-0.0193	-13	250	261	MFDLDNNGEIDR					Mascot
1438.6267	1438.6074	-0.0193	-13	250	261	MFDLDNNGEIDR					Mascot
1441.7434	1441.6571	-0.0863	-60	412	423	VNGTLTKYDFQR					Mascot
1490.775	1490.7308	-0.0442	-30	468	480	DNLVPGHAEWKPK					Mascot
1490.775	1490.7308	-0.0442	-30	468	480	DNLVPGHAEWKPK					Mascot

1818.9055	1818.8329	-0.0726	-40	349	365	DFALSMVASADISHINK					Mascot
1910.0059	1909.9011	-0.1048	-55	291	307	VLQPVENGGLLEYFFGK					Mascot
1971.8754	1971.9398	0.0644	33	250	265	MFDLDNNGEIDREEFK					Mascot
2210.1492	2210.1372	-0.012	-5	291	310	VLQPVENGGLLEYFFGK DGK					Mascot
2236.2124	2236.1377	-0.0747	-33	288	307	GLRVLQPVENGGLLEYFF GK					Mascot
2236.2124	2236.1377	-0.0747	-33	288	307	GLRVLQPVENGGLLEYFF GK					Mascot

10	Uncharacterized protein ycf45 [Triticum urartu]	gi|473741424	53468.6	7.19	14	53	0	28.456

Peptide Information

Calc. Mass	Obsrv. Mass	± da	± ppm	Start Seq.	End Seq.	Sequence	Ion Score	C. I. %	Modification	Rank	Result Type
1021.4697	1021.5018	0.0321	31	187	196	AVGEFGGDNR					Mascot
1092.583	1092.5381	-0.0449	-41	255	263	TTVMREIAR			Oxidation (M)[4]		Mascot
1113.547	1113.4602	-0.0868	-78	226	235	AVNGHVDMVR			Oxidation (M)[8]		Mascot
1252.5892	1252.5615	-0.0277	-22	418	426	MHFYNLQQR			Oxidation (M)[1]		Mascot
1377.7267	1377.6057	-0.121	-88	84	96	ASIRSVCASSALR			Carbamidomethyl (C)[7]		Mascot
1385.7206	1385.6241	-0.0965	-70	449	460	TERSVDMLLHGK					Mascot
1409.743	1409.6029	-0.1401	-99	223	235	VGRAVNGHVDMVR					Mascot
1434.745	1434.6283	-0.1167	-81	396	407	APPTFPFLIEMR			Oxidation (M)[11]		Mascot
1511.7206	1511.7238	0.0032	2	298	309	RMQVPEPSMQHR			Oxidation (M)[2]		Mascot
1562.84	1562.7413	-0.0987	-63	395	407	KAPPTFPFLIEMR			Oxidation (M)[12]		Mascot
1796.9436	1796.8546	-0.089	-50	339	355	SIAERGVMLIGTAHGER					Mascot
1796.9436	1796.8546	-0.089	-50	339	355	SIAERGVMLIGTAHGER					Mascot
1955.9683	1955.9551	-0.0132	-7	187	205	AVGEFGGDNRAGIEGTL HR					Mascot
1955.9683	1955.9551	-0.0132	-7	187	205	AVGEFGGDNRAGIEGTL HR					Mascot
1976.0634	1975.9426	-0.1208	-61	97	117	MELVLASAAAAFVGLGLG GGR			Oxidation (M)[1]		Mascot
2242.0298	2242.1096	0.0798	36	470	488	DNEFEVIIERWATYDGDG L					Mascot

Gel Idx/Pos	208/I8	Instr./Gel Origin	BA2151/Sample Project 20140814	Process Status	Analysis Succeeded
Plate [#] Name	[1] Sample Project 20140814	Instrument Sample Name		Spectra	11

Rank	Protein Name	Accession No.	Protein MW	Protein PI	Pep. Count	Protein Score	Protein Score C. I. %	Intensity Matched	Total Ion Score	Total Ion C. I. %	Confirmed
1	hypothetical protein TRIUR3_28410 [Triticum urartu]	gi|474060617	28387.9	5.53	6	297	100	36.334	274	100	

Peptide Information

Calc. Mass	Obsrv. Mass	± da	± ppm	Start Seq.	End Seq.	Sequence	Ion Score	C. I. %	Modification	Rank	Result Type
1078.535	1078.5328	-0.0022	-2	207	215	GYPLLEACR			Carbamidomethyl (C)[8]		Mascot
1078.535	1078.5328	-0.0022	-2	207	215	GYPLLEACR	71	99.987	Carbamidomethyl (C)[8]		Mascot
1113.467	1113.4681	0.0011	1	132	139	EWESCFQK			Carbamidomethyl (C)[5]		Mascot
1145.5698	1145.5669	-0.0029	-3	156	165	GHNLSLEYGR					Mascot
1145.5698	1145.5669	-0.0029	-3	156	165	GHNLSLEYGR	83	99.999			Mascot
1406.6271	1406.6235	-0.0036	-3	130	139	HREWESCFQK			Carbamidomethyl (C)[7]		Mascot
1406.6271	1406.6235	-0.0036	-3	130	139	HREWESCFQK	53	99.079	Carbamidomethyl (C)[7]		Mascot
1534.7571	1534.7111	-0.046	-30	140	152	QGLDPKPVTECYK			Carbamidomethyl (C)[11]		Mascot
1876.9222	1876.9094	-0.0128	-7	140	155	QGLDPKPVTECYKGER			Carbamidomethyl (C)[11]		Mascot
1876.9222	1876.9094	-0.0128	-7	140	155	QGLDPKPVTECYKGER	67	99.965	Carbamidomethyl (C)[11]		Mascot

Rank	Protein Name	Accession No.	Protein MW	Protein PI	Pep. Count	Protein Score	Protein Score C. I. %	Intensity Matched	Total Ion Score	Total Ion C. I. %	Confirmed
2	hypothetical protein F775_31970 [Aegilops tauschii]	gi|475578125	25615.9	6.35	3	222	100	33.833	213	100	

Peptide Information

Calc. Mass	Obsrv. Mass	± da	± ppm	Start Seq.	End Seq.	Sequence	Ion Score	C. I. %	Modification	Rank	Result Type
1078.535	1078.5328	-0.0022	-2	207	215	GYPLLEACR			Carbamidomethyl (C)[8]		Mascot
1078.535	1078.5328	-0.0022	-2	207	215	GYPLLEACR	71	99.987	Carbamidomethyl (C)[8]		Mascot
1461.722	1461.7588	0.0368	25	216	229	SLGLEADNNVYGPL					Mascot
1955.9684	1955.9619	-0.0065	-3	38	53	VHVAIYYESLCPYSVR			Carbamidomethyl (C)[11]		Mascot
1955.9684	1955.9619	-0.0065	-3	38	53	VHVAIYYESLCPYSVR	142	100	Carbamidomethyl (C)[11]		Mascot

Rank	Protein Name	Accession No.	Protein MW	Protein PI	Pep. Count	Protein Score	Protein Score C. I. %	Intensity Matched	Total Ion Score	Total Ion C. I. %	Confirmed
3	Os01g0681900 [Oryza sativa Japonica Group]	gi|113533415	238797.4	6.69	32	72	93.109	38.172			

Protein Group

RecName: Full=Glutamate synthase 1 [NADH], chloroplastic; AltName: Full=NADH-dependent glutamate synthase 1; Short=NADH-GOGAT 1; Flags: Precursor	gi|122222486	238797.4	6.6900 000572 2046

Peptide Information

Calc. Mass	Obsrv. Mass	± da	± ppm	Start Seq.	End Seq.	Sequence	Ion Score	C. I. %	Modification	Rank	Result Type
837.4465	837.4545	0.008	10	188	194	SKAEFQK					Mascot
987.4894	987.5245	0.0351	36	948	955	AAYKEYSR					Mascot
1048.5343	1048.6067	0.0724	69	1781	1789	SIECAIIDK			Carbamidomethyl (C)[4]		Mascot
1060.5634	1060.5507	-0.0127	-12	706	714	KGLEETLDR					Mascot
1076.635	1076.55	-0.085	-79	694	702	VLDITYPKK					Mascot
1230.6338	1230.5765	-0.0573	-47	26	36	RSHSSVAAPYR					Mascot
1238.5988	1238.5806	-0.0182	-15	1828	1837	MGHFVTVFER			Oxidation (M)[1]		Mascot
1273.6271	1273.5808	-0.0463	-36	537	547	DIVESVPETER					Mascot
1390.5807	1390.6548	0.0741	53	828	838	YFYASNYGMMK			Oxidation (M)[9]		Mascot
1406.5756	1406.6235	0.0479	34	828	838	YFYASNYGMMK			Oxidation (M)[9,10]		Mascot
1406.5756	1406.6235	0.0479	34	828	838	YFYASNYGMMK			Oxidation (M)[9,10]		Mascot
1410.6942	1410.6219	-0.0723	-51	1841	1853	IGGLMMYGVPNMK					Mascot
1428.7628	1428.6201	-0.1427	-100	1389	1401	AVGTMLSHEVTKR					Mascot
1444.7577	1444.7384	-0.0193	-13	1389	1401	AVGTMLSHEVTKR			Oxidation (M)[5]		Mascot
1497.8346	1497.7245	-0.1101	-74	839	852	VLAKMGISTLASYK			Oxidation (M)[5]		Mascot
1517.774	1517.7039	-0.0701	-46	114	126	RATVNDALEMLER					Mascot
1534.6705	1534.7111	0.0406	26	827	838	KYFYASNYGMMK			Oxidation (M)[10,11]		Mascot
1768.8544	1768.8678	0.0134	8	1838	1853	ADRIGGLMMYGVPNMK			Oxidation (M)[8]		Mascot
1784.8492	1784.9188	0.0696	39	1838	1853	ADRIGGLMMYGVPNMK			Oxidation (M)[8,9]		Mascot
1785.8702	1785.9167	0.0465	26	828	842	YFYASNYGMMKVLAK					Mascot
1818.879	1818.8286	-0.0504	-28	1308	1323	SDMLEVDPEVVKSNEK					Mascot
1856.9105	1856.9032	-0.0073	-4	115	130	ATVNDALEMLERMAHR					Mascot
1859.8593	1859.8853	0.026	14	98	114	DSCGVGFVAELSGDYKR			Carbamidomethyl (C)[3]		Mascot
1859.8593	1859.8853	0.026	14	98	114	DSCGVGFVAELSGDYKR			Carbamidomethyl (C)[3]		Mascot
1876.9434	1876.9094	-0.034	-18	1952	1970	VVVIGGGDTGTDCIGTSI R			Carbamidomethyl (C)[13]		Mascot
1876.9434	1876.9094	-0.034	-18	1952	1970	VVVIGGGDTGTDCIGTSI R			Carbamidomethyl (C)[13]		Mascot
1898.9872	1898.926	-0.0612	-32	138	155	NTGDGAGILVALPHNFFR					Mascot
1956.0721	1955.9619	-0.1102	-56	667	684	LALEGPLVSIDEMEAIKK					Mascot
1956.0721	1955.9619	-0.1102	-56	667	684	LALEGPLVSIDEMEAIKK					Mascot
1968.0042	1967.9226	-0.0816	-41	1291	1307	EIMSQLGFRTITEMVGR					Mascot
1972.0671	1971.9507	-0.1164	-59	667	684	LALEGPLVSIDEMEAIKK			Oxidation (M)[13]		Mascot
2013.0436	2012.9756	-0.068	-34	577	594	AFGYTVEALEMLLLPMAK			Oxidation (M)[11]		Mascot
2264.115	2264.1284	0.0134	6	1300	1319	TITEMVGRSDMLEVDPEV VK			Oxidation (M)[5]		Mascot
2323.1375	2323.1287	-0.0088	-4	972	992	FKDTADMISVDEVEPASE					Mascot

						IVK					
2356.241	2356.1257	-0.1153	-49	138	159	NTGDGAGILVALPHNFFR EVTK					Mascot
2933.3552	2933.4104	0.0552	19	398	422	SLPEAVMMMIPEAWQND VNMEPEKK			Oxidation (M)[7]		Mascot
3079.5332	3079.48	-0.0532	-17	156	183	EVTKDAGFELPQPGEYA VGMVFLPIDEK					Mascot
3079.5332	3079.48	-0.0532	-17	156	183	EVTKDAGFELPQPGEYA VGMVFLPIDEK					Mascot
3095.5283	3095.4785	-0.0498	-16	156	183	EVTKDAGFELPQPGEYA VGMVFLPIDEK			Oxidation (M)[20]		Mascot
3109.5623	3109.4917	-0.0706	-23	1863	1890	RVNLMAEEGITFVVNANV GSDPLYSIER			Oxidation (M)[5]		Mascot
3457.7073	3457.7588	0.0515	15	1271	1299	FAGEPEHVINFFFMLAEE LREIMSQLGFR					Mascot
3469.8359	3469.71	-0.1259	-36	1320	1350	SNEKLENIDLSLILKPAAEI RPGAAQYCVEK			Carbamidomethyl (C)[28]		Mascot

4 kinase domain-containing protein [Arabidopsis thaliana] gi|18394385 43718.2 8.02 14 69 87.746 24.127

Protein Group

kinase domain-containing protein [Arabidopsis thaliana] gi|332191360 43718.2 8.0200 004577 6367

Peptide Information

Calc. Mass	Obsrv. Mass	± da	± ppm	Start Seq.	End Seq.	Sequence	Ion Score	C. I. %	Modification	Rank	Result Type
951.5258	951.4813	-0.0445	-47	125	133	TLLAGGYTR					Mascot
1113.5576	1113.4681	-0.0895	-80	47	57	IGEGGFGSVYK					Mascot
1145.646	1145.5669	-0.0791	-69	303	312	LRPSMSTVVR					Mascot
1145.646	1145.5669	-0.0791	-69	303	312	LRPSMSTVVR					Mascot
1173.6587	1173.5951	-0.0636	-54	70	80	VLSAESRQGVK					Mascot
1182.5538	1182.5511	-0.0027	-2	134	143	SGIQFDWSSR					Mascot
1229.71	1229.627	-0.083	-67	169	179	DIKASNILLDK					Mascot
1231.6351	1231.5796	-0.0555	-45	292	302	IGLLCTQDSPK			Carbamidomethyl (C)[5]		Mascot
1364.5835	1364.6722	0.0887	65	100	110	LYGCCVEGNHR			Carbamidomethyl (C)[4,5]		Mascot
1461.8312	1461.7588	-0.0724	-50	172	184	ASNILLDKYLSPK					Mascot
1591.8513	1591.8413	-0.01	-6	325	338	ISRPGLISDFMDLK					Mascot
1635.8774	1635.7402	-0.1372	-84	289	302	YLKIGLLCTQDSPK			Carbamidomethyl (C)[8]		Mascot
1719.9463	1719.8309	-0.1154	-67	324	338	KISRPGLISDFMDLK					Mascot
2245.9592	2246.1194	0.1602	71	368	390	DHSNAYSSGASSANAGN TFSSTI					Mascot
3369.5693	3369.6255	0.0562	17	260	288	AWELYERNELVDLVDSG LNGVFDAEEACR			Carbamidomethyl (C)[28]		Mascot

5 PREDICTED: uncharacterized protein LOC101250579 gi|460378691 131331.7 5.48 24 69 86.564 5.098

[Solanum lycopersicum]

Peptide Information

Calc. Mass	Obsrv. Mass	± da	± ppm	Start Seq.	End Seq.	Sequence	Ion Score	C. I. %	Modification	Rank	Result Type
918.4891	918.4587	-0.0304	-33	1028	1037	LGATGSGEVK					Mascot
1048.5059	1048.6067	0.1008	96	189	197	SFSHELNSK					Mascot
1106.4961	1106.5667	0.0706	64	420	430	GNDSAETTPSK					Mascot
1127.4979	1127.5385	0.0406	36	816	823	FFYSFTCR			Carbamidomethyl (C)[7]		Mascot
1128.507	1128.5417	0.0347	31	249	257	TSESHPEWR					Mascot
1202.6263	1202.585	-0.0413	-34	2	12	STTPANLELEK					Mascot
1229.6848	1229.627	-0.0578	-47	519	528	LERVVDTLER					Mascot
1315.6853	1315.6135	-0.0718	-55	28	40	SQAPSTSPSVNLK					Mascot
1364.6661	1364.6722	0.0061	4	501	511	ICTIADRCDLK			Carbamidomethyl (C)[2,8]		Mascot
1390.7186	1390.6548	-0.0638	-46	15	27	LNPIATASHSSHR					Mascot
1410.7263	1410.6219	-0.1044	-74	364	375	YLGYEQSNLPVK					Mascot
1428.6965	1428.6201	-0.0764	-53	1154	1167	NSKESVEAANPSVP					Mascot
1444.7642	1444.7384	-0.0258	-18	2	14	STTPANLELEKNK					Mascot
1463.7224	1463.7432	0.0208	14	379	392	TNEVGDTLEVSTAK					Mascot
1497.6865	1497.7245	0.038	25	270	281	CTKMPPSEFWSK			Carbamidomethyl (C)[1]		Mascot
1517.7417	1517.7039	-0.0378	-25	702	713	YILLCGHIEDER			Carbamidomethyl (C)[5]		Mascot
1534.7903	1534.7111	-0.0792	-52	209	221	ALGRMEEIMVMVR					Mascot
1591.7996	1591.8413	0.0417	26	1	14	MSTTPANLELEKNK			Oxidation (M)[1]		Mascot
1785.9746	1785.9167	-0.0579	-32	522	536	VVDTLERILESWTPK					Mascot
1818.9133	1818.8286	-0.0847	-47	364	378	YLGYEQSNLPVKHDR					Mascot
1898.9203	1898.926	0.0057	3	118	135	SSSVVQHPAENSSEQGK K					Mascot
1955.0743	1954.9324	-0.1419	-73	613	630	SPLVTPRTGQIELMLSGR					Mascot
1978.0604	1977.9333	-0.1271	-64	1139	1156	NLSQLASINYDLIGKNSK					Mascot
2357.2458	2357.1438	-0.102	-43	631	650	ITVSEHESYQQINKLLDIA R					Mascot

6 ATP synthase beta subunit, partial [Hakea obliqua] gi|380779100 25786.5 6.75 11 66 76.65 3.013

Peptide Information

Calc. Mass	Obsrv. Mass	± da	± ppm	Start Seq.	End Seq.	Sequence	Ion Score	C. I. %	Modification	Rank	Result Type
975.5622	975.5275	-0.0347	-36	168	178	IGLFGGAGVGK					Mascot
1229.5177	1229.627	0.1093	89	208	217	EGNDLYMEMK					Mascot
1238.5868	1238.5806	-0.0062	-5	76	87	AVAMSATDGLMR			Oxidation (M)[4]		Mascot

Calc. Mass	Obsrv. Mass	± da	± ppm	Start Seq.	End Seq.	Sequence	Ion Score	C. I. %	Modification	Rank	Result Type
1390.6671	1390.6548	-0.0123	-9	232	244	VALVYGQMNEPPG			Oxidation (M)[8]		Mascot
1477.7614	1477.7103	-0.0511	-35	74	87	VRAVAMSATDGLMR					Mascot
1490.8148	1490.7308	-0.084	-56	40	52	MPNIYNALVVKGR			Oxidation (M)[1]		Mascot
1517.7441	1517.7039	-0.0402	-26	218	231	ESGVINEQNIAESK					Mascot
1785.9342	1785.9167	-0.0175	-10	2	18	RINPTTSGPGVSTLEEK					Mascot
1955.0233	1954.9324	-0.0909	-46	110	127	IFNVLGEPVDNLGPVDTR					Mascot
1983.0658	1982.9535	-0.1123	-57	128	145	TTSPIHRSAPAFIQLDTK					Mascot
2428.3997	2428.1616	-0.2381	-98	168	191	IGLFGGAGVGKTVLIMELI NNIAK					Mascot

7	hypothetical protein ARALYDRAFT_471863 [Arabidopsis lyrata subsp. lyrata]	gi|297336022	43926.2	8.29	14	65	68.502	24.261

Peptide Information

Calc. Mass	Obsrv. Mass	± da	± ppm	Start Seq.	End Seq.	Sequence	Ion Score	C. I. %	Modification	Rank	Result Type
951.5258	951.4813	-0.0445	-47	126	134	TLLAGGYTR					Mascot
1113.5576	1113.4681	-0.0895	-80	48	58	IGEGGFGSVYK					Mascot
1145.646	1145.5669	-0.0791	-69	304	313	LRPSMSTVVR					Mascot
1145.646	1145.5669	-0.0791	-69	304	313	LRPSMSTVVR					Mascot
1173.6587	1173.5951	-0.0636	-54	71	81	VLSAESRQGVK					Mascot
1182.5538	1182.5511	-0.0027	-2	135	144	SGIQFDWSSR					Mascot
1229.71	1229.627	-0.083	-67	170	180	DIKASNILLDK					Mascot
1231.6351	1231.5796	-0.0555	-45	293	303	IGLLCTQDSPK			Carbamidomethyl (C)[5]		Mascot
1343.5078	1343.6383	0.1305	97	1	10	MGCSWFSCHR			Carbamidomethyl (C)[3,8], Oxidation (M)[1]		Mascot
1364.5835	1364.6722	0.0887	65	101	111	LYGCCVEGNHR			Carbamidomethyl (C)[4,5]		Mascot
1461.8312	1461.7588	-0.0724	-50	173	185	ASNILLDKYLSPK					Mascot
1635.8774	1635.7402	-0.1372	-84	290	303	YLKIGLLCTQDSPK			Carbamidomethyl (C)[8]		Mascot
1864.9772	1864.9446	-0.0326	-17	326	341	ISRPGLISDFMDMKVR					Mascot
2245.9592	2246.1194	0.1602	71	369	391	DHSNAYSSGASSANAGN TFSSTI					Mascot
3369.5693	3369.6255	0.0562	17	261	289	AWELYERNELVDLVDSG LNGVFDAEEACR			Carbamidomethyl (C)[28]		Mascot

8	probable pyruvate kinase [Arabidopsis thaliana]	gi|332659792	54968.1	8.14	16	64	62.993	5.546

Protein Group

RecName: Full=Probable pyruvate kinase, cytosolic isozyme; Short=PK	gi|4033431	54968.1	8.1400 003433 2275
probable pyruvate kinase [Arabidopsis thaliana]	gi|15236190	54968.1	8.1400

003433
2275

Peptide Information

Calc. Mass	Obsrv. Mass	± da	± ppm	Start Seq.	End Seq.	Sequence	Ion Score	C. I. %	Modification	Rank	Result Type
918.4059	918.4587	0.0528	57	110	116	TICMSYK			Carbamidomethyl (C)[3], Oxidation (M)[4]		Mascot
978.4924	978.5208	0.0284	29	139	146	VLSCDKEK			Carbamidomethyl (C)[4]		Mascot
1046.501	1046.5059	0.0049	5	110	117	TICMSYKK			Carbamidomethyl (C)[3], Oxidation (M)[4]		Mascot
1048.5642	1048.6067	0.0425	41	28	37	LLMAGMSVAR					Mascot
1088.5881	1088.5469	-0.0412	-38	2	10	AMEQRPKTK					Mascot
1202.6892	1202.585	-0.1042	-87	438	449	GLVPVLYAGSAR					Mascot
1331.597	1331.6117	0.0147	11	106	116	GDEKTICMSYK			Carbamidomethyl (C)[7]		Mascot
1347.5919	1347.6084	0.0165	12	106	116	GDEKTICMSYK			Carbamidomethyl (C)[7], Oxidation (M)[8]		Mascot
1444.8523	1444.7384	-0.1139	-79	79	91	TGFLKDGKPIQLK					Mascot
1818.9639	1818.8286	-0.1353	-74	21	37	SVPMVEKLLMAGMSVAR					Mascot
1859.8732	1859.8853	0.0121	7	338	353	ICVEAESTLDYGDIFK			Carbamidomethyl (C)[2]		Mascot
1859.9718	1859.8853	-0.0865	-47	11	27	IVCTLGPASRSVPMVEK			Carbamidomethyl (C)[3], Oxidation (M)[14]		Mascot
1972.1127	1971.9507	-0.162	-82	432	449	HSLIYRGLVPVLYAGSAR					Mascot
2246.0979	2246.1194	0.0215	10	355	375	IMLHAAVPMSPMESLASS AVR			Oxidation (M)[2,9,12]		Mascot
2357.0581	2357.1438	0.0857	36	38	56	FNFSHGSYEYHQETLDN LR					Mascot
2933.478	2933.4104	-0.0676	-23	118	144	LAQDVNPGMVILCADGTI SLKVLSCDK			Carbamidomethyl (C)[13,25], Oxidation (M)[9]		Mascot
3499.7449	3499.6301	-0.1148	-33	221	251	TILLMSKVENQEGVANFD DILINSDAFMIAR			Oxidation (M)[5,28]		Mascot

9	pentatricopeptide repeat-containing protein [Arabidopsis thaliana]	gi|332003927	95908.7	6.98	18	64	58.478	25.997

Protein Group

RecName: Full=Putative pentatricopeptide repeat-containing protein At5g08490	gi|75171897	95908.7	6.9800 000190 7349
pentatricopeptide repeat-containing protein [Arabidopsis thaliana]	gi|15241649	95908.7	6.9800 000190 7349

Peptide Information

Calc. Mass	Obsrv. Mass	± da	± ppm	Start Seq.	End Seq.	Sequence	Ion Score	C. I. %	Modification	Rank	Result Type
918.468	918.4587	-0.0093	-10	453	460	EVHGYSVK					Mascot
978.5077	978.5208	0.0131	13	143	150	SMHSYIIK					Mascot
1046.52	1046.5059	-0.0141	-13	589	596	QCHGYIIR			Carbamidomethyl (C)[2]		Mascot
1129.6113	1129.5535	-0.0578	-51	248	256	QIHSYVVQR					Mascot
1145.6313	1145.5669	-0.0644	-56	451	460	VKEVHGYSVK					Mascot
1145.6313	1145.5669	-0.0644	-56	451	460	VKEVHGYSVK					Mascot
1230.5065	1230.5765	0.07	57	72	80	MDDCQKMFR			Carbamidomethyl (C)[4]		Mascot
1436.703	1436.6234	-0.0796	-55	620	631	HAYSVFQSDARR					Mascot
1448.7856	1448.7418	-0.0438	-30	276	288	VGRIEEAASLFTR					Mascot
1497.7618	1497.7245	-0.0373	-25	156	169	DTLVGNALVSMYAK			Oxidation (M)[11]		Mascot
1530.723	1530.7861	0.0631	41	817	829	NVFVSGDCSHPRR			Carbamidomethyl (C)[8]		Mascot
1785.9092	1785.9167	0.0075	4	830	844	DSIFDLVNALYLQMK			Oxidation (M)[14]		Mascot
1898.863	1898.926	0.063	33	379	395	FGDTSAAYWAFSLMSTK			Oxidation (M)[14]		Mascot
1926.0154	1925.9548	-0.0606	-31	829	844	RDSIFDLVNALYLQMK					Mascot
2356.1907	2356.1257	-0.065	-28	257	275	SWLQTHVFVCNSLVSFYLR			Carbamidomethyl (C)[10]		Mascot
2357.2097	2357.1438	-0.0659	-28	830	849	DSIFDLVNALYLQMKEPVVF			Oxidation (M)[14]		Mascot
3079.501	3079.48	-0.021	-7	81	107	QMDSLDPVVWNIVLTGLSVSCGRETMR			Carbamidomethyl (C)[21], Oxidation (M)[2]		Mascot
3079.501	3079.48	-0.021	-7	81	107	QMDSLDPVVWNIVLTGLSVSCGRETMR			Carbamidomethyl (C)[21], Oxidation (M)[2]		Mascot
3095.4958	3095.4785	-0.0173	-6	81	107	QMDSLDPVVWNIVLTGLSVSCGRETMR			Carbamidomethyl (C)[21], Oxidation (M)[2,26]		Mascot
3457.7024	3457.7588	0.0564	16	156	187	DTLVGNALVSMYAKFGFIFPDAYTAFDGIADK					Mascot
3469.6047	3469.71	0.1053	30	188	217	DVVSWNAIIAGFSENNMMADAFRSFCLMLK			Carbamidomethyl (C)[26], Oxidation (M)[17,18]		Mascot

10	PREDICTED: probable rhamnose biosynthetic enzyme 1-like [Fragaria vesca subsp. vesca]	gi|470115983	76130.5	6.52	17	63	50.079	3.467

Peptide Information

Calc. Mass	Obsrv. Mass	± da	± ppm	Start Seq.	End Seq.	Sequence	Ion Score	C. I. %	Modification	Rank	Result Type
978.5731	978.5208	-0.0523	-53	550	557	NFITKISR					Mascot
987.5622	987.5245	-0.0377	-38	392	401	TGWIGGVLGK					Mascot
997.4989	997.5127	0.0138	14	609	616	QYIDPSFK					Mascot
1060.5786	1060.5507	-0.0279	-26	297	305	LTVLGWSER					Mascot
1106.6205	1106.5667	-0.0538	-49	176	185	SYGLPVITTR					Mascot
1128.6161	1128.5417	-0.0744	-66	26	34	LVRSHPEYK					Mascot

1182.5316	1182.5511	0.0195	16	339	348	MLMMPGGIER	Oxidation (M)[1,3,4]	Mascot
1273.6536	1273.5808	-0.0728	-57	242	253	GEVGHVYNIGTK		Mascot
1331.7318	1331.6117	-0.1201	-90	49	60	NLLPSKSSPNFK		Mascot
1463.6914	1463.7432	0.0518	35	186	198	GNNVYGPNQFPEK		Mascot
1490.7632	1490.7308	-0.0324	-22	460	473	TNVAGTLTLADVCR	Carbamidomethyl (C)[13]	Mascot
1517.8146	1517.7039	-0.1107	-73	392	405	TGWIGGVLGKLCEK	Carbamidomethyl (C)[12]	Mascot
1620.801	1620.7921	-0.0089	-5	628	642	VIVAARSNNEMDASK	Oxidation (M)[11]	Mascot
1939.0283	1938.9674	-0.0609	-31	617	633	YSNFTIEEQAKVIVAAR		Mascot
1970.0018	1969.9535	-0.0483	-25	290	305	YFLDDQKLTVLGWSER		Mascot
2356.2153	2356.1257	-0.0896	-38	164	185	AGAEMLVMAYGRSYGLP VITTR		Mascot
3079.47	3079.48	0.01	3	322	348	NPNWWGDVSGALLPHP RMLMMPGGIER	Oxidation (M)[18,20,21]	Mascot
3079.47	3079.48	0.01	3	322	348	NPNWWGDVSGALLPHP RMLMMPGGIER	Oxidation (M)[18,20,21]	Mascot

Gel Idx/Pos	209/I9	Instr./Gel Origin	BA2151/Sample Project 20140814	Process Status	Analysis Succeeded
Plate [#] Name	[1] Sample Project 20140814	Instrument Sample Name		Spectra	11

Rank	Protein Name	Accession No.	Protein MW	Protein PI	Pep. Count	Protein Score	Protein Score C. I. %	Intensity Matched	Total Ion Score	Total Ion C. I. %	Confirmed
1	hypothetical protein F775_31970 [Aegilops tauschii]	gi|475578125	25615.9	6.35	11	493	100	47.673	417	100	

Peptide Information

Calc. Mass	Obsrv. Mass	± da	± ppm	Start Seq.	End Seq.	Sequence	Ion Score	C. I. %	Modification	Rank	Result Type
941.5567	941.5354	-0.0213	-23	54	61	FVANHLLK					Mascot
1078.535	1078.5236	-0.0114	-11	207	215	GYPLLEACR			Carbamidomethyl (C)[8]		Mascot
1078.535	1078.5236	-0.0114	-11	207	215	GYPLLEACR	71	99.985	Carbamidomethyl (C)[8]		Mascot
1117.5637	1117.5424	-0.0213	-19	159	168	GHNLSLEYGK					Mascot
1117.5637	1117.5424	-0.0213	-19	159	168	GHNLSLEYGK	67	99.962			Mascot
1562.7632	1562.7217	-0.0415	-27	143	155	QGLDPRPVTECYK			Carbamidomethyl (C)[11]		Mascot
1796.9075	1796.8391	-0.0684	-38	116	130	VHLGFIYCVSDLVMK			Carbamidomethyl (C)[8], Oxidation (M)[14]		Mascot
1796.9075	1796.8391	-0.0684	-38	116	130	VHLGFIYCVSDLVMK	84	100	Carbamidomethyl (C)[8], Oxidation (M)[14]		Mascot
1845.9594	1845.8745	-0.0849	-46	65	82	DGLLDAADLTLVPYGNAK					Mascot
1904.9283	1904.8774	-0.0509	-27	143	158	QGLDPRPVTECYKGER			Carbamidomethyl (C)[11]		Mascot
1955.9684	1955.949	-0.0194	-10	38	53	VHVAIYYESLCPYSVR			Carbamidomethyl (C)[11]		Mascot
1955.9684	1955.949	-0.0194	-10	38	53	VHVAIYYESLCPYSVR	146	100	Carbamidomethyl (C)[11]		Mascot
2236.1609	2236.1265	-0.0344	-15	62	82	AYRDGLLDAADLTLVPYG NAK					Mascot
2236.1609	2236.1265	-0.0344	-15	62	82	AYRDGLLDAADLTLVPYG NAK	48	96.724			Mascot
3399.7524	3399.7427	-0.0097	-3	169	198	QTAALVPPHQFVPWVVV DGKPLYNDYGNFK					Mascot
3675.699	3675.7107	0.0117	3	83	115	VNAHGAITCQHGPEECLL NTVEACAIDAWPDVK			Carbamidomethyl (C)[9,16,24]		Mascot

Rank	Protein Name	Accession No.	Protein MW	Protein PI	Pep. Count	Protein Score	Protein Score C. I. %	Intensity Matched	Total Ion Score	Total Ion C. I. %	Confirmed
2	hypothetical protein TRIUR3_28410 [Triticum urartu]	gi|474060617	28387.9	5.53	6	266	100	21.334	241	100	

Peptide Information

Calc. Mass	Obsrv. Mass	± da	± ppm	Start Seq.	End Seq.	Sequence	Ion Score	C. I. %	Modification	Rank	Result Type
1078.535	1078.5236	-0.0114	-11	207	215	GYPLLEACR			Carbamidomethyl (C)[8]		Mascot
1078.535	1078.5236	-0.0114	-11	207	215	GYPLLEACR	71	99.985	Carbamidomethyl (C)[8]		Mascot
1113.467	1113.45	-0.017	-15	132	139	EWESCFQK			Carbamidomethyl (C)[5]		Mascot
1406.6271	1406.6101	-0.017	-12	130	139	HREWESCFQK			Carbamidomethyl (C)[7]		Mascot
1406.6271	1406.6101	-0.017	-12	130	139	HREWESCFQK	38	68.421	Carbamidomethyl (C)[7]		Mascot

1796.9075	1796.8391	-0.0684	-38	113	127	VHLGFIYCVSDLVMK			Carbamidomethyl (C)[8], Oxidation (M)[14]		Mascot
1796.9075	1796.8391	-0.0684	-38	113	127	VHLGFIYCVSDLVMK	84	100	Carbamidomethyl (C)[8], Oxidation (M)[14]		Mascot
1845.9594	1845.8745	-0.0849	-46	62	79	DGLLDAADLTLVPYGNAK					Mascot
2236.1609	2236.1265	-0.0344	-15	59	79	AYRDGLLDAADLTLVPYG NAK					Mascot
2236.1609	2236.1265	-0.0344	-15	59	79	AYRDGLLDAADLTLVPYG NAK	48	96.724			Mascot

3	Putative LRR receptor-like serine/threonine-protein kinase [Aegilops tauschii]	gi|475600033	291010.5	5.64	33	61	17.153	13.113	

Peptide Information

Calc. Mass	Obsrv. Mass	± da	± ppm	Start Seq.	End Seq.	Sequence	Ion Score	C. I. %	Modification	Rank	Result Type
856.441	856.4855	0.0445	52	620	627	ESLPDPAK					Mascot
1021.4908	1021.4931	0.0023	2	676	686	ATGTGSSTPSR					Mascot
1127.5579	1127.4762	-0.0817	-72	458	468	GESLPEAEPAK					Mascot
1182.6378	1182.5426	-0.0952	-80	2430	2438	DKHISWIQR					Mascot
1187.599	1187.5753	-0.0237	-20	1819	1828	HCYALPVATR			Carbamidomethyl (C)[2]		Mascot
1249.6132	1249.564	-0.0492	-39	1062	1074	ANGGISGDTSSKR					Mascot
1269.6587	1269.5499	-0.1088	-86	1928	1939	INFGAPSPDPVR					Mascot
1330.5256	1330.6354	0.1098	83	58	67	YDCPFQDEEK			Carbamidomethyl (C)[3]		Mascot
1331.6074	1331.5981	-0.0093	-7	289	302	TEEKGGGDGDAPAK					Mascot
1406.6646	1406.6101	-0.0545	-39	512	524	DTDKTDSVTEPAK					Mascot
1406.6646	1406.6101	-0.0545	-39	512	524	DTDKTDSVTEPAK					Mascot
1410.5811	1410.604	0.0229	16	2646	2656	SHDMIMDNLMR			Oxidation (M)[4,6,10]		Mascot
1418.6469	1418.6205	-0.0264	-19	1294	1306	VSSPNADEVMPEK			Oxidation (M)[10]		Mascot
1421.7748	1421.6682	-0.1066	-75	2380	2391	KQFTNEVSLLSR					Mascot
1484.7228	1484.817	0.0942	63	620	633	ESLPDPAKEGAENK					Mascot
1490.7043	1490.7065	0.0022	1	359	372	DAAIVEDTPMPSTK			Oxidation (M)[10]		Mascot
1507.8479	1507.6989	-0.149	-99	1079	1092	KAADPKPQETPVVK					Mascot
1530.8058	1530.7283	-0.0775	-51	2201	2213	CSSEPQLRVVSIR			Carbamidomethyl (C)[1]		Mascot
1562.7769	1562.7217	-0.0552	-35	895	908	SVASSTEKPERDTR					Mascot
1573.7705	1573.7382	-0.0323	-21	970	983	SLPESTEDPDRVTK					Mascot
1717.9385	1717.8705	-0.068	-40	1860	1874	WSPVVVYDGARLVTR					Mascot
1796.8372	1796.8391	0.0019	1	323	338	EPEVAIDVEDSPPMPR			Oxidation (M)[14]		Mascot
1796.8372	1796.8391	0.0019	1	323	338	EPEVAIDVEDSPPMPR	3	0	Oxidation (M)[14]		Mascot
1812.9564	1812.8297	-0.1267	-70	1154	1170	MGKIAPASASFLTDFLK			Oxidation (M)[1]		Mascot
1864.9287	1864.9387	0.01	5	1438	1454	VKEEVSSPHADEAIPEK					Mascot
1867.9736	1867.9077	-0.0659	-35	1697	1715	QGGGGVCYLGGGSFLVV			Carbamidomethyl (C)[7]		Mascot

						LK					
1896.9042	1896.9003	-0.0039	-2	709	725	IIDTSMDAVDGMLSERK			Oxidation (M)[6]		Mascot
1910.8518	1910.8895	0.0377	20	2641	2656	SGTVRSHDMIMDNLMR			Oxidation (M)[9,11,15]		Mascot
1954.8342	1954.9196	0.0854	44	1171	1188	SARACTSDVEEAANTGC R			Carbamidomethyl (C)[5,17]		Mascot
1970.0488	1969.9294	-0.1194	-61	221	239	SPLAGADRLDLATAMAQ LR					Mascot
1977.9818	1977.9203	-0.0615	-31	2342	2359	NFENRIGSGGFGIVYYGK					Mascot
2036.9771	2036.9698	-0.0073	-4	1440	1458	EEVSSPHADEAIPEKAAE K					Mascot
2143.0051	2142.9604	-0.0447	-21	2657	2674	EEDESSSFPNTLRHPELR					Mascot
2235.9922	2236.1265	0.1343	60	576	597	DVAENEDSSMPSAEAID GALSK					Mascot
2235.9922	2236.1265	0.1343	60	576	597	DVAENEDSSMPSAEAID GALSK					Mascot
3187.4583	3187.5303	0.072	23	567	597	NESVPEPAKDVAENEDS SMPSAEAIDGALSK					Mascot

4	hypothetical protein ZEAMMB73_143161 [Zea mays]	gi|413934179	247668.5	4.88	33	59	0	32.504	

Peptide Information

Calc. Mass	Obsrv. Mass	± da	± ppm	Start Seq.	End Seq.	Sequence	Ion Score	C. I. %	Modification	Rank	Result Type
1021.5676	1021.4931	-0.0745	-73	341	348	LKELESFR					Mascot
1078.5415	1078.5236	-0.0179	-17	1198	1206	NEEFEVLAK					Mascot
1078.5415	1078.5236	-0.0179	-17	1198	1206	NEEFEVLAK					Mascot
1094.5729	1094.5156	-0.0573	-52	1431	1439	TEVFITQEK					Mascot
1106.5729	1106.5458	-0.0271	-24	200	209	GLTFESPEVK					Mascot
1113.5219	1113.45	-0.0719	-65	519	526	LQDECHRR			Carbamidomethyl (C)[5]		Mascot
1117.5848	1117.5424	-0.0424	-38	734	742	TELENVERK					Mascot
1117.5848	1117.5424	-0.0424	-38	734	742	TELENVERK					Mascot
1147.6218	1147.5582	-0.0636	-55	823	831	HTEEIHILR					Mascot
1182.5538	1182.5426	-0.0112	-9	1976	1986	GAAEPHEAWSK					Mascot
1187.5725	1187.5753	0.0028	2	982	991	DIISCHVSEK			Carbamidomethyl (C)[5]		Mascot
1205.6195	1205.5809	-0.0386	-32	388	397	MANEKLNELK			Oxidation (M)[1]		Mascot
1250.6012	1250.563	-0.0382	-31	45	55	LINEDADSFAR					Mascot
1312.624	1312.6602	0.0362	28	783	793	NLHSESQNEVR					Mascot
1315.5405	1315.6025	0.062	47	665	674	MYCQSQEDVR			Carbamidomethyl (C)[3]		Mascot
1319.6326	1319.59	-0.0426	-32	487	497	LQSELDETEQK					Mascot
1323.6903	1323.6306	-0.0597	-45	1735	1746	LAGIQDEHAELK					Mascot
1331.5355	1331.5981	0.0626	47	665	674	MYCQSQEDVR			Carbamidomethyl (C)[3], Oxidation (M)[1]		Mascot
1337.6444	1337.6232	-0.0212	-16	1538	1549	SVAHESENSHLK					Mascot
1340.6693	1340.6639	-0.0054	-4	549	560	TLTQDFDGSTKK					Mascot

1389.6857	1389.6681	-0.0176	-13	1525	1536	SDLDLQVDELSR						Mascot
1396.6447	1396.5894	-0.0553	-40	613	623	MQLEMEKSEQK			Oxidation (M)[1]			Mascot
1406.6766	1406.6101	-0.0665	-47	246	257	ANKAENEMQMLK						Mascot
1406.7023	1406.6101	-0.0922	-66	45	56	LINEDADSFARR						Mascot
1418.7308	1418.6205	-0.1103	-78	332	342	MQQQELEQKLK			Oxidation (M)[1]			Mascot
1420.6849	1420.6115	-0.0734	-52	237	248	QQMLSESERANK						Mascot
1422.6715	1422.608	-0.0635	-45	246	257	ANKAENEMQMLK			Oxidation (M)[8]			Mascot
1422.6715	1422.608	-0.0635	-45	246	257	ANKAENEMQMLK			Oxidation (M)[8]			Mascot
1434.6455	1434.649	0.0035	2	1829	1841	DRQDQESAESAAK						Mascot
1438.6665	1438.5996	-0.0669	-47	246	257	ANKAENEMQMLK			Oxidation (M)[8,10]			Mascot
1438.6665	1438.5996	-0.0669	-47	246	257	ANKAENEMQMLK			Oxidation (M)[8,10]			Mascot
1484.8431	1484.817	-0.0261	-18	1842	1855	LAAVSAEIQDLKAR						Mascot
1530.7329	1530.7283	-0.0046	-3	1405	1416	NQQMLEQNEQLR						Mascot
1545.7867	1545.6985	-0.0882	-57	1525	1537	SDLDLQVDELSRR						Mascot
1613.7878	1613.6996	-0.0882	-55	159	173	NGTHPEETSALSSRK						Mascot
1845.9803	1845.8745	-0.1058	-57	561	576	LSELENNKLDLESTLK						Mascot
2036.9342	2036.9698	0.0356	17	135	153	AAFDSDDLQKDGVGMSPQR						Mascot
2219.1951	2219.0229	-0.1722	-78	902	920	TTTTMNQKLIEELQITNLK						Mascot
2236.0486	2236.1265	0.0779	35	1271	1290	YAVENHSANMLVSQLMGEAR			Oxidation (M)[10]			Mascot
2236.0486	2236.1265	0.0779	35	1271	1290	YAVENHSANMLVSQLMGEAR			Oxidation (M)[10]			Mascot
3382.687	3382.7234	0.0364	11	1350	1378	DETEDVNRLMNTEFSVLSVVLLQLGMELR			Oxidation (M)[10,26]			Mascot

5	hypothetical protein SORBIDRAFT_06g016540 [Sorghum bicolor]	gi|241939010	28466.5	4.92	11	56	0	30.575

Peptide Information

Calc. Mass	Obsrv. Mass	± da	± ppm	Start Seq.	End Seq.	Sequence	Ion Score	C. I. %	Modification	Rank	Result Type
1046.4062	1046.4967	0.0905	86	79	87	DDYDGGYNK					Mascot
1113.4443	1113.45	0.0057	5	88	97	SGTNDDEYGR					Mascot
1117.5498	1117.5424	-0.0074	-7	210	218	DPEHAQRHK					Mascot
1117.5498	1117.5424	-0.0074	-7	210	218	DPEHAQRHK					Mascot
1145.4052	1145.4415	0.0363	32	168	176	DDDDSEMYR					Mascot
1230.5498	1230.5531	0.0033	3	2	12	ADEYGRSGYGR					Mascot
1269.5455	1269.5499	0.0044	3	88	98	SGTNDDEYGRR					Mascot
1273.5001	1273.5624	0.0623	49	168	177	DDDDSEMYRK					Mascot
1549.6401	1549.7122	0.0721	47	108	123	SSGDDAYTGGAGYNSK					Mascot

		Calc. Mass	Obsrv. Mass	± da	± ppm	Start Seq.	End Seq.	Sequence	Ion Score	C. I. %	Modification	Rank	Result Type
		1635.677	1635.7322	0.0552	34	124	139	SSGDDDAYTGVGYNSK					Mascot
		1678.694	1678.7368	0.0428	25	13	28	SGAGDDYDSGYNSKSR					Mascot
		1955.8003	1955.949	0.1487	76	8	26	SGYGRSGAGDDYDSGY NSK					Mascot
		1955.8003	1955.949	0.1487	76	8	26	SGYGRSGAGDDYDSGY NSK					Mascot
6	ROP interactive partner 3 isoform 3, partial [Theobroma cacao]	gi|508715860	71781.5	5.7	17	56	0	35.263					

Peptide Information

Calc. Mass	Obsrv. Mass	± da	± ppm	Start Seq.	End Seq.	Sequence	Ion Score	C. I. %	Modification	Rank	Result Type
1046.5378	1046.4967	-0.0411	-39	564	571	VQSDQWRK					Mascot
1139.5579	1139.5282	-0.0297	-26	207	216	AYESEAIQTK					Mascot
1147.6205	1147.5582	-0.0623	-54	459	468	KIEAESTELK					Mascot
1208.5906	1208.5681	-0.0225	-19	383	392	SAYEQVENIR					Mascot
1242.6801	1242.558	-0.1221	-98	80	90	SPVSEKQRPSK					Mascot
1320.6543	1320.6083	-0.046	-35	110	120	DQLTASESWKR					Mascot
1438.6921	1438.5996	-0.0925	-64	335	346	QNGENEEIQHLK					Mascot
1438.6921	1438.5996	-0.0925	-64	335	346	QNGENEEIQHLK					Mascot
1484.7228	1484.817	0.0942	63	524	536	LSYVTEEADKSSR					Mascot
1549.7527	1549.7122	-0.0405	-26	411	423	TDVEELRANLMDK			Oxidation (M)[11]		Mascot
1619.7582	1619.7397	-0.0185	-11	263	276	TQMQPETANETIEK					Mascot
1635.7531	1635.7322	-0.0209	-13	263	276	TQMQPETANETIEK			Oxidation (M)[3]		Mascot
1698.881	1698.7634	-0.1176	-69	370	382	YQEEYIRSTLQIR					Mascot
1796.8912	1796.8391	-0.0521	-29	284	299	ATEAYNTLSLELEQSK					Mascot
1796.8912	1796.8391	-0.0521	-29	284	299	ATEAYNTLSLELEQSK					Mascot
1845.9441	1845.8745	-0.0696	-38	91	106	VSELESQLTQLQDDLK					Mascot
1904.9382	1904.8774	-0.0608	-32	263	278	TQMQPETANETIEKLR			Oxidation (M)[3]		Mascot
1955.9352	1955.949	0.0138	7	572	591	AAEAAAAMLSTGNNGKH ADR					Mascot
1955.9352	1955.949	0.0138	7	572	591	AAEAAAAMLSTGNNGKH ADR					Mascot
1971.9302	1971.9243	-0.0059	-3	572	591	AAEAAAAMLSTGNNGKH ADR			Oxidation (M)[8]		Mascot
2236.0398	2236.1265	0.0867	39	541	560	VTEQLDAAQATNTEMEA ELR			Oxidation (M)[15]		Mascot
2236.0398	2236.1265	0.0867	39	541	560	VTEQLDAAQATNTEMEA ELR			Oxidation (M)[15]		Mascot
3187.4783	3187.5303	0.052	16	171	199	AWQSELEAVQNQHSMD SAALASAMNEIQK					Mascot

7	ROP interactive partner 3 isoform 1 [Theobroma cacao]	gi|508715858	69487.3	5.37	17	55	0	35.263

Peptide Information

Calc. Mass	Obsrv. Mass	± da	± ppm	Start Seq.	End Seq.	Sequence	Ion Score	C. I. %	Modification	Rank	Result Type
1046.5378	1046.4967	-0.0411	-39	547	554	VQSDQWRK					Mascot
1139.5579	1139.5282	-0.0297	-26	190	199	AYESEAIQTK					Mascot
1147.6205	1147.5582	-0.0623	-54	442	451	KIEAESTELK					Mascot
1208.5906	1208.5681	-0.0225	-19	366	375	SAYEQVENIR					Mascot
1242.6801	1242.558	-0.1221	-98	63	73	SPVSEKQRPSK					Mascot
1320.6543	1320.6083	-0.046	-35	93	103	DQLTASESWKR					Mascot
1438.6921	1438.5996	-0.0925	-64	318	329	QNGENEEIQHLK					Mascot
1438.6921	1438.5996	-0.0925	-64	318	329	QNGENEEIQHLK					Mascot
1484.7228	1484.817	0.0942	63	507	519	LSYVTEEADKSSR					Mascot
1549.7527	1549.7122	-0.0405	-26	394	406	TDVEELRANLMDK			Oxidation (M)[11]		Mascot
1619.7582	1619.7397	-0.0185	-11	246	259	TQMQPETANETIEK					Mascot
1635.7531	1635.7322	-0.0209	-13	246	259	TQMQPETANETIEK			Oxidation (M)[3]		Mascot
1698.881	1698.7634	-0.1176	-69	353	365	YQEEYIRSTLQIR					Mascot
1796.8912	1796.8391	-0.0521	-29	267	282	ATEAYNTLSLELEQSK					Mascot
1796.8912	1796.8391	-0.0521	-29	267	282	ATEAYNTLSLELEQSK					Mascot
1845.9441	1845.8745	-0.0696	-38	74	89	VSELESQLTQLQDDLK					Mascot
1904.9382	1904.8774	-0.0608	-32	246	261	TQMQPETANETIEKLR			Oxidation (M)[3]		Mascot
1955.9352	1955.949	0.0138	7	555	574	AAEAAAAMLSTGNNGKH ADR					Mascot
1955.9352	1955.949	0.0138	7	555	574	AAEAAAAMLSTGNNGKH ADR					Mascot
1971.9302	1971.9243	-0.0059	-3	555	574	AAEAAAAMLSTGNNGKH ADR			Oxidation (M)[8]		Mascot
2236.0398	2236.1265	0.0867	39	524	543	VTEQLDAAQATNTEMEA ELR			Oxidation (M)[15]		Mascot
2236.0398	2236.1265	0.0867	39	524	543	VTEQLDAAQATNTEMEA ELR			Oxidation (M)[15]		Mascot
3187.4783	3187.5303	0.052	16	154	182	AWQSELEAVQNQHSMD SAALASAMNEIQK					Mascot

8	PREDICTED: LOW QUALITY PROTEIN: kelch domain-containing protein 4-like [Cicer arietinum]	gi|502133608	76482.5	5.03	16	55	0	4.489	

Peptide Information

Calc. Mass	Obsrv. Mass	± da	± ppm	Start Seq.	End Seq.	Sequence	Ion Score	C. I. %	Modification	Rank	Result Type
1036.4703	1036.4789	0.0086	8	298	306	AGFSMCVHK			Carbamidomethyl (C)[6]		Mascot
1060.615	1060.5336	-0.0814	-77	372	380	KVEPLFTAR					Mascot
1106.5775	1106.5458	-0.0317	-29	181	189	SGHRMVLYK			Oxidation (M)[5]		Mascot
1110.5095	1110.5044	-0.0051	-5	415	425	SEGMSKESGAK					Mascot
1193.5645	1193.5795	0.015	13	257	267	EVSSDKNASEK					Mascot

1208.5664	1208.5681	0.0017	1	298	307	AGFSMCVHKR			Carbamidomethyl (C)[6], Oxidation (M)[5]		Mascot
1269.5879	1269.5499	-0.038	-30	404	414	MTIDDSETLTK			Oxidation (M)[1]		Mascot
1273.6536	1273.5624	-0.0912	-72	165	174	TNQWEQLNLK					Mascot
1312.6281	1312.6602	0.0321	24	616	626	DGFDLAEARYR					Mascot
1484.7203	1484.817	0.0967	65	268	280	GIVHSDMWSLDPK					Mascot
1549.7277	1549.7122	-0.0155	-10	451	463	DTLYIYGGMMEIK			Oxidation (M)[9]		Mascot
1559.7812	1559.7186	-0.0626	-40	56	69	EVHIEDNVPAPSPR					Mascot
1796.9324	1796.8391	-0.0933	-52	426	441	LDIQNSLPEAVKPCGR			Carbamidomethyl (C)[14]		Mascot
1796.964	1796.8391	-0.1249	-70	34	49	ISPEDDIDAILLNIQK					Mascot
1904.8464	1904.8774	0.031	16	404	420	MTIDDSETLTKSEGMSK			Oxidation (M)[1,15]		Mascot
2143.1406	2142.9604	-0.1802	-84	115	134	LISSPNSPPPRSAHQAVAWK					Mascot

9 Myosin heavy chain-related protein [Theobroma cacao] gi|508724108 101270 5.97 22 54 0 20.918

Peptide Information

Calc. Mass	Obsrv. Mass	± da	± ppm	Start Seq.	End Seq.	Sequence	Ion Score	C. I. %	Modification	Rank	Result Type
1078.5085	1078.5236	0.0151	14	482	490	EMQVDSLEK					Mascot
1078.5891	1078.5236	-0.0655	-61	219	228	KFEATVAANK					Mascot
1094.5034	1094.5156	0.0122	11	482	490	EMQVDSLEK			Oxidation (M)[2]		Mascot
1117.4983	1117.5424	0.0441	39	198	205	FYKDEQCK			Carbamidomethyl (C)[7]		Mascot
1117.4983	1117.5424	0.0441	39	198	205	FYKDEQCK			Carbamidomethyl (C)[7]		Mascot
1182.601	1182.5426	-0.0584	-49	1	10	MQKLGFPSMK			Oxidation (M)[1]		Mascot
1249.6094	1249.564	-0.0454	-36	589	599	ADKVVGEMEQK			Oxidation (M)[8]		Mascot
1250.6045	1250.563	-0.0415	-33	481	490	REMQVDSLEK			Oxidation (M)[3]		Mascot
1265.6406	1265.5752	-0.0654	-52	64	74	TDLEMANSKLK			Oxidation (M)[5]		Mascot
1319.7053	1319.59	-0.1153	-87	400	411	LESEAESLVSKK					Mascot
1320.6907	1320.6083	-0.0824	-62	815	825	TRSTVLFEDPR					Mascot
1331.6372	1331.5981	-0.0391	-29	650	660	CAQLQAENELR			Carbamidomethyl (C)[1]		Mascot
1389.7485	1389.6681	-0.0804	-58	87	98	LQNAFNENAKLK					Mascot
1421.7305	1421.6682	-0.0623	-44	434	445	SSENKMQDLLLK			Oxidation (M)[6]		Mascot
1450.6665	1450.6421	-0.0244	-17	592	603	VVGEMEQKCDQK			Carbamidomethyl (C)[9]		Mascot
1549.822	1549.7122	-0.1098	-71	265	278	GKTNLLLSNDEFAK					Mascot
1635.736	1635.7322	-0.0038	-2	315	327	FDEINALYDTCFK			Carbamidomethyl (C)[11]		Mascot
1678.8065	1678.7368	-0.0697	-42	58	72	EQASVRTDLEMANSK					Mascot
1705.7922	1705.821	0.0288	17	558	571	EISQRNDQAINDMR			Oxidation (M)[13]		Mascot
1717.8062	1717.8705	0.0643	37	712	726	VMGDKPQEEQEVNSK					Mascot

1838.9316	1838.8784	-0.0532	-29	540	554	QYDVMLESKQLELSR					Mascot
1845.9011	1845.8745	-0.0266	-14	712	727	VMGDKPQEEQEVNSKK					Mascot
1864.9335	1864.9387	0.0052	3	776	792	VENANTGSVMSIPKHHK			Oxidation (M)[10]		Mascot
2839.4836	2839.2961	-0.1875	-66	28	54	TFSVPSRPSTDSVSLGSF ANLKLTAEK					Mascot

10 hypothetical protein TRIUR3_08536 [Triticum urartu] gi|474407060 49568.3 5.63 14 53 0 20.556

Peptide Information

Calc. Mass	Obsrv. Mass	± da	± ppm	Start Seq.	End Seq.	Sequence	Ion Score	C. I. %	Modification	Rank	Result Type
1054.447	1054.4301	-0.0169	-16	330	338	DTTEQTMGR			Oxidation (M)[7]		Mascot
1078.5388	1078.5236	-0.0152	-14	33	42	GHGTGHKEQK					Mascot
1078.5388	1078.5236	-0.0152	-14	33	42	GHGTGHKEQK					Mascot
1141.575	1141.5007	-0.0743	-65	2	11	AAWHVDTVSR					Mascot
1193.5468	1193.5795	0.0327	27	377	389	DSVMGAAGGAVDK			Oxidation (M)[4]		Mascot
1249.6033	1249.564	-0.0393	-31	23	32	QHHTKQEEGR					Mascot
1312.5984	1312.6602	0.0618	47	266	276	TRDMTAQTMNK			Oxidation (M)[4]		Mascot
1410.6383	1410.604	-0.0343	-24	244	256	AEEPSFDTSTQAK					Mascot
1422.6278	1422.608	-0.0198	-14	315	329	DSVMGAAGGTADNTR					Mascot
1422.6278	1422.608	-0.0198	-14	315	329	DSVMGAAGGTADNTR					Mascot
1434.7006	1434.649	-0.0516	-36	377	391	DSVMGAAGGAVDKTR					Mascot
1438.6228	1438.5996	-0.0232	-16	315	329	DSVMGAAGGTADNTR			Oxidation (M)[4]		Mascot
1438.6228	1438.5996	-0.0232	-16	315	329	DSVMGAAGGTADNTR			Oxidation (M)[4]		Mascot
1450.6956	1450.6421	-0.0535	-37	377	391	DSVMGAAGGAVDKTR			Oxidation (M)[4]		Mascot
1651.832	1651.7448	-0.0872	-53	408	425	VVETGKSATGGGMTGTA K					Mascot
1705.8425	1705.821	-0.0215	-13	66	82	DAAMQALGITGDQTVAK			Oxidation (M)[4]		Mascot
1779.829	1779.8074	-0.0216	-12	341	358	EATGDAGNRTGSMAAQV K			Oxidation (M)[13]		Mascot
1796.9276	1796.8391	-0.0885	-49	464	481	GIVAGEEELIPVEGEAGK					Mascot
1796.9276	1796.8391	-0.0885	-49	464	481	GIVAGEEELIPVEGEAGK					Mascot
1969.9713	1969.9294	-0.0419	-21	143	162	DVTVSTGGTAAEYAKTA AEK					Mascot

Gel Idx/Pos	210/I10	Instr./Gel Origin	BA2151/Sample Project 20140814	Process Status	Analysis Succeeded
Plate [#] Name	[1] Sample Project 20140814	Instrument Sample Name		Spectra	11

Rank	Protein Name	Accession No.	Protein MW	Protein PI	Pep. Count	Protein Score	Protein Score C. I. %	Intensity Matched	Total Ion Score	Total Ion C. I. %	Confirmed
1	RecName: Full=1-Cys peroxiredoxin PER1; AltName: Full=Rehydrin homolog; AltName: Full=Thioredoxin peroxidase	gi|75324900	24178.2	6.08	13	195	100	20.187	112	100	

Peptide Information

Calc. Mass	Obsrv. Mass	± da	± ppm	Start Seq.	End Seq.	Sequence	Ion Score	C. I. %	Modification	Rank	Result Type
878.4036	878.4022	-0.0014	-2	151	157	NMDEVVR			Oxidation (M)[2]		Mascot
972.4745	972.4695	-0.005	-5	119	127	DAEGQLPSR					Mascot
979.5571	979.5389	-0.0182	-19	128	136	TLHIVGPDK					Mascot
988.5673	988.5319	-0.0354	-36	158	167	AVDSLLTAAK					Mascot
1107.5681	1107.5558	-0.0123	-11	84	93	DIEAYKPGSK					Mascot
1253.7212	1253.6019	-0.1193	-95	158	169	AVDSLLTAAKHK					Mascot
1277.6195	1277.6052	-0.0143	-11	94	104	VTYPIMADPDR					Mascot
1293.6144	1293.5979	-0.0165	-13	94	104	VTYPIMADPDR	24	0	Oxidation (M)[6]		Mascot
1300.6355	1300.6293	-0.0062	-5	140	150	LSFLYPSCTGR			Carbamidomethyl (C)[8]		Mascot
1300.6355	1300.6293	-0.0062	-5	140	150	LSFLYPSCTGR	88	100	Carbamidomethyl (C)[8]		Mascot
1471.7209	1471.7075	-0.0134	-9	67	79	LLGISCDDVQSHK			Carbamidomethyl (C)[6]		Mascot
1583.7411	1583.6924	-0.0487	-31	196	209	MFPQGFETADLPSK			Oxidation (M)[1]		Mascot
1711.8361	1711.7836	-0.0525	-31	196	210	MFPQGFETADLPSKK			Oxidation (M)[1]		Mascot
2064.0608	2064.0044	-0.0564	-27	2	21	PGLTIGDTVPNLELDSTHGK					Mascot
2144.0264	2143.9248	-0.1016	-47	140	157	LSFLYPSCTGRNMDEVVR			Carbamidomethyl (C)[8]		Mascot

Rank	Protein Name	Accession No.	Protein MW	Protein PI	Pep. Count	Protein Score	Protein Score C. I. %	Intensity Matched	Total Ion Score	Total Ion C. I. %	Confirmed
2	1-Cys peroxiredoxin PER1 [Triticum urartu]	gi|474094006	24248.3	6.08	12	188	100	19.711	112	100	

Peptide Information

Calc. Mass	Obsrv. Mass	± da	± ppm	Start Seq.	End Seq.	Sequence	Ion Score	C. I. %	Modification	Rank	Result Type
958.4589	958.4544	-0.0045	-5	119	127	DGEGQLPSR					Mascot
979.5571	979.5389	-0.0182	-19	128	136	TLHIVGPDK					Mascot
988.5673	988.5319	-0.0354	-36	159	168	AVDSLLTAAK					Mascot
1107.5681	1107.5558	-0.0123	-11	84	93	DIEAYKPGSK					Mascot
1253.7212	1253.6019	-0.1193	-95	159	170	AVDSLLTAAKHK					Mascot
1277.6195	1277.6052	-0.0143	-11	94	104	VTYPIMADPDR					Mascot

Calc. Mass	Obsrv. Mass	± da	± ppm	Start Seq.	End Seq.	Sequence	Ion Score	C. I. %	Modification	Rank	Result Type
1293.6144	1293.5979	-0.0165	-13	94	104	VTYPIMADPDR	24	0	Oxidation (M)[6]		Mascot
1300.6355	1300.6293	-0.0062	-5	140	150	LSFLYPSCTGR			Carbamidomethyl (C)[8]		Mascot
1300.6355	1300.6293	-0.0062	-5	140	150	LSFLYPSCTGR	88	100	Carbamidomethyl (C)[8]		Mascot
1471.7209	1471.7075	-0.0134	-9	67	79	LLGISCDDVQSHK			Carbamidomethyl (C)[6]		Mascot
1583.7411	1583.6924	-0.0487	-31	197	210	MFPQGFETADLPSK			Oxidation (M)[1]		Mascot
1711.8361	1711.7836	-0.0525	-31	197	211	MFPQGFETADLPSKK			Oxidation (M)[1]		Mascot
2064.0608	2064.0044	-0.0564	-27	2	21	PGLTIGDTVPNLELDSTHGK					Mascot
2143.9924	2143.9248	-0.0676	-32	109	127	QLNMVDPDEKDGEGQLPSR			Oxidation (M)[4]		Mascot

3	RecName: Full=1-Cys peroxiredoxin PER1; AltName: Full=B15C; AltName: Full=Rehydrin homolog; AltName: Full=Thioredoxin peroxidase	gi|1710077	24176.3	6.31	12	186	100	20.021	112	100	

Peptide Information

Calc. Mass	Obsrv. Mass	± da	± ppm	Start Seq.	End Seq.	Sequence	Ion Score	C. I. %	Modification	Rank	Result Type
878.4036	878.4022	-0.0014	-2	151	157	NMDEVVR			Oxidation (M)[2]		Mascot
979.5571	979.5389	-0.0182	-19	128	136	TLHIVGPDK					Mascot
988.5673	988.5319	-0.0354	-36	158	167	AVDSLLTAAK					Mascot
1107.5681	1107.5558	-0.0123	-11	84	93	DIEAYKPGSK					Mascot
1253.7212	1253.6019	-0.1193	-95	158	169	AVDSLLTAAKHK					Mascot
1277.6195	1277.6052	-0.0143	-11	94	104	VTYPIMADPDR					Mascot
1293.6144	1293.5979	-0.0165	-13	94	104	VTYPIMADPDR	24	0	Oxidation (M)[6]		Mascot
1300.6355	1300.6293	-0.0062	-5	140	150	LSFLYPSCTGR			Carbamidomethyl (C)[8]		Mascot
1300.6355	1300.6293	-0.0062	-5	140	150	LSFLYPSCTGR	88	100	Carbamidomethyl (C)[8]		Mascot
1471.7209	1471.7075	-0.0134	-9	67	79	LLGISCDDVQSHK			Carbamidomethyl (C)[6]		Mascot
1583.7411	1583.6924	-0.0487	-31	196	209	MFPQGFETADLPSK			Oxidation (M)[1]		Mascot
1711.8361	1711.7836	-0.0525	-31	196	210	MFPQGFETADLPSKK			Oxidation (M)[1]		Mascot
2064.0608	2064.0044	-0.0564	-27	2	21	PGLTIGDTVPNLELDSTHGK					Mascot
2144.0264	2143.9248	-0.1016	-47	140	157	LSFLYPSCTGRNMDEVVR			Carbamidomethyl (C)[8]		Mascot

4	1-Cys peroxiredoxin PER1 [Aegilops tauschii]	gi|475442339	24190.2	6.31	12	186	100	19.786	112	100	

Peptide Information

Calc. Mass	Obsrv. Mass	± da	± ppm	Start Seq.	End Seq.	Sequence	Ion Score	C. I. %	Modification	Rank	Result Type
878.4036	878.4022	-0.0014	-2	151	157	NMDEVVR			Oxidation (M)[2]		Mascot
957.4749	957.4382	-0.0367	-38	119	127	DGQGQLPSR					Mascot
979.5571	979.5389	-0.0182	-19	128	136	TLHIVGPDK					Mascot

988.5673	988.5319	-0.0354	-36	158	167	AVDSLLTAAK					Mascot
1253.7212	1253.6019	-0.1193	-95	158	169	AVDSLLTAAKHK					Mascot
1277.6195	1277.6052	-0.0143	-11	94	104	VTYPIMADPDR					Mascot
1293.6144	1293.5979	-0.0165	-13	94	104	VTYPIMADPDR	24	0	Oxidation (M)[6]		Mascot
1300.6355	1300.6293	-0.0062	-5	140	150	LSFLYPSCTGR			Carbamidomethyl (C)[8]		Mascot
1300.6355	1300.6293	-0.0062	-5	140	150	LSFLYPSCTGR	88	100	Carbamidomethyl (C)[8]		Mascot
1471.7209	1471.7075	-0.0134	-9	67	79	LLGISCDDVQSHK			Carbamidomethyl (C)[6]		Mascot
1583.7411	1583.6924	-0.0487	-31	196	209	MFPQGFETADLPSK			Oxidation (M)[1]		Mascot
1711.8361	1711.7836	-0.0525	-31	196	210	MFPQGFETADLPSKK			Oxidation (M)[1]		Mascot
2064.0608	2064.0044	-0.0564	-27	2	21	PGLTIGDTVPNLELDSTH GK					Mascot
2144.0264	2143.9248	-0.1016	-47	140	157	LSFLYPSCTGRNMDEVV R			Carbamidomethyl (C)[8]		Mascot

5	peroxiredoxin [Bromus inermis]	gi|478621020	24283.2	6.09	12	183	100	19.781	112	100	

Peptide Information

Calc. Mass	Obsrv. Mass	± da	± ppm	Start Seq.	End Seq.	Sequence	Ion Score	C. I. %	Modification	Rank	Result Type
878.4036	878.4022	-0.0014	-2	151	157	NMDEVVR			Oxidation (M)[2]		Mascot
972.4745	972.4695	-0.005	-5	119	127	DAEGQLPSR					Mascot
979.5571	979.5389	-0.0182	-19	128	136	TLHIVGPDK					Mascot
988.5673	988.5319	-0.0354	-36	158	167	AVDSLLTAAK					Mascot
1100.5082	1100.5117	0.0035	3	196	204	MFPQGFETK			Oxidation (M)[1]		Mascot
1107.5681	1107.5558	-0.0123	-11	84	93	DIEAYKPGSK					Mascot
1253.7212	1253.6019	-0.1193	-95	158	169	AVDSLLTAAKHK					Mascot
1277.6195	1277.6052	-0.0143	-11	94	104	VTYPIMADPDR					Mascot
1293.6144	1293.5979	-0.0165	-13	94	104	VTYPIMADPDR	24	0	Oxidation (M)[6]		Mascot
1300.6355	1300.6293	-0.0062	-5	140	150	LSFLYPSCTGR			Carbamidomethyl (C)[8]		Mascot
1300.6355	1300.6293	-0.0062	-5	140	150	LSFLYPSCTGR	88	100	Carbamidomethyl (C)[8]		Mascot
1471.7209	1471.7075	-0.0134	-9	67	79	LLGISCDDVQSHK			Carbamidomethyl (C)[6]		Mascot
2064.0608	2064.0044	-0.0564	-27	2	21	PGLTIGDTVPNLELDSTH GK					Mascot
2144.0264	2143.9248	-0.1016	-47	140	157	LSFLYPSCTGRNMDEVV R			Carbamidomethyl (C)[8]		Mascot

6	RecName: Full=Probable 1-Cys peroxiredoxin; AltName: Full=Dormancy-associated protein PBS128; AltName: Full=Rehydrin homolog; AltName: Full=Thioredoxin peroxidase	gi|1710076	22593.6	8.24	10	164	100	18.883	112	100	

Peptide Information

Calc. Mass	Obsrv. Mass	± da	± ppm	Start Seq.	End Seq.	Sequence	Ion Score	C. I. %	Modification	Rank	Result Type

878.4036	878.4022	-0.0014	-2	135	141	NMDEVVR			Oxidation (M)[2]		Mascot
972.4745	972.4695	-0.005	-5	103	111	DAEGQLPSR					Mascot
979.5571	979.5389	-0.0182	-19	112	120	TLHIVGPDK					Mascot
988.5673	988.5319	-0.0354	-36	142	151	AVDSLLTAAK					Mascot
1107.5681	1107.5558	-0.0123	-11	68	77	DIEAYKPGSK					Mascot
1253.7212	1253.6019	-0.1193	-95	142	153	AVDSLLTAAKHK					Mascot
1277.6195	1277.6052	-0.0143	-11	78	88	VTYPIMADPDR					Mascot
1293.6144	1293.5979	-0.0165	-13	78	88	VTYPIMADPDR	24	0	Oxidation (M)[6]		Mascot
1300.6355	1300.6293	-0.0062	-5	124	134	LSFLYPSCTGR			Carbamidomethyl (C)[8]		Mascot
1300.6355	1300.6293	-0.0062	-5	124	134	LSFLYPSCTGR	88	100	Carbamidomethyl (C)[8]		Mascot
1471.7209	1471.7075	-0.0134	-9	51	63	LLGISCDDVQSHK			Carbamidomethyl (C)[6]		Mascot
2144.0264	2143.9248	-0.1016	-47	124	141	LSFLYPSCTGRNMDEVVR			Carbamidomethyl (C)[8]		Mascot

7 Vicilin-like antimicrobial peptides 2-2 [Aegilops tauschii] gi|475617685 54616.7 6.23 11 129 100 12.596 93 100

Peptide Information

Calc. Mass	Obsrv. Mass	± da	± ppm	Start Seq.	End Seq.	Sequence	Ion Score	C. I. %	Modification	Rank	Result Type
805.4818	805.4109	-0.0709	-88	51	57	GLFLLDK					Mascot
1048.5674	1048.5846	0.0172	16	202	211	TSVLAGFEPK					Mascot
1060.5575	1060.5504	-0.0071	-7	397	405	AGDVFWIPR					Mascot
1187.6321	1187.5979	-0.0342	-29	406	415	HFPFVQVASR					Mascot
1187.6321	1187.5979	-0.0342	-29	406	415	HFPFVQVASR	27	0			Mascot
1324.6276	1324.5721	-0.0555	-42	489	499	EPFVMKQVAME			Oxidation (M)[5]		Mascot
1353.7122	1353.7111	-0.0011	-1	61	73	VVESEGGSVHVVR					Mascot
1438.6499	1438.62	-0.0299	-21	278	289	GSGQSTWTWSWR					Mascot
1490.7428	1490.736	-0.0068	-5	416	429	GGPFVFFGFTTSAR					Mascot
1490.7428	1490.736	-0.0068	-5	416	429	GGPFVFFGFTTSAR	67	99.953			Mascot
1557.8748	1557.8629	-0.0119	-8	431	444	NKPQFLTGPTSVLR					Mascot
1818.8446	1818.834	-0.0106	-6	310	324	APEPYNLFDHEPSFR					Mascot
1983.0653	1982.9559	-0.1094	-55	445	463	MMLGPELAAGLGVPEKELK					Mascot

8 hypothetical protein TRIUR3_28410 [Triticum urartu] gi|474060617 28387.9 5.53 3 118 100 4.641 110 100

Peptide Information

Calc. Mass	Obsrv. Mass	± da	± ppm	Start Seq.	End Seq.	Sequence	Ion Score	C. I. %	Modification	Rank	Result Type
1078.535	1078.5311	-0.0039	-4	207	215	GYPLLEACR			Carbamidomethyl (C)[8]		Mascot

1078.535	1078.5311	-0.0039	-4	207	215	GYPLLEACR	42	86.24	Carbamidomethyl (C)[8]		Mascot
1145.5698	1145.5634	-0.0064	-6	156	165	GHNLSLEYGR					Mascot
1145.5698	1145.5634	-0.0064	-6	156	165	GHNLSLEYGR	68	99.968			Mascot
1406.6271	1406.6166	-0.0105	-7	130	139	HREWESCFQK			Carbamidomethyl (C)[7]		Mascot

9 hypothetical protein F775_31970 [Aegilops tauschii] gi|475578125 25615.9 6.35 6 98 99.985 7.674 73 99.99

Peptide Information

Calc. Mass	Obsrv. Mass	± da	± ppm	Start Seq.	End Seq.	Sequence	Ion Score	C. I. %	Modification	Rank	Result Type
941.5567	941.5412	-0.0155	-16	54	61	FVANHLLK					Mascot
1078.535	1078.5311	-0.0039	-4	207	215	GYPLLEACR			Carbamidomethyl (C)[8]		Mascot
1078.535	1078.5311	-0.0039	-4	207	215	GYPLLEACR	42	86.24	Carbamidomethyl (C)[8]		Mascot
1117.5637	1117.5487	-0.015	-13	159	168	GHNLSLEYGK					Mascot
1372.6427	1372.7045	0.0618	45	133	142	HREWESCIQK			Carbamidomethyl (C)[7]		Mascot
1461.722	1461.7531	0.0311	21	216	229	SLGLEADNNVYGPL					Mascot
1955.9684	1955.9583	-0.0101	-5	38	53	VHVAIYYESLCPYSVR			Carbamidomethyl (C)[11]		Mascot
1955.9684	1955.9583	-0.0101	-5	38	53	VHVAIYYESLCPYSVR	31	0	Carbamidomethyl (C)[11]		Mascot

10 Vicilin-like antimicrobial peptides 2-2 [Triticum urartu] gi|474404647 43446.1 6.74 4 76 97.555 7.034 67 99.953

Peptide Information

Calc. Mass	Obsrv. Mass	± da	± ppm	Start Seq.	End Seq.	Sequence	Ion Score	C. I. %	Modification	Rank	Result Type
1060.5575	1060.5504	-0.0071	-7	294	302	AGDVFWIPR					Mascot
1490.7428	1490.736	-0.0068	-5	313	326	GGPFVFFGFTTSAR					Mascot
1490.7428	1490.736	-0.0068	-5	313	326	GGPFVFFGFTTSAR	67	99.953			Mascot
1557.8748	1557.8629	-0.0119	-8	328	341	NKPQFLTGPTSVLR					Mascot
1818.8446	1818.834	-0.0106	-6	207	221	APEPYNLFDHEPSFR					Mascot

Gel Idx/Pos	211/I11	Instr./Gel Origin	BA2151/Sample Project 20140814	Process Status	Analysis Succeeded
Plate [#] Name	[1] Sample Project 20140814	Instrument Sample Name		Spectra	11

Rank	Protein Name	Accession No.	Protein MW	Protein PI	Pep. Count	Protein Score	Protein Score C. I. %	Intensity Matched	Total Ion Score	Total Ion C. I. %	Confirmed
1	RecName: Full=1-Cys peroxiredoxin PER1; AltName: Full=Rehydrin homolog; AltName: Full=Thioredoxin peroxidase	gi|75324900	24178.2	6.08	10	320	100	23.538	261	100	

Peptide Information

Calc. Mass	Obsrv. Mass	± da	± ppm	Start Seq.	End Seq.	Sequence	Ion Score	C. I. %	Modification	Rank	Result Type
862.4087	862.4234	0.0147	17	151	157	NMDEVVR					Mascot
878.4036	878.4014	-0.0022	-3	151	157	NMDEVVR			Oxidation (M)[2]		Mascot
972.4745	972.4718	-0.0027	-3	119	127	DAEGQLPSR					Mascot
1277.6195	1277.613	-0.0065	-5	94	104	VTYPIMADPDR					Mascot
1293.6144	1293.5973	-0.0171	-13	94	104	VTYPIMADPDR	15	0	Oxidation (M)[6]		Mascot
1300.6355	1300.6281	-0.0074	-6	140	150	LSFLYPSCTGR			Carbamidomethyl (C)[8]		Mascot
1300.6355	1300.6281	-0.0074	-6	140	150	LSFLYPSCTGR	77	99.996	Carbamidomethyl (C)[8]		Mascot
1471.7209	1471.7009	-0.02	-14	67	79	LLGISCDDVQSHK			Carbamidomethyl (C)[6]		Mascot
1583.7411	1583.7004	-0.0407	-26	196	209	MFPQGFETADLPSK			Oxidation (M)[1]		Mascot
1711.8361	1711.7919	-0.0442	-26	196	210	MFPQGFETADLPSKK			Oxidation (M)[1]		Mascot
2064.0608	2064.0266	-0.0342	-17	2	21	PGLTIGDTVPNLELDSTH GK					Mascot
2064.0608	2064.0266	-0.0342	-17	2	21	PGLTIGDTVPNLELDSTH GK	80	99.998			Mascot
2144.0264	2143.95	-0.0764	-36	140	157	LSFLYPSCTGRNMDEVV R			Carbamidomethyl (C)[8]		Mascot
2158.0081	2157.9651	-0.043	-20	109	127	QLNMVDPDEKDAEGQLP SR			Oxidation (M)[4]		Mascot
2158.0081	2157.9651	-0.043	-20	109	127	QLNMVDPDEKDAEGQLP SR	89	100	Oxidation (M)[4]		Mascot

Rank	Protein Name	Accession No.	Protein MW	Protein PI	Pep. Count	Protein Score	Protein Score C. I. %	Intensity Matched	Total Ion Score	Total Ion C. I. %	Confirmed
2	RecName: Full=1-Cys peroxiredoxin PER1; AltName: Full=B15C; AltName: Full=Rehydrin homolog; AltName: Full=Thioredoxin peroxidase	gi|1710077	24176.3	6.31	9	222	100	22.644	172	100	

Peptide Information

Calc. Mass	Obsrv. Mass	± da	± ppm	Start Seq.	End Seq.	Sequence	Ion Score	C. I. %	Modification	Rank	Result Type
862.4087	862.4234	0.0147	17	151	157	NMDEVVR					Mascot
878.4036	878.4014	-0.0022	-3	151	157	NMDEVVR			Oxidation (M)[2]		Mascot
1277.6195	1277.613	-0.0065	-5	94	104	VTYPIMADPDR					Mascot
1293.6144	1293.5973	-0.0171	-13	94	104	VTYPIMADPDR	15	0	Oxidation (M)[6]		Mascot

1300.6355	1300.6281	-0.0074	-6	140	150	LSFLYPSCTGR			Carbamidomethyl (C)[8]		Mascot
1300.6355	1300.6281	-0.0074	-6	140	150	LSFLYPSCTGR	77	99.996	Carbamidomethyl (C)[8]		Mascot
1471.7209	1471.7009	-0.02	-14	67	79	LLGISCDDVQSHK			Carbamidomethyl (C)[6]		Mascot
1583.7411	1583.7004	-0.0407	-26	196	209	MFPQGFETADLPSK			Oxidation (M)[1]		Mascot
1711.8361	1711.7919	-0.0442	-26	196	210	MFPQGFETADLPSKK			Oxidation (M)[1]		Mascot
2064.0608	2064.0266	-0.0342	-17	2	21	PGLTIGDTVPNLELDSTH GK					Mascot
2064.0608	2064.0266	-0.0342	-17	2	21	PGLTIGDTVPNLELDSTH GK	80	99.998			Mascot
2141.0293	2140.9465	-0.0828	-39	109	127	QLNMVDPDEKDAQGQLP SR					Mascot
2141.0293	2140.9465	-0.0828	-39	109	127	QLNMVDPDEKDAQGQLP SR					Mascot
2144.0264	2143.95	-0.0764	-36	140	157	LSFLYPSCTGRNMDEVV R			Carbamidomethyl (C)[8]		Mascot

3	1-Cys peroxiredoxin PER1 [Aegilops tauschii]	gi|475442339	24190.2	6.31	9	222	100	21.624	172	100

Peptide Information

Calc. Mass	Obsrv. Mass	± da	± ppm	Start Seq.	End Seq.	Sequence	Ion Score	C. I. %	Modification	Rank	Result Type
862.4087	862.4234	0.0147	17	151	157	NMDEVVR					Mascot
878.4036	878.4014	-0.0022	-3	151	157	NMDEVVR			Oxidation (M)[2]		Mascot
1277.6195	1277.613	-0.0065	-5	94	104	VTYPIMADPDR					Mascot
1293.6144	1293.5973	-0.0171	-13	94	104	VTYPIMADPDR	15	0	Oxidation (M)[6]		Mascot
1300.6355	1300.6281	-0.0074	-6	140	150	LSFLYPSCTGR			Carbamidomethyl (C)[8]		Mascot
1300.6355	1300.6281	-0.0074	-6	140	150	LSFLYPSCTGR	77	99.996	Carbamidomethyl (C)[8]		Mascot
1471.7209	1471.7009	-0.02	-14	67	79	LLGISCDDVQSHK			Carbamidomethyl (C)[6]		Mascot
1583.7411	1583.7004	-0.0407	-26	196	209	MFPQGFETADLPSK			Oxidation (M)[1]		Mascot
1711.8361	1711.7919	-0.0442	-26	196	210	MFPQGFETADLPSKK			Oxidation (M)[1]		Mascot
2064.0608	2064.0266	-0.0342	-17	2	21	PGLTIGDTVPNLELDSTH GK					Mascot
2064.0608	2064.0266	-0.0342	-17	2	21	PGLTIGDTVPNLELDSTH GK	80	99.998			Mascot
2127.0137	2126.957	-0.0567	-27	109	127	QLNMVDPDEKDGQGQL PSR					Mascot
2144.0264	2143.95	-0.0764	-36	140	157	LSFLYPSCTGRNMDEVV R			Carbamidomethyl (C)[8]		Mascot

4	peroxiredoxin [Bromus inermis]	gi|478621020	24283.2	6.09	9	218	100	21.133	172	100

Peptide Information

Calc. Mass	Obsrv. Mass	± da	± ppm	Start Seq.	End Seq.	Sequence	Ion Score	C. I. %	Modification	Rank	Result Type
862.4087	862.4234	0.0147	17	151	157	NMDEVVR					Mascot
878.4036	878.4014	-0.0022	-3	151	157	NMDEVVR			Oxidation (M)[2]		Mascot

Calc. Mass	Obsrv. Mass	± da	± ppm	Start Seq.	End Seq.	Sequence	Ion Score	C. I. %	Modification	Rank	Result Type
972.4745	972.4718	-0.0027	-3	119	127	DAEGQLPSR					Mascot
1100.5082	1100.5155	0.0073	7	196	204	MFPQGFETK			Oxidation (M)[1]		Mascot
1277.6195	1277.613	-0.0065	-5	94	104	VTYPIMADPDR					Mascot
1293.6144	1293.5973	-0.0171	-13	94	104	VTYPIMADPDR	15	0	Oxidation (M)[6]		Mascot
1300.6355	1300.6281	-0.0074	-6	140	150	LSFLYPSCTGR			Carbamidomethyl (C)[8]		Mascot
1300.6355	1300.6281	-0.0074	-6	140	150	LSFLYPSCTGR	77	99.996	Carbamidomethyl (C)[8]		Mascot
1471.7209	1471.7009	-0.02	-14	67	79	LLGISCDDVQSHK			Carbamidomethyl (C)[6]		Mascot
1628.8466	1628.7738	-0.0728	-45	137	150	TVKLSFLYPSCTGR			Carbamidomethyl (C)[11]		Mascot
2064.0608	2064.0266	-0.0342	-17	2	21	PGLTIGDTVPNLELDSTH GK					Mascot
2064.0608	2064.0266	-0.0342	-17	2	21	PGLTIGDTVPNLELDSTH GK	80	99.998			Mascot
2144.0264	2143.95	-0.0764	-36	140	157	LSFLYPSCTGRNMDEVV R			Carbamidomethyl (C)[8]		Mascot

5	1-Cys peroxiredoxin PER1 [Triticum urartu]	gi|474094006	24248.3	6.08	8	217	100	20.702	172	100	

Peptide Information

Calc. Mass	Obsrv. Mass	± da	± ppm	Start Seq.	End Seq.	Sequence	Ion Score	C. I. %	Modification	Rank	Result Type
1277.6195	1277.613	-0.0065	-5	94	104	VTYPIMADPDR					Mascot
1293.6144	1293.5973	-0.0171	-13	94	104	VTYPIMADPDR	15	0	Oxidation (M)[6]		Mascot
1300.6355	1300.6281	-0.0074	-6	140	150	LSFLYPSCTGR			Carbamidomethyl (C)[8]		Mascot
1300.6355	1300.6281	-0.0074	-6	140	150	LSFLYPSCTGR	77	99.996	Carbamidomethyl (C)[8]		Mascot
1471.7209	1471.7009	-0.02	-14	67	79	LLGISCDDVQSHK			Carbamidomethyl (C)[6]		Mascot
1583.7411	1583.7004	-0.0407	-26	197	210	MFPQGFETADLPSK			Oxidation (M)[1]		Mascot
1711.8361	1711.7919	-0.0442	-26	197	211	MFPQGFETADLPSKK			Oxidation (M)[1]		Mascot
2064.0608	2064.0266	-0.0342	-17	2	21	PGLTIGDTVPNLELDSTH GK					Mascot
2064.0608	2064.0266	-0.0342	-17	2	21	PGLTIGDTVPNLELDSTH GK	80	99.998			Mascot
2143.9924	2143.95	-0.0424	-20	109	127	QLNMVDPDEKDGEGQLP SR			Oxidation (M)[4]		Mascot
2610.2869	2610.2419	-0.045	-17	171	195	VATPANWKPGECVVIAP GVSDDEAK			Carbamidomethyl (C)[12]		Mascot

6	RecName: Full=Probable 1-Cys peroxiredoxin; AltName: Full=Dormancy-associated protein PBS128; AltName: Full=Rehydrin homolog; AltName: Full=Thioredoxin peroxidase	gi|1710076	22593.6	8.24	7	213	100	20.811	181	100	

Peptide Information

Calc. Mass	Obsrv. Mass	± da	± ppm	Start Seq.	End Seq.	Sequence	Ion Score	C. I. %	Modification	Rank	Result Type
862.4087	862.4234	0.0147	17	135	141	NMDEVVR					Mascot

878.4036	878.4014	-0.0022	-3	135	141	NMDEVVR			Oxidation (M)[2]		Mascot
972.4745	972.4718	-0.0027	-3	103	111	DAEGQLPSR					Mascot
1277.6195	1277.613	-0.0065	-5	78	88	VTYPIMADPDR					Mascot
1293.6144	1293.5973	-0.0171	-13	78	88	VTYPIMADPDR	15	0	Oxidation (M)[6]		Mascot
1300.6355	1300.6281	-0.0074	-6	124	134	LSFLYPSCTGR			Carbamidomethyl (C)[8]		Mascot
1300.6355	1300.6281	-0.0074	-6	124	134	LSFLYPSCTGR	77	99.996	Carbamidomethyl (C)[8]		Mascot
1471.7209	1471.7009	-0.02	-14	51	63	LLGISCDDVQSHK			Carbamidomethyl (C)[6]		Mascot
2144.0264	2143.95	-0.0764	-36	124	141	LSFLYPSCTGRNMDEVV R			Carbamidomethyl (C)[8]		Mascot
2158.0081	2157.9651	-0.043	-20	93	111	QLNMVDPDEKDAEGQLP SR			Oxidation (M)[4]		Mascot
2158.0081	2157.9651	-0.043	-20	93	111	QLNMVDPDEKDAEGQLP SR	89	100	Oxidation (M)[4]		Mascot

7	hypothetical protein F775_31970 [Aegilops tauschii]	gi|475578125	25615.9	6.35	8	189	100	8.717	148	100	

Peptide Information

Calc. Mass	Obsrv. Mass	± da	± ppm	Start Seq.	End Seq.	Sequence	Ion Score	C. I. %	Modification	Rank	Result Type
941.5567	941.5426	-0.0141	-15	54	61	FVANHLLK					Mascot
1078.535	1078.5282	-0.0068	-6	207	215	GYPLLEACR			Carbamidomethyl (C)[8]		Mascot
1078.535	1078.5282	-0.0068	-6	207	215	GYPLLEACR	54	99.185	Carbamidomethyl (C)[8]		Mascot
1117.5637	1117.5507	-0.013	-12	159	168	GHNLSLEYGK					Mascot
1372.6427	1372.6962	0.0535	39	133	142	HREWESCIQK			Carbamidomethyl (C)[7]		Mascot
1562.7632	1562.7469	-0.0163	-10	143	155	QGLDPRPVTECYK			Carbamidomethyl (C)[11]		Mascot
1796.9075	1796.8629	-0.0446	-25	116	130	VHLGFIYCVSDLVMK			Carbamidomethyl (C)[8], Oxidation (M)[14]		Mascot
1955.9684	1955.9543	-0.0141	-7	38	53	VHVAIYYESLCPYSVR			Carbamidomethyl (C)[11]		Mascot
1955.9684	1955.9543	-0.0141	-7	38	53	VHVAIYYESLCPYSVR	94	100	Carbamidomethyl (C)[11]		Mascot
2236.1609	2236.1199	-0.041	-18	62	82	AYRDGLLDAADLTLVPYG NAK					Mascot

8	hypothetical protein OsI_27030 [Oryza sativa Indica Group]	gi|218200095	24228.3	5.97	5	101	99.992	4.901	80	99.998	

Protein Group

RecName: Full=1-Cys peroxiredoxin A; Short=1-Cys Prx A; AltName: Full=Protein RAB24; AltName: Full=Rice 1Cys-peroxiredoxin; Short=R1C-Prx; AltName: Full=Thioredoxin peroxidase A	gi|158517776	24228.3	5.9699997901 9165

Peptide Information

Calc. Mass	Obsrv. Mass	± da	± ppm	Start Seq.	End Seq.	Sequence	Ion Score	C. I. %	Modification	Rank	Result Type
862.4087	862.4234	0.0147	17	152	158	NMDEVVR					Mascot

878.4036	878.4014	-0.0022	-3	152	158	NMDEVVR			Oxidation (M)[2]		Mascot
987.5469	987.5217	-0.0252	-26	159	168	AVDALQTAAK					Mascot
1471.7209	1471.7009	-0.02	-14	67	79	LLGISCDDVQSHK			Carbamidomethyl (C)[6]		Mascot
2064.0608	2064.0266	-0.0342	-17	2	21	PGLTIGDTVPNLELDSTH GK					Mascot
2064.0608	2064.0266	-0.0342	-17	2	21	PGLTIGDTVPNLELDSTH GK	80	99.998			Mascot
2158.042	2157.9651	-0.0769	-36	141	158	LSFLYPSCVGRNMDEVV R			Carbamidomethyl (C)[8], Oxidation (M)[13]		Mascot
2158.042	2157.9651	-0.0769	-36	141	158	LSFLYPSCVGRNMDEVV R			Carbamidomethyl (C)[8], Oxidation (M)[13]		Mascot

9	RecName: Full=1-Cys peroxiredoxin PER1; AltName: Full=Rehydrin homolog; AltName: Full=Thioredoxin peroxidase	gi|146325682	25060.6	6.31	4	95	99.969	2.959	80	99.998	

Protein Group

1-Cys peroxiredoxin PER1 [Zea mays]	gi|162460575	25060.6	6.3099 999427 7954

Peptide Information

Calc. Mass	Obsrv. Mass	± da	± ppm	Start Seq.	End Seq.	Sequence	Ion Score	C. I. %	Modification	Rank	Result Type
1225.6576	1225.6198	-0.0378	-31	149	159	LSFLYPATTGR					Mascot
1583.7411	1583.7004	-0.0407	-26	207	220	MFPQGFETADLPSK			Oxidation (M)[1]		Mascot
1711.8361	1711.7919	-0.0442	-26	207	221	MFPQGFETADLPSKK			Oxidation (M)[1]		Mascot
2064.0608	2064.0266	-0.0342	-17	2	21	PGLTIGDTVPNLELDSTH GK					Mascot
2064.0608	2064.0266	-0.0342	-17	2	21	PGLTIGDTVPNLELDSTH GK	80	99.998			Mascot

10	Os07g0638300 [Oryza sativa Japonica Group]	gi|113611943	24198.3	5.97	4	94	99.965	2.959	80	99.998	

Protein Group

RecName: Full=1-Cys peroxiredoxin A; Short=1-Cys Prx A; AltName: Full=Protein RAB24; AltName: Full=Rice 1Cys-peroxiredoxin; Short=R1C-Prx; AltName: Full=Thioredoxin peroxidase A	gi|158517777	24198.3	5.9699 997901 9165

Peptide Information

Calc. Mass	Obsrv. Mass	± da	± ppm	Start Seq.	End Seq.	Sequence	Ion Score	C. I. %	Modification	Rank	Result Type
862.4087	862.4234	0.0147	17	152	158	NMDEVVR					Mascot
878.4036	878.4014	-0.0022	-3	152	158	NMDEVVR			Oxidation (M)[2]		Mascot

987.5469	987.5217	-0.0252	-26	159	168	AVDALQTAAK				Mascot
1471.7209	1471.7009	-0.02	-14	67	79	LLGISCDDVQSHK			Carbamidomethyl (C)[6]	Mascot
2064.0608	2064.0266	-0.0342	-17	2	21	PGLTIGDTVPNLELDSTH GK				Mascot
2064.0608	2064.0266	-0.0342	-17	2	21	PGLTIGDTVPNLELDSTH GK	80	99.998		Mascot

Gel Idx/Pos	212/I12	Instr./Gel Origin	BA2151/Sample Project 20140814	Process Status	Analysis Succeeded
Plate [#] Name	[1] Sample Project 20140814	Instrument Sample Name		Spectra	11

Rank	Protein Name	Accession No.	Protein MW	Protein PI	Pep. Count	Protein Score	Protein Score C. I. %	Intensity Matched	Total Ion Score	Total Ion C. I. %	Confirmed
1	L-ascorbate peroxidase 1, cytosolic [Triticum urartu]	gi|474311703	27561.1	5.85	15	673	100	47.949	562	100	

Protein Group

L-ascorbate peroxidase 1, cytosolic [Aegilops tauschii]	gi|475626297	27561.1	5.8499 999046 3257

Peptide Information

Calc. Mass	Obsrv. Mass	± da	± ppm	Start Seq.	End Seq.	Sequence	Ion Score	C. I. %	Modification	Rank	Result Type
911.4291	911.4224	-0.0067	-7	53	61	TGGPFGTMK			Oxidation (M)[8]		Mascot
919.4196	919.4149	-0.0047	-5	230	236	AFFEDYK					Mascot
990.4859	990.4738	-0.0121	-12	31	38	NCSPLMLR			Carbamidomethyl (C)[2]		Mascot
1006.4808	1006.4717	-0.0091	-9	31	38	NCSPLMLR			Carbamidomethyl (C)[2], Oxidation (M)[6]		Mascot
1036.4847	1036.4802	-0.0045	-4	173	181	SGFEGPWTR					Mascot
1036.4847	1036.4802	-0.0045	-4	173	181	SGFEGPWTR	50	98.165			Mascot
1249.6172	1249.6124	-0.0048	-4	120	130	EDKPQPPPEGR					Mascot
1249.6172	1249.6124	-0.0048	-4	120	130	EDKPQPPPEGR	70	99.981			Mascot
1505.7384	1505.7155	-0.0229	-15	39	52	LAWHSAGTFDVSSK					Mascot
1525.7434	1525.7373	-0.0061	-4	230	241	AFFEDYKEAHLR					Mascot
1525.7434	1525.7373	-0.0061	-4	230	241	AFFEDYKEAHLR	88	100			Mascot
1627.9418	1627.9331	-0.0087	-5	210	223	TLLTDPVFRPLVEK					Mascot
1627.9418	1627.9331	-0.0087	-5	210	223	TLLTDPVFRPLVEK	69	99.978			Mascot
1635.7538	1635.7223	-0.0315	-19	186	199	FDNSYFTELLSGDK					Mascot
1712.8378	1712.7953	-0.0425	-25	4	18	TYPVVSAEYQEAVEK					Mascot
1831.9773	1831.9717	-0.0056	-3	62	79	KPAEQAHAANAGLDIAVR					Mascot
1831.9773	1831.9717	-0.0056	-3	62	79	KPAEQAHAANAGLDIAVR	153	100			Mascot
1939.976	1939.9512	-0.0248	-13	4	20	TYPVVSAEYQEAVEKAR					Mascot
2071.0237	2070.9912	-0.0325	-16	148	167	QMGLSDQDIVALSGGHT LGR			Oxidation (M)[2]		Mascot
2071.0237	2070.9912	-0.0325	-16	148	167	QMGLSDQDIVALSGGHT LGR	132	100	Oxidation (M)[2]		Mascot
2716.3352	2716.3118	-0.0234	-9	186	209	FDNSYFTELLSGDKEGLL QLPSDK					Mascot
3580.8687	3580.8896	0.0209	6	86	119	EEIPTISYADLYQLAGVVA					Mascot

			VEVSGGPVIPFHPGR								
2	uncharacterized protein, partial [Phleum pratense]	gi|409971721	19776	5.13	7	399	100	30.438	359	100	

Peptide Information

Calc. Mass	Obsrv. Mass	± da	± ppm	Start Seq.	End Seq.	Sequence	Ion Score	C. I. %	Modification	Rank	Result Type
919.4196	919.4149	-0.0047	-5	160	166	AFFEDYK					Mascot
1249.6172	1249.6124	-0.0048	-4	50	60	EDKPQPPPEGR					Mascot
1249.6172	1249.6124	-0.0048	-4	50	60	EDKPQPPPEGR	70	99.981			Mascot
1508.7889	1508.7277	-0.0612	-41	1	15	AHAANAGLGMLEPIK			Oxidation (M)[10]		Mascot
1525.7434	1525.7373	-0.0061	-4	160	171	AFFEDYKEAHLR					Mascot
1525.7434	1525.7373	-0.0061	-4	160	171	AFFEDYKEAHLR	88	100			Mascot
1627.9418	1627.9331	-0.0087	-5	140	153	TLLTDPVFRPLVEK					Mascot
1627.9418	1627.9331	-0.0087	-5	140	153	TLLTDPVFRPLVEK	69	99.978			Mascot
1649.7694	1649.8871	0.1177	71	116	129	FDNTYFTELLSGDK					Mascot
2071.0237	2070.9912	-0.0325	-16	78	97	QMGLSDQDIVALSGGHT LGR			Oxidation (M)[2]		Mascot
2071.0237	2070.9912	-0.0325	-16	78	97	QMGLSDQDIVALSGGHT LGR	132	100	Oxidation (M)[2]		Mascot

3	uncharacterized protein, partial [Phleum pratense]	gi|409972081	19705	5.13	6	390	100	30.318	359	100

Peptide Information

Calc. Mass	Obsrv. Mass	± da	± ppm	Start Seq.	End Seq.	Sequence	Ion Score	C. I. %	Modification	Rank	Result Type
919.4196	919.4149	-0.0047	-5	159	165	AFFEDYK					Mascot
1249.6172	1249.6124	-0.0048	-4	49	59	EDKPQPPPEGR					Mascot
1249.6172	1249.6124	-0.0048	-4	49	59	EDKPQPPPEGR	70	99.981			Mascot
1525.7434	1525.7373	-0.0061	-4	159	170	AFFEDYKEAHLR					Mascot
1525.7434	1525.7373	-0.0061	-4	159	170	AFFEDYKEAHLR	88	100			Mascot
1627.9418	1627.9331	-0.0087	-5	139	152	TLLTDPVFRPLVEK					Mascot
1627.9418	1627.9331	-0.0087	-5	139	152	TLLTDPVFRPLVEK	69	99.978			Mascot
1649.7694	1649.8871	0.1177	71	115	128	FDNTYFTELLSGDK					Mascot
2071.0237	2070.9912	-0.0325	-16	77	96	QMGLSDQDIVALSGGHT LGR			Oxidation (M)[2]		Mascot
2071.0237	2070.9912	-0.0325	-16	77	96	QMGLSDQDIVALSGGHT LGR	132	100	Oxidation (M)[2]		Mascot

4	uncharacterized protein, partial [Phleum pratense]	gi|409971619	13234.6	5.15	6	329	100	28.631	289	100

Protein Group

uncharacterized protein, partial [Phleum pratense]	gi|409972029	13234.6	5.1500 000953 6743

Peptide Information

Calc. Mass	Obsrv. Mass	± da	± ppm	Start Seq.	End Seq.	Sequence	Ion Score	C. I. %	Modification	Rank	Result Type
919.4196	919.4149	-0.0047	-5	97	103	AFFEDYK					Mascot
1050.5327	1050.4625	-0.0702	-67	1	9	PQEGVDHLR					Mascot
1525.7434	1525.7373	-0.0061	-4	97	108	AFFEDYKEAHLR					Mascot
1525.7434	1525.7373	-0.0061	-4	97	108	AFFEDYKEAHLR	88	100			Mascot
1627.9418	1627.9331	-0.0087	-5	77	90	TLLTDPVFRPLVEK					Mascot
1627.9418	1627.9331	-0.0087	-5	77	90	TLLTDPVFRPLVEK	69	99.978			Mascot
1649.7694	1649.8871	0.1177	71	53	66	FDNTYFTELLSGDK					Mascot
2071.0237	2070.9912	-0.0325	-16	15	34	QMGLSDQDIVALSGGHT LGR			Oxidation (M)[2]		Mascot
2071.0237	2070.9912	-0.0325	-16	15	34	QMGLSDQDIVALSGGHT LGR	132	100	Oxidation (M)[2]		Mascot

5	uncharacterized protein, partial [Phleum pratense]	gi|409971665	14973.5	5.51	6	327	100	28.47	289	100	

Peptide Information

Calc. Mass	Obsrv. Mass	± da	± ppm	Start Seq.	End Seq.	Sequence	Ion Score	C. I. %	Modification	Rank	Result Type
919.4196	919.4149	-0.0047	-5	113	119	AFFEDYK					Mascot
1498.7761	1498.8119	0.0358	24	1	13	HLQDKPQPPPEGR					Mascot
1525.7434	1525.7373	-0.0061	-4	113	124	AFFEDYKEAHLR					Mascot
1525.7434	1525.7373	-0.0061	-4	113	124	AFFEDYKEAHLR	88	100			Mascot
1627.9418	1627.9331	-0.0087	-5	93	106	TLLTDPVFRPLVEK					Mascot
1627.9418	1627.9331	-0.0087	-5	93	106	TLLTDPVFRPLVEK	69	99.978			Mascot
1649.7694	1649.8871	0.1177	71	69	82	FDNTYFTELLSGDK					Mascot
2071.0237	2070.9912	-0.0325	-16	31	50	QMGLSDQDIVALSGGHT LGR			Oxidation (M)[2]		Mascot
2071.0237	2070.9912	-0.0325	-16	31	50	QMGLSDQDIVALSGGHT LGR	132	100	Oxidation (M)[2]		Mascot

6	hypothetical protein ZEAMMB73_022439 [Zea mays]	gi|413956090	32615.5	6.51	8	235	100	5.652	202	100	

Peptide Information

Calc. Mass	Obsrv. Mass	± da	± ppm	Start Seq.	End Seq.	Sequence	Ion Score	C. I. %	Modification	Rank	Result Type
911.4291	911.4224	-0.0067	-7	106	114	TGGPFGTMK			Oxidation (M)[8]		Mascot
1096.5997	1096.4991	-0.1006	-92	290	299	EAHLKLSELG					Mascot
1249.6172	1249.6124	-0.0048	-4	173	183	EDKPQPPPEGR					Mascot

1249.6172	1249.6124	-0.0048	-4	173	183	EDKPQPPPEGR	70	99.981			Mascot
1300.6678	1300.7076	0.0398	31	17	28	SLGLTATMHPTR			Oxidation (M)[8]		Mascot
1582.7061	1582.7583	0.0522	33	277	289	YAADEKAFFDDYK					Mascot
1583.9155	1583.8131	-0.1024	-65	263	276	ALLSDPVFRPLVEK					Mascot
1655.7992	1655.9559	0.1567	95	2	16	VQSCASKCASFLAAR			Carbamidomethyl (C)[4,8]		Mascot
2071.0237	2070.9912	-0.0325	-16	201	220	QMGLSDQDIVALSGGHT LGR			Oxidation (M)[2]		Mascot
2071.0237	2070.9912	-0.0325	-16	201	220	QMGLSDQDIVALSGGHT LGR	132	100	Oxidation (M)[2]		Mascot

7	APx1 - Cytosolic Ascorbate Peroxidase [Zea mays]	gi|226504576	27481.9	5.65	6	227	100	5.141	202	100

Protein Group

APx1 - Cytosolic Ascorbate Peroxidase [Zea mays]	gi|226530305	27461.9	5.5500 001907 3486
APx1-Cytosolic Ascorbate Peroxidase [Zea mays]	gi|413956092	27461.9	5.5500 001907 3486

Peptide Information

Calc. Mass	Obsrv. Mass	± da	± ppm	Start Seq.	End Seq.	Sequence	Ion Score	C. I. %	Modification	Rank	Result Type
911.4291	911.4224	-0.0067	-7	53	61	TGGPFGTMK			Oxidation (M)[8]		Mascot
1249.6172	1249.6124	-0.0048	-4	120	130	EDKPQPPPEGR					Mascot
1249.6172	1249.6124	-0.0048	-4	120	130	EDKPQPPPEGR	70	99.981			Mascot
1582.7061	1582.7583	0.0522	33	224	236	YAADEKAFFDDYK					Mascot
1583.9155	1583.8131	-0.1024	-65	210	223	ALLSDPVFRPLVEK					Mascot
1885.9178	1885.87	-0.0478	-25	2	18	AKNYPTVSAEYSEAVEK					Mascot
2071.0237	2070.9912	-0.0325	-16	148	167	QMGLSDQDIVALSGGHT LGR			Oxidation (M)[2]		Mascot
2071.0237	2070.9912	-0.0325	-16	148	167	QMGLSDQDIVALSGGHT LGR	132	100	Oxidation (M)[2]		Mascot

8	ascorbate peroxidase [Eleusine coracana]	gi|338760827	27561	5.79	6	225	100	5.366	202	100

Peptide Information

Calc. Mass	Obsrv. Mass	± da	± ppm	Start Seq.	End Seq.	Sequence	Ion Score	C. I. %	Modification	Rank	Result Type
911.4291	911.4224	-0.0067	-7	53	61	TGGPFGTMK			Oxidation (M)[8]		Mascot
919.4196	919.4149	-0.0047	-5	230	236	AFFEDYK					Mascot

1010.469	1010.3917	-0.0773	-76	173	181	SGFEGAWTR					Mascot
1249.6172	1249.6124	-0.0048	-4	120	130	EDKPQPPPEGR					Mascot
1249.6172	1249.6124	-0.0048	-4	120	130	EDKPQPPPEGR	70	99.981			Mascot
1610.9265	1610.8978	-0.0287	-18	210	223	ALLNDPVFRPLVEK					Mascot
2071.0237	2070.9912	-0.0325	-16	148	167	QMGLSDQDIVALSGGHT LGR			Oxidation (M)[2]		Mascot
2071.0237	2070.9912	-0.0325	-16	148	167	QMGLSDQDIVALSGGHT LGR	132	100	Oxidation (M)[2]		Mascot

9	TPA: APx1-Cytosolic Ascorbate Peroxidase [Zea mays]	gi|414866238	27467.9	5.64	5	220	100	5.059	202	100	

Protein Group

ascorbate peroxidase [Zea mays]	gi|226533502	27467.9	5.6399 998664 856

Peptide Information

Calc. Mass	Obsrv. Mass	± da	± ppm	Start Seq.	End Seq.	Sequence	Ion Score	C. I. %	Modification	Rank	Result Type
911.4291	911.4224	-0.0067	-7	53	61	TGGPFGTMK			Oxidation (M)[8]		Mascot
1249.6172	1249.6124	-0.0048	-4	120	130	EDKPQPPPEGR					Mascot
1249.6172	1249.6124	-0.0048	-4	120	130	EDKPQPPPEGR	70	99.981			Mascot
1582.7061	1582.7583	0.0522	33	224	236	YAADEKAFFDDYK					Mascot
1583.9155	1583.8131	-0.1024	-65	210	223	ALLSDPVFRPLVEK					Mascot
2071.0237	2070.9912	-0.0325	-16	148	167	QMGLSDQDIVALSGGHT LGR			Oxidation (M)[2]		Mascot
2071.0237	2070.9912	-0.0325	-16	148	167	QMGLSDQDIVALSGGHT LGR	132	100	Oxidation (M)[2]		Mascot

10	PREDICTED: L-ascorbate peroxidase 1, cytosolic-like [Setaria italica]	gi|514820260	27482.9	5.69	5	220	100	5.168	202	100	

Peptide Information

Calc. Mass	Obsrv. Mass	± da	± ppm	Start Seq.	End Seq.	Sequence	Ion Score	C. I. %	Modification	Rank	Result Type
911.4291	911.4224	-0.0067	-7	53	61	TGGPFGTMK			Oxidation (M)[8]		Mascot
1010.469	1010.3917	-0.0773	-76	173	181	SGFEGAWTR					Mascot
1249.6172	1249.6124	-0.0048	-4	120	130	EDKPQPPPEGR					Mascot
1249.6172	1249.6124	-0.0048	-4	120	130	EDKPQPPPEGR	70	99.981			Mascot
1582.7061	1582.7583	0.0522	33	224	236	YAADEKAFFDDYK					Mascot
2071.0237	2070.9912	-0.0325	-16	148	167	QMGLSDQDIVALSGGHT LGR			Oxidation (M)[2]		Mascot
2071.0237	2070.9912	-0.0325	-16	148	167	QMGLSDQDIVALSGGHT LGR	132	100	Oxidation (M)[2]		Mascot

Gel Idx/Pos	213/I13	Instr./Gel Origin	BA2151/Sample Project 20140814	Process Status	Analysis Succeeded
Plate [#] Name	[1] Sample Project 20140814	Instrument Sample Name		Spectra	11

Rank	Protein Name	Accession No.	Protein MW	Protein PI	Pep. Count	Protein Score	Protein Score C. I. %	Intensity Matched	Total Ion Score	Total Ion C. I. %	Confirmed
1	Proteasome subunit alpha type-6 [Aegilops tauschii]	gi|475538570	27533.1	6.33	14	304	100	44.046	209	100	

Peptide Information

Calc. Mass	Obsrv. Mass	± da	± ppm	Start Seq.	End Seq.	Sequence	Ion Score	C. I. %	Modification	Rank	Result Type
836.3896	836.4016	0.012	14	96	102	NEAAEFR					Mascot
946.5316	946.533	0.0014	1	34	43	SAGVTSIGVR					Mascot
946.5316	946.533	0.0014	1	34	43	SAGVTSIGVR					Mascot
1025.476	1025.4946	0.0186	18	2	11	SRGSGAGYDR					Mascot
1072.5997	1072.6053	0.0056	5	212	221	ATEIEVGVVR					Mascot
1072.5997	1072.6053	0.0056	5	212	221	ATEIEVGVVR	64	99.904			Mascot
1156.611	1156.6171	0.0061	5	12	21	HITIFSPEGR					Mascot
1156.611	1156.6171	0.0061	5	12	21	HITIFSPEGR	60	99.791			Mascot
1160.5986	1160.5919	-0.0067	-6	22	30	LYQVEYAFK					Mascot
1172.5114	1172.613	0.1016	87	1	11	MSRGSGAGYDR			Oxidation (M)[1]		Mascot
1220.6157	1220.6034	-0.0123	-10	172	181	EQEAINFLEK					Mascot
1272.5579	1272.5577	-0.0002	0	154	164	CDPAGHFFGHK			Carbamidomethyl (C)[1]		Mascot
1407.6978	1407.6737	-0.0241	-17	105	116	WGYEMPVDVLAK					Mascot
1422.7588	1422.7657	0.0069	5	75	88	YTGLLATGLTADAR					Mascot
1422.7588	1422.7657	0.0069	5	75	88	YTGLLATGLTADAR	61	99.827			Mascot
1703.9215	1703.9159	-0.0056	-3	60	74	LLDDTSITHLFSITK					Mascot
1703.9215	1703.9159	-0.0056	-3	60	74	LLDDTSITHLFSITK	25	0			Mascot
1942.9717	1942.9749	0.0032	2	229	245	SLTTEEIDQHLTAISER					Mascot
2057.9985	2058.0051	0.0066	3	229	246	SLTTEEIDQHLTAISERD					Mascot
2057.9985	2058.0051	0.0066	3	229	246	SLTTEEIDQHLTAISERD	17	0			Mascot

Rank	Protein Name	Accession No.	Protein MW	Protein PI	Pep. Count	Protein Score	Protein Score C. I. %	Intensity Matched	Total Ion Score	Total Ion C. I. %	Confirmed
2	TPA: proteasome subunit alpha type [Zea mays]	gi|414865138	27514.1	6.1	10	170	100	31.336	124	100	

Protein Group

maize 20S proteasome alpha subunit [Zea mays]	gi|162463728	27500.1	6.0999 999046 3257

Peptide Information

Calc. Mass	Obsrv. Mass	± da	± ppm	Start Seq.	End Seq.	Sequence	Ion Score	C. I. %	Modification	Rank	Result Type
807.4108	807.4277	0.0169	21	223	228	DDRVFR					Mascot
836.3896	836.4016	0.012	14	96	102	NEAAEFR					Mascot
946.5316	946.533	0.0014	1	34	43	SAGVTSIGVR					Mascot
946.5316	946.533	0.0014	1	34	43	SAGVTSIGVR					Mascot
1072.5997	1072.6053	0.0056	5	212	221	ATEIEVGVVR					Mascot
1072.5997	1072.6053	0.0056	5	212	221	ATEIEVGVVR	64	99.904			Mascot
1156.611	1156.6171	0.0061	5	12	21	HITIFSPEGR					Mascot
1156.611	1156.6171	0.0061	5	12	21	HITIFSPEGR	60	99.791			Mascot
1160.5986	1160.5919	-0.0067	-6	22	30	LYQVEYAFK					Mascot
1220.6157	1220.6034	-0.0123	-10	172	181	EQEAINFLEK					Mascot
1272.5579	1272.5577	-0.0002	0	154	164	CDPAGHFFGHK			Carbamidomethyl (C)[1]		Mascot
1407.6978	1407.6737	-0.0241	-17	105	116	WGYEMPVDVLAK					Mascot
1926.9767	1926.8766	-0.1001	-52	229	245	ALTTEEIDQHLTAISER					Mascot

3	Os03g0180400 [Oryza sativa Japonica Group]	gi|113547633	27670.2	6.19	10	168	100	31.336	124	100		

Protein Group

RecName: Full=Proteasome subunit alpha type-6; AltName: Full=20S proteasome alpha subunit A; AltName: Full=20S proteasome subunit alpha-1	gi|12229922	27670.2	6.1900 000572 2046
hypothetical protein OsI_10259 [Oryza sativa Indica Group]	gi|125542646	27670.2	6.1900 000572 2046

Peptide Information

Calc. Mass	Obsrv. Mass	± da	± ppm	Start Seq.	End Seq.	Sequence	Ion Score	C. I. %	Modification	Rank	Result Type
807.4108	807.4277	0.0169	21	223	228	DDRVFR					Mascot
836.3896	836.4016	0.012	14	96	102	NEAAEFR					Mascot
946.5316	946.533	0.0014	1	34	43	SAGVTSIGVR					Mascot
946.5316	946.533	0.0014	1	34	43	SAGVTSIGVR					Mascot
1072.5997	1072.6053	0.0056	5	212	221	ATEIEVGVVR					Mascot
1072.5997	1072.6053	0.0056	5	212	221	ATEIEVGVVR	64	99.904			Mascot
1156.611	1156.6171	0.0061	5	12	21	HITIFSPEGR					Mascot
1156.611	1156.6171	0.0061	5	12	21	HITIFSPEGR	60	99.791			Mascot

Calc. Mass	Obsrv. Mass	± da	± ppm	Start Seq.	End Seq.	Sequence	Ion Score	C. I. %	Modification	Rank	Result Type
1160.5986	1160.5919	-0.0067	-6	22	30	LYQVEYAFK					Mascot
1220.6157	1220.6034	-0.0123	-10	172	181	EQEAINFLEK					Mascot
1272.5579	1272.5577	-0.0002	0	154	164	CDPAGHFFGHK			Carbamidomethyl (C)[1]		Mascot
1407.6978	1407.6737	-0.0241	-17	105	116	WGYEMPVDVLAK					Mascot
1926.9767	1926.8766	-0.1001	-52	229	245	ALTTEEIDQHLTAISER					Mascot

4	PREDICTED: proteasome subunit alpha type-6-like [Setaria italica]	gi|514821625	27632.2	6.11	9	163	100	30.995	124	100

Peptide Information

Calc. Mass	Obsrv. Mass	± da	± ppm	Start Seq.	End Seq.	Sequence	Ion Score	C. I. %	Modification	Rank	Result Type
836.3896	836.4016	0.012	14	96	102	NEAAEFR					Mascot
946.5316	946.533	0.0014	1	34	43	SAGVTSIGVR					Mascot
946.5316	946.533	0.0014	1	34	43	SAGVTSIGVR					Mascot
1072.5997	1072.6053	0.0056	5	212	221	ATEIEVGVVR					Mascot
1072.5997	1072.6053	0.0056	5	212	221	ATEIEVGVVR	64	99.904			Mascot
1156.611	1156.6171	0.0061	5	12	21	HITIFSPEGR					Mascot
1156.611	1156.6171	0.0061	5	12	21	HITIFSPEGR	60	99.791			Mascot
1160.5986	1160.5919	-0.0067	-6	22	30	LYQVEYAFK					Mascot
1220.6157	1220.6034	-0.0123	-10	172	181	EQEAINFLEK					Mascot
1272.5579	1272.5577	-0.0002	0	154	164	CDPAGHFFGHK			Carbamidomethyl (C)[1]		Mascot
1407.6978	1407.6737	-0.0241	-17	105	116	WGYEMPVDVLAK					Mascot
1926.9767	1926.8766	-0.1001	-52	229	245	ALTTEEIDQHLTAISER					Mascot

5	uncharacterized protein LOC100191154 [Zea mays]	gi|212275456	27584.2	6.11	8	155	100	30.633	124	100

Peptide Information

Calc. Mass	Obsrv. Mass	± da	± ppm	Start Seq.	End Seq.	Sequence	Ion Score	C. I. %	Modification	Rank	Result Type
836.3896	836.4016	0.012	14	96	102	NEAAEFR					Mascot
946.5316	946.533	0.0014	1	34	43	SAGVTSIGVR					Mascot
946.5316	946.533	0.0014	1	34	43	SAGVTSIGVR					Mascot
1072.5997	1072.6053	0.0056	5	212	221	ATEIEVGVVR					Mascot
1072.5997	1072.6053	0.0056	5	212	221	ATEIEVGVVR	64	99.904			Mascot
1156.611	1156.6171	0.0061	5	12	21	HITIFSPEGR					Mascot
1156.611	1156.6171	0.0061	5	12	21	HITIFSPEGR	60	99.791			Mascot
1160.5986	1160.5919	-0.0067	-6	22	30	LYQVEYAFK					Mascot
1220.6157	1220.6034	-0.0123	-10	172	181	EQEAINFLEK					Mascot

1272.5579	1272.5577	-0.0002	0	154	164	CDPAGHFFGHK			Carbamidomethyl (C)[1]		Mascot
1407.6978	1407.6737	-0.0241	-17	105	116	WGYEMPVDVLAK					Mascot

6	hypothetical protein ZEAMMB73_334376 [Zea mays]	gi|413956824	23928.4	6.6	7	152	100	29.801	124	100	

Peptide Information

Calc. Mass	Obsrv. Mass	± da	± ppm	Start Seq.	End Seq.	Sequence	Ion Score	C. I. %	Modification	Rank	Result Type
836.3896	836.4016	0.012	14	96	102	NEAAEFR					Mascot
946.5316	946.533	0.0014	1	34	43	SAGVTSIGVR					Mascot
946.5316	946.533	0.0014	1	34	43	SAGVTSIGVR					Mascot
1072.5997	1072.6053	0.0056	5	180	189	ATEIEVGVVR					Mascot
1072.5997	1072.6053	0.0056	5	180	189	ATEIEVGVVR	64	99.904			Mascot
1156.611	1156.6171	0.0061	5	12	21	HITIFSPEGR					Mascot
1156.611	1156.6171	0.0061	5	12	21	HITIFSPEGR	60	99.791			Mascot
1160.5986	1160.5919	-0.0067	-6	22	30	LYQVEYAFK					Mascot
1272.5579	1272.5577	-0.0002	0	154	164	CDPAGHFFGHK			Carbamidomethyl (C)[1]		Mascot
1407.6978	1407.6737	-0.0241	-17	105	116	WGYEMPVDVLAK					Mascot

7	hypothetical protein CARUB_v10006215mg [Capsella rubella]	gi|482551480	27331.8	5.59	7	150	100	24.977	124	100	

Peptide Information

Calc. Mass	Obsrv. Mass	± da	± ppm	Start Seq.	End Seq.	Sequence	Ion Score	C. I. %	Modification	Rank	Result Type
836.3896	836.4016	0.012	14	96	102	NEAAEFR					Mascot
1072.5997	1072.6053	0.0056	5	212	221	ATEIEVGVVR					Mascot
1072.5997	1072.6053	0.0056	5	212	221	ATEIEVGVVR	64	99.904			Mascot
1156.611	1156.6171	0.0061	5	12	21	HITIFSPEGR					Mascot
1156.611	1156.6171	0.0061	5	12	21	HITIFSPEGR	60	99.791			Mascot
1206.6001	1206.6058	0.0057	5	172	181	EQEAVNFLEK					Mascot
1220.6304	1220.6034	-0.027	-22	44	54	GKDSVCVVTQK			Carbamidomethyl (C)[6]		Mascot
1404.7845	1404.7189	-0.0656	-47	75	88	FIGLVATGITADAR					Mascot
2480.1802	2480.2744	0.0942	38	96	116	NEAAEFRFTYGYEMPVDILAK			Oxidation (M)[14]		Mascot

8	20S proteasome subunit PAA2 [Arabidopsis thaliana]	gi|330250885	27446.9	5.75	7	146	100	25.217	123	100	

Protein Group

20S proteasome subunit PAA2 [Arabidopsis thaliana]	gi|15224993	27446.9	5.75

RecName: Full=Proteasome subunit alpha type-6-B; AltName: Full=20S proteasome subunit alpha A-2; AltName: Full=Proteasome subunit alpha type-1	gi|12229902	27446.9	5.75
hypothetical protein ARALYDRAFT_899379 [Arabidopsis lyrata subsp. lyrata]	gi|297329553	27446.9	5.75

Peptide Information

Calc. Mass	Obsrv. Mass	± da	± ppm	Start Seq.	End Seq.	Sequence	Ion Score	C. I. %	Modification	Rank	Result Type
836.3896	836.4016	0.012	14	96	102	NEAAEFR					Mascot
1025.476	1025.4946	0.0186	18	2	11	SRGSGAGYDR					Mascot
1072.5997	1072.6053	0.0056	5	212	221	ATEIEVGVVR					Mascot
1072.5997	1072.6053	0.0056	5	212	221	ATEIEVGVVR	64	99.904			Mascot
1156.611	1156.6171	0.0061	5	12	21	HITIFSPEGR					Mascot
1156.611	1156.6171	0.0061	5	12	21	HITIFSPEGR	60	99.791			Mascot
1172.5114	1172.613	0.1016	87	1	11	MSRGSGAGYDR			Oxidation (M)[1]		Mascot
1206.6001	1206.6058	0.0057	5	172	181	EQEAVNFLEK					Mascot
1220.6304	1220.6034	-0.027	-22	44	54	GKDSVCVVTQK			Carbamidomethyl (C)[6]		Mascot

9	PREDICTED: proteasome subunit alpha type-6-like [Cucumis sativus]	gi|449447912	27392.9	5.74	6	144	100	25.281	124	100

Protein Group

PREDICTED: proteasome subunit alpha type-6-like [Cucumis sativus]	gi|449480500	27392.9	5.7399 997711 1816

Peptide Information

Calc. Mass	Obsrv. Mass	± da	± ppm	Start Seq.	End Seq.	Sequence	Ion Score	C. I. %	Modification	Rank	Result Type
836.3896	836.4016	0.012	14	96	102	NEAAEFR					Mascot
848.4261	848.4168	-0.0093	-11	222	228	SDNPIFR					Mascot
1072.5997	1072.6053	0.0056	5	212	221	ATEIEVGVVR					Mascot
1072.5997	1072.6053	0.0056	5	212	221	ATEIEVGVVR	64	99.904			Mascot
1156.611	1156.6171	0.0061	5	12	21	HITIFSPEGR					Mascot

1156.611	1156.6171	0.0061	5	12	21	HITIFSPEGR	60	99.791			Mascot
1220.6157	1220.6034	-0.0123	-10	172	181	EQEAINFLEK					Mascot
1272.5579	1272.5577	-0.0002	0	154	164	CDPAGHFFGHK			Carbamidomethyl (C)[1]		Mascot

10	hypothetical protein ARALYDRAFT_493620 [Arabidopsis lyrata subsp. lyrata]	gi|297316272	27314.8	5.6	6	142	100	19.999	123	100	

Peptide Information

Calc. Mass	Obsrv. Mass	± da	± ppm	Start Seq.	End Seq.	Sequence	Ion Score	C. I. %	Modification	Rank	Result Type
1025.476	1025.4946	0.0186	18	2	11	SRGSGAGYDR					Mascot
1072.5997	1072.6053	0.0056	5	212	221	ATEIEVGVVR					Mascot
1072.5997	1072.6053	0.0056	5	212	221	ATEIEVGVVR	64	99.904			Mascot
1156.611	1156.6171	0.0061	5	12	21	HITIFSPEGR					Mascot
1156.611	1156.6171	0.0061	5	12	21	HITIFSPEGR	60	99.791			Mascot
1172.5114	1172.613	0.1016	87	1	11	MSRGSGAGYDR			Oxidation (M)[1]		Mascot
1206.6001	1206.6058	0.0057	5	172	181	EQEAVNFLEK					Mascot
1220.6304	1220.6034	-0.027	-22	44	54	GKDSVCVVTQK			Carbamidomethyl (C)[6]		Mascot

Gel Idx/Pos	214/I14	Instr./Gel Origin	BA2151/Sample Project 20140814	Process Status	Analysis Succeeded
Plate [#] Name	[1] Sample Project 20140814	Instrument Sample Name		Spectra	11

Rank	Protein Name	Accession No.	Protein MW	Protein PI	Pep. Count	Protein Score	Protein Score C. I. %	Intensity Matched	Total Ion Score	Total Ion C. I. %	Confirmed
1	hypothetical protein F775_32009 [Aegilops tauschii]	gi|475536281	35927.1	5.06	17	411	100	47.297	303	100	

Peptide Information

Calc. Mass	Obsrv. Mass	± da	± ppm	Start Seq.	End Seq.	Sequence	Ion Score	C. I. %	Modification	Rank	Result Type
842.4301	842.4733	0.0432	51	160	167	SAHVAAMR					Mascot
858.425	858.4254	0.0004	0	160	167	SAHVAAMR			Oxidation (M)[7]		Mascot
892.4022	892.4059	0.0037	4	117	122	EFMFYR					Mascot
908.3971	908.395	-0.0021	-2	117	122	EFMFYR			Oxidation (M)[3]		Mascot
908.3971	908.395	-0.0021	-2	117	122	EFMFYR	25	0	Oxidation (M)[3]		Mascot
963.5873	963.5838	-0.0035	-4	103	111	TALAFVTLK					Mascot
965.4761	965.4636	-0.0125	-13	195	202	DGIMSIWK			Oxidation (M)[4]		Mascot
987.5721	987.5706	-0.0015	-2	238	246	LLIVTDGEK					Mascot
1201.5372	1201.5342	-0.003	-2	287	296	DDSIFYNEAK					Mascot
1209.674	1209.6798	0.0058	5	184	194	LPLWPSAQAAR					Mascot
1209.674	1209.6798	0.0058	5	184	194	LPLWPSAQAAR	99	100			Mascot
1305.7202	1305.7113	-0.0089	-7	227	237	NVLSLWFEGLK					Mascot
1360.7253	1360.687	-0.0383	-28	238	249	LLIVTDGEKGCR			Carbamidomethyl (C)[11]		Mascot
1451.6948	1451.6985	0.0037	3	171	183	SAGILCSYDPNVR			Carbamidomethyl (C)[6]		Mascot
1451.6948	1451.6985	0.0037	3	171	183	SAGILCSYDPNVR	57	99.586	Carbamidomethyl (C)[6]		Mascot
1749.8517	1749.8501	-0.0016	-1	72	86	FGDDEFGHMLVEILK					Mascot
1765.8466	1765.8038	-0.0428	-24	72	86	FGDDEFGHMLVEILK			Oxidation (M)[9]		Mascot
1792.9052	1792.9064	0.0012	1	145	159	IFHYGSISLITEPCR			Carbamidomethyl (C)[14]		Mascot
1792.9052	1792.9064	0.0012	1	145	159	IFHYGSISLITEPCR	85	100	Carbamidomethyl (C)[14]		Mascot
1798.8463	1798.806	-0.0403	-22	299	314	EVLQFSNACGAICTTK			Carbamidomethyl (C)[9,13]		Mascot
1815.8192	1815.8248	0.0056	3	87	102	QNGVNAEGCLFDQHAR			Carbamidomethyl (C)[9]		Mascot
1895.1212	1895.0588	-0.0624	-33	315	333	KGAIPALPTTATALELISK					Mascot
2068.0315	2068.0317	0.0002	0	297	314	LREVLQFSNACGAICTTK			Carbamidomethyl (C)[11,15]		Mascot
2068.0315	2068.0317	0.0002	0	297	314	LREVLQFSNACGAICTTK	39	75.066	Carbamidomethyl (C)[11,15]		Mascot
2128.0955	2128.0962	0.0007	0	123	141	NPSADMLLTEAELNLDLIR					Mascot
2144.0903	2144.0696	-0.0207	-10	123	141	NPSADMLLTEAELNLDLIR			Oxidation (M)[6]		Mascot

2	hypothetical protein F775_11116 [Aegilops tauschii]	gi|475593046	35806.9	4.91	12	364	100	45.321	303	100	

Peptide Information

Calc. Mass	Obsrv. Mass	± da	± ppm	Start Seq.	End Seq.	Sequence	Ion Score	C. I. %	Modification	Rank	Result Type
858.425	858.4254	0.0004	0	157	164	SAHVSAMR					Mascot
874.4199	874.4091	-0.0108	-12	157	164	SAHVSAMR			Oxidation (M)[7]		Mascot
892.4022	892.4059	0.0037	4	114	119	EFMFYR					Mascot
908.3971	908.395	-0.0021	-2	114	119	EFMFYR			Oxidation (M)[3]		Mascot
908.3971	908.395	-0.0021	-2	114	119	EFMFYR	25	0	Oxidation (M)[3]		Mascot
963.5873	963.5838	-0.0035	-4	100	108	TALAFVTLK					Mascot
965.4761	965.4636	-0.0125	-13	192	199	DGIMSIWK			Oxidation (M)[4]		Mascot
1201.5372	1201.5342	-0.003	-2	284	293	DDSIFYNEAK					Mascot
1209.674	1209.6798	0.0058	5	181	191	LPLWPSAQAAR					Mascot
1209.674	1209.6798	0.0058	5	181	191	LPLWPSAQAAR	99	100			Mascot
1451.6948	1451.6985	0.0037	3	168	180	SAGILCSYDPNVR			Carbamidomethyl (C)[6]		Mascot
1451.6948	1451.6985	0.0037	3	168	180	SAGILCSYDPNVR	57	99.586	Carbamidomethyl (C)[6]		Mascot
1792.9052	1792.9064	0.0012	1	142	156	IFHYGSISLITEPCR			Carbamidomethyl (C)[14]		Mascot
1792.9052	1792.9064	0.0012	1	142	156	IFHYGSISLITEPCR	85	100	Carbamidomethyl (C)[14]		Mascot
1798.8463	1798.806	-0.0403	-22	296	311	EVLQFSNACGAICTTK			Carbamidomethyl (C)[9,13]		Mascot
1815.8192	1815.8248	0.0056	3	84	99	QNGVNAEGCLFDQHAR			Carbamidomethyl (C)[9]		Mascot
1895.1212	1895.0588	-0.0624	-33	312	330	KGAIPALPTTATALELISK					Mascot
2068.0315	2068.0317	0.0002	0	294	311	LREVLQFSNACGAICTTK			Carbamidomethyl (C)[11,15]		Mascot
2068.0315	2068.0317	0.0002	0	294	311	LREVLQFSNACGAICTTK	39	75.066	Carbamidomethyl (C)[11,15]		Mascot

3	hypothetical protein TRIUR3_22532 [Triticum urartu]	gi|473797438	36043.2	5.13	10	350	100	44.449	303	100	

Peptide Information

Calc. Mass	Obsrv. Mass	± da	± ppm	Start Seq.	End Seq.	Sequence	Ion Score	C. I. %	Modification	Rank	Result Type
892.4022	892.4059	0.0037	4	113	118	EFMFYR					Mascot
908.3971	908.395	-0.0021	-2	113	118	EFMFYR			Oxidation (M)[3]		Mascot
908.3971	908.395	-0.0021	-2	113	118	EFMFYR	25	0	Oxidation (M)[3]		Mascot
963.5873	963.5838	-0.0035	-4	99	107	TALAFVTLK					Mascot
965.4761	965.4636	-0.0125	-13	191	198	DGIMSIWK			Oxidation (M)[4]		Mascot
1209.674	1209.6798	0.0058	5	180	190	LPLWPSAQAAR					Mascot
1209.674	1209.6798	0.0058	5	180	190	LPLWPSAQAAR	99	100			Mascot
1451.6948	1451.6985	0.0037	3	167	179	SAGILCSYDPNVR			Carbamidomethyl (C)[6]		Mascot

Calc. Mass	Obsrv. Mass	± da	± ppm	Start Seq.	End Seq.	Sequence	Ion Score	C. I. %	Modification	Rank	Result Type
1451.6948	1451.6985	0.0037	3	167	179	SAGILCSYDPNVR	57	99.586	Carbamidomethyl (C)[6]		Mascot
1792.9052	1792.9064	0.0012	1	141	155	IFHYGSISLITEPCR			Carbamidomethyl (C)[14]		Mascot
1792.9052	1792.9064	0.0012	1	141	155	IFHYGSISLITEPCR	85	100	Carbamidomethyl (C)[14]		Mascot
1798.8463	1798.806	-0.0403	-22	295	310	EVLQFSNACGAICTTK			Carbamidomethyl (C)[9,13]		Mascot
1895.1212	1895.0588	-0.0624	-33	311	329	KGAIPALPTTATALELISK					Mascot
2068.0315	2068.0317	0.0002	0	293	310	LREVLQFSNACGAICTTK			Carbamidomethyl (C)[11,15]		Mascot
2068.0315	2068.0317	0.0002	0	293	310	LREVLQFSNACGAICTTK	39	75.066	Carbamidomethyl (C)[11,15]		Mascot
2128.0955	2128.0962	0.0007	0	119	137	NPSADMLLTEAELNLDLI R					Mascot
2144.0903	2144.0696	-0.0207	-10	119	137	NPSADMLLTEAELNLDLI R			Oxidation (M)[6]		Mascot

4	hypothetical protein F775_30923 [Aegilops tauschii]	gi|475508332	18590.4	5.19	7	235	100	24.865	195	100	

Peptide Information

Calc. Mass	Obsrv. Mass	± da	± ppm	Start Seq.	End Seq.	Sequence	Ion Score	C. I. %	Modification	Rank	Result Type
965.4761	965.4636	-0.0125	-13	30	37	DGIMSIWK			Oxidation (M)[4]		Mascot
1201.5372	1201.5342	-0.003	-2	122	131	DDSIFYNEAK					Mascot
1209.674	1209.6798	0.0058	5	19	29	LPLWPSAQAAR					Mascot
1209.674	1209.6798	0.0058	5	19	29	LPLWPSAQAAR	99	100			Mascot
1305.7202	1305.7113	-0.0089	-7	62	72	NVLSLWFEGLK					Mascot
1451.6948	1451.6985	0.0037	3	6	18	SAGILCSYDPNVR			Carbamidomethyl (C)[6]		Mascot
1451.6948	1451.6985	0.0037	3	6	18	SAGILCSYDPNVR	57	99.586	Carbamidomethyl (C)[6]		Mascot
1798.8463	1798.806	-0.0403	-22	134	149	EVLQFSNACGAICTTK			Carbamidomethyl (C)[9,13]		Mascot
2068.0315	2068.0317	0.0002	0	132	149	LREVLQFSNACGAICTTK			Carbamidomethyl (C)[11,15]		Mascot
2068.0315	2068.0317	0.0002	0	132	149	LREVLQFSNACGAICTTK	39	75.066	Carbamidomethyl (C)[11,15]		Mascot

5	RecName: Full=Fructokinase-2; AltName: Full=Fructokinase II; AltName: Full=OsFKII	gi|158513662	35893.1	5.02	13	235	100	29.924	165	100	

Protein Group

Os08g0113100 [Oryza sativa Japonica Group]	gi|113622806	35893.1	5.0199 999809 2651
RecName: Full=Fructokinase-2; AltName: Full=Fructokinase II; AltName: Full=OsFKII	gi|122234591	35893.1	5.0199 999809 2651
hypothetical protein OsI_27579 [Oryza sativa Indica Group]	gi|125559927	35822.1	5.0199 999809

Peptide Information

Calc. Mass	Obsrv. Mass	± da	± ppm	Start Seq.	End Seq.	Sequence	Ion Score	C. I. %	Modification	Rank	Result Type
842.4301	842.4733	0.0432	51	160	167	SAHVAAMR					Mascot
858.425	858.4254	0.0004	0	160	167	SAHVAAMR			Oxidation (M)[7]		Mascot
892.4022	892.4059	0.0037	4	117	122	EFMFYR					Mascot
908.3971	908.395	-0.0021	-2	117	122	EFMFYR			Oxidation (M)[3]		Mascot
908.3971	908.395	-0.0021	-2	117	122	EFMFYR	25	0	Oxidation (M)[3]		Mascot
963.5873	963.5838	-0.0035	-4	103	111	TALAFVTLK					Mascot
987.5721	987.5706	-0.0015	-2	238	246	LLIVTDGEK					Mascot
1233.5382	1233.6298	0.0916	74	287	296	DDSIFHNEEK					Mascot
1360.7253	1360.687	-0.0383	-28	238	249	LLIVTDGEKGCR			Carbamidomethyl (C)[11]		Mascot
1451.6948	1451.6985	0.0037	3	171	183	SAGILCSYDPNVR			Carbamidomethyl (C)[6]		Mascot
1451.6948	1451.6985	0.0037	3	171	183	SAGILCSYDPNVR	57	99.586	Carbamidomethyl (C)[6]		Mascot
1735.8361	1735.871	0.0349	20	72	86	FGDDEFGHMLVDILK					Mascot
1792.9052	1792.9064	0.0012	1	145	159	IFHYGSISLITEPCR			Carbamidomethyl (C)[14]		Mascot
1792.9052	1792.9064	0.0012	1	145	159	IFHYGSISLITEPCR	85	100	Carbamidomethyl (C)[14]		Mascot
1816.8395	1816.8103	-0.0292	-16	87	102	KNGVNAEGCLFDEHAR			Carbamidomethyl (C)[9]		Mascot
1816.8395	1816.8103	-0.0292	-16	87	102	KNGVNAEGCLFDEHAR			Carbamidomethyl (C)[9]		Mascot
1863.931	1863.9353	0.0043	2	72	87	FGDDEFGHMLVDILKK					Mascot
1952.8721	1952.9612	0.0891	46	209	226	VSDDEVAFLTQGDANDE K					Mascot
2128.0955	2128.0962	0.0007	0	123	141	NPSADMLLTEAELNLDLI R					Mascot
2144.0903	2144.0696	-0.0207	-10	123	141	NPSADMLLTEAELNLDLI R			Oxidation (M)[6]		Mascot

6	Fructokinase-2 [Triticum urartu]	gi|474190636	42253.2	4.78	11	212	100	27.743	164	100	

Peptide Information

Calc. Mass	Obsrv. Mass	± da	± ppm	Start Seq.	End Seq.	Sequence	Ion Score	C. I. %	Modification	Rank	Result Type
842.4301	842.4733	0.0432	51	251	258	SAHVAAMR					Mascot
858.425	858.4254	0.0004	0	251	258	SAHVAAMR			Oxidation (M)[7]		Mascot
892.4022	892.4059	0.0037	4	208	213	EFMFYR					Mascot
908.3971	908.395	-0.0021	-2	208	213	EFMFYR			Oxidation (M)[3]		Mascot
908.3971	908.395	-0.0021	-2	208	213	EFMFYR	25	0	Oxidation (M)[3]		Mascot

963.5873	963.5838	-0.0035	-4	194	202	TALAFVTLK					Mascot
965.4761	965.4636	-0.0125	-13	286	293	DGIMSIWK			Oxidation (M)[4]		Mascot
1305.7202	1305.7113	-0.0089	-7	318	328	NVLSLWFEGLK					Mascot
1451.6948	1451.6985	0.0037	3	262	274	SAGILCSYDPNVR			Carbamidomethyl (C)[6]		Mascot
1451.6948	1451.6985	0.0037	3	262	274	SAGILCSYDPNVR	57	99.586	Carbamidomethyl (C)[6]		Mascot
1735.8361	1735.871	0.0349	20	163	177	FGDDEFGHMLVDILK					Mascot
1771.8392	1771.8171	-0.0221	-12	64	80	GNVEVIHGEGAQMSATR			Oxidation (M)[13]		Mascot
1792.9052	1792.9064	0.0012	1	236	250	IFHYGSISLITEPCR			Carbamidomethyl (C)[14]		Mascot
1792.9052	1792.9064	0.0012	1	236	250	IFHYGSISLITEPCR	85	100	Carbamidomethyl (C)[14]		Mascot
2127.04	2127.0386	-0.0014	-1	61	80	WGKGNVEVIHGEGAQM SATR					Mascot
2128.0955	2128.0962	0.0007	0	214	232	NPSADMLLTEAELNLDLI R					Mascot
2144.0903	2144.0696	-0.0207	-10	214	232	NPSADMLLTEAELNLDLI R			Oxidation (M)[6]		Mascot

7 hypothetical protein TRIUR3_24487 [Triticum urartu] gi|474171341 22815.6 5.27 11 195 100 25.276 124 100

Peptide Information

Calc. Mass	Obsrv. Mass	± da	± ppm	Start Seq.	End Seq.	Sequence	Ion Score	C. I. %	Modification	Rank	Result Type
842.4301	842.4733	0.0432	51	33	40	SAHVAAMR					Mascot
858.425	858.4254	0.0004	0	33	40	SAHVAAMR			Oxidation (M)[7]		Mascot
965.4761	965.4636	-0.0125	-13	68	75	DGIMSIWK			Oxidation (M)[4]		Mascot
987.5721	987.5706	-0.0015	-2	111	119	LLIVTDGEK					Mascot
1201.5372	1201.5342	-0.003	-2	160	169	DDSIFYNEAK					Mascot
1305.7202	1305.7113	-0.0089	-7	100	110	NVLSLWFEGLK					Mascot
1360.7253	1360.687	-0.0383	-28	111	122	LLIVTDGEKGCR			Carbamidomethyl (C)[11]		Mascot
1792.9052	1792.9064	0.0012	1	18	32	IFHYGSISLITEPCR			Carbamidomethyl (C)[14]		Mascot
1792.9052	1792.9064	0.0012	1	18	32	IFHYGSISLITEPCR	85	100	Carbamidomethyl (C)[14]		Mascot
1798.8463	1798.806	-0.0403	-22	172	187	EVLQFSNACGAICTTK			Carbamidomethyl (C)[9,13]		Mascot
1815.9998	1815.8248	-0.175	-96	1	15	MLLTEAELNLDLIRR			Oxidation (M)[1]		Mascot
1895.1212	1895.0588	-0.0624	-33	188	206	KGAIPALPTTATALELISK					Mascot
2068.0315	2068.0317	0.0002	0	170	187	LREVLQFSNACGAICTTK			Carbamidomethyl (C)[11,15]		Mascot
2068.0315	2068.0317	0.0002	0	170	187	LREVLQFSNACGAICTTK	39	75.066	Carbamidomethyl (C)[11,15]		Mascot

8 hypothetical protein F775_28867 [Aegilops tauschii] gi|475566113 23025.2 9.66 11 73 95.122 23.531

Peptide Information

Calc. Mass	Obsrv. Mass	± da	± ppm	Start Seq.	End Seq.	Sequence	Ion Score	C. I. %	Modification	Rank	Result Type

873.4788	873.4371	-0.0417	-48	2	9	VIDQSGVR					Mascot
987.5258	987.5706	0.0448	45	61	68	IGVDEWLR					Mascot
1182.6378	1182.6022	-0.0356	-30	50	60	SLPFPKSHGGR					Mascot
1241.7325	1241.6672	-0.0653	-53	2	12	VIDQSGVRLVR					Mascot
1241.7325	1241.6672	-0.0653	-53	2	12	VIDQSGVRLVR					Mascot
1337.7325	1337.6886	-0.0439	-33	176	186	SAYLTRVVSWR					Mascot
1481.7609	1481.7051	-0.0558	-38	56	68	SHGGRIGVDEWLR					Mascot
1481.7609	1481.7051	-0.0558	-38	56	68	SHGGRIGVDEWLR					Mascot
1813.967	1813.8052	-0.1618	-89	189	203	LYVAINWLTTMIFGR			Oxidation (M)[11]		Mascot
1941.0018	1940.8657	-0.1361	-70	159	175	DSKGISIAGFASWFIWR					Mascot
2010.0073	2009.9468	-0.0605	-30	114	133	VMKSGGGHANSQVEVDL GPK					Mascot
2068.1162	2068.0317	-0.0845	-41	187	203	NRLYVAINWLTTMIFGR					Mascot
2068.1162	2068.0317	-0.0845	-41	187	203	NRLYVAINWLTTMIFGR					Mascot
2341.084	2341.05	-0.034	-15	69	90	VPSVPDVFAVGDCCGFL ESTGK			Carbamidomethyl (C)[13,14]		Mascot

9	putative formamidopyrimidine-DNA glycosylase 1 (ISS), partial [Ostreococcus tauri]	gi|308803987	75474.5	8.33	10	70	89.079	20.457	50	97.978	

Peptide Information

Calc. Mass	Obsrv. Mass	± da	± ppm	Start Seq.	End Seq.	Sequence	Ion Score	C. I. %	Modification	Rank	Result Type
873.5152	873.4371	-0.0781	-89	188	194	LQLKTDR					Mascot
921.5152	921.426	-0.0892	-97	487	494	AFIEASRK					Mascot
965.5275	965.4636	-0.0639	-66	109	116	ARVHPETR					Mascot
1156.6473	1156.5503	-0.097	-84	288	298	APFNALPALSR					Mascot
1241.6597	1241.6672	0.0075	6	227	240	ASSAKASPGAPAAR					Mascot
1241.6597	1241.6672	0.0075	6	227	240	ASSAKASPGAPAAR	51	98.542			Mascot
1798.8074	1798.806	-0.0014	-1	75	90	IMAGIGNWMADGMFER					Mascot
1808.9025	1808.8906	-0.0119	-7	655	671	ASSGYQKAQAALEDELK					Mascot
1814.8022	1814.8474	0.0452	25	75	90	IMAGIGNWMADGMFER			Oxidation (M)[2]		Mascot
1823.9498	1823.8079	-0.1419	-78	93	108	TDDAIASLRSTEILYR					Mascot
2009.9128	2009.9468	0.034	17	526	544	GVEMNATATVACGDGTQ RR			Carbamidomethyl (C)[12], Oxidation (M)[4]		Mascot
2197.0667	2197.1462	0.0795	36	111	129	VHPETRANEMTDSQLEAI R					Mascot

10	PREDICTED: uncharacterized protein LOC101210019 [Cucumis sativus]	gi|449447132	49986.6	10.44	16	66	77.701	12.529	

Protein Group

PREDICTED: uncharacterized LOC101210019 [Cucumis sativus]	gi|449524599	49986.6	10.439 999580

Peptide Information

Calc. Mass	Obsrv. Mass	± da	± ppm	Start Seq.	End Seq.	Sequence	Ion Score	C. I. %	Modification	Rank	Result Type
873.4577	873.4371	-0.0206	-24	90	96	QEWAAIR					Mascot
981.4645	981.4624	-0.0021	-2	1	9	MGGSGKWMK					Mascot
983.4428	983.4583	0.0155	16	406	415	SAAFSNGDSK					Mascot
997.4594	997.4595	0.0001	0	1	9	MGGSGKWMK			Oxidation (M)[1]		Mascot
1207.629	1207.6512	0.0222	18	382	391	QKTNSHLSHR					Mascot
1209.6045	1209.6798	0.0753	62	198	207	LQMRQDGAFK			Oxidation (M)[3]		Mascot
1209.6045	1209.6798	0.0753	62	198	207	LQMRQDGAFK			Oxidation (M)[3]		Mascot
1360.689	1360.687	-0.002	-1	283	294	SCIESVVSKHSK			Carbamidomethyl (C)[2]		Mascot
1589.8547	1589.7793	-0.0754	-47	90	102	QEWAAIRIQTAFR					Mascot
1749.8113	1749.8501	0.0388	22	174	188	ADLLKQAEEGWCDSK			Carbamidomethyl (C)[12]		Mascot
1776.8802	1776.8344	-0.0458	-26	231	245	TNASIYALKNYEFDK					Mascot
1806.9708	1806.8787	-0.0921	-51	223	239	AIPNSTSRTNASIYALK					Mascot
1808.9436	1808.8906	-0.053	-29	156	171	MSVEGQAVQQLLNVHR					Mascot
1815.8695	1815.8248	-0.0447	-25	392	405	VQRQSMDEYQFLQK			Oxidation (M)[6]		Mascot
2040.0656	2039.9331	-0.1325	-65	156	173	MSVEGQAVQQLLNVHRSK			Oxidation (M)[1]		Mascot
2080.1082	2080.093	-0.0152	-7	154	171	VRMSVEGQAVQQLLNVHR			Oxidation (M)[3]		Mascot
2143.9622	2144.0696	0.1074	50	240	255	NYEFDKNNWGWSWLER					Mascot
2197.0693	2197.1462	0.0769	35	272	291	TESFDVTPPSKSCIESVVSK			Carbamidomethyl (C)[13]		Mascot

Gel Idx/Pos	215/I15	Instr./Gel Origin	BA2151/Sample Project 20140814	Process Status	Analysis Succeeded
Plate [#] Name	[1] Sample Project 20140814	Instrument Sample Name		Spectra	11

Rank	Protein Name	Accession No.	Protein MW	Protein PI	Pep. Count	Protein Score	Protein Score C. I. %	Intensity Matched	Total Ion Score	Total Ion C. I. %	Confirmed
1	Late embryogenesis abundant protein Lea14-A [Triticum urartu]	gi|474359227	38327.9	5.03	14	270	100	25.559	192	100	

Peptide Information

Calc. Mass	Obsrv. Mass	± da	± ppm	Start Seq.	End Seq.	Sequence	Ion Score	C. I. %	Modification	Rank	Result Type
1178.6906	1178.6841	-0.0065	-6	77	86	IHVPHIGLHR					Mascot
1252.5879	1252.5829	-0.005	-4	296	306	IDVDTPFGNMK			Oxidation (M)[10]		Mascot
1262.6199	1262.6775	0.0576	46	274	284	DLGSAVWDMIR					Mascot
1262.6199	1262.6775	0.0576	46	274	284	DLGSAVWDMIR	18	0			Mascot
1278.6147	1278.603	-0.0117	-9	274	284	DLGSAVWDMIR			Oxidation (M)[9]		Mascot
1280.682	1280.6505	-0.0315	-25	264	273	MQIPFTFRPK			Oxidation (M)[1]		Mascot
1295.7682	1295.7664	-0.0018	-1	119	131	LVAGLIPDAGTIR					Mascot
1295.7682	1295.7664	-0.0018	-1	119	131	LVAGLIPDAGTIR	72	99.987			Mascot
1300.6896	1300.6146	-0.075	-58	50	60	VKDFIHDIGEK					Mascot
1303.6893	1303.6859	-0.0034	-3	140	150	VPITLDFDDIR					Mascot
1303.6893	1303.6859	-0.0034	-3	140	150	VPITLDFDDIR	38	67.305			Mascot
1360.7988	1360.7285	-0.0703	-52	168	179	VIFLVDVPVFGR					Mascot
1360.7988	1360.7285	-0.0703	-52	168	179	VIFLVDVPVFGR	64	99.92			Mascot
1423.8632	1423.8552	-0.008	-6	118	131	KLVAGLIPDAGTIR					Mascot
1645.8545	1645.8457	-0.0088	-5	61	76	IEEAVGFGKPSADVAR					Mascot
1907.0637	1907.0546	-0.0091	-5	151	167	STYADIKPGSIIPYLLR					Mascot
2013.0903	2013.0117	-0.0786	-39	187	204	SGEIPIPYKPDVDVDKIK					Mascot
2322.1978	2322.1709	-0.0269	-12	97	117	NPNPVPIPLVDIDYLIDSD GR					Mascot
2450.2925	2450.2693	-0.0232	-9	97	118	NPNPVPIPLVDIDYLIDSD GRK					Mascot

Rank	Protein Name	Accession No.	Protein MW	Protein PI	Pep. Count	Protein Score	Protein Score C. I. %	Intensity Matched	Total Ion Score	Total Ion C. I. %	Confirmed
2	Transaldolase [Triticum urartu]	gi|473926683	30855.8	5.24	6	265	100	6.778	242	100	

Peptide Information

Calc. Mass	Obsrv. Mass	± da	± ppm	Start Seq.	End Seq.	Sequence	Ion Score	C. I. %	Modification	Rank	Result Type
890.4578	890.4591	0.0013	1	140	147	VSTEIDAR					Mascot
1328.6958	1328.6915	-0.0043	-3	180	191	IPATWQGIEASR					Mascot

1328.6958	1328.6915	-0.0043	-3	180	191	IPATWQGIEASR	87	100			Mascot
1424.6653	1424.7144	0.0491	34	235	247	NHSGDPEIDEALK					Mascot
1588.8555	1588.8538	-0.0017	-1	124	139	ALGNVGAELAHQVPGR					Mascot
1588.8555	1588.8538	-0.0017	-1	124	139	ALGNVGAELAHQVPGR	90	100			Mascot
2092.0459	2092.043	-0.0029	-1	159	175	VHELLQIYNEHDIPSER					Mascot
2092.0459	2092.043	-0.0029	-1	159	175	VHELLQIYNEHDIPSER	65	99.939			Mascot
2230.2329	2230.1558	-0.0771	-35	71	93	FAPTAATVSSSLLLGIAGL PDTK					Mascot

3	Desiccation protectant Lea14-like protein [Aegilops tauschii]	gi|475556899	43226.4	5.49	13	182	100	22.891	120	100	

Peptide Information

Calc. Mass	Obsrv. Mass	± da	± ppm	Start Seq.	End Seq.	Sequence	Ion Score	C. I. %	Modification	Rank	Result Type
1178.6906	1178.6841	-0.0065	-6	76	85	IHVPHIGLHR					Mascot
1252.5879	1252.5829	-0.005	-4	320	330	IDVDTPFGNMK			Oxidation (M)[10]		Mascot
1262.6199	1262.6775	0.0576	46	298	308	DLGSAVWDMIR					Mascot
1262.6199	1262.6775	0.0576	46	298	308	DLGSAVWDMIR	18	0			Mascot
1278.6147	1278.603	-0.0117	-9	298	308	DLGSAVWDMIR			Oxidation (M)[9]		Mascot
1280.682	1280.6505	-0.0315	-25	288	297	MQIPFTFRPK			Oxidation (M)[1]		Mascot
1300.6896	1300.6146	-0.075	-58	49	59	VKDFIHDIGEK					Mascot
1303.6893	1303.6859	-0.0034	-3	164	174	VPITLDFDDIR					Mascot
1303.6893	1303.6859	-0.0034	-3	164	174	VPITLDFDDIR	38	67.305			Mascot
1360.7988	1360.7285	-0.0703	-52	192	203	VIFLVDVPVFGR					Mascot
1360.7988	1360.7285	-0.0703	-52	192	203	VIFLVDVPVFGR	64	99.92			Mascot
1567.7244	1567.7251	0.0007	0	356	369	NSAEPSFCCIAALK			Carbamidomethyl (C)[8,9]		Mascot
1645.8545	1645.8457	-0.0088	-5	60	75	IEEAVGFGKPSADVAR					Mascot
1907.0637	1907.0546	-0.0091	-5	175	191	STYADIKPGSIIPYLLR					Mascot
2013.0903	2013.0117	-0.0786	-39	211	228	SGEIPIPYKPDVDVDKIK					Mascot
2322.1978	2322.1709	-0.0269	-12	96	116	NPNPVPIPLVDIDYLIDSD GR					Mascot
2450.2925	2450.2693	-0.0232	-9	96	117	NPNPVPIPLVDIDYLIDSD GRK					Mascot

4	PREDICTED: transaldolase-like [Setaria italica]	gi|514793473	42909.2	5.22	8	114	100	3.149	87	100	

Peptide Information

Calc. Mass	Obsrv. Mass	± da	± ppm	Start Seq.	End Seq.	Sequence	Ion Score	C. I. %	Modification	Rank	Result Type
890.4578	890.4591	0.0013	1	154	161	VSTEIDAR					Mascot
1328.6958	1328.6915	-0.0043	-3	194	205	IPATWQGIEASR					Mascot

1328.6958	1328.6915	-0.0043	-3	194	205	IPATWQGIEASR	87	100		Mascot
1415.6658	1415.725	0.0592	42	127	137	LEDRMSCYLTK			Carbamidomethyl (C)[7]	Mascot
1424.6653	1424.7144	0.0491	34	249	261	NHSGDPEIDEALK				Mascot
1436.7897	1436.6782	-0.1115	-78	328	340	YSYAPRLTPALGK				Mascot
1812.028	1811.8757	-0.1523	-84	23	38	WSPLARSAKPVPFSLR				Mascot
1905.0732	1904.9753	-0.0979	-51	296	312	QDVFSLLGIDYIIAPLK				Mascot
2201.2175	2201.1213	-0.0962	-44	85	107	FAPTAATASSSLLLGIAGL PNTK				Mascot

5	high molecular weight glutenin subunit 1Dx [Triticum spelta]	gi|469469526	89530.2	6.88	4	110	100	4.685	110	100

Protein Group

HMW glutenin subunit 1Dx2.3*t [Aegilops tauschii]	gi|429326677	87384.2	5.96999979019165
HMW glutenin subunit 1Dx2.4*t [Aegilops tauschii]	gi|429326679	86636.8	6.09000015258789
RecName: Full=Glutenin, high molecular weight subunit DX5; Flags: Precursor	gi|300669719	90523.6	6.42999982833862
RecName: Full=Glutenin, high molecular weight subunit PW212; Flags: Precursor	gi|121453	89348.1	5.82000017166138
high molecular weight glutenin subunit 1Dx [Aegilops tauschii]	gi|469469522	91065.9	6.88000011444092
high molecular weight glutenin subunit 1Dx [Aegilops tauschii]	gi|469469520	91033	7.55999994277954
high molecular weight glutenin subunit 1Dx [Aegilops tauschii]	gi|469469518	90062.5	6.09999990463257

high molecular weight glutenin subunit 1Dx [Aegilops tauschii]	gi|469469514	88735.8	5.8200 001716 6138
high molecular weight glutenin subunit 1Dx [Aegilops tauschii]	gi|469469504	89758.3	5.8200 001716 6138
high molecular weight glutenin subunit 1Dx [Aegilops tauschii]	gi|469469516	90046.4	6.0999 999046 3257
high molecular weight glutenin subunit 1Dx [Triticum spelta]	gi|469469524	89470.2	6.0999 999046 3257
high molecular weight glutenin subunit 1Dx [Triticum spelta]	gi|469469490	90537.7	6.8800 001144 4092
high molecular weight glutenin subunit 1Dx [Triticum spelta]	gi|469469488	90521.6	6.4299 998283 3862
high molecular weight glutenin subunit 1Dx [Triticum spelta]	gi|469469492	90506.6	6.4200 000762 9395
high molecular weight glutenin subunit 1Dx [Triticum spelta]	gi|469469502	90566.7	6.4299 998283 3862
high molecular weight glutenin subunit 1Dx [Triticum spelta]	gi|469469498	90574.7	7.5500 001907 3486

high molecular weight glutenin subunit 1Dx [Triticum spelta]	gi|469469494	90722.8	8.02999973297119
high molecular weight glutenin subunit 1Dx [Triticum spelta]	gi|469469496	90647.8	6.88000011444092
high molecular weight glutenin subunit 1Dx [Triticum spelta]	gi|469469506	89435.1	5.82000017166138
high molecular weight glutenin subunit 1Dx3.1t [Aegilops tauschii]	gi|298264519	88934.9	6.42999982833862
high-molecular-weight glutenin 1Dx2.2 [Triticum aestivum]	gi|399933061	103170.4	5.61000013351 44
high-molecular-weight glutenin subunit [Triticum aestivum x Secale cereale]	gi|528080856	89445.2	5.82000017166138
high-molecular-weight glutenin subunit [Triticum aestivum]	gi|478428127	103169.4	5.75

Peptide Information

Calc. Mass	Obsrv. Mass	± da	± ppm	Start Seq.	End Seq.	Sequence	Ion Score	C. I. %	Modification	Rank	Result Type
1044.532	1044.5123	-0.0197	-19	34	41	ELQELQER					Mascot
1392.6359	1392.6281	-0.0078	-6	45	55	ACQQVMDQQLR			Carbamidomethyl (C)[2], Oxidation (M)[6]		Mascot
1473.7301	1473.7666	0.0365	25	816	828	AQQLAAQLPAMCR			Carbamidomethyl (C)[12], Oxidation (M)[11]		Mascot
1990.9618	1990.96	-0.0018	-1	83	100	GGSFYPGETTPPQQLQQR					Mascot
1990.9618	1990.96	-0.0018	-1	83	100	GGSFYPGETTPPQQLQQR	110	100			Mascot

6	high molecular weight glutenin subunit 1Dx5.3t [Aegilops tauschii]	gi|375066052	75182.4	5.24	3	110	100	4.529	110	100

Protein Group

high molecular weight glutenin subunit 1Dx [Aegilops tauschii]	gi|469469510	89972.3	5.3699 998855 5908

Peptide Information

Calc. Mass	Obsrv. Mass	± da	± ppm	Start Seq.	End Seq.	Sequence	Ion Score	C. I. %	Modification	Rank	Result Type
1392.6359	1392.6281	-0.0078	-6	45	55	ACQQVMDQQLR			Carbamidomethyl (C)[2], Oxidation (M)[6]		Mascot
1473.7301	1473.7666	0.0365	25	680	692	AQQLAAQLPAMCR			Carbamidomethyl (C)[12], Oxidation (M)[11]		Mascot
1990.9618	1990.96	-0.0018	-1	83	100	GGSFYPGETTPPQQLQQR					Mascot
1990.9618	1990.96	-0.0018	-1	83	100	GGSFYPGETTPPQQLQQR	110	100			Mascot

7	high molecular weight glutenin subunit 1Dx [Triticum spelta]	gi|469469500	90547.7	6.88	5	110	100	4.918	110	100

Peptide Information

Calc. Mass	Obsrv. Mass	± da	± ppm	Start Seq.	End Seq.	Sequence	Ion Score	C. I. %	Modification	Rank	Result Type
1044.532	1044.5123	-0.0197	-19	34	41	ELQELQER					Mascot
1392.6359	1392.6281	-0.0078	-6	45	55	ACQQVMDQQLR			Carbamidomethyl (C)[2], Oxidation (M)[6]		Mascot
1473.7301	1473.7666	0.0365	25	825	837	AQQLAAQLPAMCR			Carbamidomethyl (C)[12], Oxidation (M)[11]		Mascot
1524.7441	1524.7439	-0.0002	0	168	180	YYPTSPQQSGQLR					Mascot
1990.9618	1990.96	-0.0018	-1	83	100	GGSFYPGETTPPQQLQQR					Mascot
1990.9618	1990.96	-0.0018	-1	83	100	GGSFYPGETTPPQQLQQR	110	100			Mascot

8	HMW-glutenin subunit Glu-1Stx1 [Triticum aestivum]	gi|430801112	54146.5	6.42	3	110	100	18.085	110	100

Peptide Information

Calc. Mass	Obsrv. Mass	± da	± ppm	Start Seq.	End Seq.	Sequence	Ion Score	C. I. %	Modification	Rank	Result Type
1360.6638	1360.7285	0.0647	48	34	44	GLQERELEACR			Carbamidomethyl (C)[10]		Mascot
1360.6638	1360.7285	0.0647	48	34	44	GLQERELEACR			Carbamidomethyl (C)[10]		Mascot
1473.7301	1473.7666	0.0365	25	482	494	AQQLAAQLPAMCR			Carbamidomethyl (C)[12], Oxidation (M)[11]		Mascot
1990.9618	1990.96	-0.0018	-1	80	97	GGSFYPGETTPPQQLQQR					Mascot
1990.9618	1990.96	-0.0018	-1	80	97	GGSFYPGETTPPQQLQQR	110	100			Mascot

9	high molecular weight glutenin subunit [Elymus sibiricus]	gi|404435987	65690.6	5.6	2	110	100	4.301	110	100

Peptide Information

Calc. Mass	Obsrv. Mass	± da	± ppm	Start Seq.	End Seq.	Sequence	Ion Score	C. I. %	Modification	Rank	Result Type
1473.7301	1473.7666	0.0365	25	583	595	AQQLAAQLPAMCR			Carbamidomethyl (C)[12], Oxidation (M)[11]		Mascot
1990.9618	1990.96	-0.0018	-1	92	109	GGSFYPGETTPPQQLQQR					Mascot
1990.9618	1990.96	-0.0018	-1	92	109	GGSFYPGETTPPQQLQQR	110	100			Mascot

10	hypothetical protein OsI_27846 [Oryza sativa Indica Group]	gi|125560179	43134.3	5.18	7	110	100	2.941	87	100

Peptide Information

Calc. Mass	Obsrv. Mass	± da	± ppm	Start Seq.	End Seq.	Sequence	Ion Score	C. I. %	Modification	Rank	Result Type
890.4578	890.4591	0.0013	1	157	164	VSTEIDAR					Mascot
972.536	972.4761	-0.0599	-62	266	275	GEDAGLALVK					Mascot
1328.6958	1328.6915	-0.0043	-3	197	208	IPATWQGIEASR					Mascot
1328.6958	1328.6915	-0.0043	-3	197	208	IPATWQGIEASR	87	100			Mascot
1621.8479	1621.8403	-0.0076	-5	141	156	ALANVGAEMAHLVPGR			Oxidation (M)[9]		Mascot
1905.0732	1904.9753	-0.0979	-51	299	315	QDVFSLLGIDYIIAPLK					Mascot
2182.2078	2182.0283	-0.1795	-82	2	23	ALSISTPPTSSSLLPASLQVGR					Mascot
2230.2329	2230.1558	-0.0771	-35	88	110	FAPTAATVSSSLLLGIAGLPDTK					Mascot

Rank	Protein Name	Accession No.	Protein MW	Protein PI	Pep. Count	Protein Score	Protein Score C. I. %	Intensity Matched	Total Ion Score	Total Ion C. I. %	Confirmed
1	Adenosine kinase 2 [Aegilops tauschii]	gi|475626178	37646.9	5.02	9	382	100	17.985	340	100	

Peptide Information

Calc. Mass	Obsrv. Mass	± da	± ppm	Start Seq.	End Seq.	Sequence	Ion Score	C. I. %	Modification	Rank	Result Type
1335.625	1335.6327	0.0077	6	46	56	HLPMYDELSSK			Oxidation (M)[4]		Mascot
1424.7202	1424.714	-0.0062	-4	133	145	SLIANLSAANCYK			Carbamidomethyl (C)[11]		Mascot
1772.9177	1772.9188	0.0011	1	232	246	VRGWETENVEEIALK					Mascot
1781.9644	1781.9213	-0.0431	-24	260	277	IAVITQGADPVVVAEDGK					Mascot
1857.9017	1857.9182	0.0165	9	212	227	VLPYADYIFGNETEAR					Mascot
1857.9017	1857.9182	0.0165	9	212	227	VLPYADYIFGNETEAR	141	100			Mascot
1938.0656	1938.037	-0.0286	-15	259	277	RIAVITQGADPVVVAEDG K					Mascot
1990.9918	1990.9954	0.0036	2	191	206	VFLMNLSAPFICEFFR			Carbamidomethyl (C)[12]		Mascot
2006.9867	2006.991	0.0043	2	191	206	VFLMNLSAPFICEFFR			Carbamidomethyl (C)[12], Oxidation (M)[4]		Mascot
2703.2178	2703.2292	0.0114	4	321	344	AGCYAANVIIQQSGCTYP EKPDFN			Carbamidomethyl (C)[3,15]		Mascot
2911.2986	2911.3374	0.0388	13	105	132	AAGVTAHYYEDEAAPTG TCAVCVVGGER			Carbamidomethyl (C)[19,22]		Mascot
2911.2986	2911.3374	0.0388	13	105	132	AAGVTAHYYEDEAAPTG TCAVCVVGGER	199	100	Carbamidomethyl (C)[19,22]		Mascot

Rank	Protein Name	Accession No.	Protein MW	Protein PI	Pep. Count	Protein Score	Protein Score C. I. %	Intensity Matched	Total Ion Score	Total Ion C. I. %	Confirmed
2	Adenosine kinase 2 [Triticum urartu]	gi|474049015	37679.9	5.01	8	235	100	6.086	199	100	

Peptide Information

Calc. Mass	Obsrv. Mass	± da	± ppm	Start Seq.	End Seq.	Sequence	Ion Score	C. I. %	Modification	Rank	Result Type
1335.625	1335.6327	0.0077	6	46	56	HLPMYDELSSK			Oxidation (M)[4]		Mascot
1424.7202	1424.714	-0.0062	-4	133	145	SLIANLSAANCYK			Carbamidomethyl (C)[11]		Mascot
1772.9177	1772.9188	0.0011	1	232	246	VRGWETENVEEIALK					Mascot
1781.9644	1781.9213	-0.0431	-24	260	277	IAVITQGADPVVVAEDGK					Mascot
1938.0656	1938.037	-0.0286	-15	259	277	RIAVITQGADPVVVAEDG K					Mascot
1990.9918	1990.9954	0.0036	2	191	206	VFLMNLSAPFICEFFR			Carbamidomethyl (C)[12]		Mascot
2006.9867	2006.991	0.0043	2	191	206	VFLMNLSAPFICEFFR			Carbamidomethyl (C)[12], Oxidation (M)[4]		Mascot
2703.2178	2703.2292	0.0114	4	321	344	AGCYAANVIIQQSGCTYP EKPDFN			Carbamidomethyl (C)[3,15]		Mascot
2911.2986	2911.3374	0.0388	13	105	132	AAGVTAHYYEDEAAPTG			Carbamidomethyl (C)[19,22]		Mascot

						TCAVCVVGGER					
2911.2986	2911.3374	0.0388	13	105	132	AAGVTAHYYEDEAAPTG TCAVCVVGGER	199	100	Carbamidomethyl (C)[19,22]		Mascot

3	PREDICTED: adenosine kinase 2-like [Setaria italica]	gi|514802694	37785.1	5.4	8	211	100	25.779	180	100	

Peptide Information

Calc. Mass	Obsrv. Mass	± da	± ppm	Start Seq.	End Seq.	Sequence	Ion Score	C. I. %	Modification	Rank	Result Type
1252.6644	1252.7351	0.0707	56	247	258	ISQLPNASGTHK					Mascot
1252.6644	1252.7351	0.0707	56	247	258	ISQLPNASGTHK					Mascot
1303.6351	1303.7012	0.0661	51	46	56	HLPMYDELASK					Mascot
1335.6838	1335.6327	-0.0511	-38	321	332	AACYAANVVIQR			Carbamidomethyl (C)[3]		Mascot
1424.7202	1424.714	-0.0062	-4	133	145	SLIANLSAANCYK			Carbamidomethyl (C)[11]		Mascot
1649.8242	1649.8385	0.0143	9	57	72	GNVEYIAGGATQNSIR					Mascot
1649.8242	1649.8385	0.0143	9	57	72	GNVEYIAGGATQNSIR	97	100			Mascot
1772.9177	1772.9188	0.0011	1	232	246	VRGWETENVEEIALK					Mascot
1857.9017	1857.9182	0.0165	9	212	227	ALPYVDYIFGNETEAR					Mascot
1857.9017	1857.9182	0.0165	9	212	227	ALPYVDYIFGNETEAR	83	100			Mascot
1990.9918	1990.9954	0.0036	2	191	206	VFLMNLSAPFICEFFR			Carbamidomethyl (C)[12]		Mascot
2006.9867	2006.991	0.0043	2	191	206	VFLMNLSAPFICEFFR			Carbamidomethyl (C)[12], Oxidation (M)[4]		Mascot

4	hypothetical protein OsI_14458 [Oryza sativa Indica Group]	gi|218194174	42731.3	5.83	15	128	100	16.006	52	98.741	

Peptide Information

Calc. Mass	Obsrv. Mass	± da	± ppm	Start Seq.	End Seq.	Sequence	Ion Score	C. I. %	Modification	Rank	Result Type
853.4414	853.4326	-0.0088	-10	359	366	TQVAYGSK					Mascot
921.4524	921.455	0.0026	3	249	257	DTTTIGEGK					Mascot
937.456	937.4582	0.0022	2	258	265	MFSPGNLR			Oxidation (M)[1]		Mascot
959.5269	959.5163	-0.0106	-11	53	61	SLPSGRQSK					Mascot
1188.5532	1188.5646	0.0114	10	372	381	FEETLYGSSR					Mascot
1199.5725	1199.5977	0.0252	21	1	11	METVAAASYTR					Mascot
1225.5737	1225.5691	-0.0046	-4	94	103	LLICMGEAMR			Carbamidomethyl (C)[4], Oxidation (M)[5,9]		Mascot
1235.6631	1235.6394	-0.0237	-19	306	317	GIFTNVTSPTAK					Mascot
1588.9349	1588.8877	-0.0472	-30	322	335	LLFEVAPLGFLIEK					Mascot
1608.8091	1608.8002	-0.0089	-6	137	149	LLFEALEYSHVCK			Carbamidomethyl (C)[12]		Mascot
1649.8568	1649.8385	-0.0183	-11	289	303	YTGGMVPDVNQIIVK			Oxidation (M)[5]		Mascot
1649.8568	1649.8385	-0.0183	-11	289	303	YTGGMVPDVNQIIVK			Oxidation (M)[5]		Mascot
1671.8119	1671.7992	-0.0127	-8	1	16	METVAAASYTRGAATR			Oxidation (M)[1]		Mascot

1730.8054	1730.8131	0.0077	4	229	243	DCPGTHEFLLLDEGK			Carbamidomethyl (C)[2]		Mascot
1906.9944	1907.0732	0.0788	41	289	305	YTGGMVPDVNQIIVKEK			Oxidation (M)[5]		Mascot
1933.9801	1934.0062	0.0261	13	201	220	LTGVTGGDQVAAAMGIY GPR					Mascot
1949.975	1949.9709	-0.0041	-2	201	220	LTGVTGGDQVAAAMGIY GPR			Oxidation (M)[14]		Mascot
1949.975	1949.9709	-0.0041	-2	201	220	LTGVTGGDQVAAAMGIY GPR	52	98.741	Oxidation (M)[14]		Mascot

5	RecName: Full=Sedoheptulose-1,7-bisphosphatase, chloroplastic; AltName: Full=SED(1,7)P2ase; AltName: Full=Sedoheptulose bisphosphatase; Short=SBPase; Flags: Precursor	gi|1173347	42547.2	6.04	15	126	100	16.784	52	98.741		

Peptide Information

Calc. Mass	Obsrv. Mass	± da	± ppm	Start Seq.	End Seq.	Sequence	Ion Score	C. I. %	Modification	Rank	Result Type
853.4414	853.4326	-0.0088	-10	360	367	TQVAYGSK					Mascot
921.4611	921.455	-0.0061	-7	259	266	MFSPGNLR					Mascot
930.5255	930.532	0.0065	7	352	359	VISVLDER					Mascot
937.456	937.4582	0.0022	2	259	266	MFSPGNLR			Oxidation (M)[1]		Mascot
1185.5059	1185.5493	0.0434	37	267	276	ATFDNPDYDK					Mascot
1188.5532	1188.5646	0.0114	10	373	382	FEETLYGSSR					Mascot
1225.5737	1225.5691	-0.0046	-4	95	104	LLICMGEAMR			Carbamidomethyl (C)[4], Oxidation (M)[5,9]		Mascot
1235.6631	1235.6394	-0.0237	-19	307	318	GIFTNVTSPTAK					Mascot
1585.797	1585.8354	0.0384	24	245	258	WQHVKDTTSIGEGK					Mascot
1588.9349	1588.8877	-0.0472	-30	323	336	LLFEVAPLGFLIEK					Mascot
1608.8091	1608.8002	-0.0089	-6	138	150	LLFEALEYSHVCK			Carbamidomethyl (C)[12]		Mascot
1649.8568	1649.8385	-0.0183	-11	290	304	YTGGMVPDVNQIIVK			Oxidation (M)[5]		Mascot
1649.8568	1649.8385	-0.0183	-11	290	304	YTGGMVPDVNQIIVK			Oxidation (M)[5]		Mascot
1730.8054	1730.8131	0.0077	4	230	244	DCPGTHEFLLLDEGK			Carbamidomethyl (C)[2]		Mascot
1906.9944	1907.0732	0.0788	41	290	306	YTGGMVPDVNQIIVKEK			Oxidation (M)[5]		Mascot
1933.9801	1934.0062	0.0261	13	202	221	LTGVTGGDQVAAAMGIY GPR					Mascot
1949.975	1949.9709	-0.0041	-2	202	221	LTGVTGGDQVAAAMGIY GPR			Oxidation (M)[14]		Mascot
1949.975	1949.9709	-0.0041	-2	202	221	LTGVTGGDQVAAAMGIY GPR	52	98.741	Oxidation (M)[14]		Mascot
2064.9915	2064.97	-0.0215	-10	267	283	ATFDNPDYDKLVNYYVK					Mascot

6	Sedoheptulose-1,7-bisphosphatase, chloroplastic [Aegilops tauschii]	gi|475576723	65033.4	5.36	18	125	100	17.231	52	98.741		

Peptide Information

Calc. Mass	Obsrv. Mass	± da	± ppm	Start	End	Sequence	Ion	C. I. %	Modification	Rank	Result Type

				Seq.	Seq.		Score				
853.4414	853.4326	-0.0088	-10	545	552	TQVAYGSK					Mascot
921.4524	921.455	0.0026	3	435	443	DTTTIGEGK					Mascot
937.456	937.4582	0.0022	2	444	451	MFSPGNLR			Oxidation (M)[1]		Mascot
959.5156	959.5163	0.0007	1	537	544	VISQLDER					Mascot
1185.5059	1185.5493	0.0434	37	452	461	ATFDNPDYDK					Mascot
1188.5532	1188.5646	0.0114	10	558	567	FEETLYGSSR					Mascot
1225.5737	1225.5691	-0.0046	-4	280	289	LLICMGEAMR			Carbamidomethyl (C)[4], Oxidation (M)[5,9]		Mascot
1235.6631	1235.6394	-0.0237	-19	492	503	GIFTNVTSPTAK					Mascot
1335.6322	1335.6327	0.0005	0	90	101	NDPNTSKMLGSR			Oxidation (M)[8]		Mascot
1423.7648	1423.8723	0.1075	76	49	60	MVLMTSVLLTQR			Oxidation (M)[1,4]		Mascot
1588.9349	1588.8877	-0.0472	-30	508	521	LLFEVAPLGFLIEK					Mascot
1608.8091	1608.8002	-0.0089	-6	323	335	LLFEALEYSHVCK			Carbamidomethyl (C)[12]		Mascot
1649.8568	1649.8385	-0.0183	-11	475	489	YTGGMVPDVNQIIVK			Oxidation (M)[5]		Mascot
1649.8568	1649.8385	-0.0183	-11	475	489	YTGGMVPDVNQIIVK			Oxidation (M)[5]		Mascot
1730.8054	1730.8131	0.0077	4	415	429	DCPGTHEFLLLDEGK			Carbamidomethyl (C)[2]		Mascot
1772.9404	1772.9188	-0.0216	-12	208	221	VDLVQYYLNVFLMR					Mascot
1788.9353	1788.9181	-0.0172	-10	208	221	VDLVQYYLNVFLMR			Oxidation (M)[13]		Mascot
1906.9944	1907.0732	0.0788	41	475	491	YTGGMVPDVNQIIVKEK			Oxidation (M)[5]		Mascot
1933.9801	1934.0062	0.0261	13	387	406	LTGVTGGDQVAAAMGIY GPR					Mascot
1949.975	1949.9709	-0.0041	-2	387	406	LTGVTGGDQVAAAMGIY GPR			Oxidation (M)[14]		Mascot
1949.975	1949.9709	-0.0041	-2	387	406	LTGVTGGDQVAAAMGIY GPR	52	98.741	Oxidation (M)[14]		Mascot
2064.9915	2064.97	-0.0215	-10	452	468	ATFDNPDYDKLVNYYVK					Mascot

7	Sedoheptulose-1,7-bisphosphatase, chloroplastic [Triticum urartu]	gi|474425491	40102.1	5.74	14	123	100	16.016	52	98.741	

Peptide Information

Calc. Mass	Obsrv. Mass	± da	± ppm	Start Seq.	End Seq.	Sequence	Ion Score	C. I. %	Modification	Rank	Result Type
853.4414	853.4326	-0.0088	-10	336	343	TQVAYGSK					Mascot
921.4611	921.455	-0.0061	-7	236	243	MFSPGNLR					Mascot
930.5255	930.532	0.0065	7	328	335	VISVLDER					Mascot
937.456	937.4582	0.0022	2	236	243	MFSPGNLR			Oxidation (M)[1]		Mascot
1185.5059	1185.5493	0.0434	37	244	253	ATFDNPDYDK					Mascot
1188.5532	1188.5646	0.0114	10	349	358	FEETLYGSSR					Mascot
1225.5737	1225.5691	-0.0046	-4	72	81	LLICMGEAMR			Carbamidomethyl (C)[4], Oxidation (M)[5,9]		Mascot

Calc. Mass	Obsrv. Mass	± da	± ppm	Start Seq.	End Seq.	Sequence	Ion Score	C. I. %	Modification	Rank	Result Type
1235.6631	1235.6394	-0.0237	-19	285	296	GIFTNVTSPTAK					Mascot
1585.797	1585.8354	0.0384	24	222	235	WQHVKDTTSIGEGK					Mascot
1588.9349	1588.8877	-0.0472	-30	299	312	LLFEVAPLGFLIEK					Mascot
1608.8091	1608.8002	-0.0089	-6	115	127	LLFEALEYSHVCK			Carbamidomethyl (C)[12]		Mascot
1649.8568	1649.8385	-0.0183	-11	268	282	YTGGMVPDVNQIIVK			Oxidation (M)[5]		Mascot
1649.8568	1649.8385	-0.0183	-11	268	282	YTGGMVPDVNQIIVK			Oxidation (M)[5]		Mascot
1730.8054	1730.8131	0.0077	4	207	221	DCPGTHEFLLLDEGK			Carbamidomethyl (C)[2]		Mascot
1906.9944	1907.0732	0.0788	41	268	284	YTGGMVPDVNQIIVKEK			Oxidation (M)[5]		Mascot
1933.9801	1934.0062	0.0261	13	179	198	LTGVTGGDQVAAAMGIY GPR					Mascot
1949.975	1949.9709	-0.0041	-2	179	198	LTGVTGGDQVAAAMGIY GPR			Oxidation (M)[14]		Mascot
1949.975	1949.9709	-0.0041	-2	179	198	LTGVTGGDQVAAAMGIY GPR	52	98.741	Oxidation (M)[14]		Mascot

8 Os04g0234600 [Oryza sativa Japonica Group] gi|113563864 42731.3 5.64 14 121 100 16.006 52 98.741

Peptide Information

Calc. Mass	Obsrv. Mass	± da	± ppm	Start Seq.	End Seq.	Sequence	Ion Score	C. I. %	Modification	Rank	Result Type
853.4414	853.4326	-0.0088	-10	359	366	TQVAYGSK					Mascot
921.4611	921.455	-0.0061	-7	258	265	MFSPGNLR					Mascot
937.456	937.4582	0.0022	2	258	265	MFSPGNLR			Oxidation (M)[1]		Mascot
959.5269	959.5163	-0.0106	-11	53	61	SLPSGRQSK					Mascot
1188.5532	1188.5646	0.0114	10	372	381	FEETLYGSSR					Mascot
1199.5725	1199.5977	0.0252	21	1	11	METVAAASYTR					Mascot
1225.5737	1225.5691	-0.0046	-4	94	103	LLICMGEAMR			Carbamidomethyl (C)[4], Oxidation (M)[5,9]		Mascot
1235.6631	1235.6394	-0.0237	-19	306	317	GIFTNVTSPTAK					Mascot
1588.9349	1588.8877	-0.0472	-30	322	335	LLFEVAPLGFLIEK					Mascot
1608.8091	1608.8002	-0.0089	-6	137	149	LLFEALEYSHVCK			Carbamidomethyl (C)[12]		Mascot
1649.8568	1649.8385	-0.0183	-11	289	303	YTGGMVPDVNQIIVK			Oxidation (M)[5]		Mascot
1649.8568	1649.8385	-0.0183	-11	289	303	YTGGMVPDVNQIIVK			Oxidation (M)[5]		Mascot
1671.8119	1671.7992	-0.0127	-8	1	16	METVAAASYTRGAATR			Oxidation (M)[1]		Mascot
1730.8054	1730.8131	0.0077	4	229	243	DCPGTHEFLLLDEGK			Carbamidomethyl (C)[2]		Mascot
1906.9944	1907.0732	0.0788	41	289	305	YTGGMVPDVNQIIVKEK			Oxidation (M)[5]		Mascot
1933.9801	1934.0062	0.0261	13	201	220	LTGVTGGDQVAAAMGIY GPR					Mascot
1949.975	1949.9709	-0.0041	-2	201	220	LTGVTGGDQVAAAMGIY GPR			Oxidation (M)[14]		Mascot
1949.975	1949.9709	-0.0041	-2	201	220	LTGVTGGDQVAAAMGIY GPR	52	98.741	Oxidation (M)[14]		Mascot

9 Os02g0625500, partial [Oryza sativa Japonica Group] gi|255671104 41220 5.57 5 110 100 10.659 97 100

Peptide Information

Calc. Mass	Obsrv. Mass	± da	± ppm	Start Seq.	End Seq.	Sequence	Ion Score	C. I. %	Modification	Rank	Result Type
1303.6351	1303.7012	0.0661	51	78	88	HLPMYDELASK					Mascot
1335.6838	1335.6327	-0.0511	-38	353	364	AGCYAANVIIQR			Carbamidomethyl (C)[3]		Mascot
1649.8242	1649.8385	0.0143	9	89	104	GNVEYIAGGATQNSIR					Mascot
1649.8242	1649.8385	0.0143	9	89	104	GNVEYIAGGATQNSIR	97	100			Mascot
1772.9177	1772.9188	0.0011	1	264	278	VRGWETENVEEIALK					Mascot
1990.9918	1990.9954	0.0036	2	223	238	VFLMNLSAPFICEFFR			Carbamidomethyl (C)[12]		Mascot
2006.9867	2006.991	0.0043	2	223	238	VFLMNLSAPFICEFFR			Carbamidomethyl (C)[12], Oxidation (M)[4]		Mascot

10	hypothetical protein OsI_08139 [Oryza sativa Indica Group]	gi|125540364	34612.5	5.02	4	107	99.998	10.457	97	100	

Peptide Information

Calc. Mass	Obsrv. Mass	± da	± ppm	Start Seq.	End Seq.	Sequence	Ion Score	C. I. %	Modification	Rank	Result Type
1303.6351	1303.7012	0.0661	51	43	53	HLPMYDELASK					Mascot
1335.6838	1335.6327	-0.0511	-38	296	307	AGCYAANVIIQR			Carbamidomethyl (C)[3]		Mascot
1649.8242	1649.8385	0.0143	9	54	69	GNVEYIAGGATQNSIR					Mascot
1649.8242	1649.8385	0.0143	9	54	69	GNVEYIAGGATQNSIR	97	100			Mascot
1990.9918	1990.9954	0.0036	2	188	203	VFLMNLSAPFICEFFR			Carbamidomethyl (C)[12]		Mascot
2006.9867	2006.991	0.0043	2	188	203	VFLMNLSAPFICEFFR			Carbamidomethyl (C)[12], Oxidation (M)[4]		Mascot

Gel Idx/Pos	217/I17	Instr./Gel Origin	BA2151/Sample Project 20140814	Process Status	Analysis Succeeded
Plate [#] Name	[1] Sample Project 20140814	Instrument Sample Name		Spectra	11

Rank	Protein Name	Accession No.	Protein MW	Protein PI	Pep. Count	Protein Score	Protein Score C. I. %	Intensity Matched	Total Ion Score	Total Ion C. I. %	Confirmed
1	Adenosine kinase 2 [Aegilops tauschii]	gi|475626178	37646.9	5.02	12	551	100	45.496	485	100	

Peptide Information

Calc. Mass	Obsrv. Mass	± da	± ppm	Start Seq.	End Seq.	Sequence	Ion Score	C. I. %	Modification	Rank	Result Type
1027.655	1027.6213	-0.0337	-33	280	288	TFPVILLPK					Mascot
1213.6576	1213.661	0.0034	3	151	160	KPENWALVEK					Mascot
1424.7202	1424.7365	0.0163	11	133	145	SLIANLSAANCYK			Carbamidomethyl (C)[11]		Mascot
1517.7483	1517.7593	0.011	7	234	246	GWETENVEEIALK					Mascot
1721.8453	1721.8636	0.0183	11	57	72	ENVEYIAGGATQNSIR					Mascot
1721.8453	1721.8636	0.0183	11	57	72	ENVEYIAGGATQNSIR	71	99.987			Mascot
1772.9177	1772.938	0.0203	11	232	246	VRGWETENVEEIALK					Mascot
1772.9177	1772.938	0.0203	11	232	246	VRGWETENVEEIALK					Mascot
1857.9017	1857.9213	0.0196	11	212	227	VLPYADYIFGNETEAR					Mascot
1857.9017	1857.9213	0.0196	11	212	227	VLPYADYIFGNETEAR	144	100			Mascot
1938.0656	1938.075	0.0094	5	259	277	RIAVITQGADPVVVAEDG K					Mascot
1990.9918	1991.0117	0.0199	10	191	206	VFLMNLSAPFICEFFR			Carbamidomethyl (C)[12]		Mascot
2006.9867	2006.9976	0.0109	5	191	206	VFLMNLSAPFICEFFR			Carbamidomethyl (C)[12], Oxidation (M)[4]		Mascot
2006.9867	2006.9976	0.0109	5	191	206	VFLMNLSAPFICEFFR	70	99.984	Carbamidomethyl (C)[12], Oxidation (M)[4]		Mascot
2293.1824	2293.1672	-0.0152	-7	291	313	LVDTNGAGDAFVGGFLS QLVQGK					Mascot
2703.2178	2703.2261	0.0083	3	321	344	AGCYAANVIIQQSGCTYP EKPDFN			Carbamidomethyl (C)[3,15]		Mascot
2911.2986	2911.344	0.0454	16	105	132	AAGVTAHYYEDEAAPTG TCAVCVVGGER			Carbamidomethyl (C)[19,22]		Mascot
2911.2986	2911.344	0.0454	16	105	132	AAGVTAHYYEDEAAPTG TCAVCVVGGER	199	100	Carbamidomethyl (C)[19,22]		Mascot

Rank	Protein Name	Accession No.	Protein MW	Protein PI	Pep. Count	Protein Score	Protein Score C. I. %	Intensity Matched	Total Ion Score	Total Ion C. I. %	Confirmed
2	Adenosine kinase 2 [Triticum urartu]	gi|474049015	37679.9	5.01	12	462	100	19.39	395	100	

Peptide Information

Calc. Mass	Obsrv. Mass	± da	± ppm	Start Seq.	End Seq.	Sequence	Ion Score	C. I. %	Modification	Rank	Result Type
1027.655	1027.6213	-0.0337	-33	280	288	TFPVILLPK					Mascot
1213.6576	1213.661	0.0034	3	151	160	KPENWALVEK					Mascot
1424.7202	1424.7365	0.0163	11	133	145	SLIANLSAANCYK			Carbamidomethyl (C)[11]		Mascot

1517.7483	1517.7593	0.011	7	234	246	GWETENVEEIALK					Mascot
1707.8297	1707.8503	0.0206	12	57	72	DNVEYIAGGATQNSIR	125	100			Mascot
1772.9177	1772.938	0.0203	11	232	246	VRGWETENVEEIALK					Mascot
1772.9177	1772.938	0.0203	11	232	246	VRGWETENVEEIALK					Mascot
1938.0656	1938.075	0.0094	5	259	277	RIAVITQGADPVVVAEDG K					Mascot
1990.9918	1991.0117	0.0199	10	191	206	VFLMNLSAPFICEFFR			Carbamidomethyl (C)[12]		Mascot
2006.9867	2006.9976	0.0109	5	191	206	VFLMNLSAPFICEFFR			Carbamidomethyl (C)[12], Oxidation (M)[4]		Mascot
2006.9867	2006.9976	0.0109	5	191	206	VFLMNLSAPFICEFFR	70	99.984	Carbamidomethyl (C)[12], Oxidation (M)[4]		Mascot
2293.1824	2293.1672	-0.0152	-7	291	313	LVDTNGAGDAFVGGFLS QLVQGK					Mascot
2703.2178	2703.2261	0.0083	3	321	344	AGCYAANVIIQQSGCTYP EKPDFN			Carbamidomethyl (C)[3,15]		Mascot
2911.2986	2911.344	0.0454	16	105	132	AAGVTAHYYEDEAAPTG TCAVCVVGGER			Carbamidomethyl (C)[19,22]		Mascot
2911.2986	2911.344	0.0454	16	105	132	AAGVTAHYYEDEAAPTG TCAVCVVGGER	199	100	Carbamidomethyl (C)[19,22]		Mascot
3024.4368	3024.4753	0.0385	13	46	72	HLPMYDELSSKDNVEYIA GGATQNSIR			Oxidation (M)[4]		Mascot

3	PREDICTED: adenosine kinase 2-like [Setaria italica]	gi|514802694	37785.1	5.4	8	185	100	29.653	156	100

Peptide Information

Calc. Mass	Obsrv. Mass	± da	± ppm	Start Seq.	End Seq.	Sequence	Ion Score	C. I. %	Modification	Rank	Result Type
1027.655	1027.6213	-0.0337	-33	280	288	TFPVILLPK					Mascot
1303.6351	1303.7041	0.069	53	46	56	HLPMYDELASK					Mascot
1424.7202	1424.7365	0.0163	11	133	145	SLIANLSAANCYK			Carbamidomethyl (C)[11]		Mascot
1517.7483	1517.7593	0.011	7	234	246	GWETENVEEIALK					Mascot
1649.8242	1649.8367	0.0125	8	57	72	GNVEYIAGGATQNSIR					Mascot
1772.9177	1772.938	0.0203	11	232	246	VRGWETENVEEIALK					Mascot
1772.9177	1772.938	0.0203	11	232	246	VRGWETENVEEIALK					Mascot
1857.9017	1857.9213	0.0196	11	212	227	ALPYVDYIFGNETEAR					Mascot
1857.9017	1857.9213	0.0196	11	212	227	ALPYVDYIFGNETEAR	85	100			Mascot
1990.9918	1991.0117	0.0199	10	191	206	VFLMNLSAPFICEFFR			Carbamidomethyl (C)[12]		Mascot
2006.9867	2006.9976	0.0109	5	191	206	VFLMNLSAPFICEFFR			Carbamidomethyl (C)[12], Oxidation (M)[4]		Mascot
2006.9867	2006.9976	0.0109	5	191	206	VFLMNLSAPFICEFFR	70	99.984	Carbamidomethyl (C)[12], Oxidation (M)[4]		Mascot

4	TPA: hypothetical protein ZEAMMB73_693307 [Zea mays]	gi|414586350	29684.9	5.63	7	114	100	25.773	85	100

Peptide Information

Calc. Mass	Obsrv. Mass	± da	± ppm	Start Seq.	End Seq.	Sequence	Ion Score	C. I. %	Modification	Rank	Result Type

1027.655	1027.6213	-0.0337	-33	204	212	TFPVILLPK					Mascot
1213.6576	1213.661	0.0034	3	75	84	KPENWALVEK					Mascot
1424.7202	1424.7365	0.0163	11	57	69	SLIANLSAANCYK			Carbamidomethyl (C)[11]		Mascot
1517.7483	1517.7593	0.011	7	158	170	GWETENVEEIALK					Mascot
1772.9177	1772.938	0.0203	11	156	170	VRGWETENVEEIALK					Mascot
1772.9177	1772.938	0.0203	11	156	170	VRGWETENVEEIALK					Mascot
1857.9017	1857.9213	0.0196	11	136	151	ALPYVDYIFGNETEAR					Mascot
1857.9017	1857.9213	0.0196	11	136	151	ALPYVDYIFGNETEAR	85	100			Mascot
1937.9824	1938.075	0.0926	48	1	18	MLQIPGASSYIGCIGKDK			Carbamidomethyl (C)[13]		Mascot

5 PREDICTED: adenosine kinase 2-like [Setaria italica] gi|514715060 37511 5.15 8 113 100 28.444 82 99.999

Peptide Information

Calc. Mass	Obsrv. Mass	± da	± ppm	Start Seq.	End Seq.	Sequence	Ion Score	C. I. %	Modification	Rank	Result Type
1027.655	1027.6213	-0.0337	-33	278	286	TFPVILLPK					Mascot
1303.6351	1303.7041	0.069	53	44	54	HLPMYDELASK					Mascot
1424.7202	1424.7365	0.0163	11	131	143	SLIANLSAANCYK			Carbamidomethyl (C)[11]		Mascot
1786.9333	1786.8899	-0.0434	-24	230	244	VRGWETENIEEIALK					Mascot
1857.927	1857.9213	-0.0057	-3	210	225	VLPYVDYIFGNETEAK					Mascot
1857.927	1857.9213	-0.0057	-3	210	225	VLPYVDYIFGNETEAK	11	0			Mascot
1938.0656	1938.075	0.0094	5	257	275	RIAVITQGADPVVVAEDG K					Mascot
1990.9918	1991.0117	0.0199	10	189	204	VFLMNLSAPFICEFFR			Carbamidomethyl (C)[12]		Mascot
2006.9867	2006.9976	0.0109	5	189	204	VFLMNLSAPFICEFFR			Carbamidomethyl (C)[12], Oxidation (M)[4]		Mascot
2006.9867	2006.9976	0.0109	5	189	204	VFLMNLSAPFICEFFR	70	99.984	Carbamidomethyl (C)[12], Oxidation (M)[4]		Mascot
2293.1824	2293.1672	-0.0152	-7	289	311	LVDTNGAGDAFVGGFLS QLVQGK					Mascot

6 Os02g0625500, partial [Oryza sativa Japonica Group] gi|255671104 41220 5.57 8 97 99.982 5.872 70 99.984

Peptide Information

Calc. Mass	Obsrv. Mass	± da	± ppm	Start Seq.	End Seq.	Sequence	Ion Score	C. I. %	Modification	Rank	Result Type
1027.5895	1027.6213	0.0318	31	1	9	SLLLTSTHR					Mascot
1213.6576	1213.661	0.0034	3	183	192	KPENWALVEK					Mascot
1303.6351	1303.7041	0.069	53	78	88	HLPMYDELASK					Mascot
1517.7483	1517.7593	0.011	7	266	278	GWETENVEEIALK					Mascot
1649.8242	1649.8367	0.0125	8	89	104	GNVEYIAGGATQNSIR					Mascot
1772.9177	1772.938	0.0203	11	264	278	VRGWETENVEEIALK					Mascot
1772.9177	1772.938	0.0203	11	264	278	VRGWETENVEEIALK					Mascot

Calc. Mass	Obsrv. Mass	± da	± ppm	Start Seq.	End Seq.	Sequence	Ion Score	C. I. %	Modification	Rank	Result Type
1869.9382	1869.9657	0.0275	15	244	259	VLPFVDYIFGNETEAR					Mascot
1990.9918	1991.0117	0.0199	10	223	238	VFLMNLSAPFICEFFR			Carbamidomethyl (C)[12]		Mascot
2006.9867	2006.9976	0.0109	5	223	238	VFLMNLSAPFICEFFR			Carbamidomethyl (C)[12], Oxidation (M)[4]		Mascot
2006.9867	2006.9976	0.0109	5	223	238	VFLMNLSAPFICEFFR	70	99.984	Carbamidomethyl (C)[12], Oxidation (M)[4]		Mascot

7	hypothetical protein SORBIDRAFT_06g022730 [Sorghum bicolor]	gi|241939375	37901	5.23	5	97	99.981	24.508	85	100

Peptide Information

Calc. Mass	Obsrv. Mass	± da	± ppm	Start Seq.	End Seq.	Sequence	Ion Score	C. I. %	Modification	Rank	Result Type
1027.655	1027.6213	-0.0337	-33	280	288	TFPVILLPK					Mascot
1213.6576	1213.661	0.0034	3	151	160	KPENWALVEK					Mascot
1303.6351	1303.7041	0.069	53	46	56	HLPMYDELASK					Mascot
1424.7202	1424.7365	0.0163	11	133	145	SLIANLSAANCYK			Carbamidomethyl (C)[11]		Mascot
1857.9017	1857.9213	0.0196	11	212	227	ALPYVDYIFGNETEAR					Mascot
1857.9017	1857.9213	0.0196	11	212	227	ALPYVDYIFGNETEAR	85	100			Mascot

8	adenosine kinase [Zea mays]	gi|413937853	37539	5.15	7	94	99.965	4.711	70	99.984

Peptide Information

Calc. Mass	Obsrv. Mass	± da	± ppm	Start Seq.	End Seq.	Sequence	Ion Score	C. I. %	Modification	Rank	Result Type
1027.655	1027.6213	-0.0337	-33	278	286	TFPVILLPK					Mascot
1303.6351	1303.7041	0.069	53	44	54	HLPMYDELASK					Mascot
1424.7202	1424.7365	0.0163	11	131	143	SLIANLSAANCYK			Carbamidomethyl (C)[11]		Mascot
1786.9333	1786.8899	-0.0434	-24	230	244	VRGWETENIEEIALK					Mascot
1829.8956	1829.9071	0.0115	6	210	225	VLPYADYIFGNETEAK					Mascot
1938.0656	1938.075	0.0094	5	257	275	RIAVITQGADPVVVAEDG K					Mascot
1990.9918	1991.0117	0.0199	10	189	204	VFLMNLSAPFICEFFR			Carbamidomethyl (C)[12]		Mascot
2006.9867	2006.9976	0.0109	5	189	204	VFLMNLSAPFICEFFR			Carbamidomethyl (C)[12], Oxidation (M)[4]		Mascot
2006.9867	2006.9976	0.0109	5	189	204	VFLMNLSAPFICEFFR	70	99.984	Carbamidomethyl (C)[12], Oxidation (M)[4]		Mascot

9	hypothetical protein OsI_08139 [Oryza sativa Indica Group]	gi|125540364	34612.5	5.02	5	84	99.612	4.384	70	99.984

Peptide Information

Calc. Mass	Obsrv. Mass	± da	± ppm	Start Seq.	End Seq.	Sequence	Ion Score	C. I. %	Modification	Rank	Result Type
1027.655	1027.6213	-0.0337	-33	255	263	TFPVILLPK					Mascot

1213.6576	1213.661	0.0034	3	148	157	KPENWALVEK					Mascot
1303.6351	1303.7041	0.069	53	43	53	HLPMYDELASK					Mascot
1649.8242	1649.8367	0.0125	8	54	69	GNVEYIAGGATQNSIR					Mascot
1990.9918	1991.0117	0.0199	10	188	203	VFLMNLSAPFICEFFR			Carbamidomethyl (C)[12]		Mascot
2006.9867	2006.9976	0.0109	5	188	203	VFLMNLSAPFICEFFR			Carbamidomethyl (C)[12], Oxidation (M)[4]		Mascot
2006.9867	2006.9976	0.0109	5	188	203	VFLMNLSAPFICEFFR	70	99.984	Carbamidomethyl (C)[12], Oxidation (M)[4]		Mascot

10 hypothetical protein ZEAMMB73_112159 [Zea mays] gi|413937854 27539.6 5.17 3 79 98.687 4.161 70 99.984

Peptide Information

Calc. Mass	Obsrv. Mass	± da	± ppm	Start Seq.	End Seq.	Sequence	Ion Score	C. I. %	Modification	Rank	Result Type
1303.6351	1303.7041	0.069	53	44	54	HLPMYDELASK					Mascot
1424.7202	1424.7365	0.0163	11	131	143	SLIANLSAANCYK			Carbamidomethyl (C)[11]		Mascot
1990.9918	1991.0117	0.0199	10	189	204	VFLMNLSAPFICEFFR			Carbamidomethyl (C)[12]		Mascot
2006.9867	2006.9976	0.0109	5	189	204	VFLMNLSAPFICEFFR			Carbamidomethyl (C)[12], Oxidation (M)[4]		Mascot
2006.9867	2006.9976	0.0109	5	189	204	VFLMNLSAPFICEFFR	70	99.984	Carbamidomethyl (C)[12], Oxidation (M)[4]		Mascot

Gel Idx/Pos	218/I18	Instr./Gel Origin	BA2151/Sample Project 20140814	Process Status	Analysis Succeeded
Plate [#] Name	[1] Sample Project 20140814	Instrument Sample Name		Spectra	11

Rank	Protein Name	Accession No.	Protein MW	Protein PI	Pep. Count	Protein Score	Protein Score C. I. %	Intensity Matched	Total Ion Score	Total Ion C. I. %	Confirmed
1	40S ribosomal protein SA [Aegilops tauschii]	gi|475604728	33448.5	4.9	15	469	100	55.41	374	100	

Peptide Information

Calc. Mass	Obsrv. Mass	± da	± ppm	Start Seq.	End Seq.	Sequence	Ion Score	C. I. %	Modification	Rank	Result Type
809.4008	809.4124	0.0116	14	185	190	MVLQMR			Oxidation (M)[1,5]		Mascot
822.4832	822.4948	0.0116	14	191	198	GTILPGHK					Mascot
838.4417	838.4663	0.0246	29	133	139	TDHQPIK					Mascot
940.5826	940.6027	0.0201	21	125	132	LLILTDPR					Mascot
940.5826	940.6027	0.0201	21	125	132	LLILTDPR	61	99.864			Mascot
1099.4296	1099.4526	0.023	21	33	40	NCDFQMER			Carbamidomethyl (C)[2]		Mascot
1099.4296	1099.4526	0.023	21	33	40	NCDFQMER	29	0	Carbamidomethyl (C)[2]		Mascot
1115.4244	1115.4442	0.0198	18	33	40	NCDFQMER			Carbamidomethyl (C)[2], Oxidation (M)[6]		Mascot
1115.4244	1115.4442	0.0198	18	33	40	NCDFQMER	12	0	Carbamidomethyl (C)[2], Oxidation (M)[6]		Mascot
1192.6572	1192.6821	0.0249	21	47	57	SDGIYIINLGK					Mascot
1203.6368	1203.655	0.0182	15	160	170	YVDIGIPANNK					Mascot
1313.6709	1313.7035	0.0326	25	94	106	FAQHTGANAIAGR					Mascot
1313.6709	1313.7035	0.0326	25	94	106	FAQHTGANAIAGR	117	100			Mascot
1348.7583	1348.7883	0.03	22	46	57	RSDGIYIINLGK					Mascot
1348.7583	1348.7883	0.03	22	46	57	RSDGIYIINLGK	67	99.971			Mascot
1465.7621	1465.7946	0.0325	22	173	184	NSIGCLYWLLAR			Carbamidomethyl (C)[5]		Mascot
1465.7621	1465.7946	0.0325	22	173	184	NSIGCLYWLLAR	55	99.42	Carbamidomethyl (C)[5]		Mascot
1487.7635	1487.7911	0.0276	19	1	15	MAAAAGEAPRALSQK			Oxidation (M)[1]		Mascot
1915.9253	1915.9432	0.0179	9	16	32	EQDIQMMLAADVHLGTK			Oxidation (M)[6]		Mascot
1931.9202	1931.9381	0.0179	9	16	32	EQDIQMMLAADVHLGTK			Oxidation (M)[6,7]		Mascot
2065.9397	2065.9922	0.0525	25	107	124	HTPGTFTNQMQTSFSEP R					Mascot
2065.9397	2065.9922	0.0525	25	107	124	HTPGTFTNQMQTSFSEP R					Mascot
2081.9346	2081.9744	0.0398	19	107	124	HTPGTFTNQMQTSFSEP R			Oxidation (M)[10]		Mascot
2081.9346	2081.9744	0.0398	19	107	124	HTPGTFTNQMQTSFSEP R	44	93.996	Oxidation (M)[10]		Mascot
2195.0107	2195.0593	0.0486	22	140	159	ESALGNIPTIAFCDTDSP MR			Carbamidomethyl (C)[13]		Mascot
2466.3464	2466.4006	0.0542	22	68	89	VIVAIENPQDIIVQSARPY					Mascot

GQR

2	40S ribosomal protein SA [Triticum urartu]	gi|474222337	33930.8	4.97	14	458	100	55.328	374	100

Peptide Information

Calc. Mass	Obsrv. Mass	± da	± ppm	Start Seq.	End Seq.	Sequence	Ion Score	C. I. %	Modification	Rank	Result Type
809.4008	809.4124	0.0116	14	190	195	MVLQMR			Oxidation (M)[1,5]		Mascot
822.4832	822.4948	0.0116	14	196	203	GTILPGHK					Mascot
838.4417	838.4663	0.0246	29	138	144	TDHQPIK					Mascot
940.5826	940.6027	0.0201	21	130	137	LLILTDPR					Mascot
940.5826	940.6027	0.0201	21	130	137	LLILTDPR	61	99.864			Mascot
1099.4296	1099.4526	0.023	21	38	45	NCDFQMER			Carbamidomethyl (C)[2]		Mascot
1099.4296	1099.4526	0.023	21	38	45	NCDFQMER	29	0	Carbamidomethyl (C)[2]		Mascot
1115.4244	1115.4442	0.0198	18	38	45	NCDFQMER			Carbamidomethyl (C)[2], Oxidation (M)[6]		Mascot
1115.4244	1115.4442	0.0198	18	38	45	NCDFQMER	12	0	Carbamidomethyl (C)[2], Oxidation (M)[6]		Mascot
1192.6572	1192.6821	0.0249	21	52	62	SDGIYIINLGK					Mascot
1203.6368	1203.655	0.0182	15	165	175	YVDIGIPANNK					Mascot
1313.6709	1313.7035	0.0326	25	99	111	FAQHTGANAIAGR					Mascot
1313.6709	1313.7035	0.0326	25	99	111	FAQHTGANAIAGR	117	100			Mascot
1348.7583	1348.7883	0.03	22	51	62	RSDGIYIINLGK					Mascot
1348.7583	1348.7883	0.03	22	51	62	RSDGIYIINLGK	67	99.971			Mascot
1465.7621	1465.7946	0.0325	22	178	189	NSIGCLYWLLAR			Carbamidomethyl (C)[5]		Mascot
1465.7621	1465.7946	0.0325	22	178	189	NSIGCLYWLLAR	55	99.42	Carbamidomethyl (C)[5]		Mascot
1915.9253	1915.9432	0.0179	9	21	37	EQDIQMMLAADVHLGTK			Oxidation (M)[6]		Mascot
1931.9202	1931.9381	0.0179	9	21	37	EQDIQMMLAADVHLGTK			Oxidation (M)[6,7]		Mascot
2065.9397	2065.9922	0.0525	25	112	129	HTPGTFTNQMQTSFSEPR					Mascot
2065.9397	2065.9922	0.0525	25	112	129	HTPGTFTNQMQTSFSEPR					Mascot
2081.9346	2081.9744	0.0398	19	112	129	HTPGTFTNQMQTSFSEPR			Oxidation (M)[10]		Mascot
2081.9346	2081.9744	0.0398	19	112	129	HTPGTFTNQMQTSFSEPR	44	93.996	Oxidation (M)[10]		Mascot
2195.0107	2195.0593	0.0486	22	145	164	ESALGNIPTIAFCDTDSPMR			Carbamidomethyl (C)[13]		Mascot
2466.3464	2466.4006	0.0542	22	73	94	VIVAIENPQDIIVQSARPYGQR					Mascot

3	hypothetical protein PRUPE_ppa009383mg [Prunus persica]	gi|462395916	32506.3	5.31	10	256	100	41.359	208	100

Peptide Information

Calc. Mass	Obsrv. Mass	± da	± ppm	Start Seq.	End Seq.	Sequence	Ion Score	C. I. %	Modification	Rank	Result Type

809.4008	809.4124	0.0116	14	183	188	MVLQMR			Oxidation (M)[1,5]		Mascot
838.4417	838.4663	0.0246	29	131	137	TDHQPIK					Mascot
940.5826	940.6027	0.0201	21	123	130	LLILTDPR					Mascot
940.5826	940.6027	0.0201	21	123	130	LLILTDPR	61	99.864			Mascot
1099.4296	1099.4526	0.023	21	31	38	NCDFQMER			Carbamidomethyl (C)[2]		Mascot
1099.4296	1099.4526	0.023	21	31	38	NCDFQMER	29	0	Carbamidomethyl (C)[2]		Mascot
1115.4244	1115.4442	0.0198	18	31	38	NCDFQMER			Carbamidomethyl (C)[2], Oxidation (M)[6]		Mascot
1115.4244	1115.4442	0.0198	18	31	38	NCDFQMER	12	0	Carbamidomethyl (C)[2], Oxidation (M)[6]		Mascot
1201.6688	1201.6808	0.012	10	56	65	TWDKLQLAAR					Mascot
1203.6368	1203.655	0.0182	15	158	168	YVDIGIPANNK					Mascot
1313.6709	1313.7035	0.0326	25	92	104	FAQHTGANAIAGR					Mascot
1313.6709	1313.7035	0.0326	25	92	104	FAQHTGANAIAGR	117	100			Mascot
1412.7678	1412.7524	-0.0154	-11	1	13	MANASPPRLLSQK					Mascot
2195.0107	2195.0593	0.0486	22	138	157	EAALGNIPTIAFCDTDSPMR			Carbamidomethyl (C)[13], Oxidation (M)[19]		Mascot
2466.3828	2466.4006	0.0178	7	66	87	VIVAIENPKDIIVQSARPYGQR					Mascot

4	PREDICTED: 40S ribosomal protein SA-like [Fragaria vesca subsp. vesca]	gi|470101196	33389.8	5.4	9	248	100	42.265	208	100		

Peptide Information

Calc. Mass	Obsrv. Mass	± da	± ppm	Start Seq.	End Seq.	Sequence	Ion Score	C. I. %	Modification	Rank	Result Type
809.4008	809.4124	0.0116	14	185	190	MVLQMR			Oxidation (M)[1,5]		Mascot
838.4417	838.4663	0.0246	29	133	139	TDHQPIK					Mascot
940.5826	940.6027	0.0201	21	125	132	LLILTDPR					Mascot
940.5826	940.6027	0.0201	21	125	132	LLILTDPR	61	99.864			Mascot
1099.4296	1099.4526	0.023	21	33	40	NCDFQMER			Carbamidomethyl (C)[2]		Mascot
1099.4296	1099.4526	0.023	21	33	40	NCDFQMER	29	0	Carbamidomethyl (C)[2]		Mascot
1115.4244	1115.4442	0.0198	18	33	40	NCDFQMER			Carbamidomethyl (C)[2], Oxidation (M)[6]		Mascot
1115.4244	1115.4442	0.0198	18	33	40	NCDFQMER	12	0	Carbamidomethyl (C)[2], Oxidation (M)[6]		Mascot
1203.6368	1203.655	0.0182	15	160	170	YVDIGIPANNK					Mascot
1313.6709	1313.7035	0.0326	25	94	106	FAQHTGANAIAGR					Mascot
1313.6709	1313.7035	0.0326	25	94	106	FAQHTGANAIAGR	117	100			Mascot
1465.8121	1465.7946	-0.0175	-12	2	15	ATAAPTPPRQLSQK					Mascot
1465.8121	1465.7946	-0.0175	-12	2	15	ATAAPTPPRQLSQK					Mascot
2195.0107	2195.0593	0.0486	22	140	159	EAALGNIPTIAFCDTDSPMR			Carbamidomethyl (C)[13], Oxidation (M)[19]		Mascot
2466.3828	2466.4006	0.0178	7	68	89	VIVAIENPKDIIVQSARPYGQR					Mascot

5	hypothetical protein PRUPE_ppa008193mg [Prunus persica]	gi|462413403	37691	5.86	9	236	100	40.572	208	100	

Peptide Information

Calc. Mass	Obsrv. Mass	± da	± ppm	Start Seq.	End Seq.	Sequence	Ion Score	C. I. %	Modification	Rank	Result Type
838.4417	838.4663	0.0246	29	169	175	TDHQPIK					Mascot
940.5826	940.6027	0.0201	21	161	168	LLILTDPR					Mascot
940.5826	940.6027	0.0201	21	161	168	LLILTDPR	61	99.864			Mascot
1099.4296	1099.4526	0.023	21	69	76	NCDFQMER			Carbamidomethyl (C)[2]		Mascot
1099.4296	1099.4526	0.023	21	69	76	NCDFQMER	29	0	Carbamidomethyl (C)[2]		Mascot
1115.4244	1115.4442	0.0198	18	69	76	NCDFQMER			Carbamidomethyl (C)[2], Oxidation (M)[6]		Mascot
1115.4244	1115.4442	0.0198	18	69	76	NCDFQMER	12	0	Carbamidomethyl (C)[2], Oxidation (M)[6]		Mascot
1201.6688	1201.6808	0.012	10	94	103	TWDKLQLAAR					Mascot
1203.6368	1203.655	0.0182	15	196	206	YVDIGIPANNK					Mascot
1313.6709	1313.7035	0.0326	25	130	142	FAQHTGANAIAGR					Mascot
1313.6709	1313.7035	0.0326	25	130	142	FAQHTGANAIAGR	117	100			Mascot
1886.1085	1885.9852	-0.1233	-65	1	16	MARLPLFILVFTLAHK			Oxidation (M)[1]		Mascot
2195.0107	2195.0593	0.0486	22	176	195	EAALGNIPTIAFCDTDSPMR			Carbamidomethyl (C)[13], Oxidation (M)[19]		Mascot
2466.3828	2466.4006	0.0178	7	104	125	VIVAIENPKDIIVQSARPYGQR					Mascot

6	hypothetical protein OsI_10272 [Oryza sativa Indica Group]	gi|125542660	33265.5	4.86	10	205	100	27.439	158	100	

Peptide Information

Calc. Mass	Obsrv. Mass	± da	± ppm	Start Seq.	End Seq.	Sequence	Ion Score	C. I. %	Modification	Rank	Result Type
809.4008	809.4124	0.0116	14	185	190	MVLQMR			Oxidation (M)[1,5]		Mascot
822.4832	822.4948	0.0116	14	191	198	GTILPGHK					Mascot
838.4417	838.4663	0.0246	29	133	139	TDHQPIK					Mascot
940.5826	940.6027	0.0201	21	125	132	LLILTDPR					Mascot
940.5826	940.6027	0.0201	21	125	132	LLILTDPR	61	99.864			Mascot
1099.4296	1099.4526	0.023	21	33	40	NCDFQMER			Carbamidomethyl (C)[2]		Mascot
1099.4296	1099.4526	0.023	21	33	40	NCDFQMER	29	0	Carbamidomethyl (C)[2]		Mascot
1115.4244	1115.4442	0.0198	18	33	40	NCDFQMER			Carbamidomethyl (C)[2], Oxidation (M)[6]		Mascot
1115.4244	1115.4442	0.0198	18	33	40	NCDFQMER	12	0	Carbamidomethyl (C)[2], Oxidation (M)[6]		Mascot
1192.6572	1192.6821	0.0249	21	47	57	SDGIYIINLGK					Mascot
1203.6368	1203.655	0.0182	15	160	170	YVDIGIPANNK					Mascot

1348.7583	1348.7883	0.03	22	46	57	RSDGIYIINLGK					Mascot
1348.7583	1348.7883	0.03	22	46	57	RSDGIYIINLGK	67	99.971			Mascot
2195.0107	2195.0593	0.0486	22	140	159	ESALGNIPTIAFCDTDSP MR			Carbamidomethyl (C)[13]		Mascot
2466.3464	2466.4006	0.0542	22	68	89	VIVAIENPQDIIVQSARPY GQR					Mascot

7	40S ribosomal protein SA [Aegilops tauschii]	gi|475620366	33401	8.42	10	178	100	32.342	134	100

Peptide Information

Calc. Mass	Obsrv. Mass	± da	± ppm	Start Seq.	End Seq.	Sequence	Ion Score	C. I. %	Modification	Rank	Result Type
809.4008	809.4124	0.0116	14	184	189	MVLQMR			Oxidation (M)[1,5]		Mascot
838.4417	838.4663	0.0246	29	132	138	TDHQPIK					Mascot
847.4091	847.4722	0.0631	74	1	9	MAAEGGGVR					Mascot
940.5826	940.6027	0.0201	21	124	131	LLILTDPR					Mascot
940.5826	940.6027	0.0201	21	124	131	LLILTDPR	61	99.864			Mascot
1099.4296	1099.4526	0.023	21	32	39	NCDFQMER			Carbamidomethyl (C)[2]		Mascot
1099.4296	1099.4526	0.023	21	32	39	NCDFQMER	29	0	Carbamidomethyl (C)[2]		Mascot
1115.4244	1115.4442	0.0198	18	32	39	NCDFQMER			Carbamidomethyl (C)[2], Oxidation (M)[6]		Mascot
1115.4244	1115.4442	0.0198	18	32	39	NCDFQMER	12	0	Carbamidomethyl (C)[2], Oxidation (M)[6]		Mascot
1339.6754	1339.7114	0.036	27	93	105	FAQYTGANAIAGR					Mascot
1915.9253	1915.9432	0.0179	9	15	31	EQDIQMMLAADVHLGTK			Oxidation (M)[6]		Mascot
1931.9202	1931.9381	0.0179	9	15	31	EQDIQMMLAADVHLGTK			Oxidation (M)[6,7]		Mascot
2065.9397	2065.9922	0.0525	25	106	123	HTPGTFTNQMQTSFSEP R					Mascot
2065.9397	2065.9922	0.0525	25	106	123	HTPGTFTNQMQTSFSEP R					Mascot
2081.9346	2081.9744	0.0398	19	106	123	HTPGTFTNQMQTSFSEP R			Oxidation (M)[10]		Mascot
2081.9346	2081.9744	0.0398	19	106	123	HTPGTFTNQMQTSFSEP R	44	93.996	Oxidation (M)[10]		Mascot
2195.0107	2195.0593	0.0486	22	139	158	ESALGNIPTIAFCDTDSP MR			Carbamidomethyl (C)[13]		Mascot
2466.3464	2466.4006	0.0542	22	67	88	VIVAIENPQDIIVQSARPY GQR					Mascot

8	40S ribosomal protein SA [Triticum urartu]	gi|474262479	27262.4	4.76	8	174	100	31.221	134	100

Peptide Information

Calc. Mass	Obsrv. Mass	± da	± ppm	Start Seq.	End Seq.	Sequence	Ion Score	C. I. %	Modification	Rank	Result Type
822.4832	822.4948	0.0116	14	138	145	GTILPGHK					Mascot
847.4091	847.4722	0.0631	74	1	9	MAAEGGGVR					Mascot
940.5826	940.6027	0.0201	21	124	131	LLILTDPR					Mascot

Calc. Mass	Obsrv. Mass	± da	± ppm	Start Seq.	End Seq.	Sequence	Ion Score	C. I. %	Modification	Rank	Result Type
940.5826	940.6027	0.0201	21	124	131	LLILTDPR	61	99.864			Mascot
1099.4296	1099.4526	0.023	21	32	39	NCDFQMER			Carbamidomethyl (C)[2]		Mascot
1099.4296	1099.4526	0.023	21	32	39	NCDFQMER	29	0	Carbamidomethyl (C)[2]		Mascot
1115.4244	1115.4442	0.0198	18	32	39	NCDFQMER			Carbamidomethyl (C)[2], Oxidation (M)[6]		Mascot
1115.4244	1115.4442	0.0198	18	32	39	NCDFQMER	12	0	Carbamidomethyl (C)[2], Oxidation (M)[6]		Mascot
1339.6754	1339.7114	0.036	27	93	105	FAQYTGANAIAGR					Mascot
1915.9253	1915.9432	0.0179	9	15	31	EQDIQMMLAADVHLGTK			Oxidation (M)[6]		Mascot
1931.9202	1931.9381	0.0179	9	15	31	EQDIQMMLAADVHLGTK			Oxidation (M)[6,7]		Mascot
2065.9397	2065.9922	0.0525	25	106	123	HTPGTFTNQMQTSFSEP R					Mascot
2065.9397	2065.9922	0.0525	25	106	123	HTPGTFTNQMQTSFSEP R					Mascot
2081.9346	2081.9744	0.0398	19	106	123	HTPGTFTNQMQTSFSEP R			Oxidation (M)[10]		Mascot
2081.9346	2081.9744	0.0398	19	106	123	HTPGTFTNQMQTSFSEP R	44	93.996	Oxidation (M)[10]		Mascot
2466.3464	2466.4006	0.0542	22	67	88	VIVAIENPQDIIVQSARPY GQR					Mascot

9	hypothetical protein CARUB_v10020725mg [Capsella rubella]	gi|482571329	32421.9	5.12	8	141	100	27.237	105	100	

Peptide Information

Calc. Mass	Obsrv. Mass	± da	± ppm	Start Seq.	End Seq.	Sequence	Ion Score	C. I. %	Modification	Rank	Result Type
809.4008	809.4124	0.0116	14	185	190	MVLQMR			Oxidation (M)[1,5]		Mascot
838.4417	838.4663	0.0246	29	133	139	TDHQPIK					Mascot
940.5826	940.6027	0.0201	21	125	132	LLILTDPR					Mascot
940.5826	940.6027	0.0201	21	125	132	LLILTDPR	61	99.864			Mascot
1339.6754	1339.7114	0.036	27	94	106	FAQYTGANAIAGR					Mascot
1931.9136	1931.9381	0.0245	13	16	32	EADIRMMCAAEVHLGTK			Carbamidomethyl (C)[8]		Mascot
2065.9397	2065.9922	0.0525	25	107	124	HTPGTFTNQMQTSFSEP R					Mascot
2065.9397	2065.9922	0.0525	25	107	124	HTPGTFTNQMQTSFSEP R					Mascot
2081.9346	2081.9744	0.0398	19	107	124	HTPGTFTNQMQTSFSEP R			Oxidation (M)[10]		Mascot
2081.9346	2081.9744	0.0398	19	107	124	HTPGTFTNQMQTSFSEP R	44	93.996	Oxidation (M)[10]		Mascot
2193.0315	2193.041	0.0095	4	140	159	EGALGNIPIIAFCDTDSPM R			Carbamidomethyl (C)[13], Oxidation (M)[19]		Mascot
2466.3464	2466.4006	0.0542	22	68	89	VIVAIENPQDIIVQSARPY GQR					Mascot

10	RecName: Full=40S ribosomal protein SA; AltName: Full=Laminin receptor-like protein; AltName: Full=p40	gi|15214300	32237.9	5.14	8	140	100	26.995	105	100	

Peptide Information

Calc. Mass	Obsrv. Mass	± da	± ppm	Start	End	Sequence	Ion	C. I. %	Modification	Rank	Result Type

				Seq.	Seq.		Score			
809.4008	809.4124	0.0116	14	182	187	MVLQMR			Oxidation (M)[1,5]	Mascot
838.4417	838.4663	0.0246	29	130	136	TDHQPIK				Mascot
870.5156	870.5576	0.042	48	188	195	GTIRPAQK				Mascot
940.5826	940.6027	0.0201	21	122	129	LLILTDPR				Mascot
940.5826	940.6027	0.0201	21	122	129	LLILTDPR	61	99.882		Mascot
1339.6754	1339.7114	0.036	27	91	103	FAQYTGANAIAGR				Mascot
2065.9397	2065.9922	0.0525	25	104	121	HTPGTFTNQMQTSFSEPR				Mascot
2065.9397	2065.9922	0.0525	25	104	121	HTPGTFTNQMQTSFSEPR				Mascot
2081.9346	2081.9744	0.0398	19	104	121	HTPGTFTNQMQTSFSEPR			Oxidation (M)[10]	Mascot
2081.9346	2081.9744	0.0398	19	104	121	HTPGTFTNQMQTSFSEPR	44	94.784	Oxidation (M)[10]	Mascot
2193.0315	2193.041	0.0095	4	137	156	EGALGNIPIIAFCDTDSPMR			Carbamidomethyl (C)[13], Oxidation (M)[19]	Mascot
2466.3464	2466.4006	0.0542	22	65	86	VIVAIENPQDIIVQSARPYGQR				Mascot

Gel Idx/Pos	219/I19	Instr./Gel Origin	BA2151/Sample Project 20140814	Process Status	Analysis Succeeded
Plate [#] Name	[1] Sample Project 20140814	Instrument Sample Name		Spectra	11

Rank	Protein Name	Accession No.	Protein MW	Protein PI	Pep. Count	Protein Score	Protein Score C. I. %	Intensity Matched	Total Ion Score	Total Ion C. I. %	Confirmed
1	hypothetical protein F775_52189 [Aegilops tauschii]	gi|475624809	64143.3	8.32	15	77	98.013	12.774	25	0	

Peptide Information

Calc. Mass	Obsrv. Mass	± da	± ppm	Start Seq.	End Seq.	Sequence	Ion Score	C. I. %	Modification	Rank	Result Type
809.4152	809.4084	-0.0068	-8	524	530	DGTVVYR					Mascot
866.4618	866.447	-0.0148	-17	248	255	ASEYGLVK					Mascot
952.4887	952.5057	0.017	18	386	393	TPFFTSPR					Mascot
952.4887	952.5057	0.017	18	386	393	TPFFTSPR	25	0			Mascot
1023.5945	1023.6124	0.0179	17	421	429	IEHSIIGVR					Mascot
1266.7278	1266.6205	-0.1073	-85	421	431	IEHSIIGVRSR					Mascot
1302.6624	1302.6932	0.0308	24	407	417	EAIISHGCFLR			Carbamidomethyl (C)[8]		Mascot
1302.6624	1302.6932	0.0308	24	407	417	EAIISHGCFLR			Carbamidomethyl (C)[8]		Mascot
1306.6243	1306.662	0.0377	29	474	484	ISNCIIDMNAR			Carbamidomethyl (C)[4]		Mascot
1322.6191	1322.6451	0.026	20	474	484	ISNCIIDMNAR			Carbamidomethyl (C)[4], Oxidation (M)[8]		Mascot
1335.6978	1335.6698	-0.028	-21	1	12	MQFSSVLPLEGK					Mascot
1543.8413	1543.8767	0.0354	23	405	417	IKEAIISHGCFLR			Carbamidomethyl (C)[10]		Mascot
1646.8174	1646.8459	0.0285	17	318	331	YAELHDFGSEILPR					Mascot
1646.8174	1646.8459	0.0285	17	318	331	YAELHDFGSEILPR	3	0			Mascot
1756.9592	1756.9988	0.0396	23	205	219	SIEHILILSGDQLYR					Mascot
1838.8776	1838.976	0.0984	54	120	135	LIDIPMSNCFNSGINK			Carbamidomethyl (C)[9], Oxidation (M)[6]		Mascot
1910.8879	1910.9208	0.0329	17	495	510	EGVQEADRPEEGYYIR					Mascot
2055.9399	2055.9924	0.0525	26	229	247	HVDDNADITLSCAPVGES R			Carbamidomethyl (C)[12]		Mascot
2763.3162	2763.3708	0.0546	20	332	354	ALHDHNVQAYVFTDYWE DIGTIR					Mascot

Rank	Protein Name	Accession No.	Protein MW	Protein PI	Pep. Count	Protein Score	Protein Score C. I. %	Intensity Matched	Total Ion Score	Total Ion C. I. %	Confirmed
2	Glucose-1-phosphate adenylyltransferase large subunit 1, chloroplastic/amyloplastic [Triticum urartu]	gi|474056532	51530	8.53	13	75	96.922	12.192	25	0	

Peptide Information

Calc. Mass	Obsrv. Mass	± da	± ppm	Start Seq.	End Seq.	Sequence	Ion Score	C. I. %	Modification	Rank	Result Type
876.4897	876.5085	0.0188	21	450	458	ARAGSISSK					Mascot
925.5214	925.5168	-0.0046	-5	17	26	AAPGGQGVLR					Mascot

952.4887	952.5057	0.017	18	260	267	TPFFTSPR					Mascot
952.4887	952.5057	0.017	18	260	267	TPFFTSPR	25	0			Mascot
1023.5945	1023.6124	0.0179	17	295	303	IEHSIIGVR					Mascot
1266.7278	1266.6205	-0.1073	-85	295	305	IEHSIIGVRSR					Mascot
1302.6624	1302.6932	0.0308	24	281	291	EAIISHGCFLR			Carbamidomethyl (C)[8]		Mascot
1302.6624	1302.6932	0.0308	24	281	291	EAIISHGCFLR			Carbamidomethyl (C)[8]		Mascot
1306.6243	1306.662	0.0377	29	348	358	ISNCIIDMNAR			Carbamidomethyl (C)[4]		Mascot
1322.6191	1322.6451	0.026	20	348	358	ISNCIIDMNAR			Carbamidomethyl (C)[4], Oxidation (M)[8]		Mascot
1543.8413	1543.8767	0.0354	23	279	291	IKEAIISHGCFLR			Carbamidomethyl (C)[10]		Mascot
1646.8174	1646.8459	0.0285	17	201	214	YAELHDFGSEILPR					Mascot
1646.8174	1646.8459	0.0285	17	201	214	YAELHDFGSEILPR	3	0			Mascot
1756.9592	1756.9988	0.0396	23	157	171	SIEHILILSGDQLYR					Mascot
1838.8776	1838.976	0.0984	54	72	87	LIDIPMSNCFNSGINK			Carbamidomethyl (C)[9], Oxidation (M)[6]		Mascot
1910.8879	1910.9208	0.0329	17	369	384	EGVQEADRPEEGYYIR					Mascot
2763.3162	2763.3708	0.0546	20	215	237	ALHDHNVQAYVFTDYWE DIGTIR					Mascot

3	RecName: Full=Glucose-1-phosphate adenylyltransferase large subunit 1, chloroplastic/amyloplastic; AltName: Full=ADP-glucose pyrophosphorylase; AltName: Full=ADP-glucose synthase; AltName: Full=AGPase S; AltName: Full=Alpha-D-glucose-1-phosphate ade	gi|1707923	58580.1	6.17	13	71	91.904	12.368	25	0	

Peptide Information

Calc. Mass	Obsrv. Mass	± da	± ppm	Start Seq.	End Seq.	Sequence	Ion Score	C. I. %	Modification	Rank	Result Type
866.4618	866.447	-0.0148	-17	252	259	ASEYGLVK					Mascot
952.4887	952.5057	0.017	18	381	388	TPFFTSPR					Mascot
952.4887	952.5057	0.017	18	381	388	TPFFTSPR	25	0			Mascot
1023.5945	1023.6124	0.0179	17	416	424	IEHSIIGVR					Mascot
1266.7278	1266.6205	-0.1073	-85	416	426	IEHSIIGVRSR					Mascot
1302.6624	1302.6932	0.0308	24	402	412	EAIISHGCFLR			Carbamidomethyl (C)[8]		Mascot
1302.6624	1302.6932	0.0308	24	402	412	EAIISHGCFLR			Carbamidomethyl (C)[8]		Mascot
1306.6243	1306.662	0.0377	29	469	479	ISNCIIDMNAR			Carbamidomethyl (C)[4]		Mascot
1322.6191	1322.6451	0.026	20	469	479	ISNCIIDMNAR			Carbamidomethyl (C)[4], Oxidation (M)[8]		Mascot
1543.8413	1543.8767	0.0354	23	400	412	IKEAIISHGCFLR			Carbamidomethyl (C)[10]		Mascot
1646.8174	1646.8459	0.0285	17	322	335	YAELHDFGSEILPR					Mascot
1646.8174	1646.8459	0.0285	17	322	335	YAELHDFGSEILPR	3	0			Mascot
1756.9592	1756.9988	0.0396	23	209	223	SIEHILILSGDQLYR					Mascot
1838.8776	1838.976	0.0984	54	124	139	LIDIPMSNCFNSGINK			Carbamidomethyl (C)[9], Oxidation (M)[6]		Mascot

Calc. Mass	Obsrv. Mass	± da	± ppm	Start Seq.	End Seq.	Sequence	Ion Score	C. I. %	Modification	Rank	Result Type
1910.8879	1910.9208	0.0329	17	490	505	EGVQEADRPEEGYYIR					Mascot
2055.9399	2055.9924	0.0525	26	233	251	HVDDNADITLSCAPVGES R			Carbamidomethyl (C)[12]		Mascot
2763.3162	2763.3708	0.0546	20	336	358	ALHDHNVQAYVFTDYWE DIGTIR					Mascot

4	RecName: Full=Glucose-1-phosphate adenylyltransferase large subunit, chloroplastic/amyloplastic; AltName: Full=ADP-glucose pyrophosphorylase; AltName: Full=ADP-glucose synthase; AltName: Full=AGPase S; AltName: Full=Alpha-D-glucose-1-phosphate adeny	gi|1707930	58399.1	6.12	13	70	90.266	12.368	25	0	

Peptide Information

Calc. Mass	Obsrv. Mass	± da	± ppm	Start Seq.	End Seq.	Sequence	Ion Score	C. I. %	Modification	Rank	Result Type
866.4618	866.447	-0.0148	-17	251	258	ASEYGLVK					Mascot
952.4887	952.5057	0.017	18	380	387	TPFFTSPR					Mascot
952.4887	952.5057	0.017	18	380	387	TPFFTSPR	25	0			Mascot
1023.5945	1023.6124	0.0179	17	415	423	IEHSIIGVR					Mascot
1266.7278	1266.6205	-0.1073	-85	415	425	IEHSIIGVRSR					Mascot
1302.6624	1302.6932	0.0308	24	401	411	EAIISHGCFLR			Carbamidomethyl (C)[8]		Mascot
1302.6624	1302.6932	0.0308	24	401	411	EAIISHGCFLR			Carbamidomethyl (C)[8]		Mascot
1306.6243	1306.662	0.0377	29	468	478	ISNCIIDMNAR			Carbamidomethyl (C)[4]		Mascot
1322.6191	1322.6451	0.026	20	468	478	ISNCIIDMNAR			Carbamidomethyl (C)[4], Oxidation (M)[8]		Mascot
1543.8413	1543.8767	0.0354	23	399	411	IKEAIISHGCFLR			Carbamidomethyl (C)[10]		Mascot
1646.8174	1646.8459	0.0285	17	321	334	YAELHDFGSEILPR					Mascot
1646.8174	1646.8459	0.0285	17	321	334	YAELHDFGSEILPR	3	0			Mascot
1756.9592	1756.9988	0.0396	23	208	222	SIEHILILSGDQLYR					Mascot
1838.8776	1838.976	0.0984	54	123	138	LIDIPMSNCFNSGINK			Carbamidomethyl (C)[9], Oxidation (M)[6]		Mascot
1910.8879	1910.9208	0.0329	17	489	504	EGVQEADRPEEGYYIR					Mascot
2055.9399	2055.9924	0.0525	26	232	250	HVDDNADITLSCAPVGES R			Carbamidomethyl (C)[12]		Mascot
2763.3162	2763.3708	0.0546	20	335	357	ALHDHNVQAYVFTDYWE DIGTIR					Mascot

5	RecName: Full=Glucose-1-phosphate adenylyltransferase large subunit, chloroplastic/amyloplastic; AltName: Full=ADP-glucose pyrophosphorylase; AltName: Full=ADP-glucose synthase; AltName: Full=AGPase S; AltName: Full=Alpha-D-glucose-1-phosphate adeny	gi|121293	56037.9	6.61	11	64	56.521	9.844	25	0	

Peptide Information

Calc. Mass	Obsrv. Mass	± da	± ppm	Start Seq.	End Seq.	Sequence	Ion Score	C. I. %	Modification	Rank	Result Type

866.4618	866.447	-0.0148	-17	232	239	ASEYGLVK					Mascot
908.5424	908.486	-0.0564	-62	10	17	APLRAPQR					Mascot
952.4887	952.5057	0.017	18	361	368	TPFFTSPR					Mascot
952.4887	952.5057	0.017	18	361	368	TPFFTSPR	25	0			Mascot
1306.6243	1306.662	0.0377	29	446	456	ISNCIIDMNAR			Carbamidomethyl (C)[4]		Mascot
1322.6191	1322.6451	0.026	20	446	456	ISNCIIDMNAR			Carbamidomethyl (C)[4], Oxidation (M)[8]		Mascot
1646.8174	1646.8459	0.0285	17	302	315	YAELHDFGSEILPR					Mascot
1646.8174	1646.8459	0.0285	17	302	315	YAELHDFGSEILPR	3	0			Mascot
1745.8826	1745.9778	0.0952	55	382	395	EAIILHGCFLRECK			Carbamidomethyl (C)[8,13]		Mascot
1756.9592	1756.9988	0.0396	23	189	203	SIEHILILSGDQLYR					Mascot
1838.8776	1838.976	0.0984	54	104	119	LIDIPMSNCFNSGINK			Carbamidomethyl (C)[9], Oxidation (M)[6]		Mascot
1910.8879	1910.9208	0.0329	17	467	482	EGVQEADRPEEGYYIR					Mascot
2055.9399	2055.9924	0.0525	26	213	231	HVDDNADITLSCAPVGES R			Carbamidomethyl (C)[12]		Mascot
2763.3162	2763.3708	0.0546	20	316	338	ALHDHNVQAYVFTDYWE DIGTIR					Mascot

6	hypothetical protein CARUB_v10019644mg [Capsella rubella]	gi|482569348	315932.1	4.79	32	53	0	32.099	

Peptide Information

Calc. Mass	Obsrv. Mass	± da	± ppm	Start Seq.	End Seq.	Sequence	Ion Score	C. I. %	Modification	Rank	Result Type
809.4879	809.4084	-0.0795	-98	828	834	IHALVEK					Mascot
821.4515	821.4135	-0.038	-46	2102	2109	GGYNAVLK					Mascot
839.4733	839.4678	-0.0055	-7	719	725	GQKLHEK					Mascot
876.4308	876.5085	0.0777	89	1644	1650	IQETEEK					Mascot
936.4455	936.4868	0.0413	44	493	501	MGVKDGDSK					Mascot
949.5465	949.5086	-0.0379	-40	2102	2110	GGYNAVLKK					Mascot
952.4404	952.5057	0.0653	69	493	501	MGVKDGDSK			Oxidation (M)[1]		Mascot
952.4404	952.5057	0.0653	69	493	501	MGVKDGDSK			Oxidation (M)[1]		Mascot
972.5109	972.4847	-0.0262	-27	364	371	KPSDIQER					Mascot
1180.6433	1180.6284	-0.0149	-13	722	730	LHEKQEQIR					Mascot
1189.5947	1189.5946	-0.0001	0	1007	1016	VKEEEDIAEK					Mascot
1221.578	1221.6427	0.0647	53	1034	1044	VSDAMKGQEEK					Mascot
1237.5729	1237.6108	0.0379	31	1034	1044	VSDAMKGQEEK			Oxidation (M)[5]		Mascot
1266.6801	1266.6205	-0.0596	-47	711	721	EIGEQHIKGQK					Mascot
1302.745	1302.6932	-0.0518	-40	1	11	MELELGLKITR					Mascot
1302.745	1302.6932	-0.0518	-40	1	11	MELELGLKITR					Mascot

1345.7396	1345.646	-0.0936	-70	597	608	MIKGLVEETTPK						Mascot
1345.7396	1345.646	-0.0936	-70	597	608	MIKGLVEETTPK						Mascot
1389.6744	1389.6842	0.0098	7	1198	1209	VGEETQELVEEK						Mascot
1390.7173	1390.7068	-0.0105	-8	1146	1157	GTKIQETEETVR						Mascot
1492.7061	1492.7664	0.0603	40	1955	1967	SDDGIVRMNQETK						Mascot
1501.7856	1501.728	-0.0576	-38	697	710	VGTHSEDSSLTKLK						Mascot
1501.7856	1501.728	-0.0576	-38	697	710	VGTHSEDSSLTKLK						Mascot
1502.7122	1502.7791	0.0669	45	14	26	EDVSSSVDFRFSK						Mascot
1508.701	1508.7112	0.0102	7	1955	1967	SDDGIVRMNQETK			Oxidation (M)[8]			Mascot
1524.6669	1524.7169	0.05	33	779	791	NSVMMDINNEDVK			Oxidation (M)[4]			Mascot
1540.6619	1540.7168	0.0549	36	779	791	NSVMMDINNEDVK			Oxidation (M)[4,5]			Mascot
1543.8075	1543.8767	0.0692	45	1454	1466	TIAVEENEIVDRR						Mascot
1610.7327	1610.807	0.0743	46	1868	1881	SSRMGQTIEEESEK						Mascot
1646.8596	1646.8459	-0.0137	-8	519	533	TQVVAEKNIGETETK						Mascot
1646.8596	1646.8459	-0.0137	-8	519	533	TQVVAEKNIGETETK						Mascot
1745.848	1745.9778	0.1298	74	1574	1587	SYEDLVKEEETYLK						Mascot
1746.8617	1746.9836	0.1219	70	269	284	GSEDSSLRKPTDLEGR						Mascot
1771.9436	1772.1169	0.1733	98	876	890	INEIVEEKTLEVAER						Mascot
1809.9453	1809.9457	0.0004	0	931	945	VQEIIRQQELDEPGR						Mascot
1817.8949	1817.8987	0.0038	2	1040	1055	GQEEKDMIQALAVEEK						Mascot
1817.8949	1817.8987	0.0038	2	1040	1055	GQEEKDMIQALAVEEK						Mascot
1825.8636	1825.9316	0.068	37	112	127	ARFDDDDATLTITMPK			Oxidation (M)[14]			Mascot
1831.7941	1831.9281	0.134	73	2329	2343	TEEEKIEENEHDSSR						Mascot
1833.8899	1833.9004	0.0105	6	1040	1055	GQEEKDMIQALAVEEK			Oxidation (M)[7]			Mascot
1849.91	1849.8888	-0.0212	-11	446	461	EMEKETIDDDVVVLSK						Mascot
1849.91	1849.8888	-0.0212	-11	446	461	EMEKETIDDDVVVLSK						Mascot
1910.8502	1910.9208	0.0706	37	145	162	VDFGDVSAAGEVEEETE K						Mascot
2056.0015	2055.9924	-0.0091	-4	1305	1322	DKAHITATEEPSCQIVEK			Carbamidomethyl (C)[13]			Mascot

7	PREDICTED: uncharacterized protein LOC101263031 [Solanum lycopersicum]	gi|460390332	29998.8	9.13	10	51	0	8.01

Peptide Information

Calc. Mass	Obsrv. Mass	± da	± ppm	Start Seq.	End Seq.	Sequence	Ion Score	C. I. %	Modification	Rank	Result Type
1063.5817	1063.512	-0.0697	-66	33	42	GMVVTSNIVK			Oxidation (M)[2]		Mascot
1193.6249	1193.6833	0.0584	49	65	73	QMFARWNLK					Mascot
1233.5457	1233.6455	0.0998	81	216	225	DMTYNELYGK					Mascot

1236.6484	1236.6171	-0.0313	-25	23	32	WARVYATVDR					Mascot
1253.5765	1253.6312	0.0547	44	43	52	CINACLFEAR			Carbamidomethyl (C)[1,5]		Mascot
1276.5514	1276.6715	0.1201	94	13	22	NYLESACYEK			Carbamidomethyl (C)[7]		Mascot
1524.6431	1524.7169	0.0738	48	229	241	NSCSTCGFKGHNR			Carbamidomethyl (C)[3,6]		Mascot
1552.7101	1552.7592	0.0491	32	214	225	YRDMTYNELYGK					Mascot
1817.8929	1817.8987	0.0058	3	105	120	VVPSNNYVFSVHNEGR					Mascot
1817.8929	1817.8987	0.0058	3	105	120	VVPSNNYVFSVHNEGR					Mascot
2056.0684	2055.9924	-0.076	-37	136	152	FQIDEIPCSHAWAILKK			Carbamidomethyl (C)[8]		Mascot

8 PREDICTED: glutamine synthetase nodule isozyme-like [Cucumis sativus] gi|449436357 39264.6 5.77 7 48 0 8.344 22 0

Protein Group

PREDICTED: glutamine synthetase nodule isozyme-like [Cucumis sativus] gi|449488792 39264.6 5.7699 999809 2651

Peptide Information

Calc. Mass	Obsrv. Mass	± da	± ppm	Start Seq.	End Seq.	Sequence	Ion Score	C. I. %	Modification	Rank	Result Type
1322.7427	1322.6451	-0.0976	-74	37	49	ARTLPGPVTDPAK					Mascot
1345.6132	1345.646	0.0328	24	279	290	EHIAAYGEGNER					Mascot
1345.6132	1345.646	0.0328	24	279	290	EHIAAYGEGNER					Mascot
1501.7142	1501.728	0.0138	9	279	291	EHIAAYGEGNERR					Mascot
1501.7142	1501.728	0.0138	9	279	291	EHIAAYGEGNERR					Mascot
1610.767	1610.807	0.04	25	277	290	HKEHIAAYGEGNER					Mascot
1817.8566	1817.8987	0.0421	23	296	311	HETADINTFSWGVANR					Mascot
1817.8566	1817.8987	0.0421	23	296	311	HETADINTFSWGVANR	22	0			Mascot
2288.1167	2288.1177	0.001	0	296	316	HETADINTFSWGVANRG ASVR					Mascot
2607.3157	2607.3159	0.0002	0	333	356	RPASNMDPYVVTSMIAET TILGKP			Oxidation (M)[6]		Mascot

9 PREDICTED: uncharacterized protein LOC101224354 [Cucumis sativus] gi|449519464 13085.7 5.46 7 48 0 3.256

Peptide Information

Calc. Mass	Obsrv. Mass	± da	± ppm	Start Seq.	End Seq.	Sequence	Ion Score	C. I. %	Modification	Rank	Result Type
950.4247	950.4604	0.0357	38	108	115	LADTDMER					Mascot
982.441	982.4487	0.0077	8	1	8	MHLEDGHK			Oxidation (M)[1]		Mascot

1180.6208	1180.6284	0.0076	6	89	99	TSSYNVVSVPK					Mascot
1189.6437	1189.5946	-0.0491	-41	2	11	HLEDGHKVVR					Mascot
1253.6056	1253.6312	0.0256	20	12	22	CAGDEPLHRAK			Carbamidomethyl (C)[1]		Mascot
1345.7111	1345.646	-0.0651	-48	50	60	LYQQQTPDPKK					Mascot
1345.7111	1345.646	-0.0651	-48	50	60	LYQQQTPDPKK					Mascot
1910.8735	1910.9208	0.0473	25	100	115	SFIQMGHKLADTDMER			Oxidation (M)[5,14]		Mascot

10	glutamine synthetase cytosolic isozyme 1-3 [Arabidopsis thaliana]	gi|332642490	38798.3	5.72	7	47	0	9.983	22	0	

Protein Group

ATGSKB6 [Arabidopsis lyrata subsp. lyrata]	gi|297328929	38711.2	5.7199 997901 9165
RecName: Full=Glutamine synthetase cytosolic isozyme 1-3; Short=GS1; AltName: Full=Glutamate--ammonia ligase GLN1;3; Short=GLN1;3	gi|11386845	38798.3	5.7199 997901 9165
glutamine synthetase cytosolic isozyme 1-3 [Arabidopsis thaliana]	gi|15229530	38798.3	5.7199 997901 9165

Peptide Information

Calc. Mass	Obsrv. Mass	± da	± ppm	Start Seq.	End Seq.	Sequence	Ion Score	C. I. %	Modification	Rank	Result Type
870.5771	870.5433	-0.0338	-39	269	276	AIGKLQLK					Mascot
1345.6132	1345.646	0.0328	24	279	290	EHIAAYGEGNER					Mascot
1345.6132	1345.646	0.0328	24	279	290	EHIAAYGEGNER					Mascot
1501.7142	1501.728	0.0138	9	279	291	EHIAAYGEGNERR					Mascot
1501.7142	1501.728	0.0138	9	279	291	EHIAAYGEGNERR					Mascot
1610.767	1610.807	0.04	25	277	290	HKEHIAAYGEGNER					Mascot
1793.9255	1793.9658	0.0403	22	19	34	IIAEYIWIGGSGMDIR					Mascot
1809.9204	1809.9457	0.0253	14	19	34	IIAEYIWIGGSGMDIR			Oxidation (M)[13]		Mascot
1817.8566	1817.8987	0.0421	23	296	311	HETADINTFSWGVANR					Mascot
1817.8566	1817.8987	0.0421	23	296	311	HETADINTFSWGVANR	22	0			Mascot
2288.1167	2288.1177	0.001	0	296	316	HETADINTFSWGVANRG ASVR					Mascot

Gel Idx/Pos	220/I20	Instr./Gel Origin	BA2151/Sample Project 20140814	Process Status	Analysis Succeeded
Plate [#] Name	[1] Sample Project 20140814	Instrument Sample Name		Spectra	11

Rank	Protein Name	Accession No.	Protein MW	Protein PI	Pep. Count	Protein Score	Protein Score C. I. %	Intensity Matched	Total Ion Score	Total Ion C. I. %	Confirmed
1	cytosolic glutamine synthetase isoform [Secale cereale x Triticum durum]	gi|343465764	38990.4	5.35	11	210	100	17.285	148	100	

Peptide Information

Calc. Mass	Obsrv. Mass	± da	± ppm	Start Seq.	End Seq.	Sequence	Ion Score	C. I. %	Modification	Rank	Result Type
1173.6124	1173.6902	0.0778	66	306	316	WGVADRGASVR					Mascot
1440.7693	1440.8202	0.0509	35	39	52	TVNGPITDASQLPK					Mascot
1552.7251	1552.7704	0.0453	29	277	290	HAQHIAAYGEGNER					Mascot
1552.7251	1552.7704	0.0453	29	277	290	HAQHIAAYGEGNER	129	100			Mascot
1708.8263	1708.8668	0.0405	24	277	291	HAQHIAAYGEGNERR					Mascot
1739.8824	1739.9308	0.0484	28	291	305	RLTGHHETADINTFK					Mascot
1739.8824	1739.9308	0.0484	28	291	305	RLTGHHETADINTFK	4	0			Mascot
1789.9847	1790.0315	0.0468	26	19	34	IIVEYLWVGGSGIDIR					Mascot
1789.9847	1790.0315	0.0468	26	19	34	IIVEYLWVGGSGIDIR	19	0			Mascot
1849.8848	1849.8967	0.0119	6	2	18	ASLADLVNLDLSDCTDK			Carbamidomethyl (C)[14]		Mascot
2271.1179	2271.1506	0.0327	14	119	137	VAAEETWYGIEQEYTLLQ K					Mascot
2438.2307	2438.2896	0.0589	24	333	354	RPASNMDPYVVTSMIAET TLLL			Oxidation (M)[6]		Mascot
2454.2256	2454.2661	0.0405	17	333	354	RPASNMDPYVVTSMIAET TLLL			Oxidation (M)[6,14]		Mascot
2648.2629	2648.3406	0.0777	29	85	107	GDNLLVMCDCYTPQGVP IPTNKR			Carbamidomethyl (C)[8,10]		Mascot
2664.2578	2664.3223	0.0645	24	85	107	GDNLLVMCDCYTPQGVP IPTNKR			Carbamidomethyl (C)[8,10], Oxidation (M)[7]		Mascot
2972.3948	2972.4504	0.0556	19	53	79	WNYDGSSTGQAPGEDS EVILYPQAIFK					Mascot

Rank	Protein Name	Accession No.	Protein MW	Protein PI	Pep. Count	Protein Score	Protein Score C. I. %	Intensity Matched	Total Ion Score	Total Ion C. I. %	Confirmed
2	Glutamine synthetase cytosolic isozyme 1-2 [Aegilops tauschii]	gi|475595696	41830	6.76	9	193	100	16.5	148	100	

Peptide Information

Calc. Mass	Obsrv. Mass	± da	± ppm	Start Seq.	End Seq.	Sequence	Ion Score	C. I. %	Modification	Rank	Result Type
1440.7693	1440.8202	0.0509	35	39	52	TVNGPITDASQLPK					Mascot
1552.7251	1552.7704	0.0453	29	297	310	HAQHIAAYGEGNER					Mascot
1552.7251	1552.7704	0.0453	29	297	310	HAQHIAAYGEGNER	129	100			Mascot
1708.8263	1708.8668	0.0405	24	297	311	HAQHIAAYGEGNERR					Mascot

Calc. Mass	Obsrv. Mass	± da	± ppm	Start Seq.	End Seq.	Sequence	Ion Score	C. I. %	Modification	Rank	Result Type
1739.8824	1739.9308	0.0484	28	311	325	RLTGHHETADINTFK					Mascot
1739.8824	1739.9308	0.0484	28	311	325	RLTGHHETADINTFK	4	0			Mascot
1789.9847	1790.0315	0.0468	26	19	34	IIVEYLWVGGSGIDIR					Mascot
1789.9847	1790.0315	0.0468	26	19	34	IIVEYLWVGGSGIDIR	19	0			Mascot
1849.8848	1849.8967	0.0119	6	2	18	ASLADLVNLDLSDCTDK			Carbamidomethyl (C)[14]		Mascot
2438.2307	2438.2896	0.0589	24	353	374	RPASNMDPYVVTSMIAET TLLL			Oxidation (M)[6]		Mascot
2454.2256	2454.2661	0.0405	17	353	374	RPASNMDPYVVTSMIAET TLLL			Oxidation (M)[6,14]		Mascot
2648.2629	2648.3406	0.0777	29	85	107	GDNLLVMCDCYTPQGVP IPTNKR			Carbamidomethyl (C)[8,10]		Mascot
2664.2578	2664.3223	0.0645	24	85	107	GDNLLVMCDCYTPQGVP IPTNKR			Carbamidomethyl (C)[8,10], Oxidation (M)[7]		Mascot
2972.3948	2972.4504	0.0556	19	53	79	WNYDGSSTGQAPGEDS EVILYPQAIFK					Mascot

3	Glutamine synthetase cytosolic isozyme 1-2 [Triticum urartu]	gi|473798921	42771.6	8.08	10	179	100	15.442	129	100	

Peptide Information

Calc. Mass	Obsrv. Mass	± da	± ppm	Start Seq.	End Seq.	Sequence	Ion Score	C. I. %	Modification	Rank	Result Type
1132.5382	1132.6221	0.0839	74	146	155	SYQNGLADHK					Mascot
1370.6562	1370.7211	0.0649	47	132	141	CFKFIYYSSR			Carbamidomethyl (C)[1]		Mascot
1440.7693	1440.8202	0.0509	35	39	52	TVNGPITDASQLPK					Mascot
1552.7251	1552.7704	0.0453	29	304	317	HAQHIAAYGEGNER					Mascot
1552.7251	1552.7704	0.0453	29	304	317	HAQHIAAYGEGNER	129	100			Mascot
1708.8263	1708.8668	0.0405	24	304	318	HAQHIAAYGEGNERR					Mascot
1739.8824	1739.9308	0.0484	28	318	332	RLTGHHETADINTFK					Mascot
1739.8824	1739.9308	0.0484	28	318	332	RLTGHHETADINTFK	4	0			Mascot
1849.8848	1849.8967	0.0119	6	2	18	ASLADLVNLDLSDCTDK			Carbamidomethyl (C)[14]		Mascot
2438.2307	2438.2896	0.0589	24	360	381	RPASNMDPYVVTSMIAET TLLL			Oxidation (M)[6]		Mascot
2454.2256	2454.2661	0.0405	17	360	381	RPASNMDPYVVTSMIAET TLLL			Oxidation (M)[6,14]		Mascot
2648.2629	2648.3406	0.0777	29	85	107	GDNLLVMCDCYTPQGVP IPTNKR			Carbamidomethyl (C)[8,10]		Mascot
2664.2578	2664.3223	0.0645	24	85	107	GDNLLVMCDCYTPQGVP IPTNKR			Carbamidomethyl (C)[8,10], Oxidation (M)[7]		Mascot
2972.3948	2972.4504	0.0556	19	53	79	WNYDGSSTGQAPGEDS EVILYPQAIFK					Mascot

4	RecName: Full=Glutamine synthetase cytosolic isozyme; AltName: Full=GS1; AltName: Full=Glutamate--ammonia ligase	gi|8928127	38658.3	5.69	4	140	100	12.464	129	100	

Peptide Information

Calc. Mass	Obsrv. Mass	± da	± ppm	Start	End	Sequence	Ion	C. I. %	Modification	Rank	Result Type

Calc. Mass	Obsrv. Mass	± da	± ppm	Start Seq.	End Seq.	Sequence	Ion Score	C. I. %	Modification	Rank	Result Type
1467.8054	1467.8813	0.0759	52	38	51	TLSGPVDDPTKLPK					Mascot
1467.8054	1467.8813	0.0759	52	38	51	TLSGPVDDPTKLPK					Mascot
1552.7251	1552.7704	0.0453	29	276	289	HAQHIAAYGEGNER					Mascot
1552.7251	1552.7704	0.0453	29	276	289	HAQHIAAYGEGNER	129	100			Mascot
1708.8263	1708.8668	0.0405	24	276	290	HAQHIAAYGEGNERR					Mascot
2600.2595	2600.325	0.0655	25	84	106	GNHILVMCDTYTPAGEPIPTNKR			Carbamidomethyl (C)[8], Oxidation (M)[7]		Mascot

5	hypothetical protein CHLNCDRAFT_133631 [Chlorella variabilis]	gi|307111618	166355.1	4.96	27	67	80.579	19.049	

Peptide Information

Calc. Mass	Obsrv. Mass	± da	± ppm	Start Seq.	End Seq.	Sequence	Ion Score	C. I. %	Modification	Rank	Result Type
857.4839	857.4862	0.0023	3	536	543	VAALEQAR					Mascot
1343.7026	1343.7076	0.005	4	495	505	LSAQDEQQRLR					Mascot
1345.7144	1345.6591	-0.0553	-41	1255	1267	EMAAASALLAIER					Mascot
1433.6941	1433.7799	0.0858	60	914	925	VTQLEEEMEAVR					Mascot
1457.7343	1457.7734	0.0391	27	626	638	AAEELQRDAEAVR					Mascot
1494.7772	1494.7866	0.0094	6	695	707	HEAEGLRQQLSAR					Mascot
1501.8156	1501.7388	-0.0768	-51	1254	1267	REMAAASALLAIER					Mascot
1511.8176	1511.8696	0.052	34	303	316	LQLAEQEAAAAQLR					Mascot
1535.7305	1535.8058	0.0753	49	981	992	EVAEMQEQLRMR			Oxidation (M)[5]		Mascot
1552.8806	1552.7704	-0.1102	-71	798	811	VLAEQQAVAVQLQR					Mascot
1552.8806	1552.7704	-0.1102	-71	798	811	VLAEQQAVAVQLQR					Mascot
1577.7839	1577.7385	-0.0454	-29	893	907	ASTVEAELAVVMGER			Oxidation (M)[12]		Mascot
1580.8391	1580.7953	-0.0438	-28	481	494	HQLLQLEGELSSAR					Mascot
1598.8207	1598.8107	-0.01	-6	1462	1476	ALMPADLAAQLEEAR					Mascot
1646.853	1646.8601	0.0071	4	1255	1269	EMAAASALLAIERER			Oxidation (M)[2]		Mascot
1646.853	1646.8601	0.0071	4	1255	1269	EMAAASALLAIERER			Oxidation (M)[2]		Mascot
1708.9817	1708.8668	-0.1149	-67	797	811	RVLAEQQAVAVQLQR					Mascot
1742.947	1742.943	-0.004	-2	288	302	VVVLQEQLVVAQDMR			Oxidation (M)[14]		Mascot
1745.8916	1745.9574	0.0658	38	317	333	TAKEEAEAAAAQLEVSK					Mascot
1786.8865	1786.9889	0.1024	57	1342	1359	QEVAAAAAMAADVDAARR					Mascot
1805.9729	1806.0167	0.0438	24	819	834	ALQHQDTVAAQLRQAR					Mascot
1809.7928	1809.9603	0.1675	93	117	131	VLMSQMHNTVAEDMR			Oxidation (M)[3,6,14]		Mascot
1885.9727	1885.9873	0.0146	8	172	189	LEAQLAASAGESGRLQER					Mascot

1898.9454	1899.0466	0.1012	53	1324	1341	EEAVGEAAALDAQLEQV R					Mascot
1898.9454	1899.0466	0.1012	53	1324	1341	EEAVGEAAALDAQLEQV R					Mascot
1908.8907	1908.9158	0.0251	13	654	670	DADHARQQLAQQEGDA R					Mascot
1912.0685	1911.9385	-0.13	-68	286	302	GKVVVLQEQLVVAQDMR					Mascot
1958.9526	1958.8795	-0.0731	-37	860	879	SAQGGASELQSGLEQAA AQR					Mascot
2020.979	2020.9868	0.0078	4	462	480	IGEMGSELAMAAGDREV LR			Oxidation (M)[4]		Mascot
2573.2512	2573.2998	0.0486	19	320	344	EEAEAAAAQLEVSKEAAE AAVAQMR					Mascot

6	hypothetical protein SORBIDRAFT_04g037140 [Sorghum bicolor]	gi|241932866	140204.2	6.03	24	65	69.92	18.747	

Peptide Information

Calc. Mass	Obsrv. Mass	± da	± ppm	Start Seq.	End Seq.	Sequence	Ion Score	C. I. %	Modification	Rank	Result Type
950.5166	950.4577	-0.0589	-62	890	898	SPAHPRSAK					Mascot
1224.6372	1224.5675	-0.0697	-57	910	920	SPLHPSYQPAK					Mascot
1233.5455	1233.6418	0.0963	78	48	62	AAGGGGGDTDAGATR					Mascot
1303.6133	1303.71	0.0967	74	2	14	PAGEMAMPAAVAR			Oxidation (M)[5,7]		Mascot
1311.7163	1311.7821	0.0658	50	105	114	RYLVLLMEMK			Oxidation (M)[7]		Mascot
1389.6025	1389.7111	0.1086	78	406	416	LYLQCDDMTSK			Carbamidomethyl (C)[5], Oxidation (M)[8]		Mascot
1415.6173	1415.7003	0.083	59	424	436	DDTLTTGSSFEDK					Mascot
1467.7699	1467.8813	0.1114	76	1207	1219	KAAMVCVVDAYLK			Carbamidomethyl (C)[6]		Mascot
1467.7699	1467.8813	0.1114	76	1207	1219	KAAMVCVVDAYLK			Carbamidomethyl (C)[6]		Mascot
1540.8087	1540.7919	-0.0168	-11	2	16	PAGEMAMPAAVARLR					Mascot
1552.7603	1552.7704	0.0101	7	644	657	GSKSTQEVAQNFEK					Mascot
1552.7689	1552.7704	0.0015	1	661	672	LVCRYLDDPHHK			Carbamidomethyl (C)[3]		Mascot
1608.7072	1608.8066	0.0994	62	1036	1048	MRNDSQDHELYGK			Oxidation (M)[1]		Mascot
1623.9105	1623.8337	-0.0768	-47	750	764	SKLAVLEFANASFVK					Mascot
1666.0011	1665.9008	-0.1003	-60	235	248	QLVTPLITQLLDRR					Mascot
1666.8218	1666.9081	0.0863	52	647	660	STQEVAQNFEKVMR					Mascot
1703.839	1703.9017	0.0627	37	1	16	MPAGEMAMPAAVARLR			Oxidation (M)[1,6]		Mascot
1756.8794	1757.0048	0.1254	71	106	119	YLVLLMEMKEMNAR			Oxidation (M)[6]		Mascot
1842.0055	1841.9354	-0.0701	-38	254	268	QACHLLNFLSKELLR			Carbamidomethyl (C)[3]		Mascot
1898.9342	1899.0466	0.1124	59	424	440	DDTLTTGSSFEDKITLR					Mascot
1898.9342	1899.0466	0.1124	59	424	440	DDTLTTGSSFEDKITLR					Mascot
1909.0178	1908.9158	-0.102	-53	724	740	HANESYPIDSLLPALLR					Mascot
1926.9517	1926.9119	-0.0398	-21	536	555	ENPVEVSSGAGSSGKVG					Mascot

						THK						
2087.0742	2087.1133	0.0391	19	573	589	RPLLSKQMTNWFYASTK			Oxidation (M)[8]		Mascot
2271.2998	2271.1506	-0.1492	-66	265	283	ELLRDFEPYAELLIPVLLK					Mascot
2664.3528	2664.3223	-0.0305	-11	910	933	SPLHPSYQPAKSPLHQA YQSNSVK					Mascot

7	hypothetical protein SORBIDRAFT_03g034310 [Sorghum bicolor]	gi|241928319	103814	7.29	20	62	38.584	17.767

Peptide Information

Calc. Mass	Obsrv. Mass	± da	± ppm	Start Seq.	End Seq.	Sequence	Ion Score	C. I. %	Modification	Rank	Result Type
856.4424	856.5103	0.0679	79	710	716	NAHVPYR					Mascot
1012.5574	1012.5301	-0.0273	-27	604	612	NAFSLHPVK					Mascot
1173.6725	1173.6902	0.0177	15	302	311	LELVEVEKSK					Mascot
1303.7229	1303.71	-0.0129	-10	880	891	NGQPANPKPKPR					Mascot
1343.7166	1343.7076	-0.009	-7	221	232	VVNGILDESIER					Mascot
1360.7318	1360.7415	0.0097	7	386	397	KVEEIESASQLK					Mascot
1440.7264	1440.8202	0.0938	65	663	675	GCLHLIDLAGSER			Carbamidomethyl (C)[2]		Mascot
1552.6888	1552.7704	0.0816	53	2	15	GNVDGEHEFHAANR					Mascot
1552.7788	1552.7704	-0.0084	-5	404	416	KANILQSFMDNQK			Oxidation (M)[9]		Mascot
1577.8785	1577.7385	-0.14	-89	776	788	DIKELLEQVSYLK					Mascot
1608.772	1608.8066	0.0346	22	318	330	LGEQDMVRLMQEK			Oxidation (M)[6,10]		Mascot
1683.7292	1683.8945	0.1653	98	1	15	MGNVDGEHEFHAANR					Mascot
1708.7899	1708.8668	0.0769	45	2	16	GNVDGEHEFHAANRR					Mascot
1730.8668	1730.9242	0.0574	33	115	131	DNVTTGLGQNITNNAAK					Mascot
1817.9426	1817.9065	-0.0361	-20	613	628	STLDVLELMQIGQTNR					Mascot
1817.9426	1817.9065	-0.0361	-20	613	628	STLDVLELMQIGQTNR					Mascot
1833.9375	1833.941	0.0035	2	613	628	STLDVLELMQIGQTNR			Oxidation (M)[9]		Mascot
1849.984	1849.8967	-0.0873	-47	405	420	ANILQSFMDNQKLSIK					Mascot
1855.8253	1855.9418	0.1165	63	1	16	MGNVDGEHEFHAANRR			Oxidation (M)[1]		Mascot
1898.9714	1899.0466	0.0752	40	424	439	ISSQSIKQEMFTLQMK					Mascot
1898.9714	1899.0466	0.0752	40	424	439	ISSQSIKQEMFTLQMK					Mascot
2087.0688	2087.1133	0.0445	21	138	156	LELEESDGPIISVMTPGK R			Oxidation (M)[14]		Mascot
2550.345	2550.303	-0.042	-16	499	521	TTVIDYIGENGEILITNPFK QGK					Mascot

8	unnamed protein product [Vitis vinifera]	gi|297737496	61447	5.59	15	62	31.09	5.875

Peptide Information

Calc. Mass	Obsrv. Mass	± da	± ppm	Start Seq.	End Seq.	Sequence	Ion Score	C. I. %	Modification	Rank	Result Type

960.5109	960.5875	0.0766	80	234	241	ASEQELKR					Mascot
960.5109	960.5875	0.0766	80	234	241	ASEQELKR					Mascot
1132.611	1132.6221	0.0111	10	465	474	QNLAVGDIFR					Mascot
1233.5494	1233.6418	0.0924	75	499	507	HKQEYDEER					Mascot
1337.6696	1337.7076	0.038	28	488	498	EVENSISEFKR					Mascot
1343.7893	1343.7076	-0.0817	-61	147	158	NVQKATIESILK					Mascot
1345.7073	1345.6591	-0.0482	-36	1	11	MELYFSTTLLK					Mascot
1358.7349	1358.7225	-0.0124	-9	362	373	LLPGCTTLEQVK			Carbamidomethyl (C)[5]		Mascot
1360.736	1360.7415	0.0055	4	198	209	YDIIVDVAPEVK					Mascot
1399.8308	1399.8088	-0.022	-16	300	312	VLPGFLDSIIGIR					Mascot
1786.9374	1786.9889	0.0515	29	521	534	VLEWLKEHAEIEYK					Mascot
1821.9131	1821.9871	0.074	41	61	78	NSIHFTAFSSPASVASAK					Mascot
1827.9811	1827.9741	-0.007	-4	218	233	NLKIVVEIDSDIDAQR					Mascot
1908.8895	1908.9158	0.0263	14	179	194	IVTKFSDMEETYSSQK			Oxidation (M)[8]		Mascot
1983.9746	1983.9059	-0.0687	-35	334	349	GVHAQFTVECKELFYR			Carbamidomethyl (C)[10]		Mascot
1983.9746	1983.9059	-0.0687	-35	334	349	GVHAQFTVECKELFYR			Carbamidomethyl (C)[10]		Mascot
2648.3354	2648.3406	0.0052	2	289	312	GFHFDTEDGDKVLPGFL DSIIGIR					Mascot

9 chlororespiratory reduction 4, partial [Raphanus sativus] gi|345505234 66083.5 8.7 18 61 17.153 8.554

Peptide Information

Calc. Mass	Obsrv. Mass	± da	± ppm	Start Seq.	End Seq.	Sequence	Ion Score	C. I. %	Modification	Rank	Result Type
925.485	925.5417	0.0567	61	237	244	HGRIEDAK					Mascot
950.4764	950.4577	-0.0187	-20	245	252	GLFDVMPR			Oxidation (M)[6]		Mascot
1192.5892	1192.5765	-0.0127	-11	149	157	QVFDRMPQR			Oxidation (M)[6]		Mascot
1224.6583	1224.5675	-0.0908	-74	19	30	IVLAFASSSSSR					Mascot
1501.7944	1501.7388	-0.0556	-37	111	123	EGMQVHGFLIKSR					Mascot
1575.7771	1575.7838	0.0067	4	459	471	LQHYGCMVDILAR			Carbamidomethyl (C)[6]		Mascot
1577.7152	1577.7385	0.0233	15	158	171	DSVSYNSMIDGYVK					Mascot
1598.7883	1598.8107	0.0224	14	223	236	DLISWNSMIGGYVK			Oxidation (M)[8]		Mascot
1739.8534	1739.9308	0.0774	44	191	205	NLITWNSMIGGYAQR			Oxidation (M)[8]		Mascot
1739.8785	1739.9308	0.0523	30	253	267	RDVVTWAIMIDGYGK			Oxidation (M)[9]		Mascot
1790.9623	1791.0042	0.0419	23	106	121	LGFAKEGMQVHGFLIK			Oxidation (M)[8]		Mascot
1809.8511	1809.9603	0.1092	60	172	187	CGLVESAGELFDLMPR			Carbamidomethyl (C)[1], Oxidation (M)[14]		Mascot
1817.8309	1817.9065	0.0756	42	285	300	DVVAYNSMMSGFVQNR					Mascot
1817.8309	1817.9065	0.0756	42	285	300	DVVAYNSMMSGFVQNR					Mascot

Calc. Mass	Obsrv. Mass	± da	± ppm	Start Seq.	End Seq.	Sequence	Ion Score	C. I. %	Modification	Rank	Result Type
1833.8259	1833.941	0.1151	63	285	300	DVVAYNSMMSGFVQNR			Oxidation (M)[8]		Mascot
1849.9	1849.8967	-0.0033	-2	206	222	ADGVNVASKLFDEMPEK					Mascot
1983.9845	1983.9059	-0.0786	-40	480	495	NLIEEMPIEPNDVIWR			Oxidation (M)[6]		Mascot
1983.9845	1983.9059	-0.0786	-40	480	495	NLIEEMPIEPNDVIWR			Oxidation (M)[6]		Mascot
2000.0852	2000.0529	-0.0323	-16	49	66	SVSPGEIKDPFLWNAVIK					Mascot
2021.0774	2020.9868	-0.0906	-45	12	30	NPNLTTRIVLAFASSSSS R					Mascot
2087.0525	2087.1133	0.0608	29	455	471	IEPRLQHYGCMVDILAR			Carbamidomethyl (C)[10], Oxidation (M)[11]		Mascot

10	TPA: putative phosphatidylinositol-4-phosphate 5-kinase family protein [Zea mays]	gi|414588637	98231.1	7.84	18	60	11.227	7.534

Peptide Information

Calc. Mass	Obsrv. Mass	± da	± ppm	Start Seq.	End Seq.	Sequence	Ion Score	C. I. %	Modification	Rank	Result Type
866.4115	866.468	0.0565	65	292	300	GDQPHGAGK					Mascot
927.5985	927.5176	-0.0809	-87	715	722	AEVKVLLR					Mascot
1201.6357	1201.6725	0.0368	31	183	193	KMSQLPAPASR			Oxidation (M)[2]		Mascot
1337.6121	1337.7076	0.0955	71	424	434	YDGAWEDTRPK					Mascot
1492.7543	1492.8175	0.0632	42	43	55	YQPSAGLEKAWSR					Mascot
1535.8291	1535.8058	-0.0233	-15	838	850	IMDYSLLVGIHFK					Mascot
1598.9351	1598.8107	-0.1244	-78	147	160	QHAQARPAPVVLRR					Mascot
1809.7939	1809.9603	0.1664	92	368	383	GTQAYANGDVYDGHWR					Mascot
1817.7694	1817.9065	0.1371	75	407	423	AGDMHGCGTVIWADGD R			Carbamidomethyl (C)[7]		Mascot
1817.7694	1817.9065	0.1371	75	407	423	AGDMHGCGTVIWADGD R			Carbamidomethyl (C)[7]		Mascot
1833.7644	1833.941	0.1766	96	407	423	AGDMHGCGTVIWADGD R			Carbamidomethyl (C)[7], Oxidation (M)[4]		Mascot
1873.9246	1874.0134	0.0888	47	393	406	YIWRHGHEYIGTWR					Mascot
1911.9059	1911.9385	0.0326	17	814	828	LPGTWFEEFCRQVDK			Carbamidomethyl (C)[10]		Mascot
1927.0331	1926.9119	-0.1212	-63	592	607	GHRNYELMLNLQLGIR					Mascot
1983.9308	1983.9059	-0.0249	-13	424	440	YDGAWEDTRPKGQGTF R					Mascot
1983.9308	1983.9059	-0.0249	-13	424	440	YDGAWEDTRPKGQGTF R					Mascot
2087.0315	2087.1133	0.0818	39	762	778	FVIMGNLFCSSYAIHRR			Carbamidomethyl (C)[9], Oxidation (M)[4]		Mascot
2186.0999	2186.1926	0.0927	42	760	777	VRFVIMGNLFCSSYAIHR			Carbamidomethyl (C)[11], Oxidation (M)[6]		Mascot
2438.2092	2438.2896	0.0804	33	783	804	GSSHGRMTDKPIDQISEH TTLK					Mascot
2454.2122	2454.2661	0.0539	22	161	182	RTYTIHIHTDTPNDANVAT SAR					Mascot
2648.2468	2648.3406	0.0938	35	539	563	RSSASLDLDMLQPAAAE GGETEEVR			Oxidation (M)[10]		Mascot

Gel Idx/Pos	221/I21	Instr./Gel Origin	BA2151/Sample Project 20140814	Process Status	Analysis Succeeded
Plate [#] Name	[1] Sample Project 20140814	Instrument Sample Name		Spectra	11

Rank	Protein Name	Accession No.	Protein MW	Protein PI	Pep. Count	Protein Score	Protein Score C. I. %	Intensity Matched	Total Ion Score	Total Ion C. I. %	Confirmed
1	PREDICTED: fructose-bisphosphate aldolase, chloroplastic-like isoform X2 [Setaria italica]	gi|514807945	35897.3	5.35	10	392	100	9.768	347	100	

Peptide Information

Calc. Mass	Obsrv. Mass	± da	± ppm	Start Seq.	End Seq.	Sequence	Ion Score	C. I. %	Modification	Rank	Result Type
822.4355	822.4597	0.0242	29	168	174	TFEVAQK					Mascot
873.4577	873.501	0.0433	50	132	139	EAAWGLAR					Mascot
901.5101	901.4962	-0.0139	-15	298	306	ANSLAQLGK					Mascot
947.4979	947.5263	0.0284	30	269	276	ALQNTCLK			Carbamidomethyl (C)[6]		Mascot
1141.5007	1141.5876	0.0869	76	307	317	YTSDGEAAEAK					Mascot
1143.5906	1143.6388	0.0482	42	277	286	TWGGRPENVK					Mascot
1143.5906	1143.6388	0.0482	42	277	286	TWGGRPENVK	25	0			Mascot
1169.7001	1169.6417	-0.0584	-50	287	297	AAQDALLLRAK					Mascot
1431.7074	1431.7681	0.0607	42	12	24	LDSIGLENTEANR					Mascot
1431.7074	1431.7681	0.0607	42	12	24	LDSIGLENTEANR	94	100			Mascot
1587.8085	1587.8683	0.0598	38	11	24	RLDSIGLENTEANR					Mascot
1587.8085	1587.8683	0.0598	38	11	24	RLDSIGLENTEANR	48	96.795			Mascot
3047.5684	3047.7188	0.1504	49	140	167	YAAISQDNGLVPIVEPEIL LDGEHGIER					Mascot
3047.5684	3047.7188	0.1504	49	140	167	YAAISQDNGLVPIVEPEIL LDGEHGIER	181	100			Mascot

Rank	Protein Name	Accession No.	Protein MW	Protein PI	Pep. Count	Protein Score	Protein Score C. I. %	Intensity Matched	Total Ion Score	Total Ion C. I. %	Confirmed
2	fructose-bisphosphate aldolase, class I, partial [Silene latifolia]	gi|343173173	42784.8	6.26	10	364	100	11.96	322	100	

Peptide Information

Calc. Mass	Obsrv. Mass	± da	± ppm	Start Seq.	End Seq.	Sequence	Ion Score	C. I. %	Modification	Rank	Result Type
873.4577	873.501	0.0433	50	200	207	EAAWGLAR					Mascot
947.4979	947.5263	0.0284	30	337	344	ALQNTCLK			Carbamidomethyl (C)[6]		Mascot
1175.6168	1175.632	0.0152	13	15	24	SDWVKGQSLR					Mascot
1175.6168	1175.632	0.0152	13	15	24	SDWVKGQSLR					Mascot
1194.55	1194.5503	0.0003	0	386	395	EGMFVKGYTY					Mascot
1431.7074	1431.7681	0.0607	42	80	92	LDSIGLENTEANR					Mascot
1431.7074	1431.7681	0.0607	42	80	92	LDSIGLENTEANR	94	100			Mascot

1587.8085	1587.8683	0.0598	38	79	92	RLDSIGLENTEANR					Mascot
1587.8085	1587.8683	0.0598	38	79	92	RLDSIGLENTEANR	48	96.795			Mascot
1675.9014	1676.0194	0.118	70	278	292	ATPEQVASYTLNLLR					Mascot
2051.988	2052.1704	0.1824	89	366	385	ANALAQLGKYTGEGESD EAK					Mascot
2185.084	2185.2559	0.1719	79	25	45	QVSSLSVSKCMPSASSS LSVK			Carbamidomethyl (C)[10], Oxidation (M)[11]		Mascot
3047.5684	3047.7188	0.1504	49	208	235	YAAISQDNGLVPIVEPEIL LDGEHGIER					Mascot
3047.5684	3047.7188	0.1504	49	208	235	YAAISQDNGLVPIVEPEIL LDGEHGIER	181	100			Mascot

3	PREDICTED: fructose-bisphosphate aldolase, chloroplastic-like isoform X1 [Setaria italica]	gi|514807943	37277.3	6.23	7	347	100	6.99	322	100	

Peptide Information

Calc. Mass	Obsrv. Mass	± da	± ppm	Start Seq.	End Seq.	Sequence	Ion Score	C. I. %	Modification	Rank	Result Type
822.4355	822.4597	0.0242	29	230	236	TFEVAQK					Mascot
873.4577	873.501	0.0433	50	194	201	EAAWGLAR					Mascot
947.5594	947.5263	-0.0331	-35	1	9	MALSATLLK					Mascot
1431.7074	1431.7681	0.0607	42	74	86	LDSIGLENTEANR					Mascot
1431.7074	1431.7681	0.0607	42	74	86	LDSIGLENTEANR	94	100			Mascot
1569.8782	1569.8785	0.0003	0	24	38	QAAAPKPVTVSMVVR			Oxidation (M)[12]		Mascot
1587.8085	1587.8683	0.0598	38	73	86	RLDSIGLENTEANR					Mascot
1587.8085	1587.8683	0.0598	38	73	86	RLDSIGLENTEANR	48	96.795			Mascot
3047.5684	3047.7188	0.1504	49	202	229	YAAISQDNGLVPIVEPEIL LDGEHGIER					Mascot
3047.5684	3047.7188	0.1504	49	202	229	YAAISQDNGLVPIVEPEIL LDGEHGIER	181	100			Mascot

4	hypothetical protein SORBIDRAFT_08g004500 [Sorghum bicolor]	gi|241943586	42013.6	6.39	13	317	100	27.941	256	100	

Peptide Information

Calc. Mass	Obsrv. Mass	± da	± ppm	Start Seq.	End Seq.	Sequence	Ion Score	C. I. %	Modification	Rank	Result Type
822.4355	822.4597	0.0242	29	230	236	TFEVAQK					Mascot
873.4577	873.501	0.0433	50	194	201	EAAWGLAR					Mascot
901.5101	901.4962	-0.0139	-15	360	368	ANSLAQLGK					Mascot
947.4979	947.5263	0.0284	30	331	338	ALQNTCLK			Carbamidomethyl (C)[6]		Mascot
1141.5007	1141.5876	0.0869	76	369	379	YTSDGEAAEAK					Mascot
1156.5382	1156.5863	0.0481	42	164	173	EAAYYQQGAR					Mascot
1156.5382	1156.5863	0.0481	42	164	173	EAAYYQQGAR	75	99.994			Mascot

Calc. Mass	Obsrv. Mass	± da	± ppm	Start Seq.	End Seq.	Sequence	Ion Score	C. I. %	Modification	Rank	Result Type
1387.7175	1387.7709	0.0534	38	74	86	LASIGLENTEANR					Mascot
1507.8552	1507.7993	-0.0559	-37	1	14	MVAASLLKSSFLPK			Oxidation (M)[1]		Mascot
1510.8475	1510.8711	0.0236	16	179	193	TVVSIPNGPSELAVK					Mascot
1543.8187	1543.9049	0.0862	56	73	86	RLASIGLENTEANR					Mascot
1592.9622	1592.9935	0.0313	20	123	137	IVDILVEQGIVPGIK					Mascot
1721.0571	1721.0935	0.0364	21	122	137	KIVDILVEQGIVPGIK					Mascot
3047.5684	3047.7188	0.1504	49	202	229	YAAISQDNGLVPIVEPEIL LDGEHGIER					Mascot
3047.5684	3047.7188	0.1504	49	202	229	YAAISQDNGLVPIVEPEIL LDGEHGIER	181	100			Mascot

5	fructose-bisphosphate aldolase 3 [Camellia oleifera]	gi|432139325	43094.3	8.44	11	317	100	12.299	269	100

Peptide Information

Calc. Mass	Obsrv. Mass	± da	± ppm	Start Seq.	End Seq.	Sequence	Ion Score	C. I. %	Modification	Rank	Result Type
871.4996	871.4879	-0.0117	-13	26	33	QPSVSVVR					Mascot
873.4577	873.501	0.0433	50	202	209	EAAWGLAR					Mascot
947.4979	947.5263	0.0284	30	339	346	ALQNTCLK			Carbamidomethyl (C)[6]		Mascot
984.5836	984.6262	0.0426	43	357	365	AAQEALLIR					Mascot
984.5836	984.6262	0.0426	43	357	365	AAQEALLIR	64	99.923			Mascot
1143.5906	1143.6388	0.0482	42	347	356	TWGGRPENVK					Mascot
1143.5906	1143.6388	0.0482	42	347	356	TWGGRPENVK	25	0			Mascot
1285.6934	1285.7229	0.0295	23	34	45	CLPTAPSALTVR			Carbamidomethyl (C)[1]		Mascot
1387.7175	1387.7709	0.0534	38	82	94	LASIGLENTEANR					Mascot
1543.8187	1543.9049	0.0862	56	81	94	RLASIGLENTEANR					Mascot
2052.156	2052.1704	0.0144	7	357	376	AAQEALLIRANANSLAQL GK					Mascot
2138.175	2138.1433	-0.0317	-15	26	45	QPSVSVVRCLPTAPSALT VR			Carbamidomethyl (C)[9]		Mascot
3047.5684	3047.7188	0.1504	49	210	237	YAAISQDNGLVPIVEPEIL LDGEHGIER					Mascot
3047.5684	3047.7188	0.1504	49	210	237	YAAISQDNGLVPIVEPEIL LDGEHGIER	181	100			Mascot

6	fructose-bisphosphate aldolase [Zea mays]	gi|413916271	38407.5	6.37	11	305	100	26.592	256	100

Peptide Information

Calc. Mass	Obsrv. Mass	± da	± ppm	Start Seq.	End Seq.	Sequence	Ion Score	C. I. %	Modification	Rank	Result Type
822.4355	822.4597	0.0242	29	193	199	TFEVAQK					Mascot
873.4577	873.501	0.0433	50	157	164	EAAWGLAR					Mascot
901.5101	901.4962	-0.0139	-15	323	331	ANSLAQLGK					Mascot
947.4979	947.5263	0.0284	30	294	301	ALQNTCLK			Carbamidomethyl (C)[6]		Mascot

1141.5007	1141.5876	0.0869	76	332	342	YTSDGEAAEAK						Mascot
1156.5382	1156.5863	0.0481	42	127	136	EAAYYQQGAR						Mascot
1156.5382	1156.5863	0.0481	42	127	136	EAAYYQQGAR	75	99.994				Mascot
1169.7001	1169.6417	-0.0584	-50	312	322	AAQDALLLRAK						Mascot
1387.7175	1387.7709	0.0534	38	37	49	LASIGLENTEANR						Mascot
1510.8475	1510.8711	0.0236	16	142	156	TVVSIPNGPSELAVK						Mascot
1543.8187	1543.9049	0.0862	56	36	49	RLASIGLENTEANR						Mascot
3047.5684	3047.7188	0.1504	49	165	192	YAAISQDNGLVPIVEPEIL LDGEHGIER						Mascot
3047.5684	3047.7188	0.1504	49	165	192	YAAISQDNGLVPIVEPEIL LDGEHGIER	181	100				Mascot

7	uncharacterized protein LOC100273196 [Zea mays]	gi|226492201	41895.5	7.63	11	301	100	26.592	256	100	

Protein Group

PREDICTED: fructose-bisphosphate aldolase, chloroplastic-like [Setaria italica]	gi|514804765	41979.6	6.0799 999237 0605
fructose-bisphosphate aldolase [Zea mays]	gi|413916272	41895.5	7.6300 001144 4092

Peptide Information

Calc. Mass	Obsrv. Mass	± da	± ppm	Start Seq.	End Seq.	Sequence	Ion Score	C. I. %	Modification	Rank	Result Type
822.4355	822.4597	0.0242	29	229	235	TFEVAQK					Mascot
873.4577	873.501	0.0433	50	193	200	EAAWGLAR					Mascot
901.5101	901.4962	-0.0139	-15	359	367	ANSLAQLGK					Mascot
947.4979	947.5263	0.0284	30	330	337	ALQNTCLK			Carbamidomethyl (C)[6]		Mascot
1141.5007	1141.5876	0.0869	76	368	378	YTSDGEAAEAK					Mascot
1156.5382	1156.5863	0.0481	42	163	172	EAAYYQQGAR					Mascot
1156.5382	1156.5863	0.0481	42	163	172	EAAYYQQGAR	75	99.994			Mascot
1169.7001	1169.6417	-0.0584	-50	348	358	AAQDALLLRAK					Mascot
1387.7175	1387.7709	0.0534	38	73	85	LASIGLENTEANR					Mascot
1510.8475	1510.8711	0.0236	16	178	192	TVVSIPNGPSELAVK					Mascot
1543.8187	1543.9049	0.0862	56	72	85	RLASIGLENTEANR					Mascot
3047.5684	3047.7188	0.1504	49	201	228	YAAISQDNGLVPIVEPEIL LDGEHGIER					Mascot
3047.5684	3047.7188	0.1504	49	201	228	YAAISQDNGLVPIVEPEIL	181	100			Mascot

			LDGEHGIER									
8	unnamed protein product [Vitis vinifera]	gi|297744727	29326.1	5.87	8	218	100	5.685	181	100		

Peptide Information

Calc. Mass	Obsrv. Mass	± da	± ppm	Start Seq.	End Seq.	Sequence	Ion Score	C. I. %	Modification	Rank	Result Type
822.4355	822.4597	0.0242	29	108	114	TFEVAQK					Mascot
873.4577	873.501	0.0433	50	72	79	EAAWGLAR					Mascot
901.5101	901.4962	-0.0139	-15	238	246	ANSLAQLGK					Mascot
947.4979	947.5263	0.0284	30	209	216	ALQNTCLK			Carbamidomethyl (C)[6]		Mascot
1042.5891	1042.5854	-0.0037	-4	227	235	EAQEALLIR					Mascot
1335.6791	1335.7206	0.0415	31	150	161	ATPEQVADYTLK					Mascot
2430.1719	2430.2747	0.1028	42	19	41	GLVPLVGSNDESWCQGL DGLASR			Carbamidomethyl (C)[14]		Mascot
3047.5684	3047.7188	0.1504	49	80	107	YAAISQDNGLVPIVEPEIL LDGEHGIER					Mascot
3047.5684	3047.7188	0.1504	49	80	107	YAAISQDNGLVPIVEPEIL LDGEHGIER	181	100			Mascot

9	hypothetical protein SORBIDRAFT_05g004590 [Sorghum bicolor]	gi|241936226	42125.4	5.85	10	203	100	7.554	166	100

Peptide Information

Calc. Mass	Obsrv. Mass	± da	± ppm	Start Seq.	End Seq.	Sequence	Ion Score	C. I. %	Modification	Rank	Result Type
822.4355	822.4597	0.0242	29	229	235	TFEVAQK					Mascot
873.4577	873.501	0.0433	50	193	200	EAAWGLAR					Mascot
901.5101	901.4962	-0.0139	-15	359	367	ANSLAQLGK					Mascot
947.4979	947.5263	0.0284	30	330	337	ALQNTCLK			Carbamidomethyl (C)[6]		Mascot
1143.5906	1143.6388	0.0482	42	338	347	TWGGRPENVK					Mascot
1143.5906	1143.6388	0.0482	42	338	347	TWGGRPENVK	25	0			Mascot
1169.7001	1169.6417	-0.0584	-50	348	358	AAQDALLLRAK					Mascot
1431.7074	1431.7681	0.0607	42	73	85	LDSIGLENTEANR					Mascot
1431.7074	1431.7681	0.0607	42	73	85	LDSIGLENTEANR	94	100			Mascot
1510.8475	1510.8711	0.0236	16	178	192	TVVSIPNGPSELAVK					Mascot
1583.8938	1583.8232	-0.0706	-45	23	37	QAATPKPVTVSMVVR					Mascot
1587.8085	1587.8683	0.0598	38	72	85	RLDSIGLENTEANR					Mascot
1587.8085	1587.8683	0.0598	38	72	85	RLDSIGLENTEANR	48	96.795			Mascot

10	fructose-bisphosphate aldolase [Zea mays]	gi|413925450	42630.8	6.19	10	203	100	7.477	166	100

Protein Group

uncharacterized protein LOC100272845 [Zea mays]	gi|226494664	42630.8	6.1900

Peptide Information

Calc. Mass	Obsrv. Mass	± da	± ppm	Start Seq.	End Seq.	Sequence	Ion Score	C. I. %	Modification	Rank	Result Type
822.4355	822.4597	0.0242	29	235	241	TFEVAQK					Mascot
873.4577	873.501	0.0433	50	199	206	EAAWGLAR					Mascot
901.5101	901.4962	-0.0139	-15	365	373	ANSLAQLGK					Mascot
947.4979	947.5263	0.0284	30	336	343	ALQNTCLK			Carbamidomethyl (C)[6]		Mascot
1143.5906	1143.6388	0.0482	42	344	353	TWGGRPENVK					Mascot
1143.5906	1143.6388	0.0482	42	344	353	TWGGRPENVK	25	0			Mascot
1169.7001	1169.6417	-0.0584	-50	354	364	AAQDALLLRAK					Mascot
1431.7074	1431.7681	0.0607	42	79	91	LDSIGLENTEANR					Mascot
1431.7074	1431.7681	0.0607	42	79	91	LDSIGLENTEANR	94	100			Mascot
1566.9102	1566.8159	-0.0943	-60	128	142	IVDVLSEQGIVPGIK					Mascot
1587.8085	1587.8683	0.0598	38	78	91	RLDSIGLENTEANR					Mascot
1587.8085	1587.8683	0.0598	38	78	91	RLDSIGLENTEANR	48	97.059			Mascot
1665.9244	1665.9258	0.0014	1	1	16	MASAATTLLKSSFLPK					Mascot

Gel Idx/Pos	222/I22	Instr./Gel Origin	BA2151/Sample Project 20140814	Process Status	Analysis Succeeded
Plate [#] Name	[1] Sample Project 20140814	Instrument Sample Name		Spectra	11

Rank	Protein Name	Accession No.	Protein MW	Protein PI	Pep. Count	Protein Score	Protein Score C. I. %	Intensity Matched	Total Ion Score	Total Ion C. I. %	Confirmed
1	Fructose-bisphosphate aldolase, chloroplastic [Aegilops tauschii]	gi|475589947	42176.5	5.94	19	610	100	40.034	489	100	

Peptide Information

Calc. Mass	Obsrv. Mass	± da	± ppm	Start Seq.	End Seq.	Sequence	Ion Score	C. I. %	Modification	Rank	Result Type
822.4355	822.4487	0.0132	16	229	235	TFEVAQK					Mascot
873.4577	873.4947	0.037	42	193	200	EAAWGLAR					Mascot
901.5101	901.495	-0.0151	-17	359	367	ANSLAQLGK					Mascot
947.4979	947.5132	0.0153	16	330	337	ALQNTCLK			Carbamidomethyl (C)[6]		Mascot
1156.5382	1156.5806	0.0424	37	163	172	EAAYYQQGAR					Mascot
1156.5382	1156.5806	0.0424	37	163	172	EAAYYQQGAR	74	99.993			Mascot
1308.6682	1308.6915	0.0233	18	271	282	ATPEEVASYTLK					Mascot
1387.7175	1387.769	0.0515	37	73	85	LASIGLENTEANR					Mascot
1387.7175	1387.769	0.0515	37	73	85	LASIGLENTEANR	112	100			Mascot
1510.8475	1510.8488	0.0013	1	178	192	TVVSIPNGPSELAVK					Mascot
1543.8187	1543.8806	0.0619	40	72	85	RLASIGLENTEANR					Mascot
1579.7963	1579.8947	0.0984	62	269	282	DRATPEEVASYTLK					Mascot
1592.9622	1592.9685	0.0063	4	122	136	IVDILVEQGIVPGIK					Mascot
1592.9622	1592.9685	0.0063	4	122	136	IVDILVEQGIVPGIK	68	99.975			Mascot
1617.8452	1617.8737	0.0285	18	23	37	QAAAPKPMTVSMVVR			Oxidation (M)[8,12]		Mascot
1622.7626	1622.8186	0.056	35	58	72	GILAMDESNATCGKR			Carbamidomethyl (C)[12]		Mascot
1721.0571	1721.0658	0.0087	5	121	136	KIVDILVEQGIVPGIK					Mascot
1818.996	1819.0166	0.0206	11	271	286	ATPEEVASYTLKLLQR					Mascot
1864.7905	1864.8185	0.028	15	368	384	YTSDGEAAEASENMFVK			Oxidation (M)[14]		Mascot
2052.0986	2052.1692	0.0706	34	338	356	TWGGRPENVAAAQEALL LR					Mascot
2052.0986	2052.1692	0.0706	34	338	356	TWGGRPENVAAAQEALL LR	124	100			Mascot
2430.1719	2430.2661	0.0942	39	140	162	GLVPLVGSNDESWCQGL DGLASR			Carbamidomethyl (C)[14]		Mascot
2430.1719	2430.2661	0.0942	39	140	162	GLVPLVGSNDESWCQGL DGLASR	112	100	Carbamidomethyl (C)[14]		Mascot
3081.5198	3081.6509	0.1311	43	201	228	YAAISQDNGLVPIVEPEIM LDGEHGIER			Oxidation (M)[19]		Mascot

Rank	Protein Name	Accession No.	Protein MW	Protein PI	Pep. Count	Protein Score	Protein Score C. I. %	Intensity Matched	Total Ion Score	Total Ion C. I. %	Confirmed
2	Fructose-bisphosphate aldolase, chloroplastic [Triticum urartu]	gi|473848356	42204.5	5.94	18	600	100	39.912	489	100	

Peptide Information

Calc. Mass	Obsrv. Mass	± da	± ppm	Start Seq.	End Seq.	Sequence	Ion Score	C. I. %	Modification	Rank	Result Type
822.4355	822.4487	0.0132	16	229	235	TFEVAQK					Mascot
873.4577	873.4947	0.037	42	193	200	EAAWGLAR					Mascot
901.5101	901.495	-0.0151	-17	359	367	ANSLAQLGK					Mascot
947.4979	947.5132	0.0153	16	330	337	ALQNTCLK			Carbamidomethyl (C)[6]		Mascot
1156.5382	1156.5806	0.0424	37	163	172	EAAYYQQGAR					Mascot
1156.5382	1156.5806	0.0424	37	163	172	EAAYYQQGAR	74	99.993			Mascot
1308.6682	1308.6915	0.0233	18	271	282	ATPEEVASYTLK					Mascot
1387.7175	1387.769	0.0515	37	73	85	LASIGLENTEANR					Mascot
1387.7175	1387.769	0.0515	37	73	85	LASIGLENTEANR	112	100			Mascot
1510.8475	1510.8488	0.0013	1	178	192	TVVSIPNGPSELAVK					Mascot
1543.8187	1543.8806	0.0619	40	72	85	RLASIGLENTEANR					Mascot
1579.7963	1579.8947	0.0984	62	269	282	DRATPEEVASYTLK					Mascot
1592.9622	1592.9685	0.0063	4	122	136	IVDILVEQGIVPGIK					Mascot
1592.9622	1592.9685	0.0063	4	122	136	IVDILVEQGIVPGIK	68	99.975			Mascot
1622.7626	1622.8186	0.056	35	58	72	GILAMDESNATCGKR			Carbamidomethyl (C)[12]		Mascot
1721.0571	1721.0658	0.0087	5	121	136	KIVDILVEQGIVPGIK					Mascot
1818.996	1819.0166	0.0206	11	271	286	ATPEEVASYTLKLLQR					Mascot
1864.7905	1864.8185	0.028	15	368	384	YTSDGEAAEASENMFVK			Oxidation (M)[14]		Mascot
2052.0986	2052.1692	0.0706	34	338	356	TWGGRPENVAAAQEALL LR					Mascot
2052.0986	2052.1692	0.0706	34	338	356	TWGGRPENVAAAQEALL LR	124	100			Mascot
2430.1719	2430.2661	0.0942	39	140	162	GLVPLVGSNDESWCQGL DGLASR			Carbamidomethyl (C)[14]		Mascot
2430.1719	2430.2661	0.0942	39	140	162	GLVPLVGSNDESWCQGL DGLASR	112	100	Carbamidomethyl (C)[14]		Mascot
3081.5198	3081.6509	0.1311	43	201	228	YAAISQDNGLVPIVEPEIM LDGEHGIER			Oxidation (M)[19]		Mascot

3	hypothetical protein SORBIDRAFT_08g004500 [Sorghum bicolor]	gi|241943586	42013.6	6.39	13	316	100	33.089	254	100	

Peptide Information

Calc. Mass	Obsrv. Mass	± da	± ppm	Start Seq.	End Seq.	Sequence	Ion Score	C. I. %	Modification	Rank	Result Type
822.4355	822.4487	0.0132	16	230	236	TFEVAQK					Mascot
873.4577	873.4947	0.037	42	194	201	EAAWGLAR					Mascot
901.5101	901.495	-0.0151	-17	360	368	ANSLAQLGK					Mascot

947.4979	947.5132	0.0153	16	331	338	ALQNTCLK			Carbamidomethyl (C)[6]		Mascot
1156.5382	1156.5806	0.0424	37	164	173	EAAYYQQGAR					Mascot
1156.5382	1156.5806	0.0424	37	164	173	EAAYYQQGAR	74	99.993			Mascot
1369.8162	1369.7133	-0.1029	-75	347	359	VTAAQDALLLRAK					Mascot
1387.7175	1387.769	0.0515	37	74	86	LASIGLENTEANR					Mascot
1387.7175	1387.769	0.0515	37	74	86	LASIGLENTEANR	112	100			Mascot
1510.8475	1510.8488	0.0013	1	179	193	TVVSIPNGPSELAVK					Mascot
1543.8187	1543.8806	0.0619	40	73	86	RLASIGLENTEANR					Mascot
1592.9622	1592.9685	0.0063	4	123	137	IVDILVEQGIVPGIK					Mascot
1592.9622	1592.9685	0.0063	4	123	137	IVDILVEQGIVPGIK	68	99.975			Mascot
1622.7626	1622.8186	0.056	35	59	73	GILAMDESNATCGKR			Carbamidomethyl (C)[12]		Mascot
1721.0571	1721.0658	0.0087	5	122	137	KIVDILVEQGIVPGIK					Mascot
3047.5684	3047.7173	0.1489	49	202	229	YAAISQDNGLVPIVEPEIL LDGEHGIER					Mascot

4	unnamed protein product [Vitis vinifera]	gi|297735045	37804.6	6.77	10	266	100	6.459	223	100	

Peptide Information

Calc. Mass	Obsrv. Mass	± da	± ppm	Start Seq.	End Seq.	Sequence	Ion Score	C. I. %	Modification	Rank	Result Type
822.4355	822.4487	0.0132	16	237	243	TFEVAQK					Mascot
871.4996	871.4766	-0.023	-26	26	33	QPSVSVVR					Mascot
873.4577	873.4947	0.037	42	201	208	EAAWGLAR					Mascot
903.4894	903.4735	-0.0159	-18	339	346	ESLRSPSK					Mascot
1387.7175	1387.769	0.0515	37	81	93	LASIGLENTEANR					Mascot
1387.7175	1387.769	0.0515	37	81	93	LASIGLENTEANR	112	100			Mascot
1415.8104	1415.7927	-0.0177	-13	2	15	ASASLLKSSPVLDK					Mascot
1543.8187	1543.8806	0.0619	40	80	93	RLASIGLENTEANR					Mascot
1546.851	1546.7986	-0.0524	-34	1	15	MASASLLKSSPVLDK					Mascot
1622.7626	1622.8186	0.056	35	66	80	GILAMDESNATCGKR			Carbamidomethyl (C)[12]		Mascot
2430.1719	2430.2661	0.0942	39	148	170	GLVPLVGSNDESWCQGL DGLASR			Carbamidomethyl (C)[14]		Mascot
2430.1719	2430.2661	0.0942	39	148	170	GLVPLVGSNDESWCQGL DGLASR	112	100	Carbamidomethyl (C)[14]		Mascot

5	fructose-bisphosphate aldolase [Zea mays]	gi|413916272	41895.5	7.63	11	232	100	30.993	185	100	

Protein Group

uncharacterized protein LOC100273196 [Zea mays]	gi|226492201	41895.5	7.6300 001144 4092

Peptide Information

Calc. Mass	Obsrv. Mass	± da	± ppm	Start Seq.	End Seq.	Sequence	Ion Score	C. I. %	Modification	Rank	Result Type
822.4355	822.4487	0.0132	16	229	235	TFEVAQK					Mascot
873.4577	873.4947	0.037	42	193	200	EAAWGLAR					Mascot
901.5101	901.495	-0.0151	-17	359	367	ANSLAQLGK					Mascot
947.4979	947.5132	0.0153	16	330	337	ALQNTCLK			Carbamidomethyl (C)[6]		Mascot
1156.5382	1156.5806	0.0424	37	163	172	EAAYYQQGAR					Mascot
1156.5382	1156.5806	0.0424	37	163	172	EAAYYQQGAR	74	99.993			Mascot
1387.7175	1387.769	0.0515	37	73	85	LASIGLENTEANR					Mascot
1387.7175	1387.769	0.0515	37	73	85	LASIGLENTEANR	112	100			Mascot
1510.8475	1510.8488	0.0013	1	178	192	TVVSIPNGPSELAVK					Mascot
1543.8187	1543.8806	0.0619	40	72	85	RLASIGLENTEANR					Mascot
1579.8738	1579.8947	0.0209	13	23	37	QAAAPRPPTVSMVVR					Mascot
1622.7626	1622.8186	0.056	35	58	72	GILAMDESNATCGKR			Carbamidomethyl (C)[12]		Mascot
3047.5684	3047.7173	0.1489	49	201	228	YAAISQDNGLVPIVEPEIL LDGEHGIER					Mascot

6	PREDICTED: fructose-bisphosphate aldolase, chloroplastic-like [Setaria italica]	gi|514804765	41979.6	6.08	11	231	100	31.016	185	100	

Peptide Information

Calc. Mass	Obsrv. Mass	± da	± ppm	Start Seq.	End Seq.	Sequence	Ion Score	C. I. %	Modification	Rank	Result Type
822.4355	822.4487	0.0132	16	229	235	TFEVAQK					Mascot
873.4577	873.4947	0.037	42	193	200	EAAWGLAR					Mascot
901.5101	901.495	-0.0151	-17	359	367	ANSLAQLGK					Mascot
904.4635	904.4595	-0.004	-4	15	22	KSEWGAAR					Mascot
947.4979	947.5132	0.0153	16	330	337	ALQNTCLK			Carbamidomethyl (C)[6]		Mascot
1156.5382	1156.5806	0.0424	37	163	172	EAAYYQQGAR					Mascot
1156.5382	1156.5806	0.0424	37	163	172	EAAYYQQGAR	74	99.993			Mascot
1387.7175	1387.769	0.0515	37	73	85	LASIGLENTEANR					Mascot
1387.7175	1387.769	0.0515	37	73	85	LASIGLENTEANR	112	100			Mascot
1510.8475	1510.8488	0.0013	1	178	192	TVVSIPNGPSELAVK					Mascot
1543.8187	1543.8806	0.0619	40	72	85	RLASIGLENTEANR					Mascot
1622.7626	1622.8186	0.056	35	58	72	GILAMDESNATCGKR			Carbamidomethyl (C)[12]		Mascot
3047.5684	3047.7173	0.1489	49	201	228	YAAISQDNGLVPIVEPEIL LDGEHGIER					Mascot

7	fructose-bisphosphate aldolase [Zea mays]	gi|413916271	38407.5	6.37	10	228	100	30.89	185	100	

Peptide Information

Calc. Mass	Obsrv. Mass	± da	± ppm	Start Seq.	End Seq.	Sequence	Ion Score	C. I. %	Modification	Rank	Result Type
822.4355	822.4487	0.0132	16	193	199	TFEVAQK					Mascot
873.4577	873.4947	0.037	42	157	164	EAAWGLAR					Mascot
901.5101	901.495	-0.0151	-17	323	331	ANSLAQLGK					Mascot
947.4979	947.5132	0.0153	16	294	301	ALQNTCLK			Carbamidomethyl (C)[6]		Mascot
1156.5382	1156.5806	0.0424	37	127	136	EAAYYQQGAR					Mascot
1156.5382	1156.5806	0.0424	37	127	136	EAAYYQQGAR	74	99.993			Mascot
1387.7175	1387.769	0.0515	37	37	49	LASIGLENTEANR					Mascot
1387.7175	1387.769	0.0515	37	37	49	LASIGLENTEANR	112	100			Mascot
1510.8475	1510.8488	0.0013	1	142	156	TVVSIPNGPSELAVK					Mascot
1543.8187	1543.8806	0.0619	40	36	49	RLASIGLENTEANR					Mascot
1622.7626	1622.8186	0.056	35	22	36	GILAMDESNATCGKR			Carbamidomethyl (C)[12]		Mascot
3047.5684	3047.7173	0.1489	49	165	192	YAAISQDNGLVPIVEPEIL LDGEHGIER					Mascot

8 RecName: Full=Fructose-bisphosphate aldolase, chloroplastic; Short=ALDP; Flags: Precursor gi|78099750 42207.6 6.38 10 223 100 31.517 185 100

Protein Group

Fructose-bisphosphate aldolase, chloroplast precursor, putative, expressed [Oryza sativa Japonica Group]	gi|108864046	42207.6	6.3800 001144 4092
Fructose-bisphosphate aldolase, chloroplast precursor, putative, expressed [Oryza sativa Japonica Group]	gi|108864048	41808.3	6.0700 001716 6138
Os11g0171300 [Oryza sativa Japonica Group]	gi|113644566	42207.6	6.3800 001144 4092
hypothetical protein OsI_35277 [Oryza sativa Indica Group]	gi|125533557	42207.6	6.3800 001144 4092

Peptide Information

Calc. Mass	Obsrv. Mass	± da	± ppm	Start	End	Sequence	Ion	C. I. %	Modification	Rank	Result Type

				Seq.	Seq.		Score				
822.4355	822.4487	0.0132	16	229	235	TFEVAQK					Mascot
873.4577	873.4947	0.037	42	193	200	EAAWGLAR					Mascot
901.5101	901.495	-0.0151	-17	359	367	ANSLAQLGK					Mascot
947.4979	947.5132	0.0153	16	330	337	ALQNTCLK			Carbamidomethyl (C)[6]		Mascot
1156.5382	1156.5806	0.0424	37	163	172	EAAYYQQGAR					Mascot
1156.5382	1156.5806	0.0424	37	163	172	EAAYYQQGAR	74	99.993			Mascot
1387.7175	1387.769	0.0515	37	73	85	LASIGLENTEANR					Mascot
1387.7175	1387.769	0.0515	37	73	85	LASIGLENTEANR	112	100			Mascot
1510.8475	1510.8488	0.0013	1	178	192	TVVSIPNGPSELAVK					Mascot
1543.8187	1543.8806	0.0619	40	72	85	RLASIGLENTEANR					Mascot
1622.8021	1622.8186	0.0165	10	269	282	DRATPEQVSDYTLK					Mascot
2067.0981	2067.1934	0.0953	46	338	356	TWGGQPENVKAAQDALLLR					Mascot

9	Fructose-bisphosphate aldolase, chloroplast precursor, putative, expressed [Oryza sativa Japonica Group]	gi|108864053	23731.5	9.47	7	219	100	30.866	185	100	

Peptide Information

Calc. Mass	Obsrv. Mass	± da	± ppm	Start Seq.	End Seq.	Sequence	Ion Score	C. I. %	Modification	Rank	Result Type
873.4577	873.4947	0.037	42	193	200	EAAWGLAR					Mascot
1156.5382	1156.5806	0.0424	37	163	172	EAAYYQQGAR					Mascot
1156.5382	1156.5806	0.0424	37	163	172	EAAYYQQGAR	74	99.993			Mascot
1387.7175	1387.769	0.0515	37	73	85	LASIGLENTEANR					Mascot
1387.7175	1387.769	0.0515	37	73	85	LASIGLENTEANR	112	100			Mascot
1510.8475	1510.8488	0.0013	1	178	192	TVVSIPNGPSELAVK					Mascot
1543.8187	1543.8806	0.0619	40	72	85	RLASIGLENTEANR					Mascot
1622.7626	1622.8186	0.056	35	58	72	GILAMDESNATCGKR			Carbamidomethyl (C)[12]		Mascot
1984.0763	1983.9185	-0.1578	-80	193	210	EAAWGLARYAAISQVHIK					Mascot

10	Fructose-bisphosphate aldolase, chloroplast precursor, putative, expressed [Oryza sativa Japonica Group]	gi|108864047	39556.2	6.85	8	212	100	30.019	185	100	

Peptide Information

Calc. Mass	Obsrv. Mass	± da	± ppm	Start Seq.	End Seq.	Sequence	Ion Score	C. I. %	Modification	Rank	Result Type
822.4355	822.4487	0.0132	16	204	210	TFEVAQK					Mascot
901.5101	901.495	-0.0151	-17	334	342	ANSLAQLGK					Mascot
947.4979	947.5132	0.0153	16	305	312	ALQNTCLK			Carbamidomethyl (C)[6]		Mascot

1156.5382	1156.5806	0.0424	37	163	172	EAAYYQQGAR			Mascot
1156.5382	1156.5806	0.0424	37	163	172	EAAYYQQGAR	74	99.992	Mascot
1387.7175	1387.769	0.0515	37	73	85	LASIGLENTEANR			Mascot
1387.7175	1387.769	0.0515	37	73	85	LASIGLENTEANR	112	100	Mascot
1543.8187	1543.8806	0.0619	40	72	85	RLASIGLENTEANR			Mascot
1622.8021	1622.8186	0.0165	10	244	257	DRATPEQVSDYTLK			Mascot
2067.0981	2067.1934	0.0953	46	313	331	TWGGQPENVKAAQDALL LR			Mascot

Gel Idx/Pos	223/I23	Instr./Gel Origin	BA2151/Sample Project 20140814	Process Status	Analysis Succeeded
Plate [#] Name	[1] Sample Project 20140814	Instrument Sample Name		Spectra	11

Rank	Protein Name	Accession No.	Protein MW	Protein PI	Pep. Count	Protein Score	Protein Score C. I. %	Intensity Matched	Total Ion Score	Total Ion C. I. %	Confirmed
1	Actin-97 [Triticum urartu]	gi|474287474	41929	5.23	16	637	100	65.141	542	100	

Protein Group

Protein Name	Accession No.	Protein MW	Protein PI
Actin-97 [Aegilops tauschii]	gi|475566477	41929	5.2300 000190 7349
Os05g0106600 [Oryza sativa Japonica Group]	gi|113577970	41897	5.2300 000190 7349

Peptide Information

Calc. Mass	Obsrv. Mass	± da	± ppm	Start Seq.	End Seq.	Sequence	Ion Score	C. I. %	Modification	Rank	Result Type
895.5359	895.569	0.0331	37	331	338	VVAPPERK					Mascot
976.4483	976.486	0.0377	39	21	30	AGFAGDDAPR					Mascot
1118.5114	1118.5577	0.0463	41	199	208	GYSFTTSAER					Mascot
1118.5114	1118.5577	0.0463	41	199	208	GYSFTTSAER	22	0			Mascot
1176.55	1176.6389	0.0889	76	42	52	HTGVMVGMGQK			Oxidation (M)[5,8]		Mascot
1182.5273	1182.587	0.0597	50	53	63	DAYVGDEAQSK					Mascot
1198.7056	1198.7498	0.0442	37	31	41	AVFPSIVGRPR					Mascot
1198.7056	1198.7498	0.0442	37	31	41	AVFPSIVGRPR	43	91.308			Mascot
1487.6761	1487.7434	0.0673	45	362	374	DEYDESGPAIVHR					Mascot
1487.6761	1487.7434	0.0673	45	362	374	DEYDESGPAIVHR	118	100			Mascot
1515.7491	1515.8101	0.061	40	87	97	IWHHTFYNELR					Mascot
1515.7491	1515.8101	0.061	40	87	97	IWHHTFYNELR	75	99.995			Mascot
1547.8098	1547.8048	-0.005	-3	180	193	LDLAGRDLTDSLMK					Mascot
1547.8098	1547.8048	-0.005	-3	180	193	LDLAGRDLTDSLMK					Mascot
1563.8047	1563.7969	-0.0078	-5	180	193	LDLAGRDLTDSLMK			Oxidation (M)[13]		Mascot
1747.8861	1747.9542	0.0681	39	241	256	SYELPDGQVITIGAER					Mascot
1747.8861	1747.9542	0.0681	39	241	256	SYELPDGQVITIGAER	135	100			Mascot
1855.9324	1855.9874	0.055	30	218	233	LAYVALDYEQELETAK					Mascot

1954.0645	1954.1345	0.07	36	98	115	VAPEEHPVLLTEAPLNPK					Mascot
1954.0645	1954.1345	0.07	36	98	115	VAPEEHPVLLTEAPLNPK	97	100			Mascot
2149.9563	2149.8726	-0.0837	-39	1	20	MADGEDIQPLVCDNGTG MVK			Carbamidomethyl (C)[12]		Mascot
2197.0959	2197.1804	0.0845	38	294	314	DLYGNIVLSGGTTMFPGI ADR					Mascot
2213.0908	2213.1753	0.0845	38	294	314	DLYGNIVLSGGTTMFPGI ADR			Oxidation (M)[14]		Mascot
2325.1909	2325.2661	0.0752	32	293	314	KDLYGNIVLSGGTTMFPG IADR					Mascot
2341.1858	2341.2668	0.081	35	293	314	KDLYGNIVLSGGTTMFPG IADR			Oxidation (M)[15]		Mascot
3151.6423	3151.7881	0.1458	46	150	179	TTGIVLDSGDGVSHTVPI YEGYALPHAILR					Mascot
3151.6423	3151.7881	0.1458	46	150	179	TTGIVLDSGDGVSHTVPI YEGYALPHAILR	52	98.723			Mascot

2	PREDICTED: actin-97-like [Setaria italica]	gi|514744123	41911	5.23	15	628	100	64.876	542	100	

Peptide Information

Calc. Mass	Obsrv. Mass	± da	± ppm	Start Seq.	End Seq.	Sequence	Ion Score	C. I. %	Modification	Rank	Result Type
895.5359	895.569	0.0331	37	331	338	VVAPPERK					Mascot
976.4483	976.486	0.0377	39	21	30	AGFAGDDAPR					Mascot
1118.5114	1118.5577	0.0463	41	199	208	GYSFTTSAER					Mascot
1118.5114	1118.5577	0.0463	41	199	208	GYSFTTSAER	22	0			Mascot
1176.55	1176.6389	0.0889	76	42	52	HTGVMVGMGQK			Oxidation (M)[5,8]		Mascot
1182.5273	1182.587	0.0597	50	53	63	DAYVGDEAQSK					Mascot
1198.7056	1198.7498	0.0442	37	31	41	AVFPSIVGRPR					Mascot
1198.7056	1198.7498	0.0442	37	31	41	AVFPSIVGRPR	43	91.308			Mascot
1487.6761	1487.7434	0.0673	45	362	374	DEYDESGPAIVHR					Mascot
1487.6761	1487.7434	0.0673	45	362	374	DEYDESGPAIVHR	118	100			Mascot
1515.7491	1515.8101	0.061	40	87	97	IWHHTFYNELR					Mascot
1515.7491	1515.8101	0.061	40	87	97	IWHHTFYNELR	75	99.995			Mascot
1547.8098	1547.8048	-0.005	-3	180	193	LDLAGRDLTDSLMK					Mascot
1547.8098	1547.8048	-0.005	-3	180	193	LDLAGRDLTDSLMK					Mascot
1563.8047	1563.7969	-0.0078	-5	180	193	LDLAGRDLTDSLMK			Oxidation (M)[13]		Mascot
1747.8861	1747.9542	0.0681	39	241	256	SYELPDGQVITIGAER					Mascot
1747.8861	1747.9542	0.0681	39	241	256	SYELPDGQVITIGAER	135	100			Mascot
1954.0645	1954.1345	0.07	36	98	115	VAPEEHPVLLTEAPLNPK					Mascot
1954.0645	1954.1345	0.07	36	98	115	VAPEEHPVLLTEAPLNPK	97	100			Mascot
2149.9563	2149.8726	-0.0837	-39	1	20	MADGEDIQPLVCDNGTG MVK			Carbamidomethyl (C)[12]		Mascot
2197.0959	2197.1804	0.0845	38	294	314	DLYGNIVLSGGTTMFPGI ADR					Mascot

Calc. Mass	Obsrv. Mass	± da	± ppm	Start Seq.	End Seq.	Sequence	Ion Score	C. I. %	Modification	Rank	Result Type
2213.0908	2213.1753	0.0845	38	294	314	DLYGNIVLSGGTTMFPGI ADR			Oxidation (M)[14]		Mascot
2325.1909	2325.2661	0.0752	32	293	314	KDLYGNIVLSGGTTMFPG IADR					Mascot
2341.1858	2341.2668	0.081	35	293	314	KDLYGNIVLSGGTTMFPG IADR			Oxidation (M)[15]		Mascot
3151.6423	3151.7881	0.1458	46	150	179	TTGIVLDSGDGVSHTVPI YEGYALPHAILR					Mascot
3151.6423	3151.7881	0.1458	46	150	179	TTGIVLDSGDGVSHTVPI YEGYALPHAILR	52	98.723			Mascot

3	PREDICTED: actin-7-like isoform X4 [Setaria italica]	gi|514787600	41925	5.23	15	627	100	63.172	542	100	

Peptide Information

Calc. Mass	Obsrv. Mass	± da	± ppm	Start Seq.	End Seq.	Sequence	Ion Score	C. I. %	Modification	Rank	Result Type
895.5359	895.569	0.0331	37	331	338	VVAPPERK					Mascot
976.4483	976.486	0.0377	39	21	30	AGFAGDDAPR					Mascot
1118.5114	1118.5577	0.0463	41	199	208	GYSFTTSAER					Mascot
1118.5114	1118.5577	0.0463	41	199	208	GYSFTTSAER	22	0			Mascot
1176.55	1176.6389	0.0889	76	42	52	HTGVMVGMGQK			Oxidation (M)[5,8]		Mascot
1182.5273	1182.587	0.0597	50	53	63	DAYVGDEAQSK					Mascot
1198.7056	1198.7498	0.0442	37	31	41	AVFPSIVGRPR					Mascot
1198.7056	1198.7498	0.0442	37	31	41	AVFPSIVGRPR	43	91.308			Mascot
1487.6761	1487.7434	0.0673	45	362	374	DEYDESGPAIVHR					Mascot
1487.6761	1487.7434	0.0673	45	362	374	DEYDESGPAIVHR	118	100			Mascot
1515.7491	1515.8101	0.061	40	87	97	IWHHTFYNELR					Mascot
1515.7491	1515.8101	0.061	40	87	97	IWHHTFYNELR	75	99.995			Mascot
1547.8098	1547.8048	-0.005	-3	180	193	LDLAGRDLTDSLMK					Mascot
1547.8098	1547.8048	-0.005	-3	180	193	LDLAGRDLTDSLMK					Mascot
1563.8047	1563.7969	-0.0078	-5	180	193	LDLAGRDLTDSLMK			Oxidation (M)[13]		Mascot
1747.8861	1747.9542	0.0681	39	241	256	SYELPDGQVITIGAER					Mascot
1747.8861	1747.9542	0.0681	39	241	256	SYELPDGQVITIGAER	135	100			Mascot
1855.9324	1855.9874	0.055	30	218	233	LAYVALDYEQELETAK					Mascot
1954.0645	1954.1345	0.07	36	98	115	VAPEEHPVLLTEAPLNPK					Mascot
1954.0645	1954.1345	0.07	36	98	115	VAPEEHPVLLTEAPLNPK	97	100			Mascot
2149.9563	2149.8726	-0.0837	-39	1	20	MADGEDIQPLVCDNGTG MVK			Carbamidomethyl (C)[12]		Mascot
2183.0801	2183.1589	0.0788	36	294	314	DLYGNIVLSGGSTMFPGI ADR					Mascot
2199.0752	2199.1707	0.0955	43	294	314	DLYGNIVLSGGSTMFPGI ADR			Oxidation (M)[14]		Mascot
3151.6423	3151.7881	0.1458	46	150	179	TTGIVLDSGDGVSHTVPI YEGYALPHAILR					Mascot
3151.6423	3151.7881	0.1458	46	150	179	TTGIVLDSGDGVSHTVPI	52	98.723			Mascot

	YEGYALPHAILR										
4	PREDICTED: actin-7-like isoform X2 [Setaria italica]	gi|514787592	44106.3	5.45	13	611	100	61.668	542	100

Peptide Information

Calc. Mass	Obsrv. Mass	± da	± ppm	Start Seq.	End Seq.	Sequence	Ion Score	C. I. %	Modification	Rank	Result Type
895.5359	895.569	0.0331	37	347	354	VVAPPERK					Mascot
1118.5114	1118.5577	0.0463	41	215	224	GYSFTTSAER					Mascot
1118.5114	1118.5577	0.0463	41	215	224	GYSFTTSAER	22	0			Mascot
1176.55	1176.6389	0.0889	76	58	68	HTGVMVGMGQK			Oxidation (M)[5,8]		Mascot
1182.5273	1182.587	0.0597	50	69	79	DAYVGDEAQSK					Mascot
1198.7056	1198.7498	0.0442	37	47	57	AVFPSIVGRPR					Mascot
1198.7056	1198.7498	0.0442	37	47	57	AVFPSIVGRPR	43	91.308			Mascot
1487.6761	1487.7434	0.0673	45	378	390	DEYDESGPAIVHR					Mascot
1487.6761	1487.7434	0.0673	45	378	390	DEYDESGPAIVHR	118	100			Mascot
1515.7491	1515.8101	0.061	40	103	113	IWHHTFYNELR					Mascot
1515.7491	1515.8101	0.061	40	103	113	IWHHTFYNELR	75	99.995			Mascot
1547.8098	1547.8048	-0.005	-3	196	209	LDLAGRDLTDSLMK					Mascot
1547.8098	1547.8048	-0.005	-3	196	209	LDLAGRDLTDSLMK					Mascot
1563.8047	1563.7969	-0.0078	-5	196	209	LDLAGRDLTDSLMK			Oxidation (M)[13]		Mascot
1747.8861	1747.9542	0.0681	39	257	272	SYELPDGQVITIGAER					Mascot
1747.8861	1747.9542	0.0681	39	257	272	SYELPDGQVITIGAER	135	100			Mascot
1855.9324	1855.9874	0.055	30	234	249	LAYVALDYEQELETAK					Mascot
1954.0645	1954.1345	0.07	36	114	131	VAPEEHPVLLTEAPLNPK					Mascot
1954.0645	1954.1345	0.07	36	114	131	VAPEEHPVLLTEAPLNPK	97	100			Mascot
2183.0801	2183.1589	0.0788	36	310	330	DLYGNIVLSGGSTMFPGI ADR					Mascot
2199.0752	2199.1707	0.0955	43	310	330	DLYGNIVLSGGSTMFPGI ADR			Oxidation (M)[14]		Mascot
3151.6423	3151.7881	0.1458	46	166	195	TTGIVLDSGDGVSHTVPI YEGYALPHAILR					Mascot
3151.6423	3151.7881	0.1458	46	166	195	TTGIVLDSGDGVSHTVPI YEGYALPHAILR	52	98.723			Mascot

5	uncharacterized protein LOC101779009 [Setaria italica]	gi|526117355	41884	5.31	15	594	100	59.692	508	100

Peptide Information

Calc. Mass	Obsrv. Mass	± da	± ppm	Start Seq.	End Seq.	Sequence	Ion Score	C. I. %	Modification	Rank	Result Type
895.5359	895.569	0.0331	37	331	338	VVAPPERK					Mascot
976.4483	976.486	0.0377	39	21	30	AGFAGDDAPR					Mascot
1132.527	1132.5756	0.0486	43	199	208	GYSFTTTAER					Mascot

Calc. Mass	Obsrv. Mass	± da	± ppm	Start Seq.	End Seq.	Sequence	Ion Score	C. I. %	Modification	Rank	Result Type
1176.55	1176.6389	0.0889	76	42	52	HTGVMVGMGQK			Oxidation (M)[5,8]		Mascot
1182.5273	1182.587	0.0597	50	53	63	DAYVGDEAQSK					Mascot
1198.7056	1198.7498	0.0442	37	31	41	AVFPSIVGRPR					Mascot
1198.7056	1198.7498	0.0442	37	31	41	AVFPSIVGRPR	43	91.308			Mascot
1429.6707	1429.7329	0.0622	44	362	374	GEYDESGPAIVHR					Mascot
1429.6707	1429.7329	0.0622	44	362	374	GEYDESGPAIVHR	106	100			Mascot
1515.7491	1515.8101	0.061	40	87	97	IWHHTFYNELR					Mascot
1515.7491	1515.8101	0.061	40	87	97	IWHHTFYNELR	75	99.995			Mascot
1547.8098	1547.8048	-0.005	-3	180	193	LDLAGRDLTDSLMK					Mascot
1547.8098	1547.8048	-0.005	-3	180	193	LDLAGRDLTDSLMK					Mascot
1563.8047	1563.7969	-0.0078	-5	180	193	LDLAGRDLTDSLMK			Oxidation (M)[13]		Mascot
1747.8861	1747.9542	0.0681	39	241	256	SYELPDGQVITIGAER					Mascot
1747.8861	1747.9542	0.0681	39	241	256	SYELPDGQVITIGAER	135	100			Mascot
1855.9324	1855.9874	0.055	30	218	233	LAYVALDYEQELETAK					Mascot
1954.0645	1954.1345	0.07	36	98	115	VAPEEHPVLLTEAPLNPK					Mascot
1954.0645	1954.1345	0.07	36	98	115	VAPEEHPVLLTEAPLNPK	97	100			Mascot
2149.9563	2149.8726	-0.0837	-39	1	20	MADGEDIQPLVCDNGTG MVK			Carbamidomethyl (C)[12]		Mascot
2183.0801	2183.1589	0.0788	36	294	314	DLYGNIVLSGGSTMFPGI ADR					Mascot
2199.0752	2199.1707	0.0955	43	294	314	DLYGNIVLSGGSTMFPGI ADR			Oxidation (M)[14]		Mascot
3151.6423	3151.7881	0.1458	46	150	179	TTGIVLDSGDGVSHTVPI YEGYALPHAILR					Mascot
3151.6423	3151.7881	0.1458	46	150	179	TTGIVLDSGDGVSHTVPI YEGYALPHAILR	52	98.723			Mascot

6	Actin 7 isoform 1 [Theobroma cacao]	gi|508705536	41753	5.31	15	592	100	59.601	508	100

Peptide Information

Calc. Mass	Obsrv. Mass	± da	± ppm	Start Seq.	End Seq.	Sequence	Ion Score	C. I. %	Modification	Rank	Result Type
890.4764	890.4863	0.0099	11	208	214	EIVRDMK					Mascot
895.5359	895.569	0.0331	37	330	337	VVAPPERK					Mascot
976.4483	976.486	0.0377	39	20	29	AGFAGDDAPR					Mascot
1132.527	1132.5756	0.0486	43	198	207	GYSFTTTAER					Mascot
1176.55	1176.6389	0.0889	76	41	51	HTGVMVGMGQK			Oxidation (M)[5,8]		Mascot
1182.5273	1182.587	0.0597	50	52	62	DAYVGDEAQSK					Mascot
1198.7056	1198.7498	0.0442	37	30	40	AVFPSIVGRPR					Mascot
1198.7056	1198.7498	0.0442	37	30	40	AVFPSIVGRPR	43	91.308			Mascot
1429.6707	1429.7329	0.0622	44	361	373	GEYDESGPAIVHR					Mascot

1429.6707	1429.7329	0.0622	44	361	373	GEYDESGPAIVHR	106	100		Mascot
1515.7491	1515.8101	0.061	40	86	96	IWHHTFYNELR				Mascot
1515.7491	1515.8101	0.061	40	86	96	IWHHTFYNELR	75	99.995		Mascot
1547.8098	1547.8048	-0.005	-3	179	192	LDLAGRDLTDSLMK				Mascot
1547.8098	1547.8048	-0.005	-3	179	192	LDLAGRDLTDSLMK				Mascot
1563.8047	1563.7969	-0.0078	-5	179	192	LDLAGRDLTDSLMK			Oxidation (M)[13]	Mascot
1747.8861	1747.9542	0.0681	39	240	255	SYELPDGQVITIGAER				Mascot
1747.8861	1747.9542	0.0681	39	240	255	SYELPDGQVITIGAER	135	100		Mascot
1855.9324	1855.9874	0.055	30	217	232	LAYVALDYEQELETAK				Mascot
1954.0645	1954.1345	0.07	36	97	114	VAPEEHPVLLTEAPLNPK				Mascot
1954.0645	1954.1345	0.07	36	97	114	VAPEEHPVLLTEAPLNPK	97	100		Mascot
2183.0801	2183.1589	0.0788	36	293	313	DLYGNIVLSGGSTMFPGI ADR				Mascot
2199.0752	2199.1707	0.0955	43	293	313	DLYGNIVLSGGSTMFPGI ADR			Oxidation (M)[14]	Mascot
3151.6423	3151.7881	0.1458	46	149	178	TTGIVLDSGDGVSHTVPI YEGYALPHAILR				Mascot
3151.6423	3151.7881	0.1458	46	149	178	TTGIVLDSGDGVSHTVPI YEGYALPHAILR	52	98.723		Mascot

7	hypothetical protein OsI_35242 [Oryza sativa Indica Group]	gi|218185309	41824	5.31	14	585	100	59.427	508	100

Protein Group

Actin-7, putative, expressed [Oryza sativa Japonica Group]	gi|108864035	41824	5.3099 999427 7954
Actin-7, putative, expressed [Oryza sativa Japonica Group]	gi|108864034	41824	5.3099 999427 7954
Actin-7, putative, expressed [Oryza sativa Japonica Group]	gi|77548871	41824	5.3099 999427 7954
Actin-7, putative, expressed [Oryza sativa Japonica Group]	gi|77548870	41824	5.3099 999427 7954
Actin-7, putative, expressed [Oryza sativa Japonica Group]	gi|77548869	41824	5.3099 999427 7954

Os11g0163100 [Oryza sativa Japonica Group]	gi|113644534	41824	5.3099 999427 7954

Peptide Information

Calc. Mass	Obsrv. Mass	± da	± ppm	Start Seq.	End Seq.	Sequence	Ion Score	C. I. %	Modification	Rank	Result Type
895.5359	895.569	0.0331	37	331	338	VVAPPERK					Mascot
976.4483	976.486	0.0377	39	21	30	AGFAGDDAPR					Mascot
1132.527	1132.5756	0.0486	43	199	208	GYSFTTTAER					Mascot
1176.55	1176.6389	0.0889	76	42	52	HTGVMVGMGQK			Oxidation (M)[5,8]		Mascot
1182.5273	1182.587	0.0597	50	53	63	DAYVGDEAQSK					Mascot
1198.7056	1198.7498	0.0442	37	31	41	AVFPSIVGRPR					Mascot
1198.7056	1198.7498	0.0442	37	31	41	AVFPSIVGRPR	43	91.308			Mascot
1429.6707	1429.7329	0.0622	44	362	374	GEYDESGPAIVHR					Mascot
1429.6707	1429.7329	0.0622	44	362	374	GEYDESGPAIVHR	106	100			Mascot
1515.7491	1515.8101	0.061	40	87	97	IWHHTFYNELR					Mascot
1515.7491	1515.8101	0.061	40	87	97	IWHHTFYNELR	75	99.995			Mascot
1547.8098	1547.8048	-0.005	-3	180	193	LDLAGRDLTDSLMK					Mascot
1547.8098	1547.8048	-0.005	-3	180	193	LDLAGRDLTDSLMK					Mascot
1563.8047	1563.7969	-0.0078	-5	180	193	LDLAGRDLTDSLMK			Oxidation (M)[13]		Mascot
1747.8861	1747.9542	0.0681	39	241	256	SYELPDGQVITIGAER					Mascot
1747.8861	1747.9542	0.0681	39	241	256	SYELPDGQVITIGAER	135	100			Mascot
1954.0645	1954.1345	0.07	36	98	115	VAPEEHPVLLTEAPLNPK					Mascot
1954.0645	1954.1345	0.07	36	98	115	VAPEEHPVLLTEAPLNPK	97	100			Mascot
2149.9563	2149.8726	-0.0837	-39	1	20	MADGEDIQPLVCDNGTG MVK			Carbamidomethyl (C)[12]		Mascot
2183.0801	2183.1589	0.0788	36	294	314	DLYGNIVLSGGSTMFPGI ADR					Mascot
2199.0752	2199.1707	0.0955	43	294	314	DLYGNIVLSGGSTMFPGI ADR			Oxidation (M)[14]		Mascot
3151.6423	3151.7881	0.1458	46	150	179	TTGIVLDSGDGVSHTVPI YEGYALPHAILR					Mascot
3151.6423	3151.7881	0.1458	46	150	179	TTGIVLDSGDGVSHTVPI YEGYALPHAILR	52	98.723			Mascot

8	Actin-3 [Triticum urartu]	gi|474259583	44624.2	5.26	13	570	100	53.609	508	100

Peptide Information

Calc. Mass	Obsrv. Mass	± da	± ppm	Start	End	Sequence	Ion	C. I. %	Modification	Rank	Result Type

				Seq.	Seq.		Score				
895.5359	895.569	0.0331	37	354	361	VVAPPERK					Mascot
976.4483	976.486	0.0377	39	44	53	AGFAGDDAPR					Mascot
1132.527	1132.5756	0.0486	43	222	231	GYSFTTTAER					Mascot
1176.55	1176.6389	0.0889	76	65	75	HTGVMVGMGQK			Oxidation (M)[5,8]		Mascot
1182.5273	1182.587	0.0597	50	76	86	DAYVGDEAQSK					Mascot
1198.7056	1198.7498	0.0442	37	54	64	AVFPSIVGRPR					Mascot
1198.7056	1198.7498	0.0442	37	54	64	AVFPSIVGRPR	43	91.308			Mascot
1429.6707	1429.7329	0.0622	44	385	397	GEYDESGPAIVHR					Mascot
1429.6707	1429.7329	0.0622	44	385	397	GEYDESGPAIVHR	106	100			Mascot
1515.7491	1515.8101	0.061	40	110	120	IWHHTFYNELR					Mascot
1515.7491	1515.8101	0.061	40	110	120	IWHHTFYNELR	75	99.995			Mascot
1747.8861	1747.9542	0.0681	39	264	279	SYELPDGQVITIGAER					Mascot
1747.8861	1747.9542	0.0681	39	264	279	SYELPDGQVITIGAER	135	100			Mascot
1868.9276	1869.0087	0.0811	43	241	256	LAYVALDYEQELENAK					Mascot
1954.0645	1954.1345	0.07	36	121	138	VAPEEHPVLLTEAPLNPK					Mascot
1954.0645	1954.1345	0.07	36	121	138	VAPEEHPVLLTEAPLNPK	97	100			Mascot
2183.0801	2183.1589	0.0788	36	317	337	DLYGNIVLSGGSTMFPGI ADR					Mascot
2199.0752	2199.1707	0.0955	43	317	337	DLYGNIVLSGGSTMFPGI ADR			Oxidation (M)[14]		Mascot
3151.6423	3151.7881	0.1458	46	173	202	TTGIVLDSGDGVSHTVPI YEGYALPHAILR					Mascot
3151.6423	3151.7881	0.1458	46	173	202	TTGIVLDSGDGVSHTVPI YEGYALPHAILR	52	98.723			Mascot

9	TPA: putative actin family protein [Zea mays]	gi|414588515	41894	5.31	16	535	100	62.027	424	100	

Protein Group

putative actin family protein [Zea mays]	gi|259490134	41894	5.3099 999427 7954

Peptide Information

Calc. Mass	Obsrv. Mass	± da	± ppm	Start Seq.	End Seq.	Sequence	Ion Score	C. I. %	Modification	Rank	Result Type
895.5359	895.569	0.0331	37	331	338	VVAPPERK					Mascot
976.4483	976.486	0.0377	39	21	30	AGFAGDDAPR					Mascot
1118.5114	1118.5577	0.0463	41	199	208	GYSFTTSAER					Mascot
1118.5114	1118.5577	0.0463	41	199	208	GYSFTTSAER	22	0			Mascot

1176.55	1176.6389	0.0889	76	42	52	HTGVMVGMGQK			Oxidation (M)[5,8]		Mascot
1182.5273	1182.587	0.0597	50	53	63	DAYVGDEAQSK					Mascot
1198.7056	1198.7498	0.0442	37	31	41	AVFPSIVGRPR					Mascot
1198.7056	1198.7498	0.0442	37	31	41	AVFPSIVGRPR	43	91.308			Mascot
1429.6707	1429.7329	0.0622	44	362	374	AEYDESGPGIVHR					Mascot
1429.6707	1429.7329	0.0622	44	362	374	AEYDESGPGIVHR	14	0			Mascot
1515.7491	1515.8101	0.061	40	87	97	IWHHTFYNELR					Mascot
1515.7491	1515.8101	0.061	40	87	97	IWHHTFYNELR	75	99.995			Mascot
1547.8098	1547.8048	-0.005	-3	180	193	LDLAGRDLTDSLMK					Mascot
1547.8098	1547.8048	-0.005	-3	180	193	LDLAGRDLTDSLMK					Mascot
1563.8047	1563.7969	-0.0078	-5	180	193	LDLAGRDLTDSLMK			Oxidation (M)[13]		Mascot
1747.8861	1747.9542	0.0681	39	241	256	SYELPDGQVITIGAER					Mascot
1747.8861	1747.9542	0.0681	39	241	256	SYELPDGQVITIGAER	135	100			Mascot
1868.9276	1869.0087	0.0811	43	218	233	LAYVALEYDQELENAK					Mascot
1954.0645	1954.1345	0.07	36	98	115	VAPEEHPVLLTEAPLNPK					Mascot
1954.0645	1954.1345	0.07	36	98	115	VAPEEHPVLLTEAPLNPK	97	100			Mascot
2149.9563	2149.8726	-0.0837	-39	1	20	MADGEDIQPLVCDNGTG MVK			Carbamidomethyl (C)[12]		Mascot
2197.0959	2197.1804	0.0845	38	294	314	DLYGNIVLSGGTTMFPGI ADR					Mascot
2213.0908	2213.1753	0.0845	38	294	314	DLYGNIVLSGGTTMFPGI ADR			Oxidation (M)[14]		Mascot
2325.1909	2325.2661	0.0752	32	293	314	KDLYGNIVLSGGTTMFPG IADR					Mascot
2341.1858	2341.2668	0.081	35	293	314	KDLYGNIVLSGGTTMFPG IADR			Oxidation (M)[15]		Mascot
3151.6423	3151.7881	0.1458	46	150	179	TTGIVLDSGDGVSHTVPI YEGYALPHAILR					Mascot
3151.6423	3151.7881	0.1458	46	150	179	TTGIVLDSGDGVSHTVPI YEGYALPHAILR	52	98.723			Mascot

10	TPA: putative actin family protein [Zea mays]	gi|414873809	41928.1	5.23	13	535	100	54.298	469	100	

Protein Group

uncharacterized protein LOC100273404 [Zea mays]	gi|226494516	41928.1	5.2300 000190 7349

Peptide Information

Calc. Mass	Obsrv. Mass	± da	± ppm	Start Seq.	End Seq.	Sequence	Ion Score	C. I. %	Modification	Rank	Result Type
895.5359	895.569	0.0331	37	331	338	VVAPPERK					Mascot
976.4483	976.486	0.0377	39	21	30	AGFAGDDAPR					Mascot
1132.527	1132.5756	0.0486	43	199	208	GYSFTTTAER					Mascot

1176.55	1176.6389	0.0889	76	42	52	HTGVMVGMGQK			Oxidation (M)[5,8]	Mascot
1182.5273	1182.587	0.0597	50	53	63	DAYVGDEAQSK				Mascot
1198.7056	1198.7498	0.0442	37	31	41	AVFPSIVGRPR				Mascot
1198.7056	1198.7498	0.0442	37	31	41	AVFPSIVGRPR	43	91.308		Mascot
1487.6761	1487.7434	0.0673	45	362	374	DEYDESGPAIVHR				Mascot
1487.6761	1487.7434	0.0673	45	362	374	DEYDESGPAIVHR	118	100		Mascot
1515.7491	1515.8101	0.061	40	87	97	IWHHTFYNELR				Mascot
1515.7491	1515.8101	0.061	40	87	97	IWHHTFYNELR	75	99.995		Mascot
1747.8861	1747.9542	0.0681	39	241	256	SYELPDGQVITIGAER				Mascot
1747.8861	1747.9542	0.0681	39	241	256	SYELPDGQVITIGAER	135	100		Mascot
1954.0645	1954.1345	0.07	36	98	115	VAPEEHPVLLTEAPLNPK				Mascot
1954.0645	1954.1345	0.07	36	98	115	VAPEEHPVLLTEAPLNPK	97	100		Mascot
2149.9563	2149.8726	-0.0837	-39	1	20	MADGEDIQPLVCDNGTG MVK			Carbamidomethyl (C)[12]	Mascot
2197.0959	2197.1804	0.0845	38	294	314	DLYGNIVLSGGTTMFPGI ADR				Mascot
2213.0908	2213.1753	0.0845	38	294	314	DLYGNIVLSGGTTMFPGI ADR			Oxidation (M)[14]	Mascot
2325.1909	2325.2661	0.0752	32	293	314	KDLYGNIVLSGGTTMFPG IADR				Mascot
2341.1858	2341.2668	0.081	35	293	314	KDLYGNIVLSGGTTMFPG IADR			Oxidation (M)[15]	Mascot
