# Supplementary material for: Proteomic analysis of middle and late stages of bread wheat (Triticum aestivum L.) grain development
Source: Front Plant Sci. 2015 Sep 15;6:735. doi: 10.3389/fpls.2015.00735 (PMC4569854; doi:10.3389/fpls.2015.00735)
Supplement: Supplementary file 10 [file DataSheet9.PDF]

Analysis Information

Report Type	Protein-Peptide Summary by Spot	Analysis Type	Combined (MS+MS/MS)
Sample Set Name	Sample set_20140814	Database	NCBI_VPlant
Analysis Name	R14026-4-VP2	Creation Date	09/30/2014 08:44:24
Reported By	09/30/2014 14:51:09 - admin	Last Modified	09/30/2014 09:28:38

MS Acq. : Proc. Methods	(Unspecified) : (Unspecified)
Interpretation Method	(Unspecified)

**Gel Idx/Pos**	240/J16	**Instr./Gel Origin**	BA2151/Sample Project 20140814	**Process Status**	Analysis Succeeded
**Plate [#] Name**	[1] Sample Project 20140814	**Instrument Sample Name**		**Spectra**	11

Rank	Protein Name	Accession No.	Protein MW	Protein PI	Pep. Count	Protein Score	Protein Score C. I. %	Intensity Matched	Total Ion Score	Total Ion C. I. %	Confirmed
1	Monodehydroascorbate reductase [Triticum urartu]	gi|474291016	47953.5	5.24	9	397	100	19.728	368	100	

Peptide Information

Calc. Mass	Obsrv. Mass	± da	± ppm	Start Seq.	End Seq.	Sequence	Ion Score	C. I. %	Modification	Rank	Result Type
809.3788	809.3802	0.0014	2	319	324	IYNDER					Mascot
978.4752	978.4617	-0.0135	-14	326	333	VEHVDHSR					Mascot
1176.6161	1176.594	-0.0221	-19	54	63	AYLFPQNPAR					Mascot
1425.7485	1425.746	-0.0025	-2	9	23	YVVLGGGVSGGYAAR					Mascot
1430.7638	1430.762	-0.0018	-1	41	53	EAVAPYERPALSK					Mascot
1471.7112	1471.7186	0.0074	5	64	77	LPGFHVCVGSGGER			Carbamidomethyl (C)[7]		Mascot
1471.7112	1471.7186	0.0074	5	64	77	LPGFHVCVGSGGER	72	99.988	Carbamidomethyl (C)[7]		Mascot
1837.9708	1838.0157	0.0449	24	6	23	HFKYVVLGGGVSGGYAAR					Mascot
1983.9771	1983.9939	0.0168	8	130	147	LSDFGTQGADSNNILYLR					Mascot
1983.9771	1983.9939	0.0168	8	130	147	LSDFGTQGADSNNILYLR	146	100			Mascot
2158.9604	2158.9907	0.0303	14	347	364	EAGSAVEEYDYLPYFYSR					Mascot
2158.9604	2158.9907	0.0303	14	347	364	EAGSAVEEYDYLPYFYSR	150	100			Mascot

Rank	Protein Name	Accession No.	Protein MW	Protein PI	Pep. Count	Protein Score	Protein Score C. I. %	Intensity Matched	Total Ion Score	Total Ion C. I. %	Confirmed
2	Monodehydroascorbate reductase [Aegilops tauschii]	gi|475575533	45020	5.39	7	387	100	18.991	368	100	

Peptide Information

Calc. Mass	Obsrv. Mass	± da	± ppm	Start	End	Sequence	Ion	C. I. %	Modification	Rank	Result Type

Calc. Mass	Obsrv. Mass	± da	± ppm	Start Seq.	End Seq.	Sequence	Ion Score	C. I. %	Modification	Rank	Result Type
809.3788	809.3802	0.0014	2	306	311	IYNDER					Mascot
978.4752	978.4617	-0.0135	-14	313	320	VEHVDHSR					Mascot
1176.6161	1176.594	-0.0221	-19	54	63	AYLFPQNPAR					Mascot
1430.7638	1430.762	-0.0018	-1	41	53	EAVAPYERPALSK					Mascot
1471.7112	1471.7186	0.0074	5	64	77	LPGFHVCVGSGGER			Carbamidomethyl (C)[7]		Mascot
1471.7112	1471.7186	0.0074	5	64	77	LPGFHVCVGSGGER	72	99.988	Carbamidomethyl (C)[7]		Mascot
1983.9771	1983.9939	0.0168	8	130	147	LSDFGTQGADSNNILYLR					Mascot
1983.9771	1983.9939	0.0168	8	130	147	LSDFGTQGADSNNILYLR	146	100			Mascot
2158.9604	2158.9907	0.0303	14	334	351	EAGSAVEEYDYLPYFYSR					Mascot
2158.9604	2158.9907	0.0303	14	334	351	EAGSAVEEYDYLPYFYSR	150	100			Mascot

3	actin 7, partial [Dendrocalamus latiflorus]	gi|482667546	30315.1	4.97	13	246	100	14.636	164	100	

Peptide Information

Calc. Mass	Obsrv. Mass	± da	± ppm	Start Seq.	End Seq.	Sequence	Ion Score	C. I. %	Modification	Rank	Result Type
800.5352	800.4624	-0.0728	-91	63	69	RGILTLK					Mascot
976.4483	976.4434	-0.0049	-5	20	29	AGFAGDDAPR					Mascot
1132.527	1132.5295	0.0025	2	198	207	GYSFTTTAER					Mascot
1176.55	1176.594	0.044	37	41	51	HTGVMVGMGQK			Oxidation (M)[5,8]		Mascot
1198.7056	1198.6981	-0.0075	-6	30	40	AVFPSIVGRPR					Mascot
1515.7491	1515.7528	0.0037	2	86	96	IWHHTFYNELR					Mascot
1547.8098	1547.7478	-0.062	-40	179	192	LDLAGRDLTDSLMK					Mascot
1547.8098	1547.7478	-0.062	-40	179	192	LDLAGRDLTDSLMK					Mascot
1747.8861	1747.8982	0.0121	7	240	255	SYELPDGQVITIGAER					Mascot
1883.9385	1883.9622	0.0237	13	217	232	LAYVALDYEQELETAR					Mascot
1883.9385	1883.9622	0.0237	13	217	232	LAYVALDYEQELETAR	109	100			Mascot
1954.0645	1954.0579	-0.0066	-3	97	114	VAPEEHPVLLTEAPLNPK					Mascot
1975.91	1976.0479	0.1379	70	2	19	AEEDIQPLVCDNGTGMVK			Carbamidomethyl (C)[10]		Mascot
2141.0762	2140.9949	-0.0813	-38	215	232	EKLAYVALDYEQELETAR					Mascot
3181.6528	3181.7122	0.0594	19	149	178	TTGIVLDSGDGVSHTVPIYEGYTLPHAILR					Mascot
3181.6528	3181.7122	0.0594	19	149	178	TTGIVLDSGDGVSHTVPIYEGYTLPHAILR	55	99.44			Mascot

4	Actin-7 [Triticum urartu]	gi|474317121	41833.9	5.29	15	244	100	13.967	164	100	

Protein Group

Actin-7 [Aegilops tauschii]	gi|475561024	41833.9	5.2899

Peptide Information

Calc. Mass	Obsrv. Mass	± da	± ppm	Start Seq.	End Seq.	Sequence	Ion Score	C. I. %	Modification	Rank	Result Type
800.5352	800.4624	-0.0728	-91	63	69	RGILTLK					Mascot
976.4483	976.4434	-0.0049	-5	20	29	AGFAGDDAPR					Mascot
1098.5426	1098.516	-0.0266	-24	198	207	GYSLTTTAER					Mascot
1176.55	1176.594	0.044	37	41	51	HTGVMVGMGQK			Oxidation (M)[5,8]		Mascot
1198.7056	1198.6981	-0.0075	-6	30	40	AVFPSIVGRPR					Mascot
1515.7491	1515.7528	0.0037	2	86	96	IWHHTFYNELR					Mascot
1797.8324	1797.8264	-0.006	-3	240	255	SYEMPDGQVITIGSER			Oxidation (M)[4]		Mascot
1883.9385	1883.9622	0.0237	13	217	232	LAYVALDYEQELETAR					Mascot
1883.9385	1883.9622	0.0237	13	217	232	LAYVALDYEQELETAR	109	100			Mascot
1940.0488	1940.0363	-0.0125	-6	97	114	VAPEDHPVLLTEAPLNPK					Mascot
1963.8737	1964.0367	0.163	83	2	19	ADEDVQPIVCDNGTGMV K			Carbamidomethyl (C)[10], Oxidation (M)[16]		Mascot
1975.8855	1976.0479	0.1624	82	70	85	YPIEHGIVNNWDDMEK			Oxidation (M)[14]		Mascot
2141.0762	2140.9949	-0.0813	-38	215	232	EKLAYVALDYEQELETAR					Mascot
2169.0645	2169.092	0.0275	13	293	313	DLYGNVVLSGGSTMFPGI ADR					Mascot
2185.0596	2185.071	0.0114	5	293	313	DLYGNVVLSGGSTMFPGI ADR			Oxidation (M)[14]		Mascot
2313.1545	2313.1672	0.0127	5	292	313	KDLYGNVVLSGGSTMFP GIADR			Oxidation (M)[15]		Mascot
3181.6528	3181.7122	0.0594	19	149	178	TTGIVLDSGDGVSHTVPI YEGYTLPHAILR					Mascot
3181.6528	3181.7122	0.0594	19	149	178	TTGIVLDSGDGVSHTVPI YEGYTLPHAILR	55	99.44			Mascot

5 PREDICTED: actin-1-like isoform X2 [Setaria italica] gi|514749797 41876.9 5.22 14 236 100 13.104 164 100

Peptide Information

Calc. Mass	Obsrv. Mass	± da	± ppm	Start Seq.	End Seq.	Sequence	Ion Score	C. I. %	Modification	Rank	Result Type
800.5352	800.4624	-0.0728	-91	63	69	RGILTLK					Mascot
976.4483	976.4434	-0.0049	-5	20	29	AGFAGDDAPR					Mascot
1176.55	1176.594	0.044	37	41	51	HTGVMVGMGQK			Oxidation (M)[5,8]		Mascot
1198.7056	1198.6981	-0.0075	-6	30	40	AVFPSIVGRPR					Mascot
1515.7491	1515.7528	0.0037	2	86	96	IWHHTFYNELR					Mascot
1600.8363	1600.7819	-0.0544	-34	185	197	DLTDHLMKILTER			Oxidation (M)[7]		Mascot
1797.8324	1797.8264	-0.006	-3	240	255	SYEMPDGQVITIGSER			Oxidation (M)[4]		Mascot
1883.9385	1883.9622	0.0237	13	217	232	LAYVALDYEQELETAR					Mascot

Calc. Mass	Obsrv. Mass	± da	± ppm	Start Seq.	End Seq.	Sequence	Ion Score	C. I. %	Modification	Rank	Result Type
1883.9385	1883.9622	0.0237	13	217	232	LAYVALDYEQELETAR	109	100			Mascot
1940.0488	1940.0363	-0.0125	-6	97	114	VAPEDHPVLLTEAPLNPK					Mascot
1975.8855	1976.0479	0.1624	82	70	85	YPIEHGIVNNWDDMEK			Oxidation (M)[14]		Mascot
2141.0762	2140.9949	-0.0813	-38	215	232	EKLAYVALDYEQELETAR					Mascot
2169.0645	2169.092	0.0275	13	293	313	DLYGNVVLSGGSTMFPGI ADR					Mascot
2185.0596	2185.071	0.0114	5	293	313	DLYGNVVLSGGSTMFPGI ADR			Oxidation (M)[14]		Mascot
2313.1545	2313.1672	0.0127	5	292	313	KDLYGNVVLSGGSTMFP GIADR			Oxidation (M)[15]		Mascot
3181.6528	3181.7122	0.0594	19	149	178	TTGIVLDSGDGVSHTVPI YEGYTLPHAILR					Mascot
3181.6528	3181.7122	0.0594	19	149	178	TTGIVLDSGDGVSHTVPI YEGYTLPHAILR	55	99.44			Mascot

6	PREDICTED: actin-1-like isoform X1 [Setaria italica]	gi|514749793	51881.7	8.85	14	231	100	13.104	164	100

Peptide Information

Calc. Mass	Obsrv. Mass	± da	± ppm	Start Seq.	End Seq.	Sequence	Ion Score	C. I. %	Modification	Rank	Result Type
800.5352	800.4624	-0.0728	-91	63	69	RGILTLK					Mascot
976.4483	976.4434	-0.0049	-5	20	29	AGFAGDDAPR					Mascot
1176.55	1176.594	0.044	37	41	51	HTGVMVGMGQK			Oxidation (M)[5,8]		Mascot
1198.7056	1198.6981	-0.0075	-6	30	40	AVFPSIVGRPR					Mascot
1515.7491	1515.7528	0.0037	2	86	96	IWHHTFYNELR					Mascot
1600.8363	1600.7819	-0.0544	-34	185	197	DLTDHLMKILTER			Oxidation (M)[7]		Mascot
1797.8324	1797.8264	-0.006	-3	240	255	SYEMPDGQVITIGSER			Oxidation (M)[4]		Mascot
1883.9385	1883.9622	0.0237	13	217	232	LAYVALDYEQELETAR					Mascot
1883.9385	1883.9622	0.0237	13	217	232	LAYVALDYEQELETAR	109	100			Mascot
1940.0488	1940.0363	-0.0125	-6	97	114	VAPEDHPVLLTEAPLNPK					Mascot
1975.8855	1976.0479	0.1624	82	70	85	YPIEHGIVNNWDDMEK			Oxidation (M)[14]		Mascot
2141.0762	2140.9949	-0.0813	-38	215	232	EKLAYVALDYEQELETAR					Mascot
2169.0645	2169.092	0.0275	13	293	313	DLYGNVVLSGGSTMFPGI ADR					Mascot
2185.0596	2185.071	0.0114	5	293	313	DLYGNVVLSGGSTMFPGI ADR			Oxidation (M)[14]		Mascot
2313.1545	2313.1672	0.0127	5	292	313	KDLYGNVVLSGGSTMFP GIADR			Oxidation (M)[15]		Mascot
3181.6528	3181.7122	0.0594	19	149	178	TTGIVLDSGDGVSHTVPI YEGYTLPHAILR					Mascot
3181.6528	3181.7122	0.0594	19	149	178	TTGIVLDSGDGVSHTVPI YEGYTLPHAILR	55	99.44			Mascot

7	Os09g0567300 [Oryza sativa Japonica Group]	gi|113632193	46758.2	5.53	4	226	100	12.03	218	100

Peptide Information

Calc. Mass	Obsrv. Mass	± da	± ppm	Start Seq.	End Seq.	Sequence	Ion Score	C. I. %	Modification	Rank	Result Type

1430.7638	1430.762	-0.0018	-1	41	53	EAVAPYERPALSK					Mascot
1466.8213	1466.7603	-0.061	-42	414	426	TQPPVANIEELKK					Mascot
1471.7112	1471.7186	0.0074	5	64	77	LPGFHVCVGSGGER			Carbamidomethyl (C)[7]		Mascot
1471.7112	1471.7186	0.0074	5	64	77	LPGFHVCVGSGGER	72	99.988	Carbamidomethyl (C)[7]		Mascot
1983.9771	1983.9939	0.0168	8	130	147	LSDFGTQGADSNNILYLR					Mascot
1983.9771	1983.9939	0.0168	8	130	147	LSDFGTQGADSNNILYLR	146	100			Mascot

8	hypothetical protein OsI_32437 [Oryza sativa Indica Group]	gi|125564749	47977	5.53	3	224	100	11.137	218	100	

Peptide Information

Calc. Mass	Obsrv. Mass	± da	± ppm	Start Seq.	End Seq.	Sequence	Ion Score	C. I. %	Modification	Rank	Result Type
1466.8213	1466.7603	-0.061	-42	426	438	TQPPVANIEELKK					Mascot
1471.7112	1471.7186	0.0074	5	76	89	LPGFHVCVGSGGER			Carbamidomethyl (C)[7]		Mascot
1471.7112	1471.7186	0.0074	5	76	89	LPGFHVCVGSGGER	72	99.988	Carbamidomethyl (C)[7]		Mascot
1983.9771	1983.9939	0.0168	8	142	159	LSDFGTQGADSNNILYLR					Mascot
1983.9771	1983.9939	0.0168	8	142	159	LSDFGTQGADSNNILYLR	146	100			Mascot

9	PREDICTED: actin-7-like [Setaria italica]	gi|514784852	41871.9	5.23	11	212	100	11.527	164	100	

Peptide Information

Calc. Mass	Obsrv. Mass	± da	± ppm	Start Seq.	End Seq.	Sequence	Ion Score	C. I. %	Modification	Rank	Result Type
800.5352	800.4624	-0.0728	-91	63	69	RGILTLK					Mascot
976.4483	976.4434	-0.0049	-5	20	29	AGFAGDDAPR					Mascot
1132.527	1132.5295	0.0025	2	198	207	GYSFTTTAER					Mascot
1176.55	1176.594	0.044	37	41	51	HTGVMVGMGQK			Oxidation (M)[5,8]		Mascot
1198.7056	1198.6981	-0.0075	-6	30	40	AVFPSIVGRPR					Mascot
1515.7491	1515.7528	0.0037	2	86	96	IWHHTFYNELR					Mascot
1747.8861	1747.8982	0.0121	7	240	255	SYELPDGQVITIGAER					Mascot
1883.9385	1883.9622	0.0237	13	217	232	LAYVALDYEQELETAR					Mascot
1883.9385	1883.9622	0.0237	13	217	232	LAYVALDYEQELETAR	109	100			Mascot
1975.91	1976.0479	0.1379	70	2	19	AEEDIQPLVCDNGTGMVK			Carbamidomethyl (C)[10]		Mascot
2141.0762	2140.9949	-0.0813	-38	215	232	EKLAYVALDYEQELETAR					Mascot
3181.6528	3181.7122	0.0594	19	149	178	TTGIVLDSGDGVSHTVPIYEGYTLPHAILR					Mascot
3181.6528	3181.7122	0.0594	19	149	178	TTGIVLDSGDGVSHTVPIYEGYTLPHAILR	55	99.44			Mascot

10	actin, partial [Echinochloa phyllopogon]	gi|497157174	22355.2	4.87	7	206	100	10.583	164	100	

Peptide Information

Calc. Mass	Obsrv. Mass	± da	± ppm	Start Seq.	End Seq.	Sequence	Ion Score	C. I. %	Modification	Rank	Result Type
1515.7491	1515.7528	0.0037	2	11	21	IWHHTFYNELR					Mascot
1600.8363	1600.7819	-0.0544	-34	110	122	DLTDHLMKILTER			Oxidation (M)[7]		Mascot
1797.8324	1797.8264	-0.006	-3	165	180	SYEMPDGQVITIGSER			Oxidation (M)[4]		Mascot
1883.9385	1883.9622	0.0237	13	142	157	LAYVALDYEQELETAR					Mascot
1883.9385	1883.9622	0.0237	13	142	157	LAYVALDYEQELETAR	109	100			Mascot
1908.9347	1909.0498	0.1151	60	181	196	FRCPEVLFQPSLVGME			Carbamidomethyl (C)[3]		Mascot
2141.0762	2140.9949	-0.0813	-38	140	157	EKLAYVALDYEQELETAR					Mascot
3181.6528	3181.7122	0.0594	19	74	103	TTGIVLDSGDGVSHTVPI YEGYTLPHAILR					Mascot
3181.6528	3181.7122	0.0594	19	74	103	TTGIVLDSGDGVSHTVPI YEGYTLPHAILR	55	99.998			Mascot

Rank	Protein Name	Accession No.	Protein MW	Protein PI	Pep. Count	Protein Score	Protein Score C. I. %	Intensity Matched	Total Ion Score	Total Ion C. I. %	Confirmed
1	photosystem I assembly protein Ycf4 [Huperzia lucidula]	gi|60117184	21491.1	9.02	7	37	0	1.796			

Protein Group

RecName: Full=Photosystem I assembly protein Ycf4	gi|68053050	21491.1	9.0200 004577 6367

Peptide Information

Calc. Mass	Obsrv. Mass	± da	± ppm	Start Seq.	End Seq.	Sequence	Ion Score	C. I. %	Modification	Rank	Result Type
870.5229	870.5418	0.0189	22	141	147	VLHMKVK			Oxidation (M)[4]		Mascot
945.5363	945.5093	-0.027	-29	157	164	ISENLTLR					Mascot
962.4479	962.3818	-0.0661	-69	107	114	WGFPGENR					Mascot
1016.487	1016.5295	0.0425	42	176	184	FLHVSMEGP					Mascot
1118.5491	1118.5861	0.037	33	107	115	WGFPGENRR					Mascot
1265.5732	1265.6566	0.0834	66	1	9	MNWQSEWLR			Oxidation (M)[1]		Mascot
1627.8262	1627.8523	0.0261	16	170	184	AAELARFLHVSMEGP					Mascot

Rank	Protein Name	Accession No.	Protein MW	Protein PI	Pep. Count	Protein Score	Protein Score C. I. %	Intensity Matched	Total Ion Score	Total Ion C. I. %	Confirmed
2	hypothetical protein PRUPE_ppa008592mg [Prunus persica]	gi|462401187	36719.8	9.06	9	37	0	3.398			

Peptide Information

Calc. Mass	Obsrv. Mass	± da	± ppm	Start Seq.	End Seq.	Sequence	Ion Score	C. I. %	Modification	Rank	Result Type
800.4261	800.3522	-0.0739	-92	126	132	RPSSPEK					Mascot
838.4669	838.4088	-0.0581	-69	299	305	YLKESAK					Mascot
846.4144	846.3405	-0.0739	-87	320	326	FNVPSWP					Mascot
959.4105	959.3384	-0.0721	-75	97	104	QYGYADDK					Mascot
989.5262	989.5205	-0.0057	-6	65	72	TETIIQER					Mascot
1118.5413	1118.5861	0.0448	40	216	225	VGPMSGVNWR			Oxidation (M)[4]		Mascot
1479.6996	1479.7269	0.0273	18	286	298	LTTPTDDMETALR			Oxidation (M)[8]		Mascot
1627.8035	1627.8523	0.0488	30	266	282	NDSGSVGPTVISNAPGR					Mascot
1883.9419	1883.9219	-0.02	-11	286	301	LTTPTDDMETALRYLK			Oxidation (M)[8]		Mascot

Rank	Protein Name	Accession No.	Protein MW	Protein PI	Pep. Count	Protein Score	Protein Score C. I. %	Intensity Matched	Total Ion Score	Total Ion C. I. %	Confirmed
3	uncharacterized protein LOC100800885 [Glycine max]	gi|358249176	32003.7	9.19	8	33	0	2.03			

Peptide Information

Calc. Mass	Obsrv. Mass	± da	± ppm	Start Seq.	End Seq.	Sequence	Ion Score	C. I. %	Modification	Rank	Result Type
801.4465	801.3715	-0.075	-94	288	295	NASVSVPK					Mascot
847.438	847.4102	-0.0278	-33	281	287	SVENRSR					Mascot
945.4386	945.5093	0.0707	75	223	229	DYMPLYK			Oxidation (M)[3]		Mascot
1016.5735	1016.5295	-0.044	-43	288	297	NASVSVPKSK					Mascot
1372.6818	1372.7269	0.0451	33	223	233	DYMPLYKLGEK			Oxidation (M)[3]		Mascot
1507.7751	1507.7552	-0.0199	-13	73	85	QEDAVRVVESTFK					Mascot
1547.8251	1547.699	-0.1261	-81	1	13	MESPFRPEILKGK			Oxidation (M)[1]		Mascot
2159.1431	2158.9312	-0.2119	-98	250	268	FINGDIMIVDGGLWLSRP R					Mascot

4	hypothetical protein F775_08656 [Aegilops tauschii]	gi|475487187	7033.6	9.38	4	30	0	.731	

Peptide Information

Calc. Mass	Obsrv. Mass	± da	± ppm	Start Seq.	End Seq.	Sequence	Ion Score	C. I. %	Modification	Rank	Result Type
847.4091	847.4102	0.0011	1	54	60	INICDGR			Carbamidomethyl (C)[4]		Mascot
989.5262	989.5205	-0.0057	-6	31	39	TVVELTDGR					Mascot
1507.717	1507.7552	0.0382	25	27	39	CSDRTVVELTDGR			Carbamidomethyl (C)[1]		Mascot
2186.1775	2186.0127	-0.1648	-75	31	52	TVVELTDGRAAIAGAGTV SELR					Mascot

5	TPA: hypothetical protein ZEAMMB73_578986 [Zea mays]	gi|414867683	32454.6	7.6	7	30	0	2.452

Protein Group

uncharacterized protein LOC100382542 [Zea mays]	gi|293331465	32454.6	7.5999 999046 3257

Peptide Information

Calc. Mass	Obsrv. Mass	± da	± ppm	Start Seq.	End Seq.	Sequence	Ion Score	C. I. %	Modification	Rank	Result Type
801.4465	801.3715	-0.075	-94	125	131	EILDGVR					Mascot
1263.7897	1263.7179	-0.0718	-57	56	67	RVSALPGPVVLR					Mascot
1265.6195	1265.6566	0.0371	29	68	77	FVEEAIECLR			Carbamidomethyl (C)[8]		Mascot
1599.9469	1600.0785	0.1316	82	2	15	ASLTTVEKLILFHK					Mascot
1627.8949	1627.8523	-0.0426	-26	253	268	SAAMIPAVLNGRQTAK					Mascot
1746.9823	1746.9088	-0.0735	-42	1	15	MASLTTVEKLILFHK			Oxidation (M)[1]		Mascot
2159.1179	2158.9312	-0.1867	-86	19	36	DLFHRLVHDLAQDPVPM R					Mascot

6	uncharacterized protein LOC100776673 [Glycine max]	gi|363814278	33616.5	9.82	8	30	0	1.75

Peptide Information

Calc. Mass	Obsrv. Mass	± da	± ppm	Start Seq.	End Seq.	Sequence	Ion Score	C. I. %	Modification	Rank	Result Type
805.3621	805.3256	-0.0365	-45	138	144	MQADGQR					Mascot
809.3644	809.3536	-0.0108	-13	239	245	MMNQAAK			Oxidation (M)[1]		Mascot
856.4563	856.5344	0.0781	91	298	305	KFAGLSSF					Mascot
1050.5183	1050.5026	-0.0157	-15	237	245	TRMMNQAAK					Mascot
1479.7897	1479.7269	-0.0628	-42	270	281	ALWKGFFPTWAR					Mascot
1507.7751	1507.7552	-0.0199	-13	99	113	NVASVDNASFSIVGK					Mascot
1627.8187	1627.8523	0.0336	21	2	16	KSGYQHGGVDITHTK					Mascot
1838.8564	1838.9272	0.0708	39	183	198	AFLVNMGELACYDHAK			Carbamidomethyl (C)[11]		Mascot

7	PREDICTED: uncharacterized protein LOC101204526 isoform 2 [Cucumis sativus]	gi|449440167	20626.1	8.92	6	29	0	1.641	

Protein Group

PREDICTED: uncharacterized protein LOC101227121 isoform 2 [Cucumis sativus]	gi|449501012	20626.1	8.9200 000762 9395

Peptide Information

Calc. Mass	Obsrv. Mass	± da	± ppm	Start Seq.	End Seq.	Sequence	Ion Score	C. I. %	Modification	Rank	Result Type
805.3872	805.3256	-0.0616	-76	153	159	EAMGIER					Mascot
847.4672	847.4102	-0.057	-67	121	127	SLLDPFR					Mascot
871.3801	871.2941	-0.086	-99	128	134	FCSLGCK			Carbamidomethyl (C)[2,6]		Mascot
962.469	962.3818	-0.0872	-91	66	73	SSYHDVVR					Mascot
1118.5702	1118.5861	0.0159	14	65	73	RSSYHDVVR					Mascot
1570.7833	1570.7826	-0.0007	0	52	64	SSHHNDHQVIQIR					Mascot

8	RNA-binding (RRM/RBD/RNP motifs) family protein [Arabidopsis thaliana]	gi|334187381	30548.1	9.88	7	29	0	2.08	

Protein Group

RNA-binding (RRM/RBD/RNP motifs) family protein [Arabidopsis thaliana]	gi|332003103	30548.1	9.8800 001144 4092

Peptide Information

Calc. Mass	Obsrv. Mass	± da	± ppm	Start Seq.	End Seq.	Sequence	Ion Score	C. I. %	Modification	Rank	Result Type
925.4275	925.5131	0.0856	92	239	248	GGGFGGGNFR					Mascot
962.4286	962.3818	-0.0468	-49	38	48	GGSGSNSGPSR					Mascot
1016.4868	1016.5295	0.0427	42	26	37	GGIGGGNNTGGR					Mascot

1050.5215	1050.5026	-0.0189	-18	168	176	YNNVQLDGK					Mascot
1118.5298	1118.5861	0.0563	50	38	49	GGSGSNSGPSRR					Mascot
1736.8484	1736.7794	-0.069	-40	2	17	SGGLDMSLDDIIKSNR			Oxidation (M)[6]		Mascot
1883.8838	1883.9219	0.0381	20	1	17	MSGGLDMSLDDIIKSNR			Oxidation (M)[1,7]		Mascot

9 RNA-binding (RRM/RBD/RNP motifs) family protein [Arabidopsis thaliana] gi|30679669 30761.2 9.98 7 29 0 2.08

Protein Group

RNA-binding (RRM/RBD/RNP motifs) family protein [Arabidopsis thaliana] gi|332003102 30761.2 9.9799 995422 3633

Peptide Information

Calc. Mass	Obsrv. Mass	± da	± ppm	Start Seq.	End Seq.	Sequence	Ion Score	C. I. %	Modification	Rank	Result Type
925.4275	925.5131	0.0856	92	241	250	GGGFGGGNFR					Mascot
962.4286	962.3818	-0.0468	-49	38	48	GGSGSNSGPSR					Mascot
1016.4868	1016.5295	0.0427	42	26	37	GGIGGGNNTGGR					Mascot
1050.5215	1050.5026	-0.0189	-18	168	176	YNNVQLDGK					Mascot
1118.5298	1118.5861	0.0563	50	38	49	GGSGSNSGPSRR					Mascot
1736.8484	1736.7794	-0.069	-40	2	17	SGGLDMSLDDIIKSNR			Oxidation (M)[6]		Mascot
1883.8838	1883.9219	0.0381	20	1	17	MSGGLDMSLDDIIKSNR			Oxidation (M)[1,7]		Mascot

10 dienelactone hydrolase family protein [Arabidopsis lyrata subsp. lyrata] gi|297337024 34685.3 5.43 7 28 0 1.9

Peptide Information

Calc. Mass	Obsrv. Mass	± da	± ppm	Start Seq.	End Seq.	Sequence	Ion Score	C. I. %	Modification	Rank	Result Type
860.4182	860.4083	-0.0099	-12	251	257	DPLCEVK			Carbamidomethyl (C)[4]		Mascot
1050.5942	1050.5026	-0.0916	-87	270	278	TKVVVYEGR					Mascot
1118.6416	1118.5861	-0.0555	-50	2	13	AASLASLSSLAK					Mascot
1265.677	1265.6566	-0.0204	-16	1	13	MAASLASLSSLAK			Oxidation (M)[1]		Mascot
1570.8911	1570.7826	-0.1085	-69	2	18	AASLASLSSLAKAGAPR					Mascot
1736.8789	1736.7794	-0.0995	-57	133	147	VACNGYNVLVPDLFR			Carbamidomethyl (C)[3]		Mascot
2203.0867	2203.1479	0.0612	28	294	311	DAEEAFALMRNWLHHHLI					Mascot

Gel Idx/Pos	242/J18	Instr./Gel Origin	BA2151/Sample Project 20140814	Process Status	Analysis Succeeded
Plate [#] Name	[1] Sample Project 20140814	Instrument Sample Name		Spectra	11

Rank	Protein Name	Accession No.	Protein MW	Protein PI	Pep. Count	Protein Score	Protein Score C. I. %	Intensity Matched	Total Ion Score	Total Ion C. I. %	Confirmed
1	Serpin-Z2B [Aegilops tauschii]	gi|475621781	43026.4	5.18	15	577	100	41.952	494	100	

Protein Group

RecName: Full=Serpin-Z2B; AltName: Full=TriaeZ2b; AltName: Full=WSZ2b; AltName: Full=WZS3	gi|75279909	43011.4	5.1799 998283 3862

Peptide Information

Calc. Mass	Obsrv. Mass	± da	± ppm	Start Seq.	End Seq.	Sequence	Ion Score	C. I. %	Modification	Rank	Result Type
860.5604	860.4865	-0.0739	-86	227	233	VLKLPYK					Mascot
925.5214	925.5234	0.002	2	11	18	LSIAHQTR					Mascot
925.5214	925.5234	0.002	2	11	18	LSIAHQTR	60	99.807			Mascot
947.5156	947.5029	-0.0127	-13	2	10	ATTLATDVR					Mascot
1078.5562	1078.6249	0.0687	64	1	10	MATTLATDVR					Mascot
1137.6667	1137.6506	-0.0161	-14	172	181	LVLGNALYFK					Mascot
1192.5382	1192.5416	0.0034	3	182	191	GAWTDQFDPR					Mascot
1192.5382	1192.5416	0.0034	3	182	191	GAWTDQFDPR	44	93.094			Mascot
1223.5903	1223.5654	-0.0249	-20	127	137	AEAQSVDFQTK					Mascot
1258.7253	1258.7069	-0.0184	-15	289	300	ISLGIEASDLLK					Mascot
1372.7068	1372.7174	0.0106	8	159	171	DILPAGSIDNTTR					Mascot
1372.7068	1372.7174	0.0106	8	159	171	DILPAGSIDNTTR	99	100			Mascot
1514.7485	1514.7397	-0.0088	-6	125	137	YKAEAQSVDFQTK					Mascot
1665.8595	1665.8872	0.0277	17	261	274	LSAEPEFLEQHIPR					Mascot
1665.8595	1665.8872	0.0277	17	261	274	LSAEPEFLEQHIPR	118	100			Mascot
1922.9706	1922.9829	0.0123	6	335	353	AFVEVNETGTEAAATTIA K					Mascot
2083.1072	2083.1067	-0.0005	0	379	398	EDTSGVVLFIGHVVNPLL SS					Mascot
2838.4858	2838.5356	0.0498	18	99	124	VAFANGVFVDASLQLKPS FQELAVCK			Carbamidomethyl (C)[25]		Mascot
3751.9614	3752.0615	0.1001	27	23	61	LASAISSNPESTVNNAAF SPVSLHVALSLITAGAGG ATR					Mascot
3751.9614	3752.0615	0.1001	27	23	61	LASAISSNPESTVNNAAF SPVSLHVALSLITAGAGG ATR	174	100			Mascot

Rank	Protein Name	Accession No.	Protein MW	Protein PI	Pep. Count	Protein Score	Protein Score C. I. %	Intensity Matched	Total Ion Score	Total Ion C. I. %	Confirmed
2	Serpin-Z1B [Aegilops tauschii]	gi|475546073	43257.1	5.44	15	421	100	16.635	336	100	

Peptide Information

Calc. Mass	Obsrv. Mass	± da	± ppm	Start Seq.	End Seq.	Sequence	Ion Score	C. I. %	Modification	Rank	Result Type
806.4744	806.4302	-0.0442	-55	272	277	HIPRQR					Mascot
860.5604	860.4865	-0.0739	-86	228	234	VLKLPYK					Mascot
925.5214	925.5234	0.002	2	11	18	LSIAHQTR					Mascot
925.5214	925.5234	0.002	2	11	18	LSIAHQTR	60	99.807			Mascot
947.5156	947.5029	-0.0127	-13	2	10	ATTLATDVR					Mascot
1078.5562	1078.6249	0.0687	64	1	10	MATTLATDVR					Mascot
1151.6824	1151.661	-0.0214	-19	172	181	LVLANALYFK					Mascot
1176.5896	1176.5983	0.0087	7	262	271	LSAEPDFLER					Mascot
1176.5896	1176.5983	0.0087	7	262	271	LSAEPDFLER	82	99.999			Mascot
1292.7097	1292.6886	-0.0211	-16	290	301	ISFGIEASDLLK					Mascot
1345.6958	1345.7329	0.0371	28	159	171	NILPSGSVDNTTK					Mascot
1544.7592	1544.7599	0.0007	0	125	137	YKAETQSVDFQTK					Mascot
1561.7856	1561.7687	-0.0169	-11	138	151	AAEVTTQVNSWVEK					Mascot
2685.3955	2685.448	0.0525	20	33	61	SAASNAAFSPVSLHSALS LLAAGAGSATR					Mascot
2685.3955	2685.448	0.0525	20	33	61	SAASNAAFSPVSLHSALS LLAAGAGSATR	195	100			Mascot
2720.3525	2720.3938	0.0413	15	329	354	VSSVFHQAFVEVNEQGT EAAASTAIK					Mascot
3071.3796	3071.4233	0.0437	14	302	328	CLGLQLPFSDEADFSEM VDSPMPQGLR			Carbamidomethyl (C)[1], Oxidation (M)[17,22]		Mascot
3705.9082	3705.9927	0.0845	23	62	98	DQLVATLGTGEVEGLHAL AEQVVQFVLADASSAGG PR					Mascot

3	RecName: Full=Serpin-Z1B; AltName: Full=TriaeZ1b; AltName: Full=WSZ1b; AltName: Full=WZS2	gi|75279910	43119.9	5.44	14	408	100	16.349	336	100	

Peptide Information

Calc. Mass	Obsrv. Mass	± da	± ppm	Start Seq.	End Seq.	Sequence	Ion Score	C. I. %	Modification	Rank	Result Type
806.4744	806.4302	-0.0442	-55	272	277	HIPRQR					Mascot
860.5604	860.4865	-0.0739	-86	228	234	VLKLPYK					Mascot
925.5214	925.5234	0.002	2	11	18	LSIAHQTR					Mascot
925.5214	925.5234	0.002	2	11	18	LSIAHQTR	60	99.807			Mascot
947.5156	947.5029	-0.0127	-13	2	10	ATTLATDVR					Mascot
1078.5562	1078.6249	0.0687	64	1	10	MATTLATDVR					Mascot
1151.6824	1151.661	-0.0214	-19	172	181	LVLANALYFK					Mascot
1176.5896	1176.5983	0.0087	7	262	271	LSAEPDFLER					Mascot

Calc. Mass	Obsrv. Mass	± da	± ppm	Start Seq.	End Seq.	Sequence	Ion Score	C. I. %	Modification	Rank	Result Type
1176.5896	1176.5983	0.0087	7	262	271	LSAEPDFLER	82	99.999			Mascot
1345.6958	1345.7329	0.0371	28	159	171	NILPSGSVDNTTK					Mascot
1544.7592	1544.7599	0.0007	0	125	137	YKAETQSVDFQTK					Mascot
1561.7856	1561.7687	-0.0169	-11	138	151	AAEVTTQVNSWVEK					Mascot
1585.8295	1585.7592	-0.0703	-44	288	301	FKISFGMEASDLLK					Mascot
2685.3955	2685.448	0.0525	20	33	61	SAASNAAFSPVSLHSALS LLAAGAGSATR					Mascot
2685.3955	2685.448	0.0525	20	33	61	SAASNAAFSPVSLHSALS LLAAGAGSATR	195	100			Mascot
2720.3525	2720.3938	0.0413	15	329	354	VSSVFHQAFVEVNEQGT EAAASTAIK					Mascot
3071.3796	3071.4233	0.0437	14	302	328	CLGLQLPFSDEADFSEM VDSPMPQGLR			Carbamidomethyl (C)[1], Oxidation (M)[17,22]		Mascot

4	Serpin-Z2B [Triticum urartu]	gi|473793747	45225.7	6.03	14	290	100	36.307	221	100

Peptide Information

Calc. Mass	Obsrv. Mass	± da	± ppm	Start Seq.	End Seq.	Sequence	Ion Score	C. I. %	Modification	Rank	Result Type
860.5604	860.4865	-0.0739	-86	244	250	VLKLPYK					Mascot
925.5214	925.5234	0.002	2	11	18	LSIAHQTR					Mascot
925.5214	925.5234	0.002	2	11	18	LSIAHQTR	60	99.807			Mascot
947.5156	947.5029	-0.0127	-13	2	10	ATTLATDVR					Mascot
1078.5562	1078.6249	0.0687	64	1	10	MATTLATDVR					Mascot
1137.6667	1137.6506	-0.0161	-14	189	198	LVLGNALYFK					Mascot
1192.5382	1192.5416	0.0034	3	199	208	GAWTDQFDPR					Mascot
1192.5382	1192.5416	0.0034	3	199	208	GAWTDQFDPR	44	93.094			Mascot
1223.5903	1223.5654	-0.0249	-20	127	137	AEAQSVDFQTK					Mascot
1292.7097	1292.6886	-0.0211	-16	306	317	ISFGIEASDLLK					Mascot
1514.7485	1514.7397	-0.0088	-6	125	137	YKAEAQSVDFQTK					Mascot
1665.8595	1665.8872	0.0277	17	278	291	LSAEPEFLEQHIPR					Mascot
1665.8595	1665.8872	0.0277	17	278	291	LSAEPEFLEQHIPR	118	100			Mascot
1922.9706	1922.9829	0.0123	6	352	370	AFVEVNETGTEAAATTIA K					Mascot
2083.1072	2083.1067	-0.0005	0	396	415	EDTSGVVLFIGHVVNPLL SS					Mascot
2667.385	2667.5034	0.1184	44	146	168	YVMQLFLPKAAEVTAQV NSWVEK			Oxidation (M)[3]		Mascot
2838.4858	2838.5356	0.0498	18	99	124	VAFANGVFVDASLQLKPS FQELAVCK			Carbamidomethyl (C)[25]		Mascot

5	RecName: Full=Serpin-Z1A; AltName: Full=TriaeZ1a; AltName: Full=WSZ1a; Short=WSZ1; AltName: Full=WSZCI	gi|75282265	43262.2	5.6	11	185	100	11.72	141	100

Peptide Information

Calc. Mass	Obsrv. Mass	± da	± ppm	Start Seq.	End Seq.	Sequence	Ion Score	C. I. %	Modification	Rank	Result Type
806.4744	806.4302	-0.0442	-55	271	276	HIPRQR					Mascot
860.5604	860.4865	-0.0739	-86	227	233	VLKLPYK					Mascot
925.5214	925.5234	0.002	2	11	18	LSIAHQTR					Mascot
925.5214	925.5234	0.002	2	11	18	LSIAHQTR	60	99.807			Mascot
947.5156	947.5029	-0.0127	-13	2	10	ATTLATDVR					Mascot
1078.5562	1078.6249	0.0687	64	1	10	MATTLATDVR					Mascot
1151.6824	1151.661	-0.0214	-19	172	181	LVLANALYFK					Mascot
1176.5896	1176.5983	0.0087	7	261	270	LSAEPDFLER					Mascot
1176.5896	1176.5983	0.0087	7	261	270	LSAEPDFLER	82	99.999			Mascot
1292.7097	1292.6886	-0.0211	-16	289	300	ISFGIEASDLLK					Mascot
1544.7592	1544.7599	0.0007	0	125	137	YKAETQSVDFQTK					Mascot
1561.7856	1561.7687	-0.0169	-11	138	151	AAEVTTQVNSWVEK					Mascot
2720.3525	2720.3938	0.0413	15	328	353	VSSVFHQAFVEVNEQGT EAAASTAIK					Mascot

6 Serpin-Z1C [Triticum urartu] gi|474075261 42956 5.62 9 172 100 11.326 141 100

Peptide Information

Calc. Mass	Obsrv. Mass	± da	± ppm	Start Seq.	End Seq.	Sequence	Ion Score	C. I. %	Modification	Rank	Result Type
806.4744	806.4302	-0.0442	-55	271	276	HIPRQR					Mascot
860.5604	860.4865	-0.0739	-86	227	233	VLKLPYK					Mascot
925.5214	925.5234	0.002	2	11	18	LSIAHQTR					Mascot
925.5214	925.5234	0.002	2	11	18	LSIAHQTR	60	99.807			Mascot
947.5156	947.5029	-0.0127	-13	2	10	ATTLATDVR					Mascot
1078.5562	1078.6249	0.0687	64	1	10	MATTLATDVR					Mascot
1151.6824	1151.661	-0.0214	-19	172	181	LVLANALYFK					Mascot
1176.5896	1176.5983	0.0087	7	261	270	LSAEPDFLER					Mascot
1176.5896	1176.5983	0.0087	7	261	270	LSAEPDFLER	82	99.999			Mascot
1544.7592	1544.7599	0.0007	0	125	137	YKAETQSVDFQTK					Mascot
2720.3525	2720.3938	0.0413	15	328	353	VSSVFHQAFVEVNEQGT EAAASTAIK					Mascot

7 RecName: Full=Serpin-Z1C; AltName: Full=TriaeZ1c; AltName: Full=WSZ1c gi|75313848 42969 5.62 8 166 100 11.179 141 100

Peptide Information

Calc. Mass	Obsrv. Mass	± da	± ppm	Start Seq.	End Seq.	Sequence	Ion Score	C. I. %	Modification	Rank	Result Type

Calc. Mass	Obsrv. Mass	± da	± ppm	Start Seq.	End Seq.	Sequence	Ion Score	C. I. %	Modification	Rank	Result Type
806.4744	806.4302	-0.0442	-55	271	276	HIPRQR					Mascot
860.5604	860.4865	-0.0739	-86	227	233	VLKLPYK					Mascot
925.5214	925.5234	0.002	2	11	18	LSIAHQTR					Mascot
925.5214	925.5234	0.002	2	11	18	LSIAHQTR	60	99.807			Mascot
947.5156	947.5029	-0.0127	-13	2	10	ATTLATDVR					Mascot
1078.5562	1078.6249	0.0687	64	1	10	MATTLATDVR					Mascot
1151.6824	1151.661	-0.0214	-19	172	181	LVLANALYFK					Mascot
1176.5896	1176.5983	0.0087	7	261	270	LSAEPDFLER					Mascot
1176.5896	1176.5983	0.0087	7	261	270	LSAEPDFLER	82	99.999			Mascot
2720.3525	2720.3938	0.0413	15	328	353	VSSVFHQAFVEVNEQGT EAAASTAIK					Mascot

8 RecName: Full=Serpin-Z2A; AltName: Full=TriaeZ2a; AltName: Full=WSZ2a | gi|75313847 | 43341.5 | 5.46 | 9 | 89 | 99.883 | 7.267 | 60 | 99.807

Peptide Information

Calc. Mass	Obsrv. Mass	± da	± ppm	Start Seq.	End Seq.	Sequence	Ion Score	C. I. %	Modification	Rank	Result Type
860.5604	860.4865	-0.0739	-86	227	233	VLKLPYK					Mascot
925.5214	925.5234	0.002	2	11	18	LSIAHQTR					Mascot
925.5214	925.5234	0.002	2	11	18	LSIAHQTR	60	99.807			Mascot
947.5156	947.5029	-0.0127	-13	2	10	ATTLATDVR					Mascot
1078.5562	1078.6249	0.0687	64	1	10	MATTLATDVR					Mascot
1137.6667	1137.6506	-0.0161	-14	172	181	LVLGNALYFK					Mascot
1223.5903	1223.5654	-0.0249	-20	127	137	AEAQSVDFQTK					Mascot
1292.7097	1292.6886	-0.0211	-16	289	300	ISFGIEASDLLK					Mascot
1514.7485	1514.7397	-0.0088	-6	125	137	YKAEAQSVDFQTK					Mascot
1922.9706	1922.9829	0.0123	6	335	353	TFVEVNETGTEAAAATIA K					Mascot

9 RecName: Full=Serpin-Z4; AltName: Full=BSZ4; AltName: Full=HorvuZ4; AltName: Full=Major endosperm albumin; AltName: Full=Protein Z4; Short=Protein Z | gi|131091 | 43363.4 | 5.72 | 6 | 72 | 94.399 | 6.222 | 60 | 99.807

Peptide Information

Calc. Mass	Obsrv. Mass	± da	± ppm	Start Seq.	End Seq.	Sequence	Ion Score	C. I. %	Modification	Rank	Result Type
840.3734	840.4317	0.0583	69	190	196	FDESNTK					Mascot
907.54	907.4628	-0.0772	-85	284	290	FQLPKFK					Mascot
925.5214	925.5234	0.002	2	11	18	LSIAHQTR					Mascot
925.5214	925.5234	0.002	2	11	18	LSIAHQTR	60	99.807			Mascot

947.5156	947.5029	-0.0127	-13	2	10	ATTLATDVR					Mascot
1078.5562	1078.6249	0.0687	64	1	10	MATTLATDVR					Mascot
1151.6824	1151.661	-0.0214	-19	174	183	LILGNALYFK					Mascot

10	RecName: Full=Serpin-ZX; AltName: Full=BSZx; AltName: Full=HorvuZx	gi|75281963	42920.3	6.77	4	69	87.746	6.16	60	99.807

Peptide Information

Calc. Mass	Obsrv. Mass	± da	± ppm	Start Seq.	End Seq.	Sequence	Ion Score	C. I. %	Modification	Rank	Result Type
925.5214	925.5234	0.002	2	8	15	LSIAHQTR					Mascot
925.5214	925.5234	0.002	2	8	15	LSIAHQTR	60	99.807			Mascot
1137.6667	1137.6506	-0.0161	-14	171	180	LVLGNALYFK					Mascot
1345.7474	1345.7329	-0.0145	-11	226	237	VLKLPYQQGGDK					Mascot
2734.332	2734.4077	0.0757	28	126	150	GETQSVDFQTKAPEVAG QVNSWVEK					Mascot

Rank	Protein Name	Accession No.	Protein MW	Protein PI	Pep. Count	Protein Score	Protein Score C. I. %	Intensity Matched	Total Ion Score	Total Ion C. I. %	Confirmed
1	Vicilin-like antimicrobial peptides 2-2 [Triticum urartu]	gi|473890163	75298.3	5.79	12	131	100	9.187	117	100	

Peptide Information

Calc. Mass	Obsrv. Mass	± da	± ppm	Start Seq.	End Seq.	Sequence	Ion Score	C. I. %	Modification	Rank	Result Type
807.4359	807.3883	-0.0476	-59	660	667	KGAVFQSA					Mascot
819.3478	819.3619	0.0141	17	519	524	EEEEQR					Mascot
847.3904	847.3969	0.0065	8	607	612	EEEERR					Mascot
849.4941	849.4177	-0.0764	-90	360	366	NSVFRVK					Mascot
947.5673	947.4882	-0.0791	-83	177	184	GFDVKILR					Mascot
989.4646	989.5185	0.0539	54	543	550	EEEEAARR					Mascot
989.4646	989.5185	0.0539	54	543	550	EEEEAARR					Mascot
1017.5364	1017.5435	0.0071	7	367	375	EGDVFVVPR					Mascot
1017.5364	1017.5435	0.0071	7	367	375	EGDVFVVPR	45	93.792			Mascot
1117.5232	1117.5873	0.0641	57	541	549	QREEEEAAR					Mascot
1161.4767	1161.5367	0.06	52	590	601	EEEEGGGGQGGR					Mascot
1232.5753	1232.6104	0.0351	28	458	468	KAEQEEQEGGK					Mascot
1244.6998	1244.7128	0.013	10	365	375	VKEGDVFVVPR					Mascot
1244.6998	1244.7128	0.013	10	365	375	VKEGDVFVVPR	71	99.983			Mascot
1899.8246	1899.9703	0.1457	77	441	457	SGSTIMACVSCAEELER			Carbamidomethyl (C)[8,11]		Mascot
1915.8195	1915.8275	0.008	4	441	457	SGSTIMACVSCAEELER			Carbamidomethyl (C)[8,11], Oxidation (M)[6]		Mascot

Rank	Protein Name	Accession No.	Protein MW	Protein PI	Pep. Count	Protein Score	Protein Score C. I. %	Intensity Matched	Total Ion Score	Total Ion C. I. %	Confirmed
2	hypothetical protein ZEAMMB73_545327 [Zea mays]	gi|413956703	71377.8	6.31	11	131	100	8.415	117	100	

Peptide Information

Calc. Mass	Obsrv. Mass	± da	± ppm	Start Seq.	End Seq.	Sequence	Ion Score	C. I. %	Modification	Rank	Result Type
817.4162	817.4156	-0.0006	-1	551	556	EQEKQR					Mascot
819.3842	819.3619	-0.0223	-27	506	511	EKEEER					Mascot
847.3904	847.3969	0.0065	8	569	574	REEEER					Mascot
849.4941	849.4177	-0.0764	-90	373	379	NSVFRVK					Mascot
872.52	872.4377	-0.0823	-94	423	431	GSVLQAIGK					Mascot
876.4257	876.3801	-0.0456	-52	367	372	ARWQCR			Carbamidomethyl (C)[5]		Mascot

Calc. Mass	Obsrv. Mass	± da	± ppm	Start Seq.	End Seq.	Sequence	Ion Score	C. I. %	Modification	Rank	Result Type
890.4214	890.4382	0.0168	19	480	488	EEEGGGKGK					Mascot
936.4785	936.4963	0.0178	19	172	179	GFETDVLR					Mascot
1017.5364	1017.5435	0.0071	7	380	388	EGDVFVVPR					Mascot
1017.5364	1017.5435	0.0071	7	380	388	EGDVFVVPR	45	93.792			Mascot
1100.606	1100.536	-0.07	-64	30	39	VGGQVVEKER					Mascot
1244.6998	1244.7128	0.013	10	378	388	VKEGDVFVVPR					Mascot
1244.6998	1244.7128	0.013	10	378	388	VKEGDVFVVPR	71	99.983			Mascot

3	PREDICTED: globulin-1 S allele-like [Setaria italica]	gi|514824936	71707.9	5.75	10	124	100	8.404	117	100	

Peptide Information

Calc. Mass	Obsrv. Mass	± da	± ppm	Start Seq.	End Seq.	Sequence	Ion Score	C. I. %	Modification	Rank	Result Type
818.4002	818.401	0.0008	1	551	556	REQEEK					Mascot
819.3842	819.3619	-0.0223	-27	535	540	KEEEER					Mascot
826.3914	826.3912	-0.0002	0	348	354	GHRGDER					Mascot
834.3475	834.3426	-0.0049	-6	483	490	EEEEGGGK					Mascot
847.3904	847.3969	0.0065	8	582	587	EEEERR					Mascot
860.422	860.4653	0.0433	50	474	480	AAEEERR					Mascot
872.52	872.4377	-0.0823	-94	425	433	GSVLQAIGK					Mascot
1017.5364	1017.5435	0.0071	7	382	390	EGDVFVVPR					Mascot
1017.5364	1017.5435	0.0071	7	382	390	EGDVFVVPR	45	93.792			Mascot
1244.6998	1244.7128	0.013	10	380	390	VKEGDVFVVPR					Mascot
1244.6998	1244.7128	0.013	10	380	390	VKEGDVFVVPR	71	99.983			Mascot
1526.7333	1526.7214	-0.0119	-8	103	115	GKVTYIEEESSER					Mascot

4	Serpin-Z2B [Aegilops tauschii]	gi|475621781	43026.4	5.18	7	107	99.998	4.227	80	99.998

Protein Group

RecName: Full=Serpin-Z2B; AltName: Full=TriaeZ2b; AltName: Full=WSZ2b; AltName: Full=WZS3	gi|75279909	43011.4	5.1799 998283 3862

Peptide Information

Calc. Mass	Obsrv. Mass	± da	± ppm	Start Seq.	End Seq.	Sequence	Ion Score	C. I. %	Modification	Rank	Result Type
925.5214	925.5213	-0.0001	0	11	18	LSIAHQTR					Mascot
925.5214	925.5213	-0.0001	0	11	18	LSIAHQTR	6	0			Mascot
947.5156	947.4882	-0.0274	-29	2	10	ATTLATDVR					Mascot
1137.6667	1137.6163	-0.0504	-44	172	181	LVLGNALYFK					Mascot
1192.5382	1192.6343	0.0961	81	182	191	GAWTDQFDPR					Mascot

1372.7068	1372.7135	0.0067	5	159	171	DILPAGSIDNTTR					Mascot
1372.7068	1372.7135	0.0067	5	159	171	DILPAGSIDNTTR	22	0			Mascot
1514.7485	1514.751	0.0025	2	125	137	YKAEAQSVDFQTK					Mascot
1665.8595	1665.8821	0.0226	14	261	274	LSAEPEFLEQHIPR					Mascot
1665.8595	1665.8821	0.0226	14	261	274	LSAEPEFLEQHIPR	58	99.688			Mascot

5	Serpin-Z2B [Triticum urartu]	gi|473793747	45225.7	6.03	7	84	99.575	3.72	58	99.688

Peptide Information

Calc. Mass	Obsrv. Mass	± da	± ppm	Start Seq.	End Seq.	Sequence	Ion Score	C. I. %	Modification	Rank	Result Type
925.5214	925.5213	-0.0001	0	11	18	LSIAHQTR					Mascot
925.5214	925.5213	-0.0001	0	11	18	LSIAHQTR	6	0			Mascot
947.5156	947.4882	-0.0274	-29	2	10	ATTLATDVR					Mascot
1137.6667	1137.6163	-0.0504	-44	189	198	LVLGNALYFK					Mascot
1192.5382	1192.6343	0.0961	81	199	208	GAWTDQFDPR					Mascot
1437.7948	1437.7842	-0.0106	-7	234	246	QYISSSDGLKVLK					Mascot
1514.7485	1514.751	0.0025	2	125	137	YKAEAQSVDFQTK					Mascot
1665.8595	1665.8821	0.0226	14	278	291	LSAEPEFLEQHIPR					Mascot
1665.8595	1665.8821	0.0226	14	278	291	LSAEPEFLEQHIPR	58	99.688			Mascot

6	hypothetical protein CARUB_v10011827mg [Capsella rubella]	gi|482573368	93564.4	5.27	22	75	97.193	6.63		

Peptide Information

Calc. Mass	Obsrv. Mass	± da	± ppm	Start Seq.	End Seq.	Sequence	Ion Score	C. I. %	Modification	Rank	Result Type
818.4804	818.401	-0.0794	-97	553	559	LVMQISK					Mascot
826.369	826.3912	0.0222	27	2	9	AGEYSGSR					Mascot
829.3508	829.3928	0.042	51	251	257	MSYEGAR			Oxidation (M)[1]		Mascot
831.4869	831.4928	0.0059	7	162	168	LGVCVRK			Carbamidomethyl (C)[4]		Mascot
834.3952	834.3426	-0.0526	-63	407	414	SDGTIGER					Mascot
841.3794	841.4263	0.0469	56	446	452	ALESMMK			Oxidation (M)[5,6]		Mascot
949.4989	949.4977	-0.0012	-1	322	330	TSFSVPSPK					Mascot
963.541	963.5167	-0.0243	-25	490	496	LKFYQHK					Mascot
989.5197	989.5185	-0.0012	-1	522	530	GIGCVVQTR			Carbamidomethyl (C)[4]		Mascot
989.5513	989.5185	-0.0328	-33	434	442	LTELDSIAK	17	0			Mascot
999.5007	999.5292	0.0285	29	310	318	NFSGSFGRK					Mascot
999.5007	999.5292	0.0285	29	310	318	NFSGSFGRK					Mascot

Calc. Mass	Obsrv. Mass	± da	± ppm	Start Seq.	End Seq.	Sequence	Ion Score	C. I. %	Modification	Rank	Result Type
1016.5007	1016.5453	0.0446	44	372	379	KTEEPEQR					Mascot
1017.5873	1017.5435	-0.0438	-43	130	138	GLVRIGMQK			Oxidation (M)[7]		Mascot
1017.5873	1017.5435	-0.0438	-43	130	138	GLVRIGMQK			Oxidation (M)[7]		Mascot
1023.4888	1023.519	0.0302	30	638	647	TLANAMSSGR			Oxidation (M)[6]		Mascot
1068.5255	1068.522	-0.0035	-3	251	259	MSYEGARVR					Mascot
1176.618	1176.6007	-0.0173	-15	677	686	LEEMVIEGLK			Oxidation (M)[4]		Mascot
1194.6221	1194.6158	-0.0063	-5	443	452	QIKALESMMK			Oxidation (M)[8]		Mascot
1237.5881	1237.6204	0.0323	26	812	821	EEMLWSLSSR					Mascot
1326.7642	1326.6665	-0.0977	-74	77	87	LSLSPWRSRPK					Mascot
1423.7172	1423.7733	0.0561	39	590	602	ISSLMAIDELMGK			Oxidation (M)[5]		Mascot
1526.8359	1526.7214	-0.1145	-75	430	442	HIMRLTELDSIAK					Mascot
1900.0367	1899.9703	-0.0664	-35	214	229	FEARPFLIYLFAVDAK					Mascot

7	FZO-like protein [Arabidopsis thaliana]	gi|145335021	101181.4	5.72	19	74	96.213	5.586	23	0

Protein Group

FZO-like protein [Arabidopsis thaliana]	gi|332189416	101181.4	5.7199 997901 9165

Peptide Information

Calc. Mass	Obsrv. Mass	± da	± ppm	Start Seq.	End Seq.	Sequence	Ion Score	C. I. %	Modification	Rank	Result Type
872.5087	872.4377	-0.071	-81	244	250	EELQLLK					Mascot
904.4887	904.5279	0.0392	43	31	38	LSSFTPPR					Mascot
904.4887	904.5279	0.0392	43	31	38	LSSFTPPR	23	0			Mascot
947.5156	947.4882	-0.0274	-29	885	892	ELSDIRSK					Mascot
1013.5673	1013.4688	-0.0985	-97	1	8	MRTLISHR					Mascot
1021.5499	1021.5261	-0.0238	-23	230	237	IPIYVTCR			Carbamidomethyl (C)[7]		Mascot
1029.5623	1029.5114	-0.0509	-49	1	8	MRTLISHR			Oxidation (M)[1]		Mascot
1100.6058	1100.536	-0.0698	-63	637	646	QALSLIDNAR					Mascot
1117.6285	1117.5873	-0.0412	-37	308	316	QKLIVEMEK					Mascot
1146.5824	1146.5868	0.0044	4	581	590	LETPMAIAER			Oxidation (M)[5]		Mascot
1171.7157	1171.6182	-0.0975	-83	363	373	STVINALLGKR					Mascot
1197.6488	1197.5466	-0.1022	-85	31	40	LSSFTPPRHR					Mascot
1221.6475	1221.6074	-0.0401	-33	297	307	NSAGFIKLEDK					Mascot
1222.5369	1222.6179	0.081	66	85	95	LDADEVMSGNR			Oxidation (M)[7]		Mascot
1372.6315	1372.7135	0.082	60	627	636	MEYESISWRR			Oxidation (M)[1]		Mascot
1372.6315	1372.7135	0.082	60	627	636	MEYESISWRR			Oxidation (M)[1]		Mascot
1437.8424	1437.7842	-0.0582	-40	317	328	SVLRETIEIIHK					Mascot

Calc. Mass	Obsrv. Mass	± da	± ppm	Start Seq.	End Seq.	Sequence	Ion Score	C. I. %	Modification	Rank	Result Type
1487.6761	1487.7985	0.1224	82	539	551	DDLEIADPGSNWR					Mascot
1507.8003	1507.7479	-0.0524	-35	734	746	TQLGIDTYDLLQK					Mascot
1514.8367	1514.751	-0.0857	-57	479	490	FVFILNKSDIYR					Mascot
1851.9698	1851.9326	-0.0372	-20	734	749	TQLGIDTYDLLQKTDK					Mascot
1988.0117	1987.9363	-0.0754	-38	831	848	VNKVADALAQQLEDAMQ K			Oxidation (M)[16]		Mascot

8	hypothetical protein ARALYDRAFT_891304 [Arabidopsis lyrata subsp. lyrata]	gi|297337084	93864.4	5.24	22	74	96.213	4.959

Peptide Information

Calc. Mass	Obsrv. Mass	± da	± ppm	Start Seq.	End Seq.	Sequence	Ion Score	C. I. %	Modification	Rank	Result Type
818.4804	818.401	-0.0794	-97	553	559	LVMQISK					Mascot
826.369	826.3912	0.0222	27	2	9	AGEYSGSR					Mascot
829.3508	829.3928	0.042	51	251	257	MSYEGAR			Oxidation (M)[1]		Mascot
831.4869	831.4928	0.0059	7	162	168	LGVCVRK			Carbamidomethyl (C)[4]		Mascot
834.3952	834.3426	-0.0526	-63	407	414	SDGTIGER					Mascot
841.3794	841.4263	0.0469	56	446	452	ALESMMK			Oxidation (M)[5,6]		Mascot
886.5356	886.4831	-0.0525	-59	271	279	AKGGELALK					Mascot
949.4989	949.4977	-0.0012	-1	322	330	TSFSVPSPK					Mascot
963.541	963.5167	-0.0243	-25	490	496	LKFYQHK					Mascot
989.5197	989.5185	-0.0012	-1	522	530	GIGCVVQTR			Carbamidomethyl (C)[4]		Mascot
989.5513	989.5185	-0.0328	-33	434	442	LTELDSIAK	17	0			Mascot
1016.5007	1016.5453	0.0446	44	372	379	KTEEPEQR					Mascot
1023.4411	1023.519	0.0779	76	415	422	SVEMEEQR			Oxidation (M)[4]		Mascot
1040.5272	1040.5435	0.0163	16	310	318	NFANSFGRK					Mascot
1068.5255	1068.522	-0.0035	-3	251	259	MSYEGARVR					Mascot
1146.6075	1146.5868	-0.0207	-18	677	686	LEEMVVEGLK					Mascot
1194.6221	1194.6158	-0.0063	-5	443	452	QIKALESMMK			Oxidation (M)[8]		Mascot
1197.5463	1197.5466	0.0003	0	638	648	TMANAMSSGRR			Oxidation (M)[2]		Mascot
1205.6008	1205.6381	0.0373	31	105	116	KPEESSSGSVAK					Mascot
1326.7642	1326.6665	-0.0977	-74	77	87	LSLSPWRSRPK					Mascot
1423.7172	1423.7733	0.0561	39	590	602	ISSLMAIDELMGK			Oxidation (M)[5]		Mascot
1526.8359	1526.7214	-0.1145	-75	430	442	HIMRLTELDSIAK					Mascot

9	hypothetical protein OsI_17386 [Oryza sativa Indica Group]	gi|218195569	252768.1	4.77	38	70	90.488	11.743

Peptide Information

Calc. Mass	Obsrv. Mass	± da	± ppm	Start	End	Sequence	Ion	C. I. %	Modification	Rank	Result Type

				Seq.	Seq.		Score		
810.4144	810.3917	-0.0227	-28	1658	1663	WDAYKK			Mascot
818.4366	818.401	-0.0356	-43	214	220	RASELDK			Mascot
819.4207	819.3619	-0.0588	-72	701	707	LQSQESK			Mascot
829.4414	829.3928	-0.0486	-59	80	87	ASPIEQGK			Mascot
830.4617	830.403	-0.0587	-71	1554	1561	ELGEAIAK			Mascot
847.4156	847.3969	-0.0187	-22	130	137	GVTEAENK			Mascot
850.4516	850.4424	-0.0092	-11	1209	1216	LSSISSEK			Mascot
856.4523	856.5173	0.065	76	966	973	ALEHATSK			Mascot
860.4472	860.4653	0.0181	21	1921	1928	AATDQLNK			Mascot
890.4578	890.4382	-0.0196	-22	1754	1760	VTEEREK			Mascot
904.4622	904.5279	0.0657	73	1269	1276	EAEVTDLK			Mascot
904.4622	904.5279	0.0657	73	1269	1276	EAEVTDLK			Mascot
949.4771	949.4977	0.0206	22	1488	1495	TLDGMIQR		Oxidation (M)[5]	Mascot
963.4854	963.5167	0.0313	32	1060	1068	TKASNAESR			Mascot
1016.5622	1016.5453	-0.0169	-17	1260	1268	EVLETVAQK			Mascot
1032.5936	1032.5232	-0.0704	-68	946	954	ETSVLDKLK			Mascot
1058.5476	1058.4792	-0.0684	-65	468	476	LQLEEAEAR			Mascot
1060.527	1060.5253	-0.0017	-2	1251	1259	SAEIENELR			Mascot
1077.5721	1077.5514	-0.0207	-19	1488	1496	TLDGMIQRK		Oxidation (M)[5]	Mascot
1100.5946	1100.536	-0.0586	-53	554	562	NQLLEQEVK			Mascot
1132.5845	1132.6008	0.0163	14	1027	1038	ATAAAGEVESVK			Mascot
1161.5997	1161.5367	-0.063	-54	1279	1288	LVSLETENEK			Mascot
1171.5953	1171.6182	0.0229	20	1345	1355	SLELHSAAESK			Mascot
1224.6471	1224.5382	-0.1089	-89	1532	1541	SKYNITLEEK			Mascot
1224.6471	1224.5382	-0.1089	-89	1532	1541	SKYNITLEEK			Mascot
1232.6304	1232.6104	-0.02	-16	88	98	TAALEEQALMR			Mascot
1240.6355	1240.7271	0.0916	74	1363	1373	LHEALEMAAQK			Mascot
1243.5913	1243.681	0.0897	72	1476	1486	EGSSEEIHSLR			Mascot
1313.7059	1313.6639	-0.042	-32	1624	1634	DALEQQLLEVR			Mascot
1320.6754	1320.6121	-0.0633	-48	1792	1803	LQAKDSVESTSR			Mascot
1372.6532	1372.7135	0.0603	44	2227	2238	SPNDPFPVEWGK			Mascot
1372.6532	1372.7135	0.0603	44	2227	2238	SPNDPFPVEWGK			Mascot
1423.7064	1423.7733	0.0669	47	168	179	ELGFELESSKER			Mascot
1487.7588	1487.7985	0.0397	27	180	193	IQEIEAELAASADK			Mascot
1507.775	1507.7479	-0.0271	-18	1013	1026	LKAAEEQAASYQAK			Mascot

Calc. Mass	Obsrv. Mass	± da	± ppm	Start Seq.	End Seq.	Sequence	Ion Score	C. I. %	Modification	Rank	Result Type
1526.7485	1526.7214	-0.0271	-18	617	628	FKEVEADLEQYR					Mascot
1865.9967	1865.9263	-0.0704	-38	180	196	IQEIEAELAASADKLHK					Mascot
1885.9753	1885.969	-0.0063	-3	1515	1531	VISLSVSSTVEEHEELK					Mascot
1899.9116	1899.9703	0.0587	31	1146	1162	NAELEAQMHEALETIGK			Oxidation (M)[8]		Mascot
1915.9607	1915.8275	-0.1332	-70	361	376	QQEQILNLQTELDESK					Mascot
2407.0984	2407.3208	0.2224	92	1497	1514	EELESQLYCYYIFCSFKK			Carbamidomethyl (C)[9,14]		Mascot

10	Protein kinase APK1A, chloroplastic [Aegilops tauschii]	gi|475571926	32409.8	9.53	12	70	90.04	11.569

Peptide Information

Calc. Mass	Obsrv. Mass	± da	± ppm	Start Seq.	End Seq.	Sequence	Ion Score	C. I. %	Modification	Rank	Result Type
810.3484	810.3917	0.0433	53	194	199	CIMDQK			Carbamidomethyl (C)[1], Oxidation (M)[3]		Mascot
819.4319	819.3619	-0.07	-85	285	292	RTASANLS					Mascot
850.4669	850.4424	-0.0245	-29	101	108	LSDFGLAK					Mascot
870.5771	870.533	-0.0441	-51	61	69	LKVAIGAAK					Mascot
872.4043	872.4377	0.0334	38	219	225	CTNPQPR			Carbamidomethyl (C)[1]		Mascot
890.473	890.4382	-0.0348	-39	200	207	LSGHYSVK					Mascot
1017.5687	1017.5435	-0.0252	-25	169	177	TARAEQTIK					Mascot
1017.5687	1017.5435	-0.0252	-25	169	177	TARAEQTIK					Mascot
1068.5367	1068.522	-0.0147	-14	160	168	RAMEHVPGR			Oxidation (M)[3]		Mascot
1224.6266	1224.5382	-0.0884	-72	161	171	AMEHVPGRTAR					Mascot
1224.6266	1224.5382	-0.0884	-72	161	171	AMEHVPGRTAR					Mascot
1240.6216	1240.7271	0.1055	85	161	171	AMEHVPGRTAR			Oxidation (M)[2]		Mascot
1244.6416	1244.7128	0.0712	57	271	283	AEMKGAGTGAVPR					Mascot
1244.6416	1244.7128	0.0712	57	271	283	AEMKGAGTGAVPR	18	0			Mascot
1665.8087	1665.8821	0.0734	44	194	207	CIMDQKLSGHYSVK			Carbamidomethyl (C)[1]		Mascot
1665.8087	1665.8821	0.0734	44	194	207	CIMDQKLSGHYSVK			Carbamidomethyl (C)[1]		Mascot
1733.9446	1733.8721	-0.0725	-42	178	191	LVEWTRPYLASSRR					Mascot

Gel Idx/Pos	244/J20	Instr./Gel Origin	BA2151/Sample Project 20140814	Process Status	Analysis Succeeded
Plate [#] Name	[1] Sample Project 20140814	Instrument Sample Name		Spectra	11

Rank	Protein Name	Accession No.	Protein MW	Protein PI	Pep. Count	Protein Score	Protein Score C. I. %	Intensity Matched	Total Ion Score	Total Ion C. I. %	Confirmed
1	Glutaminyl-tRNA synthetase [Triticum urartu]	gi|474021464	90535.6	6.65	25	77	98.229	10.168			

Peptide Information

Calc. Mass	Obsrv. Mass	± da	± ppm	Start Seq.	End Seq.	Sequence	Ion Score	C. I. %	Modification	Rank	Result Type
804.421	804.3451	-0.0759	-94	550	556	SDNSLIR					Mascot
807.3454	807.3951	0.0497	62	374	379	MDSPWR			Oxidation (M)[1]		Mascot
809.3676	809.3757	0.0081	10	673	679	AEYDPSK					Mascot
813.3923	813.4012	0.0089	11	627	632	TDFRMK			Oxidation (M)[5]		Mascot
830.4519	830.3871	-0.0648	-78	560	565	LEYHIR					Mascot
856.5615	856.5229	-0.0386	-45	522	529	LLTLAGLR					Mascot
887.5197	887.4554	-0.0643	-72	594	601	VIDLDGKK					Mascot
935.4404	935.4622	0.0218	23	373	379	KMDSPWR			Oxidation (M)[2]		Mascot
947.5043	947.4869	-0.0174	-18	165	172	ATKEEIEK					Mascot
960.5434	960.4643	-0.0791	-82	1	8	MEEVIVLK					Mascot
1001.5812	1001.5284	-0.0528	-53	25	32	QMLLLDLR					Mascot
1096.4921	1096.5713	0.0792	72	325	332	WMGWEPYK					Mascot
1106.5378	1106.5371	-0.0007	-1	428	437	FTPHPHAGDK					Mascot
1182.6477	1182.6322	-0.0155	-13	247	256	AHNTKEILEK					Mascot
1193.5653	1193.611	0.0457	38	493	502	LNISHTMMSK			Oxidation (M)[7,8]		Mascot
1204.5641	1204.6403	0.0762	63	294	302	ERNGHCYLR			Carbamidomethyl (C)[6]		Mascot
1227.7168	1227.6271	-0.0897	-73	395	406	RGLIAEGAATLR					Mascot
1232.594	1232.6223	0.0283	23	133	142	ENMEAILEQR					Mascot
1251.5457	1251.663	0.1173	94	407	416	MKQDMQNDNK					Mascot
1346.7461	1346.6886	-0.0575	-43	396	408	GLIAEGAATLRMK			Oxidation (M)[12]		Mascot
1347.7168	1347.7183	0.0015	1	426	437	IKFTPHPHAGDK					Mascot
1514.8213	1514.7424	-0.0789	-52	313	324	KEYIDHIQEIVK					Mascot
1676.8544	1676.9122	0.0578	34	269	283	FPPEPNGYLHIGHAK					Mascot
1828.0062	1827.9441	-0.0621	-34	584	600	VVITNLEDGKVIDLDGK					Mascot
1990.8708	1990.9856	0.1148	58	33	49	FTHMMLTFEAGITGCDK			Carbamidomethyl (C)[15], Oxidation (M)[4,5]		Mascot

Rank	Protein Name	Accession No.	Protein MW	Protein PI	Pep. Count	Protein Score	Protein Score C. I. %	Intensity Matched	Total Ion Score	Total Ion C. I. %	Confirmed
2	PREDICTED: interactor of constitutive active ROPs 2, chloroplastic-like isoform 1 [Cucumis sativus]	gi|449440061	74482.3	5.3	25	72	94.135	9.978			

Protein Group

PREDICTED: interactor of constitutive active ROPs 2, chloroplastic-like isoform 2 [Cucumis sativus]	gi|449440063	74482.3	5.3000 001907 3486

Peptide Information

Calc. Mass	Obsrv. Mass	± da	± ppm	Start Seq.	End Seq.	Sequence	Ion Score	C. I. %	Modification	Rank	Result Type
808.3869	808.3994	0.0125	15	65	71	CLATESK			Carbamidomethyl (C)[1]		Mascot
816.4573	816.4474	-0.0099	-12	382	388	AELKEAR					Mascot
818.4366	818.3856	-0.051	-62	332	338	LEADRSK					Mascot
819.4393	819.3804	-0.0589	-72	1	7	MQTPKAK			Oxidation (M)[1]		Mascot
820.4345	820.3632	-0.0713	-87	646	652	NGNMLKK			Oxidation (M)[4]		Mascot
837.4101	837.3818	-0.0283	-34	250	256	NQFETAK					Mascot
890.5015	890.421	-0.0805	-90	518	524	MEIEKIK					Mascot
902.4479	902.4413	-0.0066	-7	592	598	VQADQWR					Mascot
960.4745	960.4643	-0.0102	-11	8	16	TGSSEVPQR					Mascot
1006.4873	1006.4957	0.0084	8	543	551	AAEQEALMK			Oxidation (M)[8]		Mascot
1016.5986	1016.5512	-0.0474	-47	479	487	SELAVELKK					Mascot
1016.5986	1016.5512	-0.0474	-47	478	486	KSELAVELK					Mascot
1023.5139	1023.5413	0.0274	27	116	124	KQLSDMSAK			Oxidation (M)[6]		Mascot
1058.5663	1058.5045	-0.0618	-58	419	427	MSQIIPVDR					Mascot
1088.5695	1088.5596	-0.0099	-9	8	17	TGSSEVPQRK					Mascot
1104.6008	1104.5818	-0.019	-17	337	347	SKSALAAAETR					Mascot
1149.582	1149.5952	0.0132	11	437	446	KLEADMGELK			Oxidation (M)[6]		Mascot
1181.6273	1181.6011	-0.0262	-22	386	395	EARADLEHLK					Mascot
1197.6296	1197.624	-0.0056	-5	361	370	IAHELVEQMK					Mascot
1232.6117	1232.6223	0.0106	9	236	246	ESETQALEVAR					Mascot
1347.7366	1347.7183	-0.0183	-14	215	226	TQLSETLSLVEK					Mascot
1665.8364	1665.8916	0.0552	33	318	331	DEIDVIKTELTSMR			Oxidation (M)[13]		Mascot
1692.8109	1692.8248	0.0139	8	400	413	EKETQLCSVVEENK			Carbamidomethyl (C)[7]		Mascot
1706.808	1706.7729	-0.0351	-21	503	517	ETELQSTTQENDALK					Mascot
1866.0266	1865.8721	-0.1545	-83	355	370	SALQIRIAHELVEQMK					Mascot

3	PREDICTED: ecotropic viral integration site 5 protein homolog isoform X3 [Setaria italica]	gi|514820221	97593.3	4.79	23	71	92.949	12.984

Peptide Information

Calc. Mass	Obsrv. Mass	± da	± ppm	Start Seq.	End Seq.	Sequence	Ion Score	C. I. %	Modification	Rank	Result Type

Calc. Mass	Obsrv. Mass	± da	± ppm	Start Seq.	End Seq.	Sequence	Ion Score	C. I. %	Modification	Rank	Result Type
807.4723	807.3951	-0.0772	-96	402	408	LLTAYAR					Mascot
808.3505	808.3994	0.0489	60	749	754	MEQEQK			Oxidation (M)[1]		Mascot
813.4253	813.4012	-0.0241	-30	27	32	FREYAK					Mascot
816.4396	816.4474	0.0078	10	311	318	GGVPMALR			Oxidation (M)[5]		Mascot
820.3318	820.3632	0.0314	38	36	41	EEEEER					Mascot
837.3585	837.3818	0.0233	28	141	147	EEETSSR					Mascot
904.4734	904.4779	0.0045	5	632	640	SKEGADGLK					Mascot
937.4957	937.4332	-0.0625	-67	223	230	MMSSRVVK					Mascot
1003.5571	1003.5351	-0.022	-22	852	860	GLGWLDKSK					Mascot
1064.5219	1064.5563	0.0344	32	139	147	VKEEETSSR					Mascot
1104.4998	1104.5818	0.082	74	2	11	GAGAFDFEYK					Mascot
1181.5546	1181.6011	0.0465	39	815	825	ANQSFTSSSPR					Mascot
1224.5742	1224.554	-0.0202	-16	33	41	IYKEEEEER					Mascot
1326.6281	1326.6709	0.0428	32	795	806	AVMAETMLEATK			Oxidation (M)[3,7]		Mascot
1347.7056	1347.7183	0.0127	9	511	521	VWDVILFEGNR					Mascot
1450.7393	1450.6801	-0.0592	-41	794	806	RAVMAETMLEATK					Mascot
1463.6794	1463.7944	0.115	79	749	760	MEQEQKVTEDAR					Mascot
1463.6794	1463.7944	0.115	79	749	760	MEQEQKVTEDAR					Mascot
1497.7478	1497.8151	0.0673	45	588	599	HRPEILTAMEER			Oxidation (M)[9]		Mascot
1497.7478	1497.8151	0.0673	45	588	599	HRPEILTAMEER			Oxidation (M)[9]		Mascot
1516.6658	1516.7666	0.1008	66	781	793	YEEAMAALSQMEK			Oxidation (M)[5]		Mascot
1516.6658	1516.7666	0.1008	66	781	793	YEEAMAALSQMEK			Oxidation (M)[5]		Mascot
1625.8428	1625.8792	0.0364	22	587	599	KHRPEILTAMEER			Oxidation (M)[10]		Mascot
1665.8806	1665.8916	0.011	7	351	365	DLVDPVLNEQISAPR					Mascot
1865.9253	1865.8721	-0.0532	-29	384	400	TFPGHPALDEDGRNALR					Mascot
1885.9152	1885.9563	0.0411	22	815	832	ANQSFTSSSPRADHVPGK					Mascot
1885.9152	1885.9563	0.0411	22	815	832	ANQSFTSSSPRADHVPGK					Mascot

4 hypothetical protein CHLNCDRAFT_59034 [Chlorella variabilis] gi|307103929 44564.8 6.15 15 68 85.603 9.315

Peptide Information

Calc. Mass	Obsrv. Mass	± da	± ppm	Start Seq.	End Seq.	Sequence	Ion Score	C. I. %	Modification	Rank	Result Type
816.421	816.4474	0.0264	32	388	394	LVEDANR					Mascot
818.4268	818.3856	-0.0412	-50	282	288	YHGGRTK					Mascot
820.4312	820.3632	-0.068	-83	100	106	FAVEQAR					Mascot
837.3771	837.3818	0.0047	6	120	126	TSCQVDK			Carbamidomethyl (C)[3]		Mascot

887.4944	887.4554	-0.039	-44	346	353	LSAELAQR					Mascot
902.4578	902.4413	-0.0165	-18	143	150	VSTEVDPR					Mascot
912.4495	912.4709	0.0214	23	164	170	QLVDMYK			Oxidation (M)[5]		Mascot
963.42	963.4664	0.0464	48	111	119	CNLEAGGDK			Carbamidomethyl (C)[1]		Mascot
1003.4744	1003.5351	0.0607	60	232	238	LHDWYNR					Mascot
1232.6998	1232.6223	-0.0775	-63	151	161	IAFDADKLLAR					Mascot
1347.6548	1347.7183	0.0635	47	183	194	MPATWAAIQACK			Carbamidomethyl (C)[11]		Mascot
1433.6511	1433.719	0.0679	47	245	257	DPNAPREMSAMAK			Oxidation (M)[8]		Mascot
1450.7213	1450.6801	-0.0412	-28	395	407	LEPYFLNLAGGQE					Mascot
1463.7345	1463.7944	0.0599	41	83	96	SVLAGIMGNPVSMR			Oxidation (M)[7,13]		Mascot
1463.7345	1463.7944	0.0599	41	83	96	SVLAGIMGNPVSMR			Oxidation (M)[7,13]		Mascot
1516.6737	1516.7666	0.0929	61	107	119	FYDKCNLEAGGDK			Carbamidomethyl (C)[5]		Mascot
1516.6737	1516.7666	0.0929	61	107	119	FYDKCNLEAGGDK			Carbamidomethyl (C)[5]		Mascot

5 kinesin heavy chain, putative [Ricinus communis] gi|223549706 120663.9 7.88 23 68 84.573 9.512

Peptide Information

Calc. Mass	Obsrv. Mass	± da	± ppm	Start Seq.	End Seq.	Sequence	Ion Score	C. I. %	Modification	Rank	Result Type
807.4359	807.3951	-0.0408	-51	895	901	GFLQQSK					Mascot
827.4118	827.3921	-0.0197	-24	506	512	QGKDSHR					Mascot
830.4617	830.3871	-0.0746	-90	329	335	QELELAK					Mascot
847.4268	847.4008	-0.026	-31	920	927	KDAGQNSK					Mascot
864.4257	864.4019	-0.0238	-28	827	833	HGMNLHR					Mascot
902.4578	902.4413	-0.0165	-18	305	311	LEEQDLR					Mascot
935.5342	935.4622	-0.072	-77	1027	1034	LSRMSLTK					Mascot
982.449	982.4701	0.0211	21	137	145	AHFDHNGGK					Mascot
1003.524	1003.5351	0.0111	11	1	8	MNIQQEIK					Mascot
1015.5604	1015.484	-0.0764	-75	82	90	LSPGLIEMR					Mascot
1058.559	1058.5045	-0.0545	-51	305	312	LEEQDLRR					Mascot
1181.6525	1181.6011	-0.0514	-43	91	101	GNIEPGPEKIK					Mascot
1193.5546	1193.611	0.0564	47	842	852	HSTESPSHSPK					Mascot
1232.6746	1232.6223	-0.0523	-42	2	11	NIQQEIKFGR					Mascot
1320.6465	1320.5986	-0.0479	-36	612	623	DLLSTVPDASMR			Oxidation (M)[11]		Mascot
1374.626	1374.6686	0.0426	31	395	404	YRSFMDHQFK			Oxidation (M)[5]		Mascot
1379.7101	1379.7412	0.0311	23	1	11	MNIQQEIKFGR			Oxidation (M)[1]		Mascot
1412.822	1412.7172	-0.1048	-74	1053	1066	KIALGSSSATRPPK					Mascot

1428.7594	1428.7281	-0.0313	-22	579	590	ALHDLFQISQTR					Mascot
1497.6898	1497.8151	0.1253	84	102	114	MFLAAMDEMGLPR			Oxidation (M)[1]		Mascot
1497.6898	1497.8151	0.1253	84	102	114	MFLAAMDEMGLPR			Oxidation (M)[1]		Mascot
1676.871	1676.9122	0.0412	25	67	81	ACLIDGTVLCSILNK			Carbamidomethyl (C)[2,10]		Mascot
1999.9944	1999.9865	-0.0079	-4	440	457	GLAEAAENYHSVLAENR R					Mascot
2664.4146	2664.3176	-0.097	-36	516	538	FNKVFGPATSQEEVFLDT RPLIR					Mascot

6 hypothetical protein CARUB_v10012830mg [Capsella rubella] gi|482565552 140840 5.9 28 68 83.47 16.032

Peptide Information

Calc. Mass	Obsrv. Mass	± da	± ppm	Start Seq.	End Seq.	Sequence	Ion Score	C. I. %	Modification	Rank	Result Type
804.421	804.3451	-0.0759	-94	858	865	TTATPASR					Mascot
816.4474	816.4474	0	0	1130	1136	WGAAQKR					Mascot
818.4254	818.3856	-0.0398	-49	62	68	LEVESNK					Mascot
840.4614	840.4031	-0.0583	-69	396	402	LFVGYNK					Mascot
864.4131	864.4019	-0.0112	-13	447	453	DKEVDMK					Mascot
870.4719	870.5371	0.0652	75	811	817	LVYFNSK					Mascot
874.4013	874.4691	0.0678	78	546	554	NGGSAESPR					Mascot
887.4806	887.4554	-0.0252	-28	910	917	NGVRTQGR					Mascot
904.4959	904.4779	-0.018	-20	914	921	TQGRSSIR					Mascot
937.4924	937.4332	-0.0592	-63	194	200	FITLCQR			Carbamidomethyl (C)[5]		Mascot
999.4854	999.4568	-0.0286	-29	661	670	GPGDQPSSVR					Mascot
1002.4963	1002.5499	0.0536	53	546	555	NGGSAESPRK					Mascot
1003.524	1003.5351	0.0111	11	555	562	KDLMPLDR			Oxidation (M)[4]		Mascot
1016.5345	1016.5512	0.0167	16	653	660	LFAEHMLR					Mascot
1016.5345	1016.5512	0.0167	16	653	660	LFAEHMLR	1	0			Mascot
1023.4589	1023.5413	0.0824	81	1240	1249	EDQTSGTSAK					Mascot
1095.5066	1095.5426	0.036	33	111	120	IYGEGTGDQR					Mascot
1104.5474	1104.5818	0.0344	31	1253	1261	SFFSLSNFR					Mascot
1117.5485	1117.6324	0.0839	75	210	218	VNQEEETIR					Mascot
1117.5485	1117.6324	0.0839	75	210	218	VNQEEETIR					Mascot
1204.6355	1204.6403	0.0048	4	898	909	KEGMKPASGVGK			Oxidation (M)[4]		Mascot
1227.644	1227.6271	-0.0169	-14	563	572	QSPLVEDRQR					Mascot
1232.6667	1232.6223	-0.0444	-36	776	785	LKAMQDILER			Oxidation (M)[4]		Mascot
1315.6602	1315.7001	0.0399	30	688	699	RPAAQDLSSDQK					Mascot
1319.6743	1319.6497	-0.0246	-19	1253	1263	SFFSLSNFRSK					Mascot

1347.6499	1347.7183	0.0684	51	1240	1252	EDQTSGTSAKAPR					Mascot
1428.7383	1428.7281	-0.0102	-7	1250	1261	APRSFFSLSNFR					Mascot
1480.7544	1480.7672	0.0128	9	69	81	NAGTWFTKGTLER					Mascot
1516.7966	1516.7666	-0.03	-20	56	68	DVQSIRLEVESNK					Mascot
1516.7966	1516.7666	-0.03	-20	56	68	DVQSIRLEVESNK					Mascot
1665.833	1665.8916	0.0586	35	940	955	GAAEATELATEFSQLK					Mascot

7	hypothetical protein F775_42730 [Aegilops tauschii]	gi|475435179	6049.8	4.49	7	67	81.875	3.271

Peptide Information

Calc. Mass	Obsrv. Mass	± da	± ppm	Start Seq.	End Seq.	Sequence	Ion Score	C. I. %	Modification	Rank	Result Type
804.3734	804.3451	-0.0283	-35	2	7	ELEEER					Mascot
837.3995	837.3818	-0.0177	-21	39	46	GSRSSMGR					Mascot
856.4424	856.5229	0.0805	94	47	53	LHVVHDH					Mascot
935.4138	935.4622	0.0484	52	1	7	MELEEER					Mascot
1067.5229	1067.5404	0.0175	16	8	18	ELGGIDGGGHR					Mascot
1374.6696	1374.6686	-0.001	-1	42	53	SSMGRLHVVHDH					Mascot
1999.9138	1999.9865	0.0727	36	1	18	MELEEERELGGIDGGGH R			Oxidation (M)[1]		Mascot

8	kinesin-like protein for actin-based chloroplast movement 1 [Ceratopteris richardii]	gi|410509304	150458.1	6.58	28	65	66.25	11.625

Peptide Information

Calc. Mass	Obsrv. Mass	± da	± ppm	Start Seq.	End Seq.	Sequence	Ion Score	C. I. %	Modification	Rank	Result Type
809.4403	809.3757	-0.0646	-80	503	508	KELYEK					Mascot
813.4617	813.4012	-0.0605	-74	1310	1315	YARLYK					Mascot
815.4006	815.3588	-0.0418	-51	1184	1190	EGPQQTR					Mascot
818.444	818.3856	-0.0584	-71	717	724	ALASPTMK					Mascot
847.438	847.4008	-0.0372	-44	1302	1309	TADGTRAR					Mascot
874.4489	874.4691	0.0202	23	875	883	GSRGSSPAR					Mascot
902.4578	902.4413	-0.0165	-18	1326	1333	SSVPDLER					Mascot
904.4734	904.4779	0.0045	5	556	564	SQSAALAEK					Mascot
960.4996	960.4643	-0.0353	-37	509	516	EKELNEAK					Mascot
963.4966	963.4664	-0.0302	-31	865	874	VGTGGSSRSR					Mascot
999.5105	999.4568	-0.0537	-54	1066	1074	SALESVEHK					Mascot
1001.5374	1001.5284	-0.009	-9	480	488	NVELSLGNR					Mascot
1003.5418	1003.5351	-0.0067	-7	656	663	LEEELSKR					Mascot

1058.5338	1058.5045	-0.0293	-28	1184	1192	EGPQQTRSR					Mascot
1064.5007	1064.5563	0.0556	52	335	343	VENPTDFSR					Mascot
1067.5225	1067.5404	0.0179	17	826	834	VMDTMLVSR			Oxidation (M)[2]		Mascot
1104.5645	1104.5818	0.0173	16	940	948	LRETTEEAR					Mascot
1117.5961	1117.6324	0.0363	32	556	566	SQSAALAEKGR					Mascot
1117.5961	1117.6324	0.0363	32	556	566	SQSAALAEKGR	1	0			Mascot
1255.5658	1255.6177	0.0519	41	383	393	LSMVDMVASDR			Oxidation (M)[3,6]		Mascot
1263.6614	1263.7039	0.0425	34	1241	1252	CGATSSLELVVK			Carbamidomethyl (C)[1]		Mascot
1315.7368	1315.7001	-0.0367	-28	1290	1301	LPEAFSQLALAR					Mascot
1347.7301	1347.7183	-0.0118	-9	712	724	VQASKALASPTMK			Oxidation (M)[12]		Mascot
1395.7955	1395.7	-0.0955	-68	87	98	LEKLHQQVSVSK					Mascot
1514.8114	1514.7424	-0.069	-46	543	555	AWKVAFTLQADHK					Mascot
1626.0135	1625.8792	-0.1343	-83	787	802	LLMLVLAAVIKAGAAR			Oxidation (M)[3]		Mascot
1665.8807	1665.8916	0.0109	7	196	211	VNTSSFGVDVGSIQKK					Mascot
1827.8444	1827.9441	0.0997	55	258	273	THTMEGSTSDRGVFFR					Mascot
1838.9607	1838.9359	-0.0248	-13	397	412	EEATGDRLTELLHINK					Mascot

9 unnamed protein product [Vitis vinifera] gi|296086345 152385.7 8.77 28 64 61.249 14.158

Peptide Information

Calc. Mass	Obsrv. Mass	± da	± ppm	Start Seq.	End Seq.	Sequence	Ion Score	C. I. %	Modification	Rank	Result Type
808.406	808.3994	-0.0066	-8	154	159	RQYGER					Mascot
820.3431	820.3632	0.0201	25	729	736	EGNGNSDK					Mascot
837.4213	837.3818	-0.0395	-47	160	167	QSAYVGGR					Mascot
838.4781	838.4029	-0.0752	-90	419	425	HTPVKEK					Mascot
840.3846	840.4031	0.0185	22	1043	1049	DGKYDSR					Mascot
841.493	841.4668	-0.0262	-31	464	470	VVVNWPK					Mascot
849.4498	849.3926	-0.0572	-67	663	669	TCQLSLK			Carbamidomethyl (C)[2]		Mascot
871.3866	871.3183	-0.0683	-78	444	450	DYLSMDK					Mascot
887.4581	887.4554	-0.0027	-3	715	722	EKNGSQPK					Mascot
912.5513	912.4709	-0.0804	-88	1276	1283	KEAPIINK					Mascot
935.468	935.4622	-0.0058	-6	682	689	DKEIDSTK					Mascot
937.4738	937.4332	-0.0406	-43	223	230	QTDYLAAR					Mascot
963.4637	963.4664	0.0027	3	1169	1177	IAEPSMGMK					Mascot
982.5316	982.4701	-0.0615	-63	1061	1068	KNLEEPPR					Mascot
1002.5214	1002.5499	0.0285	28	98	107	GVGPSTLESR					Mascot

1023.5325	1023.5413	0.0088	9	522	530	MILMSGLSR			Oxidation (M)[1]		Mascot
1067.5771	1067.5404	-0.0367	-34	455	463	LFISPEFSK					Mascot
1104.5896	1104.5818	-0.0078	-7	995	1004	QDEKIVSGTK					Mascot
1117.6212	1117.6324	0.0112	10	927	936	KVVQQGTETK					Mascot
1117.6212	1117.6324	0.0112	10	927	936	KVVQQGTETK					Mascot
1224.5565	1224.554	-0.0025	-2	108	119	SGYASAMPESPK					Mascot
1251.6072	1251.663	0.0558	45	1167	1177	QKIAEPSMGMK			Oxidation (M)[8,10]		Mascot
1370.6547	1370.7018	0.0471	34	820	832	SQQQEPSADPGVK					Mascot
1416.7329	1416.7977	0.0648	46	875	889	SDPSIAASVQGTGVK					Mascot
1450.5929	1450.6801	0.0872	60	791	803	AGTENTENEENDK					Mascot
1497.689	1497.8151	0.1261	84	1242	1255	VAGMGKEEAEEFGK			Oxidation (M)[4]		Mascot
1497.689	1497.8151	0.1261	84	1242	1255	VAGMGKEEAEEFGK			Oxidation (M)[4]		Mascot
1516.7966	1516.7666	-0.03	-20	1328	1341	ELVQSALLESNTGR					Mascot
1516.7966	1516.7666	-0.03	-20	1328	1341	ELVQSALLESNTGR					Mascot
1885.8961	1885.9563	0.0602	32	937	953	DVSEQKVEAGNPVCEPK			Carbamidomethyl (C)[14]		Mascot
1885.8961	1885.9563	0.0602	32	937	953	DVSEQKVEAGNPVCEPK			Carbamidomethyl (C)[14]		Mascot
2664.1941	2664.3176	0.1235	46	848	870	TTQDESVQPEVKIENEAQ CSEDK			Carbamidomethyl (C)[19]		Mascot

10 PREDICTED: uncharacterized protein LOC101217480 [Cucumis sativus] gi|449432958 134504.7 5.44 26 63 53.411 12.409

Peptide Information

Calc. Mass	Obsrv. Mass	± da	± ppm	Start Seq.	End Seq.	Sequence	Ion Score	C. I. %	Modification	Rank	Result Type
804.3596	804.3451	-0.0145	-18	55	60	AEMYYK					Mascot
813.4617	813.4012	-0.0605	-74	1172	1177	KYYALR					Mascot
820.3546	820.3632	0.0086	10	55	60	AEMYYK			Oxidation (M)[3]		Mascot
830.4618	830.3871	-0.0747	-90	359	365	IDELVNK					Mascot
874.4992	874.4691	-0.0301	-34	558	565	SSKLQSPK					Mascot
902.4326	902.4413	0.0087	10	655	662	NTGDDPRK					Mascot
904.437	904.4779	0.0409	45	801	807	EQNKEEK					Mascot
935.5057	935.4622	-0.0435	-46	75	82	AYRALADR					Mascot
960.4607	960.4643	0.0036	4	54	60	RAEMYYK					Mascot
963.5258	963.4664	-0.0594	-62	1011	1018	SEARPLYK					Mascot
982.4588	982.4701	0.0113	12	662	670	KDDAYGSAR					Mascot
999.5469	999.4568	-0.0901	-90	752	760	NPVDVVQTK					Mascot
1003.553	1003.5351	-0.0179	-18	607	615	KLDVNATSR					Mascot
1006.4873	1006.4957	0.0084	8	862	869	KLNEMDEK					Mascot

1016.5483	1016.5512	0.0029	3	892	899	DQEIRSLR				Mascot
1016.5483	1016.5512	0.0029	3	892	899	DQEIRSLR	8	0		Mascot
1193.5281	1193.611	0.0829	69	714	724	GENSDTDNTIK				Mascot
1197.6586	1197.624	-0.0346	-29	312	321	EKQVPNQLNK				Mascot
1204.5514	1204.6403	0.0889	74	548	557	QNEEPVTEMK				Mascot
1251.5852	1251.663	0.0778	62	43	53	LIEEDGDSFAR				Mascot
1265.6406	1265.6576	0.017	13	596	607	MDPLSLGSSSKK			Oxidation (M)[1]	Mascot
1265.6484	1265.6576	0.0092	7	1117	1127	LNDEFRLSGSK				Mascot
1315.6852	1315.7001	0.0149	11	322	332	EVDIIADEKQR				Mascot
1319.6438	1319.6497	0.0059	4	385	395	SETDELQTQIR				Mascot
1416.7441	1416.7977	0.0536	38	402	414	ASITDGKNNLQQK				Mascot
1428.7693	1428.7281	-0.0412	-29	176	187	QEALNEIDKLQK				Mascot
1665.8595	1665.8916	0.0321	19	62	74	RPELINFVEETYR				Mascot
1908.8831	1908.9042	0.0211	11	22	36	QSKWMEQNLLDMEEK				Mascot

Gel Idx/Pos	245/J21	Instr./Gel Origin	BA2151/Sample Project 20140814	Process Status	Analysis Succeeded
Plate [#] Name	[1] Sample Project 20140814	Instrument Sample Name		Spectra	11

Rank	Protein Name	Accession No.	Protein MW	Protein PI	Pep. Count	Protein Score	Protein Score C. I. %	Intensity Matched	Total Ion Score	Total Ion C. I. %	Confirmed
1	Os01g0135000 [Oryza sativa Japonica Group]	gi|113531479	9809	9.59	8	51	0	4.059			

Peptide Information

Calc. Mass	Obsrv. Mass	± da	± ppm	Start Seq.	End Seq.	Sequence	Ion Score	C. I. %	Modification	Rank	Result Type
811.4785	811.4084	-0.0701	-86	38	45	GPIGRPSK					Mascot
814.4529	814.448	-0.0049	-6	46	52	SLRHSSK					Mascot
829.4526	829.3946	-0.058	-70	2	10	IVAAGAGDR					Mascot
841.3937	841.4519	0.0582	69	31	37	SDETVYK					Mascot
963.5105	963.529	0.0185	19	63	71	LEATTTTAR					Mascot
963.5105	963.529	0.0185	19	63	71	LEATTTTAR					Mascot
1116.5942	1116.5586	-0.0356	-32	1	11	MIVAAGAGDRR					Mascot
1170.6378	1170.6189	-0.0189	-16	49	58	HSSKAYRPPK					Mascot
1245.7136	1245.6625	-0.0511	-41	53	62	AYRPPKILCK			Carbamidomethyl (C)[9]		Mascot

Rank	Protein Name	Accession No.	Protein MW	Protein PI	Pep. Count	Protein Score	Protein Score C. I. %	Intensity Matched	Total Ion Score	Total Ion C. I. %	Confirmed
2	hypothetical protein M569_00357, partial [Genlisea aurea]	gi|527209757	12123.8	11.59	9	47	0	6.511			

Peptide Information

Calc. Mass	Obsrv. Mass	± da	± ppm	Start Seq.	End Seq.	Sequence	Ion Score	C. I. %	Modification	Rank	Result Type
808.4199	808.4072	-0.0127	-16	39	45	FISEQGK					Mascot
820.4345	820.3737	-0.0608	-74	32	38	NMSLISR					Mascot
836.4294	836.3848	-0.0446	-53	32	38	NMSLISR			Oxidation (M)[2]		Mascot
887.5308	887.4655	-0.0653	-74	54	60	LTLKEQR					Mascot
963.5469	963.529	-0.0179	-19	93	101	TTTTGLITR					Mascot
963.5469	963.529	-0.0179	-19	93	101	TTTTGLITR					Mascot
1104.6161	1104.6295	0.0134	12	2	10	DKSNRPFLK					Mascot
1205.6484	1205.6827	0.0343	28	93	103	TTTTGLITRNQ					Mascot
1251.6515	1251.6506	-0.0009	-1	1	10	MDKSNRPFLK			Oxidation (M)[1]		Mascot
1251.6515	1251.6506	-0.0009	-1	1	10	MDKSNRPFLK			Oxidation (M)[1]		Mascot
1367.71	1367.8048	0.0948	69	28	38	IDYRNMSLISR					Mascot
1625.8315	1625.8925	0.061	38	32	45	NMSLISRFISEQGK			Oxidation (M)[2]		Mascot

3	hypothetical protein SORBIDRAFT_08g015300 [Sorghum bicolor]	gi|241943893	55500.7	6.68	13	46	0	9.521

Peptide Information

Calc. Mass	Obsrv. Mass	± da	± ppm	Start Seq.	End Seq.	Sequence	Ion Score	C. I. %	Modification	Rank	Result Type
820.4159	820.3737	-0.0422	-51	426	433	SDSVSGIR					Mascot
824.4485	824.404	-0.0445	-54	225	231	GREIHGR					Mascot
841.4124	841.4519	0.0395	47	330	336	IFESMSK					Mascot
856.5726	856.5277	-0.0449	-52	9	15	LSVLIRR					Mascot
963.5114	963.529	0.0176	18	436	443	MRELGMVK					Mascot
963.5114	963.529	0.0176	18	436	443	MRELGMVK					Mascot
1170.5725	1170.6189	0.0464	40	444	453	TPGCSWVHVK			Carbamidomethyl (C)[4]		Mascot
1251.658	1251.6506	-0.0074	-6	185	194	SEETLELFRK					Mascot
1251.658	1251.6506	-0.0074	-6	185	194	SEETLELFRK					Mascot
1263.6362	1263.7278	0.0916	72	194	204	KAQIESVDMAR			Oxidation (M)[9]		Mascot
1263.6362	1263.7278	0.0916	72	194	204	KAQIESVDMAR			Oxidation (M)[9]		Mascot
1319.6313	1319.6517	0.0204	15	456	466	AHAFYQGCIPR			Carbamidomethyl (C)[8]		Mascot
1326.774	1326.6749	-0.0991	-75	357	369	AGAIEEAVGIIRK					Mascot
1507.8778	1507.7622	-0.1156	-77	1	14	MPPPTAGRLSVLIR					Mascot
1657.7462	1657.8276	0.0814	49	168	181	DAVVWNCMVTGYAR			Carbamidomethyl (C)[7], Oxidation (M)[8]		Mascot
1827.9244	1827.9742	0.0498	27	438	453	ELGMVKTPGCSWVHVK			Carbamidomethyl (C)[10]		Mascot

4	hypothetical protein OsI_33249 [Oryza sativa Indica Group]	gi|218184396	100202.2	7.74	18	46	0	9.1

Peptide Information

Calc. Mass	Obsrv. Mass	± da	± ppm	Start Seq.	End Seq.	Sequence	Ion Score	C. I. %	Modification	Rank	Result Type
813.4981	813.425	-0.0731	-90	374	380	LVHLFGK					Mascot
820.4047	820.3737	-0.031	-38	771	777	GKTEEEK					Mascot
821.4073	821.4008	-0.0065	-8	605	611	EVMLDSK					Mascot
856.5502	856.5277	-0.0225	-26	658	665	VELALALK					Mascot
903.5192	903.4348	-0.0844	-93	131	137	MQRVLTR					Mascot
963.5298	963.529	-0.0008	-1	172	179	WLAEGLFK					Mascot
963.5298	963.529	-0.0008	-1	172	179	WLAEGLFK					Mascot
1052.5259	1052.5562	0.0303	29	186	195	LGDSAVESFK					Mascot
1067.5369	1067.6306	0.0937	88	381	390	FELSGSVSNK					Mascot
1069.5426	1069.5288	-0.0138	-13	550	558	YGAFQSLQR					Mascot

Calc. Mass	Obsrv. Mass	± da	± ppm	Start Seq.	End Seq.	Sequence	Ion Score	C. I. %	Modification	Rank	Result Type
1133.5685	1133.6034	0.0349	31	773	782	TEEEKAEGIK					Mascot
1172.5253	1172.6251	0.0998	85	138	148	SYTCLSGDAAK			Carbamidomethyl (C)[4]		Mascot
1173.6475	1173.6165	-0.031	-26	521	531	GDVLTALSELR					Mascot
1182.6365	1182.6575	0.021	18	511	520	ELYLSSTTIR					Mascot
1263.6222	1263.7278	0.1056	84	123	133	SNSNALARMQR			Oxidation (M)[9]		Mascot
1263.6222	1263.7278	0.1056	84	123	133	SNSNALARMQR			Oxidation (M)[9]		Mascot
1319.757	1319.6517	-0.1053	-80	329	341	YLSIGGAVTTLPK					Mascot
1373.7577	1373.7078	-0.0499	-36	646	657	LLGTWPSPFVTR					Mascot
1509.7288	1509.8456	0.1168	77	605	617	EVMLDSKMNVESK					Mascot
1657.7527	1657.8276	0.0749	45	612	625	MNVESKEYWEGAAK			Oxidation (M)[1]		Mascot

5 hypothetical protein OsI_16087 [Oryza sativa Indica Group] gi|125548498 33732.8 10.07 11 45 0 4.759

Peptide Information

Calc. Mass	Obsrv. Mass	± da	± ppm	Start Seq.	End Seq.	Sequence	Ion Score	C. I. %	Modification	Rank	Result Type
812.4988	812.4225	-0.0763	-94	80	87	GLSPAVLR					Mascot
840.4686	840.4349	-0.0337	-40	180	187	GVVPNAQR					Mascot
864.4938	864.4142	-0.0796	-92	250	256	DAKVLYR					Mascot
923.5533	923.4854	-0.0679	-74	46	52	TRLQLHR					Mascot
947.5098	947.4752	-0.0346	-37	277	284	GFLPTWAR					Mascot
1022.4757	1022.5748	0.0991	97	244	252	MMNQGKDAK					Mascot
1106.5009	1106.5505	0.0496	45	53	66	GTGGSGGGGGGVMR					Mascot
1317.5891	1317.6106	0.0215	16	2	17	SARAAGGGGDGDGGGR					Mascot
1324.7485	1324.6554	-0.0931	-70	176	187	GLWKGVVPNAQR					Mascot
1326.6722	1326.6749	0.0027	2	257	267	NSYDCLVKTVK			Carbamidomethyl (C)[5]		Mascot
1830.924	1830.9702	0.0462	25	53	73	GTGGSGGGGGGVMRVA GELVR					Mascot

6 ribosomal protein S8 [Nephroselmis olivacea] gi|110225649 14688.6 9.57 8 44 0 2.526

Peptide Information

Calc. Mass	Obsrv. Mass	± da	± ppm	Start Seq.	End Seq.	Sequence	Ion Score	C. I. %	Modification	Rank	Result Type
841.5115	841.4519	-0.0596	-71	72	78	ISRPGRR					Mascot
947.4979	947.4752	-0.0227	-24	24	31	VPCTKNTK			Carbamidomethyl (C)[3]		Mascot
1118.5048	1118.5272	0.0224	20	78	85	RVYCSYDR			Carbamidomethyl (C)[4]		Mascot
1245.7566	1245.6625	-0.0941	-76	32	41	KLLNIFLQEK					Mascot
1470.7839	1470.7897	0.0058	4	86	98	ISPEKFSIFSTSK					Mascot

1516.7101	1516.7748	0.0647	43	79	90	VYCSYDRISPEK			Carbamidomethyl (C)[3]		Mascot
1625.7853	1625.8925	0.1072	66	59	71	YEGNSPMFQRIQR					Mascot
1908.9597	1908.9491	-0.0106	-6	2	18	DRLTCLFSALQNGQSAK			Carbamidomethyl (C)[5]		Mascot

7	hypothetical protein SORBIDRAFT_03g029120 [Sorghum bicolor]	gi|241928004	26528.9	7.01	10	44	0	3.719

Peptide Information

Calc. Mass	Obsrv. Mass	± da	± ppm	Start Seq.	End Seq.	Sequence	Ion Score	C. I. %	Modification	Rank	Result Type
808.3869	808.4072	0.0203	25	77	83	AKEAMDK			Oxidation (M)[5]		Mascot
819.428	819.3803	-0.0477	-58	124	130	LDEMLAK					Mascot
821.3821	821.4008	0.0187	23	23	29	CSSKPDK			Carbamidomethyl (C)[1]		Mascot
923.4846	923.4854	0.0008	1	156	163	WIHPASGR					Mascot
1015.5432	1015.5254	-0.0178	-18	198	205	SRLEAFHR					Mascot
1096.5126	1096.5804	0.0678	62	21	29	MKCSSKPDK			Carbamidomethyl (C)[3], Oxidation (M)[1]		Mascot
1133.5797	1133.6034	0.0237	21	190	199	DDTAEVLKSR					Mascot
1461.8788	1461.869	-0.0098	-7	217	230	KGLVANLPAEKPPK					Mascot
1470.7476	1470.7897	0.0421	29	206	217	QTEPVIDYYSKK					Mascot
1991.0743	1991.006	-0.0683	-34	23	41	CSSKPDKHLILIGPPGSGK			Carbamidomethyl (C)[1]		Mascot

8	maturase K, partial (chloroplast) [Rhododendron indicum]	gi|340513016	32362.9	9.74	10	43	0	3.895

Peptide Information

Calc. Mass	Obsrv. Mass	± da	± ppm	Start Seq.	End Seq.	Sequence	Ion Score	C. I. %	Modification	Rank	Result Type
811.4573	811.4084	-0.0489	-60	257	262	FGRIYR					Mascot
813.4617	813.425	-0.0367	-45	99	104	ISFYRK					Mascot
841.4275	841.4519	0.0244	29	82	88	NQSSHLR					Mascot
856.5436	856.5277	-0.0159	-19	191	197	LKPLMVR					Mascot
923.5019	923.4854	-0.0165	-18	141	148	GTFLLMNK					Mascot
1106.6456	1106.5505	-0.0951	-86	104	112	KIELEVFTK					Mascot
1237.6761	1237.6061	-0.07	-57	141	150	GTFLLMNKWK					Mascot
1838.9316	1838.947	0.0154	8	198	213	SQMIENSFLIENASKK					Mascot
1990.9941	1991.006	0.0119	6	82	98	NQSSHLRSISSETFLER					Mascot
2723.2322	2723.3367	0.1045	38	151	169	YYLVNFWQCYFYMWSQPRR			Carbamidomethyl (C)[9], Oxidation (M)[13]		Mascot

9	hypothetical protein [Linum usitatissimum]	gi|395146509	45838.1	8.67	12	43	0	5.882

Peptide Information

Calc. Mass	Obsrv. Mass	± da	± ppm	Start Seq.	End Seq.	Sequence	Ion Score	C. I. %	Modification	Rank	Result Type
808.4498	808.4072	-0.0426	-53	338	343	FMNIKR					Mascot
810.4291	810.4418	0.0127	16	78	83	RVFTCK			Carbamidomethyl (C)[5]		Mascot
811.4421	811.4084	-0.0337	-42	178	184	HAVSELR					Mascot
824.4447	824.404	-0.0407	-49	338	343	FMNIKR			Oxidation (M)[2]		Mascot
834.4104	834.3618	-0.0486	-58	199	206	AGWNSATK					Mascot
887.4694	887.4655	-0.0039	-4	240	249	ISNVAGGGGR					Mascot
921.4756	921.4561	-0.0195	-21	343	349	RAMLVCR			Carbamidomethyl (C)[6], Oxidation (M)[3]		Mascot
1052.4756	1052.5562	0.0806	77	52	61	DDHSPPAASR					Mascot
1173.6335	1173.6165	-0.017	-14	237	249	ASKISNVAGGGGR					Mascot
1180.5991	1180.6313	0.0322	27	1	11	MASISSSVINR			Oxidation (M)[1]		Mascot
1245.6919	1245.6625	-0.0294	-24	344	354	AMLVCRVIAGR			Carbamidomethyl (C)[5]		Mascot
1335.7419	1335.6156	-0.1263	-95	195	206	IIFKAGWNSATK					Mascot
1335.7419	1335.6156	-0.1263	-95	195	206	IIFKAGWNSATK					Mascot
1838.9104	1838.947	0.0366	20	178	194	HAVSELRDGDSGNNIVR					Mascot

10	PREDICTED: uncharacterized protein LOC101220193 [Cucumis sativus]	gi|449444022	36869.9	8.92	11	43	0	4.955	

Peptide Information

Calc. Mass	Obsrv. Mass	± da	± ppm	Start Seq.	End Seq.	Sequence	Ion Score	C. I. %	Modification	Rank	Result Type
881.455	881.4303	-0.0247	-28	28	35	SGVFACLK			Carbamidomethyl (C)[6]		Mascot
1052.5007	1052.5562	0.0555	53	101	108	DQNQYEKK					Mascot
1105.6034	1105.6094	0.006	5	268	277	VALSKVSMDR					Mascot
1133.6578	1133.6034	-0.0544	-48	301	308	IVRWLQYR					Mascot
1170.6841	1170.6189	-0.0652	-56	242	253	GIGGEVAQKAIK					Mascot
1237.606	1237.6061	0.0001	0	47	57	YIPKGSSNDEK					Mascot
1244.5953	1244.6997	0.1044	84	285	294	QWMRGQDAPR					Mascot
1251.625	1251.6506	0.0256	20	261	272	ESGEGMKVALSK			Oxidation (M)[6]		Mascot
1251.625	1251.6506	0.0256	20	261	272	ESGEGMKVALSK			Oxidation (M)[6]		Mascot
1381.7145	1381.7231	0.0086	6	273	284	VSMDRLFVEASK					Mascot
1827.9535	1827.9742	0.0207	11	2	18	TAMAGFSIQASIHLQPR					Mascot
1990.9838	1991.006	0.0222	11	1	18	MTAMAGFSIQASIHLQPR			Oxidation (M)[1,4]		Mascot

Gel Idx/Pos	246/J22	Instr./Gel Origin	BA2151/Sample Project 20140814	Process Status	Analysis Succeeded
Plate [#] Name	[1] Sample Project 20140814	Instrument Sample Name		Spectra	11

Rank	Protein Name	Accession No.	Protein MW	Protein PI	Pep. Count	Protein Score	Protein Score C. I. %	Intensity Matched	Total Ion Score	Total Ion C. I. %	Confirmed
1	ATP synthase subunit beta, mitochondrial [Triticum urartu]	gi|473798701	57827	5.25	25	933	100	47.459	763	100	

Peptide Information

Calc. Mass	Obsrv. Mass	± da	± ppm	Start Seq.	End Seq.	Sequence	Ion Score	C. I. %	Modification	Rank	Result Type
832.4597	832.3896	-0.0701	-84	497	504	IMGVLDGK					Mascot
866.4003	866.4259	0.0256	30	219	225	EGNDLYR					Mascot
1100.5847	1100.6163	0.0316	29	137	145	TNSYLPIHR					Mascot
1173.6627	1173.6957	0.033	28	166	175	VVDLLAPYQR					Mascot
1173.6627	1173.6957	0.033	28	166	175	VVDLLAPYQR	47	96.79			Mascot
1278.6359	1278.6624	0.0265	21	87	98	TIAMDGTEGLVR			Oxidation (M)[4]		Mascot
1390.6863	1390.7303	0.044	32	203	216	AHGGFSVFAGVGER					Mascot
1390.6863	1390.7303	0.044	32	203	216	AHGGFSVFAGVGER	109	100			Mascot
1399.7693	1399.8136	0.0443	32	261	273	VGLTGLTVAEHFR					Mascot
1399.7693	1399.8136	0.0443	32	261	273	VGLTGLTVAEHFR	45	94.734			Mascot
1409.8112	1409.854	0.0428	30	102	115	VLNTGSPITVPVGR					Mascot
1473.8346	1473.8148	-0.0198	-13	190	202	TVLIMELINNVAK			Oxidation (M)[5]		Mascot
1492.7755	1492.8224	0.0469	31	290	303	FTQANSEVSALLGR					Mascot
1492.7755	1492.8224	0.0469	31	290	303	FTQANSEVSALLGR	71	99.988			Mascot
1513.8121	1513.8231	0.011	7	133	145	GDIKTNSYLPIHR					Mascot
1675.949	1676.0051	0.0561	33	72	86	LVLEVAQHLGENVVR					Mascot
1675.949	1676.0051	0.0561	33	72	86	LVLEVAQHLGENVVR	101	100			Mascot
1678.7676	1678.7964	0.0288	17	244	258	CALVYGQMNEPPGAR			Carbamidomethyl (C)[1], Oxidation (M)[8]		Mascot
1811.8857	1811.936	0.0503	28	386	401	MLSPHVLGEAHYNTAR			Oxidation (M)[1]		Mascot
1852.9109	1853.0294	0.1185	64	219	234	EGNDLYREMIESGVIK					Mascot
1864.944	1865.0048	0.0608	33	274	289	DAEGQDVLLFIDNIFR					Mascot
1864.944	1865.0048	0.0608	33	274	289	DAEGQDVLLFIDNIFR	117	100			Mascot
1868.9059	1869.0281	0.1222	65	219	234	EGNDLYREMIESGVIK			Oxidation (M)[9]		Mascot
2061.0498	2061.1155	0.0657	32	367	385	QISELGIYPAVDPLDSTSR					Mascot
2172.1548	2172.2068	0.052	24	146	165	EAPAFVEQATEQQILVTG IK					Mascot
2186.1453	2186.2266	0.0813	37	304	324	IPSAVGYQPTLATDLGGL QER					Mascot

2186.1453	2186.2266	0.0813	37	304	324	IPSAVGYQPTLATDLGGLQER	206	100			Mascot
2208.1343	2208.1892	0.0549	25	386	405	MLSPHVLGEAHYNTARGVQK					Mascot
2212.186	2212.2603	0.0743	34	52	71	FDEGLPPILTALEVLDNSIR					Mascot
2212.186	2212.2603	0.0743	34	52	71	FDEGLPPILTALEVLDNSIR	68	99.974			Mascot
2548.2712	2548.3699	0.0987	39	27	51	ITDDFTGAGAVGEVCQVIGAVVDVR			Carbamidomethyl (C)[15]		Mascot
2688.376	2688.4717	0.0957	36	412	435	NLQDIIAILGMDELSEDDKLTVAR			Oxidation (M)[11]		Mascot
3714.8862	3715.0525	0.1663	45	331	366	GSITSVQAIYVPADDLTDPAPATTFAHLDATTVLSR					Mascot
3842.9812	3843.1782	0.197	51	330	366	KGSITSVQAIYVPADDLTDPAPATTFAHLDATTVLSR					Mascot

2	ATP synthase subunit beta, mitochondrial [Aegilops tauschii]	gi|475548007	59152.9	5.85	24	920	100	47.342	763	100	

Peptide Information

Calc. Mass	Obsrv. Mass	± da	± ppm	Start Seq.	End Seq.	Sequence	Ion Score	C. I. %	Modification	Rank	Result Type
866.4003	866.4259	0.0256	30	264	270	EGNDLYR					Mascot
1100.5847	1100.6163	0.0316	29	182	190	TNSYLPIHR					Mascot
1173.6627	1173.6957	0.033	28	211	220	VVDLLAPYQR					Mascot
1173.6627	1173.6957	0.033	28	211	220	VVDLLAPYQR	47	96.79			Mascot
1278.6359	1278.6624	0.0265	21	132	143	TIAMDGTEGLVR			Oxidation (M)[4]		Mascot
1390.6863	1390.7303	0.044	32	248	261	AHGGFSVFAGVGER					Mascot
1390.6863	1390.7303	0.044	32	248	261	AHGGFSVFAGVGER	109	100			Mascot
1399.7693	1399.8136	0.0443	32	306	318	VGLTGLTVAEHFR					Mascot
1399.7693	1399.8136	0.0443	32	306	318	VGLTGLTVAEHFR	45	94.734			Mascot
1409.8112	1409.854	0.0428	30	147	160	VLNTGSPITVPVGR					Mascot
1473.8346	1473.8148	-0.0198	-13	235	247	TVLIMELINNVAK			Oxidation (M)[5]		Mascot
1492.7755	1492.8224	0.0469	31	335	348	FTQANSEVSALLGR					Mascot
1492.7755	1492.8224	0.0469	31	335	348	FTQANSEVSALLGR	71	99.988			Mascot
1513.8121	1513.8231	0.011	7	178	190	GDIKTNSYLPIHR					Mascot
1675.949	1676.0051	0.0561	33	117	131	LVLEVAQHLGENVVR					Mascot
1675.949	1676.0051	0.0561	33	117	131	LVLEVAQHLGENVVR	101	100			Mascot
1678.7676	1678.7964	0.0288	17	289	303	CALVYGQMNEPPGAR			Carbamidomethyl (C)[1], Oxidation (M)[8]		Mascot
1811.8857	1811.936	0.0503	28	431	446	MLSPHVLGEAHYNTAR			Oxidation (M)[1]		Mascot
1852.9109	1853.0294	0.1185	64	264	279	EGNDLYREMIESGVIK					Mascot
1864.944	1865.0048	0.0608	33	319	334	DAEGQDVLLFIDNIFR					Mascot
1864.944	1865.0048	0.0608	33	319	334	DAEGQDVLLFIDNIFR	117	100			Mascot

Calc. Mass	Obsrv. Mass	± da	± ppm	Start Seq.	End Seq.	Sequence	Ion Score	C. I. %	Modification	Rank	Result Type
1868.9059	1869.0281	0.1222	65	264	279	EGNDLYREMIESGVIK			Oxidation (M)[9]		Mascot
2061.0498	2061.1155	0.0657	32	412	430	QISELGIYPAVDPLDSTSR					Mascot
2172.1548	2172.2068	0.052	24	191	210	EAPAFVEQATEQQILVTG IK					Mascot
2186.1453	2186.2266	0.0813	37	349	369	IPSAVGYQPTLATDLGGL QER					Mascot
2186.1453	2186.2266	0.0813	37	349	369	IPSAVGYQPTLATDLGGL QER	206	100			Mascot
2208.1343	2208.1892	0.0549	25	431	450	MLSPHVLGEAHYNTARG VQK					Mascot
2212.186	2212.2603	0.0743	34	97	116	FDEGLPPILTALEVLDNSI R					Mascot
2212.186	2212.2603	0.0743	34	97	116	FDEGLPPILTALEVLDNSI R	68	99.974			Mascot
2548.2712	2548.3699	0.0987	39	72	96	ITDDFTGAGAVGEVCQVI GAVVDVR			Carbamidomethyl (C)[15]		Mascot
2688.376	2688.4717	0.0957	36	457	480	NLQDIIAILGMDELSEDDK LTVAR			Oxidation (M)[11]		Mascot
3714.8862	3715.0525	0.1663	45	376	411	GSITSVQAIYVPADDLTD PAPATTFAHLDATTVLSR					Mascot
3842.9812	3843.1782	0.197	51	375	411	KGSITSVQAIYVPADDLT DPAPATTFAHLDATTVLS R					Mascot

3 ATP synthase subunit beta, mitochondrial [Triticum urartu] gi|473990219 58081.1 5.13 19 874 100 44.222 765 100

Peptide Information

Calc. Mass	Obsrv. Mass	± da	± ppm	Start Seq.	End Seq.	Sequence	Ion Score	C. I. %	Modification	Rank	Result Type
866.4003	866.4259	0.0256	30	253	259	EGNDLYR					Mascot
1173.6627	1173.6957	0.033	28	200	209	VVDLLAPYQR					Mascot
1173.6627	1173.6957	0.033	28	200	209	VVDLLAPYQR	47	96.79			Mascot
1278.6359	1278.6624	0.0265	21	92	103	TIAMDGTEGLVR			Oxidation (M)[4]		Mascot
1390.6863	1390.7303	0.044	32	237	250	AHGGFSVFAGVGER					Mascot
1390.6863	1390.7303	0.044	32	237	250	AHGGFSVFAGVGER	109	100			Mascot
1399.7693	1399.8136	0.0443	32	295	307	VGLTGLTVAEHFR					Mascot
1399.7693	1399.8136	0.0443	32	295	307	VGLTGLTVAEHFR	45	94.734			Mascot
1473.8346	1473.8148	-0.0198	-13	224	236	TVLIMELINNVAK			Oxidation (M)[5]		Mascot
1490.7771	1490.7906	0.0135	9	260	272	EMIESGVIKLDEK					Mascot
1492.7755	1492.8224	0.0469	31	324	337	FTQANSEVSALLGR					Mascot
1492.7755	1492.8224	0.0469	31	324	337	FTQANSEVSALLGR	71	99.988			Mascot
1675.949	1676.0051	0.0561	33	77	91	LVLEVAQHLGENVVR					Mascot
1675.949	1676.0051	0.0561	33	77	91	LVLEVAQHLGENVVR	101	100			Mascot
1678.7676	1678.7964	0.0288	17	278	292	CALVYGQMNEPPGAR			Carbamidomethyl (C)[1], Oxidation (M)[8]		Mascot
1852.9109	1853.0294	0.1185	64	253	268	EGNDLYREMIESGVIK					Mascot
1864.944	1865.0048	0.0608	33	308	323	DAEGQDVLLFIDNIFR					Mascot

1864.944	1865.0048	0.0608	33	308	323	DAEGQDVLLFIDNIFR	117	100			Mascot
1868.9059	1869.0281	0.1222	65	253	268	EGNDLYREMIESGVIK			Oxidation (M)[9]		Mascot
2061.0498	2061.1155	0.0657	32	401	419	QISELGIYPAVDPLDSTSR					Mascot
2172.1548	2172.2068	0.052	24	180	199	EAPAFVEQATEQQILVTGIK					Mascot
2186.1453	2186.2266	0.0813	37	338	358	IPSAVGYQPTLATDLGGLQER					Mascot
2186.1453	2186.2266	0.0813	37	338	358	IPSAVGYQPTLATDLGGLQER	206	100			Mascot
2212.186	2212.2603	0.0743	34	57	76	FDEGLPPILTALEVLDNSIR					Mascot
2212.186	2212.2603	0.0743	34	57	76	FDEGLPPILTALEVLDNSIR	68	99.974			Mascot
2591.3135	2591.3718	0.0583	22	32	56	ITDEFTGAGSIGQVCQVIGAVVDVR			Carbamidomethyl (C)[15]		Mascot
3714.8862	3715.0525	0.1663	45	365	400	GSITSVQAIYVPADDLTDPAPATTFAHLDATTVLSR					Mascot
3842.9812	3843.1782	0.197	51	364	400	KGSITSVQAIYVPADDLTDPAPATTFAHLDATTVLSR					Mascot

4	ATP synthase subunit beta, mitochondrial [Aegilops tauschii]	gi|475568777	59080.3	5.29	17	855	100	42.169	765	100	

Peptide Information

Calc. Mass	Obsrv. Mass	± da	± ppm	Start Seq.	End Seq.	Sequence	Ion Score	C. I. %	Modification	Rank	Result Type
866.4003	866.4259	0.0256	30	228	234	EGNDLYR					Mascot
1173.6627	1173.6957	0.033	28	175	184	VVDLLAPYQR					Mascot
1173.6627	1173.6957	0.033	28	175	184	VVDLLAPYQR	47	96.79			Mascot
1278.6359	1278.6624	0.0265	21	67	78	TIAMDGTEGLVR			Oxidation (M)[4]		Mascot
1390.6863	1390.7303	0.044	32	212	225	AHGGFSVFAGVGER					Mascot
1390.6863	1390.7303	0.044	32	212	225	AHGGFSVFAGVGER	109	100			Mascot
1399.7693	1399.8136	0.0443	32	270	282	VGLTGLTVAEHFR					Mascot
1399.7693	1399.8136	0.0443	32	270	282	VGLTGLTVAEHFR	45	94.734			Mascot
1473.8346	1473.8148	-0.0198	-13	199	211	TVLIMELINNVAK			Oxidation (M)[5]		Mascot
1490.7771	1490.7906	0.0135	9	235	247	EMIESGVIKLDEK					Mascot
1492.7755	1492.8224	0.0469	31	299	312	FTQANSEVSALLGR					Mascot
1492.7755	1492.8224	0.0469	31	299	312	FTQANSEVSALLGR	71	99.988			Mascot
1675.949	1676.0051	0.0561	33	52	66	LVLEVAQHLGENVVR					Mascot
1675.949	1676.0051	0.0561	33	52	66	LVLEVAQHLGENVVR	101	100			Mascot
1678.7676	1678.7964	0.0288	17	253	267	CALVYGQMNEPPGAR			Carbamidomethyl (C)[1], Oxidation (M)[8]		Mascot
1852.9109	1853.0294	0.1185	64	228	243	EGNDLYREMIESGVIK					Mascot
1864.944	1865.0048	0.0608	33	283	298	DAEGQDVLLFIDNIFR					Mascot
1864.944	1865.0048	0.0608	33	283	298	DAEGQDVLLFIDNIFR	117	100			Mascot

Calc. Mass	Obsrv. Mass	± da	± ppm	Start Seq.	End Seq.	Sequence	Ion Score	C. I. %	Modification	Rank	Result Type
1868.9059	1869.0281	0.1222	65	228	243	EGNDLYREMIESGVIK			Oxidation (M)[9]		Mascot
2172.1548	2172.2068	0.052	24	155	174	EAPAFVEQATEQQILVTGIK					Mascot
2186.1453	2186.2266	0.0813	37	313	333	IPSAVGYQPTLATDLGGLQER					Mascot
2186.1453	2186.2266	0.0813	37	313	333	IPSAVGYQPTLATDLGGLQER	206	100			Mascot
2212.186	2212.2603	0.0743	34	32	51	FDEGLPPILTALEVLDNSIR					Mascot
2212.186	2212.2603	0.0743	34	32	51	FDEGLPPILTALEVLDNSIR	68	99.974			Mascot
3714.8862	3715.0525	0.1663	45	340	375	GSITSVQAIYVPADDLTDPAPATTFAHLDATTVLSR					Mascot
3842.9812	3843.1782	0.197	51	339	375	KGSITSVQAIYVPADDLTDPAPATTFAHLDATTVLSR					Mascot

5 hypothetical protein M569_14212, partial [Genlisea aurea] gi|527188571 57250.9 6.21 19 808 100 43.028 697 100

Peptide Information

Calc. Mass	Obsrv. Mass	± da	± ppm	Start Seq.	End Seq.	Sequence	Ion Score	C. I. %	Modification	Rank	Result Type
866.4003	866.4259	0.0256	30	262	268	EGNDLYR					Mascot
1173.6627	1173.6957	0.033	28	209	218	VVDLLAPYQR					Mascot
1173.6627	1173.6957	0.033	28	209	218	VVDLLAPYQR	47	96.79			Mascot
1390.6863	1390.7303	0.044	32	246	259	AHGGFSVFAGVGER					Mascot
1390.6863	1390.7303	0.044	32	246	259	AHGGFSVFAGVGER	109	100			Mascot
1399.7693	1399.8136	0.0443	32	304	316	VGLTGLTVAEHFR					Mascot
1399.7693	1399.8136	0.0443	32	304	316	VGLTGLTVAEHFR	45	94.734			Mascot
1409.8112	1409.854	0.0428	30	145	158	VLNTGSPITVPVGR					Mascot
1418.756	1418.7501	-0.0059	-4	269	281	EMIESGVIKLGDK					Mascot
1473.8346	1473.8148	-0.0198	-13	233	245	TVLIMELINNVAK			Oxidation (M)[5]		Mascot
1492.7755	1492.8224	0.0469	31	333	346	FTQANSEVSALLGR					Mascot
1492.7755	1492.8224	0.0469	31	333	346	FTQANSEVSALLGR	71	99.988			Mascot
1675.949	1676.0051	0.0561	33	115	129	LVLEVAQHLGENVVR					Mascot
1675.949	1676.0051	0.0561	33	115	129	LVLEVAQHLGENVVR	101	100			Mascot
1678.8	1678.7964	-0.0036	-2	130	144	CIAMDGTEGLVRGQR			Carbamidomethyl (C)[1], Oxidation (M)[4]		Mascot
1852.9109	1853.0294	0.1185	64	262	277	EGNDLYREMIESGVIK					Mascot
1864.944	1865.0048	0.0608	33	317	332	DAEGQDVLLFIDNIFR					Mascot
1864.944	1865.0048	0.0608	33	317	332	DAEGQDVLLFIDNIFR	117	100			Mascot
1868.9059	1869.0281	0.1222	65	262	277	EGNDLYREMIESGVIK			Oxidation (M)[9]		Mascot
2061.0498	2061.1155	0.0657	32	410	428	QISELGIYPAVDPLDSTSR					Mascot
2116.1033	2116.1692	0.0659	31	60	82	AAPGAALGGKITDEFTGQ					Mascot

						GAVGK					
2172.1548	2172.2068	0.052	24	189	208	EAPAFVEQATEQQILVTGIK					Mascot
2186.1453	2186.2266	0.0813	37	347	367	IPSAVGYQPTLATDLGGLQER					Mascot
2186.1453	2186.2266	0.0813	37	347	367	IPSAVGYQPTLATDLGGLQER	206	100			Mascot
2688.376	2688.4717	0.0957	36	455	478	NLQDIIAILGMDELSEDDKLTVAR			Oxidation (M)[11]		Mascot
3714.8862	3715.0525	0.1663	45	374	409	GSITSVQAIYVPADDLTDPAPATTFAHLDATTVLSR					Mascot
3842.9812	3843.1782	0.197	51	373	409	KGSITSVQAIYVPADDLTDPAPATTFAHLDATTVLSR					Mascot

6	hypothetical protein OsI_20905 [Oryza sativa Indica Group]	gi|218197235	57804.8	7.7	22	732	100	37.731	596	100

Peptide Information

Calc. Mass	Obsrv. Mass	± da	± ppm	Start Seq.	End Seq.	Sequence	Ion Score	C. I. %	Modification	Rank	Result Type
866.4003	866.4259	0.0256	30	249	255	EGNDLYR					Mascot
1173.6627	1173.6957	0.033	28	196	205	VVDLLAPYQR					Mascot
1173.6627	1173.6957	0.033	28	196	205	VVDLLAPYQR	47	96.79			Mascot
1278.6359	1278.6624	0.0265	21	117	128	TIAMDGTEGLVR			Oxidation (M)[4]		Mascot
1390.6863	1390.7303	0.044	32	233	246	AHGGFSVFAGVGER					Mascot
1390.6863	1390.7303	0.044	32	233	246	AHGGFSVFAGVGER	109	100			Mascot
1399.7693	1399.8136	0.0443	32	291	303	VGLTGLTVAEHFR					Mascot
1399.7693	1399.8136	0.0443	32	291	303	VGLTGLTVAEHFR	45	94.734			Mascot
1409.8112	1409.854	0.0428	30	132	145	VLNTGSPITVPVGR					Mascot
1418.756	1418.7501	-0.0059	-4	256	268	EMIESGVIKLGDK					Mascot
1467.6823	1467.7841	0.1018	69	43	59	AAAYATAASGEGGGASR					Mascot
1473.8346	1473.8148	-0.0198	-13	220	232	TVLIMELINNVAK			Oxidation (M)[5]		Mascot
1492.7755	1492.8224	0.0469	31	320	333	FTQANSEVSALLGR					Mascot
1492.7755	1492.8224	0.0469	31	320	333	FTQANSEVSALLGR	71	99.988			Mascot
1520.7969	1520.829	0.0321	21	163	175	GDITTNHFLPIHR					Mascot
1678.7676	1678.7964	0.0288	17	274	288	CALVYGQMNEPPGAR			Carbamidomethyl (C)[1], Oxidation (M)[8]		Mascot
1697.8871	1697.9668	0.0797	47	28	42	GPLHRPSPSGYLFNR					Mascot
1852.9109	1853.0294	0.1185	64	249	264	EGNDLYREMIESGVIK					Mascot
1864.944	1865.0048	0.0608	33	304	319	DAEGQDVLLFIDNIFR					Mascot
1864.944	1865.0048	0.0608	33	304	319	DAEGQDVLLFIDNIFR	117	100			Mascot
1868.9059	1869.0281	0.1222	65	249	264	EGNDLYREMIESGVIK			Oxidation (M)[9]		Mascot
2061.0498	2061.1155	0.0657	32	397	415	QISELGIYPAVDPLDSTSR					Mascot
2172.1548	2172.2068	0.052	24	176	195	EAPAFVEQATEQQILVTG					Mascot

Calc. Mass	Obsrv. Mass	± da	± ppm	Start Seq.	End Seq.	Sequence	Ion Score	C. I. %	Modification	Rank	Result Type
						IK					
2186.1453	2186.2266	0.0813	37	334	354	IPSAVGYQPTLATDLGGLQER					Mascot
2186.1453	2186.2266	0.0813	37	334	354	IPSAVGYQPTLATDLGGLQER	206	100			Mascot
2649.272	2649.5015	0.2295	87	93	116	SRCSTTTSASVLEVAQHLGENMVR			Carbamidomethyl (C)[3], Oxidation (M)[22]		Mascot
2688.376	2688.4717	0.0957	36	442	465	NLQDIIAILGMDELSEDDKLTVAR			Oxidation (M)[11]		Mascot
3714.8862	3715.0525	0.1663	45	361	396	GSITSVQAIYVPADDLTDPAPATTFAHLDATTVLSR					Mascot
3842.9812	3843.1782	0.197	51	360	396	KGSITSVQAIYVPADDLTDPAPATTFAHLDATTVLSR					Mascot

7 ATP synthase beta chain [Zea mays] gi|226493589 59056.9 5.9 20 712 100 37.226 596 100

Peptide Information

Calc. Mass	Obsrv. Mass	± da	± ppm	Start Seq.	End Seq.	Sequence	Ion Score	C. I. %	Modification	Rank	Result Type
866.4003	866.4259	0.0256	30	264	270	EGNDLYR					Mascot
1173.6627	1173.6957	0.033	28	211	220	VVDLLAPYQR					Mascot
1173.6627	1173.6957	0.033	28	211	220	VVDLLAPYQR	47	96.79			Mascot
1278.6359	1278.6624	0.0265	21	132	143	TIAMDGTEGLVR			Oxidation (M)[4]		Mascot
1390.6863	1390.7303	0.044	32	248	261	AHGGFSVFAGVGER					Mascot
1390.6863	1390.7303	0.044	32	248	261	AHGGFSVFAGVGER	109	100			Mascot
1399.7693	1399.8136	0.0443	32	306	318	VGLTGLTVAEHFR					Mascot
1399.7693	1399.8136	0.0443	32	306	318	VGLTGLTVAEHFR	45	94.734			Mascot
1409.8112	1409.854	0.0428	30	147	160	VLNTGSPITVPVGR					Mascot
1418.756	1418.7501	-0.0059	-4	271	283	EMIESGVIKLGDK					Mascot
1473.8346	1473.8148	-0.0198	-13	235	247	TVLIMELINNVAK			Oxidation (M)[5]		Mascot
1492.7755	1492.8224	0.0469	31	335	348	FTQANSEVSALLGR					Mascot
1492.7755	1492.8224	0.0469	31	335	348	FTQANSEVSALLGR	71	99.988			Mascot
1520.7969	1520.829	0.0321	21	178	190	GDITTNHFLPIHR					Mascot
1678.7676	1678.7964	0.0288	17	289	303	CALVYGQMNEPPGAR			Carbamidomethyl (C)[1], Oxidation (M)[8]		Mascot
1852.9109	1853.0294	0.1185	64	264	279	EGNDLYREMIESGVIK					Mascot
1864.944	1865.0048	0.0608	33	319	334	DAEGQDVLLFIDNIFR					Mascot
1864.944	1865.0048	0.0608	33	319	334	DAEGQDVLLFIDNIFR	117	100			Mascot
1868.9059	1869.0281	0.1222	65	264	279	EGNDLYREMIESGVIK			Oxidation (M)[9]		Mascot
2061.0498	2061.1155	0.0657	32	412	430	QISELGIYPAVDPLDSTSR					Mascot
2172.1548	2172.2068	0.052	24	191	210	EAPAFVEQATEQQILVTGIK					Mascot
2186.1453	2186.2266	0.0813	37	349	369	IPSAVGYQPTLATDLGGLQER					Mascot
2186.1453	2186.2266	0.0813	37	349	369	IPSAVGYQPTLATDLGGL	206	100			Mascot

						QER						
2614.3862	2614.459	0.0728	28	19	43	AASPAAPRPRGAPHRPS PAGYLFNR						Mascot
2688.376	2688.4717	0.0957	36	457	480	NLQDIIAILGMDELSEDDK LTVAR			Oxidation (M)[11]			Mascot
3714.8862	3715.0525	0.1663	45	376	411	GSITSVQAIYVPADDLTD PAPATTFAHLDATTVLSR						Mascot
3842.9812	3843.1782	0.197	51	375	411	KGSITSVQAIYVPADDLT DPAPATTFAHLDATTVLS R						Mascot

8	PREDICTED: ATP synthase subunit beta, mitochondrial-like [Cucumis sativus]	gi|449465916	60182.4	5.9	20	711	100	37.503	596	100	

Protein Group

PREDICTED: ATP synthase subunit beta, mitochondrial-like [Cucumis sativus]	gi|449519398	60182.4	5.9000 000953 6743

Peptide Information

Calc. Mass	Obsrv. Mass	± da	± ppm	Start Seq.	End Seq.	Sequence	Ion Score	C. I. %	Modification	Rank	Result Type
866.4003	866.4259	0.0256	30	271	277	EGNDLYR					Mascot
1173.6627	1173.6957	0.033	28	218	227	VVDLLAPYQR					Mascot
1173.6627	1173.6957	0.033	28	218	227	VVDLLAPYQR	47	96.79			Mascot
1278.6359	1278.6624	0.0265	21	139	150	TIAMDGTEGLVR			Oxidation (M)[4]		Mascot
1390.6863	1390.7303	0.044	32	255	268	AHGGFSVFAGVGER					Mascot
1390.6863	1390.7303	0.044	32	255	268	AHGGFSVFAGVGER	109	100			Mascot
1399.7693	1399.8136	0.0443	32	313	325	VGLTGLTVAEHFR					Mascot
1399.7693	1399.8136	0.0443	32	313	325	VGLTGLTVAEHFR	45	94.734			Mascot
1409.8112	1409.854	0.0428	30	154	167	VLNTGSPITVPVGR					Mascot
1418.756	1418.7501	-0.0059	-4	278	290	EMIESGVIKLGDK					Mascot
1473.8346	1473.8148	-0.0198	-13	242	254	TVLIMELINNVAK			Oxidation (M)[5]		Mascot
1492.7755	1492.8224	0.0469	31	342	355	FTQANSEVSALLGR					Mascot
1492.7755	1492.8224	0.0469	31	342	355	FTQANSEVSALLGR	71	99.988			Mascot
1678.7676	1678.7964	0.0288	17	296	310	CALVYGQMNEPPGAR			Carbamidomethyl (C)[1], Oxidation (M)[8]		Mascot
1852.9109	1853.0294	0.1185	64	271	286	EGNDLYREMIESGVIK					Mascot
1853.8964	1854.0549	0.1585	85	438	453	MLSPHILGEDHYNTAR					Mascot
1864.944	1865.0048	0.0608	33	326	341	DAEGQDVLLFIDNIFR					Mascot
1864.944	1865.0048	0.0608	33	326	341	DAEGQDVLLFIDNIFR	117	100			Mascot
1868.9059	1869.0281	0.1222	65	271	286	EGNDLYREMIESGVIK			Oxidation (M)[9]		Mascot
2061.0498	2061.1155	0.0657	32	419	437	QISELGIYPAVDPLDSTSR					Mascot
2172.1548	2172.2068	0.052	24	198	217	EAPAFVEQATEQQILVTG IK					Mascot
2186.1453	2186.2266	0.0813	37	356	376	IPSAVGYQPTLATDLGGL QER					Mascot

Calc. Mass	Obsrv. Mass	± da	± ppm	Start Seq.	End Seq.	Sequence	Ion Score	C. I. %	Modification	Rank	Result Type
2186.1453	2186.2266	0.0813	37	356	376	IPSAVGYQPTLATDLGGL QER	206	100			Mascot
2591.3135	2591.3718	0.0583	22	79	103	ITDEFTGAGSIGQVCQVI GAVVDVR			Carbamidomethyl (C)[15]		Mascot
2688.376	2688.4717	0.0957	36	464	487	NLQDIIAILGMDELSEDDK LTVAR			Oxidation (M)[11]		Mascot
3714.8862	3715.0525	0.1663	45	383	418	GSITSVQAIYVPADDLTD PAPATTFAHLDATTVLSR					Mascot
3842.9812	3843.1782	0.197	51	382	418	KGSITSVQAIYVPADDLT DPAPATTFAHLDATTVLS R					Mascot

9	Os05g0553000 [Oryza sativa Japonica Group]	gi|113579812	59011.8	5.95	20	710	100	37.226	596	100	

Protein Group

RecName: Full=ATP synthase subunit beta, mitochondrial; Flags: Precursor	gi|84028177	59011.8	5.9499 998092 6514

Peptide Information

Calc. Mass	Obsrv. Mass	± da	± ppm	Start Seq.	End Seq.	Sequence	Ion Score	C. I. %	Modification	Rank	Result Type
866.4003	866.4259	0.0256	30	263	269	EGNDLYR					Mascot
1173.6627	1173.6957	0.033	28	210	219	VVDLLAPYQR					Mascot
1173.6627	1173.6957	0.033	28	210	219	VVDLLAPYQR	47	96.79			Mascot
1278.6359	1278.6624	0.0265	21	131	142	TIAMDGTEGLVR			Oxidation (M)[4]		Mascot
1390.6863	1390.7303	0.044	32	247	260	AHGGFSVFAGVGER					Mascot
1390.6863	1390.7303	0.044	32	247	260	AHGGFSVFAGVGER	109	100			Mascot
1399.7693	1399.8136	0.0443	32	305	317	VGLTGLTVAEHFR					Mascot
1399.7693	1399.8136	0.0443	32	305	317	VGLTGLTVAEHFR	45	94.734			Mascot
1409.8112	1409.854	0.0428	30	146	159	VLNTGSPITVPVGR					Mascot
1418.756	1418.7501	-0.0059	-4	270	282	EMIESGVIKLGDK					Mascot
1473.8346	1473.8148	-0.0198	-13	234	246	TVLIMELINNVAK			Oxidation (M)[5]		Mascot
1492.7755	1492.8224	0.0469	31	334	347	FTQANSEVSALLGR					Mascot
1492.7755	1492.8224	0.0469	31	334	347	FTQANSEVSALLGR	71	99.988			Mascot
1520.7969	1520.829	0.0321	21	177	189	GDITTNHFLPIHR					Mascot
1678.7676	1678.7964	0.0288	17	288	302	CALVYGQMNEPPGAR			Carbamidomethyl (C)[1], Oxidation (M)[8]		Mascot
1697.8871	1697.9668	0.0797	47	28	42	GPLHRPSPSGYLFNR					Mascot
1852.9109	1853.0294	0.1185	64	263	278	EGNDLYREMIESGVIK					Mascot
1864.944	1865.0048	0.0608	33	318	333	DAEGQDVLLFIDNIFR					Mascot
1864.944	1865.0048	0.0608	33	318	333	DAEGQDVLLFIDNIFR	117	100			Mascot
1868.9059	1869.0281	0.1222	65	263	278	EGNDLYREMIESGVIK			Oxidation (M)[9]		Mascot
2061.0498	2061.1155	0.0657	32	411	429	QISELGIYPAVDPLDSTSR					Mascot
2172.1548	2172.2068	0.052	24	190	209	EAPAFVEQATEQQILVTG IK					Mascot

2186.1453	2186.2266	0.0813	37	348	368	IPSAVGYQPTLATDLGGLQER					Mascot
2186.1453	2186.2266	0.0813	37	348	368	IPSAVGYQPTLATDLGGLQER	206	100			Mascot
2688.376	2688.4717	0.0957	36	456	479	NLQDIIAILGMDELSEDDKLTVAR			Oxidation (M)[11]		Mascot
3714.8862	3715.0525	0.1663	45	375	410	GSITSVQAIYVPADDLTDPAPATTFAHLDATTVLSR					Mascot
3842.9812	3843.1782	0.197	51	374	410	KGSITSVQAIYVPADDLTDPAPATTFAHLDATTVLSR					Mascot

10	hypothetical protein OsI_03305 [Oryza sativa Indica Group]	gi|218188864	66103.4	6.69	21	709	100	39.473	596	100	

Peptide Information

Calc. Mass	Obsrv. Mass	± da	± ppm	Start Seq.	End Seq.	Sequence	Ion Score	C. I. %	Modification	Rank	Result Type
866.4003	866.4259	0.0256	30	268	274	EGNDLYR					Mascot
978.4673	978.5253	0.058	59	491	499	SLASPSTCR			Carbamidomethyl (C)[8]		Mascot
1173.6627	1173.6957	0.033	28	215	224	VVDLLAPYQR					Mascot
1173.6627	1173.6957	0.033	28	215	224	VVDLLAPYQR	47	96.79			Mascot
1278.6359	1278.6624	0.0265	21	136	147	TIAMDGTEGLVR			Oxidation (M)[4]		Mascot
1390.6863	1390.7303	0.044	32	252	265	AHGGFSVFAGVGER					Mascot
1390.6863	1390.7303	0.044	32	252	265	AHGGFSVFAGVGER	109	100			Mascot
1399.7693	1399.8136	0.0443	32	310	322	VGLTGLTVAEHFR					Mascot
1399.7693	1399.8136	0.0443	32	310	322	VGLTGLTVAEHFR	45	94.734			Mascot
1409.8112	1409.854	0.0428	30	151	164	VLNTGSPITVPVGR					Mascot
1418.756	1418.7501	-0.0059	-4	275	287	EMIESGVIKLGDK					Mascot
1473.8346	1473.8148	-0.0198	-13	239	251	TVLIMELINNVAK			Oxidation (M)[5]		Mascot
1492.7755	1492.8224	0.0469	31	339	352	FTQANSEVSALLGR					Mascot
1492.7755	1492.8224	0.0469	31	339	352	FTQANSEVSALLGR	71	99.988			Mascot
1520.7969	1520.829	0.0321	21	182	194	GDITTNHFLPIHR					Mascot
1606.8912	1606.869	-0.0222	-14	556	570	GLSVTFTTALRDGIR					Mascot
1678.7676	1678.7964	0.0288	17	293	307	CALVYGQMNEPPGAR			Carbamidomethyl (C)[1], Oxidation (M)[8]		Mascot
1852.9109	1853.0294	0.1185	64	268	283	EGNDLYREMIESGVIK					Mascot
1864.944	1865.0048	0.0608	33	323	338	DAEGQDVLLFIDNIFR					Mascot
1864.944	1865.0048	0.0608	33	323	338	DAEGQDVLLFIDNIFR	117	100			Mascot
1868.9059	1869.0281	0.1222	65	268	283	EGNDLYREMIESGVIK			Oxidation (M)[9]		Mascot
2061.0498	2061.1155	0.0657	32	416	434	QISELGIYPAVDPLDSTSR					Mascot
2172.1548	2172.2068	0.052	24	195	214	EAPAFVEQATEQQILVTGIK					Mascot
2186.1453	2186.2266	0.0813	37	353	373	IPSAVGYQPTLATDLGGLQER					Mascot

2186.1453	2186.2266	0.0813	37	353	373	IPSAVGYQPTLATDLGGL QER	206	100		Mascot
2688.376	2688.4717	0.0957	36	461	484	NLQDIIAILGMDELSEDDK LTVAR			Oxidation (M)[11]	Mascot
3714.8862	3715.0525	0.1663	45	380	415	GSITSVQAIYVPADDLTD PAPATTFAHLDATTVLSR				Mascot
3842.9812	3843.1782	0.197	51	379	415	KGSITSVQAIYVPADDLT DPAPATTFAHLDATTVLS R				Mascot

Gel Idx/Pos	247/J23	Instr./Gel Origin	BA2151/Sample Project 20140814	Process Status	Analysis Succeeded
Plate [#] Name	[1] Sample Project 20140814	Instrument Sample Name		Spectra	11

Rank	Protein Name	Accession No.	Protein MW	Protein PI	Pep. Count	Protein Score	Protein Score C. I. %	Intensity Matched	Total Ion Score	Total Ion C. I. %	Confirmed
1	RecName: Full=UTP--glucose-1-phosphate uridylyltransferase; AltName: Full=UDP-glucose pyrophosphorylase; Short=UDPGP; Short=UGPase	gi|6136111	51783.2	5.2	18	554	100	51.614	459	100	

Peptide Information

Calc. Mass	Obsrv. Mass	± da	± ppm	Start Seq.	End Seq.	Sequence	Ion Score	C. I. %	Modification	Rank	Result Type
839.5098	839.5275	0.0177	21	350	357	AIGINVPR					Mascot
918.5519	918.5631	0.0112	12	406	413	KVANFLAR					Mascot
949.5465	949.5652	0.0187	20	31	39	AGFISLVSR					Mascot
949.5465	949.5652	0.0187	20	31	39	AGFISLVSR	62	99.891			Mascot
1014.5942	1014.6111	0.0169	17	310	318	RLVDAEALK					Mascot
1052.5371	1052.5609	0.0238	23	261	270	GGTLISYEGR					Mascot
1052.5371	1052.5609	0.0238	23	261	270	GGTLISYEGR	64	99.934			Mascot
1300.7358	1300.7156	-0.0202	-16	416	427	SIPSIVELDSLK					Mascot
1312.7583	1312.7961	0.0378	29	333	345	VLQLETAAGAAIR					Mascot
1312.7583	1312.7961	0.0378	29	333	345	VLQLETAAGAAIR	110	100			Mascot
1350.7264	1350.6886	-0.0378	-28	428	441	VSGDVSFGSGVVLK					Mascot
1358.7566	1358.7612	0.0046	3	172	183	IVTEDFLPLPSK					Mascot
1390.7842	1390.6754	-0.1088	-78	346	357	FFEKAIGINVPR					Mascot
1390.7842	1390.6754	-0.1088	-78	346	357	FFEKAIGINVPR					Mascot
1428.7805	1428.7368	-0.0437	-31	2	16	AAAAVAADSKIDGLR					Mascot
1539.7325	1539.7522	0.0197	13	40	52	YLSGEAEQIEWSK					Mascot
1641.8846	1641.9037	0.0191	12	391	405	VKPSNPSIELGPEFK					Mascot
1940.0812	1940.1102	0.029	15	327	345	EVDGVKVLQLETAAGAAI R					Mascot
1967.0121	1967.0576	0.0455	23	218	235	EYVFVANSDNLGAIVDIK					Mascot
2143.9832	2144.0635	0.0803	37	189	208	DGWYPPGHGDVFPSLN NSGK					Mascot
2198.0261	2198.1274	0.1013	46	154	171	YSNSNIEIHTFNQSQYPR					Mascot
2198.0261	2198.1274	0.1013	46	154	171	YSNSNIEIHTFNQSQYPR	119	100			Mascot
2454.3127	2454.4099	0.0972	40	365	386	ATSDLLLVQSDLYTLVDG YVIR					Mascot
2454.3127	2454.4099	0.0972	40	365	386	ATSDLLLVQSDLYTLVDG YVIR	104	100			Mascot

Rank	Protein Name	Accession No.	Protein MW	Protein PI	Pep. Count	Protein Score	Protein Score C. I. %	Intensity Matched	Total Ion Score	Total Ion C. I. %	Confirmed
2	UTP--glucose-1-phosphate uridylyltransferase [Aegilops	gi|475568864	47076.7	5.15	15	554	100	51.852	478	100	

tauschii]

Peptide Information

Calc. Mass	Obsrv. Mass	± da	± ppm	Start Seq.	End Seq.	Sequence	Ion Score	C. I. %	Modification	Rank	Result Type
839.5098	839.5275	0.0177	21	306	313	AIGINVPR					Mascot
918.5519	918.5631	0.0112	12	362	369	KVANFLAR					Mascot
1014.5942	1014.6111	0.0169	17	266	274	RLVDAEALK					Mascot
1052.5371	1052.5609	0.0238	23	217	226	GGTLISYEGR					Mascot
1052.5371	1052.5609	0.0238	23	217	226	GGTLISYEGR	64	99.934			Mascot
1300.7358	1300.7156	-0.0202	-16	372	383	SIPSIVELDSLK					Mascot
1312.7583	1312.7961	0.0378	29	289	301	VLQLETAAGAAIR					Mascot
1312.7583	1312.7961	0.0378	29	289	301	VLQLETAAGAAIR	110	100			Mascot
1350.7264	1350.6886	-0.0378	-28	384	397	VSGDVSFGSGVVLK					Mascot
1358.7566	1358.7612	0.0046	3	157	168	IVTEDFLPLPSK					Mascot
1390.7842	1390.6754	-0.1088	-78	302	313	FFEKAIGINVPR					Mascot
1390.7842	1390.6754	-0.1088	-78	302	313	FFEKAIGINVPR					Mascot
1641.8846	1641.9037	0.0191	12	347	361	VKPSNPSIELGPEFK					Mascot
1940.0812	1940.1102	0.029	15	283	301	EVDGVKVLQLETAAGAAI R					Mascot
2108.1023	2108.1689	0.0666	32	227	244	VQLLEIAQVPDEHVNEFK					Mascot
2108.1023	2108.1689	0.0666	32	227	244	VQLLEIAQVPDEHVNEFK	81	99.999			Mascot
2143.9832	2144.0635	0.0803	37	174	193	DGWYPPGHGDVFPSLN NSGK					Mascot
2198.0261	2198.1274	0.1013	46	139	156	YSNSNIEIHTFNQSQYPR					Mascot
2198.0261	2198.1274	0.1013	46	139	156	YSNSNIEIHTFNQSQYPR	119	100			Mascot
2454.3127	2454.4099	0.0972	40	321	342	ATSDLLLVQSDLYTLVDG YVIR					Mascot
2454.3127	2454.4099	0.0972	40	321	342	ATSDLLLVQSDLYTLVDG YVIR	104	100			Mascot

3	UTP--glucose-1-phosphate uridylyltransferase [Triticum urartu]	gi|473993048	51082.9	5.76	18	516	100	17.784	420	100	

Peptide Information

Calc. Mass	Obsrv. Mass	± da	± ppm	Start Seq.	End Seq.	Sequence	Ion Score	C. I. %	Modification	Rank	Result Type
839.5098	839.5275	0.0177	21	334	341	AIGINVPR					Mascot
918.5519	918.5631	0.0112	12	390	397	KVANFLAR					Mascot
949.5465	949.5652	0.0187	20	27	35	AGFISLVSR					Mascot
949.5465	949.5652	0.0187	20	27	35	AGFISLVSR	62	99.891			Mascot
1014.5942	1014.6111	0.0169	17	294	302	RLVDAEALK					Mascot

Calc. Mass	Obsrv. Mass	± da	± ppm	Start Seq.	End Seq.	Sequence	Ion Score	C. I. %	Modification	Rank	Result Type
1052.5371	1052.5609	0.0238	23	245	254	GGTLISYEGR					Mascot
1052.5371	1052.5609	0.0238	23	245	254	GGTLISYEGR	64	99.934			Mascot
1114.6216	1114.548	-0.0736	-66	8	17	IDNLRDAVAK					Mascot
1173.6222	1173.6803	0.0581	50	2	12	AAADSKIDNLR					Mascot
1300.7358	1300.7156	-0.0202	-16	400	411	SIPSIVELDSLK					Mascot
1312.7583	1312.7961	0.0378	29	317	329	VLQLETAAGAAIR					Mascot
1312.7583	1312.7961	0.0378	29	317	329	VLQLETAAGAAIR	110	100			Mascot
1350.7264	1350.6886	-0.0378	-28	412	425	VSGDVSFGSGVVLK					Mascot
1358.7566	1358.7612	0.0046	3	185	196	IVTEDFLPLPSK					Mascot
1390.7842	1390.6754	-0.1088	-78	330	341	FFEKAIGINVPR					Mascot
1390.7842	1390.6754	-0.1088	-78	330	341	FFEKAIGINVPR					Mascot
1539.7325	1539.7522	0.0197	13	36	48	YLSGEAEQIEWSK					Mascot
1641.8846	1641.9037	0.0191	12	375	389	VKPSNPSIELGPEFK					Mascot
1940.0812	1940.1102	0.029	15	311	329	EVDGVKVLQLETAAGAAIR					Mascot
2108.1023	2108.1689	0.0666	32	255	272	VQLLEIAQVPDEHVNEFK					Mascot
2108.1023	2108.1689	0.0666	32	255	272	VQLLEIAQVPDEHVNEFK	81	99.999			Mascot
2143.9832	2144.0635	0.0803	37	202	221	DGWYPPGHGDVFPSLNNSGK					Mascot
2454.3127	2454.4099	0.0972	40	349	370	ATSDLLLVQSDLYTLVDGYVIR					Mascot
2454.3127	2454.4099	0.0972	40	349	370	ATSDLLLVQSDLYTLVDGYVIR	104	100			Mascot

4 uncharacterized protein LOC100191463 [Zea mays] gi|212275097 52226.5 5.37 14 497 100 49.554 436 100

Peptide Information

Calc. Mass	Obsrv. Mass	± da	± ppm	Start Seq.	End Seq.	Sequence	Ion Score	C. I. %	Modification	Rank	Result Type
839.5098	839.5275	0.0177	21	350	357	AIGINVPR					Mascot
918.5519	918.5631	0.0112	12	406	413	KVANFLAR					Mascot
949.5465	949.5652	0.0187	20	31	39	AGFISLVSR					Mascot
949.5465	949.5652	0.0187	20	31	39	AGFISLVSR	62	99.891			Mascot
1052.5371	1052.5609	0.0238	23	261	270	GGTLISYEGR					Mascot
1052.5371	1052.5609	0.0238	23	261	270	GGTLISYEGR	64	99.934			Mascot
1300.7358	1300.7156	-0.0202	-16	416	427	SIPSIVELDSLK					Mascot
1312.7583	1312.7961	0.0378	29	333	345	VLQLETAAGAAIR					Mascot
1312.7583	1312.7961	0.0378	29	333	345	VLQLETAAGAAIR	110	100			Mascot
1358.7566	1358.7612	0.0046	3	172	183	IVTEDFLPLPSK					Mascot
1539.7325	1539.7522	0.0197	13	40	52	YLSGEAEQIEWSK					Mascot
1545.8119	1545.8044	-0.0075	-5	17	30	AEVAKLSQISENEK					Mascot

Calc. Mass	Obsrv. Mass	± da	± ppm	Start Seq.	End Seq.	Sequence	Ion Score	C. I. %	Modification	Rank	Result Type
1940.0812	1940.1102	0.029	15	327	345	EVDGVKVLQLETAAGAAI R					Mascot
1967.0121	1967.0576	0.0455	23	218	235	EYVFVANSDNLGAIVDIK					Mascot
2108.1023	2108.1689	0.0666	32	271	288	VQLLEIAQVPDEHVNEFK					Mascot
2108.1023	2108.1689	0.0666	32	271	288	VQLLEIAQVPDEHVNEFK	81	99.999			Mascot
2143.9832	2144.0635	0.0803	37	189	208	DGWYPPGHGDVFPSLN NSGK					Mascot
2198.0261	2198.1274	0.1013	46	154	171	YSNSNIEIHTFNQSQYPR					Mascot
2198.0261	2198.1274	0.1013	46	154	171	YSNSNIEIHTFNQSQYPR	119	100			Mascot

5	TPA: UDP-glucose pyrophosphorylase1 isoform 2 [Zea mays]	gi|414886602	52316.6	5.23	15	444	100	50.451	374	100		

Protein Group

TPA: UDP-glucose pyrophosphorylase1 isoform 1 [Zea mays]	gi|414886601	52316.6	5.2300 000190 7349
uncharacterized protein LOC100191846 [Zea mays]	gi|212275438	52316.6	5.2300 000190 7349

Peptide Information

Calc. Mass	Obsrv. Mass	± da	± ppm	Start Seq.	End Seq.	Sequence	Ion Score	C. I. %	Modification	Rank	Result Type
839.5098	839.5275	0.0177	21	350	357	AIGINVPR					Mascot
918.5519	918.5631	0.0112	12	406	413	KVANFLAR					Mascot
965.5414	965.559	0.0176	18	31	39	SGFISLVSR					Mascot
1052.5371	1052.5609	0.0238	23	261	270	GGTLISYEGR					Mascot
1052.5371	1052.5609	0.0238	23	261	270	GGTLISYEGR	64	99.934			Mascot
1300.7358	1300.7156	-0.0202	-16	416	427	SIPSIVELDSLK					Mascot
1312.7583	1312.7961	0.0378	29	333	345	VLQLETAAGAAIR					Mascot
1312.7583	1312.7961	0.0378	29	333	345	VLQLETAAGAAIR	110	100			Mascot
1358.7566	1358.7612	0.0046	3	172	183	IVTEDFLPLPSK					Mascot
1507.7673	1507.8075	0.0402	27	1	14	MATTAVSVDEKLDK					Mascot
1539.7325	1539.7522	0.0197	13	40	52	YLSGEAEQIEWSK					Mascot
1940.0812	1940.1102	0.029	15	327	345	EVDGVKVLQLETAAGAAI R					Mascot
1967.0121	1967.0576	0.0455	23	218	235	EYVFVANSDNLGAIVDIK					Mascot
2108.1023	2108.1689	0.0666	32	271	288	VQLLEIAQVPDEHVNEFK					Mascot
2108.1023	2108.1689	0.0666	32	271	288	VQLLEIAQVPDEHVNEFK	81	99.999			Mascot
2143.9832	2144.0635	0.0803	37	189	208	DGWYPPGHGDVFPSLN NSGK					Mascot
2181.0559	2181.1753	0.1194	55	454	473	LEIPDGDVLENKDVNGPE DL					Mascot
2181.0559	2181.1753	0.1194	55	454	473	LEIPDGDVLENKDVNGPE					Mascot

Calc. Mass	Obsrv. Mass	± da	± ppm	Start Seq.	End Seq.	Sequence	Ion Score	C. I. %	Modification	Rank	Result Type
						DL					
2198.0261	2198.1274	0.1013	46	154	171	YSNSNIEIHTFNQSQYPR					Mascot
2198.0261	2198.1274	0.1013	46	154	171	YSNSNIEIHTFNQSQYPR	119	100			Mascot

6	TPA: UDP-glucose pyrophosphorylase1 [Zea mays]	gi|414886600	58101.6	5.49	15	439	100	49.781	374	100	

Peptide Information

Calc. Mass	Obsrv. Mass	± da	± ppm	Start Seq.	End Seq.	Sequence	Ion Score	C. I. %	Modification	Rank	Result Type
839.5098	839.5275	0.0177	21	350	357	AIGINVPR					Mascot
918.5519	918.5631	0.0112	12	406	413	KVANFLAR					Mascot
965.5414	965.559	0.0176	18	31	39	SGFISLVSR					Mascot
1052.5371	1052.5609	0.0238	23	261	270	GGTLISYEGR					Mascot
1052.5371	1052.5609	0.0238	23	261	270	GGTLISYEGR	64	99.934			Mascot
1300.7358	1300.7156	-0.0202	-16	416	427	SIPSIVELDSLK					Mascot
1312.7583	1312.7961	0.0378	29	333	345	VLQLETAAGAAIR					Mascot
1312.7583	1312.7961	0.0378	29	333	345	VLQLETAAGAAIR	110	100			Mascot
1358.7566	1358.7612	0.0046	3	172	183	IVTEDFLPLPSK					Mascot
1507.7673	1507.8075	0.0402	27	1	14	MATTAVSVDEKLDK					Mascot
1539.7325	1539.7522	0.0197	13	40	52	YLSGEAEQIEWSK					Mascot
1940.0812	1940.1102	0.029	15	327	345	EVDGVKVLQLETAAGAAIR					Mascot
1967.0121	1967.0576	0.0455	23	218	235	EYVFVANSDNLGAIVDIK					Mascot
2108.1023	2108.1689	0.0666	32	271	288	VQLLEIAQVPDEHVNEFK					Mascot
2108.1023	2108.1689	0.0666	32	271	288	VQLLEIAQVPDEHVNEFK	81	99.999			Mascot
2130.0933	2130.1709	0.0776	36	454	472	LEIPDGDVLENKVMSMALR					Mascot
2143.9832	2144.0635	0.0803	37	189	208	DGWYPPGHGDVFPSLNNSGK					Mascot
2146.0884	2146.1047	0.0163	8	454	472	LEIPDGDVLENKVMSMALR			Oxidation (M)[14]		Mascot
2198.0261	2198.1274	0.1013	46	154	171	YSNSNIEIHTFNQSQYPR					Mascot
2198.0261	2198.1274	0.1013	46	154	171	YSNSNIEIHTFNQSQYPR	119	100			Mascot

7	TPA: UDP-glucose pyrophosphorylase1 [Zea mays]	gi|414886599	52414.8	5.62	14	435	100	49.17	374	100	

Peptide Information

Calc. Mass	Obsrv. Mass	± da	± ppm	Start Seq.	End Seq.	Sequence	Ion Score	C. I. %	Modification	Rank	Result Type
839.5098	839.5275	0.0177	21	350	357	AIGINVPR					Mascot
918.5519	918.5631	0.0112	12	406	413	KVANFLAR					Mascot
965.5414	965.559	0.0176	18	31	39	SGFISLVSR					Mascot
1052.5371	1052.5609	0.0238	23	261	270	GGTLISYEGR					Mascot

1052.5371	1052.5609	0.0238	23	261	270	GGTLISYEGR	64	99.934			Mascot
1300.7358	1300.7156	-0.0202	-16	416	427	SIPSIVELDSLK					Mascot
1312.7583	1312.7961	0.0378	29	333	345	VLQLETAAGAAIR					Mascot
1312.7583	1312.7961	0.0378	29	333	345	VLQLETAAGAAIR	110	100			Mascot
1358.7566	1358.7612	0.0046	3	172	183	IVTEDFLPLPSK					Mascot
1507.7673	1507.8075	0.0402	27	1	14	MATTAVSVDEKLDK					Mascot
1539.7325	1539.7522	0.0197	13	40	52	YLSGEAEQIEWSK					Mascot
1940.0812	1940.1102	0.029	15	327	345	EVDGVKVLQLETAAGAAI R					Mascot
1967.0121	1967.0576	0.0455	23	218	235	EYVFVANSDNLGAIVDIK					Mascot
2108.1023	2108.1689	0.0666	32	271	288	VQLLEIAQVPDEHVNEFK					Mascot
2108.1023	2108.1689	0.0666	32	271	288	VQLLEIAQVPDEHVNEFK	81	99.999			Mascot
2143.9832	2144.0635	0.0803	37	189	208	DGWYPPGHGDVFPSLN NSGK					Mascot
2198.0261	2198.1274	0.1013	46	154	171	YSNSNIEIHTFNQSQYPR					Mascot
2198.0261	2198.1274	0.1013	46	154	171	YSNSNIEIHTFNQSQYPR	119	100			Mascot

8	hypothetical protein OsI_32322 [Oryza sativa Indica Group]	gi|218202595	51791.3	5.43	13	428	100	49.54	374	100		

Protein Group

Os09g0553200 [Oryza sativa Japonica Group]	gi|113632112	51821.3	5.4299 998283 3862

Peptide Information

Calc. Mass	Obsrv. Mass	± da	± ppm	Start Seq.	End Seq.	Sequence	Ion Score	C. I. %	Modification	Rank	Result Type
839.5098	839.5275	0.0177	21	347	354	AIGINVPR					Mascot
918.5519	918.5631	0.0112	12	403	410	KVANFLAR					Mascot
965.5414	965.559	0.0176	18	28	36	SGFISLVSR					Mascot
1052.5371	1052.5609	0.0238	23	258	267	GGTLISYEGR					Mascot
1052.5371	1052.5609	0.0238	23	258	267	GGTLISYEGR	64	99.934			Mascot
1312.7583	1312.7961	0.0378	29	330	342	VLQLETAAGAAIR					Mascot
1312.7583	1312.7961	0.0378	29	330	342	VLQLETAAGAAIR	110	100			Mascot
1358.7566	1358.7612	0.0046	3	169	180	IVTEDFLPLPSK					Mascot
1390.7842	1390.6754	-0.1088	-78	343	354	FFEKAIGINVPR					Mascot
1390.7842	1390.6754	-0.1088	-78	343	354	FFEKAIGINVPR					Mascot
1539.7325	1539.7522	0.0197	13	37	49	YLSGEAEQIEWSK					Mascot
1940.0812	1940.1102	0.029	15	324	342	EVDGVKVLQLETAAGAAI R					Mascot
1967.0121	1967.0576	0.0455	23	215	232	EYVFVANSDNLGAIVDIK					Mascot

2108.1023	2108.1689	0.0666	32	268	285	VQLLEIAQVPDEHVNEFK					Mascot
2108.1023	2108.1689	0.0666	32	268	285	VQLLEIAQVPDEHVNEFK	81	99.999			Mascot
2143.9832	2144.0635	0.0803	37	186	205	DGWYPPGHGDVFPSLN NSGK					Mascot
2198.0261	2198.1274	0.1013	46	151	168	YSNSNIEIHTFNQSQYPR					Mascot
2198.0261	2198.1274	0.1013	46	151	168	YSNSNIEIHTFNQSQYPR	119	100			Mascot

9	UDP-glucose pyrophosphorylase 2 [Theobroma cacao]	gi|508710349	51505.1	5.61	8	333	100	41.777	310	100	

Peptide Information

Calc. Mass	Obsrv. Mass	± da	± ppm	Start Seq.	End Seq.	Sequence	Ion Score	C. I. %	Modification	Rank	Result Type
1014.5942	1014.6111	0.0169	17	302	310	RLVEADALK					Mascot
1312.7583	1312.7961	0.0378	29	325	337	VLQLETAAGAAIR					Mascot
1312.7583	1312.7961	0.0378	29	325	337	VLQLETAAGAAIR	110	100			Mascot
1371.7169	1371.7751	0.0582	42	338	349	FFDHAIGVNVPR					Mascot
1614.85	1614.8572	0.0072	4	338	351	FFDHAIGVNVPRSR					Mascot
1940.0812	1940.1102	0.029	15	319	337	EVDGVKVLQLETAAGAAI R					Mascot
2055.1082	2055.0527	-0.0555	-27	4	22	LTHLKSAVASLSQISENE K					Mascot
2108.1023	2108.1689	0.0666	32	263	280	VQLLEIAQVPDEHVNEFK					Mascot
2108.1023	2108.1689	0.0666	32	263	280	VQLLEIAQVPDEHVNEFK	81	99.999			Mascot
2198.0261	2198.1274	0.1013	46	146	163	YSNSNIEIHTFNQSQYPR					Mascot
2198.0261	2198.1274	0.1013	46	146	163	YSNSNIEIHTFNQSQYPR	119	100			Mascot

10	UDP-glucose pyrophosphorylase [Gossypium hirsutum]	gi|308445439	51365.9	5.53	7	331	100	41.708	310	100	

Peptide Information

Calc. Mass	Obsrv. Mass	± da	± ppm	Start Seq.	End Seq.	Sequence	Ion Score	C. I. %	Modification	Rank	Result Type
1312.7583	1312.7961	0.0378	29	325	337	VLQLETAAGAAIR					Mascot
1312.7583	1312.7961	0.0378	29	325	337	VLQLETAAGAAIR	110	100			Mascot
1447.7275	1447.8389	0.1114	77	9	22	SAVAALSEISENEK					Mascot
1967.0121	1967.0576	0.0455	23	210	227	EYVFVANSDNLGAIVDLK					Mascot
2108.1023	2108.1689	0.0666	32	263	280	VQLLEIAQVPDEHVNEFK					Mascot
2108.1023	2108.1689	0.0666	32	263	280	VQLLEIAQVPDEHVNEFK	81	99.999			Mascot
2144.1963	2144.0635	-0.1328	-62	102	120	NGLTFLDLIVIQIENLNSK					Mascot
2198.0261	2198.1274	0.1013	46	146	163	YSNSNIEIHTFNQSQYPR					Mascot
2198.0261	2198.1274	0.1013	46	146	163	YSNSNIEIHTFNQSQYPR	119	100			Mascot
2448.2729	2448.4175	0.1446	59	9	31	SAVAALSEISENEKNGFIN LVSR					Mascot

Rank	Protein Name	Accession No.	Protein MW	Protein PI	Pep. Count	Protein Score	Protein Score C. I. %	Intensity Matched	Total Ion Score	Total Ion C. I. %	Confirmed
1	ATP synthase subunit beta, mitochondrial [Triticum urartu]	gi|473990219	58081.1	5.13	21	1,300	100	72.497	1171	100	

Peptide Information

Calc. Mass	Obsrv. Mass	± da	± ppm	Start Seq.	End Seq.	Sequence	Ion Score	C. I. %	Modification	Rank	Result Type
866.4003	866.4203	0.02	23	253	259	EGNDLYR					Mascot
1135.6007	1135.6233	0.0226	20	171	179	THDFLPIHR					Mascot
1173.6627	1173.6893	0.0266	23	200	209	VVDLLAPYQR					Mascot
1173.6627	1173.6893	0.0266	23	200	209	VVDLLAPYQR	72	99.991			Mascot
1278.6359	1278.6506	0.0147	11	92	103	TIAMDGTEGLVR			Oxidation (M)[4]		Mascot
1347.7631	1347.7711	0.008	6	155	166	IINVIGEPIDHK					Mascot
1390.6863	1390.7252	0.0389	28	237	250	AHGGFSVFAGVGER					Mascot
1390.6863	1390.7252	0.0389	28	237	250	AHGGFSVFAGVGER	130	100			Mascot
1399.7693	1399.8077	0.0384	27	295	307	VGLTGLTVAEHFR					Mascot
1399.7693	1399.8077	0.0384	27	295	307	VGLTGLTVAEHFR	37	65.748			Mascot
1473.8346	1473.8335	-0.0011	-1	224	236	TVLIMELINNVAK			Oxidation (M)[5]		Mascot
1492.7755	1492.8168	0.0413	28	324	337	FTQANSEVSALLGR					Mascot
1492.7755	1492.8168	0.0413	28	324	337	FTQANSEVSALLGR	123	100			Mascot
1675.949	1676.0007	0.0517	31	77	91	LVLEVAQHLGENVVR					Mascot
1675.949	1676.0007	0.0517	31	77	91	LVLEVAQHLGENVVR	100	100			Mascot
1678.7676	1678.7976	0.03	18	278	292	CALVYGQMNEPPGAR			Carbamidomethyl (C)[1], Oxidation (M)[8]		Mascot
1825.9014	1825.9404	0.039	21	420	435	MLSPHVLGVDHYNTAR			Oxidation (M)[1]		Mascot
1864.944	1865.0068	0.0628	34	308	323	DAEGQDVLLFIDNIFR					Mascot
1864.944	1865.0068	0.0628	34	308	323	DAEGQDVLLFIDNIFR	142	100			Mascot
2061.0498	2061.1125	0.0627	30	401	419	QISELGIYPAVDPLDSTSR					Mascot
2061.0498	2061.1125	0.0627	30	401	419	QISELGIYPAVDPLDSTSR	93	100			Mascot
2172.1548	2172.2222	0.0674	31	180	199	EAPAFVEQATEQQILVTGIK					Mascot
2186.1453	2186.2305	0.0852	39	338	358	IPSAVGYQPTLATDLGGLQER					Mascot
2186.1453	2186.2305	0.0852	39	338	358	IPSAVGYQPTLATDLGGLQER	207	100			Mascot
2212.186	2212.2642	0.0782	35	57	76	FDEGLPPILTALEVLDNSIR					Mascot
2212.186	2212.2642	0.0782	35	57	76	FDEGLPPILTALEVLDNSI	74	99.993			Mascot

Calc. Mass	Obsrv. Mass	± da	± ppm	Start Seq.	End Seq.	Sequence	Ion Score	C. I. %	Modification	Rank	Result Type
						R					
2591.3135	2591.4268	0.1133	44	32	56	ITDEFTGAGSIGQVCQVIGAVVDVR			Carbamidomethyl (C)[15]		Mascot
2591.3135	2591.4268	0.1133	44	32	56	ITDEFTGAGSIGQVCQVIGAVVDVR	194	100	Carbamidomethyl (C)[15]		Mascot
2722.3274	2722.426	0.0986	36	446	469	NLQDIIAILGMDELSEDDKMTVAR			Oxidation (M)[11,20]		Mascot
3714.8862	3715.0779	0.1917	52	365	400	GSITSVQAIYVPADDLTDPAPATTFAHLDATTVLSR					Mascot
3842.9812	3843.1807	0.1995	52	364	400	KGSITSVQAIYVPADDLTDPAPATTFAHLDATTVLSR					Mascot

2	ATP synthase subunit beta, mitochondrial [Triticum urartu]	gi|473798701	57827	5.25	21	1,110	100	63.625	976	100	

Peptide Information

Calc. Mass	Obsrv. Mass	± da	± ppm	Start Seq.	End Seq.	Sequence	Ion Score	C. I. %	Modification	Rank	Result Type
866.4003	866.4203	0.02	23	219	225	EGNDLYR					Mascot
1173.6627	1173.6893	0.0266	23	166	175	VVDLLAPYQR					Mascot
1173.6627	1173.6893	0.0266	23	166	175	VVDLLAPYQR	72	99.991			Mascot
1278.6359	1278.6506	0.0147	11	87	98	TIAMDGTEGLVR			Oxidation (M)[4]		Mascot
1347.7631	1347.7711	0.008	6	121	132	IINVIGEPIDHK					Mascot
1390.6863	1390.7252	0.0389	28	203	216	AHGGFSVFAGVGER					Mascot
1390.6863	1390.7252	0.0389	28	203	216	AHGGFSVFAGVGER	130	100			Mascot
1399.7693	1399.8077	0.0384	27	261	273	VGLTGLTVAEHFR					Mascot
1399.7693	1399.8077	0.0384	27	261	273	VGLTGLTVAEHFR	37	65.748			Mascot
1409.8112	1409.847	0.0358	25	102	115	VLNTGSPITVPVGR					Mascot
1473.8346	1473.8335	-0.0011	-1	190	202	TVLIMELINNVAK			Oxidation (M)[5]		Mascot
1492.7755	1492.8168	0.0413	28	290	303	FTQANSEVSALLGR					Mascot
1492.7755	1492.8168	0.0413	28	290	303	FTQANSEVSALLGR	123	100			Mascot
1675.949	1676.0007	0.0517	31	72	86	LVLEVAQHLGENVVR					Mascot
1675.949	1676.0007	0.0517	31	72	86	LVLEVAQHLGENVVR	100	100			Mascot
1678.7676	1678.7976	0.03	18	244	258	CALVYGQMNEPPGAR			Carbamidomethyl (C)[1], Oxidation (M)[8]		Mascot
1864.944	1865.0068	0.0628	34	274	289	DAEGQDVLLFIDNIFR					Mascot
1864.944	1865.0068	0.0628	34	274	289	DAEGQDVLLFIDNIFR	142	100			Mascot
2061.0498	2061.1125	0.0627	30	367	385	QISELGIYPAVDPLDSTSR					Mascot
2061.0498	2061.1125	0.0627	30	367	385	QISELGIYPAVDPLDSTSR	93	100			Mascot
2172.1548	2172.2222	0.0674	31	146	165	EAPAFVEQATEQQILVTGIK					Mascot
2186.1453	2186.2305	0.0852	39	304	324	IPSAVGYQPTLATDLGGLQER					Mascot
2186.1453	2186.2305	0.0852	39	304	324	IPSAVGYQPTLATDLGGLQER	207	100			Mascot

Calc. Mass	Obsrv. Mass	± da	± ppm	Start Seq.	End Seq.	Sequence	Ion Score	C. I. %	Modification	Rank	Result Type
2208.1343	2208.1965	0.0622	28	386	405	MLSPHVLGEAHYNTARG VQK					Mascot
2212.186	2212.2642	0.0782	35	52	71	FDEGLPPILTALEVLDNSI R					Mascot
2212.186	2212.2642	0.0782	35	52	71	FDEGLPPILTALEVLDNSI R	74	99.993			Mascot
2234.1663	2234.2341	0.0678	30	5	26	LSLSEAAPVTPPPTSDKH TGTK					Mascot
2548.2712	2548.3926	0.1214	48	27	51	ITDDFTGAGAVGEVCQVI GAVVDVR			Carbamidomethyl (C)[15]		Mascot
3714.8862	3715.0779	0.1917	52	331	366	GSITSVQAIYVPADDLTD PAPATTFAHLDATTVLSR					Mascot
3842.9812	3843.1807	0.1995	52	330	366	KGSITSVQAIYVPADDLT DPAPATTFAHLDATTVLS R					Mascot

3 RecName: Full=ATP synthase subunit beta, mitochondrial; Flags: Precursor gi|114421 59933.3 5.95 19 1,100 100 58.549 997 100

Peptide Information

Calc. Mass	Obsrv. Mass	± da	± ppm	Start Seq.	End Seq.	Sequence	Ion Score	C. I. %	Modification	Rank	Result Type
866.4003	866.4203	0.02	23	271	277	EGNDLYR					Mascot
1173.6627	1173.6893	0.0266	23	218	227	VVDLLAPYQR					Mascot
1173.6627	1173.6893	0.0266	23	218	227	VVDLLAPYQR	72	99.991			Mascot
1278.6359	1278.6506	0.0147	11	139	150	TIAMDGTEGLVR			Oxidation (M)[4]		Mascot
1355.8118	1355.782	-0.0298	-22	6	17	LLASLLRQSAQR					Mascot
1390.6863	1390.7252	0.0389	28	255	268	AHGGFSVFAGVGER					Mascot
1390.6863	1390.7252	0.0389	28	255	268	AHGGFSVFAGVGER	130	100			Mascot
1399.7693	1399.8077	0.0384	27	313	325	VGLTGLTVAEHFR					Mascot
1399.7693	1399.8077	0.0384	27	313	325	VGLTGLTVAEHFR	37	65.748			Mascot
1409.8112	1409.847	0.0358	25	154	167	VLNTGSPITVPVGR					Mascot
1473.8346	1473.8335	-0.0011	-1	242	254	TVLIMELINNVAK			Oxidation (M)[5]		Mascot
1492.7755	1492.8168	0.0413	28	342	355	FTQANSEVSALLGR					Mascot
1492.7755	1492.8168	0.0413	28	342	355	FTQANSEVSALLGR	123	100			Mascot
1678.7676	1678.7976	0.03	18	296	310	CALVYGQMNEPPGAR			Carbamidomethyl (C)[1], Oxidation (M)[8]		Mascot
1864.944	1865.0068	0.0628	34	326	341	DAEGQDVLLFIDNIFR					Mascot
1864.944	1865.0068	0.0628	34	326	341	DAEGQDVLLFIDNIFR	142	100			Mascot
1869.8912	1870.0319	0.1407	75	438	453	MLSPHILGEDHYNTAR			Oxidation (M)[1]		Mascot
2061.0498	2061.1125	0.0627	30	419	437	QISELGIYPAVDPLDSTSR					Mascot
2061.0498	2061.1125	0.0627	30	419	437	QISELGIYPAVDPLDSTSR	93	100			Mascot
2172.1548	2172.2222	0.0674	31	198	217	EAPAFVEQATEQQILVTG IK					Mascot
2186.1453	2186.2305	0.0852	39	356	376	IPSAVGYQPTLATDLGGL QER					Mascot
2186.1453	2186.2305	0.0852	39	356	376	IPSAVGYQPTLATDLGGL	207	100			Mascot

						QER					
2591.3499	2591.4268	0.0769	30	79	103	ITDEFTGAGSIGKVCQVIG AVVDVR			Carbamidomethyl (C)[15]		Mascot
2591.3499	2591.4268	0.0769	30	79	103	ITDEFTGAGSIGKVCQVIG AVVDVR	194	100	Carbamidomethyl (C)[15]		Mascot
2722.3274	2722.426	0.0986	36	464	487	NLQDIIAILGMDELSEDDK MTVAR			Oxidation (M)[11,20]		Mascot
3714.8862	3715.0779	0.1917	52	383	418	GSITSVQAIYVPADDLTD PAPATTFAHLDATTVLSR					Mascot
3842.9812	3843.1807	0.1995	52	382	418	KGSITSVQAIYVPADDLT DPAPATTFAHLDATTVLS R					Mascot

4	ATP synthase subunit beta, mitochondrial [Aegilops tauschii]	gi|475548007	59152.9	5.85	21	1,100	100	63.604	976	100		

Peptide Information

Calc. Mass	Obsrv. Mass	± da	± ppm	Start Seq.	End Seq.	Sequence	Ion Score	C. I. %	Modification	Rank	Result Type
866.4003	866.4203	0.02	23	264	270	EGNDLYR					Mascot
1173.6627	1173.6893	0.0266	23	211	220	VVDLLAPYQR					Mascot
1173.6627	1173.6893	0.0266	23	211	220	VVDLLAPYQR	72	99.991			Mascot
1278.6359	1278.6506	0.0147	11	132	143	TIAMDGTEGLVR			Oxidation (M)[4]		Mascot
1347.7631	1347.7711	0.008	6	166	177	IINVIGEPIDHK					Mascot
1390.6863	1390.7252	0.0389	28	248	261	AHGGFSVFAGVGER					Mascot
1390.6863	1390.7252	0.0389	28	248	261	AHGGFSVFAGVGER	130	100			Mascot
1399.7693	1399.8077	0.0384	27	306	318	VGLTGLTVAEHFR					Mascot
1399.7693	1399.8077	0.0384	27	306	318	VGLTGLTVAEHFR	37	65.748			Mascot
1409.8112	1409.847	0.0358	25	147	160	VLNTGSPITVPVGR					Mascot
1473.8346	1473.8335	-0.0011	-1	235	247	TVLIMELINNVAK			Oxidation (M)[5]		Mascot
1492.7755	1492.8168	0.0413	28	335	348	FTQANSEVSALLGR					Mascot
1492.7755	1492.8168	0.0413	28	335	348	FTQANSEVSALLGR	123	100			Mascot
1645.9286	1645.8708	-0.0578	-35	28	42	APLHRPSPAGFLLNR					Mascot
1675.949	1676.0007	0.0517	31	117	131	LVLEVAQHLGENVVR					Mascot
1675.949	1676.0007	0.0517	31	117	131	LVLEVAQHLGENVVR	100	100			Mascot
1678.7676	1678.7976	0.03	18	289	303	CALVYGQMNEPPGAR			Carbamidomethyl (C)[1], Oxidation (M)[8]		Mascot
1864.944	1865.0068	0.0628	34	319	334	DAEGQDVLLFIDNIFR					Mascot
1864.944	1865.0068	0.0628	34	319	334	DAEGQDVLLFIDNIFR	142	100			Mascot
2061.0498	2061.1125	0.0627	30	412	430	QISELGIYPAVDPLDSTSR					Mascot
2061.0498	2061.1125	0.0627	30	412	430	QISELGIYPAVDPLDSTSR	93	100			Mascot
2172.1548	2172.2222	0.0674	31	191	210	EAPAFVEQATEQQILVTG IK					Mascot
2186.1453	2186.2305	0.0852	39	349	369	IPSAVGYQPTLATDLGGL QER					Mascot
2186.1453	2186.2305	0.0852	39	349	369	IPSAVGYQPTLATDLGGL	207	100			Mascot

Calc. Mass	Obsrv. Mass	± da	± ppm	Start Seq.	End Seq.	Sequence	Ion Score	C. I. %	Modification	Rank	Result Type
						QER					
2208.1343	2208.1965	0.0622	28	431	450	MLSPHVLGEAHYNTARGVQK					Mascot
2212.186	2212.2642	0.0782	35	97	116	FDEGLPPILTALEVLDNSIR					Mascot
2212.186	2212.2642	0.0782	35	97	116	FDEGLPPILTALEVLDNSIR	74	99.993			Mascot
2548.2712	2548.3926	0.1214	48	72	96	ITDDFTGAGAVGEVCQVIGAVVDVR			Carbamidomethyl (C)[15]		Mascot
3714.8862	3715.0779	0.1917	52	376	411	GSITSVQAIYVPADDLTDPAPATTFAHLDATTVLSR					Mascot
3842.9812	3843.1807	0.1995	52	375	411	KGSITSVQAIYVPADDLTDPAPATTFAHLDATTVLSR					Mascot

5	PREDICTED: ATP synthase subunit beta, mitochondrial-like [Cucumis sativus]	gi|449434570	59885.2	5.9	18	1,100	100	58.145	997	100		

Protein Group

PREDICTED: ATP synthase subunit beta, mitochondrial-like [Cucumis sativus]	gi|449506360	59885.2	5.9000000953 6743

Peptide Information

Calc. Mass	Obsrv. Mass	± da	± ppm	Start Seq.	End Seq.	Sequence	Ion Score	C. I. %	Modification	Rank	Result Type
866.4003	866.4203	0.02	23	269	275	EGNDLYR					Mascot
1173.6627	1173.6893	0.0266	23	216	225	VVDLLAPYQR					Mascot
1173.6627	1173.6893	0.0266	23	216	225	VVDLLAPYQR	72	99.991			Mascot
1278.6359	1278.6506	0.0147	11	137	148	TIAMDGTEGLVR			Oxidation (M)[4]		Mascot
1390.6863	1390.7252	0.0389	28	253	266	AHGGFSVFAGVGER					Mascot
1390.6863	1390.7252	0.0389	28	253	266	AHGGFSVFAGVGER	130	100			Mascot
1399.7693	1399.8077	0.0384	27	311	323	VGLTGLTVAEHFR					Mascot
1399.7693	1399.8077	0.0384	27	311	323	VGLTGLTVAEHFR	37	65.748			Mascot
1409.8112	1409.847	0.0358	25	152	165	VLNTGSPITVPVGR					Mascot
1418.756	1418.7535	-0.0025	-2	276	288	EMIESGVIKLGDK					Mascot
1473.8346	1473.8335	-0.0011	-1	240	252	TVLIMELINNVAK			Oxidation (M)[5]		Mascot
1492.7755	1492.8168	0.0413	28	340	353	FTQANSEVSALLGR					Mascot
1492.7755	1492.8168	0.0413	28	340	353	FTQANSEVSALLGR	123	100			Mascot
1678.7676	1678.7976	0.03	18	294	308	CALVYGQMNEPPGAR			Carbamidomethyl (C)[1], Oxidation (M)[8]		Mascot
1864.944	1865.0068	0.0628	34	324	339	DAEGQDVLLFIDNIFR					Mascot
1864.944	1865.0068	0.0628	34	324	339	DAEGQDVLLFIDNIFR	142	100			Mascot
1869.8912	1870.0319	0.1407	75	436	451	MLSPHILGEDHYNTAR			Oxidation (M)[1]		Mascot
2061.0498	2061.1125	0.0627	30	417	435	QISELGIYPAVDPLDSTSR					Mascot
2061.0498	2061.1125	0.0627	30	417	435	QISELGIYPAVDPLDSTSR	93	100			Mascot
2186.1453	2186.2305	0.0852	39	354	374	IPSAVGYQPTLATDLGGL					Mascot

						QER					
2186.1453	2186.2305	0.0852	39	354	374	IPSAVGYQPTLATDLGGL QER	207	100			Mascot
2591.3135	2591.4268	0.1133	44	77	101	ITDEFTGAGSIGQVCQVI GAVVDVR			Carbamidomethyl (C)[15]		Mascot
2591.3135	2591.4268	0.1133	44	77	101	ITDEFTGAGSIGQVCQVI GAVVDVR	194	100	Carbamidomethyl (C)[15]		Mascot
2722.3274	2722.426	0.0986	36	462	485	NLQDIIAILGMDELSEDDK MTVAR			Oxidation (M)[11,20]		Mascot
3714.8862	3715.0779	0.1917	52	381	416	GSITSVQAIYVPADDLTD PAPATTFAHLDATTVLSR					Mascot
3842.9812	3843.1807	0.1995	52	380	416	KGSITSVQAIYVPADDLT DPAPATTFAHLDATTVLS R					Mascot

6	PREDICTED: ATP synthase subunit beta, mitochondrial-like [Cucumis sativus]	gi|449465916	60182.4	5.9	18	1,090	100	58.364	997	100

Protein Group

PREDICTED: ATP synthase subunit beta, mitochondrial-like [Cucumis sativus]	gi|449519398	60182.4	5.9000 000953 6743

Peptide Information

Calc. Mass	Obsrv. Mass	± da	± ppm	Start Seq.	End Seq.	Sequence	Ion Score	C. I. %	Modification	Rank	Result Type
866.4003	866.4203	0.02	23	271	277	EGNDLYR					Mascot
1173.6627	1173.6893	0.0266	23	218	227	VVDLLAPYQR					Mascot
1173.6627	1173.6893	0.0266	23	218	227	VVDLLAPYQR	72	99.991			Mascot
1278.6359	1278.6506	0.0147	11	139	150	TIAMDGTEGLVR			Oxidation (M)[4]		Mascot
1390.6863	1390.7252	0.0389	28	255	268	AHGGFSVFAGVGER					Mascot
1390.6863	1390.7252	0.0389	28	255	268	AHGGFSVFAGVGER	130	100			Mascot
1399.7693	1399.8077	0.0384	27	313	325	VGLTGLTVAEHFR					Mascot
1399.7693	1399.8077	0.0384	27	313	325	VGLTGLTVAEHFR	37	65.748			Mascot
1409.8112	1409.847	0.0358	25	154	167	VLNTGSPITVPVGR					Mascot
1418.756	1418.7535	-0.0025	-2	278	290	EMIESGVIKLGDK					Mascot
1473.8346	1473.8335	-0.0011	-1	242	254	TVLIMELINNVAK			Oxidation (M)[5]		Mascot
1492.7755	1492.8168	0.0413	28	342	355	FTQANSEVSALLGR					Mascot
1492.7755	1492.8168	0.0413	28	342	355	FTQANSEVSALLGR	123	100			Mascot
1678.7676	1678.7976	0.03	18	296	310	CALVYGQMNEPPGAR			Carbamidomethyl (C)[1], Oxidation (M)[8]		Mascot
1864.944	1865.0068	0.0628	34	326	341	DAEGQDVLLFIDNIFR					Mascot
1864.944	1865.0068	0.0628	34	326	341	DAEGQDVLLFIDNIFR	142	100			Mascot
1869.8912	1870.0319	0.1407	75	438	453	MLSPHILGEDHYNTAR			Oxidation (M)[1]		Mascot
2061.0498	2061.1125	0.0627	30	419	437	QISELGIYPAVDPLDSTSR					Mascot
2061.0498	2061.1125	0.0627	30	419	437	QISELGIYPAVDPLDSTSR	93	100			Mascot
2172.1548	2172.2222	0.0674	31	198	217	EAPAFVEQATEQQILVTG					Mascot

						IK					
2186.1453	2186.2305	0.0852	39	356	376	IPSAVGYQPTLATDLGGL QER					Mascot
2186.1453	2186.2305	0.0852	39	356	376	IPSAVGYQPTLATDLGGL QER	207	100			Mascot
2591.3135	2591.4268	0.1133	44	79	103	ITDEFTGAGSIGQVCQVI GAVVDVR			Carbamidomethyl (C)[15]		Mascot
2591.3135	2591.4268	0.1133	44	79	103	ITDEFTGAGSIGQVCQVI GAVVDVR	194	100	Carbamidomethyl (C)[15]		Mascot
3714.8862	3715.0779	0.1917	52	383	418	GSITSVQAIYVPADDLTD PAPATTFAHLDATTVLSR					Mascot
3842.9812	3843.1807	0.1995	52	382	418	KGSITSVQAIYVPADDLT DPAPATTFAHLDATTVLS R					Mascot

7	hypothetical protein M569_14212, partial [Genlisea aurea]	gi|527188571	57250.9	6.21	17	993	100	55.293	903	100		

Peptide Information

Calc. Mass	Obsrv. Mass	± da	± ppm	Start Seq.	End Seq.	Sequence	Ion Score	C. I. %	Modification	Rank	Result Type
866.4003	866.4203	0.02	23	262	268	EGNDLYR					Mascot
1173.6627	1173.6893	0.0266	23	209	218	VVDLLAPYQR					Mascot
1173.6627	1173.6893	0.0266	23	209	218	VVDLLAPYQR	72	99.991			Mascot
1390.6863	1390.7252	0.0389	28	246	259	AHGGFSVFAGVGER					Mascot
1390.6863	1390.7252	0.0389	28	246	259	AHGGFSVFAGVGER	130	100			Mascot
1399.7693	1399.8077	0.0384	27	304	316	VGLTGLTVAEHFR					Mascot
1399.7693	1399.8077	0.0384	27	304	316	VGLTGLTVAEHFR	37	65.748			Mascot
1409.8112	1409.847	0.0358	25	145	158	VLNTGSPITVPVGR					Mascot
1418.756	1418.7535	-0.0025	-2	269	281	EMIESGVIKLGDK					Mascot
1473.8346	1473.8335	-0.0011	-1	233	245	TVLIMELINNVAK			Oxidation (M)[5]		Mascot
1492.7755	1492.8168	0.0413	28	333	346	FTQANSEVSALLGR					Mascot
1492.7755	1492.8168	0.0413	28	333	346	FTQANSEVSALLGR	123	100			Mascot
1562.8439	1562.9657	0.1218	78	176	188	GDIKTEHFLPIHR					Mascot
1675.949	1676.0007	0.0517	31	115	129	LVLEVAQHLGENVVR					Mascot
1675.949	1676.0007	0.0517	31	115	129	LVLEVAQHLGENVVR	100	100			Mascot
1678.8	1678.7976	-0.0024	-1	130	144	CIAMDGTEGLVRGQR			Carbamidomethyl (C)[1], Oxidation (M)[4]		Mascot
1864.944	1865.0068	0.0628	34	317	332	DAEGQDVLLFIDNIFR					Mascot
1864.944	1865.0068	0.0628	34	317	332	DAEGQDVLLFIDNIFR	142	100			Mascot
2061.0498	2061.1125	0.0627	30	410	428	QISELGIYPAVDPLDSTSR					Mascot
2061.0498	2061.1125	0.0627	30	410	428	QISELGIYPAVDPLDSTSR	93	100			Mascot
2172.1548	2172.2222	0.0674	31	189	208	EAPAFVEQATEQQILVTG IK					Mascot
2186.1453	2186.2305	0.0852	39	347	367	IPSAVGYQPTLATDLGGL QER					Mascot

2186.1453	2186.2305	0.0852	39	347	367	IPSAVGYQPTLATDLGGL QER	207	100			Mascot
3714.8862	3715.0779	0.1917	52	374	409	GSITSVQAIYVPADDLTD PAPATTFAHLDATTVLSR					Mascot
3842.9812	3843.1807	0.1995	52	373	409	KGSITSVQAIYVPADDLT DPAPATTFAHLDATTVLS R					Mascot

8	ATP synthase subunit beta, mitochondrial [Aegilops tauschii]	gi|475568777	59080.3	5.29	19	990	100	60.637	884	100	

Peptide Information

Calc. Mass	Obsrv. Mass	± da	± ppm	Start Seq.	End Seq.	Sequence	Ion Score	C. I. %	Modification	Rank	Result Type
866.4003	866.4203	0.02	23	228	234	EGNDLYR					Mascot
1135.6007	1135.6233	0.0226	20	146	154	THDFLPIHR					Mascot
1173.6627	1173.6893	0.0266	23	175	184	VVDLLAPYQR					Mascot
1173.6627	1173.6893	0.0266	23	175	184	VVDLLAPYQR	72	99.991			Mascot
1278.6359	1278.6506	0.0147	11	67	78	TIAMDGTEGLVR			Oxidation (M)[4]		Mascot
1347.7631	1347.7711	0.008	6	130	141	IINVIGEPIDHK					Mascot
1390.6863	1390.7252	0.0389	28	212	225	AHGGFSVFAGVGER					Mascot
1390.6863	1390.7252	0.0389	28	212	225	AHGGFSVFAGVGER	130	100			Mascot
1399.7693	1399.8077	0.0384	27	270	282	VGLTGLTVAEHFR					Mascot
1399.7693	1399.8077	0.0384	27	270	282	VGLTGLTVAEHFR	37	65.748			Mascot
1473.8346	1473.8335	-0.0011	-1	199	211	TVLIMELINNVAK			Oxidation (M)[5]		Mascot
1492.7755	1492.8168	0.0413	28	299	312	FTQANSEVSALLGR					Mascot
1492.7755	1492.8168	0.0413	28	299	312	FTQANSEVSALLGR	123	100			Mascot
1675.949	1676.0007	0.0517	31	52	66	LVLEVAQHLGENVVR					Mascot
1675.949	1676.0007	0.0517	31	52	66	LVLEVAQHLGENVVR	100	100			Mascot
1678.7676	1678.7976	0.03	18	253	267	CALVYGQMNEPPGAR			Carbamidomethyl (C)[1], Oxidation (M)[8]		Mascot
1825.9014	1825.9404	0.039	21	423	438	MLSPHVLGVDHYNTAR			Oxidation (M)[1]		Mascot
1864.944	1865.0068	0.0628	34	283	298	DAEGQDVLLFIDNIFR					Mascot
1864.944	1865.0068	0.0628	34	283	298	DAEGQDVLLFIDNIFR	142	100			Mascot
2172.1548	2172.2222	0.0674	31	155	174	EAPAFVEQATEQQILVTG IK					Mascot
2186.1453	2186.2305	0.0852	39	313	333	IPSAVGYQPTLATDLGGL QER					Mascot
2186.1453	2186.2305	0.0852	39	313	333	IPSAVGYQPTLATDLGGL QER	207	100			Mascot
2212.186	2212.2642	0.0782	35	32	51	FDEGLPPILTALEVLDNSI R					Mascot
2212.186	2212.2642	0.0782	35	32	51	FDEGLPPILTALEVLDNSI R	74	99.993			Mascot
2722.3274	2722.426	0.0986	36	449	472	NLQDIIAILGMDELSEDDK MTVAR			Oxidation (M)[11,20]		Mascot
3714.8862	3715.0779	0.1917	52	340	375	GSITSVQAIYVPADDLTD					Mascot

3842.9812	3843.1807	0.1995	52	339	375	PAPATTFAHLDATTVLSR KGSITSVQAIYVPADDLT DPAPATTFAHLDATTVLS R					Mascot

9	hypothetical protein OsI_20905 [Oryza sativa Indica Group]	gi|218197235	57804.8	7.7	20	918	100	49.011	803	100		

Peptide Information

Calc. Mass	Obsrv. Mass	± da	± ppm	Start Seq.	End Seq.	Sequence	Ion Score	C. I. %	Modification	Rank	Result Type
866.4003	866.4203	0.02	23	249	255	EGNDLYR					Mascot
1173.6627	1173.6893	0.0266	23	196	205	VVDLLAPYQR					Mascot
1173.6627	1173.6893	0.0266	23	196	205	VVDLLAPYQR	72	99.991			Mascot
1278.6359	1278.6506	0.0147	11	117	128	TIAMDGTEGLVR			Oxidation (M)[4]		Mascot
1300.5601	1300.6714	0.1113	86	62	72	DGEGHGWRCAR			Carbamidomethyl (C)[9]		Mascot
1390.6863	1390.7252	0.0389	28	233	246	AHGGFSVFAGVGER					Mascot
1390.6863	1390.7252	0.0389	28	233	246	AHGGFSVFAGVGER	130	100			Mascot
1399.7693	1399.8077	0.0384	27	291	303	VGLTGLTVAEHFR					Mascot
1399.7693	1399.8077	0.0384	27	291	303	VGLTGLTVAEHFR	37	65.748			Mascot
1409.8112	1409.847	0.0358	25	132	145	VLNTGSPITVPVGR					Mascot
1418.756	1418.7535	-0.0025	-2	256	268	EMIESGVIKLGDK					Mascot
1473.8346	1473.8335	-0.0011	-1	220	232	TVLIMELINNVAK			Oxidation (M)[5]		Mascot
1492.7755	1492.8168	0.0413	28	320	333	FTQANSEVSALLGR					Mascot
1492.7755	1492.8168	0.0413	28	320	333	FTQANSEVSALLGR	123	100			Mascot
1520.7969	1520.8304	0.0335	22	163	175	GDITTNHFLPIHR					Mascot
1678.7676	1678.7976	0.03	18	274	288	CALVYGQMNEPPGAR			Carbamidomethyl (C)[1], Oxidation (M)[8]		Mascot
1694.8204	1694.8331	0.0127	7	43	61	AAAYATAASGEGGGASR AR					Mascot
1697.8871	1697.9658	0.0787	46	28	42	GPLHRPSPSGYLFNR					Mascot
1864.944	1865.0068	0.0628	34	304	319	DAEGQDVLLFIDNIFR					Mascot
1864.944	1865.0068	0.0628	34	304	319	DAEGQDVLLFIDNIFR	142	100			Mascot
2061.0498	2061.1125	0.0627	30	397	415	QISELGIYPAVDPLDSTSR					Mascot
2061.0498	2061.1125	0.0627	30	397	415	QISELGIYPAVDPLDSTSR	93	100			Mascot
2172.1548	2172.2222	0.0674	31	176	195	EAPAFVEQATEQQILVTG IK					Mascot
2186.1453	2186.2305	0.0852	39	334	354	IPSAVGYQPTLATDLGGL QER					Mascot
2186.1453	2186.2305	0.0852	39	334	354	IPSAVGYQPTLATDLGGL QER	207	100			Mascot
3714.8862	3715.0779	0.1917	52	361	396	GSITSVQAIYVPADDLTD PAPATTFAHLDATTVLSR					Mascot
3842.9812	3843.1807	0.1995	52	360	396	KGSITSVQAIYVPADDLT DPAPATTFAHLDATTVLS R					Mascot

10	PREDICTED: ATP synthase subunit beta, mitochondrial-like [Solanum lycopersicum]	gi|460382474	59939.2	5.94	19	912	100	56.697	803	100

Peptide Information

Calc. Mass	Obsrv. Mass	± da	± ppm	Start Seq.	End Seq.	Sequence	Ion Score	C. I. %	Modification	Rank	Result Type
866.4003	866.4203	0.02	23	273	279	EGNDLYR					Mascot
1173.6627	1173.6893	0.0266	23	220	229	VVDLLAPYQR					Mascot
1173.6627	1173.6893	0.0266	23	220	229	VVDLLAPYQR	72	99.988			Mascot
1278.6359	1278.6506	0.0147	11	141	152	TIAMDGTEGLVR			Oxidation (M)[4]		Mascot
1390.6863	1390.7252	0.0389	28	257	270	AHGGFSVFAGVGER					Mascot
1390.6863	1390.7252	0.0389	28	257	270	AHGGFSVFAGVGER	130	100			Mascot
1399.7693	1399.8077	0.0384	27	315	327	VGLTGLTVAEHFR					Mascot
1399.7693	1399.8077	0.0384	27	315	327	VGLTGLTVAEHFR	37	54.304			Mascot
1409.8112	1409.847	0.0358	25	156	169	VLNTGSPITVPVGR					Mascot
1473.8346	1473.8335	-0.0011	-1	244	256	TVLIMELINNVAK			Oxidation (M)[5]		Mascot
1492.7755	1492.8168	0.0413	28	344	357	FTQANSEVSALLGR					Mascot
1492.7755	1492.8168	0.0413	28	344	357	FTQANSEVSALLGR	123	100			Mascot
1678.7676	1678.7976	0.03	18	298	312	CALVYGQMNEPPGAR			Carbamidomethyl (C)[1], Oxidation (M)[8]		Mascot
1864.944	1865.0068	0.0628	34	328	343	DAEGQDVLLFIDNIFR					Mascot
1864.944	1865.0068	0.0628	34	328	343	DAEGQDVLLFIDNIFR	142	100			Mascot
1869.8912	1870.0319	0.1407	75	440	455	MLSPHILGEDHYNTAR			Oxidation (M)[1]		Mascot
2061.0498	2061.1125	0.0627	30	421	439	QISELGIYPAVDPLDSTSR					Mascot
2061.0498	2061.1125	0.0627	30	421	439	QISELGIYPAVDPLDSTSR	93	100			Mascot
2172.1548	2172.2222	0.0674	31	200	219	EAPAFVEQATEQQILVTG IK					Mascot
2186.1453	2186.2305	0.0852	39	358	378	IPSAVGYQPTLATDLGGL QER					Mascot
2186.1453	2186.2305	0.0852	39	358	378	IPSAVGYQPTLATDLGGL QER	207	100			Mascot
2212.1541	2212.2642	0.1101	50	18	41	GAGSGGGPISRSSVANSI ARPASR					Mascot
2212.1541	2212.2642	0.1101	50	18	41	GAGSGGGPISRSSVANSI ARPASR					Mascot
2575.355	2575.4717	0.1167	45	81	105	ITDEFTGAGAIGKVCQVIG AVVDVR			Carbamidomethyl (C)[15]		Mascot
2722.3274	2722.426	0.0986	36	466	489	NLQDIIAILGMDELSEDDK MTVAR			Oxidation (M)[11,20]		Mascot
3714.8862	3715.0779	0.1917	52	385	420	GSITSVQAIYVPADDLTD PAPATTFAHLDATTVLSR					Mascot
3842.9812	3843.1807	0.1995	52	384	420	KGSITSVQAIYVPADDLT DPAPATTFAHLDATTVLS R					Mascot

Gel Idx/Pos	249/K1	Instr./Gel Origin	BA2151/Sample Project 20140814	Process Status	Analysis Succeeded
Plate [#] Name	[1] Sample Project 20140814	Instrument Sample Name		Spectra	11

Rank	Protein Name	Accession No.	Protein MW	Protein PI	Pep. Count	Protein Score	Protein Score C. I. %	Intensity Matched	Total Ion Score	Total Ion C. I. %	Confirmed
1	UTP--glucose-1-phosphate uridylyltransferase [Aegilops tauschii]	gi|475568864	47076.7	5.15	21	575	100	48.935	449	100	

Peptide Information

Calc. Mass	Obsrv. Mass	± da	± ppm	Start Seq.	End Seq.	Sequence	Ion Score	C. I. %	Modification	Rank	Result Type
839.5098	839.5059	-0.0039	-5	306	313	AIGINVPR					Mascot
858.493	858.4778	-0.0152	-18	267	274	LVDAEALK					Mascot
872.3996	872.3157	-0.0839	-96	422	429	DINGPEDL					Mascot
918.5519	918.5441	-0.0078	-8	362	369	KVANFLAR					Mascot
918.5519	918.5441	-0.0078	-8	362	369	KVANFLAR					Mascot
957.5073	957.4853	-0.022	-23	275	282	MEIIPNPK			Oxidation (M)[1]		Mascot
1014.5942	1014.5879	-0.0063	-6	266	274	RLVDAEALK					Mascot
1052.5371	1052.5386	0.0015	1	217	226	GGTLISYEGR					Mascot
1052.5371	1052.5386	0.0015	1	217	226	GGTLISYEGR	75	99.993			Mascot
1111.745	1111.6816	-0.0634	-57	64	73	ALLDKLVVLK					Mascot
1297.6998	1297.6887	-0.0111	-9	410	421	LEIPDGAVLENK					Mascot
1300.7358	1300.7202	-0.0156	-12	372	383	SIPSIVELDSLK					Mascot
1312.7583	1312.7721	0.0138	11	289	301	VLQLETAAGAAIR					Mascot
1312.7583	1312.7721	0.0138	11	289	301	VLQLETAAGAAIR	101	100			Mascot
1350.7264	1350.7135	-0.0129	-10	384	397	VSGDVSFGSGVVLK					Mascot
1358.7566	1358.7418	-0.0148	-11	157	168	IVTEDFLPLPSK					Mascot
1358.7566	1358.7418	-0.0148	-11	157	168	IVTEDFLPLPSK	24	0			Mascot
1390.7842	1390.6921	-0.0921	-66	302	313	FFEKAIGINVPR					Mascot
1390.7842	1390.6921	-0.0921	-66	302	313	FFEKAIGINVPR					Mascot
1641.8846	1641.8799	-0.0047	-3	347	361	VKPSNPSIELGPEFK					Mascot
1679.8964	1679.8489	-0.0475	-28	211	226	TLADVKGGTLISYEGR					Mascot
1769.9796	1769.975	-0.0046	-3	347	362	VKPSNPSIELGPEFKK					Mascot
2108.1023	2108.1201	0.0178	8	227	244	VQLLEIAQVPDEHVNEFK					Mascot
2108.1023	2108.1201	0.0178	8	227	244	VQLLEIAQVPDEHVNEFK	133	100			Mascot
2143.9832	2144.0171	0.0339	16	174	193	DGWYPPGHGDVFPSLN NSGK					Mascot
2198.0261	2198.0654	0.0393	18	139	156	YSNSNIEIHTFNQSQYPR					Mascot

2198.0261	2198.0654	0.0393	18	139	156	YSNSNIEIHTFNQSQYPR	117	100			Mascot
2454.3127	2454.3491	0.0364	15	321	342	ATSDLLLVQSDLYTLVDG YVIR					Mascot

2	TPA: UDP-glucose pyrophosphorylase1 isoform 2 [Zea mays]	gi|414886602	52316.6	5.23	15	551	100	48.116	480	100	

Protein Group

TPA: UDP-glucose pyrophosphorylase1 isoform 1 [Zea mays]	gi|414886601	52316.6	5.2300 000190 7349
uncharacterized protein LOC100191846 [Zea mays]	gi|212275438	52316.6	5.2300 000190 7349

Peptide Information

Calc. Mass	Obsrv. Mass	± da	± ppm	Start Seq.	End Seq.	Sequence	Ion Score	C. I. %	Modification	Rank	Result Type
839.5098	839.5059	-0.0039	-5	350	357	AIGINVPR					Mascot
918.5519	918.5441	-0.0078	-8	406	413	KVANFLAR					Mascot
918.5519	918.5441	-0.0078	-8	406	413	KVANFLAR					Mascot
957.5073	957.4853	-0.022	-23	319	326	MEIIPNPK			Oxidation (M)[1]		Mascot
965.5414	965.5365	-0.0049	-5	31	39	SGFISLVSR					Mascot
965.5414	965.5365	-0.0049	-5	31	39	SGFISLVSR	31	0			Mascot
1052.5371	1052.5386	0.0015	1	261	270	GGTLISYEGR					Mascot
1052.5371	1052.5386	0.0015	1	261	270	GGTLISYEGR	75	99.993			Mascot
1300.7358	1300.7202	-0.0156	-12	416	427	SIPSIVELDSLK					Mascot
1312.7583	1312.7721	0.0138	11	333	345	VLQLETAAGAAIR					Mascot
1312.7583	1312.7721	0.0138	11	333	345	VLQLETAAGAAIR	101	100			Mascot
1358.7566	1358.7418	-0.0148	-11	172	183	IVTEDFLPLPSK					Mascot
1358.7566	1358.7418	-0.0148	-11	172	183	IVTEDFLPLPSK	24	0			Mascot
1507.7673	1507.7792	0.0119	8	1	14	MATTAVSVDEKLDK					Mascot
1679.8964	1679.8489	-0.0475	-28	255	270	TLADVKGGTLISYEGR					Mascot
1967.0121	1967.0215	0.0094	5	218	235	EYVFVANSDNLGAIVDIK					Mascot
2108.1023	2108.1201	0.0178	8	271	288	VQLLEIAQVPDEHVNEFK					Mascot
2108.1023	2108.1201	0.0178	8	271	288	VQLLEIAQVPDEHVNEFK	133	100			Mascot
2143.9832	2144.0171	0.0339	16	189	208	DGWYPPGHGDVFPSLN NSGK					Mascot
2181.0559	2181.1042	0.0483	22	454	473	LEIPDGDVLENKDVNGPE DL					Mascot
2198.0261	2198.0654	0.0393	18	154	171	YSNSNIEIHTFNQSQYPR					Mascot
2198.0261	2198.0654	0.0393	18	154	171	YSNSNIEIHTFNQSQYPR	117	100			Mascot

3	hypothetical protein OsI_32322 [Oryza sativa Indica Group]	gi|218202595	51791.3	5.43	15	550	100	47.381	480	100	

Protein Group

Os09g0553200 [Oryza sativa Japonica Group]	gi|113632112	51821.3	5.4299 998283 3862

Peptide Information

Calc. Mass	Obsrv. Mass	± da	± ppm	Start Seq.	End Seq.	Sequence	Ion Score	C. I. %	Modification	Rank	Result Type
839.5098	839.5059	-0.0039	-5	347	354	AIGINVPR					Mascot
872.3996	872.3157	-0.0839	-96	462	469	DINGPEDL					Mascot
918.5519	918.5441	-0.0078	-8	403	410	KVANFLAR					Mascot
918.5519	918.5441	-0.0078	-8	403	410	KVANFLAR					Mascot
957.5073	957.4853	-0.022	-23	316	323	MEIIPNPK			Oxidation (M)[1]		Mascot
965.5414	965.5365	-0.0049	-5	28	36	SGFISLVSR					Mascot
965.5414	965.5365	-0.0049	-5	28	36	SGFISLVSR	31	0			Mascot
1052.5371	1052.5386	0.0015	1	258	267	GGTLISYEGR					Mascot
1052.5371	1052.5386	0.0015	1	258	267	GGTLISYEGR	75	99.993			Mascot
1297.6998	1297.6887	-0.0111	-9	450	461	LEIPDGAVLENK					Mascot
1312.7583	1312.7721	0.0138	11	330	342	VLQLETAAGAAIR					Mascot
1312.7583	1312.7721	0.0138	11	330	342	VLQLETAAGAAIR	101	100			Mascot
1358.7566	1358.7418	-0.0148	-11	169	180	IVTEDFLPLPSK					Mascot
1358.7566	1358.7418	-0.0148	-11	169	180	IVTEDFLPLPSK	24	0			Mascot
1390.7842	1390.6921	-0.0921	-66	343	354	FFEKAIGINVPR					Mascot
1390.7842	1390.6921	-0.0921	-66	343	354	FFEKAIGINVPR					Mascot
1679.8964	1679.8489	-0.0475	-28	252	267	TLADVKGGTLISYEGR					Mascot
1967.0121	1967.0215	0.0094	5	215	232	EYVFVANSDNLGAIVDIK					Mascot
2108.1023	2108.1201	0.0178	8	268	285	VQLLEIAQVPDEHVNEFK					Mascot
2108.1023	2108.1201	0.0178	8	268	285	VQLLEIAQVPDEHVNEFK	133	100			Mascot
2143.9832	2144.0171	0.0339	16	186	205	DGWYPPGHGDVFPSLN NSGK					Mascot
2198.0261	2198.0654	0.0393	18	151	168	YSNSNIEIHTFNQSQYPR					Mascot
2198.0261	2198.0654	0.0393	18	151	168	YSNSNIEIHTFNQSQYPR	117	100			Mascot

4	TPA: UDP-glucose pyrophosphorylase1 [Zea mays]	gi|414886600	58101.6	5.49	15	546	100	47.699	480	100	

Peptide Information

Calc. Mass	Obsrv. Mass	± da	± ppm	Start Seq.	End Seq.	Sequence	Ion Score	C. I. %	Modification	Rank	Result Type
839.5098	839.5059	-0.0039	-5	350	357	AIGINVPR					Mascot
918.5519	918.5441	-0.0078	-8	406	413	KVANFLAR					Mascot

Calc. Mass	Obsrv. Mass	± da	± ppm	Start Seq.	End Seq.	Sequence	Ion Score	C. I. %	Modification	Rank	Result Type
918.5519	918.5441	-0.0078	-8	406	413	KVANFLAR					Mascot
957.5073	957.4853	-0.022	-23	319	326	MEIIPNPK			Oxidation (M)[1]		Mascot
965.5414	965.5365	-0.0049	-5	31	39	SGFISLVSR					Mascot
965.5414	965.5365	-0.0049	-5	31	39	SGFISLVSR	31	0			Mascot
1052.5371	1052.5386	0.0015	1	261	270	GGTLISYEGR					Mascot
1052.5371	1052.5386	0.0015	1	261	270	GGTLISYEGR	75	99.993			Mascot
1300.7358	1300.7202	-0.0156	-12	416	427	SIPSIVELDSLK					Mascot
1312.7583	1312.7721	0.0138	11	333	345	VLQLETAAGAAIR					Mascot
1312.7583	1312.7721	0.0138	11	333	345	VLQLETAAGAAIR	101	100			Mascot
1358.7566	1358.7418	-0.0148	-11	172	183	IVTEDFLPLPSK					Mascot
1358.7566	1358.7418	-0.0148	-11	172	183	IVTEDFLPLPSK	24	0			Mascot
1507.7673	1507.7792	0.0119	8	1	14	MATTAVSVDEKLDK					Mascot
1679.8964	1679.8489	-0.0475	-28	255	270	TLADVKGGTLISYEGR					Mascot
1967.0121	1967.0215	0.0094	5	218	235	EYVFVANSDNLGAIVDIK					Mascot
2108.1023	2108.1201	0.0178	8	271	288	VQLLEIAQVPDEHVNEFK					Mascot
2108.1023	2108.1201	0.0178	8	271	288	VQLLEIAQVPDEHVNEFK	133	100			Mascot
2130.0933	2130.1218	0.0285	13	454	472	LEIPDGDVLENKVMSMALR					Mascot
2143.9832	2144.0171	0.0339	16	189	208	DGWYPPGHGDVFPSLNNSGK					Mascot
2198.0261	2198.0654	0.0393	18	154	171	YSNSNIEIHTFNQSQYPR					Mascot
2198.0261	2198.0654	0.0393	18	154	171	YSNSNIEIHTFNQSQYPR	117	100			Mascot

5 TPA: UDP-glucose pyrophosphorylase1 [Zea mays] gi|414886599 52414.8 5.62 14 542 100 47.447 480 100

Peptide Information

Calc. Mass	Obsrv. Mass	± da	± ppm	Start Seq.	End Seq.	Sequence	Ion Score	C. I. %	Modification	Rank	Result Type
839.5098	839.5059	-0.0039	-5	350	357	AIGINVPR					Mascot
918.5519	918.5441	-0.0078	-8	406	413	KVANFLAR					Mascot
918.5519	918.5441	-0.0078	-8	406	413	KVANFLAR					Mascot
957.5073	957.4853	-0.022	-23	319	326	MEIIPNPK			Oxidation (M)[1]		Mascot
965.5414	965.5365	-0.0049	-5	31	39	SGFISLVSR					Mascot
965.5414	965.5365	-0.0049	-5	31	39	SGFISLVSR	31	0			Mascot
1052.5371	1052.5386	0.0015	1	261	270	GGTLISYEGR					Mascot
1052.5371	1052.5386	0.0015	1	261	270	GGTLISYEGR	75	99.993			Mascot
1300.7358	1300.7202	-0.0156	-12	416	427	SIPSIVELDSLK					Mascot
1312.7583	1312.7721	0.0138	11	333	345	VLQLETAAGAAIR					Mascot
1312.7583	1312.7721	0.0138	11	333	345	VLQLETAAGAAIR	101	100			Mascot

Calc. Mass	Obsrv. Mass	± da	± ppm	Start Seq.	End Seq.	Sequence	Ion Score	C. I. %	Modification	Rank	Result Type
1358.7566	1358.7418	-0.0148	-11	172	183	IVTEDFLPLPSK					Mascot
1358.7566	1358.7418	-0.0148	-11	172	183	IVTEDFLPLPSK	24	0			Mascot
1507.7673	1507.7792	0.0119	8	1	14	MATTAVSVDEKLDK					Mascot
1679.8964	1679.8489	-0.0475	-28	255	270	TLADVKGGTLISYEGR					Mascot
1967.0121	1967.0215	0.0094	5	218	235	EYVFVANSDNLGAIVDIK					Mascot
2108.1023	2108.1201	0.0178	8	271	288	VQLLEIAQVPDEHVNEFK					Mascot
2108.1023	2108.1201	0.0178	8	271	288	VQLLEIAQVPDEHVNEFK	133	100			Mascot
2143.9832	2144.0171	0.0339	16	189	208	DGWYPPGHGDVFPSLN NSGK					Mascot
2198.0261	2198.0654	0.0393	18	154	171	YSNSNIEIHTFNQSQYPR					Mascot
2198.0261	2198.0654	0.0393	18	154	171	YSNSNIEIHTFNQSQYPR	117	100			Mascot

6	uncharacterized protein LOC100191463 [Zea mays]	gi|212275097	52226.5	5.37	13	540	100	48.391	485	100

Peptide Information

Calc. Mass	Obsrv. Mass	± da	± ppm	Start Seq.	End Seq.	Sequence	Ion Score	C. I. %	Modification	Rank	Result Type
839.5098	839.5059	-0.0039	-5	350	357	AIGINVPR					Mascot
918.5519	918.5441	-0.0078	-8	406	413	KVANFLAR					Mascot
918.5519	918.5441	-0.0078	-8	406	413	KVANFLAR					Mascot
949.5465	949.542	-0.0045	-5	31	39	AGFISLVSR					Mascot
949.5465	949.542	-0.0045	-5	31	39	AGFISLVSR	36	44.553			Mascot
957.5073	957.4853	-0.022	-23	319	326	MEIIPNPK			Oxidation (M)[1]		Mascot
1052.5371	1052.5386	0.0015	1	261	270	GGTLISYEGR					Mascot
1052.5371	1052.5386	0.0015	1	261	270	GGTLISYEGR	75	99.993			Mascot
1300.7358	1300.7202	-0.0156	-12	416	427	SIPSIVELDSLK					Mascot
1312.7583	1312.7721	0.0138	11	333	345	VLQLETAAGAAIR					Mascot
1312.7583	1312.7721	0.0138	11	333	345	VLQLETAAGAAIR	101	100			Mascot
1358.7566	1358.7418	-0.0148	-11	172	183	IVTEDFLPLPSK					Mascot
1358.7566	1358.7418	-0.0148	-11	172	183	IVTEDFLPLPSK	24	0			Mascot
1679.8964	1679.8489	-0.0475	-28	255	270	TLADVKGGTLISYEGR					Mascot
1967.0121	1967.0215	0.0094	5	218	235	EYVFVANSDNLGAIVDIK					Mascot
2108.1023	2108.1201	0.0178	8	271	288	VQLLEIAQVPDEHVNEFK					Mascot
2108.1023	2108.1201	0.0178	8	271	288	VQLLEIAQVPDEHVNEFK	133	100			Mascot
2143.9832	2144.0171	0.0339	16	189	208	DGWYPPGHGDVFPSLN NSGK					Mascot
2198.0261	2198.0654	0.0393	18	154	171	YSNSNIEIHTFNQSQYPR					Mascot
2198.0261	2198.0654	0.0393	18	154	171	YSNSNIEIHTFNQSQYPR	117	100			Mascot

7	UTP--glucose-1-phosphate uridylyltransferase [Triticum urartu]	gi|473993048	51082.9	5.76	23	511	100	35.188	368	100

Peptide Information

Calc. Mass	Obsrv. Mass	± da	± ppm	Start Seq.	End Seq.	Sequence	Ion Score	C. I. %	Modification	Rank	Result Type
839.5098	839.5059	-0.0039	-5	334	341	AIGINVPR					Mascot
858.493	858.4778	-0.0152	-18	295	302	LVDAEALK					Mascot
918.5519	918.5441	-0.0078	-8	390	397	KVANFLAR					Mascot
918.5519	918.5441	-0.0078	-8	390	397	KVANFLAR					Mascot
949.5465	949.542	-0.0045	-5	27	35	AGFISLVSR					Mascot
949.5465	949.542	-0.0045	-5	27	35	AGFISLVSR	36	44.553			Mascot
957.5073	957.4853	-0.022	-23	303	310	MEIIPNPK			Oxidation (M)[1]		Mascot
1014.5942	1014.5879	-0.0063	-6	294	302	RLVDAEALK					Mascot
1018.5051	1018.5077	0.0026	3	18	26	LGEISENEK					Mascot
1052.5371	1052.5386	0.0015	1	245	254	GGTLISYEGR					Mascot
1052.5371	1052.5386	0.0015	1	245	254	GGTLISYEGR	75	99.993			Mascot
1111.745	1111.6816	-0.0634	-57	75	84	ALLDKLVVLK					Mascot
1114.6216	1114.5669	-0.0547	-49	8	17	IDNLRDAVAK					Mascot
1173.6222	1173.6573	0.0351	30	2	12	AAADSKIDNLR					Mascot
1297.6998	1297.6887	-0.0111	-9	438	449	LEIPDGAVLENK					Mascot
1300.7358	1300.7202	-0.0156	-12	400	411	SIPSIVELDSLK					Mascot
1312.7583	1312.7721	0.0138	11	317	329	VLQLETAAGAAIR					Mascot
1312.7583	1312.7721	0.0138	11	317	329	VLQLETAAGAAIR	101	100			Mascot
1350.7264	1350.7135	-0.0129	-10	412	425	VSGDVSFGSGVVLK					Mascot
1358.7566	1358.7418	-0.0148	-11	185	196	IVTEDFLPLPSK					Mascot
1358.7566	1358.7418	-0.0148	-11	185	196	IVTEDFLPLPSK	24	0			Mascot
1390.7842	1390.6921	-0.0921	-66	330	341	FFEKAIGINVPR					Mascot
1390.7842	1390.6921	-0.0921	-66	330	341	FFEKAIGINVPR					Mascot
1641.8846	1641.8799	-0.0047	-3	375	389	VKPSNPSIELGPEFK					Mascot
1679.8964	1679.8489	-0.0475	-28	239	254	TLADVKGGTLISYEGR					Mascot
1769.9796	1769.975	-0.0046	-3	375	390	VKPSNPSIELGPEFKK					Mascot
2108.1023	2108.1201	0.0178	8	255	272	VQLLEIAQVPDEHVNEFK					Mascot
2108.1023	2108.1201	0.0178	8	255	272	VQLLEIAQVPDEHVNEFK	133	100			Mascot
2143.9832	2144.0171	0.0339	16	202	221	DGWYPPGHGDVFPSLN NSGK					Mascot
2454.3127	2454.3491	0.0364	15	349	370	ATSDLLLVQSDLYTLVDG YVIR					Mascot

8	RecName: Full=UTP--glucose-1-phosphate uridylyltransferase; AltName: Full=UDP-glucose pyrophosphorylase; Short=UDPGP; Short=UGPase	gi|6136111	51783.2	5.2	24	503	100	51.584	352	100	

Peptide Information

Calc. Mass	Obsrv. Mass	± da	± ppm	Start Seq.	End Seq.	Sequence	Ion Score	C. I. %	Modification	Rank	Result Type
839.5098	839.5059	-0.0039	-5	350	357	AIGINVPR					Mascot
858.493	858.4778	-0.0152	-18	311	318	LVDAEALK					Mascot
872.3996	872.3157	-0.0839	-96	466	473	DINGPEDI					Mascot
918.5519	918.5441	-0.0078	-8	406	413	KVANFLAR					Mascot
918.5519	918.5441	-0.0078	-8	406	413	KVANFLAR					Mascot
949.5465	949.542	-0.0045	-5	31	39	AGFISLVSR					Mascot
949.5465	949.542	-0.0045	-5	31	39	AGFISLVSR	36	44.553			Mascot
957.5073	957.4853	-0.022	-23	319	326	MEIIPNPK			Oxidation (M)[1]		Mascot
1014.5942	1014.5879	-0.0063	-6	310	318	RLVDAEALK					Mascot
1018.5051	1018.5077	0.0026	3	22	30	LGEISENEK					Mascot
1052.5371	1052.5386	0.0015	1	261	270	GGTLISYEGR					Mascot
1052.5371	1052.5386	0.0015	1	261	270	GGTLISYEGR	75	99.993			Mascot
1057.6001	1057.504	-0.0961	-91	12	21	IDGLRDAVAK					Mascot
1111.745	1111.6816	-0.0634	-57	79	88	ALLDKLVVLK					Mascot
1297.6998	1297.6887	-0.0111	-9	454	465	LEIPDGAVLENK					Mascot
1300.7358	1300.7202	-0.0156	-12	416	427	SIPSIVELDSLK					Mascot
1312.7583	1312.7721	0.0138	11	333	345	VLQLETAAGAAIR					Mascot
1312.7583	1312.7721	0.0138	11	333	345	VLQLETAAGAAIR	101	100			Mascot
1350.7264	1350.7135	-0.0129	-10	428	441	VSGDVSFGSGVVLK					Mascot
1358.7566	1358.7418	-0.0148	-11	172	183	IVTEDFLPLPSK					Mascot
1358.7566	1358.7418	-0.0148	-11	172	183	IVTEDFLPLPSK	24	0			Mascot
1390.7842	1390.6921	-0.0921	-66	346	357	FFEKAIGINVPR					Mascot
1390.7842	1390.6921	-0.0921	-66	346	357	FFEKAIGINVPR					Mascot
1641.8846	1641.8799	-0.0047	-3	391	405	VKPSNPSIELGPEFK					Mascot
1679.8964	1679.8489	-0.0475	-28	255	270	TLADVKGGTLISYEGR					Mascot
1769.9796	1769.975	-0.0046	-3	391	406	VKPSNPSIELGPEFKK					Mascot
1967.0121	1967.0215	0.0094	5	218	235	EYVFVANSDNLGAIVDIK					Mascot
2143.9832	2144.0171	0.0339	16	189	208	DGWYPPGHGDVFPSLN NSGK					Mascot
2198.0261	2198.0654	0.0393	18	154	171	YSNSNIEIHTFNQSQYPR					Mascot
2198.0261	2198.0654	0.0393	18	154	171	YSNSNIEIHTFNQSQYPR	117	100			Mascot
2454.3127	2454.3491	0.0364	15	365	386	ATSDLLLVQSDLYTLVDG YVIR					Mascot

9	UDP-glucose pyrophosphorylase [Gossypium hirsutum]	gi|308445439	51365.9	5.53	11	393	100	28.921	350	100	

Peptide Information

Calc. Mass	Obsrv. Mass	± da	± ppm	Start Seq.	End Seq.	Sequence	Ion Score	C. I. %	Modification	Rank	Result Type
916.4985	916.4633	-0.0352	-38	303	310	LVEADELK					Mascot
957.5073	957.4853	-0.022	-23	311	318	MEIIPNPK			Oxidation (M)[1]		Mascot
978.5255	978.4984	-0.0271	-28	201	209	LDAFLSQGK					Mascot
1297.6998	1297.6887	-0.0111	-9	446	457	LEIPDGAVIENK					Mascot
1312.7583	1312.7721	0.0138	11	325	337	VLQLETAAGAAIR					Mascot
1312.7583	1312.7721	0.0138	11	325	337	VLQLETAAGAAIR	101	100			Mascot
1447.7275	1447.8047	0.0772	53	9	22	SAVAALSEISENEK					Mascot
1967.0121	1967.0215	0.0094	5	210	227	EYVFVANSDNLGAIVDLK					Mascot
2108.1023	2108.1201	0.0178	8	263	280	VQLLEIAQVPDEHVNEFK					Mascot
2108.1023	2108.1201	0.0178	8	263	280	VQLLEIAQVPDEHVNEFK	133	100			Mascot
2144.1963	2144.0171	-0.1792	-84	102	120	NGLTFLDLIVIQIENLNSK					Mascot
2198.0261	2198.0654	0.0393	18	146	163	YSNSNIEIHTFNQSQYPR					Mascot
2198.0261	2198.0654	0.0393	18	146	163	YSNSNIEIHTFNQSQYPR	117	100			Mascot
2454.1428	2454.3491	0.2063	84	121	141	YGCNVPLVLMNSFNTHD DTLK			Carbamidomethyl (C)[3], Oxidation (M)[10]		Mascot

10 UDP-glucose pyrophosphorylase 2 [Theobroma cacao] gi|508710349 51505.1 5.61 9 374 100 28.634 350 100

Peptide Information

Calc. Mass	Obsrv. Mass	± da	± ppm	Start Seq.	End Seq.	Sequence	Ion Score	C. I. %	Modification	Rank	Result Type
858.493	858.4778	-0.0152	-18	303	310	LVEADALK					Mascot
957.5073	957.4853	-0.022	-23	311	318	MEIIPNPK			Oxidation (M)[1]		Mascot
978.5255	978.4984	-0.0271	-28	201	209	LDAFLSQGK					Mascot
1014.5942	1014.5879	-0.0063	-6	302	310	RLVEADALK					Mascot
1297.6998	1297.6887	-0.0111	-9	446	457	LEIPDGAVIENK					Mascot
1312.7583	1312.7721	0.0138	11	325	337	VLQLETAAGAAIR					Mascot
1312.7583	1312.7721	0.0138	11	325	337	VLQLETAAGAAIR	101	100			Mascot
1371.7169	1371.731	0.0141	10	338	349	FFDHAIGVNVPR					Mascot
2108.1023	2108.1201	0.0178	8	263	280	VQLLEIAQVPDEHVNEFK					Mascot
2108.1023	2108.1201	0.0178	8	263	280	VQLLEIAQVPDEHVNEFK	133	100			Mascot
2198.0261	2198.0654	0.0393	18	146	163	YSNSNIEIHTFNQSQYPR					Mascot
2198.0261	2198.0654	0.0393	18	146	163	YSNSNIEIHTFNQSQYPR	117	100			Mascot

Gel Idx/Pos	250/K2	Instr./Gel Origin	BA2151/Sample Project 20140814	Process Status	Analysis Succeeded
Plate [#] Name	[1] Sample Project 20140814	Instrument Sample Name		Spectra	11

Rank	Protein Name	Accession No.	Protein MW	Protein PI	Pep. Count	Protein Score	Protein Score C. I. %	Intensity Matched	Total Ion Score	Total Ion C. I. %	Confirmed
1	ATP synthase CF1 beta subunit [Triticum aestivum]	gi|14017579	53880.9	5.06	25	1,160	100	53.676	977	100	

Protein Group

ATP synthase CF1 beta subunit (chloroplast) [Triticum aestivum]	gi|521301494	53937.9	5.0599 999427 7954
RecName: Full=ATP synthase subunit beta, chloroplastic; AltName: Full=ATP synthase F1 sector subunit beta; AltName: Full=F-ATPase subunit beta	gi|114574	53880.9	5.0599 999427 7954

Peptide Information

Calc. Mass	Obsrv. Mass	± da	± ppm	Start Seq.	End Seq.	Sequence	Ion Score	C. I. %	Modification	Rank	Result Type
873.5152	873.5121	-0.0031	-4	391	397	VKETLQR					Mascot
1007.5771	1007.562	-0.0151	-15	146	154	LSIFETGIK					Mascot
1201.7052	1201.7001	-0.0051	-4	155	164	VVDLLAPYRR					Mascot
1254.5818	1254.5714	-0.0104	-8	76	87	AVAMSATDGLMR			Oxidation (M)[4,11]		Mascot
1328.6707	1328.6815	0.0108	8	192	205	AHGGVSVFGGVGER					Mascot
1328.6707	1328.6815	0.0108	8	192	205	AHGGVSVFGGVGER	116	100			Mascot
1416.6866	1416.6992	0.0126	9	379	390	IVGNEHYETAQR					Mascot
1416.6866	1416.6992	0.0126	9	379	390	IVGNEHYETAQR	91	100			Mascot
1433.7748	1433.7893	0.0145	10	278	291	FVQAGSEVSALLGR					Mascot
1433.7748	1433.7893	0.0145	10	278	291	FVQAGSEVSALLGR	113	100			Mascot
1471.7614	1471.7592	-0.0022	-1	249	261	VGLTALTMAEYFR					Mascot
1487.7563	1487.7515	-0.0048	-3	249	261	VGLTALTMAEYFR			Oxidation (M)[8]		Mascot
1487.7563	1487.7515	-0.0048	-3	249	261	VGLTALTMAEYFR	30	0	Oxidation (M)[8]		Mascot
1492.8159	1492.816	0.0001	0	266	277	QDVLLFIDNIFR					Mascot
1518.6564	1518.7524	0.096	63	206	217	TREGNDLYMEMK			Oxidation (M)[9,11]		Mascot
1535.858	1535.8685	0.0105	7	40	52	LPYIYNALVVQSR					Mascot
1535.858	1535.8685	0.0105	7	40	52	LPYIYNALVVQSR	102	100			Mascot
1601.8104	1601.818	0.0076	5	232	246	VALVYGQMNEPPGAR					Mascot
1617.8054	1617.8005	-0.0049	-3	232	246	VALVYGQMNEPPGAR			Oxidation (M)[8]		Mascot
1617.8054	1617.8005	-0.0049	-3	232	246	VALVYGQMNEPPGAR	49	97.673	Oxidation (M)[8]		Mascot
1790.8928	1790.9406	0.0478	27	247	261	MRVGLTALTMAEYFR			Oxidation (M)[1,10]		Mascot
1809.0157	1808.9768	-0.0389	-22	23	39	IDQIIGPVLDVTFPPGK					Mascot

1885.955	1885.9711	0.0161	9	58	73	QINVTCEVQQLLGNNR			Carbamidomethyl (C)[6]		Mascot
1949.0491	1949.0708	0.0217	11	262	277	DVNKQDVLLFIDNIFR					Mascot
1949.0491	1949.0708	0.0217	11	262	277	DVNKQDVLLFIDNIFR	118	100			Mascot
2030.9989	2031.0533	0.0544	27	3	22	TNPTTSPPGASTIEEKSTGR					Mascot
2061.0322	2061.0659	0.0337	16	360	378	GIYPAVDPLDSTSTMLQPR					Mascot
2077.0271	2077.0442	0.0171	8	360	378	GIYPAVDPLDSTSTMLQPR			Oxidation (M)[15]		Mascot
2097.1008	2097.1152	0.0144	7	88	109	GMEVIDTGAPLSVPVGGATLGR					Mascot
2113.0959	2113.1067	0.0108	5	88	109	GMEVIDTGAPLSVPVGGATLGR			Oxidation (M)[2]		Mascot
2314.0691	2314.0925	0.0234	10	292	312	MPSAVGYQPTLSTEMGSLQER			Oxidation (M)[1,15]		Mascot
2460.1785	2460.217	0.0385	16	53	73	DTDDKQINVTCEVQQLLGNNR			Carbamidomethyl (C)[11]		Mascot
2460.1785	2460.217	0.0385	16	53	73	DTDDKQINVTCEVQQLLGNNR	190	100	Carbamidomethyl (C)[11]		Mascot
2694.3887	2694.4514	0.0627	23	110	134	IFNVLGEPVDNLGPVDSSATFPIHR					Mascot
2694.3887	2694.4514	0.0627	23	110	134	IFNVLGEPVDNLGPVDSSATFPIHR	170	100			Mascot
3325.856	3325.9202	0.0642	19	23	52	IDQIIGPVLDVTFPPGKLPYIYNALVVQSR					Mascot
3714.8862	3714.9929	0.1067	29	319	354	GSITSIQAVYVPADDLTDPAPATTFAHLDATTVLSR					Mascot
3842.9812	3843.0984	0.1172	30	318	354	KGSITSIQAVYVPADDLTDPAPATTFAHLDATTVLSR					Mascot

2	ATP synthase CF1 beta subunit (chloroplast) [Aegilops speltoides]	gi|394986499	53965	5.06	24	1,150	100	53.587	977	100		

Protein Group

ATP synthase CF1 beta subunit (chloroplast) [Aegilops speltoides]	gi|533310208	53965	5.0599 999427 7954

Peptide Information

Calc. Mass	Obsrv. Mass	± da	± ppm	Start Seq.	End Seq.	Sequence	Ion Score	C. I. %	Modification	Rank	Result Type
873.5152	873.5121	-0.0031	-4	392	398	VKETLQR					Mascot
1007.5771	1007.562	-0.0151	-15	146	154	LSIFETGIK					Mascot
1201.7052	1201.7001	-0.0051	-4	155	164	VVDLLAPYRR					Mascot
1254.5818	1254.5714	-0.0104	-8	76	87	AVAMSATDGLMR			Oxidation (M)[4,11]		Mascot
1328.6707	1328.6815	0.0108	8	193	206	AHGGVSVFGGVGER					Mascot
1328.6707	1328.6815	0.0108	8	193	206	AHGGVSVFGGVGER	116	100			Mascot
1416.6866	1416.6992	0.0126	9	380	391	IVGNEHYETAQR					Mascot
1416.6866	1416.6992	0.0126	9	380	391	IVGNEHYETAQR	91	100			Mascot
1433.7748	1433.7893	0.0145	10	279	292	FVQAGSEVSALLGR					Mascot

Calc. Mass	Obsrv. Mass	± da	± ppm	Start Seq.	End Seq.	Sequence	Ion Score	C. I. %	Modification	Rank	Result Type
1433.7748	1433.7893	0.0145	10	279	292	FVQAGSEVSALLGR	113	100			Mascot
1471.7614	1471.7592	-0.0022	-1	250	262	VGLTALTMAEYFR					Mascot
1487.7563	1487.7515	-0.0048	-3	250	262	VGLTALTMAEYFR			Oxidation (M)[8]		Mascot
1487.7563	1487.7515	-0.0048	-3	250	262	VGLTALTMAEYFR	30	0	Oxidation (M)[8]		Mascot
1492.8159	1492.816	0.0001	0	267	278	QDVLLFIDNIFR					Mascot
1518.6564	1518.7524	0.096	63	207	218	TREGNDLYMEMK			Oxidation (M)[9,11]		Mascot
1535.858	1535.8685	0.0105	7	40	52	LPYIYNALVVQSR					Mascot
1535.858	1535.8685	0.0105	7	40	52	LPYIYNALVVQSR	102	100			Mascot
1601.8104	1601.818	0.0076	5	233	247	VALVYGQMNEPPGAR					Mascot
1617.8054	1617.8005	-0.0049	-3	233	247	VALVYGQMNEPPGAR			Oxidation (M)[8]		Mascot
1617.8054	1617.8005	-0.0049	-3	233	247	VALVYGQMNEPPGAR	49	97.673	Oxidation (M)[8]		Mascot
1790.8928	1790.9406	0.0478	27	248	262	MRVGLTALTMAEYFR			Oxidation (M)[1,10]		Mascot
1809.0157	1808.9768	-0.0389	-22	23	39	IDQIIGPVLDVTFPPGK					Mascot
1885.955	1885.9711	0.0161	9	58	73	QINVTCEVQQLLGNNR			Carbamidomethyl (C)[6]		Mascot
1949.0491	1949.0708	0.0217	11	263	278	DVNKQDVLLFIDNIFR					Mascot
1949.0491	1949.0708	0.0217	11	263	278	DVNKQDVLLFIDNIFR	118	100			Mascot
2061.0322	2061.0659	0.0337	16	361	379	GIYPAVDPLDSTSTMLQPR					Mascot
2077.0271	2077.0442	0.0171	8	361	379	GIYPAVDPLDSTSTMLQPR			Oxidation (M)[15]		Mascot
2097.1008	2097.1152	0.0144	7	88	109	GMEVIDTGAPLSVPVGGATLGR					Mascot
2113.0959	2113.1067	0.0108	5	88	109	GMEVIDTGAPLSVPVGGATLGR			Oxidation (M)[2]		Mascot
2314.0691	2314.0925	0.0234	10	293	313	MPSAVGYQPTLSTEMGSLQER			Oxidation (M)[1,15]		Mascot
2460.1785	2460.217	0.0385	16	53	73	DTDDKQINVTCEVQQLLGNNR			Carbamidomethyl (C)[11]		Mascot
2460.1785	2460.217	0.0385	16	53	73	DTDDKQINVTCEVQQLLGNNR	190	100	Carbamidomethyl (C)[11]		Mascot
2694.3887	2694.4514	0.0627	23	110	134	IFNVLGEPVDNLGPVDSSATFPIHR					Mascot
2694.3887	2694.4514	0.0627	23	110	134	IFNVLGEPVDNLGPVDSSATFPIHR	170	100			Mascot
3325.856	3325.9202	0.0642	19	23	52	IDQIIGPVLDVTFPPGKLPYIYNALVVQSR					Mascot
3714.8862	3714.9929	0.1067	29	320	355	GSITSIQAVYVPADDLTDPAPATTFAHLDATTVLSR					Mascot
3842.9812	3843.0984	0.1172	30	319	355	KGSITSIQAVYVPADDLTDPAPATTFAHLDATTVLSR					Mascot

3 ATP synthase CF1 beta subunit [Brachypodium distachyon] gi|194033156 53908 5.23 24 957 100 50.946 787 100

Peptide Information

Calc. Mass	Obsrv. Mass	± da	± ppm	Start Seq.	End Seq.	Sequence	Ion Score	C. I. %	Modification	Rank	Result Type

873.5152	873.5121	-0.0031	-4	391	397	VKETLQR				Mascot
1007.5771	1007.562	-0.0151	-15	146	154	LSIFETGIK				Mascot
1201.7052	1201.7001	-0.0051	-4	155	164	VVDLLAPYRR				Mascot
1254.5818	1254.5714	-0.0104	-8	76	87	AVAMSATDGLMR			Oxidation (M)[4,11]	Mascot
1328.6707	1328.6815	0.0108	8	192	205	AHGGVSVFGGVGER				Mascot
1328.6707	1328.6815	0.0108	8	192	205	AHGGVSVFGGVGER	116	100		Mascot
1416.6866	1416.6992	0.0126	9	379	390	IVGNEHYETAQR				Mascot
1416.6866	1416.6992	0.0126	9	379	390	IVGNEHYETAQR	91	100		Mascot
1433.7748	1433.7893	0.0145	10	278	291	FVQAGSEVSALLGR				Mascot
1433.7748	1433.7893	0.0145	10	278	291	FVQAGSEVSALLGR	113	100		Mascot
1471.7614	1471.7592	-0.0022	-1	249	261	VGLTALTMAEYFR				Mascot
1487.7563	1487.7515	-0.0048	-3	249	261	VGLTALTMAEYFR			Oxidation (M)[8]	Mascot
1487.7563	1487.7515	-0.0048	-3	249	261	VGLTALTMAEYFR	30	0	Oxidation (M)[8]	Mascot
1492.8159	1492.816	0.0001	0	266	277	QDVLLFIDNIFR				Mascot
1535.858	1535.8685	0.0105	7	40	52	LPYIYNALVVQSR				Mascot
1535.858	1535.8685	0.0105	7	40	52	LPYIYNALVVQSR	102	100		Mascot
1601.8104	1601.818	0.0076	5	232	246	VALVYGQMNEPPGAR				Mascot
1617.8054	1617.8005	-0.0049	-3	232	246	VALVYGQMNEPPGAR			Oxidation (M)[8]	Mascot
1617.8054	1617.8005	-0.0049	-3	232	246	VALVYGQMNEPPGAR	49	97.673	Oxidation (M)[8]	Mascot
1790.8928	1790.9406	0.0478	27	247	261	MRVGLTALTMAEYFR			Oxidation (M)[1,10]	Mascot
1809.0157	1808.9768	-0.0389	-22	23	39	IDQIIGPVLDVTFPPGK				Mascot
1868.9825	1868.9799	-0.0026	-1	2	18	RTNPTTSRPGVSPIEEK				Mascot
1885.955	1885.9711	0.0161	9	58	73	QINVTCEVQQLLGNNR			Carbamidomethyl (C)[6]	Mascot
1949.0491	1949.0708	0.0217	11	262	277	DVNKQDVLLFIDNIFR				Mascot
1949.0491	1949.0708	0.0217	11	262	277	DVNKQDVLLFIDNIFR	118	100		Mascot
2061.0322	2061.0659	0.0337	16	360	378	GIYPAVDPLDSTSTMLQP<br>R				Mascot
2077.0271	2077.0442	0.0171	8	360	378	GIYPAVDPLDSTSTMLQP<br>R			Oxidation (M)[15]	Mascot
2097.1008	2097.1152	0.0144	7	88	109	GMEVIDTGAPLSVPVGG<br>ATLGR				Mascot
2113.0959	2113.1067	0.0108	5	88	109	GMEVIDTGAPLSVPVGG<br>ATLGR			Oxidation (M)[2]	Mascot
2314.0691	2314.0925	0.0234	10	292	312	MPSAVGYQPTLSTEMGS<br>LQER			Oxidation (M)[1,15]	Mascot
2330.218	2330.0852	-0.1328	-57	427	447	IERFLSQPFFVAEVFTGS<br>AGK				Mascot
2694.3887	2694.4514	0.0627	23	110	134	IFNVLGEPVDNLGPVDSS<br>ATFPIHR				Mascot
2694.3887	2694.4514	0.0627	23	110	134	IFNVLGEPVDNLGPVDSS<br>ATFPIHR	170	100		Mascot
3325.856	3325.9202	0.0642	19	23	52	IDQIIGPVLDVTFPPGKLP<br>YIYNALVVQSR				Mascot
3714.8862	3714.9929	0.1067	29	319	354	GSITSIQAVYVPADDLTD				Mascot

Calc. Mass	Obsrv. Mass	± da	± ppm	Start Seq.	End Seq.	Sequence	Ion Score	C. I. %	Modification	Rank	Result Type
3842.9812	3843.0984	0.1172	30	318	354	PAPATTFAHLDATTVLSR KGSITSIQAVYVPADDLT DPAPATTFAHLDATTVLS R					Mascot

4	ATP synthase CF1 beta subunit [Festuca altissima]	gi|410177773	53939.1	5.31	24	956	100	50.126	787	100	

Protein Group

ATP synthase CF1 beta subunit [Festuca altissima]	gi|427436982	53939.1	5.3099 999427 7954

Peptide Information

Calc. Mass	Obsrv. Mass	± da	± ppm	Start Seq.	End Seq.	Sequence	Ion Score	C. I. %	Modification	Rank	Result Type
873.5152	873.5121	-0.0031	-4	391	397	VKETLQR					Mascot
1007.5771	1007.562	-0.0151	-15	146	154	LSIFETGIK					Mascot
1201.7052	1201.7001	-0.0051	-4	155	164	VVDLLAPYRR					Mascot
1254.5818	1254.5714	-0.0104	-8	76	87	AVAMSATDGLMR			Oxidation (M)[4,11]		Mascot
1328.6707	1328.6815	0.0108	8	192	205	AHGGVSVFGGVGER					Mascot
1328.6707	1328.6815	0.0108	8	192	205	AHGGVSVFGGVGER	116	100			Mascot
1416.6866	1416.6992	0.0126	9	379	390	IVGNEHYETAQR					Mascot
1416.6866	1416.6992	0.0126	9	379	390	IVGNEHYETAQR	91	100			Mascot
1433.7748	1433.7893	0.0145	10	278	291	FVQAGSEVSALLGR					Mascot
1433.7748	1433.7893	0.0145	10	278	291	FVQAGSEVSALLGR	113	100			Mascot
1471.7614	1471.7592	-0.0022	-1	249	261	VGLTALTMAEYFR					Mascot
1487.7563	1487.7515	-0.0048	-3	249	261	VGLTALTMAEYFR			Oxidation (M)[8]		Mascot
1487.7563	1487.7515	-0.0048	-3	249	261	VGLTALTMAEYFR	30	0	Oxidation (M)[8]		Mascot
1492.8159	1492.816	0.0001	0	266	277	QDVLLFIDNIFR					Mascot
1518.6564	1518.7524	0.096	63	206	217	TREGNDLYMEMK			Oxidation (M)[9,11]		Mascot
1535.858	1535.8685	0.0105	7	40	52	LPYIYNALVVQSR					Mascot
1535.858	1535.8685	0.0105	7	40	52	LPYIYNALVVQSR	102	100			Mascot
1601.8104	1601.818	0.0076	5	232	246	VALVYGQMNEPPGAR					Mascot
1617.8054	1617.8005	-0.0049	-3	232	246	VALVYGQMNEPPGAR			Oxidation (M)[8]		Mascot
1617.8054	1617.8005	-0.0049	-3	232	246	VALVYGQMNEPPGAR	49	97.673	Oxidation (M)[8]		Mascot
1790.8928	1790.9406	0.0478	27	247	261	MRVGLTALTMAEYFR			Oxidation (M)[1,10]		Mascot
1809.0157	1808.9768	-0.0389	-22	23	39	IDQIIGPVLDVTFPPGK					Mascot
1885.955	1885.9711	0.0161	9	58	73	QINVTCEVQQLLGNNR			Carbamidomethyl (C)[6]		Mascot
1949.0491	1949.0708	0.0217	11	262	277	DVNKQDVLLFIDNIFR					Mascot
1949.0491	1949.0708	0.0217	11	262	277	DVNKQDVLLFIDNIFR	118	100			Mascot
2061.0322	2061.0659	0.0337	16	360	378	GIYPAVDPLDSTSTMLQP R					Mascot

2077.0271	2077.0442	0.0171	8	360	378	GIYPAVDPLDSTSTMLQPR			Oxidation (M)[15]	Mascot
2097.1008	2097.1152	0.0144	7	88	109	GMEVIDTGAPLSVPVGGATLGR				Mascot
2113.0959	2113.1067	0.0108	5	88	109	GMEVIDTGAPLSVPVGGATLGR			Oxidation (M)[2]	Mascot
2314.0691	2314.0925	0.0234	10	292	312	MPSAVGYQPTLSTEMGSLQER			Oxidation (M)[1,15]	Mascot
2330.218	2330.0852	-0.1328	-57	427	447	IERFLSQPFFVAEVFTGSAGK				Mascot
2694.3887	2694.4514	0.0627	23	110	134	IFNVLGEPVDNLGPVDSSATFPIHR				Mascot
2694.3887	2694.4514	0.0627	23	110	134	IFNVLGEPVDNLGPVDSSATFPIHR	170	100		Mascot
3325.856	3325.9202	0.0642	19	23	52	IDQIIGPVLDVTFPPGKLPYIYNALVVQSR				Mascot
3714.8862	3714.9929	0.1067	29	319	354	GSITSIQAVYVPADDLTDPAPATTFAHLDATTVLSR				Mascot
3842.9812	3843.0984	0.1172	30	318	354	KGSITSIQAVYVPADDLTDPAPATTFAHLDATTVLSR				Mascot

5	ATP synthase CF1 beta subunit (chloroplast) [Secale cereale]	gi|525782223	53924.1	5.23	23	945	100	50.025	787	100

Protein Group

ATP synthase CF1 beta subunit (chloroplast) [Aegilops tauschii]	gi|533310112	53922	5.17000007629395
ATP synthase CF1 beta subunit (chloroplast) [Aegilops tauschii]	gi|384406859	53922	5.17000007629395
ATP synthase CF1 beta subunit (chloroplast) [Hordeum vulgare subsp. spontaneum]	gi|521301117	53898	5.17000007629395
ATP synthase CF1 beta subunit (chloroplast) [Hordeum vulgare subsp. spontaneum]	gi|521301036	53898	5.17000007629395
ATP synthase CF1 beta subunit (chloroplast) [Hordeum vulgare subsp. vulgare]	gi|521300959	53898	5.17000007629395
ATP synthase CF1 beta subunit (chloroplast) [Secale cereale]	gi|521301277	53924.1	5.23000001907349
ATP synthase CF1 beta subunit (chloroplast) [Triticum monococcum subsp. aegilopoides]	gi|521301354	53865	5.17000007629395
ATP synthase CF1 beta subunit (chloroplast) [Triticum monococcum]	gi|525778512	53865	5.17000007629395
ATP synthase CF1 beta subunit (chloroplast) [Triticum monococcum]	gi|521301197	53865	5.17000007629395
ATP synthase CF1 beta subunit (chloroplast) [Triticum urartu]	gi|521301433	53865	5.17000007629395
ATP synthase CF1 beta subunit (chloroplast) [Triticum	gi|525782302	53865	5.1700

urartu]			000762 9395
ATP synthase CF1 beta subunit [Agrostis stolonifera]	gi|118430309	53839.9	5.1599 998474 1211
ATP synthase CF1 beta subunit [Festuca ovina]	gi|426406646	53910	5.2300 000190 7349
ATP synthase CF1 beta subunit [Festuca ovina]	gi|410177860	53910	5.2300 000190 7349
ATP synthase CF1 beta subunit [Hordeum vulgare subsp. vulgare]	gi|118430395	53898	5.1700 000762 9395
RecName: Full=ATP synthase subunit beta, chloroplastic; AltName: Full=ATP synthase F1 sector subunit beta; AltName: Full=F-ATPase subunit beta	gi|126302525	53898	5.1700 000762 9395
RecName: Full=ATP synthase subunit beta, chloroplastic; AltName: Full=ATP synthase F1 sector subunit beta; AltName: Full=F-ATPase subunit beta	gi|125987717	53839.9	5.1599 998474 1211
RecName: Full=ATP synthase subunit beta, chloroplastic; AltName: Full=ATP synthase F1 sector subunit beta; AltName: Full=F-ATPase subunit beta	gi|50401828	53865	5.1700 000762 9395
RecName: Full=ATP synthase subunit beta, chloroplastic; AltName: Full=ATP synthase F1 sector subunit beta; AltName: Full=F-ATPase subunit beta	gi|50401827	53865	5.1700 000762 9395

Peptide Information

Calc. Mass	Obsrv. Mass	± da	± ppm	Start Seq.	End Seq.	Sequence	Ion Score	C. I. %	Modification	Rank	Result Type
873.5152	873.5121	-0.0031	-4	391	397	VKETLQR					Mascot
1007.5771	1007.562	-0.0151	-15	146	154	LSIFETGIK					Mascot
1201.7052	1201.7001	-0.0051	-4	155	164	VVDLLAPYRR					Mascot
1254.5818	1254.5714	-0.0104	-8	76	87	AVAMSATDGLMR			Oxidation (M)[4,11]		Mascot
1328.6707	1328.6815	0.0108	8	192	205	AHGGVSVFGGVGER					Mascot
1328.6707	1328.6815	0.0108	8	192	205	AHGGVSVFGGVGER	116	100			Mascot
1416.6866	1416.6992	0.0126	9	379	390	IVGNEHYETAQR					Mascot
1416.6866	1416.6992	0.0126	9	379	390	IVGNEHYETAQR	91	100			Mascot
1433.7748	1433.7893	0.0145	10	278	291	FVQAGSEVSALLGR					Mascot
1433.7748	1433.7893	0.0145	10	278	291	FVQAGSEVSALLGR	113	100			Mascot
1471.7614	1471.7592	-0.0022	-1	249	261	VGLTALTMAEYFR					Mascot
1487.7563	1487.7515	-0.0048	-3	249	261	VGLTALTMAEYFR			Oxidation (M)[8]		Mascot
1487.7563	1487.7515	-0.0048	-3	249	261	VGLTALTMAEYFR	30	0	Oxidation (M)[8]		Mascot
1492.8159	1492.816	0.0001	0	266	277	QDVLLFIDNIFR					Mascot
1518.6564	1518.7524	0.096	63	206	217	TREGNDLYMEMK			Oxidation (M)[9,11]		Mascot
1535.858	1535.8685	0.0105	7	40	52	LPYIYNALVVQSR					Mascot

1535.858	1535.8685	0.0105	7	40	52	LPYIYNALVVQSR	102	100			Mascot
1601.8104	1601.818	0.0076	5	232	246	VALVYGQMNEPPGAR					Mascot
1617.8054	1617.8005	-0.0049	-3	232	246	VALVYGQMNEPPGAR			Oxidation (M)[8]		Mascot
1617.8054	1617.8005	-0.0049	-3	232	246	VALVYGQMNEPPGAR	49	97.673	Oxidation (M)[8]		Mascot
1790.8928	1790.9406	0.0478	27	247	261	MRVGLTALTMAEYFR			Oxidation (M)[1,10]		Mascot
1809.0157	1808.9768	-0.0389	-22	23	39	IDQIIGPVLDVTFPPGK					Mascot
1885.955	1885.9711	0.0161	9	58	73	QINVTCEVQQLLGNNR			Carbamidomethyl (C)[6]		Mascot
1949.0491	1949.0708	0.0217	11	262	277	DVNKQDVLLFIDNIFR					Mascot
1949.0491	1949.0708	0.0217	11	262	277	DVNKQDVLLFIDNIFR	118	100			Mascot
2061.0322	2061.0659	0.0337	16	360	378	GIYPAVDPLDSTSTMLQP R					Mascot
2077.0271	2077.0442	0.0171	8	360	378	GIYPAVDPLDSTSTMLQP R			Oxidation (M)[15]		Mascot
2097.1008	2097.1152	0.0144	7	88	109	GMEVIDTGAPLSVPVGG ATLGR					Mascot
2113.0959	2113.1067	0.0108	5	88	109	GMEVIDTGAPLSVPVGG ATLGR			Oxidation (M)[2]		Mascot
2314.0691	2314.0925	0.0234	10	292	312	MPSAVGYQPTLSTEMGS LQER			Oxidation (M)[1,15]		Mascot
2694.3887	2694.4514	0.0627	23	110	134	IFNVLGEPVDNLGPVDSS ATFPIHR					Mascot
2694.3887	2694.4514	0.0627	23	110	134	IFNVLGEPVDNLGPVDSS ATFPIHR	170	100			Mascot
3325.856	3325.9202	0.0642	19	23	52	IDQIIGPVLDVTFPPGKLP YIYNALVVQSR					Mascot
3714.8862	3714.9929	0.1067	29	319	354	GSITSIQAVYVPADDLTD PAPATTFAHLDATTVLSR					Mascot
3842.9812	3843.0984	0.1172	30	318	354	KGSITSIQAVYVPADDLT DPAPATTFAHLDATTVLS R					Mascot

6	ATP synthase CF1 beta subunit [Lolium multiflorum]	gi|427437225	53840	5.23	22	831	100	45.21	686	100	

Protein Group

ATP synthase CF1 beta subunit [Festuca pratensis]	gi|427437079	53840	5.2300 000190 7349
ATP synthase CF1 beta subunit [Festuca pratensis]	gi|410177947	53840	5.2300 000190 7349
ATP synthase CF1 beta subunit [Lolium multiflorum]	gi|410178034	53840	5.2300 000190 7349
RecName: Full=ATP synthase subunit beta, chloroplastic; AltName: Full=ATP synthase F1 sector subunit beta; AltName: Full=F-ATPase subunit beta	gi|190358678	53840	5.2300 000190 7349
atpB gene product (chloroplast) [Lolium perenne]	gi|159106871	53840	5.2300 000190 7349

Peptide Information

Calc. Mass	Obsrv. Mass	± da	± ppm	Start Seq.	End Seq.	Sequence	Ion Score	C. I. %	Modification	Rank	Result Type

873.5152	873.5121	-0.0031	-4	391	397	VKETLQR				Mascot
1007.5771	1007.562	-0.0151	-15	146	154	LSIFETGIK				Mascot
1201.7052	1201.7001	-0.0051	-4	155	164	VVDLLAPYRR				Mascot
1254.5818	1254.5714	-0.0104	-8	76	87	AVAMSATDGLMR			Oxidation (M)[4,11]	Mascot
1328.6707	1328.6815	0.0108	8	192	205	AHGGVSVFGGVGER				Mascot
1328.6707	1328.6815	0.0108	8	192	205	AHGGVSVFGGVGER	116	100		Mascot
1416.6866	1416.6992	0.0126	9	379	390	IVGNEHYETAQR				Mascot
1416.6866	1416.6992	0.0126	9	379	390	IVGNEHYETAQR	91	100		Mascot
1433.7748	1433.7893	0.0145	10	278	291	FVQAGSEVSALLGR				Mascot
1433.7748	1433.7893	0.0145	10	278	291	FVQAGSEVSALLGR	113	100		Mascot
1471.7614	1471.7592	-0.0022	-1	249	261	VGLTALTMAEYFR				Mascot
1487.7563	1487.7515	-0.0048	-3	249	261	VGLTALTMAEYFR			Oxidation (M)[8]	Mascot
1487.7563	1487.7515	-0.0048	-3	249	261	VGLTALTMAEYFR	30	0	Oxidation (M)[8]	Mascot
1492.8159	1492.816	0.0001	0	266	277	QDVLLFIDNIFR				Mascot
1518.6564	1518.7524	0.096	63	206	217	TREGNDLYMEMK			Oxidation (M)[9,11]	Mascot
1601.8104	1601.818	0.0076	5	232	246	VALVYGQMNEPPGAR				Mascot
1617.8054	1617.8005	-0.0049	-3	232	246	VALVYGQMNEPPGAR			Oxidation (M)[8]	Mascot
1617.8054	1617.8005	-0.0049	-3	232	246	VALVYGQMNEPPGAR	49	97.673	Oxidation (M)[8]	Mascot
1790.8928	1790.9406	0.0478	27	247	261	MRVGLTALTMAEYFR			Oxidation (M)[1,10]	Mascot
1809.0157	1808.9768	-0.0389	-22	23	39	IDQIIGPVLDVTFPPGK				Mascot
1885.955	1885.9711	0.0161	9	58	73	QINVTCEVQQLLGNNR			Carbamidomethyl (C)[6]	Mascot
1949.0491	1949.0708	0.0217	11	262	277	DVNKQDVLLFIDNIFR				Mascot
1949.0491	1949.0708	0.0217	11	262	277	DVNKQDVLLFIDNIFR	118	100		Mascot
2061.0322	2061.0659	0.0337	16	360	378	GIYPAVDPLDSTSTMLQP<br>R				Mascot
2077.0271	2077.0442	0.0171	8	360	378	GIYPAVDPLDSTSTMLQP<br>R			Oxidation (M)[15]	Mascot
2097.1008	2097.1152	0.0144	7	88	109	GMEVIDTGAPLSVPVGG<br>ATLGR				Mascot
2113.0959	2113.1067	0.0108	5	88	109	GMEVIDTGAPLSVPVGG<br>ATLGR			Oxidation (M)[2]	Mascot
2314.0691	2314.0925	0.0234	10	292	312	MPSAVGYQPTLSTEMGS<br>LQER			Oxidation (M)[1,15]	Mascot
2330.218	2330.0852	-0.1328	-57	427	447	IERFLSQPFFVAEVFTGS<br>AGK				Mascot
2694.3887	2694.4514	0.0627	23	110	134	IFNVLGEPVDNLGPVDSS<br>ATFPIHR				Mascot
2694.3887	2694.4514	0.0627	23	110	134	IFNVLGEPVDNLGPVDSS<br>ATFPIHR	170	100		Mascot
3714.8862	3714.9929	0.1067	29	319	354	GSITSIQAVYVPADDLTD<br>PAPATTFAHLDATTVLSR				Mascot
3842.9812	3843.0984	0.1172	30	318	354	KGSITSIQAVYVPADDLT<br>DPAPATTFAHLDATTVLS<br>R				Mascot

7	ATP synthase subunit beta, chloroplastic [Triticum urartu]	gi|474022890	36113.7	5.21	18	818	100	40.107	686	100

Peptide Information

Calc. Mass	Obsrv. Mass	± da	± ppm	Start Seq.	End Seq.	Sequence	Ion Score	C. I. %	Modification	Rank	Result Type
873.5152	873.5121	-0.0031	-4	303	309	VKETLQR					Mascot
1007.5771	1007.562	-0.0151	-15	58	66	LSIFETGIK					Mascot
1201.7052	1201.7001	-0.0051	-4	67	76	VVDLLAPYRR					Mascot
1328.6707	1328.6815	0.0108	8	104	117	AHGGVSVFGGVGER					Mascot
1328.6707	1328.6815	0.0108	8	104	117	AHGGVSVFGGVGER	116	100			Mascot
1416.6866	1416.6992	0.0126	9	291	302	IVGNEHYETAQR					Mascot
1416.6866	1416.6992	0.0126	9	291	302	IVGNEHYETAQR	91	100			Mascot
1433.7748	1433.7893	0.0145	10	190	203	FVQAGSEVSALLGR					Mascot
1433.7748	1433.7893	0.0145	10	190	203	FVQAGSEVSALLGR	113	100			Mascot
1471.7614	1471.7592	-0.0022	-1	161	173	VGLTALTMAEYFR					Mascot
1487.7563	1487.7515	-0.0048	-3	161	173	VGLTALTMAEYFR			Oxidation (M)[8]		Mascot
1487.7563	1487.7515	-0.0048	-3	161	173	VGLTALTMAEYFR	30	0	Oxidation (M)[8]		Mascot
1492.8159	1492.816	0.0001	0	178	189	QDVLLFIDNIFR					Mascot
1518.6564	1518.7524	0.096	63	118	129	TREGNDLYMEMK			Oxidation (M)[9,11]		Mascot
1601.8104	1601.818	0.0076	5	144	158	VALVYGQMNEPPGAR					Mascot
1617.8054	1617.8005	-0.0049	-3	144	158	VALVYGQMNEPPGAR			Oxidation (M)[8]		Mascot
1617.8054	1617.8005	-0.0049	-3	144	158	VALVYGQMNEPPGAR	49	97.673	Oxidation (M)[8]		Mascot
1790.8928	1790.9406	0.0478	27	159	173	MRVGLTALTMAEYFR			Oxidation (M)[1,10]		Mascot
1909.0389	1909.0364	-0.0025	-1	2	21	EVIDTGAPLSVPVGGATL GR					Mascot
1949.0491	1949.0708	0.0217	11	174	189	DVNKQDVLLFIDNIFR					Mascot
1949.0491	1949.0708	0.0217	11	174	189	DVNKQDVLLFIDNIFR	118	100			Mascot
2061.0322	2061.0659	0.0337	16	272	290	GIYPAVDPLDSTSTMLQP R					Mascot
2077.0271	2077.0442	0.0171	8	272	290	GIYPAVDPLDSTSTMLQP R			Oxidation (M)[15]		Mascot
2314.0691	2314.0925	0.0234	10	204	224	MPSAVGYQPTLSTEMGS LQER			Oxidation (M)[1,15]		Mascot
2694.3887	2694.4514	0.0627	23	22	46	IFNVLGEPVDNLGPVDSS ATFPIHR					Mascot
2694.3887	2694.4514	0.0627	23	22	46	IFNVLGEPVDNLGPVDSS ATFPIHR	170	100			Mascot
3714.8862	3714.9929	0.1067	29	231	266	GSITSIQAVYVPADDLTD PAPATTFAHLDATTVLSR					Mascot
3842.9812	3843.0984	0.1172	30	230	266	KGSITSIQAVYVPADDLT DPAPATTFAHLDATTVLS R					Mascot

8	ATP synthase subunit beta, chloroplastic [Triticum	gi|474124791	50614.5	5.09	18	795	100	40.107	686	100

urartu]

Peptide Information

Calc. Mass	Obsrv. Mass	± da	± ppm	Start Seq.	End Seq.	Sequence	Ion Score	C. I. %	Modification	Rank	Result Type
873.5152	873.5121	-0.0031	-4	303	309	VKETLQR					Mascot
1007.5771	1007.562	-0.0151	-15	58	66	LSIFETGIK					Mascot
1201.7052	1201.7001	-0.0051	-4	67	76	VVDLLAPYRR					Mascot
1328.6707	1328.6815	0.0108	8	104	117	AHGGVSVFGGVGER					Mascot
1328.6707	1328.6815	0.0108	8	104	117	AHGGVSVFGGVGER	116	100			Mascot
1416.6866	1416.6992	0.0126	9	291	302	IVGNEHYETAQR					Mascot
1416.6866	1416.6992	0.0126	9	291	302	IVGNEHYETAQR	91	100			Mascot
1433.7748	1433.7893	0.0145	10	190	203	FVQAGSEVSALLGR					Mascot
1433.7748	1433.7893	0.0145	10	190	203	FVQAGSEVSALLGR	113	100			Mascot
1471.7614	1471.7592	-0.0022	-1	161	173	VGLTALTMAEYFR					Mascot
1487.7563	1487.7515	-0.0048	-3	161	173	VGLTALTMAEYFR			Oxidation (M)[8]		Mascot
1487.7563	1487.7515	-0.0048	-3	161	173	VGLTALTMAEYFR	30	0	Oxidation (M)[8]		Mascot
1492.8159	1492.816	0.0001	0	178	189	QDVLLFIDNIFR					Mascot
1518.6564	1518.7524	0.096	63	118	129	TREGNDLYMEMK			Oxidation (M)[9,11]		Mascot
1601.8104	1601.818	0.0076	5	144	158	VALVYGQMNEPPGAR					Mascot
1617.8054	1617.8005	-0.0049	-3	144	158	VALVYGQMNEPPGAR			Oxidation (M)[8]		Mascot
1617.8054	1617.8005	-0.0049	-3	144	158	VALVYGQMNEPPGAR	49	97.673	Oxidation (M)[8]		Mascot
1790.8928	1790.9406	0.0478	27	159	173	MRVGLTALTMAEYFR			Oxidation (M)[1,10]		Mascot
1909.0389	1909.0364	-0.0025	-1	2	21	EVIDTGAPLSVPVGGATLGR					Mascot
1949.0491	1949.0708	0.0217	11	174	189	DVNKQDVLLFIDNIFR					Mascot
1949.0491	1949.0708	0.0217	11	174	189	DVNKQDVLLFIDNIFR	118	100			Mascot
2061.0322	2061.0659	0.0337	16	272	290	GIYPAVDPLDSTSTMLQPR					Mascot
2077.0271	2077.0442	0.0171	8	272	290	GIYPAVDPLDSTSTMLQPR			Oxidation (M)[15]		Mascot
2314.0691	2314.0925	0.0234	10	204	224	MPSAVGYQPTLSTEMGSLQER			Oxidation (M)[1,15]		Mascot
2694.3887	2694.4514	0.0627	23	22	46	IFNVLGEPVDNLGPVDSSATFPIHR					Mascot
2694.3887	2694.4514	0.0627	23	22	46	IFNVLGEPVDNLGPVDSSATFPIHR	170	100			Mascot
3714.8862	3714.9929	0.1067	29	231	266	GSITSIQAVYVPADDLTDPAPATTFAHLDATTVLSR					Mascot
3842.9812	3843.0984	0.1172	30	230	266	KGSITSIQAVYVPADDLTDPAPATTFAHLDATTVLSR					Mascot

9	AtpB [Bambusa oldhamii]	gi|253729564	53936.2	5.47	21	753	100	41.168	617	100	

Protein Group

ATP synthase CF1 beta subunit [Bambusa emeiensis]	gi|340034458	53936.2	5.4699 997901 9165

Peptide Information

Calc. Mass	Obsrv. Mass	± da	± ppm	Start Seq.	End Seq.	Sequence	Ion Score	C. I. %	Modification	Rank	Result Type
1007.5771	1007.562	-0.0151	-15	146	154	LSIFETGIK					Mascot
1201.7052	1201.7001	-0.0051	-4	155	164	VVDLLAPYRR					Mascot
1254.5818	1254.5714	-0.0104	-8	76	87	AVAMSATDGLMR			Oxidation (M)[4,11]		Mascot
1328.6707	1328.6815	0.0108	8	192	205	AHGGVSVFGGVGER					Mascot
1328.6707	1328.6815	0.0108	8	192	205	AHGGVSVFGGVGER	116	100			Mascot
1416.6866	1416.6992	0.0126	9	379	390	IVGNEHYETAQR					Mascot
1416.6866	1416.6992	0.0126	9	379	390	IVGNEHYETAQR	91	100			Mascot
1433.7748	1433.7893	0.0145	10	278	291	FVQAGSEVSALLGR					Mascot
1433.7748	1433.7893	0.0145	10	278	291	FVQAGSEVSALLGR	113	100			Mascot
1471.7614	1471.7592	-0.0022	-1	249	261	VGLTALTMAEYFR					Mascot
1487.7563	1487.7515	-0.0048	-3	249	261	VGLTALTMAEYFR			Oxidation (M)[8]		Mascot
1487.7563	1487.7515	-0.0048	-3	249	261	VGLTALTMAEYFR	30	0	Oxidation (M)[8]		Mascot
1492.8159	1492.816	0.0001	0	266	277	QDVLLFIDNIFR					Mascot
1518.6564	1518.7524	0.096	63	206	217	TREGNDLYMEMK			Oxidation (M)[9,11]		Mascot
1535.8944	1535.8685	-0.0259	-17	40	52	LPYIYNALVVKSR					Mascot
1535.8944	1535.8685	-0.0259	-17	40	52	LPYIYNALVVKSR	102	100			Mascot
1601.8104	1601.818	0.0076	5	232	246	VALVYGQMNEPPGAR					Mascot
1617.8054	1617.8005	-0.0049	-3	232	246	VALVYGQMNEPPGAR			Oxidation (M)[8]		Mascot
1617.8054	1617.8005	-0.0049	-3	232	246	VALVYGQMNEPPGAR	49	97.673	Oxidation (M)[8]		Mascot
1790.8928	1790.9406	0.0478	27	247	261	MRVGLTALTMAEYFR			Oxidation (M)[1,10]		Mascot
1885.955	1885.9711	0.0161	9	58	73	QINVTCEVQQLLGNNR			Carbamidomethyl (C)[6]		Mascot
1949.0491	1949.0708	0.0217	11	262	277	DVNKQDVLLFIDNIFR					Mascot
1949.0491	1949.0708	0.0217	11	262	277	DVNKQDVLLFIDNIFR	118	100			Mascot
2061.0322	2061.0659	0.0337	16	360	378	GIYPAVDPLDSTSTMLQP R					Mascot
2077.0271	2077.0442	0.0171	8	360	378	GIYPAVDPLDSTSTMLQP R			Oxidation (M)[15]		Mascot
2097.1008	2097.1152	0.0144	7	88	109	GMEVIDTGAPLSVPVGG ATLGR					Mascot
2113.0959	2113.1067	0.0108	5	88	109	GMEVIDTGAPLSVPVGG ATLGR			Oxidation (M)[2]		Mascot
2314.0691	2314.0925	0.0234	10	292	312	MPSAVGYQPTLSTEMGS LQER			Oxidation (M)[1,15]		Mascot
2330.218	2330.0852	-0.1328	-57	427	447	IERFLSQPFFVAEVFTGS AGK					Mascot

2722.4199	2722.4614	0.0415	15	110	134	IFNVLGEPIDNLGPVDTSATFPIHR					Mascot
3714.8862	3714.9929	0.1067	29	319	354	GSITSIQAVYVPADDLTDPAPATTFAHLDATTVLSR					Mascot
3842.9812	3843.0984	0.1172	30	318	354	KGSITSIQAVYVPADDLTDPAPATTFAHLDATTVLSR					Mascot

10 ATP synthase CF1 subunit beta [Dendrocalamus latiflorus] gi|255961390 53936.1 5.37 21 753 100 41.168 617 100

Peptide Information

Calc. Mass	Obsrv. Mass	± da	± ppm	Start Seq.	End Seq.	Sequence	Ion Score	C. I. %	Modification	Rank	Result Type
1007.5771	1007.562	-0.0151	-15	146	154	LSIFETGIK					Mascot
1201.7052	1201.7001	-0.0051	-4	155	164	VVDLLAPYRR					Mascot
1254.5818	1254.5714	-0.0104	-8	76	87	AVAMSATDGLMR			Oxidation (M)[4,11]		Mascot
1328.6707	1328.6815	0.0108	8	192	205	AHGGVSVFGGVGER					Mascot
1328.6707	1328.6815	0.0108	8	192	205	AHGGVSVFGGVGER	116	100			Mascot
1416.6866	1416.6992	0.0126	9	379	390	IVGNEHYETAQR					Mascot
1416.6866	1416.6992	0.0126	9	379	390	IVGNEHYETAQR	91	100			Mascot
1433.7748	1433.7893	0.0145	10	278	291	FVQAGSEVSALLGR					Mascot
1433.7748	1433.7893	0.0145	10	278	291	FVQAGSEVSALLGR	113	100			Mascot
1471.7614	1471.7592	-0.0022	-1	249	261	VGLTALTMAEYFR					Mascot
1487.7563	1487.7515	-0.0048	-3	249	261	VGLTALTMAEYFR			Oxidation (M)[8]		Mascot
1487.7563	1487.7515	-0.0048	-3	249	261	VGLTALTMAEYFR	30	0	Oxidation (M)[8]		Mascot
1492.8159	1492.816	0.0001	0	266	277	QDVLLFIDNIFR					Mascot
1518.6564	1518.7524	0.096	63	206	217	TREGNDLYMEMK			Oxidation (M)[9,11]		Mascot
1535.858	1535.8685	0.0105	7	40	52	LPYIYNALVVQSR					Mascot
1535.858	1535.8685	0.0105	7	40	52	LPYIYNALVVQSR	102	100			Mascot
1601.8104	1601.818	0.0076	5	232	246	VALVYGQMNEPPGAR					Mascot
1617.8054	1617.8005	-0.0049	-3	232	246	VALVYGQMNEPPGAR			Oxidation (M)[8]		Mascot
1617.8054	1617.8005	-0.0049	-3	232	246	VALVYGQMNEPPGAR	49	97.673	Oxidation (M)[8]		Mascot
1790.8928	1790.9406	0.0478	27	247	261	MRVGLTALTMAEYFR			Oxidation (M)[1,10]		Mascot
1885.955	1885.9711	0.0161	9	58	73	QINVTCEVQQLLGNNR			Carbamidomethyl (C)[6]		Mascot
1949.0491	1949.0708	0.0217	11	262	277	DVNKQDVLLFIDNIFR					Mascot
1949.0491	1949.0708	0.0217	11	262	277	DVNKQDVLLFIDNIFR	118	100			Mascot
2061.0322	2061.0659	0.0337	16	360	378	GIYPAVDPLDSTSTMLQPR					Mascot
2077.0271	2077.0442	0.0171	8	360	378	GIYPAVDPLDSTSTMLQPR			Oxidation (M)[15]		Mascot
2097.1008	2097.1152	0.0144	7	88	109	GMEVIDTGAPLSVPVGGATLGR					Mascot

2113.0959	2113.1067	0.0108	5	88	109	GMEVIDTGAPLSVPVGG ATLGR	Oxidation (M)[2]	Mascot
2314.0691	2314.0925	0.0234	10	292	312	MPSAVGYQPTLSTEMGS LQER	Oxidation (M)[1,15]	Mascot
2330.218	2330.0852	-0.1328	-57	427	447	IERFLSQPFFVAEVFTGS AGK		Mascot
2722.4199	2722.4614	0.0415	15	110	134	IFNVLGEPIDNLGPVDTSA TFPIHR		Mascot
3714.8862	3714.9929	0.1067	29	319	354	GSITSIQAVYVPADDLTD PAPATTFAHLDATTVLSR		Mascot
3842.9812	3843.0984	0.1172	30	318	354	KGSITSIQAVYVPADDLT DPAPATTFAHLDATTVLS R		Mascot

Gel Idx/Pos	251/K3	Instr./Gel Origin	BA2151/Sample Project 20140814	Process Status	Analysis Succeeded
Plate [#] Name	[1] Sample Project 20140814	Instrument Sample Name		Spectra	11

Rank	Protein Name	Accession No.	Protein MW	Protein PI	Pep. Count	Protein Score	Protein Score C. I. %	Intensity Matched	Total Ion Score	Total Ion C. I. %	Confirmed
1	PREDICTED: tubulin alpha-1 chain-like [Setaria italica]	gi|514814302	50369.6	4.89	17	479	100	22.777	386	100	

Peptide Information

Calc. Mass	Obsrv. Mass	± da	± ppm	Start Seq.	End Seq.	Sequence	Ion Score	C. I. %	Modification	Rank	Result Type
1007.454	1007.4469	-0.0071	-7	97	105	EDAANNFAR					Mascot
1132.5668	1132.5575	-0.0093	-8	113	121	EIVDLCLDR			Carbamidomethyl (C)[6]		Mascot
1299.4989	1299.472	-0.0269	-21	312	320	YMACCLMYR			Carbamidomethyl (C)[4,5], Oxidation (M)[2,7]		Mascot
1396.693	1396.7186	0.0256	18	391	401	IDHKFDLMYAK			Oxidation (M)[8]		Mascot
1473.8635	1473.854	-0.0095	-6	230	243	LVSQVISSLTASLR					Mascot
1473.8635	1473.854	-0.0095	-6	230	243	LVSQVISSLTASLR	102	100			Mascot
1589.6844	1589.6995	0.0151	9	309	320	HGKYMACCLMYR			Carbamidomethyl (C)[7,8]		Mascot
1691.8711	1691.8533	-0.0178	-11	216	229	SLDIERPTYTNLNR					Mascot
1691.8711	1691.8533	-0.0178	-11	216	229	SLDIERPTYTNLNR	67	99.964			Mascot
1701.9059	1701.8979	-0.008	-5	65	79	AVFVDLEPTVIDEVR					Mascot
1701.9059	1701.8979	-0.008	-5	65	79	AVFVDLEPTVIDEVR	71	99.986			Mascot
1808.9252	1808.8848	-0.0404	-22	265	280	IHFMLSSYAPVISAEK			Oxidation (M)[4]		Mascot
1847.9723	1847.8995	-0.0728	-39	215	229	RSLDIERPTYTNLNR					Mascot
1885.9147	1885.9192	0.0045	2	374	390	AVCMISNSTSVVEVFSR			Carbamidomethyl (C)[3]		Mascot
1901.9097	1901.9373	0.0276	15	374	390	AVCMISNSTSVVEVFSR			Carbamidomethyl (C)[3], Oxidation (M)[4]		Mascot
1977.8826	1977.8553	-0.0273	-14	41	60	TVGGGDDAFNTFFSETGAGK					Mascot
2346.0132	2346.0142	0.001	0	403	422	AFVHWYVGEGMEEGEFSEAR			Oxidation (M)[11]		Mascot
2382.8528	2383.0151	0.1623	68	431	451	DYEEVGAEFDEGEEGDEGDEY					Mascot
2385.1946	2385.2019	0.0073	3	85	105	QLFHPEQLISGKEDAANNFAR					Mascot
2395.1746	2395.2053	0.0307	13	374	394	AVCMISNSTSVVEVFSRIDHK			Carbamidomethyl (C)[3], Oxidation (M)[4]		Mascot
2408.1882	2408.2029	0.0147	6	244	264	FDGALNVDVNEFQTNLVPYPR					Mascot
2408.1882	2408.2029	0.0147	6	244	264	FDGALNVDVNEFQTNLVPYPR	145	100			Mascot

Rank	Protein Name	Accession No.	Protein MW	Protein PI	Pep. Count	Protein Score	Protein Score C. I. %	Intensity Matched	Total Ion Score	Total Ion C. I. %	Confirmed
2	Os03g0726100 [Oryza sativa Japonica Group]	gi|113549603	50528.6	4.88	17	477	100	22.224	386	100	

Protein Group

alpha tubulin [Oryza sativa Japonica Group]	gi|385717660	50528.6	4.8800

	hypothetical protein OsI_13361 [Oryza sativa Indica Group]	gi|125545580	50528.6	001144 4092 4.8800 001144 4092		

Peptide Information

Calc. Mass	Obsrv. Mass	± da	± ppm	Start Seq.	End Seq.	Sequence	Ion Score	C. I. %	Modification	Rank	Result Type
1007.454	1007.4469	-0.0071	-7	97	105	EDAANNFAR					Mascot
1132.5668	1132.5575	-0.0093	-8	113	121	EIVDLCLDR			Carbamidomethyl (C)[6]		Mascot
1299.4989	1299.472	-0.0269	-21	312	320	YMACCLMYR			Carbamidomethyl (C)[4,5], Oxidation (M)[2,7]		Mascot
1396.693	1396.7186	0.0256	18	391	401	IDHKFDLMYSK					Mascot
1423.7693	1423.6896	-0.0797	-56	85	96	QLFHPEQLINGK					Mascot
1473.8635	1473.854	-0.0095	-6	230	243	LVSQVISSLTASLR					Mascot
1473.8635	1473.854	-0.0095	-6	230	243	LVSQVISSLTASLR	102	100			Mascot
1589.6844	1589.6995	0.0151	9	309	320	HGKYMACCLMYR			Carbamidomethyl (C)[7,8]		Mascot
1691.8711	1691.8533	-0.0178	-11	216	229	SLDIERPTYTNLNR					Mascot
1691.8711	1691.8533	-0.0178	-11	216	229	SLDIERPTYTNLNR	67	99.964			Mascot
1701.9059	1701.8979	-0.008	-5	65	79	AVFVDLEPTVIDEVR					Mascot
1701.9059	1701.8979	-0.008	-5	65	79	AVFVDLEPTVIDEVR	71	99.986			Mascot
1808.9252	1808.8848	-0.0404	-22	265	280	IHFMLSSYAPVISAEK			Oxidation (M)[4]		Mascot
1847.9723	1847.8995	-0.0728	-39	215	229	RSLDIERPTYTNLNR					Mascot
1885.9147	1885.9192	0.0045	2	374	390	AVCMISNSTSVVEVFSR			Carbamidomethyl (C)[3]		Mascot
1901.9426	1901.9373	-0.0053	-3	353	370	CGINYQPPSVVPSGDLAK			Carbamidomethyl (C)[1]		Mascot
1977.8826	1977.8553	-0.0273	-14	41	60	TVGGGDDAFNTFFSETGAGK					Mascot
2346.0132	2346.0142	0.001	0	403	422	AFVHWYVGEGMEEGEFSEAR			Oxidation (M)[11]		Mascot
2395.1746	2395.2053	0.0307	13	374	394	AVCMISNSTSVVEVFSRIDHK			Carbamidomethyl (C)[3], Oxidation (M)[4]		Mascot
2408.1882	2408.2029	0.0147	6	244	264	FDGALNVDVNEFQTNLVPYPR					Mascot
2408.1882	2408.2029	0.0147	6	244	264	FDGALNVDVNEFQTNLVPYPR	145	100			Mascot

3	RecName: Full=Tubulin alpha-1 chain; AltName: Full=Alpha-1-tubulin	gi|6094429	50383.6	4.89	16	470	100	22.599	386	100		

Peptide Information

Calc. Mass	Obsrv. Mass	± da	± ppm	Start Seq.	End Seq.	Sequence	Ion Score	C. I. %	Modification	Rank	Result Type
1007.454	1007.4469	-0.0071	-7	97	105	EDAANNFAR					Mascot
1132.5668	1132.5575	-0.0093	-8	113	121	EIVDLCLDR			Carbamidomethyl (C)[6]		Mascot
1299.4989	1299.472	-0.0269	-21	312	320	YMACCLMYR			Carbamidomethyl (C)[4,5], Oxidation (M)[2,7]		Mascot

1396.693	1396.7186	0.0256	18	391	401	IDHKFDLMYAK			Oxidation (M)[8]		Mascot
1473.8635	1473.854	-0.0095	-6	230	243	LVSQVISSLTASLR					Mascot
1473.8635	1473.854	-0.0095	-6	230	243	LVSQVISSLTASLR	102	100			Mascot
1589.6844	1589.6995	0.0151	9	309	320	HGKYMACCLMYR			Carbamidomethyl (C)[7,8]		Mascot
1691.8711	1691.8533	-0.0178	-11	216	229	SLDIERPTYTNLNR					Mascot
1691.8711	1691.8533	-0.0178	-11	216	229	SLDIERPTYTNLNR	67	99.964			Mascot
1701.9059	1701.8979	-0.008	-5	65	79	AVFVDLEPTVIDEVR					Mascot
1701.9059	1701.8979	-0.008	-5	65	79	AVFVDLEPTVIDEVR	71	99.986			Mascot
1808.9252	1808.8848	-0.0404	-22	265	280	IHFMLSSYAPVISAEK			Oxidation (M)[4]		Mascot
1847.9723	1847.8995	-0.0728	-39	215	229	RSLDIERPTYTNLNR					Mascot
1885.9147	1885.9192	0.0045	2	374	390	AVCMISNSTSVVEVFSR			Carbamidomethyl (C)[3]		Mascot
1901.9097	1901.9373	0.0276	15	374	390	AVCMISNSTSVVEVFSR			Carbamidomethyl (C)[3], Oxidation (M)[4]		Mascot
2346.0132	2346.0142	0.001	0	403	422	AFVHWYVGEGMEEGEF SEAR			Oxidation (M)[11]		Mascot
2382.8528	2383.0151	0.1623	68	431	451	DYEEVGAEFDEGEEGDE GDEY					Mascot
2385.1946	2385.2019	0.0073	3	85	105	QLFHPEQLISGKEDAANN FAR					Mascot
2395.1746	2395.2053	0.0307	13	374	394	AVCMISNSTSVVEVFSRI DHK			Carbamidomethyl (C)[3], Oxidation (M)[4]		Mascot
2408.1882	2408.2029	0.0147	6	244	264	FDGALNVDVNEFQTNLV PYPR					Mascot
2408.1882	2408.2029	0.0147	6	244	264	FDGALNVDVNEFQTNLV PYPR	145	100			Mascot

4	RecName: Full=Tubulin alpha-2 chain	gi|8928422	50353.6	4.88	16	470	100	22.494	386	100	

Protein Group

RecName: Full=Tubulin alpha chain	gi|8928408	50395.6	4.8899 998664 856
RecName: Full=Tubulin alpha-3 chain	gi|8928432	50381.6	4.8899 998664 856
Tubulin alpha chain [Aegilops tauschii]	gi|475602014	50367.6	4.8899 998664 856
Tubulin alpha chain [Triticum urartu]	gi|474224323	50367.6	4.8899 998664 856
Tubulin alpha-3 chain [Aegilops tauschii]	gi|475580203	50381.6	4.8899 998664 856
Tubulin alpha-3 chain [Triticum urartu]	gi|473887549	50381.6	4.8899 998664 856

Peptide Information

Calc. Mass	Obsrv. Mass	± da	± ppm	Start Seq.	End Seq.	Sequence	Ion Score	C. I. %	Modification	Rank	Result Type

1007.454	1007.4469	-0.0071	-7	97	105	EDAANNFAR					Mascot
1132.5668	1132.5575	-0.0093	-8	113	121	EIVDLCLDR			Carbamidomethyl (C)[6]		Mascot
1299.4989	1299.472	-0.0269	-21	312	320	YMACCLMYR			Carbamidomethyl (C)[4,5], Oxidation (M)[2,7]		Mascot
1396.693	1396.7186	0.0256	18	391	401	IDHKFDLMYAK			Oxidation (M)[8]		Mascot
1473.8635	1473.854	-0.0095	-6	230	243	LVSQVISSLTASLR					Mascot
1473.8635	1473.854	-0.0095	-6	230	243	LVSQVISSLTASLR	102	100			Mascot
1589.6844	1589.6995	0.0151	9	309	320	HGKYMACCLMYR			Carbamidomethyl (C)[7,8]		Mascot
1691.8711	1691.8533	-0.0178	-11	216	229	SLDIERPTYTNLNR					Mascot
1691.8711	1691.8533	-0.0178	-11	216	229	SLDIERPTYTNLNR	67	99.964			Mascot
1701.9059	1701.8979	-0.008	-5	65	79	AVFVDLEPTVIDEVR					Mascot
1701.9059	1701.8979	-0.008	-5	65	79	AVFVDLEPTVIDEVR	71	99.986			Mascot
1808.9252	1808.8848	-0.0404	-22	265	280	IHFMLSSYAPVISAEK			Oxidation (M)[4]		Mascot
1847.9723	1847.8995	-0.0728	-39	215	229	RSLDIERPTYTNLNR					Mascot
1885.9147	1885.9192	0.0045	2	374	390	AVCMISNSTSVVEVFSR			Carbamidomethyl (C)[3]		Mascot
1901.9097	1901.9373	0.0276	15	374	390	AVCMISNSTSVVEVFSR			Carbamidomethyl (C)[3], Oxidation (M)[4]		Mascot
1977.8826	1977.8553	-0.0273	-14	41	60	TVGGGDDAFNTFFSETG AGK					Mascot
2346.0132	2346.0142	0.001	0	403	422	AFVHWYVGEGMEEGEF SEAR			Oxidation (M)[11]		Mascot
2385.1946	2385.2019	0.0073	3	85	105	QLFHPEQLISGKEDAANN FAR					Mascot
2395.1746	2395.2053	0.0307	13	374	394	AVCMISNSTSVVEVFSRI DHK			Carbamidomethyl (C)[3], Oxidation (M)[4]		Mascot
2408.1882	2408.2029	0.0147	6	244	264	FDGALNVDVNEFQTNLV PYPR					Mascot
2408.1882	2408.2029	0.0147	6	244	264	FDGALNVDVNEFQTNLV PYPR	145	100			Mascot

5	TPA: alpha tubulin [Zea mays]	gi|414872552	50529.9	4.92	15	461	100	22.137	386	100	

Peptide Information

Calc. Mass	Obsrv. Mass	± da	± ppm	Start Seq.	End Seq.	Sequence	Ion Score	C. I. %	Modification	Rank	Result Type
1132.5668	1132.5575	-0.0093	-8	114	122	EIVDLCLDR			Carbamidomethyl (C)[6]		Mascot
1299.4989	1299.472	-0.0269	-21	313	321	YMACCLMYR			Carbamidomethyl (C)[4,5], Oxidation (M)[2,7]		Mascot
1396.693	1396.7186	0.0256	18	392	402	IDHKFDLMYAK			Oxidation (M)[8]		Mascot
1473.8635	1473.854	-0.0095	-6	231	244	LVSQVISSLTASLR					Mascot
1473.8635	1473.854	-0.0095	-6	231	244	LVSQVISSLTASLR	102	100			Mascot
1589.6844	1589.6995	0.0151	9	310	321	HGKYMACCLMYR			Carbamidomethyl (C)[7,8]		Mascot
1624.874	1624.7665	-0.1075	-66	85	97	QLFHPEQLIMGRR					Mascot
1691.8711	1691.8533	-0.0178	-11	217	230	SLDIERPTYTNLNR					Mascot
1691.8711	1691.8533	-0.0178	-11	217	230	SLDIERPTYTNLNR	67	99.964			Mascot

Calc. Mass	Obsrv. Mass	± da	± ppm	Start Seq.	End Seq.	Sequence	Ion Score	C. I. %	Modification	Rank	Result Type
1701.9059	1701.8979	-0.008	-5	65	79	AVFVDLEPTVIDEVR					Mascot
1701.9059	1701.8979	-0.008	-5	65	79	AVFVDLEPTVIDEVR	71	99.986			Mascot
1808.9252	1808.8848	-0.0404	-22	266	281	IHFMLSSYAPVISAEK			Oxidation (M)[4]		Mascot
1838.0569	1837.9268	-0.1301	-71	97	113	RMQPTTLPVVTTPVIGK					Mascot
1847.9723	1847.8995	-0.0728	-39	216	230	RSLDIERPTYTNLNR					Mascot
1854.0518	1853.9102	-0.1416	-76	97	113	RMQPTTLPVVTTPVIGK			Oxidation (M)[2]		Mascot
1885.9147	1885.9192	0.0045	2	375	391	AVCMISNSTSVVEVFSR			Carbamidomethyl (C)[3]		Mascot
1901.9097	1901.9373	0.0276	15	375	391	AVCMISNSTSVVEVFSR			Carbamidomethyl (C)[3], Oxidation (M)[4]		Mascot
2346.0132	2346.0142	0.001	0	404	423	AFVHWYVGEGMEEGEF SEAR			Oxidation (M)[11]		Mascot
2395.1746	2395.2053	0.0307	13	375	395	AVCMISNSTSVVEVFSRI DHK			Carbamidomethyl (C)[3], Oxidation (M)[4]		Mascot
2408.1882	2408.2029	0.0147	6	245	265	FDGALNVDVNEFQTNLV PYPR					Mascot
2408.1882	2408.2029	0.0147	6	245	265	FDGALNVDVNEFQTNLV PYPR	145	100			Mascot

6	RecName: Full=Tubulin alpha-1 chain; AltName: Full=Alpha-1-tubulin	gi|135398	50383.6	4.89	15	461	100	22.316	386	100

Protein Group

RecName: Full=Tubulin alpha-2 chain; AltName: Full=Alpha-2-tubulin	gi|135411	50383.6	4.8899998664856
TPA: tubulin alpha-1 chain [Zea mays]	gi|414872560	50383.6	4.8899998664856
TPA: tubulin alpha-1 chain [Zea mays]	gi|414872557	50383.6	4.8899998664856
TPA: tubulin alpha-1 chain [Zea mays]	gi|414872555	50383.6	4.8899998664856
TPA: tubulin alpha-1 chain [Zea mays]	gi|414872556	50383.6	4.8899998664856
TPA: tubulin alpha-2 chain [Zea mays]	gi|414872554	50383.6	4.8899998664856
alpha tubulin4 isoform 1 [Zea mays]	gi|413933195	50369.6	4.8899998664856
alpha tubulin4 isoform 2 [Zea mays]	gi|413933196	50369.6	4.8899998664856
tubulin alpha-3 chain [Zea mays]	gi|293331323	50369.6	4.8899998664856

Peptide Information

Calc. Mass	Obsrv. Mass	± da	± ppm	Start Seq.	End Seq.	Sequence	Ion Score	C. I. %	Modification	Rank	Result Type

Calc. Mass	Obsrv. Mass	± da	± ppm	Start	End	Sequence	Ion	C. I. %	Modification	Rank	Result Type
1007.454	1007.4469	-0.0071	-7	97	105	EDAANNFAR					Mascot
1132.5668	1132.5575	-0.0093	-8	113	121	EIVDLCLDR			Carbamidomethyl (C)[6]		Mascot
1299.4989	1299.472	-0.0269	-21	312	320	YMACCLMYR			Carbamidomethyl (C)[4,5], Oxidation (M)[2,7]		Mascot
1396.693	1396.7186	0.0256	18	391	401	IDHKFDLMYAK			Oxidation (M)[8]		Mascot
1473.8635	1473.854	-0.0095	-6	230	243	LVSQVISSLTASLR					Mascot
1473.8635	1473.854	-0.0095	-6	230	243	LVSQVISSLTASLR	102	100			Mascot
1589.6844	1589.6995	0.0151	9	309	320	HGKYMACCLMYR			Carbamidomethyl (C)[7,8]		Mascot
1691.8711	1691.8533	-0.0178	-11	216	229	SLDIERPTYTNLNR					Mascot
1691.8711	1691.8533	-0.0178	-11	216	229	SLDIERPTYTNLNR	67	99.964			Mascot
1701.9059	1701.8979	-0.008	-5	65	79	AVFVDLEPTVIDEVR					Mascot
1701.9059	1701.8979	-0.008	-5	65	79	AVFVDLEPTVIDEVR	71	99.986			Mascot
1808.9252	1808.8848	-0.0404	-22	265	280	IHFMLSSYAPVISAEK			Oxidation (M)[4]		Mascot
1847.9723	1847.8995	-0.0728	-39	215	229	RSLDIERPTYTNLNR					Mascot
1885.9147	1885.9192	0.0045	2	374	390	AVCMISNSTSVVEVFSR			Carbamidomethyl (C)[3]		Mascot
1901.9097	1901.9373	0.0276	15	374	390	AVCMISNSTSVVEVFSR			Carbamidomethyl (C)[3], Oxidation (M)[4]		Mascot
2346.0132	2346.0142	0.001	0	403	422	AFVHWYVGEGMEEGEF SEAR			Oxidation (M)[11]		Mascot
2385.1946	2385.2019	0.0073	3	85	105	QLFHPEQLISGKEDAANN FAR					Mascot
2395.1746	2395.2053	0.0307	13	374	394	AVCMISNSTSVVEVFSRI DHK			Carbamidomethyl (C)[3], Oxidation (M)[4]		Mascot
2408.1882	2408.2029	0.0147	6	244	264	FDGALNVDVNEFQTNLV PYPR					Mascot
2408.1882	2408.2029	0.0147	6	244	264	FDGALNVDVNEFQTNLV PYPR	145	100			Mascot

7	alpha-tubulin [Oryza sativa Japonica Group]	gi|385717688	50389.6	4.81	15	460	100	22.273	386	100		

Protein Group

Os11g0247300 [Oryza sativa Japonica Group]	gi|113644835	50389.6	4.8099 999427 7954
RecName: Full=Tubulin alpha-2 chain	gi|75283205	50389.6	4.8099 999427 7954
Tubulin alpha-3 chain, putative, expressed [Oryza sativa Japonica Group]	gi|77549556	50389.6	4.8099 999427 7954
Tubulin alpha-3 chain, putative, expressed [Oryza sativa Japonica Group]	gi|77549555	50389.6	4.8099 999427 7954
hypothetical protein OsI_35689 [Oryza sativa Indica Group]	gi|125533963	50389.6	4.8099 999427 7954

Peptide Information

Calc. Mass	Obsrv. Mass	± da	± ppm	Start	End	Sequence	Ion	C. I. %	Modification	Rank	Result Type

				Seq.	Seq.		Score				
1007.454	1007.4469	-0.0071	-7	97	105	EDAANNFAR					Mascot
1132.5668	1132.5575	-0.0093	-8	113	121	EIVDLCLDR			Carbamidomethyl (C)[6]		Mascot
1299.4989	1299.472	-0.0269	-21	312	320	YMACCLMYR			Carbamidomethyl (C)[4,5], Oxidation (M)[2,7]		Mascot
1396.7584	1396.7186	-0.0398	-28	85	96	QLFHPEQLISGK					Mascot
1473.8635	1473.854	-0.0095	-6	230	243	LVSQVISSLTASLR					Mascot
1473.8635	1473.854	-0.0095	-6	230	243	LVSQVISSLTASLR	102	100			Mascot
1589.6844	1589.6995	0.0151	9	309	320	HGKYMACCLMYR			Carbamidomethyl (C)[7,8]		Mascot
1691.8711	1691.8533	-0.0178	-11	216	229	SLDIERPTYTNLNR					Mascot
1691.8711	1691.8533	-0.0178	-11	216	229	SLDIERPTYTNLNR	67	99.964			Mascot
1701.9059	1701.8979	-0.008	-5	65	79	AVFVDLEPTVIDEVR					Mascot
1701.9059	1701.8979	-0.008	-5	65	79	AVFVDLEPTVIDEVR	71	99.986			Mascot
1808.9252	1808.8848	-0.0404	-22	265	280	IHFMLSSYAPVISAEK			Oxidation (M)[4]		Mascot
1847.9723	1847.8995	-0.0728	-39	215	229	RSLDIERPTYTNLNR					Mascot
1885.9147	1885.9192	0.0045	2	374	390	AVCMISNSTSVVEVFSR			Carbamidomethyl (C)[3]		Mascot
1901.9097	1901.9373	0.0276	15	374	390	AVCMISNSTSVVEVFSR			Carbamidomethyl (C)[3], Oxidation (M)[4]		Mascot
1977.8826	1977.8553	-0.0273	-14	41	60	TVGGGDDAFNTFFSETG AGK					Mascot
2346.0132	2346.0142	0.001	0	403	422	AFVHWYVGEGMEEGEF SEAR			Oxidation (M)[11]		Mascot
2385.1946	2385.2019	0.0073	3	85	105	QLFHPEQLISGKEDAANN FAR					Mascot
2408.1882	2408.2029	0.0147	6	244	264	FDGALNVDVNEFQTNLV PYPR					Mascot
2408.1882	2408.2029	0.0147	6	244	264	FDGALNVDVNEFQTNLV PYPR	145	100			Mascot

8	PREDICTED: tubulin alpha chain-like [Solanum lycopersicum]	gi|460372283	50354.7	5.1	13	444	100	21.161	386	100		

Peptide Information

Calc. Mass	Obsrv. Mass	± da	± ppm	Start Seq.	End Seq.	Sequence	Ion Score	C. I. %	Modification	Rank	Result Type
1007.454	1007.4469	-0.0071	-7	97	105	EDAANNFAR					Mascot
1132.5668	1132.5575	-0.0093	-8	113	121	EIVDLCLDR			Carbamidomethyl (C)[6]		Mascot
1299.4989	1299.472	-0.0269	-21	312	320	YMACCLMYR			Carbamidomethyl (C)[4,5], Oxidation (M)[2,7]		Mascot
1396.693	1396.7186	0.0256	18	391	401	IDHKFDLMYAK			Oxidation (M)[8]		Mascot
1473.8635	1473.854	-0.0095	-6	230	243	LVSQVISSLTASLR					Mascot
1473.8635	1473.854	-0.0095	-6	230	243	LVSQVISSLTASLR	102	100			Mascot
1624.9269	1624.7665	-0.1604	-99	321	336	GDVVPKDVNAAVSIIK					Mascot
1691.8711	1691.8533	-0.0178	-11	216	229	SLDIERPTYTNLNR					Mascot
1691.8711	1691.8533	-0.0178	-11	216	229	SLDIERPTYTNLNR	67	99.964			Mascot

Calc. Mass	Obsrv. Mass	± da	± ppm	Start Seq.	End Seq.	Sequence	Ion Score	C. I. %	Modification	Rank	Result Type
1701.9059	1701.8979	-0.008	-5	65	79	AVFVDLEPTVIDEVR					Mascot
1701.9059	1701.8979	-0.008	-5	65	79	AVFVDLEPTVIDEVR	71	99.986			Mascot
1808.9252	1808.8848	-0.0404	-22	265	280	IHFMLSSYAPVISAEK			Oxidation (M)[4]		Mascot
1847.9723	1847.8995	-0.0728	-39	215	229	RSLDIERPTYTNLNR					Mascot
2346.0132	2346.0142	0.001	0	403	422	AFVHWYVGEGMEEGEF SEAR			Oxidation (M)[11]		Mascot
2385.1946	2385.2019	0.0073	3	85	105	QLFHPEQLISGKEDAANN FAR					Mascot
2408.1882	2408.2029	0.0147	6	244	264	FDGALNVDVNEFQTNLV PYPR					Mascot
2408.1882	2408.2029	0.0147	6	244	264	FDGALNVDVNEFQTNLV PYPR	145	100			Mascot

9	alpha tubulin4 [Zea mays]	gi|413933197	40963.8	4.85	13	387	100	18.181	319	100	

Peptide Information

Calc. Mass	Obsrv. Mass	± da	± ppm	Start Seq.	End Seq.	Sequence	Ion Score	C. I. %	Modification	Rank	Result Type
1007.454	1007.4469	-0.0071	-7	97	105	EDAANNFAR					Mascot
1132.5668	1132.5575	-0.0093	-8	113	121	EIVDLCLDR			Carbamidomethyl (C)[6]		Mascot
1299.4989	1299.472	-0.0269	-21	225	233	YMACCLMYR			Carbamidomethyl (C)[4,5], Oxidation (M)[2,7]		Mascot
1396.693	1396.7186	0.0256	18	304	314	IDHKFDLMYAK			Oxidation (M)[8]		Mascot
1473.8635	1473.854	-0.0095	-6	143	156	LVSQVISSLTASLR					Mascot
1473.8635	1473.854	-0.0095	-6	143	156	LVSQVISSLTASLR	102	100			Mascot
1589.6844	1589.6995	0.0151	9	222	233	HGKYMACCLMYR			Carbamidomethyl (C)[7,8]		Mascot
1701.9059	1701.8979	-0.008	-5	65	79	AVFVDLEPTVIDEVR					Mascot
1701.9059	1701.8979	-0.008	-5	65	79	AVFVDLEPTVIDEVR	71	99.986			Mascot
1808.9252	1808.8848	-0.0404	-22	178	193	IHFMLSSYAPVISAEK			Oxidation (M)[4]		Mascot
1885.9147	1885.9192	0.0045	2	287	303	AVCMISNSTSVVEVFSR			Carbamidomethyl (C)[3]		Mascot
1901.9097	1901.9373	0.0276	15	287	303	AVCMISNSTSVVEVFSR			Carbamidomethyl (C)[3], Oxidation (M)[4]		Mascot
2346.0132	2346.0142	0.001	0	316	335	AFVHWYVGEGMEEGEF SEAR			Oxidation (M)[11]		Mascot
2385.1946	2385.2019	0.0073	3	85	105	QLFHPEQLISGKEDAANN FAR					Mascot
2395.1746	2395.2053	0.0307	13	287	307	AVCMISNSTSVVEVFSRI DHK			Carbamidomethyl (C)[3], Oxidation (M)[4]		Mascot
2408.1882	2408.2029	0.0147	6	157	177	FDGALNVDVNEFQTNLV PYPR					Mascot
2408.1882	2408.2029	0.0147	6	157	177	FDGALNVDVNEFQTNLV PYPR	145	100			Mascot

10	Tubulin alpha-3 chain, putative, expressed [Oryza sativa Japonica Group]	gi|77549557	38809.2	4.9	12	376	100	13.56	315	100	

Peptide Information

Calc. Mass	Obsrv. Mass	± da	± ppm	Start Seq.	End Seq.	Sequence	Ion Score	C. I. %	Modification	Rank	Result Type

1105.6001	1105.5554	-0.0447	-40	49	58	APRLSVDYGK				Mascot
1299.4989	1299.472	-0.0269	-21	207	215	YMACCLMYR			Carbamidomethyl (C)[4,5], Oxidation (M)[2,7]	Mascot
1407.6785	1407.6097	-0.0688	-49	37	48	ASLCSTLLEEER			Carbamidomethyl (C)[4]	Mascot
1473.8635	1473.854	-0.0095	-6	125	138	LVSQVISSLTASLR				Mascot
1473.8635	1473.854	-0.0095	-6	125	138	LVSQVISSLTASLR	102	100		Mascot
1589.6844	1589.6995	0.0151	9	204	215	HGKYMACCLMYR			Carbamidomethyl (C)[7,8]	Mascot
1624.9092	1624.7665	-0.1427	-88	1	15	MQPTTLPVVTTPLAR				Mascot
1691.8711	1691.8533	-0.0178	-11	111	124	SLDIERPTYTNLNR				Mascot
1691.8711	1691.8533	-0.0178	-11	111	124	SLDIERPTYTNLNR	67	99.959		Mascot
1808.9252	1808.8848	-0.0404	-22	160	175	IHFMLSSYAPVISAEK			Oxidation (M)[4]	Mascot
1847.9723	1847.8995	-0.0728	-39	110	124	RSLDIERPTYTNLNR				Mascot
1885.9147	1885.9192	0.0045	2	269	285	AVCMISNSTSVVEVFSR			Carbamidomethyl (C)[3]	Mascot
1901.9097	1901.9373	0.0276	15	269	285	AVCMISNSTSVVEVFSR			Carbamidomethyl (C)[3], Oxidation (M)[4]	Mascot
2346.0132	2346.0142	0.001	0	298	317	AFVHWYVGEGMEEGEF SEAR			Oxidation (M)[11]	Mascot
2408.1882	2408.2029	0.0147	6	139	159	FDGALNVDVNEFQTNLV PYPR				Mascot
2408.1882	2408.2029	0.0147	6	139	159	FDGALNVDVNEFQTNLV PYPR	145	100		Mascot

Gel Idx/Pos	252/K4	Instr./Gel Origin	BA2151/Sample Project 20140814	Process Status	Analysis Succeeded
Plate [#] Name	[1] Sample Project 20140814	Instrument Sample Name		Spectra	11

Rank	Protein Name	Accession No.	Protein MW	Protein PI	Pep. Count	Protein Score	Protein Score C. I. %	Intensity Matched	Total Ion Score	Total Ion C. I. %	Confirmed
1	Beta-amylase [Aegilops tauschii]	gi|475523854	60203.5	5.07	17	269	100	25.293	190	100	

Peptide Information

Calc. Mass	Obsrv. Mass	± da	± ppm	Start Seq.	End Seq.	Sequence	Ion Score	C. I. %	Modification	Rank	Result Type
802.4305	802.4324	0.0019	2	276	282	ILDEANK					Mascot
947.5057	947.4904	-0.0153	-16	322	329	DGYRPIAR					Mascot
947.5057	947.4904	-0.0153	-16	322	329	DGYRPIAR	23	0			Mascot
1016.5564	1016.5534	-0.003	-3	412	419	LFGFTYLR					Mascot
1016.5564	1016.5534	-0.003	-3	412	419	LFGFTYLR	48	96.732			Mascot
1237.5444	1237.5525	0.0081	7	233	243	DAGTYNDTPQR					Mascot
1326.6688	1326.6642	-0.0046	-3	385	395	YDPTAYNTILR					Mascot
1326.6688	1326.6642	-0.0046	-3	385	395	YDPTAYNTILR	45	93.524			Mascot
1335.7202	1335.677	-0.0432	-32	322	332	DGYRPIARMLK			Oxidation (M)[9]		Mascot
1474.6777	1474.6841	0.0064	4	372	384	EGLNMACENALPR			Carbamidomethyl (C)[7]		Mascot
1490.6726	1490.6832	0.0106	7	372	384	EGLNMACENALPR			Carbamidomethyl (C)[7], Oxidation (M)[5]		Mascot
1589.6948	1589.6962	0.0014	1	334	346	HHASLNFTCAEMR			Carbamidomethyl (C)[9], Oxidation (M)[12]		Mascot
1646.781	1646.7871	0.0061	4	246	259	FFVDNGTYLTEQGR					Mascot
1668.7952	1668.7889	-0.0063	-4	218	232	AAAAMVGHPEWEFPR					Mascot
1671.7142	1671.6986	-0.0156	-9	148	161	SAVQMYADYMASFR			Oxidation (M)[5,10]		Mascot
1671.7142	1671.6986	-0.0156	-9	148	161	SAVQMYADYMASFR			Oxidation (M)[5,10]		Mascot
1684.79	1684.7834	-0.0066	-4	218	232	AAAAMVGHPEWEFPR			Oxidation (M)[5]		Mascot
1729.801	1729.8627	0.0617	36	333	346	RHHASLNFTCAEMR			Carbamidomethyl (C)[10]		Mascot
1752.8916	1752.866	-0.0256	-15	420	434	LSNQLVEGQNYVNFK					Mascot
1841.9678	1841.9316	-0.0362	-20	459	475	SGPELTIEMILQAAQPK			Oxidation (M)[9]		Mascot
1999.9622	1999.9746	0.0124	6	304	321	VPSHAAEVTAGYYNLHDR					Mascot
1999.9622	1999.9746	0.0124	6	304	321	VPSHAAEVTAGYYNLHDR	73	99.99			Mascot
2087.0557	2087.0625	0.0068	3	130	147	NIEYLTLGVDDQPLFHGR					Mascot
2183.0486	2183.0332	-0.0154	-7	440	458	MHANLPHDPCVDPVAPLQR			Carbamidomethyl (C)[10], Oxidation (M)[1]		Mascot

Rank	Protein Name	Accession No.	Protein MW	Protein PI	Pep. Count	Protein Score	Protein Score C. I. %	Intensity Matched	Total Ion Score	Total Ion C. I. %	Confirmed
2	beta-amylase 2, partial [Brachypodium distachyon]	gi|482677643	46785.2	5.84	13	175	100	20.181	116	100	

Peptide Information

Calc. Mass	Obsrv. Mass	± da	± ppm	Start Seq.	End Seq.	Sequence	Ion Score	C. I. %	Modification	Rank	Result Type
802.4305	802.4324	0.0019	2	266	272	ILDEANK					Mascot
947.5057	947.4904	-0.0153	-16	312	319	DGYRPIAR					Mascot
947.5057	947.4904	-0.0153	-16	312	319	DGYRPIAR	23	0			Mascot
1016.5564	1016.5534	-0.003	-3	402	409	LFGFTYLR					Mascot
1016.5564	1016.5534	-0.003	-3	402	409	LFGFTYLR	48	96.732			Mascot
1237.5444	1237.5525	0.0081	7	223	233	DAGTYNDTPQR					Mascot
1326.6688	1326.6642	-0.0046	-3	375	385	YDPTAYNTILR					Mascot
1326.6688	1326.6642	-0.0046	-3	375	385	YDPTAYNTILR	45	93.524			Mascot
1335.7202	1335.677	-0.0432	-32	312	322	DGYRPIARMLK			Oxidation (M)[9]		Mascot
1474.6777	1474.6841	0.0064	4	362	374	EGLNMACENALPR			Carbamidomethyl (C)[7]		Mascot
1490.6726	1490.6832	0.0106	7	362	374	EGLNMACENALPR			Carbamidomethyl (C)[7], Oxidation (M)[5]		Mascot
1603.7104	1603.7706	0.0602	38	324	336	HHASLNFTCTEMR			Carbamidomethyl (C)[9]		Mascot
1646.781	1646.7871	0.0061	4	236	249	FFVDNGTYLTEQGR					Mascot
1667.7734	1667.8256	0.0522	31	138	151	SAVQLYTDYMASFR			Oxidation (M)[10]		Mascot
1668.7952	1668.7889	-0.0063	-4	208	222	AAAAMVGHPEWEFPR					Mascot
1684.79	1684.7834	-0.0066	-4	208	222	AAAAMVGHPEWEFPR			Oxidation (M)[5]		Mascot
1842.0425	1841.9316	-0.1109	-60	279	293	VQLAIKISGIHWWYK					Mascot
2087.0557	2087.0625	0.0068	3	120	137	NIEYLTLGVDDQPLFHGR					Mascot

3	Beta-amylase [Triticum urartu]	gi|474451266	58995	5.34	13	166	100	20.227	116	100	

Peptide Information

Calc. Mass	Obsrv. Mass	± da	± ppm	Start Seq.	End Seq.	Sequence	Ion Score	C. I. %	Modification	Rank	Result Type
802.4305	802.4324	0.0019	2	276	282	ILDEANK					Mascot
947.5057	947.4904	-0.0153	-16	322	329	DGYRPIAR					Mascot
947.5057	947.4904	-0.0153	-16	322	329	DGYRPIAR	23	0			Mascot
1016.5564	1016.5534	-0.003	-3	412	419	LFGFTYLR					Mascot
1016.5564	1016.5534	-0.003	-3	412	419	LFGFTYLR	48	96.732			Mascot
1326.6688	1326.6642	-0.0046	-3	385	395	YDPTAYNTILR					Mascot
1326.6688	1326.6642	-0.0046	-3	385	395	YDPTAYNTILR	45	93.524			Mascot
1335.7202	1335.677	-0.0432	-32	322	332	DGYRPIARMLK			Oxidation (M)[9]		Mascot
1474.6777	1474.6841	0.0064	4	372	384	EGLNMACENALPR			Carbamidomethyl (C)[7]		Mascot
1490.6726	1490.6832	0.0106	7	372	384	EGLNMACENALPR			Carbamidomethyl (C)[7], Oxidation (M)[5]		Mascot
1646.781	1646.7871	0.0061	4	246	259	FFVDNGTYLTEQGR					Mascot
1668.7952	1668.7889	-0.0063	-4	218	232	AAAAMVGHPEWEFPR					Mascot

1684.79	1684.7834	-0.0066	-4	218	232	AAAAMVGHPEWEFPR			Oxidation (M)[5]		Mascot
1701.7247	1701.8429	0.1182	69	148	161	SAVQMYTDYMASFR			Oxidation (M)[5,10]		Mascot
1752.8916	1752.866	-0.0256	-15	420	434	LSNQLVEGQNYVNFK					Mascot
1841.9678	1841.9316	-0.0362	-20	459	475	SGPELTIEMILQAAQPK			Oxidation (M)[9]		Mascot
2087.0557	2087.0625	0.0068	3	130	147	NIEYLTLGVDDQPLFHGR					Mascot
2183.0486	2183.0332	-0.0154	-7	440	458	MHANLPHDPCVDPVAPLQR			Carbamidomethyl (C)[10], Oxidation (M)[1]		Mascot

4	RecName: Full=Beta-amylase; AltName: Full=1,4-alpha-D-glucan maltohydrolase; AltName: Full=Beta-Amy1; Flags: Precursor	gi|75107132	59886.4	5.66	11	127	100	15.78	93	100

Peptide Information

Calc. Mass	Obsrv. Mass	± da	± ppm	Start Seq.	End Seq.	Sequence	Ion Score	C. I. %	Modification	Rank	Result Type
802.4305	802.4324	0.0019	2	275	281	ILDEANK					Mascot
1016.5564	1016.5534	-0.003	-3	411	418	LFGFTYLR					Mascot
1016.5564	1016.5534	-0.003	-3	411	418	LFGFTYLR	48	96.732			Mascot
1253.6121	1253.5869	-0.0252	-20	248	258	DNGTYLTEKGR					Mascot
1285.6212	1285.6085	-0.0127	-10	61	71	GPKAYDWSAYK					Mascot
1326.6688	1326.6642	-0.0046	-3	384	394	YDPTAYNTILR					Mascot
1326.6688	1326.6642	-0.0046	-3	384	394	YDPTAYNTILR	45	93.524			Mascot
1370.6774	1370.6747	-0.0027	-2	446	457	DPYVDPMAPLPR					Mascot
1701.7247	1701.8429	0.1182	69	147	160	SAVQMYADYMTSFR			Oxidation (M)[5,10]		Mascot
1728.9221	1728.8381	-0.084	-49	257	270	GRFFLAWYSNNLIK					Mascot
1752.8916	1752.866	-0.0256	-15	419	433	LSNQLVEGQNYVNFK					Mascot
1827.9884	1827.9443	-0.0441	-24	458	474	SGPEISIEMILQAAKPK			Oxidation (M)[9]		Mascot
1842.0425	1841.9316	-0.1109	-60	288	302	VQLAIKISGIHWWYK					Mascot

5	RecName: Full=Beta-amylase; AltName: Full=1,4-alpha-D-glucan maltohydrolase	gi|113786	59894.5	5.58	10	122	100	15.445	93	100

Peptide Information

Calc. Mass	Obsrv. Mass	± da	± ppm	Start Seq.	End Seq.	Sequence	Ion Score	C. I. %	Modification	Rank	Result Type
802.4305	802.4324	0.0019	2	275	281	ILDEANK					Mascot
1016.5564	1016.5534	-0.003	-3	411	418	LFGFTYLR					Mascot
1016.5564	1016.5534	-0.003	-3	411	418	LFGFTYLR	48	96.732			Mascot
1285.6212	1285.6085	-0.0127	-10	61	71	GPKAYDWSAYK					Mascot
1326.6688	1326.6642	-0.0046	-3	384	394	YDPTAYNTILR					Mascot
1326.6688	1326.6642	-0.0046	-3	384	394	YDPTAYNTILR	45	93.524			Mascot

1370.6774	1370.6747	-0.0027	-2	446	457	DPYVDPMAPLPR					Mascot
1701.7247	1701.8429	0.1182	69	147	160	SAVQMYADYMTSFR			Oxidation (M)[5,10]		Mascot
1728.9221	1728.8381	-0.084	-49	257	270	GRFFLAWYSNNLIK					Mascot
1752.8916	1752.866	-0.0256	-15	419	433	LSNQLVEGQNYVNFK					Mascot
1827.952	1827.9443	-0.0077	-4	458	474	SGPEISIEMILQAAQPK			Oxidation (M)[9]		Mascot
1842.0425	1841.9316	-0.1109	-60	288	302	VQLAIKISGIHWWYK					Mascot

6	Chain A, Sevenfold Mutant Of Barley Beta-Amylase	gi|6729696	56504.9	5.73	9	118	100	15.321	93	100	

Peptide Information

Calc. Mass	Obsrv. Mass	± da	± ppm	Start Seq.	End Seq.	Sequence	Ion Score	C. I. %	Modification	Rank	Result Type
802.4305	802.4324	0.0019	2	271	277	ILDEANK					Mascot
1016.5564	1016.5534	-0.003	-3	407	414	LFGFTYLR					Mascot
1016.5564	1016.5534	-0.003	-3	407	414	LFGFTYLR	48	96.732			Mascot
1285.6212	1285.6085	-0.0127	-10	57	67	GPKAYDWSAYK					Mascot
1326.6688	1326.6642	-0.0046	-3	380	390	YDPTAYNTILR					Mascot
1326.6688	1326.6642	-0.0046	-3	380	390	YDPTAYNTILR	45	93.524			Mascot
1370.6774	1370.6747	-0.0027	-2	442	453	DPYVDPMAPLPR					Mascot
1701.7247	1701.8429	0.1182	69	143	156	SAVQMYADYMTSFR			Oxidation (M)[5,10]		Mascot
1728.9221	1728.8381	-0.084	-49	253	266	GRFFLAWYSNNLIK					Mascot
1752.8916	1752.866	-0.0256	-15	415	429	LSNQLVEGQNYVNFK					Mascot
1827.952	1827.9443	-0.0077	-4	454	470	SGPEISIEMILQAAQPK			Oxidation (M)[9]		Mascot

7	Chain A, Crystal Structure Of Barley Beta-Amylase Complexed With 2,3-Epoxypropyl-Alpha-D-Glucopyranoside	gi|313103508	59854.4	5.58	9	116	100	15.19	93	100	

Protein Group

Chain A, Crystal Structure Of Barley Beta-Amylase At Atomic Resolution	gi|313103503	59854.4	5.5799 999237 0605
Chain A, Crystal Structure Of Barley Beta-Amylase Complexed With 3,4- Epoxybutyl Alpha-D-Glucopyranoside	gi|313103509	59854.4	5.5799 999237 0605
Chain A, Crystal Structure Of Barley Beta-Amylase Complexed With 4-O-Alpha-D-Glucopyranosylmoranoline	gi|313103505	59854.4	5.5799 999237 0605
Chain A, Crystal Structure Of Barley Beta-Amylase Complexed With Acarbose	gi|313103502	59854.4	5.5799 999237 0605
Chain A, Crystal Structure Of Barley Beta-Amylase Complexed With Alpha-Cyclodextrin	gi|313103504	59854.4	5.5799 999237 0605

Peptide Information

Calc. Mass	Obsrv. Mass	± da	± ppm	Start Seq.	End Seq.	Sequence	Ion Score	C. I. %	Modification	Rank	Result Type
802.4305	802.4324	0.0019	2	275	281	ILDEANK					Mascot
1016.5564	1016.5534	-0.003	-3	411	418	LFGFTYLR					Mascot
1016.5564	1016.5534	-0.003	-3	411	418	LFGFTYLR	48	96.732			Mascot
1285.6212	1285.6085	-0.0127	-10	61	71	GPKAYDWSAYK					Mascot
1326.6688	1326.6642	-0.0046	-3	384	394	YDPTAYNTILR					Mascot
1326.6688	1326.6642	-0.0046	-3	384	394	YDPTAYNTILR	45	93.524			Mascot
1370.6774	1370.6747	-0.0027	-2	446	457	DPYVDPMAPLPR					Mascot
1701.7247	1701.8429	0.1182	69	147	160	SAVQMYADYMTSFR			Oxidation (M)[5,10]		Mascot
1728.9221	1728.8381	-0.084	-49	257	270	GRFFLAWYSNNLIK					Mascot
1827.952	1827.9443	-0.0077	-4	458	474	SGPEISIEMILQAAQPK			Oxidation (M)[9]		Mascot
1842.0425	1841.9316	-0.1109	-60	288	302	VQLAIKISGIHWWYK					Mascot

8	RecName: Full=Beta-amylase; AltName: Full=1,4-alpha-D-glucan maltohydrolase	gi|231540	24561.9	5.08	3	102	99.994	13.78	93	100

Peptide Information

Calc. Mass	Obsrv. Mass	± da	± ppm	Start Seq.	End Seq.	Sequence	Ion Score	C. I. %	Modification	Rank	Result Type
1016.5564	1016.5534	-0.003	-3	107	114	LFGFTYLR					Mascot
1016.5564	1016.5534	-0.003	-3	107	114	LFGFTYLR	48	96.732			Mascot
1326.6688	1326.6642	-0.0046	-3	80	90	YDPTAYNTILR					Mascot
1326.6688	1326.6642	-0.0046	-3	80	90	YDPTAYNTILR	45	93.524			Mascot
1589.6948	1589.6962	0.0014	1	29	41	HHASLNFTCAEMR			Carbamidomethyl (C)[9], Oxidation (M)[12]		Mascot

9	beta-amylase 2, partial [Milium effusum]	gi|482677645	47129.8	7.3	7	90	99.913	12.14	71	99.985

Protein Group

beta-amylase 2, partial [Milium effusum]	gi|482677647	47235.6	6.4899 997711 1816

Peptide Information

Calc. Mass	Obsrv. Mass	± da	± ppm	Start Seq.	End Seq.	Sequence	Ion Score	C. I. %	Modification	Rank	Result Type
802.4305	802.4324	0.0019	2	266	272	ILDEANK					Mascot
947.5057	947.4904	-0.0153	-16	312	319	DGYRPIAR					Mascot
947.5057	947.4904	-0.0153	-16	312	319	DGYRPIAR	23	0			Mascot
1016.5564	1016.5534	-0.003	-3	402	409	LFGFTYLR					Mascot
1016.5564	1016.5534	-0.003	-3	402	409	LFGFTYLR	48	96.732			Mascot

1335.7202	1335.677	-0.0432	-32	312	322	DGYRPIARMLK			Oxidation (M)[9]		Mascot
1603.7104	1603.7706	0.0602	38	324	336	HHATLNFTCAEMR			Carbamidomethyl (C)[9], Oxidation (M)[12]		Mascot
1729.9093	1729.8627	-0.0466	-27	386	401	NARPNGINKTGPPEHK					Mascot
2087.0557	2087.0625	0.0068	3	120	137	NIEYLTLGVDDQPLFHGR					Mascot

10	beta-amylase 2, partial [Zingeria biebersteiniana]	gi|482677623	46497.1	5.39	9	79	98.856	12.104	48	96.732

Peptide Information

Calc. Mass	Obsrv. Mass	± da	± ppm	Start Seq.	End Seq.	Sequence	Ion Score	C. I. %	Modification	Rank	Result Type
1016.5564	1016.5534	-0.003	-3	402	409	LFGFTYLR					Mascot
1016.5564	1016.5534	-0.003	-3	402	409	LFGFTYLR	48	96.732			Mascot
1285.6212	1285.6085	-0.0127	-10	52	62	GPKAYDWSAYK					Mascot
1425.6903	1425.6925	0.0022	2	362	374	EGLHVACENALGR			Carbamidomethyl (C)[7]		Mascot
1589.6948	1589.6962	0.0014	1	324	336	HHASLNFTCAEMR			Carbamidomethyl (C)[9], Oxidation (M)[12]		Mascot
1691.8275	1691.8262	-0.0013	-1	234	247	TQFFKDNGTYLTEK					Mascot
1701.7499	1701.8429	0.093	55	138	151	TAIQMYADYMTSFK			Oxidation (M)[5,10]		Mascot
1729.9093	1729.8627	-0.0466	-27	386	401	NARPTGINKNGPPEHK					Mascot
1828.0269	1827.9443	-0.0826	-45	279	293	VQLAIKVSGIHWWYK					Mascot
2087.0557	2087.0625	0.0068	3	120	137	NIEYLTLGVDDQPLFHGR					Mascot

Gel Idx/Pos	253/K5	Instr./Gel Origin	BA2151/Sample Project 20140814	Process Status	Analysis Succeeded
Plate [#] Name	[1] Sample Project 20140814	Instrument Sample Name		Spectra	11

Rank	Protein Name	Accession No.	Protein MW	Protein PI	Pep. Count	Protein Score	Protein Score C. I. %	Intensity Matched	Total Ion Score	Total Ion C. I. %	Confirmed
1	alpha-tubulin [Oryza sativa Japonica Group]	gi|385717688	50389.6	4.81	10	350	100	15.351	313	100	

Protein Group

Os11g0247300 [Oryza sativa Japonica Group]	gi|113644835	50389.6	4.8099 999427 7954
RecName: Full=Tubulin alpha-2 chain	gi|75283205	50389.6	4.8099 999427 7954
Tubulin alpha-3 chain, putative, expressed [Oryza sativa Japonica Group]	gi|77549556	50389.6	4.8099 999427 7954
Tubulin alpha-3 chain, putative, expressed [Oryza sativa Japonica Group]	gi|77549555	50389.6	4.8099 999427 7954
hypothetical protein OsI_35689 [Oryza sativa Indica Group]	gi|125533963	50389.6	4.8099 999427 7954

Peptide Information

Calc. Mass	Obsrv. Mass	± da	± ppm	Start Seq.	End Seq.	Sequence	Ion Score	C. I. %	Modification	Rank	Result Type
1007.454	1007.4495	-0.0045	-4	97	105	EDAANNFAR					Mascot
1132.5668	1132.5371	-0.0297	-26	113	121	EIVDLCLDR			Carbamidomethyl (C)[6]		Mascot
1396.7584	1396.6779	-0.0805	-58	85	96	QLFHPEQLISGK					Mascot
1473.8635	1473.8313	-0.0322	-22	230	243	LVSQVISSLTASLR					Mascot
1473.8635	1473.8313	-0.0322	-22	230	243	LVSQVISSLTASLR	71	99.987			Mascot
1589.6844	1589.6282	-0.0562	-35	309	320	HGKYMACCLMYR			Carbamidomethyl (C)[7,8]		Mascot
1691.8711	1691.8196	-0.0515	-30	216	229	SLDIERPTYTNLNR					Mascot
1691.8711	1691.8196	-0.0515	-30	216	229	SLDIERPTYTNLNR	76	99.996			Mascot
1701.9059	1701.8625	-0.0434	-26	65	79	AVFVDLEPTVIDEVR					Mascot
1701.9059	1701.8625	-0.0434	-26	65	79	AVFVDLEPTVIDEVR	76	99.995			Mascot
1808.9252	1808.8646	-0.0606	-34	265	280	IHFMLSSYAPVISAEK			Oxidation (M)[4]		Mascot
2385.1946	2385.1514	-0.0432	-18	85	105	QLFHPEQLISGKEDAANN FAR					Mascot
2408.1882	2408.1445	-0.0437	-18	244	264	FDGALNVDVNEFQTNLV PYPR					Mascot
2408.1882	2408.1445	-0.0437	-18	244	264	FDGALNVDVNEFQTNLV PYPR	90	100			Mascot

Rank	Protein Name	Accession No.	Protein MW	Protein PI	Pep. Count	Protein Score	Protein Score C. I. %	Intensity Matched	Total Ion Score	Total Ion C. I. %	Confirmed
2	RecName: Full=Tubulin alpha-3 chain	gi|8928432	50381.6	4.89	10	350	100	15.351	313	100	

Protein Group

PREDICTED: tubulin alpha-1 chain-like [Setaria italica]	gi|514814302	50369.6	4.8899998664856
RecName: Full=Tubulin alpha chain	gi|8928408	50395.6	4.8899998664856
RecName: Full=Tubulin alpha-1 chain; AltName: Full=Alpha-1-tubulin	gi|6094429	50383.6	4.8899998664856
RecName: Full=Tubulin alpha-1 chain; AltName: Full=Alpha-1-tubulin	gi|135398	50383.6	4.8899998664856
RecName: Full=Tubulin alpha-2 chain	gi|8928422	50353.6	4.88000011444092
RecName: Full=Tubulin alpha-2 chain; AltName: Full=Alpha-2-tubulin	gi|135411	50383.6	4.8899998664856
TPA: tubulin alpha-1 chain [Zea mays]	gi|414872560	50383.6	4.8899998664856
TPA: tubulin alpha-1 chain [Zea mays]	gi|414872556	50383.6	4.8899998664856
TPA: tubulin alpha-1 chain [Zea mays]	gi|414872557	50383.6	4.8899998664856
TPA: tubulin alpha-1 chain [Zea mays]	gi|414872555	50383.6	4.8899998664856
TPA: tubulin alpha-2 chain [Zea mays]	gi|414872554	50383.6	4.8899998664856
Tubulin alpha chain [Aegilops tauschii]	gi|475602014	50367.6	4.8899998664856
Tubulin alpha chain [Triticum urartu]	gi|474224323	50367.6	4.8899998664856
Tubulin alpha-3 chain [Aegilops tauschii]	gi|475580203	50381.6	4.8899998664856
Tubulin alpha-3 chain [Triticum urartu]	gi|473887549	50381.6	4.8899998664856
alpha tubulin4 isoform 1 [Zea mays]	gi|413933195	50369.6	4.8899998664856
alpha tubulin4 isoform 2 [Zea mays]	gi|413933196	50369.6	4.8899998664856
tubulin alpha-3 chain [Zea mays]	gi|293331323	50369.6	4.8899998664

Peptide Information

Calc. Mass	Obsrv. Mass	± da	± ppm	Start Seq.	End Seq.	Sequence	Ion Score	C. I. %	Modification	Rank	Result Type
1007.454	1007.4495	-0.0045	-4	97	105	EDAANNFAR					Mascot
1132.5668	1132.5371	-0.0297	-26	113	121	EIVDLCLDR			Carbamidomethyl (C)[6]		Mascot
1396.693	1396.6779	-0.0151	-11	391	401	IDHKFDLMYAK			Oxidation (M)[8]		Mascot
1473.8635	1473.8313	-0.0322	-22	230	243	LVSQVISSLTASLR					Mascot
1473.8635	1473.8313	-0.0322	-22	230	243	LVSQVISSLTASLR	71	99.987			Mascot
1589.6844	1589.6282	-0.0562	-35	309	320	HGKYMACCLMYR			Carbamidomethyl (C)[7,8]		Mascot
1691.8711	1691.8196	-0.0515	-30	216	229	SLDIERPTYTNLNR					Mascot
1691.8711	1691.8196	-0.0515	-30	216	229	SLDIERPTYTNLNR	76	99.996			Mascot
1701.9059	1701.8625	-0.0434	-26	65	79	AVFVDLEPTVIDEVR					Mascot
1701.9059	1701.8625	-0.0434	-26	65	79	AVFVDLEPTVIDEVR	76	99.995			Mascot
1808.9252	1808.8646	-0.0606	-34	265	280	IHFMLSSYAPVISAEK			Oxidation (M)[4]		Mascot
2385.1946	2385.1514	-0.0432	-18	85	105	QLFHPEQLISGKEDAANN FAR					Mascot
2408.1882	2408.1445	-0.0437	-18	244	264	FDGALNVDVNEFQTNLV PYPR					Mascot
2408.1882	2408.1445	-0.0437	-18	244	264	FDGALNVDVNEFQTNLV PYPR	90	100			Mascot

3 PREDICTED: tubulin alpha chain-like [Solanum lycopersicum] gi|460372283 50354.7 5.1 9 344 100 15.025 313 100

Peptide Information

Calc. Mass	Obsrv. Mass	± da	± ppm	Start Seq.	End Seq.	Sequence	Ion Score	C. I. %	Modification	Rank	Result Type
1007.454	1007.4495	-0.0045	-4	97	105	EDAANNFAR					Mascot
1132.5668	1132.5371	-0.0297	-26	113	121	EIVDLCLDR			Carbamidomethyl (C)[6]		Mascot
1396.693	1396.6779	-0.0151	-11	391	401	IDHKFDLMYAK			Oxidation (M)[8]		Mascot
1473.8635	1473.8313	-0.0322	-22	230	243	LVSQVISSLTASLR					Mascot
1473.8635	1473.8313	-0.0322	-22	230	243	LVSQVISSLTASLR	71	99.987			Mascot
1691.8711	1691.8196	-0.0515	-30	216	229	SLDIERPTYTNLNR					Mascot
1691.8711	1691.8196	-0.0515	-30	216	229	SLDIERPTYTNLNR	76	99.996			Mascot
1701.9059	1701.8625	-0.0434	-26	65	79	AVFVDLEPTVIDEVR					Mascot
1701.9059	1701.8625	-0.0434	-26	65	79	AVFVDLEPTVIDEVR	76	99.995			Mascot
1808.9252	1808.8646	-0.0606	-34	265	280	IHFMLSSYAPVISAEK			Oxidation (M)[4]		Mascot
2385.1946	2385.1514	-0.0432	-18	85	105	QLFHPEQLISGKEDAANN FAR					Mascot
2408.1882	2408.1445	-0.0437	-18	244	264	FDGALNVDVNEFQTNLV PYPR					Mascot

Calc. Mass	Obsrv. Mass	± da	± ppm	Start Seq.	End Seq.	Sequence	Ion Score	C. I. %	Modification	Rank	Result Type
2408.1882	2408.1445	-0.0437	-18	244	264	FDGALNVDVNEFQTNLVPYPR	90	100			Mascot

4	Os03g0726100 [Oryza sativa Japonica Group]	gi|113549603	50528.6	4.88	9	343	100	14.855	313	100	

Protein Group

alpha tubulin [Oryza sativa Japonica Group]	gi|385717660	50528.6	4.8800001144092
hypothetical protein OsI_13361 [Oryza sativa Indica Group]	gi|125545580	50528.6	4.8800001144092

Peptide Information

Calc. Mass	Obsrv. Mass	± da	± ppm	Start Seq.	End Seq.	Sequence	Ion Score	C. I. %	Modification	Rank	Result Type
1007.454	1007.4495	-0.0045	-4	97	105	EDAANNFAR					Mascot
1132.5668	1132.5371	-0.0297	-26	113	121	EIVDLCLDR			Carbamidomethyl (C)[6]		Mascot
1396.693	1396.6779	-0.0151	-11	391	401	IDHKFDLMYSK					Mascot
1473.8635	1473.8313	-0.0322	-22	230	243	LVSQVISSLTASLR					Mascot
1473.8635	1473.8313	-0.0322	-22	230	243	LVSQVISSLTASLR	71	99.987			Mascot
1589.6844	1589.6282	-0.0562	-35	309	320	HGKYMACCLMYR			Carbamidomethyl (C)[7,8]		Mascot
1691.8711	1691.8196	-0.0515	-30	216	229	SLDIERPTYTNLNR					Mascot
1691.8711	1691.8196	-0.0515	-30	216	229	SLDIERPTYTNLNR	76	99.996			Mascot
1701.9059	1701.8625	-0.0434	-26	65	79	AVFVDLEPTVIDEVR					Mascot
1701.9059	1701.8625	-0.0434	-26	65	79	AVFVDLEPTVIDEVR	76	99.995			Mascot
1808.9252	1808.8646	-0.0606	-34	265	280	IHFMLSSYAPVISAEK			Oxidation (M)[4]		Mascot
2408.1882	2408.1445	-0.0437	-18	244	264	FDGALNVDVNEFQTNLVPYPR					Mascot
2408.1882	2408.1445	-0.0437	-18	244	264	FDGALNVDVNEFQTNLVPYPR	90	100			Mascot

5	TPA: alpha tubulin [Zea mays]	gi|414872552	50529.9	4.92	8	338	100	14.351	313	100	

Peptide Information

Calc. Mass	Obsrv. Mass	± da	± ppm	Start Seq.	End Seq.	Sequence	Ion Score	C. I. %	Modification	Rank	Result Type
1132.5668	1132.5371	-0.0297	-26	114	122	EIVDLCLDR			Carbamidomethyl (C)[6]		Mascot
1396.693	1396.6779	-0.0151	-11	392	402	IDHKFDLMYAK			Oxidation (M)[8]		Mascot
1473.8635	1473.8313	-0.0322	-22	231	244	LVSQVISSLTASLR					Mascot
1473.8635	1473.8313	-0.0322	-22	231	244	LVSQVISSLTASLR	71	99.987			Mascot
1589.6844	1589.6282	-0.0562	-35	310	321	HGKYMACCLMYR			Carbamidomethyl (C)[7,8]		Mascot
1691.8711	1691.8196	-0.0515	-30	217	230	SLDIERPTYTNLNR					Mascot
1691.8711	1691.8196	-0.0515	-30	217	230	SLDIERPTYTNLNR	76	99.996			Mascot
1701.9059	1701.8625	-0.0434	-26	65	79	AVFVDLEPTVIDEVR					Mascot

1701.9059	1701.8625	-0.0434	-26	65	79	AVFVDLEPTVIDEVR	76	99.995			Mascot
1808.9252	1808.8646	-0.0606	-34	266	281	IHFMLSSYAPVISAEK			Oxidation (M)[4]		Mascot
2408.1882	2408.1445	-0.0437	-18	245	265	FDGALNVDVNEFQTNLV PYPR					Mascot
2408.1882	2408.1445	-0.0437	-18	245	265	FDGALNVDVNEFQTNLV PYPR	90	100			Mascot

6 Protein disulfide-isomerase [Aegilops tauschii] gi|475612819 51732.4 5.15 20 315 100 31.36 203 100

Peptide Information

Calc. Mass	Obsrv. Mass	± da	± ppm	Start Seq.	End Seq.	Sequence	Ion Score	C. I. %	Modification	Rank	Result Type
952.4887	952.4437	-0.045	-47	221	228	FFQTNAPK					Mascot
1023.4993	1023.471	-0.0283	-28	187	195	DFDVSALEK					Mascot
1104.5797	1104.5514	-0.0283	-26	79	87	NIQEYKGPR					Mascot
1150.5851	1150.5203	-0.0648	-56	75	84	NGGKNIQEYK					Mascot
1181.6201	1181.5607	-0.0594	-50	62	71	YEVQGFPTLK					Mascot
1182.6589	1182.6149	-0.044	-37	163	173	GDAAVERPLVR					Mascot
1182.6589	1182.6149	-0.044	-37	163	173	GDAAVERPLVR	31	0			Mascot
1210.6216	1210.5952	-0.0264	-22	211	220	NPDNHPYLLK					Mascot
1278.694	1278.6205	-0.0735	-57	88	98	EAEGIVEYLKK					Mascot
1407.6427	1407.5763	-0.0664	-47	245	257	SAYYGAVEEFSGK					Mascot
1440.7847	1440.7064	-0.0783	-54	337	349	VVVADNVHDVVFK					Mascot
1454.7373	1454.7169	-0.0204	-14	453	466	AAEPAATEPLKDEL					Mascot
1536.8308	1536.7449	-0.0859	-56	174	186	LFKPFDELVVDSK					Mascot
1647.8951	1647.7367	-0.1584	-96	24	38	SLAPEYEKAAQLLSK					Mascot
1647.8951	1647.7367	-0.1584	-96	24	38	SLAPEYEKAAQLLSK					Mascot
1654.8688	1654.7891	-0.0797	-48	196	210	FIDASSTPKVVTFDK					Mascot
1671.8436	1671.6882	-0.1554	-93	280	294	EDQAPLILIQDSDSK					Mascot
1674.9901	1674.8242	-0.1659	-99	32	47	AAQLLSKHDPAIVLAK					Mascot
1799.9386	1799.8619	-0.0767	-43	280	295	EDQAPLILIQDSDSKK					Mascot
1865.8678	1865.8357	-0.0321	-17	147	162	SDYDFGHTVHANHLPR					Mascot
1865.8678	1865.8357	-0.0321	-17	147	162	SDYDFGHTVHANHLPR	115	100			Mascot
1899.9963	1899.9279	-0.0684	-36	205	220	VVTFDKNPDNHPYLLK					Mascot
1899.9963	1899.9279	-0.0684	-36	205	220	VVTFDKNPDNHPYLLK	57	99.647			Mascot
2135.053	2135.0029	-0.0501	-23	145	162	LRSDYDFGHTVHANHLP R					Mascot

7 Protein disulfide-isomerase [Triticum urartu] gi|474046595 56482 5.92 19 300 100 30.92 203 100

Peptide Information

Calc. Mass	Obsrv. Mass	± da	± ppm	Start Seq.	End Seq.	Sequence	Ion Score	C. I. %	Modification	Rank	Result Type
1023.4993	1023.471	-0.0283	-28	241	249	DFDVSALEK					Mascot
1104.5797	1104.5514	-0.0283	-26	133	141	NIQEYKGPR					Mascot
1150.5851	1150.5203	-0.0648	-56	129	138	NGGKNIQEYK					Mascot
1181.6201	1181.5607	-0.0594	-50	116	125	YEVQGFPTLK					Mascot
1182.6589	1182.6149	-0.044	-37	217	227	GDAAVERPLVR					Mascot
1182.6589	1182.6149	-0.044	-37	217	227	GDAAVERPLVR	31	0			Mascot
1210.6216	1210.5952	-0.0264	-22	265	274	NPDNHPYLLK					Mascot
1278.694	1278.6205	-0.0735	-57	142	152	EAEGIVEYLKK					Mascot
1407.6427	1407.5763	-0.0664	-47	299	311	SAYYGAVEEFSGK					Mascot
1454.8003	1454.7169	-0.0834	-57	391	403	VVVADNIHDVVFK					Mascot
1480.714	1480.7242	0.0102	7	51	65	GRFGGVSDASVTGDR					Mascot
1536.8308	1536.7449	-0.0859	-56	228	240	LFKPFDELVVDSK					Mascot
1647.8951	1647.7367	-0.1584	-96	78	92	SLAPEYEKAAQLLSK					Mascot
1647.8951	1647.7367	-0.1584	-96	78	92	SLAPEYEKAAQLLSK					Mascot
1654.8688	1654.7891	-0.0797	-48	250	264	FIDASSTPKVVTFDK					Mascot
1671.8436	1671.6882	-0.1554	-93	334	348	EDQAPLILIQDSDSK					Mascot
1674.9901	1674.8242	-0.1659	-99	86	101	AAQLLSKHDPAIVLAK					Mascot
1799.9386	1799.8619	-0.0767	-43	334	349	EDQAPLILIQDSDSKK					Mascot
1865.8678	1865.8357	-0.0321	-17	201	216	SDYDFGHTVHANHLPR					Mascot
1865.8678	1865.8357	-0.0321	-17	201	216	SDYDFGHTVHANHLPR	115	100			Mascot
1899.9963	1899.9279	-0.0684	-36	259	274	VVTFDKNPDNHPYLLK					Mascot
1899.9963	1899.9279	-0.0684	-36	259	274	VVTFDKNPDNHPYLLK	57	99.647			Mascot
2135.053	2135.0029	-0.0501	-23	199	216	LRSDYDFGHTVHANHLPR					Mascot

8	RecName: Full=Protein disulfide-isomerase; Short=PDI; gi|1709620 Flags: Precursor	56726	4.99	18	291	100	30.747	203	100	

Peptide Information

Calc. Mass	Obsrv. Mass	± da	± ppm	Start Seq.	End Seq.	Sequence	Ion Score	C. I. %	Modification	Rank	Result Type
1023.4993	1023.471	-0.0283	-28	236	244	DFDVSALEK					Mascot
1104.5797	1104.5514	-0.0283	-26	128	136	NIQEYKGPR					Mascot
1150.599	1150.5203	-0.0787	-68	137	146	EAEGIVEYLK					Mascot
1181.6201	1181.5607	-0.0594	-50	111	120	YEVQGFPTLK					Mascot
1182.6589	1182.6149	-0.044	-37	212	222	GDAAVERPLVR					Mascot
1182.6589	1182.6149	-0.044	-37	212	222	GDAAVERPLVR	31	0			Mascot

1210.6216	1210.5952	-0.0264	-22	260	269	NPDNHPYLLK					Mascot
1278.694	1278.6205	-0.0735	-57	137	147	EAEGIVEYLKK					Mascot
1407.6427	1407.5763	-0.0664	-47	294	306	SAYYGAVEEFSGK					Mascot
1454.7373	1454.7169	-0.0204	-14	502	515	AAEPAATEPLKDEL					Mascot
1536.8308	1536.7449	-0.0859	-56	223	235	LFKPFDELVVDSK					Mascot
1647.8951	1647.7367	-0.1584	-96	73	87	SLAPEYEKAAQLLSK					Mascot
1647.8951	1647.7367	-0.1584	-96	73	87	SLAPEYEKAAQLLSK					Mascot
1654.8688	1654.7891	-0.0797	-48	245	259	FIDASSTPKVVTFDK					Mascot
1671.8436	1671.6882	-0.1554	-93	329	343	EDQAPLILIQDSDSK					Mascot
1674.9901	1674.8242	-0.1659	-99	81	96	AAQLLSKHDPAIVLAK					Mascot
1799.9386	1799.8619	-0.0767	-43	329	344	EDQAPLILIQDSDSKK					Mascot
1865.8678	1865.8357	-0.0321	-17	196	211	SDYDFGHTVHANHLPR					Mascot
1865.8678	1865.8357	-0.0321	-17	196	211	SDYDFGHTVHANHLPR	115	100			Mascot
1899.9963	1899.9279	-0.0684	-36	254	269	VVTFDKNPDNHPYLLK					Mascot
1899.9963	1899.9279	-0.0684	-36	254	269	VVTFDKNPDNHPYLLK	57	99.647			Mascot
2135.053	2135.0029	-0.0501	-23	194	211	LRSDYDFGHTVHANHLPR					Mascot

9	alpha tubulin4 [Zea mays]	gi|413933197	40963.8	4.85	9	274	100	12.468	237	100	

Peptide Information

Calc. Mass	Obsrv. Mass	± da	± ppm	Start Seq.	End Seq.	Sequence	Ion Score	C. I. %	Modification	Rank	Result Type
1007.454	1007.4495	-0.0045	-4	97	105	EDAANNFAR					Mascot
1132.5668	1132.5371	-0.0297	-26	113	121	EIVDLCLDR			Carbamidomethyl (C)[6]		Mascot
1396.693	1396.6779	-0.0151	-11	304	314	IDHKFDLMYAK			Oxidation (M)[8]		Mascot
1473.8635	1473.8313	-0.0322	-22	143	156	LVSQVISSLTASLR					Mascot
1473.8635	1473.8313	-0.0322	-22	143	156	LVSQVISSLTASLR	71	99.987			Mascot
1589.6844	1589.6282	-0.0562	-35	222	233	HGKYMACCLMYR			Carbamidomethyl (C)[7,8]		Mascot
1701.9059	1701.8625	-0.0434	-26	65	79	AVFVDLEPTVIDEVR					Mascot
1701.9059	1701.8625	-0.0434	-26	65	79	AVFVDLEPTVIDEVR	76	99.995			Mascot
1808.9252	1808.8646	-0.0606	-34	178	193	IHFMLSSYAPVISAEK			Oxidation (M)[4]		Mascot
2385.1946	2385.1514	-0.0432	-18	85	105	QLFHPEQLISGKEDAANNFAR					Mascot
2408.1882	2408.1445	-0.0437	-18	157	177	FDGALNVDVNEFQTNLVPYPR					Mascot
2408.1882	2408.1445	-0.0437	-18	157	177	FDGALNVDVNEFQTNLVPYPR	90	100			Mascot

10	hypothetical protein, partial [Scutellaria baicalensis]	gi|409894766	49106.6	6.13	9	273	100	12.516	242	100	

Peptide Information

Calc. Mass	Obsrv. Mass	± da	± ppm	Start Seq.	End Seq.	Sequence	Ion Score	C. I. %	Modification	Rank	Result Type
1007.454	1007.4495	-0.0045	-4	134	142	EDAANNFAR					Mascot
1132.5668	1132.5371	-0.0297	-26	150	158	EIVDLCLDR			Carbamidomethyl (C)[6]		Mascot
1396.7584	1396.6779	-0.0805	-58	122	133	QLFHPEQLISGK					Mascot
1589.6844	1589.6282	-0.0562	-35	346	357	HGKYMACCLMYR			Carbamidomethyl (C)[7,8]		Mascot
1691.8711	1691.8196	-0.0515	-30	253	266	SLDIERPTYTNLNR					Mascot
1691.8711	1691.8196	-0.0515	-30	253	266	SLDIERPTYTNLNR	76	99.996			Mascot
1701.9059	1701.8625	-0.0434	-26	102	116	AVFVDLEPTVIDEVR					Mascot
1701.9059	1701.8625	-0.0434	-26	102	116	AVFVDLEPTVIDEVR	76	99.996			Mascot
1808.9252	1808.8646	-0.0606	-34	302	317	IHFMLSSYAPVISAEK			Oxidation (M)[4]		Mascot
2385.1946	2385.1514	-0.0432	-18	122	142	QLFHPEQLISGKEDAANN FAR					Mascot
2408.1882	2408.1445	-0.0437	-18	281	301	FDGALNVDVNEFQTNLV PYPR					Mascot
2408.1882	2408.1445	-0.0437	-18	281	301	FDGALNVDVNEFQTNLV PYPR	90	100			Mascot

Gel Idx/Pos	254/K6	Instr./Gel Origin	BA2151/Sample Project 20140814	Process Status	Analysis Succeeded
Plate [#] Name	[1] Sample Project 20140814	Instrument Sample Name		Spectra	11

Rank	Protein Name	Accession No.	Protein MW	Protein PI	Pep. Count	Protein Score	Protein Score C. I. %	Intensity Matched	Total Ion Score	Total Ion C. I. %	Confirmed
1	Beta-amylase [Triticum urartu]	gi|474451266	58995	5.34	19	720	100	46.233	612	100	

Peptide Information

Calc. Mass	Obsrv. Mass	± da	± ppm	Start Seq.	End Seq.	Sequence	Ion Score	C. I. %	Modification	Rank	Result Type
947.5057	947.4756	-0.0301	-32	322	329	DGYRPIAR					Mascot
1016.5564	1016.5394	-0.017	-17	412	419	LFGFTYLR					Mascot
1016.5564	1016.5394	-0.017	-17	412	419	LFGFTYLR	56	99.565			Mascot
1315.5769	1315.5422	-0.0347	-26	336	346	ASLNFTCAEMR			Carbamidomethyl (C)[7], Oxidation (M)[10]		Mascot
1326.6688	1326.6444	-0.0244	-18	385	395	YDPTAYNTILR					Mascot
1326.6688	1326.6444	-0.0244	-18	385	395	YDPTAYNTILR	82	99.999			Mascot
1382.7791	1382.7256	-0.0535	-39	73	84	QLFQLVHEAGLK					Mascot
1490.6726	1490.6362	-0.0364	-24	372	384	EGLNMACENALPR			Carbamidomethyl (C)[7], Oxidation (M)[5]		Mascot
1646.781	1646.7638	-0.0172	-10	246	259	FFVDNGTYLTEQGR					Mascot
1668.7952	1668.7675	-0.0277	-17	218	232	AAAAMVGHPEWEFPR					Mascot
1684.79	1684.7556	-0.0344	-20	218	232	AAAAMVGHPEWEFPR			Oxidation (M)[5]		Mascot
1685.7299	1685.7437	0.0138	8	148	161	SAVQMYTDYMASFR			Oxidation (M)[5]		Mascot
1701.7247	1701.7063	-0.0184	-11	148	161	SAVQMYTDYMASFR			Oxidation (M)[5,10]		Mascot
1701.7247	1701.7063	-0.0184	-11	148	161	SAVQMYTDYMASFR	1	0	Oxidation (M)[5,10]		Mascot
1752.8916	1752.8329	-0.0587	-33	420	434	LSNQLVEGQNYVNFK					Mascot
1841.9678	1841.8951	-0.0727	-39	459	475	SGPELTIEMILQAAQPK			Oxidation (M)[9]		Mascot
1992.9047	1992.8866	-0.0181	-9	112	129	NVGASDPDIFYTDQHGT R					Mascot
1992.9047	1992.8866	-0.0181	-9	112	129	NVGASDPDIFYTDQHGT R	128	100			Mascot
2013.9778	2013.9647	-0.0131	-7	304	321	VPSHAAEITAGYYNLHDR					Mascot
2013.9778	2013.9647	-0.0131	-7	304	321	VPSHAAEITAGYYNLHDR	112	100			Mascot
2087.0557	2087.0425	-0.0132	-6	130	147	NIEYLTLGVDDQPLFHGR					Mascot
2087.0557	2087.0425	-0.0132	-6	130	147	NIEYLTLGVDDQPLFHGR	150	100			Mascot
2183.0486	2183.0105	-0.0381	-17	440	458	MHANLPHDPCVDPVAPL QR			Carbamidomethyl (C)[10], Oxidation (M)[1]		Mascot
2183.0486	2183.0105	-0.0381	-17	440	458	MHANLPHDPCVDPVAPL QR	85	100	Carbamidomethyl (C)[10], Oxidation (M)[1]		Mascot
2269.2075	2269.1899	-0.0176	-8	166	187	EFLDAGVIVDIEVGLGPA GELR					Mascot
2733.2786	2733.2993	0.0207	8	347	371	DSEQSSQAMSAPEELVQ					Mascot

Calc. Mass	Obsrv. Mass	± da	± ppm	Start Seq.	End Seq.	Sequence	Ion Score	C. I. %	Modification	Rank	Result Type
						QVLSAGWR					
2749.2734	2749.2756	0.0022	1	347	371	DSEQSSQAMSAPEELVQ QVLSAGWR			Oxidation (M)[9]		Mascot
2773.4077	2773.3901	-0.0176	-6	162	187	DNMKEFLDAGVIVDIEVG LGPAGELR			Oxidation (M)[3]		Mascot
2794.3916	2794.3499	-0.0417	-15	87	111	AIMSFHQCGGNVGDVVN IPIPQWVR			Carbamidomethyl (C)[8]		Mascot
2810.3865	2810.3384	-0.0481	-17	87	111	AIMSFHQCGGNVGDVVN IPIPQWVR			Carbamidomethyl (C)[8], Oxidation (M)[3]		Mascot

2 Beta-amylase [Aegilops tauschii] gi|475523854 60203.5 5.07 13 423 100 31.792 373 100

Peptide Information

Calc. Mass	Obsrv. Mass	± da	± ppm	Start Seq.	End Seq.	Sequence	Ion Score	C. I. %	Modification	Rank	Result Type
947.5057	947.4756	-0.0301	-32	322	329	DGYRPIAR					Mascot
1016.5564	1016.5394	-0.017	-17	412	419	LFGFTYLR					Mascot
1016.5564	1016.5394	-0.017	-17	412	419	LFGFTYLR	56	99.565			Mascot
1237.5444	1237.5747	0.0303	24	233	243	DAGTYNDTPQR					Mascot
1326.6688	1326.6444	-0.0244	-18	385	395	YDPTAYNTILR					Mascot
1326.6688	1326.6444	-0.0244	-18	385	395	YDPTAYNTILR	82	99.999			Mascot
1490.6726	1490.6362	-0.0364	-24	372	384	EGLNMACENALPR			Carbamidomethyl (C)[7], Oxidation (M)[5]		Mascot
1646.781	1646.7638	-0.0172	-10	246	259	FFVDNGTYLTEQGR					Mascot
1668.7952	1668.7675	-0.0277	-17	218	232	AAAAMVGHPEWEFPR					Mascot
1684.79	1684.7556	-0.0344	-20	218	232	AAAAMVGHPEWEFPR			Oxidation (M)[5]		Mascot
1729.801	1729.8262	0.0252	15	333	346	RHHASLNFTCAEMR			Carbamidomethyl (C)[10]		Mascot
1752.8916	1752.8329	-0.0587	-33	420	434	LSNQLVEGQNYVNFK					Mascot
1841.9678	1841.8951	-0.0727	-39	459	475	SGPELTIEMILQAAQPK			Oxidation (M)[9]		Mascot
2087.0557	2087.0425	-0.0132	-6	130	147	NIEYLTLGVDDQPLFHGR					Mascot
2087.0557	2087.0425	-0.0132	-6	130	147	NIEYLTLGVDDQPLFHGR	150	100			Mascot
2183.0486	2183.0105	-0.0381	-17	440	458	MHANLPHDPCVDPVAPL QR			Carbamidomethyl (C)[10], Oxidation (M)[1]		Mascot
2183.0486	2183.0105	-0.0381	-17	440	458	MHANLPHDPCVDPVAPL QR	85	100	Carbamidomethyl (C)[10], Oxidation (M)[1]		Mascot
2733.2786	2733.2993	0.0207	8	347	371	DSEQSSQAMSAPEELVQ QVLSAGWR					Mascot
2749.2734	2749.2756	0.0022	1	347	371	DSEQSSQAMSAPEELVQ QVLSAGWR			Oxidation (M)[9]		Mascot

3 beta-amylase 2, partial [Brachypodium distachyon] gi|482677643 46785.2 5.84 14 360 100 30.47 288 100

Peptide Information

Calc. Mass	Obsrv. Mass	± da	± ppm	Start Seq.	End Seq.	Sequence	Ion Score	C. I. %	Modification	Rank	Result Type
947.5057	947.4756	-0.0301	-32	312	319	DGYRPIAR					Mascot
1016.5564	1016.5394	-0.017	-17	402	409	LFGFTYLR					Mascot

Calc. Mass	Obsrv. Mass	± da	± ppm	Start Seq.	End Seq.	Sequence	Ion Score	C. I. %	Modification	Rank	Result Type
1016.5564	1016.5394	-0.017	-17	402	409	LFGFTYLR	56	99.565			Mascot
1237.5444	1237.5747	0.0303	24	223	233	DAGTYNDTPQR					Mascot
1326.6688	1326.6444	-0.0244	-18	375	385	YDPTAYNTILR					Mascot
1326.6688	1326.6444	-0.0244	-18	375	385	YDPTAYNTILR	82	99.999			Mascot
1490.6726	1490.6362	-0.0364	-24	362	374	EGLNMACENALPR			Carbamidomethyl (C)[7], Oxidation (M)[5]		Mascot
1646.781	1646.7638	-0.0172	-10	236	249	FFVDNGTYLTEQGR					Mascot
1651.7784	1651.7631	-0.0153	-9	138	151	SAVQLYTDYMASFR					Mascot
1668.7952	1668.7675	-0.0277	-17	208	222	AAAAMVGHPEWEFPR					Mascot
1684.79	1684.7556	-0.0344	-20	208	222	AAAAMVGHPEWEFPR			Oxidation (M)[5]		Mascot
1842.0425	1841.8951	-0.1474	-80	279	293	VQLAIKISGIHWWYK					Mascot
2011.9985	2011.9863	-0.0122	-6	294	311	VPSHAAEVIAGYYNLHDR					Mascot
2087.0557	2087.0425	-0.0132	-6	120	137	NIEYLTLGVDDQPLFHGR					Mascot
2087.0557	2087.0425	-0.0132	-6	120	137	NIEYLTLGVDDQPLFHGR	150	100			Mascot
2269.2075	2269.1899	-0.0176	-8	156	177	EFLDAGVIVDIEVGLGPA GELR					Mascot
2733.2786	2733.2993	0.0207	8	337	361	DSEQSSQAMSAPEELVQ QVLSAGWR					Mascot
2749.2734	2749.2756	0.0022	1	337	361	DSEQSSQAMSAPEELVQ QVLSAGWR			Oxidation (M)[9]		Mascot
2773.4077	2773.3901	-0.0176	-6	152	177	DNMKEFLDAGVIVDIEVG LGPAGELR			Oxidation (M)[3]		Mascot

4	beta-amylase 2, partial [Milium effusum]	gi|482677647	47235.6	6.49	8	342	100	27.024	318	100	

Protein Group

beta-amylase 2, partial [Milium effusum]	gi|482677645	47129.8	7.3000 001907 3486

Peptide Information

Calc. Mass	Obsrv. Mass	± da	± ppm	Start Seq.	End Seq.	Sequence	Ion Score	C. I. %	Modification	Rank	Result Type
947.5057	947.4756	-0.0301	-32	312	319	DGYRPIAR					Mascot
1016.5564	1016.5394	-0.017	-17	402	409	LFGFTYLR					Mascot
1016.5564	1016.5394	-0.017	-17	402	409	LFGFTYLR	56	99.565			Mascot
1571.8217	1571.7524	-0.0693	-44	266	278	ILDEANKVFFGYR					Mascot
1729.9093	1729.8262	-0.0831	-48	386	401	NARPNGINKTGPPEHK					Mascot
1743.8167	1743.762	-0.0547	-31	323	336	RHHATLNFTCAEMR			Carbamidomethyl (C)[10]		Mascot
2013.9778	2013.9647	-0.0131	-7	294	311	VPSHAAELTAGYYNLHD R					Mascot
2013.9778	2013.9647	-0.0131	-7	294	311	VPSHAAELTAGYYNLHD R	112	100			Mascot
2087.0557	2087.0425	-0.0132	-6	120	137	NIEYLTLGVDDQPLFHGR					Mascot
2087.0557	2087.0425	-0.0132	-6	120	137	NIEYLTLGVDDQPLFHGR	150	100			Mascot

Calc. Mass	Obsrv. Mass	± da	± ppm	Start Seq.	End Seq.	Sequence	Ion Score	C. I. %	Modification	Rank	Result Type
2733.2786	2733.2993	0.0207	8	337	361	DSEQSSQAMSAPEELVQ QVLSAGWR					Mascot
2749.2734	2749.2756	0.0022	1	337	361	DSEQSSQAMSAPEELVQ QVLSAGWR			Oxidation (M)[9]		Mascot

5	Chain A, Sevenfold Mutant Of Barley Beta-Amylase	gi|6729696	56504.9	5.73	11	290	100	23.489	249	100	

Peptide Information

Calc. Mass	Obsrv. Mass	± da	± ppm	Start Seq.	End Seq.	Sequence	Ion Score	C. I. %	Modification	Rank	Result Type
854.4301	854.4536	0.0235	28	435	441	MHANLPR			Oxidation (M)[1]		Mascot
1016.5564	1016.5394	-0.017	-17	407	414	LFGFTYLR					Mascot
1016.5564	1016.5394	-0.017	-17	407	414	LFGFTYLR	56	99.565			Mascot
1285.6212	1285.5829	-0.0383	-30	57	67	GPKAYDWSAYK					Mascot
1315.5769	1315.5422	-0.0347	-26	331	341	ASINFTCAEMR			Carbamidomethyl (C)[7], Oxidation (M)[10]		Mascot
1326.6688	1326.6444	-0.0244	-18	380	390	YDPTAYNTILR					Mascot
1326.6688	1326.6444	-0.0244	-18	380	390	YDPTAYNTILR	82	99.999			Mascot
1685.7299	1685.7437	0.0138	8	143	156	SAVQMYADYMTSFR			Oxidation (M)[5]		Mascot
1701.7247	1701.7063	-0.0184	-11	143	156	SAVQMYADYMTSFR			Oxidation (M)[5,10]		Mascot
1701.7247	1701.7063	-0.0184	-11	143	156	SAVQMYADYMTSFR			Oxidation (M)[5,10]		Mascot
1705.8181	1705.7739	-0.0442	-26	239	252	TQFFRDNGTYLSEK					Mascot
1752.8916	1752.8329	-0.0587	-33	415	429	LSNQLVEGQNYVNFK					Mascot
2013.9778	2013.9647	-0.0131	-7	299	316	VPSHAAELTAGYYNLHD R					Mascot
2013.9778	2013.9647	-0.0131	-7	299	316	VPSHAAELTAGYYNLHD R	112	100			Mascot
2773.4077	2773.3901	-0.0176	-6	157	182	ENMKDFLDAGVIVDIEVG LGPAGELR			Oxidation (M)[3]		Mascot
3098.3984	3098.4883	0.0899	29	471	500	IQPFPFQEHTDLPVGPTG GMGGQAEGPTCG			Carbamidomethyl (C)[29], Oxidation (M)[20]		Mascot

6	RecName: Full=Beta-amylase; AltName: Full=1,4-alpha-D-glucan maltohydrolase; AltName: Full=Beta-Amy1; Flags: Precursor	gi|75107132	59886.4	5.66	11	285	100	23.892	249	100	

Peptide Information

Calc. Mass	Obsrv. Mass	± da	± ppm	Start Seq.	End Seq.	Sequence	Ion Score	C. I. %	Modification	Rank	Result Type
854.4301	854.4536	0.0235	28	439	445	MHANLPR			Oxidation (M)[1]		Mascot
1016.5564	1016.5394	-0.017	-17	411	418	LFGFTYLR					Mascot
1016.5564	1016.5394	-0.017	-17	411	418	LFGFTYLR	56	99.565			Mascot
1253.6121	1253.5747	-0.0374	-30	248	258	DNGTYLTEKGR					Mascot
1285.6212	1285.5829	-0.0383	-30	61	71	GPKAYDWSAYK					Mascot
1315.5769	1315.5422	-0.0347	-26	335	345	ASINFTCAEMR			Carbamidomethyl (C)[7], Oxidation (M)[10]		Mascot
1326.6688	1326.6444	-0.0244	-18	384	394	YDPTAYNTILR					Mascot

1326.6688	1326.6444	-0.0244	-18	384	394	YDPTAYNTILR	82	99.999			Mascot
1685.7299	1685.7437	0.0138	8	147	160	SAVQMYADYMTSFR			Oxidation (M)[5]		Mascot
1701.7247	1701.7063	-0.0184	-11	147	160	SAVQMYADYMTSFR			Oxidation (M)[5,10]		Mascot
1701.7247	1701.7063	-0.0184	-11	147	160	SAVQMYADYMTSFR			Oxidation (M)[5,10]		Mascot
1752.8916	1752.8329	-0.0587	-33	419	433	LSNQLVEGQNYVNFK					Mascot
1842.0425	1841.8951	-0.1474	-80	288	302	VQLAIKISGIHWWYK					Mascot
2013.9778	2013.9647	-0.0131	-7	303	320	VPSHAAELTAGYYNLHDR					Mascot
2013.9778	2013.9647	-0.0131	-7	303	320	VPSHAAELTAGYYNLHDR	112	100			Mascot
2733.2786	2733.2993	0.0207	8	346	370	DSEQSSQAMSAPEELVQQVLSAGWR					Mascot
2749.2734	2749.2756	0.0022	1	346	370	DSEQSSQAMSAPEELVQQVLSAGWR			Oxidation (M)[9]		Mascot

7	Chain A, Crystal Structure Of Barley Beta-Amylase Complexed With 2,3-Epoxypropyl-Alpha-D-Glucopyranoside	gi|313103508	59854.4	5.58	10	279	100	23.359	249	100	

Protein Group

Chain A, Crystal Structure Of Barley Beta-Amylase At Atomic Resolution	gi|313103503	59854.4	5.5799999237 0605
Chain A, Crystal Structure Of Barley Beta-Amylase Complexed With 3,4- Epoxybutyl Alpha-D-Glucopyranoside	gi|313103509	59854.4	5.5799999237 0605
Chain A, Crystal Structure Of Barley Beta-Amylase Complexed With 4-O-Alpha-D-Glucopyranosylmoranoline	gi|313103505	59854.4	5.5799999237 0605
Chain A, Crystal Structure Of Barley Beta-Amylase Complexed With Acarbose	gi|313103502	59854.4	5.5799999237 0605
Chain A, Crystal Structure Of Barley Beta-Amylase Complexed With Alpha-Cyclodextrin	gi|313103504	59854.4	5.5799999237 0605

Peptide Information

Calc. Mass	Obsrv. Mass	± da	± ppm	Start Seq.	End Seq.	Sequence	Ion Score	C. I. %	Modification	Rank	Result Type
854.4301	854.4536	0.0235	28	439	445	MHANLPR			Oxidation (M)[1]		Mascot
1016.5564	1016.5394	-0.017	-17	411	418	LFGFTYLR					Mascot
1016.5564	1016.5394	-0.017	-17	411	418	LFGFTYLR	56	99.565			Mascot
1285.6212	1285.5829	-0.0383	-30	61	71	GPKAYDWSAYK					Mascot
1315.5769	1315.5422	-0.0347	-26	335	345	ASINFTCAEMR			Carbamidomethyl (C)[7], Oxidation (M)[10]		Mascot
1326.6688	1326.6444	-0.0244	-18	384	394	YDPTAYNTILR					Mascot
1326.6688	1326.6444	-0.0244	-18	384	394	YDPTAYNTILR	82	99.999			Mascot
1685.7299	1685.7437	0.0138	8	147	160	SAVQMYADYMTSFR			Oxidation (M)[5]		Mascot

1701.7247	1701.7063	-0.0184	-11	147	160	SAVQMYADYMTSFR			Oxidation (M)[5,10]		Mascot
1701.7247	1701.7063	-0.0184	-11	147	160	SAVQMYADYMTSFR			Oxidation (M)[5,10]		Mascot
1705.8181	1705.7739	-0.0442	-26	243	256	TQFFRDNGTYLSEK					Mascot
1842.0425	1841.8951	-0.1474	-80	288	302	VQLAIKISGIHWWYK					Mascot
2013.9778	2013.9647	-0.0131	-7	303	320	VPSHAAELTAGYYNLHD R					Mascot
2013.9778	2013.9647	-0.0131	-7	303	320	VPSHAAELTAGYYNLHD R	112	100			Mascot
2733.2786	2733.2993	0.0207	8	346	370	DSEQSSQAMSAPEELVQ QVLSAGWR					Mascot
2749.2734	2749.2756	0.0022	1	346	370	DSEQSSQAMSAPEELVQ QVLSAGWR			Oxidation (M)[9]		Mascot

8	RecName: Full=Beta-amylase; AltName: Full=1,4-alpha-D-glucan maltohydrolase	gi|113786	59894.5	5.58	10	277	100	23.329	249	100	

Peptide Information

Calc. Mass	Obsrv. Mass	± da	± ppm	Start Seq.	End Seq.	Sequence	Ion Score	C. I. %	Modification	Rank	Result Type
854.4301	854.4536	0.0235	28	439	445	MHANLPR			Oxidation (M)[1]		Mascot
1016.5564	1016.5394	-0.017	-17	411	418	LFGFTYLR					Mascot
1016.5564	1016.5394	-0.017	-17	411	418	LFGFTYLR	56	99.565			Mascot
1285.6212	1285.5829	-0.0383	-30	61	71	GPKAYDWSAYK					Mascot
1315.5769	1315.5422	-0.0347	-26	335	345	ASINFTCAEMR			Carbamidomethyl (C)[7], Oxidation (M)[10]		Mascot
1326.6688	1326.6444	-0.0244	-18	384	394	YDPTAYNTILR					Mascot
1326.6688	1326.6444	-0.0244	-18	384	394	YDPTAYNTILR	82	99.999			Mascot
1685.7299	1685.7437	0.0138	8	147	160	SAVQMYADYMTSFR			Oxidation (M)[5]		Mascot
1701.7247	1701.7063	-0.0184	-11	147	160	SAVQMYADYMTSFR			Oxidation (M)[5,10]		Mascot
1701.7247	1701.7063	-0.0184	-11	147	160	SAVQMYADYMTSFR			Oxidation (M)[5,10]		Mascot
1705.8181	1705.7739	-0.0442	-26	243	256	TQFFRDNGTYLSEK					Mascot
1752.8916	1752.8329	-0.0587	-33	419	433	LSNQLVEGQNYVNFK					Mascot
1842.0425	1841.8951	-0.1474	-80	288	302	VQLAIKISGIHWWYK					Mascot
2013.9778	2013.9647	-0.0131	-7	303	320	VPSHAAELTAGYYNLHD R					Mascot
2013.9778	2013.9647	-0.0131	-7	303	320	VPSHAAELTAGYYNLHD R	112	100			Mascot

9	hypothetical protein OsI_26372 [Oryza sativa Indica Group]	gi|125558692	55463.5	5.3	8	229	100	20.178	206	100	

Peptide Information

Calc. Mass	Obsrv. Mass	± da	± ppm	Start Seq.	End Seq.	Sequence	Ion Score	C. I. %	Modification	Rank	Result Type
1016.5564	1016.5394	-0.017	-17	411	418	LFGFTYLR					Mascot
1016.5564	1016.5394	-0.017	-17	411	418	LFGFTYLR	56	99.565			Mascot

1574.7738	1574.755	-0.0188	-12	475	488	LAPFPFDENTDLPV					Mascot
1624.7603	1624.8005	0.0402	25	111	124	DVGVNDPDIFYTNR					Mascot
1651.7251	1651.7631	0.038	23	333	345	HRACVNFTCAEMR			Carbamidomethyl (C)[4,9]		Mascot
1743.8436	1743.762	-0.0816	-47	419	433	LSDELLEGQNYSTFK					Mascot
1752.8448	1752.8329	-0.0119	-7	147	160	TAIQMYVDYMKSFR					Mascot
2087.0557	2087.0425	-0.0132	-6	129	146	NIEYLTLGVDDQPLFHGR					Mascot
2087.0557	2087.0425	-0.0132	-6	129	146	NIEYLTLGVDDQPLFHGR	150	100			Mascot
2764.3169	2764.3406	0.0237	9	161	186	ENMAEFLDAGVIVDIEVG LGPAGEMR			Oxidation (M)[3,25]		Mascot

10	beta-amylase 2, partial [Zingeria biebersteiniana subsp. trichopoda]	gi|482677639	46672	5.17	6	224	100	19.91	206	100	

Peptide Information

Calc. Mass	Obsrv. Mass	± da	± ppm	Start Seq.	End Seq.	Sequence	Ion Score	C. I. %	Modification	Rank	Result Type
1016.5564	1016.5394	-0.017	-17	402	409	LFGFTYLR					Mascot
1016.5564	1016.5394	-0.017	-17	402	409	LFGFTYLR	56	99.565			Mascot
1683.7506	1683.8013	0.0507	30	138	151	TAVQMYADYMTSFR					Mascot
1729.9093	1729.8262	-0.0831	-48	386	401	NARPTGINKNGPPEHK					Mascot
2087.0557	2087.0425	-0.0132	-6	120	137	NIEYLTLGVDDQPLFHGR					Mascot
2087.0557	2087.0425	-0.0132	-6	120	137	NIEYLTLGVDDQPLFHGR	150	100			Mascot
2118.9631	2119.0642	0.1011	48	320	336	MLTRHHDSLNFTCAEMR			Carbamidomethyl (C)[13]		Mascot
2134.958	2135.0303	0.0723	34	320	336	MLTRHHDSLNFTCAEMR			Carbamidomethyl (C)[13], Oxidation (M)[1]		Mascot
2810.2903	2810.3384	0.0481	17	208	233	AAAVKAGHPEWELPDDA GEYNDTPEK					Mascot

Gel Idx/Pos	255/K7	Instr./Gel Origin	BA2151/Sample Project 20140814	Process Status	Analysis Succeeded
Plate [#] Name	[1] Sample Project 20140814	Instrument Sample Name		Spectra	11

Rank	Protein Name	Accession No.	Protein MW	Protein PI	Pep. Count	Protein Score	Protein Score C. I. %	Intensity Matched	Total Ion Score	Total Ion C. I. %	Confirmed
1	PREDICTED: eukaryotic initiation factor 4A-like [Setaria italica]	gi|514709914	46849.9	5.39	25	1,140	100	54.198	972	100	

Peptide Information

Calc. Mass	Obsrv. Mass	± da	± ppm	Start Seq.	End Seq.	Sequence	Ion Score	C. I. %	Modification	Rank	Result Type
907.4818	907.4484	-0.0334	-37	231	237	FMNKPVR			Oxidation (M)[2]		Mascot
910.4702	910.4323	-0.0379	-42	387	393	MLFDIQK			Oxidation (M)[1]		Mascot
935.5197	935.483	-0.0367	-39	127	135	ALGDYLGVK					Mascot
952.5033	952.4671	-0.0362	-38	166	172	VFDMLRR			Oxidation (M)[4]		Mascot
976.5574	976.5386	-0.0188	-19	374	382	GVAINFVTR					Mascot
1035.5768	1035.5374	-0.0394	-38	230	237	KFMNKPVR			Oxidation (M)[3]		Mascot
1070.5953	1070.5756	-0.0197	-18	173	181	QSLRPDNIK					Mascot
1104.6525	1104.6372	-0.0153	-14	373	382	KGVAINFVTR					Mascot
1104.6525	1104.6372	-0.0153	-14	373	382	KGVAINFVTR	75	99.995			Mascot
1114.6831	1114.6646	-0.0185	-17	329	338	VLITTDLLAR					Mascot
1114.6831	1114.6646	-0.0185	-17	329	338	VLITTDLLAR	74	99.994			Mascot
1142.5736	1142.5586	-0.015	-13	136	146	VHACVGGTSVR			Carbamidomethyl (C)[4]		Mascot
1142.5736	1142.5586	-0.015	-13	136	146	VHACVGGTSVR	71	99.987	Carbamidomethyl (C)[4]		Mascot
1173.6475	1173.6292	-0.0183	-16	242	251	RDELTLEGIK					Mascot
1226.6964	1226.6692	-0.0272	-22	172	181	RQSLRPDNIK					Mascot
1401.7333	1401.6835	-0.0498	-36	74	87	GLDVIQQAQSGTGK					Mascot
1461.8538	1461.8395	-0.0143	-10	151	165	ILASGVHVVVGTPGR					Mascot
1461.8538	1461.8395	-0.0143	-10	151	165	ILASGVHVVVGTPGR	102	100			Mascot
1571.708	1571.6785	-0.0295	-19	182	194	MFVLDEADEMLSR			Oxidation (M)[1]		Mascot
1579.8367	1579.7844	-0.0523	-33	198	210	DQIYDIFQLLPSK					Mascot
1587.703	1587.6549	-0.0481	-30	182	194	MFVLDEADEMLSR			Oxidation (M)[1,10]		Mascot
1587.703	1587.6549	-0.0481	-30	182	194	MFVLDEADEMLSR	26	0	Oxidation (M)[1,10]		Mascot
1800.7566	1800.7279	-0.0287	-16	300	315	DHTVSATHGDMDQNTR			Oxidation (M)[11]		Mascot
1827.9388	1827.933	-0.0058	-3	51	66	GIYAYGFEKPSAIQQR					Mascot
1827.9388	1827.933	-0.0058	-3	51	66	GIYAYGFEKPSAIQQR	138	100			Mascot
1912.0215	1911.9354	-0.0861	-45	195	210	GFKDQIYDIFQLLPSK					Mascot

Calc. Mass	Obsrv. Mass	± da	± ppm	Start Seq.	End Seq.	Sequence	Ion Score	C. I. %	Modification	Rank	Result Type
2055.9441	2055.9917	0.0476	23	1	19	MAGLAPEGSQFDGKQYDNK					Mascot
2059.0754	2059.0776	0.0022	1	127	146	ALGDYLGVKVHACVGGTSVR			Carbamidomethyl (C)[13]		Mascot
2075.0842	2075.0544	-0.0298	-14	211	229	IQVGVFSATMPPEALEITR			Oxidation (M)[10]		Mascot
2075.0842	2075.0544	-0.0298	-14	211	229	IQVGVFSATMPPEALEITR	65	99.951	Oxidation (M)[10]		Mascot
2685.3804	2685.3704	-0.01	-4	264	286	LDTLCDLYETLAITQSVIFVNTR			Carbamidomethyl (C)[5]		Mascot
2911.4949	2911.4919	-0.003	-1	339	363	GIDVQQVSLVINYDLPTQPENYLHR					Mascot
2911.4949	2911.4919	-0.003	-1	339	363	GIDVQQVSLVINYDLPTQPENYLHR	183	100			Mascot
3124.5806	3124.575	-0.0056	-2	88	115	TATFCSGILQQLDYGLVECQALVLAPTR			Carbamidomethyl (C)[5,19]		Mascot
3124.5806	3124.575	-0.0056	-2	88	115	TATFCSGILQQLDYGLVECQALVLAPTR	237	100	Carbamidomethyl (C)[5,19]		Mascot

2	putative DEAD-box ATP-dependent RNA helicase family protein isoform 2 [Zea mays]	gi|413943217	47238.1	5.29	25	1,130	100	56.691	972	100	

Protein Group

putative DEAD-box ATP-dependent RNA helicase family protein isoform 1 [Zea mays]	gi|413943216	47238.1	5.2899 999618 5303
translational initiation factor eIF-4A [Zea mays]	gi|162458395	47237.1	5.3699 998855 5908

Peptide Information

Calc. Mass	Obsrv. Mass	± da	± ppm	Start Seq.	End Seq.	Sequence	Ion Score	C. I. %	Modification	Rank	Result Type
907.4818	907.4484	-0.0334	-37	235	241	FMNKPVR			Oxidation (M)[2]		Mascot
910.4702	910.4323	-0.0379	-42	391	397	MLFDIQK			Oxidation (M)[1]		Mascot
935.5197	935.483	-0.0367	-39	131	139	ALGDYLGVK					Mascot
952.5033	952.4671	-0.0362	-38	170	176	VFDMLRR			Oxidation (M)[4]		Mascot
976.5574	976.5386	-0.0188	-19	378	386	GVAINFVTR					Mascot
1035.5768	1035.5374	-0.0394	-38	234	241	KFMNKPVR			Oxidation (M)[3]		Mascot
1070.5953	1070.5756	-0.0197	-18	177	185	QSLRPDNIK					Mascot
1104.6525	1104.6372	-0.0153	-14	377	386	KGVAINFVTR					Mascot
1104.6525	1104.6372	-0.0153	-14	377	386	KGVAINFVTR	75	99.995			Mascot
1114.6831	1114.6646	-0.0185	-17	333	342	VLITTDLLAR					Mascot
1114.6831	1114.6646	-0.0185	-17	333	342	VLITTDLLAR	74	99.994			Mascot
1142.5736	1142.5586	-0.015	-13	140	150	VHACVGGTSVR			Carbamidomethyl (C)[4]		Mascot
1142.5736	1142.5586	-0.015	-13	140	150	VHACVGGTSVR	71	99.987	Carbamidomethyl (C)[4]		Mascot
1173.6475	1173.6292	-0.0183	-16	246	255	RDELTLEGIK					Mascot
1226.6964	1226.6692	-0.0272	-22	176	185	RQSLRPDNIK					Mascot

Calc. Mass	Obsrv. Mass	± da	± ppm	Start Seq.	End Seq.	Sequence	Ion Score	C. I. %	Modification	Rank	Result Type
1401.7333	1401.6835	-0.0498	-36	78	91	GLDVIQQAQSGTGK					Mascot
1461.8538	1461.8395	-0.0143	-10	155	169	ILASGVHVVVGTPGR					Mascot
1461.8538	1461.8395	-0.0143	-10	155	169	ILASGVHVVVGTPGR	102	100			Mascot
1571.708	1571.6785	-0.0295	-19	186	198	MFVLDEADEMLSR			Oxidation (M)[1]		Mascot
1579.8367	1579.7844	-0.0523	-33	202	214	DQIYDIFQLLPSK					Mascot
1587.703	1587.6549	-0.0481	-30	186	198	MFVLDEADEMLSR			Oxidation (M)[1,10]		Mascot
1587.703	1587.6549	-0.0481	-30	186	198	MFVLDEADEMLSR	26	0	Oxidation (M)[1,10]		Mascot
1800.7566	1800.7279	-0.0287	-16	304	319	DHTVSATHGDMDQNTR			Oxidation (M)[11]		Mascot
1827.9388	1827.933	-0.0058	-3	55	70	GIYAYGFEKPSAIQQR					Mascot
1827.9388	1827.933	-0.0058	-3	55	70	GIYAYGFEKPSAIQQR	138	100			Mascot
1912.0215	1911.9354	-0.0861	-45	199	214	GFKDQIYDIFQLLPSK					Mascot
2011.0383	2011.105	0.0667	33	247	263	DELTLEGIKQFYVNVDK					Mascot
2011.0383	2011.105	0.0667	33	247	263	DELTLEGIKQFYVNVDK					Mascot
2059.0754	2059.0776	0.0022	1	131	150	ALGDYLGVKVHACVGGT SVR			Carbamidomethyl (C)[13]		Mascot
2075.0842	2075.0544	-0.0298	-14	215	233	IQVGVFSATMPPEALEIT R			Oxidation (M)[10]		Mascot
2075.0842	2075.0544	-0.0298	-14	215	233	IQVGVFSATMPPEALEIT R	65	99.951	Oxidation (M)[10]		Mascot
2685.3804	2685.3704	-0.01	-4	268	290	LDTLCDLYETLAITQSVIF VNTR			Carbamidomethyl (C)[5]		Mascot
2911.4949	2911.4919	-0.003	-1	343	367	GIDVQQVSLVINYDLPTQ PENYLHR					Mascot
2911.4949	2911.4919	-0.003	-1	343	367	GIDVQQVSLVINYDLPTQ PENYLHR	183	100			Mascot
3124.5806	3124.575	-0.0056	-2	92	119	TATFCSGILQQLDYGLVE CQALVLAPTR			Carbamidomethyl (C)[5,19]		Mascot
3124.5806	3124.575	-0.0056	-2	92	119	TATFCSGILQQLDYGLVE CQALVLAPTR	237	100	Carbamidomethyl (C)[5,19]		Mascot

3	Eukaryotic initiation factor 4A-1 [Triticum urartu]	gi|474441074	47158	5.38	24	1,120	100	54.095	972	100

Protein Group

Eukaryotic initiation factor 4A-1 [Aegilops tauschii]	gi|475619612	47158	5.3800 001144 4092

Peptide Information

Calc. Mass	Obsrv. Mass	± da	± ppm	Start Seq.	End Seq.	Sequence	Ion Score	C. I. %	Modification	Rank	Result Type
907.4818	907.4484	-0.0334	-37	235	241	FMNKPVR			Oxidation (M)[2]		Mascot
910.4702	910.4323	-0.0379	-42	391	397	MLFDIQK			Oxidation (M)[1]		Mascot
935.5197	935.483	-0.0367	-39	131	139	ALGDYLGVK					Mascot
952.5033	952.4671	-0.0362	-38	170	176	VFDMLRR			Oxidation (M)[4]		Mascot
976.5574	976.5386	-0.0188	-19	378	386	GVAINFVTR					Mascot
1035.5768	1035.5374	-0.0394	-38	234	241	KFMNKPVR			Oxidation (M)[3]		Mascot

Calc. Mass	Obsrv. Mass	± da	± ppm	Start Seq.	End Seq.	Sequence	Ion Score	C. I. %	Modification	Rank	Result Type
1070.5953	1070.5756	-0.0197	-18	177	185	QSLRPDNIK					Mascot
1104.6525	1104.6372	-0.0153	-14	377	386	KGVAINFVTR					Mascot
1104.6525	1104.6372	-0.0153	-14	377	386	KGVAINFVTR	75	99.995			Mascot
1114.6831	1114.6646	-0.0185	-17	333	342	VLITTDLLAR					Mascot
1114.6831	1114.6646	-0.0185	-17	333	342	VLITTDLLAR	74	99.994			Mascot
1142.5736	1142.5586	-0.015	-13	140	150	VHACVGGTSVR			Carbamidomethyl (C)[4]		Mascot
1142.5736	1142.5586	-0.015	-13	140	150	VHACVGGTSVR	71	99.987	Carbamidomethyl (C)[4]		Mascot
1173.6475	1173.6292	-0.0183	-16	246	255	RDELTLEGIK					Mascot
1226.6964	1226.6692	-0.0272	-22	176	185	RQSLRPDNIK					Mascot
1401.7333	1401.6835	-0.0498	-36	78	91	GLDVIQQAQSGTGK					Mascot
1461.8538	1461.8395	-0.0143	-10	155	169	ILASGVHVVVGTPGR					Mascot
1461.8538	1461.8395	-0.0143	-10	155	169	ILASGVHVVVGTPGR	102	100			Mascot
1571.708	1571.6785	-0.0295	-19	186	198	MFVLDEADEMLSR			Oxidation (M)[1]		Mascot
1579.8367	1579.7844	-0.0523	-33	202	214	DQIYDIFQLLPSK					Mascot
1587.703	1587.6549	-0.0481	-30	186	198	MFVLDEADEMLSR			Oxidation (M)[1,10]		Mascot
1587.703	1587.6549	-0.0481	-30	186	198	MFVLDEADEMLSR	26	0	Oxidation (M)[1,10]		Mascot
1800.7566	1800.7279	-0.0287	-16	304	319	DHTVSATHGDMDQNTR			Oxidation (M)[11]		Mascot
1827.9388	1827.933	-0.0058	-3	55	70	GIYAYGFEKPSAIQQR					Mascot
1827.9388	1827.933	-0.0058	-3	55	70	GIYAYGFEKPSAIQQR	138	100			Mascot
1912.0215	1911.9354	-0.0861	-45	199	214	GFKDQIYDIFQLLPSK					Mascot
2059.0754	2059.0776	0.0022	1	131	150	ALGDYLGVKVHACVGGT SVR			Carbamidomethyl (C)[13]		Mascot
2075.0842	2075.0544	-0.0298	-14	215	233	IQVGVFSATMPPEALEIT R			Oxidation (M)[10]		Mascot
2075.0842	2075.0544	-0.0298	-14	215	233	IQVGVFSATMPPEALEIT R	65	99.951	Oxidation (M)[10]		Mascot
2685.3804	2685.3704	-0.01	-4	268	290	LDTLCDLYETLAITQSVIF VNTR			Carbamidomethyl (C)[5]		Mascot
2911.4949	2911.4919	-0.003	-1	343	367	GIDVQQVSLVINYDLPTQ PENYLHR					Mascot
2911.4949	2911.4919	-0.003	-1	343	367	GIDVQQVSLVINYDLPTQ PENYLHR	183	100			Mascot
3124.5806	3124.575	-0.0056	-2	92	119	TATFCSGILQQLDYGLVE CQALVLAPTR			Carbamidomethyl (C)[5,19]		Mascot
3124.5806	3124.575	-0.0056	-2	92	119	TATFCSGILQQLDYGLVE CQALVLAPTR	237	100	Carbamidomethyl (C)[5,19]		Mascot

4	eukaryotic initiation factor 4A [Zea mays]	gi|162460883	46848.9	5.38	23	1,120	100	53.869	972	100	

Protein Group

RecName: Full=Eukaryotic initiation factor 4A; Short=eIF-4A; AltName: Full=ATP-dependent RNA helicase eIF4A	gi|2500522	46848.9	5.3800 001144 4092

Peptide Information

Calc. Mass	Obsrv. Mass	± da	± ppm	Start Seq.	End Seq.	Sequence	Ion Score	C. I. %	Modification	Rank	Result Type

907.4818	907.4484	-0.0334	-37	231	237	FMNKPVR			Oxidation (M)[2]	Mascot
935.5197	935.483	-0.0367	-39	127	135	ALGDYLGVK				Mascot
952.5033	952.4671	-0.0362	-38	166	172	VFDMLRR			Oxidation (M)[4]	Mascot
976.5574	976.5386	-0.0188	-19	374	382	GVAINFVTR				Mascot
1035.5768	1035.5374	-0.0394	-38	230	237	KFMNKPVR			Oxidation (M)[3]	Mascot
1070.5953	1070.5756	-0.0197	-18	173	181	QSLRPDNIK				Mascot
1104.6525	1104.6372	-0.0153	-14	373	382	KGVAINFVTR				Mascot
1104.6525	1104.6372	-0.0153	-14	373	382	KGVAINFVTR	75	99.995		Mascot
1114.6831	1114.6646	-0.0185	-17	329	338	VLITTDLLAR				Mascot
1114.6831	1114.6646	-0.0185	-17	329	338	VLITTDLLAR	74	99.994		Mascot
1142.5736	1142.5586	-0.015	-13	136	146	VHACVGGTSVR			Carbamidomethyl (C)[4]	Mascot
1142.5736	1142.5586	-0.015	-13	136	146	VHACVGGTSVR	71	99.987	Carbamidomethyl (C)[4]	Mascot
1173.6475	1173.6292	-0.0183	-16	242	251	RDELTLEGIK				Mascot
1226.6964	1226.6692	-0.0272	-22	172	181	RQSLRPDNIK				Mascot
1401.7333	1401.6835	-0.0498	-36	74	87	GLDVIQQAQSGTGK				Mascot
1461.8538	1461.8395	-0.0143	-10	151	165	ILASGVHVVVGTPGR				Mascot
1461.8538	1461.8395	-0.0143	-10	151	165	ILASGVHVVVGTPGR	102	100		Mascot
1571.708	1571.6785	-0.0295	-19	182	194	MFVLDEADEMLSR			Oxidation (M)[1]	Mascot
1579.8367	1579.7844	-0.0523	-33	198	210	DQIYDIFQLLPSK				Mascot
1587.703	1587.6549	-0.0481	-30	182	194	MFVLDEADEMLSR			Oxidation (M)[1,10]	Mascot
1587.703	1587.6549	-0.0481	-30	182	194	MFVLDEADEMLSR	26	0	Oxidation (M)[1,10]	Mascot
1800.7566	1800.7279	-0.0287	-16	300	315	DHTVSATHGDMDQNTR			Oxidation (M)[11]	Mascot
1827.9388	1827.933	-0.0058	-3	51	66	GIYAYGFEKPSAIQQR				Mascot
1827.9388	1827.933	-0.0058	-3	51	66	GIYAYGFEKPSAIQQR	138	100		Mascot
1912.0215	1911.9354	-0.0861	-45	195	210	GFKDQIYDIFQLLPSK				Mascot
2059.0754	2059.0776	0.0022	1	127	146	ALGDYLGVKVHACVGGTSVR			Carbamidomethyl (C)[13]	Mascot
2075.0842	2075.0544	-0.0298	-14	211	229	IQVGVFSATMPPEALEITR			Oxidation (M)[10]	Mascot
2075.0842	2075.0544	-0.0298	-14	211	229	IQVGVFSATMPPEALEITR	65	99.951	Oxidation (M)[10]	Mascot
2685.3804	2685.3704	-0.01	-4	264	286	LDTLCDLYETLAITQSVIFVNTR			Carbamidomethyl (C)[5]	Mascot
2911.4949	2911.4919	-0.003	-1	339	363	GIDVQQVSLVINYDLPTQPENYLHR				Mascot
2911.4949	2911.4919	-0.003	-1	339	363	GIDVQQVSLVINYDLPTQPENYLHR	183	100		Mascot
3124.5806	3124.575	-0.0056	-2	88	115	TATFCSGILQQLDYGLVECQALVLAPTR			Carbamidomethyl (C)[5,19]	Mascot
3124.5806	3124.575	-0.0056	-2	88	115	TATFCSGILQQLDYGLVECQALVLAPTR	237	100	Carbamidomethyl (C)[5,19]	Mascot

5	hypothetical protein SORBIDRAFT_04g003390	gi|241931367	46949.9	5.39	23	1,110	100	53.869	972	100

[Sorghum bicolor]

Protein Group

LOC100284697 [Zea mays]	gi|226501258	46894.9	5.3800 001144 4092

Peptide Information

Calc. Mass	Obsrv. Mass	± da	± ppm	Start Seq.	End Seq.	Sequence	Ion Score	C. I. %	Modification	Rank	Result Type
907.4818	907.4484	-0.0334	-37	231	237	FMNKPVR			Oxidation (M)[2]		Mascot
935.5197	935.483	-0.0367	-39	127	135	ALGDYLGVK					Mascot
952.5033	952.4671	-0.0362	-38	166	172	VFDMLRR			Oxidation (M)[4]		Mascot
976.5574	976.5386	-0.0188	-19	374	382	GVAINFVTR					Mascot
1035.5768	1035.5374	-0.0394	-38	230	237	KFMNKPVR			Oxidation (M)[3]		Mascot
1070.5953	1070.5756	-0.0197	-18	173	181	QSLRPDNIK					Mascot
1104.6525	1104.6372	-0.0153	-14	373	382	KGVAINFVTR					Mascot
1104.6525	1104.6372	-0.0153	-14	373	382	KGVAINFVTR	75	99.995			Mascot
1114.6831	1114.6646	-0.0185	-17	329	338	VLITTDLLAR					Mascot
1114.6831	1114.6646	-0.0185	-17	329	338	VLITTDLLAR	74	99.994			Mascot
1142.5736	1142.5586	-0.015	-13	136	146	VHACVGGTSVR			Carbamidomethyl (C)[4]		Mascot
1142.5736	1142.5586	-0.015	-13	136	146	VHACVGGTSVR	71	99.987	Carbamidomethyl (C)[4]		Mascot
1173.6475	1173.6292	-0.0183	-16	242	251	RDELTLEGIK					Mascot
1226.6964	1226.6692	-0.0272	-22	172	181	RQSLRPDNIK					Mascot
1401.7333	1401.6835	-0.0498	-36	74	87	GLDVIQQAQSGTGK					Mascot
1461.8538	1461.8395	-0.0143	-10	151	165	ILASGVHVVVGTPGR					Mascot
1461.8538	1461.8395	-0.0143	-10	151	165	ILASGVHVVVGTPGR	102	100			Mascot
1571.708	1571.6785	-0.0295	-19	182	194	MFVLDEADEMLSR			Oxidation (M)[1]		Mascot
1579.8367	1579.7844	-0.0523	-33	198	210	DQIYDIFQLLPSK					Mascot
1587.703	1587.6549	-0.0481	-30	182	194	MFVLDEADEMLSR			Oxidation (M)[1,10]		Mascot
1587.703	1587.6549	-0.0481	-30	182	194	MFVLDEADEMLSR	26	0	Oxidation (M)[1,10]		Mascot
1800.7566	1800.7279	-0.0287	-16	300	315	DHTVSATHGDMDQNTR			Oxidation (M)[11]		Mascot
1827.9388	1827.933	-0.0058	-3	51	66	GIYAYGFEKPSAIQQR					Mascot
1827.9388	1827.933	-0.0058	-3	51	66	GIYAYGFEKPSAIQQR	138	100			Mascot
1912.0215	1911.9354	-0.0861	-45	195	210	GFKDQIYDIFQLLPSK					Mascot
2059.0754	2059.0776	0.0022	1	127	146	ALGDYLGVKVHACVGGT SVR			Carbamidomethyl (C)[13]		Mascot
2075.0842	2075.0544	-0.0298	-14	211	229	IQVGVFSATMPPEALEIT R			Oxidation (M)[10]		Mascot
2075.0842	2075.0544	-0.0298	-14	211	229	IQVGVFSATMPPEALEIT R	65	99.951	Oxidation (M)[10]		Mascot
2685.3804	2685.3704	-0.01	-4	264	286	LDTLCDLYETLAITQSVIF			Carbamidomethyl (C)[5]		Mascot

Calc. Mass	Obsrv. Mass	± da	± ppm	Start Seq.	End Seq.	Sequence	Ion Score	C. I. %	Modification	Rank	Result Type
						VNTR					
2911.4949	2911.4919	-0.003	-1	339	363	GIDVQQVSLVINYDLPTQ PENYLHR					Mascot
2911.4949	2911.4919	-0.003	-1	339	363	GIDVQQVSLVINYDLPTQ PENYLHR	183	100			Mascot
3124.5806	3124.575	-0.0056	-2	88	115	TATFCSGILQQLDYGLVE CQALVLAPTR			Carbamidomethyl (C)[5,19]		Mascot
3124.5806	3124.575	-0.0056	-2	88	115	TATFCSGILQQLDYGLVE CQALVLAPTR	237	100	Carbamidomethyl (C)[5,19]		Mascot

6 Eukaryotic initiation factor 4A [Aegilops tauschii] gi|475553745 47200.1 5.31 22 1,100 100 53.614 972 100

Peptide Information

Calc. Mass	Obsrv. Mass	± da	± ppm	Start Seq.	End Seq.	Sequence	Ion Score	C. I. %	Modification	Rank	Result Type
907.4818	907.4484	-0.0334	-37	235	241	FMNKPVR			Oxidation (M)[2]		Mascot
910.4702	910.4323	-0.0379	-42	391	397	MLFDIQK			Oxidation (M)[1]		Mascot
935.5197	935.483	-0.0367	-39	131	139	ALGDYLGVK					Mascot
952.5033	952.4671	-0.0362	-38	170	176	VFDMLRR			Oxidation (M)[4]		Mascot
976.5574	976.5386	-0.0188	-19	378	386	GVAINFVTR					Mascot
1035.5768	1035.5374	-0.0394	-38	234	241	KFMNKPVR			Oxidation (M)[3]		Mascot
1070.5953	1070.5756	-0.0197	-18	177	185	QSLRPDNIK					Mascot
1104.6525	1104.6372	-0.0153	-14	377	386	KGVAINFVTR					Mascot
1104.6525	1104.6372	-0.0153	-14	377	386	KGVAINFVTR	75	99.995			Mascot
1114.6831	1114.6646	-0.0185	-17	333	342	VLITTDLLAR					Mascot
1114.6831	1114.6646	-0.0185	-17	333	342	VLITTDLLAR	74	99.994			Mascot
1142.5736	1142.5586	-0.015	-13	140	150	VHACVGGTSVR			Carbamidomethyl (C)[4]		Mascot
1142.5736	1142.5586	-0.015	-13	140	150	VHACVGGTSVR	71	99.987	Carbamidomethyl (C)[4]		Mascot
1173.6475	1173.6292	-0.0183	-16	246	255	RDELTLEGIK					Mascot
1226.6964	1226.6692	-0.0272	-22	176	185	RQSLRPDNIK					Mascot
1401.7333	1401.6835	-0.0498	-36	78	91	GLDVIQQAQSGTGK					Mascot
1461.8538	1461.8395	-0.0143	-10	155	169	ILASGVHVVVGTPGR					Mascot
1461.8538	1461.8395	-0.0143	-10	155	169	ILASGVHVVVGTPGR	102	100			Mascot
1571.708	1571.6785	-0.0295	-19	186	198	MFVLDEADEMLSR			Oxidation (M)[1]		Mascot
1587.703	1587.6549	-0.0481	-30	186	198	MFVLDEADEMLSR			Oxidation (M)[1,10]		Mascot
1587.703	1587.6549	-0.0481	-30	186	198	MFVLDEADEMLSR	26	0	Oxidation (M)[1,10]		Mascot
1800.7566	1800.7279	-0.0287	-16	304	319	DHTVSATHGDMDQNTR			Oxidation (M)[11]		Mascot
1827.9388	1827.933	-0.0058	-3	55	70	GIYAYGFEKPSAIQQR					Mascot
1827.9388	1827.933	-0.0058	-3	55	70	GIYAYGFEKPSAIQQR	138	100			Mascot
2059.0754	2059.0776	0.0022	1	131	150	ALGDYLGVKVHACVGGT SVR			Carbamidomethyl (C)[13]		Mascot
2075.0842	2075.0544	-0.0298	-14	215	233	IQVGVFSATMPPEALEIT R			Oxidation (M)[10]		Mascot

2075.0842	2075.0544	-0.0298	-14	215	233	IQVGVFSATMPPEALEIT R	65	99.951	Oxidation (M)[10]		Mascot
2685.3804	2685.3704	-0.01	-4	268	290	LDTLCDLYETLAITQSVIF VNTR			Carbamidomethyl (C)[5]		Mascot
2911.4949	2911.4919	-0.003	-1	343	367	GIDVQQVSLVINYDLPTQ PENYLHR					Mascot
2911.4949	2911.4919	-0.003	-1	343	367	GIDVQQVSLVINYDLPTQ PENYLHR	183	100			Mascot
3124.5806	3124.575	-0.0056	-2	92	119	TATFCSGILQQLDYGLVE CQALVLAPTR			Carbamidomethyl (C)[5,19]		Mascot
3124.5806	3124.575	-0.0056	-2	92	119	TATFCSGILQQLDYGLVE CQALVLAPTR	237	100	Carbamidomethyl (C)[5,19]		Mascot

7	PREDICTED: eukaryotic initiation factor 4A-3-like [Setaria italica]	gi|514766903	47316.1	5.37	21	1,090	100	52.832	972	100	

Peptide Information

Calc. Mass	Obsrv. Mass	± da	± ppm	Start Seq.	End Seq.	Sequence	Ion Score	C. I. %	Modification	Rank	Result Type
907.4818	907.4484	-0.0334	-37	235	241	FMNKPVR			Oxidation (M)[2]		Mascot
935.5197	935.483	-0.0367	-39	131	139	ALGDYLGVK					Mascot
952.5033	952.4671	-0.0362	-38	170	176	VFDMLRR			Oxidation (M)[4]		Mascot
976.5574	976.5386	-0.0188	-19	378	386	GVAINFVTR					Mascot
1035.5768	1035.5374	-0.0394	-38	234	241	KFMNKPVR			Oxidation (M)[3]		Mascot
1104.6525	1104.6372	-0.0153	-14	377	386	KGVAINFVTR					Mascot
1104.6525	1104.6372	-0.0153	-14	377	386	KGVAINFVTR	75	99.995			Mascot
1114.6831	1114.6646	-0.0185	-17	333	342	VLITTDLLAR					Mascot
1114.6831	1114.6646	-0.0185	-17	333	342	VLITTDLLAR	74	99.994			Mascot
1142.5736	1142.5586	-0.015	-13	140	150	VHACVGGTSVR			Carbamidomethyl (C)[4]		Mascot
1142.5736	1142.5586	-0.015	-13	140	150	VHACVGGTSVR	71	99.987	Carbamidomethyl (C)[4]		Mascot
1173.6475	1173.6292	-0.0183	-16	246	255	RDELTLEGIK					Mascot
1401.7333	1401.6835	-0.0498	-36	78	91	GLDVIQQAQSGTGK					Mascot
1461.8538	1461.8395	-0.0143	-10	155	169	ILASGVHVVVGTPGR					Mascot
1461.8538	1461.8395	-0.0143	-10	155	169	ILASGVHVVVGTPGR	102	100			Mascot
1571.708	1571.6785	-0.0295	-19	186	198	MFVLDEADEMLSR			Oxidation (M)[1]		Mascot
1579.8367	1579.7844	-0.0523	-33	202	214	DQIYDIFQLLPSK					Mascot
1587.703	1587.6549	-0.0481	-30	186	198	MFVLDEADEMLSR			Oxidation (M)[1,10]		Mascot
1587.703	1587.6549	-0.0481	-30	186	198	MFVLDEADEMLSR	26	0	Oxidation (M)[1,10]		Mascot
1800.7566	1800.7279	-0.0287	-16	304	319	DHTVSATHGDMDQNTR			Oxidation (M)[11]		Mascot
1827.9388	1827.933	-0.0058	-3	55	70	GIYAYGFEKPSAIQQR					Mascot
1827.9388	1827.933	-0.0058	-3	55	70	GIYAYGFEKPSAIQQR	138	100			Mascot
1912.0215	1911.9354	-0.0861	-45	199	214	GFKDQIYDIFQLLPSK					Mascot
2059.0754	2059.0776	0.0022	1	131	150	ALGDYLGVKVHACVGGT			Carbamidomethyl (C)[13]		Mascot

						SVR					
2075.0842	2075.0544	-0.0298	-14	215	233	IQVGVFSATMPPEALEITR			Oxidation (M)[10]		Mascot
2075.0842	2075.0544	-0.0298	-14	215	233	IQVGVFSATMPPEALEITR	65	99.951	Oxidation (M)[10]		Mascot
2685.3804	2685.3704	-0.01	-4	268	290	LDTLCDLYETLAITQSVIFVNTR			Carbamidomethyl (C)[5]		Mascot
2911.4949	2911.4919	-0.003	-1	343	367	GIDVQQVSLVINYDLPTQPENYLHR					Mascot
2911.4949	2911.4919	-0.003	-1	343	367	GIDVQQVSLVINYDLPTQPENYLHR	183	100			Mascot
3124.5806	3124.575	-0.0056	-2	92	119	TATFCSGILQQLDYGLVECQALVLAPTR			Carbamidomethyl (C)[5,19]		Mascot
3124.5806	3124.575	-0.0056	-2	92	119	TATFCSGILQQLDYGLVECQALVLAPTR	237	100	Carbamidomethyl (C)[5,19]		Mascot

8	Os06g0701100 [Oryza sativa Japonica Group]	gi|113596521	47343.1	5.37	21	1,070	100	52.832	951	100	

Protein Group

RecName: Full=Eukaryotic initiation factor 4A-1; Short=eIF-4A-1; AltName: Full=ATP-dependent RNA helicase eIF4A-1; AltName: Full=DEAD-box ATP-dependent RNA helicase 4	gi|97536398	47343.1	5.3699998555908

Peptide Information

Calc. Mass	Obsrv. Mass	± da	± ppm	Start Seq.	End Seq.	Sequence	Ion Score	C. I. %	Modification	Rank	Result Type
907.4818	907.4484	-0.0334	-37	235	241	FMNKPVR			Oxidation (M)[2]		Mascot
935.5197	935.483	-0.0367	-39	131	139	ALGDYLGVK					Mascot
952.5033	952.4671	-0.0362	-38	170	176	VFDMLRR			Oxidation (M)[4]		Mascot
976.5574	976.5386	-0.0188	-19	378	386	GVAINFVTR					Mascot
1035.5768	1035.5374	-0.0394	-38	234	241	KFMNKPVR			Oxidation (M)[3]		Mascot
1104.6525	1104.6372	-0.0153	-14	377	386	KGVAINFVTR					Mascot
1104.6525	1104.6372	-0.0153	-14	377	386	KGVAINFVTR	75	99.995			Mascot
1114.6831	1114.6646	-0.0185	-17	333	342	VLITTDLLAR					Mascot
1114.6831	1114.6646	-0.0185	-17	333	342	VLITTDLLAR	74	99.994			Mascot
1142.5736	1142.5586	-0.015	-13	140	150	VHACVGGTSVR			Carbamidomethyl (C)[4]		Mascot
1142.5736	1142.5586	-0.015	-13	140	150	VHACVGGTSVR	71	99.987	Carbamidomethyl (C)[4]		Mascot
1173.6475	1173.6292	-0.0183	-16	246	255	RDELTLEGIK					Mascot
1401.7333	1401.6835	-0.0498	-36	78	91	GLDVIQQAQSGTGK					Mascot
1461.8538	1461.8395	-0.0143	-10	155	169	ILASGVHVVVGTPGR					Mascot
1461.8538	1461.8395	-0.0143	-10	155	169	ILASGVHVVVGTPGR	102	100			Mascot
1571.708	1571.6785	-0.0295	-19	186	198	MFVLDEADEMLSR			Oxidation (M)[1]		Mascot
1579.8367	1579.7844	-0.0523	-33	202	214	DQIYDIFQLLPSK					Mascot
1587.703	1587.6549	-0.0481	-30	186	198	MFVLDEADEMLSR			Oxidation (M)[1,10]		Mascot
1587.703	1587.6549	-0.0481	-30	186	198	MFVLDEADEMLSR	26	0	Oxidation (M)[1,10]		Mascot

1800.7566	1800.7279	-0.0287	-16	304	319	DHTVSATHGDMDQNTR			Oxidation (M)[11]		Mascot
1827.9388	1827.933	-0.0058	-3	55	70	GIYAYGFEKPSAIQQR					Mascot
1827.9388	1827.933	-0.0058	-3	55	70	GIYAYGFEKPSAIQQR	138	100			Mascot
1912.0215	1911.9354	-0.0861	-45	199	214	GFKDQIYDIFQLLPSK					Mascot
2059.0754	2059.0776	0.0022	1	131	150	ALGDYLGVKVHACVGGT SVR			Carbamidomethyl (C)[13]		Mascot
2075.0842	2075.0544	-0.0298	-14	215	233	IQVGVFSATMPPEALEIT R			Oxidation (M)[10]		Mascot
2075.0842	2075.0544	-0.0298	-14	215	233	IQVGVFSATMPPEALEIT R	65	99.951	Oxidation (M)[10]		Mascot
2685.3804	2685.3704	-0.01	-4	268	290	LDTLCDLYETLAITQSVIF VNTR			Carbamidomethyl (C)[5]		Mascot
2911.4949	2911.4919	-0.003	-1	343	367	GIDVQQVSLVINYDLPTQ PENYLHR					Mascot
2911.4949	2911.4919	-0.003	-1	343	367	GIDVQQVSLVINYDLPTQ PENYLHR	183	100			Mascot
3124.5806	3124.575	-0.0056	-2	92	119	TATFCSGILQQLDYAVVE CQALVLAPTR			Carbamidomethyl (C)[5,19]		Mascot
3124.5806	3124.575	-0.0056	-2	92	119	TATFCSGILQQLDYAVVE CQALVLAPTR	216	100	Carbamidomethyl (C)[5,19]		Mascot

9	putative DEAD-box ATP-dependent RNA helicase family protein [Zea mays]	gi|413934786	46948.9	5.29	23	1,020	100	50.586	870	100

Peptide Information

Calc. Mass	Obsrv. Mass	± da	± ppm	Start Seq.	End Seq.	Sequence	Ion Score	C. I. %	Modification	Rank	Result Type
907.4818	907.4484	-0.0334	-37	232	238	FMNKPVR			Oxidation (M)[2]		Mascot
910.4702	910.4323	-0.0379	-42	388	394	MLFDIQK			Oxidation (M)[1]		Mascot
935.5197	935.483	-0.0367	-39	128	136	ALGDYLGVK					Mascot
952.5033	952.4671	-0.0362	-38	167	173	VFDMLRR			Oxidation (M)[4]		Mascot
976.5574	976.5386	-0.0188	-19	375	383	GVAINFVTR					Mascot
1035.5768	1035.5374	-0.0394	-38	231	238	KFMNKPVR			Oxidation (M)[3]		Mascot
1070.5953	1070.5756	-0.0197	-18	174	182	QSLRPDNIK					Mascot
1104.6525	1104.6372	-0.0153	-14	374	383	KGVAINFVTR					Mascot
1104.6525	1104.6372	-0.0153	-14	374	383	KGVAINFVTR	75	99.995			Mascot
1114.6831	1114.6646	-0.0185	-17	330	339	VLITTDLLAR					Mascot
1114.6831	1114.6646	-0.0185	-17	330	339	VLITTDLLAR	74	99.994			Mascot
1142.5736	1142.5586	-0.015	-13	137	147	VHACVGGTSVR			Carbamidomethyl (C)[4]		Mascot
1142.5736	1142.5586	-0.015	-13	137	147	VHACVGGTSVR	71	99.987	Carbamidomethyl (C)[4]		Mascot
1173.6475	1173.6292	-0.0183	-16	243	252	RDELTLEGIK					Mascot
1226.6964	1226.6692	-0.0272	-22	173	182	RQSLRPDNIK					Mascot
1401.7333	1401.6835	-0.0498	-36	75	88	GLDVIQQAQSGTGK					Mascot
1571.708	1571.6785	-0.0295	-19	183	195	MFVLDEADEMLSR			Oxidation (M)[1]		Mascot
1587.703	1587.6549	-0.0481	-30	183	195	MFVLDEADEMLSR			Oxidation (M)[1,10]		Mascot

1587.703	1587.6549	-0.0481	-30	183	195	MFVLDEADEMLSR	26	0	Oxidation (M)[1,10]		Mascot
1800.7566	1800.7279	-0.0287	-16	301	316	DHTVSATHGDMDQNTR			Oxidation (M)[11]		Mascot
1810.8065	1810.9625	0.156	86	1	16	MAPEGSQFDAKHYDSK					Mascot
1826.8014	1826.9088	0.1074	59	1	16	MAPEGSQFDAKHYDSK			Oxidation (M)[1]		Mascot
1827.9388	1827.933	-0.0058	-3	52	67	GIYAYGFEKPSAIQQR					Mascot
1827.9388	1827.933	-0.0058	-3	52	67	GIYAYGFEKPSAIQQR	138	100			Mascot
2011.0383	2011.105	0.0667	33	244	260	DELTLEGIKQFYVNVDK					Mascot
2011.0383	2011.105	0.0667	33	244	260	DELTLEGIKQFYVNVDK					Mascot
2059.0754	2059.0776	0.0022	1	128	147	ALGDYLGVKVHACVGGT SVR			Carbamidomethyl (C)[13]		Mascot
2075.0842	2075.0544	-0.0298	-14	212	230	IQVGVFSATMPPEALEIT R			Oxidation (M)[10]		Mascot
2075.0842	2075.0544	-0.0298	-14	212	230	IQVGVFSATMPPEALEIT R	65	99.951	Oxidation (M)[10]		Mascot
2685.3804	2685.3704	-0.01	-4	265	287	LDTLCDLYETLAITQSVIF VNTR			Carbamidomethyl (C)[5]		Mascot
2911.4949	2911.4919	-0.003	-1	340	364	GIDVQQVSLVINYDLPTQ PENYLHR					Mascot
2911.4949	2911.4919	-0.003	-1	340	364	GIDVQQVSLVINYDLPTQ PENYLHR	183	100			Mascot
3124.5806	3124.575	-0.0056	-2	89	116	TATFCSGILQQLDYGLVE CQALVLAPTR			Carbamidomethyl (C)[5,19]		Mascot
3124.5806	3124.575	-0.0056	-2	89	116	TATFCSGILQQLDYGLVE CQALVLAPTR	237	100	Carbamidomethyl (C)[5,19]		Mascot

10 putative DEAD-box ATP-dependent RNA helicase family protein [Zea mays] gi|413934787 47208 5.29 22 998 100 49.973 870 100

Peptide Information

Calc. Mass	Obsrv. Mass	± da	± ppm	Start Seq.	End Seq.	Sequence	Ion Score	C. I. %	Modification	Rank	Result Type
907.4818	907.4484	-0.0334	-37	235	241	FMNKPVR			Oxidation (M)[2]		Mascot
910.4702	910.4323	-0.0379	-42	391	397	MLFDIQK			Oxidation (M)[1]		Mascot
935.5197	935.483	-0.0367	-39	131	139	ALGDYLGVK					Mascot
952.5033	952.4671	-0.0362	-38	170	176	VFDMLRR			Oxidation (M)[4]		Mascot
976.5574	976.5386	-0.0188	-19	378	386	GVAINFVTR					Mascot
1035.5768	1035.5374	-0.0394	-38	234	241	KFMNKPVR			Oxidation (M)[3]		Mascot
1070.5953	1070.5756	-0.0197	-18	177	185	QSLRPDNIK					Mascot
1104.6525	1104.6372	-0.0153	-14	377	386	KGVAINFVTR					Mascot
1104.6525	1104.6372	-0.0153	-14	377	386	KGVAINFVTR	75	99.995			Mascot
1114.6831	1114.6646	-0.0185	-17	333	342	VLITTDLLAR					Mascot
1114.6831	1114.6646	-0.0185	-17	333	342	VLITTDLLAR	74	99.994			Mascot
1142.5736	1142.5586	-0.015	-13	140	150	VHACVGGTSVR			Carbamidomethyl (C)[4]		Mascot
1142.5736	1142.5586	-0.015	-13	140	150	VHACVGGTSVR	71	99.987	Carbamidomethyl (C)[4]		Mascot

1173.6475	1173.6292	-0.0183	-16	246	255	RDELTLEGIK				Mascot
1226.6964	1226.6692	-0.0272	-22	176	185	RQSLRPDNIK				Mascot
1401.7333	1401.6835	-0.0498	-36	78	91	GLDVIQQAQSGTGK				Mascot
1571.708	1571.6785	-0.0295	-19	186	198	MFVLDEADEMLSR			Oxidation (M)[1]	Mascot
1587.703	1587.6549	-0.0481	-30	186	198	MFVLDEADEMLSR			Oxidation (M)[1,10]	Mascot
1587.703	1587.6549	-0.0481	-30	186	198	MFVLDEADEMLSR	26	0	Oxidation (M)[1,10]	Mascot
1800.7566	1800.7279	-0.0287	-16	304	319	DHTVSATHGDMDQNTR			Oxidation (M)[11]	Mascot
1827.9388	1827.933	-0.0058	-3	55	70	GIYAYGFEKPSAIQQR				Mascot
1827.9388	1827.933	-0.0058	-3	55	70	GIYAYGFEKPSAIQQR	138	100		Mascot
2011.0383	2011.105	0.0667	33	247	263	DELTLEGIKQFYVNVDK				Mascot
2011.0383	2011.105	0.0667	33	247	263	DELTLEGIKQFYVNVDK				Mascot
2059.0754	2059.0776	0.0022	1	131	150	ALGDYLGVKVHACVGGTSVR			Carbamidomethyl (C)[13]	Mascot
2075.0842	2075.0544	-0.0298	-14	215	233	IQVGVFSATMPPEALEITR			Oxidation (M)[10]	Mascot
2075.0842	2075.0544	-0.0298	-14	215	233	IQVGVFSATMPPEALEITR	65	99.944	Oxidation (M)[10]	Mascot
2685.3804	2685.3704	-0.01	-4	268	290	LDTLCDLYETLAITQSVIFVNTR			Carbamidomethyl (C)[5]	Mascot
2911.4949	2911.4919	-0.003	-1	343	367	GIDVQQVSLVINYDLPTQPENYLHR				Mascot
2911.4949	2911.4919	-0.003	-1	343	367	GIDVQQVSLVINYDLPTQPENYLHR	183	100		Mascot
3124.5806	3124.575	-0.0056	-2	92	119	TATFCSGILQQLDYGLVECQALVLAPTR			Carbamidomethyl (C)[5,19]	Mascot
3124.5806	3124.575	-0.0056	-2	92	119	TATFCSGILQQLDYGLVECQALVLAPTR	237	100	Carbamidomethyl (C)[5,19]	Mascot

Gel Idx/Pos	256/K8	Instr./Gel Origin	BA2151/Sample Project 20140814	Process Status	Analysis Succeeded
Plate [#] Name	[1] Sample Project 20140814	Instrument Sample Name		Spectra	11

Rank	Protein Name	Accession No.	Protein MW	Protein PI	Pep. Count	Protein Score	Protein Score C. I. %	Intensity Matched	Total Ion Score	Total Ion C. I. %	Confirmed
1	Eukaryotic initiation factor 4A [Aegilops tauschii]	gi|475553745	47200.1	5.31	25	895	100	49.505	739	100	

Peptide Information

Calc. Mass	Obsrv. Mass	± da	± ppm	Start Seq.	End Seq.	Sequence	Ion Score	C. I. %	Modification	Rank	Result Type
907.4818	907.4412	-0.0406	-45	235	241	FMNKPVR			Oxidation (M)[2]		Mascot
910.4702	910.4266	-0.0436	-48	391	397	MLFDIQK			Oxidation (M)[1]		Mascot
935.5197	935.4751	-0.0446	-48	131	139	ALGDYLGVK					Mascot
952.5033	952.4612	-0.0421	-44	170	176	VFDMLRR			Oxidation (M)[4]		Mascot
976.5574	976.5323	-0.0251	-26	378	386	GVAINFVTR					Mascot
1035.5768	1035.5188	-0.058	-56	234	241	KFMNKPVR			Oxidation (M)[3]		Mascot
1070.5953	1070.5665	-0.0288	-27	177	185	QSLRPDNIK					Mascot
1104.6525	1104.6274	-0.0251	-23	377	386	KGVAINFVTR					Mascot
1104.6525	1104.6274	-0.0251	-23	377	386	KGVAINFVTR	63	99.905			Mascot
1114.6831	1114.6573	-0.0258	-23	333	342	VLITTDLLAR					Mascot
1114.6831	1114.6573	-0.0258	-23	333	342	VLITTDLLAR	77	99.996			Mascot
1142.5736	1142.5491	-0.0245	-21	140	150	VHACVGGTSVR			Carbamidomethyl (C)[4]		Mascot
1142.5736	1142.5491	-0.0245	-21	140	150	VHACVGGTSVR	70	99.981	Carbamidomethyl (C)[4]		Mascot
1173.6475	1173.6195	-0.028	-24	246	255	RDELTLEGIK					Mascot
1226.6964	1226.662	-0.0344	-28	176	185	RQSLRPDNIK					Mascot
1401.7333	1401.6686	-0.0647	-46	78	91	GLDVIQQAQSGTGK					Mascot
1461.8538	1461.8269	-0.0269	-18	155	169	ILASGVHVVVGTPGR					Mascot
1461.8538	1461.8269	-0.0269	-18	155	169	ILASGVHVVVGTPGR	108	100			Mascot
1549.8262	1549.7515	-0.0747	-48	202	214	DQIYDIFQLLPGK					Mascot
1555.7131	1555.6875	-0.0256	-16	186	198	MFVLDEADEMLSR					Mascot
1571.708	1571.6927	-0.0153	-10	186	198	MFVLDEADEMLSR			Oxidation (M)[1]		Mascot
1587.703	1587.6492	-0.0538	-34	186	198	MFVLDEADEMLSR			Oxidation (M)[1,10]		Mascot
1598.7849	1598.726	-0.0589	-37	256	267	QFYVNVEKEEWK					Mascot
1827.9388	1827.9164	-0.0224	-12	55	70	GIYAYGFEKPSAIQQR					Mascot
1827.9388	1827.9164	-0.0224	-12	55	70	GIYAYGFEKPSAIQQR	141	100			Mascot
1882.011	1881.8552	-0.1558	-83	199	214	GFKDQIYDIFQLLPGK					Mascot
1903.8928	1903.9167	0.0239	13	186	201	MFVLDEADEMLSRGFK			Oxidation (M)[1]		Mascot

1919.8878	1919.9137	0.0259	13	186	201	MFVLDEADEMLSRGFK			Oxidation (M)[1,10]		Mascot
2059.0754	2059.063	-0.0124	-6	131	150	ALGDYLGVKVHACVGGT SVR			Carbamidomethyl (C)[13]		Mascot
2059.0894	2059.063	-0.0264	-13	215	233	IQVGVFSATMPPEALEIT R	84	100			Mascot
2075.0842	2075.033	-0.0512	-25	215	233	IQVGVFSATMPPEALEIT R			Oxidation (M)[10]		Mascot
2075.0842	2075.033	-0.0512	-25	215	233	IQVGVFSATMPPEALEIT R	66	99.949	Oxidation (M)[10]		Mascot
2685.3804	2685.3508	-0.0296	-11	268	290	LDTLCDLYETLAITQSVIF VNTR			Carbamidomethyl (C)[5]		Mascot
2911.4949	2911.4683	-0.0266	-9	343	367	GIDVQQVSLVINYDLPTQ PENYLHR					Mascot
2911.4949	2911.4683	-0.0266	-9	343	367	GIDVQQVSLVINYDLPTQ PENYLHR	197	100			Mascot
3124.5806	3124.5498	-0.0308	-10	92	119	TATFCSGILQQLDYGLVE CQALVLAPTR			Carbamidomethyl (C)[5,19]		Mascot

2	PREDICTED: eukaryotic initiation factor 4A-like [Setaria italica]	gi|514709914	46849.9	5.39	23	885	100	48.638	739	100

Peptide Information

Calc. Mass	Obsrv. Mass	± da	± ppm	Start Seq.	End Seq.	Sequence	Ion Score	C. I. %	Modification	Rank	Result Type
907.4818	907.4412	-0.0406	-45	231	237	FMNKPVR			Oxidation (M)[2]		Mascot
910.4702	910.4266	-0.0436	-48	387	393	MLFDIQK			Oxidation (M)[1]		Mascot
935.5197	935.4751	-0.0446	-48	127	135	ALGDYLGVK					Mascot
952.5033	952.4612	-0.0421	-44	166	172	VFDMLRR			Oxidation (M)[4]		Mascot
976.5574	976.5323	-0.0251	-26	374	382	GVAINFVTR					Mascot
1035.5768	1035.5188	-0.058	-56	230	237	KFMNKPVR			Oxidation (M)[3]		Mascot
1070.5953	1070.5665	-0.0288	-27	173	181	QSLRPDNIK					Mascot
1104.6525	1104.6274	-0.0251	-23	373	382	KGVAINFVTR					Mascot
1104.6525	1104.6274	-0.0251	-23	373	382	KGVAINFVTR	63	99.905			Mascot
1114.6831	1114.6573	-0.0258	-23	329	338	VLITTDLLAR					Mascot
1114.6831	1114.6573	-0.0258	-23	329	338	VLITTDLLAR	77	99.996			Mascot
1142.5736	1142.5491	-0.0245	-21	136	146	VHACVGGTSVR			Carbamidomethyl (C)[4]		Mascot
1142.5736	1142.5491	-0.0245	-21	136	146	VHACVGGTSVR	70	99.981	Carbamidomethyl (C)[4]		Mascot
1173.6475	1173.6195	-0.028	-24	242	251	RDELTLEGIK					Mascot
1226.6964	1226.662	-0.0344	-28	172	181	RQSLRPDNIK					Mascot
1401.7333	1401.6686	-0.0647	-46	74	87	GLDVIQQAQSGTGK					Mascot
1461.8538	1461.8269	-0.0269	-18	151	165	ILASGVHVVVGTPGR					Mascot
1461.8538	1461.8269	-0.0269	-18	151	165	ILASGVHVVVGTPGR	108	100			Mascot
1555.7131	1555.6875	-0.0256	-16	182	194	MFVLDEADEMLSR					Mascot
1571.708	1571.6927	-0.0153	-10	182	194	MFVLDEADEMLSR			Oxidation (M)[1]		Mascot
1587.703	1587.6492	-0.0538	-34	182	194	MFVLDEADEMLSR			Oxidation (M)[1,10]		Mascot

1827.9388	1827.9164	-0.0224	-12	51	66	GIYAYGFEKPSAIQQR					Mascot
1827.9388	1827.9164	-0.0224	-12	51	66	GIYAYGFEKPSAIQQR	141	100			Mascot
1903.8928	1903.9167	0.0239	13	182	197	MFVLDEADEMLSRGFK			Oxidation (M)[1]		Mascot
1919.8878	1919.9137	0.0259	13	182	197	MFVLDEADEMLSRGFK			Oxidation (M)[1,10]		Mascot
2059.0754	2059.063	-0.0124	-6	127	146	ALGDYLGVKVHACVGGT SVR			Carbamidomethyl (C)[13]		Mascot
2059.0894	2059.063	-0.0264	-13	211	229	IQVGVFSATMPPEALEIT R	84	100			Mascot
2071.939	2072.0264	0.0874	42	1	19	MAGLAPEGSQFDGKQYD NK			Oxidation (M)[1]		Mascot
2075.0842	2075.033	-0.0512	-25	211	229	IQVGVFSATMPPEALEIT R			Oxidation (M)[10]		Mascot
2075.0842	2075.033	-0.0512	-25	211	229	IQVGVFSATMPPEALEIT R	66	99.949	Oxidation (M)[10]		Mascot
2685.3804	2685.3508	-0.0296	-11	264	286	LDTLCDLYETLAITQSVIF VNTR			Carbamidomethyl (C)[5]		Mascot
2911.4949	2911.4683	-0.0266	-9	339	363	GIDVQQVSLVINYDLPTQ PENYLHR					Mascot
2911.4949	2911.4683	-0.0266	-9	339	363	GIDVQQVSLVINYDLPTQ PENYLHR	197	100			Mascot
3124.5806	3124.5498	-0.0308	-10	88	115	TATFCSGILQQLDYGLVE CQALVLAPTR			Carbamidomethyl (C)[5,19]		Mascot

3	putative DEAD-box ATP-dependent RNA helicase family protein isoform 2 [Zea mays]	gi|413943217	47238.1	5.29	23	878	100	50.022	739	100	

Protein Group

putative DEAD-box ATP-dependent RNA helicase family protein isoform 1 [Zea mays]	gi|413943216	47238.1	5.2899 999618 5303
translational initiation factor eIF-4A [Zea mays]	gi|162458395	47237.1	5.3699 998855 5908

Peptide Information

Calc. Mass	Obsrv. Mass	± da	± ppm	Start Seq.	End Seq.	Sequence	Ion Score	C. I. %	Modification	Rank	Result Type
907.4818	907.4412	-0.0406	-45	235	241	FMNKPVR			Oxidation (M)[2]		Mascot
910.4702	910.4266	-0.0436	-48	391	397	MLFDIQK			Oxidation (M)[1]		Mascot
935.5197	935.4751	-0.0446	-48	131	139	ALGDYLGVK					Mascot
952.5033	952.4612	-0.0421	-44	170	176	VFDMLRR			Oxidation (M)[4]		Mascot
976.5574	976.5323	-0.0251	-26	378	386	GVAINFVTR					Mascot
1035.5768	1035.5188	-0.058	-56	234	241	KFMNKPVR			Oxidation (M)[3]		Mascot
1070.5953	1070.5665	-0.0288	-27	177	185	QSLRPDNIK					Mascot
1104.6525	1104.6274	-0.0251	-23	377	386	KGVAINFVTR					Mascot
1104.6525	1104.6274	-0.0251	-23	377	386	KGVAINFVTR	63	99.905			Mascot
1114.6831	1114.6573	-0.0258	-23	333	342	VLITTDLLAR					Mascot

1114.6831	1114.6573	-0.0258	-23	333	342	VLITTDLLAR	77	99.996			Mascot
1142.5736	1142.5491	-0.0245	-21	140	150	VHACVGGTSVR			Carbamidomethyl (C)[4]		Mascot
1142.5736	1142.5491	-0.0245	-21	140	150	VHACVGGTSVR	70	99.981	Carbamidomethyl (C)[4]		Mascot
1173.6475	1173.6195	-0.028	-24	246	255	RDELTLEGIK					Mascot
1226.6964	1226.662	-0.0344	-28	176	185	RQSLRPDNIK					Mascot
1401.7333	1401.6686	-0.0647	-46	78	91	GLDVIQQAQSGTGK					Mascot
1461.8538	1461.8269	-0.0269	-18	155	169	ILASGVHVVVGTPGR					Mascot
1461.8538	1461.8269	-0.0269	-18	155	169	ILASGVHVVVGTPGR	108	100			Mascot
1555.7131	1555.6875	-0.0256	-16	186	198	MFVLDEADEMLSR					Mascot
1571.708	1571.6927	-0.0153	-10	186	198	MFVLDEADEMLSR			Oxidation (M)[1]		Mascot
1587.703	1587.6492	-0.0538	-34	186	198	MFVLDEADEMLSR			Oxidation (M)[1,10]		Mascot
1827.9388	1827.9164	-0.0224	-12	55	70	GIYAYGFEKPSAIQQR					Mascot
1827.9388	1827.9164	-0.0224	-12	55	70	GIYAYGFEKPSAIQQR	141	100			Mascot
1903.8928	1903.9167	0.0239	13	186	201	MFVLDEADEMLSRGFK			Oxidation (M)[1]		Mascot
1919.8878	1919.9137	0.0259	13	186	201	MFVLDEADEMLSRGFK			Oxidation (M)[1,10]		Mascot
2011.0383	2011.1105	0.0722	36	247	263	DELTLEGIKQFYVNVDK					Mascot
2011.0383	2011.1105	0.0722	36	247	263	DELTLEGIKQFYVNVDK					Mascot
2059.0754	2059.063	-0.0124	-6	131	150	ALGDYLGVKVHACVGGT SVR			Carbamidomethyl (C)[13]		Mascot
2059.0894	2059.063	-0.0264	-13	215	233	IQVGVFSATMPPEALEIT R	84	100			Mascot
2075.0842	2075.033	-0.0512	-25	215	233	IQVGVFSATMPPEALEIT R			Oxidation (M)[10]		Mascot
2075.0842	2075.033	-0.0512	-25	215	233	IQVGVFSATMPPEALEIT R	66	99.949	Oxidation (M)[10]		Mascot
2685.3804	2685.3508	-0.0296	-11	268	290	LDTLCDLYETLAITQSVIF VNTR			Carbamidomethyl (C)[5]		Mascot
2911.4949	2911.4683	-0.0266	-9	343	367	GIDVQQVSLVINYDLPTQ PENYLHR					Mascot
2911.4949	2911.4683	-0.0266	-9	343	367	GIDVQQVSLVINYDLPTQ PENYLHR	197	100			Mascot
3124.5806	3124.5498	-0.0308	-10	92	119	TATFCSGILQQLDYGLVE CQALVLAPTR			Carbamidomethyl (C)[5,19]		Mascot

4	Eukaryotic initiation factor 4A-1 [Triticum urartu]	gi|474441074	47158	5.38	23	875	100	48.804	739	100		

Protein Group

Eukaryotic initiation factor 4A-1 [Aegilops tauschii]	gi|475619612	47158	5.3800 001144 4092

Peptide Information

Calc. Mass	Obsrv. Mass	± da	± ppm	Start Seq.	End Seq.	Sequence	Ion Score	C. I. %	Modification	Rank	Result Type
907.4818	907.4412	-0.0406	-45	235	241	FMNKPVR			Oxidation (M)[2]		Mascot

910.4702	910.4266	-0.0436	-48	391	397	MLFDIQK			Oxidation (M)[1]	Mascot
935.5197	935.4751	-0.0446	-48	131	139	ALGDYLGVK				Mascot
952.5033	952.4612	-0.0421	-44	170	176	VFDMLRR			Oxidation (M)[4]	Mascot
976.5574	976.5323	-0.0251	-26	378	386	GVAINFVTR				Mascot
1035.5768	1035.5188	-0.058	-56	234	241	KFMNKPVR			Oxidation (M)[3]	Mascot
1070.5953	1070.5665	-0.0288	-27	177	185	QSLRPDNIK				Mascot
1104.6525	1104.6274	-0.0251	-23	377	386	KGVAINFVTR				Mascot
1104.6525	1104.6274	-0.0251	-23	377	386	KGVAINFVTR	63	99.905		Mascot
1114.6831	1114.6573	-0.0258	-23	333	342	VLITTDLLAR				Mascot
1114.6831	1114.6573	-0.0258	-23	333	342	VLITTDLLAR	77	99.996		Mascot
1142.5736	1142.5491	-0.0245	-21	140	150	VHACVGGTSVR			Carbamidomethyl (C)[4]	Mascot
1142.5736	1142.5491	-0.0245	-21	140	150	VHACVGGTSVR	70	99.981	Carbamidomethyl (C)[4]	Mascot
1173.6475	1173.6195	-0.028	-24	246	255	RDELTLEGIK				Mascot
1226.6964	1226.662	-0.0344	-28	176	185	RQSLRPDNIK				Mascot
1401.7333	1401.6686	-0.0647	-46	78	91	GLDVIQQAQSGTGK				Mascot
1461.8538	1461.8269	-0.0269	-18	155	169	ILASGVHVVVGTPGR				Mascot
1461.8538	1461.8269	-0.0269	-18	155	169	ILASGVHVVVGTPGR	108	100		Mascot
1555.7131	1555.6875	-0.0256	-16	186	198	MFVLDEADEMLSR				Mascot
1571.708	1571.6927	-0.0153	-10	186	198	MFVLDEADEMLSR			Oxidation (M)[1]	Mascot
1587.703	1587.6492	-0.0538	-34	186	198	MFVLDEADEMLSR			Oxidation (M)[1,10]	Mascot
1598.7849	1598.726	-0.0589	-37	256	267	QFYVNVEKEEWK				Mascot
1827.9388	1827.9164	-0.0224	-12	55	70	GIYAYGFEKPSAIQQR				Mascot
1827.9388	1827.9164	-0.0224	-12	55	70	GIYAYGFEKPSAIQQR	141	100		Mascot
1903.8928	1903.9167	0.0239	13	186	201	MFVLDEADEMLSRGFK			Oxidation (M)[1]	Mascot
1919.8878	1919.9137	0.0259	13	186	201	MFVLDEADEMLSRGFK			Oxidation (M)[1,10]	Mascot
2059.0754	2059.063	-0.0124	-6	131	150	ALGDYLGVKVHACVGGTSVR			Carbamidomethyl (C)[13]	Mascot
2059.0894	2059.063	-0.0264	-13	215	233	IQVGVFSATMPPEALEITR	84	100		Mascot
2075.0842	2075.033	-0.0512	-25	215	233	IQVGVFSATMPPEALEITR			Oxidation (M)[10]	Mascot
2075.0842	2075.033	-0.0512	-25	215	233	IQVGVFSATMPPEALEITR	66	99.949	Oxidation (M)[10]	Mascot
2685.3804	2685.3508	-0.0296	-11	268	290	LDTLCDLYETLAITQSVIFVNTR			Carbamidomethyl (C)[5]	Mascot
2911.4949	2911.4683	-0.0266	-9	343	367	GIDVQQVSLVINYDLPTQPENYLHR				Mascot
2911.4949	2911.4683	-0.0266	-9	343	367	GIDVQQVSLVINYDLPTQPENYLHR	197	100		Mascot
3124.5806	3124.5498	-0.0308	-10	92	119	TATFCSGILQQLDYGLVECQALVLAPTR			Carbamidomethyl (C)[5,19]	Mascot

5 eukaryotic initiation factor 4A [Zea mays] gi|162460883 46848.9 5.38 21 864 100 48.263 739 100

Protein Group

RecName: Full=Eukaryotic initiation factor 4A; Short=eIF-4A; AltName: Full=ATP-dependent RNA helicase eIF4A	gi|2500522	46848.9	5.3800 001144 4092

Peptide Information

Calc. Mass	Obsrv. Mass	± da	± ppm	Start Seq.	End Seq.	Sequence	Ion Score	C. I. %	Modification	Rank	Result Type
907.4818	907.4412	-0.0406	-45	231	237	FMNKPVR			Oxidation (M)[2]		Mascot
935.5197	935.4751	-0.0446	-48	127	135	ALGDYLGVK					Mascot
952.5033	952.4612	-0.0421	-44	166	172	VFDMLRR			Oxidation (M)[4]		Mascot
976.5574	976.5323	-0.0251	-26	374	382	GVAINFVTR					Mascot
1035.5768	1035.5188	-0.058	-56	230	237	KFMNKPVR			Oxidation (M)[3]		Mascot
1070.5953	1070.5665	-0.0288	-27	173	181	QSLRPDNIK					Mascot
1104.6525	1104.6274	-0.0251	-23	373	382	KGVAINFVTR					Mascot
1104.6525	1104.6274	-0.0251	-23	373	382	KGVAINFVTR	63	99.905			Mascot
1114.6831	1114.6573	-0.0258	-23	329	338	VLITTDLLAR					Mascot
1114.6831	1114.6573	-0.0258	-23	329	338	VLITTDLLAR	77	99.996			Mascot
1142.5736	1142.5491	-0.0245	-21	136	146	VHACVGGTSVR			Carbamidomethyl (C)[4]		Mascot
1142.5736	1142.5491	-0.0245	-21	136	146	VHACVGGTSVR	70	99.981	Carbamidomethyl (C)[4]		Mascot
1173.6475	1173.6195	-0.028	-24	242	251	RDELTLEGIK					Mascot
1226.6964	1226.662	-0.0344	-28	172	181	RQSLRPDNIK					Mascot
1401.7333	1401.6686	-0.0647	-46	74	87	GLDVIQQAQSGTGK					Mascot
1461.8538	1461.8269	-0.0269	-18	151	165	ILASGVHVVVGTPGR					Mascot
1461.8538	1461.8269	-0.0269	-18	151	165	ILASGVHVVVGTPGR	108	100			Mascot
1555.7131	1555.6875	-0.0256	-16	182	194	MFVLDEADEMLSR					Mascot
1571.708	1571.6927	-0.0153	-10	182	194	MFVLDEADEMLSR			Oxidation (M)[1]		Mascot
1587.703	1587.6492	-0.0538	-34	182	194	MFVLDEADEMLSR			Oxidation (M)[1,10]		Mascot
1827.9388	1827.9164	-0.0224	-12	51	66	GIYAYGFEKPSAIQQR					Mascot
1827.9388	1827.9164	-0.0224	-12	51	66	GIYAYGFEKPSAIQQR	141	100			Mascot
1903.8928	1903.9167	0.0239	13	182	197	MFVLDEADEMLSRGFK			Oxidation (M)[1]		Mascot
1919.8878	1919.9137	0.0259	13	182	197	MFVLDEADEMLSRGFK			Oxidation (M)[1,10]		Mascot
2059.0754	2059.063	-0.0124	-6	127	146	ALGDYLGVKVHACVGGT SVR			Carbamidomethyl (C)[13]		Mascot
2059.0894	2059.063	-0.0264	-13	211	229	IQVGVFSATMPPEALEIT R	84	100			Mascot
2075.0842	2075.033	-0.0512	-25	211	229	IQVGVFSATMPPEALEIT R			Oxidation (M)[10]		Mascot
2075.0842	2075.033	-0.0512	-25	211	229	IQVGVFSATMPPEALEIT R	66	99.949	Oxidation (M)[10]		Mascot
2685.3804	2685.3508	-0.0296	-11	264	286	LDTLCDLYETLAITQSVIF VNTR			Carbamidomethyl (C)[5]		Mascot

2911.4949	2911.4683	-0.0266	-9	339	363	GIDVQQVSLVINYDLPTQ PENYLHR					Mascot
2911.4949	2911.4683	-0.0266	-9	339	363	GIDVQQVSLVINYDLPTQ PENYLHR	197	100			Mascot
3124.5806	3124.5498	-0.0308	-10	88	115	TATFCSGILQQLDYGLVE CQALVLAPTR			Carbamidomethyl (C)[5,19]		Mascot

6	hypothetical protein SORBIDRAFT_04g003390 [Sorghum bicolor]	gi|241931367	46949.9	5.39	21	862	100	48.263	739	100	

Protein Group

LOC100284697 [Zea mays] gi|226501258 46894.9 5.3800 001144 4092

Peptide Information

Calc. Mass	Obsrv. Mass	± da	± ppm	Start Seq.	End Seq.	Sequence	Ion Score	C. I. %	Modification	Rank	Result Type
907.4818	907.4412	-0.0406	-45	231	237	FMNKPVR			Oxidation (M)[2]		Mascot
935.5197	935.4751	-0.0446	-48	127	135	ALGDYLGVK					Mascot
952.5033	952.4612	-0.0421	-44	166	172	VFDMLRR			Oxidation (M)[4]		Mascot
976.5574	976.5323	-0.0251	-26	374	382	GVAINFVTR					Mascot
1035.5768	1035.5188	-0.058	-56	230	237	KFMNKPVR			Oxidation (M)[3]		Mascot
1070.5953	1070.5665	-0.0288	-27	173	181	QSLRPDNIK					Mascot
1104.6525	1104.6274	-0.0251	-23	373	382	KGVAINFVTR					Mascot
1104.6525	1104.6274	-0.0251	-23	373	382	KGVAINFVTR	63	99.905			Mascot
1114.6831	1114.6573	-0.0258	-23	329	338	VLITTDLLAR					Mascot
1114.6831	1114.6573	-0.0258	-23	329	338	VLITTDLLAR	77	99.996			Mascot
1142.5736	1142.5491	-0.0245	-21	136	146	VHACVGGTSVR			Carbamidomethyl (C)[4]		Mascot
1142.5736	1142.5491	-0.0245	-21	136	146	VHACVGGTSVR	70	99.981	Carbamidomethyl (C)[4]		Mascot
1173.6475	1173.6195	-0.028	-24	242	251	RDELTLEGIK					Mascot
1226.6964	1226.662	-0.0344	-28	172	181	RQSLRPDNIK					Mascot
1401.7333	1401.6686	-0.0647	-46	74	87	GLDVIQQAQSGTGK					Mascot
1461.8538	1461.8269	-0.0269	-18	151	165	ILASGVHVVVGTPGR					Mascot
1461.8538	1461.8269	-0.0269	-18	151	165	ILASGVHVVVGTPGR	108	100			Mascot
1555.7131	1555.6875	-0.0256	-16	182	194	MFVLDEADEMLSR					Mascot
1571.708	1571.6927	-0.0153	-10	182	194	MFVLDEADEMLSR			Oxidation (M)[1]		Mascot
1587.703	1587.6492	-0.0538	-34	182	194	MFVLDEADEMLSR			Oxidation (M)[1,10]		Mascot
1827.9388	1827.9164	-0.0224	-12	51	66	GIYAYGFEKPSAIQQR					Mascot
1827.9388	1827.9164	-0.0224	-12	51	66	GIYAYGFEKPSAIQQR	141	100			Mascot
1903.8928	1903.9167	0.0239	13	182	197	MFVLDEADEMLSRGFK			Oxidation (M)[1]		Mascot
1919.8878	1919.9137	0.0259	13	182	197	MFVLDEADEMLSRGFK			Oxidation (M)[1,10]		Mascot

2059.0754	2059.063	-0.0124	-6	127	146	ALGDYLGVKVHACVGGTSVR			Carbamidomethyl (C)[13]		Mascot
2059.0894	2059.063	-0.0264	-13	211	229	IQVGVFSATMPPEALEITR	84	100			Mascot
2075.0842	2075.033	-0.0512	-25	211	229	IQVGVFSATMPPEALEITR			Oxidation (M)[10]		Mascot
2075.0842	2075.033	-0.0512	-25	211	229	IQVGVFSATMPPEALEITR	66	99.949	Oxidation (M)[10]		Mascot
2685.3804	2685.3508	-0.0296	-11	264	286	LDTLCDLYETLAITQSVIFVNTR			Carbamidomethyl (C)[5]		Mascot
2911.4949	2911.4683	-0.0266	-9	339	363	GIDVQQVSLVINYDLPTQPENYLHR					Mascot
2911.4949	2911.4683	-0.0266	-9	339	363	GIDVQQVSLVINYDLPTQPENYLHR	197	100			Mascot
3124.5806	3124.5498	-0.0308	-10	88	115	TATFCSGILQQLDYGLVECQALVLAPTR			Carbamidomethyl (C)[5,19]		Mascot

7 Os06g0701100 [Oryza sativa Japonica Group] gi|113596521 47343.1 5.37 20 851 100 47.437 739 100

Protein Group

RecName: Full=Eukaryotic initiation factor 4A-1; Short=eIF-4A-1; AltName: Full=ATP-dependent RNA helicase eIF4A-1; AltName: Full=DEAD-box ATP-dependent RNA helicase 4 gi|97536398 47343.1 5.3699998555908

Peptide Information

Calc. Mass	Obsrv. Mass	± da	± ppm	Start Seq.	End Seq.	Sequence	Ion Score	C. I. %	Modification	Rank	Result Type
907.4818	907.4412	-0.0406	-45	235	241	FMNKPVR			Oxidation (M)[2]		Mascot
935.5197	935.4751	-0.0446	-48	131	139	ALGDYLGVK					Mascot
952.5033	952.4612	-0.0421	-44	170	176	VFDMLRR			Oxidation (M)[4]		Mascot
976.5574	976.5323	-0.0251	-26	378	386	GVAINFVTR					Mascot
1035.5768	1035.5188	-0.058	-56	234	241	KFMNKPVR			Oxidation (M)[3]		Mascot
1104.6525	1104.6274	-0.0251	-23	377	386	KGVAINFVTR					Mascot
1104.6525	1104.6274	-0.0251	-23	377	386	KGVAINFVTR	63	99.905			Mascot
1114.6831	1114.6573	-0.0258	-23	333	342	VLITTDLLAR					Mascot
1114.6831	1114.6573	-0.0258	-23	333	342	VLITTDLLAR	77	99.996			Mascot
1142.5736	1142.5491	-0.0245	-21	140	150	VHACVGGTSVR			Carbamidomethyl (C)[4]		Mascot
1142.5736	1142.5491	-0.0245	-21	140	150	VHACVGGTSVR	70	99.981	Carbamidomethyl (C)[4]		Mascot
1173.6475	1173.6195	-0.028	-24	246	255	RDELTLEGIK					Mascot
1401.7333	1401.6686	-0.0647	-46	78	91	GLDVIQQAQSGTGK					Mascot
1461.8538	1461.8269	-0.0269	-18	155	169	ILASGVHVVVGTPGR					Mascot
1461.8538	1461.8269	-0.0269	-18	155	169	ILASGVHVVVGTPGR	108	100			Mascot
1555.7131	1555.6875	-0.0256	-16	186	198	MFVLDEADEMLSR					Mascot
1571.708	1571.6927	-0.0153	-10	186	198	MFVLDEADEMLSR			Oxidation (M)[1]		Mascot
1587.703	1587.6492	-0.0538	-34	186	198	MFVLDEADEMLSR			Oxidation (M)[1,10]		Mascot
1598.7849	1598.726	-0.0589	-37	256	267	QFYVNVEKEEWK					Mascot

1827.9388	1827.9164	-0.0224	-12	55	70	GIYAYGFEKPSAIQQR					Mascot
1827.9388	1827.9164	-0.0224	-12	55	70	GIYAYGFEKPSAIQQR	141	100			Mascot
1903.8928	1903.9167	0.0239	13	186	201	MFVLDEADEMLSRGFK			Oxidation (M)[1]		Mascot
1919.8878	1919.9137	0.0259	13	186	201	MFVLDEADEMLSRGFK			Oxidation (M)[1,10]		Mascot
2059.0754	2059.063	-0.0124	-6	131	150	ALGDYLGVKVHACVGGT SVR			Carbamidomethyl (C)[13]		Mascot
2059.0894	2059.063	-0.0264	-13	215	233	IQVGVFSATMPPEALEIT R	84	100			Mascot
2075.0842	2075.033	-0.0512	-25	215	233	IQVGVFSATMPPEALEIT R			Oxidation (M)[10]		Mascot
2075.0842	2075.033	-0.0512	-25	215	233	IQVGVFSATMPPEALEIT R	66	99.949	Oxidation (M)[10]		Mascot
2685.3804	2685.3508	-0.0296	-11	268	290	LDTLCDLYETLAITQSVIF VNTR			Carbamidomethyl (C)[5]		Mascot
2911.4949	2911.4683	-0.0266	-9	343	367	GIDVQQVSLVINYDLPTQ PENYLHR					Mascot
2911.4949	2911.4683	-0.0266	-9	343	367	GIDVQQVSLVINYDLPTQ PENYLHR	197	100			Mascot
3124.5806	3124.5498	-0.0308	-10	92	119	TATFCSGILQQLDYAVVE CQALVLAPTR			Carbamidomethyl (C)[5,19]		Mascot

8	PREDICTED: eukaryotic initiation factor 4A-3-like [Setaria italica]	gi|514766903	47316.1	5.37	19	843	100	47.151	739	100	

Peptide Information

Calc. Mass	Obsrv. Mass	± da	± ppm	Start Seq.	End Seq.	Sequence	Ion Score	C. I. %	Modification	Rank	Result Type
907.4818	907.4412	-0.0406	-45	235	241	FMNKPVR			Oxidation (M)[2]		Mascot
935.5197	935.4751	-0.0446	-48	131	139	ALGDYLGVK					Mascot
952.5033	952.4612	-0.0421	-44	170	176	VFDMLRR			Oxidation (M)[4]		Mascot
976.5574	976.5323	-0.0251	-26	378	386	GVAINFVTR					Mascot
1035.5768	1035.5188	-0.058	-56	234	241	KFMNKPVR			Oxidation (M)[3]		Mascot
1104.6525	1104.6274	-0.0251	-23	377	386	KGVAINFVTR					Mascot
1104.6525	1104.6274	-0.0251	-23	377	386	KGVAINFVTR	63	99.905			Mascot
1114.6831	1114.6573	-0.0258	-23	333	342	VLITTDLLAR					Mascot
1114.6831	1114.6573	-0.0258	-23	333	342	VLITTDLLAR	77	99.996			Mascot
1142.5736	1142.5491	-0.0245	-21	140	150	VHACVGGTSVR			Carbamidomethyl (C)[4]		Mascot
1142.5736	1142.5491	-0.0245	-21	140	150	VHACVGGTSVR	70	99.981	Carbamidomethyl (C)[4]		Mascot
1173.6475	1173.6195	-0.028	-24	246	255	RDELTLEGIK					Mascot
1401.7333	1401.6686	-0.0647	-46	78	91	GLDVIQQAQSGTGK					Mascot
1461.8538	1461.8269	-0.0269	-18	155	169	ILASGVHVVVGTPGR					Mascot
1461.8538	1461.8269	-0.0269	-18	155	169	ILASGVHVVVGTPGR	108	100			Mascot
1555.7131	1555.6875	-0.0256	-16	186	198	MFVLDEADEMLSR					Mascot
1571.708	1571.6927	-0.0153	-10	186	198	MFVLDEADEMLSR			Oxidation (M)[1]		Mascot

1587.703	1587.6492	-0.0538	-34	186	198	MFVLDEADEMLSR			Oxidation (M)[1,10]		Mascot
1827.9388	1827.9164	-0.0224	-12	55	70	GIYAYGFEKPSAIQQR					Mascot
1827.9388	1827.9164	-0.0224	-12	55	70	GIYAYGFEKPSAIQQR	141	100			Mascot
1903.8928	1903.9167	0.0239	13	186	201	MFVLDEADEMLSRGFK			Oxidation (M)[1]		Mascot
1919.8878	1919.9137	0.0259	13	186	201	MFVLDEADEMLSRGFK			Oxidation (M)[1,10]		Mascot
2059.0754	2059.063	-0.0124	-6	131	150	ALGDYLGVKVHACVGGT SVR			Carbamidomethyl (C)[13]		Mascot
2059.0894	2059.063	-0.0264	-13	215	233	IQVGVFSATMPPEALEIT R	84	100			Mascot
2075.0842	2075.033	-0.0512	-25	215	233	IQVGVFSATMPPEALEIT R			Oxidation (M)[10]		Mascot
2075.0842	2075.033	-0.0512	-25	215	233	IQVGVFSATMPPEALEIT R	66	99.949	Oxidation (M)[10]		Mascot
2685.3804	2685.3508	-0.0296	-11	268	290	LDTLCDLYETLAITQSVIF VNTR			Carbamidomethyl (C)[5]		Mascot
2911.4949	2911.4683	-0.0266	-9	343	367	GIDVQQVSLVINYDLPTQ PENYLHR					Mascot
2911.4949	2911.4683	-0.0266	-9	343	367	GIDVQQVSLVINYDLPTQ PENYLHR	197	100			Mascot
3124.5806	3124.5498	-0.0308	-10	92	119	TATFCSGILQQLDYGLVE CQALVLAPTR			Carbamidomethyl (C)[5,19]		Mascot

9	Os02g0146600 [Oryza sativa Japonica Group]	gi|113535409	47393.1	5.43	18	831	100	45.889	739	100	

Protein Group

RecName: Full=Eukaryotic initiation factor 4A-3; Short=eIF-4A-3; AltName: Full=ATP-dependent RNA helicase eIF4A-3; AltName: Full=DEAD-box ATP-dependent RNA helicase 23; AltName: Full=eIF4A-2	gi|75325389	47393.1	5.4299 998283 3862
hypothetical protein OsI_05839 [Oryza sativa Indica Group]	gi|125538069	47393.1	5.4299 998283 3862

Peptide Information

Calc. Mass	Obsrv. Mass	± da	± ppm	Start Seq.	End Seq.	Sequence	Ion Score	C. I. %	Modification	Rank	Result Type
907.4818	907.4412	-0.0406	-45	235	241	FMNKPVR			Oxidation (M)[2]		Mascot
935.5197	935.4751	-0.0446	-48	131	139	ALGDYLGVK					Mascot
952.5033	952.4612	-0.0421	-44	170	176	VFDMLRR			Oxidation (M)[4]		Mascot
976.5574	976.5323	-0.0251	-26	378	386	GVAINFVTR					Mascot
1035.5768	1035.5188	-0.058	-56	234	241	KFMNKPVR			Oxidation (M)[3]		Mascot
1104.6525	1104.6274	-0.0251	-23	377	386	KGVAINFVTR					Mascot
1104.6525	1104.6274	-0.0251	-23	377	386	KGVAINFVTR	63	99.905			Mascot
1114.6831	1114.6573	-0.0258	-23	333	342	VLITTDLLAR					Mascot
1114.6831	1114.6573	-0.0258	-23	333	342	VLITTDLLAR	77	99.996			Mascot
1142.5736	1142.5491	-0.0245	-21	140	150	VHACVGGTSVR			Carbamidomethyl (C)[4]		Mascot

Calc. Mass	Obsrv. Mass	± da	± ppm	Start Seq.	End Seq.	Sequence	Ion Score	C. I. %	Modification	Rank	Result Type
1142.5736	1142.5491	-0.0245	-21	140	150	VHACVGGTSVR	70	99.981	Carbamidomethyl (C)[4]		Mascot
1173.6475	1173.6195	-0.028	-24	246	255	RDELTLEGIK					Mascot
1401.7333	1401.6686	-0.0647	-46	78	91	GLDVIQQAQSGTGK					Mascot
1461.8538	1461.8269	-0.0269	-18	155	169	ILASGVHVVVGTPGR					Mascot
1461.8538	1461.8269	-0.0269	-18	155	169	ILASGVHVVVGTPGR	108	100			Mascot
1555.7131	1555.6875	-0.0256	-16	186	198	MFVLDEADEMLSR					Mascot
1571.708	1571.6927	-0.0153	-10	186	198	MFVLDEADEMLSR			Oxidation (M)[1]		Mascot
1587.703	1587.6492	-0.0538	-34	186	198	MFVLDEADEMLSR			Oxidation (M)[1,10]		Mascot
1827.9388	1827.9164	-0.0224	-12	55	70	GIYAYGFEKPSAIQQR					Mascot
1827.9388	1827.9164	-0.0224	-12	55	70	GIYAYGFEKPSAIQQR	141	100			Mascot
1903.8928	1903.9167	0.0239	13	186	201	MFVLDEADEMLSRGFK			Oxidation (M)[1]		Mascot
1919.8878	1919.9137	0.0259	13	186	201	MFVLDEADEMLSRGFK			Oxidation (M)[1,10]		Mascot
2059.0754	2059.063	-0.0124	-6	131	150	ALGDYLGVKVHACVGGT SVR			Carbamidomethyl (C)[13]		Mascot
2059.0894	2059.063	-0.0264	-13	215	233	IQVGVFSATMPPEALEIT R	84	100			Mascot
2075.0842	2075.033	-0.0512	-25	215	233	IQVGVFSATMPPEALEIT R			Oxidation (M)[10]		Mascot
2075.0842	2075.033	-0.0512	-25	215	233	IQVGVFSATMPPEALEIT R	66	99.949	Oxidation (M)[10]		Mascot
2685.3804	2685.3508	-0.0296	-11	268	290	LDTLCDLYETLAITQSVIF VNTR			Carbamidomethyl (C)[5]		Mascot
2911.4949	2911.4683	-0.0266	-9	343	367	GIDVQQVSLVINYDLPTQ PENYLHR					Mascot
2911.4949	2911.4683	-0.0266	-9	343	367	GIDVQQVSLVINYDLPTQ PENYLHR	197	100			Mascot

10	putative DEAD-box ATP-dependent RNA helicase family protein [Zea mays]	gi|413934786	46948.9	5.29	23	777	100	46.035	630	100	

Peptide Information

Calc. Mass	Obsrv. Mass	± da	± ppm	Start Seq.	End Seq.	Sequence	Ion Score	C. I. %	Modification	Rank	Result Type
907.4818	907.4412	-0.0406	-45	232	238	FMNKPVR			Oxidation (M)[2]		Mascot
910.4702	910.4266	-0.0436	-48	388	394	MLFDIQK			Oxidation (M)[1]		Mascot
935.5197	935.4751	-0.0446	-48	128	136	ALGDYLGVK					Mascot
952.5033	952.4612	-0.0421	-44	167	173	VFDMLRR			Oxidation (M)[4]		Mascot
976.5574	976.5323	-0.0251	-26	375	383	GVAINFVTR					Mascot
1035.5768	1035.5188	-0.058	-56	231	238	KFMNKPVR			Oxidation (M)[3]		Mascot
1070.5953	1070.5665	-0.0288	-27	174	182	QSLRPDNIK					Mascot
1104.6525	1104.6274	-0.0251	-23	374	383	KGVAINFVTR					Mascot
1104.6525	1104.6274	-0.0251	-23	374	383	KGVAINFVTR	63	99.905			Mascot
1114.6831	1114.6573	-0.0258	-23	330	339	VLITTDLLAR					Mascot
1114.6831	1114.6573	-0.0258	-23	330	339	VLITTDLLAR	77	99.996			Mascot

1142.5736	1142.5491	-0.0245	-21	137	147	VHACVGGTSVR			Carbamidomethyl (C)[4]	Mascot
1142.5736	1142.5491	-0.0245	-21	137	147	VHACVGGTSVR	70	99.982	Carbamidomethyl (C)[4]	Mascot
1173.6475	1173.6195	-0.028	-24	243	252	RDELTLEGIK				Mascot
1226.6964	1226.662	-0.0344	-28	173	182	RQSLRPDNIK				Mascot
1401.7333	1401.6686	-0.0647	-46	75	88	GLDVIQQAQSGTGK				Mascot
1555.7131	1555.6875	-0.0256	-16	183	195	MFVLDEADEMLSR				Mascot
1571.708	1571.6927	-0.0153	-10	183	195	MFVLDEADEMLSR			Oxidation (M)[1]	Mascot
1587.703	1587.6492	-0.0538	-34	183	195	MFVLDEADEMLSR			Oxidation (M)[1,10]	Mascot
1810.8065	1810.9457	0.1392	77	1	16	MAPEGSQFDAKHYDSK				Mascot
1826.8014	1826.8981	0.0967	53	1	16	MAPEGSQFDAKHYDSK			Oxidation (M)[1]	Mascot
1827.9388	1827.9164	-0.0224	-12	52	67	GIYAYGFEKPSAIQQR				Mascot
1827.9388	1827.9164	-0.0224	-12	52	67	GIYAYGFEKPSAIQQR	141	100		Mascot
1903.8928	1903.9167	0.0239	13	183	198	MFVLDEADEMLSRGFK			Oxidation (M)[1]	Mascot
1919.8878	1919.9137	0.0259	13	183	198	MFVLDEADEMLSRGFK			Oxidation (M)[1,10]	Mascot
2011.0383	2011.1105	0.0722	36	244	260	DELTLEGIKQFYVNVDK				Mascot
2011.0383	2011.1105	0.0722	36	244	260	DELTLEGIKQFYVNVDK				Mascot
2059.0754	2059.063	-0.0124	-6	128	147	ALGDYLGVKVHACVGGTSVR			Carbamidomethyl (C)[13]	Mascot
2059.0894	2059.063	-0.0264	-13	212	230	IQVGVFSATMPPEALEITR	84	100		Mascot
2075.0842	2075.033	-0.0512	-25	212	230	IQVGVFSATMPPEALEITR			Oxidation (M)[10]	Mascot
2075.0842	2075.033	-0.0512	-25	212	230	IQVGVFSATMPPEALEITR	66	99.95	Oxidation (M)[10]	Mascot
2685.3804	2685.3508	-0.0296	-11	265	287	LDTLCDLYETLAITQSVIFVNTR			Carbamidomethyl (C)[5]	Mascot
2911.4949	2911.4683	-0.0266	-9	340	364	GIDVQQVSLVINYDLPTQPENYLHR				Mascot
2911.4949	2911.4683	-0.0266	-9	340	364	GIDVQQVSLVINYDLPTQPENYLHR	197	100		Mascot
3124.5806	3124.5498	-0.0308	-10	89	116	TATFCSGILQQLDYGLVECQALVLAPTR			Carbamidomethyl (C)[5,19]	Mascot

Gel Idx/Pos	257/K9	Instr./Gel Origin	BA2151/Sample Project 20140814	Process Status	Analysis Succeeded
Plate [#] Name	[1] Sample Project 20140814	Instrument Sample Name		Spectra	11

Rank	Protein Name	Accession No.	Protein MW	Protein PI	Pep. Count	Protein Score	Protein Score C. I. %	Intensity Matched	Total Ion Score	Total Ion C. I. %	Confirmed
1	RecName: Full=Beta-amylase; AltName: Full=1,4-alpha-D-glucan maltohydrolase; AltName: Full=Beta-Amy1; Flags: Precursor	gi|75107132	59886.4	5.66	9	279	100	27.322	254	100	

Peptide Information

Calc. Mass	Obsrv. Mass	± da	± ppm	Start Seq.	End Seq.	Sequence	Ion Score	C. I. %	Modification	Rank	Result Type
1016.5564	1016.526	-0.0304	-30	411	418	LFGFTYLR					Mascot
1016.5564	1016.526	-0.0304	-30	411	418	LFGFTYLR	60	99.821			Mascot
1253.6121	1253.5538	-0.0583	-47	248	258	DNGTYLTEKGR					Mascot
1326.6688	1326.6288	-0.04	-30	384	394	YDPTAYNTILR					Mascot
1326.6688	1326.6288	-0.04	-30	384	394	YDPTAYNTILR	79	99.998			Mascot
1442.7057	1442.6742	-0.0315	-22	371	383	EGLNVACENALPR			Carbamidomethyl (C)[7]		Mascot
1442.7057	1442.6742	-0.0315	-22	371	383	EGLNVACENALPR	115	100	Carbamidomethyl (C)[7]		Mascot
1669.7349	1669.7363	0.0014	1	147	160	SAVQMYADYMTSFR					Mascot
1752.8916	1752.8192	-0.0724	-41	419	433	LSNQLVEGQNYVNFK					Mascot
1827.9884	1827.9044	-0.084	-46	458	474	SGPEISIEMILQAAKPK			Oxidation (M)[9]		Mascot
1827.9884	1827.9044	-0.084	-46	458	474	SGPEISIEMILQAAKPK			Oxidation (M)[9]		Mascot
1842.0425	1841.882	-0.1605	-87	288	302	VQLAIKISGIHWWYK					Mascot
2013.9778	2013.9648	-0.013	-6	303	320	VPSHAAELTAGYYNLHDR					Mascot

Rank	Protein Name	Accession No.	Protein MW	Protein PI	Pep. Count	Protein Score	Protein Score C. I. %	Intensity Matched	Total Ion Score	Total Ion C. I. %	Confirmed
2	RecName: Full=Beta-amylase; AltName: Full=1,4-alpha-D-glucan maltohydrolase	gi|113786	59894.5	5.58	8	275	100	26.501	254	100	

Peptide Information

Calc. Mass	Obsrv. Mass	± da	± ppm	Start Seq.	End Seq.	Sequence	Ion Score	C. I. %	Modification	Rank	Result Type
1016.5564	1016.526	-0.0304	-30	411	418	LFGFTYLR					Mascot
1016.5564	1016.526	-0.0304	-30	411	418	LFGFTYLR	60	99.821			Mascot
1326.6688	1326.6288	-0.04	-30	384	394	YDPTAYNTILR					Mascot
1326.6688	1326.6288	-0.04	-30	384	394	YDPTAYNTILR	79	99.998			Mascot
1442.7057	1442.6742	-0.0315	-22	371	383	EGLNVACENALPR			Carbamidomethyl (C)[7]		Mascot
1442.7057	1442.6742	-0.0315	-22	371	383	EGLNVACENALPR	115	100	Carbamidomethyl (C)[7]		Mascot
1669.7349	1669.7363	0.0014	1	147	160	SAVQMYADYMTSFR					Mascot

1752.8916	1752.8192	-0.0724	-41	419	433	LSNQLVEGQNYVNFK					Mascot
1827.952	1827.9044	-0.0476	-26	458	474	SGPEISIEMILQAAQPK			Oxidation (M)[9]		Mascot
1827.952	1827.9044	-0.0476	-26	458	474	SGPEISIEMILQAAQPK			Oxidation (M)[9]		Mascot
1842.0425	1841.882	-0.1605	-87	288	302	VQLAIKISGIHWWYK					Mascot
2013.9778	2013.9648	-0.013	-6	303	320	VPSHAAELTAGYYNLHDR					Mascot

3	Chain A, Crystal Structure Of Barley Beta-Amylase Complexed With 2,3-Epoxypropyl-Alpha-D-Glucopyranoside	gi|313103508	59854.4	5.58	7	270	100	25.92	254	100	

Protein Group

Chain A, Crystal Structure Of Barley Beta-Amylase At Atomic Resolution	gi|313103503	59854.4	5.5799999237 0605
Chain A, Crystal Structure Of Barley Beta-Amylase Complexed With 3,4- Epoxybutyl Alpha-D-Glucopyranoside	gi|313103509	59854.4	5.5799999237 0605
Chain A, Crystal Structure Of Barley Beta-Amylase Complexed With 4-O-Alpha-D-Glucopyranosylmoranoline	gi|313103505	59854.4	5.5799999237 0605
Chain A, Crystal Structure Of Barley Beta-Amylase Complexed With Acarbose	gi|313103502	59854.4	5.5799999237 0605
Chain A, Crystal Structure Of Barley Beta-Amylase Complexed With Alpha-Cyclodextrin	gi|313103504	59854.4	5.5799999237 0605

Peptide Information

Calc. Mass	Obsrv. Mass	± da	± ppm	Start Seq.	End Seq.	Sequence	Ion Score	C. I. %	Modification	Rank	Result Type
1016.5564	1016.526	-0.0304	-30	411	418	LFGFTYLR					Mascot
1016.5564	1016.526	-0.0304	-30	411	418	LFGFTYLR	60	99.821			Mascot
1326.6688	1326.6288	-0.04	-30	384	394	YDPTAYNTILR					Mascot
1326.6688	1326.6288	-0.04	-30	384	394	YDPTAYNTILR	79	99.998			Mascot
1442.7057	1442.6742	-0.0315	-22	371	383	EGLNVACENALPR			Carbamidomethyl (C)[7]		Mascot
1442.7057	1442.6742	-0.0315	-22	371	383	EGLNVACENALPR	115	100	Carbamidomethyl (C)[7]		Mascot
1669.7349	1669.7363	0.0014	1	147	160	SAVQMYADYMTSFR					Mascot
1827.952	1827.9044	-0.0476	-26	458	474	SGPEISIEMILQAAQPK			Oxidation (M)[9]		Mascot
1827.952	1827.9044	-0.0476	-26	458	474	SGPEISIEMILQAAQPK			Oxidation (M)[9]		Mascot
1842.0425	1841.882	-0.1605	-87	288	302	VQLAIKISGIHWWYK					Mascot
2013.9778	2013.9648	-0.013	-6	303	320	VPSHAAELTAGYYNLHDR					Mascot

4	Beta-amylase [Aegilops tauschii]	gi|475523854	60203.5	5.07	12	198	100	25.14	157	100

Peptide Information

Calc. Mass	Obsrv. Mass	± da	± ppm	Start Seq.	End Seq.	Sequence	Ion Score	C. I. %	Modification	Rank	Result Type
947.5057	947.4587	-0.047	-50	322	329	DGYRPIAR					Mascot
1016.5564	1016.526	-0.0304	-30	412	419	LFGFTYLR					Mascot
1016.5564	1016.526	-0.0304	-30	412	419	LFGFTYLR	60	99.821			Mascot
1237.5444	1237.5571	0.0127	10	233	243	DAGTYNDTPQR					Mascot
1326.6688	1326.6288	-0.04	-30	385	395	YDPTAYNTILR					Mascot
1326.6688	1326.6288	-0.04	-30	385	395	YDPTAYNTILR	79	99.998			Mascot
1589.6948	1589.6394	-0.0554	-35	334	346	HHASLNFTCAEMR			Carbamidomethyl (C)[9], Oxidation (M)[12]		Mascot
1646.781	1646.7448	-0.0362	-22	246	259	FFVDNGTYLTEQGR					Mascot
1655.7192	1655.6982	-0.021	-13	148	161	SAVQMYADYMASFR			Oxidation (M)[5]		Mascot
1671.7142	1671.6472	-0.067	-40	148	161	SAVQMYADYMASFR			Oxidation (M)[5,10]		Mascot
1671.7142	1671.6472	-0.067	-40	148	161	SAVQMYADYMASFR	18	0	Oxidation (M)[5,10]		Mascot
1729.801	1729.8262	0.0252	15	333	346	RHHASLNFTCAEMR			Carbamidomethyl (C)[10]		Mascot
1752.8916	1752.8192	-0.0724	-41	420	434	LSNQLVEGQNYVNFK					Mascot
1841.9678	1841.882	-0.0858	-47	459	475	SGPELTIEMILQAAQPK			Oxidation (M)[9]		Mascot
2087.0557	2087.0181	-0.0376	-18	130	147	NIEYLTLGVDDQPLFHGR					Mascot
2183.0486	2182.9871	-0.0615	-28	440	458	MHANLPHDPCVDPVAPL QR			Carbamidomethyl (C)[10], Oxidation (M)[1]		Mascot

5	Eukaryotic initiation factor 4A [Aegilops tauschii]	gi|475553745	47200.1	5.31	16	180	100	15.504	106	100

Peptide Information

Calc. Mass	Obsrv. Mass	± da	± ppm	Start Seq.	End Seq.	Sequence	Ion Score	C. I. %	Modification	Rank	Result Type
976.5574	976.53	-0.0274	-28	378	386	GVAINFVTR					Mascot
1070.5953	1070.5638	-0.0315	-29	177	185	QSLRPDNIK					Mascot
1104.6525	1104.6251	-0.0274	-25	377	386	KGVAINFVTR					Mascot
1114.6831	1114.6538	-0.0293	-26	333	342	VLITTDLLAR					Mascot
1142.5736	1142.5458	-0.0278	-24	140	150	VHACVGGTSVR			Carbamidomethyl (C)[4]		Mascot
1173.6475	1173.6141	-0.0334	-28	246	255	RDELTLEGIK					Mascot
1461.8538	1461.8214	-0.0324	-22	155	169	ILASGVHVVVGTPGR					Mascot
1571.708	1571.6755	-0.0325	-21	186	198	MFVLDEADEMLSR			Oxidation (M)[1]		Mascot
1587.703	1587.6487	-0.0543	-34	186	198	MFVLDEADEMLSR			Oxidation (M)[1,10]		Mascot
1800.7566	1800.8596	0.103	57	304	319	DHTVSATHGDMDQNTR			Oxidation (M)[11]		Mascot
1827.9388	1827.9044	-0.0344	-19	55	70	GIYAYGFEKPSAIQQR					Mascot

1827.9388	1827.9044	-0.0344	-19	55	70	GIYAYGFEKPSAIQQR	106	100			Mascot
1882.011	1881.8931	-0.1179	-63	199	214	GFKDQIYDIFQLLPGK					Mascot
2013.8792	2013.9648	0.0856	43	302	319	GRDHTVSATHGDMDQNT R			Oxidation (M)[13]		Mascot
2075.0842	2075.022	-0.0622	-30	215	233	IQVGVFSATMPPEALEIT R			Oxidation (M)[10]		Mascot
2685.3804	2685.3381	-0.0423	-16	268	290	LDTLCDLYETLAITQSVIF VNTR			Carbamidomethyl (C)[5]		Mascot
2911.4949	2911.4631	-0.0318	-11	343	367	GIDVQQVSLVINYDLPTQ PENYLHR					Mascot
3124.5806	3124.5454	-0.0352	-11	92	119	TATFCSGILQQLDYGLVE CQALVLAPTR			Carbamidomethyl (C)[5,19]		Mascot

6	translational initiation factor eIF-4A [Zea mays]	gi|162458395	47237.1	5.37	16	179	100	15.911	106	100	

Peptide Information

Calc. Mass	Obsrv. Mass	± da	± ppm	Start Seq.	End Seq.	Sequence	Ion Score	C. I. %	Modification	Rank	Result Type
976.5574	976.53	-0.0274	-28	378	386	GVAINFVTR					Mascot
1070.5953	1070.5638	-0.0315	-29	177	185	QSLRPDNIK					Mascot
1104.6525	1104.6251	-0.0274	-25	377	386	KGVAINFVTR					Mascot
1114.6831	1114.6538	-0.0293	-26	333	342	VLITTDLLAR					Mascot
1142.5736	1142.5458	-0.0278	-24	140	150	VHACVGGTSVR			Carbamidomethyl (C)[4]		Mascot
1173.6475	1173.6141	-0.0334	-28	246	255	RDELTLEGIK					Mascot
1424.6838	1424.6653	-0.0185	-13	387	397	DNERMLFDIQK			Oxidation (M)[5]		Mascot
1461.8538	1461.8214	-0.0324	-22	155	169	ILASGVHVVVGTPGR					Mascot
1571.708	1571.6755	-0.0325	-21	186	198	MFVLDEADEMLSR			Oxidation (M)[1]		Mascot
1587.703	1587.6487	-0.0543	-34	186	198	MFVLDEADEMLSR			Oxidation (M)[1,10]		Mascot
1800.7566	1800.8596	0.103	57	304	319	DHTVSATHGDMDQNTR			Oxidation (M)[11]		Mascot
1827.9388	1827.9044	-0.0344	-19	55	70	GIYAYGFEKPSAIQQR					Mascot
1827.9388	1827.9044	-0.0344	-19	55	70	GIYAYGFEKPSAIQQR	106	100			Mascot
2011.0383	2011.0721	0.0338	17	247	263	DELTLEGIKQFYVNVDK					Mascot
2075.0842	2075.022	-0.0622	-30	215	233	IQVGVFSATMPPEALEIT R			Oxidation (M)[10]		Mascot
2685.3804	2685.3381	-0.0423	-16	268	290	LDTLCDLYETLAITQSVIF VNTR			Carbamidomethyl (C)[5]		Mascot
2911.4949	2911.4631	-0.0318	-11	343	367	GIDVQQVSLVINYDLPTQ PENYLHR					Mascot
3124.5806	3124.5454	-0.0352	-11	92	119	TATFCSGILQQLDYGLVE CQALVLAPTR			Carbamidomethyl (C)[5,19]		Mascot

7	Beta-amylase [Triticum urartu]	gi|474451266	58995	5.34	11	177	100	22.988	139	100	

Peptide Information

Calc. Mass	Obsrv. Mass	± da	± ppm	Start Seq.	End Seq.	Sequence	Ion Score	C. I. %	Modification	Rank	Result Type

Calc. Mass	Obsrv. Mass	± da	± ppm	Start Seq.	End Seq.	Sequence	Ion Score	C. I. %	Modification	Rank	Result Type
947.5057	947.4587	-0.047	-50	322	329	DGYRPIAR					Mascot
1016.5564	1016.526	-0.0304	-30	412	419	LFGFTYLR					Mascot
1016.5564	1016.526	-0.0304	-30	412	419	LFGFTYLR	60	99.821			Mascot
1326.6688	1326.6288	-0.04	-30	385	395	YDPTAYNTILR					Mascot
1326.6688	1326.6288	-0.04	-30	385	395	YDPTAYNTILR	79	99.998			Mascot
1646.781	1646.7448	-0.0362	-22	246	259	FFVDNGTYLTEQGR					Mascot
1669.7349	1669.7363	0.0014	1	148	161	SAVQMYTDYMASFR					Mascot
1752.8916	1752.8192	-0.0724	-41	420	434	LSNQLVEGQNYVNFK					Mascot
1841.9678	1841.882	-0.0858	-47	459	475	SGPELTIEMILQAAQPK			Oxidation (M)[9]		Mascot
2013.9778	2013.9648	-0.013	-6	304	321	VPSHAAEITAGYYNLHDR					Mascot
2087.0557	2087.0181	-0.0376	-18	130	147	NIEYLTLGVDDQPLFHGR					Mascot
2183.0486	2182.9871	-0.0615	-28	440	458	MHANLPHDPCVDPVAPL QR			Carbamidomethyl (C)[10], Oxidation (M)[1]		Mascot
2269.2075	2269.1658	-0.0417	-18	166	187	EFLDAGVIVDIEVGLGPA GELR					Mascot

8	putative DEAD-box ATP-dependent RNA helicase family protein [Zea mays]	gi|413934786	46948.9	5.29	15	176	100	14.971	106	100	

Peptide Information

Calc. Mass	Obsrv. Mass	± da	± ppm	Start Seq.	End Seq.	Sequence	Ion Score	C. I. %	Modification	Rank	Result Type
976.5574	976.53	-0.0274	-28	375	383	GVAINFVTR					Mascot
1070.5953	1070.5638	-0.0315	-29	174	182	QSLRPDNIK					Mascot
1104.6525	1104.6251	-0.0274	-25	374	383	KGVAINFVTR					Mascot
1114.6831	1114.6538	-0.0293	-26	330	339	VLITTDLLAR					Mascot
1142.5736	1142.5458	-0.0278	-24	137	147	VHACVGGTSVR			Carbamidomethyl (C)[4]		Mascot
1173.6475	1173.6141	-0.0334	-28	243	252	RDELTLEGIK					Mascot
1571.708	1571.6755	-0.0325	-21	183	195	MFVLDEADEMLSR			Oxidation (M)[1]		Mascot
1587.703	1587.6487	-0.0543	-34	183	195	MFVLDEADEMLSR			Oxidation (M)[1,10]		Mascot
1800.7566	1800.8596	0.103	57	301	316	DHTVSATHGDMDQNTR			Oxidation (M)[11]		Mascot
1810.8065	1810.9182	0.1117	62	1	16	MAPEGSQFDAKHYDSK					Mascot
1826.8014	1826.8784	0.077	42	1	16	MAPEGSQFDAKHYDSK			Oxidation (M)[1]		Mascot
1827.9388	1827.9044	-0.0344	-19	52	67	GIYAYGFEKPSAIQQR					Mascot
1827.9388	1827.9044	-0.0344	-19	52	67	GIYAYGFEKPSAIQQR	106	100			Mascot
2011.0383	2011.0721	0.0338	17	244	260	DELTLEGIKQFYVNVDK					Mascot
2075.0842	2075.022	-0.0622	-30	212	230	IQVGVFSATMPPEALEIT R			Oxidation (M)[10]		Mascot
2685.3804	2685.3381	-0.0423	-16	265	287	LDTLCDLYETLAITQSVIF VNTR			Carbamidomethyl (C)[5]		Mascot
2911.4949	2911.4631	-0.0318	-11	340	364	GIDVQQVSLVINYDLPTQ PENYLHR					Mascot

3124.5806	3124.5454	-0.0352	-11	89	116	TATFCSGILQQLDYGLVE CQALVLAPTR			Carbamidomethyl (C)[5,19]		Mascot

9	Eukaryotic initiation factor 4A-1 [Triticum urartu]	gi|474441074	47158	5.38	15	173	100	15.39	106	100	

Protein Group

Eukaryotic initiation factor 4A-1 [Aegilops tauschii]	gi|475619612	47158	5.3800 001144 4092

Peptide Information

Calc. Mass	Obsrv. Mass	± da	± ppm	Start Seq.	End Seq.	Sequence	Ion Score	C. I. %	Modification	Rank	Result Type
976.5574	976.53	-0.0274	-28	378	386	GVAINFVTR					Mascot
1070.5953	1070.5638	-0.0315	-29	177	185	QSLRPDNIK					Mascot
1104.6525	1104.6251	-0.0274	-25	377	386	KGVAINFVTR					Mascot
1114.6831	1114.6538	-0.0293	-26	333	342	VLITTDLLAR					Mascot
1142.5736	1142.5458	-0.0278	-24	140	150	VHACVGGTSVR			Carbamidomethyl (C)[4]		Mascot
1173.6475	1173.6141	-0.0334	-28	246	255	RDELTLEGIK					Mascot
1461.8538	1461.8214	-0.0324	-22	155	169	ILASGVHVVVGTPGR					Mascot
1571.708	1571.6755	-0.0325	-21	186	198	MFVLDEADEMLSR			Oxidation (M)[1]		Mascot
1587.703	1587.6487	-0.0543	-34	186	198	MFVLDEADEMLSR			Oxidation (M)[1,10]		Mascot
1800.7566	1800.8596	0.103	57	304	319	DHTVSATHGDMDQNTR			Oxidation (M)[11]		Mascot
1827.9388	1827.9044	-0.0344	-19	55	70	GIYAYGFEKPSAIQQR					Mascot
1827.9388	1827.9044	-0.0344	-19	55	70	GIYAYGFEKPSAIQQR	106	100			Mascot
2013.8792	2013.9648	0.0856	43	302	319	GRDHTVSATHGDMDQNT R			Oxidation (M)[13]		Mascot
2075.0842	2075.022	-0.0622	-30	215	233	IQVGVFSATMPPEALEIT R			Oxidation (M)[10]		Mascot
2685.3804	2685.3381	-0.0423	-16	268	290	LDTLCDLYETLAITQSVIF VNTR			Carbamidomethyl (C)[5]		Mascot
2911.4949	2911.4631	-0.0318	-11	343	367	GIDVQQVSLVINYDLPTQ PENYLHR					Mascot
3124.5806	3124.5454	-0.0352	-11	92	119	TATFCSGILQQLDYGLVE CQALVLAPTR			Carbamidomethyl (C)[5,19]		Mascot

10	putative DEAD-box ATP-dependent RNA helicase family protein isoform 1 [Zea mays]	gi|413943216	47238.1	5.29	15	172	100	15.67	106	100	

Protein Group

putative DEAD-box ATP-dependent RNA helicase family protein isoform 2 [Zea mays]	gi|413943217	47238.1	5.2899 999618 5303

Peptide Information

Calc. Mass	Obsrv. Mass	± da	± ppm	Start Seq.	End Seq.	Sequence	Ion Score	C. I. %	Modification	Rank	Result Type

976.5574	976.53	-0.0274	-28	378	386	GVAINFVTR				Mascot
1070.5953	1070.5638	-0.0315	-29	177	185	QSLRPDNIK				Mascot
1104.6525	1104.6251	-0.0274	-25	377	386	KGVAINFVTR				Mascot
1114.6831	1114.6538	-0.0293	-26	333	342	VLITTDLLAR				Mascot
1142.5736	1142.5458	-0.0278	-24	140	150	VHACVGGTSVR			Carbamidomethyl (C)[4]	Mascot
1173.6475	1173.6141	-0.0334	-28	246	255	RDELTLEGIK				Mascot
1461.8538	1461.8214	-0.0324	-22	155	169	ILASGVHVVVGTPGR				Mascot
1571.708	1571.6755	-0.0325	-21	186	198	MFVLDEADEMLSR			Oxidation (M)[1]	Mascot
1587.703	1587.6487	-0.0543	-34	186	198	MFVLDEADEMLSR			Oxidation (M)[1,10]	Mascot
1800.7566	1800.8596	0.103	57	304	319	DHTVSATHGDMDQNTR			Oxidation (M)[11]	Mascot
1827.9388	1827.9044	-0.0344	-19	55	70	GIYAYGFEKPSAIQQR				Mascot
1827.9388	1827.9044	-0.0344	-19	55	70	GIYAYGFEKPSAIQQR	106	100		Mascot
2011.0383	2011.0721	0.0338	17	247	263	DELTLEGIKQFYVNVDK				Mascot
2075.0842	2075.022	-0.0622	-30	215	233	IQVGVFSATMPPEALEIT R			Oxidation (M)[10]	Mascot
2685.3804	2685.3381	-0.0423	-16	268	290	LDTLCDLYETLAITQSVIF VNTR			Carbamidomethyl (C)[5]	Mascot
2911.4949	2911.4631	-0.0318	-11	343	367	GIDVQQVSLVINYDLPTQ PENYLHR				Mascot
3124.5806	3124.5454	-0.0352	-11	92	119	TATFCSGILQQLDYGLVE CQALVLAPTR			Carbamidomethyl (C)[5,19]	Mascot

Gel Idx/Pos	258/K10	Instr./Gel Origin	BA2151/Sample Project 20140814	Process Status	Analysis Succeeded
Plate [#] Name	[1] Sample Project 20140814	Instrument Sample Name		Spectra	11

Rank	Protein Name	Accession No.	Protein MW	Protein PI	Pep. Count	Protein Score	Protein Score C. I. %	Intensity Matched	Total Ion Score	Total Ion C. I. %	Confirmed
1	enolase [Triticum aestivum]	gi|461744058	48459.5	5.49	17	627	100	54.224	531	100	

Peptide Information

Calc. Mass	Obsrv. Mass	± da	± ppm	Start Seq.	End Seq.	Sequence	Ion Score	C. I. %	Modification	Rank	Result Type
806.4519	806.4372	-0.0147	-18	418	423	YNQLLR					Mascot
918.5077	918.4749	-0.0328	-36	347	354	SCNALLLK			Carbamidomethyl (C)[2]		Mascot
978.5043	978.4792	-0.0251	-26	439	446	FRAPVEPY					Mascot
978.5043	978.4792	-0.0251	-26	439	446	FRAPVEPY	39	81.27			Mascot
1189.6034	1189.614	0.0106	9	190	199	MGVEVYHNLK					Mascot
1205.5984	1205.5643	-0.0341	-28	190	199	MGVEVYHNLK			Oxidation (M)[1]		Mascot
1238.5848	1238.552	-0.0328	-26	373	383	HAGWGVMTSHR					Mascot
1254.5797	1254.5375	-0.0422	-34	373	383	HAGWGVMTSHR			Oxidation (M)[7]		Mascot
1551.8643	1551.8042	-0.0601	-39	134	147	IPLYQHIANLAGNK					Mascot
1573.8433	1573.7914	-0.0519	-33	355	369	VNQIGSVTESIEAVK					Mascot
1577.8058	1577.7406	-0.0652	-41	424	438	IEEELGDAAVYAGLK					Mascot
1790.9283	1790.9041	-0.0242	-14	36	53	AAVPSGASTGVYEALELR					Mascot
1790.9283	1790.9041	-0.0242	-14	36	53	AAVPSGASTGVYEALELR	155	100			Mascot
1901.8512	1901.8148	-0.0364	-19	264	279	TYDLNFKEENNDGSQK					Mascot
1916.9497	1916.8655	-0.0842	-44	169	185	LAMQEFMILPTGATSFK			Oxidation (M)[3,7]		Mascot
1983.8536	1983.8239	-0.0297	-15	18	35	GNPTVEVDVCCSDGTFA R			Carbamidomethyl (C)[10,11]		Mascot
1983.8536	1983.8239	-0.0297	-15	18	35	GNPTVEVDVCCSDGTFA R	157	100	Carbamidomethyl (C)[10,11]		Mascot
2016.9584	2016.8817	-0.0767	-38	246	263	VVIGMDVAASEFYNDKD K			Oxidation (M)[5]		Mascot
2132.1611	2132.0872	-0.0739	-35	148	168	QLVLPVPAFNVINGGSHA GNK					Mascot
2251.0547	2251.0205	-0.0342	-15	83	102	DPTAQTELDNFMVQQLD GTK					Mascot
2252.1294	2252.0425	-0.0869	-39	384	405	SGETEDTFIADLAVGLST GQIK					Mascot
2267.0496	2266.9934	-0.0562	-25	83	102	DPTAQTELDNFMVQQLD GTK			Oxidation (M)[12]		Mascot
2575.2378	2575.2151	-0.0227	-9	316	338	MTEECGVEVQIVGDDLLV TNPTR			Carbamidomethyl (C)[5]		Mascot
2575.2378	2575.2151	-0.0227	-9	316	338	MTEECGVEVQIVGDDLLV TNPTR	171	100	Carbamidomethyl (C)[5]		Mascot
2591.2329	2591.1909	-0.042	-16	316	338	MTEECGVEVQIVGDDLLV TNPTR			Carbamidomethyl (C)[5], Oxidation (M)[1]		Mascot

Calc. Mass	Obsrv. Mass	± da	± ppm	Start Seq.	End Seq.	Sequence	Ion Score	C. I. %	Modification	Rank	Result Type
2591.2329	2591.1909	-0.042	-16	316	338	MTEECGVEVQIVGDDLLV TNPTR	180	100	Carbamidomethyl (C)[5], Oxidation (M)[1]		Mascot

2	enolase [Triticum aestivum]	gi|461744056	48416.5	5.59	17	625	100	54.29	531	100	

Protein Group

enolase [Triticum aestivum]	gi|461744078	48416.5	5.5900 001525 8789

Peptide Information

Calc. Mass	Obsrv. Mass	± da	± ppm	Start Seq.	End Seq.	Sequence	Ion Score	C. I. %	Modification	Rank	Result Type
806.4519	806.4372	-0.0147	-18	418	423	YNQLLR					Mascot
918.5077	918.4749	-0.0328	-36	347	354	SCNALLLK			Carbamidomethyl (C)[2]		Mascot
978.5043	978.4792	-0.0251	-26	439	446	FRAPVEPY					Mascot
978.5043	978.4792	-0.0251	-26	439	446	FRAPVEPY	39	81.27			Mascot
1189.6034	1189.614	0.0106	9	190	199	MGVEVYHNLK					Mascot
1205.5984	1205.5643	-0.0341	-28	190	199	MGVEVYHNLK			Oxidation (M)[1]		Mascot
1238.5848	1238.552	-0.0328	-26	373	383	HAGWGVMTSHR					Mascot
1254.5797	1254.5375	-0.0422	-34	373	383	HAGWGVMTSHR			Oxidation (M)[7]		Mascot
1551.8643	1551.8042	-0.0601	-39	134	147	IPLYQHIANLAGNK					Mascot
1573.8433	1573.7914	-0.0519	-33	355	369	VNQIGSVTESIEAVK					Mascot
1790.9283	1790.9041	-0.0242	-14	36	53	AAVPSGASTGVYEALELR					Mascot
1790.9283	1790.9041	-0.0242	-14	36	53	AAVPSGASTGVYEALELR	155	100			Mascot
1886.9391	1886.8562	-0.0829	-44	169	185	LAMQEFMILPTGAASFK			Oxidation (M)[3,7]		Mascot
1901.8512	1901.8148	-0.0364	-19	264	279	TYDLNFKEENNDGSQK					Mascot
1983.8536	1983.8239	-0.0297	-15	18	35	GNPTVEVDVCCSDGTFA R			Carbamidomethyl (C)[10,11]		Mascot
1983.8536	1983.8239	-0.0297	-15	18	35	GNPTVEVDVCCSDGTFA R	157	100	Carbamidomethyl (C)[10,11]		Mascot
2016.9584	2016.8817	-0.0767	-38	246	263	VVIGMDVAASEFYNDKD K			Oxidation (M)[5]		Mascot
2132.1611	2132.0872	-0.0739	-35	148	168	QLVLPVPAFNVINGGSHA GNK					Mascot
2252.1294	2252.0425	-0.0869	-39	384	405	SGETEDTFIADLAVGLST GQIK					Mascot
2267.0496	2266.9934	-0.0562	-25	83	102	DPTAQTELDNYMVQQLD GTK					Mascot
2346.1543	2345.9802	-0.1741	-74	169	189	LAMQEFMILPTGAASFKE AMK			Oxidation (M)[3,7]		Mascot
2575.2378	2575.2151	-0.0227	-9	316	338	MTEECGVEVQIVGDDLLV TNPTR			Carbamidomethyl (C)[5]		Mascot
2575.2378	2575.2151	-0.0227	-9	316	338	MTEECGVEVQIVGDDLLV TNPTR	171	100	Carbamidomethyl (C)[5]		Mascot
2591.2329	2591.1909	-0.042	-16	316	338	MTEECGVEVQIVGDDLLV TNPTR			Carbamidomethyl (C)[5], Oxidation (M)[1]		Mascot
2591.2329	2591.1909	-0.042	-16	316	338	MTEECGVEVQIVGDDLLV	180	100	Carbamidomethyl (C)[5], Oxidation (M)[1]		Mascot

TNPTR

3	Enolase [Aegilops tauschii]	gi|475605231	51992.1	5.4	13	554	100	33.217	492	100

Peptide Information

Calc. Mass	Obsrv. Mass	± da	± ppm	Start Seq.	End Seq.	Sequence	Ion Score	C. I. %	Modification	Rank	Result Type
918.5077	918.4749	-0.0328	-36	366	373	SCNALLLK			Carbamidomethyl (C)[2]		Mascot
1189.6034	1189.614	0.0106	9	209	218	MGVEVYHNLK					Mascot
1205.5984	1205.5643	-0.0341	-28	209	218	MGVEVYHNLK			Oxidation (M)[1]		Mascot
1238.5848	1238.552	-0.0328	-26	392	402	HAGWGVMTSHR					Mascot
1254.5797	1254.5375	-0.0422	-34	392	402	HAGWGVMTSHR			Oxidation (M)[7]		Mascot
1573.8433	1573.7914	-0.0519	-33	374	388	VNQIGSVTESIEAVK					Mascot
1790.9283	1790.9041	-0.0242	-14	88	105	AAVPSGASTGVYEALELR					Mascot
1790.9283	1790.9041	-0.0242	-14	88	105	AAVPSGASTGVYEALELR	155	100			Mascot
1901.8512	1901.8148	-0.0364	-19	283	298	TYDLNFKEENNDGSQK					Mascot
1983.8536	1983.8239	-0.0297	-15	70	87	GNPTVEVDVCCSDGTFA R			Carbamidomethyl (C)[10,11]		Mascot
1983.8536	1983.8239	-0.0297	-15	70	87	GNPTVEVDVCCSDGTFA R	157	100	Carbamidomethyl (C)[10,11]		Mascot
2016.9584	2016.8817	-0.0767	-38	265	282	VVIGMDVAASEFYNDKD K			Oxidation (M)[5]		Mascot
2154.1667	2154.0757	-0.091	-42	186	204	KIPLYQEFMILPTGAASFK					Mascot
2252.1294	2252.0425	-0.0869	-39	403	424	SGETEDTFIADLAVGLST GQIK					Mascot
2324.0425	2323.9866	-0.0559	-24	225	246	YGQDATNVGDEGGFAPN IQENK					Mascot
2452.1375	2452.0972	-0.0403	-16	224	246	KYGQDATNVGDEGGFAP NIQENK					Mascot
2575.2378	2575.2151	-0.0227	-9	335	357	MTEECGVEVQIVGDDLLV TNPTR			Carbamidomethyl (C)[5]		Mascot
2575.2378	2575.2151	-0.0227	-9	335	357	MTEECGVEVQIVGDDLLV TNPTR	171	100	Carbamidomethyl (C)[5]		Mascot
2591.2329	2591.1909	-0.042	-16	335	357	MTEECGVEVQIVGDDLLV TNPTR			Carbamidomethyl (C)[5], Oxidation (M)[1]		Mascot
2591.2329	2591.1909	-0.042	-16	335	357	MTEECGVEVQIVGDDLLV TNPTR	180	100	Carbamidomethyl (C)[5], Oxidation (M)[1]		Mascot

4	RecName: Full=Enolase; AltName: Full=2-phospho-D-glycerate hydro-lyase; AltName: Full=2-phosphoglycerate dehydratase; AltName: Full=OSE1	gi|90110845	48284.5	5.41	19	462	100	51.591	352	100

Peptide Information

Calc. Mass	Obsrv. Mass	± da	± ppm	Start Seq.	End Seq.	Sequence	Ion Score	C. I. %	Modification	Rank	Result Type
806.4519	806.4372	-0.0147	-18	418	423	YNQLLR					Mascot
918.5077	918.4749	-0.0328	-36	347	354	SCNALLLK			Carbamidomethyl (C)[2]		Mascot

978.5043	978.4792	-0.0251	-26	439	446	FRAPVEPY					Mascot
978.5043	978.4792	-0.0251	-26	439	446	FRAPVEPY	39	81.27			Mascot
1015.6259	1015.5479	-0.078	-77	2	11	AATIVSVKAR					Mascot
1117.5208	1117.585	0.0642	57	374	383	AGWGVMTSHR			Oxidation (M)[6]		Mascot
1146.6664	1146.5768	-0.0896	-78	1	11	MAATIVSVKAR					Mascot
1189.6034	1189.614	0.0106	9	190	199	MGVEVYHNLK					Mascot
1205.5984	1205.5643	-0.0341	-28	190	199	MGVEVYHNLK			Oxidation (M)[1]		Mascot
1551.8643	1551.8042	-0.0601	-39	134	147	IPLYQHIANLAGNK					Mascot
1573.8433	1573.7914	-0.0519	-33	355	369	VNQIGSVTESIEAVK					Mascot
1770.0306	1769.8848	-0.1458	-82	110	126	QKLGANAILAVSLAICK			Carbamidomethyl (C)[16]		Mascot
1790.9283	1790.9041	-0.0242	-14	36	53	AAVPSGASTGVYEALELR					Mascot
1790.9283	1790.9041	-0.0242	-14	36	53	AAVPSGASTGVYEALELR	155	100			Mascot
1886.9391	1886.8562	-0.0829	-44	169	185	LAMQEFMILPTGAASFK			Oxidation (M)[3,7]		Mascot
1901.8512	1901.8148	-0.0364	-19	264	279	TYDLNFKEENNDGSQK					Mascot
1983.8536	1983.8239	-0.0297	-15	18	35	GNPTVEVDVCCSDGTFA R			Carbamidomethyl (C)[10,11]		Mascot
1983.8536	1983.8239	-0.0297	-15	18	35	GNPTVEVDVCCSDGTFA R	157	100	Carbamidomethyl (C)[10,11]		Mascot
2016.9584	2016.8817	-0.0767	-38	246	263	VVIGMDVAASEFYNDKD K			Oxidation (M)[5]		Mascot
2132.1611	2132.0872	-0.0739	-35	148	168	QLVLPVPAFNVINGGSHA GNK					Mascot
2324.0425	2323.9866	-0.0559	-24	206	227	YGQDATNVGDEGGFAPN IQENK					Mascot
2346.1543	2345.9802	-0.1741	-74	169	189	LAMQEFMILPTGAASFKE AMK			Oxidation (M)[3,7]		Mascot
2452.1375	2452.0972	-0.0403	-16	205	227	KYGQDATNVGDEGGFAP NIQENK					Mascot

5 PREDICTED: enolase 2-like isoform X2 [Setaria italica] gi|514752421 48210.4 5.59 18 455 100 52.51 352 100

Peptide Information

Calc. Mass	Obsrv. Mass	± da	± ppm	Start Seq.	End Seq.	Sequence	Ion Score	C. I. %	Modification	Rank	Result Type
806.4519	806.4372	-0.0147	-18	417	422	YNQLLR					Mascot
918.5077	918.4749	-0.0328	-36	346	353	SCNALLLK			Carbamidomethyl (C)[2]		Mascot
978.5043	978.4792	-0.0251	-26	438	445	FRAPVEPY					Mascot
978.5043	978.4792	-0.0251	-26	438	445	FRAPVEPY	39	81.27			Mascot
1189.6034	1189.614	0.0106	9	189	198	MGVEVYHNLK					Mascot
1205.5984	1205.5643	-0.0341	-28	189	198	MGVEVYHNLK			Oxidation (M)[1]		Mascot
1238.5848	1238.552	-0.0328	-26	372	382	HAGWGVMTSHR					Mascot
1254.5797	1254.5375	-0.0422	-34	372	382	HAGWGVMTSHR			Oxidation (M)[7]		Mascot
1551.8643	1551.8042	-0.0601	-39	133	146	IPLYQHIANLAGNK					Mascot

Calc. Mass	Obsrv. Mass	± da	± ppm	Start Seq.	End Seq.	Sequence	Ion Score	C. I. %	Modification	Rank	Result Type
1573.8433	1573.7914	-0.0519	-33	354	368	VNQIGSVTESIEAVK					Mascot
1790.9283	1790.9041	-0.0242	-14	35	52	AAVPSGASTGVYEALELR					Mascot
1790.9283	1790.9041	-0.0242	-14	35	52	AAVPSGASTGVYEALELR	155	100			Mascot
1886.9391	1886.8562	-0.0829	-44	168	184	LAMQEFMILPTGAASFK			Oxidation (M)[3,7]		Mascot
1901.8512	1901.8148	-0.0364	-19	263	278	TYDLNFKEENNDGSQK					Mascot
1983.8536	1983.8239	-0.0297	-15	17	34	GNPTVEVDVCCSDGTFAR			Carbamidomethyl (C)[10,11]		Mascot
1983.8536	1983.8239	-0.0297	-15	17	34	GNPTVEVDVCCSDGTFAR	157	100	Carbamidomethyl (C)[10,11]		Mascot
2016.9584	2016.8817	-0.0767	-38	245	262	VVIGMDVAASEFYNDKDK			Oxidation (M)[5]		Mascot
2132.1611	2132.0872	-0.0739	-35	147	167	QLVLPVPAFNVINGGSHAGNK					Mascot
2251.0547	2251.0205	-0.0342	-15	82	101	DPTAQTEIDNFMVQQLDGTK					Mascot
2267.0496	2266.9934	-0.0562	-25	82	101	DPTAQTEIDNFMVQQLDGTK			Oxidation (M)[12]		Mascot
2324.0425	2323.9866	-0.0559	-24	205	226	YGQDATNVGDEGGFAPNIQENK					Mascot
2346.1543	2345.9802	-0.1741	-74	168	188	LAMQEFMILPTGAASFKEAMK			Oxidation (M)[3,7]		Mascot
2452.1375	2452.0972	-0.0403	-16	204	226	KYGQDATNVGDEGGFAPNIQENK					Mascot
2573.2764	2573.2383	-0.0381	-15	315	337	MTEEIGEQVQIVGDDLLVTNPTR			Oxidation (M)[1]		Mascot

6 Os10g0167300 [Oryza sativa Japonica Group] gi|113638832 48226.5 5.5 18 452 100 51.214 352 100

Peptide Information

Calc. Mass	Obsrv. Mass	± da	± ppm	Start Seq.	End Seq.	Sequence	Ion Score	C. I. %	Modification	Rank	Result Type
806.4519	806.4372	-0.0147	-18	418	423	YNQLLR					Mascot
918.5077	918.4749	-0.0328	-36	347	354	SCNALLLK			Carbamidomethyl (C)[2]		Mascot
978.5043	978.4792	-0.0251	-26	439	446	FRAPVEPY					Mascot
978.5043	978.4792	-0.0251	-26	439	446	FRAPVEPY	39	81.27			Mascot
1015.6259	1015.5479	-0.078	-77	2	11	AATIVSVKAR					Mascot
1117.5208	1117.585	0.0642	57	374	383	AGWGVMTSHR			Oxidation (M)[6]		Mascot
1146.6664	1146.5768	-0.0896	-78	1	11	MAATIVSVKAR					Mascot
1189.6034	1189.614	0.0106	9	190	199	MGVEVYHNLK					Mascot
1205.5984	1205.5643	-0.0341	-28	190	199	MGVEVYHNLK			Oxidation (M)[1]		Mascot
1551.8643	1551.8042	-0.0601	-39	134	147	IPLYQHIANLAGNK					Mascot
1573.8433	1573.7914	-0.0519	-33	355	369	VNQIGSVTESIEAVK					Mascot
1770.0306	1769.8848	-0.1458	-82	110	126	QKLGANAILAVSLAICK			Carbamidomethyl (C)[16]		Mascot
1790.9283	1790.9041	-0.0242	-14	36	53	AAVPSGASTGVYEALELR					Mascot
1790.9283	1790.9041	-0.0242	-14	36	53	AAVPSGASTGVYEALELR	155	100			Mascot
1812.9387	1812.8508	-0.0879	-48	169	185	LAMQAFMILPTGAASFK			Oxidation (M)[3]		Mascot

1901.8512	1901.8148	-0.0364	-19	264	279	TYDLNFKEENNDGSQK					Mascot
1983.8536	1983.8239	-0.0297	-15	18	35	GNPTVEVDVCCSDGTFAR			Carbamidomethyl (C)[10,11]		Mascot
1983.8536	1983.8239	-0.0297	-15	18	35	GNPTVEVDVCCSDGTFAR	157	100	Carbamidomethyl (C)[10,11]		Mascot
2016.9584	2016.8817	-0.0767	-38	246	263	VVIGMDVAASEFYNDKDK			Oxidation (M)[5]		Mascot
2132.1611	2132.0872	-0.0739	-35	148	168	QLVLPVPAFNVINGGSHAGNK					Mascot
2324.0425	2323.9866	-0.0559	-24	206	227	YGQDATNVGDEGGFAPNIQENK					Mascot
2452.1375	2452.0972	-0.0403	-16	205	227	KYGQDATNVGDEGGFAPNIQENK					Mascot

7	hypothetical protein OsI_32842 [Oryza sativa Indica Group]	gi|218184182	48355.5	5.41	17	443	100	51.055	352	100	

Peptide Information

Calc. Mass	Obsrv. Mass	± da	± ppm	Start Seq.	End Seq.	Sequence	Ion Score	C. I. %	Modification	Rank	Result Type
806.4519	806.4372	-0.0147	-18	419	424	YNQLLR					Mascot
918.5077	918.4749	-0.0328	-36	348	355	SCNALLLK			Carbamidomethyl (C)[2]		Mascot
978.5043	978.4792	-0.0251	-26	440	447	FRAPVEPY					Mascot
978.5043	978.4792	-0.0251	-26	440	447	FRAPVEPY	39	81.27			Mascot
1015.6259	1015.5479	-0.078	-77	2	11	AATIVSVKAR					Mascot
1117.5208	1117.585	0.0642	57	375	384	AGWGVMTSHR			Oxidation (M)[6]		Mascot
1146.6664	1146.5768	-0.0896	-78	1	11	MAATIVSVKAR					Mascot
1189.6034	1189.614	0.0106	9	191	200	MGVEVYHNLK					Mascot
1205.5984	1205.5643	-0.0341	-28	191	200	MGVEVYHNLK			Oxidation (M)[1]		Mascot
1551.8643	1551.8042	-0.0601	-39	134	147	IPLYQHIANLAGNK					Mascot
1573.8433	1573.7914	-0.0519	-33	356	370	VNQIGSVTESIEAVK					Mascot
1770.0306	1769.8848	-0.1458	-82	110	126	QKLGANAILAVSLAICK			Carbamidomethyl (C)[16]		Mascot
1790.9283	1790.9041	-0.0242	-14	36	53	AAVPSGASTGVYEALELR					Mascot
1790.9283	1790.9041	-0.0242	-14	36	53	AAVPSGASTGVYEALELR	155	100			Mascot
1901.8512	1901.8148	-0.0364	-19	265	280	TYDLNFKEENNDGSQK					Mascot
1983.8536	1983.8239	-0.0297	-15	18	35	GNPTVEVDVCCSDGTFAR			Carbamidomethyl (C)[10,11]		Mascot
1983.8536	1983.8239	-0.0297	-15	18	35	GNPTVEVDVCCSDGTFAR	157	100	Carbamidomethyl (C)[10,11]		Mascot
2016.9584	2016.8817	-0.0767	-38	247	264	VVIGMDVAASEFYNDKDK			Oxidation (M)[5]		Mascot
2132.1611	2132.0872	-0.0739	-35	148	168	QLVLPVPAFNVINGGSHAGNK					Mascot
2324.0425	2323.9866	-0.0559	-24	207	228	YGQDATNVGDEGGFAPNIQENK					Mascot
2452.1375	2452.0972	-0.0403	-16	206	228	KYGQDATNVGDEGGFAPNIQENK					Mascot

8	uncharacterized protein, partial [Phleum pratense]	gi|409972433	46491.5	5.18	16	440	100	51.149	352	100	

Peptide Information

Calc. Mass	Obsrv. Mass	± da	± ppm	Start Seq.	End Seq.	Sequence	Ion Score	C. I. %	Modification	Rank	Result Type
806.4519	806.4372	-0.0147	-18	401	406	YNQLLR					Mascot
918.5077	918.4749	-0.0328	-36	330	337	SCNALLLK			Carbamidomethyl (C)[2]		Mascot
978.5043	978.4792	-0.0251	-26	422	429	FRAPVEPY					Mascot
978.5043	978.4792	-0.0251	-26	422	429	FRAPVEPY	39	81.27			Mascot
1117.5208	1117.585	0.0642	57	357	366	AGWGVMTSHR			Oxidation (M)[6]		Mascot
1189.6034	1189.614	0.0106	9	173	182	MGVEVYHNLK					Mascot
1205.5984	1205.5643	-0.0341	-28	173	182	MGVEVYHNLK			Oxidation (M)[1]		Mascot
1551.8643	1551.8042	-0.0601	-39	117	130	IPLYQHIANLAGNK					Mascot
1573.8433	1573.7914	-0.0519	-33	338	352	VNQIGSVTESIEAVK					Mascot
1790.9283	1790.9041	-0.0242	-14	19	36	AAVPSGASTGVYEALELR					Mascot
1790.9283	1790.9041	-0.0242	-14	19	36	AAVPSGASTGVYEALELR	155	100			Mascot
1886.9391	1886.8562	-0.0829	-44	152	168	LAMQEFMILPTGASSFK			Oxidation (M)[3]		Mascot
1983.8536	1983.8239	-0.0297	-15	1	18	GNPTVEVDVCCSDGTFA R			Carbamidomethyl (C)[10,11]		Mascot
1983.8536	1983.8239	-0.0297	-15	1	18	GNPTVEVDVCCSDGTFA R	157	100	Carbamidomethyl (C)[10,11]		Mascot
2132.1611	2132.0872	-0.0739	-35	131	151	QLVLPVPAFNVINGGSHA GNK					Mascot
2252.1294	2252.0425	-0.0869	-39	367	388	SGETEDTFIADLAVGLST GQIK					Mascot
2324.0425	2323.9866	-0.0559	-24	189	210	YGQDATNVGDEGGFAPN IQENK					Mascot
2346.1543	2345.9802	-0.1741	-74	152	172	LAMQEFMILPTGASSFKE AMK			Oxidation (M)[3]		Mascot
2452.1375	2452.0972	-0.0403	-16	188	210	KYGQDATNVGDEGGFAP NIQENK					Mascot
2573.2764	2573.2383	-0.0381	-15	299	321	MTEEIGEQVQIVGDDLLV TNPTR			Oxidation (M)[1]		Mascot

9	Enolase [Triticum urartu]	gi|474188401	43643.1	5.54	12	394	100	22.623	335	100	

Peptide Information

Calc. Mass	Obsrv. Mass	± da	± ppm	Start Seq.	End Seq.	Sequence	Ion Score	C. I. %	Modification	Rank	Result Type
918.5077	918.4749	-0.0328	-36	294	301	SCNALLLK			Carbamidomethyl (C)[2]		Mascot
1189.6034	1189.614	0.0106	9	137	146	MGVEVYHNLK					Mascot
1205.5984	1205.5643	-0.0341	-28	137	146	MGVEVYHNLK			Oxidation (M)[1]		Mascot
1238.5848	1238.552	-0.0328	-26	320	330	HAGWGVMTSHR					Mascot
1254.5797	1254.5375	-0.0422	-34	320	330	HAGWGVMTSHR			Oxidation (M)[7]		Mascot

Calc. Mass	Obsrv. Mass	± da	± ppm	Start Seq.	End Seq.	Sequence	Ion Score	C. I. %	Modification	Rank	Result Type
1573.8433	1573.7914	-0.0519	-33	302	316	VNQIGSVTESIEAVK					Mascot
1790.9283	1790.9041	-0.0242	-14	34	51	AAVPSGASTGVYEALELR					Mascot
1790.9283	1790.9041	-0.0242	-14	34	51	AAVPSGASTGVYEALELR	155	100			Mascot
1901.8512	1901.8148	-0.0364	-19	211	226	TYDLNFKEENNDGSQK					Mascot
2016.9584	2016.8817	-0.0767	-38	193	210	VVIGMDVAASEFYNDKD K			Oxidation (M)[5]		Mascot
2154.1667	2154.0757	-0.091	-42	114	132	KIPLYQEFMILPTGAASFK					Mascot
2252.1294	2252.0425	-0.0869	-39	331	352	SGETEDTFIADLAVGLST GQIK					Mascot
2324.0425	2323.9866	-0.0559	-24	153	174	YGQDATNVGDEGGFAPN IQENK					Mascot
2452.1375	2452.0972	-0.0403	-16	152	174	KYGQDATNVGDEGGFAP NIQENK					Mascot
2575.2378	2575.2151	-0.0227	-9	263	285	MTEECGVEVQIVGDDLLV TNPTR			Carbamidomethyl (C)[5]		Mascot
2575.2378	2575.2151	-0.0227	-9	263	285	MTEECGVEVQIVGDDLLV TNPTR	171	100	Carbamidomethyl (C)[5]		Mascot
2591.2329	2591.1909	-0.042	-16	263	285	MTEECGVEVQIVGDDLLV TNPTR			Carbamidomethyl (C)[5], Oxidation (M)[1]		Mascot
2591.2329	2591.1909	-0.042	-16	263	285	MTEECGVEVQIVGDDLLV TNPTR	180	100	Carbamidomethyl (C)[5], Oxidation (M)[1]		Mascot

10	uncharacterized protein, partial [Phleum pratense]	gi|409972085	48323.5	5.4	15	391	100	35.364	312	100	

Peptide Information

Calc. Mass	Obsrv. Mass	± da	± ppm	Start Seq.	End Seq.	Sequence	Ion Score	C. I. %	Modification	Rank	Result Type
806.4519	806.4372	-0.0147	-18	423	428	YNQLLR					Mascot
918.5077	918.4749	-0.0328	-36	352	359	SCNALLLK			Carbamidomethyl (C)[2]		Mascot
1189.6034	1189.614	0.0106	9	195	204	MGVEVYHNLK					Mascot
1205.5984	1205.5643	-0.0341	-28	195	204	MGVEVYHNLK			Oxidation (M)[1]		Mascot
1407.7665	1407.6544	-0.1121	-80	1	14	PPPPAMAATIQSVK					Mascot
1551.8643	1551.8042	-0.0601	-39	139	152	IPLYQHIANLAGNK					Mascot
1573.8433	1573.7914	-0.0519	-33	360	374	VNQIGSVTESIEAVK					Mascot
1790.9283	1790.9041	-0.0242	-14	41	58	AAVPSGASTGVYEALELR					Mascot
1790.9283	1790.9041	-0.0242	-14	41	58	AAVPSGASTGVYEALELR	155	100			Mascot
1886.9391	1886.8562	-0.0829	-44	174	190	LAMQEFMILPTGASSFK			Oxidation (M)[3]		Mascot
1983.8536	1983.8239	-0.0297	-15	23	40	GNPTVEVDVCCSDGTFA R			Carbamidomethyl (C)[10,11]		Mascot
1983.8536	1983.8239	-0.0297	-15	23	40	GNPTVEVDVCCSDGTFA R	157	100	Carbamidomethyl (C)[10,11]		Mascot
2132.1611	2132.0872	-0.0739	-35	153	173	QLVLPVPAFNVINGGSHA GNK					Mascot
2252.1294	2252.0425	-0.0869	-39	389	410	SGETEDTFIADLAVGLST GQIK					Mascot
2324.0425	2323.9866	-0.0559	-24	211	232	YGQDATNVGDEGGFAPN IQENK					Mascot
2346.1543	2345.9802	-0.1741	-74	174	194	LAMQEFMILPTGASSFKE AMK			Oxidation (M)[3]		Mascot

2452.1375	2452.0972	-0.0403	-16	210	232	KYGQDATNVGDEGGFAP NIQENK		Mascot
2573.2764	2573.2383	-0.0381	-15	321	343	MTEEIGEQVQIVGDDLLV TNPTR	Oxidation (M)[1]	Mascot

Gel Idx/Pos	259/K11	Instr./Gel Origin	BA2151/Sample Project 20140814	Process Status	Analysis Succeeded
Plate [#] Name	[1] Sample Project 20140814	Instrument Sample Name		Spectra	11

Rank	Protein Name	Accession No.	Protein MW	Protein PI	Pep. Count	Protein Score	Protein Score C. I. %	Intensity Matched	Total Ion Score	Total Ion C. I. %	Confirmed
1	Serpin-Z1B [Aegilops tauschii]	gi|475546073	43257.1	5.44	12	640	100	65.017	574	100	

Peptide Information

Calc. Mass	Obsrv. Mass	± da	± ppm	Start Seq.	End Seq.	Sequence	Ion Score	C. I. %	Modification	Rank	Result Type
925.5214	925.5015	-0.0199	-22	11	18	LSIAHQTR					Mascot
925.5214	925.5015	-0.0199	-22	11	18	LSIAHQTR	31	0			Mascot
1151.6824	1151.6327	-0.0497	-43	172	181	LVLANALYFK					Mascot
1176.5896	1176.572	-0.0176	-15	262	271	LSAEPDFLER					Mascot
1176.5896	1176.572	-0.0176	-15	262	271	LSAEPDFLER	76	99.996			Mascot
1292.7097	1292.6455	-0.0642	-50	290	301	ISFGIEASDLLK					Mascot
1345.6958	1345.7191	0.0233	17	159	171	NILPSGSVDNTTK					Mascot
1475.6438	1475.641	-0.0028	-2	182	194	GAWTDQFDSYGTK					Mascot
2062.0564	2061.9919	-0.0645	-31	138	156	AAEVTTQVNSWVEKVTS GR					Mascot
2685.3955	2685.3792	-0.0163	-6	33	61	SAASNAAFSPVSLHSALS LLAAGAGSATR					Mascot
2685.3955	2685.3792	-0.0163	-6	33	61	SAASNAAFSPVSLHSALS LLAAGAGSATR	240	100			Mascot
2720.3525	2720.3101	-0.0424	-16	329	354	VSSVFHQAFVEVNEQGT EAAASTAIK					Mascot
2720.3525	2720.3101	-0.0424	-16	329	354	VSSVFHQAFVEVNEQGT EAAASTAIK	39	81.911			Mascot
2941.5215	2941.5994	0.0779	26	355	379	MVPQQARPPSVMDFIAD HPFLFLLR			Oxidation (M)[1]		Mascot
2957.5166	2957.4834	-0.0332	-11	355	379	MVPQQARPPSVMDFIAD HPFLFLLR			Oxidation (M)[1,12]		Mascot
3055.3845	3055.3315	-0.053	-17	302	328	CLGLQLPFSDEADFSEM VDSPMPQGLR			Carbamidomethyl (C)[1], Oxidation (M)[17]		Mascot
3071.3796	3071.325	-0.0546	-18	302	328	CLGLQLPFSDEADFSEM VDSPMPQGLR			Carbamidomethyl (C)[1], Oxidation (M)[17,22]		Mascot
3705.9082	3705.8901	-0.0181	-5	62	98	DQLVATLGTGEVEGLHAL AEQVVQFVLADASSAGG PR					Mascot
3705.9082	3705.8901	-0.0181	-5	62	98	DQLVATLGTGEVEGLHAL AEQVVQFVLADASSAGG PR	189	100			Mascot

Rank	Protein Name	Accession No.	Protein MW	Protein PI	Pep. Count	Protein Score	Protein Score C. I. %	Intensity Matched	Total Ion Score	Total Ion C. I. %	Confirmed
2	RecName: Full=Serpin-Z1B; AltName: Full=TriaeZ1b; AltName: Full=WSZ1b; AltName: Full=WZS2	gi|75279910	43119.9	5.44	11	440	100	63.593	385	100	

Peptide Information

Calc. Mass	Obsrv. Mass	± da	± ppm	Start Seq.	End Seq.	Sequence	Ion Score	C. I. %	Modification	Rank	Result Type

Calc. Mass	Obsrv. Mass	± da	± ppm	Start Seq.	End Seq.	Sequence	Ion Score	C. I. %	Modification	Rank	Result Type
925.5214	925.5015	-0.0199	-22	11	18	LSIAHQTR					Mascot
925.5214	925.5015	-0.0199	-22	11	18	LSIAHQTR	31	0			Mascot
1151.6824	1151.6327	-0.0497	-43	172	181	LVLANALYFK					Mascot
1176.5896	1176.572	-0.0176	-15	262	271	LSAEPDFLER					Mascot
1176.5896	1176.572	-0.0176	-15	262	271	LSAEPDFLER	76	99.996			Mascot
1345.6958	1345.7191	0.0233	17	159	171	NILPSGSVDNTTK					Mascot
1475.6438	1475.641	-0.0028	-2	182	194	GAWTDQFDSYGTK					Mascot
2062.0564	2061.9919	-0.0645	-31	138	156	AAEVTTQVNSWVEKVTSGR					Mascot
2113.0999	2113.0837	-0.0162	-8	380	399	EDISGVVLFMGHVVNPLLSS					Mascot
2129.0947	2128.9883	-0.1064	-50	380	399	EDISGVVLFMGHVVNPLLSS			Oxidation (M)[10]		Mascot
2685.3955	2685.3792	-0.0163	-6	33	61	SAASNAAFSPVSLHSALSLLAAGAGSATR					Mascot
2685.3955	2685.3792	-0.0163	-6	33	61	SAASNAAFSPVSLHSALSLLAAGAGSATR	240	100			Mascot
2720.3525	2720.3101	-0.0424	-16	329	354	VSSVFHQAFVEVNEQGTEAAASTAIK					Mascot
2720.3525	2720.3101	-0.0424	-16	329	354	VSSVFHQAFVEVNEQGTEAAASTAIK	39	81.911			Mascot
2941.5215	2941.5994	0.0779	26	355	379	MVPQQARPPSVMDFIADHPFLFLLR			Oxidation (M)[1]		Mascot
2957.5166	2957.4834	-0.0332	-11	355	379	MVPQQARPPSVMDFIADHPFLFLLR			Oxidation (M)[1,12]		Mascot
3055.3845	3055.3315	-0.053	-17	302	328	CLGLQLPFSDEADFSEMVDSPMPQGLR			Carbamidomethyl (C)[1], Oxidation (M)[17]		Mascot
3071.3796	3071.325	-0.0546	-18	302	328	CLGLQLPFSDEADFSEMVDSPMPQGLR			Carbamidomethyl (C)[1], Oxidation (M)[17,22]		Mascot

3	RecName: Full=Serpin-Z1A; AltName: Full=TriaeZ1a; AltName: Full=WSZ1a; Short=WSZ1; AltName: Full=WSZCI	gi|75282265	43262.2	5.6	9	181	100	17.136	145	100

Peptide Information

Calc. Mass	Obsrv. Mass	± da	± ppm	Start Seq.	End Seq.	Sequence	Ion Score	C. I. %	Modification	Rank	Result Type
925.5214	925.5015	-0.0199	-22	11	18	LSIAHQTR					Mascot
925.5214	925.5015	-0.0199	-22	11	18	LSIAHQTR	31	0			Mascot
1151.6824	1151.6327	-0.0497	-43	172	181	LVLANALYFK					Mascot
1176.5896	1176.572	-0.0176	-15	261	270	LSAEPDFLER					Mascot
1176.5896	1176.572	-0.0176	-15	261	270	LSAEPDFLER	76	99.996			Mascot
1292.7097	1292.6455	-0.0642	-50	289	300	ISFGIEASDLLK					Mascot
1611.8953	1611.766	-0.1293	-80	157	171	IKDILPPGSIDNTTK					Mascot
2062.0564	2061.9919	-0.0645	-31	138	156	AAEVTTQVNSWVEKVTSGR					Mascot
2113.0999	2113.0837	-0.0162	-8	379	398	EDISGVVLFMGHVVNPLL					Mascot

Calc. Mass	Obsrv. Mass	± da	± ppm	Start Seq.	End Seq.	Sequence	Ion Score	C. I. %	Modification	Rank	Result Type
						SS					
2129.0947	2128.9883	-0.1064	-50	379	398	EDISGVVLFMGHVVNPLL SS			Oxidation (M)[10]		Mascot
2720.3525	2720.3101	-0.0424	-16	328	353	VSSVFHQAFVEVNEQGT EAAASTAIK					Mascot
2720.3525	2720.3101	-0.0424	-16	328	353	VSSVFHQAFVEVNEQGT EAAASTAIK	39	81.911			Mascot
2943.5374	2943.7	0.1626	55	354	378	MVLQQARPPSVMDFIAD HPFLFLVR			Oxidation (M)[1]		Mascot
2943.5374	2943.7	0.1626	55	354	378	MVLQQARPPSVMDFIAD HPFLFLVR			Oxidation (M)[1]		Mascot

4	RecName: Full=Serpin-Z1C; AltName: Full=TriaeZ1c; AltName: Full=WSZ1c	gi|75313848	42969	5.62	7	170	100	15.837	145	100		

Peptide Information

Calc. Mass	Obsrv. Mass	± da	± ppm	Start Seq.	End Seq.	Sequence	Ion Score	C. I. %	Modification	Rank	Result Type
925.5214	925.5015	-0.0199	-22	11	18	LSIAHQTR					Mascot
925.5214	925.5015	-0.0199	-22	11	18	LSIAHQTR	31	0			Mascot
1151.6824	1151.6327	-0.0497	-43	172	181	LVLANALYFK					Mascot
1176.5896	1176.572	-0.0176	-15	261	270	LSAEPDFLER					Mascot
1176.5896	1176.572	-0.0176	-15	261	270	LSAEPDFLER	76	99.996			Mascot
1239.5852	1239.6814	0.0962	78	127	137	ADTQSVDFQTK					Mascot
2113.0999	2113.0837	-0.0162	-8	379	398	EDISGVVLFMGHVVNPLL SS					Mascot
2129.0947	2128.9883	-0.1064	-50	379	398	EDISGVVLFMGHVVNPLL SS			Oxidation (M)[10]		Mascot
2720.3525	2720.3101	-0.0424	-16	328	353	VSSVFHQAFVEVNEQGT EAAASTAIK					Mascot
2720.3525	2720.3101	-0.0424	-16	328	353	VSSVFHQAFVEVNEQGT EAAASTAIK	39	81.911			Mascot
2725.4631	2725.325	-0.1381	-51	33	61	SAASNAVFSPVSLHVALS LLAAGAGSATR					Mascot

5	Serpin-Z1C [Triticum urartu]	gi|474075261	42956	5.62	6	165	100	15.724	145	100		

Peptide Information

Calc. Mass	Obsrv. Mass	± da	± ppm	Start Seq.	End Seq.	Sequence	Ion Score	C. I. %	Modification	Rank	Result Type
925.5214	925.5015	-0.0199	-22	11	18	LSIAHQTR					Mascot
925.5214	925.5015	-0.0199	-22	11	18	LSIAHQTR	31	0			Mascot
1151.6824	1151.6327	-0.0497	-43	172	181	LVLANALYFK					Mascot
1176.5896	1176.572	-0.0176	-15	261	270	LSAEPDFLER					Mascot
1176.5896	1176.572	-0.0176	-15	261	270	LSAEPDFLER	76	99.996			Mascot
2113.0999	2113.0837	-0.0162	-8	379	398	EDISGVVLFMGHVVNPLL SS					Mascot
2129.0947	2128.9883	-0.1064	-50	379	398	EDISGVVLFMGHVVNPLL SS			Oxidation (M)[10]		Mascot

2720.3525	2720.3101	-0.0424	-16	328	353	VSSVFHQAFVEVNEQGT EAAASTAIK					Mascot
2720.3525	2720.3101	-0.0424	-16	328	353	VSSVFHQAFVEVNEQGT EAAASTAIK	39	81.911			Mascot
2725.4631	2725.325	-0.1381	-51	33	61	SAASNAVFSPVSLHVALS LLAAGAGSATR					Mascot

6 Presequence protease 2 isoform 4 [Theobroma cacao] gi|508706322 96255.9 5.86 17 59 0 50.445

Peptide Information

Calc. Mass	Obsrv. Mass	± da	± ppm	Start Seq.	End Seq.	Sequence	Ion Score	C. I. %	Modification	Rank	Result Type
837.4981	837.4443	-0.0538	-64	150	156	VFGIVFR					Mascot
974.5557	974.511	-0.0447	-46	184	191	EPFVELLK					Mascot
989.556	989.5076	-0.0484	-49	1	8	MERTALLR					Mascot
989.556	989.5076	-0.0484	-49	1	8	MERTALLR	9	0			Mascot
1189.6477	1189.6082	-0.0395	-33	20	29	FLFSAPKHSR					Mascot
1225.5994	1225.6278	0.0284	23	728	737	EDPCSHIIVR			Carbamidomethyl (C)[4]		Mascot
1292.713	1292.6455	-0.0675	-52	466	476	VEELIMSSLKK			Oxidation (M)[6]		Mascot
1296.6484	1296.7017	0.0533	41	813	822	VTWNSYKGWR					Mascot
1341.6692	1341.74	0.0708	53	784	797	GSGHGIAAARMDAK					Mascot
1611.6803	1611.766	0.0857	53	135	149	TGAEVMSVSNDDENK			Oxidation (M)[6]		Mascot
1648.8112	1648.7787	-0.0325	-20	798	812	LNVSGWISEQMGGVR			Oxidation (M)[11]		Mascot
2065.0317	2065.0388	0.0071	3	218	234	DFYNLVDVYLDAVFFPK					Mascot
2204.1707	2204.0789	-0.0918	-42	679	697	ELLPLVPLFCQSLLEMGT K			Carbamidomethyl (C)[10], Oxidation (M)[16]		Mascot
2223.1228	2223.1047	-0.0181	-8	262	281	GVVFNEMKGVYSQPDNL LGR					Mascot
2527.24	2527.2747	0.0347	14	798	819	LNVSGWISEQMGGVRVT WNSYK			Oxidation (M)[11]		Mascot
2685.3706	2685.3792	0.0086	3	520	540	WIYDMDPFEPLKYEKPL MILK			Oxidation (M)[5]		Mascot
2685.3706	2685.3792	0.0086	3	520	540	WIYDMDPFEPLKYEKPL MILK			Oxidation (M)[5]		Mascot
2689.2661	2689.0911	-0.175	-65	476	499	KLAEEGFDTDAVEASMN TIEFSLR			Oxidation (M)[16]		Mascot
2713.2913	2713.3625	0.0712	26	341	364	ILSEYLDMFDASTAPDES KVEPQK					Mascot

7 RNAligase isoform 1 [Theobroma cacao] gi|508785842 127191.4 8.61 22 56 0 15.761

Peptide Information

Calc. Mass	Obsrv. Mass	± da	± ppm	Start Seq.	End Seq.	Sequence	Ion Score	C. I. %	Modification	Rank	Result Type
804.4574	804.4279	-0.0295	-37	117	123	RSVDLSK					Mascot
819.4359	819.3906	-0.0453	-55	612	619	IWGTSAGK					Mascot
836.4777	836.4518	-0.0259	-31	468	474	RFPAAFK					Mascot

837.4208	837.4443	0.0235	28	166	172	MIEMVSK					Mascot
887.4944	887.5195	0.0251	28	536	543	ERAAEIAK					Mascot
901.4448	901.4322	-0.0126	-14	1020	1026	HMEDILK			Oxidation (M)[2]		Mascot
989.5374	989.5076	-0.0298	-30	435	443	NADRSVLSK					Mascot
989.5374	989.5076	-0.0298	-30	435	443	NADRSVLSK					Mascot
1016.5483	1016.5355	-0.0128	-13	158	165	SDQEIRIR					Mascot
1176.6082	1176.572	-0.0362	-31	1018	1026	YKHMEDILK					Mascot
1176.6082	1176.572	-0.0362	-31	1018	1026	YKHMEDILK					Mascot
1189.6544	1189.6082	-0.0462	-39	457	465	LQEMIRLMR					Mascot
1292.59	1292.6455	0.0555	43	396	406	EICAANRSDEK			Carbamidomethyl (C)[3]		Mascot
1306.6638	1306.6001	-0.0637	-49	679	690	TEDFLAIVEGGR					Mascot
1384.6791	1384.6703	-0.0088	-6	494	505	MVIHVHSDSGFR					Mascot
1593.8635	1593.7354	-0.1281	-80	589	603	NGLSILFKDGPAAYK					Mascot
2061.9229	2061.9919	0.069	33	786	801	NAPNEEVWRQIENMCR			Carbamidomethyl (C)[15], Oxidation (M)[14]		Mascot
2151.1768	2151.0164	-0.1604	-75	350	369	VQGEILEGLVARIVSHES SK					Mascot
2204.0845	2204.0789	-0.0056	-3	847	865	ASQNAGYVLLMFYHLYE GK					Mascot
2655.4023	2655.3911	-0.0112	-4	739	764	ELLTAPGGLGDDLSVQSL MGDLIKGR					Mascot
2655.4023	2655.5725	0.1702	64	739	764	ELLTAPGGLGDDLSVQSL MGDLIKGR					Mascot
2725.304	2725.325	0.021	8	482	505	AESVSSDNLFYKMVIHVH SDSGFR					Mascot
2748.3774	2748.2622	-0.1152	-42	370	395	HMEEVLKDHPPPPADGA GIDLGPSLR					Mascot
2974.4888	2974.4536	-0.0352	-12	18	42	STPFTFLRSSPSYYFPFS RPLSFSR					Mascot
3055.4719	3055.3315	-0.1404	-46	805	833	ASAVPVIPDSEGTDSNPF SLDALGVFMFR			Oxidation (M)[27]		Mascot

8	PREDICTED: proline-rich receptor-like protein kinase PERK12-like [Solanum lycopersicum]	gi|460384098	63722.4	6.04	13	55	0	58.066

Peptide Information

Calc. Mass	Obsrv. Mass	± da	± ppm	Start Seq.	End Seq.	Sequence	Ion Score	C. I. %	Modification	Rank	Result Type
804.4396	804.4279	-0.0117	-15	460	466	KAIDAMR					Mascot
814.4781	814.3985	-0.0796	-98	122	129	ITAGTPVR					Mascot
1042.6255	1042.5957	-0.0298	-29	281	290	LKDGQVIAAK					Mascot
1151.5803	1151.6327	0.0524	46	31	39	DHPEQEIKR					Mascot
1176.6736	1176.572	-0.1016	-86	484	493	LALHELIDPR					Mascot
1176.6736	1176.572	-0.1016	-86	484	493	LALHELIDPR	5	0			Mascot
1255.6351	1255.6111	-0.024	-19	240	249	TELYIKDSMR					Mascot
2064.925	2065.0388	0.1138	55	2	21	ASSMGNNSSHTDVDSLIT			Oxidation (M)[4]		Mascot

						GR					
2087.0781	2087.0217	-0.0564	-27	467	483	EDSHHSLRQWAIPLIER					Mascot
2655.2153	2655.3911	0.1758	66	66	87	YLHPMGYQMEAEPLSMF GTHKR			Oxidation (M)[5,9]		Mascot
2685.407	2685.3792	-0.0278	-10	387	410	DLRPSNILLTHDYVPMLG DFGLAK					Mascot
2685.407	2685.3792	-0.0278	-10	387	410	DLRPSNILLTHDYVPMLG DFGLAK					Mascot
2702.3857	2702.3669	-0.0188	-7	176	198	VVRPYSIVDADNIEQKLY FSMSK					Mascot
2846.2529	2846.4429	0.19	67	97	121	IDAYVNMLMQSAQDCEG EGVDIEVK			Carbamidomethyl (C)[15], Oxidation (M)[7,9]		Mascot
2942.3582	2942.6294	0.2712	92	96	121	KIDAYVNMLMQSAQDCE GEGVDIEVK			Carbamidomethyl (C)[16]		Mascot
2974.3479	2974.4536	0.1057	36	96	121	KIDAYVNMLMQSAQDCE GEGVDIEVK			Carbamidomethyl (C)[16], Oxidation (M)[8,10]		Mascot

9	ARM repeat superfamily protein isoform 1 [Theobroma cacao]	gi|508709137	94427.2	5.48	17	54	0	3.266

Peptide Information

Calc. Mass	Obsrv. Mass	± da	± ppm	Start Seq.	End Seq.	Sequence	Ion Score	C. I. %	Modification	Rank	Result Type
868.4523	868.4282	-0.0241	-28	37	44	KNSSFSAK					Mascot
887.5746	887.5195	-0.0551	-62	699	705	LLCKILK			Carbamidomethyl (C)[3]		Mascot
917.4574	917.4333	-0.0241	-26	374	381	NVDIEEAK					Mascot
1115.642	1115.5707	-0.0713	-64	286	295	IDVEGSKVIR					Mascot
1173.7089	1173.6484	-0.0605	-52	677	686	LLTKLLESEK					Mascot
1175.6267	1175.5756	-0.0511	-43	773	783	IISEGVVDSTR					Mascot
1281.6256	1281.5765	-0.0491	-38	114	124	MLGLDHDPLDR					Mascot
1306.6387	1306.6001	-0.0386	-30	407	417	KSQAEFPTDQR					Mascot
1341.7347	1341.74	0.0053	4	418	429	LALLPWMDGVAR					Mascot
1646.7439	1646.7543	0.0104	6	445	459	AAESIADSSINEHMR			Oxidation (M)[14]		Mascot
1966.0215	1966.0315	0.01	5	706	722	SDIPLHNKDWVAACLVK			Carbamidomethyl (C)[14]		Mascot
2113.1614	2113.0837	-0.0777	-37	313	332	ILVIEEGLVPVPMVGADA YK					Mascot
2129.1562	2128.9883	-0.1679	-79	313	332	ILVIEEGLVPVPMVGADA YK			Oxidation (M)[13]		Mascot
2151.0752	2151.0164	-0.0588	-27	565	583	LDAFVGLTEDRPVSIMES R			Oxidation (M)[16]		Mascot
2656.4404	2656.5151	0.0747	28	185	210	GLVAESGAIEEITGLLSRP SLTSEVK					Mascot
2698.2817	2698.3271	0.0454	17	333	355	SFRPQLYSWPTMPDGTE IEQTSK					Mascot
2982.5742	2982.437	-0.1372	-46	756	783	LSLSPEAQEAAVVELNRII SEGVVDSTR					Mascot
3055.4717	3055.3315	-0.1402	-46	723	749	LSSFSGPNVDFENPVNM EVTLYETIPR					Mascot
3071.4668	3071.325	-0.1418	-46	723	749	LSSFSGPNVDFENPVNM EVTLYETIPR			Oxidation (M)[17]		Mascot

10	ARM repeat superfamily protein, putative isoform 3 [Theobroma cacao]	gi|508709139	71137.6	5.22	15	53	0	2.83

Peptide Information

Calc. Mass	Obsrv. Mass	± da	± ppm	Start Seq.	End Seq.	Sequence	Ion Score	C. I. %	Modification	Rank	Result Type
887.5746	887.5195	-0.0551	-62	486	492	LLCKILK			Carbamidomethyl (C)[3]		Mascot
917.4574	917.4333	-0.0241	-26	161	168	NVDIEEAK					Mascot
1115.642	1115.5707	-0.0713	-64	73	82	IDVEGSKVIR					Mascot
1173.7089	1173.6484	-0.0605	-52	464	473	LLTKLLESEK					Mascot
1175.6267	1175.5756	-0.0511	-43	560	570	IISEGVVDSTR					Mascot
1281.6508	1281.5765	-0.0743	-58	468	477	LLESEKFCQK			Carbamidomethyl (C)[8]		Mascot
1306.6387	1306.6001	-0.0386	-30	194	204	KSQAEFPTDQR					Mascot
1341.7347	1341.74	0.0053	4	205	216	LALLPWMDGVAR					Mascot
1646.7439	1646.7543	0.0104	6	232	246	AAESIADSSINEHMR			Oxidation (M)[14]		Mascot
1966.0215	1966.0315	0.01	5	493	509	SDIPLHNKDWVAACLVK			Carbamidomethyl (C)[14]		Mascot
2113.1614	2113.0837	-0.0777	-37	100	119	ILVIEEGLVPVPMVGADA YK					Mascot
2129.1562	2128.9883	-0.1679	-79	100	119	ILVIEEGLVPVPMVGADA YK			Oxidation (M)[13]		Mascot
2151.0752	2151.0164	-0.0588	-27	352	370	LDAFVGLTEDRPVSIMES R			Oxidation (M)[16]		Mascot
2698.2817	2698.3271	0.0454	17	120	142	SFRPQLYSWPTMPDGTE IEQTSK					Mascot
2982.5742	2982.437	-0.1372	-46	543	570	LSLSPEAQEAAVVELNRII SEGVVDSTR					Mascot
3055.4717	3055.3315	-0.1402	-46	510	536	LSSFSGPNVDFENPVNM EVTLYETIPR					Mascot
3071.4668	3071.325	-0.1418	-46	510	536	LSSFSGPNVDFENPVNM EVTLYETIPR			Oxidation (M)[17]		Mascot

Gel Idx/Pos	260/K12	Instr./Gel Origin	BA2151/Sample Project 20140814	Process Status	Analysis Succeeded
Plate [#] Name	[1] Sample Project 20140814	Instrument Sample Name		Spectra	11

Rank	Protein Name	Accession No.	Protein MW	Protein PI	Pep. Count	Protein Score	Protein Score C. I. %	Intensity Matched	Total Ion Score	Total Ion C. I. %	Confirmed
1	Bifunctional polymyxin resistance protein ArnA [Triticum urartu]	gi|474224464	43507.3	7.53	16	307	100	20.033	220	100	

Peptide Information

Calc. Mass	Obsrv. Mass	± da	± ppm	Start Seq.	End Seq.	Sequence	Ion Score	C. I. %	Modification	Rank	Result Type
842.4519	842.4771	0.0252	30	345	351	QLGWNPK					Mascot
862.5032	862.4528	-0.0504	-58	140	147	TIGSFLPK					Mascot
933.3908	933.4069	0.0161	17	40	48	EGEEAGDAR					Mascot
960.4745	960.4437	-0.0308	-32	246	254	LVDGGESQR					Mascot
960.4745	960.4437	-0.0308	-32	246	254	LVDGGESQR	50	97.875			Mascot
1216.6719	1216.6477	-0.0242	-20	335	344	RIPDMTLINK			Oxidation (M)[5]		Mascot
1306.6936	1306.6619	-0.0317	-24	230	240	VLACFSNNLLR			Carbamidomethyl (C)[4]		Mascot
1306.6936	1306.6619	-0.0317	-24	230	240	VLACFSNNLLR	87	100	Carbamidomethyl (C)[4]		Mascot
1427.7489	1427.6968	-0.0521	-36	242	254	EPLKLVDGGESQR					Mascot
1453.7145	1453.6626	-0.0519	-36	128	139	LIHFSTCEVYGK			Carbamidomethyl (C)[7]		Mascot
1461.7584	1461.6963	-0.0621	-42	356	367	DLLETTLTYQHK					Mascot
1524.7628	1524.6855	-0.0773	-51	177	188	WSYACAKQLIER			Carbamidomethyl (C)[5]		Mascot
1580.65	1580.6161	-0.0339	-21	323	335	EFYGEGYDDSDKR					Mascot
1654.8833	1654.8354	-0.0479	-29	261	275	DAIEAVLLMIENPAR					Mascot
1670.8782	1670.8124	-0.0658	-39	261	275	DAIEAVLLMIENPAR			Oxidation (M)[9]		Mascot
1673.7362	1673.7976	0.0614	37	31	48	ASPGGDAGKEGEEAGDA R					Mascot
1688.7949	1688.7306	-0.0643	-38	214	229	MDFIPGVDGPSEGVPR			Oxidation (M)[1]		Mascot
1688.7949	1688.7306	-0.0643	-38	214	229	MDFIPGVDGPSEGVPR	83	99.999	Oxidation (M)[1]		Mascot
1765.9219	1765.8307	-0.0912	-52	306	322	VSGEPPLEEPVIDVSAK					Mascot
1952.9574	1952.915	-0.0424	-22	276	293	ANGHIFNVGNPDNEVTV R					Mascot

Rank	Protein Name	Accession No.	Protein MW	Protein PI	Pep. Count	Protein Score	Protein Score C. I. %	Intensity Matched	Total Ion Score	Total Ion C. I. %	Confirmed
2	Bifunctional polymyxin resistance protein arnA [Aegilops tauschii]	gi|475528612	39324.8	5.12	13	286	100	19.565	220	100	

Peptide Information

Calc. Mass	Obsrv. Mass	± da	± ppm	Start Seq.	End Seq.	Sequence	Ion Score	C. I. %	Modification	Rank	Result Type
842.4519	842.4771	0.0252	30	307	313	QLGWNPK					Mascot

Calc. Mass	Obsrv. Mass	± da	± ppm	Start Seq.	End Seq.	Sequence	Ion Score	C. I. %	Modification	Rank	Result Type
862.5032	862.4528	-0.0504	-58	102	109	TIGSFLPK					Mascot
960.4745	960.4437	-0.0308	-32	208	216	LVDGGESQR					Mascot
960.4745	960.4437	-0.0308	-32	208	216	LVDGGESQR	50	97.875			Mascot
1216.6719	1216.6477	-0.0242	-20	297	306	RIPDMTLINK			Oxidation (M)[5]		Mascot
1306.6936	1306.6619	-0.0317	-24	192	202	VLACFSNNLLR			Carbamidomethyl (C)[4]		Mascot
1306.6936	1306.6619	-0.0317	-24	192	202	VLACFSNNLLR	87	100	Carbamidomethyl (C)[4]		Mascot
1427.7489	1427.6968	-0.0521	-36	204	216	EPLKLVDGGESQR					Mascot
1453.7145	1453.6626	-0.0519	-36	90	101	LIHFSTCEVYGK			Carbamidomethyl (C)[7]		Mascot
1461.7584	1461.6963	-0.0621	-42	318	329	DLLETTLTYQHK					Mascot
1524.7628	1524.6855	-0.0773	-51	139	150	WSYACAKQLIER			Carbamidomethyl (C)[5]		Mascot
1580.65	1580.6161	-0.0339	-21	285	297	EFYGEGYDDSDKR					Mascot
1654.8833	1654.8354	-0.0479	-29	223	237	DAIEAVLLMIENPAR					Mascot
1670.8782	1670.8124	-0.0658	-39	223	237	DAIEAVLLMIENPAR			Oxidation (M)[9]		Mascot
1688.7949	1688.7306	-0.0643	-38	176	191	MDFIPGVDGPSEGVPR			Oxidation (M)[1]		Mascot
1688.7949	1688.7306	-0.0643	-38	176	191	MDFIPGVDGPSEGVPR	83	99.999	Oxidation (M)[1]		Mascot
1952.9574	1952.915	-0.0424	-22	238	255	ANGHIFNVGNPDNEVTVR					Mascot

3	hypothetical protein ZEAMMB73_292856 [Zea mays]	gi|413951217	19083.5	4.85	7	210	100	14.855	170	100

Peptide Information

Calc. Mass	Obsrv. Mass	± da	± ppm	Start Seq.	End Seq.	Sequence	Ion Score	C. I. %	Modification	Rank	Result Type
842.4519	842.4771	0.0252	30	132	138	QLGWNPK					Mascot
1216.6719	1216.6477	-0.0242	-20	122	131	RIPDMTIINK			Oxidation (M)[5]		Mascot
1306.6936	1306.6619	-0.0317	-24	17	27	VLACFSNNLLR			Carbamidomethyl (C)[4]		Mascot
1306.6936	1306.6619	-0.0317	-24	17	27	VLACFSNNLLR	87	100	Carbamidomethyl (C)[4]		Mascot
1461.7584	1461.6963	-0.0621	-42	143	154	DLLETTLTYQHK					Mascot
1541.7595	1541.719	-0.0405	-26	2	16	DFIPGVDGPSEGVPR					Mascot
1688.7949	1688.7306	-0.0643	-38	1	16	MDFIPGVDGPSEGVPR			Oxidation (M)[1]		Mascot
1688.7949	1688.7306	-0.0643	-38	1	16	MDFIPGVDGPSEGVPR	83	99.999	Oxidation (M)[1]		Mascot
1952.9574	1952.915	-0.0424	-22	63	80	ANGHIFNVGNPDNEVTVR					Mascot

4	bifunctional polymyxin resistance arnA protein [Zea mays]	gi|413951216	44450.5	5.67	10	206	100	16.238	170	100

Protein Group

bifunctional polymyxin resistance arnA protein [Zea mays]	gi|226502716	44450.5	5.6700000762 9395

Peptide Information

Calc. Mass	Obsrv. Mass	± da	± ppm	Start Seq.	End Seq.	Sequence	Ion Score	C. I. %	Modification	Rank	Result Type
842.4519	842.4771	0.0252	30	357	363	QLGWNPK					Mascot
862.5032	862.4528	-0.0504	-58	152	159	TIGSFLPK					Mascot
1216.6719	1216.6477	-0.0242	-20	347	356	RIPDMTIINK			Oxidation (M)[5]		Mascot
1306.6936	1306.6619	-0.0317	-24	242	252	VLACFSNNLLR			Carbamidomethyl (C)[4]		Mascot
1306.6936	1306.6619	-0.0317	-24	242	252	VLACFSNNLLR	87	100	Carbamidomethyl (C)[4]		Mascot
1417.7145	1417.6949	-0.0196	-14	49	60	HVVLAVDVYCDK			Carbamidomethyl (C)[10]		Mascot
1453.7145	1453.6626	-0.0519	-36	140	151	LIHFSTCEVYGK			Carbamidomethyl (C)[7]		Mascot
1461.7584	1461.6963	-0.0621	-42	368	379	DLLETTLTYQHK					Mascot
1524.7628	1524.6855	-0.0773	-51	189	200	WSYACAKQLIER			Carbamidomethyl (C)[5]		Mascot
1688.7949	1688.7306	-0.0643	-38	226	241	MDFIPGVDGPSEGVPR			Oxidation (M)[1]		Mascot
1688.7949	1688.7306	-0.0643	-38	226	241	MDFIPGVDGPSEGVPR	83	99.999	Oxidation (M)[1]		Mascot
1952.9574	1952.915	-0.0424	-22	288	305	ANGHIFNVGNPDNEVTVR					Mascot

5	TPA: DTDP-glucose 4,6-dehydratase [Zea mays]	gi|414878185	44359.4	5.85	10	206	100	17.119	170	100	

Peptide Information

Calc. Mass	Obsrv. Mass	± da	± ppm	Start Seq.	End Seq.	Sequence	Ion Score	C. I. %	Modification	Rank	Result Type
842.4519	842.4771	0.0252	30	355	361	QLGWNPK					Mascot
862.5032	862.4528	-0.0504	-58	150	157	TIGSFLPK					Mascot
960.4745	960.4437	-0.0308	-32	2	11	SSSPSPAATR					Mascot
960.4745	960.4437	-0.0308	-32	2	11	SSSPSPAATR	1	0			Mascot
1216.6719	1216.6477	-0.0242	-20	345	354	RIPDMTIINK			Oxidation (M)[5]		Mascot
1306.6936	1306.6619	-0.0317	-24	240	250	VLACFSNNLLR			Carbamidomethyl (C)[4]		Mascot
1306.6936	1306.6619	-0.0317	-24	240	250	VLACFSNNLLR	87	100	Carbamidomethyl (C)[4]		Mascot
1453.7145	1453.6626	-0.0519	-36	138	149	LIHFSTCEVYGK			Carbamidomethyl (C)[7]		Mascot
1461.7584	1461.6963	-0.0621	-42	366	377	DLLETTLTYQHK					Mascot
1524.7628	1524.6855	-0.0773	-51	187	198	WSYACAKQLIER			Carbamidomethyl (C)[5]		Mascot
1688.7949	1688.7306	-0.0643	-38	224	239	MDFIPGVDGPSEGVPR			Oxidation (M)[1]		Mascot
1688.7949	1688.7306	-0.0643	-38	224	239	MDFIPGVDGPSEGVPR	83	99.999	Oxidation (M)[1]		Mascot
1951.9734	1951.8915	-0.0819	-42	286	303	ANGHIFNVGNPNNEVTVR					Mascot

6	hypothetical protein SORBIDRAFT_03g047200 [Sorghum bicolor]	gi|241931146	44620.6	5.78	10	204	100	16.238	169	100	

Peptide Information

Calc. Mass	Obsrv. Mass	± da	± ppm	Start Seq.	End Seq.	Sequence	Ion Score	C. I. %	Modification	Rank	Result Type
842.4519	842.4771	0.0252	30	358	364	QLGWNPK					Mascot
862.5032	862.4528	-0.0504	-58	153	160	TIGSFLPK					Mascot
1216.6719	1216.6477	-0.0242	-20	348	357	RIPDMTIINK			Oxidation (M)[5]		Mascot
1306.6936	1306.6619	-0.0317	-24	243	253	VLACFSNNLLR			Carbamidomethyl (C)[4]		Mascot
1306.6936	1306.6619	-0.0317	-24	243	253	VLACFSNNLLR	87	100	Carbamidomethyl (C)[4]		Mascot
1417.7145	1417.6949	-0.0196	-14	50	61	HVVLAVDVYCDK			Carbamidomethyl (C)[10]		Mascot
1453.7145	1453.6626	-0.0519	-36	141	152	LIHFSTCEVYGK			Carbamidomethyl (C)[7]		Mascot
1461.7584	1461.6963	-0.0621	-42	369	380	DLLETTLTYQHK					Mascot
1524.7628	1524.6855	-0.0773	-51	190	201	WSYACAKQLIER			Carbamidomethyl (C)[5]		Mascot
1688.7949	1688.7306	-0.0643	-38	227	242	MDFIPGVDGPSEGVPR			Oxidation (M)[1]		Mascot
1688.7949	1688.7306	-0.0643	-38	227	242	MDFIPGVDGPSEGVPR	83	99.999	Oxidation (M)[1]		Mascot
1952.9574	1952.915	-0.0424	-22	289	306	ANGHIFNVGNPDNEVTVR					Mascot

7	PREDICTED: UDP-glucuronic acid decarboxylase 1-like [Setaria italica]	gi|514787733	44200.4	5.78	10	204	100	15.63	170	100

Peptide Information

Calc. Mass	Obsrv. Mass	± da	± ppm	Start Seq.	End Seq.	Sequence	Ion Score	C. I. %	Modification	Rank	Result Type
842.4519	842.4771	0.0252	30	354	360	QLGWNPK					Mascot
862.5032	862.4528	-0.0504	-58	149	156	TIGSFLPK					Mascot
896.3778	896.3991	0.0213	24	385	393	QMSQASASS					Mascot
1016.4829	1016.5134	0.0305	30	1	10	MSSPSPAPAR			Oxidation (M)[1]		Mascot
1216.6719	1216.6477	-0.0242	-20	344	353	RIPDMTIINK			Oxidation (M)[5]		Mascot
1306.6936	1306.6619	-0.0317	-24	239	249	VLACFSNNLLR			Carbamidomethyl (C)[4]		Mascot
1306.6936	1306.6619	-0.0317	-24	239	249	VLACFSNNLLR	87	100	Carbamidomethyl (C)[4]		Mascot
1453.7145	1453.6626	-0.0519	-36	137	148	LIHFSTCEVYGK			Carbamidomethyl (C)[7]		Mascot
1461.7584	1461.6963	-0.0621	-42	365	376	DLLETTLTYQHK					Mascot
1524.7628	1524.6855	-0.0773	-51	186	197	WSYACAKQLIER			Carbamidomethyl (C)[5]		Mascot
1688.7949	1688.7306	-0.0643	-38	223	238	MDFIPGVDGPSEGVPR			Oxidation (M)[1]		Mascot
1688.7949	1688.7306	-0.0643	-38	223	238	MDFIPGVDGPSEGVPR	83	99.999	Oxidation (M)[1]		Mascot

8	LOC542159 [Zea mays]	gi|162462229	44514.4	5.85	9	202	100	16.062	170	100

Peptide Information

Calc. Mass	Obsrv. Mass	± da	± ppm	Start Seq.	End Seq.	Sequence	Ion Score	C. I. %	Modification	Rank	Result Type

842.4519	842.4771	0.0252	30	356	362	QLGWNPK					Mascot
862.5032	862.4528	-0.0504	-58	150	157	TIGSFLPK					Mascot
960.4745	960.4437	-0.0308	-32	2	11	SSSPSPAATR					Mascot
960.4745	960.4437	-0.0308	-32	2	11	SSSPSPAATR	1	0			Mascot
1216.6719	1216.6477	-0.0242	-20	346	355	RIPDMTIINK			Oxidation (M)[5]		Mascot
1306.6936	1306.6619	-0.0317	-24	240	250	VLACFSNNLLR			Carbamidomethyl (C)[4]		Mascot
1306.6936	1306.6619	-0.0317	-24	240	250	VLACFSNNLLR	87	100	Carbamidomethyl (C)[4]		Mascot
1461.7584	1461.6963	-0.0621	-42	367	378	DLLETTLTYQHK					Mascot
1524.7628	1524.6855	-0.0773	-51	187	198	WSYACAKQLIER			Carbamidomethyl (C)[5]		Mascot
1688.7949	1688.7306	-0.0643	-38	224	239	MDFIPGVDGPSEGVPR			Oxidation (M)[1]		Mascot
1688.7949	1688.7306	-0.0643	-38	224	239	MDFIPGVDGPSEGVPR	83	99.999	Oxidation (M)[1]		Mascot
1951.9734	1951.8915	-0.0819	-42	286	303	ANGHIFNVGNPNNEVTVR					Mascot

9	Os01g0969100 [Oryza sativa Japonica Group]	gi|113535054	44817.6	5.85	9	200	100	15.396	170	100	

Protein Group

hypothetical protein OsI_05369 [Oryza sativa Indica Group]	gi|125529268	45688	5.7399997111816

Peptide Information

Calc. Mass	Obsrv. Mass	± da	± ppm	Start Seq.	End Seq.	Sequence	Ion Score	C. I. %	Modification	Rank	Result Type
842.4519	842.4771	0.0252	30	359	365	QLGWNPK					Mascot
896.3778	896.3991	0.0213	24	390	398	QMSQASASS					Mascot
1216.6719	1216.6477	-0.0242	-20	349	358	RIPDMTIINK			Oxidation (M)[5]		Mascot
1306.6936	1306.6619	-0.0317	-24	244	254	VLACFSNNLLR			Carbamidomethyl (C)[4]		Mascot
1306.6936	1306.6619	-0.0317	-24	244	254	VLACFSNNLLR	87	100	Carbamidomethyl (C)[4]		Mascot
1377.6427	1377.588	-0.0547	-40	1	15	MSSSSSPPAASAAAR					Mascot
1453.7145	1453.6626	-0.0519	-36	142	153	LIHFSTCEVYGK			Carbamidomethyl (C)[7]		Mascot
1461.7584	1461.6963	-0.0621	-42	370	381	DLLETTLTYQHK					Mascot
1524.7628	1524.6855	-0.0773	-51	191	202	WSYACAKQLIER			Carbamidomethyl (C)[5]		Mascot
1688.7949	1688.7306	-0.0643	-38	228	243	MDFIPGVDGPSEGVPR			Oxidation (M)[1]		Mascot
1688.7949	1688.7306	-0.0643	-38	228	243	MDFIPGVDGPSEGVPR	83	99.999	Oxidation (M)[1]		Mascot

10	PREDICTED: bifunctional polymyxin resistance protein ArnA-like [Solanum lycopersicum]	gi|460391381	43655.2	5.76	11	178	100	18.299	137	100	

Peptide Information

Calc. Mass	Obsrv. Mass	± da	± ppm	Start Seq.	End Seq.	Sequence	Ion Score	C. I. %	Modification	Rank	Result Type

856.4675	856.4612	-0.0063	-7	348	354	QLAWNPK				Mascot
857.3458	857.3925	0.0467	54	123	129	YCSENGK			Carbamidomethyl (C)[2]	Mascot
960.4745	960.4437	-0.0308	-32	249	257	LVDGGESQR				Mascot
960.4745	960.4437	-0.0308	-32	249	257	LVDGGESQR	50	98.652		Mascot
1216.6719	1216.6477	-0.0242	-20	338	347	RIPDMTIINK			Oxidation (M)[5]	Mascot
1306.6936	1306.6619	-0.0317	-24	233	243	VLACFSNNLLR			Carbamidomethyl (C)[4]	Mascot
1306.6936	1306.6619	-0.0317	-24	233	243	VLACFSNNLLR	87	100	Carbamidomethyl (C)[4]	Mascot
1427.7489	1427.6968	-0.0521	-36	245	257	EPLKLVDGGESQR				Mascot
1524.7628	1524.6855	-0.0773	-51	180	191	WSYACAKQLIER			Carbamidomethyl (C)[5]	Mascot
1580.65	1580.6161	-0.0339	-21	326	338	EFYGEGYDDSDKR				Mascot
1623.8312	1623.8596	0.0284	17	130	142	RLIHFSTCEIYGK			Carbamidomethyl (C)[8]	Mascot
1623.8312	1623.8596	0.0284	17	130	142	RLIHFSTCEIYGK			Carbamidomethyl (C)[8]	Mascot
1654.8833	1654.8354	-0.0479	-29	264	278	DAIEAVLLMIENPAR				Mascot
1670.8782	1670.8124	-0.0658	-39	264	278	DAIEAVLLMIENPAR			Oxidation (M)[9]	Mascot
1951.9734	1951.8915	-0.0819	-42	279	296	ANGHIFNVGNPNNEVTV R				Mascot

Gel Idx/Pos	261/K13	Instr./Gel Origin	BA2151/Sample Project 20140814	Process Status	Analysis Succeeded
Plate [#] Name	[1] Sample Project 20140814	Instrument Sample Name		Spectra	11

Rank	Protein Name	Accession No.	Protein MW	Protein PI	Pep. Count	Protein Score	Protein Score C. I. %	Intensity Matched	Total Ion Score	Total Ion C. I. %	Confirmed
1	Malate dehydrogenase, cytoplasmic [Aegilops tauschii]	gi|475581489	39692.3	6.41	11	363	100	28.694	313	100	

Peptide Information

Calc. Mass	Obsrv. Mass	± da	± ppm	Start Seq.	End Seq.	Sequence	Ion Score	C. I. %	Modification	Rank	Result Type
863.4404	863.4255	-0.0149	-17	189	195	NISCLTR			Carbamidomethyl (C)[4]		Mascot
873.4788	873.4628	-0.016	-18	201	208	ALGQISER					Mascot
1360.7545	1360.6942	-0.0603	-44	93	104	MELIDAAFPLLK					Mascot
1376.7494	1376.6934	-0.056	-41	93	104	MELIDAAFPLLK			Oxidation (M)[1]		Mascot
1508.7928	1508.6887	-0.1041	-69	196	208	LDHNRALGQISER					Mascot
1645.7963	1645.7635	-0.0328	-20	278	293	LSSALSAASSACDHIR			Carbamidomethyl (C)[12]		Mascot
1649.9949	1649.85	-0.1449	-88	164	179	VLVVANPANTNALILK					Mascot
1773.8912	1773.8575	-0.0337	-19	277	293	KLSSALSAASSACDHIR			Carbamidomethyl (C)[13]		Mascot
2390.1624	2390.1448	-0.0176	-7	249	268	ELVQDDEWLNGEFIATV QQR					Mascot
2390.1624	2390.1448	-0.0176	-7	249	268	ELVQDDEWLNGEFIATV QQR	187	100			Mascot
2438.1848	2438.1343	-0.0505	-21	218	239	NAIIWGNHSSSQYPDVN HATVK					Mascot
2438.1848	2438.1343	-0.0505	-21	218	239	NAIIWGNHSSSQYPDVN HATVK					Mascot
2605.3113	2605.2822	-0.0291	-11	105	130	GVVATTDVVEACTGVNV AVMVGGFPR			Carbamidomethyl (C)[12]		Mascot
2605.3113	2605.2822	-0.0291	-11	105	130	GVVATTDVVEACTGVNV AVMVGGFPR	126	100	Carbamidomethyl (C)[12]		Mascot
2621.3064	2621.2654	-0.041	-16	105	130	GVVATTDVVEACTGVNV AVMVGGFPR			Carbamidomethyl (C)[12], Oxidation (M)[20]		Mascot
2621.3064	2621.2654	-0.041	-16	105	130	GVVATTDVVEACTGVNV AVMVGGFPR	87	100	Carbamidomethyl (C)[12], Oxidation (M)[20]		Mascot
2648.5022	2648.2666	-0.2356	-89	164	188	VLVVANPANTNALILKEFA PSIPEK					Mascot

Rank	Protein Name	Accession No.	Protein MW	Protein PI	Pep. Count	Protein Score	Protein Score C. I. %	Intensity Matched	Total Ion Score	Total Ion C. I. %	Confirmed
2	malate dehydrogenase, cytoplasmic [Zea mays]	gi|162464321	35909.3	5.77	9	263	100	20.705	222	100	

Protein Group

RecName: Full=Malate dehydrogenase, cytoplasmic	gi|18202485	35909.3	5.7699 999809 2651
TPA: malate dehydrogenase4 [Zea mays]	gi|414871066	35909.3	5.7699 999809 2651

Peptide Information

Calc. Mass	Obsrv. Mass	± da	± ppm	Start Seq.	End Seq.	Sequence	Ion Score	C. I. %	Modification	Rank	Result Type
863.4404	863.4255	-0.0149	-17	152	158	NVTCLTR			Carbamidomethyl (C)[4]		Mascot
873.4788	873.4628	-0.016	-18	164	171	ALGQISER					Mascot
1373.7424	1373.7098	-0.0326	-24	299	310	IVQGLPIDEFSR					Mascot
1508.7928	1508.6887	-0.1041	-69	159	171	LDHNRALGQISER					Mascot
1649.9949	1649.85	-0.1449	-88	127	142	VLVVANPANTNALILK					Mascot
2000.1361	2000.0997	-0.0364	-18	8	27	VLVTGAAGQIGYALVPMI AR					Mascot
2000.1361	2000.0997	-0.0364	-18	8	27	VLVTGAAGQIGYALVPMI AR	44	94.944			Mascot
2016.1311	2016.0746	-0.0565	-28	8	27	VLVTGAAGQIGYALVPMI AR			Oxidation (M)[17]		Mascot
2016.1311	2016.0746	-0.0565	-28	8	27	VLVTGAAGQIGYALVPMI AR	96	100	Oxidation (M)[17]		Mascot
2466.2161	2466.2424	0.0263	11	181	202	NVIIWGNHSSSQYPDVN HATVK					Mascot
2605.3113	2605.2822	-0.0291	-11	68	93	GVVATTDVVEACTGVNV AVMVGGFPR			Carbamidomethyl (C)[12]		Mascot
2605.3113	2605.2822	-0.0291	-11	68	93	GVVATTDVVEACTGVNV AVMVGGFPR	126	100	Carbamidomethyl (C)[12]		Mascot
2621.3064	2621.2654	-0.041	-16	68	93	GVVATTDVVEACTGVNV AVMVGGFPR			Carbamidomethyl (C)[12], Oxidation (M)[20]		Mascot
2621.3064	2621.2654	-0.041	-16	68	93	GVVATTDVVEACTGVNV AVMVGGFPR	87	100	Carbamidomethyl (C)[12], Oxidation (M)[20]		Mascot
2648.5022	2648.2666	-0.2356	-89	127	151	VLVVANPANTNALILKEFA PSIPEK					Mascot

3	TPA: malate dehydrogenase4 [Zea mays]	gi|414871065	30570.8	6.45	8	260	100	20.617	222	100	

Peptide Information

Calc. Mass	Obsrv. Mass	± da	± ppm	Start Seq.	End Seq.	Sequence	Ion Score	C. I. %	Modification	Rank	Result Type
863.4404	863.4255	-0.0149	-17	152	158	NVTCLTR			Carbamidomethyl (C)[4]		Mascot
873.4788	873.4628	-0.016	-18	164	171	ALGQISER					Mascot
1508.7928	1508.6887	-0.1041	-69	159	171	LDHNRALGQISER					Mascot
1649.9949	1649.85	-0.1449	-88	127	142	VLVVANPANTNALILK					Mascot
2000.1361	2000.0997	-0.0364	-18	8	27	VLVTGAAGQIGYALVPMI AR					Mascot
2000.1361	2000.0997	-0.0364	-18	8	27	VLVTGAAGQIGYALVPMI AR	44	94.944			Mascot
2016.1311	2016.0746	-0.0565	-28	8	27	VLVTGAAGQIGYALVPMI AR			Oxidation (M)[17]		Mascot
2016.1311	2016.0746	-0.0565	-28	8	27	VLVTGAAGQIGYALVPMI AR	96	100	Oxidation (M)[17]		Mascot
2466.2161	2466.2424	0.0263	11	181	202	NVIIWGNHSSSQYPDVN HATVK					Mascot
2605.3113	2605.2822	-0.0291	-11	68	93	GVVATTDVVEACTGVNV AVMVGGFPR			Carbamidomethyl (C)[12]		Mascot

Calc. Mass	Obsrv. Mass	± da	± ppm	Start Seq.	End Seq.	Sequence	Ion Score	C. I. %	Modification	Rank	Result Type
2605.3113	2605.2822	-0.0291	-11	68	93	GVVATTDVVEACTGVNVAVMVGGFPR	126	100	Carbamidomethyl (C)[12]		Mascot
2621.3064	2621.2654	-0.041	-16	68	93	GVVATTDVVEACTGVNVAVMVGGFPR			Carbamidomethyl (C)[12], Oxidation (M)[20]		Mascot
2621.3064	2621.2654	-0.041	-16	68	93	GVVATTDVVEACTGVNVAVMVGGFPR	87	100	Carbamidomethyl (C)[12], Oxidation (M)[20]		Mascot
2648.5022	2648.2666	-0.2356	-89	127	151	VLVVANPANTNALILKEFAPSIPEK					Mascot

4	PREDICTED: malate dehydrogenase, cytoplasmic-like [Setaria italica]	gi|514816242	35803.2	5.76	8	255	100	20.602	222	100

Peptide Information

Calc. Mass	Obsrv. Mass	± da	± ppm	Start Seq.	End Seq.	Sequence	Ion Score	C. I. %	Modification	Rank	Result Type
863.4404	863.4255	-0.0149	-17	152	158	NVTCLTR			Carbamidomethyl (C)[4]		Mascot
873.4788	873.4628	-0.016	-18	164	171	ALGQISER					Mascot
1373.7424	1373.7098	-0.0326	-24	299	310	IVQGLPIDEFSR					Mascot
1508.7928	1508.6887	-0.1041	-69	159	171	LDHNRALGQISER					Mascot
1649.9949	1649.85	-0.1449	-88	127	142	VLVVANPANTNALILK					Mascot
2000.1361	2000.0997	-0.0364	-18	8	27	VLVTGAAGQIGYALVPMIAR					Mascot
2000.1361	2000.0997	-0.0364	-18	8	27	VLVTGAAGQIGYALVPMIAR	44	94.944			Mascot
2016.1311	2016.0746	-0.0565	-28	8	27	VLVTGAAGQIGYALVPMIAR			Oxidation (M)[17]		Mascot
2016.1311	2016.0746	-0.0565	-28	8	27	VLVTGAAGQIGYALVPMIAR	96	100	Oxidation (M)[17]		Mascot
2466.2161	2466.2424	0.0263	11	181	202	NVIIWGNHSSSQYPDVNHATVK					Mascot
2605.3113	2605.2822	-0.0291	-11	68	93	GVVATTDVVEACTGVNVAVMVGGFPR			Carbamidomethyl (C)[12]		Mascot
2605.3113	2605.2822	-0.0291	-11	68	93	GVVATTDVVEACTGVNVAVMVGGFPR	126	100	Carbamidomethyl (C)[12]		Mascot
2621.3064	2621.2654	-0.041	-16	68	93	GVVATTDVVEACTGVNVAVMVGGFPR			Carbamidomethyl (C)[12], Oxidation (M)[20]		Mascot
2621.3064	2621.2654	-0.041	-16	68	93	GVVATTDVVEACTGVNVAVMVGGFPR	87	100	Carbamidomethyl (C)[12], Oxidation (M)[20]		Mascot

5	malate dehydrogenase, putative [Ricinus communis]	gi|223526678	35984.5	6.4	6	244	100	20.47	222	100

Peptide Information

Calc. Mass	Obsrv. Mass	± da	± ppm	Start Seq.	End Seq.	Sequence	Ion Score	C. I. %	Modification	Rank	Result Type
873.4788	873.4628	-0.016	-18	164	171	ALGQISER					Mascot
1508.7928	1508.6887	-0.1041	-69	159	171	LDHNRALGQISER					Mascot
1645.7963	1645.7635	-0.0328	-20	241	256	LSSALSAASSACDHIR			Carbamidomethyl (C)[12]		Mascot
1773.8912	1773.8575	-0.0337	-19	240	256	KLSSALSAASSACDHIR			Carbamidomethyl (C)[13]		Mascot
2000.1361	2000.0997	-0.0364	-18	8	27	VLVTGAAGQIGYALVPMIAR					Mascot

2000.1361	2000.0997	-0.0364	-18	8	27	VLVTGAAGQIGYALVPMIAR	44	94.944			Mascot
2016.1311	2016.0746	-0.0565	-28	8	27	VLVTGAAGQIGYALVPMIAR			Oxidation (M)[17]		Mascot
2016.1311	2016.0746	-0.0565	-28	8	27	VLVTGAAGQIGYALVPMIAR	96	100	Oxidation (M)[17]		Mascot
2605.3113	2605.2822	-0.0291	-11	68	93	GVVATTDVVEACTGVNVAVMVGGFPR			Carbamidomethyl (C)[12]		Mascot
2605.3113	2605.2822	-0.0291	-11	68	93	GVVATTDVVEACTGVNVAVMVGGFPR	126	100	Carbamidomethyl (C)[12]		Mascot
2621.3064	2621.2654	-0.041	-16	68	93	GVVATTDVVEACTGVNVAVMVGGFPR			Carbamidomethyl (C)[12], Oxidation (M)[20]		Mascot
2621.3064	2621.2654	-0.041	-16	68	93	GVVATTDVVEACTGVNVAVMVGGFPR	87	100	Carbamidomethyl (C)[12], Oxidation (M)[20]		Mascot

6	malate dehydrogenase, cytoplasmic [Zea mays]	gi|226503019	35846.3	5.76	6	242	100	20.301	222	100

Peptide Information

Calc. Mass	Obsrv. Mass	± da	± ppm	Start Seq.	End Seq.	Sequence	Ion Score	C. I. %	Modification	Rank	Result Type
863.4404	863.4255	-0.0149	-17	152	158	NVTCLTR			Carbamidomethyl (C)[4]		Mascot
873.4788	873.4628	-0.016	-18	164	171	ALGQISER					Mascot
1508.7928	1508.6887	-0.1041	-69	159	171	LDHNRALGQISER					Mascot
1649.9949	1649.85	-0.1449	-88	127	142	VLVVANPANTNALILK					Mascot
2000.1361	2000.0997	-0.0364	-18	8	27	VLVTGAAGQIGYALVPMIAR					Mascot
2000.1361	2000.0997	-0.0364	-18	8	27	VLVTGAAGQIGYALVPMIAR	44	94.944			Mascot
2016.1311	2016.0746	-0.0565	-28	8	27	VLVTGAAGQIGYALVPMIAR			Oxidation (M)[17]		Mascot
2016.1311	2016.0746	-0.0565	-28	8	27	VLVTGAAGQIGYALVPMIAR	96	100	Oxidation (M)[17]		Mascot
2605.3113	2605.2822	-0.0291	-11	68	93	GVVATTDVVEACTGVNVAVMVGGFPR			Carbamidomethyl (C)[12]		Mascot
2605.3113	2605.2822	-0.0291	-11	68	93	GVVATTDVVEACTGVNVAVMVGGFPR	126	100	Carbamidomethyl (C)[12]		Mascot
2621.3064	2621.2654	-0.041	-16	68	93	GVVATTDVVEACTGVNVAVMVGGFPR			Carbamidomethyl (C)[12], Oxidation (M)[20]		Mascot
2621.3064	2621.2654	-0.041	-16	68	93	GVVATTDVVEACTGVNVAVMVGGFPR	87	100	Carbamidomethyl (C)[12], Oxidation (M)[20]		Mascot

7	putative NAD-dependent dehydrogenase 1 [Erythroxylum coca]	gi|392056683	39915.4	7.55	10	203	100	21.072	153	100

Peptide Information

Calc. Mass	Obsrv. Mass	± da	± ppm	Start Seq.	End Seq.	Sequence	Ion Score	C. I. %	Modification	Rank	Result Type
873.4788	873.4628	-0.016	-18	164	171	ALGQISER					Mascot
1508.7928	1508.6887	-0.1041	-69	159	171	LDHNRALGQISER					Mascot
1645.7963	1645.7635	-0.0328	-20	241	256	LSSALSAASSACDHIR			Carbamidomethyl (C)[12]		Mascot
1649.9949	1649.85	-0.1449	-88	127	142	VLVVANPANTNALILK					Mascot

1773.8912	1773.8575	-0.0337	-19	240	256	KLSSALSAASSACDHIR			Carbamidomethyl (C)[13]		Mascot
2000.1361	2000.0997	-0.0364	-18	8	27	VLVTGAAGQIGYALVPMI AR					Mascot
2000.1361	2000.0997	-0.0364	-18	8	27	VLVTGAAGQIGYALVPMI AR	44	94.944			Mascot
2016.1311	2016.0746	-0.0565	-28	8	27	VLVTGAAGQIGYALVPMI AR			Oxidation (M)[17]		Mascot
2016.1311	2016.0746	-0.0565	-28	8	27	VLVTGAAGQIGYALVPMI AR	96	100	Oxidation (M)[17]		Mascot
2402.3337	2402.1431	-0.1906	-79	120	142	HAAANCKVLVVANPANT NALILK			Carbamidomethyl (C)[6]		Mascot
2605.3113	2605.2822	-0.0291	-11	68	93	GVVATTDVVDACTGVNIA VMVGGFPR			Carbamidomethyl (C)[12]		Mascot
2605.3113	2605.2822	-0.0291	-11	68	93	GVVATTDVVDACTGVNIA VMVGGFPR	56	99.698	Carbamidomethyl (C)[12]		Mascot
2621.3064	2621.2654	-0.041	-16	68	93	GVVATTDVVDACTGVNIA VMVGGFPR			Carbamidomethyl (C)[12], Oxidation (M)[20]		Mascot
2621.3064	2621.2654	-0.041	-16	68	93	GVVATTDVVDACTGVNIA VMVGGFPR	49	98.459	Carbamidomethyl (C)[12], Oxidation (M)[20]		Mascot
2648.5022	2648.2666	-0.2356	-89	127	151	VLVVANPANTNALILKEFA PSIPEK					Mascot
2958.5791	2958.3923	-0.1868	-63	28	55	GVMLGLDQPVIIHMLDIQ PAAEALNGVK			Oxidation (M)[3]		Mascot

8	PREDICTED: malate dehydrogenase, cytoplasmic-like [Fragaria vesca subsp. vesca]	gi|470120564	35979.5	6.01	12	159	100	15.711	96	100	

Peptide Information

Calc. Mass	Obsrv. Mass	± da	± ppm	Start Seq.	End Seq.	Sequence	Ion Score	C. I. %	Modification	Rank	Result Type
863.4404	863.4255	-0.0149	-17	152	158	NISCLTR			Carbamidomethyl (C)[4]		Mascot
873.4788	873.4628	-0.016	-18	164	171	ALGQISER					Mascot
970.5316	970.508	-0.0236	-24	203	211	TPSGEKPVR					Mascot
1360.7545	1360.6942	-0.0603	-44	56	67	MELIDAAFPLLK					Mascot
1373.7424	1373.7098	-0.0326	-24	299	310	IVQGLPIDEFSR					Mascot
1376.7494	1376.6934	-0.056	-41	56	67	MELIDAAFPLLK			Oxidation (M)[1]		Mascot
1508.7928	1508.6887	-0.1041	-69	159	171	LDHNRALGQISER					Mascot
1645.7963	1645.7635	-0.0328	-20	241	256	LSSALSAASSACDHIR			Carbamidomethyl (C)[12]		Mascot
1649.9949	1649.85	-0.1449	-88	127	142	VLVVANPANTNALILK					Mascot
1773.8912	1773.8575	-0.0337	-19	240	256	KLSSALSAASSACDHIR			Carbamidomethyl (C)[13]		Mascot
2000.1361	2000.0997	-0.0364	-18	8	27	VLVTGAAGQIGYALVPMI AR					Mascot
2000.1361	2000.0997	-0.0364	-18	8	27	VLVTGAAGQIGYALVPMI AR	44	94.944			Mascot
2016.1311	2016.0746	-0.0565	-28	8	27	VLVTGAAGQIGYALVPMI AR			Oxidation (M)[17]		Mascot
2016.1311	2016.0746	-0.0565	-28	8	27	VLVTGAAGQIGYALVPMI AR	96	100	Oxidation (M)[17]		Mascot
2466.2161	2466.2424	0.0263	11	181	202	NVIIWGNHSSSQYPDVN HATVK					Mascot
2648.5022	2648.2666	-0.2356	-89	127	151	VLVVANPANTNALILKEFA					Mascot

PSIPEK

9	malate dehydrogenase, partial [Genlisea aurea]	gi|527204987	35852.6	6.33	10	148	100	14.644	96	100

Peptide Information

Calc. Mass	Obsrv. Mass	± da	± ppm	Start Seq.	End Seq.	Sequence	Ion Score	C. I. %	Modification	Rank	Result Type
873.4788	873.4628	-0.016	-18	164	171	ALGQISER					Mascot
1508.7928	1508.6887	-0.1041	-69	159	171	LDHNRALGQISER					Mascot
1645.7963	1645.7635	-0.0328	-20	241	256	LSSALSAASSACDHIR			Carbamidomethyl (C)[12]		Mascot
1649.9949	1649.85	-0.1449	-88	127	142	VLVVANPANTNALILK					Mascot
1773.8912	1773.8575	-0.0337	-19	240	256	KLSSALSAASSACDHIR			Carbamidomethyl (C)[13]		Mascot
2000.1361	2000.0997	-0.0364	-18	8	27	VLVTGAAGQIGYALVPMI AR					Mascot
2000.1361	2000.0997	-0.0364	-18	8	27	VLVTGAAGQIGYALVPMI AR	44	94.944			Mascot
2016.1311	2016.0746	-0.0565	-28	8	27	VLVTGAAGQIGYALVPMI AR			Oxidation (M)[17]		Mascot
2016.1311	2016.0746	-0.0565	-28	8	27	VLVTGAAGQIGYALVPMI AR	96	100	Oxidation (M)[17]		Mascot
2388.2559	2388.1274	-0.1285	-54	212	231	VLVADDKWLNEDFIVTVQ QR					Mascot
2402.3337	2402.1431	-0.1906	-79	120	142	HAAANCKVLVVANPANT NALILK			Carbamidomethyl (C)[6]		Mascot
2633.3428	2633.3242	-0.0186	-7	68	93	GVIATTDVVEACTGVNIA VMVGGFPR			Carbamidomethyl (C)[12]		Mascot
2648.5022	2648.2666	-0.2356	-89	127	151	VLVVANPANTNALILKEFA PSIPEK					Mascot
2649.3376	2649.293	-0.0446	-17	68	93	GVIATTDVVEACTGVNIA VMVGGFPR			Carbamidomethyl (C)[12], Oxidation (M)[20]		Mascot

10	RecName: Full=Malate dehydrogenase, cytoplasmic 1	gi|11133509	35890.4	6.11	10	143	100	15.357	96	100

Protein Group

hypothetical protein ARALYDRAFT_470418 [Arabidopsis lyrata subsp. lyrata]	gi|297338058	35945.5	6.1100 001335 144
malate dehydrogenase [Arabidopsis thaliana]	gi|15219721	35890.4	6.1100 001335 144
malate dehydrogenase [Arabidopsis thaliana]	gi|332189573	35890.4	6.1100 001335 144

Peptide Information

Calc. Mass	Obsrv. Mass	± da	± ppm	Start Seq.	End Seq.	Sequence	Ion Score	C. I. %	Modification	Rank	Result Type
863.4404	863.4255	-0.0149	-17	152	158	NISCLTR			Carbamidomethyl (C)[4]		Mascot
873.4788	873.4628	-0.016	-18	164	171	ALGQISER					Mascot

1360.7545	1360.6942	-0.0603	-44	56	67	MELIDAAFPLLK					Mascot
1376.7494	1376.6934	-0.056	-41	56	67	MELIDAAFPLLK				Oxidation (M)[1]	Mascot
1508.7928	1508.6887	-0.1041	-69	159	171	LDHNRALGQISER					Mascot
1645.7963	1645.7635	-0.0328	-20	241	256	LSSALSAASSACDHIR				Carbamidomethyl (C)[12]	Mascot
1649.9949	1649.85	-0.1449	-88	127	142	VLVVANPANTNALILK					Mascot
1773.8912	1773.8575	-0.0337	-19	240	256	KLSSALSAASSACDHIR				Carbamidomethyl (C)[13]	Mascot
2000.1361	2000.0997	-0.0364	-18	8	27	VLVTGAAGQIGYALVPMI AR					Mascot
2000.1361	2000.0997	-0.0364	-18	8	27	VLVTGAAGQIGYALVPMI AR		44	94.293		Mascot
2016.1311	2016.0746	-0.0565	-28	8	27	VLVTGAAGQIGYALVPMI AR				Oxidation (M)[17]	Mascot
2016.1311	2016.0746	-0.0565	-28	8	27	VLVTGAAGQIGYALVPMI AR		96	100	Oxidation (M)[17]	Mascot
2563.2644	2563.2583	-0.0061	-2	68	93	GVVATTDAVEGCTGVNV AVMVGGFPR				Carbamidomethyl (C)[12]	Mascot
2648.5022	2648.2666	-0.2356	-89	127	151	VLVVANPANTNALILKEFA PSIPEK					Mascot

Gel Idx/Pos	262/K14	Instr./Gel Origin	BA2151/Sample Project 20140814	Process Status	Analysis Succeeded
Plate [#] Name	[1] Sample Project 20140814	Instrument Sample Name		Spectra	11

Rank	Protein Name	Accession No.	Protein MW	Protein PI	Pep. Count	Protein Score	Protein Score C. I. %	Intensity Matched	Total Ion Score	Total Ion C. I. %	Confirmed
1	Beta-amylase [Triticum urartu]	gi|474451266	58995	5.34	17	549	100	26.762	469	100	

Peptide Information

Calc. Mass	Obsrv. Mass	± da	± ppm	Start Seq.	End Seq.	Sequence	Ion Score	C. I. %	Modification	Rank	Result Type
947.5057	947.4637	-0.042	-44	322	329	DGYRPIAR					Mascot
993.4999	993.4631	-0.0368	-37	28	35	FEKGDELR					Mascot
1239.6328	1239.5414	-0.0914	-74	272	282	HGDKILDEANK					Mascot
1299.582	1299.552	-0.03	-23	336	346	ASLNFTCAEMR			Carbamidomethyl (C)[7]		Mascot
1313.6273	1313.6583	0.031	24	62	72	GPRAYDWSAYK					Mascot
1315.5769	1315.5256	-0.0513	-39	336	346	ASLNFTCAEMR			Carbamidomethyl (C)[7], Oxidation (M)[10]		Mascot
1335.7202	1335.6029	-0.1173	-88	322	332	DGYRPIARMLK			Oxidation (M)[9]		Mascot
1382.7791	1382.7128	-0.0663	-48	73	84	QLFQLVHEAGLK					Mascot
1515.7994	1515.6556	-0.1438	-95	260	271	FFLAWYSNNLIK					Mascot
1592.7421	1592.6691	-0.073	-46	334	346	HRASLNFTCAEMR			Carbamidomethyl (C)[9]		Mascot
1623.9581	1623.8064	-0.1517	-93	73	86	QLFQLVHEAGLKLK					Mascot
1646.781	1646.7518	-0.0292	-18	246	259	FFVDNGTYLTEQGR					Mascot
1646.781	1646.7518	-0.0292	-18	246	259	FFVDNGTYLTEQGR	84	100			Mascot
1668.7952	1668.7633	-0.0319	-19	218	232	AAAAMVGHPEWEFPR					Mascot
1669.7349	1669.7563	0.0214	13	148	161	SAVQMYTDYMASFR					Mascot
1684.79	1684.7444	-0.0456	-27	218	232	AAAAMVGHPEWEFPR			Oxidation (M)[5]		Mascot
1701.7247	1701.6964	-0.0283	-17	148	161	SAVQMYTDYMASFR			Oxidation (M)[5,10]		Mascot
1992.9047	1992.8723	-0.0324	-16	112	129	NVGASDPDIFYTDQHGTR					Mascot
1992.9047	1992.8723	-0.0324	-16	112	129	NVGASDPDIFYTDQHGTR	146	100			Mascot
2013.9778	2013.9503	-0.0275	-14	304	321	VPSHAAEITAGYYNLHDR					Mascot
2013.9778	2013.9503	-0.0275	-14	304	321	VPSHAAEITAGYYNLHDR	118	100			Mascot
2087.0557	2087.0286	-0.0271	-13	130	147	NIEYLTLGVDDQPLFHGR					Mascot
2087.0557	2087.0286	-0.0271	-13	130	147	NIEYLTLGVDDQPLFHGR	121	100			Mascot
2269.2075	2269.1716	-0.0359	-16	166	187	EFLDAGVIVDIEVGLGPAGELR					Mascot

Rank	Protein Name	Accession No.	Protein MW	Protein PI	Pep. Count	Protein Score	Protein Score C. I. %	Intensity Matched	Total Ion Score	Total Ion C. I. %	Confirmed
2	beta-amylase 2, partial [Milium effusum]	gi|482677647	47235.6	6.49	8	261	100	14.436	239	100	

Peptide Information

Calc. Mass	Obsrv. Mass	± da	± ppm	Start Seq.	End Seq.	Sequence	Ion Score	C. I. %	Modification	Rank	Result Type
947.5057	947.4637	-0.042	-44	312	319	DGYRPIAR					Mascot
1239.6328	1239.5414	-0.0914	-74	262	272	HGDKILDEANK					Mascot
1283.6201	1283.6833	0.0632	49	208	219	AAAAMVGHPEWK			Oxidation (M)[5]		Mascot
1313.6273	1313.6583	0.031	24	52	62	GPRAYDWSAYK					Mascot
1335.7202	1335.6029	-0.1173	-88	312	322	DGYRPIARMLK			Oxidation (M)[9]		Mascot
1571.8217	1571.7612	-0.0605	-38	266	278	ILDEANKVFFGYR					Mascot
2013.9778	2013.9503	-0.0275	-14	294	311	VPSHAAELTAGYYNLHDR					Mascot
2013.9778	2013.9503	-0.0275	-14	294	311	VPSHAAELTAGYYNLHDR	118	100			Mascot
2087.0557	2087.0286	-0.0271	-13	120	137	NIEYLTLGVDDQPLFHGR					Mascot
2087.0557	2087.0286	-0.0271	-13	120	137	NIEYLTLGVDDQPLFHGR	121	100			Mascot

3 beta-amylase 2, partial [Milium effusum] gi|482677645 47129.8 7.3 8 260 100 14.436 239 100

Peptide Information

Calc. Mass	Obsrv. Mass	± da	± ppm	Start Seq.	End Seq.	Sequence	Ion Score	C. I. %	Modification	Rank	Result Type
947.5057	947.4637	-0.042	-44	312	319	DGYRPIAR					Mascot
1239.6328	1239.5414	-0.0914	-74	262	272	HGDKILDEANK					Mascot
1283.6201	1283.6833	0.0632	49	208	219	AAAAMVGHPEWK			Oxidation (M)[5]		Mascot
1313.6273	1313.6583	0.031	24	52	62	GPRAYDWSAYK					Mascot
1335.7202	1335.6029	-0.1173	-88	312	322	DGYRPIARMLK			Oxidation (M)[9]		Mascot
1571.8217	1571.7612	-0.0605	-38	266	278	ILDEANKVFFGYR					Mascot
2013.9778	2013.9503	-0.0275	-14	294	311	VPSHAAELTAGYYNLHDR					Mascot
2013.9778	2013.9503	-0.0275	-14	294	311	VPSHAAELTAGYYNLHDR	118	100			Mascot
2087.0557	2087.0286	-0.0271	-13	120	137	NIEYLTLGVDDQPLFHGR					Mascot
2087.0557	2087.0286	-0.0271	-13	120	137	NIEYLTLGVDDQPLFHGR	121	100			Mascot

4 beta-amylase 2, partial [Brachypodium distachyon] gi|482677643 46785.2 5.84 10 243 100 15.264 205 100

Peptide Information

Calc. Mass	Obsrv. Mass	± da	± ppm	Start Seq.	End Seq.	Sequence	Ion Score	C. I. %	Modification	Rank	Result Type
947.5057	947.4637	-0.042	-44	312	319	DGYRPIAR					Mascot
993.4999	993.4631	-0.0368	-37	18	25	FEKGDELR					Mascot
1239.6328	1239.5414	-0.0914	-74	262	272	HGDKILDEANK					Mascot
1335.7202	1335.6029	-0.1173	-88	312	322	DGYRPIARMLK			Oxidation (M)[9]		Mascot

Calc. Mass	Obsrv. Mass	± da	± ppm	Start Seq.	End Seq.	Sequence	Ion Score	C. I. %	Modification	Rank	Result Type
1515.7994	1515.6556	-0.1438	-95	250	261	FFLAWYSNNLIK					Mascot
1646.781	1646.7518	-0.0292	-18	236	249	FFVDNGTYLTEQGR					Mascot
1646.781	1646.7518	-0.0292	-18	236	249	FFVDNGTYLTEQGR	84	100			Mascot
1651.7784	1651.7557	-0.0227	-14	138	151	SAVQLYTDYMASFR					Mascot
1667.7734	1667.8126	0.0392	24	138	151	SAVQLYTDYMASFR			Oxidation (M)[10]		Mascot
1668.7952	1668.7633	-0.0319	-19	208	222	AAAAMVGHPEWEFPR					Mascot
1684.79	1684.7444	-0.0456	-27	208	222	AAAAMVGHPEWEFPR			Oxidation (M)[5]		Mascot
2087.0557	2087.0286	-0.0271	-13	120	137	NIEYLTLGVDDQPLFHGR					Mascot
2087.0557	2087.0286	-0.0271	-13	120	137	NIEYLTLGVDDQPLFHGR	121	100			Mascot
2269.2075	2269.1716	-0.0359	-16	156	177	EFLDAGVIVDIEVGLGPA GELR					Mascot

5	Alpha-1,4-glucan-protein synthase (UDP-forming) [Aegilops tauschii]	gi|475618083	41955	5.82	13	239	100	22.866	175	100	

Peptide Information

Calc. Mass	Obsrv. Mass	± da	± ppm	Start Seq.	End Seq.	Sequence	Ion Score	C. I. %	Modification	Rank	Result Type
839.441	839.4164	-0.0246	-29	159	165	GYPFSLR					Mascot
989.5414	989.4857	-0.0557	-56	276	284	ASNPFVNLK					Mascot
1180.6321	1180.5765	-0.0556	-47	123	132	DINALEQHIK					Mascot
1201.6365	1201.5807	-0.0558	-46	266	275	TGLPYLWHSK					Mascot
1283.7206	1283.6833	-0.0373	-29	20	30	DELDIVIPTIR					Mascot
1401.625	1401.5948	-0.0302	-22	83	94	ASCISFKDSACR			Carbamidomethyl (C)[3,11]		Mascot
1483.7461	1483.6969	-0.0492	-33	315	326	CYISLSEQVKEK			Carbamidomethyl (C)[1]		Mascot
1501.6958	1501.6659	-0.0299	-20	61	72	VPEGFDYELYNR					Mascot
1501.6958	1501.6659	-0.0299	-20	61	72	VPEGFDYELYNR	99	100			Mascot
1592.7302	1592.6691	-0.0611	-38	105	117	YVFTIDDDCFVAK			Carbamidomethyl (C)[9]		Mascot
1745.7809	1745.7543	-0.0266	-15	209	223	GTLFPMCGMNLAFDR			Carbamidomethyl (C)[7], Oxidation (M)[6]		Mascot
1761.7758	1761.7183	-0.0575	-33	209	223	GTLFPMCGMNLAFDR			Carbamidomethyl (C)[7], Oxidation (M)[6,9]		Mascot
2086.9785	2087.0286	0.0501	24	308	324	ECDTVQKCYISLSEQVK			Carbamidomethyl (C)[2,8]		Mascot
2086.9785	2087.0286	0.0501	24	308	324	ECDTVQKCYISLSEQVK			Carbamidomethyl (C)[2,8]		Mascot
2137.0569	2136.9932	-0.0637	-30	224	243	QLIGPAMYFGLMGDGQPI GR			Oxidation (M)[7]		Mascot
2153.0518	2152.9929	-0.0589	-27	224	243	QLIGPAMYFGLMGDGQPI GR			Oxidation (M)[7,12]		Mascot
2292.1335	2292.1003	-0.0332	-14	133	151	NLLSPSTPFFFNTLYDPY R					Mascot
2292.1335	2292.1003	-0.0332	-14	133	151	NLLSPSTPFFFNTLYDPY R	76	99.996			Mascot

6	Beta-amylase [Aegilops tauschii]	gi|475523854	60203.5	5.07	9	227	100	14.837	205	100	

Peptide Information

Calc. Mass	Obsrv. Mass	± da	± ppm	Start Seq.	End Seq.	Sequence	Ion Score	C. I. %	Modification	Rank	Result Type
947.5057	947.4637	-0.042	-44	322	329	DGYRPIAR					Mascot
993.4999	993.4631	-0.0368	-37	28	35	FEKGDELR					Mascot
1239.6328	1239.5414	-0.0914	-74	272	282	HGDKILDEANK					Mascot
1335.7202	1335.6029	-0.1173	-88	322	332	DGYRPIARMLK			Oxidation (M)[9]		Mascot
1515.7994	1515.6556	-0.1438	-95	260	271	FFLAWYSNNLIK					Mascot
1646.781	1646.7518	-0.0292	-18	246	259	FFVDNGTYLTEQGR					Mascot
1646.781	1646.7518	-0.0292	-18	246	259	FFVDNGTYLTEQGR	84	100			Mascot
1668.7952	1668.7633	-0.0319	-19	218	232	AAAAMVGHPEWEFPR					Mascot
1684.79	1684.7444	-0.0456	-27	218	232	AAAAMVGHPEWEFPR			Oxidation (M)[5]		Mascot
1745.7959	1745.7543	-0.0416	-24	333	346	RHHASLNFTCAEMR			Carbamidomethyl (C)[10], Oxidation (M)[13]		Mascot
2087.0557	2087.0286	-0.0271	-13	130	147	NIEYLTLGVDDQPLFHGR					Mascot
2087.0557	2087.0286	-0.0271	-13	130	147	NIEYLTLGVDDQPLFHGR	121	100			Mascot

7	hypothetical protein OsI_26792 [Oryza sativa Indica Group]	gi|218199984	41624.9	6.01	11	222	100	15.279	175	100

Peptide Information

Calc. Mass	Obsrv. Mass	± da	± ppm	Start Seq.	End Seq.	Sequence	Ion Score	C. I. %	Modification	Rank	Result Type
839.441	839.4164	-0.0246	-29	157	163	GYPFSLR					Mascot
989.5414	989.4857	-0.0557	-56	274	282	ASNPFVNLK					Mascot
1180.6321	1180.5765	-0.0556	-47	121	130	DINALEQHIK					Mascot
1201.6365	1201.5807	-0.0558	-46	264	273	TGLPYIWHSK					Mascot
1283.7206	1283.6833	-0.0373	-29	18	28	DELDIVIPTIR					Mascot
1401.625	1401.5948	-0.0302	-22	81	92	ASCISFKDSACR			Carbamidomethyl (C)[3,11]		Mascot
1501.6958	1501.6659	-0.0299	-20	59	70	VPEGFDYELYNR					Mascot
1501.6958	1501.6659	-0.0299	-20	59	70	VPEGFDYELYNR	99	100			Mascot
1667.8309	1667.8126	-0.0183	-11	306	319	EADTVQKCYLELAK			Carbamidomethyl (C)[8]		Mascot
1745.7809	1745.7543	-0.0266	-15	207	221	GTLFPMCGMNLAFDR			Carbamidomethyl (C)[7], Oxidation (M)[6]		Mascot
1761.7758	1761.7183	-0.0575	-33	207	221	GTLFPMCGMNLAFDR			Carbamidomethyl (C)[7], Oxidation (M)[6,9]		Mascot
2114.9778	2114.9851	0.0073	3	59	75	VPEGFDYELYNRDDINR					Mascot
2292.1335	2292.1003	-0.0332	-14	131	149	NLLSPSTPFFFNTLYDPYR					Mascot
2292.1335	2292.1003	-0.0332	-14	131	149	NLLSPSTPFFFNTLYDPYR	76	99.996			Mascot

8	Alpha-1,4-glucan-protein synthase [UDP-forming] 1 [Triticum urartu]	gi|474042704	63955.1	5.55	13	217	100	16.945	175	100

Peptide Information

Calc. Mass	Obsrv. Mass	± da	± ppm	Start Seq.	End Seq.	Sequence	Ion Score	C. I. %	Modification	Rank	Result Type
839.441	839.4164	-0.0246	-29	150	156	GYPFSLR					Mascot
989.5414	989.4857	-0.0557	-56	267	275	ASNPFVNLK					Mascot
1180.6321	1180.5765	-0.0556	-47	114	123	DINALEQHIK					Mascot
1201.6365	1201.5807	-0.0558	-46	257	266	TGLPYIWHSK					Mascot
1271.5275	1271.5276	0.0001	0	550	560	YVDLTSDTNDE					Mascot
1283.7206	1283.6833	-0.0373	-29	11	21	DELDIVIPTIR					Mascot
1401.625	1401.5948	-0.0302	-22	74	85	ASCISFKDSACR			Carbamidomethyl (C)[3,11]		Mascot
1501.6958	1501.6659	-0.0299	-20	52	63	VPEGFDYELYNR					Mascot
1501.6958	1501.6659	-0.0299	-20	52	63	VPEGFDYELYNR	99	100			Mascot
1574.8285	1574.7452	-0.0833	-53	374	388	AGLETSAQSRAIGWK					Mascot
1745.7809	1745.7543	-0.0266	-15	200	214	GTLFPMCGMNLAFDR			Carbamidomethyl (C)[7], Oxidation (M)[6]		Mascot
1761.7758	1761.7183	-0.0575	-33	200	214	GTLFPMCGMNLAFDR			Carbamidomethyl (C)[7], Oxidation (M)[6,9]		Mascot
1789.777	1789.8464	0.0694	39	429	447	LSCGSSSAGSSFAGASN SR			Carbamidomethyl (C)[3]		Mascot
2136.0278	2135.9727	-0.0551	-26	514	531	EEEVNDVLFEEGLARVC K			Carbamidomethyl (C)[17]		Mascot
2292.1335	2292.1003	-0.0332	-14	124	142	NLLSPSTPFFFNTLYDPY R					Mascot
2292.1335	2292.1003	-0.0332	-14	124	142	NLLSPSTPFFFNTLYDPY R	76	99.996			Mascot

9	RecName: Full=Alpha-1,4-glucan-protein synthase [UDP-forming]; AltName: Full=Reversibly glycosylated polypeptide 1; AltName: Full=UDP-glucose:protein transglucosylase; Short=UPTG	gi|34582497	42059	5.73	10	215	100	16.914	175	100	

Peptide Information

Calc. Mass	Obsrv. Mass	± da	± ppm	Start Seq.	End Seq.	Sequence	Ion Score	C. I. %	Modification	Rank	Result Type
839.441	839.4164	-0.0246	-29	152	158	GYPFSLR					Mascot
989.5414	989.4857	-0.0557	-56	269	277	ASNPFVNLK					Mascot
1201.6365	1201.5807	-0.0558	-46	259	268	TGLPYIWHSK					Mascot
1283.7206	1283.6833	-0.0373	-29	13	23	DELDIVIPTIR					Mascot
1401.625	1401.5948	-0.0302	-22	76	87	ASCISFKDSACR			Carbamidomethyl (C)[3,11]		Mascot
1501.6958	1501.6659	-0.0299	-20	54	65	VPEGFDYELYNR					Mascot
1501.6958	1501.6659	-0.0299	-20	54	65	VPEGFDYELYNR	99	100			Mascot
1701.8555	1701.6964	-0.1591	-93	111	125	DPTGHEINALEQHIK					Mascot
1714.7863	1714.8383	0.052	30	202	216	GSLFPMCGMNLAFNR			Carbamidomethyl (C)[7]		Mascot

1730.8088	1730.8306	0.0218	13	301	314	DCTSVQKCYIELSK			Carbamidomethyl (C)[2,8]		Mascot
2292.1335	2292.1003	-0.0332	-14	126	144	NLLSPSTPFFFNTLYDPY R					Mascot
2292.1335	2292.1003	-0.0332	-14	126	144	NLLSPSTPFFFNTLYDPY R	76	99.996			Mascot

10	RecName: Full=Alpha-1,4-glucan-protein synthase [UDP-forming]; AltName: Full=Amylogenin; AltName: Full=Golgi-associated protein se-wap41; AltName: Full=Reversibly glycosylated polypeptide; Short=RGP; AltName: Full=UDP-glucose:protein transglucosylas	gi|34588146	41690.8	5.75	10	213	100	15.261	173	100	

Protein Group

alpha-1,4-glucan-protein synthase [UDP-forming] [Zea mays]	gi|162463414	41690.8	5.75

Peptide Information

Calc. Mass	Obsrv. Mass	± da	± ppm	Start Seq.	End Seq.	Sequence	Ion Score	C. I. %	Modification	Rank	Result Type
839.441	839.4164	-0.0246	-29	159	165	GYPFSLR					Mascot
989.5414	989.4857	-0.0557	-56	276	284	ASNPFVNLK					Mascot
1180.6321	1180.5765	-0.0556	-47	123	132	DINALEQHIK					Mascot
1201.6365	1201.5807	-0.0558	-46	266	275	TGLPYIWHSK					Mascot
1223.5878	1223.5668	-0.021	-17	31	39	NLDFLEMWR					Mascot
1239.5826	1239.5414	-0.0412	-33	31	39	NLDFLEMWR			Oxidation (M)[7]		Mascot
1283.7206	1283.6833	-0.0373	-29	20	30	DELDIVIPTIR					Mascot
1401.625	1401.5948	-0.0302	-22	83	94	ASCISFKDSACR			Carbamidomethyl (C)[3,11]		Mascot
1501.6958	1501.6659	-0.0299	-20	61	72	VPEGFDYELYNR					Mascot
1501.6958	1501.6659	-0.0299	-20	61	72	VPEGFDYELYNR	99	100			Mascot
1745.7809	1745.7543	-0.0266	-15	209	223	GTLFPMCGMNLAFDR			Carbamidomethyl (C)[7], Oxidation (M)[6]		Mascot
1761.7758	1761.7183	-0.0575	-33	209	223	GTLFPMCGMNLAFDR			Carbamidomethyl (C)[7], Oxidation (M)[6,9]		Mascot
2292.1335	2292.1003	-0.0332	-14	133	151	NLLSPSTPFFFNTLYDPY R					Mascot
2292.1335	2292.1003	-0.0332	-14	133	151	NLLSPSTPFFFNTLYDPY R	76	99.996			Mascot

Gel Idx/Pos	263/K15	Instr./Gel Origin	BA2151/Sample Project 20140814	Process Status	Analysis Succeeded
Plate [#] Name	[1] Sample Project 20140814	Instrument Sample Name		Spectra	11

Rank	Protein Name	Accession No.	Protein MW	Protein PI	Pep. Count	Protein Score	Protein Score C. I. %	Intensity Matched	Total Ion Score	Total Ion C. I. %	Confirmed
1	Glutelin type-A 1 [Triticum urartu]	gi|474023259	33443.4	6.23	16	627	100	53.945	521	100	

Peptide Information

Calc. Mass	Obsrv. Mass	± da	± ppm	Start Seq.	End Seq.	Sequence	Ion Score	C. I. %	Modification	Rank	Result Type
877.5142	877.4645	-0.0497	-57	12	19	VAYVLQGK					Mascot
981.5364	981.5081	-0.0283	-29	132	140	LPVPVDADR					Mascot
981.5364	981.5081	-0.0283	-29	132	140	LPVPVDADR	56	99.396			Mascot
1028.5735	1028.5447	-0.0288	-28	175	184	EVGLGADLVR					Mascot
1028.5735	1028.5447	-0.0288	-28	175	184	EVGLGADLVR	85	100			Mascot
1054.6005	1054.5669	-0.0336	-32	213	222	VQVVGPDGKR					Mascot
1055.5481	1055.5266	-0.0215	-20	152	161	LDVDIPNGGR					Mascot
1109.6313	1109.5995	-0.0318	-29	132	141	LPVPVDADRK					Mascot
1109.6313	1109.5995	-0.0318	-29	132	141	LPVPVDADRK	44	90.074			Mascot
1184.6886	1184.616	-0.0726	-61	20	31	GTVGIVLPEATK					Mascot
1185.6838	1185.6259	-0.0579	-49	114	125	LVSSQPASGIVK					Mascot
1187.6783	1187.6514	-0.0269	-23	228	238	IEGGSLFIVPR					Mascot
1187.6783	1187.6514	-0.0269	-23	228	238	IEGGSLFIVPR	101	100			Mascot
1291.6497	1291.6804	0.0307	24	141	151	KDMALNCLQAK			Carbamidomethyl (C)[7]		Mascot
1291.6497	1291.6804	0.0307	24	141	151	KDMALNCLQAK			Carbamidomethyl (C)[7]		Mascot
1308.6583	1308.626	-0.0323	-25	300	310	RLDSEIFFAPN					Mascot
1308.6583	1308.626	-0.0323	-25	300	310	RLDSEIFFAPN	90	100			Mascot
1379.8621	1379.7728	-0.0893	-65	162	174	VVVLNTANLPLVK					Mascot
2109.0056	2108.918	-0.0876	-42	276	294	AISPEVLEASFNTTPEME K			Oxidation (M)[17]		Mascot
2694.3333	2694.3499	0.0166	6	271	294	TSVWKAISPEVLEASFNT TPEMEK					Mascot
2764.2166	2764.1853	-0.0313	-11	185	208	IDAHSMCSPGFSCDSAY QVTYIVR			Carbamidomethyl (C)[7,13]		Mascot
2780.2114	2780.1611	-0.0503	-18	185	208	IDAHSMCSPGFSCDSAY QVTYIVR			Carbamidomethyl (C)[7,13], Oxidation (M)[6]		Mascot
2780.2114	2780.1611	-0.0503	-18	185	208	IDAHSMCSPGFSCDSAY QVTYIVR	145	100	Carbamidomethyl (C)[7,13], Oxidation (M)[6]		Mascot
3116.5337	3116.499	-0.0347	-11	74	102	GHRPGQFTNFQLTGASG IFTGFSTEFVGR					Mascot

Rank	Protein Name	Accession No.	Protein MW	Protein PI	Pep. Count	Protein Score	Protein Score C. I. %	Intensity Matched	Total Ion Score	Total Ion C. I. %	Confirmed
2	Glutelin type-A 2 [Aegilops tauschii]	gi|475560198	38139.6	5.6	16	514	100	26.02	418	100	

Peptide Information

Calc. Mass	Obsrv. Mass	± da	± ppm	Start Seq.	End Seq.	Sequence	Ion Score	C. I. %	Modification	Rank	Result Type
877.5142	877.4645	-0.0497	-57	60	67	VAYVLQGK					Mascot
981.5364	981.5081	-0.0283	-29	180	188	LPVPVDADR					Mascot
981.5364	981.5081	-0.0283	-29	180	188	LPVPVDADR	56	99.396			Mascot
1028.5735	1028.5447	-0.0288	-28	223	232	EVGLGADLVR					Mascot
1028.5735	1028.5447	-0.0288	-28	223	232	EVGLGADLVR	85	100			Mascot
1055.5481	1055.5266	-0.0215	-20	200	209	LDVDIPNGGR					Mascot
1109.6313	1109.5995	-0.0318	-29	180	189	LPVPVDADRK					Mascot
1109.6313	1109.5995	-0.0318	-29	180	189	LPVPVDADRK	44	90.074			Mascot
1180.5337	1180.5558	0.0221	19	190	199	DMALNCLEAK			Carbamidomethyl (C)[6], Oxidation (M)[2]		Mascot
1184.6886	1184.616	-0.0726	-61	68	79	GTVGIVLPEATK					Mascot
1185.6838	1185.6259	-0.0579	-49	162	173	LVSSQPASGIVK					Mascot
1186.6063	1186.6293	0.023	19	257	269	GSGSVQVVGPDGK					Mascot
1308.6287	1308.626	-0.0027	-2	189	199	KDMALNCLEAK			Carbamidomethyl (C)[7], Oxidation (M)[3]		Mascot
1308.6583	1308.626	-0.0323	-25	348	358	RLDSEIFFAPN	90	100			Mascot
1379.8621	1379.7728	-0.0893	-65	210	222	VVVLNTANLPLVK					Mascot
2109.0056	2108.918	-0.0876	-42	324	342	AISPEVLEASFNTTPEME K			Oxidation (M)[17]		Mascot
2694.3333	2694.3499	0.0166	6	319	342	TSVWKAISPEVLEASFNT TPEMEK					Mascot
2764.2166	2764.1853	-0.0313	-11	233	256	IDAHSMCSPGFSCDSAY QVTYIVR			Carbamidomethyl (C)[7,13]		Mascot
2780.2114	2780.1611	-0.0503	-18	233	256	IDAHSMCSPGFSCDSAY QVTYIVR			Carbamidomethyl (C)[7,13], Oxidation (M)[6]		Mascot
2780.2114	2780.1611	-0.0503	-18	233	256	IDAHSMCSPGFSCDSAY QVTYIVR	145	100	Carbamidomethyl (C)[7,13], Oxidation (M)[6]		Mascot
3116.5337	3116.499	-0.0347	-11	122	150	GHRPGQFTNFQLTGASG IFTGFSTEFVGR					Mascot

3	putative rmlC-like cupins superfamily protein [Zea mays] gi|413942236	38282.8	6.23	8	362	100	32.309	331	100

Protein Group

LOC542653 [Zea mays]	gi|162458978	38282.8	6.2300 000190 7349

Peptide Information

Calc. Mass	Obsrv. Mass	± da	± ppm	Start Seq.	End Seq.	Sequence	Ion Score	C. I. %	Modification	Rank	Result Type
1028.5735	1028.5447	-0.0288	-28	225	234	EVGLGADLVR					Mascot
1028.5735	1028.5447	-0.0288	-28	225	234	EVGLGADLVR	85	100			Mascot
1185.6838	1185.6259	-0.0579	-49	163	174	LVSSQPASGIVK					Mascot

1187.6783	1187.6514	-0.0269	-23	278	288	IEGGSLFIVPR						Mascot
1187.6783	1187.6514	-0.0269	-23	278	288	IEGGSLFIVPR	101	100				Mascot
1407.8683	1407.8065	-0.0618	-44	212	224	VVVLNTANLPLVR						Mascot
1423.6741	1423.7172	0.0431	30	351	363	LDSEIFFAPPSSN						Mascot
1561.8333	1561.7097	-0.1236	-79	175	190	LPASAAALPAPSPQDR						Mascot
1736.9065	1736.8242	-0.0823	-47	43	60	LSLAAGGLSLPSYSDSAK						Mascot
2764.2166	2764.1853	-0.0313	-11	235	258	IDAHSMCSPGFSCDSAY QVTYIVR			Carbamidomethyl (C)[7,13]			Mascot
2780.2114	2780.1611	-0.0503	-18	235	258	IDAHSMCSPGFSCDSAY QVTYIVR			Carbamidomethyl (C)[7,13], Oxidation (M)[6]			Mascot
2780.2114	2780.1611	-0.0503	-18	235	258	IDAHSMCSPGFSCDSAY QVTYIVR	145	100	Carbamidomethyl (C)[7,13], Oxidation (M)[6]			Mascot

4 putative rmlC-like cupins superfamily protein [Zea mays] gi|413942237 32493.8 6.1 7 359 100 32.198 331 100

Peptide Information

Calc. Mass	Obsrv. Mass	± da	± ppm	Start Seq.	End Seq.	Sequence	Ion Score	C. I. %	Modification	Rank	Result Type
1028.5735	1028.5447	-0.0288	-28	225	234	EVGLGADLVR					Mascot
1028.5735	1028.5447	-0.0288	-28	225	234	EVGLGADLVR	85	100			Mascot
1185.6838	1185.6259	-0.0579	-49	163	174	LVSSQPASGIVK					Mascot
1187.6783	1187.6514	-0.0269	-23	278	288	IEGGSLFIVPR					Mascot
1187.6783	1187.6514	-0.0269	-23	278	288	IEGGSLFIVPR	101	100			Mascot
1407.8683	1407.8065	-0.0618	-44	212	224	VVVLNTANLPLVR					Mascot
1561.8333	1561.7097	-0.1236	-79	175	190	LPASAAALPAPSPQDR					Mascot
1736.9065	1736.8242	-0.0823	-47	43	60	LSLAAGGLSLPSYSDSAK					Mascot
2764.2166	2764.1853	-0.0313	-11	235	258	IDAHSMCSPGFSCDSAY QVTYIVR			Carbamidomethyl (C)[7,13]		Mascot
2780.2114	2780.1611	-0.0503	-18	235	258	IDAHSMCSPGFSCDSAY QVTYIVR			Carbamidomethyl (C)[7,13], Oxidation (M)[6]		Mascot
2780.2114	2780.1611	-0.0503	-18	235	258	IDAHSMCSPGFSCDSAY QVTYIVR	145	100	Carbamidomethyl (C)[7,13], Oxidation (M)[6]		Mascot

5 uncharacterized protein, partial [Phleum pratense] gi|409972347 9279.7 6.92 4 359 100 31.866 331 100

Peptide Information

Calc. Mass	Obsrv. Mass	± da	± ppm	Start Seq.	End Seq.	Sequence	Ion Score	C. I. %	Modification	Rank	Result Type
1028.5735	1028.5447	-0.0288	-28	2	11	EVGLGADLVR					Mascot
1028.5735	1028.5447	-0.0288	-28	2	11	EVGLGADLVR	85	100			Mascot
1054.6005	1054.5669	-0.0336	-32	40	49	VQVVGPDGKR					Mascot
1187.6783	1187.6514	-0.0269	-23	55	65	IEGGSLFIVPR					Mascot
1187.6783	1187.6514	-0.0269	-23	55	65	IEGGSLFIVPR	101	100			Mascot

2764.2166	2764.1853	-0.0313	-11	12	35	IDAHSMCSPGFSCDSAY QVTYIVR			Carbamidomethyl (C)[7,13]		Mascot
2780.2114	2780.1611	-0.0503	-18	12	35	IDAHSMCSPGFSCDSAY QVTYIVR			Carbamidomethyl (C)[7,13], Oxidation (M)[6]		Mascot
2780.2114	2780.1611	-0.0503	-18	12	35	IDAHSMCSPGFSCDSAY QVTYIVR	145	100	Carbamidomethyl (C)[7,13], Oxidation (M)[6]		Mascot

6	PREDICTED: 13S globulin seed storage protein 1-like [Setaria italica]	gi|514743857	38190.7	6.14	7	345	100	22.259	320	100		

Peptide Information

Calc. Mass	Obsrv. Mass	± da	± ppm	Start Seq.	End Seq.	Sequence	Ion Score	C. I. %	Modification	Rank	Result Type
877.5142	877.4645	-0.0497	-57	60	67	VAYVLQGK					Mascot
1028.5735	1028.5447	-0.0288	-28	223	232	EVGLGADLVR					Mascot
1028.5735	1028.5447	-0.0288	-28	223	232	EVGLGADLVR	85	100			Mascot
1185.6838	1185.6259	-0.0579	-49	162	173	LVSSQPASGIVK					Mascot
1308.6583	1308.626	-0.0323	-25	348	358	RLDSEIFFAPN					Mascot
1308.6583	1308.626	-0.0323	-25	348	358	RLDSEIFFAPN	90	100			Mascot
1703.8851	1703.7708	-0.1143	-67	324	339	AISPEVLEASFNTTPK					Mascot
1736.9065	1736.8242	-0.0823	-47	42	59	LSLAAGGLSLPSYSDSAK					Mascot
2764.2166	2764.1853	-0.0313	-11	233	256	IDAHSMCSPGFSCDSAY QVTYIVR			Carbamidomethyl (C)[7,13]		Mascot
2780.2114	2780.1611	-0.0503	-18	233	256	IDAHSMCSPGFSCDSAY QVTYIVR			Carbamidomethyl (C)[7,13], Oxidation (M)[6]		Mascot
2780.2114	2780.1611	-0.0503	-18	233	256	IDAHSMCSPGFSCDSAY QVTYIVR	145	100	Carbamidomethyl (C)[7,13], Oxidation (M)[6]		Mascot

7	uncharacterized protein, partial [Phleum pratense]	gi|409972479	7727.8	6.92	3	268	100	28.361	247	100		

Peptide Information

Calc. Mass	Obsrv. Mass	± da	± ppm	Start Seq.	End Seq.	Sequence	Ion Score	C. I. %	Modification	Rank	Result Type
1054.6005	1054.5669	-0.0336	-32	29	38	VQVVGPDGKR					Mascot
1187.6783	1187.6514	-0.0269	-23	44	54	IEGGSLFIVPR					Mascot
1187.6783	1187.6514	-0.0269	-23	44	54	IEGGSLFIVPR	101	100			Mascot
2764.2166	2764.1853	-0.0313	-11	1	24	IDAHSMCSPGFSCDSAY QVTYIVR			Carbamidomethyl (C)[7,13]		Mascot
2780.2114	2780.1611	-0.0503	-18	1	24	IDAHSMCSPGFSCDSAY QVTYIVR			Carbamidomethyl (C)[7,13], Oxidation (M)[6]		Mascot
2780.2114	2780.1611	-0.0503	-18	1	24	IDAHSMCSPGFSCDSAY QVTYIVR	145	100	Carbamidomethyl (C)[7,13], Oxidation (M)[6]		Mascot

8	TPA: putative rmlC-like cupins superfamily protein [Zea mays]	gi|414877747	40217.5	5.83	7	252	100	7.009	230	100		

Protein Group

legumin-like protein [Zea mays]	gi|162459523	40233.5	5.8299 999237

Peptide Information

Calc. Mass	Obsrv. Mass	± da	± ppm	Start Seq.	End Seq.	Sequence	Ion Score	C. I. %	Modification	Rank	Result Type
1028.5735	1028.5447	-0.0288	-28	236	245	EVGLGADLVR					Mascot
1028.5735	1028.5447	-0.0288	-28	236	245	EVGLGADLVR	85	100			Mascot
1056.4891	1056.5052	0.0161	15	189	197	DGHKMPEAR			Oxidation (M)[5]		Mascot
1056.4891	1056.5052	0.0161	15	189	197	DGHKMPEAR			Oxidation (M)[5]		Mascot
1134.4845	1134.5701	0.0856	75	193	201	MPEARDEDR			Oxidation (M)[1]		Mascot
1233.5747	1233.5961	0.0214	17	164	174	AWDLTQDDAAK					Mascot
1400.7605	1400.6851	-0.0754	-54	270	283	GSGRVQVVGIDGTR					Mascot
1487.8468	1487.7085	-0.1383	-93	80	93	GIFGVVLPEATKEK					Mascot
2764.2166	2764.1853	-0.0313	-11	246	269	IDAHSMCSPGFSCDSAY QVTYIVR			Carbamidomethyl (C)[7,13]		Mascot
2780.2114	2780.1611	-0.0503	-18	246	269	IDAHSMCSPGFSCDSAY QVTYIVR			Carbamidomethyl (C)[7,13], Oxidation (M)[6]		Mascot
2780.2114	2780.1611	-0.0503	-18	246	269	IDAHSMCSPGFSCDSAY QVTYIVR	145	100	Carbamidomethyl (C)[7,13], Oxidation (M)[6]		Mascot

9	PREDICTED: 13S globulin seed storage protein 1-like [Setaria italica]	gi|514810879	44253.6	8.12	7	250	100	7.388	230	100	

Peptide Information

Calc. Mass	Obsrv. Mass	± da	± ppm	Start Seq.	End Seq.	Sequence	Ion Score	C. I. %	Modification	Rank	Result Type
863.4192	863.4173	-0.0019	-2	1	6	MEKHFR			Oxidation (M)[1]		Mascot
981.5146	981.5081	-0.0065	-7	18	25	MTTRTSLR			Oxidation (M)[1]		Mascot
981.5146	981.5081	-0.0065	-7	18	25	MTTRTSLR			Oxidation (M)[1]		Mascot
1028.5735	1028.5447	-0.0288	-28	272	281	EVGLGADLVR					Mascot
1028.5735	1028.5447	-0.0288	-28	272	281	EVGLGADLVR	85	100			Mascot
1185.5898	1185.6259	0.0361	30	200	210	AWDLPQDAAAK					Mascot
1327.5769	1327.6813	0.1044	79	225	235	DGHKMPEPCEK			Carbamidomethyl (C)[9]		Mascot
1400.7605	1400.6851	-0.0754	-54	306	319	GSGRVQVVGIDGTR					Mascot
2764.2166	2764.1853	-0.0313	-11	282	305	IDAHSMCSPGFSCDSAY QVTYIVR			Carbamidomethyl (C)[7,13]		Mascot
2780.2114	2780.1611	-0.0503	-18	282	305	IDAHSMCSPGFSCDSAY QVTYIVR			Carbamidomethyl (C)[7,13], Oxidation (M)[6]		Mascot
2780.2114	2780.1611	-0.0503	-18	282	305	IDAHSMCSPGFSCDSAY QVTYIVR	145	100	Carbamidomethyl (C)[7,13], Oxidation (M)[6]		Mascot

10	legumin-like protein [Zea mays]	gi|162459030	38046.6	5.79	6	248	100	5.877	230	100	

Protein Group

legumin-like protein [Zea mays]	gi|525343211	38076.6	5.7899

		999618 5303
putative rmlC-like cupins superfamily protein [Zea mays] gi|413950180	38046.6	5.7899 999618 5303

Peptide Information

Calc. Mass	Obsrv. Mass	± da	± ppm	Start Seq.	End Seq.	Sequence	Ion Score	C. I. %	Modification	Rank	Result Type
1028.5735	1028.5447	-0.0288	-28	223	232	EVGLGADLVR					Mascot
1028.5735	1028.5447	-0.0288	-28	223	232	EVGLGADLVR	85	100			Mascot
1166.6165	1166.609	-0.0075	-6	175	186	ASSSPLPAPSPR					Mascot
1233.6991	1233.5961	-0.103	-83	276	286	VEGGFLFIVPR					Mascot
1336.642	1336.6443	0.0023	2	349	360	LDSEIFFAPPSN					Mascot
1379.8621	1379.7728	-0.0893	-65	210	222	VVVLNTANLPLVK					Mascot
2764.2166	2764.1853	-0.0313	-11	233	256	IDAHSMCSPGFSCDSAY QVTYIVR			Carbamidomethyl (C)[7,13]		Mascot
2780.2114	2780.1611	-0.0503	-18	233	256	IDAHSMCSPGFSCDSAY QVTYIVR			Carbamidomethyl (C)[7,13], Oxidation (M)[6]		Mascot
2780.2114	2780.1611	-0.0503	-18	233	256	IDAHSMCSPGFSCDSAY QVTYIVR	145	100	Carbamidomethyl (C)[7,13], Oxidation (M)[6]		Mascot

Gel Idx/Pos	264/K16	Instr./Gel Origin	BA2151/Sample Project 20140814	Process Status	Analysis Succeeded
Plate [#] Name	[1] Sample Project 20140814	Instrument Sample Name		Spectra	11

Rank	Protein Name	Accession No.	Protein MW	Protein PI	Pep. Count	Protein Score	Protein Score C. I. %	Intensity Matched	Total Ion Score	Total Ion C. I. %	Confirmed
1	Serpin-Z2B [Triticum urartu]	gi|473793747	45225.7	6.03	12	318	100	26.106	267	100	

Peptide Information

Calc. Mass	Obsrv. Mass	± da	± ppm	Start Seq.	End Seq.	Sequence	Ion Score	C. I. %	Modification	Rank	Result Type
925.5214	925.4976	-0.0238	-26	11	18	LSIAHQTR					Mascot
925.5214	925.4976	-0.0238	-26	11	18	LSIAHQTR	37	67.412			Mascot
1137.6667	1137.6052	-0.0615	-54	189	198	LVLGNALYFK					Mascot
1192.5382	1192.5138	-0.0244	-20	199	208	GAWTDQFDPR					Mascot
1223.5903	1223.5265	-0.0638	-52	127	137	AEAQSVDFQTK					Mascot
1292.7097	1292.651	-0.0587	-45	306	317	ISFGIEASDLLK					Mascot
1385.7019	1385.6683	-0.0336	-24	176	188	DILPAGSIDNNTR					Mascot
1385.7019	1385.6683	-0.0336	-24	176	188	DILPAGSIDNNTR	84	100			Mascot
1446.7965	1446.6721	-0.1244	-86	11	22	LSIAHQTRFAFR					Mascot
1483.7614	1483.7064	-0.055	-37	257	268	QFSMYILLPEAR			Oxidation (M)[4]		Mascot
1483.7614	1483.7064	-0.055	-37	257	268	QFSMYILLPEAR	36	58.785	Oxidation (M)[4]		Mascot
1514.7485	1514.6949	-0.0536	-35	125	137	YKAEAQSVDFQTK					Mascot
1665.8595	1665.8331	-0.0264	-16	278	291	LSAEPEFLEQHIPR					Mascot
1665.8595	1665.8331	-0.0264	-16	278	291	LSAEPEFLEQHIPR	109	100			Mascot
1922.9706	1922.9031	-0.0675	-35	352	370	AFVEVNETGTEAAATTIA K					Mascot
2083.1072	2083.0286	-0.0786	-38	396	415	EDTSGVVLFIGHVVNPLL SS					Mascot

Rank	Protein Name	Accession No.	Protein MW	Protein PI	Pep. Count	Protein Score	Protein Score C. I. %	Intensity Matched	Total Ion Score	Total Ion C. I. %	Confirmed
2	Serpin-Z2B [Aegilops tauschii]	gi|475621781	43026.4	5.18	9	178	100	20.611	146	100	

Protein Group

RecName: Full=Serpin-Z2B; AltName: Full=TriaeZ2b; AltName: Full=WSZ2b; AltName: Full=WZS3	gi|75279909	43011.4	5.1799 998283 3862

Peptide Information

Calc. Mass	Obsrv. Mass	± da	± ppm	Start Seq.	End Seq.	Sequence	Ion Score	C. I. %	Modification	Rank	Result Type
925.5214	925.4976	-0.0238	-26	11	18	LSIAHQTR					Mascot
925.5214	925.4976	-0.0238	-26	11	18	LSIAHQTR	37	67.412			Mascot

Calc. Mass	Obsrv. Mass	± da	± ppm	Start Seq.	End Seq.	Sequence	Ion Score	C. I. %	Modification	Rank	Result Type
1137.6667	1137.6052	-0.0615	-54	172	181	LVLGNALYFK					Mascot
1192.5382	1192.5138	-0.0244	-20	182	191	GAWTDQFDPR					Mascot
1223.5903	1223.5265	-0.0638	-52	127	137	AEAQSVDFQTK					Mascot
1446.7965	1446.6721	-0.1244	-86	11	22	LSIAHQTRFAFR					Mascot
1514.7485	1514.6949	-0.0536	-35	125	137	YKAEAQSVDFQTK					Mascot
1665.8595	1665.8331	-0.0264	-16	261	274	LSAEPEFLEQHIPR					Mascot
1665.8595	1665.8331	-0.0264	-16	261	274	LSAEPEFLEQHIPR	109	100			Mascot
1922.9706	1922.9031	-0.0675	-35	335	353	AFVEVNETGTEAAATTIA K					Mascot
2083.1072	2083.0286	-0.0786	-38	379	398	EDTSGVVLFIGHVVNPLL SS					Mascot

3	hypothetical protein SORBIDRAFT_04g034830 [Sorghum bicolor]	gi|241932737	174673.8	8.8	26	70	89.079	9.352

Peptide Information

Calc. Mass	Obsrv. Mass	± da	± ppm	Start Seq.	End Seq.	Sequence	Ion Score	C. I. %	Modification	Rank	Result Type
809.445	809.3937	-0.0513	-63	775	780	MYAIRR					Mascot
833.4185	833.3911	-0.0274	-33	896	902	MDDLALR					Mascot
856.4999	856.4688	-0.0311	-36	1107	1113	SRLPVER					Mascot
877.489	877.4555	-0.0335	-38	1224	1231	SPSKFVGR					Mascot
893.4575	893.4229	-0.0346	-39	336	343	DTDSSKIK					Mascot
948.4633	948.4809	0.0176	19	416	424	SIGQDVDSK					Mascot
972.5836	972.5	-0.0836	-86	30	38	AVVVLTSQR					Mascot
1022.5186	1022.4725	-0.0461	-45	970	978	KIMSAEAEK			Oxidation (M)[3]		Mascot
1022.5186	1022.4725	-0.0461	-45	970	978	KIMSAEAEK	19	0	Oxidation (M)[3]		Mascot
1028.5524	1028.5383	-0.0141	-14	600	608	FSSVASRFK					Mascot
1056.5685	1056.5651	-0.0034	-3	242	250	VVQITDPER					Mascot
1103.5878	1103.5602	-0.0276	-25	719	728	AGQIAILDMR			Oxidation (M)[9]		Mascot
1133.6096	1133.5016	-0.108	-95	301	310	RAMDIVGISR			Oxidation (M)[3]		Mascot
1174.5885	1174.5302	-0.0583	-50	1416	1425	QDLCPSLSVR			Carbamidomethyl (C)[4]		Mascot
1205.6385	1205.566	-0.0725	-60	1200	1210	SNGLFATPSRR					Mascot
1252.5991	1252.5382	-0.0609	-49	703	712	MELENFQLGR			Oxidation (M)[1]		Mascot
1330.6526	1330.6229	-0.0297	-22	551	561	VTYQTDSFLEK					Mascot
1419.6863	1419.7271	0.0408	29	344	356	DSTSNFHLQTAAK					Mascot
1563.7683	1563.7039	-0.0644	-41	971	984	IMSAEAEKENSNLK					Mascot
1563.7683	1563.7039	-0.0644	-41	971	984	IMSAEAEKENSNLK					Mascot
1644.8236	1644.8073	-0.0163	-10	1050	1064	AFNMPTMNNLPVAPK					Mascot
1714.9052	1714.8389	-0.0663	-39	425	439	VQIGVLDIYGFESFK					Mascot

Calc. Mass	Obsrv. Mass	± da	± ppm	Start Seq.	End Seq.	Sequence	Ion Score	C. I. %	Modification	Rank	Result Type
1763.8381	1763.8708	0.0327	19	564	578	DYIVAEHCNLLSSSR			Carbamidomethyl (C)[8]		Mascot
1777.8037	1777.946	0.1423	80	1426	1438	QIYRICSMYWDDK			Carbamidomethyl (C)[6]		Mascot
1861.8174	1861.8522	0.0348	19	440	454	NNSFEQFCINFANEK			Carbamidomethyl (C)[8]		Mascot
1861.8174	1861.8522	0.0348	19	440	454	NNSFEQFCINFANEK			Carbamidomethyl (C)[8]		Mascot
1923.0083	1922.9031	-0.1052	-55	216	233	FVEIQFDGSGRISGAAIR					Mascot
2187.1292	2187.1333	0.0041	2	184	203	TVEQQVLESNPLLEAFGN AK					Mascot
3765.7363	3765.9622	0.2259	60	466	495	MEQEEYKSEEINWSYIEF IDNQDVLDLIEK			Oxidation (M)[1]		Mascot

4	hypothetical protein SORBIDRAFT_03g035320 [Sorghum bicolor]	gi|241930510	40473.3	6.53	8	69	87.461	2.565	42	87.496	

Peptide Information

Calc. Mass	Obsrv. Mass	± da	± ppm	Start Seq.	End Seq.	Sequence	Ion Score	C. I. %	Modification	Rank	Result Type
839.441	839.4224	-0.0186	-22	146	152	GYPFSLR					Mascot
989.5414	989.491	-0.0504	-51	263	271	ASNPFVNLK					Mascot
1209.6296	1209.5206	-0.109	-90	302	311	CYIALSQQVK			Carbamidomethyl (C)[1]		Mascot
1446.5818	1446.6721	0.0903	62	231	241	YDDMWAGWCVK			Carbamidomethyl (C)[9], Oxidation (M)[4]		Mascot
1466.7672	1466.6937	-0.0735	-50	302	313	CYIALSQQVKEK			Carbamidomethyl (C)[1]		Mascot
1501.6958	1501.6715	-0.0243	-16	48	59	VPEGFDYELYNR					Mascot
1501.6958	1501.6715	-0.0243	-16	48	59	VPEGFDYELYNR	42	87.496			Mascot
1621.8545	1621.7931	-0.0614	-38	105	119	DPTGKDIDALAQHIK					Mascot
1843.9225	1843.8583	-0.0642	-35	45	59	TIKVPEGFDYELYNR					Mascot

5	PREDICTED: alpha-1,4-glucan-protein synthase [UDP-forming]-like [Cucumis sativus]	gi|449452847	41231.7	5.83	7	65	69.92	2.9	42	87.496	

Protein Group

PREDICTED: alpha-1,4-glucan-protein synthase [UDP-forming]-like [Cucumis sativus]	gi|449523313	41231.7	5.8299 999237 0605

Peptide Information

Calc. Mass	Obsrv. Mass	± da	± ppm	Start Seq.	End Seq.	Sequence	Ion Score	C. I. %	Modification	Rank	Result Type
839.441	839.4224	-0.0186	-22	153	159	GYPFSLR					Mascot
989.5414	989.491	-0.0504	-51	270	278	ASNPFVNLK					Mascot
1419.7882	1419.7271	-0.0611	-43	319	330	EKLGPIDPYFLK					Mascot
1446.5818	1446.6721	0.0903	62	238	248	YDDMWAGWCVK			Carbamidomethyl (C)[9], Oxidation (M)[4]		Mascot
1501.6958	1501.6715	-0.0243	-16	55	66	VPEGFDYELYNR					Mascot

Calc. Mass	Obsrv. Mass	± da	± ppm	Start Seq.	End Seq.	Sequence	Ion Score	C. I. %	Modification	Rank	Result Type
1501.6958	1501.6715	-0.0243	-16	55	66	VPEGFDYELYNR	42	87.496			Mascot
1843.9225	1843.8583	-0.0642	-35	52	66	TIKVPEGFDYELYNR					Mascot
3017.4792	3017.637	0.1578	52	127	152	NLLSPATPNFFNTLYDPY REGADFVR					Mascot

6	Alpha-1,4-glucan-protein synthase (UDP-forming) [Aegilops tauschii]	gi|475618083	41955	5.82	7	63	54.472	3.745	42	87.496	

Peptide Information

Calc. Mass	Obsrv. Mass	± da	± ppm	Start Seq.	End Seq.	Sequence	Ion Score	C. I. %	Modification	Rank	Result Type
839.441	839.4224	-0.0186	-22	159	165	GYPFSLR					Mascot
989.5414	989.491	-0.0504	-51	276	284	ASNPFVNLK					Mascot
1446.5818	1446.6721	0.0903	62	244	254	YDDMWAGWCVK			Carbamidomethyl (C)[9], Oxidation (M)[4]		Mascot
1483.7461	1483.7064	-0.0397	-27	315	326	CYISLSEQVKEK			Carbamidomethyl (C)[1]		Mascot
1483.7461	1483.7064	-0.0397	-27	315	326	CYISLSEQVKEK			Carbamidomethyl (C)[1]		Mascot
1501.6958	1501.6715	-0.0243	-16	61	72	VPEGFDYELYNR					Mascot
1501.6958	1501.6715	-0.0243	-16	61	72	VPEGFDYELYNR	42	87.496			Mascot
1720.8252	1720.951	0.1258	73	104	117	KYVFTIDDDCFVAK			Carbamidomethyl (C)[10]		Mascot
2292.1335	2292.0925	-0.041	-18	133	151	NLLSPSTPFFFNTLYDPY R					Mascot

7	RecName: Full=Alpha-1,4-glucan-protein synthase [UDP-forming]; AltName: Full=Amylogenin; AltName: Full=Golgi-associated protein se-wap41; AltName: Full=Reversibly glycosylated polypeptide; Short=RGP; AltName: Full=UDP-glucose:protein transglucosylas	gi|34588146	41690.8	5.75	7	63	52.326	2.244	42	87.496	

Protein Group

alpha-1,4-glucan-protein synthase [UDP-forming] [Zea mays]	gi|162463414	41690.8	5.75

Peptide Information

Calc. Mass	Obsrv. Mass	± da	± ppm	Start Seq.	End Seq.	Sequence	Ion Score	C. I. %	Modification	Rank	Result Type
839.441	839.4224	-0.0186	-22	159	165	GYPFSLR					Mascot
989.5414	989.491	-0.0504	-51	276	284	ASNPFVNLK					Mascot
1223.5878	1223.5265	-0.0613	-50	31	39	NLDFLEMWR					Mascot
1446.5818	1446.6721	0.0903	62	244	254	YDDMWAGWCVK			Carbamidomethyl (C)[9], Oxidation (M)[4]		Mascot
1501.6958	1501.6715	-0.0243	-16	61	72	VPEGFDYELYNR					Mascot
1501.6958	1501.6715	-0.0243	-16	61	72	VPEGFDYELYNR	42	87.496			Mascot
1843.9225	1843.8583	-0.0642	-35	58	72	TIKVPEGFDYELYNR					Mascot
2292.1335	2292.0925	-0.041	-18	133	151	NLLSPSTPFFFNTLYDPY					Mascot

R

8 RecName: Full=Alpha-1,4-glucan-protein synthase [UDP-forming]; AltName: Full=Reversibly glycosylated polypeptide; Short=RGP; AltName: Full=UDP-glucose:protein transglucosylase; Short=UPTG gi|229891797 6899.6 7.82 3 63 47.727 1.423 42 87.496

Peptide Information

Calc. Mass	Obsrv. Mass	± da	± ppm	Start Seq.	End Seq.	Sequence	Ion Score	C. I. %	Modification	Rank	Result Type
989.5414	989.491	-0.0504	-51	52	60	ASNPFVNLK					Mascot
1501.6958	1501.6715	-0.0243	-16	4	15	VPEGFDYELYNR					Mascot
1501.6958	1501.6715	-0.0243	-16	4	15	VPEGFDYELYNR	42	87.496			Mascot
1843.9225	1843.8583	-0.0642	-35	1	15	TIKVPEGFDYELYNR					Mascot

9 hypothetical protein PRUPE_ppa006155mg [Prunus persica] gi|462404903 47978 6.52 13 60 7.044 3.719

Peptide Information

Calc. Mass	Obsrv. Mass	± da	± ppm	Start Seq.	End Seq.	Sequence	Ion Score	C. I. %	Modification	Rank	Result Type
839.3893	839.4224	0.0331	39	69	75	VFDESSR					Mascot
1148.5769	1148.5723	-0.0046	-4	99	107	QLFEEMPVR					Mascot
1292.5875	1292.651	0.0635	49	283	293	HYGCMADLLGR			Carbamidomethyl (C)[4]		Mascot
1308.5824	1308.6528	0.0704	54	283	293	HYGCMADLLGR			Carbamidomethyl (C)[4], Oxidation (M)[5]		Mascot
1557.7255	1557.7008	-0.0247	-16	267	279	LFDDMETVYGVPR			Oxidation (M)[5]		Mascot
1612.8475	1612.7401	-0.1074	-67	382	396	ISGCSLINLNGSIHK			Carbamidomethyl (C)[4]		Mascot
1662.8091	1662.8546	0.0455	27	280	293	ELKHYGCMADLLGR			Carbamidomethyl (C)[7]		Mascot
1703.9255	1703.7787	-0.1468	-86	177	191	LSSYFLTALVDLYAK					Mascot
1727.8785	1727.7576	-0.1209	-70	76	90	RDVVSYNAMLDAFVK					Mascot
1763.9156	1763.8708	-0.0448	-25	4	18	FSLPPDFHTFPFALK					Mascot
1920.0167	1919.9197	-0.097	-51	3	18	RFSLPPDFHTFPFALK					Mascot
2083.0364	2083.0286	-0.0078	-4	209	227	NVFTWNAMLVGLAMHGH GK					Mascot
2187.0752	2187.1333	0.0581	27	77	96	DVVSYNAMLDAFVKAGE VSR			Oxidation (M)[8]		Mascot
3765.7412	3765.9622	0.221	59	335	368	AAEHVMELDPEDGGVYS IMANVYANAEKWDDVVK					Mascot

10 conserved hypothetical protein [Ricinus communis] gi|223505130 36536.3 5.95 13 60 7.044 3.625

Peptide Information

Calc. Mass	Obsrv. Mass	± da	± ppm	Start Seq.	End Seq.	Sequence	Ion Score	C. I. %	Modification	Rank	Result Type

832.3471	832.3566	0.0095	11	1	6	YSDEYR		Mascot
856.4271	856.4688	0.0417	49	219	226	NGQPSTPR		Mascot
877.4261	877.4555	0.0294	34	87	93	DQKETEK		Mascot
1088.5331	1088.46	-0.0731	-67	260	270	ESLGQGAAAER		Mascot
1126.5262	1126.5195	-0.0067	-6	231	240	DAYSTIEEAK		Mascot
1446.774	1446.6721	-0.1019	-70	271	282	KLYNTYLASAFR		Mascot
1510.7649	1510.7201	-0.0448	-30	77	89	AAFEAYVNVRDQK		Mascot
1514.7921	1514.6949	-0.0972	-64	256	270	GQLKESLGQGAAAER		Mascot
1544.8279	1544.7765	-0.0514	-33	15	29	LLKDAANATDNASLK		Mascot
1693.8214	1693.8617	0.0403	24	100	113	HMQELENNLPLDPK	Oxidation (M)[2]	Mascot
1693.8214	1693.8617	0.0403	24	100	113	HMQELENNLPLDPK	Oxidation (M)[2]	Mascot
1727.8025	1727.7576	-0.0449	-26	307	323	GGFVSHGDGTFSVDFAK		Mascot
1821.9415	1821.8529	-0.0886	-49	167	182	NVQEAKFEATLMPISK	Oxidation (M)[12]	Mascot
2187.0825	2187.1333	0.0508	23	241	259	ADVTGLWALTYMMEKGQ LK	Oxidation (M)[12,13]	Mascot

Rank	Protein Name	Accession No.	Protein MW	Protein PI	Pep. Count	Protein Score	Protein Score C. I. %	Intensity Matched	Total Ion Score	Total Ion C. I. %	Confirmed
1	RecName: Full=Serpin-Z1C; AltName: Full=TriaeZ1c; AltName: Full=WSZ1c	gi|75313848	42969	5.62	10	148	100	10.664	105	100	

Peptide Information

Calc. Mass	Obsrv. Mass	± da	± ppm	Start Seq.	End Seq.	Sequence	Ion Score	C. I. %	Modification	Rank	Result Type
925.5214	925.5001	-0.0213	-23	11	18	LSIAHQTR					Mascot
925.5214	925.5001	-0.0213	-23	11	18	LSIAHQTR	35	42.239			Mascot
947.5156	947.4678	-0.0478	-50	2	10	ATTLATDVR					Mascot
1151.6824	1151.5846	-0.0978	-85	172	181	LVLANALYFK					Mascot
1176.5896	1176.5691	-0.0205	-17	261	270	LSAEPDFLER					Mascot
1176.5896	1176.5691	-0.0205	-17	261	270	LSAEPDFLER	70	99.981			Mascot
1239.5852	1239.6824	0.0972	78	127	137	ADTQSVDFQTK					Mascot
1352.6945	1352.6407	-0.0538	-40	289	300	ISFETEASDLLK					Mascot
1399.6125	1399.6244	0.0119	9	182	194	GAWTDQFDSSGTK					Mascot
2129.0947	2129.0059	-0.0888	-42	379	398	EDISGVVLFMGHVVNPLLSS			Oxidation (M)[10]		Mascot
2720.3525	2720.302	-0.0505	-19	328	353	VSSVFHQAFVEVNEQGTEAAASTAIK					Mascot
2725.4631	2725.4294	-0.0337	-12	33	61	SAASNAVFSPVSLHVALSLLAAGAGSATR					Mascot

Rank	Protein Name	Accession No.	Protein MW	Protein PI	Pep. Count	Protein Score	Protein Score C. I. %	Intensity Matched	Total Ion Score	Total Ion C. I. %	Confirmed
2	Serpin-Z1C [Triticum urartu]	gi|474075261	42956	5.62	9	141	100	10.43	105	100	

Peptide Information

Calc. Mass	Obsrv. Mass	± da	± ppm	Start Seq.	End Seq.	Sequence	Ion Score	C. I. %	Modification	Rank	Result Type
925.5214	925.5001	-0.0213	-23	11	18	LSIAHQTR					Mascot
925.5214	925.5001	-0.0213	-23	11	18	LSIAHQTR	35	42.239			Mascot
947.5156	947.4678	-0.0478	-50	2	10	ATTLATDVR					Mascot
1151.6824	1151.5846	-0.0978	-85	172	181	LVLANALYFK					Mascot
1176.5896	1176.5691	-0.0205	-17	261	270	LSAEPDFLER					Mascot
1176.5896	1176.5691	-0.0205	-17	261	270	LSAEPDFLER	70	99.981			Mascot
1352.6945	1352.6407	-0.0538	-40	289	300	ISFETEASDLLK					Mascot
1399.6125	1399.6244	0.0119	9	182	194	GAWTDQFDSSGTK					Mascot
2129.0947	2129.0059	-0.0888	-42	379	398	EDISGVVLFMGHVVNPLL			Oxidation (M)[10]		Mascot

						SS					
2720.3525	2720.302	-0.0505	-19	328	353	VSSVFHQAFVEVNEQGT EAAASTAIK					Mascot
2725.4631	2725.4294	-0.0337	-12	33	61	SAASNAVFSPVSLHVALS LLAAGAGSATR					Mascot

3	RecName: Full=Serpin-Z1B; AltName: Full=TriaeZ1b; AltName: Full=WSZ1b; AltName: Full=WZS2	gi|75279910	43119.9	5.44	7	128	100	9.708	105	100

Peptide Information

Calc. Mass	Obsrv. Mass	± da	± ppm	Start Seq.	End Seq.	Sequence	Ion Score	C. I. %	Modification	Rank	Result Type
925.5214	925.5001	-0.0213	-23	11	18	LSIAHQTR					Mascot
925.5214	925.5001	-0.0213	-23	11	18	LSIAHQTR	35	42.239			Mascot
947.5156	947.4678	-0.0478	-50	2	10	ATTLATDVR					Mascot
1151.6824	1151.5846	-0.0978	-85	172	181	LVLANALYFK					Mascot
1176.5896	1176.5691	-0.0205	-17	262	271	LSAEPDFLER					Mascot
1176.5896	1176.5691	-0.0205	-17	262	271	LSAEPDFLER	70	99.981			Mascot
1585.8295	1585.7083	-0.1212	-76	288	301	FKISFGMEASDLLK					Mascot
1601.8243	1601.7025	-0.1218	-76	288	301	FKISFGMEASDLLK			Oxidation (M)[7]		Mascot
2129.0947	2129.0059	-0.0888	-42	380	399	EDISGVVLFMGHVVNPLL SS			Oxidation (M)[10]		Mascot
2720.3525	2720.302	-0.0505	-19	329	354	VSSVFHQAFVEVNEQGT EAAASTAIK					Mascot

4	RecName: Full=Serpin-Z1A; AltName: Full=TriaeZ1a; AltName: Full=WSZ1a; Short=WSZ1; AltName: Full=WSZCI	gi|75282265	43262.2	5.6	6	123	100	9.412	105	100

Peptide Information

Calc. Mass	Obsrv. Mass	± da	± ppm	Start Seq.	End Seq.	Sequence	Ion Score	C. I. %	Modification	Rank	Result Type
925.5214	925.5001	-0.0213	-23	11	18	LSIAHQTR					Mascot
925.5214	925.5001	-0.0213	-23	11	18	LSIAHQTR	35	42.239			Mascot
947.5156	947.4678	-0.0478	-50	2	10	ATTLATDVR					Mascot
1151.6824	1151.5846	-0.0978	-85	172	181	LVLANALYFK					Mascot
1176.5896	1176.5691	-0.0205	-17	261	270	LSAEPDFLER					Mascot
1176.5896	1176.5691	-0.0205	-17	261	270	LSAEPDFLER	70	99.981			Mascot
2129.0947	2129.0059	-0.0888	-42	379	398	EDISGVVLFMGHVVNPLL SS			Oxidation (M)[10]		Mascot
2720.3525	2720.302	-0.0505	-19	328	353	VSSVFHQAFVEVNEQGT EAAASTAIK					Mascot

5	Serpin-Z1B [Aegilops tauschii]	gi|475546073	43257.1	5.44	5	118	100	9.286	105	100

Peptide Information

Calc. Mass	Obsrv. Mass	± da	± ppm	Start	End	Sequence	Ion	C. I. %	Modification	Rank	Result Type

				Seq.	Seq.		Score				
925.5214	925.5001	-0.0213	-23	11	18	LSIAHQTR					Mascot
925.5214	925.5001	-0.0213	-23	11	18	LSIAHQTR	35	42.239			Mascot
947.5156	947.4678	-0.0478	-50	2	10	ATTLATDVR					Mascot
1151.6824	1151.5846	-0.0978	-85	172	181	LVLANALYFK					Mascot
1176.5896	1176.5691	-0.0205	-17	262	271	LSAEPDFLER					Mascot
1176.5896	1176.5691	-0.0205	-17	262	271	LSAEPDFLER	70	99.981			Mascot
2720.3525	2720.302	-0.0505	-19	329	354	VSSVFHQAFVEVNEQGT EAAASTAIK					Mascot

6 hypothetical protein PRUPE_ppa018471mg [Prunus persica] gi|462402107 36092.4 8.79 14 77 97.966 8.086

Peptide Information

Calc. Mass	Obsrv. Mass	± da	± ppm	Start Seq.	End Seq.	Sequence	Ion Score	C. I. %	Modification	Rank	Result Type
804.421	804.364	-0.057	-71	264	271	GALTSNNK					Mascot
809.4012	809.3904	-0.0108	-13	316	321	RDRPHE					Mascot
814.457	814.3973	-0.0597	-73	36	42	TWAAPIR					Mascot
823.4308	823.4119	-0.0189	-23	213	220	AAATQFSK					Mascot
925.5254	925.5001	-0.0253	-27	45	52	LWIHAASK					Mascot
925.5254	925.5001	-0.0253	-27	45	52	LWIHAASK	1	0			Mascot
947.3927	947.4678	0.0751	79	61	67	AMEDFYR			Oxidation (M)[2]		Mascot
963.5621	963.4927	-0.0694	-72	150	157	KIYEAAIR					Mascot
1033.4922	1033.5057	0.0135	13	3	11	RGHSSGNYR					Mascot
1198.5997	1198.5815	-0.0182	-15	303	313	GSISPRGEHMK					Mascot
1516.8087	1516.8213	0.0126	8	89	101	LLGCVEVVGCVRR			Carbamidomethyl (C)[4,10]		Mascot
1909.0138	1908.9419	-0.0719	-38	201	220	SSSLTAAIAGARAAATQF SK					Mascot
1909.0138	1908.9419	-0.0719	-38	201	220	SSSLTAAIAGARAAATQF SK					Mascot
2186.1313	2186.1833	0.052	24	132	149	LLIPFEMRGYQGVYNLEK			Oxidation (M)[7]		Mascot
2186.1313	2186.1833	0.052	24	132	149	LLIPFEMRGYQGVYNLEK			Oxidation (M)[7]		Mascot
2219.0688	2219.0793	0.0105	5	61	79	AMEDFYREIYAVDGIADL K					Mascot
2725.3049	2725.4294	0.1245	46	239	263	TLPLKSESFEEDTTPSAN LSETSNK					Mascot

7 putative protein kinase superfamily protein [Zea mays] gi|413949299 26615.5 9.08 12 66 73.191 7.954

Peptide Information

Calc. Mass	Obsrv. Mass	± da	± ppm	Start Seq.	End Seq.	Sequence	Ion Score	C. I. %	Modification	Rank	Result Type

Calc. Mass	Obsrv. Mass	± da	± ppm	Start Seq.	End Seq.	Sequence	Ion Score	C. I. %	Modification	Rank	Result Type
809.3974	809.3904	-0.007	-9	5	10	QYMPVR			Oxidation (M)[3]		Mascot
816.4573	816.3769	-0.0804	-98	210	217	LSAADALR					Mascot
832.4927	832.4158	-0.0769	-92	189	196	LLSPAGFK					Mascot
875.4767	875.4155	-0.0612	-70	165	171	EKVACLR			Carbamidomethyl (C)[5]		Mascot
889.5155	889.4562	-0.0593	-67	218	223	KPWFRR					Mascot
949.5036	949.4415	-0.0621	-65	5	11	QYMPVRR					Mascot
1176.6306	1176.5691	-0.0615	-52	2	10	NLRQYMPVR					Mascot
1176.6306	1176.5691	-0.0615	-52	2	10	NLRQYMPVR					Mascot
1232.6495	1232.5883	-0.0612	-50	28	38	QLLAGAEHTHR					Mascot
1323.666	1323.6239	-0.0421	-32	1	10	MNLRQYMPVR			Oxidation (M)[1]		Mascot
1377.6646	1377.7163	0.0517	38	83	95	DDSGLLASPSPYR					Mascot
1516.7504	1516.8213	0.0709	47	11	23	RLNGQPFGEDEVR					Mascot
1733.8865	1733.8257	-0.0608	-35	24	38	DVMRQLLAGAEHTHR					Mascot

8	hypothetical protein PRUPE_ppa000985mg [Prunus persica]	gi|462402872	107334.6	6.46	15	64	65.463	11.454	33	5.019

Peptide Information

Calc. Mass	Obsrv. Mass	± da	± ppm	Start Seq.	End Seq.	Sequence	Ion Score	C. I. %	Modification	Rank	Result Type
810.3423	810.3907	0.0484	60	625	630	CSNHHR			Carbamidomethyl (C)[1]		Mascot
856.4788	856.4639	-0.0149	-17	379	385	KGHVPYR					Mascot
862.4451	862.4264	-0.0187	-22	31	37	SVCDIIR			Carbamidomethyl (C)[3]		Mascot
904.4921	904.4564	-0.0357	-39	584	591	NSCLVALK			Carbamidomethyl (C)[3]		Mascot
925.4751	925.5001	0.025	27	666	672	SHFHRNK					Mascot
925.4751	925.5001	0.025	27	666	672	SHFHRNK					Mascot
963.4741	963.4927	0.0186	19	847	855	EDSGISLSR					Mascot
981.4272	981.4249	-0.0023	-2	743	750	DFDNIDSR					Mascot
981.4272	981.4249	-0.0023	-2	743	750	DFDNIDSR					Mascot
984.5043	984.4881	-0.0162	-16	683	690	KHICNGQK			Carbamidomethyl (C)[4]		Mascot
1176.5677	1176.5691	0.0014	1	317	327	ISVTCSGAPER			Carbamidomethyl (C)[5]		Mascot
1176.5677	1176.5691	0.0014	1	317	327	ISVTCSGAPER	33	5.019	Carbamidomethyl (C)[5]		Mascot
1198.6177	1198.5815	-0.0362	-30	902	911	ALFMEYVTPK					Mascot
1323.6772	1323.6239	-0.0533	-40	526	535	FMRPTICSRR			Carbamidomethyl (C)[7]		Mascot
1585.8043	1585.7083	-0.096	-61	899	911	SQRALFMEYVTPK			Oxidation (M)[7]		Mascot
1906.0909	1905.925	-0.1659	-87	14	30	IHSLLGLKSHLTSSWVK					Mascot
2253.126	2253.0725	-0.0535	-24	877	896	EDLDTLFQSSAEGIRHGL HK					Mascot
2758.4519	2758.2742	-0.1777	-64	243	267	DLLIPQPAKAMDPLPPCL			Carbamidomethyl (C)[17]		Mascot

SIQTDPK

9	Cyclophilin-type peptidyl-prolyl cis-trans isomerase (ISS) [Ostreococcus tauri]	gi|308811751	58110.9	5.87	17	63	52.326	4.101

Peptide Information

Calc. Mass	Obsrv. Mass	± da	± ppm	Start Seq.	End Seq.	Sequence	Ion Score	C. I. %	Modification	Rank	Result Type
820.3981	820.3947	-0.0034	-4	337	342	EMKEQR					Mascot
822.3484	822.4172	0.0688	84	32	38	EAPMACK			Carbamidomethyl (C)[6], Oxidation (M)[4]		Mascot
860.4472	860.4457	-0.0015	-2	324	330	EIERDAK					Mascot
862.4265	862.4264	-0.0001	0	74	83	GGTSALGDGK					Mascot
904.4734	904.4564	-0.017	-19	400	406	EKETLER					Mascot
963.4014	963.4927	0.0913	95	270	278	ETEEGADGR					Mascot
1091.4963	1091.4911	-0.0052	-5	269	278	KETEEGADGR					Mascot
1151.5804	1151.5846	0.0042	4	156	165	DDRPIDPAPR					Mascot
1507.7289	1507.7097	-0.0192	-13	84	95	ETFADEFHSRLR					Mascot
1516.7603	1516.8213	0.061	40	373	386	DEEAALLTSGQVQR					Mascot
1563.7068	1563.7043	-0.0025	-2	314	327	EMGDVDGGTREIER					Mascot
1582.7418	1582.7272	-0.0146	-9	1	14	MSQIYVNEPATEGK			Oxidation (M)[1]		Mascot
1812.8293	1812.8483	0.019	10	151	165	CETDRDDRPIDPAPR			Carbamidomethyl (C)[1]		Mascot
2005.0125	2005.0156	0.0031	2	207	225	LALLSFGEEAADEEQALAK					Mascot
2213.1094	2213.1084	-0.001	0	131	150	HTIFGKLVGATMYNAMEIGK			Oxidation (M)[12,16]		Mascot
2252.949	2253.0725	0.1235	55	39	56	SFVQHCLNGYYDDCAFTR			Carbamidomethyl (C)[6,14]		Mascot
2329.2034	2329.134	-0.0694	-30	207	228	LALLSFGEEAADEEQALAKAPR					Mascot

10	uncharacterized protein [Arabidopsis thaliana]	gi|334187111	72010.2	6.55	18	62	45.263	6.744

Protein Group

uncharacterized protein AT4G32970 [Arabidopsis thaliana]	gi|332660753	72010.2	6.5500 001907 3486

Peptide Information

Calc. Mass	Obsrv. Mass	± da	± ppm	Start Seq.	End Seq.	Sequence	Ion Score	C. I. %	Modification	Rank	Result Type
810.3628	810.3907	0.0279	34	89	95	EFSNDAK					Mascot
832.4384	832.4158	-0.0226	-27	128	134	TSRAQNR					Mascot
834.4138	834.3884	-0.0254	-30	586	591	CELERK			Carbamidomethyl (C)[1]		Mascot
857.4224	857.4324	0.01	12	429	435	EDRQGPR					Mascot

870.5043	870.4689	-0.0354	-41	251	258	GAKQPLEK		Mascot
904.4557	904.4564	0.0007	1	236	243	KPAVSCDK	Carbamidomethyl (C)[6]	Mascot
984.4969	984.4881	-0.0088	-9	66	73	LSRNSDHR		Mascot
1058.559	1058.5388	-0.0202	-19	436	444	EAAVKEQQR		Mascot
1158.6226	1158.5581	-0.0645	-56	40	50	TTGTNGKPLNR		Mascot
1323.709	1323.6239	-0.0851	-64	224	235	CAQVDLPKPPAK	Carbamidomethyl (C)[1]	Mascot
1352.7542	1352.6407	-0.1135	-84	135	144	ILRVILMYCR	Carbamidomethyl (C)[9], Oxidation (M)[7]	Mascot
1431.671	1431.5555	-0.1155	-81	420	431	QEDVVANEKEDR		Mascot
1472.7931	1472.6735	-0.1196	-81	349	361	QSKMLSPFPPNVK		Mascot
1507.7169	1507.7097	-0.0072	-5	2	15	EEDRATMTGAVATR		Mascot
1623.7723	1623.8994	0.1271	78	299	312	AYYEAVVISYCSAK	Carbamidomethyl (C)[11]	Mascot
1623.7723	1623.8994	0.1271	78	299	312	AYYEAVVISYCSAK	Carbamidomethyl (C)[11]	Mascot
1664.8503	1664.7168	-0.1335	-80	145	158	SSVRPTHDWPINQK		Mascot
1908.916	1908.9419	0.0259	14	299	314	AYYEAVVISYCSAKER	Carbamidomethyl (C)[11]	Mascot
1908.916	1908.9419	0.0259	14	299	314	AYYEAVVISYCSAKER	Carbamidomethyl (C)[11]	Mascot
2209.1467	2209.1145	-0.0322	-15	224	243	CAQVDLPKPPAKKPAVS CDK	Carbamidomethyl (C)[1,18]	Mascot

Gel Idx/Pos	266/K18	Instr./Gel Origin	BA2151/Sample Project 20140814	Process Status	Analysis Succeeded
Plate [#] Name	[1] Sample Project 20140814	Instrument Sample Name		Spectra	11

Rank	Protein Name	Accession No.	Protein MW	Protein PI	Pep. Count	Protein Score	Protein Score C. I. %	Intensity Matched	Total Ion Score	Total Ion C. I. %	Confirmed
1	RecName: Full=Phosphoglycerate kinase, cytosolic	gi|129916	42152.7	5.64	19	651	100	57.398	530	100	

Peptide Information

Calc. Mass	Obsrv. Mass	± da	± ppm	Start Seq.	End Seq.	Sequence	Ion Score	C. I. %	Modification	Rank	Result Type
1030.6409	1030.6362	-0.0047	-5	75	83	FSLKPLVAR					Mascot
1030.6409	1030.6362	-0.0047	-5	75	83	FSLKPLVAR	46	95.667			Mascot
1089.5786	1089.5455	-0.0331	-30	6	16	SVGTLGEADLK					Mascot
1096.5521	1096.5519	-0.0002	0	307	316	TFAEALDTTK					Mascot
1245.6798	1245.6925	0.0127	10	5	16	RSVGTLGEADLK					Mascot
1274.6951	1274.6445	-0.0506	-40	6	18	SVGTLGEADLKGK					Mascot
1298.6587	1298.6355	-0.0232	-18	24	35	ADLNVPLDDAQK					Mascot
1375.8196	1375.7859	-0.0337	-24	44	55	ASIPTIKYLLEK					Mascot
1388.7421	1388.708	-0.0341	-25	179	191	ELDYLVGAVANPK					Mascot
1443.8782	1443.7881	-0.0901	-62	203	216	VSSKIGVIESLLAK					Mascot
1493.8297	1493.8295	-0.0002	0	166	178	FLRPSVAGFLMQK					Mascot
1509.8247	1509.8046	-0.0201	-13	166	178	FLRPSVAGFLMQK			Oxidation (M)[11]		Mascot
1509.8247	1509.8046	-0.0201	-13	166	178	FLRPSVAGFLMQK	25	0	Oxidation (M)[11]		Mascot
1573.8433	1573.7906	-0.0527	-33	350	366	GVTTIIGGGDSVAAVEK					Mascot
1720.9956	1721.0099	0.0143	8	106	122	LAAALPDGGVLLLENVR					Mascot
1720.9956	1721.0099	0.0143	8	106	122	LAAALPDGGVLLLENVR	152	100			Mascot
1769.8568	1769.8281	-0.0287	-16	317	331	TVIWNGPMGVFEFEK			Oxidation (M)[8]		Mascot
1919.9611	1919.9753	0.0142	7	138	155	LASVADLYVNDAFGTAH R					Mascot
1919.9611	1919.9753	0.0142	7	138	155	LASVADLYVNDAFGTAH R	151	100			Mascot
2048.0559	2048.0669	0.011	5	137	155	KLASVADLYVNDAFGTAH R					Mascot
2048.0559	2048.0669	0.011	5	137	155	KLASVADLYVNDAFGTAH R	155	100			Mascot
2089.1274	2089.0889	-0.0385	-18	241	259	SLVEEDKLELATSLIETAK					Mascot
2159.2224	2159.1467	-0.0757	-35	106	125	LAAALPDGGVLLLENVRF YK					Mascot
2446.2092	2446.1875	-0.0217	-9	84	105	LSELLGLEVVMAPDCIGE EVEK			Carbamidomethyl (C)[15], Oxidation (M)[11]		Mascot
2878.4866	2878.4949	0.0083	3	373	401	MSHISTGGGASLELLEGK PLPGVLALDEA			Oxidation (M)[1]		Mascot

2	Phosphoglycerate kinase, cytosolic [Triticum urartu]	gi|473781647	45286.1	5.9	18	642	100	57.478	540	100

Peptide Information

Calc. Mass	Obsrv. Mass	± da	± ppm	Start Seq.	End Seq.	Sequence	Ion Score	C. I. %	Modification	Rank	Result Type
1056.6565	1056.6521	-0.0044	-4	75	83	FSLKPLVPR					Mascot
1056.6565	1056.6521	-0.0044	-4	75	83	FSLKPLVPR	56	99.586			Mascot
1089.5786	1089.5455	-0.0331	-30	6	16	SVGTLGEADLK					Mascot
1096.5521	1096.5519	-0.0002	0	307	316	TFAEALDTTK					Mascot
1245.6798	1245.6925	0.0127	10	5	16	RSVGTLGEADLK					Mascot
1274.6951	1274.6445	-0.0506	-40	6	18	SVGTLGEADLKGK					Mascot
1298.6587	1298.6355	-0.0232	-18	24	35	ADLNVPLDDAQK					Mascot
1375.8196	1375.7859	-0.0337	-24	44	55	ASIPTIKYLLEK					Mascot
1388.7421	1388.708	-0.0341	-25	179	191	ELDYLVGAVANPK					Mascot
1443.8782	1443.7881	-0.0901	-62	203	216	VSSKIGVIESLLAK					Mascot
1493.8297	1493.8295	-0.0002	0	166	178	FLRPSVAGFLMQK					Mascot
1509.8247	1509.8046	-0.0201	-13	166	178	FLRPSVAGFLMQK			Oxidation (M)[11]		Mascot
1509.8247	1509.8046	-0.0201	-13	166	178	FLRPSVAGFLMQK	25	0	Oxidation (M)[11]		Mascot
1573.8433	1573.7906	-0.0527	-33	362	378	GVTTIIGGGDSVAAVEK					Mascot
1720.9956	1721.0099	0.0143	8	106	122	LAAALPDGGVLLLENVR					Mascot
1720.9956	1721.0099	0.0143	8	106	122	LAAALPDGGVLLLENVR	152	100			Mascot
1769.8568	1769.8281	-0.0287	-16	317	331	TVIWNGPMGVFEFEK			Oxidation (M)[8]		Mascot
1919.9611	1919.9753	0.0142	7	138	155	LASVADLYVNDAFGTAH R					Mascot
1919.9611	1919.9753	0.0142	7	138	155	LASVADLYVNDAFGTAH R	151	100			Mascot
2048.0559	2048.0669	0.011	5	137	155	KLASVADLYVNDAFGTAH R					Mascot
2048.0559	2048.0669	0.011	5	137	155	KLASVADLYVNDAFGTAH R	155	100			Mascot
2089.1274	2089.0889	-0.0385	-18	241	259	SLVEEDKLELATSLIETAK					Mascot
2159.2224	2159.1467	-0.0757	-35	106	125	LAAALPDGGVLLLENVRF YK					Mascot
2446.2092	2446.1875	-0.0217	-9	84	105	LSELLGLEVVMAPDCIGE EVEK			Carbamidomethyl (C)[15], Oxidation (M)[11]		Mascot

3	Phosphoglycerate kinase, cytosolic [Aegilops tauschii]	gi|475614782	52949.9	6.75	18	604	100	49.592	515	100

Peptide Information

Calc. Mass	Obsrv. Mass	± da	± ppm	Start Seq.	End Seq.	Sequence	Ion Score	C. I. %	Modification	Rank	Result Type
1056.6565	1056.6521	-0.0044	-4	75	83	FSLKPLVPR					Mascot
1056.6565	1056.6521	-0.0044	-4	75	83	FSLKPLVPR	56	99.586			Mascot

1089.5786	1089.5455	-0.0331	-30	6	16	SVGTLGEADLK					Mascot
1096.5521	1096.5519	-0.0002	0	307	316	TFAEALDTTK					Mascot
1245.6798	1245.6925	0.0127	10	5	16	RSVGTLGEADLK					Mascot
1274.6951	1274.6445	-0.0506	-40	6	18	SVGTLGEADLKGK					Mascot
1298.6587	1298.6355	-0.0232	-18	24	35	ADLNVPLDDAQK					Mascot
1375.8196	1375.7859	-0.0337	-24	44	55	ASIPTIKYLLEK					Mascot
1388.7421	1388.708	-0.0341	-25	179	191	ELDYLVGAVANPK					Mascot
1416.6974	1416.6963	-0.0011	-1	436	446	MKLQHDLMEQK			Oxidation (M)[1]		Mascot
1443.8782	1443.7881	-0.0901	-62	203	216	VSSKIGVIESLLAK					Mascot
1573.8433	1573.7906	-0.0527	-33	350	366	GVTTIIGGGDSVAAVEK					Mascot
1720.9956	1721.0099	0.0143	8	106	122	LAAALPDGGVLLLENVR					Mascot
1720.9956	1721.0099	0.0143	8	106	122	LAAALPDGGVLLLENVR	152	100			Mascot
1769.8568	1769.8281	-0.0287	-16	317	331	TVIWNGPMGVFEFEK			Oxidation (M)[8]		Mascot
1919.9611	1919.9753	0.0142	7	138	155	LASVADLYVNDAFGTAHR					Mascot
1919.9611	1919.9753	0.0142	7	138	155	LASVADLYVNDAFGTAHR	151	100			Mascot
2048.0559	2048.0669	0.011	5	137	155	KLASVADLYVNDAFGTAHR					Mascot
2048.0559	2048.0669	0.011	5	137	155	KLASVADLYVNDAFGTAHR	155	100			Mascot
2089.1274	2089.0889	-0.0385	-18	241	259	SLVEEDKLELATSLIETAK					Mascot
2159.2224	2159.1467	-0.0757	-35	106	125	LAAALPDGGVLLLENVRFYK					Mascot
2446.2092	2446.1875	-0.0217	-9	84	105	LSELLGLEVVMAPDCIGEEVEK			Carbamidomethyl (C)[15], Oxidation (M)[11]		Mascot

4 Phosphoglycerate kinase, cytosolic [Triticum urartu] gi|473995124 55052.6 5.45 9 505 100 54.801 475 100

Peptide Information

Calc. Mass	Obsrv. Mass	± da	± ppm	Start Seq.	End Seq.	Sequence	Ion Score	C. I. %	Modification	Rank	Result Type
1493.8297	1493.8295	-0.0002	0	209	221	YLRPAVAGFLMQK					Mascot
1509.8247	1509.8046	-0.0201	-13	209	221	YLRPAVAGFLMQK			Oxidation (M)[11]		Mascot
1509.8247	1509.8046	-0.0201	-13	209	221	YLRPAVAGFLMQK	16	0	Oxidation (M)[11]		Mascot
1536.8269	1536.8265	-0.0004	0	506	520	VVFLNSQLVSAATTS					Mascot
1573.8433	1573.7906	-0.0527	-33	393	409	GVTTIIGGGDSVAAVEK					Mascot
1720.9956	1721.0099	0.0143	8	149	165	LAAALPDGGVLLLENVR					Mascot
1720.9956	1721.0099	0.0143	8	149	165	LAAALPDGGVLLLENVR	152	100			Mascot
1769.8568	1769.8281	-0.0287	-16	360	374	TVIWNGPMGVFEFEK			Oxidation (M)[8]		Mascot
1919.9611	1919.9753	0.0142	7	181	198	LASVADLYVNDAFGTAHR					Mascot
1919.9611	1919.9753	0.0142	7	181	198	LASVADLYVNDAFGTAH	151	100			Mascot

						R					
2048.0559	2048.0669	0.011	5	180	198	KLASVADLYVNDAFGTAH R					Mascot
2048.0559	2048.0669	0.011	5	180	198	KLASVADLYVNDAFGTAH R	155	100			Mascot
2159.2224	2159.1467	-0.0757	-35	149	168	LAAALPDGGVLLLENVRF YK					Mascot
2382.2373	2382.208	-0.0293	-12	328	350	IVPASAIPDGWMGLDVGP DSIKK			Oxidation (M)[12]		Mascot

5 uncharacterized protein, partial [Phleum pratense] gi|409971693 38020.5 7.71 14 459 100 30.748 388 100

Peptide Information

Calc. Mass	Obsrv. Mass	± da	± ppm	Start Seq.	End Seq.	Sequence	Ion Score	C. I. %	Modification	Rank	Result Type
1056.6565	1056.6521	-0.0044	-4	101	109	FSLKPLVPR					Mascot
1056.6565	1056.6521	-0.0044	-4	101	109	FSLKPLVPR	56	99.586			Mascot
1089.5786	1089.5455	-0.0331	-30	32	42	SVGTLGEADLK					Mascot
1175.6241	1175.6033	-0.0208	-18	20	30	FLHAAVAMATK			Oxidation (M)[8]		Mascot
1245.6798	1245.6925	0.0127	10	31	42	RSVGTLGEADLK					Mascot
1274.6951	1274.6445	-0.0506	-40	32	44	SVGTLGEADLKGK					Mascot
1298.6587	1298.6355	-0.0232	-18	50	61	ADLNVPLDDAQK					Mascot
1388.7421	1388.708	-0.0341	-25	205	217	ELDYLVGAVANPK					Mascot
1443.8782	1443.7881	-0.0901	-62	229	242	VSSKIGVIESLLAK					Mascot
1493.8297	1493.8295	-0.0002	0	192	204	FLRPSVAGFLMQK					Mascot
1509.8247	1509.8046	-0.0201	-13	192	204	FLRPSVAGFLMQK			Oxidation (M)[11]		Mascot
1509.8247	1509.8046	-0.0201	-13	192	204	FLRPSVAGFLMQK	25	0	Oxidation (M)[11]		Mascot
1525.8837	1525.8105	-0.0732	-48	288	302	GVSLLLPTDVVVADK					Mascot
1706.9047	1706.9015	-0.0032	-2	16	30	FDLRFLHAAVAMATK			Oxidation (M)[12]		Mascot
1919.9611	1919.9753	0.0142	7	164	181	LASVADLYVNDAFGTAH R					Mascot
1919.9611	1919.9753	0.0142	7	164	181	LASVADLYVNDAFGTAH R	151	100			Mascot
2048.0559	2048.0669	0.011	5	163	181	KLASVADLYVNDAFGTAH R					Mascot
2048.0559	2048.0669	0.011	5	163	181	KLASVADLYVNDAFGTAH R	155	100			Mascot
2089.1274	2089.0889	-0.0385	-18	267	285	SLVEEDKLELATSLIETAK					Mascot

6 uncharacterized protein, partial [Phleum pratense] gi|409972135 37649.2 7.7 14 458 100 30.739 388 100

Peptide Information

Calc. Mass	Obsrv. Mass	± da	± ppm	Start Seq.	End Seq.	Sequence	Ion Score	C. I. %	Modification	Rank	Result Type
1056.6565	1056.6521	-0.0044	-4	91	99	FSLKPLVPR					Mascot
1056.6565	1056.6521	-0.0044	-4	91	99	FSLKPLVPR	56	99.586			Mascot

1089.5786	1089.5455	-0.0331	-30	22	32	SVGTLGEADLK					Mascot
1175.6241	1175.6033	-0.0208	-18	10	20	FLHAAVAMATK			Oxidation (M)[8]		Mascot
1245.6798	1245.6925	0.0127	10	21	32	RSVGTLGEADLK					Mascot
1274.6951	1274.6445	-0.0506	-40	22	34	SVGTLGEADLKGK					Mascot
1298.6587	1298.6355	-0.0232	-18	40	51	ADLNVPLDDAQK					Mascot
1388.7421	1388.708	-0.0341	-25	195	207	ELDYLVGAVANPK					Mascot
1443.8782	1443.7881	-0.0901	-62	219	232	VSSKIGVIESLLAK					Mascot
1493.8297	1493.8295	-0.0002	0	182	194	FLRPSVAGFLMQK					Mascot
1509.8247	1509.8046	-0.0201	-13	182	194	FLRPSVAGFLMQK			Oxidation (M)[11]		Mascot
1509.8247	1509.8046	-0.0201	-13	182	194	FLRPSVAGFLMQK	25	0	Oxidation (M)[11]		Mascot
1525.8837	1525.8105	-0.0732	-48	278	292	GVSLLLPTDVVVADK					Mascot
1769.8568	1769.8281	-0.0287	-16	333	347	TVIWNGPMGVFEFEK			Oxidation (M)[8]		Mascot
1919.9611	1919.9753	0.0142	7	154	171	LASVADLYVNDAFGTAH R					Mascot
1919.9611	1919.9753	0.0142	7	154	171	LASVADLYVNDAFGTAH R	151	100			Mascot
2048.0559	2048.0669	0.011	5	153	171	KLASVADLYVNDAFGTAH R					Mascot
2048.0559	2048.0669	0.011	5	153	171	KLASVADLYVNDAFGTAH R	155	100			Mascot
2089.1274	2089.0889	-0.0385	-18	257	275	SLVEEDKLELATSLIETAK					Mascot

7	uncharacterized protein, partial [Phleum pratense]	gi|409972371	35863.2	6.09	13	455	100	30.651	388	100	

Peptide Information

Calc. Mass	Obsrv. Mass	± da	± ppm	Start Seq.	End Seq.	Sequence	Ion Score	C. I. %	Modification	Rank	Result Type
1056.6565	1056.6521	-0.0044	-4	76	84	FSLKPLVPR					Mascot
1056.6565	1056.6521	-0.0044	-4	76	84	FSLKPLVPR	56	99.586			Mascot
1089.5786	1089.5455	-0.0331	-30	7	17	SVGTLGEADLK					Mascot
1245.6798	1245.6925	0.0127	10	6	17	RSVGTLGEADLK					Mascot
1274.6951	1274.6445	-0.0506	-40	7	19	SVGTLGEADLKGK					Mascot
1298.6587	1298.6355	-0.0232	-18	25	36	ADLNVPLDDAQK					Mascot
1388.7421	1388.708	-0.0341	-25	180	192	ELDYLVGAVANPK					Mascot
1443.8782	1443.7881	-0.0901	-62	204	217	VSSKIGVIESLLAK					Mascot
1493.8297	1493.8295	-0.0002	0	167	179	FLRPSVAGFLMQK					Mascot
1509.8247	1509.8046	-0.0201	-13	167	179	FLRPSVAGFLMQK			Oxidation (M)[11]		Mascot
1509.8247	1509.8046	-0.0201	-13	167	179	FLRPSVAGFLMQK	25	0	Oxidation (M)[11]		Mascot
1525.8837	1525.8105	-0.0732	-48	263	277	GVSLLLPTDVVVADK					Mascot
1769.8568	1769.8281	-0.0287	-16	318	332	TVIWNGPMGVFEFEK			Oxidation (M)[8]		Mascot
1919.9611	1919.9753	0.0142	7	139	156	LASVADLYVNDAFGTAH					Mascot

Calc. Mass	Obsrv. Mass	± da	± ppm	Start Seq.	End Seq.	Sequence	Ion Score	C. I. %	Modification	Rank	Result Type
						R					
1919.9611	1919.9753	0.0142	7	139	156	LASVADLYVNDAFGTAHR	151	100			Mascot
2048.0559	2048.0669	0.011	5	138	156	KLASVADLYVNDAFGTAHR					Mascot
2048.0559	2048.0669	0.011	5	138	156	KLASVADLYVNDAFGTAHR	155	100			Mascot
2089.1274	2089.0889	-0.0385	-18	242	260	SLVEEDKLELATSLIETAK					Mascot

8	uncharacterized protein, partial [Phleum pratense]	gi|409971689	37945.3	7.05	13	452	100	30.651	388	100

Protein Group

uncharacterized protein, partial [Phleum pratense]	gi|409971711	37945.3	7.0500 001907 3486

Peptide Information

Calc. Mass	Obsrv. Mass	± da	± ppm	Start Seq.	End Seq.	Sequence	Ion Score	C. I. %	Modification	Rank	Result Type
1056.6565	1056.6521	-0.0044	-4	94	102	FSLKPLVPR					Mascot
1056.6565	1056.6521	-0.0044	-4	94	102	FSLKPLVPR	56	99.586			Mascot
1089.5786	1089.5455	-0.0331	-30	25	35	SVGTLGEADLK					Mascot
1245.6798	1245.6925	0.0127	10	24	35	RSVGTLGEADLK					Mascot
1274.6951	1274.6445	-0.0506	-40	25	37	SVGTLGEADLKGK					Mascot
1298.6587	1298.6355	-0.0232	-18	43	54	ADLNVPLDDAQK					Mascot
1388.7421	1388.708	-0.0341	-25	198	210	ELDYLVGAVANPK					Mascot
1443.8782	1443.7881	-0.0901	-62	222	235	VSSKIGVIESLLAK					Mascot
1493.8297	1493.8295	-0.0002	0	185	197	FLRPSVAGFLMQK					Mascot
1509.8247	1509.8046	-0.0201	-13	185	197	FLRPSVAGFLMQK			Oxidation (M)[11]		Mascot
1509.8247	1509.8046	-0.0201	-13	185	197	FLRPSVAGFLMQK	25	0	Oxidation (M)[11]		Mascot
1525.8837	1525.8105	-0.0732	-48	281	295	GVSLLLPTDVVVADK					Mascot
1769.8568	1769.8281	-0.0287	-16	336	350	TVIWNGPMGVFEFEK			Oxidation (M)[8]		Mascot
1919.9611	1919.9753	0.0142	7	157	174	LASVADLYVNDAFGTAHR					Mascot
1919.9611	1919.9753	0.0142	7	157	174	LASVADLYVNDAFGTAHR	151	100			Mascot
2048.0559	2048.0669	0.011	5	156	174	KLASVADLYVNDAFGTAHR					Mascot
2048.0559	2048.0669	0.011	5	156	174	KLASVADLYVNDAFGTAHR	155	100			Mascot
2089.1274	2089.0889	-0.0385	-18	260	278	SLVEEDKLELATSLIETAK					Mascot

9	uncharacterized protein, partial [Phleum pratense]	gi|409972391	26729.7	9.4	12	451	100	30.147	388	100

Peptide Information

Calc. Mass	Obsrv. Mass	± da	± ppm	Start Seq.	End Seq.	Sequence	Ion Score	C. I. %	Modification	Rank	Result Type

1056.6565	1056.6521	-0.0044	-4	93	101	FSLKPLVPR					Mascot
1056.6565	1056.6521	-0.0044	-4	93	101	FSLKPLVPR	56	99.586			Mascot
1089.5786	1089.5455	-0.0331	-30	24	34	SVGTLGEADLK					Mascot
1175.6241	1175.6033	-0.0208	-18	12	22	FLHAAVAMATK			Oxidation (M)[8]		Mascot
1245.6798	1245.6925	0.0127	10	23	34	RSVGTLGEADLK					Mascot
1274.6951	1274.6445	-0.0506	-40	24	36	SVGTLGEADLKGK					Mascot
1298.6587	1298.6355	-0.0232	-18	42	53	ADLNVPLDDAQK					Mascot
1388.7421	1388.708	-0.0341	-25	197	209	ELDYLVGAVANPK					Mascot
1443.8782	1443.7881	-0.0901	-62	221	234	VSSKIGVIESLLAK					Mascot
1493.8297	1493.8295	-0.0002	0	184	196	FLRPSVAGFLMQK					Mascot
1509.8247	1509.8046	-0.0201	-13	184	196	FLRPSVAGFLMQK			Oxidation (M)[11]		Mascot
1509.8247	1509.8046	-0.0201	-13	184	196	FLRPSVAGFLMQK	25	0	Oxidation (M)[11]		Mascot
1706.9047	1706.9015	-0.0032	-2	8	22	FDLRFLHAAVAMATK			Oxidation (M)[12]		Mascot
1919.9611	1919.9753	0.0142	7	156	173	LASVADLYVNDAFGTAHR					Mascot
1919.9611	1919.9753	0.0142	7	156	173	LASVADLYVNDAFGTAHR	151	100			Mascot
2048.0559	2048.0669	0.011	5	155	173	KLASVADLYVNDAFGTAHR					Mascot
2048.0559	2048.0669	0.011	5	155	173	KLASVADLYVNDAFGTAHR	155	100			Mascot

10	uncharacterized protein, partial [Phleum pratense]	gi|409972297	37972.3	6.21	13	450	100	30.537	388	100

Peptide Information

Calc. Mass	Obsrv. Mass	± da	± ppm	Start Seq.	End Seq.	Sequence	Ion Score	C. I. %	Modification	Rank	Result Type
1056.6565	1056.6521	-0.0044	-4	92	100	FSLKPLVPR					Mascot
1056.6565	1056.6521	-0.0044	-4	92	100	FSLKPLVPR	56	99.586			Mascot
1089.5786	1089.5455	-0.0331	-30	23	33	SVGTLGEADLK					Mascot
1245.6798	1245.6925	0.0127	10	22	33	RSVGTLGEADLK					Mascot
1274.6951	1274.6445	-0.0506	-40	23	35	SVGTLGEADLKGK					Mascot
1298.6587	1298.6355	-0.0232	-18	41	52	ADLNVPLDDAQK					Mascot
1388.7421	1388.708	-0.0341	-25	196	208	ELDYLVGAVANPK					Mascot
1443.8782	1443.7881	-0.0901	-62	220	233	VSSKIGVIESLLAK					Mascot
1493.8297	1493.8295	-0.0002	0	183	195	FLRPSVAGFLMQK					Mascot
1509.8247	1509.8046	-0.0201	-13	183	195	FLRPSVAGFLMQK			Oxidation (M)[11]		Mascot
1509.8247	1509.8046	-0.0201	-13	183	195	FLRPSVAGFLMQK	25	0	Oxidation (M)[11]		Mascot
1525.8837	1525.8105	-0.0732	-48	279	293	GVSLLLPTDVVVADK					Mascot
1747.9313	1747.8895	-0.0418	-24	7	22	FDLGFLHAAVAMATKR					Mascot

1769.8568	1769.8281	-0.0287	-16	334	348	TVIWNGPMGVFEFEK			Oxidation (M)[8]	Mascot
1919.9611	1919.9753	0.0142	7	155	172	LASVADLYVNDAFGTAH R				Mascot
1919.9611	1919.9753	0.0142	7	155	172	LASVADLYVNDAFGTAH R	151	100		Mascot
2048.0559	2048.0669	0.011	5	154	172	KLASVADLYVNDAFGTAH R				Mascot
2048.0559	2048.0669	0.011	5	154	172	KLASVADLYVNDAFGTAH R	155	100		Mascot

Gel Idx/Pos	267/K19	Instr./Gel Origin	BA2151/Sample Project 20140814	Process Status	Analysis Succeeded
Plate [#] Name	[1] Sample Project 20140814	Instrument Sample Name		Spectra	11

Rank	Protein Name	Accession No.	Protein MW	Protein PI	Pep. Count	Protein Score	Protein Score C. I. %	Intensity Matched	Total Ion Score	Total Ion C. I. %	Confirmed
1	RecName: Full=Serpin-Z1C; AltName: Full=TriaeZ1c; AltName: Full=WSZ1c	gi|75313848	42969	5.62	14	345	100	60.817	269	100	

Peptide Information

Calc. Mass	Obsrv. Mass	± da	± ppm	Start Seq.	End Seq.	Sequence	Ion Score	C. I. %	Modification	Rank	Result Type
806.4744	806.4313	-0.0431	-53	271	276	HIPRQR					Mascot
925.5214	925.5156	-0.0058	-6	11	18	LSIAHQTR					Mascot
925.5214	925.5156	-0.0058	-6	11	18	LSIAHQTR	43	91.593			Mascot
947.5156	947.4764	-0.0392	-41	2	10	ATTLATDVR					Mascot
1078.5562	1078.6202	0.064	59	1	10	MATTLATDVR					Mascot
1151.6824	1151.6451	-0.0373	-32	172	181	LVLANALYFK					Mascot
1176.5896	1176.5881	-0.0015	-1	261	270	LSAEPDFLER					Mascot
1176.5896	1176.5881	-0.0015	-1	261	270	LSAEPDFLER	69	99.98			Mascot
1352.6945	1352.6516	-0.0429	-32	289	300	ISFETEASDLLK					Mascot
1399.6125	1399.5831	-0.0294	-21	182	194	GAWTDQFDSSGTK					Mascot
1530.7435	1530.7631	0.0196	13	125	137	YKADTQSVDFQTK					Mascot
2129.0947	2129.0332	-0.0615	-29	379	398	EDISGVVLFMGHVVNPLLSS			Oxidation (M)[10]		Mascot
2720.3525	2720.3455	-0.007	-3	328	353	VSSVFHQAFVEVNEQGTEAAASTAIK					Mascot
2725.4631	2725.4707	0.0076	3	33	61	SAASNAVFSPVSLHVALSLLAAGAGSATR					Mascot
2725.4631	2725.4707	0.0076	3	33	61	SAASNAVFSPVSLHVALSLLAAGAGSATR	120	100			Mascot
2930.542	2930.5276	-0.0144	-5	354	378	MALLQARPPSVMDFIADHPFLFLLR			Oxidation (M)[1,12]		Mascot
3053.3801	3053.3704	-0.0097	-3	301	327	CLGLQLPFSNEADFSEMVDSPMAHGLR			Carbamidomethyl (C)[1], Oxidation (M)[17,22]		Mascot
3053.3801	3053.3704	-0.0097	-3	301	327	CLGLQLPFSNEADFSEMVDSPMAHGLR	37	70.985	Carbamidomethyl (C)[1], Oxidation (M)[17,22]		Mascot

Rank	Protein Name	Accession No.	Protein MW	Protein PI	Pep. Count	Protein Score	Protein Score C. I. %	Intensity Matched	Total Ion Score	Total Ion C. I. %	Confirmed
2	Serpin-Z1C [Triticum urartu]	gi|474075261	42956	5.62	13	337	100	60.708	269	100	

Peptide Information

Calc. Mass	Obsrv. Mass	± da	± ppm	Start Seq.	End Seq.	Sequence	Ion Score	C. I. %	Modification	Rank	Result Type
806.4744	806.4313	-0.0431	-53	271	276	HIPRQR					Mascot
925.5214	925.5156	-0.0058	-6	11	18	LSIAHQTR					Mascot

Calc. Mass	Obsrv. Mass	± da	± ppm	Start Seq.	End Seq.	Sequence	Ion Score	C. I. %	Modification	Rank	Result Type
925.5214	925.5156	-0.0058	-6	11	18	LSIAHQTR	43	91.593			Mascot
947.5156	947.4764	-0.0392	-41	2	10	ATTLATDVR					Mascot
1078.5562	1078.6202	0.064	59	1	10	MATTLATDVR					Mascot
1151.6824	1151.6451	-0.0373	-32	172	181	LVLANALYFK					Mascot
1176.5896	1176.5881	-0.0015	-1	261	270	LSAEPDFLER					Mascot
1176.5896	1176.5881	-0.0015	-1	261	270	LSAEPDFLER	69	99.98			Mascot
1352.6945	1352.6516	-0.0429	-32	289	300	ISFETEASDLLK					Mascot
1399.6125	1399.5831	-0.0294	-21	182	194	GAWTDQFDSSGTK					Mascot
2129.0947	2129.0332	-0.0615	-29	379	398	EDISGVVLFMGHVVNPLLSS			Oxidation (M)[10]		Mascot
2720.3525	2720.3455	-0.007	-3	328	353	VSSVFHQAFVEVNEQGTEAAASTAIK					Mascot
2725.4631	2725.4707	0.0076	3	33	61	SAASNAVFSPVSLHVALSLLAAGAGSATR					Mascot
2725.4631	2725.4707	0.0076	3	33	61	SAASNAVFSPVSLHVALSLLAAGAGSATR	120	100			Mascot
2930.542	2930.5276	-0.0144	-5	354	378	MALLQARPPSVMDFIADHPFLFLLR			Oxidation (M)[1,12]		Mascot
3053.3801	3053.3704	-0.0097	-3	301	327	CLGLQLPFSNEADFSEMVDSPMAHGLR			Carbamidomethyl (C)[1], Oxidation (M)[17,22]		Mascot
3053.3801	3053.3704	-0.0097	-3	301	327	CLGLQLPFSNEADFSEMVDSPMAHGLR	37	70.985	Carbamidomethyl (C)[1], Oxidation (M)[17,22]		Mascot

3 RecName: Full=Serpin-Z1B; AltName: Full=TriaeZ1b; AltName: Full=WSZ1b; AltName: Full=WZS2 gi|75279910 43119.9 5.44 10 151 100 26.805 112 100

Peptide Information

Calc. Mass	Obsrv. Mass	± da	± ppm	Start Seq.	End Seq.	Sequence	Ion Score	C. I. %	Modification	Rank	Result Type
806.4744	806.4313	-0.0431	-53	272	277	HIPRQR					Mascot
925.5214	925.5156	-0.0058	-6	11	18	LSIAHQTR					Mascot
925.5214	925.5156	-0.0058	-6	11	18	LSIAHQTR	43	91.593			Mascot
947.5156	947.4764	-0.0392	-41	2	10	ATTLATDVR					Mascot
1078.5562	1078.6202	0.064	59	1	10	MATTLATDVR					Mascot
1151.6824	1151.6451	-0.0373	-32	172	181	LVLANALYFK					Mascot
1176.5896	1176.5881	-0.0015	-1	262	271	LSAEPDFLER					Mascot
1176.5896	1176.5881	-0.0015	-1	262	271	LSAEPDFLER	69	99.98			Mascot
1585.8295	1585.7332	-0.0963	-61	288	301	FKISFGMEASDLLK					Mascot
1601.8243	1601.7195	-0.1048	-65	288	301	FKISFGMEASDLLK			Oxidation (M)[7]		Mascot
2062.0564	2061.9585	-0.0979	-47	138	156	AAEVTTQVNSWVEKVTSGR					Mascot
2129.0947	2129.0332	-0.0615	-29	380	399	EDISGVVLFMGHVVNPLLSS			Oxidation (M)[10]		Mascot
2720.3525	2720.3455	-0.007	-3	329	354	VSSVFHQAFVEVNEQGTEAAASTAIK					Mascot

4 RecName: Full=Serpin-Z1A; AltName: Full=TriaeZ1a; gi|75282265 43262.2 5.6 9 145 100 26.392 112 100

AltName: Full=WSZ1a; Short=WSZ1; AltName: Full=WSZCI

Peptide Information

Calc. Mass	Obsrv. Mass	± da	± ppm	Start Seq.	End Seq.	Sequence	Ion Score	C. I. %	Modification	Rank	Result Type
806.4744	806.4313	-0.0431	-53	271	276	HIPRQR					Mascot
925.5214	925.5156	-0.0058	-6	11	18	LSIAHQTR					Mascot
925.5214	925.5156	-0.0058	-6	11	18	LSIAHQTR	43	91.593			Mascot
947.5156	947.4764	-0.0392	-41	2	10	ATTLATDVR					Mascot
1078.5562	1078.6202	0.064	59	1	10	MATTLATDVR					Mascot
1151.6824	1151.6451	-0.0373	-32	172	181	LVLANALYFK					Mascot
1176.5896	1176.5881	-0.0015	-1	261	270	LSAEPDFLER					Mascot
1176.5896	1176.5881	-0.0015	-1	261	270	LSAEPDFLER	69	99.98			Mascot
2062.0564	2061.9585	-0.0979	-47	138	156	AAEVTTQVNSWVEKVTSGR					Mascot
2129.0947	2129.0332	-0.0615	-29	379	398	EDISGVVLFMGHVVNPLLSS			Oxidation (M)[10]		Mascot
2720.3525	2720.3455	-0.007	-3	328	353	VSSVFHQAFVEVNEQGTEAAASTAIK					Mascot

5	Serpin-Z1B [Aegilops tauschii]	gi|475546073	43257.1	5.44	8	138	100	26.013	112	100	

Peptide Information

Calc. Mass	Obsrv. Mass	± da	± ppm	Start Seq.	End Seq.	Sequence	Ion Score	C. I. %	Modification	Rank	Result Type
806.4744	806.4313	-0.0431	-53	272	277	HIPRQR					Mascot
925.5214	925.5156	-0.0058	-6	11	18	LSIAHQTR					Mascot
925.5214	925.5156	-0.0058	-6	11	18	LSIAHQTR	43	91.593			Mascot
947.5156	947.4764	-0.0392	-41	2	10	ATTLATDVR					Mascot
1078.5562	1078.6202	0.064	59	1	10	MATTLATDVR					Mascot
1151.6824	1151.6451	-0.0373	-32	172	181	LVLANALYFK					Mascot
1176.5896	1176.5881	-0.0015	-1	262	271	LSAEPDFLER					Mascot
1176.5896	1176.5881	-0.0015	-1	262	271	LSAEPDFLER	69	99.98			Mascot
2062.0564	2061.9585	-0.0979	-47	138	156	AAEVTTQVNSWVEKVTSGR					Mascot
2720.3525	2720.3455	-0.007	-3	329	354	VSSVFHQAFVEVNEQGTEAAASTAIK					Mascot

6	hypothetical protein CARUB_v10013379mg [Capsella rubella]	gi|482566068	62713.1	5.8	19	70	90.266	7.551	

Peptide Information

Calc. Mass	Obsrv. Mass	± da	± ppm	Start Seq.	End Seq.	Sequence	Ion Score	C. I. %	Modification	Rank	Result Type

811.442	811.3983	-0.0437	-54	454	459	KLEHER					Mascot
851.3563	851.4385	0.0822	97	217	223	VMADEDR			Oxidation (M)[2]		Mascot
860.422	860.4617	0.0397	46	435	441	EAEEARR					Mascot
867.5046	867.4333	-0.0713	-82	381	387	QLPPEKR					Mascot
900.4608	900.4388	-0.022	-24	87	93	HCETLLK			Carbamidomethyl (C)[2]		Mascot
947.4325	947.4764	0.0439	46	79	86	SFGMSKMK			Oxidation (M)[4,7]		Mascot
962.4174	962.3524	-0.065	-68	372	380	DGNSAQNEK					Mascot
985.4922	985.5208	0.0286	29	543	550	SHSQQRSR					Mascot
1042.5752	1042.611	0.0358	34	333	341	SRSHITSVR					Mascot
1078.5198	1078.6202	0.1004	93	217	225	VMADEDRVK			Oxidation (M)[2]		Mascot
1175.5912	1175.5703	-0.0209	-18	85	93	MKHCETLLK			Carbamidomethyl (C)[4], Oxidation (M)[1]		Mascot
1188.5321	1188.6119	0.0798	67	5	14	YGGFPVDYDR					Mascot
1399.694	1399.5831	-0.1109	-79	95	105	LMSHQHGWLFK			Oxidation (M)[2]		Mascot
1717.8604	1717.8729	0.0125	7	342	358	TDIASVGGLNQLEDASK					Mascot
2061.8521	2061.9585	0.1064	52	526	542	HDEDEDEDEVDLLAFDR					Mascot
2062.99	2062.9014	-0.0886	-43	361	380	LSHVEGVGGHKDGNSAQ NEK					Mascot
2087.1682	2087.0862	-0.082	-39	39	56	MVLPLSGLELQQLRSFQ K					Mascot
2331.2417	2331.2705	0.0288	12	113	132	LNIPDYFTIIKHPMDLGTV K			Oxidation (M)[14]		Mascot
2331.2417	2331.2705	0.0288	12	113	132	LNIPDYFTIIKHPMDLGTV K			Oxidation (M)[14]		Mascot
2720.251	2720.3455	0.0945	35	135	159	LTSGTYSNPSDFSADVY NMAHTLTK					Mascot

7 hypothetical protein ARALYDRAFT_900393 [Arabidopsis lyrata subsp. lyrata] gi|297330040 36668.4 8.86 10 58 0 23.282

Peptide Information

Calc. Mass	Obsrv. Mass	± da	± ppm	Start Seq.	End Seq.	Sequence	Ion Score	C. I. %	Modification	Rank	Result Type
813.4505	813.4012	-0.0493	-61	166	172	FPLPDPK					Mascot
823.4308	823.4111	-0.0197	-24	209	216	AAATQFSK					Mascot
842.4519	842.4411	-0.0108	-13	32	38	SWPSPIR					Mascot
925.5254	925.5156	-0.0098	-11	41	48	LWIHAASK					Mascot
925.5254	925.5156	-0.0098	-11	41	48	LWIHAASK	4	0			Mascot
1034.5703	1034.5166	-0.0537	-52	128	135	LIIPFEMR			Oxidation (M)[7]		Mascot
1176.5604	1176.5881	0.0277	24	297	306	EDRNTSIGER					Mascot
1176.5604	1176.5881	0.0277	24	297	306	EDRNTSIGER	12	0			Mascot
1381.7985	1381.6917	-0.1068	-77	315	327	IMAAAIRNLKPPS					Mascot

Calc. Mass	Obsrv. Mass	± da	± ppm	Start Seq.	End Seq.	Sequence	Ion Score	C. I. %	Modification	Rank	Result Type
1972.9723	1973.0472	0.0749	38	218	234	GQSLQTNNIFDYTTRSK					Mascot
2045.0736	2045.0061	-0.0675	-33	173	190	DPFSLKPGSIPCTIQEKK			Carbamidomethyl (C)[12]		Mascot
3053.4683	3053.3704	-0.0979	-32	112	135	LEGQTNFCWLCEQPQKL IIPFEMR			Carbamidomethyl (C)[8,11], Oxidation (M)[23]		Mascot
3053.4683	3053.3704	-0.0979	-32	112	135	LEGQTNFCWLCEQPQKL IIPFEMR			Carbamidomethyl (C)[8,11], Oxidation (M)[23]		Mascot

8	PREDICTED: formimidoyltransferase-cyclodeaminase-like [Fragaria vesca subsp. vesca]	gi|470140559	52185.4	5.32	14	56	0	3.798		

Peptide Information

Calc. Mass	Obsrv. Mass	± da	± ppm	Start Seq.	End Seq.	Sequence	Ion Score	C. I. %	Modification	Rank	Result Type
853.4414	853.4337	-0.0077	-9	176	182	VYISESR					Mascot
860.4948	860.4617	-0.0331	-38	310	316	TLDSIRR					Mascot
866.4982	866.443	-0.0552	-64	214	221	VGYTLVSK					Mascot
871.347	871.3071	-0.0399	-46	169	175	SMLGCCK			Carbamidomethyl (C)[5,6], Oxidation (M)[2]		Mascot
904.4921	904.465	-0.0271	-30	112	120	AVCASLGVK			Carbamidomethyl (C)[3]		Mascot
985.485	985.5208	0.0358	36	121	128	LEYNGHPR					Mascot
999.5469	999.4797	-0.0672	-67	346	354	GPLQVTQEK					Mascot
1078.5641	1078.6202	0.0561	52	66	74	VDPTRFSTR					Mascot
1642.8646	1642.83	-0.0346	-21	26	41	DGLVAGKEEAEQVGLK					Mascot
1844.9072	1845.0026	0.0954	52	55	70	GCVDVWNSAIRVDPTR			Carbamidomethyl (C)[2]		Mascot
2004.9584	2005.0356	0.0772	39	1	17	MDDIFGESVNLEEIHIK			Oxidation (M)[1]		Mascot
2044.9611	2045.0061	0.045	22	197	213	FSEAPVVNKFEDETYNR					Mascot
2240.1572	2240.2458	0.0886	40	364	382	WVDNYNVPVHSTDIAVV RR					Mascot
2742.3005	2742.3484	0.0479	17	2	25	DDIFGESVNLEEIHIKQGF DDGHK					Mascot

9	RecName: Full=Serpin-Z4; AltName: Full=BSZ4; AltName: Full=HorvuZ4; AltName: Full=Major endosperm albumin; AltName: Full=Protein Z4; Short=Protein Z	gi|131091	43363.4	5.72	6	56	0	6.717	43	91.593

Peptide Information

Calc. Mass	Obsrv. Mass	± da	± ppm	Start Seq.	End Seq.	Sequence	Ion Score	C. I. %	Modification	Rank	Result Type
840.3734	840.427	0.0536	64	190	196	FDESNTK					Mascot
925.5214	925.5156	-0.0058	-6	11	18	LSIAHQTR					Mascot
925.5214	925.5156	-0.0058	-6	11	18	LSIAHQTR	43	91.593			Mascot
947.5156	947.4764	-0.0392	-41	2	10	ATTLATDVR					Mascot
1078.5562	1078.6202	0.064	59	1	10	MATTLATDVR					Mascot

1151.6824	1151.6451	-0.0373	-32	174	183	LILGNALYFK	Mascot
1282.6638	1282.6517	-0.0121	-9	218	228	KQYISSSDNLK	Mascot

10	hypothetical protein OsI_24456 [Oryza sativa Indica Group]	gi|125556746	13607.5	8.46	9	55	0	1.43

Peptide Information

Calc. Mass	Obsrv. Mass	± da	± ppm	Start Seq.	End Seq.	Sequence	Ion Score	C. I. %	Modification	Rank	Result Type
823.4308	823.4111	-0.0197	-24	7	13	YVEVASR					Mascot
860.3971	860.4617	0.0646	75	45	50	DIFMYR			Oxidation (M)[4]		Mascot
1116.6273	1116.5483	-0.079	-71	105	114	RPTIANFAAR					Mascot
1453.6967	1453.5785	-0.1182	-81	27	37	LHFLESCFLCK			Carbamidomethyl (C)[7,10]		Mascot
1530.7369	1530.7631	0.0262	17	38	50	SSIAGDRDIFMYR					Mascot
1642.7378	1642.83	0.0922	56	1	13	MEEEERYVEVASR			Oxidation (M)[1]		Mascot
1871.948	1871.798	-0.15	-80	99	114	SPMMHRRPTIANFAAR			Oxidation (M)[3]		Mascot
1887.9429	1887.7928	-0.1501	-80	99	114	SPMMHRRPTIANFAAR			Oxidation (M)[3,4]		Mascot
1906.8746	1906.9467	0.0721	38	62	77	QEQMDMDEALQAVARR			Oxidation (M)[4]		Mascot
2989.218	2989.4011	0.1831	61	51	76	GDAAFCSDDCRQEQMD MDEALQAVAR			Carbamidomethyl (C)[6,10]		Mascot

Gel Idx/Pos	268/K20	Instr./Gel Origin	BA2151/Sample Project 20140814	Process Status	Analysis Succeeded
Plate [#] Name	[1] Sample Project 20140814	Instrument Sample Name		Spectra	11

Rank	Protein Name	Accession No.	Protein MW	Protein PI	Pep. Count	Protein Score	Protein Score C. I. %	Intensity Matched	Total Ion Score	Total Ion C. I. %	Confirmed
1	Beta-amylase [Triticum urartu]	gi|474451266	58995	5.34	17	445	100	26.36	362	100	

Peptide Information

Calc. Mass	Obsrv. Mass	± da	± ppm	Start Seq.	End Seq.	Sequence	Ion Score	C. I. %	Modification	Rank	Result Type
947.5057	947.4819	-0.0238	-25	322	329	DGYRPIAR					Mascot
1016.5564	1016.5454	-0.011	-11	412	419	LFGFTYLR					Mascot
1016.5564	1016.5454	-0.011	-11	412	419	LFGFTYLR	50	98.118			Mascot
1189.6154	1189.5902	-0.0252	-21	295	303	ISGIHWWYK					Mascot
1299.582	1299.5822	0.0002	0	336	346	ASLNFTCAEMR			Carbamidomethyl (C)[7]		Mascot
1315.5769	1315.552	-0.0249	-19	336	346	ASLNFTCAEMR			Carbamidomethyl (C)[7], Oxidation (M)[10]		Mascot
1326.6688	1326.6537	-0.0151	-11	385	395	YDPTAYNTILR					Mascot
1326.6688	1326.6537	-0.0151	-11	385	395	YDPTAYNTILR	66	99.947			Mascot
1474.6777	1474.698	0.0203	14	372	384	EGLNMACENALPR			Carbamidomethyl (C)[7]		Mascot
1490.6726	1490.6471	-0.0255	-17	372	384	EGLNMACENALPR			Carbamidomethyl (C)[7], Oxidation (M)[5]		Mascot
1511.8329	1511.7593	-0.0736	-49	276	288	ILDEANKVFLGHR					Mascot
1623.9581	1623.8678	-0.0903	-56	73	86	QLFQLVHEAGLKLK					Mascot
1646.781	1646.777	-0.004	-2	246	259	FFVDNGTYLTEQGR					Mascot
1646.781	1646.777	-0.004	-2	246	259	FFVDNGTYLTEQGR	95	100			Mascot
1668.7952	1668.797	0.0018	1	218	232	AAAAMVGHPEWEFPR					Mascot
1669.7349	1669.7777	0.0428	26	148	161	SAVQMYTDYMASFR					Mascot
1684.79	1684.7666	-0.0234	-14	218	232	AAAAMVGHPEWEFPR			Oxidation (M)[5]		Mascot
1685.7299	1685.7469	0.017	10	148	161	SAVQMYTDYMASFR			Oxidation (M)[5]		Mascot
1701.7247	1701.7129	-0.0118	-7	148	161	SAVQMYTDYMASFR			Oxidation (M)[5,10]		Mascot
1738.9095	1738.8765	-0.033	-19	396	411	NARPHGINKSGPPEHK					Mascot
1752.8916	1752.8417	-0.0499	-28	420	434	LSNQLVEGQNYVNFK					Mascot
2013.9778	2013.9774	-0.0004	0	304	321	VPSHAAEITAGYYNLHDR					Mascot
2013.9778	2013.9774	-0.0004	0	304	321	VPSHAAEITAGYYNLHDR	150	100			Mascot
2087.0557	2087.041	-0.0147	-7	130	147	NIEYLTLGVDDQPLFHGR					Mascot
2167.0537	2167.0452	-0.0085	-4	440	458	MHANLPHDPCVDPVAPLQR			Carbamidomethyl (C)[10]		Mascot
2183.0486	2183.0181	-0.0305	-14	440	458	MHANLPHDPCVDPVAPLQR			Carbamidomethyl (C)[10], Oxidation (M)[1]		Mascot
2269.2075	2269.1758	-0.0317	-14	166	187	EFLDAGVIVDIEVGLGPA					Mascot

GELR

2	RecName: Full=Beta-amylase; AltName: Full=1,4-alpha-D-glucan maltohydrolase; AltName: Full=Beta-Amy1; Flags: Precursor	gi|75107132	59886.4	5.66	11	303	100	18.164	266	100	

Peptide Information

Calc. Mass	Obsrv. Mass	± da	± ppm	Start Seq.	End Seq.	Sequence	Ion Score	C. I. %	Modification	Rank	Result Type
1016.5564	1016.5454	-0.011	-11	411	418	LFGFTYLR					Mascot
1016.5564	1016.5454	-0.011	-11	411	418	LFGFTYLR	50	98.118			Mascot
1189.6154	1189.5902	-0.0252	-21	294	302	ISGIHWWYK					Mascot
1253.6121	1253.5831	-0.029	-23	248	258	DNGTYLTEKGR					Mascot
1299.582	1299.5822	0.0002	0	335	345	ASINFTCAEMR			Carbamidomethyl (C)[7]		Mascot
1315.5769	1315.552	-0.0249	-19	335	345	ASINFTCAEMR			Carbamidomethyl (C)[7], Oxidation (M)[10]		Mascot
1326.6688	1326.6537	-0.0151	-11	384	394	YDPTAYNTILR					Mascot
1326.6688	1326.6537	-0.0151	-11	384	394	YDPTAYNTILR	66	99.947			Mascot
1669.7349	1669.7777	0.0428	26	147	160	SAVQMYADYMTSFR					Mascot
1685.7299	1685.7469	0.017	10	147	160	SAVQMYADYMTSFR			Oxidation (M)[5]		Mascot
1701.7247	1701.7129	-0.0118	-7	147	160	SAVQMYADYMTSFR			Oxidation (M)[5,10]		Mascot
1738.8732	1738.8765	0.0033	2	395	410	NARPHGINQSGPPEHK					Mascot
1752.8916	1752.8417	-0.0499	-28	419	433	LSNQLVEGQNYVNFK					Mascot
1827.9884	1827.9347	-0.0537	-29	458	474	SGPEISIEMILQAAKPK			Oxidation (M)[9]		Mascot
2013.9778	2013.9774	-0.0004	0	303	320	VPSHAAELTAGYYNLHDR					Mascot
2013.9778	2013.9774	-0.0004	0	303	320	VPSHAAELTAGYYNLHDR	150	100			Mascot
2267.1489	2267.0903	-0.0586	-26	7	26	GNYVQVYVMLPLDAVSVNNR			Oxidation (M)[9]		Mascot

3	RecName: Full=Beta-amylase; AltName: Full=1,4-alpha-D-glucan maltohydrolase	gi|113786	59894.5	5.58	10	298	100	17.581	266	100	

Peptide Information

Calc. Mass	Obsrv. Mass	± da	± ppm	Start Seq.	End Seq.	Sequence	Ion Score	C. I. %	Modification	Rank	Result Type
1016.5564	1016.5454	-0.011	-11	411	418	LFGFTYLR					Mascot
1016.5564	1016.5454	-0.011	-11	411	418	LFGFTYLR	50	98.118			Mascot
1189.6154	1189.5902	-0.0252	-21	294	302	ISGIHWWYK					Mascot
1299.582	1299.5822	0.0002	0	335	345	ASINFTCAEMR			Carbamidomethyl (C)[7]		Mascot
1315.5769	1315.552	-0.0249	-19	335	345	ASINFTCAEMR			Carbamidomethyl (C)[7], Oxidation (M)[10]		Mascot
1326.6688	1326.6537	-0.0151	-11	384	394	YDPTAYNTILR					Mascot
1326.6688	1326.6537	-0.0151	-11	384	394	YDPTAYNTILR	66	99.947			Mascot

1669.7349	1669.7777	0.0428	26	147	160	SAVQMYADYMTSFR					Mascot
1685.7299	1685.7469	0.017	10	147	160	SAVQMYADYMTSFR			Oxidation (M)[5]		Mascot
1701.7247	1701.7129	-0.0118	-7	147	160	SAVQMYADYMTSFR			Oxidation (M)[5,10]		Mascot
1738.8732	1738.8765	0.0033	2	395	410	NARPHGINQSGPPEHK					Mascot
1752.8916	1752.8417	-0.0499	-28	419	433	LSNQLVEGQNYVNFK					Mascot
1827.952	1827.9347	-0.0173	-9	458	474	SGPEISIEMILQAAQPK			Oxidation (M)[9]		Mascot
2013.9778	2013.9774	-0.0004	0	303	320	VPSHAAELTAGYYNLHD R					Mascot
2013.9778	2013.9774	-0.0004	0	303	320	VPSHAAELTAGYYNLHD R	150	100			Mascot
2267.1489	2267.0903	-0.0586	-26	7	26	GNYVQVYVMLPLDAVSV NNR			Oxidation (M)[9]		Mascot

4	Chain A, Sevenfold Mutant Of Barley Beta-Amylase	gi|6729696	56504.9	5.73	9	295	100	17.4	266	100	

Peptide Information

Calc. Mass	Obsrv. Mass	± da	± ppm	Start Seq.	End Seq.	Sequence	Ion Score	C. I. %	Modification	Rank	Result Type
1016.5564	1016.5454	-0.011	-11	407	414	LFGFTYLR					Mascot
1016.5564	1016.5454	-0.011	-11	407	414	LFGFTYLR	50	98.118			Mascot
1299.582	1299.5822	0.0002	0	331	341	ASINFTCAEMR			Carbamidomethyl (C)[7]		Mascot
1315.5769	1315.552	-0.0249	-19	331	341	ASINFTCAEMR			Carbamidomethyl (C)[7], Oxidation (M)[10]		Mascot
1326.6688	1326.6537	-0.0151	-11	380	390	YDPTAYNTILR					Mascot
1326.6688	1326.6537	-0.0151	-11	380	390	YDPTAYNTILR	66	99.947			Mascot
1669.7349	1669.7777	0.0428	26	143	156	SAVQMYADYMTSFR					Mascot
1685.7299	1685.7469	0.017	10	143	156	SAVQMYADYMTSFR			Oxidation (M)[5]		Mascot
1701.7247	1701.7129	-0.0118	-7	143	156	SAVQMYADYMTSFR			Oxidation (M)[5,10]		Mascot
1738.8732	1738.8765	0.0033	2	391	406	NARPHGINQSGPPEHK					Mascot
1752.8916	1752.8417	-0.0499	-28	415	429	LSNQLVEGQNYVNFK					Mascot
1827.952	1827.9347	-0.0173	-9	454	470	SGPEISIEMILQAAQPK			Oxidation (M)[9]		Mascot
2013.9778	2013.9774	-0.0004	0	299	316	VPSHAAELTAGYYNLHD R					Mascot
2013.9778	2013.9774	-0.0004	0	299	316	VPSHAAELTAGYYNLHD R	150	100			Mascot
2267.1489	2267.0903	-0.0586	-26	3	22	GNYVQVYVMLPLDAVSV NNR			Oxidation (M)[9]		Mascot

5	Chain A, Crystal Structure Of Barley Beta-Amylase Complexed With 2,3-Epoxypropyl-Alpha-D-Glucopyranoside	gi|313103508	59854.4	5.58	9	292	100	17.203	266	100	

Protein Group

Chain A, Crystal Structure Of Barley Beta-Amylase At Atomic Resolution	gi|313103503	59854.4	5.5799 999237 0605

Chain A, Crystal Structure Of Barley Beta-Amylase Complexed With 3,4- Epoxybutyl Alpha-D-Glucopyranoside   gi|313103509   59854.4   5.5799999237 0605

Chain A, Crystal Structure Of Barley Beta-Amylase Complexed With 4-O- Alpha-D-Glucopyranosylmoranoline   gi|313103505   59854.4   5.5799999237 0605

Chain A, Crystal Structure Of Barley Beta-Amylase Complexed With Acarbose   gi|313103502   59854.4   5.5799999237 0605

Chain A, Crystal Structure Of Barley Beta-Amylase Complexed With Alpha-Cyclodextrin   gi|313103504   59854.4   5.5799999237 0605

Peptide Information

Calc. Mass	Obsrv. Mass	± da	± ppm	Start Seq.	End Seq.	Sequence	Ion Score	C. I. %	Modification	Rank	Result Type
1016.5564	1016.5454	-0.011	-11	411	418	LFGFTYLR					Mascot
1016.5564	1016.5454	-0.011	-11	411	418	LFGFTYLR	50	98.118			Mascot
1189.6154	1189.5902	-0.0252	-21	294	302	ISGIHWWYK					Mascot
1299.582	1299.5822	0.0002	0	335	345	ASINFTCAEMR			Carbamidomethyl (C)[7]		Mascot
1315.5769	1315.552	-0.0249	-19	335	345	ASINFTCAEMR			Carbamidomethyl (C)[7], Oxidation (M)[10]		Mascot
1326.6688	1326.6537	-0.0151	-11	384	394	YDPTAYNTILR					Mascot
1326.6688	1326.6537	-0.0151	-11	384	394	YDPTAYNTILR	66	99.947			Mascot
1669.7349	1669.7777	0.0428	26	147	160	SAVQMYADYMTSFR					Mascot
1685.7299	1685.7469	0.017	10	147	160	SAVQMYADYMTSFR			Oxidation (M)[5]		Mascot
1701.7247	1701.7129	-0.0118	-7	147	160	SAVQMYADYMTSFR			Oxidation (M)[5,10]		Mascot
1738.8732	1738.8765	0.0033	2	395	410	NARPHGINQSGPPEHK					Mascot
1827.952	1827.9347	-0.0173	-9	458	474	SGPEISIEMILQAAQPK			Oxidation (M)[9]		Mascot
2013.9778	2013.9774	-0.0004	0	303	320	VPSHAAELTAGYYNLHDR					Mascot
2013.9778	2013.9774	-0.0004	0	303	320	VPSHAAELTAGYYNLHDR	150	100			Mascot
2267.1489	2267.0903	-0.0586	-26	7	26	GNYVQVYVMLPLDAVSVNNR			Oxidation (M)[9]		Mascot

6   Beta-amylase [Aegilops tauschii]   gi|475523854   60203.5   5.07   13   260   100   19.98   211   100

Peptide Information

Calc. Mass	Obsrv. Mass	± da	± ppm	Start Seq.	End Seq.	Sequence	Ion Score	C. I. %	Modification	Rank	Result Type
947.5057	947.4819	-0.0238	-25	322	329	DGYRPIAR					Mascot
1016.5564	1016.5454	-0.011	-11	412	419	LFGFTYLR					Mascot

1016.5564	1016.5454	-0.011	-11	412	419	LFGFTYLR	50	98.118			Mascot
1189.6154	1189.5902	-0.0252	-21	295	303	ISGIHWWYK					Mascot
1326.6688	1326.6537	-0.0151	-11	385	395	YDPTAYNTILR					Mascot
1326.6688	1326.6537	-0.0151	-11	385	395	YDPTAYNTILR	66	99.947			Mascot
1474.6777	1474.698	0.0203	14	372	384	EGLNMACENALPR			Carbamidomethyl (C)[7]		Mascot
1490.6726	1490.6471	-0.0255	-17	372	384	EGLNMACENALPR			Carbamidomethyl (C)[7], Oxidation (M)[5]		Mascot
1573.6998	1573.7653	0.0655	42	334	346	HHASLNFTCAEMR			Carbamidomethyl (C)[9]		Mascot
1646.781	1646.777	-0.004	-2	246	259	FFVDNGTYLTEQGR					Mascot
1646.781	1646.777	-0.004	-2	246	259	FFVDNGTYLTEQGR	95	100			Mascot
1668.7952	1668.797	0.0018	1	218	232	AAAAMVGHPEWEFPR					Mascot
1671.7142	1671.7775	0.0633	38	148	161	SAVQMYADYMASFR			Oxidation (M)[5,10]		Mascot
1684.79	1684.7666	-0.0234	-14	218	232	AAAAMVGHPEWEFPR			Oxidation (M)[5]		Mascot
1738.9095	1738.8765	-0.033	-19	396	411	NARPHGINKSGPPEHK					Mascot
1752.8916	1752.8417	-0.0499	-28	420	434	LSNQLVEGQNYVNFK					Mascot
2087.0557	2087.041	-0.0147	-7	130	147	NIEYLTLGVDDQPLFHGR					Mascot
2167.0537	2167.0452	-0.0085	-4	440	458	MHANLPHDPCVDPVAPL QR			Carbamidomethyl (C)[10]		Mascot
2183.0486	2183.0181	-0.0305	-14	440	458	MHANLPHDPCVDPVAPL QR			Carbamidomethyl (C)[10], Oxidation (M)[1]		Mascot

7 beta-amylase 2, partial [Brachypodium distachyon] gi|482677643 46785.2 5.84 11 257 100 19.213 211 100

Peptide Information

Calc. Mass	Obsrv. Mass	± da	± ppm	Start Seq.	End Seq.	Sequence	Ion Score	C. I. %	Modification	Rank	Result Type
947.5057	947.4819	-0.0238	-25	312	319	DGYRPIAR					Mascot
1016.5564	1016.5454	-0.011	-11	402	409	LFGFTYLR					Mascot
1016.5564	1016.5454	-0.011	-11	402	409	LFGFTYLR	50	98.118			Mascot
1189.6154	1189.5902	-0.0252	-21	285	293	ISGIHWWYK					Mascot
1326.6688	1326.6537	-0.0151	-11	375	385	YDPTAYNTILR					Mascot
1326.6688	1326.6537	-0.0151	-11	375	385	YDPTAYNTILR	66	99.947			Mascot
1474.6777	1474.698	0.0203	14	362	374	EGLNMACENALPR			Carbamidomethyl (C)[7]		Mascot
1490.6726	1490.6471	-0.0255	-17	362	374	EGLNMACENALPR			Carbamidomethyl (C)[7], Oxidation (M)[5]		Mascot
1511.8329	1511.7593	-0.0736	-49	266	278	ILDEANKVFLGHR					Mascot
1646.781	1646.777	-0.004	-2	236	249	FFVDNGTYLTEQGR					Mascot
1646.781	1646.777	-0.004	-2	236	249	FFVDNGTYLTEQGR	95	100			Mascot
1668.7952	1668.797	0.0018	1	208	222	AAAAMVGHPEWEFPR					Mascot
1684.79	1684.7666	-0.0234	-14	208	222	AAAAMVGHPEWEFPR			Oxidation (M)[5]		Mascot
2087.0557	2087.041	-0.0147	-7	120	137	NIEYLTLGVDDQPLFHGR					Mascot

2139.9839	2140.0823	0.0984	46	138	155	SAVQLYTDYMASFRDNM K					Mascot
2269.2075	2269.1758	-0.0317	-14	156	177	EFLDAGVIVDIEVGLGPA GELR					Mascot

8	beta-amylase 2, partial [Milium effusum]	gi|482677645	47129.8	7.3	5	212	100	12.288	200	100

Protein Group

beta-amylase 2, partial [Milium effusum]	gi|482677647	47235.6	6.4899 997711 1816

Peptide Information

Calc. Mass	Obsrv. Mass	± da	± ppm	Start Seq.	End Seq.	Sequence	Ion Score	C. I. %	Modification	Rank	Result Type
947.5057	947.4819	-0.0238	-25	312	319	DGYRPIAR					Mascot
1016.5564	1016.5454	-0.011	-11	402	409	LFGFTYLR					Mascot
1016.5564	1016.5454	-0.011	-11	402	409	LFGFTYLR	50	98.118			Mascot
1529.8516	1529.7906	-0.061	-40	250	261	FFLAWYSNKLIK					Mascot
2013.9778	2013.9774	-0.0004	0	294	311	VPSHAAELTAGYYNLHD R					Mascot
2013.9778	2013.9774	-0.0004	0	294	311	VPSHAAELTAGYYNLHD R	150	100			Mascot
2087.0557	2087.041	-0.0147	-7	120	137	NIEYLTLGVDDQPLFHGR					Mascot

9	putative cinnamyl alcohol dehydrogenase [Triticum urartu]	gi|474360235	39042.5	5.62	9	192	100	5.769	156	100

Peptide Information

Calc. Mass	Obsrv. Mass	± da	± ppm	Start Seq.	End Seq.	Sequence	Ion Score	C. I. %	Modification	Rank	Result Type
1128.5983	1128.5863	-0.012	-11	175	184	HFGLMTPGLR					Mascot
1144.5933	1144.5651	-0.0282	-25	175	184	HFGLMTPGLR			Oxidation (M)[5]		Mascot
1253.5944	1253.5831	-0.0113	-9	109	118	ANVEQYCNKK			Carbamidomethyl (C)[7]		Mascot
1272.5677	1272.5724	0.0047	4	328	337	MDYVNQAFER					Mascot
1288.5627	1288.5341	-0.0286	-22	328	337	MDYVNQAFER			Oxidation (M)[1]		Mascot
1517.7563	1517.7496	-0.0067	-4	88	102	AGDVVGVGVIVGCCR			Carbamidomethyl (C)[13,14]		Mascot
1517.7563	1517.7496	-0.0067	-4	88	102	AGDVVGVGVIVGCCR	85	100	Carbamidomethyl (C)[13,14]		Mascot
1594.786	1594.7784	-0.0076	-5	18	31	DATGHLSPYTYTLR					Mascot
1594.786	1594.7784	-0.0076	-5	18	31	DATGHLSPYTYTLR	71	99.984			Mascot
1611.8271	1611.7799	-0.0472	-29	203	217	SMGHHVTVISSSNKK					Mascot
1665.9469	1665.8182	-0.1287	-77	185	202	GGILGLGGVGHMGVKVA K			Oxidation (M)[12]		Mascot
1738.8065	1738.8765	0.07	40	1	17	MGSVDASETTVTGWAAR					Mascot
1797.9276	1797.9135	-0.0141	-8	200	216	VAKSMGHHVTVISSSNK			Oxidation (M)[5]		Mascot

10	RecName: Full=Beta-amylase; AltName: Full=1,4-alpha-D-glucan maltohydrolase	gi|231540	24561.9	5.08	3	125	100	10.35	116	100	

Peptide Information

Calc. Mass	Obsrv. Mass	± da	± ppm	Start Seq.	End Seq.	Sequence	Ion Score	C. I. %	Modification	Rank	Result Type
1016.5564	1016.5454	-0.011	-11	107	114	LFGFTYLR					Mascot
1016.5564	1016.5454	-0.011	-11	107	114	LFGFTYLR	50	98.118			Mascot
1326.6688	1326.6537	-0.0151	-11	80	90	YDPTAYNTILR					Mascot
1326.6688	1326.6537	-0.0151	-11	80	90	YDPTAYNTILR	66	99.947			Mascot
1573.6998	1573.7653	0.0655	42	29	41	HHASLNFTCAEMR			Carbamidomethyl (C)[9]		Mascot

Gel Idx/Pos	269/K21	Instr./Gel Origin	BA2151/Sample Project 20140814	Process Status	Analysis Succeeded
Plate [#] Name	[1] Sample Project 20140814	Instrument Sample Name		Spectra	11

Rank	Protein Name	Accession No.	Protein MW	Protein PI	Pep. Count	Protein Score	Protein Score C. I. %	Intensity Matched	Total Ion Score	Total Ion C. I. %	Confirmed
1	putative NADP-dependent oxidoreductase P1 [Triticum urartu]	gi|473799043	38359.4	5.53	12	348	100	19.046	287	100	

Peptide Information

Calc. Mass	Obsrv. Mass	± da	± ppm	Start Seq.	End Seq.	Sequence	Ion Score	C. I. %	Modification	Rank	Result Type
885.5152	885.502	-0.0132	-15	339	346	QLVAVARE					Mascot
1008.5546	1008.5253	-0.0293	-29	271	278	NLFCIITK			Carbamidomethyl (C)[4]		Mascot
1188.6219	1188.6544	0.0325	27	212	221	EQDLDATLKR					Mascot
1223.5369	1223.5138	-0.0231	-19	201	210	FGFDDAFNYK					Mascot
1360.6454	1360.6564	0.011	8	296	306	KFEEEMAGYLK			Oxidation (M)[6]		Mascot
1413.6831	1413.6804	-0.0027	-2	49	59	NLYLSCDPYLR			Carbamidomethyl (C)[6]		Mascot
1413.6831	1413.6804	-0.0027	-2	49	59	NLYLSCDPYLR	65	99.945	Carbamidomethyl (C)[6]		Mascot
1528.6948	1528.6642	-0.0306	-20	179	193	ISGCYVVGSAGSDEK			Carbamidomethyl (C)[4]		Mascot
1656.8163	1656.7595	-0.0568	-34	49	61	NLYLSCDPYLRSR			Carbamidomethyl (C)[6]		Mascot
1688.7738	1688.8044	0.0306	18	282	295	MEGFIVTDHYGTYR					Mascot
1704.7687	1704.7454	-0.0233	-14	282	295	MEGFIVTDHYGTYR			Oxidation (M)[1]		Mascot
1704.7687	1704.7454	-0.0233	-14	282	295	MEGFIVTDHYGTYR	75	99.995	Oxidation (M)[1]		Mascot
2113.9746	2113.9597	-0.0149	-7	17	35	YVTGFPSEDDMELVPATAR			Oxidation (M)[11]		Mascot
2138.0547	2138.0662	0.0115	5	252	270	VSVCGLISQYNLEQSEGVR			Carbamidomethyl (C)[4]		Mascot
2138.0547	2138.0662	0.0115	5	252	270	VSVCGLISQYNLEQSEGVR	147	100	Carbamidomethyl (C)[4]		Mascot
2279.2395	2279.2256	-0.0139	-6	156	178	KGEYVFVSAASGAVGQLVGQLAK					Mascot

Rank	Protein Name	Accession No.	Protein MW	Protein PI	Pep. Count	Protein Score	Protein Score C. I. %	Intensity Matched	Total Ion Score	Total Ion C. I. %	Confirmed
2	Putative NADP-dependent oxidoreductase P1 [Aegilops tauschii]	gi|475581762	41112.8	5.74	10	254	100	16.654	212	100	

Peptide Information

Calc. Mass	Obsrv. Mass	± da	± ppm	Start Seq.	End Seq.	Sequence	Ion Score	C. I. %	Modification	Rank	Result Type
1008.5546	1008.5253	-0.0293	-29	268	275	NLFCIITK			Carbamidomethyl (C)[4]		Mascot
1188.6219	1188.6544	0.0325	27	209	218	EQDLDATLKR					Mascot
1223.5369	1223.5138	-0.0231	-19	198	207	FGFDDAFNYK					Mascot
1360.6454	1360.6564	0.011	8	293	303	KFEEEMAGYLK			Oxidation (M)[6]		Mascot

Calc. Mass	Obsrv. Mass	± da	± ppm	Start Seq.	End Seq.	Sequence	Ion Score	C. I. %	Modification	Rank	Result Type
1413.6831	1413.6804	-0.0027	-2	46	56	NLYLSCDPYLR			Carbamidomethyl (C)[6]		Mascot
1413.6831	1413.6804	-0.0027	-2	46	56	NLYLSCDPYLR	65	99.945	Carbamidomethyl (C)[6]		Mascot
1528.6948	1528.6642	-0.0306	-20	176	190	ISGCYVVGSAGSDEK			Carbamidomethyl (C)[4]		Mascot
1641.8993	1641.8018	-0.0975	-59	336	350	QLAHIMLVAGESTKK			Oxidation (M)[6]		Mascot
1641.8993	1642.0579	0.1586	97	336	350	QLAHIMLVAGESTKK			Oxidation (M)[6]		Mascot
1656.8163	1656.7595	-0.0568	-34	46	58	NLYLSCDPYLRSR			Carbamidomethyl (C)[6]		Mascot
2138.0547	2138.0662	0.0115	5	249	267	VSVCGLISQYNLEQSEGV R			Carbamidomethyl (C)[4]		Mascot
2138.0547	2138.0662	0.0115	5	249	267	VSVCGLISQYNLEQSEGV R	147	100	Carbamidomethyl (C)[4]		Mascot
2279.2395	2279.2256	-0.0139	-6	153	175	KGEYVFVSAASGAVGQL VGQLAK					Mascot

3	putative NADP-dependent oxidoreductase P1 [Triticum urartu]	gi|474246662	38736.6	5.75	6	82	99.427	12.735	65	99.945

Peptide Information

Calc. Mass	Obsrv. Mass	± da	± ppm	Start Seq.	End Seq.	Sequence	Ion Score	C. I. %	Modification	Rank	Result Type
885.5152	885.502	-0.0132	-15	342	349	QLVAVARE					Mascot
1223.5369	1223.5138	-0.0231	-19	204	213	FGFDDAFNYK					Mascot
1360.6454	1360.6564	0.011	8	299	309	KFEEEMAGYLK			Oxidation (M)[6]		Mascot
1413.6831	1413.6804	-0.0027	-2	52	62	NLYLSCDPYLR			Carbamidomethyl (C)[6]		Mascot
1413.6831	1413.6804	-0.0027	-2	52	62	NLYLSCDPYLR	65	99.945	Carbamidomethyl (C)[6]		Mascot
1528.6948	1528.6642	-0.0306	-20	182	196	ISGCYVVGSAGSDEK			Carbamidomethyl (C)[4]		Mascot
2046.9524	2047.0533	0.1009	49	283	298	IRMEGFIVMDHYSNYR			Oxidation (M)[3]		Mascot

4	unnamed protein product [Vitis vinifera]	gi|296084352	31108.7	6.45	4	75	97.06	11.198	65	99.945

Peptide Information

Calc. Mass	Obsrv. Mass	± da	± ppm	Start Seq.	End Seq.	Sequence	Ion Score	C. I. %	Modification	Rank	Result Type
856.5363	856.5144	-0.0219	-26	14	20	NKQVVLR					Mascot
944.4724	944.493	0.0206	22	21	28	DYVSGYLK					Mascot
1375.7001	1375.759	0.0589	43	266	276	LYVSYCMIILT			Carbamidomethyl (C)[6]		Mascot
1413.6831	1413.6804	-0.0027	-2	55	65	NLYLSCDPYIR			Carbamidomethyl (C)[6]		Mascot
1413.6831	1413.6804	-0.0027	-2	55	65	NLYLSCDPYIR	65	99.945	Carbamidomethyl (C)[6]		Mascot

5	oxidoreductase, zinc-binding dehydrogenase family protein [Oryza sativa Japonica Group]	gi|108862362	35522.1	5.29	4	74	96.384	11.234	65	99.945

Peptide Information

Calc. Mass	Obsrv. Mass	± da	± ppm	Start Seq.	End Seq.	Sequence	Ion Score	C. I. %	Modification	Rank	Result Type

1145.6161	1145.6022	-0.0139	-12	188	197	ETDLEAALKR					Mascot
1223.5369	1223.5138	-0.0231	-19	177	186	FGFDDAFNYK					Mascot
1372.6202	1372.6547	0.0345	25	272	282	QFEEEMAGYLR					Mascot
1413.6831	1413.6804	-0.0027	-2	52	62	NLYISCDPYLR			Carbamidomethyl (C)[6]		Mascot
1413.6831	1413.6804	-0.0027	-2	52	62	NLYISCDPYLR	65	99.945	Carbamidomethyl (C)[6]		Mascot

6	hypothetical protein PRUPE_ppa008058mg [Prunus persica]	gi|462401937	38616.6	5.94	4	74	96.035	11.12	65	99.945

Peptide Information

Calc. Mass	Obsrv. Mass	± da	± ppm	Start Seq.	End Seq.	Sequence	Ion Score	C. I. %	Modification	Rank	Result Type
856.5251	856.5144	-0.0107	-12	37	44	LKVPQGSK					Mascot
1360.6301	1360.6564	0.0263	19	25	36	ESDMYVTTSSIK					Mascot
1372.66	1372.6547	-0.0053	-4	180	192	LMGCYVVGSVGSK			Carbamidomethyl (C)[4], Oxidation (M)[2]		Mascot
1413.6831	1413.6804	-0.0027	-2	50	60	NLYLSCDPYLR			Carbamidomethyl (C)[6]		Mascot
1413.6831	1413.6804	-0.0027	-2	50	60	NLYLSCDPYLR	65	99.945	Carbamidomethyl (C)[6]		Mascot

7	PREDICTED: NADP-dependent alkenal double bond reductase P2-like [Fragaria vesca subsp. vesca]	gi|470101421	38164.4	6.67	3	72	93.266	11.059	65	99.945

Peptide Information

Calc. Mass	Obsrv. Mass	± da	± ppm	Start Seq.	End Seq.	Sequence	Ion Score	C. I. %	Modification	Rank	Result Type
1175.5912	1175.6577	0.0665	57	236	245	MLDAVLPNMR			Oxidation (M)[1]		Mascot
1413.6831	1413.6804	-0.0027	-2	47	57	NLYLSCDPYLR			Carbamidomethyl (C)[6]		Mascot
1413.6831	1413.6804	-0.0027	-2	47	57	NLYLSCDPYLR	65	99.945	Carbamidomethyl (C)[6]		Mascot
1528.7499	1528.6642	-0.0857	-56	1	13	MASEMSNKQVIFK			Oxidation (M)[1]		Mascot

8	hypothetical protein M569_01914, partial [Genlisea aurea]	gi|527207621	28164.9	5.24	12	71	92.616	18.579		

Peptide Information

Calc. Mass	Obsrv. Mass	± da	± ppm	Start Seq.	End Seq.	Sequence	Ion Score	C. I. %	Modification	Rank	Result Type
800.4009	800.3734	-0.0275	-34	146	153	ATHASTGR					Mascot
917.5163	917.5063	-0.01	-11	19	26	DLSIASRR					Mascot
960.536	960.4789	-0.0571	-59	209	216	TSLEELLR					Mascot
1107.6157	1107.5645	-0.0512	-46	44	53	DVSIGKYLGR					Mascot
1253.696	1253.6788	-0.0172	-14	207	216	HRTSLEELLR					Mascot

1263.6229	1263.632	0.0091	7	57	66	DTHFSRPFTR					Mascot
1322.5529	1322.6727	0.1198	91	220	230	QTEENCEVGEK			Carbamidomethyl (C)[6]		Mascot
1322.5529	1322.6727	0.1198	91	220	230	QTEENCEVGEK			Carbamidomethyl (C)[6]		Mascot
1413.6758	1413.6804	0.0046	3	33	43	LSTDDRQYYPR					Mascot
1413.6758	1413.6804	0.0046	3	33	43	LSTDDRQYYPR					Mascot
1551.6956	1551.7861	0.0905	58	218	230	TKQTEENCEVGEK			Carbamidomethyl (C)[8]		Mascot
1703.8558	1703.7974	-0.0584	-34	11	25	ENNNETLKDLSIASR					Mascot
1704.8698	1704.7454	-0.1244	-73	189	204	RSVSVTEQTGGPLSMR					Mascot
1704.8698	1704.7454	-0.1244	-73	189	204	RSVSVTEQTGGPLSMR					Mascot
1720.8647	1720.7665	-0.0982	-57	189	204	RSVSVTEQTGGPLSMR			Oxidation (M)[15]		Mascot
2113.9409	2113.9597	0.0188	9	114	131	ETQVEEDEPELNNDEPVK					Mascot

9 Os12g0226300 [Oryza sativa Japonica Group] gi|255670159 16680.4 5.3 1 69 87.746 10.444 65 99.945

Peptide Information

Calc. Mass	Obsrv. Mass	± da	± ppm	Start Seq.	End Seq.	Sequence	Ion Score	C. I. %	Modification	Rank	Result Type
1413.6831	1413.6804	-0.0027	-2	52	62	NLYISCDPYLR			Carbamidomethyl (C)[6]		Mascot
1413.6831	1413.6804	-0.0027	-2	52	62	NLYISCDPYLR	65	99.945	Carbamidomethyl (C)[6]		Mascot

10 retrotransposon protein, putative, unclassified [Oryza sativa Japonica Group] gi|108864531 96272.9 5.46 21 68 83.846 12.769

Peptide Information

Calc. Mass	Obsrv. Mass	± da	± ppm	Start Seq.	End Seq.	Sequence	Ion Score	C. I. %	Modification	Rank	Result Type
813.3598	813.4261	0.0663	82	193	201	GHDGAGGSR					Mascot
815.4482	815.4124	-0.0358	-44	668	674	RNLEGAR					Mascot
819.4028	819.3983	-0.0045	-5	585	591	AMALEER					Mascot
846.4679	846.4564	-0.0115	-14	651	658	LAESASLR					Mascot
857.5454	857.4737	-0.0717	-84	505	512	KVLGEIAK					Mascot
885.4676	885.502	0.0344	39	794	800	LPEELER					Mascot
905.4323	905.4454	0.0131	14	567	575	AAETEAASR					Mascot
1188.5854	1188.6544	0.069	58	640	650	ALEEAEATAQR					Mascot
1193.5262	1193.6071	0.0809	68	135	143	WDWAPEDFK					Mascot
1205.5757	1205.6465	0.0708	59	369	380	QAEAATTSEAAR					Mascot
1262.5972	1262.6157	0.0185	15	700	713	SGGAATGESDLAAR					Mascot
1290.6802	1290.6154	-0.0648	-50	234	245	KQEGTPPPSPPR					Mascot
1424.7281	1424.6902	-0.0379	-27	57	69	LPSQPPAQASSWR					Mascot

1474.7609	1474.7662	0.0053	4	367	380	LRQAEAATTSEAAR		Mascot
1476.7917	1476.7444	-0.0473	-32	770	783	TVKYAANQGGLAQR		Mascot
1633.785	1633.8066	0.0216	13	578	591	EEALEARAMALEER	Oxidation (M)[9]	Mascot
1656.8665	1656.7595	-0.107	-65	550	565	LDTARGVLDAAAAQER		Mascot
1704.7783	1704.7454	-0.0329	-19	314	329	SGQSEAEDPATTEARR		Mascot
1704.7783	1704.7454	-0.0329	-19	314	329	SGQSEAEDPATTEARR		Mascot
2016.0542	2016.1077	0.0535	27	174	192	ASILTIMQAVGASEERAP R	Oxidation (M)[7]	Mascot
2138.1792	2138.0662	-0.113	-53	85	104	LAGLRSLGLIGAMVYGDY LR		Mascot
2138.1792	2138.0662	-0.113	-53	85	104	LAGLRSLGLIGAMVYGDY LR		Mascot
2279.156	2279.2256	0.0696	31	773	793	YAANQGGLAQRLSEMVG TLQR	Oxidation (M)[15]	Mascot

Gel Idx/Pos	270/K22	Instr./Gel Origin	BA2151/Sample Project 20140814	Process Status	Analysis Succeeded
Plate [#] Name	[1] Sample Project 20140814	Instrument Sample Name		Spectra	11

Rank	Protein Name	Accession No.	Protein MW	Protein PI	Pep. Count	Protein Score	Protein Score C. I. %	Intensity Matched	Total Ion Score	Total Ion C. I. %	Confirmed
1	Phosphoglycerate kinase, cytosolic [Triticum urartu]	gi|473781647	45286.1	5.9	18	646	100	56.039	539	100	

Peptide Information

Calc. Mass	Obsrv. Mass	± da	± ppm	Start Seq.	End Seq.	Sequence	Ion Score	C. I. %	Modification	Rank	Result Type
1056.6565	1056.6548	-0.0017	-2	75	83	FSLKPLVPR					Mascot
1056.6565	1056.6548	-0.0017	-2	75	83	FSLKPLVPR	60	99.837			Mascot
1074.6306	1074.6031	-0.0275	-26	192	202	KPFAAIVGGSK					Mascot
1089.5786	1089.5549	-0.0237	-22	6	16	SVGTLGEADLK					Mascot
1298.6587	1298.6323	-0.0264	-20	24	35	ADLNVPLDDAQK					Mascot
1335.6063	1335.6732	0.0669	50	126	136	EEEKNDPEFAK					Mascot
1388.7421	1388.7196	-0.0225	-16	179	191	ELDYLVGAVANPK					Mascot
1509.8247	1509.8083	-0.0164	-11	166	178	FLRPSVAGFLMQK			Oxidation (M)[11]		Mascot
1509.8247	1509.8083	-0.0164	-11	166	178	FLRPSVAGFLMQK	16	0	Oxidation (M)[11]		Mascot
1573.8433	1573.8103	-0.033	-21	362	378	GVTTIIGGGDSVAAVEK					Mascot
1720.9956	1721.0206	0.025	15	106	122	LAAALPDGGVLLLENVR					Mascot
1720.9956	1721.0206	0.025	15	106	122	LAAALPDGGVLLLENVR	152	100			Mascot
1769.8568	1769.8505	-0.0063	-4	317	331	TVIWNGPMGVFEFEK			Oxidation (M)[8]		Mascot
1802.9762	1802.8641	-0.1121	-62	217	232	VDILILGGGMIFTFYK			Oxidation (M)[10]		Mascot
1919.9611	1919.9871	0.026	14	138	155	LASVADLYVNDAFGTAH R					Mascot
1919.9611	1919.9871	0.026	14	138	155	LASVADLYVNDAFGTAH R	148	100			Mascot
2048.0559	2048.0833	0.0274	13	137	155	KLASVADLYVNDAFGTAH R					Mascot
2048.0559	2048.0833	0.0274	13	137	155	KLASVADLYVNDAFGTAH R	163	100			Mascot
2089.1274	2089.1179	-0.0095	-5	241	259	SLVEEDKLELATSLIETAK					Mascot
2102.1379	2102.135	-0.0029	-1	265	284	LLLPTDVVVADKFAADAE SK					Mascot
2159.2224	2159.1672	-0.0552	-26	106	125	LAAALPDGGVLLLENVRF YK					Mascot
2268.1582	2268.1665	0.0083	4	285	306	IVPATAIPDGWMGLDVGP DSIK			Oxidation (M)[12]		Mascot
2446.2092	2446.2239	0.0147	6	84	105	LSELLGLEVVMAPDCIGE EVEK			Carbamidomethyl (C)[15], Oxidation (M)[11]		Mascot

Rank	Protein Name	Accession No.	Protein MW	Protein PI	Pep. Count	Protein Score	Protein Score C. I. %	Intensity Matched	Total Ion Score	Total Ion C. I. %	Confirmed
2	Phosphoglycerate kinase, cytosolic [Aegilops tauschii]	gi|475614782	52949.9	6.75	19	627	100	55.841	524	100	

Peptide Information

Calc. Mass	Obsrv. Mass	± da	± ppm	Start Seq.	End Seq.	Sequence	Ion Score	C. I. %	Modification	Rank	Result Type
1056.6565	1056.6548	-0.0017	-2	75	83	FSLKPLVPR					Mascot
1056.6565	1056.6548	-0.0017	-2	75	83	FSLKPLVPR	60	99.837			Mascot
1074.6306	1074.6031	-0.0275	-26	192	202	KPFAAIVGGSK					Mascot
1089.5786	1089.5549	-0.0237	-22	6	16	SVGTLGEADLK					Mascot
1298.6587	1298.6323	-0.0264	-20	24	35	ADLNVPLDDAQK					Mascot
1335.6063	1335.6732	0.0669	50	126	136	EEEKNDPEFAK					Mascot
1388.7421	1388.7196	-0.0225	-16	179	191	ELDYLVGAVANPK					Mascot
1416.6974	1416.774	0.0766	54	436	446	MKLQHDLMEQK			Oxidation (M)[1]		Mascot
1500.759	1500.7413	-0.0177	-12	166	178	FLMPSVAGFLMQK			Oxidation (M)[3,11]		Mascot
1573.8433	1573.8103	-0.033	-21	350	366	GVTTIIGGGDSVAAVEK					Mascot
1720.9956	1721.0206	0.025	15	106	122	LAAALPDGGVLLLENVR					Mascot
1720.9956	1721.0206	0.025	15	106	122	LAAALPDGGVLLLENVR	152	100			Mascot
1769.8568	1769.8505	-0.0063	-4	317	331	TVIWNGPMGVFEFEK			Oxidation (M)[8]		Mascot
1802.9762	1802.8641	-0.1121	-62	217	232	VDILILGGGMIFTFYK			Oxidation (M)[10]		Mascot
1919.9611	1919.9871	0.026	14	138	155	LASVADLYVNDAFGTAH R					Mascot
1919.9611	1919.9871	0.026	14	138	155	LASVADLYVNDAFGTAH R	148	100			Mascot
2048.0559	2048.0833	0.0274	13	137	155	KLASVADLYVNDAFGTAH R					Mascot
2048.0559	2048.0833	0.0274	13	137	155	KLASVADLYVNDAFGTAH R	163	100			Mascot
2089.1274	2089.1179	-0.0095	-5	241	259	SLVEEDKLELATSLIETAK					Mascot
2102.1379	2102.135	-0.0029	-1	265	284	LLLPTDVVVADKFAADAE SK					Mascot
2159.2224	2159.1672	-0.0552	-26	106	125	LAAALPDGGVLLLENVRF YK					Mascot
2268.1582	2268.1665	0.0083	4	285	306	IVPATAIPDGWMGLDVGP DSIK			Oxidation (M)[12]		Mascot
2446.2092	2446.2239	0.0147	6	84	105	LSELLGLEVVMAPDCIGE EVEK			Carbamidomethyl (C)[15], Oxidation (M)[11]		Mascot

3	RecName: Full=Phosphoglycerate kinase, cytosolic	gi|129916	42152.7	5.64	19	602	100	52.168	479	100	

Peptide Information

Calc. Mass	Obsrv. Mass	± da	± ppm	Start Seq.	End Seq.	Sequence	Ion Score	C. I. %	Modification	Rank	Result Type
1030.6409	1030.6354	-0.0055	-5	75	83	FSLKPLVAR					Mascot
1074.6306	1074.6031	-0.0275	-26	192	202	KPFAAIVGGSK					Mascot
1089.5786	1089.5549	-0.0237	-22	6	16	SVGTLGEADLK					Mascot
1298.6587	1298.6323	-0.0264	-20	24	35	ADLNVPLDDAQK					Mascot

Calc. Mass	Obsrv. Mass	± da	± ppm	Start Seq.	End Seq.	Sequence	Ion Score	C. I. %	Modification	Rank	Result Type
1335.6063	1335.6732	0.0669	50	126	136	EEEKNDPEFAK					Mascot
1388.7421	1388.7196	-0.0225	-16	179	191	ELDYLVGAVANPK					Mascot
1509.8247	1509.8083	-0.0164	-11	166	178	FLRPSVAGFLMQK			Oxidation (M)[11]		Mascot
1509.8247	1509.8083	-0.0164	-11	166	178	FLRPSVAGFLMQK	16	0	Oxidation (M)[11]		Mascot
1573.8433	1573.8103	-0.033	-21	350	366	GVTTIIGGGDSVAAVEK					Mascot
1720.9956	1721.0206	0.025	15	106	122	LAAALPDGGVLLLENVR					Mascot
1720.9956	1721.0206	0.025	15	106	122	LAAALPDGGVLLLENVR	152	100			Mascot
1769.8568	1769.8505	-0.0063	-4	317	331	TVIWNGPMGVFEFEK			Oxidation (M)[8]		Mascot
1802.9762	1802.8641	-0.1121	-62	217	232	VDILILGGGMIFTFYK			Oxidation (M)[10]		Mascot
1919.9611	1919.9871	0.026	14	138	155	LASVADLYVNDAFGTAHR					Mascot
1919.9611	1919.9871	0.026	14	138	155	LASVADLYVNDAFGTAHR	148	100			Mascot
2048.0559	2048.0833	0.0274	13	137	155	KLASVADLYVNDAFGTAHR					Mascot
2048.0559	2048.0833	0.0274	13	137	155	KLASVADLYVNDAFGTAHR	163	100			Mascot
2089.1274	2089.1179	-0.0095	-5	241	259	SLVEEDKLELATSLIETAK					Mascot
2102.1379	2102.135	-0.0029	-1	265	284	LLLPTDVVVADKFAADAESK					Mascot
2159.2224	2159.1672	-0.0552	-26	106	125	LAAALPDGGVLLLENVRFYK					Mascot
2268.1582	2268.1665	0.0083	4	285	306	IVPATAIPDGWMGLDVGPDSIK			Oxidation (M)[12]		Mascot
2446.2092	2446.2239	0.0147	6	84	105	LSELLGLEVVMAPDCIGEEVEK			Carbamidomethyl (C)[15], Oxidation (M)[11]		Mascot
2878.4866	2878.5269	0.0403	14	373	401	MSHISTGGGASLELLEGKPLPGVLALDEA			Oxidation (M)[1]		Mascot

4 Phosphoglycerate kinase, cytosolic [Triticum urartu] gi|473995124 55052.6 5.45 12 519 100 50.49 463 100

Peptide Information

Calc. Mass	Obsrv. Mass	± da	± ppm	Start Seq.	End Seq.	Sequence	Ion Score	C. I. %	Modification	Rank	Result Type
1074.5765	1074.6031	0.0266	25	463	472	APSPIVMFGR					Mascot
1176.7212	1176.6947	-0.0265	-23	102	112	VVLASHLGRPK					Mascot
1335.6063	1335.6732	0.0669	50	169	179	EEEKNDPEFAK					Mascot
1509.8247	1509.8083	-0.0164	-11	209	221	YLRPAVAGFLMQK			Oxidation (M)[11]		Mascot
1509.8247	1509.8083	-0.0164	-11	209	221	YLRPAVAGFLMQK	9	0	Oxidation (M)[11]		Mascot
1573.8433	1573.8103	-0.033	-21	393	409	GVTTIIGGGDSVAAVEK					Mascot
1720.9956	1721.0206	0.025	15	149	165	LAAALPDGGVLLLENVR					Mascot
1720.9956	1721.0206	0.025	15	149	165	LAAALPDGGVLLLENVR	152	100			Mascot
1769.8568	1769.8505	-0.0063	-4	360	374	TVIWNGPMGVFEFEK			Oxidation (M)[8]		Mascot
1802.9762	1802.8641	-0.1121	-62	260	275	VDILILGGGMIYTFYK					Mascot
1919.9611	1919.9871	0.026	14	181	198	LASVADLYVNDAFGTAH					Mascot

Calc. Mass	Obsrv. Mass	± da	± ppm	Start Seq.	End Seq.	Sequence	Ion Score	C. I. %	Modification	Rank	Result Type
						R					
1919.9611	1919.9871	0.026	14	181	198	LASVADLYVNDAFGTAHR	148	100			Mascot
2048.0559	2048.0833	0.0274	13	180	198	KLASVADLYVNDAFGTAHR					Mascot
2048.0559	2048.0833	0.0274	13	180	198	KLASVADLYVNDAFGTAHR	163	100			Mascot
2159.2224	2159.1672	-0.0552	-26	149	168	LAAALPDGGVLLLENVRFYK					Mascot
2382.2373	2382.2368	-0.0005	0	328	350	IVPASAIPDGWMGLDVGPDSIKK			Oxidation (M)[12]		Mascot

5	uncharacterized protein, partial [Phleum pratense]	gi|409972135	37649.2	7.7	13	452	100	26.252	387	100

Peptide Information

Calc. Mass	Obsrv. Mass	± da	± ppm	Start Seq.	End Seq.	Sequence	Ion Score	C. I. %	Modification	Rank	Result Type
1056.6565	1056.6548	-0.0017	-2	91	99	FSLKPLVPR					Mascot
1056.6565	1056.6548	-0.0017	-2	91	99	FSLKPLVPR	60	99.837			Mascot
1074.6306	1074.6031	-0.0275	-26	208	218	KPFAAIVGGSK					Mascot
1089.5786	1089.5549	-0.0237	-22	22	32	SVGTLGEADLK					Mascot
1298.6587	1298.6323	-0.0264	-20	40	51	ADLNVPLDDAQK					Mascot
1335.6063	1335.6732	0.0669	50	142	152	EEEKNDPEFAK					Mascot
1388.7421	1388.7196	-0.0225	-16	195	207	ELDYLVGAVANPK					Mascot
1509.8247	1509.8083	-0.0164	-11	182	194	FLRPSVAGFLMQK			Oxidation (M)[11]		Mascot
1509.8247	1509.8083	-0.0164	-11	182	194	FLRPSVAGFLMQK	16	0	Oxidation (M)[11]		Mascot
1769.8568	1769.8505	-0.0063	-4	333	347	TVIWNGPMGVFEFEK			Oxidation (M)[8]		Mascot
1802.9762	1802.8641	-0.1121	-62	233	248	VDILILGGGMIFTFYK			Oxidation (M)[10]		Mascot
1919.9611	1919.9871	0.026	14	154	171	LASVADLYVNDAFGTAHR					Mascot
1919.9611	1919.9871	0.026	14	154	171	LASVADLYVNDAFGTAHR	148	100			Mascot
2048.0559	2048.0833	0.0274	13	153	171	KLASVADLYVNDAFGTAHR					Mascot
2048.0559	2048.0833	0.0274	13	153	171	KLASVADLYVNDAFGTAHR	163	100			Mascot
2089.1274	2089.1179	-0.0095	-5	257	275	SLVEEDKLELATSLIETAK					Mascot
2270.1011	2270.1543	0.0532	23	301	322	TVPADAIPDGWMGLDVGPDSIK			Oxidation (M)[12]		Mascot

6	uncharacterized protein, partial [Phleum pratense]	gi|409972371	35863.2	6.09	12	445	100	26.146	387	100

Peptide Information

Calc. Mass	Obsrv. Mass	± da	± ppm	Start Seq.	End Seq.	Sequence	Ion Score	C. I. %	Modification	Rank	Result Type
1056.6565	1056.6548	-0.0017	-2	76	84	FSLKPLVPR					Mascot
1056.6565	1056.6548	-0.0017	-2	76	84	FSLKPLVPR	60	99.837			Mascot

1074.6306	1074.6031	-0.0275	-26	193	203	KPFAAIVGGSK					Mascot
1089.5786	1089.5549	-0.0237	-22	7	17	SVGTLGEADLK					Mascot
1298.6587	1298.6323	-0.0264	-20	25	36	ADLNVPLDDAQK					Mascot
1335.6063	1335.6732	0.0669	50	127	137	EEEKNDPEFAK					Mascot
1388.7421	1388.7196	-0.0225	-16	180	192	ELDYLVGAVANPK					Mascot
1509.8247	1509.8083	-0.0164	-11	167	179	FLRPSVAGFLMQK			Oxidation (M)[11]		Mascot
1509.8247	1509.8083	-0.0164	-11	167	179	FLRPSVAGFLMQK	16	0	Oxidation (M)[11]		Mascot
1769.8568	1769.8505	-0.0063	-4	318	332	TVIWNGPMGVFEFEK			Oxidation (M)[8]		Mascot
1802.9762	1802.8641	-0.1121	-62	218	233	VDILILGGGMIFTFYK			Oxidation (M)[10]		Mascot
1919.9611	1919.9871	0.026	14	139	156	LASVADLYVNDAFGTAHR					Mascot
1919.9611	1919.9871	0.026	14	139	156	LASVADLYVNDAFGTAHR	148	100			Mascot
2048.0559	2048.0833	0.0274	13	138	156	KLASVADLYVNDAFGTAHR					Mascot
2048.0559	2048.0833	0.0274	13	138	156	KLASVADLYVNDAFGTAHR	163	100			Mascot
2089.1274	2089.1179	-0.0095	-5	242	260	SLVEEDKLELATSLIETAK					Mascot

7	uncharacterized protein, partial [Phleum pratense]	gi|409971693	38020.5	7.71	12	445	100	26.275	387	100	

Peptide Information

Calc. Mass	Obsrv. Mass	± da	± ppm	Start Seq.	End Seq.	Sequence	Ion Score	C. I. %	Modification	Rank	Result Type
1056.6565	1056.6548	-0.0017	-2	101	109	FSLKPLVPR					Mascot
1056.6565	1056.6548	-0.0017	-2	101	109	FSLKPLVPR	60	99.837			Mascot
1074.6306	1074.6031	-0.0275	-26	218	228	KPFAAIVGGSK					Mascot
1089.5786	1089.5549	-0.0237	-22	32	42	SVGTLGEADLK					Mascot
1298.6587	1298.6323	-0.0264	-20	50	61	ADLNVPLDDAQK					Mascot
1335.6063	1335.6732	0.0669	50	152	162	EEEKNDPEFAK					Mascot
1388.7421	1388.7196	-0.0225	-16	205	217	ELDYLVGAVANPK					Mascot
1509.8247	1509.8083	-0.0164	-11	192	204	FLRPSVAGFLMQK			Oxidation (M)[11]		Mascot
1509.8247	1509.8083	-0.0164	-11	192	204	FLRPSVAGFLMQK	16	0	Oxidation (M)[11]		Mascot
1690.9098	1691.001	0.0912	54	16	30	FDLRFLHAAVAMATK					Mascot
1802.9762	1802.8641	-0.1121	-62	243	258	VDILILGGGMIFTFYK			Oxidation (M)[10]		Mascot
1919.9611	1919.9871	0.026	14	164	181	LASVADLYVNDAFGTAHR					Mascot
1919.9611	1919.9871	0.026	14	164	181	LASVADLYVNDAFGTAHR	148	100			Mascot
2048.0559	2048.0833	0.0274	13	163	181	KLASVADLYVNDAFGTAHR					Mascot
2048.0559	2048.0833	0.0274	13	163	181	KLASVADLYVNDAFGTAHR	163	100			Mascot
2089.1274	2089.1179	-0.0095	-5	267	285	SLVEEDKLELATSLIETAK					Mascot

8	uncharacterized protein, partial [Phleum pratense]	gi|409972295	37534.1	6.92	12	445	100	25.689	387	100

Peptide Information

Calc. Mass	Obsrv. Mass	± da	± ppm	Start Seq.	End Seq.	Sequence	Ion Score	C. I. %	Modification	Rank	Result Type
1056.6565	1056.6548	-0.0017	-2	91	99	FSLKPLVPR					Mascot
1056.6565	1056.6548	-0.0017	-2	91	99	FSLKPLVPR	60	99.837			Mascot
1074.6306	1074.6031	-0.0275	-26	208	218	KPFAAIVGGSK					Mascot
1089.5786	1089.5549	-0.0237	-22	22	32	SVGTLGEADLK					Mascot
1298.6587	1298.6323	-0.0264	-20	40	51	ADLNVPLDDAQK					Mascot
1335.6063	1335.6732	0.0669	50	142	152	EEEKNDPEFAK					Mascot
1388.7421	1388.7196	-0.0225	-16	195	207	ELDYLVGAVANPK					Mascot
1509.8247	1509.8083	-0.0164	-11	182	194	FLRPSVAGFLMQK			Oxidation (M)[11]		Mascot
1509.8247	1509.8083	-0.0164	-11	182	194	FLRPSVAGFLMQK	16	0	Oxidation (M)[11]		Mascot
1769.8568	1769.8505	-0.0063	-4	333	347	TVIWNGPMGVFEFEK			Oxidation (M)[8]		Mascot
1802.9762	1802.8641	-0.1121	-62	233	248	VDILILGGGMIFTFYK			Oxidation (M)[10]		Mascot
1919.9611	1919.9871	0.026	14	154	171	LASVADLYVNDAFGTAH R					Mascot
1919.9611	1919.9871	0.026	14	154	171	LASVADLYVNDAFGTAH R	148	100			Mascot
2048.0559	2048.0833	0.0274	13	153	171	KLASVADLYVNDAFGTAH R					Mascot
2048.0559	2048.0833	0.0274	13	153	171	KLASVADLYVNDAFGTAH R	163	100			Mascot
2270.1011	2270.1543	0.0532	23	301	322	TVPADAIPDGWMGLDVG PDSIK			Oxidation (M)[12]		Mascot

9	uncharacterized protein, partial [Phleum pratense]	gi|409971689	37945.3	7.05	12	444	100	26.146	387	100

Protein Group

uncharacterized protein, partial [Phleum pratense]	gi|409971711	37945.3	7.0500 001907 3486

Peptide Information

Calc. Mass	Obsrv. Mass	± da	± ppm	Start Seq.	End Seq.	Sequence	Ion Score	C. I. %	Modification	Rank	Result Type
1056.6565	1056.6548	-0.0017	-2	94	102	FSLKPLVPR					Mascot
1056.6565	1056.6548	-0.0017	-2	94	102	FSLKPLVPR	60	99.837			Mascot
1074.6306	1074.6031	-0.0275	-26	211	221	KPFAAIVGGSK					Mascot
1089.5786	1089.5549	-0.0237	-22	25	35	SVGTLGEADLK					Mascot
1298.6587	1298.6323	-0.0264	-20	43	54	ADLNVPLDDAQK					Mascot
1335.6063	1335.6732	0.0669	50	145	155	EEEKNDPEFAK					Mascot
1388.7421	1388.7196	-0.0225	-16	198	210	ELDYLVGAVANPK					Mascot

1509.8247	1509.8083	-0.0164	-11	185	197	FLRPSVAGFLMQK			Oxidation (M)[11]		Mascot
1509.8247	1509.8083	-0.0164	-11	185	197	FLRPSVAGFLMQK	16	0	Oxidation (M)[11]		Mascot
1769.8568	1769.8505	-0.0063	-4	336	350	TVIWNGPMGVFEFEK			Oxidation (M)[8]		Mascot
1802.9762	1802.8641	-0.1121	-62	236	251	VDILILGGGMIFTFYK			Oxidation (M)[10]		Mascot
1919.9611	1919.9871	0.026	14	157	174	LASVADLYVNDAFGTAH R					Mascot
1919.9611	1919.9871	0.026	14	157	174	LASVADLYVNDAFGTAH R	148	100			Mascot
2048.0559	2048.0833	0.0274	13	156	174	KLASVADLYVNDAFGTAH R					Mascot
2048.0559	2048.0833	0.0274	13	156	174	KLASVADLYVNDAFGTAH R	163	100			Mascot
2089.1274	2089.1179	-0.0095	-5	260	278	SLVEEDKLELATSLIETAK					Mascot

10	uncharacterized protein, partial [Phleum pratense]	gi|409972297	37972.3	6.21	12	442	100	25.686	387	100	

Peptide Information

Calc. Mass	Obsrv. Mass	± da	± ppm	Start Seq.	End Seq.	Sequence	Ion Score	C. I. %	Modification	Rank	Result Type
1056.6565	1056.6548	-0.0017	-2	92	100	FSLKPLVPR					Mascot
1056.6565	1056.6548	-0.0017	-2	92	100	FSLKPLVPR	60	99.837			Mascot
1074.6306	1074.6031	-0.0275	-26	209	219	KPFAAIVGGSK					Mascot
1089.5786	1089.5549	-0.0237	-22	23	33	SVGTLGEADLK					Mascot
1298.6587	1298.6323	-0.0264	-20	41	52	ADLNVPLDDAQK					Mascot
1335.6063	1335.6732	0.0669	50	143	153	EEEKNDPEFAK					Mascot
1388.7421	1388.7196	-0.0225	-16	196	208	ELDYLVGAVANPK					Mascot
1509.8247	1509.8083	-0.0164	-11	183	195	FLRPSVAGFLMQK			Oxidation (M)[11]		Mascot
1509.8247	1509.8083	-0.0164	-11	183	195	FLRPSVAGFLMQK	16	0	Oxidation (M)[11]		Mascot
1747.9313	1747.8907	-0.0406	-23	7	22	FDLGFLHAAVAMATKR					Mascot
1769.8568	1769.8505	-0.0063	-4	334	348	TVIWNGPMGVFEFEK			Oxidation (M)[8]		Mascot
1802.9762	1802.8641	-0.1121	-62	234	249	VDILILGGGMIFTFYK			Oxidation (M)[10]		Mascot
1919.9611	1919.9871	0.026	14	155	172	LASVADLYVNDAFGTAH R					Mascot
1919.9611	1919.9871	0.026	14	155	172	LASVADLYVNDAFGTAH R	148	100			Mascot
2048.0559	2048.0833	0.0274	13	154	172	KLASVADLYVNDAFGTAH R					Mascot
2048.0559	2048.0833	0.0274	13	154	172	KLASVADLYVNDAFGTAH R	163	100			Mascot

Gel Idx/Pos	271/K23	Instr./Gel Origin	BA2151/Sample Project 20140814	Process Status	Analysis Succeeded
Plate [#] Name	[1] Sample Project 20140814	Instrument Sample Name		Spectra	11

Rank	Protein Name	Accession No.	Protein MW	Protein PI	Pep. Count	Protein Score	Protein Score C. I. %	Intensity Matched	Total Ion Score	Total Ion C. I. %	Confirmed
1	Beta-amylase [Aegilops tauschii]	gi|475523854	60203.5	5.07	13	620	100	39.587	571	100	

Peptide Information

Calc. Mass	Obsrv. Mass	± da	± ppm	Start Seq.	End Seq.	Sequence	Ion Score	C. I. %	Modification	Rank	Result Type
884.4512	884.4348	-0.0164	-19	211	217	YLQADFK					Mascot
947.5057	947.4902	-0.0155	-16	322	329	DGYRPIAR					Mascot
947.5057	947.4902	-0.0155	-16	322	329	DGYRPIAR	26	0			Mascot
993.4999	993.4896	-0.0103	-10	28	35	FEKGDELR					Mascot
1237.5444	1237.5508	0.0064	5	233	243	DAGTYNDTPQR					Mascot
1237.5444	1237.5508	0.0064	5	233	243	DAGTYNDTPQR	54	99.213			Mascot
1325.7941	1325.7498	-0.0443	-33	283	294	VFLGHTVQLAIK					Mascot
1335.7202	1335.637	-0.0832	-62	322	332	DGYRPIARMLK			Oxidation (M)[9]		Mascot
1589.6948	1589.6837	-0.0111	-7	334	346	HHASLNFTCAEMR			Carbamidomethyl (C)[9], Oxidation (M)[12]		Mascot
1646.781	1646.7966	0.0156	9	246	259	FFVDNGTYLTEQGR					Mascot
1646.781	1646.7966	0.0156	9	246	259	FFVDNGTYLTEQGR	88	100			Mascot
1668.7952	1668.7944	-0.0008	0	218	232	AAAAMVGHPEWEFPR					Mascot
1671.7142	1671.6985	-0.0157	-9	148	161	SAVQMYADYMASFR			Oxidation (M)[5,10]		Mascot
1671.7142	1671.6985	-0.0157	-9	148	161	SAVQMYADYMASFR			Oxidation (M)[5,10]		Mascot
1684.79	1684.7838	-0.0062	-4	218	232	AAAAMVGHPEWEFPR			Oxidation (M)[5]		Mascot
1999.9622	1999.9877	0.0255	13	304	321	VPSHAAEVTAGYYNLHD R					Mascot
1999.9622	1999.9877	0.0255	13	304	321	VPSHAAEVTAGYYNLHD R	142	100			Mascot
2020.936	2020.953	0.017	8	112	129	NVGVSDPDIFYTDQHGT R					Mascot
2020.936	2020.953	0.017	8	112	129	NVGVSDPDIFYTDQHGT R	128	100			Mascot
2087.0557	2087.0823	0.0266	13	130	147	NIEYLTLGVDDQPLFHGR					Mascot
2087.0557	2087.0823	0.0266	13	130	147	NIEYLTLGVDDQPLFHGR	132	100			Mascot

Rank	Protein Name	Accession No.	Protein MW	Protein PI	Pep. Count	Protein Score	Protein Score C. I. %	Intensity Matched	Total Ion Score	Total Ion C. I. %	Confirmed
2	beta-amylase 2, partial [Brachypodium distachyon]	gi|482677643	46785.2	5.84	10	337	100	23.834	300	100	

Peptide Information

Calc. Mass	Obsrv. Mass	± da	± ppm	Start Seq.	End Seq.	Sequence	Ion Score	C. I. %	Modification	Rank	Result Type

Calc. Mass	Obsrv. Mass	± da	± ppm	Start Seq.	End Seq.	Sequence	Ion Score	C. I. %	Modification	Rank	Result Type
884.4512	884.4348	-0.0164	-19	201	207	YLQADFK					Mascot
947.5057	947.4902	-0.0155	-16	312	319	DGYRPIAR					Mascot
947.5057	947.4902	-0.0155	-16	312	319	DGYRPIAR	26	0			Mascot
993.4999	993.4896	-0.0103	-10	18	25	FEKGDELR					Mascot
1237.5444	1237.5508	0.0064	5	223	233	DAGTYNDTPQR					Mascot
1237.5444	1237.5508	0.0064	5	223	233	DAGTYNDTPQR	54	99.213			Mascot
1335.7202	1335.637	-0.0832	-62	312	322	DGYRPIARMLK			Oxidation (M)[9]		Mascot
1603.7104	1603.7758	0.0654	41	324	336	HHASLNFTCTEMR			Carbamidomethyl (C)[9]		Mascot
1646.781	1646.7966	0.0156	9	236	249	FFVDNGTYLTEQGR					Mascot
1646.781	1646.7966	0.0156	9	236	249	FFVDNGTYLTEQGR	88	100			Mascot
1651.7784	1651.8032	0.0248	15	138	151	SAVQLYTDYMASFR					Mascot
1668.7952	1668.7944	-0.0008	0	208	222	AAAAMVGHPEWEFPR					Mascot
1684.79	1684.7838	-0.0062	-4	208	222	AAAAMVGHPEWEFPR			Oxidation (M)[5]		Mascot
2087.0557	2087.0823	0.0266	13	120	137	NIEYLTLGVDDQPLFHGR					Mascot
2087.0557	2087.0823	0.0266	13	120	137	NIEYLTLGVDDQPLFHGR	132	100			Mascot

3 Beta-amylase [Triticum urartu] gi|474451266 58995 5.34 10 276 100 23.259 247 100

Peptide Information

Calc. Mass	Obsrv. Mass	± da	± ppm	Start Seq.	End Seq.	Sequence	Ion Score	C. I. %	Modification	Rank	Result Type
884.4512	884.4348	-0.0164	-19	211	217	YLQADFK					Mascot
947.5057	947.4902	-0.0155	-16	322	329	DGYRPIAR					Mascot
947.5057	947.4902	-0.0155	-16	322	329	DGYRPIAR	26	0			Mascot
993.4999	993.4896	-0.0103	-10	28	35	FEKGDELR					Mascot
1335.7202	1335.637	-0.0832	-62	322	332	DGYRPIARMLK			Oxidation (M)[9]		Mascot
1623.9581	1623.9006	-0.0575	-35	73	86	QLFQLVHEAGLKLK					Mascot
1646.781	1646.7966	0.0156	9	246	259	FFVDNGTYLTEQGR					Mascot
1646.781	1646.7966	0.0156	9	246	259	FFVDNGTYLTEQGR	88	100			Mascot
1668.7952	1668.7944	-0.0008	0	218	232	AAAAMVGHPEWEFPR					Mascot
1684.79	1684.7838	-0.0062	-4	218	232	AAAAMVGHPEWEFPR			Oxidation (M)[5]		Mascot
1701.7247	1701.8016	0.0769	45	148	161	SAVQMYTDYMASFR			Oxidation (M)[5,10]		Mascot
2087.0557	2087.0823	0.0266	13	130	147	NIEYLTLGVDDQPLFHGR					Mascot
2087.0557	2087.0823	0.0266	13	130	147	NIEYLTLGVDDQPLFHGR	132	100			Mascot
2173.9351	2174.0701	0.135	62	148	165	SAVQMYTDYMASFRDNMK			Oxidation (M)[5]		Mascot

4 beta-amylase 2, partial [Milium effusum] gi|482677645 47129.8 7.3 6 173 100 13.63 159 100

Protein Group

	beta-amylase 2, partial [Milium effusum]	gi|482677647	47235.6	6.4899 997711 1816	

Peptide Information

Calc. Mass	Obsrv. Mass	± da	± ppm	Start Seq.	End Seq.	Sequence	Ion Score	C. I. %	Modification	Rank	Result Type
884.4512	884.4348	-0.0164	-19	201	207	YLQADFK					Mascot
947.5057	947.4902	-0.0155	-16	312	319	DGYRPIAR					Mascot
947.5057	947.4902	-0.0155	-16	312	319	DGYRPIAR	26	0			Mascot
1335.7202	1335.637	-0.0832	-62	312	322	DGYRPIARMLK			Oxidation (M)[9]		Mascot
1603.7104	1603.7758	0.0654	41	324	336	HHATLNFTCAEMR			Carbamidomethyl (C)[9], Oxidation (M)[12]		Mascot
1851.931	1851.9681	0.0371	20	138	152	TAIQLYVDYMTSFRK			Oxidation (M)[10]		Mascot
2087.0557	2087.0823	0.0266	13	120	137	NIEYLTLGVDDQPLFHGR					Mascot
2087.0557	2087.0823	0.0266	13	120	137	NIEYLTLGVDDQPLFHGR	132	100			Mascot

5	beta-amylase 2, partial [Deschampsia antarctica]	gi|482677599	46584.9	4.94	6	148	100	12.138	132	100	

Peptide Information

Calc. Mass	Obsrv. Mass	± da	± ppm	Start Seq.	End Seq.	Sequence	Ion Score	C. I. %	Modification	Rank	Result Type
1024.4581	1024.5244	0.0663	65	239	247	DNGTYPTEK					Mascot
1325.71	1325.7498	0.0398	30	201	212	YLEADFKAAAVK					Mascot
1589.6948	1589.6837	-0.0111	-7	324	336	HHASLNFTCAEMR			Carbamidomethyl (C)[9], Oxidation (M)[12]		Mascot
1675.7963	1675.7578	-0.0385	-23	234	247	TQFFKDNGTYPTEK					Mascot
1998.9517	1999.0538	0.1021	51	102	119	DIGATDPDIFYTNRSGTR					Mascot
2087.0557	2087.0823	0.0266	13	120	137	NIEYLTLGVDDQPLFHGR					Mascot
2087.0557	2087.0823	0.0266	13	120	137	NIEYLTLGVDDQPLFHGR	132	100			Mascot

6	beta-amylase 2, partial [Zingeria biebersteiniana]	gi|482677623	46497.1	5.39	6	148	100	12.388	132	100	

Peptide Information

Calc. Mass	Obsrv. Mass	± da	± ppm	Start Seq.	End Seq.	Sequence	Ion Score	C. I. %	Modification	Rank	Result Type
1285.6212	1285.6163	-0.0049	-4	52	62	GPKAYDWSAYK					Mascot
1325.71	1325.7498	0.0398	30	201	212	YLEADFKAAAVK					Mascot
1553.7595	1553.7815	0.022	14	102	115	AIGATDPDIFYTNR					Mascot
1589.6948	1589.6837	-0.0111	-7	324	336	HHASLNFTCAEMR			Carbamidomethyl (C)[9], Oxidation (M)[12]		Mascot
1701.7499	1701.8016	0.0517	30	138	151	TAIQMYADYMTSFK			Oxidation (M)[5,10]		Mascot
2087.0557	2087.0823	0.0266	13	120	137	NIEYLTLGVDDQPLFHGR					Mascot

2087.0557	2087.0823	0.0266	13	120	137	NIEYLTLGVDDQPLFHGR	132	100			Mascot

7	Beta-amylase [Triticum urartu]	gi|474019719	63864.1	5.29	7	147	100	13.115	132	100		

Peptide Information

Calc. Mass	Obsrv. Mass	± da	± ppm	Start Seq.	End Seq.	Sequence	Ion Score	C. I. %	Modification	Rank	Result Type
1285.6212	1285.6163	-0.0049	-4	61	71	GPKAYDWSAYK					Mascot
1449.7307	1449.6873	-0.0434	-30	487	497	KQWPYVMNDLR					Mascot
1607.6512	1607.7347	0.0835	52	333	345	HHASMNFTCAEMR			Carbamidomethyl (C)[9], Oxidation (M)[5]		Mascot
1686.8949	1686.7914	-0.1035	-61	552	566	YYGETKTVLSDVLAK					Mascot
1696.948	1696.7971	-0.1509	-89	472	486	ESAQDILNLKPLIDK					Mascot
2087.0557	2087.0823	0.0266	13	129	146	NIEYLTLGVDDQPLFHGR					Mascot
2087.0557	2087.0823	0.0266	13	129	146	NIEYLTLGVDDQPLFHGR	132	100			Mascot
2124.9194	2125.0215	0.1021	48	329	345	MLTRHHASMNFTCAEMR			Carbamidomethyl (C)[13], Oxidation (M)[1,9]		Mascot

8	beta-amylase 1, partial [Bromus sterilis]	gi|482677609	46635.2	5.47	5	145	100	12.148	132	100

Peptide Information

Calc. Mass	Obsrv. Mass	± da	± ppm	Start Seq.	End Seq.	Sequence	Ion Score	C. I. %	Modification	Rank	Result Type
884.4512	884.4348	-0.0164	-19	201	207	YLQADFK					Mascot
1285.6212	1285.6163	-0.0049	-4	52	62	GPKAYDWSAYK					Mascot
1589.6948	1589.6837	-0.0111	-7	324	336	HHASLNFTCAEMR			Carbamidomethyl (C)[9], Oxidation (M)[12]		Mascot
2087.0557	2087.0823	0.0266	13	120	137	NIEYLTLGVDDQPLFHGR					Mascot
2087.0557	2087.0823	0.0266	13	120	137	NIEYLTLGVDDQPLFHGR	132	100			Mascot
3048.4966	3048.6245	0.1279	42	75	101	LQAIMSCHQCGGNVGDV VNIPIPQWVR			Carbamidomethyl (C)[7,10]		Mascot

9	Beta-amylase [Aegilops tauschii]	gi|475619509	56860.2	5.24	5	142	100	12.516	132	100

Protein Group

RecName: Full=Beta-amylase; AltName: Full=1,4-alpha-D-glucan maltohydrolase	gi|3334120	56860.2	5.2399 997711 1816

Peptide Information

Calc. Mass	Obsrv. Mass	± da	± ppm	Start Seq.	End Seq.	Sequence	Ion Score	C. I. %	Modification	Rank	Result Type
993.4999	993.4896	-0.0103	-10	27	34	FEKGDEIR					Mascot
1285.6212	1285.6163	-0.0049	-4	61	71	GPKAYDWSAYK					Mascot
1607.6512	1607.7347	0.0835	52	333	345	HHASMNFTCAEMR			Carbamidomethyl (C)[9], Oxidation (M)[5]		Mascot
2087.0557	2087.0823	0.0266	13	129	146	NIEYLTLGVDDQPLFHGR					Mascot

2087.0557	2087.0823	0.0266	13	129	146	NIEYLTLGVDDQPLFHGR	132	100			Mascot
2124.9194	2125.0215	0.1021	48	329	345	MLTRHHASMNFTCAEMR			Carbamidomethyl (C)[13], Oxidation (M)[1,9]		Mascot

10	hypothetical protein OsI_26372 [Oryza sativa Indica Group]	gi|125558692	55463.5	5.3	5	142	100	11.416	132	100		

Peptide Information

Calc. Mass	Obsrv. Mass	± da	± ppm	Start Seq.	End Seq.	Sequence	Ion Score	C. I. %	Modification	Rank	Result Type
989.4794	989.5106	0.0312	32	458	466	SMPEMPIGK					Mascot
1421.6584	1421.7081	0.0497	35	245	256	FFTDNGTYVTEK					Mascot
1559.8621	1559.7656	-0.0965	-62	259	270	FFLTWYSNKLIK					Mascot
1651.7251	1651.8032	0.0781	47	333	345	HRACVNFTCAEMR			Carbamidomethyl (C)[4,9]		Mascot
2087.0557	2087.0823	0.0266	13	129	146	NIEYLTLGVDDQPLFHGR					Mascot
2087.0557	2087.0823	0.0266	13	129	146	NIEYLTLGVDDQPLFHGR	132	100			Mascot

Gel Idx/Pos	272/K24	Instr./Gel Origin	BA2151/Sample Project 20140814	Process Status	Analysis Succeeded
Plate [#] Name	[1] Sample Project 20140814	Instrument Sample Name		Spectra	11

Rank	Protein Name	Accession No.	Protein MW	Protein PI	Pep. Count	Protein Score	Protein Score C. I. %	Intensity Matched	Total Ion Score	Total Ion C. I. %	Confirmed
1	Putative NADP-dependent oxidoreductase P1 [Aegilops tauschii]	gi|475581762	41112.8	5.74	15	536	100	41.506	449	100	

Peptide Information

Calc. Mass	Obsrv. Mass	± da	± ppm	Start Seq.	End Seq.	Sequence	Ion Score	C. I. %	Modification	Rank	Result Type
1008.5546	1008.5222	-0.0324	-32	268	275	NLFCIITK			Carbamidomethyl (C)[4]		Mascot
1032.5209	1032.5051	-0.0158	-15	209	217	EQDLDATLK					Mascot
1188.6219	1188.6204	-0.0015	-1	209	218	EQDLDATLKR					Mascot
1223.5369	1223.5114	-0.0255	-21	198	207	FGFDDAFNYK					Mascot
1223.5369	1223.5114	-0.0255	-21	198	207	FGFDDAFNYK	58	99.703			Mascot
1232.5504	1232.6123	0.0619	50	294	303	FEEEMAGYLK			Oxidation (M)[5]		Mascot
1250.7467	1250.6512	-0.0955	-76	33	45	LAVPPGSNAVVVK					Mascot
1360.6454	1360.6233	-0.0221	-16	293	303	KFEEEMAGYLK			Oxidation (M)[6]		Mascot
1413.6831	1413.6869	0.0038	3	46	56	NLYLSCDPYLR			Carbamidomethyl (C)[6]		Mascot
1413.6831	1413.6869	0.0038	3	46	56	NLYLSCDPYLR	85	100	Carbamidomethyl (C)[6]		Mascot
1528.6948	1528.6652	-0.0296	-19	176	190	ISGCYVVGSAGSDEK			Carbamidomethyl (C)[4]		Mascot
1674.7581	1674.774	0.0159	9	279	292	MEGFIVTDHYGSYR					Mascot
1690.7531	1690.7365	-0.0166	-10	279	292	MEGFIVTDHYGSYR			Oxidation (M)[1]		Mascot
1690.7531	1690.7365	-0.0166	-10	279	292	MEGFIVTDHYGSYR	55	99.49	Oxidation (M)[1]		Mascot
2081.9849	2082.0254	0.0405	19	14	32	YVTGFPAEDDMELVPAT AR					Mascot
2097.9797	2097.9773	-0.0024	-1	14	32	YVTGFPAEDDMELVPAT AR			Oxidation (M)[11]		Mascot
2097.9797	2097.9773	-0.0024	-1	14	32	YVTGFPAEDDMELVPAT AR	81	99.999	Oxidation (M)[11]		Mascot
2138.0547	2138.0818	0.0271	13	249	267	VSVCGLISQYNLEQSEGV R			Carbamidomethyl (C)[4]		Mascot
2138.0547	2138.0818	0.0271	13	249	267	VSVCGLISQYNLEQSEGV R	170	100	Carbamidomethyl (C)[4]		Mascot
2279.2395	2279.2351	-0.0044	-2	153	175	KGEYVFVSAASGAVGQL VGQLAK					Mascot
2759.3193	2759.342	0.0227	8	59	84	MSGNDEPSHVPDFVQGE VLTTLGVSK			Oxidation (M)[1]		Mascot
2760.4131	2760.4214	0.0083	3	307	331	ITYVEDVAEGIESFPTALI GLFYGR					Mascot

Rank	Protein Name	Accession No.	Protein MW	Protein PI	Pep. Count	Protein Score	Protein Score C. I. %	Intensity Matched	Total Ion Score	Total Ion C. I. %	Confirmed
2	putative NADP-dependent oxidoreductase P1 [Triticum urartu]	gi|473799043	38359.4	5.53	15	430	100	36.195	340	100	

Peptide Information

Calc. Mass	Obsrv. Mass	± da	± ppm	Start Seq.	End Seq.	Sequence	Ion Score	C. I. %	Modification	Rank	Result Type
860.4472	860.4519	0.0047	5	2	10	AAAAEVSNK					Mascot
1008.5546	1008.5222	-0.0324	-32	271	278	NLFCIITK			Carbamidomethyl (C)[4]		Mascot
1032.5209	1032.5051	-0.0158	-15	212	220	EQDLDATLK					Mascot
1188.6219	1188.6204	-0.0015	-1	212	221	EQDLDATLKR					Mascot
1223.5369	1223.5114	-0.0255	-21	201	210	FGFDDAFNYK					Mascot
1223.5369	1223.5114	-0.0255	-21	201	210	FGFDDAFNYK	58	99.703			Mascot
1232.5504	1232.6123	0.0619	50	297	306	FEEEMAGYLK			Oxidation (M)[5]		Mascot
1360.6454	1360.6233	-0.0221	-16	296	306	KFEEEMAGYLK			Oxidation (M)[6]		Mascot
1413.6831	1413.6869	0.0038	3	49	59	NLYLSCDPYLR			Carbamidomethyl (C)[6]		Mascot
1413.6831	1413.6869	0.0038	3	49	59	NLYLSCDPYLR	85	100	Carbamidomethyl (C)[6]		Mascot
1528.6948	1528.6652	-0.0296	-19	179	193	ISGCYVVGSAGSDEK			Carbamidomethyl (C)[4]		Mascot
1704.7687	1704.751	-0.0177	-10	282	295	MEGFIVTDHYGTYR			Oxidation (M)[1]		Mascot
2097.9797	2097.9773	-0.0024	-1	17	35	YVTGFPSEDDMELVPAT AR					Mascot
2097.9797	2097.9773	-0.0024	-1	17	35	YVTGFPSEDDMELVPAT AR	28	0			Mascot
2113.9746	2113.9873	0.0127	6	17	35	YVTGFPSEDDMELVPAT AR			Oxidation (M)[11]		Mascot
2138.0547	2138.0818	0.0271	13	252	270	VSVCGLISQYNLEQSEGV R			Carbamidomethyl (C)[4]		Mascot
2138.0547	2138.0818	0.0271	13	252	270	VSVCGLISQYNLEQSEGV R	170	100	Carbamidomethyl (C)[4]		Mascot
2279.2395	2279.2351	-0.0044	-2	156	178	KGEYVFVSAASGAVGQL VGQLAK					Mascot
2759.3193	2759.342	0.0227	8	62	87	MSGNDEPSHVPDFVQGE VLTTLGVSK			Oxidation (M)[1]		Mascot
2760.4131	2760.4214	0.0083	3	310	334	ITYVEDVAEGIESFPTALI GLFYGR					Mascot

3	putative NADP-dependent oxidoreductase P1 [Triticum urartu]	gi|474246662	38736.6	5.75	6	160	100	18.834	142	100	

Peptide Information

Calc. Mass	Obsrv. Mass	± da	± ppm	Start Seq.	End Seq.	Sequence	Ion Score	C. I. %	Modification	Rank	Result Type
1223.5369	1223.5114	-0.0255	-21	204	213	FGFDDAFNYK					Mascot
1223.5369	1223.5114	-0.0255	-21	204	213	FGFDDAFNYK	58	99.703			Mascot
1232.5504	1232.6123	0.0619	50	300	309	FEEEMAGYLK			Oxidation (M)[5]		Mascot
1360.6454	1360.6233	-0.0221	-16	299	309	KFEEEMAGYLK			Oxidation (M)[6]		Mascot
1413.6831	1413.6869	0.0038	3	52	62	NLYLSCDPYLR			Carbamidomethyl (C)[6]		Mascot
1413.6831	1413.6869	0.0038	3	52	62	NLYLSCDPYLR	85	100	Carbamidomethyl (C)[6]		Mascot

Calc. Mass	Obsrv. Mass	± da	± ppm	Start Seq.	End Seq.	Sequence	Ion Score	C. I. %	Modification	Rank	Result Type
1528.6948	1528.6652	-0.0296	-19	182	196	ISGCYVVGSAGSDEK			Carbamidomethyl (C)[4]		Mascot
2046.9524	2047.0742	0.1218	60	283	298	IRMEGFIVMDHYSNYR			Oxidation (M)[3]		Mascot
2046.9524	2047.0742	0.1218	60	283	298	IRMEGFIVMDHYSNYR			Oxidation (M)[3]		Mascot

4	oxidoreductase, zinc-binding dehydrogenase family protein [Oryza sativa Japonica Group]	gi|108862362	35522.1	5.29	3	149	100	16.569	142	100

Peptide Information

Calc. Mass	Obsrv. Mass	± da	± ppm	Start Seq.	End Seq.	Sequence	Ion Score	C. I. %	Modification	Rank	Result Type
1145.6161	1145.5896	-0.0265	-23	188	197	ETDLEAALKR					Mascot
1223.5369	1223.5114	-0.0255	-21	177	186	FGFDDAFNYK					Mascot
1223.5369	1223.5114	-0.0255	-21	177	186	FGFDDAFNYK	58	99.703			Mascot
1413.6831	1413.6869	0.0038	3	52	62	NLYISCDPYLR			Carbamidomethyl (C)[6]		Mascot
1413.6831	1413.6869	0.0038	3	52	62	NLYISCDPYLR	85	100	Carbamidomethyl (C)[6]		Mascot

5	unnamed protein product [Vitis vinifera]	gi|296084352	31108.7	6.45	5	100	99.989	15.88	85	100

Peptide Information

Calc. Mass	Obsrv. Mass	± da	± ppm	Start Seq.	End Seq.	Sequence	Ion Score	C. I. %	Modification	Rank	Result Type
802.4603	802.449	-0.0113	-14	258	264	IAACVLR			Carbamidomethyl (C)[4]		Mascot
844.5138	844.4587	-0.0551	-65	198	204	EKVDLLK					Mascot
856.5363	856.5088	-0.0275	-32	14	20	NKQVVLR					Mascot
1413.6831	1413.6869	0.0038	3	55	65	NLYLSCDPYIR			Carbamidomethyl (C)[6]		Mascot
1413.6831	1413.6869	0.0038	3	55	65	NLYLSCDPYIR	85	100	Carbamidomethyl (C)[6]		Mascot
1567.8149	1567.7993	-0.0156	-10	185	199	LLGCYVVGSAGSKEK			Carbamidomethyl (C)[4]		Mascot

6	hypothetical protein PRUPE_ppa008058mg [Prunus persica]	gi|462401937	38616.6	5.94	5	96	99.978	16.106	85	100

Peptide Information

Calc. Mass	Obsrv. Mass	± da	± ppm	Start Seq.	End Seq.	Sequence	Ion Score	C. I. %	Modification	Rank	Result Type
844.5138	844.4587	-0.0551	-65	193	199	EKVDLLK					Mascot
856.5251	856.5088	-0.0163	-19	37	44	LKVPQGSK					Mascot
1128.5685	1128.549	-0.0195	-17	89	98	VLDSGHPEFK					Mascot
1360.6301	1360.6233	-0.0068	-5	25	36	ESDMYVTTSSIK					Mascot
1413.6831	1413.6869	0.0038	3	50	60	NLYLSCDPYLR			Carbamidomethyl (C)[6]		Mascot
1413.6831	1413.6869	0.0038	3	50	60	NLYLSCDPYLR	85	100	Carbamidomethyl (C)[6]		Mascot

7	PREDICTED: NADP-dependent alkenal double bond	gi|470101423	38289.5	7.01	3	91	99.928	15.363	85	100

reductase P1-like [Fragaria vesca subsp. vesca]

Peptide Information

Calc. Mass	Obsrv. Mass	± da	± ppm	Start Seq.	End Seq.	Sequence	Ion Score	C. I. %	Modification	Rank	Result Type
844.441	844.4587	0.0177	21	89	96	VLESGDPK					Mascot
1413.6831	1413.6869	0.0038	3	50	60	NLYLSCDPYLR			Carbamidomethyl (C)[6]		Mascot
1413.6831	1413.6869	0.0038	3	50	60	NLYLSCDPYLR	85	100	Carbamidomethyl (C)[6]		Mascot
1426.736	1426.7145	-0.0215	-15	180	193	LTGCYVVGSAGSKK			Carbamidomethyl (C)[4]		Mascot

8 PREDICTED: NADP-dependent alkenal double bond reductase P2-like [Fragaria vesca subsp. vesca] gi|470101421 38164.4 6.67 3 91 99.926 15.818 85 100

Peptide Information

Calc. Mass	Obsrv. Mass	± da	± ppm	Start Seq.	End Seq.	Sequence	Ion Score	C. I. %	Modification	Rank	Result Type
844.441	844.4587	0.0177	21	86	93	VLESGDPK					Mascot
1413.6831	1413.6869	0.0038	3	47	57	NLYLSCDPYLR			Carbamidomethyl (C)[6]		Mascot
1413.6831	1413.6869	0.0038	3	47	57	NLYLSCDPYLR	85	100	Carbamidomethyl (C)[6]		Mascot
1528.7499	1528.6652	-0.0847	-55	1	13	MASEMSNKQVIFK			Oxidation (M)[1]		Mascot

9 Os12g0226300 [Oryza sativa Japonica Group] gi|255670159 16680.4 5.3 1 89 99.872 15.076 85 100

Peptide Information

Calc. Mass	Obsrv. Mass	± da	± ppm	Start Seq.	End Seq.	Sequence	Ion Score	C. I. %	Modification	Rank	Result Type
1413.6831	1413.6869	0.0038	3	52	62	NLYISCDPYLR			Carbamidomethyl (C)[6]		Mascot
1413.6831	1413.6869	0.0038	3	52	62	NLYISCDPYLR	85	100	Carbamidomethyl (C)[6]		Mascot

10 hypothetical protein CARUB_v10011078mg [Capsella rubella] gi|482574614 38930.7 5.94 7 82 99.372 5.385 58 99.703

Peptide Information

Calc. Mass	Obsrv. Mass	± da	± ppm	Start Seq.	End Seq.	Sequence	Ion Score	C. I. %	Modification	Rank	Result Type
844.5138	844.4587	-0.0551	-65	197	203	EKVDLLK					Mascot
1129.6464	1129.574	-0.0724	-64	40	50	LPEGSTSVLVK					Mascot
1223.5369	1223.5114	-0.0255	-21	206	215	FGFDDAFNYK					Mascot
1223.5369	1223.5114	-0.0255	-21	206	215	FGFDDAFNYK	58	99.703			Mascot
1236.5712	1236.5498	-0.0214	-17	1	12	MANAEAAMVSNK					Mascot
1626.8818	1626.939	0.0572	35	243	256	MLDAVLINMKLHGR			Oxidation (M)[1]		Mascot

1626.8818	1626.939	0.0572	35	243	256	MLDAVLINMKLHGR	Oxidation (M)[1]	Mascot
1642.8768	1642.754	-0.1228	-75	243	256	MLDAVLINMKLHGR	Oxidation (M)[1,9]	Mascot
1660.9098	1660.7534	-0.1564	-94	299	311	YSKFLDFVLPYIR		Mascot
2760.3372	2760.4214	0.0842	31	1	25	MANAEAAMVSNKQIIFPD YVSGFPK	Oxidation (M)[1,8]	Mascot

Gel Idx/Pos	273/L1	Instr./Gel Origin	BA2151/Sample Project 20140814	Process Status	Analysis Succeeded
Plate [#] Name	[1] Sample Project 20140814	Instrument Sample Name		Spectra	11

Rank	Protein Name	Accession No.	Protein MW	Protein PI	Pep. Count	Protein Score	Protein Score C. I. %	Intensity Matched	Total Ion Score	Total Ion C. I. %	Confirmed
1	Globulin-1 S allele [Triticum urartu]	gi|474411419	57108.4	9.1	12	284	100	6.944	248	100	

Peptide Information

Calc. Mass	Obsrv. Mass	± da	± ppm	Start Seq.	End Seq.	Sequence	Ion Score	C. I. %	Modification	Rank	Result Type
818.4003	818.4104	0.0101	12	226	232	ASEEQVR					Mascot
823.4645	823.4	-0.0645	-78	269	275	IANRHGR					Mascot
837.4101	837.4008	-0.0093	-11	276	282	LYEADAR					Mascot
906.468	906.4623	-0.0057	-6	457	463	EVQEVFR					Mascot
990.5214	990.4847	-0.0367	-37	195	203	AALKTSDER					Mascot
1187.5917	1187.6055	0.0138	12	273	282	HGRLYEADAR					Mascot
1280.6495	1280.6497	0.0002	0	283	293	SFHALAQHDVR					Mascot
1280.6495	1280.6497	0.0002	0	283	293	SFHALAQHDVR	51	98.524			Mascot
1320.5452	1320.6154	0.0702	53	349	358	WGEEEEDDRR					Mascot
1791.8984	1791.7439	-0.1545	-86	254	268	GDSRDTYNLLEQRPK					Mascot
1822.8752	1822.8926	0.0174	10	411	426	GSSNLQVVCFEINAER			Carbamidomethyl (C)[9]		Mascot
1822.8752	1822.8926	0.0174	10	411	426	GSSNLQVVCFEINAER	110	100	Carbamidomethyl (C)[9]		Mascot
1906.0182	1906.0371	0.0189	10	392	410	GSAFVVPPGHPVVEIASS R					Mascot
1906.0182	1906.0371	0.0189	10	392	410	GSAFVVPPGHPVVEIASS R	87	100			Mascot
2176.1431	2176.1775	0.0344	16	316	335	LAVVLEGEGEVEIVCPHL GR			Carbamidomethyl (C)[15]		Mascot

Rank	Protein Name	Accession No.	Protein MW	Protein PI	Pep. Count	Protein Score	Protein Score C. I. %	Intensity Matched	Total Ion Score	Total Ion C. I. %	Confirmed
2	PREDICTED: uncharacterized protein LOC101253008 [Solanum lycopersicum]	gi|460398290	304186.6	4.82	44	75	96.85	14.986			

Peptide Information

Calc. Mass	Obsrv. Mass	± da	± ppm	Start Seq.	End Seq.	Sequence	Ion Score	C. I. %	Modification	Rank	Result Type
800.4189	800.4175	-0.0014	-2	1423	1428	ETFYLK					Mascot
803.4258	803.4163	-0.0095	-12	2030	2037	SGVDQLGK					Mascot
806.3712	806.3817	0.0105	13	2377	2382	IEEMER					Mascot
809.4338	809.4114	-0.0224	-28	386	391	FLLTCR			Carbamidomethyl (C)[5]		Mascot
813.4213	813.4298	0.0085	10	2137	2143	QHSNSLK					Mascot
816.4574	816.4218	-0.0356	-44	40	47	TDLLAAGR					Mascot

818.473	818.4104	-0.0626	-76	1338	1344	VSSLREK		Mascot
821.4185	821.4147	-0.0038	-5	2313	2318	LMEKER	Oxidation (M)[2]	Mascot
850.4305	850.4283	-0.0022	-3	1513	1519	FEELQGK		Mascot
851.4257	851.438	0.0123	14	2220	2226	FTEEGIR		Mascot
854.444	854.4067	-0.0373	-44	1140	1146	IYGNLMK	Oxidation (M)[6]	Mascot
856.5614	856.4946	-0.0668	-78	2383	2389	LLLQKNK		Mascot
866.356	866.4195	0.0635	73	170	176	MVNEDDK	Oxidation (M)[1]	Mascot
870.5519	870.5239	-0.028	-32	1352	1359	GKGLIVQR		Mascot
887.3676	887.4447	0.0771	87	812	818	HMEEADR		Mascot
888.4785	888.4429	-0.0356	-40	2099	2106	EIGSINQK		Mascot
906.4778	906.4623	-0.0155	-17	660	667	SLLSETEK		Mascot
944.5523	944.4589	-0.0934	-99	40	48	TDLLAAGRK		Mascot
999.5105	999.4688	-0.0417	-42	392	401	DSAPNAPSLK		Mascot
1023.5105	1023.508	-0.0025	-2	1209	1216	DFEELSKR		Mascot
1037.5449	1037.5135	-0.0314	-30	2555	2562	EFQLTMLR		Mascot
1053.5398	1053.4933	-0.0465	-44	2555	2562	EFQLTMLR	Oxidation (M)[6]	Mascot
1057.5272	1057.5293	0.0021	2	2010	2018	LSSIHSEER		Mascot
1060.5997	1060.5491	-0.0506	-48	569	578	KLGTDLAQSK		Mascot
1081.5269	1081.5199	-0.007	-6	755	763	IMELSETMK		Mascot
1177.5518	1177.5564	0.0046	4	1664	1673	LNEMLGAEER	Oxidation (M)[4]	Mascot
1187.6379	1187.6055	-0.0324	-27	1263	1272	EAIEDVRLSR		Mascot
1212.5604	1212.574	0.0136	11	778	788	EEAGHQLEGSR		Mascot
1212.5604	1212.574	0.0136	11	778	788	EEAGHQLEGSR		Mascot
1232.6229	1232.6024	-0.0205	-17	979	989	QEVATKAENSR		Mascot
1259.6968	1259.6522	-0.0446	-35	1674	1683	IHHLEGEIRR		Mascot
1262.666	1262.6086	-0.0574	-45	2342	2352	VKSLEDMLEAK		Mascot
1280.6667	1280.6497	-0.017	-13	2553	2562	DKEFQLTMLR		Mascot
1280.6667	1280.6497	-0.017	-13	2553	2562	DKEFQLTMLR		Mascot
1331.6987	1331.6564	-0.0423	-32	2155	2165	EKDAELLMVQR		Mascot
1333.7145	1333.6558	-0.0587	-44	2144	2154	TDLMRLESIQK		Mascot
1414.79	1414.7664	-0.0236	-17	2256	2268	AAIASLQKELQDK		Mascot
1427.7676	1427.7773	0.0097	7	1001	1012	LPILQNQLGEMR	Oxidation (M)[11]	Mascot
1471.8367	1471.7223	-0.1144	-78	1770	1782	KLEDALNDLLSLK		Mascot
1513.8009	1513.7412	-0.0597	-39	1416	1428	NSATALRETFYLK		Mascot
1655.7866	1655.8416	0.055	33	755	768	IMELSETMKTESPK	Oxidation (M)[2,8]	Mascot
1674.8657	1674.775	-0.0907	-54	1783	1797	EEKESTALANQSLVR		Mascot
1835.9135	1835.9231	0.0096	5	495	510	GEIGEFSDRINELQTK		Mascot

Calc. Mass	Obsrv. Mass	± da	± ppm	Start Seq.	End Seq.	Sequence	Ion Score	C. I. %	Modification	Rank	Result Type
1905.9475	1906.0371	0.0896	47	184	202	GTIASDMSTISPATDVPVK			Oxidation (M)[7]		Mascot
1905.9475	1906.0371	0.0896	47	184	202	GTIASDMSTISPATDVPVK			Oxidation (M)[7]		Mascot
2117.116	2117.0945	-0.0215	-10	2503	2520	EMIEKQVVAVISELEDLR			Oxidation (M)[2]		Mascot
2176.0703	2176.1775	0.1072	49	2292	2309	SYLQELQIAKSEMDDLHR					Mascot
2317.9912	2318.0894	0.0982	42	1017	1036	EMGSCISNQVEGLYEEVSDR			Carbamidomethyl (C)[5], Oxidation (M)[2]		Mascot

3	P-loop containing nucleoside triphosphate hydrolases superfamily protein, putative isoform 3 [Theobroma cacao]	gi|508723775	95022	5.79	25	68	86.251	6.263

Peptide Information

Calc. Mass	Obsrv. Mass	± da	± ppm	Start Seq.	End Seq.	Sequence	Ion Score	C. I. %	Modification	Rank	Result Type
821.4437	821.4147	-0.029	-35	661	667	MLSETLK					Mascot
837.4386	837.4008	-0.0378	-45	661	667	MLSETLK			Oxidation (M)[1]		Mascot
851.4621	851.438	-0.0241	-28	255	261	EGKYINK					Mascot
865.5029	865.4307	-0.0722	-83	61	67	VYELLTK					Mascot
872.4836	872.428	-0.0556	-64	533	539	QELQNLK					Mascot
874.4265	874.411	-0.0155	-18	759	766	TVSESSHK					Mascot
888.4938	888.4429	-0.0509	-57	458	466	AAPNAFVAK					Mascot
905.4873	905.4537	-0.0336	-37	378	384	LRNDMLK			Oxidation (M)[5]		Mascot
944.5411	944.4589	-0.0822	-87	767	774	LTLLNDQK					Mascot
990.5731	990.4847	-0.0884	-89	575	582	QEALFVRK					Mascot
1003.5306	1003.5089	-0.0217	-22	789	796	KDLEEEIK					Mascot
1031.548	1031.5179	-0.0301	-29	707	715	LSNELNSVR					Mascot
1033.5598	1033.5012	-0.0586	-57	566	574	LMGELTELK					Mascot
1049.5547	1049.4989	-0.0558	-53	566	574	LMGELTELK			Oxidation (M)[2]		Mascot
1053.4703	1053.4933	0.023	22	587	595	LCESVASCK			Carbamidomethyl (C)[2,8]		Mascot
1057.6	1057.5293	-0.0707	-67	354	361	LEIEELRR					Mascot
1081.5413	1081.5199	-0.0214	-20	596	604	DIYEDVLSK					Mascot
1177.5405	1177.5564	0.0159	14	392	400	LEMELEEER					Mascot
1187.6816	1187.6055	-0.0761	-64	262	272	SLMVLGNVINK					Mascot
1192.5164	1192.5261	0.0097	8	187	196	HFGETNMNAR			Oxidation (M)[7]		Mascot
1240.7008	1240.6106	-0.0902	-73	823	833	LRAENPVSVQK					Mascot
1333.6416	1333.6558	0.0142	11	392	401	LEMELEEERR					Mascot
1404.704	1404.7167	0.0127	9	775	786	ENVLMDLNTEVK					Mascot
1430.7485	1430.6456	-0.1029	-72	754	766	DSLLKTVSESSHK					Mascot
1447.7726	1447.689	-0.0836	-58	200	211	SHTIFRMVIESK					Mascot

1471.8115	1471.7223	-0.0892	-61	229	242	VSVLNLVDLAGSER					Mascot
1565.7233	1565.6531	-0.0702	-45	583	595	MPQRLCESVASCK			Carbamidomethyl (C)[6,12]		Mascot

4 PREDICTED: serine/threonine-protein kinase TOR-like [Solanum lycopersicum] gi|460369660 279995.8 6.66 36 68 84.924 10.532

Peptide Information

Calc. Mass	Obsrv. Mass	± da	± ppm	Start Seq.	End Seq.	Sequence	Ion Score	C. I. %	Modification	Rank	Result Type
809.39	809.4114	0.0214	26	2151	2156	EYRDAR					Mascot
814.5032	814.4454	-0.0578	-71	1298	1305	LLGALAEK					Mascot
816.4574	816.4218	-0.0356	-44	38	45	DGAALTLR					Mascot
818.4744	818.4104	-0.064	-78	1114	1119	HRLQHK					Mascot
819.3955	819.3972	0.0017	2	1187	1194	TAGEASQR					Mascot
823.4349	823.4	-0.0349	-42	914	919	EFITWK					Mascot
825.3784	825.409	0.0306	37	447	453	SHHAMSR					Mascot
829.489	829.4117	-0.0773	-93	2	9	AATVQAIR					Mascot
834.4025	834.3798	-0.0227	-27	907	913	ICEDGLK			Carbamidomethyl (C)[2]		Mascot
850.4781	850.4283	-0.0498	-59	114	120	VAFETKR					Mascot
851.3927	851.438	0.0453	53	654	660	CKEESAK			Carbamidomethyl (C)[1]		Mascot
856.4523	856.4946	0.0423	49	1214	1221	ESPSPALR					Mascot
865.3621	865.4307	0.0686	79	1567	1572	NMWNER			Oxidation (M)[2]		Mascot
874.4629	874.411	-0.0519	-59	1036	1043	VDASVEVR					Mascot
906.4825	906.4623	-0.0202	-22	2399	2406	MSNKLTGR					Mascot
928.5462	928.4558	-0.0904	-97	278	285	EVAEIVLR					Mascot
1057.5426	1057.5293	-0.0133	-13	493	501	GHDLLEFAR					Mascot
1177.5881	1177.5564	-0.0317	-27	1670	1679	LQDLAMDLSR			Oxidation (M)[6]		Mascot
1212.6696	1212.574	-0.0956	-79	34	45	GNPKDGAALTLR					Mascot
1212.6696	1212.574	-0.0956	-79	34	45	GNPKDGAALTLR					Mascot
1228.6797	1228.6349	-0.0448	-36	153	162	VALEWLRGER					Mascot
1228.6797	1228.6349	-0.0448	-36	153	162	VALEWLRGER					Mascot
1240.5627	1240.6106	0.0479	39	237	247	MFEATQDGLGR			Oxidation (M)[1]		Mascot
1259.6743	1259.6522	-0.0221	-18	777	787	LLNGELAWSTR					Mascot
1262.5801	1262.6086	0.0285	23	1653	1662	YQWSLGEDHK					Mascot
1273.6107	1273.6464	0.0357	28	1732	1742	NATHCATKWGK			Carbamidomethyl (C)[5]		Mascot
1344.6688	1344.6713	0.0025	2	1214	1225	ESPSPALRTCAR			Carbamidomethyl (C)[10]		Mascot
1486.8199	1486.7845	-0.0354	-24	793	806	VLGIMGALDPHVHK					Mascot
1513.7758	1513.7412	-0.0346	-23	1116	1127	LQHKEFEEIQGR					Mascot

1523.79	1523.6859	-0.1041	-68	2239	2251	HPSNLMLHRFSGK					Mascot
1554.7554	1554.7832	0.0278	18	447	460	SHHAMSRQSAALSR			Oxidation (M)[5]		Mascot
1655.8898	1655.8416	-0.0482	-29	373	388	GRPSLEALACVGNIAK			Carbamidomethyl (C)[10]		Mascot
1714.8728	1714.8402	-0.0326	-19	2297	2310	STCENVMQVLRLHK			Carbamidomethyl (C)[3]		Mascot
1791.8872	1791.7439	-0.1433	-80	47	62	LVEEEARDLSGEAFAR					Mascot
2120.002	2120.0562	0.0542	26	231	247	VQWYYRMFEATQDGLG R					Mascot
2176.0776	2176.1775	0.0999	46	981	998	YLPDILPCCIQVLTDAER			Carbamidomethyl (C)[8,9]		Mascot
2303.1853	2303.0713	-0.114	-49	1801	1820	LLTLWFNHGATSEVQMA LQK			Oxidation (M)[16]		Mascot
2318.1558	2318.0894	-0.0664	-29	1384	1405	AYTAKASQASSPHLCLDA TLGR			Carbamidomethyl (C)[15]		Mascot

5 P-loop containing nucleoside triphosphate hydrolases superfamily protein isoform 1 [Theobroma cacao] gi|508723773 95468.1 5.79 25 68 83.846 6.263

Peptide Information

Calc. Mass	Obsrv. Mass	± da	± ppm	Start Seq.	End Seq.	Sequence	Ion Score	C. I. %	Modification	Rank	Result Type
821.4437	821.4147	-0.029	-35	661	667	MLSETLK					Mascot
837.4386	837.4008	-0.0378	-45	661	667	MLSETLK			Oxidation (M)[1]		Mascot
851.4621	851.438	-0.0241	-28	255	261	EGKYINK					Mascot
865.5029	865.4307	-0.0722	-83	61	67	VYELLTK					Mascot
872.4836	872.428	-0.0556	-64	533	539	QELQNLK					Mascot
874.4265	874.411	-0.0155	-18	763	770	TVSESSHK					Mascot
888.4938	888.4429	-0.0509	-57	458	466	AAPNAFVAK					Mascot
905.4873	905.4537	-0.0336	-37	378	384	LRNDMLK			Oxidation (M)[5]		Mascot
944.5411	944.4589	-0.0822	-87	771	778	LTLLNDQK					Mascot
990.5731	990.4847	-0.0884	-89	575	582	QEALFVRK					Mascot
1003.5306	1003.5089	-0.0217	-22	793	800	KDLEEEIK					Mascot
1031.548	1031.5179	-0.0301	-29	711	719	LSNELNSVR					Mascot
1033.5598	1033.5012	-0.0586	-57	566	574	LMGELTELK					Mascot
1049.5547	1049.4989	-0.0558	-53	566	574	LMGELTELK			Oxidation (M)[2]		Mascot
1053.4703	1053.4933	0.023	22	587	595	LCESVASCK			Carbamidomethyl (C)[2,8]		Mascot
1057.6	1057.5293	-0.0707	-67	354	361	LEIEELRR					Mascot
1081.5413	1081.5199	-0.0214	-20	596	604	DIYEDVLSK					Mascot
1177.5405	1177.5564	0.0159	14	392	400	LEMELEEER					Mascot
1187.6816	1187.6055	-0.0761	-64	262	272	SLMVLGNVINK					Mascot
1192.5164	1192.5261	0.0097	8	187	196	HFGETNMNAR			Oxidation (M)[7]		Mascot
1240.7008	1240.6106	-0.0902	-73	827	837	LRAENPVSVQK					Mascot

1333.6416	1333.6558	0.0142	11	392	401	LEMELEEERR					Mascot
1404.704	1404.7167	0.0127	9	779	790	ENVLMDLNTEVK					Mascot
1430.7485	1430.6456	-0.1029	-72	758	770	DSLLKTVSESSHK					Mascot
1447.7726	1447.689	-0.0836	-58	200	211	SHTIFRMVIESK					Mascot
1471.8115	1471.7223	-0.0892	-61	229	242	VSVLNLVDLAGSER					Mascot
1565.7233	1565.6531	-0.0702	-45	583	595	MPQRLCESVASCK			Carbamidomethyl (C)[6,12]		Mascot

6	hypothetical protein ZEAMMB73_139941 [Zea mays]	gi|413916567	95608.9	8.54	22	68	83.47	6.896

Peptide Information

Calc. Mass	Obsrv. Mass	± da	± ppm	Start Seq.	End Seq.	Sequence	Ion Score	C. I. %	Modification	Rank	Result Type
809.4152	809.4114	-0.0038	-5	653	659	SSPSIYR					Mascot
812.4148	812.4257	0.0109	13	150	156	SFSESKK					Mascot
818.4366	818.4104	-0.0262	-32	272	278	QNNLTTK					Mascot
827.3893	827.4085	0.0192	23	817	823	YEATSTR					Mascot
856.4523	856.4946	0.0423	49	663	671	GNATPATPK					Mascot
887.4944	887.4447	-0.0497	-56	354	360	VLEENRK					Mascot
900.4131	900.4651	0.052	58	461	468	TYTMSGPK			Oxidation (M)[4]		Mascot
944.5411	944.4589	-0.0822	-87	607	616	VASVELGAAK					Mascot
999.4022	999.4688	0.0666	67	182	189	NCDAFMNK			Carbamidomethyl (C)[2]		Mascot
1003.5167	1003.5089	-0.0078	-8	542	551	AVGSTAINDR					Mascot
1037.531	1037.5135	-0.0175	-17	370	377	GNIRVYCR			Carbamidomethyl (C)[7]		Mascot
1049.5626	1049.4989	-0.0637	-61	253	260	VIQEYELR					Mascot
1060.5382	1060.5491	0.0109	10	617	625	QNKEGSEIR					Mascot
1262.7103	1262.6086	-0.1017	-81	360	369	KLYNQIQDLK					Mascot
1273.6608	1273.6464	-0.0144	-11	540	551	NRAVGSTAINDR					Mascot
1333.6819	1333.6558	-0.0261	-20	542	554	AVGSTAINDRSSR					Mascot
1340.5835	1340.6417	0.0582	43	182	192	NCDAFMNKNAR			Carbamidomethyl (C)[2]		Mascot
1353.7448	1353.6858	-0.059	-44	399	410	TITIMTPTKYGK					Mascot
1360.624	1360.7036	0.0796	59	12	25	WGGGSVGDDNVAAR					Mascot
1360.624	1360.7036	0.0796	59	12	25	WGGGSVGDDNVAAR					Mascot
1380.6802	1380.6982	0.018	13	555	566	SHSCLTVHVQGR			Carbamidomethyl (C)[4]		Mascot
1447.7904	1447.689	-0.1014	-70	603	616	FAERVASVELGAAK					Mascot
2120.0117	2120.0562	0.0445	21	441	460	SVLDGFNVCIFAYGQTGSGK			Carbamidomethyl (C)[9]		Mascot

7	Globulin-1 S allele [Aegilops tauschii]	gi|475588764	62687.5	9.44	9	66	74.98	3.642	51	98.524	

Peptide Information

Calc. Mass	Obsrv. Mass	± da	± ppm	Start Seq.	End Seq.	Sequence	Ion Score	C. I. %	Modification	Rank	Result Type
803.4046	803.4163	0.0117	15	150	156	TDHGFVK					Mascot
823.4645	823.4	-0.0645	-78	353	359	IANRHGR					Mascot
837.4101	837.4008	-0.0093	-11	360	366	LYEADAR					Mascot
906.468	906.4623	-0.0057	-6	523	529	EVQEVFR					Mascot
990.5214	990.4847	-0.0367	-37	279	287	AALKTSDER					Mascot
1187.5917	1187.6055	0.0138	12	357	366	HGRLYEADAR					Mascot
1280.6495	1280.6497	0.0002	0	367	377	SFHALAQHDVR					Mascot
1280.6495	1280.6497	0.0002	0	367	377	SFHALAQHDVR	51	98.524			Mascot
1684.8766	1684.8519	-0.0247	-15	508	522	LDNPAQELTFGRPAR					Mascot
1791.8984	1791.7439	-0.1545	-86	338	352	GDSRDTYNLLEQRPK					Mascot

8 conserved hypothetical protein [Ricinus communis] gi|223542582 9997.9 11.55 10 65 67.769 3.631

Peptide Information

Calc. Mass	Obsrv. Mass	± da	± ppm	Start Seq.	End Seq.	Sequence	Ion Score	C. I. %	Modification	Rank	Result Type
816.4686	816.4218	-0.0468	-57	42	48	VQQTRGK					Mascot
818.3825	818.4104	0.0279	34	12	18	DCTPVAR			Carbamidomethyl (C)[2]		Mascot
821.4008	821.4147	0.0139	17	50	56	MMPLASR			Oxidation (M)[1]		Mascot
836.3931	836.4023	0.0092	11	1	7	MSKEDAR					Mascot
837.3957	837.4008	0.0051	6	50	56	MMPLASR			Oxidation (M)[1,2]		Mascot
874.4854	874.411	-0.0744	-85	40	46	SRVQQTR					Mascot
1033.4844	1033.5012	0.0168	16	4	11	EDARQQMR					Mascot
1049.4792	1049.4989	0.0197	19	4	11	EDARQQMR			Oxidation (M)[7]		Mascot
1057.4653	1057.5293	0.064	61	82	90	LDTTAMMSR			Oxidation (M)[6,7]		Mascot
1228.6393	1228.6349	-0.0044	-4	70	80	SSHQLAKNSTR					Mascot
1228.6393	1228.6349	-0.0044	-4	70	80	SSHQLAKNSTR	9	0			Mascot
1380.6722	1380.6982	0.026	19	50	62	MMPLASRSSSASR					Mascot
2175.0347	2175.1843	0.1496	69	19	39	SQSSCEVAGHSTQAISEL VGK			Carbamidomethyl (C)[5]		Mascot

9 PREDICTED: myosin-H heavy chain-like [Solanum lycopersicum] gi|460389666 173093.9 8.34 35 65 66.25 12.213

Peptide Information

Calc. Mass	Obsrv. Mass	± da	± ppm	Start Seq.	End Seq.	Sequence	Ion Score	C. I. %	Modification	Rank	Result Type
800.4737	800.4175	-0.0562	-70	869	875	VARGELR					Mascot

803.4734	803.4163	-0.0571	-71	1285	1292	GSVLKSGR		Mascot
809.3345	809.4114	0.0769	95	913	919	ADMEEAK	Oxidation (M)[3]	Mascot
818.4254	818.4104	-0.015	-18	975	981	LSVENEK		Mascot
820.4424	820.4069	-0.0355	-43	824	829	NEVRFR		Mascot
830.4843	830.4454	-0.0389	-47	731	737	RTEVLGR		Mascot
851.5025	851.438	-0.0645	-76	1241	1247	YPALLFK		Mascot
856.457	856.4946	0.0376	44	746	752	VRSHMAR		Mascot
872.4519	872.428	-0.0239	-27	746	752	VRSHMAR	Oxidation (M)[5]	Mascot
888.4785	888.4429	-0.0356	-40	883	891	ETGALQAAK		Mascot
900.5374	900.4651	-0.0723	-80	227	235	GRISGAAIR		Mascot
928.4999	928.4558	-0.0441	-47	271	277	YKLDHPR		Mascot
990.5466	990.4847	-0.0619	-62	984	992	SLVSSLEQK		Mascot
1003.5101	1003.5089	-0.0012	-1	819	827	GMAARNEVR		Mascot
1023.4966	1023.508	0.0114	11	209	217	NNNSSRFGK		Mascot
1037.5739	1037.5135	-0.0604	-58	236	243	TYLLERSR		Mascot
1049.4932	1049.4989	0.0057	5	337	346	GKEMDSSAPK		Mascot
1060.5422	1060.5491	0.0069	7	897	904	QVEELTWR		Mascot
1091.5514	1091.5485	-0.0029	-3	367	375	ALEDSLCKR	Carbamidomethyl (C)[7]	Mascot
1177.5782	1177.5564	-0.0218	-19	857	866	AAITTQCAWR	Carbamidomethyl (C)[7]	Mascot
1212.579	1212.574	-0.005	-4	1026	1034	TTMQRFQER	Oxidation (M)[3]	Mascot
1212.579	1212.574	-0.005	-4	1026	1034	TTMQRFQER	Oxidation (M)[3]	Mascot
1228.6321	1228.6349	0.0028	2	387	397	WLDPEAAAISR		Mascot
1228.6321	1228.6349	0.0028	2	387	397	WLDPEAAAISR		Mascot
1302.6688	1302.6412	-0.0276	-21	599	610	QSKFSSIGSSFK		Mascot
1320.6511	1320.6154	-0.0357	-27	765	775	IQSMCRGELAR	Carbamidomethyl (C)[5]	Mascot
1331.7029	1331.6564	-0.0465	-35	1319	1329	ENFMPPILVQK	Oxidation (M)[4]	Mascot
1333.6893	1333.6558	-0.0335	-25	879	891	MAARETGALQAAK	Oxidation (M)[1]	Mascot
1367.6624	1367.6431	-0.0193	-14	942	952	EMFVQERETAK		Mascot
1404.7166	1404.7167	0.0001	0	857	868	AAITTQCAWRAR	Carbamidomethyl (C)[7]	Mascot
1430.7638	1430.6456	-0.1182	-83	894	904	LEKQVEELTWR		Mascot
1507.6193	1507.745	0.1257	83	1351	1362	ECCTFSNAEYVK	Carbamidomethyl (C)[2,3]	Mascot
1513.7799	1513.7412	-0.0387	-26	215	226	FGKFVEIQFDQR		Mascot
1603.8955	1603.8362	-0.0593	-37	70	83	LAYLHEPGVLHNLK		Mascot
1655.8864	1655.8416	-0.0448	-27	565	578	NKDYVVAEHQALLR		Mascot
1684.9414	1684.8519	-0.0895	-53	1267	1281	KELGSLLSLCIQAPR	Carbamidomethyl (C)[10]	Mascot
1905.9099	1906.0371	0.1272	67	107	121	RLPHLYDSHMMAQYK	Oxidation (M)[10]	Mascot

1905.9099	1906.0371	0.1272	67	107	121	RLPHLYDSHMMAQYK			Oxidation (M)[10]		Mascot
2303.1667	2303.0713	-0.0954	-41	408	427	LFDWLVDKINNSIGQDPN SK					Mascot

10	predicted protein [Bathycoccus prasinos]	gi|412988322	58704.4	5.55	19	63	54.472	8.247

Peptide Information

Calc. Mass	Obsrv. Mass	± da	± ppm	Start Seq.	End Seq.	Sequence	Ion Score	C. I. %	Modification	Rank	Result Type
803.4145	803.4163	0.0018	2	204	210	ELDIEGK					Mascot
863.4655	863.4218	-0.0437	-51	1	8	MTVAAKDK					Mascot
865.3971	865.4307	0.0336	39	387	393	LSEEEMK					Mascot
888.5037	888.4429	-0.0608	-68	34	41	GTLEEVIK					Mascot
990.4924	990.4847	-0.0077	-8	72	80	DVAAVDICK			Carbamidomethyl (C)[8]		Mascot
1023.5027	1023.508	0.0053	5	150	158	ETADVLMTK			Oxidation (M)[7]		Mascot
1031.5004	1031.5179	0.0175	17	492	500	LDENNADLK					Mascot
1037.5197	1037.5135	-0.0062	-6	512	519	RAFMQQEK					Mascot
1053.5146	1053.4933	-0.0213	-20	512	519	RAFMQQEK			Oxidation (M)[4]		Mascot
1091.5415	1091.5485	0.007	6	431	439	VWANRSACK			Carbamidomethyl (C)[8]		Mascot
1187.5579	1187.6055	0.0476	40	411	420	YQDALDAYTK					Mascot
1280.7004	1280.6497	-0.0507	-40	60	70	GRGCLHLIAQR			Carbamidomethyl (C)[4]		Mascot
1280.7004	1280.6497	-0.0507	-40	60	70	GRGCLHLIAQR			Carbamidomethyl (C)[4]		Mascot
1344.7733	1344.6713	-0.102	-76	30	41	SIQKGTLEEVIK					Mascot
1360.7068	1360.7036	-0.0032	-2	48	59	DQTEVLSARDVK					Mascot
1360.7068	1360.7036	-0.0032	-2	48	59	DQTEVLSARDVK					Mascot
1404.7152	1404.7167	0.0015	1	94	107	DADGATALLIAMSR					Mascot
1507.7421	1507.745	0.0029	2	146	158	EQARETADVLMTK			Oxidation (M)[11]		Mascot
1603.7268	1603.8362	0.1094	68	387	399	LSEEEMKEAEQHK			Oxidation (M)[6]		Mascot
1622.8571	1622.7574	-0.0997	-61	34	47	GTLEEVIKSCLAFR			Carbamidomethyl (C)[10]		Mascot
1622.8571	1622.7574	-0.0997	-61	34	47	GTLEEVIKSCLAFR			Carbamidomethyl (C)[10]		Mascot
2303.2856	2303.0713	-0.2143	-93	108	129	ANALVLEYLLVDGKADAL VTSK					Mascot
2318.167	2318.0894	-0.0776	-33	247	268	IDANQRAEGGMSALHVL ASHPK			Oxidation (M)[11]		Mascot

Gel Idx/Pos	274/L2	Instr./Gel Origin	BA2151/Sample Project 20140814	Process Status	Analysis Succeeded
Plate [#] Name	[1] Sample Project 20140814	Instrument Sample Name		Spectra	11

Rank	Protein Name	Accession No.	Protein MW	Protein PI	Pep. Count	Protein Score	Protein Score C. I. %	Intensity Matched	Total Ion Score	Total Ion C. I. %	Confirmed
1	hypothetical protein F775_29537 [Aegilops tauschii]	gi|475620773	33107.5	5.96	12	823	100	36.839	751	100	

Peptide Information

Calc. Mass	Obsrv. Mass	± da	± ppm	Start Seq.	End Seq.	Sequence	Ion Score	C. I. %	Modification	Rank	Result Type
872.4737	872.4738	0.0001	0	110	116	RFVGHEK					Mascot
1248.6583	1248.6611	0.0028	2	99	109	LWDLSTGVTTR					Mascot
1335.6903	1335.699	0.0087	7	117	128	DVLSVAFSIDNR					Mascot
1335.6903	1335.699	0.0087	7	117	128	DVLSVAFSIDNR	99	100			Mascot
1455.6937	1455.6965	0.0028	2	261	272	YWLCAATQDSIK			Carbamidomethyl (C)[4]		Mascot
1602.8486	1602.8632	0.0146	9	228	241	DGVTLLWDLTEGKR					Mascot
1717.9596	1717.9836	0.024	14	280	294	HIVQDLRPEVPVSTK					Mascot
1717.9596	1717.9836	0.024	14	280	294	HIVQDLRPEVPVSTK	65	99.943			Mascot
1894.9083	1894.9457	0.0374	20	171	187	FSPNNFAPTIVSGSWDR					Mascot
1894.9083	1894.9457	0.0374	20	171	187	FSPNNFAPTIVSGSWDR	131	100			Mascot
2127.054	2127.1038	0.0498	23	242	260	LYSLDAGSIINSLCFSPNR			Carbamidomethyl (C)[14]		Mascot
2127.054	2127.1038	0.0498	23	242	260	LYSLDAGSIINSLCFSPNR	125	100	Carbamidomethyl (C)[14]		Mascot
2178.0034	2178.0469	0.0435	20	150	170	YTIGGDLGGGEGHTGWV SCVR			Carbamidomethyl (C)[19]		Mascot
2178.0034	2178.0469	0.0435	20	150	170	YTIGGDLGGGEGHTGWV SCVR	160	100	Carbamidomethyl (C)[19]		Mascot
2209.1038	2209.0549	-0.0489	-22	171	190	FSPNNFAPTIVSGSWDRS VK					Mascot
2497.2683	2497.333	0.0647	26	16	39	GHNDVVTAIATPIDNSPFI VSSSR					Mascot
2497.2683	2497.333	0.0647	26	16	39	GHNDVVTAIATPIDNSPFI VSSSR	172	100			Mascot
2923.4473	2923.5444	0.0971	33	42	67	SLLVWDLTNPIQATQDSS SEYGVPFR					Mascot

Rank	Protein Name	Accession No.	Protein MW	Protein PI	Pep. Count	Protein Score	Protein Score C. I. %	Intensity Matched	Total Ion Score	Total Ion C. I. %	Confirmed
2	Guanine nucleotide-binding protein subunit beta-like protein A [Triticum urartu]	gi|473957859	27532.7	6.29	9	706	100	32.98	652	100	

Peptide Information

Calc. Mass	Obsrv. Mass	± da	± ppm	Start Seq.	End Seq.	Sequence	Ion Score	C. I. %	Modification	Rank	Result Type
1455.6937	1455.6965	0.0028	2	211	222	YWLCAATQDSIK			Carbamidomethyl (C)[4]		Mascot
1602.8486	1602.8632	0.0146	9	178	191	DGVTLLWDLTEGKR					Mascot

1717.9596	1717.9836	0.024	14	230	244	HIVQDLRPEVPVSTK					Mascot
1717.9596	1717.9836	0.024	14	230	244	HIVQDLRPEVPVSTK	65	99.943			Mascot
1894.9083	1894.9457	0.0374	20	121	137	FSPNNFAPTIVSGSWDR					Mascot
1894.9083	1894.9457	0.0374	20	121	137	FSPNNFAPTIVSGSWDR	131	100			Mascot
2127.054	2127.1038	0.0498	23	192	210	LYSLDAGSIINSLCFSPNR			Carbamidomethyl (C)[14]		Mascot
2127.054	2127.1038	0.0498	23	192	210	LYSLDAGSIINSLCFSPNR	125	100	Carbamidomethyl (C)[14]		Mascot
2178.0034	2178.0469	0.0435	20	100	120	YTIGGDLGGGEGHTGWVSCVR			Carbamidomethyl (C)[19]		Mascot
2178.0034	2178.0469	0.0435	20	100	120	YTIGGDLGGGEGHTGWVSCVR	160	100	Carbamidomethyl (C)[19]		Mascot
2209.1038	2209.0549	-0.0489	-22	121	140	FSPNNFAPTIVSGSWDRSVK					Mascot
2497.2683	2497.333	0.0647	26	16	39	GHNDVVTAIATPIDNSPFIVSSSR					Mascot
2497.2683	2497.333	0.0647	26	16	39	GHNDVVTAIATPIDNSPFIVSSSR	172	100			Mascot
2923.4473	2923.5444	0.0971	33	42	67	SLLVWDLTNPIQATQDSSSEYGVPFR					Mascot

3	PREDICTED: guanine nucleotide-binding protein subunit beta-like protein A-like [Setaria italica]	gi|514780383	36641.3	6.35	5	112	100	3.576	99	100

Peptide Information

Calc. Mass	Obsrv. Mass	± da	± ppm	Start Seq.	End Seq.	Sequence	Ion Score	C. I. %	Modification	Rank	Result Type
872.4737	872.4738	0.0001	0	112	118	RFVGHEK					Mascot
1262.674	1262.6654	-0.0086	-7	101	111	LWDLSTGLTTR					Mascot
1335.6903	1335.699	0.0087	7	119	130	DVLSVAFSIDNR					Mascot
1335.6903	1335.699	0.0087	7	119	130	DVLSVAFSIDNR	99	100			Mascot
1455.6937	1455.6965	0.0028	2	263	274	YWLCAATQDSIK			Carbamidomethyl (C)[4]		Mascot
2134.075	2134.0605	-0.0145	-7	244	262	LYALDAGSIIHSLCFSPNR			Carbamidomethyl (C)[14]		Mascot

4	receptor for activated protein kinase C, putative [Ricinus communis]	gi|223530969	36436.5	7.59	4	109	99.999	4.015	99	100

Peptide Information

Calc. Mass	Obsrv. Mass	± da	± ppm	Start Seq.	End Seq.	Sequence	Ion Score	C. I. %	Modification	Rank	Result Type
1335.6903	1335.699	0.0087	7	107	118	DVLSVAFSIDNR					Mascot
1335.6903	1335.699	0.0087	7	107	118	DVLSVAFSIDNR	99	100			Mascot
1403.7861	1403.7765	-0.0096	-7	1	12	MAERLILLGTMR					Mascot
1455.6937	1455.6965	0.0028	2	247	258	YWLCAATENSIK			Carbamidomethyl (C)[4]		Mascot
2035.8927	2036.0845	0.1918	94	140	156	YTIQDGDAHNDWVSCVR			Carbamidomethyl (C)[15]		Mascot

5	RecName: Full=Guanine nucleotide-binding protein subunit beta-like protein	gi|3023857	36157.3	8.05	3	107	99.998	3.282	99	100

Peptide Information

Calc. Mass	Obsrv. Mass	± da	± ppm	Start Seq.	End Seq.	Sequence	Ion Score	C. I. %	Modification	Rank	Result Type
1335.6903	1335.699	0.0087	7	107	118	DVLSVAFSLDNR					Mascot
1335.6903	1335.699	0.0087	7	107	118	DVLSVAFSLDNR	99	100			Mascot
1422.6707	1422.6573	-0.0134	-9	278	292	AEAEKSDGSGTAATK					Mascot
2193.1221	2193.0671	-0.055	-25	158	177	FSPNTLQPTIVSASCDKTVK			Carbamidomethyl (C)[15]		Mascot

6	heterotrimeric guanine nucleotide-binding protein subunit beta [Eschscholzia californica]	gi|410073999	35041.6	7.64	3	106	99.998	3.359	99	100	

Peptide Information

Calc. Mass	Obsrv. Mass	± da	± ppm	Start Seq.	End Seq.	Sequence	Ion Score	C. I. %	Modification	Rank	Result Type
1335.6903	1335.699	0.0087	7	107	118	DVLSVAFSIDNR					Mascot
1335.6903	1335.699	0.0087	7	107	118	DVLSVAFSIDNR	99	100			Mascot
1433.7496	1433.7699	0.0203	14	89	100	LWDLNTGTTTRR					Mascot
2166.1277	2166.041	-0.0867	-40	228	246	LYSLPAGHIIHALCFSPNR			Carbamidomethyl (C)[14]		Mascot

7	hypothetical protein CARUB_v10009720mg [Capsella rubella]	gi|482574050	36181.4	7.62	3	106	99.998	3.296	99	100	

Peptide Information

Calc. Mass	Obsrv. Mass	± da	± ppm	Start Seq.	End Seq.	Sequence	Ion Score	C. I. %	Modification	Rank	Result Type
1262.576	1262.6654	0.0894	71	140	151	YTISEGGGEGHR					Mascot
1335.6903	1335.699	0.0087	7	107	118	DVLSVAFSLDNR					Mascot
1335.6903	1335.699	0.0087	7	107	118	DVLSVAFSLDNR	99	100			Mascot
2164.9829	2165.041	0.0581	27	140	158	YTISEGGGEGHRDWVSCVR			Carbamidomethyl (C)[17]		Mascot

8	RecName: Full=Guanine nucleotide-binding protein subunit beta-like protein	gi|3023847	36043.2	7.07	2	105	99.997	3.202	99	100	

Protein Group

PREDICTED: guanine nucleotide-binding protein subunit beta-like protein-like [Cucumis sativus]	gi|449497541	36407.3	6.7100000381 4697
PREDICTED: guanine nucleotide-binding protein subunit beta-like protein-like [Cucumis sativus]	gi|449439103	36407.3	6.7100000381 4697
uncharacterized protein LOC100778205 [Glycine max]	gi|359806172	36125.2	7.6199998855

Peptide Information

Calc. Mass	Obsrv. Mass	± da	± ppm	Start Seq.	End Seq.	Sequence	Ion Score	C. I. %	Modification	Rank	Result Type
1335.6903	1335.699	0.0087	7	107	118	DVLSVAFSIDNR					Mascot
1335.6903	1335.699	0.0087	7	107	118	DVLSVAFSIDNR	99	100			Mascot
2134.075	2134.0605	-0.0145	-7	228	246	LYSLDAGSIIHALCFSPNR			Carbamidomethyl (C)[14]		Mascot

9	PREDICTED: guanine nucleotide-binding protein subunit beta-like protein-like [Fragaria vesca subsp. vesca]	gi|470127169	36526.5	7.04	2	104	99.996	3.147	99	100	

Peptide Information

Calc. Mass	Obsrv. Mass	± da	± ppm	Start Seq.	End Seq.	Sequence	Ion Score	C. I. %	Modification	Rank	Result Type
1335.6903	1335.699	0.0087	7	108	119	DVLSVAFSIDNR					Mascot
1335.6903	1335.699	0.0087	7	108	119	DVLSVAFSIDNR	99	100			Mascot
2132.1135	2132.1035	-0.01	-5	40	57	SIILWQLNKDENSYGVPR					Mascot

10	RecName: Full=Guanine nucleotide-binding protein subunit beta-like protein	gi|3023858	35985.2	7.62	2	104	99.996	3.159	99	100	

Protein Group

guanine nucleotide-binding protein subunit beta-like protein [Glycine max]	gi|351727433	35985.2	7.6199 998855 5908

Peptide Information

Calc. Mass	Obsrv. Mass	± da	± ppm	Start Seq.	End Seq.	Sequence	Ion Score	C. I. %	Modification	Rank	Result Type
1335.6903	1335.699	0.0087	7	107	118	DVLSVAFSIDNR					Mascot
1335.6903	1335.699	0.0087	7	107	118	DVLSVAFSIDNR	99	100			Mascot
1747.8457	1747.854	0.0083	5	273	290	VDLKTEADATSGGGNAN K					Mascot

Gel Idx/Pos	275/L3	Instr./Gel Origin	BA2151/Sample Project 20140814	Process Status	Analysis Succeeded
Plate [#] Name	[1] Sample Project 20140814	Instrument Sample Name		Spectra	11

Rank	Protein Name	Accession No.	Protein MW	Protein PI	Pep. Count	Protein Score	Protein Score C. I. %	Intensity Matched	Total Ion Score	Total Ion C. I. %	Confirmed
1	PREDICTED: ATP synthase subunit alpha, mitochondrial-like [Setaria italica]	gi|514825289	55564.9	5.7	21	760	100	55.198	641	100	

Peptide Information

Calc. Mass	Obsrv. Mass	± da	± ppm	Start Seq.	End Seq.	Sequence	Ion Score	C. I. %	Modification	Rank	Result Type
815.4621	815.4589	-0.0032	-4	167	173	ELIIGDR					Mascot
860.5022	860.4783	-0.0239	-28	283	289	QMSLLLR					Mascot
876.4971	876.4769	-0.0202	-23	283	289	QMSLLLR			Oxidation (M)[2]		Mascot
884.4221	884.4244	0.0023	3	121	128	GALSDHER					Mascot
892.4886	892.4926	0.004	4	395	401	LELAQYR					Mascot
972.5473	972.5458	-0.0015	-2	33	42	VVSVGDGIAR					Mascot
1026.5942	1026.593	-0.0012	-1	154	163	AVDSLVPIGR					Mascot
1026.5942	1026.593	-0.0012	-1	154	163	AVDSLVPIGR	70	99.983			Mascot
1203.658	1203.6637	0.0057	5	7	17	AAELTTLLESR					Mascot
1242.6147	1242.5919	-0.0228	-18	143	153	SVHEPMQTGLK			Oxidation (M)[6]		Mascot
1300.7471	1300.7046	-0.0425	-33	178	189	TAIAIDTILNQK					Mascot
1341.705	1341.667	-0.038	-28	433	443	QPQYEPLPIEK					Mascot
1438.8489	1438.8525	0.0036	3	363	376	GIRPAINVGLSVSR					Mascot
1438.8489	1438.8525	0.0036	3	363	376	GIRPAINVGLSVSR	54	99.307			Mascot
1537.7434	1537.7633	0.0199	13	295	307	EAFPGDVFYLHSR					Mascot
1537.7434	1537.7633	0.0199	13	295	307	EAFPGDVFYLHSR	109	100			Mascot
1638.8268	1638.778	-0.0488	-30	388	401	QVCGSSKLELAQYR			Carbamidomethyl (C)[3]		Mascot
1690.8357	1690.8682	0.0325	19	495	509	MEPDASLKENALPYL					Mascot
1704.8262	1704.8168	-0.0094	-6	262	276	DNGMHALIIYDDLSK					Mascot
1724.8789	1724.9037	0.0248	14	444	458	QIVVIYAAVNGFCDR			Carbamidomethyl (C)[13]		Mascot
1834.8429	1834.8728	0.0299	16	18	32	MTNFYTNFQVDEIGR					Mascot
1834.8429	1834.8728	0.0299	16	18	32	MTNFYTNFQVDEIGR	130	100			Mascot
1850.8378	1850.8463	0.0085	5	18	32	MTNFYTNFQVDEIGR			Oxidation (M)[1]		Mascot
1850.8378	1850.8463	0.0085	5	18	32	MTNFYTNFQVDEIGR	167	100	Oxidation (M)[1]		Mascot
2031.9692	2031.9708	0.0016	1	195	212	GTNESETLYCVYVAIGQK			Carbamidomethyl (C)[10]		Mascot
2141.0583	2141.0762	0.0179	8	43	62	VYGLNEIQAGEMVEFAS GVK					Mascot

2157.0532	2157.0676	0.0144	7	43	62	VYGLNEIQAGEMVEFAS GVK			Oxidation (M)[12]		Mascot
2308.1567	2308.2153	0.0586	25	402	423	EVAAFAQFGSDLDAATQ ALLNR					Mascot
2308.1567	2308.2153	0.0586	25	402	423	EVAAFAQFGSDLDAATQ ALLNR	241	100			Mascot
2373.2661	2373.2607	-0.0054	-2	63	85	GIALNLENENVGIVVFGS DTAIK					Mascot

2	atp1 [Triticum aestivum]	gi|81176509	55556.8	5.7	21	759	100	55.254	641	100

Peptide Information

Calc. Mass	Obsrv. Mass	± da	± ppm	Start Seq.	End Seq.	Sequence	Ion Score	C. I. %	Modification	Rank	Result Type
815.4621	815.4589	-0.0032	-4	167	173	ELIIGDR					Mascot
860.5022	860.4783	-0.0239	-28	283	289	QMSLLLR					Mascot
876.4971	876.4769	-0.0202	-23	283	289	QMSLLLR			Oxidation (M)[2]		Mascot
884.4221	884.4244	0.0023	3	121	128	GALSDHER					Mascot
892.4886	892.4926	0.004	4	395	401	LELAQYR					Mascot
972.5473	972.5458	-0.0015	-2	33	42	VVSVGDGIAR					Mascot
1026.5942	1026.593	-0.0012	-1	154	163	AVDSLVPIGR					Mascot
1026.5942	1026.593	-0.0012	-1	154	163	AVDSLVPIGR	70	99.983			Mascot
1203.658	1203.6637	0.0057	5	7	17	AAELTTLLESR					Mascot
1242.6147	1242.5919	-0.0228	-18	143	153	SVHEPMQTGLK			Oxidation (M)[6]		Mascot
1300.7471	1300.7046	-0.0425	-33	178	189	TAIAIDTILNQK					Mascot
1326.7627	1326.7194	-0.0433	-33	470	481	AILSTINPELQK					Mascot
1341.705	1341.667	-0.038	-28	433	443	QPQYEPLPIEK					Mascot
1438.8489	1438.8525	0.0036	3	363	376	GIRPAINVGLSVSR					Mascot
1438.8489	1438.8525	0.0036	3	363	376	GIRPAINVGLSVSR	54	99.307			Mascot
1537.7434	1537.7633	0.0199	13	295	307	EAFPGDVFYLHSR					Mascot
1537.7434	1537.7633	0.0199	13	295	307	EAFPGDVFYLHSR	109	100			Mascot
1638.8268	1638.778	-0.0488	-30	388	401	QVCGSSKLELAQYR			Carbamidomethyl (C)[3]		Mascot
1704.8262	1704.8168	-0.0094	-6	262	276	DNGMHALIIYDDLSK					Mascot
1724.8789	1724.9037	0.0248	14	444	458	QIVVIYAAVNGFCDR			Carbamidomethyl (C)[13]		Mascot
1834.8429	1834.8728	0.0299	16	18	32	MTNFYTNFQVDEIGR					Mascot
1834.8429	1834.8728	0.0299	16	18	32	MTNFYTNFQVDEIGR	130	100			Mascot
1850.8378	1850.8463	0.0085	5	18	32	MTNFYTNFQVDEIGR			Oxidation (M)[1]		Mascot
1850.8378	1850.8463	0.0085	5	18	32	MTNFYTNFQVDEIGR	167	100	Oxidation (M)[1]		Mascot
2031.9692	2031.9708	0.0016	1	195	212	GTNESETLYCVYVAIGQK			Carbamidomethyl (C)[10]		Mascot
2141.0583	2141.0762	0.0179	8	43	62	VYGLNEIQAGEMVEFAS GVK					Mascot
2157.0532	2157.0676	0.0144	7	43	62	VYGLNEIQAGEMVEFAS			Oxidation (M)[12]		Mascot

						GVK						
2308.1567	2308.2153	0.0586	25	402	423	EVAAFAQFGSDLDAATQ ALLNR						Mascot
2308.1567	2308.2153	0.0586	25	402	423	EVAAFAQFGSDLDAATQ ALLNR	241	100				Mascot
2373.2661	2373.2607	-0.0054	-2	63	85	GIALNLENENVGIVVFGS DTAIK						Mascot

3	ATP synthase F0 subunit 1 [Oryza sativa Indica Group]	gi|89280711	55531.9	5.85	20	750	100	55.064	641	100		

Protein Group

ATP synthase F0 subunit 1 (mitochondrion) [Oryza sativa Indica Group]	gi|353685290	55531.9	5.8499 999046 3257
ATP synthase F0 subunit 1 (mitochondrion) [Oryza sativa Indica Group]	gi|353685289	55531.9	5.8499 999046 3257
ATP synthase F0 subunit 1 (mitochondrion) [Oryza sativa Indica Group]	gi|353685221	55531.9	5.8499 999046 3257
ATP synthase F0 subunit 1 (mitochondrion) [Oryza sativa Indica Group]	gi|353685220	55531.9	5.8499 999046 3257
ATP synthase F0 subunit 1 [Oryza rufipogon]	gi|289065052	55531.9	5.8499 999046 3257

Peptide Information

Calc. Mass	Obsrv. Mass	± da	± ppm	Start Seq.	End Seq.	Sequence	Ion Score	C. I. %	Modification	Rank	Result Type
815.4621	815.4589	-0.0032	-4	167	173	ELIIGDR					Mascot
860.5022	860.4783	-0.0239	-28	283	289	QMSLLLR					Mascot
876.4971	876.4769	-0.0202	-23	283	289	QMSLLLR			Oxidation (M)[2]		Mascot
884.4221	884.4244	0.0023	3	121	128	GALSDHER					Mascot
892.4886	892.4926	0.004	4	395	401	LELAQYR					Mascot
972.5473	972.5458	-0.0015	-2	33	42	VVSVGDGIAR					Mascot
1026.5942	1026.593	-0.0012	-1	154	163	AVDSLVPIGR					Mascot
1026.5942	1026.593	-0.0012	-1	154	163	AVDSLVPIGR	70	99.983			Mascot
1203.658	1203.6637	0.0057	5	7	17	AAELTTLLESR					Mascot
1242.6147	1242.5919	-0.0228	-18	143	153	SVHEPMQTGLK			Oxidation (M)[6]		Mascot
1300.7107	1300.7046	-0.0061	-5	495	506	IEPDASLKQTAK					Mascot
1341.705	1341.667	-0.038	-28	433	443	QPQYEPLPIEK					Mascot
1438.8489	1438.8525	0.0036	3	363	376	GIRPAINVGLSVSR					Mascot

1438.8489	1438.8525	0.0036	3	363	376	GIRPAINVGLSVSR	54	99.307			Mascot
1537.7434	1537.7633	0.0199	13	295	307	EAFPGDVFYLHSR					Mascot
1537.7434	1537.7633	0.0199	13	295	307	EAFPGDVFYLHSR	109	100			Mascot
1638.8268	1638.778	-0.0488	-30	388	401	QVCGSSKLELAQYR			Carbamidomethyl (C)[3]		Mascot
1704.8262	1704.8168	-0.0094	-6	262	276	DNGMHALIIYDDLSK					Mascot
1724.8789	1724.9037	0.0248	14	444	458	QIVVIYAAVNGFCDR			Carbamidomethyl (C)[13]		Mascot
1834.8429	1834.8728	0.0299	16	18	32	MTNFYTNFQVDEIGR					Mascot
1834.8429	1834.8728	0.0299	16	18	32	MTNFYTNFQVDEIGR	130	100			Mascot
1850.8378	1850.8463	0.0085	5	18	32	MTNFYTNFQVDEIGR			Oxidation (M)[1]		Mascot
1850.8378	1850.8463	0.0085	5	18	32	MTNFYTNFQVDEIGR	167	100	Oxidation (M)[1]		Mascot
2031.9692	2031.9708	0.0016	1	195	212	GTNESETLYCVYVAIGQK			Carbamidomethyl (C)[10]		Mascot
2141.0583	2141.0762	0.0179	8	43	62	VYGLNEIQAGEMVEFAS GVK					Mascot
2157.0532	2157.0676	0.0144	7	43	62	VYGLNEIQAGEMVEFAS GVK			Oxidation (M)[12]		Mascot
2308.1567	2308.2153	0.0586	25	402	423	EVAAFAQFGSDLDAATQ ALLNR					Mascot
2308.1567	2308.2153	0.0586	25	402	423	EVAAFAQFGSDLDAATQ ALLNR	241	100			Mascot
2373.2661	2373.2607	-0.0054	-2	63	85	GIALNLENENVGIVVFGS DTAIK					Mascot

4 ATPase subunit 1 [Sorghum bicolor] gi|115278526 55933.1 5.71 20 750 100 55.064 641 100

Peptide Information

Calc. Mass	Obsrv. Mass	± da	± ppm	Start Seq.	End Seq.	Sequence	Ion Score	C. I. %	Modification	Rank	Result Type
815.4621	815.4589	-0.0032	-4	167	173	ELIIGDR					Mascot
860.5022	860.4783	-0.0239	-28	283	289	QMSLLLR					Mascot
876.4971	876.4769	-0.0202	-23	283	289	QMSLLLR			Oxidation (M)[2]		Mascot
884.4221	884.4244	0.0023	3	121	128	GALSDHER					Mascot
892.4886	892.4926	0.004	4	395	401	LELAQYR					Mascot
972.5473	972.5458	-0.0015	-2	33	42	VVSVGDGIAR					Mascot
1026.5942	1026.593	-0.0012	-1	154	163	AVDSLVPIGR					Mascot
1026.5942	1026.593	-0.0012	-1	154	163	AVDSLVPIGR	70	99.983			Mascot
1203.658	1203.6637	0.0057	5	7	17	AAELTTLLESR					Mascot
1242.6147	1242.5919	-0.0228	-18	143	153	SVHEPMQTGLK			Oxidation (M)[6]		Mascot
1300.7471	1300.7046	-0.0425	-33	178	189	TAIAIDTILNQK					Mascot
1341.705	1341.667	-0.038	-28	433	443	QPQYEPLPIEK					Mascot
1438.8489	1438.8525	0.0036	3	363	376	GIRPAINVGLSVSR					Mascot
1438.8489	1438.8525	0.0036	3	363	376	GIRPAINVGLSVSR	54	99.307			Mascot
1537.7434	1537.7633	0.0199	13	295	307	EAFPGDVFYLHSR					Mascot

1537.7434	1537.7633	0.0199	13	295	307	EAFPGDVFYLHSR	109	100			Mascot
1638.8268	1638.778	-0.0488	-30	388	401	QVCGSSKLELAQYR			Carbamidomethyl (C)[3]		Mascot
1704.8262	1704.8168	-0.0094	-6	262	276	DNGMHALIIYDDLSK					Mascot
1724.8789	1724.9037	0.0248	14	444	458	QIVVIYAAVNGFCDR			Carbamidomethyl (C)[13]		Mascot
1834.8429	1834.8728	0.0299	16	18	32	MTNFYTNFQVDEIGR					Mascot
1834.8429	1834.8728	0.0299	16	18	32	MTNFYTNFQVDEIGR	130	100			Mascot
1850.8378	1850.8463	0.0085	5	18	32	MTNFYTNFQVDEIGR			Oxidation (M)[1]		Mascot
1850.8378	1850.8463	0.0085	5	18	32	MTNFYTNFQVDEIGR	167	100	Oxidation (M)[1]		Mascot
2031.9692	2031.9708	0.0016	1	195	212	GTNESETLYCVYVAIGQK			Carbamidomethyl (C)[10]		Mascot
2141.0583	2141.0762	0.0179	8	43	62	VYGLNEIQAGEMVEFAS GVK					Mascot
2157.0532	2157.0676	0.0144	7	43	62	VYGLNEIQAGEMVEFAS GVK			Oxidation (M)[12]		Mascot
2308.1567	2308.2153	0.0586	25	402	423	EVAAFAQFGSDLDAATQ ALLNR					Mascot
2308.1567	2308.2153	0.0586	25	402	423	EVAAFAQFGSDLDAATQ ALLNR	241	100			Mascot
2373.2661	2373.2607	-0.0054	-2	63	85	GIALNLENENVGIVVFGS DTAIK					Mascot

5	RecName: Full=ATP synthase subunit alpha, mitochondrial	gi|148886791	55624	5.85	19	741	100	54.9	641	100	

Protein Group

ATP synthase F0 subunit 1 [Oryza sativa Japonica Group]	gi|194033257	55624	5.8499 999046 3257
RecName: Full=ATP synthase subunit alpha, mitochondrial	gi|148886790	55624	5.8499 999046 3257
RecName: Full=ATP synthase subunit alpha, mitochondrial	gi|148886792	55624	5.8499 999046 3257

Peptide Information

Calc. Mass	Obsrv. Mass	± da	± ppm	Start Seq.	End Seq.	Sequence	Ion Score	C. I. %	Modification	Rank	Result Type
815.4621	815.4589	-0.0032	-4	167	173	ELIIGDR					Mascot
860.5022	860.4783	-0.0239	-28	283	289	QMSLLLR					Mascot
876.4971	876.4769	-0.0202	-23	283	289	QMSLLLR			Oxidation (M)[2]		Mascot
884.4221	884.4244	0.0023	3	121	128	GALSDHER					Mascot
892.4886	892.4926	0.004	4	395	401	LELAQYR					Mascot
972.5473	972.5458	-0.0015	-2	33	42	VVSVGDGIAR					Mascot
1026.5942	1026.593	-0.0012	-1	154	163	AVDSLVPIGR					Mascot

1026.5942	1026.593	-0.0012	-1	154	163	AVDSLVPIGR	70	99.983			Mascot
1203.658	1203.6637	0.0057	5	7	17	AAELTTLLESR					Mascot
1242.6147	1242.5919	-0.0228	-18	143	153	SVHEPMQTGLK			Oxidation (M)[6]		Mascot
1300.7471	1300.7046	-0.0425	-33	178	189	TAIAIDTILNQK					Mascot
1341.705	1341.667	-0.038	-28	433	443	QPQYEPLPIEK					Mascot
1438.8489	1438.8525	0.0036	3	363	376	GIRPAINVGLSVSR					Mascot
1438.8489	1438.8525	0.0036	3	363	376	GIRPAINVGLSVSR	54	99.307			Mascot
1537.7434	1537.7633	0.0199	13	295	307	EAFPGDVFYLHSR					Mascot
1537.7434	1537.7633	0.0199	13	295	307	EAFPGDVFYLHSR	109	100			Mascot
1704.8262	1704.8168	-0.0094	-6	262	276	DNGMHALIIYDDLSK					Mascot
1724.8789	1724.9037	0.0248	14	444	458	QIVVIYAAVNGFCDR			Carbamidomethyl (C)[13]		Mascot
1834.8429	1834.8728	0.0299	16	18	32	MTNFYTNFQVDEIGR					Mascot
1834.8429	1834.8728	0.0299	16	18	32	MTNFYTNFQVDEIGR	130	100			Mascot
1850.8378	1850.8463	0.0085	5	18	32	MTNFYTNFQVDEIGR			Oxidation (M)[1]		Mascot
1850.8378	1850.8463	0.0085	5	18	32	MTNFYTNFQVDEIGR	167	100	Oxidation (M)[1]		Mascot
2031.9692	2031.9708	0.0016	1	195	212	GTNESETLYCVYVAIGQK			Carbamidomethyl (C)[10]		Mascot
2141.0583	2141.0762	0.0179	8	43	62	VYGLNEIQAGEMVEFAS GVK					Mascot
2157.0532	2157.0676	0.0144	7	43	62	VYGLNEIQAGEMVEFAS GVK			Oxidation (M)[12]		Mascot
2308.1567	2308.2153	0.0586	25	402	423	EVAAFAQFGSDLDAATQ ALLNR					Mascot
2308.1567	2308.2153	0.0586	25	402	423	EVAAFAQFGSDLDAATQ ALLNR	241	100			Mascot
2373.2661	2373.2607	-0.0054	-2	63	85	GIALNLENENVGIVVFGS DTAIK					Mascot

6 ATP synthase subunit alpha, mitochondrial [Aegilops tauschii] gi|475624600 54469.2 5.67 19 741 100 53.45 641 100

Peptide Information

Calc. Mass	Obsrv. Mass	± da	± ppm	Start Seq.	End Seq.	Sequence	Ion Score	C. I. %	Modification	Rank	Result Type
815.4621	815.4589	-0.0032	-4	157	163	ELIIGDR					Mascot
892.4886	892.4926	0.004	4	385	391	LELAQYR					Mascot
972.5473	972.5458	-0.0015	-2	16	25	VVSVGDGIAR					Mascot
1010.5126	1010.4449	-0.0677	-67	132	140	GALGDHERR					Mascot
1026.5942	1026.593	-0.0012	-1	144	153	AVDSLVPIGR					Mascot
1026.5942	1026.593	-0.0012	-1	144	153	AVDSLVPIGR	70	99.983			Mascot
1259.7107	1259.603	-0.1077	-85	46	56	GIALNLENFLR					Mascot
1300.7471	1300.7046	-0.0425	-33	168	179	TAIAIDTILNQK					Mascot
1326.7627	1326.7194	-0.0433	-33	460	471	AILSTINPELQK					Mascot

1341.705	1341.667	-0.038	-28	423	433	QPQYEPLPIEK					Mascot
1438.8489	1438.8525	0.0036	3	353	366	GIRPAINVGLSVSR					Mascot
1438.8489	1438.8525	0.0036	3	353	366	GIRPAINVGLSVSR	54	99.307			Mascot
1537.7434	1537.7633	0.0199	13	285	297	EAFPGDVFYLHSR					Mascot
1537.7434	1537.7633	0.0199	13	285	297	EAFPGDVFYLHSR	109	100			Mascot
1573.8632	1573.8478	-0.0154	-10	267	279	QAVAYRHMSLLLR			Oxidation (M)[8]		Mascot
1626.8561	1626.7968	-0.0593	-36	59	73	KIMGIFPSEFASGVK			Oxidation (M)[3]		Mascot
1638.8268	1638.778	-0.0488	-30	378	391	QVCGSSKLELAQYR			Carbamidomethyl (C)[3]		Mascot
1703.8025	1703.8191	0.0166	10	2	15	TNFYTNFQVDEIGR					Mascot
1724.8789	1724.9037	0.0248	14	434	448	QIVVIYAAVNGFCDR			Carbamidomethyl (C)[13]		Mascot
1834.8429	1834.8728	0.0299	16	1	15	MTNFYTNFQVDEIGR					Mascot
1834.8429	1834.8728	0.0299	16	1	15	MTNFYTNFQVDEIGR	130	100			Mascot
1850.8378	1850.8463	0.0085	5	1	15	MTNFYTNFQVDEIGR			Oxidation (M)[1]		Mascot
1850.8378	1850.8463	0.0085	5	1	15	MTNFYTNFQVDEIGR	167	100	Oxidation (M)[1]		Mascot
2141.0583	2141.0762	0.0179	8	26	45	VYGLNEIQAGEMVEFAS GVK					Mascot
2157.0532	2157.0676	0.0144	7	26	45	VYGLNEIQAGEMVEFAS GVK			Oxidation (M)[12]		Mascot
2308.1567	2308.2153	0.0586	25	392	413	EVAAFAQFGSDLDAATQ ALLNR					Mascot
2308.1567	2308.2153	0.0586	25	392	413	EVAAFAQFGSDLDAATQ ALLNR	241	100			Mascot

7	ATP synthase subunits 1 (mitochondrion) [Lolium perenne]	gi|472833568	55526.8	5.7	19	741	100	54.84	641	100	

Peptide Information

Calc. Mass	Obsrv. Mass	± da	± ppm	Start Seq.	End Seq.	Sequence	Ion Score	C. I. %	Modification	Rank	Result Type
815.4621	815.4589	-0.0032	-4	167	173	ELIIGDR					Mascot
860.5022	860.4783	-0.0239	-28	283	289	QMSLLLR					Mascot
876.4971	876.4769	-0.0202	-23	283	289	QMSLLLR			Oxidation (M)[2]		Mascot
884.4221	884.4244	0.0023	3	121	128	GALSDHER					Mascot
892.4886	892.4926	0.004	4	395	401	LELAQYR					Mascot
972.5473	972.5458	-0.0015	-2	33	42	VVSVGDGIAR					Mascot
1026.5942	1026.593	-0.0012	-1	154	163	AVDSLVPIGR					Mascot
1026.5942	1026.593	-0.0012	-1	154	163	AVDSLVPIGR	70	99.983			Mascot
1203.658	1203.6637	0.0057	5	7	17	AAELTTLLESR					Mascot
1242.6147	1242.5919	-0.0228	-18	143	153	SVHEPMQTGLK			Oxidation (M)[6]		Mascot
1300.7471	1300.7046	-0.0425	-33	178	189	TAIAIDTILNQK					Mascot
1341.705	1341.667	-0.038	-28	433	443	QPQYEPLPIEK					Mascot

1438.8489	1438.8525	0.0036	3	363	376	GIRPAINVGLSVSR					Mascot
1438.8489	1438.8525	0.0036	3	363	376	GIRPAINVGLSVSR	54	99.307			Mascot
1537.7434	1537.7633	0.0199	13	295	307	EAFPGDVFYLHSR					Mascot
1537.7434	1537.7633	0.0199	13	295	307	EAFPGDVFYLHSR	109	100			Mascot
1638.8268	1638.778	-0.0488	-30	388	401	QVCGSSKLELAQYR			Carbamidomethyl (C)[3]		Mascot
1704.8262	1704.8168	-0.0094	-6	262	276	DNGMHALIIYDDLSK					Mascot
1724.8789	1724.9037	0.0248	14	444	458	QIVVIYAAVNGFCDR			Carbamidomethyl (C)[13]		Mascot
1834.8429	1834.8728	0.0299	16	18	32	MTNFYTNFQVDEIGR					Mascot
1834.8429	1834.8728	0.0299	16	18	32	MTNFYTNFQVDEIGR	130	100			Mascot
1850.8378	1850.8463	0.0085	5	18	32	MTNFYTNFQVDEIGR			Oxidation (M)[1]		Mascot
1850.8378	1850.8463	0.0085	5	18	32	MTNFYTNFQVDEIGR	167	100	Oxidation (M)[1]		Mascot
2141.0583	2141.0762	0.0179	8	43	62	VYGLNEIQAGEMVEFAS GVK					Mascot
2157.0532	2157.0676	0.0144	7	43	62	VYGLNEIQAGEMVEFAS GVK			Oxidation (M)[12]		Mascot
2308.1567	2308.2153	0.0586	25	402	423	EVAAFAQFGSDLDAATQ ALLNR					Mascot
2308.1567	2308.2153	0.0586	25	402	423	EVAAFAQFGSDLDAATQ ALLNR	241	100			Mascot
2373.2661	2373.2607	-0.0054	-2	63	85	GIALNLENENVGIVVFGS DTAIK					Mascot

8	RecName: Full=ATP synthase subunit alpha, mitochondrial	gi|114411	55595.1	6.51	16	715	100	53.484	641	100	

Peptide Information

Calc. Mass	Obsrv. Mass	± da	± ppm	Start Seq.	End Seq.	Sequence	Ion Score	C. I. %	Modification	Rank	Result Type
815.4621	815.4589	-0.0032	-4	167	173	ELIIGDR					Mascot
860.5022	860.4783	-0.0239	-28	283	289	QMSLLLR					Mascot
876.4971	876.4769	-0.0202	-23	283	289	QMSLLLR			Oxidation (M)[2]		Mascot
884.4221	884.4244	0.0023	3	121	128	GALSDHER					Mascot
892.4886	892.4926	0.004	4	395	401	LELAQYR					Mascot
972.5473	972.5458	-0.0015	-2	33	42	VVSVGDGIAR					Mascot
1026.5942	1026.593	-0.0012	-1	154	163	AVDSLVPIGR					Mascot
1026.5942	1026.593	-0.0012	-1	154	163	AVDSLVPIGR	70	99.983			Mascot
1203.658	1203.6637	0.0057	5	7	17	AAELTTLLESR					Mascot
1242.6147	1242.5919	-0.0228	-18	143	153	SVHEPMQTGLK			Oxidation (M)[6]		Mascot
1300.7471	1300.7046	-0.0425	-33	178	189	TAIAIDTILNQK					Mascot
1438.8489	1438.8525	0.0036	3	363	376	GIRPAINVGLSVSR					Mascot
1438.8489	1438.8525	0.0036	3	363	376	GIRPAINVGLSVSR	54	99.307			Mascot
1537.7434	1537.7633	0.0199	13	295	307	EAFPGDVFYLHSR					Mascot

1537.7434	1537.7633	0.0199	13	295	307	EAFPGDVFYLHSR	109	100			Mascot
1704.8262	1704.8168	-0.0094	-6	262	276	DNGMHALIIYDDLSK					Mascot
1834.8429	1834.8728	0.0299	16	18	32	MTNFYTNFQVDEIGR					Mascot
1834.8429	1834.8728	0.0299	16	18	32	MTNFYTNFQVDEIGR	130	100			Mascot
1850.8378	1850.8463	0.0085	5	18	32	MTNFYTNFQVDEIGR			Oxidation (M)[1]		Mascot
1850.8378	1850.8463	0.0085	5	18	32	MTNFYTNFQVDEIGR	167	100	Oxidation (M)[1]		Mascot
2141.0583	2141.0762	0.0179	8	43	62	VYGLNEIQAGEMVEFAS GVK					Mascot
2157.0532	2157.0676	0.0144	7	43	62	VYGLNEIQAGEMVEFAS GVK			Oxidation (M)[12]		Mascot
2308.1567	2308.2153	0.0586	25	402	423	EVAAFAQFGSDLDAATQ ALLNR					Mascot
2308.1567	2308.2153	0.0586	25	402	423	EVAAFAQFGSDLDAATQ ALLNR	241	100			Mascot
2373.2661	2373.2607	-0.0054	-2	63	85	GIALNLENENVGIVVFGS DTAIK					Mascot

9	ATP synthase subunit alpha, mitochondrial [Aegilops tauschii]	gi|475624598	41077.1	5.93	14	592	100	48.784	517	100	

Peptide Information

Calc. Mass	Obsrv. Mass	± da	± ppm	Start Seq.	End Seq.	Sequence	Ion Score	C. I. %	Modification	Rank	Result Type
860.5022	860.4783	-0.0239	-28	190	196	QMSLLLR					Mascot
876.4971	876.4769	-0.0202	-23	190	196	QMSLLLR			Oxidation (M)[2]		Mascot
892.4886	892.4926	0.004	4	257	263	LELAQYR					Mascot
972.5473	972.5458	-0.0015	-2	16	25	VVSVGDGIAR					Mascot
1326.7627	1326.7194	-0.0433	-33	332	343	AILSTINPELQK					Mascot
1341.705	1341.667	-0.038	-28	295	305	QPQYEPLPIEK					Mascot
1537.7434	1537.7633	0.0199	13	202	214	EAFPGDVFYLHSR					Mascot
1537.7434	1537.7633	0.0199	13	202	214	EAFPGDVFYLHSR	109	100			Mascot
1638.8268	1638.778	-0.0488	-30	250	263	QVCGSSKLELAQYR			Carbamidomethyl (C)[3]		Mascot
1703.8025	1703.8191	0.0166	10	2	15	TNFYTNFQVDEIGR					Mascot
1704.8262	1704.8168	-0.0094	-6	169	183	DNGMHALIIYDDLSK					Mascot
1724.8789	1724.9037	0.0248	14	306	320	QIVVIYAAVNGFCDR			Carbamidomethyl (C)[13]		Mascot
1834.8429	1834.8728	0.0299	16	1	15	MTNFYTNFQVDEIGR					Mascot
1834.8429	1834.8728	0.0299	16	1	15	MTNFYTNFQVDEIGR	130	100			Mascot
1850.8378	1850.8463	0.0085	5	1	15	MTNFYTNFQVDEIGR			Oxidation (M)[1]		Mascot
1850.8378	1850.8463	0.0085	5	1	15	MTNFYTNFQVDEIGR	167	100	Oxidation (M)[1]		Mascot
2031.9692	2031.9708	0.0016	1	102	119	GTNESETLYCVYVAIGQK			Carbamidomethyl (C)[10]		Mascot
2308.1567	2308.2153	0.0586	25	264	285	EVAAFAQFGSDLDAATQ ALLNR					Mascot
2308.1567	2308.2153	0.0586	25	264	285	EVAAFAQFGSDLDAATQ	241	100			Mascot

2373.2661	2373.2607	-0.0054	-2	46	68	ALLNR GIALNLENENVGIVVFGS DTAIK					Mascot

10 ATPase subunit 1 [Zea mays subsp. mays] gi|94502566 55430.9 5.85 19 572 100 47.542 474 100

Protein Group

ATPase subunit 1 [Tripsacum dactyloides]	gi|115278595	55412.9	6.0199 999809 2651
ATPase subunit 1 [Zea luxurians]	gi|114151591	55430.9	5.8499 999046 3257
ATPase subunit 1 [Zea mays subsp. mays]	gi|94502565	55430.9	5.8499 999046 3257
ATPase subunit 1 [Zea mays subsp. parviglumis]	gi|114151624	55430.9	5.8499 999046 3257
ATPase subunit 1 [Zea perennis]	gi|114151558	55430.9	5.8499 999046 3257
RecName: Full=ATP synthase subunit alpha, mitochondrial	gi|114405	55430.9	5.8499 999046 3257

Peptide Information

Calc. Mass	Obsrv. Mass	± da	± ppm	Start Seq.	End Seq.	Sequence	Ion Score	C. I. %	Modification	Rank	Result Type
815.4621	815.4589	-0.0032	-4	167	173	ELIIGDR					Mascot
860.5022	860.4783	-0.0239	-28	283	289	QMSLLLR					Mascot
876.4971	876.4769	-0.0202	-23	283	289	QMSLLLR			Oxidation (M)[2]		Mascot
884.4221	884.4244	0.0023	3	121	128	GALSDHER					Mascot
892.4886	892.4926	0.004	4	395	401	LELAQYR					Mascot
972.5473	972.5458	-0.0015	-2	33	42	VVSVGDGIAR					Mascot
1026.5942	1026.593	-0.0012	-1	154	163	AVDSLVPIGR					Mascot
1026.5942	1026.593	-0.0012	-1	154	163	AVDSLVPIGR	70	99.983			Mascot
1203.658	1203.6637	0.0057	5	7	17	AAELTTLLESR					Mascot
1242.6147	1242.5919	-0.0228	-18	143	153	SVHEPMQTGLK			Oxidation (M)[6]		Mascot
1300.7471	1300.7046	-0.0425	-33	178	189	TAIAIDTILNQK					Mascot
1341.705	1341.667	-0.038	-28	433	443	QPQYEPLPIEK					Mascot
1438.8489	1438.8525	0.0036	3	363	376	GIRPAINVGLSVSR					Mascot

1438.8489	1438.8525	0.0036	3	363	376	GIRPAINVGLSVSR	54	99.307		Mascot
1537.7434	1537.7633	0.0199	13	295	307	EAFPGDVFYLHSR				Mascot
1537.7434	1537.7633	0.0199	13	295	307	EAFPGDVFYLHSR	109	100		Mascot
1638.8268	1638.778	-0.0488	-30	388	401	QVCGSSKLELAQYR			Carbamidomethyl (C)[3]	Mascot
1704.8262	1704.8168	-0.0094	-6	262	276	DNGMHALIIYDDLSK				Mascot
1724.8789	1724.9037	0.0248	14	444	458	QIVVIYAAVNGFCDR			Carbamidomethyl (C)[13]	Mascot
2031.9692	2031.9708	0.0016	1	195	212	GTNESETLYCVYVAIGQK			Carbamidomethyl (C)[10]	Mascot
2141.0583	2141.0762	0.0179	8	43	62	VYGLNEIQAGEMVEFAS GVK				Mascot
2157.0532	2157.0676	0.0144	7	43	62	VYGLNEIQAGEMVEFAS GVK			Oxidation (M)[12]	Mascot
2308.1567	2308.2153	0.0586	25	402	423	EVAAFAQFGSDLDAATQ ALLNR				Mascot
2308.1567	2308.2153	0.0586	25	402	423	EVAAFAQFGSDLDAATQ ALLNR	241	100		Mascot
2373.2661	2373.2607	-0.0054	-2	63	85	GIALNLENENVGIVVFGS DTAIK				Mascot

Gel Idx/Pos	276/L4	Instr./Gel Origin	BA2151/Sample Project 20140814	Process Status	Analysis Succeeded
Plate [#] Name	[1] Sample Project 20140814	Instrument Sample Name		Spectra	11

Rank	Protein Name	Accession No.	Protein MW	Protein PI	Pep. Count	Protein Score	Protein Score C. I. %	Intensity Matched	Total Ion Score	Total Ion C. I. %	Confirmed
1	Beta-amylase [Triticum urartu]	gi|474451266	58995	5.34	15	449	100	34.98	384	100	

Peptide Information

Calc. Mass	Obsrv. Mass	± da	± ppm	Start Seq.	End Seq.	Sequence	Ion Score	C. I. %	Modification	Rank	Result Type
947.5057	947.4861	-0.0196	-21	322	329	DGYRPIAR					Mascot
1016.5564	1016.5525	-0.0039	-4	412	419	LFGFTYLR					Mascot
1016.5564	1016.5525	-0.0039	-4	412	419	LFGFTYLR	59	99.75			Mascot
1234.5448	1234.5884	0.0436	35	233	243	DAGQYNDAPQR					Mascot
1234.5448	1234.5884	0.0436	35	233	243	DAGQYNDAPQR					Mascot
1315.5769	1315.5629	-0.014	-11	336	346	ASLNFTCAEMR			Carbamidomethyl (C)[7], Oxidation (M)[10]		Mascot
1326.6688	1326.6639	-0.0049	-4	385	395	YDPTAYNTILR					Mascot
1326.6688	1326.6639	-0.0049	-4	385	395	YDPTAYNTILR	81	99.999			Mascot
1382.7791	1382.7528	-0.0263	-19	73	84	QLFQLVHEAGLK					Mascot
1490.6726	1490.6649	-0.0077	-5	372	384	EGLNMACENALPR			Carbamidomethyl (C)[7], Oxidation (M)[5]		Mascot
1646.781	1646.793	0.012	7	246	259	FFVDNGTYLTEQGR					Mascot
1668.7952	1668.7999	0.0047	3	218	232	AAAAMVGHPEWEFPR					Mascot
1684.79	1684.7843	-0.0057	-3	218	232	AAAAMVGHPEWEFPR			Oxidation (M)[5]		Mascot
1701.7247	1701.7289	0.0042	2	148	161	SAVQMYTDYMASFR			Oxidation (M)[5,10]		Mascot
1752.8916	1752.8677	-0.0239	-14	420	434	LSNQLVEGQNYVNFK					Mascot
1992.9047	1992.9198	0.0151	8	112	129	NVGASDPDIFYTDQHGT R					Mascot
1992.9047	1992.9198	0.0151	8	112	129	NVGASDPDIFYTDQHGT R	117	100			Mascot
2013.9778	2013.9973	0.0195	10	304	321	VPSHAAEITAGYYNLHDR					Mascot
2013.9778	2013.9973	0.0195	10	304	321	VPSHAAEITAGYYNLHDR	127	100			Mascot
2087.0557	2087.0767	0.021	10	130	147	NIEYLTLGVDDQPLFHGR					Mascot
2167.0537	2167.0732	0.0195	9	440	458	MHANLPHDPCVDPVAPL QR			Carbamidomethyl (C)[10]		Mascot
2183.0486	2183.0481	-0.0005	0	440	458	MHANLPHDPCVDPVAPL QR			Carbamidomethyl (C)[10], Oxidation (M)[1]		Mascot

Rank	Protein Name	Accession No.	Protein MW	Protein PI	Pep. Count	Protein Score	Protein Score C. I. %	Intensity Matched	Total Ion Score	Total Ion C. I. %	Confirmed
2	Chain A, Sevenfold Mutant Of Barley Beta-Amylase	gi|6729696	56504.9	5.73	10	299	100	19.682	267	100	

Peptide Information

Calc. Mass	Obsrv. Mass	± da	± ppm	Start Seq.	End Seq.	Sequence	Ion Score	C. I. %	Modification	Rank	Result Type

Calc. Mass	Obsrv. Mass	± da	± ppm	Start Seq.	End Seq.	Sequence	Ion Score	C. I. %	Modification	Rank	Result Type
1016.5564	1016.5525	-0.0039	-4	407	414	LFGFTYLR					Mascot
1016.5564	1016.5525	-0.0039	-4	407	414	LFGFTYLR	59	99.75			Mascot
1026.4739	1026.4773	0.0034	3	244	252	DNGTYLSEK					Mascot
1285.6212	1285.5966	-0.0246	-19	57	67	GPKAYDWSAYK					Mascot
1315.5769	1315.5629	-0.014	-11	331	341	ASINFTCAEMR			Carbamidomethyl (C)[7], Oxidation (M)[10]		Mascot
1326.6688	1326.6639	-0.0049	-4	380	390	YDPTAYNTILR					Mascot
1326.6688	1326.6639	-0.0049	-4	380	390	YDPTAYNTILR	81	99.999			Mascot
1701.7247	1701.7289	0.0042	2	143	156	SAVQMYADYMTSFR			Oxidation (M)[5,10]		Mascot
1705.8181	1705.8896	0.0715	42	239	252	TQFFRDNGTYLSEK					Mascot
1752.8916	1752.8677	-0.0239	-14	415	429	LSNQLVEGQNYVNFK					Mascot
2013.9778	2013.9973	0.0195	10	299	316	VPSHAAELTAGYYNLHD R					Mascot
2013.9778	2013.9973	0.0195	10	299	316	VPSHAAELTAGYYNLHD R	127	100			Mascot
2255.1919	2255.1514	-0.0405	-18	161	182	DFLDAGVIVDIEVGLGPA GELR					Mascot

3	Aldehyde dehydrogenase family 2 member B4, mitochondrial [Aegilops tauschii]	gi|475507148	59569.8	6.4	14	298	100	11.545	243	100	

Peptide Information

Calc. Mass	Obsrv. Mass	± da	± ppm	Start Seq.	End Seq.	Sequence	Ion Score	C. I. %	Modification	Rank	Result Type
900.5513	900.5527	0.0014	2	300	307	VILELSAR					Mascot
917.5203	917.5028	-0.0175	-19	378	385	VVGDPFRK					Mascot
932.4836	932.4709	-0.0127	-14	85	92	TFPTVDPR					Mascot
1028.4935	1028.5205	0.027	26	362	369	VYDEFVEK					Mascot
1074.5004	1074.5442	0.0438	41	118	126	AFDEGPWPR					Mascot
1202.5953	1202.5991	0.0038	3	117	126	KAFDEGPWPR					Mascot
1247.6743	1247.6477	-0.0266	-21	458	467	FNDLNEVIKR					Mascot
1507.8003	1507.7488	-0.0515	-34	241	254	TAEQTPLSALYVSK					Mascot
1589.7443	1589.7802	0.0359	23	386	399	GVEQGPQIDDEQFK					Mascot
1705.916	1705.8896	-0.0264	-15	443	457	IAQEEIFGPVQSIFK					Mascot
1977.9263	1977.917	-0.0093	-5	426	442	GYYIQPTIFSDVQDGMK			Oxidation (M)[16]		Mascot
1993.1124	1992.9198	-0.1926	-97	17	35	CLLSRPAASPAAVPSALR R			Carbamidomethyl (C)[1]		Mascot
1993.1124	1992.9198	-0.1926	-97	17	35	CLLSRPAASPAAVPSALR R			Carbamidomethyl (C)[1]		Mascot
2583.2798	2583.3201	0.0403	16	468	492	ANASQYGLAAGVFTNNL DTANTLTR					Mascot
2583.2798	2583.3201	0.0403	16	468	492	ANASQYGLAAGVFTNNL DTANTLTR	243	100			Mascot
2739.3809	2739.4189	0.038	14	467	492	RANASQYGLAAGVFTNN					Mascot

LDTANTLTR

4	RecName: Full=Beta-amylase; AltName: Full=1,4-alpha-D-glucan maltohydrolase	gi|113786	59894.5	5.58	9	290	100	19.536	267	100

Peptide Information

Calc. Mass	Obsrv. Mass	± da	± ppm	Start Seq.	End Seq.	Sequence	Ion Score	C. I. %	Modification	Rank	Result Type
1016.5564	1016.5525	-0.0039	-4	411	418	LFGFTYLR					Mascot
1016.5564	1016.5525	-0.0039	-4	411	418	LFGFTYLR	59	99.75			Mascot
1026.4739	1026.4773	0.0034	3	248	256	DNGTYLSEK					Mascot
1285.6212	1285.5966	-0.0246	-19	61	71	GPKAYDWSAYK					Mascot
1315.5769	1315.5629	-0.014	-11	335	345	ASINFTCAEMR			Carbamidomethyl (C)[7], Oxidation (M)[10]		Mascot
1326.6688	1326.6639	-0.0049	-4	384	394	YDPTAYNTILR					Mascot
1326.6688	1326.6639	-0.0049	-4	384	394	YDPTAYNTILR	81	99.999			Mascot
1701.7247	1701.7289	0.0042	2	147	160	SAVQMYADYMTSFR			Oxidation (M)[5,10]		Mascot
1705.8181	1705.8896	0.0715	42	243	256	TQFFRDNGTYLSEK					Mascot
1752.8916	1752.8677	-0.0239	-14	419	433	LSNQLVEGQNYVNFK					Mascot
2013.9778	2013.9973	0.0195	10	303	320	VPSHAAELTAGYYNLHD R					Mascot
2013.9778	2013.9973	0.0195	10	303	320	VPSHAAELTAGYYNLHD R	127	100			Mascot

5	Aldehyde dehydrogenase family 2 member B7, mitochondrial [Triticum urartu]	gi|473987280	59912.1	5.84	13	290	100	9.32	243	100

Peptide Information

Calc. Mass	Obsrv. Mass	± da	± ppm	Start Seq.	End Seq.	Sequence	Ion Score	C. I. %	Modification	Rank	Result Type
900.5513	900.5527	0.0014	2	301	308	VILELSAR					Mascot
917.5203	917.5028	-0.0175	-19	379	386	VVGDPFRK					Mascot
932.4836	932.4709	-0.0127	-14	86	93	TFPTVDPR					Mascot
1028.4935	1028.5205	0.027	26	363	370	VYDEFVEK					Mascot
1074.5004	1074.5442	0.0438	41	119	127	AFDEGPWPR					Mascot
1202.5953	1202.5991	0.0038	3	118	127	KAFDEGPWPR					Mascot
1247.6743	1247.6477	-0.0266	-21	459	468	FNDLNEVIKR					Mascot
1507.8003	1507.7488	-0.0515	-34	242	255	TAEQTPLSALYVSK					Mascot
1589.7443	1589.7802	0.0359	23	387	400	GVEQGPQIDDEQFK					Mascot
1705.916	1705.8896	-0.0264	-15	444	458	IAQEEIFGPVQSIFK					Mascot
1977.9263	1977.917	-0.0093	-5	427	443	GYYIQPTIFSDVQDGMK			Oxidation (M)[16]		Mascot
2583.2798	2583.3201	0.0403	16	469	493	ANASQYGLAAGVFTNNL DTANTLTR					Mascot
2583.2798	2583.3201	0.0403	16	469	493	ANASQYGLAAGVFTNNL	243	100			Mascot

	2739.3809	2739.4189	0.038	14	468	493	DTANTLTR RANASQYGLAAGVFTNN LDTANTLTR									Mascot
6	RecName: Full=Beta-amylase; AltName: Full=1,4-alpha-D-glucan maltohydrolase; AltName: Full=Beta-Amy1; Flags: Precursor	gi|75107132	59886.4	5.66	8	286	100	20.235	267	100						

Peptide Information

Calc. Mass	Obsrv. Mass	± da	± ppm	Start Seq.	End Seq.	Sequence	Ion Score	C. I. %	Modification	Rank	Result Type
1016.5564	1016.5525	-0.0039	-4	411	418	LFGFTYLR					Mascot
1016.5564	1016.5525	-0.0039	-4	411	418	LFGFTYLR	59	99.75			Mascot
1253.6121	1253.5848	-0.0273	-22	248	258	DNGTYLTEKGR					Mascot
1285.6212	1285.5966	-0.0246	-19	61	71	GPKAYDWSAYK					Mascot
1315.5769	1315.5629	-0.014	-11	335	345	ASINFTCAEMR			Carbamidomethyl (C)[7], Oxidation (M)[10]		Mascot
1326.6688	1326.6639	-0.0049	-4	384	394	YDPTAYNTILR					Mascot
1326.6688	1326.6639	-0.0049	-4	384	394	YDPTAYNTILR	81	99.999			Mascot
1701.7247	1701.7289	0.0042	2	147	160	SAVQMYADYMTSFR			Oxidation (M)[5,10]		Mascot
1752.8916	1752.8677	-0.0239	-14	419	433	LSNQLVEGQNYVNFK					Mascot
2013.9778	2013.9973	0.0195	10	303	320	VPSHAAELTAGYYNLHD R					Mascot
2013.9778	2013.9973	0.0195	10	303	320	VPSHAAELTAGYYNLHD R	127	100			Mascot

7	Chain A, Crystal Structure Of Barley Beta-Amylase Complexed With 2,3-Epoxypropyl-Alpha-D-Glucopyranoside	gi|313103508	59854.4	5.58	8	285	100	19.156	267	100	

Protein Group

Chain A, Crystal Structure Of Barley Beta-Amylase At Atomic Resolution	gi|313103503	59854.4	5.5799 999237 0605
Chain A, Crystal Structure Of Barley Beta-Amylase Complexed With 3,4- Epoxybutyl Alpha-D-Glucopyranoside	gi|313103509	59854.4	5.5799 999237 0605
Chain A, Crystal Structure Of Barley Beta-Amylase Complexed With 4-O-Alpha-D-Glucopyranosylmoranoline	gi|313103505	59854.4	5.5799 999237 0605
Chain A, Crystal Structure Of Barley Beta-Amylase Complexed With Acarbose	gi|313103502	59854.4	5.5799 999237 0605
Chain A, Crystal Structure Of Barley Beta-Amylase Complexed With Alpha-Cyclodextrin	gi|313103504	59854.4	5.5799 999237 0605

Peptide Information

Calc. Mass	Obsrv. Mass	± da	± ppm	Start Seq.	End Seq.	Sequence	Ion Score	C. I. %	Modification	Rank	Result Type
1016.5564	1016.5525	-0.0039	-4	411	418	LFGFTYLR					Mascot
1016.5564	1016.5525	-0.0039	-4	411	418	LFGFTYLR	59	99.75			Mascot
1026.4739	1026.4773	0.0034	3	248	256	DNGTYLSEK					Mascot
1285.6212	1285.5966	-0.0246	-19	61	71	GPKAYDWSAYK					Mascot
1315.5769	1315.5629	-0.014	-11	335	345	ASINFTCAEMR			Carbamidomethyl (C)[7], Oxidation (M)[10]		Mascot
1326.6688	1326.6639	-0.0049	-4	384	394	YDPTAYNTILR					Mascot
1326.6688	1326.6639	-0.0049	-4	384	394	YDPTAYNTILR	81	99.999			Mascot
1701.7247	1701.7289	0.0042	2	147	160	SAVQMYADYMTSFR			Oxidation (M)[5,10]		Mascot
1705.8181	1705.8896	0.0715	42	243	256	TQFFRDNGTYLSEK					Mascot
2013.9778	2013.9973	0.0195	10	303	320	VPSHAAELTAGYYNLHDR					Mascot
2013.9778	2013.9973	0.0195	10	303	320	VPSHAAELTAGYYNLHDR	127	100			Mascot

8 beta-amylase 2, partial [Milium effusum] gi|482677647 47235.6 6.49 7 204 100 16.549 186 100

Peptide Information

Calc. Mass	Obsrv. Mass	± da	± ppm	Start Seq.	End Seq.	Sequence	Ion Score	C. I. %	Modification	Rank	Result Type
947.5057	947.4861	-0.0196	-21	312	319	DGYRPIAR					Mascot
1016.5564	1016.5525	-0.0039	-4	402	409	LFGFTYLR					Mascot
1016.5564	1016.5525	-0.0039	-4	402	409	LFGFTYLR	59	99.75			Mascot
1729.9093	1729.874	-0.0353	-20	386	401	NARPNGINKTGPPEHK					Mascot
1743.8167	1743.8541	0.0374	21	323	336	RHHATLNFTCAEMR			Carbamidomethyl (C)[10]		Mascot
1835.936	1836.0403	0.1043	57	138	152	TAIQLYVDYMTSFRK					Mascot
2013.9778	2013.9973	0.0195	10	294	311	VPSHAAELTAGYYNLHDR					Mascot
2013.9778	2013.9973	0.0195	10	294	311	VPSHAAELTAGYYNLHDR	127	100			Mascot
2087.0557	2087.0767	0.021	10	120	137	NIEYLTLGVDDQPLFHGR					Mascot

9 beta-amylase 2, partial [Milium effusum] gi|482677645 47129.8 7.3 7 203 100 16.549 186 100

Peptide Information

Calc. Mass	Obsrv. Mass	± da	± ppm	Start Seq.	End Seq.	Sequence	Ion Score	C. I. %	Modification	Rank	Result Type
947.5057	947.4861	-0.0196	-21	312	319	DGYRPIAR					Mascot
1016.5564	1016.5525	-0.0039	-4	402	409	LFGFTYLR					Mascot
1016.5564	1016.5525	-0.0039	-4	402	409	LFGFTYLR	59	99.75			Mascot

Calc. Mass	Obsrv. Mass	± da	± ppm	Start Seq.	End Seq.	Sequence	Ion Score	C. I. %	Modification	Rank	Result Type
1729.9093	1729.874	-0.0353	-20	386	401	NARPNGINKTGPPEHK					Mascot
1743.8167	1743.8541	0.0374	21	323	336	RHHATLNFTCAEMR			Carbamidomethyl (C)[10]		Mascot
1835.936	1836.0403	0.1043	57	138	152	TAIQLYVDYMTSFRK					Mascot
2013.9778	2013.9973	0.0195	10	294	311	VPSHAAELTAGYYNLHD R					Mascot
2013.9778	2013.9973	0.0195	10	294	311	VPSHAAELTAGYYNLHD R	127	100			Mascot
2087.0557	2087.0767	0.021	10	120	137	NIEYLTLGVDDQPLFHGR					Mascot

10 Beta-amylase [Aegilops tauschii] gi|475523854 60203.5 5.07 11 176 100 20.767 140 100

Peptide Information

Calc. Mass	Obsrv. Mass	± da	± ppm	Start Seq.	End Seq.	Sequence	Ion Score	C. I. %	Modification	Rank	Result Type
947.5057	947.4861	-0.0196	-21	322	329	DGYRPIAR					Mascot
1016.5564	1016.5525	-0.0039	-4	412	419	LFGFTYLR					Mascot
1016.5564	1016.5525	-0.0039	-4	412	419	LFGFTYLR	59	99.75			Mascot
1326.6688	1326.6639	-0.0049	-4	385	395	YDPTAYNTILR					Mascot
1326.6688	1326.6639	-0.0049	-4	385	395	YDPTAYNTILR	81	99.999			Mascot
1490.6726	1490.6649	-0.0077	-5	372	384	EGLNMACENALPR			Carbamidomethyl (C)[7], Oxidation (M)[5]		Mascot
1573.6998	1573.8138	0.114	72	334	346	HHASLNFTCAEMR			Carbamidomethyl (C)[9]		Mascot
1589.6948	1589.7802	0.0854	54	334	346	HHASLNFTCAEMR			Carbamidomethyl (C)[9], Oxidation (M)[12]		Mascot
1646.781	1646.793	0.012	7	246	259	FFVDNGTYLTEQGR					Mascot
1668.7952	1668.7999	0.0047	3	218	232	AAAAMVGHPEWEFPR					Mascot
1684.79	1684.7843	-0.0057	-3	218	232	AAAAMVGHPEWEFPR			Oxidation (M)[5]		Mascot
1729.801	1729.874	0.073	42	333	346	RHHASLNFTCAEMR			Carbamidomethyl (C)[10]		Mascot
1752.8916	1752.8677	-0.0239	-14	420	434	LSNQLVEGQNYVNFK					Mascot
2087.0557	2087.0767	0.021	10	130	147	NIEYLTLGVDDQPLFHGR					Mascot
2167.0537	2167.0732	0.0195	9	440	458	MHANLPHDPCVDPVAPL QR			Carbamidomethyl (C)[10]		Mascot
2183.0486	2183.0481	-0.0005	0	440	458	MHANLPHDPCVDPVAPL QR			Carbamidomethyl (C)[10], Oxidation (M)[1]		Mascot

Gel Idx/Pos	277/L5	Instr./Gel Origin	BA2151/Sample Project 20140814	Process Status	Analysis Succeeded
Plate [#] Name	[1] Sample Project 20140814	Instrument Sample Name		Spectra	11

Rank	Protein Name	Accession No.	Protein MW	Protein PI	Pep. Count	Protein Score	Protein Score C. I. %	Intensity Matched	Total Ion Score	Total Ion C. I. %	Confirmed
1	Adenosylhomocysteinase [Aegilops tauschii]	gi|475434162	53905.2	5.62	18	563	100	24.824	473	100	

Peptide Information

Calc. Mass	Obsrv. Mass	± da	± ppm	Start Seq.	End Seq.	Sequence	Ion Score	C. I. %	Modification	Rank	Result Type
827.5713	827.5648	-0.0065	-8	176	182	IVLTIIR					Mascot
1008.4745	1008.4741	-0.0004	0	18	26	DLSQADFGR					Mascot
1008.4745	1008.4741	-0.0004	0	18	26	DLSQADFGR	75	99.995			Mascot
1028.6099	1028.6066	-0.0033	-3	385	394	TGIIVLAEGR					Mascot
1028.6099	1028.6066	-0.0033	-3	385	394	TGIIVLAEGR	13	0			Mascot
1041.5146	1041.5033	-0.0113	-11	246	254	HSLPDGLMR			Oxidation (M)[8]		Mascot
1044.4568	1044.4551	-0.0017	-2	238	245	FDNLYGCR			Carbamidomethyl (C)[7]		Mascot
1044.4568	1044.4551	-0.0017	-2	238	245	FDNLYGCR	30	0	Carbamidomethyl (C)[7]		Mascot
1071.6157	1071.6138	-0.0019	-2	369	377	ITIKPQTDR					Mascot
1137.5576	1137.5415	-0.0161	-14	44	53	TEFGPSQPFK					Mascot
1161.5392	1161.538	-0.0012	-1	329	337	DIIMVDHMR			Oxidation (M)[4,8]		Mascot
1235.6379	1235.6345	-0.0034	-3	16	26	VKDLSQADFGR					Mascot
1259.5837	1259.5884	0.0047	4	236	245	SKFDNLYGCR			Carbamidomethyl (C)[9]		Mascot
1259.5837	1259.5884	0.0047	4	236	245	SKFDNLYGCR	44	93.971	Carbamidomethyl (C)[9]		Mascot
1758.7428	1758.7628	0.02	11	111	123	GETLEEYWWCTER			Carbamidomethyl (C)[10]		Mascot
1758.7428	1758.7628	0.02	11	111	123	GETLEEYWWCTER	79	99.998	Carbamidomethyl (C)[10]		Mascot
1916.0641	1916.0217	-0.0424	-22	378	394	WVFPETKTGIIVLAEGR					Mascot
1978.9282	1978.9274	-0.0008	0	27	43	LELDLAEVEMPGLMACR			Carbamidomethyl (C)[16], Oxidation (M)[10,14]		Mascot
2178.9807	2179.0144	0.0337	15	83	101	WCSCNIFSTQDHAAAAIA R			Carbamidomethyl (C)[2,4]		Mascot
2178.9807	2179.0144	0.0337	15	83	101	WCSCNIFSTQDHAAAAIA R	114	100	Carbamidomethyl (C)[2,4]		Mascot
2241.1187	2241.1587	0.04	18	465	484	SQSDYISIPVEGPYKPAA YR					Mascot
2241.1187	2241.1587	0.04	18	465	484	SQSDYISIPVEGPYKPAA YR	116	100			Mascot
2583.3455	2583.3264	-0.0191	-7	462	484	LTKSQSDYISIPVEGPYKP AAYR					Mascot
2584.2964	2584.322	0.0256	10	213	235	LYQMQESGTLLFPAINVN DSVTK			Oxidation (M)[4]		Mascot
2740.4915	2740.4712	-0.0203	-7	57	82	ISGSLHMTIQTAVLIETLT ALGAEVR			Oxidation (M)[7]		Mascot

2	hypothetical protein OsI_35998 [Oryza sativa Indica Group]	gi|125534270	53860.2	5.62	14	481	100	18.037	425	100		

Peptide Information

Calc. Mass	Obsrv. Mass	± da	± ppm	Start Seq.	End Seq.	Sequence	Ion Score	C. I. %	Modification	Rank	Result Type
827.5713	827.5648	-0.0065	-8	176	182	IVLTIIR					Mascot
1008.4745	1008.4741	-0.0004	0	18	26	DLSQADFGR					Mascot
1008.4745	1008.4741	-0.0004	0	18	26	DLSQADFGR	75	99.995			Mascot
1041.5146	1041.5033	-0.0113	-11	246	254	HSLPDGLMR			Oxidation (M)[8]		Mascot
1044.4568	1044.4551	-0.0017	-2	238	245	FDNLYGCR			Carbamidomethyl (C)[7]		Mascot
1044.4568	1044.4551	-0.0017	-2	238	245	FDNLYGCR	30	0	Carbamidomethyl (C)[7]		Mascot
1071.6157	1071.6138	-0.0019	-2	369	377	ITIKPQTDR					Mascot
1107.547	1107.5271	-0.0199	-18	44	53	AEFGPSQPFK					Mascot
1161.5392	1161.538	-0.0012	-1	329	337	DIIMVDHMR			Oxidation (M)[4,8]		Mascot
1235.6379	1235.6345	-0.0034	-3	16	26	VKDLSQADFGR					Mascot
1259.5837	1259.5884	0.0047	4	236	245	SKFDNLYGCR			Carbamidomethyl (C)[9]		Mascot
1259.5837	1259.5884	0.0047	4	236	245	SKFDNLYGCR	44	93.971	Carbamidomethyl (C)[9]		Mascot
1758.7428	1758.7628	0.02	11	111	123	GETLEEYWWCTER			Carbamidomethyl (C)[10]		Mascot
1758.7428	1758.7628	0.02	11	111	123	GETLEEYWWCTER	79	99.998	Carbamidomethyl (C)[10]		Mascot
1902.0121	1902.0322	0.0201	11	378	394	WVFPETNTGIIVLAEGR					Mascot
1902.0121	1902.0322	0.0201	11	378	394	WVFPETNTGIIVLAEGR	81	99.999			Mascot
1992.9438	1992.9331	-0.0107	-5	27	43	LEIELAEVEMPGLMACR			Carbamidomethyl (C)[16], Oxidation (M)[10,14]		Mascot
2178.9807	2179.0144	0.0337	15	83	101	WCSCNIFSTQDHAAAAIA R			Carbamidomethyl (C)[2,4]		Mascot
2178.9807	2179.0144	0.0337	15	83	101	WCSCNIFSTQDHAAAAIA R	114	100	Carbamidomethyl (C)[2,4]		Mascot
2740.4915	2740.4712	-0.0203	-7	57	82	ISGSLHMTIQTAVLIETLT ALGAEVR			Oxidation (M)[7]		Mascot

3	Os11g0455500, partial [Oryza sativa Japonica Group]	gi|255680068	57302.1	6.62	14	479	100	18.037	425	100		

Peptide Information

Calc. Mass	Obsrv. Mass	± da	± ppm	Start Seq.	End Seq.	Sequence	Ion Score	C. I. %	Modification	Rank	Result Type
827.5713	827.5648	-0.0065	-8	206	212	IVLTIIR					Mascot
1008.4745	1008.4741	-0.0004	0	48	56	DLSQADFGR					Mascot
1008.4745	1008.4741	-0.0004	0	48	56	DLSQADFGR	75	99.995			Mascot
1041.5146	1041.5033	-0.0113	-11	276	284	HSLPDGLMR			Oxidation (M)[8]		Mascot
1044.4568	1044.4551	-0.0017	-2	268	275	FDNLYGCR			Carbamidomethyl (C)[7]		Mascot
1044.4568	1044.4551	-0.0017	-2	268	275	FDNLYGCR	30	0	Carbamidomethyl (C)[7]		Mascot

1071.6157	1071.6138	-0.0019	-2	399	407	ITIKPQTDR					Mascot
1107.547	1107.5271	-0.0199	-18	74	83	AEFGPSQPFK					Mascot
1161.5392	1161.538	-0.0012	-1	359	367	DIIMVDHMR			Oxidation (M)[4,8]		Mascot
1235.6379	1235.6345	-0.0034	-3	46	56	VKDLSQADFGR					Mascot
1259.5837	1259.5884	0.0047	4	266	275	SKFDNLYGCR			Carbamidomethyl (C)[9]		Mascot
1259.5837	1259.5884	0.0047	4	266	275	SKFDNLYGCR	44	93.971	Carbamidomethyl (C)[9]		Mascot
1758.7428	1758.7628	0.02	11	141	153	GETLEEYWWCTER			Carbamidomethyl (C)[10]		Mascot
1758.7428	1758.7628	0.02	11	141	153	GETLEEYWWCTER	79	99.998	Carbamidomethyl (C)[10]		Mascot
1902.0121	1902.0322	0.0201	11	408	424	WVFPETNTGIIVLAEGR					Mascot
1902.0121	1902.0322	0.0201	11	408	424	WVFPETNTGIIVLAEGR	81	99.999			Mascot
1992.9438	1992.9331	-0.0107	-5	57	73	LEIELAEVEMPGLMACR			Carbamidomethyl (C)[16], Oxidation (M)[10,14]		Mascot
2178.9807	2179.0144	0.0337	15	113	131	WCSCNIFSTQDHAAAAIA R			Carbamidomethyl (C)[2,4]		Mascot
2178.9807	2179.0144	0.0337	15	113	131	WCSCNIFSTQDHAAAAIA R	114	100	Carbamidomethyl (C)[2,4]		Mascot
2740.4915	2740.4712	-0.0203	-7	87	112	ISGSLHMTIQTAVLIETLT ALGAEVR			Oxidation (M)[7]		Mascot

4 adenosylhomocysteinase [Zea mays] gi|226491362 53898.3 5.63 13 475 100 17.877 425 100

Protein Group

PREDICTED: adenosylhomocysteinase-like [Setaria italica] — gi|514794242 — 53974.2 — 5.6799998283 3862

adenosyl homocysteine hydrolase1 isoform 1 [Zea mays] — gi|413920900 — 53884.3 — 5.6199998855 5908

adenosyl homocysteine hydrolase1 isoform 2 [Zea mays] — gi|413920901 — 53884.3 — 5.6199998855 5908

Peptide Information

Calc. Mass	Obsrv. Mass	± da	± ppm	Start Seq.	End Seq.	Sequence	Ion Score	C. I. %	Modification	Rank	Result Type
827.5713	827.5648	-0.0065	-8	176	182	IVLTIIR					Mascot
1008.4745	1008.4741	-0.0004	0	18	26	DLSQADFGR					Mascot
1008.4745	1008.4741	-0.0004	0	18	26	DLSQADFGR	75	99.995			Mascot
1041.5146	1041.5033	-0.0113	-11	246	254	HSLPDGLMR			Oxidation (M)[8]		Mascot
1044.4568	1044.4551	-0.0017	-2	238	245	FDNLYGCR			Carbamidomethyl (C)[7]		Mascot
1044.4568	1044.4551	-0.0017	-2	238	245	FDNLYGCR	30	0	Carbamidomethyl (C)[7]		Mascot
1071.6157	1071.6138	-0.0019	-2	369	377	ITIKPQTDR					Mascot
1161.5392	1161.538	-0.0012	-1	329	337	DIIMVDHMR			Oxidation (M)[4,8]		Mascot

Calc. Mass	Obsrv. Mass	± da	± ppm	Start Seq.	End Seq.	Sequence	Ion Score	C. I. %	Modification	Rank	Result Type
1235.6379	1235.6345	-0.0034	-3	16	26	VKDLSQADFGR					Mascot
1259.5837	1259.5884	0.0047	4	236	245	SKFDNLYGCR			Carbamidomethyl (C)[9]		Mascot
1259.5837	1259.5884	0.0047	4	236	245	SKFDNLYGCR	44	93.971	Carbamidomethyl (C)[9]		Mascot
1758.7428	1758.7628	0.02	11	111	123	GETLEEYWWCTER			Carbamidomethyl (C)[10]		Mascot
1758.7428	1758.7628	0.02	11	111	123	GETLEEYWWCTER	79	99.998	Carbamidomethyl (C)[10]		Mascot
1902.0121	1902.0322	0.0201	11	378	394	WVFPETNTGIIVLAEGR					Mascot
1902.0121	1902.0322	0.0201	11	378	394	WVFPETNTGIIVLAEGR	81	99.999			Mascot
1992.9438	1992.9331	-0.0107	-5	27	43	LEIELAEVEMPGLMACR			Carbamidomethyl (C)[16], Oxidation (M)[10,14]		Mascot
2178.9807	2179.0144	0.0337	15	83	101	WCSCNIFSTQDHAAAAIA R			Carbamidomethyl (C)[2,4]		Mascot
2178.9807	2179.0144	0.0337	15	83	101	WCSCNIFSTQDHAAAAIA R	114	100	Carbamidomethyl (C)[2,4]		Mascot
2740.4915	2740.4712	-0.0203	-7	57	82	ISGSLHMTIQTAVLIETLT ALGAEVR			Oxidation (M)[7]		Mascot

5	PREDICTED: adenosylhomocysteinase-like [Setaria italica]	gi|514716433	54002.3	5.68	14	412	100	17.203	357	100

Peptide Information

Calc. Mass	Obsrv. Mass	± da	± ppm	Start Seq.	End Seq.	Sequence	Ion Score	C. I. %	Modification	Rank	Result Type
827.5713	827.5648	-0.0065	-8	176	182	IVLTIIR					Mascot
1008.4745	1008.4741	-0.0004	0	18	26	DLSQADFGR					Mascot
1008.4745	1008.4741	-0.0004	0	18	26	DLSQADFGR	75	99.995			Mascot
1028.6099	1028.6066	-0.0033	-3	385	394	TGIIVLAEGR					Mascot
1028.6099	1028.6066	-0.0033	-3	385	394	TGIIVLAEGR	13	0			Mascot
1041.5146	1041.5033	-0.0113	-11	246	254	HSLPDGLMR			Oxidation (M)[8]		Mascot
1044.4568	1044.4551	-0.0017	-2	238	245	FDNLYGCR			Carbamidomethyl (C)[7]		Mascot
1044.4568	1044.4551	-0.0017	-2	238	245	FDNLYGCR	30	0	Carbamidomethyl (C)[7]		Mascot
1071.6157	1071.6138	-0.0019	-2	369	377	ITIKPQTDR					Mascot
1161.5392	1161.538	-0.0012	-1	329	337	DIIMVDHMR			Oxidation (M)[4,8]		Mascot
1235.6379	1235.6345	-0.0034	-3	16	26	VKDLSQADFGR					Mascot
1259.5837	1259.5884	0.0047	4	236	245	SKFDNLYGCR			Carbamidomethyl (C)[9]		Mascot
1259.5837	1259.5884	0.0047	4	236	245	SKFDNLYGCR	44	93.971	Carbamidomethyl (C)[9]		Mascot
1758.7428	1758.7628	0.02	11	111	123	GETLEEYWWCTER			Carbamidomethyl (C)[10]		Mascot
1758.7428	1758.7628	0.02	11	111	123	GETLEEYWWCTER	79	99.998	Carbamidomethyl (C)[10]		Mascot
1916.0641	1916.0217	-0.0424	-22	378	394	WVFPETKTGIIVLAEGR					Mascot
1992.9438	1992.9331	-0.0107	-5	27	43	LEIELAEVEMPGLMACR			Carbamidomethyl (C)[16], Oxidation (M)[10,14]		Mascot
2178.9807	2179.0144	0.0337	15	83	101	WCSCNIFSTQDHAAAAIA R			Carbamidomethyl (C)[2,4]		Mascot
2178.9807	2179.0144	0.0337	15	83	101	WCSCNIFSTQDHAAAAIA	114	100	Carbamidomethyl (C)[2,4]		Mascot

						R					
2740.4915	2740.4712	-0.0203	-7	57	82	ISGSLHMTIQTAVLIETLT ALGAEVR			Oxidation (M)[7]		Mascot

6	Adenosylhomocysteinase, putative, expressed [Oryza sativa Japonica Group]	gi|108864354	43453.6	5.1	11	396	100	14.5	349	100

Peptide Information

Calc. Mass	Obsrv. Mass	± da	± ppm	Start Seq.	End Seq.	Sequence	Ion Score	C. I. %	Modification	Rank	Result Type
827.5713	827.5648	-0.0065	-8	141	147	IVLTIIR					Mascot
1041.5146	1041.5033	-0.0113	-11	211	219	HSLPDGLMR			Oxidation (M)[8]		Mascot
1044.4568	1044.4551	-0.0017	-2	203	210	FDNLYGCR			Carbamidomethyl (C)[7]		Mascot
1044.4568	1044.4551	-0.0017	-2	203	210	FDNLYGCR	30	0	Carbamidomethyl (C)[7]		Mascot
1071.6157	1071.6138	-0.0019	-2	334	342	ITIKPQTDR					Mascot
1107.547	1107.5271	-0.0199	-18	9	18	AEFGPSQPFK					Mascot
1161.5392	1161.538	-0.0012	-1	294	302	DIIMVDHMR			Oxidation (M)[4,8]		Mascot
1259.5837	1259.5884	0.0047	4	201	210	SKFDNLYGCR			Carbamidomethyl (C)[9]		Mascot
1259.5837	1259.5884	0.0047	4	201	210	SKFDNLYGCR	44	93.971	Carbamidomethyl (C)[9]		Mascot
1758.7428	1758.7628	0.02	11	76	88	GETLEEYWWCTER			Carbamidomethyl (C)[10]		Mascot
1758.7428	1758.7628	0.02	11	76	88	GETLEEYWWCTER	79	99.998	Carbamidomethyl (C)[10]		Mascot
1902.0121	1902.0322	0.0201	11	343	359	WVFPETNTGIIVLAEGR					Mascot
1902.0121	1902.0322	0.0201	11	343	359	WVFPETNTGIIVLAEGR	81	99.999			Mascot
2178.9807	2179.0144	0.0337	15	48	66	WCSCNIFSTQDHAAAAIA R			Carbamidomethyl (C)[2,4]		Mascot
2178.9807	2179.0144	0.0337	15	48	66	WCSCNIFSTQDHAAAAIA R	114	100	Carbamidomethyl (C)[2,4]		Mascot
2740.4915	2740.4712	-0.0203	-7	22	47	ISGSLHMTIQTAVLIETLT ALGAEVR			Oxidation (M)[7]		Mascot

7	Adenosylhomocysteinase, putative, expressed [Oryza sativa Japonica Group]	gi|77550703	49951.3	5.88	11	391	100	14.5	349	100

Protein Group

Adenosylhomocysteinase, putative, expressed [Oryza sativa Japonica Group]	gi|77550704	49951.3	5.8800 001144 4092

Peptide Information

Calc. Mass	Obsrv. Mass	± da	± ppm	Start Seq.	End Seq.	Sequence	Ion Score	C. I. %	Modification	Rank	Result Type
827.5713	827.5648	-0.0065	-8	141	147	IVLTIIR					Mascot
1041.5146	1041.5033	-0.0113	-11	211	219	HSLPDGLMR			Oxidation (M)[8]		Mascot
1044.4568	1044.4551	-0.0017	-2	203	210	FDNLYGCR			Carbamidomethyl (C)[7]		Mascot
1044.4568	1044.4551	-0.0017	-2	203	210	FDNLYGCR	30	0	Carbamidomethyl (C)[7]		Mascot

1071.6157	1071.6138	-0.0019	-2	334	342	ITIKPQTDR					Mascot
1107.547	1107.5271	-0.0199	-18	9	18	AEFGPSQPFK					Mascot
1161.5392	1161.538	-0.0012	-1	294	302	DIIMVDHMR			Oxidation (M)[4,8]		Mascot
1259.5837	1259.5884	0.0047	4	201	210	SKFDNLYGCR			Carbamidomethyl (C)[9]		Mascot
1259.5837	1259.5884	0.0047	4	201	210	SKFDNLYGCR	44	93.971	Carbamidomethyl (C)[9]		Mascot
1758.7428	1758.7628	0.02	11	76	88	GETLEEYWWCTER			Carbamidomethyl (C)[10]		Mascot
1758.7428	1758.7628	0.02	11	76	88	GETLEEYWWCTER	79	99.998	Carbamidomethyl (C)[10]		Mascot
1902.0121	1902.0322	0.0201	11	343	359	WVFPETNTGIIVLAEGR					Mascot
1902.0121	1902.0322	0.0201	11	343	359	WVFPETNTGIIVLAEGR	81	99.999			Mascot
2178.9807	2179.0144	0.0337	15	48	66	WCSCNIFSTQDHAAAAIAR			Carbamidomethyl (C)[2,4]		Mascot
2178.9807	2179.0144	0.0337	15	48	66	WCSCNIFSTQDHAAAAIAR	114	100	Carbamidomethyl (C)[2,4]		Mascot
2740.4915	2740.4712	-0.0203	-7	22	47	ISGSLHMTIQTAVLIETLTALGAEVR			Oxidation (M)[7]		Mascot

8	adenosylhomocysteinase-like [Cicer arietinum]	gi|525314123	53923.3	5.53	11	383	100	13.697	346	100	

Peptide Information

Calc. Mass	Obsrv. Mass	± da	± ppm	Start Seq.	End Seq.	Sequence	Ion Score	C. I. %	Modification	Rank	Result Type
1008.4745	1008.4741	-0.0004	0	18	26	DLSQADFGR					Mascot
1008.4745	1008.4741	-0.0004	0	18	26	DLSQADFGR	75	99.995			Mascot
1041.5146	1041.5033	-0.0113	-11	246	254	HSLPDGLMR			Oxidation (M)[8]		Mascot
1044.4568	1044.4551	-0.0017	-2	238	245	FDNLYGCR			Carbamidomethyl (C)[7]		Mascot
1044.4568	1044.4551	-0.0017	-2	238	245	FDNLYGCR	30	0	Carbamidomethyl (C)[7]		Mascot
1071.6157	1071.6138	-0.0019	-2	369	377	ITIKPQTDR					Mascot
1107.5173	1107.5271	0.0098	9	329	337	DIIMVDDMR					Mascot
1137.5576	1137.5415	-0.0161	-14	44	53	TEFGPSQPFK					Mascot
1235.6379	1235.6345	-0.0034	-3	16	26	VKDLSQADFGR					Mascot
1259.5837	1259.5884	0.0047	4	236	245	SKFDNLYGCR			Carbamidomethyl (C)[9]		Mascot
1259.5837	1259.5884	0.0047	4	236	245	SKFDNLYGCR	44	93.971	Carbamidomethyl (C)[9]		Mascot
1902.0121	1902.0322	0.0201	11	378	394	WVFPETNTGIIVLAEGR					Mascot
1902.0121	1902.0322	0.0201	11	378	394	WVFPETNTGIIVLAEGR	81	99.999			Mascot
1916.0045	1916.0217	0.0172	9	27	43	LEIELAEIEMPGLISSR			Oxidation (M)[10]		Mascot
2178.9807	2179.0144	0.0337	15	83	101	WCSCNIFSTQDHAAAAIAR			Carbamidomethyl (C)[2,4]		Mascot
2178.9807	2179.0144	0.0337	15	83	101	WCSCNIFSTQDHAAAAIAR	114	100	Carbamidomethyl (C)[2,4]		Mascot

9	Adenosylhomocysteinase [Triticum urartu]	gi|474154141	46127.7	6.48	16	354	100	20.902	280	100	

Peptide Information

Calc. Mass	Obsrv. Mass	± da	± ppm	Start Seq.	End Seq.	Sequence	Ion Score	C. I. %	Modification	Rank	Result Type
827.5713	827.5648	-0.0065	-8	107	113	IVLTIIR					Mascot
1008.4745	1008.4741	-0.0004	0	18	26	DLSQADFGR					Mascot
1008.4745	1008.4741	-0.0004	0	18	26	DLSQADFGR	75	99.995			Mascot
1028.6099	1028.6066	-0.0033	-3	316	325	TGIIVLAEGR					Mascot
1028.6099	1028.6066	-0.0033	-3	316	325	TGIIVLAEGR	13	0			Mascot
1041.5146	1041.5033	-0.0113	-11	177	185	HSLPDGLMR			Oxidation (M)[8]		Mascot
1044.4568	1044.4551	-0.0017	-2	169	176	FDNLYGCR			Carbamidomethyl (C)[7]		Mascot
1044.4568	1044.4551	-0.0017	-2	169	176	FDNLYGCR	30	0	Carbamidomethyl (C)[7]		Mascot
1071.6157	1071.6138	-0.0019	-2	300	308	ITIKPQTDR					Mascot
1137.5576	1137.5415	-0.0161	-14	44	53	TEFGPSQPFK					Mascot
1161.5392	1161.538	-0.0012	-1	260	268	DIIMVDHMR			Oxidation (M)[4,8]		Mascot
1235.6379	1235.6345	-0.0034	-3	16	26	VKDLSQADFGR					Mascot
1259.5837	1259.5884	0.0047	4	167	176	SKFDNLYGCR			Carbamidomethyl (C)[9]		Mascot
1259.5837	1259.5884	0.0047	4	167	176	SKFDNLYGCR	44	93.971	Carbamidomethyl (C)[9]		Mascot
1916.0641	1916.0217	-0.0424	-22	309	325	WVFPETKTGIIVLAEGR					Mascot
1978.9282	1978.9274	-0.0008	0	27	43	LELDLAEVEMPGLMACR			Carbamidomethyl (C)[16], Oxidation (M)[10,14]		Mascot
2241.1187	2241.1587	0.04	18	396	415	SQSDYISIPVEGPYKPAAYR					Mascot
2241.1187	2241.1587	0.04	18	396	415	SQSDYISIPVEGPYKPAAYR	116	100			Mascot
2583.3455	2583.3264	-0.0191	-7	393	415	LTKSQSDYISIPVEGPYKPAAYR					Mascot
2584.2964	2584.322	0.0256	10	144	166	LYQMQESGTLLFPAINVNDSVTK			Oxidation (M)[4]		Mascot
2740.3975	2740.4712	0.0737	27	143	166	RLYQMQESGTLLFPAINVNDSVTK			Oxidation (M)[5]		Mascot

10	adenosyl homocysteine hydrolase1 [Zea mays]	gi|413920902	28835.5	4.85	8	308	100	9.454	269	100		

Peptide Information

Calc. Mass	Obsrv. Mass	± da	± ppm	Start Seq.	End Seq.	Sequence	Ion Score	C. I. %	Modification	Rank	Result Type
827.5713	827.5648	-0.0065	-8	176	182	IVLTIIR					Mascot
1008.4745	1008.4741	-0.0004	0	18	26	DLSQADFGR					Mascot
1008.4745	1008.4741	-0.0004	0	18	26	DLSQADFGR	75	99.995			Mascot
1235.6379	1235.6345	-0.0034	-3	16	26	VKDLSQADFGR					Mascot
1647.8927	1647.7847	-0.108	-66	238	251	VILACLSLDPFPFR			Carbamidomethyl (C)[5]		Mascot
1758.7428	1758.7628	0.02	11	111	123	GETLEEYWWCTER			Carbamidomethyl (C)[10]		Mascot

1758.7428	1758.7628	0.02	11	111	123	GETLEEYWWCTER	79	99.998	Carbamidomethyl (C)[10]	Mascot
1992.9438	1992.9331	-0.0107	-5	27	43	LEIELAEVEMPGLMACR			Carbamidomethyl (C)[16], Oxidation (M)[10,14]	Mascot
2178.9807	2179.0144	0.0337	15	83	101	WCSCNIFSTQDHAAAAIA R			Carbamidomethyl (C)[2,4]	Mascot
2178.9807	2179.0144	0.0337	15	83	101	WCSCNIFSTQDHAAAAIA R	114	100	Carbamidomethyl (C)[2,4]	Mascot
2740.4915	2740.4712	-0.0203	-7	57	82	ISGSLHMTIQTAVLIETLT ALGAEVR			Oxidation (M)[7]	Mascot

Gel Idx/Pos	278/L6	Instr./Gel Origin	BA2151/Sample Project 20140814	Process Status	Analysis Succeeded
Plate [#] Name	[1] Sample Project 20140814	Instrument Sample Name		Spectra	11

Rank	Protein Name	Accession No.	Protein MW	Protein PI	Pep. Count	Protein Score	Protein Score C. I. %	Intensity Matched	Total Ion Score	Total Ion C. I. %	Confirmed
1	atp1 [Triticum aestivum]	gi|81176509	55556.8	5.7	19	837	100	47.484	739	100	

Peptide Information

Calc. Mass	Obsrv. Mass	± da	± ppm	Start Seq.	End Seq.	Sequence	Ion Score	C. I. %	Modification	Rank	Result Type
815.4621	815.4484	-0.0137	-17	167	173	ELIIGDR					Mascot
860.5022	860.4489	-0.0533	-62	283	289	QMSLLLR					Mascot
874.4741	874.4857	0.0116	13	487	494	GGLTNERK					Mascot
876.4971	876.4717	-0.0254	-29	283	289	QMSLLLR			Oxidation (M)[2]		Mascot
884.4221	884.4128	-0.0093	-11	121	128	GALSDHER					Mascot
892.4886	892.4791	-0.0095	-11	395	401	LELAQYR					Mascot
972.5473	972.5329	-0.0144	-15	33	42	VVSVGDGIAR					Mascot
972.5473	972.5329	-0.0144	-15	33	42	VVSVGDGIAR	72	99.986			Mascot
1026.5942	1026.5762	-0.018	-18	154	163	AVDSLVPIGR					Mascot
1026.5942	1026.5762	-0.018	-18	154	163	AVDSLVPIGR	73	99.991			Mascot
1203.658	1203.6455	-0.0125	-10	7	17	AAELTTLLESR					Mascot
1203.658	1203.6455	-0.0125	-10	7	17	AAELTTLLESR	74	99.993			Mascot
1242.6147	1242.5756	-0.0391	-31	143	153	SVHEPMQTGLK			Oxidation (M)[6]		Mascot
1300.7471	1300.6886	-0.0585	-45	178	189	TAIAIDTILNQK					Mascot
1326.7627	1326.7087	-0.054	-41	470	481	AILSTINPELQK					Mascot
1341.705	1341.649	-0.056	-42	433	443	QPQYEPLPIEK					Mascot
1438.8489	1438.8287	-0.0202	-14	363	376	GIRPAINVGLSVSR					Mascot
1438.8489	1438.8287	-0.0202	-14	363	376	GIRPAINVGLSVSR	45	93.846			Mascot
1537.7434	1537.7349	-0.0085	-6	295	307	EAFPGDVFYLHSR					Mascot
1537.7434	1537.7349	-0.0085	-6	295	307	EAFPGDVFYLHSR	108	100			Mascot
1720.821	1720.9739	0.1529	89	262	276	DNGMHALIIYDDLSK			Oxidation (M)[4]		Mascot
1724.8789	1724.8748	-0.0041	-2	444	458	QIVVIYAAVNGFCDR			Carbamidomethyl (C)[13]		Mascot
1834.8429	1834.851	0.0081	4	18	32	MTNFYTNFQVDEIGR					Mascot
1850.8378	1850.8162	-0.0216	-12	18	32	MTNFYTNFQVDEIGR			Oxidation (M)[1]		Mascot
1850.8378	1850.8162	-0.0216	-12	18	32	MTNFYTNFQVDEIGR	133	100	Oxidation (M)[1]		Mascot
2157.0532	2157.0288	-0.0244	-11	43	62	VYGLNEIQAGEMVEFAS GVK			Oxidation (M)[12]		Mascot
2308.1567	2308.1646	0.0079	3	402	423	EVAAFAQFGSDLDAATQ					Mascot

2308.1567	2308.1646	0.0079	3	402	423	ALLNR EVAAFAQFGSDLDAATQ ALLNR	233	100			Mascot

2	RecName: Full=ATP synthase subunit alpha, mitochondrial	gi|148886791	55624	5.85	19	837	100	47.013	739	100	

Protein Group

ATP synthase F0 subunit 1 [Oryza sativa Japonica Group]	gi|194033257	55624	5.8499 999046 3257
RecName: Full=ATP synthase subunit alpha, mitochondrial	gi|148886790	55624	5.8499 999046 3257
RecName: Full=ATP synthase subunit alpha, mitochondrial	gi|148886792	55624	5.8499 999046 3257

Peptide Information

Calc. Mass	Obsrv. Mass	± da	± ppm	Start Seq.	End Seq.	Sequence	Ion Score	C. I. %	Modification	Rank	Result Type
815.4621	815.4484	-0.0137	-17	167	173	ELIIGDR					Mascot
860.5022	860.4489	-0.0533	-62	283	289	QMSLLLR					Mascot
874.4741	874.4857	0.0116	13	487	494	GGLTNERK					Mascot
876.4971	876.4717	-0.0254	-29	283	289	QMSLLLR			Oxidation (M)[2]		Mascot
884.4221	884.4128	-0.0093	-11	121	128	GALSDHER					Mascot
892.4886	892.4791	-0.0095	-11	395	401	LELAQYR					Mascot
972.5473	972.5329	-0.0144	-15	33	42	VVSVGDGIAR					Mascot
972.5473	972.5329	-0.0144	-15	33	42	VVSVGDGIAR	72	99.986			Mascot
1026.5942	1026.5762	-0.018	-18	154	163	AVDSLVPIGR					Mascot
1026.5942	1026.5762	-0.018	-18	154	163	AVDSLVPIGR	73	99.991			Mascot
1137.5756	1137.5282	-0.0474	-42	385	394	AMKQVCGSLK			Carbamidomethyl (C)[6], Oxidation (M)[2]		Mascot
1203.658	1203.6455	-0.0125	-10	7	17	AAELTTLLESR					Mascot
1203.658	1203.6455	-0.0125	-10	7	17	AAELTTLLESR	74	99.993			Mascot
1242.6147	1242.5756	-0.0391	-31	143	153	SVHEPMQTGLK			Oxidation (M)[6]		Mascot
1300.7471	1300.6886	-0.0585	-45	178	189	TAIAIDTILNQK					Mascot
1341.705	1341.649	-0.056	-42	433	443	QPQYEPLPIEK					Mascot
1438.8489	1438.8287	-0.0202	-14	363	376	GIRPAINVGLSVSR					Mascot
1438.8489	1438.8287	-0.0202	-14	363	376	GIRPAINVGLSVSR	45	93.846			Mascot
1537.7434	1537.7349	-0.0085	-6	295	307	EAFPGDVFYLHSR					Mascot
1537.7434	1537.7349	-0.0085	-6	295	307	EAFPGDVFYLHSR	108	100			Mascot
1720.821	1720.9739	0.1529	89	262	276	DNGMHALIIYDDLSK			Oxidation (M)[4]		Mascot

Calc. Mass	Obsrv. Mass	± da	± ppm	Start Seq.	End Seq.	Sequence	Ion Score	C. I. %	Modification	Rank	Result Type
1724.8789	1724.8748	-0.0041	-2	444	458	QIVVIYAAVNGFCDR			Carbamidomethyl (C)[13]		Mascot
1834.8429	1834.851	0.0081	4	18	32	MTNFYTNFQVDEIGR					Mascot
1850.8378	1850.8162	-0.0216	-12	18	32	MTNFYTNFQVDEIGR			Oxidation (M)[1]		Mascot
1850.8378	1850.8162	-0.0216	-12	18	32	MTNFYTNFQVDEIGR	133	100	Oxidation (M)[1]		Mascot
2157.0532	2157.0288	-0.0244	-11	43	62	VYGLNEIQAGEMVEFAS GVK			Oxidation (M)[12]		Mascot
2308.1567	2308.1646	0.0079	3	402	423	EVAAFAQFGSDLDAATQ ALLNR					Mascot
2308.1567	2308.1646	0.0079	3	402	423	EVAAFAQFGSDLDAATQ ALLNR	233	100			Mascot

3 ATP synthase subunits 1 (mitochondrion) [Lolium perenne] gi|472833568 55526.8 5.7 18 829 100 46.843 739 100

Protein Group

PREDICTED: ATP synthase subunit alpha, mitochondrial-like [Setaria italica] gi|514825289 55564.9 5.6999998092 6514

Peptide Information

Calc. Mass	Obsrv. Mass	± da	± ppm	Start Seq.	End Seq.	Sequence	Ion Score	C. I. %	Modification	Rank	Result Type
815.4621	815.4484	-0.0137	-17	167	173	ELIIGDR					Mascot
860.5022	860.4489	-0.0533	-62	283	289	QMSLLLR					Mascot
874.4741	874.4857	0.0116	13	487	494	GGLTNERK					Mascot
876.4971	876.4717	-0.0254	-29	283	289	QMSLLLR			Oxidation (M)[2]		Mascot
884.4221	884.4128	-0.0093	-11	121	128	GALSDHER					Mascot
892.4886	892.4791	-0.0095	-11	395	401	LELAQYR					Mascot
972.5473	972.5329	-0.0144	-15	33	42	VVSVGDGIAR					Mascot
972.5473	972.5329	-0.0144	-15	33	42	VVSVGDGIAR	72	99.986			Mascot
1026.5942	1026.5762	-0.018	-18	154	163	AVDSLVPIGR					Mascot
1026.5942	1026.5762	-0.018	-18	154	163	AVDSLVPIGR	73	99.991			Mascot
1203.658	1203.6455	-0.0125	-10	7	17	AAELTTLLESR					Mascot
1203.658	1203.6455	-0.0125	-10	7	17	AAELTTLLESR	74	99.993			Mascot
1242.6147	1242.5756	-0.0391	-31	143	153	SVHEPMQTGLK			Oxidation (M)[6]		Mascot
1300.7471	1300.6886	-0.0585	-45	178	189	TAIAIDTILNQK					Mascot
1341.705	1341.649	-0.056	-42	433	443	QPQYEPLPIEK					Mascot
1438.8489	1438.8287	-0.0202	-14	363	376	GIRPAINVGLSVSR					Mascot
1438.8489	1438.8287	-0.0202	-14	363	376	GIRPAINVGLSVSR	45	93.846			Mascot
1537.7434	1537.7349	-0.0085	-6	295	307	EAFPGDVFYLHSR					Mascot
1537.7434	1537.7349	-0.0085	-6	295	307	EAFPGDVFYLHSR	108	100			Mascot
1720.821	1720.9739	0.1529	89	262	276	DNGMHALIIYDDLSK			Oxidation (M)[4]		Mascot

1724.8789	1724.8748	-0.0041	-2	444	458	QIVVIYAAVNGFCDR			Carbamidomethyl (C)[13]		Mascot
1834.8429	1834.851	0.0081	4	18	32	MTNFYTNFQVDEIGR					Mascot
1850.8378	1850.8162	-0.0216	-12	18	32	MTNFYTNFQVDEIGR			Oxidation (M)[1]		Mascot
1850.8378	1850.8162	-0.0216	-12	18	32	MTNFYTNFQVDEIGR	133	100	Oxidation (M)[1]		Mascot
2157.0532	2157.0288	-0.0244	-11	43	62	VYGLNEIQAGEMVEFAS GVK			Oxidation (M)[12]		Mascot
2308.1567	2308.1646	0.0079	3	402	423	EVAAFAQFGSDLDAATQ ALLNR					Mascot
2308.1567	2308.1646	0.0079	3	402	423	EVAAFAQFGSDLDAATQ ALLNR	233	100			Mascot

4	ATP synthase F0 subunit 1 [Oryza sativa Indica Group]	gi|89280711	55531.9	5.85	18	829	100	46.843	739	100	

Protein Group

ATP synthase F0 subunit 1 (mitochondrion) [Oryza sativa Indica Group]	gi|353685290	55531.9	5.8499 999046 3257
ATP synthase F0 subunit 1 (mitochondrion) [Oryza sativa Indica Group]	gi|353685289	55531.9	5.8499 999046 3257
ATP synthase F0 subunit 1 (mitochondrion) [Oryza sativa Indica Group]	gi|353685221	55531.9	5.8499 999046 3257
ATP synthase F0 subunit 1 (mitochondrion) [Oryza sativa Indica Group]	gi|353685220	55531.9	5.8499 999046 3257
ATP synthase F0 subunit 1 [Oryza rufipogon]	gi|289065052	55531.9	5.8499 999046 3257

Peptide Information

Calc. Mass	Obsrv. Mass	± da	± ppm	Start Seq.	End Seq.	Sequence	Ion Score	C. I. %	Modification	Rank	Result Type
815.4621	815.4484	-0.0137	-17	167	173	ELIIGDR					Mascot
860.5022	860.4489	-0.0533	-62	283	289	QMSLLLR					Mascot
874.4741	874.4857	0.0116	13	487	494	GGLTNERK					Mascot
876.4971	876.4717	-0.0254	-29	283	289	QMSLLLR			Oxidation (M)[2]		Mascot
884.4221	884.4128	-0.0093	-11	121	128	GALSDHER					Mascot
892.4886	892.4791	-0.0095	-11	395	401	LELAQYR					Mascot
972.5473	972.5329	-0.0144	-15	33	42	VVSVGDGIAR					Mascot
972.5473	972.5329	-0.0144	-15	33	42	VVSVGDGIAR	72	99.986			Mascot
1026.5942	1026.5762	-0.018	-18	154	163	AVDSLVPIGR					Mascot
1026.5942	1026.5762	-0.018	-18	154	163	AVDSLVPIGR	73	99.991			Mascot

Calc. Mass	Obsrv. Mass	± da	± ppm	Start Seq.	End Seq.	Sequence	Ion Score	C. I. %	Modification	Rank	Result Type
1203.658	1203.6455	-0.0125	-10	7	17	AAELTTLLESR					Mascot
1203.658	1203.6455	-0.0125	-10	7	17	AAELTTLLESR	74	99.993			Mascot
1242.6147	1242.5756	-0.0391	-31	143	153	SVHEPMQTGLK			Oxidation (M)[6]		Mascot
1300.7107	1300.6886	-0.0221	-17	495	506	IEPDASLKQTAK					Mascot
1341.705	1341.649	-0.056	-42	433	443	QPQYEPLPIEK					Mascot
1438.8489	1438.8287	-0.0202	-14	363	376	GIRPAINVGLSVSR					Mascot
1438.8489	1438.8287	-0.0202	-14	363	376	GIRPAINVGLSVSR	45	93.846			Mascot
1537.7434	1537.7349	-0.0085	-6	295	307	EAFPGDVFYLHSR					Mascot
1537.7434	1537.7349	-0.0085	-6	295	307	EAFPGDVFYLHSR	108	100			Mascot
1720.821	1720.9739	0.1529	89	262	276	DNGMHALIIYDDLSK			Oxidation (M)[4]		Mascot
1724.8789	1724.8748	-0.0041	-2	444	458	QIVVIYAAVNGFCDR			Carbamidomethyl (C)[13]		Mascot
1834.8429	1834.851	0.0081	4	18	32	MTNFYTNFQVDEIGR					Mascot
1850.8378	1850.8162	-0.0216	-12	18	32	MTNFYTNFQVDEIGR			Oxidation (M)[1]		Mascot
1850.8378	1850.8162	-0.0216	-12	18	32	MTNFYTNFQVDEIGR	133	100	Oxidation (M)[1]		Mascot
2157.0532	2157.0288	-0.0244	-11	43	62	VYGLNEIQAGEMVEFAS GVK			Oxidation (M)[12]		Mascot
2308.1567	2308.1646	0.0079	3	402	423	EVAAFAQFGSDLDAATQ ALLNR					Mascot
2308.1567	2308.1646	0.0079	3	402	423	EVAAFAQFGSDLDAATQ ALLNR	233	100			Mascot

5 ATPase subunit 1 [Sorghum bicolor] gi|115278526 55933.1 5.71 18 828 100 46.843 739 100

Peptide Information

Calc. Mass	Obsrv. Mass	± da	± ppm	Start Seq.	End Seq.	Sequence	Ion Score	C. I. %	Modification	Rank	Result Type
815.4621	815.4484	-0.0137	-17	167	173	ELIIGDR					Mascot
860.5022	860.4489	-0.0533	-62	283	289	QMSLLLR					Mascot
874.4741	874.4857	0.0116	13	487	494	GGLTNERK					Mascot
876.4971	876.4717	-0.0254	-29	283	289	QMSLLLR			Oxidation (M)[2]		Mascot
884.4221	884.4128	-0.0093	-11	121	128	GALSDHER					Mascot
892.4886	892.4791	-0.0095	-11	395	401	LELAQYR					Mascot
972.5473	972.5329	-0.0144	-15	33	42	VVSVGDGIAR					Mascot
972.5473	972.5329	-0.0144	-15	33	42	VVSVGDGIAR	72	99.986			Mascot
1026.5942	1026.5762	-0.018	-18	154	163	AVDSLVPIGR					Mascot
1026.5942	1026.5762	-0.018	-18	154	163	AVDSLVPIGR	73	99.991			Mascot
1203.658	1203.6455	-0.0125	-10	7	17	AAELTTLLESR					Mascot
1203.658	1203.6455	-0.0125	-10	7	17	AAELTTLLESR	74	99.993			Mascot
1242.6147	1242.5756	-0.0391	-31	143	153	SVHEPMQTGLK			Oxidation (M)[6]		Mascot
1300.7471	1300.6886	-0.0585	-45	178	189	TAIAIDTILNQK					Mascot

1341.705	1341.649	-0.056	-42	433	443	QPQYEPLPIEK					Mascot
1438.8489	1438.8287	-0.0202	-14	363	376	GIRPAINVGLSVSR					Mascot
1438.8489	1438.8287	-0.0202	-14	363	376	GIRPAINVGLSVSR	45	93.846			Mascot
1537.7434	1537.7349	-0.0085	-6	295	307	EAFPGDVFYLHSR					Mascot
1537.7434	1537.7349	-0.0085	-6	295	307	EAFPGDVFYLHSR	108	100			Mascot
1720.821	1720.9739	0.1529	89	262	276	DNGMHALIIYDDLSK			Oxidation (M)[4]		Mascot
1724.8789	1724.8748	-0.0041	-2	444	458	QIVVIYAAVNGFCDR			Carbamidomethyl (C)[13]		Mascot
1834.8429	1834.851	0.0081	4	18	32	MTNFYTNFQVDEIGR					Mascot
1850.8378	1850.8162	-0.0216	-12	18	32	MTNFYTNFQVDEIGR			Oxidation (M)[1]		Mascot
1850.8378	1850.8162	-0.0216	-12	18	32	MTNFYTNFQVDEIGR	133	100	Oxidation (M)[1]		Mascot
2157.0532	2157.0288	-0.0244	-11	43	62	VYGLNEIQAGEMVEFAS GVK			Oxidation (M)[12]		Mascot
2308.1567	2308.1646	0.0079	3	402	423	EVAAFAQFGSDLDAATQ ALLNR					Mascot
2308.1567	2308.1646	0.0079	3	402	423	EVAAFAQFGSDLDAATQ ALLNR	233	100			Mascot

6	RecName: Full=ATP synthase subunit alpha, mitochondrial	gi|114411	55595.1	6.51	16	813	100	45.707	739	100	

Peptide Information

Calc. Mass	Obsrv. Mass	± da	± ppm	Start Seq.	End Seq.	Sequence	Ion Score	C. I. %	Modification	Rank	Result Type
815.4621	815.4484	-0.0137	-17	167	173	ELIIGDR					Mascot
860.5022	860.4489	-0.0533	-62	283	289	QMSLLLR					Mascot
876.4971	876.4717	-0.0254	-29	283	289	QMSLLLR			Oxidation (M)[2]		Mascot
884.4221	884.4128	-0.0093	-11	121	128	GALSDHER					Mascot
892.4886	892.4791	-0.0095	-11	395	401	LELAQYR					Mascot
972.5473	972.5329	-0.0144	-15	33	42	VVSVGDGIAR					Mascot
972.5473	972.5329	-0.0144	-15	33	42	VVSVGDGIAR	72	99.986			Mascot
1026.5942	1026.5762	-0.018	-18	154	163	AVDSLVPIGR					Mascot
1026.5942	1026.5762	-0.018	-18	154	163	AVDSLVPIGR	73	99.991			Mascot
1203.658	1203.6455	-0.0125	-10	7	17	AAELTTLLESR					Mascot
1203.658	1203.6455	-0.0125	-10	7	17	AAELTTLLESR	74	99.993			Mascot
1242.6147	1242.5756	-0.0391	-31	143	153	SVHEPMQTGLK			Oxidation (M)[6]		Mascot
1300.7471	1300.6886	-0.0585	-45	178	189	TAIAIDTILNQK					Mascot
1438.8489	1438.8287	-0.0202	-14	363	376	GIRPAINVGLSVSR					Mascot
1438.8489	1438.8287	-0.0202	-14	363	376	GIRPAINVGLSVSR	45	93.846			Mascot
1537.7434	1537.7349	-0.0085	-6	295	307	EAFPGDVFYLHSR					Mascot
1537.7434	1537.7349	-0.0085	-6	295	307	EAFPGDVFYLHSR	108	100			Mascot

1712.0204	1711.9003	-0.1201	-70	470	484	DILTTIKPELLQSLK					Mascot
1720.821	1720.9739	0.1529	89	262	276	DNGMHALIIYDDLSK			Oxidation (M)[4]		Mascot
1834.8429	1834.851	0.0081	4	18	32	MTNFYTNFQVDEIGR					Mascot
1850.8378	1850.8162	-0.0216	-12	18	32	MTNFYTNFQVDEIGR			Oxidation (M)[1]		Mascot
1850.8378	1850.8162	-0.0216	-12	18	32	MTNFYTNFQVDEIGR	133	100	Oxidation (M)[1]		Mascot
2157.0532	2157.0288	-0.0244	-11	43	62	VYGLNEIQAGEMVEFAS GVK			Oxidation (M)[12]		Mascot
2308.1567	2308.1646	0.0079	3	402	423	EVAAFAQFGSDLDAATQ ALLNR					Mascot
2308.1567	2308.1646	0.0079	3	402	423	EVAAFAQFGSDLDAATQ ALLNR	233	100			Mascot

7	ATP synthase subunit alpha, mitochondrial [Aegilops tauschii]	gi|475624600	54469.2	5.67	17	747	100	43.895	664	100	

Peptide Information

Calc. Mass	Obsrv. Mass	± da	± ppm	Start Seq.	End Seq.	Sequence	Ion Score	C. I. %	Modification	Rank	Result Type
815.4621	815.4484	-0.0137	-17	157	163	ELIIGDR					Mascot
874.4741	874.4857	0.0116	13	477	484	GGLTNERK					Mascot
892.4886	892.4791	-0.0095	-11	385	391	LELAQYR					Mascot
972.5473	972.5329	-0.0144	-15	16	25	VVSVGDGIAR					Mascot
972.5473	972.5329	-0.0144	-15	16	25	VVSVGDGIAR	72	99.986			Mascot
1010.5126	1010.4536	-0.059	-58	132	140	GALGDHERR					Mascot
1026.5942	1026.5762	-0.018	-18	144	153	AVDSLVPIGR					Mascot
1026.5942	1026.5762	-0.018	-18	144	153	AVDSLVPIGR	73	99.991			Mascot
1300.7471	1300.6886	-0.0585	-45	168	179	TAIAIDTILNQK					Mascot
1326.7627	1326.7087	-0.054	-41	460	471	AILSTINPELQK					Mascot
1341.705	1341.649	-0.056	-42	423	433	QPQYEPLPIEK					Mascot
1438.8489	1438.8287	-0.0202	-14	353	366	GIRPAINVGLSVSR					Mascot
1438.8489	1438.8287	-0.0202	-14	353	366	GIRPAINVGLSVSR	45	93.846			Mascot
1537.7434	1537.7349	-0.0085	-6	285	297	EAFPGDVFYLHSR					Mascot
1537.7434	1537.7349	-0.0085	-6	285	297	EAFPGDVFYLHSR	108	100			Mascot
1573.8632	1573.8185	-0.0447	-28	267	279	QAVAYRHMSLLLR			Oxidation (M)[8]		Mascot
1703.8025	1703.7719	-0.0306	-18	2	15	TNFYTNFQVDEIGR					Mascot
1724.8789	1724.8748	-0.0041	-2	434	448	QIVVIYAAVNGFCDR			Carbamidomethyl (C)[13]		Mascot
1834.8429	1834.851	0.0081	4	1	15	MTNFYTNFQVDEIGR					Mascot
1850.8378	1850.8162	-0.0216	-12	1	15	MTNFYTNFQVDEIGR			Oxidation (M)[1]		Mascot
1850.8378	1850.8162	-0.0216	-12	1	15	MTNFYTNFQVDEIGR	133	100	Oxidation (M)[1]		Mascot
2157.0532	2157.0288	-0.0244	-11	26	45	VYGLNEIQAGEMVEFAS GVK			Oxidation (M)[12]		Mascot

Calc. Mass	Obsrv. Mass	± da	± ppm	Start Seq.	End Seq.	Sequence	Ion Score	C. I. %	Modification	Rank	Result Type
2308.1567	2308.1646	0.0079	3	392	413	EVAAFAQFGSDLDAATQ ALLNR					Mascot
2308.1567	2308.1646	0.0079	3	392	413	EVAAFAQFGSDLDAATQ ALLNR	233	100			Mascot

8	ATPase subunit 1 [Silene latifolia]	gi|307101720	56130.3	6.02	18	694	100	42.776	606	100	

Peptide Information

Calc. Mass	Obsrv. Mass	± da	± ppm	Start Seq.	End Seq.	Sequence	Ion Score	C. I. %	Modification	Rank	Result Type
815.4621	815.4484	-0.0137	-17	167	173	ELIIGDR					Mascot
860.5022	860.4489	-0.0533	-62	283	289	QMSLLLR					Mascot
876.4971	876.4717	-0.0254	-29	283	289	QMSLLLR			Oxidation (M)[2]		Mascot
884.4221	884.4128	-0.0093	-11	121	128	GALSDHER					Mascot
892.4886	892.4791	-0.0095	-11	395	401	LELAQYR					Mascot
972.5473	972.5329	-0.0144	-15	33	42	VVSVGDGIAR					Mascot
972.5473	972.5329	-0.0144	-15	33	42	VVSVGDGIAR	72	99.986			Mascot
1010.4975	1010.4536	-0.0439	-43	501	508	ECALNYLK			Carbamidomethyl (C)[2]		Mascot
1026.5942	1026.5762	-0.018	-18	154	163	AVDSLVPIGR					Mascot
1026.5942	1026.5762	-0.018	-18	154	163	AVDSLVPIGR	73	99.991			Mascot
1110.5864	1110.549	-0.0374	-34	492	500	KMELDSFLK					Mascot
1137.5756	1137.5282	-0.0474	-42	385	394	AMKQVCGSLK			Carbamidomethyl (C)[6], Oxidation (M)[2]		Mascot
1203.658	1203.6455	-0.0125	-10	7	17	AAELTTLLESR					Mascot
1203.658	1203.6455	-0.0125	-10	7	17	AAELTTLLESR	74	99.993			Mascot
1242.6147	1242.5756	-0.0391	-31	143	153	SVHEPMQTGLK			Oxidation (M)[6]		Mascot
1300.7471	1300.6886	-0.0585	-45	178	189	TAIAIDTILNQK					Mascot
1438.8489	1438.8287	-0.0202	-14	363	376	GIRPAINVGLSVSR					Mascot
1438.8489	1438.8287	-0.0202	-14	363	376	GIRPAINVGLSVSR	45	93.846			Mascot
1537.7434	1537.7349	-0.0085	-6	295	307	EAFPGDVFYLHSR					Mascot
1537.7434	1537.7349	-0.0085	-6	295	307	EAFPGDVFYLHSR	108	100			Mascot
1720.821	1720.9739	0.1529	89	262	276	DNGMHALIIYDDLSK			Oxidation (M)[4]		Mascot
1802.8708	1802.8431	-0.0277	-15	18	32	ISNFYTNFQVDEIGR					Mascot
2157.0532	2157.0288	-0.0244	-11	43	62	VYGLNEIQAGEMVEFAS GVK			Oxidation (M)[12]		Mascot
2308.1567	2308.1646	0.0079	3	402	423	EVAAFAQFGSDLDAATQ ALLNR					Mascot
2308.1567	2308.1646	0.0079	3	402	423	EVAAFAQFGSDLDAATQ ALLNR	233	100			Mascot

9	ATPase subunit 1 (mitochondrion) [Lotus japonicus]	gi|357197351	55158	6.01	17	687	100	42.612	606	100	

Protein Group

ATPase subunit 1 (mitochondrion) [Lotus japonicus]	gi|372450305	55158	6.0100 002288

Peptide Information

Calc. Mass	Obsrv. Mass	± da	± ppm	Start Seq.	End Seq.	Sequence	Ion Score	C. I. %	Modification	Rank	Result Type
815.4621	815.4484	-0.0137	-17	167	173	ELIIGDR					Mascot
860.5022	860.4489	-0.0533	-62	283	289	QMSLLLR					Mascot
876.4971	876.4717	-0.0254	-29	283	289	QMSLLLR			Oxidation (M)[2]		Mascot
884.4221	884.4128	-0.0093	-11	121	128	GALSDHER					Mascot
892.4886	892.4791	-0.0095	-11	395	401	LELAQYR					Mascot
972.5473	972.5329	-0.0144	-15	33	42	VVSVGDGIAR					Mascot
972.5473	972.5329	-0.0144	-15	33	42	VVSVGDGIAR	72	99.986			Mascot
1026.5942	1026.5762	-0.018	-18	154	163	AVDSLVPIGR					Mascot
1026.5942	1026.5762	-0.018	-18	154	163	AVDSLVPIGR	73	99.991			Mascot
1137.5756	1137.5282	-0.0474	-42	385	394	AMKQVCGSLK			Carbamidomethyl (C)[6], Oxidation (M)[2]		Mascot
1203.658	1203.6455	-0.0125	-10	7	17	AAELTTLLESR					Mascot
1203.658	1203.6455	-0.0125	-10	7	17	AAELTTLLESR	74	99.993			Mascot
1242.6147	1242.5756	-0.0391	-31	143	153	SVHEPMQTGLK			Oxidation (M)[6]		Mascot
1300.7471	1300.6886	-0.0585	-45	178	189	TAIAIDTILNQK					Mascot
1363.7039	1363.6704	-0.0335	-25	459	469	MPLDKIAQYER					Mascot
1438.8489	1438.8287	-0.0202	-14	363	376	GIRPAINVGLSVSR					Mascot
1438.8489	1438.8287	-0.0202	-14	363	376	GIRPAINVGLSVSR	45	93.846			Mascot
1537.7434	1537.7349	-0.0085	-6	295	307	EAFPGDVFYLHSR					Mascot
1537.7434	1537.7349	-0.0085	-6	295	307	EAFPGDVFYLHSR	108	100			Mascot
1720.821	1720.9739	0.1529	89	262	276	DNGMHALIIYDDLSK			Oxidation (M)[4]		Mascot
1835.9862	1835.8602	-0.126	-69	2	17	EFSLRAAELTTLLESR					Mascot
2157.0532	2157.0288	-0.0244	-11	43	62	VYGLNEIQAGEMVEFAS GVK			Oxidation (M)[12]		Mascot
2308.1567	2308.1646	0.0079	3	402	423	EVAAFAQFGSDLDAATQ ALLNR					Mascot
2308.1567	2308.1646	0.0079	3	402	423	EVAAFAQFGSDLDAATQ ALLNR	233	100			Mascot

10	ATPase subunit 1 [Zea mays subsp. mays]	gi|94502566	55430.9	5.85	17	686	100	43.434	606	100		

Protein Group

ATPase subunit 1 [Tripsacum dactyloides]	gi|115278595	55412.9	6.0199 999809 2651
ATPase subunit 1 [Zea luxurians]	gi|114151591	55430.9	5.8499 999046 3257

ATPase subunit 1 [Zea mays subsp. mays]	gi|94502565	55430.9	5.8499<br>999046<br>3257
ATPase subunit 1 [Zea mays subsp. parviglumis]	gi|114151624	55430.9	5.8499<br>999046<br>3257
ATPase subunit 1 [Zea perennis]	gi|114151558	55430.9	5.8499<br>999046<br>3257
RecName: Full=ATP synthase subunit alpha, mitochondrial	gi|114405	55430.9	5.8499<br>999046<br>3257

Peptide Information

Calc. Mass	Obsrv. Mass	± da	± ppm	Start Seq.	End Seq.	Sequence	Ion Score	C. I. %	Modification	Rank	Result Type
815.4621	815.4484	-0.0137	-17	167	173	ELIIGDR					Mascot
860.5022	860.4489	-0.0533	-62	283	289	QMSLLLR					Mascot
874.4741	874.4857	0.0116	13	487	494	GGLTNERK					Mascot
876.4971	876.4717	-0.0254	-29	283	289	QMSLLLR			Oxidation (M)[2]		Mascot
884.4221	884.4128	-0.0093	-11	121	128	GALSDHER					Mascot
892.4886	892.4791	-0.0095	-11	395	401	LELAQYR					Mascot
972.5473	972.5329	-0.0144	-15	33	42	VVSVGDGIAR					Mascot
972.5473	972.5329	-0.0144	-15	33	42	VVSVGDGIAR	72	99.987			Mascot
1026.5942	1026.5762	-0.018	-18	154	163	AVDSLVPIGR					Mascot
1026.5942	1026.5762	-0.018	-18	154	163	AVDSLVPIGR	73	99.991			Mascot
1203.658	1203.6455	-0.0125	-10	7	17	AAELTTLLESR					Mascot
1203.658	1203.6455	-0.0125	-10	7	17	AAELTTLLESR	74	99.993			Mascot
1242.6147	1242.5756	-0.0391	-31	143	153	SVHEPMQTGLK			Oxidation (M)[6]		Mascot
1300.7471	1300.6886	-0.0585	-45	178	189	TAIAIDTILNQK					Mascot
1341.705	1341.649	-0.056	-42	433	443	QPQYEPLPIEK					Mascot
1438.8489	1438.8287	-0.0202	-14	363	376	GIRPAINVGLSVSR					Mascot
1438.8489	1438.8287	-0.0202	-14	363	376	GIRPAINVGLSVSR	45	94.027			Mascot
1537.7434	1537.7349	-0.0085	-6	295	307	EAFPGDVFYLHSR					Mascot
1537.7434	1537.7349	-0.0085	-6	295	307	EAFPGDVFYLHSR	108	100			Mascot
1720.821	1720.9739	0.1529	89	262	276	DNGMHALIIYDDLSK			Oxidation (M)[4]		Mascot
1724.8789	1724.8748	-0.0041	-2	444	458	QIVVIYAAVNGFCDR			Carbamidomethyl (C)[13]		Mascot
2157.0532	2157.0288	-0.0244	-11	43	62	VYGLNEIQAGEMVEFAS GVK			Oxidation (M)[12]		Mascot

2308.1567	2308.1646	0.0079	3	402	423	EVAAFAQFGSDLDAATQ ALLNR			Mascot
2308.1567	2308.1646	0.0079	3	402	423	EVAAFAQFGSDLDAATQ ALLNR	233	100	Mascot

Rank	Protein Name	Accession No.	Protein MW	Protein PI	Pep. Count	Protein Score	Protein Score C. I. %	Intensity Matched	Total Ion Score	Total Ion C. I. %	Confirmed
1	6-phosphogluconate dehydrogenase, decarboxylating [Aegilops tauschii]	gi|475592570	66295.1	5.4	22	622	100	39.81	511	100	

Peptide Information

Calc. Mass	Obsrv. Mass	± da	± ppm	Start Seq.	End Seq.	Sequence	Ion Score	C. I. %	Modification	Rank	Result Type
846.4316	846.4282	-0.0034	-4	155	161	VDETVQR					Mascot
908.4142	908.4077	-0.0065	-7	231	237	EKAMEER			Oxidation (M)[4]		Mascot
943.5207	943.5165	-0.0042	-4	436	443	AQLIEDVR					Mascot
1052.5524	1052.5509	-0.0015	-1	142	150	GFPISVYNR					Mascot
1052.5524	1052.5509	-0.0015	-1	142	150	GFPISVYNR	70	99.979			Mascot
1064.5735	1064.5676	-0.0059	-6	408	416	FLSGLKDER					Mascot
1064.5735	1064.5676	-0.0059	-6	408	416	FLSGLKDER	43	89.644			Mascot
1071.6157	1071.6086	-0.0071	-7	436	444	AQLIEDVRK					Mascot
1109.6426	1109.641	-0.0016	-1	557	566	LPANLVQAQR					Mascot
1185.6376	1185.6371	-0.0005	0	469	479	GWGLNLGELAR					Mascot
1258.5488	1258.5519	0.0031	2	567	576	DYFGAHTYER					Mascot
1258.5488	1258.5519	0.0031	2	567	576	DYFGAHTYER	79	99.998			Mascot
1263.6539	1263.6343	-0.0196	-16	151	161	TTSKVDETVQR					Mascot
1380.7706	1380.7672	-0.0034	-2	555	566	DRLPANLVQAQR					Mascot
1380.7706	1380.7672	-0.0034	-2	555	566	DRLPANLVQAQR	66	99.95			Mascot
1544.7228	1544.8306	0.1078	70	422	435	IFQGDYSSGETVNK					Mascot
1665.8265	1665.834	0.0075	5	238	254	GLLYLGMGVSGGEEGAR					Mascot
1681.8214	1681.806	-0.0154	-9	238	254	GLLYLGMGVSGGEEGAR			Oxidation (M)[7]		Mascot
1681.8214	1681.806	-0.0154	-9	238	254	GLLYLGMGVSGGEEGAR	102	100	Oxidation (M)[7]		Mascot
1777.879	1777.8411	-0.0379	-21	278	294	VSAQVPDSGPCVTYIGK			Carbamidomethyl (C)[11]		Mascot
2012.1208	2012.077	-0.0438	-22	122	141	IGLAGLAVMGQNLALNIA EK			Oxidation (M)[9]		Mascot
2304.1177	2304.1191	0.0014	1	502	521	NGELANLLIDPEFAQEIM DR			Oxidation (M)[18]		Mascot
2443.2463	2443.2715	0.0252	10	385	407	WTVQQAAELSVAAPTIEA SLDSR					Mascot
2699.394	2699.4307	0.0367	14	164	187	LEGNLPLYGFHDPASFVN SIQKPR					Mascot
2699.394	2699.4307	0.0367	14	164	187	LEGNLPLYGFHDPASFVN SIQKPR	152	100			Mascot

Calc. Mass	Obsrv. Mass	± da	± ppm	Start Seq.	End Seq.	Sequence	Ion Score	C. I. %	Modification	Rank	Result Type
2808.3333	2808.3503	0.017	6	528	553	VVCLAINNGVSTPGMSAS LAYFDSYR			Carbamidomethyl (C)[3], Oxidation (M)[15]		Mascot
2809.3462	2809.3469	0.0007	0	498	521	AYDRNGELANLLIDPEFA QEIMDR			Oxidation (M)[22]		Mascot
2880.427	2880.4761	0.0491	17	303	328	MVHNGIEYGDMQLIAEAY DVLKSVGK					Mascot
2898.5261	2898.5608	0.0347	12	162	187	AKLEGNLPLYGFHDPASF VNSIQKPR					Mascot

2 6-phosphogluconate dehydrogenase, decarboxylating [Aegilops tauschii] gi|475626608 108521.5 5.63 23 595 100 39.909 511 100

Peptide Information

Calc. Mass	Obsrv. Mass	± da	± ppm	Start Seq.	End Seq.	Sequence	Ion Score	C. I. %	Modification	Rank	Result Type
826.4781	826.4362	-0.0419	-51	974	981	SPPGLSLR					Mascot
846.4316	846.4282	-0.0034	-4	39	45	VDETVQR					Mascot
908.4142	908.4077	-0.0065	-7	115	121	EKAMEER			Oxidation (M)[4]		Mascot
943.5207	943.5165	-0.0042	-4	320	327	AQLIEDVR					Mascot
1052.5524	1052.5509	-0.0015	-1	26	34	GFPISVYNR					Mascot
1052.5524	1052.5509	-0.0015	-1	26	34	GFPISVYNR	70	99.979			Mascot
1064.5735	1064.5676	-0.0059	-6	292	300	FLSGLKDER					Mascot
1064.5735	1064.5676	-0.0059	-6	292	300	FLSGLKDER	43	89.644			Mascot
1071.6157	1071.6086	-0.0071	-7	320	328	AQLIEDVRK					Mascot
1109.6426	1109.641	-0.0016	-1	441	450	LPANLVQAQR					Mascot
1185.6376	1185.6371	-0.0005	0	353	363	GWGLNLGELAR					Mascot
1258.5488	1258.5519	0.0031	2	451	460	DYFGAHTYER					Mascot
1258.5488	1258.5519	0.0031	2	451	460	DYFGAHTYER	79	99.998			Mascot
1263.6539	1263.6343	-0.0196	-16	35	45	TTSKVDETVQR					Mascot
1380.7706	1380.7672	-0.0034	-2	439	450	DRLPANLVQAQR					Mascot
1380.7706	1380.7672	-0.0034	-2	439	450	DRLPANLVQAQR	66	99.95			Mascot
1665.8265	1665.834	0.0075	5	122	138	GLLYLGMGVSGGEEGAR					Mascot
1681.8214	1681.806	-0.0154	-9	122	138	GLLYLGMGVSGGEEGAR			Oxidation (M)[7]		Mascot
1681.8214	1681.806	-0.0154	-9	122	138	GLLYLGMGVSGGEEGAR	102	100	Oxidation (M)[7]		Mascot
1777.879	1777.8411	-0.0379	-21	162	178	VSAQVPDSGPCVTYIGK			Carbamidomethyl (C)[11]		Mascot
2012.1208	2012.077	-0.0438	-22	6	25	IGLAGLAVMGQNLALNIA EK			Oxidation (M)[9]		Mascot
2304.1177	2304.1191	0.0014	1	386	405	NGELANLLIDPEFAQEIM DR			Oxidation (M)[18]		Mascot
2443.2463	2443.2715	0.0252	10	269	291	WTVQQAAELSVAAPTIEA SLDSR					Mascot
2470.2097	2470.2358	0.0261	11	306	327	IFQGDYSSGETVDKAQLI EDVR					Mascot
2699.394	2699.4307	0.0367	14	48	71	LEGNLPLYGFHDPASFVN SIQKPR					Mascot

2699.394	2699.4307	0.0367	14	48	71	LEGNLPLYGFHDPASFVN SIQKPR	152	100			Mascot
2808.3333	2808.3503	0.017	6	412	437	VVCLAINNGVSTPGMSAS LAYFDSYR			Carbamidomethyl (C)[3], Oxidation (M)[15]		Mascot
2809.3462	2809.3469	0.0007	0	382	405	AYDRNGELANLLIDPEFA QEIMDR			Oxidation (M)[22]		Mascot
2880.427	2880.4761	0.0491	17	187	212	MVHNGIEYGDMQLIAEAY DVLKSVGK					Mascot
2898.5261	2898.5608	0.0347	12	46	71	AKLEGNLPLYGFHDPASF VNSIQKPR					Mascot

3	6-phosphogluconate dehydrogenase, decarboxylating [Triticum urartu]	gi|474156904	53712.4	9.45	19	500	100	33.803	409	100

Peptide Information

Calc. Mass	Obsrv. Mass	± da	± ppm	Start Seq.	End Seq.	Sequence	Ion Score	C. I. %	Modification	Rank	Result Type
846.4316	846.4282	-0.0034	-4	140	146	VDETVQR					Mascot
943.5207	943.5165	-0.0042	-4	321	328	AQLIEDVR					Mascot
1052.5524	1052.5509	-0.0015	-1	127	135	GFPISVYNR					Mascot
1052.5524	1052.5509	-0.0015	-1	127	135	GFPISVYNR	70	99.979			Mascot
1064.5735	1064.5676	-0.0059	-6	293	301	FLSGLKDER					Mascot
1064.5735	1064.5676	-0.0059	-6	293	301	FLSGLKDER	43	89.644			Mascot
1071.6157	1071.6086	-0.0071	-7	321	329	AQLIEDVRK					Mascot
1075.5742	1075.5322	-0.042	-39	140	148	VDETVQRTK					Mascot
1109.6426	1109.641	-0.0016	-1	442	451	LPANLVQAQR					Mascot
1185.6376	1185.6371	-0.0005	0	354	364	GWGLNLGELAR					Mascot
1258.5488	1258.5519	0.0031	2	452	461	DYFGAHTYER					Mascot
1258.5488	1258.5519	0.0031	2	452	461	DYFGAHTYER	79	99.998			Mascot
1263.6539	1263.6343	-0.0196	-16	136	146	TTSKVDETVQR					Mascot
1380.7706	1380.7672	-0.0034	-2	440	451	DRLPANLVQAQR					Mascot
1380.7706	1380.7672	-0.0034	-2	440	451	DRLPANLVQAQR	66	99.95			Mascot
1439.6848	1439.724	0.0392	27	24	34	MQWIRGENYSR					Mascot
1709.9255	1709.822	-0.1035	-61	91	106	VKLGLASSAYEMALTR					Mascot
1777.9232	1777.8411	-0.0821	-46	62	77	RSDPPLELPAGHFVDK					Mascot
2012.1208	2012.077	-0.0438	-22	107	126	IGLAGLAVMGQNLALNIA EK			Oxidation (M)[9]		Mascot
2304.1177	2304.1191	0.0014	1	387	406	NGELANLLIDPEFAQEIM DR			Oxidation (M)[18]		Mascot
2470.2097	2470.2358	0.0261	11	307	328	IFQGDYSSGETVDKAQLI EDVR					Mascot
2699.394	2699.4307	0.0367	14	149	172	LEGNLPLYGFHDPASFVN SIQKPR					Mascot
2699.394	2699.4307	0.0367	14	149	172	LEGNLPLYGFHDPASFVN SIQKPR	152	100			Mascot
2809.3462	2809.3469	0.0007	0	383	406	AYDRNGELANLLIDPEFA QEIMDR			Oxidation (M)[22]		Mascot

4	6-phosphogluconate dehydrogenase, decarboxylating [Triticum urartu]	gi|474379872	81632	8.56	20	498	100	36.703	406	100

Peptide Information

Calc. Mass	Obsrv. Mass	± da	± ppm	Start Seq.	End Seq.	Sequence	Ion Score	C. I. %	Modification	Rank	Result Type
846.4316	846.4282	-0.0034	-4	481	487	VDETVQR					Mascot
908.4142	908.4077	-0.0065	-7	115	121	EKAMEER			Oxidation (M)[4]		Mascot
943.5207	943.5165	-0.0042	-4	578	585	AQLIEDVR					Mascot
1052.5524	1052.5509	-0.0015	-1	468	476	GFPISVYNR					Mascot
1052.5524	1052.5509	-0.0015	-1	26	34	GFPISVYNR	70	99.979			Mascot
1064.5735	1064.5676	-0.0059	-6	271	279	FLSGLKDER					Mascot
1064.5735	1064.5676	-0.0059	-6	271	279	FLSGLKDER	43	89.644			Mascot
1071.6157	1071.6086	-0.0071	-7	578	586	AQLIEDVRK					Mascot
1109.6426	1109.641	-0.0016	-1	699	708	LPANLVQAQR					Mascot
1185.6376	1185.6371	-0.0005	0	611	621	GWGLNLGELAR					Mascot
1258.5488	1258.5519	0.0031	2	709	718	DYFGAHTYER					Mascot
1258.5488	1258.5519	0.0031	2	709	718	DYFGAHTYER	79	99.998			Mascot
1263.6539	1263.6343	-0.0196	-16	477	487	TTSKVDETVQR					Mascot
1380.7706	1380.7672	-0.0034	-2	697	708	DRLPANLVQAQR					Mascot
1380.7706	1380.7672	-0.0034	-2	697	708	DRLPANLVQAQR	66	99.95			Mascot
2012.1208	2012.077	-0.0438	-22	448	467	IGLAGLAVMGQNLALNIAEK			Oxidation (M)[9]		Mascot
2304.1177	2304.1191	0.0014	1	644	663	NGELANLLIDPEFAQEIMDR			Oxidation (M)[18]		Mascot
2443.2463	2443.2715	0.0252	10	248	270	WTVQQAAELSVAAPTIEASLDSR					Mascot
2470.2097	2470.2358	0.0261	11	564	585	IFQGDYSSGETVDKAQLIEDVR					Mascot
2596.2998	2596.3823	0.0825	32	117	140	AMEERGLLYLGMGVSGGEEDILLK			Oxidation (M)[2]		Mascot
2699.394	2699.4307	0.0367	14	490	513	LEGNLPLYGFHDPASFVNSIQKPR					Mascot
2699.394	2699.4307	0.0367	14	48	71	LEGNLPLYGFHDPASFVNSIQKPR	152	100			Mascot
2809.3462	2809.3469	0.0007	0	640	663	AYDRNGELANLLIDPEFAQEIMDR			Oxidation (M)[22]		Mascot
2880.427	2880.4761	0.0491	17	166	191	MVHNGIEYGDMQLIAEAYDVLKSVGK					Mascot
2898.5261	2898.5608	0.0347	12	488	513	AKLEGNLPLYGFHDPASFVNSIQKPR					Mascot

5	TPA: 6-phosphogluconate dehydrogenase2 [Zea mays]	gi|414878798	52908	6	16	433	100	33.976	359	100

Peptide Information

Calc. Mass	Obsrv. Mass	± da	± ppm	Start Seq.	End Seq.	Sequence	Ion Score	C. I. %	Modification	Rank	Result Type

Calc. Mass	Obsrv. Mass	± da	± ppm	Start Seq.	End Seq.	Sequence	Ion Score	C. I. %	Modification	Rank	Result Type
846.4316	846.4282	-0.0034	-4	39	45	VDETVQR					Mascot
908.4142	908.4077	-0.0065	-7	115	121	EKAMEER			Oxidation (M)[4]		Mascot
943.5207	943.5165	-0.0042	-4	320	327	AQLIEDVR					Mascot
1052.5524	1052.5509	-0.0015	-1	26	34	GFPISVYNR					Mascot
1052.5524	1052.5509	-0.0015	-1	26	34	GFPISVYNR	70	99.979			Mascot
1064.5735	1064.5676	-0.0059	-6	292	300	FLSGLKDER					Mascot
1064.5735	1064.5676	-0.0059	-6	292	300	FLSGLKDER	43	89.644			Mascot
1109.6426	1109.641	-0.0016	-1	441	450	LPANLVQAQR					Mascot
1185.6376	1185.6371	-0.0005	0	353	363	GWGLNLGELAR					Mascot
1258.5488	1258.5519	0.0031	2	451	460	DYFGAHTYER					Mascot
1258.5488	1258.5519	0.0031	2	451	460	DYFGAHTYER	79	99.998			Mascot
1263.6539	1263.6343	-0.0196	-16	35	45	TTSKVDETVQR					Mascot
1380.7706	1380.7672	-0.0034	-2	439	450	DRLPANLVQAQR					Mascot
1380.7706	1380.7672	-0.0034	-2	439	450	DRLPANLVQAQR	66	99.95			Mascot
1580.7203	1580.7152	-0.0051	-3	461	473	VDMPGSFHTEWFK					Mascot
1665.8265	1665.834	0.0075	5	122	138	GLLYLGMGVSGGEEGAR					Mascot
1681.8214	1681.806	-0.0154	-9	122	138	GLLYLGMGVSGGEEGAR			Oxidation (M)[7]		Mascot
1681.8214	1681.806	-0.0154	-9	122	138	GLLYLGMGVSGGEEGAR	102	100	Oxidation (M)[7]		Mascot
2012.1208	2012.077	-0.0438	-22	6	25	IGLAGLAVMGQNLALNIA EK			Oxidation (M)[9]		Mascot
2443.2463	2443.2715	0.0252	10	269	291	WTVQQAAELSVAAPTIEA SLDSR					Mascot
2808.3333	2808.3503	0.017	6	412	437	VVCLAINNGVSTPGMSAS LAYFDSYR			Carbamidomethyl (C)[3], Oxidation (M)[15]		Mascot
2880.427	2880.4761	0.0491	17	187	212	MVHNGIEYGDMQLIAEAY DVLKSVGK					Mascot

6	PREDICTED: 6-phosphogluconate dehydrogenase, decarboxylating 1-like [Setaria italica]	gi|514786876	52978	5.65	16	432	100	33.208	359	100	

Peptide Information

Calc. Mass	Obsrv. Mass	± da	± ppm	Start Seq.	End Seq.	Sequence	Ion Score	C. I. %	Modification	Rank	Result Type
846.4316	846.4282	-0.0034	-4	39	45	VDETVQR					Mascot
908.4142	908.4077	-0.0065	-7	115	121	EKAMEER			Oxidation (M)[4]		Mascot
943.5207	943.5165	-0.0042	-4	320	327	AQLIEDVR					Mascot
1052.5524	1052.5509	-0.0015	-1	26	34	GFPISVYNR					Mascot
1052.5524	1052.5509	-0.0015	-1	26	34	GFPISVYNR	70	99.979			Mascot
1064.5735	1064.5676	-0.0059	-6	292	300	FLSGLKDER					Mascot
1064.5735	1064.5676	-0.0059	-6	292	300	FLSGLKDER	43	89.644			Mascot

1109.6426	1109.641	-0.0016	-1	441	450	LPANLVQAQR					Mascot
1199.6532	1199.6263	-0.0269	-22	353	363	GWALNLGELAR					Mascot
1258.5488	1258.5519	0.0031	2	451	460	DYFGAHTYER					Mascot
1258.5488	1258.5519	0.0031	2	451	460	DYFGAHTYER	79	99.998			Mascot
1263.6539	1263.6343	-0.0196	-16	35	45	TTSKVDETVQR					Mascot
1380.7706	1380.7672	-0.0034	-2	439	450	DRLPANLVQAQR					Mascot
1380.7706	1380.7672	-0.0034	-2	439	450	DRLPANLVQAQR	66	99.95			Mascot
1580.7203	1580.7152	-0.0051	-3	461	473	VDMPGSFHTEWFK					Mascot
1665.8265	1665.834	0.0075	5	122	138	GLLYLGMGVSGGEEGAR					Mascot
1681.8214	1681.806	-0.0154	-9	122	138	GLLYLGMGVSGGEEGAR			Oxidation (M)[7]		Mascot
1681.8214	1681.806	-0.0154	-9	122	138	GLLYLGMGVSGGEEGAR	102	100	Oxidation (M)[7]		Mascot
2012.1208	2012.077	-0.0438	-22	6	25	IGLAGLAVMGQNLALNIAEK			Oxidation (M)[9]		Mascot
2443.2463	2443.2715	0.0252	10	269	291	WTVQQAAELSVAAPTIEASLDSR					Mascot
2808.3333	2808.3503	0.017	6	412	437	VVCLAINNGVSTPGMSASLAYFDSYR			Carbamidomethyl (C)[3], Oxidation (M)[15]		Mascot
2880.427	2880.4761	0.0491	17	187	212	MVHNGIEYGDMQLIAEAYDVLKSVGK					Mascot

7	NADPH producing dehydrogenase of the oxidative pentose phosphate pathway [Zea mays]	gi|162463282	53307.3	5.92	15	426	100	33.686	359	100

Peptide Information

Calc. Mass	Obsrv. Mass	± da	± ppm	Start Seq.	End Seq.	Sequence	Ion Score	C. I. %	Modification	Rank	Result Type
846.4316	846.4282	-0.0034	-4	39	45	VDETVQR					Mascot
908.4142	908.4077	-0.0065	-7	115	121	EKAMEER			Oxidation (M)[4]		Mascot
943.5207	943.5165	-0.0042	-4	320	327	AQLIEDVR					Mascot
1052.5524	1052.5509	-0.0015	-1	26	34	GFPISVYNR					Mascot
1052.5524	1052.5509	-0.0015	-1	26	34	GFPISVYNR	70	99.979			Mascot
1064.5735	1064.5676	-0.0059	-6	292	300	FLSGLKDER					Mascot
1064.5735	1064.5676	-0.0059	-6	292	300	FLSGLKDER	43	89.644			Mascot
1109.6426	1109.641	-0.0016	-1	441	450	LPANLVQAQR					Mascot
1185.6376	1185.6371	-0.0005	0	353	363	GWGLNLGELAR					Mascot
1258.5488	1258.5519	0.0031	2	451	460	DYFGAHTYER					Mascot
1258.5488	1258.5519	0.0031	2	451	460	DYFGAHTYER	79	99.998			Mascot
1263.6539	1263.6343	-0.0196	-16	35	45	TTSKVDETVQR					Mascot
1380.7706	1380.7672	-0.0034	-2	439	450	DRLPANLVQAQR					Mascot
1380.7706	1380.7672	-0.0034	-2	439	450	DRLPANLVQAQR	66	99.95			Mascot
1665.8265	1665.834	0.0075	5	122	138	GLLYLGMGVSGGEEGAR					Mascot

1681.8214	1681.806	-0.0154	-9	122	138	GLLYLGMGVSGGEEGAR			Oxidation (M)[7]		Mascot
1681.8214	1681.806	-0.0154	-9	122	138	GLLYLGMGVSGGEEGAR	102	100	Oxidation (M)[7]		Mascot
2012.1208	2012.077	-0.0438	-22	6	25	IGLAGLAVMGQNLALNIA EK			Oxidation (M)[9]		Mascot
2443.2463	2443.2715	0.0252	10	269	291	WTVQQAAELSVAAPTIEA SLDSR					Mascot
2808.3333	2808.3503	0.017	6	412	437	VVCLAINNGVSTPGMSAS LAYFDSYR			Carbamidomethyl (C)[3], Oxidation (M)[15]		Mascot
2880.427	2880.4761	0.0491	17	187	212	MVHNGIEYGDMQLIAEAY DVLKSVGK					Mascot

8	PREDICTED: 6-phosphogluconate dehydrogenase, decarboxylating 1-like isoform X1 [Setaria italica]	gi|514759593	52948.9	5.53	15	426	100	33.383	359	100

Protein Group

PREDICTED: 6-phosphogluconate dehydrogenase, decarboxylating 1-like isoform X2 [Setaria italica]	gi|514759597	52948.9	5.5300 002098 0835

Peptide Information

Calc. Mass	Obsrv. Mass	± da	± ppm	Start Seq.	End Seq.	Sequence	Ion Score	C. I. %	Modification	Rank	Result Type
846.4316	846.4282	-0.0034	-4	39	45	VDETVQR					Mascot
943.5207	943.5165	-0.0042	-4	320	327	AQLIEDVR					Mascot
1052.5524	1052.5509	-0.0015	-1	26	34	GFPISVYNR					Mascot
1052.5524	1052.5509	-0.0015	-1	26	34	GFPISVYNR	70	99.979			Mascot
1064.5735	1064.5676	-0.0059	-6	292	300	FLSGLKDER					Mascot
1064.5735	1064.5676	-0.0059	-6	292	300	FLSGLKDER	43	89.644			Mascot
1109.6426	1109.641	-0.0016	-1	441	450	LPANLVQAQR					Mascot
1185.6376	1185.6371	-0.0005	0	353	363	GWGLNLGELAR					Mascot
1258.5488	1258.5519	0.0031	2	451	460	DYFGAHTYER					Mascot
1258.5488	1258.5519	0.0031	2	451	460	DYFGAHTYER	79	99.998			Mascot
1263.6539	1263.6343	-0.0196	-16	35	45	TTSKVDETVQR					Mascot
1380.7706	1380.7672	-0.0034	-2	439	450	DRLPANLVQAQR					Mascot
1380.7706	1380.7672	-0.0034	-2	439	450	DRLPANLVQAQR	66	99.95			Mascot
1580.7203	1580.7152	-0.0051	-3	461	473	VDMPGSFHTEWFK					Mascot
1665.8265	1665.834	0.0075	5	122	138	GLLYLGMGVSGGEEGAR					Mascot
1681.8214	1681.806	-0.0154	-9	122	138	GLLYLGMGVSGGEEGAR			Oxidation (M)[7]		Mascot
1681.8214	1681.806	-0.0154	-9	122	138	GLLYLGMGVSGGEEGAR	102	100	Oxidation (M)[7]		Mascot
2012.1208	2012.077	-0.0438	-22	6	25	IGLAGLAVMGQNLALNIA EK			Oxidation (M)[9]		Mascot
2443.2463	2443.2715	0.0252	10	269	291	WTVQQAAELSVAAPTIEA SLDSR					Mascot
2808.3333	2808.3503	0.017	6	412	437	VVCLAINNGVSTPGMSAS LAYFDSYR			Carbamidomethyl (C)[3], Oxidation (M)[15]		Mascot

2880.427	2880.4761	0.0491	17	187	212	MVHNGIEYGDMQLIAEAY DVLKSVGK					Mascot

9	6-phosphogluconate dehydrogenase isoenzyme B isoform 3 [Zea mays]	gi|413942605	53102.9	5.93	14	415	100	33.345	359	100		

Protein Group

6-phosphogluconate dehydrogenase isoenzyme B isoform 1 [Zea mays]	gi|413942603	53102.9	5.9299 998283 3862
6-phosphogluconate dehydrogenase isoenzyme B isoform 1 [Zea mays]	gi|525342897	53102.9	5.9299 998283 3862
6-phosphogluconate dehydrogenase isoenzyme B isoform 2 [Zea mays]	gi|413942604	53102.9	5.9299 998283 3862

Peptide Information

Calc. Mass	Obsrv. Mass	± da	± ppm	Start Seq.	End Seq.	Sequence	Ion Score	C. I. %	Modification	Rank	Result Type
846.4316	846.4282	-0.0034	-4	39	45	VDETVQR					Mascot
908.4142	908.4077	-0.0065	-7	115	121	EKAMEER			Oxidation (M)[4]		Mascot
1052.5524	1052.5509	-0.0015	-1	26	34	GFPISVYNR					Mascot
1052.5524	1052.5509	-0.0015	-1	26	34	GFPISVYNR	70	99.979			Mascot
1064.5735	1064.5676	-0.0059	-6	292	300	FLSGLKDER					Mascot
1064.5735	1064.5676	-0.0059	-6	292	300	FLSGLKDER	43	89.644			Mascot
1109.6426	1109.641	-0.0016	-1	442	451	LPANLVQAQR					Mascot
1185.6376	1185.6371	-0.0005	0	354	364	GWGLNLGELAR					Mascot
1258.5488	1258.5519	0.0031	2	452	461	DYFGAHTYER					Mascot
1258.5488	1258.5519	0.0031	2	452	461	DYFGAHTYER	79	99.998			Mascot
1263.6539	1263.6343	-0.0196	-16	35	45	TTSKVDETVQR					Mascot
1380.7706	1380.7672	-0.0034	-2	440	451	DRLPANLVQAQR					Mascot
1380.7706	1380.7672	-0.0034	-2	440	451	DRLPANLVQAQR	66	99.95			Mascot
1580.7203	1580.7152	-0.0051	-3	462	474	VDMPGSFHTEWFK					Mascot
1665.8265	1665.834	0.0075	5	122	138	GLLYLGMGVSGGEEGAR					Mascot
1681.8214	1681.806	-0.0154	-9	122	138	GLLYLGMGVSGGEEGAR			Oxidation (M)[7]		Mascot
1681.8214	1681.806	-0.0154	-9	122	138	GLLYLGMGVSGGEEGAR	102	100	Oxidation (M)[7]		Mascot
2012.1208	2012.077	-0.0438	-22	6	25	IGLAGLAVMGQNLALNIA EK			Oxidation (M)[9]		Mascot
2443.2463	2443.2715	0.0252	10	269	291	WTVQQAAELSVAAPTIEA SLDSR					Mascot
2808.3333	2808.3503	0.017	6	413	438	VVCLAINNGVSTPGMSAS LAYFDSYR			Carbamidomethyl (C)[3], Oxidation (M)[15]		Mascot

10	Os06g0111500 [Oryza sativa Japonica Group]	gi|113594626	52972.9	5.85	14	415	100	32.959	359	100

Protein Group

RecName: Full=6-phosphogluconate dehydrogenase, decarboxylating 1; Short=OsG6PGH1	gi|75311185	52972.9	5.8499 999046 3257

Peptide Information

Calc. Mass	Obsrv. Mass	± da	± ppm	Start Seq.	End Seq.	Sequence	Ion Score	C. I. %	Modification	Rank	Result Type
846.4316	846.4282	-0.0034	-4	39	45	VDETVQR					Mascot
908.4142	908.4077	-0.0065	-7	115	121	EKAMEER			Oxidation (M)[4]		Mascot
943.5207	943.5165	-0.0042	-4	320	327	AQLIEDVR					Mascot
1052.5524	1052.5509	-0.0015	-1	26	34	GFPISVYNR					Mascot
1052.5524	1052.5509	-0.0015	-1	26	34	GFPISVYNR	70	99.981			Mascot
1064.5735	1064.5676	-0.0059	-6	292	300	FLSGLKDER					Mascot
1064.5735	1064.5676	-0.0059	-6	292	300	FLSGLKDER	43	90.6			Mascot
1109.6426	1109.641	-0.0016	-1	441	450	LPANLVQAQR					Mascot
1258.5488	1258.5519	0.0031	2	451	460	DYFGAHTYER					Mascot
1258.5488	1258.5519	0.0031	2	451	460	DYFGAHTYER	79	99.998			Mascot
1263.6539	1263.6343	-0.0196	-16	35	45	TTSKVDETVQR					Mascot
1380.7706	1380.7672	-0.0034	-2	439	450	DRLPANLVQAQR					Mascot
1380.7706	1380.7672	-0.0034	-2	439	450	DRLPANLVQAQR	66	99.955			Mascot
1580.7203	1580.7152	-0.0051	-3	461	473	VDMPGSFHTEWFK					Mascot
1665.8265	1665.834	0.0075	5	122	138	GLLYLGMGVSGGEEGAR					Mascot
1681.8214	1681.806	-0.0154	-9	122	138	GLLYLGMGVSGGEEGAR			Oxidation (M)[7]		Mascot
1681.8214	1681.806	-0.0154	-9	122	138	GLLYLGMGVSGGEEGAR	102	100	Oxidation (M)[7]		Mascot
2012.1208	2012.077	-0.0438	-22	6	25	IGLAGLAVMGQNLALNIA EK			Oxidation (M)[9]		Mascot
2443.2463	2443.2715	0.0252	10	269	291	WTVQQAAELSVAAPTIEA SLDSR					Mascot
2808.3333	2808.3503	0.017	6	412	437	VVCLAINNGVSTPGMSAS LAYFDSYR			Carbamidomethyl (C)[3], Oxidation (M)[15]		Mascot

Gel Idx/Pos	280/L8	Instr./Gel Origin	BA2151/Sample Project 20140814	Process Status	Analysis Succeeded
Plate [#] Name	[1] Sample Project 20140814	Instrument Sample Name		Spectra	11

Rank	Protein Name	Accession No.	Protein MW	Protein PI	Pep. Count	Protein Score	Protein Score C. I. %	Intensity Matched	Total Ion Score	Total Ion C. I. %	Confirmed
1	waxy protein [Triticum aestivum]	gi|399153338	67223.3	8.1	23	660	100	28.707	558	100	

Peptide Information

Calc. Mass	Obsrv. Mass	± da	± ppm	Start Seq.	End Seq.	Sequence	Ion Score	C. I. %	Modification	Rank	Result Type
817.46	817.4323	-0.0277	-34	113	119	VMVISPR			Oxidation (M)[2]		Mascot
821.3723	821.3473	-0.025	-30	513	519	TGFHMGR			Oxidation (M)[5]		Mascot
908.5173	908.4616	-0.0557	-61	53	59	QSRKPHR					Mascot
917.3974	917.3704	-0.027	-29	146	151	YFHCYK			Carbamidomethyl (C)[4]		Mascot
971.6036	971.574	-0.0296	-30	394	402	VPLVAFIGR					Mascot
1099.6986	1099.6765	-0.0221	-20	393	402	KVPLVAFIGR					Mascot
1123.4871	1123.4641	-0.023	-20	330	338	GCELDNIMR			Carbamidomethyl (C)[2], Oxidation (M)[8]		Mascot
1346.6774	1346.636	-0.0414	-31	545	556	VVGTPAYHEMVK			Oxidation (M)[10]		Mascot
1362.7264	1362.6519	-0.0745	-55	125	136	DAWDTSVISKIK					Mascot
1390.6825	1390.6514	-0.0311	-22	157	167	VFVDHPCFLEK			Carbamidomethyl (C)[7]		Mascot
1396.7432	1396.7229	-0.0203	-15	380	392	EALQAEVGLPVDR					Mascot
1503.7988	1503.7808	-0.018	-12	299	311	INWMKAGILQADK			Oxidation (M)[4]		Mascot
1503.7988	1503.7808	-0.018	-12	192	204	FSLLCQAALEVPR	88	100	Carbamidomethyl (C)[5]		Mascot
1524.838	1524.7905	-0.0475	-31	380	393	EALQAEVGLPVDRK					Mascot
1564.7611	1564.7469	-0.0142	-9	479	491	FEPCGLIQLQGMR			Carbamidomethyl (C)[4], Oxidation (M)[12]		Mascot
1564.7689	1564.7469	-0.022	-14	253	265	VAFCIHNISYQGR	67	99.966	Carbamidomethyl (C)[4]		Mascot
1684.7966	1684.7849	-0.0117	-7	266	279	FSFDDFAQLNLPDR					Mascot
1684.7966	1684.7849	-0.0117	-7	266	279	FSFDDFAQLNLPDR	119	100			Mascot
1831.8497	1831.8289	-0.0208	-11	282	297	SSFDFIDGYDKPVEGR					Mascot
1831.8497	1831.8289	-0.0208	-11	282	297	SSFDFIDGYDKPVEGR	113	100			Mascot
1841.7937	1841.7733	-0.0204	-11	176	191	IYGPDAGTDYEDNQQR					Mascot
1841.7937	1841.7733	-0.0204	-11	176	191	IYGPDAGTDYEDNQQR	68	99.972			Mascot
1913.9855	1913.9971	0.0116	6	359	375	DKFLTVNYDVTTALEGK					Mascot
1997.0226	1997.0088	-0.0138	-7	312	329	VLTVSPYYAEELISGEAR					Mascot
1997.0226	1997.0088	-0.0138	-7	312	329	VLTVSPYYAEELISGEAR	106	100			Mascot
2098.9312	2098.9155	-0.0157	-7	174	191	EKIYGPDAGTDYEDNQQ R					Mascot
2232.0901	2232.0498	-0.0403	-18	458	478	FNAPLAHQMMAGADVLA			Oxidation (M)[9,10]		Mascot

		VTSR									
2	waxy protein [Triticum dicoccoides]	gi|399153332	67165.2	7.85	23	660	100	29.323	558	100	

Peptide Information

Calc. Mass	Obsrv. Mass	± da	± ppm	Start Seq.	End Seq.	Sequence	Ion Score	C. I. %	Modification	Rank	Result Type
817.46	817.4323	-0.0277	-34	113	119	VMVISPR			Oxidation (M)[2]		Mascot
821.3723	821.3473	-0.025	-30	513	519	TGFHMGR			Oxidation (M)[5]		Mascot
908.5173	908.4616	-0.0557	-61	53	59	QSRKPHR					Mascot
917.3974	917.3704	-0.027	-29	146	151	YFHCYK			Carbamidomethyl (C)[4]		Mascot
971.6036	971.574	-0.0296	-30	394	402	VPLVAFIGR					Mascot
1099.6986	1099.6765	-0.0221	-20	393	402	KVPLVAFIGR					Mascot
1123.4871	1123.4641	-0.023	-20	330	338	GCELDNIMR			Carbamidomethyl (C)[2], Oxidation (M)[8]		Mascot
1346.6774	1346.636	-0.0414	-31	545	556	VVGTPAYHEMVK			Oxidation (M)[10]		Mascot
1390.6825	1390.6514	-0.0311	-22	157	167	VFVDHPCFLEK			Carbamidomethyl (C)[7]		Mascot
1396.7432	1396.7229	-0.0203	-15	380	392	EALQAEVGLPVDR					Mascot
1500.78	1500.7559	-0.0241	-16	408	421	GPDVMIAAIPEIMK			Oxidation (M)[5]		Mascot
1503.7988	1503.7808	-0.018	-12	299	311	INWMKAGILQADK			Oxidation (M)[4]		Mascot
1503.7988	1503.7808	-0.018	-12	192	204	FSLLCQAALEVPR	88	100	Carbamidomethyl (C)[5]		Mascot
1524.838	1524.7905	-0.0475	-31	380	393	EALQAEVGLPVDRK					Mascot
1564.7611	1564.7469	-0.0142	-9	479	491	FEPCGLIQLQGMR			Carbamidomethyl (C)[4], Oxidation (M)[12]		Mascot
1564.7689	1564.7469	-0.022	-14	253	265	VAFCIHNISYQGR	67	99.966	Carbamidomethyl (C)[4]		Mascot
1684.7966	1684.7849	-0.0117	-7	266	279	FSFDDFAQLNLPDR					Mascot
1684.7966	1684.7849	-0.0117	-7	266	279	FSFDDFAQLNLPDR	119	100			Mascot
1831.8497	1831.8289	-0.0208	-11	282	297	SSFDFIDGYDKPVEGR					Mascot
1831.8497	1831.8289	-0.0208	-11	282	297	SSFDFIDGYDKPVEGR	113	100			Mascot
1841.7937	1841.7733	-0.0204	-11	176	191	IYGPDAGTDYEDNQQR					Mascot
1841.7937	1841.7733	-0.0204	-11	176	191	IYGPDAGTDYEDNQQR	68	99.972			Mascot
1913.9855	1913.9971	0.0116	6	359	375	DKFLTVNYDVTTALEGK					Mascot
1997.0226	1997.0088	-0.0138	-7	312	329	VLTVSPYYAEELISGEAR					Mascot
1997.0226	1997.0088	-0.0138	-7	312	329	VLTVSPYYAEELISGEAR	106	100			Mascot
2098.9312	2098.9155	-0.0157	-7	174	191	EKIYGPDAGTDYEDNQQ R					Mascot
2232.0901	2232.0498	-0.0403	-18	458	478	FNAPLAHQMMAGADVLA VTSR			Oxidation (M)[9,10]		Mascot

3	waxy protein [Aegilops umbellulata]	gi|452113209	67100.2	7.85	23	659	100	28.584	558	100

Protein Group

waxy protein [Aegilops umbellulata]	gi|452113211	67100.2	7.8499 999046

Peptide Information

Calc. Mass	Obsrv. Mass	± da	± ppm	Start Seq.	End Seq.	Sequence	Ion Score	C. I. %	Modification	Rank	Result Type
817.46	817.4323	-0.0277	-34	114	120	VMVISPR			Oxidation (M)[2]		Mascot
821.3723	821.3473	-0.025	-30	514	520	TGFHMGR			Oxidation (M)[5]		Mascot
832.4709	832.4615	-0.0094	-11	65	71	CLSVVVR			Carbamidomethyl (C)[1]		Mascot
908.4836	908.4616	-0.022	-24	138	144	VVDKYER					Mascot
917.3974	917.3704	-0.027	-29	147	152	YFHCYK			Carbamidomethyl (C)[4]		Mascot
971.6036	971.574	-0.0296	-30	395	403	VPLVAFIGR					Mascot
1099.6986	1099.6765	-0.0221	-20	394	403	KVPLVAFIGR					Mascot
1123.4871	1123.4641	-0.023	-20	331	339	GCELDNIMR			Carbamidomethyl (C)[2], Oxidation (M)[8]		Mascot
1346.6774	1346.636	-0.0414	-31	546	557	VVGTPAYHEMVK			Oxidation (M)[10]		Mascot
1390.6825	1390.6514	-0.0311	-22	158	168	VFVDHPCFLEK			Carbamidomethyl (C)[7]		Mascot
1396.7432	1396.7229	-0.0203	-15	381	393	EALQAEVGLPVDR					Mascot
1503.7988	1503.7808	-0.018	-12	300	312	INWMKAGILQADK			Oxidation (M)[4]		Mascot
1503.7988	1503.7808	-0.018	-12	193	205	FSLLCQAALEVPR	88	100	Carbamidomethyl (C)[5]		Mascot
1524.838	1524.7905	-0.0475	-31	381	394	EALQAEVGLPVDRK					Mascot
1564.7611	1564.7469	-0.0142	-9	480	492	FEPCGLIQLQGMR			Carbamidomethyl (C)[4], Oxidation (M)[12]		Mascot
1564.7689	1564.7469	-0.022	-14	254	266	VAFCIHNISYQGR	67	99.966	Carbamidomethyl (C)[4]		Mascot
1684.7966	1684.7849	-0.0117	-7	267	280	FSFDDFAQLNLPDR					Mascot
1684.7966	1684.7849	-0.0117	-7	267	280	FSFDDFAQLNLPDR	119	100			Mascot
1831.8497	1831.8289	-0.0208	-11	283	298	SSFDFIDGYDKPVEGR					Mascot
1831.8497	1831.8289	-0.0208	-11	283	298	SSFDFIDGYDKPVEGR	113	100			Mascot
1841.7937	1841.7733	-0.0204	-11	177	192	IYGPDAGTDYEDNQQR					Mascot
1841.7937	1841.7733	-0.0204	-11	177	192	IYGPDAGTDYEDNQQR	68	99.972			Mascot
1869.9592	1869.8092	-0.15	-80	360	376	DKFLAANYDITTALEGK					Mascot
1997.0226	1997.0088	-0.0138	-7	313	330	VLTVSPYYAEELISGEAR					Mascot
1997.0226	1997.0088	-0.0138	-7	313	330	VLTVSPYYAEELISGEAR	106	100			Mascot
2098.9312	2098.9155	-0.0157	-7	175	192	EKIYGPDAGTDYEDNQQ R					Mascot
2232.0901	2232.0498	-0.0403	-18	459	479	FNAPLAHQMMAGADVLA VTSR			Oxidation (M)[9,10]		Mascot

4	waxy protein [Triticum aestivum]	gi|399153330	67152.2	7.85	22	653	100	28.567	558	100	

Protein Group

waxy protein [Triticum durum]	gi|399153336	67133.2	7.8499 999046 3257

Peptide Information

Calc. Mass	Obsrv. Mass	± da	± ppm	Start Seq.	End Seq.	Sequence	Ion Score	C. I. %	Modification	Rank	Result Type
817.46	817.4323	-0.0277	-34	113	119	VMVISPR			Oxidation (M)[2]		Mascot
821.3723	821.3473	-0.025	-30	513	519	TGFHMGR			Oxidation (M)[5]		Mascot
908.5173	908.4616	-0.0557	-61	53	59	QSRKPHR					Mascot
917.3974	917.3704	-0.027	-29	146	151	YFHCYK			Carbamidomethyl (C)[4]		Mascot
971.6036	971.574	-0.0296	-30	394	402	VPLVAFIGR					Mascot
1099.6986	1099.6765	-0.0221	-20	393	402	KVPLVAFIGR					Mascot
1123.4871	1123.4641	-0.023	-20	330	338	GCELDNIMR			Carbamidomethyl (C)[2], Oxidation (M)[8]		Mascot
1346.6774	1346.636	-0.0414	-31	545	556	VVGTPAYHEMVK			Oxidation (M)[10]		Mascot
1390.6825	1390.6514	-0.0311	-22	157	167	VFVDHPCFLEK			Carbamidomethyl (C)[7]		Mascot
1396.7432	1396.7229	-0.0203	-15	380	392	EALQAEVGLPVDR					Mascot
1503.7988	1503.7808	-0.018	-12	299	311	INWMKAGILQADK			Oxidation (M)[4]		Mascot
1503.7988	1503.7808	-0.018	-12	192	204	FSLLCQAALEVPR	88	100	Carbamidomethyl (C)[5]		Mascot
1524.838	1524.7905	-0.0475	-31	380	393	EALQAEVGLPVDRK					Mascot
1564.7611	1564.7469	-0.0142	-9	479	491	FEPCGLIQLQGMR			Carbamidomethyl (C)[4], Oxidation (M)[12]		Mascot
1564.7689	1564.7469	-0.022	-14	253	265	VAFCIHNISYQGR	67	99.966	Carbamidomethyl (C)[4]		Mascot
1684.7966	1684.7849	-0.0117	-7	266	279	FSFDDFAQLNLPDR					Mascot
1684.7966	1684.7849	-0.0117	-7	266	279	FSFDDFAQLNLPDR	119	100			Mascot
1831.8497	1831.8289	-0.0208	-11	282	297	SSFDFIDGYDKPVEGR					Mascot
1831.8497	1831.8289	-0.0208	-11	282	297	SSFDFIDGYDKPVEGR	113	100			Mascot
1841.7937	1841.7733	-0.0204	-11	176	191	IYGPDAGTDYEDNQQR					Mascot
1841.7937	1841.7733	-0.0204	-11	176	191	IYGPDAGTDYEDNQQR	68	99.972			Mascot
1913.9855	1913.9971	0.0116	6	359	375	DKFLTVNYDVTTALEGK					Mascot
1997.0226	1997.0088	-0.0138	-7	312	329	VLTVSPYYAEELISGEAR					Mascot
1997.0226	1997.0088	-0.0138	-7	312	329	VLTVSPYYAEELISGEAR	106	100			Mascot
2098.9312	2098.9155	-0.0157	-7	174	191	EKIYGPDAGTDYEDNQQ R					Mascot
2232.0901	2232.0498	-0.0403	-18	458	478	FNAPLAHQMMAGADVLA VTSR			Oxidation (M)[9,10]		Mascot

5 waxy protein [Aegilops markgrafii] gi|452113217 67032.1 7.5 22 653 100 28.461 558 100

Peptide Information

Calc. Mass	Obsrv. Mass	± da	± ppm	Start Seq.	End Seq.	Sequence	Ion Score	C. I. %	Modification	Rank	Result Type
817.46	817.4323	-0.0277	-34	114	120	VMVISPR			Oxidation (M)[2]		Mascot
821.3723	821.3473	-0.025	-30	514	520	TGFHMGR			Oxidation (M)[5]		Mascot

Calc. Mass	Obsrv. Mass	± da	± ppm	Start Seq.	End Seq.	Sequence	Ion Score	C. I. %	Modification	Rank	Result Type
908.4836	908.4616	-0.022	-24	138	144	VVDKYER					Mascot
917.3974	917.3704	-0.027	-29	147	152	YFHCYK			Carbamidomethyl (C)[4]		Mascot
971.6036	971.574	-0.0296	-30	395	403	VPLVAFIGR					Mascot
1099.6986	1099.6765	-0.0221	-20	394	403	KVPLVAFIGR					Mascot
1123.4871	1123.4641	-0.023	-20	331	339	GCELDNIMR			Carbamidomethyl (C)[2], Oxidation (M)[8]		Mascot
1346.6774	1346.636	-0.0414	-31	546	557	VVGTPAYHEMVK			Oxidation (M)[10]		Mascot
1390.6825	1390.6514	-0.0311	-22	158	168	VFVDHPCFLEK			Carbamidomethyl (C)[7]		Mascot
1396.7432	1396.7229	-0.0203	-15	381	393	EALQAEVGLPVDR					Mascot
1503.7988	1503.7808	-0.018	-12	300	312	INWMKAGILQADK			Oxidation (M)[4]		Mascot
1503.7988	1503.7808	-0.018	-12	193	205	FSLLCQAALEVPR	88	100	Carbamidomethyl (C)[5]		Mascot
1524.838	1524.7905	-0.0475	-31	381	394	EALQAEVGLPVDRK					Mascot
1564.7611	1564.7469	-0.0142	-9	480	492	FEPCGLIQLQGMR			Carbamidomethyl (C)[4], Oxidation (M)[12]		Mascot
1564.7689	1564.7469	-0.022	-14	254	266	VAFCIHNISYQGR	67	99.966	Carbamidomethyl (C)[4]		Mascot
1684.7966	1684.7849	-0.0117	-7	267	280	FSFDDFAQLNLPDR					Mascot
1684.7966	1684.7849	-0.0117	-7	267	280	FSFDDFAQLNLPDR	119	100			Mascot
1831.8497	1831.8289	-0.0208	-11	283	298	SSFDFIDGYDKPVEGR					Mascot
1831.8497	1831.8289	-0.0208	-11	283	298	SSFDFIDGYDKPVEGR	113	100			Mascot
1841.7937	1841.7733	-0.0204	-11	177	192	IYGPDAGTDYEDNQQR					Mascot
1841.7937	1841.7733	-0.0204	-11	177	192	IYGPDAGTDYEDNQQR	68	99.972			Mascot
1869.9592	1869.8092	-0.15	-80	360	376	DKFLAANYDITTALEGK					Mascot
1997.0226	1997.0088	-0.0138	-7	313	330	VLTVSPYYAEELISGEAR					Mascot
1997.0226	1997.0088	-0.0138	-7	313	330	VLTVSPYYAEELISGEAR	106	100			Mascot
2098.9312	2098.9155	-0.0157	-7	175	192	EKIYGPDAGTDYEDNQQ R					Mascot
2232.0901	2232.0498	-0.0403	-18	459	479	FNAPLAHQMMAGADVLA VTSR			Oxidation (M)[9,10]		Mascot

6	waxy protein [Aegilops markgrafii]	gi|452113215	67029.1	7.5	22	652	100	28.461	558	100

Peptide Information

Calc. Mass	Obsrv. Mass	± da	± ppm	Start Seq.	End Seq.	Sequence	Ion Score	C. I. %	Modification	Rank	Result Type
817.46	817.4323	-0.0277	-34	114	120	VMVISPR			Oxidation (M)[2]		Mascot
821.3723	821.3473	-0.025	-30	514	520	TGFHMGR			Oxidation (M)[5]		Mascot
908.4836	908.4616	-0.022	-24	138	144	VVDKYER					Mascot
917.3974	917.3704	-0.027	-29	147	152	YFHCYK			Carbamidomethyl (C)[4]		Mascot
971.6036	971.574	-0.0296	-30	395	403	VPLVAFIGR					Mascot
1099.6986	1099.6765	-0.0221	-20	394	403	KVPLVAFIGR					Mascot
1123.4871	1123.4641	-0.023	-20	331	339	GCELDNIMR			Carbamidomethyl (C)[2], Oxidation (M)[8]		Mascot

1346.6774	1346.636	-0.0414	-31	546	557	VVGTPAYHEMVK			Oxidation (M)[10]		Mascot
1390.6825	1390.6514	-0.0311	-22	158	168	VFVDHPCFLEK			Carbamidomethyl (C)[7]		Mascot
1396.7432	1396.7229	-0.0203	-15	381	393	EALQAEVGLPVDR					Mascot
1503.7988	1503.7808	-0.018	-12	300	312	INWMKAGILQADK			Oxidation (M)[4]		Mascot
1503.7988	1503.7808	-0.018	-12	193	205	FSLLCQAALEVPR	88	100	Carbamidomethyl (C)[5]		Mascot
1524.838	1524.7905	-0.0475	-31	381	394	EALQAEVGLPVDRK					Mascot
1564.7611	1564.7469	-0.0142	-9	480	492	FEPCGLIQLQGMR			Carbamidomethyl (C)[4], Oxidation (M)[12]		Mascot
1564.7689	1564.7469	-0.022	-14	254	266	VAFCIHNISYQGR	67	99.966	Carbamidomethyl (C)[4]		Mascot
1684.7966	1684.7849	-0.0117	-7	267	280	FSFDDFAQLNLPDR					Mascot
1684.7966	1684.7849	-0.0117	-7	267	280	FSFDDFAQLNLPDR	119	100			Mascot
1831.8497	1831.8289	-0.0208	-11	283	298	SSFDFIDGYDKPVEGR					Mascot
1831.8497	1831.8289	-0.0208	-11	283	298	SSFDFIDGYDKPVEGR	113	100			Mascot
1841.7937	1841.7733	-0.0204	-11	177	192	IYGPDAGTDYEDNQQR					Mascot
1841.7937	1841.7733	-0.0204	-11	177	192	IYGPDAGTDYEDNQQR	68	99.972			Mascot
1869.9592	1869.8092	-0.15	-80	360	376	DKFLAANYDITTALEGK					Mascot
1997.0226	1997.0088	-0.0138	-7	313	330	VLTVSPYYAEELISGEAR					Mascot
1997.0226	1997.0088	-0.0138	-7	313	330	VLTVSPYYAEELISGEAR	106	100			Mascot
2098.9312	2098.9155	-0.0157	-7	175	192	EKIYGPDAGTDYEDNQQ R					Mascot
2232.0901	2232.0498	-0.0403	-18	459	479	FNAPLAHQMMAGADVLA VTSR			Oxidation (M)[9,10]		Mascot

7	waxy protein [Aegilops umbellulata]	gi|452113213	67090.1	7.85	22	651	100	28.461	558	100	

Peptide Information

Calc. Mass	Obsrv. Mass	± da	± ppm	Start Seq.	End Seq.	Sequence	Ion Score	C. I. %	Modification	Rank	Result Type
817.46	817.4323	-0.0277	-34	114	120	VMVISPR			Oxidation (M)[2]		Mascot
821.3723	821.3473	-0.025	-30	514	520	TGFHMGR			Oxidation (M)[5]		Mascot
908.4836	908.4616	-0.022	-24	138	144	VVDKYER					Mascot
917.3974	917.3704	-0.027	-29	147	152	YFHCYK			Carbamidomethyl (C)[4]		Mascot
971.6036	971.574	-0.0296	-30	395	403	VPLVAFIGR					Mascot
1099.6986	1099.6765	-0.0221	-20	394	403	KVPLVAFIGR					Mascot
1123.4871	1123.4641	-0.023	-20	331	339	GCELDNIMR			Carbamidomethyl (C)[2], Oxidation (M)[8]		Mascot
1346.6774	1346.636	-0.0414	-31	546	557	VVGTPAYHEMVK			Oxidation (M)[10]		Mascot
1390.6825	1390.6514	-0.0311	-22	158	168	VFVDHPCFLEK			Carbamidomethyl (C)[7]		Mascot
1396.7432	1396.7229	-0.0203	-15	381	393	EALQAEVGLPVDR					Mascot
1503.7988	1503.7808	-0.018	-12	300	312	INWMKAGILQADK			Oxidation (M)[4]		Mascot
1503.7988	1503.7808	-0.018	-12	193	205	FSLLCQAALEVPR	88	100	Carbamidomethyl (C)[5]		Mascot

1524.838	1524.7905	-0.0475	-31	381	394	EALQAEVGLPVDRK					Mascot
1564.7611	1564.7469	-0.0142	-9	480	492	FEPCGLIQLQGMR			Carbamidomethyl (C)[4], Oxidation (M)[12]		Mascot
1564.7689	1564.7469	-0.022	-14	254	266	VAFCIHNISYQGR	67	99.966	Carbamidomethyl (C)[4]		Mascot
1684.7966	1684.7849	-0.0117	-7	267	280	FSFDDFAQLNLPDR					Mascot
1684.7966	1684.7849	-0.0117	-7	267	280	FSFDDFAQLNLPDR	119	100			Mascot
1831.8497	1831.8289	-0.0208	-11	283	298	SSFDFIDGYDKPVEGR					Mascot
1831.8497	1831.8289	-0.0208	-11	283	298	SSFDFIDGYDKPVEGR	113	100			Mascot
1841.7937	1841.7733	-0.0204	-11	177	192	IYGPDAGTDYEDNQQR					Mascot
1841.7937	1841.7733	-0.0204	-11	177	192	IYGPDAGTDYEDNQQR	68	99.972			Mascot
1869.9592	1869.8092	-0.15	-80	360	376	DKFLAANYDITTALEGK					Mascot
1997.0226	1997.0088	-0.0138	-7	313	330	VLTVSPYYAEELISGEAR					Mascot
1997.0226	1997.0088	-0.0138	-7	313	330	VLTVSPYYAEELISGEAR	106	100			Mascot
2098.9312	2098.9155	-0.0157	-7	175	192	EKIYGPDAGTDYEDNQQ R					Mascot
2232.0901	2232.0498	-0.0403	-18	459	479	FNAPLAHQMMAGADVLA VTSR			Oxidation (M)[9,10]		Mascot

8 waxy protein [Triticum durum] gi|399153334 67132.3 8.27 21 648 100 28.567 560 100

Peptide Information

Calc. Mass	Obsrv. Mass	± da	± ppm	Start Seq.	End Seq.	Sequence	Ion Score	C. I. %	Modification	Rank	Result Type
817.46	817.4323	-0.0277	-34	113	119	VMVISPR			Oxidation (M)[2]		Mascot
821.3723	821.3473	-0.025	-30	513	519	TGFHMGR			Oxidation (M)[5]		Mascot
908.5173	908.4616	-0.0557	-61	53	59	QSRKPHR					Mascot
917.3974	917.3704	-0.027	-29	146	151	YFHCYK			Carbamidomethyl (C)[4]		Mascot
971.6036	971.574	-0.0296	-30	394	402	VPLVAFIGR					Mascot
1099.6986	1099.6765	-0.0221	-20	393	402	KVPLVAFIGR					Mascot
1123.4871	1123.4641	-0.023	-20	330	338	GCELDNIMR			Carbamidomethyl (C)[2], Oxidation (M)[8]		Mascot
1346.6774	1346.636	-0.0414	-31	545	556	VVGTPAYHEMVK			Oxidation (M)[10]		Mascot
1390.6825	1390.6514	-0.0311	-22	157	167	VFVDHPCFLEK			Carbamidomethyl (C)[7]		Mascot
1396.7432	1396.7229	-0.0203	-15	380	392	EALQAEVGLPVDR					Mascot
1503.7988	1503.7808	-0.018	-12	299	311	INWMKAGILQADK			Oxidation (M)[4]		Mascot
1503.7988	1503.7808	-0.018	-12	192	204	FSLLCQAALEVPR	88	100	Carbamidomethyl (C)[5]		Mascot
1524.838	1524.7905	-0.0475	-31	380	393	EALQAEVGLPVDRK					Mascot
1564.7689	1564.7469	-0.022	-14	253	265	VAFCIHNISYQGR			Carbamidomethyl (C)[4]		Mascot
1564.7689	1564.7469	-0.022	-14	253	265	VAFCIHNISYQGR	67	99.966	Carbamidomethyl (C)[4]		Mascot
1684.7966	1684.7849	-0.0117	-7	266	279	FSFDDFAQLNLPDR					Mascot
1684.7966	1684.7849	-0.0117	-7	266	279	FSFDDFAQLNLPDR	119	100			Mascot

1831.8497	1831.8289	-0.0208	-11	282	297	SSFDFIDGYDKPVEGR					Mascot
1831.8497	1831.8289	-0.0208	-11	282	297	SSFDFIDGYDKPVEGR	113	100			Mascot
1841.7937	1841.7733	-0.0204	-11	176	191	IYGPDAGTDYEDNQQR					Mascot
1841.7937	1841.7733	-0.0204	-11	176	191	IYGPDAGTDYEDNQQR	68	99.972			Mascot
1913.9855	1913.9971	0.0116	6	359	375	DKFLTVNYDVTTALEGK					Mascot
1997.0226	1997.0088	-0.0138	-7	312	329	VLTVSPYYAEELISGEAR					Mascot
1997.0226	1997.0088	-0.0138	-7	312	329	VLTVSPYYAEELISGEAR	106	100			Mascot
2098.9312	2098.9155	-0.0157	-7	174	191	EKIYGPDAGTDYEDNQQR					Mascot
2232.0901	2232.0498	-0.0403	-18	458	478	FNAPLAHQMMAGADVLAVTSR			Oxidation (M)[9,10]		Mascot

9 RecName: Full=Granule-bound starch synthase 1, chloroplastic/amyloplastic; AltName: Full=Granule-bound starch synthase I; Short=GBSS-I; Flags: Precursor | gi|136765 | 68506 | 8.25 | 22 | 647 | 100 | 28.567 | 558 | 100

Peptide Information

Calc. Mass	Obsrv. Mass	± da	± ppm	Start Seq.	End Seq.	Sequence	Ion Score	C. I. %	Modification	Rank	Result Type
817.46	817.4323	-0.0277	-34	113	119	VMVISPR			Oxidation (M)[2]		Mascot
821.3723	821.3473	-0.025	-30	524	530	TGFHMGR			Oxidation (M)[5]		Mascot
908.5173	908.4616	-0.0557	-61	53	59	QSRKPHR					Mascot
917.3974	917.3704	-0.027	-29	146	151	YFHCYK			Carbamidomethyl (C)[4]		Mascot
971.6036	971.574	-0.0296	-30	405	413	VPLVAFIGR					Mascot
1099.6986	1099.6765	-0.0221	-20	404	413	KVPLVAFIGR					Mascot
1123.4871	1123.4641	-0.023	-20	341	349	GCELDNIMR			Carbamidomethyl (C)[2], Oxidation (M)[8]		Mascot
1346.6774	1346.636	-0.0414	-31	556	567	VVGTPAYHEMVK			Oxidation (M)[10]		Mascot
1390.6825	1390.6514	-0.0311	-22	157	167	VFVDHPCFLEK			Carbamidomethyl (C)[7]		Mascot
1396.7432	1396.7229	-0.0203	-15	391	403	EALQAEVGLPVDR					Mascot
1503.7988	1503.7808	-0.018	-12	310	322	INWMKAGILQADK			Oxidation (M)[4]		Mascot
1503.7988	1503.7808	-0.018	-12	192	204	FSLLCQAALEVPR	88	100	Carbamidomethyl (C)[5]		Mascot
1524.838	1524.7905	-0.0475	-31	391	404	EALQAEVGLPVDRK					Mascot
1564.7611	1564.7469	-0.0142	-9	490	502	FEPCGLIQLQGMR			Carbamidomethyl (C)[4], Oxidation (M)[12]		Mascot
1564.7689	1564.7469	-0.022	-14	264	276	VAFCIHNISYQGR	67	99.966	Carbamidomethyl (C)[4]		Mascot
1684.7966	1684.7849	-0.0117	-7	277	290	FSFDDFAQLNLPDR					Mascot
1684.7966	1684.7849	-0.0117	-7	277	290	FSFDDFAQLNLPDR	119	100			Mascot
1831.8497	1831.8289	-0.0208	-11	293	308	SSFDFIDGYDKPVEGR					Mascot
1831.8497	1831.8289	-0.0208	-11	293	308	SSFDFIDGYDKPVEGR	113	100			Mascot
1841.7937	1841.7733	-0.0204	-11	176	191	IYGPDAGTDYEDNQQR					Mascot
1841.7937	1841.7733	-0.0204	-11	176	191	IYGPDAGTDYEDNQQR	68	99.972			Mascot

Calc. Mass	Obsrv. Mass	± da	± ppm	Start Seq.	End Seq.	Sequence	Ion Score	C. I. %	Modification	Rank	Result Type
1913.9855	1913.9971	0.0116	6	370	386	DKFLTVNYDVTTALEGK					Mascot
1997.0226	1997.0088	-0.0138	-7	323	340	VLTVSPYYAEELISGEAR					Mascot
1997.0226	1997.0088	-0.0138	-7	323	340	VLTVSPYYAEELISGEAR	106	100			Mascot
2098.9312	2098.9155	-0.0157	-7	174	191	EKIYGPDAGTDYEDNQQR					Mascot
2232.0688	2232.0498	-0.019	-9	205	223	ILDLNNNPHFSGPYAMLCR			Carbamidomethyl (C)[18]		Mascot

10	Granule-bound starch synthase 1, chloroplastic/amyloplastic [Aegilops tauschii]	gi|475495669	66968.1	7.85	21	641	100	28.342	558	100

Peptide Information

Calc. Mass	Obsrv. Mass	± da	± ppm	Start Seq.	End Seq.	Sequence	Ion Score	C. I. %	Modification	Rank	Result Type
817.46	817.4323	-0.0277	-34	113	119	VMVISPR			Oxidation (M)[2]		Mascot
821.3723	821.3473	-0.025	-30	513	519	TGFHMGR			Oxidation (M)[5]		Mascot
908.4836	908.4616	-0.022	-24	137	143	VVDKYER					Mascot
917.3974	917.3704	-0.027	-29	146	151	YFHCYK			Carbamidomethyl (C)[4]		Mascot
971.6036	971.574	-0.0296	-30	394	402	VPLVAFIGR					Mascot
1099.6986	1099.6765	-0.0221	-20	393	402	KVPLVAFIGR					Mascot
1123.4871	1123.4641	-0.023	-20	330	338	GCELDNIMR			Carbamidomethyl (C)[2], Oxidation (M)[8]		Mascot
1346.6774	1346.636	-0.0414	-31	545	556	VVGTPAYHEMVK			Oxidation (M)[10]		Mascot
1390.6825	1390.6514	-0.0311	-22	157	167	VFVDHPCFLEK			Carbamidomethyl (C)[7]		Mascot
1396.7432	1396.7229	-0.0203	-15	380	392	EALQAEVGLPVDR					Mascot
1503.7988	1503.7808	-0.018	-12	299	311	INWMKAGILQADK			Oxidation (M)[4]		Mascot
1503.7988	1503.7808	-0.018	-12	192	204	FSLLCQAALEVPR	88	100	Carbamidomethyl (C)[5]		Mascot
1524.838	1524.7905	-0.0475	-31	380	393	EALQAEVGLPVDRK					Mascot
1564.7611	1564.7469	-0.0142	-9	479	491	FEPCGLIQLQGMR			Carbamidomethyl (C)[4], Oxidation (M)[12]		Mascot
1564.7689	1564.7469	-0.022	-14	253	265	VAFCIHNISYQGR	67	99.966	Carbamidomethyl (C)[4]		Mascot
1684.7966	1684.7849	-0.0117	-7	266	279	FSFDDFAQLNLPDR					Mascot
1684.7966	1684.7849	-0.0117	-7	266	279	FSFDDFAQLNLPDR	119	100			Mascot
1831.8497	1831.8289	-0.0208	-11	282	297	SSFDFIDGYDKPVEGR					Mascot
1831.8497	1831.8289	-0.0208	-11	282	297	SSFDFIDGYDKPVEGR	113	100			Mascot
1841.7937	1841.7733	-0.0204	-11	176	191	IYGPDAGTDYEDNQQR					Mascot
1841.7937	1841.7733	-0.0204	-11	176	191	IYGPDAGTDYEDNQQR	68	99.972			Mascot
1997.0226	1997.0088	-0.0138	-7	312	329	VLTVSPYYAEELISGEAR					Mascot
1997.0226	1997.0088	-0.0138	-7	312	329	VLTVSPYYAEELISGEAR	106	100			Mascot
2098.9312	2098.9155	-0.0157	-7	174	191	EKIYGPDAGTDYEDNQQR					Mascot
2232.0901	2232.0498	-0.0403	-18	458	478	FNAPLAHQMMAGADVLAVTSR			Oxidation (M)[9,10]		Mascot

Gel Idx/Pos	281/L9	Instr./Gel Origin	BA2151/Sample Project 20140814	Process Status	Analysis Succeeded
Plate [#] Name	[1] Sample Project 20140814	Instrument Sample Name		Spectra	11

Rank	Protein Name	Accession No.	Protein MW	Protein PI	Pep. Count	Protein Score	Protein Score C. I. %	Intensity Matched	Total Ion Score	Total Ion C. I. %	Confirmed
1	Chain A, Ketol-Acid Reductoisomerase (Kari) In Complex With Mg2+	gi|226887786	57566.1	5.46	12	324	100	18.924	279	100	

Protein Group

Protein Name	Accession No.	Protein MW	Protein PI
Chain A, Rice Ketolacid Reductoisomerase In Complex With Mg2+-Nadph	gi|226887789	57566.1	5.4600000381 4697
Chain B, Ketol-Acid Reductoisomerase (Kari) In Complex With Mg2+	gi|226887787	57566.1	5.4600000381 4697
Chain B, Rice Ketolacid Reductoisomerase In Complex With Mg2+-Nadph	gi|226887790	57566.1	5.4600000381 4697

Peptide Information

Calc. Mass	Obsrv. Mass	± da	± ppm	Start Seq.	End Seq.	Sequence	Ion Score	C. I. %	Modification	Rank	Result Type
823.3945	823.3724	-0.0221	-27	243	249	SDIFGER					Mascot
863.5098	863.4813	-0.0285	-33	189	195	RLYVQGK					Mascot
877.46	877.456	-0.004	-5	370	376	MWKVGEK					Mascot
1099.6833	1099.6649	-0.0184	-17	82	91	SDIVVKIGLR					Mascot
1288.73	1288.6642	-0.0658	-51	42	52	NLFPLLPEAFK					Mascot
1372.7219	1372.7006	-0.0213	-16	27	38	VSLAGHEEYIVR					Mascot
1372.7219	1372.7006	-0.0213	-16	27	38	VSLAGHEEYIVR	102	100			Mascot
1528.6884	1528.6809	-0.0075	-5	438	451	GVAFMVDNCSTTAR			Carbamidomethyl (C)[9]		Mascot
1544.6832	1544.6357	-0.0475	-31	438	451	GVAFMVDNCSTTAR			Carbamidomethyl (C)[9], Oxidation (M)[5]		Mascot
1544.6832	1544.6357	-0.0475	-31	438	451	GVAFMVDNCSTTAR	39	77.388	Carbamidomethyl (C)[9], Oxidation (M)[5]		Mascot
1664.9847	1664.9518	-0.0329	-20	250	265	GILLGAVHGIVEALFR					Mascot
1713.8364	1713.8126	-0.0238	-14	297	311	GMLEVYNSLTEEGKK			Oxidation (M)[2]		Mascot
1980.041	1980.0227	-0.0183	-9	56	74	QIGVIGWGSQGPAQAQN LR					Mascot
1980.041	1980.0227	-0.0183	-9	56	74	QIGVIGWGSQGPAQAQN LR	80	99.998			Mascot
2085.9949	2085.9739	-0.021	-10	196	215	EINGAGINSSFAVHQDVD GR					Mascot
2085.9949	2085.9739	-0.021	-10	196	215	EINGAGINSSFAVHQDVD GR	59	99.796			Mascot
2947.3892	2947.3635	-0.0257	-9	412	437	GHSYSEIINESVIESVDSL			Oxidation (M)[23]		Mascot

NPFMHAR

2	Os05g0573700 [Oryza sativa Japonica Group]	gi|113579935	62679.9	6.01	12	320	100	18.924	279	100

Protein Group

RecName: Full=Ketol-acid reductoisomerase, chloroplastic; AltName: Full=Acetohydroxy-acid reductoisomerase; AltName: Full=Alpha-keto-beta-hydroxylacyl reductoisomerase; AltName: Full=Protein KARI; Flags: Precursor	gi|75115272	62679.9	6.0100 002288 8184
hypothetical protein OsI_21076 [Oryza sativa Indica Group]	gi|218197310	62651.8	6.0100 002288 8184

Peptide Information

Calc. Mass	Obsrv. Mass	± da	± ppm	Start Seq.	End Seq.	Sequence	Ion Score	C. I. %	Modification	Rank	Result Type
823.3945	823.3724	-0.0221	-27	296	302	SDIFGER					Mascot
863.5098	863.4813	-0.0285	-33	242	248	RLYVQGK					Mascot
877.46	877.456	-0.004	-5	423	429	MWKVGEK					Mascot
1099.6833	1099.6649	-0.0184	-17	135	144	SDIVVKIGLR					Mascot
1288.73	1288.6642	-0.0658	-51	95	105	NLFPLLPEAFK					Mascot
1372.7219	1372.7006	-0.0213	-16	80	91	VSLAGHEEYIVR					Mascot
1372.7219	1372.7006	-0.0213	-16	80	91	VSLAGHEEYIVR	102	100			Mascot
1528.6884	1528.6809	-0.0075	-5	491	504	GVAFMVDNCSTTAR			Carbamidomethyl (C)[9]		Mascot
1544.6832	1544.6357	-0.0475	-31	491	504	GVAFMVDNCSTTAR			Carbamidomethyl (C)[9], Oxidation (M)[5]		Mascot
1544.6832	1544.6357	-0.0475	-31	491	504	GVAFMVDNCSTTAR	39	77.388	Carbamidomethyl (C)[9], Oxidation (M)[5]		Mascot
1664.9847	1664.9518	-0.0329	-20	303	318	GILLGAVHGIVEALFR					Mascot
1713.8364	1713.8126	-0.0238	-14	350	364	GMLEVYNSLTEEGKK			Oxidation (M)[2]		Mascot
1980.041	1980.0227	-0.0183	-9	109	127	QIGVIGWGSQGPAQAQN LR					Mascot
1980.041	1980.0227	-0.0183	-9	109	127	QIGVIGWGSQGPAQAQN LR	80	99.998			Mascot
2085.9949	2085.9739	-0.021	-10	249	268	EINGAGINSSFAVHQDVD GR					Mascot
2085.9949	2085.9739	-0.021	-10	249	268	EINGAGINSSFAVHQDVD GR	59	99.796			Mascot
2947.3892	2947.3635	-0.0257	-9	465	490	GHSYSEIINESVIESVDSL NPFMHAR			Oxidation (M)[23]		Mascot

3	hypothetical protein SORBIDRAFT_09g029170 [Sorghum bicolor]	gi|241946836	63644.2	5.88	12	260	100	18.204	220	100

Peptide Information

Calc. Mass	Obsrv. Mass	± da	± ppm	Start Seq.	End Seq.	Sequence	Ion Score	C. I. %	Modification	Rank	Result Type
823.3945	823.3724	-0.0221	-27	305	311	SDIFGER					Mascot

Calc. Mass	Obsrv. Mass	± da	± ppm	Start Seq.	End Seq.	Sequence	Ion Score	C. I. %	Modification	Rank	Result Type
863.5098	863.4813	-0.0285	-33	251	257	RLYVQGK					Mascot
877.46	877.456	-0.004	-5	432	438	MWKVGEK					Mascot
1099.6833	1099.6649	-0.0184	-17	144	153	SDIVVKIGLR					Mascot
1288.73	1288.6642	-0.0658	-51	104	114	NLFPLLPEAFK					Mascot
1372.7219	1372.7006	-0.0213	-16	89	100	VSLAGHEEYIVR					Mascot
1372.7219	1372.7006	-0.0213	-16	89	100	VSLAGHEEYIVR	102	100			Mascot
1528.6884	1528.6809	-0.0075	-5	500	513	GVAFMVDNCSTTAR			Carbamidomethyl (C)[9]		Mascot
1544.6832	1544.6357	-0.0475	-31	500	513	GVAFMVDNCSTTAR			Carbamidomethyl (C)[9], Oxidation (M)[5]		Mascot
1544.6832	1544.6357	-0.0475	-31	500	513	GVAFMVDNCSTTAR	39	77.388	Carbamidomethyl (C)[9], Oxidation (M)[5]		Mascot
1664.9847	1664.9518	-0.0329	-20	312	327	GILLGAVHGIVEALFR					Mascot
1699.8208	1699.7753	-0.0455	-27	359	373	GMLEVYNSLSEEGKK			Oxidation (M)[2]		Mascot
1980.041	1980.0227	-0.0183	-9	118	136	QIGVIGWGSQGPAQAQN LR					Mascot
1980.041	1980.0227	-0.0183	-9	118	136	QIGVIGWGSQGPAQAQN LR	80	99.998			Mascot
2069.1138	2068.9917	-0.1221	-59	2	24	AASTLAFGHPKTLGAAAA SAGAK					Mascot
2947.3892	2947.3635	-0.0257	-9	474	499	GHSYSEIINESVIESVDSL NPFMHAR			Oxidation (M)[23]		Mascot

4	PREDICTED: ketol-acid reductoisomerase, chloroplastic-like [Setaria italica]	gi|514746589	63351.2	6.01	12	260	100	18.25	220	100	

Peptide Information

Calc. Mass	Obsrv. Mass	± da	± ppm	Start Seq.	End Seq.	Sequence	Ion Score	C. I. %	Modification	Rank	Result Type
823.3945	823.3724	-0.0221	-27	302	308	SDIFGER					Mascot
863.5098	863.4813	-0.0285	-33	248	254	RLYVQGK					Mascot
877.46	877.456	-0.004	-5	429	435	MWKVGEK					Mascot
1099.6833	1099.6649	-0.0184	-17	141	150	SDIVVKIGLR					Mascot
1288.73	1288.6642	-0.0658	-51	101	111	NLFPLLPEAFK					Mascot
1372.7219	1372.7006	-0.0213	-16	86	97	VSLAGHEEYIVR					Mascot
1372.7219	1372.7006	-0.0213	-16	86	97	VSLAGHEEYIVR	102	100			Mascot
1528.6884	1528.6809	-0.0075	-5	497	510	GVAFMVDNCSTTAR			Carbamidomethyl (C)[9]		Mascot
1544.6832	1544.6357	-0.0475	-31	497	510	GVAFMVDNCSTTAR			Carbamidomethyl (C)[9], Oxidation (M)[5]		Mascot
1544.6832	1544.6357	-0.0475	-31	497	510	GVAFMVDNCSTTAR	39	77.388	Carbamidomethyl (C)[9], Oxidation (M)[5]		Mascot
1664.9847	1664.9518	-0.0329	-20	309	324	GILLGAVHGIVEALFR					Mascot
1699.8208	1699.7753	-0.0455	-27	356	370	GMLEVYNSLSEEGKK			Oxidation (M)[2]		Mascot
1713.8378	1713.8126	-0.0252	-15	1	17	MAAHTSAASTLAFGHPK			Oxidation (M)[1]		Mascot
1980.041	1980.0227	-0.0183	-9	115	133	QIGVIGWGSQGPAQAQN LR					Mascot
1980.041	1980.0227	-0.0183	-9	115	133	QIGVIGWGSQGPAQAQN	80	99.998			Mascot

						LR					
2947.3892	2947.3635	-0.0257	-9	471	496	GHSYSEIINESVIESVDSL NPFMHAR			Oxidation (M)[23]		Mascot

5	TPA: ketol-acid reductoisomerase [Zea mays]	gi|414881183	63305.2	6.31	9	242	100	17.483	220	100	

Protein Group

ketol-acid reductoisomerase [Zea mays]	gi|212722020	63305.2	6.3099 999427 7954

Peptide Information

Calc. Mass	Obsrv. Mass	± da	± ppm	Start Seq.	End Seq.	Sequence	Ion Score	C. I. %	Modification	Rank	Result Type
823.3945	823.3724	-0.0221	-27	297	303	SDIFGER					Mascot
863.5098	863.4813	-0.0285	-33	243	249	RLYVQGK					Mascot
1062.5289	1062.4911	-0.0378	-36	409	418	EGLPAFPMGK			Oxidation (M)[8]		Mascot
1099.6833	1099.6649	-0.0184	-17	136	145	SDIVVKIGLR					Mascot
1368.7118	1368.583	-0.1288	-94	2	15	AAATSSSTYLAIGR					Mascot
1372.7219	1372.7006	-0.0213	-16	81	92	VSLAGHEEYIVR					Mascot
1372.7219	1372.7006	-0.0213	-16	81	92	VSLAGHEEYIVR	102	100			Mascot
1528.6884	1528.6809	-0.0075	-5	492	505	GVAFMVDNCSTTAR			Carbamidomethyl (C)[9]		Mascot
1544.6832	1544.6357	-0.0475	-31	492	505	GVAFMVDNCSTTAR			Carbamidomethyl (C)[9], Oxidation (M)[5]		Mascot
1544.6832	1544.6357	-0.0475	-31	492	505	GVAFMVDNCSTTAR	39	77.388	Carbamidomethyl (C)[9], Oxidation (M)[5]		Mascot
1713.8364	1713.8126	-0.0238	-14	351	365	GMLEVYNSLTEEGKK			Oxidation (M)[2]		Mascot
1980.041	1980.0227	-0.0183	-9	110	128	QIGVIGWGSQGPAQAQN LR					Mascot
1980.041	1980.0227	-0.0183	-9	110	128	QIGVIGWGSQGPAQAQN LR	80	99.998			Mascot

6	PREDICTED: ketol-acid reductoisomerase, chloroplastic-like [Setaria italica]	gi|514779378	63161.2	6.82	8	237	100	17.365	220	100	

Peptide Information

Calc. Mass	Obsrv. Mass	± da	± ppm	Start Seq.	End Seq.	Sequence	Ion Score	C. I. %	Modification	Rank	Result Type
823.3945	823.3724	-0.0221	-27	299	305	SDIFGER					Mascot
863.5098	863.4813	-0.0285	-33	245	251	RLYVQGK					Mascot
1062.5289	1062.4911	-0.0378	-36	411	420	EGLPAFPMGK			Oxidation (M)[8]		Mascot
1099.6833	1099.6649	-0.0184	-17	138	147	SDIVVKIGLR					Mascot
1372.7219	1372.7006	-0.0213	-16	83	94	VSLAGHEEYIVR					Mascot
1372.7219	1372.7006	-0.0213	-16	83	94	VSLAGHEEYIVR	102	100			Mascot
1528.6884	1528.6809	-0.0075	-5	494	507	GVAFMVDNCSTTAR			Carbamidomethyl (C)[9]		Mascot
1544.6832	1544.6357	-0.0475	-31	494	507	GVAFMVDNCSTTAR			Carbamidomethyl (C)[9], Oxidation (M)[5]		Mascot

1544.6832	1544.6357	-0.0475	-31	494	507	GVAFMVDNCSTTAR	39	77.388	Carbamidomethyl (C)[9], Oxidation (M)[5]		Mascot
1713.8364	1713.8126	-0.0238	-14	353	367	GMLEVYNSLTEEGKK			Oxidation (M)[2]		Mascot
1980.041	1980.0227	-0.0183	-9	112	130	QIGVIGWGSQGPAQAQNLR					Mascot
1980.041	1980.0227	-0.0183	-9	112	130	QIGVIGWGSQGPAQAQNLR	80	99.998			Mascot

7	hypothetical protein SORBIDRAFT_03g029720 [Sorghum bicolor]	gi|241928040	63171.2	6.13	6	230	100	17.09	219	100

Peptide Information

Calc. Mass	Obsrv. Mass	± da	± ppm	Start Seq.	End Seq.	Sequence	Ion Score	C. I. %	Modification	Rank	Result Type
823.3945	823.3724	-0.0221	-27	297	303	SDIFGER					Mascot
863.5098	863.4813	-0.0285	-33	243	249	RLYVQGK					Mascot
1372.7219	1372.7006	-0.0213	-16	81	92	VSLAGHEEYIVR					Mascot
1372.7219	1372.7006	-0.0213	-16	81	92	VSLAGHEEYIVR	102	100			Mascot
1528.6884	1528.6809	-0.0075	-5	492	505	GVAFMVDNCSTTAR			Carbamidomethyl (C)[9]		Mascot
1544.6832	1544.6357	-0.0475	-31	492	505	GVAFMVDNCSTTAR			Carbamidomethyl (C)[9], Oxidation (M)[5]		Mascot
1544.6832	1544.6357	-0.0475	-31	492	505	GVAFMVDNCSTTAR	39	77.388	Carbamidomethyl (C)[9], Oxidation (M)[5]		Mascot
1713.8364	1713.8126	-0.0238	-14	351	365	GMLEVYNSLTEEGKK			Oxidation (M)[2]		Mascot
1980.041	1980.0227	-0.0183	-9	110	128	QIGVIGWGSQGPAQAQNLR					Mascot
1980.041	1980.0227	-0.0183	-9	110	128	QIGVIGWGSQGPAQAQNLR	80	99.998			Mascot

8	ketol-acid reductoisomerase, chloroplast precursor, putative [Ricinus communis]	gi|223535574	63712.1	6.35	9	218	100	17.892	193	100

Peptide Information

Calc. Mass	Obsrv. Mass	± da	± ppm	Start Seq.	End Seq.	Sequence	Ion Score	C. I. %	Modification	Rank	Result Type
823.3945	823.3724	-0.0221	-27	301	307	SDIFGER					Mascot
863.5098	863.4813	-0.0285	-33	247	253	RLYVQGK					Mascot
1099.6833	1099.6649	-0.0184	-17	140	149	SDIVVKIGLR					Mascot
1372.7219	1372.7006	-0.0213	-16	85	96	ISLAGHDEYIVR					Mascot
1372.7219	1372.7006	-0.0213	-16	85	96	ISLAGHDEYIVR	33	12.23			Mascot
1544.6832	1544.6357	-0.0475	-31	496	509	GVSFMVDNCSTTAR			Carbamidomethyl (C)[9]		Mascot
1544.6832	1544.6357	-0.0475	-31	496	509	GVSFMVDNCSTTAR	21	0	Carbamidomethyl (C)[9]		Mascot
1980.041	1980.0227	-0.0183	-9	114	132	QIGVIGWGSQGPAQAQNLR					Mascot
1980.041	1980.0227	-0.0183	-9	114	132	QIGVIGWGSQGPAQAQNLR	80	99.998			Mascot
2027.9954	2028.0043	0.0089	4	350	368	TISTQGMLAVYNSLSEEGK					Mascot

Calc. Mass	Obsrv. Mass	± da	± ppm	Start Seq.	End Seq.	Sequence	Ion Score	C. I. %	Modification	Rank	Result Type
2085.9949	2085.9739	-0.021	-10	254	273	EINGAGINSSFAVHQDVD GR					Mascot
2085.9949	2085.9739	-0.021	-10	254	273	EINGAGINSSFAVHQDVD GR	59	99.796			Mascot
2947.3892	2947.3635	-0.0257	-9	470	495	GHSYSEIINESVIESVDSL NPFMHAR			Oxidation (M)[23]		Mascot

9	hypothetical protein PRUPE_ppa003271mg [Prunus persica]	gi|462407261	63730.4	6.22	11	213	100	10.632	176	100

Peptide Information

Calc. Mass	Obsrv. Mass	± da	± ppm	Start Seq.	End Seq.	Sequence	Ion Score	C. I. %	Modification	Rank	Result Type
823.3945	823.3724	-0.0221	-27	305	311	SDIFGER					Mascot
863.5098	863.4813	-0.0285	-33	251	257	RLYVQGK					Mascot
1099.6833	1099.6649	-0.0184	-17	144	153	SDIVVKIGLR					Mascot
1288.6604	1288.6642	0.0038	3	50	64	GVNSVSGGGSALGAR					Mascot
1528.6884	1528.6809	-0.0075	-5	500	513	GVAFMVDNCSTTAR			Carbamidomethyl (C)[9]		Mascot
1544.6832	1544.6357	-0.0475	-31	500	513	GVAFMVDNCSTTAR			Carbamidomethyl (C)[9], Oxidation (M)[5]		Mascot
1544.6832	1544.6357	-0.0475	-31	500	513	GVAFMVDNCSTTAR	39	77.388	Carbamidomethyl (C)[9], Oxidation (M)[5]		Mascot
1615.7826	1615.7245	-0.0581	-36	413	426	FYEKDGLPAFPMGK			Oxidation (M)[12]		Mascot
1699.8208	1699.7753	-0.0455	-27	359	373	GMLEIYNSLSEDGKK			Oxidation (M)[2]		Mascot
1980.041	1980.0227	-0.0183	-9	118	136	QIGVIGWGSQGPAQAQN LR					Mascot
1980.041	1980.0227	-0.0183	-9	118	136	QIGVIGWGSQGPAQAQN LR	80	99.998			Mascot
1995.0393	1995.0007	-0.0386	-19	2	22	AAATSSFSPALSALQLSS ASK					Mascot
1995.0393	1995.0007	-0.0386	-19	2	22	AAATSSFSPALSALQLSS ASK					Mascot
2085.9949	2085.9739	-0.021	-10	258	277	EINGAGINSSFAVHQDVD GR					Mascot
2085.9949	2085.9739	-0.021	-10	258	277	EINGAGINSSFAVHQDVD GR	59	99.796			Mascot
2947.3892	2947.3635	-0.0257	-9	474	499	GHSYSEIINESVIESVDSL NPFMHAR			Oxidation (M)[23]		Mascot

10	TPA: hypothetical protein ZEAMMB73_673532 [Zea mays]	gi|414881182	51364.2	7.15	8	203	100	16.19	182	100

Peptide Information

Calc. Mass	Obsrv. Mass	± da	± ppm	Start Seq.	End Seq.	Sequence	Ion Score	C. I. %	Modification	Rank	Result Type
823.3945	823.3724	-0.0221	-27	297	303	SDIFGER					Mascot
863.5098	863.4813	-0.0285	-33	243	249	RLYVQGK					Mascot
1062.5289	1062.4911	-0.0378	-36	409	418	EGLPAFPMGK			Oxidation (M)[8]		Mascot
1099.6833	1099.6649	-0.0184	-17	136	145	SDIVVKIGLR					Mascot
1368.7118	1368.583	-0.1288	-94	2	15	AAATSSSTYLAIGR					Mascot

1372.7219	1372.7006	-0.0213	-16	81	92	VSLAGHEEYIVR					Mascot
1372.7219	1372.7006	-0.0213	-16	81	92	VSLAGHEEYIVR		102	100		Mascot
1713.8364	1713.8126	-0.0238	-14	351	365	GMLEVYNSLTEEGKK				Oxidation (M)[2]	Mascot
1980.041	1980.0227	-0.0183	-9	110	128	QIGVIGWGSQGPAQAQN LR					Mascot
1980.041	1980.0227	-0.0183	-9	110	128	QIGVIGWGSQGPAQAQN LR		80	99.998		Mascot

Gel Idx/Pos	282/L10	Instr./Gel Origin	BA2151/Sample Project 20140814	Process Status	Analysis Succeeded
Plate [#] Name	[1] Sample Project 20140814	Instrument Sample Name		Spectra	11

Rank	Protein Name	Accession No.	Protein MW	Protein PI	Pep. Count	Protein Score	Protein Score C. I. %	Intensity Matched	Total Ion Score	Total Ion C. I. %	Confirmed
1	Betaine aldehyde dehydrogenase 1, chloroplastic [Aegilops tauschii]	gi|475544878	55319.1	5.52	14	532	100	14.747	465	100	

Peptide Information

Calc. Mass	Obsrv. Mass	± da	± ppm	Start Seq.	End Seq.	Sequence	Ion Score	C. I. %	Modification	Rank	Result Type
1149.5687	1149.5292	-0.0395	-34	11	19	QLFIDGDWR					Mascot
1161.5205	1161.4916	-0.0289	-25	324	333	VSDPLEEGCR			Carbamidomethyl (C)[9]		Mascot
1229.6161	1229.5562	-0.0599	-49	235	246	VAFTGSYATGQK					Mascot
1276.6606	1276.6068	-0.0538	-42	393	403	EEVFGPVLCVK			Carbamidomethyl (C)[9]		Mascot
1305.6686	1305.6112	-0.0574	-44	334	345	LGPVVSEGQYEK					Mascot
1385.7748	1385.7404	-0.0344	-25	355	368	SEGATILTGGVRPK					Mascot
1385.7748	1385.7404	-0.0344	-25	355	368	SEGATILTGGVRPK	29	0			Mascot
1457.7859	1457.7318	-0.0541	-37	133	145	RQNAAVALPENFK					Mascot
1461.7472	1461.6838	-0.0634	-43	266	278	SPIVVFDDVDIDK					Mascot
1507.7639	1507.7036	-0.0603	-40	471	484	ELGEGGIDNYLSIK					Mascot
1507.7639	1507.7036	-0.0603	-40	471	484	ELGEGGIDNYLSIK					Mascot
1911.0984	1911.0066	-0.0918	-48	247	265	IMVAAAPTVKPVTLELGG K			Oxidation (M)[2]		Mascot
2012.0084	2012.0045	-0.0039	-2	466	484	SGFGRELGEGGIDNYLSI K					Mascot
2994.3962	2994.3801	-0.0161	-5	404	431	EFSTEEEAIELANDTHYG LAGAVISGDR					Mascot
2994.3962	2994.3801	-0.0161	-5	404	431	EFSTEEEAIELANDTHYG LAGAVISGDR	268	100			Mascot
3035.5532	3035.5315	-0.0217	-7	27	57	LPVINPTTEASIGEIPAGT SEDVDAAVAAAR					Mascot
3035.5532	3035.5315	-0.0217	-7	27	57	LPVINPTTEASIGEIPAGT SEDVDAAVAAAR	168	100			Mascot
3191.6543	3191.6328	-0.0215	-7	26	57	RLPVINPTTEASIGEIPAG TSEDVDAAVAAAR					Mascot

Rank	Protein Name	Accession No.	Protein MW	Protein PI	Pep. Count	Protein Score	Protein Score C. I. %	Intensity Matched	Total Ion Score	Total Ion C. I. %	Confirmed
2	Betaine aldehyde dehydrogenase 1, chloroplastic [Triticum urartu]	gi|474447706	46073	4.97	13	362	100	11.653	297	100	

Peptide Information

Calc. Mass	Obsrv. Mass	± da	± ppm	Start Seq.	End Seq.	Sequence	Ion Score	C. I. %	Modification	Rank	Result Type
823.4164	823.3699	-0.0465	-56	1	6	MMIERK			Oxidation (M)[1]		Mascot
1161.5205	1161.4916	-0.0289	-25	239	248	VSDPLEEGCR			Carbamidomethyl (C)[9]		Mascot

1229.6161	1229.5562	-0.0599	-49	150	161	VAFTGSYATGQK				Mascot
1276.6606	1276.6068	-0.0538	-42	308	318	EEVFGPVLCVK			Carbamidomethyl (C)[9]	Mascot
1305.6686	1305.6112	-0.0574	-44	249	260	LGPVVSEGQYEK				Mascot
1385.7748	1385.7404	-0.0344	-25	270	283	SEGATILTGGVRPK				Mascot
1385.7748	1385.7404	-0.0344	-25	270	283	SEGATILTGGVRPK	29	0		Mascot
1457.7859	1457.7318	-0.0541	-37	48	60	RQNAAVALPENFK				Mascot
1461.7472	1461.6838	-0.0634	-43	181	193	SPIVVFDDVDIDK				Mascot
1507.7639	1507.7036	-0.0603	-40	386	399	ELGEGGIDNYLSIK				Mascot
1507.7639	1507.7036	-0.0603	-40	386	399	ELGEGGIDNYLSIK				Mascot
1911.0984	1911.0066	-0.0918	-48	162	180	IMVAAAPTVKPVTLELGG K			Oxidation (M)[2]	Mascot
2012.0084	2012.0045	-0.0039	-2	381	399	SGFGRELGEGGIDNYLSI K				Mascot
2994.3962	2994.3801	-0.0161	-5	319	346	EFSTEEEAIELANDTHYG LAGAVISGDR				Mascot
2994.3962	2994.3801	-0.0161	-5	319	346	EFSTEEEAIELANDTHYG LAGAVISGDR	268	100		Mascot
3056.5898	3056.5515	-0.0383	-13	119	149	EIGLPSGVLNIVTGLGNE AGAPLSSHPDVDK				Mascot

3	hypothetical protein OsI_29289 [Oryza sativa Indica Group]	gi|218201175	55446.2	5.36	6	283	100	6.905	268	100	

Protein Group

Os08g0424500 [Oryza sativa Japonica Group]	gi|113623802	55446.2	5.3600 001335 144

Peptide Information

Calc. Mass	Obsrv. Mass	± da	± ppm	Start Seq.	End Seq.	Sequence	Ion Score	C. I. %	Modification	Rank	Result Type
1161.5205	1161.4916	-0.0289	-25	323	332	VSDPLEEGCR			Carbamidomethyl (C)[9]		Mascot
1276.6606	1276.6068	-0.0538	-42	392	402	EEVFGPVLCVK			Carbamidomethyl (C)[9]		Mascot
1305.6686	1305.6112	-0.0574	-44	333	344	LGPVVSEGQYEK					Mascot
1461.7472	1461.6838	-0.0634	-43	265	277	SPIVVFDDVDVEK					Mascot
1490.7308	1490.7133	-0.0175	-12	132	144	QNAPVSLPMENFK			Oxidation (M)[9]		Mascot
2994.3962	2994.3801	-0.0161	-5	403	430	EFSTEEEAIELANDTHYG LAGAVLSGDR					Mascot
2994.3962	2994.3801	-0.0161	-5	403	430	EFSTEEEAIELANDTHYG LAGAVLSGDR	268	100			Mascot

4	granule-bound starch synthase, partial [Triticum aestivum]	gi|417381855	12867.4	6.75	7	205	100	6.072	153	100	

Protein Group

granule-bound starch synthase, partial [Triticum aestivum]	gi|417381851	12867.4	6.75

granule-bound starch synthase, partial [Triticum aestivum]	gi|417381843	12867.4	6.75
granule-bound starch synthase, partial [Triticum aestivum]	gi|417381857	12867.4	6.75

Peptide Information

Calc. Mass	Obsrv. Mass	± da	± ppm	Start Seq.	End Seq.	Sequence	Ion Score	C. I. %	Modification	Rank	Result Type
1107.4922	1107.5118	0.0196	18	99	107	GCELDNIMR			Carbamidomethyl (C)[2]		Mascot
1201.5597	1201.5629	0.0032	3	9	18	SNYQSNGIYR					Mascot
1503.7988	1503.7635	-0.0353	-23	68	80	INWMKAGILQADK			Oxidation (M)[4]		Mascot
1564.7689	1564.7291	-0.0398	-25	22	34	VAFCIHNISYQGR			Carbamidomethyl (C)[4]		Mascot
1684.7966	1684.7695	-0.0271	-16	35	48	FSFDDFAQLNLPDR					Mascot
1684.7966	1684.7695	-0.0271	-16	35	48	FSFDDFAQLNLPDR	91	100			Mascot
1831.8497	1831.8109	-0.0388	-21	51	66	SSFDFIDGYDKPVEGR					Mascot
1997.0226	1996.9845	-0.0381	-19	81	98	VLTVSPYYAEELISGEAR					Mascot
1997.0226	1996.9845	-0.0381	-19	81	98	VLTVSPYYAEELISGEAR	63	99.909			Mascot

5	RecName: Full=Granule-bound starch synthase 1, chloroplastic/amyloplastic; AltName: Full=Granule-bound starch synthase I; Short=GBSS-I; Flags: Precursor	gi|136755	66853	7.06	15	198	100	9.56	153	100

Peptide Information

Calc. Mass	Obsrv. Mass	± da	± ppm	Start Seq.	End Seq.	Sequence	Ion Score	C. I. %	Modification	Rank	Result Type
971.6036	971.5504	-0.0532	-55	393	401	VPLVAFIGR					Mascot
1099.6986	1099.6608	-0.0378	-34	392	401	KVPLVAFIGR					Mascot
1107.4922	1107.5118	0.0196	18	329	337	GCELDNIMR			Carbamidomethyl (C)[2]		Mascot
1136.5695	1136.5132	-0.0563	-50	136	144	VADEYERVR					Mascot
1201.5597	1201.5629	0.0032	3	239	248	SNYQSNGIYR					Mascot
1363.6741	1363.7002	0.0261	19	124	135	DAWDTSVISEIK					Mascot
1396.7432	1396.7061	-0.0371	-27	379	391	EALQAEVGLPVDR					Mascot
1500.7516	1500.7078	-0.0438	-29	112	123	VMVVSPRYDQYK			Oxidation (M)[2]		Mascot
1503.7988	1503.7635	-0.0353	-23	298	310	INWMKAGILQADK			Oxidation (M)[4]		Mascot
1544.7626	1544.6477	-0.1149	-74	519	532	LSVDCNVVEPADVK			Carbamidomethyl (C)[5]		Mascot
1564.7611	1564.7291	-0.032	-20	478	490	FEPCGLIQLQGMR			Carbamidomethyl (C)[4], Oxidation (M)[12]		Mascot

Calc. Mass	Obsrv. Mass	± da	± ppm	Start Seq.	End Seq.	Sequence	Ion Score	C. I. %	Modification	Rank	Result Type
1684.7966	1684.7695	-0.0271	-16	265	278	FSFDDFAQLNLPDR					Mascot
1684.7966	1684.7695	-0.0271	-16	265	278	FSFDDFAQLNLPDR	91	100			Mascot
1831.8497	1831.8109	-0.0388	-21	281	296	SSFDFIDGYDKPVEGR					Mascot
1841.7937	1841.7545	-0.0392	-21	175	190	IYGPDAGTDYEDNQQR					Mascot
1997.0226	1996.9845	-0.0381	-19	311	328	VLTVSPYYAEELISGEAR					Mascot
1997.0226	1996.9845	-0.0381	-19	311	328	VLTVSPYYAEELISGEAR	63	99.909			Mascot

6	waxy protein [Triticum dicoccoides]	gi|399153332	67165.2	7.85	15	197	100	9.754	153	100	

Peptide Information

Calc. Mass	Obsrv. Mass	± da	± ppm	Start Seq.	End Seq.	Sequence	Ion Score	C. I. %	Modification	Rank	Result Type
971.6036	971.5504	-0.0532	-55	394	402	VPLVAFIGR					Mascot
1099.6986	1099.6608	-0.0378	-34	393	402	KVPLVAFIGR					Mascot
1107.4922	1107.5118	0.0196	18	330	338	GCELDNIMR			Carbamidomethyl (C)[2]		Mascot
1201.5597	1201.5629	0.0032	3	240	249	SNYQSNGIYR					Mascot
1363.6741	1363.7002	0.0261	19	125	136	DAWDTSVISEIK					Mascot
1390.6825	1390.6661	-0.0164	-12	157	167	VFVDHPCFLEK			Carbamidomethyl (C)[7]		Mascot
1396.7432	1396.7061	-0.0371	-27	380	392	EALQAEVGLPVDR					Mascot
1500.78	1500.7078	-0.0722	-48	408	421	GPDVMIAAIPEIMK			Oxidation (M)[5]		Mascot
1503.7988	1503.7635	-0.0353	-23	299	311	INWMKAGILQADK			Oxidation (M)[4]		Mascot
1544.7626	1544.6477	-0.1149	-74	520	533	LSVDCNVVEPADVK			Carbamidomethyl (C)[5]		Mascot
1564.7611	1564.7291	-0.032	-20	479	491	FEPCGLIQLQGMR			Carbamidomethyl (C)[4], Oxidation (M)[12]		Mascot
1684.7966	1684.7695	-0.0271	-16	266	279	FSFDDFAQLNLPDR					Mascot
1684.7966	1684.7695	-0.0271	-16	266	279	FSFDDFAQLNLPDR	91	100			Mascot
1831.8497	1831.8109	-0.0388	-21	282	297	SSFDFIDGYDKPVEGR					Mascot
1841.7937	1841.7545	-0.0392	-21	176	191	IYGPDAGTDYEDNQQR					Mascot
1997.0226	1996.9845	-0.0381	-19	312	329	VLTVSPYYAEELISGEAR					Mascot
1997.0226	1996.9845	-0.0381	-19	312	329	VLTVSPYYAEELISGEAR	63	99.909			Mascot

7	waxy protein [Aegilops comosa]	gi|452113205	66998	7.13	14	195	100	8.829	153	100	

Peptide Information

Calc. Mass	Obsrv. Mass	± da	± ppm	Start Seq.	End Seq.	Sequence	Ion Score	C. I. %	Modification	Rank	Result Type
971.6036	971.5504	-0.0532	-55	395	403	VPLVAFIGR					Mascot
1099.6986	1099.6608	-0.0378	-34	394	403	KVPLVAFIGR					Mascot
1107.4922	1107.5118	0.0196	18	331	339	GCELDNIMR			Carbamidomethyl (C)[2]		Mascot
1136.606	1136.5132	-0.0928	-82	138	146	VVDTYERVR					Mascot

Calc. Mass	Obsrv. Mass	± da	± ppm	Start Seq.	End Seq.	Sequence	Ion Score	C. I. %	Modification	Rank	Result Type
1201.5597	1201.5629	0.0032	3	241	250	SNYQSNGIYR					Mascot
1390.6825	1390.6661	-0.0164	-12	158	168	VFVDHPCFLEK			Carbamidomethyl (C)[7]		Mascot
1396.7432	1396.7061	-0.0371	-27	381	393	EALQAEVGLPVDR					Mascot
1503.7988	1503.7635	-0.0353	-23	300	312	INWMKAGILQADK			Oxidation (M)[4]		Mascot
1544.7626	1544.6477	-0.1149	-74	521	534	LSVDCNVVEPADVK			Carbamidomethyl (C)[5]		Mascot
1564.7611	1564.7291	-0.032	-20	480	492	FEPCGLIQLQGMR			Carbamidomethyl (C)[4], Oxidation (M)[12]		Mascot
1684.7966	1684.7695	-0.0271	-16	267	280	FSFDDFAQLNLPDR					Mascot
1684.7966	1684.7695	-0.0271	-16	267	280	FSFDDFAQLNLPDR	91	100			Mascot
1831.8497	1831.8109	-0.0388	-21	283	298	SSFDFIDGYDKPVEGR					Mascot
1841.7937	1841.7545	-0.0392	-21	177	192	IYGPDAGTDYEDNQQR					Mascot
1997.0226	1996.9845	-0.0381	-19	313	330	VLTVSPYYAEELISGEAR					Mascot
1997.0226	1996.9845	-0.0381	-19	313	330	VLTVSPYYAEELISGEAR	63	99.909			Mascot

8	waxy protein [Triticum durum]	gi|399153334	67132.3	8.27	14	193	100	9.538	153	100	

Peptide Information

Calc. Mass	Obsrv. Mass	± da	± ppm	Start Seq.	End Seq.	Sequence	Ion Score	C. I. %	Modification	Rank	Result Type
971.6036	971.5504	-0.0532	-55	394	402	VPLVAFIGR					Mascot
1099.6986	1099.6608	-0.0378	-34	393	402	KVPLVAFIGR					Mascot
1107.4922	1107.5118	0.0196	18	330	338	GCELDNIMR			Carbamidomethyl (C)[2]		Mascot
1201.5597	1201.5629	0.0032	3	240	249	SNYQSNGIYR					Mascot
1363.6741	1363.7002	0.0261	19	125	136	DAWDTSVISEIK					Mascot
1390.6825	1390.6661	-0.0164	-12	157	167	VFVDHPCFLEK			Carbamidomethyl (C)[7]		Mascot
1396.7432	1396.7061	-0.0371	-27	380	392	EALQAEVGLPVDR					Mascot
1503.7988	1503.7635	-0.0353	-23	299	311	INWMKAGILQADK			Oxidation (M)[4]		Mascot
1544.7626	1544.6477	-0.1149	-74	520	533	LSVDCNVVEPADVK			Carbamidomethyl (C)[5]		Mascot
1564.7689	1564.7291	-0.0398	-25	253	265	VAFCIHNISYQGR			Carbamidomethyl (C)[4]		Mascot
1684.7966	1684.7695	-0.0271	-16	266	279	FSFDDFAQLNLPDR					Mascot
1684.7966	1684.7695	-0.0271	-16	266	279	FSFDDFAQLNLPDR	91	100			Mascot
1831.8497	1831.8109	-0.0388	-21	282	297	SSFDFIDGYDKPVEGR					Mascot
1841.7937	1841.7545	-0.0392	-21	176	191	IYGPDAGTDYEDNQQR					Mascot
1997.0226	1996.9845	-0.0381	-19	312	329	VLTVSPYYAEELISGEAR					Mascot
1997.0226	1996.9845	-0.0381	-19	312	329	VLTVSPYYAEELISGEAR	63	99.909			Mascot

9	waxy protein [Triticum aestivum]	gi|399153330	67152.2	7.85	14	192	100	9.538	153	100	

Protein Group

waxy protein [Triticum durum]	gi|399153336	67133.2	7.8499 999046

Peptide Information

Calc. Mass	Obsrv. Mass	± da	± ppm	Start Seq.	End Seq.	Sequence	Ion Score	C. I. %	Modification	Rank	Result Type
971.6036	971.5504	-0.0532	-55	394	402	VPLVAFIGR					Mascot
1099.6986	1099.6608	-0.0378	-34	393	402	KVPLVAFIGR					Mascot
1107.4922	1107.5118	0.0196	18	330	338	GCELDNIMR			Carbamidomethyl (C)[2]		Mascot
1201.5597	1201.5629	0.0032	3	240	249	SNYQSNGIYR					Mascot
1363.6741	1363.7002	0.0261	19	125	136	DAWDTSVISEIK					Mascot
1390.6825	1390.6661	-0.0164	-12	157	167	VFVDHPCFLEK			Carbamidomethyl (C)[7]		Mascot
1396.7432	1396.7061	-0.0371	-27	380	392	EALQAEVGLPVDR					Mascot
1503.7988	1503.7635	-0.0353	-23	299	311	INWMKAGILQADK			Oxidation (M)[4]		Mascot
1544.7626	1544.6477	-0.1149	-74	520	533	LSVDCNVVEPADVK			Carbamidomethyl (C)[5]		Mascot
1564.7611	1564.7291	-0.032	-20	479	491	FEPCGLIQLQGMR			Carbamidomethyl (C)[4], Oxidation (M)[12]		Mascot
1684.7966	1684.7695	-0.0271	-16	266	279	FSFDDFAQLNLPDR					Mascot
1684.7966	1684.7695	-0.0271	-16	266	279	FSFDDFAQLNLPDR	91	100			Mascot
1831.8497	1831.8109	-0.0388	-21	282	297	SSFDFIDGYDKPVEGR					Mascot
1841.7937	1841.7545	-0.0392	-21	176	191	IYGPDAGTDYEDNQQR					Mascot
1997.0226	1996.9845	-0.0381	-19	312	329	VLTVSPYYAEELISGEAR					Mascot
1997.0226	1996.9845	-0.0381	-19	312	329	VLTVSPYYAEELISGEAR	63	99.909			Mascot

10	waxy protein [Aegilops searsii]	gi|452113219	66836.9	6.81	13	191	100	8	153	100	

Peptide Information

Calc. Mass	Obsrv. Mass	± da	± ppm	Start Seq.	End Seq.	Sequence	Ion Score	C. I. %	Modification	Rank	Result Type
971.6036	971.5504	-0.0532	-55	394	402	VPLVAFIGR					Mascot
1099.6986	1099.6608	-0.0378	-34	393	402	KVPLVAFIGR					Mascot
1107.4922	1107.5118	0.0196	18	330	338	GCELDNIMR			Carbamidomethyl (C)[2]		Mascot
1136.5695	1136.5132	-0.0563	-50	137	145	VADEYERVR					Mascot
1390.6825	1390.6661	-0.0164	-12	157	167	VFVDHPCFLEK			Carbamidomethyl (C)[7]		Mascot
1396.7432	1396.7061	-0.0371	-27	380	392	EALQAEVGLPVDR					Mascot
1503.7988	1503.7635	-0.0353	-23	299	311	INWMKAGILQADK			Oxidation (M)[4]		Mascot
1544.7626	1544.6477	-0.1149	-74	520	533	LSVDCNVVEPADVK			Carbamidomethyl (C)[5]		Mascot
1564.7611	1564.7291	-0.032	-20	479	491	FEPCGLIQLQGMR			Carbamidomethyl (C)[4], Oxidation (M)[12]		Mascot
1684.7966	1684.7695	-0.0271	-16	266	279	FSFDDFAQLNLPDR					Mascot
1684.7966	1684.7695	-0.0271	-16	266	279	FSFDDFAQLNLPDR	91	100			Mascot

1831.8497	1831.8109	-0.0388	-21	282	297	SSFDFIDGYDKPVEGR				Mascot
1997.0226	1996.9845	-0.0381	-19	312	329	VLTVSPYYAEELISGEAR				Mascot
1997.0226	1996.9845	-0.0381	-19	312	329	VLTVSPYYAEELISGEAR	63	99.908		Mascot
3022.4299	3022.4058	-0.0241	-8	240	265	SNYQSNGIYMTAKVAFCI HNISYQGR			Carbamidomethyl (C)[17]	Mascot

Gel Idx/Pos	283/L11	Instr./Gel Origin	BA2151/Sample Project 20140814	Process Status	Analysis Succeeded
Plate [#] Name	[1] Sample Project 20140814	Instrument Sample Name		Spectra	11

Rank	Protein Name	Accession No.	Protein MW	Protein PI	Pep. Count	Protein Score	Protein Score C. I. %	Intensity Matched	Total Ion Score	Total Ion C. I. %	Confirmed
1	Chain A, Ketol-Acid Reductoisomerase (Kari) In Complex With Mg2+	gi|226887786	57566.1	5.46	11	327	100	21.377	289	100	

Protein Group

Protein Name	Accession No.	Protein MW	Protein PI
Chain A, Rice Ketolacid Reductoisomerase In Complex With Mg2+-Nadph	gi|226887789	57566.1	5.4600 000381 4697
Chain B, Ketol-Acid Reductoisomerase (Kari) In Complex With Mg2+	gi|226887787	57566.1	5.4600 000381 4697
Chain B, Rice Ketolacid Reductoisomerase In Complex With Mg2+-Nadph	gi|226887790	57566.1	5.4600 000381 4697

Peptide Information

Calc. Mass	Obsrv. Mass	± da	± ppm	Start Seq.	End Seq.	Sequence	Ion Score	C. I. %	Modification	Rank	Result Type
823.3945	823.3763	-0.0182	-22	243	249	SDIFGER					Mascot
863.5098	863.4956	-0.0142	-16	189	195	RLYVQGK					Mascot
877.46	877.4656	0.0056	6	370	376	MWKVGEK					Mascot
1288.73	1288.6733	-0.0567	-44	42	52	NLFPLLPEAFK					Mascot
1372.7219	1372.7041	-0.0178	-13	27	38	VSLAGHEEYIVR					Mascot
1372.7219	1372.7041	-0.0178	-13	27	38	VSLAGHEEYIVR	98	100			Mascot
1528.6884	1528.6823	-0.0061	-4	438	451	GVAFMVDNCSTTAR			Carbamidomethyl (C)[9]		Mascot
1544.6832	1544.6385	-0.0447	-29	438	451	GVAFMVDNCSTTAR			Carbamidomethyl (C)[9], Oxidation (M)[5]		Mascot
1544.6832	1544.6385	-0.0447	-29	438	451	GVAFMVDNCSTTAR	61	99.871	Carbamidomethyl (C)[9], Oxidation (M)[5]		Mascot
1664.9847	1664.9684	-0.0163	-10	250	265	GILLGAVHGIVEALFR					Mascot
1713.8364	1713.8142	-0.0222	-13	297	311	GMLEVYNSLTEEGKK			Oxidation (M)[2]		Mascot
1980.041	1980.0363	-0.0047	-2	56	74	QIGVIGWGSQGPAQAQN LR					Mascot
1980.041	1980.0363	-0.0047	-2	56	74	QIGVIGWGSQGPAQAQN LR	130	100			Mascot
2085.9949	2085.9619	-0.033	-16	196	215	EINGAGINSSFAVHQDVD GR					Mascot
2947.3892	2947.3728	-0.0164	-6	412	437	GHSYSEIINESVIESVDSL NPFMHAR			Oxidation (M)[23]		Mascot

Rank	Protein Name	Accession No.	Protein MW	Protein PI	Pep. Count	Protein Score	Protein Score C. I. %	Intensity Matched	Total Ion Score	Total Ion C. I. %	Confirmed
2	Os05g0573700 [Oryza sativa Japonica Group]	gi|113579935	62679.9	6.01	11	324	100	21.377	289	100	

Protein Group

RecName: Full=Ketol-acid reductoisomerase, chloroplastic; AltName: Full=Acetohydroxy-acid reductoisomerase; AltName: Full=Alpha-keto-beta-hydroxylacyl reductoisomerase; AltName: Full=Protein KARI; Flags: Precursor	gi|75115272	62679.9	6.0100 002288 8184
hypothetical protein OsI_21076 [Oryza sativa Indica Group]	gi|218197310	62651.8	6.0100 002288 8184

Peptide Information

Calc. Mass	Obsrv. Mass	± da	± ppm	Start Seq.	End Seq.	Sequence	Ion Score	C. I. %	Modification	Rank	Result Type
823.3945	823.3763	-0.0182	-22	296	302	SDIFGER					Mascot
863.5098	863.4956	-0.0142	-16	242	248	RLYVQGK					Mascot
877.46	877.4656	0.0056	6	423	429	MWKVGEK					Mascot
1288.73	1288.6733	-0.0567	-44	95	105	NLFPLLPEAFK					Mascot
1372.7219	1372.7041	-0.0178	-13	80	91	VSLAGHEEYIVR					Mascot
1372.7219	1372.7041	-0.0178	-13	80	91	VSLAGHEEYIVR	98	100			Mascot
1528.6884	1528.6823	-0.0061	-4	491	504	GVAFMVDNCSTTAR			Carbamidomethyl (C)[9]		Mascot
1544.6832	1544.6385	-0.0447	-29	491	504	GVAFMVDNCSTTAR			Carbamidomethyl (C)[9], Oxidation (M)[5]		Mascot
1544.6832	1544.6385	-0.0447	-29	491	504	GVAFMVDNCSTTAR	61	99.871	Carbamidomethyl (C)[9], Oxidation (M)[5]		Mascot
1664.9847	1664.9684	-0.0163	-10	303	318	GILLGAVHGIVEALFR					Mascot
1713.8364	1713.8142	-0.0222	-13	350	364	GMLEVYNSLTEEGKK			Oxidation (M)[2]		Mascot
1980.041	1980.0363	-0.0047	-2	109	127	QIGVIGWGSQGPAQAQN LR					Mascot
1980.041	1980.0363	-0.0047	-2	109	127	QIGVIGWGSQGPAQAQN LR	130	100			Mascot
2085.9949	2085.9619	-0.033	-16	249	268	EINGAGINSSFAVHQDVD GR					Mascot
2947.3892	2947.3728	-0.0164	-6	465	490	GHSYSEIINESVIESVDSL NPFMHAR			Oxidation (M)[23]		Mascot

3	PREDICTED: ketol-acid reductoisomerase, chloroplastic-like [Setaria italica]	gi|514746589	63351.2	6.01	10	317	100	20.503	289	100	

Peptide Information

Calc. Mass	Obsrv. Mass	± da	± ppm	Start Seq.	End Seq.	Sequence	Ion Score	C. I. %	Modification	Rank	Result Type
823.3945	823.3763	-0.0182	-22	302	308	SDIFGER					Mascot
863.5098	863.4956	-0.0142	-16	248	254	RLYVQGK					Mascot
877.46	877.4656	0.0056	6	429	435	MWKVGEK					Mascot
1288.73	1288.6733	-0.0567	-44	101	111	NLFPLLPEAFK					Mascot
1372.7219	1372.7041	-0.0178	-13	86	97	VSLAGHEEYIVR					Mascot

Calc. Mass	Obsrv. Mass	± da	± ppm	Start Seq.	End Seq.	Sequence	Ion Score	C. I. %	Modification	Rank	Result Type
1372.7219	1372.7041	-0.0178	-13	86	97	VSLAGHEEYIVR	98	100			Mascot
1528.6884	1528.6823	-0.0061	-4	497	510	GVAFMVDNCSTTAR			Carbamidomethyl (C)[9]		Mascot
1544.6832	1544.6385	-0.0447	-29	497	510	GVAFMVDNCSTTAR			Carbamidomethyl (C)[9], Oxidation (M)[5]		Mascot
1544.6832	1544.6385	-0.0447	-29	497	510	GVAFMVDNCSTTAR	61	99.871	Carbamidomethyl (C)[9], Oxidation (M)[5]		Mascot
1664.9847	1664.9684	-0.0163	-10	309	324	GILLGAVHGIVEALFR					Mascot
1713.8378	1713.8142	-0.0236	-14	1	17	MAAHTSAASTLAFGHPK			Oxidation (M)[1]		Mascot
1980.041	1980.0363	-0.0047	-2	115	133	QIGVIGWGSQGPAQAQN LR					Mascot
1980.041	1980.0363	-0.0047	-2	115	133	QIGVIGWGSQGPAQAQN LR	130	100			Mascot
2947.3892	2947.3728	-0.0164	-6	471	496	GHSYSEIINESVIESVDSL NPFMHAR			Oxidation (M)[23]		Mascot

4	hypothetical protein SORBIDRAFT_09g029170 [Sorghum bicolor]	gi|241946836	63644.2	5.88	9	312	100	20.338	289	100	

Peptide Information

Calc. Mass	Obsrv. Mass	± da	± ppm	Start Seq.	End Seq.	Sequence	Ion Score	C. I. %	Modification	Rank	Result Type
823.3945	823.3763	-0.0182	-22	305	311	SDIFGER					Mascot
863.5098	863.4956	-0.0142	-16	251	257	RLYVQGK					Mascot
877.46	877.4656	0.0056	6	432	438	MWKVGEK					Mascot
1288.73	1288.6733	-0.0567	-44	104	114	NLFPLLPEAFK					Mascot
1372.7219	1372.7041	-0.0178	-13	89	100	VSLAGHEEYIVR					Mascot
1372.7219	1372.7041	-0.0178	-13	89	100	VSLAGHEEYIVR	98	100			Mascot
1528.6884	1528.6823	-0.0061	-4	500	513	GVAFMVDNCSTTAR			Carbamidomethyl (C)[9]		Mascot
1544.6832	1544.6385	-0.0447	-29	500	513	GVAFMVDNCSTTAR			Carbamidomethyl (C)[9], Oxidation (M)[5]		Mascot
1544.6832	1544.6385	-0.0447	-29	500	513	GVAFMVDNCSTTAR	61	99.871	Carbamidomethyl (C)[9], Oxidation (M)[5]		Mascot
1664.9847	1664.9684	-0.0163	-10	312	327	GILLGAVHGIVEALFR					Mascot
1980.041	1980.0363	-0.0047	-2	118	136	QIGVIGWGSQGPAQAQN LR					Mascot
1980.041	1980.0363	-0.0047	-2	118	136	QIGVIGWGSQGPAQAQN LR	130	100			Mascot
2947.3892	2947.3728	-0.0164	-6	474	499	GHSYSEIINESVIESVDSL NPFMHAR			Oxidation (M)[23]		Mascot

5	TPA: ketol-acid reductoisomerase [Zea mays]	gi|414881183	63305.2	6.31	7	303	100	19.358	289	100	

Protein Group

PREDICTED: ketol-acid reductoisomerase, chloroplastic-like [Setaria italica]	gi|514779378	63161.2	6.8200 001716 6138
ketol-acid reductoisomerase [Zea mays]	gi|212722020	63305.2	6.3099 999427 7954

Peptide Information

Calc. Mass	Obsrv. Mass	± da	± ppm	Start Seq.	End Seq.	Sequence	Ion Score	C. I. %	Modification	Rank	Result Type
823.3945	823.3763	-0.0182	-22	297	303	SDIFGER					Mascot
863.5098	863.4956	-0.0142	-16	243	249	RLYVQGK					Mascot
1372.7219	1372.7041	-0.0178	-13	81	92	VSLAGHEEYIVR					Mascot
1372.7219	1372.7041	-0.0178	-13	81	92	VSLAGHEEYIVR	98	100			Mascot
1528.6884	1528.6823	-0.0061	-4	492	505	GVAFMVDNCSTTAR			Carbamidomethyl (C)[9]		Mascot
1544.6832	1544.6385	-0.0447	-29	492	505	GVAFMVDNCSTTAR			Carbamidomethyl (C)[9], Oxidation (M)[5]		Mascot
1544.6832	1544.6385	-0.0447	-29	492	505	GVAFMVDNCSTTAR	61	99.871	Carbamidomethyl (C)[9], Oxidation (M)[5]		Mascot
1698.936	1698.8505	-0.0855	-50	304	319	GILLGAVHGMVEALFR			Oxidation (M)[10]		Mascot
1713.8364	1713.8142	-0.0222	-13	351	365	GMLEVYNSLTEEGKK			Oxidation (M)[2]		Mascot
1980.041	1980.0363	-0.0047	-2	110	128	QIGVIGWGSQGPAQAQN LR					Mascot
1980.041	1980.0363	-0.0047	-2	110	128	QIGVIGWGSQGPAQAQN LR	130	100			Mascot

6 hypothetical protein SORBIDRAFT_03g029720 [Sorghum bicolor] gi|241928040 63171.2 6.13 7 302 100 19.358 288 100

Peptide Information

Calc. Mass	Obsrv. Mass	± da	± ppm	Start Seq.	End Seq.	Sequence	Ion Score	C. I. %	Modification	Rank	Result Type
823.3945	823.3763	-0.0182	-22	297	303	SDIFGER					Mascot
863.5098	863.4956	-0.0142	-16	243	249	RLYVQGK					Mascot
1372.7219	1372.7041	-0.0178	-13	81	92	VSLAGHEEYIVR					Mascot
1372.7219	1372.7041	-0.0178	-13	81	92	VSLAGHEEYIVR	98	100			Mascot
1528.6884	1528.6823	-0.0061	-4	492	505	GVAFMVDNCSTTAR			Carbamidomethyl (C)[9]		Mascot
1544.6832	1544.6385	-0.0447	-29	492	505	GVAFMVDNCSTTAR			Carbamidomethyl (C)[9], Oxidation (M)[5]		Mascot
1544.6832	1544.6385	-0.0447	-29	492	505	GVAFMVDNCSTTAR	61	99.871	Carbamidomethyl (C)[9], Oxidation (M)[5]		Mascot
1698.936	1698.8505	-0.0855	-50	304	319	GILLGAVHGMVEALFR			Oxidation (M)[10]		Mascot
1713.8364	1713.8142	-0.0222	-13	351	365	GMLEVYNSLTEEGKK			Oxidation (M)[2]		Mascot
1980.041	1980.0363	-0.0047	-2	110	128	QIGVIGWGSQGPAQAQN LR					Mascot
1980.041	1980.0363	-0.0047	-2	110	128	QIGVIGWGSQGPAQAQN LR	130	100			Mascot

7 TPA: hypothetical protein ZEAMMB73_673532 [Zea mays] gi|414881182 51364.2 7.15 6 241 100 18 228 100

Peptide Information

Calc. Mass	Obsrv. Mass	± da	± ppm	Start Seq.	End Seq.	Sequence	Ion Score	C. I. %	Modification	Rank	Result Type

Calc. Mass	Obsrv. Mass	± da	± ppm	Start Seq.	End Seq.	Sequence	Ion Score	C. I. %	Modification	Rank	Result Type
823.3945	823.3763	-0.0182	-22	297	303	SDIFGER					Mascot
863.5098	863.4956	-0.0142	-16	243	249	RLYVQGK					Mascot
1372.7219	1372.7041	-0.0178	-13	81	92	VSLAGHEEYIVR					Mascot
1372.7219	1372.7041	-0.0178	-13	81	92	VSLAGHEEYIVR	98	100			Mascot
1698.936	1698.8505	-0.0855	-50	304	319	GILLGAVHGMVEALFR			Oxidation (M)[10]		Mascot
1713.8364	1713.8142	-0.0222	-13	351	365	GMLEVYNSLTEEGKK			Oxidation (M)[2]		Mascot
1980.041	1980.0363	-0.0047	-2	110	128	QIGVIGWGSQGPAQAQN LR					Mascot
1980.041	1980.0363	-0.0047	-2	110	128	QIGVIGWGSQGPAQAQN LR	130	100			Mascot

8	ketol-acid reductoisomerase, chloroplast precursor, putative [Ricinus communis]	gi|223535574	63712.1	6.35	8	228	100	20.252	207	100	

Peptide Information

Calc. Mass	Obsrv. Mass	± da	± ppm	Start Seq.	End Seq.	Sequence	Ion Score	C. I. %	Modification	Rank	Result Type
823.3945	823.3763	-0.0182	-22	301	307	SDIFGER					Mascot
863.5098	863.4956	-0.0142	-16	247	253	RLYVQGK					Mascot
1372.7219	1372.7041	-0.0178	-13	85	96	ISLAGHDEYIVR					Mascot
1372.7219	1372.7041	-0.0178	-13	85	96	ISLAGHDEYIVR	33	12.258			Mascot
1544.6832	1544.6385	-0.0447	-29	496	509	GVSFMVDNCSTTAR			Carbamidomethyl (C)[9]		Mascot
1544.6832	1544.6385	-0.0447	-29	496	509	GVSFMVDNCSTTAR	44	93.375	Carbamidomethyl (C)[9]		Mascot
1560.6782	1560.6761	-0.0021	-1	496	509	GVSFMVDNCSTTAR			Carbamidomethyl (C)[9], Oxidation (M)[5]		Mascot
1980.041	1980.0363	-0.0047	-2	114	132	QIGVIGWGSQGPAQAQN LR					Mascot
1980.041	1980.0363	-0.0047	-2	114	132	QIGVIGWGSQGPAQAQN LR	130	100			Mascot
2027.9954	2028.0066	0.0112	6	350	368	TISTQGMLAVYNSLSEEG K					Mascot
2085.9949	2085.9619	-0.033	-16	254	273	EINGAGINSSFAVHQDVD GR					Mascot
2947.3892	2947.3728	-0.0164	-6	470	495	GHSYSEIINESVIESVDSL NPFMHAR			Oxidation (M)[23]		Mascot

9	Ketol-acid reductoisomerase [Theobroma cacao]	gi|508700932	64136.5	7.18	8	225	100	19.544	207	100	

Peptide Information

Calc. Mass	Obsrv. Mass	± da	± ppm	Start Seq.	End Seq.	Sequence	Ion Score	C. I. %	Modification	Rank	Result Type
823.3945	823.3763	-0.0182	-22	305	311	SDIFGER					Mascot
863.5098	863.4956	-0.0142	-16	251	257	RLYVQGK					Mascot
1354.6342	1354.6958	0.0616	45	342	353	NTVECVTGIMSK			Carbamidomethyl (C)[5], Oxidation (M)[10]		Mascot
1372.7219	1372.7041	-0.0178	-13	89	100	ISLAGHDEYIVR					Mascot
1372.7219	1372.7041	-0.0178	-13	89	100	ISLAGHDEYIVR	33	12.258			Mascot

1544.6832	1544.6385	-0.0447	-29	500	513	GVSFMVDNCSTTAR			Carbamidomethyl (C)[9]		Mascot
1544.6832	1544.6385	-0.0447	-29	500	513	GVSFMVDNCSTTAR	44	93.375	Carbamidomethyl (C)[9]		Mascot
1560.6782	1560.6761	-0.0021	-1	500	513	GVSFMVDNCSTTAR			Carbamidomethyl (C)[9], Oxidation (M)[5]		Mascot
1615.7826	1615.7275	-0.0551	-34	413	426	FYEKDGLPAFPMGK			Oxidation (M)[12]		Mascot
1977.0652	1977.005	-0.0602	-30	23	41	THKTLATPSTLGFLSSTSK					Mascot
1980.041	1980.0363	-0.0047	-2	118	136	QIGVIGWGSQGPAQAQNLR					Mascot
1980.041	1980.0363	-0.0047	-2	118	136	QIGVIGWGSQGPAQAQNLR	130	100			Mascot

10	Ketol-acid reductoisomerase isoform 1 [Theobroma cacao]	gi|508780765	64920.9	7.64	7	222	100	36.54	207	100	

Peptide Information

Calc. Mass	Obsrv. Mass	± da	± ppm	Start Seq.	End Seq.	Sequence	Ion Score	C. I. %	Modification	Rank	Result Type
823.3945	823.3763	-0.0182	-22	313	319	SDIFGER					Mascot
863.5098	863.4956	-0.0142	-16	259	265	RLYVQGK					Mascot
1372.7219	1372.7041	-0.0178	-13	97	108	ISLAGHDEYIVR					Mascot
1372.7219	1372.7041	-0.0178	-13	97	108	ISLAGHDEYIVR	33	12.258			Mascot
1544.6832	1544.6385	-0.0447	-29	508	521	GVSFMVDNCSTTAR			Carbamidomethyl (C)[9]		Mascot
1544.6832	1544.6385	-0.0447	-29	508	521	GVSFMVDNCSTTAR	44	93.375	Carbamidomethyl (C)[9]		Mascot
1560.6782	1560.6761	-0.0021	-1	508	521	GVSFMVDNCSTTAR			Carbamidomethyl (C)[9], Oxidation (M)[5]		Mascot
1980.041	1980.0363	-0.0047	-2	126	144	QIGVIGWGSQGPAQAQNLR					Mascot
1980.041	1980.0363	-0.0047	-2	126	144	QIGVIGWGSQGPAQAQNLR	130	100			Mascot
2011.9945	2012.0264	0.0319	16	266	285	EINGAGINASFAVHQGVDGR					Mascot
2011.9945	2012.0264	0.0319	16	266	285	EINGAGINASFAVHQGVDGR					Mascot
2028.0317	2028.0066	-0.0251	-12	362	380	TISTKGMLAVYNSLSEEGK					Mascot

Gel Idx/Pos	284/L12	Instr./Gel Origin	BA2151/Sample Project 20140814	Process Status	Analysis Succeeded
Plate [#] Name	[1] Sample Project 20140814	Instrument Sample Name		Spectra	11

Rank	Protein Name	Accession No.	Protein MW	Protein PI	Pep. Count	Protein Score	Protein Score C. I. %	Intensity Matched	Total Ion Score	Total Ion C. I. %	Confirmed
1	Glucose-1-phosphate adenylyltransferase small subunit, chloroplastic/amyloplastic [Aegilops tauschii]	gi|475605779	52345.5	5.53	21	943	100	41.603	814	100	

Peptide Information

Calc. Mass	Obsrv. Mass	± da	± ppm	Start Seq.	End Seq.	Sequence	Ion Score	C. I. %	Modification	Rank	Result Type
854.444	854.4305	-0.0135	-16	200	207	ATAFGLMK			Oxidation (M)[7]		Mascot
947.4833	947.4744	-0.0089	-9	9	17	TFPSPSPSK					Mascot
972.4673	972.4487	-0.0186	-19	448	455	ETDGYFIK					Mascot
1009.5975	1009.5825	-0.015	-15	260	267	HVMLQLLR					Mascot
1017.5952	1017.5868	-0.0084	-8	366	374	IHHSVVGLR					Mascot
1025.5925	1025.5709	-0.0216	-21	260	267	HVMLQLLR			Oxidation (M)[3]		Mascot
1029.5	1029.5046	0.0046	4	110	119	AYGSNIGGYK					Mascot
1032.5472	1032.5397	-0.0075	-7	330	338	SAPIYTQPR					Mascot
1032.5472	1032.5397	-0.0075	-7	330	338	SAPIYTQPR	45	94.677			Mascot
1074.6194	1074.5847	-0.0347	-32	214	222	IIEFAEKPK					Mascot
1256.7109	1256.7004	-0.0105	-8	64	75	AKPAVPLGANYR					Mascot
1256.7109	1256.7004	-0.0105	-8	64	75	AKPAVPLGANYR	29	0			Mascot
1384.6896	1384.6841	-0.0055	-4	319	329	KPIPDFSFYDR					Mascot
1384.6896	1384.6841	-0.0055	-4	319	329	KPIPDFSFYDR	88	100			Mascot
1412.8121	1412.6833	-0.1288	-91	63	75	RAKPAVPLGANYR					Mascot
1621.7925	1621.7832	-0.0093	-6	228	242	AMMVDTTILGLDDAR					Mascot
1625.8646	1625.8612	-0.0034	-2	92	105	IYVLTQFNSASLNR	114	100			Mascot
1637.7874	1637.786	-0.0014	-1	228	242	AMMVDTTILGLDDAR			Oxidation (M)[2]		Mascot
1653.7822	1653.77	-0.0122	-7	228	242	AMMVDTTILGLDDAR			Oxidation (M)[2,3]		Mascot
1733.8489	1733.8354	-0.0135	-8	245	259	EMPYIASMGIYVISK			Oxidation (M)[2,8]		Mascot
1786.9368	1786.9095	-0.0273	-15	76	91	LIDIPVSNCLNSNISK			Carbamidomethyl (C)[9]		Mascot
1875.864	1875.8551	-0.0089	-5	183	199	ETDADITVAALPMDEER					Mascot
1889.9525	1889.9269	-0.0256	-14	345	362	VLDADVTDSVIGEGCVIK			Carbamidomethyl (C)[15]		Mascot
1891.859	1891.8402	-0.0188	-10	183	199	ETDADITVAALPMDEER			Oxidation (M)[13]		Mascot
1970.9965	1970.9867	-0.0098	-5	430	447	IGDNVMIINVDNVQEAAR					Mascot
1970.9965	1970.9867	-0.0098	-5	430	447	IGDNVMIINVDNVQEAAR	164	100			Mascot

1986.9913	1986.968	-0.0233	-12	430	447	IGDNVMIINVDNVQEAAR			Oxidation (M)[6]		Mascot
2355.2163	2355.2185	0.0022	1	32	55	HADLNPHANDSVLGIILG GGAGTR					Mascot
2355.2163	2355.2185	0.0022	1	32	55	HADLNPHANDSVLGIILG GGAGTR	153	100			Mascot
2368.0874	2368.0959	0.0085	4	268	290	EQFPGANDFGSEVIPGAT STGMR					Mascot
3061.4287	3061.4373	0.0086	3	120	147	NEGFVEVLAAQQSPDNP DWFQGTADAVR					Mascot
3061.4287	3061.4373	0.0086	3	120	147	NEGFVEVLAAQQSPDNP DWFQGTADAVR	221	100			Mascot

2	RecName: Full=Glucose-1-phosphate adenylyltransferase small subunit, chloroplastic/amyloplastic; AltName: Full=ADP-glucose pyrophosphorylase; AltName: Full=ADP-glucose synthase; AltName: Full=AGPase B; AltName: Full=Alpha-D-glucose-1-phosphate adeny	gi|1707940	56412.8	6.11	19	761	100	35.022	661	100

Peptide Information

Calc. Mass	Obsrv. Mass	± da	± ppm	Start Seq.	End Seq.	Sequence	Ion Score	C. I. %	Modification	Rank	Result Type
854.444	854.4305	-0.0135	-16	240	247	ATAFGLMK			Oxidation (M)[7]		Mascot
964.605	964.5187	-0.0863	-89	47	55	GLVARPVPR					Mascot
972.4673	972.4487	-0.0186	-19	488	495	ETDGYFIK					Mascot
1009.5975	1009.5825	-0.015	-15	300	307	HVMLQLLR					Mascot
1017.5952	1017.5868	-0.0084	-8	406	414	IHHSVVGLR					Mascot
1025.5925	1025.5709	-0.0216	-21	300	307	HVMLQLLR			Oxidation (M)[3]		Mascot
1029.5	1029.5046	0.0046	4	150	159	AYGSNIGGYK					Mascot
1032.5472	1032.5397	-0.0075	-7	370	378	SAPIYTQPR					Mascot
1032.5472	1032.5397	-0.0075	-7	370	378	SAPIYTQPR	45	94.677			Mascot
1074.6194	1074.5847	-0.0347	-32	254	262	IIEFAEKPK					Mascot
1256.7109	1256.7004	-0.0105	-8	104	115	AKPAVPLGANYR					Mascot
1256.7109	1256.7004	-0.0105	-8	104	115	AKPAVPLGANYR	29	0			Mascot
1384.6896	1384.6841	-0.0055	-4	359	369	KPIPDFSFYDR					Mascot
1384.6896	1384.6841	-0.0055	-4	359	369	KPIPDFSFYDR	88	100			Mascot
1412.8121	1412.6833	-0.1288	-91	103	115	RAKPAVPLGANYR					Mascot
1625.8646	1625.8612	-0.0034	-2	132	145	IYVLTQFNSASLNR	114	100			Mascot
1733.8489	1733.8354	-0.0135	-8	285	299	EMPYIASMGIYVISK			Oxidation (M)[2,8]		Mascot
1786.9368	1786.9095	-0.0273	-15	116	131	LIDIPVSNCLNSNISK			Carbamidomethyl (C)[9]		Mascot
1875.864	1875.8551	-0.0089	-5	223	239	ETDADITVAALPMDEER					Mascot
1889.9525	1889.9269	-0.0256	-14	385	402	VLDADVTDSVIGEGCVIK			Carbamidomethyl (C)[15]		Mascot
1891.859	1891.8402	-0.0188	-10	223	239	ETDADITVAALPMDEER			Oxidation (M)[13]		Mascot
1970.9965	1970.9867	-0.0098	-5	470	487	IGDNVMIINVDNVQEAAR					Mascot

Calc. Mass	Obsrv. Mass	± da	± ppm	Start Seq.	End Seq.	Sequence	Ion Score	C. I. %	Modification	Rank	Result Type
1970.9965	1970.9867	-0.0098	-5	470	487	IGDNVMIINVDNVQEAAR	164	100			Mascot
1986.9913	1986.968	-0.0233	-12	470	487	IGDNVMIINVDNVQEAAR			Oxidation (M)[6]		Mascot
2368.0874	2368.0959	0.0085	4	308	330	EQFPGANDFGSEVIPGAT STGMR					Mascot
3061.4287	3061.4373	0.0086	3	160	187	NEGFVEVLAAQQSPDNP DWFQGTADAVR					Mascot
3061.4287	3061.4373	0.0086	3	160	187	NEGFVEVLAAQQSPDNP DWFQGTADAVR	221	100			Mascot

3	ADP glucose pyrophosphorylase [Triticum aestivum]	gi|469952290	52399.6	5.54	21	673	100	35.931	546	100		

Protein Group

RecName: Full=Glucose-1-phosphate adenylyltransferase small subunit, chloroplastic/amyloplastic; AltName: Full=ADP-glucose pyrophosphorylase; AltName: Full=ADP-glucose synthase; AltName: Full=AGPase B; AltName: Full=Alpha-D-glucose-1-phosphate adeny	gi|232172	52399.6	5.5399 999618 5303

Peptide Information

Calc. Mass	Obsrv. Mass	± da	± ppm	Start Seq.	End Seq.	Sequence	Ion Score	C. I. %	Modification	Rank	Result Type
854.444	854.4305	-0.0135	-16	200	207	ATAFGLMK			Oxidation (M)[7]		Mascot
947.4833	947.4744	-0.0089	-9	9	17	TFPSPSPSK					Mascot
972.4673	972.4487	-0.0186	-19	448	455	ETDGYFIK					Mascot
1009.5975	1009.5825	-0.015	-15	260	267	HVMLQLLR					Mascot
1017.5952	1017.5868	-0.0084	-8	366	374	IHHSVVGLR					Mascot
1025.5925	1025.5709	-0.0216	-21	260	267	HVMLQLLR			Oxidation (M)[3]		Mascot
1029.5	1029.5046	0.0046	4	110	119	AYGSNIGGYK					Mascot
1032.5472	1032.5397	-0.0075	-7	330	338	SAPIYTQPR					Mascot
1032.5472	1032.5397	-0.0075	-7	330	338	SAPIYTQPR	45	94.677			Mascot
1074.6194	1074.5847	-0.0347	-32	214	222	IIEFAEKPK					Mascot
1256.7109	1256.7004	-0.0105	-8	64	75	AKPAVPLGANYR					Mascot
1256.7109	1256.7004	-0.0105	-8	64	75	AKPAVPLGANYR	29	0			Mascot
1384.6896	1384.6841	-0.0055	-4	319	329	KPIPDFSFYDR					Mascot
1384.6896	1384.6841	-0.0055	-4	319	329	KPIPDFSFYDR	88	100			Mascot
1412.8121	1412.6833	-0.1288	-91	63	75	RAKPAVPLGANYR					Mascot
1621.7925	1621.7832	-0.0093	-6	228	242	AMMVDTTILGLDDAR					Mascot
1637.7874	1637.786	-0.0014	-1	228	242	AMMVDTTILGLDDAR			Oxidation (M)[2]		Mascot
1653.7822	1653.77	-0.0122	-7	228	242	AMMVDTTILGLDDAR			Oxidation (M)[2,3]		Mascot
1733.8489	1733.8354	-0.0135	-8	245	259	EMPYIASMGIYVISK			Oxidation (M)[2,8]		Mascot
1786.9368	1786.9095	-0.0273	-15	76	91	LIDIPVSNCLNSNISK			Carbamidomethyl (C)[9]		Mascot
1804.915	1804.9409	0.0259	14	1	17	MDVPLASKTFPSPSPSK			Oxidation (M)[1]		Mascot

Calc. Mass	Obsrv. Mass	± da	± ppm	Start Seq.	End Seq.	Sequence	Ion Score	C. I. %	Modification	Rank	Result Type
1875.864	1875.8551	-0.0089	-5	183	199	ETDADITVAALPMDEER					Mascot
1889.9525	1889.9269	-0.0256	-14	345	362	VLDADVTDSVIGEGCVIK			Carbamidomethyl (C)[15]		Mascot
1891.859	1891.8402	-0.0188	-10	183	199	ETDADITVAALPMDEER			Oxidation (M)[13]		Mascot
1970.9965	1970.9867	-0.0098	-5	430	447	IGDNVMIINVDNVQEAAR					Mascot
1970.9965	1970.9867	-0.0098	-5	430	447	IGDNVMIINVDNVQEAAR	164	100			Mascot
1986.9913	1986.968	-0.0233	-12	430	447	IGDNVMIINVDNVQEAAR			Oxidation (M)[6]		Mascot
2318.2537	2318.1902	-0.0635	-27	76	95	LIDIPVSNCLNSNISKIYVR			Carbamidomethyl (C)[9]		Mascot
2368.0874	2368.0959	0.0085	4	268	290	EQFPGANDFGSEVIPGAT STGMR					Mascot
3061.4287	3061.4373	0.0086	3	120	147	NEGFVEVLAAQQSPDNP DWFQGTADAVR					Mascot
3061.4287	3061.4373	0.0086	3	120	147	NEGFVEVLAAQQSPDNP DWFQGTADAVR	221	100			Mascot

4	Glucose-1-phosphate adenylyltransferase small subunit, chloroplastic/amyloplastic [Triticum urartu]	gi|474108293	65309.1	7.9	18	582	100	32.514	497	100	

Peptide Information

Calc. Mass	Obsrv. Mass	± da	± ppm	Start Seq.	End Seq.	Sequence	Ion Score	C. I. %	Modification	Rank	Result Type
854.444	854.4305	-0.0135	-16	241	248	ATAFGLMK			Oxidation (M)[7]		Mascot
1009.5975	1009.5825	-0.015	-15	301	308	HVMLQLLR					Mascot
1017.5952	1017.5868	-0.0084	-8	407	415	IHHSVVGLR					Mascot
1025.5925	1025.5709	-0.0216	-21	301	308	HVMLQLLR			Oxidation (M)[3]		Mascot
1029.5	1029.5046	0.0046	4	151	160	AYGSNIGGYK					Mascot
1032.5472	1032.5397	-0.0075	-7	371	379	SAPIYTQPR					Mascot
1032.5472	1032.5397	-0.0075	-7	371	379	SAPIYTQPR	45	94.677			Mascot
1074.6194	1074.5847	-0.0347	-32	255	263	IIEFAEKPK					Mascot
1256.7109	1256.7004	-0.0105	-8	105	116	AKPAVPLGANYR					Mascot
1256.7109	1256.7004	-0.0105	-8	105	116	AKPAVPLGANYR	29	0			Mascot
1384.6896	1384.6841	-0.0055	-4	360	370	KPIPDFSFYDR					Mascot
1384.6896	1384.6841	-0.0055	-4	360	370	KPIPDFSFYDR	88	100			Mascot
1412.8121	1412.6833	-0.1288	-91	104	116	RAKPAVPLGANYR					Mascot
1621.7925	1621.7832	-0.0093	-6	269	283	AMMVDTTILGLDDAR					Mascot
1625.8646	1625.8612	-0.0034	-2	133	146	IYVLTQFNSASLNR	114	100			Mascot
1637.7874	1637.786	-0.0014	-1	269	283	AMMVDTTILGLDDAR			Oxidation (M)[2]		Mascot
1653.7822	1653.77	-0.0122	-7	269	283	AMMVDTTILGLDDAR			Oxidation (M)[2,3]		Mascot
1733.8489	1733.8354	-0.0135	-8	286	300	EMPYIASMGIYVISK			Oxidation (M)[2,8]		Mascot
1786.9368	1786.9095	-0.0273	-15	117	132	LIDIPVSNCLNSNISK			Carbamidomethyl (C)[9]		Mascot
1875.864	1875.8551	-0.0089	-5	224	240	ETDADITVAALPMDEER					Mascot
1889.9525	1889.9269	-0.0256	-14	386	403	VLDADVTDSVIGEGCVIK			Carbamidomethyl (C)[15]		Mascot

1891.859	1891.8402	-0.0188	-10	224	240	ETDADITVAALPMDEER			Oxidation (M)[13]		Mascot
2366.2246	2366.0994	-0.1252	-53	12	34	ILIPPHRASAATAAASTSC DSLR			Carbamidomethyl (C)[19]		Mascot
2368.0874	2368.0959	0.0085	4	309	331	EQFPGANDFGSEVIPGAT STGMR					Mascot
3061.4287	3061.4373	0.0086	3	161	188	NEGFVEVLAAQQSPDNP DWFQGTADAVR					Mascot
3061.4287	3061.4373	0.0086	3	161	188	NEGFVEVLAAQQSPDNP DWFQGTADAVR	221	100			Mascot

5	PREDICTED: glucose-1-phosphate adenylyltransferase small subunit, chloroplastic/amyloplastic-like [Setaria italica]	gi|514795259	56535.9	6.62	13	325	100	24.009	276	100	

Peptide Information

Calc. Mass	Obsrv. Mass	± da	± ppm	Start Seq.	End Seq.	Sequence	Ion Score	C. I. %	Modification	Rank	Result Type
854.444	854.4305	-0.0135	-16	242	249	ATAFGLMK			Oxidation (M)[7]		Mascot
972.4673	972.4487	-0.0186	-19	490	497	ETDGYFIK					Mascot
1017.5952	1017.5868	-0.0084	-8	408	416	IHHSVVGLR					Mascot
1029.5	1029.5046	0.0046	4	152	161	AYGSNIGGYK					Mascot
1032.5472	1032.5397	-0.0075	-7	372	380	SAPIYTQPR					Mascot
1032.5472	1032.5397	-0.0075	-7	372	380	SAPIYTQPR	45	94.677			Mascot
1074.6194	1074.5847	-0.0347	-32	256	264	IIEFAEKPK					Mascot
1256.7109	1256.7004	-0.0105	-8	106	117	AKPAVPLGANYR					Mascot
1256.7109	1256.7004	-0.0105	-8	106	117	AKPAVPLGANYR	29	0			Mascot
1384.6896	1384.6841	-0.0055	-4	361	371	KPIPDFSFYDR					Mascot
1384.6896	1384.6841	-0.0055	-4	361	371	KPIPDFSFYDR	88	100			Mascot
1412.8121	1412.6833	-0.1288	-91	105	117	RAKPAVPLGANYR					Mascot
1625.8646	1625.8612	-0.0034	-2	134	147	IYVLTQFNSASLNR	114	100			Mascot
1786.9368	1786.9095	-0.0273	-15	118	133	LIDIPVSNCLNSNISK			Carbamidomethyl (C)[9]		Mascot
1903.9681	1903.8861	-0.082	-43	387	404	ILDADVTDSVIGEGCVIK			Carbamidomethyl (C)[15]		Mascot
3214.5039	3214.5359	0.032	10	2	33	AMAAMASPSSMTLIPAR HHGAAPSTSGDSSLR			Oxidation (M)[2,5,11]		Mascot

6	TPA: glucose-1-phosphate adenylyltransferase small subunit (ADP-glucose pyrophosphorylase) isoform 1 [Zea mays]	gi|414870680	56789	6.48	12	316	100	23.97	276	100	

Protein Group

TPA: glucose-1-phosphate adenylyltransferase small subunit (ADP-glucose pyrophosphorylase) isoform 2 [Zea mays]	gi|414870681	56789	6.4800 000190 7349

Peptide Information

Calc. Mass	Obsrv. Mass	± da	± ppm	Start Seq.	End Seq.	Sequence	Ion Score	C. I. %	Modification	Rank	Result Type
854.444	854.4305	-0.0135	-16	244	251	ATAFGLMK			Oxidation (M)[7]		Mascot
972.4673	972.4487	-0.0186	-19	492	499	ETDGYFIK					Mascot
1017.5952	1017.5868	-0.0084	-8	410	418	IHHSVVGLR					Mascot
1032.5472	1032.5397	-0.0075	-7	374	382	SAPIYTQPR					Mascot
1032.5472	1032.5397	-0.0075	-7	374	382	SAPIYTQPR	45	94.677			Mascot
1074.6194	1074.5847	-0.0347	-32	258	266	IIEFAEKPK					Mascot
1256.7109	1256.7004	-0.0105	-8	108	119	AKPAVPLGANYR					Mascot
1256.7109	1256.7004	-0.0105	-8	108	119	AKPAVPLGANYR	29	0			Mascot
1384.6896	1384.6841	-0.0055	-4	363	373	KPIPDFSFYDR					Mascot
1384.6896	1384.6841	-0.0055	-4	363	373	KPIPDFSFYDR	88	100			Mascot
1412.8121	1412.6833	-0.1288	-91	107	119	RAKPAVPLGANYR					Mascot
1625.8646	1625.8612	-0.0034	-2	136	149	IYVLTQFNSASLNR	114	100			Mascot
1663.7783	1663.8035	0.0252	15	19	35	HHGAAPSPSTSGDSSLR					Mascot
1786.9368	1786.9095	-0.0273	-15	120	135	LIDIPVSNCLNSNISK			Carbamidomethyl (C)[9]		Mascot
1889.9525	1889.9269	-0.0256	-14	389	406	VLDADVTDSVIGEGCVIK			Carbamidomethyl (C)[15]		Mascot

7 glucose-1-phosphate adenylyltransferase, putative [Ricinus communis] gi|223536136 57474.1 6.48 14 286 100 21.779 231 100

Peptide Information

Calc. Mass	Obsrv. Mass	± da	± ppm	Start Seq.	End Seq.	Sequence	Ion Score	C. I. %	Modification	Rank	Result Type
854.444	854.4305	-0.0135	-16	248	255	ATAFGLMK			Oxidation (M)[7]		Mascot
972.4673	972.4487	-0.0186	-19	496	503	ETDGYFIK					Mascot
1017.5952	1017.5868	-0.0084	-8	414	422	IHHSVVGLR					Mascot
1074.6194	1074.5847	-0.0347	-32	262	270	IIEFAEKPK					Mascot
1256.7109	1256.7004	-0.0105	-8	112	123	AKPAVPLGANYR					Mascot
1256.7109	1256.7004	-0.0105	-8	112	123	AKPAVPLGANYR	29	0			Mascot
1270.7478	1270.7382	-0.0096	-8	90	103	SVLGIILGGGAGTR					Mascot
1384.6896	1384.6841	-0.0055	-4	367	377	KPIPDFSFYDR					Mascot
1384.6896	1384.6841	-0.0055	-4	367	377	KPIPDFSFYDR	88	100			Mascot
1412.8121	1412.6833	-0.1288	-91	111	123	RAKPAVPLGANYR					Mascot
1570.7432	1570.8472	0.104	66	154	167	HLSRAYASNMGGYK			Oxidation (M)[10]		Mascot
1625.8646	1625.8612	-0.0034	-2	140	153	IYVLTQFNSASLNR	114	100			Mascot
1733.9221	1733.8354	-0.0867	-50	378	392	SSPIYTQPRYLPPSK					Mascot

1786.9368	1786.9095	-0.0273	-15	124	139	LIDIPVSNCLNSNISK			Carbamidomethyl (C)[9]		Mascot
1921.9246	1922.0004	0.0758	39	393	410	MLDADVTDSVIGEGCVIK			Carbamidomethyl (C)[15]		Mascot
2348.1882	2348.1313	-0.0569	-24	316	338	DKFPGANDFGSEVIPGAT SIGLR					Mascot

8	PREDICTED: glucose-1-phosphate adenylyltransferase small subunit, chloroplastic-like [Fragaria vesca subsp. vesca]	gi|470136248	57662.4	7.04	14	286	100	20.379	231	100	

Peptide Information

Calc. Mass	Obsrv. Mass	± da	± ppm	Start Seq.	End Seq.	Sequence	Ion Score	C. I. %	Modification	Rank	Result Type
854.444	854.4305	-0.0135	-16	251	258	ATAFGLMK			Oxidation (M)[7]		Mascot
972.4673	972.4487	-0.0186	-19	499	506	ETDGYFIK					Mascot
1074.6194	1074.5847	-0.0347	-32	265	273	IIEFAEKPK					Mascot
1256.7109	1256.7004	-0.0105	-8	115	126	AKPAVPLGANYR					Mascot
1256.7109	1256.7004	-0.0105	-8	115	126	AKPAVPLGANYR	29	0			Mascot
1270.7478	1270.7382	-0.0096	-8	93	106	SVLGIILGGGAGTR					Mascot
1384.6896	1384.6841	-0.0055	-4	370	380	KPIPDFSFYDR					Mascot
1384.6896	1384.6841	-0.0055	-4	370	380	KPIPDFSFYDR	88	100			Mascot
1406.8114	1406.7175	-0.0939	-67	458	471	GSVPIGIGKNSHIK					Mascot
1412.8121	1412.6833	-0.1288	-91	114	126	RAKPAVPLGANYR					Mascot
1508.7196	1508.6846	-0.035	-23	1	15	MASSSMAANGVPTLR			Oxidation (M)[1]		Mascot
1570.7432	1570.8472	0.104	66	157	170	HLSRAYASNMGGYK			Oxidation (M)[10]		Mascot
1625.8646	1625.8612	-0.0034	-2	143	156	IYVLTQFNSASLNR	114	100			Mascot
1733.9221	1733.8354	-0.0867	-50	381	395	SSPIYTQPRYLPPSK					Mascot
1986.025	1985.9537	-0.0713	-36	481	498	IGDNVKIINSDNVQETAR					Mascot
2338.1089	2338.1716	0.0627	27	396	416	MLDADITDSVIGEGCVIKN CK			Carbamidomethyl (C)[15,20]		Mascot

9	ADP glucose pyrophosphorylase [Actinidia chinensis]	gi|398363561	55762.7	7.62	16	275	100	10.817	208	100	

Peptide Information

Calc. Mass	Obsrv. Mass	± da	± ppm	Start Seq.	End Seq.	Sequence	Ion Score	C. I. %	Modification	Rank	Result Type
818.4804	818.4702	-0.0102	-12	1	8	MAAIGVLK			Oxidation (M)[1]		Mascot
854.444	854.4305	-0.0135	-16	232	239	ATAFGLMK			Oxidation (M)[7]		Mascot
972.4673	972.4487	-0.0186	-19	480	487	ETDGYFIK					Mascot
1017.5952	1017.5868	-0.0084	-8	398	406	IHHSVVGLR					Mascot
1032.5472	1032.5397	-0.0075	-7	362	370	SAPIYTQPR					Mascot
1032.5472	1032.5397	-0.0075	-7	362	370	SAPIYTQPR	45	94.677			Mascot

1074.6194	1074.5847	-0.0347	-32	246	254	IIEFAEKPK					Mascot
1256.7109	1256.7004	-0.0105	-8	96	107	AKPAVPLGANYR					Mascot
1256.7109	1256.7004	-0.0105	-8	96	107	AKPAVPLGANYR	29	0			Mascot
1270.7478	1270.7382	-0.0096	-8	74	87	SVLGIILGGGAGTR					Mascot
1406.8114	1406.7175	-0.0939	-67	439	452	GSVPIGIGKNSHIK					Mascot
1412.8121	1412.6833	-0.1288	-91	95	107	RAKPAVPLGANYR					Mascot
1570.7432	1570.8472	0.104	66	138	151	HLSRAYASNMGGYK			Oxidation (M)[10]		Mascot
1625.8646	1625.8612	-0.0034	-2	124	137	IYVLTQFNSASLNR	114	100			Mascot
1786.9368	1786.9095	-0.0273	-15	108	123	LIDIPVSNCLNSNISK			Carbamidomethyl (C)[9]		Mascot
1921.9246	1922.0004	0.0758	39	377	394	MLDADVTDSVIGEGCVIK			Carbamidomethyl (C)[15]		Mascot
1971.0142	1970.9867	-0.0275	-14	462	479	IGDDVKIINSDNIQEAAR					Mascot
1971.0142	1970.9867	-0.0275	-14	462	479	IGDDVKIINSDNIQEAAR	20	0			Mascot
2366.1445	2366.0994	-0.0451	-19	300	322	DKFPGANDFGSEVIPGAT SIGMR					Mascot

10 brittle endosperm2 [Zea mays] gi|413921885 56981.3 7.63 12 272 100 22.809 231 100

Peptide Information

Calc. Mass	Obsrv. Mass	± da	± ppm	Start Seq.	End Seq.	Sequence	Ion Score	C. I. %	Modification	Rank	Result Type
854.444	854.4305	-0.0135	-16	244	251	ATAFGLMK			Oxidation (M)[7]		Mascot
1016.5557	1016.5454	-0.0103	-10	49	58	GVSISMPVAR					Mascot
1029.5	1029.5046	0.0046	4	154	163	AYGSNIGGYK					Mascot
1032.5507	1032.5397	-0.011	-11	49	58	GVSISMPVAR			Oxidation (M)[6]		Mascot
1032.5507	1032.5397	-0.011	-11	49	58	GVSISMPVAR			Oxidation (M)[6]		Mascot
1074.6194	1074.5847	-0.0347	-32	258	266	IIEFAEKPK					Mascot
1256.7109	1256.7004	-0.0105	-8	108	119	AKPAVPLGANYR					Mascot
1256.7109	1256.7004	-0.0105	-8	108	119	AKPAVPLGANYR	29	0			Mascot
1384.6896	1384.6841	-0.0055	-4	363	373	KPIPDFSFYDR					Mascot
1384.6896	1384.6841	-0.0055	-4	363	373	KPIPDFSFYDR	88	100			Mascot
1412.8121	1412.6833	-0.1288	-91	107	119	RAKPAVPLGANYR					Mascot
1441.7944	1441.7061	-0.0883	-61	451	464	GGIPIGIGKNSCIR			Carbamidomethyl (C)[12]		Mascot
1625.8646	1625.8612	-0.0034	-2	136	149	IYVLTQFNSASLNR	114	100			Mascot
1733.8263	1733.8354	0.0091	5	227	242	ETNADITVAALPMDEK			Oxidation (M)[13]		Mascot
1786.9368	1786.9095	-0.0273	-15	120	135	LIDIPVSNCLNSNISK			Carbamidomethyl (C)[9]		Mascot
1873.9324	1873.859	-0.0734	-39	227	243	ETNADITVAALPMDEKR					Mascot
1889.9274	1889.9269	-0.0005	0	227	243	ETNADITVAALPMDEKR			Oxidation (M)[13]		Mascot

Gel Idx/Pos	285/L13	Instr./Gel Origin	BA2151/Sample Project 20140814	Process Status	Analysis Succeeded
Plate [#] Name	[1] Sample Project 20140814	Instrument Sample Name		Spectra	11

Rank	Protein Name	Accession No.	Protein MW	Protein PI	Pep. Count	Protein Score	Protein Score C. I. %	Intensity Matched	Total Ion Score	Total Ion C. I. %	Confirmed
1	2,3-bisphosphoglycerate-independent phosphoglycerate mutase [Aegilops tauschii]	gi|475612558	66379	5.93	18	718	100	28.307	646	100	

Peptide Information

Calc. Mass	Obsrv. Mass	± da	± ppm	Start Seq.	End Seq.	Sequence	Ion Score	C. I. %	Modification	Rank	Result Type
801.4464	801.4352	-0.0112	-14	453	459	ALEIAER					Mascot
924.5261	924.4982	-0.0279	-30	195	202	LHILTDGR					Mascot
963.4928	963.4769	-0.0159	-17	398	406	TNMGQVLGK			Oxidation (M)[3]		Mascot
974.4214	974.399	-0.0224	-23	424	431	SGYFDETR					Mascot
1165.6576	1165.5593	-0.0983	-84	21	31	LADPIVPNQAK					Mascot
1186.6831	1186.6573	-0.0258	-22	363	372	YLVSPPLIER					Mascot
1186.6831	1186.6573	-0.0258	-22	363	372	YLVSPPLIER	46	96.465			Mascot
1403.6511	1403.5999	-0.0512	-36	347	358	YAGMLQYDGELK			Oxidation (M)[4]		Mascot
1407.7015	1407.6813	-0.0202	-14	256	268	GWDAQVLGEAPHK					Mascot
1481.7074	1481.6864	-0.021	-14	412	423	FGHVTFFWNGNR					Mascot
1499.7166	1499.6884	-0.0282	-19	328	339	ALEFPDFDKFDR					Mascot
1499.7166	1499.6884	-0.0282	-19	328	339	ALEFPDFDKFDR	82	100			Mascot
1513.725	1513.6738	-0.0512	-34	232	245	IASGGGRMYVTMDR					Mascot
1563.8027	1563.7573	-0.0454	-29	255	268	RGWDAQVLGEAPHK					Mascot
1731.9501	1731.9226	-0.0275	-16	158	174	GTLHLIGLLSDGGVHSR					Mascot
1731.9501	1731.9226	-0.0275	-16	158	174	GTLHLIGLLSDGGVHSR	117	100			Mascot
1792.9229	1792.8931	-0.0298	-17	302	318	SVGPIVDGDAVVTFNFR					Mascot
1792.9229	1792.8931	-0.0298	-17	302	318	SVGPIVDGDAVVTFNFR	121	100			Mascot
2064.1377	2064.1187	-0.019	-9	363	380	YLVSPPLIERTSGEYLVK					Mascot
2721.2283	2721.1909	-0.0374	-14	97	124	AHGTAVGLPSDDDMGNS EVGHNALGAGR			Oxidation (M)[14]		Mascot
2888.6106	2888.5979	-0.0127	-4	542	570	SGSIQILTSHTLQPVPVAI GGPGLHPGVR					Mascot
2888.6106	2888.5979	-0.0127	-4	542	570	SGSIQILTSHTLQPVPVAI GGPGLHPGVR	186	100			Mascot
3137.4221	3137.4116	-0.0105	-3	424	450	SGYFDETREEYVEIPSDS GITFNEQPK					Mascot
3137.4221	3137.4116	-0.0105	-3	424	450	SGYFDETREEYVEIPSDS GITFNEQPK	94	100			Mascot

Rank	Protein Name	Accession No.	Protein MW	Protein PI	Pep. Count	Protein Score	Protein Score C. I. %	Intensity Matched	Total Ion Score	Total Ion C. I. %	Confirmed
2	2,3-bisphosphoglycerate-independent phosphoglycerate mutase [Triticum urartu]	gi|473886714	57764.3	5.28	15	657	100	21.43	600	100	

Peptide Information

Calc. Mass	Obsrv. Mass	± da	± ppm	Start Seq.	End Seq.	Sequence	Ion Score	C. I. %	Modification	Rank	Result Type
801.4464	801.4352	-0.0112	-14	374	380	ALEIAER					Mascot
924.5261	924.4982	-0.0279	-30	161	168	LHILTDGR					Mascot
974.4214	974.399	-0.0224	-23	345	352	SGYFDETR					Mascot
1403.6511	1403.5999	-0.0512	-36	313	324	YAGMLQYDGELK			Oxidation (M)[4]		Mascot
1407.7015	1407.6813	-0.0202	-14	222	234	GWDAQVLGEAPHK					Mascot
1481.7074	1481.6864	-0.021	-14	333	344	FGHVTFFWNGNR					Mascot
1499.7166	1499.6884	-0.0282	-19	294	305	ALEFPDFDKFDR					Mascot
1499.7166	1499.6884	-0.0282	-19	294	305	ALEFPDFDKFDR	82	100			Mascot
1513.725	1513.6738	-0.0512	-34	198	211	IASGGGRMYVTMDR					Mascot
1563.8027	1563.7573	-0.0454	-29	221	234	RGWDAQVLGEAPHK					Mascot
1731.9501	1731.9226	-0.0275	-16	124	140	GTLHLIGLLSDGGVHSR					Mascot
1731.9501	1731.9226	-0.0275	-16	124	140	GTLHLIGLLSDGGVHSR	117	100			Mascot
1792.9229	1792.8931	-0.0298	-17	268	284	SVGPIVDGDAVVTFNFR					Mascot
1792.9229	1792.8931	-0.0298	-17	268	284	SVGPIVDGDAVVTFNFR	121	100			Mascot
2350.1729	2350.0681	-0.1048	-45	325	344	LPSNETVKFGHVTFFWN GNR					Mascot
2721.2283	2721.1909	-0.0374	-14	63	90	AHGTAVGLPSDDDMGNS EVGHNALGAGR			Oxidation (M)[14]		Mascot
2888.6106	2888.5979	-0.0127	-4	463	491	SGSIQILTSHTLQPVPVAI GGPGLHPGVR					Mascot
2888.6106	2888.5979	-0.0127	-4	463	491	SGSIQILTSHTLQPVPVAI GGPGLHPGVR	186	100			Mascot
3137.4221	3137.4116	-0.0105	-3	345	371	SGYFDETREEYVEIPSDS GITFNEQPK					Mascot
3137.4221	3137.4116	-0.0105	-3	345	371	SGYFDETREEYVEIPSDS GITFNEQPK	94	100			Mascot

3 uncharacterized protein, partial [Phleum pratense] gi|409971907 17282.9 7.96 6 144 100 4.689 121 100

Peptide Information

Calc. Mass	Obsrv. Mass	± da	± ppm	Start Seq.	End Seq.	Sequence	Ion Score	C. I. %	Modification	Rank	Result Type
924.4356	924.4982	0.0626	68	145	152	NGVRTFAC			Carbamidomethyl (C)[8]		Mascot
1128.5289	1128.4897	-0.0392	-35	1	9	GRMYVTMDR					Mascot
1323.6328	1323.6229	-0.0099	-7	10	19	YENDWDVVKR					Mascot
1403.6511	1403.5999	-0.0512	-36	111	122	YAGMLQYDGELK			Oxidation (M)[4]		Mascot
1792.9229	1792.8931	-0.0298	-17	66	82	SVGPIVDGDAVVTFNFR					Mascot
1792.9229	1792.8931	-0.0298	-17	66	82	SVGPIVDGDAVVTFNFR	121	100			Mascot
2063.9202	2064.1187	0.1985	96	3	18	MYVTMDRYENDWDVVK					Mascot

4	uncharacterized protein, partial [Phleum pratense]	gi|409972321	25660.4	5.75	6	143	100	4.765	121	100	

Peptide Information

Calc. Mass	Obsrv. Mass	± da	± ppm	Start Seq.	End Seq.	Sequence	Ion Score	C. I. %	Modification	Rank	Result Type
924.5261	924.4982	-0.0279	-30	43	50	LHILTDGR					Mascot
1323.6328	1323.6229	-0.0099	-7	94	103	YENDWDVVKR					Mascot
1403.6511	1403.5999	-0.0512	-36	195	206	YAGMLQYDGELK			Oxidation (M)[4]		Mascot
1792.9229	1792.8931	-0.0298	-17	150	166	SVGPIVDGDAVVTFNFR					Mascot
1792.9229	1792.8931	-0.0298	-17	150	166	SVGPIVDGDAVVTFNFR	121	100			Mascot
1985.9169	1985.8885	-0.0284	-14	75	93	GVDAQVASGGGRMYVT MDR			Oxidation (M)[13]		Mascot
2063.9202	2064.1187	0.1985	96	87	102	MYVTMDRYENDWDVVK					Mascot

5	uncharacterized protein, partial [Phleum pratense]	gi|409971861	34093.5	5.39	7	143	100	5.018	121	100	

Peptide Information

Calc. Mass	Obsrv. Mass	± da	± ppm	Start Seq.	End Seq.	Sequence	Ion Score	C. I. %	Modification	Rank	Result Type
924.5261	924.4982	-0.0279	-30	37	44	LHILTDGR					Mascot
1323.6328	1323.6229	-0.0099	-7	88	97	YENDWDVVKR					Mascot
1403.6511	1403.5999	-0.0512	-36	189	200	YAGMLQYDGELK			Oxidation (M)[4]		Mascot
1481.7074	1481.6864	-0.021	-14	236	247	FGHVTFFWNGNR					Mascot
1792.9229	1792.8931	-0.0298	-17	144	160	SVGPIVDGDAVVTFNFR					Mascot
1792.9229	1792.8931	-0.0298	-17	144	160	SVGPIVDGDAVVTFNFR	121	100			Mascot
1985.9169	1985.8885	-0.0284	-14	69	87	GVDAQVASGGGRMYVT MDR			Oxidation (M)[13]		Mascot
2063.9202	2064.1187	0.1985	96	81	96	MYVTMDRYENDWDVVK					Mascot

6	uncharacterized protein, partial [Phleum pratense]	gi|409971901	20517.6	5.19	5	142	100	4.447	121	100	

Peptide Information

Calc. Mass	Obsrv. Mass	± da	± ppm	Start Seq.	End Seq.	Sequence	Ion Score	C. I. %	Modification	Rank	Result Type
1323.6328	1323.6229	-0.0099	-7	45	54	YENDWDVVKR					Mascot
1403.6511	1403.5999	-0.0512	-36	146	157	YAGMLQYDGELK			Oxidation (M)[4]		Mascot
1792.9229	1792.8931	-0.0298	-17	101	117	SVGPIVDGDAVVTFNFR					Mascot
1792.9229	1792.8931	-0.0298	-17	101	117	SVGPIVDGDAVVTFNFR	121	100			Mascot
1985.9169	1985.8885	-0.0284	-14	26	44	GVDAQVASGGGRMYVT MDR			Oxidation (M)[13]		Mascot
2063.9202	2064.1187	0.1985	96	38	53	MYVTMDRYENDWDVVK					Mascot

7	uncharacterized protein, partial [Phleum pratense]	gi|409971779	32252.6	5.68	6	141	100	4.7	121	100

Peptide Information

Calc. Mass	Obsrv. Mass	± da	± ppm	Start Seq.	End Seq.	Sequence	Ion Score	C. I. %	Modification	Rank	Result Type
1323.6328	1323.6229	-0.0099	-7	73	82	YENDWDVVKR					Mascot
1403.6511	1403.5999	-0.0512	-36	174	185	YAGMLQYDGELK			Oxidation (M)[4]		Mascot
1481.7074	1481.6864	-0.021	-14	221	232	FGHVTFFWNGNR					Mascot
1792.9229	1792.8931	-0.0298	-17	129	145	SVGPIVDGDAVVTFNFR					Mascot
1792.9229	1792.8931	-0.0298	-17	129	145	SVGPIVDGDAVVTFNFR	121	100			Mascot
1985.9169	1985.8885	-0.0284	-14	54	72	GVDAQVASGGGRMYVT MDR			Oxidation (M)[13]		Mascot
2063.9202	2064.1187	0.1985	96	66	81	MYVTMDRYENDWDVVK					Mascot

8	uncharacterized protein, partial [Phleum pratense]	gi|409972221	25974.2	5.68	5	140	100	4.478	121	100

Peptide Information

Calc. Mass	Obsrv. Mass	± da	± ppm	Start Seq.	End Seq.	Sequence	Ion Score	C. I. %	Modification	Rank	Result Type
1323.6328	1323.6229	-0.0099	-7	15	24	YENDWDVVKR					Mascot
1403.6511	1403.5999	-0.0512	-36	116	127	YAGMLQYDGELK			Oxidation (M)[4]		Mascot
1481.7074	1481.6864	-0.021	-14	163	174	FGHVTFFWNGNR					Mascot
1792.9229	1792.8931	-0.0298	-17	71	87	SVGPIVDGDAVVTFNFR					Mascot
1792.9229	1792.8931	-0.0298	-17	71	87	SVGPIVDGDAVVTFNFR	121	100			Mascot
2063.9202	2064.1187	0.1985	96	8	23	MYVTMDRYENDWDVVK					Mascot

9	PREDICTED: LOW QUALITY PROTEIN: 2,3-bisphosphoglycerate-independent phosphoglycerate mutase-like [Cucumis sativus]	gi|449524762	52813	5.55	9	140	100	5.931	121	100

Peptide Information

Calc. Mass	Obsrv. Mass	± da	± ppm	Start Seq.	End Seq.	Sequence	Ion Score	C. I. %	Modification	Rank	Result Type
928.441	928.4516	0.0106	11	33	40	IYDGEGFK					Mascot
960.5182	960.4462	-0.072	-75	377	384	MILDAIER					Mascot
1128.5208	1128.4897	-0.0311	-28	219	227	ALEYENFDK					Mascot
1338.651	1338.6427	-0.0083	-6	2	15	GNSEVGHNALGAGR					Mascot
1403.6511	1403.5999	-0.0512	-36	238	249	YAGMLQYDGELK			Oxidation (M)[4]		Mascot
1407.7015	1407.6813	-0.0202	-14	147	159	GWDAQVLGEAPHK					Mascot
1481.7074	1481.6864	-0.021	-14	285	296	FGHVTFFWNGNR					Mascot
1563.8027	1563.7573	-0.0454	-29	146	159	RGWDAQVLGEAPHK					Mascot

1792.9229	1792.8931	-0.0298	-17	193	209	SVGPIVDGDAVVTFNFR					Mascot
1792.9229	1792.8931	-0.0298	-17	193	209	SVGPIVDGDAVVTFNFR	121	100			Mascot

10	PREDICTED: 2,3-bisphosphoglycerate-independent phosphoglycerate mutase-like [Cicer arietinum]	gi|502104660	61421.2	5.34	8	139	100	7.434	121	100

Peptide Information

Calc. Mass	Obsrv. Mass	± da	± ppm	Start Seq.	End Seq.	Sequence	Ion Score	C. I. %	Modification	Rank	Result Type
924.5261	924.4982	-0.0279	-30	163	170	LHILTDGR					Mascot
980.506	980.4362	-0.0698	-71	54	61	KGAPEHWR					Mascot
1036.5278	1036.4984	-0.0294	-28	287	295	ADRMTMLAK					Mascot
1052.5227	1052.5006	-0.0221	-21	287	295	ADRMTMLAK			Oxidation (M)[4]		Mascot
1068.5177	1068.4869	-0.0308	-29	287	295	ADRMTMLAK			Oxidation (M)[4,6]		Mascot
1068.5177	1068.4869	-0.0308	-29	287	295	ADRMTMLAK			Oxidation (M)[4,6]		Mascot
1481.7074	1481.6864	-0.021	-14	362	373	FGHVTFFWNGNR					Mascot
1497.6969	1497.7029	0.006	4	296	307	ALENENFDKFDR					Mascot
1513.725	1513.6738	-0.0512	-34	200	213	IASGGGRMYVTMDR					Mascot
1657.8254	1657.7684	-0.057	-34	313	326	IRYAGMLEYDGELK					Mascot
1792.9229	1792.8931	-0.0298	-17	270	286	SVGPIVDGDAVVTFNFR					Mascot
1792.9229	1792.8931	-0.0298	-17	270	286	SVGPIVDGDAVVTFNFR	121	100			Mascot

Gel Idx/Pos	286/L14	Instr./Gel Origin	BA2151/Sample Project 20140814	Process Status	Analysis Succeeded
Plate [#] Name	[1] Sample Project 20140814	Instrument Sample Name		Spectra	11

Rank	Protein Name	Accession No.	Protein MW	Protein PI	Pep. Count	Protein Score	Protein Score C. I. %	Intensity Matched	Total Ion Score	Total Ion C. I. %	Confirmed
1	2,3-bisphosphoglycerate-independent phosphoglycerate mutase [Triticum urartu]	gi|473886714	57764.3	5.28	21	1,020	100	38.67	920	100	

Peptide Information

Calc. Mass	Obsrv. Mass	± da	± ppm	Start Seq.	End Seq.	Sequence	Ion Score	C. I. %	Modification	Rank	Result Type
801.4464	801.4344	-0.012	-15	374	380	ALEIAER					Mascot
924.5261	924.4944	-0.0317	-34	161	168	LHILTDGR					Mascot
947.3961	947.4342	0.0381	40	205	211	MYVTMDR			Oxidation (M)[1,5]		Mascot
974.4214	974.3896	-0.0318	-33	345	352	SGYFDETR					Mascot
974.4214	974.3896	-0.0318	-33	345	352	SGYFDETR	48	98.023			Mascot
1019.552	1019.5002	-0.0518	-51	235	243	FQNALEAVK					Mascot
1139.5369	1139.5072	-0.0297	-26	212	220	YENDWSVVK					Mascot
1403.6511	1403.5919	-0.0592	-42	313	324	YAGMLQYDGELK			Oxidation (M)[4]		Mascot
1407.7015	1407.6548	-0.0467	-33	222	234	GWDAQVLGEAPHK					Mascot
1413.6686	1413.6185	-0.0501	-35	108	118	IWEDEGFNYIK					Mascot
1481.7074	1481.6821	-0.0253	-17	333	344	FGHVTFFWNGNR					Mascot
1499.7166	1499.6847	-0.0319	-21	294	305	ALEFPDFDKFDR					Mascot
1499.7166	1499.6847	-0.0319	-21	294	305	ALEFPDFDKFDR	103	100			Mascot
1513.725	1513.6747	-0.0503	-33	198	211	IASGGGRMYVTMDR					Mascot
1563.8027	1563.7623	-0.0404	-26	221	234	RGWDAQVLGEAPHK					Mascot
1731.9501	1731.92	-0.0301	-17	124	140	GTLHLIGLLSDGGVHSR					Mascot
1731.9501	1731.92	-0.0301	-17	124	140	GTLHLIGLLSDGGVHSR	124	100			Mascot
1792.9229	1792.894	-0.0289	-16	268	284	SVGPIVDGDAVVTFNFR					Mascot
1792.9229	1792.894	-0.0289	-16	268	284	SVGPIVDGDAVVTFNFR	137	100			Mascot
1891.9436	1891.8815	-0.0621	-33	251	267	ANDQYLPPFVIVDESGK					Mascot
2035.9253	2035.931	0.0057	3	205	220	MYVTMDRYENDWSVVK					Mascot
2377.1882	2377.1641	-0.0241	-10	169	190	DVLDGSSVGFVETIENDL AQLR					Mascot
2377.1882	2377.1641	-0.0241	-10	169	190	DVLDGSSVGFVETIENDL AQLR	191	100			Mascot
2721.2283	2721.1895	-0.0388	-14	63	90	AHGTAVGLPSDDDMGNS EVGHNALGAGR			Oxidation (M)[14]		Mascot
2888.6106	2888.5957	-0.0149	-5	463	491	SGSIQILTSHTLQPVPVAI GGPGLHPGVR					Mascot
2888.6106	2888.5957	-0.0149	-5	463	491	SGSIQILTSHTLQPVPVAI	191	100			Mascot

						GGPGLHPGVR					
3137.4221	3137.4119	-0.0102	-3	345	371	SGYFDETREEYVEIPSDS GITFNEQPK					Mascot
3137.4221	3137.4119	-0.0102	-3	345	371	SGYFDETREEYVEIPSDS GITFNEQPK	125	100			Mascot

2	2,3-bisphosphoglycerate-independent phosphoglycerate mutase [Aegilops tauschii]	gi|475612558	66379	5.93	24	906	100	50.282	798	100	

Peptide Information

Calc. Mass	Obsrv. Mass	± da	± ppm	Start Seq.	End Seq.	Sequence	Ion Score	C. I. %	Modification	Rank	Result Type
801.4464	801.4344	-0.012	-15	453	459	ALEIAER					Mascot
896.4724	896.4312	-0.0412	-46	373	380	TSGEYLVK					Mascot
924.5261	924.4944	-0.0317	-34	195	202	LHILTDGR					Mascot
947.3961	947.4342	0.0381	40	239	245	MYVTMDR			Oxidation (M)[1,5]		Mascot
974.4214	974.3896	-0.0318	-33	424	431	SGYFDETR					Mascot
974.4214	974.3896	-0.0318	-33	424	431	SGYFDETR	48	98.023			Mascot
1019.552	1019.5002	-0.0518	-51	269	277	FQNALEAVK					Mascot
1139.5369	1139.5072	-0.0297	-26	246	254	YENDWSVVK					Mascot
1186.6831	1186.6544	-0.0287	-24	363	372	YLVSPPLIER					Mascot
1186.6831	1186.6544	-0.0287	-24	363	372	YLVSPPLIER	69	99.984			Mascot
1322.7063	1322.6094	-0.0969	-73	373	384	TSGEYLVKNGVR					Mascot
1403.6511	1403.5919	-0.0592	-42	347	358	YAGMLQYDGELK			Oxidation (M)[4]		Mascot
1407.7015	1407.6548	-0.0467	-33	256	268	GWDAQVLGEAPHK					Mascot
1413.6686	1413.6185	-0.0501	-35	142	152	IWEDEGFNYIK					Mascot
1481.7074	1481.6821	-0.0253	-17	412	423	FGHVTFFWNGNR					Mascot
1499.7166	1499.6847	-0.0319	-21	328	339	ALEFPDFDKFDR					Mascot
1499.7166	1499.6847	-0.0319	-21	328	339	ALEFPDFDKFDR	103	100			Mascot
1513.725	1513.6747	-0.0503	-33	232	245	IASGGGRMYVTMDR					Mascot
1563.8027	1563.7623	-0.0404	-26	255	268	RGWDAQVLGEAPHK					Mascot
1611.9469	1611.788	-0.1589	-99	359	372	LPSKYLVSPPLIER					Mascot
1731.9501	1731.92	-0.0301	-17	158	174	GTLHLIGLLSDGGVHSR					Mascot
1731.9501	1731.92	-0.0301	-17	158	174	GTLHLIGLLSDGGVHSR	124	100			Mascot
1792.9229	1792.894	-0.0289	-16	302	318	SVGPIVDGDAVVTFNFR					Mascot
1792.9229	1792.894	-0.0289	-16	302	318	SVGPIVDGDAVVTFNFR	137	100			Mascot
1891.9436	1891.8815	-0.0621	-33	285	301	ANDQYLPPFVIVDESGK					Mascot
2035.9253	2035.931	0.0057	3	239	254	MYVTMDRYENDWSVVK					Mascot
2721.2283	2721.1895	-0.0388	-14	97	124	AHGTAVGLPSDDDMGNS EVGHNALGAGR			Oxidation (M)[14]		Mascot
2888.6106	2888.5957	-0.0149	-5	542	570	SGSIQILTSHTLQPVPVAI					Mascot

Calc. Mass	Obsrv. Mass	± da	± ppm	Start Seq.	End Seq.	Sequence	Ion Score	C. I. %	Modification	Rank	Result Type
						GGPGLHPGVR					
2888.6106	2888.5957	-0.0149	-5	542	570	SGSIQILTSHTLQPVPVAI GGPGLHPGVR	191	100			Mascot
3137.4221	3137.4119	-0.0102	-3	424	450	SGYFDETREEYVEIPSDS GITFNEQPK					Mascot
3137.4221	3137.4119	-0.0102	-3	424	450	SGYFDETREEYVEIPSDS GITFNEQPK	125	100			Mascot

3	uncharacterized protein, partial [Phleum pratense]	gi|409971861	34093.5	5.39	12	381	100	11.993	328	100	

Peptide Information

Calc. Mass	Obsrv. Mass	± da	± ppm	Start Seq.	End Seq.	Sequence	Ion Score	C. I. %	Modification	Rank	Result Type
896.4724	896.4312	-0.0412	-46	215	222	TSGEYLVK					Mascot
924.5261	924.4944	-0.0317	-34	37	44	LHILTDGR					Mascot
947.3961	947.4342	0.0381	40	81	87	MYVTMDR			Oxidation (M)[1,5]		Mascot
1030.5891	1030.5607	-0.0284	-28	277	285	ALEIAEKTR					Mascot
1042.4874	1042.4586	-0.0288	-28	227	235	TFACSETVK			Carbamidomethyl (C)[4]		Mascot
1322.7063	1322.6094	-0.0969	-73	215	226	TSGEYLVKNGVR					Mascot
1403.6511	1403.5919	-0.0592	-42	189	200	YAGMLQYDGELK			Oxidation (M)[4]		Mascot
1473.7009	1473.6963	-0.0046	-3	170	181	ALEFADFDKFDR					Mascot
1481.7074	1481.6821	-0.0253	-17	236	247	FGHVTFFWNGNR					Mascot
1792.9229	1792.894	-0.0289	-16	144	160	SVGPIVDGDAVVTFNFR					Mascot
1792.9229	1792.894	-0.0289	-16	144	160	SVGPIVDGDAVVTFNFR	137	100			Mascot
1985.9169	1985.8677	-0.0492	-25	69	87	GVDAQVASGGGRMYVT MDR			Oxidation (M)[13]		Mascot
2377.1882	2377.1641	-0.0241	-10	45	66	DVLDGSSVGFVETLENDL AQLR					Mascot
2377.1882	2377.1641	-0.0241	-10	45	66	DVLDGSSVGFVETLENDL AQLR	191	100			Mascot

4	uncharacterized protein, partial [Phleum pratense]	gi|409971779	32252.6	5.68	11	379	100	11.088	328	100	

Peptide Information

Calc. Mass	Obsrv. Mass	± da	± ppm	Start Seq.	End Seq.	Sequence	Ion Score	C. I. %	Modification	Rank	Result Type
896.4724	896.4312	-0.0412	-46	200	207	TSGEYLVK					Mascot
947.3961	947.4342	0.0381	40	66	72	MYVTMDR			Oxidation (M)[1,5]		Mascot
1030.5891	1030.5607	-0.0284	-28	262	270	ALEIAEKTR					Mascot
1042.4874	1042.4586	-0.0288	-28	212	220	TFACSETVK			Carbamidomethyl (C)[4]		Mascot
1322.7063	1322.6094	-0.0969	-73	200	211	TSGEYLVKNGVR					Mascot
1403.6511	1403.5919	-0.0592	-42	174	185	YAGMLQYDGELK			Oxidation (M)[4]		Mascot
1473.7009	1473.6963	-0.0046	-3	155	166	ALEFADFDKFDR					Mascot
1481.7074	1481.6821	-0.0253	-17	221	232	FGHVTFFWNGNR					Mascot

Calc. Mass	Obsrv. Mass	± da	± ppm	Start Seq.	End Seq.	Sequence	Ion Score	C. I. %	Modification	Rank	Result Type
1792.9229	1792.894	-0.0289	-16	129	145	SVGPIVDGDAVVTFNFR					Mascot
1792.9229	1792.894	-0.0289	-16	129	145	SVGPIVDGDAVVTFNFR	137	100			Mascot
1985.9169	1985.8677	-0.0492	-25	54	72	GVDAQVASGGGRMYVT MDR			Oxidation (M)[13]		Mascot
2377.1882	2377.1641	-0.0241	-10	30	51	DVLDGSSVGFVETLENDL AQLR					Mascot
2377.1882	2377.1641	-0.0241	-10	30	51	DVLDGSSVGFVETLENDL AQLR	191	100			Mascot

5	PREDICTED: 2,3-bisphosphoglycerate-independent phosphoglycerate mutase-like [Cucumis sativus]	gi|449459570	61265.3	5.7	14	373	100	11.619	328	100	

Peptide Information

Calc. Mass	Obsrv. Mass	± da	± ppm	Start Seq.	End Seq.	Sequence	Ion Score	C. I. %	Modification	Rank	Result Type
947.3961	947.4342	0.0381	40	207	213	MYVTMDR			Oxidation (M)[1,5]		Mascot
1002.4349	1002.4324	-0.0025	-2	1	9	MGSSGFSWK			Oxidation (M)[1]		Mascot
1042.4874	1042.4586	-0.0288	-28	353	361	TFACSETVK			Carbamidomethyl (C)[4]		Mascot
1358.7023	1358.6671	-0.0352	-26	193	206	EKGIDAQIASGGGR					Mascot
1403.6511	1403.5919	-0.0592	-42	315	326	YAGMLQYDGELK			Oxidation (M)[4]		Mascot
1407.7015	1407.6548	-0.0467	-33	224	236	GWDAQVLGEAPHK					Mascot
1481.7074	1481.6821	-0.0253	-17	362	373	FGHVTFFWNGNR					Mascot
1497.6969	1497.6793	-0.0176	-12	296	307	ALENENFDKFDR					Mascot
1563.8027	1563.7623	-0.0404	-26	223	236	RGWDAQVLGEAPHK					Mascot
1646.8418	1646.7578	-0.084	-51	447	461	AADDAVKMILDAIER			Oxidation (M)[8]		Mascot
1682.865	1682.8103	-0.0547	-33	224	238	GWDAQVLGEAPHKFK					Mascot
1792.9229	1792.894	-0.0289	-16	270	286	SVGPIVDGDAVVTFNFR					Mascot
1792.9229	1792.894	-0.0289	-16	270	286	SVGPIVDGDAVVTFNFR	137	100			Mascot
1825.9443	1825.8073	-0.137	-75	21	37	TIGLIVLDGWGEANPDR					Mascot
2377.2246	2377.1641	-0.0605	-25	171	192	DVLDGSSVGFVETLENDL AKLR					Mascot
2377.2246	2377.1641	-0.0605	-25	171	192	DVLDGSSVGFVETLENDL AKLR	191	100			Mascot

6	PREDICTED: LOW QUALITY PROTEIN: 2,3-bisphosphoglycerate-independent phosphoglycerate mutase-like [Cucumis sativus]	gi|449524762	52813	5.55	13	372	100	11.273	328	100	

Peptide Information

Calc. Mass	Obsrv. Mass	± da	± ppm	Start Seq.	End Seq.	Sequence	Ion Score	C. I. %	Modification	Rank	Result Type
947.3961	947.4342	0.0381	40	130	136	MYVTMDR			Oxidation (M)[1,5]		Mascot
1042.4874	1042.4586	-0.0288	-28	276	284	TFACSETVK			Carbamidomethyl (C)[4]		Mascot
1338.651	1338.6401	-0.0109	-8	2	15	GNSEVGHNALGAGR					Mascot

Calc. Mass	Obsrv. Mass	± da	± ppm	Start Seq.	End Seq.	Sequence	Ion Score	C. I. %	Modification	Rank	Result Type
1358.7023	1358.6671	-0.0352	-26	116	129	EKGIDAQIASGGGR					Mascot
1403.6511	1403.5919	-0.0592	-42	238	249	YAGMLQYDGELK			Oxidation (M)[4]		Mascot
1407.7015	1407.6548	-0.0467	-33	147	159	GWDAQVLGEAPHK					Mascot
1481.7074	1481.6821	-0.0253	-17	285	296	FGHVTFFWNGNR					Mascot
1485.6863	1485.6726	-0.0137	-9	1	15	MGNSEVGHNALGAGR			Oxidation (M)[1]		Mascot
1563.8027	1563.7623	-0.0404	-26	146	159	RGWDAQVLGEAPHK					Mascot
1646.8418	1646.7578	-0.084	-51	370	384	AADDAVKMILDAIER			Oxidation (M)[8]		Mascot
1682.865	1682.8103	-0.0547	-33	147	161	GWDAQVLGEAPHKFK					Mascot
1792.9229	1792.894	-0.0289	-16	193	209	SVGPIVDGDAVVTFNFR					Mascot
1792.9229	1792.894	-0.0289	-16	193	209	SVGPIVDGDAVVTFNFR	137	100			Mascot
2377.2246	2377.1641	-0.0605	-25	94	115	DVLDGSSVGFVETLENDL AKLR					Mascot
2377.2246	2377.1641	-0.0605	-25	94	115	DVLDGSSVGFVETLENDL AKLR	191	100			Mascot

7 uncharacterized protein, partial [Phleum pratense] gi|409972321 25660.4 5.75 9 370 100 11.023 328 100

Peptide Information

Calc. Mass	Obsrv. Mass	± da	± ppm	Start Seq.	End Seq.	Sequence	Ion Score	C. I. %	Modification	Rank	Result Type
896.4724	896.4312	-0.0412	-46	221	228	TSGEYLVK					Mascot
924.5261	924.4944	-0.0317	-34	43	50	LHILTDGR					Mascot
947.3961	947.4342	0.0381	40	87	93	MYVTMDR			Oxidation (M)[1,5]		Mascot
1322.7063	1322.6094	-0.0969	-73	221	232	TSGEYLVKNGVR					Mascot
1403.6511	1403.5919	-0.0592	-42	195	206	YAGMLQYDGELK			Oxidation (M)[4]		Mascot
1473.7009	1473.6963	-0.0046	-3	176	187	ALEFADFDKFDR					Mascot
1792.9229	1792.894	-0.0289	-16	150	166	SVGPIVDGDAVVTFNFR					Mascot
1792.9229	1792.894	-0.0289	-16	150	166	SVGPIVDGDAVVTFNFR	137	100			Mascot
1985.9169	1985.8677	-0.0492	-25	75	93	GVDAQVASGGGRMYVT MDR			Oxidation (M)[13]		Mascot
2377.1882	2377.1641	-0.0241	-10	51	72	DVLDGSSVGFVETLENDL AQLR					Mascot
2377.1882	2377.1641	-0.0241	-10	51	72	DVLDGSSVGFVETLENDL AQLR	191	100			Mascot

8 uncharacterized protein, partial [Phleum pratense] gi|409971901 20517.6 5.19 8 369 100 10.118 328 100

Peptide Information

Calc. Mass	Obsrv. Mass	± da	± ppm	Start Seq.	End Seq.	Sequence	Ion Score	C. I. %	Modification	Rank	Result Type
896.4724	896.4312	-0.0412	-46	172	179	TSGEYLVK					Mascot
947.3961	947.4342	0.0381	40	38	44	MYVTMDR			Oxidation (M)[1,5]		Mascot
1322.7063	1322.6094	-0.0969	-73	172	183	TSGEYLVKNGVR					Mascot

Calc. Mass	Obsrv. Mass	± da	± ppm	Start Seq.	End Seq.	Sequence	Ion Score	C. I. %	Modification	Rank	Result Type
1403.6511	1403.5919	-0.0592	-42	146	157	YAGMLQYDGELK			Oxidation (M)[4]		Mascot
1473.7009	1473.6963	-0.0046	-3	127	138	ALEFADFDKFDR					Mascot
1792.9229	1792.894	-0.0289	-16	101	117	SVGPIVDGDAVVTFNFR					Mascot
1792.9229	1792.894	-0.0289	-16	101	117	SVGPIVDGDAVVTFNFR	137	100			Mascot
1985.9169	1985.8677	-0.0492	-25	26	44	GVDAQVASGGGRMYVT MDR			Oxidation (M)[13]		Mascot
2377.1882	2377.1641	-0.0241	-10	2	23	DVLDGSSVGFVETLENDL AQLR					Mascot
2377.1882	2377.1641	-0.0241	-10	2	23	DVLDGSSVGFVETLENDL AQLR	191	100			Mascot

9	uncharacterized protein, partial [Phleum pratense]	gi|409972263	14472.5	5.62	4	211	100	3.432	191	100	

Peptide Information

Calc. Mass	Obsrv. Mass	± da	± ppm	Start Seq.	End Seq.	Sequence	Ion Score	C. I. %	Modification	Rank	Result Type
924.5261	924.4944	-0.0317	-34	44	51	LHILTDGR					Mascot
947.3961	947.4342	0.0381	40	88	94	MYVTMDR			Oxidation (M)[1,5]		Mascot
1985.9169	1985.8677	-0.0492	-25	76	94	GVDAQVASGGGRMYVT MDR			Oxidation (M)[13]		Mascot
2377.1882	2377.1641	-0.0241	-10	52	73	DVLDGSSVGFVETLENDL AQLR					Mascot
2377.1882	2377.1641	-0.0241	-10	52	73	DVLDGSSVGFVETLENDL AQLR	191	100			Mascot

10	RecName: Full=2,3-bisphosphoglycerate-independent phosphoglycerate mutase; Short=BPG-independent PGAM; Short=Phosphoglyceromutase; AltName: Full=PGAM-I	gi|3914394	61316.1	5.39	10	211	100	3.913	191	100	

Peptide Information

Calc. Mass	Obsrv. Mass	± da	± ppm	Start Seq.	End Seq.	Sequence	Ion Score	C. I. %	Modification	Rank	Result Type
947.3961	947.4342	0.0381	40	207	213	MYVTMDR			Oxidation (M)[1,5]		Mascot
1042.4874	1042.4586	-0.0288	-28	353	361	TFACSETVK			Carbamidomethyl (C)[4]		Mascot
1139.5369	1139.5072	-0.0297	-26	214	222	YENDWSVVK					Mascot
1403.6511	1403.5919	-0.0592	-42	315	326	YAGMLQYDGELK			Oxidation (M)[4]		Mascot
1481.7074	1481.6821	-0.0253	-17	362	373	FGHVTFFWNGNR					Mascot
1682.9225	1682.8103	-0.1122	-67	327	340	LPNRYLVSPPEIER					Mascot
1749.8265	1749.843	0.0165	9	1	15	MGSTEFSWKLADHPK			Oxidation (M)[1]		Mascot
1999.9325	1999.9305	-0.002	-1	195	213	GVDAQIASGGGRMYVTM DR			Oxidation (M)[13]		Mascot
2035.9253	2035.931	0.0057	3	207	222	MYVTMDRYENDWSVVK					Mascot
2377.1882	2377.1641	-0.0241	-10	171	192	DVLDGSSVGFVETLENDL AQLR					Mascot
2377.1882	2377.1641	-0.0241	-10	171	192	DVLDGSSVGFVETLENDL AQLR	191	100			Mascot

Gel Idx/Pos	287/L15	Instr./Gel Origin	BA2151/Sample Project 20140814	Process Status	Analysis Succeeded
Plate [#] Name	[1] Sample Project 20140814	Instrument Sample Name		Spectra	11

Rank	Protein Name	Accession No.	Protein MW	Protein PI	Pep. Count	Protein Score	Protein Score C. I. %	Intensity Matched	Total Ion Score	Total Ion C. I. %	Confirmed
1	2,3-bisphosphoglycerate-independent phosphoglycerate mutase [Aegilops tauschii]	gi|475612558	66379	5.93	19	804	100	51.2	732	100	

Peptide Information

Calc. Mass	Obsrv. Mass	± da	± ppm	Start Seq.	End Seq.	Sequence	Ion Score	C. I. %	Modification	Rank	Result Type
801.4464	801.4226	-0.0238	-30	453	459	ALEIAER					Mascot
896.4724	896.4455	-0.0269	-30	373	380	TSGEYLVK					Mascot
924.5261	924.5121	-0.014	-15	195	202	LHILTDGR					Mascot
947.3961	947.4465	0.0504	53	239	245	MYVTMDR			Oxidation (M)[1,5]		Mascot
974.4214	974.4093	-0.0121	-12	424	431	SGYFDETR					Mascot
974.4214	974.4093	-0.0121	-12	424	431	SGYFDETR	27	0			Mascot
1139.5369	1139.5653	0.0284	25	246	254	YENDWSVVK					Mascot
1186.6831	1186.6746	-0.0085	-7	363	372	YLVSPPLIER					Mascot
1186.6831	1186.6746	-0.0085	-7	363	372	YLVSPPLIER	68	99.973			Mascot
1403.6511	1403.6001	-0.051	-36	347	358	YAGMLQYDGELK			Oxidation (M)[4]		Mascot
1413.6686	1413.6373	-0.0313	-22	142	152	IWEDEGFNYIK					Mascot
1481.7074	1481.6935	-0.0139	-9	412	423	FGHVTFFWNGNR					Mascot
1499.7166	1499.7074	-0.0092	-6	328	339	ALEFPDFDKFDR					Mascot
1499.7166	1499.7074	-0.0092	-6	328	339	ALEFPDFDKFDR	100	100			Mascot
1513.725	1513.6871	-0.0379	-25	232	245	IASGGGRMYVTMDR					Mascot
1731.9501	1731.9423	-0.0078	-5	158	174	GTLHLIGLLSDGGVHSR					Mascot
1731.9501	1731.9423	-0.0078	-5	158	174	GTLHLIGLLSDGGVHSR	126	100			Mascot
1792.9229	1792.9136	-0.0093	-5	302	318	SVGPIVDGDAVVTFNFR					Mascot
1792.9229	1792.9136	-0.0093	-5	302	318	SVGPIVDGDAVVTFNFR	136	100			Mascot
1891.9436	1891.907	-0.0366	-19	285	301	ANDQYLPPFVIVDESGK					Mascot
2035.9253	2035.9226	-0.0027	-1	239	254	MYVTMDRYENDWSVVK					Mascot
2721.2283	2721.22	-0.0083	-3	97	124	AHGTAVGLPSDDDMGNS EVGHNALGAGR			Oxidation (M)[14]		Mascot
2888.6106	2888.6157	0.0051	2	542	570	SGSIQILTSHTLQPVPVAI GGPGLHPGVR					Mascot
2888.6106	2888.6157	0.0051	2	542	570	SGSIQILTSHTLQPVPVAI GGPGLHPGVR	169	100			Mascot
3137.4221	3137.4365	0.0144	5	424	450	SGYFDETREEYVEIPSDS GITFNEQPK					Mascot
3137.4221	3137.4365	0.0144	5	424	450	SGYFDETREEYVEIPSDS	106	100			Mascot

GITFNEQPK

2 2,3-bisphosphoglycerate-independent phosphoglycerate mutase [Triticum urartu] gi|473886714 57764.3 5.28 18 744 100 38.98 664 100

Peptide Information

Calc. Mass	Obsrv. Mass	± da	± ppm	Start Seq.	End Seq.	Sequence	Ion Score	C. I. %	Modification	Rank	Result Type
801.4464	801.4226	-0.0238	-30	374	380	ALEIAER					Mascot
924.5261	924.5121	-0.014	-15	161	168	LHILTDGR					Mascot
947.3961	947.4465	0.0504	53	205	211	MYVTMDR			Oxidation (M)[1,5]		Mascot
974.4214	974.4093	-0.0121	-12	345	352	SGYFDETR					Mascot
974.4214	974.4093	-0.0121	-12	345	352	SGYFDETR	27	0			Mascot
1139.5369	1139.5653	0.0284	25	212	220	YENDWSVVK					Mascot
1403.6511	1403.6001	-0.051	-36	313	324	YAGMLQYDGELK			Oxidation (M)[4]		Mascot
1413.6686	1413.6373	-0.0313	-22	108	118	IWEDEGFNYIK					Mascot
1481.7074	1481.6935	-0.0139	-9	333	344	FGHVTFFWNGNR					Mascot
1499.7166	1499.7074	-0.0092	-6	294	305	ALEFPDFDKFDR					Mascot
1499.7166	1499.7074	-0.0092	-6	294	305	ALEFPDFDKFDR	100	100			Mascot
1513.725	1513.6871	-0.0379	-25	198	211	IASGGGRMYVTMDR					Mascot
1731.9501	1731.9423	-0.0078	-5	124	140	GTLHLIGLLSDGGVHSR					Mascot
1731.9501	1731.9423	-0.0078	-5	124	140	GTLHLIGLLSDGGVHSR	126	100			Mascot
1792.9229	1792.9136	-0.0093	-5	268	284	SVGPIVDGDAVVTFNFR					Mascot
1792.9229	1792.9136	-0.0093	-5	268	284	SVGPIVDGDAVVTFNFR	136	100			Mascot
1891.9436	1891.907	-0.0366	-19	251	267	ANDQYLPPFVIVDESGK					Mascot
2035.9253	2035.9226	-0.0027	-1	205	220	MYVTMDRYENDWSVVK					Mascot
2377.1882	2377.1819	-0.0063	-3	169	190	DVLDGSSVGFVETIENDL AQLR					Mascot
2721.2283	2721.22	-0.0083	-3	63	90	AHGTAVGLPSDDDMGNS EVGHNALGAGR			Oxidation (M)[14]		Mascot
2888.6106	2888.6157	0.0051	2	463	491	SGSIQILTSHTLQPVPVAI GGPGLHPGVR					Mascot
2888.6106	2888.6157	0.0051	2	463	491	SGSIQILTSHTLQPVPVAI GGPGLHPGVR	169	100			Mascot
3137.4221	3137.4365	0.0144	5	345	371	SGYFDETREEYVEIPSDS GITFNEQPK					Mascot
3137.4221	3137.4365	0.0144	5	345	371	SGYFDETREEYVEIPSDS GITFNEQPK	106	100			Mascot

3 uncharacterized protein, partial [Phleum pratense] gi|409971861 34093.5 5.39 12 190 100 10.208 136 100

Peptide Information

Calc. Mass	Obsrv. Mass	± da	± ppm	Start Seq.	End Seq.	Sequence	Ion Score	C. I. %	Modification	Rank	Result Type
896.4724	896.4455	-0.0269	-30	215	222	TSGEYLVK					Mascot

Calc. Mass	Obsrv. Mass	± da	± ppm	Start Seq.	End Seq.	Sequence	Ion Score	C. I. %	Modification	Rank	Result Type
924.5261	924.5121	-0.014	-15	37	44	LHILTDGR					Mascot
947.3961	947.4465	0.0504	53	81	87	MYVTMDR			Oxidation (M)[1,5]		Mascot
1042.4874	1042.4751	-0.0123	-12	227	235	TFACSETVK			Carbamidomethyl (C)[4]		Mascot
1323.6328	1323.6545	0.0217	16	88	97	YENDWDVVKR					Mascot
1403.6511	1403.6001	-0.051	-36	189	200	YAGMLQYDGELK			Oxidation (M)[4]		Mascot
1481.7074	1481.6935	-0.0139	-9	236	247	FGHVTFFWNGNR					Mascot
1589.8071	1589.7869	-0.0202	-13	97	110	RGWDAQVLGEAPYK					Mascot
1744.8649	1744.8921	0.0272	16	164	178	MVMLAKALEFADFDK			Oxidation (M)[1]		Mascot
1792.9229	1792.9136	-0.0093	-5	144	160	SVGPIVDGDAVVTFNFR					Mascot
1792.9229	1792.9136	-0.0093	-5	144	160	SVGPIVDGDAVVTFNFR	136	100			Mascot
1985.9169	1985.921	0.0041	2	69	87	GVDAQVASGGGRMYVT MDR			Oxidation (M)[13]		Mascot
2377.1882	2377.1819	-0.0063	-3	45	66	DVLDGSSVGFVETLENDL AQLR					Mascot

4	uncharacterized protein, partial [Phleum pratense]	gi|409971779	32252.6	5.68	11	187	100	9.348	136	100

Peptide Information

Calc. Mass	Obsrv. Mass	± da	± ppm	Start Seq.	End Seq.	Sequence	Ion Score	C. I. %	Modification	Rank	Result Type
896.4724	896.4455	-0.0269	-30	200	207	TSGEYLVK					Mascot
947.3961	947.4465	0.0504	53	66	72	MYVTMDR			Oxidation (M)[1,5]		Mascot
1042.4874	1042.4751	-0.0123	-12	212	220	TFACSETVK			Carbamidomethyl (C)[4]		Mascot
1323.6328	1323.6545	0.0217	16	73	82	YENDWDVVKR					Mascot
1403.6511	1403.6001	-0.051	-36	174	185	YAGMLQYDGELK			Oxidation (M)[4]		Mascot
1481.7074	1481.6935	-0.0139	-9	221	232	FGHVTFFWNGNR					Mascot
1589.8071	1589.7869	-0.0202	-13	82	95	RGWDAQVLGEAPYK					Mascot
1744.8649	1744.8921	0.0272	16	149	163	MVMLAKALEFADFDK			Oxidation (M)[1]		Mascot
1792.9229	1792.9136	-0.0093	-5	129	145	SVGPIVDGDAVVTFNFR					Mascot
1792.9229	1792.9136	-0.0093	-5	129	145	SVGPIVDGDAVVTFNFR	136	100			Mascot
1985.9169	1985.921	0.0041	2	54	72	GVDAQVASGGGRMYVT MDR			Oxidation (M)[13]		Mascot
2377.1882	2377.1819	-0.0063	-3	30	51	DVLDGSSVGFVETLENDL AQLR					Mascot

5	uncharacterized protein, partial [Phleum pratense]	gi|409972321	25660.4	5.75	10	186	100	9.93	136	100

Peptide Information

Calc. Mass	Obsrv. Mass	± da	± ppm	Start Seq.	End Seq.	Sequence	Ion Score	C. I. %	Modification	Rank	Result Type
896.4724	896.4455	-0.0269	-30	221	228	TSGEYLVK					Mascot
924.5261	924.5121	-0.014	-15	43	50	LHILTDGR					Mascot

947.3961	947.4465	0.0504	53	87	93	MYVTMDR			Oxidation (M)[1,5]		Mascot
1323.6328	1323.6545	0.0217	16	94	103	YENDWDVVKR					Mascot
1403.6511	1403.6001	-0.051	-36	195	206	YAGMLQYDGELK			Oxidation (M)[4]		Mascot
1589.8071	1589.7869	-0.0202	-13	103	116	RGWDAQVLGEAPYK					Mascot
1744.8649	1744.8921	0.0272	16	170	184	MVMLAKALEFADFDK			Oxidation (M)[1]		Mascot
1792.9229	1792.9136	-0.0093	-5	150	166	SVGPIVDGDAVVTFNFR					Mascot
1792.9229	1792.9136	-0.0093	-5	150	166	SVGPIVDGDAVVTFNFR	136	100			Mascot
1985.9169	1985.921	0.0041	2	75	93	GVDAQVASGGGRMYVT MDR			Oxidation (M)[13]		Mascot
2377.1882	2377.1819	-0.0063	-3	51	72	DVLDGSSVGFVETLENDL AQLR					Mascot

6 uncharacterized protein, partial [Phleum pratense] gi|409971901 20517.6 5.19 9 186 100 9.069 136 100

Peptide Information

Calc. Mass	Obsrv. Mass	± da	± ppm	Start Seq.	End Seq.	Sequence	Ion Score	C. I. %	Modification	Rank	Result Type
896.4724	896.4455	-0.0269	-30	172	179	TSGEYLVK					Mascot
947.3961	947.4465	0.0504	53	38	44	MYVTMDR			Oxidation (M)[1,5]		Mascot
1323.6328	1323.6545	0.0217	16	45	54	YENDWDVVKR					Mascot
1403.6511	1403.6001	-0.051	-36	146	157	YAGMLQYDGELK			Oxidation (M)[4]		Mascot
1589.8071	1589.7869	-0.0202	-13	54	67	RGWDAQVLGEAPYK					Mascot
1744.8649	1744.8921	0.0272	16	121	135	MVMLAKALEFADFDK			Oxidation (M)[1]		Mascot
1792.9229	1792.9136	-0.0093	-5	101	117	SVGPIVDGDAVVTFNFR					Mascot
1792.9229	1792.9136	-0.0093	-5	101	117	SVGPIVDGDAVVTFNFR	136	100			Mascot
1985.9169	1985.921	0.0041	2	26	44	GVDAQVASGGGRMYVT MDR			Oxidation (M)[13]		Mascot
2377.1882	2377.1819	-0.0063	-3	2	23	DVLDGSSVGFVETLENDL AQLR					Mascot

7 uncharacterized protein, partial [Phleum pratense] gi|409971907 17282.9 7.96 8 172 100 9.497 136 100

Peptide Information

Calc. Mass	Obsrv. Mass	± da	± ppm	Start Seq.	End Seq.	Sequence	Ion Score	C. I. %	Modification	Rank	Result Type
896.4724	896.4455	-0.0269	-30	137	144	TSGEYLVK					Mascot
924.4356	924.5121	0.0765	83	145	152	NGVRTFAC			Carbamidomethyl (C)[8]		Mascot
947.3961	947.4465	0.0504	53	3	9	MYVTMDR			Oxidation (M)[1,5]		Mascot
1323.6328	1323.6545	0.0217	16	10	19	YENDWDVVKR					Mascot
1403.6511	1403.6001	-0.051	-36	111	122	YAGMLQYDGELK			Oxidation (M)[4]		Mascot
1589.8071	1589.7869	-0.0202	-13	19	32	RGWDAQVLGEAPYK					Mascot
1744.8649	1744.8921	0.0272	16	86	100	MVMLAKALEFADFDK			Oxidation (M)[1]		Mascot

1792.9229	1792.9136	-0.0093	-5	66	82	SVGPIVDGDAVVTFNFR					Mascot
1792.9229	1792.9136	-0.0093	-5	66	82	SVGPIVDGDAVVTFNFR	136	100			Mascot

8 PREDICTED: LOW QUALITY PROTEIN: 2,3-bisphosphoglycerate-independent phosphoglycerate mutase-like [Cucumis sativus] | gi|449524762 | 52813 | 5.55 | 11 | 171 | 100 | 9.356 | 136 | 100

Peptide Information

Calc. Mass	Obsrv. Mass	± da	± ppm	Start Seq.	End Seq.	Sequence	Ion Score	C. I. %	Modification	Rank	Result Type
947.3961	947.4465	0.0504	53	130	136	MYVTMDR			Oxidation (M)[1,5]		Mascot
1042.4874	1042.4751	-0.0123	-12	276	284	TFACSETVK			Carbamidomethyl (C)[4]		Mascot
1338.651	1338.6641	0.0131	10	2	15	GNSEVGHNALGAGR					Mascot
1403.6511	1403.6001	-0.051	-36	238	249	YAGMLQYDGELK			Oxidation (M)[4]		Mascot
1481.7074	1481.6935	-0.0139	-9	285	296	FGHVTFFWNGNR					Mascot
1646.8418	1646.7733	-0.0685	-42	370	384	AADDAVKMILDAIER			Oxidation (M)[8]		Mascot
1682.865	1682.848	-0.017	-10	147	161	GWDAQVLGEAPHKFK					Mascot
1792.9229	1792.9136	-0.0093	-5	193	209	SVGPIVDGDAVVTFNFR					Mascot
1792.9229	1792.9136	-0.0093	-5	193	209	SVGPIVDGDAVVTFNFR	136	100			Mascot
1801.8864	1801.8964	0.01	6	213	227	MVMIAKALEYENFDK					Mascot
1974.959	1974.9341	-0.0249	-13	385	403	VGGIYVVTADHGNAEDM VK					Mascot
2377.2246	2377.1819	-0.0427	-18	94	115	DVLDGSSVGFVETLENDL AKLR					Mascot

9 uncharacterized protein, partial [Phleum pratense] | gi|409972221 | 25974.2 | 5.68 | 9 | 170 | 100 | 8.915 | 136 | 100

Peptide Information

Calc. Mass	Obsrv. Mass	± da	± ppm	Start Seq.	End Seq.	Sequence	Ion Score	C. I. %	Modification	Rank	Result Type
896.4724	896.4455	-0.0269	-30	142	149	TSGEYLVK					Mascot
947.3961	947.4465	0.0504	53	8	14	MYVTMDR			Oxidation (M)[1,5]		Mascot
1042.4874	1042.4751	-0.0123	-12	154	162	TFACSETVK			Carbamidomethyl (C)[4]		Mascot
1323.6328	1323.6545	0.0217	16	15	24	YENDWDVVKR					Mascot
1403.6511	1403.6001	-0.051	-36	116	127	YAGMLQYDGELK			Oxidation (M)[4]		Mascot
1481.7074	1481.6935	-0.0139	-9	163	174	FGHVTFFWNGNR					Mascot
1589.8071	1589.7869	-0.0202	-13	24	37	RGWDAQVLGEAPYK					Mascot
1744.8649	1744.8921	0.0272	16	91	105	MVMLAKALEFADFDK			Oxidation (M)[1]		Mascot
1792.9229	1792.9136	-0.0093	-5	71	87	SVGPIVDGDAVVTFNFR					Mascot
1792.9229	1792.9136	-0.0093	-5	71	87	SVGPIVDGDAVVTFNFR	136	100			Mascot

10 PREDICTED: 2,3-bisphosphoglycerate-independent | gi|502104660 | 61421.2 | 5.34 | 12 | 162 | 100 | 10.387 | 136 | 100

phosphoglycerate mutase-like [Cicer arietinum]

Peptide Information

Calc. Mass	Obsrv. Mass	± da	± ppm	Start Seq.	End Seq.	Sequence	Ion Score	C. I. %	Modification	Rank	Result Type
924.5261	924.5121	-0.014	-15	163	170	LHILTDGR					Mascot
947.3961	947.4465	0.0504	53	207	213	MYVTMDR			Oxidation (M)[1,5]		Mascot
1042.4874	1042.4751	-0.0123	-12	353	361	TFACSETVK			Carbamidomethyl (C)[4]		Mascot
1139.5369	1139.5653	0.0284	25	214	222	YENDWSVVK					Mascot
1481.7074	1481.6935	-0.0139	-9	362	373	FGHVTFFWNGNR					Mascot
1497.6969	1497.7102	0.0133	9	296	307	ALENENFDKFDR					Mascot
1513.725	1513.6871	-0.0379	-25	200	213	IASGGGRMYVTMDR					Mascot
1589.8071	1589.7869	-0.0202	-13	223	236	RGWDAQVLGEAPYK					Mascot
1652.937	1652.7756	-0.1614	-98	19	34	GKPIAVIVLDGWGEAK					Mascot
1792.9229	1792.9136	-0.0093	-5	270	286	SVGPIVDGDAVVTFNFR					Mascot
1792.9229	1792.9136	-0.0093	-5	270	286	SVGPIVDGDAVVTFNFR	136	100			Mascot
1891.9436	1891.907	-0.0366	-19	253	269	ANDQYLPPFVIVDESGK					Mascot
2035.9253	2035.9226	-0.0027	-1	207	222	MYVTMDRYENDWSVVK					Mascot

Gel Idx/Pos	288/L16	Instr./Gel Origin	BA2151/Sample Project 20140814	Process Status	Analysis Succeeded
Plate [#] Name	[1] Sample Project 20140814	Instrument Sample Name		Spectra	11

Rank	Protein Name	Accession No.	Protein MW	Protein PI	Pep. Count	Protein Score	Protein Score C. I. %	Intensity Matched	Total Ion Score	Total Ion C. I. %	Confirmed
1	Alanine aminotransferase 2 [Aegilops tauschii]	gi|475595366	56452	6.2	20	439	100	36.801	327	100	

Peptide Information

Calc. Mass	Obsrv. Mass	± da	± ppm	Start Seq.	End Seq.	Sequence	Ion Score	C. I. %	Modification	Rank	Result Type
915.5258	915.5275	0.0017	2	391	399	DGILASLAR					Mascot
960.5109	960.5087	-0.0022	-2	123	132	DAIASGIASR					Mascot
980.6251	980.6287	0.0036	4	99	107	QILALIPGR					Mascot
1022.5741	1022.5752	0.0011	1	23	31	GEIVIHAQR					Mascot
1023.5258	1023.5353	0.0095	9	445	453	APDAFYALR					Mascot
1023.5258	1023.5353	0.0095	9	445	453	APDAFYALR	64	99.941			Mascot
1089.4783	1089.488	0.0097	9	326	334	GYYGECGKR			Carbamidomethyl (C)[6]		Mascot
1096.5634	1096.5681	0.0047	4	87	96	TLFSADSISR					Mascot
1243.7004	1243.7025	0.0021	2	388	399	AEKDGILASLAR					Mascot
1407.7379	1407.7445	0.0066	5	441	453	AANKAPDAFYALR					Mascot
1484.7203	1484.7324	0.0121	8	335	348	GGYMEITGFSAPVR					Mascot
1500.7152	1500.7115	-0.0037	-2	335	348	GGYMEITGFSAPVR			Oxidation (M)[4]		Mascot
1500.7152	1500.7115	-0.0037	-2	335	348	GGYMEITGFSAPVR	74	99.993	Oxidation (M)[4]		Mascot
1522.7935	1522.7615	-0.032	-21	69	81	EVLALCDHPDLLK			Carbamidomethyl (C)[6]		Mascot
1640.8214	1640.8143	-0.0071	-4	334	348	RGGYMEITGFSAPVR					Mascot
1650.8884	1650.7999	-0.0885	-54	69	82	EVLALCDHPDLLKK			Carbamidomethyl (C)[6]		Mascot
1656.8163	1656.8071	-0.0092	-6	334	348	RGGYMEITGFSAPVR			Oxidation (M)[5]		Mascot
1677.7731	1677.8005	0.0274	16	495	507	FTVFHETFMAEYR					Mascot
1693.7679	1693.7909	0.023	14	495	507	FTVFHETFMAEYR			Oxidation (M)[9]		Mascot
1792.8	1792.8221	0.0221	12	495	508	FTVFHETFMAEYRD					Mascot
1792.8	1792.8221	0.0221	12	495	508	FTVFHETFMAEYRD	55	99.448			Mascot
1808.7949	1808.8041	0.0092	5	495	508	FTVFHETFMAEYRD			Oxidation (M)[9]		Mascot
1808.7949	1808.8041	0.0092	5	495	508	FTVFHETFMAEYRD	36	56.936	Oxidation (M)[9]		Mascot
1868.031	1867.9723	-0.0587	-31	479	494	CTILPQEEKIPAIISR			Carbamidomethyl (C)[1]		Mascot
1881.9238	1881.8962	-0.0276	-15	419	434	AEGAMYLFPQICLPQK			Carbamidomethyl (C)[12], Oxidation (M)[5]		Mascot
2538.2722	2538.3054	0.0332	13	276	297	NEGLVLLADEVYQENIYA DNKK					Mascot
2640.3933	2640.4429	0.0496	19	454	478	LLESTGIVVVPGSGFGQV					Mascot

Calc. Mass	Obsrv. Mass	± da	± ppm	Start Seq.	End Seq.	Sequence	Ion Score	C. I. %	Modification	Rank	Result Type
						PGTWHFR					
2640.3933	2640.4429	0.0496	19	454	478	LLESTGIVVVPGSGFGQV PGTWHFR	134	100			Mascot
3146.5286	3146.6016	0.073	23	133	161	DGFPANADDIFLTDGASP GVHLMMQLLIR			Oxidation (M)[23,24]		Mascot

2 Alanine aminotransferase 2 [Triticum urartu] gi|473789790 57750.7 6.77 20 437 100 36.801 327 100

Peptide Information

Calc. Mass	Obsrv. Mass	± da	± ppm	Start Seq.	End Seq.	Sequence	Ion Score	C. I. %	Modification	Rank	Result Type
915.5258	915.5275	0.0017	2	402	410	DGILASLAR					Mascot
960.5109	960.5087	-0.0022	-2	134	143	DAIASGIASR					Mascot
980.6251	980.6287	0.0036	4	110	118	QILALIPGR					Mascot
1022.5741	1022.5752	0.0011	1	34	42	GEIVIHAQR					Mascot
1023.5258	1023.5353	0.0095	9	456	464	APDAFYALR					Mascot
1023.5258	1023.5353	0.0095	9	456	464	APDAFYALR	64	99.941			Mascot
1089.4783	1089.488	0.0097	9	337	345	GYYGECGKR			Carbamidomethyl (C)[6]		Mascot
1096.5634	1096.5681	0.0047	4	98	107	TLFSADSISR					Mascot
1243.7004	1243.7025	0.0021	2	399	410	AEKDGILASLAR					Mascot
1407.7379	1407.7445	0.0066	5	452	464	AANKAPDAFYALR					Mascot
1484.7203	1484.7324	0.0121	8	346	359	GGYMEITGFSAPVR					Mascot
1500.7152	1500.7115	-0.0037	-2	346	359	GGYMEITGFSAPVR			Oxidation (M)[4]		Mascot
1500.7152	1500.7115	-0.0037	-2	346	359	GGYMEITGFSAPVR	74	99.993	Oxidation (M)[4]		Mascot
1522.7935	1522.7615	-0.032	-21	80	92	EVLALCDHPDLLK			Carbamidomethyl (C)[6]		Mascot
1640.8214	1640.8143	-0.0071	-4	345	359	RGGYMEITGFSAPVR					Mascot
1650.8884	1650.7999	-0.0885	-54	80	93	EVLALCDHPDLLKK			Carbamidomethyl (C)[6]		Mascot
1656.8163	1656.8071	-0.0092	-6	345	359	RGGYMEITGFSAPVR			Oxidation (M)[5]		Mascot
1677.7731	1677.8005	0.0274	16	506	518	FTVFHETFMAEYR					Mascot
1693.7679	1693.7909	0.023	14	506	518	FTVFHETFMAEYR			Oxidation (M)[9]		Mascot
1792.8	1792.8221	0.0221	12	506	519	FTVFHETFMAEYRD					Mascot
1792.8	1792.8221	0.0221	12	506	519	FTVFHETFMAEYRD	55	99.448			Mascot
1808.7949	1808.8041	0.0092	5	506	519	FTVFHETFMAEYRD			Oxidation (M)[9]		Mascot
1808.7949	1808.8041	0.0092	5	506	519	FTVFHETFMAEYRD	36	56.936	Oxidation (M)[9]		Mascot
1868.031	1867.9723	-0.0587	-31	490	505	CTILPQEEKIPAIISR			Carbamidomethyl (C)[1]		Mascot
1881.9238	1881.8962	-0.0276	-15	430	445	AEGAMYLFPQICLPQK			Carbamidomethyl (C)[12], Oxidation (M)[5]		Mascot
2538.2722	2538.3054	0.0332	13	287	308	NEGLVLLADEVYQENIYA DNKK					Mascot
2640.3933	2640.4429	0.0496	19	465	489	LLESTGIVVVPGSGFGQV PGTWHFR					Mascot
2640.3933	2640.4429	0.0496	19	465	489	LLESTGIVVVPGSGFGQV PGTWHFR	134	100			Mascot

3146.5286	3146.6016	0.073	23	144	172	DGFPANADDIFLTDGASP GVHLMMQLLIR			Oxidation (M)[23,24]		Mascot

3	RecName: Full=Alanine aminotransferase 2; Short=ALAAT-2; AltName: Full=Glutamate pyruvate transaminase 2; Short=GPT; AltName: Full=Glutamic--alanine transaminase 2; AltName: Full=Glutamic--pyruvic transaminase 2	gi|1703227	53357.3	5.93	14	320	100	23.979	260	100

Peptide Information

Calc. Mass	Obsrv. Mass	± da	± ppm	Start Seq.	End Seq.	Sequence	Ion Score	C. I. %	Modification	Rank	Result Type
915.5258	915.5275	0.0017	2	365	373	DGILASLAR					Mascot
960.5109	960.5087	-0.0022	-2	123	132	DAIASGIASR					Mascot
1022.5741	1022.5752	0.0011	1	23	31	GEIVIHAQR					Mascot
1023.5258	1023.5353	0.0095	9	419	427	APDAFYALR					Mascot
1023.5258	1023.5353	0.0095	9	419	427	APDAFYALR	64	99.941			Mascot
1089.4783	1089.488	0.0097	9	300	308	GYYGECGKR			Carbamidomethyl (C)[6]		Mascot
1096.5634	1096.5681	0.0047	4	87	96	TLFSADSISR					Mascot
1197.7136	1197.5985	-0.1151	-96	97	107	AKQILAMIPGR					Mascot
1213.7086	1213.5964	-0.1122	-92	97	107	AKQILAMIPGR			Oxidation (M)[7]		Mascot
1243.7004	1243.7025	0.0021	2	362	373	AEKDGILASLAR					Mascot
1407.7379	1407.7445	0.0066	5	415	427	AANKAPDAFYALR					Mascot
1500.7482	1500.7115	-0.0367	-24	309	322	GGYFEITGFSAPVR					Mascot
1500.7482	1500.7115	-0.0367	-24	309	322	GGYFEITGFSAPVR	61	99.883			Mascot
1656.8494	1656.8071	-0.0423	-26	308	322	RGGYFEITGFSAPVR					Mascot
1778.7843	1778.8208	0.0365	21	469	482	FTVFHEAFMSEYRD					Mascot
2640.3933	2640.4429	0.0496	19	428	452	LLESTGIVVVPGSGFGQV PGTWHFR					Mascot
2640.3933	2640.4429	0.0496	19	428	452	LLESTGIVVVPGSGFGQV PGTWHFR	134	100			Mascot
3146.5286	3146.6016	0.073	23	133	161	DGFPANADDIFLTDGASP GVHLMMQLLIR			Oxidation (M)[23,24]		Mascot

4	Chain A, Crystal Structure Of Alanine Aminotransferase From Hordeum Vulgare	gi|395759273	55614.1	6.06	14	319	100	23.979	260	100

Protein Group

Chain B, Crystal Structure Of Alanine Aminotransferase From Hordeum Vulgare	gi|395759274	55614.1	6.0599 999427 7954

Peptide Information

Calc. Mass	Obsrv. Mass	± da	± ppm	Start Seq.	End Seq.	Sequence	Ion Score	C. I. %	Modification	Rank	Result Type

915.5258	915.5275	0.0017	2	383	391	DGILASLAR					Mascot
960.5109	960.5087	-0.0022	-2	141	150	DAIASGIASR					Mascot
1022.5741	1022.5752	0.0011	1	41	49	GEIVIHAQR					Mascot
1023.5258	1023.5353	0.0095	9	437	445	APDAFYALR					Mascot
1023.5258	1023.5353	0.0095	9	437	445	APDAFYALR	64	99.941			Mascot
1089.4783	1089.488	0.0097	9	318	326	GYYGECGKR			Carbamidomethyl (C)[6]		Mascot
1096.5634	1096.5681	0.0047	4	105	114	TLFSADSISR					Mascot
1197.7136	1197.5985	-0.1151	-96	115	125	AKQILAMIPGR					Mascot
1213.7086	1213.5964	-0.1122	-92	115	125	AKQILAMIPGR			Oxidation (M)[7]		Mascot
1243.7004	1243.7025	0.0021	2	380	391	AEKDGILASLAR					Mascot
1407.7379	1407.7445	0.0066	5	433	445	AANKAPDAFYALR					Mascot
1500.7482	1500.7115	-0.0367	-24	327	340	GGYFEITGFSAPVR					Mascot
1500.7482	1500.7115	-0.0367	-24	327	340	GGYFEITGFSAPVR	61	99.883			Mascot
1656.8494	1656.8071	-0.0423	-26	326	340	RGGYFEITGFSAPVR					Mascot
1778.7843	1778.8208	0.0365	21	487	500	FTVFHEAFMSEYRD					Mascot
2640.3933	2640.4429	0.0496	19	446	470	LLESTGIVVVPGSGFGQV PGTWHFR					Mascot
2640.3933	2640.4429	0.0496	19	446	470	LLESTGIVVVPGSGFGQV PGTWHFR	134	100			Mascot
3146.5286	3146.6016	0.073	23	151	179	DGFPANADDIFLTDGASP GVHLMMQLLIR			Oxidation (M)[23,24]		Mascot

5	PREDICTED: alanine aminotransferase 2-like [Setaria italica]	gi|514816727	53256.3	5.99	8	161	100	22.132	138	100

Peptide Information

Calc. Mass	Obsrv. Mass	± da	± ppm	Start Seq.	End Seq.	Sequence	Ion Score	C. I. %	Modification	Rank	Result Type
1022.5741	1022.5752	0.0011	1	23	31	GEIVIHAQR					Mascot
1023.5258	1023.5353	0.0095	9	419	427	APDAFYALR					Mascot
1023.5258	1023.5353	0.0095	9	419	427	APDAFYALR	64	99.941			Mascot
1089.4783	1089.488	0.0097	9	300	308	GYYGECGKR			Carbamidomethyl (C)[6]		Mascot
1484.7203	1484.7324	0.0121	8	309	322	GGYMEITGFSAPVR					Mascot
1500.7152	1500.7115	-0.0037	-2	309	322	GGYMEITGFSAPVR			Oxidation (M)[4]		Mascot
1500.7152	1500.7115	-0.0037	-2	309	322	GGYMEITGFSAPVR	74	99.993	Oxidation (M)[4]		Mascot
1640.8214	1640.8143	-0.0071	-4	308	322	RGGYMEITGFSAPVR					Mascot
1656.8163	1656.8071	-0.0092	-6	308	322	RGGYMEITGFSAPVR			Oxidation (M)[5]		Mascot
1808.8484	1808.8041	-0.0443	-24	377	392	ALEDAFNNLEGITCNK			Carbamidomethyl (C)[14]		Mascot
1808.8484	1808.8041	-0.0443	-24	377	392	ALEDAFNNLEGITCNK			Carbamidomethyl (C)[14]		Mascot
1868.031	1867.9723	-0.0587	-31	453	468	CTILPQEEKIPAVITR			Carbamidomethyl (C)[1]		Mascot

3146.5286	3146.6016	0.073	23	133	161	DGFPANADDIFITDGASP GVHLMMQLLIR			Oxidation (M)[23,24]		Mascot

6	hypothetical protein OsI_33417 [Oryza sativa Indica Group]	gi|218184449	53130.1	6.23	8	159	100	16.675	138	100	

Protein Group

Os10g0390500 [Oryza sativa Japonica Group]	gi|113639113	53130.1	6.2300 000190 7349

Peptide Information

Calc. Mass	Obsrv. Mass	± da	± ppm	Start Seq.	End Seq.	Sequence	Ion Score	C. I. %	Modification	Rank	Result Type
1022.5741	1022.5752	0.0011	1	24	32	GEIVIHAQR					Mascot
1023.5258	1023.5353	0.0095	9	420	428	APDAFYALR					Mascot
1023.5258	1023.5353	0.0095	9	420	428	APDAFYALR	64	99.941			Mascot
1089.4783	1089.488	0.0097	9	301	309	GYYGECGKR			Carbamidomethyl (C)[6]		Mascot
1407.7379	1407.7445	0.0066	5	416	428	AANKAPDAFYALR					Mascot
1484.7203	1484.7324	0.0121	8	310	323	GGYMEITGFSAPVR					Mascot
1500.7152	1500.7115	-0.0037	-2	310	323	GGYMEITGFSAPVR			Oxidation (M)[4]		Mascot
1500.7152	1500.7115	-0.0037	-2	310	323	GGYMEITGFSAPVR	74	99.993	Oxidation (M)[4]		Mascot
1640.8214	1640.8143	-0.0071	-4	309	323	RGGYMEITGFSAPVR					Mascot
1656.8163	1656.8071	-0.0092	-6	309	323	RGGYMEITGFSAPVR			Oxidation (M)[5]		Mascot
1868.031	1867.9723	-0.0587	-31	454	469	CTILPQEEKIPAIISR			Carbamidomethyl (C)[1]		Mascot
2408.2529	2408.2119	-0.041	-17	394	415	TEGAMYLFPQLSLPQKAI DAAK			Oxidation (M)[5]		Mascot

7	RecName: Full=Alanine aminotransferase 2; Short=ALAAT-2; AltName: Full=Glutamate pyruvate transaminase 2; Short=GPT; AltName: Full=Glutamic--alanine transaminase 2; AltName: Full=Glutamic--pyruvic transaminase 2	gi|461498	53276.1	5.82	7	154	100	14.486	138	100	

Peptide Information

Calc. Mass	Obsrv. Mass	± da	± ppm	Start Seq.	End Seq.	Sequence	Ion Score	C. I. %	Modification	Rank	Result Type
1023.5258	1023.5353	0.0095	9	419	427	APDAFYALR					Mascot
1023.5258	1023.5353	0.0095	9	419	427	APDAFYALR	64	99.941			Mascot
1089.4783	1089.488	0.0097	9	300	308	GYYGECGKR			Carbamidomethyl (C)[6]		Mascot
1373.7094	1373.7268	0.0174	13	1	13	MAATVAVENLNPK			Oxidation (M)[1]		Mascot
1407.7379	1407.7445	0.0066	5	415	427	AANKAPDAFYALR					Mascot

1484.7203	1484.7324	0.0121	8	309	322	GGYMEITGFSAPVR					Mascot
1500.7152	1500.7115	-0.0037	-2	309	322	GGYMEITGFSAPVR			Oxidation (M)[4]		Mascot
1500.7152	1500.7115	-0.0037	-2	309	322	GGYMEITGFSAPVR	74	99.993	Oxidation (M)[4]		Mascot
1640.8214	1640.8143	-0.0071	-4	308	322	RGGYMEITGFSAPVR					Mascot
1656.8163	1656.8071	-0.0092	-6	308	322	RGGYMEITGFSAPVR			Oxidation (M)[5]		Mascot
1714.8986	1714.823	-0.0756	-44	393	407	AEGAMYLFPQIHLPK					Mascot

8 hypothetical protein ZEAMMB73_492015 [Zea mays] gi|413934298 22164.3 6.74 4 153 100 13.953 138 100

Peptide Information

Calc. Mass	Obsrv. Mass	± da	± ppm	Start Seq.	End Seq.	Sequence	Ion Score	C. I. %	Modification	Rank	Result Type
1023.5258	1023.5353	0.0095	9	138	146	APDAFYALR					Mascot
1023.5258	1023.5353	0.0095	9	138	146	APDAFYALR	64	99.941			Mascot
1089.4783	1089.488	0.0097	9	19	27	GYYGECGKR			Carbamidomethyl (C)[6]		Mascot
1484.7203	1484.7324	0.0121	8	28	41	GGYMEITGFSAPVR					Mascot
1500.7152	1500.7115	-0.0037	-2	28	41	GGYMEITGFSAPVR			Oxidation (M)[4]		Mascot
1500.7152	1500.7115	-0.0037	-2	28	41	GGYMEITGFSAPVR	74	99.993	Oxidation (M)[4]		Mascot
1640.8214	1640.8143	-0.0071	-4	27	41	RGGYMEITGFSAPVR					Mascot
1656.8163	1656.8071	-0.0092	-6	27	41	RGGYMEITGFSAPVR			Oxidation (M)[5]		Mascot

9 alanine aminotransferase [Zea mays] gi|413934299 53391.3 6.07 6 150 100 15.837 138 100

Peptide Information

Calc. Mass	Obsrv. Mass	± da	± ppm	Start Seq.	End Seq.	Sequence	Ion Score	C. I. %	Modification	Rank	Result Type
1022.5741	1022.5752	0.0011	1	23	31	GEIVIHAQR					Mascot
1023.5258	1023.5353	0.0095	9	419	427	APDAFYALR					Mascot
1023.5258	1023.5353	0.0095	9	419	427	APDAFYALR	64	99.941			Mascot
1089.4783	1089.488	0.0097	9	300	308	GYYGECGKR			Carbamidomethyl (C)[6]		Mascot
1373.7094	1373.7268	0.0174	13	1	13	MAASVTVENLNPK					Mascot
1484.7203	1484.7324	0.0121	8	309	322	GGYMEITGFSAPVR					Mascot
1500.7152	1500.7115	-0.0037	-2	309	322	GGYMEITGFSAPVR			Oxidation (M)[4]		Mascot
1500.7152	1500.7115	-0.0037	-2	309	322	GGYMEITGFSAPVR	74	99.993	Oxidation (M)[4]		Mascot
1640.8214	1640.8143	-0.0071	-4	308	322	RGGYMEITGFSAPVR					Mascot
1656.8163	1656.8071	-0.0092	-6	308	322	RGGYMEITGFSAPVR			Oxidation (M)[5]		Mascot

10 LOC100282849 [Zea mays] gi|226493655 53570.4 6.23 5 72 93.998 12.003 64 99.941

Peptide Information

Calc. Mass	Obsrv. Mass	± da	± ppm	Start	End	Sequence	Ion	C. I. %	Modification	Rank	Result Type

				Seq.	Seq.		Score			
1022.5741	1022.5752	0.0011	1	23	31	GEIVIHAQR				Mascot
1023.5258	1023.5353	0.0095	9	419	427	APDAFYALR				Mascot
1023.5258	1023.5353	0.0095	9	419	427	APDAFYALR	64	99.914		Mascot
1089.4783	1089.488	0.0097	9	300	308	GYYGECGKR			Carbamidomethyl (C)[6]	Mascot
1373.7094	1373.7268	0.0174	13	1	13	MAASVTVENLNPK				Mascot
1656.8281	1656.8071	-0.021	-13	469	481	FRAFHEAFLAEYR				Mascot

Gel Idx/Pos	289/L17	Instr./Gel Origin	BA2151/Sample Project 20140814	Process Status	Analysis Succeeded
Plate [#] Name	[1] Sample Project 20140814	Instrument Sample Name		Spectra	11

Rank	Protein Name	Accession No.	Protein MW	Protein PI	Pep. Count	Protein Score	Protein Score C. I. %	Intensity Matched	Total Ion Score	Total Ion C. I. %	Confirmed
1	ribulose 1,5-bisphosphate carboxylase/oxygenase large subunit (chloroplast) [Aegilops speltoides]	gi|394986500	53444.6	6.22	28	858	100	58.535	655	100	

Protein Group

RecName: Full=Ribulose bisphosphate carboxylase large chain; Short=RuBisCO large subunit; Flags: Precursor	gi|132065	53444.6	6.2199997901 9165
ribulose 1,5-bisphosphate carboxylase/oxygenase large subunit (chloroplast) [Aegilops speltoides]	gi|533310209	53444.6	6.2199997901 9165
ribulose-1,5-bisphosphate carboxylase/oxygenase large subunit (chloroplast) [Triticum aestivum]	gi|521301495	53444.6	6.2199997901 9165
ribulose-1,5-bisphosphate carboxylase/oxygenase large subunit [Triticum aestivum]	gi|14017580	53444.6	6.2199997901 9165

Peptide Information

Calc. Mass	Obsrv. Mass	± da	± ppm	Start Seq.	End Seq.	Sequence	Ion Score	C. I. %	Modification	Rank	Result Type
830.4366	830.4283	-0.0083	-10	440	446	EGNEIIR					Mascot
898.41	898.4123	0.0023	3	306	312	NHGMHFR					Mascot
912.472	912.4646	-0.0074	-8	296	303	AMHAVIDR					Mascot
914.405	914.3882	-0.0168	-18	306	312	NHGMHFR			Oxidation (M)[4]		Mascot
928.4669	928.441	-0.0259	-28	296	303	AMHAVIDR			Oxidation (M)[2]		Mascot
971.4073	971.4023	-0.005	-5	188	194	ACYECLR			Carbamidomethyl (C)[2,5]		Mascot
971.4073	971.4023	-0.005	-5	188	194	ACYECLR	24	0	Carbamidomethyl (C)[2,5]		Mascot
1021.5312	1021.5198	-0.0114	-11	33	41	DTDILAAFR					Mascot
1021.5312	1021.5198	-0.0114	-11	33	41	DTDILAAFR	56	99.427			Mascot
1037.4899	1037.4851	-0.0048	-5	351	358	DDFIEKDR					Mascot

1116.583	1116.5812	-0.0018	-2	422	431	VALEACVQAR			Carbamidomethyl (C)[6]	Mascot
1170.5586	1170.6375	0.0789	67	304	312	QKNHGMHFR			Oxidation (M)[6]	Mascot
1170.5586	1170.6375	0.0789	67	304	312	QKNHGMHFR			Oxidation (M)[6]	Mascot
1187.6644	1187.6611	-0.0033	-3	286	295	DNGLLLHIHR				Mascot
1247.6129	1247.5988	-0.0141	-11	218	227	FVFCAEAIYK			Carbamidomethyl (C)[4]	Mascot
1285.6859	1285.5776	-0.1083	-84	436	446	DLAREGNEIIR				Mascot
1293.6871	1293.6863	-0.0008	-1	340	350	EMTLGFVDLLR	90	100		Mascot
1309.6821	1309.6604	-0.0217	-17	340	350	EMTLGFVDLLR			Oxidation (M)[2]	Mascot
1325.6896	1325.6711	-0.0185	-14	306	316	NHGMHFRVLAK			Oxidation (M)[4]	Mascot
1339.6417	1339.6206	-0.0211	-16	467	477	FEFEPVDTIDK				Mascot
1407.6678	1407.6458	-0.022	-16	22	32	LTYYTPEYETK				Mascot
1451.6219	1451.6394	0.0175	12	202	213	DDENVNSQPFMR				Mascot
1465.7546	1465.765	0.0104	7	147	159	TFQGPPHGIQVER				Mascot
1465.7546	1465.765	0.0104	7	147	159	TFQGPPHGIQVER	102	100		Mascot
1502.8512	1502.849	-0.0022	-1	165	177	YGRPLLGCTIKPK			Carbamidomethyl (C)[8]	Mascot
1502.8512	1502.849	-0.0022	-1	165	177	YGRPLLGCTIKPK	6	0	Carbamidomethyl (C)[8]	Mascot
1518.741	1518.7408	-0.0002	0	216	227	DRFVFCAEAIYK			Carbamidomethyl (C)[6]	Mascot
1546.736	1546.7114	-0.0246	-16	451	463	WSPELAAACEVWK			Carbamidomethyl (C)[9]	Mascot
1651.8578	1651.8324	-0.0254	-15	464	477	AIKFEFEPVDTIDK				Mascot
1893.9739	1893.9507	-0.0232	-12	335	350	LEGEREMTLGFVDLLR			Oxidation (M)[7]	Mascot
1950.916	1950.9291	0.0131	7	237	253	GHYLNATAGTCEEMIKR			Carbamidomethyl (C)[11]	Mascot
1966.911	1966.9098	-0.0012	-1	237	253	GHYLNATAGTCEEMIKR			Carbamidomethyl (C)[11], Oxidation (M)[14]	Mascot
2041.0311	2041.0352	0.0041	2	340	356	EMTLGFVDLLRDDFIEK				Mascot
2057.0261	2057.0132	-0.0129	-6	340	356	EMTLGFVDLLRDDFIEK			Oxidation (M)[2]	Mascot
2169.9871	2169.999	0.0119	5	195	213	GGLDFTKDDENVNSQPF MR				Mascot
2169.9871	2169.999	0.0119	5	195	213	GGLDFTKDDENVNSQPF MR	93	100		Mascot
2185.9819	2185.9814	-0.0005	0	195	213	GGLDFTKDDENVNSQPF MR			Oxidation (M)[18]	Mascot
2252.1824	2252.1777	-0.0047	-2	140	159	IPPTYSKTFQGPPHGIQV ER				Mascot
3036.4707	3036.5229	0.0522	17	259	285	ELGVPIVMHDYLTGGFTA NTTLAHYCR			Carbamidomethyl (C)[26]	Mascot
3036.4707	3036.5229	0.0522	17	259	285	ELGVPIVMHDYLTGGFTA NTTLAHYCR	159	100	Carbamidomethyl (C)[26]	Mascot
3052.4656	3052.5239	0.0583	19	259	285	ELGVPIVMHDYLTGGFTA NTTLAHYCR			Carbamidomethyl (C)[26], Oxidation (M)[8]	Mascot
3840.8564	3840.968	0.1116	29	42	79	VSPQPGVPPEEAGAAVA AESSTGTWTTVWTDGLT SLDR				Mascot
3840.8564	3840.968	0.1116	29	42	79	VSPQPGVPPEEAGAAVA AESSTGTWTTVWTDGLT SLDR	131	100		Mascot

2	ribulose-1,5-bisphosphate carboxylase/oxygenase large subunit (chloroplast) [Hordeum vulgare subsp. spontaneum]	gi|521301037	53671.8	6.22	28	856	100	58.535	655	100

Protein Group

RecName: Full=Ribulose bisphosphate carboxylase large chain; Short=RuBisCO large subunit; Flags: Precursor	gi|126302590	53671.8	6.2199997901<br>9165
ribulose 1,5-bisphosphate carboxylase/oxygenase large subunit [Hordeum vulgare subsp. vulgare]	gi|118430396	53671.8	6.2199997901<br>9165
ribulose-1,5-bisphosphate carboxylase/oxygenase large subunit (chloroplast) [Hordeum vulgare subsp. spontaneum]	gi|521301118	53671.8	6.2199997901<br>9165
ribulose-1,5-bisphosphate carboxylase/oxygenase large subunit (chloroplast) [Hordeum vulgare subsp. vulgare]	gi|521300960	53671.8	6.2199997901<br>9165

Peptide Information

Calc. Mass	Obsrv. Mass	± da	± ppm	Start Seq.	End Seq.	Sequence	Ion Score	C. I. %	Modification	Rank	Result Type
830.4366	830.4283	-0.0083	-10	440	446	EGNEIIR					Mascot
898.41	898.4123	0.0023	3	306	312	NHGMHFR					Mascot
912.472	912.4646	-0.0074	-8	296	303	AMHAVIDR					Mascot
914.405	914.3882	-0.0168	-18	306	312	NHGMHFR			Oxidation (M)[4]		Mascot
928.4669	928.441	-0.0259	-28	296	303	AMHAVIDR			Oxidation (M)[2]		Mascot
971.4073	971.4023	-0.005	-5	188	194	ACYECLR			Carbamidomethyl (C)[2,5]		Mascot
971.4073	971.4023	-0.005	-5	188	194	ACYECLR	24	0	Carbamidomethyl (C)[2,5]		Mascot
1021.5312	1021.5198	-0.0114	-11	33	41	DTDILAAFR					Mascot
1021.5312	1021.5198	-0.0114	-11	33	41	DTDILAAFR	56	99.427			Mascot
1037.4899	1037.4851	-0.0048	-5	351	358	DDFIEKDR					Mascot
1116.583	1116.5812	-0.0018	-2	422	431	VALEACVQAR			Carbamidomethyl (C)[6]		Mascot
1170.5586	1170.6375	0.0789	67	304	312	QKNHGMHFR			Oxidation (M)[6]		Mascot
1170.5586	1170.6375	0.0789	67	304	312	QKNHGMHFR			Oxidation (M)[6]		Mascot
1187.6644	1187.6611	-0.0033	-3	286	295	DNGLLLHIHR					Mascot

Calc. Mass	Obsrv. Mass	± da	± ppm	Start Seq.	End Seq.	Sequence	Ion Score	C. I. %	Modification	Rank	Result Type
1247.6129	1247.5988	-0.0141	-11	218	227	FVFCAEAIYK			Carbamidomethyl (C)[4]		Mascot
1285.6859	1285.5776	-0.1083	-84	436	446	DLAREGNEIIR					Mascot
1293.6871	1293.6863	-0.0008	-1	340	350	EMTLGFVDLLR	90	100			Mascot
1309.6821	1309.6604	-0.0217	-17	340	350	EMTLGFVDLLR			Oxidation (M)[2]		Mascot
1325.6896	1325.6711	-0.0185	-14	306	316	NHGMHFRVLAK			Oxidation (M)[4]		Mascot
1339.6417	1339.6206	-0.0211	-16	467	477	FEFEPVDTIDK					Mascot
1407.6678	1407.6458	-0.022	-16	22	32	LTYYTPEYETK					Mascot
1451.6219	1451.6394	0.0175	12	202	213	DDENVNSQPFMR					Mascot
1465.7546	1465.765	0.0104	7	147	159	TFQGPPHGIQVER					Mascot
1465.7546	1465.765	0.0104	7	147	159	TFQGPPHGIQVER	102	100			Mascot
1502.8512	1502.849	-0.0022	-1	165	177	YGRPLLGCTIKPK			Carbamidomethyl (C)[8]		Mascot
1502.8512	1502.849	-0.0022	-1	165	177	YGRPLLGCTIKPK	6	0	Carbamidomethyl (C)[8]		Mascot
1518.741	1518.7408	-0.0002	0	216	227	DRFVFCAEAIYK			Carbamidomethyl (C)[6]		Mascot
1546.736	1546.7114	-0.0246	-16	451	463	WSPELAAACEVWK			Carbamidomethyl (C)[9]		Mascot
1651.8578	1651.8324	-0.0254	-15	464	477	AIKFEFEPVDTIDK					Mascot
1893.9739	1893.9507	-0.0232	-12	335	350	LEGEREMTLGFVDLLR			Oxidation (M)[7]		Mascot
1950.916	1950.9291	0.0131	7	237	253	GHYLNATAGTCEEMIKR			Carbamidomethyl (C)[11]		Mascot
1966.911	1966.9098	-0.0012	-1	237	253	GHYLNATAGTCEEMIKR			Carbamidomethyl (C)[11], Oxidation (M)[14]		Mascot
2041.0311	2041.0352	0.0041	2	340	356	EMTLGFVDLLRDDFIEK					Mascot
2057.0261	2057.0132	-0.0129	-6	340	356	EMTLGFVDLLRDDFIEK			Oxidation (M)[2]		Mascot
2169.9871	2169.999	0.0119	5	195	213	GGLDFTKDDENVNSQPF MR					Mascot
2169.9871	2169.999	0.0119	5	195	213	GGLDFTKDDENVNSQPF MR	93	100			Mascot
2185.9819	2185.9814	-0.0005	0	195	213	GGLDFTKDDENVNSQPF MR			Oxidation (M)[18]		Mascot
2252.1824	2252.1777	-0.0047	-2	140	159	IPPTYSKTFQGPPHGIQV ER					Mascot
3036.4707	3036.5229	0.0522	17	259	285	ELGVPIVMHDYLTGGFTA NTTLAHYCR			Carbamidomethyl (C)[26]		Mascot
3036.4707	3036.5229	0.0522	17	259	285	ELGVPIVMHDYLTGGFTA NTTLAHYCR	159	100	Carbamidomethyl (C)[26]		Mascot
3052.4656	3052.5239	0.0583	19	259	285	ELGVPIVMHDYLTGGFTA NTTLAHYCR			Carbamidomethyl (C)[26], Oxidation (M)[8]		Mascot
3840.8564	3840.968	0.1116	29	42	79	VSPQPGVPPEEAGAAVA AESSTGTWTTVWTDGLT SLDR					Mascot
3840.8564	3840.968	0.1116	29	42	79	VSPQPGVPPEEAGAAVA AESSTGTWTTVWTDGLT SLDR	131	100			Mascot

3 ribulose-1,5-bisphosphate carboxylase/oxygenase large subunit, partial (chloroplast) [Bromus ciliatus] gi|424949903 51579.6 6.23 27 853 100 58.232 654 100

Peptide Information

Calc. Mass	Obsrv. Mass	± da	± ppm	Start Seq.	End Seq.	Sequence	Ion Score	C. I. %	Modification	Rank	Result Type

830.4366	830.4283	-0.0083	-10	443	449	EGNEIIR				Mascot
898.41	898.4123	0.0023	3	309	315	NHGMHFR				Mascot
912.472	912.4646	-0.0074	-8	299	306	AMHAVIDR				Mascot
914.405	914.3882	-0.0168	-18	309	315	NHGMHFR			Oxidation (M)[4]	Mascot
928.4669	928.441	-0.0259	-28	299	306	AMHAVIDR			Oxidation (M)[2]	Mascot
971.4073	971.4023	-0.005	-5	191	197	ACYECLR			Carbamidomethyl (C)[2,5]	Mascot
971.4073	971.4023	-0.005	-5	191	197	ACYECLR	24	0	Carbamidomethyl (C)[2,5]	Mascot
1021.5312	1021.5198	-0.0114	-11	36	44	DTDILAAFR				Mascot
1021.5312	1021.5198	-0.0114	-11	36	44	DTDILAAFR	56	99.427		Mascot
1037.4899	1037.4851	-0.0048	-5	354	361	DDFIEKDR				Mascot
1116.583	1116.5812	-0.0018	-2	425	434	VALEACVQAR			Carbamidomethyl (C)[6]	Mascot
1170.5586	1170.6375	0.0789	67	307	315	QKNHGMHFR			Oxidation (M)[6]	Mascot
1170.5586	1170.6375	0.0789	67	307	315	QKNHGMHFR			Oxidation (M)[6]	Mascot
1187.6644	1187.6611	-0.0033	-3	289	298	DNGLLLHIHR				Mascot
1247.6129	1247.5988	-0.0141	-11	221	230	FVFCAEAIYK			Carbamidomethyl (C)[4]	Mascot
1285.6859	1285.5776	-0.1083	-84	439	449	DLAREGNEIIR				Mascot
1293.6871	1293.6863	-0.0008	-1	343	353	EMTLGFVDLLR	90	100		Mascot
1309.6821	1309.6604	-0.0217	-17	343	353	EMTLGFVDLLR			Oxidation (M)[2]	Mascot
1325.6896	1325.6711	-0.0185	-14	309	319	NHGMHFRVLAK			Oxidation (M)[4]	Mascot
1339.7006	1339.6206	-0.08	-60	12	24	AGVGFQAGVKDYK				Mascot
1407.6678	1407.6458	-0.022	-16	25	35	LTYYTPEYETK				Mascot
1451.6219	1451.6394	0.0175	12	205	216	DDENVNSQPFMR				Mascot
1464.661	1464.7468	0.0858	59	450	462	AACKWSPELAATC			Carbamidomethyl (C)[3,13]	Mascot
1465.7546	1465.765	0.0104	7	150	162	TFQGPPHGIQVER				Mascot
1465.7546	1465.765	0.0104	7	150	162	TFQGPPHGIQVER	102	100		Mascot
1502.8512	1502.849	-0.0022	-1	168	180	YGRPLLGCTIKPK			Carbamidomethyl (C)[8]	Mascot
1502.8512	1502.849	-0.0022	-1	168	180	YGRPLLGCTIKPK	6	0	Carbamidomethyl (C)[8]	Mascot
1518.741	1518.7408	-0.0002	0	219	230	DRFVFCAEAIYK			Carbamidomethyl (C)[6]	Mascot
1893.9739	1893.9507	-0.0232	-12	338	353	LEGEREMTLGFVDLLR			Oxidation (M)[7]	Mascot
1950.916	1950.9291	0.0131	7	240	256	GHYLNATAGTCEEMIKR			Carbamidomethyl (C)[11]	Mascot
1966.911	1966.9098	-0.0012	-1	240	256	GHYLNATAGTCEEMIKR			Carbamidomethyl (C)[11], Oxidation (M)[14]	Mascot
2041.0311	2041.0352	0.0041	2	343	359	EMTLGFVDLLRDDFIEK				Mascot
2057.0261	2057.0132	-0.0129	-6	343	359	EMTLGFVDLLRDDFIEK			Oxidation (M)[2]	Mascot
2169.9871	2169.999	0.0119	5	198	216	GGLDFTKDDENVNSQPF MR				Mascot
2169.9871	2169.999	0.0119	5	198	216	GGLDFTKDDENVNSQPF MR	93	100		Mascot

2185.9819	2185.9814	-0.0005	0	198	216	GGLDFTKDDENVNSQPFMR			Oxidation (M)[18]	Mascot
2252.1824	2252.1777	-0.0047	-2	143	162	IPPTYSKTFQGPPHGIQVER				Mascot
3036.4707	3036.5229	0.0522	17	262	288	ELGVPIVMHDYLTGGFTANTTLAHYCR			Carbamidomethyl (C)[26]	Mascot
3036.4707	3036.5229	0.0522	17	262	288	ELGVPIVMHDYLTGGFTANTTLAHYCR	159	100	Carbamidomethyl (C)[26]	Mascot
3052.4656	3052.5239	0.0583	19	262	288	ELGVPIVMHDYLTGGFTANTTLAHYCR			Carbamidomethyl (C)[26], Oxidation (M)[8]	Mascot
3840.8564	3840.968	0.1116	29	45	82	VSPQPGVPPEEAGAAVAAESSTGTWTTVWTDGLTSLDR				Mascot
3840.8564	3840.968	0.1116	29	45	82	VSPQPGVPPEEAGAAVAAESSTGTWTTVWTDGLTSLDR	131	100		Mascot

4	ribulose-1,5-bisphosphate carboxylase/oxygenase large subunit (chloroplast) [Secale cereale]	gi|525782224	53343.5	6.04	27	850	100	58.301	655	100

Protein Group

ribulose 1,5-bisphosphate carboxylase/oxygenase large subunit (chloroplast) [Aegilops tauschii]	gi|533310113	53343.5	6.03999996185303
ribulose 1,5-bisphosphate carboxylase/oxygenase large subunit (chloroplast) [Aegilops tauschii]	gi|384406860	53343.5	6.03999996185303
ribulose-1,5-bisphosphate carboxylase/oxygenase large subunit (chloroplast) [Secale cereale]	gi|521301278	53343.5	6.03999996185303
ribulose-1,5-bisphosphate carboxylase/oxygenase large subunit (chloroplast) [Triticum monococcum subsp. aegilopoides]	gi|521301355	53343.5	6.03999996185303
ribulose-1,5-bisphosphate carboxylase/oxygenase large subunit (chloroplast) [Triticum monococcum]	gi|525778513	53343.5	6.03999996185303
ribulose-1,5-bisphosphate carboxylase/oxygenase large subunit (chloroplast) [Triticum monococcum]	gi|521301198	53343.5	6.03999996185303

ribulose-1,5-bisphosphate carboxylase/oxygenase large subunit (chloroplast) [Triticum urartu]	gi|521301434	53343.5	6.0399 999618 5303
ribulose-1,5-bisphosphate carboxylase/oxygenase large subunit (chloroplast) [Triticum urartu]	gi|525782303	53343.5	6.0399 999618 5303

Peptide Information

Calc. Mass	Obsrv. Mass	± da	± ppm	Start Seq.	End Seq.	Sequence	Ion Score	C. I. %	Modification	Rank	Result Type
830.4366	830.4283	-0.0083	-10	440	446	EGNEIIR					Mascot
898.41	898.4123	0.0023	3	306	312	NHGMHFR					Mascot
912.472	912.4646	-0.0074	-8	296	303	AMHAVIDR					Mascot
914.405	914.3882	-0.0168	-18	306	312	NHGMHFR			Oxidation (M)[4]		Mascot
928.4669	928.441	-0.0259	-28	296	303	AMHAVIDR			Oxidation (M)[2]		Mascot
971.4073	971.4023	-0.005	-5	188	194	ACYECLR			Carbamidomethyl (C)[2,5]		Mascot
971.4073	971.4023	-0.005	-5	188	194	ACYECLR	24	0	Carbamidomethyl (C)[2,5]		Mascot
1021.5312	1021.5198	-0.0114	-11	33	41	DTDILAAFR					Mascot
1021.5312	1021.5198	-0.0114	-11	33	41	DTDILAAFR	56	99.427			Mascot
1037.4899	1037.4851	-0.0048	-5	351	358	DDFIEKDR					Mascot
1116.583	1116.5812	-0.0018	-2	422	431	VALEACVQAR			Carbamidomethyl (C)[6]		Mascot
1170.5586	1170.6375	0.0789	67	304	312	QKNHGMHFR			Oxidation (M)[6]		Mascot
1170.5586	1170.6375	0.0789	67	304	312	QKNHGMHFR			Oxidation (M)[6]		Mascot
1187.6644	1187.6611	-0.0033	-3	286	295	DNGLLLHIHR					Mascot
1247.6129	1247.5988	-0.0141	-11	218	227	FVFCAEAIYK			Carbamidomethyl (C)[4]		Mascot
1285.6859	1285.5776	-0.1083	-84	436	446	DLAREGNEIIR					Mascot
1293.6871	1293.6863	-0.0008	-1	340	350	EMTLGFVDLLR	90	100			Mascot
1309.6821	1309.6604	-0.0217	-17	340	350	EMTLGFVDLLR			Oxidation (M)[2]		Mascot
1325.6896	1325.6711	-0.0185	-14	306	316	NHGMHFRVLAK			Oxidation (M)[4]		Mascot
1339.7006	1339.6206	-0.08	-60	9	21	AGVGFQAGVKDYK					Mascot
1407.6678	1407.6458	-0.022	-16	22	32	LTYYTPEYETK					Mascot
1451.6219	1451.6394	0.0175	12	202	213	DDENVNSQPFMR					Mascot
1465.7546	1465.765	0.0104	7	147	159	TFQGPPHGIQVER					Mascot
1465.7546	1465.765	0.0104	7	147	159	TFQGPPHGIQVER	102	100			Mascot
1502.8512	1502.849	-0.0022	-1	165	177	YGRPLLGCTIKPK			Carbamidomethyl (C)[8]		Mascot
1502.8512	1502.849	-0.0022	-1	165	177	YGRPLLGCTIKPK	6	0	Carbamidomethyl (C)[8]		Mascot

1518.741	1518.7408	-0.0002	0	216	227	DRFVFCAEAIYK			Carbamidomethyl (C)[6]		Mascot
1546.736	1546.7114	-0.0246	-16	451	463	WSPELAAACEVWK			Carbamidomethyl (C)[9]		Mascot
1893.9739	1893.9507	-0.0232	-12	335	350	LEGEREMTLGFVDLLR			Oxidation (M)[7]		Mascot
1950.916	1950.9291	0.0131	7	237	253	GHYLNATAGTCEEMIKR			Carbamidomethyl (C)[11]		Mascot
1966.911	1966.9098	-0.0012	-1	237	253	GHYLNATAGTCEEMIKR			Carbamidomethyl (C)[11], Oxidation (M)[14]		Mascot
2041.0311	2041.0352	0.0041	2	340	356	EMTLGFVDLLRDDFIEK					Mascot
2057.0261	2057.0132	-0.0129	-6	340	356	EMTLGFVDLLRDDFIEK			Oxidation (M)[2]		Mascot
2169.9871	2169.999	0.0119	5	195	213	GGLDFTKDDENVNSQPFMR					Mascot
2169.9871	2169.999	0.0119	5	195	213	GGLDFTKDDENVNSQPFMR	93	100			Mascot
2185.9819	2185.9814	-0.0005	0	195	213	GGLDFTKDDENVNSQPFMR			Oxidation (M)[18]		Mascot
2252.1824	2252.1777	-0.0047	-2	140	159	IPPTYSKTFQGPPHGIQVER					Mascot
3036.4707	3036.5229	0.0522	17	259	285	ELGVPIVMHDYLTGGFTANTTLAHYCR			Carbamidomethyl (C)[26]		Mascot
3036.4707	3036.5229	0.0522	17	259	285	ELGVPIVMHDYLTGGFTANTTLAHYCR	159	100	Carbamidomethyl (C)[26]		Mascot
3052.4656	3052.5239	0.0583	19	259	285	ELGVPIVMHDYLTGGFTANTTLAHYCR			Carbamidomethyl (C)[26], Oxidation (M)[8]		Mascot
3840.8564	3840.968	0.1116	29	42	79	VSPQPGVPPEEAGAAVAAESSTGTWTTVWTDGLTSLDR					Mascot
3840.8564	3840.968	0.1116	29	42	79	VSPQPGVPPEEAGAAVAAESSTGTWTTVWTDGLTSLDR	131	100			Mascot

5 ribulose-bisphosphate carboxylase, partial (chloroplast) [Boissiera squarrosa] gi|406034800 50220 6.46 26 847 100 58.114 655 100

Peptide Information

Calc. Mass	Obsrv. Mass	± da	± ppm	Start Seq.	End Seq.	Sequence	Ion Score	C. I. %	Modification	Rank	Result Type
830.4366	830.4283	-0.0083	-10	433	439	EGNEIIR					Mascot
898.41	898.4123	0.0023	3	299	305	NHGMHFR					Mascot
912.472	912.4646	-0.0074	-8	289	296	AMHAVIDR					Mascot
914.405	914.3882	-0.0168	-18	299	305	NHGMHFR			Oxidation (M)[4]		Mascot
928.4669	928.441	-0.0259	-28	289	296	AMHAVIDR			Oxidation (M)[2]		Mascot
971.4073	971.4023	-0.005	-5	181	187	ACYECLR			Carbamidomethyl (C)[2,5]		Mascot
971.4073	971.4023	-0.005	-5	181	187	ACYECLR	24	0	Carbamidomethyl (C)[2,5]		Mascot
1021.5312	1021.5198	-0.0114	-11	26	34	DTDILAAFR					Mascot
1021.5312	1021.5198	-0.0114	-11	26	34	DTDILAAFR	56	99.427			Mascot
1037.4899	1037.4851	-0.0048	-5	344	351	DDFIEKDR					Mascot
1116.583	1116.5812	-0.0018	-2	415	424	VALEACVQAR			Carbamidomethyl (C)[6]		Mascot
1170.5586	1170.6375	0.0789	67	297	305	QKNHGMHFR			Oxidation (M)[6]		Mascot

Calc. Mass	Obsrv. Mass	± da	± ppm	Start	End	Sequence	Ion	C. I. %	Modification	Rank	Result Type
1170.5586	1170.6375	0.0789	67	297	305	QKNHGMHFR			Oxidation (M)[6]		Mascot
1187.6644	1187.6611	-0.0033	-3	279	288	DNGLLLHIHR					Mascot
1247.6129	1247.5988	-0.0141	-11	211	220	FVFCAEAIYK			Carbamidomethyl (C)[4]		Mascot
1285.6859	1285.5776	-0.1083	-84	429	439	DLAREGNEIIR					Mascot
1293.6871	1293.6863	-0.0008	-1	333	343	EMTLGFVDLLR	90	100			Mascot
1309.6821	1309.6604	-0.0217	-17	333	343	EMTLGFVDLLR			Oxidation (M)[2]		Mascot
1325.6896	1325.6711	-0.0185	-14	299	309	NHGMHFRVLAK			Oxidation (M)[4]		Mascot
1339.7006	1339.6206	-0.08	-60	2	14	AGVGFQAGVKDYK					Mascot
1407.6678	1407.6458	-0.022	-16	15	25	LTYYTPEYETK					Mascot
1451.6219	1451.6394	0.0175	12	195	206	DDENVNSQPFMR					Mascot
1465.7546	1465.765	0.0104	7	140	152	TFQGPPHGIQVER					Mascot
1465.7546	1465.765	0.0104	7	140	152	TFQGPPHGIQVER	102	100			Mascot
1502.8512	1502.849	-0.0022	-1	158	170	YGRPLLGCTIKPK			Carbamidomethyl (C)[8]		Mascot
1502.8512	1502.849	-0.0022	-1	158	170	YGRPLLGCTIKPK	6	0	Carbamidomethyl (C)[8]		Mascot
1518.741	1518.7408	-0.0002	0	209	220	DRFVFCAEAIYK			Carbamidomethyl (C)[6]		Mascot
1893.9739	1893.9507	-0.0232	-12	328	343	LEGEREMTLGFVDLLR			Oxidation (M)[7]		Mascot
1950.916	1950.9291	0.0131	7	230	246	GHYLNATAGTCEEMIKR			Carbamidomethyl (C)[11]		Mascot
1966.911	1966.9098	-0.0012	-1	230	246	GHYLNATAGTCEEMIKR			Carbamidomethyl (C)[11], Oxidation (M)[14]		Mascot
2041.0311	2041.0352	0.0041	2	333	349	EMTLGFVDLLRDDFIEK					Mascot
2057.0261	2057.0132	-0.0129	-6	333	349	EMTLGFVDLLRDDFIEK			Oxidation (M)[2]		Mascot
2169.9871	2169.999	0.0119	5	188	206	GGLDFTKDDENVNSQPF MR					Mascot
2169.9871	2169.999	0.0119	5	188	206	GGLDFTKDDENVNSQPF MR	93	100			Mascot
2185.9819	2185.9814	-0.0005	0	188	206	GGLDFTKDDENVNSQPF MR			Oxidation (M)[18]		Mascot
2252.1824	2252.1777	-0.0047	-2	133	152	IPPTYSKTFQGPPHGIQV ER					Mascot
3036.4707	3036.5229	0.0522	17	252	278	ELGVPIVMHDYLTGGFTA NTTLAHYCR			Carbamidomethyl (C)[26]		Mascot
3036.4707	3036.5229	0.0522	17	252	278	ELGVPIVMHDYLTGGFTA NTTLAHYCR	159	100	Carbamidomethyl (C)[26]		Mascot
3052.4656	3052.5239	0.0583	19	252	278	ELGVPIVMHDYLTGGFTA NTTLAHYCR			Carbamidomethyl (C)[26], Oxidation (M)[8]		Mascot
3840.8564	3840.968	0.1116	29	35	72	VSPQPGVPPEEAGAAVA AESSTGTWTTVWTDGLT SLDR					Mascot
3840.8564	3840.968	0.1116	29	35	72	VSPQPGVPPEEAGAAVA AESSTGTWTTVWTDGLT SLDR	131	100			Mascot

6	ribulose-1,5-bisphosphate carboxylase/oxygenase large subunit, partial (chloroplast) [Hordeum jubatum]	gi|424949923	50832.3	6.32	26	844	100	58.114	655	100	

Peptide Information

Calc. Mass	Obsrv. Mass	± da	± ppm	Start	End	Sequence	Ion	C. I. %	Modification	Rank	Result Type

				Seq.	Seq.		Score			
830.4366	830.4283	-0.0083	-10	439	445	EGNEIIR				Mascot
898.41	898.4123	0.0023	3	305	311	NHGMHFR				Mascot
912.472	912.4646	-0.0074	-8	295	302	AMHAVIDR				Mascot
914.405	914.3882	-0.0168	-18	305	311	NHGMHFR			Oxidation (M)[4]	Mascot
928.4669	928.441	-0.0259	-28	295	302	AMHAVIDR			Oxidation (M)[2]	Mascot
971.4073	971.4023	-0.005	-5	187	193	ACYECLR			Carbamidomethyl (C)[2,5]	Mascot
971.4073	971.4023	-0.005	-5	187	193	ACYECLR	24	0	Carbamidomethyl (C)[2,5]	Mascot
1021.5312	1021.5198	-0.0114	-11	32	40	DTDILAAFR				Mascot
1021.5312	1021.5198	-0.0114	-11	32	40	DTDILAAFR	56	99.427		Mascot
1037.4899	1037.4851	-0.0048	-5	350	357	DDFIEKDR				Mascot
1116.583	1116.5812	-0.0018	-2	421	430	VALEACVQAR			Carbamidomethyl (C)[6]	Mascot
1170.5586	1170.6375	0.0789	67	303	311	QKNHGMHFR			Oxidation (M)[6]	Mascot
1170.5586	1170.6375	0.0789	67	303	311	QKNHGMHFR			Oxidation (M)[6]	Mascot
1187.6644	1187.6611	-0.0033	-3	285	294	DNGLLLHIHR				Mascot
1247.6129	1247.5988	-0.0141	-11	217	226	FVFCAEAIYK			Carbamidomethyl (C)[4]	Mascot
1285.6859	1285.5776	-0.1083	-84	435	445	DLAREGNEIIR				Mascot
1293.6871	1293.6863	-0.0008	-1	339	349	EMTLGFVDLLR	90	100		Mascot
1309.6821	1309.6604	-0.0217	-17	339	349	EMTLGFVDLLR			Oxidation (M)[2]	Mascot
1325.6896	1325.6711	-0.0185	-14	305	315	NHGMHFRVLAK			Oxidation (M)[4]	Mascot
1339.7006	1339.6206	-0.08	-60	8	20	AGVGFQAGVKDYK				Mascot
1407.6678	1407.6458	-0.022	-16	21	31	LTYYTPEYETK				Mascot
1451.6219	1451.6394	0.0175	12	201	212	DDENVNSQPFMR				Mascot
1465.7546	1465.765	0.0104	7	146	158	TFQGPPHGIQVER				Mascot
1465.7546	1465.765	0.0104	7	146	158	TFQGPPHGIQVER	102	100		Mascot
1502.8512	1502.849	-0.0022	-1	164	176	YGRPLLGCTIKPK			Carbamidomethyl (C)[8]	Mascot
1502.8512	1502.849	-0.0022	-1	164	176	YGRPLLGCTIKPK	6	0	Carbamidomethyl (C)[8]	Mascot
1518.741	1518.7408	-0.0002	0	215	226	DRFVFCAEAIYK			Carbamidomethyl (C)[6]	Mascot
1893.9739	1893.9507	-0.0232	-12	334	349	LEGEREMTLGFVDLLR			Oxidation (M)[7]	Mascot
1950.916	1950.9291	0.0131	7	236	252	GHYLNATAGTCEEMIKR			Carbamidomethyl (C)[11]	Mascot
1966.911	1966.9098	-0.0012	-1	236	252	GHYLNATAGTCEEMIKR			Carbamidomethyl (C)[11], Oxidation (M)[14]	Mascot
2041.0311	2041.0352	0.0041	2	339	355	EMTLGFVDLLRDDFIEK				Mascot
2057.0261	2057.0132	-0.0129	-6	339	355	EMTLGFVDLLRDDFIEK			Oxidation (M)[2]	Mascot
2169.9871	2169.999	0.0119	5	194	212	GGLDFTKDDENVNSQPF<br>MR				Mascot
2169.9871	2169.999	0.0119	5	194	212	GGLDFTKDDENVNSQPF<br>MR	93	100		Mascot
2185.9819	2185.9814	-0.0005	0	194	212	GGLDFTKDDENVNSQPF			Oxidation (M)[18]	Mascot

Calc. Mass	Obsrv. Mass	± da	± ppm	Start Seq.	End Seq.	Sequence	Ion Score	C. I. %	Modification	Rank	Result Type
						MR					
2252.1824	2252.1777	-0.0047	-2	139	158	IPPTYSKTFQGPPHGIQVER					Mascot
3036.4707	3036.5229	0.0522	17	258	284	ELGVPIVMHDYLTGGFTANTTLAHYCR			Carbamidomethyl (C)[26]		Mascot
3036.4707	3036.5229	0.0522	17	258	284	ELGVPIVMHDYLTGGFTANTTLAHYCR	159	100	Carbamidomethyl (C)[26]		Mascot
3052.4656	3052.5239	0.0583	19	258	284	ELGVPIVMHDYLTGGFTANTTLAHYCR			Carbamidomethyl (C)[26], Oxidation (M)[8]		Mascot
3840.8564	3840.968	0.1116	29	41	78	VSPQPGVPPEEAGAAVAAESSTGTWTTVWTDGLTSLDR					Mascot
3840.8564	3840.968	0.1116	29	41	78	VSPQPGVPPEEAGAAVAAESSTGTWTTVWTDGLTSLDR	131	100			Mascot

7	ribulose-1,5-bisphosphate carboxylase/oxygenase large subunit, partial (chloroplast) [Bromus inermis]	gi|424949905	51708.7	6.14	26	841	100	58.114	654	100	

Peptide Information

Calc. Mass	Obsrv. Mass	± da	± ppm	Start Seq.	End Seq.	Sequence	Ion Score	C. I. %	Modification	Rank	Result Type
830.4366	830.4283	-0.0083	-10	443	449	EGNEIIR					Mascot
898.41	898.4123	0.0023	3	309	315	NHGMHFR					Mascot
912.472	912.4646	-0.0074	-8	299	306	AMHAVIDR					Mascot
914.405	914.3882	-0.0168	-18	309	315	NHGMHFR			Oxidation (M)[4]		Mascot
928.4669	928.441	-0.0259	-28	299	306	AMHAVIDR			Oxidation (M)[2]		Mascot
971.4073	971.4023	-0.005	-5	191	197	ACYECLR			Carbamidomethyl (C)[2,5]		Mascot
971.4073	971.4023	-0.005	-5	191	197	ACYECLR	24	0	Carbamidomethyl (C)[2,5]		Mascot
1021.5312	1021.5198	-0.0114	-11	36	44	DTDILAAFR					Mascot
1021.5312	1021.5198	-0.0114	-11	36	44	DTDILAAFR	56	99.427			Mascot
1037.4899	1037.4851	-0.0048	-5	354	361	DDFIEKDR					Mascot
1116.583	1116.5812	-0.0018	-2	425	434	VALEACVQAR			Carbamidomethyl (C)[6]		Mascot
1170.5586	1170.6375	0.0789	67	307	315	QKNHGMHFR			Oxidation (M)[6]		Mascot
1170.5586	1170.6375	0.0789	67	307	315	QKNHGMHFR			Oxidation (M)[6]		Mascot
1187.6644	1187.6611	-0.0033	-3	289	298	DNGLLLHIHR					Mascot
1247.6129	1247.5988	-0.0141	-11	221	230	FVFCAEAIYK			Carbamidomethyl (C)[4]		Mascot
1285.6859	1285.5776	-0.1083	-84	439	449	DLAREGNEIIR					Mascot
1293.6871	1293.6863	-0.0008	-1	343	353	EMTLGFVDLLR	90	100			Mascot
1309.6821	1309.6604	-0.0217	-17	343	353	EMTLGFVDLLR			Oxidation (M)[2]		Mascot
1325.6896	1325.6711	-0.0185	-14	309	319	NHGMHFRVLAK			Oxidation (M)[4]		Mascot
1339.7006	1339.6206	-0.08	-60	12	24	AGVGFQAGVKDYK					Mascot
1407.6678	1407.6458	-0.022	-16	25	35	LTYYTPEYETK					Mascot
1451.6219	1451.6394	0.0175	12	205	216	DDENVNSQPFMR					Mascot

1465.7546	1465.765	0.0104	7	150	162	TFQGPPHGIQVER					Mascot
1465.7546	1465.765	0.0104	7	150	162	TFQGPPHGIQVER	102	100			Mascot
1502.8512	1502.849	-0.0022	-1	168	180	YGRPLLGCTIKPK			Carbamidomethyl (C)[8]		Mascot
1502.8512	1502.849	-0.0022	-1	168	180	YGRPLLGCTIKPK	6	0	Carbamidomethyl (C)[8]		Mascot
1518.741	1518.7408	-0.0002	0	219	230	DRFVFCAEAIYK			Carbamidomethyl (C)[6]		Mascot
1893.9739	1893.9507	-0.0232	-12	338	353	LEGEREMTLGFVDLLR			Oxidation (M)[7]		Mascot
1950.916	1950.9291	0.0131	7	240	256	GHYLNATAGTCEEMIKR			Carbamidomethyl (C)[11]		Mascot
1966.911	1966.9098	-0.0012	-1	240	256	GHYLNATAGTCEEMIKR			Carbamidomethyl (C)[11], Oxidation (M)[14]		Mascot
2041.0311	2041.0352	0.0041	2	343	359	EMTLGFVDLLRDDFIEK					Mascot
2057.0261	2057.0132	-0.0129	-6	343	359	EMTLGFVDLLRDDFIEK			Oxidation (M)[2]		Mascot
2169.9871	2169.999	0.0119	5	198	216	GGLDFTKDDENVNSQPF MR					Mascot
2169.9871	2169.999	0.0119	5	198	216	GGLDFTKDDENVNSQPF MR	93	100			Mascot
2185.9819	2185.9814	-0.0005	0	198	216	GGLDFTKDDENVNSQPF MR			Oxidation (M)[18]		Mascot
2252.1824	2252.1777	-0.0047	-2	143	162	IPPTYSKTFQGPPHGIQV ER					Mascot
3036.4707	3036.5229	0.0522	17	262	288	ELGVPIVMHDYLTGGFTA NTTLAHYCR			Carbamidomethyl (C)[26]		Mascot
3036.4707	3036.5229	0.0522	17	262	288	ELGVPIVMHDYLTGGFTA NTTLAHYCR	159	100	Carbamidomethyl (C)[26]		Mascot
3052.4656	3052.5239	0.0583	19	262	288	ELGVPIVMHDYLTGGFTA NTTLAHYCR			Carbamidomethyl (C)[26], Oxidation (M)[8]		Mascot
3840.8564	3840.968	0.1116	29	45	82	VSPQPGVPPEEAGAAVA AESSTGTWTTVWTDGLT SLDR					Mascot
3840.8564	3840.968	0.1116	29	45	82	VSPQPGVPPEEAGAAVA AESSTGTWTTVWTDGLT SLDR	131	100			Mascot

8	ribulose-1,5-bisphosphate carboxylase/oxygenase large subunit, partial (chloroplast) [Agropyron cristatum]	gi|424949889	51264.5	6.32	26	840	100	58.062	655	100	

Protein Group

ribulose-1,5-bisphosphate carboxylase/oxygenase large subunit, partial (chloroplast) [Agropyron cristatum subsp. pectinatum]	gi|424949891	50874.4	6.4400 000572 2046

Peptide Information

Calc. Mass	Obsrv. Mass	± da	± ppm	Start Seq.	End Seq.	Sequence	Ion Score	C. I. %	Modification	Rank	Result Type
830.4366	830.4283	-0.0083	-10	439	445	EGNEIIR					Mascot
898.41	898.4123	0.0023	3	305	311	NHGMHFR					Mascot
912.472	912.4646	-0.0074	-8	295	302	AMHAVIDR					Mascot
914.405	914.3882	-0.0168	-18	305	311	NHGMHFR			Oxidation (M)[4]		Mascot

928.4669	928.441	-0.0259	-28	295	302	AMHAVIDR			Oxidation (M)[2]	Mascot
971.4073	971.4023	-0.005	-5	187	193	ACYECLR			Carbamidomethyl (C)[2,5]	Mascot
971.4073	971.4023	-0.005	-5	187	193	ACYECLR	24	0	Carbamidomethyl (C)[2,5]	Mascot
1021.5312	1021.5198	-0.0114	-11	32	40	DTDILAAFR				Mascot
1021.5312	1021.5198	-0.0114	-11	32	40	DTDILAAFR	56	99.427		Mascot
1037.4899	1037.4851	-0.0048	-5	350	357	DDFIEKDR				Mascot
1116.583	1116.5812	-0.0018	-2	421	430	VALEACVQAR			Carbamidomethyl (C)[6]	Mascot
1170.5586	1170.6375	0.0789	67	303	311	QKNHGMHFR			Oxidation (M)[6]	Mascot
1170.5586	1170.6375	0.0789	67	303	311	QKNHGMHFR			Oxidation (M)[6]	Mascot
1187.6644	1187.6611	-0.0033	-3	285	294	DNGLLLHIHR				Mascot
1247.6129	1247.5988	-0.0141	-11	217	226	FVFCAEAIYK			Carbamidomethyl (C)[4]	Mascot
1285.6859	1285.5776	-0.1083	-84	435	445	DLAREGNEIIR				Mascot
1293.6871	1293.6863	-0.0008	-1	339	349	EMTLGFVDLLR	90	100		Mascot
1309.6821	1309.6604	-0.0217	-17	339	349	EMTLGFVDLLR			Oxidation (M)[2]	Mascot
1325.6896	1325.6711	-0.0185	-14	305	315	NHGMHFRVLAK			Oxidation (M)[4]	Mascot
1407.6678	1407.6458	-0.022	-16	21	31	LTYYTPEYETK				Mascot
1451.6219	1451.6394	0.0175	12	201	212	DDENVNSQPFMR				Mascot
1465.7546	1465.765	0.0104	7	146	158	TFQGPPHGIQVER				Mascot
1465.7546	1465.765	0.0104	7	146	158	TFQGPPHGIQVER	102	100		Mascot
1483.6959	1483.7828	0.0869	59	319	333	MSGGDHVHSGTVVGK			Oxidation (M)[1]	Mascot
1502.8512	1502.849	-0.0022	-1	164	176	YGRPLLGCTIKPK			Carbamidomethyl (C)[8]	Mascot
1502.8512	1502.849	-0.0022	-1	164	176	YGRPLLGCTIKPK	6	0	Carbamidomethyl (C)[8]	Mascot
1518.741	1518.7408	-0.0002	0	215	226	DRFVFCAEAIYK			Carbamidomethyl (C)[6]	Mascot
1893.9739	1893.9507	-0.0232	-12	334	349	LEGEREMTLGFVDLLR			Oxidation (M)[7]	Mascot
1950.916	1950.9291	0.0131	7	236	252	GHYLNATAGTCEEMIKR			Carbamidomethyl (C)[11]	Mascot
1966.911	1966.9098	-0.0012	-1	236	252	GHYLNATAGTCEEMIKR			Carbamidomethyl (C)[11], Oxidation (M)[14]	Mascot
2041.0311	2041.0352	0.0041	2	339	355	EMTLGFVDLLRDDFIEK				Mascot
2057.0261	2057.0132	-0.0129	-6	339	355	EMTLGFVDLLRDDFIEK			Oxidation (M)[2]	Mascot
2169.9871	2169.999	0.0119	5	194	212	GGLDFTKDDENVNSQPF MR				Mascot
2169.9871	2169.999	0.0119	5	194	212	GGLDFTKDDENVNSQPF MR	93	100		Mascot
2185.9819	2185.9814	-0.0005	0	194	212	GGLDFTKDDENVNSQPF MR			Oxidation (M)[18]	Mascot
2252.1824	2252.1777	-0.0047	-2	139	158	IPPTYSKTFQGPPHGIQV ER				Mascot
3036.4707	3036.5229	0.0522	17	258	284	ELGVPIVMHDYLTGGFTA NTTLAHYCR			Carbamidomethyl (C)[26]	Mascot
3036.4707	3036.5229	0.0522	17	258	284	ELGVPIVMHDYLTGGFTA NTTLAHYCR	159	100	Carbamidomethyl (C)[26]	Mascot
3052.4656	3052.5239	0.0583	19	258	284	ELGVPIVMHDYLTGGFTA NTTLAHYCR			Carbamidomethyl (C)[26], Oxidation (M)[8]	Mascot

3840.8564	3840.968	0.1116	29	41	78	VSPQPGVPPEEAGAAVA AESSTGTWTTVWTDGLT SLDR					Mascot
3840.8564	3840.968	0.1116	29	41	78	VSPQPGVPPEEAGAAVA AESSTGTWTTVWTDGLT SLDR	131	100			Mascot

9	ribulose-1,5-bisphosphate carboxylase/oxygenase large subunit, partial (chloroplast) [Bromus porteri]	gi|424949907	50051	6.33	25	837	100	58.189	655	100	

Peptide Information

Calc. Mass	Obsrv. Mass	± da	± ppm	Start Seq.	End Seq.	Sequence	Ion Score	C. I. %	Modification	Rank	Result Type
830.4366	830.4283	-0.0083	-10	430	436	EGNEIIR					Mascot
898.41	898.4123	0.0023	3	296	302	NHGMHFR					Mascot
912.472	912.4646	-0.0074	-8	286	293	AMHAVIDR					Mascot
914.405	914.3882	-0.0168	-18	296	302	NHGMHFR			Oxidation (M)[4]		Mascot
928.4669	928.441	-0.0259	-28	286	293	AMHAVIDR			Oxidation (M)[2]		Mascot
971.4073	971.4023	-0.005	-5	178	184	ACYECLR			Carbamidomethyl (C)[2,5]		Mascot
971.4073	971.4023	-0.005	-5	178	184	ACYECLR	24	0	Carbamidomethyl (C)[2,5]		Mascot
1021.5312	1021.5198	-0.0114	-11	23	31	DTDILAAFR					Mascot
1021.5312	1021.5198	-0.0114	-11	23	31	DTDILAAFR	56	99.427			Mascot
1037.4899	1037.4851	-0.0048	-5	341	348	DDFIEKDR					Mascot
1116.583	1116.5812	-0.0018	-2	412	421	VALEACVQAR			Carbamidomethyl (C)[6]		Mascot
1170.5586	1170.6375	0.0789	67	294	302	QKNHGMHFR			Oxidation (M)[6]		Mascot
1170.5586	1170.6375	0.0789	67	294	302	QKNHGMHFR			Oxidation (M)[6]		Mascot
1187.6644	1187.6611	-0.0033	-3	276	285	DNGLLLHIHR					Mascot
1247.6129	1247.5988	-0.0141	-11	208	217	FVFCAEAIYK			Carbamidomethyl (C)[4]		Mascot
1285.6859	1285.5776	-0.1083	-84	426	436	DLAREGNEIIR					Mascot
1293.6871	1293.6863	-0.0008	-1	330	340	EMTLGFVDLLR	90	100			Mascot
1309.6821	1309.6604	-0.0217	-17	330	340	EMTLGFVDLLR			Oxidation (M)[2]		Mascot
1325.6896	1325.6711	-0.0185	-14	296	306	NHGMHFRVLAK			Oxidation (M)[4]		Mascot
1407.6678	1407.6458	-0.022	-16	12	22	LTYYTPEYETK					Mascot
1447.8156	1447.754	-0.0616	-43	125	136	LEDLRIPLTYSK					Mascot
1451.6219	1451.6394	0.0175	12	192	203	DDENVNSQPFMR					Mascot
1465.7546	1465.765	0.0104	7	137	149	TFQGPPHGIQVER					Mascot
1465.7546	1465.765	0.0104	7	137	149	TFQGPPHGIQVER	102	100			Mascot
1502.8512	1502.849	-0.0022	-1	155	167	YGRPLLGCTIKPK			Carbamidomethyl (C)[8]		Mascot
1502.8512	1502.849	-0.0022	-1	155	167	YGRPLLGCTIKPK	6	0	Carbamidomethyl (C)[8]		Mascot
1518.741	1518.7408	-0.0002	0	206	217	DRFVFCAEAIYK			Carbamidomethyl (C)[6]		Mascot
1893.9739	1893.9507	-0.0232	-12	325	340	LEGEREMTLGFVDLLR			Oxidation (M)[7]		Mascot

1950.916	1950.9291	0.0131	7	227	243	GHYLNATAGTCEEMIKR			Carbamidomethyl (C)[11]		Mascot
1966.911	1966.9098	-0.0012	-1	227	243	GHYLNATAGTCEEMIKR			Carbamidomethyl (C)[11], Oxidation (M)[14]		Mascot
2041.0311	2041.0352	0.0041	2	330	346	EMTLGFVDLLRDDFIEK					Mascot
2057.0261	2057.0132	-0.0129	-6	330	346	EMTLGFVDLLRDDFIEK			Oxidation (M)[2]		Mascot
2169.9871	2169.999	0.0119	5	185	203	GGLDFTKDDENVNSQPF MR					Mascot
2169.9871	2169.999	0.0119	5	185	203	GGLDFTKDDENVNSQPF MR	93	100			Mascot
2185.9819	2185.9814	-0.0005	0	185	203	GGLDFTKDDENVNSQPF MR			Oxidation (M)[18]		Mascot
3036.4707	3036.5229	0.0522	17	249	275	ELGVPIVMHDYLTGGFTA NTTLAHYCR			Carbamidomethyl (C)[26]		Mascot
3036.4707	3036.5229	0.0522	17	249	275	ELGVPIVMHDYLTGGFTA NTTLAHYCR	159	100	Carbamidomethyl (C)[26]		Mascot
3052.4656	3052.5239	0.0583	19	249	275	ELGVPIVMHDYLTGGFTA NTTLAHYCR			Carbamidomethyl (C)[26], Oxidation (M)[8]		Mascot
3840.8564	3840.968	0.1116	29	32	69	VSPQPGVPPEEAGAAVA AESSTGTWTTVWTDGLT SLDR					Mascot
3840.8564	3840.968	0.1116	29	32	69	VSPQPGVPPEEAGAAVA AESSTGTWTTVWTDGLT SLDR	131	100			Mascot

10	ribulose-1,5-bisphosphate carboxylase/oxygenase large subunit, partial (chloroplast) [Bromus hordeaceus]	gi|340510952	49532.7	6.34	25	837	100	57.954	655	100	

Peptide Information

Calc. Mass	Obsrv. Mass	± da	± ppm	Start Seq.	End Seq.	Sequence	Ion Score	C. I. %	Modification	Rank	Result Type
830.4366	830.4283	-0.0083	-10	425	431	EGNEIIR					Mascot
898.41	898.4123	0.0023	3	291	297	NHGMHFR					Mascot
912.472	912.4646	-0.0074	-8	281	288	AMHAVIDR					Mascot
914.405	914.3882	-0.0168	-18	291	297	NHGMHFR			Oxidation (M)[4]		Mascot
928.4669	928.441	-0.0259	-28	281	288	AMHAVIDR			Oxidation (M)[2]		Mascot
971.4073	971.4023	-0.005	-5	173	179	ACYECLR			Carbamidomethyl (C)[2,5]		Mascot
971.4073	971.4023	-0.005	-5	173	179	ACYECLR	24	0	Carbamidomethyl (C)[2,5]		Mascot
1021.5312	1021.5198	-0.0114	-11	18	26	DTDILAAFR					Mascot
1021.5312	1021.5198	-0.0114	-11	18	26	DTDILAAFR	56	99.427			Mascot
1037.4899	1037.4851	-0.0048	-5	336	343	DDFIEKDR					Mascot
1116.583	1116.5812	-0.0018	-2	407	416	VALEACVQAR			Carbamidomethyl (C)[6]		Mascot
1170.5586	1170.6375	0.0789	67	289	297	QKNHGMHFR			Oxidation (M)[6]		Mascot
1170.5586	1170.6375	0.0789	67	289	297	QKNHGMHFR			Oxidation (M)[6]		Mascot
1187.6644	1187.6611	-0.0033	-3	271	280	DNGLLLHIHR					Mascot
1247.6129	1247.5988	-0.0141	-11	203	212	FVFCAEAIYK			Carbamidomethyl (C)[4]		Mascot
1285.6859	1285.5776	-0.1083	-84	421	431	DLAREGNEIIR					Mascot

1293.6871	1293.6863	-0.0008	-1	325	335	EMTLGFVDLLR	90	100		Mascot
1309.6821	1309.6604	-0.0217	-17	325	335	EMTLGFVDLLR			Oxidation (M)[2]	Mascot
1325.6896	1325.6711	-0.0185	-14	291	301	NHGMHFRVLAK			Oxidation (M)[4]	Mascot
1407.6678	1407.6458	-0.022	-16	7	17	LTYYTPEYETK				Mascot
1451.6219	1451.6394	0.0175	12	187	198	DDENVNSQPFMR				Mascot
1465.7546	1465.765	0.0104	7	132	144	TFQGPPHGIQVER				Mascot
1465.7546	1465.765	0.0104	7	132	144	TFQGPPHGIQVER	102	100		Mascot
1502.8512	1502.849	-0.0022	-1	150	162	YGRPLLGCTIKPK			Carbamidomethyl (C)[8]	Mascot
1502.8512	1502.849	-0.0022	-1	150	162	YGRPLLGCTIKPK	6	0	Carbamidomethyl (C)[8]	Mascot
1518.741	1518.7408	-0.0002	0	201	212	DRFVFCAEAIYK			Carbamidomethyl (C)[6]	Mascot
1893.9739	1893.9507	-0.0232	-12	320	335	LEGEREMTLGFVDLLR			Oxidation (M)[7]	Mascot
1950.916	1950.9291	0.0131	7	222	238	GHYLNATAGTCEEMIKR			Carbamidomethyl (C)[11]	Mascot
1966.911	1966.9098	-0.0012	-1	222	238	GHYLNATAGTCEEMIKR			Carbamidomethyl (C)[11], Oxidation (M)[14]	Mascot
2041.0311	2041.0352	0.0041	2	325	341	EMTLGFVDLLRDDFIEK				Mascot
2057.0261	2057.0132	-0.0129	-6	325	341	EMTLGFVDLLRDDFIEK			Oxidation (M)[2]	Mascot
2169.9871	2169.999	0.0119	5	180	198	GGLDFTKDDENVNSQPF MR				Mascot
2169.9871	2169.999	0.0119	5	180	198	GGLDFTKDDENVNSQPF MR	93	100		Mascot
2185.9819	2185.9814	-0.0005	0	180	198	GGLDFTKDDENVNSQPF MR			Oxidation (M)[18]	Mascot
2252.1824	2252.1777	-0.0047	-2	125	144	IPPTYSKTFQGPPHGIQV ER				Mascot
3036.4707	3036.5229	0.0522	17	244	270	ELGVPIVMHDYLTGGFTA NTTLAHYCR			Carbamidomethyl (C)[26]	Mascot
3036.4707	3036.5229	0.0522	17	244	270	ELGVPIVMHDYLTGGFTA NTTLAHYCR	159	100	Carbamidomethyl (C)[26]	Mascot
3052.4656	3052.5239	0.0583	19	244	270	ELGVPIVMHDYLTGGFTA NTTLAHYCR			Carbamidomethyl (C)[26], Oxidation (M)[8]	Mascot
3840.8564	3840.968	0.1116	29	27	64	VSPQPGVPPEEAGAAVA AESSTGTWTTVWTDGLT SLDR				Mascot
3840.8564	3840.968	0.1116	29	27	64	VSPQPGVPPEEAGAAVA AESSTGTWTTVWTDGLT SLDR	131	100		Mascot

Rank	Protein Name	Accession No.	Protein MW	Protein PI	Pep. Count	Protein Score	Protein Score C. I. %	Intensity Matched	Total Ion Score	Total Ion C. I. %	Confirmed
1	RecName: Full=Glucose-1-phosphate adenylyltransferase large subunit, chloroplastic/amyloplastic; AltName: Full=ADP-glucose pyrophosphorylase; AltName: Full=ADP-glucose synthase; AltName: Full=AGPase S; AltName: Full=Alpha-D-glucose-1-phosphate adeny	gi|1707930	58399.1	6.12	29	1,140	100	63.89	955	100	

Peptide Information

Calc. Mass	Obsrv. Mass	± da	± ppm	Start Seq.	End Seq.	Sequence	Ion Score	C. I. %	Modification	Rank	Result Type
802.424	802.4251	0.0011	1	16	22	ACISPVR			Carbamidomethyl (C)[2]		Mascot
820.4159	820.4044	-0.0115	-14	33	40	VGDSSSIR					Mascot
847.452	847.4308	-0.0212	-25	426	433	LNSGSELK					Mascot
866.4618	866.4388	-0.023	-27	251	258	ASEYGLVK					Mascot
927.5873	927.5493	-0.038	-41	311	318	DVLLNLLK					Mascot
945.4352	945.4153	-0.0199	-21	373	379	FEFYDPK					Mascot
952.4887	952.4767	-0.012	-13	380	387	TPFFTSPR					Mascot
952.4887	952.4767	-0.012	-13	380	387	TPFFTSPR	51	98.47			Mascot
1013.5626	1013.5337	-0.0289	-29	458	467	VPIGVGENTK					Mascot
1023.5945	1023.5873	-0.0072	-7	415	423	IEHSIIGVR					Mascot
1023.5945	1023.5873	-0.0072	-7	415	423	IEHSIIGVR	53	98.956			Mascot
1061.595	1061.5651	-0.0299	-28	31	40	LKVGDSSSIR					Mascot
1170.7205	1170.6298	-0.0907	-77	311	320	DVLLNLLKSR					Mascot
1261.6359	1261.6375	0.0016	1	111	122	ATPAVPIGGCYR			Carbamidomethyl (C)[10]		Mascot
1261.6359	1261.6375	0.0016	1	111	122	ATPAVPIGGCYR	76	99.995	Carbamidomethyl (C)[10]		Mascot
1302.6624	1302.6641	0.0017	1	401	411	EAIISHGCFLR			Carbamidomethyl (C)[8]		Mascot
1302.6624	1302.6641	0.0017	1	401	411	EAIISHGCFLR	83	100	Carbamidomethyl (C)[8]		Mascot
1322.6191	1322.5989	-0.0202	-15	468	478	ISNCIIDMNAR			Carbamidomethyl (C)[4], Oxidation (M)[8]		Mascot
1322.6191	1322.5989	-0.0202	-15	468	478	ISNCIIDMNAR	37	61.744	Carbamidomethyl (C)[4], Oxidation (M)[8]		Mascot
1503.7882	1503.7552	-0.033	-22	195	205	KFIWVLEDYYK					Mascot
1553.7709	1553.7338	-0.0371	-24	297	309	YPYIASMGVYVFK			Oxidation (M)[7]		Mascot
1643.821	1643.8031	-0.0179	-11	139	152	IFVMTQFNSASLNR			Oxidation (M)[4]		Mascot
1646.8174	1646.8174	0	0	321	334	YAELHDFGSEILPR					Mascot
1646.8174	1646.8174	0	0	321	334	YAELHDFGSEILPR	108	100			Mascot

1652.8167	1652.7986	-0.0181	-11	282	296	VDTSFLNFAIDDPAK					Mascot
1674.8732	1674.8409	-0.0323	-19	452	467	LMSEGKVPIGVGENTK			Oxidation (M)[2]		Mascot
1709.8719	1709.8036	-0.0683	-40	297	310	YPYIASMGVYVFKR			Oxidation (M)[7]		Mascot
1756.9592	1756.9644	0.0052	3	208	222	SIEHILILSGDQLYR					Mascot
1756.9592	1756.9644	0.0052	3	208	222	SIEHILILSGDQLYR	110	100			Mascot
1770.7826	1770.7554	-0.0272	-15	358	372	SFFDANMALCEQPPK			Carbamidomethyl (C)[10], Oxidation (M)[7]		Mascot
1838.8776	1838.8567	-0.0209	-11	123	138	LIDIPMSNCFNSGINK			Carbamidomethyl (C)[9], Oxidation (M)[6]		Mascot
1910.8879	1910.8832	-0.0047	-2	489	504	EGVQEADRPEEGYYIR					Mascot
2055.9399	2055.9438	0.0039	2	232	250	HVDDNADITLSCAPVGES R			Carbamidomethyl (C)[12]		Mascot
2371.1924	2371.2029	0.0105	4	54	77	GPAATGAQCVLTSDASP ADTLVLR			Carbamidomethyl (C)[9]		Mascot
2371.1924	2371.2029	0.0105	4	54	77	GPAATGAQCVLTSDASP ADTLVLR	198	100	Carbamidomethyl (C)[9]		Mascot
2763.3162	2763.3372	0.021	8	335	357	ALHDHNVQAYVFTDYWE DIGTIR					Mascot
2862.4634	2862.4819	0.0185	6	83	110	NYADPNEVAAVILGGGT GTQLFPLTSTR					Mascot
2862.4634	2862.4819	0.0185	6	83	110	NYADPNEVAAVILGGGT GTQLFPLTSTR	239	100			Mascot

2 hypothetical protein F775_52189 [Aegilops tauschii] gi|475624809 64143.3 8.32 28 1,130 100 63.559 955 100

Peptide Information

Calc. Mass	Obsrv. Mass	± da	± ppm	Start Seq.	End Seq.	Sequence	Ion Score	C. I. %	Modification	Rank	Result Type
820.4159	820.4044	-0.0115	-14	30	37	VGDSSSIR					Mascot
847.452	847.4308	-0.0212	-25	432	439	LNSGSELK					Mascot
866.4618	866.4388	-0.023	-27	248	255	ASEYGLVK					Mascot
927.5873	927.5493	-0.038	-41	308	315	DVLLNLLK					Mascot
945.4352	945.4153	-0.0199	-21	379	385	FEFYDPK					Mascot
952.4887	952.4767	-0.012	-13	386	393	TPFFTSPR					Mascot
952.4887	952.4767	-0.012	-13	386	393	TPFFTSPR	51	98.47			Mascot
1013.5626	1013.5337	-0.0289	-29	464	473	VPIGVGENTK					Mascot
1023.5945	1023.5873	-0.0072	-7	421	429	IEHSIIGVR					Mascot
1023.5945	1023.5873	-0.0072	-7	421	429	IEHSIIGVR	53	98.956			Mascot
1061.595	1061.5651	-0.0299	-28	28	37	LKVGDSSSIR					Mascot
1170.7205	1170.6298	-0.0907	-77	308	317	DVLLNLLKSR					Mascot
1204.6572	1204.6145	-0.0427	-35	2	12	QFSSVLPLEGK					Mascot
1261.6359	1261.6375	0.0016	1	108	119	ATPAVPIGGCYR			Carbamidomethyl (C)[10]		Mascot
1261.6359	1261.6375	0.0016	1	108	119	ATPAVPIGGCYR	76	99.995	Carbamidomethyl (C)[10]		Mascot
1302.6624	1302.6641	0.0017	1	407	417	EAIISHGCFLR			Carbamidomethyl (C)[8]		Mascot

Calc. Mass	Obsrv. Mass	± da	± ppm	Start Seq.	End Seq.	Sequence	Ion Score	C. I. %	Modification	Rank	Result Type
1302.6624	1302.6641	0.0017	1	407	417	EAIISHGCFLR	83	100	Carbamidomethyl (C)[8]		Mascot
1322.6191	1322.5989	-0.0202	-15	474	484	ISNCIIDMNAR			Carbamidomethyl (C)[4], Oxidation (M)[8]		Mascot
1322.6191	1322.5989	-0.0202	-15	474	484	ISNCIIDMNAR	37	61.744	Carbamidomethyl (C)[4], Oxidation (M)[8]		Mascot
1503.7882	1503.7552	-0.033	-22	192	202	KFIWVLEDYYK					Mascot
1553.7709	1553.7338	-0.0371	-24	294	306	YPYIASMGVYVFK			Oxidation (M)[7]		Mascot
1643.821	1643.8031	-0.0179	-11	136	149	IFVMTQFNSASLNR			Oxidation (M)[4]		Mascot
1646.8174	1646.8174	0	0	318	331	YAELHDFGSEILPR					Mascot
1646.8174	1646.8174	0	0	318	331	YAELHDFGSEILPR	108	100			Mascot
1652.8167	1652.7986	-0.0181	-11	279	293	VDTSFLNFAIDDPAK					Mascot
1674.8732	1674.8409	-0.0323	-19	458	473	LMSEGKVPIGVGENTK			Oxidation (M)[2]		Mascot
1709.8719	1709.8036	-0.0683	-40	294	307	YPYIASMGVYVFKR			Oxidation (M)[7]		Mascot
1756.9592	1756.9644	0.0052	3	205	219	SIEHILILSGDQLYR					Mascot
1756.9592	1756.9644	0.0052	3	205	219	SIEHILILSGDQLYR	110	100			Mascot
1838.8776	1838.8567	-0.0209	-11	120	135	LIDIPMSNCFNSGINK			Carbamidomethyl (C)[9], Oxidation (M)[6]		Mascot
1910.8879	1910.8832	-0.0047	-2	495	510	EGVQEADRPEEGYYIR					Mascot
2055.9399	2055.9438	0.0039	2	229	247	HVDDNADITLSCAPVGES R			Carbamidomethyl (C)[12]		Mascot
2371.1924	2371.2029	0.0105	4	51	74	GPAATGAQCVLTSDASP ADTLVLR			Carbamidomethyl (C)[9]		Mascot
2371.1924	2371.2029	0.0105	4	51	74	GPAATGAQCVLTSDASP ADTLVLR	198	100	Carbamidomethyl (C)[9]		Mascot
2763.3162	2763.3372	0.021	8	332	354	ALHDHNVQAYVFTDYWE DIGTIR					Mascot
2862.4634	2862.4819	0.0185	6	80	107	NYADPNEVAAVILGGGT GTQLFPLTSTR					Mascot
2862.4634	2862.4819	0.0185	6	80	107	NYADPNEVAAVILGGGT GTQLFPLTSTR	239	100			Mascot

3	RecName: Full=Glucose-1-phosphate adenylyltransferase large subunit 1, chloroplastic/amyloplastic; AltName: Full=ADP-glucose pyrophosphorylase; AltName: Full=ADP-glucose synthase; AltName: Full=AGPase S; AltName: Full=Alpha-D-glucose-1-phosphate ade	gi|1707923	58580.1	6.17	27	1,120	100	62.895	955	100	

Peptide Information

Calc. Mass	Obsrv. Mass	± da	± ppm	Start Seq.	End Seq.	Sequence	Ion Score	C. I. %	Modification	Rank	Result Type
808.3254	808.3248	-0.0006	-1	24	30	EGSACER			Carbamidomethyl (C)[5]		Mascot
847.452	847.4308	-0.0212	-25	427	434	LNSGSELK					Mascot
866.4618	866.4388	-0.023	-27	252	259	ASEYGLVK					Mascot
927.5873	927.5493	-0.038	-41	312	319	DVLLNLLK					Mascot
945.4352	945.4153	-0.0199	-21	374	380	FEFYDPK					Mascot
952.4887	952.4767	-0.012	-13	381	388	TPFFTSPR					Mascot

952.4887	952.4767	-0.012	-13	381	388	TPFFTSPR	51	98.47			Mascot
1013.5626	1013.5337	-0.0289	-29	459	468	VPIGVGENTK					Mascot
1023.5945	1023.5873	-0.0072	-7	416	424	IEHSIIGVR					Mascot
1023.5945	1023.5873	-0.0072	-7	416	424	IEHSIIGVR	53	98.956			Mascot
1170.7205	1170.6298	-0.0907	-77	312	321	DVLLNLLKSR					Mascot
1261.6359	1261.6375	0.0016	1	112	123	ATPAVPIGGCYR			Carbamidomethyl (C)[10]		Mascot
1261.6359	1261.6375	0.0016	1	112	123	ATPAVPIGGCYR	76	99.995	Carbamidomethyl (C)[10]		Mascot
1302.6624	1302.6641	0.0017	1	402	412	EAIISHGCFLR			Carbamidomethyl (C)[8]		Mascot
1302.6624	1302.6641	0.0017	1	402	412	EAIISHGCFLR	83	100	Carbamidomethyl (C)[8]		Mascot
1322.6191	1322.5989	-0.0202	-15	469	479	ISNCIIDMNAR			Carbamidomethyl (C)[4], Oxidation (M)[8]		Mascot
1322.6191	1322.5989	-0.0202	-15	469	479	ISNCIIDMNAR	37	61.744	Carbamidomethyl (C)[4], Oxidation (M)[8]		Mascot
1503.7882	1503.7552	-0.033	-22	196	206	KFIWVLEDYYK					Mascot
1553.7709	1553.7338	-0.0371	-24	298	310	YPYIASMGVYVFK			Oxidation (M)[7]		Mascot
1643.821	1643.8031	-0.0179	-11	140	153	IFVMTQFNSASLNR			Oxidation (M)[4]		Mascot
1646.8174	1646.8174	0	0	322	335	YAELHDFGSEILPR					Mascot
1646.8174	1646.8174	0	0	322	335	YAELHDFGSEILPR	108	100			Mascot
1652.8167	1652.7986	-0.0181	-11	283	297	VDTSFLNFAIDDPAK					Mascot
1674.8732	1674.8409	-0.0323	-19	453	468	LMSEGKVPIGVGENTK			Oxidation (M)[2]		Mascot
1709.8719	1709.8036	-0.0683	-40	298	311	YPYIASMGVYVFKR			Oxidation (M)[7]		Mascot
1756.9592	1756.9644	0.0052	3	209	223	SIEHILILSGDQLYR					Mascot
1756.9592	1756.9644	0.0052	3	209	223	SIEHILILSGDQLYR	110	100			Mascot
1770.7826	1770.7554	-0.0272	-15	359	373	SFFDANMALCEQPPK			Carbamidomethyl (C)[10], Oxidation (M)[7]		Mascot
1838.8776	1838.8567	-0.0209	-11	124	139	LIDIPMSNCFNSGINK			Carbamidomethyl (C)[9], Oxidation (M)[6]		Mascot
1910.8879	1910.8832	-0.0047	-2	490	505	EGVQEADRPEEGYYIR					Mascot
2055.9399	2055.9438	0.0039	2	233	251	HVDDNADITLSCAPVGES R			Carbamidomethyl (C)[12]		Mascot
2371.1924	2371.2029	0.0105	4	55	78	GPAATGAQCVLTSDASP ADTLVLR			Carbamidomethyl (C)[9]		Mascot
2371.1924	2371.2029	0.0105	4	55	78	GPAATGAQCVLTSDASP ADTLVLR	198	100	Carbamidomethyl (C)[9]		Mascot
2763.3162	2763.3372	0.021	8	336	358	ALHDHNVQAYVFTDYWE DIGTIR					Mascot
2862.4634	2862.4819	0.0185	6	84	111	NYADPNEVAAVILGGGT GTQLFPLTSTR					Mascot
2862.4634	2862.4819	0.0185	6	84	111	NYADPNEVAAVILGGGT GTQLFPLTSTR	239	100			Mascot

4	Glucose-1-phosphate adenylyltransferase large subunit 1, chloroplastic/amyloplastic [Triticum urartu]	gi|474056532	51530	8.53	18	851	100	55.153	758	100	

Peptide Information

Calc. Mass	Obsrv. Mass	± da	± ppm	Start Seq.	End Seq.	Sequence	Ion Score	C. I. %	Modification	Rank	Result Type

847.452	847.4308	-0.0212	-25	306	313	LNSGSELK					Mascot
945.4352	945.4153	-0.0199	-21	253	259	FEFYDPK					Mascot
952.4887	952.4767	-0.012	-13	260	267	TPFFTSPR					Mascot
952.4887	952.4767	-0.012	-13	260	267	TPFFTSPR	51	98.47			Mascot
1013.5626	1013.5337	-0.0289	-29	338	347	VPIGVGENTK					Mascot
1023.5945	1023.5873	-0.0072	-7	295	303	IEHSIIGVR					Mascot
1023.5945	1023.5873	-0.0072	-7	295	303	IEHSIIGVR	53	98.956			Mascot
1261.6359	1261.6375	0.0016	1	60	71	ATPAVPIGGCYR			Carbamidomethyl (C)[10]		Mascot
1261.6359	1261.6375	0.0016	1	60	71	ATPAVPIGGCYR	76	99.995	Carbamidomethyl (C)[10]		Mascot
1302.6624	1302.6641	0.0017	1	281	291	EAIISHGCFLR			Carbamidomethyl (C)[8]		Mascot
1302.6624	1302.6641	0.0017	1	281	291	EAIISHGCFLR	83	100	Carbamidomethyl (C)[8]		Mascot
1322.6191	1322.5989	-0.0202	-15	348	358	ISNCIIDMNAR			Carbamidomethyl (C)[4], Oxidation (M)[8]		Mascot
1322.6191	1322.5989	-0.0202	-15	348	358	ISNCIIDMNAR	37	61.744	Carbamidomethyl (C)[4], Oxidation (M)[8]		Mascot
1503.7882	1503.7552	-0.033	-22	144	154	KFIWVLEDYYK					Mascot
1643.821	1643.8031	-0.0179	-11	88	101	IFVMTQFNSASLNR			Oxidation (M)[4]		Mascot
1646.8174	1646.8174	0	0	201	214	YAELHDFGSEILPR					Mascot
1646.8174	1646.8174	0	0	201	214	YAELHDFGSEILPR	108	100			Mascot
1674.8732	1674.8409	-0.0323	-19	332	347	LMSEGKVPIGVGENTK			Oxidation (M)[2]		Mascot
1756.9592	1756.9644	0.0052	3	157	171	SIEHILILSGDQLYR					Mascot
1756.9592	1756.9644	0.0052	3	157	171	SIEHILILSGDQLYR	110	100			Mascot
1770.7826	1770.7554	-0.0272	-15	238	252	SFFDANMALCEQPPK			Carbamidomethyl (C)[10], Oxidation (M)[7]		Mascot
1838.8776	1838.8567	-0.0209	-11	72	87	LIDIPMSNCFNSGINK			Carbamidomethyl (C)[9], Oxidation (M)[6]		Mascot
1910.8879	1910.8832	-0.0047	-2	369	384	EGVQEADRPEEGYYIR					Mascot
2763.3162	2763.3372	0.021	8	215	237	ALHDHNVQAYVFTDYWE DIGTIR					Mascot
2862.4634	2862.4819	0.0185	6	32	59	NYADPNEVAAVILGGGT GTQLFPLTSTR					Mascot
2862.4634	2862.4819	0.0185	6	32	59	NYADPNEVAAVILGGGT GTQLFPLTSTR	239	100			Mascot

5 PREDICTED: glucose-1-phosphate adenylyltransferase large subunit, chloroplastic/amyloplastic-like [Setaria italica] gi|514746428 58156.9 6.11 19 498 100 28.72 404 100

Peptide Information

Calc. Mass	Obsrv. Mass	± da	± ppm	Start Seq.	End Seq.	Sequence	Ion Score	C. I. %	Modification	Rank	Result Type
908.403	908.4543	0.0513	56	270	277	GADLEEMK			Oxidation (M)[7]		Mascot
927.5873	927.5493	-0.038	-41	307	314	DVLLNLLK					Mascot
945.4352	945.4153	-0.0199	-21	369	375	FEFYDPK					Mascot
952.4887	952.4767	-0.012	-13	376	383	TPFFTSPR					Mascot

952.4887	952.4767	-0.012	-13	376	383	TPFFTSPR	51	98.47			Mascot
1013.5626	1013.5337	-0.0289	-29	454	463	VPIGVGENTK					Mascot
1170.7205	1170.6298	-0.0907	-77	307	316	DVLLNLLKSR					Mascot
1210.5668	1210.6331	0.0663	55	30	39	MRIGDCSSIR			Carbamidomethyl (C)[6], Oxidation (M)[1]		Mascot
1261.6359	1261.6375	0.0016	1	108	119	ATPAVPIGGCYR			Carbamidomethyl (C)[10]		Mascot
1261.6359	1261.6375	0.0016	1	108	119	ATPAVPIGGCYR	76	99.995	Carbamidomethyl (C)[10]		Mascot
1322.6191	1322.5989	-0.0202	-15	464	474	ISNCIIDMNAR			Carbamidomethyl (C)[4], Oxidation (M)[8]		Mascot
1322.6191	1322.5989	-0.0202	-15	464	474	ISNCIIDMNAR	37	61.744	Carbamidomethyl (C)[4], Oxidation (M)[8]		Mascot
1385.677	1385.642	-0.035	-25	1	12	MQFSSVFPLEGK			Oxidation (M)[1]		Mascot
1503.7882	1503.7552	-0.033	-22	191	201	KFIWVLEDYYK					Mascot
1553.7709	1553.7338	-0.0371	-24	293	305	YPYIASMGVYVFK			Oxidation (M)[7]		Mascot
1643.821	1643.8031	-0.0179	-11	136	149	IFVMTQFNSASLNR			Oxidation (M)[4]		Mascot
1668.8116	1668.8027	-0.0089	-5	278	292	VDTSFLNFAIDDPSK					Mascot
1709.8719	1709.8036	-0.0683	-40	293	306	YPYIASMGVYVFKR			Oxidation (M)[7]		Mascot
1770.7826	1770.7554	-0.0272	-15	354	368	SFFDANMALCEQPPK			Carbamidomethyl (C)[10], Oxidation (M)[7]		Mascot
1838.8776	1838.8567	-0.0209	-11	120	135	LIDIPMSNCFNSGINK			Carbamidomethyl (C)[9], Oxidation (M)[6]		Mascot
2055.9399	2055.9438	0.0039	2	228	246	HVDDNADITLSCAPVGES R			Carbamidomethyl (C)[12]		Mascot
2862.4634	2862.4819	0.0185	6	80	107	NYADPNEVAAVILGGGT GTQLFPLTSTR					Mascot
2862.4634	2862.4819	0.0185	6	80	107	NYADPNEVAAVILGGGT GTQLFPLTSTR	239	100			Mascot

6 RecName: Full=Glucose-1-phosphate adenylyltransferase large subunit, chloroplastic/amyloplastic; AltName: Full=ADP-glucose pyrophosphorylase; AltName: Full=ADP-glucose synthase; AltName: Full=AGPase S; AltName: Full=Alpha-D-glucose-1-phosphate adeny   gi|121293   56037.9   6.61   22   496   100   47.287   383   100

Peptide Information

Calc. Mass	Obsrv. Mass	± da	± ppm	Start Seq.	End Seq.	Sequence	Ion Score	C. I. %	Modification	Rank	Result Type
847.452	847.4308	-0.0212	-25	404	411	LNSGSELK					Mascot
866.4618	866.4388	-0.023	-27	232	239	ASEYGLVK					Mascot
908.5424	908.4543	-0.0881	-97	10	17	APLRAPQR					Mascot
927.5873	927.5493	-0.038	-41	292	299	DVLLNLLK					Mascot
945.4352	945.4153	-0.0199	-21	354	360	FEFYDPK					Mascot
952.4887	952.4767	-0.012	-13	361	368	TPFFTSPR					Mascot
952.4887	952.4767	-0.012	-13	361	368	TPFFTSPR	51	98.47			Mascot
1013.5626	1013.5337	-0.0289	-29	436	445	VPIGVGENTK					Mascot
1061.5626	1061.5651	0.0025	2	246	254	VVQFSEQPK					Mascot

Calc. Mass	Obsrv. Mass	± da	± ppm	Start Seq.	End Seq.	Sequence	Ion Score	C. I. %	Modification	Rank	Result Type
1170.7205	1170.6298	-0.0907	-77	292	301	DVLLNLLKSR					Mascot
1261.6359	1261.6375	0.0016	1	92	103	ATPAVPIGGCYR			Carbamidomethyl (C)[10]		Mascot
1261.6359	1261.6375	0.0016	1	92	103	ATPAVPIGGCYR	76	99.995	Carbamidomethyl (C)[10]		Mascot
1322.6191	1322.5989	-0.0202	-15	446	456	ISNCIIDMNAR			Carbamidomethyl (C)[4], Oxidation (M)[8]		Mascot
1322.6191	1322.5989	-0.0202	-15	446	456	ISNCIIDMNAR	37	61.744	Carbamidomethyl (C)[4], Oxidation (M)[8]		Mascot
1553.7709	1553.7338	-0.0371	-24	278	290	YPYIASMGVYVFK			Oxidation (M)[7]		Mascot
1643.821	1643.8031	-0.0179	-11	120	133	IFVMTQFNSASLNR			Oxidation (M)[4]		Mascot
1646.8174	1646.8174	0	0	302	315	YAELHDFGSEILPR					Mascot
1646.8174	1646.8174	0	0	302	315	YAELHDFGSEILPR	108	100			Mascot
1652.8167	1652.7986	-0.0181	-11	263	277	VDTSFLNFAIDDPAK					Mascot
1674.8732	1674.8409	-0.0323	-19	430	445	LMSEGKVPIGVGENTK			Oxidation (M)[2]		Mascot
1709.8719	1709.8036	-0.0683	-40	278	291	YPYIASMGVYVFKR			Oxidation (M)[7]		Mascot
1756.9592	1756.9644	0.0052	3	189	203	SIEHILILSGDQLYR					Mascot
1756.9592	1756.9644	0.0052	3	189	203	SIEHILILSGDQLYR	110	100			Mascot
1838.8776	1838.8567	-0.0209	-11	104	119	LIDIPMSNCFNSGINK			Carbamidomethyl (C)[9], Oxidation (M)[6]		Mascot
1910.8879	1910.8832	-0.0047	-2	467	482	EGVQEADRPEEGYYIR					Mascot
2055.9399	2055.9438	0.0039	2	213	231	HVDDNADITLSCAPVGES R			Carbamidomethyl (C)[12]		Mascot
2763.3162	2763.3372	0.021	8	316	338	ALHDHNVQAYVFTDYWE DIGTIR					Mascot

7 hypothetical protein SORBIDRAFT_09g029610 [Sorghum bicolor] gi|241945598 58275.1 6.4 16 471 100 27.183 404 100

Peptide Information

Calc. Mass	Obsrv. Mass	± da	± ppm	Start Seq.	End Seq.	Sequence	Ion Score	C. I. %	Modification	Rank	Result Type
820.3804	820.4044	0.024	29	13	19	ACMSPVR			Carbamidomethyl (C)[2]		Mascot
906.4462	906.4552	0.009	10	32	39	IGNSCSIR			Carbamidomethyl (C)[5]		Mascot
945.4352	945.4153	-0.0199	-21	370	376	FEFYDPK					Mascot
952.4887	952.4767	-0.012	-13	377	384	TPFFTSPR					Mascot
952.4887	952.4767	-0.012	-13	377	384	TPFFTSPR	51	98.47			Mascot
1013.5626	1013.5337	-0.0289	-29	455	464	VPIGVGENTK					Mascot
1261.6359	1261.6375	0.0016	1	108	119	ATPAVPIGGCYR			Carbamidomethyl (C)[10]		Mascot
1261.6359	1261.6375	0.0016	1	108	119	ATPAVPIGGCYR	76	99.995	Carbamidomethyl (C)[10]		Mascot
1322.6191	1322.5989	-0.0202	-15	465	475	ISNCIIDMNAR			Carbamidomethyl (C)[4], Oxidation (M)[8]		Mascot
1322.6191	1322.5989	-0.0202	-15	465	475	ISNCIIDMNAR	37	61.744	Carbamidomethyl (C)[4], Oxidation (M)[8]		Mascot
1385.677	1385.642	-0.035	-25	1	12	MQFSSVFPLEGK			Oxidation (M)[1]		Mascot
1503.7882	1503.7552	-0.033	-22	192	202	KFIWVLEDYYK					Mascot
1553.7709	1553.7338	-0.0371	-24	294	306	YPYIASMGVYVFK			Oxidation (M)[7]		Mascot

1643.821	1643.8031	-0.0179	-11	136	149	IFVMTQFNSASLNR			Oxidation (M)[4]		Mascot
1709.8719	1709.8036	-0.0683	-40	294	307	YPYIASMGVYVFKR			Oxidation (M)[7]		Mascot
1770.7826	1770.7554	-0.0272	-15	355	369	SFFDANMALCEQPPK			Carbamidomethyl (C)[10], Oxidation (M)[7]		Mascot
1838.8776	1838.8567	-0.0209	-11	120	135	LIDIPMSNCFNSGINK			Carbamidomethyl (C)[9], Oxidation (M)[6]		Mascot
2055.9399	2055.9438	0.0039	2	229	247	HVDDNADITLSCAPVGES R			Carbamidomethyl (C)[12]		Mascot
2862.4634	2862.4819	0.0185	6	80	107	NYADPNEVAAVILGGGT GTQLFPLTSTR					Mascot
2862.4634	2862.4819	0.0185	6	80	107	NYADPNEVAAVILGGGT GTQLFPLTSTR	239	100			Mascot

8	glucose-1-phosphate adenylyltransferase large subunit 2, /amyloplastic Precursor(ADP-glucose pyrophosphorylase) isoform 1 [Zea mays]	gi|413946675	58212	6.4	13	452	100	26.557	404	100	

Protein Group

glucose-1-phosphate adenylyltransferase large subunit 2, /amyloplastic Precursor(ADP-glucose pyrophosphorylase) isoform 2 [Zea mays]	gi|413946676	58212	6.4000 000953 6743

Peptide Information

Calc. Mass	Obsrv. Mass	± da	± ppm	Start Seq.	End Seq.	Sequence	Ion Score	C. I. %	Modification	Rank	Result Type
820.3804	820.4044	0.024	29	13	19	ACMSPVR			Carbamidomethyl (C)[2]		Mascot
945.4352	945.4153	-0.0199	-21	369	375	FEFYDPK					Mascot
952.4887	952.4767	-0.012	-13	376	383	TPFFTSPR					Mascot
952.4887	952.4767	-0.012	-13	376	383	TPFFTSPR	51	98.47			Mascot
1013.5626	1013.5337	-0.0289	-29	454	463	VPIGVGENTK					Mascot
1204.6572	1204.6145	-0.0427	-35	2	12	QFSSVLPLEGK					Mascot
1261.6359	1261.6375	0.0016	1	107	118	ATPAVPIGGCYR			Carbamidomethyl (C)[10]		Mascot
1261.6359	1261.6375	0.0016	1	107	118	ATPAVPIGGCYR	76	99.995	Carbamidomethyl (C)[10]		Mascot
1322.6191	1322.5989	-0.0202	-15	464	474	ISNCIIDMNAR			Carbamidomethyl (C)[4], Oxidation (M)[8]		Mascot
1322.6191	1322.5989	-0.0202	-15	464	474	ISNCIIDMNAR	37	61.744	Carbamidomethyl (C)[4], Oxidation (M)[8]		Mascot
1503.7882	1503.7552	-0.033	-22	191	201	KFIWVLEDYYK					Mascot
1643.821	1643.8031	-0.0179	-11	135	148	IFVMTQFNSASLNR			Oxidation (M)[4]		Mascot
1770.7826	1770.7554	-0.0272	-15	354	368	SFFDANMALCEQPPK			Carbamidomethyl (C)[10], Oxidation (M)[7]		Mascot
1838.8776	1838.8567	-0.0209	-11	119	134	LIDIPMSNCFNSGINK			Carbamidomethyl (C)[9], Oxidation (M)[6]		Mascot
2055.9399	2055.9438	0.0039	2	228	246	HVDDNADITLSCAPVGES R			Carbamidomethyl (C)[12]		Mascot
2862.4634	2862.4819	0.0185	6	79	106	NYADPNEVAAVILGGGT GTQLFPLTSTR					Mascot
2862.4634	2862.4819	0.0185	6	79	106	NYADPNEVAAVILGGGT GTQLFPLTSTR	239	100			Mascot

9	ADP-glucose pyrophosphorylase large subunit [Zea mays]	gi|413946674	59961.9	6.4	12	439	100	26.368	404	100

Peptide Information

Calc. Mass	Obsrv. Mass	± da	± ppm	Start Seq.	End Seq.	Sequence	Ion Score	C. I. %	Modification	Rank	Result Type
820.3804	820.4044	0.024	29	13	19	ACMSPVR			Carbamidomethyl (C)[2]		Mascot
945.4352	945.4153	-0.0199	-21	385	391	FEFYDPK					Mascot
952.4887	952.4767	-0.012	-13	392	399	TPFFTSPR					Mascot
952.4887	952.4767	-0.012	-13	392	399	TPFFTSPR	51	98.47			Mascot
1013.5626	1013.5337	-0.0289	-29	470	479	VPIGVGENTK					Mascot
1204.6572	1204.6145	-0.0427	-35	2	12	QFSSVLPLEGK					Mascot
1261.6359	1261.6375	0.0016	1	107	118	ATPAVPIGGCYR			Carbamidomethyl (C)[10]		Mascot
1261.6359	1261.6375	0.0016	1	107	118	ATPAVPIGGCYR	76	99.995	Carbamidomethyl (C)[10]		Mascot
1322.6191	1322.5989	-0.0202	-15	480	490	ISNCIIDMNAR			Carbamidomethyl (C)[4], Oxidation (M)[8]		Mascot
1322.6191	1322.5989	-0.0202	-15	480	490	ISNCIIDMNAR	37	61.744	Carbamidomethyl (C)[4], Oxidation (M)[8]		Mascot
1503.7882	1503.7552	-0.033	-22	191	201	KFIWVLEDYYK					Mascot
1643.821	1643.8031	-0.0179	-11	135	148	IFVMTQFNSASLNR			Oxidation (M)[4]		Mascot
1838.8776	1838.8567	-0.0209	-11	119	134	LIDIPMSNCFNSGINK			Carbamidomethyl (C)[9], Oxidation (M)[6]		Mascot
2055.9399	2055.9438	0.0039	2	228	246	HVDDNADITLSCAPVGES R			Carbamidomethyl (C)[12]		Mascot
2862.4634	2862.4819	0.0185	6	79	106	NYADPNEVAAVILGGGT GTQLFPLTSTR					Mascot
2862.4634	2862.4819	0.0185	6	79	106	NYADPNEVAAVILGGGT GTQLFPLTSTR	239	100			Mascot

10	RecName: Full=Glucose-1-phosphate adenylyltransferase large subunit 2, chloroplastic/amyloplastic; AltName: Full=ADP-glucose pyrophosphorylase; AltName: Full=ADP-glucose synthase; AltName: Full=AGPase S; AltName: Full=Alpha-D-glucose-1-phosphate ade	gi|1707928	58863.4	8.37	14	419	100	25.454	367	100

Protein Group

glucose-1-phosphate adenylyltransferase large subunit 2, chloroplastic/amyloplastic [Zea mays]	gi|162458350	58863.4	8.3699 998855 5908

Peptide Information

Calc. Mass	Obsrv. Mass	± da	± ppm	Start Seq.	End Seq.	Sequence	Ion Score	C. I. %	Modification	Rank	Result Type
820.3804	820.4044	0.024	29	13	19	ACMSPVR			Carbamidomethyl (C)[2]		Mascot
945.4352	945.4153	-0.0199	-21	371	377	FEFYDPK					Mascot

952.4887	952.4767	-0.012	-13	378	385	TPFFTSPR				Mascot
952.4887	952.4767	-0.012	-13	378	385	TPFFTSPR	51	98.544		Mascot
1013.5626	1013.5337	-0.0289	-29	456	465	VPIGVGENTK				Mascot
1204.6572	1204.6145	-0.0427	-35	2	12	QFSSVLPLEGK				Mascot
1261.6359	1261.6375	0.0016	1	108	119	ATPAVPIGGCYR			Carbamidomethyl (C)[10]	Mascot
1261.6359	1261.6375	0.0016	1	108	119	ATPAVPIGGCYR	76	99.996	Carbamidomethyl (C)[10]	Mascot
1503.7882	1503.7552	-0.033	-22	192	202	KFIWVLEDYYK				Mascot
1553.774	1553.7338	-0.0402	-26	410	423	ECAIEHSIVGVPSR			Carbamidomethyl (C)[2]	Mascot
1643.821	1643.8031	-0.0179	-11	136	149	IFVMTQFNSASLNR			Oxidation (M)[4]	Mascot
1738.771	1738.9294	0.1584	91	466	479	ISNCIIDMNCQGWK			Carbamidomethyl (C)[4,10]	Mascot
1770.7826	1770.7554	-0.0272	-15	356	370	SFFDANMALCEQPPK			Carbamidomethyl (C)[10], Oxidation (M)[7]	Mascot
1838.8776	1838.8567	-0.0209	-11	120	135	LIDIPMSNCFNSGINK			Carbamidomethyl (C)[9], Oxidation (M)[6]	Mascot
2055.9399	2055.9438	0.0039	2	229	247	HVDDNADITLSCAPVGES R			Carbamidomethyl (C)[12]	Mascot
2862.4634	2862.4819	0.0185	6	80	107	NYADPNEVAAVILGGGT GTQLFPLTSTR				Mascot
2862.4634	2862.4819	0.0185	6	80	107	NYADPNEVAAVILGGGT GTQLFPLTSTR	239	100		Mascot

Gel Idx/Pos	291/L19	Instr./Gel Origin	BA2151/Sample Project 20140814	Process Status	Analysis Succeeded
Plate [#] Name	[1] Sample Project 20140814	Instrument Sample Name		Spectra	11

Rank	Protein Name	Accession No.	Protein MW	Protein PI	Pep. Count	Protein Score	Protein Score C. I. %	Intensity Matched	Total Ion Score	Total Ion C. I. %	Confirmed
1	ATP synthase CF1 alpha subunit (chloroplast) [Secale cereale]	gi|525782213	55318.3	6.11	27	932	100	63.84	754	100	

Protein Group

Protein Name	Accession No.	Protein MW	Protein PI
ATP synthase CF1 alpha subunit (chloroplast) [Aegilops speltoides]	gi|533310198	55318.3	6.1100001335144
ATP synthase CF1 alpha subunit (chloroplast) [Aegilops speltoides]	gi|394986489	55318.3	6.1100001335144
ATP synthase CF1 alpha subunit (chloroplast) [Aegilops tauschii]	gi|533310102	55318.3	6.1100001335144
ATP synthase CF1 alpha subunit (chloroplast) [Aegilops tauschii]	gi|384406849	55318.3	6.1100001335144
ATP synthase CF1 alpha subunit (chloroplast) [Secale cereale]	gi|521301267	55318.3	6.1100001335144
ATP synthase CF1 alpha subunit (chloroplast) [Triticum aestivum]	gi|521301484	55318.3	6.1100001335144
ATP synthase CF1 alpha subunit (chloroplast) [Triticum monococcum subsp. aegilopoides]	gi|521301345	55318.3	6.1100001335144
ATP synthase CF1 alpha subunit (chloroplast) [Triticum urartu]	gi|521301423	55318.3	6.1100001335

			144
ATP synthase CF1 alpha subunit (chloroplast) [Triticum urartu]	gi|525782292	55318.3	6.1100 001335 144
ATP synthase CF1 alpha subunit [Triticum aestivum]	gi|14017569	55318.3	6.1100 001335 144
RecName: Full=ATP synthase subunit alpha, chloroplastic; AltName: Full=ATP synthase F1 sector subunit alpha; AltName: Full=F-ATPase subunit alpha	gi|17371040	55318.3	6.1100 001335 144

Peptide Information

Calc. Mass	Obsrv. Mass	± da	± ppm	Start Seq.	End Seq.	Sequence	Ion Score	C. I. %	Modification	Rank	Result Type
815.4621	815.4515	-0.0106	-13	166	172	ELIIGDR					Mascot
822.4104	822.4001	-0.0103	-13	17	22	IEQYNR					Mascot
857.4839	857.4806	-0.0033	-4	24	31	VGIENIGR					Mascot
876.4971	876.4763	-0.0208	-24	273	279	QMSLLLR			Oxidation (M)[2]		Mascot
917.4799	917.4731	-0.0068	-7	406	413	TSQNQLAR					Mascot
920.52	920.5038	-0.0162	-18	457	463	FLDELRK					Mascot
961.4949	961.4859	-0.009	-9	120	128	GEIIASESR					Mascot
985.5789	985.571	-0.0079	-8	23	31	KVGIENIGR					Mascot
1013.5739	1013.5659	-0.008	-8	32	41	VVQVGDGIAR					Mascot
1107.5542	1107.5417	-0.0125	-11	15	22	ERIEQYNR					Mascot
1115.5691	1115.5665	-0.0026	-2	492	500	EAIQEQLER					Mascot
1115.5691	1115.5665	-0.0026	-2	492	500	EAIQEQLER	52	98.945			Mascot
1274.6951	1274.6492	-0.0459	-36	177	188	TAVATDTILNQK					Mascot
1282.7366	1282.7344	-0.0022	-2	129	140	LIESPAPSIISR					Mascot
1282.7366	1282.7344	-0.0022	-2	129	140	LIESPAPSIISR	72	99.988			Mascot
1292.7097	1292.6729	-0.0368	-28	481	491	TFTEQAEILLK					Mascot
1317.705	1317.6711	-0.0339	-26	256	266	HTLIIYDDLSK					Mascot
1416.7845	1416.7905	0.006	4	95	107	IAQIPVSEAYLGR					Mascot
1416.7845	1416.7905	0.006	4	95	107	IAQIPVSEAYLGR	124	100			Mascot

1438.8376	1438.7535	-0.0841	-58	129	141	LIESPAPSIISRR					Mascot
1507.7639	1507.7301	-0.0338	-22	444	456	GYLDSLEIEQVNK					Mascot
1507.7639	1507.7301	-0.0338	-22	444	456	GYLDSLEIEQVNK	66	99.955			Mascot
1519.8051	1519.8083	0.0032	2	189	202	GQGVICVYVAIGQR			Carbamidomethyl (C)[6]		Mascot
1519.8051	1519.8083	0.0032	2	189	202	GQGVICVYVAIGQR	55	99.445	Carbamidomethyl (C)[6]		Mascot
1553.7384	1553.7443	0.0059	4	285	297	EAYPGDVFYLHSR					Mascot
1553.7384	1553.7443	0.0059	4	285	297	EAYPGDVFYLHSR	103	100			Mascot
1593.853	1593.7144	-0.1386	-87	267	279	QAQAYRQMSLLLR			Oxidation (M)[8]		Mascot
1607.8275	1607.8008	-0.0267	-17	467	480	DTKPQFQEIISSSK					Mascot
2121.0896	2121.1057	0.0161	8	42	61	IIGLGEIMSGELVEFAEGTR					Mascot
2137.0845	2137.0872	0.0027	1	42	61	IIGLGEIMSGELVEFAEGTR			Oxidation (M)[8]		Mascot
2137.0845	2137.0872	0.0027	1	42	61	IIGLGEIMSGELVEFAEGTR	132	100	Oxidation (M)[8]		Mascot
2225.2136	2225.2644	0.0508	23	120	140	GEIIASESRLIESPAPSIISR					Mascot
2289.2158	2289.2336	0.0178	8	142	162	SVYEPLQTGLIAIDSMIPIGR			Oxidation (M)[16]		Mascot
2316.1831	2316.2073	0.0242	10	423	443	QSQANPLPVEEQIATIYTGTR					Mascot
2316.1831	2316.2073	0.0242	10	423	443	QSQANPLPVEEQIATIYTGTR	148	100			Mascot
2445.3171	2445.3088	-0.0083	-3	141	162	RSVYEPLQTGLIAIDSMIPIGR			Oxidation (M)[17]		Mascot

2	ATP synthase CF1 alpha subunit (chloroplast) [Triticum monococcum]	gi|521301187	55318.3	6.11	27	932	100	63.84	754	100	

Protein Group

ATP synthase CF1 alpha subunit (chloroplast) [Triticum monococcum]	gi|525778502	55318.3	6.1100001335144

Peptide Information

Calc. Mass	Obsrv. Mass	± da	± ppm	Start Seq.	End Seq.	Sequence	Ion Score	C. I. %	Modification	Rank	Result Type
815.4621	815.4515	-0.0106	-13	166	172	ELIIGDR					Mascot
822.4104	822.4001	-0.0103	-13	17	22	IEQYNR					Mascot
857.4839	857.4806	-0.0033	-4	24	31	VGIENIGR					Mascot
876.4971	876.4763	-0.0208	-24	273	279	QMSLLLR			Oxidation (M)[2]		Mascot
917.4799	917.4731	-0.0068	-7	406	413	TSQNQLAR					Mascot
920.52	920.5038	-0.0162	-18	457	463	FLDELRK					Mascot
961.4949	961.4859	-0.009	-9	120	128	GEILASESR					Mascot
985.5789	985.571	-0.0079	-8	23	31	KVGIENIGR					Mascot

1013.5739	1013.5659	-0.008	-8	32	41	VVQVGDGIAR					Mascot
1107.5542	1107.5417	-0.0125	-11	15	22	ERIEQYNR					Mascot
1115.5691	1115.5665	-0.0026	-2	492	500	EAIQEQLER					Mascot
1115.5691	1115.5665	-0.0026	-2	492	500	EAIQEQLER	52	98.945			Mascot
1274.6951	1274.6492	-0.0459	-36	177	188	TAVATDTILNQK					Mascot
1282.7366	1282.7344	-0.0022	-2	129	140	LIESPAPSIISR					Mascot
1282.7366	1282.7344	-0.0022	-2	129	140	LIESPAPSIISR	72	99.988			Mascot
1292.7097	1292.6729	-0.0368	-28	481	491	TFTEQAEILLK					Mascot
1317.705	1317.6711	-0.0339	-26	256	266	HTLIIYDDLSK					Mascot
1416.7845	1416.7905	0.006	4	95	107	IAQIPVSEAYLGR					Mascot
1416.7845	1416.7905	0.006	4	95	107	IAQIPVSEAYLGR	124	100			Mascot
1438.8376	1438.7535	-0.0841	-58	129	141	LIESPAPSIISRR					Mascot
1507.7639	1507.7301	-0.0338	-22	444	456	GYLDSLEIEQVNK					Mascot
1507.7639	1507.7301	-0.0338	-22	444	456	GYLDSLEIEQVNK	66	99.955			Mascot
1519.8051	1519.8083	0.0032	2	189	202	GQGVICVYVAIGQR			Carbamidomethyl (C)[6]		Mascot
1519.8051	1519.8083	0.0032	2	189	202	GQGVICVYVAIGQR	55	99.445	Carbamidomethyl (C)[6]		Mascot
1553.7384	1553.7443	0.0059	4	285	297	EAYPGDVFYLHSR					Mascot
1553.7384	1553.7443	0.0059	4	285	297	EAYPGDVFYLHSR	103	100			Mascot
1593.853	1593.7144	-0.1386	-87	267	279	QAQAYRQMSLLLR			Oxidation (M)[8]		Mascot
1607.8275	1607.8008	-0.0267	-17	467	480	DTKPQFQEIISSSK					Mascot
2121.0896	2121.1057	0.0161	8	42	61	IIGLGEIMSGELVEFAEGTR					Mascot
2137.0845	2137.0872	0.0027	1	42	61	IIGLGEIMSGELVEFAEGTR			Oxidation (M)[8]		Mascot
2137.0845	2137.0872	0.0027	1	42	61	IIGLGEIMSGELVEFAEGTR	132	100	Oxidation (M)[8]		Mascot
2225.2136	2225.2644	0.0508	23	120	140	GEILASESRLIESPAPSIISR					Mascot
2289.2158	2289.2336	0.0178	8	142	162	SVYEPLQTGLIAIDSMIPIGR			Oxidation (M)[16]		Mascot
2316.1831	2316.2073	0.0242	10	423	443	QSQANPLPVEEQIATIYTGTR					Mascot
2316.1831	2316.2073	0.0242	10	423	443	QSQANPLPVEEQIATIYTGTR	148	100			Mascot
2445.3171	2445.3088	-0.0083	-3	141	162	RSVYEPLQTGLIAIDSMIPIGR			Oxidation (M)[17]		Mascot

3	ATP synthase subunit alpha, chloroplastic [Triticum urartu]	gi|473750243	58399.8	6.03	27	927	100	63.84	754	100	

Peptide Information

Calc. Mass	Obsrv. Mass	± da	± ppm	Start Seq.	End Seq.	Sequence	Ion Score	C. I. %	Modification	Rank	Result Type
815.4621	815.4515	-0.0106	-13	166	172	ELIIGDR					Mascot

822.4104	822.4001	-0.0103	-13	17	22	IEQYNR				Mascot
857.4839	857.4806	-0.0033	-4	24	31	VGIENIGR				Mascot
876.4971	876.4763	-0.0208	-24	298	304	QMSLLLR			Oxidation (M)[2]	Mascot
917.4799	917.4731	-0.0068	-7	431	438	TSQNQLAR				Mascot
920.52	920.5038	-0.0162	-18	482	488	FLDELRK				Mascot
961.4949	961.4859	-0.009	-9	120	128	GEIIASESR				Mascot
985.5789	985.571	-0.0079	-8	23	31	KVGIENIGR				Mascot
1013.5739	1013.5659	-0.008	-8	32	41	VVQVGDGIAR				Mascot
1107.5542	1107.5417	-0.0125	-11	15	22	ERIEQYNR				Mascot
1115.5691	1115.5665	-0.0026	-2	517	525	EAIQEQLER				Mascot
1115.5691	1115.5665	-0.0026	-2	517	525	EAIQEQLER	52	98.945		Mascot
1274.6951	1274.6492	-0.0459	-36	177	188	TAVATDTILNQK				Mascot
1282.7366	1282.7344	-0.0022	-2	129	140	LIESPAPSIISR				Mascot
1282.7366	1282.7344	-0.0022	-2	129	140	LIESPAPSIISR	72	99.988		Mascot
1292.7097	1292.6729	-0.0368	-28	506	516	TFTEQAEILLK				Mascot
1317.705	1317.6711	-0.0339	-26	281	291	HTLIIYDDLSK				Mascot
1416.7845	1416.7905	0.006	4	95	107	IAQIPVSEAYLGR				Mascot
1416.7845	1416.7905	0.006	4	95	107	IAQIPVSEAYLGR	124	100		Mascot
1438.8376	1438.7535	-0.0841	-58	129	141	LIESPAPSIISRR				Mascot
1507.7639	1507.7301	-0.0338	-22	469	481	GYLDSLEIEQVNK				Mascot
1507.7639	1507.7301	-0.0338	-22	469	481	GYLDSLEIEQVNK	66	99.955		Mascot
1519.8051	1519.8083	0.0032	2	189	202	GQGVICVYVAIGQR			Carbamidomethyl (C)[6]	Mascot
1519.8051	1519.8083	0.0032	2	189	202	GQGVICVYVAIGQR	55	99.445	Carbamidomethyl (C)[6]	Mascot
1553.7384	1553.7443	0.0059	4	310	322	EAYPGDVFYLHSR				Mascot
1553.7384	1553.7443	0.0059	4	310	322	EAYPGDVFYLHSR	103	100		Mascot
1593.853	1593.7144	-0.1386	-87	292	304	QAQAYRQMSLLLR			Oxidation (M)[8]	Mascot
1607.8275	1607.8008	-0.0267	-17	492	505	DTKPQFQEIISSSK				Mascot
2121.0896	2121.1057	0.0161	8	42	61	IIGLGEIMSGELVEFAEGTR				Mascot
2137.0845	2137.0872	0.0027	1	42	61	IIGLGEIMSGELVEFAEGTR			Oxidation (M)[8]	Mascot
2137.0845	2137.0872	0.0027	1	42	61	IIGLGEIMSGELVEFAEGTR	132	100	Oxidation (M)[8]	Mascot
2225.2136	2225.2644	0.0508	23	120	140	GEIIASESRLIESPAPSIISR				Mascot
2289.2158	2289.2336	0.0178	8	142	162	SVYEPLQTGLIAIDSMIPIGR			Oxidation (M)[16]	Mascot
2316.1831	2316.2073	0.0242	10	448	468	QSQANPLPVEEQIATIYTGTR				Mascot
2316.1831	2316.2073	0.0242	10	448	468	QSQANPLPVEEQIATIYTGTR	148	100		Mascot
2445.3171	2445.3088	-0.0083	-3	141	162	RSVYEPLQTGLIAIDSMIPIGR			Oxidation (M)[17]	Mascot

4	ATP synthase subunit alpha, chloroplastic [Triticum urartu]	gi|473826750	61894.6	6.2	27	920	100	63.84	754	100

Protein Group

ATP synthase subunit alpha, chloroplastic [Triticum urartu]	gi|474384779	61894.6	6.1999998092 6514

Peptide Information

Calc. Mass	Obsrv. Mass	± da	± ppm	Start Seq.	End Seq.	Sequence	Ion Score	C. I. %	Modification	Rank	Result Type
815.4621	815.4515	-0.0106	-13	230	236	ELIIGDR					Mascot
822.4104	822.4001	-0.0103	-13	81	86	IEQYNR					Mascot
857.4839	857.4806	-0.0033	-4	88	95	VGIENIGR					Mascot
876.4971	876.4763	-0.0208	-24	337	343	QMSLLLR			Oxidation (M)[2]		Mascot
917.4799	917.4731	-0.0068	-7	470	477	TSQNQLAR					Mascot
920.52	920.5038	-0.0162	-18	521	527	FLDELRK					Mascot
961.4949	961.4859	-0.009	-9	184	192	GEIIASESR					Mascot
985.5789	985.571	-0.0079	-8	87	95	KVGIENIGR					Mascot
1013.5739	1013.5659	-0.008	-8	96	105	VVQVGDGIAR					Mascot
1107.5542	1107.5417	-0.0125	-11	79	86	ERIEQYNR					Mascot
1115.5691	1115.5665	-0.0026	-2	556	564	EAIQEQLER					Mascot
1115.5691	1115.5665	-0.0026	-2	556	564	EAIQEQLER	52	98.945			Mascot
1274.6951	1274.6492	-0.0459	-36	241	252	TAVATDTILNQK					Mascot
1282.7366	1282.7344	-0.0022	-2	193	204	LIESPAPSIISR					Mascot
1282.7366	1282.7344	-0.0022	-2	193	204	LIESPAPSIISR	72	99.988			Mascot
1292.7097	1292.6729	-0.0368	-28	545	555	TFTEQAEILLK					Mascot
1317.705	1317.6711	-0.0339	-26	320	330	HTLIIYDDLSK					Mascot
1416.7845	1416.7905	0.006	4	159	171	IAQIPVSEAYLGR					Mascot
1416.7845	1416.7905	0.006	4	159	171	IAQIPVSEAYLGR	124	100			Mascot
1438.8376	1438.7535	-0.0841	-58	193	205	LIESPAPSIISRR					Mascot
1507.7639	1507.7301	-0.0338	-22	508	520	GYLDSLEIEQVNK					Mascot
1507.7639	1507.7301	-0.0338	-22	508	520	GYLDSLEIEQVNK	66	99.955			Mascot
1519.8051	1519.8083	0.0032	2	253	266	GQGVICVYVAIGQR			Carbamidomethyl (C)[6]		Mascot
1519.8051	1519.8083	0.0032	2	253	266	GQGVICVYVAIGQR	55	99.445	Carbamidomethyl (C)[6]		Mascot
1553.7384	1553.7443	0.0059	4	349	361	EAYPGDVFYLHSR					Mascot
1553.7384	1553.7443	0.0059	4	349	361	EAYPGDVFYLHSR	103	100			Mascot
1593.853	1593.7144	-0.1386	-87	331	343	QAQAYRQMSLLLR			Oxidation (M)[8]		Mascot

Calc. Mass	Obsrv. Mass	± da	± ppm	Start Seq.	End Seq.	Sequence	Ion Score	C. I. %	Modification	Rank	Result Type
1607.8275	1607.8008	-0.0267	-17	531	544	DTKPQFQEIISSSK					Mascot
2121.0896	2121.1057	0.0161	8	106	125	IIGLGEIMSGELVEFAEGTR					Mascot
2137.0845	2137.0872	0.0027	1	106	125	IIGLGEIMSGELVEFAEGTR			Oxidation (M)[8]		Mascot
2137.0845	2137.0872	0.0027	1	106	125	IIGLGEIMSGELVEFAEGTR	132	100	Oxidation (M)[8]		Mascot
2225.2136	2225.2644	0.0508	23	184	204	GEIIASESRLIESPAPSIISR					Mascot
2289.2158	2289.2336	0.0178	8	206	226	SVYEPLQTGLIAIDSMIPIGR			Oxidation (M)[16]		Mascot
2316.1831	2316.2073	0.0242	10	487	507	QSQANPLPVEEQIATIYTGTR					Mascot
2316.1831	2316.2073	0.0242	10	487	507	QSQANPLPVEEQIATIYTGTR	148	100			Mascot
2445.3171	2445.3088	-0.0083	-3	205	226	RSVYEPLQTGLIAIDSMIPIGR			Oxidation (M)[17]		Mascot

5	ATP synthase subunit alpha, chloroplastic [Aegilops tauschii]	gi|475593610	55249.3	6.11	24	902	100	62.532	754	100		

Peptide Information

Calc. Mass	Obsrv. Mass	± da	± ppm	Start Seq.	End Seq.	Sequence	Ion Score	C. I. %	Modification	Rank	Result Type
815.4621	815.4515	-0.0106	-13	166	172	ELIIGDR					Mascot
822.4104	822.4001	-0.0103	-13	17	22	IEQYNR					Mascot
857.4839	857.4806	-0.0033	-4	24	31	VGIENIGR					Mascot
917.4799	917.4731	-0.0068	-7	406	413	TSQNQLAR					Mascot
920.52	920.5038	-0.0162	-18	457	463	FLDELRK					Mascot
961.4949	961.4859	-0.009	-9	120	128	GEIIASESR					Mascot
985.5789	985.571	-0.0079	-8	23	31	KVGIENIGR					Mascot
1013.5739	1013.5659	-0.008	-8	32	41	VVQVGDGIAR					Mascot
1115.5691	1115.5665	-0.0026	-2	492	500	EAIQEQLER					Mascot
1115.5691	1115.5665	-0.0026	-2	492	500	EAIQEQLER	52	98.945			Mascot
1274.6951	1274.6492	-0.0459	-36	177	188	TAVATDTILNQK					Mascot
1282.7366	1282.7344	-0.0022	-2	129	140	LIESPAPSIISR					Mascot
1282.7366	1282.7344	-0.0022	-2	129	140	LIESPAPSIISR	72	99.988			Mascot
1317.705	1317.6711	-0.0339	-26	256	266	HTLIIYDDLSK					Mascot
1384.7729	1384.6361	-0.1368	-99	273	284	QMSLLLTRPPGR			Oxidation (M)[2]		Mascot
1416.7845	1416.7905	0.006	4	95	107	IAQIPVSEAYLGR					Mascot
1416.7845	1416.7905	0.006	4	95	107	IAQIPVSEAYLGR	124	100			Mascot
1438.8376	1438.7535	-0.0841	-58	129	141	LIESPAPSIISRR					Mascot
1507.7639	1507.7301	-0.0338	-22	444	456	GYLDSLEIEQVNK					Mascot
1507.7639	1507.7301	-0.0338	-22	444	456	GYLDSLEIEQVNK	66	99.955			Mascot

Calc. Mass	Obsrv. Mass	± da	± ppm	Start Seq.	End Seq.	Sequence	Ion Score	C. I. %	Modification	Rank	Result Type
1519.8051	1519.8083	0.0032	2	189	202	GQGVICVYVAIGQR			Carbamidomethyl (C)[6]		Mascot
1519.8051	1519.8083	0.0032	2	189	202	GQGVICVYVAIGQR	55	99.445	Carbamidomethyl (C)[6]		Mascot
1553.7384	1553.7443	0.0059	4	285	297	EAYPGDVFYLHSR					Mascot
1553.7384	1553.7443	0.0059	4	285	297	EAYPGDVFYLHSR	103	100			Mascot
1607.8275	1607.8008	-0.0267	-17	467	480	DTKPQFQEIISSSK					Mascot
2121.0896	2121.1057	0.0161	8	42	61	IIGLGEIMSGELVEFAEGTR					Mascot
2137.0845	2137.0872	0.0027	1	42	61	IIGLGEIMSGELVEFAEGTR			Oxidation (M)[8]		Mascot
2137.0845	2137.0872	0.0027	1	42	61	IIGLGEIMSGELVEFAEGTR	132	100	Oxidation (M)[8]		Mascot
2225.2136	2225.2644	0.0508	23	120	140	GEIIASESRLIESPAPSIISR					Mascot
2289.2158	2289.2336	0.0178	8	142	162	SVYEPLQTGLIAIDSMIPIGR			Oxidation (M)[16]		Mascot
2316.1831	2316.2073	0.0242	10	423	443	QSQANPLPVEEQIATIYTGTR					Mascot
2316.1831	2316.2073	0.0242	10	423	443	QSQANPLPVEEQIATIYTGTR	148	100			Mascot
2445.3171	2445.3088	-0.0083	-3	141	162	RSVYEPLQTGLIAIDSMIPIGR			Oxidation (M)[17]		Mascot

6	ATP synthase CF1 alpha subunit [Festuca altissima]	gi|410177763	55773.5	6.11	26	865	100	62.063	699	100	

Protein Group

ATP synthase CF1 alpha subunit [Festuca altissima]	gi|427436972	55773.5	6.1100001335144

Peptide Information

Calc. Mass	Obsrv. Mass	± da	± ppm	Start Seq.	End Seq.	Sequence	Ion Score	C. I. %	Modification	Rank	Result Type
815.4621	815.4515	-0.0106	-13	166	172	ELIIGDR					Mascot
822.4104	822.4001	-0.0103	-13	17	22	IEQYNR					Mascot
857.4839	857.4806	-0.0033	-4	24	31	VGIENIGR					Mascot
876.4971	876.4763	-0.0208	-24	273	279	QMSLLLR			Oxidation (M)[2]		Mascot
917.4799	917.4731	-0.0068	-7	406	413	TSQNQLAR					Mascot
920.52	920.5038	-0.0162	-18	457	463	FLDELRK					Mascot
961.4949	961.4859	-0.009	-9	120	128	GEIIASESR					Mascot
985.5789	985.571	-0.0079	-8	23	31	KVGIENIGR					Mascot
1013.5739	1013.5659	-0.008	-8	32	41	VVQVGDGIAR					Mascot
1107.5542	1107.5417	-0.0125	-11	15	22	ERIEQYNR					Mascot
1115.5691	1115.5665	-0.0026	-2	492	500	EAIQEQLER					Mascot
1115.5691	1115.5665	-0.0026	-2	492	500	EAIQEQLER	52	98.945			Mascot

1274.6951	1274.6492	-0.0459	-36	177	188	TAVATDTILNQK				Mascot
1282.7366	1282.7344	-0.0022	-2	129	140	LIESPAPSIISR				Mascot
1282.7366	1282.7344	-0.0022	-2	129	140	LIESPAPSIISR	72	99.988		Mascot
1292.7097	1292.6729	-0.0368	-28	481	491	TFTEQAEILLK				Mascot
1317.705	1317.6711	-0.0339	-26	256	266	HTLIIYDDLSK				Mascot
1416.7845	1416.7905	0.006	4	95	107	IAQIPVSEAYLGR				Mascot
1416.7845	1416.7905	0.006	4	95	107	IAQIPVSEAYLGR	124	100		Mascot
1438.8376	1438.7535	-0.0841	-58	129	141	LIESPAPSIISRR				Mascot
1507.7639	1507.7301	-0.0338	-22	444	456	GYLDSLEIEQVNK				Mascot
1507.7639	1507.7301	-0.0338	-22	444	456	GYLDSLEIEQVNK	66	99.955		Mascot
1553.7384	1553.7443	0.0059	4	285	297	EAYPGDVFYLHSR				Mascot
1553.7384	1553.7443	0.0059	4	285	297	EAYPGDVFYLHSR	103	100		Mascot
1593.853	1593.7144	-0.1386	-87	267	279	QAQAYRQMSLLLR			Oxidation (M)[8]	Mascot
1607.8275	1607.8008	-0.0267	-17	467	480	DTKPQFQEILSSSK				Mascot
2121.0896	2121.1057	0.0161	8	42	61	IIGLGEIMSGELVEFAEGTR				Mascot
2137.0845	2137.0872	0.0027	1	42	61	IIGLGEIMSGELVEFAEGTR			Oxidation (M)[8]	Mascot
2137.0845	2137.0872	0.0027	1	42	61	IIGLGEIMSGELVEFAEGTR	132	100	Oxidation (M)[8]	Mascot
2225.2136	2225.2644	0.0508	23	120	140	GEIIASESRLIESPAPSIISR				Mascot
2289.2158	2289.2336	0.0178	8	142	162	SVYEPLQTGLIAIDSMIPIGR			Oxidation (M)[16]	Mascot
2316.1831	2316.2073	0.0242	10	423	443	QSQANPLPVEEQIATIYTGTR				Mascot
2316.1831	2316.2073	0.0242	10	423	443	QSQANPLPVEEQIATIYTGTR	148	100		Mascot
2445.3171	2445.3088	-0.0083	-3	141	162	RSVYEPLQTGLIAIDSMIPIGR			Oxidation (M)[17]	Mascot

7	ATP synthase CF1 alpha subunit [Lolium multiflorum]	gi|427437215	55761.4	6.11	25	855	100	61.03	699	100		

Protein Group

ATP synthase CF1 alpha subunit [Lolium multiflorum]	gi|410178024	55761.4	6.1100001335144
RecName: Full=ATP synthase subunit alpha, chloroplastic; AltName: Full=ATP synthase F1 sector subunit alpha; AltName: Full=F-ATPase subunit alpha	gi|190358183	55761.4	6.1100001335144
atpA gene product (chloroplast) [Lolium perenne]	gi|159106861	55761.4	6.1100001335144

Peptide Information

Calc. Mass	Obsrv. Mass	± da	± ppm	Start Seq.	End Seq.	Sequence	Ion Score	C. I. %	Modification	Rank	Result Type
815.4621	815.4515	-0.0106	-13	166	172	ELIIGDR					Mascot
822.4104	822.4001	-0.0103	-13	17	22	IEQYNR					Mascot
857.4839	857.4806	-0.0033	-4	24	31	VGIENIGR					Mascot
876.4971	876.4763	-0.0208	-24	273	279	QMSLLLR			Oxidation (M)[2]		Mascot
917.4799	917.4731	-0.0068	-7	406	413	TSQNQLAR					Mascot
961.4949	961.4859	-0.009	-9	120	128	GEIIASESR					Mascot
985.5789	985.571	-0.0079	-8	23	31	KVGIENIGR					Mascot
1013.5739	1013.5659	-0.008	-8	32	41	VVQVGDGIAR					Mascot
1107.5542	1107.5417	-0.0125	-11	15	22	ERIEQYNR					Mascot
1115.5691	1115.5665	-0.0026	-2	492	500	EAIQEQLER					Mascot
1115.5691	1115.5665	-0.0026	-2	492	500	EAIQEQLER	52	98.945			Mascot
1274.6951	1274.6492	-0.0459	-36	177	188	TAVATDTILNQK					Mascot
1282.7366	1282.7344	-0.0022	-2	129	140	LIESPAPSIISR					Mascot
1282.7366	1282.7344	-0.0022	-2	129	140	LIESPAPSIISR	72	99.988			Mascot
1292.7097	1292.6729	-0.0368	-28	481	491	TFTEQAEILLK					Mascot
1317.705	1317.6711	-0.0339	-26	256	266	HTLIIYDDLSK					Mascot
1416.7845	1416.7905	0.006	4	95	107	IAQIPVSEAYLGR					Mascot
1416.7845	1416.7905	0.006	4	95	107	IAQIPVSEAYLGR	124	100			Mascot
1438.8376	1438.7535	-0.0841	-58	129	141	LIESPAPSIISRR					Mascot
1507.7639	1507.7301	-0.0338	-22	444	456	GYLDSLEIEQVNK					Mascot
1507.7639	1507.7301	-0.0338	-22	444	456	GYLDSLEIEQVNK	66	99.955			Mascot
1553.7384	1553.7443	0.0059	4	285	297	EAYPGDVFYLHSR					Mascot
1553.7384	1553.7443	0.0059	4	285	297	EAYPGDVFYLHSR	103	100			Mascot
1593.853	1593.7144	-0.1386	-87	267	279	QAQAYRQMSLLLR			Oxidation (M)[8]		Mascot
1607.8275	1607.8008	-0.0267	-17	467	480	DTKPQFQEILSSSK					Mascot
2121.0896	2121.1057	0.0161	8	42	61	IIGLGEIMSGELVEFAEGT R					Mascot
2137.0845	2137.0872	0.0027	1	42	61	IIGLGEIMSGELVEFAEGT R			Oxidation (M)[8]		Mascot
2137.0845	2137.0872	0.0027	1	42	61	IIGLGEIMSGELVEFAEGT R	132	100	Oxidation (M)[8]		Mascot
2225.2136	2225.2644	0.0508	23	120	140	GEIIASESRLIESPAPSIIS R					Mascot
2289.2158	2289.2336	0.0178	8	142	162	SVYEPLQTGLIAIDSMIPI GR			Oxidation (M)[16]		Mascot
2316.1831	2316.2073	0.0242	10	423	443	QSQANPLPVEEQIATIYT GTR					Mascot

2316.1831	2316.2073	0.0242	10	423	443	QSQANPLPVEEQIATIYT GTR	148	100			Mascot
2445.3171	2445.3088	-0.0083	-3	141	162	RSVYEPLQTGLIAIDSMIPI GR			Oxidation (M)[17]		Mascot

8 ATP synthase CF1 alpha subunit (chloroplast) [Hordeum vulgare subsp. spontaneum] gi|521301026 55317.3 6.32 26 787 100 60.526 622 100

Protein Group

ATP synthase CF1 alpha subunit (chloroplast) [Hordeum vulgare subsp. spontaneum]	gi|521301107	55317.3	6.3200 001716 6138
ATP synthase CF1 alpha subunit (chloroplast) [Hordeum vulgare subsp. vulgare]	gi|521300949	55317.3	6.3200 001716 6138
ATP synthase CF1 alpha subunit [Hordeum vulgare subsp. vulgare]	gi|118430385	55317.3	6.3200 001716 6138
RecName: Full=ATP synthase subunit alpha, chloroplastic; AltName: Full=ATP synthase F1 sector subunit alpha; AltName: Full=F-ATPase subunit alpha	gi|190358176	55317.3	6.3200 001716 6138

Peptide Information

Calc. Mass	Obsrv. Mass	± da	± ppm	Start Seq.	End Seq.	Sequence	Ion Score	C. I. %	Modification	Rank	Result Type
815.4621	815.4515	-0.0106	-13	166	172	ELIIGDR					Mascot
822.4104	822.4001	-0.0103	-13	17	22	IEQYNR					Mascot
857.4839	857.4806	-0.0033	-4	24	31	VGIENIGR					Mascot
876.4971	876.4763	-0.0208	-24	273	279	QMSLLLR			Oxidation (M)[2]		Mascot
917.4799	917.4731	-0.0068	-7	406	413	TSQNQLAR					Mascot
920.52	920.5038	-0.0162	-18	457	463	FLDELRK					Mascot
961.4949	961.4859	-0.009	-9	120	128	GEIIASESR					Mascot
985.5789	985.571	-0.0079	-8	23	31	KVGIENIGR					Mascot
1013.5739	1013.5659	-0.008	-8	32	41	VVQVGDGIAR					Mascot
1107.5542	1107.5417	-0.0125	-11	15	22	ERIEQYNR					Mascot
1115.5691	1115.5665	-0.0026	-2	492	500	EAIQEQLER					Mascot
1115.5691	1115.5665	-0.0026	-2	492	500	EAIQEQLER	52	98.945			Mascot

1274.6951	1274.6492	-0.0459	-36	177	188	TAVATDTILNQK					Mascot
1282.7366	1282.7344	-0.0022	-2	129	140	LIESPAPSIISR					Mascot
1282.7366	1282.7344	-0.0022	-2	129	140	LIESPAPSIISR	72	99.988			Mascot
1292.7097	1292.6729	-0.0368	-28	481	491	TFTEQAEILLK					Mascot
1317.705	1317.6711	-0.0339	-26	256	266	HTLIIYDDLSK					Mascot
1416.7845	1416.7905	0.006	4	95	107	IAQIPVSEAYLGR					Mascot
1416.7845	1416.7905	0.006	4	95	107	IAQIPVSEAYLGR	124	100			Mascot
1438.8376	1438.7535	-0.0841	-58	129	141	LIESPAPSIISRR					Mascot
1507.7639	1507.7301	-0.0338	-22	444	456	GYLDSLEIEQVNK					Mascot
1507.7639	1507.7301	-0.0338	-22	444	456	GYLDSLEIEQVNK	66	99.955			Mascot
1519.8051	1519.8083	0.0032	2	189	202	GQGVICVYVAIGQR			Carbamidomethyl (C)[6]		Mascot
1519.8051	1519.8083	0.0032	2	189	202	GQGVICVYVAIGQR	55	99.445	Carbamidomethyl (C)[6]		Mascot
1553.7384	1553.7443	0.0059	4	285	297	EAYPGDVFYLHSR					Mascot
1553.7384	1553.7443	0.0059	4	285	297	EAYPGDVFYLHSR	103	100			Mascot
1593.853	1593.7144	-0.1386	-87	267	279	QAQAYRQMSLLLR			Oxidation (M)[8]		Mascot
1607.8275	1607.8008	-0.0267	-17	467	480	DTKPQFQEIISSSK					Mascot
2225.2136	2225.2644	0.0508	23	120	140	GEIIASESRLIESPAPSIIS R					Mascot
2289.2158	2289.2336	0.0178	8	142	162	SVYEPLQTGLIAIDSMIPI GR			Oxidation (M)[16]		Mascot
2316.1831	2316.2073	0.0242	10	423	443	QSQANPLPVEEQIATIYT GTR					Mascot
2316.1831	2316.2073	0.0242	10	423	443	QSQANPLPVEEQIATIYT GTR	148	100			Mascot
2445.3171	2445.3088	-0.0083	-3	141	162	RSVYEPLQTGLIAIDSMIPI GR			Oxidation (M)[17]		Mascot

9	ATP synthase CF1 alpha subunit [Festuca ovina]	gi|410177850	55374.3	6.11	23	768	100	59.57	633	100	

Protein Group

ATP synthase CF1 alpha subunit [Festuca ovina]	gi|426406636	55374.3	6.1100 001335 144

Peptide Information

Calc. Mass	Obsrv. Mass	± da	± ppm	Start Seq.	End Seq.	Sequence	Ion Score	C. I. %	Modification	Rank	Result Type
815.4621	815.4515	-0.0106	-13	166	172	ELIIGDR					Mascot
822.4104	822.4001	-0.0103	-13	17	22	IEQYNR					Mascot
857.4839	857.4806	-0.0033	-4	24	31	VGIENIGR					Mascot
876.4971	876.4763	-0.0208	-24	273	279	QMSLLLR			Oxidation (M)[2]		Mascot
917.4799	917.4731	-0.0068	-7	406	413	TSQNQLAR					Mascot

920.52	920.5038	-0.0162	-18	457	463	FLDELRK					Mascot
985.5789	985.571	-0.0079	-8	23	31	KVGIENIGR					Mascot
1013.5739	1013.5659	-0.008	-8	32	41	VVQVGDGIAR					Mascot
1107.5542	1107.5417	-0.0125	-11	15	22	ERIEQYNR					Mascot
1115.5691	1115.5665	-0.0026	-2	492	500	EAIQEQLER					Mascot
1115.5691	1115.5665	-0.0026	-2	492	500	EAIQEQLER	52	98.945			Mascot
1274.6951	1274.6492	-0.0459	-36	177	188	TAVATDTILNQK					Mascot
1282.7366	1282.7344	-0.0022	-2	129	140	LIESPAPSIISR					Mascot
1282.7366	1282.7344	-0.0022	-2	129	140	LIESPAPSIISR	72	99.988			Mascot
1292.7097	1292.6729	-0.0368	-28	481	491	TFTEQAEILLK					Mascot
1317.705	1317.6711	-0.0339	-26	256	266	HTLIIYDDLSK					Mascot
1416.7845	1416.7905	0.006	4	95	107	IAQIPVSEAYLGR					Mascot
1416.7845	1416.7905	0.006	4	95	107	IAQIPVSEAYLGR	124	100			Mascot
1438.8376	1438.7535	-0.0841	-58	129	141	LIESPAPSIISRR					Mascot
1553.7384	1553.7443	0.0059	4	285	297	EAYPGDVFYLHSR					Mascot
1553.7384	1553.7443	0.0059	4	285	297	EAYPGDVFYLHSR	103	100			Mascot
1593.853	1593.7144	-0.1386	-87	267	279	QAQAYRQMSLLLR			Oxidation (M)[8]		Mascot
1607.8275	1607.8008	-0.0267	-17	467	480	DTKPQFQEILSSSK					Mascot
2121.0896	2121.1057	0.0161	8	42	61	IIGLGEIMSGELVEFAEGT R					Mascot
2137.0845	2137.0872	0.0027	1	42	61	IIGLGEIMSGELVEFAEGT R			Oxidation (M)[8]		Mascot
2137.0845	2137.0872	0.0027	1	42	61	IIGLGEIMSGELVEFAEGT R	132	100	Oxidation (M)[8]		Mascot
2289.2158	2289.2336	0.0178	8	142	162	SVYEPLQTGLIAIDSMIPI GR			Oxidation (M)[16]		Mascot
2316.1831	2316.2073	0.0242	10	423	443	QSQANPLPVEEQIATIYT GTR					Mascot
2316.1831	2316.2073	0.0242	10	423	443	QSQANPLPVEEQIATIYT GTR	148	100			Mascot
2445.3171	2445.3088	-0.0083	-3	141	162	RSVYEPLQTGLIAIDSMIPI GR			Oxidation (M)[17]		Mascot

10 ATP synthase CF1 alpha subunit [Agrostis stolonifera] gi|118430299 55491.4 6.11 23 761 100 58.911 627 100

Protein Group

RecName: Full=ATP synthase subunit alpha, chloroplastic; AltName: Full=ATP synthase F1 sector subunit alpha; AltName: Full=F-ATPase subunit alpha gi|125987712 55491.4 6.1100001335144

Peptide Information

Calc. Mass	Obsrv. Mass	± da	± ppm	Start Seq.	End Seq.	Sequence	Ion Score	C. I. %	Modification	Rank	Result Type
815.4621	815.4515	-0.0106	-13	166	172	ELIIGDR					Mascot

822.4104	822.4001	-0.0103	-13	17	22	IEQYNR				Mascot
857.4839	857.4806	-0.0033	-4	24	31	VGIENIGR				Mascot
876.4971	876.4763	-0.0208	-24	273	279	QMSLLLR			Oxidation (M)[2]	Mascot
917.4799	917.4731	-0.0068	-7	406	413	TSQNQLAR				Mascot
920.52	920.5038	-0.0162	-18	457	463	FLDELRK				Mascot
961.4949	961.4859	-0.009	-9	120	128	GEIIASESR				Mascot
985.5789	985.571	-0.0079	-8	23	31	KVGIENIGR				Mascot
1013.5739	1013.5659	-0.008	-8	32	41	VVQVGDGIAR				Mascot
1107.5542	1107.5417	-0.0125	-11	15	22	ERIEQYNR				Mascot
1115.5691	1115.5665	-0.0026	-2	492	500	EAIQEQLER				Mascot
1115.5691	1115.5665	-0.0026	-2	492	500	EAIQEQLER	52	99.091		Mascot
1274.6951	1274.6492	-0.0459	-36	177	188	TAVATDTILNQK				Mascot
1292.7097	1292.6729	-0.0368	-28	481	491	TFTEQAEILLK				Mascot
1317.705	1317.6711	-0.0339	-26	256	266	HTLIIYDDLSK				Mascot
1416.7845	1416.7905	0.006	4	95	107	IAQIPVSEAYLGR				Mascot
1416.7845	1416.7905	0.006	4	95	107	IAQIPVSEAYLGR	124	100		Mascot
1507.7639	1507.7301	-0.0338	-22	444	456	GYLDSLEIEQVNK				Mascot
1507.7639	1507.7301	-0.0338	-22	444	456	GYLDSLEIEQVNK	66	99.961		Mascot
1553.7384	1553.7443	0.0059	4	285	297	EAYPGDVFYLHSR				Mascot
1553.7384	1553.7443	0.0059	4	285	297	EAYPGDVFYLHSR	103	100		Mascot
1593.853	1593.7144	-0.1386	-87	267	279	QAQAYRQMSLLLR			Oxidation (M)[8]	Mascot
1607.8275	1607.8008	-0.0267	-17	467	480	DTKPQFQEIISSSK				Mascot
2121.0896	2121.1057	0.0161	8	42	61	IIGLGEIMSGELVEFAEGT R				Mascot
2137.0845	2137.0872	0.0027	1	42	61	IIGLGEIMSGELVEFAEGT R			Oxidation (M)[8]	Mascot
2137.0845	2137.0872	0.0027	1	42	61	IIGLGEIMSGELVEFAEGT R	132	100	Oxidation (M)[8]	Mascot
2289.2158	2289.2336	0.0178	8	142	162	SVYEPLQTGLIAIDSMIPI GR			Oxidation (M)[16]	Mascot
2316.1831	2316.2073	0.0242	10	423	443	QSQANPLPVEEQIATIYT GTR				Mascot
2316.1831	2316.2073	0.0242	10	423	443	QSQANPLPVEEQIATIYT GTR	148	100		Mascot
2445.3171	2445.3088	-0.0083	-3	141	162	RSVYEPLQTGLIAIDSMIPI GR			Oxidation (M)[17]	Mascot

Gel Idx/Pos	292/L20	Instr./Gel Origin	BA2151/Sample Project 20140814	Process Status	Analysis Succeeded
Plate [#] Name	[1] Sample Project 20140814	Instrument Sample Name		Spectra	11

Rank	Protein Name	Accession No.	Protein MW	Protein PI	Pep. Count	Protein Score	Protein Score C. I. %	Intensity Matched	Total Ion Score	Total Ion C. I. %	Confirmed
1	12S seed storage globulin 1 [Aegilops tauschii]	gi|475608251	62758.7	6.43	10	508	100	72.448	478	100	

Peptide Information

Calc. Mass	Obsrv. Mass	± da	± ppm	Start Seq.	End Seq.	Sequence	Ion Score	C. I. %	Modification	Rank	Result Type
826.3512	826.3635	0.0123	15	22	27	ECTFNR			Carbamidomethyl (C)[2]		Mascot
885.5152	885.5341	0.0189	21	28	35	LQASTPLR					Mascot
923.4767	923.4944	0.0177	19	56	63	CTGVFAIR			Carbamidomethyl (C)[1]		Mascot
1116.5472	1116.5741	0.0269	24	180	188	EFLFAGNYR					Mascot
1116.5472	1116.5741	0.0269	24	180	188	EFLFAGNYR	67	99.968			Mascot
1263.7208	1263.7505	0.0297	24	237	246	FLKPVFTQQR					Mascot
1263.7208	1263.7505	0.0297	24	237	246	FLKPVFTQQR	56	99.646			Mascot
1381.7012	1381.7372	0.036	26	178	188	HKEFLFAGNYR					Mascot
1381.7012	1381.7372	0.036	26	178	188	HKEFLFAGNYR	65	99.951			Mascot
1908.9199	1908.9667	0.0468	25	189	205	SSQLHSSQNIFSGFDVR					Mascot
1908.9199	1908.9667	0.0468	25	189	205	SSQLHSSQNIFSGFDVR	137	100			Mascot
2062.8989	2062.9482	0.0493	24	39	55	SQAGLTEYFDEENEQFR					Mascot
2062.8989	2062.9482	0.0493	24	39	55	SQAGLTEYFDEENEQFR	152	100			Mascot
2107.9204	2107.9399	0.0195	9	247	264	EPESYPHTQYEEGQSQA K					Mascot
3353.7634	3353.6753	-0.0881	-26	486	518	TNANSMVSHIAGKNSILG ALPVDVIANAYGISR					Mascot

Rank	Protein Name	Accession No.	Protein MW	Protein PI	Pep. Count	Protein Score	Protein Score C. I. %	Intensity Matched	Total Ion Score	Total Ion C. I. %	Confirmed
2	12S seed storage globulin 1 [Triticum urartu]	gi|474246751	64229.5	6.62	9	498	100	71.519	476	100	

Peptide Information

Calc. Mass	Obsrv. Mass	± da	± ppm	Start Seq.	End Seq.	Sequence	Ion Score	C. I. %	Modification	Rank	Result Type
826.3512	826.3635	0.0123	15	44	49	ECTFNR			Carbamidomethyl (C)[2]		Mascot
885.5152	885.5341	0.0189	21	50	57	LQASTPLR					Mascot
1116.5472	1116.5741	0.0269	24	202	210	EFLFAGNYR					Mascot
1116.5472	1116.5741	0.0269	24	202	210	EFLFAGNYR	67	99.968			Mascot
1263.7208	1263.7505	0.0297	24	259	268	FLKPVFTQQR					Mascot
1263.7208	1263.7505	0.0297	24	259	268	FLKPVFTQQR	56	99.646			Mascot
1381.7012	1381.7372	0.036	26	200	210	HKEFLFAGNYR					Mascot

1381.7012	1381.7372	0.036	26	200	210	HKEFLFAGNYR	65	99.951			Mascot
1666.7966	1666.7762	-0.0204	-12	312	326	HIQGEQPQMGQSQAK					Mascot
1908.9199	1908.9667	0.0468	25	211	227	SSQLHSSQNIFSGFDVR					Mascot
1908.9199	1908.9667	0.0468	25	211	227	SSQLHSSQNIFSGFDVR	137	100			Mascot
2062.8989	2062.9482	0.0493	24	61	77	SQAGLTEYFDEENEQFR					Mascot
2062.8989	2062.9482	0.0493	24	61	77	SQAGLTEYFDEENEQFR	152	100			Mascot
2107.9204	2107.9399	0.0195	9	269	286	EPESYPHTQYEEGQSQAK					Mascot

3	Mitochondrial transcription termination factor family protein, putative isoform 1 [Theobroma cacao]	gi|508712975	51008.1	9.32	14	53	0	22.004

Protein Group

Mitochondrial transcription termination factor family protein, putative isoform 1 [Theobroma cacao]	gi|508712974	51008.1	9.3199996948 2422
Mitochondrial transcription termination factor family protein, putative isoform 1 [Theobroma cacao]	gi|508712973	51008.1	9.3199996948 2422
Mitochondrial transcription termination factor family protein, putative isoform 1 [Theobroma cacao]	gi|508712976	51008.1	9.3199996948 2422

Peptide Information

Calc. Mass	Obsrv. Mass	± da	± ppm	Start Seq.	End Seq.	Sequence	Ion Score	C. I. %	Modification	Rank	Result Type
822.4066	822.3866	-0.02	-24	433	439	SFMEPLV					Mascot
866.4651	866.4651	0	0	1	8	MAVTSLTK			Oxidation (M)[1]		Mascot
885.5516	885.5341	-0.0175	-20	275	282	AGLEVKLR					Mascot
1058.5015	1058.5083	0.0068	6	398	406	FVEDAGHQR					Mascot
1098.5314	1098.5603	0.0289	26	421	429	YTESKEDVK					Mascot
1263.6732	1263.7505	0.0773	61	180	190	VEFFEGIGIPR					Mascot
1263.6732	1263.7505	0.0773	61	180	190	VEFFEGIGIPR					Mascot
1364.7817	1364.7655	-0.0162	-12	1	13	MAVTSLTKATTLK					Mascot
1380.7767	1380.7087	-0.068	-49	1	13	MAVTSLTKATTLK			Oxidation (M)[1]		Mascot
1438.7577	1438.7573	-0.0004	0	16	28	LSSTQFFSTVPPK					Mascot
1547.7668	1547.7542	-0.0126	-8	236	249	DPRVLGMALGEMSR			Oxidation (M)[7]		Mascot

1634.9265	1634.7838	-0.1427	-87	346	358	QIVPRYNVIEYLK					Mascot
1664.8003	1664.807	0.0067	4	45	58	CGFPPSQFHTFLAR			Carbamidomethyl (C)[1]		Mascot
2119.0681	2119.031	-0.0371	-18	378	393	LRFYNLYVKPYPECEK			Carbamidomethyl (C)[14]		Mascot
2194.2119	2194.0408	-0.1711	-78	351	370	YNVIEYLKSNGALGLEIGL K					Mascot
2194.2119	2194.0408	-0.1711	-78	351	370	YNVIEYLKSNGALGLEIGL K					Mascot
2248.186	2247.9897	-0.1963	-87	212	230	LKPLLEEFVELGFSENEV R					Mascot

4	hypothetical protein ARALYDRAFT_321053 [Arabidopsis lyrata subsp. lyrata]	gi|297327110	200219.9	5.45	24	52	0	38.662

Peptide Information

Calc. Mass	Obsrv. Mass	± da	± ppm	Start Seq.	End Seq.	Sequence	Ion Score	C. I. %	Modification	Rank	Result Type
822.3951	822.3866	-0.0085	-10	609	615	SREESSK					Mascot
866.473	866.4651	-0.0079	-9	763	769	VLTYQSR					Mascot
1003.5054	1003.4968	-0.0086	-9	923	930	TQEVEELR					Mascot
1057.4507	1057.5122	0.0615	58	486	493	MEQEEYTK					Mascot
1116.626	1116.5741	-0.0519	-46	938	947	LQLGETQVTK					Mascot
1116.626	1116.5741	-0.0519	-46	938	947	LQLGETQVTK					Mascot
1261.6688	1261.7255	0.0567	45	1369	1379	KPPQPTSFFGR					Mascot
1262.7579	1262.7115	-0.0464	-37	61	72	TVVAKVNAVHPK					Mascot
1291.6576	1291.7385	0.0809	63	859	869	KAAITTQCGWR			Carbamidomethyl (C)[8]		Mascot
1320.7675	1320.7614	-0.0061	-5	1565	1575	KNYVPLFLAQK					Mascot
1393.6682	1393.7296	0.0614	44	235	245	FVEIQFNHMGR			Oxidation (M)[9]		Mascot
1438.6818	1438.7573	0.0755	52	1218	1229	MSPQMSFTRPPK			Oxidation (M)[1,5]		Mascot
1534.7869	1534.8468	0.0599	39	1217	1229	KMSPQMSFTRPPK					Mascot
1678.8364	1678.8494	0.013	8	384	397	ALENSLCNRVMVTR			Carbamidomethyl (C)[7], Oxidation (M)[11]		Mascot
1890.9531	1891.0233	0.0702	37	124	138	RLPHLYGNEIMEQYK					Mascot
1906.948	1907.0146	0.0666	35	124	138	RLPHLYGNEIMEQYK			Oxidation (M)[11]		Mascot
1946.9852	1946.9208	-0.0644	-33	160	179	MINEGVSQAILVSGESGA GK					Mascot
2046.0702	2046.0138	-0.0564	-28	235	252	FVEIQFNHMGRISGAAIR					Mascot
2062.0652	2061.988	-0.0772	-37	235	252	FVEIQFNHMGRISGAAIR			Oxidation (M)[9]		Mascot
2063.0193	2062.9482	-0.0711	-34	533	550	STHDTLAEKLYQTFGSHK					Mascot
2063.0193	2062.9482	-0.0711	-34	533	550	STHDTLAEKLYQTFGSHK					Mascot
2091.075	2090.9734	-0.1016	-49	159	179	KMINEGVSQAILVSGESG AGK			Oxidation (M)[2]		Mascot
2091.075	2090.9734	-0.1016	-49	159	179	KMINEGVSQAILVSGESG AGK			Oxidation (M)[2]		Mascot
2119.0461	2119.031	-0.0151	-7	738	757	AGQMAELDAHRAEVLGH					Mascot

						SAR					
2129.9292	2129.9443	0.0151	7	1067	1085	ESATSDYEMLSNLAAENER					Mascot
2135.041	2134.9883	-0.0527	-25	738	757	AGQMAELDAHRAEVLGHSAR			Oxidation (M)[4]		Mascot
2145.9241	2146.085	0.1609	75	1067	1085	ESATSDYEMLSNLAAENER			Oxidation (M)[9]		Mascot
2145.9241	2146.085	0.1609	75	1067	1085	ESATSDYEMLSNLAAENER			Oxidation (M)[9]		Mascot
2159.0979	2158.979	-0.1189	-55	203	222	SVEQQVLESNPVLEAFGNAK					Mascot
2258.1704	2258.0017	-0.1687	-75	1411	1429	QQLTAYIETIYGIFQENVK					Mascot
2265.0935	2265.0088	-0.0847	-37	139	159	GTDFGELSPHPFAVADSAYRK					Mascot
3618.6499	3618.6265	-0.0234	-6	1054	1085	HYVTSSNISDQLKESATSDYEMLSNLAAENER			Oxidation (M)[22]		Mascot
3618.6499	3618.6265	-0.0234	-6	1054	1085	HYVTSSNISDQLKESATSDYEMLSNLAAENER			Oxidation (M)[22]		Mascot

5 hypothetical protein F775_27450 [Aegilops tauschii] gi|475592631 83361.6 10.05 16 52 0 31.549

Peptide Information

Calc. Mass	Obsrv. Mass	± da	± ppm	Start Seq.	End Seq.	Sequence	Ion Score	C. I. %	Modification	Rank	Result Type
906.5156	906.4699	-0.0457	-50	503	510	HADVVPLR					Mascot
979.4592	979.4737	0.0145	15	743	751	YGEAAGQQR					Mascot
1057.5889	1057.5122	-0.0767	-73	573	581	ENTLKLDPK					Mascot
1269.6508	1269.6855	0.0347	27	614	624	TSKPEMKSYAK					Mascot
1285.6456	1285.724	0.0784	61	614	624	TSKPEMKSYAK			Oxidation (M)[6]		Mascot
1320.7535	1320.7614	0.0079	6	683	694	VPNPPPPPPPRR					Mascot
1364.6917	1364.7655	0.0738	54	397	409	EPAPNNANIATPR					Mascot
1380.7845	1380.7087	-0.0758	-55	249	261	GPESAKPIVINQK					Mascot
1403.7754	1403.7128	-0.0626	-45	372	384	ELAPKHANVATPR					Mascot
1661.7548	1661.7866	0.0318	19	156	170	DHNESISNGISTMTR					Mascot
1677.7498	1677.7709	0.0211	13	156	170	DHNESISNGISTMTR			Oxidation (M)[13]		Mascot
1906.8344	1907.0146	0.1802	95	197	213	CGQDDLVMPNEATMTPK			Carbamidomethyl (C)[1]		Mascot
1922.8293	1922.9498	0.1205	63	197	213	CGQDDLVMPNEATMTPK			Carbamidomethyl (C)[1], Oxidation (M)[8]		Mascot
2062.9355	2062.9482	0.0127	6	197	214	CGQDDLVMPNEATMTPKR			Carbamidomethyl (C)[1]		Mascot
2062.9355	2062.9482	0.0127	6	197	214	CGQDDLVMPNEATMTPKR			Carbamidomethyl (C)[1]		Mascot
2078.9304	2078.9392	0.0088	4	197	214	CGQDDLVMPNEATMTPKR			Carbamidomethyl (C)[1], Oxidation (M)[8]		Mascot
2106.9404	2106.9263	-0.0141	-7	322	341	DGSRNPAGMASSCRPSTAER			Carbamidomethyl (C)[13]		Mascot
2129.1436	2128.9453	-0.1983	-93	428	446	HANIATPQRPSTAERRPAR					Mascot
2192.0435	2192.1018	0.0583	27	588	609	AMPGDSNGAMATAATPQKAITR			Oxidation (M)[2,10]		Mascot

Calc. Mass	Obsrv. Mass	± da	± ppm	Start Seq.	End Seq.	Sequence	Ion Score	C. I. %	Modification	Rank	Result Type
2210.0078	2210.0166	0.0088	4	523	542	DTGLKHANVGNPCCPSTPER			Carbamidomethyl (C)[13,14]		Mascot
2210.0078	2210.0166	0.0088	4	523	542	DTGLKHANVGNPCCPSTPER			Carbamidomethyl (C)[13,14]		Mascot
2242.1687	2242.0032	-0.1655	-74	422	442	EPAAPKHANIATPQRPSTAER					Mascot

6	hypothetical protein CARUB_v10021033mg, partial [Capsella rubella]	gi|482571614	18862.7	5.89	8	51	0	21.409

Peptide Information

Calc. Mass	Obsrv. Mass	± da	± ppm	Start Seq.	End Seq.	Sequence	Ion Score	C. I. %	Modification	Rank	Result Type
940.4743	940.506	0.0317	34	121	127	YVCVLMR			Carbamidomethyl (C)[3]		Mascot
1116.6372	1116.5741	-0.0631	-57	1	12	VLVTGASSGIGR					Mascot
1116.6372	1116.5741	-0.0631	-57	1	12	VLVTGASSGIGR					Mascot
1363.6709	1363.7332	0.0623	46	13	24	EICLDLGKAGCK			Carbamidomethyl (C)[3,11]		Mascot
1678.8503	1678.8494	-0.0009	-1	160	175	GGVDTITRMMAIELGV			Oxidation (M)[9]		Mascot
1908.8611	1908.9667	0.1056	55	91	106	GNVKSSLDWSEEEWDK					Mascot
1908.8611	1908.9667	0.1056	55	91	106	GNVKSSLDWSEEEWDK					Mascot
2045.106	2045.01	-0.096	-47	1	20	VLVTGASSGIGREICLDLGK			Carbamidomethyl (C)[15]		Mascot
2106.1013	2105.9856	-0.1157	-55	147	167	GLLPGGLAYACSKGGVDTITR			Carbamidomethyl (C)[11]		Mascot
2144.1135	2144.0625	-0.051	-24	72	90	EAWEIFGNIDVLINNAGIR					Mascot

7	multi-sensor hybrid histidine kinase [Bathycoccus prasinos]	gi|412990839	117830.8	6.44	21	51	0	39.128

Peptide Information

Calc. Mass	Obsrv. Mass	± da	± ppm	Start Seq.	End Seq.	Sequence	Ion Score	C. I. %	Modification	Rank	Result Type
866.389	866.4651	0.0761	88	990	996	EQYPDSK					Mascot
977.4469	977.4518	0.0049	5	1050	1058	QRATSPSAC			Carbamidomethyl (C)[9]		Mascot
1058.5114	1058.5083	-0.0031	-3	484	493	SHELSEATGK					Mascot
1204.6168	1204.566	-0.0508	-42	766	777	STDGGSAEKKPK					Mascot
1219.6793	1219.7335	0.0542	44	907	918	APESAGPPPKLR					Mascot
1263.6362	1263.7505	0.1143	90	527	538	DTGIGLSANMIR			Oxidation (M)[10]		Mascot
1263.6362	1263.7505	0.1143	90	527	538	DTGIGLSANMIR			Oxidation (M)[10]		Mascot
1269.7526	1269.6855	-0.0671	-53	76	85	LQEEILQLRK					Mascot
1320.7271	1320.7614	0.0343	26	555	567	FDGSGLGLAISRK					Mascot
1340.6239	1340.655	0.0311	23	430	440	LAHMCPHFSPK			Carbamidomethyl (C)[5], Oxidation (M)[4]		Mascot
1363.6383	1363.7332	0.0949	70	649	660	VNMSIGDENGRR			Oxidation (M)[3]		Mascot
1380.6503	1380.7087	0.0584	42	703	714	DVAAHPEDEKNR					Mascot

1677.8088	1677.7709	-0.0379	-23	934	948	AVLGKCGCDDVVWAK			Carbamidomethyl (C)[6,8]		Mascot
1678.9011	1678.8494	-0.0517	-31	738	753	QKLVSEDGSTFLGAVK					Mascot
1890.9266	1891.0233	0.0967	51	413	429	VNSSLAMNDDWITPLSK					Mascot
1906.9215	1907.0146	0.0931	49	413	429	VNSSLAMNDDWITPLSK			Oxidation (M)[7]		Mascot
1908.8831	1908.9667	0.0836	44	568	585	LCEAMGGTIWAESEGLGK			Carbamidomethyl (C)[2]		Mascot
1908.8831	1908.9667	0.0836	44	568	585	LCEAMGGTIWAESEGLGK			Carbamidomethyl (C)[2]		Mascot
1930.944	1930.9395	-0.0045	-2	165	180	NCRFLQGPETEPEVVR			Carbamidomethyl (C)[2]		Mascot
2019.0038	2018.9517	-0.0521	-26	184	200	YAVMNGLSITCQLKNYK			Carbamidomethyl (C)[11], Oxidation (M)[4]		Mascot
2091.0789	2090.9734	-0.1055	-50	96	115	TSTSSANANQTALSNNLLRK					Mascot
2091.0789	2090.9734	-0.1055	-50	96	115	TSTSSANANQTALSNNLLRK					Mascot
2100.9744	2100.8896	-0.0848	-40	1012	1028	DNCMRDGFTGWLTKPFR			Carbamidomethyl (C)[3]		Mascot
2146.1326	2146.085	-0.0476	-22	847	865	LCEAILAQSNFEAVTKPVR			Carbamidomethyl (C)[2]		Mascot
2146.1326	2146.085	-0.0476	-22	847	865	LCEAILAQSNFEAVTKPVR			Carbamidomethyl (C)[2]		Mascot
2232.1692	2232.0066	-0.1626	-73	94	114	LRTSTSSANANQTALSNNLLR					Mascot

8	unknown protein [Bathycoccus prasinos]	gi|412987805	39698.6	6.88	13	51	0	2.423

Peptide Information

Calc. Mass	Obsrv. Mass	± da	± ppm	Start Seq.	End Seq.	Sequence	Ion Score	C. I. %	Modification	Rank	Result Type
822.3661	822.3866	0.0205	25	90	96	MEENVGK			Oxidation (M)[1]		Mascot
866.473	866.4651	-0.0079	-9	292	298	FRELSSK					Mascot
876.4495	876.4258	-0.0237	-27	193	199	IDMEQLK					Mascot
929.3934	929.4495	0.0561	60	123	129	TSGCWYR			Carbamidomethyl (C)[4]		Mascot
951.4564	951.4329	-0.0235	-25	146	153	EARSMVDK			Oxidation (M)[5]		Mascot
979.4955	979.4737	-0.0218	-22	163	170	SSQWTRSK					Mascot
1547.8693	1547.7542	-0.1151	-74	235	248	THLPHEVAPPPPKK					Mascot
1600.6917	1600.7362	0.0445	28	171	183	MIPGADLCTQCYR			Carbamidomethyl (C)[8,11], Oxidation (M)[1]		Mascot
1664.765	1664.807	0.042	25	31	44	DQIVAYDPEDKEDK					Mascot
1678.901	1678.8494	-0.0516	-31	258	272	RPVIIPDISPEDAEK					Mascot
2061.1702	2061.0049	-0.1653	-80	258	275	RPVIIPDISPEDAEKLLR					Mascot
2193.9741	2194.0408	0.0667	30	210	233	KSDDAEEAESGASDATAAGGIGGK					Mascot
2193.9741	2194.0408	0.0667	30	210	233	KSDDAEEAESGASDATAAGGIGGK					Mascot
2227.1089	2227.021	-0.0879	-39	47	67	EADGGNPLTKEQESEALPTLK					Mascot

9	TPA: hypothetical protein ZEAMMB73_104357 [Zea	gi|414880998	39868.6	7.09	11	50	0	19.444

mays]

Peptide Information

Calc. Mass	Obsrv. Mass	± da	± ppm	Start Seq.	End Seq.	Sequence	Ion Score	C. I. %	Modification	Rank	Result Type
906.4427	906.4699	0.0272	30	25	31	SWNSREK					Mascot
1098.579	1098.5603	-0.0187	-17	343	352	EQPNKIAIEG					Mascot
1634.7333	1634.7838	0.0505	31	248	260	TSILDDFDFYENR					Mascot
1661.8951	1661.7866	-0.1085	-65	188	200	WNGFFSVKWHIIK					Mascot
1664.8391	1664.807	-0.0321	-19	116	130	SVKYNVWASTPNGNK					Mascot
1696.9269	1696.7806	-0.1463	-86	271	285	LLTTSFDHPLPKAEK					Mascot
1890.8868	1891.0233	0.1365	72	248	262	TSILDDFDFYENRQK					Mascot
1908.8969	1908.9667	0.0698	37	306	323	SDEGQGNNVGMVLDTTK K			Oxidation (M)[11]		Mascot
1908.8969	1908.9667	0.0698	37	306	323	SDEGQGNNVGMVLDTTK K			Oxidation (M)[11]		Mascot
2100.9907	2100.8896	-0.1011	-48	225	242	DTQEVNFSQGTEMLNIFK					Mascot
2192.002	2192.1018	0.0998	46	179	195	NMNFWQQDKWNGFFSV K			Oxidation (M)[2]		Mascot
2242.1562	2242.0032	-0.153	-68	62	85	SDVVGLSSAGDANASIPS PVAISK					Mascot

10 hypothetical protein F775_03321 [Aegilops tauschii] gi|475615600 45746.9 10.4 11 50 0 1.47

Peptide Information

Calc. Mass	Obsrv. Mass	± da	± ppm	Start Seq.	End Seq.	Sequence	Ion Score	C. I. %	Modification	Rank	Result Type
875.4621	875.4527	-0.0094	-11	225	231	ITPPEYR					Mascot
1003.5432	1003.4968	-0.0464	-46	324	333	GRGGPAAVYR					Mascot
1340.6667	1340.655	-0.0117	-9	214	224	ELEPPPVFPCR			Carbamidomethyl (C)[10]		Mascot
1547.8475	1547.7542	-0.0933	-60	148	162	RGLQGVAGPLAPHMK			Oxidation (M)[14]		Mascot
1930.8926	1930.9395	0.0469	24	379	395	FSCVYVPSVVDPSMESK			Carbamidomethyl (C)[3]		Mascot
1946.8875	1946.9208	0.0333	17	379	395	FSCVYVPSVVDPSMESK			Carbamidomethyl (C)[3], Oxidation (M)[14]		Mascot
2129.0669	2128.9453	-0.1216	-57	326	346	GGPAAVYRMHVQVGQS ASGTR					Mascot
2144.0151	2144.0625	0.0474	22	377	395	GRFSCVYVPSVVDPSME SK			Carbamidomethyl (C)[5]		Mascot
2151.9587	2152.0068	0.0481	22	279	296	GTCGYDVDRGVWEMLH EK			Carbamidomethyl (C)[3]		Mascot
2162.2332	2162.0422	-0.191	-88	238	258	IASYATVGSHILLSVAVHV PK					Mascot
2216.0728	2216.0227	-0.0501	-23	379	397	FSCVYVPSVVDPSMESK LR			Carbamidomethyl (C)[3], Oxidation (M)[14]		Mascot
3347.5613	3347.646	0.0847	25	186	213	GAFGLDYLPWFEQISFED GRPRCAEVCK			Carbamidomethyl (C)[23,27]		Mascot

Gel Idx/Pos	293/L21	Instr./Gel Origin	BA2151/Sample Project 20140814	Process Status	Analysis Succeeded
Plate [#] Name	[1] Sample Project 20140814	Instrument Sample Name		Spectra	11

Rank	Protein Name	Accession No.	Protein MW	Protein PI	Pep. Count	Protein Score	Protein Score C. I. %	Intensity Matched	Total Ion Score	Total Ion C. I. %	Confirmed
1	12S seed storage globulin 1 [Aegilops tauschii]	gi|475608251	62758.7	6.43	12	572	100	72.035	529	100	

Peptide Information

Calc. Mass	Obsrv. Mass	± da	± ppm	Start Seq.	End Seq.	Sequence	Ion Score	C. I. %	Modification	Rank	Result Type
826.3512	826.3522	0.001	1	22	27	ECTFNR			Carbamidomethyl (C)[2]		Mascot
885.5152	885.5197	0.0045	5	28	35	LQASTPLR					Mascot
923.4767	923.4773	0.0006	1	56	63	CTGVFAIR			Carbamidomethyl (C)[1]		Mascot
1116.5472	1116.5555	0.0083	7	180	188	EFLFAGNYR					Mascot
1116.5472	1116.5555	0.0083	7	180	188	EFLFAGNYR	61	99.862			Mascot
1263.7208	1263.7356	0.0148	12	237	246	FLKPVFTQQR					Mascot
1263.7208	1263.7356	0.0148	12	237	246	FLKPVFTQQR	67	99.968			Mascot
1381.7012	1381.718	0.0168	12	178	188	HKEFLFAGNYR					Mascot
1381.7012	1381.718	0.0168	12	178	188	HKEFLFAGNYR	91	100			Mascot
1639.7671	1639.7712	0.0041	3	110	124	YGQSQSVQGQSQSQK					Mascot
1728.7759	1728.7894	0.0135	8	300	314	HTQGEQPQMGWSQAK			Oxidation (M)[9]		Mascot
1908.9199	1908.9552	0.0353	18	189	205	SSQLHSSQNIFSGFDVR					Mascot
1908.9199	1908.9552	0.0353	18	189	205	SSQLHSSQNIFSGFDVR	142	100			Mascot
2062.8989	2062.9451	0.0462	22	39	55	SQAGLTEYFDEENEQFR					Mascot
2062.8989	2062.9451	0.0462	22	39	55	SQAGLTEYFDEENEQFR	146	100			Mascot
2107.9204	2107.9377	0.0173	8	247	264	EPESYPHTQYEEGQSQA K					Mascot
2107.9204	2107.9377	0.0173	8	247	264	EPESYPHTQYEEGQSQA K	23	0			Mascot
3353.7634	3353.7017	-0.0617	-18	486	518	TNANSMVSHIAGKNSILG ALPVDVIANAYGISR					Mascot

Rank	Protein Name	Accession No.	Protein MW	Protein PI	Pep. Count	Protein Score	Protein Score C. I. %	Intensity Matched	Total Ion Score	Total Ion C. I. %	Confirmed
2	12S seed storage globulin 1 [Triticum urartu]	gi|474246751	64229.5	6.62	9	550	100	71.136	528	100	

Peptide Information

Calc. Mass	Obsrv. Mass	± da	± ppm	Start Seq.	End Seq.	Sequence	Ion Score	C. I. %	Modification	Rank	Result Type
826.3512	826.3522	0.001	1	44	49	ECTFNR			Carbamidomethyl (C)[2]		Mascot
885.5152	885.5197	0.0045	5	50	57	LQASTPLR					Mascot
1116.5472	1116.5555	0.0083	7	202	210	EFLFAGNYR					Mascot
1116.5472	1116.5555	0.0083	7	202	210	EFLFAGNYR	61	99.862			Mascot

Calc. Mass	Obsrv. Mass	± da	± ppm	Start Seq.	End Seq.	Sequence	Ion Score	C. I. %	Modification	Rank	Result Type
1263.7208	1263.7356	0.0148	12	259	268	FLKPVFTQQR					Mascot
1263.7208	1263.7356	0.0148	12	259	268	FLKPVFTQQR	67	99.968			Mascot
1381.7012	1381.718	0.0168	12	200	210	HKEFLFAGNYR					Mascot
1381.7012	1381.718	0.0168	12	200	210	HKEFLFAGNYR	91	100			Mascot
1666.7966	1666.7633	-0.0333	-20	312	326	HIQGEQPQMGQSQAK					Mascot
1908.9199	1908.9552	0.0353	18	211	227	SSQLHSSQNIFSGFDVR					Mascot
1908.9199	1908.9552	0.0353	18	211	227	SSQLHSSQNIFSGFDVR	142	100			Mascot
2062.8989	2062.9451	0.0462	22	61	77	SQAGLTEYFDEENEQFR					Mascot
2062.8989	2062.9451	0.0462	22	61	77	SQAGLTEYFDEENEQFR	146	100			Mascot
2107.9204	2107.9377	0.0173	8	269	286	EPESYPHTQYEEGQSQA K					Mascot
2107.9204	2107.9377	0.0173	8	269	286	EPESYPHTQYEEGQSQA K	23	0			Mascot

3 TPA: hypothetical protein ZEAMMB73_104357 [Zea mays] gi|414880998 39868.6 7.09 12 59 0 20.897

Peptide Information

Calc. Mass	Obsrv. Mass	± da	± ppm	Start Seq.	End Seq.	Sequence	Ion Score	C. I. %	Modification	Rank	Result Type
904.4669	904.4637	-0.0032	-4	261	267	QKVMQDR					Mascot
906.4427	906.4564	0.0137	15	25	31	SWNSREK					Mascot
1098.579	1098.5452	-0.0338	-31	343	352	EQPNKIAIEG					Mascot
1634.7333	1634.7667	0.0334	20	248	260	TSILDDFDFYENR					Mascot
1661.8951	1661.7749	-0.1202	-72	188	200	WNGFFSVKWHIIK					Mascot
1664.8391	1664.7537	-0.0854	-51	116	130	SVKYNVWASTPNGNK					Mascot
1696.9269	1696.7863	-0.1406	-83	271	285	LLTTSFDHPLPKAEK					Mascot
1890.8868	1890.9977	0.1109	59	248	262	TSILDDFDFYENRQK					Mascot
1908.8969	1908.9552	0.0583	31	306	323	SDEGQGNNVGMVLDTTK K			Oxidation (M)[11]		Mascot
1908.8969	1908.9552	0.0583	31	306	323	SDEGQGNNVGMVLDTTK K			Oxidation (M)[11]		Mascot
2100.9907	2100.8921	-0.0986	-47	225	242	DTQEVNFSQGTEMLNIFK					Mascot
2176.0071	2176.1079	0.1008	46	179	195	NMNFWQQDKWNGFFSV K					Mascot
2192.002	2192.0518	0.0498	23	179	195	NMNFWQQDKWNGFFSV K			Oxidation (M)[2]		Mascot
2242.1562	2242.0107	-0.1455	-65	62	85	SDVVGLSSAGDANASIPS PVAISK					Mascot
2242.1562	2242.0107	-0.1455	-65	62	85	SDVVGLSSAGDANASIPS PVAISK	2	0			Mascot

4 conserved hypothetical protein [Ricinus communis] gi|223536470 7738.9 6.9 7 57 0 26.329

Peptide Information

Calc. Mass	Obsrv. Mass	± da	± ppm	Start	End	Sequence	Ion	C. I. %	Modification	Rank	Result Type

				Seq.	Seq.		Score				
826.3512	826.3522	0.001	1	1	7	MDGQYGR					Mascot
842.4189	842.463	0.0441	52	32	38	VLHGECK			Carbamidomethyl (C)[6]		Mascot
936.4897	936.4298	-0.0599	-64	2	9	DGQYGRIK					Mascot
1098.6167	1098.5452	-0.0715	-65	58	66	HKPNTFKAR					Mascot
1116.5433	1116.5555	0.0122	11	21	31	VGVGDDWGVGR					Mascot
1116.5433	1116.5555	0.0122	11	21	31	VGVGDDWGVGR					Mascot
2062.9565	2062.9451	-0.0114	-6	39	57	DEDVSKAHTSPFESIGTD K					Mascot
2062.9565	2062.9451	-0.0114	-6	39	57	DEDVSKAHTSPFESIGTD K					Mascot
2242.1252	2242.0107	-0.1145	-51	45	64	AHTSPFESIGTDKHKPNT FK					Mascot
2242.1252	2242.0107	-0.1145	-51	45	64	AHTSPFESIGTDKHKPNT FK					Mascot

5 PREDICTED: phospholipase D delta-like [Setaria italica] gi|514822940 99635 8.09 19 56 0 28.984

Peptide Information

Calc. Mass	Obsrv. Mass	± da	± ppm	Start Seq.	End Seq.	Sequence	Ion Score	C. I. %	Modification	Rank	Result Type
842.4155	842.463	0.0475	56	752	758	GQVYGYR					Mascot
904.4886	904.4637	-0.0249	-28	544	551	SIHSAYVK					Mascot
923.5057	923.4773	-0.0284	-31	111	119	VEVAHAAAR					Mascot
979.5458	979.4662	-0.0796	-81	406	413	DLDTIFKK					Mascot
1244.7109	1244.6582	-0.0527	-42	544	554	SIHSAYVKAIR					Mascot
1380.6682	1380.6949	0.0267	19	177	187	YEPIEDNPLYK					Mascot
1381.5909	1381.718	0.1271	92	27	37	CLPNMDIMSER			Carbamidomethyl (C)[1], Oxidation (M)[5]		Mascot
1381.5909	1381.718	0.1271	92	27	37	CLPNMDIMSER			Carbamidomethyl (C)[1], Oxidation (M)[5]		Mascot
1403.6697	1403.6797	0.01	7	368	380	CVIVDSQAAGNNR			Carbamidomethyl (C)[1]		Mascot
1507.7574	1507.7385	-0.0189	-13	382	395	ITAFIGGLDLCDGR			Carbamidomethyl (C)[11]		Mascot
1639.7421	1639.7712	0.0291	18	729	742	DTEIAMGAYQPHYK			Oxidation (M)[6]		Mascot
1715.9227	1715.7756	-0.1471	-86	467	480	VASWHYDTLIKLNR					Mascot
1875.9137	1875.9746	0.0609	32	413	428	KDFHNPTFPVNSYGPR					Mascot
1878.9919	1879.0388	0.0469	25	4	21	GSAELEAAVLLHGDLDIR					Mascot
1905.9231	1906.0125	0.0894	47	22	37	IVEAKCLPNMDIMSER			Carbamidomethyl (C)[6]		Mascot
1966.0393	1965.9669	-0.0724	-37	126	144	DNDVFGAQLIGVASVPVH K					Mascot
2018.0376	2017.9053	-0.1323	-66	520	537	GFPKLVQEAESQNLVCA K			Carbamidomethyl (C)[16]		Mascot
2062.9651	2062.9451	-0.02	-10	313	329	FLFKTDGVMHTHDEETR					Mascot

	2062.9651	2062.9451	-0.02	-10	313	329	FLFKTDGVMHTHDEETR					Mascot
	2090.019	2089.9851	-0.0339	-16	177	195	YEPIEDNPLYKDGVGAGP R					Mascot
	2195.1489	2195.0439	-0.105	-48	1	21	MGKGSAELEAAVLLHGD LDIR					Mascot

6	NB-ARC domain-containing disease resistance protein, putative isoform 1 [Theobroma cacao]	gi|508718026	252016.1	5.84	28	56	0	35.057

Protein Group

NB-ARC domain-containing disease resistance protein, putative isoform 1 [Theobroma cacao]	gi|508718027	252016.1	5.8400 001525 8789

Peptide Information

Calc. Mass	Obsrv. Mass	± da	± ppm	Start Seq.	End Seq.	Sequence	Ion Score	C. I. %	Modification	Rank	Result Type
808.3691	808.3516	-0.0175	-22	2122	2127	ECPKMK			Carbamidomethyl (C)[2], Oxidation (M)[5]		Mascot
842.473	842.463	-0.01	-12	996	1003	AAVPSLER					Mascot
936.5189	936.4298	-0.0891	-95	2110	2117	FEFPVLGK					Mascot
1116.4813	1116.5555	0.0742	66	1403	1410	MTIQYCER			Carbamidomethyl (C)[6], Oxidation (M)[1]		Mascot
1116.4813	1116.5555	0.0742	66	1403	1410	MTIQYCER			Carbamidomethyl (C)[6], Oxidation (M)[1]		Mascot
1248.7198	1248.7161	-0.0037	-3	2	13	DFVVGVVSSIVK					Mascot
1269.6547	1269.6744	0.0197	16	52	62	ERVQHSVDTAK					Mascot
1301.7577	1301.6593	-0.0984	-76	841	851	QIVFPQLTSLR					Mascot
1381.6846	1381.718	0.0334	24	113	124	YKLSVDAEEEAK					Mascot
1381.6846	1381.718	0.0334	24	113	124	YKLSVDAEEEAK					Mascot
1409.8224	1409.7151	-0.1073	-76	1973	1984	HALTLTRLSELR					Mascot
1507.8003	1507.7385	-0.0618	-41	1680	1692	LYDLDLSSINVQK					Mascot
1547.8217	1547.7727	-0.049	-32	364	375	LFEWENALRELK					Mascot
1678.837	1678.8386	0.0016	1	1907	1920	GTWHSQVLSTKCFK			Carbamidomethyl (C)[12]		Mascot
1696.8225	1696.7863	-0.0362	-21	485	498	DHHMFVLRDGDVQK					Mascot
1728.8561	1728.7894	-0.0667	-39	1730	1743	HFMQLHSLSILDCK			Carbamidomethyl (C)[13]		Mascot
1733.8164	1733.8439	0.0275	16	1608	1622	IESCSNLTSFYLGSR			Carbamidomethyl (C)[4]		Mascot
1890.9395	1890.9977	0.0582	31	1186	1200	CFYPGQHTILWPMLK			Carbamidomethyl (C)[1]		Mascot
1907.8754	1908.0073	0.1319	69	101	114	CFLGLCPCFWTRYK			Carbamidomethyl (C)[1,6,8]		Mascot
1908.9373	1908.9552	0.0179	9	1411	1428	MTAIVANEGDENVITFGK					Mascot
1908.9373	1908.9552	0.0179	9	1411	1428	MTAIVANEGDENVITFGK					Mascot
2019.0343	2018.9275	-0.1068	-53	1186	1201	CFYPGQHTILWPMLKK			Carbamidomethyl (C)[1]		Mascot
2046.0325	2046.0022	-0.0303	-15	1608	1625	IESCSNLTSFYLGSRALK			Carbamidomethyl (C)[4]		Mascot

Calc. Mass	Obsrv. Mass	± da	± ppm	Start Seq.	End Seq.	Sequence	Ion Score	C. I. %	Modification	Rank	Result Type
2062.0273	2062.0437	0.0164	8	1303	1321	LVFCEGDVGEKPDAGILSR			Carbamidomethyl (C)[4]		Mascot
2091.1121	2090.9656	-0.1465	-70	1229	1246	GQSLFFGEEVIPKLEELR					Mascot
2091.1121	2090.9656	-0.1465	-70	1229	1246	GQSLFFGEEVIPKLEELR					Mascot
2108.0811	2107.9377	-0.1434	-68	306	323	NFAVGLLNENEAWDLFKK					Mascot
2108.0811	2107.9377	-0.1434	-68	306	323	NFAVGLLNENEAWDLFKK					Mascot
2163.1909	2163.3882	0.1973	91	203	222	EGKLFDSVVIATVTQTLDVK					Mascot
2178.0073	2178.0786	0.0713	33	710	727	IFIGDGAWDRFSVYECSR			Carbamidomethyl (C)[16]		Mascot
3528.804	3528.6387	-0.1653	-47	1028	1058	LKEMTVSFCDELLALFPSNMLGVLQSLESLK			Carbamidomethyl (C)[9], Oxidation (M)[4]		Mascot
3618.7561	3618.656	-0.1001	-28	1730	1759	HFMQLHSLSILDCKIMEEVILMEGLTEEEK			Carbamidomethyl (C)[13], Oxidation (M)[3]		Mascot
3618.7561	3618.656	-0.1001	-28	1730	1759	HFMQLHSLSILDCKIMEEVILMEGLTEEEK			Carbamidomethyl (C)[13], Oxidation (M)[3]		Mascot
3646.728	3646.6821	-0.0459	-13	1744	1774	IMEEVILMEGLTEEEKMSSMLLIVNCDSLNK			Carbamidomethyl (C)[26], Oxidation (M)[2,8,17]		Mascot

7 conserved hypothetical protein [Ricinus communis] gi|223530466 68101.5 6.35 15 54 0 3.749

Peptide Information

Calc. Mass	Obsrv. Mass	± da	± ppm	Start Seq.	End Seq.	Sequence	Ion Score	C. I. %	Modification	Rank	Result Type
826.4305	826.3522	-0.0783	-95	372	378	EKATSYK					Mascot
904.5032	904.4637	-0.0395	-44	1	7	METVRLR					Mascot
922.4628	922.4489	-0.0139	-15	418	425	EGDVIAYR					Mascot
929.3781	929.4438	0.0657	71	448	454	YDMQSNR			Oxidation (M)[3]		Mascot
936.4996	936.4298	-0.0698	-75	218	226	SESSKALSK					Mascot
1098.5651	1098.5452	-0.0199	-18	521	529	VPVRDQDNR					Mascot
1248.6431	1248.7161	0.073	58	277	287	KEQSSQTLAEK					Mascot
1269.7162	1269.6744	-0.0418	-33	513	524	SVAGEIDKVPVR					Mascot
1285.5953	1285.6833	0.088	68	445	454	ISRYDMQSNR			Oxidation (M)[6]		Mascot
1931.0961	1930.9198	-0.1763	-91	8	24	LVFDQILSKVQNTQGLK					Mascot
2145.9326	2146.0811	0.1485	69	530	549	TGCRSNNGNESHVSAQENGK			Carbamidomethyl (C)[3]		Mascot
2159.1455	2158.9331	-0.2124	-98	314	332	DSDLEDDIVPIVIRPGHIR					Mascot
2162.1492	2162.0366	-0.1126	-52	426	444	LIELSPSWTPEISSYRVGK					Mascot
2176	2176.1079	0.1079	50	230	251	ISQPQENGDASVDASPSTSGTK					Mascot
2226.1248	2226.0222	-0.1026	-46	147	166	QEEEAVHVVENASEVKTISK					Mascot

8 hypothetical protein SORBIDRAFT_06g028210 [Sorghum bicolor] gi|241938263 70718.7 6.49 15 54 0 11.045

Peptide Information

Calc. Mass	Obsrv. Mass	± da	± ppm	Start Seq.	End Seq.	Sequence	Ion Score	C. I. %	Modification	Rank	Result Type
808.4199	808.3516	-0.0683	-84	284	289	YQQELK					Mascot
840.3702	840.4207	0.0505	60	479	485	LMSTCGR			Carbamidomethyl (C)[5], Oxidation (M)[2]		Mascot
922.5104	922.4489	-0.0615	-67	111	117	VLDRTYR					Mascot
1244.6958	1244.6582	-0.0376	-30	397	407	ASLLQDRNVTK					Mascot
1286.6998	1286.6631	-0.0367	-29	524	533	LQKPIDTCRR			Carbamidomethyl (C)[8]		Mascot
1376.6991	1376.693	-0.0061	-4	297	307	ECKLDQLPFAR			Carbamidomethyl (C)[2]		Mascot
1381.791	1381.718	-0.073	-53	264	275	HTSRDILTLAVR					Mascot
1381.791	1381.718	-0.073	-53	264	275	HTSRDILTLAVR					Mascot
1664.8311	1664.7537	-0.0774	-46	19	35	AISSSSSRLSSGGNAQR					Mascot
1875.7921	1875.9746	0.1825	97	123	138	DEEIMLDAATCAMAFR			Carbamidomethyl (C)[11], Oxidation (M)[5,13]		Mascot
1965.9011	1965.9669	0.0658	33	222	237	EVEHALDCPFYTTLDR			Carbamidomethyl (C)[8]		Mascot
2089.9211	2089.9851	0.064	31	560	577	VCYFFYSGTDGFSSPVE K			Carbamidomethyl (C)[2]		Mascot
2162.124	2162.0366	-0.0874	-40	600	617	VVWEREEPSLNDVVHLIQ					Mascot
2176.1721	2176.1079	-0.0642	-30	580	599	EVDAVINEPLKLQGSHAS LR					Mascot
2226.0715	2226.0222	-0.0493	-22	123	141	DEEIMLDAATCAMAFRIL R			Carbamidomethyl (C)[11]		Mascot
2242.0664	2242.0107	-0.0557	-25	123	141	DEEIMLDAATCAMAFRIL R			Carbamidomethyl (C)[11], Oxidation (M)[5]		Mascot
2242.0664	2242.0107	-0.0557	-25	123	141	DEEIMLDAATCAMAFRIL R	3	0	Carbamidomethyl (C)[11], Oxidation (M)[13]		Mascot
2258.0613	2258.0088	-0.0525	-23	123	141	DEEIMLDAATCAMAFRIL R			Carbamidomethyl (C)[11], Oxidation (M)[5,13]		Mascot
3647.8591	3647.668	-0.1911	-52	372	403	IEFYSEQVEIVFSSIYNSV NQLGAKASLLQDR					Mascot

9 hypothetical protein ZEAMMB73_466394 [Zea mays] gi|413925140 21686.8 5.97 9 54 0 .947

Protein Group

hypothetical protein ZEAMMB73_466394 [Zea mays] gi|413925141 21686.8 5.9699 997901 9165

Peptide Information

Calc. Mass	Obsrv. Mass	± da	± ppm	Start Seq.	End Seq.	Sequence	Ion Score	C. I. %	Modification	Rank	Result Type
904.5284	904.4637	-0.0647	-72	1	8	MVQKIASK					Mascot
1057.5942	1057.5154	-0.0788	-75	131	138	LHVAWLYR					Mascot
1204.7161	1204.6118	-0.1043	-87	94	104	HRLPLLAAADK					Mascot
1261.6357	1261.6919	0.0562	45	51	60	SMFVHDLREK					Mascot

1262.6886	1262.6959	0.0073	6	5	16	IASKSALGCLSR			Carbamidomethyl (C)[9]		Mascot
1432.7657	1432.6985	-0.0672	-47	144	155	KGCLTYLFVFGK			Carbamidomethyl (C)[3]		Mascot
2130.0747	2129.9331	-0.1416	-66	17	36	MLADSIHADVTINTTDGV LK			Oxidation (M)[1]		Mascot
2194.9592	2195.0439	0.0847	39	112	130	DCCEESLLEDIGSGNVLE R			Carbamidomethyl (C)[2,3]		Mascot
3647.5964	3647.668	0.0716	20	161	189	DEMHGFFHHADRELMHD MFQEVLSVWKPM			Oxidation (M)[3,15,18]		Mascot

10 small zinc finger-like protein [Solanum lycopersicum] gi|350537255 9668.6 6.56 8 53 0 22.752

Peptide Information

Calc. Mass	Obsrv. Mass	± da	± ppm	Start Seq.	End Seq.	Sequence	Ion Score	C. I. %	Modification	Rank	Result Type
906.4502	906.4564	0.0062	7	31	37	LTHTCFK			Carbamidomethyl (C)[5]		Mascot
940.4556	940.485	0.0294	31	39	45	CVENKYK			Carbamidomethyl (C)[1]		Mascot
1098.5137	1098.5452	0.0315	29	11	19	EQIFSMAEK			Oxidation (M)[6]		Mascot
1507.6556	1507.7385	0.0829	55	20	30	EMEYRVEMFNK			Oxidation (M)[2,8]		Mascot
1639.6687	1639.7712	0.1025	63	46	59	DSELNMGENSCIDR			Carbamidomethyl (C)[11]		Mascot
1930.8269	1930.9198	0.0929	48	44	59	YKDSELNMGENSCIDR			Carbamidomethyl (C)[13]		Mascot
1946.8219	1946.8938	0.0719	37	44	59	YKDSELNMGENSCIDR			Carbamidomethyl (C)[13], Oxidation (M)[8]		Mascot
2063.0742	2062.9451	-0.1291	-63	64	81	YWQVTNLVGTLLGNTRP M					Mascot
2063.0742	2062.9451	-0.1291	-63	64	81	YWQVTNLVGTLLGNTRP M					Mascot
2129.8896	2129.9331	0.0435	20	46	63	DSELNMGENSCIDRCVS K			Carbamidomethyl (C)[11,15], Oxidation (M)[6]		Mascot

Gel Idx/Pos	294/L22	Instr./Gel Origin	BA2151/Sample Project 20140814	Process Status	Analysis Succeeded
Plate [#] Name	[1] Sample Project 20140814	Instrument Sample Name		Spectra	11

Rank	Protein Name	Accession No.	Protein MW	Protein PI	Pep. Count	Protein Score	Protein Score C. I. %	Intensity Matched	Total Ion Score	Total Ion C. I. %	Confirmed
1	unnamed protein product [Ostreococcus tauri]	gi|308811316	108797.3	5.42	34	74	95.848	13.981			

Peptide Information

Calc. Mass	Obsrv. Mass	± da	± ppm	Start Seq.	End Seq.	Sequence	Ion Score	C. I. %	Modification	Rank	Result Type
809.3788	809.4311	0.0523	65	435	441	FAEATDR					Mascot
817.4778	817.4589	-0.0189	-23	552	558	VEISTLR					Mascot
823.3614	823.4265	0.0651	79	568	574	AMTSEER					Mascot
832.3948	832.4409	0.0461	55	649	655	NSAHFEK					Mascot
837.4312	837.4022	-0.029	-35	28	35	ASSSTKEK					Mascot
839.3563	839.431	0.0747	89	568	574	AMTSEER			Oxidation (M)[2]		Mascot
846.493	846.5141	0.0211	25	697	703	ESLEKIK					Mascot
847.4631	847.481	0.0179	21	909	915	EKINSTR					Mascot
851.3927	851.4108	0.0181	21	848	854	KNSEMDK					Mascot
864.4244	864.4678	0.0434	50	828	834	KMEVADR			Oxidation (M)[2]		Mascot
870.5043	870.5388	0.0345	40	501	508	LHSTLTAK					Mascot
890.469	890.4818	0.0128	14	320	326	ESLERTR					Mascot
891.4352	891.4639	0.0287	32	233	239	QDLMNVR			Oxidation (M)[4]		Mascot
893.4574	893.4639	0.0065	7	605	612	SSVESEKK					Mascot
921.4709	921.4852	0.0143	16	374	381	TDEVAMKK					Mascot
932.4683	932.4739	0.0056	6	492	499	SEALDELR					Mascot
946.4839	946.5076	0.0237	25	240	247	AVETEIER					Mascot
960.4897	960.5273	0.0376	39	649	656	NSAHFEKK					Mascot
963.4854	963.4975	0.0121	13	81	89	EQSRASASK					Mascot
1001.5374	1001.5231	-0.0143	-14	676	684	AKVNEGDLR					Mascot
1021.5346	1021.5029	-0.0317	-31	721	729	KDVSMLQGK			Oxidation (M)[5]		Mascot
1027.4547	1027.5166	0.0619	60	919	928	TDAGGMKGMK			Oxidation (M)[6,9]		Mascot
1033.571	1033.5222	-0.0488	-47	669	677	SDLMQLKAK					Mascot
1049.566	1049.5669	0.0009	1	669	677	SDLMQLKAK			Oxidation (M)[4]		Mascot
1066.447	1066.55	0.103	97	132	140	GMQSEDDLR			Oxidation (M)[2]		Mascot
1147.6205	1147.5923	-0.0282	-25	381	390	KVEEELATTK					Mascot
1199.5837	1199.646	0.0623	52	63	73	VEAEAVMHAAR			Oxidation (M)[7]		Mascot

1216.6182	1216.5809	-0.0373	-31	267	275	QTWQNERVR					Mascot
1216.6182	1216.5809	-0.0373	-31	267	275	QTWQNERVR					Mascot
1232.637	1232.623	-0.014	-11	953	963	DLLQQSITSID					Mascot
1340.6338	1340.7075	0.0737	55	541	551	RAFEMEMADLK					Mascot
1372.6235	1372.6788	0.0553	40	541	551	RAFEMEMADLK			Oxidation (M)[5,7]		Mascot
1407.7413	1407.715	-0.0263	-19	593	604	QASFQGRLLEMK					Mascot
1455.6823	1455.7073	0.025	17	802	813	DQHDTNAEEIKR					Mascot
1458.8163	1458.7604	-0.0559	-38	656	668	KLEITAEQLSQAK					Mascot
1463.7523	1463.7532	0.0009	1	459	471	LNETIALTNSTMR					Mascot
1479.7472	1479.7737	0.0265	18	459	471	LNETIALTNSTMR			Oxidation (M)[12]		Mascot
1496.8795	1496.7445	-0.135	-90	789	801	QLAALNKEIDILR					Mascot
1818.9015	1818.9351	0.0336	18	233	247	QDLMNVRAVETEIER			Oxidation (M)[4]		Mascot

2 PREDICTED: LOW QUALITY PROTEIN: kinesin-like protein 1-like [Setaria italica] gi|514707837 146676.3 6.28 36 72 93.569 13.068

Peptide Information

Calc. Mass	Obsrv. Mass	± da	± ppm	Start Seq.	End Seq.	Sequence	Ion Score	C. I. %	Modification	Rank	Result Type
818.4254	818.4578	0.0324	40	597	603	QELEATK					Mascot
831.457	831.471	0.014	17	708	715	VVTNAAEK					Mascot
832.3795	832.4409	0.0614	74	460	466	QAIDDDR					Mascot
835.4268	835.4343	0.0075	9	1192	1199	TSSAGKER					Mascot
839.437	839.431	-0.006	-7	958	964	EAQHINK					Mascot
847.4421	847.481	0.0389	46	215	221	NGQIPYR					Mascot
856.4424	856.5267	0.0843	98	980	986	NAHVPYR					Mascot
858.5229	858.5036	-0.0193	-22	53	59	LLCALLR			Carbamidomethyl (C)[3]		Mascot
890.4214	890.4818	0.0604	68	1064	1070	KDDEIDR					Mascot
922.4298	922.4679	0.0381	41	324	330	EKEDMVR			Oxidation (M)[5]		Mascot
946.5203	946.5076	-0.0127	-13	597	604	QELEATKK					Mascot
960.4745	960.5273	0.0528	55	459	466	KQAIDDDR					Mascot
991.5492	991.5438	-0.0054	-5	346	353	VDMLLKEK			Oxidation (M)[3]		Mascot
1001.501	1001.5231	0.0221	22	1226	1235	DPSAPRSSGK					Mascot
1014.624	1014.5282	-0.0958	-94	53	60	LLCALLRR			Carbamidomethyl (C)[3]		Mascot
1021.5095	1021.5029	-0.0066	-6	139	146	NMESKLQR			Oxidation (M)[2]		Mascot
1033.571	1033.5222	-0.0488	-47	435	442	EDIIRLMK			Oxidation (M)[7]		Mascot
1037.531	1037.5398	0.0088	8	735	742	GNIRVYCR			Carbamidomethyl (C)[7]		Mascot
1087.6106	1087.604	-0.0066	-6	595	603	LRQELEATK					Mascot

1101.6262	1101.5842	-0.042	-38	553	561	LKQDLESLR					Mascot
1141.5637	1141.4982	-0.0655	-57	777	786	EGGKNFTFNK					Mascot
1155.535	1155.6075	0.0725	63	383	391	EKEDMFNVK			Oxidation (M)[5]		Mascot
1158.6014	1158.6189	0.0175	15	716	725	YHAALADNRK					Mascot
1189.6497	1189.5776	-0.0721	-61	1049	1058	ELMDQLALLK			Oxidation (M)[3]		Mascot
1193.6082	1193.6187	0.0105	9	425	434	EEMISSLKEK					Mascot
1216.6355	1216.5809	-0.0546	-45	264	274	VLEALACGVER			Carbamidomethyl (C)[7]		Mascot
1216.6355	1216.5809	-0.0546	-45	264	274	VLEALACGVER			Carbamidomethyl (C)[7]		Mascot
1357.6669	1357.6873	0.0204	15	544	554	EDYSNTIMKLK			Oxidation (M)[8]		Mascot
1372.7617	1372.6788	-0.0829	-60	586	596	EMNENIILKLR					Mascot
1415.804	1415.7048	-0.0992	-70	144	156	LQRVLSSPIMSGK					Mascot
1458.8024	1458.7604	-0.042	-29	673	685	NAQDLRLSSVSIR					Mascot
1463.7411	1463.7532	0.0121	8	630	641	EIELMLEDSTKR					Mascot
1473.8346	1473.7614	-0.0732	-50	98	111	GELSAVVTCILALK			Carbamidomethyl (C)[9]		Mascot
1479.736	1479.7737	0.0377	25	630	641	EIELMLEDSTKR			Oxidation (M)[5]		Mascot
1549.7163	1549.7593	0.043	28	445	458	EDMVNLAGVEVSDR			Oxidation (M)[3]		Mascot
1661.8164	1661.824	0.0076	5	445	459	EDMVNLAGVEVSDRK					Mascot
1699.8394	1699.8628	0.0234	14	291	303	LMQYEEDLVRLMK			Oxidation (M)[2,12]		Mascot
1818.9756	1818.9351	-0.0405	-22	1215	1231	VGPASMRPKPRDPSAPR					Mascot

3	hypothetical protein TRIUR3_30168 [Triticum urartu]	gi|473888425	75325.4	5.21	26	64	59.423	9.817

Peptide Information

Calc. Mass	Obsrv. Mass	± da	± ppm	Start Seq.	End Seq.	Sequence	Ion Score	C. I. %	Modification	Rank	Result Type
819.3843	819.433	0.0487	59	112	118	SEAEVER					Mascot
823.4494	823.4265	-0.0229	-28	1	7	MAVIAYR					Mascot
831.4359	831.471	0.0351	42	53	58	VWELER					Mascot
832.441	832.4409	-0.0001	0	146	152	LESENLK					Mascot
839.4443	839.431	-0.0133	-16	1	7	MAVIAYR			Oxidation (M)[1]		Mascot
846.4567	846.5141	0.0574	68	451	457	VEEIQTK					Mascot
921.4458	921.4852	0.0394	43	295	301	EMDNLKR			Oxidation (M)[2]		Mascot
946.4588	946.5076	0.0488	52	541	548	NKDVEADR					Mascot
960.4897	960.5273	0.0376	39	90	97	SFKAENHK					Mascot
988.5421	988.5034	-0.0387	-39	146	153	LESENLKR					Mascot
991.4942	991.5438	0.0496	50	100	108	SEVSIAEEK					Mascot
995.5455	995.6255	0.08	80	1	8	MAVIAYRR			Oxidation (M)[1]		Mascot
995.5455	995.6255	0.08	80	1	8	MAVIAYRR			Oxidation (M)[1]		Mascot

1004.5445	1004.5174	-0.0271	-27	60	67	ITQLICEK			Carbamidomethyl (C)[6]		Mascot
1021.5173	1021.5029	-0.0144	-14	8	16	RAGYDVGQR					Mascot
1023.5105	1023.606	0.0955	93	85	92	EEVERSFK					Mascot
1049.5626	1049.5669	0.0043	4	205	214	EAAAKAFDVK					Mascot
1106.5259	1106.5123	-0.0136	-12	257	265	CRQSEAEVK			Carbamidomethyl (C)[1]		Mascot
1118.6125	1118.5353	-0.0772	-69	417	426	MELVALVEAK			Oxidation (M)[1]		Mascot
1164.5895	1164.5743	-0.0152	-13	279	289	EAAAKAFEAEK					Mascot
1189.6609	1189.5776	-0.0833	-70	60	69	ITQLICEKGK			Carbamidomethyl (C)[6]		Mascot
1193.5468	1193.6187	0.0719	60	577	586	LNDEMNSVQK			Oxidation (M)[5]		Mascot
1199.6201	1199.646	0.0259	22	479	488	HAMSELEVKR					Mascot
1260.6794	1260.6345	-0.0449	-36	98	108	LRSEVSIAEEK					Mascot
1289.7134	1289.6633	-0.0501	-39	489	500	LKMDLAALAEAK			Oxidation (M)[3]		Mascot
1455.6971	1455.7073	0.0102	7	653	665	CSILSFLSPCGSK			Carbamidomethyl (C)[1,10]		Mascot
1463.7158	1463.7532	0.0374	26	183	194	QNMSEAEIERLK			Oxidation (M)[3]		Mascot
1479.6744	1479.7737	0.0993	67	181	192	EKQNMSEAEIER			Oxidation (M)[5]		Mascot

4	hypothetical protein ZEAMMB73_523136 [Zea mays]	gi|413924587	131334	5.21	32	64	58.478	12.019

Peptide Information

Calc. Mass	Obsrv. Mass	± da	± ppm	Start Seq.	End Seq.	Sequence	Ion Score	C. I. %	Modification	Rank	Result Type
804.421	804.4183	-0.0027	-3	592	599	ITDAAASR					Mascot
807.4029	807.4246	0.0217	27	577	582	KDMELR			Oxidation (M)[3]		Mascot
818.4003	818.4578	0.0575	70	878	885	AVSDAEAR					Mascot
831.4934	831.471	-0.0224	-27	478	484	NLKSELK					Mascot
832.441	832.4409	-0.0001	0	134	140	DAEIKEK					Mascot
847.452	847.481	0.029	34	687	693	TELESLR					Mascot
858.4791	858.5036	0.0245	29	782	788	RALDEVR					Mascot
863.4217	863.4628	0.0411	48	723	730	ESNSASLR					Mascot
890.4366	890.4818	0.0452	51	871	877	ELNNSWK					Mascot
893.4727	893.4639	-0.0088	-10	737	743	LVVDYER					Mascot
921.4458	921.4852	0.0394	43	864	870	QVEDMRK			Oxidation (M)[5]		Mascot
932.5159	932.4739	-0.042	-45	591	599	KITDAAASR					Mascot
946.4839	946.5076	0.0237	25	1124	1131	EKDDALQK					Mascot
991.5207	991.5438	0.0231	23	699	707	AVLEADFAR					Mascot
993.5251	993.5377	0.0126	13	583	590	ESFQIELK					Mascot
1014.569	1014.5282	-0.0408	-40	24	32	ADAAIRELR					Mascot

Calc. Mass	Obsrv. Mass	± da	± ppm	Start Seq.	End Seq.	Sequence	Ion Score	C. I. %	Modification	Rank	Result Type
1023.5391	1023.606	0.0669	65	762	770	LSMEVSTLK			Oxidation (M)[3]		Mascot
1049.5739	1049.5669	-0.007	-7	737	744	LVVDYERR					Mascot
1107.5317	1107.5498	0.0181	16	1132	1140	YNELNDQVI					Mascot
1109.5586	1109.5552	-0.0034	-3	666	674	KAYEQVSER					Mascot
1158.6365	1158.6189	-0.0176	-15	311	320	DLEKEAVNLK					Mascot
1302.7263	1302.6438	-0.0825	-63	1112	1123	SSAEQEKIGLLK					Mascot
1316.5684	1316.5812	0.0128	10	451	461	MVEAYAMMDQK					Mascot
1320.7191	1320.6062	-0.1129	-85	762	773	LSMEVSTLKNAK					Mascot
1320.7191	1320.6062	-0.1129	-85	762	773	LSMEVSTLKNAK					Mascot
1384.689	1384.764	0.075	54	565	576	NQIHMLSADLDK					Mascot
1384.6968	1384.764	0.0672	49	710	721	LNGFAAELEHQR					Mascot
1433.723	1433.7382	0.0152	11	70	83	SKAEAAELTASAER					Mascot
1458.7911	1458.7604	-0.0307	-21	151	164	IVNLAETSAGKEAR					Mascot
1479.6863	1479.7737	0.0874	59	406	417	YQEATDAFLHER					Mascot
1496.818	1496.7445	-0.0735	-49	552	564	DINGLVQQNVQLR					Mascot
1661.7911	1661.824	0.0329	20	1098	1111	QTADALKNENECLR			Carbamidomethyl (C)[12]		Mascot
1818.894	1818.9351	0.0411	23	634	650	ARSNVESAPTTLQDSSR					Mascot

5	hypothetical protein PRUPE_ppa005253mg [Prunus persica]	gi|462411480	53939.6	9.25	17	62	38.584	6.4	

Peptide Information

Calc. Mass	Obsrv. Mass	± da	± ppm	Start Seq.	End Seq.	Sequence	Ion Score	C. I. %	Modification	Rank	Result Type
804.4106	804.4183	0.0077	10	289	295	MNLAPMK					Mascot
807.428	807.4246	-0.0034	-4	167	173	LMIAESK			Oxidation (M)[2]		Mascot
815.4846	815.5005	0.0159	19	2	9	AVAVRGSR					Mascot
818.4267	818.4578	0.0311	38	435	440	ERFNPR					Mascot
832.4563	832.4409	-0.0154	-18	395	401	DLTGLWK					Mascot
858.5407	858.5036	-0.0371	-43	108	114	LKLEISR					Mascot
946.5251	946.5076	-0.0175	-18	1	9	MAVAVRGSR					Mascot
1001.5414	1001.5231	-0.0183	-18	230	237	ISHPEKYK					Mascot
1021.604	1021.5029	-0.1011	-99	82	89	TRLDLIYK					Mascot
1109.5851	1109.5552	-0.0299	-27	280	288	HVFHVVTDR					Mascot
1155.5542	1155.6075	0.0533	46	10	22	GGGGSGFGGFSLR					Mascot
1164.6372	1164.5743	-0.0629	-54	201	212	KNGAFASSIAAK					Mascot
1455.7089	1455.7073	-0.0016	-1	7	22	GSRGGGGSGFGGFSLR					Mascot
1461.806	1461.7346	-0.0714	-49	202	216	NGAFASSIAAKSIPK					Mascot

1473.7948	1473.7614	-0.0334	-23	395	407	DLTGLWKIDLDGK					Mascot
1549.8156	1549.7593	-0.0563	-36	300	312	MRPVERGAYVEVK			Oxidation (M)[1]		Mascot
2246.0581	2246.2185	0.1604	71	118	136	MFDDLASNISNLQMKPSY R			Oxidation (M)[1]		Mascot

6	unnamed protein product [Vitis vinifera]	gi|296087303	109526.4	5.5	30	61	22.682	10.091

Peptide Information

Calc. Mass	Obsrv. Mass	± da	± ppm	Start Seq.	End Seq.	Sequence	Ion Score	C. I. %	Modification	Rank	Result Type
804.4144	804.4183	0.0039	5	395	400	LCARER			Carbamidomethyl (C)[2]		Mascot
809.4039	809.4311	0.0272	34	234	240	SFEVEAK					Mascot
817.4665	817.4589	-0.0076	-9	511	517	EDLLSLK					Mascot
831.4822	831.471	-0.0112	-13	2	8	ELLSEIK					Mascot
832.4523	832.4409	-0.0114	-14	491	497	SIRAEEK					Mascot
839.4145	839.431	0.0165	20	207	213	SEYAEIK					Mascot
845.4727	845.4951	0.0224	26	461	467	EQALEKK					Mascot
847.4407	847.481	0.0403	48	431	437	SLEEELK					Mascot
893.5091	893.4639	-0.0452	-51	331	337	IFTQKEK					Mascot
908.4836	908.4781	-0.0055	-6	567	573	ELFSKER					Mascot
921.457	921.4852	0.0282	31	808	815	SDSVMARR					Mascot
932.4796	932.4739	-0.0057	-6	261	268	KSQEVDAR					Mascot
946.558	946.5076	-0.0504	-53	835	841	YNLRRPK					Mascot
960.4996	960.5273	0.0277	29	338	345	DLEEAQKK					Mascot
1004.5081	1004.5174	0.0093	9	193	200	QCVLDLEK			Carbamidomethyl (C)[2]		Mascot
1027.5895	1027.5166	-0.0729	-71	662	670	QAIVEPSKR					Mascot
1106.6125	1106.5123	-0.1002	-91	1	9	MELLSEIKK			Oxidation (M)[1]		Mascot
1109.599	1109.5552	-0.0438	-39	576	584	FIAFVEQQK					Mascot
1115.6306	1115.6151	-0.0155	-14	525	533	VEIEEQKLK					Mascot
1164.5784	1164.5743	-0.0041	-4	420	428	EFELEIEQK					Mascot
1199.6995	1199.646	-0.0535	-45	154	164	QALVDVKDALK					Mascot
1316.7111	1316.5812	-0.1299	-99	46	56	KVWSGWSLTPR					Mascot
1320.6794	1320.6062	-0.0732	-55	420	429	EFELEIEQKR					Mascot
1320.6794	1320.6062	-0.0732	-55	419	428	REFELEIEQK					Mascot
1433.7821	1433.7382	-0.0439	-31	34	45	VVEIVVMFTPQR			Oxidation (M)[7]		Mascot
1463.6649	1463.7532	0.0883	60	671	683	LGSTEDEPEPSFR					Mascot
1507.8479	1507.7626	-0.0853	-57	406	418	LVDEHNIILDAKK					Mascot
1507.8479	1507.7626	-0.0853	-57	406	418	LVDEHNIILDAKK					Mascot
1549.7316	1549.7593	0.0277	18	447	459	ETEFNHMEAKVAK			Oxidation (M)[7]		Mascot

1628.8966	1628.8672	-0.0294	-18	842	857	TTVTVAAAKSSTNLHK					Mascot
1707.9164	1707.7964	-0.12	-70	762	777	AILGESLELKSSFADK					Mascot

7 PREDICTED: BTB/POZ domain-containing protein At1g30440-like [Cucumis sativus] gi|449433732 71364.1 5.88 17 61 19.038 6.499

Peptide Information

Calc. Mass	Obsrv. Mass	± da	± ppm	Start Seq.	End Seq.	Sequence	Ion Score	C. I. %	Modification	Rank	Result Type
825.4175	825.4743	0.0568	69	81	87	TFEMVAK					Mascot
831.545	831.471	-0.074	-89	309	315	LLFGLLR					Mascot
832.4675	832.4409	-0.0266	-32	256	262	YIPGLNR					Mascot
835.4924	835.4343	-0.0581	-70	447	453	AIDIYLK					Mascot
988.5687	988.5034	-0.0653	-66	256	263	YIPGLNRR					Mascot
991.524	991.5438	0.0198	20	556	563	MRVSELEK					Mascot
993.522	993.5377	0.0157	16	1	9	MACVKLGSK			Carbamidomethyl (C)[3]		Mascot
1106.5986	1106.5123	-0.0863	-78	230	238	RLISVMESR			Oxidation (M)[6]		Mascot
1107.5793	1107.5498	-0.0295	-27	6	15	LGSKNDAFQK					Mascot
1109.5013	1109.5552	0.0539	49	549	557	VGMDNMRMR					Mascot
1141.4911	1141.4982	0.0071	6	549	557	VGMDNMRMR			Oxidation (M)[3,6]		Mascot
1232.7031	1232.623	-0.0801	-65	231	241	LISVMESRGIK					Mascot
1260.6252	1260.6345	0.0093	7	322	332	VNPSCISNLEK			Carbamidomethyl (C)[5]		Mascot
1366.7213	1366.6744	-0.0469	-34	242	254	QEIIAGSVTSYAK					Mascot
1366.7213	1366.6744	-0.0469	-34	242	254	QEIIAGSVTSYAK					Mascot
1372.7692	1372.6788	-0.0904	-66	297	308	MLPMQKSLVPTK					Mascot
1433.6213	1433.7382	0.1169	82	56	67	MVAEAPEEQEER			Oxidation (M)[1]		Mascot
1496.6654	1496.7445	0.0791	53	464	474	EQLCRLMDCQK			Carbamidomethyl (C)[4,9], Oxidation (M)[7]		Mascot
1549.7462	1549.7593	0.0131	8	543	555	ENQVLKVGMDNMR			Oxidation (M)[9]		Mascot

8 adenylyl cyclase-associated protein [Genlisea aurea] gi|527190306 51775.3 7.14 16 60 11.227 5.902

Peptide Information

Calc. Mass	Obsrv. Mass	± da	± ppm	Start Seq.	End Seq.	Sequence	Ion Score	C. I. %	Modification	Rank	Result Type
811.5036	811.4601	-0.0435	-54	216	223	TVAPAPKK					Mascot
817.489	817.4589	-0.0301	-37	269	276	GVTSGLRK					Mascot
832.4523	832.4409	-0.0114	-14	112	119	VSALTEGR					Mascot
846.4502	846.5141	0.0639	75	315	321	LELQMGR					Mascot
863.4291	863.4628	0.0337	39	1	7	MDESLIR					Mascot

Calc. Mass	Obsrv. Mass	± da	± ppm	Start Seq.	End Seq.	Sequence	Ion Score	C. I. %	Modification	Rank	Result Type
960.5513	960.5273	-0.024	-25	190	197	ELYIPGLR					Mascot
988.5534	988.5034	-0.05	-51	112	120	VSALTEGRR					Mascot
1051.5969	1051.6747	0.0778	74	369	377	CTKLGVVFK			Carbamidomethyl (C)[1]		Mascot
1101.6263	1101.5842	-0.0421	-38	359	368	GKVNNITIDK					Mascot
1107.4913	1107.5498	0.0585	53	27	37	GSDAAEDSLSR					Mascot
1118.5411	1118.5353	-0.0058	-5	323	331	WMVENQVGR					Mascot
1127.5626	1127.5483	-0.0143	-13	350	358	CNESVLHIR			Carbamidomethyl (C)[1]		Mascot
1216.6532	1216.5809	-0.0723	-59	16	26	LEALDLTTANR					Mascot
1216.6532	1216.5809	-0.0723	-59	16	26	LEALDLTTANR					Mascot
1312.6791	1312.634	-0.0451	-34	350	360	CNESVLHIRGK			Carbamidomethyl (C)[1]		Mascot
1482.6893	1482.7701	0.0808	54	255	268	DGMAAVFQEINSGK			Oxidation (M)[3]		Mascot
1707.8661	1707.7964	-0.0697	-41	285	299	NQVDRSFLVTASDQK					Mascot

9	Dihydroflavonol-4-reductase [Aegilops tauschii]	gi|475410009	26877.6	8.92	11	60	.395	4.383

Peptide Information

Calc. Mass	Obsrv. Mass	± da	± ppm	Start Seq.	End Seq.	Sequence	Ion Score	C. I. %	Modification	Rank	Result Type
815.4482	815.5005	0.0523	64	2	9	AADAGVRR					Mascot
863.4621	863.4628	0.0007	1	179	185	YTNQPLK					Mascot
868.4271	868.4468	0.0197	23	118	125	DAAEAHVR					Mascot
946.4887	946.5076	0.0189	20	1	9	MAADAGVRR					Mascot
960.5221	960.5273	0.0052	5	214	223	ASGNKGLASR					Mascot
1004.4294	1004.5174	0.088	88	47	53	NWYCYAK			Carbamidomethyl (C)[4]		Mascot
1049.5739	1049.5669	-0.007	-7	209	218	GFIQKASGNK					Mascot
1141.5895	1141.4982	-0.0913	-80	143	151	TLHRGELCR			Carbamidomethyl (C)[8]		Mascot
1407.7301	1407.715	-0.0151	-11	179	190	YTNQPLKDLGMK					Mascot
1516.7543	1516.7252	-0.0291	-19	105	117	AYVNESHAYVHVK					Mascot
2839.5234	2839.3608	-0.1626	-57	66	93	ALGLDLAGGIPVVTLGEL LQPSMNTSTR			Oxidation (M)[23]		Mascot
2839.5234	2839.3608	-0.1626	-57	66	93	ALGLDLAGGIPVVTLGEL LQPSMNTSTR			Oxidation (M)[23]		Mascot

10	PREDICTED: SEC14 cytosolic factor-like isoform X1 [Cicer arietinum]	gi|502153342	64376	6.21	18	60	0	6.67

Protein Group

PREDICTED: SEC14 cytosolic factor-like isoform X2 [Cicer arietinum]	gi|502153346	64376	6.2100 000381 4697

Peptide Information

Calc. Mass	Obsrv. Mass	± da	± ppm	Start Seq.	End Seq.	Sequence	Ion Score	C. I. %	Modification	Rank	Result Type
817.4202	817.4589	0.0387	47	303	309	SDKGPWK					Mascot
823.3944	823.4265	0.0321	39	310	315	DQEIYR					Mascot
831.457	831.471	0.014	17	149	156	LGQVDATK					Mascot
835.3865	835.4343	0.0478	57	485	491	MAELEDK					Mascot
851.3815	851.4108	0.0293	34	485	491	MAELEDK			Oxidation (M)[1]		Mascot
891.4756	891.4639	-0.0117	-13	247	253	MLWNTVK					Mascot
893.4332	893.4639	0.0307	34	435	441	MTRNMPK			Oxidation (M)[1]		Mascot
991.4877	991.5438	0.0561	57	484	491	RMAELEDK					Mascot
1066.5714	1066.55	-0.0214	-20	182	191	FAACSIAAKK			Carbamidomethyl (C)[4]		Mascot
1107.6045	1107.5498	-0.0547	-49	254	263	SFLDPKTTAK					Mascot
1118.5953	1118.5353	-0.06	-54	140	148	EGRPVYIER					Mascot
1155.5376	1155.6075	0.0699	60	337	347	TTTSEVSESSK					Mascot
1199.5626	1199.646	0.0834	70	75	83	HDDYHMLLR					Mascot
1212.6194	1212.5834	-0.036	-30	236	246	MFIINAGSGFR					Mascot
1463.7411	1463.7532	0.0121	8	516	528	ADALEQELMATKK			Oxidation (M)[9]		Mascot
2450.1902	2450.2244	0.0342	14	506	527	EEMLNAAISRADALEQEL MATK			Oxidation (M)[3]		Mascot
2564.3179	2564.2705	-0.0474	-18	63	83	QALVLDELLPEKHDDYH MLLR			Oxidation (M)[18]		Mascot
2839.2915	2839.3608	0.0693	24	492	515	MVNMNQITCMPPEKEEM LNAAISR			Carbamidomethyl (C)[9], Oxidation (M)[1,4]		Mascot
2839.4668	2839.3608	-0.106	-37	410	434	IHEKVNSQIFTGVMAFVM GIVTMVR			Oxidation (M)[14,18]		Mascot

Gel Idx/Pos	295/L23	Instr./Gel Origin	BA2151/Sample Project 20140814	Process Status	Analysis Succeeded
Plate [#] Name	[1] Sample Project 20140814	Instrument Sample Name		Spectra	11

Rank	Protein Name	Accession No.	Protein MW	Protein PI	Pep. Count	Protein Score	Protein Score C. I. %	Intensity Matched	Total Ion Score	Total Ion C. I. %	Confirmed
1	uncharacterized protein [Arabidopsis thaliana]	gi|240254562	148695.8	4.74	33	84	99.585	10.402			

Protein Group

uncharacterized protein AT2G32240 [Arabidopsis thaliana]	gi|330253560	148695.8	4.7399 997711 1816

Peptide Information

Calc. Mass	Obsrv. Mass	± da	± ppm	Start Seq.	End Seq.	Sequence	Ion Score	C. I. %	Modification	Rank	Result Type
802.4669	802.4499	-0.017	-21	831	837	DLEGKIK					Mascot
813.4465	813.408	-0.0385	-47	31	38	AVNGEVPK					Mascot
816.4574	816.4243	-0.0331	-41	857	863	LEQTLGR					Mascot
824.4261	824.441	0.0149	18	327	333	ASESRFK					Mascot
830.473	830.432	-0.041	-49	829	835	LRDLEGK					Mascot
831.4207	831.4301	0.0094	11	151	157	IVEGEER					Mascot
891.4418	891.4423	0.0005	1	1059	1066	QLTSEGEK					Mascot
918.4639	918.4467	-0.0172	-19	209	216	RSAEEAQK					Mascot
927.4054	927.4841	0.0787	85	428	435	TSDENFSK					Mascot
999.4741	999.4961	0.022	22	87	94	ELHESQEK					Mascot
1015.553	1015.516	-0.037	-36	926	933	RLEEAIER					Mascot
1016.5622	1016.5214	-0.0408	-40	1090	1098	EELQSVIAK					Mascot
1032.5571	1032.5116	-0.0455	-44	121	129	DELLSAKEK					Mascot
1049.5878	1049.5485	-0.0393	-37	235	243	ALEFSELLK					Mascot
1054.5276	1054.5325	0.0049	5	622	630	HEDAEGRLK					Mascot
1060.5521	1060.5697	0.0176	17	748	756	LESIENDLK					Mascot
1095.4841	1095.5701	0.086	79	42	50	EEEDGEFIK					Mascot
1101.5171	1101.616	0.0989	90	656	665	KHGETEADSK					Mascot
1171.6932	1171.5842	-0.109	-93	199	208	KLIELEEGLK					Mascot
1199.6267	1199.6605	0.0338	28	31	41	AVNGEVPKEEK					Mascot
1201.6675	1201.6339	-0.0336	-28	631	640	DLELLLQTEK					Mascot
1232.6117	1232.6223	0.0106	9	1150	1161	ENVENAATASVK					Mascot

1262.6475	1262.642	-0.0055	-4	1099	1109	LEEQLTVESSK					Mascot
1290.5961	1290.6887	0.0926	72	950	959	THENQIEEYK					Mascot
1323.6461	1323.6731	0.027	20	1253	1264	TKETEAMDVGVK			Oxidation (M)[7]		Mascot
1323.6461	1323.6731	0.027	20	1253	1264	TKETEAMDVGVK			Oxidation (M)[7]		Mascot
1363.6852	1363.7101	0.0249	18	874	884	LKQEFDQAQEK					Mascot
1366.6155	1366.6907	0.0752	55	265	275	ELNEKMSENEK			Oxidation (M)[6]		Mascot
1366.7074	1366.6907	-0.0167	-12	589	600	IALQKGAEHEDR					Mascot
1381.7209	1381.7578	0.0369	27	715	726	KLEATVDEYSVK					Mascot
1463.7336	1463.802	0.0684	47	484	497	SSSQAAEEAKSQIK					Mascot
1475.7588	1475.7709	0.0121	8	17	30	GDVDDLKTADISVK					Mascot
1621.7426	1621.8745	0.1319	81	594	607	GAEHEDRANTTHQR					Mascot
1657.8755	1657.8401	-0.0354	-21	580	593	NSELEEDLRIALQK					Mascot

2 Cleavage stimulation factor subunit 1 [Triticum urartu] gi|474365350 70971.2 9.27 19 68 84.214 7.25

Peptide Information

Calc. Mass	Obsrv. Mass	± da	± ppm	Start Seq.	End Seq.	Sequence	Ion Score	C. I. %	Modification	Rank	Result Type
816.4686	816.4243	-0.0443	-54	271	277	ASEVVRR					Mascot
822.462	822.4008	-0.0612	-74	638	643	SVRFWK					Mascot
826.4893	826.4271	-0.0622	-75	290	297	GLGPRQAK					Mascot
849.4424	849.4384	-0.004	-5	2	9	DTSTRAAK					Mascot
861.4828	861.4442	-0.0386	-45	322	329	GPSKTFPK					Mascot
1015.5319	1015.516	-0.0159	-16	326	333	TFPKHETR					Mascot
1068.6161	1068.5432	-0.0729	-68	262	270	LQVNQNKPK					Mascot
1081.575	1081.5223	-0.0527	-49	435	443	VIQDTHNVR					Mascot
1095.4922	1095.5701	0.0779	71	54	63	MQVASGEAMR			Oxidation (M)[1]		Mascot
1106.6205	1106.5417	-0.0788	-71	166	175	YKGGIDVVQK					Mascot
1187.6816	1187.6211	-0.0605	-51	168	178	GGIDVVQKIMK					Mascot
1232.7362	1232.6223	-0.1139	-92	65	75	NALATFKNILK					Mascot
1235.5984	1235.6146	0.0162	13	54	64	MQVASGEAMRR					Mascot
1259.7107	1259.6743	-0.0364	-29	85	97	GFGTVITGAIPAR					Mascot
1262.6311	1262.642	0.0109	9	155	165	LMVQGYSGHVR			Oxidation (M)[2]		Mascot
1325.7648	1325.6694	-0.0954	-72	260	270	TRLQVNQNKPK					Mascot
1367.6149	1367.7141	0.0992	73	491	503	YSGTGSMYVTASK			Oxidation (M)[7]		Mascot
1384.7042	1384.7765	0.0723	52	213	224	MIWSALGHLNDK					Mascot
1384.7042	1384.7765	0.0723	52	213	224	MIWSALGHLNDK					Mascot
1443.8319	1443.7413	-0.0906	-63	72	84	NILKVDGVPGLYR					Mascot

3	uncharacterized protein LOC100191878 [Zea mays]	gi|212276328	60398.4	9.72	17	63	54.472	8.672

Peptide Information

Calc. Mass	Obsrv. Mass	± da	± ppm	Start Seq.	End Seq.	Sequence	Ion Score	C. I. %	Modification	Rank	Result Type
822.4138	822.4008	-0.013	-16	371	377	NMSTTLR					Mascot
832.373	832.3424	-0.0306	-37	14	19	REDCPR			Carbamidomethyl (C)[4]		Mascot
835.4342	835.4055	-0.0287	-34	204	210	VSDMKQK					Mascot
982.5066	982.4589	-0.0477	-49	433	439	MWFELLK			Oxidation (M)[1]		Mascot
1025.4755	1025.5587	0.0832	81	469	477	MVSIAGMER			Oxidation (M)[1,7]		Mascot
1106.6178	1106.5417	-0.0761	-69	443	452	KPASAHPSRR					Mascot
1107.4744	1107.5583	0.0839	76	511	519	CMNMPIEGR			Carbamidomethyl (C)[1]		Mascot
1139.4642	1139.5697	0.1055	93	511	519	CMNMPIEGR			Carbamidomethyl (C)[1], Oxidation (M)[2,4]		Mascot
1141.6034	1141.5438	-0.0596	-52	1	11	MPAPVAPVTSR			Oxidation (M)[1]		Mascot
1199.6168	1199.6605	0.0437	36	527	538	GQRAAFPPTPSA					Mascot
1235.5693	1235.6146	0.0453	37	511	520	CMNMPIEGRK			Carbamidomethyl (C)[1]		Mascot
1325.6333	1325.6694	0.0361	27	481	493	DSGVEVTAASFSR					Mascot
1363.6635	1363.7101	0.0466	34	453	465	GLGSQITAMTDNR					Mascot
1366.7876	1366.6907	-0.0969	-71	1	13	MPAPVAPVTSRLK					Mascot
1366.7876	1366.6907	-0.0969	-71	1	13	MPAPVAPVTSRLK					Mascot
1367.6658	1367.7141	0.0483	35	469	480	MVSIAGMERTEK			Oxidation (M)[1]		Mascot
1379.6584	1379.7222	0.0638	46	453	465	GLGSQITAMTDNR			Oxidation (M)[9]		Mascot
1384.6382	1384.7765	0.1383	100	466	477	SCKMVSIAGMER			Carbamidomethyl (C)[2], Oxidation (M)[4]		Mascot
1384.6382	1384.7765	0.1383	100	466	477	SCKMVSIAGMER			Carbamidomethyl (C)[2], Oxidation (M)[4]		Mascot
1519.7645	1519.7815	0.017	11	452	465	RGLGSQITAMTDNR					Mascot
1812.9636	1812.8986	-0.065	-36	222	239	GSGPANTSPSIIKSMLPR					Mascot

4	hypothetical protein SORBIDRAFT_07g016530 [Sorghum bicolor]	gi|241940584	110576.9	5.68	22	63	54.472	7.248

Peptide Information

Calc. Mass	Obsrv. Mass	± da	± ppm	Start Seq.	End Seq.	Sequence	Ion Score	C. I. %	Modification	Rank	Result Type
810.4216	810.4192	-0.0024	-3	778	785	QPGGQAPR					Mascot
811.3614	811.4166	0.0552	68	591	597	SSTMTER					Mascot
820.3948	820.4149	0.0201	25	517	523	GVPYNDR					Mascot
877.4639	877.4401	-0.0238	-27	250	256	DIHISHR					Mascot
891.4907	891.4423	-0.0484	-54	313	319	HPPTRQR					Mascot

Calc. Mass	Obsrv. Mass	± da	± ppm	Start Seq.	End Seq.	Sequence	Ion Score	C. I. %	Modification	Rank	Result Type
1006.5164	1006.4911	-0.0253	-25	489	498	ITSSPSTGTR					Mascot
1025.5336	1025.5587	0.0251	24	29	36	VLTCLYEK			Carbamidomethyl (C)[4]		Mascot
1032.532	1032.5116	-0.0204	-20	558	567	ASQQTLADAK					Mascot
1033.5214	1033.5253	0.0039	4	202	209	WWNTISAR					Mascot
1114.5852	1114.5632	-0.022	-20	470	479	DPSGVLNREK					Mascot
1126.6216	1126.5381	-0.0835	-74	786	796	NTGVLSSVPPR					Mascot
1141.6073	1141.5438	-0.0635	-56	548	557	QGADIRTPQR					Mascot
1187.6379	1187.6211	-0.0168	-14	398	408	SGVQDVQKQAK					Mascot
1262.6699	1262.642	-0.0279	-22	969	979	ITSDTQQVKSR					Mascot
1265.5968	1265.6455	0.0487	38	605	617	ASDSQGTSASVQK					Mascot
1316.6918	1316.5952	-0.0966	-73	369	381	TGQRQAGALADTK					Mascot
1366.74	1366.6907	-0.0493	-36	29	39	VLTCLYEKNVK			Carbamidomethyl (C)[4]		Mascot
1366.74	1366.6907	-0.0493	-36	29	39	VLTCLYEKNVK			Carbamidomethyl (C)[4]		Mascot
1379.6359	1379.7222	0.0863	63	654	666	MATEPTSETLGDK					Mascot
1407.675	1407.7302	0.0552	39	948	959	DLPTNADKNYEK					Mascot
1584.7612	1584.7711	0.0099	6	383	397	ATEADQDRAPSPAQK					Mascot
1812.9524	1812.8986	-0.0538	-30	667	684	STIAAMVDPSTALPAPVR			Oxidation (M)[6]		Mascot
2148.1409	2148.2754	0.1345	63	786	805	NTGVLSSVPPRVKPPED NNK					Mascot

5 hypersensitive to PI starvation 4 [Arabidopsis thaliana] gi|334183420 302087.8 8.66 44 62 41.348 16.286

Protein Group

hypersensitive to PI starvation 4 [Arabidopsis thaliana] gi|332195403 302087.8 8.6599 998474 1211

Peptide Information

Calc. Mass	Obsrv. Mass	± da	± ppm	Start Seq.	End Seq.	Sequence	Ion Score	C. I. %	Modification	Rank	Result Type
807.4029	807.4188	0.0159	20	2494	2501	SVTGMQGK					Mascot
808.4199	808.4236	0.0037	5	1833	1838	EFEQKK					Mascot
810.4944	810.4192	-0.0752	-93	947	953	HILSGKR					Mascot
811.442	811.4166	-0.0254	-31	1054	1059	EEIHKR					Mascot
812.4777	812.4122	-0.0655	-81	419	424	LQPWLR					Mascot
816.4032	816.4243	0.0211	26	2202	2208	NCLPNAK			Carbamidomethyl (C)[2]		Mascot
823.4421	823.4351	-0.007	-9	1730	1735	QYTQRK					Mascot
831.4683	831.4301	-0.0382	-46	953	959	RESSKPK					Mascot
833.4363	833.372	-0.0643	-77	1494	1501	SGNLSLDK					Mascot

835.4421	835.4055	-0.0366	-44	2554	2560	GLFNTQR		Mascot
836.4625	836.4198	-0.0427	-51	780	786	SLVYAQR		Mascot
845.4727	845.4816	0.0089	11	603	610	TVSVQPSK		Mascot
852.4461	852.4339	-0.0122	-14	2109	2115	ELTYAQK		Mascot
861.4611	861.4442	-0.0169	-20	2341	2347	SNVICLR	Carbamidomethyl (C)[5]	Mascot
877.4349	877.4401	0.0052	6	1877	1883	SFHSIMR		Mascot
909.4611	909.4614	0.0003	0	412	418	CNLFISR	Carbamidomethyl (C)[1]	Mascot
909.4611	909.4614	0.0003	0	412	418	CNLFISR	Carbamidomethyl (C)[1]	Mascot
913.5101	913.4289	-0.0812	-89	2230	2238	VDAKQGAPK		Mascot
946.5329	946.478	-0.0549	-58	2613	2619	FRPRSQR		Mascot
1013.6102	1013.5228	-0.0874	-86	2403	2411	EIKPVKSGR		Mascot
1016.4755	1016.5214	0.0459	45	2409	2417	SGRSSHEEK		Mascot
1023.5444	1023.628	0.0836	82	29	36	LFAWMLSR		Mascot
1025.5599	1025.5587	-0.0012	-1	119	127	SHKPRTSGR		Mascot
1026.5111	1026.5347	0.0236	23	1502	1509	VMTEFMLR		Mascot
1032.5295	1032.5116	-0.0179	-17	2462	2469	LLMDTFHR		Mascot
1037.5415	1037.5352	-0.0063	-6	1396	1403	EKVFDPFR		Mascot
1049.4907	1049.5485	0.0578	55	2433	2440	MMEFHNIK		Mascot
1065.4856	1065.5458	0.0602	57	2433	2440	MMEFHNIK	Oxidation (M)[1]	Mascot
1081.4806	1081.5223	0.0417	39	2433	2440	MMEFHNIK	Oxidation (M)[1,2]	Mascot
1095.5793	1095.5701	-0.0092	-8	2109	2117	ELTYAQKSR		Mascot
1101.6163	1101.616	-0.0003	0	780	788	SLVYAQRHK		Mascot
1106.5437	1106.5417	-0.002	-2	2348	2358	SSTGASAQELR		Mascot
1107.6157	1107.5583	-0.0574	-52	2187	2196	DIGVARFTTK		Mascot
1171.6504	1171.5842	-0.0662	-57	1258	1267	NPDMVQVIKK		Mascot
1187.6453	1187.6211	-0.0242	-20	1258	1267	NPDMVQVIKK	Oxidation (M)[4]	Mascot
1193.6273	1193.6241	-0.0032	-3	1320	1330	QSDGRVFVSAK		Mascot
1199.6644	1199.6605	-0.0039	-3	1641	1650	LLAWQEAGRR		Mascot
1212.5856	1212.6089	0.0233	19	547	557	TSSDEVGFRSK		Mascot
1235.6188	1235.6146	-0.0042	-3	372	382	EADSSILEIMK		Mascot
1259.6703	1259.6743	0.004	3	810	820	INQSNSVNKQK		Mascot
1262.6448	1262.642	-0.0028	-2	2348	2359	SSTGASAQELRR		Mascot
1265.6526	1265.6455	-0.0071	-6	1707	1718	DAVWSFVGGISK		Mascot
1277.6519	1277.7323	0.0804	63	700	710	DKLSTQMELGR		Mascot
1316.7256	1316.5952	-0.1304	-99	1873	1883	VLARSFHSIMR		Mascot
1338.782	1338.6897	-0.0923	-69	1839	1848	IYYWNLLLLK		Mascot

1384.7543	1384.7765	0.0222	16	280	293	TSATAASSAKKPHK					Mascot
1384.7543	1384.7765	0.0222	16	280	293	TSATAASSAKKPHK					Mascot
1605.8153	1605.7699	-0.0454	-28	925	938	AIDDSIEEMLRGLK			Oxidation (M)[9]		Mascot
3133.7134	3133.6973	-0.0161	-5	2	28	AASPAKFFFGFLIVSIVLW MIFILCSR			Carbamidomethyl (C)[25]		Mascot

6 retrotransposon protein, putative, Ty3-gypsy subclass [Oryza sativa Japonica Group] gi|77550683 151273.7 8.96 26 62 32.659 10.803

Peptide Information

Calc. Mass	Obsrv. Mass	± da	± ppm	Start Seq.	End Seq.	Sequence	Ion Score	C. I. %	Modification	Rank	Result Type
807.3665	807.4188	0.0523	65	89	95	GMEDVTR					Mascot
816.4686	816.4243	-0.0443	-54	1142	1148	VAQSRQK					Mascot
819.3777	819.4213	0.0436	53	321	327	TQTGCPR			Carbamidomethyl (C)[5]		Mascot
822.4719	822.4008	-0.0711	-86	671	677	SFEALKK					Mascot
823.3615	823.4351	0.0736	89	89	95	GMEDVTR			Oxidation (M)[2]		Mascot
828.4838	828.4448	-0.039	-47	100	105	QRHFLK					Mascot
831.457	831.4301	-0.0269	-32	1255	1261	ILETAER					Mascot
849.4577	849.4384	-0.0193	-23	379	385	GERVNFK					Mascot
870.5771	870.5538	-0.0233	-27	570	576	LVLEKLR					Mascot
877.4196	877.4401	0.0205	23	363	370	AVLDCGSR			Carbamidomethyl (C)[5]		Mascot
891.4781	891.4423	-0.0358	-40	371	378	SITLENSK					Mascot
937.4416	937.4431	0.0015	2	49	55	MMMQQLR					Mascot
999.5582	999.4961	-0.0621	-62	799	808	ANIVADALGR					Mascot
1033.5499	1033.5253	-0.0246	-24	1119	1127	MVFGPDVIR					Mascot
1049.5449	1049.5485	0.0036	3	1119	1127	MVFGPDVIR			Oxidation (M)[1]		Mascot
1060.502	1060.5697	0.0677	64	538	545	VFMEYLDK			Oxidation (M)[3]		Mascot
1095.5364	1095.5701	0.0337	31	923	930	MYQDLRNR					Mascot
1110.5248	1110.5577	0.0329	30	810	818	AYCNNVEIK			Carbamidomethyl (C)[3]		Mascot
1127.6532	1127.5436	-0.1096	-97	799	809	ANIVADALGRK					Mascot
1262.5697	1262.642	0.0723	57	584	592	FSKCEFWMK			Carbamidomethyl (C)[4]		Mascot
1265.7465	1265.6455	-0.101	-80	893	903	LVVPDQPELKK					Mascot
1308.6287	1308.6779	0.0492	38	703	714	TGLGCVLMQEGK			Carbamidomethyl (C)[5], Oxidation (M)[8]		Mascot
1316.623	1316.5952	-0.0278	-21	1108	1118	TPLNWNETGER					Mascot
1320.6517	1320.6177	-0.034	-26	630	640	SFLGMAGYYRR					Mascot
1379.6624	1379.7222	0.0598	43	756	766	CEVYTDHKSLK			Carbamidomethyl (C)[1]		Mascot
1507.7244	1507.7723	0.0479	32	308	320	CSLLIQTPGSEMR			Carbamidomethyl (C)[1], Oxidation (M)[12]		Mascot
1507.7244	1507.7723	0.0479	32	308	320	CSLLIQTPGSEMR			Carbamidomethyl (C)[1], Oxidation (M)[12]		Mascot

1584.8024	1584.7711	-0.0313	-20	147	160	KMNQQASGPSQRPR						Mascot
1657.8771	1657.8401	-0.037	-22	76	88	MFEFLRTKPPTFK				Oxidation (M)[1]		Mascot

7	PREDICTED: DNA repair endonuclease UVH1-like [Solanum lycopersicum]	gi|460400619	109059.4	6.32	27	61	29.485	7.916

Peptide Information

Calc. Mass	Obsrv. Mass	± da	± ppm	Start Seq.	End Seq.	Sequence	Ion Score	C. I. %	Modification	Rank	Result Type
802.4669	802.4499	-0.017	-21	277	283	QLVSDLK					Mascot
810.3484	810.4192	0.0708	87	918	924	AIMDGCK			Carbamidomethyl (C)[6], Oxidation (M)[3]		Mascot
814.3651	814.4427	0.0776	95	638	643	EVEMYK			Oxidation (M)[4]		Mascot
820.441	820.4149	-0.0261	-32	503	510	AEVSSISK					Mascot
835.3792	835.4055	0.0263	31	413	420	QASSGEEK					Mascot
836.4811	836.4198	-0.0613	-73	179	184	CLFLRK			Carbamidomethyl (C)[1]		Mascot
837.4352	837.4028	-0.0324	-39	255	261	SFDEIVK					Mascot
864.4686	864.4208	-0.0478	-55	667	673	FEASVRR					Mascot
891.5298	891.4423	-0.0875	-98	288	294	LLDYLVR					Mascot
946.5026	946.478	-0.0246	-26	937	945	LAELMGGQK					Mascot
948.536	948.4582	-0.0778	-82	502	510	KAEVSSISK					Mascot
1033.6252	1033.5253	-0.0999	-97	345	354	LGLSKTVSTK					Mascot
1095.5906	1095.5701	-0.0205	-19	149	157	STNRSLYVR					Mascot
1110.4595	1110.5577	0.0982	88	456	463	VMCEEWEK			Carbamidomethyl (C)[3]		Mascot
1126.4543	1126.5381	0.0838	74	456	463	VMCEEWEK			Carbamidomethyl (C)[3], Oxidation (M)[2]		Mascot
1199.5652	1199.6605	0.0953	79	588	598	HDLGLDAESSR					Mascot
1232.6667	1232.6223	-0.0444	-36	937	948	LAELMGGQKAAK			Oxidation (M)[5]		Mascot
1277.6597	1277.7323	0.0726	57	907	917	RLPGVTDSNYR					Mascot
1316.6521	1316.5952	-0.0569	-43	315	325	SVWIFAESSYK					Mascot
1325.5977	1325.6694	0.0717	54	789	798	LYHQVEMMSR			Oxidation (M)[7,8]		Mascot
1360.6963	1360.7316	0.0353	26	925	936	SLAELAMLPMER					Mascot
1367.7062	1367.7141	0.0079	6	211	222	VPMSTYMIGIQK					Mascot
1443.7227	1443.7413	0.0186	13	303	314	YLDSLRASESFR					Mascot
1463.8118	1463.802	-0.0098	-7	263	274	QLDPIWHTLGKR					Mascot
1487.7336	1487.7836	0.05	34	527	539	INKDIVGEDDNQK					Mascot
1584.7865	1584.7711	-0.0154	-10	879	893	VGVPSEEGVVENDVR					Mascot
1605.8887	1605.7699	-0.1188	-74	603	616	LPPVVFYALESDKK					Mascot
1791.8306	1791.7716	-0.059	-33	789	801	LYHQVEMMSRYYR			Oxidation (M)[7]		Mascot

8	hypothetical protein F775_19889 [Aegilops tauschii]	gi|475578522	49342.9	9.64	17	61	27.843	6.217

Peptide Information

Calc. Mass	Obsrv. Mass	± da	± ppm	Start Seq.	End Seq.	Sequence	Ion Score	C. I. %	Modification	Rank	Result Type
802.4716	802.4499	-0.0217	-27	379	384	QMRLVR					Mascot
807.4723	807.4188	-0.0535	-66	71	77	LTLTGFR					Mascot
813.4465	813.408	-0.0385	-47	33	41	SLGGPTGPK					Mascot
877.4639	877.4401	-0.0238	-27	207	213	DQHPRPK					Mascot
891.5046	891.4423	-0.0623	-70	408	414	QIFQSLR					Mascot
918.4891	918.4467	-0.0424	-46	159	167	KEATDGAVK					Mascot
1013.585	1013.5228	-0.0622	-61	347	356	GRVAVGEGLR					Mascot
1114.5739	1114.5632	-0.0107	-10	228	237	QEPAIGEKDK					Mascot
1212.5864	1212.6089	0.0225	19	394	403	CYVVSCLVGR			Carbamidomethyl (C)[1,6]		Mascot
1232.6456	1232.6223	-0.0233	-19	357	367	VSAPVATMVWR			Oxidation (M)[8]		Mascot
1262.641	1262.642	0.001	1	260	272	AKAAATPTAMDAK			Oxidation (M)[10]		Mascot
1316.6958	1316.5952	-0.1006	-76	303	313	WADNTLEIARK					Mascot
1325.7059	1325.6694	-0.0365	-28	194	205	QEEEKKPAPAAK					Mascot
1384.8101	1384.7765	-0.0336	-24	10	21	LHLFLPTPPPPR					Mascot
1384.8101	1384.7765	-0.0336	-24	10	21	LHLFLPTPPPPR					Mascot
1463.7336	1463.802	0.0684	47	160	173	EATDGAVKESTLSR					Mascot
1487.7714	1487.7836	0.0122	8	95	110	GATRPGAGGPGSALYR					Mascot
1657.785	1657.8401	0.0551	33	262	277	AAATPTAMDAKAEHEK			Oxidation (M)[8]		Mascot

9	RNA polymerase beta' subunit [Liriodendron tulipifera]	gi|114107036	157839.1	9.19	28	61	17.153	9.447

Protein Group

RecName: Full=DNA-directed RNA polymerase subunit beta''; AltName: Full=PEP; AltName: Full=Plastid-encoded RNA polymerase subunit beta''; Short=RNA polymerase subunit beta''	gi|122239950	157839.1	9.1899995803833

Peptide Information

Calc. Mass	Obsrv. Mass	± da	± ppm	Start Seq.	End Seq.	Sequence	Ion Score	C. I. %	Modification	Rank	Result Type
814.4781	814.4427	-0.0354	-43	357	364	APSNGKIK					Mascot
816.4574	816.4243	-0.0331	-41	192	199	GVVDTAVR					Mascot
819.4359	819.4213	-0.0146	-18	947	954	IGPFNGSK					Mascot
822.3774	822.4008	0.0234	28	222	228	TDCGTIR			Carbamidomethyl (C)[3]		Mascot
824.4849	824.441	-0.0439	-53	1324	1330	GLVHRSR					Mascot
835.4632	835.4055	-0.0577	-69	624	631	RSSSGITK					Mascot

847.4341	847.4644	0.0303	36	2	8	EVLMAER					Mascot
856.5615	856.5396	-0.0219	-26	1263	1270	AILLGITR					Mascot
864.4495	864.4208	-0.0287	-33	17	24	VIDATAMK			Oxidation (M)[7]		Mascot
909.4247	909.4614	0.0367	40	296	302	STSWICR			Carbamidomethyl (C)[6]		Mascot
909.4247	909.4614	0.0367	40	296	302	STSWICR			Carbamidomethyl (C)[6]		Mascot
918.4713	918.4467	-0.0246	-27	589	595	EKELMPR			Oxidation (M)[5]		Mascot
937.4625	937.4431	-0.0194	-21	644	650	EDLIEYR					Mascot
1010.4645	1010.5079	0.0434	43	1	8	MEVLMAER			Oxidation (M)[1,5]		Mascot
1015.5418	1015.516	-0.0258	-25	1133	1142	SGDITQGLPK					Mascot
1065.5575	1065.5458	-0.0117	-11	643	650	KEDLIEYR					Mascot
1110.5248	1110.5577	0.0329	30	1254	1262	ALEEGICYR			Carbamidomethyl (C)[7]		Mascot
1114.6256	1114.5632	-0.0624	-56	487	496	SSIVPFSLHK					Mascot
1139.5804	1139.5697	-0.0107	-9	200	209	TSDAGYLTRR					Mascot
1171.6218	1171.5842	-0.0376	-32	947	957	IGPFNGSKSHK					Mascot
1193.6161	1193.6241	0.008	7	644	653	EDLIEYRGAK					Mascot
1265.6559	1265.6455	-0.0104	-8	251	261	VLADDIYMGLR					Mascot
1290.7893	1290.6887	-0.1006	-78	210	220	LVEVVQHIVVR					Mascot
1320.6543	1320.6177	-0.0366	-28	538	548	ERILDYSGPDR					Mascot
1325.6372	1325.6694	0.0322	24	611	621	DTILAYFDDPR					Mascot
1381.7546	1381.7578	0.0032	2	913	924	IQSLTQHQGTIR					Mascot
1443.7512	1443.7413	-0.0099	-7	961	972	ESIKEDPMIPIR			Oxidation (M)[8]		Mascot
1605.9111	1605.7699	-0.1412	-88	811	824	VVNYILYGNGKPIR					Mascot
1657.7599	1657.8401	0.0802	48	497	510	DQDQMNVHSLSVER					Mascot

10 PREDICTED: uncharacterized protein LOC101763465 [Setaria italica] gi|514805500 84883 5.9 19 60 11.227 6.63

Peptide Information

Calc. Mass	Obsrv. Mass	± da	± ppm	Start Seq.	End Seq.	Sequence	Ion Score	C. I. %	Modification	Rank	Result Type
812.3528	812.4122	0.0594	73	411	416	IEMMEK			Oxidation (M)[3,4]		Mascot
813.3811	813.408	0.0269	33	244	250	SYADMVK					Mascot
830.4883	830.432	-0.0563	-68	78	84	QLFGLPR					Mascot
845.4628	845.4816	0.0188	22	85	91	AHISYVR					Mascot
861.4577	861.4442	-0.0135	-16	417	423	KQAWSNK					Mascot
870.5156	870.5538	0.0382	44	2	8	QTPKIQR					Mascot
1015.5353	1015.516	-0.0193	-19	598	605	NQPMLEKR					Mascot
1016.5483	1016.5214	-0.0269	-26	559	569	GSLAGVSVNGR					Mascot

1025.4576	1025.5587	0.1011	99	644	652	MLPMMSAGR	Oxidation (M)[1,4]	Mascot
1110.579	1110.5577	-0.0213	-19	512	522	SYASTVALNGK		Mascot
1139.5514	1139.5697	0.0183	16	136	145	TSYPAQVAMR	Oxidation (M)[9]	Mascot
1141.5493	1141.5438	-0.0055	-5	750	758	RCYCSAIVL	Carbamidomethyl (C)[2,4]	Mascot
1212.6624	1212.6089	-0.0535	-44	221	231	ELVSSPVWAPK		Mascot
1302.6875	1302.6768	-0.0107	-8	595	604	WIKNQPMLEK	Oxidation (M)[7]	Mascot
1308.6655	1308.6779	0.0124	9	424	434	EVQHLQGVNER		Mascot
1338.6392	1338.6897	0.0505	38	406	416	ELSGRIEMMEK	Oxidation (M)[8]	Mascot
1360.7179	1360.7316	0.0137	10	41	52	STRPTNLSISER		Mascot
1363.6311	1363.7101	0.079	58	546	556	DDWITCPSLTR	Carbamidomethyl (C)[6]	Mascot
1621.8431	1621.8745	0.0314	19	721	734	AEADTILDVVERYK		Mascot

Gel Idx/Pos	296/L24	Instr./Gel Origin	BA2151/Sample Project 20140814	Process Status	Analysis Succeeded
Plate [#] Name	[1] Sample Project 20140814	Instrument Sample Name		Spectra	11

Rank	Protein Name	Accession No.	Protein MW	Protein PI	Pep. Count	Protein Score	Protein Score C. I. %	Intensity Matched	Total Ion Score	Total Ion C. I. %	Confirmed
1	5-methyltetrahydropteroyltriglutamate--homocysteine methyltransferase [Triticum urartu]	gi|473993302	84841.4	5.74	20	1,120	100	30.702	1030	100	

Protein Group

5-methyltetrahydropteroyltriglutamate--homocysteine methyltransferase [Aegilops tauschii]	gi|475526839	84841.4	5.7399 997711 1816

Peptide Information

Calc. Mass	Obsrv. Mass	± da	± ppm	Start Seq.	End Seq.	Sequence	Ion Score	C. I. %	Modification	Rank	Result Type
1096.5786	1096.6073	0.0287	26	287	296	YLFAGVVDGR					Mascot
1096.5786	1096.6073	0.0287	26	287	296	YLFAGVVDGR	86	100			Mascot
1199.5732	1199.6453	0.0721	60	19	28	FALESFWDGK					Mascot
1373.7019	1373.7046	0.0027	2	389	400	SSPRVTNEEVQK					Mascot
1456.7505	1456.765	0.0145	10	577	588	FETCYQIALAIK			Carbamidomethyl (C)[4]		Mascot
1540.739	1540.7914	0.0524	34	373	387	DEAYFAANAAALASR					Mascot
1540.739	1540.7914	0.0524	34	373	387	DEAYFAANAAALASR	58	99.792			Mascot
1790.9623	1790.9556	-0.0067	-4	556	571	GMLTGPVTILNWSFVR					Mascot
1806.9572	1807.0002	0.043	24	556	571	GMLTGPVTILNWSFVR			Oxidation (M)[2]		Mascot
1859.9386	1860.0032	0.0646	35	254	270	TLTSLSSVTAYGFDLER					Mascot
1859.9386	1860.0032	0.0646	35	254	270	TLTSLSSVTAYGFDLER	130	100			Mascot
1864.8766	1864.9762	0.0996	53	618	632	SEHAFYLDWAVHSFR					Mascot
1991.0081	1991.0825	0.0744	37	477	493	IQEELDIDVLVHGEPER					Mascot
1991.0081	1991.0825	0.0744	37	477	493	IQEELDIDVLVHGEPER	123	100			Mascot
2043.0295	2043.1112	0.0817	40	685	704	EGVVYGAGIGPGVYDIHS PR					Mascot
2043.0295	2043.1112	0.0817	40	685	704	EGVVYGAGIGPGVYDIHS PR	167	100			Mascot
2067.0327	2067.125	0.0923	45	572	588	NDQPRFETCYQIALAIK			Carbamidomethyl (C)[9]		Mascot
2098.9539	2099.0269	0.073	35	82	99	YSWTGGEINLSTYFSMA R			Oxidation (M)[16]		Mascot
2109.0723	2109.1504	0.0781	37	367	387	ALAGQKDEAYFAANAAA LASR					Mascot
2109.0723	2109.1504	0.0781	37	367	387	ALAGQKDEAYFAANAAA LASR	91	100			Mascot
2165.0703	2165.1357	0.0654	30	112	129	WFDTNYHFIVPELAPSTK					Mascot

2268.1719	2268.2661	0.0942	42	590	610	EVEDLEAGGIQVIQIDEAALR					Mascot
2268.1719	2268.2661	0.0942	42	590	610	EVEDLEAGGIQVIQIDEAALR	232	100			Mascot
2296.2913	2296.3796	0.0883	38	427	447	LNLPVLPTTTIGSFPQTVELR					Mascot
2296.2913	2296.3796	0.0883	38	427	447	LNLPVLPTTTIGSFPQTVELR	23	0			Mascot
2396.2668	2396.3687	0.1019	43	589	610	KEVEDLEAGGIQVIQIDEAALR					Mascot
2424.3862	2424.4856	0.0994	41	426	447	KLNLPVLPTTTIGSFPQTVELR					Mascot
2424.3862	2424.4856	0.0994	41	426	447	KLNLPVLPTTTIGSFPQTVELR	119	100			Mascot
2707.3735	2707.4761	0.1026	38	522	544	CVKPPIIYGDVSRPNPMTVFWSK			Carbamidomethyl (C)[1], Oxidation (M)[17]		Mascot
2937.3975	2937.5366	0.1391	47	56	81	YIPSNTFSYYDQVLDTTAMLGAVPDR					Mascot
2953.3926	2953.5283	0.1357	46	56	81	YIPSNTFSYYDQVLDTTAMLGAVPDR			Oxidation (M)[19]		Mascot

2	5-methyltetrahydropteroyltriglutamate-homocysteine methyltransferase, putative, expressed [Oryza sativa Japonica Group]	gi|108862992	84924.6	5.93	16	642	100	15.899	583	100

Protein Group

5-methyltetrahydropteroyltriglutamate-homocysteine methyltransferase, putative, expressed [Oryza sativa Japonica Group] gi|108862993 84924.6 5.9299998283862

Peptide Information

Calc. Mass	Obsrv. Mass	± da	± ppm	Start Seq.	End Seq.	Sequence	Ion Score	C. I. %	Modification	Rank	Result Type
1096.5786	1096.6073	0.0287	26	288	297	YLFAGVVDGR					Mascot
1096.5786	1096.6073	0.0287	26	288	297	YLFAGVVDGR	86	100			Mascot
1199.5732	1199.6453	0.0721	60	19	28	FALESFWDGK					Mascot
1373.7019	1373.7046	0.0027	2	390	401	SSPRVTNEEVQK					Mascot
1456.7505	1456.765	0.0145	10	578	589	FETCYQIALAIK			Carbamidomethyl (C)[4]		Mascot
1485.7908	1485.7977	0.0069	5	706	718	IPSTEEIADRINK					Mascot
1499.8176	1499.7708	-0.0468	-31	394	407	VTNEEVQKAAAALR					Mascot
1764.9564	1764.9193	-0.0371	-21	741	756	YTEVKPALTNMVLAAK			Oxidation (M)[11]		Mascot
1790.9623	1790.9556	-0.0067	-4	557	572	GMLTGPVTILNWSFVR					Mascot
1806.9572	1807.0002	0.043	24	557	572	GMLTGPVTILNWSFVR			Oxidation (M)[2]		Mascot
1991.0081	1991.0825	0.0744	37	478	494	IQEELDIDVLVHGEPER					Mascot
1991.0081	1991.0825	0.0744	37	478	494	IQEELDIDVLVHGEPER	123	100			Mascot
2067.0327	2067.125	0.0923	45	573	589	NDQPRFETCYQIALAIK			Carbamidomethyl (C)[9]		Mascot

2131.2307	2131.0383	-0.1924	-90	741	759	YTEVKPALTNMVLAAKLI R					Mascot
2268.1719	2268.2661	0.0942	42	591	611	EVEDLEAGGIQVIQIDEAA LR					Mascot
2268.1719	2268.2661	0.0942	42	591	611	EVEDLEAGGIQVIQIDEAA LR	232	100			Mascot
2296.2913	2296.3796	0.0883	38	428	448	LNLPVLPTTTIGSFPQTVE LR					Mascot
2296.2913	2296.3796	0.0883	38	428	448	LNLPVLPTTTIGSFPQTVE LR	23	0			Mascot
2396.2668	2396.3687	0.1019	43	590	611	KEVEDLEAGGIQVIQIDEA ALR					Mascot
2424.3862	2424.4856	0.0994	41	427	448	KLNLPVLPTTTIGSFPQTV ELR					Mascot
2424.3862	2424.4856	0.0994	41	427	448	KLNLPVLPTTTIGSFPQTV ELR	119	100			Mascot
2937.3975	2937.5366	0.1391	47	56	81	YIPSNTFSYYDQVLDTAA MLGAVPER			Oxidation (M)[19]		Mascot

3	Os12g0624000 [Oryza sativa Japonica Group]	gi|113649821	84954.6	5.93	15	634	100	15.73	583	100

Protein Group

5-methyltetrahydropteroyltriglutamate-homocysteine methyltransferase [Oryza sativa Japonica Group]	gi|385717666	84954.6	5.9299 998283 3862
5-methyltetrahydropteroyltriglutamate-homocysteine methyltransferase, putative, expressed [Oryza sativa Japonica Group]	gi|77556633	84954.6	5.9299 998283 3862
5-methyltetrahydropteroyltriglutamate-homocysteine methyltransferase, putative, expressed [Oryza sativa Japonica Group]	gi|77556632	84954.6	5.9299 998283 3862
hypothetical protein OsI_39180 [Oryza sativa Indica Group]	gi|218187282	84954.6	5.9299 998283 3862

Peptide Information

Calc. Mass	Obsrv. Mass	± da	± ppm	Start Seq.	End Seq.	Sequence	Ion Score	C. I. %	Modification	Rank	Result Type
1096.5786	1096.6073	0.0287	26	288	297	YLFAGVVDGR					Mascot
1096.5786	1096.6073	0.0287	26	288	297	YLFAGVVDGR	86	100			Mascot
1199.5732	1199.6453	0.0721	60	19	28	FALESFWDGK					Mascot
1373.7019	1373.7046	0.0027	2	390	401	SSPRVTNEEVQK					Mascot

1456.7505	1456.765	0.0145	10	578	589	FETCYQIALAIK			Carbamidomethyl (C)[4]	Mascot
1485.7908	1485.7977	0.0069	5	706	718	IPSTEEIADRINK				Mascot
1499.8176	1499.7708	-0.0468	-31	394	407	VTNEEVQKAAAALR				Mascot
1764.9564	1764.9193	-0.0371	-21	741	756	YTEVKPALTNMVLAAK			Oxidation (M)[11]	Mascot
1790.9623	1790.9556	-0.0067	-4	557	572	GMLTGPVTILNWSFVR				Mascot
1806.9572	1807.0002	0.043	24	557	572	GMLTGPVTILNWSFVR			Oxidation (M)[2]	Mascot
1991.0081	1991.0825	0.0744	37	478	494	IQEELDIDVLVHGEPER				Mascot
1991.0081	1991.0825	0.0744	37	478	494	IQEELDIDVLVHGEPER	123	100		Mascot
2067.0327	2067.125	0.0923	45	573	589	NDQPRFETCYQIALAIK			Carbamidomethyl (C)[9]	Mascot
2131.2307	2131.0383	-0.1924	-90	741	759	YTEVKPALTNMVLAAKLI R				Mascot
2268.1719	2268.2661	0.0942	42	591	611	EVEDLEAGGIQVIQIDEAA LR				Mascot
2268.1719	2268.2661	0.0942	42	591	611	EVEDLEAGGIQVIQIDEAA LR	232	100		Mascot
2296.2913	2296.3796	0.0883	38	428	448	LNLPVLPTTTIGSFPQTVE LR				Mascot
2296.2913	2296.3796	0.0883	38	428	448	LNLPVLPTTTIGSFPQTVE LR	23	0		Mascot
2396.2668	2396.3687	0.1019	43	590	611	KEVEDLEAGGIQVIQIDEA ALR				Mascot
2424.3862	2424.4856	0.0994	41	427	448	KLNLPVLPTTTIGSFPQTV ELR				Mascot
2424.3862	2424.4856	0.0994	41	427	448	KLNLPVLPTTTIGSFPQTV ELR	119	100		Mascot

4	Os12g0623900 [Oryza sativa Japonica Group]	gi|113649820	84873.6	5.93	14	628	100	15.32	582	100

Protein Group

5-methyltetrahydropteroyltriglutamate-homocysteine methyltransferase [Oryza sativa Japonica Group]	gi|385717668	84873.6	5.9299 998283 3862
5-methyltetrahydropteroyltriglutamate-homocysteine methyltransferase, putative, expressed [Oryza sativa Japonica Group]	gi|108862991	84873.6	5.9299 998283 3862
5-methyltetrahydropteroyltriglutamate-homocysteine methyltransferase, putative, expressed [Oryza sativa Japonica Group]	gi|77556631	84873.6	5.9299 998283 3862
hypothetical protein OsI_39179 [Oryza sativa Indica Group]	gi|125537469	84873.6	5.9299 998283 3862

Peptide Information

Calc. Mass	Obsrv. Mass	± da	± ppm	Start Seq.	End Seq.	Sequence	Ion Score	C. I. %	Modification	Rank	Result Type
1096.5786	1096.6073	0.0287	26	288	297	YLFAGVVDGR	86	100			Mascot
1096.5858	1096.6073	0.0215	20	402	412	AAAALKGSDHR					Mascot
1199.5732	1199.6453	0.0721	60	19	28	FALESFWDGK					Mascot
1373.7019	1373.7046	0.0027	2	390	401	SSPRVTNEEVQK					Mascot
1456.7505	1456.765	0.0145	10	578	589	FETCYQIALAIK			Carbamidomethyl (C)[4]		Mascot
1790.9623	1790.9556	-0.0067	-4	557	572	GMLTGPVTILNWSFVR					Mascot
1806.9572	1807.0002	0.043	24	557	572	GMLTGPVTILNWSFVR			Oxidation (M)[2]		Mascot
1991.0081	1991.0825	0.0744	37	478	494	IQEELDIDVLVHGEPER					Mascot
1991.0081	1991.0825	0.0744	37	478	494	IQEELDIDVLVHGEPER	123	100			Mascot
2067.0327	2067.125	0.0923	45	573	589	NDQPRFETCYQIALAIK			Carbamidomethyl (C)[9]		Mascot
2268.1719	2268.2661	0.0942	42	591	611	EVEDLEAGGIQVIQIDEAA LR					Mascot
2268.1719	2268.2661	0.0942	42	591	611	EVEDLEAGGIQVIQIDEAA LR	232	100			Mascot
2296.2913	2296.3796	0.0883	38	428	448	LNLPVLPTTTIGSFPQTVE LR					Mascot
2296.2913	2296.3796	0.0883	38	428	448	LNLPVLPTTTIGSFPQTVE LR	23	0			Mascot
2396.2668	2396.3687	0.1019	43	590	611	KEVEDLEAGGIQVIQIDEA ALR					Mascot
2424.3862	2424.4856	0.0994	41	427	448	KLNLPVLPTTTIGSFPQTV ELR					Mascot
2424.3862	2424.4856	0.0994	41	427	448	KLNLPVLPTTTIGSFPQTV ELR	119	100			Mascot
2707.3735	2707.4761	0.1026	38	523	545	CVKPPIIYGDVSRPNPMT VFWSK			Carbamidomethyl (C)[1], Oxidation (M)[17]		Mascot
2937.3975	2937.5366	0.1391	47	56	81	YIPSNTFSYYDQVLDTAA MLGAVPER			Oxidation (M)[19]		Mascot

5	5-methyltetrahydropteroyltriglutamate-homocysteine methyltransferase, putative, expressed [Oryza sativa Japonica Group]	gi|108862990	79272	7.19	12	619	100	15.004	582	100	

Peptide Information

Calc. Mass	Obsrv. Mass	± da	± ppm	Start Seq.	End Seq.	Sequence	Ion Score	C. I. %	Modification	Rank	Result Type
1096.5786	1096.6073	0.0287	26	238	247	YLFAGVVDGR	86	100			Mascot
1096.5858	1096.6073	0.0215	20	352	362	AAAALKGSDHR					Mascot
1373.7019	1373.7046	0.0027	2	340	351	SSPRVTNEEVQK					Mascot
1456.7505	1456.765	0.0145	10	528	539	FETCYQIALAIK			Carbamidomethyl (C)[4]		Mascot
1790.9623	1790.9556	-0.0067	-4	507	522	GMLTGPVTILNWSFVR					Mascot
1806.9572	1807.0002	0.043	24	507	522	GMLTGPVTILNWSFVR			Oxidation (M)[2]		Mascot

1991.0081	1991.0825	0.0744	37	428	444	IQEELDIDVLVHGEPER					Mascot
1991.0081	1991.0825	0.0744	37	428	444	IQEELDIDVLVHGEPER	123	100			Mascot
2067.0327	2067.125	0.0923	45	523	539	NDQPRFETCYQIALAIK			Carbamidomethyl (C)[9]		Mascot
2268.1719	2268.2661	0.0942	42	541	561	EVEDLEAGGIQVIQIDEAALR					Mascot
2268.1719	2268.2661	0.0942	42	541	561	EVEDLEAGGIQVIQIDEAALR	232	100			Mascot
2296.2913	2296.3796	0.0883	38	378	398	LNLPVLPTTTIGSFPQTVELR					Mascot
2296.2913	2296.3796	0.0883	38	378	398	LNLPVLPTTTIGSFPQTVELR	23	0			Mascot
2396.2668	2396.3687	0.1019	43	540	561	KEVEDLEAGGIQVIQIDEAALR					Mascot
2424.3862	2424.4856	0.0994	41	377	398	KLNLPVLPTTTIGSFPQTVELR					Mascot
2424.3862	2424.4856	0.0994	41	377	398	KLNLPVLPTTTIGSFPQTVELR	119	100			Mascot
2707.3735	2707.4761	0.1026	38	473	495	CVKPPIIYGDVSRPNPMTVFWSK			Carbamidomethyl (C)[1], Oxidation (M)[17]		Mascot

6	PREDICTED: 5-methyltetrahydropteroyltriglutamate--homocysteine methyltransferase-like [Setaria italica]	gi|514814998	84774.6	6.05	16	555	100	15.815	499	100	

Peptide Information

Calc. Mass	Obsrv. Mass	± da	± ppm	Start Seq.	End Seq.	Sequence	Ion Score	C. I. %	Modification	Rank	Result Type
1096.5786	1096.6073	0.0287	26	287	296	YLFAGVVDGR	86	100			Mascot
1096.5858	1096.6073	0.0215	20	401	411	AAAALKGSDHR					Mascot
1199.5732	1199.6453	0.0721	60	19	28	FALESFWDGK					Mascot
1456.7505	1456.765	0.0145	10	577	588	FETCYQIALAIK			Carbamidomethyl (C)[4]		Mascot
1485.7908	1485.7977	0.0069	5	705	717	IPSTEEIADRINK					Mascot
1516.743	1516.7673	0.0243	16	130	142	FSYASHKAVSEYK					Mascot
1540.739	1540.7914	0.0524	34	373	387	DEAYFAANAAALASR					Mascot
1540.739	1540.7914	0.0524	34	373	387	DEAYFAANAAALASR	58	99.792			Mascot
1786.9124	1786.9355	0.0231	13	280	296	SSFPSGKYLFAGVVDGR					Mascot
1790.9623	1790.9556	-0.0067	-4	556	571	GMLTGPVTILNWSFVR					Mascot
1806.9572	1807.0002	0.043	24	556	571	GMLTGPVTILNWSFVR			Oxidation (M)[2]		Mascot
1864.8766	1864.9762	0.0996	53	618	632	SEHAFYLDWAVHSFR					Mascot
1881.015	1880.959	-0.056	-30	739	755	KYTEVKPALTNMVSATK					Mascot
1897.01	1896.9429	-0.0671	-35	739	755	KYTEVKPALTNMVSATK			Oxidation (M)[12]		Mascot
1991.0081	1991.0825	0.0744	37	477	493	IQEELDIDVLVHGEPER					Mascot
1991.0081	1991.0825	0.0744	37	477	493	IQEELDIDVLVHGEPER	123	100			Mascot
2067.0327	2067.125	0.0923	45	572	588	NDQPRFETCYQIALAIK			Carbamidomethyl (C)[9]		Mascot

2109.0723	2109.1504	0.0781	37	367	387	ALAGQKDEAYFAANAAA LASR					Mascot
2109.0723	2109.1504	0.0781	37	367	387	ALAGQKDEAYFAANAAA LASR	91	100			Mascot
2296.2913	2296.3796	0.0883	38	427	447	LNLPVLPTTTIGSFPQTVE LR					Mascot
2296.2913	2296.3796	0.0883	38	427	447	LNLPVLPTTTIGSFPQTVE LR	23	0			Mascot
2424.3862	2424.4856	0.0994	41	426	447	KLNLPVLPTTTIGSFPQTV ELR					Mascot
2424.3862	2424.4856	0.0994	41	426	447	KLNLPVLPTTTIGSFPQTV ELR	119	100			Mascot

7	5-methyltetrahydropteroyltriglutamate--homocysteine methyltransferase [Aegilops tauschii]	gi|475620440	84650.5	5.97	18	511	100	14.726	440	100	

Peptide Information

Calc. Mass	Obsrv. Mass	± da	± ppm	Start Seq.	End Seq.	Sequence	Ion Score	C. I. %	Modification	Rank	Result Type
857.4952	857.4821	-0.0131	-15	413	420	RATPVSAR					Mascot
1096.5786	1096.6073	0.0287	26	288	297	YLFAGVVDGR	86	100			Mascot
1096.5858	1096.6073	0.0215	20	402	412	AAAALKGSDHR					Mascot
1199.5732	1199.6453	0.0721	60	19	28	FALESFWDGK					Mascot
1456.7505	1456.765	0.0145	10	578	589	FETCYQIALAIK			Carbamidomethyl (C)[4]		Mascot
1503.7649	1503.8326	0.0677	45	29	42	SSAEDLEKVAADLR					Mascot
1516.743	1516.7673	0.0243	16	130	142	FSYASHKAVSEYK					Mascot
1790.9623	1790.9556	-0.0067	-4	557	572	GMLTGPVTILNWSFVR					Mascot
1806.9572	1807.0002	0.043	24	557	572	GMLTGPVTILNWSFVR			Oxidation (M)[2]		Mascot
1864.8766	1864.9762	0.0996	53	619	633	SEHAFYLDWAVHSFR					Mascot
1991.0081	1991.0825	0.0744	37	478	494	IQEELDIDVLVHGEPER					Mascot
1991.0081	1991.0825	0.0744	37	478	494	IQEELDIDVLVHGEPER	123	100			Mascot
2031.9559	2032.1113	0.1554	76	82	99	YSWTGGEIGHSTYFSIAR					Mascot
2067.0327	2067.125	0.0923	45	573	589	NDQPRFETCYQIALAIK			Carbamidomethyl (C)[9]		Mascot
2109.0723	2109.1504	0.0781	37	368	388	ALAGLKDEAYFAANAAA QASR					Mascot
2109.0723	2109.1504	0.0781	37	368	388	ALAGLKDEAYFAANAAA QASR					Mascot
2165.0703	2165.1357	0.0654	30	112	129	WFDTNYHFIVPELSPATK					Mascot
2268.1719	2268.2661	0.0942	42	591	611	EVEDLEAGGIQVIQIDEAA LR					Mascot
2268.1719	2268.2661	0.0942	42	591	611	EVEDLEAGGIQVIQIDEAA LR	232	100			Mascot
2396.2668	2396.3687	0.1019	43	590	611	KEVEDLEAGGIQVIQIDEA ALR					Mascot
2707.3735	2707.4761	0.1026	38	523	545	CVKPPIIYGDVSRPNPMT VFWSK			Carbamidomethyl (C)[1], Oxidation (M)[17]		Mascot
2937.3975	2937.5366	0.1391	47	56	81	YIPSNTFSYYDQVLDTTA MLGAVPDR					Mascot

Calc. Mass	Obsrv. Mass	± da	± ppm	Start Seq.	End Seq.	Sequence	Ion Score	C. I. %	Modification	Rank	Result Type
2953.3926	2953.5283	0.1357	46	56	81	YIPSNTFSYYDQVLDTTA MLGAVPDR			Oxidation (M)[19]		Mascot

8	5-methyltetrahydropteroyltriglutamate--homocysteine methyltransferase [Triticum urartu]	gi|473954466	82012.1	6.17	17	510	100	14.749	441	100

Peptide Information

Calc. Mass	Obsrv. Mass	± da	± ppm	Start Seq.	End Seq.	Sequence	Ion Score	C. I. %	Modification	Rank	Result Type
857.4952	857.4821	-0.0131	-15	387	394	RATPVSAR					Mascot
1096.5786	1096.6073	0.0287	26	256	265	YLFAGVVDGR					Mascot
1096.5786	1096.6073	0.0287	26	256	265	YLFAGVVDGR	86	100			Mascot
1199.5732	1199.6453	0.0721	60	19	28	FALESFWDGK					Mascot
1456.7505	1456.765	0.0145	10	552	563	FETCYQIALAIK			Carbamidomethyl (C)[4]		Mascot
1503.7649	1503.8326	0.0677	45	29	42	SSAEDLEKVAADLR					Mascot
1516.743	1516.7673	0.0243	16	130	142	FSYASHKAVSEYK					Mascot
1790.9623	1790.9556	-0.0067	-4	531	546	GMLTGPVTILNWSFVR					Mascot
1806.9572	1807.0002	0.043	24	531	546	GMLTGPVTILNWSFVR			Oxidation (M)[2]		Mascot
1864.8766	1864.9762	0.0996	53	593	607	SEHAFYLDWAVHSFR					Mascot
1991.0081	1991.0825	0.0744	37	452	468	IQEELDIDVLVHGEPER					Mascot
1991.0081	1991.0825	0.0744	37	452	468	IQEELDIDVLVHGEPER	123	100			Mascot
2067.0327	2067.125	0.0923	45	547	563	NDQPRFETCYQIALAIK			Carbamidomethyl (C)[9]		Mascot
2109.0723	2109.1504	0.0781	37	336	356	ALAGLKDEAYFAANAAA QASR					Mascot
2109.0723	2109.1504	0.0781	37	336	356	ALAGLKDEAYFAANAAA QASR					Mascot
2165.0703	2165.1357	0.0654	30	112	129	WFDTNYHFIVPELSPATK					Mascot
2268.1719	2268.2661	0.0942	42	565	585	EVEDLEAGGIQVIQIDEAA LR					Mascot
2268.1719	2268.2661	0.0942	42	565	585	EVEDLEAGGIQVIQIDEAA LR	232	100			Mascot
2396.2668	2396.3687	0.1019	43	564	585	KEVEDLEAGGIQVIQIDEA ALR					Mascot
2707.3735	2707.4761	0.1026	38	497	519	CVKPPIIYGDVSRPNPMT VFWSK			Carbamidomethyl (C)[1], Oxidation (M)[17]		Mascot
2937.3975	2937.5366	0.1391	47	56	81	YIPSNTFSYYDQVLDTTA MLGAVPDR					Mascot
2953.3926	2953.5283	0.1357	46	56	81	YIPSNTFSYYDQVLDTTA MLGAVPDR			Oxidation (M)[19]		Mascot
3312.4758	3312.4648	-0.011	-3	82	111	YSWTGGEIGHSTYFSMA RGNATVPAMEMTK			Oxidation (M)[16,26]		Mascot

9	TPA: putative methionine synthase family protein isoform 1 [Zea mays]	gi|414869037	84781.4	5.54	13	479	100	13.294	440	100

Protein Group

TPA: putative methionine synthase family protein isoform 2 [Zea mays]	gi|414869038	84781.4	5.5399 999618 5303

Peptide Information

Calc. Mass	Obsrv. Mass	± da	± ppm	Start Seq.	End Seq.	Sequence	Ion Score	C. I. %	Modification	Rank	Result Type
1096.5786	1096.6073	0.0287	26	288	297	YLFAGVVDGR	86	100			Mascot
1096.5858	1096.6073	0.0215	20	402	412	AAAALKGSDHR					Mascot
1199.5732	1199.6453	0.0721	60	19	28	FALESFWDGK					Mascot
1373.7019	1373.7046	0.0027	2	390	401	SSPRVTNEEVQK					Mascot
1456.7642	1456.765	0.0008	1	706	718	IPSAEEIADRIDK					Mascot
1790.9623	1790.9556	-0.0067	-4	557	572	GMLTGPVTILNWSFVR					Mascot
1806.9572	1807.0002	0.043	24	557	572	GMLTGPVTILNWSFVR			Oxidation (M)[2]		Mascot
1991.0081	1991.0825	0.0744	37	478	494	LQEELDIDVLVHGEPER					Mascot
1991.0081	1991.0825	0.0744	37	478	494	LQEELDIDVLVHGEPER	123	100			Mascot
2067.0327	2067.125	0.0923	45	573	589	NDQPRFETCYQIALAIK			Carbamidomethyl (C)[9]		Mascot
2268.1719	2268.2661	0.0942	42	591	611	EVEDLEAGGIQVIQIDEAA LR					Mascot
2268.1719	2268.2661	0.0942	42	591	611	EVEDLEAGGIQVIQIDEAA LR	232	100			Mascot
2310.3069	2310.3999	0.093	40	428	448	LNLPILPTTTIGSFPQTVE LR					Mascot
2396.2668	2396.3687	0.1019	43	590	611	KEVEDLEAGGIQVIQIDEA ALR					Mascot
2438.4019	2438.5037	0.1018	42	427	448	KLNLPILPTTTIGSFPQTV ELR					Mascot
2707.3735	2707.4761	0.1026	38	523	545	CVKPPIIYGDVSRPNPMT VFWSK			Carbamidomethyl (C)[1], Oxidation (M)[17]		Mascot

10	TPA: putative methionine synthase family protein [Zea mays]	gi|414869036	86475	5.49	13	478	100	13.294	440	100	

Peptide Information

Calc. Mass	Obsrv. Mass	± da	± ppm	Start Seq.	End Seq.	Sequence	Ion Score	C. I. %	Modification	Rank	Result Type
1096.5786	1096.6073	0.0287	26	288	297	YLFAGVVDGR	86	100			Mascot
1096.5858	1096.6073	0.0215	20	402	412	AAAALKGSDHR					Mascot
1199.5732	1199.6453	0.0721	60	19	28	FALESFWDGK					Mascot
1373.7019	1373.7046	0.0027	2	390	401	SSPRVTNEEVQK					Mascot
1456.7642	1456.765	0.0008	1	706	718	IPSAEEIADRIDK					Mascot
1790.9623	1790.9556	-0.0067	-4	557	572	GMLTGPVTILNWSFVR					Mascot
1806.9572	1807.0002	0.043	24	557	572	GMLTGPVTILNWSFVR			Oxidation (M)[2]		Mascot
1991.0081	1991.0825	0.0744	37	478	494	LQEELDIDVLVHGEPER					Mascot
1991.0081	1991.0825	0.0744	37	478	494	LQEELDIDVLVHGEPER	123	100			Mascot

2067.0327	2067.125	0.0923	45	573	589	NDQPRFETCYQIALAIK			Carbamidomethyl (C)[9]	Mascot
2268.1719	2268.2661	0.0942	42	591	611	EVEDLEAGGIQVIQIDEAALR				Mascot
2268.1719	2268.2661	0.0942	42	591	611	EVEDLEAGGIQVIQIDEAALR	232	100		Mascot
2310.3069	2310.3999	0.093	40	428	448	LNLPILPTTTIGSFPQTVELR				Mascot
2396.2668	2396.3687	0.1019	43	590	611	KEVEDLEAGGIQVIQIDEAALR				Mascot
2438.4019	2438.5037	0.1018	42	427	448	KLNLPILPTTTIGSFPQTVELR				Mascot
2707.3735	2707.4761	0.1026	38	523	545	CVKPPIIYGDVSRPNPMTVFWSK			Carbamidomethyl (C)[1], Oxidation (M)[17]	Mascot

Gel Idx/Pos	299/M1	Instr./Gel Origin	BA2151/Sample Project 20140814	Process Status	Analysis Succeeded
Plate [#] Name	[1] Sample Project 20140814	Instrument Sample Name		Spectra	11

Rank	Protein Name	Accession No.	Protein MW	Protein PI	Pep. Count	Protein Score	Protein Score C. I. %	Intensity Matched	Total Ion Score	Total Ion C. I. %	Confirmed
1	Elongation factor 2 [Triticum urartu]	gi|474416088	97317.3	5.96	23	611	100	24.977	528	100	

Peptide Information

Calc. Mass	Obsrv. Mass	± da	± ppm	Start Seq.	End Seq.	Sequence	Ion Score	C. I. %	Modification	Rank	Result Type
865.4414	865.4394	-0.002	-2	4	10	FTAEELR					Mascot
890.5095	890.5068	-0.0027	-3	504	511	FSVSPVVR					Mascot
955.532	955.5226	-0.0094	-10	723	732	GGGQVIPTAR					Mascot
959.4615	959.4772	0.0157	16	493	500	EVDACPIR			Carbamidomethyl (C)[5]		Mascot
1039.6622	1039.6492	-0.013	-13	172	180	IRPVLTVNK					Mascot
1135.5049	1135.5085	0.0036	3	695	704	EGALAEENMR			Oxidation (M)[9]		Mascot
1188.6194	1188.5979	-0.0215	-18	342	351	VMQTWLPASR					Mascot
1204.6144	1204.6097	-0.0047	-4	342	351	VMQTWLPASR			Oxidation (M)[2]		Mascot
1220.682	1220.6019	-0.0801	-66	501	511	AMKFSVSPVVR					Mascot
1236.677	1236.5984	-0.0786	-64	501	511	AMKFSVSPVVR			Oxidation (M)[2]		Mascot
1315.7191	1315.6771	-0.042	-32	432	443	IMGPNFVPGQKK					Mascot
1346.7791	1346.7725	-0.0066	-5	734	745	VIYASQLTAKPR					Mascot
1543.7727	1543.7751	0.0024	2	290	301	RGFVQFCYEPIK			Carbamidomethyl (C)[7]		Mascot
1745.8965	1745.9255	0.029	17	352	366	ALLEMMIFHLPSPSK			Oxidation (M)[5,6]		Mascot
1745.8965	1745.9255	0.029	17	352	366	ALLEMMIFHLPSPSK			Oxidation (M)[5,6]		Mascot
1786.9375	1786.9601	0.0226	13	792	807	AYLPVIESFGFSSTLR					Mascot
1786.9375	1786.9601	0.0226	13	792	807	AYLPVIESFGFSSTLR	107	100			Mascot
1830.9015	1830.9303	0.0288	16	485	500	NATLTNEKEVDACPIR			Carbamidomethyl (C)[13]		Mascot
2065.0237	2065.0598	0.0361	17	372	389	VENLYEGPLDDIYANAIR					Mascot
2065.0237	2065.0598	0.0361	17	372	389	VENLYEGPLDDIYANAIR	112	100			Mascot
2132.0593	2132.0872	0.0279	13	774	791	GHVFEEMQRPGTPLYNI K			Oxidation (M)[7]		Mascot
2206.0122	2206.0483	0.0361	16	184	201	CFLELQVEGEEAYQTFS R			Carbamidomethyl (C)[1]		Mascot
2206.0122	2206.0483	0.0361	16	184	201	CFLELQVEGEEAYQTFS R	92	100	Carbamidomethyl (C)[1]		Mascot
2257.2034	2257.2478	0.0444	20	54	76	STLTDSLVAAAGIIAQEVA GDVR					Mascot
2257.2034	2257.2478	0.0444	20	54	76	STLTDSLVAAAGIIAQEVA GDVR	136	100			Mascot
2264.0864	2264.1226	0.0362	16	608	627	LYMEARPLEEGLAEAIDD			Oxidation (M)[3]		Mascot

Calc. Mass	Obsrv. Mass	± da	± ppm	Start Seq.	End Seq.	Sequence	Ion Score	C. I. %	Modification	Rank	Result Type
						GR					
2384.188	2384.2336	0.0456	19	370	389	YRVENLYEGPLDDIYANAIR					Mascot
2384.188	2384.2336	0.0456	19	370	389	YRVENLYEGPLDDIYANAIR	82	99.999			Mascot
2406.2009	2406.2437	0.0428	18	565	586	DLQEDFMGGAEIIVSPPVVSFR					Mascot
2422.196	2422.2366	0.0406	17	565	586	DLQEDFMGGAEIIVSPPVVSFR			Oxidation (M)[7]		Mascot
2646.3479	2646.4104	0.0625	24	142	165	ITDGALVVVDCIEGVCVQTETVLR			Carbamidomethyl (C)[11,16]		Mascot
2933.4163	2933.4939	0.0776	26	115	141	DGDEYLINLIDSPGHVDFSSEVTAALR					Mascot

2	Elongation factor 2 [Triticum urartu]	gi|473786548	94753.9	5.85	23	607	100	24.537	528	100	

Protein Group

Elongation factor 2 [Aegilops tauschii]	gi|475617126	94770.9	5.84000015258789

Peptide Information

Calc. Mass	Obsrv. Mass	± da	± ppm	Start Seq.	End Seq.	Sequence	Ion Score	C. I. %	Modification	Rank	Result Type
865.4414	865.4394	-0.002	-2	4	10	FTAEELR					Mascot
890.5095	890.5068	-0.0027	-3	483	490	FSVSPVVR					Mascot
955.532	955.5226	-0.0094	-10	702	711	GGGQVIPTAR					Mascot
959.4615	959.4772	0.0157	16	472	479	EVDACPIR			Carbamidomethyl (C)[5]		Mascot
1039.6622	1039.6492	-0.013	-13	151	159	IRPVLTVNK					Mascot
1135.5049	1135.5085	0.0036	3	674	683	EGALAEENMR			Oxidation (M)[9]		Mascot
1148.5154	1148.5072	-0.0082	-7	753	761	GHVFEEMQR			Oxidation (M)[7]		Mascot
1188.6194	1188.5979	-0.0215	-18	321	330	VMQTWLPASR					Mascot
1204.6144	1204.6097	-0.0047	-4	321	330	VMQTWLPASR			Oxidation (M)[2]		Mascot
1220.682	1220.6019	-0.0801	-66	480	490	AMKFSVSPVVR					Mascot
1236.677	1236.5984	-0.0786	-64	480	490	AMKFSVSPVVR			Oxidation (M)[2]		Mascot
1315.7191	1315.6771	-0.042	-32	411	422	IMGPNFVPGQKK					Mascot
1346.7791	1346.7725	-0.0066	-5	713	724	VIYASQLTAKPR					Mascot
1543.7727	1543.7751	0.0024	2	269	280	RGFVQFCYEPIK			Carbamidomethyl (C)[7]		Mascot
1745.8965	1745.9255	0.029	17	331	345	ALLEMMIFHLPSPSK			Oxidation (M)[5,6]		Mascot
1745.8965	1745.9255	0.029	17	331	345	ALLEMMIFHLPSPSK			Oxidation (M)[5,6]		Mascot
1786.9375	1786.9601	0.0226	13	771	786	AYLPVIESFGFSSTLR					Mascot
1786.9375	1786.9601	0.0226	13	771	786	AYLPVIESFGFSSTLR	107	100			Mascot
1830.9015	1830.9303	0.0288	16	464	479	NATLTNEKEVDACPIR			Carbamidomethyl (C)[13]		Mascot

Calc. Mass	Obsrv. Mass	± da	± ppm	Start Seq.	End Seq.	Sequence	Ion Score	C. I. %	Modification	Rank	Result Type
2065.0237	2065.0598	0.0361	17	351	368	VENLYEGPLDDIYANAIR					Mascot
2065.0237	2065.0598	0.0361	17	351	368	VENLYEGPLDDIYANAIR	112	100			Mascot
2206.0122	2206.0483	0.0361	16	163	180	CFLELQVEGEEAYQTFSR			Carbamidomethyl (C)[1]		Mascot
2206.0122	2206.0483	0.0361	16	163	180	CFLELQVEGEEAYQTFSR	92	100	Carbamidomethyl (C)[1]		Mascot
2257.2034	2257.2478	0.0444	20	33	55	STLTDSLVAAAGIIAQEVAGDVR					Mascot
2257.2034	2257.2478	0.0444	20	33	55	STLTDSLVAAAGIIAQEVAGDVR	136	100			Mascot
2264.0864	2264.1226	0.0362	16	587	606	LYMEARPLEEGLAEAIDDGR			Oxidation (M)[3]		Mascot
2384.188	2384.2336	0.0456	19	349	368	YRVENLYEGPLDDIYANAIR					Mascot
2384.188	2384.2336	0.0456	19	349	368	YRVENLYEGPLDDIYANAIR	82	99.999			Mascot
2408.1804	2408.2285	0.0481	20	544	565	DLQDDFMGGAEIIVSPPVVSFR			Oxidation (M)[7]		Mascot
2646.3479	2646.4104	0.0625	24	121	144	ITDGALVVVDCIEGVCVQTETVLR			Carbamidomethyl (C)[11,16]		Mascot
2933.4163	2933.4939	0.0776	26	94	120	DGDEYLINLIDSPGHVDFSSEVTAALR					Mascot

3 Elongation factor 2 [Triticum urartu] gi|474224670 94757.9 5.85 22 601 100 24.364 528 100

Peptide Information

Calc. Mass	Obsrv. Mass	± da	± ppm	Start Seq.	End Seq.	Sequence	Ion Score	C. I. %	Modification	Rank	Result Type
865.4414	865.4394	-0.002	-2	4	10	FTAEELR					Mascot
890.5095	890.5068	-0.0027	-3	483	490	FSVSPVVR					Mascot
955.532	955.5226	-0.0094	-10	702	711	GGGQVIPTAR					Mascot
959.4615	959.4772	0.0157	16	472	479	EVDACPIR			Carbamidomethyl (C)[5]		Mascot
1039.6622	1039.6492	-0.013	-13	151	159	IRPVLTVNK					Mascot
1135.5049	1135.5085	0.0036	3	674	683	EGALAEENMR			Oxidation (M)[9]		Mascot
1148.5154	1148.5072	-0.0082	-7	753	761	GHVFEEMQR			Oxidation (M)[7]		Mascot
1188.6194	1188.5979	-0.0215	-18	321	330	VMQTWLPASR					Mascot
1204.6144	1204.6097	-0.0047	-4	321	330	VMQTWLPASR			Oxidation (M)[2]		Mascot
1220.682	1220.6019	-0.0801	-66	480	490	AMKFSVSPVVR					Mascot
1236.677	1236.5984	-0.0786	-64	480	490	AMKFSVSPVVR			Oxidation (M)[2]		Mascot
1315.7191	1315.6771	-0.042	-32	411	422	IMGPNFVPGQKK					Mascot
1346.7791	1346.7725	-0.0066	-5	713	724	VIYASQLTAKPR					Mascot
1543.7727	1543.7751	0.0024	2	269	280	RGFVQFCYEPIK			Carbamidomethyl (C)[7]		Mascot
1745.8965	1745.9255	0.029	17	331	345	ALLEMMIFHLPSPSK			Oxidation (M)[5,6]		Mascot
1745.8965	1745.9255	0.029	17	331	345	ALLEMMIFHLPSPSK			Oxidation (M)[5,6]		Mascot
1786.9375	1786.9601	0.0226	13	771	786	AYLPVIESFGFSSTLR					Mascot
1786.9375	1786.9601	0.0226	13	771	786	AYLPVIESFGFSSTLR	107	100			Mascot

2065.0237	2065.0598	0.0361	17	351	368	VENLYEGPLDDIYANAIR					Mascot
2065.0237	2065.0598	0.0361	17	351	368	VENLYEGPLDDIYANAIR	112	100			Mascot
2206.0122	2206.0483	0.0361	16	163	180	CFLELQVEGEEAYQTFSR			Carbamidomethyl (C)[1]		Mascot
2206.0122	2206.0483	0.0361	16	163	180	CFLELQVEGEEAYQTFSR	92	100	Carbamidomethyl (C)[1]		Mascot
2257.2034	2257.2478	0.0444	20	33	55	STLTDSLVAAAGIIAQEVAGDVR					Mascot
2257.2034	2257.2478	0.0444	20	33	55	STLTDSLVAAAGIIAQEVAGDVR	136	100			Mascot
2264.0864	2264.1226	0.0362	16	587	606	LYMEARPLEEGLAEAIDDGR			Oxidation (M)[3]		Mascot
2384.188	2384.2336	0.0456	19	349	368	YRVENLYEGPLDDIYANAIR					Mascot
2384.188	2384.2336	0.0456	19	349	368	YRVENLYEGPLDDIYANAIR	82	99.999			Mascot
2408.1804	2408.2285	0.0481	20	544	565	DLQDDFMGGAEIIVSPPVVSFR			Oxidation (M)[7]		Mascot
2646.3479	2646.4104	0.0625	24	121	144	ITDGALVVVDCIEGVCVQTETVLR			Carbamidomethyl (C)[11,16]		Mascot
2933.4163	2933.4939	0.0776	26	94	120	DGDEYLINLIDSPGHVDFSSEVTAALR					Mascot

4	Elongation factor 2 [Aegilops tauschii]	gi|475585009	84595.6	5.98	18	581	100	22.184	528	100	

Peptide Information

Calc. Mass	Obsrv. Mass	± da	± ppm	Start Seq.	End Seq.	Sequence	Ion Score	C. I. %	Modification	Rank	Result Type
865.4414	865.4394	-0.002	-2	4	10	FTAEELR					Mascot
955.532	955.5226	-0.0094	-10	610	619	GGGQVIPTAR					Mascot
1039.6622	1039.6492	-0.013	-13	151	159	IRPVLTVNK					Mascot
1135.5049	1135.5085	0.0036	3	582	591	EGALAEENMR			Oxidation (M)[9]		Mascot
1188.6194	1188.5979	-0.0215	-18	321	330	VMQTWLPASR					Mascot
1204.6144	1204.6097	-0.0047	-4	321	330	VMQTWLPASR			Oxidation (M)[2]		Mascot
1315.7191	1315.6771	-0.042	-32	411	422	IMGPNFVPGQKK					Mascot
1346.7791	1346.7725	-0.0066	-5	621	632	VIYASQLTAKPR					Mascot
1543.7727	1543.7751	0.0024	2	269	280	RGFVQFCYEPIK			Carbamidomethyl (C)[7]		Mascot
1745.8965	1745.9255	0.029	17	331	345	ALLEMMIFHLPSPSK			Oxidation (M)[5,6]		Mascot
1745.8965	1745.9255	0.029	17	331	345	ALLEMMIFHLPSPSK			Oxidation (M)[5,6]		Mascot
1786.9375	1786.9601	0.0226	13	679	694	AYLPVIESFGFSSTLR					Mascot
1786.9375	1786.9601	0.0226	13	679	694	AYLPVIESFGFSSTLR	107	100			Mascot
2065.0237	2065.0598	0.0361	17	351	368	VENLYEGPLDDIYANAIR					Mascot
2065.0237	2065.0598	0.0361	17	351	368	VENLYEGPLDDIYANAIR	112	100			Mascot
2132.0593	2132.0872	0.0279	13	661	678	GHVFEEMQRPGTPLYNIK			Oxidation (M)[7]		Mascot
2206.0122	2206.0483	0.0361	16	163	180	CFLELQVEGEEAYQTFSR			Carbamidomethyl (C)[1]		Mascot

2206.0122	2206.0483	0.0361	16	163	180	CFLELQVEGEEAYQTFSR	92	100	Carbamidomethyl (C)[1]		Mascot
2257.2034	2257.2478	0.0444	20	33	55	STLTDSLVAAAGIIAQEVAGDVR					Mascot
2257.2034	2257.2478	0.0444	20	33	55	STLTDSLVAAAGIIAQEVAGDVR	136	100			Mascot
2264.0864	2264.1226	0.0362	16	495	514	LYMEARPLEEGLAEAIDDGR			Oxidation (M)[3]		Mascot
2384.188	2384.2336	0.0456	19	349	368	YRVENLYEGPLDDIYANAIR					Mascot
2384.188	2384.2336	0.0456	19	349	368	YRVENLYEGPLDDIYANAIR	82	99.999			Mascot
2646.3479	2646.4104	0.0625	24	121	144	ITDGALVVVDCIEGVCVQTETVLR			Carbamidomethyl (C)[11,16]		Mascot
2933.4163	2933.4939	0.0776	26	94	120	DGDEYLINLIDSPGHVDFSSEVTAALR					Mascot

5	Elongation factor 2 [Triticum urartu]	gi|474416089	94980	5.85	21	499	100	22.937	436	100

Peptide Information

Calc. Mass	Obsrv. Mass	± da	± ppm	Start Seq.	End Seq.	Sequence	Ion Score	C. I. %	Modification	Rank	Result Type
865.4414	865.4394	-0.002	-2	4	10	FTAEELR					Mascot
890.5095	890.5068	-0.0027	-3	483	490	FSVSPVVR					Mascot
955.532	955.5226	-0.0094	-10	702	711	GGGQVIPTAR					Mascot
959.4615	959.4772	0.0157	16	472	479	EVDACPIR			Carbamidomethyl (C)[5]		Mascot
1039.6622	1039.6492	-0.013	-13	151	159	IRPVLTVNK					Mascot
1148.5154	1148.5072	-0.0082	-7	753	761	GHVFEEMQR			Oxidation (M)[7]		Mascot
1188.6194	1188.5979	-0.0215	-18	321	330	VMQTWLPASR					Mascot
1204.6144	1204.6097	-0.0047	-4	321	330	VMQTWLPASR			Oxidation (M)[2]		Mascot
1220.682	1220.6019	-0.0801	-66	480	490	AMKFSVSPVVR					Mascot
1236.677	1236.5984	-0.0786	-64	480	490	AMKFSVSPVVR			Oxidation (M)[2]		Mascot
1315.7191	1315.6771	-0.042	-32	411	422	IMGPNFVPGQKK					Mascot
1346.7791	1346.7725	-0.0066	-5	713	724	VIYASQLTAKPR					Mascot
1543.7727	1543.7751	0.0024	2	269	280	RGFVQFCYEPIK			Carbamidomethyl (C)[7]		Mascot
1745.8965	1745.9255	0.029	17	331	345	ALLEMMIFHLPSPSK			Oxidation (M)[5,6]		Mascot
1745.8965	1745.9255	0.029	17	331	345	ALLEMMIFHLPSPSK			Oxidation (M)[5,6]		Mascot
1786.9375	1786.9601	0.0226	13	771	786	AYLPVIESFGFSSTLR					Mascot
1786.9375	1786.9601	0.0226	13	771	786	AYLPVIESFGFSSTLR	107	100			Mascot
1830.9015	1830.9303	0.0288	16	464	479	NATLTNEKEVDACPIR			Carbamidomethyl (C)[13]		Mascot
2065.0237	2065.0598	0.0361	17	351	368	VENLYEGPLDDIYANAIR					Mascot
2065.0237	2065.0598	0.0361	17	351	368	VENLYEGPLDDIYANAIR	112	100			Mascot
2257.2034	2257.2478	0.0444	20	33	55	STLTDSLVAAAGIIAQEVAGDVR					Mascot
2257.2034	2257.2478	0.0444	20	33	55	STLTDSLVAAAGIIAQEVA	136	100			Mascot

						GDVR					
2264.0864	2264.1226	0.0362	16	587	606	LYMEARPLEEGLAEAIDD GR			Oxidation (M)[3]		Mascot
2384.188	2384.2336	0.0456	19	349	368	YRVENLYEGPLDDIYANA IR					Mascot
2384.188	2384.2336	0.0456	19	349	368	YRVENLYEGPLDDIYANA IR	82	99.999			Mascot
2408.1804	2408.2285	0.0481	20	544	565	DLQDDFMGGAEIIVSPPV VSFR			Oxidation (M)[7]		Mascot
2646.3479	2646.4104	0.0625	24	121	144	ITDGALVVVDCIEGVCVQ TETVLR			Carbamidomethyl (C)[11,16]		Mascot
2933.4163	2933.4939	0.0776	26	94	120	DGDEYLINLIDSPGHVDF SSEVTAALR					Mascot

6	PREDICTED: elongation factor 2-like [Cucumis sativus]	gi|449450860	95031.9	5.97	19	294	100	16.59	243	100	

Peptide Information

Calc. Mass	Obsrv. Mass	± da	± ppm	Start Seq.	End Seq.	Sequence	Ion Score	C. I. %	Modification	Rank	Result Type
865.4414	865.4394	-0.002	-2	4	10	FTAEELR					Mascot
890.5095	890.5068	-0.0027	-3	483	490	FSVSPVVR					Mascot
955.532	955.5226	-0.0094	-10	702	711	GGGQVIPTAR					Mascot
1021.5425	1021.5178	-0.0247	-24	4	11	FTAEELRR					Mascot
1039.6622	1039.6492	-0.013	-13	151	159	IRPVLTVNK					Mascot
1135.5049	1135.5085	0.0036	3	674	683	EGALAEENMR			Oxidation (M)[9]		Mascot
1188.6592	1188.5979	-0.0613	-52	311	320	DLMGKPLMKR					Mascot
1204.6031	1204.6097	0.0066	5	411	421	IMGPNYVPGEK					Mascot
1220.598	1220.6019	0.0039	3	411	421	IMGPNYVPGEK			Oxidation (M)[2]		Mascot
1236.677	1236.5984	-0.0786	-64	480	490	AMKFSVSPVVR			Oxidation (M)[2]		Mascot
1346.7791	1346.7725	-0.0066	-5	713	724	VIYASQLTAKPR					Mascot
1452.7369	1452.7728	0.0359	25	617	628	SKILSEEFAWDK					Mascot
1507.7495	1507.806	0.0565	37	307	319	SDEKDLMGKPLMK			Oxidation (M)[7]		Mascot
1543.7727	1543.7751	0.0024	2	269	280	RGFVQFCYEPIK			Carbamidomethyl (C)[7]		Mascot
1786.9375	1786.9601	0.0226	13	771	786	AYLPVIESFGFSSTLR					Mascot
1786.9375	1786.9601	0.0226	13	771	786	AYLPVIESFGFSSTLR	107	100			Mascot
2132.0593	2132.0872	0.0279	13	753	770	GHVFEEMQRPGTPLYNI K			Oxidation (M)[7]		Mascot
2257.2034	2257.2478	0.0444	20	33	55	STLTDSLVAAAGIIAQEVA GDVR					Mascot
2257.2034	2257.2478	0.0444	20	33	55	STLTDSLVAAAGIIAQEVA GDVR	136	100			Mascot
2335.1738	2335.2424	0.0686	29	67	87	GITIKSTGISLYYEMSDES LK					Mascot
2358.136	2358.2539	0.1179	50	349	368	YRVENLYEGPQDDVYAS AIR					Mascot
2646.3479	2646.4104	0.0625	24	121	144	ITDGALVVVDCIEGVCVQ TETVLR			Carbamidomethyl (C)[11,16]		Mascot

7	hypothetical protein SORBIDRAFT_03g034200 [Sorghum bicolor]	gi|241928310	94856	5.93	20	288	100	12.155	228	100	

Peptide Information

Calc. Mass	Obsrv. Mass	± da	± ppm	Start Seq.	End Seq.	Sequence	Ion Score	C. I. %	Modification	Rank	Result Type
865.4414	865.4394	-0.002	-2	4	10	FTAEELR					Mascot
890.5095	890.5068	-0.0027	-3	483	490	FSVSPVVR					Mascot
955.532	955.5226	-0.0094	-10	702	711	GGGQVIPTAR					Mascot
959.4615	959.4772	0.0157	16	472	479	EVDACPIR			Carbamidomethyl (C)[5]		Mascot
1039.6622	1039.6492	-0.013	-13	151	159	IRPVLTVNK					Mascot
1135.5049	1135.5085	0.0036	3	674	683	EGALAEENMR			Oxidation (M)[9]		Mascot
1148.598	1148.5072	-0.0908	-79	301	310	LNVTMKADEK					Mascot
1164.5929	1164.6117	0.0188	16	301	310	LNVTMKADEK			Oxidation (M)[5]		Mascot
1172.6497	1172.6288	-0.0209	-18	292	300	EKLWPMLQK					Mascot
1188.6445	1188.5979	-0.0466	-39	292	300	EKLWPMLQK			Oxidation (M)[6]		Mascot
1220.682	1220.6019	-0.0801	-66	480	490	AMKFSVSPVVR					Mascot
1236.677	1236.5984	-0.0786	-64	480	490	AMKFSVSPVVR			Oxidation (M)[2]		Mascot
1346.7791	1346.7725	-0.0066	-5	713	724	VIYASQLTAKPR					Mascot
1543.7727	1543.7751	0.0024	2	269	280	RGFVQFCYEPIK			Carbamidomethyl (C)[7]		Mascot
1725.8364	1725.9689	0.1325	77	181	195	VIENANVIMATYEDK			Oxidation (M)[9]		Mascot
1767.8469	1767.9607	0.1138	64	828	842	GLKEQMTPLSEFEDK			Oxidation (M)[6]		Mascot
1813.9484	1813.8201	-0.1283	-71	771	786	AYLPVIESFGFSSQLR					Mascot
1830.9015	1830.9303	0.0288	16	464	479	NATLTNEKEVDACPIR			Carbamidomethyl (C)[13]		Mascot
2132.0593	2132.0872	0.0279	13	753	770	GHVFEEMQRPGTPLYNI K			Oxidation (M)[7]		Mascot
2206.0122	2206.0483	0.0361	16	163	180	CFLELQVEGEEAYQTFS R			Carbamidomethyl (C)[1]		Mascot
2206.0122	2206.0483	0.0361	16	163	180	CFLELQVEGEEAYQTFS R	92	100	Carbamidomethyl (C)[1]		Mascot
2257.2034	2257.2478	0.0444	20	33	55	STLTDSLVAAAGIIAQEVA GDVR					Mascot
2257.2034	2257.2478	0.0444	20	33	55	STLTDSLVAAAGIIAQEVA GDVR	136	100			Mascot
2406.2009	2406.2437	0.0428	18	544	565	DLQEDFMGGAEIIVSPPV VSFR					Mascot
2422.196	2422.2366	0.0406	17	544	565	DLQEDFMGGAEIIVSPPV VSFR			Oxidation (M)[7]		Mascot
2646.3479	2646.4104	0.0625	24	121	144	ITDGALVVVDCIEGVCVQ TETVLR			Carbamidomethyl (C)[11,16]		Mascot

8	TPA: putative translation elongation factor family protein [Zea mays]	gi|414873820	94813	6	20	288	100	12.726	228	100	

Peptide Information

Calc. Mass	Obsrv. Mass	± da	± ppm	Start	End	Sequence	Ion	C. I. %	Modification	Rank	Result Type

Calc. Mass	Obsrv. Mass	± da	± ppm	Start Seq.	End Seq.	Sequence	Ion Score	C. I. %	Modification	Rank	Result Type
865.4414	865.4394	-0.002	-2	4	10	FTAEELR					Mascot
890.5095	890.5068	-0.0027	-3	483	490	FSVSPVVR					Mascot
955.532	955.5226	-0.0094	-10	702	711	GGGQVIPTAR					Mascot
961.4407	961.4987	0.058	60	472	479	ETDACPIR			Carbamidomethyl (C)[5]		Mascot
1039.6622	1039.6492	-0.013	-13	151	159	IRPVLTVNK					Mascot
1135.5049	1135.5085	0.0036	3	674	683	EGALAEENMR			Oxidation (M)[9]		Mascot
1148.598	1148.5072	-0.0908	-79	301	310	LNVTMKADEK					Mascot
1164.5929	1164.6117	0.0188	16	301	310	LNVTMKADEK			Oxidation (M)[5]		Mascot
1172.6497	1172.6288	-0.0209	-18	292	300	EKLWPMLQK					Mascot
1188.6445	1188.5979	-0.0466	-39	292	300	EKLWPMLQK			Oxidation (M)[6]		Mascot
1220.682	1220.6019	-0.0801	-66	480	490	AMKFSVSPVVR					Mascot
1236.677	1236.5984	-0.0786	-64	480	490	AMKFSVSPVVR			Oxidation (M)[2]		Mascot
1346.7791	1346.7725	-0.0066	-5	713	724	VIYASQLTAKPR					Mascot
1543.7727	1543.7751	0.0024	2	269	280	RGFVQFCYEPIK			Carbamidomethyl (C)[7]		Mascot
1594.767	1594.8578	0.0908	57	831	843	EQMIPLSEFEDKL			Oxidation (M)[3]		Mascot
1725.8364	1725.9689	0.1325	77	181	195	VIENANVIMATYEDK			Oxidation (M)[9]		Mascot
1813.9484	1813.8201	-0.1283	-71	771	786	AYLPVIESFGFSSQLR					Mascot
2132.0593	2132.0872	0.0279	13	753	770	GHVFEEMQRPGTPLYNIK			Oxidation (M)[7]		Mascot
2206.0122	2206.0483	0.0361	16	163	180	CFLELQVEGEEAYQTFSR			Carbamidomethyl (C)[1]		Mascot
2206.0122	2206.0483	0.0361	16	163	180	CFLELQVEGEEAYQTFSR	92	100	Carbamidomethyl (C)[1]		Mascot
2257.2034	2257.2478	0.0444	20	33	55	STLTDSLVAAAGIIAQEVAGDVR					Mascot
2257.2034	2257.2478	0.0444	20	33	55	STLTDSLVAAAGIIAQEVAGDVR	136	100			Mascot
2264.0864	2264.1226	0.0362	16	587	606	LYMEARPLEEGLAEAIDDGR			Oxidation (M)[3]		Mascot
2406.2009	2406.2437	0.0428	18	544	565	DLQEDFMGGAEIIVSPPVVSFR					Mascot
2422.196	2422.2366	0.0406	17	544	565	DLQEDFMGGAEIIVSPPVVSFR			Oxidation (M)[7]		Mascot
2646.3479	2646.4104	0.0625	24	121	144	ITDGALVVVDCIEGVCVQTETVLR			Carbamidomethyl (C)[11,16]		Mascot

9	putative translation elongation factor family protein [Zea mays]	gi|413952473	94872	6	20	286	100	12.157	228	100	

Peptide Information

Calc. Mass	Obsrv. Mass	± da	± ppm	Start Seq.	End Seq.	Sequence	Ion Score	C. I. %	Modification	Rank	Result Type
865.4414	865.4394	-0.002	-2	4	10	FTAEELR					Mascot
890.5095	890.5068	-0.0027	-3	483	490	FSVSPVVR					Mascot

955.532	955.5226	-0.0094	-10	702	711	GGGQVIPTAR				Mascot
959.4615	959.4772	0.0157	16	472	479	EVDACPIR			Carbamidomethyl (C)[5]	Mascot
1039.6622	1039.6492	-0.013	-13	151	159	IRPVLTVNK				Mascot
1135.5049	1135.5085	0.0036	3	674	683	EGALAEENMR			Oxidation (M)[9]	Mascot
1148.598	1148.5072	-0.0908	-79	301	310	LNVTMKADEK				Mascot
1164.5929	1164.6117	0.0188	16	301	310	LNVTMKADEK			Oxidation (M)[5]	Mascot
1172.6497	1172.6288	-0.0209	-18	292	300	EKLWPMLQK				Mascot
1188.6445	1188.5979	-0.0466	-39	292	300	EKLWPMLQK			Oxidation (M)[6]	Mascot
1220.682	1220.6019	-0.0801	-66	480	490	AMKFSVSPVVR				Mascot
1236.677	1236.5984	-0.0786	-64	480	490	AMKFSVSPVVR			Oxidation (M)[2]	Mascot
1346.7791	1346.7725	-0.0066	-5	713	724	VIYASQLTAKPR				Mascot
1543.7727	1543.7751	0.0024	2	269	280	RGFVQFCYEPIK			Carbamidomethyl (C)[7]	Mascot
1725.8364	1725.9689	0.1325	77	181	195	VIENANVIMATYEDK			Oxidation (M)[9]	Mascot
1767.8469	1767.9607	0.1138	64	828	842	GLKEQMTPLSEFEDK			Oxidation (M)[6]	Mascot
1830.9015	1830.9303	0.0288	16	464	479	NATLTNEKEVDACPIR			Carbamidomethyl (C)[13]	Mascot
2132.0593	2132.0872	0.0279	13	753	770	GHVFEEMQRPGTPLYNI K			Oxidation (M)[7]	Mascot
2206.0122	2206.0483	0.0361	16	163	180	CFLELQVEGEEAYQTFS R			Carbamidomethyl (C)[1]	Mascot
2206.0122	2206.0483	0.0361	16	163	180	CFLELQVEGEEAYQTFS R	92	100	Carbamidomethyl (C)[1]	Mascot
2257.2034	2257.2478	0.0444	20	33	55	STLTDSLVAAAGIIAQEVA GDVR				Mascot
2257.2034	2257.2478	0.0444	20	33	55	STLTDSLVAAAGIIAQEVA GDVR	136	100		Mascot
2264.0864	2264.1226	0.0362	16	587	606	LYMEARPLEEGLAEAIDD GR			Oxidation (M)[3]	Mascot
2406.2009	2406.2437	0.0428	18	544	565	DLQEDFMGGAEIIVSPPV VSFR				Mascot
2422.196	2422.2366	0.0406	17	544	565	DLQEDFMGGAEIIVSPPV VSFR			Oxidation (M)[7]	Mascot
2646.3479	2646.4104	0.0625	24	121	144	ITDGALVVVDCIEGVCVQ TETVLR			Carbamidomethyl (C)[11,16]	Mascot

10	PREDICTED: elongation factor 2-like isoform X1 [Setaria italica]	gi|514825371	94876	5.93	19	283	100	11.408	228	100

Protein Group

PREDICTED: elongation factor 2-like isoform X1 [Setaria italica]	gi|514781700	94876	5.9299 998283 3862
PREDICTED: elongation factor 2-like isoform X2 [Setaria italica]	gi|514781704	94876	5.9299 998283 3862

PREDICTED: elongation factor 2-like isoform X2 [Setaria italica]	gi|514825373	94876	5.9299998283 3862

Peptide Information

Calc. Mass	Obsrv. Mass	± da	± ppm	Start Seq.	End Seq.	Sequence	Ion Score	C. I. %	Modification	Rank	Result Type
865.4414	865.4394	-0.002	-2	4	10	FTAEELR					Mascot
890.5095	890.5068	-0.0027	-3	483	490	FSVSPVVR					Mascot
955.532	955.5226	-0.0094	-10	702	711	GGGQVIPTAR					Mascot
959.4615	959.4772	0.0157	16	472	479	EVDACPIR			Carbamidomethyl (C)[5]		Mascot
1039.6622	1039.6492	-0.013	-13	151	159	IRPVLTVNK					Mascot
1135.5049	1135.5085	0.0036	3	674	683	EGALAEENMR			Oxidation (M)[9]		Mascot
1164.5929	1164.6117	0.0188	16	301	310	LNVTMKSDEK					Mascot
1220.682	1220.6019	-0.0801	-66	480	490	AMKFSVSPVVR					Mascot
1236.677	1236.5984	-0.0786	-64	480	490	AMKFSVSPVVR			Oxidation (M)[2]		Mascot
1346.7791	1346.7725	-0.0066	-5	713	724	VIYASQLTAKPR					Mascot
1543.7727	1543.7751	0.0024	2	269	280	RGFVQFCYEPIK			Carbamidomethyl (C)[7]		Mascot
1725.8364	1725.9689	0.1325	77	181	195	VIENANVIMATYEDK			Oxidation (M)[9]		Mascot
1767.8469	1767.9607	0.1138	64	828	842	GLKEQMTPLSEFEDK			Oxidation (M)[6]		Mascot
1813.9484	1813.8201	-0.1283	-71	771	786	AYLPVIESFGFSSQLR					Mascot
1830.9015	1830.9303	0.0288	16	464	479	NATLTNEKEVDACPIR			Carbamidomethyl (C)[13]		Mascot
2132.0593	2132.0872	0.0279	13	753	770	GHVFEEMQRPGTPLYNIK			Oxidation (M)[7]		Mascot
2206.0122	2206.0483	0.0361	16	163	180	CFLELQVEGEEAYQTFSR			Carbamidomethyl (C)[1]		Mascot
2206.0122	2206.0483	0.0361	16	163	180	CFLELQVEGEEAYQTFSR	92	100	Carbamidomethyl (C)[1]		Mascot
2257.2034	2257.2478	0.0444	20	33	55	STLTDSLVAAAGIIAQEVAGDVR					Mascot
2257.2034	2257.2478	0.0444	20	33	55	STLTDSLVAAAGIIAQEVAGDVR	136	100			Mascot
2406.2009	2406.2437	0.0428	18	544	565	DLQEDFMGGAEIIVSPPVVSFR					Mascot
2422.196	2422.2366	0.0406	17	544	565	DLQEDFMGGAEIIVSPPVVSFR			Oxidation (M)[7]		Mascot
2646.3479	2646.4104	0.0625	24	121	144	ITDGALVVVDCIEGVCVQTETVLR			Carbamidomethyl (C)[11,16]		Mascot

Gel Idx/Pos	300/M2	Instr./Gel Origin	BA2151/Sample Project 20140814	Process Status	Analysis Succeeded
Plate [#] Name	[1] Sample Project 20140814	Instrument Sample Name		Spectra	11

Rank	Protein Name	Accession No.	Protein MW	Protein PI	Pep. Count	Protein Score	Protein Score C. I. %	Intensity Matched	Total Ion Score	Total Ion C. I. %	Confirmed
1	Chlorophyll a-b binding protein 1, chloroplastic [Triticum urartu]	gi|473965828	30439.1	5.25	6	149	100	8.616	129	100	

Peptide Information

Calc. Mass	Obsrv. Mass	± da	± ppm	Start Seq.	End Seq.	Sequence	Ion Score	C. I. %	Modification	Rank	Result Type
804.3998	804.4075	0.0077	10	23	30	AAPFGEGR					Mascot
983.4985	983.468	-0.0305	-31	125	132	FGEAVWFK					Mascot
1055.5303	1055.5291	-0.0012	-1	96	103	ELEVIHCR			Carbamidomethyl (C)[7]		Mascot
1055.5303	1055.5291	-0.0012	-1	96	103	ELEVIHCR	42	88.381	Carbamidomethyl (C)[7]		Mascot
1325.6743	1325.6754	0.0011	1	94	103	NRELEVIHCR			Carbamidomethyl (C)[9]		Mascot
1382.6335	1382.6498	0.0163	12	42	54	VAASSSPWYGSDR					Mascot
1382.6335	1382.6498	0.0163	12	42	54	VAASSSPWYGSDR	87	100			Mascot
1919.9871	1920.0138	0.0267	14	104	120	WAMLGALGCVFPELLAR			Carbamidomethyl (C)[9], Oxidation (M)[3]		Mascot

Rank	Protein Name	Accession No.	Protein MW	Protein PI	Pep. Count	Protein Score	Protein Score C. I. %	Intensity Matched	Total Ion Score	Total Ion C. I. %	Confirmed
2	hypothetical protein F775_26875 [Aegilops tauschii]	gi|475582903	26622.7	5.01	6	118	100	3.327	95	100	

Peptide Information

Calc. Mass	Obsrv. Mass	± da	± ppm	Start Seq.	End Seq.	Sequence	Ion Score	C. I. %	Modification	Rank	Result Type
816.4362	816.4263	-0.0099	-12	201	207	LAAWAER					Mascot
1051.5089	1051.4957	-0.0132	-13	134	142	MEETLSGLR			Oxidation (M)[1]		Mascot
1135.618	1135.5906	-0.0274	-24	114	123	LFPSCTGILK			Carbamidomethyl (C)[5]		Mascot
1192.7048	1192.7067	0.0019	2	186	197	LAGLGPIIDPAR					Mascot
1192.7048	1192.7067	0.0019	2	186	197	LAGLGPIIDPAR	68	99.971			Mascot
1327.6165	1327.635	0.0185	14	32	42	GLSYDYLPEDR					Mascot
1327.6165	1327.635	0.0185	14	32	42	GLSYDYLPEDR	27	0			Mascot
1465.8083	1465.6833	-0.125	-85	114	126	LFPSCTGILKTTK			Carbamidomethyl (C)[5]		Mascot

Rank	Protein Name	Accession No.	Protein MW	Protein PI	Pep. Count	Protein Score	Protein Score C. I. %	Intensity Matched	Total Ion Score	Total Ion C. I. %	Confirmed
3	hypothetical protein F775_23920 [Aegilops tauschii]	gi|475582904	21946.3	4.84	4	82	99.413	1.902	68	99.971	

Peptide Information

Calc. Mass	Obsrv. Mass	± da	± ppm	Start Seq.	End Seq.	Sequence	Ion Score	C. I. %	Modification	Rank	Result Type
816.4362	816.4263	-0.0099	-12	159	165	LAAWAER					Mascot

Calc. Mass	Obsrv. Mass	± da	± ppm	Start Seq.	End Seq.	Sequence	Ion Score	C. I. %	Modification	Rank	Result Type
1065.5364	1065.5117	-0.0247	-23	63	71	VWAAYVNDK					Mascot
1135.618	1135.5906	-0.0274	-24	72	81	LFPSCTGILK			Carbamidomethyl (C)[5]		Mascot
1192.7048	1192.7067	0.0019	2	144	155	LAGLGPLIDPAR					Mascot
1192.7048	1192.7067	0.0019	2	144	155	LAGLGPLIDPAR	68	99.971			Mascot

4	Chlorophyll a-b binding protein, chloroplastic [Aegilops tauschii]	gi|475471123	27994.2	5.46	8	75	97.193	9.41	42	88.381

Peptide Information

Calc. Mass	Obsrv. Mass	± da	± ppm	Start Seq.	End Seq.	Sequence	Ion Score	C. I. %	Modification	Rank	Result Type
964.5098	964.5188	0.009	9	2	11	SLSSPAFAGK					Mascot
983.4985	983.468	-0.0305	-31	121	128	FGEAVWFK					Mascot
1055.5303	1055.5291	-0.0012	-1	92	99	ELEVIHCR			Carbamidomethyl (C)[7]		Mascot
1055.5303	1055.5291	-0.0012	-1	92	99	ELEVIHCR	42	88.381	Carbamidomethyl (C)[7]		Mascot
1325.6743	1325.6754	0.0011	1	90	99	NRELEVIHCR			Carbamidomethyl (C)[9]		Mascot
1337.6848	1337.6776	-0.0072	-5	15	26	NLPSSFLFGEAR					Mascot
1382.7103	1382.6498	-0.0605	-44	117	128	DGVKFGEAVWFK					Mascot
1382.7103	1382.6498	-0.0605	-44	117	128	DGVKFGEAVWFK					Mascot
1409.6445	1409.6514	0.0069	5	38	50	QVASGSPWYGSDR					Mascot
1919.9871	1920.0138	0.0267	14	100	116	WAMLGALGCVFPELLAR			Carbamidomethyl (C)[9], Oxidation (M)[3]		Mascot

5	hypothetical protein F775_27020 [Aegilops tauschii]	gi|475532296	26469.8	6.71	2	74	95.751	1.68	68	99.971

Peptide Information

Calc. Mass	Obsrv. Mass	± da	± ppm	Start Seq.	End Seq.	Sequence	Ion Score	C. I. %	Modification	Rank	Result Type
816.4362	816.4263	-0.0099	-12	187	193	LAAWAER					Mascot
1192.7048	1192.7067	0.0019	2	172	183	LAGLGPIIDPAR					Mascot
1192.7048	1192.7067	0.0019	2	172	183	LAGLGPIIDPAR	68	99.971			Mascot

6	RecName: Full=Chlorophyll a-b binding protein 1, chloroplastic; AltName: Full=LHCII type I CAB-1; Short=LHCP; Flags: Precursor	gi|115778	28328.2	5.17	7	72	94.135	6.232	42	88.381

Peptide Information

Calc. Mass	Obsrv. Mass	± da	± ppm	Start Seq.	End Seq.	Sequence	Ion Score	C. I. %	Modification	Rank	Result Type
983.4985	983.468	-0.0305	-31	125	132	FGEAVWFK					Mascot
1055.5303	1055.5291	-0.0012	-1	96	103	ELEVIHCR			Carbamidomethyl (C)[7]		Mascot
1055.5303	1055.5291	-0.0012	-1	96	103	ELEVIHCR	42	88.381	Carbamidomethyl (C)[7]		Mascot

Calc. Mass	Obsrv. Mass	± da	± ppm	Start Seq.	End Seq.	Sequence	Ion Score	C. I. %	Modification	Rank	Result Type
1325.6743	1325.6754	0.0011	1	94	103	NRELEVIHCR			Carbamidomethyl (C)[9]		Mascot
1409.7094	1409.6514	-0.058	-41	2	16	AASTMALSSPAFAGK					Mascot
1575.849	1575.8531	0.0041	3	17	32	AVKLSPGASEVFGTGR					Mascot
1870.9402	1871.043	0.1028	55	1	19	MAASTMALSSPAFAGKAVK			Oxidation (M)[1,6]		Mascot
1919.9871	1920.0138	0.0267	14	104	120	WAMLGALGCVFPELLAR			Carbamidomethyl (C)[9], Oxidation (M)[3]		Mascot

7	chlorophyll a/b binding protein [Salicornia brachiata]	gi|478694386	28485.4	5.31	7	72	94.135	6.493	42	88.381	

Peptide Information

Calc. Mass	Obsrv. Mass	± da	± ppm	Start Seq.	End Seq.	Sequence	Ion Score	C. I. %	Modification	Rank	Result Type
983.4985	983.468	-0.0305	-31	127	134	FGEAVWFK					Mascot
1055.5303	1055.5291	-0.0012	-1	98	105	ELEVIHCR			Carbamidomethyl (C)[7]		Mascot
1055.5303	1055.5291	-0.0012	-1	98	105	ELEVIHCR	42	88.381	Carbamidomethyl (C)[7]		Mascot
1325.6743	1325.6754	0.0011	1	96	105	NRELEVIHCR			Carbamidomethyl (C)[9]		Mascot
1419.6652	1419.7238	0.0586	41	44	56	QVASGSPWYGPDR					Mascot
1761.9415	1761.9556	0.0141	8	2	19	ASSTMALSSPSLAGKPIK			Oxidation (M)[5]		Mascot
1876.9871	1877.0045	0.0174	9	1	19	MASSTMALSSPSLAGKPIK					Mascot
1919.9871	1920.0138	0.0267	14	106	122	WAMLGALGCVFPELLAR			Carbamidomethyl (C)[9], Oxidation (M)[3]		Mascot

8	Chlorophyll a-b binding protein, chloroplastic [Aegilops tauschii]	gi|475549279	34057.4	9.33	7	72	93.419	6.261	41	83.887	

Peptide Information

Calc. Mass	Obsrv. Mass	± da	± ppm	Start Seq.	End Seq.	Sequence	Ion Score	C. I. %	Modification	Rank	Result Type
832.4384	832.4073	-0.0311	-37	288	295	ARQGGSTR					Mascot
998.5853	998.5137	-0.0716	-72	276	283	QQQRLGLR					Mascot
1055.5303	1055.5291	-0.0012	-1	142	149	ELEVIHCR			Carbamidomethyl (C)[7]		Mascot
1055.5303	1055.5291	-0.0012	-1	97	104	ELEVIHCR	42	88.381	Carbamidomethyl (C)[7]		Mascot
1325.6743	1325.6754	0.0011	1	140	149	NRELEVIHCR			Carbamidomethyl (C)[9]		Mascot
1439.72	1439.6439	-0.0761	-53	2	16	ATTAMALSPSSFAGK					Mascot
1455.7148	1455.6631	-0.0517	-36	2	16	ATTAMALSPSSFAGK			Oxidation (M)[5]		Mascot
1593.7656	1593.8019	0.0363	23	41	55	AKPVSSGSPWYGSDR					Mascot
1919.9871	1920.0138	0.0267	14	150	166	WAMLGALGCVFPELLAR			Carbamidomethyl (C)[9], Oxidation (M)[3]		Mascot

9	uncharacterized protein LOC100306448 [Glycine max]	gi|351722452	16732.6	6.31	11	71	92.949	1.506	

Peptide Information

Calc. Mass	Obsrv. Mass	± da	± ppm	Start Seq.	End Seq.	Sequence	Ion Score	C. I. %	Modification	Rank	Result Type

Calc. Mass	Obsrv. Mass	± da	± ppm	Start Seq.	End Seq.	Sequence	Ion Score	C. I. %	Modification	Rank	Result Type
816.4825	816.4263	-0.0562	-69	57	63	VDDKVLK					Mascot
832.4523	832.4073	-0.045	-54	54	60	SIRVDDK					Mascot
844.5039	844.4474	-0.0565	-67	107	112	WLKELR					Mascot
944.4836	944.5554	0.0718	76	77	84	AITSAYYR					Mascot
1065.6051	1065.5117	-0.0934	-88	27	35	SNLLSRFTK					Mascot
1436.7856	1436.6473	-0.1383	-96	44	56	STIGVEFATRSIR					Mascot
1454.6758	1454.6672	-0.0086	-6	135	147	AVSTEETTNFAER					Mascot
1593.7769	1593.8019	0.025	16	64	76	AQIWDTAGQERYR					Mascot
1625.8317	1625.778	-0.0537	-33	113	127	DHTDANIVVMLVGNK					Mascot
1725.7755	1725.8616	0.0861	50	2	15	GAYRADEDYDYLFK					Mascot
1860.9199	1860.983	0.0631	34	132	147	HLRAVSTEETTNFAER					Mascot

10	Chlorophyll a-b binding protein, chloroplastic [Triticum urartu]	gi|474413347	28531.3	5.29	7	70	90.916	6.502	42	88.381

Peptide Information

Calc. Mass	Obsrv. Mass	± da	± ppm	Start Seq.	End Seq.	Sequence	Ion Score	C. I. %	Modification	Rank	Result Type
983.4985	983.468	-0.0305	-31	126	133	FGEAVWFK					Mascot
1055.5303	1055.5291	-0.0012	-1	97	104	ELEVIHCR			Carbamidomethyl (C)[7]		Mascot
1055.5303	1055.5291	-0.0012	-1	97	104	ELEVIHCR	42	88.381	Carbamidomethyl (C)[7]		Mascot
1325.6743	1325.6754	0.0011	1	95	104	NRELEVIHCR			Carbamidomethyl (C)[9]		Mascot
1439.72	1439.6439	-0.0761	-53	2	16	AATTMSLSSPAFAGK					Mascot
1455.7148	1455.6631	-0.0517	-36	2	16	AATTMSLSSPAFAGK			Oxidation (M)[5]		Mascot
1469.6656	1469.6794	0.0138	9	43	55	QVTSSSPWYGSDR					Mascot
1575.849	1575.8531	0.0041	3	17	31	AVKNLPSSSLFGEAR					Mascot
1919.9871	1920.0138	0.0267	14	105	121	WAMLGALGCVFPELLAR			Carbamidomethyl (C)[9], Oxidation (M)[3]		Mascot

Gel Idx/Pos	301/M3	Instr./Gel Origin	BA2151/Sample Project 20140814	Process Status	Analysis Succeeded
Plate [#] Name	[1] Sample Project 20140814	Instrument Sample Name		Spectra	11

Rank	Protein Name	Accession No.	Protein MW	Protein PI	Pep. Count	Protein Score	Protein Score C. I. %	Intensity Matched	Total Ion Score	Total Ion C. I. %	Confirmed
1	20 kDa chaperonin, chloroplastic [Triticum urartu]	gi|474407512	29805.7	6.77	8	389	100	6.672	348	100	

Peptide Information

Calc. Mass	Obsrv. Mass	± da	± ppm	Start Seq.	End Seq.	Sequence	Ion Score	C. I. %	Modification	Rank	Result Type
943.5207	943.5082	-0.0125	-13	142	149	DLKPLSDR					Mascot
1122.579	1122.5712	-0.0078	-7	257	266	GSEGEYIVLR					Mascot
1122.579	1122.5712	-0.0078	-7	257	266	GSEGEYIVLR	70	99.983			Mascot
1135.6106	1135.5966	-0.014	-12	42	51	YTSVKPLGDR					Mascot
1569.8846	1569.8328	-0.0518	-33	90	105	SLGSNIVEISVPVGAK					Mascot
1904.9388	1905.0001	0.0613	32	250	266	YAGSEFKGSEGEYIVLR					Mascot
2094.0828	2094.0974	0.0146	7	213	234	EKPSVGTVVAVGPGPLG DDGSR					Mascot
2094.0828	2094.0974	0.0146	7	213	234	EKPSVGTVVAVGPGPLG DDGSR	175	100			Mascot
2618.467	2618.5288	0.0618	24	1	25	MAKPALADGLRIPTSSSA RPLRPGR					Mascot
2635.4163	2635.4487	0.0324	12	63	89	TAGGILLPTTAQSRPQAG EVVAVGEGR					Mascot
2635.4163	2635.4487	0.0324	12	63	89	TAGGILLPTTAQSRPQAG EVVAVGEGR	103	100			Mascot

Rank	Protein Name	Accession No.	Protein MW	Protein PI	Pep. Count	Protein Score	Protein Score C. I. %	Intensity Matched	Total Ion Score	Total Ion C. I. %	Confirmed
2	20 kDa chaperonin, chloroplastic [Aegilops tauschii]	gi|475545030	32118.9	8.57	7	380	100	6.036	348	100	

Peptide Information

Calc. Mass	Obsrv. Mass	± da	± ppm	Start Seq.	End Seq.	Sequence	Ion Score	C. I. %	Modification	Rank	Result Type
943.5207	943.5082	-0.0125	-13	208	215	DLKPLSDR					Mascot
1122.579	1122.5712	-0.0078	-7	283	292	GSEGEYIVLR					Mascot
1122.579	1122.5712	-0.0078	-7	283	292	GSEGEYIVLR	70	99.983			Mascot
1904.9388	1905.0001	0.0613	32	276	292	YAGSEFKGSEGEYIVLR					Mascot
1932.9761	1932.9668	-0.0093	-5	220	238	VAESEEETAGGLLLTQAS K					Mascot
1934.9364	1934.9846	0.0482	25	89	104	WQGTNLCLTCGVAWLR			Carbamidomethyl (C)[7,10]		Mascot
2094.0828	2094.0974	0.0146	7	239	260	EKPSVGTVVAVGPGPLG DDGSR					Mascot
2094.0828	2094.0974	0.0146	7	239	260	EKPSVGTVVAVGPGPLG DDGSR	175	100			Mascot
2635.4163	2635.4487	0.0324	12	129	155	TAGGILLPTTAQSRPQAG EVVAVGEGR					Mascot

2635.4163	2635.4487	0.0324	12	129	155	TAGGILLPTTAQSRPQAGEVVAVGEGR	103	100			Mascot

3	Chlorophyll a-b binding protein, chloroplastic [Aegilops tauschii]	gi|475549279	34057.4	9.33	6	219	100	14.946	188	100	

Peptide Information

Calc. Mass	Obsrv. Mass	± da	± ppm	Start Seq.	End Seq.	Sequence	Ion Score	C. I. %	Modification	Rank	Result Type
998.5853	998.4989	-0.0864	-87	276	283	QQQRLGLR					Mascot
1055.5303	1055.5221	-0.0082	-8	142	149	ELEVIHCR			Carbamidomethyl (C)[7]		Mascot
1055.5303	1055.5221	-0.0082	-8	97	104	ELEVIHCR	58	99.736	Carbamidomethyl (C)[7]		Mascot
1325.6743	1325.6655	-0.0088	-7	140	149	NRELEVIHCR			Carbamidomethyl (C)[9]		Mascot
1325.6743	1325.6655	-0.0088	-7	95	104	NRELEVIHCR	64	99.922	Carbamidomethyl (C)[9]		Mascot
1439.72	1439.6511	-0.0689	-48	2	16	ATTAMALSPSSFAGK					Mascot
1593.7656	1593.7772	0.0116	7	41	55	AKPVSSGSPWYGSDR					Mascot
1593.7656	1593.7772	0.0116	7	41	55	AKPVSSGSPWYGSDR	69	99.976			Mascot
1903.9922	1904.0045	0.0123	6	150	166	WAMLGALGCVFPELLAR			Carbamidomethyl (C)[9]		Mascot
1919.9871	1919.9934	0.0063	3	150	166	WAMLGALGCVFPELLAR			Carbamidomethyl (C)[9], Oxidation (M)[3]		Mascot

4	unnamed protein product [Vitis vinifera]	gi|302143545	16026.2	9.17	6	164	100	15.25	135	100	

Peptide Information

Calc. Mass	Obsrv. Mass	± da	± ppm	Start Seq.	End Seq.	Sequence	Ion Score	C. I. %	Modification	Rank	Result Type
1055.5303	1055.5221	-0.0082	-8	41	48	ELEVIHCR			Carbamidomethyl (C)[7]		Mascot
1055.5303	1055.5221	-0.0082	-8	41	48	ELEVIHCR	58	99.736	Carbamidomethyl (C)[7]		Mascot
1325.6743	1325.6655	-0.0088	-7	39	48	NRELEVIHCR			Carbamidomethyl (C)[9]		Mascot
1325.6743	1325.6655	-0.0088	-7	39	48	NRELEVIHCR	64	99.922	Carbamidomethyl (C)[9]		Mascot
1593.7656	1593.7772	0.0116	7	8	22	SKAVSSGSPWYGPDR					Mascot
1593.7656	1593.7772	0.0116	7	8	22	SKAVSSGSPWYGPDR	13	0			Mascot
1605.802	1605.7825	-0.0195	-12	10	24	AVSSGSPWYGPDRVK					Mascot
1812.8916	1812.943	0.0514	28	25	40	YLGPFSADPETFAKNR					Mascot
1919.9871	1919.9934	0.0063	3	49	65	WAMLGALGCVFPELLSR			Carbamidomethyl (C)[9]		Mascot
1935.9819	1935.9849	0.003	2	49	65	WAMLGALGCVFPELLSR			Carbamidomethyl (C)[9], Oxidation (M)[3]		Mascot

5	RecName: Full=Chlorophyll a-b binding protein, chloroplastic; AltName: Full=LHCII type I CAB; Short=LHCP; Flags: Precursor	gi|115780	28577.4	5.31	8	159	100	10.651	122	100	

Peptide Information

Calc. Mass	Obsrv. Mass	± da	± ppm	Start Seq.	End Seq.	Sequence	Ion Score	C. I. %	Modification	Rank	Result Type

Calc. Mass	Obsrv. Mass	± da	± ppm	Start Seq.	End Seq.	Sequence	Ion Score	C. I. %	Modification	Rank	Result Type
983.4985	983.4614	-0.0371	-38	127	134	FGEAVWFK					Mascot
1055.5303	1055.5221	-0.0082	-8	98	105	ELEVIHCR			Carbamidomethyl (C)[7]		Mascot
1055.5303	1055.5221	-0.0082	-8	98	105	ELEVIHCR	58	99.736	Carbamidomethyl (C)[7]		Mascot
1325.6743	1325.6655	-0.0088	-7	96	105	NRELEVIHCR			Carbamidomethyl (C)[9]		Mascot
1325.6743	1325.6655	-0.0088	-7	96	105	NRELEVIHCR	64	99.922	Carbamidomethyl (C)[9]		Mascot
1423.7097	1423.6359	-0.0738	-52	2	16	ASSTMALSSPSLAGK			Oxidation (M)[5]		Mascot
1554.7502	1554.734	-0.0162	-10	1	16	MASSTMALSSPSLAGK			Oxidation (M)[1]		Mascot
1597.8909	1597.7753	-0.1156	-72	17	32	AVKLGPTASEIIGEGR					Mascot
1705.9153	1705.9233	0.008	5	2	19	ASSTMALSSPSLAGKAVK					Mascot
1903.9922	1904.0045	0.0123	6	106	122	WAMLGALGCVFPELLAR			Carbamidomethyl (C)[9]		Mascot
1919.9871	1919.9934	0.0063	3	106	122	WAMLGALGCVFPELLAR			Carbamidomethyl (C)[9], Oxidation (M)[3]		Mascot

6 unnamed protein product [Vitis vinifera] gi|297740653 15867.1 8.64 6 157 100 10.012 122 100

Peptide Information

Calc. Mass	Obsrv. Mass	± da	± ppm	Start Seq.	End Seq.	Sequence	Ion Score	C. I. %	Modification	Rank	Result Type
983.4985	983.4614	-0.0371	-38	59	66	FGEAVWFK					Mascot
1055.5303	1055.5221	-0.0082	-8	30	37	ELEVIHCR			Carbamidomethyl (C)[7]		Mascot
1055.5303	1055.5221	-0.0082	-8	30	37	ELEVIHCR	58	99.736	Carbamidomethyl (C)[7]		Mascot
1325.6743	1325.6655	-0.0088	-7	28	37	NRELEVIHCR			Carbamidomethyl (C)[9]		Mascot
1325.6743	1325.6655	-0.0088	-7	28	37	NRELEVIHCR	64	99.922	Carbamidomethyl (C)[9]		Mascot
1662.8732	1662.8262	-0.047	-28	2	19	AASTMALSSPSLAGTAVK					Mascot
1825.9034	1825.9045	0.0011	1	1	19	MAASTMALSSPSLAGTAVK			Oxidation (M)[1,6]		Mascot
1919.9871	1919.9934	0.0063	3	38	54	WAMLGALGCVFPELLSR			Carbamidomethyl (C)[9]		Mascot
1935.9819	1935.9849	0.003	2	38	54	WAMLGALGCVFPELLSR			Carbamidomethyl (C)[9], Oxidation (M)[3]		Mascot

7 Chlorophyll a-b binding protein, chloroplastic [Aegilops tauschii] gi|475471123 27994.2 5.46 8 156 100 11.178 122 100

Peptide Information

Calc. Mass	Obsrv. Mass	± da	± ppm	Start Seq.	End Seq.	Sequence	Ion Score	C. I. %	Modification	Rank	Result Type
964.5098	964.5096	-0.0002	0	2	11	SLSSPAFAGK					Mascot
983.4985	983.4614	-0.0371	-38	121	128	FGEAVWFK					Mascot
1055.5303	1055.5221	-0.0082	-8	92	99	ELEVIHCR			Carbamidomethyl (C)[7]		Mascot
1055.5303	1055.5221	-0.0082	-8	92	99	ELEVIHCR	58	99.736	Carbamidomethyl (C)[7]		Mascot
1325.6743	1325.6655	-0.0088	-7	90	99	NRELEVIHCR			Carbamidomethyl (C)[9]		Mascot

Calc. Mass	Obsrv. Mass	± da	± ppm	Start Seq.	End Seq.	Sequence	Ion Score	C. I. %	Modification	Rank	Result Type
1325.6743	1325.6655	-0.0088	-7	90	99	NRELEVIHCR	64	99.922	Carbamidomethyl (C)[9]		Mascot
1337.6848	1337.6588	-0.026	-19	15	26	NLPSSFLFGEAR					Mascot
1382.7103	1382.6367	-0.0736	-53	117	128	DGVKFGEAVWFK					Mascot
1608.7765	1608.7917	0.0152	9	36	50	AKQVASGSPWYGSDR					Mascot
1903.9922	1904.0045	0.0123	6	100	116	WAMLGALGCVFPELLAR			Carbamidomethyl (C)[9]		Mascot
1919.9871	1919.9934	0.0063	3	100	116	WAMLGALGCVFPELLAR			Carbamidomethyl (C)[9], Oxidation (M)[3]		Mascot

8	Chlorophyll a-b binding protein 3C, chloroplastic [Aegilops tauschii]	gi|475478904	27933.1	5.13	6	155	100	17.709	132	100	

Peptide Information

Calc. Mass	Obsrv. Mass	± da	± ppm	Start Seq.	End Seq.	Sequence	Ion Score	C. I. %	Modification	Rank	Result Type
983.4985	983.4614	-0.0371	-38	123	130	FGEAVWFK					Mascot
1055.5303	1055.5221	-0.0082	-8	94	101	ELEVIHCR			Carbamidomethyl (C)[7]		Mascot
1055.5303	1055.5221	-0.0082	-8	94	101	ELEVIHCR	58	99.736	Carbamidomethyl (C)[7]		Mascot
1325.6743	1325.6655	-0.0088	-7	92	101	NRELEVIHCR			Carbamidomethyl (C)[9]		Mascot
1325.6743	1325.6655	-0.0088	-7	92	101	NRELEVIHCR	64	99.922	Carbamidomethyl (C)[9]		Mascot
1589.7708	1589.7832	0.0124	8	38	52	AKPAASSSPWYGPDR					Mascot
1593.7975	1593.7772	-0.0203	-13	1	17	MAASTMALSSPGLAAAK			Oxidation (M)[1]		Mascot
1593.7975	1593.7772	-0.0203	-13	1	17	MAASTMALSSPGLAAAK	10	0	Oxidation (M)[6]		Mascot
1609.7924	1609.7708	-0.0216	-13	1	17	MAASTMALSSPGLAAAK			Oxidation (M)[1,6]		Mascot
1609.7924	1609.7708	-0.0216	-13	1	17	MAASTMALSSPGLAAAK			Oxidation (M)[1,6]		Mascot
1903.9922	1904.0045	0.0123	6	102	118	WAMLGALGCVFPELLAR			Carbamidomethyl (C)[9]		Mascot
1919.9871	1919.9934	0.0063	3	102	118	WAMLGALGCVFPELLAR			Carbamidomethyl (C)[9], Oxidation (M)[3]		Mascot

9	Chlorophyll a-b binding protein, chloroplastic [Triticum urartu]	gi|473952980	28455.3	5.15	7	151	100	10.215	122	100	

Peptide Information

Calc. Mass	Obsrv. Mass	± da	± ppm	Start Seq.	End Seq.	Sequence	Ion Score	C. I. %	Modification	Rank	Result Type
983.4985	983.4614	-0.0371	-38	126	133	FGEAVWFK					Mascot
1055.5303	1055.5221	-0.0082	-8	97	104	ELEVIHCR			Carbamidomethyl (C)[7]		Mascot
1055.5303	1055.5221	-0.0082	-8	97	104	ELEVIHCR	58	99.736	Carbamidomethyl (C)[7]		Mascot
1325.6743	1325.6655	-0.0088	-7	95	104	NRELEVIHCR			Carbamidomethyl (C)[9]		Mascot
1325.6743	1325.6655	-0.0088	-7	95	104	NRELEVIHCR	64	99.922	Carbamidomethyl (C)[9]		Mascot
1439.7318	1439.6511	-0.0807	-56	122	133	NDVKFGEAVWFK					Mascot
1592.7295	1592.7844	0.0549	34	1	16	MAAATMSLSSSTFAGK			Oxidation (M)[1,6]		Mascot
1608.7765	1608.7917	0.0152	9	41	55	AKQVSSGSPWYGADR					Mascot

1903.9922	1904.0045	0.0123	6	105	121	WAMLGALGCVFPELLAR			Carbamidomethyl (C)[9]		Mascot
1919.9871	1919.9934	0.0063	3	105	121	WAMLGALGCVFPELLAR			Carbamidomethyl (C)[9], Oxidation (M)[3]		Mascot

10	RecName: Full=Chlorophyll a-b binding protein 40, chloroplastic; AltName: Full=LHCII type I CAB-40; Short=LHCP; Flags: Precursor	gi|115805	28450.2	5.48	7	150	100	10.613	122	100	

Peptide Information

Calc. Mass	Obsrv. Mass	± da	± ppm	Start Seq.	End Seq.	Sequence	Ion Score	C. I. %	Modification	Rank	Result Type
983.4985	983.4614	-0.0371	-38	127	134	FGEAVWFK					Mascot
1055.5303	1055.5221	-0.0082	-8	98	105	ELEVIHCR			Carbamidomethyl (C)[7]		Mascot
1055.5303	1055.5221	-0.0082	-8	98	105	ELEVIHCR	58	99.736	Carbamidomethyl (C)[7]		Mascot
1325.6743	1325.6655	-0.0088	-7	96	105	NRELEVIHCR			Carbamidomethyl (C)[9]		Mascot
1325.6743	1325.6655	-0.0088	-7	96	105	NRELEVIHCR	64	99.922	Carbamidomethyl (C)[9]		Mascot
1441.6992	1441.6422	-0.057	-40	2	16	AASTMALSSPSFAGK			Oxidation (M)[5]		Mascot
1607.7812	1607.7842	0.003	2	42	56	AKTVSSGSPWYGPDR					Mascot
1870.9402	1871.0072	0.067	36	1	19	MAASTMALSSPSFAGKAVK			Oxidation (M)[1]		Mascot
1903.9922	1904.0045	0.0123	6	106	122	WAMLGALGCVFPELLAR			Carbamidomethyl (C)[9]		Mascot
1919.9871	1919.9934	0.0063	3	106	122	WAMLGALGCVFPELLAR			Carbamidomethyl (C)[9], Oxidation (M)[3]		Mascot

Gel Idx/Pos	302/M4	Instr./Gel Origin	BA2151/Sample Project 20140814	Process Status	Analysis Succeeded
Plate [#] Name	[1] Sample Project 20140814	Instrument Sample Name		Spectra	11

Rank	Protein Name	Accession No.	Protein MW	Protein PI	Pep. Count	Protein Score	Protein Score C. I. %	Intensity Matched	Total Ion Score	Total Ion C. I. %	Confirmed
1	Chlorophyll a-b binding protein 1B, chloroplastic [Aegilops tauschii]	gi|475598488	27090.7	5.12	5	164	100	14.611	147	100	

Peptide Information

Calc. Mass	Obsrv. Mass	± da	± ppm	Start Seq.	End Seq.	Sequence	Ion Score	C. I. %	Modification	Rank	Result Type
983.4985	983.463	-0.0355	-36	115	122	FGEAVWFK					Mascot
983.4985	983.463	-0.0355	-36	115	122	FGEAVWFK	25	0			Mascot
1055.5303	1055.5171	-0.0132	-13	86	93	ELEVIHCR			Carbamidomethyl (C)[7]		Mascot
1055.5303	1055.5171	-0.0132	-13	86	93	ELEVIHCR	48	96.317	Carbamidomethyl (C)[7]		Mascot
1325.6743	1325.652	-0.0223	-17	84	93	NRELEVIHCR			Carbamidomethyl (C)[9]		Mascot
1407.6288	1407.6229	-0.0059	-4	32	44	ASASNSPWYGPDR					Mascot
1407.6288	1407.6229	-0.0059	-4	32	44	ASASNSPWYGPDR	74	99.992			Mascot
1919.9871	1919.9635	-0.0236	-12	94	110	WAMLGALGCVFPELLAR			Carbamidomethyl (C)[9], Oxidation (M)[3]		Mascot

Rank	Protein Name	Accession No.	Protein MW	Protein PI	Pep. Count	Protein Score	Protein Score C. I. %	Intensity Matched	Total Ion Score	Total Ion C. I. %	Confirmed
2	Chlorophyll a-b binding protein, chloroplastic [Aegilops tauschii]	gi|475542040	28717.3	5.14	7	103	99.995	23.534	73	99.989	

Peptide Information

Calc. Mass	Obsrv. Mass	± da	± ppm	Start Seq.	End Seq.	Sequence	Ion Score	C. I. %	Modification	Rank	Result Type
983.4985	983.463	-0.0355	-36	126	133	FGEAVWFK					Mascot
983.4985	983.463	-0.0355	-36	126	133	FGEAVWFK	25	0			Mascot
1055.5303	1055.5171	-0.0132	-13	97	104	ELEVIHCR			Carbamidomethyl (C)[7]		Mascot
1055.5303	1055.5171	-0.0132	-13	97	104	ELEVIHCR	48	96.317	Carbamidomethyl (C)[7]		Mascot
1325.6743	1325.652	-0.0223	-17	95	104	NRELEVIHCR			Carbamidomethyl (C)[9]		Mascot
1423.6602	1423.6178	-0.0424	-30	43	55	QVASSSPWYGADR					Mascot
1423.6602	1423.6178	-0.0424	-30	43	55	QVASSSPWYGADR					Mascot
1576.7346	1576.7382	0.0036	2	1	16	MAATTMSLSSSAFAGK			Oxidation (M)[1]		Mascot
1919.9871	1919.9635	-0.0236	-12	105	121	WAMLGALGCVFPELLSR			Carbamidomethyl (C)[9]		Mascot
2056.0283	2055.9363	-0.092	-45	220	237	LAMFSMFSFFVQAIVTGK			Oxidation (M)[3,6]		Mascot

Rank	Protein Name	Accession No.	Protein MW	Protein PI	Pep. Count	Protein Score	Protein Score C. I. %	Intensity Matched	Total Ion Score	Total Ion C. I. %	Confirmed
3	Chlorophyll a-b binding protein, chloroplastic [Triticum urartu]	gi|473952980	28455.3	5.15	7	102	99.994	19.083	73	99.989	

Peptide Information

Calc. Mass	Obsrv. Mass	± da	± ppm	Start Seq.	End Seq.	Sequence	Ion Score	C. I. %	Modification	Rank	Result Type
983.4985	983.463	-0.0355	-36	126	133	FGEAVWFK					Mascot
983.4985	983.463	-0.0355	-36	126	133	FGEAVWFK	25	0			Mascot
1055.5303	1055.5171	-0.0132	-13	97	104	ELEVIHCR			Carbamidomethyl (C)[7]		Mascot
1055.5303	1055.5171	-0.0132	-13	97	104	ELEVIHCR	48	96.317	Carbamidomethyl (C)[7]		Mascot
1273.6899	1273.6294	-0.0605	-47	20	31	NVPSLALFGEAR					Mascot
1325.6743	1325.652	-0.0223	-17	95	104	NRELEVIHCR			Carbamidomethyl (C)[9]		Mascot
1439.7318	1439.6124	-0.1194	-83	122	133	NDVKFGEAVWFK					Mascot
1439.7318	1439.6124	-0.1194	-83	122	133	NDVKFGEAVWFK					Mascot
1576.7346	1576.7382	0.0036	2	1	16	MAAATMSLSSSTFAGK			Oxidation (M)[1]		Mascot
1919.9871	1919.9635	-0.0236	-12	105	121	WAMLGALGCVFPELLAR			Carbamidomethyl (C)[9], Oxidation (M)[3]		Mascot

4	Chlorophyll a-b binding protein, chloroplastic [Aegilops tauschii]	gi|475471123	27994.2	5.46	7	100	99.989	10.802	73	99.989	

Peptide Information

Calc. Mass	Obsrv. Mass	± da	± ppm	Start Seq.	End Seq.	Sequence	Ion Score	C. I. %	Modification	Rank	Result Type
964.5098	964.5062	-0.0036	-4	2	11	SLSSPAFAGK					Mascot
964.5098	964.5062	-0.0036	-4	2	11	SLSSPAFAGK					Mascot
983.4985	983.463	-0.0355	-36	121	128	FGEAVWFK					Mascot
983.4985	983.463	-0.0355	-36	121	128	FGEAVWFK	25	0			Mascot
1055.5303	1055.5171	-0.0132	-13	92	99	ELEVIHCR			Carbamidomethyl (C)[7]		Mascot
1055.5303	1055.5171	-0.0132	-13	92	99	ELEVIHCR	48	96.317	Carbamidomethyl (C)[7]		Mascot
1325.6743	1325.652	-0.0223	-17	90	99	NRELEVIHCR			Carbamidomethyl (C)[9]		Mascot
1337.6848	1337.6472	-0.0376	-28	15	26	NLPSSFLFGEAR					Mascot
1382.7103	1382.626	-0.0843	-61	117	128	DGVKFGEAVWFK					Mascot
1919.9871	1919.9635	-0.0236	-12	100	116	WAMLGALGCVFPELLAR			Carbamidomethyl (C)[9], Oxidation (M)[3]		Mascot

5	unnamed protein product [Vitis vinifera]	gi|297740653	15867.1	8.64	5	98	99.983	9.673	73	99.989	

Peptide Information

Calc. Mass	Obsrv. Mass	± da	± ppm	Start Seq.	End Seq.	Sequence	Ion Score	C. I. %	Modification	Rank	Result Type
983.4985	983.463	-0.0355	-36	59	66	FGEAVWFK					Mascot
983.4985	983.463	-0.0355	-36	59	66	FGEAVWFK	25	0			Mascot
1055.5303	1055.5171	-0.0132	-13	30	37	ELEVIHCR			Carbamidomethyl (C)[7]		Mascot
1055.5303	1055.5171	-0.0132	-13	30	37	ELEVIHCR	48	96.317	Carbamidomethyl (C)[7]		Mascot

Calc. Mass	Obsrv. Mass	± da	± ppm	Start Seq.	End Seq.	Sequence	Ion Score	C. I. %	Modification	Rank	Result Type
1117.6113	1117.5077	-0.1036	-93	20	29	LGPNTFAKNR					Mascot
1325.6743	1325.652	-0.0223	-17	28	37	NRELEVIHCR			Carbamidomethyl (C)[9]		Mascot
1919.9871	1919.9635	-0.0236	-12	38	54	WAMLGALGCVFPELLSR			Carbamidomethyl (C)[9]		Mascot

6	Chlorophyll a-b binding protein 3C, chloroplastic [Aegilops tauschii]	gi|475478904	27933.1	5.13	6	96	99.974	9.897	73	99.989	

Peptide Information

Calc. Mass	Obsrv. Mass	± da	± ppm	Start Seq.	End Seq.	Sequence	Ion Score	C. I. %	Modification	Rank	Result Type
983.4985	983.463	-0.0355	-36	123	130	FGEAVWFK					Mascot
983.4985	983.463	-0.0355	-36	123	130	FGEAVWFK	25	0			Mascot
1055.5303	1055.5171	-0.0132	-13	94	101	ELEVIHCR			Carbamidomethyl (C)[7]		Mascot
1055.5303	1055.5171	-0.0132	-13	94	101	ELEVIHCR	48	96.317	Carbamidomethyl (C)[7]		Mascot
1178.6165	1178.5184	-0.0981	-83	18	28	IISSNVFGEGR					Mascot
1325.6743	1325.652	-0.0223	-17	92	101	NRELEVIHCR			Carbamidomethyl (C)[9]		Mascot
1593.7975	1593.7512	-0.0463	-29	1	17	MAASTMALSSPGLAAAK			Oxidation (M)[1]		Mascot
1919.9871	1919.9635	-0.0236	-12	102	118	WAMLGALGCVFPELLAR			Carbamidomethyl (C)[9], Oxidation (M)[3]		Mascot

7	RecName: Full=Chlorophyll a-b binding protein, chloroplastic; AltName: Full=LHCII type I CAB; Short=LHCP; Flags: Precursor	gi|115786	28148.2	5.29	6	96	99.974	9.878	73	99.989	

Peptide Information

Calc. Mass	Obsrv. Mass	± da	± ppm	Start Seq.	End Seq.	Sequence	Ion Score	C. I. %	Modification	Rank	Result Type
983.4985	983.463	-0.0355	-36	125	132	FGEAVWFK					Mascot
983.4985	983.463	-0.0355	-36	125	132	FGEAVWFK	25	0			Mascot
1055.5303	1055.5171	-0.0132	-13	96	103	ELEVIHCR			Carbamidomethyl (C)[7]		Mascot
1055.5303	1055.5171	-0.0132	-13	96	103	ELEVIHCR	48	96.317	Carbamidomethyl (C)[7]		Mascot
1320.6543	1320.611	-0.0433	-33	17	29	AVNVPSSSFGEAR					Mascot
1325.6743	1325.652	-0.0223	-17	94	103	NRELEVIHCR			Carbamidomethyl (C)[9]		Mascot
1576.7346	1576.7382	0.0036	2	1	16	MASSTMALSSTAFAGK			Oxidation (M)[1]		Mascot
1919.9871	1919.9635	-0.0236	-12	104	120	WAMLGALGCVFPELLAR			Carbamidomethyl (C)[9], Oxidation (M)[3]		Mascot

8	Chlorophyll a-b binding protein, chloroplastic [Triticum urartu]	gi|474413347	28531.3	5.29	6	96	99.974	19.332	73	99.989	

Peptide Information

Calc. Mass	Obsrv. Mass	± da	± ppm	Start Seq.	End Seq.	Sequence	Ion Score	C. I. %	Modification	Rank	Result Type
983.4985	983.463	-0.0355	-36	126	133	FGEAVWFK					Mascot

Calc. Mass	Obsrv. Mass	± da	± ppm	Start Seq.	End Seq.	Sequence	Ion Score	C. I. %	Modification	Rank	Result Type
983.4985	983.463	-0.0355	-36	126	133	FGEAVWFK	25	0			Mascot
1055.5303	1055.5171	-0.0132	-13	97	104	ELEVIHCR			Carbamidomethyl (C)[7]		Mascot
1055.5303	1055.5171	-0.0132	-13	97	104	ELEVIHCR	48	96.317	Carbamidomethyl (C)[7]		Mascot
1325.6743	1325.652	-0.0223	-17	95	104	NRELEVIHCR			Carbamidomethyl (C)[9]		Mascot
1411.7369	1411.6213	-0.1156	-82	122	133	NSVKFGEAVWFK					Mascot
1439.72	1439.6124	-0.1076	-75	2	16	AATTMSLSSPAFAGK					Mascot
1439.72	1439.6124	-0.1076	-75	2	16	AATTMSLSSPAFAGK					Mascot
1455.7148	1455.6071	-0.1077	-74	2	16	AATTMSLSSPAFAGK			Oxidation (M)[5]		Mascot
1919.9871	1919.9635	-0.0236	-12	105	121	WAMLGALGCVFPELLAR			Carbamidomethyl (C)[9], Oxidation (M)[3]		Mascot

9	Chlorophyll a-b binding protein, chloroplastic [Aegilops tauschii]	gi|475614191	28466.3	5.27	6	95	99.971	19.086	73	99.989

Peptide Information

Calc. Mass	Obsrv. Mass	± da	± ppm	Start Seq.	End Seq.	Sequence	Ion Score	C. I. %	Modification	Rank	Result Type
983.4985	983.463	-0.0355	-36	126	133	FGEAVWFK					Mascot
983.4985	983.463	-0.0355	-36	126	133	FGEAVWFK	25	0			Mascot
1055.5303	1055.5171	-0.0132	-13	97	104	ELEVIHCR			Carbamidomethyl (C)[7]		Mascot
1055.5303	1055.5171	-0.0132	-13	97	104	ELEVIHCR	48	96.317	Carbamidomethyl (C)[7]		Mascot
1325.6743	1325.652	-0.0223	-17	95	104	NRELEVIHCR			Carbamidomethyl (C)[9]		Mascot
1437.6758	1437.6115	-0.0643	-45	43	55	QVVSGSPWYGSDR					Mascot
1439.72	1439.6124	-0.1076	-75	2	16	AATTMSLSSPAFAGK					Mascot
1439.72	1439.6124	-0.1076	-75	2	16	AATTMSLSSPAFAGK					Mascot
1455.7148	1455.6071	-0.1077	-74	2	16	AATTMSLSSPAFAGK			Oxidation (M)[5]		Mascot
1919.9871	1919.9635	-0.0236	-12	105	121	WAMLGALGCVFPELLAR			Carbamidomethyl (C)[9], Oxidation (M)[3]		Mascot

10	chlorophyll A/B binding protein, putative [Ricinus communis]	gi|223545686	28509.3	5.29	6	95	99.971	19.054	73	99.989

Peptide Information

Calc. Mass	Obsrv. Mass	± da	± ppm	Start Seq.	End Seq.	Sequence	Ion Score	C. I. %	Modification	Rank	Result Type
983.4985	983.463	-0.0355	-36	127	134	FGEAVWFK					Mascot
983.4985	983.463	-0.0355	-36	127	134	FGEAVWFK	25	0			Mascot
1023.5	1023.4998	-0.0002	0	29	37	SSNGRVTMR			Oxidation (M)[8]		Mascot
1055.5303	1055.5171	-0.0132	-13	98	105	ELEVIHCR			Carbamidomethyl (C)[7]		Mascot
1055.5303	1055.5171	-0.0132	-13	98	105	ELEVIHCR	48	96.317	Carbamidomethyl (C)[7]		Mascot
1325.6743	1325.652	-0.0223	-17	96	105	NRELEVIHCR			Carbamidomethyl (C)[9]		Mascot

1391.6545	1391.6296	-0.0249	-18	1	13	MATMTLSSTFTGK	Oxidation (M)[1]	Mascot
1391.6545	1391.6296	-0.0249	-18	1	13	MATMTLSSTFTGK	Oxidation (M)[1]	Mascot
1407.6495	1407.6229	-0.0266	-19	1	13	MATMTLSSTFTGK	Oxidation (M)[1,4]	Mascot
1407.6495	1407.6229	-0.0266	-19	1	13	MATMTLSSTFTGK	Oxidation (M)[1,4]	Mascot
1919.9871	1919.9635	-0.0236	-12	106	122	WAMLGALGCVFPELLAR	Carbamidomethyl (C)[9], Oxidation (M)[3]	Mascot

Gel Idx/Pos	303/M5	Instr./Gel Origin	BA2151/Sample Project 20140814	Process Status	Analysis Succeeded
Plate [#] Name	[1] Sample Project 20140814	Instrument Sample Name		Spectra	11

Rank	Protein Name	Accession No.	Protein MW	Protein PI	Pep. Count	Protein Score	Protein Score C. I. %	Intensity Matched	Total Ion Score	Total Ion C. I. %	Confirmed
1	hypothetical protein F775_28748 [Aegilops tauschii]	gi|475569529	22813.2	6.2	2	94	99.965	23.294	87	100	

Protein Group

Protein Name	Accession No.	Protein MW	Protein PI
Avenin-3 [Triticum urartu]	gi|474329936	22657.1	6.3499 999046 3257
alpha-amylase inhibitor 1 [Aegilops tauschii]	gi|442614142	22813.2	6.1999 998092 6514

Peptide Information

Calc. Mass	Obsrv. Mass	± da	± ppm	Start Seq.	End Seq.	Sequence	Ion Score	C. I. %	Modification	Rank	Result Type
1950.9413	1950.9327	-0.0086	-4	60	76	DALLQQCSPVADMSFLR			Carbamidomethyl (C)[7]		Mascot
1950.9413	1950.9327	-0.0086	-4	60	76	DALLQQCSPVADMSFLR	87	100	Carbamidomethyl (C)[7]		Mascot
1966.9362	1966.9016	-0.0346	-18	60	76	DALLQQCSPVADMSFLR			Carbamidomethyl (C)[7], Oxidation (M)[13]		Mascot
1966.9362	1966.9016	-0.0346	-18	60	76	DALLQQCSPVADMSFLR	81	99.999	Carbamidomethyl (C)[7], Oxidation (M)[13]		Mascot
2635.1885	2635.1743	-0.0142	-5	77	97	SQVVQHSSCLVMWEQC CQQLK			Carbamidomethyl (C)[9,16,17]		Mascot
2651.1833	2651.157	-0.0263	-10	77	97	SQVVQHSSCLVMWEQC CQQLK			Carbamidomethyl (C)[9,16,17], Oxidation (M)[12]		Mascot

Rank	Protein Name	Accession No.	Protein MW	Protein PI	Pep. Count	Protein Score	Protein Score C. I. %	Intensity Matched	Total Ion Score	Total Ion C. I. %	Confirmed
2	conserved hypothetical protein [Ricinus communis]	gi|223506873	9263.4	5.58	7	60	4.878	1.867			

Peptide Information

Calc. Mass	Obsrv. Mass	± da	± ppm	Start Seq.	End Seq.	Sequence	Ion Score	C. I. %	Modification	Rank	Result Type
945.4458	945.4924	0.0466	49	46	53	GDICEVPR			Carbamidomethyl (C)[4]		Mascot
1234.5997	1234.6174	0.0177	14	34	43	EALRMWDAAR			Oxidation (M)[5]		Mascot
1786.9554	1786.8433	-0.1121	-63	1	17	MPMVTVSISPLQAAGIR			Oxidation (M)[1]		Mascot
1859.8552	1859.9543	0.0991	53	46	63	GDICEVPRAANDGGETA K			Carbamidomethyl (C)[4]		Mascot
1919.8011	1919.9287	0.1276	66	64	79	NGRCVADMFADYEAER			Carbamidomethyl (C)[4], Oxidation (M)[8]		Mascot
3233.6836	3233.54	-0.1436	-44	2	33	PMVTVSISPLQAAGIRAA VDTGTYASSSEVVR					Mascot
3249.6785	3249.533	-0.1455	-45	2	33	PMVTVSISPLQAAGIRAA			Oxidation (M)[2]		Mascot

Calc. Mass	Obsrv. Mass	± da	± ppm	Start Seq.	End Seq.	Sequence	Ion Score	C. I. %	Modification	Rank	Result Type
						VDTGTYASSSEVVR					
3249.6785	3249.533	-0.1455	-45	2	33	PMVTVSISPLQAAGIRAA VDTGTYASSSEVVR			Oxidation (M)[2]		Mascot
3396.7139	3396.6963	-0.0176	-5	1	33	MPMVTVSISPLQAAGIRA AVDTGTYASSSEVVR			Oxidation (M)[1,3]		Mascot

3 hypothetical protein ARALYDRAFT_496034 [Arabidopsis lyrata subsp. lyrata] gi|297310444 45997.6 6.17 11 50 0 1.822

Peptide Information

Calc. Mass	Obsrv. Mass	± da	± ppm	Start Seq.	End Seq.	Sequence	Ion Score	C. I. %	Modification	Rank	Result Type
836.4584	836.3863	-0.0721	-86	23	29	TTTTTRR					Mascot
945.555	945.4924	-0.0626	-66	283	291	VLGVLAMSR					Mascot
1051.6007	1051.5031	-0.0976	-93	164	171	ERLHELVR					Mascot
1550.719	1550.7734	0.0544	35	191	205	MDMEVVALNADGAAK			Oxidation (M)[1]		Mascot
1786.9844	1786.8433	-0.1411	-79	379	394	ACEEASLLLTRLALAR			Carbamidomethyl (C)[2]		Mascot
1851.8688	1851.8918	0.023	12	342	358	MCLSGKANGQLSTSPER			Carbamidomethyl (C)[2], Oxidation (M)[1]		Mascot
1919.8838	1919.9287	0.0449	23	359	378	EMTGVGAGNVMVGGGD LPDK			Oxidation (M)[2]		Mascot
1932.0525	1931.96	-0.0925	-48	275	291	VIYWDGPRVLGVLAMSR					Mascot
2467.1223	2467.1868	0.0645	26	1	21	MAEICYENETMMTKTTAT VVK			Carbamidomethyl (C)[5], Oxidation (M)[1]		Mascot
3163.5068	3163.5439	0.0371	12	359	389	EMTGVGAGNVMVGGGD LPDKACEEASLLLTR			Carbamidomethyl (C)[22], Oxidation (M)[2]		Mascot
3163.5068	3163.5439	0.0371	12	359	389	EMTGVGAGNVMVGGGD LPDKACEEASLLLTR			Carbamidomethyl (C)[22], Oxidation (M)[2]		Mascot
3404.8611	3404.7297	-0.1314	-39	283	313	VLGVLAMSRAIGDNYLKP YVISKPEVTVTDR					Mascot

4 o-methyltransferase, putative [Ricinus communis] gi|223533072 26651.9 5.85 8 49 0 14.255

Peptide Information

Calc. Mass	Obsrv. Mass	± da	± ppm	Start Seq.	End Seq.	Sequence	Ion Score	C. I. %	Modification	Rank	Result Type
800.4875	800.4221	-0.0654	-82	202	208	AILEINK					Mascot
812.4335	812.4256	-0.0079	-10	1	7	MNPLPPK			Oxidation (M)[1]		Mascot
1903.0066	1902.9875	-0.0191	-10	216	232	IEISQVSVGDGITLCRR			Carbamidomethyl (C)[15]		Mascot
1903.0066	1902.9875	-0.0191	-10	216	232	IEISQVSVGDGITLCRR			Carbamidomethyl (C)[15]		Mascot
1949.0487	1948.9728	-0.0759	-39	47	64	SIMSVPVDEGLFIAMLVK					Mascot
1950.8756	1950.9327	0.0571	29	143	158	EEYDFAFVDADKPNYK					Mascot
1950.8756	1950.9327	0.0571	29	143	158	EEYDFAFVDADKPNYK					Mascot
1981.0385	1980.912	-0.1265	-64	47	64	SIMSVPVDEGLFIAMLVK			Oxidation (M)[3,15]		Mascot
3163.5305	3163.5439	0.0134	4	170	197	VGGIIAYDNTLWFGFVAQ EENAVPEHMR					Mascot
3163.5305	3163.5439	0.0134	4	170	197	VGGIIAYDNTLWFGFVAQ EENAVPEHMR					Mascot

	3389.6536	3389.6851	0.0315	9	130	158	SVLSELLSNDKPREEYDF AFVDADKPNYK					Mascot
	3476.8523	3476.7288	-0.1235	-36	70	102	RTLEIGVFTGYSLLATALA LPDDGQVTAIDIDK					Mascot

5 pentatricopeptide repeat protein, partial [Arabidopsis lyrata subsp. petraea] gi|404352362 23057.2 5.8 9 49 0 4.8

Peptide Information

Calc. Mass	Obsrv. Mass	± da	± ppm	Start Seq.	End Seq.	Sequence	Ion Score	C. I. %	Modification	Rank	Result Type
1105.4806	1105.525	0.0444	40	149	156	QYFDMMVR			Oxidation (M)[5]		Mascot
1507.7131	1507.702	-0.0111	-7	51	63	NCDVDVAMDVLIK			Carbamidomethyl (C)[2], Oxidation (M)[8]		Mascot
1859.9221	1859.9543	0.0322	17	90	106	DLASWNAMILGFATHGR					Mascot
1875.9171	1875.8892	-0.0279	-15	90	106	DLASWNAMILGFATHGR			Oxidation (M)[8]		Mascot
1875.9171	1875.8892	-0.0279	-15	90	106	DLASWNAMILGFATHGR			Oxidation (M)[8]		Mascot
1885.8718	1885.9667	0.0949	50	73	88	CGSLRMAEQVFQGMQK			Carbamidomethyl (C)[1], Oxidation (M)[6]		Mascot
1918.7803	1918.9216	0.1413	74	107	122	AEEAMNCFDCMVKEGK			Carbamidomethyl (C)[7,10]		Mascot
1934.7751	1934.9537	0.1786	92	107	122	AEEAMNCFDCMVKEGK			Carbamidomethyl (C)[7,10], Oxidation (M)[5]		Mascot
1940.9647	1940.9358	-0.0289	-15	1	16	VGEYDSALQLFRQMQR					Mascot
1950.77	1950.9327	0.1627	83	107	122	AEEAMNCFDCMVKEGK			Carbamidomethyl (C)[7,10], Oxidation (M)[5,11]		Mascot
1950.77	1950.9327	0.1627	83	107	122	AEEAMNCFDCMVKEGK			Carbamidomethyl (C)[7,10], Oxidation (M)[5,11]		Mascot
2467.3237	2467.1868	-0.1369	-55	120	141	EGKNVKPNSVTFVALLIA CNHR			Carbamidomethyl (C)[19]		Mascot
3374.6006	3374.6655	0.0649	19	149	176	QYFDMMVRDYGIEPALE HYGCIIDLVAR			Carbamidomethyl (C)[21]		Mascot
3445.5254	3445.6912	0.1658	48	90	119	DLASWNAMILGFATHGR AEEAMNCFDCMVK			Carbamidomethyl (C)[24,27]		Mascot

6 PREDICTED: phospholipase D delta-like [Solanum lycopersicum] gi|460375555 98352.2 6.67 16 49 0 2.769

Peptide Information

Calc. Mass	Obsrv. Mass	± da	± ppm	Start Seq.	End Seq.	Sequence	Ion Score	C. I. %	Modification	Rank	Result Type
812.4988	812.4256	-0.0732	-90	132	138	VKIPAER					Mascot
1234.6791	1234.6174	-0.0617	-50	550	560	SIEAAYIQAIR					Mascot
1351.6311	1351.6569	0.0258	19	728	739	DTEIAMGAYQPR					Mascot
1507.7574	1507.702	-0.0554	-37	374	387	ITAFLGGLDLCDGR			Carbamidomethyl (C)[11]		Mascot
1550.7544	1550.7734	0.019	12	530	543	STDVTEEQNLISSK					Mascot
1885.8411	1885.9667	0.1256	67	674	691	EANAQSSSDADKVSDSF K					Mascot
1891.9661	1891.9006	-0.0655	-35	794	809	RYTADNFTELNGHLLK					Mascot
1916.9501	1916.9415	-0.0086	-4	217	232	FPEIKLENGTNFEHNK					Mascot

Calc. Mass	Obsrv. Mass	± da	± ppm	Start Seq.	End Seq.	Sequence	Ion Score	C. I. %	Modification	Rank	Result Type
1919.9233	1919.9287	0.0054	3	489	504	ERTEIPEDDPELYVSK					Mascot
1924.8977	1924.9072	0.0095	5	90	104	VISNSQYPFWDEHFR					Mascot
1955.9055	1955.9598	0.0543	28	305	321	FFITTEGLMGTHDEETK					Mascot
1995.058	1994.9181	-0.1399	-70	585	603	DAGADHLVPMELALKIAS K			Oxidation (M)[10]		Mascot
2683.2366	2683.1504	-0.0862	-32	36	59	CFTFGGICGKPHHSTVER AGGDHR			Carbamidomethyl (C)[1,8]		Mascot
3401.5884	3401.7397	0.1513	44	626	652	STTMQEILYWQSQTMQM MYQVIARELK			Oxidation (M)[4,15,17,18]		Mascot
3445.748	3445.6912	-0.0568	-16	90	117	VISNSQYPFWDEHFRIPL AHPLAYLEFR					Mascot
3458.7178	3458.6831	-0.0347	-10	233	260	CWEDICYAIAEAHHLIYIV GWSVFHKIK			Carbamidomethyl (C)[1,6]		Mascot

7 PREDICTED: putative disease resistance protein RGA4-like isoform X1 [Setaria italica] gi|514814721 32475.5 5.02 10 48 0 2.177

Protein Group

PREDICTED: putative disease resistance protein RGA4-like isoform X2 [Setaria italica]	gi|514814723	32475.5	5.0199 999809 2651

Peptide Information

Calc. Mass	Obsrv. Mass	± da	± ppm	Start Seq.	End Seq.	Sequence	Ion Score	C. I. %	Modification	Rank	Result Type
800.4261	800.4221	-0.004	-5	198	205	VDAPVGSR					Mascot
818.3865	818.3765	-0.01	-12	104	109	CWDIPK			Carbamidomethyl (C)[1]		Mascot
1051.4691	1051.5031	0.034	32	237	244	VEENFEER					Mascot
1263.6151	1263.6095	-0.0056	-4	100	109	TTSRCWDIPK			Carbamidomethyl (C)[5]		Mascot
1859.8229	1859.9543	0.1314	71	230	244	MVFGDSRVEENFEER			Oxidation (M)[1]		Mascot
1916.9924	1916.9415	-0.0509	-27	37	53	LETTLRDLSDILGDAER					Mascot
1919.9611	1919.9287	-0.0324	-17	237	251	VEENFEERIWLSVNR					Mascot
1980.0437	1979.9415	-0.1022	-52	252	269	EFNEINVLQSLIASFGAK					Mascot
3249.4272	3249.533	0.1058	33	70	99	DVMYDADDVLDLCQLME GGEDPPAPTSAPK			Carbamidomethyl (C)[13]		Mascot
3249.4272	3249.533	0.1058	33	70	99	DVMYDADDVLDLCQLME GGEDPPAPTSAPK			Carbamidomethyl (C)[13]		Mascot
3265.4221	3265.5474	0.1253	38	70	99	DVMYDADDVLDLCQLME GGEDPPAPTSAPK			Carbamidomethyl (C)[13], Oxidation (M)[3]		Mascot
3458.7122	3458.6831	-0.0291	-8	252	282	EFNEINVLQSLIASFGAKH EGCAGNQDLLQR			Carbamidomethyl (C)[22]		Mascot

8 Os07g0160000 [Oryza sativa Japonica Group] gi|255677531 102944.3 5.3 15 46 0 22.112

Peptide Information

Calc. Mass	Obsrv. Mass	± da	± ppm	Start Seq.	End Seq.	Sequence	Ion Score	C. I. %	Modification	Rank	Result Type

Calc. Mass	Obsrv. Mass	± da	± ppm	Start Seq.	End Seq.	Sequence	Ion Score	C. I. %	Modification	Rank	Result Type
818.4155	818.3765	-0.039	-48	321	326	LWNTER					Mascot
890.5193	890.4451	-0.0742	-83	146	153	ETSILTVK					Mascot
1507.7639	1507.702	-0.0619	-41	506	518	YLEEPSDSVSLLR					Mascot
1550.8761	1550.7734	-0.1027	-66	112	125	VLARQNSSLALHNK					Mascot
1786.8236	1786.8433	0.0197	11	1	19	MATAATAQASASSSTAS TR			Oxidation (M)[1]		Mascot
1859.8956	1859.9543	0.0587	32	732	748	GNEDSVALHGCLEAFLK			Carbamidomethyl (C)[11]		Mascot
1873.908	1873.9329	0.0249	13	286	300	LVDYFLGDYDRDINR					Mascot
1875.9977	1875.8892	-0.1085	-58	321	335	LWNTERKPVSPHHFK					Mascot
1875.9977	1875.8892	-0.1085	-58	321	335	LWNTERKPVSPHHFK					Mascot
1884.8585	1884.9646	0.1061	56	662	676	EAHMEEFEFYLINGR					Mascot
1900.8534	1900.9346	0.0812	43	662	676	EAHMEEFEFYLINGR			Oxidation (M)[4]		Mascot
1916.9283	1916.9415	0.0132	7	20	38	GSPAASSSSHSAVCLVP FR			Carbamidomethyl (C)[14]		Mascot
2005.0098	2004.8306	-0.1792	-89	91	111	GTGAGAGGRSYALVPAD ELSR					Mascot
3278.699	3278.5632	-0.1358	-41	698	725	LLINVHWQQNAVGQYDT SMLKSLPEIHK			Oxidation (M)[19]		Mascot
3375.592	3375.6782	0.0862	26	883	911	LDGHQSFMCNKVSWTN CVLNDNSIPQVVI			Carbamidomethyl (C)[9,17]		Mascot
3389.6372	3389.6851	0.0479	14	170	197	IFNIDSQPVHVWDFSGQT NLILMNEWNR			Oxidation (M)[23]		Mascot
3419.554	3419.7341	0.1801	53	829	858	LYAISNHYGNMGGGHYT ASIYHEEGKGWYK			Oxidation (M)[11]		Mascot
3419.554	3419.7341	0.1801	53	829	858	LYAISNHYGNMGGGHYT ASIYHEEGKGWYK			Oxidation (M)[11]		Mascot

9	hypothetical protein ZEAMMB73_829906 [Zea mays]	gi|413932657	43590.2	8.53	11	45	0	8.926	

Peptide Information

Calc. Mass	Obsrv. Mass	± da	± ppm	Start Seq.	End Seq.	Sequence	Ion Score	C. I. %	Modification	Rank	Result Type
810.3563	810.3385	-0.0178	-22	211	216	FVNDCR			Carbamidomethyl (C)[5]		Mascot
820.4531	820.3776	-0.0755	-92	2	7	CMLLRK			Carbamidomethyl (C)[1]		Mascot
832.3795	832.3108	-0.0687	-83	261	267	DNEEAVR					Mascot
836.4481	836.3863	-0.0618	-74	2	7	CMLLRK			Carbamidomethyl (C)[1], Oxidation (M)[2]		Mascot
859.4706	859.395	-0.0756	-88	33	39	IPTELMR					Mascot
1144.6144	1144.5598	-0.0546	-48	31	39	ERIPTELMR					Mascot
1234.553	1234.6174	0.0644	52	236	245	GFMRMTGFCK			Carbamidomethyl (C)[9]		Mascot
1507.7396	1507.702	-0.0376	-25	268	280	AYGIHLGTEMCKK			Carbamidomethyl (C)[11]		Mascot
1812.0643	1811.9572	-0.1071	-59	330	344	VKEVVRPIFWASRPK					Mascot
1812.0643	1811.9572	-0.1071	-59	330	344	VKEVVRPIFWASRPK					Mascot
2635.3557	2635.1743	-0.1814	-69	217	239	QIGITCPIVPGIMPINNYK GFMR			Carbamidomethyl (C)[6], Oxidation (M)[13]		Mascot
2651.3506	2651.157	-0.1936	-73	217	239	QIGITCPIVPGIMPINNYK			Carbamidomethyl (C)[6], Oxidation (M)[13,22]		Mascot

						GFMR					
3233.595	3233.54	-0.055	-17	350	377	TLGWDQYPHEGGVILET HHMEHLGIVHK					Mascot
3249.5898	3249.533	-0.0568	-17	350	377	TLGWDQYPHEGGVILET HHMEHLGIVHK			Oxidation (M)[20]		Mascot
3249.5898	3249.533	-0.0568	-17	350	377	TLGWDQYPHEGGVILET HHMEHLGIVHK			Oxidation (M)[20]		Mascot
3389.696	3389.6851	-0.0109	-3	349	377	RTLGWDQYPHEGGVILE THHMEHLGIVHK					Mascot
3405.6909	3405.6726	-0.0183	-5	349	377	RTLGWDQYPHEGGVILE THHMEHLGIVHK			Oxidation (M)[21]		Mascot

10	PREDICTED: psbP domain-containing protein 2, chloroplastic-like isoform 2 [Fragaria vesca subsp. vesca]	gi|470103453	21018.6	9.26	7	45	0	2.904	

Peptide Information

Calc. Mass	Obsrv. Mass	± da	± ppm	Start Seq.	End Seq.	Sequence	Ion Score	C. I. %	Modification	Rank	Result Type
1144.4775	1144.5598	0.0823	72	174	181	CRGYWSCR			Carbamidomethyl (C)[1,7]		Mascot
1226.5735	1226.5923	0.0188	15	176	184	GYWSCREIR			Carbamidomethyl (C)[5]		Mascot
1857.8445	1857.9189	0.0744	40	1	15	MSLHICNCFALSNYK			Carbamidomethyl (C)[6,8]		Mascot
1873.8394	1873.9329	0.0935	50	1	15	MSLHICNCFALSNYK			Carbamidomethyl (C)[6,8], Oxidation (M)[1]		Mascot
1876.0327	1875.8892	-0.1435	-76	85	100	EGFTLLRPSSWIKVDK					Mascot
1876.0327	1875.8892	-0.1435	-76	85	100	EGFTLLRPSSWIKVDK					Mascot
2467.1428	2467.1868	0.044	18	1	20	MSLHICNCFALSNYKQAH TR			Carbamidomethyl (C)[6,8], Oxidation (M)[1]		Mascot
3277.8447	3277.5522	-0.2925	-89	50	79	SLAFSLVAPLLLNGVLPN LPIFAEELELER					Mascot
3433.9458	3433.7078	-0.238	-69	49	79	RSLAFSLVAPLLLNGVLP NLPIFAEELELER					Mascot

Rank	Protein Name	Accession No.	Protein MW	Protein PI	Pep. Count	Protein Score	Protein Score C. I. %	Intensity Matched	Total Ion Score	Total Ion C. I. %	Confirmed
1	hypothetical protein OsI_18213 [Oryza sativa Indica Group]	gi|218195985	17371.9	5.56	5	225	100	27.524	199	100	

Peptide Information

Calc. Mass	Obsrv. Mass	± da	± ppm	Start Seq.	End Seq.	Sequence	Ion Score	C. I. %	Modification	Rank	Result Type
944.5662	944.4809	-0.0853	-90	26	33	VLLTLEEK					Mascot
1490.8076	1490.7255	-0.0821	-55	103	114	IFSCFITFLKSK			Carbamidomethyl (C)[4]		Mascot
1607.9003	1607.8101	-0.0902	-56	123	136	ALLTELQALEEHLK					Mascot
1827.8444	1827.8322	-0.0122	-7	9	25	AAVGHPDTLGDCPFSQR			Carbamidomethyl (C)[12]		Mascot
1827.8444	1827.8322	-0.0122	-7	9	25	AAVGHPDTLGDCPFSQR	147	100	Carbamidomethyl (C)[12]		Mascot
1892.9641	1892.8965	-0.0676	-36	85	102	YPTPSLVTPPEYASVGSK					Mascot
1892.9641	1892.8965	-0.0676	-36	85	102	YPTPSLVTPPEYASVGSK	52	98.807			Mascot

Rank	Protein Name	Accession No.	Protein MW	Protein PI	Pep. Count	Protein Score	Protein Score C. I. %	Intensity Matched	Total Ion Score	Total Ion C. I. %	Confirmed
2	Os05g0116100 [Oryza sativa Japonica Group]	gi|113578021	23726.3	5.81	5	220	100	28.19	199	100	

Peptide Information

Calc. Mass	Obsrv. Mass	± da	± ppm	Start Seq.	End Seq.	Sequence	Ion Score	C. I. %	Modification	Rank	Result Type
944.5662	944.4809	-0.0853	-90	26	33	VLLTLEEK					Mascot
1497.8213	1497.7782	-0.0431	-29	158	169	LYHLQVALEHFK					Mascot
1607.9003	1607.8101	-0.0902	-56	123	136	ALLTELQALEEHLK					Mascot
1827.8444	1827.8322	-0.0122	-7	9	25	AAVGHPDTLGDCPFSQR			Carbamidomethyl (C)[12]		Mascot
1827.8444	1827.8322	-0.0122	-7	9	25	AAVGHPDTLGDCPFSQR	147	100	Carbamidomethyl (C)[12]		Mascot
1892.9641	1892.8965	-0.0676	-36	85	102	YPTPSLVTPPEYASVGSK					Mascot
1892.9641	1892.8965	-0.0676	-36	85	102	YPTPSLVTPPEYASVGSK	52	98.807			Mascot

Rank	Protein Name	Accession No.	Protein MW	Protein PI	Pep. Count	Protein Score	Protein Score C. I. %	Intensity Matched	Total Ion Score	Total Ion C. I. %	Confirmed
3	Glutathione S-transferase DHAR2 [Triticum urartu]	gi|474023258	45260	8.3	9	173	100	28.695	147	100	

Peptide Information

Calc. Mass	Obsrv. Mass	± da	± ppm	Start Seq.	End Seq.	Sequence	Ion Score	C. I. %	Modification	Rank	Result Type
909.4862	909.4286	-0.0576	-63	78	84	KVPYQMK			Oxidation (M)[6]		Mascot
943.4744	943.4584	-0.016	-17	297	304	ARSSFGYR					Mascot
944.5662	944.4809	-0.0853	-90	70	77	VLLTLEEK					Mascot

1202.682	1202.6294	-0.0526	-44	147	156	IFSTFVTFLK					Mascot
1497.8213	1497.7782	-0.0431	-29	202	213	LYHLQVALEHFK					Mascot
1574.781	1574.7878	0.0068	4	325	338	LSLGTFETVHEADR					Mascot
1607.8639	1607.8101	-0.0538	-33	167	180	ALVDELQALEEHLK					Mascot
1827.8444	1827.8322	-0.0122	-7	53	69	AAVGHPDTLGDCPFSQR			Carbamidomethyl (C)[12]		Mascot
1827.8444	1827.8322	-0.0122	-7	53	69	AAVGHPDTLGDCPFSQR	147	100	Carbamidomethyl (C)[12]		Mascot
2038.9242	2038.9896	0.0654	32	386	402	RQCCLLITEADELAMAE			Carbamidomethyl (C)[3,4], Oxidation (M)[15]		Mascot

4 hypothetical protein F775_52341 [Aegilops tauschii] gi|475525121 22836.3 6.63 1 81 99.19 3.971 81 99.998

Peptide Information

Calc. Mass	Obsrv. Mass	± da	± ppm	Start Seq.	End Seq.	Sequence	Ion Score	C. I. %	Modification	Rank	Result Type
1592.7228	1592.705	-0.0178	-11	148	160	ADFITYYEDAQTR					Mascot
1592.7228	1592.705	-0.0178	-11	148	160	ADFITYYEDAQTR	81	99.998			Mascot

5 hypothetical protein CHLNCDRAFT_144253 [Chlorella variabilis] gi|307108334 37491.6 8.66 10 68 85.603 13.096 29 0

Peptide Information

Calc. Mass	Obsrv. Mass	± da	± ppm	Start Seq.	End Seq.	Sequence	Ion Score	C. I. %	Modification	Rank	Result Type
856.5436	856.5048	-0.0388	-45	144	150	VLPRLMK					Mascot
904.4921	904.5524	0.0603	67	42	49	AVADLLMR			Oxidation (M)[7]		Mascot
909.4247	909.4286	0.0039	4	30	36	CNSLYPR			Carbamidomethyl (C)[1]		Mascot
928.4847	928.4445	-0.0402	-43	238	247	GDPVVGAGTR					Mascot
960.4745	960.4383	-0.0362	-38	304	312	DSELGNAVR					Mascot
960.4745	960.4383	-0.0362	-38	304	312	DSELGNAVR					Mascot
974.436	974.4619	0.0259	27	313	322	MPGSPAGDSR					Mascot
1130.5371	1130.5435	0.0064	6	313	323	MPGSPAGDSRR					Mascot
1130.5371	1130.5435	0.0064	6	313	323	MPGSPAGDSRR	29	0			Mascot
1152.5896	1152.5459	-0.0437	-38	134	143	VDYASDVQKK					Mascot
1505.7529	1505.6194	-0.1335	-89	30	41	CNSLYPRQDKPK			Carbamidomethyl (C)[1]		Mascot
1736.9054	1736.8196	-0.0858	-49	225	237	RPFKCRPLDAWYK			Carbamidomethyl (C)[5]		Mascot
1736.9054	1736.8196	-0.0858	-49	225	237	RPFKCRPLDAWYK			Carbamidomethyl (C)[5]		Mascot

6 unknown protein [Bathycoccus prasinos] gi|412985490 29822.9 6.43 12 62 41.348 3.578

Peptide Information

Calc. Mass	Obsrv. Mass	± da	± ppm	Start Seq.	End Seq.	Sequence	Ion Score	C. I. %	Modification	Rank	Result Type

837.4577	837.4003	-0.0574	-69	120	126	SAYVRNK					Mascot
1033.4983	1033.5018	0.0035	3	1	9	MDPLATTER					Mascot
1078.5375	1078.5182	-0.0193	-18	97	106	LENVTNSSSK					Mascot
1081.4718	1081.5464	0.0746	69	135	143	TLAEMEDEK			Oxidation (M)[5]		Mascot
1135.5413	1135.5653	0.024	21	228	236	LSEDVEKCR			Carbamidomethyl (C)[8]		Mascot
1438.7206	1438.7139	-0.0067	-5	107	119	MSSISEETKASLR					Mascot
1481.7053	1481.7222	0.0169	11	1	12	MDPLATTERFER			Oxidation (M)[1]		Mascot
1620.6732	1620.7412	0.068	42	175	189	DGREEEESEGVGGDR					Mascot
1884.955	1884.8516	-0.1034	-55	16	33	EALSFNSSLSSSSDKVVK					Mascot
1990.9353	1991.0398	0.1045	52	13	30	EFREALSFNSSLSSSSDK					Mascot
1990.9353	1991.0398	0.1045	52	13	30	EFREALSFNSSLSSSSDK					Mascot
1995.8455	1995.965	0.1195	60	57	72	DDEHYPFDSDEKELEK					Mascot
2070.9861	2070.9636	-0.0225	-11	97	115	LENVTNSSSKMSSISEET K					Mascot

7 Os03g0119700, partial [Oryza sativa Japonica Group] gi|113547252 47925.7 9.38 15 61 29.485 28.749

Peptide Information

Calc. Mass	Obsrv. Mass	± da	± ppm	Start Seq.	End Seq.	Sequence	Ion Score	C. I. %	Modification	Rank	Result Type
870.5156	870.5151	-0.0005	-1	153	159	RVPLTER					Mascot
928.3862	928.4445	0.0583	63	145	152	AMESMSTR			Oxidation (M)[2]		Mascot
1068.4924	1068.4933	0.0009	1	145	153	AMESMSTRR					Mascot
1081.5095	1081.5464	0.0369	34	242	249	EVFDEMRR					Mascot
1152.5969	1152.5459	-0.051	-44	315	323	ILDQMFLEK			Oxidation (M)[5]		Mascot
1323.6515	1323.6266	-0.0249	-19	5	15	VHSPLSFSMFR			Oxidation (M)[9]		Mascot
1644.7469	1644.7634	0.0165	10	344	357	IDDACRYVSAMSTR			Carbamidomethyl (C)[5]		Mascot
1782.7865	1782.8169	0.0304	17	234	248	TGDFHGAREVFDEMR			Oxidation (M)[14]		Mascot
1809.8888	1809.8578	-0.031	-17	1	15	SCVRVHSPLSFSMFR			Carbamidomethyl (C)[2]		Mascot
1825.8837	1825.8855	0.0018	1	1	15	SCVRVHSPLSFSMFR			Carbamidomethyl (C)[2], Oxidation (M)[13]		Mascot
1827.8802	1827.8322	-0.048	-26	309	323	MDSAMRILDQMFLEK					Mascot
1827.8802	1827.8322	-0.048	-26	309	323	MDSAMRILDQMFLEK					Mascot
1843.8751	1843.7957	-0.0794	-43	309	323	MDSAMRILDQMFLEK			Oxidation (M)[1]		Mascot
1849.8531	1849.7875	-0.0656	-35	269	284	NGRVAEACELVEAMER			Carbamidomethyl (C)[8], Oxidation (M)[14]		Mascot
1885.9978	1885.8545	-0.1433	-76	47	67	TSSVASAAAPAAAAVSDA IVR					Mascot
1908.9922	1908.8068	-0.1854	-97	376	392	AGMIVDAGRILYELMEK					Mascot
2037.0852	2037.0267	-0.0585	-29	5	21	VHSPLSFSMFRLPPLHR			Oxidation (M)[9]		Mascot

2080.0378	2080.0234	-0.0144	-7	200	219	ALASIGEFDAAMSVIQETAR					Mascot

8	hypothetical protein CHLNCDRAFT_143537 [Chlorella variabilis]	gi|307108722	71539.4	5.17	15	61	19.038	8.582

Peptide Information

Calc. Mass	Obsrv. Mass	± da	± ppm	Start Seq.	End Seq.	Sequence	Ion Score	C. I. %	Modification	Rank	Result Type
928.521	928.4445	-0.0765	-82	442	450	LEAQLAGAR					Mascot
960.5043	960.4383	-0.066	-69	434	441	NMALERAR					Mascot
960.5043	960.4383	-0.066	-69	434	441	NMALERAR					Mascot
976.4992	976.4539	-0.0453	-46	434	441	NMALERAR			Oxidation (M)[2]		Mascot
999.4676	999.4207	-0.0469	-47	481	489	GHNMADLNK					Mascot
1240.7008	1240.6077	-0.0931	-75	397	407	TVTETHGRLVK					Mascot
1490.6758	1490.7255	0.0497	33	408	420	ANTYTYTAEDVSR					Mascot
1505.7377	1505.6194	-0.1183	-79	298	311	VVAAEQGSDMSQKR					Mascot
1620.8527	1620.7412	-0.1115	-69	311	323	RYVLCQVVAVEER			Carbamidomethyl (C)[5]		Mascot
1629.802	1629.8071	0.0051	3	337	350	SPYPFGPNQEKTHK					Mascot
1811.0439	1810.8842	-0.1597	-88	611	627	KLLGPTWRPAPPPPGAR					Mascot
1838.845	1838.9059	0.0609	33	491	506	NADMNFKNALDNVSNR			Oxidation (M)[4]		Mascot
1885.9688	1885.8545	-0.1143	-61	458	476	VAELEAQMAQLEAGLAGGK					Mascot
1989.9135	1989.9581	0.0446	22	93	108	LEIDEEDLIEDEEDRK					Mascot
1990.9829	1991.0398	0.0569	29	507	526	VGAAAGVQDATTSQLDPFSR					Mascot
1990.9829	1991.0398	0.0569	29	507	526	VGAAAGVQDATTSQLDPFSR	6	0			Mascot
1995.7898	1995.965	0.1752	88	224	242	EGSEEGALDSEDDLSGDDR					Mascot
2717.2935	2717.0793	-0.2142	-79	451	476	ENGEEEKVAELEAQMAQLEAGLAGGK			Oxidation (M)[15]		Mascot

9	hypothetical protein OsI_15784 [Oryza sativa Indica Group]	gi|218194807	21158.7	5.95	10	60	7.044	7.866

Peptide Information

Calc. Mass	Obsrv. Mass	± da	± ppm	Start Seq.	End Seq.	Sequence	Ion Score	C. I. %	Modification	Rank	Result Type
952.5172	952.4574	-0.0598	-63	169	176	VEFTVVMK					Mascot
968.5121	968.4633	-0.0488	-50	169	176	VEFTVVMK			Oxidation (M)[7]		Mascot
999.504	999.4207	-0.0833	-83	14	21	LHGEIMQR			Oxidation (M)[6]		Mascot
1034.5564	1034.4974	-0.059	-57	121	129	MIPFSAGRR					Mascot
1130.5623	1130.5435	-0.0188	-17	4	13	VMDNPSIQAR					Mascot
1130.5623	1130.5435	-0.0188	-17	4	13	VMDNPSIQAR					Mascot

1885.9446	1885.8545	-0.0901	-48	130	147	ICPGVGMGTAHIALMVAR			Carbamidomethyl (C)[2], Oxidation (M)[7,15]		Mascot
1908.9603	1908.8068	-0.1535	-80	50	67	HPPTYFALSHAAVEPGSK					Mascot
1996.0209	1995.965	-0.0559	-28	32	48	DTDGMPYLQAFVKELLR					Mascot
2037.0553	2037.0267	-0.0286	-14	49	67	KHPPTYFALSHAAVEPGS K					Mascot
2080.0354	2080.0234	-0.012	-6	14	31	LHGEIMQRVGDARPVDD R			Oxidation (M)[6]		Mascot
2119.1768	2118.9736	-0.2032	-96	169	186	VEFTVVMKRPLLAMVTP R			Oxidation (M)[7,14]		Mascot

10 PRENDICTED: uncharacterized protein LOC101488930 [Cicer arietinum] gi|502160126 50054.9 6.53 14 58 0 3.135

Peptide Information

Calc. Mass	Obsrv. Mass	± da	± ppm	Start Seq.	End Seq.	Sequence	Ion Score	C. I. %	Modification	Rank	Result Type
968.5047	968.4633	-0.0414	-43	62	70	KAYISSDGK					Mascot
1102.6401	1102.5439	-0.0962	-87	13	21	LLIRMSTPR			Oxidation (M)[5]		Mascot
1202.674	1202.6294	-0.0446	-37	386	396	QKIGSSNLEVK					Mascot
1270.7002	1270.6686	-0.0316	-25	112	123	GLPVLELTGTDR					Mascot
1302.7198	1302.6364	-0.0834	-64	307	317	VIQCLKASVER			Carbamidomethyl (C)[4]		Mascot
1320.63	1320.576	-0.054	-41	208	217	RLHQMMFADR			Oxidation (M)[5]		Mascot
1487.7449	1487.7212	-0.0237	-16	294	306	DGTLISSEPERQR					Mascot
1505.7628	1505.6194	-0.1434	-95	329	341	TEDKQGLLAEVMR			Oxidation (M)[12]		Mascot
1588.8177	1588.7958	-0.0219	-14	319	332	ASEGIQLELRTEDK					Mascot
1809.8284	1809.8578	0.0294	16	161	176	DCNSGTTIEDSQKINK			Carbamidomethyl (C)[2]		Mascot
1849.9331	1849.7875	-0.1456	-79	2	16	EWPASTDEYEKLLIR					Mascot
1908.9451	1908.8068	-0.1383	-72	227	242	FTSDNTIVTVQNWVER					Mascot
1980.9735	1980.9403	-0.0332	-17	1	16	MEWPASTDEYEKLLIR					Mascot
2340.2307	2340.272	0.0413	18	406	428	AESQDQAIGVGGAVLWSI GNLVR					Mascot

Gel Idx/Pos	305/M7	Instr./Gel Origin	BA2151/Sample Project 20140814	Process Status	Analysis Succeeded
Plate [#] Name	[1] Sample Project 20140814	Instrument Sample Name		Spectra	11

Rank	Protein Name	Accession No.	Protein MW	Protein PI	Pep. Count	Protein Score	Protein Score C. I. %	Intensity Matched	Total Ion Score	Total Ion C. I. %	Confirmed
1	Glutathione S-transferase DHAR2 [Triticum urartu]	gi|474023258	45260	8.3	11	425	100	49.034	382	100	

Peptide Information

Calc. Mass	Obsrv. Mass	± da	± ppm	Start Seq.	End Seq.	Sequence	Ion Score	C. I. %	Modification	Rank	Result Type
909.4862	909.4279	-0.0583	-64	78	84	KVPYQMK			Oxidation (M)[6]		Mascot
944.5662	944.5034	-0.0628	-66	70	77	VLLTLEEK					Mascot
1202.682	1202.6251	-0.0569	-47	147	156	IFSTFVTFLK					Mascot
1497.8213	1497.7751	-0.0462	-31	202	213	LYHLQVALEHFK					Mascot
1497.8213	1497.7751	-0.0462	-31	202	213	LYHLQVALEHFK	82	99.999			Mascot
1607.8639	1607.8123	-0.0516	-32	167	180	ALVDELQALEEHLK					Mascot
1645.8881	1645.7777	-0.1104	-67	21	34	EHASPQIDRARPLR					Mascot
1722.8381	1722.7915	-0.0466	-27	347	360	LFRTSLHMNFDDAR					Mascot
1827.8444	1827.8257	-0.0187	-10	53	69	AAVGHPDTLGDCPFSQR			Carbamidomethyl (C)[12]		Mascot
1827.8444	1827.8257	-0.0187	-10	53	69	AAVGHPDTLGDCPFSQR	148	100	Carbamidomethyl (C)[12]		Mascot
1866.8282	1866.8588	0.0306	16	387	402	QCCLLITEADELAMAE			Carbamidomethyl (C)[2,3]		Mascot
2021.0338	2021.0204	-0.0134	-7	217	234	VPETLTSVHAYTEALFSR					Mascot
2021.0338	2021.0204	-0.0134	-7	217	234	VPETLTSVHAYTEALFSR	151	100			Mascot
2108.1135	2108.0623	-0.0512	-24	181	201	AHGPYINGANISAVDLSL APK					Mascot

Rank	Protein Name	Accession No.	Protein MW	Protein PI	Pep. Count	Protein Score	Protein Score C. I. %	Intensity Matched	Total Ion Score	Total Ion C. I. %	Confirmed
2	Os05g0116100 [Oryza sativa Japonica Group]	gi|113578021	23726.3	5.81	5	251	100	28.393	230	100	

Peptide Information

Calc. Mass	Obsrv. Mass	± da	± ppm	Start Seq.	End Seq.	Sequence	Ion Score	C. I. %	Modification	Rank	Result Type
944.5662	944.5034	-0.0628	-66	26	33	VLLTLEEK					Mascot
1497.8213	1497.7751	-0.0462	-31	158	169	LYHLQVALEHFK					Mascot
1497.8213	1497.7751	-0.0462	-31	158	169	LYHLQVALEHFK	82	99.999			Mascot
1607.9003	1607.8123	-0.088	-55	123	136	ALLTELQALEEHLK					Mascot
1827.8444	1827.8257	-0.0187	-10	9	25	AAVGHPDTLGDCPFSQR			Carbamidomethyl (C)[12]		Mascot
1827.8444	1827.8257	-0.0187	-10	9	25	AAVGHPDTLGDCPFSQR	148	100	Carbamidomethyl (C)[12]		Mascot
1892.9641	1892.8944	-0.0697	-37	85	102	YPTPSLVTPPEYASVGSK					Mascot

Rank	Protein Name	Accession No.	Protein MW	Protein PI	Pep. Count	Protein Score	Protein Score C. I. %	Intensity Matched	Total Ion Score	Total Ion C. I. %	Confirmed
3	hypothetical protein OsI_18213 [Oryza sativa Indica	gi|218195985	17371.9	5.56	4	168	100	26.926	148	100	

Group]

Peptide Information

Calc. Mass	Obsrv. Mass	± da	± ppm	Start Seq.	End Seq.	Sequence	Ion Score	C. I. %	Modification	Rank	Result Type
944.5662	944.5034	-0.0628	-66	26	33	VLLTLEEK					Mascot
1607.9003	1607.8123	-0.088	-55	123	136	ALLTELQALEEHLK					Mascot
1827.8444	1827.8257	-0.0187	-10	9	25	AAVGHPDTLGDCPFSQR			Carbamidomethyl (C)[12]		Mascot
1827.8444	1827.8257	-0.0187	-10	9	25	AAVGHPDTLGDCPFSQR	148	100	Carbamidomethyl (C)[12]		Mascot
1892.9641	1892.8944	-0.0697	-37	85	102	YPTPSLVTPPEYASVGSK					Mascot

4	PREDICTED: probable methyltransferase PMT26-like isoform X1 [Cicer arietinum]	gi|502139819	93030.2	5	20	70	91.325	9.284

Protein Group

PREDICTED: probable methyltransferase PMT26-like isoform X2 [Cicer arietinum]	gi|502139821	93030.2	5

Peptide Information

Calc. Mass	Obsrv. Mass	± da	± ppm	Start Seq.	End Seq.	Sequence	Ion Score	C. I. %	Modification	Rank	Result Type
837.4577	837.4064	-0.0513	-61	2	8	AQAKYTR					Mascot
846.4316	846.407	-0.0246	-29	599	606	APVSSTER					Mascot
870.5407	870.5077	-0.033	-38	496	502	LLLELNR					Mascot
909.4788	909.4279	-0.0509	-56	6	12	YTRIDNK					Mascot
968.4982	968.4636	-0.0346	-36	1	8	MAQAKYTR					Mascot
1078.5602	1078.5234	-0.0368	-34	434	443	DVLAMSFAPK					Mascot
1135.5114	1135.4432	-0.0682	-60	115	124	SDEKSPEDTK					Mascot
1167.5125	1167.5629	0.0504	43	188	198	NSSGSDESEKK					Mascot
1195.5186	1195.5596	0.041	34	187	197	RNSSGSDESEK					Mascot
1195.5186	1195.5596	0.041	34	187	197	RNSSGSDESEK					Mascot
1300.7406	1300.6162	-0.1244	-96	457	469	GIPAISAVMGTKR					Mascot
1606.8336	1606.8026	-0.031	-19	655	668	SYLNGLGIQWSNVR					Mascot
1606.8336	1606.8026	-0.031	-19	655	668	SYLNGLGIQWSNVR					Mascot
1786.9486	1786.8242	-0.1244	-70	728	742	SYDLLHADHLFSKLK					Mascot
1815.7263	1815.8209	0.0946	52	173	187	YESNDNEQSDSDEKR					Mascot
1822.8351	1822.9509	0.1158	64	789	803	MTYSKDTEGFLCVQK			Carbamidomethyl (C)[12], Oxidation (M)[1]		Mascot
1884.8796	1884.8418	-0.0378	-20	804	819	SMWRPKESETVDYAIG			Oxidation (M)[2]		Mascot

1928.0601	1927.9122	-0.1479	-77	747	763	FEAVVAEVDRILRPEGK					Mascot
2020.0975	2019.9525	-0.145	-72	651	668	VVSKSYLNGLGIQWSNV R					Mascot
2043.0369	2042.972	-0.0649	-32	415	433	VILDVGCGVASFGGFLFE R			Carbamidomethyl (C)[7]		Mascot
2048.978	2049.0408	0.0628	31	290	307	VCNVTAGPDYIPCLDNLK			Carbamidomethyl (C)[2,13]		Mascot
2234.9966	2235.1409	0.1443	65	710	727	GLFGIYHDWCESFSTYPR			Carbamidomethyl (C)[10]		Mascot

5 PREDICTED: uncharacterized protein LOC101222929 [Cucumis sativus] gi|449438703 118575.8 8.26 22 67 80.126 27.258

Peptide Information

Calc. Mass	Obsrv. Mass	± da	± ppm	Start Seq.	End Seq.	Sequence	Ion Score	C. I. %	Modification	Rank	Result Type
944.4546	944.5034	0.0488	52	579	586	VMGAYFEK					Mascot
960.4495	960.441	-0.0085	-9	579	586	VMGAYFEK			Oxidation (M)[2]		Mascot
1078.4912	1078.5234	0.0322	30	438	447	HHSSAGPEEK					Mascot
1629.8595	1629.8051	-0.0544	-33	950	964	SPKPGSFELQLEAAR					Mascot
1644.844	1644.7655	-0.0785	-48	457	471	QSDLLLTSKPGDENK					Mascot
1693.7235	1693.7559	0.0324	19	311	325	SSTAGFVHDEDMNQR					Mascot
1706.8816	1706.7218	-0.1598	-94	1	15	MKAMATLQDLIQEAK			Oxidation (M)[1]		Mascot
1722.8765	1722.7915	-0.085	-49	1	15	MKAMATLQDLIQEAK			Oxidation (M)[1,4]		Mascot
1765.8677	1765.9045	0.0368	21	103	118	IDSPAVEAAMKDFIDK			Oxidation (M)[10]		Mascot
1768.9341	1768.8822	-0.0519	-29	169	183	DVVDLVGDHLDLFRR					Mascot
1770.9861	1770.8192	-0.1669	-94	1028	1043	NLILEIHGKSHVSQPV					Mascot
1783.9008	1783.8257	-0.0751	-42	415	430	NYGTSIMQPATKTTVR			Oxidation (M)[7]		Mascot
1785.9606	1785.843	-0.1176	-66	950	965	SPKPGSFELQLEAARR					Mascot
1826.8848	1826.8519	-0.0329	-18	122	136	DFVVDLWYSEITPDK					Mascot
1834.8715	1834.8837	0.0122	7	376	390	TEVLMPENLENMWTK					Mascot
1866.8612	1866.8588	-0.0024	-1	376	390	TEVLMPENLENMWTK			Oxidation (M)[5,12]		Mascot
1892.9019	1892.8944	-0.0075	-4	184	201	NQAAIGVDVMGTLSSEE R			Oxidation (M)[10]		Mascot
1914.8135	1914.9091	0.0956	50	723	740	VVGSTSPDEACASSNYD R			Carbamidomethyl (C)[11]		Mascot
1946.0602	1945.9729	-0.0873	-45	38	54	SMWMNVPLAILLVSALR			Oxidation (M)[2,4]		Mascot
1990.9725	1990.9972	0.0247	12	375	390	RTEVLMPENLENMWTK					Mascot
2020.8627	2021.0204	0.1577	78	345	360	EISSDYMFQDEPLQMR			Oxidation (M)[7,15]		Mascot
2020.8627	2021.0204	0.1577	78	345	360	EISSDYMFQDEPLQMR			Oxidation (M)[7,15]		Mascot
2042.9084	2042.972	0.0636	31	723	741	VVGSTSPDEACASSNYD RK			Carbamidomethyl (C)[11]		Mascot
2049.0029	2049.0408	0.0379	18	183	201	RNQAAIGVDVMGTLSSE ER			Oxidation (M)[11]		Mascot
2131.1487	2131.0413	-0.1074	-50	913	929	WVQDVLWPNGIFFIQLR					Mascot

Calc. Mass	Obsrv. Mass	± da	± ppm	Start Seq.	End Seq.	Sequence	Ion Score	C. I. %	Modification	Rank	Result Type
2252.1743	2252.085	-0.0893	-40	137	156	EFPEQIHALIMDALGEIAVR					Mascot
2252.1743	2252.085	-0.0893	-40	137	156	EFPEQIHALIMDALGEIAVR					Mascot

6	Actin-binding FH2 protein isoform 4 [Theobroma cacao]	gi|508774688	116747.8	6.76	21	65	69.219	30.069

Peptide Information

Calc. Mass	Obsrv. Mass	± da	± ppm	Start Seq.	End Seq.	Sequence	Ion Score	C. I. %	Modification	Rank	Result Type
909.387	909.4279	0.0409	45	497	503	LEEMEDK			Oxidation (M)[4]		Mascot
944.5345	944.5034	-0.0311	-33	306	313	ANILMLNR					Mascot
960.4897	960.441	-0.0487	-51	400	406	EQKDVWR					Mascot
1008.4666	1008.4695	0.0029	3	452	460	MDSDLNAVK			Oxidation (M)[1]		Mascot
1167.6593	1167.5629	-0.0964	-83	1020	1031	GLSRPAAATAPR					Mascot
1168.6473	1168.5311	-0.1162	-99	924	935	SGAPPPPPPPVR					Mascot
1347.6937	1347.6158	-0.0779	-58	449	460	LNKMDSDLNAVK					Mascot
1578.7086	1578.7942	0.0856	54	1058	1069	HGEPQMYFIFFY					Mascot
1589.8105	1589.8545	0.044	28	221	234	GCTPVIRVYGQDPK			Carbamidomethyl (C)[2]		Mascot
1606.8489	1606.8026	-0.0463	-29	877	891	HGVAPPPPPPPPQYR					Mascot
1606.8489	1606.8026	-0.0463	-29	877	891	HGVAPPPPPPPPQYR					Mascot
1768.9493	1768.8822	-0.0671	-38	967	986	GAPPPPVGGAPPPPPPPGGR					Mascot
1781.9408	1781.8245	-0.1163	-65	727	743	MSLAAPPPPPPPPPPYR					Mascot
1785.9606	1785.843	-0.1176	-66	1043	1057	VTRAIQGSLWEELQR					Mascot
1893.0455	1892.8944	-0.1511	-80	130	146	GGWPVLAFMLAGLLLYR			Oxidation (M)[9]		Mascot
1931.0308	1930.8986	-0.1322	-68	332	348	TEVLFMDPDAVVPRLTK					Mascot
1946.1069	1945.9729	-0.134	-69	166	182	ELLQLLSPLNPQPSQLR					Mascot
2021.1405	2021.0204	-0.1201	-59	130	147	GGWPVLAFMLAGLLLYRK			Oxidation (M)[9]		Mascot
2021.1405	2021.0204	-0.1201	-59	130	147	GGWPVLAFMLAGLLLYRK			Oxidation (M)[9]		Mascot
2049.0886	2049.0408	-0.0478	-23	297	313	VMFHTAFVRANILMLNR			Oxidation (M)[2]		Mascot
2146.8618	2147.0247	0.1629	76	407	425	EDVDPPTFQDCASDDGNHK			Carbamidomethyl (C)[11]		Mascot
2252.1646	2252.085	-0.0796	-35	987	1010	APCPPPPPGTPGGAPPPPPPLGAR			Carbamidomethyl (C)[3]		Mascot
2252.1646	2252.085	-0.0796	-35	987	1010	APCPPPPPGTPGGAPPPPPPLGAR			Carbamidomethyl (C)[3]		Mascot
2615.3286	2615.1821	-0.1465	-56	190	211	RNLSSDWPPSDTPLFLDCLILR			Carbamidomethyl (C)[18]		Mascot

7	hypothetical protein ZEAMMB73_804372 [Zea mays]	gi|413936704	18391.8	9.06	10	64	62.131	2.765

Protein Group

hypothetical protein [Zea mays]	gi|293332647	18391.8	9.0600

Peptide Information

Calc. Mass	Obsrv. Mass	± da	± ppm	Start Seq.	End Seq.	Sequence	Ion Score	C. I. %	Modification	Rank	Result Type
979.522	979.4794	-0.0426	-43	102	108	WPDHRLR					Mascot
1152.5038	1152.5452	0.0414	36	1	9	MAARWDCSR			Carbamidomethyl (C)[7]		Mascot
1168.4987	1168.5311	0.0324	28	1	9	MAARWDCSR			Carbamidomethyl (C)[7], Oxidation (M)[1]		Mascot
1195.608	1195.5596	-0.0484	-40	126	138	AAGHGVPAGHHGK					Mascot
1195.608	1195.5596	-0.0484	-40	126	138	AAGHGVPAGHHGK					Mascot
1519.6144	1519.7643	0.1499	99	148	162	DEGERGEEEEAVGGG					Mascot
1572.7877	1572.736	-0.0517	-33	79	91	ESSQPWITQGQRR					Mascot
1677.7902	1677.8026	0.0124	7	110	124	SCTHGAAYLELAEQK			Carbamidomethyl (C)[2]		Mascot
1694.7439	1694.7759	0.032	19	23	38	TGSHMGEGAYVQEANK			Oxidation (M)[5]		Mascot
1833.8912	1833.8317	-0.0595	-32	110	125	SCTHGAAYLELAEQKR			Carbamidomethyl (C)[2]		Mascot
1834.8501	1834.8837	0.0336	18	22	38	RTGSHMGEGAYVQEAN K					Mascot
1990.9175	1990.9972	0.0797	40	39	55	EPRPWGELTCSSSDLEK			Carbamidomethyl (C)[10]		Mascot

8 Actin-binding FH2 protein isoform 2 [Theobroma cacao] gi|508774686 154634.6 7.23 24 63 50.079 30.645

Peptide Information

Calc. Mass	Obsrv. Mass	± da	± ppm	Start Seq.	End Seq.	Sequence	Ion Score	C. I. %	Modification	Rank	Result Type
909.387	909.4279	0.0409	45	497	503	LEEMEDK			Oxidation (M)[4]		Mascot
944.5345	944.5034	-0.0311	-33	306	313	ANILMLNR					Mascot
960.4897	960.441	-0.0487	-51	400	406	EQKDVWR					Mascot
1008.4666	1008.4695	0.0029	3	452	460	MDSDLNAVK			Oxidation (M)[1]		Mascot
1167.6593	1167.5629	-0.0964	-83	1020	1031	GLSRPAAATAPR					Mascot
1168.6473	1168.5311	-0.1162	-99	924	935	SGAPPPPPPPVR					Mascot
1228.5524	1228.5286	-0.0238	-19	1272	1280	MTLMHYLCK			Carbamidomethyl (C)[8], Oxidation (M)[1,4]		Mascot
1347.6937	1347.6158	-0.0779	-58	449	460	LNKMDSDLNAVK					Mascot
1589.8105	1589.8545	0.044	28	221	234	GCTPVIRVYGQDPK			Carbamidomethyl (C)[2]		Mascot
1592.7698	1592.7111	-0.0587	-37	1210	1223	KSLNTVNSACDEVR			Carbamidomethyl (C)[10]		Mascot
1606.8489	1606.8026	-0.0463	-29	877	891	HGVAPPPPPPPPQYR					Mascot
1606.8489	1606.8026	-0.0463	-29	877	891	HGVAPPPPPPPPQYR					Mascot
1622.7809	1622.7886	0.0077	5	1369	1383	NADALALYFGEDPAR					Mascot

Calc. Mass	Obsrv. Mass	± da	± ppm	Start Seq.	End Seq.	Sequence	Ion Score	C. I. %	Modification	Rank	Result Type
1694.879	1694.7759	-0.1031	-61	1272	1285	MTLMHYLCKVLAAK			Carbamidomethyl (C)[8], Oxidation (M)[1]		Mascot
1768.9493	1768.8822	-0.0671	-38	967	986	GAPPPPVGGAPPPPPPP GGR					Mascot
1781.9408	1781.8245	-0.1163	-65	727	743	MSLAAPPPPPPPPPPYR					Mascot
1785.9606	1785.843	-0.1176	-66	1043	1057	VTRAIQGSLWEELQR					Mascot
1893.0455	1892.8944	-0.1511	-80	130	146	GGWPVLAFMLAGLLLYR			Oxidation (M)[9]		Mascot
1931.0308	1930.8986	-0.1322	-68	332	348	TEVLFMDPDAVVPRLTK					Mascot
1946.1069	1945.9729	-0.134	-69	166	182	ELLQLLSPLNPQPSQLR					Mascot
2021.1405	2021.0204	-0.1201	-59	130	147	GGWPVLAFMLAGLLLYR K			Oxidation (M)[9]		Mascot
2021.1405	2021.0204	-0.1201	-59	130	147	GGWPVLAFMLAGLLLYR K			Oxidation (M)[9]		Mascot
2049.0886	2049.0408	-0.0478	-23	297	313	VMFHTAFVRANILMLNR			Oxidation (M)[2]		Mascot
2146.8618	2147.0247	0.1629	76	407	425	EDVDPPTFQDCASDDGN HK			Carbamidomethyl (C)[11]		Mascot
2252.1646	2252.085	-0.0796	-35	987	1010	APCPPPPPGTPGGAPPP PPPLGAR			Carbamidomethyl (C)[3]		Mascot
2252.1646	2252.085	-0.0796	-35	987	1010	APCPPPPPGTPGGAPPP PPPLGAR			Carbamidomethyl (C)[3]		Mascot
2615.3286	2615.1821	-0.1465	-56	190	211	RNLSSDWPPSDTPLFLD CLILR			Carbamidomethyl (C)[18]		Mascot

9	J-domain protein required for chloroplast accumulation response 1, putative isoform 1 [Theobroma cacao]	gi|508722801	80607.1	8.69	18	62	42.684	34.684	

Protein Group

J-domain protein required for chloroplast accumulation response 1, putative isoform 1 [Theobroma cacao]	gi|508722802	80607.1	8.6899 995803 833

Peptide Information

Calc. Mass	Obsrv. Mass	± da	± ppm	Start Seq.	End Seq.	Sequence	Ion Score	C. I. %	Modification	Rank	Result Type
960.5109	960.441	-0.0699	-73	381	389	NVSGDTVLR					Mascot
968.473	968.4636	-0.0094	-10	1	7	MQRFSQR			Oxidation (M)[1]		Mascot
1034.4758	1034.498	0.0222	21	556	563	IHMTNMQK			Oxidation (M)[3,6]		Mascot
1130.5809	1130.5438	-0.0371	-33	556	564	IHMTNMQKK					Mascot
1130.5809	1130.5438	-0.0371	-33	556	564	IHMTNMQKK					Mascot
1135.5338	1135.4432	-0.0906	-80	113	123	GNESSSSSPRK					Mascot
1202.6991	1202.6251	-0.074	-62	456	466	LSEILDGKSIK					Mascot
1481.6965	1481.7145	0.018	12	229	242	ELSTGSEESTNLSK					Mascot
1572.8203	1572.736	-0.0843	-54	681	693	SYQKALLCLHPDK			Carbamidomethyl (C)[8]		Mascot
1606.9528	1606.8026	-0.1502	-93	666	680	TVPLVDIIEGPAVKR					Mascot
1606.9528	1606.8026	-0.1502	-93	666	680	TVPLVDIIEGPAVKR					Mascot

1607.7483	1607.8123	0.064	40	484	498	TSVKGSPMNSWDNGK					Mascot
1622.7446	1622.7886	0.044	27	33	47	NSDIDFSDVFGGPPR					Mascot
1634.7981	1634.7471	-0.051	-31	381	396	NVSGDTVLRSSGGEEK					Mascot
1785.8589	1785.843	-0.0159	-9	167	182	GMDLPTFGSPTRSTYR					Mascot
1930.885	1930.8986	0.0136	7	340	357	NENGSLIDSRNGDAEPS R					Mascot
1945.9363	1945.9729	0.0366	19	332	349	SFGVEHGKNENGSLIDS R					Mascot
2019.9519	2019.9525	0.0006	0	29	47	SPGRNSDIDFSDVFGGP PR					Mascot
2020.9781	2021.0204	0.0423	21	566	585	SSSDIPTNGASEKNVNSS VK					Mascot
2020.9781	2021.0204	0.0423	21	566	585	SSSDIPTNGASEKNVNSS VK					Mascot
2049.0076	2049.0408	0.0332	16	710	727	VFDILQDAWTHFNSLGSV					Mascot

10	RecName: Full=DNA polymerase epsilon catalytic subunit A; AltName: Full=DNA polymerase 2 a; Short=AtPOL2a; AltName: Full=DNA polymerase II subunit a; AltName: Full=Protein ABA OVERLY SENSITIVE a; AltName: Full=Protein EARLY IN SHORT DAYS 7; AltName:	gi|426019656	251873.9	6.19	33	62	38.584	12.985

Protein Group

DNA polymerase epsilon catalytic subunit A [Arabidopsis thaliana]	gi|334182383	251873.9	6.1900 000572 2046
DNA polymerase epsilon catalytic subunit A [Arabidopsis thaliana]	gi|332190145	251873.9	6.1900 000572 2046

Peptide Information

Calc. Mass	Obsrv. Mass	± da	± ppm	Start Seq.	End Seq.	Sequence	Ion Score	C. I. %	Modification	Rank	Result Type
837.4135	837.4064	-0.0071	-8	1022	1029	SCAVTTAK			Carbamidomethyl (C)[2]		Mascot
846.4753	846.407	-0.0683	-81	910	917	AMIIPASK			Oxidation (M)[2]		Mascot
870.4719	870.5077	0.0358	41	1331	1337	VFYINSK					Mascot
960.4608	960.441	-0.0198	-21	698	704	YCQKAYK			Carbamidomethyl (C)[2]		Mascot
1129.5269	1129.5466	0.0197	17	2133	2141	MEIFMDIAK			Oxidation (M)[1,5]		Mascot
1152.5354	1152.5452	0.0098	9	1434	1442	TTTECHYLK			Carbamidomethyl (C)[5]		Mascot
1195.5994	1195.5596	-0.0398	-33	1338	1347	VPIDEYFQGK					Mascot
1195.5994	1195.5596	-0.0398	-33	1338	1347	VPIDEYFQGK					Mascot

1300.5726	1300.6162	0.0436	34	562	572	MDSISNYDEVK		Mascot
1438.7358	1438.6042	-0.1316	-91	98	108	MELELEAYLRR	Oxidation (M)[1]	Mascot
1519.7573	1519.7643	0.007	5	1556	1568	EGIKALDDFPCVR	Carbamidomethyl (C)[11]	Mascot
1587.7141	1587.7635	0.0494	31	560	572	MRMDSISNYDEVK		Mascot
1603.7091	1603.7729	0.0638	40	560	572	MRMDSISNYDEVK	Oxidation (M)[1]	Mascot
1607.8826	1607.8123	-0.0703	-44	1939	1952	EQIGSTFINMLVKK		Mascot
1628.9292	1628.8	-0.1292	-79	910	924	AMIIPASKEEGILIK	Oxidation (M)[2]	Mascot
1629.802	1629.8051	0.0031	2	723	735	ENPFYVDTVRSFR		Mascot
1634.8822	1634.7471	-0.1351	-83	243	256	VCAFDIETTKLPLK	Carbamidomethyl (C)[2]	Mascot
1693.8942	1693.7559	-0.1383	-82	141	154	ISFDTVQQLMEVKR		Mascot
1735.8466	1735.8351	-0.0115	-7	2075	2088	EQMESSLLEMVRQR		Mascot
1736.803	1736.8229	0.0199	11	2089	2101	ERMYHMQDVVCIR	Carbamidomethyl (C)[11]	Mascot
1736.803	1736.8229	0.0199	11	2089	2101	ERMYHMQDVVCIR	Carbamidomethyl (C)[11]	Mascot
1768.7928	1768.8822	0.0894	51	2089	2101	ERMYHMQDVVCIR	Carbamidomethyl (C)[11], Oxidation (M)[3,6]	Mascot
1783.865	1783.8257	-0.0393	-22	45	59	LGWLLTFSSSSWEDR		Mascot
1785.8212	1785.843	0.0218	12	562	576	MDSISNYDEVKDEIK		Mascot
1809.8186	1809.8734	0.0548	30	799	814	WYSMEMAGVVTYTGAK	Oxidation (M)[4]	Mascot
1817.8483	1817.8241	-0.0242	-13	469	484	GSGTLCEMLLMVEAYK	Carbamidomethyl (C)[6], Oxidation (M)[8]	Mascot
1822.9442	1822.9509	0.0067	4	859	874	LTISYPCVMLNVDVAK	Carbamidomethyl (C)[7]	Mascot
1825.8636	1825.8992	0.0356	19	661	677	QIESEFVDAGANIMSSK		Mascot
1826.976	1826.8519	-0.1241	-68	1443	1458	RSIPLVYLYNSTSTGR		Mascot
1833.8431	1833.8317	-0.0114	-6	469	484	GSGTLCEMLLMVEAYK	Carbamidomethyl (C)[6], Oxidation (M)[8,11]	Mascot
1841.8586	1841.821	-0.0376	-20	661	677	QIESEFVDAGANIMSSK	Oxidation (M)[14]	Mascot
1843.8967	1843.7876	-0.1091	-59	182	196	EQRPQDCLDSIVDLR	Carbamidomethyl (C)[7]	Mascot
1843.8967	1843.7876	-0.1091	-59	182	196	EQRPQDCLDSIVDLR	Carbamidomethyl (C)[7]	Mascot
1855.9094	1855.8334	-0.076	-41	1590	1604	IAMFRCAAAFQWLDR	Carbamidomethyl (C)[6]	Mascot
1888.7953	1888.8826	0.0873	46	2060	2074	EWSCADTQCGKIYDR	Carbamidomethyl (C)[4,9]	Mascot
1904.9462	1904.8815	-0.0647	-34	875	890	NNTNDQYQTLVDPVRK		Mascot
1945.9689	1945.9729	0.004	2	403	419	AKLGYDPLEVNPEDMVR		Mascot
1961.9637	1961.9678	0.0041	2	403	419	AKLGYDPLEVNPEDMVR	Oxidation (M)[15]	Mascot
2003.0413	2003.072	0.0307	15	706	722	VLDKPITEVREAGICMR	Carbamidomethyl (C)[15], Oxidation (M)[16]	Mascot
2077.9834	2078.0337	0.0503	24	796	814	GARWYSMEMAGVVTYT GAK		Mascot
2108.0195	2108.0623	0.0428	20	197	213	EYDVPYHVRFAIDNDVR		Mascot

Gel Idx/Pos	306/M8	Instr./Gel Origin	BA2151/Sample Project 20140814	Process Status	Analysis Succeeded
Plate [#] Name	[1] Sample Project 20140814	Instrument Sample Name		Spectra	11

Rank	Protein Name	Accession No.	Protein MW	Protein PI	Pep. Count	Protein Score	Protein Score C. I. %	Intensity Matched	Total Ion Score	Total Ion C. I. %	Confirmed
1	RecName: Full=Oxygen-evolving enhancer protein 2, chloroplastic; Short=OEE2; AltName: Full=23 kDa subunit of oxygen evolving system of photosystem II; AltName: Full=23 kDa thylakoid membrane protein; AltName: Full=OEC 23 kDa subunit; Flags: Precurso	gi|131394	27423.9	8.84	15	525	100	50.016	411	100	

Peptide Information

Calc. Mass	Obsrv. Mass	± da	± ppm	Start Seq.	End Seq.	Sequence	Ion Score	C. I. %	Modification	Rank	Result Type
849.3585	849.3956	0.0371	44	208	216	TADGDEGGK					Mascot
895.4706	895.4012	-0.0694	-78	28	34	TQLFVCK			Carbamidomethyl (C)[6]		Mascot
945.5152	945.4871	-0.0281	-30	114	121	EFPGQVLR					Mascot
945.5152	945.4871	-0.0281	-30	114	121	EFPGQVLR	59	99.766			Mascot
1230.6589	1230.6284	-0.0305	-25	112	121	EREFPGQVLR					Mascot
1243.6682	1243.6417	-0.0265	-21	198	207	QYYSITVLTR					Mascot
1243.6682	1243.6417	-0.0265	-21	198	207	QYYSITVLTR	67	99.963			Mascot
1253.6848	1253.6121	-0.0727	-58	217	228	HQLITATVADGK					Mascot
1326.6688	1326.6298	-0.039	-29	246	258	KFVENAAGSFSVA					Mascot
1434.6537	1434.5995	-0.0542	-38	88	100	NTDFVAYSGEGFK					Mascot
1434.6537	1434.5995	-0.0542	-38	88	100	NTDFVAYSGEGFK	91	100			Mascot
1562.7487	1562.6932	-0.0555	-36	87	100	KNTDFVAYSGEGFK					Mascot
1562.7487	1562.6932	-0.0555	-36	87	100	KNTDFVAYSGEGFK	82	99.999			Mascot
2084.0254	2083.97	-0.0554	-27	208	228	TADGDEGGKHQLITATVA DGK					Mascot
2242.0874	2242.0212	-0.0662	-30	122	141	YEDNFDATSNLSVIINPTT K					Mascot
2242.0874	2242.0212	-0.0662	-30	122	141	YEDNFDATSNLSVIINPTT K	112	100			Mascot
2752.2795	2752.23	-0.0495	-18	170	197	TDSEGGFESDAVATANV LESSAPVVDGK					Mascot
2921.4204	2921.3787	-0.0417	-14	143	169	TITDYGSPEEFLSQVGFL LGQQSYGGK					Mascot
2943.6301	2943.3691	-0.261	-89	53	84	AALSLLAGAAAIAVKVSP AAAAYGEAANVFGK					Mascot
3049.5154	3049.4673	-0.0481	-16	142	169	KTITDYGSPEEFLSQVGF LLGQQSYGGK					Mascot

Rank	Protein Name	Accession No.	Protein MW	Protein PI	Pep. Count	Protein Score	Protein Score C. I. %	Intensity Matched	Total Ion Score	Total Ion C. I. %	Confirmed
2	Oxygen-evolving enhancer protein 2, chloroplastic [Triticum urartu]	gi|474077556	25713.9	8.94	13	505	100	49.823	411	100	

Peptide Information

Calc. Mass	Obsrv. Mass	± da	± ppm	Start Seq.	End Seq.	Sequence	Ion Score	C. I. %	Modification	Rank	Result Type
849.3585	849.3956	0.0371	44	187	195	TADGDEGGK					Mascot
945.5152	945.4871	-0.0281	-30	93	100	EFPGQVLR					Mascot
945.5152	945.4871	-0.0281	-30	93	100	EFPGQVLR	59	99.766			Mascot
1230.6589	1230.6284	-0.0305	-25	91	100	EREFPGQVLR					Mascot
1243.6682	1243.6417	-0.0265	-21	177	186	QYYSITVLTR					Mascot
1243.6682	1243.6417	-0.0265	-21	177	186	QYYSITVLTR	67	99.963			Mascot
1253.6848	1253.6121	-0.0727	-58	196	207	HQLITATVADGK					Mascot
1326.6688	1326.6298	-0.039	-29	226	238	KFVENAAGSFSVA					Mascot
1434.6537	1434.5995	-0.0542	-38	67	79	NTDFVAYSGEGFK					Mascot
1434.6537	1434.5995	-0.0542	-38	67	79	NTDFVAYSGEGFK	91	100			Mascot
1562.7487	1562.6932	-0.0555	-36	66	79	KNTDFVAYSGEGFK					Mascot
1562.7487	1562.6932	-0.0555	-36	66	79	KNTDFVAYSGEGFK	82	99.999			Mascot
2084.0254	2083.97	-0.0554	-27	187	207	TADGDEGGKHQLITATVA DGK					Mascot
2242.0874	2242.0212	-0.0662	-30	101	120	YEDNFDATSNLSVIINPTT K					Mascot
2242.0874	2242.0212	-0.0662	-30	101	120	YEDNFDATSNLSVIINPTT K	112	100			Mascot
2752.2795	2752.23	-0.0495	-18	149	176	TDSEGGFESDAVATANV LESSAPVVDGK					Mascot
2921.4204	2921.3787	-0.0417	-14	122	148	TITDYGSPEEFLSQVGFL LGQQSYGGK					Mascot
3049.5154	3049.4673	-0.0481	-16	121	148	KTITDYGSPEEFLSQVGF LLGQQSYGGK					Mascot

3 Oxygen-evolving enhancer protein 2, chloroplastic [Aegilops tauschii] gi|475616831 31444 8.54 12 482 100 49.416 411 100

Peptide Information

Calc. Mass	Obsrv. Mass	± da	± ppm	Start Seq.	End Seq.	Sequence	Ion Score	C. I. %	Modification	Rank	Result Type
849.3585	849.3956	0.0371	44	249	257	TADGDEGGK					Mascot
945.5152	945.4871	-0.0281	-30	147	154	EFPGQVLR					Mascot
945.5152	945.4871	-0.0281	-30	147	154	EFPGQVLR	59	99.766			Mascot
1230.6589	1230.6284	-0.0305	-25	145	154	EREFPGQVLR					Mascot
1243.6682	1243.6417	-0.0265	-21	239	248	QYYSITVLTR					Mascot
1243.6682	1243.6417	-0.0265	-21	239	248	QYYSITVLTR	67	99.963			Mascot
1253.6848	1253.6121	-0.0727	-58	258	269	HQLITATVADGK					Mascot
1326.6688	1326.6298	-0.039	-29	288	300	KFVENAAGSFSVA					Mascot
1434.6537	1434.5995	-0.0542	-38	121	133	NTDFVAYSGEGFK					Mascot
1434.6537	1434.5995	-0.0542	-38	121	133	NTDFVAYSGEGFK	91	100			Mascot

Calc. Mass	Obsrv. Mass	± da	± ppm	Start Seq.	End Seq.	Sequence	Ion Score	C. I. %	Modification	Rank	Result Type
1562.7487	1562.6932	-0.0555	-36	120	133	KNTDFVAYSGEGFK					Mascot
1562.7487	1562.6932	-0.0555	-36	120	133	KNTDFVAYSGEGFK	82	99.999			Mascot
2084.0254	2083.97	-0.0554	-27	249	269	TADGDEGGKHQLITATVADGK					Mascot
2242.0874	2242.0212	-0.0662	-30	155	174	YEDNFDATSNLSVIINPTTK					Mascot
2242.0874	2242.0212	-0.0662	-30	155	174	YEDNFDATSNLSVIINPTTK	112	100			Mascot
2921.4204	2921.3787	-0.0417	-14	176	202	TITDYGSPEEFLSQVGFLLGQQSYGGK					Mascot
3049.5154	3049.4673	-0.0481	-16	175	202	KTITDYGSPEEFLSQVGFLLGQQSYGGK					Mascot

4	TPA: hypothetical protein ZEAMMB73_321092 [Zea mays]	gi|414592172	27411	8.97	6	150	100	45.155	127	100	

Peptide Information

Calc. Mass	Obsrv. Mass	± da	± ppm	Start Seq.	End Seq.	Sequence	Ion Score	C. I. %	Modification	Rank	Result Type
849.3585	849.3956	0.0371	44	207	215	TADGDEGGK					Mascot
945.5152	945.4871	-0.0281	-30	113	120	EFPGQVLR					Mascot
945.5152	945.4871	-0.0281	-30	113	120	EFPGQVLR	59	99.766			Mascot
1230.6589	1230.6284	-0.0305	-25	111	120	EREFPGQVLR					Mascot
1243.6682	1243.6417	-0.0265	-21	197	206	QYYSITVLTR					Mascot
1243.6682	1243.6417	-0.0265	-21	197	206	QYYSITVLTR	67	99.963			Mascot
1279.6827	1279.6042	-0.0785	-61	28	38	SAQLFVCKAQK			Carbamidomethyl (C)[7]		Mascot
2369.1619	2369.0166	-0.1453	-61	121	141	YEDNFDANSNVSVIIQPTSKK					Mascot

5	Chain A, Structure Of Psbp Protein From Spinacia Oleracea At 1.98 A Resolution	gi|409974117	20666.3	6.19	3	136	100	41.76	127	100	

Peptide Information

Calc. Mass	Obsrv. Mass	± da	± ppm	Start Seq.	End Seq.	Sequence	Ion Score	C. I. %	Modification	Rank	Result Type
849.3585	849.3956	0.0371	44	139	147	TADGDEGGK					Mascot
945.5152	945.4871	-0.0281	-30	45	52	EFPGQVLR					Mascot
945.5152	945.4871	-0.0281	-30	45	52	EFPGQVLR	59	99.766			Mascot
1243.6682	1243.6417	-0.0265	-21	129	138	QYYSITVLTR					Mascot
1243.6682	1243.6417	-0.0265	-21	129	138	QYYSITVLTR	67	99.963			Mascot

6	RecName: Full=Oxygen-evolving enhancer protein 2, chloroplastic; Short=OEE2; AltName: Full=23 kDa subunit of oxygen evolving system of photosystem II; AltName: Full=23 kDa thylakoid membrane protein; AltName: Full=OEC 23 kDa subunit; Flags: Precurso	gi|131392	28614.4	8.58	3	134	100	41.76	127	100	

Peptide Information

Calc. Mass	Obsrv. Mass	± da	± ppm	Start Seq.	End Seq.	Sequence	Ion Score	C. I. %	Modification	Rank	Result Type
849.3585	849.3956	0.0371	44	216	224	TADGDEGGK					Mascot
945.5152	945.4871	-0.0281	-30	122	129	EFPGQVLR					Mascot
945.5152	945.4871	-0.0281	-30	122	129	EFPGQVLR	59	99.766			Mascot
1243.6682	1243.6417	-0.0265	-21	206	215	QYYSITVLTR					Mascot
1243.6682	1243.6417	-0.0265	-21	206	215	QYYSITVLTR	67	99.963			Mascot

7	TPA: hypothetical protein ZEAMMB73_927356 [Zea mays]	gi|414883503	32072.5	9.5	8	96	99.973	31.671	59	99.766	

Peptide Information

Calc. Mass	Obsrv. Mass	± da	± ppm	Start Seq.	End Seq.	Sequence	Ion Score	C. I. %	Modification	Rank	Result Type
849.3585	849.3956	0.0371	44	252	260	TADGDEGGK					Mascot
945.5152	945.4871	-0.0281	-30	158	165	EFPGQVLR					Mascot
945.5152	945.4871	-0.0281	-30	158	165	EFPGQVLR	59	99.766			Mascot
1145.646	1145.5449	-0.1011	-88	72	81	SAQLLVCKAR			Carbamidomethyl (C)[7]		Mascot
1145.646	1145.5449	-0.1011	-88	72	81	SAQLLVCKAR			Carbamidomethyl (C)[7]		Mascot
1199.642	1199.6095	-0.0325	-27	242	251	QYYAVSVLTR					Mascot
1230.6589	1230.6284	-0.0305	-25	156	165	EREFPGQVLR					Mascot
2369.1619	2369.0166	-0.1453	-61	166	186	YEDNFDANSNVSVIIQPT SKK					Mascot
2501.2559	2501.2036	-0.0523	-21	186	207	KTIADYGSPEEFLSQVDY LLGR					Mascot
2752.3159	2752.23	-0.0859	-31	214	241	TDSEGGFETGAVATANV LESSTPVIDGK					Mascot

8	oxygen-evolving enhancer protein, partial [Silene latifolia]	gi|343172527	18583.2	5.2	6	91	99.919	30.113	59	99.766	

Peptide Information

Calc. Mass	Obsrv. Mass	± da	± ppm	Start Seq.	End Seq.	Sequence	Ion Score	C. I. %	Modification	Rank	Result Type
849.3585	849.3956	0.0371	44	119	127	TADGDEGGK					Mascot
945.5152	945.4871	-0.0281	-30	25	32	EFPGQVLR					Mascot
945.5152	945.4871	-0.0281	-30	25	32	EFPGQVLR	59	99.766			Mascot
1230.6589	1230.6284	-0.0305	-25	23	32	EREFPGQVLR					Mascot
1242.6841	1242.6104	-0.0737	-59	12	22	LSVPSKWNPSK					Mascot
1826.9243	1826.7615	-0.1628	-89	119	136	TADGDEGGKHQLISATVK					Mascot
1875.8947	1875.8774	-0.0173	-9	1	17	DFMAYNGDGFKLSVPSK					Mascot

9	hypothetical protein OsI_24850 [Oryza sativa Indica Group]	gi|125557196	27093.8	8.66	7	89	99.877	30.662	59	99.766

Protein Group

Os07g0141400 [Oryza sativa Japonica Group]	gi|113610399	27093.8	8.6599 998474 1211

Peptide Information

Calc. Mass	Obsrv. Mass	± da	± ppm	Start Seq.	End Seq.	Sequence	Ion Score	C. I. %	Modification	Rank	Result Type
849.3585	849.3956	0.0371	44	203	211	TADGDEGGK					Mascot
898.4927	898.4531	-0.0396	-44	28	34	THLLVCR			Carbamidomethyl (C)[6]		Mascot
945.5152	945.4871	-0.0281	-30	109	116	EFPGQVLR					Mascot
945.5152	945.4871	-0.0281	-30	109	116	EFPGQVLR	59	99.766			Mascot
1225.6835	1225.6605	-0.023	-19	28	37	THLLVCRAQK			Carbamidomethyl (C)[6]		Mascot
1230.6589	1230.6284	-0.0305	-25	107	116	EREFPGQVLR					Mascot
1396.8523	1396.7544	-0.0979	-70	47	62	AALALLAGATAAVGVK					Mascot
2369.1619	2369.0166	-0.1453	-61	117	137	YEDNFDANSNVSVIINPT TKK					Mascot

10	hypothetical protein OsI_24852 [Oryza sativa Indica Group]	gi|125557198	20892.6	8.87	6	88	99.838	30.474	59	99.766

Peptide Information

Calc. Mass	Obsrv. Mass	± da	± ppm	Start Seq.	End Seq.	Sequence	Ion Score	C. I. %	Modification	Rank	Result Type
898.4927	898.4531	-0.0396	-44	28	34	THLLVCR			Carbamidomethyl (C)[6]		Mascot
945.5152	945.4871	-0.0281	-30	109	116	EFPGQVLR					Mascot
945.5152	945.4871	-0.0281	-30	109	116	EFPGQVLR	59	99.766			Mascot
1225.6835	1225.6605	-0.023	-19	28	37	THLLVCRAQK			Carbamidomethyl (C)[6]		Mascot
1230.6589	1230.6284	-0.0305	-25	107	116	EREFPGQVLR					Mascot
1396.8523	1396.7544	-0.0979	-70	47	62	AALALLAGATAAVGVK					Mascot
2369.1619	2369.0166	-0.1453	-61	117	137	YEDNFDANSNVSVIINPT TKK					Mascot

Gel Idx/Pos	307/M9	Instr./Gel Origin	BA2151/Sample Project 20140814	Process Status	Analysis Succeeded
Plate [#] Name	[1] Sample Project 20140814	Instrument Sample Name		Spectra	11

Rank	Protein Name	Accession No.	Protein MW	Protein PI	Pep. Count	Protein Score	Protein Score C. I. %	Intensity Matched	Total Ion Score	Total Ion C. I. %	Confirmed
1	Tropinone reductase 1 [Triticum urartu]	gi|473901715	26007.3	5.54	3	64	64.659	1.65	56	99.688	

Peptide Information

Calc. Mass	Obsrv. Mass	± da	± ppm	Start Seq.	End Seq.	Sequence	Ion Score	C. I. %	Modification	Rank	Result Type
1112.6423	1112.5942	-0.0481	-43	85	94	LDILVNNVGR					Mascot
1384.6777	1384.6011	-0.0766	-55	73	84	LMETAKQTFDGK			Oxidation (M)[2]		Mascot
1590.8962	1590.8569	-0.0393	-25	27	42	GIGHAIVEELVGLGAR					Mascot
1590.8962	1590.8569	-0.0393	-25	27	42	GIGHAIVEELVGLGAR	56	99.688			Mascot

Rank	Protein Name	Accession No.	Protein MW	Protein PI	Pep. Count	Protein Score	Protein Score C. I. %	Intensity Matched	Total Ion Score	Total Ion C. I. %	Confirmed
2	PREDICTED: Golgi SNAP receptor complex member 1-2-like [Fragaria vesca subsp. vesca]	gi|470142494	26866.9	9.3	12	58	0	5.523			

Peptide Information

Calc. Mass	Obsrv. Mass	± da	± ppm	Start Seq.	End Seq.	Sequence	Ion Score	C. I. %	Modification	Rank	Result Type
843.541	843.488	-0.053	-63	206	213	GLLGSIKR					Mascot
869.3887	869.3177	-0.071	-82	135	141	DDISEYK					Mascot
945.5298	945.5183	-0.0115	-12	150	156	MQILRER					Mascot
952.4557	952.437	-0.0187	-20	185	193	AMFGDVQGK					Mascot
1163.5725	1163.522	-0.0505	-43	75	84	LLDINDSMSR					Mascot
1433.7352	1433.6365	-0.0987	-69	142	154	ASGSMSPRMQILR					Mascot
1447.6635	1447.666	0.0025	2	104	114	DILHECTQEFR			Carbamidomethyl (C)[6]		Mascot
1453.6913	1453.7041	0.0128	9	63	74	SMEMEIQSLLEK			Oxidation (M)[2]		Mascot
1642.7378	1642.7039	-0.0339	-21	135	149	DDISEYKASGSMSPR					Mascot
1838.9027	1838.8755	-0.0272	-15	60	74	SWKSMEMEIQSLLEK					Mascot
2166.0132	2166.1221	0.1089	50	1	18	MTDSSLELQESGWEELR R					Mascot
2266.1211	2266.0044	-0.1167	-51	37	59	LGARFTQGGGYVESGSP PVGSSR					Mascot
2266.1211	2266.0044	-0.1167	-51	37	59	LGARFTQGGGYVESGSP PVGSSR					Mascot

Rank	Protein Name	Accession No.	Protein MW	Protein PI	Pep. Count	Protein Score	Protein Score C. I. %	Intensity Matched	Total Ion Score	Total Ion C. I. %	Confirmed
3	Pre-mRNA-splicing factor SF2 [Triticum urartu]	gi|474319872	39635.6	10.08	12	55	0	2.244			

Peptide Information

Calc. Mass	Obsrv. Mass	± da	± ppm	Start Seq.	End Seq.	Sequence	Ion Score	C. I. %	Modification	Rank	Result Type

Calc. Mass	Obsrv. Mass	± da	± ppm	Start Seq.	End Seq.	Sequence	Ion Score	C. I. %	Modification	Rank	Result Type
822.4216	822.3672	-0.0544	-66	156	163	VEPAHGGR					Mascot
869.386	869.3177	-0.0683	-79	164	172	GSGGPSHDR					Mascot
885.3995	885.3408	-0.0587	-66	263	269	CGRSYSR			Carbamidomethyl (C)[1]		Mascot
1177.5696	1177.5364	-0.0332	-28	342	352	SSGKPEETQSK					Mascot
1210.5197	1210.5563	0.0366	30	173	188	SSSFGGGGGGGGGGGR					Mascot
1286.5835	1286.5712	-0.0123	-10	206	216	AGDVCFAEVYR			Carbamidomethyl (C)[5]		Mascot
1450.5951	1450.7036	0.1085	75	194	204	HTDYRGMDHMR			Oxidation (M)[7,10]		Mascot
1672.7899	1672.782	-0.0079	-5	156	172	VEPAHGGRGSGGPSHD R					Mascot
1795.828	1795.8834	0.0554	31	337	352	WCSGKSSGKPEETQSK			Carbamidomethyl (C)[2]		Mascot
1947.8641	1947.9255	0.0614	32	217	234	EGGGTIGIVDYTNYDDMK					Mascot
2002.7944	2002.8453	0.0509	25	136	153	DAEDACAGRDGYNFDGN R			Carbamidomethyl (C)[6]		Mascot
2839.4448	2839.2639	-0.1809	-64	312	336	SPSSCLWNFLQITSKIQIP NSFSGK			Carbamidomethyl (C)[5]		Mascot

4	TPA: hypothetical protein ZEAMMB73_174918 [Zea mays]	gi|414589618	44231.7	9.23	12	53	0	11.761

Peptide Information

Calc. Mass	Obsrv. Mass	± da	± ppm	Start Seq.	End Seq.	Sequence	Ion Score	C. I. %	Modification	Rank	Result Type
825.4114	825.3993	-0.0121	-15	222	228	HGHGVYR					Mascot
842.4267	842.4541	0.0274	33	212	217	YRGQYR					Mascot
993.5224	993.4419	-0.0805	-81	142	151	ADKAAAPHGR					Mascot
1030.4371	1030.3969	-0.0402	-39	1	12	MDGHGGGAGAGK			Oxidation (M)[1]		Mascot
1049.4575	1049.5248	0.0673	64	281	289	YAGEYFGDK					Mascot
1068.4634	1068.4587	-0.0047	-4	189	197	YEGDWVDGK					Mascot
1384.675	1384.6011	-0.0739	-53	1	15	MDGHGGGAGAGKLTR					Mascot
1491.6499	1491.649	-0.0009	-1	277	289	NGDRYAGEYFGDK					Mascot
1517.743	1517.7074	-0.0356	-23	264	276	CGVKHGIGSYHFR			Carbamidomethyl (C)[1]		Mascot
1564.6962	1564.6432	-0.053	-34	314	326	QGFGMYTFRNGDR			Oxidation (M)[5]		Mascot
2002.0829	2001.8856	-0.1973	-99	338	356	TPLPPADPSVQRAVQAA QR					Mascot
2002.0829	2001.8856	-0.1973	-99	338	356	TPLPPADPSVQRAVQAA QR					Mascot
2379.9573	2380.0464	0.0891	37	155	174	EGVEFYSNGDCYEGEFH QGR			Carbamidomethyl (C)[11]		Mascot
2379.9573	2380.0464	0.0891	37	155	174	EGVEFYSNGDCYEGEFH QGR	2	0	Carbamidomethyl (C)[11]		Mascot

5	PREDICTED: transcription repressor MYB4-like [Setaria italica]	gi|514808040	31715.7	8.63	10	51	0	7.101

Peptide Information

Calc. Mass	Obsrv. Mass	± da	± ppm	Start Seq.	End Seq.	Sequence	Ion Score	C. I. %	Modification	Rank	Result Type
805.3322	805.3553	0.0231	29	180	186	SSDDEPR					Mascot
817.3951	817.3801	-0.015	-18	263	268	QEQWAR					Mascot
945.5298	945.5183	-0.0115	-12	43	51	AAGLLRCGK			Carbamidomethyl (C)[7]		Mascot
1184.6423	1184.5255	-0.1168	-99	187	198	SATPPPPPAPPR					Mascot
1219.5702	1219.5609	-0.0093	-8	15	24	GAWTKEEDQR					Mascot
1425.6804	1425.5509	-0.1295	-91	31	42	AHGEGCWRSLPR			Carbamidomethyl (C)[6]		Mascot
1425.6804	1425.5509	-0.1295	-91	31	42	AHGEGCWRSLPR			Carbamidomethyl (C)[6]		Mascot
1536.7428	1536.5942	-0.1486	-97	68	80	GNFTEEEDELIIK					Mascot
1536.7428	1536.5942	-0.1486	-97	68	80	GNFTEEEDELIIK					Mascot
1774.9388	1774.8676	-0.0712	-40	81	95	FHELFGNKWSLIAGR					Mascot
1874.8273	1874.7969	-0.0304	-16	4	19	SPCCEQAHTNKGAWTK			Carbamidomethyl (C)[3,4]		Mascot
3103.4299	3103.1431	-0.2868	-92	152	179	LHAAAAPGHHHQQDHFE VISNSPEACSR			Carbamidomethyl (C)[26]		Mascot

6 hypothetical protein ZEAMMB73_366211 [Zea mays] gi|413957179 11689.7 6.03 6 51 0 2.285 13 0

Peptide Information

Calc. Mass	Obsrv. Mass	± da	± ppm	Start Seq.	End Seq.	Sequence	Ion Score	C. I. %	Modification	Rank	Result Type
950.4578	950.3837	-0.0741	-78	78	86	IAWSSDGSK					Mascot
956.4142	956.5038	0.0896	94	87	94	FMSVSDDR					Mascot
956.4142	956.5038	0.0896	94	87	94	FMSVSDDR	13	0			Mascot
1049.5415	1049.5248	-0.0167	-16	96	104	LVYFNLGSH					Mascot
1112.5154	1112.5942	0.0788	71	87	95	FMSVSDDRR					Mascot
1946.0997	1945.9729	-0.1268	-65	10	26	EFLVASGTILNEILIWK					Mascot
2062.032	2061.8323	-0.1997	-97	52	69	IMNPDDSQHMAVHLGRL K					Mascot

7 TCP-1/cpn60 chaperonin family protein isoform 1 [Theobroma cacao] gi|508719317 59749.5 5.59 14 50 0 10.944

Peptide Information

Calc. Mass	Obsrv. Mass	± da	± ppm	Start Seq.	End Seq.	Sequence	Ion Score	C. I. %	Modification	Rank	Result Type
993.3466	993.4419	0.0953	96	541	549	DQPAGMDED			Oxidation (M)[6]		Mascot
1019.4826	1019.4836	0.001	1	44	53	TSLGPDGMNK					Mascot
1149.4478	1149.5146	0.0668	58	540	549	RDQPAGMDED			Oxidation (M)[7]		Mascot
1247.6379	1247.6019	-0.036	-29	469	479	LYEKHASGNTK					Mascot

Calc. Mass	Obsrv. Mass	± da	± ppm	Start Seq.	End Seq.	Sequence	Ion Score	C. I. %	Modification	Rank	Result Type
1259.7094	1259.6461	-0.0633	-50	138	148	TIEVLSELVEK					Mascot
1302.6761	1302.6205	-0.0556	-43	362	373	VTVVRSEEGGNR					Mascot
1320.6278	1320.5996	-0.0282	-21	381	392	GSTDSILDDLER					Mascot
1410.7952	1410.663	-0.1322	-94	410	423	IVPGAAATEIELAR					Mascot
1577.7113	1577.6318	-0.0795	-50	480	495	VGIDLGGGDSEDGVCK			Carbamidomethyl (C)[15]		Mascot
1672.8574	1672.782	-0.0754	-45	453	468	TLAENAGLNPMDVISK					Mascot
1795.9436	1795.8834	-0.0602	-34	239	256	AKVAVFASGIDTSATETK					Mascot
1947.9415	1947.9255	-0.016	-8	292	309	VIVSGGAVGEMALHFCE R			Carbamidomethyl (C)[16], Oxidation (M)[11]		Mascot
2001.9733	2001.8856	-0.0877	-44	44	61	TSLGPDGMNKMVINHLD K			Oxidation (M)[8,11]		Mascot
2001.9733	2001.8856	-0.0877	-44	44	61	TSLGPDGMNKMVINHLD K			Oxidation (M)[8,11]		Mascot
2380.2507	2380.0464	-0.2043	-86	62	83	LFVTNDAATIVNELEVQH PAAK					Mascot
2380.2507	2380.0464	-0.2043	-86	62	83	LFVTNDAATIVNELEVQH PAAK					Mascot

8	hypothetical protein TRIUR3_10241 [Triticum urartu]	gi|473750146	32718.3	8.53	11	49	0	2.188

Peptide Information

Calc. Mass	Obsrv. Mass	± da	± ppm	Start Seq.	End Seq.	Sequence	Ion Score	C. I. %	Modification	Rank	Result Type
941.4839	941.4282	-0.0557	-59	33	40	YIAFNSAR					Mascot
993.5112	993.4419	-0.0693	-70	223	230	GLESFRER					Mascot
1068.4667	1068.4587	-0.008	-7	139	146	FLENECEK			Carbamidomethyl (C)[6]		Mascot
1184.6058	1184.5255	-0.0803	-68	33	42	YIAFNSARDK					Mascot
1219.5413	1219.5609	0.0196	16	149	159	GFASESTMAYR					Mascot
1302.7389	1302.6205	-0.1184	-91	2	12	HNIKNIHTGLR					Mascot
1433.7794	1433.6365	-0.1429	-100	1	12	MHNIKNIHTGLR					Mascot
1446.7046	1446.5999	-0.1047	-72	147	159	VKGFASESTMAYR					Mascot
1491.7261	1491.649	-0.0771	-52	127	138	EVSEHFKESIMR					Mascot
1507.7209	1507.693	-0.0279	-19	127	138	EVSEHFKESIMR			Oxidation (M)[11]		Mascot
1684.767	1684.756	-0.011	-7	134	146	ESIMRFLENECEK			Carbamidomethyl (C)[11]		Mascot
2361.9973	2362.1111	0.1138	48	258	277	NNKNNNNNNNNNNEAF CPK			Carbamidomethyl (C)[18]		Mascot

9	glycosyltransferase family protein 64 [Arabidopsis thaliana]	gi|145327757	38923.3	6.17	11	49	0	6.904

Protein Group

glycosyltransferase family protein 64 [Arabidopsis thaliana]	gi|332198263	38923.3	6.1700 000762 9395

Peptide Information

Calc. Mass	Obsrv. Mass	± da	± ppm	Start Seq.	End Seq.	Sequence	Ion Score	C. I. %	Modification	Rank	Result Type
809.3974	809.3481	-0.0493	-61	331	336	LVFCDR			Carbamidomethyl (C)[4]		Mascot
1019.5091	1019.4836	-0.0255	-25	298	305	EFHRVMGK			Oxidation (M)[6]		Mascot
1069.429	1069.5214	0.0924	86	219	227	GGVEMEEMR			Oxidation (M)[5,8]		Mascot
1177.6324	1177.5364	-0.096	-82	132	141	FLPRSSVDTR					Mascot
1238.5392	1238.4939	-0.0453	-37	1	12	MPSASEADMGVK			Oxidation (M)[1]		Mascot
1271.689	1271.6239	-0.0651	-51	251	262	AGPIMVGAERVR			Oxidation (M)[5]		Mascot
1433.7053	1433.6365	-0.0688	-48	2	15	PSASEADMGVKSVR					Mascot
1491.7772	1491.649	-0.1282	-86	197	208	YSIVLTKFMMMK					Mascot
1507.7722	1507.693	-0.0792	-53	197	208	YSIVLTKFMMMK			Oxidation (M)[9]		Mascot
1536.726	1536.5942	-0.1318	-86	302	314	VMGKMPLMYSYGK			Oxidation (M)[2,5]		Mascot
1536.726	1536.5942	-0.1318	-86	302	314	VMGKMPLMYSYGK			Oxidation (M)[2,5]		Mascot
1564.7458	1564.6432	-0.1026	-66	1	15	MPSASEADMGVKSVR					Mascot
1646.7075	1646.7739	0.0664	40	32	46	TLSGDPDPCDATNQR			Carbamidomethyl (C)[9]		Mascot

10 hypothetical protein F775_04877 [Aegilops tauschii] gi|475626980 39302.9 8.62 11 48 0 3.197

Peptide Information

Calc. Mass	Obsrv. Mass	± da	± ppm	Start Seq.	End Seq.	Sequence	Ion Score	C. I. %	Modification	Rank	Result Type
807.3818	807.3825	0.0007	1	341	346	HSKYIC			Carbamidomethyl (C)[6]		Mascot
832.3795	832.3033	-0.0762	-92	94	101	DADADAVR					Mascot
1027.4084	1027.4468	0.0384	37	158	165	GSCQPYCR			Carbamidomethyl (C)[3,7]		Mascot
1068.5507	1068.4587	-0.092	-86	285	293	ANMALNKYK			Oxidation (M)[3]		Mascot
1259.6842	1259.6461	-0.0381	-30	274	284	LETNSDILVQK					Mascot
1302.743	1302.6205	-0.1225	-94	147	157	AGYAVIFLHRR					Mascot
1756.9667	1756.835	-0.1317	-75	292	306	YKMNIVVANLLATYK			Oxidation (M)[3]		Mascot
1795.9054	1795.8834	-0.022	-12	1	17	MAAGPLLQCPGPAARNR			Carbamidomethyl (C)[9], Oxidation (M)[1]		Mascot
1946.0454	1945.9729	-0.0725	-37	104	121	LQEFIARNSIISAGGVDR					Mascot
2002.0637	2001.8856	-0.1781	-89	274	291	LETNSDILVQKANMALNK					Mascot
2002.0637	2001.8856	-0.1781	-89	274	291	LETNSDILVQKANMALNK					Mascot
2362.1748	2362.1111	-0.0637	-27	72	93	KSPTMLGAEDPESFFATA PPLR					Mascot

Gel Idx/Pos	308/M10	Instr./Gel Origin	BA2151/Sample Project 20140814	Process Status	Analysis Succeeded
Plate [#] Name	[1] Sample Project 20140814	Instrument Sample Name		Spectra	11

Rank	Protein Name	Accession No.	Protein MW	Protein PI	Pep. Count	Protein Score	Protein Score C. I. %	Intensity Matched	Total Ion Score	Total Ion C. I. %	Confirmed
1	hypothetical protein PRUPE_ppa018596mg [Prunus persica]	gi|462423202	11763.6	6.71	7	49	0	1.899			

Peptide Information

Calc. Mass	Obsrv. Mass	± da	± ppm	Start Seq.	End Seq.	Sequence	Ion Score	C. I. %	Modification	Rank	Result Type
1106.551	1106.4727	-0.0783	-71	1	10	MGILTDSQNK					Mascot
1125.4895	1125.4442	-0.0453	-40	26	34	AAYHNCFSR			Carbamidomethyl (C)[6]		Mascot
1194.5096	1194.5653	0.0557	47	48	56	EECVSEWQK			Carbamidomethyl (C)[3]		Mascot
1262.6522	1262.5723	-0.0799	-63	1	11	MGILTDSQNKR					Mascot
1308.5914	1308.6113	0.0199	15	91	103	TTKTDDAPTGSSQ					Mascot
1450.6591	1450.5955	-0.0636	-44	12	25	SPSSSSTSPCANLR			Carbamidomethyl (C)[10]		Mascot
1491.7261	1491.6946	-0.0315	-21	57	68	YKACLSQHLEDK			Carbamidomethyl (C)[4]		Mascot

Rank	Protein Name	Accession No.	Protein MW	Protein PI	Pep. Count	Protein Score	Protein Score C. I. %	Intensity Matched	Total Ion Score	Total Ion C. I. %	Confirmed
2	VP1/ABI3-like protein 3, partial [Arabidopsis lyrata]	gi|410516138	15865.8	9.55	8	46	0	2.571			

Peptide Information

Calc. Mass	Obsrv. Mass	± da	± ppm	Start Seq.	End Seq.	Sequence	Ion Score	C. I. %	Modification	Rank	Result Type
808.3869	808.3643	-0.0226	-28	43	49	LMSENSK					Mascot
824.3818	824.3006	-0.0812	-98	43	49	LMSENSK			Oxidation (M)[2]		Mascot
851.3749	851.3115	-0.0634	-74	25	32	ATACPMGK			Carbamidomethyl (C)[4], Oxidation (M)[6]		Mascot
1037.4867	1037.4777	-0.009	-9	34	42	RGMNDMIGK			Oxidation (M)[3]		Mascot
1106.5082	1106.4727	-0.0355	-32	23	32	DRATACPMGK			Carbamidomethyl (C)[6]		Mascot
1193.6022	1193.5719	-0.0303	-25	1	10	SALQFDSRNR					Mascot
1252.5991	1252.5413	-0.0578	-46	43	52	LMSENSKHYK			Oxidation (M)[2]		Mascot
1265.5757	1265.583	0.0073	6	11	22	GGNNEFSQPTSK					Mascot
1716.7823	1716.8031	0.0208	12	112	127	LHNDLNGGSDSPFESK					Mascot

Rank	Protein Name	Accession No.	Protein MW	Protein PI	Pep. Count	Protein Score	Protein Score C. I. %	Intensity Matched	Total Ion Score	Total Ion C. I. %	Confirmed
3	hypothetical protein OsI_16644 [Oryza sativa Indica Group]	gi|125549027	16055.9	11.13	8	46	0	1.621			

Peptide Information

Calc. Mass	Obsrv. Mass	± da	± ppm	Start Seq.	End Seq.	Sequence	Ion Score	C. I. %	Modification	Rank	Result Type

828.3781	828.308	-0.0701	-85	150	156	GCRTPTH			Carbamidomethyl (C)[2]		Mascot
1042.5276	1042.46	-0.0676	-65	141	149	RGLEEEPGR					Mascot
1104.5215	1104.491	-0.0305	-28	80	92	SGGAAAAGVAGCR			Carbamidomethyl (C)[12]		Mascot
1259.5797	1259.6371	0.0574	46	142	152	GLEEEPGRGCR			Carbamidomethyl (C)[10]		Mascot
1276.6202	1276.5819	-0.0383	-30	1	14	MAAASAAPTSSAPK			Oxidation (M)[1]		Mascot
1493.6545	1493.6898	0.0353	24	43	56	CSPSAAMTPMSTPR			Carbamidomethyl (C)[1]		Mascot
1890.9338	1890.8998	-0.034	-18	124	141	LDQAVADLAEAMASGTR R			Oxidation (M)[12]		Mascot
2380.1133	2380.0764	-0.0369	-16	19	42	SPPCSTGAPSTGATAPP CSPARPR			Carbamidomethyl (C)[4,18]		Mascot

4	PREDICTED: uncharacterized protein LOC101252286 [Solanum lycopersicum]	gi|460388536	49776.7	6.07	12	46	0	3.307	

Peptide Information

Calc. Mass	Obsrv. Mass	± da	± ppm	Start Seq.	End Seq.	Sequence	Ion Score	C. I. %	Modification	Rank	Result Type
809.361	809.2975	-0.0635	-78	38	43	SWQNMK			Oxidation (M)[5]		Mascot
1262.6488	1262.5723	-0.0765	-61	199	210	ISAGSDGKVSWR					Mascot
1265.6267	1265.583	-0.0437	-35	273	283	ARSSSNVEIMR			Oxidation (M)[10]		Mascot
1323.6515	1323.6052	-0.0463	-35	38	47	SWQNMKSWIK			Oxidation (M)[5]		Mascot
1355.6624	1355.5521	-0.1103	-81	103	114	INDHPIETSMAK					Mascot
1390.6459	1390.6479	0.002	1	349	360	FGENSEGHTRTR					Mascot
1479.6632	1479.6942	0.031	21	430	443	GAAKIMAIDEDSQD			Oxidation (M)[6]		Mascot
1493.7206	1493.6898	-0.0308	-21	170	183	NGAGEMGGFVLWQK					Mascot
1579.7495	1579.5938	-0.1557	-99	376	389	GLSLDCFLPPSDMK			Carbamidomethyl (C)[6]		Mascot
1707.8445	1707.7307	-0.1138	-67	376	390	GLSLDCFLPPSDMKK			Carbamidomethyl (C)[6]		Mascot
1837.9	1837.8822	-0.0178	-10	144	161	MIASEFIEGDGLGLNSGK					Mascot
2139.0427	2138.9399	-0.1028	-48	359	375	TRMEEVWTIEEVDFNIK					Mascot

5	hypothetical protein OsI_16194 [Oryza sativa Indica Group]	gi|218194989	33615.4	9.42	5	45	0	1.966	31	0

Peptide Information

Calc. Mass	Obsrv. Mass	± da	± ppm	Start Seq.	End Seq.	Sequence	Ion Score	C. I. %	Modification	Rank	Result Type
809.3505	809.2975	-0.053	-65	156	161	NRCMGR			Carbamidomethyl (C)[3], Oxidation (M)[4]		Mascot
1037.4833	1037.4777	-0.0056	-5	147	155	SSHGLYMSR					Mascot
1067.5369	1067.4608	-0.0761	-71	281	289	ESFVQDSKK					Mascot
1277.6736	1277.6653	-0.0083	-6	162	172	LVEALEEVGYR	31	0			Mascot
1323.6223	1323.6052	-0.0171	-13	147	157	SSHGLYMSRNR			Oxidation (M)[7]		Mascot

6	hypothetical protein OsI_28385 [Oryza sativa Indica Group]	gi|125560703	44686.5	7.7	15	44	0	4.851

Peptide Information

Calc. Mass	Obsrv. Mass	± da	± ppm	Start Seq.	End Seq.	Sequence	Ion Score	C. I. %	Modification	Rank	Result Type
930.3478	930.3394	-0.0084	-9	290	296	SMQMDMR			Oxidation (M)[2,4]		Mascot
1014.5037	1014.4041	-0.0996	-98	201	208	TQQIHEMK					Mascot
1060.5569	1060.511	-0.0459	-43	192	200	MVIGNQQVR			Oxidation (M)[1]		Mascot
1067.5442	1067.4608	-0.0834	-78	265	273	MIVDAYEVK					Mascot
1118.5549	1118.4744	-0.0805	-72	127	135	ESTNDLRQR					Mascot
1263.6436	1263.6121	-0.0315	-25	235	245	SGMEIMNLLQK					Mascot
1277.6519	1277.6653	0.0134	10	136	146	MQSDISRLEAK					Mascot
1320.6543	1320.5498	-0.1045	-79	182	191	LQQERDEFQK					Mascot
1320.6543	1320.5498	-0.1045	-79	182	191	LQQERDEFQK					Mascot
1383.6542	1383.6151	-0.0391	-28	286	296	ALLRSMQMDMR			Oxidation (M)[6,8]		Mascot
1390.7107	1390.6479	-0.0628	-45	329	340	TARMTQLQDAQK					Mascot
1490.7268	1490.7118	-0.015	-10	274	285	KQELMQENADLR			Oxidation (M)[5]		Mascot
1605.8087	1605.6647	-0.144	-90	235	248	SGMEIMNLLQKEGR					Mascot
1657.8391	1657.7584	-0.0807	-49	386	401	LSGLDAEREAVAAQEV					Mascot
1707.8483	1707.7307	-0.1176	-69	187	200	DEFQKMVIGNQQVR			Oxidation (M)[6]		Mascot
2831.3386	2831.1521	-0.1865	-66	290	315	SMQMDMREFLNAPNGV PQPAVVGNGR			Oxidation (M)[2]		Mascot

7	hypothetical protein SORBIDRAFT_04g037320 [Sorghum bicolor]	gi|241932874	64467.3	9.24	14	44	0	4.065

Peptide Information

Calc. Mass	Obsrv. Mass	± da	± ppm	Start Seq.	End Seq.	Sequence	Ion Score	C. I. %	Modification	Rank	Result Type
998.4915	998.435	-0.0565	-57	572	578	YEHRHTR					Mascot
1033.5175	1033.4742	-0.0433	-42	77	85	DHQPAPNVR					Mascot
1067.5632	1067.4608	-0.1024	-96	258	266	RAFNLFGDK					Mascot
1106.5623	1106.4727	-0.0896	-81	100	110	SLSAALAACSR			Carbamidomethyl (C)[9]		Mascot
1107.5139	1107.5071	-0.0068	-6	291	299	NLFDEMPNK					Mascot
1118.5221	1118.4744	-0.0477	-43	461	469	KMFLSMSDK			Oxidation (M)[2,6]		Mascot
1140.6049	1140.5194	-0.0855	-75	259	268	AFNLFGDKTK					Mascot
1235.6783	1235.562	-0.1163	-94	374	384	VATSFVHIYAK					Mascot
1332.6075	1332.5144	-0.0931	-70	272	282	IWSVMMSGYAR			Oxidation (M)[5,6]		Mascot
1373.7537	1373.6219	-0.1318	-96	546	557	LTLSGLFSDHKR					Mascot

1434.6617	1434.7098	0.0481	34	385	396	HGCIQTAMDVFR			Carbamidomethyl (C)[3]		Mascot
1450.6566	1450.5955	-0.0611	-42	385	396	HGCIQTAMDVFR			Carbamidomethyl (C)[3], Oxidation (M)[8]		Mascot
1475.8104	1475.7074	-0.103	-70	533	545	QILGLDATYKPEK					Mascot
1890.9341	1890.8998	-0.0343	-18	462	477	MFLSMSDKYGVKPDIK			Oxidation (M)[1,5]		Mascot
2136.1045	2135.9487	-0.1558	-73	231	249	HTLFDSAEVVIGLVDMYVK					Mascot

8	putative protein phosphatase 2C family protein [Zea mays]	gi|413948637	19894	6.22	9	43	0	2.538

Peptide Information

Calc. Mass	Obsrv. Mass	± da	± ppm	Start Seq.	End Seq.	Sequence	Ion Score	C. I. %	Modification	Rank	Result Type
905.4873	905.3986	-0.0887	-98	101	109	VGGVLAMSR			Oxidation (M)[7]		Mascot
1104.5143	1104.491	-0.0233	-21	3	11	HPEFMTNTK					Mascot
1107.6157	1107.5071	-0.1086	-98	12	20	LAINETYRK					Mascot
1118.4783	1118.4744	-0.0039	-3	135	144	MEEDPEAAAR					Mascot
1232.6093	1232.5566	-0.0527	-43	2	11	KHPEFMTNTK					Mascot
1262.5681	1262.5723	0.0042	3	135	145	MEEDPEAAARK			Oxidation (M)[1]		Mascot
1379.6447	1379.6514	0.0067	5	1	11	MKHPEFMTNTK			Oxidation (M)[1]		Mascot
1434.7634	1434.7098	-0.0536	-37	101	114	VGGVLAMSRAFGNR					Mascot
1837.9152	1837.8822	-0.033	-18	68	83	AIALSEDHKPNRSDER					Mascot

9	hypothetical protein PRUPE_ppa018702mg [Prunus persica]	gi|462406525	31006.9	4.8	9	42	0	3.517

Peptide Information

Calc. Mass	Obsrv. Mass	± da	± ppm	Start Seq.	End Seq.	Sequence	Ion Score	C. I. %	Modification	Rank	Result Type
1020.4479	1020.4179	-0.03	-29	60	67	EEIEEESR					Mascot
1060.4921	1060.511	0.0189	18	258	266	SFFACFPGK			Carbamidomethyl (C)[5]		Mascot
1235.6552	1235.562	-0.0932	-75	167	177	VTVETMLIDSK					Mascot
1265.6484	1265.583	-0.0654	-52	142	151	LYINEESNKR					Mascot
1320.6682	1320.5498	-0.1184	-90	2	12	EEKEYVPVEAK					Mascot
1320.6682	1320.5498	-0.1184	-90	2	12	EEKEYVPVEAK					Mascot
1373.7384	1373.6219	-0.1165	-85	130	141	LSKGQLTEDQVR					Mascot
1487.7417	1487.6934	-0.0483	-32	5	16	EYVPVEAKDFYK					Mascot
2136.0566	2135.9487	-0.1079	-51	133	150	GQLTEDQVRLYINEESNK					Mascot
2510.2092	2510.0449	-0.1643	-65	236	257	KVEADQQVNGPIMPANTHYPER			Oxidation (M)[13]		Mascot

10	hypothetical protein TRIUR3_32194 [Triticum urartu]	gi|474207867	19544.8	9.83	8	42	0	3.101

Peptide Information

Calc. Mass	Obsrv. Mass	± da	± ppm	Start Seq.	End Seq.	Sequence	Ion Score	C. I. %	Modification	Rank	Result Type
808.3505	808.3643	0.0138	17	115	121	TSEPSCK			Carbamidomethyl (C)[6]		Mascot
1042.5428	1042.46	-0.0828	-79	19	27	SGYLYRGAR					Mascot
1276.6016	1276.5819	-0.0197	-15	60	71	DGDDNGTVKIDK					Mascot
1323.6692	1323.6052	-0.064	-48	14	24	NEPPKSGYLYR					Mascot
1487.7067	1487.6934	-0.0133	-9	152	163	FDFSKHPYAYGR					Mascot
1490.7961	1490.7118	-0.0843	-57	46	59	LAVADEIHTHVASK					Mascot
1837.8837	1837.8822	-0.0015	-1	137	151	CKCFSPHISGQTFLR			Carbamidomethyl (C)[1,3]		Mascot
1908.9379	1908.7632	-0.1747	-92	28	45	RPAGLSCSGTTTIRCGSK			Carbamidomethyl (C)[7,15]		Mascot

Gel Idx/Pos	309/M11	Instr./Gel Origin	BA2151/Sample Project 20140814	Process Status	Analysis Succeeded
Plate [#] Name	[1] Sample Project 20140814	Instrument Sample Name		Spectra	11

Rank	Protein Name	Accession No.	Protein MW	Protein PI	Pep. Count	Protein Score	Protein Score C. I. %	Intensity Matched	Total Ion Score	Total Ion C. I. %	Confirmed
1	alpha purothionin, partial [Triticum aestivum]	gi|402485682	12724.6	5.04	4	204	100	6.815	183	100	

Peptide Information

Calc. Mass	Obsrv. Mass	± da	± ppm	Start Seq.	End Seq.	Sequence	Ion Score	C. I. %	Modification	Rank	Result Type
835.3913	835.3531	-0.0382	-46	25	31	LCAGVCR			Carbamidomethyl (C)[2,6]		Mascot
841.3477	841.3763	0.0286	34	1	6	MKSCCR			Carbamidomethyl (C)[4,5]		Mascot
999.4135	999.3727	-0.0408	-41	12	18	NCYNLCR			Carbamidomethyl (C)[2,6]		Mascot
999.4135	999.3727	-0.0408	-41	12	18	NCYNLCR	41	88.704	Carbamidomethyl (C)[2,6]		Mascot
2456.0706	2455.999	-0.0716	-29	47	67	LALESNSDEPDTIEYCNL GCR			Carbamidomethyl (C)[16,20]		Mascot
2456.0706	2455.999	-0.0716	-29	47	67	LALESNSDEPDTIEYCNL GCR	142	100	Carbamidomethyl (C)[16,20]		Mascot

Rank	Protein Name	Accession No.	Protein MW	Protein PI	Pep. Count	Protein Score	Protein Score C. I. %	Intensity Matched	Total Ion Score	Total Ion C. I. %	Confirmed
2	RecName: Full=Alpha-1-purothionin; AltName: Full=Purothionin A-II; Contains: RecName: Full=Alpha-1-purothionin; Contains: RecName: Full=Acidic protein; Flags: Precursor	gi|401182	14371.6	4.76	3	197	100	6.536	183	100	

Peptide Information

Calc. Mass	Obsrv. Mass	± da	± ppm	Start Seq.	End Seq.	Sequence	Ion Score	C. I. %	Modification	Rank	Result Type
835.3913	835.3531	-0.0382	-46	40	46	LCAGVCR			Carbamidomethyl (C)[2,6]		Mascot
999.4135	999.3727	-0.0408	-41	27	33	NCYNLCR			Carbamidomethyl (C)[2,6]		Mascot
999.4135	999.3727	-0.0408	-41	27	33	NCYNLCR	41	88.704	Carbamidomethyl (C)[2,6]		Mascot
2456.0706	2455.999	-0.0716	-29	62	82	LALESNSDEPDTIEYCNL GCR			Carbamidomethyl (C)[16,20]		Mascot
2456.0706	2455.999	-0.0716	-29	62	82	LALESNSDEPDTIEYCNL GCR	142	100	Carbamidomethyl (C)[16,20]		Mascot

Rank	Protein Name	Accession No.	Protein MW	Protein PI	Pep. Count	Protein Score	Protein Score C. I. %	Intensity Matched	Total Ion Score	Total Ion C. I. %	Confirmed
3	RecName: Full=Alpha-2-purothionin; Contains: RecName: Full=Alpha-2-purothionin; Contains: RecName: Full=Acidic protein; Flags: Precursor	gi|401184	15403.1	5.13	2	192	100	5.567	183	100	

Peptide Information

Calc. Mass	Obsrv. Mass	± da	± ppm	Start Seq.	End Seq.	Sequence	Ion Score	C. I. %	Modification	Rank	Result Type
999.4135	999.3727	-0.0408	-41	38	44	NCYNLCR			Carbamidomethyl (C)[2,6]		Mascot
999.4135	999.3727	-0.0408	-41	38	44	NCYNLCR	41	88.704	Carbamidomethyl (C)[2,6]		Mascot

2456.0706	2455.999	-0.0716	-29	73	93	LALESNSDEPDTIEYCNL GCR			Carbamidomethyl (C)[16,20]		Mascot
2456.0706	2455.999	-0.0716	-29	73	93	LALESNSDEPDTIEYCNL GCR	142	100	Carbamidomethyl (C)[16,20]		Mascot

4	Alpha-2-purothionin [Aegilops tauschii]	gi|475460813	19408.2	6.1	2	191	100	5.567	183	100

Peptide Information

Calc. Mass	Obsrv. Mass	± da	± ppm	Start Seq.	End Seq.	Sequence	Ion Score	C. I. %	Modification	Rank	Result Type
999.4135	999.3727	-0.0408	-41	74	80	NCYNLCR			Carbamidomethyl (C)[2,6]		Mascot
999.4135	999.3727	-0.0408	-41	74	80	NCYNLCR	41	88.704	Carbamidomethyl (C)[2,6]		Mascot
2456.0706	2455.999	-0.0716	-29	109	129	LALESNSDEPDTIEYCNL GCR			Carbamidomethyl (C)[16,20]		Mascot
2456.0706	2455.999	-0.0716	-29	109	129	LALESNSDEPDTIEYCNL GCR	142	100	Carbamidomethyl (C)[16,20]		Mascot

5	PREDICTED: 24.1 kDa heat shock protein, mitochondrial-like isoform X1 [Setaria italica]	gi|514718387	24158.4	6.78	4	91	99.917	2.915	78	99.998

Protein Group

PREDICTED: 24.1 kDa heat shock protein, mitochondrial-like isoform X2 [Setaria italica]	gi|514718391	24102.4	6.2399 997711 1816

Peptide Information

Calc. Mass	Obsrv. Mass	± da	± ppm	Start Seq.	End Seq.	Sequence	Ion Score	C. I. %	Modification	Rank	Result Type
810.4025	810.3514	-0.0511	-63	1	8	MASTVASK			Oxidation (M)[1]		Mascot
1111.5378	1111.4634	-0.0744	-67	126	134	EDEEALHLR					Mascot
1140.4851	1140.4991	0.014	12	66	74	EYENCRGGR			Carbamidomethyl (C)[5]		Mascot
1174.5852	1174.5254	-0.0598	-51	89	99	DPFSAPQSLGR					Mascot
1174.5852	1174.5254	-0.0598	-51	89	99	DPFSAPQSLGR	78	99.998			Mascot

6	hypothetical protein SORBIDRAFT_04g027330 [Sorghum bicolor]	gi|241934070	24208.5	6.79	3	87	99.787	2.639	78	99.998

Peptide Information

Calc. Mass	Obsrv. Mass	± da	± ppm	Start Seq.	End Seq.	Sequence	Ion Score	C. I. %	Modification	Rank	Result Type
1061.5878	1061.4905	-0.0973	-92	178	186	IELAPEVYK					Mascot
1111.5378	1111.4634	-0.0744	-67	125	133	EDEEALHLR					Mascot
1174.5852	1174.5254	-0.0598	-51	88	98	DPFSAPQSLGR					Mascot
1174.5852	1174.5254	-0.0598	-51	88	98	DPFSAPQSLGR	78	99.998			Mascot

7	Os02g0758000 [Oryza sativa Japonica Group]	gi|113537706	24082.4	6.88	1	78	98.385	2.174	78	99.998

Protein Group

RecName: Full=24.1 kDa heat shock protein, mitochondrial; Short=OsHsp24.1; Flags: Precursor	gi|75294195	24082.4	6.8800 001144 4092
hypothetical protein OsI_09007 [Oryza sativa Indica Group]	gi|125541201	24082.4	6.8800 001144 4092

Peptide Information

Calc. Mass	Obsrv. Mass	± da	± ppm	Start Seq.	End Seq.	Sequence	Ion Score	C. I. %	Modification	Rank	Result Type
1174.5852	1174.5254	-0.0598	-51	87	97	DPFSAPQSLGR					Mascot
1174.5852	1174.5254	-0.0598	-51	87	97	DPFSAPQSLGR	78	99.998			Mascot

8	RecName: Full=Alpha-hordothionin; AltName: Full=Purothionin II; Contains: RecName: Full=Alpha-hordothionin; Contains: RecName: Full=Acidic protein; Flags: Precursor	gi|135788	14442.7	6.18	5	67	81.875	5.673	41	88.704

Peptide Information

Calc. Mass	Obsrv. Mass	± da	± ppm	Start Seq.	End Seq.	Sequence	Ion Score	C. I. %	Modification	Rank	Result Type
806.3865	806.3486	-0.0379	-47	57	63	CPTGFPK			Carbamidomethyl (C)[1]		Mascot
835.3913	835.3531	-0.0382	-46	42	48	LCAGVCR			Carbamidomethyl (C)[2,6]		Mascot
942.392	942.3607	-0.0313	-33	78	84	YCNLGCR			Carbamidomethyl (C)[2,6]		Mascot
999.4135	999.3727	-0.0408	-41	29	35	NCYNLCR			Carbamidomethyl (C)[2,6]		Mascot
999.4135	999.3727	-0.0408	-41	29	35	NCYNLCR	41	88.704	Carbamidomethyl (C)[2,6]		Mascot
1379.6989	1379.6552	-0.0437	-32	51	63	LTSSGKCPTGFPK			Carbamidomethyl (C)[7]		Mascot

9	Chain A, Crystal Structure Of Beta Hordothionin	gi|60593930	5379.6	9.75	4	63	52.326	4.647	41	88.704

Protein Group

Chain B, Crystal Structure Of Beta Hordothionin	gi|60593931	5379.6	9.75

Peptide Information

Calc. Mass	Obsrv. Mass	± da	± ppm	Start Seq.	End Seq.	Sequence	Ion Score	C. I. %	Modification	Rank	Result Type

Calc. Mass	Obsrv. Mass	± da	± ppm	Start Seq.	End Seq.	Sequence	Ion Score	C. I. %	Modification	Rank	Result Type
822.3814	822.343	-0.0384	-47	39	45	CPSSFPK			Carbamidomethyl (C)[1]		Mascot
864.3814	864.3704	-0.011	-13	24	30	LCANACR			Carbamidomethyl (C)[2,6]		Mascot
999.4135	999.3727	-0.0408	-41	11	17	NCYNLCR			Carbamidomethyl (C)[2,6]		Mascot
999.4135	999.3727	-0.0408	-41	11	17	NCYNLCR	41	88.704	Carbamidomethyl (C)[2,6]		Mascot
1152.5071	1152.4904	-0.0167	-14	24	32	LCANACRCK			Carbamidomethyl (C)[2,6,8]		Mascot

10	uncharacterized protein LOC100382724 [Zea mays]	gi|293334519	34505.8	11.32	16	59	0	8.422

Peptide Information

Calc. Mass	Obsrv. Mass	± da	± ppm	Start Seq.	End Seq.	Sequence	Ion Score	C. I. %	Modification	Rank	Result Type
802.3914	802.3693	-0.0221	-28	294	302	GEGGGGGRR					Mascot
807.3665	807.3603	-0.0062	-8	140	146	EMEIGGR			Oxidation (M)[2]		Mascot
817.3911	817.3696	-0.0215	-26	283	289	EQGDGRR					Mascot
818.3751	818.3601	-0.015	-18	158	164	GDREDAR					Mascot
820.3907	820.3546	-0.0361	-44	115	123	QGSGASSAR					Mascot
834.4064	834.3403	-0.0661	-79	2	8	SERESAR					Mascot
848.3897	848.3549	-0.0348	-41	198	205	GWGGSEQK					Mascot
954.4388	954.3843	-0.0545	-57	243	254	GAEGGAGHGGGK					Mascot
982.4813	982.396	-0.0853	-87	209	217	RAEGAEGHR					Mascot
999.4537	999.3727	-0.081	-81	255	263	GAGRAHDCR			Carbamidomethyl (C)[8]		Mascot
999.4537	999.3727	-0.081	-81	255	263	GAGRAHDCR			Carbamidomethyl (C)[8]		Mascot
1063.5127	1063.4937	-0.019	-18	113	123	DKQGSGASSAR					Mascot
1106.4757	1106.4612	-0.0145	-13	290	301	GSCRGEGGGGGR			Carbamidomethyl (C)[3]		Mascot
1155.6117	1155.5016	-0.1101	-95	22	31	SKLEQQHTGK					Mascot
1158.5175	1158.5287	0.0112	10	232	242	SGAPWEGDPSR					Mascot
1323.6475	1323.5927	-0.0548	-41	57	67	RQTAAMYDVPR			Oxidation (M)[6]		Mascot
1339.6396	1339.5789	-0.0607	-45	259	270	AHDCRGSRPAGR			Carbamidomethyl (C)[4]		Mascot

Gel Idx/Pos	310/M12	Instr./Gel Origin	BA2151/Sample Project 20140814	Process Status	Analysis Succeeded
Plate [#] Name	[1] Sample Project 20140814	Instrument Sample Name		Spectra	11

Rank	Protein Name	Accession No.	Protein MW	Protein PI	Pep. Count	Protein Score	Protein Score C. I. %	Intensity Matched	Total Ion Score	Total Ion C. I. %	Confirmed
1	RecName: Full=Alpha-amylase/trypsin inhibitor CM2; AltName: Full=Chloroform/methanol-soluble protein CM2; Flags: Precursor	gi|123956	16019.7	6.86	5	312	100	31.26	282	100	

Peptide Information

Calc. Mass	Obsrv. Mass	± da	± ppm	Start Seq.	End Seq.	Sequence	Ion Score	C. I. %	Modification	Rank	Result Type
814.3876	814.3585	-0.0291	-36	107	113	DLPGCPR			Carbamidomethyl (C)[5]		Mascot
1017.5211	1017.4822	-0.0389	-38	97	106	TSDPNSGVLK					Mascot
1278.5532	1278.514	-0.0392	-31	77	86	ELYDASQHCR			Carbamidomethyl (C)[9]		Mascot
1278.5532	1278.514	-0.0392	-31	77	86	ELYDASQHCR	83	100	Carbamidomethyl (C)[9]		Mascot
2703.3044	2703.2476	-0.0568	-21	46	71	EYVAQQTCGVGIVGSPV STEPGNTPR			Carbamidomethyl (C)[8]		Mascot
2703.3044	2703.2476	-0.0568	-21	46	71	EYVAQQTCGVGIVGSPV STEPGNTPR	199	100	Carbamidomethyl (C)[8]		Mascot
2726.3101	2726.219	-0.0911	-33	122	145	VLVTPGHCNVMTVHNTP YCLGLDI			Carbamidomethyl (C)[8,19], Oxidation (M)[11]		Mascot

Rank	Protein Name	Accession No.	Protein MW	Protein PI	Pep. Count	Protein Score	Protein Score C. I. %	Intensity Matched	Total Ion Score	Total Ion C. I. %	Confirmed
2	dimeric alpha-amylase inhibitor, partial [Triticum durum]	gi|386877064	14502.8	4.78	3	162	100	2.522	146	100	

Peptide Information

Calc. Mass	Obsrv. Mass	± da	± ppm	Start Seq.	End Seq.	Sequence	Ion Score	C. I. %	Modification	Rank	Result Type
1570.8007	1570.7483	-0.0524	-33	32	45	LQCNGSQVPEAVLR			Carbamidomethyl (C)[3]		Mascot
1677.8517	1677.7589	-0.0928	-55	107	122	LPIVIDASGDGAYVCK			Carbamidomethyl (C)[15]		Mascot
1887.8403	1887.7843	-0.056	-30	73	90	EHGVQEGQAGTGAFPSC R			Carbamidomethyl (C)[17]		Mascot
1887.8403	1887.7843	-0.056	-30	73	90	EHGVQEGQAGTGAFPSC R	146	100	Carbamidomethyl (C)[17]		Mascot

Rank	Protein Name	Accession No.	Protein MW	Protein PI	Pep. Count	Protein Score	Protein Score C. I. %	Intensity Matched	Total Ion Score	Total Ion C. I. %	Confirmed
3	RecName: Full=Chymotrypsin inhibitor WCI; AltName: Full=Chloroform/methanol-soluble protein WCI	gi|75107149	13505.5	7.42	3	144	100	4.549	129	100	

Peptide Information

Calc. Mass	Obsrv. Mass	± da	± ppm	Start Seq.	End Seq.	Sequence	Ion Score	C. I. %	Modification	Rank	Result Type
889.456	889.452	-0.004	-4	87	93	CPTEVKR			Carbamidomethyl (C)[1]		Mascot
1120.5415	1120.5042	-0.0373	-33	47	56	ELAAISSNCR			Carbamidomethyl (C)[9]		Mascot
1986.0193	1985.9745	-0.0448	-23	67	86	AFPPSQSQGGGPPQPPL APR					Mascot

Calc. Mass	Obsrv. Mass	± da	± ppm	Start Seq.	End Seq.	Sequence	Ion Score	C. I. %	Modification	Rank	Result Type
1986.0193	1985.9745	-0.0448	-23	67	86	AFPPSQSQGGGPPQPPL APR	129	100			Mascot

4 Trypsin inhibitor CMc [Aegilops tauschii] gi|475430040 15876.7 7.48 3 142 100 4.549 129 100

Peptide Information

Calc. Mass	Obsrv. Mass	± da	± ppm	Start Seq.	End Seq.	Sequence	Ion Score	C. I. %	Modification	Rank	Result Type
889.456	889.452	-0.004	-4	111	117	CPTEVKR			Carbamidomethyl (C)[1]		Mascot
1120.5415	1120.5042	-0.0373	-33	71	80	ELAAISSNCR			Carbamidomethyl (C)[9]		Mascot
1986.0193	1985.9745	-0.0448	-23	91	110	AFPPSQSQGGGPPQPPL APR					Mascot
1986.0193	1985.9745	-0.0448	-23	91	110	AFPPSQSQGGGPPQPPL APR	129	100			Mascot

5 Alpha-amylase/trypsin inhibitor CM1 [Aegilops tauschii] gi|475582216 16086.8 7.49 6 118 100 24.495 83 100

Protein Group

RecName: Full=Alpha-amylase/trypsin inhibitor CM1; AltName: Full=Chloroform/methanol-soluble protein CM1; Flags: Precursor gi|123955 16076.8 7.4899 997711 1816

Peptide Information

Calc. Mass	Obsrv. Mass	± da	± ppm	Start Seq.	End Seq.	Sequence	Ion Score	C. I. %	Modification	Rank	Result Type
811.4573	811.4189	-0.0384	-47	92	97	YFIGRR					Mascot
814.3876	814.3585	-0.0291	-36	107	113	DLPGCPR			Carbamidomethyl (C)[5]		Mascot
1102.5852	1102.5223	-0.0629	-57	97	106	RSDPNSSVLK					Mascot
1278.5532	1278.514	-0.0392	-31	77	86	ELYDASQHCR			Carbamidomethyl (C)[9]		Mascot
1278.5532	1278.514	-0.0392	-31	77	86	ELYDASQHCR	83	100	Carbamidomethyl (C)[9]		Mascot
1741.8538	1741.7435	-0.1103	-63	98	113	SDPNSSVLKDLPGCPR			Carbamidomethyl (C)[14]		Mascot
2709.2786	2709.2214	-0.0572	-21	46	71	EYVAQQTCGISISGSAVS TEPGNTPR			Carbamidomethyl (C)[8]		Mascot

6 hypothetical protein F775_13286 [Aegilops tauschii] gi|475530535 16397.1 8.7 8 113 100 6.943 67 99.972

Peptide Information

Calc. Mass	Obsrv. Mass	± da	± ppm	Start Seq.	End Seq.	Sequence	Ion Score	C. I. %	Modification	Rank	Result Type
961.5465	961.514	-0.0325	-34	112	120	EFIAGIVGR					Mascot
961.5465	961.514	-0.0325	-34	112	120	EFIAGIVGR	67	99.972			Mascot
1323.6395	1323.6251	-0.0144	-11	97	108	GSLLQDMSGCKK			Carbamidomethyl (C)[10]		Mascot
1341.6104	1341.5671	-0.0433	-32	34	44	EITYDSLNACR			Carbamidomethyl (C)[10]		Mascot
1344.6399	1344.611	-0.0289	-21	78	88	CEVLHVLMDGR			Carbamidomethyl (C)[1], Oxidation (M)[8]		Mascot

1347.5999	1347.5624	-0.0375	-28	50	60	QTCGYYLSAER			Carbamidomethyl (C)[3]		Mascot
1353.6104	1353.5784	-0.032	-24	121	131	EECNLETVFGR			Carbamidomethyl (C)[3]		Mascot
1723.8354	1723.8367	0.0013	1	92	107	EGVVKGSLLQDMSGCK			Carbamidomethyl (C)[15], Oxidation (M)[12]		Mascot
1887.9017	1887.7843	-0.1174	-62	34	49	EITYDSLNACRGYAVR			Carbamidomethyl (C)[10]		Mascot
1887.9017	1887.7843	-0.1174	-62	34	49	EITYDSLNACRGYAVR	1	0	Carbamidomethyl (C)[10]		Mascot

7	hypothetical protein F775_31509 [Aegilops tauschii]	gi|475546491	16904.4	6.81	4	85	99.67	2.766	67	99.972

Peptide Information

Calc. Mass	Obsrv. Mass	± da	± ppm	Start Seq.	End Seq.	Sequence	Ion Score	C. I. %	Modification	Rank	Result Type
845.3934	845.4031	0.0097	11	27	33	EQCVPGR			Carbamidomethyl (C)[3]		Mascot
961.5465	961.514	-0.0325	-34	113	121	EFIAGIVGR					Mascot
961.5465	961.514	-0.0325	-34	113	121	EFIAGIVGR	67	99.972			Mascot
1347.5999	1347.5624	-0.0375	-28	50	60	QTCGYYLSAER			Carbamidomethyl (C)[3]		Mascot
1358.6555	1358.5756	-0.0799	-59	78	88	CEVLHILMDGR			Carbamidomethyl (C)[1], Oxidation (M)[8]		Mascot

8	Trypsin/alpha-amylase inhibitor CMX1/CMX3 [Triticum urartu]	gi|474202113	16973.5	8.59	4	85	99.67	4.133	67	99.972

Peptide Information

Calc. Mass	Obsrv. Mass	± da	± ppm	Start Seq.	End Seq.	Sequence	Ion Score	C. I. %	Modification	Rank	Result Type
808.3505	808.4156	0.0651	81	65	71	GCDELSK			Carbamidomethyl (C)[2]		Mascot
961.5465	961.514	-0.0325	-34	112	120	EFIAGIVGR					Mascot
961.5465	961.514	-0.0325	-34	112	120	EFIAGIVGR	67	99.972			Mascot
1347.5999	1347.5624	-0.0375	-28	50	60	QTCGYYLSAER			Carbamidomethyl (C)[3]		Mascot
1353.6104	1353.5784	-0.032	-24	121	131	EECNLETVFGR			Carbamidomethyl (C)[3]		Mascot

9	RecName: Full=Trypsin/alpha-amylase inhibitor CMX2; AltName: Full=ITRL-2; Flags: Precursor	gi|3913903	14279.3	9.08	4	78	98.56	4.195	67	99.972

Protein Group

RecName: Full=Trypsin/alpha-amylase inhibitor CMX1/CMX3; AltName: Full=ITRL-1/ITRL-3; Flags: Precursor	gi|3913904	14221.3	9.2299995422 3633

Peptide Information

Calc. Mass	Obsrv. Mass	± da	± ppm	Start Seq.	End Seq.	Sequence	Ion Score	C. I. %	Modification	Rank	Result Type
845.3934	845.4031	0.0097	11	27	33	EQCVPGR			Carbamidomethyl (C)[3]		Mascot

961.5465	961.514	-0.0325	-34	112	120	EFIAGIVGR					Mascot
961.5465	961.514	-0.0325	-34	112	120	EFIAGIVGR	67	99.972			Mascot
1347.5999	1347.5624	-0.0375	-28	50	60	QTCGYYLSAER			Carbamidomethyl (C)[3]		Mascot
1351.6964	1351.5908	-0.1056	-78	34	44	EITYESLNARR					Mascot
1351.6964	1351.5908	-0.1056	-78	34	44	EITYESLNARR					Mascot

10 hypothetical protein F775_20718 [Aegilops tauschii] gi|475584715 9219.5 9.25 9 62 32.659 2.651

Peptide Information

Calc. Mass	Obsrv. Mass	± da	± ppm	Start Seq.	End Seq.	Sequence	Ion Score	C. I. %	Modification	Rank	Result Type
807.4029	807.3776	-0.0253	-31	28	36	AAASTAMGK					Mascot
834.4315	834.3725	-0.059	-71	21	27	RISDSEK					Mascot
1055.4528	1055.5166	0.0638	60	47	55	ESFTGEEEK					Mascot
1120.5303	1120.5042	-0.0261	-23	37	46	AMAGEEEKQK					Mascot
1323.6111	1323.6251	0.014	11	56	66	KEACNSWTTAR			Carbamidomethyl (C)[4]		Mascot
1375.7118	1375.5874	-0.1244	-90	67	80	VHPWGSSPAAAAPK					Mascot
1571.7345	1571.7396	0.0051	3	1	14	MSSMGFSYAQVHVK					Mascot
1571.7345	1571.7396	0.0051	3	1	14	MSSMGFSYAQVHVK					Mascot
1869.8912	1869.8026	-0.0886	-47	2	17	SSMGFSYAQVHVKQER			Oxidation (M)[3]		Mascot
1984.9368	1984.951	0.0142	7	1	17	MSSMGFSYAQVHVKQE R					Mascot

Gel Idx/Pos	311/M13	Instr./Gel Origin	BA2151/Sample Project 20140814	Process Status	Analysis Succeeded
Plate [#] Name	[1] Sample Project 20140814	Instrument Sample Name		Spectra	11

Rank	Protein Name	Accession No.	Protein MW	Protein PI	Pep. Count	Protein Score	Protein Score C. I. %	Intensity Matched	Total Ion Score	Total Ion C. I. %	Confirmed
1	RecName: Full=Alpha-amylase/trypsin inhibitor CM2; AltName: Full=Chloroform/methanol-soluble protein CM2; Flags: Precursor	gi|123956	16019.7	6.86	4	302	100	17.919	282	100	

Peptide Information

Calc. Mass	Obsrv. Mass	± da	± ppm	Start Seq.	End Seq.	Sequence	Ion Score	C. I. %	Modification	Rank	Result Type
814.3876	814.3555	-0.0321	-39	107	113	DLPGCPR			Carbamidomethyl (C)[5]		Mascot
1017.5211	1017.4763	-0.0448	-44	97	106	TSDPNSGVLK					Mascot
1278.5532	1278.5017	-0.0515	-40	77	86	ELYDASQHCR			Carbamidomethyl (C)[9]		Mascot
1278.5532	1278.5017	-0.0515	-40	77	86	ELYDASQHCR	78	99.998	Carbamidomethyl (C)[9]		Mascot
2703.3044	2703.2388	-0.0656	-24	46	71	EYVAQQTCGVGIVGSPV STEPGNTPR			Carbamidomethyl (C)[8]		Mascot
2703.3044	2703.2388	-0.0656	-24	46	71	EYVAQQTCGVGIVGSPV STEPGNTPR	204	100	Carbamidomethyl (C)[8]		Mascot

Rank	Protein Name	Accession No.	Protein MW	Protein PI	Pep. Count	Protein Score	Protein Score C. I. %	Intensity Matched	Total Ion Score	Total Ion C. I. %	Confirmed
2	dimeric alpha-amylase inhibitor, partial [Triticum durum]	gi|386877064	14502.8	4.78	3	148	100	3.028	133	100	

Peptide Information

Calc. Mass	Obsrv. Mass	± da	± ppm	Start Seq.	End Seq.	Sequence	Ion Score	C. I. %	Modification	Rank	Result Type
1570.8007	1570.7379	-0.0628	-40	32	45	LQCNGSQVPEAVLR			Carbamidomethyl (C)[3]		Mascot
1677.8517	1677.755	-0.0967	-58	107	122	LPIVIDASGDGAYVCK			Carbamidomethyl (C)[15]		Mascot
1887.8403	1887.771	-0.0693	-37	73	90	EHGVQEGQAGTGAFPSC R			Carbamidomethyl (C)[17]		Mascot
1887.8403	1887.771	-0.0693	-37	73	90	EHGVQEGQAGTGAFPSC R	133	100	Carbamidomethyl (C)[17]		Mascot

Rank	Protein Name	Accession No.	Protein MW	Protein PI	Pep. Count	Protein Score	Protein Score C. I. %	Intensity Matched	Total Ion Score	Total Ion C. I. %	Confirmed
3	Alpha-amylase/trypsin inhibitor CM1 [Aegilops tauschii]	gi|475582216	16086.8	7.49	4	98	99.985	11.162	78	99.998	

Protein Group

RecName: Full=Alpha-amylase/trypsin inhibitor CM1; AltName: Full=Chloroform/methanol-soluble protein CM1; Flags: Precursor	gi|123955	16076.8	7.4899 997711 1816

Peptide Information

Calc. Mass	Obsrv. Mass	± da	± ppm	Start Seq.	End Seq.	Sequence	Ion Score	C. I. %	Modification	Rank	Result Type
811.4573	811.3989	-0.0584	-72	92	97	YFIGRR					Mascot

Calc. Mass	Obsrv. Mass	± da	± ppm	Start Seq.	End Seq.	Sequence	Ion Score	C. I. %	Modification	Rank	Result Type
814.3876	814.3555	-0.0321	-39	107	113	DLPGCPR			Carbamidomethyl (C)[5]		Mascot
1278.5532	1278.5017	-0.0515	-40	77	86	ELYDASQHCR			Carbamidomethyl (C)[9]		Mascot
1278.5532	1278.5017	-0.0515	-40	77	86	ELYDASQHCR	78	99.998	Carbamidomethyl (C)[9]		Mascot
2709.2786	2709.21	-0.0686	-25	46	71	EYVAQQTCGISISGSAVS TEPGNTPR			Carbamidomethyl (C)[8]		Mascot

4	RecName: Full=Chymotrypsin inhibitor WCI; AltName: Full=Chloroform/methanol-soluble protein WCI	gi|75107149	13505.5	7.42	1	96	99.973	1.031	90	100

Peptide Information

Calc. Mass	Obsrv. Mass	± da	± ppm	Start Seq.	End Seq.	Sequence	Ion Score	C. I. %	Modification	Rank	Result Type
1986.0193	1985.9674	-0.0519	-26	67	86	AFPPSQSQGGGPPQPPL APR					Mascot
1986.0193	1985.9674	-0.0519	-26	67	86	AFPPSQSQGGGPPQPPL APR	90	100			Mascot

5	Trypsin inhibitor CMc [Aegilops tauschii]	gi|475430040	15876.7	7.48	1	95	99.972	1.031	90	100

Peptide Information

Calc. Mass	Obsrv. Mass	± da	± ppm	Start Seq.	End Seq.	Sequence	Ion Score	C. I. %	Modification	Rank	Result Type
1986.0193	1985.9674	-0.0519	-26	91	110	AFPPSQSQGGGPPQPPL APR					Mascot
1986.0193	1985.9674	-0.0519	-26	91	110	AFPPSQSQGGGPPQPPL APR	90	100			Mascot

6	PREDICTED: protein slowmo homolog 2-like isoform X1 [Cicer arietinum]	gi|502081729	21629.8	8.45	12	58	0	3.384		

Protein Group

PREDICTED: protein slowmo homolog 2-like isoform X2 [Cicer arietinum]	gi|502081732	21629.8	8.4499998092 6514

Peptide Information

Calc. Mass	Obsrv. Mass	± da	± ppm	Start Seq.	End Seq.	Sequence	Ion Score	C. I. %	Modification	Rank	Result Type
806.4155	806.3808	-0.0347	-43	18	24	VTSASWR					Mascot
807.4359	807.3705	-0.0654	-81	156	162	FLQNSAK					Mascot
830.4341	830.3638	-0.0703	-85	65	70	CPWLVR			Carbamidomethyl (C)[1]		Mascot
1033.4844	1033.4762	-0.0082	-8	148	155	VEQRCADR			Carbamidomethyl (C)[5]		Mascot
1050.5071	1050.5188	0.0117	11	165	172	DVMERICK			Carbamidomethyl (C)[7]		Mascot
1050.5691	1050.5188	-0.0503	-48	156	164	FLQNSAKSR					Mascot
1127.5626	1127.4806	-0.082	-73	90	98	SRSMQLSYR					Mascot

Calc. Mass	Obsrv. Mass	± da	± ppm	Start Seq.	End Seq.	Sequence	Ion Score	C. I. %	Modification	Rank	Result Type
1232.5681	1232.5525	-0.0156	-13	173	183	YLEAESSSFSL					Mascot
1309.6318	1309.6202	-0.0116	-9	152	162	CADRFLQNSAK			Carbamidomethyl (C)[1]		Mascot
1480.837	1480.722	-0.115	-78	34	46	VLSHILEVDTLNK					Mascot
1599.7618	1599.7491	-0.0127	-8	92	104	SMQLSYRNVNMEK					Mascot
1867.8855	1867.8192	-0.0663	-35	99	113	NVNMEKFIEVEENTR			Oxidation (M)[4]		Mascot

7	Alpha-amylase inhibitor 0.28 [Aegilops tauschii]	gi|475578459	17187.3	7.45	6	56	0	2.933	26	0	

Peptide Information

Calc. Mass	Obsrv. Mass	± da	± ppm	Start Seq.	End Seq.	Sequence	Ion Score	C. I. %	Modification	Rank	Result Type
830.4189	830.3638	-0.0551	-66	107	113	EVLPGCR			Carbamidomethyl (C)[6]		Mascot
863.4404	863.4083	-0.0321	-37	45	52	VSALTGCR			Carbamidomethyl (C)[7]		Mascot
1050.5579	1050.5188	-0.0391	-37	95	103	SVYQELGVR					Mascot
1050.5579	1050.5188	-0.0391	-37	95	103	SVYQELGVR	26	0			Mascot
1308.6763	1308.6606	-0.0157	-12	45	56	VSALTGCRAMVK			Carbamidomethyl (C)[7], Oxidation (M)[10]		Mascot
1555.8262	1555.7734	-0.0528	-34	57	70	LQCVGSQVPEAVLR			Carbamidomethyl (C)[3]		Mascot
1677.855	1677.755	-0.1	-60	115	129	EVMKLTAASVPEVCK			Carbamidomethyl (C)[14], Oxidation (M)[3]		Mascot

8	PREDICTED: exosome complex component RRP41-like isoform X1 [Cicer arietinum]	gi|502113879	26815.6	5.62	12	56	0	14.874	

Protein Group

PREDICTED: exosome complex component RRP41-like isoform X2 [Cicer arietinum]	gi|502113882	26815.6	5.6199 998855 5908

Peptide Information

Calc. Mass	Obsrv. Mass	± da	± ppm	Start Seq.	End Seq.	Sequence	Ion Score	C. I. %	Modification	Rank	Result Type
819.3964	819.3623	-0.0341	-42	15	20	RPMEMR					Mascot
835.3913	835.3594	-0.0319	-38	15	20	RPMEMR			Oxidation (M)[3]		Mascot
864.4686	864.4855	0.0169	20	234	239	QLEYRR					Mascot
959.5408	959.4924	-0.0484	-50	226	233	EILLENTK					Mascot
1050.55	1050.5188	-0.0312	-30	192	200	VTLLQMDSK			Oxidation (M)[6]		Mascot
1050.55	1050.5188	-0.0312	-30	192	200	VTLLQMDSK			Oxidation (M)[6]		Mascot
1207.5776	1207.5278	-0.0498	-41	1	10	MEYVNPEGLR					Mascot
1232.6351	1232.5525	-0.0826	-67	15	23	RPMEMRQIR			Oxidation (M)[3]		Mascot
1278.6042	1278.5017	-0.1025	-80	11	20	VDGRRPMEMR			Oxidation (M)[7,9]		Mascot

1278.6042	1278.5017	-0.1025	-80	11	20	VDGRRPMEMR			Oxidation (M)[7,9]		Mascot
1340.6151	1340.6097	-0.0054	-4	32	44	ADGSAIFEMGNTK					Mascot
1390.7611	1390.6445	-0.1166	-84	189	200	LDKVTLLQMDSK					Mascot
1553.5996	1553.6785	0.0789	51	70	82	CEYSMANFSTGDR			Carbamidomethyl (C)[1], Oxidation (M)[5]		Mascot
1571.8652	1571.7329	-0.1323	-84	45	58	VIAAVYGPREVQNR					Mascot
1571.8652	1571.7329	-0.1323	-84	45	58	VIAAVYGPREVQNR					Mascot
2731.394	2731.2498	-0.1442	-53	92	114	STEISLVIRQTMEACILTH LMPR			Carbamidomethyl (C)[15], Oxidation (M)[12,21]		Mascot

9	RecName: Full=Alpha-amylase inhibitor 0.28; AltName: Full=CIII; AltName: Full=WMAI-1; Flags: Precursor	gi|110282974	17358.3	7.45	6	56	0	2.933	26	0	

Peptide Information

Calc. Mass	Obsrv. Mass	± da	± ppm	Start Seq.	End Seq.	Sequence	Ion Score	C. I. %	Modification	Rank	Result Type
830.4189	830.3638	-0.0551	-66	107	113	EVLPGCR			Carbamidomethyl (C)[6]		Mascot
863.4404	863.4083	-0.0321	-37	45	52	VSALTGCR			Carbamidomethyl (C)[7]		Mascot
1050.5579	1050.5188	-0.0391	-37	95	103	SVYQELGVR					Mascot
1050.5579	1050.5188	-0.0391	-37	95	103	SVYQELGVR	26	0			Mascot
1308.6763	1308.6606	-0.0157	-12	45	56	VSALTGCRAMVK			Carbamidomethyl (C)[7], Oxidation (M)[10]		Mascot
1555.8262	1555.7734	-0.0528	-34	57	70	LQCVGSQVPEAVLR			Carbamidomethyl (C)[3]		Mascot
1677.855	1677.755	-0.1	-60	115	129	EVMKLTAASVPEVCK			Carbamidomethyl (C)[14], Oxidation (M)[3]		Mascot

10	hypothetical protein ZEAMMB73_923732 [Zea mays]	gi|413918838	21404.7	9.85	12	55	0	3.829			

Peptide Information

Calc. Mass	Obsrv. Mass	± da	± ppm	Start Seq.	End Seq.	Sequence	Ion Score	C. I. %	Modification	Rank	Result Type
807.3665	807.3705	0.004	5	32	39	GSSGPQMK			Oxidation (M)[7]		Mascot
812.4122	812.3823	-0.0299	-37	103	109	EGARHSR					Mascot
819.4207	819.3623	-0.0584	-71	144	150	NSKDEVK					Mascot
836.3268	836.4036	0.0768	92	136	143	DDAGSEDK					Mascot
1006.4986	1006.4109	-0.0877	-87	30	39	AKGSSGPQMK			Oxidation (M)[9]		Mascot
1033.5095	1033.4762	-0.0333	-32	32	42	GSSGPQMKGGK					Mascot
1107.4437	1107.5004	0.0567	51	126	135	DEPDSTDNSK					Mascot
1351.5973	1351.5817	-0.0156	-12	147	159	DEVKSSEGNDGSK					Mascot
1379.5808	1379.6326	0.0518	38	169	180	LEENEGESSEEK					Mascot
1480.6398	1480.722	0.0822	56	122	135	ASSKDEPDSTDNSK					Mascot
1507.6758	1507.6919	0.0161	11	168	180	KLEENEGESSEEK					Mascot
1507.6758	1507.6919	0.0161	11	168	180	KLEENEGESSEEK					Mascot

2242.0339	2241.8916	-0.1423	-63	62	82	GMHDSNNMPNADSSVTP ARLK	Mascot

Gel Idx/Pos	312/M14	Instr./Gel Origin	BA2151/Sample Project 20140814	Process Status	Analysis Succeeded
Plate [#] Name	[1] Sample Project 20140814	Instrument Sample Name		Spectra	11

Rank	Protein Name	Accession No.	Protein MW	Protein PI	Pep. Count	Protein Score	Protein Score C. I. %	Intensity Matched	Total Ion Score	Total Ion C. I. %	Confirmed
1	dimeric alpha-amylase inhibitor, partial [Triticum durum]	gi|386877064	14502.8	4.78	5	182	100	25.443	152	100	

Peptide Information

Calc. Mass	Obsrv. Mass	± da	± ppm	Start Seq.	End Seq.	Sequence	Ion Score	C. I. %	Modification	Rank	Result Type
1554.6637	1554.719	0.0553	36	60	72	CGALYSMLDSMYK			Carbamidomethyl (C)[1], Oxidation (M)[7]		Mascot
1570.8007	1570.7502	-0.0505	-32	32	45	LQCNGSQVPEAVLR			Carbamidomethyl (C)[3]		Mascot
1677.8517	1677.7567	-0.095	-57	107	122	LPIVIDASGDGAYVCK			Carbamidomethyl (C)[15]		Mascot
1887.8403	1887.7791	-0.0612	-32	73	90	EHGVQEGGQAGTGAFPSCR			Carbamidomethyl (C)[17]		Mascot
1887.8403	1887.7791	-0.0612	-32	73	90	EHGVQEGGQAGTGAFPSCR	152	100	Carbamidomethyl (C)[17]		Mascot
2043.9414	2043.8724	-0.069	-34	73	91	EHGVQEGGQAGTGAFPSCRR			Carbamidomethyl (C)[17]		Mascot

Rank	Protein Name	Accession No.	Protein MW	Protein PI	Pep. Count	Protein Score	Protein Score C. I. %	Intensity Matched	Total Ion Score	Total Ion C. I. %	Confirmed
2	dimeric alpha-amylase inhibitor [Kengyilia batalinii]	gi|534313896	15685.5	4.72	3	62	34.192	14.31	48	97.942	

Protein Group

dimeric alpha-amylase inhibitor [Kengyilia pamirica]	gi|534313976	15643.5	5.0700001716138

Peptide Information

Calc. Mass	Obsrv. Mass	± da	± ppm	Start Seq.	End Seq.	Sequence	Ion Score	C. I. %	Modification	Rank	Result Type
1565.6797	1565.6198	-0.0599	-38	71	83	CGALYSMLDNMYK			Carbamidomethyl (C)[1]		Mascot
1571.7847	1571.7378	-0.0469	-30	43	56	LQCNGSEVPEAVLR			Carbamidomethyl (C)[3]		Mascot
1571.7847	1571.7378	-0.0469	-30	43	56	LQCNGSEVPEAVLR	48	97.942	Carbamidomethyl (C)[3]		Mascot
1597.6696	1597.6624	-0.0072	-5	71	83	CGALYSMLDNMYK			Carbamidomethyl (C)[1], Oxidation (M)[7,11]		Mascot
1677.8517	1677.7567	-0.095	-57	118	133	LPIVIDASGDGAYVCK			Carbamidomethyl (C)[15]		Mascot

Rank	Protein Name	Accession No.	Protein MW	Protein PI	Pep. Count	Protein Score	Protein Score C. I. %	Intensity Matched	Total Ion Score	Total Ion C. I. %	Confirmed
3	dimeric alpha-amylase inhibitor [Kengyilia tahelacana]	gi|534313994	15728.5	4.88	3	60	9.16	14.31	47	97.146	

Protein Group

dimeric alpha-amylase inhibitor [Agropyron cristatum]	gi|534313874	15712.5	4.86999988555908

dimeric alpha-amylase inhibitor [Kengyilia alatavica]	gi|534313889	15731.5	4.8800 001144 4092
dimeric alpha-amylase inhibitor [Kengyilia kaschgarica]	gi|534313925	15731.5	4.8800 001144 4092
dimeric alpha-amylase inhibitor [Kengyilia zhaosuensis]	gi|534314015	15731.5	4.8800 001144 4092

Peptide Information

Calc. Mass	Obsrv. Mass	± da	± ppm	Start Seq.	End Seq.	Sequence	Ion Score	C. I. %	Modification	Rank	Result Type
1565.6797	1565.6198	-0.0599	-38	71	83	CGALYSMLDNMYK			Carbamidomethyl (C)[1]		Mascot
1571.7847	1571.7378	-0.0469	-30	43	56	LQCNGSEVPEAVLR			Carbamidomethyl (C)[3]		Mascot
1571.7847	1571.7378	-0.0469	-30	43	56	LQCNGSEVPEAVLR	48	97.942	Carbamidomethyl (C)[3]		Mascot
1597.6696	1597.6624	-0.0072	-5	71	83	CGALYSMLDNMYK			Carbamidomethyl (C)[1], Oxidation (M)[7,11]		Mascot
1677.8517	1677.7567	-0.095	-57	118	133	LPIVIDASGDGAYVCK			Carbamidomethyl (C)[15]		Mascot

4	dimeric alpha-amylase inhibitor [Kengyilia gobicola]	gi	534313905	15659.5	4.88	2	56	0	13.779	48	97.942

Peptide Information

Calc. Mass	Obsrv. Mass	± da	± ppm	Start Seq.	End Seq.	Sequence	Ion Score	C. I. %	Modification	Rank	Result Type
1565.6797	1565.6198	-0.0599	-38	71	83	CGALYSMLDNMYK			Carbamidomethyl (C)[1]		Mascot
1571.7847	1571.7378	-0.0469	-30	43	56	LQCNGSEVPEAVLR			Carbamidomethyl (C)[3]		Mascot
1571.7847	1571.7378	-0.0469	-30	43	56	LQCNGSEVPEAVLR	48	97.942	Carbamidomethyl (C)[3]		Mascot
1597.6696	1597.6624	-0.0072	-5	71	83	CGALYSMLDNMYK			Carbamidomethyl (C)[1], Oxidation (M)[7,11]		Mascot

5	dimeric alpha-amylase inhibitor [Kengyilia batalinii var. nana]	gi|534313967	15703.5	4.88	2	54	0	13.779	47	97.146

Peptide Information

Calc. Mass	Obsrv. Mass	± da	± ppm	Start Seq.	End Seq.	Sequence	Ion Score	C. I. %	Modification	Rank	Result Type
1565.6797	1565.6198	-0.0599	-38	71	83	CGALYSMLDNMYK			Carbamidomethyl (C)[1]		Mascot
1571.7847	1571.7378	-0.0469	-30	43	56	LQCNGSEVPEAVLR			Carbamidomethyl (C)[3]		Mascot

Calc. Mass	Obsrv. Mass	± da	± ppm	Start Seq.	End Seq.	Sequence	Ion Score	C. I. %	Modification	Rank	Result Type
1571.7847	1571.7378	-0.0469	-30	43	56	LQCNGSEVPEAVLR	48	97.942	Carbamidomethyl (C)[3]		Mascot
1597.6696	1597.6624	-0.0072	-5	71	83	CGALYSMLDNMYK			Carbamidomethyl (C)[1], Oxidation (M)[7,11]		Mascot

6	TPA: hypothetical protein ZEAMMB73_602074 [Zea mays]	gi|414883797	5368.4	8.69	5	49	0	1.455			

Peptide Information

Calc. Mass	Obsrv. Mass	± da	± ppm	Start Seq.	End Seq.	Sequence	Ion Score	C. I. %	Modification	Rank	Result Type
1565.7417	1565.6198	-0.1219	-78	30	43	IGYPSFGLCSHIQS			Carbamidomethyl (C)[9]		Mascot
1705.6372	1705.6868	0.0496	29	15	27	VSCGCCSNMCWLR			Carbamidomethyl (C)[3,5,6,10], Oxidation (M)[9]		Mascot
1767.8458	1767.7109	-0.1349	-76	2	14	ISMWLHCQNFLFR			Carbamidomethyl (C)[7], Oxidation (M)[3]		Mascot
1882.8914	1882.7079	-0.1835	-97	1	14	MISMWLHCQNFLFR			Carbamidomethyl (C)[8]		Mascot
1898.8864	1898.719	-0.1674	-88	1	14	MISMWLHCQNFLFR			Carbamidomethyl (C)[8], Oxidation (M)[1]		Mascot
1914.8812	1914.7145	-0.1667	-87	1	14	MISMWLHCQNFLFR			Carbamidomethyl (C)[8], Oxidation (M)[1,4]		Mascot
1958.8275	1958.7292	-0.0983	-50	15	29	VSCGCCSNMCWLRLR			Carbamidomethyl (C)[3,5,6,10]		Mascot

7	hypothetical protein TRIUR3_30584 [Triticum urartu]	gi|472904814	9464.2	7.56	6	46	0	1.114			

Peptide Information

Calc. Mass	Obsrv. Mass	± da	± ppm	Start Seq.	End Seq.	Sequence	Ion Score	C. I. %	Modification	Rank	Result Type
1583.7668	1583.7166	-0.0502	-32	68	82	MGKTAAPYVNSGMTR					Mascot
1599.7618	1599.7566	-0.0052	-3	68	82	MGKTAAPYVNSGMTR			Oxidation (M)[1]		Mascot
1599.7618	1599.7566	-0.0052	-3	68	82	MGKTAAPYVNSGMTR			Oxidation (M)[1]		Mascot
1759.7238	1759.7499	0.0261	15	1	15	MTTGVGTGFYSCCYR			Carbamidomethyl (C)[12,13]		Mascot
1810.7926	1810.7228	-0.0698	-39	52	67	NRGFDMSAGWEANAER					Mascot
1827.8153	1827.7357	-0.0796	-44	2	17	TTGVGTGFYSCCYRAK			Carbamidomethyl (C)[11,12]		Mascot
1856.8054	1856.7228	-0.0826	-44	54	70	GFDMSAGWEANAERMGK					Mascot
1958.8558	1958.7292	-0.1266	-65	1	17	MTTGVGTGFYSCCYRAK			Carbamidomethyl (C)[12,13]		Mascot

8	Autoimmune regulator [Aegilops tauschii]	gi|475587117	218351.5	7.18	13	44	0	3.581	36	67.976	

Peptide Information

Calc. Mass	Obsrv. Mass	± da	± ppm	Start Seq.	End Seq.	Sequence	Ion Score	C. I. %	Modification	Rank	Result Type
959.425	959.5172	0.0922	96	980	988	GEGTAMEHK					Mascot
1215.658	1215.717	0.059	49	1609	1619	LDGNITTKPEK					Mascot
1289.7001	1289.761	0.0609	47	1735	1744	WKQFVEPSIR					Mascot
1320.6399	1320.7251	0.0852	65	872	883	LPSGSNQMSMLR					Mascot

Calc. Mass	Obsrv. Mass	± da	± ppm	Start Seq.	End Seq.	Sequence	Ion Score	C. I. %	Modification	Rank	Result Type
1480.7247	1480.7294	0.0047	3	871	883	KLPSGSNQMSMLR			Oxidation (M)[9,11]		Mascot
1480.7247	1480.7294	0.0047	3	871	883	KLPSGSNQMSMLR	36	67.976	Oxidation (M)[9,11]		Mascot
1553.7555	1553.7299	-0.0256	-16	938	952	EPTAHTISEPASSAR					Mascot
1611.7246	1611.5731	-0.1515	-94	792	806	EPLEHTTSGPDSDAR					Mascot
1651.7415	1651.6121	-0.1294	-78	601	615	VQSTPVIMGSDCGER			Carbamidomethyl (C)[12], Oxidation (M)[8]		Mascot
1704.7839	1704.7001	-0.0838	-49	503	515	HPMEWSDVDKFWK					Mascot
1738.829	1738.6759	-0.1531	-88	623	638	SQAVPSCHVLEGGQNR			Carbamidomethyl (C)[7]		Mascot
1779.8364	1779.722	-0.1144	-64	600	615	KVQSTPVIMGSDCGER			Carbamidomethyl (C)[13], Oxidation (M)[9]		Mascot
1944.9603	1944.776	-0.1843	-95	360	376	EYPHFSKPAYDAAVPPR					Mascot
1990.0096	1989.984	-0.0256	-13	823	840	LPSESNQMIMLRGEGTV K					Mascot

9 Sulfotransferase 17 [Theobroma cacao] gi|508717762 41787 8.69 10 41 0 23.638

Peptide Information

Calc. Mass	Obsrv. Mass	± da	± ppm	Start Seq.	End Seq.	Sequence	Ion Score	C. I. %	Modification	Rank	Result Type
1055.464	1055.5541	0.0901	85	257	266	YAGDSEATNK					Mascot
1249.5729	1249.6886	0.1157	93	13	22	EEVSPEKMER			Oxidation (M)[8]		Mascot
1526.7573	1526.7119	-0.0454	-30	166	178	DAFVSMFHFAAKR					Mascot
1553.7053	1553.7299	0.0246	16	25	37	EFMSTLPAENGWR			Oxidation (M)[3]		Mascot
1597.7428	1597.6624	-0.0804	-50	274	286	VGDWQNYLTMAQR			Oxidation (M)[10]		Mascot
1724.8466	1724.8496	0.003	2	163	177	EPKDAFVSMFHFAAK					Mascot
1792.88	1792.7035	-0.1765	-98	23	37	VREFMSTLPAENGWR					Mascot
1887.8818	1887.7791	-0.1027	-54	2	19	EPSGAADSLDKEEVSPE K					Mascot
1887.8818	1887.7791	-0.1027	-54	2	19	EPSGAADSLDKEEVSPE K					Mascot
1898.908	1898.719	-0.189	-100	336	350	WYHLAQGSDLRHCQK			Carbamidomethyl (C)[13]		Mascot
1919.9181	1919.7516	-0.1665	-87	331	346	SCAQKWYHLAQGSDLR			Carbamidomethyl (C)[2]		Mascot

10 Os06g0658200 [Oryza sativa Japonica Group] gi|255677289 16009.9 9.5 7 41 0 1.527

Peptide Information

Calc. Mass	Obsrv. Mass	± da	± ppm	Start Seq.	End Seq.	Sequence	Ion Score	C. I. %	Modification	Rank	Result Type
1289.6824	1289.761	0.0786	61	79	88	WIWMVQASLR					Mascot
1479.7196	1479.7424	0.0228	15	49	61	EAVGMVTAMGWRR			Oxidation (M)[5]		Mascot
1491.6306	1491.614	-0.0166	-11	147	162	ETRGDDGQSAEGAAAG					Mascot
1704.8486	1704.7001	-0.1485	-87	123	138	SGATWLARPASAETMR					Mascot
1724.8458	1724.8496	0.0038	2	45	60	VSAKEAVGMVTAMGWR			Oxidation (M)[9,13]		Mascot

1917.9712	1918.005	0.0338	18	123	140	SGATWLARPASAETMRG R	Mascot
2039.9781	2039.8225	-0.1556	-76	93	113	LAGWQEDGVAVGDGGD GPALR	Mascot

Gel Idx/Pos	313/M15	Instr./Gel Origin	BA2151/Sample Project 20140814	Process Status	Analysis Succeeded
Plate [#] Name	[1] Sample Project 20140814	Instrument Sample Name		Spectra	11

Rank	Protein Name	Accession No.	Protein MW	Protein PI	Pep. Count	Protein Score	Protein Score C. I. %	Intensity Matched	Total Ion Score	Total Ion C. I. %	Confirmed
1	RecName: Full=Alpha-amylase inhibitor 0.28; AltName: Full=CIII; AltName: Full=WMAI-1; Flags: Precursor	gi|110282974	17358.3	7.45	7	355	100	58.353	317	100	

Protein Group

Alpha-amylase inhibitor 0.28 [Aegilops tauschii]	gi|475578459	17187.3	7.4499 998092 6514

Peptide Information

Calc. Mass	Obsrv. Mass	± da	± ppm	Start Seq.	End Seq.	Sequence	Ion Score	C. I. %	Modification	Rank	Result Type
830.4189	830.389	-0.0299	-36	107	113	EVLPGCR			Carbamidomethyl (C)[6]		Mascot
863.4404	863.4152	-0.0252	-29	45	52	VSALTGCR			Carbamidomethyl (C)[7]		Mascot
1050.5579	1050.522	-0.0359	-34	95	103	SVYQELGVR					Mascot
1050.5579	1050.522	-0.0359	-34	95	103	SVYQELGVR	72	99.99			Mascot
1054.4657	1054.4099	-0.0558	-53	86	94	CGDLSSMLR			Carbamidomethyl (C)[1], Oxidation (M)[7]		Mascot
1174.6136	1174.5415	-0.0721	-61	119	129	LTAASVPEVCK			Carbamidomethyl (C)[10]		Mascot
1555.8262	1555.7826	-0.0436	-28	57	70	LQCVGSQVPEAVLR			Carbamidomethyl (C)[3]		Mascot
1555.8262	1555.7826	-0.0436	-28	57	70	LQCVGSQVPEAVLR	113	100	Carbamidomethyl (C)[3]		Mascot
1981.795	1981.7411	-0.0539	-27	71	85	DCCQQLADINNEWCR			Carbamidomethyl (C)[2,3,14]		Mascot
1981.795	1981.7411	-0.0539	-27	71	85	DCCQQLADINNEWCR	132	100	Carbamidomethyl (C)[2,3,14]		Mascot

Rank	Protein Name	Accession No.	Protein MW	Protein PI	Pep. Count	Protein Score	Protein Score C. I. %	Intensity Matched	Total Ion Score	Total Ion C. I. %	Confirmed
2	dimeric alpha-amylase inhibitor, partial [Triticum durum]	gi|386877064	14502.8	4.78	4	136	100	2.294	115	100	

Peptide Information

Calc. Mass	Obsrv. Mass	± da	± ppm	Start Seq.	End Seq.	Sequence	Ion Score	C. I. %	Modification	Rank	Result Type
821.3676	821.4019	0.0343	42	123	130	DVAAYPDA					Mascot
1554.6637	1554.7639	0.1002	64	60	72	CGALYSMLDSMYK			Carbamidomethyl (C)[1], Oxidation (M)[7]		Mascot
1570.8007	1570.78	-0.0207	-13	32	45	LQCNGSQVPEAVLR			Carbamidomethyl (C)[3]		Mascot
1887.8403	1887.7812	-0.0591	-31	73	90	EHGVQEGQAGTGAFPSC R			Carbamidomethyl (C)[17]		Mascot
1887.8403	1887.7812	-0.0591	-31	73	90	EHGVQEGQAGTGAFPSC R	115	100	Carbamidomethyl (C)[17]		Mascot

Rank	Protein Name	Accession No.	Protein MW	Protein PI	Pep. Count	Protein Score	Protein Score C. I. %	Intensity Matched	Total Ion Score	Total Ion C. I. %	Confirmed
3	dimeric alpha-amylase inhibitor [Elymus tibeticus]	gi|534314066	15479.2	5.61	4	133	100	26.889	113	100	

Peptide Information

Calc. Mass	Obsrv. Mass	± da	± ppm	Start Seq.	End Seq.	Sequence	Ion Score	C. I. %	Modification	Rank	Result Type
1032.5581	1032.5063	-0.0518	-50	1	10	MLVATPMAAK					Mascot
1174.6136	1174.5415	-0.0721	-61	106	116	LTAASVPEVCK			Carbamidomethyl (C)[10]		Mascot
1555.8262	1555.7826	-0.0436	-28	44	57	LQCVGSQVPEAVLR			Carbamidomethyl (C)[3]		Mascot
1555.8262	1555.7826	-0.0436	-28	44	57	LQCVGSQVPEAVLR	113	100	Carbamidomethyl (C)[3]		Mascot
1894.7993	1894.724	-0.0753	-40	58	72	DCCQQLAGIVNDWCR			Carbamidomethyl (C)[2,3,14]		Mascot

4	dimeric alpha-amylase inhibitor [Elymus ciliaris]	gi|534314045	15097	4.71	3	126	100	29.009	113	100	

Protein Group

dimeric alpha-amylase inhibitor [Elymus brevipes]	gi|534314033	15281.1	5.0700 001716 6138
dimeric alpha-amylase inhibitor [Elymus tibeticus]	gi|534314063	15129	4.7100 000381 4697
dimeric alpha-amylase inhibitor [Kengyilia grandiglumis]	gi|534313917	15132	4.7100 000381 4697
dimeric alpha-amylase inhibitor [Kengyilia kokonorica]	gi|534313934	15129	4.7100 000381 4697
dimeric alpha-amylase inhibitor [Kengyilia longiglumis]	gi|534313943	15101	4.5599 999427 7954
dimeric alpha-amylase inhibitor [Kengyilia melanthera]	gi|534313952	15187	4.5900 001525 8789
dimeric alpha-amylase inhibitor [Kengyilia mutica]	gi|534313961	15129	4.7100 000381 4697

dimeric alpha-amylase inhibitor [Kengyilia rigidula]	gi|534313988	15129	4.7100 000381 4697
dimeric alpha-amylase inhibitor [Kengyilia thoroldiana]	gi|534314006	15129	4.7100 000381 4697
dimeric alpha-amylase inhibitor [Pseudoroegneria strigosa]	gi|534314029	15421.1	4.6199 998855 5908

Peptide Information

Calc. Mass	Obsrv. Mass	± da	± ppm	Start Seq.	End Seq.	Sequence	Ion Score	C. I. %	Modification	Rank	Result Type
863.4404	863.4152	-0.0252	-29	32	39	VSALTGCR			Carbamidomethyl (C)[7]		Mascot
1555.8262	1555.7826	-0.0436	-28	44	57	LQCVGSQVPEAVLR			Carbamidomethyl (C)[3]		Mascot
1555.8262	1555.7826	-0.0436	-28	44	57	LQCVGSQVPEAVLR	113	100	Carbamidomethyl (C)[3]		Mascot
1968.7997	1968.741	-0.0587	-30	58	72	DCCQQLADITNEWCR			Carbamidomethyl (C)[2,3,14]		Mascot

5	dimeric alpha-amylase inhibitor [Elymus grandis]	gi|534314051	15377.1	4.85	2	121	100	27.046	113	100

Peptide Information

Calc. Mass	Obsrv. Mass	± da	± ppm	Start Seq.	End Seq.	Sequence	Ion Score	C. I. %	Modification	Rank	Result Type
1537.7501	1537.7701	0.02	13	1	15	MLVATPMAAEYGAGR					Mascot
1555.8262	1555.7826	-0.0436	-28	44	57	LQCVGSQVPEAVLR			Carbamidomethyl (C)[3]		Mascot
1555.8262	1555.7826	-0.0436	-28	44	57	LQCVGSQVPEAVLR	113	100	Carbamidomethyl (C)[3]		Mascot
1569.74	1569.7878	0.0478	30	1	15	MLVATPMAAEYGAGR			Oxidation (M)[1,7]		Mascot

6	dimeric alpha-amylase inhibitor [Elymus spicatus]	gi|534314025	15220	5.2	2	121	100	27.054	113	100

Peptide Information

Calc. Mass	Obsrv. Mass	± da	± ppm	Start Seq.	End Seq.	Sequence	Ion Score	C. I. %	Modification	Rank	Result Type
1306.6494	1306.7256	0.0762	58	32	43	VSPLTGCQAMVK			Carbamidomethyl (C)[7], Oxidation (M)[10]		Mascot
1555.8262	1555.7826	-0.0436	-28	44	57	LQCVGSQVPEAVLR			Carbamidomethyl (C)[3]		Mascot

1555.8262	1555.7826	-0.0436	-28	44	57	LQCVGSQVPEAVLR	113	100	Carbamidomethyl (C)[3]		Mascot

7 dimeric alpha-amylase inhibitor [Elymus ciliaris] gi|534314048 15424.1 5.27 2 121 100 26.625 113 100

Protein Group

dimeric alpha-amylase inhibitor [Elymus grandis] gi|534314054 15496.2 5.0599 999427 7954

Peptide Information

Calc. Mass	Obsrv. Mass	± da	± ppm	Start Seq.	End Seq.	Sequence	Ion Score	C. I. %	Modification	Rank	Result Type
1174.6136	1174.5415	-0.0721	-61	106	116	LTAASVPEVCK			Carbamidomethyl (C)[10]		Mascot
1555.8262	1555.7826	-0.0436	-28	44	57	LQCVGSQVPEAVLR			Carbamidomethyl (C)[3]		Mascot
1555.8262	1555.7826	-0.0436	-28	44	57	LQCVGSQVPEAVLR	113	100	Carbamidomethyl (C)[3]		Mascot

8 dimeric alpha-amylase inhibitor [Elymus caucasicus] gi|534314039 15448.2 5.29 1 118 100 26.542 113 100

Protein Group

dimeric alpha-amylase inhibitor [Elymus caucasicus] gi|534314042 15412.1 5.2899 999618 5303

Peptide Information

Calc. Mass	Obsrv. Mass	± da	± ppm	Start Seq.	End Seq.	Sequence	Ion Score	C. I. %	Modification	Rank	Result Type
1555.8262	1555.7826	-0.0436	-28	44	57	LQCVGSQVPEAVLR			Carbamidomethyl (C)[3]		Mascot
1555.8262	1555.7826	-0.0436	-28	44	57	LQCVGSQVPEAVLR	113	100	Carbamidomethyl (C)[3]		Mascot

9 dimeric alpha-amylase inhibitor [Kengyilia zhaosuensis] gi|534314012 15451.2 5.06 3 75 96.922 2.832 62 99.885

Peptide Information

Calc. Mass	Obsrv. Mass	± da	± ppm	Start Seq.	End Seq.	Sequence	Ion Score	C. I. %	Modification	Rank	Result Type
1174.6136	1174.5415	-0.0721	-61	106	116	LTAASVPEVCK			Carbamidomethyl (C)[10]		Mascot
1583.8574	1583.8069	-0.0505	-32	44	57	LQCVGSQVPEVVLR			Carbamidomethyl (C)[3]		Mascot
1583.8574	1583.8069	-0.0505	-32	44	57	LQCVGSQVPEVVLR	62	99.885	Carbamidomethyl (C)[3]		Mascot
1869.8008	1869.762	-0.0388	-21	58	72	DCYQQLAGIANDWCR			Carbamidomethyl (C)[2,14]		Mascot

10 dimeric alpha-amylase inhibitor [Kengyilia gobicola] gi|534313912 15451.2 5.3 3 75 96.922 2.832 62 99.885

Peptide Information

Calc. Mass	Obsrv. Mass	± da	± ppm	Start Seq.	End Seq.	Sequence	Ion Score	C. I. %	Modification	Rank	Result Type
1174.6136	1174.5415	-0.0721	-61	106	116	LTAASVPEVCK			Carbamidomethyl (C)[10]		Mascot
1583.8574	1583.8069	-0.0505	-32	44	57	LQCVGSQVPEVVLR			Carbamidomethyl (C)[3]		Mascot
1583.8574	1583.8069	-0.0505	-32	44	57	LQCVGSQVPEVVLR	62	99.885	Carbamidomethyl (C)[3]		Mascot
1869.8372	1869.762	-0.0752	-40	58	72	DCYQKLAGIANDWCR			Carbamidomethyl (C)[2,14]		Mascot
